# CausalCellInfer: Prioritizing Cell‐Type‐Specific Disease Associations From Genetically Predicted Transcriptomes in Biobank Cohorts

**DOI:** 10.1002/advs.78197

**Published:** 2026-10-14

**Authors:** Liangying Yin, Yujia Shi, Ruoyu Zhang, Yong Xiang, Jinghong Qiu, Pak‐Chung Sham, Hon‐Cheong So

**Affiliations:** ^1^ School of Biomedical Sciences The Chinese University of Hong Kong Shatin Hong Kong SAR China; ^2^ Eric and Wendy Schmidt Center Broad Institute of MIT and Harvard Cambridge Massachusetts USA; ^3^ Department of Psychiatry The University of Hong Kong Hong Kong Island Hong Kong SAR China; ^4^ KIZ‐CUHK Joint Laboratory of Bioresources and Molecular Research of Common Diseases Kunming Institute of Zoology and The Chinese University of Hong Kong Kunming China; ^5^ Department of Psychiatry The Chinese University of Hong Kong Hong Kong SAR China; ^6^ CUHK Shenzhen Research Institute Shenzhen China; ^7^ Margaret K.L. Cheung Research Centre for Management of Parkinsonism The Chinese University of Hong Kong Hong Kong SAR China; ^8^ Brain and Mind Institute The Chinese University of Hong Kong Hong Kong Hong Kong SAR China; ^9^ Hong Kong Branch of the Chinese Academy of Sciences Center for Excellence in Animal Evolution and Genetics The Chinese University of Hong Kong Hong Kong Hong Kong SAR China

**Keywords:** causal principle informed marker (gene) finding, cell deconvolution, cell type specific expression inference, deep learning, regularized matrix completion

## Abstract

Integrating single‐cell RNA sequencing(scRNA‐seq) with the phenotypic depth of population‐scale biobanks is essential for elucidating the cellular basis of complex diseases. However, this is hindered by limited scRNA‐seq sample size and the lack of cellular resolution in biobanks. We present CausalCellInfer, a scalable computational framework bringing cellular resolution to bulk and genotype‐imputed transcriptomes. CausalCellInfer employs an invariant causal prediction‐inspired method(scRNA‐seq‐based invariant generalized covariance measure, scI‐GCM) to select environment‐stable marker genes, a parsimonious deep neural network for cell‐fraction deconvolution, and regularized matrix completion to reconstruct cell‐type‐specific (CTS) expression profiles. This design is optimized for biobank‐scale data with technical heterogeneity and limited gene overlap. Validated across simulated data, pseudo‐bulk and real peripheral blood mononuclear cell (PBMC) datasets, CausalCellInfer demonstrated superior accuracy and efficiency over existing methods. Applied to ∼500 000 UK Biobank (UKBB) participants across 29 traits, it captured known pathological shifts, such as reduced pancreatic β‐cell proportions in type 2 diabetes, and identified novel biological signals, including disrupted excitatory neuron‐oligodendrocyte interactions in depression. Inferred CTS differential expression patterns showed significant concordance with independent single‐cell studies and were enriched for Open Targets disease genes. Overall, CausalCellInfer bridges single‐cell insights and population‐scale genomics, enabling systematic discovery of putative disease mechanisms at cellular resolution.

## Graphical abstract

Bulk and genotype‐imputed transcriptomes obscure the cellular drivers of complex disease. CausalCellInfer resolves them by integrating invariant marker selection, deep learning deconvolution, and regularized matrix completion. Applied to ∼500 000 UK Biobank participants across 29 traits, it uncovers hallmark cell‐type proportion shifts, including pancreatic β‐cell depletion in type 2 diabetes, and pinpoints cell‐type‐specific genes linked to disease risk and prognosis.

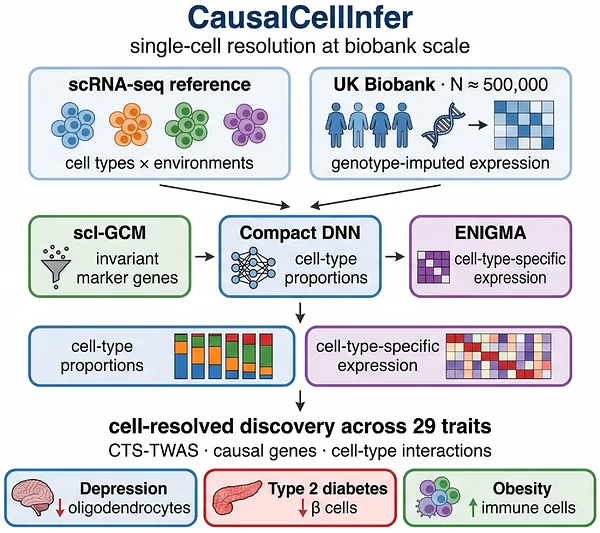

## 1 Introduction

### 1.1 Background: Bridging Cellular Resolution and Biobank‐Scale Data

Single‐cell RNA‐sequencing (scRNA‐seq) has transformed our understanding of cellular diversity and disease biology by enabling gene expression profiling at single‐cell resolution [1, 2, 3, 4]. However, routine application of scRNA‐seq in large cohorts remains limited by cost, technical complexity, and often sparse clinical data on symptoms, prognosis, and comorbidities. Most single‐cell studies are designed for a specific disease or question, and therefore often include limited subject‐level clinical information (e.g., symptoms, prognosis, comorbidities, medications) and small numbers of individuals. These constraints make it difficult to connect cellular mechanisms to diverse clinical outcomes, to systematically assess effect modifiers, or to distinguish causes from consequences of disease.

In contrast, bulk RNA‐seq and genotype‐imputed transcriptome data are available at population scale. Resources such as The Cancer Genome Atlas (TCGA) and Genotype‐Tissue Expression (GTEx) offer bulk expression across tissues, and large biobanks like the UK Biobank (UKBB) provide extensive genotype data together with deep clinical and biological phenotypes. Genotype data further enable the prediction of individual‐level gene expression, creating transcriptome‐like measurements at biobank scale [5, 6, 7]. However, both measured bulk expression and genetically predicted expression lack cellular resolution. Bulk expression profiles aggregate signals across heterogeneous cell types, obscuring the cell‐type‐specific (CTS) drivers of complex traits. Bridging the mechanistic resolution of scRNA‐seq with the statistical power and phenotypic breadth of biobanks would enable cell‐type–resolved discovery in large cohorts and across a variety of clinical outcomes.

### 1.2 The CausalCellInfer Framework

We address this gap with **CausalCellInfer**, a framework inspired by the concept of invariant causal prediction (ICP), as proposed by Peters et al. [8]. CausalCellInfer uses scRNA‐seq reference data to identify robust, cell‐defining features and then transfers this information to bulk transcriptomes, including genotype/genome‐wide association study(GWAS)‐imputed expression in large cohorts, to infer *(i) cell‐type proportions and (ii) CTS expression*.

The design goal is biobank readiness: the proposed framework is scalable to very large sample sizes and reliable under distribution shifts. By anchoring inference at the cell‐type level, CausalCellInfer enables biobank‐scale analyses of how cellular composition and CTS expression relate to risk factors, disease onset/progression, prognosis, and treatment response. Although the framework can be used with any bulk transcriptome data, one of its most powerful applications arises in biobanks where broad phenotyping, large sample size, and individual‐level covariates enable rigorous, cell‐type‐resolved association analyses.

### 1.3 Downstream Applications for Cellular‐Level Discovery

Using inferred cell‐type proportions and CTS expression, we support an application suite that is difficult to implement with conventional single‐cell study designs because of limited subject‐level phenotyping and small sample sizes:

1. Cell‐type‐specific transcriptome‐wide association study (CTS‐TWAS): We test whether genetically predicted CTS expression is associated with complex traits/diseases, localizing gene–trait associations to the most relevant cell types.
2. CTS‐TWAS with putative causal gene prioritization (CTS‐TWAS‐Causal): By integrating causal feature selection, we prioritize genes whose CTS expression is most consistent with a direct effect on traits, helping to distinguish core drivers from correlated signals.
3. Cell‐type proportion association studies (CPAS): We assess whether genetically inferred cell‐type abundances relate to disease risk or clinical phenotypes. This highlights cellular contributors to pathophysiology (e.g., expansion or depletion of specific immune or stromal populations) and nominates candidate cell types for therapeutic targeting.
4. Cell‐type proportion interaction analyses: We evaluate interactions within and across tissues (e.g., immune–parenchymal crosstalk) to identify synergistic or antagonistic relationships that affect disease expression, prognosis, or treatment response.
5. Conditional transcriptome‐wide association study (TWAS): We adjust bulk or imputed expression analyses for inferred cell‐type proportions to reduce confounding by cellular composition when identifying differentially expressed genes. This improves interpretability by separating compositional shifts from CTS expression changes.

### 1.4 Advantages and Application to the UKBB

These analyses operate on individual‐level genotype and imputed expression, and also scale readily to thousands of participants and thousands of phenotypes, typical in biobanks. This scale yields several practical advantages. First, comprehensive covariates available in biobanks (e.g., age, sex, body mass index (BMI), smoking, medications, socioeconomic factors) can be included to reduce confounding and to test effect modification. Second, large samples naturally support interaction models, stratified analyses, and prediction frameworks that are underpowered in typical single‐cell cohorts. Third, when expression and cell‐type measures are genetically predicted (from germline variations), associations are less susceptible to reverse causation and time‐varying confounders (e.g., disease severity, treatment), strengthening causal interpretation relative to observational single‐cell measurements. While not definitive proof of causality, this approach helps infer whether cellular features are more likely antecedents rather than consequences of a disease.

Applying CausalCellInfer to UKBB dataset, we connect cell‐type proportions and CTS expression to a wide spectrum of clinical phenotypes, from cardiometabolic traits and immune disorders to neurological diseases. This biobank‐scale setting enables us to: (i) map cell types most strongly linked to specific outcomes; (ii) nominate CTS genes whose dysregulation associates with disease liability; and (iii) reveal cellular interactions that may underlie comorbidities or variable treatment response. We further assess biological plausibility by demonstrating significant overlaps between CTS differentially expressed genes inferred from UKBB and those reported in independent scRNA‐seq studies, providing external validation across diseases and tissues.

### 1.5 Comparison With Existing Deconvolution Methods

Here we highlight key differences and strengths of our proposed approach relative to several existing deconvolution methods. Signature‐based deconvolution methods (e.g., CIBERSORTx, DWLS) rely on reference gene expression profiles for marker genes derived from scRNA‐seq [9, 10, 11, 12, 13, 14]. However, applying signature‐based approaches to genotype‐imputed transcriptomes is often challenging because reliable imputed expression is available for only a *subset* of genes (typically on the order of a few thousand, depending on tissue and prediction models), which can remove or weaken key markers and reduce identifiability of cell types. Also, while signature‐based methods can perform well when the reference and target data are well matched, in practice their accuracy can degrade under technical and biological mismatches (e.g., batch effects, platform differences, disease states, subject heterogeneity, etc.) because the marker set and reference profiles may not transfer very well across settings.

Signature‐free, deep learning–based methods (e.g., Scaden, TAPE) improve robustness by learning directly from single‐cell‐derived training data [15, 16, 17], but they typically require high‐dimensional features as input and substantial computational resources. Also, their performance may be sensitive to how well the training distribution matches the target cohort, creating risks of overfitting or reduced generalization under distribution shifts [18, 19, 20, 21].

Additionally, the aforementioned methods are susceptible to confounding effects in reference profiles, as such confounders may influence the construction of signature profiles and the identification of marker genes. For example, Dong et al. demonstrated that different single‐cell reference datasets can yield substantially different deconvolution estimates due to both biological variation and technical noise [22], highlighting the importance of accounting for reference‐specific variability when establishing prior knowledge for deconvolution.

Our framework is designed for the biobank use case: it focuses on identifying a parsimonious set of cell‐defining features that are stable across environments (motivated by invariance ideas [8, 23]), and it explicitly targets transfer to bulk and genotype‐imputed expression where feature availability may be constrained. By prioritizing transferable, compact feature sets and emphasizing out‐of‐cohort reliability, our framework is intended to remain accurate across cohorts with different characteristics (e.g., demographics, technologies, disease burdens) while scaling to very large samples and thousands of phenotypes. These design choices are driven not by methodological novelty per se, but by the practical need to execute cell‐type‐resolved analyses across very large samples and many phenotypes.

### 1.6 Summary of Core Objectives

In summary, the major objective of CausalCellInfer is to bring cellular resolution to biobank‐scale discovery. By inferring cell‐type proportions and CTS expression from bulk and genotype‐imputed transcriptomes, we enable:

1. Systematic **CTS‐TWAS** to localize gene–trait associations to the most relevant cell types;
2. Prioritization of putatively **causal CTS genes** for mechanistic studies and therapeutic target nomination;
3. Association and interaction analyses of **cell‐type abundances** within and across tissues; and
4. Confounding‐aware differential expression through **conditional TWAS**.

Together, these capabilities allow investigators to interrogate putative disease mechanisms at the cellular level while retaining the statistical power, phenotypic breadth, and covariate control afforded by large cohorts.

### 1.7 Novelty and Contributions of CausalCellInfer

The contributions of CausalCellInfer are summarized below.

1. Environment‐stable marker selection using scI‐GCM.
  CausalCellInfer introduces scI‐GCM to identify a compact and parsimonious set of cell‐type markers from reference single‐cell RNA‐sequencing data. Grounded in the principle of invariant causal prediction, scI‐GCM prioritizes genes whose relationships with cell identity remain stable across heterogeneous environments. The selected markers are therefore intended to be less sensitive to technical batch effects, disease‐related expression changes, and other environment‐specific perturbations than markers selected solely through conventional differential‐expression analysis.
2. A parsimonious deconvolution design for genotype‐imputed transcriptomes.
  CausalCellInfer incorporates the environment‐stable markers into a compact deep neural network(DNN)‐based deconvolution model. We do not claim that the general use of a DNN for cell‐type deconvolution is itself novel. Rather, the contribution lies in adapting the deconvolution framework to operate with a highly restricted and causally selected feature set. This design addresses an important constraint of genotype‐imputed transcriptomes, for which reliably predicted expression may be available for substantially fewer genes than in conventional bulk RNA‐sequencing data. In contrast to approaches that rely on thousands of highly variable genes, CausalCellInfer is designed to retain deconvolution accuracy under limited gene availability.
3. Integration of calibration, deconvolution, and CTS reconstruction.
  CausalCellInfer provides an integrated workflow for transforming standardized PrediXcan‐imputed expression profiles into a pseudo‐bulk‐compatible expression space, estimating individual‐level cell‐type proportions, and reconstructing individualized CTS expression profiles. Although the individual components are based on existing methods, their integration addresses the distinct statistical and computational challenges associated with applying cellular deconvolution to genotype‐imputed transcriptomes.
4. Population‐scale cellular characterization: application to the UKBB to uncover disease associations with cellular level data.
  An important contribution of CausalCellInfer is its application to large biobank cohorts, including the UKBB, to derive individual‐level cell‐type proportions and CTS expression profiles from genetically predicted expression data. These cellular readouts enable population‐scale downstream analyses, including CTS‐TWAS, cellular abundance association analyses, cell‐type interaction modeling, and conditional TWAS.

## 2 Methods

### 2.1 Overview of CausalCellInfer

In this study, we introduce an integrated framework, CausalCellInfer, that combines invariant causal prediction and DNNs with scRNA data to estimate cell proportions and expression profiles from bulk or genotype‐imputed expression data across large cohorts.

Given (1) a reference scRNA‐seq dataset with cell‐type labels and (optionally) ‘environment’ annotations and (2) a target cohort with bulk or genetically‐predicted expression, CausalCellInfer produces two outputs for each target individual: **estimated cell‐type proportions** and **CTS expression profiles**.

The proposed workflow consists of three main steps (Figure 1):

1. **Invariant cell‐marker identification** from the scRNA‐seq reference using an invariant causal prediction (ICP)‐inspired feature selection procedure (scI‐GCM).
2. **Cell‐type deconvolution** in the target cohort using a deep neural network (DNN) trained on pseudo‐bulk mixtures generated from the scRNA‐seq reference, restricted to the selected marker genes.
3. **CTS expression reconstruction** using a regularized matrix completion approach (ENIGMA) informed by the estimated cell proportions and scRNA‐seq reference profiles.

**FIGURE 1:**
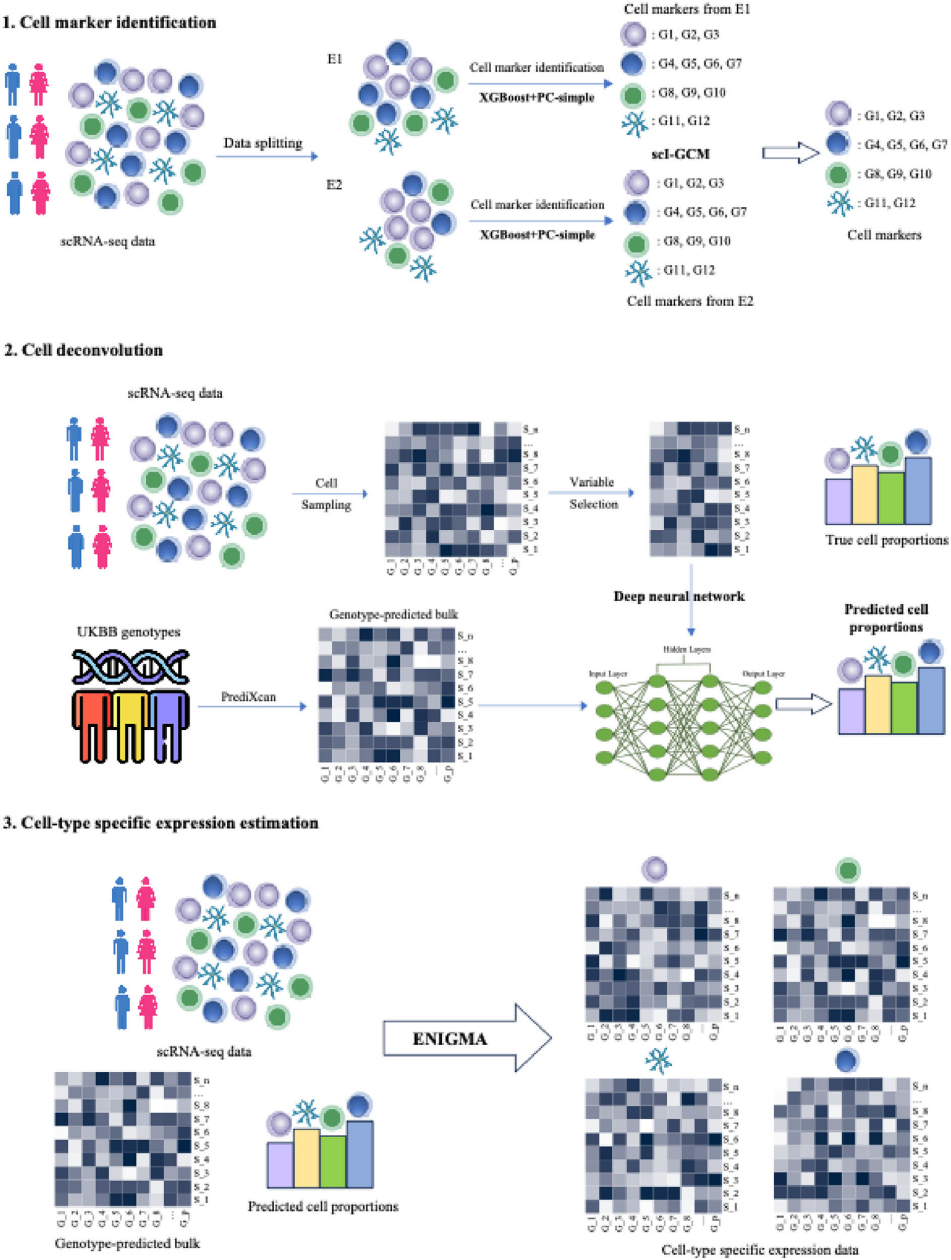
Workflow of our proposed framework for cell decomposition and cell‐type‐specific expression estimation. 1. **Cell marker identification**: We proposed scRNA‐seq‐based invariant generalized covariance measure (scI‐GCM) to identify cell markers for each cell type from the reference scRNA‐seq data; 2. **Cell deconvolution**: We modified Scaden, a DNN‐based deconvolutional method, to estimate cell proportions from bulk expression data using a small yet informative gene set; 3. **Cell‐type‐specific expression estimation**: We employed a regularized matrix completion method (ENIGMA) to reconstruct cell‐type‐specific expression data by leveraging information from the reference RNA‐seq data and the estimated cell proportions from step 2.

Subsequent sections will provide a detailed description of each step.

***Step 1: Cell marker identification***

Genes used as “markers” in scRNA‐seq are usually cell‐type enriched, but they can still vary substantially with disease states, age, sex, technical batch, or other sample‐specific factors. For deconvolution and transfer to external cohorts, we seek a *parsimonious* set of cell‐defining genes whose relationship to cell identity is stable across environments.

Robust cell markers tend to exhibit stability across diverse environments. In this context, we define “environments” (*E*) as subject‐related or experimental background features that may shift expression distributions. These environments may include variations in disease pathology, demographics, spatiotemporal factors, and batch effects, alongside experimental perturbations like gene knockdown or overexpression. However, the “environment” should not include the outcome variable and its descendants [8]. According to the theory of Invariant Causal Prediction (ICP), the conditional distribution of a target variable *Y* is invariant across environments when conditioned on its direct causes. Notably, tissue type represents the baseline biological domain within which single‐cell reference profiles are defined, rather than an environmental variable.

Operationally, we seek to identify a gene set *X^S^* such that the association between cell‐type *Y* and *E* disappears after conditioning on *X^S^*, satisfying the invariance criterion:

$$E⊥Y|X^{S}$$

Under standard ICP assumptions, this independence ensures that *X^S^* captures the fundamental, stable signals defining cell identity.

To identify such markers within large‐scale scRNA‐seq data, we introduce an *adapted* and improved Invariant Generalized Covariance Measure (I‐GCM) framework for single‐cell analysis, which we also refer to as “scI‐GCM” below. The presented approach refines our previously proposed I‐GCM framework [24], originally developed to identify clinical and genetic risk factors in epidemiological cohorts [24]. Here we optimized the I‐GCM pipeline to tackle the high‐dimensional challenges inherent in single‐cell transcriptomic data. The resulting framework is grounded in causal graphical models and invariant prediction principles, operating under standard causal assumptions such as partial faithfulness, unconfoundedness, and stable causal mechanisms. Crucially, the method incorporates a scalable, hierarchical feature selection process to ensure computational efficiency.

Below we summarize the key steps and then describe adaptations specific to cell marker selection:

## 3 Candidate Marker Identification via Two‐Stage Hierarchical Screening

To address the computational intensity of standard causal discovery, we implemented a two‐stage hierarchical screening process:

1. ***Initial Screening (XGBoost)***: The scRNA‐seq data was first partitioned into training and testing sets. The training set was then subdivided into subsets based on selected environmental variables, specifically disease status and gender for this study. Within each environment stratum, we trained an XGBoost model (max tree depth 2, learning rate 1) to predict the binary cell type (*Y*) from gene expression data (*X*). This step performs a “coarse” reduction, retaining genes with positive importance scores.
2. ***Causal Refinement (PC‐simple)***: To eliminate spurious associations captured by XGBoost, we refine the retained genes using the PC‐simple algorithm within each environment stratum. We set the maximum conditioning order to 3 and significance level (α) to 0.05. The union of genes across all environments formed the initial candidate set to be analyzed in the next stage.

Regarding the principles of PC‐simple, it is a causal inference algorithm that generalizes ordered correlation screening to select putative direct causal variables. A candidate gene *X^j^* is retained if the null hypothesis of conditional independence between *Y* and *X^j^* given a conditioning set *X^S^* is rejected using Fisher's Z‐transformed partial correlation:

(1) $${\left(n-|s|-3\right)}^{1/2}\left|Z\left(Y,X^{j}|X^{S}\right)\right|>\phi^{-1}\left(1-\alpha /2\right)$$

where $Z\;\left(Y,X^{j}|X^{s}\right)=\frac{1}{2}\log\left\{\frac{1+\hat{\rho }\left(Y,X^{j}|X^{s}\right)}{1-\hat{\rho }\left(Y,X^{j}|X^{s}\right)}\right\}$ denotes Fisher's Z‐transform of the partial correlation, *n* denotes the sample size, |*s*| is the conditioning set size, $\phi^{-1}$ indicates the standard normal quantile function.

## 4 Invariant Marker Identification Via Generalized Covariance Measure (GCM)

The final stage identifies markers that define cell identity amidst environmental noise. We employed the generalized covariance measure (GCM) (*T^n^*) [25] to test whether the conditional association between the environment variable(*E*) and cell type(*Y*) remains stable when conditioning on a specific gene set (*E*⊥*Y*|*X^S^*). The GCM statistic(*T^n^*) tests independence based on residuals (*R*):

(2) $$\begin{array}{ccc} T^{n} & = & \frac{\sqrt{n}.\frac{1}{n}\sum_{i=1}^{n}R_{i}}{{\left(\frac{1}{n}\sum_{i=1}^{n}R_{i}^{2}-{\left(\frac{1}{n}\sum_{r=1}^{n}R_{r}\right)}^{2}\right)}^{1/2}}\\ & = & \frac{\frac{1}{n}\sum_{i=1}^{n}R_{i}}{\left[{\left(\frac{1}{n}\sum_{i=1}^{n}R_{i}^{2}-{\left(\frac{1}{n}\sum_{r=1}^{n}R_{r}\right)}^{2}\right)}^{1/2}\right]/\sqrt{n}} \end{array}$$

here $R_{i}=\left(e_{i}^{j}-f\left(x_{i}^{S}\right)\right)\left(y_{i}^{j}-f\left(x_{i}^{S}\right)\right)$ represents the product between residuals from the prediction functions of *E* and Y using *X^S^* for each observation. We utilize XGBoost as the prediction model to calculate this conditional independence; genes are iteratively removed if their inclusion does not contribute to improving the invariance. Other machine learning methods can also be used as substitutes for XGBoost, offering high flexibility. Significant deviation from the null hypothesis(indicating a change in GCM distance between consecutive conditional gene sets) is rejected if:

(3) $$\frac{\Delta T_{S_{j-1},S_{j}}^{n}}{sqrt\left(\mathrm{Var}\left(\Delta T_{S_{j-1},S_{j}}^{n}\right)\right)}>\phi^{-1}\left(1-\alpha \right)$$

$\Delta T_{S_{j-1},S_{j}}^{n}$ defines the change in the distance of GCM from zero between two consecutive conditional sets, with α  =  0.05. For more technical details, please refer to Supporting Information.

***Step 2: Cell‐type deconvolution with ensemble DNN***

### 4.1 Training Data Generation (Pseudo‐Bulk Mixtures)

In our framework, we employ a deep neural network (DNN) model to estimate cell proportions. It uses simulated bulk gene counts for the selected marker genes derived from reference scRNA‐seq data to estimate cell proportions in bulk expression samples.

We train a DNN to map bulk expression of the selected marker genes to cell‐type fractions. Because the target cohort may be bulk RNA‐seq or genotype‐imputed expression, we train using pseudo‐bulk mixtures generated from the scRNA‐seq reference, following established pseudo‐bulk simulation procedures.

To train the DNN models, we simulated 5000 pseudo‐bulk samples with random fractions of different cell types ($f_{c}\;\varepsilon \;\left[0,1\right]$ *for all* cε{1,2,..C}, *where* $\sum_{1}^{C}f_{c}=1$) by subsampling cells from the reference RNA‐seq data, following the pseudo‐bulk data generation approach by Chen et al. [16]. Only genes selected by our proposed scI‐GCM approach were included as model features. Each simulated bulk sample was constructed from 500 randomly sampled cells. The number of cells for each cell type in each bulk sample was $r_{c}=f_{c}\cdot 500$, where *r_c_* refers to the cell number of cell type *c* and *f_c_* refers to the fraction of cell type *c*. We generated the final pseudo‐bulk expression profile by summing up the single‐cell expression levels for each sample.

### 4.2 Model Architecture and Training

We used the MinMaxScaler() class from Sklearn to scale the input data to the range [0,1]. We trained an ensemble of 3 DNN models with distinct architectures and degrees of dropout regularization, as the Ensemble DNN model has been shown to outperform individual models [15]. Each model outputs a *C*‐dimensional fraction vector via a Softmax output layer.

We optimize the mean absolute error (L1 loss):

(4) $$L1\left(y_{i},\hat{y_{i}}\right)=\frac{1}{n}\left|y_{i}-\hat{y_{i}}\right|$$

where *y_i_* is the vector of ground‐truth cell fractions of the pseudo‐bulk sample *i* , $\hat{y_{i}}$ is the vector of predicted fractions of the sample *i*, and *n* is the sample size. The final estimated proportions are the ensemble average:

(5) $$\hat{y_{c}}=\frac{\hat{y_{c}^{1}}+\hat{y_{c}^{2}}+\hat{y_{c}^{3}}}{3}$$

Models were implemented in PyTorch. Trained models are then applied to target bulk (or calibrated imputed) expression to estimate cell‐type proportions per individual.

***Step 3: CTS expression estimation***

We employed a regularized matrix completion‐based approach called ENIGMA [26] to estimate cell‐type‐specific expression profiles based on predicted cell proportions and genotype‐imputed bulk expression profiles. ENIGMA is developed based on two assumptions. The first assumption is that the expectation of each CTS expression profile aligns with the reference profile matrix from the external scRNA‐seq data [27]. The second assumption is that observation noise is consistent across different samples. We estimate the CTS expression by the trace norm model, following [26]:

(6) $$\begin{array}{ccc} {\hat{\left(X_{i}\right)}}_{i=1,2,..C} & = & argmin\left(\frac{1}{2}\alpha ∥O-\sum_{i=1}^{C}X_{i}diag\left(\theta_{i}\right){∥}_{F}^{2}\right.\\ & & +\left.\;\frac{1}{2}\left(1-\alpha \right)\sum_{i=1}^{C}∥X_{i}a-R_{i}{∥}_{2}^{2}+\beta \sum_{i=1}^{C}\hat{\theta_{i}}∥X_{i}{∥}_{*}\;\right)\; \end{array}$$

where *i* denotes the cell type index, $O\in R^{m\times n}$ denotes the bulk count expression matrix for *m* genes and *n* subjects, *X_i_* denotes the true CTS expression for the *i*th cell type, θ_*i*_ denotes the cell proportion of the *i*th cell type, *R_i_* denotes the reference expression vector for the *i*th cell type, || || [2] denotes the *L* ^2^ norm of a matrix,

α ε(0, 1) denotes the weights of the aggregated CTS expression profiles (note that 1 − α represents the ‘agreement weight’ between the aggregated CTS expression profiles and its corresponding reference expression vector),

β∈(0,+∞) denotes the regularization strength,

$a={\left(\frac{1}{n},\text{…},\frac{1}{n}\right)}^{T}\;\varepsilon R^{n}$ with *n* indicating bulk sample size.

To reduce the computational complexity of the optimization problem, we employed the maximum *L*_2_ norm model to deconvolute bulk samples [27]:

(7) $$\begin{array}{ccc} {\hat{\left(X_{i}\right)}}_{i=1,2,..C} & = & \left(\frac{1}{2}\alpha ∥O-\sum_{i=1}^{C}X_{i}diag\left(\theta_{i}\right){∥}_{F}^{2}\right.\\ & & +\left.\;\frac{1}{2}\left(1-\alpha \right)\;\sum_{i=1}^{C}\;∥X_{i}a-R_{i}{∥}_{2}^{2}+\beta \;\sum_{i=1}^{C}\hat{\theta_{i}}∥t\left(X_{i}\right){∥}_{2,max}^{2}\;\;\right) \end{array}$$

where $∥.{∥}_{2,max}^{2}$ indicates the square of the maximum *L*_2_ row norm of a matrix and *t*(*X_i_*) denotes the transpose of the CTS expression for the *i*th cell type. The optimization procedure was described in [27].

### 4.3 Calibrating PrediXcan‐imputed Expression

PrediXcan‐imputed expression values are typically standardized per gene across individuals (often reported as Z‐like scores). This per‐gene standardization preserves between‐individual variation for a given gene, but obscures cross‐gene magnitudes *within* an individual. In other words, PrediXcan outputs are suitable for comparing a single gene across individuals (e.g., a value of 1 for a gene in person B indicates higher expression than a value of 0 for the same gene in person A), but they do not reflect actual expression differences between genes within the same individual (e.g., a value of 0 for gene A and −0.3 for gene B in the same person cannot be directly compared). Consequently, the scale of the PrediXcan data is incompatible with scRNA‐seq reference data, preventing direct inference of cell composition and CTS expression.

To mitigate this limitation, we mapped the PrediXcan‐imputed values back into the expression space of the scRNA‐seq reference, using a heuristic pseudo‐bulk calibration. Specifically, we generated 1,000 pseudo‐bulk RNA‐seq samples from the control‐group scRNA‐seq data using a procedure similar to that used in the TAPE approach [16]:

1. We took the reference cell‐type proportions as a baseline. For each pseudo‐bulk sample, we perturbed these proportions by adding Gaussian noise to each cell type, clipped negatives to zero, and renormalized to sum to one.
2. We then sampled 1000 cells from the scRNA‐seq reference with probabilities given by the perturbed proportions and summed their profiles to form one pseudo‐bulk sample.

From these 1000 pseudo‐bulk samples, we computed, for each gene *g*, the mean µ_*g*_ and standard deviation σ_*g*_. For each subject *s*, let *Z_g,s_* denote the PrediXcan‐imputed standardized value for gene *g*. We define the calibrated pseudo‐bulk‐like value:

$$\begin{array}{cccc} & \;{\tilde{O}}_{g,s}=Z_{g,s}\;\;\sigma_{g}+\mu_{g}. & & \end{array}$$

We use $\tilde{O}$ as the bulk input *O* for both DNN deconvolution and ENIGMA reconstruction.

After obtaining the re‐scaled bulk RNA‐seq data (*O*), we can derive the reference profile matrix *R* by averaging the expression profiles of cells within each cell type in the scRNA‐seq reference. Subsequently, the inferred cell type proportion matrix(θ), reference profile matrix (*R*), and the re‐scaled bulk RNA‐seq data (*O*) were plugged into the above formula to infer the final CTS expression profile. The quality of the inferred CTS expression profiles can be significantly influenced by the reference CTS expression profiles. To ensure the reliability of these estimated profiles for subsequent analyses, we recommend using those inferred from the cell types in the reference scRNA‐seq dataset with a total cell count exceeding 2000. Additionally, we aimed to balance the reconstructed CTS so that it resembles the reference scRNA‐seq dataset, while ensuring the reconstructed bulk RNA‐seq data approximates the observed bulk data. Therefore, we set α to 0.5 in our analysis and kept β at the default setting.

## 5 Evaluation of the Proposed Framework

### 5.1 Ablation Analysis to Evaluate scI‐GCM for Single‐Cell Analysis

To validate the advantages of the scI‐GCM method, we first established a baseline simulation using simulated scRNA‐seq datasets confounded by environmental variables. We compared scI‐GCM's performance against analogous algorithms (PC‐simple) and state‐of‐the‐art deconvolution methods (DWLS, Scaden). Notably, the analogous PC‐simple method refers to our approach but only replacing scI‐GCM in step 1 with PC‐simple. Our objective was to test whether scI‐GCM more accurately identifies cell‐type‐specific gene markers in the presence of confounding factors, thereby leading to more robust predictions of cell type proportions.

We utilized scDesign3 [28] to create scRNA‐seq data based on a publicly available peripheral blood mononuclear cells (PBMC) dataset. scDesign3 is a statistical simulator that generates realistic single‐cell data using a unified probabilistic model, the parameters of which are derived from actual data. This model allows us to manipulate interpretable parameters, introducing environmental effects that lead to confounding, and subsequently simulating new scRNA‐seq and bulk data impacted by these effects. We conducted simulations on bulk samples comprising four cell types with varying confounding effects. For more details, please refer to the Supporting Information.

### 5.2 Evaluation of the Proposed Approach on Real and Pseudo‐Bulk Data

To verify the reliability of our proposed framework, we compared the performance of our method with several existing deconvolution approaches across various evaluation metrics, including root mean square error (RMSE), mean absolute error (MAE), and Lin's Concordance Correlation Coefficient (CCC).

We used pseudo‐bulk expression profiles generated from reference scRNA‐seq data of four different tissues: the frontal cortex, pancreas islet, and subcutaneous and visceral adipose tissues. These pseudo‐bulk expression profiles were simulated in silico from scRNA‐seq data with randomly generated cell type proportions, essentially a summation of single‐cell expression profiles [16]. We added noise to each generated pseudo‐bulk expression profile to mimic the measurement errors in real RNA‐seq data [16]. The noise was randomly generated from a Gaussian distribution based on the cells used to create the corresponding pseudo‐bulk profile. We only included genes that could be reliably predicted from genotypes by PrediXcan for model training.

Additionally, we conducted benchmarking on real datasets, using available bulk PBMC RNA‐seq data and their corresponding cell proportions assessed by flow cytometry from Monaco et al. [29]. To generate pseudo‐bulk datasets for data training and to perform scI‐GCM to identify cell markers, we merged five PBMC scRNA‐seq datasets obtained from different individuals, and these datasets were all downloaded from the 10x Genomics database.

To mitigate overfitting, we partitioned each reference scRNA‐seq dataset into two subsets based on subject ID: a training set and a testing set. We initially trained our deep neural network model using the training set and subsequently tested it with pseudo‐bulk samples generated from the testing set. The training set was also used to obtain references for MuSiC [30], DWLS [31], and Scaden [15].

### 5.3 Validating Estimated CTS Expression Profiles via Differential Expression Analysis

To further validate the robustness of our proposed framework, we performed a comparative analysis on its ability to detect differentially expressed genes across different cell types, especially when cell‐type‐specific (CTS) expression profiles were imputed from genotypes.

Specifically, we identified differentially expressed (DE) genes from both actual scRNA‐seq data and genotype‐predicted CTS expression profiles. For raw scRNA‐seq data, we utilized the FindMarkers() function from the Seurat [32] package to detect differentially expressed genes (DEGs) using the default settings. This function by default employs the Wilcoxon Rank Sum test to identify genes differentially expressed between two cell groups. For our genotype‐predicted CTS expression profiles, we applied the same test to detect DEGs. We used positive predictive value (PPV) for the evaluation, considering the DEGs (adjusted *p*‐value ≤0.05) identified from the actual scRNA‐seq dataset as the reference set.

## 6 UKBB Analyses

### 6.1 Reference Datasets, Target Cohorts and Pipeline Specifications

Detailed specifications of all reference single‐cell datasets, chosen environmental variables, target bulk imputation models and pipeline across all four tissue applications are systematically summarized in Table 1. Briefly, reference scRNA‐seq datasets were curated from public repositories, including Human Pancreas Analysis Program (HPAP), the NCBI Gene Expression Omnibus (GEO: GSE144136) and the Broad Institute Single Cell Portal (SCP1376). For each tissue, primary disease status (e.g., cases vs. controls) was designated as the environmental variable *E* within our scI‐GCM framework to select disease‐invariant cell‐type marker genes across distinct pathological conditions. For biobank‐scale applications, target bulk transcriptomes were genetically predicted using tissue‐specific prediction models (i.e., PrediXcan trained on GTEx v7) applied to individual‐level genotype data from UKBB. Notably, we imposed an additional restriction prior to cell marker screening: we retain only genes present in both the reference scRNA‐seq dataset and the genotype‐imputed target transcriptome and run scI‐GCM exclusively on this shared gene set. This ensures that every invariant cell marker identified can, by construction, also be quantified in the target cohort. Cell composition and CTS expression profiles were subsequently derived using a deep neural network (DNN) architecture integrated with regularized matrix completion.

**TABLE 1:** Overview of single‐cell reference datasets, environmental variables, target cohorts, and pipeline specifications across tissue analyses.

Specification	Pancreas	Frontal cortex	Subcutaneous adipose tissue (SAT)	Visceral adipose tissue (VAT)
Target Outcome	Type 2 Diabetes Mellitus (T2DM)	Major Depressive Disorder (MDD)	Obesity	Obesity
Environmental Variable	Disease status (Case vs. Control)	Disease status (Case vs. Control)	Disease status (Case vs. Control)	Disease status (Case vs. Control)
sc/snRNA‐seq Reference	HPAP Database	GEO: GSE144136	Single Cell Portal (SCP1376)	Single Cell Portal (SCP1376)
Imputation model	GTEx Pancreas (v7)	GTEx Frontal Cortex (BA9) (v7)	GTEx Adipose Subcutaneous (v7)	GTEx Adipose Visceral Omentum (v7)
Imputation model sample size	N = 253	N = 158	N = 491	N = 401
Number of Imputed Genes	3,958	2,857	6,339	4,928
Deconvolution Pipeline	scI‐GCM + DNN + Matrix Completion	scI‐GCM + DNN + Matrix Completion	scI‐GCM + DNN + Matrix Completion	scI‐GCM + DNN + Matrix Completion
Primary Model Purpose	Derive invariant pancreatic markers, infer cell composition & CTS expression	Derive invariant cortical markers infer cell composition & CTS expression	Derive invariant SAT markers infer cell composition & CTS expression	Derive invariant VAT markers infer cell composition & CTS expression
Covariates Adjusted	Top 10 PCs; Top 10 PCs + Age + Sex	Top 10 PCs; Top 10 PCs + Age + Sex	Top 10 PCs; Top 10 PCs + Age + Sex	Top 10 PCs; Top 10 PCs + Age + Sex

*Note*: PC, Principal Components; CTS, Cell‐Type‐Specific; DNN, Deep Neural Network.

### 6.2 Hyperparameter Stability Analysis

We assessed the sensitivity of scI‐GCM to its hyperparameters (the XGBoost pre‐selection tree depth (max_depth) and learning rate (η), and the PC‐simple significance threshold (α) and maximum conditioning‐set order (max_ord)) using a one‐at‐a‐time sweep around the default operating point (α=0.05,max_ord=3,max_depth=2,η=1). This analysis was repeated on four single‐cell datasets (pancreas/type 2 diabetes mellitus (T2DM), subcutaneous and visceral adipose/obesity, frontal cortex/depression; 45 cell types). The identified cell markers were robust to PC‐simple parameters, whereas the XGBoost preselection parameters primarily governed the size of the candidate gene pool (Figure S1), with *nrounds* fixed, larger values lead to more candidate genes. These patterns were consistent across four datasets. Therefore, we recommend using the default parameters: α=0.05,max_ord=3,max_depth=2,η=1 throughout. The default parameters provide a stable operating point, where downstream PC‐simple and I‐GCM steps prune the candidate pool to a compact, high‐confidence marker set. For more details, please refer to Supporting Information.

### 6.3 Cell‐Type Proportion Association and Interaction Analyses With Clinical Phenotypes

We tested associations between inferred cell‐type proportions from imputed gene expression profiles and disease status/phenotypes using regression models, adjusting for relevant covariates and controlling false discovery rate (FDR) at 0.05 via the Benjamini–Hochberg procedure. Besides, we examined whether interactions between cell types, including cell types *across* different tissues, could affect disease phenotypes using regression models.

### 6.4 Identifying Cell‐Type‐specific Genes Involved in Disease Susceptibility (CTS‐TWAS) and Causal Gene Prioritization

Upon acquiring the CTS expression profiles for each target disease/trait from UKBB, we identified genes contributing to disease susceptibility for each cell type. This may be considered as a CTS‐TWAS. To mitigate potential confounding effects from population stratification, the top 10 principal components (PCs) derived from the corresponding genotype dataset were included as covariates. Specifically, individual‐level CTS‐TWAS association tests were performed using multivariable linear regression modeling:

$$Y_{i}=\beta_{0}+\beta_{g,k}^{CTS}{\hat{S}}_{ikg}+Z_{i}\alpha +\varepsilon_{i}$$

where *Y_i_* represents the target outcome/trait for individual *i*, ${\hat{S}}_{ikg}$ is the inferred CTS expression for gene *g* in cell type *k*, *Z_i_* represents the vector of top 10 PCs for individual *i*, and $\beta_{g,k}^{CTS}$ quantifies the CTS gene‐trait association, ε_*i*_ is the error term. Notably, for binary traits, individual‐level association testing was performed using linear regression. In large biobank cohorts (N >100 000), linear regression yields P‐values asymptotically equivalent to logistic regression while providing substantial computational efficiency for high‐dimensional cell‐type‐specific transcriptomic screening.

Subsequently, we utilized the PC‐simple algorithm to identify putative direct causal genes for the outcome variable. For binary and count data, we applied PC‐simple using linear regression‐based partial correlations as an approximation, treating the outcome as a continuous variable. This follows common practice in high‐dimensional causal discovery [5, 33]. Following ref. [33], we considered an alpha threshold of 0.05 and an order of 3 for the correlation tests. For a more comprehensive description of the algorithm, please refer to ref. [5, 33] and the Supporting Information.

To evaluate the sensitivity and robustness of the proposed CTS‐TWAS and causal gene prioritization pipelines, we performed four additional analyses. The first two analyses extended the PCs‐only baseline model to assess the robustness of the empirical findings to alternative analytical settings. First, for covariate adjustment, we expanded the baseline model by additionally including sex and age as covariates across all tissues. Second, for ancestry restriction, we repeated the discovery analysis in a European‐ancestry subset (n = 472 714 of 502 524 participants) and compared the results with those obtained from the full, covariate‐adjusted, multi‐ancestry cohort. Robustness was quantified using the Pearson correlation of gene‐level Z‐scores between (i) the covariate‐adjusted and basic PCs‐only models and (ii) the full multi‐ancestry cohort and the European‐ancestry subset.

We further performed two simulation‐based analyses to examine the robustness of the pipeline under conditions that may affect its practical performance. Specifically, we varied the proportion of gene‐expression variance explained by cis‐expression quantitative trait locus(cis‐eQTL) to assess how gene‐expression prediction accuracy influences cell‐type deconvolution and downstream association analyses. Finally, we evaluated the ability of the proposed pipeline to recover signals from rare cell types across a range of cell‐type proportions. Further details of these sensitivity analyses are provided in the Supporting Information.

### 6.5 Uncovering Cell‐Type‐Specific Genes Involved in Disease Prognosis

UKBB is a comprehensive database that provides extensive phenotypic and prognostic information on various diseases. With the CTS profiles, we could probe the involvement of cell‐specific genes in disease *prognoses*, such as mortality and hospitalization frequency. Our application to UKBB mainly focused on 4 diseases/outcomes with imputed expression in relevant tissues (i.e., depression in frontal cortex, T2DM in pancreas, and obesity in subcutaneous and visceral adipose).

We concentrated on key prognostic variables of clinical significance for these diseases. Specifically, we focused on treatment resistance and psychiatric hospitalization frequency for depression (tissue studied: frontal cortex); mortality and related hospitalization frequency for T2DM (tissue studied: pancreas); coronary artery disease (CAD) mortality and related hospitalization frequency for obesity (tissue studied: subcutaneous and visceral adipose). Please refer to the Supporting Information for more details.

Moreover, we conducted analyses for 12 other related disorders/phenotypes, including psychosis, bipolar disorder, anxiety (tissue studied: frontal cortex); type 1 diabetes mellitus (T1DM), prediabetes, T2DM‐related hospitalization, T2DM requiring insulin (tissue studied: pancreas); CAD, T2DM, heart failure, hypertension, stroke (tissue studied: subcutaneous and visceral adipose). We adjusted the CTS profiles and target prognosis variables by the top 10 PCs to mitigate population stratification. We then employed PC‐simple to identify putative direct causal genes contributing to disease outcomes.

### 6.6 Enrichment Analysis

We further validated our methodology by assessing whether the identified gene set was significantly enriched among disease genes listed in an independent, established database. We leveraged the Open Targets database [34], which provides an overall association score for each gene, aggregating seven types of evidence: genetic associations, somatic mutations, known drugs, affected pathways, RNA expression, literature support, and animal models. Enrichment was performed at the disease level by aggregating CTS genes across cell types.

For further functional interpretation of prioritized (putatively causal) genes, we performed pathway over‐representation analysis using ConsensusPathDB [35] with hypergeometric testing.

### 6.7 Conditional TWAS Controlling for Cell‐Type Proportions

We first performed a standard TWAS analysis on the tissue‐specific expression data for each target trait. Then, we examined whether controlling for the estimated cell proportions would significantly affect the effect size of trait‐associated genes. Cell proportion is a potential confounder that may lead to spurious associations between imputed expression and outcomes.

For each gene, we fit two univariate regression models: one adjusting for the top 10 genotype PCs and one adjusting for PCs plus estimated cell fractions. We compared coefficients using the method of Clogg et al. [36].

## 7 Results

### 7.1 Overview of the Proposed Framework

We proposed CausalCellInfer, a framework which integrates scI‐GCM with a deep neural network to deconvolute cell proportions and employs a regularized matrix completion algorithm (ENIGMA) to estimate the corresponding CTS profiles. CausalCellInfer can reliably and efficiently deconvolute cell compositions and estimate the corresponding expression profiles from large‐scale bulk expression data, including GWAS‐predicted expression.

We verified the efficacy of our proposed method by comparing it against existing state‐of‐the‐art cell deconvolution methods, including NNLS, DWLS, MuSiC, Scaden, and DISSECT across various real and pseudo‐bulk samples. Finally, we applied our proposed framework to deconvolute four GWAS‐predicted tissue‐specific expression profiles in the UKBB, i.e., frontal cortex, pancreas, subcutaneous adipose tissue (SAT), and visceral adipose tissue (VAT), using disease status as environment variable. With the deconvoluted cell compositions and corresponding CTS expression profiles, we conducted a wide range of analyses for 29 clinically relevant phenotypes (4 ‘main’ disorders, 8 prognostic traits pertaining to the 4 main disorders, 17 other related disorders) in the UKBB, including cell type association analysis, CTS‐TWAS and CTS‐TWAS‐causal, and (tissue‐level) TWAS controlled for cell proportions.

### 7.2 Ablation Analysis Results of scI‐GCM for Marker Selection and Cell‐Type Deconvolution Under Confounding

To evaluate marker selection under environment‐induced confounding, we compared scI‐GCM against another causal‐screening approach (PC‐simple) and three representative state‐of‐the‐art methods (DWLS, MuSiC, and Scaden) using simulated scRNA‐seq and bulk transcriptomic data. We considered two scenarios with different confounding effect sizes (modest and substantial) to mimic realistic settings in which factors such as disease status impose strong confounding effects on cell‐specific expression profiles.

Despite using the smallest marker gene set, scI‐GCM outperformed all competitors in terms of concordance correlation coefficient (CCC), root mean square error (RMSE), and mean absolute error (MAE) in simulations with four cell types (Figure 2). Notably, as the magnitude of confounding increased, the performance advantage of scI‐GCM became more pronounced. In contrast, signature‐based methods, such as DWLS, struggled to deconvolute samples simulated from diseased conditions (Figure 2, the median of CCC, MAE and RMSE is comparable to other methods), although it achieved good performance on control samples. This discrepancy likely arises because DWLS constructs its deconvolution signature matrix from control samples, which limits its generalizability to samples affected by confounding factors such as disease. Notably, our DNN is trained on multi‐sample pseudo‐bulk data, it learns broader transcriptional variation across batches and disease states. Combining this architecture with scI‐GCM feature selection yields high prediction accuracy with significantly reduced data requirements.

**FIGURE 2:**
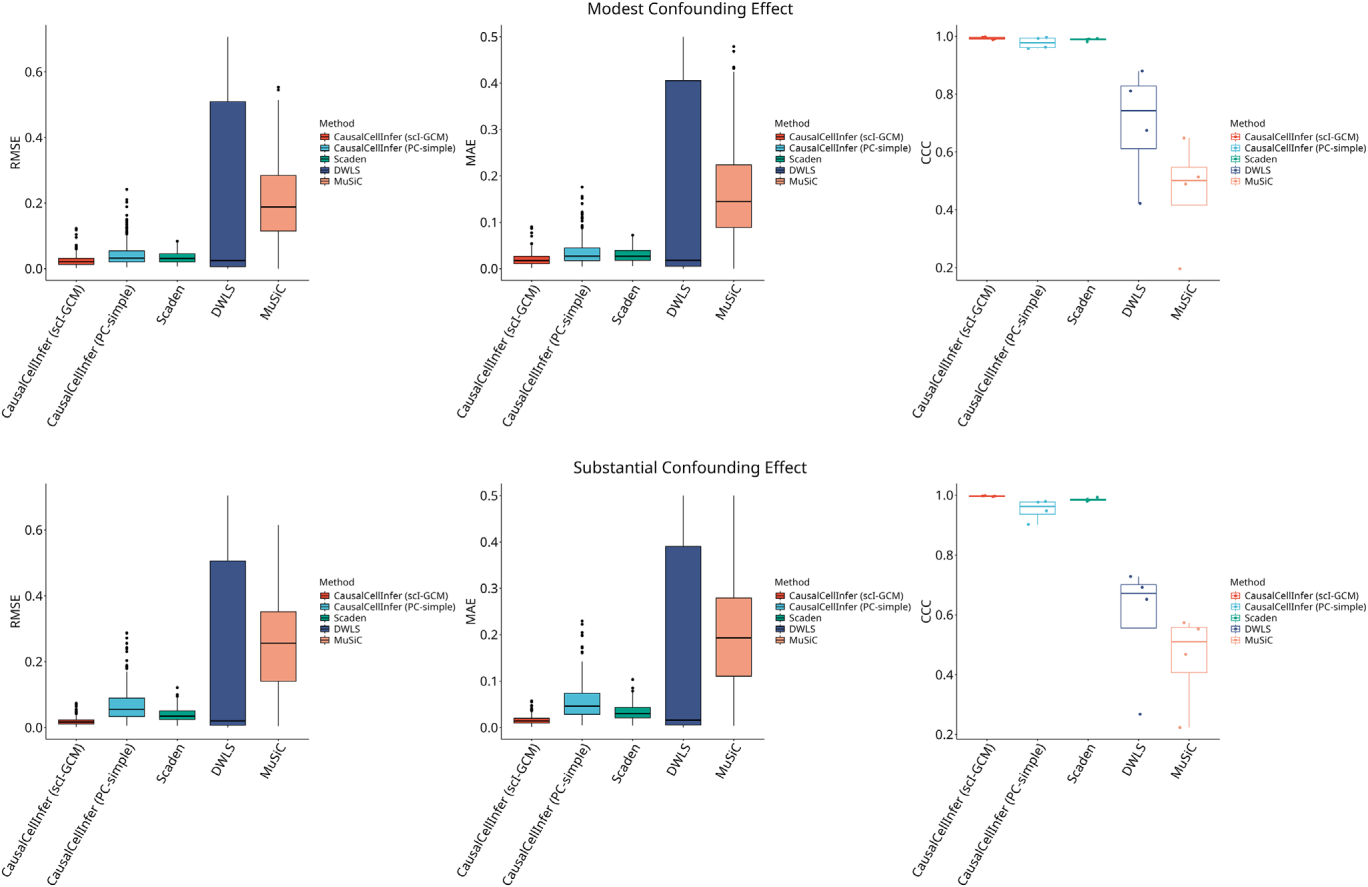
Ablation analysis of scI‐GCM across methods on simulated bulk samples comprising 4 cell types with varied confounding effects.

We also assessed cell marker quality by different algorithms. scI‐GCM also demonstrated superior performance in identifying highly ranked DEGs relative to PC‐simple. Genes were ranked by the mean inter‐cell‐type difference in decreasing order; the top 100 were considered DEGs. Marker gene sets identified by scI‐GCM achieved higher PPV for DEGs than those derived from PC‐simple (Table 2). Moreover, as confounding effects intensified, scI‐GCM more reliably identified trustworthy DEGs for downstream deconvolution.

**TABLE 2:** Comparison of scI‐GCM and PC‐simple in identifying DEGs.

Scenario	Positive predictive value (PPV)	
	scI‐GCM	PC‐simple
4 cell types with modest confounding effects	0.476	0.207
4 cell types with substantial confounding effects	0.627	0.208

## 8 Deconvolution Accuracy on Pseudo‐Bulk Mixtures

We next evaluated cell‐type deconvolution accuracy using pseudo‐bulk mixtures generated from scRNA‐seq reference datasets representing four tissues: frontal cortex, pancreas, SAT, and VAT. To minimize information leakage and provide a more stringent assessment of generalizability, each scRNA‐seq reference dataset was partitioned by subject into non‐overlapping training and testing subsets. The training subset was used to construct the reference profiles or pseudo‐bulk training data required by the respective methods, whereas the testing subset was used exclusively to generate the evaluation mixtures. We compared two implementations of CausalCellInfer—using either scI‐GCM or PC‐simple for marker selection—with five existing deconvolution methods: Scaden, MuSiC, DWLS, NNLS, and DISSECT. These methods differ in both their input representations and modeling strategies. NNLS, DWLS, and MuSiC estimate cell fractions using cell‐type reference expression profiles derived from the training scRNA‐seq data, whereas Scaden, DISSECT, and CausalCellInfer learn prediction models from simulated pseudo‐bulk mixtures (see Supporting Information method for more details). All methods were run using their recommended settings, with the learning‐based models trained for a maximum of 5000 steps. Performance was assessed using Lin's concordance correlation coefficient (CCC), for which higher values indicate better agreement, and RMSE and MAE, for which lower values indicate greater accuracy.

Overall, both CausalCellInfer implementations demonstrated consistently strong, robust deconvolution performance across tissues in both single‐cell reference gene set (Full) and UKBB restricted gene set (UKBB) scenarios (Table S1). Under the single‐cell reference gene‐set scenario, CausalCellInfer with scI‐GCM achieved the highest concordance correlation coefficient (CCC) in two of the four tissues, i.e., SAT (0.8921), and VAT (0.8982), while yielding the lowest errors in both adipose tissues (SAT: RMSE 0.0397, MAE 0.0234; VAT: RMSE 0.0411, MAE 0.0227) (Figures 3 and 4 and Table 3). In the cortex, CausalCellInfer with scI‐GCM achieved slightly lower MAE (0.0236), though CausalCellInfer with PC‐simple maintained a slightly higher overall concordance (0.9847 vs. 0.9834). For pancreas, we observed that CausalCellInfer with scI‐GCM achieved the smallest RMSE (0.0382) and MAE (0.0256), while the CCC is slightly lower than that in CausalCellInfer with PC‐simple (0.9530 vs. 0.9624) (Figures 3 and 4 and Table 3). We observed a similar pattern under the UKBB‐restricted gene‐set scenario (Table S1), although performance was slightly reduced due to the exclusion of potentially informative genes. These findings confirm that the compact, environment‐stable marker set selected by scI‐GCM retains sufficient information for highly accurate deconvolution without relying on a much larger expression feature set.

**FIGURE 3:**
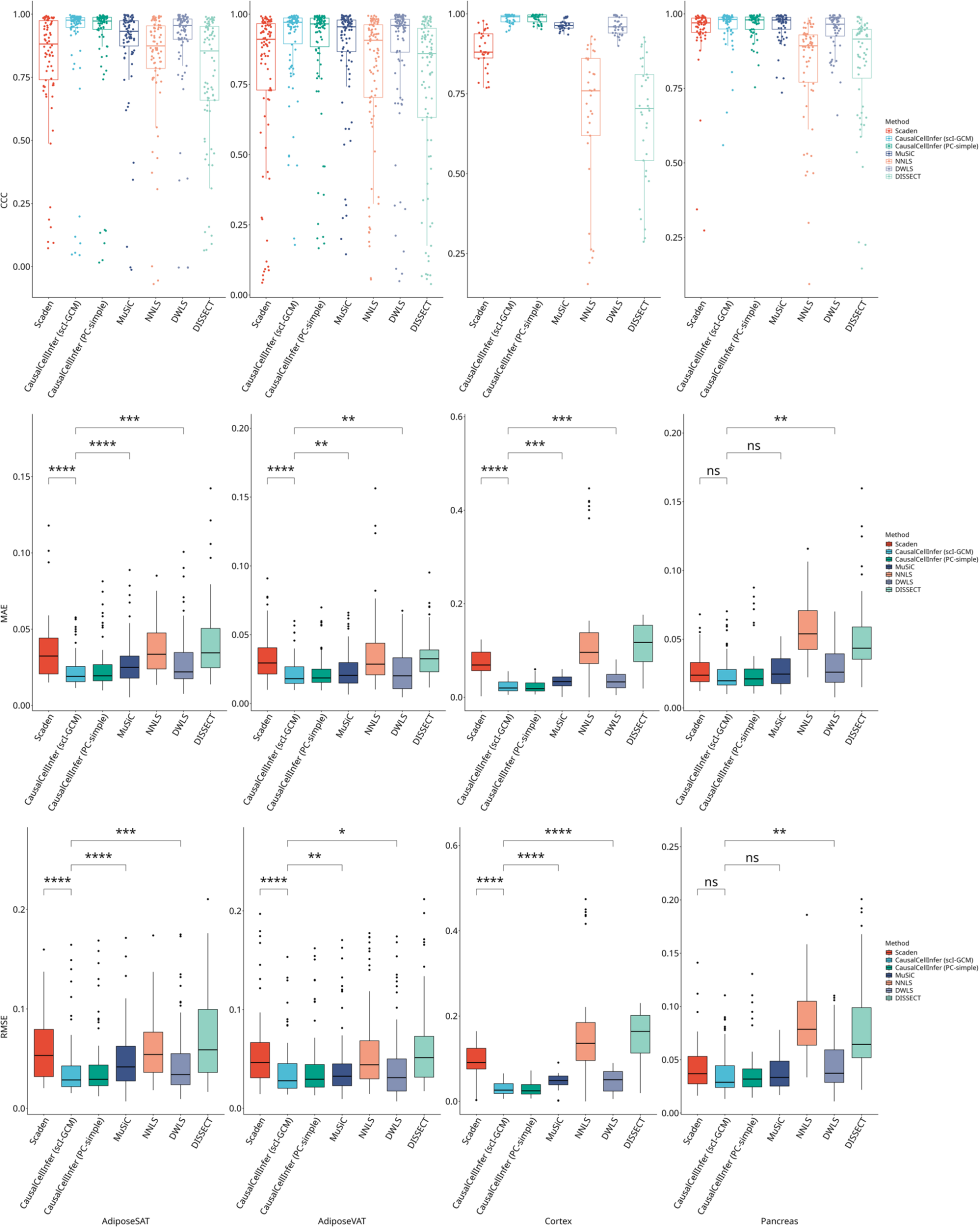
Benchmarking of cell deconvolution accuracy on pseudo‐bulk data constructed by combining reference scRNA‐seq examples.

**FIGURE 4:**
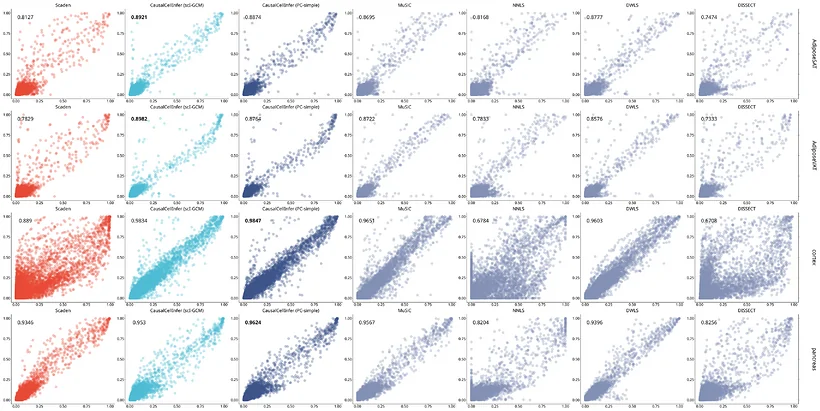
Benchmarking of cell deconvolution accuracy on pseudo‐bulk data constructed by combining reference scRNA‐seq examples with Lin's Concordance Correlation Coefficient (CCC).

**TABLE 3:** Comparison of cell deconvolution accuracy on pseudo‐bulk data constructed by combining reference scRNA‐seq examples.

Methods	Cortex			Adipose SAT			Adipose VAT			Pancreas		
	CCC	RMSE	MAE	CCC	RMSE	MAE	CCC	RMSE	MAE	CCC	RMSE	MAE
Scaden	0.8890	0.0932	0.0704	0.8127	0.0606	0.0353	0.7829	0.0556	0.0331	0.9346	0.0426	0.0279
CausalCellInfer (scI‐GCM)	0.9834	0.0309	**0.0236**	**0.8921**	**0.0397**	**0.0234**	**0.8982**	**0.0378**	**0.0218**	0.9530	**0.0382**	**0.0256**
CausalCellInfer (PC‐simple)	**0.9847**	**0.0302**	0.0237	0.8874	0.0414	0.0245	0.8764	0.0411	0.0227	0. **9624**	0.0385	0.0264
MuSiC	0.9651	0.0460	0.0342	0.8695	0.0490	0.0278	0.8722	0.0428	0.0247	0.9567	0.0385	0.0271
NNLS	0.6784	0.1749	0.1417	0.8168	0.0596	0.0376	0.7833	0.0591	0.0367	0.8204	0.0870	0.0578
DWLS	0.9603	0.0482	0.0368	0.8777	0.0479	0.0284	0.8576	0.0433	0.0252	0.9396	0.0463	0.0298
DISSECT	0.6708	0.1512	0.1110	0.7474	0.0721	0.0415	0.7333	0.0618	0.0340	0.8256	0.0801	0.0514

The top 2 performing methods are underlined. The best‐performing method is also bolded, while the second‐best methods are in italics.

Note: CCC is the abbreviation for Lin's Concordance Correlation Coefficient, RMSE is the abbreviation for Root Mean Square Error, MAE is the abbreviation for Mean Absolute Error.

Among benchmark methods, MuSiC and DWLS demonstrated intermediate, competitive performance, whereas NNLS declined sharply in the pancreas (CCC 0.8204) and DISSECT exhibited the weakest overall accuracy (cortex CCC 0.6708). Altogether, while tissue complexity and transcriptional similarity influence relative performance, CausalCellInfer emerges as one of the most consistently accurate strategies, highlighting the advantage of pairing biologically informed invariant markers with non‐linear deconvolution models.

Additionally, we performed sensitivity analyses to evaluate the effect of the number of cells used to construct each pseudo‐bulk sample on deconvolution accuracy. Specifically, we considered fixed pseudo‐bulk sizes of 100, 500, 1000, and 2000 cells. Deconvolution performance improved as the number of cells increased and became largely stable when 500 or more cells were used per pseudo‐bulk sample (Figure S2).

### 8.1 Evaluation on Real‐World Datasets

In the evaluation of real‐world datasets, our method exhibited comparable performance to Scaden, as indicated by analogous RMSE and MAE values. Importantly, our methodology outperformed alternative approaches by attaining a superior CCC across most cell types. Upon application to the Monaco and GSE107572 datasets, our proposed method (CausalCellInfer(scI‐GCM)) excelled, surpassing all other methods in terms of both MAE and RMSE. Furthermore, it achieved the highest average CCC among all evaluated methods (Figure 5). Interestingly, this pattern is consistent with prior benchmarking on the same dataset [17], where MuSiC underperformed in the real‐world PBMC dataset.

**FIGURE 5:**
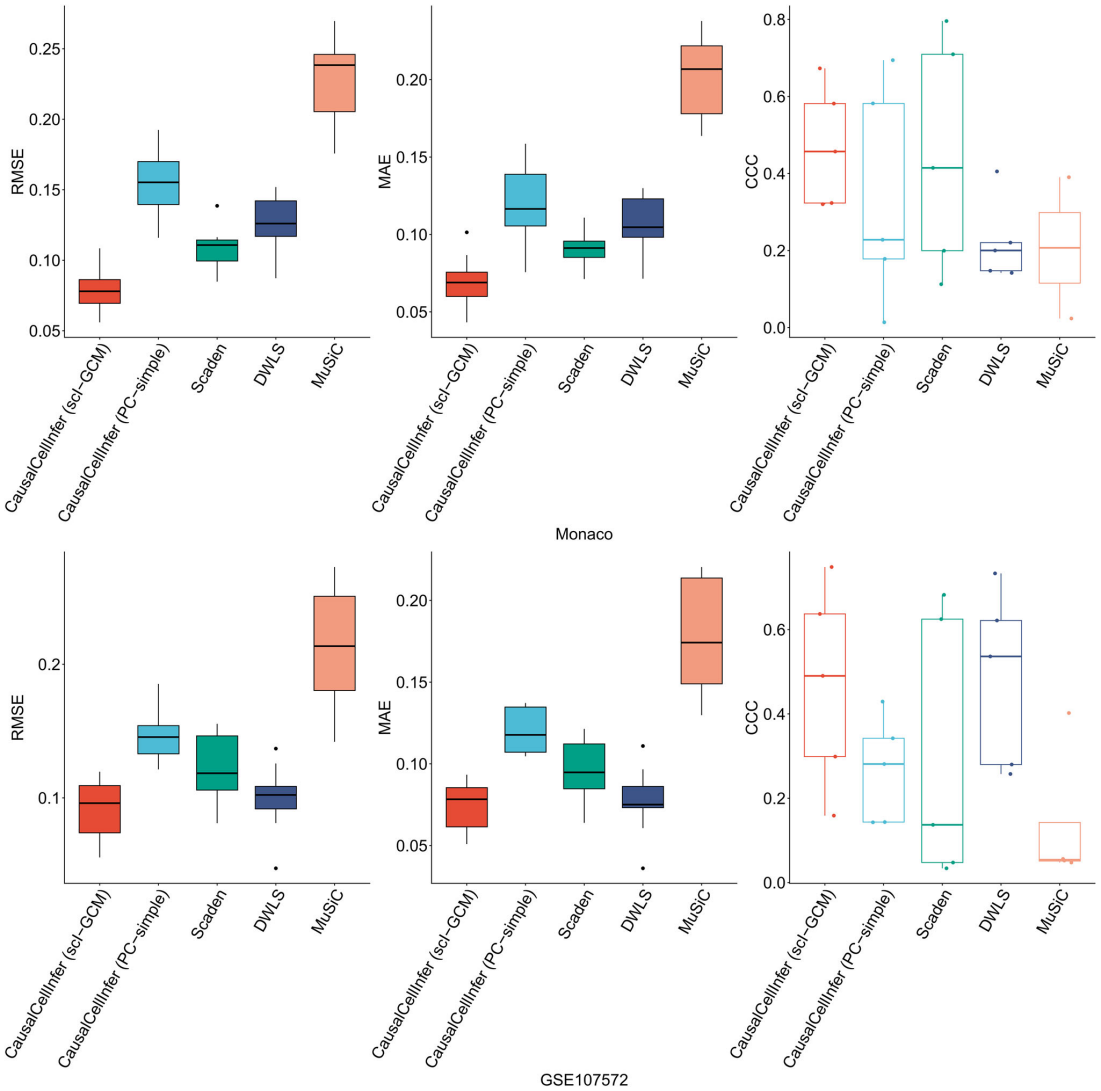
Benchmarking of cell deconvolution accuracy on real bulk data.

### 8.2 Simulation Validation of PrediXcan Expression Calibration and Downstream Cell‐Type‐Specific (CTS) Expression Signal Recovery

As a primary goal of our framework is its application to PrediXcan‐imputed expression profiles derived from large‐scale GWAS biobank cohorts, we conducted simulation analyses to systematically evaluate the validity and robustness of the proposed calibration procedure. Across the evaluated simulation settings, both the mean–standard deviation(mean‐SD) and quantile calibration methods substantially improved recovery of the underlying biological signals relative to uncalibrated standardized expression profiles. Specifically, the mean absolute errors of the estimated cell fractions were consistently lower after mean–SD or quantile calibration than those obtained without calibration (Figure S3), demonstrating that the transformation into a pseudo‐bulk‐like expression space improves downstream cell‐fraction estimation.

We further evaluated whether the calibrated expression profiles supported accurate recovery of CTS expression signals using ENIGMA. Under the same simulation framework, ENIGMA was applied to the calibrated profiles using alternative sources of cell‐fraction information. As expected, ENIGMA supplied with the true cell fractions (which are not available in practice) achieved the lowest CTS‐expression mean absolute error, whereas ENIGMA supplied with cell fractions estimated by CausalCellInfer achieved the second‐lowest error (Figure S4). By contrast, the baseline approach, which assigned the same population‐average CTS expression profile to every participant, showed the poorest performance. Together, these findings support the validity and robustness of the proposed calibration procedure and demonstrate that the ENIGMA component of CausalCellInfer recovers meaningful interindividual variation in CTS expression rather than merely reproducing population‐average signals.

Furthermore, we evaluated how variation in the proportion of gene‐expression variance explained by cis‐eQTLs affects deconvolution accuracy and downstream association performance. We found that the Pearson correlation between estimated and true cell fractions decreased from nearly 1 under the ideal scenario (*R* ^2^ =  1) to approximately 0.6 when using the observed heterogeneous gene‐level (*R* ^2^) values (Figure S5). However, when evaluating downstream analyses, including CTS‐TWAS and CPAS, imperfect prediction accuracy had little apparent effect on type I error control, whereas statistical power increased progressively with improving prediction accuracy (Figures S6–S8).

We also evaluated the ability of CausalCellInfer to recover signals from rare cell types. Specifically, we varied the abundance of each target cell type across 0%, 0.1%, 0.5%, 1%, 2%, 3%, 4%, 5%, and 10% in pseudo‐bulk samples generated from independent control‐validation cells. Across the investigated settings, our results suggest that a cell‐type abundance of approximately 5% represents a practical empirical benchmark for reliable evaluation of most cell types using CausalCellInfer (Figure S9).

### 8.3 Validity of Inferred UKBB Cell Fraction and Cell‐Type‐Specific Expression Profile

To evaluate the validity of the inferred cell fractions and CTS expression profiles in UKBB, we employed two complementary validation strategies. First, we compared cell‐type‐specific DEGs identified from the inferred CTS profiles derived from GWAS‐predicted expression with those identified from reference scRNA‐seq datasets for comparable phenotypes. Second, we performed cross‐center analyses to evaluate the consistency of CPAS and TWAS results across different UKBB assessment centers.

For the first validation, we compared cell‐type‐specific DEGs identified from the inferred CTS profiles with those derived from the corresponding reference scRNA‐seq datasets. If the inferred CTS expression profiles reliably capture cell‐type‐specific transcriptional changes, substantial overlap between the DEGs identified from these two data sources would be expected. We therefore treated the DEGs identified from the observed scRNA‐seq data as the reference and evaluated the positive predictive value (PPV) of the DEGs identified from the inferred CTS profiles.

We found that DEGs identified from the inferred CTS expression profiles showed substantial overlap with those derived from the reference scRNA‐seq data, with satisfactory PPVs for the majority of cell types. For example, in the pancreas, the primary cell types, including acinar, alpha, beta, and delta cells, achieved PPVs of approximately 0.5 or higher, substantially exceeding expectation (Table S2). In adipose tissue, the two predominant cell types, adipocytes and adipose‐derived stem and progenitor cells (ASPCs), exhibited the highest PPVs among all evaluated cell types, with values exceeding 0.8 in SAT and 0.6 in VAT. These findings support the reliability of our proposed method for inferring CTS expression profiles.

As a second validation, we performed pairwise cross‐center analyses of the CPAS and TWAS results obtained from different UKBB assessment centers. Details of the methodology are provided in the Supporting Information Methods. Briefly, effect estimates showed significant positive Pearson correlations across center pairs, indicating consistent effect patterns between centers. Directional concordance was also consistently high, with a mean concordance exceeding 60%. Together, these cross‐center results provide additional evidence for the robustness and reproducibility of the inferred cell fractions and CTS expression profiles in downstream association analyses (Figures S10–S13).

## 9 UKBB Application: Cell‐Type Proportion and Cell‐Type‐Specific Expression Estimation at Biobank Scale

We applied CausalCellInfer to PrediXcan‐imputed tissue‐specific gene expression profiles across ∼500,000 UKBB participants. Binary traits (except obesity) were defined using self‐reported diagnoses and International Classification of Diseases (ICD) codes. Obesity was defined as BMI > 30.

We targeted primary disease‐susceptible tissues representing the key pathological and genetic epicenters for each trait: the frontal cortex for major depression [37], the pancreas for T2DM [38], and SAT and VAT for obesity [39]. Because CausalCellInfer operates on tissue‐imputed transcriptomes, all derived cell‐type proportions and CTS expression signals reflect these specific tissue microenvironments.

### 9.1 Distinguishing Cell Types Associated With Various Diseases

To elucidate the relationship between the estimated cell proportions and diseases/traits, we conducted univariate association tests for each cell type via logistic/linear regression, with the trait/disease of interest as the outcome. We revealed that the estimated proportions of various cell types were associated with different diseases, as detailed below.

### 9.2 Depression

We deconvoluted the frontal cortex into six distinct cell types and discovered that the estimated cell proportions for astrocytes, micro/macro cells, and oligodendrocytes were significantly associated with risk of depression (Figure 6 and Table S3). Notably, a lower inferred proportion of microglial/macrophage cells and oligodendrocytes were associated with depression, while the proportion of neurons was elevated. These observations are consistent with existing literature; for instance, Rajkowska et al. [40] observed a decrease in both the density and ultrastructure of oligodendrocytes in the prefrontal cortex in depression.

**FIGURE 6:**
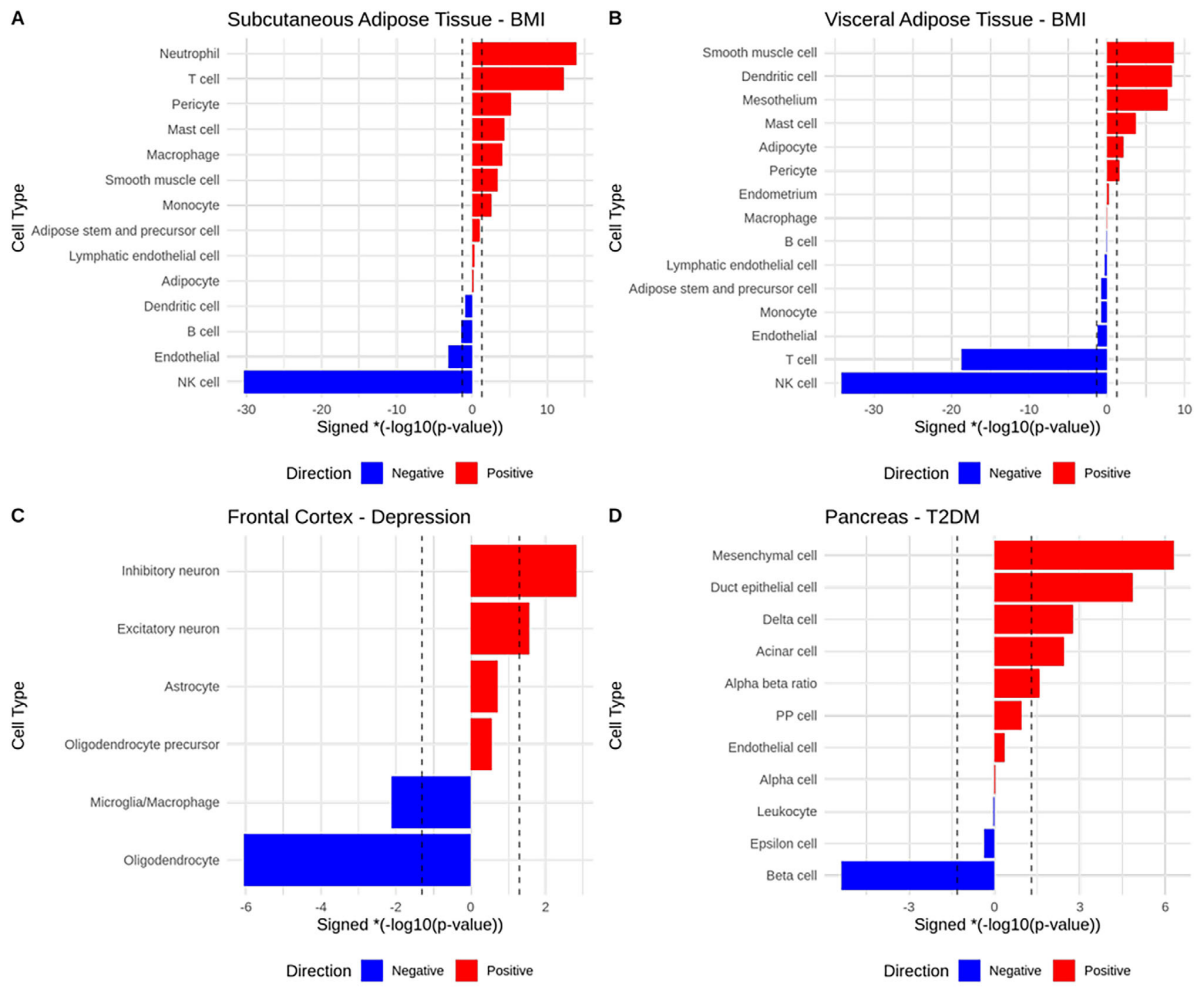
Associations between estimated cell proportions and disease onset.

### 9.3 Type 2 Diabetes

In our analysis of ten distinct cell types within the pancreas, we observed that the inferred proportions of six cell types, including beta cells, exhibited a significant association with T2DM risks. Notably, significantly lower inferred proportions of beta cells were observed in T2DM. Conversely, we detected associations of elevated proportions of acinar, delta, duct epithelial, and mesenchymal cells with T2DM, with the increase in mesenchymal cell proportions being especially prominent (Figure 6). We also observed significant alpha beta ratio increase in T2DM patients. Numerous studies have underscored the link between T2DM and a decrease in beta‐cell mass and function [41, 42]. Also, previous GWAS have pinpointed *IRX3* as a susceptibility gene for T2DM, and knockdown of the *IRX3* orthologue in zebrafish led to a decrease in insulin‐producing beta cells [43].

### 9.4 Obesity

In the SAT and VAT, we deconvoluted 14 and 15 distinct cell types respectively, and tested associations with obesity status. More than half of these cell types, including smooth muscle cells (SMC) and adipocyte, were found to be associated with obesity. Interestingly, the involvement of immune‐related cell types in obesity was observed to vary between adipose tissues. Some cell types, such as natural killer cells (NK cells) and T cells, were associated with obesity in both tissues (SAT and VAT), while others, like neutrophils and macrophage, were associated with obesity in only one tissue.

Compared to normal subjects, there was a significant increase in the estimated proportions of various cell types, including SMCs, macrophage, mast, monocyte, neutrophil, pericyte and T cells in SAT. Conversely, a significant decrease in NK, endothelial and B cell proportion was noted.

Notably, a significant increase in pericyte proportions was also observed in both adipose tissues. Supporting our findings, Tang et al. [44] demonstrated that pericytes are one of the precursor cells of adipocytes, underscoring their crucial role in obesity development. Watanabe et al. [45]. also showed that mice fed with a high‐fat diet (HFD) exhibit a higher level of pericyte count than those on a chow diet.

### 9.5 Interactions Between Cell Types and Their Associations With Disease

We tested the effects of cell type interactions on 4 tissue‐specific outcome variables using multivariate regression analyses, including depression in the frontal cortex, T2DM in the pancreas, BMI in the SAT, and BMI in the VAT.

Our results revealed that interactions between certain cell types were significantly associated with disease risks (Table 4 and Table S4). For example, the interaction between astrocytes and microglia/macrophages was linked to depression (based on expression from the frontal cortex). This is consistent with the literature suggesting a disrupted structural communication between excitatory neuron and oligodendrocyte in the frontal cortex, which contributes to both cognitive retardation and emotional instability commonly seen in depression [46].

**TABLE 4:** Cell type interactions significantly associated with diseases.

Tissue	Trait	Cell type interaction	*p*‐value
**Frontal cortex**	Depression	Excitatory * Oligodendrocyte	1.11E‐02
**Pancreas**	T2DM	Duct epithelial cell * Mesenchymal cell	3.22E‐03
		Leukocyte * Beta cell	3.19E‐03
		Mesenchymal cell * Alpha cell	3.06E‐02
		Mesenchymal cell * PP cell	5.65E‐03
		Alpha cell * PP cell	3.28E‐03
**SAT**	Obesity	Adipose stem and precursor cell * Lymphatic endothelial cell	4.73E‐03
		Adipose stem and precursor cell * Mast cell	1.78E‐03
		Adipose stem and precursor cell * NK cell	3.17E‐04
		Adipose stem and precursor cell * Pericyte	1.43E‐03
		Adipose stem and precursor cell * T cell	4.13E‐07
		Lymphatic endothelial cell * Smooth muscle cell	1.81E‐02
		Lymphatic endothelial cell * Adipocyte	1.88E‐02
		Lymphatic endothelial cell * B cell	2.52E‐04
		Lymphatic endothelial cell * Dendritic cell	1.76E‐03
		Lymphatic endothelial cell * Mast cell	9.74E‐03
		Lymphatic endothelial cell * NK cell	9.25E‐06
		Lymphatic endothelial cell * Pericyte	4.09E‐02
		Lymphatic endothelial cell * T cell	4.59E‐03
		Smooth muscle cell * Adipocyte	4.13E‐03
		Smooth muscle cell * Macrophage	9.21E‐03
		Smooth muscle cell * Mast cell	2.89E‐02
		Smooth muscle cell * Neutrophil	2.40E‐02
		Smooth muscle cell * NK cell	4.25E‐05
		Smooth muscle cell * Pericyte	1.31E‐06
		Smooth muscle cell * T cell	3.46E‐03
		Adipocyte * Mast cell	4.24E‐03
		Adipocyte * T cell	6.89E‐03
		B cell * Mast cell	4.21E‐03
		B cell * Neutrophil	3.84E‐02
		B cell * NK cell	1.05E‐02
		B cell * Pericyte	7.65E‐03
		B cell * T cell	1.97E‐04
		Dendritic cell * Neutrophil	2.39E‐03
		Macrophage * NK cell	4.25E‐03
		Macrophage * Pericyte	1.24E‐02
		Macrophage * T cell	2.48E‐04
		Mast cell * NK cell	1.32E‐03
		Mast cell * Pericyte	2.82E‐02
		Neutrophil * NK cell	7.85E‐07
		Neutrophil * Pericyte	1.37E‐03
		NK cell * Pericyte	2.46E‐02
		Pericyte * T cell	2.23E‐02
**VAT**	Obesity	Adipose stem and precursor cell * Adipocyte	7.95E‐03
		Adipose stem and precursor cell * B cell	1.36E‐02
		Adipose stem and precursor cell * Endometrium	5.41E‐03
		Adipose stem and precursor cell * Mesothelium	3.07E‐02
		Lymphatic endothelial cell * Adipocyte	9.66E‐03
		Lymphatic endothelial cell * Endometrium	5.57E‐08
		Lymphatic endothelial cell * Macrophage	4.76E‐02
		Lymphatic endothelial cell * Mesothelium	5.68E‐03
		Lymphatic endothelial cell * NK cell	3.75E‐03
		Lymphatic endothelial cell * Pericyte	1.67E‐04
		Smooth muscle cell * Endometrium	1.03E‐04
		Smooth muscle cell * NK cell	1.28E‐04
		Smooth muscle cell * Pericyte	3.45E‐05
		Adipocyte * Endometrium	2.78E‐09
		Adipocyte * Mesothelium	2.23E‐02
		Adipocyte * NK cell	3.19E‐05
		Adipocyte * Pericyte	2.73E‐05
		B cell * Endometrium	2.91E‐08
		B cell * Mesothelium	1.70E‐04
		B cell * NK cell	1.46E‐06
		B cell * Pericyte	1.22E‐07
		Dendritic cell * Endometrium	1.53E‐02
		Dendritic cell * Macrophage	4.10E‐02
		Dendritic cell * Mesothelium	7.23E‐03
		Dendritic cell * Pericyte	1.41E‐03
		Endometrium * Macrophage	5.64E‐04
		Endometrium * NK cell	2.82E‐07
		Macrophage * Pericyte	1.90E‐02
		Mast cell * Monocyte	2.42E‐02
		Mesothelium * NK cell	1.27E‐04
		NK cell * Pericyte	9.47E‐04

*Note*: PP cell indicates pancreatic polypeptide cell.

In addition, we found that interaction of cell types *across different tissues* may contribute to disease risks. For example, the interaction between B cells on SAT and dendritic cells in VAT was significantly associated with obesity. Both cell types play crucial roles in maintaining immune homeostasis, and previous studies [47, 48] have revealed significant involvement of inflammation in obesity. To conclude, interactions between certain cell types may play an important role in disease pathogenesis.

## 10 Cell‐Type‐Specific Genes Linked to Disease Susceptibility (focusing on Putative Direct Causal Genes as Prioritized by PC‐Simple)

Putative direct causal effect genes for disease susceptibility were found to exhibit variation across different cell types (Figure 7 and Table S5). We refer to genes selected by PC‐simple as putative direct causal genes. We define genes detected in exactly one cell type as cell‐type‐specific and genes detected in ≥2 cell types as cell‐type‐common.

**FIGURE 7:**
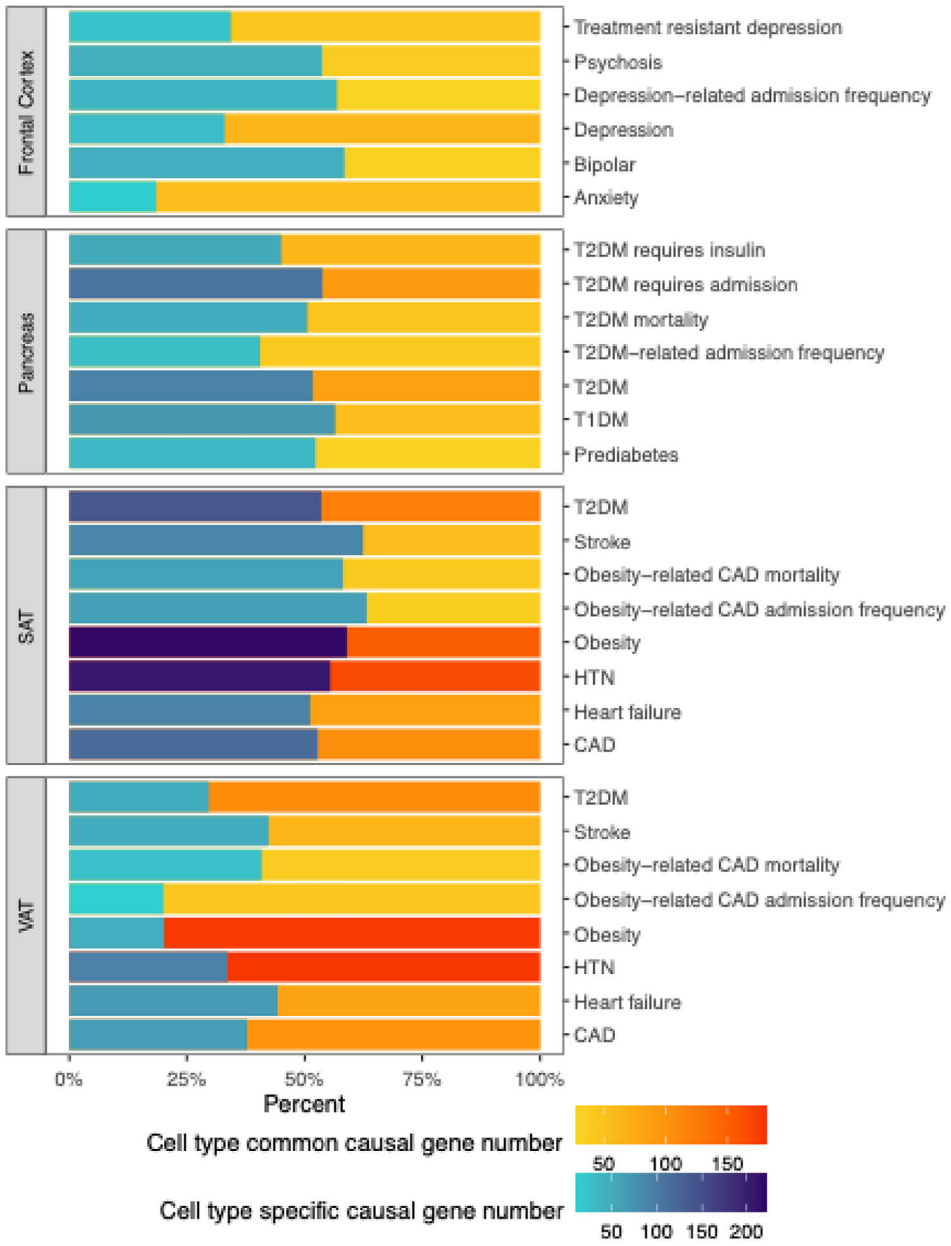
Cell specificity comparison of the identified putative direct causal genes by PC‐simple for disease onset and prognoses.

### 10.1 Depression

For depression, approximately one‐third of the 94 prioritized genes were cell‐type‐specific. A notable example is *KLC3*, identified as a causal gene for depression susceptibility exclusively within excitatory neurons (Table 5 and Table S5). This finding aligns with a previous study [49] reporting neuronal hypomethylated changes of *KLC3* in the prefrontal cortex of bipolar patients, suggesting a potential epigenetic link to mood disorders.

**TABLE 5:** Top putative direct causal genes by PC‐simple (ranked by *p*‐value) from inferred cell‐type‐specific (CTS) expression profiles.

Tissue	Disorder	Genes
**Frontal cortex**	Depression	*KLC3, ZSCAN9, RP9, CCDC64B, ZSCAN21, SPATC1L*
**Pancreas**	T2DM	*JAZF1, IRX3, CLEC18A, HLA‐DQA2, CEP68, NOTCH2, UBA7, CILP2, MAN2C1, OPRL1*
**SAT**	Obesity	*LMOD1, HSD17B12, C1QTNF4, ITIH4, TRIM73, CLEC18A, ATP8B3, UHRF1BP1, PRSS16, EIF3C, ADORA2B, SERPINC1, SLC5A11, SULT1A2*
**VAT**	Obesity	*SULT1A2, CENPO, CLEC18A, TRIM73, RNF123, CTSW, DNAH10, ATP8B3, SLC5A11, SERPINC1, C1QTNF4, RBM6, HSD17B12, TTLL6, ADORA2B*

Our analysis revealed a nuanced relationship between cell‐type alterations and disease status. Some cell types exhibited both altered proportions and changes in gene expression, while others showed only gene expression alterations without significant proportional changes. For instance, despite no significant association between oligodendrocyte precursor cells (OPCs) proportion and depression susceptibility, this cell type harbored 16 putative direct‐effect genes.

### 10.2 T2DM

For T2DM, we identified 178 putative direct‐effect genes associated with disease risk, with varying involvement across different cell types. In contrast to depression, where cell‐type‐specificity was more prominent, nearly half (86 genes, ∼48.3%) of the T2DM‐associated genes were common, detected in two or more cell types (Figure 7 and Table S5). For instance, *JAZF1* was identified as a putative direct causal gene in 6 distinct cell types, such as acinar, alpha, beta, and delta cells (Table S5). The association of *JAZF1* with increased T2DM risk is well‐established [50, 51].

### 10.3 Obesity

Our analysis of obesity revealed a substantial number of putative direct‐effect genes in both subcutaneous and visceral adipose tissues, but with a large difference in the distribution of cell‐type‐specific versus common genes. In SAT, a high degree of cell‐type‐specificity was observed, with approximately 69.7% of causal genes being specific to a single cell type (only 77 genes, or 30.3% of ∼254 identified genes, were cell‐type‐common). Conversely, VAT displayed a much higher proportion of common genes, with 178 genes (79.8% of ∼223 identified genes) contributing to obesity across multiple cell types. This suggests a broader, multi‐cellular involvement of causal mechanisms in VAT.

A notable cell‐type‐specific example in SAT is *PRSS53* (also known as *POL3S*), identified as a causal gene exclusively in adipocytes. This finding is supported by a previous study [52] showing *PRSS53* downregulation in obesity. Given *PRSS53*’s critical role in maintaining mitochondrial health and function for metabolism [53], its potential causal role in obesity development within SAT is further substantiated.

### 10.4 Cell‐Type‐Specific Genes Involved in Disease Prognosis (Focusing on Putative Direct Causal Genes as Prioritized by PC‐Simple)

Identifying specific genes that contribute to disease outcomes or prognosis is crucial for developing personalized treatments. Leveraging detailed prognosis data from the UKBB, which are often absent in single‐cell studies, we conducted analyses to pinpoint putative direct‐effect genes associated with disease‐specific mortality and hospitalization frequencies.

### 10.5 Depression

For depression, our analysis revealed distinct patterns for treatment resistance and hospitalization frequency. A substantial proportion (∼65.7%) of putative direct‐effect genes linked to treatment resistance were common, identified in at least two cell types (Table S5a). Conversely, genes associated with the frequency of hospital admissions were predominantly cell‐type‐specific (∼56.9%) (Table S5a).

### 10.6 Type 2 Diabetes Mellitus

In T2DM, we observed that a majority (∼59.4%) of putative direct‐effect genes influencing hospitalization frequencies were common across multiple cell types (Table S5b). However, for T2DM‐related mortality, a slightly different distribution emerged, with approximately half (∼49.5%) of the causal genes being cell‐type common (Table S5b). For example, we identified the gene *LIMK1* as a determinant of mortality in T2DM within beta cells. This finding aligns with a recent study indicating a relationship between genetic polymorphisms of *LIMK1* and glucose dysregulation and insulin exocytosis [54].

### 10.7 Obesity

Given obesity's established role as a risk factor for coronary artery disease (CAD) [55], we investigated genes influencing CAD‐related mortality and hospitalization frequency in obese patients. A notable divergence was observed between adipose tissues: SAT showed a higher proportion of cell‐type‐specific genes, while VAT exhibited a higher proportion of common genes influencing these outcomes (Table S5c,d).

### 10.8 Other Disorders

The distribution of identified causal genes varied considerably across tissues. In SAT, for most obesity‐related traits (CAD, T2DM, hypertension, heart failure, stroke), cell‐type‐specific causal genes predominated. The inverse pattern was consistently found for these same traits in VAT.

Similarly, for traits associated with the frontal cortex (anxiety, bipolar disorder, psychosis) and pancreas (prediabetes, T1DM, T2DM requires admission, T2DM requires insulin), the majority of identified causal genes were found to be cell‐type‐specific.

### 10.9 Open Targets Enrichment Results

We conducted enrichment analyses for depression, T2DM, and obesity. For obesity, we performed four sets of enrichment analyses for each pair of tissues associated with obesity. Specifically, we investigated whether the genes we identified were significantly overrepresented as targets for BMI/obesity/weight/WHR (waist‐hip‐ratio) in the Open Targets database.

Encouragingly, the sets of causal genes identified for all the studied diseases were significantly enriched among the disease targets catalogued in Open Targets (Table 6). This observation provides further validation for the usefulness of our proposed framework in estimating cell proportions and CTS expression profiles.

**TABLE 6:** Open Targets enrichment results based on gene‐sets identified for disease susceptibility.

Tissue	Trait	Enrichment *p*‐value
Pancreas	T2DM	1.034E‐14
	T2DM requires admission	1.092E‐13
	T2DM requires insulin	1.535E‐04
SAT	Obesity	1.207E‐02
	Body weight	4.568E‐21
	BMI	2.699E‐35
	WHR	1.566E‐10
VAT	Obesity	3.965E‐04
	Body weight	4.370E‐18
	BMI	6.224E‐32
	WHR	2.377E‐11
Frontal cortex	Depression	4.654E‐02

### 10.10 Validation of Causally Prioritized Genes

To test whether the genes prioritized by our causal analysis reflect genuine disease biology rather than statistical artifacts, we cross‐validated the top‐ranked genes per trait (selected by zMin) against three independent lines of evidence beyond Open Targets: published gene–trait associations (PubMed/Europe PMC), tissue‐matched cis‐eQTL (GTEx v8), and Mendelian Randomization (MR; defined by published studies explicitly utilizing MR to prioritize or evaluate the candidate gene for the target trait (≥ 1 explicit MR paper). Candidates without such published MR studies were treated as unconfirmed for this specific line of evidence). Of the 210 top‐ranked (≤10 per trait × tissue) cell type‐specific gene‐trait pairs, 199(95%) corresponded to a significant *cis*‐eGene in the disease‐relevant tissue, more encouragingly, and all of them carry at least one line of supporting evidence. Corroboration was strongest for metabolic traits: prioritized type‐2‐diabetes genes included the established susceptibility loci ***JAZF1*** and ***NOTCH2*,** together with ***IRS1*** and the FTO‐locus effector ***IRX3***, while *FES* was prioritized for hypertension. Importantly, the framework also corroborates less‐studied candidates through tissue‐matched genetic regulation: for example, ***KLC3***, prioritized in frontal cortex for depression, is a significant *cis*‐eQTL gene in cortical tissue (GTEx frontal cortex, *q* ≈ 0.014), providing orthogonal genetic support for a gene not previously prominent in the psychiatric literature and nominating it for functional follow‐up. Together, these analyses indicate that the causal prioritization recovers bona fide disease genes while providing an auditable, per‐gene evidence trail rather than relying on a single enrichment resource (Figure S14).

### 10.11 Results of Further Pathway Enrichment Analyses

We combined the identified cell‐type‐specific causal genes (prioritized by the PC‐simple algorithm) for pathway enrichment analysis. The enriched pathways were shown in Table S6. For instance, “Pyrophosphate hydrolysis” was significantly enriched for depression, consistent with Sha et al. [56]. work suggesting its protective role in astrocytes against depression via lysosomal function.

For T2DM, pathways like “Vitamin B3 (nicotinate and nicotinamide) metabolism”, “Pre‐NOTCH Processing in the Endoplasmic Reticulum”, and “Pre‐NOTCH Processing in Golgi” were significantly enriched. This aligns with Lu et al. [57]. findings on dysregulated Notch signaling worsening T2DM through hepatic fat deposition and gluconeogenesis.

We also identified enriched pathways based on gene sets associated with disease prognosis (Table S6). For instance, the “Apoptotic cleavage of cellular proteins” pathway was significantly enriched in treatment‐resistant depression (TRD). This aligns with Kosten et al. [58] who showed that antidepressant treatment can differentially regulate apoptotic genes in the brain structures implicated in the etiology and treatment of depressive disorder. Fries et al. [59]. also showed that apoptotic events may be responsible for the chronic low‐grade inflammation in depressive patients. The “Ceramide signaling pathway” was enriched in relation to the frequency of depression‐related admissions. Gulbins et al. [60] reported that increased ceramide concentration can lead to the reversal of stress‐induced MDD via activation of autophagy.

“Role of mitochondria in apoptotic signaling” emerged as a significantly enriched pathway in relation to T2DM‐related mortality, supporting existing literature that apoptotic signaling drives cell death in diabetic complications like cardiovascular diseases (CVD) [61] and beta cell failure [62], increasing T2DM mortality.

### 10.12 Results from Conditional TWAS (Controlled for Cell‐Type Proportions)

We compared trait‐associated genes identified from two different linear models using bulk expression data, one with estimated cell proportions as covariates (conditional TWAS) and one without. Genes from the adjusted TWAS are shown in Table 7 and Table S7. Adjusting for estimated cell proportions when performing TWAS analysis led to significant coefficient changes for numerous genes (Table 8 and Tables S8 and S9), consistent with cell composition acting as a confounder or mediator of bulk‐level associations. For example, adjusting for cell proportions resulted in significant effect size changes for ∼59.7% of T1DM‐associated genes. Some genes were significant only in the standard TWAS (e.g., *EFEMP2* in SAT obesity), illustrating that bulk‐level associations can be sensitive to compositional variation.

**TABLE 7:** Examples of top (ranked by *p*‐value) trait‐associated genes identified from TWAS after adjustment for estimated cell‐type proportions.

Tissue	Disorder	Genes
Frontal cortex	Depression	*ZSCAN9, BTN3A2, C4A*
Pancreas	T2DM	*IRX3, NOTCH4, CD84*
SAT	Obesity	*RTN4, TPCN2, SEMA3F*
VAT	Obesity	*TBL2, NOVA1, SULT1A2*

**TABLE 8:** Trait‐associated genes from a standard TWAS analysis with significant coefficient changes after adjusting for estimated cell‐type proportions.

Tissue	Disorder	Genes	Estimates (standard TWAS)	*p*‐value (standard TWAS)	Estimates (conditional TWAS)	*p*‐value (conditional TWAS)	Coeff_diff_P	*p*‐value_BH (standard TWAS)	*p*‐value_BH (conditional TWAS)
Pancreas	T1DM	*ZSCAN31*	0.00118	2.76E‐07	0.00110	1.96E‐06	6.28E‐08	2.03E‐05	1.44E‐04
		*TRIM26*	−0.00124	2.19E‐02	−0.00187	6.77E‐04	2.57E‐11	3.66E‐01	3.48E‐02
	T2DM	*BAK1*	0.01193	2.30E‐07	0.01102	2.02E‐06	1.00E‐04	3.94E‐05	4.32E‐04
		*CD84*	0.00218	1.17E‐01	0.00591	4.36E‐04	7.60E‐05	5.11E‐01	2.02E‐02
	T2DM requires admission	*HLA‐L*	0.00598	7.37E‐07	0.00564	3.27E‐06	5.17E‐04	8.60E‐05	3.51E‐04
		*AGER*	0.01238	3.16E‐05	0.01050	5.04E‐04	3.66E‐04	1.93E‐03	1.58E‐02
	T2DM requires insulin	*HLA‐DMA*	0.00139	2.21E‐05	0.00150	6.49E‐06	4.34E‐02	5.54E‐03	2.08E‐03
		*SKIV2L*	−0.00096	2.68E‐03	−0.00145	1.79E‐05	6.51E‐06	1.56E‐01	4.61E‐03
SAT	CAD	*GRK4*	0.00482	2.06E‐04	0.00479	2.22E‐04	1.76E‐02	2.05E‐02	2.10E‐02
		*C6orf106*	0.01231	1.38E‐06	0.01241	1.14E‐06	1.82E‐02	6.75E‐04	6.25E‐04
	HTN	*BANF1*	0.00542	1.21E‐01	0.01031	4.81E‐03	5.56E‐06	3.70E‐01	4.44E‐02
		*SNX31*	−0.02702	2.85E‐05	−0.02690	3.09E‐05	3.13E‐02	8.19E‐04	8.44E‐04
	Heart failure	*HLA‐S*	−0.00242	8.23E‐02	−0.00530	2.47E‐04	1.35E‐13	5.92E‐01	2.72E‐02
		*TSLP*	0.01451	7.15E‐06	0.01444	7.88E‐06	2.41E‐03	1.24E‐03	2.58E‐03
	Obesity	*RTN4*	−0.01358	6.42E‐03	−0.01438	3.92E‐03	2.52E‐05	5.83E‐02	4.44E‐02
		*CENPC*	−0.01467	9.49E‐06	−0.01476	8.36E‐06	7.65E‐04	2.76E‐04	2.98E‐04
	T2DM	*SPTA1*	−0.00482	1.60E‐03	−0.00470	2.10E‐03	5.85E‐03	3.62E‐02	5.00E‐02
		*NPC1*	−0.01650	6.72E‐06	−0.01643	7.27E‐06	1.64E‐02	5.01E‐04	6.28E‐04
VAT	CAD	*ITPR3*	0.01946	6.84E‐05	0.02006	4.21E‐05	4.92E‐02	1.72E‐02	1.25E‐02
		*ADIPOQ*	−0.00925	3.12E‐03	−0.01174	3.38E‐04	9.65E‐03	1.08E‐01	3.89E‐02
	HTN	*MLXIPL*	−0.01251	4.89E‐02	−0.01985	2.63E‐03	3.90E‐05	2.23E‐01	3.06E‐02
		*WASH2P*	−0.01919	5.32E‐06	−0.01910	5.94E‐06	1.42E‐02	2.26E‐04	2.52E‐04
	Heart failure	*MAST2*	0.01151	8.62E‐05	0.01158	7.90E‐05	7.87E‐03	1.13E‐02	1.15E‐02
		*MICB*	0.00245	2.31E‐03	0.00279	5.78E‐04	1.56E‐03	1.12E‐01	4.58E‐02
	Obesity	*TBL2*	0.00999	9.80E‐03	0.01204	1.95E‐03	1.70E‐08	7.27E‐02	2.39E‐02
		*ERAP1*	−0.01853	5.76E‐06	−0.01861	5.22E‐06	1.07E‐02	2.01E‐04	2.18E‐04
	T2DM	*HLA‐C*	−0.00229	3.32E‐02	−0.00393	7.07E‐04	1.58E‐04	2.55E‐01	2.47E‐02
		*HLA‐L*	−0.01872	4.50E‐21	−0.01607	2.53E‐14	1.68E‐04	1.07E‐17	6.98E‐11

*Note*: “Coeff_diff_P” indicates the calculated *p*‐value for coefficient change between standard and conditional TWAS, standard TWAS estimates are used as the reference for the calculation of coefficient differences, HTN is the acronym for hypertension.

### 10.13 Results from CTS‐TWAS (Univariate Association Tests)

Here we report the results from CTS‐TWAS based on univariate association tests (distinct from PC‐simple, which involves repeated conditional tests). Genes identified in at least two cell types were classified as “cell‐type common”, while those in only one were ‘cell‐type‐specific’.

Similar to putative direct causal gene analysis by PC‐simple, the majority of genes identified from CTS‐TWAS were cell‐type‐specific (Table 9 and Tables S10 and S11). The number of significant genes for each trait is listed in Table S10, with FDR<0.05 considered as ‘significant’ genes. Among traits with significant genes, T2DM‐related traits typically showed higher cell type specificity, whereas most obesity‐related traits showed broader sharing across cell types.

**TABLE 9:** Top‐ranked (by absolute effect size from linear regression) trait‐associated genes for T2DM (tissue studied: Pancreas) identified from CTS‐TWAS (univariate tests).

Cell type	Genes	Estimates	*p*‐value	Q‐value
acinar cell	*PAX9*	−0.0959	1.97E‐04	4.10E‐02
	*MYRFL*	0.0499	2.22E‐04	4.37E‐02
	*LINGO4*	−0.0329	8.22E‐06	5.12E‐03
alpha cell	*EXTL1*	0.0600	9.78E‐05	1.79E‐02
	*ESPNL*	−0.0432	4.30E‐04	3.50E‐02
	*ZNF215*	0.0355	8.14E‐06	3.78E‐03
beta cell	*USP6*	−0.0546	5.26E‐04	4.19E‐02
	*HLA‐DQB2*	−0.0538	4.24E‐04	3.78E‐02
	*IP6K3*	−0.0506	7.12E‐04	4.93E‐02
delta cell	*RMI2*	0.0339	3.61E‐04	4.83E‐02
	*TNNI3K*	−0.0303	4.43E‐04	4.89E‐02
	*FAM153A*	0.0290	1.38E‐05	6.47E‐03
duct epithelial cell	*SLC25A41*	−0.0471	7.26E‐05	1.51E‐02
	*IRX3*	−0.0216	4.86E‐04	4.91E‐02
	*GPRC5A*	0.0213	1.73E‐08	6.47E‐05
endothelial cell	*GNA15*	−0.0349	3.13E‐06	2.86E‐03
	*CRHR2*	−0.0328	2.69E‐05	1.12E‐02
	*RNF122*	0.0291	9.44E‐05	1.77E‐02
epsilon cell	*PRAP1*	−0.0737	1.12E‐04	1.14E‐02
	*WNT10B*	−0.0530	3.60E‐04	2.47E‐02
	*CLNK*	−0.0485	3.43E‐04	2.42E‐02
Leukocyte	*GYS2*	−0.0616	1.93E‐04	2.38E‐02
	*NAALAD2*	0.0579	1.43E‐06	8.92E‐04
	*TDRKH*	−0.0225	2.09E‐04	2.38E‐02
mesenchymal cell	*PAX9*	0.2298	2.39E‐19	8.93E‐16
	*HCG9*	0.0546	7.89E‐06	3.67E‐03
	*SLC6A19*	−0.0394	4.78E‐04	4.97E‐02
PP cell	*PCDHA2*	−0.0599	1.06E‐04	1.04E‐02
	*C10orf62*	−0.0541	4.79E‐05	6.57E‐03
	*CDC25A*	−0.0466	1.02E‐05	2.95E‐03

*Note*: Q‐value indicated false discovery rate which are calculated from *p*‐value after correcting for multiple testing by Benjamini‐Hochberg method, PP cell indicates pancreatic polypeptide cell.

Open Targets enrichment analysis of CTS‐TWAS genes for 4 tissue‐disorder pair with ≥5 genes (including T2DM and hospitalization frequency (pancreas), obesity (SAT/VAT)) confirmed significant or nominal enrichment among known disease targets listed in Open Targets (Table 10).

**TABLE 10:** Open Targets enrichment results based on gene‐sets identified from CTS‐TWAS using inferred CTS expression profiles.

Tissue	Trait	*p*‐value
**Pancreas**	T2DM	9.405E‐19
	T2DM requires admission	6.044E‐19
	T2DM requires insulin	7.659E‐03
**SAT**	Obesity	1.514E‐64
	Body weight	9.696E‐42
	BMI	3.201E‐60
	WHR	9.026E‐28
**VAT**	Obesity	1.639E‐98
	Body weight	6.682E‐54
	BMI	4.919E‐63
	WHR	1.559E‐38
**Cortex**	Depression	1.204E‐02

Furthermore, we compared the genes identified from a causal inference approach (PC‐simple, which prioritizes putative direct‐effect genes) and univariate association analysis (TWAS) using inferred CTS expression profiles. For most traits, we identified considerably more associated genes from univariate TWAS (Table S10). As PC‐simple relies on repeated conditional independence testing, it is more stringent than univariate testing. Interestingly, PC‐simple also uncovered novel associations not found in standard association tests (Table S12). We hypothesize that some TWAS‐identified genes may be “peripheral” (putative indirect causal) genes acting through the putative direct causal genes identified by PC‐simple. As expected, if we relax the multiple testing correction criteria, there is an increased overlap between PC‐simple and standard association analyses (Table S12).

We observed high concordance of identified trait‐associated genes across covariate adjustment and ancestry restriction analyses (Table S13, See Supporting Information for more details). Taken together, we conclude that the reported gene‐discovery results are generally robust and stable, and are not substantially driven by residual demographic confounding, population stratification or differential cross‐ancestry portability of the expression‐prediction models.

## 11 Discussion

In this study, we introduced CausalCellInfer, a comprehensive framework that integrates invariant causal prediction, deep learning, and regularized matrix completion, with applications to GWAS‐imputed transcriptome profiles. This framework is designed to deconvolute cell compositions and estimate CTS expression profiles from bulk and genotype‐imputed transcriptomes at biobank scale (*N*∼500K). As a fully data‐driven method, CausalCellInfer first identifies marker genes from the reference scRNA‐seq dataset, leveraging the invariant causal prediction principle. It then trains a deep neural network‐based deconvolution model using these identified marker genes. Finally, it employs a regularized matrix completion algorithm, ENIGMA, to estimate the corresponding CTS profiles.

We validated the robustness and accuracy of CausalCellInfer in cell deconvolution by comparing it against five state‐of‐the‐art deconvolution methods, namely DWLS, NNLS, Scaden, DISSECT, and MuSiC. This comparison was conducted on four simulated and two real peripheral blood mononuclear cell (PBMC) datasets. To further demonstrate the reliability of CausalCellInfer in estimating CTS profiles, we have conducted a wide range of enrichment analyses, including DEGs enrichment analysis, Open Targets enrichment analysis, and pathway analyses.

Moreover, we applied CausalCellInfer to GWAS‐imputed expression datasets from the UKBB (four different tissues) and performed cell‐type‐level analyses for 29 clinical phenotypes, including susceptibility, prognosis‐related outcomes, interaction analyses between cell types, CTS‐TWAS, and composition‐adjusted (conditional) TWAS. These applications illustrate a central advantage of the approach: it enables cell‐type‐resolved hypothesis testing in cohorts where the scale and phenotypic depth are far beyond what is typically feasible for single‐cell studies.

### 11.1 Strengths of the Framework

The framework presented in this study exhibits several strengths. First, a notable advantage is that CausalCellInfer incorporated a novel marker‐gene informed and deep learning‐based cell deconvolution model. Marker genes are learned from scRNA‐seq reference data rather than relying on predefined marker lists.

Secondly, our framework does not require a constant marker‐gene expression assumption as required in classical signature‐based methods (i.e., a given marker gene is expressed at a constant level across samples within a specific cell type) for accurate estimation, making it more robust to inter‐subject heterogeneities or other technical variabilities across samples.

Thirdly, our method is likely more resistant to the influence of confounders and selection bias than signature‐based methods. As also reported in ref. [16], deep learning models can learn latent representations of cell types that integrate information from multiple genes rather than relying solely on predefined marker genes. Consequently, the learned representations may capture higher‐order molecular patterns and reduce the reliance on individual genes that are vulnerable to technical variation or context‐dependent biological changes. This property provides an opportunity to identify more stable gene features for cell‐type deconvolution. For example, GFAP is a well‐established marker of astrocytes in the central nervous system; however, its expression can be markedly altered in reactive astrocytes under conditions such as inflammation or neurodegeneration. Therefore, although GFAP remains biologically informative, its expression level may not serve as a consistently reliable feature for cell‐type identification across heterogeneous biological contexts, potentially reducing deconvolution accuracy when used as a primary marker. In contrast, our framework can identify and prioritize more stable features by jointly modeling information across genes, thereby reducing the impact of context‐dependent or confounded markers such as GFAP on deconvolution performance. This advantage is supported by our simulation analyses, in which the proposed framework showed improved robustness under scenarios involving confounding effects.

Fourth, our incorporation of the invariance principle further enhances the robustness of the method to confounding and improves generalizability to new samples.

Fifth, despite being based on deep neural networks, our method is time‐ and resource‐efficient, as it is trained on a compact set of informative markers. It operates much faster than most competing methods under study.

Sixth, as shown in real data applications, the proposed framework is scalable to large biobanks and genotype‐imputed transcriptome data. It is designed to operate when only a subset of genes is available and when expression is derived from genetic prediction, enabling cell‐type‐resolved analyses at sample sizes that are typically infeasible for scRNA‐seq.

Finally, a variety of downstream analyses are enabled by ***individual‐level*** inference. Once cell‐type proportions and CTS profiles are inferred per person under our framework, many analyses become possible, including CTS‐TWAS, putative CTS direct causal gene detection via PC‐simple, interaction models, prognosis modeling, and composition‐adjusted TWAS.

### 11.2 Two Complementary Applications: TWAS‐type (E.g., UKBB) and Bulk RNA‐seq

We wish to highlight that CausalCellInfer supports two complementary application modes that differ in their inferential objectives. In biobank‐scale TWAS‐type applications (e.g., UKBB), the primary goal is to identify and prioritize putative causal associations between genetically predicted cell‐type fractions or CTS expression and clinical outcomes, rather than to recover exact individual‐level cellular readouts. In this setting, the genetic variants used to predict cellular phenotypes function analogously to instrumental variables in MR [63, 64]: validity requires that the instruments capture sufficient variance in the exposure, not that prediction be perfect. Imperfect prediction accuracy primarily reduces statistical power and attenuates effect estimates *without inflating type I error*, provided the instrument‐exposure association is non‐trivial [65, 66]; the proper control of type I error was also confirmed in our simulations. Even when genetic instruments are weak, the well‐characterized consequence in two‐sample MR (single nucleotide polymorphism(SNP)‐exposure and SNP‐outcome associations being measured in independent datasets) is bias toward the null (i.e., conservative attenuation), not inflation of false positives [67]. This constraint is inherent to all TWAS‐type analyses, which have been generally restricted to genes with sufficient cis‐heritability, yet have produced numerous valid and replicable gene‐trait associations [6, 68, 69]. In contrast, when CausalCellInfer is applied to directly measured bulk or single‐cell RNA‐seq data, the full gene set is available, canonical markers are retained, and the objective expands to include accurate individual‐level estimation of cell‐type proportions and CTS expression. In this mode, the concerns regarding SNP‐predictability do not apply.

### 11.3 Related Approaches

Numerous methods have been proposed to deconvolute cell compositions and estimate CTS expression profiles from bulk samples. However, these methodologies differ substantially from our current work. For instance, CIBERSORT/CIBERSORTx^9^ can infer cell compositions and CTS expression profiles from bulk transcriptomes. It utilizes reference gene expression profiles (GEPs) of predetermined marker genes for cell deconvolution. However, classical signature‐matrix methods typically rely on the assumption of constant marker gene expression levels within cell types across samples, which might not hold in practice.

Another similar line of work is deep learning‐based cell deconvolution and CTS expression estimation methods, such as Scaden and TAPE. Menden et al. [15]. introduced Scaden, a deep neural network‐based cell deconvolution model designed to dissect bulk samples. Following a similar approach to Scaden, Chen et al. [16] proposed an autoencoder‐based model, TAPE, to infer cell compositions and CTS profiles from bulk samples. Rather than relying on GEPs of marker genes, they trained their deconvolution model directly on the reference scRNA‐seq using typically ∼10 000 highly variable genes, making them computationally intensive and prone to learning dataset‐specific artifacts or noise. CausalCellInfer occupies a strategic middle ground that combines the non‐linear flexibility of deep neural networks with the rigorous, parsimonious feature selection of causal inference methods. Leveraging the invariance principle, the identified invariant marker genes from scI‐GCM were likely more resistant to data bias and noise, and more generalizable to new datasets.

Another important distinction of our work from all previous studies on cell deconvolution is that we pioneered applications to genotype‐imputed expression data, especially from large biobanks. Our work helps to bridge the gap between GWAS/sequencing and scRNA‐seq studies and enables a wide variety of individual‐level analysis with cell proportions and CTS expression and their relationship with diverse clinical phenotypes in a large sample. Such analyses are generally not possible in conventional single‐cell studies due to the limited number of subjects and phenotypic information available.

More broadly, several frameworks link scRNA‐seq and GWAS to identify disease‐relevant cell types or to interpret GWAS signals in a cellular context. For example, Jagadeesh et al. [4] integrated GWAS summary statistics, epigenomics, and scRNA‐seq data to detect the underlying cell types and processes by which genetic variants influence the target traits, and utilized stratified linkage disequilibrium score regression (LDSC) to calculate heritability enrichment. In another study, scGWAS was developed to leverage scRNA‐seq to interrogate CTS transcriptional changes induced by genetic variants [70]. It identified associations between traits and cell types by examining whether genes implicated by GWAS exhibit concordant activation in a particular cell type using proportion tests. Additionally, Zhou et al. [71] proposed a CTS transcriptome‐wide association framework (this work was released after our preprint was first posted in Oct 2024 [72]). The approach leveraged external single‐cell transcriptome and genotype data, and used Enformer‐inferred epigenomics features to predict CTS expression; then a linearized model was built and association tests performed using GWAS summary statistics.

The current work is different from the aforementioned methods, which mainly operate with and focus on GWAS summary data. Our objective is fundamentally different: we infer ***individual‐level*** cell‐type proportions and CTS expression from bulk or genotype‐imputed transcriptomes, enabling biobank‐scale, cell‐type‐resolved association analyses across a wide phenotype spectrum. Individual‐level data also allows for more diverse types of analysis, such as interaction modeling, cell‐type‐composition‐adjusted TWAS, prioritization of potential direct causal genes via PC‐simple, adjustment for other genetic/clinical covariates, flexible definition of outcome phenotypes, and the use of non‐linear or machine learning models, which are generally not feasible with GWAS summary statistics alone. Moreover, our approach is methodologically novel and distinct, combining causal inference and invariance principles with deep neural networks and regularized matrix completion to estimate cell compositions and corresponding CTS expression profiles, a comprehensive strategy not previously employed.

Beyond conventional deconvolutional models, recent advances in cross‐modal alignment and generative representation learning offer promising new paradigms for expanding single‐cell and biobank integration. For example, Wang et al. introduced AlzCLIP [73], a contrastive learning framework that jointly embeds multi‐modal genetic profiles and structural neuroimaging features into a unified representation space to discover cross‐modal disease biomarkers. In cell deconvolution and CTS prediction, extending such contrastive alignment paradigms could allow models to jointly align high‐dimensional genomic variants, single‐cell references, and bulk profiles into a shared latent space. Li et al. proposed SpaIM [74], a style transfer framework designed to impute unmeasured spatial transcriptomic profiles by disentangling underlying biological content from modality‐specific “styles” (e.g., technical noise or sequencing platform effects). Incorporating style transfer and explicit domain disentanglement into deconvolution frameworks holds great promise for overcoming platform‐specific batch effects between reference scRNA‐seq datasets and bulk target cohorts, thereby improving cross‐study generalizability and enabling accurate spatial or single‐cell imputation at scale.

### 11.4 Limitations

Several limitations are worth noting but are likely increasingly addressable in practice. First, as with all reference‐based methods, performance depends on the coverage and representativeness of the scRNA‐seq reference. Mismatch of reference scRNA‐seq with the target tissue could lead to misleading results. Rare cell types with few cells can yield less stable markers and less accurate cell fraction estimates. Additionally, it is also possible that the target sample may have more cell types than the number of cell types present in the reference profile. These challenges are expected to diminish as the volume of publicly available single‐cell data grows, and it may be further mitigated by integrating multiple reference datasets and employing appropriate harmonization and batch correction methods. Furthermore, like most reference‐based approaches, differences in factors such as ancestry, age, disease burden, and technical platforms between the reference and target cohorts (e.g., UKBB) can degrade transferability. Our proposed invariance‐based marker selection aims to improve robustness and transferability across diverse environments, though it cannot entirely eliminate the effects of such mismatches.

Second, a key assumption of scI‐GCM is that the environmental variable (*E*) is not a descendant of the target. Using disease status as *E* may theoretically violate this assumption, because cell‐type composition may influence disease risk while disease processes can also remodel tissue composition. In practice, however, the violation is likely modest: although cell‐type proportions can affect disease susceptibility, reported effect sizes are generally small, so this violation is unlikely to be severe. Also, disease status is used here as an illustrative environment under our framework; investigators are encouraged to substitute other environments as appropriate to their question. In addition, here we retained only those gene‐cell‐type relationships that are empirically invariant across the observed disease states, which at least strengthens the stability of our results, despite that the identified genes may not be perfectly causal. The same bidirectional relationship could also raise concerns for downstream biobank analyses that relate inferred cell compositions to disease. Our application, however, estimates compositions from genetically predicted expressions and thereby follows the logic of MR: germline predictors predate disease onset and are robust to reverse causation, so inferred associations run from cell proportion to disease rather than the reverse. Formal accommodation of cyclic or feedback relationships will be an important area for future work.

Third, genotype‐imputed expression reflects only the genetically regulated component of expression, and typically has reduced dynamic range relative to measured bulk RNA‐seq. Our pseudo‐bulk calibration provides a pragmatic way to align scales for deconvolution and CTS reconstruction, but future work could further refine this step or incorporate uncertainty in imputation.

Fourth, similar to the principles of MR, our analysis is based on genetically predicted expression, and focuses on how expression influences outcomes. The approach does not capture reverse causality, where phenotypic traits might induce changes in gene expression. Also, our characterization of identified genes and markers as “causal” is contingent upon satisfying the standard assumptions of causal graphical models and invariant prediction. Consequently, while our findings are promising, these outputs should be interpreted as hypothesis‐generating and putative, requiring experimental validation for definitive confirmation. It is worth noting, however, that markers identified as invariant are expected to be less sensitive to context‐induced shifts. Therefore, even if they are not strictly causal, they are likely to be more transferable and robust across different cohorts and conditions. Despite the above limitations, our proposed framework is highly flexible and holds potential for extension to address these challenges in future developments.

Fifth, the validation of inferred CTS expression remains constrained by data availability and access limitations. At the single‐cell level, although we utilized comprehensive tissue‐relevant reference datasets and split them into non‐overlapping training and validation subsets, both subsets originated from the same cohort, which may not fully capture platform‐ or protocol‐specific batch effects across independent single‐cell studies. At the population level, our findings lack direct replication in an independent external biobank. Although large‐scale cohorts with relevant data exist (e.g., *All of Us*, *FinnGen*), current data access constraints precluded their inclusion within the scope of this revision. To mitigate this, we conducted an internal cross‐assessment‐center replication within the UKBB, demonstrating that the identified CTS associations are reproducible across geographically distinct participant subgroups and not driven by center‐specific sampling artifacts. Nonetheless, cross‐center evaluation within a single cohort cannot fully replace external replication or direct experimental confirmation. Future studies leveraging accessible external single‐cell datasets and independent biobank cohorts will be essential to further validate the accuracy, generalizability, and biological mechanisms of the reconstructed CTS profiles.

Lastly, our UKBB phenotypes are ascertained from ICD‐code‐based hospital records and self‐report, a common source of misclassification in biobank‐scale genetic studies [75]. Because such misclassification is governed by clinical coding practice and reporting behavior rather than by germline genotype, it should be largely non‐differential with respect to our genetic instrument; under standard measurement‐error theory, this is expected to attenuate effect estimates and reduce power rather than generate spurious associations [76, 77], a pattern also observed empirically in UKBB [78]. Nonetheless, more precise or validated phenotype definitions could further improve statistical power and specificity in future applications.

## 12 Conclusions

In summary, CausalCellInfer offers a scalable strategy for converting bulk and genotype‐imputed transcriptomes into *cell‐type‐resolved* readouts. This enables a wide variety of analyses at biobank scale, such as systematic CTS‐TWAS, cell type composition‐aware inference, interaction analyses, and causal gene prioritization. Through benchmarking and application to the UKBB, we demonstrate that cellular hypotheses can be tested with rich phenotypic depth, facilitating the translation of genomic associations into mechanistic and clinically actionable insights.

## Author Contributions

**Liangying Yin**: conceptualization, Writing – original draft, methodology, validation, software, data curation, project administration, formal analysis, Writing – review and editing, visualization, investigation, resources. **Yujia Shi**: conceptualization, writing – original draft, methodology, validation, software, data curation, project administration, formal analysis, writing – review and editing, visualization, investigation. **Ruoyu Zhang**: data curation, resources. **Yong Xiang**: data curation. **Jinghong Qiu**: visualization. **Pak‐Chung Sham**: conceptualization, supervision, resources. **Hon‐Cheong So**: conceptualization, writing – original draft, writing – review and editing, funding acquisition, supervision, resources.

## Conflicts of Interest

The authors declare no conflicts of interest.

## Supporting information




**Supporting File 1**: advs78197‐sup‐0001‐SuppMat.docx.

**Supporting File 2**: advs78197‐sup‐0002‐SuppMat.zip.

## Data Availability

The data that support the findings of this study are available from UK Biobank. Restrictions apply to the availability of these data, which were used under license for this study. UK Biobank data is available to any researchers who formally apply for the data. However, the data is not publicly available due to privacy concerns. Reference scRNA‐seq datasets are publicly available at the following sites: frontal cortex: https://www.ncbi.nlm.nih.gov/geo/query/acc.cgi?acc = GSE144136 adipose tissues: https://singlecell.broadinstitute.org/single_cell/study/SCP1376/a‐single‐cell‐atlas‐of‐human‐and‐mouse‐white‐adipose‐tissue?cluster = Human%20WAT&spatialGroups = –&annotation = fat__type–group–study&subsample = 100000#study‐summary pancreas: https://hpap.pmacs.upenn.edu/.

## References

[1] F. A. Wolf , P. Angerer , and F. J. Theis , “SCANPY: Large‐scale Single‐cell Gene Expression Data Analysis,” Genome Biology 19, no. 1 (2018): 15, 10.1186/s13059-017-1382-0. PMC5802054 29409532

[2] Y. Zheng , P. Lu , Y. Deng , et al., “Single‐cell Transcriptomics Reveal Immune Mechanisms of the Onset and Progression of IgA Nephropathy,” Cell Reports 33, no. 12 (2020): 108525, 10.1016/j.celrep.2020.108525. 33357427

[3] S. Nomura , “Single‐cell Genomics to Understand Disease Pathogenesis,” Journal of Human Genetics 66, no. 1 (2021): 75–84, 10.1038/s10038-020-00844-3. PMC7728598 32951011

[4] K. A. Jagadeesh , K. K. Dey , D. T. Montoro , et al., “Identifying Disease‐critical Cell Types and Cellular Processes by Integrating Single‐cell RNA‐sequencing and human Genetics,” Nature genetics 54, no. 10 (2022): 1479–1492, 10.1038/s41588-022-01187-9. PMC9910198 36175791

[5] L. Yin , Y. Feng , Y. Shi , et al., “Estimation of Causal Effects of Genes on Complex Traits Using a Bayesian‐network‐based Framework Applied to GWAS Data,” Nature Machine Intelligence 6, no. 10 (2024): 1231–1244, 10.1038/s42256-024-00906-7.

[6] E. R. Gamazon , H. E. Wheeler , K. P. Shah , et al., “A Gene‐based Association Method for Mapping Traits Using Reference Transcriptome Data,” Nature Genetics 47, no. 9 (2015): 1091–1098. 10.1038/ng.3367 PMC4552594 26258848

[7] L. Yin , C. K. Chau , P. C. Sham , and H. C. So , “Integrating Clinical Data and Imputed Transcriptome From GWAS to Uncover Complex Disease Subtypes: Applications in Psychiatry and Cardiology,” The American Journal of Human Genetics 105, no. 6 (2019): 1193–1212, 10.1016/j.ajhg.2019.10.012. PMC6904812 31785786

[8] J. Peters , P. Bühlmann , and N. Meinshausen , “Causal Inference by Using Invariant Prediction: Identification and Confidence Intervals,” Journal of the Royal Statistical Society Series B: Statistical Methodology 78, no. 5 (2016): 947–1012, 10.1111/rssb.12167.

[9] A. M. Newman , C. L. Liu , M. R. Green , et al., “Robust Enumeration of Cell Subsets From Tissue Expression Profiles,” Nature Methods 12, no. 5 (2015): 453–457, 10.1038/nmeth.3337. PMC4739640 25822800

[10] B. Chen , M. S. Khodadoust , C. L. Liu , A. M. Newman , and A. A. Alizadeh , “Profiling Tumor Infiltrating Immune Cells with CIBERSORT,” in Cancer Systems Biology: Methods and Protocols (Springer, 2018), 243–259, 10.1007/978-1-4939-7493-110.1007/978-1-4939-7493-1_12. PMC5895181 29344893

[11] T. Gong and J. D. Szustakowski , “DeconRNASeq: a Statistical Framework for Deconvolution of Heterogeneous Tissue Samples Based on mRNA‐Seq Data,” Bioinformatics 29, no. 8 (2013): 1083–1085, 10.1093/bioinformatics/btt090. 23428642

[12] D. Tsoucas , R. Dong , H. Chen , Q. Zhu , G. Guo , and G. C. Yuan , “Accurate Estimation of Cell‐type Composition From Gene Expression Data,” Nature Communications 10, no. 1 (2019): 2975, 10.1038/s41467-019-10802-z. PMC6611906 31278265

[13] M. I. Love , W. Huber , and S. Anders , “Moderated Estimation of Fold Change and Dispersion for RNA‐seq Data with DESeq2,” Genome Biology 15, no. 12 (2014): 550, 10.1186/s13059-014-0550-8. PMC4302049 25516281

[14] M. E. Ritchie , B. Phipson , D. Wu , et al., “limma Powers Differential Expression Analyses for RNA‐sequencing and Microarray Studies,” Nucleic Acids Research 43, no. 7 (2015): 47, 10.1093/nar/gkv007. PMC4402510 25605792

[15] K. Menden , M. Marouf , S. Oller , et al., “Deep Learning–based Cell Composition Analysis From Tissue Expression Profiles,” Science Advances 6, no. 30 (2020): aba2619, 10.1126/sciadv.aba2619. PMC7439569 32832661

[16] Y. Chen , Y. Wang , Y. Chen , et al., “Deep Autoencoder for Interpretable Tissue‐Adaptive Deconvolution and Cell‐Type‐Specific Gene Analysis,” Nature Communications 13, no. 1 (2022): 6735, 10.1038/s41467-022-34550-9. PMC9641692 36347853

[17] K. A. Tran , V. Addala , R. L. Johnston , et al., “Performance of Tumour Microenvironment Deconvolution Methods in Breast Cancer Using Single‐cell Simulated Bulk Mixtures,” Nature Communications 14, no. 1 (2023): 5758, 10.1038/s41467-023-41385-5. PMC10505141 37717006

[18] A. Thakkar and R. Lohiya , “Fusion of Statistical Importance for Feature Selection in Deep Neural Network‐Based Intrusion Detection System,” Information Fusion 90 (2023): 353–363.

[19] L. H. Cheng , T. C. Hsu , and C. Lin , “Integrating Ensemble Systems Biology Feature Selection and Bimodal Deep Neural Network for Breast Cancer Prognosis Prediction,” Scientific Reports 11, no. 1 (2021): 14914, 10.1038/s41598-021-92864-y. PMC8295302 34290286

[20] Z. Chen , M. Pang , Z. Zhao , et al., “Feature Selection May Improve Deep Neural Networks for the Bioinformatics Problems,” Bioinformatics 36, no. 5 (2020): 1542–1552, 10.1093/bioinformatics/btz763. 31591638

[21] P. Cui and S. Athey , “Stable Learning Establishes some Common Ground between Causal Inference and Machine Learning,” Nature Machine Intelligence 4, no. 2 (2022): 110–115, 10.1038/s42256-022-00445-z.

[22] M. Dong , A. Thennavan , E. Urrutia , et al., “SCDC: Bulk Gene Expression Deconvolution by Multiple Single‐Cell RNA Sequencing References,” Briefings in Bioinformatics 22, no. 1 (2021): 416–427, 10.1093/bib/bbz166. PMC7820884 31925417

[23] M. Arjovsky , L. Bottou , I. Gulrajani , and L. P. David , “Invariant Risk Minimization,” arXiv (2019): 190702893.

[24] L. Yin , M. Liu , Y. Shi , J. Qiu , and S. H. Cheong , “Direct Causal Variable Discovery Leveraging the Invariance Principle: Application in Biomedical Studies,” medRxiv (2024): 2008, 10.1101/2024.08.29.24312763.

[25] R. D. Shah and J. Peters , “The Hardness of Conditional Independence Testing and the Generalised Covariance Measure,” The Annals of Statistics 48, no. 3 (2020): 1514–1538, 10.1214/19-AOS1857.

[26] W. Wang , X. Zhou , J. Wang , et al., “Approximate Estimation of Cell‐Type Resolution Transcriptome in Bulk Tissue Through Matrix Completion,” Briefings in Bioinformatics 24, no. 5 (2023): bbad273, 10.1093/bib/bbad273. 37529921

[27] J. Wang , K. Roeder , and B. Devlin , “Bayesian Estimation of Cell Type–Specific Gene Expression with Prior Derived From Single‐Cell Data,” Genome Research 31, no. 10 (2021): 1807–1818, 10.1101/gr.268722.120. PMC8494232 33837133

[28] D. Song , Q. Wang , G. Yan , T. Liu , T. Sun , and J. J. Li , “scDesign3 generates Realistic in Silico Data for Multimodal Single‐Cell and Spatial Omics,” Nature Biotechnology 42, no. 2 (2024): 247–252, 10.1038/s41587-023-01772-1. PMC11182337 37169966

[29] G. Monaco , B. Lee , W. Xu , et al., “RNA‐Seq Signatures Normalized by mRNA Abundance Allow Absolute Deconvolution of Human Immune Cell Types,” Cell Reports 26, no. 6 (2019): 1627–1640.e7, 10.1016/j.celrep.2019.01.041. PMC6367568 30726743

[30] X. Wang , J. Park , K. Susztak , N. R. Zhang , and M. Li , “Bulk Tissue Cell Type Deconvolution with Multi‐subject Single‐Cell Expression Reference,” Nature Communications 10, no. 1 (2019): 380, 10.1038/s41467-018-08023-x. PMC6342984 30670690

[31] Y. Hao , S. Hao , E. Andersen‐Nissen , et al., “Integrated Analysis of Multimodal Single‐Cell Data,” Cell 184, no. 13 (2021): 3573–3587.e29, 10.1016/j.cell.2021.04.048. PMC8238499 34062119

[32] A. Butler , P. Hoffman , P. Smibert , E. Papalexi , and R. Satija , “Integrating Single‐cell Transcriptomic Data across Different Conditions, Technologies, and Species,” Nature Biotechnology 36, no. 5 (2018): 411–420, 10.1038/nbt.4096. PMC6700744 29608179

[33] P. Bühlmann , M. Kalisch , and M. H. Maathuis , “Variable Selection in High‐Dimensional Linear Models: Partially Faithful Distributions and the PC‐Simple Algorithm,” Biometrika 97, no. 2 (2010): 261–278, 10.1093/biomet/asq008.

[34] G. Koscielny , P. An , D. Carvalho‐Silva , et al., “Open Targets: a Platform for Therapeutic Target Identification and Validation,” Nucleic Acids Research 45, no. D1 (2017): D985–D994, 10.1093/nar/gkw1055. PMC5210543 27899665

[35] A. Kamburov , U. Stelzl , H. Lehrach , and R. Herwig , “The ConsensusPathDB Interaction Database: 2013 Update,” Nucleic Acids Research 41, no. D1 (2013): D793–D800, 10.1093/nar/gks1055. PMC3531102 23143270

[36] C. C. Clogg , E. Petkova , and A. Haritou , “Statistical Methods for Comparing Regression Coefficients between Models,” American Journal of Sociology 100, no. 5 (1995): 1261–1293, 10.1086/230638.

[37] D. M. Howard , M. J. Adams , T.‐K. Clarke , et al., “Genome‐wide Meta‐analysis of Depression Identifies 102 Independent Variants and Highlights the Importance of the Prefrontal Brain Regions,” Nature Neuroscience 22, no. 3 (2019): 343–352, 10.1038/s41593-018-0326-7. PMC6522363 30718901

[38] A. Mahajan , D. Taliun , M. Thurner , et al., “Fine‐mapping Type 2 Diabetes Loci to Single‐Variant Resolution Using High‐Density Imputation and Islet‐specific Epigenome Maps,” Nature Genetics 50, no. 11 (2018): 1505–1513, 10.1038/s41588-018-0241-6. PMC6287706 30297969

[39] A. E. Locke , B. Kahali , S. I. Berndt , et al., “Genetic Studies of Body Mass Index Yield New Insights for Obesity Biology,” Nature 518, no. 7538 (2015): 197–206, 10.1038/nature14177. PMC4382211 25673413

[40] G. Rajkowska and J. J. Miguel‐Hidalgo , “Gliogenesis and Glial Pathology in Depression,” CNS & Neurological Disorders—Drug Targets 6, no. 3: 219–233, 10.2174/187152707780619326. PMC2918806 17511618

[41] M. M. Holter , M. Saikia , and B. P. Cummings , “Alpha‐cell Paracrine Signaling in the Regulation of Beta‐Cell Insulin Secretion,” Frontiers in Endocrinology 13 (2022): 934775, 10.3389/fendo.2022.934775. PMC9360487 35957816

[42] Y. Saisho , “β‐cell Dysfunction: Its Critical Role in Prevention and Management of Type 2 Diabetes,” World Journal of Diabetes 6, no. 1 (2015): 109–124, 10.4239/wjd.v6.i1.109. PMC4317303 25685282

[43] A. Ragvin , E. Moro , D. Fredman , et al., “Long‐range Gene Regulation Links Genomic Type 2 Diabetes and Obesity Risk Regions to HHEX, SOX4, and IRX3,” Proceedings of the National Academy of Sciences 107, no. 2 (2010): 775–780, 10.1073/pnas.0911591107. PMC2818943 20080751

[44] W. Tang , D. Zeve , J. M. Suh , et al., “White Fat Progenitor Cells Reside in the Adipose Vasculature,” Science 322, no. 5901 (2008): 583–586, 10.1126/science.1156232. PMC2597101 18801968

[45] E. Watanabe , T. Wada , A. Okekawa , et al., “Stromal Cell‐derived Factor 1 (SDF1) Attenuates Platelet‐derived Growth Factor‐B (PDGF‐B)‐induced Vascular Remodeling for Adipose Tissue Expansion in Obesity,” Angiogenesis 23, no. 4 (2020): 667–684, 10.1007/s10456-020-09738-6. 32699964

[46] B. Zhou , Z. Zhu , B. R. Ransom , and X. Tong , “Oligodendrocyte Lineage Cells and Depression,” Molecular Psychiatry 26, no. 1 (2021): 103–117, 10.1038/s41380-020-00930-0. PMC7815509 33144710

[47] M. S. Ellulu , I. Patimah , H. Khaza'ai , A. Rahmat , and Y. Abed , “Obesity and Inflammation: the Linking Mechanism and the Complications,” Archives of Medical Science 4, no. 4 (2017): 851–863, 10.5114/aoms.2016.58928. PMC5507106 28721154

[48] T. Hägglöf , C. Vanz , A. Kumagai , et al., “T‐bet^+^ B Cells Accumulate in Adipose Tissue and Exacerbate Metabolic Disorder During Obesity,” Cell Metabolism 34, no. 8 (2022): 1121–1136, 10.1016/j.cmet.2022.07.002. PMC9357106 35868310

[49] M. Bundo , J. Ueda , Y. Nakachi , K. Kasai , T. Kato , and K. Iwamoto , “Decreased DNA Methylation at Promoters and Gene‐Specific Neuronal Hypermethylation in the Prefrontal Cortex of Patients with Bipolar Disorder,” Molecular Psychiatry 26, no. 7 (2021): 3407–3418, 10.1038/s41380-021-01079-0. PMC8505249 33875800

[50] Z. Liao , Y. Wang , X. Qi , and X. Xiao , “JAZF1, a Relevant Metabolic Regulator in Type 2 Diabetes,” Diabetes/Metabolism Research and Reviews 35, no. 5 (2019): 3148, 10.1002/dmrr.3148. 30838734

[51] H.‐Y. Lee , H. R. Jang , H. Li , et al., “Deletion of Jazf1 Gene Causes Early Growth Retardation and Insulin Resistance in Mice,” Proceedings of the National Academy of Sciences 119, no. 49 (2022): 2213628119, 10.1073/pnas.2213628119. PMC9894197 36442127

[52] K. Hrovatin , A. Bastidas‐Ponce , M. Bakhti , et al., “Delineating Mouse β‐Cell Identity During Lifetime and in Diabetes with a Single Cell Atlas,” Nature Metabolism 5, no. 9 (2023): 1615–1637, 10.1038/s42255-023-00876-x. PMC10513934 37697055

[53] N. Mizusawa , N. Harada , T. Iwata , I. Ohigashi , M. Itakura , and K. Yoshimoto , “Identification of Protease Serine S1 family Member 53 as a Mitochondrial Protein in Murine Islet Beta Cells,” Islets 14, no. 1 (2022): 1–13, 10.1080/19382014.2021.1982325. PMC8812782 34636707

[54] I. J. J. Muffels , T. Carter , H. Rehmann , et al., “LIMK1 variants Are Associated with Divergent Endocrinological Phenotypes and Altered Exocytosis Dynamics,” Iscience 28, no. 6 (2025): 112585, 10.1016/j.isci.2025.112585. PMC12146536 40491492

[55] E. Jahangir , A. De Schutter , and C. J. Lavie , “The Relationship between Obesity and Coronary Artery Disease,” Translational Research 164, no. 4 (2014): 336–344, 10.1016/j.trsl.2014.03.010. 24726461

[56] L. Sha , J. Li , H. Shen , et al., “LHPP‐Mediated Inorganic Pyrophosphate Hydrolysis‐Driven Lysosomal Acidification in Astrocytes Regulates Adult Neurogenesis,” Cell Reports 42, no. 8 (2023): 112975, 10.1016/j.celrep.2023.112975. 37573508

[57] X. Lu , Q. Xie , X. Pan , et al., “Type 2 Diabetes Mellitus in Adults: Pathogenesis, Prevention and Therapy,” Signal Transduction and Targeted Therapy 9, no. 1 (2024): 262, 10.1038/s41392-024-01951-9. PMC11445387 39353925

[58] T. A. Kosten , M. P. Galloway , R. S. Duman , D. S. Russell , and C. D'Sa , “Repeated Unpredictable Stress and Antidepressants Differentially Regulate Expression of the Bcl‐2 Family of Apoptotic Genes in Rat Cortical, Hippocampal, and Limbic Brain Structures,” Neuropsychopharmacology 33, no. 7 (2008): 1545–1558, 10.1038/sj.npp.1301527. 17700647

[59] G. R. Fries , V. A. Saldana , J. Finnstein , and T. Rein , “Molecular Pathways of Major Depressive Disorder Converge on the Synapse,” Molecular Psychiatry 28, no. 1 (2023): 284–297, 10.1038/s41380-022-01806-1. PMC9540059 36203007

[60] A. Gulbins , F. Schumacher , K. A. Becker , et al., “Antidepressants Act by Inducing Autophagy Controlled by Sphingomyelin–Ceramide,” Molecular Psychiatry 23, no. 12 (2018): 2324–2346, 10.1038/s41380-018-0090-9. PMC6294742 30038230

[61] C. M. O. Volpe , P. H. Villar‐Delfino , P. M. F. dos Anjos , and J. A. Nogueira‐Machado , “Cellular Death, Reactive Oxygen Species (ROS) and Diabetic Complications,” Cell Death & Disease 9, no. 2 (2018): 119, 10.1038/s41419-017-0135-z. PMC5833737 29371661

[62] S. C. Lee and S. Pervaiz , “Apoptosis in the Pathophysiology of Diabetes Mellitus,” The International Journal of Biochemistry & Cell Biology 39, no. 3 (2007): 497–504, 10.1016/j.biocel.2006.09.007. 17074529

[63] G. Davey Smith and G. Hemani , “Mendelian Randomization: Genetic Anchors for Causal Inference in Epidemiological Studies,” Human Molecular Genetics 23, no. R1 (2014): R89–R98, 10.1093/hmg/ddu328. PMC4170722 25064373

[64] D. A. Lawlor , R. M. Harbord , J. A. C. Sterne , N. Timpson , and G. Davey Smith , “Mendelian Randomization: Using Genes as Instruments for Making Causal Inferences in Epidemiology,” Statistics in Medicine 27, no. 8 (2008): 1133–1163, 10.1002/sim.3034. 17886233

[65] S. Burgess and S. G. Thompson , “Bias in Causal Estimates From Mendelian Randomization Studies With Weak Instruments,” Statistics in Medicine 30, no. 11 (2011): 1312–1323, 10.1002/sim.4197. 21432888

[66] M. Wainberg , N. Sinnott‐Armstrong , N. Mancuso , et al., “Opportunities and Challenges for Transcriptome‐Wide Association Studies,” Nature Genetics 51, no. 4 (2019): 592–599, 10.1038/s41588-019-0385-z. PMC6777347 30926968

[67] B. L. Pierce and S. Burgess , “Efficient Design for Mendelian Randomization Studies: Subsample and 2‐Sample Instrumental Variable Estimators,” American Journal of Epidemiology 178, no. 7 (2013): 1177–1184, 10.1093/aje/kwt084. PMC3783091 23863760

[68] A. Gusev , A. Ko , H. Shi , et al., “Integrative Approaches for Large‐Scale Transcriptome‐Wide Association Studies,” Nature Genetics 48, no. 3 (2016): 245–252, 10.1038/ng.3506. PMC4767558 26854917

[69] A. N. Barbeira , S. P. Dickinson , R. Bonazzola , et al., “Exploring the Phenotypic Consequences of Tissue Specific Gene Expression Variation Inferred From GWAS Summary Statistics,” Nature Communications 9, no. 1 (2018): 1825, 10.1038/s41467-018-03621-1. PMC5940825 29739930

[70] P. Jia , R. Hu , F. Yan , Y. Dai , and Z. Zhao , “scGWAS: Landscape of Trait‐Cell Type Associations by Integrating Single‐Cell Transcriptomics‐Wide and Genome‐Wide Association Studies,” Genome Biology 23, no. 1 (2022): 220, 10.1186/s13059-022-02785-w. PMC9575201 36253801

[71] Y. Zhou , T. Adeluwa , L. Zhu , et al., “scPrediXcan Integrates Deep Learning Methods and Single‐cell Data into a Cell‐Type‐Specific Transcriptome‐Wide Association Study Framework,” Cell Genomics 5, no. 5 (2025): 100875, 10.1016/j.xgen.2025.100875. PMC12143334 40373737

[72] L. Yin , Y. Shi , R. Zhang , et al., “Estimating Cell Compositions and Cell‐Type‐Specific Expressions from GWAS Data Using Invariant Causal Prediction, Deep Learning and Regularized Matrix Completion,” medRxiv 10, no. 17 (2024): 24315646, 10.1101/2024.10.17.24315646.

[73] Y. Wang , Q. Wang , M. Zhou , et al., “Integration of Genetic and Imaging Data for Alzheimer's Disease Diagnosis and Interpretation,” Advanced Science 12, no. 41 (2025): 07629, 10.1002/advs.202507629. PMC12591116 40788127

[74] B. Li , Z. Tang , A. Budhkar , et al., “SpaIM: Single‐Cell Spatial Transcriptomics Imputation via Style Transfer,” Nature Communications 16, no. 1 (2025): 7861, 10.1038/s41467-025-63185-9. PMC12375071 40849313

[75] M. Garg , M. Karpinski , D. Matelska , et al., “Disease Prediction with Multi‐Omics and Biomarkers Empowers Case–control Genetic Discoveries in the UK Biobank,” Nature Genetics 56, no. 9 (2024): 1821–1831, 10.1038/s41588-024-01898-1. PMC11390475 39261665

[76] S. Wacholder , P. Hartge , J. H. Lubin , and M. Dosemeci , “Non‐differential Misclassification and Bias towards the Null: A Clarification,” Occupational and Environmental Medicine 52, no. 8 (1995): 557–558, 10.1136/oem.52.8.557. PMC1128296 7663646

[77] B. L. Pierce and T. J. VanderWeele , “The Effect of Non‐differential Measurement Error on Bias, Precision and Power in Mendelian Randomization Studies,” International Journal of Epidemiology 43, no. 6 (2014): 1999–1999, 10.1093/ije/dyu226. 23045203

[78] T. Schoeler , J. B. Pingault , and Z. Kutalik , “The Impact of Self‐Report Inaccuracy in the UK Biobank and Its Interplay with Selective Participation,” Nature Human Behaviour 9, no. 3 (2024): 584–594, 10.1038/s41562-024-02061-w. PMC11936832 39695248

