# Supplementary material for: CausalCellInfer: Prioritizing Cell‐Type‐Specific Disease Associations From Genetically Predicted Transcriptomes in Biobank Cohorts
Source: Adv Sci (Weinh). 2026 Oct 14:e78197. Online ahead of print. doi: 10.1002/advs.78197 (PMC13669230; doi:10.1002/advs.78197)
Supplement: Supplementary file 1 [file ADVS-9999-e78197-s001.docx]

**CausalCellInfer: prioritizing cell-type-specific disease associations from genetically predicted transcriptomes in biobank cohorts**

**Supplementary Text**

**UK Biobank data and phenotype definitions**

UKBB is a comprehensive database that provides extensive prognostic information on various diseases.  Using cell-type-specific (CTS) expression profiles, we investigated the relationship between cell-specific genes and disease prognoses, including mortality and hospitalization frequency. Our application to UKBB focused on 4 primary tissue-specific diseases/outcomes (i.e., major depression in the frontal cortex, type 2 diabetes mellitus (T2DM) in the pancreas, and obesity in subcutaneous and visceral adipose tissues). We also studied 8 prognosis-related variables and 17 related disorders or traits. Here we concentrated on prognosis variables that are of high clinical significance for the target diseases.

More specifically, we focused on treatment resistance status and depression-related hospitalization frequency for patients with depression (tissue studied: frontal cortex); T2DM-related mortality and hospitalization frequency (tissue studied: pancreas) in T2DM subjects; coronary artery disease (CAD) mortality, and CAD-related hospitalization frequency for obesity patients (tissue studied: subcutaneous and visceral adipose tissues (subcutaneous adipose tissue (SAT) / visceral adipose tissue (VAT))). We also studied other related disorders, including anxiety disorders, bipolar disorder, and psychosis (frontal cortex); prediabetes, type 1 diabetes mellitus (T1DM), T2DM requires hospitalization, T2DM requires insulin (pancreas); CAD, T2DM, hypertension, heart failure, stroke (SAT/VAT) respectively.

With the UKBB summary data of hospital inpatient episodes (Data-Field 41270) and their corresponding diagnosis dates (Data-Field 41280), we extracted relevant phenotypes based on the International Classification of Diseases, 10th Revision (ICD-10) code. For each disorder, cases were defined as participants with either an ICD-10 coded primary diagnosis or a corresponding self-reported diagnosis; controls were participants without any record of that code or related self-report.

***Depression, depression related disorders and prognosis variables***

Depression and related disorders were directly extracted from the UKBB with a combination of self-reported data and ICD-10 codes. Depression is characterized by ICD-10 codes F32-33; psychosis is characterized by ICD-10 codes F20-25; bipolar disorder is characterized by F31; anxiety disorders are characterized by ICD-10 code F41.

Using the general practitioner (GP) records, we also extracted treatment-resistance status and admission frequency of depression patients. Specifically, treatment-resistant depression (TRD) was defined as depression patients who tried at least two different anti-depressant drugs for adequate durations. Following the definition in Fabbri et al.^1^, the time interval between two drugs should be no longer than 14 weeks and each drug should be prescribed for at least 6 weeks.

***T2DM, T2DM-related disorders and prognosis variables***

The prediabetes phenotype is specifically characterized by codes starting with R73, whereas the ICD-10 codes for T1DM and T2DM begin with E10 and E11 respectively. For each patient with available hospital records, the earliest diagnosis dates for prediabetes, T1DM, and T2DM were extracted for data analysis. T2DM-related admission was defined as T2DM patients who had at least one hospitalization record related to T2DM.

Besides, by restricting the Anatomical Therapeutic Chemical (ATC) code starting with A10A, we obtained the issue date of insulin from General Practice (GP) records, which contain prescription data from two electronic health record (EHR) systems (TPP or EMIS) for UKBB participants. In our “T2DM requiring insulin” dataset, once insulin is prescribed after the earliest T2DM diagnosis, the variable “T2DM requiring insulin” was coded as 1, otherwise it was coded as 0.

***Obesity, obesity related disorders and prognosis variables***

For obesity, we adhered to the World Health Organization (WHO) definition, which classifies individuals with a Body Mass Index (BMI) exceeding 30 as obese. CAD, hypertension, stroke, heart failure and T2DM were directly extracted from the UKBB with a combination of self-reported data and ICD-10 codes.

**Methods**

***Invariant Generalized Covariance Measure (I-GCM)***

Briefly, I-GCM is a two-stage approach, which firstly conducts feature selection using PC-simple, then leverages the principle of causal invariance across different environments to identify robust direct causal variables. Briefly, PC-simple can be regarded as a generalization of ordered-correlation screening, which is used to preselect the direct causal variables. Suppose $X=[X^{1},X^{2},\ldots, X^{p}]$ is an $n\times p$ matrix for $n$ observations with $p$ variables, $Y$ is the target variable. Variable $X^{j}$ is independent of Y if:

$$\exists s\subseteq S,\rho\left( {Y,X}^{j} | X^{s} \right)=0$$

(1)

$S=\{1,2,\ldots,j-1,j+1,\ldots,p\}$ defines the set including variables excluding $X^{j}$, $s$ is a subset of $S$, $\rho\left( {Y,X}^{j} | X^{S} \right)$ denotes the partial correlation between $X^{j}$ and $Y$. The null hypothesis of independence would be rejected if

$${(n-\left| s \right|-3)}^{1/2}\left| Z\left( Y,X^{j} | X^{S} \right) \right|>\phi^{-1}(1-\alpha/2)$$

(2)

Where $Z\left( Y,X^{j} | X^{s} \right)= \frac{1}{2}\{\frac{1+\hat{\rho}\left( Y,X^{j} | X^{s} \right)}{1-\hat{\rho}\left( Y,X^{j} | X^{s} \right)}\}$ denotes Fisher’s Z-transform of the partial correlation, $n$ denotes the sample size, $\left| s \right|$ is the cardinality of the set, $\phi$ indicates the inverse cumulative function for normal distribution. In our implementation, $Y$ denotes the cell-type indicator, $X$ indicates the gene expression matrix. Recursively performing partial correlation screening with increased order could exclude irrelevant variables from previous candidate variables set until they do not vary anymore. In this study, we set the maximum order of $\left| s \right|$ to 3 and $\alpha$ to 0.05 for the feature screening process. This implies that variables which survive the third-order partial correlation screening will be retained for further analysis. The corresponding P-value of the independence test can be used to indicate the reliability of the identified causal variable. The smaller the P-value, the more reliable the identified causal relationship is. As shown by Shah and Peters.^2^, generalized covariance measure (GCM) (a.k.a., $T^{n}$) can also be used to test whether $X^{j}$ and Y are conditionally independent given variable(s) $X^{S}$ :

$$T^{n}=\frac{\sqrt{n}.\frac{1}{n}\sum_{i=1}^{n} R_{i}}{{(\frac{1}{n}\sum_{i=1}^{n} R_{i}^{2}-{(\frac{1}{n}\sum_{r=1}^{n} R_{r})}^{2})}^{\frac{1}{2}}}=\frac{\frac{1}{n}\sum_{i=1}^{n} R_{i}}{[{(\frac{1}{n}\sum_{i=1}^{n} R_{i}^{2}-{(\frac{1}{n}\sum_{r=1}^{n} R_{r})}^{2})}^{\frac{1}{2}}]/\sqrt{n}}$$

(3)

Where $R_{i}=(X_{i}^{j}-f(X_{i}^{S}))(Y_{i}-f(X_{i}^{S}))$ defines the product between residuals(*R*) from the prediction functions of $X^{j}$ and Y using $X^{S}$ for each observation. In this study, XGBoost was employed to build prediction models. XGBoost^3^, a supervised machine learning method, is composed of a sequence of simpler, weaker decision trees. It operates by iteratively adding new trees, each one designed to correct the errors made by its predecessors, until the model achieves an optimal fit to the data. The complexity of the trees is penalized to prevent overfitting and enhance the model’s generalizability.

For a given environment variable E(i.e., disease status, sex), the computed GCM between *E* and the target should be constant or similarly close to zero when conditioned on the full set of direct causes, even if some other (non-directly causal) variables are included. However, if we exclude some direct causes from the conditional set, the calculated GCM between *E* and the target is expected to divert away from the null. In other words, significant change of the calculated GCM would be observed between the reduced conditional set and full conditional set. If we exclude some irrelevant variables in the conditional set, the calculated GCM should remain stable. While disease status was selected as the environment variable for pancreas, frontal cortex, subcutaneous adipose tissue, visceral adipose tissue, batch was selected for peripheral blood mononuclear cells tissue. The change in the distance of GCM from zero between two consecutive conditional sets ($\Delta T_{S_{j-1},S_{j}}^{n}$) could be represented as follows:

$$\Delta T_{S_{j-1},S_{j}}^{n}=|T_{S_{j-1}}^{n}|-|T_{S_{j}}^{n}|$$

(4)

In this study, $\alpha$ was set to 0.05. The variance of the distance change of GCM (Var($\Delta T_{S_{j-1},S_{j}}^{n})$) under the null can be estimated from the following:

| Var($\Delta T_{S_{j-1},S_{j}}^{n})=Var\left( \left\vert\left\vert T_{S_{j-1}}^{n} \right\vert\right\vert\right)+Var\left( \left\vert T_{S_{j}}^{n} \right\vert\right)-2\left[ E\left( \left\vert T_{S_{j-1}}^{n}*T_{S_{j}}^{n} \right\vert\right)-E\left( \left\vert T_{S_{j-1}}^{n} \right\vert\right)*E\left( \left\vert T_{S_{j}}^{n} \right\vert\right) \right]$  $=2-\frac{4}{\pi}(\rho*arcsin\rho+ \sqrt{1-\rho^{2}})$ (see refs^4,5^) | (5) |
| --- | --- |

where $\rho=Cor(T_{S_{j-1}}^{n},T_{S_{j}}^{n})$ indicates the correlation between product of residuals calculated from conditional set $S_{j-1}$ and $S_{j}$, $S_{j-1}$ and $S_{j}$ respectively denote two consecutive conditional sets with $j-1$ and $j$ variables.

The null hypothesis of no significant distance change in GCM between two consecutive conditional sets would be rejected if:

$$\frac{\Delta T_{S_{j-1},S_{j}}^{n}}{sqrt\left( \mathrm{Var}\left( \Delta T_{S_{j-1},S_{j}}^{n} \right) \right)}>\phi^{-1}(1-\alpha)$$

(6)

***scRNA-seq-based Invariant Generalized Covariance Measure (scI-GCM)***

In this study, we proposed to employ a modified I-GCM method designed for single cell data (scI-GCM) to identify cell markers. Algorithm S1 summarizes how we identify the corresponding cell markers for each target cell type using scI-GCM.

Algorithm S1: scI-GCM for cell marker identification

Input: $\boldsymbol{X\in}\boldsymbol{R}^{\boldsymbol{n\times q}}$, $\boldsymbol{Y\in}\boldsymbol{R}^{\boldsymbol{n}}$(cell type labels)*, E* $\boldsymbol{\in}\boldsymbol{R}^{\boldsymbol{n}}$, $\boldsymbol{\alpha}$*, T*

For each cell type $\boldsymbol{m=c}_{\boldsymbol{1}}\boldsymbol{:}\boldsymbol{c}_{\boldsymbol{m}}$

*If* $\boldsymbol{Y}_{\boldsymbol{i}}\boldsymbol{=m}$ set $\boldsymbol{Y}_{\boldsymbol{i}}\boldsymbol{=1}$ otherwise set $\boldsymbol{Y}_{\boldsymbol{i}}\boldsymbol{=0}$

For each environment $\boldsymbol{e}$:

Identify candidate marker genes using XGBoost(environment variable e excluded)

Further remove redundant marker genes using PC-simple to get marker gene set for environment $\boldsymbol{e}$: $\boldsymbol{G}_{\boldsymbol{e}}$

end Merge and re-rank $\boldsymbol{G}_{\boldsymbol{e}}$ based on the derived Z-score from PC-simple in descending order to get the candidate marker gene set: $\boldsymbol{G}_{\boldsymbol{q}}$

For j = q$\boldsymbol{:2}$, set $\boldsymbol{S}_{\boldsymbol{j}}\boldsymbol{=}\left\{ \boldsymbol{1,2,\ldots j} \right\}\boldsymbol{,}\boldsymbol{S}_{\boldsymbol{j-1}}\boldsymbol{=}\left\{ \boldsymbol{1,2,\ldots j-1} \right\}$

1. Calculate the GCM between $\boldsymbol{E}$ and *Y* by conditioning on $\boldsymbol{S}_{\boldsymbol{j}}$ ($\boldsymbol{T}_{\boldsymbol{S}_{\boldsymbol{j}}}^{\boldsymbol{n}}$) and $\boldsymbol{S}_{\boldsymbol{j}\mathbf{-1}}$($\boldsymbol{T}_{\boldsymbol{S}_{\boldsymbol{j}\mathbf{-1}}}^{\boldsymbol{n}}$) respectively based on equations 3

2. Compute the distance change between $\boldsymbol{T}_{\boldsymbol{S}_{\boldsymbol{j}\mathbf{-1}}}^{\boldsymbol{n}}\boldsymbol{and}\boldsymbol{T}_{\boldsymbol{S}_{\boldsymbol{j}}}^{\boldsymbol{n}}$, and the corresponding variance based on equations 4-5

3. Test the null hypothesis of no significant distance change. If inequality holds(inequality 6) and $\boldsymbol{\Delta}\boldsymbol{T}^{\boldsymbol{n}}\mathbf{>}\boldsymbol{T}$ stop the testing

end

Output: marker gene set for cell type $\boldsymbol{m}$ : $\boldsymbol{S}_{\boldsymbol{m}}$

$\boldsymbol{X\in}\boldsymbol{R}^{\boldsymbol{n\times q}}$ : gene expression matrix with n indicates cell number, q indicates gene number; $\boldsymbol{Y\in}\boldsymbol{R}^{\boldsymbol{n}}$: cell type labels; *E* $\boldsymbol{\in}\boldsymbol{R}^{\boldsymbol{n}}$: environment variable; $\boldsymbol{m}$ is the number of total cell types; $\boldsymbol{S}_{\boldsymbol{m}}$: identified cell markers for the corresponding cell type $m$; after ranking, indices $1,\ldots,q$ correspond to genes from most to least strongly associated. $\boldsymbol{S}_{\boldsymbol{j}}$ thus denotes the top j genes in this ranked list; $T$ is set to 0 for this task.

**Evaluation of the proposed scI-GCM approach**

To evaluate the performance of the scI-GCM approach in handling scRNA-seq datasets confounded by environmental variables, we designed a simulation experiment to deconvolute a series of synthetic bulk samples comprising four cell types with varying confounding effect sizes. We utilized scDesign3^6^ to generate synthetic scRNA-seq data based on a publicly available peripheral blood mononuclear cell (PBMC) dataset containing known batch effects. To reduce computational cost, we selected four cell types from the PBMC dataset and restricted the simulation to 200 genes. Using the reference PBMC data, we fitted an interpretable parametric model in scDesign3, capturing both marginal distributional parameters and pairwise gene correlations. A key advantage of scDesign3 is its flexibility; it allows for the modification of fitted parameters to reflect specific hypotheses and generate corresponding in silico data that retain real-world characteristics. Leveraging this, we generated a baseline scRNA-seq dataset free of batch effects by setting the batch covariate coefficients to zero. Subsequently, we generated datasets with batch effects by adjusting these coefficients to specific constants. We simulated two scenarios representing moderate and strong batch effects by setting the coefficient magnitudes to 8 and 16, respectively. Finally, following the TAPE approach, we simulated bulk samples derived from both the batch-free and batch-affected scRNA-seq datasets. Comprehensive simulation code and scripts are available in the accompanying GitHub repository.

**PC-simple algorithm**

We employed PC-simple to detect cell-type specific genes that are likely directly causal to the studied phenotypes. PC-simple is capable of distinguishing direct causal variables from indirect causal and spuriously associated genes. It can be considered a localized version of the PC algorithm, where an outcome (*Y*) and a predictor are deemed causally linked if they remain correlated, even when conditioned on any subset of the other covariates.

Suppose *Z* and *Y* are two random variables, and *S* is a variable set excluding *Z* and *Y.* *Z* and *Y* are conditionally independent if:

$$\exists s\subseteq S, \rho\left( Z,Y | S \right)=0$$

$\rho\left( Z,Y | S \right)$ denotes the partial correlation between *Z* and *Y* given S. Let $X=[X^{1},X^{2},\ldots X^{p}]$ be a $n\times p$ matrix of adjusted gene expression data for *p* genes, *Y* be a vector of the corresponding adjusted phenotypes for *n* subjects. We could identify the gene sets that are strongly associated with the target *Y* given other variables at a specified significance level *α* by ordered independence screening.

Initially, the candidate set includes all input variables. We iteratively update the candidate set by removing independent variables via conditional independence tests. The conditional independence tests are done level by level with an increasing order of the conditional set, starting with an empty conditional set. As shown in Algorithm S1, the initial candidate set $\boldsymbol{G}^{\mathbf{0}}$ is the complete set of input variables. Candidate set $\boldsymbol{G}^{\mathbf{1}}$ is firstly set to $\boldsymbol{G}^{\mathbf{0}}$ and then updated by removing independent variables via independence tests (conditioned on the null set). Similarly, candidate set $\boldsymbol{G}^{\mathbf{2}}$ is initially set to $\boldsymbol{G}^{\mathbf{1}}$, and is updated by removing variables that are independent of *Y* given any other single variable in $\boldsymbol{G}^{\mathbf{1}}$. Through recursively performing independence screening with an increasing order of the conditional set, we exclude genes from the previous candidate gene set until it does not change anymore.

To improve computational efficiency, we set the maximum order for partial correlation screening to 3. Fisher’s Z-transformation was employed to convert the skewed partial correlation into a normally distributed variable, and we tested whether the partial correlation is significantly different from zero:

$$Z_{score}\left( Y,Z | S \right)= \frac{1}{2}\{\frac{1+\hat{\rho}\left( Y,Z | S \right)}{1-\hat{\rho}\left( Y,Z | S \right)}\}$$

As suggested by Buhlmann et al.^7^, the null hypothesis $\hat{\rho}\left( Y,Z | S \right)=0$ would be rejected if

$${(n-\left| S \right|-3)}^{1/2}\left| Zscore\left( Y,Z | S \right) \right|>\phi^{-1}(1-\alpha/2)$$

where |*S*| defines the order of the conditional set, *α* and *ϕ* respectively denote the significance level and standard normal cumulative distribution function. In this study, we set α=0.05. The derived vector $Z_{p\times1}$ indicates the reliability of the inferred causal relationships between variables. We could convert it into a *P-value* vector by $P=2*\phi(-abs(Z))$.

**Calibrating PrediXcan-imputed expression**

The scale of the PrediXcan data is incompatible with scRNA-seq reference data, preventing direct inference of cell composition and cell-type-specific expression.

To mitigate this limitation, we mapped the PrediXcan-imputed values back into the expression space of the scRNA-seq reference, using a heuristic pseudo-bulk calibration. Specifically, we generated 1,000 pseudo-bulk RNA-seq samples from the control-group scRNA-seq data using a procedure similar to that used in the TAPE approach^8^:

1. We took the reference cell-type proportions as a baseline. For each pseudo-bulk sample, we perturbed these proportions by adding Gaussian noise to each cell type, clipped negatives to zero, and renormalized to sum to one.
2. We then sampled 1,000 cells from the scRNA-seq reference with probabilities given by the perturbed proportions and summed their profiles to form one pseudo-bulk sample.

From these 1,000 pseudo-bulk samples, we computed the mean, $\mu_{g}$, and standard deviation, $\sigma_{g}$, for each gene $g$. For each subject *s*, let *Z_g,s_* denote the latent standardized PrediXcan-imputed value for gene $g$*.* We then defined the corresponding observed standardized PrediXcan-imputed value as:

$$\hat{Z}_{g, s}=\sqrt{R_{g}^{2}}Z_{g,s}+\sqrt{1-R_{g}^{2}}\epsilon_{g}+\Delta_{g}$$

where $\epsilon_{g}\sim N\left( 0, 1 \right)$ and $\Delta_{g}\sim N\left( 0,b^{2} \right)$. In this formulation, $R_{g}^{2}$ represents the gene-specific PrediXcan-like expression-prediction $R^{2}$, whereas $b$ controls the magnitude of the gene-level cross-platform shift. In the absence of such a shift ($b=0$), both $Z_{g,s}$ and the simulated observed predictor $\hat{Z}_{g, s}$ have approximately unit variance, with $Corr^{2}(\hat{Z}_{g,s},Z_{g,s})\approx R_{g}^{2}$. This framework therefore allows us to simulate the PrediXcan-like standardized expression value with imperfect gene expression prediction, governed by gene-level $R^{2}$, while simultaneously incorporating gene-level cross-platform shifts. Using the simulated observed standardized PrediXcan-imputed values, we further defined the calibrated pseudo-bulk-like expression value as:

$$\begin{matrix} & \tilde{O}_{g,s}=\hat{Z}_{g, s}\text{ }\sigma_{g}+\mu_{g}. & & \end{matrix}$$

We use $\tilde{O}$ as the bulk-expression input $O$ for both deep neural network (DNN)-based deconvolution and ENIGMA reconstruction.

This approach relies on two key assumptions. First, we assume that standardized PrediXcan imputations, trained on normal-tissue RNA-seq data (e.g., Genotype-Tissue Expression (GTEx)), accurately reflect genetically regulated expression and preserve the hierarchy of gene expression across individuals within a tissue. Second, we assume that the cell proportion ratios and cell-type-specific expression profiles derived from reference single-cell RNA-seq (scRNA-seq) data of healthy individuals follow a distribution similar to that of the normal-tissue RNA-seq data used for PrediXcan training. Under these assumptions, mapping PrediXcan-imputed values onto a pseudo-bulk scale estimated from control scRNA-seq data allows for the recovery of both between-individual variation and within-individual gene expression rankings. For instance, housekeeping genes remain highly expressed, while lowly expressed genes remain relatively silent; similarly, genes highly expressed in specific population subgroups retain their elevated status after mapping. The resulting rescaled bulk RNA-seq data are subsequently used for cell type proportion estimation and the inference of cell-type-specific expression profiles.

We note that this mapping procedure serves as a heuristic calibration step: it posits that the standardized PrediXcan-predicted values for each gene exist on a latent scale comparable to the pseudo-bulk expression distribution estimated from the scRNA-seq reference. Nevertheless, this offers a practical means to generate bulk-like expression magnitudes compatible with ENIGMA or other cell deconvolution approaches, all while preserving the population-wide gene expression hierarchy described above.

**Identifying cell-type-specific genes involved in disease susceptibility (CTS-TWAS) and causal gene prioritization**

For the two PC-only(i.e., population stratification adjusted) CTS-TWAS and causal gene prioritization analyses, we conducted two additional analyses: (1) **covariate adjustment**: adding sex, age to the PC-only specification; and (2) **ancestry restriction**: repeating discovery in a European-only subsample (n = 472,714 of 502,524) versus the full, covariate-adjusted, all-ancestry sample. To better understand the robustness of the proposed gene discovery methods, we compared the genes identified from covariate adjustment analysis against those identified basic PC-only analysis, identified from full analysis against those identified from European-only analysis.

**Comparative deconvolution methods and benchmarking settings**

A primary objective of our proposed framework is to estimate cellular composition from bulk RNA-seq expression profiles. We therefore benchmarked its deconvolution performance against four well-established methods representing complementary methodological strategies: Scaden and DISSECT, two deep-learning-based methods, and DWLS and MuSiC, two signature-based regression methods. Brief descriptions of these methods and the corresponding implementation settings used in our benchmark are provided below.

***Scaden***

Scaden is a deep-learning-based method developed by Menden et al. for estimating cell-type proportions from bulk RNA-seq data. Scaden first generates pseudo-bulk training samples by sampling and aggregating cells from a reference single-cell RNA-sequencing dataset. The simulated pseudo-bulk profiles and their corresponding cell-type proportions are then used to train an ensemble of three deep neural networks with different architectures. For each bulk expression profile, the final cell-type proportion estimates are obtained by averaging the predictions from the three trained models.

In our benchmark, Scaden was implemented largely using the default settings recommended by Menden et al.^9^. The three neural-network architectures were M256, with hidden-layer sizes of 256, 128, 64, and 32 and no dropout; M512, with hidden-layer sizes of 512, 256, 128, and 64 and dropout rates of 0, 0.3, 0.2, and 0.1, respectively; and M1024, with hidden-layer sizes of 1,024, 512, 256, and 128 and dropout rates of 0, 0.6, 0.3, and 0.1, respectively. All three models were trained using the L1 loss function. Rectified linear unit (ReLU) activation functions were applied to the hidden layers, whereas a softmax activation function was used in the output layer to ensure that the estimated cell-type proportions summed to one.

***DISSECT***

DISSECT is a deep-learning-based deconvolution method developed by Khatri et al.^10^. For cell-type proportion estimation, DISSECT uses an ensemble of multilayer perceptrons (MLPs), broadly resembling the neural-network-based framework employed by Scaden. A key distinction, however, lies in the training strategy. Whereas Scaden is trained exclusively on labelled pseudo-bulk samples simulated from a single-cell RNA-sequencing reference, DISSECT adopts a semi-supervised learning framework that incorporates both labelled pseudo-bulk samples and the unlabelled target bulk RNA-sequencing samples. Specifically, DISSECT generates mixtures of simulated and real bulk expression profiles and optimizes a composite objective comprising a supervised Kullback–Leibler divergence loss on the simulated samples and a consistency loss on the mixed samples. This consistency constraint encourages the estimated cell-type proportions of each mixed expression profile to agree with the corresponding weighted combination of the proportions predicted for the real bulk sample and the known proportions of the simulated sample, thereby reducing the effect of domain shift between simulated and real bulk expression data.

In our benchmark, we followed the default settings recommended by the DISSECT authors. The MLP model contained four hidden layers with 512, 256, 128, and 64 units, respectively, followed by a linear output layer with one unit per cell type. ReLU activation functions were applied to the hidden layers, and a softmax transformation was applied to the output layer to constrain the estimated cell-type proportions to be non-negative and to sum to one.

***DWLS***

DWLS is a signature-based deconvolution method that estimates cell-type proportions using a dampened weighted least-squares framework^11^. Following the default DWLS procedure, marker genes were defined as genes with a false-discovery-rate-adjusted (P)-value of less than 0.01, as determined using the hurdle model implemented in the MAST R package, and a log2 mean fold change greater than 0.5.

To construct the signature matrix, we generated 151 candidate matrices containing between 50 and 200 marker genes per cell type. For each candidate marker set, gene expression values were averaged across cells belonging to the same cell type, resulting in an ($n \times k$) signature matrix, where (n) denotes the number of selected genes and (k) denotes the number of cell types. Following a strategy similar to that used in CIBERSORT, the candidate matrix with the lowest condition number was selected as the final signature matrix. This selection procedure was intended to reduce collinearity among cell-type-specific expression signatures and improve the numerical stability of the deconvolution, following the suggestion from the original DWLS study.

***MuSiC***

MuSiC is a signature-based deconvolution method designed to account for cross-subject variation in cell-type-specific gene expression when the reference single-cell RNA-sequencing dataset contains samples from multiple donors^12^. Unlike methods that construct a single reference profile without explicitly accounting for donor heterogeneity, MuSiC incorporates both cell-type-specific expression consistency and cross-subject variability into its weighting scheme. Genes with stable cell-type-specific expression across subjects receive greater weights, whereas genes exhibiting substantial cross-subject variation receive lower weights. This approach reduces sensitivity to the particular subjects included in the reference dataset and helps mitigate subject-selection bias.

In our benchmark, MuSiC was implemented using the default settings provided in the official MuSiC tutorial, with the single-cell reference profiles, donor identifiers, and cell-type annotations supplied as inputs.

**Results**

***Hyperparameter stability analysis***

To test sensitivity of scI-GCM hyperparameters (the XGBoost pre-selection tree depth ($max\_depth$) and learning rate ($\eta$), and the PC-simple significance threshold ($\alpha$) and maximum conditioning-set order ($max\_ord$)), we conducted a one-at-a-time sweep around the default operating point. scI-GCM has tunable hyperparameters spanning its learning steps: the XGBoost variable pre-selection (tree depth $max\_depth$, learning rate $\eta$) and the PC-simple causal screening (significance threshold $\alpha$, maximum conditioning-set order max_ord); the remaining settings (XGBoost $nrounds = 10$, I-GCM change-detection $\alpha= 0.05$) were held fixed. We varied each hyperparameter one-at-a-time around the default operating point ($\alpha= 0.05$, $max\_depth = 2$, $\eta= 1$, $max\_ord = 3$) and quantified, for each setting, (i) the number of identified markers per cell type and (ii) the Jaccard stability of the marker set relative to the default. To assess robustness across datasets, the full sweep was repeated on four independent single-cell datasets spanning four tissues, i.e., type-2 diabetes (pancreas), obesity (subcutaneous and visceral adipose), and depression (frontal cortex), 45 cell types in total.

The marker sets are robust to the two PC-simple parameters: varying $\alpha$ over two orders of magnitude (0.001–0.2) or $max\_ord$ over 2–5 changed the marker set only marginally (mean Jaccard ≥ 0.91 and ≥ 0.82, respectively), with near-constant marker counts (Figure S1). In contrast, the two XGBoost pre-selection parameters ($max\_depth$, $\eta$) act primarily as controls on the size of the candidate pool with deeper trees or a larger $\eta$ admit more distinct genes (from ~9 to ~52 markers per cell type across $max\_depth$ 1→6, and ≈ 9→21 across $\eta$ 0.1→0.9) and therefore have a larger effect on the exact marker set (mean Jaccard 0.33–0.55 and 0.36–0.68). These patterns were consistent across all four datasets (per-dataset mean Jaccard vs default 0.64–0.70), indicating dataset-independent behavior. Accordingly, we recommend and use the default parameters throughout: $\alpha= 0.05, max\_depth = 2, \eta= 1, max\_ord = 3$. Because the marker set is insensitive to $\alpha$ and $max\_ord$, in practice only the two pre-selection parameters need be considered, with $nrounds$ fixed they set the size of the candidate gene pool, which the downstream PC-simple and I-GCM steps then causally prune to a compact, high-confidence marker set. The default ($max\_depth$ = 2, η = 1) provides a stable operating point in this regime.

***Sensitivity analysis evaluating the impact of different fixed cell numbers on deconvolution accuracy***

We performed a series of sensitivity analyses to evaluate the effect of the number of cells used to construct each pseudo-bulk sample on deconvolution accuracy. Specifically, we considered fixed pseudo-bulk sizes of 100, 500, 1,000, and 2,000 cells. As shown in Figure S2, deconvolution performance improved as the number of cells increased and became largely stable at 500 cells or more, supporting the robustness of our original choice of 500 cells under the evaluated conditions. We additionally examined a randomized-cell-number setting, in which the number of cells contributing to each pseudo-bulk sample was independently sampled from a predefined range. This strategy achieved performance comparable to the best-performing fixed-cell-number settings, indicating that variation in pseudo-bulk cell numbers can be incorporated without compromising deconvolution accuracy. Moreover, this design more closely reflects the variability in cellular input and effective sequencing depth encountered in real-world bulk transcriptomic datasets.

***Sensitivity analysis evaluating the impact of different proportions of variance explained by cis-eQTLs on deconvolution accuracy and downstream analysis performance***

In genome-wide association study (GWAS)-based transcriptome analyses, gene-expression prediction accuracy is inherently heterogeneous because the proportion of expression variance explained by cis-expression quantitative trait locus (cis-eQTLs) varies substantially across genes. Such gene-specific variation in prediction performance cannot be fully addressed by calibration alone. Mean–standard deviation (SD) or quantile calibration can transform standardized PrediXcan-imputed expression values onto a pseudo-bulk-like expression scale, but it cannot recover between-individual expression variation that was not captured by the underlying genotype-based prediction model. We therefore performed additional simulations to evaluate how heterogeneous gene-level prediction accuracy affects cell-type fraction estimation and downstream cell proportion association study (CPAS) and cell-type-specific transcriptome-wide association study (CTS-TWAS) analyses.

To mimic our primary application, in which cell-type deconvolution is performed using standardized gene expression predicted from genotype data, we generated PrediXcan-like expression profiles from the single-cell reference datasets. Ground-truth pseudo-bulk expression profiles were first generated from the single-cell data and subsequently standardized. Controlled prediction noise was then introduced to reproduce the imperfect gene-expression prediction expected from cis-eQTL-based models. The magnitude of this noise was determined by gene-level $R^{2}$under four settings: (i) perfect prediction $R^{2}=1$; (ii) a constant mean-$R^{2}$ scenario; (iii) the empirically observed heterogeneous gene-level $R^{2}$ distribution; and (iv) a permuted heterogeneous-$R^{2}$ scenario. The resulting PrediXcan-like expression profiles were then subjected to the alternative calibration strategies, and performance was evaluated for recovery of both expression signals and cell-type fractions.

As expected, imperfect upstream gene-expression prediction reduced deconvolution accuracy because expression variation not captured by the genotype-based prediction model cannot be recovered through post hoc calibration. Under the empirically observed heterogeneous gene-specific $R^{2}$ distribution, the mean concordance correlation coefficient (CCC) of estimated cell-type fractions decreased from 0.976 to 0.548 in cortex, from 0.869 to 0.423 in pancreas, from 0.944 to 0.439 in subcutaneous adipose tissue, and from 0.895 to 0.399 in visceral adipose tissue (Figure S5). A similar pattern can also be observed for Pearson R, decreasing from nearly 1 under the ideal scenario ($R^{2}=1$) to approximately 0.6 when using the observed heterogeneous gene-level ($R^{2}$) values (Figure S5). Comparisons among the observed heterogeneous-$R^{2}$, constant mean-$R^{2}$, and permuted heterogeneous-$R^{2}$ scenarios further indicated that deconvolution performance depends not only on the overall amount of predictive information retained, but also on which marker genes are predicted accurately. Filtering marker genes according to their prediction $R^{2}$ improved deconvolution performance in pancreas and both adipose tissues, whereas it produced a slight reduction in performance in cortex. This tissue-specific pattern likely reflects a trade-off between excluding poorly predicted markers and inadvertently removing informative markers with relatively low prediction accuracy. Accordingly, $R^{2}$-based marker filtering was treated as a sensitivity analysis rather than as a universally applicable correction strategy.

Because the primary objective of the framework is to support downstream association analyses rather than to achieve perfect reconstruction of individual-level expression or cell-type fractions, we next examined whether imperfect prediction accuracy compromised CPAS and TWAS inference. These simulations were conducted under an idealized setting designed to isolate the effect of prediction accuracy itself. Type I error and statistical power were evaluated using correctly specified linear models, with no pleiotropy or population structure and with predictor and residual terms generated independently under the null model.

Across prediction $R^{2}$ values ranging from 0.01 to 1.00, the empirical type I error of TWAS remained close to the nominal level, ranging from 0.039 to 0.058 at (N=500) and from 0.041 to 0.057 at (N=2000) (Figure S6). A similar pattern was observed for CPAS, for which the empirical type I error ranged from 0.0435 to 0.061 across cell-fraction prediction $R^{2}$ values from 0.01 to 1.00 (Figure S7). In contrast, statistical power decreased markedly as prediction $R^{2}$ declined. For TWAS at (N=2000), when true gene expression explained 0.5% of the trait variance, power was 0.068, 0.306, 0.589, and 0.868 at prediction $R^{2}$ values of 0.01, 0.20, 0.50, and 1.00, respectively (Figure S6). Similarly, for CPAS at (N=2000), when the true cell-type fraction explained 0.5% of the trait variance, power was 0.061, 0.296, 0.608, and 0.879 at prediction $R^{2}$ values of 0.01, 0.20, 0.50, and 1.00, respectively (Figure S7).

We further evaluated CPAS across multiple gene-expression prediction-accuracy scenarios to better reflect heterogeneous gene-level performance. Across all tissue and scenario summaries, the null rejection rate remained well calibrated, ranging from 0.0486 to 0.0521. Under the empirically observed heterogeneous-$R^{2}$ scenario, CPAS power ranged from 0.149 to 0.163, compared with an oracle power of 0.334 to 0.342, when the true cell-type fraction explained 1% of the outcome variance. When the true cell-type fraction explained 5% of the outcome variance, power increased to 0.513–0.566, compared with 0.941–0.943 under the oracle setting. Despite this loss of power, effect-direction accuracy remained high, ranging from 0.968 to 0.981 when the true cell-type fraction explained 5% of the outcome variance (Figure S8). These results indicate that heterogeneous gene-level prediction error primarily reduces statistical power rather than compromising type I error control, while the direction of detected associations remains highly consistent for stronger underlying effects.

Overall, under these idealized simulation assumptions, reduced prediction $R^{2}$ primarily decreases statistical efficiency and power rather than inflating type I error. These findings support the use of genetically predicted gene expression and derived cell-type fractions for downstream association discovery, while emphasizing that limited upstream prediction accuracy can substantially reduce the ability to detect true associations.

***Sensitivity analysis of CausalCellInfer for recovering rare cell-type signals***

Because our framework is primarily designed for application to biobank-scale imputed expression profiles, its ability to recover signals from very rare cell types may be limited by the reduced contribution of these cell populations to the overall expression profile. To systematically evaluate the performance of CausalCellInfer under rare-cell scenarios, we performed a held-out rare-cell titration analysis across the four tissues examined in this study: frontal cortex, pancreas, subcutaneous adipose tissue, and visceral adipose tissue, including 45 cell types in total.

Following the DNN architecture and training strategy used in the manuscript, we varied the abundance of each target cell type across 0%, 0.1%, 0.5%, 1%, 2%, 3%, 4%, 5%, and 10% in pseudo-bulk samples generated from independent control-validation cells. For each abundance level, we generated 80 pseudo-bulk mixtures and performed three independent simulation/model replicates. We evaluated two expression settings: (i) measured bulk expression generated using the complete production marker panel and (ii) calibrated PrediXcan-like expression generated by applying the training-derived calibration procedure to Z-standardized expression profiles.

Detection thresholds were determined relative to the cell-type-specific 95th percentile of estimated abundance under the 0% condition. A cell type was considered reliably detectable at a given abundance level if the detection sensitivity reached at least 80% at that abundance and remained above this threshold at all higher tested fractions. Under the measured bulk-expression setting, 41 of 45 cell types (91.1%) were reliably detectable at 10% abundance, with a median sustained detection threshold of 5% (interquartile range [IQR], 2–5%) (Figure S9). Among the 21 cell types represented by at least 2,000 cells in the training reference dataset, 20 (95.2%) were detectable at 10% abundance, although the median detection threshold remained approximately 5%. At 2% abundance, tissue-level median detection sensitivity ranged from approximately 38% to 84%, whereas at 5% abundance, sensitivity increased to approximately 94–100%.

Under the calibrated PrediXcan-like expression setting, 35 of 45 cell types were reliably detectable at 10% abundance, with a median sustained detection threshold of 5% (IQR, 5–10%). These results suggest that an abundance of approximately 5% represents a practical empirical benchmark for evaluating most cell types using CausalCellInfer under the investigated conditions. However, this value should not be interpreted as a universal detection cutoff. Cell types below 5% abundance may still be recoverable in favorable settings, but their estimation accuracy is expected to depend more strongly on factors including reference-cell representation, marker-gene availability, and the prediction accuracy of the corresponding genes in PrediXcan models.

***Cross-center reproducibility of cell-type fraction/CTS gene–phenotype associations***

To assess the robustness of the identified associations across geographic recruitment sites, we conducted a cross-assessment-center replication analysis using UK Biobank (UKBB) participants from the five centers with the largest numbers of eligible individuals: Leeds, Bristol, Newcastle, Nottingham, and Liverpool. BMI distributions were highly comparable across centers, with similar medians and interquartile ranges (Figure S10). In contrast, the binary outcomes were relatively uncommon and showed greater between-center variability in prevalence, with prevalence generally below 10% across the five centers. Because stratification by assessment center substantially reduced the available sample size—and particularly the number of cases for binary outcomes—statistical power for cross-center replication of binary phenotype associations was limited. This consideration is especially relevant for transcriptome- and cell type-wide association analyses based on genetically predicted molecular profiles, for which effect sizes are generally modest and relatively large sample sizes are required for reliable detection. We therefore focused the cross-center replication analysis on BMI, an objectively measured continuous phenotype with consistent distributions across centers and greater statistical information under the available sample-size constraints.

The estimated cell type fraction–BMI associations showed generally good pairwise agreement across centers in both subcutaneous adipose tissue (SAT) and visceral adipose tissue (VAT). Across all pairwise center comparisons, Pearson correlations ranged from 0.435 to 0.753 for SAT, with a median correlation of 0.687, and from 0.230 to 0.780 for VAT, with a median correlation of 0.423 (Figure S11). The corresponding median directional concordance of cell type effects was 78.6% for SAT and 73.3% for VAT, indicating that the majority of estimated cell type effects were consistent in direction across independently recruited center subsets.

We further assessed the reproducibility of BMI associations for established gene–cell type pairs. For each gene–cell type pair, such as for gene LEP in adipocytes, the association with BMI was estimated separately within each of the five centers. We performed the same analysis for all available genes across all included cell types, and followed by pairwise Pearson correlation calculation of the resulting effect estimates for all gene-cell type pairs. Overall, the median pairwise Pearson correlation was 0.462 for SAT (range: 0.390–0.651) and 0.357 for VAT (range: 0.117–0.581). The corresponding median directional concordance was 67.2% for SAT and 62.5% for VAT (Figure S12), and the Pearson correlations are significantly greater than 0. Concordance plots comparing the two largest centers, Leeds and Bristol, further showed that the estimated effects for most genes were broadly consistent in both magnitude and direction (Figure S13).

Together, these results demonstrate generally consistent cross-center reproducibility of the BMI-associated cell type fraction and CTS gene-level signals. The observation that similar association patterns were obtained across independently recruited assessment-center subsets reduces the likelihood that the identified BMI-associated signals are primarily driven by center-specific sampling characteristics or technical factors. Nevertheless, this cross-center analysis should be interpreted as an assessment of the reproducibility of downstream associations within the UK Biobank rather than as independent external validation.

***Identifying cell type-specific genes involved in disease susceptibility (CTS-TWAS) and causal gene prioritization***

For the covariate sensitivity analysis, CTS-TWAS Z-score rankings were highly concordant between the basic (top-10- principal components (PCs)) and adjusted (top-10-PCs + age + sex) models across all four tissues. Causal gene sets were similarly stable in frontal cortex, SAT, and pancreas (0.99, 0.94, and 0.99), with VAT showing modestly lower concordance (0.69). For the ancestry-stratification analysis, comparing European-ancestry-only results against the full multi-ancestry cohort (both using the adjusted model), CTS-TWAS z-score rankings remained highly consistent across all tissues, as did causal gene sets. The majority of tissue–trait combinations show strong concordance under both robustness checks (please refer to Supplementary Table S13 for more details). Taken together, we conclude that the reported gene-discovery results are not substantially artifacts of residual population stratification or of differential cross-ancestry portability of the expression-prediction models, and that the top-10-PC adjustment used throughout the original analysis remains an adequate safeguard against both pathways.

**References**

1.	Fabbri, C. *et al.* Genetic and clinical characteristics of treatment-resistant depression using primary care records in two UK cohorts. *Mol Psychiatry* **26**, 3363–3373 (2021).

2.	Shah, R. D. & Peters, J. The hardness of conditional independence testing and the generalised covariance measure. *Ann. Statist.* **48**, (2020).

3.	Chen, T. & Guestrin, C. XGBoost: A Scalable Tree Boosting System. in *Proceedings of the 22nd ACM SIGKDD International Conference on Knowledge Discovery and Data Mining* 785–794 (ACM, San Francisco California USA, 2016). doi:10.1145/2939672.2939785.

4.	Haas, M. A note on the absolute moments of the bivariate normal distribution. *Economics Bulletin* **38**, 650–656 (2018).

5.	Shojaie, S. R. H., Aminghafari, M. & Mohammadpour, A. On the Expected Absolute Value of a Bivariate Normal Distribution. *Journal of Statistical Theory and Applications* **11**, 371–377 (2012).

6.	Song, D. *et al.* scDesign3 generates realistic in silico data for multimodal single-cell and spatial omics. *Nature Biotechnology* **42**, 247–252 (2024).

7.	Buhlmann, P., Kalisch, M. & Maathuis, M. H. Variable selection in high-dimensional linear models: partially faithful distributions and the PC-simple algorithm. *Biometrika* **97**, 261–278 (2010).

8.	Chen, Y. *et al.* Deep autoencoder for interpretable tissue-adaptive deconvolution and cell-type-specific gene analysis. *Nature Communications* **13**, 6735 (2022).

9.	Menden, K. *et al.* Deep learning–based cell composition analysis from tissue expression profiles. *Sci. Adv.* **6**, eaba2619 (2020).

10.	Khatri, R., Machart, P. & Bonn, S. DISSECT: deep semi-supervised consistency regularization for accurate cell type fraction and gene expression estimation. *Genome Biol* **25**, 112 (2024).

11.	Tsoucas, D. *et al.* Accurate estimation of cell-type composition from gene expression data. *Nat Commun* **10**, 2975 (2019).

12.	Wang, X., Park, J., Susztak, K., Zhang, N. R. & Li, M. Bulk tissue cell type deconvolution with multi-subject single-cell expression reference. *Nat Commun* **10**, 380 (2019).

**Supplementary Tables**

Table S1 Performance benchmark analysis across full and UKBB specific gene panels

Table S2 Comparison of differentially expressed genes identified

Table S3 Estimated associations between estimated cell proportions and disease onset

Table S4 Cell type interaction effects on disorder status

Table S5 Identified causal genes from inferred CTS expression profiles

Table S6 Enriched pathways based on identified putative causal genes for studied traits from UK Biobank

Table S7 Genes identified from TWAS analysis with adjustment for estimated cell proportions

Table S8 Number of genes with significant coefficient changes after adjusting for estimated cell proportions

Table S9 TWAS and conditional TWAS analyses results

Table S10 Cell-type common and unique associated genes identified from TWAS analysis, based on estimated CTS expression profiles of all cell types in the target tissue

Table S11 Identified trait-associated genes from CTS-TWAS analysis

Table S12 Comparison of genes identified from causal inference and CTS-TWAS analysis

Table S13 Sensitivity and robustness of the analyses

**Supplementary Figures**

Figure S1 Hyperparameter stability analysis for scI-GCM

Figure S2 Sensitivity of deconvolution accuracy to training cellularity

Figure S3 MAE of the cell fraction across different calibration methods

Figure S4 Sample-Level CTS recovery against sampled-cell truth

Figure S5 Gene-specific prediction reliability propagates to deconvolution

Figure S6 Type I error and power for CTS-TWAS analysis under different gene-level PrediXcan-like R²

Figure S7 Type I error and power for CPAS analysis under different gene-level PrediXcan-like R²

Figure S8 Type I error, power and direction accuracy comparison across 1% and 5% explained outcome variances

Figure S9 Rare Cell-Type Detection Sensitivity in Measured and Calibrated PrediXcan-like Bulk Expression

Figure S10 Distributions of disease outcomes and BMI across the five largest UK Biobank assessment centers

Figure S11 Cross-center agreement of cell-type proportion associations

Figure S12 Cross-center agreement of CTS associations

Figure S13 Leeds-Bristol CTS association concordance for BMI

Figure S14 Gene validation evidence
