# Supplementary material for: CausalCellInfer: Prioritizing Cell‐Type‐Specific Disease Associations From Genetically Predicted Transcriptomes in Biobank Cohorts
Source: Adv Sci (Weinh). 2026 Oct 14:e78197. Online ahead of print. doi: 10.1002/advs.78197 (PMC13669230; doi:10.1002/advs.78197)
Supplement: Supplementary file 2 [file ADVS-9999-e78197-s002.zip › advs78197-sup-00010-S9.pdf]

Table S9 TWAS and conditional TWAS analyses results

Tissue	Disorder	Genes	Estimates	Pvalues(standard TWAS)	SD(standard TWAS)	Residual variance(standard TWAS)	Estimates(conditional TWAS)	Pvalues(conditional TWAS)	SD(conditional TWAS)	Residual variance(conditional TWAS)	coefficient change	pvalue of coefficient change	Pvalue Bonferroni(standard TWAS)	Pvalue BH(standard TWAS)	Pvalue Bonferroni(conditional TWAS)	Pvalue BH(conditional TWAS)	gene_id	version	gene_name
Frontal_Cortex	Anxiety	ENSG00000006016.6	0.001670908	0.293181019	0.001589569	0.022786724	0.00165484	0.297843647	0.001589556	0.022785788	1.61E-05	0.046159201	1	0.947573237	1	0.953162532	ENSG00000000457	9	SCYL3
Frontal_Cortex	Anxiety	ENSG00000011590.9	0.000357985	0.744130591	0.001096805	0.022786797	0.000370957	0.735199999	0.001096802	0.022785859	-1.30E-05	0.047354966	1	0.978113594	1	0.977376329	ENSG00000000457	9	SCYL3
Frontal_Cortex	Anxiety	ENSG00000057468.6	-0.000333607	0.688372135	0.000831802	0.022786793	-0.000345427	0.67793979	0.000831801	0.022785855	1.18E-05	0.02246455	1	0.975684776	1	0.977376329	ENSG00000001460	13	STPG1
Frontal_Cortex	Anxiety	ENSG00000062038.9	0.00225482	0.216490814	0.001824412	0.022786693	0.002285599	0.21028472	0.001824414	0.022785753	-3.08E-05	0.0096653	1	0.903356609	1	0.892631846	ENSG00000001461	12	NIPAL3
Frontal_Cortex	Anxiety	ENSG00000064309.10	-0.000520596	0.79521483	0.002005802	0.0227868	-0.000548261	0.784594886	0.00200581	0.022785862	2.77E-05	0.048438773	1	0.982406987	1	0.985862728	ENSG00000001561	6	ENPP4
Frontal_Cortex	Anxiety	ENSG00000067141.12	-0.000867002	0.718632083	0.002406415	0.022786795	-0.000834727	0.728684151	0.002406409	0.022785859	-3.23E-05	0.024923512	1	0.975684776	1	0.977376329	ENSG00000001561	6	ENPP4
Frontal_Cortex	Anxiety	ENSG00000069275.12	-0.002655992	0.05467061	0.001382255	0.022786535	-0.002671586	0.053263151	0.001382244	0.022785595	1.56E-05	0.027187821	1	0.732084207	1	0.754532031	ENSG00000001561	6	ENPP4
Frontal_Cortex	Anxiety	ENSG00000070785.12	0.000528103	0.801321271	0.002098666	0.0227868	0.000652836	0.755782947	0.002098993	0.02278586	-0.000124733	0.001548793	1	0.982406987	1	0.982577711	ENSG00000001561	6	ENPP4
Frontal_Cortex	Anxiety	ENSG00000074054.13	0.00435098	0.072659814	0.002423988	0.022786569	0.004305207	0.075722032	0.002424005	0.022785637	4.58E-05	0.010907812	1	0.791500747	1	0.810468204	ENSG00000001561	6	ENPP4
Frontal_Cortex	Anxiety	ENSG00000078018.15	-0.000378436	0.692798559	0.00095792	0.022786793	-0.001815428	0.101316289	0.001107975	0.022785672	0.001436992	0.009859026	1	0.975684776	1	0.827410035	ENSG00000001561	6	ENPP4
Frontal_Cortex	Anxiety	ENSG00000085733.11	0.001442453	0.344241812	0.001525084	0.022786739	0.001424273	0.350355336	0.001525078	0.022785804	1.82E-05	0.038163628	1	0.954139853	1	0.960761585	ENSG00000001617	7	SEMA3F
Frontal_Cortex	Anxiety	ENSG00000085741.8	-0.00136753	0.437017341	0.001759468	0.02278676	-0.00139044	0.429374678	0.001759465	0.022785822	2.29E-05	0.032809492	1	0.974419575	1	0.976777864	ENSG00000002919	10	SNX11
Frontal_Cortex	Anxiety	ENSG00000087365.10	-0.00082041	0.68950032	0.002053419	0.022786793	-0.000785813	0.701953581	0.002053419	0.022785857	-3.46E-05	0.00849836	1	0.975684776	1	0.977376329	ENSG00000002919	10	SNX11
Frontal_Cortex	Anxiety	ENSG00000092969.7	-0.001498493	0.487347481	0.002157549	0.022786769	-0.001464195	0.497369098	0.002157556	0.022785834	-3.43E-05	0.021514182	1	0.975684776	1	0.977376329	ENSG00000002919	10	SNX11
Frontal_Cortex	Anxiety	ENSG00000099282.5	-6.57E-05	0.967990253	0.001636309	0.022786804	-8.64E-05	0.957898773	0.001636307	0.022785867	2.07E-05	0.042418125	1	0.996527112	1	0.995692427	ENSG00000002919	10	SNX11
Frontal_Cortex	Anxiety	ENSG00000099331.9	0.000691669	0.775918026	0.002429954	0.022786799	0.000723757	0.765819462	0.002429955	0.022785861	-3.21E-05	0.042215464	1	0.982406987	1	0.982577711	ENSG00000003056	3	M6PR
Frontal_Cortex	Anxiety	ENSG00000100099.16	-0.000381453	0.66449982	0.000879514	0.022786791	-0.00039426	0.653960221	0.000879519	0.022785853	1.28E-05	0.04860299	1	0.975684776	1	0.977376329	ENSG00000003056	3	M6PR
Frontal_Cortex	Anxiety	ENSG00000100197.16	-0.000725162	0.514516461	0.001112515	0.022786773	-0.000707282	0.524939745	0.001112516	0.022785838	-1.79E-05	0.013376137	1	0.975684776	1	0.977376329	ENSG00000003249	9	DBNDD1
Frontal_Cortex	Anxiety	ENSG00000100726.10	-0.000713129	0.210210963	0.000569143	0.02278669	-0.000706385	0.214553742	0.00056914	0.022785755	-6.74E-06	0.035910329	1	0.89263614	1	0.89933777	ENSG00000003249	9	DBNDD1
Frontal_Cortex	Anxiety	ENSG00000104140.6	-0.004364746	0.054107698	0.002266246	0.022786534	-0.004388377	0.052817103	0.002266231	0.022785594	2.36E-05	0.049658898	1	0.732084207	1	0.754532031	ENSG00000004809	9	SLC22A16
Frontal_Cortex	Anxiety	ENSG00000104979.4	0.002834983	0.180848517	0.002118584	0.022786674	0.002861863	0.176747911	0.002118581	0.022785734	-2.69E-05	0.041766883	1	0.879106816	1	0.887019637	ENSG00000004809	9	SLC22A16
Frontal_Cortex	Anxiety	ENSG00000105640.8	0.002928076	0.026014652	0.001315396	0.022786443	0.003251814	0.013783719	0.001320338	0.022785425	-0.000323739	0.004683021	1	0.732084207	1	0.746228219	ENSG00000004809	9	SLC22A16
Frontal_Cortex	Anxiety	ENSG00000105889.10	0.001001653	0.512517041	0.001529418	0.022786773	0.001022114	0.503939409	0.001529413	0.022785835	-2.05E-05	0.023128845	1	0.975684776	1	0.977376329	ENSG00000004809	9	SLC22A16
Frontal_Cortex	Anxiety	ENSG00000107164.15	-0.000178717	0.872043671	0.00110961	0.022786803	-0.000190225	0.86388138	0.001109603	0.022785865	1.15E-05	0.048358759	1	0.983816039	1	0.988452375	ENSG00000004897	7	CDC27
Frontal_Cortex	Anxiety	ENSG00000112473.12	2.45E-05	0.983930396	0.001215311	0.022786804	0.000452608	0.71159749	0.001224219	0.022785858	-0.00042813	0.003728897	1	0.999327591	1	0.977376329	ENSG00000005102	8	MEOX1
Frontal_Cortex	Anxiety	ENSG00000112763.11	7.21E-05	0.968626824	0.001832007	0.022786804	-5.34E-05	0.976768403	0.001832494	0.022785867	0.000125417	0.004248055	1	0.996527112	1	0.998606721	ENSG00000005102	8	MEOX1
Frontal_Cortex	Anxiety	ENSG00000114021.7	0.001097273	0.496720683	0.001614446	0.022786771	0.001126142	0.485467256	0.001614454	0.022785832	-2.89E-05	0.01261057	1	0.975684776	1	0.977376329	ENSG00000005102	8	MEOX1
Frontal_Cortex	Anxiety	ENSG00000119411.10	0.000204424	0.772325094	0.000706531	0.022786798	0.000214018	0.7619557	0.000706532	0.022785861	-9.59E-06	0.038159926	1	0.982406987	1	0.982577711	ENSG00000005156	7	LIG3
Frontal_Cortex	Anxiety	ENSG00000119844.10	-0.001774895	0.502747227	0.002648408	0.022786772	-0.001735964	0.512163269	0.002648411	0.022785836	-3.89E-05	0.025462979	1	0.975684776	1	0.977376329	ENSG00000005156	7	LIG3
Frontal_Cortex	Anxiety	ENSG00000121406.4	-0.003340557	0.038914418	0.001617625	0.022786493	-0.003367549	0.037363565	0.001617631	0.022785551	2.70E-05	0.016981543	1	0.732084207	1	0.754532031	ENSG00000005156	7	LIG3
Frontal_Cortex	Anxiety	ENSG00000122085.12	-0.003040879	0.049411809	0.001547499	0.022786523	-0.003057345	0.048191649	0.001547488	0.022785583	1.65E-05	0.043340381	1	0.732084207	1	0.754532031	ENSG00000005156	7	LIG3
Frontal_Cortex	Anxiety	ENSG00000124508.12	-0.000531724	0.693261838	0.001348072	0.022786793	-0.000628175	0.64133107	0.001348481	0.022785852	9.65E-05	0.00495967	1	0.975684776	1	0.977376329	ENSG00000005189	15	AC004381.6
Frontal_Cortex	Anxiety	ENSG00000124613.4	0.000871764	0.558439749	0.001489785	0.02278678	0.000931727	0.531735562	0.001489907	0.022785839	-6.00E-05	0.004970063	1	0.975684776	1	0.977376329	ENSG00000005243	5	COPZ2
Frontal_Cortex	Anxiety	ENSG00000128891.11	0.000635472	0.51970682	0.000987068	0.022786774	0.000617798	0.531388529	0.000987074	0.022785839	1.77E-05	0.014185771	1	0.975684776	1	0.977376329	ENSG00000005243	5	COPZ2
Frontal_Cortex	Anxiety	ENSG00000134160.9	-0.000185413	0.935961379	0.002307654	0.022786804	-0.000146423	0.949407412	0.00230766	0.022785867	-3.90E-05	0.013355873	1	0.99194522	1	0.995681012	ENSG00000005243	5	COPZ2
Frontal_Cortex	Anxiety	ENSG00000134222.12	0.002486058	0.07917927	0.001416175	0.02278658	0.00250436	0.076995099	0.001416173	0.022785639	-1.83E-05	0.038592778	1	0.791500747	1	0.812107305	ENSG00000005243	5	COPZ2
Frontal_Cortex	Anxiety	ENSG00000134324.7	0.001163959	0.530923193	0.001857585	0.022786776	0.001183831	0.523930239	0.001857574	0.022785838	-1.99E-05	0.046306871	1	0.975684776	1	0.977376329	ENSG00000005513	9	SOX8
Frontal_Cortex	Anxiety	ENSG00000134815.14	-0.002338423	0.189874995	0.00178376	0.022786679	-0.002306224	0.196047218	0.001783763	0.022785745	-3.22E-05	0.006760069	1	0.879106816	1	0.888056607	ENSG00000005513	9	SOX8
Frontal_Cortex	Anxiety	ENSG00000136492.4	0.000600203	0.811856198	0.002521519	0.0227868	0.00062575	0.804006256	0.002521501	0.022785863	-2.55E-05	0.04902638	1	0.983816039	1	0.986980224	ENSG00000005513	9	SOX8
Frontal_Cortex	Anxiety	ENSG00000136950.9	0.00217071	0.120154012	0.001396735	0.022786628	0.002189716	0.116942773	0.001396734	0.022785688	-1.90E-05	0.030704626	1	0.831449405	1	0.837078333	ENSG00000005801	12	ZNF195
Frontal_Cortex	Anxiety	ENSG00000137185.7	-0.002847426	0.000132231	0.000744948	0.022785738	-0.002777419	0.000194437	0.00074538	0.022784854	-7.00E-05	0.006601883	0.26604846	0.13302423	0.391207122	0.195603561	ENSG00000005801	12	ZNF195
Frontal_Cortex	Anxiety	ENSG00000137288.5	-0.001082379	0.330391782	0.001112041	0.022786735	-0.000894439	0.422054534	0.001114062	0.02278582	-0.00018794	0.00532259	1	0.954139853	1	0.97340047	ENSG00000005801	12	ZNF195
Frontal_Cortex	Anxiety	ENSG00000137411.12	0.000375595	0.467153429	0.000516552	0.022786766	0.000351038	0.49684182	0.000516636	0.022785834	2.46E-05	0.01293654	1	0.975684776	1	0.977376329	ENSG00000005955	8	GGNBP2
Frontal_Cortex	Anxiety	ENSG00000138346.10	-0.003145505	0.120259715	0.00202454	0.022786628	-0.003170031	0.117394284	0.002024532	0.022785689	2.45E-05	0.034440889	1	0.831449405	1	0.837078333	ENSG00000005955	8	GGNBP2
Frontal_Cortex	Anxiety	ENSG00000138472.6	0.000416857	0.706586332	0.001107351	0.022786794	0.000435941	0.693821785	0.001107364	0.022785856	-1.91E-05	0.033255067	1	0.975684776	1	0.977376329	ENSG00000005981	8	ASB4
Frontal_Cortex	Anxiety	ENSG00000140265.8	-0.002374604	0.268009497	0.002143802	0.022786715	-0.002400269	0.262869828	0.002143794	0.022785776	2.57E-05	0.03978872	1	0.942322745	1	0.948868815	ENSG00000005981	8	ASB4
Frontal_Cortex	Anxiety	ENSG00000140876.7	-0.003243017	0.15067775	0.002256569	0.022786654	-0.00327751	0.146382343	0.002256566	0.022785714	3.45E-05	0.01377728	1	0.870476788	1	0.875203335	ENSG00000005981	8	ASB4
Frontal_Cortex	Anxiety	ENSG00000143740.10	-1.52E-06	0.999428767	0.00211829	0.022786805	-3.78E-05	0.985761882	0.002118292	0.022785868	3.63E-05	0.008689849	1	0.999973246	1	0.999485958	ENSG00000006016	6	CRLF1
Frontal_Cortex	Anxiety	ENSG00000146733.9	0.000828372	0.591403684	0.001543157	0.022786783	0.000853641	0.580145449	0.001543172	0.022785845	-2.53E-05	0.03557899	1	0.975684776	1	0.977376329	ENSG00000006071	7	ABCC8
Frontal_Cortex	Anxiety	ENSG00000147576.11	-0.000716741	0.383845638	0.000823051	0.022786749	-0.000707513	0.389996246	0.000823048	0.022785814	-9.23E-06	0.049936376	1	0.962964367	1	0.970014921	ENSG00000006071	7	ABCC8
Frontal_Cortex	Anxiety	ENSG00000148655.10	0.004031796	0.124805105	0.002626718	0.022786632	0.002867818	0.27948781	0.002651771	0.022785782	0.001163978	0.001385308	1	0.839825657	1	0.953162532	ENSG00000006282	15	SPATA20
Frontal_Cortex	Anxiety	ENSG00000154263.13	0.001410842	0.569170342	0.002478317	0.022786781	0.001449465	0.558644522	0.002478324	0.022785843	-3.86E-05	0.023441118	1	0.975684776	1	0.977376329	ENSG00000006432	11	MAP3K9
Frontal_Cortex	Anxiety	ENSG00000155957.12	0.001047792	0.486090508	0.001504279	0.022786769	0.001066999	0.478131093	0.001504272	0.022785831	-1.92E-05	0.025016133	1	0.975684776	1	0.977376329	ENSG00000006530	11	AGK
Frontal_Cortex	Anxiety	ENSG00000156253.2	0.00023493	0.599455956	0.00044733	0.022786784	0.000241167	0.589800643	0.000447329	0.022785846	-6.24E-06	0.024207941	1	0.975684776	1	0.977376329	ENSG00000006704	6	GTF2IRD1
Frontal_Cortex	Anxiety	ENSG00000160948.9	0.001161767	0.573592421	0.002064375	0.022786781	0.001187918	0.564995571	0.002064372	0.022785843	-2.62E-05	0.039975349	1	0.975684776	1	0.977376329	ENSG00000006715	11	VPS41
Frontal_Cortex	Anxiety	ENSG00000162461.7	0.001714547	0.306470754	0.001676561	0.022786728	0.001695639	0.31183291	0.001676551	0.022785793	1.89E-05	0.035994682	1	0.947573237	1	0.953162532	ENSG00000006715	11	VPS41
Frontal_Cortex	Anxiety	ENSG00000163138.14	-0.00057351	0.817348444	0.002483193	0.022786801	-0.000601103	0.808726285	0.00248318	0.022785863	2.76E-05	0.044759122	1	0.983816039	1	0.988452375	ENSG00000006715	11	VPS41
Frontal_Cortex	Anxiety	ENSG00000163354.10	-0.004084609	0.040303854	0.00199188	0.022786497	-0.004059046	0.041570739	0.001991876	0.022785564	-2.56E-05	0.03429987	1	0.732084207	1	0.754532031	ENSG00000006744	14	ELAC2
Frontal_Cortex	Anxiety	ENSG00000164111.10	0.001773418	0.349490198	0.001895528	0.022786741	0.00174283	0.357867205	0.001895538	0.022785806	3.06E-05	0.024957939	1	0.954139853	1	0.960761585	ENSG00000006744	14	ELAC2
Frontal_Cortex	Anxiety	ENSG00000165733.7	0.001746212	0.293650091	0.001662822	0.022786724	0.001723015	0.300108581	0.001662817	0.022785789	2.32E-05	0.018736452	1	0.947573237	1	0.953162532	ENSG00000007341	14	ST7L
Frontal_Cortex	Anxiety	ENSG00000166529.10	-0.000637729	0.440630125	0.000827005	0.022786761	-0.000628046	0.447598475	0.000827002	0.022785825	-9.68E-06	0.042591235	1	0.974419575	1	0.977376329	ENSG00000007341	14	ST7L
Frontal_Cortex	Anxiety	ENSG00000166770.6	-0.00141706	0.346249071	0.001504484	0.02278674	-0.001434036	0.340500181	0.001504477	0.022785801	1.70E-05	0.045078042	1	0.954139853	1	0.960761585	ENSG00000007372	16	PAX6
Frontal_Cortex	Anxiety	ENSG00000166821.4	0.002421912	0.200781702	0.001893109	0.022786685	0.002443168	0.19685549	0.0018931	0.022785746	-2.13E-05	0.044139354	1	0.88859291	1	0.888056607	ENSG00000007372	16	PAX6
Frontal_Cortex	Anxiety	ENSG00000168778.7	-0.003318621	0.063362458	0.001787428	0.022786553	-0.003343368	0.061415623	0.001787421	0.022785612	2.47E-05	0.01732634	1	0.749913332	1	0.767504561	ENSG00000007541	10	PIGQ
Frontal_Cortex	Anxiety	ENSG00000171103.6	-0.001876779	0.132140734	0.001246435	0.022786639	-0.001895524	0.128321583	0.001246434	0.022785699	1.87E-05	0.016103928	1	0.846710687	1	0.856073301	ENSG00000007541	10	PIGQ
Frontal_Cortex	Anxiety	ENSG00000171357.5	-0.001316844	0.572767819	0.002334911	0.022786781	-0.001267906	0.587119674	0.002334936	0.022785846	-4.89E-05	0.008031266	1	0.975684776	1	0.977376329	ENSG00000008324	6	SS18L2
Frontal_Cortex	Anxiety	ENSG00000172113.4	-0.003242797	0.017578072	0.001365733	0.022786393	-0.003221496	0.018334392	0.001365731	0.022785461	-2.13E-05	0.011318474	1	0.732084207	1	0.754532031	ENSG00000008324	6	SS18L2
Frontal_Cortex	Anxiety	ENSG00000172171.6	-2.37E-05	0.978709941	0.000888236	0.022786804	-3.55E-05	0.968146776	0.000888237	0.022785867	1.18E-05	0.043360118	1	0.998018934	1	0.995959201	ENSG00000008394	8	MGST1
Frontal_Cortex	Anxiety	ENSG00000173209.18	-0.002680091	0.159434529	0.001904855	0.02278666	-0.002646713	0.164699105	0.001904874	0.022785727	-3.34E-05	0.024813069	1	0.875855548	1	0.887019637	ENSG00000008394	8	MGST1
Frontal_Cortex	Anxiety	ENSG00000173540.8	0.000354801	0.756192016	0.00114273	0.022786797	0.000333022	0.770725948	0.001142735	0.022785861	2.18E-05	0.006778774	1	0.982406987	1	0.982577711	ENSG00000008394	8	MGST1
Frontal_Cortex	Anxiety	ENSG00000176998.3	0.000210836	0.775182647	0.000738213	0.022786799	9.63E-05	0.896338798	0.000739433	0.022785866	0.000114497	0.007363793	1	0.982406987	1	0.990584607	ENSG00000008394	8	MGST1
Frontal_Cortex	Anxiety	ENSG00000177370.4	-0.000529765	0.696122232	0.001356422	0.022786793	-0.000497719	0.713671781	0.001356442	0.022785858	-3.20E-05	0.004749935	1	0.975684776	1	0.977376329	ENSG00000008441	12	NFIX
Frontal_Cortex	Anxiety	ENSG00000177409.7	0.001681103	0.394381892	0.001973824	0.022786752	0.001714741	0.384989555	0.001973823	0.022785812	-3.36E-05	0.007712329	1	0.965610146	1	0.969930908	ENSG00000008710	13	PKD1
Frontal_Cortex	Anxiety	ENSG00000179344.12	0.001888505	0.224272876	0.00155409	0.022786697	0.00055245	0.735580495	0.001637031	0.022785859	0.00133566	0.009471699	1	0.903356609	1	0.977376329	ENSG00000008853	12	RHOBTB2
Frontal_Cortex	Anxiety	ENSG00000179639.6	0.002477184	0.321957849	0.002501084	0.022786733	0.002510055	0.315578995	0.002501086	0.022785794	-3.29E-05	0.044572755	1	0.954139853	1	0.954804417	ENSG00000008853	12	RHOBTB2
Frontal_Cortex	Anxiety	ENSG00000181126.9	-0.000653562	0.443622431	0.000853112	0.022786762	-0.000518629	0.543866942	0.000854446	0.022785841	-0.000134933	0.004980815	1	0.974419575	1	0.977376329	ENSG00000008853	12	RHOBTB2
Frontal_Cortex	Anxiety	ENSG00000182771.13	0.000886678	0.73776822	0.002648297	0.022786796	0.001045561	0.693101781	0.002649341	0.022785856	-0.000158883	0.037223651	1	0.975684776	1	0.977376329	ENSG00000008853	12	RHOBTB2
Frontal_Cortex	Anxiety	ENSG00000183688.4	0.000650041	0.718139232	0.00180093	0.022786795	0.000627224	0.727631744	0.001800931	0.022785859	2.28E-05	0.049949756	1	0.975684776	1	0.977376329	ENSG00000008853	12	RHOBTB2
Frontal_Cortex	Anxiety	ENSG00000185290.3	-0.000783271	0.409062379	0.000948791	0.022786755	-0.000767366	0.418640956	0.000948791	0.02278582	-1.59E-05	0.008919793	1	0.96914464	1	0.97340047	ENSG00000009413	11	REV3L
Frontal_Cortex	Anxiety	ENSG00000185684.8	0.002524252	0.161156428	0.001801498	0.022786661	0.002556162	0.155929551	0.001801511	0.022785721	-3.19E-05	0.017951906	1	0.875855548	1	0.880880974	ENSG00000009413	11	REV3L
Frontal_Cortex	Anxiety	ENSG00000186230.6	-0.000245808	0.667574136	0.000572339	0.022786791	-0.00025473	0.656269935	0.000572338	0.022785853	8.92E-06	0.014179861	1	0.975684776	1	0.977376329	ENSG00000009413	11	REV3L
Frontal_Cortex	Anxiety	ENSG00000186470.9	0.001349495	0.055491434	0.000704691	0.022786537	0.001271631	0.071387995	0.000705283	0.02278563	7.79E-05	0.007725625	1	0.732084207	1	0.810468204	ENSG00000009830	7	POMT2
Frontal_Cortex	Anxiety	ENSG00000186567.8	0.002165015	0.051606185	0.001112311	0.022786528	0.002177732	0.050247901	0.001112305	0.022785588	-1.27E-05	0.038402606	1	0.732084207	1	0.754532031	ENSG00000009830	7	POMT2
Frontal_Cortex	Anxiety	ENSG00000187837.2	-0.004724534	0.030397065	0.002182346	0.022786462	-0.004451334	0.041598579	0.00218468	0.022785564	-0.0002732	0.007324394	1	0.732084207	1	0.754532031	ENSG00000009950	11	MLXIPL
Frontal_Cortex	Anxiety	ENSG00000187987.5	-0.002138079	0.112435166	0.001346951	0.022786621	-0.002005044	0.1368258	0.00134773	0.022785706	-0.000133035	0.004292134	1	0.831449405	1	0.874752649	ENSG00000009950	11	MLXIPL
Frontal_Cortex	Anxiety	ENSG00000188234.9	-0.002739982	0.265988262	0.002463236	0.022786714	-0.002773385	0.260201912	0.002463226	0.022785775	3.34E-05	0.018319142	1	0.942197859	1	0.948868815	ENSG00000009950	11	MLXIPL
Frontal_Cortex	Anxiety	ENSG00000189298.9	0.003311552	0.000100365	0.000851352	0.0227857	0.003246697	0.000137873	0.000851704	0.022784807	6.49E-05	0.009728474	0.20193404	0.13302423	0.277401391	0.195603561	ENSG00000009950	11	MLXIPL
Frontal_Cortex	Anxiety	ENSG00000196126.6	0.002247675	0.040671388	0.001098102	0.022786499	0.000434765	0.759593584	0.001420731	0.022785861	0.00181291	0.044323519	1	0.732084207	1	0.982577711	ENSG00000010219	9	DYRK4
Frontal_Cortex	Anxiety	ENSG00000196151.6	0.001593673	0.429305232	0.002016332	0.022786759	0.001617345	0.42248109	0.002016325	0.022785821	-2.37E-05	0.045513751	1	0.973801722	1	0.97340047	ENSG00000010219	9	DYRK4
Frontal_Cortex	Anxiety	ENSG00000196787.2	0.000472465	0.732886055	0.001384355	0.022786796	0.00041087	0.766645486	0.001384493	0.022785861	6.16E-05	0.004178642	1	0.975684776	1	0.982577711	ENSG00000010292	8	NCAPD2
Frontal_Cortex	Anxiety	ENSG00000197062.7	0.002278325	0.037518784	0.001095305	0.022786489	0.002120589	0.05317977	0.001096781	0.022785595	0.000157736	0.00590922	1	0.732084207	1	0.754532031	ENSG00000010292	8	NCAPD2
Frontal_Cortex	Anxiety	ENSG00000197457.5	7.22E-05	0.974621887	0.00227025	0.022786804	0.000107422	0.962260481	0.002270262	0.022785867	-3.52E-05	0.030837662	1	0.997933454	1	0.995959201	ENSG00000010539	7	ZNF200
Frontal_Cortex	Anxiety	ENSG00000197914.2	0.004932974	0.009286334	0.001896316	0.02278631	0.004575881	0.01608039	0.001900994	0.022785444	0.000357092	0.007607114	1	0.584946729	1	0.754532031	ENSG00000010704	14	HFE
Frontal_Cortex	Anxiety	ENSG00000198270.8	-0.001237223	0.229013282	0.001028528	0.022786699	-0.001247648	0.225110908	0.00102852	0.02278576	1.04E-05	0.048879873	1	0.903356609	1	0.908010471	ENSG00000010704	14	HFE
Frontal_Cortex	Anxiety	ENSG00000203403.2	0.002489794	0.022231302	0.001088956	0.022786423	0.002502286	0.021568844	0.00108895	0.022785482	-1.25E-05	0.036887442	1	0.732084207	1	0.754532031	ENSG00000010803	12	SCMH1
Frontal_Cortex	Anxiety	ENSG00000203756.3	0.001711118	0.398459247	0.002026485	0.022786752	0.001668783	0.410236488	0.002026504	0.022785818	4.23E-05	0.007084574	1	0.968693242	1	0.97340047	ENSG00000010803	12	SCMH1
Frontal_Cortex	Anxiety	ENSG00000204084.8	-0.000509474	0.439808925	0.000659499	0.022786761	-0.000501823	0.446705349	0.000659496	0.022785825	-7.65E-06	0.038345267	1	0.974419575	1	0.977376329	ENSG00000010803	12	SCMH1
Frontal_Cortex	Anxiety	ENSG00000204248.6	5.32E-05	0.950263977	0.000852436	0.022786804	-0.000240169	0.779698467	0.000858628	0.022785862	0.00029334	0.004428932	1	0.99542388	1	0.982928143	ENSG00000011105	7	TSPAN9
Frontal_Cortex	Anxiety	ENSG00000204256.8	-0.001679468	0.021309575	0.000729422	0.022786417	-0.001466815	0.045649139	0.00073391	0.022785576	-0.000212654	0.008789578	1	0.732084207	1	0.754532031	ENSG00000011105	7	TSPAN9
Frontal_Cortex	Anxiety	ENSG00000204257.10	-0.00217883	0.01432337	0.000889658	0.022786367	-0.002093744	0.018675188	0.000890211	0.022785464	-8.51E-05	0.007550402	1	0.719280595	1	0.754532031	ENSG00000011114	10	BTBD7
Frontal_Cortex	Anxiety	ENSG00000204267.9	-0.000849073	0.419337293	0.001051389	0.022786757	-0.000696344	0.508308766	0.001052715	0.022785836	-0.000152729	0.004118809	1	0.96914464	1	0.977376329	ENSG00000011114	10	BTBD7
Frontal_Cortex	Anxiety	ENSG00000204308.6	0.000472855	0.580440733	0.000855472	0.022786782	6.08E-06	0.994438127	0.000871617	0.022785868	0.000466779	0.005207341	1	0.975684776	1	0.999485958	ENSG00000011376	5	LARS2
Frontal_Cortex	Anxiety	ENSG00000204351.7	-0.000119921	0.854617841	0.000654485	0.022786802	5.74E-05	0.930467013	0.000657427	0.022785867	-0.000177287	0.004411915	1	0.983816039	1	0.99421287	ENSG00000011376	5	LARS2
Frontal_Cortex	Anxiety	ENSG00000204439.3	-0.001443263	0.290659578	0.00136585	0.022786723	-0.000621694	0.656842386	0.001399338	0.022785853	-0.000821569	0.006958894	1	0.947573237	1	0.977376329	ENSG00000011485	10	PPP5C
Frontal_Cortex	Anxiety	ENSG00000204463.8	0.000368463	0.568084525	0.000645436	0.022786781	0.000490929	0.447842046	0.000646795	0.022785825	-0.000122466	0.003638555	1	0.975684776	1	0.977376329	ENSG00000011485	10	PPP5C
Frontal_Cortex	Anxiety	ENSG00000204516.5	-0.000557275	0.443302484	0.000726915	0.022786762	-0.000483776	0.506089914	0.000727549	0.022785835	-7.35E-05	0.016736784	1	0.974419575	1	0.977376329	ENSG00000011523	9	CEP68
Frontal_Cortex	Anxiety	ENSG00000204525.10	-0.000692862	0.161823252	0.000495266	0.022786662	-0.000767505	0.121698353	0.000495904	0.022785693	7.46E-05	0.003240462	1	0.875855548	1	0.837078333	ENSG00000011523	9	CEP68
Frontal_Cortex	Anxiety	ENSG00000204657.2	-0.001779408	0.462120803	0.002419778	0.022786765	-0.001630147	0.500609303	0.002420295	0.022785834	-0.000149262	0.004355564	1	0.975684776	1	0.977376329	ENSG00000011523	9	CEP68
Frontal_Cortex	Anxiety	ENSG00000204681.6	0.000642715	0.611855146	0.001266614	0.022786786	0.000392543	0.757247243	0.001269964	0.022785861	0.000250172	0.006858578	1	0.975684776	1	0.982577711	ENSG00000011523	9	CEP68
Frontal_Cortex	Anxiety	ENSG00000204789.3	-0.000905559	0.228544805	0.000752051	0.022786699	-0.000888003	0.237710677	0.000752079	0.022785766	-1.76E-05	0.029498361	1	0.903356609	1	0.914522914	ENSG00000011523	9	CEP68
Frontal_Cortex	Anxiety	ENSG00000205704.5	0.000650262	0.493830512	0.000950355	0.02278677	0.00063913	0.50125306	0.00095035	0.022785835	1.11E-05	0.03271001	1	0.975684776	1	0.977376329	ENSG00000011523	9	CEP68
Frontal_Cortex	Anxiety	ENSG00000205822.6	-0.000243342	0.876445632	0.001565136	0.022786803	-0.000267639	0.864224858	0.001565152	0.022785865	2.43E-05	0.047845025	1	0.983816039	1	0.988452375	ENSG00000011523	9	CEP68
Frontal_Cortex	Anxiety	ENSG00000206341.6	0.000254506	0.798944713	0.000999186	0.0227868	0.000380148	0.703896682	0.001000216	0.022785857	-0.000125642	0.006128383	1	0.982406987	1	0.977376329	ENSG00000011523	9	CEP68
Frontal_Cortex	Anxiety	ENSG00000206344.6	0.000132302	0.799002092	0.000519567	0.0227868	0.000194687	0.708124991	0.000520031	0.022785857	-6.24E-05	0.005002832	1	0.982406987	1	0.977376329	ENSG00000011590	9	ZBTB32
Frontal_Cortex	Anxiety	ENSG00000206503.7	0.000814736	0.599973227	0.001553537	0.022786784	0.000635488	0.682748424	0.001554841	0.022785855	0.000179248	0.005405565	1	0.975684776	1	0.977376329	ENSG00000012061	11	ERCC1
Frontal_Cortex	Anxiety	ENSG00000206562.7	-0.000867951	0.348900723	0.000926581	0.02278674	-0.000854237	0.356569648	0.000926582	0.022785805	-1.37E-05	0.026220436	1	0.954139853	1	0.960761585	ENSG00000012061	11	ERCC1
Frontal_Cortex	Anxiety	ENSG00000213315.4	0.001110979	0.559983023	0.001906059	0.02278678	0.001138983	0.5501339	0.001906057	0.022785841	-2.80E-05	0.018402999	1	0.975684776	1	0.977376329	ENSG00000012061	11	ERCC1
Frontal_Cortex	Anxiety	ENSG00000214491.4	0.00167847	0.265053192	0.001505991	0.022786714	0.001660794	0.2701153	0.001505981	0.022785779	1.77E-05	0.025779033	1	0.942197859	1	0.953162532	ENSG00000012232	4	EXTL3
Frontal_Cortex	Anxiety	ENSG00000215452.3	-0.000468166	0.69199774	0.001181802	0.022786793	-0.000449538	0.70366218	0.001181807	0.022785857	-1.86E-05	0.024335182	1	0.975684776	1	0.977376329	ENSG00000012232	4	EXTL3
Frontal_Cortex	Anxiety	ENSG00000216775.2	-0.001777338	0.282788088	0.001654755	0.02278672	-0.001758145	0.288016478	0.001654749	0.022785785	-1.92E-05	0.045089374	1	0.947573237	1	0.953162532	ENSG00000012232	4	EXTL3
Frontal_Cortex	Anxiety	ENSG00000216901.1	-0.001167627	0.00919111	0.000448246	0.022786309	-0.001104319	0.013882058	0.000448853	0.022785426	-6.33E-05	0.007039041	1	0.584946729	1	0.746228219	ENSG00000012232	4	EXTL3
Frontal_Cortex	Anxiety	ENSG00000219392.1	0.002847555	0.00062163	0.000832124	0.02278595	0.00274994	0.00096139	0.000832906	0.022785072	9.76E-05	0.007411476	1	0.250143712	1	0.386863274	ENSG00000012963	9	UBR7
Frontal_Cortex	Anxiety	ENSG00000221983.3	0.00340446	0.106440562	0.002108801	0.022786614	0.004098277	0.053546468	0.002122915	0.022785595	-0.000693817	0.004593122	1	0.821912463	1	0.754532031	ENSG00000012963	9	UBR7
Frontal_Cortex	Anxiety	ENSG00000223823.1	0.000411405	0.693448637	0.001043698	0.022786793	0.000433926	0.677590002	0.001043707	0.022785855	-2.25E-05	0.00503671	1	0.975684776	1	0.977376329	ENSG00000012963	9	UBR7
Frontal_Cortex	Anxiety	ENSG00000224557.3	0.000708882	0.174053646	0.000521506	0.02278667	0.00073504	0.158774508	0.000521598	0.022785723	-2.62E-05	0.011368776	1	0.879106816	1	0.884914984	ENSG00000013306	11	SLC25A39
Frontal_Cortex	Anxiety	ENSG00000224635.1	-0.000694322	0.575802763	0.00124091	0.022786782	-0.000728809	0.55700379	0.001240953	0.022785842	3.45E-05	0.008024142	1	0.975684776	1	0.977376329	ENSG00000013306	11	SLC25A39
Frontal_Cortex	Anxiety	ENSG00000225849.1	0.003143329	0.011914227	0.001249987	0.022786343	0.003126268	0.012383679	0.001249991	0.022785411	1.71E-05	0.045625721	1	0.665872926	1	0.746228219	ENSG00000013523	5	ANGEL1
Frontal_Cortex	Anxiety	ENSG00000226314.3	-0.001389762	0.00168778	0.000442554	0.022786084	-0.001356587	0.002183157	0.00044273	0.022785182	-3.32E-05	0.009510701	1	0.40325044	1	0.403493216	ENSG00000013573	12	DDX11
Frontal_Cortex	Anxiety	ENSG00000227262.3	0.000983473	0.365420892	0.001086608	0.022786745	0.000954338	0.379810403	0.00108664	0.022785811	2.91E-05	0.007025277	1	0.956084178	1	0.96652945	ENSG00000013810	14	TACC3
Frontal_Cortex	Anxiety	ENSG00000227958.1	0.002528038	0.072136506	0.001405823	0.022786568	0.002511692	0.073996017	0.001405816	0.022785634	1.63E-05	0.034692946	1	0.791500747	1	0.810468204	ENSG00000013810	14	TACC3
Frontal_Cortex	Anxiety	ENSG00000228022.1	0.00108296	0.077756942	0.000613972	0.022786577	0.001042709	0.089568037	0.000614194	0.022785657	4.03E-05	0.01764599	1	0.791500747	1	0.825400182	ENSG00000014164	6	ZC3H3
Frontal_Cortex	Anxiety	ENSG00000228078.1	0.000178353	0.905736818	0.001506132	0.022786803	0.000148752	0.921327479	0.001506167	0.022785867	2.96E-05	0.036089497	1	0.990619017	1	0.993905606	ENSG00000014164	6	ZC3H3
Frontal_Cortex	Anxiety	ENSG00000228418.2	0.000627512	0.731457563	0.001828481	0.022786796	0.000650612	0.72197405	0.001828475	0.022785858	-2.31E-05	0.030370357	1	0.975684776	1	0.977376329	ENSG00000014914	15	MTMR11
Frontal_Cortex	Anxiety	ENSG00000228789.2	0.000279118	0.785939666	0.001027728	0.022786799	0.000445909	0.664936246	0.001029556	0.022785854	-0.000166792	0.006843497	1	0.982406987	1	0.977376329	ENSG00000014914	15	MTMR11

Frontal_Cortex	Anxiety	ENSG00000229391.3	-0.001786345	0.045690762	0.000893956	0.022786513	-0.000923745	0.343455307	0.000975071	0.022785802	-0.0008626	0.026745374	1	0.732084207	1	0.960761585	ENSG00000015133	14	CCDC88C
Frontal_Cortex	Anxiety	ENSG00000230795.2	0.000233975	0.733090794	0.000686108	0.022786796	0.000174883	0.798909256	0.000686465	0.022785863	5.91E-05	0.00882665	1	0.975684776	1	0.98622766	ENSG00000015676	13	NUDCD3
Frontal_Cortex	Anxiety	ENSG00000231500.2	0.003838973	0.082661641	0.002212087	0.022786585	0.003522945	0.111718496	0.002214963	0.022785683	0.000316028	0.00543926	1	0.800053168	1	0.837078333	ENSG00000016864	12	GLT8D1
Frontal_Cortex	Anxiety	ENSG00000231799.2	-0.001123039	0.570938501	0.001981821	0.022786781	-0.001165507	0.556471406	0.001981846	0.022785842	4.25E-05	0.008639752	1	0.975684776	1	0.977376329	ENSG00000016864	12	GLT8D1
Frontal_Cortex	Anxiety	ENSG00000232561.2	-0.000921136	0.597997287	0.001746948	0.022786784	-0.000945773	0.588243436	0.001746956	0.022785846	2.46E-05	0.045896343	1	0.975684776	1	0.977376329	ENSG00000016864	12	GLT8D1
Frontal_Cortex	Anxiety	ENSG00000232629.4	-0.000539928	0.277256272	0.00049694	0.022786718	-0.000213548	0.676272504	0.000511424	0.022785855	-0.00032638	0.006938025	1	0.947278828	1	0.977376329	ENSG00000016864	12	GLT8D1
Frontal_Cortex	Anxiety	ENSG00000234745.5	0.000526402	0.459986999	0.000712443	0.022786765	0.00106049	0.14790449	0.000732901	0.022785715	-0.000534088	0.001904388	1	0.975684776	1	0.875203335	ENSG00000018189	8	RUFY3
Frontal_Cortex	Anxiety	ENSG00000235109.3	-0.001701541	0.003400861	0.00058093	0.022786178	-0.001623034	0.005263069	0.00058163	0.022785299	-7.85E-05	0.006298994	1	0.40325044	1	0.423571825	ENSG00000018189	8	RUFY3
Frontal_Cortex	Anxiety	ENSG00000235290.1	0.001683966	0.016351332	0.000701363	0.022786384	0.001613505	0.021510853	0.000701857	0.022785482	7.05E-05	0.00833203	1	0.728293434	1	0.754532031	ENSG00000018280	12	SLC11A1
Frontal_Cortex	Anxiety	ENSG00000235370.5	0.007050282	0.002804551	0.002359208	0.022786152	0.007010924	0.002961505	0.002359207	0.022785223	3.94E-05	0.007990495	1	0.40325044	1	0.403493216	ENSG00000018280	12	SLC11A1
Frontal_Cortex	Anxiety	ENSG00000235374.1	0.003738441	0.100369137	0.002275276	0.022786607	0.003708292	0.103139824	0.002275272	0.022785674	3.01E-05	0.030326105	1	0.812485947	1	0.827410035	ENSG00000018510	8	AGPS
Frontal_Cortex	Anxiety	ENSG00000235821.1	0.001755171	0.154781445	0.001233563	0.022786657	0.001602871	0.194280494	0.001234856	0.022785745	0.0001523	0.007586534	1	0.870476788	1	0.888056607	ENSG00000018510	8	AGPS
Frontal_Cortex	Anxiety	ENSG00000236411.1	0.001789929	0.4096897	0.002171082	0.022786755	0.001765746	0.416042545	0.002171069	0.022785819	2.42E-05	0.03867666	1	0.96914464	1	0.97340047	ENSG00000018510	8	AGPS
Frontal_Cortex	Anxiety	ENSG00000236485.1	-0.000395182	0.576062316	0.00070676	0.022786782	-0.000387879	0.583131703	0.000706755	0.022785846	-7.30E-06	0.041324788	1	0.975684776	1	0.977376329	ENSG00000018699	7	TTC27
Frontal_Cortex	Anxiety	ENSG00000236603.1	-0.005740874	0.005226492	0.002055633	0.022786235	-0.006232399	0.002507776	0.002062052	0.022785201	0.000491525	0.002590716	1	0.420628052	1	0.403493216	ENSG00000018699	7	TTC27
Frontal_Cortex	Anxiety	ENSG00000236624.4	0.000131543	0.799809709	0.000518716	0.0227868	0.000107697	0.835536434	0.000518758	0.022785864	2.38E-05	0.001214006	1	0.982406987	1	0.988452375	ENSG00000019169	9	MARCO
Frontal_Cortex	Anxiety	ENSG00000237541.3	-0.001051122	0.250054349	0.000913845	0.022786708	0.000145744	0.886486041	0.00102096	0.022785866	-0.001196867	0.008566956	1	0.928246033	1	0.988543414	ENSG00000020922	8	MRE11A
Frontal_Cortex	Anxiety	ENSG00000237669.1	-0.000572481	0.327496095	0.000584659	0.022786735	-0.000528088	0.36661055	0.000584917	0.022785808	-4.44E-05	0.012555932	1	0.954139853	1	0.96212728	ENSG00000020922	8	MRE11A
Frontal_Cortex	Anxiety	ENSG00000237887.1	0.002646373	0.333968256	0.002739082	0.022786736	0.002607978	0.341028189	0.002739079	0.022785801	3.84E-05	0.023483132	1	0.954139853	1	0.960761585	ENSG00000020922	8	MRE11A
Frontal_Cortex	Anxiety	ENSG00000240053.8	0.001172059	0.034360162	0.000553948	0.022786478	0.00101794	0.067654485	0.000557071	0.022785624	0.000154119	0.008998844	1	0.732084207	1	0.79602821	ENSG00000020922	8	MRE11A
Frontal_Cortex	Anxiety	ENSG00000241106.2	0.000338957	0.449775401	0.00044848	0.022786763	0.000429495	0.339362953	0.000449533	0.022785801	-9.05E-05	0.00337355	1	0.974440587	1	0.960761585	ENSG00000020922	8	MRE11A
Frontal_Cortex	Anxiety	ENSG00000241634.1	-0.000472233	0.671107713	0.001112109	0.022786791	-0.000486586	0.661722535	0.001112104	0.022785854	1.44E-05	0.021246046	1	0.975684776	1	0.977376329	ENSG00000022556	11	NLRP2
Frontal_Cortex	Anxiety	ENSG00000243444.3	-0.000304107	0.798897065	0.001193629	0.0227868	0.000908729	0.496739946	0.001337096	0.022785834	-0.001212836	0.044152274	1	0.982406987	1	0.977376329	ENSG00000022556	11	NLRP2
Frontal_Cortex	Anxiety	ENSG00000244119.1	0.00164555	0.427513769	0.002073906	0.022786759	0.001622586	0.433988725	0.002073896	0.022785823	2.30E-05	0.047371012	1	0.971929609	1	0.976777864	ENSG00000022556	11	NLRP2
Frontal_Cortex	Anxiety	ENSG00000244731.3	0.001430028	0.161030923	0.001020271	0.022786661	0.001116198	0.277349501	0.001027528	0.022785781	0.00031383	0.010143294	1	0.875855548	1	0.953162532	ENSG00000022556	11	NLRP2
Frontal_Cortex	Anxiety	ENSG00000249626.1	-0.000479701	0.767759709	0.001624417	0.022786798	-0.000456751	0.778573436	0.001624413	0.022785862	-2.30E-05	0.019579855	1	0.982406987	1	0.982741376	ENSG00000022556	11	NLRP2
Frontal_Cortex	Anxiety	ENSG00000253444.1	-0.000991978	0.28305256	0.000924069	0.02278672	-0.001007163	0.275749065	0.000924071	0.022785781	1.52E-05	0.014590584	1	0.947573237	1	0.953162532	ENSG00000023041	7	ZDHHC6
Frontal_Cortex	Anxiety	ENSG00000256594.3	0.00175418	0.506469444	0.002640462	0.022786772	0.001796139	0.496355638	0.002640465	0.022785834	-4.20E-05	0.015844074	1	0.975684776	1	0.977376329	ENSG00000023041	7	ZDHHC6
Frontal_Cortex	Anxiety	ENSG00000258181.1	0.001228883	0.590747472	0.002285215	0.022786783	0.001197071	0.60039329	0.002285204	0.022785847	3.18E-05	0.013497013	1	0.975684776	1	0.977376329	ENSG00000023041	7	ZDHHC6
Frontal_Cortex	Anxiety	ENSG00000259362.2	0.000222541	0.858289945	0.001246371	0.022786802	0.000239192	0.847811562	0.001246366	0.022785865	-1.67E-05	0.022643683	1	0.983816039	1	0.988452375	ENSG00000023191	12	RNH1
Frontal_Cortex	Anxiety	ENSG00000260306.1	3.56E-05	0.989910452	0.002817794	0.022786804	-4.92E-07	0.999860687	0.002817793	0.022785868	3.61E-05	0.044321121	1	0.999849312	1	0.999860687	ENSG00000023191	12	RNH1
Frontal_Cortex	Anxiety	ENSG00000261353.1	-0.001843942	0.04152817	0.00090468	0.022786501	-0.001799599	0.046709808	0.000904808	0.022785579	-4.43E-05	0.006415781	1	0.732084207	1	0.754532031	ENSG00000023572	4	GLRX2
Frontal_Cortex	Anxiety	ENSG00000261584.1	-7.28E-06	0.994909617	0.001141739	0.022786805	-8.65E-05	0.939638677	0.001142044	0.022785867	7.92E-05	0.003792643	1	0.999973246	1	0.99421287	ENSG00000023572	4	GLRX2
Frontal_Cortex	Anxiety	ENSG00000261786.1	0.001810401	0.417404523	0.00223249	0.022786756	0.001844367	0.408723414	0.002232503	0.022785818	-3.40E-05	0.03652099	1	0.96914464	1	0.97340047	ENSG00000025156	8	HSF2
Frontal_Cortex	Anxiety	ENSG00000268658.1	0.00277256	0.174732002	0.002042916	0.02278667	0.002817854	0.167805398	0.00204297	0.022785729	-4.53E-05	0.021955597	1	0.879106816	1	0.887019637	ENSG00000025708	8	TYMP
Frontal_Cortex	Anxiety	ENSG00000270604.1	0.003144131	0.026563494	0.001417627	0.022786445	0.002594357	0.070549172	0.001434634	0.022785629	0.000549775	0.012621141	1	0.732084207	1	0.810468204	ENSG00000025800	9	KPNA6
Frontal_Cortex	Anxiety	ENSG00000271755.1	0.000457827	0.642796061	0.000987135	0.022786789	0.000495467	0.615752297	0.000987222	0.022785849	-3.76E-05	0.009889497	1	0.975684776	1	0.977376329	ENSG00000025800	9	KPNA6
Frontal_Cortex	Anxiety	ENSG00000272221.1	-0.000136746	0.875561581	0.000873226	0.022786803	-0.000174624	0.841514651	0.00087331	0.022785865	3.79E-05	0.004522349	1	0.983816039	1	0.988452375	ENSG00000026297	11	RNASET2
Frontal_Cortex	Anxiety	ENSG00000272462.2	-0.00135395	0.032065978	0.000631625	0.022786469	-0.0012987	0.039888434	0.000631998	0.022785559	-5.53E-05	0.012296222	1	0.732084207	1	0.754532031	ENSG00000026297	11	RNASET2
Frontal_Cortex	Anxiety	ENSG00000272983.1	0.000300676	0.648754158	0.00066011	0.022786789	0.000294313	0.655700365	0.000660105	0.022785853	6.36E-06	0.046797677	1	0.975684776	1	0.977376329	ENSG00000026950	12	BTN3A1
Frontal_Cortex	Depression	ENSG00000070785.12	0.000849714	0.830405725	0.003967261	0.082019693	0.001003516	0.800339573	0.003967911	0.082018088	-0.000153802	0.037428979	1	0.996212728	1	0.989691378	ENSG00000026950	12	BTN3A1
Frontal_Cortex	Depression	ENSG00000106853.12	-0.001021195	0.639867634	0.00218258	0.082019648	-0.000940962	0.6664265	0.002182912	0.082018057	-8.02E-05	0.04101176	1	0.965289072	1	0.966991696	ENSG00000026950	12	BTN3A1
Frontal_Cortex	Depression	ENSG00000116675.11	0.00411902	0.365432429	0.004551084	0.082019491	0.004775356	0.295289028	0.004562772	0.082017819	-0.000656336	0.044748914	1	0.948993265	1	0.950067467	ENSG00000026950	12	BTN3A1
Frontal_Cortex	Depression	ENSG00000148655.10	0.000558198	0.910493102	0.004965439	0.082019702	-0.000834093	0.867846695	0.005012747	0.082018098	0.001392291	0.042822757	1	0.996212728	1	0.997190658	ENSG00000026950	12	BTN3A1
Frontal_Cortex	Depression	ENSG00000171357.5	-0.000902941	0.837987644	0.004416042	0.082019694	-0.000831508	0.850650558	0.004416136	0.082018096	-7.14E-05	0.039515495	1	0.996212728	1	0.997190658	ENSG00000026950	12	BTN3A1
Frontal_Cortex	Depression	ENSG00000236624.4	8.45E-05	0.931365821	0.000980814	0.082019703	5.50E-05	0.955299205	0.000980901	0.082018104	2.95E-05	0.031775457	1	0.996212728	1	0.997190658	ENSG00000029725	12	RABEP1
Frontal_Cortex	Psychosis	ENSG00000007541.10	-0.00029054	0.597567361	0.000550367	0.003450919	-0.000299588	0.586207109	0.000550369	0.003450787	9.05E-06	0.011670804	1	0.996311095	1	0.996713278	ENSG00000030110	8	BAK1
Frontal_Cortex	Psychosis	ENSG00000047936.6	0.001035876	0.087529149	0.000606278	0.00345089	0.001042842	0.085419436	0.000606277	0.003450758	-6.97E-06	0.049673707	1	0.836091108	1	0.838360512	ENSG00000030110	8	BAK1
Frontal_Cortex	Psychosis	ENSG00000048545.9	-0.000531326	0.320930092	0.000535314	0.003450911	-0.000522289	0.329233129	0.000535318	0.00345078	-9.04E-06	0.022327112	1	0.950491103	1	0.951230466	ENSG00000030110	8	BAK1
Frontal_Cortex	Psychosis	ENSG00000067365.10	-0.000351886	0.686368618	0.000871462	0.00345092	-0.000361498	0.67827475	0.000871459	0.003450789	9.61E-06	0.045564666	1	0.996561629	1	0.996713278	ENSG00000030110	8	BAK1
Frontal_Cortex	Psychosis	ENSG00000078295.11	-2.10E-05	0.983010244	0.000987196	0.003450922	0.000140332	0.887115744	0.000988568	0.00345079	-0.000161355	0.002082002	1	0.998166036	1	0.997262509	ENSG00000030110	8	BAK1
Frontal_Cortex	Psychosis	ENSG00000081913.9	0.00016595	0.707970535	0.000443026	0.00345092	4.68E-05	0.916517051	0.000446575	0.00345079	0.000119139	0.03418111	1	0.996561629	1	0.998402312	ENSG00000030110	8	BAK1
Frontal_Cortex	Psychosis	ENSG00000087266.11	0.001545284	0.069031419	0.000849888	0.003450885	0.001558187	0.066743452	0.000849888	0.003450753	-1.29E-05	0.012227998	1	0.808945891	1	0.822157607	ENSG00000030110	8	BAK1
Frontal_Cortex	Psychosis	ENSG00000097046.8	-0.000391027	0.452293367	0.000520261	0.003450916	-0.000381771	0.46306844	0.000520263	0.003450785	-9.26E-06	0.008269305	1	0.977814099	1	0.981288252	ENSG00000030110	8	BAK1
Frontal_Cortex	Psychosis	ENSG00000104044.11	-0.000899726	0.289165822	0.000848834	0.003450909	-0.000889267	0.294806301	0.000848831	0.003450778	-1.05E-05	0.025318208	1	0.945359859	1	0.950813143	ENSG00000030110	8	BAK1
Frontal_Cortex	Psychosis	ENSG00000106853.12	9.93E-05	0.82532789	0.000449771	0.003450921	7.63E-05	0.865340488	0.000449834	0.00345079	2.30E-05	0.004263594	1	0.998166036	1	0.997262509	ENSG00000031691	6	CENPQ
Frontal_Cortex	Psychosis	ENSG00000107242.13	0.000711906	0.186236767	0.000538589	0.003450902	0.001014368	0.066828881	0.000553444	0.003450753	-0.000302462	0.017601872	1	0.927900596	1	0.822157607	ENSG00000031691	6	CENPQ
Frontal_Cortex	Psychosis	ENSG00000111850.6	-0.00042363	0.346154772	0.000449677	0.003450912	-0.000429928	0.339033358	0.000449679	0.00345078	6.30E-06	0.036118326	1	0.955368176	1	0.951230466	ENSG00000032444	11	PNPLA6
Frontal_Cortex	Psychosis	ENSG00000112599.8	0.000428378	0.330307713	0.000440041	0.003450911	0.00043469	0.323233534	0.000440042	0.00345078	-6.31E-06	0.035542609	1	0.950491103	1	0.951230466	ENSG00000032742	13	IFT88
Frontal_Cortex	Psychosis	ENSG00000114547.5	-0.000690748	0.440465293	0.000895437	0.003450915	-0.000679076	0.448228899	0.000895439	0.003450784	-1.17E-05	0.046916702	1	0.977814099	1	0.981288252	ENSG00000032742	13	IFT88
Frontal_Cortex	Psychosis	ENSG00000115255.6	-0.000840276	0.354374363	0.00090729	0.003450912	-0.000828305	0.36127113	0.000907287	0.003450781	-1.20E-05	0.022029854	1	0.958388154	1	0.959429486	ENSG00000033100	10	CHPF2
Frontal_Cortex	Psychosis	ENSG00000116675.11	0.000834638	0.373577898	0.000938014	0.003450913	0.000647806	0.490916699	0.000940414	0.003450785	0.000186832	0.005559264	1	0.966142504	1	0.981288252	ENSG00000033178	8	UBA6
Frontal_Cortex	Psychosis	ENSG00000117682.12	0.000160692	0.852689003	0.000865383	0.003450921	0.000173016	0.841536493	0.000865387	0.00345079	-1.23E-05	0.040209742	1	0.998166036	1	0.997262509	ENSG00000033178	8	UBA6
Frontal_Cortex	Psychosis	ENSG00000118197.9	1.58E-05	0.971230903	0.000439227	0.003450922	9.26E-06	0.983182819	0.000439229	0.003450791	6.58E-06	0.023795209	1	0.998166036	1	0.998725145	ENSG00000033178	8	UBA6
Frontal_Cortex	Psychosis	ENSG00000121289.13	-2.12E-05	0.966989939	0.000513474	0.003450922	-1.53E-05	0.976274916	0.000513472	0.003450791	-5.98E-06	0.040335864	1	0.998166036	1	0.998725145	ENSG00000033178	8	UBA6
Frontal_Cortex	Psychosis	ENSG00000121417.9	-0.000123253	0.887755902	0.000873237	0.003450922	-0.000133601	0.878402432	0.000873236	0.00345079	1.03E-05	0.049252857	1	0.998166036	1	0.997262509	ENSG00000033800	9	PIAS1
Frontal_Cortex	Psychosis	ENSG00000122218.10	-0.000768312	0.163241576	0.000551055	0.0034509	-0.000761707	0.166888609	0.000551053	0.003450769	-6.61E-06	0.029184867	1	0.923358565	1	0.921921371	ENSG00000034533	7	ASTE1
Frontal_Cortex	Psychosis	ENSG00000122678.10	-2.90E-06	0.994160924	0.000395666	0.003450922	2.53E-06	0.994893984	0.000395667	0.003450791	-5.43E-06	0.034186907	1	0.998166036	1	0.998725145	ENSG00000034533	7	ASTE1
Frontal_Cortex	Psychosis	ENSG00000125991.14	-0.000527075	0.408771881	0.00063806	0.003450914	-0.000535808	0.401053378	0.000638063	0.003450783	8.73E-06	0.043713025	1	0.972923465	1	0.980415059	ENSG00000034533	7	ASTE1
Frontal_Cortex	Psychosis	ENSG00000127337.2	-5.06E-05	0.842601867	0.000254893	0.003450921	-4.69E-05	0.854070945	0.000254893	0.00345079	-3.73E-06	0.021403313	1	0.998166036	1	0.997262509	ENSG00000034533	7	ASTE1
Frontal_Cortex	Psychosis	ENSG00000127957.12	-6.23E-05	0.75235166	0.000197402	0.003450921	-6.56E-05	0.739831071	0.000197403	0.003450789	3.26E-06	0.021830657	1	0.998166036	1	0.997262509	ENSG00000035115	17	SH3YL1
Frontal_Cortex	Psychosis	ENSG00000132613.10	-2.08E-05	0.979456915	0.000806072	0.003450922	0.000136006	0.86633608	0.000808049	0.00345079	-0.000156763	0.005701566	1	0.998166036	1	0.997262509	ENSG00000035115	17	SH3YL1
Frontal_Cortex	Psychosis	ENSG00000138592.9	0.000355428	0.583877953	0.000648911	0.003450919	0.000345614	0.594308328	0.000648914	0.003450787	9.81E-06	0.026455123	1	0.996311095	1	0.996713278	ENSG00000035115	17	SH3YL1
Frontal_Cortex	Psychosis	ENSG00000138777.15	-0.000217062	0.827240435	0.000994593	0.003450921	-0.000202978	0.838290054	0.000994594	0.00345079	-1.41E-05	0.027734961	1	0.998166036	1	0.997262509	ENSG00000035115	17	SH3YL1
Frontal_Cortex	Psychosis	ENSG00000141569.6	-0.001135483	0.068888237	0.00062418	0.003450885	-0.001127904	0.070760158	0.000624179	0.003450754	-7.58E-06	0.037940997	1	0.808945891	1	0.822157607	ENSG00000035115	17	SH3YL1
Frontal_Cortex	Psychosis	ENSG00000141959.12	-0.00012272	0.657345575	0.000276657	0.00345092	-0.0001259	0.649053219	0.000276656	0.003450788	3.18E-06	0.043627053	1	0.996561629	1	0.996713278	ENSG00000037042	8	TUBG2
Frontal_Cortex	Psychosis	ENSG00000141994.11	0.00098384	0.009553684	0.000379627	0.003450847	0.000991076	0.009038044	0.000379631	0.003450715	-7.24E-06	0.013316193	1	0.685039569	1	0.717109479	ENSG00000037474	10	NSUN2
Frontal_Cortex	Psychosis	ENSG00000143119.8	-0.00048458	0.22776842	0.000401764	0.003450906	-0.00125836	0.016336415	0.000524027	0.003450727	0.00077378	0.02145739	1	0.927900596	1	0.777758283	ENSG00000037474	10	NSUN2
Frontal_Cortex	Psychosis	ENSG00000143811.12	0.00018513	0.831334611	0.000869196	0.003450921	0.000196153	0.821456325	0.000869194	0.00345079	-1.10E-05	0.029551218	1	0.998166036	1	0.997262509	ENSG00000037474	10	NSUN2
Frontal_Cortex	Psychosis	ENSG00000143891.12	-0.000345057	0.524252747	0.000541858	0.003450917	-0.000336963	0.534034047	0.000541862	0.003450786	-8.09E-06	0.044551059	1	0.984848837	1	0.981288252	ENSG00000037749	7	MFAP3
Frontal_Cortex	Psychosis	ENSG00000145217.9	-0.000427851	0.455280786	0.000573034	0.003450916	-0.000419371	0.464265575	0.000573035	0.003450785	-8.48E-06	0.021227359	1	0.977814099	1	0.981288252	ENSG00000037749	7	MFAP3
Frontal_Cortex	Psychosis	ENSG00000148386.5	0.000216129	0.737403522	0.000644596	0.003450921	0.00022437	0.727781265	0.000644596	0.003450789	-8.24E-06	0.035169529	1	0.998166036	1	0.997262509	ENSG00000037757	9	MRI1
Frontal_Cortex	Psychosis	ENSG00000150893.9	8.44E-05	0.843680341	0.000428069	0.003450921	8.98E-05	0.83378565	0.00042807	0.00345079	-5.42E-06	0.048392084	1	0.998166036	1	0.997262509	ENSG00000037757	9	MRI1
Frontal_Cortex	Psychosis	ENSG00000152778.7	-0.001120127	0.031082671	0.000519534	0.00345087	-0.001127113	0.030047933	0.000519534	0.003450738	6.99E-06	0.031193657	1	0.808945891	1	0.822157607	ENSG00000037757	9	MRI1
Frontal_Cortex	Psychosis	ENSG00000153201.11	-0.001151321	0.118977756	0.00073846	0.003450895	-0.001159984	0.116225509	0.000738458	0.003450763	8.66E-06	0.036591804	1	0.863116848	1	0.867850871	ENSG00000037757	9	MRI1
Frontal_Cortex	Psychosis	ENSG00000154079.5	0.000553047	0.600051249	0.001054775	0.003450919	0.000565985	0.591547706	0.001054772	0.003450787	-1.29E-05	0.03194102	1	0.996311095	1	0.996713278	ENSG00000037757	9	MRI1
Frontal_Cortex	Psychosis	ENSG00000157445.10	-0.000279375	0.706409982	0.000741672	0.00345092	-0.000267677	0.718167106	0.000741673	0.003450789	-1.17E-05	0.013786431	1	0.996561629	1	0.997262509	ENSG00000037757	9	MRI1
Frontal_Cortex	Psychosis	ENSG00000160200.13	-0.000437799	0.538778079	0.000712259	0.003450918	-0.000447909	0.529442849	0.000712259	0.003450786	1.01E-05	0.020800896	1	0.984848837	1	0.981288252	ENSG00000038219	8	BOD1L1
Frontal_Cortex	Psychosis	ENSG00000160886.9	2.95E-05	0.976089332	0.000984129	0.003450922	4.31E-05	0.965048216	0.000984132	0.003450791	-1.36E-05	0.03397524	1	0.998166036	1	0.998725145	ENSG00000038219	8	BOD1L1
Frontal_Cortex	Psychosis	ENSG00000163444.7	0.00056532	0.395460948	0.000665272	0.003450914	0.00057465	0.387708389	0.000665272	0.003450782	-9.33E-06	0.021319699	1	0.972923465	1	0.980415059	ENSG00000038219	8	BOD1L1
Frontal_Cortex	Psychosis	ENSG00000163625.11	0.000285992	0.779100132	0.001019606	0.003450921	0.000252102	0.804728582	0.001019697	0.00345079	3.39E-05	0.023669728	1	0.998166036	1	0.997262509	ENSG00000038219	8	BOD1L1
Frontal_Cortex	Psychosis	ENSG00000163864.10	-0.000839753	0.175057161	0.000619225	0.003450901	-0.000832628	0.178744497	0.000619223	0.003450771	-7.13E-06	0.035132202	1	0.926881598	1	0.926773198	ENSG00000040199	14	PHLPP2
Frontal_Cortex	Psychosis	ENSG00000163938.12	0.000108597	0.711306382	0.000293425	0.00345092	0.000104543	0.721627233	0.000293426	0.003450789	4.05E-06	0.045309882	1	0.996561629	1	0.997262509	ENSG00000040199	14	PHLPP2
Frontal_Cortex	Psychosis	ENSG00000165794.5	0.000128955	0.847543829	0.000670758	0.003450921	0.000137997	0.836999982	0.000670758	0.00345079	-9.04E-06	0.032823364	1	0.998166036	1	0.997262509	ENSG00000040531	10	CTNS
Frontal_Cortex	Psychosis	ENSG00000167700.4	-0.000662454	0.385280745	0.000763013	0.003450914	-0.000676436	0.375333451	0.000763016	0.003450782	1.40E-05	0.007581829	1	0.972923465	1	0.967528421	ENSG00000040531	10	CTNS
Frontal_Cortex	Psychosis	ENSG00000169385.2	0.000534482	0.584033371	0.000976218	0.003450919	0.000606983	0.5342356	0.000976556	0.003450786	-7.25E-05	0.006073889	1	0.996311095	1	0.981288252	ENSG00000040531	10	CTNS
Frontal_Cortex	Psychosis	ENSG00000169442.4	-0.000254321	0.372978877	0.000285463	0.003450913	-0.000260306	0.36184469	0.000285468	0.003450781	5.99E-06	0.017697628	1	0.966142504	1	0.959429486	ENSG00000040531	10	CTNS
Frontal_Cortex	Psychosis	ENSG00000170209.4	-0.0011044	0.063521229	0.000595193	0.003450884	-0.001114377	0.061169784	0.0005952	0.003450752	9.98E-06	0.032712187	1	0.808945891	1	0.822157607	ENSG00000040933	11	INPP4A
Frontal_Cortex	Psychosis	ENSG00000171450.4	-0.000522278	0.54818745	0.000869769	0.003450918	-0.000511575	0.556414375	0.000869765	0.003450787	-1.07E-05	0.026071471	1	0.984848837	1	0.988534574	ENSG00000041353	5	RAB27B
Frontal_Cortex	Psychosis	ENSG00000171962.13	-0.001473709	0.070717963	0.000815425	0.003450886	-0.001463573	0.072676223	0.000815422	0.003450755	-1.01E-05	0.028973026	1	0.808945891	1	0.822157607	ENSG00000041353	5	RAB27B
Frontal_Cortex	Psychosis	ENSG00000172548.10	0.000990441	0.32654215	0.001009516	0.003450911	0.000974448	0.334414779	0.001009518	0.00345078	1.60E-05	0.013551308	1	0.950491103	1	0.951230466	ENSG00000041353	5	RAB27B
Frontal_Cortex	Psychosis	ENSG00000174125.3	0.001700046	0.105879471	0.001051359	0.003450893	0.001717054	0.102435269	0.001051366	0.003450761	-1.70E-05	0.024974624	1	0.858989904	1	0.857620674	ENSG00000041353	5	RAB27B
Frontal_Cortex	Psychosis	ENSG00000175130.6	0.000108965	0.835463963	0.000524631	0.003450921	-8.34E-05	0.875215488	0.000530813	0.00345079	0.000192323	0.017357496	1	0.998166036	1	0.997262509	ENSG00000041353	5	RAB27B
Frontal_Cortex	Psychosis	ENSG00000175662.13	-0.000624494	0.031359569	0.000290127	0.003450871	-0.000620259	0.032526596	0.000290127	0.00345074	-4.24E-06	0.020736229	1	0.808945891	1	0.822157607	ENSG00000041802	6	LSG1
Frontal_Cortex	Psychosis	ENSG00000176268.5	-0.00040664	0.507188738	0.000613127	0.003450917	-0.000397402	0.516885589	0.000613128	0.003450786	-9.24E-06	0.019928216	1	0.984848837	1	0.981288252	ENSG00000041802	6	LSG1
Frontal_Cortex	Psychosis	ENSG00000176909.7	0.000164122	0.679197971	0.000396854	0.00345092	0.000172274	0.664218832	0.000396858	0.003450788	-8.15E-06	0.00507065	1	0.996561629	1	0.996713278	ENSG00000041802	6	LSG1
Frontal_Cortex	Psychosis	ENSG00000178802.13	-0.000317295	0.525581744	0.000499861	0.003450917	-0.000311617	0.533016047	0.00049986	0.003450786	-5.68E-06	0.041685869	1	0.984848837	1	0.981288252	ENSG00000042429	6	MED17
Frontal_Cortex	Psychosis	ENSG00000180535.3	-0.000158471	0.820245433	0.000697404	0.003450921	-0.00016891	0.80862818	0.000697408	0.00345079	1.04E-05	0.032146343	1	0.998166036	1	0.997262509	ENSG00000042429	6	MED17
Frontal_Cortex	Psychosis	ENSG00000181396.8	-0.000151312	0.765051191	0.000506306	0.003450921	-0.000157407	0.75588239	0.000506305	0.00345079	6.09E-06	0.035682112	1	0.998166036	1	0.997262509	ENSG00000042493	11	CAPG
Frontal_Cortex	Psychosis	ENSG00000182670.9	-0.001719158	0.01873771	0.000731332	0.003450861	-0.001731059	0.017934329	0.000731335	0.003450728	1.19E-05	0.016270967	1	0.808945891	1	0.801863787	ENSG00000042493	11	CAPG
Frontal_Cortex	Psychosis	ENSG00000182986.8	1.48E-05	0.959913324	0.000293653	0.003450922	1.86E-05	0.949375677	0.000293654	0.003450791	-3.88E-06	0.049171365	1	0.998166036	1	0.998725145	ENSG00000043514	11	TRIT1
Frontal_Cortex	Psychosis	ENSG00000188878.12	-0.000879018	0.14503557	0.000603184	0.003450898	-0.00086791	0.150187319	0.000603187	0.003450768	-1.11E-05	0.007063024	1	0.896136687	1	0.910171345	ENSG00000043514	11	TRIT1
Frontal_Cortex	Psychosis	ENSG00000198478.6	0.001604639	0.032605294	0.000750912	0.003450871	0.001613304	0.031677371	0.000750909	0.003450739	-8.67E-06	0.035522111	1	0.808945891	1	0.822157607	ENSG00000044090	4	CUL7
Frontal_Cortex	Psychosis	ENSG00000204169.6	0.000979452	0.215887855	0.000791452	0.003450905	0.000963183	0.2236172	0.000791461	0.003450774	1.63E-05	0.009479759	1	0.927900596	1	0.932819306	ENSG00000044090	4	CUL7
Frontal_Cortex	Psychosis	ENSG00000205356.5	-6.42E-05	0.936245952	0.000803081	0.003450922	-5.34E-05	0.946947546	0.00080308	0.003450791	-1.08E-05	0.020873077	1	0.998166036	1	0.998725145	ENSG00000044090	4	CUL7
Frontal_Cortex	Psychosis	ENSG00000205918.5	0.00060096	0.093681918	0.000358505	0.003450891	0.000595532	0.096687433	0.000358509	0.00345076	5.43E-06	0.04655432	1	0.83985898	1	0.853224191	ENSG00000047936	6	ROS1
Frontal_Cortex	Psychosis	ENSG00000213465.3	0.001360824	0.011970313	0.000541507	0.003450852	0.001353645	0.012427606	0.000541506	0.003450721	7.18E-06	0.025829149	1	0.752633419	1	0.777758283	ENSG00000048140	13	TSPAN17
Frontal_Cortex	Psychosis	ENSG00000215237.5	0.000310686	0.72382603	0.000879257	0.00345092	0.000296297	0.736129127	0.000879261	0.003450789	1.44E-05	0.017234691	1	0.997218736	1	0.997262509	ENSG00000048140	13	TSPAN17
Frontal_Cortex	Psychosis	ENSG00000215915.5	-0.000382745	0.547651034	0.000636546	0.003450918	-0.000375176	0.555596598	0.000636544	0.003450787	-7.57E-06	0.03274261	1	0.984848837	1	0.988534574	ENSG00000048140	13	TSPAN17
Frontal_Cortex	Psychosis	ENSG00000219201.3	-0.00104374	0.164916784	0.00075158	0.0034509	-0.001030587	0.17030746	0.000751584	0.00345077	-1.32E-05	0.011447255	1	0.923358565	1	0.921921371	ENSG00000048540	10	LMO3
Frontal_Cortex	Psychosis	ENSG00000223496.1	-0.001199084	0.255482632	0.001054478	0.003450908	-0.001165241	0.269163009	0.001054523	0.003450777	-3.38E-05	0.003648478	1	0.936726396	1	0.940460229	ENSG00000048544	5	MRPS10
Frontal_Cortex	Psychosis	ENSG00000225872.2	8.37E-05	0.897969922	0.000652825	0.003450922	9.08E-05	0.889326007	0.000652822	0.00345079	-7.14E-06	0.041834778	1	0.998166036	1	0.997262509	ENSG00000048544	5	MRPS10
Frontal_Cortex	Psychosis	ENSG00000226232.4	-0.000427549	0.627330112	0.000880658	0.003450919	-0.000453552	0.60655815	0.000880695	0.003450788	2.60E-05	0.007726708	1	0.996311095	1	0.996713278	ENSG00000048544	5	MRPS10
Frontal_Cortex	Psychosis	ENSG00000232063.1	0.000117075	0.856056059	0.00064541	0.003450922	0.000123662	0.848052678	0.000645406	0.00345079	-6.59E-06	0.047904991	1	0.998166036	1	0.997262509	ENSG00000048544	5	MRPS10
Frontal_Cortex	Psychosis	ENSG00000232859.5	0.0001241	0.85257802	0.000667813	0.003450921	0.000113082	0.865537085	0.000667819	0.00345079	1.10E-05	0.028633498	1	0.998166036	1	0.997262509	ENSG00000048545	9	GUCA1A
Frontal_Cortex	Psychosis	ENSG00000235021.1	4.99E-05	0.910441045	0.000443364	0.003450922	5.48E-05	0.901715732	0.000443363	0.00345079	-4.88E-06	0.049461462	1	0.998166036	1	0.997262509	ENSG00000049089	9	COL9A2
Frontal_Cortex	Psychosis	ENSG00000235994.4	0.001414568	0.169366355	0.001029341	0.003450901	0.001402259	0.173106353	0.001029337	0.00345077	1.23E-05	0.027700677	1	0.923358565	1	0.923878279	ENSG00000049192	10	ADAMTS6
Frontal_Cortex	Psychosis	ENSG00000236155.2	-4.44E-05	0.922690436	0.000457362	0.003450922	-5.13E-05	0.910633486	0.000457367	0.00345079	6.95E-06	0.049682364	1	0.998166036	1	0.997262509	ENSG00000049247	9	UTS2
Frontal_Cortex	Psychosis	ENSG00000236834.1	0.000256357	0.644716668	0.000555951	0.00345092	0.000249654	0.653389051	0.00055595	0.003450788	6.70E-06	0.032691909	1	0.996561629	1	0.996713278	ENSG00000049449	4	RCN1
Frontal_Cortex	Psychosis	ENSG00000242616.3	-0.000857651	0.210635215	0.000685121	0.003450904	-0.000867396	0.205498017	0.000685122	0.003450773	9.74E-06	0.027634975	1	0.927900596	1	0.930914275	ENSG00000049449	4	RCN1
Frontal_Cortex	Psychosis	ENSG00000243444.3	0.000374972	0.420128162	0.00046511	0.003450915	-0.000201228	0.699329419	0.00052101	0.003450789	0.000576199	0.014128249	1	0.972923465	1	0.996713278	ENSG00000049449	4	RCN1
Frontal_Cortex	Psychosis	ENSG00000243646.4	-4.55E-05	0.901217195	0.000366298	0.003450922	-5.08E-05	0.88968202	0.0003663	0.00345079	5.34E-06	0.030211179	1	0.998166036	1	0.997262509	ENSG00000049449	4	RCN1
Frontal_Cortex	Psychosis	ENSG00000243759.1	0.000510793	0.451007291	0.00067768	0.003450916	0.000500805	0.459907476	0.000677679	0.003450785	9.99E-06	0.015114468	1	0.977814099	1	0.981288252	ENSG00000050130	13	JKAMP
Frontal_Cortex	Psychosis	ENSG00000248161.1	0.000463222	0.322405601	0.000468124	0.003450911	0.000468134	0.317298046	0.000468121	0.003450779	-4.91E-06	0.045412107	1	0.950491103	1	0.951230466	ENSG00000050130	13	JKAMP
Frontal_Cortex	Psychosis	ENSG00000249109.1	-0.001564397	0.131867887	0.001038238	0.003450897	-0.001575489	0.129148493	0.001038233	0.003450765	1.11E-05	0.041118086	1	0.88836141	1	0.880701241	ENSG00000050130	13	JKAMP
Frontal_Cortex	Psychosis	ENSG00000250974.1	-0.000321553	0.718343741	0.000891534	0.00345092	-0.000307533	0.730136343	0.000891543	0.003450789	-1.40E-05	0.039716551	1	0.997197795	1	0.997262509	ENSG00000050130	13	JKAMP
Frontal_Cortex	Psychosis	ENSG00000251350.1	-0.000388649	0.622257224	0.000788888	0.003450919	-0.000379604	0.630380932	0.000788885	0.003450788	-9.04E-06	0.034079705	1	0.996311095	1	0.996713278	ENSG00000050130	13	JKAMP

Frontal_Cortex	Psychosis	ENSG00000251365.1	0.000185358	0.828704354	0.000856698	0.003450921	0.000202759	0.812910727	0.000856704	0.00345079	-1.74E-05	0.005654761	1	0.998166036	1	0.997262509	ENSG00000050393	7	MCUR1
Frontal_Cortex	Psychosis	ENSG00000251600.1	-0.000258069	0.50970444	0.00039143	0.003450917	-0.000252993	0.518065277	0.00039143	0.003450786	-5.08E-06	0.038987733	1	0.984848837	1	0.981288252	ENSG00000050393	7	MCUR1
Frontal_Cortex	Psychosis	ENSG00000253395.1	-0.00022772	0.804239059	0.000918725	0.003450921	-0.000218082	0.812365513	0.00091872	0.00345079	-9.64E-06	0.043556868	1	0.998166036	1	0.997262509	ENSG00000050393	7	MCUR1
Frontal_Cortex	Psychosis	ENSG00000258561.1	0.001410317	0.095992939	0.000847239	0.003450891	0.00142379	0.092861634	0.000847243	0.003450759	-1.35E-05	0.022218146	1	0.83985898	1	0.841610847	ENSG00000050405	9	LIMA1
Frontal_Cortex	Psychosis	ENSG00000259205.2	-7.63E-05	0.811319956	0.000319526	0.003450921	-7.09E-05	0.824424848	0.000319527	0.00345079	-5.39E-06	0.013632246	1	0.998166036	1	0.997262509	ENSG00000050405	9	LIMA1
Frontal_Cortex	Psychosis	ENSG00000260088.1	5.56E-05	0.767826235	0.000188287	0.003450921	5.86E-05	0.755734729	0.000188288	0.00345079	-2.99E-06	0.017086706	1	0.998166036	1	0.997262509	ENSG00000050555	13	LAMC3
Frontal_Cortex	Psychosis	ENSG00000260404.2	-6.59E-05	0.882395797	0.000445388	0.003450922	-7.20E-05	0.871626393	0.00044539	0.00345079	6.08E-06	0.043123136	1	0.998166036	1	0.997262509	ENSG00000050555	13	LAMC3
Frontal_Cortex	Psychosis	ENSG00000260844.2	0.000686475	0.291776158	0.00065116	0.00345091	0.000693384	0.286944636	0.000651157	0.003450778	-6.91E-06	0.047269745	1	0.945359859	1	0.950813143	ENSG00000050555	13	LAMC3
Frontal_Cortex	Psychosis	ENSG00000261705.1	0.000258311	0.565565045	0.000449553	0.003450918	0.000250119	0.577959404	0.000449556	0.003450787	8.19E-06	0.012048449	1	0.994682579	1	0.996713278	ENSG00000050555	13	LAMC3
Frontal_Cortex	Psychosis	ENSG00000267336.1	-0.000313238	0.756990259	0.001012288	0.003450921	-0.000300283	0.766742952	0.001012286	0.00345079	-1.30E-05	0.031016199	1	0.998166036	1	0.997262509	ENSG00000051382	4	PIK3CB
Frontal_Cortex	Psychosis	ENSG00000267422.1	0.000170849	0.857576499	0.000952017	0.003450922	0.000182185	0.848238186	0.000952016	0.00345079	-1.13E-05	0.047583617	1	0.998166036	1	0.997262509	ENSG00000051620	6	HEBP2
Frontal_Cortex	Psychosis	ENSG00000268225.1	0.000229686	0.49679294	0.000337999	0.003450917	0.000234713	0.487423901	0.000338002	0.003450785	-5.03E-06	0.042953134	1	0.984848837	1	0.981288252	ENSG00000053372	4	MRTO4
Frontal_Cortex	Psychosis	ENSG00000270614.1	0.000445757	0.437403132	0.000573995	0.003450915	0.00045534	0.427614816	0.000573996	0.003450784	-9.58E-06	0.011705634	1	0.977814099	1	0.981288252	ENSG00000053900	6	ANAPC4
Frontal_Cortex	Psychosis	ENSG00000271265.1	0.000258752	0.7606504	0.000849405	0.003450921	0.000269958	0.750620945	0.000849404	0.003450789	-1.12E-05	0.026887819	1	0.998166036	1	0.997262509	ENSG00000053900	6	ANAPC4
Frontal_Cortex	Psychosis	ENSG00000271396.1	0.000472711	0.514160911	0.000724601	0.003450917	0.000482024	0.505906575	0.000724602	0.003450786	-9.31E-06	0.03986657	1	0.984848837	1	0.981288252	ENSG00000053900	6	ANAPC4
Frontal_Cortex	Psychosis	ENSG00000271553.1	0.000113999	0.894385147	0.000858699	0.003450922	0.00010065	0.906692469	0.000858704	0.00345079	1.33E-05	0.027936389	1	0.998166036	1	0.997262509	ENSG00000054277	8	OPN3
Frontal_Cortex	Psychosis	ENSG00000272444.1	-0.000345845	0.637003034	0.000732887	0.003450919	-0.000358395	0.624835416	0.0007329	0.003450788	1.26E-05	0.045204485	1	0.996311095	1	0.996713278	ENSG00000054277	8	OPN3
Frontal_Cortex	Psychosis	ENSG00000272942.1	0.001012984	0.255577722	0.000890999	0.003450908	0.000998235	0.26256727	0.000891006	0.003450777	1.47E-05	0.025312849	1	0.936726396	1	0.940460229	ENSG00000054282	11	SDCCAG8
Pancreas	Prediabetes	ENSG00000008394.8	-0.000229905	0.720258092	0.000641988	0.002176049	0.00104959	0.194553328	0.000809102	0.002175991	-0.001279495	0.009370972	1	0.980154834	1	0.933545778	ENSG00000054523	12	KIF1B
Pancreas	Prediabetes	ENSG00000066294.10	0.000251681	0.361082334	0.000275571	0.002176046	0.000644747	0.052702938	0.000332798	0.002175982	-0.000393066	0.035158106	1	0.931097229	1	0.931226496	ENSG00000054938	11	CHRDL2
Pancreas	Prediabetes	ENSG00000093144.14	-0.000161981	0.684791813	0.000399033	0.002176049	-0.000156164	0.695538391	0.000399039	0.002175998	-5.82E-06	0.043940017	1	0.975612224	1	0.975423656	ENSG00000054938	11	CHRDL2
Pancreas	Prediabetes	ENSG00000101452.10	0.000700088	0.205361186	0.000552806	0.002176042	0.000719322	0.193245348	0.000552882	0.002175991	-1.92E-05	0.043481357	1	0.931097229	1	0.933545778	ENSG00000054938	11	CHRDL2
Pancreas	Prediabetes	ENSG00000111450.9	0.000183833	0.622093117	0.000372972	0.002176049	0.00018917	0.612022559	0.000372977	0.002175998	-5.34E-06	0.04028706	1	0.968763492	1	0.964723844	ENSG00000054983	12	GALC
Pancreas	Prediabetes	ENSG00000136425.8	-0.000479168	0.022136681	0.000209425	0.002176026	-0.000475123	0.023291382	0.000209432	0.002175976	-4.04E-06	0.044910734	1	0.926755475	1	0.931226496	ENSG00000055147	13	FAM114A2
Pancreas	Prediabetes	ENSG00000138286.10	-6.67E-05	0.801680766	0.000265705	0.002176049	-7.03E-05	0.791296448	0.000265707	0.002175999	3.58E-06	0.030832539	1	0.982693856	1	0.981881037	ENSG00000055147	13	FAM114A2
Pancreas	Prediabetes	ENSG00000140105.13	0.000143018	0.751837849	0.000452278	0.002176049	0.000149306	0.741311999	0.000452282	0.002175999	-6.29E-06	0.034124062	1	0.980154834	1	0.981881037	ENSG00000055211	8	GINM1
Pancreas	Prediabetes	ENSG00000141127.10	0.000196678	0.671133419	0.000463216	0.002176049	0.000204634	0.658663576	0.000463225	0.002175998	-7.96E-06	0.028904513	1	0.975612224	1	0.973329377	ENSG00000055211	8	GINM1
Pancreas	Prediabetes	ENSG00000141994.11	0.000106021	0.65426481	0.000236736	0.002176049	0.000109305	0.644286216	0.000236738	0.002175998	-3.28E-06	0.029105158	1	0.974245837	1	0.9720762	ENSG00000055950	12	MRPL43
Pancreas	Prediabetes	ENSG00000158158.7	-0.000749981	0.009188245	0.000287903	0.002176019	-0.00075525	0.00871063	0.00028791	0.002175968	5.27E-06	0.029768736	1	0.926755475	1	0.931226496	ENSG00000055955	11	ITIH4
Pancreas	Prediabetes	ENSG00000164118.8	0.000491648	0.364190175	0.000541814	0.002176046	0.000502118	0.354080923	0.000541832	0.002175995	-1.05E-05	0.041562892	1	0.931097229	1	0.933545778	ENSG00000055955	11	ITIH4
Pancreas	Prediabetes	ENSG00000164308.12	5.67E-05	0.564056024	9.83E-05	0.002176048	5.51E-05	0.575333679	9.83E-05	0.002175998	1.63E-06	0.01940657	1	0.96510431	1	0.964589659	ENSG00000056661	9	PCGF2
Pancreas	Prediabetes	ENSG00000166321.9	0.000101763	0.762559955	0.000336829	0.002176049	0.000106101	0.752764959	0.000336832	0.002175999	-4.34E-06	0.037350225	1	0.980154834	1	0.981881037	ENSG00000056661	9	PCGF2
Pancreas	Prediabetes	ENSG00000168653.6	0.001123634	0.063871487	0.00060636	0.002176034	0.001110739	0.066989037	0.000606381	0.002175984	1.29E-05	0.02644547	1	0.926755475	1	0.931226496	ENSG00000057468	6	MSH4
Pancreas	Prediabetes	ENSG00000171862.5	5.49E-05	0.87658077	0.000353541	0.00217605	4.89E-05	0.889891332	0.000353547	0.002175999	5.96E-06	0.025331249	1	0.983178195	1	0.985769389	ENSG00000057468	6	MSH4
Pancreas	Prediabetes	ENSG00000176974.13	-9.25E-05	0.759761759	0.000302382	0.002176049	-8.86E-05	0.769589899	0.000302385	0.002175999	-3.89E-06	0.048953617	1	0.980154834	1	0.981881037	ENSG00000057468	6	MSH4
Pancreas	Prediabetes	ENSG00000179021.5	0.000260628	0.595786573	0.000491316	0.002176048	0.00026756	0.586047585	0.000491321	0.002175998	-6.93E-06	0.033399173	1	0.96510431	1	0.964589659	ENSG00000057608	12	GDI2
Pancreas	Prediabetes	ENSG00000182685.3	0.000221881	0.689936815	0.000556174	0.002176049	0.000231345	0.67744824	0.000556188	0.002175998	-9.46E-06	0.044486578	1	0.975612224	1	0.974993264	ENSG00000057608	12	GDI2
Pancreas	Prediabetes	ENSG00000205578.4	-0.000208181	0.523627913	0.000326423	0.002176048	-0.000203813	0.532380394	0.000326426	0.002175997	-4.37E-06	0.035770065	1	0.96510431	1	0.964589659	ENSG00000057608	12	GDI2
Pancreas	Prediabetes	ENSG00000224635.1	-0.000398182	0.392803968	0.000465958	0.002176046	-0.000313779	0.502326974	0.000467745	0.002175997	-8.44E-05	0.039076195	1	0.941475052	1	0.964589659	ENSG00000058063	11	ATP11B
Pancreas	Prediabetes	ENSG00000244219.2	-0.000692078	0.164150561	0.000497451	0.002176041	-0.000684683	0.16870958	0.000497458	0.002175991	-7.40E-06	0.033100399	1	0.931097229	1	0.933545778	ENSG00000058085	10	LAMC2
Pancreas	Prediabetes	ENSG00000248905.4	-0.00067793	0.202237395	0.000531624	0.002176042	-0.000685993	0.196930007	0.000531633	0.002175992	8.06E-06	0.043931424	1	0.931097229	1	0.933545778	ENSG00000058085	10	LAMC2
Pancreas	Prediabetes	ENSG00000258647.1	0.00032754	0.526639067	0.000517322	0.002176048	0.000320858	0.535110767	0.000517327	0.002175997	6.68E-06	0.046094748	1	0.96510431	1	0.964589659	ENSG00000058085	10	LAMC2
Pancreas	T1DM	ENSG00000005102.8	-8.70E-05	0.926232476	0.000940201	0.008204775	-0.00011864	0.899589056	0.000940242	0.00820389	3.16E-05	0.015772547	1	0.997898334	1	0.994038165	ENSG00000058085	10	LAMC2
Pancreas	T1DM	ENSG00000005513.9	0.000302992	0.736780576	0.00090144	0.008204774	0.000289813	0.747823517	0.000901411	0.008203888	1.32E-05	0.024418123	1	0.975415042	1	0.968082788	ENSG00000058453	12	CROCC
Pancreas	T1DM	ENSG00000005801.12	-0.000182328	0.806555627	0.000744584	0.008204775	-0.000194925	0.793477084	0.000744564	0.008203889	1.26E-05	0.019756276	1	0.98201188	1	0.978923745	ENSG00000058453	12	CROCC
Pancreas	T1DM	ENSG00000005981.8	0.000710182	0.528101569	0.001125653	0.008204769	0.000686589	0.541882355	0.001125612	0.008203884	2.36E-05	0.000460206	1	0.919664009	1	0.928420568	ENSG00000058453	12	CROCC
Pancreas	T1DM	ENSG00000006715.11	-0.000375735	0.620822424	0.000759542	0.008204771	-0.000393132	0.604733261	0.00075952	0.008203885	1.74E-05	0.000979919	1	0.95220407	1	0.939938439	ENSG00000058453	12	CROCC
Pancreas	T1DM	ENSG00000009413.11	1.99E-05	0.987496499	0.001271343	0.008204776	5.04E-05	0.968365903	0.001271307	0.00820389	-3.05E-05	0.000724632	1	0.997898334	1	0.99966149	ENSG00000058453	12	CROCC
Pancreas	T1DM	ENSG00000012232.4	-0.000373201	0.6385104	0.000794411	0.008204772	-0.000346369	0.662824744	0.000794394	0.008203887	-2.68E-05	2.85E-05	1	0.960781248	1	0.961035047	ENSG00000059573	4	ALDH18A1
Pancreas	T1DM	ENSG00000012963.9	6.58E-06	0.992607705	0.000709783	0.008204776	2.18E-05	0.975527042	0.00070976	0.00820389	-1.52E-05	0.000990099	1	0.998452063	1	0.99966149	ENSG00000059573	4	ALDH18A1
Pancreas	T1DM	ENSG00000013523.5	0.000293677	0.703781363	0.000772385	0.008204773	0.000279614	0.717333545	0.00077236	0.008203888	1.41E-05	0.00605114	1	0.974645207	1	0.968082788	ENSG00000060558	3	GNA15
Pancreas	T1DM	ENSG00000020922.8	-1.79E-05	0.932704322	0.000212393	0.008204775	-1.16E-05	0.956320427	0.000212396	0.00820389	-6.30E-06	0.011717587	1	0.997898334	1	0.999388266	ENSG00000060558	3	GNA15
Pancreas	T1DM	ENSG00000022556.11	-0.0014297	0.1234426	0.000928084	0.008204736	-0.001447034	0.11894591	0.000928052	0.008203849	1.73E-05	0.003045661	1	0.680843282	1	0.666556371	ENSG00000060642	6	PIGV
Pancreas	T1DM	ENSG00000029725.12	-0.000435317	0.501707348	0.00064798	0.008204768	-0.000426116	0.510777039	0.000647958	0.008203883	-9.20E-06	0.025670187	1	0.914783379	1	0.915335266	ENSG00000061455	10	PRDM6
Pancreas	T1DM	ENSG00000030110.8	-0.001141157	0.198170146	0.000886828	0.008204748	-0.001732625	0.051929547	0.000891395	0.008203826	0.000591468	6.67E-11	1	0.761827748	1	0.509067603	ENSG00000061455	10	PRDM6
Pancreas	T1DM	ENSG00000032444.11	-0.001556812	0.157545632	0.001101484	0.008204742	-0.001541253	0.161725368	0.001101451	0.008203857	-1.56E-05	0.038723559	1	0.719888227	1	0.723580189	ENSG00000061455	10	PRDM6
Pancreas	T1DM	ENSG00000037757.9	-0.00046209	0.516669377	0.000712564	0.008204768	-0.000492157	0.489753937	0.000712548	0.008203882	3.01E-05	7.65E-08	1	0.914783379	1	0.914417995	ENSG00000061938	12	TNK2
Pancreas	T1DM	ENSG00000038219.8	0.000223751	0.586991106	0.000411911	0.008204771	0.000230561	0.575649402	0.000411899	0.008203885	-6.81E-06	0.017118442	1	0.937588291	1	0.936315417	ENSG00000062038	9	CDH3
Pancreas	T1DM	ENSG00000049089.9	-0.001131456	0.12882902	0.000744997	0.008204737	-0.001943017	0.015975836	0.000806407	0.008203792	0.000811561	0.008578067	1	0.68791014	1	0.301309092	ENSG00000062370	12	ZNF112
Pancreas	T1DM	ENSG00000051620.6	0.001195078	0.329492963	0.00122555	0.00820476	0.001217714	0.320396737	0.001225504	0.008203873	-2.26E-05	0.001366205	1	0.83462449	1	0.830335577	ENSG00000063046	13	EIF4B
Pancreas	T1DM	ENSG00000053372.4	-0.000839768	0.39406325	0.000985328	0.008204763	-0.0008522	0.387081753	0.000985291	0.008203877	1.24E-05	0.029048621	1	0.873903579	1	0.867022428	ENSG00000063046	13	EIF4B
Pancreas	T1DM	ENSG00000058085.10	0.000620171	0.528071803	0.000982912	0.008204769	0.000598498	0.542580632	0.000982891	0.008203884	2.17E-05	0.005797114	1	0.919664009	1	0.928420568	ENSG00000063046	13	EIF4B
Pancreas	T1DM	ENSG00000061455.10	0.000967032	0.293110817	0.000919824	0.008204757	0.000980812	0.286271963	0.000919795	0.008203871	-1.38E-05	0.025688523	1	0.810235282	1	0.801351316	ENSG00000063046	13	EIF4B
Pancreas	T1DM	ENSG00000062370.12	-0.001000739	0.13328891	0.000666599	0.008204738	-0.000991587	0.136860411	0.000666574	0.008203853	-9.15E-06	0.014457551	1	0.692886478	1	0.692461116	ENSG00000063046	13	EIF4B
Pancreas	T1DM	ENSG00000063046.13	0.00014468	0.742814915	0.000440925	0.008204774	0.000152587	0.729288574	0.000440911	0.008203888	-7.91E-06	0.008941009	1	0.975415042	1	0.968082788	ENSG00000063046	13	EIF4B
Pancreas	T1DM	ENSG00000063854.8	-0.000601748	0.60318421	0.001157591	0.008204771	-0.000562649	0.626923488	0.001157566	0.008203886	-3.91E-05	2.65E-05	1	0.941627387	1	0.953755638	ENSG00000063854	8	HAGH
Pancreas	T1DM	ENSG00000064687.8	-0.000715477	0.380989588	0.000816684	0.008204763	-0.00070112	0.390605698	0.000816661	0.008203878	-1.44E-05	0.012544694	1	0.862440233	1	0.868463578	ENSG00000063854	8	HAGH
Pancreas	T1DM	ENSG00000066185.8	0.001148324	0.059885745	0.000610279	0.008204716	0.001134589	0.063003539	0.000610266	0.008203832	1.37E-05	0.005521953	1	0.527104393	1	0.528735579	ENSG00000063854	8	HAGH
Pancreas	T1DM	ENSG00000066294.10	0.000112087	0.834081759	0.000535098	0.008204775	0.002053123	0.001486705	0.000646188	0.00820372	-0.001941036	8.44E-08	1	0.984098328	1	0.064162591	ENSG00000063854	8	HAGH
Pancreas	T1DM	ENSG00000069966.14	0.000722708	0.491656298	0.00105094	0.008204768	0.000739287	0.481758812	0.001050904	0.008203882	-1.66E-05	0.0115784	1	0.914693561	1	0.913030722	ENSG00000064225	8	ST3GAL6
Pancreas	T1DM	ENSG00000069974.11	0.001007627	0.214369406	0.000811528	0.00820475	0.001018056	0.209646791	0.000811498	0.008203863	-1.04E-05	0.029531004	1	0.772090651	1	0.769778216	ENSG00000064270	8	ATP2C2
Pancreas	T1DM	ENSG00000070182.13	0.001765808	0.026653535	0.000796643	0.008204693	0.00177863	0.025567974	0.000796621	0.008203806	-1.28E-05	0.026538311	1	0.395643231	1	0.374802485	ENSG00000064270	8	ATP2C2
Pancreas	T1DM	ENSG00000071462.7	-0.001051108	0.319437124	0.001055736	0.008204759	-0.00103967	0.324712616	0.001055694	0.008203874	-1.14E-05	0.045627896	1	0.829307456	1	0.830335577	ENSG00000064309	10	CDON
Pancreas	T1DM	ENSG00000072858.6	0.0004284	0.469847572	0.000592754	0.008204767	0.000420042	0.478538821	0.000592731	0.008203882	8.36E-06	0.010606694	1	0.908712989	1	0.911454131	ENSG00000064309	10	CDON
Pancreas	T1DM	ENSG00000073849.10	0.000562288	0.556698674	0.000956674	0.00820477	0.00054701	0.567454509	0.000956638	0.008203884	1.53E-05	0.00539663	1	0.93270646	1	0.936315417	ENSG00000064313	7	TAF2
Pancreas	T1DM	ENSG00000074706.9	9.42E-05	0.928092728	0.001043453	0.008204775	0.000108948	0.916840058	0.001043413	0.00820389	-1.48E-05	0.011617259	1	0.997898334	1	0.997092545	ENSG00000064313	7	TAF2
Pancreas	T1DM	ENSG00000075826.12	0.001074193	0.08186078	0.000617354	0.008204725	0.001065012	0.084496085	0.000617334	0.00820384	9.18E-06	0.023220308	1	0.587627507	1	0.580557814	ENSG00000064607	12	SUGP2
Pancreas	T1DM	ENSG00000076662.5	-0.001331314	0.12252765	0.000862109	0.008204735	-0.001315603	0.126994963	0.00086209	0.008203851	-1.57E-05	0.022083737	1	0.680843282	1	0.673807988	ENSG00000064607	12	SUGP2
Pancreas	T1DM	ENSG00000077080.5	0.001843035	0.075831502	0.001038092	0.008204722	0.001829484	0.077998867	0.001038052	0.008203838	1.36E-05	0.016802929	1	0.57107125	1	0.562287494	ENSG00000064687	8	ABCA7
Pancreas	T1DM	ENSG00000077238.9	0.000403097	0.701074828	0.001050088	0.008204773	0.000419348	0.689628887	0.001050052	0.008203887	-1.63E-05	0.014498348	1	0.974645207	1	0.966685034	ENSG00000064687	8	ABCA7
Pancreas	T1DM	ENSG00000079257.3	0.000353366	0.601635494	0.000676881	0.008204771	0.000362659	0.592096038	0.000676854	0.008203885	-9.29E-06	0.012230851	1	0.940352982	1	0.937577789	ENSG00000064687	8	ABCA7
Pancreas	T1DM	ENSG00000079335.13	0.000448987	0.670236671	0.001054398	0.008204773	0.000431789	0.682154297	0.001054363	0.008203887	1.72E-05	0.011804409	1	0.963860935	1	0.966338775	ENSG00000064703	7	DDX20
Pancreas	T1DM	ENSG00000079459.8	-0.001536529	0.031763821	0.000715538	0.008204698	-0.001523611	0.033221326	0.000715511	0.008203814	-1.29E-05	0.001459671	1	0.427517623	1	0.43087053	ENSG00000064995	12	TAF11
Pancreas	T1DM	ENSG00000079689.9	0.001201371	0.203369713	0.000944466	0.008204748	0.001068324	0.258093619	0.000944659	0.008203868	0.000133047	5.67E-10	1	0.766891956	1	0.788955599	ENSG00000064995	12	TAF11
Pancreas	T1DM	ENSG00000081791.4	-2.65E-05	0.950202489	0.000424894	0.008204775	-3.36E-05	0.936934667	0.00042488	0.00820389	7.08E-06	0.011296863	1	0.997898334	1	0.999388266	ENSG00000065057	3	NTHL1
Pancreas	T1DM	ENSG00000084693.11	-0.000353767	0.655764546	0.00079361	0.008204772	-0.000367722	0.643098834	0.000793581	0.008203886	1.40E-05	0.003149444	1	0.962467202	1	0.960161515	ENSG00000065060	12	UHRF1BP1
Pancreas	T1DM	ENSG00000084774.9	0.001649236	0.084292709	0.000955354	0.008204725	0.001636802	0.08664718	0.000955317	0.008203841	1.24E-05	0.018207069	1	0.589819284	1	0.580557814	ENSG00000065060	12	UHRF1BP1
Pancreas	T1DM	ENSG00000085117.7	-0.001118391	0.148649427	0.000774337	0.00820474	-0.001106204	0.153111989	0.000774313	0.008203856	-1.22E-05	0.018459746	1	0.712186059	1	0.711161998	ENSG00000065060	12	UHRF1BP1
Pancreas	T1DM	ENSG00000085491.11	0.001013868	0.375422313	0.001143852	0.008204762	0.001033721	0.366126393	0.001143808	0.008203876	-1.99E-05	0.002095696	1	0.860999343	1	0.850635855	ENSG00000065060	12	UHRF1BP1
Pancreas	T1DM	ENSG00000085982.9	-0.000648911	0.353446015	0.000699312	0.008204761	-0.000658546	0.346326083	0.000699286	0.008203875	9.64E-06	0.015591862	1	0.854061442	1	0.847091708	ENSG00000065060	12	UHRF1BP1
Pancreas	T1DM	ENSG00000086232.8	0.000701934	0.26754324	0.000633092	0.008204755	0.000693001	0.273664071	0.000633072	0.00820387	8.93E-06	0.03202559	1	0.798582374	1	0.79588826	ENSG00000065060	12	UHRF1BP1
Pancreas	T1DM	ENSG00000087191.8	-0.001465713	0.035977312	0.000698894	0.008204702	-0.001476938	0.034573842	0.000698871	0.008203815	1.12E-05	0.013228217	1	0.443514005	1	0.43733359	ENSG00000065060	12	UHRF1BP1
Pancreas	T1DM	ENSG00000087206.12	0.000424607	0.736582748	0.001262276	0.008204774	0.000440479	0.727112906	0.001262231	0.008203888	-1.59E-05	0.037009391	1	0.975415042	1	0.968082788	ENSG00000065268	6	WDR18
Pancreas	T1DM	ENSG00000088451.6	-0.00037867	0.455087924	0.000506947	0.008204766	-0.000455521	0.370088718	0.00050822	0.008203876	7.69E-05	0.034376052	1	0.899832468	1	0.851044876	ENSG00000065268	6	WDR18
Pancreas	T1DM	ENSG00000089060.7	0.000378668	0.636827613	0.000802025	0.008204772	0.000386585	0.629784195	0.000801991	0.008203886	-7.92E-06	0.040576672	1	0.960748332	1	0.955974304	ENSG00000065457	6	ADAT1
Pancreas	T1DM	ENSG00000089177.13	0.001753765	0.012702629	0.000703755	0.008204671	0.001766354	0.012074454	0.000703735	0.008203784	-1.26E-05	0.011189782	1	0.276443652	1	0.258393321	ENSG00000065665	16	SEC61A2
Pancreas	T1DM	ENSG00000090054.9	-0.000130328	0.805787362	0.000530083	0.008204775	-0.000121788	0.818278115	0.000530067	0.008203889	-8.54E-06	0.020564672	1	0.98201188	1	0.981810269	ENSG00000065665	16	SEC61A2
Pancreas	T1DM	ENSG00000090238.7	-0.000161401	0.728995875	0.000465856	0.008204774	-0.000151417	0.745151443	0.000465843	0.008203888	-9.98E-06	0.002210113	1	0.975415042	1	0.968082788	ENSG00000065665	16	SEC61A2
Pancreas	T1DM	ENSG00000090686.11	-0.000130163	0.788575996	0.000485391	0.008204774	-0.00014952	0.758057145	0.000485403	0.008203888	1.94E-05	0.001523767	1	0.981920629	1	0.968082788	ENSG00000066185	8	ZMYND12
Pancreas	T1DM	ENSG00000092200.8	0.001042317	0.396750105	0.001229959	0.008204763	0.001028551	0.402996653	0.001229909	0.008203878	1.38E-05	0.027292914	1	0.874941428	1	0.877030004	ENSG00000066185	8	ZMYND12
Pancreas	T1DM	ENSG00000093134.9	0.001516205	0.184921318	0.001143653	0.008204746	0.001498476	0.190097269	0.001143619	0.008203861	1.77E-05	0.024544301	1	0.746326771	1	0.756852382	ENSG00000066185	8	ZMYND12
Pancreas	T1DM	ENSG00000094916.9	-0.000400827	0.6343312	0.000842712	0.008204772	-0.000383581	0.648972828	0.000842684	0.008203886	-1.72E-05	0.001462247	1	0.959806249	1	0.961035047	ENSG00000066185	8	ZMYND12
Pancreas	T1DM	ENSG00000095209.7	0.000207337	0.8179642	0.000900821	0.008204775	0.000191113	0.831980961	0.000900787	0.008203889	1.62E-05	0.001775148	1	0.982079052	1	0.984766677	ENSG00000066230	6	SLC9A3
Pancreas	T1DM	ENSG00000099956.13	-0.000430259	0.626189752	0.000883313	0.008204772	-0.00041692	0.636917011	0.000883279	0.008203886	-1.33E-05	0.007962407	1	0.953769445	1	0.955974304	ENSG00000066294	10	CD84
Pancreas	T1DM	ENSG00000099991.12	-0.000179241	0.889366855	0.001288525	0.008204775	-0.000163025	0.89931587	0.001288474	0.00820389	-1.62E-05	0.019452199	1	0.99200447	1	0.994038165	ENSG00000066294	10	CD84
Pancreas	T1DM	ENSG00000100099.16	-0.000250343	0.804257193	0.001010093	0.008204775	-0.000222298	0.825806446	0.001010061	0.008203889	-2.80E-05	3.45E-05	1	0.98201188	1	0.984037376	ENSG00000066294	10	CD84
Pancreas	T1DM	ENSG00000100209.5	0.000519648	0.520087124	0.000807895	0.008204769	0.000508535	0.529034903	0.000807865	0.008203883	1.11E-05	0.016305309	1	0.914783379	1	0.920313283	ENSG00000066294	10	CD84
Pancreas	T1DM	ENSG00000100243.16	0.000239645	0.784468381	0.000876212	0.008204774	0.000250683	0.774795403	0.000876182	0.008203889	-1.10E-05	0.042809469	1	0.981920629	1	0.972640681	ENSG00000066294	10	CD84
Pancreas	T1DM	ENSG00000100258.13	-0.00051296	0.23001115	0.000427349	0.008204751	-0.000518846	0.2246907	0.000427333	0.008203865	5.89E-06	0.013549121	1	0.785512902	1	0.780702981	ENSG00000066379	10	ZNRD1
Pancreas	T1DM	ENSG00000100290.2	0.000490968	0.639127102	0.001047018	0.008204772	0.000502201	0.631464097	0.001046977	0.008203886	-1.12E-05	0.048275164	1	0.96093583	1	0.955974304	ENSG00000066379	10	ZNRD1
Pancreas	T1DM	ENSG00000100299.13	2.90E-05	0.923045773	0.000300001	0.008204775	3.34E-05	0.911230922	0.000299993	0.00820389	-4.47E-06	0.038578721	1	0.997898334	1	0.996366272	ENSG00000066379	10	ZNRD1
Pancreas	T1DM	ENSG00000100461.13	0.000283034	0.604494056	0.000546452	0.008204771	0.000293492	0.591196336	0.000546435	0.008203885	-1.05E-05	0.004167682	1	0.941811128	1	0.937577789	ENSG00000066379	10	ZNRD1
Pancreas	T1DM	ENSG00000100731.11	0.000541241	0.580763707	0.000980031	0.00820477	0.000521842	0.594383427	0.000979994	0.008203885	1.94E-05	0.000665394	1	0.937588291	1	0.937577789	ENSG00000066379	10	ZNRD1
Pancreas	T1DM	ENSG00000100908.9	-1.01E-06	0.998309284	0.000477462	0.008204776	-1.19E-05	0.980143645	0.000477465	0.00820389	1.09E-05	0.0370828	1	0.999322433	1	0.99966149	ENSG00000066379	10	ZNRD1
Pancreas	T1DM	ENSG00000101230.5	0.00085473	0.511357497	0.001301502	0.008204768	0.000878553	0.49964071	0.001301453	0.008203882	-2.38E-05	0.001456798	1	0.914783379	1	0.914417995	ENSG00000066427	17	ATXN3
Pancreas	T1DM	ENSG00000101307.11	0.000226303	0.820109949	0.00099516	0.008204775	0.000246042	0.804717648	0.000995127	0.008203889	-1.97E-05	0.002183227	1	0.982562562	1	0.979350575	ENSG00000066855	11	MTFR1
Pancreas	T1DM	ENSG00000101333.12	0.000289433	0.589223333	0.000536025	0.008204771	0.000904981	0.134316297	0.000604409	0.008203852	-0.000615548	0.027543344	1	0.937588291	1	0.685736609	ENSG00000066926	6	FECH
Pancreas	T1DM	ENSG00000101361.10	-0.000616192	0.529715674	0.00098051	0.008204769	-0.000632142	0.519103609	0.000980478	0.008203883	1.59E-05	0.012889284	1	0.920284891	1	0.915978195	ENSG00000066926	6	FECH
Pancreas	T1DM	ENSG00000101452.10	0.000259482	0.808987651	0.001073427	0.008204775	0.000314462	0.769580563	0.001073529	0.008203889	-5.50E-05	0.002953965	1	0.98201188	1	0.972640681	ENSG00000066933	11	MYO9A
Pancreas	T1DM	ENSG00000101457.8	-0.001335606	0.268768943	0.001207706	0.008204755	-0.001360401	0.259967614	0.001207669	0.008203869	2.48E-05	0.002366221	1	0.798582374	1	0.788955599	ENSG00000066933	11	MYO9A
Pancreas	T1DM	ENSG00000102575.6	-0.001065666	0.238425863	0.000903925	0.008204752	-0.000303428	0.744170269	0.000929803	0.008203888	-0.000762238	0.000472389	1	0.785512902	1	0.968082788	ENSG00000067141	12	NEO1
Pancreas	T1DM	ENSG00000102837.6	-0.00044046	0.563862825	0.000763211	0.00820477	-0.000421068	0.581140364	0.000763191	0.008203885	-1.94E-05	0.000599084	1	0.936396473	1	0.936315417	ENSG00000067167	3	TRAM1
Pancreas	T1DM	ENSG00000103351.8	0.000601874	0.299082193	0.000579614	0.008204757	0.000611452	0.291441258	0.000579594	0.008203871	-9.58E-06	0.008259867	1	0.817067098	1	0.805533316	ENSG00000067365	10	METTL22
Pancreas	T1DM	ENSG00000103479.10	-0.00029614	0.666833282	0.0006879	0.008204772	-0.000284556	0.679114314	0.000687879	0.008203887	-1.16E-05	0.01398634	1	0.963860935	1	0.964582721	ENSG00000067533	5	RRP15
Pancreas	T1DM	ENSG00000103671.5	-0.000394599	0.662061063	0.000902829	0.008204772	-0.000383126	0.671290077	0.000902795	0.008203887	-1.15E-05	0.025538653	1	0.962467202	1	0.961035047	ENSG00000067533	5	RRP15
Pancreas	T1DM	ENSG00000104518.6	-0.000408234	0.212209593	0.000327238	0.008204749	-0.000412285	0.207691587	0.000327226	0.008203863	4.05E-06	0.025485076	1	0.772090651	1	0.769778216	ENSG00000067533	5	RRP15
Pancreas	T1DM	ENSG00000104774.8	-0.000303158	0.683138454	0.000742699	0.008204773	-0.000258012	0.728319874	0.000742777	0.008203888	-4.51E-05	0.000657588	1	0.967740456	1	0.968082788	ENSG00000067596	6	DHX8
Pancreas	T1DM	ENSG00000104823.4	-0.002170213	0.011570811	0.000859497	0.008204668	-0.002151684	0.012299543	0.00085949	0.008203784	-1.85E-05	0.024847952	1	0.260075765	1	0.258895305	ENSG00000067715	9	SYT1
Pancreas	T1DM	ENSG00000104901.2	0.000572447	0.435276555	0.000733724	0.008204765	0.000559483	0.445733859	0.000733702	0.00820388	1.30E-05	0.011833693	1	0.89813429	1	0.896838446	ENSG00000068028	13	RASSF1
Pancreas	T1DM	ENSG00000104953.14	-0.000617121	0.571305568	0.001090069	0.00820477	-0.000633549	0.561089944	0.001090027	0.008203884	1.64E-05	0.005365192	1	0.937588291	1	0.936315417	ENSG00000068028	13	RASSF1
Pancreas	T1DM	ENSG00000105290.7	6.45E-05	0.867438209	0.000386536	0.008204775	7.52E-05	0.845770454	0.000386552	0.008203889	-1.07E-05	0.045498743	1	0.99183698	1	0.986098414	ENSG00000068078	13	FGFR3
Pancreas	T1DM	ENSG00000105355.4	-0.001029209	0.15418469	0.000722299	0.008204741	-0.001336228	0.069745778	0.000736798	0.008203835	0.000307019	0.035029549	1	0.717060869	1	0.538522335	ENSG00000068078	13	FGFR3
Pancreas	T1DM	ENSG00000106299.7	2.45E-05	0.952157927	0.000407634	0.008204776	3.06E-05	0.940132263	0.00040762	0.00820389	-6.16E-06	0.020728218	1	0.997898334	1	0.999388266	ENSG00000068079	3	IFI35
Pancreas	T1DM	ENSG00000106460.14	0.001918235	0.126193516	0.001254331	0.008204736	0.001934908	0.122919237	0.001254285	0.00820385	-1.67E-05	0.022689332	1	0.684395521	1	0.671609788	ENSG00000068654	11	POLR1A
Pancreas	T1DM	ENSG00000106688.7	0.000719123	0.393648214	0.000843031	0.008204763	0.000705312	0.402779535	0.000843001	0.008203878	1.38E-05	0.006512192	1	0.873903579	1	0.877030004	ENSG00000068654	11	POLR1A
Pancreas	T1DM	ENSG00000107099.11	-7.02E-05	0.934008135	0.000848299	0.008204775	-5.90E-05	0.944527428	0.000848268	0.00820389	-1.12E-05	0.023887522	1	0.997898334	1	0.999388266	ENSG00000069275	12	NUCKS1
Pancreas	T1DM	ENSG00000107672.10	0.000775186	0.143244232	0.000529564	0.008204739	0.000782211	0.139640701	0.000529547	0.008203853	-7.03E-06	0.041091121	1	0.706048346	1	0.693219864	ENSG00000069493	10	CLEC2D

Pancreas	T1DM	ENSG00000107954.6	-0.000686894	0.521059655	0.001070406	0.008204769	-0.000698591	0.513970627	0.001070363	0.008203883	1.17E-05	0.03322963	1	0.914876612	1	0.915381179	ENSG00000069869	11	NEDD4
Pancreas	T1DM	ENSG00000108187.11	2.24E-05	0.962916549	0.000481059	0.008204776	3.09E-05	0.948780881	0.000481043	0.00820389	-8.53E-06	0.005855287	1	0.997898334	1	0.999388266	ENSG00000069869	11	NEDD4
Pancreas	T1DM	ENSG00000108264.12	-0.000101247	0.795266989	0.000390197	0.008204774	-9.31E-05	0.811444492	0.000390184	0.008203889	-8.16E-06	0.000976117	1	0.981920629	1	0.981619417	ENSG00000069869	11	NEDD4
Pancreas	T1DM	ENSG00000108509.16	-0.000410558	0.634570345	0.000863779	0.008204772	-0.000421201	0.625801973	0.000863747	0.008203886	1.06E-05	0.034090308	1	0.959806249	1	0.953755638	ENSG00000069966	14	GNB5
Pancreas	T1DM	ENSG00000108846.11	-0.00041426	0.556639169	0.000704715	0.00820477	-0.000439534	0.532842486	0.000704751	0.008203883	2.53E-05	0.013230416	1	0.93270646	1	0.920352808	ENSG00000069966	14	GNB5
Pancreas	T1DM	ENSG00000108924.9	-0.000293214	0.787729514	0.001088963	0.008204774	-0.000277853	0.798597207	0.001088923	0.008203889	-1.54E-05	0.01730699	1	0.981920629	1	0.978923745	ENSG00000069966	14	GNB5
Pancreas	T1DM	ENSG00000109332.15	0.002417565	0.008296655	0.000915826	0.008204658	0.002429157	0.007989422	0.00091579	0.008203772	-1.16E-05	0.016819757	1	0.221935509	1	0.204622746	ENSG00000069966	14	GNB5
Pancreas	T1DM	ENSG00000109794.9	2.43E-05	0.977670847	0.000866786	0.008204776	9.93E-06	0.990859777	0.000866752	0.00820389	1.43E-05	0.00258485	1	0.997898334	1	0.99966149	ENSG00000069966	14	GNB5
Pancreas	T1DM	ENSG00000110042.3	0.000332239	0.727119388	0.000952084	0.008204774	0.000316563	0.739508932	0.000952058	0.008203888	1.57E-05	0.025504112	1	0.975415042	1	0.968082788	ENSG00000069974	11	RAB27A
Pancreas	T1DM	ENSG00000110074.6	0.00110406	0.28504016	0.001032733	0.008204756	0.001091361	0.290599572	0.001032695	0.008203871	1.27E-05	0.03802037	1	0.804377068	1	0.805533316	ENSG00000069998	8	CECR5
Pancreas	T1DM	ENSG00000110455.9	0.000422144	0.406683003	0.000508763	0.008204764	0.000415506	0.414083829	0.000508744	0.008203879	6.64E-06	0.027770893	1	0.88063625	1	0.883839257	ENSG00000069998	8	CECR5
Pancreas	T1DM	ENSG00000110888.13	0.000788115	0.190513909	0.000602046	0.008204747	0.00077799	0.196255431	0.000602022	0.008203862	1.01E-05	0.002043977	1	0.752840936	1	0.762792436	ENSG00000070182	13	SPTB
Pancreas	T1DM	ENSG00000110944.4	0.000345058	0.477016176	0.000485239	0.008204767	0.000354533	0.464991214	0.000485227	0.008203881	-9.47E-06	0.012076623	1	0.911441622	1	0.903250709	ENSG00000070182	13	SPTB
Pancreas	T1DM	ENSG00000111215.7	0.000869393	0.177666192	0.000644961	0.008204745	0.000861357	0.181690406	0.000644935	0.00820386	8.04E-06	0.018609907	1	0.732338331	1	0.739805892	ENSG00000070182	13	SPTB
Pancreas	T1DM	ENSG00000111321.6	-0.000666631	0.505706486	0.001001642	0.008204768	-0.000679234	0.497679454	0.001001604	0.008203882	1.26E-05	0.026801779	1	0.914783379	1	0.914417995	ENSG00000070190	8	DAPP1
Pancreas	T1DM	ENSG00000111364.11	-0.000462427	0.519900555	0.000718613	0.008204769	-0.000471076	0.512105968	0.000718584	0.008203883	8.65E-06	0.017499881	1	0.914783379	1	0.915381179	ENSG00000070190	8	DAPP1
Pancreas	T1DM	ENSG00000111615.8	-8.52E-05	0.901833543	0.000691019	0.008204775	-7.45E-05	0.914185041	0.000690995	0.00820389	-1.08E-05	0.011693526	1	0.99317359	1	0.996869293	ENSG00000070413	14	DGCR2
Pancreas	T1DM	ENSG00000111786.4	-0.000856852	0.385882533	0.000988171	0.008204763	-0.000872302	0.377356214	0.000988132	0.008203877	1.54E-05	0.003776681	1	0.865581925	1	0.858326624	ENSG00000070501	7	POLB
Pancreas	T1DM	ENSG00000111886.9	0.00020495	0.807628394	0.000841728	0.008204775	0.000191844	0.819704523	0.000841699	0.008203889	1.31E-05	0.014004428	1	0.98201188	1	0.981810269	ENSG00000070540	8	WIPI1
Pancreas	T1DM	ENSG00000112031.11	-0.00048126	0.323799239	0.000487756	0.008204759	-0.000475199	0.329913228	0.000487739	0.008203874	-6.06E-06	0.042205625	1	0.832533491	1	0.832236906	ENSG00000070540	8	WIPI1
Pancreas	T1DM	ENSG00000112149.5	3.08E-05	0.959716235	0.00060972	0.008204776	0.001073224	0.089125591	0.000631297	0.008203841	-0.001042428	1.95E-10	1	0.997898334	1	0.585431045	ENSG00000070785	12	EIF2B3
Pancreas	T1DM	ENSG00000112294.8	-0.000684021	0.537131049	0.00110834	0.008204769	-0.000717098	0.51762459	0.001108323	0.008203883	3.31E-05	0.000671713	1	0.924272254	1	0.915978195	ENSG00000070785	12	EIF2B3
Pancreas	T1DM	ENSG00000112343.8	-0.000626238	0.392891204	0.000732968	0.008204763	-0.000598262	0.414385695	0.000732983	0.008203879	-2.80E-05	0.0017201	1	0.873903579	1	0.883839257	ENSG00000071462	7	WBSCR22
Pancreas	T1DM	ENSG00000112667.8	0.001460508	0.01995432	0.000627578	0.008204684	0.001450942	0.020775731	0.000627559	0.0082038	9.57E-06	0.024948685	1	0.346717973	1	0.339822154	ENSG00000071462	7	WBSCR22
Pancreas	T1DM	ENSG00000112874.5	0.000340808	0.757725907	0.00110484	0.008204774	0.000360915	0.74391147	0.001104803	0.008203888	-2.01E-05	0.005014531	1	0.976337245	1	0.968082788	ENSG00000071462	7	WBSCR22
Pancreas	T1DM	ENSG00000112977.11	0.000310031	0.554783971	0.000524936	0.00820477	0.000317564	0.54519401	0.000524918	0.008203884	-7.53E-06	0.025302306	1	0.93270646	1	0.928420568	ENSG00000071575	7	TRIB2
Pancreas	T1DM	ENSG00000113328.14	0.001153519	0.243075991	0.000988165	0.008204753	0.001137831	0.249530419	0.000988136	0.008203868	1.57E-05	0.023466731	1	0.787779958	1	0.788955599	ENSG00000071575	7	TRIB2
Pancreas	T1DM	ENSG00000113593.7	0.001746102	0.098272024	0.001056137	0.00820473	0.001764309	0.094802231	0.001056096	0.008203843	-1.82E-05	0.00206278	1	0.633704355	1	0.605602312	ENSG00000071575	7	TRIB2
Pancreas	T1DM	ENSG00000113734.13	9.57E-05	0.802184984	0.000382072	0.008204775	9.03E-05	0.813189899	0.000382058	0.008203889	5.43E-06	0.021187459	1	0.98201188	1	0.981619417	ENSG00000071967	7	CYBRD1
Pancreas	T1DM	ENSG00000114383.5	-0.000955528	0.412767414	0.001166653	0.008204764	-0.000941918	0.41943545	0.001166604	0.008203879	-1.36E-05	0.018873995	1	0.883403823	1	0.888704815	ENSG00000071967	7	CYBRD1
Pancreas	T1DM	ENSG00000114395.6	-0.000248896	0.438275518	0.000321111	0.008204765	-0.000253361	0.4300849	0.000321098	0.00820388	4.47E-06	0.007335331	1	0.89813429	1	0.890691955	ENSG00000072134	11	EPN2
Pancreas	T1DM	ENSG00000114446.4	0.001080707	0.359951899	0.00118051	0.008204761	0.001099768	0.351522208	0.001180463	0.008203875	-1.91E-05	0.001886732	1	0.854209198	1	0.847508521	ENSG00000072364	8	AFF4
Pancreas	T1DM	ENSG00000114771.9	-0.000247663	0.783954475	0.00090332	0.008204774	-0.000235803	0.794055066	0.00090329	0.008203889	-1.19E-05	0.039497143	1	0.981920629	1	0.978923745	ENSG00000072736	14	NFATC3
Pancreas	T1DM	ENSG00000115648.9	-0.000652302	0.43630758	0.000837956	0.008204765	-0.000669323	0.424419212	0.000837933	0.008203879	1.70E-05	0.005507918	1	0.89813429	1	0.889250668	ENSG00000072858	6	SIDT1
Pancreas	T1DM	ENSG00000115718.13	0.001291097	0.187886178	0.000980436	0.008204746	0.002063326	0.042046131	0.001014883	0.00820382	-0.000772229	0.00324806	1	0.750693896	1	0.471816773	ENSG00000072864	8	NDE1
Pancreas	T1DM	ENSG00000116157.5	-0.000518802	0.087853856	0.000303956	0.008204727	-0.000512037	0.09205993	0.000303946	0.008203842	-6.76E-06	0.000455637	1	0.595273618	1	0.598506077	ENSG00000072864	8	NDE1
Pancreas	T1DM	ENSG00000116459.6	0.000251617	0.711901226	0.000681328	0.008204773	0.000244674	0.719499838	0.0006813	0.008203888	6.94E-06	0.048754124	1	0.975415042	1	0.968082788	ENSG00000072864	8	NDE1
Pancreas	T1DM	ENSG00000116771.5	7.78E-05	0.890378559	0.000564128	0.008204775	8.46E-05	0.880723325	0.000564107	0.00820389	-6.89E-06	0.036458887	1	0.99200447	1	0.993128823	ENSG00000072958	4	AP1M1
Pancreas	T1DM	ENSG00000117122.9	0.000214867	0.659534917	0.000487717	0.008204772	0.000203266	0.676836651	0.0004877	0.008203887	1.16E-05	8.22E-05	1	0.962467202	1	0.96294544	ENSG00000073008	10	PVR
Pancreas	T1DM	ENSG00000117505.8	0.000567751	0.654498281	0.001268655	0.008204772	0.000546712	0.666501259	0.001268607	0.008203887	2.10E-05	0.003158774	1	0.962467202	1	0.961035047	ENSG00000073146	11	MOV10L1
Pancreas	T1DM	ENSG00000117748.5	-0.000489232	0.509452928	0.000741609	0.008204768	-0.000500864	0.49942125	0.00074158	0.008203882	1.16E-05	0.004308947	1	0.914783379	1	0.914417995	ENSG00000073331	13	ALPK1
Pancreas	T1DM	ENSG00000118690.8	0.000302579	0.743554177	0.000924893	0.008204774	0.000288698	0.754923885	0.000924857	0.008203888	1.39E-05	0.007803943	1	0.975415042	1	0.968082788	ENSG00000073350	9	LLGL2
Pancreas	T1DM	ENSG00000118997.9	0.000198596	0.720149952	0.000554336	0.008204773	0.000206748	0.709165629	0.000554318	0.008203888	-8.15E-06	0.023436003	1	0.975415042	1	0.968082788	ENSG00000073417	10	PDE8A
Pancreas	T1DM	ENSG00000119013.4	-0.000886371	0.494125494	0.001296313	0.008204768	-0.00086793	0.503139341	0.001296271	0.008203882	-1.84E-05	0.030019971	1	0.914693561	1	0.914417995	ENSG00000073578	12	SDHA
Pancreas	T1DM	ENSG00000119203.9	-0.000755074	0.231989404	0.000631728	0.008204752	-0.000762821	0.227217968	0.000631705	0.008203865	7.75E-06	0.041496248	1	0.785512902	1	0.780702981	ENSG00000073584	14	SMARCE1
Pancreas	T1DM	ENSG00000119227.3	-0.000706438	0.241155839	0.000602709	0.008204752	-0.000715599	0.23509371	0.00060269	0.008203866	9.16E-06	0.021526584	1	0.786897324	1	0.780702981	ENSG00000073670	9	ADAM11
Pancreas	T1DM	ENSG00000119715.10	0.000787222	0.38263922	0.000901698	0.008204763	0.000775063	0.390017596	0.00090167	0.008203878	1.22E-05	0.047526292	1	0.864072637	1	0.867930487	ENSG00000073849	10	ST6GAL1
Pancreas	T1DM	ENSG00000120457.7	-0.000283075	0.395292088	0.000333006	0.008204763	0.000809872	0.164926047	0.000583189	0.008203858	-0.001092947	0.022443223	1	0.873903579	1	0.726605951	ENSG00000073849	10	ST6GAL1
Pancreas	T1DM	ENSG00000120539.10	0.000101554	0.747409786	0.000315328	0.008204774	0.000105403	0.738170077	0.000315317	0.008203888	-3.85E-06	0.04162637	1	0.975415042	1	0.968082788	ENSG00000073849	10	ST6GAL1
Pancreas	T1DM	ENSG00000120675.4	2.17E-05	0.985749482	0.001217373	0.008204776	9.10E-06	0.994036843	0.001217323	0.00820389	1.26E-05	0.038679977	1	0.997898334	1	0.999770584	ENSG00000074054	13	CLASP1
Pancreas	T1DM	ENSG00000120725.8	2.76E-05	0.971031999	0.000761063	0.008204776	1.22E-05	0.987262157	0.000761036	0.00820389	1.55E-05	0.001010872	1	0.997898334	1	0.99966149	ENSG00000074706	9	IPCEF1
Pancreas	T1DM	ENSG00000120756.8	-0.000684516	0.485206211	0.000980747	0.008204767	-0.000662593	0.499280119	0.000980713	0.008203882	-2.19E-05	0.000321745	1	0.914693561	1	0.914417995	ENSG00000074706	9	IPCEF1
Pancreas	T1DM	ENSG00000120800.4	2.61E-05	0.981443232	0.001120712	0.008204776	4.04E-06	0.997121781	0.001120677	0.00820389	2.20E-05	0.003305501	1	0.997898334	1	0.999770584	ENSG00000074706	9	IPCEF1
Pancreas	T1DM	ENSG00000120860.6	0.000350257	0.719103506	0.000973864	0.008204773	0.000366201	0.706886317	0.000973832	0.008203888	-1.59E-05	0.011776901	1	0.975415042	1	0.968082788	ENSG00000075151	15	EIF4G3
Pancreas	T1DM	ENSG00000121454.4	-0.001006324	0.292035992	0.000955069	0.008204757	-0.000994405	0.297772862	0.000955036	0.008203872	-1.19E-05	0.045818474	1	0.810235282	1	0.810652485	ENSG00000075151	15	EIF4G3
Pancreas	T1DM	ENSG00000121898.8	-0.000582108	0.489861774	0.000842988	0.008204768	-0.000569133	0.499575994	0.000842963	0.008203882	-1.30E-05	0.026653187	1	0.914693561	1	0.914417995	ENSG00000075461	5	CACNG4
Pancreas	T1DM	ENSG00000122176.10	3.72E-05	0.974729086	0.001174306	0.008204776	5.12E-05	0.965216377	0.001174262	0.00820389	-1.40E-05	0.035757709	1	0.997898334	1	0.999388266	ENSG00000075461	5	CACNG4
Pancreas	T1DM	ENSG00000122477.8	-0.000437908	0.331588398	0.000451024	0.00820476	-0.000430974	0.339288772	0.000451011	0.008203875	-6.93E-06	0.029735527	1	0.83462449	1	0.839770819	ENSG00000075461	5	CACNG4
Pancreas	T1DM	ENSG00000122481.12	-0.001015791	0.317868148	0.001016961	0.008204759	-0.000994605	0.32805234	0.001016931	0.008203874	-2.12E-05	0.00291871	1	0.829307456	1	0.830795913	ENSG00000075643	5	MOCOS
Pancreas	T1DM	ENSG00000122483.13	-0.000141934	0.871988052	0.000880845	0.008204775	-0.00012675	0.885578936	0.000880813	0.00820389	-1.52E-05	0.002990139	1	0.99200447	1	0.993128823	ENSG00000075643	5	MOCOS
Pancreas	T1DM	ENSG00000122783.12	-0.000710844	0.50825913	0.001074511	0.008204768	-0.00068731	0.522386766	0.001074473	0.008203883	-2.35E-05	0.000389448	1	0.914783379	1	0.917005275	ENSG00000075673	7	ATP12A
Pancreas	T1DM	ENSG00000122863.5	0.000620067	0.465297849	0.00084923	0.008204767	0.000604496	0.476562242	0.000849198	0.008203881	1.56E-05	0.001091923	1	0.908712989	1	0.911454131	ENSG00000075826	12	SEC31B
Pancreas	T1DM	ENSG00000123179.9	3.29E-06	0.996896842	0.000844799	0.008204776	-1.37E-05	0.987095813	0.000844767	0.00820389	1.69E-05	0.000502356	1	0.998452063	1	0.99966149	ENSG00000075826	12	SEC31B
Pancreas	T1DM	ENSG00000123191.9	-9.33E-05	0.859348072	0.00052671	0.008204775	-8.44E-05	0.872665253	0.000526691	0.00820389	-8.92E-06	0.00380453	1	0.990317162	1	0.993128823	ENSG00000075975	11	MKRN2
Pancreas	T1DM	ENSG00000123453.12	0.000429069	0.657816713	0.000968706	0.008204772	0.00041438	0.668811971	0.000968677	0.008203887	1.47E-05	0.028742242	1	0.962467202	1	0.961035047	ENSG00000076242	10	MLH1
Pancreas	T1DM	ENSG00000123643.8	0.000277292	0.572921297	0.000491866	0.00820477	0.000296059	0.547237113	0.000491869	0.008203884	-1.88E-05	0.000477088	1	0.937588291	1	0.929364427	ENSG00000076242	10	MLH1
Pancreas	T1DM	ENSG00000123815.7	-0.000168072	0.858928668	0.000945615	0.008204775	-0.000149169	0.874653525	0.000945599	0.00820389	-1.89E-05	0.01987623	1	0.990317162	1	0.993128823	ENSG00000076242	10	MLH1
Pancreas	T1DM	ENSG00000124104.14	-0.001764949	0.132018264	0.001171794	0.008204737	-0.001791024	0.126390095	0.001171756	0.008203851	2.61E-05	0.000645639	1	0.690950163	1	0.673807988	ENSG00000076258	5	FMO4
Pancreas	T1DM	ENSG00000124116.14	-0.000263724	0.590431913	0.000490002	0.008204771	-0.000274645	0.575128008	0.000489987	0.008203885	1.09E-05	0.000890531	1	0.937680367	1	0.936315417	ENSG00000076344	11	RGS11
Pancreas	T1DM	ENSG00000124549.10	-0.000899671	0.005618459	0.000324877	0.008204646	-0.000939852	0.003843222	0.000325122	0.008203749	4.02E-05	0.002114768	1	0.179978441	1	0.129860443	ENSG00000076344	11	RGS11
Pancreas	T1DM	ENSG00000124570.13	3.57E-05	0.959043238	0.000694757	0.008204776	5.17E-05	0.940673647	0.000694747	0.00820389	-1.60E-05	0.009687191	1	0.997898334	1	0.999388266	ENSG00000076344	11	RGS11
Pancreas	T1DM	ENSG00000124613.4	0.000539347	0.173776254	0.000396529	0.008204744	0.000472014	0.234081206	0.000396679	0.008203866	6.73E-05	7.91E-09	1	0.729268002	1	0.780702981	ENSG00000076554	11	TPD52
Pancreas	T1DM	ENSG00000125257.9	-0.001537856	0.220873746	0.001256203	0.00820475	-0.001505135	0.230848308	0.001256186	0.008203866	-3.27E-05	0.003842161	1	0.780835021	1	0.780702981	ENSG00000076554	11	TPD52
Pancreas	T1DM	ENSG00000125611.11	-5.19E-05	0.943649503	0.000734806	0.008204775	-4.00E-05	0.956614744	0.000734782	0.00820389	-1.20E-05	0.012187931	1	0.997898334	1	0.999388266	ENSG00000076662	5	ICAM3
Pancreas	T1DM	ENSG00000125735.6	-9.95E-05	0.847268785	0.000516462	0.008204775	-8.78E-05	0.864977574	0.000516447	0.008203889	-1.17E-05	0.001012161	1	0.989913489	1	0.990577333	ENSG00000076662	5	ICAM3
Pancreas	T1DM	ENSG00000125826.15	0.00148391	0.083504357	0.0008574	0.008204725	0.001470063	0.086415878	0.00085737	0.00820384	1.38E-05	0.008718723	1	0.589819284	1	0.580557814	ENSG00000076662	5	ICAM3
Pancreas	T1DM	ENSG00000126457.16	-0.001504976	0.137413457	0.001013115	0.008204738	-0.001491034	0.141080043	0.00101308	0.008203854	-1.39E-05	0.027783784	1	0.69738343	1	0.693219864	ENSG00000076662	5	ICAM3
Pancreas	T1DM	ENSG00000126562.12	-0.000332199	0.649284994	0.000730499	0.008204772	-0.000314697	0.666609506	0.000730484	0.008203887	-1.75E-05	0.003414596	1	0.962467202	1	0.961035047	ENSG00000076685	14	NT5C2
Pancreas	T1DM	ENSG00000127418.10	-0.000202305	0.758125883	0.000656957	0.008204774	-0.000191673	0.770464977	0.000656937	0.008203889	-1.06E-05	0.016941434	1	0.976362722	1	0.972640681	ENSG00000076685	14	NT5C2
Pancreas	T1DM	ENSG00000128604.14	0.000464378	0.467497101	0.000639149	0.008204767	0.000453094	0.478368463	0.000639123	0.008203882	1.13E-05	0.001061706	1	0.908712989	1	0.911454131	ENSG00000076685	14	NT5C2
Pancreas	T1DM	ENSG00000128699.9	0.000202242	0.645686481	0.000439883	0.008204772	0.000194261	0.65875862	0.000439875	0.008203887	7.98E-06	0.032873626	1	0.961788216	1	0.961035047	ENSG00000076685	14	NT5C2
Pancreas	T1DM	ENSG00000128965.7	-0.000404394	0.743822652	0.001237453	0.008204774	-0.000387691	0.754046006	0.001237406	0.008203888	-1.67E-05	0.017545243	1	0.975415042	1	0.968082788	ENSG00000077080	5	ACTL6B
Pancreas	T1DM	ENSG00000129204.12	0.000581286	0.380069575	0.000662231	0.008204763	0.000597283	0.367084995	0.000662215	0.008203876	-1.60E-05	0.001477302	1	0.862207304	1	0.850635855	ENSG00000077147	10	TM9SF3
Pancreas	T1DM	ENSG00000129355.6	0.000590576	0.481039923	0.000838135	0.008204767	0.00061565	0.462602216	0.00083811	0.008203881	-2.51E-05	1.65E-05	1	0.913691214	1	0.901757398	ENSG00000077238	9	IL4R
Pancreas	T1DM	ENSG00000129933.16	-0.001854648	0.014209628	0.000756404	0.008204674	-0.001864486	0.013700416	0.000756374	0.008203788	9.84E-06	0.013940132	1	0.2919226	1	0.277028884	ENSG00000077420	11	APBB1IP
Pancreas	T1DM	ENSG00000130147.11	-0.000252676	0.699630708	0.000654908	0.008204773	-0.000264786	0.685976223	0.000654889	0.008203887	1.21E-05	0.010159595	1	0.974470668	1	0.966338775	ENSG00000077585	9	GPR137B
Pancreas	T1DM	ENSG00000130299.12	-0.000534424	0.230617073	0.00044581	0.008204751	-0.000557158	0.211506337	0.000445927	0.008203864	2.27E-05	0.043199695	1	0.785512902	1	0.769778216	ENSG00000077585	9	GPR137B
Pancreas	T1DM	ENSG00000130332.10	0.000388518	0.423325991	0.000485244	0.008204765	0.000396336	0.414042308	0.000485229	0.008203879	-7.82E-06	0.017599201	1	0.889410424	1	0.883839257	ENSG00000077585	9	GPR137B
Pancreas	T1DM	ENSG00000130363.7	0.001652188	0.181521923	0.001236588	0.008204746	0.001626113	0.188494278	0.001236542	0.008203861	2.61E-05	0.000241839	1	0.737576421	1	0.753977114	ENSG00000077585	9	GPR137B
Pancreas	T1DM	ENSG00000130414.7	-0.000458013	0.284836181	0.000428242	0.008204756	-0.000452582	0.290567788	0.000428226	0.008203871	-5.43E-06	0.031441908	1	0.804377068	1	0.805533316	ENSG00000077616	6	NAALAD2
Pancreas	T1DM	ENSG00000131401.7	-0.000547041	0.137312684	0.000368161	0.008204738	-0.000541975	0.140976297	0.000368148	0.008203853	-5.07E-06	0.022425979	1	0.69738343	1	0.693219864	ENSG00000077616	6	NAALAD2
Pancreas	T1DM	ENSG00000132274.11	0.000257372	0.796553225	0.000998298	0.008204774	0.000272725	0.784702469	0.000998274	0.008203889	-1.54E-05	0.046289227	1	0.981920629	1	0.976789113	ENSG00000078018	15	MAP2
Pancreas	T1DM	ENSG00000132305.16	-0.000800948	0.304082205	0.000779345	0.008204758	-0.0008133	0.296671121	0.000779322	0.008203872	1.24E-05	0.021667642	1	0.82025536	1	0.809618957	ENSG00000078070	7	MCCC1
Pancreas	T1DM	ENSG00000132330.12	0.000665742	0.469833389	0.000921122	0.008204767	0.000648384	0.481477474	0.000921094	0.008203882	1.74E-05	0.005313078	1	0.908712989	1	0.913030722	ENSG00000078070	7	MCCC1
Pancreas	T1DM	ENSG00000132507.13	-0.002656127	0.004651242	0.000938475	0.008204641	-0.002673956	0.004381181	0.000938447	0.008203753	1.78E-05	0.006578463	1	0.151194791	1	0.138899658	ENSG00000078269	9	SYNJ2
Pancreas	T1DM	ENSG00000132881.7	-7.41E-05	0.935168321	0.000911021	0.008204775	-6.26E-05	0.945226393	0.00091099	0.00820389	-1.15E-05	0.041364769	1	0.997898334	1	0.999388266	ENSG00000078295	11	ADCY2
Pancreas	T1DM	ENSG00000132906.13	0.000537225	0.375990118	0.000606821	0.008204762	0.000530457	0.382014746	0.000606798	0.008203877	6.77E-06	0.044973072	1	0.860999343	1	0.860591675	ENSG00000078618	15	NRD1
Pancreas	T1DM	ENSG00000133028.6	0.002642411	0.038488242	0.00127676	0.008204703	0.002624793	0.039793175	0.001276715	0.008203819	1.76E-05	0.026170232	1	0.459710727	1	0.458621792	ENSG00000079150	13	FKBP7
Pancreas	T1DM	ENSG00000133316.11	9.90E-05	0.85550284	0.000543386	0.008204775	8.85E-05	0.870594175	0.000543366	0.00820389	1.04E-05	0.000566081	1	0.990317162	1	0.992315064	ENSG00000079150	13	FKBP7
Pancreas	T1DM	ENSG00000134056.7	-0.000144796	0.831176476	0.000679179	0.008204775	-0.00013726	0.83983486	0.000679152	0.008203889	-7.54E-06	0.033142885	1	0.984098328	1	0.98524254	ENSG00000079150	13	FKBP7
Pancreas	T1DM	ENSG00000134201.6	-0.000914895	0.358676047	0.000996735	0.008204761	-0.00090067	0.36617861	0.000996696	0.008203876	-1.42E-05	0.008775806	1	0.854209198	1	0.850635855	ENSG00000079150	13	FKBP7
Pancreas	T1DM	ENSG00000134222.12	0.000173029	0.694594293	0.000440695	0.008204773	0.000166348	0.705818867	0.000440682	0.008203888	6.68E-06	0.028125872	1	0.972276902	1	0.968082788	ENSG00000079150	13	FKBP7
Pancreas	T1DM	ENSG00000134460.11	-0.00105241	0.410447484	0.001278582	0.008204764	-0.001033335	0.418966357	0.001278538	0.008203879	-1.91E-05	0.017473264	1	0.88063625	1	0.888443934	ENSG00000079257	3	LXN
Pancreas	T1DM	ENSG00000134463.10	-0.000346753	0.658215865	0.000783839	0.008204772	-0.000356972	0.648799138	0.000783811	0.008203886	1.02E-05	0.030434049	1	0.962467202	1	0.961035047	ENSG00000079257	3	LXN
Pancreas	T1DM	ENSG00000134698.10	-0.000189691	0.857695787	0.001057904	0.008204775	-0.000204493	0.84671824	0.001057863	0.008203889	1.48E-05	0.011702719	1	0.990317162	1	0.986098414	ENSG00000079257	3	LXN
Pancreas	T1DM	ENSG00000134852.10	-0.000355798	0.511138224	0.000541494	0.008204768	-0.000342427	0.527128364	0.000541475	0.008203883	-1.34E-05	9.33E-05	1	0.914783379	1	0.920313283	ENSG00000079257	3	LXN
Pancreas	T1DM	ENSG00000134905.12	-0.000749232	0.257149133	0.000661191	0.008204754	-0.000756429	0.252588631	0.000661165	0.008203868	7.20E-06	0.045412353	1	0.79720158	1	0.788955599	ENSG00000079257	3	LXN
Pancreas	T1DM	ENSG00000135269.13	0.000868505	0.436495516	0.001116151	0.008204765	0.000685602	0.54035752	0.001119765	0.008203884	0.000182903	0.043606063	1	0.89813429	1	0.928420568	ENSG00000079263	14	SP140
Pancreas	T1DM	ENSG00000136104.14	-0.000122184	0.910567467	0.001087791	0.008204775	-0.000137517	0.899397851	0.001087757	0.00820389	1.53E-05	0.034698803	1	0.995698473	1	0.994038165	ENSG00000079263	14	SP140
Pancreas	T1DM	ENSG00000136144.7	0.000380564	0.232048938	0.000318437	0.008204752	0.000374626	0.239399323	0.000318427	0.008203867	5.94E-06	0.003832229	1	0.785512902	1	0.780702981	ENSG00000079263	14	SP140
Pancreas	T1DM	ENSG00000136449.9	0.000157112	0.750042153	0.000493157	0.008204774	0.000149764	0.761361999	0.000493143	0.008203888	7.35E-06	0.036706758	1	0.975415042	1	0.968355942	ENSG00000079335	13	CDC14A
Pancreas	T1DM	ENSG00000136490.4	0.001507355	0.032821152	0.000706263	0.008204699	0.001520877	0.031281635	0.000706242	0.008203812	-1.35E-05	0.006950098	1	0.434431116	1	0.417001664	ENSG00000079335	13	CDC14A
Pancreas	T1DM	ENSG00000136710.5	0.000189971	0.849446058	0.001000768	0.008204775	0.000177023	0.859591988	0.001000733	0.008203889	1.29E-05	0.033560969	1	0.989913489	1	0.990577333	ENSG00000079335	13	CDC14A
Pancreas	T1DM	ENSG00000136717.10	0.00069815	0.512875512	0.001066909	0.008204768	0.000673495	0.52786791	0.001066899	0.008203883	2.47E-05	0.014336587	1	0.914783379	1	0.920313283	ENSG00000079459	8	FDFT1
Pancreas	T1DM	ENSG00000136888.6	0.000991492	0.385938981	0.001143581	0.008204763	0.001007229	0.378427546	0.001143541	0.008203877	-1.57E-05	0.028256572	1	0.865581925	1	0.858482278	ENSG00000079689	9	SCGN
Pancreas	T1DM	ENSG00000136932.9	0.000125828	0.898778743	0.00098918	0.008204775	0.000112721	0.909270813	0.000989144	0.00820389	1.31E-05	0.02501854	1	0.99281063	1	0.996366272	ENSG00000079689	9	SCGN
Pancreas	T1DM	ENSG00000137070.13	0.000664581	0.106568907	0.000411808	0.008204732	0.000654562	0.11195248	0.000411808	0.008203847	1.00E-05	0.019106463	1	0.661051879	1	0.666335944	ENSG00000079689	9	SCGN
Pancreas	T1DM	ENSG00000137288.5	0.003197771	8.88E-06	0.000719771	0.008204443	0.002643162	0.000293663	0.000729983	0.008203669	0.000554609	5.35E-06	0.022811571	0.000543133	0.754127073	0.016394067	ENSG00000079689	9	SCGN
Pancreas	T1DM	ENSG00000137310.7	0.00079295	0.10340664	0.000486902	0.008204731	0.000743768	0.126838879	0.000487177	0.008203851	4.92E-05	0.004052413	1	0.654059731	1	0.673807988	ENSG00000079950	9	STX7
Pancreas	T1DM	ENSG00000137392.5	0.000939376	0.361242675	0.001028889	0.008204762	0.000954637	0.353476656	0.001028851	0.008203875	-1.53E-05	0.01128258	1	0.854209198	1	0.847990418	ENSG00000079950	9	STX7
Pancreas	T1DM	ENSG00000137478.10	0.000727922	0.216223252	0.000588631	0.00820475	0.000747939	0.20384305	0.000588613	0.008203863	-2.00E-05	1.01E-06	1	0.77457828	1	0.76794547	ENSG00000080345	13	RIF1
Pancreas	T1DM	ENSG00000137509.6	0.000629403	0.23809428	0.000533499	0.008204752	0.000621229	0.244233062	0.000533485	0.008203867	8.17E-06	0.041236596	1	0.785512902	1	0.78207287	ENSG00000080815	14	PSEN1
Pancreas	T1DM	ENSG00000137672.8	0.000440018	0.732007083	0.001284886	0.008204774	0.00046394	0.718034933	0.001284844	0.008203888	-2.39E-05	0.004693324	1	0.975415042	1	0.968082788	ENSG00000081665	9	ZNF506
Pancreas	T1DM	ENSG00000137673.4	0.000193062	0.826963659	0.000883184	0.008204775	-0.00033173	0.717535271	0.000917001	0.008203888	0.000524792	0.033546645	1	0.984098328	1	0.968082788	ENSG00000081791	4	KIAA0141
Pancreas	T1DM	ENSG00000137709.5	0.000317371	0.741545578	0.000962293	0.008204774	0.000357701	0.710130918	0.000962388	0.008203888	-4.03E-05	0.016703516	1	0.975415042	1	0.968082788	ENSG00000081913	9	PHLPP1
Pancreas	T1DM	ENSG00000137752.18	0.001063149	0.363590485	0.001170166	0.008204762	0.001022088	0.382401784	0.001170132	0.008203877	4.11E-05	7.11E-07	1	0.854831234	1	0.860591675	ENSG00000082516	8	GEMIN5
Pancreas	T1DM	ENSG00000137831.10	0.001146333	0.352874629	0.001233905	0.008204761	0.001167713	0.343948716	0.001233856	0.008203875	-2.14E-05	0.00130294	1	0.854061442	1	0.846034773	ENSG00000082516	8	GEMIN5
Pancreas	T1DM	ENSG00000137843.7	-0.000224187	0.637985445	0.000476469	0.008204772	-0.000217559	0.647940829	0.000476449	0.008203886	-6.63E-06	0.004739601	1	0.960781248	1	0.961035047	ENSG00000082516	8	GEMIN5
Pancreas	T1DM	ENSG00000137857.13	0.000129832	0.871328984	0.000801579	0.008204775	0.000112047	0.888828209	0.000801553	0.00820389	1.78E-05	0.000611504	1	0.99200447	1	0.993128823	ENSG00000082516	8	GEMIN5
Pancreas	T1DM	ENSG00000137876.5	0.000289981	0.738044526	0.000867054	0.008204774	0.000300708	0.728720539	0.000867022	0.008203888	-1.07E-05	0.036637615	1	0.975415042	1	0.968082788	ENSG00000082684	10	SEMA5B
Pancreas	T1DM	ENSG00000138119.12	0.000262747	0.578214206	0.00047257	0.00820477	0.000252532	0.593069474	0.000472559	0.008203885	1.02E-05	0.005023471	1	0.937588291	1	0.937577789	ENSG00000083097	10	DOPEY1
Pancreas	T1DM	ENSG00000138286.10	-0.00065959	0.201100006	0.000515939	0.008204748	-0.000666152	0.19663886	0.000515921	0.008203862	6.56E-06	0.041270833	1	0.7650738	1	0.762792436	ENSG00000083097	10	DOPEY1
Pancreas	T1DM	ENSG00000138346.10	-0.000241396	0.652927041	0.000536794	0.008204772	-0.000247289	0.645017611	0.000536774	0.008203886	5.89E-06	0.046169391	1	0.962467202	1	0.961035047	ENSG00000083097	10	DOPEY1
Pancreas	T1DM	ENSG00000138613.9	0.000646852	0.60983979	0.001267584	0.008204771	0.000679452	0.591934376	0.001267551	0.008203885	-3.26E-05	0.000580792	1	0.943530179	1	0.937577789	ENSG00000083097	10	DOPEY1
Pancreas	T1DM	ENSG00000138686.5	-9.19E-06	0.994258331	0.001277017	0.008204776	-6.34E-05	0.960391043	0.001277188	0.00820389	5.42E-05	0.028590631	1	0.998452063	1	0.999388266	ENSG00000083099	6	LYRM2
Pancreas	T1DM	ENSG00000138760.4	-0.000679227	0.245625802	0.000585016	0.008204753	-0.000688903	0.23894801	0.000584995	0.008203867	9.68E-06	0.007039465	1	0.788479116	1	0.780702981	ENSG00000083099	6	LYRM2
Pancreas	T1DM	ENSG00000138777.15	-0.000234016	0.816802659	0.001010171	0.008204775	-0.000213108	0.832910133	0.001010132	0.008203889	-2.09E-05	0.000210485	1	0.98201188	1	0.984766677	ENSG00000083099	6	LYRM2

Pancreas	T1DM	ENSG00000138834.8	0.000844118	0.454952367	0.001129731	0.008204766	0.000875226	0.438506312	0.001129734	0.00820388	-3.11E-05	0.009706188	1	0.899832468	1	0.896263433	ENSG00000083290	15	ULK2
Pancreas	T1DM	ENSG00000139192.7	-0.000259991	0.307636281	0.000254844	0.008204758	-0.000256222	0.31468764	0.000254837	0.008203873	-3.77E-06	0.036744247	1	0.824645062	1	0.826271144	ENSG00000083720	8	OXCT1
Pancreas	T1DM	ENSG00000139651.9	-0.000486087	0.678755564	0.001173664	0.008204773	-0.000465797	0.691452774	0.001173633	0.008203887	-2.03E-05	0.020006521	1	0.966210802	1	0.966923215	ENSG00000083828	11	ZNF586
Pancreas	T1DM	ENSG00000139998.10	0.000221191	0.792415945	0.000840473	0.008204774	0.000208139	0.804402604	0.000840444	0.008203889	1.31E-05	0.012489161	1	0.981920629	1	0.979350575	ENSG00000083828	11	ZNF586
Pancreas	T1DM	ENSG00000140009.14	0.000380714	0.651803184	0.000843672	0.008204772	0.000363644	0.66644021	0.000843644	0.008203887	1.71E-05	0.001811708	1	0.962467202	1	0.961035047	ENSG00000083845	4	RPS5
Pancreas	T1DM	ENSG00000140264.15	-0.000244958	0.7859343	0.000901926	0.008204774	-0.000233012	0.796131263	0.000901897	0.008203889	-1.19E-05	0.042685041	1	0.981920629	1	0.978923745	ENSG00000083937	4	CHMP2B
Pancreas	T1DM	ENSG00000140470.9	3.28E-05	0.975419844	0.001063216	0.008204776	5.39E-05	0.959560501	0.001063186	0.00820389	-2.11E-05	0.005487693	1	0.997898334	1	0.999388266	ENSG00000084072	12	PPIE
Pancreas	T1DM	ENSG00000140575.8	0.000260877	0.669618667	0.000611423	0.008204772	0.000249442	0.683286193	0.000611403	0.008203887	1.14E-05	0.00328313	1	0.963860935	1	0.966338775	ENSG00000084072	12	PPIE
Pancreas	T1DM	ENSG00000140839.7	0.000420798	0.675756583	0.001006066	0.008204773	0.00043355	0.666503155	0.001006028	0.008203887	-1.28E-05	0.029236868	1	0.963860935	1	0.961035047	ENSG00000084092	6	NOA1
Pancreas	T1DM	ENSG00000140955.6	-0.001158488	0.359040063	0.001263075	0.008204761	-0.001182026	0.349341584	0.001263026	0.008203875	2.35E-05	0.000749911	1	0.854209198	1	0.847508521	ENSG00000084092	6	NOA1
Pancreas	T1DM	ENSG00000141127.10	-3.81E-05	0.966245054	0.000899461	0.008204776	-1.88E-05	0.983298747	0.00089944	0.00820389	-1.92E-05	0.006517581	1	0.997898334	1	0.99966149	ENSG00000084092	6	NOA1
Pancreas	T1DM	ENSG00000141198.9	0.000112732	0.893408749	0.000841335	0.008204775	0.000123823	0.882989833	0.000841303	0.00820389	-1.11E-05	0.016723735	1	0.99200447	1	0.993128823	ENSG00000084207	11	GSTP1
Pancreas	T1DM	ENSG00000141560.10	-0.000319848	0.283685347	0.000298344	0.008204756	-0.000311859	0.295869959	0.000298335	0.008203872	-7.99E-06	7.86E-05	1	0.804088269	1	0.809618957	ENSG00000084207	11	GSTP1
Pancreas	T1DM	ENSG00000141562.13	-0.00021513	0.755594163	0.000691131	0.008204774	-0.00020573	0.765946913	0.000691108	0.008203888	-9.40E-06	0.031697947	1	0.975415042	1	0.971814067	ENSG00000084207	11	GSTP1
Pancreas	T1DM	ENSG00000141569.6	0.000164903	0.813110788	0.000697514	0.008204775	0.000177324	0.799318201	0.000697497	0.008203889	-1.24E-05	0.021281045	1	0.98201188	1	0.978923745	ENSG00000084693	11	AGBL5
Pancreas	T1DM	ENSG00000141664.5	0.000580643	0.596859712	0.001097786	0.008204771	0.000597293	0.586369575	0.001097754	0.008203885	-1.66E-05	0.029378614	1	0.938170574	1	0.936945142	ENSG00000084774	9	CAD
Pancreas	T1DM	ENSG00000142102.11	-0.000191122	0.849803406	0.001009254	0.008204775	-0.000155401	0.877640567	0.001009356	0.00820389	-3.57E-05	0.044462721	1	0.989913489	1	0.993128823	ENSG00000085063	10	CD59
Pancreas	T1DM	ENSG00000142178.7	-0.000714813	0.248643879	0.000619609	0.008204753	-0.000724721	0.242127131	0.000619585	0.008203867	9.91E-06	0.003175046	1	0.789267592	1	0.780845092	ENSG00000085117	7	CD82
Pancreas	T1DM	ENSG00000142188.12	0.000229469	0.444100538	0.000299847	0.008204766	0.000224916	0.453177522	0.000299836	0.008203881	4.55E-06	0.014126067	1	0.898699907	1	0.899350755	ENSG00000085117	7	CD82
Pancreas	T1DM	ENSG00000142197.8	0.0006362	0.596170359	0.001200568	0.008204771	0.000620887	0.605030467	0.001200524	0.008203885	1.53E-05	0.029058385	1	0.938170574	1	0.939938439	ENSG00000085117	7	CD82
Pancreas	T1DM	ENSG00000142207.5	-0.000130068	0.697546843	0.00033468	0.008204773	-0.000134962	0.686747767	0.000334668	0.008203887	4.89E-06	0.018010521	1	0.974062149	1	0.966338775	ENSG00000085117	7	CD82
Pancreas	T1DM	ENSG00000142273.6	0.000211638	0.807484963	0.000868537	0.008204775	0.000229027	0.792008652	0.000868503	0.008203889	-1.74E-05	0.000283038	1	0.98201188	1	0.978923745	ENSG00000085117	7	CD82
Pancreas	T1DM	ENSG00000142937.7	-0.000572624	0.584540797	0.001047295	0.008204771	-0.000529189	0.613377385	0.001047363	0.008203886	-4.34E-05	0.007066927	1	0.937588291	1	0.946218353	ENSG00000085117	7	CD82
Pancreas	T1DM	ENSG00000143155.8	0.000154728	0.809427442	0.000641586	0.008204775	0.000165314	0.796660334	0.000641569	0.008203889	-1.06E-05	0.024977669	1	0.98201188	1	0.978923745	ENSG00000085117	7	CD82
Pancreas	T1DM	ENSG00000143622.6	0.000517063	0.308184157	0.000507401	0.008204758	0.000525392	0.300440446	0.000507385	0.008203872	-8.33E-06	0.011268513	1	0.825252257	1	0.812137964	ENSG00000085117	7	CD82
Pancreas	T1DM	ENSG00000144118.9	-0.002546889	0.048833978	0.001292809	0.00820471	-0.002574902	0.046395761	0.001292764	0.008203823	2.80E-05	0.000572885	1	0.507019677	1	0.490305822	ENSG00000085117	7	CD82
Pancreas	T1DM	ENSG00000144362.7	0.000227931	0.693255435	0.000577858	0.008204773	0.000219622	0.703890808	0.000577839	0.008203888	8.31E-06	0.028136221	1	0.971848816	1	0.968082788	ENSG00000085117	7	CD82
Pancreas	T1DM	ENSG00000144559.6	0.000684064	0.4395478	0.000884996	0.008204766	0.000700369	0.428706199	0.000884965	0.008203879	-1.63E-05	0.003132904	1	0.89813429	1	0.890691955	ENSG00000085117	7	CD82
Pancreas	T1DM	ENSG00000144834.8	0.000151516	0.870104515	0.000926558	0.008204775	0.000163816	0.859659597	0.000926521	0.008203889	-1.23E-05	0.01388643	1	0.99200447	1	0.990577333	ENSG00000085365	13	SCAMP1
Pancreas	T1DM	ENSG00000145358.2	0.000341518	0.768302505	0.001159276	0.008204774	0.000358003	0.757452879	0.001159233	0.008203888	-1.65E-05	0.014619908	1	0.977622335	1	0.968082788	ENSG00000085365	13	SCAMP1
Pancreas	T1DM	ENSG00000146425.6	0.000268515	0.826252941	0.001223247	0.008204775	0.000248767	0.838844776	0.001223213	0.008203889	1.97E-05	0.025750463	1	0.984098328	1	0.98524254	ENSG00000085365	13	SCAMP1
Pancreas	T1DM	ENSG00000146457.10	-0.000608417	0.207980663	0.000483201	0.008204749	-0.000602086	0.212733713	0.000483182	0.008203864	-6.33E-06	0.018037498	1	0.770698909	1	0.769778216	ENSG00000085491	11	SLC25A24
Pancreas	T1DM	ENSG00000146535.9	-0.000133101	0.894978465	0.001008281	0.008204775	-0.000111904	0.911626391	0.001008263	0.00820389	-2.12E-05	0.01335978	1	0.99200447	1	0.996366272	ENSG00000085514	11	PILRA
Pancreas	T1DM	ENSG00000146574.11	-0.00032772	0.507641835	0.000494662	0.008204768	-0.000335504	0.497598835	0.000494645	0.008203882	7.78E-06	0.01391215	1	0.914783379	1	0.914417995	ENSG00000085644	9	ZNF213
Pancreas	T1DM	ENSG00000146733.9	-0.000475274	0.395487266	0.000559337	0.008204763	-0.000483449	0.387391774	0.000559316	0.008203877	8.18E-06	0.009062018	1	0.873903579	1	0.867022428	ENSG00000085644	9	ZNF213
Pancreas	T1DM	ENSG00000146833.11	-0.000264147	0.245862036	0.000227623	0.008204753	-0.00026008	0.253195105	0.000227617	0.008203868	-4.07E-06	0.017795751	1	0.788479116	1	0.788955599	ENSG00000085733	11	CTTN
Pancreas	T1DM	ENSG00000147955.12	-0.000812699	0.22959434	0.000676457	0.008204751	-0.000838373	0.215203895	0.000676443	0.008203864	2.57E-05	4.45E-06	1	0.785512902	1	0.77077211	ENSG00000085741	8	WNT11
Pancreas	T1DM	ENSG00000148218.11	0.001287456	0.117092668	0.000821554	0.008204734	0.001275105	0.120636555	0.00082153	0.008203849	1.24E-05	0.030321699	1	0.67493056	1	0.669849249	ENSG00000085982	9	USP40
Pancreas	T1DM	ENSG00000149260.10	0.000189094	0.691919513	0.000477206	0.008204773	0.000177902	0.709290132	0.000477193	0.008203888	1.12E-05	0.001549042	1	0.971486774	1	0.968082788	ENSG00000086015	16	MAST2
Pancreas	T1DM	ENSG00000149328.10	0.000799552	0.336130467	0.000831273	0.00820476	0.000783435	0.34595488	0.000831261	0.008203875	1.61E-05	0.029448828	1	0.838041785	1	0.847091708	ENSG00000086015	16	MAST2
Pancreas	T1DM	ENSG00000149577.11	-0.001270828	0.135737347	0.000851843	0.008204738	-0.001239307	0.145700252	0.000851822	0.008203854	-3.15E-05	1.07E-06	1	0.694369535	1	0.700862587	ENSG00000086205	12	FOLH1
Pancreas	T1DM	ENSG00000149591.12	-0.001533325	0.091915565	0.000909779	0.008204728	-0.001502554	0.098615629	0.000909753	0.008203844	-3.08E-05	2.17E-06	1	0.607172104	1	0.623755999	ENSG00000086232	8	EIF2AK1
Pancreas	T1DM	ENSG00000151176.3	0.000286707	0.635138566	0.000604221	0.008204772	0.000292597	0.628190694	0.000604196	0.008203886	-5.89E-06	0.042959912	1	0.95999755	1	0.954552486	ENSG00000086300	11	SNX10
Pancreas	T1DM	ENSG00000151276.19	-0.000455439	0.55876046	0.000778949	0.00820477	-0.00044023	0.571956367	0.00077893	0.008203885	-1.52E-05	0.011253118	1	0.93270646	1	0.936315417	ENSG00000086300	11	SNX10
Pancreas	T1DM	ENSG00000152078.5	-0.001010341	0.312447924	0.001000239	0.008204758	-0.000993762	0.320440483	0.001000209	0.008203873	-1.66E-05	0.017422637	1	0.828095461	1	0.830335577	ENSG00000086475	10	SEPHS1
Pancreas	T1DM	ENSG00000152270.4	-0.00110066	0.180908058	0.000822637	0.008204745	-0.001113307	0.175930573	0.000822604	0.008203859	1.26E-05	0.004516832	1	0.736247058	1	0.729595175	ENSG00000086475	10	SEPHS1
Pancreas	T1DM	ENSG00000152348.11	1.28E-05	0.963435335	0.000278144	0.008204776	1.82E-05	0.94770575	0.000278137	0.00820389	-5.49E-06	0.012856295	1	0.997898334	1	0.999388266	ENSG00000086544	2	ITPKC
Pancreas	T1DM	ENSG00000152439.8	0.000624005	0.166686146	0.000451218	0.008204743	0.000614239	0.173406981	0.000451202	0.008203859	9.77E-06	0.000290078	1	0.729268002	1	0.728578649	ENSG00000086598	6	TMED2
Pancreas	T1DM	ENSG00000152492.9	6.21E-06	0.989748066	0.000483671	0.008204776	-1.10E-06	0.998190543	0.000483651	0.00820389	7.31E-06	0.003364686	1	0.997898334	1	0.999770584	ENSG00000086598	6	TMED2
Pancreas	T1DM	ENSG00000152689.13	-0.000133667	0.832899087	0.000633537	0.008204775	-0.000144618	0.819432767	0.000633524	0.008203889	1.10E-05	0.03795823	1	0.984098328	1	0.981810269	ENSG00000086619	9	ERO1LB
Pancreas	T1DM	ENSG00000152749.7	0.001058792	0.262027171	0.000943989	0.008204754	0.001118938	0.236104431	0.000944426	0.008203866	-6.01E-05	0.047399824	1	0.79720158	1	0.780702981	ENSG00000087008	11	ACOX3
Pancreas	T1DM	ENSG00000152990.9	0.000905107	0.402162926	0.001080382	0.008204764	0.000887026	0.411612664	0.001080343	0.008203879	1.81E-05	0.005256356	1	0.87893991	1	0.88342201	ENSG00000087191	8	PSMC5
Pancreas	T1DM	ENSG00000153822.9	-0.000203905	0.704675541	0.000537985	0.008204773	-0.000212129	0.693346836	0.000537964	0.008203887	8.22E-06	0.006819661	1	0.974645207	1	0.968082788	ENSG00000087191	8	PSMC5
Pancreas	T1DM	ENSG00000154265.11	0.00111653	0.27252271	0.001017551	0.008204755	0.00108985	0.284129865	0.001017516	0.008203871	2.67E-05	3.33E-05	1	0.798582374	1	0.799151365	ENSG00000087206	12	UIMC1
Pancreas	T1DM	ENSG00000154710.11	-0.000663969	0.174016315	0.000488423	0.008204744	-0.000657388	0.178305054	0.000488403	0.008203859	-6.58E-06	0.00850491	1	0.729268002	1	0.731367165	ENSG00000087263	12	OGFOD1
Pancreas	T1DM	ENSG00000154719.9	0.001408802	0.016870034	0.000589571	0.008204679	0.001419845	0.016026311	0.000589557	0.008203792	-1.10E-05	0.01569707	1	0.328198842	1	0.301309092	ENSG00000087263	12	OGFOD1
Pancreas	T1DM	ENSG00000154768.4	0.000115978	0.886244361	0.000810706	0.008204775	0.000106357	0.895620498	0.000810674	0.00820389	9.62E-06	0.031857545	1	0.99200447	1	0.993128823	ENSG00000087266	11	SH3BP2
Pancreas	T1DM	ENSG00000154864.7	0.001155225	0.290503988	0.00109291	0.008204757	0.001169641	0.284507916	0.001092868	0.008203871	-1.44E-05	0.02062253	1	0.809076913	1	0.799151365	ENSG00000087301	4	TXNDC16
Pancreas	T1DM	ENSG00000155542.7	-0.000734573	0.125059281	0.000478898	0.008204736	-0.00074678	0.118895554	0.00047888	0.008203849	1.22E-05	1.04E-05	1	0.680843282	1	0.666556371	ENSG00000087301	4	TXNDC16
Pancreas	T1DM	ENSG00000156042.13	0.000356509	0.604952365	0.000689182	0.008204771	0.000371777	0.589568601	0.000689163	0.008203885	-1.53E-05	0.002223719	1	0.941811128	1	0.937577789	ENSG00000087301	4	TXNDC16
Pancreas	T1DM	ENSG00000156219.12	-0.000483441	0.071251678	0.000268001	0.008204721	-0.000488102	0.068557118	0.000267992	0.008203834	4.66E-06	0.003538467	1	0.557848504	1	0.536516198	ENSG00000087301	4	TXNDC16
Pancreas	T1DM	ENSG00000156413.9	-0.000723266	0.270442045	0.000656294	0.008204755	-0.000716323	0.275047698	0.000656266	0.00820387	-6.94E-06	0.029170566	1	0.798582374	1	0.795892573	ENSG00000087365	10	SF3B2
Pancreas	T1DM	ENSG00000156711.12	-0.000606125	0.239309457	0.000515099	0.008204752	-0.000589158	0.252707205	0.000515089	0.008203868	-1.70E-05	5.83E-05	1	0.785512902	1	0.788955599	ENSG00000087365	10	SF3B2
Pancreas	T1DM	ENSG00000157214.9	-0.000255933	0.827329744	0.001173316	0.008204775	-0.00028226	0.809884941	0.001173275	0.008203889	2.63E-05	0.000279881	1	0.984098328	1	0.981245796	ENSG00000087365	10	SF3B2
Pancreas	T1DM	ENSG00000158158.7	0.000561516	0.315177973	0.000559046	0.008204759	0.000549538	0.325602775	0.000559036	0.008203874	1.20E-05	0.010947983	1	0.829307456	1	0.830335577	ENSG00000087365	10	SF3B2
Pancreas	T1DM	ENSG00000158793.9	0.000451488	0.503748544	0.00067527	0.008204768	0.000334062	0.621060782	0.000675763	0.008203886	0.000117427	1.11E-05	1	0.914783379	1	0.950611338	ENSG00000087448	5	KLHL42
Pancreas	T1DM	ENSG00000159055.3	0.000627802	0.257804987	0.000554794	0.008204754	0.00063785	0.250252604	0.000554779	0.008203868	-1.00E-05	0.013918958	1	0.79720158	1	0.788955599	ENSG00000087448	5	KLHL42
Pancreas	T1DM	ENSG00000159128.10	0.000224123	0.729049985	0.000647025	0.008204774	0.000211835	0.743359778	0.000647006	0.008203888	1.23E-05	0.006674424	1	0.975415042	1	0.968082788	ENSG00000087448	5	KLHL42
Pancreas	T1DM	ENSG00000159173.14	0.000592436	0.295996602	0.000566893	0.008204757	0.000602363	0.287963816	0.000566876	0.008203871	-9.93E-06	0.013611829	1	0.81296179	1	0.801351316	ENSG00000087495	12	PHACTR3
Pancreas	T1DM	ENSG00000160072.15	-0.000451482	0.678698709	0.001089906	0.008204773	-0.000435459	0.689485781	0.001089866	0.008203887	-1.60E-05	0.011113436	1	0.966210802	1	0.966685034	ENSG00000087884	10	AAMDC
Pancreas	T1DM	ENSG00000160200.13	0.000433878	0.277321354	0.000399388	0.008204756	0.000441039	0.269452293	0.000399374	0.008203869	-7.16E-06	0.004137334	1	0.798582374	1	0.795306713	ENSG00000088053	7	GP6
Pancreas	T1DM	ENSG00000160695.10	-0.002006953	0.002087299	0.000652129	0.008204616	-0.002026855	0.001883754	0.000652143	0.008203727	1.99E-05	0.013225722	1	0.083752862	1	0.078023894	ENSG00000088205	8	DDX18
Pancreas	T1DM	ENSG00000160766.10	-6.33E-06	0.990277623	0.000519667	0.008204776	5.45E-06	0.991638436	0.000519652	0.00820389	-1.18E-05	0.001585304	1	0.997898334	1	0.99966149	ENSG00000088298	8	EDEM2
Pancreas	T1DM	ENSG00000160828.13	-0.000324876	0.681028528	0.000790338	0.008204773	-0.000316066	0.689210269	0.000790308	0.008203887	-8.81E-06	0.046773609	1	0.967301581	1	0.966685034	ENSG00000088298	8	EDEM2
Pancreas	T1DM	ENSG00000160932.6	-0.000308732	0.725886516	0.000880574	0.008204773	-0.000296182	0.736597993	0.000880546	0.008203888	-1.25E-05	0.030518822	1	0.975415042	1	0.968082788	ENSG00000088298	8	EDEM2
Pancreas	T1DM	ENSG00000161021.7	0.000567922	0.609053924	0.001110471	0.008204771	0.000587461	0.596777225	0.001110426	0.008203885	-1.95E-05	0.00062791	1	0.943530179	1	0.939046515	ENSG00000088325	11	TPX2
Pancreas	T1DM	ENSG00000161618.5	0.000686446	0.262130713	0.000612149	0.008204754	0.000675654	0.269691003	0.00061213	0.008203869	1.08E-05	0.009058337	1	0.79720158	1	0.795306713	ENSG00000088325	11	TPX2
Pancreas	T1DM	ENSG00000161888.7	-0.001928532	0.090710892	0.001140021	0.008204727	-0.001913481	0.093247168	0.001139984	0.008203843	-1.51E-05	0.044638421	1	0.603485936	1	0.60170043	ENSG00000088325	11	TPX2
Pancreas	T1DM	ENSG00000161896.6	7.74E-05	0.868392087	0.000466888	0.008204775	0.000174795	0.708506581	0.000467537	0.008203888	-9.74E-05	0.00010356	1	0.99183698	1	0.968082788	ENSG00000088325	11	TPX2
Pancreas	T1DM	ENSG00000161904.7	-0.00094246	0.122357382	0.000610023	0.008204735	-0.001128906	0.064617972	0.000610914	0.008203832	0.000186446	2.85E-08	1	0.680843282	1	0.536001791	ENSG00000088325	11	TPX2
Pancreas	T1DM	ENSG00000161970.8	-0.00151384	0.204788307	0.001193853	0.008204748	-0.001530855	0.19973074	0.001193816	0.008203862	1.70E-05	0.035868839	1	0.769258416	1	0.764823318	ENSG00000088340	11	FER1L4
Pancreas	T1DM	ENSG00000162066.10	0.001340425	0.172342305	0.000982199	0.008204744	0.00135201	0.168647359	0.000982162	0.008203858	-1.16E-05	0.035069561	1	0.729268002	1	0.726605951	ENSG00000088451	6	TGDS
Pancreas	T1DM	ENSG00000162300.7	0.000601536	0.587803167	0.001109798	0.008204771	0.000615305	0.579270618	0.001109756	0.008203885	-1.38E-05	0.027853268	1	0.937588291	1	0.936315417	ENSG00000088451	6	TGDS
Pancreas	T1DM	ENSG00000162341.11	0.000846231	0.384659941	0.000973414	0.008204763	0.000862979	0.375305344	0.000973379	0.008203877	-1.67E-05	0.004160125	1	0.865581925	1	0.855936166	ENSG00000088451	6	TGDS
Pancreas	T1DM	ENSG00000162734.8	1.44E-06	0.998965386	0.001109845	0.008204776	-5.78E-05	0.9584421	0.001109953	0.00820389	5.93E-05	0.002194308	1	0.999322433	1	0.999388266	ENSG00000088538	12	DOCK3
Pancreas	T1DM	ENSG00000162817.6	-0.000111539	0.740801815	0.000337188	0.008204774	-0.000106482	0.752149959	0.000337176	0.008203888	-5.06E-06	0.009439015	1	0.975415042	1	0.968082788	ENSG00000088538	12	DOCK3
Pancreas	T1DM	ENSG00000162836.7	-0.000634061	0.466244697	0.000870242	0.008204767	-0.00064727	0.456994725	0.000870215	0.008203881	1.32E-05	0.023551967	1	0.908712989	1	0.899776609	ENSG00000088833	13	NSFL1C
Pancreas	T1DM	ENSG00000162877.8	-0.000195477	0.795180983	0.000753025	0.008204774	-0.000176969	0.814193675	0.000752998	0.008203889	-1.85E-05	6.46E-05	1	0.981920629	1	0.981619417	ENSG00000088833	13	NSFL1C
Pancreas	T1DM	ENSG00000163161.8	0.00163715	0.194061357	0.001260646	0.008204747	0.001721084	0.172325425	0.001261078	0.008203859	-8.39E-05	0.018212792	1	0.757378621	1	0.728578649	ENSG00000088833	13	NSFL1C
Pancreas	T1DM	ENSG00000163281.7	0.000912249	0.009407721	0.000351286	0.008204662	0.000918052	0.008962488	0.000351274	0.008203775	-5.80E-06	0.006837679	1	0.232298348	1	0.208629512	ENSG00000088876	7	ZNF343
Pancreas	T1DM	ENSG00000163389.6	-0.000412995	0.249529387	0.00035866	0.008204753	-0.000419313	0.242341721	0.000358646	0.008203867	6.32E-06	0.0015197	1	0.789394255	1	0.780845092	ENSG00000088876	7	ZNF343
Pancreas	T1DM	ENSG00000163395.12	-0.000642799	0.547822722	0.001069502	0.008204769	-0.000623373	0.559972751	0.001069466	0.008203884	-1.94E-05	0.004767004	1	0.930855143	1	0.936315417	ENSG00000088888	13	MAVS
Pancreas	T1DM	ENSG00000163486.8	0.002632351	0.004265555	0.000921098	0.008204638	0.002653552	0.003964987	0.000921069	0.00820375	-2.12E-05	0.000575145	1	0.142259026	1	0.132234879	ENSG00000089048	10	ESF1
Pancreas	T1DM	ENSG00000163492.9	-0.000582537	0.49383333	0.000851382	0.008204768	-0.000569035	0.503888726	0.000851359	0.008203882	-1.35E-05	0.030430452	1	0.914693561	1	0.914417995	ENSG00000089050	10	RBBP9
Pancreas	T1DM	ENSG00000163545.7	9.78E-05	0.914516698	0.000910794	0.008204775	7.63E-05	0.933208433	0.000910763	0.00820389	2.14E-05	0.000169795	1	0.995870671	1	0.999388266	ENSG00000089060	7	SLC8B1
Pancreas	T1DM	ENSG00000163576.13	-6.15E-05	0.852155439	0.000330002	0.008204775	-6.72E-05	0.838630339	0.000329991	0.008203889	5.70E-06	0.005129694	1	0.989913489	1	0.98524254	ENSG00000089063	10	TMEM230
Pancreas	T1DM	ENSG00000163596.12	-2.94E-05	0.930068504	0.000334859	0.008204775	-2.51E-05	0.94033212	0.000334847	0.00820389	-4.32E-06	0.027457839	1	0.997898334	1	0.999388266	ENSG00000089063	10	TMEM230
Pancreas	T1DM	ENSG00000163618.13	0.000658787	0.405194308	0.000791449	0.008204764	0.000524684	0.508840855	0.0007942	0.008203883	0.000134104	0.043891191	1	0.88063625	1	0.914417995	ENSG00000089177	13	KIF16B
Pancreas	T1DM	ENSG00000163788.9	0.000611827	0.630653935	0.0012725	0.008204772	0.000627533	0.621895268	0.001272456	0.008203886	-1.57E-05	0.047483836	1	0.957728744	1	0.950611338	ENSG00000089335	16	ZNF302
Pancreas	T1DM	ENSG00000163913.7	0.000447766	0.459966216	0.000605988	0.008204766	0.000455174	0.452557105	0.000605962	0.00820388	-7.41E-06	0.012154003	1	0.903743873	1	0.899052624	ENSG00000089335	16	ZNF302
Pancreas	T1DM	ENSG00000163945.11	0.000496484	0.377215773	0.000562245	0.008204762	0.000484612	0.388720549	0.000562234	0.008203877	1.19E-05	0.009835411	1	0.860999343	1	0.867275734	ENSG00000089558	4	KCNH4
Pancreas	T1DM	ENSG00000163947.7	0.000820901	0.492460024	0.001195948	0.008204768	0.00083546	0.484801458	0.001195904	0.008203882	-1.46E-05	0.040444243	1	0.914693561	1	0.914417995	ENSG00000089693	6	MLF2
Pancreas	T1DM	ENSG00000164002.7	0.00221566	0.050996993	0.001135357	0.008204711	0.002256071	0.046915178	0.001135376	0.008203824	-4.04E-05	0.002815994	1	0.511511249	1	0.491747656	ENSG00000089916	13	GPATCH2L
Pancreas	T1DM	ENSG00000164081.8	0.000278422	0.777107285	0.000983506	0.008204774	0.000305463	0.756107257	0.000983472	0.008203888	-2.70E-05	1.12E-05	1	0.979738629	1	0.968082788	ENSG00000090054	9	SPTLC1
Pancreas	T1DM	ENSG00000164111.10	-0.000410166	0.474811031	0.000573921	0.008204767	-0.000496752	0.387931764	0.000575361	0.008203877	8.66E-05	0.035243232	1	0.911384054	1	0.867022428	ENSG00000090054	9	SPTLC1
Pancreas	T1DM	ENSG00000164171.6	0.000322685	0.687195499	0.000801375	0.008204773	0.00033525	0.675685307	0.000801348	0.008203887	-1.26E-05	0.013052696	1	0.968560944	1	0.962373748	ENSG00000090054	9	SPTLC1
Pancreas	T1DM	ENSG00000164181.9	-0.000332561	0.644907297	0.000721627	0.008204772	-0.000342602	0.634944702	0.000721602	0.008203886	1.00E-05	0.025363362	1	0.961788216	1	0.955974304	ENSG00000090054	9	SPTLC1
Pancreas	T1DM	ENSG00000164307.8	0.000383579	0.287260481	0.000360455	0.008204756	0.000388976	0.280514496	0.000360442	0.00820387	-5.40E-06	0.010995407	1	0.809076913	1	0.797009688	ENSG00000090054	9	SPTLC1
Pancreas	T1DM	ENSG00000164620.4	0.00059405	0.48292956	0.000846708	0.008204767	0.00058425	0.490162159	0.000846676	0.008203882	9.80E-06	0.038989193	1	0.914693561	1	0.914417995	ENSG00000090054	9	SPTLC1
Pancreas	T1DM	ENSG00000164733.16	0.001025173	0.182530763	0.000769069	0.008204746	0.001100974	0.152513499	0.000769529	0.008203856	-7.58E-05	0.00635907	1	0.739746982	1	0.711161998	ENSG00000090054	9	SPTLC1
Pancreas	T1DM	ENSG00000164941.9	0.000764531	0.13612757	0.00051298	0.008204738	0.000757028	0.139999674	0.000512963	0.008203853	7.50E-06	0.020475641	1	0.694981311	1	0.693219864	ENSG00000090054	9	SPTLC1
Pancreas	T1DM	ENSG00000165055.11	0.000612594	0.15851381	0.000434436	0.008204742	0.000605825	0.163150014	0.000434421	0.008203857	6.77E-06	0.011258676	1	0.720466306	1	0.726116528	ENSG00000090238	7	YPEL3
Pancreas	T1DM	ENSG00000165171.6	3.59E-05	0.85448368	0.000195868	0.008204775	2.44E-05	0.900891362	0.000195898	0.00820389	1.15E-05	0.003781724	1	0.990317162	1	0.99419382	ENSG00000090238	7	YPEL3
Pancreas	T1DM	ENSG00000165730.10	0.000532002	0.117600025	0.000339953	0.008204734	0.000536306	0.114647643	0.00033994	0.008203848	-4.30E-06	0.036267371	1	0.67493056	1	0.666335944	ENSG00000090238	7	YPEL3
Pancreas	T1DM	ENSG00000165934.8	0.000504526	0.162584862	0.000361296	0.008204743	0.000512766	0.155817231	0.000361285	0.008203856	-8.24E-06	0.000750543	1	0.727383146	1	0.713429173	ENSG00000090238	7	YPEL3
Pancreas	T1DM	ENSG00000165985.8	0.00216611	0.060101595	0.001152154	0.008204716	0.00217992	0.05847622	0.001152108	0.00820383	-1.38E-05	0.021304385	1	0.527104393	1	0.510771882	ENSG00000090238	7	YPEL3
Pancreas	T1DM	ENSG00000166037.6	-0.000109556	0.862551844	0.000632808	0.008204775	-0.000117823	0.8522892	0.000632782	0.008203889	8.27E-06	0.010235023	1	0.990623048	1	0.989215428	ENSG00000090238	7	YPEL3
Pancreas	T1DM	ENSG00000166228.4	-0.000918942	0.171278678	0.000671688	0.008204744	-0.000906402	0.177181809	0.000671665	0.008203859	-1.25E-05	0.002837131	1	0.729268002	1	0.730341709	ENSG00000090263	11	MRPS33
Pancreas	T1DM	ENSG00000166268.6	-0.000482156	0.140429794	0.000327064	0.008204739	-0.000486329	0.13701259	0.000327052	0.008203853	4.17E-06	0.028108415	1	0.703643077	1	0.692461116	ENSG00000090263	11	MRPS33
Pancreas	T1DM	ENSG00000166321.9	0.000953102	0.145050189	0.000654045	0.00820474	0.000962018	0.141312254	0.000654023	0.008203854	-8.92E-06	0.027535434	1	0.70950264	1	0.693219864	ENSG00000090316	11	MAEA
Pancreas	T1DM	ENSG00000166402.4	-0.000626464	0.546135343	0.001037944	0.008204769	-0.000605688	0.559514396	0.001037912	0.008203884	-2.08E-05	0.003158168	1	0.930023581	1	0.936315417	ENSG00000090316	11	MAEA
Pancreas	T1DM	ENSG00000166435.11	-0.000387943	0.329667898	0.000397979	0.00820476	-0.000392664	0.323799548	0.000397964	0.008203874	4.72E-06	0.045007647	1	0.83462449	1	0.830335577	ENSG00000090316	11	MAEA
Pancreas	T1DM	ENSG00000166532.11	-0.000370843	0.663230346	0.000851617	0.008204772	-0.000359105	0.673251037	0.000851584	0.008203887	-1.17E-05	0.011059851	1	0.962467202	1	0.961035047	ENSG00000090316	11	MAEA
Pancreas	T1DM	ENSG00000166669.9	-0.001963796	0.096211863	0.001180516	0.008204729	-0.001978994	0.093653029	0.001180475	0.008203843	1.52E-05	0.037228724	1	0.625289471	1	0.60170043	ENSG00000090432	5	MUL1
Pancreas	T1DM	ENSG00000166902.4	0.000243846	0.681858419	0.000594851	0.008204773	0.000253973	0.669403861	0.000594832	0.008203887	-1.01E-05	0.010224441	1	0.967410176	1	0.961035047	ENSG00000090615	8	GOLGA3
Pancreas	T1DM	ENSG00000167083.2	-0.001752897	0.088930859	0.001030469	0.008204727	-0.001936972	0.060998625	0.001033873	0.008203831	0.000184075	0.029425106	1	0.5974078	1	0.519575945	ENSG00000090621	9	PABPC4
Pancreas	T1DM	ENSG00000167257.6	0.001807925	0.092536626	0.001074758	0.008204728	0.001825703	0.089364864	0.001074726	0.008203841	-1.78E-05	0.017761895	1	0.60775973	1	0.585431045	ENSG00000090621	9	PABPC4
Pancreas	T1DM	ENSG00000167434.5	0.000973964	0.343719963	0.001028645	0.00820476	0.000957635	0.35185692	0.001028615	0.008203875	1.63E-05	0.023416314	1	0.85026773	1	0.847508521	ENSG00000090621	9	PABPC4
Pancreas	T1DM	ENSG00000167670.11	0.000201608	0.738828455	0.000604695	0.008204774	0.000212636	0.725097684	0.000604674	0.008203888	-1.10E-05	0.003997827	1	0.975415042	1	0.968082788	ENSG00000090686	11	USP48
Pancreas	T1DM	ENSG00000167840.9	0.00043785	0.320151138	0.000440427	0.008204759	0.000447889	0.3091686	0.000440415	0.008203873	-1.00E-05	0.001579728	1	0.829307456	1	0.82064652	ENSG00000090686	11	USP48
Pancreas	T1DM	ENSG00000167895.10	-1.04E-05	0.987931589	0.00068613	0.008204776	-1.91E-05	0.977763416	0.000686102	0.00820389	8.75E-06	0.014512794	1	0.997898334	1	0.99966149	ENSG00000090686	11	USP48
Pancreas	T1DM	ENSG00000168026.12	-0.00026359	0.645471614	0.000572945	0.008204772	-0.000272578	0.634241031	0.000572925	0.008203886	8.99E-06	0.01082818	1	0.961788216	1	0.955974304	ENSG00000090686	11	USP48
Pancreas	T1DM	ENSG00000168028.9	4.15E-05	0.890947859	0.000302387	0.008204775	3.34E-05	0.912151318	0.000302377	0.00820389	8.10E-06	1.99E-05	1	0.99200447	1	0.996366272	ENSG00000091542	8	ALKBH5
Pancreas	T1DM	ENSG00000168405.10	0.000743951	0.271888403	0.000677104	0.008204755	0.000770941	0.254942899	0.000677199	0.008203868	-2.70E-05	0.04285227	1	0.798582374	1	0.788955599	ENSG00000091640	3	SPAG7
Pancreas	T1DM	ENSG00000168612.4	-0.001478863	0.045855096	0.000740643	0.008204708	-0.001493327	0.043765882	0.000740616	0.008203822	1.45E-05	0.001003939	1	0.494772636	1	0.47422272	ENSG00000091640	3	SPAG7
Pancreas	T1DM	ENSG00000168653.6	-0.001385006	0.239472026	0.001177417	0.008204752	-0.001421115	0.227437988	0.001177408	0.008203865	3.61E-05	0.001369074	1	0.785512902	1	0.780702981	ENSG00000091640	3	SPAG7
Pancreas	T1DM	ENSG00000168778.7	-0.000293792	0.357756882	0.000319461	0.008204761	-0.000287767	0.367681386	0.000319448	0.008203876	-6.02E-06	0.000705482	1	0.854209198	1	0.850635855	ENSG00000091640	3	SPAG7

Pancreas	T1DM	ENSG00000169129.10	0.00088117	0.278520788	0.00081315	0.008204756	0.000891076	0.273134211	0.000813178	0.00820387	-9.91E-06	0.029203669	1	0.798679956	1	0.79588826	ENSG00000092094	6	OSGEP
Pancreas	T1DM	ENSG00000169203.12	-0.001679691	0.030776231	0.000777649	0.008204697	-0.001670807	0.031664846	0.000777618	0.008203812	-8.88E-06	0.030082419	1	0.424384765	1	0.417001664	ENSG00000092108	16	SCFD1
Pancreas	T1DM	ENSG00000169231.9	-0.000166521	0.793470027	0.000636042	0.008204774	-0.000183881	0.772499162	0.000636031	0.008203886	1.74E-05	0.001345382	1	0.981920629	1	0.972640681	ENSG00000092108	16	SCFD1
Pancreas	T1DM	ENSG00000169248.8	0.000528292	0.049914739	0.000269441	0.008204711	0.000533166	0.047832092	0.000269431	0.008203824	-4.87E-06	0.001928895	1	0.507019677	1	0.498304494	ENSG00000092200	8	RPGRIP1
Pancreas	T1DM	ENSG00000169330.4	-0.001722801	0.14908391	0.001194086	0.008204741	-0.001739661	0.145131044	0.001194044	0.00820385	1.69E-05	0.019365588	1	0.71293758	1	0.700862587	ENSG00000092295	7	TGM1
Pancreas	T1DM	ENSG00000169967.12	-0.000416631	0.67385491	0.000989944	0.008204773	-0.000488698	0.621633627	0.000990196	0.008203886	7.21E-05	0.003392611	1	0.963860935	1	0.950611338	ENSG00000092295	7	TGM1
Pancreas	T1DM	ENSG00000169972.7	9.76E-06	0.993007118	0.001113674	0.008204776	-5.25E-06	0.99623902	0.001113631	0.00820389	1.50E-05	0.014767842	1	0.998452063	1	0.999770584	ENSG00000092295	7	TGM1
Pancreas	T1DM	ENSG00000170439.5	-0.00010654	0.918361716	0.001039442	0.008204775	-8.24E-05	0.936786816	0.001039419	0.00820389	-2.41E-05	0.003391812	1	0.997611204	1	0.999388266	ENSG00000092931	7	MFSD11
Pancreas	T1DM	ENSG00000170632.9	0.000460427	0.62219259	0.000934411	0.008204771	0.00047311	0.612620349	0.000934378	0.008203886	-1.27E-05	0.027066896	1	0.95220407	1	0.946218353	ENSG00000092969	7	TGFB2
Pancreas	T1DM	ENSG00000170837.2	-0.001427086	0.180575127	0.001065795	0.008204745	-0.001409634	0.185949147	0.001065757	0.008203861	-1.75E-05	0.007544871	1	0.736247058	1	0.748740319	ENSG00000093000	14	NUP50
Pancreas	T1DM	ENSG00000170915.8	-0.001620481	0.105544336	0.001001192	0.008204731	-0.001556821	0.120042473	0.00100143	0.008203849	-6.37E-05	0.008484607	1	0.661051879	1	0.669849249	ENSG00000093000	14	NUP50
Pancreas	T1DM	ENSG00000171130.13	-0.000228451	0.735452043	0.000676127	0.008204774	-0.000243499	0.718743755	0.000676125	0.008203888	1.50E-05	0.026643793	1	0.975415042	1	0.968082788	ENSG00000093000	14	NUP50
Pancreas	T1DM	ENSG00000171357.5	-0.000891021	0.202802801	0.000699604	0.008204748	-0.000905794	0.195402681	0.000699583	0.008203862	1.48E-05	0.002464903	1	0.766675365	1	0.762604991	ENSG00000093000	14	NUP50
Pancreas	T1DM	ENSG00000171862.5	-0.001785977	0.009279422	0.000686493	0.008204662	-0.001797583	0.008830262	0.000686476	0.008203775	1.16E-05	0.024943996	1	0.231681817	1	0.208037741	ENSG00000093000	14	NUP50
Pancreas	T1DM	ENSG00000171928.9	0.001069455	0.293989643	0.0010191	0.008204757	0.001050848	0.302455516	0.00101907	0.008203872	1.86E-05	0.009010543	1	0.810918801	1	0.81428005	ENSG00000093072	11	CECR1
Pancreas	T1DM	ENSG00000171953.11	-9.09E-05	0.88965131	0.000655131	0.008204775	-7.21E-05	0.912358757	0.000655115	0.00820389	-1.88E-05	0.000195484	1	0.99200447	1	0.996366272	ENSG00000093072	11	CECR1
Pancreas	T1DM	ENSG00000171984.10	-0.000551317	0.558501572	0.00094231	0.00820477	-0.000535901	0.569543779	0.000942286	0.008203885	-1.54E-05	0.029134732	1	0.93270646	1	0.936315417	ENSG00000093134	9	VNN3
Pancreas	T1DM	ENSG00000172000.3	-0.000296893	0.74039579	0.000896064	0.008204775	-0.000282641	0.752430956	0.000896032	0.008203888	-1.43E-05	0.008068262	1	0.975415042	1	0.968082788	ENSG00000093144	14	ECHDC1
Pancreas	T1DM	ENSG00000172113.4	0.001859945	0.116442163	0.001184764	0.008204734	0.001875903	0.113329915	0.001184727	0.008203848	-1.60E-05	0.046832112	1	0.67493056	1	0.666335944	ENSG00000094916	9	CBX5
Pancreas	T1DM	ENSG00000172123.8	0.001177947	0.160789029	0.000839936	0.008204742	0.001191958	0.15585427	0.000839906	0.008203856	-1.40E-05	0.006073817	1	0.724477654	1	0.713429173	ENSG00000094916	9	CBX5
Pancreas	T1DM	ENSG00000172322.9	9.02E-05	0.900865391	0.000723935	0.008204775	0.00010682	0.882690399	0.00072391	0.00820389	-1.66E-05	0.000219746	1	0.99317359	1	0.993128823	ENSG00000094916	9	CBX5
Pancreas	T1DM	ENSG00000172572.6	-7.39E-05	0.910880258	0.000660168	0.008204775	-5.36E-05	0.935271268	0.000660163	0.00820389	-2.03E-05	0.001462201	1	0.995698473	1	0.999388266	ENSG00000094963	9	FMO2
Pancreas	T1DM	ENSG00000172613.3	-0.000626208	0.341875517	0.000658843	0.00820476	-0.000635512	0.334734896	0.00065882	0.008203874	9.30E-06	0.026023101	1	0.847428888	1	0.835993972	ENSG00000094963	9	FMO2
Pancreas	T1DM	ENSG00000172780.12	-7.98E-05	0.914880878	0.000746562	0.008204775	-6.07E-05	0.935214061	0.000746537	0.00820389	-1.91E-05	5.46E-05	1	0.995870671	1	0.999388266	ENSG00000094963	9	FMO2
Pancreas	T1DM	ENSG00000174652.13	-0.000160593	0.518378511	0.000248655	0.008204769	-0.000157759	0.525770583	0.000248645	0.008203883	-2.83E-06	0.034795991	1	0.914783379	1	0.920313283	ENSG00000094963	9	FMO2
Pancreas	T1DM	ENSG00000174780.11	6.47E-05	0.957013232	0.001199805	0.008204776	8.13E-05	0.945978319	0.00119976	0.00820389	-1.66E-05	0.014731287	1	0.997898334	1	0.999388266	ENSG00000095209	7	TMEM38B
Pancreas	T1DM	ENSG00000174808.7	-0.000537693	0.657959033	0.001214486	0.008204772	-0.000513647	0.67233216	0.00121444	0.008203887	-2.40E-05	0.000536237	1	0.962467202	1	0.961035047	ENSG00000095485	12	CWF19L1
Pancreas	T1DM	ENSG00000174891.8	0.00032101	0.76385843	0.001068544	0.008204774	0.000337197	0.752324224	0.00106851	0.008203888	-1.62E-05	0.023155512	1	0.977622335	1	0.968082788	ENSG00000095485	12	CWF19L1
Pancreas	T1DM	ENSG00000175220.7	-0.000281268	0.484037443	0.000401913	0.008204767	-0.000287583	0.474266061	0.000401902	0.008203881	6.32E-06	0.033834337	1	0.914693561	1	0.911454131	ENSG00000095485	12	CWF19L1
Pancreas	T1DM	ENSG00000175283.7	-0.000474676	0.55405127	0.000802223	0.00820477	-0.000486017	0.544609825	0.000802198	0.008203884	1.13E-05	0.033418728	1	0.93270646	1	0.928420568	ENSG00000095485	12	CWF19L1
Pancreas	T1DM	ENSG00000175305.12	0.001055901	0.242754944	0.000903975	0.00820475	0.00106719	0.237764579	0.000903943	0.008203867	-1.12E-05	0.039700253	1	0.787779958	1	0.780702981	ENSG00000095485	12	CWF19L1
Pancreas	T1DM	ENSG00000175344.12	-0.001903245	0.026201675	0.000856077	0.008204692	-0.001915049	0.025281703	0.000856049	0.008203806	1.18E-05	0.034285528	1	0.393484808	1	0.374802485	ENSG00000095713	9	CRTAC1
Pancreas	T1DM	ENSG00000175356.8	-0.000605254	0.108307621	0.000376906	0.008204732	-0.000598456	0.112316225	0.000376892	0.008203848	-6.80E-06	0.003043652	1	0.661051879	1	0.666335944	ENSG00000095713	9	CRTAC1
Pancreas	T1DM	ENSG00000175581.9	-0.000319985	0.642378106	0.000689062	0.008204772	-0.000305186	0.657827287	0.00068904	0.008203887	-1.48E-05	0.000945306	1	0.961788216	1	0.961035047	ENSG00000095787	17	WAC
Pancreas	T1DM	ENSG00000175911.4	-0.000207023	0.808763027	0.000855386	0.008204775	-0.000216281	0.800380043	0.000855353	0.008203889	9.26E-06	0.046121637	1	0.98201188	1	0.978923745	ENSG00000095917	9	TPSD1
Pancreas	T1DM	ENSG00000176148.11	0.0008166	0.187698865	0.000619848	0.008204746	0.000801504	0.195974598	0.000619828	0.008203862	1.51E-05	0.000175097	1	0.750693896	1	0.762792436	ENSG00000096433	6	ITPR3
Pancreas	T1DM	ENSG00000176171.7	-0.000889487	0.378245735	0.00100948	0.008204762	-0.000905377	0.369768429	0.001009442	0.008203876	1.59E-05	0.000630479	1	0.860999343	1	0.851044876	ENSG00000096433	6	ITPR3
Pancreas	T1DM	ENSG00000176915.10	0.002097007	0.008476586	0.000796589	0.008204659	0.002106853	0.008170587	0.000796557	0.008203772	-9.85E-06	0.020848601	1	0.222121144	1	0.204622746	ENSG00000096433	6	ITPR3
Pancreas	T1DM	ENSG00000177628.11	-0.000744109	0.342959718	0.000784648	0.00820476	-0.00072013	0.358724414	0.000784627	0.008203876	-2.40E-05	3.46E-05	1	0.849296581	1	0.850635855	ENSG00000096433	6	ITPR3
Pancreas	T1DM	ENSG00000177990.7	-0.000117723	0.815751215	0.000505222	0.008204775	-0.000130628	0.795971221	0.000505205	0.008203889	1.29E-05	0.000111815	1	0.98201188	1	0.978923745	ENSG00000096433	6	ITPR3
Pancreas	T1DM	ENSG00000178015.4	0.000428283	0.494202545	0.000626474	0.008204768	0.000439665	0.482787224	0.000626457	0.008203882	-1.14E-05	0.011681638	1	0.914693561	1	0.91349461	ENSG00000096872	11	IFT74
Pancreas	T1DM	ENSG00000178078.7	-0.001260174	0.076280067	0.000710881	0.008204723	-0.001269508	0.074118962	0.000710858	0.008203836	9.33E-06	0.044638175	1	0.571784974	1	0.550108364	ENSG00000096996	11	IL12RB1
Pancreas	T1DM	ENSG00000178248.10	-1.07E-05	0.989282728	0.000797421	0.008204776	4.39E-07	0.999560723	0.000797394	0.00820389	-1.11E-05	0.028123673	1	0.997898334	1	0.999800859	ENSG00000096996	11	IL12RB1
Pancreas	T1DM	ENSG00000178623.7	0.000109496	0.871676829	0.000677871	0.008204775	0.000121155	0.858145099	0.000677845	0.008203889	-1.17E-05	0.002659519	1	0.99200447	1	0.990577333	ENSG00000097046	8	CDC7
Pancreas	T1DM	ENSG00000179021.5	0.000562484	0.555465334	0.000954024	0.00820477	0.000577566	0.544901146	0.000953994	0.008203884	-1.51E-05	0.017142863	1	0.93270646	1	0.928420568	ENSG00000097046	8	CDC7
Pancreas	T1DM	ENSG00000179163.11	0.000150717	0.839457375	0.00074396	0.008204775	0.000124442	0.867154116	0.000743941	0.008203889	2.63E-05	2.97E-06	1	0.986602535	1	0.990577333	ENSG00000097046	8	CDC7
Pancreas	T1DM	ENSG00000179406.6	-0.000937343	0.12739937	0.000614877	0.008204736	-0.000930399	0.130227277	0.000614851	0.008203851	-6.94E-06	0.024411753	1	0.684395521	1	0.676232745	ENSG00000097046	8	CDC7
Pancreas	T1DM	ENSG00000179774.7	7.68E-06	0.986366474	0.000449446	0.008204776	1.12E-07	0.999800859	0.000449429	0.00820389	7.57E-06	0.003592288	1	0.997898334	1	0.999800859	ENSG00000099246	12	RAB18
Pancreas	T1DM	ENSG00000179954.10	0.001585797	0.189734618	0.001209271	0.008204747	0.001605644	0.184236463	0.001209229	0.00820386	-1.98E-05	0.007840426	1	0.752840936	1	0.747990804	ENSG00000099256	14	PRTFDC1
Pancreas	T1DM	ENSG00000180185.7	-0.000415224	0.127657887	0.000272563	0.008204736	-0.000407814	0.13458382	0.000272554	0.008203852	-7.41E-06	1.83E-05	1	0.684395521	1	0.685736609	ENSG00000099256	14	PRTFDC1
Pancreas	T1DM	ENSG00000180211.5	0.000831021	0.442593726	0.00108231	0.008204766	0.000849374	0.432566876	0.001082271	0.00820388	-1.84E-05	0.004475112	1	0.89813429	1	0.890803318	ENSG00000099282	5	TSPAN15
Pancreas	T1DM	ENSG00000180481.6	-3.91E-05	0.97131832	0.001088328	0.008204776	-2.43E-05	0.982153738	0.00108829	0.00820389	-1.48E-05	0.02948026	1	0.997898334	1	0.99966149	ENSG00000099330	4	OCEL1
Pancreas	T1DM	ENSG00000181027.6	-0.001766267	0.043812324	0.000876174	0.008204707	-0.001756488	0.044984316	0.000876138	0.008203822	-9.78E-06	0.032746282	1	0.48081217	1	0.485049395	ENSG00000099330	4	OCEL1
Pancreas	T1DM	ENSG00000181126.9	0.000460439	0.116028188	0.000292962	0.008204734	0.00036119	0.218909892	0.000293786	0.008203865	9.92E-05	7.75E-06	1	0.67493056	1	0.775393934	ENSG00000099330	4	OCEL1
Pancreas	T1DM	ENSG00000181626.10	-5.10E-05	0.961900202	0.001066622	0.008204776	-3.69E-05	0.972393551	0.00106658	0.00820389	-1.40E-05	0.01510388	1	0.997898334	1	0.99966149	ENSG00000099331	9	MYO9B
Pancreas	T1DM	ENSG00000182230.7	-0.001113591	0.318031431	0.00111525	0.008204759	-0.001099332	0.324250458	0.00111521	0.008203874	-1.43E-05	0.033087004	1	0.829307456	1	0.830335577	ENSG00000099338	18	CATSPERG
Pancreas	T1DM	ENSG00000182512.4	0.000396197	0.275845275	0.000363583	0.008204756	0.000400805	0.270283113	0.000363571	0.00820387	-4.61E-06	0.045385601	1	0.798582374	1	0.795306713	ENSG00000099783	7	HNRNPM
Pancreas	T1DM	ENSG00000182858.9	-0.00084167	0.21021077	0.000671741	0.008204749	-0.000857972	0.201507202	0.000671723	0.008203863	1.63E-05	0.000913357	1	0.771198903	1	0.766509931	ENSG00000099810	14	MTAP
Pancreas	T1DM	ENSG00000182919.10	-0.000539809	0.177551098	0.000400352	0.008204745	-0.000488483	0.223099797	0.000400945	0.008203865	-5.13E-05	0.020662898	1	0.732338331	1	0.78054534	ENSG00000099810	14	MTAP
Pancreas	T1DM	ENSG00000182952.4	-0.000129845	0.814310538	0.000552841	0.008204775	-0.000204661	0.711374154	0.00055312	0.008203888	7.48E-05	5.15E-05	1	0.98201188	1	0.968082788	ENSG00000099814	11	CEP170B
Pancreas	T1DM	ENSG00000183426.11	0.000275603	0.424503786	0.000345094	0.008204765	0.000271297	0.431760397	0.000345081	0.00820388	4.31E-06	0.025987288	1	0.889410424	1	0.890803318	ENSG00000099940	7	SNAP29
Pancreas	T1DM	ENSG00000183431.7	0.000904178	0.12945897	0.000596329	0.008204737	0.000911169	0.126507322	0.000596306	0.008203851	-6.99E-06	0.038011709	1	0.688303313	1	0.673807988	ENSG00000099940	7	SNAP29
Pancreas	T1DM	ENSG00000183444.10	-0.002350875	0.025384996	0.00105161	0.008204691	-0.002365038	0.024510004	0.001051574	0.008203805	1.42E-05	0.029617873	1	0.392702831	1	0.374802485	ENSG00000099949	14	LZTR1
Pancreas	T1DM	ENSG00000183506.12	-0.000279221	0.625799389	0.000572588	0.008204772	-0.000270534	0.636575286	0.000572567	0.008203886	-8.69E-06	0.010159859	1	0.953740553	1	0.955974304	ENSG00000099949	14	LZTR1
Pancreas	T1DM	ENSG00000183562.2	0.001868999	0.092016623	0.001109292	0.008204728	0.001890853	0.088267987	0.001109259	0.008203841	-2.19E-05	0.00509926	1	0.607172104	1	0.582754369	ENSG00000099949	14	LZTR1
Pancreas	T1DM	ENSG00000183793.9	-0.000577854	0.202730791	0.000453642	0.008204748	-0.000571888	0.207414584	0.000453624	0.008203865	-5.97E-06	0.015385079	1	0.766675365	1	0.769778216	ENSG00000099949	14	LZTR1
Pancreas	T1DM	ENSG00000183833.12	-0.000512008	0.164982197	0.000368746	0.008204743	-0.000517598	0.160402911	0.000368733	0.008203857	5.59E-06	0.011445451	1	0.729268002	1	0.723297686	ENSG00000099956	13	SMARCB1
Pancreas	T1DM	ENSG00000184110.10	2.77E-05	0.968139831	0.000694471	0.008204776	1.74E-05	0.979981967	0.00069445	0.00820389	1.03E-05	0.031045716	1	0.997898334	1	0.99966149	ENSG00000099956	13	SMARCB1
Pancreas	T1DM	ENSG00000184164.10	-0.000772982	0.333006782	0.000798472	0.00820476	-0.000797249	0.31804026	0.000798451	0.008203873	2.43E-05	4.83E-05	1	0.83462449	1	0.830006722	ENSG00000099956	13	SMARCB1
Pancreas	T1DM	ENSG00000184389.8	-0.000954088	0.174946229	0.000703354	0.008204745	-0.000970469	0.16764257	0.00070333	0.008203858	1.64E-05	0.002221843	1	0.729321291	1	0.726605951	ENSG00000099956	13	SMARCB1
Pancreas	T1DM	ENSG00000184465.11	-0.000518211	0.469384763	0.000716274	0.008204767	-0.000529181	0.460015922	0.000716251	0.008203881	1.10E-05	0.018418075	1	0.908712989	1	0.901310005	ENSG00000099974	7	DDTL
Pancreas	T1DM	ENSG00000184608.4	0.000998341	0.310081085	0.000983536	0.008204758	0.000974796	0.321618907	0.000983512	0.008203873	2.35E-05	0.002095	1	0.826025131	1	0.830335577	ENSG00000099974	7	DDTL
Pancreas	T1DM	ENSG00000184945.9	-3.15E-05	0.939421726	0.000414207	0.008204775	-3.96E-05	0.923889654	0.000414193	0.00820389	8.09E-06	0.002368383	1	0.997898334	1	0.999388266	ENSG00000099974	7	DDTL
Pancreas	T1DM	ENSG00000184967.2	-0.00107922	0.24762865	0.000933476	0.008204753	-0.001096182	0.240257474	0.000933441	0.008203867	1.70E-05	0.001389004	1	0.788479116	1	0.780702981	ENSG00000099977	9	DDT
Pancreas	T1DM	ENSG00000184983.5	0.000576768	0.064620654	0.000312125	0.008204718	0.000567658	0.06895197	0.000312116	0.008203834	9.11E-06	8.26E-05	1	0.544685729	1	0.536516198	ENSG00000099984	6	GSTT2
Pancreas	T1DM	ENSG00000185101.8	0.001812203	0.023112301	0.000797772	0.008204689	0.001833324	0.021560333	0.00079778	0.008203801	-2.11E-05	0.019264292	1	0.382918644	1	0.349355274	ENSG00000099984	6	GSTT2
Pancreas	T1DM	ENSG00000185340.11	-0.000500131	0.625182767	0.00102377	0.008204772	-0.000485318	0.635451462	0.00102373	0.008203886	-1.48E-05	0.008882936	1	0.953366595	1	0.955974304	ENSG00000099985	3	OSM
Pancreas	T1DM	ENSG00000185684.8	-0.000131664	0.739461467	0.000395902	0.008204774	-0.000139982	0.723646224	0.000395888	0.008203888	8.32E-06	0.000608356	1	0.975415042	1	0.968082788	ENSG00000099991	12	CABIN1
Pancreas	T1DM	ENSG00000185917.9	-5.19E-05	0.966958691	0.001253907	0.008204776	-3.65E-05	0.976773582	0.001253861	0.00820389	-1.54E-05	0.037836986	1	0.997898334	1	0.99966149	ENSG00000099991	12	CABIN1
Pancreas	T1DM	ENSG00000186364.7	-0.001346063	0.289720349	0.001271386	0.008204757	-0.001361536	0.284194934	0.001271342	0.008203871	1.55E-05	0.049223431	1	0.809076913	1	0.799151365	ENSG00000099994	10	SUSD2
Pancreas	T1DM	ENSG00000187010.14	-0.000278782	0.651627359	0.000617453	0.008204772	-0.000288066	0.640819547	0.000617434	0.008203886	9.28E-06	0.029139688	1	0.962467202	1	0.957315064	ENSG00000100014	15	SPECC1L
Pancreas	T1DM	ENSG00000187013.2	0.00082409	0.296719187	0.000789745	0.008204757	0.000812425	0.303596014	0.000789718	0.008203872	1.17E-05	0.016445221	1	0.813206908	1	0.815517326	ENSG00000100014	15	SPECC1L
Pancreas	T1DM	ENSG00000187741.10	-0.000817319	0.140838217	0.000554988	0.008204739	-0.000832139	0.13376351	0.000554971	0.008203852	1.48E-05	0.001147149	1	0.703643077	1	0.685736609	ENSG00000100014	15	SPECC1L
Pancreas	T1DM	ENSG00000187815.5	0.002211815	0.082937297	0.001275634	0.008204725	0.00224076	0.078982925	0.001275611	0.008203838	-2.90E-05	0.007709429	1	0.589329992	1	0.56498092	ENSG00000100014	15	SPECC1L
Pancreas	T1DM	ENSG00000187987.5	0.002660471	0.031797456	0.001239184	0.008204698	0.002371499	0.055945612	0.001240672	0.008203828	0.000288972	3.23E-06	1	0.427517623	1	0.510771882	ENSG00000100014	15	SPECC1L
Pancreas	T1DM	ENSG00000188321.9	-0.000120678	0.650258237	0.00026616	0.008204772	-0.000115673	0.663840497	0.000266149	0.008203887	-5.00E-06	0.000901143	1	0.962467202	1	0.961035047	ENSG00000100023	13	PPIL2
Pancreas	T1DM	ENSG00000188629.7	-0.000284057	0.514211778	0.000435473	0.008204768	-0.000276826	0.524961686	0.000435456	0.008203883	-7.23E-06	0.001826405	1	0.914783379	1	0.919578178	ENSG00000100023	13	PPIL2
Pancreas	T1DM	ENSG00000188677.10	-0.000224546	0.59218393	0.000419184	0.008204771	-0.000231411	0.580900363	0.00041917	0.008203885	6.87E-06	0.011884471	1	0.938170574	1	0.936315417	ENSG00000100024	10	UPB1
Pancreas	T1DM	ENSG00000188760.6	0.000791113	0.522056258	0.001235768	0.008204769	0.000807946	0.513225483	0.001235724	0.008203883	-1.68E-05	0.024577729	1	0.915738026	1	0.915381179	ENSG00000100024	10	UPB1
Pancreas	T1DM	ENSG00000188786.9	0.00026527	0.766743839	0.000894257	0.008204774	0.000253285	0.776988985	0.000894226	0.008203889	1.20E-05	0.031019011	1	0.977622335	1	0.972830721	ENSG00000100033	12	PRODH
Pancreas	T1DM	ENSG00000188820.8	0.001170642	0.172351293	0.000857809	0.008204744	0.001184555	0.167296042	0.000857785	0.008203858	-1.39E-05	0.025177236	1	0.729268002	1	0.726605951	ENSG00000100033	12	PRODH
Pancreas	T1DM	ENSG00000189011.9	0.000325328	0.710590798	0.000876747	0.008204773	0.000369879	0.673137876	0.000876811	0.008203887	-4.46E-05	0.001482998	1	0.975415042	1	0.961035047	ENSG00000100068	7	LRP5L
Pancreas	T1DM	ENSG00000189190.7	0.001276739	0.075810066	0.000719072	0.008204722	0.001285493	0.07381264	0.000719046	0.008203836	-8.75E-06	0.035203766	1	0.57107125	1	0.550108364	ENSG00000100068	7	LRP5L
Pancreas	T1DM	ENSG00000189298.9	-0.005086132	4.91E-10	0.000817428	0.008204124	-0.004961783	1.41E-09	0.000819543	0.008203273	-0.00012435	0.036397457	1.26E-06	5.48E-08	3.62E-06	1.58E-07	ENSG00000100079	5	LGALS2
Pancreas	T1DM	ENSG00000189306.6	-8.93E-05	0.922299043	0.000915685	0.008204775	-0.000109878	0.904483553	0.000915653	0.00820389	2.06E-05	0.000285034	1	0.997898334	1	0.995067586	ENSG00000100079	5	LGALS2
Pancreas	T1DM	ENSG00000196126.6	-0.00264574	1.22E-09	0.000435334	0.008204154	-0.001615162	0.008356584	0.000612519	0.008203773	-0.001030577	0.016770568	3.14E-06	1.31E-07	1	0.204622746	ENSG00000100083	14	GGA1
Pancreas	T1DM	ENSG00000196151.6	-0.000802161	0.221662278	0.000656368	0.00820475	-0.000826973	0.207717244	0.000656396	0.00820386	2.48E-05	0.00649261	1	0.780835021	1	0.769778216	ENSG00000100099	16	HPS4
Pancreas	T1DM	ENSG00000196260.3	-0.002886511	2.21E-09	0.000482535	0.008204173	-0.003202614	3.94E-11	0.00048477	0.008203155	0.000316103	1.44E-11	5.67E-06	2.18E-07	1.01E-07	5.06E-09	ENSG00000100099	16	HPS4
Pancreas	T1DM	ENSG00000196458.6	0.00139306	0.110469699	0.000872796	0.008204733	0.001405427	0.107328676	0.000872763	0.008203846	-1.24E-05	0.011375218	1	0.665930015	1	0.652153441	ENSG00000100099	16	HPS4
Pancreas	T1DM	ENSG00000196735.7	-0.000439896	0.353577714	0.000474193	0.008204761	0.001595421	0.004219253	0.000557586	0.008203752	-0.002035317	3.99E-12	1	0.854061442	1	0.135438011	ENSG00000100099	16	HPS4
Pancreas	T1DM	ENSG00000196743.4	-0.000889548	0.394508381	0.00104472	0.008204763	0.000689613	0.510137705	0.001047051	0.008203883	-0.00199935	0.004666178	1	0.873903579	1	0.915335266	ENSG00000100099	16	HPS4
Pancreas	T1DM	ENSG00000196821.5	0.000190855	0.51969723	0.000296445	0.008204769	0.000202192	0.495208521	0.000296447	0.008203883	-1.13E-05	0.000653004	1	0.914783379	1	0.914417995	ENSG00000100106	15	TRIOBP
Pancreas	T1DM	ENSG00000196839.8	-0.001107314	0.241061519	0.000944534	0.008204752	-0.001121921	0.234895638	0.000944502	0.008203866	1.46E-05	0.014858715	1	0.786897324	1	0.780702981	ENSG00000100109	12	TFIP11
Pancreas	T1DM	ENSG00000196859.3	0.000553593	0.237461431	0.000468608	0.008204752	0.0005462	0.24376667	0.00046859	0.008203866	7.39E-06	0.005482304	1	0.785512902	1	0.78207287	ENSG00000100109	12	TFIP11
Pancreas	T1DM	ENSG00000196912.8	0.000375297	0.625123904	0.000768104	0.008204772	0.000387543	0.613868158	0.000768082	0.008203886	-1.22E-05	0.026185163	1	0.953366595	1	0.946218353	ENSG00000100129	13	EIF3L
Pancreas	T1DM	ENSG00000197054.7	-0.000391422	0.710637177	0.001055052	0.008204773	-0.000455849	0.665709469	0.001055098	0.008203887	6.44E-05	1.23E-05	1	0.975415042	1	0.961035047	ENSG00000100129	13	EIF3L
Pancreas	T1DM	ENSG00000197062.10	-0.002236417	5.09E-09	0.000382665	0.008204201	-0.002154522	2.03E-08	0.0003841	0.008203336	-8.19E-05	0.014202592	1.31E-05	4.84E-07	5.22E-05	2.01E-06	ENSG00000100129	13	EIF3L
Pancreas	T1DM	ENSG00000197146.2	-0.000216733	0.778734578	0.000771377	0.008204774	-0.000230409	0.765162	0.000771348	0.008203888	1.37E-05	0.001852027	1	0.979738629	1	0.971298081	ENSG00000100147	9	CCDC134
Pancreas	T1DM	ENSG00000197157.6	-2.01E-05	0.980575869	0.000824887	0.008204776	-3.11E-06	0.996992451	0.000824859	0.00820389	-1.70E-05	0.001309443	1	0.997898334	1	0.999770584	ENSG00000100151	11	PICK1
Pancreas	T1DM	ENSG00000197363.5	-0.002883199	0.014048883	0.001173931	0.008204674	-0.002867104	0.014590495	0.00117389	0.00820379	-1.61E-05	0.025383337	1	0.2919226	1	0.288218403	ENSG00000100162	10	CENPM
Pancreas	T1DM	ENSG00000197415.7	-0.000277771	0.80603252	0.001131232	0.008204775	-0.000251085	0.824341644	0.001131194	0.008203889	-2.67E-05	0.000221223	1	0.98201188	1	0.984037376	ENSG00000100181	17	TPTEP1
Pancreas	T1DM	ENSG00000197568.9	0.001664289	0.051459012	0.000854517	0.008204712	0.001652289	0.053155451	0.000854487	0.008203827	1.20E-05	0.023711032	1	0.512196676	1	0.510771882	ENSG00000100181	17	TPTEP1
Pancreas	T1DM	ENSG00000197580.7	-0.0006187	0.385450618	0.000712872	0.008204763	-0.000607405	0.394169938	0.00071285	0.008203878	-1.13E-05	0.01877078	1	0.865581925	1	0.871859088	ENSG00000100196	6	KDELR3
Pancreas	T1DM	ENSG00000197586.8	-0.000736872	0.239813908	0.000626883	0.008204752	-0.000745324	0.234447989	0.000626859	0.008203866	8.45E-06	0.019192647	1	0.785512902	1	0.780702981	ENSG00000100196	6	KDELR3
Pancreas	T1DM	ENSG00000197728.5	0.001184472	0.000448646	0.000337485	0.008204568	0.00118086	0.000466793	0.000337471	0.008203684	3.61E-06	0.033916602	1	0.022156213	1	0.024973406	ENSG00000100197	16	CYP2D6
Pancreas	T1DM	ENSG00000197816.9	-0.000341064	0.705674775	0.000903071	0.008204773	-0.000327395	0.716941107	0.000903043	0.008203887	-1.37E-05	0.025967888	1	0.974645207	1	0.968082788	ENSG00000100197	16	CYP2D6
Pancreas	T1DM	ENSG00000197822.6	-0.000721454	0.330748605	0.000741772	0.00820476	-0.000732269	0.323534892	0.000741748	0.008203874	1.08E-05	0.025433747	1	0.83462449	1	0.830335577	ENSG00000100201	14	DDX17
Pancreas	T1DM	ENSG00000197903.6	0.000670958	0.288555714	0.000632206	0.008204757	0.000773936	0.221187809	0.000632622	0.008203865	-0.000102978	1.62E-05	1	0.809076913	1	0.776261656	ENSG00000100201	14	DDX17
Pancreas	T1DM	ENSG00000197935.6	-0.000943382	0.073871611	0.000527792	0.00820472	-0.001212178	0.022096416	0.000529633	0.008203802	0.000268796	1.50E-09	1	0.563394551	1	0.350269115	ENSG00000100201	14	DDX17
Pancreas	T1DM	ENSG00000197969.7	0.000993824	0.30537611	0.00096961	0.008204758	0.000979342	0.312459938	0.000969574	0.008203873	1.45E-05	0.000906343	1	0.822018711	1	0.825219435	ENSG00000100209	5	HSCB
Pancreas	T1DM	ENSG00000198053.7	-0.000688288	0.35955525	0.00075123	0.008204761	-0.000678665	0.36629375	0.000751203	0.008203876	-9.62E-06	0.029181835	1	0.854209198	1	0.850635855	ENSG00000100211	6	CBY1
Pancreas	T1DM	ENSG00000198171.8	0.001988148	0.117435393	0.001269869	0.008204734	0.002007162	0.11395463	0.001269819	0.008203848	-1.90E-05	0.005257596	1	0.67493056	1	0.666335944	ENSG00000100211	6	CBY1
Pancreas	T1DM	ENSG00000198468.3	0.000571097	0.156941239	0.000403478	0.008204742	0.000565756	0.160826705	0.000403464	0.008203857	5.32E-06	0.028706533	1	0.719687683	1	0.723297686	ENSG00000100211	6	CBY1
Pancreas	T1DM	ENSG00000198624.8	-0.000394853	0.584220079	0.000721546	0.008204771	-0.000273609	0.705010869	0.000722752	0.008203888	-0.000121244	0.00424502	1	0.937588291	1	0.968082788	ENSG00000100211	6	CBY1
Pancreas	T1DM	ENSG00000198756.6	0.000427397	0.544015019	0.0007044	0.008204769	0.000404777	0.565526582	0.000704385	0.008203884	2.26E-05	6.56E-05	1	0.929438478	1	0.936315417	ENSG00000100220	7	RTCB
Pancreas	T1DM	ENSG00000198885.5	4.37E-05	0.943373185	0.000615468	0.008204775	3.56E-05	0.953901599	0.000615446	0.00820389	8.14E-06	0.027789416	1	0.997898334	1	0.999388266	ENSG00000100225	13	FBXO7
Pancreas	T1DM	ENSG00000198925.6	-0.000235589	0.617825082	0.000472183	0.008204771	-0.000242747	0.607173693	0.000472167	0.008203886	7.16E-06	0.016839082	1	0.950614027	1	0.940990975	ENSG00000100225	13	FBXO7
Pancreas	T1DM	ENSG00000198961.5	0.000299301	0.655186433	0.000670222	0.008204772	0.000307431	0.646438505	0.000670199	0.008203886	-8.13E-06	0.047784506	1	0.962467202	1	0.961035047	ENSG00000100225	13	FBXO7
Pancreas	T1DM	ENSG00000204140.8	0.000514339	0.50055935	0.000763555	0.008204768	0.000541237	0.478415042	0.000763538	0.008203883	-2.69E-05	6.76E-06	1	0.914783379	1	0.911454131	ENSG00000100243	16	CYB5R3
Pancreas	T1DM	ENSG00000204228.3	0.004910809	4.88E-30	0.000431254	0.008202593	0.0047664	3.40E-28	0.000432867	0.008201849	0.000144409	0.000121084	1.25E-26	1.79E-27	8.73E-25	1.25E-25	ENSG00000100258	13	LMF2
Pancreas	T1DM	ENSG00000204301.5	-0.007024958	1.13E-29	0.0006209	0.008202621	-0.006629097	9.03E-26	0.00063156	0.008202036	-0.000395861	0.000620636	2.89E-26	3.62E-27	2.32E-22	2.32E-23	ENSG00000100258	13	LMF2
Pancreas	T1DM	ENSG00000204305.9	0.004690598	2.39E-06	0.000994321	0.008204401	0.003713229	0.000235442	0.001009681	0.008203662	0.000977369	2.66E-08	0.006136418	0.00015341	0.604614121	0.01374123	ENSG00000100258	13	LMF2
Pancreas	T1DM	ENSG00000204316.8	-0.00018094	0.864220972	0.001058107	0.008204775	-0.000162653	0.87782677	0.001058081	0.00820389	-1.83E-05	0.025051467	1	0.990858518	1	0.993128823	ENSG00000100284	16	TOM1
Pancreas	T1DM	ENSG00000204338.4	-0.003354602	1.85E-27	0.000308967	0.008202791	-0.003504122	4.50E-29	0.000313095	0.008201782	0.00014952	0.003239236	4.75E-24	4.75E-25	1.16E-25	1.93E-26	ENSG00000100284	16	TOM1
Pancreas	T1DM	ENSG00000204351.7	-0.00203178	4.26E-14	0.000268999	0.008203815	-0.002830754	2.10E-23	0.000283966	0.008202217	0.000798974	1.71E-18	1.09E-10	6.83E-12	5.40E-20	4.91E-21	ENSG00000100284	16	TOM1
Pancreas	T1DM	ENSG00000204371.7	0.004136711	9.16E-05	0.001057448	0.008204518	0.004453456	2.63E-05	0.001059575	0.008203593	-0.000316745	3.24E-06	0.235115938	0.005002467	0.067635975	0.001690899	ENSG00000100290	2	BIK
Pancreas	T1DM	ENSG00000204516.5	0.000708123	0.033326686	0.00033274	0.008204699	0.000608904	0.068135351	0.000333808	0.008203834	9.92E-05	0.000219671	1	0.434431116	1	0.536516198	ENSG00000100296	9	THOC5

Pancreas	T1DM	ENSG00000204525.10	0.000710321	0.001797712	0.000227537	0.008204611	0.000970859	3.03E-05	0.000232771	0.008203597	-0.000260538	1.15E-07	1	0.075680736	0.077936275	0.001900885	ENSG00000100296	9	THOC5
Pancreas	T1DM	ENSG00000204531.11	0.001882674	0.000308418	0.000521784	0.008204556	0.00168047	0.001308986	0.000522856	0.008203716	0.000202204	2.41E-09	0.792016331	0.015529732	1	0.05795648	ENSG00000100296	9	THOC5
Pancreas	T1DM	ENSG00000204560.5	0.002509043	0.026825859	0.001133237	0.008204693	0.002789045	0.014012056	0.00113516	0.008203788	-0.000280002	3.01E-05	1	0.395912673	1	0.281029074	ENSG00000100299	13	ARSA
Pancreas	T1DM	ENSG00000204616.6	-0.002245114	4.32E-08	0.000409903	0.00820427	-0.002149228	1.71E-07	0.00041104	0.00820343	-9.59E-05	0.001864791	0.000111035	3.58E-06	0.000438408	1.51E-05	ENSG00000100300	13	TSPO
Pancreas	T1DM	ENSG00000204618.4	0.004451946	8.74E-14	0.000596871	0.008203839	0.004892466	6.37E-16	0.000605336	0.00820279	-0.00044052	1.32E-05	2.25E-10	1.25E-11	1.64E-12	1.02E-13	ENSG00000100300	13	TSPO
Pancreas	T1DM	ENSG00000204622.6	0.00101445	0.190886824	0.000775596	0.008204747	0.000839992	0.279864139	0.00077732	0.00820387	0.000174458	0.000862237	1	0.752840936	1	0.797009688	ENSG00000100300	13	TSPO
Pancreas	T1DM	ENSG00000204625.6	0.001775607	0.010242653	0.00069156	0.008204665	0.00119589	0.086467829	0.000697582	0.008203841	0.000579717	2.61E-10	1	0.24354753	1	0.580557814	ENSG00000100300	13	TSPO
Pancreas	T1DM	ENSG00000204632.7	0.000738628	0.151725073	0.000515274	0.008204741	0.00077949	0.130394119	0.000515348	0.008203851	-4.09E-05	6.69E-05	1	0.717060869	1	0.676232745	ENSG00000100302	6	RASD2
Pancreas	T1DM	ENSG00000204642.9	-0.000902069	0.003996583	0.000313388	0.008204636	-0.000777125	0.013392186	0.000314222	0.008203787	-0.000124944	6.33E-08	1	0.135042429	1	0.275576512	ENSG00000100302	6	RASD2
Pancreas	T1DM	ENSG00000204789.3	0.000497807	0.111746007	0.000313008	0.008204733	0.000434657	0.165434031	0.000313373	0.008203858	6.31E-05	4.46E-05	1	0.670476041	1	0.726605951	ENSG00000100330	11	MTMR3
Pancreas	T1DM	ENSG00000204792.2	-0.00012187	0.914029623	0.001128869	0.008204775	-0.00010703	0.924461722	0.001128826	0.00820389	-1.48E-05	0.019329492	1	0.995870671	1	0.999388266	ENSG00000100336	13	APOL4
Pancreas	T1DM	ENSG00000204959.3	-0.001307787	0.085008097	0.000759307	0.008204726	-0.001317751	0.082646932	0.000759277	0.008203839	9.96E-06	0.01475093	1	0.590002147	1	0.578303331	ENSG00000100350	10	FOXRED2
Pancreas	T1DM	ENSG00000204991.6	-0.000216831	0.699736726	0.000562209	0.008204773	-0.000230768	0.681455731	0.000562193	0.008203887	1.39E-05	0.000625978	1	0.974470668	1	0.966338775	ENSG00000100350	10	FOXRED2
Pancreas	T1DM	ENSG00000205045.4	0.000313907	0.607924635	0.000611859	0.008204771	0.000326414	0.593692158	0.000611842	0.008203885	-1.25E-05	0.003924735	1	0.943530179	1	0.937577789	ENSG00000100372	10	SLC25A17
Pancreas	T1DM	ENSG00000205476.4	-0.000191613	0.842723932	0.000965738	0.008204775	-0.000202901	0.833583566	0.0009657	0.008203889	1.13E-05	0.027527212	1	0.987729373	1	0.985109341	ENSG00000100376	7	FAM118A
Pancreas	T1DM	ENSG00000205517.8	0.000604592	0.417846521	0.000746259	0.008204765	0.000567672	0.446871616	0.000746307	0.00820388	3.69E-05	0.001263198	1	0.886967947	1	0.896838446	ENSG00000100395	10	L3MBTL2
Pancreas	T1DM	ENSG00000205560.8	-0.000725032	0.249913368	0.000630155	0.008204753	-0.000739179	0.240777555	0.000630136	0.008203867	1.41E-05	0.001172792	1	0.789394255	1	0.780702981	ENSG00000100413	12	POLR3H
Pancreas	T1DM	ENSG00000205565.6	0.000618591	0.275246055	0.000566962	0.008204756	0.000628807	0.267381899	0.000566945	0.008203869	-1.02E-05	0.009929491	1	0.798582374	1	0.795306713	ENSG00000100429	13	HDAC10
Pancreas	T1DM	ENSG00000205578.4	-0.000587844	0.353702022	0.000633839	0.008204761	-0.000574159	0.36500293	0.000633818	0.008203876	-1.37E-05	0.000705081	1	0.854061442	1	0.850635855	ENSG00000100429	13	HDAC10
Pancreas	T1DM	ENSG00000205978.5	0.001025825	0.292738186	0.000974994	0.008204757	0.001056298	0.27866902	0.00097506	0.00820387	-3.05E-05	0.045870968	1	0.810235282	1	0.796020071	ENSG00000100429	13	HDAC10
Pancreas	T1DM	ENSG00000206013.2	3.08E-05	0.97035886	0.000827759	0.008204776	1.12E-05	0.989197603	0.000827756	0.00820389	1.96E-05	0.018496236	1	0.997898334	1	0.99966149	ENSG00000100441	5	KHNYN
Pancreas	T1DM	ENSG00000206341.6	0.00093338	0.030303433	0.000430902	0.008204697	0.000731396	0.090589145	0.00043219	0.008203842	0.000201984	1.91E-09	1	0.424384765	1	0.590438896	ENSG00000100441	5	KHNYN
Pancreas	T1DM	ENSG00000206344.6	0.00255688	8.16E-09	0.000443502	0.008204216	0.002092894	3.72E-06	0.000452357	0.00820353	0.000463986	1.95E-07	2.10E-05	7.23E-07	0.009546279	0.000260615	ENSG00000100445	12	SDR39U1
Pancreas	T1DM	ENSG00000206503.7	0.000615738	0.120676219	0.000396753	0.008204735	0.001102708	0.006356598	0.000404099	0.008203765	-0.000486969	2.31E-10	1	0.677664852	1	0.186420941	ENSG00000100445	12	SDR39U1
Pancreas	T1DM	ENSG00000212127.5	0.000804727	0.138499046	0.000543219	0.008204739	0.000796172	0.14272718	0.000543198	0.008203854	8.56E-06	0.004938267	1	0.69738343	1	0.696812542	ENSG00000100445	12	SDR39U1
Pancreas	T1DM	ENSG00000213221.4	0.000449854	0.202122142	0.00035268	0.008204748	0.000445742	0.206260144	0.000352667	0.008203863	4.11E-06	0.04138566	1	0.766675365	1	0.769778216	ENSG00000100445	12	SDR39U1
Pancreas	T1DM	ENSG00000213588.4	0.00194083	0.015571065	0.000802385	0.008204677	0.002120752	0.008315004	0.000803615	0.008203773	-0.000179921	6.95E-05	1	0.309972834	1	0.204622746	ENSG00000100461	13	RBM23
Pancreas	T1DM	ENSG00000213676.6	0.002281016	7.87E-11	0.00035075	0.008204064	0.002074921	4.48E-09	0.000353759	0.008203311	0.000206095	8.03E-06	2.02E-07	9.62E-09	1.15E-05	4.80E-07	ENSG00000100461	13	RBM23
Pancreas	T1DM	ENSG00000213760.6	-0.001065077	0.085719432	0.000619797	0.008204726	-0.002161277	0.000760322	0.000641924	0.008203699	0.0010962	5.56E-11	1	0.592504613	1	0.038284453	ENSG00000100461	13	RBM23
Pancreas	T1DM	ENSG00000213761.4	0.000796424	0.416439292	0.000980075	0.008204764	0.000814264	0.406060451	0.000980041	0.008203878	-1.78E-05	0.002900864	1	0.886746354	1	0.882202402	ENSG00000100478	10	AP4S1
Pancreas	T1DM	ENSG00000213901.6	0.002015448	0.109052243	0.001257713	0.008204732	0.002040559	0.104698621	0.00125767	0.008203846	-2.51E-05	0.001361345	1	0.661051879	1	0.646312642	ENSG00000100478	10	AP4S1
Pancreas	T1DM	ENSG00000214376.5	-0.000889844	0.266660499	0.000801093	0.008204755	-0.002134883	0.01509487	0.000878515	0.008203791	0.001245039	0.00055727	1	0.797304864	1	0.293663832	ENSG00000100483	9	VCPKMT
Pancreas	T1DM	ENSG00000215305.5	0.001098734	0.257961223	0.000971279	0.008204754	0.001113174	0.25174084	0.000971244	0.008203868	-1.44E-05	0.013578644	1	0.79720158	1	0.788955599	ENSG00000100505	9	TRIM9
Pancreas	T1DM	ENSG00000219159.3	3.88E-05	0.932631508	0.000459251	0.008204775	2.95E-05	0.948795638	0.000459233	0.00820389	9.33E-06	0.000444993	1	0.997898334	1	0.999388266	ENSG00000100505	9	TRIM9
Pancreas	T1DM	ENSG00000221944.3	-0.000143838	0.860542188	0.000818728	0.008204775	-0.000135927	0.868133962	0.000818694	0.00820389	-7.91E-06	0.049128286	1	0.990511029	1	0.990577333	ENSG00000100528	7	CNIH1
Pancreas	T1DM	ENSG00000221988.8	0.007009714	1.09E-13	0.000943467	0.008203846	0.00623609	8.79E-11	0.000961389	0.008203182	0.000773625	2.88E-05	2.80E-10	1.47E-11	2.26E-07	1.03E-08	ENSG00000100554	7	ATP6V1D
Pancreas	T1DM	ENSG00000222019.3	-0.000386833	0.659896957	0.000879055	0.008204772	-0.000406534	0.643736964	0.00087903	0.008203886	1.97E-05	0.001820355	1	0.962467202	1	0.960555796	ENSG00000100558	4	PLEK2
Pancreas	T1DM	ENSG00000223756.2	-0.000376242	0.555709166	0.000638534	0.00820477	-0.000387822	0.543598121	0.000638515	0.008203884	1.16E-05	0.009866343	1	0.93270646	1	0.928420568	ENSG00000100564	4	PIGH
Pancreas	T1DM	ENSG00000223865.6	-0.001066126	0.008764201	0.000406743	0.00820466	-0.000475023	0.299093973	0.000457466	0.008203872	-0.000591102	0.004761386	1	0.225064674	1	0.810652485	ENSG00000100564	4	PIGH
Pancreas	T1DM	ENSG00000224389.4	0.00589385	5.07E-07	0.001173243	0.008204351	0.005486048	3.86E-06	0.001187707	0.008203531	0.000407802	0.027640292	0.001303041	3.62E-05	0.009903386	0.000260615	ENSG00000100580	7	TMED8
Pancreas	T1DM	ENSG00000224635.1	-0.001349911	0.135707628	0.000904784	0.008204738	-0.001157801	0.202377487	0.000908216	0.008203863	-0.00019211	0.015573678	1	0.694369535	1	0.766509931	ENSG00000100580	7	TMED8
Pancreas	T1DM	ENSG00000224796.1	0.005772603	8.88E-17	0.000693893	0.00820361	0.005930985	1.33E-17	0.000694387	0.008202662	-0.000158383	6.09E-09	2.28E-13	1.63E-14	3.42E-14	2.28E-15	ENSG00000100580	7	TMED8
Pancreas	T1DM	ENSG00000225294.1	0.000152366	0.895962183	0.001165196	0.008204775	0.000180394	0.876961921	0.001165173	0.00820389	-2.80E-05	0.0035103	1	0.99200447	1	0.993128823	ENSG00000100580	7	TMED8
Pancreas	T1DM	ENSG00000225784.5	0.001354953	0.145431883	0.000930689	0.00820474	0.001344232	0.148626776	0.00093065	0.008203855	1.07E-05	0.019052584	1	0.709552898	1	0.706802888	ENSG00000100614	13	PPM1A
Pancreas	T1DM	ENSG00000226686.3	-0.000421231	0.417793848	0.000519875	0.008204765	-0.000410313	0.429950332	0.000519859	0.008203879	-1.09E-05	0.001824704	1	0.886967947	1	0.890691955	ENSG00000100614	13	PPM1A
Pancreas	T1DM	ENSG00000226913.1	0.000163312	0.842230267	0.00082049	0.008204775	0.000175448	0.830672449	0.000820465	0.008203889	-1.21E-05	0.028446894	1	0.987601519	1	0.984297508	ENSG00000100726	10	TELO2
Pancreas	T1DM	ENSG00000227262.3	0.001877649	0.001899047	0.000604601	0.008204613	0.001797001	0.002968927	0.000604857	0.008203741	8.06E-05	1.54E-05	1	0.078657282	1	0.112120641	ENSG00000100731	11	PCNX
Pancreas	T1DM	ENSG00000227755.1	-0.000480383	0.622487952	0.000975739	0.008204771	-0.000466882	0.632289226	0.000975705	0.008203886	-1.35E-05	0.027559675	1	0.95220407	1	0.955974304	ENSG00000100731	11	PCNX
Pancreas	T1DM	ENSG00000228065.6	0.000188293	0.794754071	0.000723806	0.008204774	0.000207573	0.774273507	0.000723782	0.008203889	-1.93E-05	3.07E-05	1	0.981920629	1	0.972640681	ENSG00000100731	11	PCNX
Pancreas	T1DM	ENSG00000228106.1	-0.000784493	0.422723236	0.000978529	0.008204765	-0.000770761	0.430871009	0.000978493	0.00820388	-1.37E-05	0.016765051	1	0.889410424	1	0.890803318	ENSG00000100731	11	PCNX
Pancreas	T1DM	ENSG00000228262.4	-0.000954923	0.369781727	0.001064713	0.008204762	-0.000970596	0.361961702	0.001064676	0.008203876	1.57E-05	0.018924232	1	0.856244109	1	0.850635855	ENSG00000100731	11	PCNX
Pancreas	T1DM	ENSG00000228672.2	-0.002133208	0.045101875	0.00106463	0.008204708	-0.002150725	0.043360108	0.001064594	0.008203821	1.75E-05	0.009838765	1	0.490769555	1	0.471816773	ENSG00000100731	11	PCNX
Pancreas	T1DM	ENSG00000228782.3	0.000777921	0.473839874	0.001086108	0.008204767	0.000756758	0.485947707	0.001086096	0.008203882	2.12E-05	0.03395431	1	0.911384054	1	0.914417995	ENSG00000100731	11	PCNX
Pancreas	T1DM	ENSG00000229089.2	0.001352507	0.271834962	0.001230841	0.008204755	0.001331006	0.279515023	0.001230806	0.00820387	2.15E-05	0.013724392	1	0.798582374	1	0.797009688	ENSG00000100908	9	EMC9
Pancreas	T1DM	ENSG00000229091.2	-0.000381112	0.645376379	0.000828154	0.008204772	-0.00037143	0.653775972	0.000828118	0.008203887	-9.68E-06	0.01347867	1	0.961788216	1	0.961035047	ENSG00000100908	9	EMC9
Pancreas	T1DM	ENSG00000229212.3	-0.000888004	0.467500002	0.001222215	0.008204767	-0.000913217	0.454938685	0.001222173	0.008203881	2.52E-05	0.001068549	1	0.908712989	1	0.899776609	ENSG00000100908	9	EMC9
Pancreas	T1DM	ENSG00000229390.1	-0.001709263	0.011503191	0.00067639	0.008204668	-0.001967553	0.003697345	0.000677788	0.008203748	0.00025829	4.69E-09	1	0.260075765	1	0.128265071	ENSG00000100908	9	EMC9
Pancreas	T1DM	ENSG00000229391.3	0.005122867	5.98E-59	0.000316388	0.008200364	0.005878019	1.51E-53	0.000381513	0.008199895	-0.000755153	0.000397251	1.54E-55	1.54E-55	3.88E-50	3.88E-50	ENSG00000100979	10	PLTP
Pancreas	T1DM	ENSG00000229759.1	-0.000985625	0.001025772	0.000300186	0.008204594	-0.000990963	0.00096253	0.000300176	0.008203707	5.34E-06	0.005733977	1	0.047038972	1	0.045773668	ENSG00000100994	7	PYGB
Pancreas	T1DM	ENSG00000230310.1	0.000244935	0.748053599	0.000762546	0.008204774	0.00022557	0.767373932	0.000762544	0.008203889	1.94E-05	0.012498543	1	0.975415042	1	0.972640681	ENSG00000100994	7	PYGB
Pancreas	T1DM	ENSG00000230438.5	-5.74E-05	0.911560401	0.000516375	0.008204775	-6.76E-05	0.895780752	0.000516367	0.00820389	1.03E-05	0.021923025	1	0.995698473	1	0.993128823	ENSG00000100997	14	ABHD12
Pancreas	T1DM	ENSG00000230454.1	-0.000543256	0.405464882	0.000653029	0.008204764	-0.000553429	0.396709297	0.000653004	0.008203878	1.02E-05	0.004211125	1	0.88063625	1	0.871863634	ENSG00000101000	4	PROCR
Pancreas	T1DM	ENSG00000230795.2	-2.18E-05	0.938743813	0.000283945	0.008204775	-0.000114269	0.688377092	0.000284918	0.008203887	9.24E-05	9.59E-05	1	0.997898334	1	0.966685034	ENSG00000101000	4	PROCR
Pancreas	T1DM	ENSG00000231007.4	-0.001126019	0.026107633	0.000506164	0.008204692	-0.001116062	0.027453998	0.000506149	0.008203808	-9.96E-06	0.004092989	1	0.393484808	1	0.393865178	ENSG00000101000	4	PROCR
Pancreas	T1DM	ENSG00000231205.7	-0.000277874	0.714872875	0.000760632	0.008204773	-0.000257351	0.735100102	0.000760609	0.008203888	-2.05E-05	7.97E-05	1	0.975415042	1	0.968082788	ENSG00000101019	17	UQCC1
Pancreas	T1DM	ENSG00000231402.1	0.001752144	2.00E-09	0.000292109	0.00820417	0.001939407	6.79E-11	0.000297211	0.008203173	-0.000187263	0.000651418	5.13E-06	2.05E-07	1.74E-07	8.30E-09	ENSG00000101052	8	IFT52
Pancreas	T1DM	ENSG00000231443.2	0.001189497	0.14208444	0.000810242	0.008204739	0.001202657	0.137711216	0.000810213	0.008203853	-1.32E-05	0.006697566	1	0.705750175	1	0.692461116	ENSG00000101052	8	IFT52
Pancreas	T1DM	ENSG00000231500.2	-0.000244714	0.733599279	0.000719023	0.008204774	-0.000584094	0.418267373	0.00072161	0.008203879	0.000339381	3.44E-08	1	0.975415042	1	0.88842896	ENSG00000101052	8	IFT52
Pancreas	T1DM	ENSG00000232040.2	0.000521515	0.361884422	0.000571974	0.008204762	0.000336832	0.556430399	0.000572695	0.008203884	0.000184683	3.04E-10	1	0.854831234	1	0.93515266	ENSG00000101052	8	IFT52
Pancreas	T1DM	ENSG00000232210.1	-4.86E-05	0.926609199	0.000527781	0.008204775	-3.75E-05	0.943317993	0.000527777	0.00820389	-1.11E-05	0.029081205	1	0.997898334	1	0.999388266	ENSG00000101181	13	MTG2
Pancreas	T1DM	ENSG00000232615.4	-0.000724301	0.564996623	0.001258699	0.00820477	-0.000691977	0.582473849	0.001258654	0.008203885	-3.23E-05	1.78E-05	1	0.936676131	1	0.936315417	ENSG00000101181	13	MTG2
Pancreas	T1DM	ENSG00000233590.1	4.93E-05	0.899244942	0.000389653	0.008204775	4.28E-05	0.912559763	0.000389639	0.00820389	6.55E-06	0.003897409	1	0.99281063	1	0.996366272	ENSG00000101190	8	TCFL5
Pancreas	T1DM	ENSG00000233609.3	2.16E-05	0.977933636	0.000781152	0.008204776	-4.45E-06	0.995451665	0.000781161	0.00820389	2.61E-05	0.003509354	1	0.997898334	1	0.999770584	ENSG00000101191	12	DIDO1
Pancreas	T1DM	ENSG00000233922.1	0.000379619	0.752885572	0.001205762	0.008204774	0.000395957	0.742609483	0.001205715	0.008203888	-1.63E-05	0.013675767	1	0.975415042	1	0.968082788	ENSG00000101197	8	BIRC7
Pancreas	T1DM	ENSG00000234127.4	-0.001239381	0.021921401	0.000540805	0.008204687	-0.001865492	0.000676758	0.00054886	0.008203695	0.000626111	2.57E-11	1	0.366243427	1	0.034758299	ENSG00000101197	8	BIRC7
Pancreas	T1DM	ENSG00000234745.5	0.00146043	4.65E-05	0.000358581	0.008204496	0.000962971	0.009528653	0.000371446	0.008203777	0.000497458	2.90E-07	0.11930418	0.002651204	1	0.216544969	ENSG00000101198	10	NKAIN4
Pancreas	T1DM	ENSG00000234882.1	-0.000809632	0.456221979	0.001086634	0.008204766	-0.000827787	0.446170952	0.001086599	0.00820388	1.82E-05	0.011583075	1	0.899832468	1	0.896838446	ENSG00000101224	13	CDC25B
Pancreas	T1DM	ENSG00000235109.3	0.001183503	2.76E-07	0.000230302	0.008204331	0.001098206	1.96E-06	0.00023083	0.008203509	8.53E-05	6.28E-08	0.00070991	2.03E-05	0.005031361	0.000143753	ENSG00000101224	13	CDC25B
Pancreas	T1DM	ENSG00000235241.1	-9.53E-05	0.813415736	0.000403627	0.008204775	-8.80E-05	0.827462334	0.000403615	0.008203889	-7.29E-06	0.010609469	1	0.98201188	1	0.984037376	ENSG00000101224	13	CDC25B
Pancreas	T1DM	ENSG00000235290.1	0.001153289	0.04770598	0.000582474	0.00820471	0.001372884	0.018656361	0.000583626	0.008203797	-0.000219595	3.45E-09	1	0.50640868	1	0.329178568	ENSG00000101230	5	ISM1
Pancreas	T1DM	ENSG00000235477.2	-0.002518486	0.00347586	0.000861846	0.008204632	-0.002552613	0.00306095	0.000861914	0.008203742	3.41E-05	0.014893725	1	0.123976875	1	0.112293137	ENSG00000101282	4	RSPO4
Pancreas	T1DM	ENSG00000235677.1	0.00085416	0.240744466	0.000728104	0.008204752	0.000844635	0.246011427	0.000728075	0.008203867	9.53E-06	0.015234452	1	0.786897324	1	0.784493531	ENSG00000101307	11	SIRPB1
Pancreas	T1DM	ENSG00000235821.1	3.95E-05	0.968924943	0.001012731	0.008204776	0.000364097	0.719708278	0.001014625	0.008203888	-0.000324644	2.40E-07	1	0.997898334	1	0.968082788	ENSG00000101310	10	SEC23B
Pancreas	T1DM	ENSG00000235863.2	-0.002725338	5.93E-08	0.000502731	0.008204281	-0.001878393	0.000528441	0.000541959	0.008203688	-0.000846945	2.88E-05	0.000152211	4.76E-06	1	0.027694627	ENSG00000101310	10	SEC23B
Pancreas	T1DM	ENSG00000236417.2	-0.00111696	0.257718642	0.000986887	0.008204754	-0.001130075	0.252156268	0.000986854	0.008203868	1.31E-05	0.0362861	1	0.79720158	1	0.788955599	ENSG00000101310	10	SEC23B
Pancreas	T1DM	ENSG00000236624.4	0.000919359	0.098707844	0.000556798	0.00820473	0.000910377	0.102032855	0.000556778	0.008203845	8.98E-06	0.008153658	1	0.633704355	1	0.637519152	ENSG00000101310	10	SEC23B
Pancreas	T1DM	ENSG00000236682.1	0.001089748	0.076371591	0.000614933	0.008204723	0.001021151	0.097117144	0.000615523	0.008203844	6.86E-05	0.013276791	1	0.571784974	1	0.617318874	ENSG00000101333	12	PLCB4
Pancreas	T1DM	ENSG00000236699.4	0.00075968	0.357385442	0.000825416	0.008204761	0.000773341	0.348791615	0.000825393	0.008203875	-1.37E-05	0.02047818	1	0.854209198	1	0.847508521	ENSG00000101333	12	PLCB4
Pancreas	T1DM	ENSG00000237669.1	0.001805601	8.42E-06	0.000405371	0.008204442	0.002010631	7.94E-07	0.000407271	0.00820348	-0.000205029	2.15E-07	0.021629374	0.000527546	0.002039598	6.37E-05	ENSG00000101335	5	MYL9
Pancreas	T1DM	ENSG00000238034.1	0.000253395	0.722127215	0.000712549	0.008204773	0.000263553	0.711468379	0.000712528	0.008203888	-1.02E-05	0.039811249	1	0.975415042	1	0.968082788	ENSG00000101335	5	MYL9
Pancreas	T1DM	ENSG00000238105.3	0.000886374	0.322283326	0.000895529	0.008204759	0.000910082	0.309496296	0.000895503	0.008203873	-2.37E-05	0.000176319	1	0.832533491	1	0.82064652	ENSG00000101343	10	CRNKL1
Pancreas	T1DM	ENSG00000238142.1	0.000982921	0.258194566	0.000869326	0.008204754	0.000998596	0.250663323	0.000869296	0.008203868	-1.57E-05	0.00353551	1	0.79720158	1	0.788955599	ENSG00000101353	10	MROH8
Pancreas	T1DM	ENSG00000239257.1	-5.93E-05	0.890871112	0.000432216	0.008204775	-0.000236733	0.584753415	0.000433216	0.008203885	0.000177432	2.48E-09	1	0.99200447	1	0.936772783	ENSG00000101361	10	NOP56
Pancreas	T1DM	ENSG00000239272.1	-0.000187442	0.779973857	0.000670985	0.008204774	-0.000178473	0.790242753	0.000670962	0.008203889	-8.97E-06	0.034641258	1	0.980404234	1	0.97879227	ENSG00000101361	10	NOP56
Pancreas	T1DM	ENSG00000240053.8	-0.000302148	0.493962824	0.000441724	0.008204768	-4.71E-05	0.916140551	0.000447489	0.00820389	-0.000255029	0.000378443	1	0.914693561	1	0.997092545	ENSG00000101363	8	MANBAL
Pancreas	T1DM	ENSG00000240065.3	0.008406519	2.42E-21	0.000886254	0.008203261	0.008117937	6.51E-20	0.000888578	0.008202485	0.000288582	8.46E-06	6.22E-18	5.19E-19	1.67E-16	1.29E-17	ENSG00000101363	8	MANBAL
Pancreas	T1DM	ENSG00000240344.4	0.000196572	0.518951653	0.00030478	0.008204769	0.000191691	0.529368633	0.00030477	0.008203883	4.88E-06	0.014442056	1	0.914783379	1	0.920313283	ENSG00000101363	8	MANBAL
Pancreas	T1DM	ENSG00000241549.4	3.82E-05	0.889587231	0.00027522	0.008204775	2.53E-05	0.926903285	0.000275246	0.00820389	1.30E-05	0.006361831	1	0.99200447	1	0.999388266	ENSG00000101417	7	PXMP4
Pancreas	T1DM	ENSG00000241837.2	-0.000251117	0.740992035	0.000759718	0.008204774	-0.000235563	0.756504685	0.000759698	0.008203888	-1.56E-05	0.005922047	1	0.975415042	1	0.968082788	ENSG00000101425	8	BPI
Pancreas	T1DM	ENSG00000241839.5	0.000791347	0.329413134	0.00081139	0.00820476	0.000775192	0.339365834	0.000811363	0.008203875	1.62E-05	0.001753246	1	0.83462449	1	0.839770819	ENSG00000101452	10	DHX35
Pancreas	T1DM	ENSG00000243056.1	-0.001702261	0.082540202	0.000980487	0.008204725	-0.001688888	0.084969397	0.000980455	0.00820384	-1.34E-05	0.032385119	1	0.589329992	1	0.580557814	ENSG00000101452	10	DHX35
Pancreas	T1DM	ENSG00000243753.1	0.00148345	0.000240012	0.000403911	0.008204548	0.001306587	0.001262876	0.000405231	0.008203715	0.000176863	7.84E-08	0.616352091	0.012840669	1	0.056895894	ENSG00000101452	10	DHX35
Pancreas	T1DM	ENSG00000244219.2	-0.001080709	0.263218586	0.000965939	0.008204754	-0.001061323	0.271864514	0.00096591	0.00820387	-1.94E-05	0.004019711	1	0.79720158	1	0.79588826	ENSG00000101457	8	DNTTIP1
Pancreas	T1DM	ENSG00000244414.2	0.000468557	0.71388598	0.001277969	0.008204773	0.000491517	0.700519152	0.001277929	0.008203887	-2.30E-05	0.008417052	1	0.975415042	1	0.968082788	ENSG00000101457	8	DNTTIP1
Pancreas	T1DM	ENSG00000247157.2	0.000294935	0.351234257	0.000316386	0.008204761	0.000289365	0.360387484	0.000316375	0.008203876	5.57E-06	0.002724456	1	0.854061442	1	0.850635855	ENSG00000101457	8	DNTTIP1
Pancreas	T1DM	ENSG00000247596.4	0.001145022	0.118050197	0.000732574	0.008204734	0.001137052	0.120615832	0.000732544	0.008203849	7.97E-06	0.032643502	1	0.67493056	1	0.669849249	ENSG00000101457	8	DNTTIP1
Pancreas	T1DM	ENSG00000248202.1	-0.000679228	0.469926343	0.000939979	0.008204767	-0.000693951	0.460337815	0.000939942	0.008203881	1.47E-05	0.003982042	1	0.908712989	1	0.901310005	ENSG00000101460	8	MAP1LC3A
Pancreas	T1DM	ENSG00000248778.1	-2.54E-05	0.976570716	0.000864626	0.008204776	-1.16E-05	0.989323816	0.000864601	0.00820389	-1.38E-05	0.022632414	1	0.997898334	1	0.99966149	ENSG00000101464	6	PIGU
Pancreas	T1DM	ENSG00000248905.4	-0.000770922	0.455182853	0.001032296	0.008204766	-0.000804081	0.436012726	0.00103227	0.00820388	3.32E-05	1.98E-05	1	0.899832468	1	0.894313643	ENSG00000101546	8	RBFA
Pancreas	T1DM	ENSG00000248927.1	-0.000113185	0.893446593	0.000845014	0.008204775	-9.18E-05	0.913475053	0.000844986	0.00820389	-2.14E-05	6.91E-05	1	0.99200447	1	0.996818865	ENSG00000101639	14	CEP192
Pancreas	T1DM	ENSG00000249780.1	0.000137355	0.873912995	0.000865555	0.008204775	0.000154373	0.858443052	0.00086553	0.008203889	-1.70E-05	0.005275693	1	0.99200447	1	0.990577333	ENSG00000101751	6	POLI
Pancreas	T1DM	ENSG00000250376.1	-1.45E-05	0.988763035	0.001026609	0.008204776	-2.91E-05	0.977363819	0.001026571	0.00820389	1.47E-05	0.012165248	1	0.997898334	1	0.99966149	ENSG00000101751	6	POLI
Pancreas	T1DM	ENSG00000250506.2	-0.000532276	0.197693578	0.000413209	0.008204748	-0.000538335	0.192622862	0.000413195	0.008203861	6.06E-06	0.021052791	1	0.761135096	1	0.761003345	ENSG00000101751	6	POLI
Pancreas	T1DM	ENSG00000250535.1	0.000856703	0.026075995	0.00038502	0.008204692	0.000800378	0.037826469	0.000385402	0.008203817	5.63E-05	0.001372155	1	0.393484808	1	0.449714688	ENSG00000102575	6	ACP5
Pancreas	T1DM	ENSG00000250656.2	0.001058632	0.405036864	0.001271385	0.008204764	0.001079724	0.395725782	0.00127134	0.008203878	-2.11E-05	0.006501137	1	0.88063625	1	0.871863634	ENSG00000102575	6	ACP5
Pancreas	T1DM	ENSG00000250986.1	-0.000561109	0.435906661	0.000720179	0.008204765	-0.000573393	0.425910794	0.000720153	0.008203879	1.23E-05	0.00497619	1	0.89813429	1	0.889527368	ENSG00000102699	5	PARP4
Pancreas	T1DM	ENSG00000251008.1	-0.000169862	0.877046613	0.001097905	0.008204775	-0.000186403	0.865177656	0.001097862	0.008203889	1.65E-05	0.005802518	1	0.99200447	1	0.990577333	ENSG00000102760	12	RGCC
Pancreas	T1DM	ENSG00000251226.1	-0.000750896	0.49658219	0.001104458	0.008204768	-0.000731599	0.507699366	0.001104426	0.008203883	-1.93E-05	0.0145484	1	0.914783379	1	0.914417995	ENSG00000102780	12	DGKH
Pancreas	T1DM	ENSG00000251417.1	-0.001085838	0.330363558	0.00111553	0.00820476	-0.001063943	0.340193967	0.001115498	0.008203875	-2.19E-05	0.005450065	1	0.83462449	1	0.840825897	ENSG00000102780	12	DGKH
Pancreas	T1DM	ENSG00000251468.2	-0.001641867	0.188823597	0.001249452	0.008204746	-0.001658462	0.184376238	0.001249404	0.00820386	1.66E-05	0.017888367	1	0.751239289	1	0.747990804	ENSG00000102837	6	OLFM4
Pancreas	T1DM	ENSG00000251580.1	0.000458175	0.662372888	0.00104932	0.008204772	0.000442137	0.67348364	0.00104928	0.008203887	1.60E-05	0.005686269	1	0.962467202	1	0.961035047	ENSG00000102837	6	OLFM4
Pancreas	T1DM	ENSG00000251639.2	-0.000862755	0.450438053	0.001143198	0.008204766	-0.000848245	0.458076952	0.00114316	0.008203881	-1.45E-05	0.046014923	1	0.899061289	1	0.900031838	ENSG00000102837	6	OLFM4
Pancreas	T1DM	ENSG00000253816.2	0.000763963	0.306140065	0.000746527	0.008204758	0.00077668	0.298144052	0.000746504	0.008203872	-1.27E-05	0.012668172	1	0.822290036	1	0.810652485	ENSG00000102858	8	MGRN1
Pancreas	T1DM	ENSG00000254731.1	0.000245061	0.810247562	0.001020631	0.008204775	0.000258455	0.80008376	0.001020599	0.008203889	-1.34E-05	0.048938385	1	0.98201188	1	0.978923745	ENSG00000102967	7	DHODH
Pancreas	T1DM	ENSG00000254811.1	0.00021151	0.850898636	0.001125219	0.008204775	0.000195722	0.86190698	0.001125181	0.008203889	1.58E-05	0.024674025	1	0.989913489	1	0.990577333	ENSG00000102977	9	ACD
Pancreas	T1DM	ENSG00000254851.1	0.0006701	0.071875172	0.000372296	0.008204721	0.000659704	0.07638796	0.000372284	0.008203837	1.04E-05	2.80E-05	1	0.560516232	1	0.562075305	ENSG00000102996	4	MMP15
Pancreas	T1DM	ENSG00000254872.2	0.002688338	0.001797366	0.000861139	0.008204611	0.00266918	0.001937575	0.000861122	0.008203728	1.92E-05	0.006411044	1	0.075680736	1	0.078979241	ENSG00000102996	4	MMP15
Pancreas	T1DM	ENSG00000254999.2	-0.000369866	0.235190515	0.000311572	0.008204752	-0.000375448	0.228185223	0.000311562	0.008203866	5.58E-06	0.00743254	1	0.785512902	1	0.780702981	ENSG00000103148	11	NPRL3
Pancreas	T1DM	ENSG00000255065.1	7.65E-05	0.932663148	0.000905817	0.008204775	6.21E-05	0.945376623	0.000905786	0.00820389	1.45E-05	0.009245703	1	0.997898334	1	0.999388266	ENSG00000103148	11	NPRL3
Pancreas	T1DM	ENSG00000255394.2	0.000546917	0.662338242	0.001252421	0.008204772	0.000597204	0.63347999	0.001252435	0.008203886	-5.03E-05	0.000422047	1	0.962467202	1	0.955974304	ENSG00000103148	11	NPRL3
Pancreas	T1DM	ENSG00000255423.1	-0.001140452	0.209748111	0.000909263	0.008204749	-0.001130595	0.213696023	0.000909227	0.008203864	-9.86E-06	0.045631431	1	0.771198903	1	0.769778216	ENSG00000103168	12	TAF1C
Pancreas	T1DM	ENSG00000255533.1	0.000259217	0.585559814	0.000475381	0.008204771	0.000246415	0.604227101	0.0004754	0.008203885	1.28E-05	0.049187396	1	0.937588291	1	0.939938439	ENSG00000103168	12	TAF1C
Pancreas	T1DM	ENSG00000255595.1	0.000920482	0.260904897	0.000818748	0.008204754	0.000934136	0.253882864	0.00081872	0.008203868	-1.37E-05	0.009064687	1	0.79720158	1	0.788955599	ENSG00000103168	12	TAF1C
Pancreas	T1DM	ENSG00000255837.1	0.000887597	0.12513942	0.000578783	0.008204736	0.000879716	0.128513203	0.000578763	0.008203851	7.88E-06	0.023504386	1	0.680843282	1	0.676232745	ENSG00000103174	7	NAGPA
Pancreas	T1DM	ENSG00000256073.2	-9.89E-05	0.83254263	0.000467733	0.008204775	-9.07E-05	0.846244202	0.000467715	0.008203889	-8.20E-06	0.002501772	1	0.984098328	1	0.986098414	ENSG00000103174	7	NAGPA
Pancreas	T1DM	ENSG00000256166.1	0.00471818	1.50E-25	0.000451575	0.008202938	0.004916826	6.19E-27	0.000457522	0.008201946	-0.000198646	0.007027382	3.86E-22	3.51E-23	1.59E-23	1.84E-24	ENSG00000103196	7	CRISPLD2
Pancreas	T1DM	ENSG00000256433.1	-0.00015256	0.855132148	0.000835606	0.008204775	-0.000161648	0.846600938	0.000835573	0.008203889	9.09E-06	0.046734146	1	0.990317162	1	0.986098414	ENSG00000103196	7	CRISPLD2

Pancreas	T1DM	ENSG00000256799.1	-0.000150167	0.832045851	0.000708072	0.008204775	-0.000160846	0.820292245	0.000708043	0.008203889	1.07E-05	0.003412607	1	0.984098328	1	0.98205617	ENSG00000103199	9	ZNF500
Pancreas	T1DM	ENSG00000257048.1	-0.001434722	0.222724202	0.00117666	0.008204751	-0.001408048	0.23142748	0.001176617	0.008203866	-2.67E-05	0.000139973	1	0.783501029	1	0.780702981	ENSG00000103226	13	NOMO3
Pancreas	T1DM	ENSG00000257218.1	-0.00211421	0.087504839	0.001237312	0.008204726	-0.002133375	0.084661727	0.00123727	0.00820384	1.92E-05	0.015945015	1	0.595273618	1	0.580557814	ENSG00000103248	13	MTHFSD
Pancreas	T1DM	ENSG00000257949.2	-0.001403248	0.067100106	0.00076638	0.008204719	-0.001412185	0.065367531	0.000766351	0.008203833	8.94E-06	0.036058565	1	0.548630246	1	0.536001791	ENSG00000103269	9	RHBDL1
Pancreas	T1DM	ENSG00000258301.2	-0.000344835	0.582688343	0.000627586	0.00820477	-0.000355395	0.57118371	0.000627563	0.008203885	1.06E-05	0.004201821	1	0.937588291	1	0.936315417	ENSG00000103269	9	RHBDL1
Pancreas	T1DM	ENSG00000259205.2	-0.000168309	0.671320216	0.000396641	0.008204773	-0.000173297	0.662161812	0.000396625	0.008203887	4.99E-06	0.01686453	1	0.963860935	1	0.961035047	ENSG00000103269	9	RHBDL1
Pancreas	T1DM	ENSG00000259363.1	-0.001376841	0.135567666	0.000922504	0.008204738	-0.001394987	0.130478003	0.000922475	0.008203851	1.81E-05	0.003508336	1	0.694369535	1	0.676232745	ENSG00000103275	14	UBE2I
Pancreas	T1DM	ENSG00000259433.2	0.000857829	0.033891261	0.000404375	0.0082047	0.000851696	0.035180896	0.000404361	0.008203815	6.13E-06	0.014279517	1	0.436429495	1	0.43733359	ENSG00000103351	8	CLUAP1
Pancreas	T1DM	ENSG00000259726.1	-0.000132091	0.891877232	0.000971765	0.008204775	-0.000145656	0.880849148	0.000971733	0.00820389	1.36E-05	0.030787093	1	0.99200447	1	0.993128823	ENSG00000103351	8	CLUAP1
Pancreas	T1DM	ENSG00000259982.1	7.19E-06	0.985126293	0.000385784	0.008204776	1.45E-06	0.997008559	0.000385773	0.00820389	5.75E-06	0.035348063	1	0.997898334	1	0.999770584	ENSG00000103356	11	EARS2
Pancreas	T1DM	ENSG00000260302.1	0.00336409	1.27E-06	0.000694403	0.00820438	0.003374929	1.17E-06	0.00069438	0.008203492	-1.08E-05	0.016274433	0.003259257	8.63E-05	0.00301007	9.12E-05	ENSG00000103356	11	EARS2
Pancreas	T1DM	ENSG00000260539.1	0.000681607	0.588847034	0.001261049	0.008204771	0.000656893	0.602415463	0.001261	0.008203885	2.47E-05	0.000335155	1	0.937588291	1	0.939938439	ENSG00000103356	11	EARS2
Pancreas	T1DM	ENSG00000260804.2	0.000916801	0.246840422	0.00079167	0.008204753	0.000942388	0.233886249	0.00079165	0.008203866	-2.56E-05	2.14E-05	1	0.788479116	1	0.780702981	ENSG00000103356	11	EARS2
Pancreas	T1DM	ENSG00000261584.1	-0.000939859	0.020750815	0.000406426	0.008204686	-0.000901806	0.026515993	0.00040648	0.008203807	-3.81E-05	1.14E-06	1	0.35290128	1	0.382545334	ENSG00000103356	11	EARS2
Pancreas	T1DM	ENSG00000261652.2	-0.000138042	0.915599996	0.00130256	0.008204775	-0.000165061	0.899159874	0.001302534	0.00820389	2.70E-05	0.012230987	1	0.995870671	1	0.994038165	ENSG00000103415	7	HMOX2
Pancreas	T1DM	ENSG00000262855.1	0.000582968	0.370105514	0.000650434	0.008204762	0.000595102	0.360213432	0.000650413	0.008203876	-1.21E-05	0.00576012	1	0.856244109	1	0.850635855	ENSG00000103415	7	HMOX2
Pancreas	T1DM	ENSG00000262943.3	-0.000340931	0.675252624	0.000813774	0.008204773	-0.000330163	0.684939951	0.000813748	0.008203887	-1.08E-05	0.044769729	1	0.963860935	1	0.966338775	ENSG00000103415	7	HMOX2
Pancreas	T1DM	ENSG00000263006.2	-0.001523212	0.17436548	0.001121401	0.008204744	-0.001550908	0.166649978	0.001121367	0.008203858	2.77E-05	0.000343969	1	0.729268002	1	0.726605951	ENSG00000103472	5	RRN3P2
Pancreas	T1DM	ENSG00000264469.1	0.000552632	0.36317565	0.000607734	0.008204762	0.000537563	0.376409473	0.000607738	0.008203877	1.51E-05	0.02354872	1	0.854831234	1	0.857692571	ENSG00000103472	5	RRN3P2
Pancreas	T1DM	ENSG00000266402.2	0.001108064	0.15981502	0.000788267	0.008204742	0.001088511	0.167297849	0.000788239	0.008203858	1.96E-05	3.37E-05	1	0.724477654	1	0.726605951	ENSG00000103472	5	RRN3P2
Pancreas	T1DM	ENSG00000266904.1	0.000529321	0.403865143	0.000634115	0.008204764	0.000539576	0.39480465	0.000634096	0.008203878	-1.03E-05	0.01685294	1	0.879665554	1	0.871863634	ENSG00000103479	10	RBL2
Pancreas	T1DM	ENSG00000266995.1	-0.000101643	0.850808891	0.000540407	0.008204775	-9.24E-05	0.864183854	0.000540386	0.008203889	-9.21E-06	0.002280723	1	0.989913489	1	0.990577333	ENSG00000103479	10	RBL2
Pancreas	T1DM	ENSG00000267648.1	0.00126068	0.148544256	0.000872627	0.00820474	0.001279922	0.142431914	0.000872596	0.008203854	-1.92E-05	0.000344558	1	0.712186059	1	0.696695532	ENSG00000103550	9	KNOP1
Pancreas	T1DM	ENSG00000268362.1	-2.38E-05	0.961353034	0.000491947	0.008204776	-1.64E-05	0.973367387	0.000491932	0.00820389	-7.41E-06	0.022655139	1	0.997898334	1	0.99966149	ENSG00000103550	9	KNOP1
Pancreas	T1DM	ENSG00000268822.1	-0.000118201	0.868178372	0.000712171	0.008204775	-0.00013198	0.852972529	0.000712145	0.008203889	1.38E-05	0.000910303	1	0.99183698	1	0.989215428	ENSG00000103550	9	KNOP1
Pancreas	T1DM	ENSG00000269893.2	-0.00138849	0.200339641	0.001084262	0.008204748	-0.00140403	0.195333024	0.001084223	0.008203862	1.55E-05	0.017613739	1	0.7650738	1	0.762604991	ENSG00000103671	5	TRIP4
Pancreas	T1DM	ENSG00000269990.1	0.000515611	0.618276427	0.00103475	0.008204771	0.000502061	0.627522382	0.001034713	0.008203886	1.36E-05	0.028408873	1	0.95073884	1	0.954101526	ENSG00000103742	7	IGDCC4
Pancreas	T1DM	ENSG00000270081.1	0.000122945	0.860240302	0.000698281	0.008204775	0.000230429	0.741808038	0.000699412	0.008203888	-0.000107483	0.007842431	1	0.990511029	1	0.968082788	ENSG00000103742	7	IGDCC4
Pancreas	T1DM	ENSG00000270127.1	-0.000101892	0.725661778	0.000290372	0.008204773	-0.000106353	0.714156914	0.000290361	0.008203888	4.46E-06	0.004703015	1	0.975415042	1	0.968082788	ENSG00000103811	11	CTSH
Pancreas	T1DM	ENSG00000270237.1	0.000177476	0.879664065	0.001172267	0.008204775	0.000199315	0.864986506	0.001172236	0.008203889	-2.18E-05	0.011896548	1	0.99200447	1	0.990577333	ENSG00000103811	11	CTSH
Pancreas	T1DM	ENSG00000270917.1	-0.000409911	0.66179306	0.000937069	0.008204772	-0.000423944	0.650956909	0.000937031	0.008203887	1.40E-05	0.004605483	1	0.962467202	1	0.961035047	ENSG00000103811	11	CTSH
Pancreas	T1DM	ENSG00000271821.1	0.004981549	1.08E-10	0.000771607	0.008204074	0.003872823	1.56E-06	0.000806248	0.008203502	0.001108726	2.13E-06	2.76E-07	1.26E-08	0.004004901	0.000117791	ENSG00000103811	11	CTSH
Pancreas	T1DM	ENSG00000271889.1	0.000937929	0.221310826	0.000766876	0.00820475	0.000926409	0.227020271	0.00076685	0.008203865	1.15E-05	0.015102417	1	0.780835021	1	0.780702981	ENSG00000103811	11	CTSH
Pancreas	T1DM	ENSG00000272097.1	0.001379181	0.174067392	0.001014662	0.008204744	0.001367173	0.177830207	0.001014623	0.008203859	1.20E-05	0.038162405	1	0.729268002	1	0.731367165	ENSG00000103932	7	RPAP1
Pancreas	T1DM	ENSG00000272221.1	0.001054874	1.58E-06	0.000219696	0.008204387	0.001179828	9.10E-08	0.000220777	0.008203409	-0.000124954	1.24E-08	0.004045142	0.000103722	0.000233599	8.34E-06	ENSG00000103932	7	RPAP1
Pancreas	T1DM	ENSG00000272541.1	0.001031465	0.215199232	0.000832232	0.00820475	0.00102124	0.219765285	0.000832202	0.008203865	1.02E-05	0.044234835	1	0.773993876	1	0.776261656	ENSG00000104044	11	OCA2
Pancreas	T1DM	ENSG00000272733.1	0.000326959	0.581277709	0.000592834	0.00820477	0.000333959	0.573196934	0.000592809	0.008203885	-7.00E-06	0.021631784	1	0.937588291	1	0.936315417	ENSG00000104140	6	RHOV
Pancreas	T1DM	ENSG00000272799.1	2.51E-05	0.965067058	0.000573188	0.008204776	3.24E-05	0.954871115	0.000573167	0.00820389	-7.33E-06	0.028397922	1	0.997898334	1	0.999388266	ENSG00000104219	8	ZDHHC2
Pancreas	T1DM	ENSG00000273234.1	-0.000492003	0.630120898	0.001021693	0.008204772	-0.000477039	0.640550648	0.001021652	0.008203886	-1.50E-05	0.006368559	1	0.95754589	1	0.957315064	ENSG00000104219	8	ZDHHC2
Pancreas	T1DM	ENSG00000273340.1	-0.001708305	6.75E-09	0.000294678	0.00820421	-0.001647747	2.43E-08	0.000295384	0.008203366	-6.06E-05	0.003328182	1.73E-05	6.19E-07	6.24E-05	2.31E-06	ENSG00000104231	6	ZFAND1
Pancreas	T2DM_admission	ENSG00000002919.10	0.001249152	0.33262339	0.001289322	0.073378338	0.001227799	0.340952034	0.001289317	0.07337387	2.14E-05	0.022647671	1	0.708860469	1	0.715170664	ENSG00000104324	11	CPQ
Pancreas	T2DM_admission	ENSG00000005102.8	0.005294099	0.059717802	0.002811709	0.073377945	0.005187869	0.065042229	0.002811896	0.073373494	0.000106229	0.006637847	1	0.322853295	1	0.338114259	ENSG00000104331	4	IMPAD1
Pancreas	T2DM_admission	ENSG00000005513.9	0.004103606	0.127953577	0.002695796	0.07337813	0.004061112	0.131945914	0.002695771	0.073373665	4.25E-05	0.015248037	1	0.483297266	1	0.490357609	ENSG00000104388	10	RAB2A
Pancreas	T2DM_admission	ENSG00000005981.8	0.003074695	0.361048536	0.00336632	0.073378354	0.003022825	0.369200562	0.003366277	0.073373885	5.19E-05	0.010021531	1	0.723010568	1	0.727627319	ENSG00000104388	10	RAB2A
Pancreas	T2DM_admission	ENSG00000009413.11	-0.00129455	0.733487233	0.003802015	0.073378462	-0.001205638	0.751162698	0.003801994	0.073373991	-8.89E-05	0.000982518	1	0.919728783	1	0.925863522	ENSG00000104442	5	ARMC1
Pancreas	T2DM_admission	ENSG00000011114.10	0.00352401	0.331658926	0.003630085	0.073378337	0.003599353	0.321425729	0.003630088	0.073373859	-7.53E-05	0.008676559	1	0.707897253	1	0.706943978	ENSG00000104442	5	ARMC1
Pancreas	T2DM_admission	ENSG00000012232.4	-0.001840316	0.438556732	0.002375726	0.073378389	-0.001802116	0.448120769	0.002375731	0.07337392	-3.82E-05	0.046322742	1	0.774562372	1	0.779994504	ENSG00000104442	5	ARMC1
Pancreas	T2DM_admission	ENSG00000012963.9	0.004244308	0.045549621	0.002122634	0.073377877	0.004282629	0.043631314	0.002122614	0.073373394	-3.83E-05	0.005489301	1	0.285670854	1	0.28152064	ENSG00000104442	5	ARMC1
Pancreas	T2DM_admission	ENSG00000022556.11	0.000256711	0.926306962	0.002775488	0.073378478	0.000217506	0.937535644	0.002775458	0.073374006	3.92E-05	0.025028505	1	0.979054639	1	0.98183872	ENSG00000104442	5	ARMC1
Pancreas	T2DM_admission	ENSG00000030110.8	0.016024865	1.52E-09	0.002652009	0.073372982	0.015031688	1.71E-08	0.002665748	0.073369219	0.000993178	0.000246949	3.90E-06	4.87E-07	4.40E-05	5.50E-06	ENSG00000104442	5	ARMC1
Pancreas	T2DM_admission	ENSG00000037757.9	-0.001877259	0.378347296	0.002130957	0.073378362	-0.001951203	0.359853134	0.002130958	0.07337388	7.39E-05	9.87E-06	1	0.733271857	1	0.72195535	ENSG00000104450	8	SPAG1
Pancreas	T2DM_admission	ENSG00000038219.8	0.000782718	0.525164807	0.001231841	0.073378418	0.00080257	0.514707885	0.001231833	0.073373943	-1.99E-05	0.02012302	1	0.827884115	1	0.81978735	ENSG00000104518	6	GSDMD
Pancreas	T2DM_admission	ENSG00000049449.4	-0.000327736	0.90086644	0.002631006	0.073378477	-0.000375703	0.886450704	0.002631035	0.073374003	4.80E-05	0.044855867	1	0.972507979	1	0.972260921	ENSG00000104522	11	TSTA3
Pancreas	T2DM_admission	ENSG00000061455.10	0.005622553	0.040954942	0.002750772	0.07337785	0.005670307	0.03926847	0.00275075	0.073373367	-4.78E-05	0.009730729	1	0.274649	1	0.27460182	ENSG00000104679	6	R3HCC1
Pancreas	T2DM_admission	ENSG00000063046.13	0.000709474	0.590544244	0.001318607	0.073378436	0.000739876	0.574724173	0.001318598	0.073373959	-3.04E-05	0.000776641	1	0.858891717	1	0.84967857	ENSG00000104689	5	TNFRSF10A
Pancreas	T2DM_admission	ENSG00000063854.8	-0.010218324	0.003160101	0.003461804	0.073377167	-0.010134706	0.00341634	0.003461811	0.073372716	-8.36E-05	0.002660116	1	0.057554185	1	0.062665442	ENSG00000104689	5	TNFRSF10A
Pancreas	T2DM_admission	ENSG00000064270.8	0.000302326	0.889633692	0.002178649	0.073378476	0.000256225	0.90637876	0.002178644	0.073374004	4.61E-05	0.004812447	1	0.972507979	1	0.972260921	ENSG00000104689	5	TNFRSF10A
Pancreas	T2DM_admission	ENSG00000064687.8	-0.001038041	0.670822654	0.002442337	0.073378452	-0.000993793	0.684078142	0.002442323	0.073373982	-4.42E-05	0.010092476	1	0.894197754	1	0.899034119	ENSG00000104714	9	ERICH1
Pancreas	T2DM_admission	ENSG00000066294.10	0.002385396	0.136052744	0.001600234	0.073378145	0.007160392	0.000211199	0.001932495	0.073371939	-0.004774996	1.05E-05	1	0.496989255	0.542359703	0.00904011	ENSG00000104731	9	KLHDC4
Pancreas	T2DM_admission	ENSG00000069966.14	0.005094226	0.105044432	0.003142881	0.073378084	0.005142243	0.101803815	0.003142846	0.073373603	-4.80E-05	0.014483039	1	0.438624554	1	0.434949328	ENSG00000104731	9	KLHDC4
Pancreas	T2DM_admission	ENSG00000070182.13	0.007528394	0.001577677	0.002382388	0.073376976	0.007574605	0.001475735	0.002382377	0.073372484	-4.62E-05	0.007511067	1	0.03523021	1	0.033836506	ENSG00000104774	8	MAN2B1
Pancreas	T2DM_admission	ENSG00000073849.10	0.01083172	0.000153067	0.00286094	0.073376321	0.010792528	0.000161688	0.0028609	0.073371864	3.92E-05	0.017000187	0.393075034	0.00680233	0.415215244	0.007414558	ENSG00000104774	8	MAN2B1
Pancreas	T2DM_admission	ENSG00000074706.9	-0.005431895	0.081733365	0.003120488	0.073378023	-0.005385141	0.084390828	0.003120442	0.073373558	-4.68E-05	0.007608974	1	0.383490365	1	0.395012569	ENSG00000104774	8	MAN2B1
Pancreas	T2DM_admission	ENSG00000076662.5	-0.00226732	0.379171014	0.002578183	0.073378363	-0.002220832	0.389022041	0.002578186	0.073373895	-4.65E-05	0.023537009	1	0.733271857	1	0.743863441	ENSG00000104774	8	MAN2B1
Pancreas	T2DM_admission	ENSG00000077585.9	-0.001451856	0.560180005	0.002492137	0.073378428	-0.00149315	0.549073526	0.002492117	0.073373952	4.13E-05	0.01281163	1	0.847939375	1	0.84635103	ENSG00000104774	8	MAN2B1
Pancreas	T2DM_admission	ENSG00000079689.9	0.000419846	0.881833452	0.002824476	0.073378476	0.000173814	0.950941506	0.00282512	0.073374006	0.000246031	0.000126464	1	0.972507979	1	0.98349488	ENSG00000104774	8	MAN2B1
Pancreas	T2DM_admission	ENSG00000085117.7	-5.36E-05	0.981532143	0.002315699	0.073378479	-1.81E-05	0.993759643	0.00231568	0.073374007	-3.55E-05	0.021755768	1	0.994701872	1	0.999041616	ENSG00000104823	4	ECH1
Pancreas	T2DM_admission	ENSG00000088451.6	-0.000218062	0.885630306	0.001516052	0.073378476	-0.000551693	0.716618783	0.001519893	0.073373987	0.000333631	0.002132901	1	0.972507979	1	0.913596366	ENSG00000104823	4	ECH1
Pancreas	T2DM_admission	ENSG00000090686.11	-0.000433316	0.765312446	0.001451584	0.073378466	-0.000492501	0.734407568	0.001451655	0.073373989	5.92E-05	0.001191238	1	0.927632086	1	0.922046709	ENSG00000104823	4	ECH1
Pancreas	T2DM_admission	ENSG00000094916.9	-0.00269989	0.284029375	0.00252017	0.073378306	-0.002647307	0.293508085	0.002520145	0.07337384	-5.26E-05	0.001177961	1	0.679218679	1	0.689809485	ENSG00000104835	10	SARS2
Pancreas	T2DM_admission	ENSG00000100099.16	0.002654587	0.379515913	0.003020732	0.073378363	0.002710927	0.369480539	0.003020707	0.073373885	-5.63E-05	0.005400968	1	0.733271857	1	0.727627319	ENSG00000104835	10	SARS2
Pancreas	T2DM_admission	ENSG00000100258.13	0.001625625	0.203373976	0.001278008	0.073378236	0.001608935	0.208045941	0.001277989	0.073373768	1.67E-05	0.019225593	1	0.584188335	1	0.592308178	ENSG00000104835	10	SARS2
Pancreas	T2DM_admission	ENSG00000100558.4	0.002646962	0.428921254	0.003346181	0.073378385	0.002610656	0.435272362	0.003346128	0.073373915	3.63E-05	0.045207829	1	0.767595622	1	0.771947117	ENSG00000104852	10	SNRNP70
Pancreas	T2DM_admission	ENSG00000100731.11	0.002457134	0.401820595	0.002930829	0.073378373	0.002407513	0.411387311	0.002930789	0.073373905	4.96E-05	0.003603246	1	0.754055489	1	0.758117931	ENSG00000104866	6	PPP1R37
Pancreas	T2DM_admission	ENSG00000100908.9	0.001271809	0.373089351	0.001427872	0.07337836	0.001237148	0.38627012	0.001427914	0.073373894	3.47E-05	0.026243464	1	0.730660228	1	0.740807819	ENSG00000104866	6	PPP1R37
Pancreas	T2DM_admission	ENSG00000101457.8	-0.001900598	0.598727088	0.003611709	0.073378437	-0.001953673	0.588554933	0.003611682	0.073373962	5.31E-05	0.029565634	1	0.862328189	1	0.856807861	ENSG00000104866	6	PPP1R37
Pancreas	T2DM_admission	ENSG00000102575.6	-0.002966475	0.272475719	0.002703233	0.073378298	-0.001626673	0.55855496	0.002780687	0.073373955	-0.001339802	0.039905731	1	0.670227632	1	0.847111521	ENSG00000104866	6	PPP1R37
Pancreas	T2DM_admission	ENSG00000102837.6	0.005563028	0.014795657	0.002282407	0.073377585	0.005624561	0.013727733	0.0022824	0.073373092	-6.15E-05	0.000271043	1	0.163069734	1	0.152590546	ENSG00000104866	6	PPP1R37
Pancreas	T2DM_admission	ENSG00000102996.4	0.001675917	0.614394862	0.003326484	0.073378441	0.001579471	0.634939519	0.003326699	0.073373973	9.64E-05	0.035578484	1	0.871212593	1	0.880942324	ENSG00000104870	8	FCGRT
Pancreas	T2DM_admission	ENSG00000104774.8	-0.000458181	0.836566141	0.002221078	0.073378473	-0.000368391	0.86828296	0.002221364	0.073374002	-8.98E-05	0.023476416	1	0.958120026	1	0.967511091	ENSG00000104899	4	AMH
Pancreas	T2DM_admission	ENSG00000104823.4	0.00026145	0.918981898	0.002570385	0.073378478	0.000313636	0.9028856	0.002570426	0.073374004	-5.22E-05	0.034596773	1	0.977203112	1	0.972260921	ENSG00000104901	2	DKKL1
Pancreas	T2DM_admission	ENSG00000105479.11	0.000549574	0.876974473	0.00355009	0.073378476	0.000602454	0.865244772	0.003550077	0.073374002	-5.29E-05	0.042657618	1	0.970211524	1	0.965225271	ENSG00000104901	2	DKKL1
Pancreas	T2DM_admission	ENSG00000106992.13	0.000178438	0.93073588	0.002052917	0.073378478	0.000150069	0.941725829	0.002052895	0.073374006	2.84E-05	0.027742581	1	0.979119408	1	0.981898834	ENSG00000104901	2	DKKL1
Pancreas	T2DM_admission	ENSG00000108264.12	0.000790186	0.49830216	0.001166904	0.07337841	0.000810033	0.487569755	0.001166892	0.073373934	-1.98E-05	0.007352523	1	0.808874808	1	0.804739043	ENSG00000104953	14	TLE6
Pancreas	T2DM_admission	ENSG00000109063.10	-0.001987865	0.523249639	0.003114094	0.073378418	-0.001945963	0.532040938	0.00311407	0.073373948	-4.19E-05	0.045460393	1	0.825853281	1	0.834115463	ENSG00000104953	14	TLE6
Pancreas	T2DM_admission	ENSG00000109511.6	0.000482456	0.865086029	0.002839586	0.073378475	0.000525346	0.85322109	0.002839553	0.073374001	-4.29E-05	0.014060785	1	0.966374076	1	0.961211992	ENSG00000104953	14	TLE6
Pancreas	T2DM_admission	ENSG00000109794.9	0.006696785	0.009780967	0.002592148	0.073377474	0.006663016	0.010155464	0.002592109	0.073373012	3.38E-05	0.017589675	1	0.120179531	1	0.123598259	ENSG00000104953	14	TLE6
Pancreas	T2DM_admission	ENSG00000110436.7	-0.001412892	0.711173994	0.00381574	0.073378459	-0.001186036	0.756022104	0.003817194	0.073373992	-0.000226857	0.038221367	1	0.912258576	1	0.926713491	ENSG00000104953	14	TLE6
Pancreas	T2DM_admission	ENSG00000110888.13	0.002276359	0.206112245	0.001800447	0.073378238	0.002253559	0.210685099	0.001800419	0.073373771	2.28E-05	0.02022533	1	0.590494782	1	0.597756217	ENSG00000104967	6	NOVA2
Pancreas	T2DM_admission	ENSG00000111725.6	-0.000443703	0.861637279	0.002545769	0.073378475	-0.000408653	0.872469006	0.002545754	0.073374003	-3.51E-05	0.049546427	1	0.965890282	1	0.967818751	ENSG00000104967	6	NOVA2
Pancreas	T2DM_admission	ENSG00000112149.5	-0.004668042	0.010464694	0.001823387	0.073377492	-0.002468677	0.191015653	0.001887971	0.073373749	-0.002199364	7.10E-06	1	0.125576324	1	0.570204108	ENSG00000104979	4	C19orf53
Pancreas	T2DM_admission	ENSG00000112294.8	0.002762611	0.404573797	0.003314545	0.073378375	0.002684894	0.417924779	0.003314572	0.073373908	7.77E-05	0.007543522	1	0.755550584	1	0.763867723	ENSG00000104983	4	CCDC61
Pancreas	T2DM_admission	ENSG00000112343.8	0.000551183	0.801463074	0.00219198	0.07337847	0.000614829	0.77911102	0.002192076	0.073373995	-6.36E-05	0.017098792	1	0.939717987	1	0.936079501	ENSG00000105072	4	C19orf44
Pancreas	T2DM_admission	ENSG00000112874.5	0.001685977	0.609861451	0.003304077	0.07337844	0.001731792	0.600179377	0.003304046	0.073373965	-4.58E-05	0.032514065	1	0.868723317	1	0.863271188	ENSG00000105088	4	OLFM2
Pancreas	T2DM_admission	ENSG00000112977.11	0.001122667	0.474520155	0.001569846	0.073378402	0.001146254	0.465282436	0.001569831	0.073373926	-2.36E-05	0.019187727	1	0.795049308	1	0.789197685	ENSG00000105088	4	OLFM2
Pancreas	T2DM_admission	ENSG00000113734.13	0.000455187	0.690352963	0.001142604	0.073378455	0.0004367	0.702311884	0.001142591	0.073373985	1.85E-05	0.008719955	1	0.903120941	1	0.908125337	ENSG00000105088	4	OLFM2
Pancreas	T2DM_admission	ENSG00000114446.4	0.006374746	0.070967417	0.003530366	0.073377988	0.006415495	0.069177432	0.003530306	0.073373509	-4.07E-05	0.026324015	1	0.35594595	1	0.35006171	ENSG00000105088	4	OLFM2
Pancreas	T2DM_admission	ENSG00000114638.3	0.001469613	0.665697215	0.003401402	0.073378451	0.00142526	0.675197028	0.00340136	0.07337398	4.44E-05	0.030456347	1	0.891995883	1	0.894675443	ENSG00000105088	4	OLFM2
Pancreas	T2DM_admission	ENSG00000115718.13	0.004785605	0.102643048	0.00293204	0.073378078	0.006331542	0.036971202	0.003035127	0.073373351	-0.001545937	0.048816228	1	0.431403186	1	0.266843516	ENSG00000105088	4	OLFM2
Pancreas	T2DM_admission	ENSG00000116157.5	-0.002034419	0.025214933	0.000908994	0.073377725	-0.00201968	0.026289944	0.000908984	0.073373263	-1.47E-05	0.010648467	1	0.207485894	1	0.213574989	ENSG00000105088	4	OLFM2
Pancreas	T2DM_admission	ENSG00000116771.5	-0.002599082	0.12341253	0.001687048	0.073378122	-0.002577609	0.126537642	0.001687026	0.073373655	-2.15E-05	0.02937284	1	0.477599265	1	0.479275315	ENSG00000105136	15	ZNF419
Pancreas	T2DM_admission	ENSG00000117122.9	0.002007397	0.168727722	0.001458541	0.073378194	0.001988294	0.172812485	0.001458523	0.073373727	1.91E-05	0.030133153	1	0.548084313	1	0.553785383	ENSG00000105146	8	AURKC
Pancreas	T2DM_admission	ENSG00000119013.4	0.000911192	0.814174982	0.003876691	0.073378471	0.000966942	0.803030733	0.003876656	0.073373997	-5.58E-05	0.02826986	1	0.946063961	1	0.939682535	ENSG00000105146	8	AURKC
Pancreas	T2DM_admission	ENSG00000119227.3	0.001360666	0.450306323	0.001802434	0.073378393	0.001337127	0.458177523	0.001802418	0.073373924	2.35E-05	0.048261028	1	0.781199104	1	0.786497245	ENSG00000105146	8	AURKC
Pancreas	T2DM_admission	ENSG00000119715.10	-0.002071409	0.44239024	0.002696572	0.07337839	-0.002110712	0.433777355	0.002696552	0.073373914	3.93E-05	0.032211939	1	0.776526409	1	0.771947117	ENSG00000105185	7	PDCD5
Pancreas	T2DM_admission	ENSG00000120451.6	-0.000970287	0.307457301	0.000950728	0.073378322	-0.000985969	0.299701693	0.000950722	0.073373845	1.57E-05	0.017409646	1	0.702883653	1	0.696408377	ENSG00000105185	7	PDCD5
Pancreas	T2DM_admission	ENSG00000120675.4	-0.001529069	0.674483413	0.003640614	0.073378453	-0.00157384	0.665517502	0.003640549	0.073373978	4.48E-05	0.01438008	1	0.895591213	1	0.88982611	ENSG00000105193	4	RPS16
Pancreas	T2DM_admission	ENSG00000120725.8	-0.001455783	0.52241675	0.002275994	0.073378418	-0.001490091	0.512657084	0.002275968	0.073373942	3.43E-05	0.01488395	1	0.825071473	1	0.81978735	ENSG00000105193	4	RPS16
Pancreas	T2DM_admission	ENSG00000120756.8	0.00141158	0.630318105	0.002932973	0.073378444	0.001447602	0.621612512	0.002932941	0.07337397	-3.60E-05	0.048125555	1	0.879226992	1	0.875160807	ENSG00000105258	4	POLR2I
Pancreas	T2DM_admission	ENSG00000120868.9	0.001418535	0.60406492	0.002735506	0.073378439	0.001383466	0.613032513	0.002735476	0.073373968	3.51E-05	0.040063624	1	0.865912508	1	0.870241843	ENSG00000105258	4	POLR2I
Pancreas	T2DM_admission	ENSG00000121898.8	0.001685034	0.503878605	0.002520997	0.073378412	0.001736183	0.491016553	0.002520981	0.073373935	-5.11E-05	0.003480925	1	0.813942548	1	0.805191895	ENSG00000105258	4	POLR2I
Pancreas	T2DM_admission	ENSG00000122483.13	0.001208938	0.646279279	0.002634212	0.073378447	0.001246726	0.636007425	0.002634176	0.073373973	-3.78E-05	0.013497982	1	0.886308052	1	0.880942324	ENSG00000105258	4	POLR2I
Pancreas	T2DM_admission	ENSG00000122692.7	-6.43E-06	0.99803215	0.002606562	0.073378479	-4.27E-05	0.986920843	0.002606525	0.073374007	3.63E-05	0.015161072	1	0.99984869	1	0.997626211	ENSG00000105258	4	POLR2I
Pancreas	T2DM_admission	ENSG00000122783.12	0.002580997	0.421857351	0.003213377	0.073378382	0.002631553	0.412817415	0.00321334	0.073373906	-5.06E-05	0.010835947	1	0.764941163	1	0.758851196	ENSG00000105270	10	CLIP3
Pancreas	T2DM_admission	ENSG00000123179.9	0.002370647	0.348067043	0.00252641	0.073378347	0.002339724	0.354384461	0.002526375	0.073373877	3.09E-05	0.033777204	1	0.714946894	1	0.720077577	ENSG00000105270	10	CLIP3
Pancreas	T2DM_admission	ENSG00000123453.12	-6.96E-05	0.980826051	0.002896963	0.073378479	-0.000126085	0.965284416	0.002896944	0.073374006	5.65E-05	0.004939641	1	0.994701872	1	0.988657083	ENSG00000105287	8	PRKD2
Pancreas	T2DM_admission	ENSG00000123643.8	-7.03E-05	0.96190825	0.001470952	0.073378479	-2.68E-05	0.985463436	0.001470994	0.073374007	-4.34E-05	0.006842684	1	0.987677084	1	0.997569125	ENSG00000105290	7	APLP1
Pancreas	T2DM_admission	ENSG00000123815.7	0.006667655	0.018383103	0.002827892	0.073377642	0.006719201	0.017500401	0.00282791	0.073373156	-5.15E-05	0.033731521	1	0.180872825	1	0.174867821	ENSG00000105329	5	TGFB1
Pancreas	T2DM_admission	ENSG00000124104.14	-0.00112174	0.748890966	0.003504317	0.073378464	-0.001173102	0.737804638	0.003504285	0.07337399	5.14E-05	0.024636358	1	0.923704131	1	0.922650112	ENSG00000105355	4	PLIN3
Pancreas	T2DM_admission	ENSG00000124116.14	-0.000628743	0.667875396	0.001465376	0.073378451	-0.00066006	0.652392485	0.001465364	0.073373976	3.13E-05	0.001440568	1	0.893283342	1	0.887364354	ENSG00000105364	9	MRPL4
Pancreas	T2DM_admission	ENSG00000124422.7	0.004155986	0.111286615	0.002609823	0.073378097	0.004097732	0.11640028	0.00260991	0.073373635	5.83E-05	0.047743183	1	0.454346625	1	0.469231995	ENSG00000105364	9	MRPL4
Pancreas	T2DM_admission	ENSG00000124613.4	0.000151216	0.898530582	0.001185841	0.073378477	2.66E-05	0.98209306	0.001186318	0.073374006	0.000124589	0.000356797	1	0.972507979	1	0.996056468	ENSG00000105383	10	CD33
Pancreas	T2DM_admission	ENSG00000125257.9	-0.000245587	0.94787761	0.003756742	0.073378479	-0.000138505	0.970590331	0.00375678	0.073374006	-0.000107082	0.001559461	1	0.983097618	1	0.991832858	ENSG00000105447	8	GRWD1
Pancreas	T2DM_admission	ENSG00000125734.9	0.000847568	0.769228882	0.00288894	0.073378466	0.000882115	0.76010237	0.002888898	0.073373993	-3.45E-05	0.034712296	1	0.93002814	1	0.927749352	ENSG00000105447	8	GRWD1
Pancreas	T2DM_admission	ENSG00000127418.10	-0.002605584	0.184765493	0.001964661	0.073378214	-0.002575316	0.189915961	0.001964646	0.073373748	-3.03E-05	0.023026457	1	0.565800753	1	0.570204108	ENSG00000105447	8	GRWD1
Pancreas	T2DM_admission	ENSG00000128604.14	0.002163275	0.257730581	0.001911404	0.073378286	0.002141951	0.262444052	0.001911373	0.073373817	2.13E-05	0.038603424	1	0.654004082	1	0.658289049	ENSG00000105479	11	CCDC114
Pancreas	T2DM_admission	ENSG00000129204.12	0.000982777	0.619723367	0.001980437	0.073378442	0.001013087	0.608967739	0.001980434	0.073373967	-3.03E-05	0.044001569	1	0.874422861	1	0.867518185	ENSG00000105479	11	CCDC114
Pancreas	T2DM_admission	ENSG00000129355.6	-0.001529027	0.541843319	0.002506485	0.073378423	-0.001482157	0.554297795	0.002506469	0.073373954	-4.69E-05	0.007089086	1	0.838486276	1	0.846590114	ENSG00000105479	11	CCDC114
Pancreas	T2DM_admission	ENSG00000130300.4	-0.001588797	0.679533418	0.003846033	0.073378453	0.002495191	0.567025339	0.004358883	0.073373957	-0.004083988	0.046514008	1	0.899069186	1	0.849476828	ENSG00000105479	11	CCDC114
Pancreas	T2DM_admission	ENSG00000130332.10	0.002011804	0.165638589	0.001451145	0.07337819	0.002032045	0.161419517	0.001451134	0.073373711	-2.02E-05	0.039862354	1	0.54463495	1	0.53974651	ENSG00000105497	3	ZNF175
Pancreas	T2DM_admission	ENSG00000130363.7	0.004037785	0.274895385	0.003698081	0.0733783	0.003981757	0.281604085	0.003698029	0.073373832	5.60E-05	0.008355734	1	0.670309634	1	0.677115441	ENSG00000105501	7	SIGLEC5
Pancreas	T2DM_admission	ENSG00000131653.8	0.001717064	0.651245684	0.003798541	0.073378448	0.001843891	0.627395636	0.003798734	0.073373971	-0.000126826	0.008873568	1	0.887189213	1	0.877200348	ENSG00000105519	8	CAPS
Pancreas	T2DM_admission	ENSG00000132274.11	-0.001913943	0.521465446	0.002985458	0.073378417	-0.001847426	0.536042575	0.002985456	0.073373949	-6.65E-05	0.003890841	1	0.824583291	1	0.836849651	ENSG00000105618	9	PRPF31
Pancreas	T2DM_admission	ENSG00000132305.16	0.004163402	0.074042003	0.002330666	0.073377999	0.004126058	0.076670057	0.00233065	0.073373535	3.73E-05	0.020269514	1	0.360189008	1	0.370788522	ENSG00000105640	8	RPL18A
Pancreas	T2DM_admission	ENSG00000132507.13	-0.003702405	0.187106178	0.002806575	0.073378217	-0.003751967	0.181269807	0.002806557	0.073373737	4.96E-05	0.011541346	1	0.567450741	1	0.561218928	ENSG00000105640	8	RPL18A
Pancreas	T2DM_admission	ENSG00000133316.11	-0.002199509	0.175888105	0.001625021	0.073378203	-0.002220769	0.171742034	0.001624997	0.073373725	2.13E-05	0.018853248	1	0.557630438	1	0.552673613	ENSG00000105640	8	RPL18A
Pancreas	T2DM_admission	ENSG00000133519.8	-0.000797147	0.810509989	0.003324657	0.073378471	-0.000845153	0.799333262	0.00332462	0.073373997	4.80E-05	0.020328766	1	0.943940886	1	0.939682535	ENSG00000105656	8	ELL

Pancreas	T2DM_admission	ENSG00000133706.13	-0.001364491	0.374719439	0.001537162	0.073378361	-0.001343595	0.382070897	0.001537139	0.073373892	-2.09E-05	0.015314062	1	0.730660228	1	0.736052561	ENSG00000105656	8	ELL
Pancreas	T2DM_admission	ENSG00000134285.6	0.002088081	0.452352684	0.002778554	0.073378394	0.002043545	0.462051386	0.002778542	0.073373925	4.45E-05	0.026974461	1	0.781199104	1	0.787880451	ENSG00000105695	10	MAG
Pancreas	T2DM_admission	ENSG00000134460.11	-0.005051143	0.186494034	0.003823658	0.073378216	-0.004997862	0.191178246	0.003823617	0.073373749	-5.33E-05	0.026430389	1	0.567450741	1	0.570204108	ENSG00000105699	12	LSR
Pancreas	T2DM_admission	ENSG00000134852.10	-0.000273965	0.865654791	0.001619365	0.073378475	-0.000242692	0.880866982	0.001619348	0.073374003	-3.13E-05	0.002244091	1	0.966374076	1	0.971621772	ENSG00000105699	12	LSR
Pancreas	T2DM_admission	ENSG00000134873.5	0.003995998	0.245296079	0.003439334	0.073378276	0.001838448	0.601458778	0.00351988	0.073373965	0.00215755	0.003976933	1	0.636926523	1	0.86327687	ENSG00000105699	12	LSR
Pancreas	T2DM_admission	ENSG00000135269.13	0.000170424	0.959279996	0.003337906	0.073378479	-0.00043237	0.897269061	0.003348794	0.073374004	0.000602794	0.026168489	1	0.986823205	1	0.972260921	ENSG00000105750	10	ZNF85
Pancreas	T2DM_admission	ENSG00000135778.7	0.000395881	0.908129261	0.00343055	0.073378477	0.000348894	0.918992511	0.003430519	0.073374005	4.70E-05	0.036901788	1	0.972507979	1	0.978433155	ENSG00000105755	3	ETHE1
Pancreas	T2DM_admission	ENSG00000135828.7	0.001206148	0.669932435	0.002829732	0.073378452	0.001244084	0.660189837	0.002829705	0.073373977	-3.79E-05	0.038178951	1	0.89370727	1	0.888091933	ENSG00000105755	3	ETHE1
Pancreas	T2DM_admission	ENSG00000136490.4	0.002582743	0.221398105	0.00211212	0.073378254	0.002614671	0.215737484	0.002112108	0.073373776	-3.19E-05	0.033071554	1	0.610032546	1	0.602188978	ENSG00000105771	9	SMG9
Pancreas	T2DM_admission	ENSG00000137288.5	0.001859037	0.387784307	0.002152552	0.073378367	0.000988814	0.650596627	0.002183131	0.073373976	0.000870223	0.016965779	1	0.740290513	1	0.885860095	ENSG00000105880	4	DLX5
Pancreas	T2DM_admission	ENSG00000137310.7	0.001275846	0.380920243	0.001456108	0.073378364	0.001095883	0.451949371	0.001456963	0.073373921	0.000179962	0.000437344	1	0.733835847	1	0.783134943	ENSG00000105880	4	DLX5
Pancreas	T2DM_admission	ENSG00000137478.10	0.001764029	0.316295412	0.001760331	0.073378328	0.0018033	0.305639404	0.00176032	0.073373849	-3.93E-05	0.001338836	1	0.705815487	1	0.703146938	ENSG00000105889	10	STEAP1B
Pancreas	T2DM_admission	ENSG00000137672.8	-0.005359457	0.163082456	0.003842507	0.073378186	-0.005285868	0.168933145	0.003842474	0.073373722	-7.36E-05	0.003633766	1	0.541071967	1	0.54913964	ENSG00000105928	9	DFNA5
Pancreas	T2DM_admission	ENSG00000137673.4	0.002652436	0.315257131	0.002641203	0.073378327	0.000588408	0.830111133	0.002742403	0.073374	0.002064028	0.005185582	1	0.705815487	1	0.955502191	ENSG00000105928	9	DFNA5
Pancreas	T2DM_admission	ENSG00000137752.18	-0.000696362	0.842268845	0.003499443	0.073378473	-0.000769134	0.826035826	0.003499424	0.073373999	7.28E-05	0.003300647	1	0.959017468	1	0.954095567	ENSG00000105968	14	H2AFV
Pancreas	T2DM_admission	ENSG00000137831.10	0.000264028	0.942959195	0.003690057	0.073378478	0.000303326	0.934485965	0.003689998	0.073374006	-3.93E-05	0.04812715	1	0.982923376	1	0.98183872	ENSG00000105968	14	H2AFV
Pancreas	T2DM_admission	ENSG00000137843.7	-0.002383801	0.094335927	0.001424901	0.073378058	-0.002369779	0.096283215	0.001424874	0.07337359	-1.40E-05	0.045749087	1	0.41411053	1	0.419076774	ENSG00000105968	14	H2AFV
Pancreas	T2DM_admission	ENSG00000137857.13	0.002332804	0.330477722	0.002397161	0.073378337	0.002282176	0.341076211	0.002397138	0.07337387	5.06E-05	0.001108407	1	0.707406446	1	0.715170664	ENSG00000105968	14	H2AFV
Pancreas	T2DM_admission	ENSG00000138119.12	0.002011575	0.154627593	0.001413242	0.073378174	0.001982574	0.160660266	0.001413241	0.07337371	2.90E-05	0.00773254	1	0.531571161	1	0.538610395	ENSG00000105982	12	RNF32
Pancreas	T2DM_admission	ENSG00000138613.9	0.000532973	0.888188124	0.003790775	0.073378476	0.000625154	0.869011008	0.003790765	0.073374002	-9.22E-05	0.001142543	1	0.972507979	1	0.967645366	ENSG00000105982	12	RNF32
Pancreas	T2DM_admission	ENSG00000138660.7	0.002182284	0.54204713	0.003579154	0.073378423	0.002116681	0.55425968	0.003579165	0.073373954	6.56E-05	0.025553076	1	0.838486276	1	0.846590114	ENSG00000105982	12	RNF32
Pancreas	T2DM_admission	ENSG00000138686.5	-0.004701285	0.218311102	0.003818975	0.073378251	-0.004858955	0.20333158	0.003819577	0.073373763	0.000157671	0.033350007	1	0.607038146	1	0.585376119	ENSG00000105983	15	LMBR1
Pancreas	T2DM_admission	ENSG00000138834.8	-0.001049234	0.756135054	0.003378518	0.073378465	-0.000954902	0.777459145	0.003378607	0.073373995	-9.43E-05	0.008735663	1	0.926431379	1	0.935113451	ENSG00000106009	11	BRAT1
Pancreas	T2DM_admission	ENSG00000139651.9	0.001254718	0.720732833	0.0035099	0.07337846	0.001315347	0.707843206	0.00350989	0.073373985	-6.06E-05	0.020112564	1	0.915352085	1	0.91023603	ENSG00000106009	11	BRAT1
Pancreas	T2DM_admission	ENSG00000140575.8	-0.000411049	0.822133185	0.001828492	0.073378472	-0.000435712	0.811654041	0.001828474	0.073373998	2.47E-05	0.033987046	1	0.951436692	1	0.945273278	ENSG00000106009	11	BRAT1
Pancreas	T2DM_admission	ENSG00000140955.6	-0.003111909	0.410026926	0.00377729	0.073378377	-0.003152855	0.403887182	0.003777232	0.073373902	4.09E-05	0.049918138	1	0.756489657	1	0.755027733	ENSG00000106049	4	HIBADH
Pancreas	T2DM_admission	ENSG00000141084.6	0.001303482	0.452372732	0.001734586	0.073378394	0.001283598	0.459292705	0.001734562	0.073373924	1.99E-05	0.047910795	1	0.781199104	1	0.78659096	ENSG00000106077	14	ABHD11
Pancreas	T2DM_admission	ENSG00000141560.10	-0.002714941	0.002342802	0.000892205	0.073377085	-0.002701629	0.002461443	0.000892199	0.073372626	-1.33E-05	0.027813426	1	0.04685311	1	0.048622958	ENSG00000106077	14	ABHD11
Pancreas	T2DM_admission	ENSG00000141569.6	-0.001727442	0.407595524	0.002085948	0.073378376	-0.001693549	0.41685788	0.002085947	0.073373907	-3.39E-05	0.035629088	1	0.755743904	1	0.763545674	ENSG00000106077	14	ABHD11
Pancreas	T2DM_admission	ENSG00000142273.6	-0.005728586	0.02741833	0.00259739	0.073377747	-0.005694872	0.028338845	0.00259735	0.073373283	-3.37E-05	0.018595329	1	0.216737649	1	0.223585926	ENSG00000106113	14	CRHR2
Pancreas	T2DM_admission	ENSG00000144834.8	0.003366925	0.224330079	0.002770913	0.073378257	0.003401534	0.219595522	0.002770868	0.07337378	-3.46E-05	0.02062606	1	0.614157401	1	0.606366989	ENSG00000106123	7	EPHB6
Pancreas	T2DM_admission	ENSG00000146457.10	-0.001072481	0.457977234	0.001445037	0.073378396	-0.001056614	0.464649561	0.001445015	0.073373926	-1.59E-05	0.047491355	1	0.783534669	1	0.788724539	ENSG00000106125	14	FAM188B
Pancreas	T2DM_admission	ENSG00000146833.11	-0.000989099	0.146216622	0.000680716	0.073378161	-0.000976846	0.15127922	0.000680714	0.073373696	-1.23E-05	0.016947998	1	0.516484575	1	0.529993244	ENSG00000106125	14	FAM188B
Pancreas	T2DM_admission	ENSG00000147955.12	-0.003599379	0.075199114	0.002022973	0.073378002	-0.003632579	0.07254974	0.002022981	0.073373521	3.32E-05	0.04720457	1	0.362991211	1	0.361061527	ENSG00000106133	13	NSUN5P2
Pancreas	T2DM_admission	ENSG00000149328.10	0.000899689	0.717421526	0.002485965	0.073378459	0.000841302	0.73504873	0.002485988	0.073373989	5.84E-05	0.00834794	1	0.915352085	1	0.922046709	ENSG00000106133	13	NSUN5P2
Pancreas	T2DM_admission	ENSG00000149577.11	-0.005903298	0.02048685	0.00254747	0.073377671	-0.005843437	0.021801076	0.002547466	0.073373214	-5.99E-05	0.0019461	1	0.192167217	1	0.19439293	ENSG00000106133	13	NSUN5P2
Pancreas	T2DM_admission	ENSG00000149591.12	-0.007280603	0.007451403	0.002720728	0.073377401	-0.007223066	0.007934832	0.002720715	0.073372945	-5.75E-05	0.003061914	1	0.103016032	1	0.109202996	ENSG00000106133	13	NSUN5P2
Pancreas	T2DM_admission	ENSG00000151725.7	0.003887492	0.285384025	0.003638938	0.073378307	0.003926512	0.280568557	0.003638879	0.073373831	-3.90E-05	0.044961316	1	0.679838752	1	0.675257783	ENSG00000106153	12	CHCHD2
Pancreas	T2DM_admission	ENSG00000152439.8	0.002757142	0.041027798	0.001349388	0.073377851	0.00273315	0.042816647	0.001349371	0.073373389	2.40E-05	0.002908069	1	0.274649	1	0.28152064	ENSG00000106153	12	CHCHD2
Pancreas	T2DM_admission	ENSG00000152749.7	6.55E-05	0.981480237	0.00282305	0.073378479	0.000326978	0.907836415	0.002824422	0.073374005	-0.000261446	0.00395359	1	0.994701872	1	0.972600715	ENSG00000106178	2	CCL24
Pancreas	T2DM_admission	ENSG00000153822.9	0.001059855	0.510051818	0.001608869	0.073378414	0.001040726	0.517711683	0.001608846	0.073373944	1.91E-05	0.03534523	1	0.815555259	1	0.821175789	ENSG00000106178	2	CCL24
Pancreas	T2DM_admission	ENSG00000154265.11	-0.001627679	0.592728925	0.003043038	0.073378436	-0.001681636	0.580521658	0.003043006	0.073373961	5.40E-05	0.005011995	1	0.858891717	1	0.853336931	ENSG00000106236	3	NPTX2
Pancreas	T2DM_admission	ENSG00000154710.11	-0.001556679	0.286541335	0.001460654	0.073378308	-0.001539891	0.291761561	0.001460629	0.073373839	-1.68E-05	0.024807941	1	0.680701339	1	0.68737953	ENSG00000106263	13	EIF3B
Pancreas	T2DM_admission	ENSG00000154864.7	0.00260911	0.424706658	0.003268402	0.073378383	0.0026555	0.415976847	0.003268355	0.073373907	-4.94E-05	0.007940662	1	0.765366105	1	0.762475763	ENSG00000106263	13	EIF3B
Pancreas	T2DM_admission	ENSG00000155542.7	-0.002747234	0.05508167	0.001432167	0.073377925	-0.002774121	0.052741457	0.001432147	0.073373442	2.69E-05	0.001168429	1	0.312941876	1	0.309931493	ENSG00000106263	13	EIF3B
Pancreas	T2DM_admission	ENSG00000155957.12	-0.000963967	0.578099217	0.001733244	0.073378433	-0.000934539	0.589761663	0.001733253	0.073373963	-2.94E-05	0.044802337	1	0.85368533	1	0.857158565	ENSG00000106263	13	EIF3B
Pancreas	T2DM_admission	ENSG00000156219.12	-0.001367511	0.087962277	0.000801472	0.073378041	-0.001377022	0.085771951	0.000801462	0.073373562	9.51E-06	0.046571646	1	0.395599172	1	0.396836678	ENSG00000106290	10	TAF6
Pancreas	T2DM_admission	ENSG00000156711.12	0.001460345	0.765061131	0.001540431	0.073378466	0.000501954	0.744536436	0.001540436	0.073373991	-4.16E-05	0.000980508	1	0.927632086	1	0.92339769	ENSG00000106299	7	WASL
Pancreas	T2DM_admission	ENSG00000156931.11	0.00356573	0.345937467	0.00378327	0.073378345	0.003637579	0.336300951	0.00378323	0.073373867	-7.18E-05	0.002749894	1	0.714946894	1	0.712394628	ENSG00000106330	7	MOSPD3
Pancreas	T2DM_admission	ENSG00000157214.9	0.005184465	0.139531911	0.003508852	0.07337815	0.0051409	0.142881846	0.003508812	0.073373683	4.35E-05	0.044499516	1	0.500672845	1	0.511031449	ENSG00000106344	4	RBM28
Pancreas	T2DM_admission	ENSG00000158793.9	0.002254707	0.264205429	0.002019428	0.073378291	0.002028152	0.315588813	0.002020948	0.073373855	0.000226554	0.004855629	1	0.661240615	1	0.706943978	ENSG00000106344	4	RBM28
Pancreas	T2DM_admission	ENSG00000159055.3	-0.002119099	0.201521882	0.001659137	0.073378234	-0.002093344	0.20705358	0.001659132	0.073373767	-2.58E-05	0.035039109	1	0.581469879	1	0.591085559	ENSG00000106348	12	IMPDH1
Pancreas	T2DM_admission	ENSG00000159128.10	-0.002662469	0.168827032	0.001934955	0.073378194	-0.002695596	0.163586182	0.001934994	0.073373714	3.31E-05	0.014473537	1	0.548084313	1	0.54345319	ENSG00000106460	14	TMEM106B
Pancreas	T2DM_admission	ENSG00000160072.15	-0.000936617	0.773839103	0.003259419	0.073378467	-0.000891606	0.7844295	0.003259374	0.073373995	-4.50E-05	0.017079588	1	0.930781647	1	0.936079501	ENSG00000106477	14	CEP41
Pancreas	T2DM_admission	ENSG00000160194.13	0.000289294	0.644943321	0.00062781	0.073378447	0.000298047	0.634969339	0.000627804	0.073373973	-8.75E-06	0.031539085	1	0.885748011	1	0.880942324	ENSG00000106536	5	RARRES2
Pancreas	T2DM_admission	ENSG00000160209.14	-0.005201246	0.049455556	0.002647419	0.073377898	-0.005170134	0.05082933	0.002647383	0.073373432	-3.11E-05	0.042994484	1	0.296041653	1	0.304975981	ENSG00000106565	13	TMEM176B
Pancreas	T2DM_admission	ENSG00000160229.7	0.002137906	0.557069346	0.003640846	0.073378427	0.002080491	0.567704669	0.003640814	0.073373957	5.74E-05	0.016977483	1	0.847939375	1	0.849476828	ENSG00000106591	3	MRPL32
Pancreas	T2DM_admission	ENSG00000160766.10	-0.002451238	0.114730433	0.001554086	0.073378105	-0.002421799	0.119150871	0.001554079	0.073373641	-2.94E-05	0.008295776	1	0.463251181	1	0.472809902	ENSG00000106603	13	COA1
Pancreas	T2DM_admission	ENSG00000161021.7	0.002592097	0.435075567	0.003320917	0.073378387	0.002630466	0.428300448	0.00332086	0.073373912	-3.84E-05	0.024751773	1	0.771915116	1	0.769192559	ENSG00000106603	13	COA1
Pancreas	T2DM_admission	ENSG00000161542.12	-0.003321407	0.169285472	0.002416438	0.073378195	-0.003292311	0.173045879	0.002416404	0.07337727	-2.91E-05	0.034924093	1	0.548084313	1	0.553785383	ENSG00000106603	13	COA1
Pancreas	T2DM_admission	ENSG00000161896.6	0.003206127	0.021662048	0.001396245	0.073377685	0.003495952	0.012409419	0.001398217	0.073373065	-0.000289824	0.000112732	1	0.193802603	1	0.143546788	ENSG00000106610	10	STAG3L4
Pancreas	T2DM_admission	ENSG00000161904.7	0.00127816	0.483536182	0.001824309	0.073378405	0.000980336	0.591559757	0.001827017	0.073373963	0.000297824	0.000300032	1	0.799048209	1	0.857294328	ENSG00000106610	10	STAG3L4
Pancreas	T2DM_admission	ENSG00000162341.11	0.003377017	0.246020085	0.002911041	0.073378277	0.003414497	0.240811788	0.002911005	0.073373799	-3.75E-05	0.031997955	1	0.63757234	1	0.627263575	ENSG00000106610	10	STAG3L4
Pancreas	T2DM_admission	ENSG00000162734.8	-0.001105471	0.739082246	0.003319045	0.073378462	-0.001229477	0.711094989	0.003319448	0.073373986	0.000124006	0.032168678	1	0.920031049	1	0.912283108	ENSG00000106635	3	BCL7B
Pancreas	T2DM_admission	ENSG00000162817.6	0.000981304	0.330478644	0.001008378	0.073378337	0.000994577	0.323972861	0.001008364	0.07337386	-1.33E-05	0.022721109	1	0.707406446	1	0.706943978	ENSG00000106635	3	BCL7B
Pancreas	T2DM_admission	ENSG00000162877.8	-0.001397982	0.534741113	0.002251957	0.073378421	-0.001367787	0.543596396	0.002251931	0.073373951	-3.02E-05	0.029287233	1	0.832756324	1	0.842968325	ENSG00000106635	3	BCL7B
Pancreas	T2DM_admission	ENSG00000163281.7	0.001237718	0.238730624	0.001050545	0.07337827	0.001252828	0.233040684	0.001050533	0.073373792	-1.51E-05	0.018523318	1	0.627977804	1	0.619840368	ENSG00000106638	11	TBL2
Pancreas	T2DM_admission	ENSG00000163389.6	-0.0010583	0.323769614	0.00107259	0.073378333	-0.001071097	0.317977804	0.001072574	0.073373856	1.27E-05	0.032686258	1	0.707406446	1	0.706943978	ENSG00000106638	11	TBL2
Pancreas	T2DM_admission	ENSG00000163545.7	0.000922521	0.734841676	0.002723744	0.073378462	0.000886708	0.744767021	0.002723744	0.073373991	3.58E-05	0.035686477	1	0.919728783	1	0.92339769	ENSG00000106638	11	TBL2
Pancreas	T2DM_admission	ENSG00000163788.9	-0.002684339	0.480568665	0.003805474	0.073378404	-0.002621606	0.490879264	0.003805432	0.073373935	-6.27E-05	0.00811841	1	0.797318048	1	0.805191895	ENSG00000106638	11	TBL2
Pancreas	T2DM_admission	ENSG00000163945.11	0.002965644	0.077770541	0.001681419	0.073378011	0.002928397	0.081576027	0.001681424	0.07337355	3.72E-05	0.006765159	1	0.371908286	1	0.386507818	ENSG00000106688	7	SLC1A1
Pancreas	T2DM_admission	ENSG00000164081.8	-0.00357966	0.223579335	0.002941219	0.073378256	-0.003539723	0.228782833	0.002941187	0.073373788	-3.99E-05	0.030040634	1	0.613012193	1	0.615924595	ENSG00000106688	7	SLC1A1
Pancreas	T2DM_admission	ENSG00000164111.10	-0.000651236	0.704366106	0.001716339	0.073378458	-0.000914535	0.595076182	0.001720687	0.073373964	0.000263298	0.032279195	1	0.909407823	1	0.859705602	ENSG00000106688	7	SLC1A1
Pancreas	T2DM_admission	ENSG00000164733.16	-0.000595566	0.795675398	0.002299942	0.073378469	-0.000373184	0.871182276	0.002301372	0.073374003	-0.000222382	0.007433288	1	0.938428627	1	0.967645366	ENSG00000106733	16	NMRK1
Pancreas	T2DM_admission	ENSG00000164941.9	0.001902998	0.214820078	0.001534094	0.073378245	0.001883267	0.219589771	0.001534078	0.07337378	1.97E-05	0.041550444	1	0.60288425	1	0.606366989	ENSG00000106733	16	NMRK1
Pancreas	T2DM_admission	ENSG00000165055.11	0.002029868	0.118195297	0.001299202	0.073378112	0.002012798	0.12131616	0.001299187	0.073373645	1.71E-05	0.032582848	1	0.469127545	1	0.473464893	ENSG00000106733	16	NMRK1
Pancreas	T2DM_admission	ENSG00000165171.6	-0.000524408	0.37064313	0.000585754	0.073378359	-0.000556385	0.342267986	0.000585857	0.073373871	3.20E-05	0.00722327	1	0.728887861	1	0.715170664	ENSG00000106733	16	NMRK1
Pancreas	T2DM_admission	ENSG00000165410.10	0.002743189	0.412648325	0.003348445	0.073378378	0.002791211	0.404510872	0.00334841	0.073373902	-4.80E-05	0.023942388	1	0.757001665	1	0.755027733	ENSG00000106733	16	NMRK1
Pancreas	T2DM_admission	ENSG00000165934.8	0.000619819	0.566202782	0.001080476	0.07337843	0.000637018	0.555474853	0.001080468	0.073373954	-1.72E-05	0.018644925	1	0.850297512	1	0.846590114	ENSG00000106853	12	PTGR1
Pancreas	T2DM_admission	ENSG00000166037.6	-0.005985048	0.001563578	0.001892425	0.073376973	-0.006004083	0.001510123	0.001892391	0.073372491	1.90E-05	0.048048565	1	0.035221657	1	0.034318558	ENSG00000106853	12	PTGR1
Pancreas	T2DM_admission	ENSG00000166224.12	-0.006829173	0.000604667	0.001991283	0.073376708	-0.006799718	0.000638376	0.001991258	0.073372251	-2.95E-05	0.014071952	1	0.017666591	1	0.018419672	ENSG00000106853	12	PTGR1
Pancreas	T2DM_admission	ENSG00000166228.4	-0.008293634	3.61E-05	0.002008684	0.073375909	-0.008258884	3.93E-05	0.002008663	0.073371461	-3.95E-05	0.001676631	0.092670575	0.00215513	0.100905808	0.00252645	ENSG00000106868	12	SUSD1
Pancreas	T2DM_admission	ENSG00000166268.6	-0.00027934	0.775189557	0.000978103	0.073378467	-0.000292612	0.764812916	0.000978089	0.073373993	1.33E-05	0.019553844	1	0.931097653	1	0.928829506	ENSG00000106992	13	AK1
Pancreas	T2DM_admission	ENSG00000166436.11	0.002176182	0.084509962	0.001261481	0.073378031	0.002193145	0.082112486	0.00126147	0.073373551	-1.70E-05	0.04224197	1	0.391029878	1	0.386704232	ENSG00000106992	13	AK1
Pancreas	T2DM_admission	ENSG00000166902.4	0.001317355	0.458977333	0.00177893	0.073378397	0.001353834	0.446630439	0.001778915	0.073373919	-3.65E-05	0.001979107	1	0.784014077	1	0.779994504	ENSG00000106992	13	AK1
Pancreas	T2DM_admission	ENSG00000167257.6	0.000128099	0.968208685	0.003214127	0.073378479	0.00018573	0.953918934	0.003214107	0.073374006	-5.76E-05	0.010180364	1	0.989399086	1	0.984195992	ENSG00000107020	5	PLGRKT
Pancreas	T2DM_admission	ENSG00000167757.9	-0.000793591	0.715671082	0.002178696	0.073378459	-0.000820598	0.706433509	0.002178673	0.073373985	2.70E-05	0.049601426	1	0.914349919	1	0.909378635	ENSG00000107020	5	PLGRKT
Pancreas	T2DM_admission	ENSG00000167800.9	0.004515197	0.11525075	0.002866742	0.073378106	0.004550069	0.112463969	0.002866695	0.073373627	-3.49E-05	0.021993411	1	0.46462155	1	0.461815776	ENSG00000107099	11	DOCK8
Pancreas	T2DM_admission	ENSG00000167972.9	-0.004919588	0.055951181	0.002573828	0.073377929	-0.004999315	0.052104401	0.002573948	0.073373439	7.97E-05	0.012582656	1	0.313835611	1	0.308304381	ENSG00000107099	11	DOCK8
Pancreas	T2DM_admission	ENSG00000168028.9	0.000658535	0.466477061	0.000904303	0.073378399	0.000644091	0.47630448	0.000904293	0.07337393	1.44E-05	0.010963424	1	0.790180141	1	0.79570852	ENSG00000107099	11	DOCK8
Pancreas	T2DM_admission	ENSG00000168385.13	0.000581145	0.668733887	0.001358175	0.073378452	0.00060196	0.657610078	0.001358167	0.073373977	-2.08E-05	0.027909811	1	0.893500844	1	0.887614942	ENSG00000107099	11	DOCK8
Pancreas	T2DM_admission	ENSG00000168778.7	0.000494701	0.604587666	0.000955363	0.073378439	0.000508357	0.594645554	0.000955349	0.073373964	-1.37E-05	0.010241433	1	0.865912508	1	0.859705602	ENSG00000107099	11	DOCK8
Pancreas	T2DM_admission	ENSG00000169231.9	0.00359699	0.058617706	0.001902108	0.073377941	0.003540358	0.062706681	0.00190212	0.073373485	5.66E-05	0.000470263	1	0.320228732	1	0.334088709	ENSG00000107099	11	DOCK8
Pancreas	T2DM_admission	ENSG00000169609.9	-0.001028505	0.52724445	0.00162682	0.073378419	-0.001011439	0.534114066	0.001626791	0.073373948	-1.71E-05	0.038621828	1	0.82963465	1	0.835325774	ENSG00000107099	11	DOCK8
Pancreas	T2DM_admission	ENSG00000170439.5	-0.001389398	0.654899157	0.003108502	0.073378449	-0.0013358	0.66744782	0.003108505	0.073373979	-5.38E-05	0.028727333	1	0.889360674	1	0.88982611	ENSG00000107130	6	NCS1
Pancreas	T2DM_admission	ENSG00000170632.9	0.002483643	0.374114993	0.002794401	0.07337836	0.002524796	0.366245055	0.002794368	0.073373884	-4.12E-05	0.016469794	1	0.730660228	1	0.725397099	ENSG00000107130	6	NCS1
Pancreas	T2DM_admission	ENSG00000170915.8	0.002374418	0.427762543	0.00299412	0.073378384	0.002516349	0.400790631	0.002994902	0.07337339	-0.000141931	0.049729275	1	0.767595622	1	0.753462913	ENSG00000107130	6	NCS1
Pancreas	T2DM_admission	ENSG00000171357.5	0.002961061	0.156984956	0.002092198	0.073378178	0.002925563	0.162015032	0.002092185	0.073373712	3.55E-05	0.014986687	1	0.53381119	1	0.540033938	ENSG00000107164	15	FUBP3
Pancreas	T2DM_admission	ENSG00000172000.3	0.001300009	0.627588987	0.002679725	0.073378444	0.001339169	0.617253143	0.002679691	0.073373969	-3.92E-05	0.01493197	1	0.879226992	1	0.872396545	ENSG00000107242	13	PIP5K1B
Pancreas	T2DM_admission	ENSG00000172322.9	-0.001864134	0.389212935	0.002164963	0.073378368	-0.001825463	0.399164671	0.002164939	0.073373899	-3.87E-05	0.004086693	1	0.741467965	1	0.752527081	ENSG00000107443	11	CCNJ
Pancreas	T2DM_admission	ENSG00000172493.16	-0.00203128	0.592576137	0.003796026	0.073378436	-0.002091336	0.581680705	0.003795997	0.073373961	6.01E-05	0.019178876	1	0.858891717	1	0.854282157	ENSG00000107443	11	CCNJ
Pancreas	T2DM_admission	ENSG00000172780.12	-0.002436581	0.275119297	0.002232629	0.07337873	-0.002400611	0.282263125	0.002232606	0.073373832	-3.60E-05	0.01110782	1	0.670309634	1	0.678065206	ENSG00000107443	11	CCNJ
Pancreas	T2DM_admission	ENSG00000174808.7	0.006767385	0.062423088	0.003631969	0.073377956	0.006825661	0.060196657	0.003631917	0.073373475	-5.83E-05	0.005023546	1	0.32978603	1	0.32708278	ENSG00000107443	11	CCNJ
Pancreas	T2DM_admission	ENSG00000175220.7	-0.001097179	0.36132712	0.00120194	0.073378354	-0.001115927	0.35317945	0.001201936	0.073373877	1.87E-05	0.035160971	1	0.723010568	1	0.71888913	ENSG00000107537	9	PHYH
Pancreas	T2DM_admission	ENSG00000175390.8	-0.001543418	0.449620448	0.002041427	0.073378393	-0.001569618	0.441958628	0.002041406	0.073373918	2.62E-05	0.04189656	1	0.781199104	1	0.777362848	ENSG00000107537	9	PHYH
Pancreas	T2DM_admission	ENSG00000175581.9	0.002383078	0.247495099	0.002060673	0.073378278	0.002422877	0.239683037	0.002060653	0.073373798	-3.98E-05	0.002946183	1	0.638493224	1	0.626788227	ENSG00000107554	11	DNMBP
Pancreas	T2DM_admission	ENSG00000177570.9	0.000677222	0.781837422	0.002445503	0.07337847	0.000708904	0.771904632	0.002445471	0.073373994	-3.17E-05	0.02800114	1	0.934710661	1	0.931343937	ENSG00000107554	11	DNMBP
Pancreas	T2DM_admission	ENSG00000177628.11	0.001523294	0.516229728	0.002346531	0.073378416	0.001575774	0.501879781	0.002346523	0.073373939	-5.25E-05	0.002444013	1	0.821323036	1	0.814167579	ENSG00000107593	15	PKD2L1
Pancreas	T2DM_admission	ENSG00000177990.7	0.000882975	0.558946795	0.00151089	0.073378428	0.000910234	0.546872215	0.001510877	0.073373952	-2.73E-05	0.006357042	1	0.847939375	1	0.845484919	ENSG00000107593	15	PKD2L1
Pancreas	T2DM_admission	ENSG00000178623.7	-0.00049044	0.808835345	0.002027206	0.07337847	-0.000465132	0.81852071	0.002027178	0.073373999	-2.53E-05	0.029207054	1	0.942917767	1	0.949824303	ENSG00000107672	10	NSMCE4A
Pancreas	T2DM_admission	ENSG00000179163.11	0.00198957	0.371188331	0.002224846	0.073378359	0.001939781	0.383277736	0.002224842	0.073373892	4.98E-05	0.003067562	1	0.729283118	1	0.737196508	ENSG00000107742	8	SPOCK2
Pancreas	T2DM_admission	ENSG00000179406.6	-0.00222443	0.226392923	0.001838821	0.073378259	-0.002205778	0.230302201	0.001838788	0.07337379	-1.87E-05	0.04325359	1	0.617173065	1	0.617989606	ENSG00000107742	8	SPOCK2
Pancreas	T2DM_admission	ENSG00000180185.7	-0.000301818	0.71117655	0.000815115	0.073378459	-0.000285074	0.726534781	0.000815106	0.073373988	-1.67E-05	0.001207851	1	0.912258576	1	0.917883561	ENSG00000107779	7	BMPR1A
Pancreas	T2DM_admission	ENSG00000180211.5	0.000217298	0.946473898	0.003236704	0.073378479	0.000256646	0.936799298	0.003236663	0.073374006	-3.93E-05	0.041571421	1	0.982923376	1	0.98183872	ENSG00000107779	7	BMPR1A
Pancreas	T2DM_admission	ENSG00000181104.6	0.006946571	0.042011293	0.003416211	0.073377857	0.007001218	0.040420661	0.003416179	0.073373374	-5.46E-05	0.013795004	1	0.277250207	1	0.277540798	ENSG00000107798	13	LIPA
Pancreas	T2DM_admission	ENSG00000181126.9	0.000579311	0.508468893	0.000876119	0.073378413	0.000361662	0.680608427	0.000878603	0.073373981	0.000217649	0.00104118	1	0.815555259	1	0.898151305	ENSG00000107798	13	LIPA
Pancreas	T2DM_admission	ENSG00000182858.9	0.003292359	0.101232952	0.002008873	0.073378075	0.00326257	0.104358141	0.002008865	0.073373609	2.98E-05	0.042750172	1	0.429184223	1	0.440775833	ENSG00000107890	12	ANKRD26
Pancreas	T2DM_admission	ENSG00000182952.4	0.000746686	0.651532695	0.001653298	0.073378448	0.000601446	0.71616171	0.00165417	0.073373987	0.00014524	0.000858411	1	0.887189213	1	0.913596366	ENSG00000107890	12	ANKRD26
Pancreas	T2DM_admission	ENSG00000183426.11	0.001477071	0.152360734	0.00103201	0.073378171	0.001465104	0.155704421	0.001032003	0.073373703	1.20E-05	0.028574629	1	0.52858693	1	0.534225295	ENSG00000107890	12	ANKRD26
Pancreas	T2DM_admission	ENSG00000183444.10	-0.001325917	0.673311704	0.003144908	0.073378452	-0.001369446	0.663233467	0.003144873	0.073373978	4.35E-05	0.025391432	1	0.895424369	1	0.889390884	ENSG00000107890	12	ANKRD26
Pancreas	T2DM_admission	ENSG00000184110.10	-0.006731407	0.00119037	0.002076828	0.073376897	-0.006701508	0.001251815	0.002076814	0.073372439	2.99E-05	0.035789966	1	0.027789735	1	0.029224201	ENSG00000107954	6	NEURL1
Pancreas	T2DM_admission	ENSG00000184164.10	0.003390942	0.155587705	0.002387868	0.073378176	0.003346342	0.161096156	0.002387862	0.073373711	4.46E-05	0.012509163	1	0.532022937	1	0.539367574	ENSG00000108107	8	RPL28
Pancreas	T2DM_admission	ENSG00000184389.8	-0.00378113	0.072238268	0.002103412	0.073377993	-0.003812237	0.069921066	0.00210339	0.073373512	3.11E-05	0.019039753	1	0.357418609	1	0.352073131	ENSG00000108107	8	RPL28
Pancreas	T2DM_admission	ENSG00000184608.4	0.002992835	0.308907987	0.002941312	0.073378323	0.002925157	0.319976168	0.002941311	0.073373858	6.77E-05	0.003107361	1	0.702883653	1	0.706943978	ENSG00000108187	11	PBLD
Pancreas	T2DM_admission	ENSG00000184674.8	-0.002208341	0.314885236	0.002197306	0.073378327	-0.002170256	0.32330217	0.002197292	0.07337386	-3.81E-05	0.012905913	1	0.705815487	1	0.706943978	ENSG00000108187	11	PBLD
Pancreas	T2DM_admission	ENSG00000184945.9	0.001024086	0.408385447	0.001238705	0.073378376	0.001000592	0.419217325	0.001238693	0.073373908	2.35E-05	0.003167182	1	0.756116674	1	0.763867723	ENSG00000108244	17	KRT23
Pancreas	T2DM_admission	ENSG00000184983.5	-0.002233557	0.016717531	0.000933422	0.073377617	-0.002218527	0.017465127	0.000933419	0.073373156	1.50E-05	0.029868839	1	0.173120979	1	0.174867821	ENSG00000108264	12	TADA2A
Pancreas	T2DM_admission	ENSG00000185101.8	-0.001300337	0.585730137	0.002385788	0.073378434	-0.001242156	0.602624448	0.002385869	0.073373966	-5.82E-05	0.031109608	1	0.856580292	1	0.863582357	ENSG00000108264	12	TADA2A
Pancreas	T2DM_admission	ENSG00000185340.11	-0.001026891	0.737318968	0.003061635	0.073378462	-0.000992844	0.745718726	0.003061589	0.073373991	-3.40E-05	0.044328756	1	0.919728783	1	0.923784058	ENSG00000108264	12	TADA2A
Pancreas	T2DM_admission	ENSG00000185986.10	-0.002226331	0.426848947	0.00280184	0.073378384	-0.002194388	0.43350663	0.002801802	0.073373914	-3.19E-05	0.048745908	1	0.767595622	1	0.771947117	ENSG00000108264	12	TADA2A
Pancreas	T2DM_admission	ENSG00000187010.14	0.002530758	0.170514119	0.00184652	0.073378196	0.00250374	0.17512115	0.001846508	0.07337373	2.70E-05	0.033767886	1	0.548084313	1	0.556573159	ENSG00000108264	12	TADA2A
Pancreas	T2DM_admission	ENSG00000187741.10	-0.001160796	0.922820617	0.001659723	0.073378478	-0.000199066	0.904530597	0.001659712	0.073374004	3.83E-05	0.000866841	1	0.978091879	1	0.972260921	ENSG00000108264	12	TADA2A
Pancreas	T2DM_admission	ENSG00000187987.5	0.005033356	0.174359079	0.003705852	0.073378201	0.004483213	0.226937327	0.003710387	0.073373787	0.000550143	0.00303858	1	0.553580425	1	0.614988604	ENSG00000108272	9	DHRS11
Pancreas	T2DM_admission	ENSG00000188321.9	0.000591044	0.457754378	0.000795963	0.073378396	0.000600542	0.450552029	0.000795951	0.073373921	-9.50E-06	0.035140285	1	0.783534669	1	0.78249589	ENSG00000108272	9	DHRS11
Pancreas	T2DM_admission	ENSG00000188489.9	0.004659215	0.060464892	0.002481748	0.073377948	-0.001002397	0.758367642	0.003258502	0.073373992	0.005661612	0.007537545	1	0.324840676	1	0.92717566	ENSG00000108298	9	DHRS11
Pancreas	T2DM_admission	ENSG00000188529.10	0.003410811	0.231438201	0.002850267	0.073378264	0.003366567	0.237547153	0.002850266	0.073373796	4.42E-05	0.045573929	1	0.619742754	1	0.623743445	ENSG00000108298	5	RPL19
Pancreas	T2DM_admission	ENSG00000188611.10	-0.006754057	0.029895706	0.003110353	0.073377769	-0.006699365	0.03124919	0.003110354	0.073373308	-5.47E-05	0.025032839	1	0.229023436	1	0.238833099	ENSG00000108384	10	RAD51C

Pancreas	T2DM_admission	ENSG00000188629.7	0.000995723	0.444518817	0.001302304	0.073378391	0.0010098	0.438099391	0.001302283	0.073373916	-1.41E-05	0.042428101	1	0.777745586	1	0.774133008	ENSG00000108384	10	RAD51C
Pancreas	T2DM_admission	ENSG00000188786.9	0.000382656	0.886222716	0.00267432	0.073378476	0.000349951	0.895888006	0.00267429	0.073374004	3.27E-05	0.049052533	1	0.972507979	1	0.972260921	ENSG00000108384	10	RAD51C
Pancreas	T2DM_admission	ENSG00000189011.9	0.001659556	0.526768803	0.002621953	0.073378419	0.001777593	0.497835785	0.002622208	0.073373937	-0.000118037	0.004870276	1	0.829394412	1	0.809993529	ENSG00000108384	10	RAD51C
Pancreas	T2DM_admission	ENSG00000196151.6	0.000358391	0.855126195	0.001962904	0.073378474	0.000297651	0.879480236	0.001963033	0.073374003	6.07E-05	0.025879969	1	0.965328541	1	0.971621772	ENSG00000108387	10	SEPT4
Pancreas	T2DM_admission	ENSG00000196260.3	0.001031803	0.474614715	0.001443098	0.073378402	0.000437064	0.763064398	0.001449828	0.073373993	0.000594739	2.15E-05	1	0.795049308	1	0.928625715	ENSG00000108439	5	PNPO
Pancreas	T2DM_admission	ENSG00000196535.10	-0.001857032	0.240447462	0.001581973	0.073378272	-0.001971317	0.213009266	0.001582961	0.073373773	0.000114285	0.045959099	1	0.629908248	1	0.601107468	ENSG00000108439	5	PNPO
Pancreas	T2DM_admission	ENSG00000196666.3	0.000959125	0.549017417	0.001600587	0.073378425	0.000942038	0.556151683	0.00160056	0.073373954	1.71E-05	0.041900534	1	0.841622385	1	0.846590114	ENSG00000108465	10	CDK5RAP3
Pancreas	T2DM_admission	ENSG00000196735.7	-0.004072321	0.004082908	0.001418088	0.073377237	-0.001292418	0.438313627	0.00166754	0.073373916	-0.002779903	0.001532861	1	0.068540999	1	0.774133008	ENSG00000108465	10	CDK5RAP3
Pancreas	T2DM_admission	ENSG00000196743.4	0.001391234	0.656105889	0.003124289	0.073378449	0.002043153	0.514087596	0.003131333	0.073373942	-0.000651919	0.002037763	1	0.890058068	1	0.81978735	ENSG00000108468	10	CBX1
Pancreas	T2DM_admission	ENSG00000196912.8	0.000994091	0.665183629	0.002297054	0.073378451	0.001028966	0.654187676	0.002297043	0.073373976	-3.49E-05	0.034234378	1	0.891995883	1	0.887614942	ENSG00000108469	10	RECQL5
Pancreas	T2DM_admission	ENSG00000197054.7	-0.00167443	0.595632887	0.003155185	0.073378437	-0.001793316	0.569809217	0.003155397	0.073373958	0.000118886	0.006989081	1	0.859482295	1	0.849574534	ENSG00000108509	16	CAMTA2
Pancreas	T2DM_admission	ENSG00000197157.6	0.000505656	0.83758849	0.002466865	0.073378473	0.000540025	0.826717578	0.002466841	0.073373999	-3.44E-05	0.029561521	1	0.958120026	1	0.954095567	ENSG00000108509	16	CAMTA2
Pancreas	T2DM_admission	ENSG00000197415.7	-0.002032567	0.547961988	0.003383003	0.073378425	-0.00198267	0.557826136	0.003382969	0.073373955	-4.99E-05	0.020934737	1	0.841622385	1	0.847111521	ENSG00000108556	7	CHRNE
Pancreas	T2DM_admission	ENSG00000197568.9	-0.001752497	0.492852859	0.002555484	0.073378408	-0.001784986	0.484864798	0.002555456	0.073373933	3.25E-05	0.040603296	1	0.805630899	1	0.803311485	ENSG00000108556	7	CHRNE
Pancreas	T2DM_admission	ENSG00000197580.7	-0.001012805	0.634733048	0.00213188	0.073378445	-0.000974437	0.647612238	0.002131863	0.073373975	-3.84E-05	0.00759989	1	0.881724325	1	0.884341453	ENSG00000108556	7	CHRNE
Pancreas	T2DM_admission	ENSG00000197816.9	0.001272656	0.637472543	0.002700677	0.073378446	0.001312714	0.62691626	0.002700657	0.073373971	-4.01E-05	0.029114354	1	0.8820202	1	0.877200348	ENSG00000108556	7	CHRNE
Pancreas	T2DM_admission	ENSG00000197903.6	0.002742997	0.146827949	0.00189064	0.073378162	0.00296845	0.116647311	0.001891931	0.073373636	-0.000225452	0.001595194	1	0.517931557	1	0.469231995	ENSG00000108556	7	CHRNE
Pancreas	T2DM_admission	ENSG00000197915.5	0.000852029	0.306421206	0.000833067	0.073378322	0.000837567	0.314707286	0.000833073	0.073373854	1.45E-05	0.043797303	1	0.702883653	1	0.706943978	ENSG00000108592	12	FTSJ3
Pancreas	T2DM_admission	ENSG00000197935.6	-0.001367991	0.3861076	0.001578393	0.073378366	-0.001908754	0.228176841	0.001583936	0.073373788	0.000540763	4.77E-05	1	0.737741307	1	0.615685781	ENSG00000108602	13	ALDH3A1
Pancreas	T2DM_admission	ENSG00000198624.8	0.000916581	0.671003449	0.00215782	0.073378452	0.001302128	0.546891501	0.002161477	0.073373952	-0.000385548	0.002362922	1	0.894197754	1	0.845484919	ENSG00000108602	13	ALDH3A1
Pancreas	T2DM_admission	ENSG00000198756.6	-0.000805931	0.702028259	0.002106545	0.073378457	-0.000862842	0.682099615	0.002106549	0.073373981	5.69E-05	0.000784608	1	0.908467044	1	0.898858571	ENSG00000108639	3	SYNGR2
Pancreas	T2DM_admission	ENSG00000204140.8	-0.0023891	0.295437642	0.002283449	0.073378314	-0.002331341	0.30726755	0.002283449	0.07337385	-5.78E-05	0.001229837	1	0.689712604	1	0.703146938	ENSG00000108639	3	SYNGR2
Pancreas	T2DM_admission	ENSG00000204147.5	0.004242144	0.035177935	0.002014017	0.073377811	0.004199828	0.037042898	0.00201402	0.073373352	4.23E-05	0.008453073	1	0.260336995	1	0.266843516	ENSG00000108639	3	SYNGR2
Pancreas	T2DM_admission	ENSG00000204149.5	0.004385182	0.006614914	0.001614785	0.073377369	0.004411657	0.00629459	0.001614785	0.073372883	-2.65E-05	0.037203192	1	0.094172779	1	0.092368612	ENSG00000108654	7	DDX5
Pancreas	T2DM_admission	ENSG00000204228.3	0.000532127	0.67993856	0.001289857	0.073378454	0.00024968	0.847078568	0.001294703	0.073374001	0.000282447	0.011949475	1	0.899069186	1	0.961211992	ENSG00000108654	7	DDX5
Pancreas	T2DM_admission	ENSG00000204301.5	-0.011072802	2.48E-09	0.001857009	0.073373126	-0.010163301	7.43E-08	0.001888914	0.073369648	-0.000909501	0.008562442	6.38E-06	7.08E-07	0.000190837	1.59E-05	ENSG00000108666	5	C17orf75
Pancreas	T2DM_admission	ENSG00000204305.9	0.012376063	3.16E-05	0.002973581	0.073375871	0.010503577	0.000504287	0.003019578	0.073372185	0.001872486	0.000365934	0.0810249	0.001929164	1	0.015792794	ENSG00000108666	5	C17orf75
Pancreas	T2DM_admission	ENSG00000204351.7	-0.000543803	0.499071758	0.0008045	0.07337841	-0.001687163	0.046979143	0.000849317	0.073373412	0.00114336	2.69E-05	1	0.809101183	1	0.292821452	ENSG00000108669	12	CYTH1
Pancreas	T2DM_admission	ENSG00000204371.7	-0.0041664	0.187677349	0.003162394	0.073378218	-0.003641943	0.25043345	0.00316884	0.073373808	-0.000524458	0.009960072	1	0.567450741	1	0.641749029	ENSG00000108669	12	CYTH1
Pancreas	T2DM_admission	ENSG00000204516.5	0.00073224	0.461820734	0.000995091	0.073378398	0.000520711	0.601949236	0.000998296	0.073373966	0.00021153	0.008432103	1	0.785921567	1	0.86327687	ENSG00000108733	5	PEX12
Pancreas	T2DM_admission	ENSG00000204525.10	-0.002087796	0.002153586	0.000680461	0.073377062	-0.001731655	0.012864226	0.000696139	0.073373075	-0.00035614	0.015399722	1	0.044962667	1	0.145219373	ENSG00000108771	8	DHX58
Pancreas	T2DM_admission	ENSG00000204531.11	0.004537075	0.003642473	0.001560427	0.073377206	0.004170265	0.007653994	0.001563669	0.073372936	0.000366809	0.000295025	1	0.064286245	1	0.107944917	ENSG00000108774	10	RAB5C
Pancreas	T2DM_admission	ENSG00000204560.5	0.002817196	0.405820216	0.003389017	0.073378375	0.003303269	0.330541954	0.003394849	0.073373864	-0.000486073	0.015425547	1	0.75562027	1	0.707173382	ENSG00000108774	10	RAB5C
Pancreas	T2DM_admission	ENSG00000204616.6	-0.002643152	0.031072721	0.001225869	0.073377779	-0.002379582	0.052901089	0.001229293	0.073373442	-0.00026357	0.004245147	1	0.234690436	1	0.310159807	ENSG00000108786	6	HSD17B1
Pancreas	T2DM_admission	ENSG00000204618.4	0.006737938	0.000160248	0.001785047	0.073376334	0.007550476	3.04E-05	0.001810418	0.073371388	-0.000812538	0.007207645	0.411516019	0.006974848	0.078039375	0.002053668	ENSG00000108823	11	SGCA
Pancreas	T2DM_admission	ENSG00000204625.6	0.000298897	0.885087476	0.002068159	0.073378476	-0.000971168	0.641560578	0.002086209	0.073373974	0.001270065	3.66E-06	1	0.972507979	1	0.883436051	ENSG00000108823	11	SGCA
Pancreas	T2DM_admission	ENSG00000204642.9	-0.00014606	0.876154576	0.00093721	0.073378476	0.000133859	0.886728139	0.000939723	0.073374003	-0.00027992	5.07E-05	1	0.970211524	1	0.972260921	ENSG00000108846	11	ABCC3
Pancreas	T2DM_admission	ENSG00000204789.3	-0.000357433	0.702576148	0.000936068	0.073378457	-0.000509788	0.586470357	0.000937182	0.073373962	0.000152355	0.000990901	1	0.908467044	1	0.855368726	ENSG00000108846	11	ABCC3
Pancreas	T2DM_admission	ENSG00000204991.6	-0.000241657	0.885713049	0.001681314	0.073378476	-0.000278526	0.86842409	0.001681307	0.073374002	3.69E-05	0.002483861	1	0.972507979	1	0.967511091	ENSG00000108846	11	ABCC3
Pancreas	T2DM_admission	ENSG00000205517.8	-0.000748205	0.737430515	0.002231727	0.073378462	-0.000822019	0.712648875	0.002231921	0.073373986	7.38E-05	0.031125456	1	0.919728783	1	0.912759257	ENSG00000108846	11	ABCC3
Pancreas	T2DM_admission	ENSG00000205560.8	-0.000133954	0.943332844	0.001884512	0.073378478	-0.000165637	0.929960694	0.0018845	0.073374005	3.17E-05	0.01508656	1	0.982923376	1	0.981537798	ENSG00000108924	9	HLF
Pancreas	T2DM_admission	ENSG00000205937.7	0.001824245	0.436675823	0.002345332	0.073378388	0.001872159	0.424737537	0.002345388	0.073373911	-4.79E-05	0.04980182	1	0.77336794	1	0.768116897	ENSG00000108924	9	HLF
Pancreas	T2DM_admission	ENSG00000205978.5	0.00652243	0.025289363	0.002915755	0.073377726	0.006633862	0.022908236	0.002916023	0.073373227	-0.000111432	0.01463471	1	0.207485894	1	0.20026655	ENSG00000108924	9	HLF
Pancreas	T2DM_admission	ENSG00000206341.6	0.002478199	0.054465726	0.001288635	0.073377922	0.002067117	0.109755553	0.001292517	0.073373621	0.000411082	4.37E-05	1	0.311854896	1	0.455334832	ENSG00000108924	9	HLF
Pancreas	T2DM_admission	ENSG00000206344.6	0.007140656	7.30E-08	0.001326319	0.073374115	0.006293701	3.28E-06	0.001352828	0.073370748	0.000846955	0.00149171	0.000187355	1.17E-05	0.008433577	0.000351399	ENSG00000109063	10	MYH3
Pancreas	T2DM_admission	ENSG00000206503.7	0.000248047	0.83440465	0.001186512	0.073378473	0.001369545	0.257110768	0.001208513	0.073373813	-0.001121497	1.05E-06	1	0.958120026	1	0.651827256	ENSG00000109084	9	TMEM97
Pancreas	T2DM_admission	ENSG00000212127.5	0.004665016	0.004083634	0.001624512	0.073377237	0.004644	0.004253357	0.001624488	0.073372776	2.10E-05	0.02094745	1	0.068540999	1	0.073306173	ENSG00000109171	10	SLAIN2
Pancreas	T2DM_admission	ENSG00000213221.4	0.003597034	0.000648504	0.001054696	0.073376728	0.003583132	0.000680445	0.001054681	0.073372269	1.39E-05	0.021130899	1	0.018300632	1	0.019202006	ENSG00000109265	8	KIAA1211
Pancreas	T2DM_admission	ENSG00000213588.4	7.77E-05	0.974178753	0.002399588	0.073378479	0.000573046	0.811540648	0.002403324	0.073373998	-0.000495377	0.000250056	1	0.991496562	1	0.945273278	ENSG00000109265	8	KIAA1211
Pancreas	T2DM_admission	ENSG00000213676.6	0.004774112	5.33E-06	0.001048959	0.07337536	0.004333867	4.20E-05	0.001057978	0.07337148	0.000440245	0.001428732	0.013695565	0.000456519	0.107791619	0.002629064	ENSG00000109265	8	KIAA1211
Pancreas	T2DM_admission	ENSG00000213760.6	0.003712728	0.045172001	0.001853533	0.073377875	0.001999338	0.297669492	0.001919772	0.073373843	0.00171339	0.000612779	1	0.285670854	1	0.694291785	ENSG00000109321	6	AREG
Pancreas	T2DM_admission	ENSG00000213901.6	0.003622807	0.335452838	0.00376126	0.073378339	0.003679286	0.327967749	0.003761218	0.073373862	-5.65E-05	0.016009956	1	0.71074304	1	0.706943978	ENSG00000109321	6	AREG
Pancreas	T2DM_admission	ENSG00000214376.5	-0.001143021	0.633282967	0.002395711	0.073378445	-0.003496377	0.183262466	0.002627316	0.07337374	0.002353355	0.029143891	1	0.881247101	1	0.565681828	ENSG00000109332	15	UBE2D3
Pancreas	T2DM_admission	ENSG00000219159.3	0.000110261	0.936012659	0.001373411	0.073378478	8.53E-05	0.95047603	0.001373393	0.073374006	2.50E-05	0.001681946	1	0.980909228	1	0.983433635	ENSG00000109472	9	CPE
Pancreas	T2DM_admission	ENSG00000221988.8	0.01276871	6.03E-06	0.002821584	0.073375396	0.011155718	0.000104495	0.002875228	0.07337174	0.001612993	0.003546609	0.015486148	0.000499553	0.268344037	0.005063095	ENSG00000109511	6	ANXA10
Pancreas	T2DM_admission	ENSG00000222019.3	0.001187277	0.651534008	0.002628857	0.073378448	0.001134444	0.666078159	0.002628845	0.073373979	5.28E-05	0.005173609	1	0.887189213	1	0.88982611	ENSG00000109511	6	ANXA10
Pancreas	T2DM_admission	ENSG00000224796.1	0.000619143	0.765440962	0.002075268	0.073378466	0.00094586	0.648792561	0.002076803	0.073373975	-0.000326717	6.07E-05	1	0.927632086	1	0.884341453	ENSG00000109771	11	LRP2BP
Pancreas	T2DM_admission	ENSG00000227057.3	0.002285495	0.134399433	0.001526738	0.073378142	0.001864976	0.22510162	0.001537395	0.073373785	0.000420519	0.020220019	1	0.495887562	1	0.614988604	ENSG00000109775	6	UFSP2
Pancreas	T2DM_admission	ENSG00000227262.3	0.000857858	0.635178724	0.001808107	0.073378445	0.000661097	0.714763334	0.001808913	0.073373986	0.000196761	0.000420779	1	0.881724325	1	0.913596366	ENSG00000109794	9	FAM149A
Pancreas	T2DM_admission	ENSG00000227825.3	-0.00302392	0.323354576	0.003061922	0.073378332	-0.0030627	0.317182574	0.003061887	0.073373856	3.88E-05	0.03942211	1	0.707406446	1	0.706943978	ENSG00000109794	9	FAM149A
Pancreas	T2DM_admission	ENSG00000228065.6	0.001801145	0.405352681	0.002164577	0.073378375	0.00183394	0.39685092	0.002164555	0.073373898	-3.28E-05	0.0177652	1	0.755550584	1	0.751558379	ENSG00000109854	9	HTATIP2
Pancreas	T2DM_admission	ENSG00000228262.4	0.002761997	0.385701212	0.003184077	0.073378366	0.002718837	0.393163783	0.003184043	0.073373897	4.32E-05	0.030678623	1	0.737741307	1	0.748994506	ENSG00000109854	9	HTATIP2
Pancreas	T2DM_admission	ENSG00000228672.2	-0.000307115	0.923154932	0.003183843	0.073378478	-0.000358835	0.91026364	0.00318381	0.073374005	5.17E-05	0.010815264	1	0.978091879	1	0.97369587	ENSG00000109854	9	HTATIP2
Pancreas	T2DM_admission	ENSG00000229089.2	0.008236264	0.02524878	0.003680877	0.073377725	0.008182086	0.026224706	0.003680858	0.073373263	5.42E-05	0.037850709	1	0.207485894	1	0.213574989	ENSG00000109861	11	CTSC
Pancreas	T2DM_admission	ENSG00000229212.3	-0.005095525	0.1632908	0.003655088	0.073378187	-0.0051749	0.156828245	0.00365505	0.073373705	7.94E-05	0.000572724	1	0.541071967	1	0.534414035	ENSG00000109917	6	ZNF259
Pancreas	T2DM_admission	ENSG00000229390.1	-0.001414751	0.484298592	0.002022789	0.073378406	-0.001860338	0.358739915	0.002027021	0.07337388	0.000445587	0.000727207	1	0.799279425	1	0.721412765	ENSG00000110011	9	DNAJC4
Pancreas	T2DM_admission	ENSG00000230454.1	-0.001207973	0.536214322	0.001952917	0.073378422	-0.001234027	0.527453138	0.001952886	0.073373946	2.61E-05	0.014256611	1	0.833893716	1	0.829727017	ENSG00000110011	9	DNAJC4
Pancreas	T2DM_admission	ENSG00000230795.2	-0.000150123	0.859672685	0.000849153	0.073378474	-0.000360185	0.672505194	0.00085208	0.07337398	0.000210062	0.003039582	1	0.965890282	1	0.893214295	ENSG00000110011	9	DNAJC4
Pancreas	T2DM_admission	ENSG00000231205.7	0.000982269	0.665870759	0.002274707	0.073378451	0.001019303	0.654076866	0.002274691	0.073373976	-3.70E-05	0.017281355	1	0.891995883	1	0.887614942	ENSG00000110025	8	SNX15
Pancreas	T2DM_admission	ENSG00000231464.1	0.007558535	0.029965683	0.003482313	0.07337777	0.00750734	0.031094687	0.003482287	0.073373307	5.12E-05	0.029588519	1	0.229023436	1	0.238361663	ENSG00000110025	8	SNX15
Pancreas	T2DM_admission	ENSG00000231500.2	0.004849205	0.024123182	0.002150263	0.073377713	0.004153519	0.054272724	0.002158055	0.073373449	0.000695687	0.00015575	1	0.203396248	1	0.314289811	ENSG00000110025	8	SNX15
Pancreas	T2DM_admission	ENSG00000232040.2	-0.000599986	0.72576652	0.001710517	0.073378461	-0.00100873	0.555883942	0.001712712	0.073373954	0.000408745	3.17E-06	1	0.915856719	1	0.846590114	ENSG00000110025	8	SNX15
Pancreas	T2DM_admission	ENSG00000232615.4	-0.003212575	0.39340759	0.0037642	0.07337837	-0.003143624	0.403635017	0.003764153	0.073373902	-6.90E-05	0.002210346	1	0.746137882	1	0.755027733	ENSG00000110025	8	SNX15
Pancreas	T2DM_admission	ENSG00000232653.4	0.002584962	0.200640549	0.002019927	0.073378233	0.002553805	0.206115434	0.002019902	0.073373766	3.12E-05	0.010648358	1	0.580230776	1	0.590082981	ENSG00000110025	8	SNX15
Pancreas	T2DM_admission	ENSG00000233609.3	0.007807652	0.000831084	0.002336047	0.073376797	0.007748022	0.00091124	0.002336128	0.07337235	5.96E-05	0.025509518	1	0.021777795	1	0.023400641	ENSG00000110042	3	DTX4
Pancreas	T2DM_admission	ENSG00000234127.4	-0.002841693	0.078909509	0.001617307	0.073378014	-0.004093603	0.012634859	0.001641441	0.07337307	0.00125191	8.23E-06	1	0.375066777	1	0.144849635	ENSG00000110060	4	PUS3
Pancreas	T2DM_admission	ENSG00000234745.5	0.005618334	1.61E-07	0.001072342	0.073374346	0.004756387	1.85E-05	0.001110842	0.073371246	0.000861947	0.002956379	0.000414074	2.44E-05	0.047619377	0.001443011	ENSG00000110074	6	FOXRED1
Pancreas	T2DM_admission	ENSG00000234882.1	-0.002850795	0.380341944	0.003249631	0.073378363	-0.002895952	0.372837916	0.003249603	0.073373887	4.52E-05	0.035752333	1	0.733271857	1	0.730903468	ENSG00000110074	6	FOXRED1
Pancreas	T2DM_admission	ENSG00000235109.3	0.00157975	0.02181034	0.000688746	0.073377687	0.001397517	0.04292998	0.000690338	0.073373389	0.000182234	0.000111023	1	0.193802603	1	0.28152064	ENSG00000110074	6	FOXRED1
Pancreas	T2DM_admission	ENSG00000235290.1	0.001402766	0.42064851	0.001741923	0.073378382	0.001969061	0.259263181	0.001745411	0.073373815	-0.000566295	3.49E-07	1	0.764941163	1	0.654717006	ENSG00000110169	6	HPX
Pancreas	T2DM_admission	ENSG00000235821.1	-0.002612769	0.388306589	0.003028619	0.073378367	-0.001920178	0.526856329	0.003034356	0.073373946	-0.000692591	0.00022912	1	0.740290513	1	0.829556081	ENSG00000110169	6	HPX
Pancreas	T2DM_admission	ENSG00000235863.2	-0.003284045	0.028940954	0.00150348	0.073377761	-0.001516166	0.349563965	0.001620811	0.073373875	-0.001767879	0.00350673	1	0.224532842	1	0.717365997	ENSG00000110203	4	FOLR3
Pancreas	T2DM_admission	ENSG00000236417.2	0.001587137	0.590737164	0.002951339	0.073378436	0.00154316	0.60106265	0.002951308	0.073373965	4.40E-05	0.018899362	1	0.858891717	1	0.863271188	ENSG00000110436	7	SLC1A2
Pancreas	T2DM_admission	ENSG00000236624.4	-0.001165483	0.483969799	0.001665136	0.073378405	-0.001185632	0.476439186	0.001665116	0.07337393	2.01E-05	0.047188342	1	0.799250446	1	0.79570852	ENSG00000110455	9	ACCS
Pancreas	T2DM_admission	ENSG00000237669.1	0.001051561	0.38571983	0.001212305	0.073378366	0.001535191	0.207526096	0.00121802	0.073373767	-0.00048363	4.31E-05	1	0.737741307	1	0.591483923	ENSG00000110455	9	ACCS
Pancreas	T2DM_admission	ENSG00000238034.1	0.001199136	0.573616619	0.002130913	0.073378432	0.001237935	0.56127787	0.002130899	0.073373956	-3.88E-05	0.008655094	1	0.851343964	1	0.848858403	ENSG00000110455	9	ACCS
Pancreas	T2DM_admission	ENSG00000239257.1	0.001416926	0.272984895	0.001292562	0.073378298	0.00101676	0.432577123	0.001295583	0.073373914	0.000400166	6.90E-06	1	0.670309634	1	0.771947117	ENSG00000110455	9	ACCS
Pancreas	T2DM_admission	ENSG00000240053.8	-0.001443037	0.274664401	0.001320996	0.0733783	-0.000990789	0.459087121	0.001338269	0.073373924	-0.000452247	0.035049813	1	0.670309634	1	0.78659086	ENSG00000110628	9	SLC22A18
Pancreas	T2DM_admission	ENSG00000240065.3	0.00343994	0.194362162	0.002650626	0.073378226	0.002718742	0.306309483	0.002657623	0.073373849	0.000721197	0.000198375	1	0.572793457	1	0.703146938	ENSG00000110628	9	SLC22A18
Pancreas	T2DM_admission	ENSG00000240929.2	0.003681853	0.288530856	0.003469025	0.07337831	0.003630344	0.295324612	0.00346899	0.073373842	5.15E-05	0.019931289	1	0.682535343	1	0.690198968	ENSG00000110660	10	SLC35F2
Pancreas	T2DM_admission	ENSG00000241258.2	-0.002825475	0.262564234	0.002521952	0.07337829	-0.002790648	0.26848617	0.00252192	0.073373822	-3.48E-05	0.020203822	1	0.660396625	1	0.663592381	ENSG00000110665	7	C11orf21
Pancreas	T2DM_admission	ENSG00000241484.5	0.001364017	0.395037134	0.001603748	0.07337837	0.001384045	0.388127538	0.001603725	0.073373894	-2.00E-05	0.030689306	1	0.747572115	1	0.742706048	ENSG00000110700	2	RPS13
Pancreas	T2DM_admission	ENSG00000241549.4	-0.000442171	0.591109809	0.000823058	0.073378436	-0.000473714	0.56496383	0.000823156	0.073373957	3.15E-05	0.02633781	1	0.858891717	1	0.849476828	ENSG00000110700	2	RPS13
Pancreas	T2DM_admission	ENSG00000241839.5	0.001414357	0.559975355	0.002426504	0.073378428	0.001379469	0.569691413	0.002426479	0.073373958	3.49E-05	0.023842904	1	0.847939375	1	0.849574534	ENSG00000110700	2	RPS13
Pancreas	T2DM_admission	ENSG00000242252.1	-0.004909743	0.038377557	0.002370935	0.073377833	-0.004875279	0.03975451	0.002370903	0.07337337	-3.45E-05	0.012725844	1	0.265643038	1	0.275174077	ENSG00000110711	5	AIP
Pancreas	T2DM_admission	ENSG00000243056.1	-0.004833139	0.099291852	0.002932197	0.07337807	-0.004780392	0.103033072	0.002932168	0.073373606	-5.27E-05	0.004771574	1	0.426555097	1	0.436615395	ENSG00000110711	5	AIP
Pancreas	T2DM_admission	ENSG00000243753.1	0.005980814	7.37E-07	0.001207902	0.073374788	0.005638929	3.27E-06	0.001211877	0.073370747	0.000341884	0.000517412	0.001892709	8.60E-05	0.008400931	0.000351399	ENSG00000110711	5	AIP
Pancreas	T2DM_admission	ENSG00000244165.1	0.004908529	0.003499632	0.00168096	0.073377195	0.004942627	0.003278413	0.001680957	0.073372705	-3.41E-05	0.007593206	1	0.062610489	1	0.061006986	ENSG00000110768	7	GTF2H1
Pancreas	T2DM_admission	ENSG00000244414.2	-0.002541482	0.506056265	0.003821827	0.073378413	-0.002482233	0.516019944	0.003821799	0.073373943	-5.92E-05	0.022989616	1	0.814511821	1	0.820393199	ENSG00000110768	7	GTF2H1
Pancreas	T2DM_admission	ENSG00000247157.2	0.002228509	0.018507697	0.000946164	0.073377644	0.002215334	0.019211204	0.000946152	0.073373181	1.32E-05	0.017753188	1	0.181403685	1	0.183399155	ENSG00000110768	7	GTF2H1
Pancreas	T2DM_admission	ENSG00000247596.4	-0.002343457	0.284763599	0.002190801	0.073378307	-0.002365531	0.280242719	0.002190762	0.073373831	2.21E-05	0.047871865	1	0.679621676	1	0.675106288	ENSG00000110768	7	GTF2H1
Pancreas	T2DM_admission	ENSG00000248202.1	-0.000177749	0.949581547	0.002811054	0.073378479	-0.000211005	0.940164101	0.00281101	0.073374006	3.33E-05	0.029637988	1	0.984069981	1	0.98183872	ENSG00000110888	13	CAPRIN2
Pancreas	T2DM_admission	ENSG00000248927.1	0.001586434	0.530148662	0.002527056	0.07337842	0.001643509	0.515452482	0.00252703	0.073373943	-5.71E-05	0.000380367	1	0.831148818	1	0.820125138	ENSG00000110888	13	CAPRIN2
Pancreas	T2DM_admission	ENSG00000250376.1	0.005405326	0.078302408	0.003070116	0.073378012	0.00536552	0.08051921	0.003070073	0.073373547	3.98E-05	0.022905949	1	0.373062306	1	0.383122792	ENSG00000110888	13	CAPRIN2
Pancreas	T2DM_admission	ENSG00000250535.1	0.001703719	0.138964611	0.001151426	0.07337815	0.001560457	0.175779794	0.001152593	0.073373731	0.000143262	0.006490227	1	0.500672845	1	0.556849121	ENSG00000110888	13	CAPRIN2
Pancreas	T2DM_admission	ENSG00000250656.2	0.008654302	0.022835912	0.003802123	0.073377699	0.008708424	0.021996359	0.003802078	0.073373217	-5.41E-05	0.019545775	1	0.198567397	1	0.195455536	ENSG00000110906	8	KCTD10
Pancreas	T2DM_admission	ENSG00000250986.1	0.001251422	0.56120821	0.002153731	0.073378428	0.001223865	0.569858343	0.002153705	0.073373958	2.76E-05	0.035138289	1	0.847939375	1	0.849574534	ENSG00000110944	4	IL23A
Pancreas	T2DM_admission	ENSG00000251008.1	0.009501762	0.003804359	0.00328331	0.073377218	0.009461067	0.003956686	0.003283259	0.073372756	4.07E-05	0.023239385	1	0.065967883	1	0.069941797	ENSG00000111058	3	ACSS3
Pancreas	T2DM_admission	ENSG00000251417.1	-0.016163669	1.26E-06	0.003335971	0.073374944	-0.016108067	1.38E-06	0.003335953	0.073370496	-5.56E-05	0.01827569	0.00324801	0.00012992	0.003531518	0.000196195	ENSG00000111058	3	ACSS3
Pancreas	T2DM_admission	ENSG00000254851.1	0.002963488	0.007774102	0.001113366	0.073377412	0.00294359	0.008196261	0.001113357	0.073372954	1.99E-05	0.00734396	1	0.105629064	1	0.109202996	ENSG00000111142	9	METAP2
Pancreas	T2DM_admission	ENSG00000255394.2	0.005365271	0.152004775	0.00374542	0.07337817	0.005497829	0.142151007	0.003745549	0.073373682	-0.000132559	0.001884796	1	0.528211449	1	0.50983769	ENSG00000111144	5	LTA4H
Pancreas	T2DM_admission	ENSG00000255595.1	0.003370121	0.168698597	0.002448505	0.073378194	0.003404631	0.164375237	0.00244848	0.073373715	-3.45E-05	0.027409372	1	0.548084313	1	0.544665301	ENSG00000111144	5	LTA4H
Pancreas	T2DM_admission	ENSG00000255837.1	0.005887506	0.000670265	0.001730864	0.073376737	0.005865702	0.000701718	0.001730843	0.073372277	2.18E-05	0.036143935	1	0.018709131	1	0.019587077	ENSG00000111215	7	PRR4
Pancreas	T2DM_admission	ENSG00000257048.1	-0.006295981	0.073580993	0.003518853	0.073377997	-0.006248218	0.075789099	0.003518809	0.073373532	-4.78E-05	0.022601575	1	0.360189008	1	0.369310068	ENSG00000111215	7	PRR4
Pancreas	T2DM_admission	ENSG00000257218.1	-0.009141318	0.013493795	0.00370023	0.07337756	-0.009195552	0.012949858	0.003700193	0.073373077	5.42E-05	0.022568635	1	0.151982746	1	0.145219373	ENSG00000111254	3	AKAP3
Pancreas	T2DM_admission	ENSG00000259205.2	-0.000807958	0.495778682	0.001186173	0.073378409	-0.000822329	0.488137766	0.001186153	0.073373934	1.44E-05	0.021330542	1	0.807330155	1	0.804739043	ENSG00000111321	6	LTBR
Pancreas	T2DM_admission	ENSG00000259363.1	-0.002299365	0.404582407	0.002758798	0.073378375	-0.002342317	0.395858451	0.002758777	0.073373898	4.30E-05	0.020857451	1	0.755550584	1	0.751558379	ENSG00000111321	6	LTBR
Pancreas	T2DM_admission	ENSG00000259726.1	0.004306323	0.138388984	0.002906105	0.073378149	0.004260332	0.142646874	0.002906078	0.073373683	4.60E-05	0.014338475	1	0.500672845	1	0.510902611	ENSG00000111341	5	MGP
Pancreas	T2DM_admission	ENSG00000259982.1	0.003784703	0.001036275	0.001153694	0.073376859	0.00376819	0.001090039	0.001153688	0.0733724	1.65E-05	0.043144875	1	0.025105219	1	0.02640773	ENSG00000111341	5	MGP
Pancreas	T2DM_admission	ENSG00000260302.1	0.005951037	0.004161691	0.002076678	0.073377243	0.005980856	0.003976442	0.002076659	0.073372758	-2.98E-05	0.027088373	1	0.069397554	1	0.069941797	ENSG00000111341	5	MGP
Pancreas	T2DM_admission	ENSG00000260539.1	-0.002371075	0.52952777	0.003771229	0.07337842	-0.002414241	0.522053642	0.00377117	0.073373945	4.32E-05	0.036204643	1	0.830895322	1	0.825005386	ENSG00000111364	11	DDX55
Pancreas	T2DM_admission	ENSG00000260804.2	0.0025675	0.278159768	0.00236753	0.073378302	0.002626444	0.267274443	0.002367526	0.073373821	-5.89E-05	0.001060649	1	0.671348012	1	0.663456699	ENSG00000111450	9	STX2
Pancreas	T2DM_admission	ENSG00000261349.1	0.000834519	0.675658697	0.001994574	0.073378453	0.000807321	0.68565198	0.001994552	0.073373982	2.72E-05	0.027921227	1	0.896564018	1	0.900181127	ENSG00000111450	9	STX2
Pancreas	T2DM_admission	ENSG00000261584.1	-0.000934031	0.442208679	0.001215444	0.07337839	-0.000844707	0.487136558	0.001215632	0.073373934	-8.93E-05	0.000133821	1	0.776526409	1	0.804739043	ENSG00000111450	9	STX2
Pancreas	T2DM_admission	ENSG00000263006.2	-0.000299959	0.928729401	0.003353612	0.073378478	-0.000346007	0.917823956	0.00335359	0.073374005	4.60E-05	0.046572996	1	0.979054639	1	0.978402623	ENSG00000111530	8	CAND1
Pancreas	T2DM_admission	ENSG00000264469.1	-0.002620321	0.149372924	0.001817456	0.073378166	-0.002676783	0.140812229	0.001817509	0.07337368	5.65E-05	0.004550966	1	0.521890706	1	0.506450707	ENSG00000111530	8	CAND1
Pancreas	T2DM_admission	ENSG00000266402.2	0.006264612	0.007872722	0.002357341	0.073377416	0.006219012	0.008335485	0.002357311	0.073372959	4.56E-05	0.001222627	1	0.105848958	1	0.109202996	ENSG00000111615	8	KRR1
Pancreas	T2DM_admission	ENSG00000266995.1	-0.001768904	0.273717428	0.001616112	0.073378299	-0.001746645	0.279792741	0.001616088	0.073373831	-2.23E-05	0.013676918	1	0.670309634	1	0.674655172	ENSG00000111615	8	KRR1
Pancreas	T2DM_admission	ENSG00000268822.1	0.000616758	0.772131875	0.002129782	0.073378467	0.000577944	0.786109031	0.002129753	0.073373995	3.88E-05	0.001782414	1	0.930781647	1	0.936079501	ENSG00000111639	3	MRPL51
Pancreas	T2DM_admission	ENSG00000270127.1	-0.000561587	0.517818092	0.000868371	0.073378416	-0.000574033	0.508576716	0.000868358	0.073373941	1.24E-05	0.00836034	1	0.821852201	1	0.817366177	ENSG00000111639	3	MRPL51
Pancreas	T2DM_admission	ENSG00000271821.1	0.00855494	0.000209505	0.002307594	0.07337641	0.006221137	0.00987817	0.002411223	0.073373004	0.002333803	0.000848257	0.538010056	0.008674644	1	0.122546576	ENSG00000111664	6	GNB3
Pancreas	T2DM_admission	ENSG00000272221.1	-0.001954919	0.002925999	0.000657022	0.073377146	-0.001743235	0.008286746	0.000660274	0.073372957	-0.000211685	0.001258796	1	0.054846454	1	0.109202996	ENSG00000111666	6	CHPT1
Pancreas	T2DM_admission	ENSG00000272799.1	-0.001924627	0.261526622	0.001714144	0.073378289	-0.001896827	0.268472279	0.001714121	0.073373822	-2.78E-05	0.005462602	1	0.659077885	1	0.663592381	ENSG00000111666	6	CHPT1
Pancreas	T2DM_admission	ENSG00000273340.1	-0.000905549	0.304166799	0.000881278	0.07337832	-0.000780272	0.377100893	0.000883409	0.073373889	-0.000125277	0.042288775	1	0.701687643	1	0.733034226	ENSG00000111666	6	CHPT1
Pancreas	T2DM_insulin	ENSG00000012232.4	0.000677951	0.472376495	0.000943409	0.011571118	0.000694263	0.461792539	0.000943421	0.011570676	-1.63E-05	0.032153147	1	0.890734922	1	0.886973253	ENSG00000111676	10	ATN1
Pancreas	T2DM_insulin	ENSG00000030110.8	0.003374198	0.001355836	0.00105315	0.011570886	0.003063946	0.00380025	0.001058615	0.01157049	0.000310252	0.003935307	1	0.112315715	1	0.17977037	ENSG00000111696	7	NT5DC3
Pancreas	T2DM_insulin	ENSG00000034533.7	-0.000120448	0.837862935	0.000588621	0.011571129	-0.000110898	0.850560643	0.000588623	0.011570688	-9.55E-06	0.014793264	1	0.97568398	1	0.979057591	ENSG00000111725	6	PRKAB1

Pancreas	T2DM_insulin	ENSG00000037757.9	0.000853033	0.313424833	0.00084621	0.011571106	0.000839097	0.321401734	0.00084622	0.011570666	1.39E-05	0.035926996	1	0.835397773	1	0.835647835	ENSG00000111725	6	PRKAB1
Pancreas	T2DM_insulin	ENSG00000048540.10	0.000319651	0.607041406	0.000621524	0.011571123	-0.002227176	0.086607146	0.001299724	0.011570619	0.002546827	0.025672092	1	0.927884944	1	0.596265823	ENSG00000111725	6	PRKAB1
Pancreas	T2DM_insulin	ENSG00000066294.10	0.000890588	0.161068483	0.000635458	0.011571083	0.002385046	0.001884275	0.000767412	0.01157046	-0.001494459	0.000514159	1	0.6998417	1	0.15121308	ENSG00000111786	4	SRSF9
Pancreas	T2DM_insulin	ENSG00000076662.5	-0.001937488	0.058432517	0.001023802	0.011571045	-0.001919282	0.060843298	0.001023815	0.011570606	-1.82E-05	0.025526452	1	0.530577607	1	0.538777894	ENSG00000111788	9	RP11-22B23.1
Pancreas	T2DM_insulin	ENSG00000079689.9	0.000359666	0.748460766	0.001121609	0.011571127	0.000297058	0.791173844	0.001121877	0.011570687	6.26E-05	0.014034239	1	0.965153689	1	0.967426237	ENSG00000111801	11	BTN3A3
Pancreas	T2DM_insulin	ENSG00000085117.7	-0.000903164	0.326022556	0.000919571	0.011571107	-0.000888651	0.33385835	0.000919574	0.011570667	-1.45E-05	0.018139914	1	0.835554813	1	0.837432686	ENSG00000111801	11	BTN3A3
Pancreas	T2DM_insulin	ENSG00000099783.7	-0.000620689	0.549231371	0.00103636	0.011571121	-0.000633102	0.541272577	0.001036359	0.01157068	1.24E-05	0.04913899	1	0.907039349	1	0.902870944	ENSG00000111801	11	BTN3A3
Pancreas	T2DM_insulin	ENSG00000100099.16	-0.001464722	0.222060991	0.001199542	0.011571094	-0.001446651	0.227818602	0.001199546	0.011570654	-1.81E-05	0.024631637	1	0.763006274	1	0.769631524	ENSG00000111801	11	BTN3A3
Pancreas	T2DM_insulin	ENSG00000100201.14	-5.10E-05	0.956094245	0.000927008	0.01157113	-3.69E-05	0.968222456	0.000927012	0.011570689	-1.41E-05	0.027327601	1	0.989356544	1	0.988759842	ENSG00000111801	11	BTN3A3
Pancreas	T2DM_insulin	ENSG00000100731.11	0.000687813	0.554531102	0.001163842	0.011571121	0.000674273	0.562351858	0.00116384	0.011570681	1.35E-05	0.045479932	1	0.910322362	1	0.908714845	ENSG00000111801	11	BTN3A3
Pancreas	T2DM_insulin	ENSG00000104331.4	-0.002417369	0.073708268	0.001351676	0.011571054	-0.002392372	0.076740818	0.001351683	0.011570615	-2.50E-05	0.00775162	1	0.582072897	1	0.587816164	ENSG00000111850	6	SMIM8
Pancreas	T2DM_insulin	ENSG00000106153.12	-5.31E-05	0.942970551	0.000742259	0.01157113	-6.18E-05	0.933595466	0.000742257	0.011570689	8.75E-06	0.039572284	1	0.989356544	1	0.988759842	ENSG00000111850	6	SMIM8
Pancreas	T2DM_insulin	ENSG00000106992.13	-0.000654018	0.422403375	0.00081522	0.011571114	-0.000665198	0.414515403	0.00081522	0.011570673	1.12E-05	0.028935205	1	0.870194075	1	0.86606442	ENSG00000111879	14	FAM184A
Pancreas	T2DM_insulin	ENSG00000108924.9	0.000720445	0.577459361	0.001293205	0.011571122	0.000735661	0.569446292	0.001293203	0.011570681	-1.52E-05	0.04711356	1	0.917471363	1	0.911776571	ENSG00000111879	14	FAM184A
Pancreas	T2DM_insulin	ENSG00000111254.3	8.53E-05	0.893664944	0.000638207	0.011571129	7.67E-05	0.904284137	0.000638209	0.011570689	8.56E-06	0.045145999	1	0.988687703	1	0.988759842	ENSG00000111879	14	FAM184A
Pancreas	T2DM_insulin	ENSG00000112149.5	-0.000145757	0.840464294	0.000724078	0.011571129	0.000562059	0.45344639	0.000749729	0.011570676	-0.000707815	0.000273279	1	0.97568398	1	0.880237961	ENSG00000111886	9	GABRR2
Pancreas	T2DM_insulin	ENSG00000112294.8	8.42E-05	0.949012333	0.001316218	0.01157113	5.85E-05	0.964526293	0.001316244	0.011570689	2.56E-05	0.026498293	1	0.989356544	1	0.988759842	ENSG00000112031	11	MTRF1L
Pancreas	T2DM_insulin	ENSG00000112977.11	0.000460789	0.459806838	0.000623391	0.011571117	0.000468749	0.452092016	0.000623392	0.011570676	-7.96E-06	0.046586257	1	0.886474445	1	0.880222754	ENSG00000112031	11	MTRF1L
Pancreas	T2DM_insulin	ENSG00000120333.4	-0.001066551	0.189633519	0.000813127	0.011571089	-0.001056751	0.193733067	0.000813126	0.011570649	-9.80E-06	0.039219247	1	0.728954236	1	0.731489476	ENSG00000112031	11	MTRF1L
Pancreas	T2DM_insulin	ENSG00000120725.8	-0.001141899	0.206432723	0.000903804	0.011571092	-0.001154689	0.201395516	0.000903804	0.01157065	1.28E-05	0.022256801	1	0.751302486	1	0.743080008	ENSG00000112031	11	MTRF1L
Pancreas	T2DM_insulin	ENSG00000122481.12	-0.001970093	0.102831746	0.001207697	0.011571067	-0.001953338	0.105793116	0.001207704	0.011570627	-1.68E-05	0.047479641	1	0.616990479	1	0.614110331	ENSG00000112041	8	TULP1
Pancreas	T2DM_insulin	ENSG00000123643.8	-9.45E-05	0.871457312	0.00058412	0.011571129	-7.52E-05	0.897576686	0.000584143	0.011570689	-1.93E-05	0.002457475	1	0.984420735	1	0.988759842	ENSG00000112041	8	TULP1
Pancreas	T2DM_insulin	ENSG00000124116.14	-0.000453552	0.435728843	0.000581905	0.011571115	-0.000462508	0.426721697	0.000581907	0.011570674	8.96E-06	0.021752904	1	0.875147029	1	0.870390246	ENSG00000112053	9	SLC26A8
Pancreas	T2DM_insulin	ENSG00000124549.10	-0.000795455	0.039229923	0.000385811	0.011571019	-0.000833964	0.030782473	0.000386116	0.011570578	3.85E-05	0.013122436	1	0.489907645	1	0.440318545	ENSG00000112053	9	SLC26A8
Pancreas	T2DM_insulin	ENSG00000124613.4	0.000481531	0.306509772	0.000470901	0.011571105	0.000440285	0.349996527	0.000471096	0.011570668	4.12E-05	0.002917185	1	0.835397773	1	0.842353403	ENSG00000112053	9	SLC26A8
Pancreas	T2DM_insulin	ENSG00000128829.7	0.000398035	0.769763499	0.001359947	0.011571128	0.000374569	0.782989929	0.001359966	0.011570687	2.35E-05	0.034821821	1	0.965153689	1	0.965819649	ENSG00000112053	9	SLC26A8
Pancreas	T2DM_insulin	ENSG00000128886.7	-7.03E-05	0.953470156	0.001205159	0.01157113	-5.32E-05	0.964801822	0.001205161	0.011570689	-1.71E-05	0.026347094	1	0.989356544	1	0.988759842	ENSG00000112053	9	SLC26A8
Pancreas	T2DM_insulin	ENSG00000132819.12	-0.002732398	0.06173312	0.001462574	0.011571047	-0.002710711	0.063830074	0.001462586	0.011570607	-2.17E-05	0.04391999	1	0.546657422	1	0.551424497	ENSG00000112053	9	SLC26A8
Pancreas	T2DM_insulin	ENSG00000132824.9	0.000927343	0.232406889	0.00077655	0.011571096	0.000915733	0.238309974	0.000776557	0.011570656	1.16E-05	0.04379621	1	0.7689997	1	0.772107424	ENSG00000112110	5	MRPL18
Pancreas	T2DM_insulin	ENSG00000134460.11	-0.000474136	0.754841312	0.001518839	0.011571127	-0.000453745	0.765067848	0.001518391	0.011570687	-2.04E-05	0.032408532	1	0.965153689	1	0.965703051	ENSG00000112110	5	MRPL18
Pancreas	T2DM_insulin	ENSG00000136874.6	4.41E-05	0.959939999	0.000877127	0.01157113	5.65E-05	0.9486546	0.00087713	0.011570689	-1.24E-05	0.03272454	1	0.989356544	1	0.988759842	ENSG00000112110	5	MRPL18
Pancreas	T2DM_insulin	ENSG00000137310.7	0.000774585	0.180378962	0.000578225	0.011571087	0.000726038	0.209522079	0.000578571	0.011570652	4.85E-05	0.016905538	1	0.724903247	1	0.751903832	ENSG00000112130	12	RNF8
Pancreas	T2DM_insulin	ENSG00000137392.5	0.001506417	0.217619212	0.001221863	0.011571094	0.001522156	0.212849766	0.001221861	0.011570652	-1.57E-05	0.02777792	1	0.762155249	1	0.756014105	ENSG00000112130	12	RNF8
Pancreas	T2DM_insulin	ENSG00000137478.10	0.001367293	0.050467852	0.000699031	0.011571039	0.001376961	0.048861496	0.000699035	0.011570597	-9.67E-06	0.046741574	1	0.5082124	1	0.5082859	ENSG00000112130	12	RNF8
Pancreas	T2DM_insulin	ENSG00000137752.18	0.000170579	0.90230481	0.00138964	0.011571129	0.000144982	0.91690734	0.001389649	0.011570689	2.56E-05	0.009253046	1	0.988687703	1	0.988759842	ENSG00000112149	5	CD83
Pancreas	T2DM_insulin	ENSG00000137831.10	0.001427332	0.330023899	0.001465332	0.011571107	0.00144328	0.324646724	0.001465326	0.011570666	-1.59E-05	0.043422461	1	0.836625244	1	0.835647835	ENSG00000112149	5	CD83
Pancreas	T2DM_insulin	ENSG00000138030.8	0.001064439	0.467696755	0.0014657	0.011571117	0.001043381	0.47655037	0.001465706	0.011570677	2.11E-05	0.03433609	1	0.890322658	1	0.890022702	ENSG00000112149	5	CD83
Pancreas	T2DM_insulin	ENSG00000138613.9	0.000253472	0.866281934	0.001505329	0.011571129	0.000276873	0.854070934	0.001505343	0.011570688	-2.34E-05	0.037577549	1	0.982534385	1	0.980006326	ENSG00000112149	5	CD83
Pancreas	T2DM_insulin	ENSG00000140264.15	9.40E-05	0.930093101	0.001071089	0.01157113	0.000109095	0.918872859	0.001071091	0.011570689	-1.51E-05	0.030655343	1	0.988687703	1	0.988759842	ENSG00000112159	7	MDN1
Pancreas	T2DM_insulin	ENSG00000142798.12	-0.001566616	0.022897871	0.000688858	0.011571007	-0.001579382	0.021810216	0.000688585	0.011570564	1.28E-05	0.011236409	1	0.407644283	1	0.397935172	ENSG00000112218	7	GPR63
Pancreas	T2DM_insulin	ENSG00000146386.7	-0.0012922	0.248974073	0.001126903	0.011571098	-0.001280618	0.255792428	0.001126915	0.011570658	-1.86E-05	0.030326606	1	0.785376024	1	0.792479149	ENSG00000112290	8	WASF1
Pancreas	T2DM_insulin	ENSG00000146425.6	-0.002277254	0.116967903	0.001452672	0.011571071	-0.002302659	0.112942433	0.001452682	0.011570629	2.54E-05	0.01571211	1	0.637653295	1	0.627975408	ENSG00000112290	8	WASF1
Pancreas	T2DM_insulin	ENSG00000147955.12	-0.000388296	0.628842586	0.000803332	0.011571124	-0.000403021	0.615893821	0.000803344	0.011570683	1.47E-05	0.026668353	1	0.931641109	1	0.926645564	ENSG00000112293	10	GPLD1
Pancreas	T2DM_insulin	ENSG00000148357.12	-0.000354562	0.721317064	0.000994009	0.011571127	-0.000343694	0.729581411	0.000994004	0.011570686	-1.09E-05	0.039477282	1	0.957777777	1	0.960804329	ENSG00000112293	10	GPLD1
Pancreas	T2DM_insulin	ENSG00000148834.8	-0.000132815	0.925304539	0.001416631	0.01157113	-0.000292302	0.836745186	0.001418539	0.011570688	0.000159487	0.031282863	1	0.988687703	1	0.974053326	ENSG00000112294	8	ALDH5A1
Pancreas	T2DM_insulin	ENSG00000149177.8	-0.000354732	0.702922111	0.00093013	0.011571126	-0.000340754	0.714105763	0.000930139	0.011570686	-1.40E-05	0.046121147	1	0.951524626	1	0.953885959	ENSG00000112294	8	ALDH5A1
Pancreas	T2DM_insulin	ENSG00000149485.12	-8.59E-05	0.788995992	0.000321106	0.011571128	-9.02E-05	0.778706494	0.000321105	0.011570687	4.30E-06	0.021881376	1	0.967242173	1	0.965819649	ENSG00000112294	8	ALDH5A1
Pancreas	T2DM_insulin	ENSG00000149577.11	-0.002087248	0.039085368	0.00101162	0.011571029	-0.002071933	0.040547413	0.00101162	0.011570589	-1.53E-05	0.045924702	1	0.489907645	1	0.486639905	ENSG00000112294	8	ALDH5A1
Pancreas	T2DM_insulin	ENSG00000151276.19	0.000741091	0.423051088	0.000925046	0.011571115	0.000755656	0.413999366	0.000925055	0.011570673	-1.46E-05	0.040951315	1	0.870194075	1	0.86606442	ENSG00000112294	8	ALDH5A1
Pancreas	T2DM_insulin	ENSG00000152348.11	9.75E-05	0.767868664	0.000330311	0.011571128	0.000104315	0.752150292	0.000330316	0.011570687	-6.82E-06	0.009288335	1	0.965153689	1	0.965703051	ENSG00000112299	7	VNN1
Pancreas	T2DM_insulin	ENSG00000152689.13	-0.000412433	0.583565062	0.000752361	0.011571123	-0.000427783	0.569641993	0.000752372	0.011570681	1.54E-05	0.014304318	1	0.920512947	1	0.911776571	ENSG00000112308	8	C6orf62
Pancreas	T2DM_insulin	ENSG00000155542.7	-0.001487316	0.008917387	0.000568716	0.011570967	-0.001493769	0.008625334	0.000568715	0.011570525	6.45E-06	0.049751873	1	0.260225561	1	0.255611981	ENSG00000112308	8	C6orf62
Pancreas	T2DM_insulin	ENSG00000157214.9	-0.000702177	0.614305099	0.001393379	0.011571124	-0.00072164	0.604523927	0.001393379	0.011570683	1.95E-05	0.023719848	1	0.927999789	1	0.925226219	ENSG00000112308	8	C6orf62
Pancreas	T2DM_insulin	ENSG00000158793.9	0.000394973	0.622343253	0.000801922	0.011571124	0.000328408	0.682383531	0.000802535	0.011570685	6.66E-05	0.035956519	1	0.928033725	1	0.945452933	ENSG00000112308	8	C6orf62
Pancreas	T2DM_insulin	ENSG00000159055.3	-0.000133027	0.839989096	0.00065885	0.011571129	-0.000122516	0.852481609	0.000658855	0.011570688	-1.05E-05	0.030956135	1	0.97568398	1	0.979057591	ENSG00000112339	10	HBS1L
Pancreas	T2DM_insulin	ENSG00000160172.6	-0.000793314	0.145739472	0.000545327	0.011571078	-0.000787056	0.14894172	0.000545326	0.011570639	-6.26E-06	0.043472696	1	0.676779319	1	0.685443348	ENSG00000112339	10	HBS1L
Pancreas	T2DM_insulin	ENSG00000161896.6	6.05E-05	0.913130036	0.000554457	0.011571129	0.000123725	0.82366918	0.000555247	0.011570688	-6.32E-05	0.033864467	1	0.988687703	1	0.971625322	ENSG00000112339	10	HBS1L
Pancreas	T2DM_insulin	ENSG00000164171.6	-0.000577241	0.544149306	0.000951678	0.011571121	-0.000562686	0.554348946	0.000951679	0.011570681	-1.46E-05	0.015472344	1	0.906527682	1	0.905957993	ENSG00000112339	10	HBS1L
Pancreas	T2DM_insulin	ENSG00000167535.3	-0.000582707	0.615950915	0.001161702	0.011571124	-0.000604002	0.603117613	0.001161715	0.011570683	2.13E-05	0.017880102	1	0.928033725	1	0.925226219	ENSG00000112343	8	TRIM38
Pancreas	T2DM_insulin	ENSG00000168028.9	-0.00015832	0.659301985	0.000359102	0.011571125	-0.000163214	0.649464568	0.000359102	0.011570684	4.89E-06	0.029950115	1	0.936442199	1	0.931745816	ENSG00000112343	8	TRIM38
Pancreas	T2DM_insulin	ENSG00000168405.10	0.000875019	0.276508514	0.0008041	0.011571102	0.000909836	0.257929782	0.00080424	0.011570659	-3.48E-05	0.027822037	1	0.821426426	1	0.795356813	ENSG00000112343	8	TRIM38
Pancreas	T2DM_insulin	ENSG00000168778.7	0.000396219	0.296304591	0.000379378	0.011571104	0.000401358	0.290082959	0.000379376	0.011570662	-5.14E-06	0.014971436	1	0.8274401	1	0.823130431	ENSG00000112357	8	PEX7
Pancreas	T2DM_insulin	ENSG00000168803.10	-0.000699449	0.604085267	0.001348895	0.011571123	-0.000715988	0.595559729	0.001348892	0.011570682	1.65E-05	0.036414576	1	0.927884944	1	0.922435093	ENSG00000112357	8	PEX7
Pancreas	T2DM_insulin	ENSG00000171421.8	-0.000700085	0.602995546	0.001346063	0.011571123	-0.000715843	0.594861051	0.001346062	0.011570682	1.58E-05	0.049112677	1	0.927884944	1	0.921947305	ENSG00000112357	8	PEX7
Pancreas	T2DM_insulin	ENSG00000172322.9	0.000244625	0.77599549	0.000859715	0.011571128	0.000256237	0.765665939	0.000859715	0.011570687	-1.16E-05	0.029906896	1	0.965153689	1	0.965703051	ENSG00000112378	11	PERP
Pancreas	T2DM_insulin	ENSG00000172493.16	-0.002450919	0.103968873	0.001507411	0.011571067	-0.002471828	0.101052093	0.001507417	0.011570625	2.09E-05	0.040035591	1	0.618037191	1	0.609159095	ENSG00000112378	11	PERP
Pancreas	T2DM_insulin	ENSG00000172992.7	7.92E-05	0.914975886	0.000742021	0.011571129	9.28E-05	0.900483976	0.000742024	0.011570689	-1.36E-05	0.008020866	1	0.988687703	1	0.988759842	ENSG00000112378	11	PERP
Pancreas	T2DM_insulin	ENSG00000174851.10	-6.82E-05	0.904490579	0.00054532	0.011571129	-5.87E-05	0.914246573	0.000545326	0.011570689	-9.46E-06	0.02618616	1	0.988687703	1	0.988759842	ENSG00000112378	11	PERP
Pancreas	T2DM_insulin	ENSG00000177628.11	0.000447259	0.631236839	0.000931815	0.011571124	0.000461325	0.620544985	0.000931823	0.011570683	-1.41E-05	0.040829703	1	0.931641109	1	0.926645564	ENSG00000112473	12	SLC39A7
Pancreas	T2DM_insulin	ENSG00000179163.11	-0.000745606	0.39870946	0.000883494	0.011571113	-0.000759458	0.390010489	0.000883502	0.011570671	1.39E-05	0.038046536	1	0.863490577	1	0.863051851	ENSG00000112514	11	CUTA
Pancreas	T2DM_insulin	ENSG00000182858.9	-0.000199343	0.80267484	0.000797732	0.011571128	-0.00021346	0.789021254	0.000797738	0.011570687	1.41E-05	0.015604522	1	0.969074255	1	0.96624062	ENSG00000112514	11	CUTA
Pancreas	T2DM_insulin	ENSG00000183562.2	0.000508763	0.699347272	0.001317351	0.011571126	0.000533293	0.685610235	0.001317358	0.011570685	-2.45E-05	0.008123349	1	0.949787479	1	0.94546011	ENSG00000112584	9	FAM120B
Pancreas	T2DM_insulin	ENSG00000184164.10	0.000139281	0.883222766	0.000948232	0.011571129	0.000118184	0.900812968	0.000948241	0.011570689	2.11E-05	0.002929816	1	0.985280653	1	0.988759842	ENSG00000112599	8	GUCA1B
Pancreas	T2DM_insulin	ENSG00000184608.4	0.000108041	0.926300475	0.001168006	0.01157113	8.84E-05	0.939694888	0.001168019	0.011570689	1.97E-05	0.030398367	1	0.988687703	1	0.988759842	ENSG00000112619	6	PRPH2
Pancreas	T2DM_insulin	ENSG00000185340.11	0.000512549	0.673333068	0.001215785	0.011571126	0.000526241	0.665129567	0.001215781	0.011570684	-1.37E-05	0.041688065	1	0.941892795	1	0.937945164	ENSG00000112619	6	PRPH2
Pancreas	T2DM_insulin	ENSG00000185624.10	0.001901175	0.197992459	0.001476877	0.011571109	0.001880989	0.202794812	0.001476871	0.01157065	2.02E-05	0.012806424	1	0.740790404	1	0.743967252	ENSG00000112619	6	PRPH2
Pancreas	T2DM_insulin	ENSG00000187715.9	0.000588463	0.610530177	0.001155395	0.011571124	0.000572951	0.619970754	0.001155395	0.011570683	1.55E-05	0.030124608	1	0.927884944	1	0.926645564	ENSG00000112619	6	PRPH2
Pancreas	T2DM_insulin	ENSG00000188312.9	3.82E-05	0.952543848	0.000642555	0.01157113	4.65E-05	0.94231791	0.000642554	0.011570689	-8.25E-06	0.027275009	1	0.989356544	1	0.988759842	ENSG00000112667	8	DNPH1
Pancreas	T2DM_insulin	ENSG00000188659.5	0.000365099	0.218151951	0.000296477	0.011571094	0.000369444	0.212726664	0.000296479	0.011570652	-4.34E-06	0.041544105	1	0.762155249	1	0.756014105	ENSG00000112667	8	DNPH1
Pancreas	T2DM_insulin	ENSG00000196247.7	-0.000437023	0.296561293	0.00041867	0.011571104	-0.000443192	0.289797491	0.000418672	0.011570662	6.17E-06	0.032867775	1	0.8274401	1	0.823130431	ENSG00000112667	8	DNPH1
Pancreas	T2DM_insulin	ENSG00000196260.3	-0.001338148	0.019538166	0.000573056	0.0115710	-0.001524375	0.008104227	0.000575734	0.011570522	0.000186227	0.000807245	1	0.377248201	1	0.255611981	ENSG00000112667	8	DNPH1
Pancreas	T2DM_insulin	ENSG00000196655.5	0.000756031	0.154831302	0.000531416	0.011571082	0.000764406	0.150313497	0.000531418	0.01157064	-8.38E-06	0.022086703	1	0.692694745	1	0.686843532	ENSG00000112667	8	DNPH1
Pancreas	T2DM_insulin	ENSG00000196735.7	-0.000767252	0.173048873	0.000563131	0.011571086	0.000183425	0.781783889	0.000662194	0.011570687	-0.000950676	0.000361222	1	0.716757267	1	0.965819649	ENSG00000112697	11	TMEM30A
Pancreas	T2DM_insulin	ENSG00000196743.4	-0.000864794	0.485777831	0.001240665	0.011571118	-0.000634902	0.609641132	0.001243477	0.011570683	-0.000229892	0.006158154	1	0.89524472	1	0.925226219	ENSG00000112763	11	BTN2A1
Pancreas	T2DM_insulin	ENSG00000197363.5	-0.00284319	0.041408159	0.001394113	0.011571031	-0.002826318	0.042629009	0.001394113	0.011570591	-1.69E-05	0.048460841	1	0.490028351	1	0.494154798	ENSG00000112763	11	BTN2A1
Pancreas	T2DM_insulin	ENSG00000197903.6	0.000991668	0.186551954	0.00075078	0.011571088	0.001080122	0.150528517	0.000751301	0.01157064	-8.85E-05	0.001815275	1	0.726940181	1	0.686843532	ENSG00000112763	11	BTN2A1
Pancreas	T2DM_insulin	ENSG00000197935.6	-0.001068679	0.088191241	0.000626784	0.011571061	-0.00125261	0.046431271	0.000628992	0.011570595	0.000183931	0.000495907	1	0.597559646	1	0.504240075	ENSG00000112763	11	BTN2A1
Pancreas	T2DM_insulin	ENSG00000198624.12	-0.000543684	0.525758776	0.000856877	0.01157112	-0.000406695	0.635631637	0.00085834	0.011570684	-0.000136989	0.00652133	1	0.904925293	1	0.928352537	ENSG00000112812	15	PRSS16
Pancreas	T2DM_insulin	ENSG00000198756.6	0.000901025	0.281428591	0.000836515	0.011571102	0.000885408	0.289858325	0.000836526	0.011570662	1.56E-05	0.020310864	1	0.82273396	1	0.823130431	ENSG00000112812	15	PRSS16
Pancreas	T2DM_insulin	ENSG00000203722.3	0.00223207	0.118651269	0.00143039	0.011571072	0.00220401	0.123357857	0.0014304	0.011570633	2.81E-05	0.006580572	1	0.637653295	1	0.639285058	ENSG00000112812	15	PRSS16
Pancreas	T2DM_insulin	ENSG00000204140.8	0.000580077	0.522354161	0.000906766	0.01157112	0.000598389	0.509313394	0.000906776	0.011570679	-1.83E-05	0.009876355	1	0.902196499	1	0.895220396	ENSG00000112852	4	PCDHB2
Pancreas	T2DM_insulin	ENSG00000204169.6	-0.001389219	0.224895834	0.001144696	0.011571095	-0.001374795	0.229747079	0.001144697	0.011570655	-1.44E-05	0.04345084	1	0.763931881	1	0.769631524	ENSG00000112874	5	NUDT12
Pancreas	T2DM_insulin	ENSG00000204257.10	0.001389163	2.21E-05	0.000327449	0.011570702	0.001495763	6.49E-06	0.000331669	0.011570206	-0.000106601	0.043441293	0.056804435	0.005539781	0.016667122	0.00208339	ENSG00000112874	5	NUDT12
Pancreas	T2DM_insulin	ENSG00000204305.9	0.003702295	0.001716662	0.001180828	0.011570896	0.003278703	0.00625163	0.001199104	0.011570511	0.000423592	0.004235611	1	0.131838761	1	0.231056694	ENSG00000112874	5	NUDT12
Pancreas	T2DM_insulin	ENSG00000204338.4	-0.001120077	0.002270769	0.000366957	0.011570909	-0.001275678	0.000602734	0.000371874	0.01157041	0.0001556	0.009893663	1	0.138841298	1	0.064492585	ENSG00000112964	9	GHR
Pancreas	T2DM_insulin	ENSG00000204351.7	-0.000958943	0.002684916	0.000319467	0.011570916	-0.001446573	1.79E-05	0.000337266	0.011570252	0.00048763	6.51E-06	1	0.155835303	0.046073901	0.00460739	ENSG00000112964	9	GHR
Pancreas	T2DM_insulin	ENSG00000204371.7	-0.001016391	0.418309398	0.001255798	0.011571114	-0.000770038	0.540583231	0.001258372	0.01157068	-0.000246353	0.002299716	1	0.870194075	1	0.902870944	ENSG00000112977	11	DAP
Pancreas	T2DM_insulin	ENSG00000204525.10	-0.000154325	0.567919361	0.000270216	0.011571122	3.08E-05	0.911361341	0.000276444	0.011570689	-0.0001851	0.001519103	1	0.915166981	1	0.988759842	ENSG00000112977	11	DAP
Pancreas	T2DM_insulin	ENSG00000204531.11	0.001536777	0.01313623	0.000619652	0.011570984	0.001419019	0.02229845	0.000620946	0.011570565	0.000117758	0.003431372	1	0.318243773	1	0.400436509	ENSG00000112977	11	DAP
Pancreas	T2DM_insulin	ENSG00000204536.9	0.001438313	0.042972564	0.000710635	0.011571032	0.001659498	0.020973953	0.000718877	0.011570562	-0.000221185	0.001746035	1	0.494856299	1	0.393901236	ENSG00000112977	11	DAP
Pancreas	T2DM_insulin	ENSG00000204540.6	-0.001377838	0.00021222	0.000371983	0.011570804	-0.001441721	0.000111166	0.000373033	0.011570334	6.39E-05	0.022853586	0.544979856	0.034061241	0.285475097	0.0202407	ENSG00000112977	11	DAP
Pancreas	T2DM_insulin	ENSG00000204560.5	0.001057272	0.432093627	0.001348579	0.011571115	0.001304215	0.333328858	0.001348121	0.011570667	-0.000246942	0.001942688	1	0.874402233	1	0.837432686	ENSG00000112977	11	DAP
Pancreas	T2DM_insulin	ENSG00000204618.4	0.002218398	0.001750739	0.000708851	0.011570897	0.002611841	0.000280247	0.000718934	0.011570376	-0.000393442	0.001051029	1	0.131838761	0.719673768	0.039981876	ENSG00000112977	11	DAP
Pancreas	T2DM_insulin	ENSG00000204622.6	0.00061444	0.504711077	0.000921066	0.011571119	0.000442399	0.631775017	0.000923145	0.011570683	0.00017204	0.005661337	1	0.895546057	1	0.928352537	ENSG00000112977	11	DAP
Pancreas	T2DM_insulin	ENSG00000204625.6	-4.63E-05	0.955032728	0.000821273	0.01157113	-0.000408146	0.622251399	0.00082845	0.011570683	0.000361836	0.000895046	1	0.989356544	1	0.926830112	ENSG00000112992	12	NNT
Pancreas	T2DM_insulin	ENSG00000204642.9	-0.000492151	0.1860399	0.000372168	0.011571088	-0.000401684	0.281745746	0.000373171	0.011570661	-9.05E-05	0.00097398	1	0.726940181	1	0.822541329	ENSG00000113068	5	PFDN1
Pancreas	T2DM_insulin	ENSG00000205517.8	-0.001000584	0.258881352	0.000886225	0.011571099	-0.001027417	0.246373347	0.000886312	0.011570657	2.68E-05	0.048473349	1	0.796330558	1	0.782060264	ENSG00000113119	8	TMCO6
Pancreas	T2DM_insulin	ENSG00000206341.6	0.000435658	0.39457232	0.000511723	0.011571113	0.000313597	0.541213485	0.00051327	0.01157068	0.000122061	0.00224343	1	0.863490577	1	0.902870944	ENSG00000113119	8	TMCO6
Pancreas	T2DM_insulin	ENSG00000206344.6	0.0016944	0.001295303	0.000526696	0.011570884	0.001399168	0.009203064	0.000537227	0.011570528	0.000295232	0.005301854	1	0.110877909	1	0.262793788	ENSG00000113141	11	IK
Pancreas	T2DM_insulin	ENSG00000206503.7	0.000512365	0.276843782	0.000471167	0.011571102	0.000834925	0.081902358	0.000479909	0.011570617	-0.00032256	0.000406871	1	0.821426426	1	0.587816164	ENSG00000113141	11	IK
Pancreas	T2DM_insulin	ENSG00000213676.6	0.00138007	0.000922741	0.000416549	0.011570869	0.001215498	0.0038146	0.000420135	0.01157049	0.000164572	0.002684418	1	0.087762956	1	0.17977037	ENSG00000113141	11	IK
Pancreas	T2DM_insulin	ENSG00000213760.6	-0.000328015	0.655854337	0.000736047	0.011571125	-0.001033978	0.175006705	0.000762356	0.011570645	0.000705963	0.000378548	1	0.936254796	1	0.717520656	ENSG00000113248	3	PCDHB15
Pancreas	T2DM_insulin	ENSG00000216906.2	0.001322319	0.099627323	0.000803028	0.011571065	0.001306792	0.103671185	0.000803036	0.011570626	1.55E-05	0.011649081	1	0.61011476	1	0.612137828	ENSG00000113248	3	PCDHB15
Pancreas	T2DM_insulin	ENSG00000223572.5	-0.000244678	0.854145383	0.001330988	0.011571129	-0.000260263	0.844969341	0.001330986	0.011570688	1.56E-05	0.046408055	1	0.982534385	1	0.975833338	ENSG00000113249	8	HAVCR1
Pancreas	T2DM_insulin	ENSG00000228065.6	-0.00072898	0.396391311	0.000859561	0.011571113	-0.000716787	0.404338902	0.000859562	0.011570672	-1.22E-05	0.026456425	1	0.863490577	1	0.86606442	ENSG00000113249	8	HAVCR1
Pancreas	T2DM_insulin	ENSG00000229212.3	-0.003740746	0.009958877	0.001451441	0.011570972	-0.003761851	0.009547734	0.001451442	0.011570529	2.11E-05	0.021101903	1	0.272068047	1	0.262793788	ENSG00000113273	11	ARSB
Pancreas	T2DM_insulin	ENSG00000229390.1	-0.000580203	0.470102043	0.000803256	0.011571117	-0.000733334	0.362277231	0.000804946	0.011570669	0.000153131	0.00345209	1	0.890734922	1	0.846603325	ENSG00000113318	9	MSH3
Pancreas	T2DM_insulin	ENSG00000229836.1	0.001081786	0.00728235	0.000403101	0.011570959	0.001172173	0.003850222	0.000405569	0.011570491	-9.04E-05	0.043400934	1	0.242871117	1	0.17977037	ENSG00000113328	14	CCNG1
Pancreas	T2DM_insulin	ENSG00000230795.2	-0.000111335	0.741269118	0.000337201	0.011571127	-0.000195077	0.564261259	0.000338368	0.011570681	8.37E-05	0.002927554	1	0.965153689	1	0.908714845	ENSG00000113360	12	DROSHA
Pancreas	T2DM_insulin	ENSG00000231205.7	7.65E-05	0.932474808	0.000903294	0.01157113	9.16E-05	0.919232738	0.000903298	0.011570689	-1.51E-05	0.014790819	1	0.988687703	1	0.988759842	ENSG00000113361	8	CDH6
Pancreas	T2DM_insulin	ENSG00000231402.1	0.000412182	0.234772494	0.000346909	0.011571096	0.000590833	0.094164101	0.000352982	0.011570622	-0.000178651	0.000170415	1	0.7689997	1	0.601947903	ENSG00000113520	6	IL4
Pancreas	T2DM_insulin	ENSG00000231500.2	-9.29E-05	0.913363938	0.000853881	0.011571129	-0.000334761	0.696072953	0.000856984	0.011570685	0.000241862	0.000930295	1	0.988687703	1	0.948517485	ENSG00000113593	7	PPWD1
Pancreas	T2DM_insulin	ENSG00000232040.2	-0.000375894	0.579994158	0.000679252	0.011571122	-0.000483707	0.476962447	0.000680131	0.011570677	0.000107814	0.001967259	1	0.918264486	1	0.890145032	ENSG00000113593	7	PPWD1
Pancreas	T2DM_insulin	ENSG00000234127.4	-0.000982731	0.125976607	0.000642239	0.011571074	-0.001385595	0.033528843	0.00065183	0.011570582	0.000402863	0.000302131	1	0.650322205	1	0.451379305	ENSG00000113593	7	PPWD1
Pancreas	T2DM_insulin	ENSG00000235290.1	0.000511484	0.459644001	0.000691724	0.011571117	0.000632842	0.361223148	0.000693117	0.011570669	-0.000121358	0.005966695	1	0.886474445	1	0.846567342	ENSG00000113593	7	PPWD1
Pancreas	T2DM_insulin	ENSG00000236624.4	0.000410877	0.534348941	0.00066231	0.011571121	0.000402015	0.54320109	0.000661231	0.01157068	8.86E-06	0.027953086	1	0.906527682	1	0.902870944	ENSG00000113597	13	TRAPPC13
Pancreas	T2DM_insulin	ENSG00000237669.1	0.000881945	0.066905875	0.000481409	0.011571051	0.001037087	0.032021871	0.000483684	0.011570581	-0.000155142	0.000952699	1	0.558070981	1	0.446913945	ENSG00000113597	13	TRAPPC13
Pancreas	T2DM_insulin	ENSG00000238034.1	0.000500906	0.553882302	0.000846192	0.01157112	0.000517249	0.541025791	0.000846197	0.01157068	-1.63E-05	0.005357183	1	0.910322362	1	0.902870944	ENSG00000113597	13	TRAPPC13
Pancreas	T2DM_insulin	ENSG00000240053.8	-0.000625903	0.232803829	0.000524572	0.011571096	-0.000387641	0.465744497	0.000531437	0.011570676	-0.000238261	0.000516783	1	0.7689997	1	0.887263998	ENSG00000113597	13	TRAPPC13
Pancreas	T2DM_insulin	ENSG00000243753.1	0.00073748	0.124180246	0.000479673	0.011571074	0.000642816	0.181645838	0.000481256	0.011570647	9.47E-05	0.015497207	1	0.646084789	1	0.720968335	ENSG00000113734	13	BNIP1
Pancreas	T2DM_insulin	ENSG00000244165.1	0.000732781	0.272306863	0.00066752	0.011571101	0.000745346	0.264174748	0.000667526	0.011570659	-1.26E-05	0.013238708	1	0.817876053	1	0.801805531	ENSG00000113734	13	BNIP1
Pancreas	T2DM_insulin	ENSG00000244693.1	0.001047328	0.079782746	0.000597806	0.011571057	0.001038124	0.082469665	0.000597813	0.011570617	9.20E-06	0.046400708	1	0.592408703	1	0.587816164	ENSG00000113734	13	BNIP1
Pancreas	T2DM_insulin	ENSG00000244731.3	-0.00240558	5.28E-07	0.000479573	0.011570532	-0.002611987	9.79E-08	0.000489997	0.011570014	0.000206407	0.040158114	0.001354727	0.000169341	0.000251466	3.59E-05	ENSG00000113734	13	BNIP1
Pancreas	T2DM_insulin	ENSG00000248098.6	0.001923681	0.157214993	0.00135997	0.011571082	0.001941872	0.153328016	0.001359972	0.011570641	-1.82E-05	0.037674289	1	0.696168424	1	0.688839634	ENSG00000113734	13	BNIP1
Pancreas	T2DM_insulin	ENSG00000250535.1	0.000708597	0.121203792	0.000457235	0.011571073	0.000649578	0.155838316	0.000457704	0.011570641	5.90E-05	0.004746466	1	0.63982072	1	0.689930464	ENSG00000113971	14	NPHP3
Pancreas	T2DM_insulin	ENSG00000250986.1	-0.000931157	0.276264737	0.000855253	0.011571102	-0.000942277	0.270572115	0.000855252	0.01157066	1.11E-05	0.03235337	1	0.821426426	1	0.815055965	ENSG00000113971	14	NPHP3
Pancreas	T2DM_insulin	ENSG00000251639.2	-0.001482415	0.274865253	0.001357612	0.011571101	-0.001460154	0.282138242	0.001357614	0.011570661	-2.23E-05	0.00995234	1	0.821426426	1	0.822541329	ENSG00000113971	14	NPHP3

Pancreas	T2DM_insulin	ENSG00000256166.1	0.00196412	0.000250097	0.000536324	0.011570811	0.002186259	5.74E-05	0.000543408	0.011570305	-0.00022214	0.011151126	0.642248041	0.037403048	0.147433792	0.011341061	ENSG00000113971	14	NPHP3
Pancreas	T2DM_insulin	ENSG00000258289.3	0.00010261	0.89962266	0.000813471	0.011571129	9.36E-05	0.908368668	0.000813468	0.011570689	8.98E-06	0.04834875	1	0.988687703	1	0.988759842	ENSG00000114021	7	NIT2
Pancreas	T2DM_insulin	ENSG00000259205.2	-0.000431005	0.360181542	0.000471033	0.01157111	-0.000435942	0.354703278	0.000471031	0.011570669	4.94E-06	0.046428851	1	0.848639286	1	0.843405573	ENSG00000114023	11	FAM162A
Pancreas	T2DM_insulin	ENSG00000260091.1	8.61E-05	0.93123212	0.00099809	0.01157113	7.04E-05	0.943778525	0.000998102	0.011570689	1.57E-05	0.043916997	1	0.988687703	1	0.988759842	ENSG00000114054	9	PCCB
Pancreas	T2DM_insulin	ENSG00000260302.1	0.002151593	0.009078976	0.000824657	0.011570968	0.002163587	0.00870046	0.000824659	0.011570526	-1.20E-05	0.025170488	1	0.261507236	1	0.255611981	ENSG00000114125	9	RNF7
Pancreas	T2DM_insulin	ENSG00000261584.1	-0.000490649	0.309363617	0.000482656	0.011571105	-0.000459611	0.341049502	0.000482737	0.011570667	-3.10E-05	0.000831891	1	0.835397773	1	0.838317574	ENSG00000114125	9	RNF7
Pancreas	T2DM_insulin	ENSG00000264469.1	-0.000775209	0.282771742	0.000721718	0.011571102	-0.000793502	0.271586519	0.000721748	0.01157066	1.83E-05	0.020618769	1	0.82273396	1	0.815055965	ENSG00000114200	5	BCHE
Pancreas	T2DM_insulin	ENSG00000270127.1	-0.000277223	0.421435409	0.000344833	0.011571114	-0.000281362	0.414535183	0.000344832	0.011570673	4.14E-06	0.027226752	1	0.870194075	1	0.86606442	ENSG00000114200	5	BCHE
Pancreas	T2DM_insulin	ENSG00000271821.1	0.002589936	0.004708597	0.000916359	0.01157094	0.001899157	0.047321596	0.000957518	0.011570596	0.00069078	0.012885163	1	0.191931378	1	0.504240075	ENSG00000114302	11	PRKAR2A
Pancreas	T2DM_insulin	ENSG00000272221.1	0.000345472	0.185464751	0.000260908	0.011571088	0.000435648	0.096612783	0.000262201	0.011570623	-9.02E-05	0.000540601	1	0.726940181	1	0.605592158	ENSG00000114302	11	PRKAR2A
Pancreas	T2DM_insulin	ENSG00000272799.1	-0.000288636	0.671541876	0.000680693	0.011571125	-0.000280572	0.680203127	0.000680692	0.011570685	-8.06E-06	0.042382127	1	0.941892795	1	0.945452933	ENSG00000114378	12	HYAL1
Pancreas	T2DM	ENSG00000002919.10	0.001101838	0.325884304	0.001121533	0.055522567	0.001085424	0.333142444	0.001121532	0.055519517	1.64E-05	0.043987851	1	0.723936757	1	0.735008377	ENSG00000114383	5	TUSC2
Pancreas	T2DM	ENSG00000005102.8	0.003638725	0.136819772	0.002445805	0.055522425	0.003542856	0.147493749	0.002445975	0.055519385	9.59E-05	0.004859027	1	0.542179906	1	0.55791981	ENSG00000114383	5	TUSC2
Pancreas	T2DM	ENSG00000005513.9	0.002074875	0.376255929	0.002344977	0.055522588	0.002039064	0.384546254	0.002344962	0.055519538	3.58E-05	0.018737599	1	0.763882089	1	0.772390956	ENSG00000114395	6	CYB561D2
Pancreas	T2DM	ENSG00000005981.8	0.004271553	0.144635202	0.002928233	0.055522435	0.004232103	0.148377268	0.002928205	0.055519386	3.94E-05	0.024354402	1	0.554532985	1	0.55791981	ENSG00000114405	6	C3orf14
Pancreas	T2DM	ENSG00000009413.11	-0.004744713	0.151387299	0.003307225	0.055522443	-0.004679508	0.157087291	0.003307218	0.055519396	-6.52E-05	0.005463617	1	0.556171292	1	0.561838668	ENSG00000114405	6	C3orf14
Pancreas	T2DM	ENSG00000011114.10	0.000579837	0.854305831	0.00315768	0.055522673	0.000632558	0.841228248	0.003157692	0.05551962	-5.27E-05	0.034751053	1	0.97245451	1	0.968298585	ENSG00000114446	4	IFT57
Pancreas	T2DM	ENSG00000012232.4	-0.000274724	0.894242365	0.002066557	0.055522675	-0.000236654	0.908829388	0.002066568	0.055519623	-3.81E-05	0.022445366	1	0.981247774	1	0.985211345	ENSG00000114446	4	IFT57
Pancreas	T2DM	ENSG00000012963.9	0.003720054	0.04393032	0.0018464	0.055522215	0.003746137	0.042469028	0.001846388	0.055519155	-2.61E-05	0.02979463	1	0.311795766	1	0.31228686	ENSG00000114446	4	IFT57
Pancreas	T2DM	ENSG00000022556.11	-0.001473088	0.541760674	0.002414293	0.055522635	-0.001505798	0.532820918	0.002414274	0.05551958	3.27E-05	0.031604649	1	0.860225195	1	0.854992848	ENSG00000114503	6	NCBP2
Pancreas	T2DM	ENSG00000030110.8	0.011934087	2.30E-07	0.002306908	0.055519628	0.011017341	2.02E-06	0.002318864	0.055517052	0.000916745	0.000100475	0.000591188	3.94E-05	0.00519481	0.000431985	ENSG00000114503	6	NCBP2
Pancreas	T2DM	ENSG00000037757.9	-0.001143924	0.537154134	0.001853642	0.055522634	-0.001202505	0.516517418	0.001853648	0.055519576	5.86E-05	5.68E-05	1	0.858495882	1	0.848610147	ENSG00000114503	6	NCBP2
Pancreas	T2DM	ENSG00000049449.4	0.000720066	0.75304297	0.002288615	0.055522666	0.000662953	0.772068676	0.002288646	0.055519615	5.71E-05	0.006035528	1	0.935675707	1	0.943354401	ENSG00000114544	11	SLC41A3
Pancreas	T2DM	ENSG00000054277.8	0.002797242	0.231149613	0.002336088	0.055522514	0.002766521	0.236309751	0.002336071	0.055519464	3.07E-05	0.038633283	1	0.648026426	1	0.655938795	ENSG00000114544	11	SLC41A3
Pancreas	T2DM	ENSG00000061455.10	0.004740686	0.047565649	0.002392795	0.05552223	0.00478506	0.045523627	0.002392783	0.055519168	-4.44E-05	0.00574995	1	0.325729564	1	0.321166687	ENSG00000114547	5	ROPN1B
Pancreas	T2DM	ENSG00000063046.13	1.74E-05	0.987925094	0.001147008	0.055522677	3.92E-05	0.972740473	0.001147003	0.055519624	-2.18E-05	0.005518622	1	0.997207295	1	0.996011776	ENSG00000114638	3	UPK1B
Pancreas	T2DM	ENSG00000063854.8	-0.007178164	0.017138136	0.003011305	0.05552203	-0.007111635	0.018194969	0.003011319	0.055518989	-6.65E-05	0.005994954	1	0.200048793	1	0.212384905	ENSG00000114638	3	UPK1B
Pancreas	T2DM	ENSG00000064270.8	0.000363645	0.847832843	0.001895126	0.055522673	0.000326178	0.863347979	0.001895128	0.055519621	3.75E-05	0.008436691	1	0.970153451	1	0.974539608	ENSG00000114735	5	HEMK1
Pancreas	T2DM	ENSG00000064687.8	-0.001597647	0.452044464	0.002124497	0.055522613	-0.00156496	0.461348848	0.002124492	0.055519562	-3.27E-05	0.028908463	1	0.807173989	1	0.814816947	ENSG00000114742	9	WDR48
Pancreas	T2DM	ENSG00000066294.10	0.002183167	0.116791917	0.001391984	0.055522397	0.005912261	0.000436359	0.001681013	0.055518215	-0.003729094	7.60E-05	1	0.510742285	1	0.020225759	ENSG00000114742	9	WDR48
Pancreas	T2DM	ENSG00000069966.14	0.003384382	0.21573838	0.002733879	0.055522503	0.003425983	0.210144975	0.002733857	0.055519445	-4.16E-05	0.014879011	1	0.621094348	1	0.620720671	ENSG00000114742	9	WDR48
Pancreas	T2DM	ENSG00000070182.13	0.006092444	0.003283685	0.002072354	0.055521692	0.006123793	0.00312686	0.002072351	0.055518629	-3.13E-05	0.037087816	1	0.074942369	1	0.072830925	ENSG00000114771	9	AADAC
Pancreas	T2DM	ENSG00000073849.10	0.008285735	0.0008703	0.002488634	0.055521414	0.008251104	0.000914691	0.002488607	0.055518372	3.46E-05	0.015329531	1	0.034383558	1	0.035306133	ENSG00000114796	11	KLHL24
Pancreas	T2DM	ENSG00000074706.9	-0.003651337	0.178570121	0.0027144	0.055522471	-0.00361982	0.182343082	0.002714369	0.055519422	-3.15E-05	0.03860933	1	0.581719513	1	0.586401239	ENSG00000114796	11	KLHL24
Pancreas	T2DM	ENSG00000075673.7	-0.002436898	0.147878898	0.001684026	0.055522439	-0.002464331	0.14336935	0.001684022	0.05551938	2.74E-05	0.021232617	1	0.556171292	1	0.552027703	ENSG00000114796	11	KLHL24
Pancreas	T2DM	ENSG00000076662.5	0.000367509	0.869832593	0.002242668	0.055522674	0.000404549	0.856849185	0.002242677	0.05551962	-3.70E-05	0.038053255	1	0.976734957	1	0.973048371	ENSG00000114841	13	DNAH1
Pancreas	T2DM	ENSG00000077585.9	0.000569526	0.792767817	0.002167819	0.055522669	0.000538639	0.803769486	0.002167807	0.055519617	3.09E-05	0.032341692	1	0.949920498	1	0.951905176	ENSG00000114988	7	LMAN2L
Pancreas	T2DM	ENSG00000079689.9	-0.001679367	0.494272858	0.002456906	0.055522624	-0.001882584	0.443637874	0.002457473	0.055519557	0.000203217	0.000272786	1	0.838924455	1	0.801344566	ENSG00000114988	7	LMAN2L
Pancreas	T2DM	ENSG00000085117.7	-0.002119663	0.2926682	0.002014338	0.055522551	-0.002093005	0.298777934	0.002014328	0.055519501	-2.67E-05	0.047560462	1	0.703060747	1	0.709510872	ENSG00000115084	8	SLC35F5
Pancreas	T2DM	ENSG00000088451.6	0.00031139	0.813336616	0.001318758	0.055522671	8.12E-05	0.951022028	0.001322103	0.055519624	0.000230182	0.014858487	1	0.957217428	1	0.991564989	ENSG00000115084	8	SLC35F5
Pancreas	T2DM	ENSG00000090686.11	0.000504038	0.689759533	0.00126268	0.055522659	0.00044965	0.721773882	0.001262745	0.05551961	5.44E-05	0.000617366	1	0.91152931	1	0.921688379	ENSG00000115084	8	SLC35F5
Pancreas	T2DM	ENSG00000094916.9	-0.003036084	0.16606901	0.0021922	0.055522459	-0.002995594	0.171786712	0.002192186	0.055519411	-4.05E-05	0.00408166	1	0.570140665	1	0.578934746	ENSG00000115084	8	SLC35F5
Pancreas	T2DM	ENSG00000099974.7	-0.001233052	0.514123424	0.001889935	0.055522629	-0.001261564	0.504441196	0.00188993	0.055519573	2.85E-05	0.033724602	1	0.848731838	1	0.844462185	ENSG00000115163	10	CENPA
Pancreas	T2DM	ENSG00000099984.6	-0.000488504	0.839340424	0.00240954	0.055522672	-0.000525198	0.827454992	0.002409529	0.055519619	3.67E-05	0.024086725	1	0.966558838	1	0.961060344	ENSG00000115221	6	ITGB6
Pancreas	T2DM	ENSG00000100099.16	0.002501381	0.341119799	0.002627622	0.055522574	0.002548429	0.332113636	0.002627609	0.055519517	-4.70E-05	0.007565637	1	0.738900364	1	0.735008377	ENSG00000115221	6	ITGB6
Pancreas	T2DM	ENSG00000100201.14	-0.002132792	0.293575051	0.002030627	0.055522551	-0.002103307	0.300297843	0.002030619	0.055519502	-2.95E-05	0.035230964	1	0.703265608	1	0.709510872	ENSG00000115221	6	ITGB6
Pancreas	T2DM	ENSG00000100258.13	0.001290977	0.245531849	0.001111692	0.055522523	0.001278459	0.250134127	0.001111679	0.055519473	1.25E-05	0.043523717	1	0.658561546	1	0.666460318	ENSG00000115255	6	REEP6
Pancreas	T2DM	ENSG00000100731.11	0.001798388	0.480555104	0.00254942	0.05552262	0.001754126	0.491417684	0.002549393	0.05551957	4.43E-05	0.002835339	1	0.827121653	1	0.834196451	ENSG00000115274	10	INO80B
Pancreas	T2DM	ENSG00000101333.12	-0.000248541	0.858532513	0.001394398	0.055522674	0.001246123	0.428051983	0.001572336	0.055519553	-0.001494665	0.039689926	1	0.975540847	1	0.793088295	ENSG00000115274	10	INO80B
Pancreas	T2DM	ENSG00000101361.10	0.004371888	0.086525315	0.00255066	0.055522342	0.004337597	0.089020967	0.002550645	0.055519295	3.43E-05	0.03985555	1	0.45439061	1	0.455804017	ENSG00000115275	7	MOGS
Pancreas	T2DM	ENSG00000101452.10	-0.002095767	0.452935248	0.002792378	0.055522613	-0.001999482	0.474014441	0.002792716	0.055519566	-9.63E-05	0.045385456	1	0.807173989	1	0.821923758	ENSG00000115275	7	MOGS
Pancreas	T2DM	ENSG00000101457.8	-0.001110162	0.723815559	0.003141693	0.055522663	-0.001154429	0.713279003	0.003141678	0.055519609	4.43E-05	0.036955237	1	0.921476762	1	0.920472548	ENSG00000115310	13	RTN4
Pancreas	T2DM	ENSG00000102837.6	0.003542648	0.074365606	0.001985387	0.055522314	0.003592705	0.070362454	0.001985387	0.055519251	-5.01E-05	0.000660411	1	0.424379724	1	0.410660871	ENSG00000115310	13	RTN4
Pancreas	T2DM	ENSG00000102996.4	0.002291923	0.428319757	0.002893584	0.055522606	0.002208041	0.445445821	0.00289378	0.055519558	8.39E-05	0.035606221	1	0.792249323	1	0.8027301	ENSG00000115423	14	DNAH6
Pancreas	T2DM	ENSG00000104823.4	-0.00025594	0.908865637	0.002235883	0.055522676	-0.000205052	0.92693007	0.002235925	0.055519623	-5.09E-05	0.017846869	1	0.98382859	1	0.987209256	ENSG00000115423	14	DNAH6
Pancreas	T2DM	ENSG00000105479.11	-0.002563672	0.406437008	0.003088089	0.055522599	-0.002511886	0.415982638	0.003088088	0.055519549	-5.18E-05	0.022482225	1	0.784169975	1	0.786638662	ENSG00000115423	14	DNAH6
Pancreas	T2DM	ENSG00000106538.5	-2.77E-05	0.988853073	0.001981754	0.055522677	-0.001456492	0.487333672	0.00209701	0.055519569	0.001428805	0.037213806	1	0.997207295	1	0.831484518	ENSG00000115423	14	DNAH6
Pancreas	T2DM	ENSG00000106992.13	-0.000732814	0.681537355	0.001785756	0.055522658	-0.000756303	0.671914043	0.001785742	0.055519604	2.35E-05	0.036183755	1	0.91019995	1	0.907798476	ENSG00000115457	5	IGFBP2
Pancreas	T2DM	ENSG00000108264.12	0.000634783	0.531726335	0.001015047	0.055522633	0.000651256	0.521128583	0.001015039	0.055519577	-1.65E-05	0.010539985	1	0.857709315	1	0.848610147	ENSG00000115486	7	GGCX
Pancreas	T2DM	ENSG00000108384.10	-0.002338405	0.41062199	0.002842008	0.0555226	-0.00238075	0.402197501	0.002841995	0.055519544	4.23E-05	0.028050425	1	0.785561726	1	0.782159733	ENSG00000115486	7	GGCX
Pancreas	T2DM	ENSG00000109511.6	-0.000310028	0.900115927	0.002470051	0.055522675	-0.000275146	0.911304174	0.002470029	0.055519623	-3.49E-05	0.021676186	1	0.981944648	1	0.985399068	ENSG00000115486	7	GGCX
Pancreas	T2DM	ENSG00000109775.6	0.001182018	0.511039106	0.001798509	0.055522628	0.001156792	0.520097041	0.001798505	0.055519577	2.52E-05	0.048582176	1	0.848731838	1	0.848610147	ENSG00000115486	7	GGCX
Pancreas	T2DM	ENSG00000110074.6	0.001799618	0.502941229	0.00268652	0.055522626	0.001766614	0.510801342	0.002686493	0.055519575	3.30E-05	0.038206024	1	0.846716275	1	0.847548645	ENSG00000115486	7	GGCX
Pancreas	T2DM	ENSG00000110888.13	0.002202102	0.159704464	0.001566142	0.055522452	0.002183666	0.163223508	0.001566122	0.055519403	1.84E-05	0.030880478	1	0.563353108	1	0.568944307	ENSG00000115561	10	CHMP3
Pancreas	T2DM	ENSG00000112149.5	-0.003374963	0.033351076	0.0015861	0.055522161	-0.00157581	0.337294929	0.001642282	0.055519519	-0.001799153	2.41E-05	1	0.280805126	1	0.739686916	ENSG00000115561	10	CHMP3
Pancreas	T2DM	ENSG00000112294.8	0.001324029	0.646075032	0.002883202	0.055522653	0.001242131	0.666605403	0.002883233	0.055519603	8.19E-05	0.001208681	1	0.898449905	1	0.905736865	ENSG00000115561	10	CHMP3
Pancreas	T2DM	ENSG00000112343.8	0.001299631	0.49548915	0.001906721	0.055522624	0.001347771	0.479678814	0.00190681	0.055519567	-4.81E-05	0.038128082	1	0.84043338	1	0.827056253	ENSG00000115568	11	ZNF142
Pancreas	T2DM	ENSG00000112874.5	0.001597958	0.578220365	0.002874094	0.055522642	0.00164436	0.567230622	0.002874076	0.055519587	-4.64E-05	0.012796978	1	0.878208132	1	0.868486338	ENSG00000115568	11	ZNF142
Pancreas	T2DM	ENSG00000112977.11	0.002862478	0.036063657	0.001365546	0.055522176	0.002882141	0.034804681	0.001365536	0.055519117	-1.97E-05	0.024806301	1	0.285894549	1	0.288681949	ENSG00000115568	11	ZNF142
Pancreas	T2DM	ENSG00000113734.13	-5.17E-05	0.958551378	0.000993909	0.055522677	-6.92E-05	0.94449752	0.000993901	0.055519624	1.75E-05	0.004227304	1	0.990168921	1	0.987209256	ENSG00000115648	9	MLPH
Pancreas	T2DM	ENSG00000114383.5	-0.001885299	0.534463556	0.003034895	0.055522633	-0.001854822	0.54108446	0.003034849	0.055519582	-3.05E-05	0.043263362	1	0.858495882	1	0.858355187	ENSG00000115677	12	HDLBP
Pancreas	T2DM	ENSG00000114446.4	0.008286376	0.006968969	0.003070922	0.055521848	0.008319014	0.006748846	0.003070879	0.055518788	-3.26E-05	0.040812505	1	0.122577481	1	0.121196067	ENSG00000115677	12	HDLBP
Pancreas	T2DM	ENSG00000114638.3	0.0007897	0.789544016	0.002958754	0.055522669	0.000750994	0.799632034	0.002958727	0.055519617	3.87E-05	0.029926447	1	0.949920498	1	0.95067364	ENSG00000115687	9	PASK
Pancreas	T2DM	ENSG00000116157.5	-0.001822233	0.021190394	0.0007907	0.055522072	-0.001808769	0.022162883	0.000790694	0.055519028	-1.35E-05	0.007320419	1	0.223020208	1	0.237142843	ENSG00000115687	9	PASK
Pancreas	T2DM	ENSG00000116771.5	-0.00233522	0.111544738	0.0014675	0.055522389	-0.002317062	0.114351929	0.001467485	0.05551934	-1.82E-05	0.034194657	1	0.503138201	1	0.506303024	ENSG00000115687	9	PASK
Pancreas	T2DM	ENSG00000117122.9	0.002309158	0.068750929	0.001268729	0.0555223	0.00229223	0.070804894	0.001268717	0.055519252	1.69E-05	0.027180809	1	0.406108759	1	0.412306048	ENSG00000115718	13	PROC
Pancreas	T2DM	ENSG00000119013.4	0.002251965	0.504258358	0.003372189	0.055522626	0.002304524	0.494357524	0.003372169	0.055519571	-5.26E-05	0.017439511	1	0.846916588	1	0.835942127	ENSG00000115718	13	PROC
Pancreas	T2DM	ENSG00000119599.12	0.001117164	0.549982127	0.001868831	0.055522636	0.001139777	0.54193237	0.001868812	0.055519582	-2.26E-05	0.039690157	1	0.865271405	1	0.859063164	ENSG00000115750	12	TAF1B
Pancreas	T2DM	ENSG00000119715.10	-0.001345168	0.566324069	0.002345649	0.05552264	-0.001379699	0.55639991	0.002345638	0.055519585	3.45E-05	0.030526272	1	0.872395717	1	0.864388971	ENSG00000115750	12	TAF1B
Pancreas	T2DM	ENSG00000120451.6	-0.000859235	0.298817273	0.000827003	0.055522554	-0.000871936	0.291729434	0.000827	0.055519497	1.27E-05	0.026818582	1	0.705945498	1	0.702726916	ENSG00000115825	5	PRKD3
Pancreas	T2DM	ENSG00000120675.4	-0.006972319	0.027688514	0.00316682	0.055522125	-0.007005858	0.026946259	0.003166773	0.055519067	3.35E-05	0.035036903	1	0.254853419	1	0.254404388	ENSG00000115840	9	SLC25A12
Pancreas	T2DM	ENSG00000120725.8	-0.002052521	0.299862016	0.001979802	0.055522555	-0.002081655	0.293050186	0.001979785	0.055519498	2.91E-05	0.017439786	1	0.707112634	1	0.702726916	ENSG00000115840	9	SLC25A12
Pancreas	T2DM	ENSG00000121898.8	-0.000808916	0.712220448	0.002192923	0.055522662	-0.000765758	0.726941896	0.002192915	0.05551961	-4.32E-05	0.004595705	1	0.921421644	1	0.922488007	ENSG00000115947	9	ORC4
Pancreas	T2DM	ENSG00000122483.13	-5.07E-05	0.982357678	0.002291404	0.055522677	-2.41E-05	0.991597337	0.002291379	0.055519624	-2.65E-05	0.04609251	1	0.99701908	1	0.998538835	ENSG00000115947	9	ORC4
Pancreas	T2DM	ENSG00000122692.7	-0.00162937	0.472373677	0.00226735	0.055522618	-0.001656701	0.464971955	0.002267325	0.055519563	2.73E-05	0.035554634	1	0.817750308	1	0.815865042	ENSG00000115947	9	ORC4
Pancreas	T2DM	ENSG00000122783.12	0.002201835	0.430860463	0.002795198	0.055522606	0.002240944	0.422716068	0.002795174	0.055519551	-3.91E-05	0.023461829	1	0.792249323	1	0.791410966	ENSG00000116044	11	NFE2L2
Pancreas	T2DM	ENSG00000123453.12	-0.000347742	0.890244431	0.002519961	0.055522675	-0.000388986	0.877323977	0.002519952	0.055519621	4.12E-05	0.018247596	1	0.981247774	1	0.980763999	ENSG00000116127	13	ALMS1
Pancreas	T2DM	ENSG00000123643.8	-0.000349741	0.784594961	0.001279526	0.055522669	-0.000321009	0.80191232	0.001279567	0.055519617	-2.87E-05	0.039796935	1	0.949163351	1	0.951905176	ENSG00000116138	8	DNAJC16
Pancreas	T2DM	ENSG00000123815.7	0.004038571	0.100637972	0.002459886	0.05552237	0.004091512	0.096257483	0.002459909	0.055519309	-5.29E-05	0.012177264	1	0.483403189	1	0.472157259	ENSG00000116151	9	MORN1
Pancreas	T2DM	ENSG00000124104.14	0.002946157	0.333794347	0.003048273	0.055522571	0.00289869	0.3416381	0.003048254	0.055519521	4.75E-05	0.016972015	1	0.733262517	1	0.741897611	ENSG00000116151	9	MORN1
Pancreas	T2DM	ENSG00000124116.14	-0.000269679	0.832445343	0.001274676	0.055522672	-0.000295383	0.81674546	0.00127467	0.055519618	2.57E-05	0.002642429	1	0.964161038	1	0.956844134	ENSG00000116151	9	MORN1
Pancreas	T2DM	ENSG00000124422.7	0.001413157	0.53362466	0.002270193	0.055522633	0.001358278	0.549648132	0.002270275	0.055519583	5.49E-05	0.032038127	1	0.858495882	1	0.863653712	ENSG00000116157	5	GPX7
Pancreas	T2DM	ENSG00000124549.10	-0.000483381	0.567349818	0.000845131	0.05552264	-0.00055814	0.509316692	0.000845792	0.055519575	7.48E-05	0.027928291	1	0.872395717	1	0.847548645	ENSG00000116157	5	GPX7
Pancreas	T2DM	ENSG00000124613.4	-0.000213503	0.836026358	0.001031519	0.055522672	-0.000331571	0.747976278	0.001031937	0.055519612	0.000118068	0.000100483	1	0.964161038	1	0.933447328	ENSG00000116157	5	GPX7
Pancreas	T2DM	ENSG00000125257.9	0.000811149	0.803963657	0.003267851	0.05552267	0.000897749	0.783533184	0.003267894	0.055519616	-8.66E-05	0.0032712	1	0.952792712	1	0.945477902	ENSG00000116157	5	GPX7
Pancreas	T2DM	ENSG00000125734.9	0.002178316	0.386037813	0.00251298	0.055522592	0.002206507	0.379914862	0.002512951	0.055519536	-2.82E-05	0.047595347	1	0.770733172	1	0.768582324	ENSG00000116157	5	GPX7
Pancreas	T2DM	ENSG00000125818.13	-0.001330486	0.39598565	0.001567467	0.055522595	-0.001351724	0.388485243	0.001567455	0.055519539	2.12E-05	0.034172266	1	0.776325713	1	0.772390956	ENSG00000116157	5	GPX7
Pancreas	T2DM	ENSG00000127418.10	-0.002990945	0.080096308	0.001708983	0.055522328	-0.002963799	0.082873541	0.001708976	0.055519281	-2.71E-05	0.019102068	1	0.438568358	1	0.449543964	ENSG00000116157	5	GPX7
Pancreas	T2DM	ENSG00000129204.12	0.000177895	0.917752885	0.001722709	0.055522676	0.000205665	0.904970781	0.001722712	0.055519623	-2.78E-05	0.033886907	1	0.9849439	1	0.984551279	ENSG00000116157	5	GPX7
Pancreas	T2DM	ENSG00000129355.6	-0.002869986	0.188064177	0.002180295	0.05552248	-0.002839298	0.192828657	0.002180288	0.055519431	-3.07E-05	0.042687924	1	0.591234665	1	0.602109922	ENSG00000116157	5	GPX7
Pancreas	T2DM	ENSG00000130299.12	-0.002785141	0.016324644	0.00115971	0.05552202	-0.002843138	0.014251028	0.001160047	0.05551894	5.80E-05	0.047399015	1	0.197743801	1	0.18767507	ENSG00000116198	8	CEP104
Pancreas	T2DM	ENSG00000130300.4	-0.000952809	0.775796393	0.003345522	0.055522668	0.002978494	0.432136113	0.003791641	0.055519554	-0.003931303	0.027597918	1	0.948363389	1	0.793088295	ENSG00000116198	8	CEP104
Pancreas	T2DM	ENSG00000130363.7	0.001703888	0.59633415	0.003216827	0.055522645	0.001652563	0.60744075	0.003216791	0.055519594	5.13E-05	0.005479361	1	0.88286336	1	0.882490377	ENSG00000116221	11	MRPL37
Pancreas	T2DM	ENSG00000131653.8	0.00026467	0.936157211	0.003304211	0.055522676	0.00036174	0.912827629	0.003304389	0.055519623	-9.71E-05	0.021303271	1	0.987512886	1	0.985865795	ENSG00000116221	11	MRPL37
Pancreas	T2DM	ENSG00000132274.11	-0.001329987	0.608555747	0.002596939	0.055522647	-0.001288603	0.619753997	0.002596945	0.055519596	-4.14E-05	0.038937383	1	0.884921381	1	0.888839757	ENSG00000116221	11	MRPL37
Pancreas	T2DM	ENSG00000132305.16	-0.000665391	0.742757171	0.002027366	0.055522665	-0.000694865	0.731791048	0.002027359	0.055519611	2.95E-05	0.035180373	1	0.930893321	1	0.924390297	ENSG00000116459	6	ATP5F1
Pancreas	T2DM	ENSG00000132330.12	0.004598824	0.054954662	0.00239617	0.055522257	0.004562306	0.056909771	0.002396159	0.055519211	3.65E-05	0.024178057	1	0.360929851	1	0.375107923	ENSG00000116489	8	CAPZA1
Pancreas	T2DM	ENSG00000132507.13	-0.000665253	0.785241397	0.002441339	0.055522669	-0.000716292	0.769215021	0.002441332	0.055519614	5.10E-05	0.002787463	1	0.949163351	1	0.943143698	ENSG00000116539	6	ASH1L
Pancreas	T2DM	ENSG00000133316.11	-0.001437053	0.309330259	0.001413547	0.055522559	-0.00145493	0.303344837	0.00141353	0.055519503	1.79E-05	0.023202542	1	0.715658284	1	0.712707723	ENSG00000116539	6	ASH1L
Pancreas	T2DM	ENSG00000133706.13	-0.001920713	0.150873002	0.001337119	0.055522442	-0.001905253	0.154183433	0.001337103	0.055519393	-1.55E-05	0.039163757	1	0.556171292	1	0.561621356	ENSG00000116539	6	ASH1L
Pancreas	T2DM	ENSG00000134285.6	0.00033878	0.888526987	0.002416963	0.055522675	0.000297808	0.90193614	0.00241696	0.055519622	4.10E-05	0.019314443	1	0.981247774	1	0.984551279	ENSG00000116661	9	FBXO2
Pancreas	T2DM	ENSG00000134852.10	0.000576017	0.682597725	0.001408625	0.055522658	0.000598177	0.671087542	0.001408615	0.055519604	-2.22E-05	0.012796324	1	0.91019995	1	0.907798476	ENSG00000116663	6	FBXO6
Pancreas	T2DM	ENSG00000134873.5	0.0024912	0.405020318	0.002991752	0.055522598	0.001006608	0.742336582	0.003061823	0.055519612	0.001484592	0.022716971	1	0.783229955	1	0.930820479	ENSG00000116667	8	C1orf21
Pancreas	T2DM	ENSG00000135828.7	-0.00017128	0.944524616	0.00246148	0.055522677	-0.000133248	0.95682687	0.002461464	0.055519624	-3.80E-05	0.016889969	1	0.989794375	1	0.991898954	ENSG00000116675	11	DNAJC6
Pancreas	T2DM	ENSG00000136490.4	0.002923348	0.111576113	0.001837253	0.055522389	0.002950182	0.108327069	0.001837248	0.05551933	-2.68E-05	0.03947978	1	0.503138201	1	0.498212091	ENSG00000116675	11	DNAJC6
Pancreas	T2DM	ENSG00000137288.5	0.001509949	0.420004709	0.001872425	0.055522603	0.000753056	0.691701326	0.001899031	0.055519606	0.000756894	0.016977941	1	0.7882025	1	0.913082753	ENSG00000116679	11	IVNS1ABP
Pancreas	T2DM	ENSG00000137310.7	0.000669411	0.597149677	0.001266615	0.055522645	0.000520354	0.681380338	0.001267362	0.055519605	0.000149057	0.000813098	1	0.88286336	1	0.910080116	ENSG00000116688	12	MFN2
Pancreas	T2DM	ENSG00000137478.10	0.001178799	0.441401635	0.001531248	0.05552261	0.001215055	0.427481588	0.001531242	0.055519552	-3.63E-05	0.00066327	1	0.797847021	1	0.793088295	ENSG00000116701	10	NCF2
Pancreas	T2DM	ENSG00000137672.8	-0.003858201	0.248377402	0.003342457	0.055522525	-0.003807868	0.254599951	0.003342438	0.055519476	-5.03E-05	0.022207437	1	0.659767299	1	0.670900145	ENSG00000116771	5	AGMAT
Pancreas	T2DM	ENSG00000137752.18	-0.001979244	0.515560671	0.003044035	0.055522629	-0.002032468	0.504331556	0.003044027	0.055519573	5.32E-05	0.013493164	1	0.848731838	1	0.844462185	ENSG00000116771	5	AGMAT
Pancreas	T2DM	ENSG00000137857.13	0.00158431	0.447382023	0.002085202	0.055522611	0.001548004	0.457856888	0.002085188	0.055519561	3.63E-05	0.007172973	1	0.803972733	1	0.812561499	ENSG00000116771	5	AGMAT
Pancreas	T2DM	ENSG00000138119.12	0.001753376	0.153785336	0.001229326	0.055522445	0.001731276	0.159040166	0.001229329	0.055519398	2.21E-05	0.019629813	1	0.558586622	1	0.567025251	ENSG00000116785	9	CFHR3
Pancreas	T2DM	ENSG00000138613.9	-3.46E-05	0.991637599	0.003297455	0.055522677	2.25E-05	0.99455717	0.003297456	0.055519624	-5.71E-05	0.020639733	1	0.997347815	1	0.998538835	ENSG00000116791	9	CRYZ
Pancreas	T2DM	ENSG00000138834.8	-0.002749492	0.34949573	0.002938846	0.055522577	-0.002666958	0.364164976	0.002938931	0.05551953	-8.25E-05	0.008351208	1	0.743483984	1	0.758455523	ENSG00000116857	12	TMEM9
Pancreas	T2DM	ENSG00000140955.6	-0.001790545	0.585790464	0.003285726	0.055522643	-0.001827918	0.57798708	0.003285685	0.055519589	3.74E-05	0.03965952	1	0.87968741	1	0.873565759	ENSG00000116857	12	TMEM9
Pancreas	T2DM	ENSG00000141314.8	-0.001335829	0.40143666	0.001592056	0.055522597	-0.001303088	0.413084234	0.001592085	0.055519548	-3.27E-05	0.031283656	1	0.781568873	1	0.7881136	ENSG00000116857	12	TMEM9
Pancreas	T2DM	ENSG00000141560.10	-0.000702706	0.365239011	0.000776103	0.055522584	-0.00068845	0.37504512	0.0007761	0.055519534	-1.43E-05	0.006759231	1	0.75545002	1	0.766201963	ENSG00000116857	12	TMEM9
Pancreas	T2DM	ENSG00000142273.6	-0.002950434	0.191600903	0.00225938	0.055522483	-0.002925379	0.1953946	0.002259353	0.055519433	-2.51E-05	0.044354056	1	0.59495903	1	0.60527543	ENSG00000116874	7	WARS2
Pancreas	T2DM	ENSG00000142621.15	-0.001426756	0.430062665	0.001808114	0.055522606	-0.001455354	0.420878677	0.001808121	0.05551955	2.86E-05	0.044772804	1	0.792249323	1	0.790339598	ENSG00000116874	7	WARS2
Pancreas	T2DM	ENSG00000143067.4	0.001704002	0.380760326	0.001944105	0.05552259	0.001682979	0.38665839	0.001944081	0.055519539	2.10E-05	0.049988352	1	0.767612694	1	0.772390956	ENSG00000116874	7	WARS2
Pancreas	T2DM	ENSG00000146263.7	-6.28E-05	0.978029635	0.002279259	0.055522677	-9.03E-05	0.968411273	0.002279235	0.055519624	2.75E-05	0.037613349	1	0.99701908	1	0.995018895	ENSG00000116874	7	WARS2
Pancreas	T2DM	ENSG00000146833.11	-0.001166765	0.048786406	0.000592128	0.055522235	-0.001156054	0.050895111	0.000592129	0.05551919	-1.07E-05	0.016419394	1	0.329693399	1	0.344851303	ENSG00000116883	8	RP11-268J15.5
Pancreas	T2DM	ENSG00000148824.14	-0.000304751	0.796523839	0.001181899	0.05552267	-0.000404122	0.732595311	0.001182767	0.055519611	9.94E-05	0.031293991	1	0.949920498	1	0.924390297	ENSG00000116906	7	GNPAT
Pancreas	T2DM	ENSG00000149328.10	0.002413461	0.264388945	0.002162447	0.055522535	0.002370501	0.272992127	0.002162473	0.055519487	4.30E-05	0.025676651	1	0.67759562	1	0.68377529	ENSG00000116977	14	LGALS8
Pancreas	T2DM	ENSG00000149577.11	-0.002867908	0.195594632	0.002215958	0.055522486	-0.00282453	0.20244098	0.002215961	0.055519439	-4.34E-05	0.009848045	1	0.599883083	1	0.615373581	ENSG00000117115	8	PADI2
Pancreas	T2DM	ENSG00000149591.12	-0.003921639	0.097514565	0.002366671	0.055522364	-0.003882249	0.10092541	0.002366667	0.055519318	-3.94E-05	0.019767899	1	0.478030203	1	0.486257122	ENSG00000117115	8	PADI2
Pancreas	T2DM	ENSG00000152439.8	0.000874452	0.456281656	0.001173787	0.055522614	0.000855547	0.466072436	0.001173775	0.055519564	1.89E-05	0.006997541	1	0.809765924	1	0.815865042	ENSG00000117115	8	PADI2

Pancreas	T2DM	ENSG00000152689.13	0.000673771	0.682667405	0.001648062	0.055522658	0.000642671	0.696571664	0.001648074	0.055519607	3.11E-05	0.023477347	1	0.91019995	1	0.9151214	ENSG00000117115	8	PADI2
Pancreas	T2DM	ENSG00000152749.7	-0.002454058	0.317627765	0.002455664	0.055522563	-0.002274496	0.35456511	0.002456865	0.055519526	-0.000179562	0.022884385	1	0.722957701	1	0.750019112	ENSG00000117122	9	MFAP2
Pancreas	T2DM	ENSG00000153774.4	0.003710133	0.117262883	0.00236862	0.055522398	0.003820657	0.10681976	0.002369169	0.055519328	-0.000110524	0.040615557	1	0.510742285	1	0.494470247	ENSG00000117122	9	MFAP2
Pancreas	T2DM	ENSG00000154265.11	0.001014651	0.701484649	0.002647027	0.05552266	0.00096797	0.714600498	0.002647007	0.055519609	4.67E-05	0.005253769	1	0.917424714	1	0.920472548	ENSG00000117122	9	MFAP2
Pancreas	T2DM	ENSG00000154316.10	-0.00385111	0.040759395	0.001882286	0.0555222	-0.003817299	0.042562461	0.001882313	0.055519156	-3.38E-05	0.048760426	1	0.304588118	1	0.31228686	ENSG00000117122	9	MFAP2
Pancreas	T2DM	ENSG00000154710.11	-0.001494656	0.239448386	0.001270569	0.055522519	-0.001480164	0.244028082	0.00127055	0.05551947	-1.45E-05	0.025921755	1	0.651353501	1	0.662902761	ENSG00000117122	9	MFAP2
Pancreas	T2DM	ENSG00000154864.7	-0.000431182	0.879454329	0.002843064	0.055522675	-0.000394816	0.889551716	0.002843032	0.055519622	-3.64E-05	0.024788893	1	0.980887799	1	0.981479231	ENSG00000117122	9	MFAP2
Pancreas	T2DM	ENSG00000155542.7	-0.002093852	0.092813543	0.00124579	0.055522355	-0.002115542	0.089476068	0.001245776	0.055519296	2.17E-05	0.002606514	1	0.467725388	1	0.455804017	ENSG00000117153	11	KLHL12
Pancreas	T2DM	ENSG00000156711.12	0.001513342	0.258733165	0.001339962	0.055522532	0.001546294	0.248510625	0.00133997	0.055519472	-3.30E-05	0.002693661	1	0.675917364	1	0.666460318	ENSG00000117174	6	ZNHIT6
Pancreas	T2DM	ENSG00000156931.11	-0.001237275	0.706942894	0.003290929	0.055522661	-0.001180574	0.719790817	0.003290905	0.055519609	-5.67E-05	0.006596104	1	0.918274836	1	0.92146093	ENSG00000117174	6	ZNHIT6
Pancreas	T2DM	ENSG00000157214.9	0.007455189	0.014584025	0.003052208	0.055521997	0.007417863	0.015084743	0.003052183	0.055518951	3.73E-05	0.047688254	1	0.186327245	1	0.192724475	ENSG00000117174	6	ZNHIT6
Pancreas	T2DM	ENSG00000158793.9	0.002682015	0.126811108	0.001756624	0.055522412	0.002516057	0.152360964	0.001757951	0.055519391	0.000165958	0.01697553	1	0.527160606	1	0.558149724	ENSG00000117174	6	ZNHIT6
Pancreas	T2DM	ENSG00000159055.3	-0.002285409	0.113297309	0.001443221	0.055522391	-0.002262174	0.117010448	0.00144322	0.055519344	-2.32E-05	0.028803921	1	0.505335944	1	0.512769334	ENSG00000117222	9	RBBP5
Pancreas	T2DM	ENSG00000159128.10	-0.001304724	0.438240327	0.001683148	0.055522609	-0.001339741	0.426046624	0.001683143	0.055519552	3.50E-05	0.002963591	1	0.796485955	1	0.793088295	ENSG00000117222	9	RBBP5
Pancreas	T2DM	ENSG00000160072.15	-0.001944987	0.492711753	0.002835247	0.055522624	-0.001908307	0.500901318	0.002835216	0.055519573	-3.67E-05	0.025460689	1	0.837381722	1	0.842380212	ENSG00000117226	7	GBP3
Pancreas	T2DM	ENSG00000160209.14	-0.006418876	0.005314758	0.002302882	0.055521792	-0.006388851	0.00553204	0.002302858	0.055518747	-3.00E-05	0.024750028	1	0.10498691	1	0.10972011	ENSG00000117226	7	GBP3
Pancreas	T2DM	ENSG00000160229.7	0.005239212	0.098067498	0.003167029	0.055522365	0.005192248	0.101114215	0.003167011	0.055519318	4.70E-05	0.024783184	1	0.479690164	1	0.486257122	ENSG00000117450	9	PRDX1
Pancreas	T2DM	ENSG00000160766.10	-0.001935922	0.152126634	0.001351842	0.055522443	-0.001912812	0.157078281	0.00135184	0.055519396	-2.31E-05	0.017199488	1	0.557291293	1	0.561838668	ENSG00000117450	9	PRDX1
Pancreas	T2DM	ENSG00000160948.9	0.003856836	0.026847217	0.001742227	0.055522119	0.003836508	0.027659091	0.001742209	0.055519072	2.03E-05	0.044207889	1	0.251619173	1	0.256314825	ENSG00000117450	9	PRDX1
Pancreas	T2DM	ENSG00000161021.7	0.002407565	0.404602557	0.002888743	0.055522598	0.002440804	0.398139902	0.002888701	0.055519543	-3.32E-05	0.025349592	1	0.783229955	1	0.778692512	ENSG00000117450	9	PRDX1
Pancreas	T2DM	ENSG00000161542.12	-0.004256836	0.042850488	0.002101965	0.05552221	-0.004230811	0.044135076	0.002101942	0.055519163	-2.60E-05	0.030093723	1	0.311721277	1	0.31658904	ENSG00000117477	8	CCDC181
Pancreas	T2DM	ENSG00000161888.7	-0.002965088	0.3173979	0.002965621	0.055522563	-0.002921599	0.324545116	0.002965603	0.055519514	-4.35E-05	0.025716613	1	0.722957701	1	0.729254023	ENSG00000117480	11	FAAH
Pancreas	T2DM	ENSG00000161896.6	0.001429515	0.239198112	0.001214547	0.055522519	0.001683829	0.166229098	0.001216266	0.055519406	-0.000254314	9.81E-05	1	0.651353501	1	0.572425504	ENSG00000117480	11	FAAH
Pancreas	T2DM	ENSG00000161904.7	0.000104001	0.94774624	0.001586899	0.055522677	-0.000135937	0.931836441	0.00158926	0.055519623	0.000239938	0.006036893	1	0.989794375	1	0.987209256	ENSG00000117480	11	FAAH
Pancreas	T2DM	ENSG00000162341.11	0.004166166	0.099914135	0.002532203	0.055522369	0.004200803	0.097123008	0.002532179	0.055519311	-3.46E-05	0.022711767	1	0.483200563	1	0.472157259	ENSG00000117481	6	NSUN4
Pancreas	T2DM	ENSG00000162817.6	0.000863958	0.324644401	0.000877151	0.055522567	0.000875285	0.318336368	0.000877141	0.055519511	-1.13E-05	0.025426465	1	0.723686476	1	0.729012597	ENSG00000117481	6	NSUN4
Pancreas	T2DM	ENSG00000163281.7	0.000157237	0.863387353	0.000913832	0.055522674	0.000171711	0.850952102	0.000913824	0.05551962	-1.45E-05	0.009507463	1	0.976495788	1	0.97099298	ENSG00000117505	8	DR1
Pancreas	T2DM	ENSG00000163395.12	0.001365568	0.623547857	0.002782168	0.05552265	0.001406806	0.613099447	0.002782149	0.055519595	-4.12E-05	0.021269443	1	0.89259216	1	0.885013704	ENSG00000117523	11	PRRC2C
Pancreas	T2DM	ENSG00000163545.7	0.000924	0.696546296	0.00236931	0.05552266	0.00088917	0.707445523	0.002369291	0.055519608	3.48E-05	0.018852179	1	0.916358037	1	0.919392765	ENSG00000117523	11	PRRC2C
Pancreas	T2DM	ENSG00000163576.13	0.000363282	0.672164277	0.000858457	0.055522657	0.000350442	0.683108006	0.00085845	0.055519605	1.28E-05	0.015352232	1	0.909524436	1	0.910080116	ENSG00000117533	10	VAMP4
Pancreas	T2DM	ENSG00000163596.12	-0.002742729	0.001640399	0.000871083	0.055521548	-0.002732557	0.001706974	0.000871074	0.055518503	-1.02E-05	0.046084922	1	0.048420063	1	0.050971036	ENSG00000117533	10	VAMP4
Pancreas	T2DM	ENSG00000163788.9	-0.001930328	0.559800702	0.003310242	0.055522638	-0.001886007	0.568845024	0.003310215	0.055519587	-4.43E-05	0.031559102	1	0.86976803	1	0.868486338	ENSG00000117569	14	PTBP2
Pancreas	T2DM	ENSG00000164081.8	-0.001960986	0.44339617	0.002558459	0.05552261	-0.001925454	0.451697181	0.002558439	0.05551956	-3.55E-05	0.026482341	1	0.799538512	1	0.805155685	ENSG00000117569	14	PTBP2
Pancreas	T2DM	ENSG00000164733.16	-0.001228004	0.539342952	0.002000634	0.055522634	-0.001022681	0.609449787	0.002001884	0.055519594	-0.000205323	0.004494747	1	0.858495882	1	0.883228336	ENSG00000117595	6	IRF6
Pancreas	T2DM	ENSG00000164941.9	0.00136915	0.304891526	0.001334452	0.055522557	0.001351987	0.310990245	0.001334442	0.055519507	1.72E-05	0.04156034	1	0.711360218	1	0.721874686	ENSG00000117595	6	IRF6
Pancreas	T2DM	ENSG00000165055.11	0.001855024	0.100708998	0.001130127	0.05552237	0.001839461	0.103595009	0.001130118	0.055519322	1.56E-05	0.025092576	1	0.483403189	1	0.486323959	ENSG00000117595	6	IRF6
Pancreas	T2DM	ENSG00000165171.6	-0.000393348	0.440121803	0.000509526	0.055522609	-0.000420338	0.409479302	0.000509617	0.055519547	2.70E-05	0.009144689	1	0.797847021	1	0.785871608	ENSG00000117595	6	IRF6
Pancreas	T2DM	ENSG00000165410.10	0.001842036	0.527114205	0.002912689	0.055522632	0.001890247	0.516355206	0.002912667	0.055519576	-4.82E-05	0.009158739	1	0.856215034	1	0.848610147	ENSG00000117682	12	DHDDS
Pancreas	T2DM	ENSG00000165934.8	-8.65E-05	0.926668997	0.000939866	0.055522676	-7.15E-05	0.939346365	0.000939862	0.055519623	-1.50E-05	0.018449059	1	0.986516064	1	0.987209256	ENSG00000117697	10	NSL1
Pancreas	T2DM	ENSG00000166224.12	-0.004993864	0.003938683	0.001732149	0.05552173	-0.004969242	0.004119756	0.001732133	0.055518686	-2.46E-05	0.01829558	1	0.083591224	1	0.088903632	ENSG00000117697	10	NSL1
Pancreas	T2DM	ENSG00000166228.4	-0.005824005	0.000858711	0.001747291	0.055521411	-0.005793195	0.000914678	0.001747277	0.055518372	-3.08E-05	0.004817152	1	0.034383558	1	0.035306133	ENSG00000117748	5	RPA2
Pancreas	T2DM	ENSG00000166902.4	0.001560702	0.313176433	0.001547424	0.055522561	0.001590308	0.304082799	0.001547416	0.055519504	-2.96E-05	0.003900563	1	0.719997385	1	0.713788508	ENSG00000117868	11	ESYT2
Pancreas	T2DM	ENSG00000167257.6	0.00097048	0.728505361	0.002795849	0.055522663	0.001021502	0.714839193	0.002795841	0.055519609	-5.10E-05	0.008916102	1	0.923852724	1	0.920472548	ENSG00000118017	3	A4GNT
Pancreas	T2DM	ENSG00000167800.9	0.002798512	0.261759309	0.002493676	0.055522534	0.002826693	0.256979661	0.002493642	0.055519478	-2.82E-05	0.033343233	1	0.67693646	1	0.672705168	ENSG00000118017	3	A4GNT
Pancreas	T2DM	ENSG00000167972.9	-0.002875846	0.198967556	0.002238883	0.055522489	-0.002936412	0.189693663	0.002238993	0.055519428	6.06E-05	0.029311659	1	0.606827415	1	0.59849	ENSG00000118096	3	IFT46
Pancreas	T2DM	ENSG00000168028.9	-4.45E-05	0.954911395	0.00078662	0.055522677	-5.49E-05	0.944362042	0.000786614	0.055519624	1.04E-05	0.034861373	1	0.989794375	1	0.987209256	ENSG00000118197	9	DDX59
Pancreas	T2DM	ENSG00000168385.13	0.000158611	0.893201882	0.001181426	0.055522675	0.000178158	0.880133642	0.001181423	0.055519622	-1.95E-05	0.017622106	1	0.981247774	1	0.980763999	ENSG00000118197	9	DDX59
Pancreas	T2DM	ENSG00000168778.7	0.000164271	0.843303037	0.000831036	0.055522673	0.000175258	0.832970695	0.000831026	0.055519619	-1.10E-05	0.017560588	1	0.96894953	1	0.962244149	ENSG00000118197	9	DDX59
Pancreas	T2DM	ENSG00000169231.9	0.003605659	0.029316569	0.001654572	0.055522136	0.003560783	0.031392454	0.001654586	0.055519096	4.49E-05	0.001443884	1	0.264157714	1	0.275139321	ENSG00000118197	9	DDX59
Pancreas	T2DM	ENSG00000169609.9	-9.18E-05	0.948281103	0.001415111	0.055522677	-7.61E-05	0.957136141	0.00141509	0.055519624	-1.57E-05	0.028383699	1	0.989794375	1	0.991898954	ENSG00000118197	9	DDX59
Pancreas	T2DM	ENSG00000170632.9	0.003665992	0.13150953	0.002430742	0.055522418	0.003706374	0.127308543	0.002430721	0.055519359	-4.04E-05	0.006821596	1	0.531854333	1	0.53156386	ENSG00000118200	10	CAMSAP2
Pancreas	T2DM	ENSG00000170667.10	-0.002537355	0.126250557	0.001659423	0.055522411	-0.002557751	0.123227863	0.001659403	0.055519353	2.04E-05	0.028297417	1	0.526317257	1	0.521333034	ENSG00000118307	14	CASC1
Pancreas	T2DM	ENSG00000171357.5	0.000517256	0.776243511	0.001819929	0.055522668	0.000488673	0.7883046	0.001819924	0.055519616	2.86E-05	0.024331323	1	0.948363389	1	0.947789023	ENSG00000118307	14	CASC1
Pancreas	T2DM	ENSG00000172000.3	0.001841811	0.429446326	0.002330992	0.055522606	0.001880405	0.419836827	0.00233097	0.05551955	-3.86E-05	0.005819781	1	0.792249323	1	0.789846866	ENSG00000118307	14	CASC1
Pancreas	T2DM	ENSG00000172322.9	-0.003024156	0.108308546	0.001883218	0.055522383	-0.002993008	0.111989224	0.001883202	0.055519336	-3.11E-05	0.007841299	1	0.49934712	1	0.500338797	ENSG00000118307	14	CASC1
Pancreas	T2DM	ENSG00000172493.16	-0.000359974	0.913189746	0.003302024	0.055522676	-0.000417246	0.899446086	0.003302008	0.055519622	5.73E-05	0.010239892	1	0.98441119	1	0.984551279	ENSG00000118308	10	LRMP
Pancreas	T2DM	ENSG00000172780.12	-0.000693552	0.721004169	0.001942084	0.055522663	-0.000669395	0.730333953	0.001942069	0.055519611	-2.42E-05	0.049946004	1	0.921476762	1	0.924345782	ENSG00000118402	5	ELOVL4
Pancreas	T2DM	ENSG00000174808.7	0.007456977	0.018259595	0.003159308	0.055522042	0.00750216	0.017566094	0.003159273	0.055518982	-4.52E-05	0.01239789	1	0.206641906	1	0.209456125	ENSG00000118407	10	FILIP1
Pancreas	T2DM	ENSG00000175213.2	-0.004440494	0.06348675	0.002392802	0.055522285	-0.004475137	0.061448663	0.002392792	0.055519226	3.46E-05	0.033703455	1	0.386336431	1	0.38581948	ENSG00000118407	10	FILIP1
Pancreas	T2DM	ENSG00000175220.7	-0.000154234	0.882722626	0.001045524	0.055522675	-0.000171845	0.869445822	0.001045524	0.055519621	1.76E-05	0.022920516	1	0.980887799	1	0.976273228	ENSG00000118507	11	AKAP7
Pancreas	T2DM	ENSG00000175390.8	-0.001340961	0.450161379	0.001775762	0.055522612	-0.001363084	0.442718852	0.001775749	0.055519557	2.21E-05	0.048268883	1	0.806146738	1	0.800635219	ENSG00000118507	11	AKAP7
Pancreas	T2DM	ENSG00000175575.8	0.000832856	0.317063627	0.000832431	0.055522563	0.000845223	0.309931166	0.000832431	0.055519507	-1.24E-05	0.044775264	1	0.722957701	1	0.720926844	ENSG00000118507	11	AKAP7
Pancreas	T2DM	ENSG00000175581.9	0.002046541	0.253570041	0.001792503	0.055522529	0.002074224	0.247202061	0.001792492	0.055519472	-2.77E-05	0.017427548	1	0.66855017	1	0.664032315	ENSG00000118507	11	AKAP7
Pancreas	T2DM	ENSG00000176171.7	-0.000585454	0.823580321	0.00262603	0.055522671	-0.000615813	0.814592253	0.002626002	0.055519618	3.04E-05	0.046161783	1	0.960390921	1	0.956268122	ENSG00000118514	9	ALDH8A1
Pancreas	T2DM	ENSG00000177628.11	0.002316489	0.256422276	0.002041159	0.05552253	0.002363015	0.246993682	0.002041158	0.055519471	-4.65E-05	0.002012639	1	0.67153808	1	0.664032315	ENSG00000118514	9	ALDH8A1
Pancreas	T2DM	ENSG00000177990.7	0.001068366	0.416276367	0.001314267	0.055522602	0.001089385	0.407163308	0.001314259	0.055519546	-2.10E-05	0.015568664	1	0.786828099	1	0.784981512	ENSG00000118514	9	ALDH8A1
Pancreas	T2DM	ENSG00000179163.11	-0.000182288	0.924957769	0.001935313	0.055522676	-0.000225588	0.907205736	0.001935315	0.055519623	4.33E-05	0.003074394	1	0.986312425	1	0.985211345	ENSG00000118514	9	ALDH8A1
Pancreas	T2DM	ENSG00000180185.7	1.71E-05	0.980760835	0.000709038	0.055522677	2.95E-05	0.966763674	0.000709033	0.055519624	-1.24E-05	0.005675189	1	0.99701908	1	0.99385473	ENSG00000118579	7	MED28
Pancreas	T2DM	ENSG00000180979.5	0.003769288	0.10283518	0.002310654	0.055522374	0.003734178	0.106077679	0.002310639	0.055519327	3.51E-05	0.018870118	1	0.487300933	1	0.493491813	ENSG00000118579	7	MED28
Pancreas	T2DM	ENSG00000181126.9	0.000358488	0.63807414	0.000762103	0.055522652	0.000197356	0.796229722	0.000764267	0.055519617	0.000161132	0.00525703	1	0.898449905	1	0.948825024	ENSG00000118579	7	MED28
Pancreas	T2DM	ENSG00000182952.4	0.001241699	0.387915184	0.001438142	0.055522592	0.001142467	0.427205479	0.001438906	0.055519552	9.92E-05	0.03899189	1	0.770733172	1	0.793088295	ENSG00000118600	7	TMEM5
Pancreas	T2DM	ENSG00000183426.11	0.000879311	0.327334419	0.000897716	0.055522568	0.000868918	0.333078405	0.000897705	0.055519517	1.04E-05	0.038894491	1	0.725275917	1	0.735008377	ENSG00000118640	6	VAMP8
Pancreas	T2DM	ENSG00000184164.10	0.002420148	0.243960325	0.002077119	0.055522522	0.002386184	0.250641336	0.00207712	0.055519474	3.40E-05	0.028787765	1	0.656811468	1	0.666460318	ENSG00000118640	6	VAMP8
Pancreas	T2DM	ENSG00000184389.8	-0.001655181	0.365663698	0.001829685	0.055522584	-0.001682342	0.357846203	0.001829671	0.055519528	2.72E-05	0.018597619	1	0.75545002	1	0.752004132	ENSG00000118690	8	ARMC2
Pancreas	T2DM	ENSG00000184608.4	0.003089306	0.227259353	0.002558537	0.055522511	0.00303355	0.235759192	0.002558544	0.055519464	5.58E-05	0.005102935	1	0.6413209	1	0.655938795	ENSG00000118900	10	UBN1
Pancreas	T2DM	ENSG00000184674.8	-0.000887522	0.642403363	0.001911356	0.055522653	-0.000856229	0.654174515	0.00191135	0.055519601	-3.13E-05	0.018865622	1	0.898449905	1	0.900271628	ENSG00000118900	10	UBN1
Pancreas	T2DM	ENSG00000184945.9	0.000459079	0.670065828	0.001077504	0.055522656	0.000441535	0.681968828	0.001077497	0.055519605	1.75E-05	0.011294982	1	0.908515863	1	0.910080116	ENSG00000118900	10	UBN1
Pancreas	T2DM	ENSG00000184983.5	0.001336172	0.099840528	0.000811951	0.055522369	0.00132395	0.102980174	0.000811951	0.055519321	1.22E-05	0.042326108	1	0.483200563	1	0.486323959	ENSG00000118900	10	UBN1
Pancreas	T2DM	ENSG00000185101.8	-0.001644604	0.428091362	0.002075308	0.055522606	-0.001578416	0.446931408	0.002075384	0.055519558	-6.62E-05	0.004813564	1	0.792249323	1	0.8027301	ENSG00000118961	10	C2orf43
Pancreas	T2DM	ENSG00000185627.13	-0.002327406	0.018227452	0.000985783	0.055522042	-0.002519006	0.010968884	0.000990293	0.055518887	0.0001916	0.043043385	1	0.206641906	1	0.16247674	ENSG00000118961	10	C2orf43
Pancreas	T2DM	ENSG00000187741.10	-0.001052241	0.466103126	0.001443731	0.055522617	-0.001083939	0.452777824	0.001443726	0.05551956	3.17E-05	0.001517944	1	0.815461018	1	0.806333878	ENSG00000118961	10	C2orf43
Pancreas	T2DM	ENSG00000187987.5	0.004043275	0.209741102	0.003223584	0.055522498	0.003617152	0.262409508	0.003227538	0.055519481	0.000426123	0.008312403	1	0.61697039	1	0.677451547	ENSG00000118961	10	C2orf43
Pancreas	T2DM	ENSG00000188321.9	-1.81E-05	0.979094739	0.000692379	0.055522677	-9.28E-06	0.989306689	0.000692371	0.055519624	-8.86E-06	0.023816934	1	0.99701908	1	0.998538835	ENSG00000118961	10	C2orf43
Pancreas	T2DM	ENSG00000188488.9	0.00234185	0.278010939	0.002158786	0.055522543	-0.001677034	0.554078908	0.002834458	0.055519584	0.004018884	0.028675925	1	0.688459105	1	0.864388971	ENSG00000118961	10	C2orf43
Pancreas	T2DM	ENSG00000188629.7	-1.72E-05	0.98790529	0.001132827	0.055522677	-4.56E-06	0.996789086	0.001132812	0.055519624	-1.26E-05	0.036580676	1	0.997207295	1	0.998538835	ENSG00000118997	9	DNAH7
Pancreas	T2DM	ENSG00000188707.4	-0.000493399	0.795714155	0.001905767	0.05552267	-0.001049907	0.585605189	0.001925672	0.05551959	0.000556508	0.044165898	1	0.949920498	1	0.876360212	ENSG00000119013	4	NDUFB3
Pancreas	T2DM	ENSG00000189011.9	0.001177058	0.605794638	0.00228074	0.055522647	0.001267165	0.578527088	0.002280969	0.055519589	-9.01E-05	0.013480526	1	0.883541492	1	0.873565759	ENSG00000119013	4	NDUFB3
Pancreas	T2DM	ENSG00000196151.6	-9.78E-05	0.954336415	0.001707457	0.055522677	-0.000147569	0.931132541	0.001707575	0.055519623	4.98E-05	0.03574147	1	0.989794375	1	0.987209256	ENSG00000119013	4	NDUFB3
Pancreas	T2DM	ENSG00000196260.3	0.001082671	0.388423093	0.001255297	0.055522592	0.000564483	0.654447642	0.001261155	0.055519601	0.000518187	2.08E-05	1	0.770733172	1	0.900271628	ENSG00000119203	9	CPSF3
Pancreas	T2DM	ENSG00000196535.10	-0.001631548	0.235768192	0.0013761	0.055522517	-0.001735346	0.20757235	0.001376963	0.055519443	0.000103798	0.037177142	1	0.65041537	1	0.617666041	ENSG00000119203	9	CPSF3
Pancreas	T2DM	ENSG00000196735.7	-0.004343645	0.000429489	0.001233537	0.055521264	-0.002095232	0.148611492	0.001450534	0.055519386	-0.002248413	0.003219071	1	0.020102425	1	0.55791981	ENSG00000119203	9	CPSF3
Pancreas	T2DM	ENSG00000196743.4	-0.001136666	0.675768156	0.002717703	0.055522657	-0.000732575	0.787968695	0.00272384	0.055519616	-0.000404091	0.027943931	1	0.909524436	1	0.947789023	ENSG00000119203	9	CPSF3
Pancreas	T2DM	ENSG00000196912.8	0.000794212	0.691014214	0.001998122	0.055522659	0.000826573	0.679111933	0.001998119	0.055519605	-3.24E-05	0.023910968	1	0.91152931	1	0.909733669	ENSG00000119227	3	PIGZ
Pancreas	T2DM	ENSG00000197054.7	-0.00333837	0.223851339	0.002744575	0.055522509	-0.003418967	0.212900485	0.002744767	0.055519447	8.06E-05	0.035534977	1	0.636627966	1	0.621677611	ENSG00000119227	3	PIGZ
Pancreas	T2DM	ENSG00000197157.6	0.001166176	0.586813067	0.002145834	0.055522643	0.001202746	0.575134073	0.002145819	0.055519588	-3.66E-05	0.007775168	1	0.87968741	1	0.87300225	ENSG00000119227	3	PIGZ
Pancreas	T2DM	ENSG00000197816.9	0.001676391	0.475477901	0.002349218	0.055522619	0.001713469	0.465768671	0.002349208	0.055519564	-3.71E-05	0.020246698	1	0.821134667	1	0.815865042	ENSG00000119333	7	WDR34
Pancreas	T2DM	ENSG00000197903.6	0.003547413	0.031005031	0.001644594	0.055522147	0.003767599	0.022060578	0.001645721	0.055519027	-0.000220186	0.000393676	1	0.269901423	1	0.237035833	ENSG00000119333	7	WDR34
Pancreas	T2DM	ENSG00000197935.6	-0.001116731	0.416012338	0.001372986	0.055522602	-0.001621377	0.239285256	0.001377812	0.055519466	0.000504645	1.29E-05	1	0.786828099	1	0.655938795	ENSG00000119411	10	BSPRY
Pancreas	T2DM	ENSG00000198502.5	-0.002554607	2.85E-06	0.000545723	0.055520181	-0.001891736	0.001806254	0.000606251	0.055518515	-0.000662871	0.012067198	0.007328071	0.000407115	1	0.05167457	ENSG00000119431	5	HDHD3
Pancreas	T2DM	ENSG00000198624.8	-0.000244342	0.896426807	0.001877008	0.055522675	-4.35E-06	0.998152931	0.001880195	0.055519624	-0.00023999	0.029581708	1	0.981247774	1	0.998541772	ENSG00000119431	5	HDHD3
Pancreas	T2DM	ENSG00000198756.6	-0.000944471	0.606255082	0.001832405	0.055522647	-0.000999833	0.58531571	0.001832414	0.05551959	5.54E-05	0.000173014	1	0.883541492	1	0.876360212	ENSG00000119431	5	HDHD3
Pancreas	T2DM	ENSG00000198885.5	0.000221983	0.889728874	0.001601058	0.055522675	0.000202141	0.899529706	0.001601043	0.055519622	1.98E-05	0.039229125	1	0.981247774	1	0.984551279	ENSG00000119431	5	HDHD3
Pancreas	T2DM	ENSG00000204140.8	-0.002081178	0.294743789	0.001986287	0.055522552	-0.002033617	0.305918516	0.001986294	0.055519505	-4.76E-05	0.002218077	1	0.703918761	1	0.714830528	ENSG00000119431	5	HDHD3
Pancreas	T2DM	ENSG00000204147.5	0.004545899	0.009464388	0.001751914	0.05552191	0.004515255	0.00995741	0.001751922	0.055518867	3.06E-05	0.028353185	1	0.144669926	1	0.154039932	ENSG00000119471	10	HSDL2
Pancreas	T2DM	ENSG00000204228.3	0.001144713	0.307612528	0.001121997	0.055522559	0.000934856	0.406490719	0.001126217	0.055519546	0.000209857	0.031791658	1	0.715533489	1	0.784273604	ENSG00000119471	10	HSDL2
Pancreas	T2DM	ENSG00000204301.5	-0.009227531	1.11E-08	0.001615348	0.055518959	-0.008497019	2.33E-07	0.001643105	0.055516577	-0.000730512	0.015200963	2.86E-05	3.58E-06	0.00059716	5.43E-05	ENSG00000119471	10	HSDL2
Pancreas	T2DM	ENSG00000204305.9	0.006540707	0.011450431	0.002586637	0.055521949	0.004839405	0.065413667	0.00262665	0.055519237	0.001701302	0.000197596	1	0.161564328	1	0.398062316	ENSG00000119471	10	HSDL2
Pancreas	T2DM	ENSG00000204351.7	-0.000511655	0.464694221	0.000699805	0.055522616	-0.001581279	0.032326248	0.000738792	0.055519102	0.001069624	6.32E-06	1	0.815461018	1	0.280452045	ENSG00000119509	8	INVS
Pancreas	T2DM	ENSG00000204371.7	-0.002909236	0.290248886	0.002750851	0.05552255	-0.002371027	0.389696311	0.002756466	0.05551954	-0.00053821	0.002362183	1	0.700376625	1	0.772390956	ENSG00000119509	8	INVS
Pancreas	T2DM	ENSG00000204516.5	0.001381367	0.110519425	0.000865591	0.055522387	0.001233648	0.155425201	0.000868382	0.055519394	0.000147719	0.034445774	1	0.503138201	1	0.561765877	ENSG00000119599	12	DCAF4
Pancreas	T2DM	ENSG00000204525.10	-0.001480412	0.012381934	0.00059191	0.055521964	-0.001102088	0.068762241	0.000605549	0.055519247	-0.000378324	0.003088531	1	0.169131954	1	0.405934333	ENSG00000119599	12	DCAF4
Pancreas	T2DM	ENSG00000204531.11	0.00335196	0.013531726	0.001357361	0.055521982	0.003014369	0.026681917	0.001360185	0.055519065	0.000337591	0.000128313	1	0.175502385	1	0.252838238	ENSG00000119614	2	VSX2
Pancreas	T2DM	ENSG00000204560.5	0.001267427	0.667246568	0.002947982	0.055522656	0.001775548	0.547669661	0.002953064	0.055519583	-0.000508121	0.003603745	1	0.90774704	1	0.863361381	ENSG00000119673	10	ACOT2
Pancreas	T2DM	ENSG00000204616.6	-0.002362129	0.026748192	0.001066337	0.055522118	-0.002183761	0.041132549	0.001069319	0.055519149	-0.000178368	0.026113185	1	0.251609367	1	0.31158816	ENSG00000119673	10	ACOT2
Pancreas	T2DM	ENSG00000204618.4	0.005046817	0.001153156	0.001552752	0.055521474	0.00587238	0.000192334	0.001574826	0.05551804	-0.000825563	0.001697569	1	0.039484046	0.493914241	0.011759863	ENSG00000119711	8	ALDH6A1
Pancreas	T2DM	ENSG00000204622.6	-0.000291945	0.884949226	0.002017615	0.055522675	-0.000645616	0.749520401	0.002022152	0.055519613	0.000353671	0.009416	1	0.981184043	1	0.933447328	ENSG00000119715	10	ESRRB
Pancreas	T2DM	ENSG00000204625.6	0.001000043	0.57829048	0.001799014	0.055522642	-0.000105159	0.953790212	0.001814722	0.055519624	0.001105202	3.63E-06	1	0.878208132	1	0.991898954	ENSG00000119715	10	ESRRB
Pancreas	T2DM	ENSG00000204642.9	-0.000263458	0.746570664	0.000815244	0.055522665	-3.65E-06	0.996434562	0.000817433	0.055519624	-0.000259805	1.53E-05	1	0.932263247	1	0.998538835	ENSG00000119715	10	ESRRB
Pancreas	T2DM	ENSG00000204789.3	-0.000269374	0.740777553	0.000814251	0.055522665	-0.000381629	0.639692745	0.000815223	0.055519599	0.000112255	0.005281361	1	0.930574024	1	0.895174073	ENSG00000119715	10	ESRRB
Pancreas	T2DM	ENSG00000204991.6	-0.00051811	0.72314398	0.001462513	0.055522663	-0.000547259	0.708262224	0.001462511	0.055519608	2.91E-05	0.005967389	1	0.921476762	1	0.919473235	ENSG00000119715	10	ESRRB
Pancreas	T2DM	ENSG00000205978.5	0.004477673	0.077492724	0.002536312	0.055522322	0.004569888	0.071606902	0.002536553	0.055519254	-9.22E-05	0.020206063	1	0.429808455	1	0.414158837	ENSG00000119715	10	ESRRB
Pancreas	T2DM	ENSG00000206341.6	0.001319648	0.239088329	0.001120939	0.055522519	0.00094315	0.401546012	0.001124318	0.055519544	0.000376497	1.68E-05	1	0.651353501	1	0.782159733	ENSG00000119718	6	EIF2B2
Pancreas	T2DM	ENSG00000206344.6	0.005575874	1.35E-06	0.001153723	0.055520016	0.004772884	5.00E-05	0.001176785	0.05551775	0.00080299	0.000536353	0.00345579	0.000215987	0.128287656	0.005131506	ENSG00000119718	6	EIF2B2
Pancreas	T2DM	ENSG00000206503.7	-0.0002805	0.785794892	0.001032103	0.055522669	0.000671714	0.522842579	0.001051245	0.055519578	-0.000952214	1.89E-06	1	0.949163351	1	0.850322826	ENSG00000119718	6	EIF2B2
Pancreas	T2DM	ENSG00000212127.5	0.004614728	0.001092097	0.001413099	0.055521462	0.004598987	0.001135688	0.001413083	0.055518417	1.57E-05	0.046800895	1	0.038951466	1	0.040083532	ENSG00000119718	6	EIF2B2
Pancreas	T2DM	ENSG00000212694.4	-0.004985887	0.023665375	0.002203682	0.055522094	-0.004956912	0.024487496	0.002203659	0.055519048	-2.90E-05	0.024822354	1	0.236493759	1	0.245759311	ENSG00000119772	12	DNMT3A
Pancreas	T2DM	ENSG00000213221.4	0.003482854	0.0001469	0.000917438	0.055521035	0.003469077	0.000156025	0.000917428	0.055517995	1.38E-05	0.008620228	0.377238052	0.009200928	0.400672926	0.010998318	ENSG00000119844	10	AFTPH
Pancreas	T2DM	ENSG00000213523.5	0.001828533	0.248135127	0.001583293	0.055522525	0.001804442	0.254420299	0.001583288	0.055519476	2.41E-05	0.029374411	1	0.659767299	1	0.670900145	ENSG00000119865	4	CNRIP1
Pancreas	T2DM	ENSG00000213588.4	0.000899478	0.666522098	0.002087312	0.055522656	0.001315047	0.529324292	0.002090568	0.055519579	-0.00041557	0.000412715	1	0.90774704	1	0.854617121	ENSG00000119865	4	CNRIP1
Pancreas	T2DM	ENSG00000213676.6	0.003729919	4.36E-05	0.000912454	0.055520773	0.003302169	0.000333084	0.000920302	0.055518157	0.00042775	0.000368358	0.111855854	0.003857098	0.855358434	0.018199116	ENSG00000119878	5	CRIPT
Pancreas	T2DM	ENSG00000213760.6	0.003463129	0.031720729	0.001612319	0.055522151	0.001854023	0.266899987	0.001669943	0.055519484	0.001609106	0.000216698	1	0.272437568	1	0.682076264	ENSG00000119878	5	CRIPT
Pancreas	T2DM	ENSG00000213901.6	0.002549955	0.435757431	0.003271782	0.055522608	0.002591779	0.428263187	0.003271755	0.055519553	-4.18E-05	0.040310094	1	0.795009083	1	0.793088295	ENSG00000119899	11	SLC17A5
Pancreas	T2DM	ENSG00000214376.5	-0.001273613	0.541096642	0.00208394	0.055522635	-0.003571992	0.118063999	0.00228541	0.055519346	0.00229838	0.014312843	1	0.860225195	1	0.514751016	ENSG00000119900	7	OGFRL1
Pancreas	T2DM	ENSG00000215146.4	-0.000302325	0.778206039	0.001073378	0.055522668	-0.000318785	0.766471927	0.001073377	0.055519614	1.65E-05	0.033807803	1	0.948363389	1	0.941828222	ENSG00000119900	7	OGFRL1
Pancreas	T2DM	ENSG00000218537.1	0.000451033	0.685675043	0.001114401	0.055522658	0.000466004	0.675824583	0.001114396	0.055519604	-1.50E-05	0.046716905	1	0.91019995	1	0.907798476	ENSG00000119943	6	PYROXD2
Pancreas	T2DM	ENSG00000219159.3	-2.82E-05	0.98119396	0.00119468	0.055522677	-4.69E-05	0.968671042	0.001194667	0.055519624	1.88E-05	0.006645653	1	0.99701908	1	0.995018895	ENSG00000119943	6	PYROXD2
Pancreas	T2DM	ENSG00000221988.8	0.011817598	1.47E-06	0.002454385	0.055520036	0.010536438	2.52E-05	0.002501055	0.055517602	0.001281161	0.007755739	0.003783263	0.000222545	0.064783756	0.003239188	ENSG00000119977	16	TCTN3

Pancreas	T2DM	ENSG00000222019.3	-0.001843325	0.420190507	0.002286745	0.055522603	-0.001891666	0.408105857	0.002286741	0.055519546	4.83E-05	0.003271893	1	0.7882025	1	0.785840676	ENSG00000119977	16	TCTN3
Pancreas	T2DM	ENSG00000224796.1	0.002824547	0.117659184	0.001805194	0.055522398	0.003099772	0.086187059	0.001806534	0.055519289	-0.000275225	0.000102921	1	0.510920333	1	0.452744404	ENSG00000119977	16	TCTN3
Pancreas	T2DM	ENSG00000225282.1	0.000152481	0.91445956	0.001419547	0.055522676	0.000171056	0.904086048	0.001419539	0.055519622	-1.86E-05	0.046651785	1	0.9849439	1	0.984551279	ENSG00000120055	5	C10orf95
Pancreas	T2DM	ENSG00000227057.3	0.001923811	0.147451496	0.001328053	0.055522438	0.001539772	0.249576276	0.001337327	0.055519473	0.000384039	0.014766456	1	0.556171292	1	0.666460318	ENSG00000120055	5	C10orf95
Pancreas	T2DM	ENSG00000227262.3	-0.00020769	0.89494441	0.001572806	0.055522675	-0.000354224	0.821888563	0.001573511	0.055519618	0.000146534	0.00253305	1	0.981247774	1	0.958932226	ENSG00000120071	8	KANSL1
Pancreas	T2DM	ENSG00000227825.3	-0.002325415	0.38261847	0.002663453	0.05552259	-0.00235923	0.375733218	0.002663431	0.055519535	3.38E-05	0.038945534	1	0.76943166	1	0.766997539	ENSG00000120071	8	KANSL1
Pancreas	T2DM	ENSG00000228065.6	0.000969629	0.60657463	0.001882886	0.055522647	0.000996759	0.596540829	0.001882873	0.055519592	-2.71E-05	0.024171163	1	0.883541492	1	0.879499402	ENSG00000120093	7	HOXB3
Pancreas	T2DM	ENSG00000228672.2	0.000310775	0.910654423	0.002769507	0.055522676	0.000273515	0.921328641	0.002769487	0.055519623	3.73E-05	0.034794463	1	0.98382859	1	0.987209256	ENSG00000120159	7	CAAP1
Pancreas	T2DM	ENSG00000229212.3	-0.006419279	0.043486746	0.003179419	0.055522213	-0.006488599	0.041268409	0.003179395	0.05551915	6.93E-05	0.000544225	1	0.311795766	1	0.311697862	ENSG00000120314	14	WDR55
Pancreas	T2DM	ENSG00000229390.1	-0.002312886	0.188686001	0.001759547	0.05552248	-0.002724783	0.12226623	0.001763233	0.055519352	0.000411896	0.000329503	1	0.591234665	1	0.519878374	ENSG00000120314	14	WDR55
Pancreas	T2DM	ENSG00000230454.1	-0.001909562	0.260977059	0.001698768	0.055522533	-0.001931093	0.255633037	0.001698747	0.055519477	2.15E-05	0.019899359	1	0.676275568	1	0.671232759	ENSG00000120322	2	PCDHB8
Pancreas	T2DM	ENSG00000230606.6	-4.18E-06	0.997366516	0.001267095	0.055522677	1.37E-05	0.99135995	0.001267089	0.055519624	-1.79E-05	0.038481598	1	0.99892247	1	0.998538835	ENSG00000120328	4	PCDHB12
Pancreas	T2DM	ENSG00000230795.2	-0.000343295	0.64210185	0.000738647	0.055522653	-0.000549651	0.458346028	0.000741195	0.055519561	0.000206355	0.000817023	1	0.898449905	1	0.812867817	ENSG00000120328	4	PCDHB12
Pancreas	T2DM	ENSG00000231205.7	-0.001611536	0.415387994	0.001978682	0.055522602	-0.001579951	0.424586607	0.001978674	0.055519551	-3.16E-05	0.019586911	1	0.786828099	1	0.792397097	ENSG00000120333	4	MRPS14
Pancreas	T2DM	ENSG00000231402.1	0.000668723	0.378859102	0.000759912	0.055522589	0.000973168	0.208172278	0.000773209	0.055519444	-0.000304445	0.033129145	1	0.766674685	1	0.61801897	ENSG00000120437	7	ACAT2
Pancreas	T2DM	ENSG00000231500.2	0.003992597	0.032795653	0.001870435	0.055522158	0.003340479	0.075161278	0.001877219	0.055519263	0.000652118	4.60E-05	1	0.277951279	1	0.426361119	ENSG00000120437	7	ACAT2
Pancreas	T2DM	ENSG00000232040.2	-8.02E-05	0.957027614	0.001487916	0.055522677	-0.000430648	0.772536723	0.00148983	0.055519615	0.000350473	4.36E-06	1	0.990168921	1	0.943354401	ENSG00000120437	7	ACAT2
Pancreas	T2DM	ENSG00000232615.4	-0.000310311	0.924497029	0.003274341	0.055522676	-0.000255691	0.937756561	0.00327431	0.055519623	-5.46E-05	0.005319075	1	0.986312425	1	0.987209256	ENSG00000120451	6	SNX19
Pancreas	T2DM	ENSG00000232653.4	0.001845625	0.293532544	0.001757061	0.055522551	0.001824242	0.29915671	0.001757045	0.055519501	2.14E-05	0.043898777	1	0.703265608	1	0.709510872	ENSG00000120451	6	SNX19
Pancreas	T2DM	ENSG00000233609.3	0.00651977	0.001334464	0.002032042	0.055521504	0.006472533	0.001447058	0.002032119	0.055518468	4.72E-05	0.041940999	1	0.041791518	1	0.045626317	ENSG00000120457	7	KCNJ5
Pancreas	T2DM	ENSG00000234127.4	-0.002218439	0.114819361	0.001406836	0.055522394	-0.003358316	0.018671419	0.001427834	0.055518994	0.001139876	3.05E-06	1	0.50643167	1	0.215982899	ENSG00000120539	10	MASTL
Pancreas	T2DM	ENSG00000234745.5	0.004353564	3.05E-06	0.000932796	0.055520195	0.003641763	0.00016404	0.000966287	0.055518006	0.000711801	0.004777311	0.007842296	0.000412752	0.421253663	0.010998318	ENSG00000120539	10	MASTL
Pancreas	T2DM	ENSG00000234981.1	-0.00075722	0.763266448	0.002514058	0.055522667	-0.000727491	0.772296143	0.002514025	0.055519615	-2.97E-05	0.027742077	1	0.94281578	1	0.943354401	ENSG00000120539	10	MASTL
Pancreas	T2DM	ENSG00000235109.3	0.00080907	0.176876133	0.000599117	0.055522469	0.00066029	0.271523717	0.000600503	0.055519486	0.00014878	0.000285979	1	0.581719513	1	0.68377529	ENSG00000120555	9	SEPT7P9
Pancreas	T2DM	ENSG00000235290.1	0.000739563	0.625490042	0.001515235	0.05552265	0.001194509	0.431426136	0.001518274	0.055519554	-0.000454946	2.53E-06	1	0.89259216	1	0.793088295	ENSG00000120555	9	SEPT7P9
Pancreas	T2DM	ENSG00000235821.1	-0.002155382	0.413276437	0.002634484	0.055522601	-0.001628218	0.537320545	0.002639481	0.055519581	-0.000527164	0.001263973	1	0.785561726	1	0.856222273	ENSG00000120555	9	SEPT7P9
Pancreas	T2DM	ENSG00000235863.2	-0.003838492	0.003335209	0.001307816	0.055521696	-0.002299427	0.102905415	0.001409885	0.055519321	-0.001539065	0.003480112	1	0.075129978	1	0.486323959	ENSG00000120669	11	SOHLH2
Pancreas	T2DM	ENSG00000237669.1	0.000142979	0.892150309	0.00105454	0.055522675	0.000562922	0.59521026	0.001059515	0.055519592	-0.000419943	4.45E-05	1	0.981247774	1	0.879499402	ENSG00000120675	4	DNAJC15
Pancreas	T2DM	ENSG00000238034.1	-6.84E-05	0.970552382	0.001853603	0.055522677	-3.62E-05	0.984417492	0.001853596	0.055519624	-3.22E-05	0.01218646	1	0.994696741	1	0.997232395	ENSG00000120675	4	DNAJC15
Pancreas	T2DM	ENSG00000239257.1	0.001048472	0.351072458	0.001124352	0.055522578	0.000735215	0.514160122	0.001126983	0.055519576	0.000313257	5.19E-05	1	0.743483984	1	0.847609136	ENSG00000120675	4	DNAJC15
Pancreas	T2DM	ENSG00000240053.8	-0.00041498	0.717995925	0.001149087	0.055522662	8.75E-05	0.9400928	0.001164115	0.055519623	-0.000502467	0.007098585	1	0.921476762	1	0.987209256	ENSG00000120725	8	SIL1
Pancreas	T2DM	ENSG00000240065.3	0.003113355	0.176921162	0.002305681	0.055522469	0.00254367	0.271197407	0.002311774	0.055519486	0.000569685	0.000727309	1	0.581719513	1	0.68377529	ENSG00000120725	8	SIL1
Pancreas	T2DM	ENSG00000240972.1	0.000112959	0.94819611	0.001738576	0.055522677	0.000138939	0.936304618	0.001738577	0.055519623	-2.60E-05	0.045946079	1	0.989794375	1	0.987209256	ENSG00000120725	8	SIL1
Pancreas	T2DM	ENSG00000241258.2	-0.002846504	0.194442464	0.002193752	0.055522485	-0.002817574	0.199011166	0.00219373	0.055519436	-2.89E-05	0.026562777	1	0.599023	1	0.610586229	ENSG00000120725	8	SIL1
Pancreas	T2DM	ENSG00000241484.5	-0.000364153	0.794066543	0.001395042	0.055522669	-0.000347763	0.803138179	0.001395027	0.055519617	-1.64E-05	0.042043105	1	0.949920498	1	0.951905176	ENSG00000120756	8	PLS1
Pancreas	T2DM	ENSG00000242252.1	-0.00457988	0.026373801	0.002062387	0.055522115	-0.004553223	0.027260976	0.002062366	0.055519069	-2.67E-05	0.026740815	1	0.251609367	1	0.255898044	ENSG00000120756	8	PLS1
Pancreas	T2DM	ENSG00000243056.1	-0.002609958	0.306182319	0.002550614	0.055522558	-0.002567058	0.314197664	0.002550596	0.055519509	-4.29E-05	0.008325224	1	0.712852398	1	0.725505793	ENSG00000120784	11	ZFP30
Pancreas	T2DM	ENSG00000243753.1	0.00425696	5.09E-05	0.001050718	0.055520807	0.003968251	0.000167031	0.001054178	0.05551801	0.000288709	0.000751196	0.130719261	0.004357309	0.428934408	0.010998318	ENSG00000120784	11	ZFP30
Pancreas	T2DM	ENSG00000244165.1	0.003847184	0.008511672	0.001462207	0.055521888	0.003873344	0.008074064	0.001462209	0.055518825	-2.62E-05	0.018541609	1	0.13747153	1	0.13385914	ENSG00000120784	11	ZFP30
Pancreas	T2DM	ENSG00000244414.2	3.06E-05	0.992649205	0.003324467	0.055522677	8.43E-05	0.979762646	0.003324453	0.055519624	-5.37E-05	0.017836597	1	0.997347815	1	0.9961668	ENSG00000120784	11	ZFP30
Pancreas	T2DM	ENSG00000248927.1	-0.000468324	0.831288381	0.002198193	0.055522672	-0.000427527	0.845790894	0.002198177	0.05551962	-4.08E-05	0.003501522	1	0.963990872	1	0.969100518	ENSG00000120800	4	UTP20
Pancreas	T2DM	ENSG00000250376.1	0.004603244	0.084765066	0.002670581	0.055522339	0.004568333	0.087149521	0.002670551	0.055519291	3.49E-05	0.021800108	1	0.449552839	1	0.453955316	ENSG00000120805	9	ARL1
Pancreas	T2DM	ENSG00000250535.1	0.001432449	0.152664447	0.001001583	0.055522444	0.001285087	0.199930076	0.001002602	0.055519437	0.000147362	0.001287769	1	0.558464815	1	0.611164195	ENSG00000120805	9	ARL1
Pancreas	T2DM	ENSG00000250656.2	0.004838428	0.143484995	0.003307337	0.055522433	0.004890675	0.139208172	0.003307307	0.055519375	-5.22E-05	0.009562346	1	0.553257458	1	0.548928759	ENSG00000120805	9	ARL1
Pancreas	T2DM	ENSG00000251417.1	-0.011321098	9.57E-05	0.002901862	0.055520943	-0.011279226	0.000101545	0.002901855	0.055517903	-4.19E-05	0.041045994	0.245710472	0.006466065	0.260767811	0.007669642	ENSG00000120805	9	ARL1
Pancreas	T2DM	ENSG00000254851.1	0.001667709	0.085072689	0.00096848	0.055522339	0.001654991	0.087476944	0.000968475	0.055519291	1.27E-05	0.048862714	1	0.449552839	1	0.454738448	ENSG00000120860	6	CCDC53
Pancreas	T2DM	ENSG00000254872.2	0.004474255	0.045793424	0.002240154	0.055522223	0.004430565	0.047953755	0.002240167	0.055519178	4.37E-05	0.01686092	1	0.319558457	1	0.331035598	ENSG00000120860	6	CCDC53
Pancreas	T2DM	ENSG00000255394.2	0.002343607	0.471933306	0.003258008	0.055522618	0.002457635	0.450664295	0.003258129	0.055519559	-0.000114028	0.002117119	1	0.817750308	1	0.805155685	ENSG00000120868	9	APAF1
Pancreas	T2DM	ENSG00000255595.1	0.003500784	0.100245619	0.002129862	0.055522369	0.003535594	0.096910504	0.002129847	0.05551931	-3.48E-05	0.010539645	1	0.483403189	1	0.472157259	ENSG00000120907	13	ADRA1A
Pancreas	T2DM	ENSG00000256092.2	9.14E-05	0.937133826	0.001159103	0.055522676	0.000111711	0.923220792	0.0011591	0.055519623	-2.03E-05	0.012929221	1	0.987914477	1	0.987209256	ENSG00000120907	13	ADRA1A
Pancreas	T2DM	ENSG00000257218.1	-0.003401702	0.290579328	0.003218709	0.05552255	-0.003445189	0.28445294	0.003218687	0.055519494	4.35E-05	0.035526366	1	0.700376625	1	0.694923899	ENSG00000120915	9	EPHX2
Pancreas	T2DM	ENSG00000259205.2	0.000239882	0.816160042	0.001031808	0.055522671	0.000229167	0.824231868	0.001031794	0.055519619	1.07E-05	0.048480965	1	0.958017935	1	0.959648084	ENSG00000120915	9	EPHX2
Pancreas	T2DM	ENSG00000259363.1	0.001473705	0.539149022	0.002399777	0.055522634	0.00143658	0.549417582	0.002399766	0.055519583	3.71E-05	0.021685092	1	0.858495882	1	0.863653712	ENSG00000120915	9	EPHX2
Pancreas	T2DM	ENSG00000259726.1	0.004014248	0.112293871	0.002527913	0.05552239	0.00397987	0.115400176	0.002527896	0.055519342	3.44E-05	0.035362881	1	0.50414451	1	0.508315014	ENSG00000120963	7	ZNF706
Pancreas	T2DM	ENSG00000260804.2	0.0035403	0.085601507	0.002059423	0.05552234	0.00357907	0.082229505	0.002059425	0.05551928	-3.88E-05	0.013302319	1	0.45138536	1	0.448334114	ENSG00000120963	7	ZNF706
Pancreas	T2DM	ENSG00000261584.1	-0.000881041	0.404666218	0.001057269	0.055522598	-0.000796782	0.451147877	0.001057436	0.055519559	-8.43E-05	3.45E-05	1	0.783229955	1	0.805155685	ENSG00000121064	8	SCPEP1
Pancreas	T2DM	ENSG00000262855.1	0.001063148	0.529787118	0.001692018	0.055522632	0.001086892	0.520635066	0.00169201	0.055519577	-2.37E-05	0.037799308	1	0.856215034	1	0.848610147	ENSG00000121064	8	SCPEP1
Pancreas	T2DM	ENSG00000263006.2	-0.001396662	0.632101804	0.002917183	0.055522651	-0.001441466	0.621213578	0.002917172	0.055519596	4.48E-05	0.026008571	1	0.89608366	1	0.888839757	ENSG00000121068	9	TBX2
Pancreas	T2DM	ENSG00000264469.1	-0.001998606	0.206162648	0.001580938	0.055522495	-0.002047829	0.195223208	0.00158099	0.055519433	4.92E-05	0.004461983	1	0.612052808	1	0.60527543	ENSG00000121101	11	TEX14
Pancreas	T2DM	ENSG00000266402.2	0.006449132	0.001660611	0.002050557	0.05552155	0.006414145	0.001759923	0.002050538	0.055518509	3.50E-05	0.004342366	1	0.048459653	1	0.05167457	ENSG00000121101	11	TEX14
Pancreas	T2DM	ENSG00000266995.1	0.000154951	0.912232593	0.001405797	0.055522676	0.000172122	0.902551521	0.001405781	0.055519622	-1.72E-05	0.028768399	1	0.984291302	1	0.984551279	ENSG00000121289	13	CEP89
Pancreas	T2DM	ENSG00000267800.1	-0.002749551	0.158144051	0.00194818	0.05552245	-0.002778163	0.153858884	0.001948173	0.055519392	2.86E-05	0.034552435	1	0.562662048	1	0.561235248	ENSG00000121350	11	PYROXD1
Pancreas	T2DM	ENSG00000268822.1	0.000110101	0.952609495	0.001852619	0.055522677	8.14E-05	0.964947883	0.001852599	0.055519624	2.87E-05	0.007938611	1	0.989794375	1	0.99332874	ENSG00000121350	11	PYROXD1
Pancreas	T2DM	ENSG00000270127.1	0.00018337	0.808193453	0.000755364	0.05552267	0.000174154	0.81765708	0.000755355	0.055519618	9.22E-06	0.024775884	1	0.955103906	1	0.957038916	ENSG00000121350	11	PYROXD1
Pancreas	T2DM	ENSG00000271821.1	0.006188043	0.002050948	0.002007299	0.055521594	0.003960008	0.059024717	0.002097447	0.055519218	0.002228036	0.00025039	1	0.055440362	1	0.378938685	ENSG00000121350	11	PYROXD1
Pancreas	T2DM	ENSG00000272221.1	-0.001492574	0.009012675	0.000571521	0.0555219	-0.001284731	0.025297221	0.000574351	0.055519054	-0.000207843	0.000272196	1	0.142867588	1	0.248901391	ENSG00000121406	4	ZNF549
Pancreas	T2DM	ENSG00000272787.1	-0.001076283	0.554539678	0.001821209	0.055522637	-0.001102015	0.545110606	0.001821202	0.055519582	2.57E-05	0.039125712	1	0.867798838	1	0.861442484	ENSG00000121413	8	ZSCAN18
Pancreas	T2DM	ENSG00000272799.1	-0.001432652	0.33664279	0.001491071	0.055522572	-0.001411328	0.343879004	0.001491056	0.055519522	-2.13E-05	0.014282842	1	0.734309489	1	0.743948203	ENSG00000121417	9	ZNF211
Pancreas	T2DM	ENSG00000272849.1	-0.003561481	0.080096791	0.002034983	0.055522328	-0.003533235	0.08251827	0.002034967	0.055519281	-2.82E-05	0.027681045	1	0.438568358	1	0.448955377	ENSG00000121454	4	LHX4
Pancreas	T2DM	ENSG00000273295.1	0.000621682	0.555543313	0.001054638	0.055522638	0.00063614	0.546384952	0.001054633	0.055519583	-1.45E-05	0.042549105	1	0.868305521	1	0.862394934	ENSG00000121552	3	CSTA
SAT	CAD	ENSG00000008853.12	-0.00283586	0.506616824	0.004270132	0.138947771	-0.00274797	0.519889427	0.004270242	0.138942021	-8.79E-05	0.032850908	1	0.832236789	1	0.837215552	ENSG00000121552	3	CSTA
SAT	CAD	ENSG00000010803.12	0.000236297	0.961302676	0.004870198	0.138947896	0.00031916	0.947750197	0.004870274	0.138942138	-8.29E-05	0.04586508	1	0.991640191	1	0.985387556	ENSG00000121552	3	CSTA
SAT	CAD	ENSG00000026950.12	0.001908181	0.270567	0.00173194	0.13894755	0.001993502	0.249800948	0.001732223	0.138941762	-8.53E-05	0.010322541	1	0.669985153	1	0.656684558	ENSG00000121552	3	CSTA
SAT	CAD	ENSG00000041353.5	-0.009326129	0.067351198	0.005098119	0.138946942	-0.01978308	0.001009338	0.006016879	0.138939057	0.010456951	0.001067759	1	0.415234417	1	0.058098932	ENSG00000121552	3	CSTA
SAT	CAD	ENSG00000050405.9	0.00240232	0.429097847	0.003038077	0.138947718	0.0015643	0.608938597	0.003057725	0.138942065	0.00083802	0.015618152	1	0.794212752	1	0.869331532	ENSG00000121577	9	POPDC2
SAT	CAD	ENSG00000087448.5	-0.003627023	0.398629372	0.004297053	0.138947693	-0.003693584	0.390034217	0.004297083	0.138941929	6.66E-05	0.038003758	1	0.776664471	1	0.772767069	ENSG00000121594	7	CD80
SAT	CAD	ENSG00000088325.11	0.001560433	0.57793247	0.002804478	0.138947808	0.001610976	0.565683115	0.00280452	0.138942045	-5.05E-05	0.032529203	1	0.864959129	1	0.853336231	ENSG00000121691	4	CAT
SAT	CAD	ENSG00000100181.17	-0.001631532	0.625284124	0.003340733	0.138947828	-0.00170808	0.609156508	0.003340803	0.138942065	7.65E-05	0.012278148	1	0.878643356	1	0.869331532	ENSG00000121691	4	CAT
SAT	CAD	ENSG00000101000.4	-0.005355433	0.039402292	0.002599767	0.138946687	-0.005305693	0.041271653	0.002599816	0.138940952	-4.97E-05	0.030794032	1	0.338426494	1	0.349902564	ENSG00000121716	14	PILRB
SAT	CAD	ENSG00000105088.4	0.000978885	0.766918646	0.003302494	0.138947871	0.001049275	0.750700004	0.00330255	0.13894211	-7.04E-05	0.014108188	1	0.930280564	1	0.927351925	ENSG00000121898	8	CPXM2
SAT	CAD	ENSG00000105968.14	-0.006567851	0.207621473	0.005212024	0.138947444	-0.006469475	0.214518204	0.005212113	0.1389417	-9.84E-05	0.029574031	1	0.623552391	1	0.633044396	ENSG00000121898	8	CPXM2
SAT	CAD	ENSG00000107443.11	0.007412605	0.169650382	0.005397541	0.138947359	0.007501787	0.164579615	0.00539761	0.138941589	-8.92E-05	0.043975304	1	0.579212177	1	0.576272733	ENSG00000121898	8	CPXM2
SAT	CAD	ENSG00000107798.13	0.006951097	0.038261192	0.003354694	0.138946672	0.007024234	0.036277485	0.003354763	0.138940889	-7.31E-05	0.01675441	1	0.337586455	1	0.340087662	ENSG00000121957	8	GPSM2
SAT	CAD	ENSG00000112667.8	0.001105245	0.632312909	0.002309936	0.138947831	0.00113926	0.621874183	0.002309952	0.13894207	-3.40E-05	0.04899668	1	0.880256991	1	0.872418743	ENSG00000122085	12	MTERFD2
SAT	CAD	ENSG00000119718.6	0.006674678	0.000427677	0.001894917	0.138944359	0.006710809	0.000398018	0.001894949	0.138938563	-3.61E-05	0.028752325	1	0.031133309	1	0.031912061	ENSG00000122085	12	MTERFD2
SAT	CAD	ENSG00000119900.7	-0.0029734	0.407502866	0.003589777	0.138947701	-0.003030044	0.398631308	0.003589806	0.138941936	5.66E-05	0.038477077	1	0.78509507	1	0.776668242	ENSG00000122085	12	MTERFD2
SAT	CAD	ENSG00000121552.3	-1.82E-06	0.999706633	0.004953546	0.138947896	-7.76E-05	0.987498905	0.004953587	0.138942139	7.58E-05	0.044506289	1	0.999715369	1	0.997619483	ENSG00000122176	10	FMOD
SAT	CAD	ENSG00000122417.11	0.005517646	0.107734173	0.003430381	0.138947159	0.005567798	0.104573885	0.0034304	0.138941388	-5.02E-05	0.043025115	1	0.501109823	1	0.506174025	ENSG00000122218	10	COPA
SAT	CAD	ENSG00000122912.10	0.00623342	0.222468023	0.005109389	0.138947472	0.006309748	0.216860095	0.00510943	0.138941704	-7.63E-05	0.049004501	1	0.633338537	1	0.633679313	ENSG00000122367	15	LDB3
SAT	CAD	ENSG00000125388.15	0.004818505	0.000206239	0.001298342	0.138943969	0.004794537	0.00022184	0.001298354	0.138938251	2.40E-05	0.017641472	0.676671469	0.020521943	0.727855892	0.021008952	ENSG00000122376	7	FAM35A
SAT	CAD	ENSG00000129480.8	-0.004155457	0.206255376	0.003287724	0.138947441	-0.004216229	0.199702125	0.003287758	0.13894167	6.08E-05	0.019152623	1	0.623552391	1	0.619657947	ENSG00000122376	7	FAM35A
SAT	CAD	ENSG00000134201.6	0.00357279	0.383073996	0.004096075	0.13894768	0.003654292	0.372327332	0.004096174	0.138941912	-8.15E-05	0.035732128	1	0.7654481	1	0.756881026	ENSG00000122376	7	FAM35A
SAT	CAD	ENSG00000136279.14	0.002254101	0.63035977	0.00468413	0.13894783	0.002175085	0.642399885	0.004684188	0.138942078	7.90E-05	0.038261571	1	0.879715187	1	0.881519876	ENSG00000122376	7	FAM35A
SAT	CAD	ENSG00000137404.10	0.00624555	0.158170507	0.004425533	0.138947329	0.006906668	0.119499015	0.004436216	0.138941448	-0.000661118	0.032407465	1	0.568778433	1	0.518715438	ENSG00000122417	11	ODF2L
SAT	CAD	ENSG00000137509.6	0.007161061	0.064779481	0.003877591	0.138946924	0.007109954	0.066714046	0.003877593	0.138941181	5.11E-05	0.043818279	1	0.412224502	1	0.422893011	ENSG00000122417	11	ODF2L
SAT	CAD	ENSG00000139624.8	-0.005559453	0.188124041	0.004224024	0.138947403	-0.005681154	0.17866578	0.004224294	0.138941624	0.000121701	0.026904105	1	0.599839629	1	0.593087199	ENSG00000122417	11	ODF2L
SAT	CAD	ENSG00000146555.14	0.000216712	0.930559356	0.002486904	0.138947894	0.001211376	0.631519737	0.002525862	0.138942074	-0.000994664	0.024491357	1	0.980888267	1	0.877853708	ENSG00000122477	8	LRRC39
SAT	CAD	ENSG00000147457.9	0.004037462	0.330193472	0.004146408	0.138947626	0.004279782	0.30213165	0.004147582	0.138941836	-0.00024232	0.017775945	1	0.730754359	1	0.705646132	ENSG00000122477	8	LRRC39
SAT	CAD	ENSG00000149489.4	-0.000976192	0.684903217	0.002405708	0.13894785	-0.000877164	0.715440927	0.002406099	0.138942101	-9.90E-05	0.031446406	1	0.903579074	1	0.911950925	ENSG00000122477	8	LRRC39
SAT	CAD	ENSG00000153714.5	-0.005599754	0.153035795	0.003918947	0.138947314	-0.005534787	0.157862689	0.003918992	0.138941571	-6.50E-05	0.038270596	1	0.56761721	1	0.575497204	ENSG00000122477	8	LRRC39
SAT	CAD	ENSG00000154240.12	0.005341101	0.25037092	0.004646657	0.13894752	0.004928371	0.289344346	0.004651328	0.138941819	0.00041273	0.049918068	1	0.652182137	1	0.691433941	ENSG00000122481	12	RWDD3
SAT	CAD	ENSG00000156603.10	0.004305926	0.012499823	0.001723943	0.138946118	0.004464766	0.009680011	0.001725803	0.138940231	-0.000158841	0.049601477	1	0.219747192	1	0.194847333	ENSG00000122481	12	RWDD3
SAT	CAD	ENSG00000157540.15	0.004626933	0.42064541	0.005745583	0.138947712	0.004388879	0.445016493	0.005746485	0.138941973	0.000238055	0.027939679	1	0.791817322	1	0.801408187	ENSG00000122483	13	CCDC18
SAT	CAD	ENSG00000161813.16	0.010042488	0.013180057	0.004051227	0.138946144	0.009414926	0.020412167	0.004060447	0.138940606	0.000627562	0.022329621	1	0.222647204	1	0.275231745	ENSG00000122483	13	CCDC18
SAT	CAD	ENSG00000161996.13	0.000131727	0.952528287	0.002212697	0.138947895	0.000167933	0.939503451	0.002212724	0.138942138	-3.62E-05	0.043991116	1	0.988062414	1	0.984697834	ENSG00000122483	13	CCDC18
SAT	CAD	ENSG00000163281.7	0.001842795	0.211676676	0.001475452	0.138947452	0.001868489	0.205383253	0.001475475	0.138941682	-2.57E-05	0.040657014	1	0.625685743	1	0.623946715	ENSG00000122507	16	BBS9
SAT	CAD	ENSG00000166348.13	-0.001058529	0.75513997	0.003394144	0.138947869	-0.000433604	0.898722554	0.003406823	0.138942135	-0.000624925	0.033811448	1	0.930016551	1	0.971567941	ENSG00000122565	14	CBX3
SAT	CAD	ENSG00000166669.9	-0.005127122	0.304037104	0.004988367	0.138947595	-0.005046403	0.311719463	0.004988429	0.138941847	-8.07E-05	0.046702112	1	0.706076325	1	0.711630554	ENSG00000122674	8	CCZ1
SAT	CAD	ENSG00000166801.11	-0.002212667	0.216981788	0.00179222	0.138947462	-0.001948865	0.278199397	0.001797225	0.138941804	-0.000263802	0.049868097	1	0.627239866	1	0.684502895	ENSG00000122678	10	POLM
SAT	CAD	ENSG00000168000.10	-0.002779491	0.122561953	0.001800058	0.138947217	-0.002666286	0.138715736	0.001800823	0.138941514	-0.000113205	0.035161326	1	0.520011049	1	0.544767853	ENSG00000122678	10	POLM
SAT	CAD	ENSG00000168702.12	0.003590249	0.443227085	0.004682389	0.138947729	0.001051925	0.828060931	0.004843357	0.138942126	0.002538324	0.040434387	1	0.800421792	1	0.953956431	ENSG00000122692	7	SMU1
SAT	CAD	ENSG00000169519.15	-0.002561811	0.456289306	0.003438804	0.138947738	-0.002512569	0.464992856	0.00343881	0.138941987	-4.92E-05	0.032552587	1	0.807488986	1	0.810911439	ENSG00000122692	7	SMU1
SAT	CAD	ENSG00000171502.10	0.001108136	0.625738753	0.002272011	0.138947829	0.001068259	0.638228535	0.002272034	0.138942076	3.99E-05	0.02502442	1	0.878873651	1	0.87989599	ENSG00000122696	8	SLC25A51
SAT	CAD	ENSG00000173530.5	-0.000872846	0.759078911	0.002846013	0.13894787	-0.002082385	0.473562357	0.002905537	0.138941993	0.00120954	0.038813191	1	0.930057215	1	0.811850954	ENSG00000122696	8	SLC25A51
SAT	CAD	ENSG00000175445.10	-0.018302836	7.19E-07	0.003692923	0.138940893	-0.020132155	8.84E-08	0.003763553	0.138933981	0.001829319	0.011765093	0.002359439	0.000471888	0.00028992	7.25E-05	ENSG00000122696	8	SLC25A51
SAT	CAD	ENSG00000176933.4	0.000424441	0.905501244	0.003575296	0.138947892	0.000879786	0.80580301	0.003578635	0.138942122	-0.000455345	0.003566669	1	0.972806019	1	0.945895506	ENSG00000122696	8	SLC25A51
SAT	CAD	ENSG00000181092.5	-0.007485109	0.012191397	0.002986202	0.138946105	-0.00967918	0.002071165	0.003142744	0.138939435	0.002194071	0.025125208	1	0.217391164	1	0.090606557	ENSG00000122696	8	SLC25A51
SAT	CAD	ENSG00000181126.9	0.003113916	0.086078126	0.001814148	0.138947056	0.002627906	0.14996908	0.001825387	0.138941548	0.00048601	0.016432398	1	0.456355415	1	0.570769052	ENSG00000122696	8	SLC25A51
SAT	CAD	ENSG00000182240.11	-0.003176849	0.244159462	0.002727718	0.13894751	-0.004323825	0.116485216	0.002754552	0.138941437	0.001146976	0.002816364	1	0.651832164	1	0.516412287	ENSG00000122733	11	KIAA1045
SAT	CAD	ENSG00000182534.9	-0.002784091	0.129559691	0.001836662	0.138947241	-0.002755611	0.133530299	0.001836673	0.138941497	-2.85E-05	0.03472134	1	0.530136906	1	0.539205888	ENSG00000122733	11	KIAA1045
SAT	CAD	ENSG00000183486.8	-0.002178298	0.627477281	0.004488736	0.138947829	-0.001300153	0.773110271	0.00450958	0.138942116	-0.000878145	0.043055588	1	0.879075282	1	0.931084711	ENSG00000122783	12	C7orf49
SAT	CAD	ENSG00000184574.5	-0.00926804	0.021784878	0.004039937	0.138946396	-0.0091782	0.023099809	0.00404008	0.138940668	-8.98E-05	0.035577533	1	0.272809861	1	0.288176704	ENSG00000122783	12	C7orf49
SAT	CAD	ENSG00000185344.9	0.000778616	0.85884548	0.004378081	0.138947887	0.000847965	0.846424494	0.004378119	0.138942129	-6.93E-05	0.038843169	1	0.965404111	1	0.958221905	ENSG00000122783	12	C7orf49
SAT	CAD	ENSG00000185499.12	0.00790488	0.041641646	0.003880478	0.138946713	0.007990161	0.039490991	0.003880528	0.13894093	-8.53E-05	0.007496864	1	0.345015756	1	0.346299459	ENSG00000122783	12	C7orf49
SAT	CAD	ENSG00000185684.8	-0.000723642	0.596179577	0.001365612	0.138947816	-0.000746037	0.584860305	0.001365619	0.138942054	2.24E-05	0.021662353	1	0.871671099	1	0.86205151	ENSG00000122783	12	C7orf49
SAT	CAD	ENSG00000186766.7	-0.002557469	0.353057425	0.002753887	0.138947651	-0.002514846	0.361143377	0.002753915	0.138941902	-4.26E-05	0.049120674	1	0.747968053	1	0.750418885	ENSG00000122863	5	CHST3
SAT	CAD	ENSG00000196301.3	0.00250371	0.152876548	0.001751524	0.138947314	9.85E-06	0.996111975	0.002021113	0.138942139	0.002493861	0.013409004	1	0.56761721	1	0.999893579	ENSG00000122873	7	CISD1
SAT	CAD	ENSG00000196821.5	0.012308291	1.38E-06	0.002549715	0.138941252	0.012407021	1.14E-06	0.002550004	0.13893539	-9.87E-05	0.018183008	0.004541695	0.000674511	0.003747179	0.00062453	ENSG00000122873	7	CISD1
SAT	CAD	ENSG00000196968.6	0.002555329	0.557512574	0.004356612	0.138947798	0.002684534	0.53779347	0.004356923	0.138942031	-0.000129204	0.029070513	1	0.856342959	1	0.846069608	ENSG00000122912	10	SLC25A16
SAT	CAD	ENSG00000197217.8	-0.00372103	0.367639824	0.004130329	0.138947665	-0.003809936	0.35631993	0.004130455	0.138941897	8.89E-05	0.033496495	1	0.751266179	1	0.748429484	ENSG00000122912	10	SLC25A16
SAT	CAD	ENSG00000198315.6	-0.003590563	0.440266645	0.004652533	0.138947727	-0.003937231	0.397561139	0.004654016	0.138941935	0.000346668	0.004249313	1	0.799399481	1	0.776418397	ENSG00000122912	10	SLC25A16
SAT	CAD	ENSG00000203705.6	0.000695599	0.734744051	0.002052994	0.138947864	0.00066168	0.747228359	0.002053017	0.13894211	3.39E-05	0.037666001	1	0.921273782	1	0.925296786	ENSG00000122912	10	SLC25A16
SAT	CAD	ENSG00000204227.4	-0.001741266	0.683013118	0.004264098	0.138947849	-0.002672328	0.532514511	0.004281392	0.138942028	0.000931061	0.015703825	1	0.903565193	1	0.844456312	ENSG00000122912	10	SLC25A16
SAT	CAD	ENSG00000204228.3	-0.000466305	0.895546698	0.00355174	0.138947892	-0.001533922	0.668088882	0.003577473	0.138942087	0.001067618	0.012809164	1	0.970661333	1	0.897319103	ENSG00000122952	12	ZWINT
SAT	CAD	ENSG00000204314.6	0.007507119	0.018389926	0.003184113	0.138946312	0.005850467	0.074134146	0.003276126	0.13894123	0.001656652	0.031708684	1	0.25766667	1	0.439050783	ENSG00000123009	3	NME2P1
SAT	CAD	ENSG00000204344.10	-0.002191519	0.332103073	0.002259561	0.138947628	-0.001617334	0.476569624	0.002272075	0.138941995	-0.000574186	0.016093629	1	0.731123848	1	0.811850954	ENSG00000123096	7	SSPN
SAT	CAD	ENSG00000204410.10	0.007410801	0.044338383	0.003685347	0.138946743	0.005405201	0.156560517	0.003815247	0.138941567	0.002005599	0.042222422	1	0.353926215	1	0.574768189	ENSG00000123096	7	SSPN

SAT	CAD	ENSG00000204427.7	-0.007142202	0.013304448	0.002885128	0.138946149	-0.008442926	0.003792174	0.002916417	0.13893975	0.001300724	0.002290483	1	0.222713752	1	0.123298635	ENSG00000123096	7	SSPN
SAT	CAD	ENSG00000204463.8	0.006127681	0.00143178	0.001921997	0.138944998	0.004956171	0.0130285	0.001996043	0.138940381	0.00117151	0.029664113	1	0.071330396	1	0.233587473	ENSG00000123143	8	PKN1
SAT	CAD	ENSG00000204520.8	0.000433978	0.714118468	0.001184662	0.138947858	7.68E-05	0.948613161	0.001191463	0.138942138	0.00035719	0.005035063	1	0.912127964	1	0.985387556	ENSG00000123143	8	PKN1
SAT	CAD	ENSG00000204540.6	0.001241723	0.13531404	0.000831433	0.13894726	0.000971295	0.248810348	0.000842228	0.13894176	0.000270428	0.044388103	1	0.535543265	1	0.656525015	ENSG00000123179	9	EBPL
SAT	CAD	ENSG00000204622.6	-0.000715445	0.691459944	0.001802697	0.138947852	-0.001455651	0.425459086	0.001826443	0.138941958	0.000740205	0.01175202	1	0.904577383	1	0.794497018	ENSG00000123179	9	EBPL
SAT	CAD	ENSG00000206341.6	0.001985428	0.360005967	0.002169026	0.138947658	0.001397851	0.521998587	0.002183227	0.138942022	0.000587577	0.018284524	1	0.750654992	1	0.838314911	ENSG00000123179	9	EBPL
SAT	CAD	ENSG00000206344.6	0.00291739	0.009696312	0.001127936	0.138945989	0.002422701	0.035072893	0.00114955	0.138940873	0.000494689	0.025840009	1	0.189366663	1	0.340042469	ENSG00000123191	9	ATP7B
SAT	CAD	ENSG00000213676.6	0.00466321	0.005455483	0.001678112	0.138945695	0.003812417	0.027468109	0.001729139	0.138940753	0.000850793	0.041366394	1	0.146128675	1	0.310609304	ENSG00000123191	9	ATP7B
SAT	CAD	ENSG00000213760.6	0.002110286	0.212760974	0.001693634	0.138947454	0.001231389	0.48073529	0.001746352	0.138941998	0.000878897	0.039092398	1	0.626499199	1	0.814411031	ENSG00000123191	9	ATP7B
SAT	CAD	ENSG00000214087.4	0.001333989	0.118111318	0.000853615	0.1389472	0.001348444	0.114183884	0.000853626	0.138941428	-1.45E-05	0.038909404	1	0.511544451	1	0.516412287	ENSG00000123213	18	NLN
SAT	CAD	ENSG00000223865.6	0.001270849	0.709641368	0.00341316	0.138947857	0.002502868	0.470136528	0.003465341	0.138941991	-0.001232019	0.039876809	1	0.911825726	1	0.811850954	ENSG00000123213	18	NLN
SAT	CAD	ENSG00000224796.1	-0.001632355	0.114322303	0.001033749	0.138947186	-0.001085636	0.308717331	0.001066525	0.138941844	-0.000546719	0.037238618	1	0.511544451	1	0.709397581	ENSG00000123213	18	NLN
SAT	CAD	ENSG00000227766.1	-0.002506043	0.262287905	0.00223554	0.138947538	-0.003165128	0.159323675	0.002248995	0.138941575	0.000659085	0.007398291	1	0.659183967	1	0.575903653	ENSG00000123243	10	ITIH5
SAT	CAD	ENSG00000229390.1	1.49E-05	0.995993452	0.002971627	0.138947896	-0.000623616	0.834265159	0.002980464	0.138942127	0.000638538	0.005527651	1	0.999715369	1	0.954845447	ENSG00000123374	6	CDK2
SAT	CAD	ENSG00000230521.1	0.002810558	0.036733164	0.001345587	0.138946652	0.002914524	0.030384141	0.001346168	0.138940803	-0.000103966	0.010259593	1	0.337152794	1	0.323670021	ENSG00000123374	6	CDK2
SAT	CAD	ENSG00000231925.7	0.0003086	0.894658258	0.002330596	0.138947891	0.000857748	0.713695548	0.00233784	0.138942101	-0.000549148	0.002918219	1	0.970661333	1	0.911950925	ENSG00000123415	10	SMUG1
SAT	CAD	ENSG00000233265.1	0.001341371	0.359539199	0.001463988	0.138947657	0.001030439	0.482892619	0.001468577	0.138941999	0.000310932	0.007546212	1	0.750414105	1	0.814411031	ENSG00000123415	10	SMUG1
SAT	CAD	ENSG00000233892.1	-0.00785316	0.056422538	0.004116425	0.138946859	-0.008734037	0.034407299	0.004129038	0.138940864	0.000880877	0.006489808	1	0.382484189	1	0.336986116	ENSG00000123415	10	SMUG1
SAT	CAD	ENSG00000233916.1	-0.004878657	0.037885516	0.002349918	0.138946667	-0.005380504	0.022597717	0.002359689	0.138940657	0.000501847	0.019627	1	0.337586455	1	0.287074915	ENSG00000123427	11	METTL21B
SAT	CAD	ENSG00000234127.4	-0.00445109	0.118046047	0.00284773	0.1389472	-0.005552498	0.053620597	0.002877101	0.138941077	0.001101408	0.007292043	1	0.511544451	1	0.38135058	ENSG00000123427	11	METTL21B
SAT	CAD	ENSG00000234745.5	0.007457169	0.00035946	0.00208989	0.138944266	0.006646833	0.001725514	0.002120994	0.138939339	0.000810336	0.025237075	1	0.028765591	1	0.085131734	ENSG00000123453	12	SARDH
SAT	CAD	ENSG00000236710.1	0.002442282	0.600758502	0.004666985	0.138947818	0.002373189	0.611101501	0.00466702	0.138942066	6.91E-05	0.04804071	1	0.871671099	1	0.869331532	ENSG00000123453	12	SARDH
SAT	CAD	ENSG00000237984.2	0.00157825	0.756205953	0.00508347	0.138947869	0.001659558	0.744077114	0.005083509	0.138942109	-8.13E-05	0.033456109	1	0.930016551	1	0.924790073	ENSG00000123453	12	SARDH
SAT	CAD	ENSG00000241106.2	-0.001584536	0.172000104	0.001160146	0.138947365	-0.001852952	0.11244666	0.001167365	0.138941421	0.000268417	0.038720186	1	0.580279266	1	0.516412287	ENSG00000123454	6	DBH
SAT	CAD	ENSG00000241549.4	-0.000567078	0.685188886	0.001398836	0.13894785	-0.000490485	0.725933462	0.001399226	0.138942104	-7.66E-05	0.025295129	1	0.903579074	1	0.917446451	ENSG00000123454	6	DBH
SAT	CAD	ENSG00000253304.1	0.006763865	0.141291099	0.004598133	0.138947279	0.00640042	0.164163833	0.004600629	0.138941587	0.000363446	0.018545035	1	0.545530242	1	0.576272733	ENSG00000123454	6	DBH
SAT	CAD	ENSG00000256087.2	0.003645696	0.351338003	0.0039117	0.138947649	0.003707894	0.343187102	0.003911745	0.138941883	-6.22E-05	0.047657794	1	0.745627419	1	0.742742007	ENSG00000123545	5	NDUFAF4
SAT	CAD	ENSG00000260947.1	0.000245779	0.834224795	0.001174365	0.138947884	0.000266217	0.82066642	0.001174376	0.138942125	-2.04E-05	0.025294694	1	0.956013789	1	0.951114986	ENSG00000123545	5	NDUFAF4
SAT	CAD	ENSG00000261586.2	0.00115196	0.73593201	0.00341579	0.138947864	0.00132846	0.697403069	0.00341657	0.138942096	-0.000176501	0.020672756	1	0.921952243	1	0.905849293	ENSG00000123545	5	NDUFAF4
SAT	CAD	ENSG00000262370.1	0.004853072	0.015368844	0.002002442	0.138946222	0.004824808	0.015976944	0.002002452	0.138940484	2.83E-05	0.04878891	1	0.241268794	1	0.26048278	ENSG00000123545	5	NDUFAF4
SAT	CAD	ENSG00000272003.1	-0.000678167	0.89581302	0.005178732	0.138947892	-0.000763496	0.882794232	0.00517877	0.138942133	8.53E-05	0.027938342	1	0.970661333	1	0.966771654	ENSG00000123643	8	SLC36A1
SAT	CAD	ENSG00000272810.1	0.003424596	0.133049956	0.002279743	0.138947253	0.003192389	0.161751011	0.002281567	0.138941581	0.000232207	0.011941262	1	0.533294064	1	0.576272733	ENSG00000123643	8	SLC36A1
SAT	Heart_Failure	ENSG00000000457.9	0.004567263	0.134131938	0.003048892	0.076055143	0.004494403	0.140408654	0.003048555	0.076035001	7.29E-05	0.000291797	1	0.659800431	1	0.655068096	ENSG00000123643	8	SLC36A1
SAT	Heart_Failure	ENSG00000001561.6	-0.00305141	0.062748954	0.001639688	0.076054953	-0.003085676	0.059826321	0.001639507	0.076034787	3.43E-05	0.001818478	1	0.515988262	1	0.504627254	ENSG00000123643	8	SLC36A1
SAT	Heart_Failure	ENSG00000001617.7	-0.013232957	9.42E-07	0.00269868	0.076051741	-0.013173139	1.05E-06	0.002698406	0.076031622	-5.98E-05	0.004825078	0.003089965	0.000233648	0.003449752	0.000581811	ENSG00000123643	8	SLC36A1
SAT	Heart_Failure	ENSG00000004809.9	0.003058205	0.409107228	0.00370482	0.076055387	0.002974887	0.421948702	0.003704505	0.07603524	8.33E-05	0.021078539	1	0.870429337	1	0.873390936	ENSG00000123815	7	ADCK4
SAT	Heart_Failure	ENSG00000004897.7	0.001149478	0.367916714	0.001276654	0.076055366	0.00116673	0.36071871	0.001276514	0.07603521	-1.73E-05	0.043817774	1	0.853098755	1	0.848283315	ENSG00000123815	7	ADCK4
SAT	Heart_Failure	ENSG00000005243.5	0.003076803	0.199831098	0.002399937	0.076055236	0.003029419	0.206797629	0.002399688	0.076035091	4.74E-05	0.009114902	1	0.734105989	1	0.73263258	ENSG00000123815	7	ADCK4
SAT	Heart_Failure	ENSG00000005801.12	-0.000570225	0.785017296	0.002090369	0.076055481	-0.000623786	0.765366292	0.002090145	0.076035326	5.36E-05	0.000320848	1	0.972730696	1	0.965963214	ENSG00000123815	7	ADCK4
SAT	Heart_Failure	ENSG00000005955.8	-0.004545118	0.068513208	0.002495096	0.076054975	-0.004590538	0.065767075	0.002494841	0.076034812	4.54E-05	0.019419391	1	0.537536555	1	0.515872519	ENSG00000123815	7	ADCK4
SAT	Heart_Failure	ENSG00000006704.6	0.002448139	0.47029459	0.003390772	0.076055412	0.002541269	0.453534066	0.003390453	0.076035252	-9.31E-05	0.001688897	1	0.893630105	1	0.886796943	ENSG00000123815	7	ADCK4
SAT	Heart_Failure	ENSG00000006744.14	0.002718464	0.365926188	0.003006713	0.076055365	0.002654612	0.377242412	0.003006392	0.076035218	6.39E-05	0.002992921	1	0.85158824	1	0.854967426	ENSG00000123870	9	ZNF137P
SAT	Heart_Failure	ENSG00000007341.14	-0.002792347	0.008490783	0.001060956	0.076054412	-0.00281035	0.008069339	0.001060843	0.076034245	1.80E-05	0.01884834	1	0.207897455	1	0.209225934	ENSG00000123870	9	ZNF137P
SAT	Heart_Failure	ENSG00000008324.6	-0.002183536	0.555004099	0.003699162	0.076055439	-0.002235297	0.54561867	0.003698749	0.076035283	5.18E-05	0.030262966	1	0.921542737	1	0.912597963	ENSG00000123989	9	CHPF
SAT	Heart_Failure	ENSG00000008710.13	0.001629777	0.549598545	0.00272373	0.076055437	0.001586787	0.560135246	0.002723439	0.076035287	4.30E-05	0.027529248	1	0.918172602	1	0.917983887	ENSG00000123989	9	CHPF
SAT	Heart_Failure	ENSG00000008853.12	0.001419368	0.653232032	0.003159226	0.076055462	0.001481845	0.639003084	0.003158954	0.076035306	-6.25E-05	0.040312447	1	0.962760695	1	0.949533115	ENSG00000124098	9	FAM210B
SAT	Heart_Failure	ENSG00000010219.9	-0.001820299	0.63650251	0.003851711	0.076055458	-0.001764398	0.646859034	0.003851301	0.076035307	-5.59E-05	0.043803552	1	0.959428651	1	0.95317119	ENSG00000124104	14	SNX21
SAT	Heart_Failure	ENSG00000010803.12	-0.002840419	0.430516063	0.003603177	0.076055396	-0.002683984	0.456293075	0.003602831	0.076035254	-0.000156435	3.48E-07	1	0.878825835	1	0.887431878	ENSG00000124104	14	SNX21
SAT	Heart_Failure	ENSG00000011376.5	0.002606592	0.490517508	0.003780483	0.076055419	0.002483206	0.511233943	0.003780085	0.076035273	0.000123386	9.00E-06	1	0.898909993	1	0.902750226	ENSG00000124104	14	SNX21
SAT	Heart_Failure	ENSG00000011485.10	0.00072956	0.825143407	0.003302153	0.076055485	0.000780319	0.813175575	0.003301799	0.076035331	-5.08E-05	0.030860685	1	0.976669362	1	0.972791643	ENSG00000124116	14	WFDC3
SAT	Heart_Failure	ENSG00000011523.9	-0.000397812	0.796519124	0.001542776	0.076055483	-0.000354695	0.818144683	0.001542608	0.076035332	-4.31E-05	4.99E-05	1	0.976456935	1	0.972791643	ENSG00000124116	14	WFDC3
SAT	Heart_Failure	ENSG00000013810.14	0.002185777	0.566557965	0.003813764	0.076055442	0.00206008	0.589042367	0.003813377	0.076035295	0.000125696	2.90E-05	1	0.927165893	1	0.934989637	ENSG00000124116	14	WFDC3
SAT	Heart_Failure	ENSG00000014164.6	0.004270483	0.052093403	0.002198599	0.076054904	0.004321713	0.049314796	0.002198378	0.076034737	-5.12E-05	0.003677801	1	0.491119403	1	0.480049081	ENSG00000124116	14	WFDC3
SAT	Heart_Failure	ENSG00000014914.15	0.000258642	0.853060529	0.001396444	0.076055488	0.000162302	0.907490302	0.001396689	0.076035338	9.63E-05	0.005474731	1	0.976669362	1	0.982067422	ENSG00000124116	14	WFDC3
SAT	Heart_Failure	ENSG00000015676.13	0.001157644	0.49518791	0.001697224	0.07605542	0.001184371	0.485237873	0.001697041	0.076035264	-2.67E-05	0.026199616	1	0.898909993	1	0.894608312	ENSG00000124116	14	WFDC3
SAT	Heart_Failure	ENSG00000016864.12	-0.008015698	0.004399542	0.002814491	0.076054227	-0.008103568	0.0039829	0.002814202	0.076034046	8.79E-05	5.27E-05	1	0.154647076	1	0.161355485	ENSG00000124116	14	WFDC3
SAT	Heart_Failure	ENSG00000018189.8	0.001047339	0.63370775	0.002197913	0.076055458	0.001090325	0.619804511	0.00219767	0.076035302	-4.30E-05	0.003133932	1	0.959428651	1	0.94798204	ENSG00000124140	8	SLC12A5
SAT	Heart_Failure	ENSG00000019169.9	-0.004623183	0.122556062	0.002994026	0.076055121	-0.004544778	0.128987669	0.002993711	0.076034981	-7.84E-05	0.00038047	1	0.646585619	1	0.649215182	ENSG00000124164	11	VAPB
SAT	Heart_Failure	ENSG00000022556.11	0.00230165	0.495615855	0.003377802	0.076055421	0.002245519	0.506144477	0.003377463	0.076035271	5.61E-05	0.038137846	1	0.898909993	1	0.900571724	ENSG00000124164	11	VAPB
SAT	Heart_Failure	ENSG00000023041.7	0.000584135	0.854847251	0.003193088	0.076055488	0.000633935	0.842611116	0.003192745	0.076035334	-4.98E-05	0.026881631	1	0.976669362	1	0.972791643	ENSG00000124191	13	TOX2
SAT	Heart_Failure	ENSG00000023191.12	0.000150691	0.937251425	0.001914147	0.076055492	0.000194228	0.91916868	0.001913939	0.076035339	-4.35E-05	0.000993173	1	0.990058572	1	0.984094962	ENSG00000124191	13	TOX2
SAT	Heart_Failure	ENSG00000023572.4	0.00142793	0.5596253	0.002447606	0.07605544	0.001334579	0.585544082	0.002447399	0.076035294	9.34E-05	9.74E-05	1	0.923606947	1	0.933194299	ENSG00000124191	13	TOX2
SAT	Heart_Failure	ENSG00000025708.8	-0.005256494	0.005587046	0.001896902	0.076054295	-0.005205621	0.006061813	0.001896794	0.076034165	-5.09E-05	0.028889585	1	0.164958288	1	0.182466145	ENSG00000124207	12	CSE1L
SAT	Heart_Failure	ENSG00000026297.11	0.001901419	0.589751841	0.003526396	0.076055448	0.001959859	0.578329146	0.003526022	0.076035292	-5.84E-05	0.022571097	1	0.941136084	1	0.929235028	ENSG00000124226	7	RNF114
SAT	Heart_Failure	ENSG00000026950.12	0.000387168	0.762535351	0.001281364	0.076055479	0.000627829	0.624173842	0.001281431	0.076035303	-0.000240661	1.38E-22	1	0.969898847	1	0.94798204	ENSG00000124226	7	RNF114
SAT	Heart_Failure	ENSG00000037042.8	0.000707921	0.617342382	0.001416917	0.076055454	0.000683372	0.629559775	0.001416764	0.076035304	2.45E-05	0.013034655	1	0.954216727	1	0.94798204	ENSG00000124257	6	NEURL2
SAT	Heart_Failure	ENSG00000040531.10	0.000187699	0.883181091	0.001277404	0.07605549	0.000157532	0.901841312	0.001277264	0.076035338	3.02E-05	0.000471844	1	0.979223495	1	0.980373283	ENSG00000124406	12	ATP8A1
SAT	Heart_Failure	ENSG00000040933.11	0.001435264	0.325018336	0.001458307	0.076055342	0.001457967	0.317373628	0.001458156	0.076035184	-2.27E-05	0.039932277	1	0.832463044	1	0.823487262	ENSG00000124422	7	USP22
SAT	Heart_Failure	ENSG00000041353.5	0.001201978	0.749974257	0.003771818	0.076055477	-0.013430079	0.002550661	0.004451053	0.07603392	0.014632058	6.05E-10	1	0.966658386	1	0.122791633	ENSG00000124422	7	USP22
SAT	Heart_Failure	ENSG00000042493.11	-0.000166328	0.958035525	0.003160998	0.076055493	-5.89E-05	0.985133672	0.003160764	0.07603534	-0.000107433	0.001695771	1	0.994671121	1	0.999768899	ENSG00000124508	12	BTN2A2
SAT	Heart_Failure	ENSG00000048544.5	0.000337694	0.646780814	0.000736939	0.07605546	0.000350107	0.63469302	0.000736862	0.076035305	-1.24E-05	0.023859732	1	0.961525986	1	0.94798204	ENSG00000124508	12	BTN2A2
SAT	Heart_Failure	ENSG00000049449.4	-0.005561554	0.106856364	0.003449051	0.076055087	-0.005630068	0.102570088	0.003448692	0.076034924	6.85E-05	0.008562879	1	0.638670167	1	0.614882715	ENSG00000124508	12	BTN2A2
SAT	Heart_Failure	ENSG00000050130.13	0.00202791	0.383435299	0.002326692	0.076055374	0.001710271	0.462351835	0.002326962	0.076035256	0.000317638	9.09E-10	1	0.85759885	1	0.892844363	ENSG00000124508	12	BTN2A2
SAT	Heart_Failure	ENSG00000050405.9	1.39E-05	0.995066858	0.0022477	0.076055493	-0.000724388	0.748782841	0.002261982	0.076035324	0.000738285	0.003985786	1	0.999479422	1	0.962415493	ENSG00000124508	12	BTN2A2
SAT	Heart_Failure	ENSG00000053900.6	0.000973634	0.750031649	0.003055997	0.076055477	0.001086299	0.722212967	0.003055667	0.07603532	-0.000112665	1.61E-07	1	0.966658386	1	0.958028875	ENSG00000124532	10	MRS2
SAT	Heart_Failure	ENSG00000054282.11	0.005935203	0.001385088	0.001856051	0.076053897	0.005971097	0.001293564	0.001855863	0.076033725	-3.59E-05	0.015194877	1	0.077908515	1	0.081932833	ENSG00000124532	10	MRS2
SAT	Heart_Failure	ENSG00000054983.12	0.004040528	0.169446678	0.002940734	0.076055198	0.003995679	0.1741869	0.002940432	0.076035052	4.48E-05	0.04795248	1	0.699678071	1	0.700325828	ENSG00000124532	10	MRS2
SAT	Heart_Failure	ENSG00000055950.12	-0.00441185	0.053664244	0.002286476	0.076054912	-0.004323943	0.058589405	0.002286269	0.076034782	-8.79E-05	2.66E-05	1	0.497196222	1	0.503576399	ENSG00000124532	10	MRS2
SAT	Heart_Failure	ENSG00000055955.11	0.000420186	0.701909992	0.001097826	0.07605547	0.000376923	0.731320671	0.001097719	0.076035322	4.33E-05	2.68E-06	1	0.966658386	1	0.958028875	ENSG00000124532	10	MRS2
SAT	Heart_Failure	ENSG00000056661.9	0.001706105	0.196057155	0.001319628	0.076055232	0.00168372	0.201943284	0.001319492	0.076035086	2.24E-05	0.028313791	1	0.726878652	1	0.728906397	ENSG00000124532	10	MRS2
SAT	Heart_Failure	ENSG00000057468.6	-0.001510863	0.353812797	0.001629455	0.076055359	-0.001472252	0.366204152	0.001629305	0.076035213	-3.86E-05	0.008382042	1	0.848363544	1	0.849290727	ENSG00000124549	10	BTN2A3P
SAT	Heart_Failure	ENSG00000064225.8	-0.003137685	0.242639543	0.002685417	0.07605528	-0.003077473	0.251749911	0.002685145	0.076035135	-6.02E-05	0.00436838	1	0.759865642	1	0.765423527	ENSG00000124549	10	BTN2A3P
SAT	Heart_Failure	ENSG00000064313.7	-0.00867937	0.000962078	0.00262899	0.076053792	-0.008738436	0.000886737	0.00262872	0.076033616	5.91E-05	0.003606746	1	0.05739234	1	0.06612236	ENSG00000124549	10	BTN2A3P
SAT	Heart_Failure	ENSG00000064607.12	-0.002373291	0.436790142	0.003051972	0.076055399	-0.002440298	0.423907683	0.003051666	0.07603524	6.70E-05	0.006293415	1	0.880339889	1	0.873390936	ENSG00000124549	10	BTN2A3P
SAT	Heart_Failure	ENSG00000065268.6	-0.003069696	0.120831458	0.001978795	0.076055117	-0.003015976	0.127435123	0.001978601	0.076034978	-5.37E-05	0.001086062	1	0.646585619	1	0.647874406	ENSG00000124549	10	BTN2A3P
SAT	Heart_Failure	ENSG00000065457.6	-0.000681547	0.627927674	0.001406278	0.076055456	-0.000654954	0.641370097	0.001406131	0.076035306	-2.66E-05	0.011239999	1	0.958246837	1	0.951372499	ENSG00000124549	10	BTN2A3P
SAT	Heart_Failure	ENSG00000066185.8	0.004230695	0.097373613	0.002552109	0.076055064	0.004168724	0.102339947	0.002551837	0.076034924	6.20E-05	0.000727828	1	0.61676221	1	0.614882715	ENSG00000124562	5	SNRPC
SAT	Heart_Failure	ENSG00000066230.6	0.000929224	0.583257981	0.001693709	0.076055446	0.000895207	0.597081501	0.001693536	0.076035297	3.40E-05	0.01013479	1	0.937265914	1	0.938230079	ENSG00000124562	5	SNRPC
SAT	Heart_Failure	ENSG00000066379.10	-0.002484597	0.216675469	0.002011138	0.076055255	-0.003908221	0.057104361	0.002054238	0.076034775	0.001423624	0.00069772	1	0.745333464	1	0.503576399	ENSG00000124562	5	SNRPC
SAT	Heart_Failure	ENSG00000067533.5	-0.00146868	0.202085643	0.001151334	0.076055239	-0.001505525	0.190949892	0.00115121	0.076035073	3.68E-05	7.47E-06	1	0.735350411	1	0.720122525	ENSG00000124562	5	SNRPC
SAT	Heart_Failure	ENSG00000067596.6	-0.003921893	0.10820947	0.002441575	0.07605509	-0.003881224	0.111880356	0.002441327	0.076034946	-4.07E-05	0.033962061	1	0.640599412	1	0.640177219	ENSG00000124562	5	SNRPC
SAT	Heart_Failure	ENSG00000067715.9	-0.002551367	0.450299291	0.003379666	0.076055404	-0.002613326	0.439326924	0.003379311	0.076035247	6.20E-05	0.013405248	1	0.887019168	1	0.879941679	ENSG00000124562	5	SNRPC
SAT	Heart_Failure	ENSG00000068028.13	0.00587648	0.050483208	0.003004563	0.076054896	0.005825159	0.052506045	0.003004261	0.076034754	5.13E-05	0.031715848	1	0.48962926	1	0.490345005	ENSG00000124562	5	SNRPC
SAT	Heart_Failure	ENSG00000068079.3	0.001110418	0.568985561	0.001949653	0.076055442	0.001143518	0.557480813	0.001949441	0.076035286	-3.31E-05	0.013543608	1	0.92942386	1	0.915531921	ENSG00000124570	13	SERPINB6
SAT	Heart_Failure	ENSG00000069869.11	-0.000101692	0.977014127	0.003529454	0.076055493	-0.000158744	0.964121721	0.003529066	0.07603534	5.71E-05	0.016425336	1	0.998920999	1	0.996055789	ENSG00000124575	5	HIST1H1D
SAT	Heart_Failure	ENSG00000069966.14	-0.000133222	0.955045017	0.002363234	0.076055493	-8.74E-05	0.97049246	0.002363005	0.07603534	-4.58E-05	0.02144464	1	0.994131568	1	0.99755193	ENSG00000124575	5	HIST1H1D
SAT	Heart_Failure	ENSG00000069998.8	0.003060918	0.194086633	0.002357116	0.07605523	0.003024686	0.199369878	0.002356866	0.076035083	3.62E-05	0.035198196	1	0.726878652	1	0.726007291	ENSG00000124575	5	HIST1H1D
SAT	Heart_Failure	ENSG00000071967.7	0.004183342	0.189357396	0.003187346	0.076055224	0.00423513	0.183893279	0.003187029	0.076035065	-5.18E-05	0.045490116	1	0.722420486	1	0.715224819	ENSG00000124587	9	PEX6
SAT	Heart_Failure	ENSG00000072364.8	-0.000757262	0.798170561	0.002961345	0.076055483	-0.000687688	0.816346676	0.002961027	0.076035332	-6.96E-05	0.000885754	1	0.976669362	1	0.972791643	ENSG00000124587	9	PEX6
SAT	Heart_Failure	ENSG00000072864.8	-0.001360002	0.697524081	0.003499161	0.076055469	-0.001266663	0.717334847	0.003498845	0.07603532	-9.33E-05	0.003709865	1	0.966658386	1	0.958028875	ENSG00000124587	9	PEX6
SAT	Heart_Failure	ENSG00000073008.10	0.002988902	0.23385553	0.002510655	0.076055272	0.002938816	0.241734409	0.002510383	0.076035126	5.01E-05	0.004342788	1	0.754244409	1	0.753454838	ENSG00000124613	4	ZNF391
SAT	Heart_Failure	ENSG00000073417.10	0.003694631	0.234480127	0.003107608	0.076055272	0.003519413	0.257513483	0.003108228	0.07603514	0.000175218	0.028677901	1	0.754244409	1	0.770781077	ENSG00000124613	4	ZNF391
SAT	Heart_Failure	ENSG00000073584.14	0.001585613	0.64538377	0.003445601	0.07605546	0.001693222	0.623097415	0.00344525	0.076035302	-0.000107608	6.41E-05	1	0.960776086	1	0.94798204	ENSG00000124613	4	ZNF391
SAT	Heart_Failure	ENSG00000075151.15	6.32E-05	0.985498562	0.003475668	0.076055493	0.000140844	0.967672862	0.00347531	0.07603534	-7.77E-05	0.003598079	1	0.998924529	1	0.99755193	ENSG00000124613	4	ZNF391
SAT	Heart_Failure	ENSG00000076242.10	-0.000774803	0.768509587	0.002632472	0.076055479	-0.000691014	0.792917402	0.002632193	0.076035329	-8.38E-05	1.21E-05	1	0.971467105	1	0.970635778	ENSG00000124613	4	ZNF391
SAT	Heart_Failure	ENSG00000076685.14	0.002419669	0.523126846	0.003789417	0.076055429	0.002521902	0.505683141	0.003789059	0.076035271	-0.000102233	0.001933522	1	0.906724424	1	0.900571724	ENSG00000124613	4	ZNF391
SAT	Heart_Failure	ENSG00000077147.10	-0.004535782	0.148679718	0.00314066	0.076055167	-0.004590067	0.143834844	0.003140309	0.076035007	5.43E-05	0.007463831	1	0.674894985	1	0.659101398	ENSG00000124613	4	ZNF391
SAT	Heart_Failure	ENSG00000077616.6	0.001573637	0.690014464	0.003945577	0.076055468	0.001470552	0.709336339	0.003945165	0.076035318	0.000103085	0.000497306	1	0.966658386	1	0.958028875	ENSG00000124613	4	ZNF391
SAT	Heart_Failure	ENSG00000079150.13	-0.00149339	0.36782241	0.001658289	0.076055366	-0.001455333	0.380103231	0.001658108	0.07603522	-3.81E-05	0.000768441	1	0.853098755	1	0.854967426	ENSG00000124613	4	ZNF391
SAT	Heart_Failure	ENSG00000079257.3	0.002337701	0.528763572	0.003711255	0.076055431	0.002223654	0.549021327	0.003710866	0.076035284	0.000114047	3.63E-05	1	0.907158447	1	0.91411374	ENSG00000124613	4	ZNF391
SAT	Heart_Failure	ENSG00000079335.13	-0.002979047	0.203100623	0.002340603	0.07605524	-0.002555689	0.27569769	0.002344591	0.076035155	-0.000423358	0.002845235	1	0.735350411	1	0.785211912	ENSG00000124635	7	HIST1H2BJ
SAT	Heart_Failure	ENSG00000079950.9	0.001093729	0.704041704	0.002879215	0.07605547	0.000965363	0.737389792	0.002878998	0.076035323	0.000128366	3.14E-05	1	0.966658386	1	0.960499534	ENSG00000124635	7	HIST1H2BJ
SAT	Heart_Failure	ENSG00000080345.13	0.000953504	0.552319529	0.001604446	0.076055438	0.000998148	0.533823697	0.001604275	0.07603528	-4.46E-05	0.000109971	1	0.920018143	1	0.908441675	ENSG00000124641	10	MED20
SAT	Heart_Failure	ENSG00000082516.8	0.000140407	0.962414372	0.00297951	0.076055493	0.000186815	0.95000018	0.002979199	0.07603534	-4.64E-05	0.037418439	1	0.995788523	1	0.990755659	ENSG00000124641	10	MED20
SAT	Heart_Failure	ENSG00000083099.6	-0.001141517	0.46385963	0.00155837	0.076055409	-0.001163854	0.455110774	0.001558199	0.076035253	2.23E-05	0.032614847	1	0.890605014	1	0.886989673	ENSG00000124641	10	MED20
SAT	Heart_Failure	ENSG00000083290.15	-0.000993898	0.751230219	0.003135149	0.076055477	-0.001043878	0.739136494	0.003134794	0.076035323	5.00E-05	0.010265387	1	0.966658386	1	0.960499534	ENSG00000124659	5	TBCC
SAT	Heart_Failure	ENSG00000083828.11	-0.007471402	0.016610468	0.003119288	0.076054598	-0.007559457	0.015363648	0.003118974	0.076034424	8.81E-05	0.0004094	1	0.287798638	1	0.276579386	ENSG00000124659	5	TBCC
SAT	Heart_Failure	ENSG00000084072.12	0.001668641	0.522324317	0.0026082	0.076055429	0.001701301	0.51416663	0.002607902	0.076035274	-3.27E-05	0.037177761	1	0.906724424	1	0.903395669	ENSG00000124678	13	TCP11
SAT	Heart_Failure	ENSG00000085117.7	0.00427832	0.149252189	0.002966564	0.076055168	0.004211528	0.155661307	0.002966245	0.076035026	6.68E-05	0.001466662	1	0.675317996	1	0.669469928	ENSG00000124678	13	TCP11
SAT	Heart_Failure	ENSG00000086300.11	-8.20E-06	0.99225804	0.00084495	0.076055493	-3.37E-05	0.968228696	0.000844865	0.07603534	2.55E-05	0.000170132	1	0.999479422	1	0.99755193	ENSG00000124678	13	TCP11
SAT	Heart_Failure	ENSG00000086475.10	0.002544857	0.533896297	0.004090952	0.076055433	0.002443768	0.550228113	0.004090545	0.076035284	0.000101089	0.002376456	1	0.908781401	1	0.914465015	ENSG00000124678	13	TCP11
SAT	Heart_Failure	ENSG00000087008.11	0.009661948	0.010763432	0.003788558	0.076054478	0.009742199	0.010118984	0.003788164	0.076034308	-8.03E-05	0.005051281	1	0.232334342	1	0.230558242	ENSG00000124678	13	TCP11
SAT	Heart_Failure	ENSG00000087191.8	0.004330566	0.037216579	0.002078619	0.076054816	0.004260946	0.040369073	0.002078553	0.076034684	6.96E-05	0.01800951	1	0.436153525	1	0.452859815	ENSG00000124713	5	GNMT
SAT	Heart_Failure	ENSG00000087448.5	-0.001020678	0.748169388	0.003179144	0.076055477	-0.001143061	0.719155699	0.003178812	0.07603532	0.000122383	2.51E-07	1	0.966658386	1	0.958028875	ENSG00000124713	5	GNMT
SAT	Heart_Failure	ENSG00000088298.8	-0.002334306	0.186661883	0.001767717	0.076055221	-0.002237239	0.20561039	0.001767548	0.07603509	-9.71E-05	1.77E-10	1	0.720656885	1	0.731678624	ENSG00000124713	5	GNMT
SAT	Heart_Failure	ENSG00000088325.11	-0.000516339	0.803474373	0.002074873	0.076055483	-0.000424857	0.837741823	0.002074672	0.076035334	-9.15E-05	1.69E-07	1	0.976669362	1	0.972791643	ENSG00000124713	5	GNMT
SAT	Heart_Failure	ENSG00000088538.12	-0.006544938	0.074664892	0.003671731	0.076054997	-0.006502063	0.076553372	0.003671308	0.076034851	-4.29E-05	0.048246619	1	0.564459699	1	0.560403102	ENSG00000124713	5	GNMT
SAT	Heart_Failure	ENSG00000089063.10	-0.006588938	0.057551806	0.003469504	0.07605493	-0.006498764	0.061029189	0.003469174	0.076034793	-9.02E-05	0.002665849	1	0.503931718	1	0.505632969	ENSG00000124713	5	GNMT
SAT	Heart_Failure	ENSG00000089558.4	0.008326065	0.004741784	0.002948235	0.076054248	0.00841109	0.004328019	0.002947927	0.07603407	-8.50E-05	0.00011856	1	0.157149427	1	0.161355485	ENSG00000124733	3	MEA1
SAT	Heart_Failure	ENSG00000090054.9	0.000300058	0.908104878	0.002599498	0.076055491	0.00042242	0.870900036	0.002599263	0.076035336	-0.000122362	2.83E-07	1	0.983702811	1	0.974665278	ENSG00000124733	3	MEA1
SAT	Heart_Failure	ENSG00000090238.7	0.000179575	0.809811908	0.00074615	0.076055484	0.000160278	0.82990419	0.000746086	0.076035333	1.93E-05	0.006709967	1	0.976669362	1	0.972791643	ENSG00000124733	3	MEA1
SAT	Heart_Failure	ENSG00000090316.11	0.000203719	0.888535289	0.001453494	0.07605549	0.000156692	0.914143099	0.001453355	0.076035338	4.70E-05	0.000157615	1	0.979270459	1	0.98402346	ENSG00000124767	6	GLO1
SAT	Heart_Failure	ENSG00000090615.8	0.000169506	0.959617324	0.003347682	0.076055493	0.000224091	0.946624345	0.003347319	0.076035339	-5.46E-05	0.019011894	1	0.994780111	1	0.990755659	ENSG00000124767	6	GLO1
SAT	Heart_Failure	ENSG00000090621.9	-0.006633774	8.19E-05	0.001684171	0.076053072	-0.006657589	7.70E-05	0.001683982	0.076032901	2.38E-05	0.026661498	0.268610782	0.008953693	0.252746332	0.012637317	ENSG00000124784	4	RIOK1
SAT	Heart_Failure	ENSG00000090686.11	0.005406533	0.024378385	0.002401708	0.076054702	0.0053625	0.025548509	0.002401463	0.076034562	4.40E-05	0.019008513	1	0.336073452	1	0.354978312	ENSG00000124787	9	RPP40
SAT	Heart_Failure	ENSG00000091640.3	0.000903307	0.53081311	0.001441219	0.076055432	0.00094547	0.511769406	0.001441078	0.076035273	-4.22E-05	0.000416513	1	0.908320477	1	0.902750226	ENSG00000124787	9	RPP40
SAT	Heart_Failure	ENSG00000092108.16	0.000880535	0.753744894	0.002806885	0.076055478	0.000776034	0.782161749	0.002806602	0.076035328	0.000104501	2.83E-06	1	0.966658386	1	0.968039494	ENSG00000124787	9	RPP40

SAT	Heart_Failure	ENSG00000094963.9	0.002116719	0.520497137	0.003294096	0.076055429	0.002033029	0.537081006	0.003293776	0.076035281	8.37E-05	0.002551023	1	0.906724424	1	0.909319572	ENSG00000124920	9	MYRF
SAT	Heart_Failure	ENSG00000099246.12	-0.002578068	0.225867583	0.002128745	0.076055264	-0.002652784	0.212683029	0.002128658	0.076035098	7.47E-05	0.00969501	1	0.751217604	1	0.733958243	ENSG00000124920	9	MYRF
SAT	Heart_Failure	ENSG00000099330.4	1.65E-05	0.996321599	0.003583551	0.076055493	-6.68E-05	0.985124474	0.003583171	0.07603534	8.33E-05	0.001429001	1	0.999479422	1	0.999768899	ENSG00000124920	9	MYRF
SAT	Heart_Failure	ENSG00000099814.11	-0.002696219	0.333742915	0.002789375	0.076055347	-0.002771689	0.320342519	0.002789106	0.076035186	7.55E-05	0.001463509	1	0.841293164	1	0.825446748	ENSG00000124920	9	MYRF
SAT	Heart_Failure	ENSG00000099940.7	-0.000952361	0.346505888	0.001011655	0.076055355	-0.000971648	0.336776312	0.001011548	0.076035196	1.93E-05	0.009885677	1	0.847011103	1	0.837075007	ENSG00000124939	4	SCGB2A1
SAT	Heart_Failure	ENSG00000099956.13	0.003452816	0.024249803	0.001532436	0.076054701	0.003406536	0.026203778	0.001532277	0.076034569	4.63E-05	7.28E-05	1	0.336073452	1	0.356741057	ENSG00000124939	4	SCGB2A1
SAT	Heart_Failure	ENSG00000100014.15	0.001432894	0.457160175	0.001927146	0.076055407	0.001473977	0.444312662	0.00192694	0.076035249	-4.11E-05	0.002961436	1	0.88796436	1	0.882462767	ENSG00000124939	4	SCGB2A1
SAT	Heart_Failure	ENSG00000100083.14	-0.001943897	0.383163075	0.002229026	0.076055374	-0.001989265	0.372107336	0.002228784	0.076035216	4.54E-05	0.003459644	1	0.85759885	1	0.850197891	ENSG00000124939	4	SCGB2A1
SAT	Heart_Failure	ENSG00000100109.12	0.002444504	0.143276823	0.001670086	0.076055159	0.002404281	0.149932972	0.001669905	0.076035017	4.02E-05	0.00053503	1	0.669181587	1	0.665121824	ENSG00000125107	12	CNOT1
SAT	Heart_Failure	ENSG00000100181.17	0.001725534	0.485090006	0.002471616	0.076055417	0.001649247	0.504558356	0.002471392	0.076035271	7.63E-05	0.000742443	1	0.897349195	1	0.900571724	ENSG00000125107	12	CNOT1
SAT	Heart_Failure	ENSG00000100225.13	0.003539522	0.147147879	0.002441585	0.076055165	0.003579519	0.142590886	0.002441336	0.076035005	-4.00E-05	0.036168814	1	0.674290771	1	0.658003792	ENSG00000125124	7	BBS2
SAT	Heart_Failure	ENSG00000100284.16	-0.004280376	0.126286254	0.002799617	0.076055128	-0.004244196	0.129478331	0.002799297	0.076034981	-3.62E-05	0.031407238	1	0.64967372	1	0.649215182	ENSG00000125124	7	BBS2
SAT	Heart_Failure	ENSG00000100296.9	0.003428568	0.192116498	0.002628574	0.076055227	0.003481491	0.185297411	0.002628292	0.076035066	-5.29E-05	0.004482631	1	0.722912434	1	0.715224819	ENSG00000125257	9	ABCC4
SAT	Heart_Failure	ENSG00000100300.13	-7.61E-05	0.976817088	0.002617239	0.076055493	-3.65E-05	0.988885572	0.002616954	0.07603534	-3.96E-05	0.027053191	1	0.998920999	1	0.999768899	ENSG00000125257	9	ABCC4
SAT	Heart_Failure	ENSG00000100350.10	-0.000933075	0.663526143	0.002144752	0.076055463	-0.000974249	0.649614946	0.002144518	0.076035308	4.12E-05	0.005145747	1	0.962994646	1	0.953700363	ENSG00000125257	9	ABCC4
SAT	Heart_Failure	ENSG00000100429.13	0.001083406	0.710257927	0.002916223	0.076055471	0.00101493	0.727790661	0.002915913	0.076035321	6.85E-05	0.001123171	1	0.966658386	1	0.958028875	ENSG00000125388	15	GRK4
SAT	Heart_Failure	ENSG00000100445.12	-0.000325559	0.815963569	0.00139881	0.076055485	-0.000302013	0.829042755	0.001398667	0.076035333	-2.35E-05	0.030688715	1	0.976669362	1	0.972791643	ENSG00000125388	15	GRK4
SAT	Heart_Failure	ENSG00000100483.9	-0.001240235	0.556779861	0.002110566	0.076055439	-0.001194784	0.571289569	0.002110352	0.07603529	-4.55E-05	0.006547972	1	0.922001323	1	0.924404562	ENSG00000125388	15	GRK4
SAT	Heart_Failure	ENSG00000100528.7	0.000388561	0.890950883	0.002834118	0.07605549	0.000428402	0.879837112	0.002833798	0.076035337	-3.98E-05	0.024539839	1	0.979270459	1	0.974665278	ENSG00000125388	15	GRK4
SAT	Heart_Failure	ENSG00000100564.4	0.000338601	0.889578838	0.002438837	0.07605549	0.000284965	0.906973562	0.002438578	0.076035338	5.36E-05	0.002436846	1	0.979270459	1	0.982067422	ENSG00000125388	15	GRK4
SAT	Heart_Failure	ENSG00000100580.7	-0.0006083	0.818538691	0.002651413	0.076055485	-0.000651899	0.805762491	0.002651112	0.076035331	4.36E-05	0.007383747	1	0.976669362	1	0.972791643	ENSG00000125409	8	TEKT3
SAT	Heart_Failure	ENSG00000100614.13	0.000132374	0.96492559	0.003010306	0.076055493	4.35E-05	0.988481822	0.003010057	0.07603534	8.89E-05	0.002993081	1	0.996432082	1	0.999768899	ENSG00000125409	8	TEKT3
SAT	Heart_Failure	ENSG00000100731.11	0.000166091	0.896202158	0.001273115	0.07605549	0.000129159	0.919183394	0.001272975	0.076035339	3.69E-05	1.26E-05	1	0.979542669	1	0.984094962	ENSG00000125450	6	NUP85
SAT	Heart_Failure	ENSG00000101000.4	-0.001501816	0.434918737	0.001923426	0.076055398	-0.001397832	0.467343634	0.001923246	0.076035258	-0.000103985	1.04E-09	1	0.880339889	1	0.892844363	ENSG00000125450	6	NUP85
SAT	Heart_Failure	ENSG00000101052.8	-0.002967252	0.365967515	0.003282164	0.076055365	-0.003058683	0.35133189	0.003281814	0.076035205	9.14E-05	0.000106385	1	0.85158824	1	0.843366096	ENSG00000125450	6	NUP85
SAT	Heart_Failure	ENSG00000101181.13	0.003351185	0.396016066	0.003948337	0.076055381	0.003245583	0.411020538	0.003947919	0.076035235	0.000105602	0.000231577	1	0.866797005	1	0.867451005	ENSG00000125450	6	NUP85
SAT	Heart_Failure	ENSG00000101190.8	-0.001012913	0.654194293	0.002261253	0.076055462	-0.001045753	0.643711542	0.002261009	0.076035307	3.28E-05	0.038364962	1	0.962948172	1	0.953076521	ENSG00000125450	6	NUP85
SAT	Heart_Failure	ENSG00000101224.13	-0.001198467	0.71179645	0.00324397	0.076055472	-0.001302156	0.688092085	0.003243665	0.076035315	0.000103688	0.000268415	1	0.966658386	1	0.956878334	ENSG00000125485	13	DDX31
SAT	Heart_Failure	ENSG00000101310.10	0.001874303	0.285878671	0.001756268	0.076055315	0.001845037	0.293419794	0.001756091	0.076035168	2.93E-05	0.035625371	1	0.800157	1	0.807642907	ENSG00000125498	15	KIR2DL1
SAT	Heart_Failure	ENSG00000101335.5	0.009287858	0.018526472	0.003944002	0.076054627	0.008376678	0.034099769	0.003953322	0.07603464	0.00091118	0.001079115	1	0.296513921	1	0.413317418	ENSG00000125510	11	OPRL1
SAT	Heart_Failure	ENSG00000101363.8	0.001074972	0.3364147	0.001118278	0.076055349	0.00102776	0.358034928	0.001118204	0.076035208	4.72E-05	0.000234008	1	0.841293164	1	0.846989344	ENSG00000125510	11	OPRL1
SAT	Heart_Failure	ENSG00000101457.8	-0.000896707	0.504775498	0.001344398	0.076055424	-0.000916072	0.49557269	0.001344254	0.076035268	1.94E-05	0.043217076	1	0.9005699	1	0.894608312	ENSG00000125534	5	PPDPF
SAT	Heart_Failure	ENSG00000101460.8	-0.001118411	0.758234119	0.003633557	0.076055478	-0.001243494	0.732158618	0.003633232	0.076035322	0.000125083	0.000201883	1	0.967625883	1	0.958028875	ENSG00000125534	5	PPDPF
SAT	Heart_Failure	ENSG00000101751.6	0.001374359	0.124428845	0.000894503	0.076055125	0.001319839	0.1400527	0.000894444	0.076035	5.45E-05	1.23E-07	1	0.647388543	1	0.654576795	ENSG00000125534	5	PPDPF
SAT	Heart_Failure	ENSG00000102858.8	0.001804026	0.363540766	0.001985414	0.076055364	0.001773513	0.371660591	0.001985199	0.076035216	3.05E-05	0.026301994	1	0.85040303	1	0.849768918	ENSG00000125611	11	CHCHD5
SAT	Heart_Failure	ENSG00000102977.9	0.001165275	0.686202917	0.002884247	0.076055468	0.001098782	0.703204828	0.002883957	0.076035317	6.65E-05	0.004078305	1	0.966658386	1	0.956953563	ENSG00000125611	11	CHCHD5
SAT	Heart_Failure	ENSG00000103148.11	0.001752389	0.426881	0.002205536	0.076055394	0.001789049	0.417221859	0.002205296	0.076035237	-3.67E-05	0.014934883	1	0.878482333	1	0.867451005	ENSG00000125611	11	CHCHD5
SAT	Heart_Failure	ENSG00000103168.12	-0.000134118	0.882973066	0.00091112	0.07605549	-0.000171435	0.850745129	0.000911076	0.076035335	3.73E-05	0.001585424	1	0.979223495	1	0.972791643	ENSG00000125611	11	CHCHD5
SAT	Heart_Failure	ENSG00000103356.11	-0.000101367	0.94839158	0.001566077	0.076055492	-0.000124769	0.936492668	0.001565903	0.076035339	2.34E-05	0.021560918	1	0.992559098	1	0.988983601	ENSG00000125630	11	POLR1B
SAT	Heart_Failure	ENSG00000103415.7	0.000105721	0.954415991	0.001849486	0.076055493	0.000177962	0.923336305	0.001849304	0.076035339	-7.22E-05	2.15E-06	1	0.994131568	1	0.985235111	ENSG00000125648	10	SLC25A23
SAT	Heart_Failure	ENSG00000103472.5	0.002105623	0.216783008	0.00170478	0.076055255	0.002038334	0.231781323	0.0017046	0.076035117	6.73E-05	7.42E-08	1	0.745333464	1	0.750242469	ENSG00000125648	10	SLC25A23
SAT	Heart_Failure	ENSG00000103479.10	0.002492426	0.093350448	0.001485368	0.076055054	0.002451641	0.098797711	0.0014852	0.076034915	4.08E-05	1.35E-05	1	0.604759516	1	0.610461938	ENSG00000125656	4	CLPP
SAT	Heart_Failure	ENSG00000103550.9	0.00148573	0.554609543	0.002514489	0.076055439	0.001423241	0.571347248	0.002514254	0.07603529	6.25E-05	0.004861519	1	0.921542737	1	0.924404562	ENSG00000125656	4	CLPP
SAT	Heart_Failure	ENSG00000103742.7	6.99E-05	0.954109142	0.001214814	0.076055493	9.10E-05	0.940263976	0.001214685	0.076035339	-2.11E-05	0.016915938	1	0.994131568	1	0.988983601	ENSG00000125734	9	GPR108
SAT	Heart_Failure	ENSG00000103811.11	-0.002176666	0.004033832	0.000756965	0.076054203	-0.002189387	0.003820581	0.000756887	0.076034035	1.27E-05	0.02879651	1	0.1521008	1	0.159899049	ENSG00000125734	9	GPR108
SAT	Heart_Failure	ENSG00000104219.8	-0.00314576	0.068793061	0.001728648	0.076054976	-0.003179509	0.065844215	0.001728476	0.076034812	3.37E-05	0.01641193	1	0.537536555	1	0.515872519	ENSG00000125735	6	TNFSF14
SAT	Heart_Failure	ENSG00000104324.11	0.003892794	0.058704224	0.002059237	0.076054935	0.00392358	0.056708618	0.002059022	0.076034774	-3.08E-05	0.047734751	1	0.506864626	1	0.501512062	ENSG00000125818	13	PSMF1
SAT	Heart_Failure	ENSG00000104388.10	-0.001963703	0.476109325	0.002755786	0.076055414	-0.00205749	0.455254681	0.002755502	0.076035253	9.38E-05	9.60E-06	1	0.895715287	1	0.886989673	ENSG00000125818	13	PSMF1
SAT	Heart_Failure	ENSG00000104442.5	0.000891568	0.531305864	0.001424198	0.076055432	0.000972228	0.49489939	0.001424432	0.076035267	-8.07E-05	0.020047615	1	0.908320477	1	0.894608312	ENSG00000125821	7	DTD1
SAT	Heart_Failure	ENSG00000104689.5	0.001969853	0.362817194	0.002164648	0.076055364	0.001942609	0.369439223	0.002164406	0.076035214	2.72E-05	0.049799178	1	0.85040303	1	0.849425432	ENSG00000125821	7	DTD1
SAT	Heart_Failure	ENSG00000104731.9	-0.00083254	0.645890186	0.00181192	0.07605546	-0.000804628	0.656955073	0.001811731	0.076035309	-2.79E-05	0.040042641	1	0.960776086	1	0.956286421	ENSG00000125821	7	DTD1
SAT	Heart_Failure	ENSG00000104774.8	0.004383119	0.047064154	0.002207311	0.076054878	0.004434764	0.044503219	0.002207091	0.07603471	-5.16E-05	0.003894669	1	0.476597187	1	0.456297071	ENSG00000125821	7	DTD1
SAT	Heart_Failure	ENSG00000104835.10	0.002506325	0.372465812	0.002810207	0.076055369	0.002607415	0.353443248	0.00280992	0.076035206	-0.000101091	4.29E-06	1	0.857586195	1	0.843366096	ENSG00000125826	15	RBCK1
SAT	Heart_Failure	ENSG00000104866.6	0.004534335	0.222350561	0.003715745	0.076055261	0.004445656	0.231481324	0.003715385	0.076035117	8.87E-05	0.004726086	1	0.751090303	1	0.750242469	ENSG00000125844	11	RRBP1
SAT	Heart_Failure	ENSG00000104899.4	0.003827495	0.252335462	0.003343676	0.076055289	0.003767736	0.259766708	0.003343321	0.076035142	5.98E-05	0.013624691	1	0.767444733	1	0.773899606	ENSG00000125863	13	MKKS
SAT	Heart_Failure	ENSG00000104901.2	-0.000648619	0.858921785	0.003649113	0.076055488	-0.000588896	0.871780005	0.003648718	0.076035336	-5.97E-05	0.018457962	1	0.976669362	1	0.974665278	ENSG00000125863	13	MKKS
SAT	Heart_Failure	ENSG00000105072.4	0.001034	0.513960367	0.001584228	0.076055427	0.001079511	0.49556656	0.001584062	0.076035268	-4.55E-05	0.000115604	1	0.905155107	1	0.894608312	ENSG00000125885	9	MCM8
SAT	Heart_Failure	ENSG00000105088.4	-5.88E-05	0.980804696	0.002443325	0.076055493	7.08E-07	0.999768899	0.002443094	0.07603534	-5.95E-05	0.0050412	1	0.998920999	1	0.999768899	ENSG00000125901	5	MRPS26
SAT	Heart_Failure	ENSG00000105270.10	0.000129045	0.923499766	0.001343849	0.076055492	0.000154495	0.908464125	0.001343712	0.076035338	-2.55E-05	0.015742469	1	0.988739396	1	0.982067422	ENSG00000125901	5	MRPS26
SAT	Heart_Failure	ENSG00000105447.8	-0.004733264	0.016833564	0.001980171	0.076054601	-0.004691373	0.01781714	0.00197998	0.076034464	-4.19E-05	0.01253838	1	0.287798638	1	0.299784809	ENSG00000125912	6	NCLN
SAT	Heart_Failure	ENSG00000105479.11	-0.00303027	0.179332255	0.002256654	0.076055212	-0.002985672	0.185773693	0.002256424	0.076035067	-4.46E-05	0.011800675	1	0.714462461	1	0.715224819	ENSG00000125965	4	GDF5
SAT	Heart_Failure	ENSG00000105755.3	0.000210961	0.935372952	0.002601672	0.076055492	0.000265838	0.918605442	0.002601394	0.076035339	-5.49E-05	0.003330486	1	0.989966131	1	0.984094962	ENSG00000125965	4	GDF5
SAT	Heart_Failure	ENSG00000105880.4	-0.004749646	0.210527136	0.003793287	0.076055248	-0.00465011	0.220197887	0.0037929	0.076035106	-9.95E-05	0.000777573	1	0.739879495	1	0.742028061	ENSG00000125966	8	MMP24
SAT	Heart_Failure	ENSG00000105928.9	0.000907537	0.779883314	0.003247334	0.076055481	0.000818832	0.800900341	0.003246988	0.07603533	8.87E-05	0.000140556	1	0.972664863	1	0.972791643	ENSG00000125966	8	MMP24
SAT	Heart_Failure	ENSG00000105982.12	-0.000223726	0.924326631	0.002355377	0.076055492	-0.000188673	0.936148573	0.002355123	0.076035339	-3.51E-05	0.034698518	1	0.988739396	1	0.988983601	ENSG00000125966	8	MMP24
SAT	Heart_Failure	ENSG00000105983.15	0.005116452	0.06762312	0.002799678	0.076054972	0.005074424	0.069878912	0.002799375	0.076034827	4.20E-05	0.030496779	1	0.535424809	1	0.529994954	ENSG00000125991	14	ERGIC3
SAT	Heart_Failure	ENSG00000106178.2	0.003365882	0.222680368	0.002760202	0.076055261	0.003418739	0.215454982	0.002759928	0.076035101	-5.29E-05	0.018795185	1	0.751090303	1	0.736362287	ENSG00000126091	15	ST3GAL3
SAT	Heart_Failure	ENSG00000106263.13	-0.00093025	0.787960414	0.003458691	0.076055482	-0.00084327	0.80735891	0.003458365	0.076035331	-8.70E-05	0.004143498	1	0.974113835	1	0.972791643	ENSG00000126091	15	ST3GAL3
SAT	Heart_Failure	ENSG00000106348.12	-0.000375102	0.878490714	0.002453524	0.076055489	-0.000415243	0.865591092	0.002453264	0.076035336	4.01E-05	0.02457632	1	0.979223495	1	0.974123167	ENSG00000126215	9	XRCC3
SAT	Heart_Failure	ENSG00000106477.14	-0.002730718	0.32152572	0.002754604	0.07605534	-0.002664579	0.333335326	0.002754318	0.076035194	-6.61E-05	0.001487356	1	0.828692762	1	0.836656731	ENSG00000126226	17	PCID2
SAT	Heart_Failure	ENSG00000106610.10	-0.008357823	0.008727173	0.003186881	0.07605442	-0.008431481	0.00814622	0.00318655	0.076034248	7.37E-05	0.00224533	1	0.210020691	1	0.209225934	ENSG00000126226	17	PCID2
SAT	Heart_Failure	ENSG00000106853.12	0.002511517	0.322614189	0.002539191	0.07605534	0.002567829	0.31183051	0.002538912	0.076035181	-5.63E-05	0.000909982	1	0.829398936	1	0.821155716	ENSG00000126246	5	IGFLR1
SAT	Heart_Failure	ENSG00000106868.12	0.00018575	0.909048133	0.001625975	0.076055491	0.000155806	0.923653427	0.00162581	0.076035339	2.99E-05	0.019566576	1	0.983702811	1	0.985235111	ENSG00000126247	6	CAPNS1
SAT	Heart_Failure	ENSG00000107020.5	0.001561353	0.335654385	0.0016217	0.076055348	0.001539035	0.342554456	0.00162152	0.0760352	2.23E-05	0.036077389	1	0.841293164	1	0.839311387	ENSG00000126247	6	CAPNS1
SAT	Heart_Failure	ENSG00000107099.11	-0.000612577	0.811290521	0.002565644	0.076055484	-0.000193182	0.940123762	0.00257184	0.076035339	-0.000419395	0.022095151	1	0.976669362	1	0.988983601	ENSG00000126247	6	CAPNS1
SAT	Heart_Failure	ENSG00000107443.11	0.000798817	0.841451324	0.003993338	0.076055487	0.000901937	0.821292597	0.003992943	0.076035332	-0.00010312	0.001641194	1	0.976669362	1	0.972791643	ENSG00000126251	5	GPR42
SAT	Heart_Failure	ENSG00000107537.9	-0.000239439	0.856226221	0.001321554	0.076055488	-0.000262144	0.842746253	0.001321411	0.076035334	2.27E-05	0.014712199	1	0.976669362	1	0.972791643	ENSG00000126251	5	GPR42
SAT	Heart_Failure	ENSG00000107779.7	0.001249516	0.590797115	0.002323895	0.076055448	0.000457499	0.845938795	0.002354568	0.076035334	0.000792016	0.037487328	1	0.941376309	1	0.972791643	ENSG00000126251	5	GPR42
SAT	Heart_Failure	ENSG00000107798.13	0.00229679	0.354760486	0.002481954	0.076055359	0.002421758	0.329146541	0.002481728	0.076035192	-0.000124968	3.29E-08	1	0.848363544	1	0.833382337	ENSG00000126457	16	PRMT1
SAT	Heart_Failure	ENSG00000107890.12	-0.001059539	0.681236812	0.002579359	0.076055467	-0.000997697	0.698876123	0.002579107	0.076035317	-6.18E-05	0.00393868	1	0.966658386	1	0.956878334	ENSG00000126464	9	PRR12
SAT	Heart_Failure	ENSG00000108107.8	-5.60E-05	0.969269069	0.00145379	0.076055493	-9.03E-05	0.95044991	0.001453635	0.07603534	3.43E-05	0.000943044	1	0.996609664	1	0.990755659	ENSG00000126500	3	FLRT1
SAT	Heart_Failure	ENSG00000108264.12	-0.001496144	0.0732649	0.000835284	0.076054992	-0.001457763	0.080916706	0.000835205	0.076034865	-3.84E-05	1.32E-07	1	0.559028222	1	0.580120808	ENSG00000126500	3	FLRT1
SAT	Heart_Failure	ENSG00000108387.10	-0.004764141	0.214587204	0.003838793	0.076055253	-0.004832931	0.207993948	0.003838394	0.076035093	6.88E-05	0.01757662	1	0.745086527	1	0.733958243	ENSG00000126562	12	WNK4
SAT	Heart_Failure	ENSG00000108469.10	-0.004392669	0.148469107	0.003039989	0.076055167	-0.004340872	0.153268619	0.00303965	0.076035022	-5.18E-05	0.008257833	1	0.674894985	1	0.667541522	ENSG00000126602	6	TRAP1
SAT	Heart_Failure	ENSG00000108556.7	-0.00178436	0.248845115	0.001547365	0.076055285	-0.001818381	0.239887525	0.001547204	0.076035125	3.40E-05	0.003367988	1	0.765246076	1	0.752162449	ENSG00000126602	6	TRAP1
SAT	Heart_Failure	ENSG00000108639.3	-0.005075415	0.055530924	0.002650758	0.076054921	-0.004986379	0.059932977	0.00265051	0.076034788	-8.90E-05	0.000138316	1	0.503306524	1	0.504627254	ENSG00000126698	6	DNAJC8
SAT	Heart_Failure	ENSG00000108654.7	0.000784352	0.720456384	0.002191851	0.076055473	0.000504776	0.817985863	0.002193376	0.076035332	0.000279576	0.00172296	1	0.966658386	1	0.972791643	ENSG00000126698	6	DNAJC8
SAT	Heart_Failure	ENSG00000108669.12	0.000213613	0.95343757	0.003658343	0.076055492	0.000129726	0.971709479	0.003657933	0.07603534	8.39E-05	0.000330917	1	0.994131568	1	0.998061182	ENSG00000126746	13	ZNF384
SAT	Heart_Failure	ENSG00000108774.10	0.007134404	0.015696989	0.002953111	0.076054582	0.007208057	0.014643221	0.002952799	0.07603441	-7.37E-05	0.000655978	1	0.276891502	1	0.272970533	ENSG00000126790	7	L3HYPDH
SAT	Heart_Failure	ENSG00000108786.6	-0.001475092	0.219903846	0.001202404	0.076055258	-0.001499453	0.212335984	0.001202285	0.076035097	2.44E-05	0.013220137	1	0.749908476	1	0.733958243	ENSG00000126790	7	L3HYPDH
SAT	Heart_Failure	ENSG00000108823.11	0.000691694	0.719859374	0.00192862	0.076055473	0.000740575	0.700955226	0.001928421	0.076035317	-4.89E-05	0.000970307	1	0.966658386	1	0.956878334	ENSG00000126790	7	L3HYPDH
SAT	Heart_Failure	ENSG00000109321.6	0.00229048	0.468148731	0.003157127	0.076055411	0.002027322	0.520786954	0.003157167	0.076035276	0.000263157	1.01E-06	1	0.893397133	1	0.904369708	ENSG00000126858	12	RHOT1
SAT	Heart_Failure	ENSG00000110074.6	0.000996398	0.699888602	0.002584884	0.07605547	0.000947588	0.713901862	0.002584654	0.076035319	4.88E-05	0.042560714	1	0.966658386	1	0.958028875	ENSG00000126870	11	WDR60
SAT	Heart_Failure	ENSG00000110203.4	-4.07E-05	0.977902662	0.001470843	0.076055493	-6.55E-05	0.964483159	0.001470698	0.07603534	2.47E-05	0.041592746	1	0.998920999	1	0.996055789	ENSG00000126870	11	WDR60
SAT	Heart_Failure	ENSG00000110628.9	0.000219216	0.844714689	0.00111921	0.076055487	0.000246849	0.825418767	0.001119087	0.076035333	-2.76E-05	0.000253659	1	0.976669362	1	0.972791643	ENSG00000126870	11	WDR60
SAT	Heart_Failure	ENSG00000110665.7	0.005890389	0.120696539	0.003795698	0.076055117	0.005826805	0.124717113	0.003795286	0.076034972	6.36E-05	0.01545436	1	0.646585619	1	0.647874406	ENSG00000127220	5	ABHD8
SAT	Heart_Failure	ENSG00000111058.3	-0.000214858	0.927719494	0.002368507	0.076055492	-0.000419396	0.859491786	0.002369183	0.076035335	0.000204538	0.002814382	1	0.988739396	1	0.972791643	ENSG00000127220	5	ABHD8
SAT	Heart_Failure	ENSG00000111142.9	0.001080612	0.7764573	0.003805789	0.07605548	0.001018427	0.788985747	0.00380538	0.076035329	6.22E-05	0.021432309	1	0.972664863	1	0.970635778	ENSG00000127337	2	YEATS4
SAT	Heart_Failure	ENSG00000111530.8	-0.001468441	0.450847407	0.001947525	0.076055404	-0.00151144	0.437652643	0.001947321	0.076035246	4.30E-05	0.003127505	1	0.887019168	1	0.879864169	ENSG00000127337	2	YEATS4
SAT	Heart_Failure	ENSG00000111696.7	0.002376216	0.374897606	0.002677917	0.07605537	0.002421126	0.365888725	0.002677639	0.076035213	-4.49E-05	0.026607922	1	0.85759885	1	0.849290727	ENSG00000127337	2	YEATS4
SAT	Heart_Failure	ENSG00000111725.6	-0.000628296	0.723308856	0.001774646	0.076055473	-0.000597873	0.736167209	0.001774454	0.076035322	-3.04E-05	0.013994629	1	0.966658386	1	0.960001834	ENSG00000127337	2	YEATS4
SAT	Heart_Failure	ENSG00000111801.11	-0.000389905	0.662389156	0.000893014	0.076055463	-0.00027389	0.759397845	0.00089427	0.076035326	-0.000116016	0.019206567	1	0.962994646	1	0.963228667	ENSG00000127337	2	YEATS4
SAT	Heart_Failure	ENSG00000112053.9	0.001787476	0.320582507	0.001799601	0.076055339	0.001912447	0.287894768	0.001799523	0.076035164	-0.000124971	2.03E-07	1	0.828692762	1	0.799139368	ENSG00000127337	2	YEATS4
SAT	Heart_Failure	ENSG00000112130.12	-0.001435107	0.540656792	0.002345634	0.076055435	-0.001493515	0.524264305	0.002345395	0.076035277	5.84E-05	0.001388127	1	0.913035829	1	0.904369708	ENSG00000127337	2	YEATS4
SAT	Heart_Failure	ENSG00000112218.7	-0.002558291	0.4297361	0.003239804	0.076055396	-0.002473039	0.445222035	0.003239483	0.076035249	-8.53E-05	0.001332178	1	0.878482333	1	0.882462767	ENSG00000127337	2	YEATS4
SAT	Heart_Failure	ENSG00000112290.8	-0.000793617	0.7073418	0.002113895	0.076055471	-0.000854679	0.685952835	0.002113693	0.076035315	6.11E-05	0.000777755	1	0.966658386	1	0.956878334	ENSG00000127418	10	FGFRL1
SAT	Heart_Failure	ENSG00000112293.10	0.008798877	0.014917226	0.003614406	0.076054568	0.009008611	0.012682119	0.003614168	0.076034371	-0.000209734	5.30E-07	1	0.270426545	1	0.253719716	ENSG00000127418	10	FGFRL1
SAT	Heart_Failure	ENSG00000112299.7	0.000668441	0.8373327	0.003255818	0.076055486	0.000619732	0.849021013	0.003255446	0.076035334	4.87E-05	0.013668614	1	0.976669362	1	0.972791643	ENSG00000127418	10	FGFRL1
SAT	Heart_Failure	ENSG00000112308.8	0.001327405	0.732704508	0.003886637	0.076055475	0.001707756	0.660547021	0.003888702	0.07603531	-0.000380351	0.00723645	1	0.966658386	1	0.956878334	ENSG00000127419	12	TMEM175
SAT	Heart_Failure	ENSG00000112584.9	0.003995445	0.016992797	0.001673925	0.076054604	0.003974558	0.017565109	0.001673732	0.07603446	2.09E-05	0.03142404	1	0.288877546	1	0.297067641	ENSG00000127463	9	EMC1
SAT	Heart_Failure	ENSG00000112619.6	-0.005077357	0.114462046	0.003216665	0.076055104	-0.00501395	0.119017983	0.003216315	0.076034961	-6.34E-05	0.004102832	1	0.641850091	1	0.64481576	ENSG00000127481	10	UBR4
SAT	Heart_Failure	ENSG00000112667.8	-0.001471045	0.389365358	0.001708988	0.076055377	-0.001415348	0.407520461	0.001708809	0.076035233	-5.57E-05	1.32E-05	1	0.861555536	1	0.867451005	ENSG00000127507	13	EMR2
SAT	Heart_Failure	ENSG00000112697.11	0.006141858	0.062441642	0.00329649	0.076054951	0.006069202	0.065575744	0.00329612	0.076034811	7.27E-05	0.000540444	1	0.515554877	1	0.515872519	ENSG00000127616	13	SMARCA4
SAT	Heart_Failure	ENSG00000112812.11	0.00179013	0.44588518	0.002348348	0.076055402	0.002918865	0.225043309	0.002405867	0.07603511	-0.001128735	0.031343446	1	0.88490557	1	0.747837725	ENSG00000127616	13	SMARCA4
SAT	Heart_Failure	ENSG00000113248.3	-0.000351935	0.802798561	0.001409278	0.076055483	-0.000326277	0.816890023	0.001409118	0.076035332	-2.57E-05	0.00360793	1	0.976669362	1	0.972791643	ENSG00000127616	13	SMARCA4
SAT	Heart_Failure	ENSG00000113249.8	-0.003277282	0.269614749	0.002968683	0.076055303	-0.003342604	0.260133425	0.002968362	0.076035142	6.53E-05	0.001540121	1	0.782624692	1	0.773899606	ENSG00000127616	13	SMARCA4
SAT	Heart_Failure	ENSG00000113318.9	0.001133508	0.665121384	0.002618688	0.076055464	0.001062331	0.684951478	0.002618409	0.076035314	7.12E-05	0.000159963	1	0.962994646	1	0.956878334	ENSG00000127903	12	ZNF835
SAT	Heart_Failure	ENSG00000113361.8	-0.000443615	0.764451343	0.001480489	0.076055479	-0.003274198	0.100375617	0.00199277	0.076034919	0.002830583	0.033864254	1	0.970642331	1	0.614666997	ENSG00000127903	12	ZNF835
SAT	Heart_Failure	ENSG00000113520.6	-0.009919875	0.009832191	0.003842402	0.076054453	-0.009988084	0.009330764	0.003842012	0.076034286	6.82E-05	0.024163472	1	0.227178996	1	0.221316258	ENSG00000127903	12	ZNF835
SAT	Heart_Failure	ENSG00000113593.7	0.000635244	0.606501129	0.001233306	0.076055452	0.0006644	0.590046824	0.001233183	0.076035295	-2.92E-05	0.003424126	1	0.948489135	1	0.934989637	ENSG00000127903	12	ZNF835
SAT	Heart_Failure	ENSG00000113597.13	-0.001692419	0.627833187	0.003491113	0.076055456	-0.001622963	0.641980711	0.003490756	0.076035306	-6.95E-05	0.010709222	1	0.958246837	1	0.951372499	ENSG00000127952	12	STYXL1
SAT	Heart_Failure	ENSG00000113734.13	0.002756468	0.011167134	0.001086321	0.076054488	0.00273229	0.011888625	0.001086206	0.076034353	2.42E-05	0.00248451	1	0.236383009	1	0.246278972	ENSG00000127952	12	STYXL1
SAT	Heart_Failure	ENSG00000114023.11	-0.002306514	0.526164158	0.003638766	0.07605543	-0.002242185	0.537721216	0.003638356	0.076035281	-6.43E-05	0.005165045	1	0.906724424	1	0.909319572	ENSG00000127957	12	PMS2P3
SAT	Heart_Failure	ENSG00000114125.9	0.000895738	0.585775372	0.00164365	0.076055447	0.00092101	0.575205693	0.001643482	0.076035291	-2.53E-05	0.047868528	1	0.939818579	1	0.927160988	ENSG00000128039	6	SRD5A3
SAT	Heart_Failure	ENSG00000114200.5	-0.007137204	0.032865066	0.003344936	0.076054782	-0.007064339	0.034674318	0.003344624	0.076034644	-7.29E-05	0.013720054	1	0.408448042	1	0.418258961	ENSG00000128039	6	SRD5A3
SAT	Heart_Failure	ENSG00000114302.11	0.003802962	0.184702265	0.002867094	0.076055218	0.003720911	0.19431157	0.002866801	0.076035077	8.21E-05	0.000235101	1	0.720580417	1	0.723506187	ENSG00000128185	5	DGCR6L
SAT	Heart_Failure	ENSG00000114378.12	0.001325611	0.408193663	0.001602765	0.076055386	0.001350044	0.399558257	0.001602596	0.076035229	-2.44E-05	0.036786529	1	0.870429337	1	0.865885497	ENSG00000128185	5	DGCR6L
SAT	Heart_Failure	ENSG00000114796.11	0.005730545	0.104660968	0.003531557	0.076055082	0.005786551	0.10127729	0.003531193	0.076034921	-5.60E-05	0.039457949	1	0.632398959	1	0.614882715	ENSG00000128185	5	DGCR6L
SAT	Heart_Failure	ENSG00000115084.8	-0.000335374	0.875421539	0.002139194	0.076055489	-0.000283023	0.894733086	0.002138964	0.076035337	-5.24E-05	0.000566838	1	0.979223495	1	0.97886604	ENSG00000128218	7	VPREB3
SAT	Heart_Failure	ENSG00000115310.13	0.000645507	0.840239813	0.003202129	0.076055487	0.000897493	0.779391054	0.00320404	0.076035328	-0.000251986	0.039375141	1	0.976669362	1	0.966957854	ENSG00000128218	7	VPREB3
SAT	Heart_Failure	ENSG00000115423.14	0.00208492	0.062219493	0.00111808	0.07605495	0.002559245	0.024083042	0.001134513	0.076034546	-0.000474325	0.014118617	1	0.515510499	1	0.342062594	ENSG00000128262	4	POM121L9P
SAT	Heart_Failure	ENSG00000115486.7	0.001396578	0.379982472	0.001590764	0.076055373	0.001354654	0.394421446	0.001590665	0.076035227	4.19E-05	0.026487371	1	0.85759885	1	0.862334842	ENSG00000128262	4	POM121L9P
SAT	Heart_Failure	ENSG00000115561.10	-0.002237536	0.147751175	0.001545769	0.076055166	-0.002209095	0.152925734	0.001545605	0.076035021	-2.84E-05	0.011611826	1	0.674894985	1	0.667541522	ENSG00000128262	4	POM121L9P
SAT	Heart_Failure	ENSG00000115568.11	-0.001218164	0.669533185	0.002854253	0.076055465	-0.001166323	0.682784129	0.002853968	0.076035314	-5.18E-05	0.024683852	1	0.96567764	1	0.956878334	ENSG00000128271	15	ADORA2A
SAT	Heart_Failure	ENSG00000115840.9	0.000923791	0.791314545	0.00349122	0.076055482	0.000987604	0.777245621	0.003490872	0.076035328	-6.38E-05	0.023966644	1	0.9760395	1	0.965963214	ENSG00000128274	11	A4GALT
SAT	Heart_Failure	ENSG00000116127.13	-0.001408829	0.658679925	0.003189297	0.076055463	-0.001354238	0.671079441	0.00318894	0.076035312	-5.46E-05	0.007592877	1	0.962994646	1	0.956878334	ENSG00000128408	7	RIBC2
SAT	Heart_Failure	ENSG00000116151.9	0.001739741	0.616332454	0.003472155	0.076055454	0.001792315	0.605679909	0.003471796	0.076035299	-5.26E-05	0.047645876	1	0.954216727	1	0.942350873	ENSG00000128408	7	RIBC2

SAT	Heart_Failure	ENSG00000116157.5	0.000645771	0.570858587	0.001139352	0.076055443	0.000681248	0.549851192	0.001139243	0.076035284	-3.55E-05	0.000261762	1	0.930445615	1	0.914465015	ENSG00000128463	8	EMC4
SAT	Heart_Failure	ENSG00000116489.8	-0.00900808	0.010737512	0.003531006	0.076054477	-0.00906673	0.010228369	0.003530628	0.076034311	5.87E-05	0.02034722	1	0.232334342	1	0.230669232	ENSG00000128463	8	EMC4
SAT	Heart_Failure	ENSG00000116857.12	-0.000704133	0.716542818	0.001939314	0.076055472	-0.000664468	0.731849992	0.001939115	0.076035322	-3.97E-05	0.00782894	1	0.966658386	1	0.958028875	ENSG00000128563	9	PRKRIP1
SAT	Heart_Failure	ENSG00000116874.7	-0.003124374	0.155887394	0.002201749	0.076055179	-0.00316312	0.150777359	0.002201513	0.076035018	3.87E-05	0.013855751	1	0.677064618	1	0.665121824	ENSG00000128591	11	FLNC
SAT	Heart_Failure	ENSG00000116883.8	-0.006535635	4.04E-05	0.001592128	0.076052863	-0.006508555	4.34E-05	0.001591965	0.076032732	-2.71E-05	0.027729187	0.132696315	0.005529013	0.142549737	0.009503316	ENSG00000128604	14	IRF5
SAT	Heart_Failure	ENSG00000117174.6	-0.002341425	0.183932447	0.001762132	0.076055217	-0.002377708	0.177184945	0.00176195	0.076035056	3.63E-05	0.007214842	1	0.720580417	1	0.704659157	ENSG00000128604	14	IRF5
SAT	Heart_Failure	ENSG00000117226.7	-0.00043054	0.505856252	0.000647132	0.076055424	-0.000418092	0.518192822	0.000647067	0.076035275	-1.24E-05	0.015647082	1	0.9005699	1	0.903395669	ENSG00000128604	14	IRF5
SAT	Heart_Failure	ENSG00000117450.9	0.003414699	0.308011945	0.003349703	0.076055331	0.003355581	0.316410055	0.003349339	0.076035184	5.91E-05	0.010680513	1	0.816225947	1	0.823487262	ENSG00000128604	14	IRF5
SAT	Heart_Failure	ENSG00000117480.11	0.001170411	0.467010211	0.001609138	0.07605541	0.00113816	0.479326382	0.001608963	0.076035262	3.23E-05	0.003913948	1	0.892925701	1	0.894608312	ENSG00000128604	14	IRF5
SAT	Heart_Failure	ENSG00000117533.10	-0.003974961	0.159504169	0.002825648	0.076055184	-0.00390346	0.167101768	0.002825362	0.076035042	-7.15E-05	0.001380042	1	0.678956178	1	0.684786616	ENSG00000128604	14	IRF5
SAT	Heart_Failure	ENSG00000117569.14	0.002019603	0.28554793	0.00189112	0.076055315	0.001971494	0.297131725	0.001890931	0.076035171	4.81E-05	0.001659627	1	0.800157	1	0.810520426	ENSG00000128604	14	IRF5
SAT	Heart_Failure	ENSG00000117595.6	0.001506092	0.528727415	0.002390811	0.076055431	0.001404831	0.5567649	0.002390575	0.076035286	0.000101261	2.41E-07	1	0.907158447	1	0.915531921	ENSG00000128617	2	OPN1SW
SAT	Heart_Failure	ENSG00000117697.10	0.006922058	0.016841615	0.00289607	0.076054601	0.006991073	0.015768503	0.002895767	0.076034431	-6.90E-05	0.00147358	1	0.287798638	1	0.278152999	ENSG00000128645	11	HOXD1
SAT	Heart_Failure	ENSG00000118017.3	0.000356268	0.832817386	0.001687756	0.076055486	0.000394385	0.815219027	0.001687578	0.076035332	-3.81E-05	0.002030459	1	0.976669362	1	0.972791643	ENSG00000128652	7	HOXD3
SAT	Heart_Failure	ENSG00000118197.9	-0.001202413	0.49863478	0.001777036	0.076055422	-0.00116585	0.511738952	0.00177685	0.076035273	-3.66E-05	0.006015935	1	0.899263643	1	0.902750226	ENSG00000128655	12	PDE11A
SAT	Heart_Failure	ENSG00000118200.10	0.001226358	0.736302428	0.003641701	0.076055475	0.001144554	0.753275333	0.003641322	0.076035325	8.18E-05	0.002902714	1	0.966658386	1	0.962420704	ENSG00000128655	12	PDE11A
SAT	Heart_Failure	ENSG00000118308.10	-0.000803429	0.778165817	0.002851971	0.076055481	-0.000849051	0.765904029	0.002851683	0.076035326	4.56E-05	0.043956713	1	0.972664863	1	0.965963214	ENSG00000128655	12	PDE11A
SAT	Heart_Failure	ENSG00000118407.10	0.003916127	0.242172279	0.003348331	0.07605528	0.003859984	0.248937625	0.003347963	0.076035133	5.61E-05	0.012501016	1	0.759865642	1	0.764045228	ENSG00000128699	9	ORMDL1
SAT	Heart_Failure	ENSG00000118507.11	-0.001647031	0.44702993	0.002166073	0.076055403	-0.001587105	0.463706587	0.002165934	0.076035256	-5.99E-05	0.017974283	1	0.884975994	1	0.892844363	ENSG00000128699	9	ORMDL1
SAT	Heart_Failure	ENSG00000118900.10	0.000580718	0.87810257	0.003786259	0.076055489	0.000521365	0.890466414	0.00378585	0.076035337	5.94E-05	0.025313285	1	0.979223495	1	0.977805317	ENSG00000128709	10	HOXD9
SAT	Heart_Failure	ENSG00000119227.3	-0.000811108	0.605662714	0.001571078	0.076055451	-0.000857934	0.584971387	0.001570911	0.076035294	4.68E-05	3.63E-05	1	0.947817475	1	0.933194299	ENSG00000128789	16	PSMG2
SAT	Heart_Failure	ENSG00000119333.7	6.00E-05	0.982758128	0.002778228	0.076055493	1.03E-05	0.997055904	0.002777946	0.07603534	4.98E-05	0.023201163	1	0.998920999	1	0.999768899	ENSG00000128815	13	WDFY4
SAT	Heart_Failure	ENSG00000119431.5	0.000439854	0.712776319	0.001194833	0.076055472	0.000464427	0.697468355	0.001194698	0.076035317	-2.46E-05	0.001045332	1	0.966658386	1	0.956878334	ENSG00000128815	13	WDFY4
SAT	Heart_Failure	ENSG00000119614.2	0.002802209	0.18677419	0.00212259	0.076055221	0.002764905	0.192658657	0.00212235	0.076035075	3.73E-05	0.004966407	1	0.720656885	1	0.72324148	ENSG00000128829	7	EIF2AK4
SAT	Heart_Failure	ENSG00000119673.10	0.001143491	0.318599671	0.00114654	0.076055338	0.00111977	0.328697597	0.001146434	0.076035191	2.37E-05	0.02182673	1	0.827258183	1	0.833382337	ENSG00000128829	7	EIF2AK4
SAT	Heart_Failure	ENSG00000119718.6	0.001623556	0.246837957	0.001401956	0.076055284	0.001690563	0.227827919	0.001401823	0.076035113	-6.70E-05	4.19E-08	1	0.761161032	1	0.747837725	ENSG00000128829	7	EIF2AK4
SAT	Heart_Failure	ENSG00000119865.4	0.002881191	0.296132483	0.002757743	0.076055323	0.002795703	0.310645991	0.002757461	0.07603518	8.55E-05	6.57E-05	1	0.813063328	1	0.821155716	ENSG00000128886	7	ELL3
SAT	Heart_Failure	ENSG00000119900.7	-0.000389999	0.883255102	0.002655871	0.07605549	-0.000466999	0.860408074	0.002655596	0.076035335	7.70E-05	0.000142763	1	0.979223495	1	0.972791643	ENSG00000128891	11	C15orf57
SAT	Heart_Failure	ENSG00000119977.16	-0.001799587	0.635472923	0.003796291	0.076055458	-0.00188146	0.620136018	0.003795884	0.076035302	8.19E-05	0.002401192	1	0.959428651	1	0.94798204	ENSG00000128891	11	C15orf57
SAT	Heart_Failure	ENSG00000120071.8	0.001156747	0.652927437	0.002572267	0.076055461	0.001248903	0.627267837	0.002572002	0.076035303	-9.22E-05	2.91E-06	1	0.962760695	1	0.94798204	ENSG00000128928	4	IVD
SAT	Heart_Failure	ENSG00000120093.7	0.002056706	0.420811835	0.002554869	0.076055392	0.00212637	0.405206044	0.002554628	0.076035232	-6.97E-05	0.001770846	1	0.876068293	1	0.867451005	ENSG00000128944	9	KNSTRN
SAT	Heart_Failure	ENSG00000120322.2	-7.96E-05	0.962984756	0.001714509	0.076055493	-5.14E-05	0.976064283	0.001714324	0.07603534	-2.81E-05	0.01947219	1	0.995788523	1	0.999521508	ENSG00000128965	7	CHAC1
SAT	Heart_Failure	ENSG00000120784.11	0.001270785	0.357250697	0.001380362	0.076055361	0.001246962	0.366288988	0.001380227	0.076035213	2.38E-05	0.036887428	1	0.848363544	1	0.849290727	ENSG00000129003	11	VPS13C
SAT	Heart_Failure	ENSG00000120805.9	0.002137015	0.432620672	0.002723299	0.076055397	0.002100968	0.440373129	0.002722999	0.076035247	3.60E-05	0.047959076	1	0.879986926	1	0.879941679	ENSG00000129003	11	VPS13C
SAT	Heart_Failure	ENSG00000120963.7	0.002308345	0.460818657	0.003129958	0.076055408	0.002407521	0.441732941	0.003129615	0.076035248	-9.92E-05	2.60E-06	1	0.890157253	1	0.880611614	ENSG00000129003	11	VPS13C
SAT	Heart_Failure	ENSG00000121552.3	-0.000726594	0.842841383	0.003664844	0.076055487	-0.000887635	0.808603858	0.003664464	0.076035331	0.000161041	7.88E-09	1	0.976669362	1	0.972791643	ENSG00000129055	8	ANAPC13
SAT	Heart_Failure	ENSG00000122417.11	0.002359802	0.352471091	0.002537947	0.076055358	0.002444555	0.335395233	0.002537676	0.076035195	-8.48E-05	3.79E-06	1	0.848363544	1	0.836830236	ENSG00000129055	8	ANAPC13
SAT	Heart_Failure	ENSG00000122477.8	0.000608074	0.72766858	0.001746192	0.076055474	0.000653745	0.708093445	0.001746028	0.076035318	-4.57E-05	0.002984851	1	0.966658386	1	0.958028875	ENSG00000129055	8	ANAPC13
SAT	Heart_Failure	ENSG00000122565.14	0.000379184	0.896247209	0.002907777	0.07605549	0.000293444	0.919608398	0.002907484	0.076035339	8.57E-05	0.00021578	1	0.979542669	1	0.984094962	ENSG00000129055	8	ANAPC13
SAT	Heart_Failure	ENSG00000122912.10	0.005584592	0.139582376	0.003780141	0.076055152	0.005675587	0.133206777	0.003779748	0.076034988	-9.10E-05	0.001511832	1	0.666622671	1	0.649215182	ENSG00000129055	8	ANAPC13
SAT	Heart_Failure	ENSG00000123096.7	0.001465075	0.63682594	0.003103041	0.076055458	0.001408129	0.649946362	0.003102719	0.076035308	5.69E-05	0.015702586	1	0.959428651	1	0.953700363	ENSG00000129187	10	DCTD
SAT	Heart_Failure	ENSG00000123143.8	0.000800479	0.808450868	0.003301966	0.076055484	0.000923004	0.779814228	0.003301615	0.076035328	-0.000122524	2.99E-07	1	0.976669362	1	0.966957854	ENSG00000129187	10	DCTD
SAT	Heart_Failure	ENSG00000123179.9	0.000204013	0.88062377	0.001358471	0.076055489	0.000221593	0.870409865	0.001358318	0.076035336	-1.76E-05	0.039752222	1	0.979223495	1	0.974665278	ENSG00000129197	10	RPAIN
SAT	Heart_Failure	ENSG00000123191.9	-0.00013013	0.894324342	0.000979637	0.07605549	-9.98E-05	0.918816407	0.000979537	0.076035339	-3.03E-05	6.49E-05	1	0.979270459	1	0.984094962	ENSG00000129204	12	USP6
SAT	Heart_Failure	ENSG00000123213.18	-0.002180071	0.345413983	0.002310565	0.076055354	-0.002222146	0.336133288	0.00231032	0.076035196	4.21E-05	0.012933578	1	0.847011103	1	0.837075007	ENSG00000129204	12	USP6
SAT	Heart_Failure	ENSG00000123545.5	0.001521746	0.298907576	0.001464936	0.076055325	0.001496574	0.306921532	0.001464782	0.076035177	2.52E-05	0.020672632	1	0.814547972	1	0.815392345	ENSG00000129204	12	USP6
SAT	Heart_Failure	ENSG00000123870.9	-0.002658655	0.384002152	0.003054006	0.076055375	-0.002605695	0.393493388	0.003053669	0.076035227	-5.30E-05	0.009043186	1	0.858250041	1	0.862334842	ENSG00000129204	12	USP6
SAT	Heart_Failure	ENSG00000123989.9	-0.001357213	0.621434236	0.002748402	0.076055455	-0.001318239	0.631447604	0.002748103	0.076035304	-3.90E-05	0.039687528	1	0.954216727	1	0.94798204	ENSG00000129204	12	USP6
SAT	Heart_Failure	ENSG00000124098.9	-0.004956065	0.122785197	0.00321157	0.076055121	-0.00489182	0.127671792	0.003211225	0.076034978	-6.42E-05	0.004620035	1	0.646585619	1	0.647874406	ENSG00000129204	12	USP6
SAT	Heart_Failure	ENSG00000124140.8	-0.002417244	0.138340591	0.001631068	0.07605515	-0.002384368	0.143739999	0.001630887	0.076035007	-3.29E-05	0.00239269	1	0.664561462	1	0.659101398	ENSG00000129219	9	PLD2
SAT	Heart_Failure	ENSG00000124226.7	0.00297941	0.427351873	0.003753673	0.076055395	0.003063643	0.414350235	0.003753255	0.076035236	-8.42E-05	0.000567152	1	0.878482333	1	0.867451005	ENSG00000129219	9	PLD2
SAT	Heart_Failure	ENSG00000124406.12	0.001126527	0.731410552	0.003281942	0.076055475	0.001175911	0.720092695	0.003281595	0.07603532	-4.94E-05	0.039448202	1	0.966658386	1	0.958028875	ENSG00000129317	10	PUS7L
SAT	Heart_Failure	ENSG00000124508.12	0.000984096	0.450670349	0.00130465	0.076055404	0.001142358	0.38204321	0.001306839	0.076035221	-0.000158262	0.043874246	1	0.887019168	1	0.854967426	ENSG00000129317	10	PUS7L
SAT	Heart_Failure	ENSG00000124532.10	0.005799644	0.04488301	0.002891503	0.076054865	0.005867448	0.04241647	0.0028912	0.076034697	-6.78E-05	0.001697117	1	0.469267596	1	0.45628996	ENSG00000129317	10	PUS7L
SAT	Heart_Failure	ENSG00000124562.5	0.002143471	0.142264994	0.001460717	0.076055157	0.002204589	0.131208206	0.001460614	0.076034985	-6.11E-05	0.000186596	1	0.669181587	1	0.649215182	ENSG00000129347	15	KRI1
SAT	Heart_Failure	ENSG00000124587.9	-0.000158538	0.884848236	0.001094676	0.07605549	-0.000177351	0.871282528	0.001094556	0.076035336	1.88E-05	0.011991013	1	0.979270459	1	0.974665278	ENSG00000129354	7	AP1M2
SAT	Heart_Failure	ENSG00000124641.10	-0.007986919	0.011923296	0.003176451	0.076054506	-0.007936967	0.01245656	0.003176122	0.076034366	-5.00E-05	0.038394622	1	0.24440108	1	0.252283786	ENSG00000129355	6	CDKN2D
SAT	Heart_Failure	ENSG00000124659.5	-0.007215493	0.005092145	0.002575896	0.076054268	-0.007140948	0.005562795	0.002575626	0.076034141	-7.45E-05	9.86E-05	1	0.162207064	1	0.179428249	ENSG00000129355	6	CDKN2D
SAT	Heart_Failure	ENSG00000124678.13	0.004374063	0.119717384	0.00281116	0.076055115	0.004306998	0.125463353	0.002810912	0.076034974	6.71E-05	0.011200334	1	0.646585619	1	0.647874406	ENSG00000129355	6	CDKN2D
SAT	Heart_Failure	ENSG00000124713.5	-0.001733801	0.290793614	0.001641261	0.076055319	-0.001698303	0.300731128	0.001641082	0.076035173	-3.55E-05	0.001623881	1	0.803109301	1	0.810520426	ENSG00000129451	7	KLK10
SAT	Heart_Failure	ENSG00000124787.9	0.000414242	0.901782594	0.003356633	0.076055491	0.000479885	0.886305444	0.003356294	0.076035337	-6.56E-05	0.013748156	1	0.981342849	1	0.975781755	ENSG00000129465	11	RIPK3
SAT	Heart_Failure	ENSG00000124920.9	-0.003347244	0.220661455	0.002732951	0.076055259	-0.003236527	0.236290526	0.002732835	0.076035121	-0.000110717	0.002532339	1	0.750422029	1	0.752162449	ENSG00000129473	5	BCL2L2
SAT	Heart_Failure	ENSG00000124939.4	-0.002750025	0.30242988	0.002666723	0.076055327	-0.002540614	0.340929815	0.002667786	0.076035199	-0.000209411	0.015977274	1	0.816013516	1	0.83914552	ENSG00000129473	5	BCL2L2
SAT	Heart_Failure	ENSG00000125124.7	-0.000776465	0.391614339	0.00090635	0.076055378	-0.000799452	0.377696022	0.000906254	0.076035219	2.30E-05	0.00049987	1	0.862642401	1	0.854967426	ENSG00000129480	8	DTD2
SAT	Heart_Failure	ENSG00000125388.15	0.002181496	0.023145757	0.000960577	0.076054688	0.002148146	0.025317073	0.000960479	0.07603456	3.34E-05	8.06E-06	1	0.327199936	1	0.353469426	ENSG00000129480	8	DTD2
SAT	Heart_Failure	ENSG00000125510.11	-0.002714242	0.377175701	0.003073494	0.076055371	-0.002786477	0.36456234	0.003073184	0.076035212	7.22E-05	0.003209219	1	0.85759885	1	0.849290727	ENSG00000129480	8	DTD2
SAT	Heart_Failure	ENSG00000125648.10	-0.004773946	0.067035217	0.00260666	0.07605497	-0.004715019	0.070445736	0.002606369	0.076034829	-5.89E-05	0.000452113	1	0.534977442	1	0.532563273	ENSG00000129484	9	PARP2
SAT	Heart_Failure	ENSG00000125656.4	-0.000435882	0.885574476	0.003028927	0.07605549	-0.000540666	0.858316299	0.003028638	0.076035335	0.000104784	5.70E-05	1	0.979270459	1	0.972791643	ENSG00000129484	9	PARP2
SAT	Heart_Failure	ENSG00000125818.13	0.000868176	0.695891463	0.002221121	0.076055469	0.000927572	0.676198325	0.002220896	0.076035313	-5.94E-05	0.000698871	1	0.966658386	1	0.956878334	ENSG00000129484	9	PARP2
SAT	Heart_Failure	ENSG00000125965.4	-0.00210129	0.484306755	0.003004448	0.076055417	-0.002041358	0.496818445	0.003004185	0.076035268	-5.99E-05	0.034901056	1	0.897349195	1	0.894608312	ENSG00000129562	6	DAD1
SAT	Heart_Failure	ENSG00000125966.8	-0.006245438	0.10103496	0.003808519	0.076055073	-0.006173651	0.104976747	0.00380809	0.07603493	-7.18E-05	0.002619981	1	0.622638992	1	0.620855876	ENSG00000129562	6	DAD1
SAT	Heart_Failure	ENSG00000126091.15	0.008645909	0.00542871	0.003109545	0.076054286	0.008545103	0.005990657	0.003109227	0.076034162	0.000100806	2.85E-05	1	0.164958288	1	0.182466145	ENSG00000129562	6	DAD1
SAT	Heart_Failure	ENSG00000126226.17	0.000965522	0.803310753	0.00387659	0.076055483	0.001034296	0.789595343	0.003876146	0.076035329	-6.88E-05	0.003121355	1	0.976669362	1	0.970635778	ENSG00000129562	6	DAD1
SAT	Heart_Failure	ENSG00000126251.5	0.000267015	0.880223469	0.001771995	0.076055489	0.000235326	0.894338869	0.001771815	0.076035337	3.17E-05	0.022190237	1	0.979223495	1	0.97886604	ENSG00000129657	10	SEC14L1
SAT	Heart_Failure	ENSG00000126464.9	3.82E-05	0.983763997	0.001874809	0.076055493	-3.31E-06	0.998589554	0.001874604	0.07603534	4.15E-05	0.001172563	1	0.998924529	1	0.999768899	ENSG00000129657	10	SEC14L1
SAT	Heart_Failure	ENSG00000126602.6	-0.000412112	0.530177749	0.000656506	0.076055432	-0.000401475	0.540802131	0.000656432	0.076035282	-1.06E-05	0.010616822	1	0.908320477	1	0.911336308	ENSG00000129657	10	SEC14L1
SAT	Heart_Failure	ENSG00000126698.6	0.002022392	0.500833518	0.003004239	0.076055422	0.001865029	0.534691024	0.003003937	0.07603528	0.000157363	4.88E-11	1	0.900549813	1	0.90897474	ENSG00000129667	8	RHBDF2
SAT	Heart_Failure	ENSG00000126790.7	-0.001651979	0.416717123	0.00203413	0.07605539	-0.001420294	0.48507373	0.002034321	0.076035264	-0.000231685	8.74E-08	1	0.87398914	1	0.894608312	ENSG00000129667	8	RHBDF2
SAT	Heart_Failure	ENSG00000126870.11	-0.003102669	0.308561659	0.003047072	0.076055331	-0.003190211	0.295059337	0.003046745	0.076035169	8.75E-05	4.74E-05	1	0.816225947	1	0.808088218	ENSG00000129673	5	AANAT
SAT	Heart_Failure	ENSG00000127337.2	-0.000810192	0.517607563	0.001252155	0.076055428	-0.000792054	0.526982304	0.001252023	0.076035278	-1.81E-05	0.048264416	1	0.90671138	1	0.906199653	ENSG00000129933	16	MAU2
SAT	Heart_Failure	ENSG00000127419.12	-0.001088843	0.718336917	0.003018839	0.076055473	-0.001157263	0.701433421	0.00301853	0.076035317	6.84E-05	0.003350335	1	0.966658386	1	0.956878334	ENSG00000130035	2	GALNT8
SAT	Heart_Failure	ENSG00000127463.9	0.00109189	0.68441654	0.002686446	0.076055467	0.00104237	0.697977878	0.002686163	0.076035317	4.95E-05	0.012305895	1	0.966658386	1	0.956878334	ENSG00000130035	2	GALNT8
SAT	Heart_Failure	ENSG00000127481.10	9.67E-05	0.944671817	0.001392983	0.076055492	5.42E-05	0.968986608	0.001392834	0.07603534	4.25E-05	1.76E-05	1	0.992414898	1	0.99755193	ENSG00000130147	11	SH3BP4
SAT	Heart_Failure	ENSG00000127616.13	-0.003351544	0.272099346	0.003051735	0.076055305	-0.003250991	0.286701073	0.003051463	0.076035163	-0.000100553	0.000397085	1	0.785878482	1	0.799139368	ENSG00000130158	9	DOCK6
SAT	Heart_Failure	ENSG00000127903.12	-0.00141587	0.650144893	0.003121675	0.076055461	-0.001471291	0.637381402	0.003121352	0.076035305	5.54E-05	0.020017125	1	0.962760695	1	0.94798204	ENSG00000130177	10	CDC16
SAT	Heart_Failure	ENSG00000128185.5	-0.001966329	0.556313863	0.003342245	0.076055439	-0.002016764	0.546190011	0.003341893	0.076035283	5.04E-05	0.040215289	1	0.922001323	1	0.912597963	ENSG00000130177	10	CDC16
SAT	Heart_Failure	ENSG00000128408.7	0.004224543	0.075844495	0.002379585	0.076055001	0.004098684	0.084977024	0.002379477	0.076034877	0.000125859	6.03E-05	1	0.57074722	1	0.58987835	ENSG00000130193	7	THEM6
SAT	Heart_Failure	ENSG00000128563.9	0.000288972	0.923381345	0.003004637	0.076055492	0.00035832	0.905066891	0.003004444	0.076035338	-6.93E-05	0.048246331	1	0.988739396	1	0.981985605	ENSG00000130244	8	FAM98C
SAT	Heart_Failure	ENSG00000128591.11	0.003182036	0.413265109	0.003889249	0.076055389	0.003096891	0.425826414	0.003888835	0.076035241	8.51E-05	0.002389652	1	0.872873333	1	0.873465455	ENSG00000130244	8	FAM98C
SAT	Heart_Failure	ENSG00000128604.14	-0.000474654	0.856832006	0.002630998	0.076055488	-0.000551086	0.834072985	0.002630725	0.076035333	7.64E-05	0.000131912	1	0.976669362	1	0.972791643	ENSG00000130270	12	ATP8B3
SAT	Heart_Failure	ENSG00000128789.16	-0.000358194	0.840180592	0.001776207	0.076055487	-0.000397242	0.823014246	0.00177602	0.076035332	3.90E-05	0.002898333	1	0.976669362	1	0.972791643	ENSG00000130270	12	ATP8B3
SAT	Heart_Failure	ENSG00000128829.7	-0.001288024	0.392778424	0.001507183	0.076055379	-0.001315175	0.382826877	0.001507018	0.076035221	2.72E-05	0.007879255	1	0.863144683	1	0.854967426	ENSG00000130299	12	GTPBP3
SAT	Heart_Failure	ENSG00000129003.11	-0.001598451	0.504399805	0.002394383	0.076055423	-0.001531681	0.522325402	0.002394129	0.076035276	-6.68E-05	0.000128801	1	0.9005699	1	0.904369708	ENSG00000130299	12	GTPBP3
SAT	Heart_Failure	ENSG00000129204.12	-0.000689358	0.623388053	0.001403831	0.076055455	-0.000668012	0.634144493	0.001403676	0.076035305	-2.13E-05	0.023286202	1	0.955683491	1	0.94798204	ENSG00000130300	4	PLVAP
SAT	Heart_Failure	ENSG00000129317.10	-0.003151207	0.223992168	0.00259149	0.076055262	-0.003086624	0.23359362	0.002591288	0.076035119	-6.46E-05	0.016762258	1	0.751217604	1	0.750248244	ENSG00000130300	4	PLVAP
SAT	Heart_Failure	ENSG00000129480.8	-0.002488857	0.306208748	0.0024324	0.07605533	-0.002531311	0.297982914	0.002432153	0.076035171	4.25E-05	0.02695793	1	0.816225947	1	0.810520426	ENSG00000130300	4	PLVAP
SAT	Heart_Failure	ENSG00000129484.9	0.000108043	0.975051031	0.00345471	0.076055493	0.000164029	0.962127019	0.00345437	0.07603534	-5.60E-05	0.049544659	1	0.998920999	1	0.99592587	ENSG00000130300	4	PLVAP
SAT	Heart_Failure	ENSG00000129562.6	0.000627649	0.814100981	0.002669269	0.076055484	0.000675419	0.800221989	0.002669006	0.07603533	-4.78E-05	0.029878265	1	0.976669362	1	0.972791643	ENSG00000130312	2	MRPL34
SAT	Heart_Failure	ENSG00000129657.10	0.001647223	0.520974097	0.002566387	0.076055429	0.00171915	0.502893418	0.002566108	0.07603527	-7.19E-05	4.45E-05	1	0.906724424	1	0.900571724	ENSG00000130312	2	MRPL34
SAT	Heart_Failure	ENSG00000130177.10	-0.000309786	0.866177825	0.001838326	0.076055489	-0.000337608	0.854273643	0.001838136	0.076035335	2.78E-05	0.047549223	1	0.978259692	1	0.972791643	ENSG00000130332	10	LSM7
SAT	Heart_Failure	ENSG00000130244.8	0.002415952	0.346741047	0.002567621	0.076055355	0.002348948	0.360227777	0.002567347	0.07603521	6.70E-05	0.000264666	1	0.847011103	1	0.848283315	ENSG00000130332	10	LSM7
SAT	Heart_Failure	ENSG00000130270.12	-0.000352894	0.824196712	0.001588536	0.076055485	-0.000323763	0.838484186	0.001588373	0.076035334	-2.91E-05	0.017227737	1	0.976669362	1	0.972791643	ENSG00000130332	10	LSM7
SAT	Heart_Failure	ENSG00000130363.7	-0.000782225	0.576403103	0.001400213	0.076055444	-0.00081107	0.5623794	0.001400058	0.076035288	2.88E-05	0.001754647	1	0.932993874	1	0.918907775	ENSG00000130348	7	QRSL1
SAT	Heart_Failure	ENSG00000130656.4	0.003381002	0.238994485	0.002871327	0.076055277	0.003293511	0.251318301	0.002871027	0.076035135	8.75E-05	4.45E-05	1	0.758532873	1	0.765423527	ENSG00000130348	7	QRSL1
SAT	Heart_Failure	ENSG00000130684.9	0.00155464	0.3567559	0.001686956	0.07605536	0.00157717	0.349774474	0.001686763	0.076035204	-2.25E-05	0.026969866	1	0.848363544	1	0.842583404	ENSG00000130363	7	RSPH3
SAT	Heart_Failure	ENSG00000130695.9	0.001361145	0.467717321	0.001874343	0.076055411	0.001335137	0.476215374	0.001874131	0.076035261	2.60E-05	0.026802329	1	0.893397133	1	0.894608312	ENSG00000130363	7	RSPH3
SAT	Heart_Failure	ENSG00000130714.11	-0.00228231	0.53820593	0.003707885	0.076055434	-0.002346147	0.526856463	0.003707492	0.076035278	6.38E-05	0.017926831	1	0.912011429	1	0.906199653	ENSG00000130363	7	RSPH3
SAT	Heart_Failure	ENSG00000130770.13	0.001124111	0.513540538	0.001720573	0.076055426	0.00103596	0.547067711	0.001720406	0.076035284	8.82E-05	1.16E-09	1	0.905155107	1	0.912597963	ENSG00000130363	7	RSPH3
SAT	Heart_Failure	ENSG00000131100.8	-0.00277356	0.48225139	0.003947079	0.076055416	-0.002716671	0.491234686	0.00394666	0.076035266	-5.69E-05	0.046315378	1	0.897349195	1	0.894608312	ENSG00000130363	7	RSPH3
SAT	Heart_Failure	ENSG00000131165.10	0.008828029	0.000408947	0.00249784	0.076053544	0.008755673	0.00045558	0.002497608	0.076033423	7.24E-05	0.001125138	1	0.031946583	1	0.039533553	ENSG00000130413	11	STK33
SAT	Heart_Failure	ENSG00000131401.7	-0.002153574	0.582850897	0.003921111	0.076055446	-0.002235487	0.568561057	0.003920721	0.076035289	8.19E-05	0.010412594	1	0.937265914	1	0.923489519	ENSG00000130414	7	NDUFA10
SAT	Heart_Failure	ENSG00000131437.11	0.002765978	0.302816808	0.002684339	0.076055327	0.002685456	0.317063785	0.002684084	0.076035184	8.05E-05	0.000518628	1	0.816145643	1	0.823487262	ENSG00000130414	7	NDUFA10
SAT	Heart_Failure	ENSG00000131626.11	0.000710704	0.849992928	0.003757801	0.076055487	0.000785113	0.834485909	0.003757388	0.076035333	-7.44E-05	0.003187419	1	0.976669362	1	0.972791643	ENSG00000130511	11	SSBP4
SAT	Heart_Failure	ENSG00000131711.10	0.002843965	0.041740042	0.001396765	0.076054846	0.002818433	0.043586063	0.001396608	0.076034705	2.55E-05	0.003913555	1	0.461108001	1	0.456297071	ENSG00000130511	11	SSBP4
SAT	Heart_Failure	ENSG00000131849.10	0.001485599	0.652557495	0.003299773	0.076055461	0.001373738	0.677149007	0.003299424	0.076035313	0.000111861	3.30E-06	1	0.962760695	1	0.956878334	ENSG00000130517	9	PGPEP1
SAT	Heart_Failure	ENSG00000131943.13	0.001161394	0.748987039	0.003629633	0.076055477	0.001071041	0.767909462	0.003629284	0.076035327	9.04E-05	0.003565737	1	0.966658386	1	0.965963214	ENSG00000130517	9	PGPEP1
SAT	Heart_Failure	ENSG00000131944.5	-0.003492781	0.162389667	0.002500055	0.076055188	-0.003449073	0.167665669	0.00249979	0.076035043	-4.37E-05	0.016121091	1	0.686431329	1	0.685924016	ENSG00000130518	12	KIAA1683
SAT	Heart_Failure	ENSG00000132002.3	-0.007540214	0.053795995	0.003909926	0.076054913	-0.007592057	0.052143043	0.00390949	0.076034752	5.18E-05	0.041954669	1	0.497196222	1	0.49020437	ENSG00000130518	12	KIAA1683
SAT	Heart_Failure	ENSG00000132139.8	-0.002261918	0.091748126	0.001341389	0.076055049	-0.002301261	0.086209234	0.00134126	0.076034881	3.93E-05	0.000597605	1	0.60325772	1	0.59547894	ENSG00000130520	6	LSM4
SAT	Heart_Failure	ENSG00000132199.14	0.001813232	0.635387939	0.003824115	0.076055458	0.001751075	0.646985508	0.00382369	0.076035307	6.22E-05	0.012728173	1	0.959428651	1	0.95317119	ENSG00000130520	6	LSM4
SAT	Heart_Failure	ENSG00000132205.6	0.004728319	0.167935308	0.003429125	0.076055196	0.004830895	0.158857216	0.003428774	0.07603503	-0.000102576	0.00012753	1	0.694824396	1	0.670798618	ENSG00000130520	6	LSM4
SAT	Heart_Failure	ENSG00000132313.10	0.00408498	0.00519247	0.001461606	0.076054274	0.004024597	0.005898504	0.001461692	0.076034157	6.04E-05	0.034468392	1	0.163002345	1	0.182466145	ENSG00000130520	6	LSM4
SAT	Heart_Failure	ENSG00000132432.9	0.002399849	0.193547966	0.001845815	0.076055229	0.002437759	0.186565299	0.001845655	0.076035068	-3.79E-05	0.031443416	1	0.726250291	1	0.715930698	ENSG00000130529	11	TRPM4
SAT	Heart_Failure	ENSG00000132563.11	-0.007332752	5.96E-05	0.001826686	0.076052978	-0.007308444	6.30E-05	0.001826475	0.076032842	-2.43E-05	0.023079532	0.195701696	0.007106166	0.206637533	0.011955478	ENSG00000130640	9	TUBGCP2
SAT	Heart_Failure	ENSG00000132840.5	-0.00297638	0.087687507	0.001742889	0.076055038	-0.002947731	0.090746004	0.001742695	0.076034894	-2.86E-05	0.010983355	1	0.592308253	1	0.598355674	ENSG00000130656	4	HBZ
SAT	Heart_Failure	ENSG00000132906.13	0.000770718	0.578138961	0.00138592	0.076055445	0.000798801	0.564323887	0.001385769	0.076035288	-2.81E-05	0.003240736	1	0.933960577	1	0.92032393	ENSG00000130684	9	ZNF337
SAT	Heart_Failure	ENSG00000133048.8	0.003017268	0.049316966	0.001534845	0.07605489	0.002981797	0.052025435	0.001534693	0.076034751	3.55E-05	0.004659076	1	0.483011842	1	0.49020437	ENSG00000130695	9	CEP85
SAT	Heart_Failure	ENSG00000133243.4	-0.009932722	0.003186408	0.003367969	0.076054136	-0.00997971	0.003042215	0.003367593	0.07603397	4.70E-05	0.031586796	1	0.127495168	1	0.134046431	ENSG00000130714	11	POMT1
SAT	Heart_Failure	ENSG00000133661.11	-0.000500328	0.671857286	0.001181131	0.076055465	-0.000466627	0.692763743	0.001181013	0.076035316	-3.37E-05	0.000353043	1	0.965985276	1	0.956878334	ENSG00000130714	11	POMT1
SAT	Heart_Failure	ENSG00000133731.5	-0.000378941	0.708982672	0.001015321	0.076055471	-0.000404594	0.690237499	0.001015209	0.076035315	2.57E-05	0.000176994	1	0.966658386	1	0.956878334	ENSG00000130724	4	CHMP2A
SAT	Heart_Failure	ENSG00000133739.11	-0.00161732	0.454695127	0.002163317	0.076055406	-0.001573395	0.466993719	0.0021631	0.076035258	-4.39E-05	0.011381298	1	0.887019168	1	0.892844363	ENSG00000130733	6	YIPF2
SAT	Heart_Failure	ENSG00000133816.9	0.001989139	0.347004688	0.00211517	0.076055355	0.001935033	0.360225393	0.002114937	0.07603521	5.41E-05	0.000137583	1	0.847011103	1	0.848283315	ENSG00000130733	6	YIPF2
SAT	Heart_Failure	ENSG00000133983.10	-0.001676771	0.54092453	0.002742439	0.076055435	-0.001621171	0.554382003	0.002742138	0.076035286	-5.56E-05	0.002542718	1	0.913035829	1	0.915531921	ENSG00000130733	6	YIPF2
SAT	Heart_Failure	ENSG00000133997.7	-0.002079645	0.495732181	0.003052821	0.076055421	-0.002023059	0.507484957	0.003052479	0.076035272	-5.66E-05	0.003733818	1	0.898909993	1	0.901005489	ENSG00000130758	3	MAP3K10
SAT	Heart_Failure	ENSG00000134077.11	-0.004125567	0.169534918	0.003003248	0.076055198	-0.004190869	0.162841218	0.003002956	0.076035036	6.53E-05	0.009519355	1	0.699678071	1	0.679583582	ENSG00000130758	3	MAP3K10
SAT	Heart_Failure	ENSG00000134184.8	0.001593424	0.228497481	0.001323178	0.076055267	0.001653385	0.21141561	0.001323042	0.076035096	-6.00E-05	4.79E-09	1	0.751217604	1	0.733958243	ENSG00000130764	5	LRRC47
SAT	Heart_Failure	ENSG00000134201.6	0.002447187	0.419360327	0.003030451	0.076055391	0.00261904	0.387414196	0.003030185	0.076035224	-0.000171854	2.16E-09	1	0.874438427	1	0.858921514	ENSG00000130770	13	ATPIF1

SAT	Heart_Failure	ENSG00000134202.6	0.001078777	0.387963612	0.001249572	0.076055377	0.001118818	0.370543123	0.001249438	0.076035215	-4.00E-05	6.59E-06	1	0.86096131	1	0.8495821	ENSG00000130770	13	ATPIF1
SAT	Heart_Failure	ENSG00000134216.14	0.000109171	0.966527937	0.002601581	0.076055493	0.000148096	0.954599569	0.002601296	0.07603534	-3.89E-05	0.027591481	1	0.996432082	1	0.991911131	ENSG00000130803	10	ZNF317
SAT	Heart_Failure	ENSG00000134245.13	-0.001153325	0.668589884	0.002694153	0.076055464	-0.001213142	0.652470913	0.00269388	0.076035308	5.98E-05	0.004937111	1	0.965492279	1	0.954583686	ENSG00000130856	11	ZNF236
SAT	Heart_Failure	ENSG00000134262.8	-0.001639782	0.652111232	0.00363724	0.076055461	-0.001566376	0.666693447	0.003636893	0.076035311	-7.34E-05	0.019452609	1	0.962760695	1	0.956878334	ENSG00000130921	3	C12orf65
SAT	Heart_Failure	ENSG00000134283.13	0.001153017	0.683340085	0.002826648	0.076055467	0.00108891	0.700039387	0.002826373	0.076035317	6.41E-05	0.007015741	1	0.966658386	1	0.956878334	ENSG00000130943	5	PKDREJ
SAT	Heart_Failure	ENSG00000134285.6	0.000161862	0.954328818	0.002826218	0.076055493	0.000212787	0.939977298	0.002825921	0.076035339	-5.09E-05	0.015034793	1	0.994131568	1	0.988983601	ENSG00000130943	5	PKDREJ
SAT	Heart_Failure	ENSG00000134574.7	0.006542464	0.085243214	0.003801448	0.076055031	0.006658865	0.079814159	0.003801223	0.076034861	-0.000116401	0.011564839	1	0.592308253	1	0.576806732	ENSG00000131100	8	ATP6V1E1
SAT	Heart_Failure	ENSG00000134775.11	0.001461047	0.66266135	0.003349167	0.076055463	0.001376678	0.681003371	0.00334881	0.076035314	8.44E-05	0.000475899	1	0.962994646	1	0.956878334	ENSG00000131165	10	CHMP1A
SAT	Heart_Failure	ENSG00000134809.4	0.00593309	0.069573452	0.003269493	0.076054979	0.006114801	0.061489654	0.003270013	0.076034795	-0.000181711	0.021384541	1	0.537536555	1	0.505632969	ENSG00000131165	10	CHMP1A
SAT	Heart_Failure	ENSG00000134815.14	-0.005937781	0.045081795	0.002963119	0.076054866	-0.00584861	0.048395436	0.002962995	0.076034732	-8.92E-05	0.02535976	1	0.469267596	1	0.476833109	ENSG00000131351	10	HAUS8
SAT	Heart_Failure	ENSG00000135164.14	-0.000900649	0.698023336	0.002321317	0.07605547	-0.000943459	0.68439325	0.002321071	0.076035314	4.28E-05	0.011242844	1	0.966658386	1	0.956878334	ENSG00000131401	7	NAPSB
SAT	Heart_Failure	ENSG00000135213.8	-0.016293676	9.59E-07	0.003325287	0.076051746	-0.016235475	1.05E-06	0.003324967	0.07603162	-5.82E-05	0.039327579	0.00314652	0.000233648	0.003430974	0.000581811	ENSG00000131401	7	NAPSB
SAT	Heart_Failure	ENSG00000135269.13	-0.000977808	0.683854792	0.002401246	0.076055467	-0.000907032	0.705599284	0.002400995	0.076035318	-7.08E-05	8.49E-05	1	0.966658386	1	0.958028875	ENSG00000131401	7	NAPSB
SAT	Heart_Failure	ENSG00000135480.10	0.001128549	0.7528201	0.003583569	0.076055478	0.001228246	0.731765887	0.003583214	0.076035322	-9.97E-05	0.000677784	1	0.966658386	1	0.958028875	ENSG00000131437	11	KIF3A
SAT	Heart_Failure	ENSG00000135541.16	-0.002156204	0.023023197	0.000948592	0.076054687	-0.002135659	0.024344785	0.000948486	0.076034549	-2.05E-05	0.000665949	1	0.327199936	1	0.342903392	ENSG00000131437	11	KIF3A
SAT	Heart_Failure	ENSG00000135587.4	0.003534599	0.123744235	0.002296314	0.076055123	0.003616593	0.115230578	0.002296082	0.076034953	-8.20E-05	6.37E-06	1	0.647388543	1	0.64481576	ENSG00000131437	11	KIF3A
SAT	Heart_Failure	ENSG00000135636.9	9.60E-05	0.976305485	0.00323306	0.076055493	1.24E-05	0.996934651	0.003232709	0.07603534	8.36E-05	0.000189244	1	0.998920999	1	0.999768899	ENSG00000131437	11	KIF3A
SAT	Heart_Failure	ENSG00000135747.7	0.001506142	0.45658091	0.002023054	0.076055407	0.001472034	0.466793544	0.00202284	0.076035258	3.41E-05	0.021606854	1	0.88796436	1	0.892844363	ENSG00000131473	12	ACLY
SAT	Heart_Failure	ENSG00000135750.10	-0.000126073	0.927464445	0.001384878	0.076055492	-9.36E-05	0.946114995	0.001384733	0.076035339	-3.25E-05	0.00156375	1	0.988739396	1	0.990755659	ENSG00000131473	12	ACLY
SAT	Heart_Failure	ENSG00000135778.7	0.001853046	0.630573135	0.003853122	0.076055457	0.001763231	0.647197843	0.00385272	0.076035307	8.98E-05	0.001917047	1	0.959161083	1	0.95317119	ENSG00000131495	4	NDUFA2
SAT	Heart_Failure	ENSG00000135828.7	-0.001255325	0.534023329	0.002018608	0.076055433	-0.001219335	0.545769155	0.00201839	0.076035283	-3.60E-05	0.011184579	1	0.908781401	1	0.912597963	ENSG00000131495	4	NDUFA2
SAT	Heart_Failure	ENSG00000135845.5	-0.00179907	0.547520067	0.002991068	0.076055437	-0.001914293	0.522126792	0.002990752	0.076035276	0.000115223	1.40E-07	1	0.917005278	1	0.904369708	ENSG00000131495	4	NDUFA2
SAT	Heart_Failure	ENSG00000135913.6	0.006097639	0.021618152	0.002654589	0.07605467	0.006047499	0.022706038	0.00265433	0.07603453	5.01E-05	0.023870538	1	0.317837587	1	0.334074035	ENSG00000131558	10	EXOC4
SAT	Heart_Failure	ENSG00000135926.8	-0.003480989	0.17246318	0.00255142	0.076055203	-0.003445544	0.176828437	0.002551146	0.076035056	-3.54E-05	0.048663933	1	0.704583343	1	0.704659157	ENSG00000131558	10	EXOC4
SAT	Heart_Failure	ENSG00000135972.4	0.001308006	0.372171101	0.001465693	0.076055369	0.001338752	0.360985391	0.001465535	0.07603521	-3.07E-05	0.003187	1	0.857509398	1	0.848283315	ENSG00000131626	11	PPFIA1
SAT	Heart_Failure	ENSG00000136048.9	0.002621343	0.347532062	0.00279048	0.076055355	0.00258085	0.354979756	0.002790186	0.076035207	4.05E-05	0.048922143	1	0.847141677	1	0.843366096	ENSG00000131626	11	PPFIA1
SAT	Heart_Failure	ENSG00000136098.12	-0.000588468	0.561262052	0.001012909	0.07605544	-0.00056603	0.576249935	0.001012809	0.076035291	-2.24E-05	0.006394324	1	0.924448189	1	0.9277115	ENSG00000131653	8	TRAF7
SAT	Heart_Failure	ENSG00000136100.8	-0.001824577	0.409808599	0.002213671	0.076055387	-0.0017453	0.430405435	0.002213446	0.076035243	-7.93E-05	5.66E-06	1	0.870429337	1	0.876213234	ENSG00000131653	8	TRAF7
SAT	Heart_Failure	ENSG00000136104.14	-0.002030294	0.427439125	0.002558392	0.076055395	-0.001981467	0.43858837	0.002558119	0.076035247	-4.88E-05	0.008004552	1	0.878482333	1	0.879941679	ENSG00000131697	13	NPHP4
SAT	Heart_Failure	ENSG00000136143.9	-0.001436635	0.560397154	0.002467376	0.07605544	-0.00151957	0.537942483	0.002467123	0.076035281	8.29E-05	1.39E-05	1	0.923681045	1	0.909319572	ENSG00000131711	10	MAP1B
SAT	Heart_Failure	ENSG00000136213.7	-0.002885079	0.36286915	0.003170722	0.076055364	-0.00282258	0.373308472	0.003170391	0.076035216	-6.25E-05	0.008621983	1	0.85040303	1	0.851164069	ENSG00000131711	10	MAP1B
SAT	Heart_Failure	ENSG00000136247.10	0.008929145	0.02110007	0.003871806	0.076054663	0.008839826	0.022409097	0.003871397	0.076034527	8.93E-05	0.001608556	1	0.315614662	1	0.332367548	ENSG00000131711	10	MAP1B
SAT	Heart_Failure	ENSG00000136319.7	0.003901635	0.077009462	0.002206419	0.076055005	0.003938211	0.074247498	0.00220617	0.076034843	-3.66E-05	0.008887224	1	0.574245555	1	0.55673323	ENSG00000131797	8	CLUHP3
SAT	Heart_Failure	ENSG00000136371.5	0.000763499	0.599224519	0.001452855	0.07605545	0.000744682	0.608215161	0.001452689	0.076035299	1.88E-05	0.033568863	1	0.944941904	1	0.943970645	ENSG00000131845	10	ZNF304
SAT	Heart_Failure	ENSG00000136425.8	-0.001724059	0.59699245	0.003260751	0.076055449	-0.001795886	0.581762793	0.003260434	0.076035293	7.18E-05	0.008612201	1	0.944127995	1	0.931104255	ENSG00000131845	10	ZNF304
SAT	Heart_Failure	ENSG00000136448.7	0.004892308	0.000623825	0.001430056	0.076053666	0.004854534	0.000686288	0.001429898	0.076033542	3.78E-05	6.76E-05	1	0.04264103	1	0.054919768	ENSG00000131845	10	ZNF304
SAT	Heart_Failure	ENSG00000136715.13	0.000322683	0.928355779	0.003588804	0.076055492	0.000418844	0.907082101	0.003588452	0.076035338	-9.62E-05	0.001205885	1	0.988739396	1	0.982067422	ENSG00000131849	10	ZNF132
SAT	Heart_Failure	ENSG00000136783.9	-0.000948873	0.795891609	0.003668305	0.076055483	-0.000833679	0.820198082	0.003667902	0.076035332	-0.000115194	2.80E-06	1	0.976456935	1	0.972791643	ENSG00000131849	10	ZNF132
SAT	Heart_Failure	ENSG00000136856.13	0.000996441	0.738874315	0.002989223	0.076055476	0.000953978	0.749594204	0.00298889	0.076035324	4.25E-05	0.02831516	1	0.966658386	1	0.962415493	ENSG00000131849	10	ZNF132
SAT	Heart_Failure	ENSG00000136982.5	-0.000983178	0.195628208	0.000759733	0.076055232	-0.000995874	0.189872484	0.000759654	0.076035072	1.27E-05	0.024043555	1	0.726878652	1	0.720122525	ENSG00000131849	10	ZNF132
SAT	Heart_Failure	ENSG00000137054.11	0.000121969	0.946883031	0.001830779	0.076055492	8.28E-05	0.963933143	0.001830592	0.07603534	3.92E-05	0.005603878	1	0.992414898	1	0.996055789	ENSG00000131941	3	RHPN2
SAT	Heart_Failure	ENSG00000137106.13	0.002092657	0.285268881	0.001958392	0.076055315	0.002147171	0.272855335	0.001958183	0.076035153	-5.45E-05	0.000122165	1	0.800157	1	0.783093703	ENSG00000131943	13	C19orf12
SAT	Heart_Failure	ENSG00000137185.7	0.000319409	0.840205495	0.001584127	0.076055487	0.001122452	0.484846162	0.001606877	0.076035264	-0.000803043	0.003008225	1	0.976669362	1	0.894608312	ENSG00000131943	13	C19orf12
SAT	Heart_Failure	ENSG00000137216.14	0.003227854	0.232761422	0.002705033	0.076055271	0.003275541	0.225880554	0.002704733	0.076035111	-4.77E-05	0.007655861	1	0.753146179	1	0.747837725	ENSG00000131944	5	C19orf40
SAT	Heart_Failure	ENSG00000137285.9	-0.001618263	0.526928641	0.002557702	0.076055431	-0.001695107	0.507453146	0.002557459	0.076035272	7.68E-05	0.000551649	1	0.906724424	1	0.901005489	ENSG00000131944	5	C19orf40
SAT	Heart_Failure	ENSG00000137310.7	-0.008275913	0.017783664	0.003491799	0.076054616	-0.011932909	0.001553435	0.003770832	0.076033778	0.003656996	0.010264159	1	0.290924976	1	0.09221864	ENSG00000132002	3	DNAJB1
SAT	Heart_Failure	ENSG00000137312.10	0.006788396	0.002646539	0.002258223	0.076054083	0.009188357	6.79E-05	0.002306666	0.076032864	-0.002399961	3.63E-07	1	0.113128813	0.222937851	0.011955478	ENSG00000132139	8	GAS2L2
SAT	Heart_Failure	ENSG00000137404.10	-0.002201354	0.501372287	0.003274203	0.076055422	-0.000278908	0.932271156	0.003281743	0.076035339	-0.001922446	4.12E-17	1	0.900549813	1	0.988742522	ENSG00000132139	8	GAS2L2
SAT	Heart_Failure	ENSG00000137411.12	-0.000496688	0.463089465	0.000676898	0.076055409	-0.001160942	0.095017936	0.000695381	0.076034905	0.000664255	3.17E-05	1	0.890529906	1	0.60300551	ENSG00000132139	8	GAS2L2
SAT	Heart_Failure	ENSG00000137509.6	-0.00013957	0.961197655	0.002868817	0.076055493	-0.000208956	0.941929284	0.002868498	0.076035339	6.94E-05	0.000215938	1	0.995482799	1	0.988983601	ENSG00000132141	9	CCT6B
SAT	Heart_Failure	ENSG00000137561.4	-0.000865101	0.679616997	0.002094747	0.076055466	-0.000897665	0.668230669	0.00209452	0.076035311	3.26E-05	0.025643925	1	0.966658386	1	0.956878334	ENSG00000132141	9	CCT6B
SAT	Heart_Failure	ENSG00000137563.7	-0.001669299	0.159460518	0.001186517	0.076055184	-0.001694176	0.153291078	0.001186394	0.076035022	2.49E-05	0.00592776	1	0.678956178	1	0.667541522	ENSG00000132141	9	CCT6B
SAT	Heart_Failure	ENSG00000137692.7	0.004298671	0.204911676	0.003390966	0.076055242	0.004359535	0.19852749	0.003390629	0.076035082	-6.09E-05	0.027428964	1	0.735350411	1	0.725655076	ENSG00000132141	9	CCT6B
SAT	Heart_Failure	ENSG00000137841.7	-0.001599811	0.353943882	0.001725855	0.076055359	-0.001566641	0.363959481	0.001725666	0.076035212	-3.32E-05	0.004789246	1	0.848363544	1	0.849290727	ENSG00000132199	14	ENOSF1
SAT	Heart_Failure	ENSG00000137876.5	-0.002507079	0.141401379	0.001704806	0.076055155	-0.002532644	0.13734576	0.001704623	0.076034996	2.56E-05	0.033909524	1	0.669181587	1	0.649215182	ENSG00000132205	6	EMILIN2
SAT	Heart_Failure	ENSG00000138036.14	0.002487681	0.361896327	0.002728443	0.076055363	0.002444701	0.37019983	0.002728156	0.076035215	4.30E-05	0.033300014	1	0.85040303	1	0.84956238	ENSG00000132205	6	EMILIN2
SAT	Heart_Failure	ENSG00000138172.6	0.004847633	0.21444437	0.003904851	0.076055252	0.004915429	0.208057932	0.003904464	0.076035093	-6.78E-05	0.034006927	1	0.745086527	1	0.733958243	ENSG00000132205	6	EMILIN2
SAT	Heart_Failure	ENSG00000138175.8	0.003491213	0.15829016	0.00247455	0.076055182	0.003443714	0.163985335	0.002474298	0.076035038	4.75E-05	0.014532669	1	0.67888891	1	0.679583582	ENSG00000132205	6	EMILIN2
SAT	Heart_Failure	ENSG00000138286.10	8.31E-05	0.973020798	0.002457826	0.076055493	1.14E-06	0.999628555	0.002457813	0.07603534	8.20E-05	0.036399614	1	0.99766005	1	0.999768899	ENSG00000132274	11	TRIM22
SAT	Heart_Failure	ENSG00000138380.13	0.003731137	0.09030693	0.002202841	0.076055045	0.003691977	0.09370287	0.002202609	0.076034902	3.92E-05	0.015989281	1	0.598579873	1	0.600153846	ENSG00000132274	11	TRIM22
SAT	Heart_Failure	ENSG00000138459.4	0.002456812	0.020440504	0.001059807	0.076054654	0.002434757	0.021584809	0.001059694	0.076034516	2.21E-05	0.004025303	1	0.312755508	1	0.326358332	ENSG00000132274	11	TRIM22
SAT	Heart_Failure	ENSG00000138592.9	0.001168676	0.648114392	0.002560732	0.076055461	0.001232486	0.630264894	0.002560455	0.076035304	-6.38E-05	0.000360717	1	0.962760695	1	0.94798204	ENSG00000132286	7	TIMM10B
SAT	Heart_Failure	ENSG00000138621.7	-0.001507301	0.13753994	0.001015006	0.076055149	-0.001525388	0.132839397	0.001014895	0.076034988	1.81E-05	0.008657954	1	0.663104757	1	0.649215182	ENSG00000132286	7	TIMM10B
SAT	Heart_Failure	ENSG00000138744.10	0.000835873	0.693735191	0.002122628	0.076055469	0.000880582	0.678217621	0.002122411	0.076035313	-4.47E-05	0.006844058	1	0.966658386	1	0.956878334	ENSG00000132305	16	IMMT
SAT	Heart_Failure	ENSG00000138757.10	0.005088956	0.022053355	0.00222278	0.076054675	0.004957127	0.025727198	0.002222616	0.076034564	0.000131829	3.91E-08	1	0.321586925	1	0.354978312	ENSG00000132305	16	IMMT
SAT	Heart_Failure	ENSG00000138777.15	-0.000413179	0.856955419	0.002292238	0.076055488	-0.000271004	0.905893137	0.002292363	0.076035338	-0.000142175	0.001356933	1	0.976669362	1	0.982067422	ENSG00000132305	16	IMMT
SAT	Heart_Failure	ENSG00000138801.4	0.003555405	0.177836375	0.00263862	0.07605521	0.00363043	0.168818529	0.002638371	0.076035045	-7.50E-05	0.001176145	1	0.713302136	1	0.688922379	ENSG00000132305	16	IMMT
SAT	Heart_Failure	ENSG00000139168.3	0.000782378	0.759713899	0.002557988	0.076055478	0.000733065	0.774411635	0.002557715	0.076035327	4.93E-05	0.007018546	1	0.969137365	1	0.965963214	ENSG00000132305	16	IMMT
SAT	Heart_Failure	ENSG00000139187.5	-0.00156001	0.290673673	0.001476379	0.076055319	-0.001491027	0.312595991	0.00147657	0.076035181	-6.90E-05	0.041110345	1	0.803109301	1	0.821155716	ENSG00000132305	16	IMMT
SAT	Heart_Failure	ENSG00000139197.6	0.000212177	0.951449395	0.003484771	0.076055492	0.000520337	0.881392418	0.003487409	0.076035337	-0.00030816	0.036050016	1	0.994131568	1	0.974665278	ENSG00000132313	10	MRPL35
SAT	Heart_Failure	ENSG00000139343.6	0.000872849	0.624500276	0.001783207	0.076055456	0.000814418	0.647842656	0.001783026	0.076035308	5.84E-05	3.16E-05	1	0.955683491	1	0.95317119	ENSG00000132313	10	MRPL35
SAT	Heart_Failure	ENSG00000139505.10	-0.001542326	0.424065006	0.00192938	0.076055393	-0.001572142	0.415113448	0.001929177	0.076035237	2.98E-05	0.037095119	1	0.876565321	1	0.867451005	ENSG00000132313	10	MRPL35
SAT	Heart_Failure	ENSG00000139508.10	-0.002569239	0.424256547	0.00321533	0.076055393	-0.002617653	0.415528865	0.003214987	0.076035237	4.84E-05	0.035868219	1	0.876565321	1	0.867451005	ENSG00000132330	12	SCLY
SAT	Heart_Failure	ENSG00000139537.6	-0.000769996	0.739078226	0.002311785	0.076055476	-0.000707695	0.759485694	0.002311547	0.076035326	-6.23E-05	0.000464305	1	0.966658386	1	0.963228667	ENSG00000132330	12	SCLY
SAT	Heart_Failure	ENSG00000139620.8	0.000241298	0.893211092	0.001797481	0.07605549	0.000275295	0.878263317	0.001797304	0.076035336	-3.40E-05	0.022315337	1	0.979270459	1	0.974665278	ENSG00000132382	10	MYBBP1A
SAT	Heart_Failure	ENSG00000139624.8	-0.005488452	0.07904742	0.003125108	0.076055012	-0.00561357	0.072436925	0.003124959	0.076034837	0.000125118	0.002102349	1	0.580653917	1	0.545104471	ENSG00000132382	10	MYBBP1A
SAT	Heart_Failure	ENSG00000139835.9	-0.000918273	0.703538405	0.002413024	0.07605547	-0.000866004	0.719648869	0.00241275	0.07603532	-5.23E-05	0.000415436	1	0.966658386	1	0.958028875	ENSG00000132383	7	RPA1
SAT	Heart_Failure	ENSG00000140319.6	0.002230588	0.441821696	0.002900177	0.076055401	0.002278753	0.431976688	0.002899863	0.076035244	-4.82E-05	0.016898785	1	0.882529682	1	0.876305924	ENSG00000132406	7	TMEM128
SAT	Heart_Failure	ENSG00000140386.8	-0.000245865	0.911269801	0.002206309	0.076055491	-0.000274428	0.901000911	0.002206062	0.076035338	2.86E-05	0.041693186	1	0.984491733	1	0.980373283	ENSG00000132432	9	SEC61G
SAT	Heart_Failure	ENSG00000140553.12	0.001255656	0.518480199	0.001944672	0.076055428	0.001284892	0.508744692	0.001944466	0.076035272	-2.92E-05	0.039625393	1	0.906724424	1	0.902291622	ENSG00000132432	9	SEC61G
SAT	Heart_Failure	ENSG00000140575.8	-0.003159543	0.022563671	0.001385309	0.076054681	-0.003196991	0.020998974	0.001385172	0.076034509	3.74E-05	0.000958115	1	0.324699143	1	0.320454116	ENSG00000132432	9	SEC61G
SAT	Heart_Failure	ENSG00000140995.12	0.004021842	0.086409569	0.002345573	0.076055034	0.003967805	0.090684173	0.002345312	0.076034894	5.40E-05	0.000394643	1	0.592308253	1	0.598355674	ENSG00000132507	13	EIF5A
SAT	Heart_Failure	ENSG00000141012.8	-0.003687109	0.285887906	0.003454979	0.076055315	-0.003796111	0.271835039	0.003454629	0.076035152	0.000109002	6.37E-05	1	0.800157	1	0.783093703	ENSG00000132507	13	EIF5A
SAT	Heart_Failure	ENSG00000141013.10	0.001326292	0.265779784	0.001191813	0.0760553	0.001301686	0.274697147	0.001191681	0.076035154	2.46E-05	0.001783254	1	0.777897832	1	0.784122993	ENSG00000132507	13	EIF5A
SAT	Heart_Failure	ENSG00000141076.13	0.003432469	0.038860594	0.001661677	0.076054827	0.00347367	0.036556936	0.001661499	0.076034658	-4.12E-05	0.000564825	1	0.445276442	1	0.42836895	ENSG00000132563	11	REEP2
SAT	Heart_Failure	ENSG00000141127.10	-0.001367644	0.618167976	0.002743801	0.076055454	-0.001311891	0.632520856	0.002743498	0.076035304	-5.58E-05	0.002138568	1	0.954216727	1	0.94798204	ENSG00000132563	11	REEP2
SAT	Heart_Failure	ENSG00000141219.11	-0.00013697	0.947633875	0.002085458	0.076055492	-8.06E-05	0.969173689	0.002085259	0.07603534	-5.64E-05	0.001729699	1	0.992414898	1	0.99755193	ENSG00000132600	12	PRMT7
SAT	Heart_Failure	ENSG00000141258.8	-0.00123046	0.633886662	0.002583569	0.076055458	-0.001300566	0.614645914	0.002583292	0.076035301	7.01E-05	0.000135733	1	0.959428651	1	0.945897394	ENSG00000132600	12	PRMT7
SAT	Heart_Failure	ENSG00000141295.9	-0.000129334	0.926300306	0.001398191	0.076055492	-0.000101786	0.941960912	0.001398047	0.076035339	-2.75E-05	0.010540886	1	0.988739396	1	0.988983601	ENSG00000132613	10	MTSS1L
SAT	Heart_Failure	ENSG00000141391.9	0.000153751	0.933741656	0.001849336	0.076055492	0.000200558	0.91363004	0.001849127	0.076035338	-4.68E-05	5.25E-05	1	0.989642217	1	0.98402346	ENSG00000132819	12	RBM38
SAT	Heart_Failure	ENSG00000141452.5	-0.003274805	0.208805887	0.00260555	0.076055246	-0.003194255	0.220172197	0.002605275	0.076035106	-8.05E-05	2.64E-05	1	0.739840299	1	0.742028061	ENSG00000132824	9	SERINC3
SAT	Heart_Failure	ENSG00000141458.8	-0.003240448	0.360102267	0.003540811	0.076055362	-0.003147467	0.374000047	0.003540432	0.076035217	-9.30E-05	0.000240778	1	0.85040303	1	0.852148719	ENSG00000132824	9	SERINC3
SAT	Heart_Failure	ENSG00000141519.10	0.001787314	0.617673204	0.003580708	0.076055454	0.001876281	0.600241298	0.00358032	0.076035297	-8.90E-05	0.000363081	1	0.954216727	1	0.938338302	ENSG00000132824	9	SERINC3
SAT	Heart_Failure	ENSG00000141569.6	0.003733222	0.055213631	0.00194723	0.076054919	0.003641023	0.06148105	0.001947046	0.076034794	9.22E-05	5.09E-08	1	0.503210899	1	0.505632969	ENSG00000132837	10	DMGDH
SAT	Heart_Failure	ENSG00000141576.10	-0.000859242	0.707735949	0.002291932	0.076055471	-0.000926495	0.686005973	0.002291709	0.076035315	6.73E-05	0.000473176	1	0.966658386	1	0.956878334	ENSG00000132837	10	DMGDH
SAT	Heart_Failure	ENSG00000141577.9	-0.000584278	0.876344981	0.003754893	0.076055489	-0.000654516	0.861606984	0.003754488	0.076035335	7.02E-05	0.00783878	1	0.979223495	1	0.972791643	ENSG00000132837	10	DMGDH
SAT	Heart_Failure	ENSG00000141580.11	-0.003133355	0.220104195	0.002555228	0.076055258	-0.003191017	0.211686363	0.002554974	0.076035097	5.77E-05	0.005390615	1	0.749908476	1	0.733958243	ENSG00000132837	10	DMGDH
SAT	Heart_Failure	ENSG00000141639.7	-0.005502929	0.110301778	0.003446134	0.076055095	-0.005442087	0.114254001	0.003445753	0.076034951	-6.08E-05	0.007544093	1	0.640599412	1	0.64481576	ENSG00000132840	5	BHMT2
SAT	Heart_Failure	ENSG00000141759.10	-0.001110865	0.402132614	0.001325901	0.076055383	-0.001064047	0.422210187	0.001325761	0.07603524	-4.68E-05	1.56E-06	1	0.870429337	1	0.873390936	ENSG00000132840	5	BHMT2
SAT	Heart_Failure	ENSG00000141956.9	-0.00091676	0.543176713	0.001507787	0.076055435	-0.000887833	0.555937474	0.001507647	0.076035286	-2.89E-05	0.030702589	1	0.913779466	1	0.915531921	ENSG00000132840	5	BHMT2
SAT	Heart_Failure	ENSG00000141959.12	0.001564207	0.42833476	0.001974896	0.076055395	0.00160946	0.415050065	0.001974701	0.076035236	-4.53E-05	0.005256542	1	0.878482333	1	0.867451005	ENSG00000132881	7	RSG1
SAT	Heart_Failure	ENSG00000141994.11	-0.00341284	0.031401426	0.001585926	0.07605477	-0.0033773	0.033189886	0.001585747	0.076034632	-3.55E-05	0.000364248	1	0.396261842	1	0.407850241	ENSG00000132906	13	CASP9
SAT	Heart_Failure	ENSG00000142185.12	-0.000619272	0.806110058	0.002523033	0.076055484	-0.000507039	0.840710817	0.002522777	0.076035334	-0.000112233	1.38E-08	1	0.976669362	1	0.972791643	ENSG00000132906	13	CASP9
SAT	Heart_Failure	ENSG00000142675.13	0.000380754	0.708100411	0.001016949	0.076055471	0.000398895	0.694843431	0.001016834	0.076035316	-1.81E-05	0.004689622	1	0.966658386	1	0.956878334	ENSG00000132906	13	CASP9
SAT	Heart_Failure	ENSG00000142677.3	0.003219621	0.400499077	0.00382955	0.076055383	0.00329899	0.388936754	0.003829142	0.076035224	-7.94E-05	0.004148974	1	0.870429337	1	0.858921514	ENSG00000132906	13	CASP9
SAT	Heart_Failure	ENSG00000142684.7	0.00108751	0.730021116	0.00315131	0.076055474	0.001142402	0.716935623	0.003150954	0.07603532	-5.49E-05	0.005651272	1	0.966658386	1	0.958028875	ENSG00000133028	6	SCO1
SAT	Heart_Failure	ENSG00000142798.12	-0.001922506	0.100553412	0.001170704	0.076055072	-0.002519016	0.031805324	0.001173352	0.076034621	0.000596509	1.89E-13	1	0.622495351	1	0.401948008	ENSG00000133048	8	CHI3L1
SAT	Heart_Failure	ENSG00000142973.8	-3.29E-06	0.998951098	0.002502299	0.076055493	-4.59E-05	0.985375884	0.002502027	0.07603534	4.26E-05	0.013615371	1	0.999875583	1	0.999768899	ENSG00000133048	8	CHI3L1
SAT	Heart_Failure	ENSG00000143028.7	0.006561862	0.029681029	0.003017881	0.076054755	0.006497797	0.031294237	0.003017575	0.076034617	6.41E-05	0.007044907	1	0.380908976	1	0.397970509	ENSG00000133059	12	DSTYK
SAT	Heart_Failure	ENSG00000143303.7	0.005066722	0.024680375	0.002255509	0.076054705	0.005146585	0.022488752	0.002255279	0.076034528	-7.99E-05	5.78E-06	1	0.336323411	1	0.332367548	ENSG00000133059	12	DSTYK
SAT	Heart_Failure	ENSG00000143367.11	-0.000962508	0.743576439	0.002942359	0.076055476	-0.000550901	0.851680513	0.00294639	0.076035335	-0.000411607	0.01074267	1	0.966658386	1	0.972791643	ENSG00000133069	10	TMCC2
SAT	Heart_Failure	ENSG00000143418.15	0.002210548	0.481804685	0.003142646	0.076055416	0.00308132	0.330265997	0.00316494	0.076035192	-0.000870771	0.02140778	1	0.897349195	1	0.834182246	ENSG00000133069	10	TMCC2
SAT	Heart_Failure	ENSG00000143437.16	-0.007324629	0.025883812	0.003287598	0.076054718	-0.006653023	0.043926481	0.003302081	0.076034707	-0.000671606	0.03217013	1	0.348052412	1	0.456297071	ENSG00000133243	4	BTBD2
SAT	Heart_Failure	ENSG00000143442.17	0.000509131	0.823620489	0.002284214	0.076055485	0.000594315	0.794703903	0.002284003	0.07603533	-8.52E-05	3.11E-05	1	0.976669362	1	0.970635778	ENSG00000133316	11	WDR74
SAT	Heart_Failure	ENSG00000143452.11	0.000211928	0.826820377	0.000968675	0.076055486	0.000539943	0.580793851	0.000977758	0.076035293	-0.000328015	0.014293121	1	0.976669362	1	0.930810832	ENSG00000133316	11	WDR74
SAT	Heart_Failure	ENSG00000143486.11	-0.002363978	0.35694446	0.002566183	0.076055361	-0.002305445	0.368930818	0.002565943	0.076035214	-5.85E-05	0.009653062	1	0.848363544	1	0.849425432	ENSG00000133316	11	WDR74
SAT	Heart_Failure	ENSG00000143631.10	-0.005342412	0.00066766	0.001570121	0.076053686	-0.005291242	0.000751165	0.001570003	0.076033568	-5.12E-05	0.002341759	1	0.04470599	1	0.05868032	ENSG00000133433	6	GSTT2B
SAT	Heart_Failure	ENSG00000143702.11	0.009909371	0.001400976	0.003102041	0.0760539	0.009976148	0.001298539	0.003101726	0.076033726	-6.68E-05	0.006467385	1	0.077908515	1	0.081932833	ENSG00000133460	15	SLC2A11
SAT	Heart_Failure	ENSG00000144021.2	-0.000285955	0.93474588	0.003492562	0.076055492	-0.000215058	0.950895938	0.003492237	0.07603534	-7.09E-05	0.022228178	1	0.989642217	1	0.990755659	ENSG00000133519	8	ZDHHC8P1
SAT	Heart_Failure	ENSG00000144182.12	-0.002574431	0.205073339	0.00203154	0.076055242	-0.002636867	0.194258107	0.002031349	0.076035077	6.24E-05	0.000444163	1	0.735350411	1	0.723506187	ENSG00000133597	5	ADCK2
SAT	Heart_Failure	ENSG00000144230.12	-0.003134893	0.046362711	0.001573677	0.076054874	-0.003162218	0.044468723	0.001573516	0.07603471	2.73E-05	0.025304228	1	0.475002578	1	0.456297071	ENSG00000133597	5	ADCK2
SAT	Heart_Failure	ENSG00000144407.5	-0.007027887	0.05494654	0.003661686	0.076054918	-0.00692416	0.058600476	0.003661284	0.076034782	-0.000103727	2.50E-05	1	0.502171577	1	0.503576399	ENSG00000133661	11	SFTPD
SAT	Heart_Failure	ENSG00000144426.14	0.003073219	0.087115418	0.001796345	0.076055036	0.003040811	0.090464736	0.001796156	0.076034893	3.24E-05	0.014685212	1	0.592308253	1	0.598355674	ENSG00000133661	11	SFTPD
SAT	Heart_Failure	ENSG00000144677.10	-0.001918881	0.608122007	0.003742285	0.076055452	-0.001791611	0.632093003	0.003742009	0.076035304	-0.000127269	0.001660364	1	0.949666018	1	0.94798204	ENSG00000133661	11	SFTPD
SAT	Heart_Failure	ENSG00000144843.7	-0.001245507	0.476113216	0.001747912	0.076055414	-0.001292865	0.459463423	0.00174775	0.076035255	4.74E-05	0.00242847	1	0.895715287	1	0.890430886	ENSG00000133661	11	SFTPD
SAT	Heart_Failure	ENSG00000145020.10	-0.001748265	0.058904451	0.000925542	0.076054936	-0.001719009	0.063241996	0.000925447	0.076034802	-2.93E-05	4.31E-05	1	0.507258544	1	0.511069956	ENSG00000133706	13	LARS
SAT	Heart_Failure	ENSG00000145029.7	-0.002949328	0.056373051	0.001545652	0.076054925	-0.002901544	0.060460731	0.001545493	0.07603479	-4.78E-05	6.02E-05	1	0.503931718	1	0.50513473	ENSG00000133706	13	LARS
SAT	Heart_Failure	ENSG00000145103.8	-0.002557168	0.298404716	0.002459143	0.076055324	-0.002421307	0.324772311	0.002458931	0.076035189	-0.000135861	9.07E-09	1	0.814547972	1	0.828598718	ENSG00000133706	13	LARS
SAT	Heart_Failure	ENSG00000145113.17	0.002416407	0.128590746	0.00159007	0.076055133	0.002442792	0.12442996	0.001589899	0.076034972	-2.64E-05	0.018471127	1	0.649888404	1	0.647874406	ENSG00000133706	13	LARS
SAT	Heart_Failure	ENSG00000145241.6	0.000364108	0.864212822	0.002129111	0.076055488	0.000408033	0.848005373	0.002128896	0.076035334	-4.39E-05	0.009250901	1	0.978183841	1	0.972791643	ENSG00000133731	5	IMPA1
SAT	Heart_Failure	ENSG00000145692.10	-0.005960893	0.000503146	0.001713344	0.076053604	-0.005991572	0.00046992	0.001713167	0.076033432	3.07E-05	0.02024726	1	0.03682027	1	0.039533553	ENSG00000133731	5	IMPA1
SAT	Heart_Failure	ENSG00000145736.10	-0.000375781	0.904348198	0.00312707	0.076055491	-0.000461279	0.882715936	0.003126738	0.076035337	8.55E-05	0.000162134	1	0.981517198	1	0.974665278	ENSG00000133739	11	LRRCC1
SAT	Heart_Failure	ENSG00000145777.10	0.014512329	7.15E-06	0.00323277	0.076052348	0.014444087	7.88E-06	0.00323242	0.076032225	6.82E-05	0.002414828	0.023469109	0.001235216	0.025849598	0.00258496	ENSG00000133739	11	LRRCC1
SAT	Heart_Failure	ENSG00000145781.4	0.001032381	0.685598419	0.002550123	0.076055467	0.001125304	0.658986048	0.002549897	0.07603531	-9.29E-05	9.84E-05	1	0.966658386	1	0.956791995	ENSG00000133816	9	MICAL2
SAT	Heart_Failure	ENSG00000145990.6	0.000317127	0.893425721	0.002367132	0.07605549	-0.000546821	0.818564372	0.002383783	0.076035332	0.000863947	0.0023402	1	0.979270459	1	0.972791643	ENSG00000133816	9	MICAL2
SAT	Heart_Failure	ENSG00000146205.9	0.001126478	0.524869196	0.001771588	0.07605543	0.001179692	0.505433333	0.001771401	0.076035271	-5.32E-05	4.60E-05	1	0.906724424	1	0.900571724	ENSG00000133816	9	MICAL2
SAT	Heart_Failure	ENSG00000146263.7	-0.004016817	0.271057678	0.003649557	0.076055304	-0.003914436	0.283412172	0.003649188	0.076035161	-0.000102381	0.00039441	1	0.784141183	1	0.795445114	ENSG00000133816	9	MICAL2

SAT	Heart_Failure	ENSG00000146386.7	-0.003279883	0.291326864	0.003108256	0.076055319	-0.003220301	0.300126341	0.003107908	0.076035173	-5.96E-05	0.002776039	1	0.803905332	1	0.810520426	ENSG00000133884	5	DPF2
SAT	Heart_Failure	ENSG00000146453.8	-0.00093704	0.802395962	0.00374445	0.076055483	-0.000879887	0.814201444	0.00374405	0.076035332	-5.72E-05	0.033361558	1	0.976669362	1	0.972791643	ENSG00000133884	5	DPF2
SAT	Heart_Failure	ENSG00000146469.8	-0.001174615	0.696001111	0.003006249	0.076055469	-0.001225702	0.683450414	0.003005942	0.076035314	5.11E-05	0.029291942	1	0.966658386	1	0.956878334	ENSG00000133884	5	DPF2
SAT	Heart_Failure	ENSG00000146555.14	-0.001619669	0.378699903	0.001839916	0.076055372	-0.000595278	0.750044625	0.001868531	0.076035324	-0.001024391	0.001739174	1	0.85759885	1	0.962415493	ENSG00000133983	10	COX16
SAT	Heart_Failure	ENSG00000146707.10	0.000190906	0.93206895	0.002239576	0.076055492	0.000136061	0.951550731	0.002239336	0.07603534	5.48E-05	0.000591048	1	0.989362092	1	0.991047336	ENSG00000133983	10	COX16
SAT	Heart_Failure	ENSG00000146918.15	0.002411406	0.347220339	0.002565338	0.076055355	0.002456581	0.338214359	0.002565083	0.076035197	-4.52E-05	0.030977718	1	0.847011103	1	0.837495329	ENSG00000133983	10	COX16
SAT	Heart_Failure	ENSG00000147457.9	0.003154281	0.303843443	0.003067689	0.076055328	0.003320093	0.279211802	0.003068214	0.076035157	-0.000165811	0.028347154	1	0.816145643	1	0.791037893	ENSG00000133983	10	COX16
SAT	Heart_Failure	ENSG00000147509.9	0.005946682	0.039706894	0.002891243	0.076054833	0.006007387	0.037708169	0.002890921	0.076034666	-6.07E-05	0.001221114	1	0.448303633	1	0.434107027	ENSG00000133997	7	MED6
SAT	Heart_Failure	ENSG00000147576.11	-0.002675129	0.162630011	0.001915893	0.076055189	-0.00150373	0.443870247	0.001963928	0.076035249	-0.001171399	0.006800434	1	0.686431329	1	0.882462767	ENSG00000133997	7	MED6
SAT	Heart_Failure	ENSG00000148290.5	0.00075044	0.659685154	0.001704198	0.076055463	0.000773628	0.649825491	0.001704007	0.076035308	-2.32E-05	0.034158486	1	0.962994646	1	0.953700363	ENSG00000133997	7	MED6
SAT	Heart_Failure	ENSG00000148396.14	0.001795481	0.484039949	0.002565632	0.076055417	0.001848224	0.471246625	0.002565365	0.076035259	-5.27E-05	0.006473559	1	0.897349195	1	0.894608312	ENSG00000133997	7	MED6
SAT	Heart_Failure	ENSG00000148484.13	0.003948134	0.281693336	0.003667482	0.076055312	0.003895048	0.28815952	0.003667071	0.076035164	5.31E-05	0.022776072	1	0.796074522	1	0.799198129	ENSG00000134056	7	MRPS36
SAT	Heart_Failure	ENSG00000148843.9	0.001355897	0.720212539	0.003785571	0.076055473	0.001296491	0.7319612	0.003785175	0.076035322	5.94E-05	0.035251633	1	0.966658386	1	0.958028875	ENSG00000134077	11	THUMPD3
SAT	Heart_Failure	ENSG00000149054.10	-0.001458261	0.307999901	0.001430468	0.076055331	-0.001426184	0.318714202	0.001430326	0.076035185	-3.21E-05	0.00543291	1	0.816225947	1	0.823562856	ENSG00000134160	9	TRPM1
SAT	Heart_Failure	ENSG00000149100.8	-0.000782153	0.79377586	0.002992045	0.076055482	-0.000833651	0.780514027	0.002991755	0.076035328	5.15E-05	0.041222228	1	0.976267425	1	0.967288536	ENSG00000134184	8	GSTM1
SAT	Heart_Failure	ENSG00000149308.12	0.000889546	0.711492645	0.002405137	0.076055472	0.00094646	0.693908789	0.002404891	0.076035316	-5.69E-05	0.002295738	1	0.966658386	1	0.956878334	ENSG00000134184	8	GSTM1
SAT	Heart_Failure	ENSG00000149380.7	0.001723866	0.576351391	0.00308537	0.076055444	0.001825138	0.554113858	0.00308505	0.076035286	-0.000101273	1.60E-05	1	0.932993874	1	0.915531921	ENSG00000134201	6	GSTM5
SAT	Heart_Failure	ENSG00000149480.2	-0.002300468	0.442643359	0.002996424	0.076055401	-0.002172831	0.468328299	0.002996173	0.076035258	-0.000127636	1.58E-05	1	0.882758633	1	0.892844363	ENSG00000134201	6	GSTM5
SAT	Heart_Failure	ENSG00000149489.4	0.005053643	0.004520101	0.001779831	0.076054235	0.005163903	0.003717536	0.00177992	0.076034027	-0.00011026	0.001203233	1	0.154647076	1	0.158405666	ENSG00000134201	6	GSTM5
SAT	Heart_Failure	ENSG00000149679.7	-0.004251877	0.111431009	0.002671118	0.076055098	-0.00419711	0.11607574	0.00267083	0.076034955	-5.48E-05	0.003453369	1	0.640599412	1	0.64481576	ENSG00000134201	6	GSTM5
SAT	Heart_Failure	ENSG00000150656.10	0.001190522	0.686119719	0.002945913	0.076055468	0.001260378	0.668736471	0.002945612	0.076035312	-6.99E-05	0.002364437	1	0.966658386	1	0.956878334	ENSG00000134201	6	GSTM5
SAT	Heart_Failure	ENSG00000151117.4	-0.00197183	0.359318295	0.002151089	0.076055362	-0.002040385	0.342809682	0.002150877	0.0760352	6.86E-05	0.000111875	1	0.85040303	1	0.839311387	ENSG00000134201	6	GSTM5
SAT	Heart_Failure	ENSG00000151148.9	0.003311742	0.142711223	0.002259385	0.076055158	0.003372121	0.135529915	0.002259153	0.076034992	-6.04E-05	0.000553984	1	0.669181587	1	0.649215182	ENSG00000134202	6	GSTM3
SAT	Heart_Failure	ENSG00000151176.3	-0.001659859	0.563942085	0.002876723	0.076055441	-0.001703866	0.553612296	0.002876421	0.076035285	4.40E-05	0.039184911	1	0.925856651	1	0.915531921	ENSG00000134202	6	GSTM3
SAT	Heart_Failure	ENSG00000151532.9	-0.004589969	0.1705896	0.003349575	0.0760552	-0.004538946	0.175346366	0.003349218	0.076035054	-5.10E-05	0.034232224	1	0.702264088	1	0.700744733	ENSG00000134216	14	CHIA
SAT	Heart_Failure	ENSG00000151552.7	-0.000855188	0.546771833	0.001419151	0.076055436	-0.000892164	0.529526178	0.001418994	0.076035278	3.70E-05	7.34E-05	1	0.916219808	1	0.908441675	ENSG00000134222	12	PSRC1
SAT	Heart_Failure	ENSG00000151640.8	0.002360872	0.019381434	0.001009731	0.07605464	0.002378772	0.018468683	0.001009626	0.076034474	-1.79E-05	0.018596772	1	0.302151987	1	0.304501243	ENSG00000134222	12	PSRC1
SAT	Heart_Failure	ENSG00000151725.7	0.000717711	0.745038464	0.002207062	0.076055477	0.000753954	0.732618852	0.002206839	0.076035322	-3.62E-05	0.039126419	1	0.966658386	1	0.958028875	ENSG00000134222	12	PSRC1
SAT	Heart_Failure	ENSG00000151849.10	0.003192672	0.326755553	0.0032556	0.076055343	0.003140626	0.334655486	0.003255277	0.076035195	5.20E-05	0.049914993	1	0.833010855	1	0.836830236	ENSG00000134222	12	PSRC1
SAT	Heart_Failure	ENSG00000151883.12	-0.002840401	0.316328051	0.002834637	0.076055336	-0.00292622	0.301881107	0.002834362	0.076035174	8.58E-05	0.000312604	1	0.824393135	1	0.810625291	ENSG00000134245	13	WNT2B
SAT	Heart_Failure	ENSG00000152061.17	-0.005851359	0.004046262	0.002035577	0.076054203	-0.005819362	0.004248255	0.002035363	0.076034065	-3.20E-05	0.033277363	1	0.1521008	1	0.161355485	ENSG00000134262	8	AP4B1
SAT	Heart_Failure	ENSG00000152253.4	-0.003890584	0.060305666	0.00207105	0.076054942	-0.003537111	0.088575875	0.002077035	0.076034888	-0.000353473	0.028241466	1	0.508644962	1	0.596493272	ENSG00000134262	8	AP4B1
SAT	Heart_Failure	ENSG00000152291.9	-0.004796471	0.124505386	0.003122424	0.076055125	-0.004513728	0.148395841	0.003123205	0.076035014	-0.000282743	0.001058349	1	0.647388543	1	0.665121824	ENSG00000134283	13	PPHLN1
SAT	Heart_Failure	ENSG00000152348.11	-1.37E-05	0.984838343	0.000723093	0.076055493	3.94E-06	0.995647559	0.000723018	0.07603534	-1.77E-05	0.001224757	1	0.998924529	1	0.999768899	ENSG00000134283	13	PPHLN1
SAT	Heart_Failure	ENSG00000152359.10	-0.003748325	0.017828758	0.00158213	0.076054617	-0.003771544	0.017121416	0.001581958	0.076034453	2.32E-05	0.034270233	1	0.290924976	1	0.294111855	ENSG00000134285	6	FKBP11
SAT	Heart_Failure	ENSG00000152465.13	-0.003421442	0.382339864	0.003916513	0.076055374	-0.00335486	0.391620926	0.003916108	0.076035226	-6.66E-05	0.025483942	1	0.85759885	1	0.861776162	ENSG00000134285	6	FKBP11
SAT	Heart_Failure	ENSG00000152492.9	-0.002760286	0.079107681	0.001572015	0.076055012	-0.002800715	0.074784768	0.001571858	0.076034845	4.04E-05	0.001595074	1	0.580653917	1	0.557368838	ENSG00000134285	6	FKBP11
SAT	Heart_Failure	ENSG00000152689.13	-0.003928287	0.077405674	0.002224467	0.076055006	-0.002466529	0.292983482	0.0023455	0.076035168	-0.001461759	0.049624527	1	0.575515143	1	0.807119064	ENSG00000134324	7	LPIN1
SAT	Heart_Failure	ENSG00000152749.7	-0.006171068	0.108154027	0.003841196	0.07605509	-0.006277769	0.102157981	0.003840827	0.076034923	0.000106701	0.001180443	1	0.640599412	1	0.614882715	ENSG00000134440	7	NARS
SAT	Heart_Failure	ENSG00000152784.11	-0.003136127	0.427921902	0.003955993	0.076055395	-0.003039758	0.442218936	0.003955689	0.076035248	-9.64E-05	0.020766656	1	0.878482333	1	0.880611614	ENSG00000134440	7	NARS
SAT	Heart_Failure	ENSG00000153044.5	-0.001521213	0.556966359	0.002589943	0.076055439	-0.001463308	0.572036262	0.002589672	0.07603529	-5.79E-05	0.002613197	1	0.922001323	1	0.924557132	ENSG00000134440	7	NARS
SAT	Heart_Failure	ENSG00000153406.9	0.000256933	0.822829685	0.0011475	0.076055485	0.000303774	0.791200117	0.001147389	0.076035329	-4.68E-05	1.39E-06	1	0.976669362	1	0.970635778	ENSG00000134440	7	NARS
SAT	Heart_Failure	ENSG00000153446.11	0.003138357	0.295865195	0.003002229	0.076055322	0.003081302	0.304679555	0.003001899	0.076035176	5.71E-05	0.004737433	1	0.813063328	1	0.812640695	ENSG00000134460	11	IL2RA
SAT	Heart_Failure	ENSG00000153531.8	0.000980868	0.741109154	0.002968861	0.076055476	0.001065339	0.719687157	0.002968534	0.07603532	-8.45E-05	2.02E-05	1	0.966658386	1	0.958028875	ENSG00000134460	11	IL2RA
SAT	Heart_Failure	ENSG00000153714.5	-0.004628336	0.110420922	0.002899402	0.076055095	-0.00455052	0.116502865	0.002899111	0.076034956	-7.78E-05	0.000794273	1	0.640599412	1	0.64481576	ENSG00000134460	11	IL2RA
SAT	Heart_Failure	ENSG00000153814.7	-0.005371146	0.08772947	0.003145617	0.076055038	-0.005581485	0.076018731	0.003145788	0.076034849	0.000210339	0.000548104	1	0.592308253	1	0.560403102	ENSG00000134463	10	ECHDC3
SAT	Heart_Failure	ENSG00000154134.10	-0.004288174	0.223319425	0.00352139	0.076055262	-0.004207135	0.232140838	0.003521016	0.076035117	-8.10E-05	0.001525278	1	0.751217604	1	0.750242469	ENSG00000134490	9	TMEM241
SAT	Heart_Failure	ENSG00000154222.10	-0.001750922	0.245372675	0.001507254	0.076055282	-0.001787085	0.235706849	0.001507087	0.076035121	3.62E-05	0.000290636	1	0.760933598	1	0.752162449	ENSG00000134531	5	EMP1
SAT	Heart_Failure	ENSG00000154240.12	-0.001113238	0.746072191	0.003437799	0.076055477	-0.001642165	0.633182204	0.003440869	0.076035305	0.000528926	0.000682349	1	0.966658386	1	0.94798204	ENSG00000134531	5	EMP1
SAT	Heart_Failure	ENSG00000154518.5	0.003231227	0.226633017	0.002672469	0.076055265	0.003137627	0.240337954	0.002672268	0.076035125	9.36E-05	0.001023627	1	0.751217604	1	0.752432087	ENSG00000134574	7	DDB2
SAT	Heart_Failure	ENSG00000154719.9	0.000729983	0.543365967	0.00120116	0.076055435	0.000749068	0.532832081	0.001201028	0.076035279	-1.91E-05	0.017561849	1	0.913779466	1	0.908441675	ENSG00000134698	10	AGO4
SAT	Heart_Failure	ENSG00000154743.13	-0.008258533	0.019335798	0.003530795	0.076054639	-0.008174156	0.020594474	0.003530427	0.076034504	-8.44E-05	0.001421426	1	0.302151987	1	0.320239196	ENSG00000134744	9	ZCCHC11
SAT	Heart_Failure	ENSG00000154803.8	0.004574271	0.156420293	0.003227645	0.07605518	0.00462639	0.151710999	0.003227302	0.076035019	-5.21E-05	0.026141739	1	0.677064618	1	0.666350453	ENSG00000134775	11	FHOD3
SAT	Heart_Failure	ENSG00000154856.8	0.002690552	0.450066153	0.003562204	0.076055404	0.002558977	0.472483843	0.003561832	0.07603526	0.000131575	7.42E-07	1	0.887019168	1	0.894608312	ENSG00000134809	4	TIMM10
SAT	Heart_Failure	ENSG00000155189.7	0.00059632	0.869049534	0.003616996	0.076055489	0.00066882	0.853285768	0.003616655	0.076035335	-7.25E-05	0.021999624	1	0.979223495	1	0.972791643	ENSG00000134809	4	TIMM10
SAT	Heart_Failure	ENSG00000155393.8	0.001702278	0.118278055	0.001089775	0.076055112	0.001679687	0.123202144	0.001089663	0.076034969	2.26E-05	0.0065894	1	0.646149939	1	0.647747917	ENSG00000134809	4	TIMM10
SAT	Heart_Failure	ENSG00000156136.5	0.00654105	0.085092165	0.003798787	0.07605503	0.006484368	0.087795368	0.003798365	0.076034885	5.67E-05	0.02243421	1	0.592308253	1	0.596493272	ENSG00000134809	4	TIMM10
SAT	Heart_Failure	ENSG00000156162.12	-0.007406857	0.051037247	0.003796108	0.076054899	-0.007338601	0.05318985	0.003795734	0.076034757	-6.83E-05	0.029157004	1	0.48962926	1	0.490345005	ENSG00000134815	14	DHX34
SAT	Heart_Failure	ENSG00000156172.5	0.002251153	0.410141199	0.002733156	0.076055387	0.002320826	0.39576005	0.002732896	0.076035228	-6.97E-05	0.003198065	1	0.870429337	1	0.862334842	ENSG00000134815	14	DHX34
SAT	Heart_Failure	ENSG00000156232.6	-0.004965755	0.002527922	0.001644293	0.07605407	-0.005023774	0.002246522	0.001644141	0.076033883	5.80E-05	9.15E-05	1	0.113128813	1	0.11516936	ENSG00000134825	9	TMEM258
SAT	Heart_Failure	ENSG00000156253.2	0.001571214	0.410445689	0.001908874	0.076055387	0.00154759	0.417465753	0.001908657	0.076035238	2.36E-05	0.042799507	1	0.870429337	1	0.867451005	ENSG00000134825	9	TMEM258
SAT	Heart_Failure	ENSG00000156414.14	0.002876138	0.255145061	0.00252749	0.076055291	0.002832098	0.262441631	0.002527215	0.076035144	4.40E-05	0.011118066	1	0.770621765	1	0.774729353	ENSG00000134851	8	TMEM165
SAT	Heart_Failure	ENSG00000156968.8	-5.76E-05	0.9791978	0.002207639	0.076055493	-8.86E-05	0.967965702	0.002207388	0.07603534	3.11E-05	0.022038686	1	0.998920999	1	0.99755193	ENSG00000134851	8	TMEM165
SAT	Heart_Failure	ENSG00000157322.12	-0.00200024	0.173729937	0.001470421	0.076055204	-0.001951278	0.18446479	0.001470294	0.076035065	-4.90E-05	0.000525271	1	0.704583343	1	0.715224819	ENSG00000134852	10	CLOCK
SAT	Heart_Failure	ENSG00000157601.9	-0.00127811	0.204207677	0.001006658	0.076055241	-0.001293676	0.198704387	0.001006553	0.076035082	1.56E-05	0.038516854	1	0.735350411	1	0.725655076	ENSG00000134852	10	CLOCK
SAT	Heart_Failure	ENSG00000157833.8	0.002407688	0.157703081	0.001704146	0.076055181	0.002472123	0.146835239	0.001703968	0.076035012	-6.44E-05	4.14E-07	1	0.678274948	1	0.664505404	ENSG00000134852	10	CLOCK
SAT	Heart_Failure	ENSG00000157837.11	0.006665018	0.017896017	0.002814892	0.076054618	0.006620389	0.018664057	0.002814574	0.076034477	4.46E-05	0.011113078	1	0.290924976	1	0.304660546	ENSG00000134873	5	CLDN10
SAT	Heart_Failure	ENSG00000157870.10	-0.001156921	0.705624684	0.003062752	0.076055471	-0.0012007	0.695002046	0.003062415	0.076035316	4.38E-05	0.033107161	1	0.966658386	1	0.956878334	ENSG00000134873	5	CLDN10
SAT	Heart_Failure	ENSG00000157895.7	0.005474168	0.036944658	0.002623762	0.076054814	0.005517556	0.03545422	0.002623488	0.07603465	-4.34E-05	0.0266	1	0.436153525	1	0.423805321	ENSG00000134874	13	DZIP1
SAT	Heart_Failure	ENSG00000158023.5	0.001512081	0.520830099	0.002355021	0.076055429	0.001445866	0.539205903	0.002354774	0.076035281	6.62E-05	0.000149159	1	0.906724424	1	0.91051702	ENSG00000134874	13	DZIP1
SAT	Heart_Failure	ENSG00000158104.7	0.007323192	0.059242007	0.003882104	0.076054938	0.007269461	0.061102147	0.003881684	0.076034793	5.37E-05	0.045975296	1	0.507375885	1	0.505632969	ENSG00000134905	12	CARS2
SAT	Heart_Failure	ENSG00000158161.11	-0.00325607	0.157868007	0.00230554	0.076055182	-0.003193241	0.166002098	0.002305311	0.076035041	-6.28E-05	0.000794357	1	0.678274948	1	0.68372624	ENSG00000134905	12	CARS2
SAT	Heart_Failure	ENSG00000158186.8	0.000615883	0.87080857	0.003786984	0.076055489	0.000677323	0.858036456	0.003786585	0.076035335	-6.14E-05	0.028022665	1	0.979223495	1	0.972791643	ENSG00000134905	12	CARS2
SAT	Heart_Failure	ENSG00000158201.5	0.005954646	0.037731959	0.002865897	0.076054819	0.006004522	0.036138335	0.00286561	0.076034655	-4.99E-05	0.030885499	1	0.439253432	1	0.428050103	ENSG00000134905	12	CARS2
SAT	Heart_Failure	ENSG00000158411.6	0.003085431	0.179291717	0.002297518	0.076055212	0.003158504	0.169171479	0.002297306	0.076035045	-7.31E-05	0.000399369	1	0.714462461	1	0.689505122	ENSG00000135052	12	GOLM1
SAT	Heart_Failure	ENSG00000158457.4	-0.001059738	0.616346832	0.002115099	0.076055454	-0.001112757	0.598782386	0.002114894	0.076035297	5.30E-05	0.002949812	1	0.954216727	1	0.938318026	ENSG00000135052	12	GOLM1
SAT	Heart_Failure	ENSG00000158623.10	-0.000485462	0.664242416	0.001118411	0.076055464	-0.000510902	0.647772182	0.00111829	0.076035308	2.54E-05	0.001152392	1	0.962994646	1	0.95317119	ENSG00000135074	11	ADAM19
SAT	Heart_Failure	ENSG00000159110.15	-0.00150257	0.334557886	0.001557108	0.076055348	-0.00146624	0.346322024	0.001556934	0.076035202	-3.63E-05	0.000310397	1	0.841293164	1	0.840027851	ENSG00000135074	11	ADAM19
SAT	Heart_Failure	ENSG00000159231.5	0.000137399	0.956763348	0.002534307	0.076055493	0.000202782	0.93621899	0.002534046	0.076035339	-6.54E-05	0.000736936	1	0.994657967	1	0.988983601	ENSG00000135074	11	ADAM19
SAT	Heart_Failure	ENSG00000159579.9	0.001399503	0.698356733	0.003611248	0.07605547	0.001520072	0.67377712	0.003610878	0.076035312	-0.000120569	1.65E-05	1	0.966658386	1	0.956878334	ENSG00000135093	8	USP30
SAT	Heart_Failure	ENSG00000159588.10	0.006369146	0.087523179	0.003727669	0.076055037	0.006546592	0.079023333	0.003727311	0.076034859	-0.000177446	2.53E-08	1	0.592308253	1	0.572352216	ENSG00000135164	14	DMTF1
SAT	Heart_Failure	ENSG00000159733.9	0.000873734	0.718123026	0.002420526	0.076055473	0.000829583	0.731774095	0.002420256	0.076035322	4.42E-05	0.004788778	1	0.966658386	1	0.958028875	ENSG00000135211	5	TMEM60
SAT	Heart_Failure	ENSG00000159917.10	0.00091718	0.783948768	0.003345212	0.076055481	0.000965804	0.772777935	0.003344853	0.076035327	-4.86E-05	0.041032724	1	0.972730696	1	0.965963214	ENSG00000135213	8	POM121C
SAT	Heart_Failure	ENSG00000160049.7	0.001016405	0.72658749	0.002906767	0.076055474	0.001109576	0.702641144	0.002906491	0.076035317	-9.32E-05	0.000211011	1	0.966658386	1	0.956878334	ENSG00000135213	8	POM121C
SAT	Heart_Failure	ENSG00000160179.14	-0.000528148	0.836481737	0.002558909	0.076055486	-0.000490323	0.848027424	0.002558619	0.076035334	-3.78E-05	0.017455808	1	0.976669362	1	0.972791643	ENSG00000135213	8	POM121C
SAT	Heart_Failure	ENSG00000160194.13	0.000327244	0.676161228	0.000783428	0.076055466	0.000349865	0.655142644	0.000783344	0.076035309	-2.26E-05	3.69E-05	1	0.966658386	1	0.95563262	ENSG00000135269	13	TES
SAT	Heart_Failure	ENSG00000160200.13	-0.003328115	0.14009862	0.002255698	0.076055153	-0.003398449	0.131873258	0.002255471	0.076034986	7.03E-05	9.85E-05	1	0.668115657	1	0.649215182	ENSG00000135269	13	TES
SAT	Heart_Failure	ENSG00000160201.7	-0.003831756	0.191073047	0.002930792	0.076055226	-0.003752737	0.200338785	0.002930478	0.076035084	-7.90E-05	0.000150641	1	0.722912434	1	0.727919771	ENSG00000135269	13	TES
SAT	Heart_Failure	ENSG00000160223.12	-0.001829924	0.409397399	0.00221821	0.076055387	-0.001794511	0.418469704	0.002217965	0.076035238	-3.54E-05	0.015753855	1	0.870429337	1	0.868986772	ENSG00000135269	13	TES
SAT	Heart_Failure	ENSG00000160256.8	-0.00147585	0.630169375	0.003065181	0.076055457	-0.001555772	0.611722158	0.003064853	0.0760353	7.99E-05	0.000264078	1	0.959161083	1	0.945324871	ENSG00000135297	11	MTO1
SAT	Heart_Failure	ENSG00000160285.10	-0.004948572	0.188605323	0.00376398	0.076055223	-0.004800046	0.202191973	0.003763759	0.076035086	-0.000148526	0.001146653	1	0.722191019	1	0.729002049	ENSG00000135314	8	KHDC1
SAT	Heart_Failure	ENSG00000160299.12	-0.0016987	0.25223347	0.001483655	0.076055288	-0.001668912	0.260600831	0.001483513	0.076035143	-2.98E-05	0.018221804	1	0.767444733	1	0.773899606	ENSG00000135318	7	NT5E
SAT	Heart_Failure	ENSG00000160321.10	-0.002110679	0.591807206	0.003936224	0.076055448	-0.002206717	0.575023208	0.003935861	0.076035291	9.60E-05	0.006653347	1	0.942124911	1	0.927160988	ENSG00000135424	11	ITGA7
SAT	Heart_Failure	ENSG00000160323.14	0.000759587	0.820264905	0.003343188	0.076055485	0.000652145	0.845324238	0.003342828	0.076035334	0.000107442	5.27E-06	1	0.976669362	1	0.972791643	ENSG00000135480	10	KRT7
SAT	Heart_Failure	ENSG00000160339.11	0.000256626	0.914861543	0.002400429	0.076055491	0.000534309	0.824089138	0.002403669	0.076035332	-0.000277682	0.033674188	1	0.985971321	1	0.972791643	ENSG00000135480	10	KRT7
SAT	Heart_Failure	ENSG00000160856.16	-0.008342933	0.002468433	0.00275599	0.076054063	-0.008464572	0.002128898	0.002755707	0.076033868	0.000121639	1.37E-08	1	0.113128813	1	0.111957631	ENSG00000135480	10	KRT7
SAT	Heart_Failure	ENSG00000160932.6	-0.003777039	0.328977484	0.003869217	0.076055344	-0.00386332	0.31800011	0.003868825	0.076035185	8.63E-05	0.004698424	1	0.836086663	1	0.823487262	ENSG00000135480	10	KRT7
SAT	Heart_Failure	ENSG00000161036.6	-0.002167588	0.260286205	0.001925519	0.076055295	-0.00220362	0.252401554	0.001925338	0.076035136	3.60E-05	0.03215501	1	0.774632399	1	0.765423527	ENSG00000135525	14	MAP7
SAT	Heart_Failure	ENSG00000161180.6	-0.001255596	0.73114904	0.003654263	0.076055475	-0.001319176	0.718076472	0.003653913	0.07603532	6.36E-05	0.042679646	1	0.966658386	1	0.958028875	ENSG00000135541	16	AHI1
SAT	Heart_Failure	ENSG00000161204.7	-0.000467346	0.779969904	0.001672925	0.076055481	-0.000434332	0.795132165	0.001672746	0.07603533	-3.30E-05	0.005971931	1	0.972664863	1	0.970635778	ENSG00000135541	16	AHI1
SAT	Heart_Failure	ENSG00000161243.4	-0.001069152	0.719301655	0.002974887	0.076055473	-0.001013162	0.733399365	0.002974574	0.076035322	-5.60E-05	0.010824131	1	0.966658386	1	0.958028875	ENSG00000135587	4	SMPD2
SAT	Heart_Failure	ENSG00000161249.16	-0.002833989	0.30713794	0.002775028	0.07605533	-0.002876673	0.299859468	0.002774741	0.076035172	4.27E-05	0.043416816	1	0.816225947	1	0.810520426	ENSG00000135587	4	SMPD2
SAT	Heart_Failure	ENSG00000161281.6	0.000845485	0.669587513	0.001981384	0.076055465	0.000888938	0.653658366	0.001981202	0.076035309	-4.35E-05	0.015094149	1	0.96567764	1	0.954876713	ENSG00000135596	13	MICAL1
SAT	Heart_Failure	ENSG00000161664.2	-0.0028505	0.352410042	0.003065299	0.076055358	-0.0029045	0.343309671	0.00306496	0.0760352	5.40E-05	0.007934368	1	0.848363544	1	0.839311387	ENSG00000135636	9	DYSF
SAT	Heart_Failure	ENSG00000161813.16	0.005117755	0.087735998	0.00299728	0.076055038	0.004575904	0.127661967	0.003003765	0.076034978	0.000541851	0.00766156	1	0.592308253	1	0.647874406	ENSG00000135747	7	ZNF670
SAT	Heart_Failure	ENSG00000161904.7	0.004110574	0.116131856	0.002616164	0.076055108	0.003627036	0.165773758	0.002617072	0.07603504	0.000483537	2.33E-09	1	0.642397685	1	0.68372624	ENSG00000135747	7	ZNF670
SAT	Heart_Failure	ENSG00000162244.6	0.003713802	0.194587457	0.002863094	0.07605523	0.003637991	0.203805451	0.002862789	0.076035088	7.58E-05	0.000217543	1	0.726878652	1	0.729840368	ENSG00000135747	7	ZNF670
SAT	Heart_Failure	ENSG00000162384.9	0.001512361	0.619164303	0.003042762	0.076055454	0.001565854	0.606783884	0.003042442	0.076035299	-5.35E-05	0.017153525	1	0.954216727	1	0.943534562	ENSG00000135750	10	KCNK1
SAT	Heart_Failure	ENSG00000162402.8	0.004155511	0.071897599	0.002308912	0.076054987	0.004109448	0.075075985	0.002308674	0.076034846	4.61E-05	0.009066596	1	0.549874178	1	0.557368838	ENSG00000135778	7	NTPCR
SAT	Heart_Failure	ENSG00000162600.7	-0.001299322	0.454966379	0.001739011	0.076055406	-0.001239425	0.47597548	0.001738834	0.076035261	-5.99E-05	1.02E-05	1	0.887019168	1	0.894608312	ENSG00000135778	7	NTPCR
SAT	Heart_Failure	ENSG00000162631.14	7.35E-05	0.980151418	0.002954458	0.076055493	0.000196203	0.947048146	0.002954231	0.076035339	-0.000122698	8.29E-05	1	0.998920999	1	0.990755659	ENSG00000135828	7	RNASEL
SAT	Heart_Failure	ENSG00000162722.8	-0.002731376	0.457744583	0.003678292	0.076055407	-0.002785947	0.448761908	0.003677908	0.076035251	5.46E-05	0.048524427	1	0.888149011	1	0.882462767	ENSG00000135828	7	RNASEL
SAT	Heart_Failure	ENSG00000162885.8	-0.001242939	0.565841744	0.002164696	0.076055442	-0.00131869	0.542363261	0.002164471	0.076035282	7.58E-05	3.81E-06	1	0.927165893	1	0.912245255	ENSG00000135828	7	RNASEL
SAT	Heart_Failure	ENSG00000163125.11	-0.00418207	0.196286036	0.003236377	0.076055232	-0.004581608	0.157161804	0.003238609	0.076035028	0.000399539	0.002338284	1	0.726878652	1	0.669469928	ENSG00000135845	5	PIGC
SAT	Heart_Failure	ENSG00000163131.6	0.001611598	0.301683493	0.00156037	0.076055327	0.002491462	0.118006922	0.001593825	0.076034959	-0.000879864	0.006928956	1	0.815859931	1	0.64481576	ENSG00000135845	5	PIGC
SAT	Heart_Failure	ENSG00000163138.14	-0.003453075	0.07979031	0.001971037	0.076055014	-0.003410199	0.08357008	0.001970825	0.076034873	-4.29E-05	0.002125315	1	0.582544121	1	0.586427958	ENSG00000135913	6	USP37
SAT	Heart_Failure	ENSG00000163281.7	0.000398014	0.715399977	0.001091604	0.076055472	0.000437015	0.688876026	0.001091499	0.076035315	-3.90E-05	2.66E-05	1	0.966658386	1	0.956878334	ENSG00000135913	6	USP37
SAT	Heart_Failure	ENSG00000163297.12	0.005375726	0.155723648	0.003786778	0.076055178	0.006154267	0.105021338	0.00379662	0.07603493	-0.000778541	0.005441729	1	0.677064618	1	0.620855876	ENSG00000135917	9	SLC19A3
SAT	Heart_Failure	ENSG00000163389.6	-0.000386627	0.641853687	0.00083126	0.076055459	-0.000331761	0.689822283	0.000831281	0.076035315	-5.49E-05	0.000194988	1	0.960292726	1	0.956878334	ENSG00000135917	9	SLC19A3
SAT	Heart_Failure	ENSG00000163431.11	-0.004752363	0.119466806	0.00305222	0.076055115	-0.006373313	0.040960114	0.003118152	0.076034688	0.00162095	0.011290399	1	0.646585619	1	0.453733817	ENSG00000135926	8	TMBIM1
SAT	Heart_Failure	ENSG00000163521.11	-0.0020577	0.446565913	0.002703398	0.076055403	-0.002108603	0.435351649	0.002703104	0.076035245	5.09E-05	0.006251396	1	0.88490557	1	0.877927942	ENSG00000135926	8	TMBIM1
SAT	Heart_Failure	ENSG00000163576.13	0.001754853	0.004112231	0.000611567	0.076054208	0.001733569	0.004584132	0.000611509	0.076034086	2.13E-05	4.77E-05	1	0.1521008	1	0.163212403	ENSG00000135951	10	TSGA10
SAT	Heart_Failure	ENSG00000163596.12	-0.003929334	0.085472227	0.002284783	0.076055031	-0.003885618	0.088975804	0.002284544	0.076034889	-4.37E-05	0.010585864	1	0.592308253	1	0.596493272	ENSG00000135951	10	TSGA10
SAT	Heart_Failure	ENSG00000163743.9	-0.006651194	0.009592291	0.00256783	0.076054446	-0.006852273	0.007619783	0.002567856	0.076034229	0.000201079	3.70E-06	1	0.224802197	1	0.202724069	ENSG00000135968	15	GCC2
SAT	Heart_Failure	ENSG00000163807.4	0.000217144	0.858970213	0.001222069	0.076055488	0.000240139	0.844201058	0.001221939	0.076035334	-2.30E-05	0.009235996	1	0.976669362	1	0.972791643	ENSG00000135968	15	GCC2
SAT	Heart_Failure	ENSG00000164024.7	-0.00381456	0.308537528	0.00374602	0.076055331	-0.003743611	0.317569402	0.00374561	0.076035184	-7.09E-05	0.005269769	1	0.816225947	1	0.823487262	ENSG00000135972	4	MRPS9
SAT	Heart_Failure	ENSG00000164062.8	-0.00509743	0.01199286	0.002028938	0.076054508	-0.005027959	0.013198096	0.002028722	0.076034382	-6.95E-05	1.79E-06	1	0.24440108	1	0.258578925	ENSG00000135972	4	MRPS9
SAT	Heart_Failure	ENSG00000164068.11	0.004967505	5.60E-06	0.001093929	0.076052275	0.004947231	6.10E-06	0.001093818	0.076032148	2.03E-05	0.018865977	0.018376036	0.001020891	0.020018014	0.002502252	ENSG00000136014	7	USP44
SAT	Heart_Failure	ENSG00000164074.10	0.003885372	0.172705616	0.002849425	0.076055203	0.003791137	0.183317641	0.00284917	0.076035064	9.42E-05	0.000361398	1	0.704583343	1	0.715224819	ENSG00000136014	7	USP44
SAT	Heart_Failure	ENSG00000164078.8	0.005791965	0.033747542	0.002728094	0.07605479	0.005747848	0.03510573	0.002727793	0.076034647	4.41E-05	0.014932703	1	0.411793489	1	0.42191172	ENSG00000136048	9	DRAM1
SAT	Heart_Failure	ENSG00000164080.9	0.00492383	0.110704368	0.003086967	0.076055096	0.004839678	0.116896937	0.003086657	0.076034957	8.42E-05	0.000661877	1	0.640599412	1	0.64481576	ENSG00000136048	9	DRAM1
SAT	Heart_Failure	ENSG00000164082.10	-0.002298561	0.10599293	0.001421962	0.076055085	-0.002319715	0.102779553	0.001421805	0.076034925	2.12E-05	0.026807108	1	0.636928212	1	0.614882715	ENSG00000136098	12	NEK3
SAT	Heart_Failure	ENSG00000164253.8	0.001616345	0.608904774	0.003159158	0.076055452	0.00167626	0.595652357	0.003158802	0.076035296	-5.99E-05	0.002553746	1	0.9499841	1	0.937611754	ENSG00000136098	12	NEK3
SAT	Heart_Failure	ENSG00000164323.8	-0.00485745	0.142001422	0.003308031	0.076055157	-0.005004795	0.130272549	0.003307795	0.076034983	0.000147345	5.77E-05	1	0.669181587	1	0.649215182	ENSG00000136100	8	VPS36
SAT	Heart_Failure	ENSG00000164346.5	-0.000541021	0.416897143	0.000666433	0.07605539	-0.0005282	0.427973376	0.00066636	0.076035242	-1.28E-05	0.004850972	1	0.87398914	1	0.875416354	ENSG00000136104	14	RNASEH2B
SAT	Heart_Failure	ENSG00000164615.3	-0.001191263	0.613064446	0.00235565	0.076055453	-0.001247383	0.596399086	0.002355392	0.076035296	5.61E-05	0.000406998	1	0.954205146	1	0.937611754	ENSG00000136104	14	RNASEH2B
SAT	Heart_Failure	ENSG00000164659.10	-0.002367257	0.210369036	0.001889945	0.076055248	-0.002450637	0.194713796	0.001889813	0.076035078	8.34E-05	7.74E-05	1	0.739879495	1	0.723506187	ENSG00000136104	14	RNASEH2B

SAT	Heart_Failure	ENSG00000164669.8	0.001427273	0.352482288	0.001535055	0.076055358	0.001382154	0.367860131	0.001534892	0.076035214	4.51E-05	4.84E-05	1	0.848363544	1	0.849290727	ENSG00000136143	9	SUCLA2
SAT	Heart_Failure	ENSG00000164825.3	-0.000367286	0.915924905	0.003479127	0.076055491	-0.000274715	0.937057719	0.003478793	0.076035339	-9.26E-05	0.00183301	1	0.986559779	1	0.988983601	ENSG00000136143	9	SUCLA2
SAT	Heart_Failure	ENSG00000164830.13	-0.002998364	0.399361793	0.003557782	0.076055382	-0.002892592	0.416153378	0.003557427	0.076035237	-0.000105772	0.000247349	1	0.870429337	1	0.867451005	ENSG00000136144	7	RCBTB1
SAT	Heart_Failure	ENSG00000165118.10	-0.002073246	0.503318252	0.003097734	0.076055423	-0.002014963	0.515353449	0.003097442	0.076035274	-5.83E-05	0.031068603	1	0.900549813	1	0.903395669	ENSG00000136146	10	MED4
SAT	Heart_Failure	ENSG00000165202.2	0.001641808	0.439424783	0.002123488	0.0760554	0.001597138	0.451928173	0.002123275	0.076035252	4.47E-05	0.00860693	1	0.880728596	1	0.88576842	ENSG00000136146	10	MED4
SAT	Heart_Failure	ENSG00000165233.13	-0.000843952	0.761171333	0.002776676	0.076055479	-0.000891371	0.748169852	0.002776394	0.076035324	4.74E-05	0.029521199	1	0.969898847	1	0.962415493	ENSG00000136146	10	MED4
SAT	Heart_Failure	ENSG00000165632.7	-0.00422376	0.229893527	0.003517939	0.076055268	-0.004282543	0.223427561	0.003517588	0.076035109	5.88E-05	0.03850854	1	0.751389909	1	0.747263842	ENSG00000136169	12	SETDB2
SAT	Heart_Failure	ENSG00000165650.7	0.001096765	0.475847553	0.001538244	0.076055414	0.000142778	0.926926173	0.00155679	0.076035339	0.000953987	7.48E-05	1	0.895715287	1	0.987053153	ENSG00000136169	12	SETDB2
SAT	Heart_Failure	ENSG00000165684.3	-0.000585767	0.688726418	0.001462281	0.076055468	-0.00055306	0.70524062	0.001462132	0.076035318	-3.27E-05	0.004382976	1	0.966658386	1	0.958028875	ENSG00000136197	8	C7orf25
SAT	Heart_Failure	ENSG00000165730.10	0.002067949	0.378242663	0.002346902	0.076055372	0.002025705	0.388011238	0.002346656	0.076035224	4.22E-05	0.015583336	1	0.85759885	1	0.858921514	ENSG00000136213	7	CHST12
SAT	Heart_Failure	ENSG00000165806.15	1.48E-05	0.991115203	0.001326177	0.076055493	-1.09E-05	0.993445012	0.001326034	0.07603534	2.57E-05	0.006454395	1	0.999479422	1	0.999768899	ENSG00000136213	7	CHST12
SAT	Heart_Failure	ENSG00000165916.4	-0.002066149	0.351784797	0.00221896	0.076055358	-0.002133197	0.336331362	0.002218752	0.076035196	6.70E-05	0.000592111	1	0.848363544	1	0.837075007	ENSG00000136213	7	CHST12
SAT	Heart_Failure	ENSG00000166037.6	-0.001755289	0.454335328	0.002345992	0.076055406	-0.001787319	0.446093233	0.002345733	0.07603525	3.20E-05	0.039777743	1	0.887019168	1	0.882462767	ENSG00000136213	7	CHST12
SAT	Heart_Failure	ENSG00000166166.8	-0.006402607	0.089368643	0.003769031	0.076055043	-0.006556861	0.081885552	0.00376863	0.076034868	0.000154254	1.74E-08	1	0.597756036	1	0.581529209	ENSG00000136247	10	ZDHHC4
SAT	Heart_Failure	ENSG00000166220.8	0.00165004	0.498487502	0.002437743	0.076055422	0.00168448	0.489518366	0.002437475	0.076035266	-3.44E-05	0.035602284	1	0.899263643	1	0.894608312	ENSG00000136247	10	ZDHHC4
SAT	Heart_Failure	ENSG00000166262.11	0.002023932	0.489261265	0.002926937	0.076055418	0.002104421	0.472102938	0.00292662	0.076035259	-8.05E-05	7.59E-05	1	0.898909993	1	0.894608312	ENSG00000136247	10	ZDHHC4
SAT	Heart_Failure	ENSG00000166275.11	-0.000238224	0.777641855	0.000843588	0.076055481	-0.000260948	0.757044784	0.000843499	0.076035325	2.27E-05	0.000243871	1	0.972664863	1	0.962699472	ENSG00000136247	10	ZDHHC4
SAT	Heart_Failure	ENSG00000166278.10	0.015750908	1.69E-05	0.003661154	0.076052604	0.000675242	0.866081304	0.004004086	0.076035336	0.015075666	1.50E-20	0.055503379	0.002522881	1	0.974123167	ENSG00000136279	14	DBNL
SAT	Heart_Failure	ENSG00000166394.10	0.001495694	0.679234924	0.00361709	0.076055466	0.001579084	0.662393529	0.003616682	0.07603531	-8.34E-05	0.000242421	1	0.966658386	1	0.956878334	ENSG00000136279	14	DBNL
SAT	Heart_Failure	ENSG00000166446.10	0.00055497	0.730807882	0.001613043	0.076055475	0.00052706	0.74383334	0.001612884	0.076035323	2.79E-05	0.034610167	1	0.966658386	1	0.962349048	ENSG00000136279	14	DBNL
SAT	Heart_Failure	ENSG00000166669.9	0.004632491	0.209402371	0.003690605	0.076055247	0.004748672	0.198157614	0.003690237	0.076035082	-0.00011618	0.00010887	1	0.739879495	1	0.725655076	ENSG00000136279	14	DBNL
SAT	Heart_Failure	ENSG00000166762.12	-0.00210836	0.417660964	0.002601351	0.07605539	-0.0020561	0.429245412	0.002601064	0.076035243	-5.23E-05	0.002575295	1	0.87398914	1	0.875416354	ENSG00000136319	7	TTC5
SAT	Heart_Failure	ENSG00000166763.7	0.000615914	0.82308105	0.002754731	0.076055485	0.000552503	0.841023742	0.002754468	0.076035334	6.34E-05	0.007452301	1	0.976669362	1	0.972791643	ENSG00000136371	5	MTHFS
SAT	Heart_Failure	ENSG00000166796.7	0.001937632	0.407817909	0.002340869	0.076055386	0.001899302	0.417108248	0.00234063	0.076035237	3.83E-05	0.035374335	1	0.870429337	1	0.867451005	ENSG00000136371	5	MTHFS
SAT	Heart_Failure	ENSG00000166839.12	0.005563327	0.154041327	0.003902985	0.076055176	0.005513761	0.157696835	0.003902547	0.076035029	4.96E-05	0.046082225	1	0.677064618	1	0.669469928	ENSG00000136371	5	MTHFS
SAT	Heart_Failure	ENSG00000167103.7	-0.000872835	0.569422201	0.001534243	0.076055443	-0.000897136	0.558678381	0.001534074	0.076035287	2.43E-05	0.018844012	1	0.92942386	1	0.916248766	ENSG00000136371	5	MTHFS
SAT	Heart_Failure	ENSG00000167123.14	6.04E-05	0.980104003	0.002420775	0.076055493	1.89E-05	0.993777617	0.002420513	0.07603534	4.15E-05	0.013979582	1	0.998920999	1	0.999768899	ENSG00000136371	5	MTHFS
SAT	Heart_Failure	ENSG00000167136.6	0.0038122	0.137633142	0.002567717	0.076055149	0.003850736	0.133656711	0.002567432	0.076034989	-3.85E-05	0.023432524	1	0.663104757	1	0.649215182	ENSG00000136371	5	MTHFS
SAT	Heart_Failure	ENSG00000167311.9	-0.001142565	0.66545827	0.002642443	0.076055464	-0.001059684	0.688371891	0.002642169	0.076035315	-8.29E-05	3.86E-05	1	0.962994646	1	0.956878334	ENSG00000136425	8	CIB2
SAT	Heart_Failure	ENSG00000167325.10	-0.000626813	0.651332252	0.001387024	0.076055461	-0.000664931	0.631621896	0.001386874	0.076035304	3.81E-05	8.11E-05	1	0.962760695	1	0.94798204	ENSG00000136425	8	CIB2
SAT	Heart_Failure	ENSG00000167332.7	0.004510811	0.21582402	0.00364448	0.076055254	0.004613795	0.205477894	0.0036441	0.07603509	-0.000102985	0.000180316	1	0.745333464	1	0.731678624	ENSG00000136448	7	NMT1
SAT	Heart_Failure	ENSG00000167528.8	0.000170144	0.899144254	0.001342438	0.076055491	0.000197953	0.882759677	0.00134231	0.076035337	-2.78E-05	0.016855028	1	0.980097109	1	0.974665278	ENSG00000136448	7	NMT1
SAT	Heart_Failure	ENSG00000167644.7	-0.00189122	0.252384294	0.001652329	0.076055289	-0.001933178	0.241961113	0.001652147	0.076035127	4.20E-05	0.000160896	1	0.767444733	1	0.753454838	ENSG00000136448	7	NMT1
SAT	Heart_Failure	ENSG00000167671.7	-0.007460915	0.030552651	0.003449564	0.076054763	-0.007371836	0.032579386	0.00344924	0.076034627	-8.91E-05	0.003209399	1	0.390051553	1	0.406437132	ENSG00000136449	9	MYCBPAP
SAT	Heart_Failure	ENSG00000167695.10	0.003939019	0.088958341	0.002315814	0.076055041	0.003980014	0.085650525	0.00231557	0.076034879	-4.10E-05	0.016412735	1	0.597756036	1	0.592867877	ENSG00000136449	9	MYCBPAP
SAT	Heart_Failure	ENSG00000167711.9	-0.000164092	0.959360038	0.003220228	0.076055493	-0.000107753	0.973304191	0.003219923	0.07603534	-5.63E-05	0.044347974	1	0.994780111	1	0.998213938	ENSG00000136490	4	LIMD2
SAT	Heart_Failure	ENSG00000167840.9	-0.000368044	0.738669208	0.001103194	0.076055476	-0.000346765	0.753246011	0.001103073	0.076035325	-2.13E-05	0.004086698	1	0.966658386	1	0.962420704	ENSG00000136490	4	LIMD2
SAT	Heart_Failure	ENSG00000167920.4	-0.002789899	0.164630237	0.002007601	0.076055192	-0.002754021	0.170080015	0.002007379	0.076035046	-3.59E-05	0.007235616	1	0.689582559	1	0.692348051	ENSG00000136490	4	LIMD2
SAT	Heart_Failure	ENSG00000167930.11	0.000215369	0.94360509	0.003044529	0.076055492	0.000172051	0.954929448	0.0030442	0.07603534	4.33E-05	0.041030332	1	0.992414898	1	0.991911131	ENSG00000136492	4	BRIP1
SAT	Heart_Failure	ENSG00000167972.9	-0.001436399	0.660271946	0.003267976	0.076055463	-0.001497021	0.646855027	0.003267634	0.076035307	6.06E-05	0.013342604	1	0.962994646	1	0.95317119	ENSG00000136710	5	CCDC115
SAT	Heart_Failure	ENSG00000168000.10	-0.000501232	0.706644021	0.001331763	0.076055471	-0.000400935	0.763443672	0.00133218	0.076035326	-0.000100297	0.011640823	1	0.966658386	1	0.965635578	ENSG00000136715	13	SAP130
SAT	Heart_Failure	ENSG00000168016.9	-0.003769685	0.308516012	0.003701787	0.076055331	-0.003870254	0.295739226	0.003701413	0.07603517	0.000100569	0.000610101	1	0.816225947	1	0.809274729	ENSG00000136715	13	SAP130
SAT	Heart_Failure	ENSG00000168116.9	0.000873872	0.822062279	0.003885715	0.076055485	0.000806748	0.835510385	0.003885329	0.076035333	6.71E-05	0.033308583	1	0.976669362	1	0.972791643	ENSG00000136715	13	SAP130
SAT	Heart_Failure	ENSG00000168159.9	0.00177486	0.532745526	0.002845144	0.076055432	0.001844944	0.516651358	0.002844869	0.076035275	-7.01E-05	0.003573493	1	0.908491721	1	0.903395669	ENSG00000136715	13	SAP130
SAT	Heart_Failure	ENSG00000168291.8	-0.00013364	0.970148438	0.00357114	0.076055493	-7.56E-05	0.983110455	0.003570753	0.07603534	-5.80E-05	0.019856771	1	0.996886008	1	0.999768899	ENSG00000136717	10	BIN1
SAT	Heart_Failure	ENSG00000168297.11	-0.00191071	0.526417229	0.003016187	0.07605543	-0.001962673	0.515187186	0.003015866	0.076035274	5.20E-05	0.017085598	1	0.906724424	1	0.903395669	ENSG00000136783	9	NIPSNAP3A
SAT	Heart_Failure	ENSG00000168394.9	-0.003294555	0.397302777	0.003892208	0.076055381	0.003971338	0.323564233	0.00402299	0.076035188	-0.007265894	1.02E-12	1	0.86729901	1	0.828598718	ENSG00000136783	9	NIPSNAP3A
SAT	Heart_Failure	ENSG00000168404.8	0.000256309	0.836712579	0.00124361	0.076055486	0.000303714	0.807042244	0.001243487	0.076035331	-4.74E-05	2.90E-06	1	0.976669362	1	0.972791643	ENSG00000136816	11	TOR1B
SAT	Heart_Failure	ENSG00000168569.7	-0.006131396	0.128270962	0.004031272	0.076055132	-0.005989433	0.137345326	0.004031246	0.076034996	-0.000141963	0.026600812	1	0.649888404	1	0.649215182	ENSG00000136816	11	TOR1B
SAT	Heart_Failure	ENSG00000168672.3	-0.004346298	0.172354377	0.003184849	0.076055202	-0.004270526	0.179911188	0.003184511	0.07603506	-7.58E-05	0.001055382	1	0.704583343	1	0.709481499	ENSG00000136816	11	TOR1B
SAT	Heart_Failure	ENSG00000168679.13	0.001468092	0.577125479	0.002632929	0.076055444	0.001546371	0.556952171	0.002632681	0.076035286	-7.83E-05	0.000685768	1	0.933702513	1	0.915531921	ENSG00000136856	13	SLC2A8
SAT	Heart_Failure	ENSG00000168702.12	-0.000199585	0.954056874	0.003464233	0.076055493	-0.004065115	0.256550817	0.003582917	0.076035139	0.00386553	2.46E-05	1	0.994131568	1	0.770781077	ENSG00000136856	13	SLC2A8
SAT	Heart_Failure	ENSG00000168765.11	0.00471074	0.018162268	0.001994132	0.076054622	0.00478972	0.016293457	0.001993925	0.07603444	-7.92E-05	1.68E-07	1	0.292109804	1	0.285876102	ENSG00000136856	13	SLC2A8
SAT	Heart_Failure	ENSG00000168778.7	0.00123112	0.152856034	0.000861214	0.076055174	0.001245127	0.148193986	0.000861118	0.076035014	-1.40E-05	0.012651031	1	0.677064618	1	0.665121824	ENSG00000136856	13	SLC2A8
SAT	Heart_Failure	ENSG00000168803.10	-3.99E-05	0.967328137	0.000975134	0.076055493	-2.41E-05	0.980291619	0.000975033	0.07603534	-1.59E-05	0.031605969	1	0.996432082	1	0.999768899	ENSG00000136866	9	ZFP37
SAT	Heart_Failure	ENSG00000168827.10	-0.000388602	0.816672914	0.001676262	0.076055485	-0.000341223	0.838679602	0.001676089	0.076035334	-4.74E-05	0.000209029	1	0.976669362	1	0.972791643	ENSG00000136874	6	STX17
SAT	Heart_Failure	ENSG00000169085.7	0.001341731	0.103534697	0.000824181	0.076055079	0.000839346	0.320818322	0.000845451	0.076035186	0.000502385	0.007832121	1	0.629069151	1	0.825572481	ENSG00000136875	8	PRPF4
SAT	Heart_Failure	ENSG00000169116.7	-0.005674692	0.145635351	0.003899798	0.076055163	-0.002011933	0.612077737	0.003967448	0.0760353	-0.003662759	5.67E-07	1	0.672999418	1	0.945324871	ENSG00000136875	8	PRPF4
SAT	Heart_Failure	ENSG00000169220.13	-0.004651528	0.002220424	0.001520573	0.076054033	-0.004622258	0.00236481	0.00152041	0.076033898	-2.93E-05	0.006728188	1	0.10407443	1	0.119368322	ENSG00000136875	8	PRPF4
SAT	Heart_Failure	ENSG00000169313.9	-0.002597182	0.401692333	0.003097032	0.076055383	-0.002695297	0.384096917	0.003096714	0.076035222	9.81E-05	4.05E-05	1	0.870429337	1	0.854967426	ENSG00000136888	6	ATP6V1G1
SAT	Heart_Failure	ENSG00000169442.4	-0.00620257	0.085342643	0.003605102	0.076055031	-0.006138566	0.088586797	0.003604765	0.076034888	-6.40E-05	0.044040404	1	0.592308253	1	0.596493272	ENSG00000136932	9	C9orf156
SAT	Heart_Failure	ENSG00000169519.15	-0.001325036	0.602498074	0.002544174	0.076055451	-0.001265807	0.618775845	0.002543894	0.076035302	-5.92E-05	0.000509753	1	0.945538861	1	0.94798204	ENSG00000136932	9	C9orf156
SAT	Heart_Failure	ENSG00000169609.9	-0.000126141	0.942899443	0.001761105	0.076055492	-8.60E-06	0.996104796	0.001761319	0.07603534	-0.000117543	0.003047083	1	0.992414898	1	0.999768899	ENSG00000136932	9	C9orf156
SAT	Heart_Failure	ENSG00000169612.3	0.000726405	0.704846718	0.001917717	0.076055471	0.000787663	0.681256582	0.001917625	0.076035314	-6.13E-05	0.014067874	1	0.966658386	1	0.956878334	ENSG00000136950	9	ARPC5L
SAT	Heart_Failure	ENSG00000169683.3	-0.00130023	0.502622498	0.001939572	0.076055423	-0.001334713	0.491312539	0.001939361	0.076035266	3.45E-05	0.010281988	1	0.900549813	1	0.894608312	ENSG00000136982	5	DSCC1
SAT	Heart_Failure	ENSG00000169914.5	-0.000433159	0.772665634	0.001499392	0.07605548	-0.000484673	0.746482332	0.001499231	0.076035324	5.15E-05	1.48E-06	1	0.971681082	1	0.962415493	ENSG00000136982	5	DSCC1
SAT	Heart_Failure	ENSG00000170209.4	-0.000336757	0.860811403	0.001920578	0.076055488	-0.000293813	0.878400144	0.001920374	0.076035336	-4.29E-05	0.002018423	1	0.976669362	1	0.974665278	ENSG00000137054	11	POLR1E
SAT	Heart_Failure	ENSG00000170430.9	-0.003221149	0.114216366	0.002039315	0.076055104	-0.003173052	0.119686326	0.002039113	0.076034962	-4.81E-05	0.00379405	1	0.641850091	1	0.64481576	ENSG00000137054	11	POLR1E
SAT	Heart_Failure	ENSG00000170454.5	-0.000531549	0.839809571	0.002629645	0.076055487	-0.000583392	0.824411598	0.002629376	0.076035332	5.18E-05	0.011180186	1	0.976669362	1	0.972791643	ENSG00000137070	13	IL11RA
SAT	Heart_Failure	ENSG00000170545.12	-0.002346753	0.525750311	0.003698546	0.07605543	-0.002206837	0.550689037	0.003698227	0.076035285	-0.000139916	8.53E-05	1	0.906724424	1	0.914465015	ENSG00000137070	13	IL11RA
SAT	Heart_Failure	ENSG00000170632.9	-0.001239909	0.59803221	0.002351731	0.07605545	-0.001305229	0.578848818	0.002351479	0.076035292	6.53E-05	0.000103102	1	0.944700858	1	0.929614769	ENSG00000137070	13	IL11RA
SAT	Heart_Failure	ENSG00000170633.12	0.002141156	0.41678579	0.002636857	0.07605539	0.002206326	0.402698905	0.002636586	0.076035231	-6.52E-05	0.00136262	1	0.87398914	1	0.867451005	ENSG00000137070	13	IL11RA
SAT	Heart_Failure	ENSG00000170667.10	-0.006047184	0.069537359	0.003331935	0.076054979	-0.006116912	0.066353692	0.003331595	0.076034814	6.97E-05	0.007414671	1	0.537536555	1	0.516964783	ENSG00000137106	13	GRHPR
SAT	Heart_Failure	ENSG00000170776.15	0.004093533	0.266619509	0.003684938	0.0760553	0.004288786	0.244472941	0.003684902	0.076035129	-0.000195253	0.000720093	1	0.779024202	1	0.757427498	ENSG00000137106	13	GRHPR
SAT	Heart_Failure	ENSG00000170837.2	-0.002545461	0.447747684	0.003352926	0.076055403	-0.002622308	0.434113248	0.003352592	0.076035245	7.68E-05	0.004869993	1	0.884975994	1	0.877927942	ENSG00000137185	7	ZSCAN9
SAT	Heart_Failure	ENSG00000170866.7	-0.000807402	0.753202436	0.002567911	0.076055478	-0.000761257	0.766862449	0.002567638	0.076035326	-4.61E-05	0.013416189	1	0.966658386	1	0.965963214	ENSG00000137185	7	ZSCAN9
SAT	Heart_Failure	ENSG00000170955.9	0.002383044	0.332458016	0.002458835	0.076055346	0.002333224	0.342617035	0.002458593	0.0760352	4.98E-05	0.013689098	1	0.840365755	1	0.839311387	ENSG00000137185	7	ZSCAN9
SAT	Heart_Failure	ENSG00000171045.10	-0.00019057	0.891623486	0.001398675	0.07605549	-0.000169056	0.903785062	0.001398531	0.076035338	-2.15E-05	0.046027889	1	0.979270459	1	0.981243808	ENSG00000137185	7	ZSCAN9
SAT	Heart_Failure	ENSG00000171084.11	0.001230049	0.505867213	0.0018489	0.076055424	0.001183368	0.522100924	0.001848693	0.076035276	4.67E-05	7.61E-05	1	0.9005699	1	0.904369708	ENSG00000137216	14	TMEM63B
SAT	Heart_Failure	ENSG00000171298.8	-0.001387855	0.198903996	0.00108031	0.076055235	-0.001370539	0.204518093	0.001080198	0.076035089	-1.73E-05	0.033860838	1	0.732439966	1	0.729840368	ENSG00000137216	14	TMEM63B
SAT	Heart_Failure	ENSG00000171310.6	0.000228041	0.942263236	0.00314862	0.076055492	0.000313111	0.920777465	0.003148279	0.076035339	-8.51E-05	0.000107209	1	0.992414898	1	0.984731983	ENSG00000137225	8	CAPN11
SAT	Heart_Failure	ENSG00000171314.8	-0.000440472	0.650120447	0.00097107	0.076055461	-0.000420263	0.665140219	0.000970971	0.076035311	-2.02E-05	0.00800492	1	0.962760695	1	0.956878334	ENSG00000137225	8	CAPN11
SAT	Heart_Failure	ENSG00000171421.8	-0.004945334	0.201168743	0.003868891	0.076055238	-0.005024083	0.194041941	0.0038685	0.076035077	7.87E-05	0.010307431	1	0.735350411	1	0.723506187	ENSG00000137225	8	CAPN11
SAT	Heart_Failure	ENSG00000171502.10	-0.001931269	0.250585486	0.001680929	0.076055287	-0.002001396	0.233744341	0.001680757	0.076035119	7.01E-05	9.96E-08	1	0.766457153	1	0.750248244	ENSG00000137265	10	IRF4
SAT	Heart_Failure	ENSG00000171552.8	0.004502565	0.107168078	0.002794791	0.076055088	0.004576889	0.101460738	0.002794508	0.076034922	-7.43E-05	0.000779706	1	0.639306298	1	0.614882715	ENSG00000137274	8	BPHL
SAT	Heart_Failure	ENSG00000171574.13	0.000547144	0.744416795	0.001678303	0.076055476	0.000486541	0.771868902	0.001678126	0.076035327	6.06E-05	8.80E-07	1	0.966658386	1	0.965963214	ENSG00000137275	9	RIPK1
SAT	Heart_Failure	ENSG00000171723.11	-0.002862426	0.2678371	0.002583281	0.076055301	-0.003000181	0.245440071	0.002583028	0.07603513	0.000137755	1.57E-10	1	0.780722709	1	0.758328312	ENSG00000137285	9	TUBB2B
SAT	Heart_Failure	ENSG00000171763.13	-0.00114058	0.494796317	0.001670688	0.07605542	-0.001095184	0.512084398	0.001670517	0.076035273	-4.54E-05	0.000491767	1	0.898909993	1	0.902820479	ENSG00000137285	9	TUBB2B
SAT	Heart_Failure	ENSG00000171772.11	0.002024638	0.627667406	0.004174401	0.076055456	0.002132378	0.609438857	0.004173973	0.076035299	-0.00010774	0.000864486	1	0.958246837	1	0.944452141	ENSG00000137285	9	TUBB2B
SAT	Heart_Failure	ENSG00000172183.10	-0.0019812	0.382789707	0.002270018	0.076055374	-0.002033625	0.370275588	0.002269777	0.076035215	5.24E-05	0.001498832	1	0.85759885	1	0.84956238	ENSG00000137285	9	TUBB2B
SAT	Heart_Failure	ENSG00000172247.3	-0.001112574	0.228376071	0.00092364	0.076055267	-0.001096752	0.235011013	0.000923541	0.07603512	-1.58E-05	0.016511451	1	0.751217604	1	0.751870773	ENSG00000137288	5	UQCC2
SAT	Heart_Failure	ENSG00000172340.10	-0.001180586	0.62894249	0.002443183	0.076055457	-0.001099332	0.652707093	0.002442932	0.076035309	-8.13E-05	1.62E-05	1	0.95890349	1	0.954583686	ENSG00000137288	5	UQCC2
SAT	Heart_Failure	ENSG00000172671.15	0.001860554	0.638188758	0.003956664	0.076055459	0.001911985	0.628892845	0.003956219	0.076035304	-5.14E-05	0.039855628	1	0.959428651	1	0.94798204	ENSG00000137288	5	UQCC2
SAT	Heart_Failure	ENSG00000172687.9	-0.000287376	0.782497683	0.001040971	0.076055481	-0.000330704	0.7507264	0.00104099	0.076035324	4.33E-05	0.016516239	1	0.972730696	1	0.962420704	ENSG00000137288	5	UQCC2
SAT	Heart_Failure	ENSG00000172728.11	0.001481551	0.272485077	0.001350106	0.076055305	0.001501649	0.2659807	0.001349957	0.076035147	-2.01E-05	0.025329175	1	0.786300384	1	0.774729353	ENSG00000137310	7	TCF19
SAT	Heart_Failure	ENSG00000172789.3	-0.001417277	0.711622531	0.003833815	0.076055472	-0.001296709	0.735163772	0.003833414	0.076035322	-0.000120568	2.59E-05	1	0.966658386	1	0.959837778	ENSG00000137310	7	TCF19
SAT	Heart_Failure	ENSG00000172803.13	-0.002862256	0.228847183	0.002378602	0.076055267	-0.012510897	0.007661623	0.004691633	0.076034231	0.009648641	0.017041087	1	0.751217604	1	0.202724069	ENSG00000137310	7	TCF19
SAT	Heart_Failure	ENSG00000172955.13	-0.000864776	0.724722748	0.002455675	0.076055474	-0.000819381	0.738602655	0.002455409	0.076035323	-4.54E-05	0.007812786	1	0.966658386	1	0.960499534	ENSG00000137310	7	TCF19
SAT	Heart_Failure	ENSG00000173258.8	0.000320611	0.851756763	0.001715623	0.076055488	0.000264839	0.877306212	0.001715446	0.076035336	5.58E-05	2.25E-05	1	0.976669362	1	0.974665278	ENSG00000137310	7	TCF19
SAT	Heart_Failure	ENSG00000173531.11	-0.001905572	0.338014843	0.001988915	0.07605535	-0.001878159	0.344957335	0.001988698	0.076035201	-2.74E-05	0.043505754	1	0.842725455	1	0.83966685	ENSG00000137310	7	TCF19
SAT	Heart_Failure	ENSG00000173805.11	0.000185952	0.89771036	0.001446487	0.07605549	0.000154832	0.914748162	0.001446333	0.076035338	3.11E-05	0.002723224	1	0.979990566	1	0.984094962	ENSG00000137310	7	TCF19
SAT	Heart_Failure	ENSG00000173905.4	0.011245539	0.000313956	0.003120701	0.076053466	0.011184706	0.000337862	0.003120368	0.076033335	6.08E-05	0.006514629	1	0.02641253	1	0.033591688	ENSG00000137310	7	TCF19
SAT	Heart_Failure	ENSG00000173917.9	0.000509957	0.83019191	0.002377913	0.076055486	0.000553958	0.81577535	0.002377686	0.076035332	-4.40E-05	0.031645761	1	0.976669362	1	0.972791643	ENSG00000137310	7	TCF19
SAT	Heart_Failure	ENSG00000174004.5	-0.009722441	0.000505002	0.002795321	0.076053605	-0.009783529	0.000464676	0.002795009	0.076033428	6.11E-05	0.000717112	1	0.03682027	1	0.039533553	ENSG00000137310	7	TCF19
SAT	Heart_Failure	ENSG00000174007.7	-0.005044599	0.03856122	0.002438366	0.076054825	-0.00507573	0.037357205	0.002438093	0.076034664	3.11E-05	0.046719167	1	0.44392759	1	0.431580953	ENSG00000137312	10	FLOT1
SAT	Heart_Failure	ENSG00000174226.4	5.62E-05	0.987648174	0.003632013	0.076055493	0.000122436	0.973105627	0.003631665	0.07603534	-6.62E-05	0.033272057	1	0.999440735	1	0.998213938	ENSG00000137312	10	FLOT1
SAT	Heart_Failure	ENSG00000174891.8	-0.003483859	0.028127314	0.001586809	0.076054741	-0.003458142	0.029291988	0.001586638	0.076034599	-2.57E-05	0.021148119	1	0.369142867	1	0.383605048	ENSG00000137312	10	FLOT1
SAT	Heart_Failure	ENSG00000174915.7	4.94E-05	0.984512806	0.002543949	0.076055493	8.88E-05	0.972145224	0.002543685	0.07603534	-3.94E-05	0.040911243	1	0.998924529	1	0.998061182	ENSG00000137312	10	FLOT1
SAT	Heart_Failure	ENSG00000175387.11	0.002322202	0.261173089	0.002066707	0.076055296	0.002387134	0.248026446	0.002066501	0.076035132	-6.49E-05	9.63E-05	1	0.774632399	1	0.763390965	ENSG00000137312	10	FLOT1
SAT	Heart_Failure	ENSG00000175395.11	-0.001102064	0.405340869	0.001324403	0.076055385	-0.001137254	0.390459724	0.00132426	0.076035225	3.52E-05	0.000133353	1	0.870429337	1	0.859797554	ENSG00000137312	10	FLOT1
SAT	Heart_Failure	ENSG00000175643.7	-0.00296507	0.167790508	0.002149629	0.076055196	-0.002889494	0.178845226	0.002149407	0.076035058	-7.56E-05	4.33E-06	1	0.694824396	1	0.706126577	ENSG00000137392	5	CLPS
SAT	Heart_Failure	ENSG00000175730.7	-0.004306762	0.068663215	0.002365529	0.076054976	-0.004350327	0.065879484	0.002365276	0.076034812	4.36E-05	0.009992041	1	0.537536555	1	0.515872519	ENSG00000137392	5	CLPS
SAT	Heart_Failure	ENSG00000175764.10	-0.003066407	0.057336998	0.001613272	0.076054929	-0.00310095	0.054561595	0.001613099	0.076034764	3.45E-05	0.002593303	1	0.503931718	1	0.492997393	ENSG00000137404	10	NRM
SAT	Heart_Failure	ENSG00000175832.8	0.00085785	0.813971364	0.003645681	0.076055484	0.000785337	0.829424467	0.003645273	0.076035333	7.25E-05	0.001986153	1	0.976669362	1	0.972791643	ENSG00000137404	10	NRM
SAT	Heart_Failure	ENSG00000175854.7	-0.0018356	0.520791523	0.002858629	0.076055429	-0.001734514	0.543969315	0.002858356	0.076035283	-0.000101087	4.23E-05	1	0.906724424	1	0.912455686	ENSG00000137404	10	NRM
SAT	Heart_Failure	ENSG00000176142.8	-0.00277148	0.388225025	0.003212038	0.076055377	-0.002831446	0.377999628	0.003211753	0.076035219	6.00E-05	0.046029912	1	0.86096131	1	0.854967426	ENSG00000137411	12	VARS2
SAT	Heart_Failure	ENSG00000176148.11	0.001837062	0.225473291	0.001515598	0.076055264	0.001813771	0.231360137	0.001515436	0.076035117	2.33E-05	0.03080691	1	0.751217604	1	0.750242469	ENSG00000137411	12	VARS2
SAT	Heart_Failure	ENSG00000176194.13	-0.000178271	0.919714922	0.001768685	0.076055491	-0.000205907	0.90731105	0.001768489	0.076035338	2.76E-05	0.018524825	1	0.988399823	1	0.982067422	ENSG00000137411	12	VARS2
SAT	Heart_Failure	ENSG00000176390.10	0.006727515	0.061228955	0.00359406	0.076054946	0.006800128	0.058458333	0.003593674	0.076034781	-7.26E-05	0.004469819	1	0.510326031	1	0.503576399	ENSG00000137411	12	VARS2
SAT	Heart_Failure	ENSG00000176393.6	0.003124141	0.216944059	0.002530291	0.076055255	0.003051545	0.227781818	0.002530105	0.076035113	7.26E-05	0.008277737	1	0.745333464	1	0.747837725	ENSG00000137411	12	VARS2
SAT	Heart_Failure	ENSG00000176472.6	-0.000353101	0.694375645	0.000898648	0.076055469	-0.000338133	0.706688338	0.000898555	0.076035318	-1.50E-05	0.02814697	1	0.966658386	1	0.958028875	ENSG00000137411	12	VARS2
SAT	Heart_Failure	ENSG00000176485.6	0.00066982	0.77069273	0.002298082	0.07605548	-0.001222834	0.605735444	0.00236905	0.076035299	0.001892654	0.00103191	1	0.971467105	1	0.942350873	ENSG00000137411	12	VARS2
SAT	Heart_Failure	ENSG00000176531.6	-0.002356489	0.338437181	0.002461706	0.07605535	-0.002301531	0.349772354	0.002461447	0.076035204	-5.50E-05	0.002517527	1	0.84294423	1	0.842583404	ENSG00000137474	15	MYO7A
SAT	Heart_Failure	ENSG00000176783.10	0.000472908	0.811281845	0.001980577	0.076055484	0.000526679	0.790277739	0.001980366	0.076035329	-5.38E-05	0.000160606	1	0.976669362	1	0.970635778	ENSG00000137478	10	FCHSD2
SAT	Heart_Failure	ENSG00000176933.4	-0.003273798	0.215842823	0.002645152	0.076055254	-0.002081807	0.431645133	0.002647329	0.076035244	-0.001191991	6.20E-25	1	0.745333464	1	0.876305924	ENSG00000137478	10	FCHSD2
SAT	Heart_Failure	ENSG00000176946.7	0.005632262	0.147947819	0.003892854	0.076055166	0.005719111	0.141752748	0.003892412	0.076035003	-8.68E-05	0.000286957	1	0.674894985	1	0.657837012	ENSG00000137478	10	FCHSD2
SAT	Heart_Failure	ENSG00000176974.13	0.001756222	0.574404032	0.003127299	0.076055444	0.001832508	0.557857764	0.003127003	0.076035287	-7.63E-05	0.004973038	1	0.932980015	1	0.915623473	ENSG00000137478	10	FCHSD2
SAT	Heart_Failure	ENSG00000177042.10	9.02E-05	0.965170726	0.00206585	0.076055493	0.000122661	0.952647874	0.002065621	0.07603534	-3.25E-05	0.016865458	1	0.996432082	1	0.991228627	ENSG00000137501	12	SYTL2
SAT	Heart_Failure	ENSG00000177156.6	0.004915945	0.150132105	0.003416063	0.07605517	0.004829644	0.157377016	0.003415709	0.076035028	8.63E-05	0.000864378	1	0.6766256	1	0.669469928	ENSG00000137509	6	PRCP
SAT	Heart_Failure	ENSG00000177294.6	0.004707783	0.009808579	0.001822944	0.076054452	0.00474939	0.009171066	0.001822748	0.076034281	-4.16E-05	0.001172801	1	0.227178996	1	0.221316258	ENSG00000137509	6	PRCP
SAT	Heart_Failure	ENSG00000177311.6	0.009385883	0.003747479	0.00323798	0.076054182	0.009433806	0.003570734	0.003237628	0.076034015	-4.79E-05	0.033059851	1	0.146649141	1	0.154152365	ENSG00000137509	6	PRCP
SAT	Heart_Failure	ENSG00000177409.7	-0.001341714	0.270018638	0.0012164	0.076055303	-0.001366324	0.261281269	0.001216273	0.076035143	2.46E-05	0.006986757	1	0.782624692	1	0.774402751	ENSG00000137509	6	PRCP
SAT	Heart_Failure	ENSG00000177989.9	-0.008409378	0.008083753	0.003175073	0.076054398	-0.008302218	0.008925632	0.003174966	0.076034273	-0.00010716	0.016263516	1	0.203936797	1	0.218544776	ENSG00000137509	6	PRCP
SAT	Heart_Failure	ENSG00000178149.12	0.00070752	0.814558277	0.003016501	0.076055484	0.000777244	0.796646579	0.003016202	0.07603533	-6.97E-05	0.00463922	1	0.976669362	1	0.971094631	ENSG00000137561	4	TTPA
SAT	Heart_Failure	ENSG00000178188.10	-0.002567598	0.202204507	0.002013332	0.076055239	-0.002509408	0.212571325	0.002013119	0.076035098	-5.82E-05	6.49E-05	1	0.735350411	1	0.733958243	ENSG00000137561	4	TTPA
SAT	Heart_Failure	ENSG00000178386.8	-0.000485501	0.640381753	0.001039249	0.076055459	-0.00045571	0.660990878	0.001039138	0.07603531	-2.98E-05	5.97E-05	1	0.959428651	1	0.956878334	ENSG00000137563	7	GGH
SAT	Heart_Failure	ENSG00000178467.13	-0.005048236	0.188341419	0.003837494	0.076055223	-0.004944872	0.197501429	0.003837088	0.076035081	-0.000103363	0.00022447	1	0.722191019	1	0.725655076	ENSG00000137563	7	GGH

SAT	Heart_Failure	ENSG00000178538.5	-0.000580547	0.872177747	0.003608278	0.076055489	-0.000519035	0.885610815	0.003607912	0.076035337	-6.15E-05	0.02992715	1	0.979223495	1	0.975390764	ENSG00000137563	7	GGH
SAT	Heart_Failure	ENSG00000178685.9	0.000892331	0.77796173	0.003164559	0.076055481	0.001001805	0.751545879	0.003164243	0.076035324	-0.000109474	1.95E-05	1	0.972664863	1	0.962420704	ENSG00000137574	6	TGS1
SAT	Heart_Failure	ENSG00000178695.4	0.000460032	0.853188767	0.002485966	0.076055488	0.00054679	0.825891272	0.002485701	0.076035333	-8.68E-05	1.16E-06	1	0.976669362	1	0.972791643	ENSG00000137574	6	TGS1
SAT	Heart_Failure	ENSG00000178852.11	0.0017645	0.344369945	0.001866076	0.076055353	0.001799017	0.334963024	0.001865878	0.076035195	-3.45E-05	0.011470987	1	0.847011103	1	0.836830236	ENSG00000137672	8	TRPC6
SAT	Heart_Failure	ENSG00000178922.12	-0.001447616	0.378492248	0.001643751	0.076055372	-0.001419104	0.38790579	0.001643581	0.076035224	-2.85E-05	0.022946978	1	0.85759885	1	0.858921514	ENSG00000137672	8	TRPC6
SAT	Heart_Failure	ENSG00000178952.4	0.000786845	0.59738181	0.001489757	0.076055449	0.000743622	0.617632655	0.001489603	0.076035301	4.32E-05	0.000129358	1	0.944127995	1	0.947942988	ENSG00000137672	8	TRPC6
SAT	Heart_Failure	ENSG00000178966.11	0.002773691	0.277440172	0.002553834	0.076055309	0.002714569	0.287759003	0.002553563	0.076035164	5.91E-05	0.001370295	1	0.793619184	1	0.799139368	ENSG00000137673	4	MMP7
SAT	Heart_Failure	ENSG00000178971.9	0.001600121	0.321029092	0.001612462	0.076055339	0.00157559	0.328453128	0.001612293	0.076035191	2.45E-05	0.041555428	1	0.828692762	1	0.833382337	ENSG00000137673	4	MMP7
SAT	Heart_Failure	ENSG00000179023.7	-0.002822188	0.007048596	0.001047369	0.07605436	-0.002843259	0.006628857	0.001047261	0.07603419	2.11E-05	0.009528222	1	0.182097987	1	0.191383111	ENSG00000137675	4	MMP27
SAT	Heart_Failure	ENSG00000179163.11	-0.002062104	0.516252138	0.0031767	0.076055427	-0.001982859	0.532459439	0.003176353	0.076035279	-7.92E-05	0.000246504	1	0.906069117	1	0.908441675	ENSG00000137692	7	DCUN1D5
SAT	Heart_Failure	ENSG00000179299.12	0.000736321	0.691961081	0.00185848	0.076055469	0.000654551	0.724670221	0.001858335	0.076035321	8.18E-05	2.61E-05	1	0.966658386	1	0.958028875	ENSG00000137692	7	DCUN1D5
SAT	Heart_Failure	ENSG00000179361.13	0.003073596	0.411591754	0.003743283	0.076055388	0.003175859	0.396158315	0.003742897	0.076035228	-0.000102263	0.000355764	1	0.870429337	1	0.862334842	ENSG00000137700	12	SLC37A4
SAT	Heart_Failure	ENSG00000179978.10	-0.000681056	0.781802275	0.002458941	0.076055481	-0.000736234	0.764602668	0.002458684	0.076035326	5.52E-05	0.002787344	1	0.972730696	1	0.965679451	ENSG00000137709	5	POU2F3
SAT	Heart_Failure	ENSG00000179979.7	-0.000709024	0.521268165	0.001105445	0.076055429	-0.000691244	0.531728017	0.001105334	0.076035279	-1.78E-05	0.046874549	1	0.906724424	1	0.908441675	ENSG00000137709	5	POU2F3
SAT	Heart_Failure	ENSG00000180537.8	-0.001060594	0.784719133	0.003882477	0.076055481	-0.000897184	0.817234747	0.003882181	0.076035332	-0.00016341	7.22E-05	1	0.972730696	1	0.972791643	ENSG00000137709	5	POU2F3
SAT	Heart_Failure	ENSG00000180785.8	0.000914193	0.704807868	0.002413145	0.076055471	0.00095937	0.690922391	0.002412879	0.076035315	-4.52E-05	0.005117811	1	0.966658386	1	0.956878334	ENSG00000137709	5	POU2F3
SAT	Heart_Failure	ENSG00000180953.7	-0.000304793	0.730789708	0.000885832	0.076055475	-0.000274669	0.756484747	0.000885741	0.076035325	-3.01E-05	9.58E-06	1	0.966658386	1	0.962699472	ENSG00000137709	5	POU2F3
SAT	Heart_Failure	ENSG00000180992.5	0.003210185	0.228387856	0.002665107	0.076055267	0.003141224	0.238489565	0.002664827	0.076035123	6.90E-05	0.000471798	1	0.751217604	1	0.752162449	ENSG00000137752	18	CASP1
SAT	Heart_Failure	ENSG00000181027.6	0.00445417	0.004373679	0.001562929	0.076054225	0.004497801	0.004001389	0.001562786	0.076034048	-4.36E-05	0.002035712	1	0.154647076	1	0.161355485	ENSG00000137752	18	CASP1
SAT	Heart_Failure	ENSG00000181126.9	-0.000107081	0.936411782	0.001342188	0.076055492	-0.001645344	0.223050262	0.001350349	0.076035108	0.001538264	1.01E-24	1	0.989966131	1	0.746763174	ENSG00000137752	18	CASP1
SAT	Heart_Failure	ENSG00000181218.4	0.00154404	0.694826159	0.003935722	0.076055469	0.001676567	0.670087961	0.00393535	0.076035312	-0.000132527	0.000111421	1	0.966658386	1	0.956878334	ENSG00000137752	18	CASP1
SAT	Heart_Failure	ENSG00000181222.10	-0.00559271	0.130094679	0.003694652	0.076055135	-0.005511504	0.135726417	0.003694289	0.076034993	-8.12E-05	0.007731935	1	0.649888404	1	0.649215182	ENSG00000137767	9	SQRDL
SAT	Heart_Failure	ENSG00000181315.6	-0.000532041	0.804658265	0.002151196	0.076055483	-0.000791987	0.713098882	0.002153909	0.076035319	0.000259947	0.022134043	1	0.976669362	1	0.958028875	ENSG00000137806	4	NDUFAF1
SAT	Heart_Failure	ENSG00000181619.11	0.003131546	0.383356371	0.003592336	0.076055374	0.002477043	0.490644509	0.003593643	0.076035266	0.000654503	7.29E-09	1	0.85759885	1	0.894608312	ENSG00000137806	4	NDUFAF1
SAT	Heart_Failure	ENSG00000181625.13	0.000803008	0.655878041	0.001802037	0.076055462	0.000856779	0.634432789	0.001801858	0.076035305	-5.38E-05	0.00026348	1	0.962994646	1	0.94798204	ENSG00000137806	4	NDUFAF1
SAT	Heart_Failure	ENSG00000181754.6	0.001268941	0.434937661	0.001625242	0.076055398	0.001230591	0.448896787	0.001625065	0.076035251	3.84E-05	0.000639586	1	0.880339889	1	0.882462767	ENSG00000137831	10	UACA
SAT	Heart_Failure	ENSG00000182048.7	0.003794411	0.293141158	0.003609405	0.076055321	0.003693045	0.30617457	0.003609016	0.076035177	0.000101366	6.93E-05	1	0.806875957	1	0.814926763	ENSG00000137831	10	UACA
SAT	Heart_Failure	ENSG00000182077.6	-0.001357566	0.196162078	0.001050288	0.076055232	-0.001384264	0.187476735	0.00105021	0.076035069	2.67E-05	0.018698171	1	0.726878652	1	0.7171788	ENSG00000137831	10	UACA
SAT	Heart_Failure	ENSG00000182141.5	0.001182276	0.726809196	0.003383989	0.076055474	0.001092311	0.746832411	0.003383663	0.076035324	9.00E-05	0.001743797	1	0.966658386	1	0.962415493	ENSG00000137841	7	PLCB2
SAT	Heart_Failure	ENSG00000182154.7	0.000252759	0.938535936	0.003277893	0.076055492	0.000379566	0.907805695	0.00327758	0.076035338	-0.000126807	6.69E-06	1	0.990777479	1	0.982067422	ENSG00000137841	7	PLCB2
SAT	Heart_Failure	ENSG00000182240.11	0.000531892	0.792116934	0.002018084	0.076055482	-0.001305939	0.521597695	0.002037711	0.076035276	0.001837831	9.80E-11	1	0.9760395	1	0.904369708	ENSG00000137843	7	PAK6
SAT	Heart_Failure	ENSG00000182362.9	0.00078984	0.30059309	0.00076301	0.076055326	0.000766669	0.314949327	0.000762936	0.076035183	2.32E-05	0.000319838	1	0.814702237	1	0.823487262	ENSG00000137843	7	PAK6
SAT	Heart_Failure	ENSG00000182389.14	-0.001334023	0.679525702	0.003229213	0.076055466	-0.0014065	0.663125667	0.003228864	0.076035311	7.25E-05	0.001317019	1	0.966658386	1	0.956878334	ENSG00000137857	13	DUOX1
SAT	Heart_Failure	ENSG00000182534.9	-0.002000618	0.140941234	0.001358841	0.076055155	-0.00195422	0.150348542	0.001358698	0.076035017	-4.64E-05	3.31E-06	1	0.669181587	1	0.665121824	ENSG00000137857	13	DUOX1
SAT	Heart_Failure	ENSG00000182841.8	0.000676098	0.861397619	0.003872371	0.076055488	0.000762301	0.843923512	0.003871956	0.076035334	-8.62E-05	0.0017731	1	0.97692554	1	0.972791643	ENSG00000137857	13	DUOX1
SAT	Heart_Failure	ENSG00000183172.8	0.000646647	0.400640795	0.00076938	0.076055383	0.000625905	0.415872423	0.000769299	0.076035237	2.07E-05	0.000249326	1	0.870429337	1	0.867451005	ENSG00000137876	5	RSL24D1
SAT	Heart_Failure	ENSG00000183309.7	-0.01070211	0.004641689	0.003780441	0.076054242	-0.010853437	0.004089179	0.003780088	0.076034054	0.000151327	6.27E-06	1	0.15540184	1	0.161355485	ENSG00000137876	5	RSL24D1
SAT	Heart_Failure	ENSG00000183346.6	0.00087987	0.326551707	0.000896834	0.076055343	0.000895717	0.317861773	0.000896737	0.076035184	-1.58E-05	0.012198395	1	0.833010855	1	0.823487262	ENSG00000137876	5	RSL24D1
SAT	Heart_Failure	ENSG00000183431.7	-0.00238399	0.370156036	0.002660163	0.076055368	-0.002334217	0.380183472	0.002659897	0.07603522	-4.98E-05	0.020481651	1	0.855871709	1	0.854967426	ENSG00000137876	5	RSL24D1
SAT	Heart_Failure	ENSG00000183486.8	0.000866262	0.794210609	0.003320958	0.076055482	0.001979561	0.552918627	0.003336006	0.076035285	-0.001113299	0.000525775	1	0.976267425	1	0.915531921	ENSG00000137877	8	SPTBN5
SAT	Heart_Failure	ENSG00000183506.12	0.000466594	0.736527815	0.001386794	0.076055475	0.000432196	0.755279089	0.001386639	0.076035325	3.44E-05	0.000115084	1	0.966658386	1	0.962699472	ENSG00000137992	10	DBT
SAT	Heart_Failure	ENSG00000183617.4	0.000479371	0.744285145	0.001469633	0.076055476	0.000511091	0.727986772	0.001469476	0.076035321	-3.17E-05	0.002546544	1	0.966658386	1	0.958028875	ENSG00000137992	10	DBT
SAT	Heart_Failure	ENSG00000183628.8	0.000654319	0.545176268	0.001081512	0.076055436	0.00068194	0.528296141	0.001081398	0.076035278	-2.76E-05	0.000577075	1	0.91464723	1	0.907507664	ENSG00000137996	8	RTCA
SAT	Heart_Failure	ENSG00000183762.8	-0.000825892	0.499300783	0.001222473	0.076055422	-0.000805172	0.510077876	0.001222334	0.076035272	-2.07E-05	0.005226462	1	0.899263643	1	0.902678269	ENSG00000137996	8	RTCA
SAT	Heart_Failure	ENSG00000183833.12	0.000136711	0.961642647	0.002842677	0.076055493	5.76E-05	0.983838691	0.0028424	0.07603534	7.91E-05	0.000874163	1	0.995629386	1	0.999768899	ENSG00000137996	8	RTCA
SAT	Heart_Failure	ENSG00000183844.12	-0.001401495	0.185855018	0.001059377	0.07605522	-0.001493989	0.158486805	0.00105943	0.07603503	9.25E-05	5.16E-06	1	0.720656885	1	0.670798618	ENSG00000138030	8	KHK
SAT	Heart_Failure	ENSG00000183891.5	-4.01E-05	0.989707951	0.003108655	0.076055493	4.68E-05	0.987990567	0.003108313	0.07603534	-8.69E-05	2.96E-05	1	0.999440735	1	0.999768899	ENSG00000138030	8	KHK
SAT	Heart_Failure	ENSG00000184110.10	0.001785149	0.381414616	0.002039486	0.076055373	0.001714806	0.400410099	0.002039276	0.07603523	7.03E-05	7.45E-06	1	0.85759885	1	0.867158768	ENSG00000138030	8	KHK
SAT	Heart_Failure	ENSG00000184117.7	-0.001494345	0.638993128	0.003185507	0.076055459	-0.001549724	0.626581274	0.003185164	0.076035303	5.54E-05	0.013545513	1	0.959428651	1	0.94798204	ENSG00000138036	14	DYNC2LI1
SAT	Heart_Failure	ENSG00000184232.4	-0.003555964	0.203531134	0.002796543	0.076055241	-0.003470217	0.214596172	0.002796245	0.0760351	-8.57E-05	1.99E-05	1	0.735350411	1	0.736362287	ENSG00000138036	14	DYNC2LI1
SAT	Heart_Failure	ENSG00000184277.8	-0.001056787	0.731129708	0.003075422	0.076055475	-0.001000645	0.744876132	0.003075092	0.076035324	-5.61E-05	0.010443708	1	0.966658386	1	0.962415493	ENSG00000138085	12	ATRAID
SAT	Heart_Failure	ENSG00000184292.5	-0.001223218	0.47694513	0.001719874	0.076055414	-0.001144428	0.5057434	0.001719701	0.076035271	-7.88E-05	9.37E-09	1	0.895739537	1	0.900571724	ENSG00000138085	12	ATRAID
SAT	Heart_Failure	ENSG00000184371.9	0.006081321	0.007817399	0.002286325	0.076054389	0.003972451	0.087643342	0.002325838	0.076034885	0.00210887	8.57E-07	1	0.198828569	1	0.596493272	ENSG00000138111	10	TMEM180
SAT	Heart_Failure	ENSG00000184381.14	0.004000209	0.278706988	0.003692853	0.07605531	0.003927953	0.28742991	0.003692456	0.076035164	7.23E-05	0.005609717	1	0.793905695	1	0.799139368	ENSG00000138119	12	MYOF
SAT	Heart_Failure	ENSG00000184385.2	-0.000568886	0.718001642	0.00157529	0.076055473	-0.000545395	0.729150417	0.001575121	0.076035321	-2.35E-05	0.037029039	1	0.966658386	1	0.958028875	ENSG00000138119	12	MYOF
SAT	Heart_Failure	ENSG00000184508.6	-0.000812516	0.837382753	0.003958812	0.076055486	-0.000742932	0.851123077	0.003958384	0.076035335	-6.96E-05	0.01167313	1	0.976669362	1	0.972791643	ENSG00000138119	12	MYOF
SAT	Heart_Failure	ENSG00000184574.5	-0.001901929	0.524565691	0.002988932	0.07605543	-0.00181006	0.544758113	0.002988703	0.076035283	-9.19E-05	0.003669816	1	0.906724424	1	0.912597963	ENSG00000138119	12	MYOF
SAT	Heart_Failure	ENSG00000184674.8	0.001533581	0.363891712	0.001689011	0.076055364	0.001495009	0.376033812	0.001688839	0.076035218	3.86E-05	0.003437753	1	0.85040303	1	0.854967426	ENSG00000138119	12	MYOF
SAT	Heart_Failure	ENSG00000184743.8	-0.001950421	0.489323992	0.002821035	0.076055418	-0.001599376	0.571016281	0.00282298	0.07603529	-0.000351046	0.002145794	1	0.898909993	1	0.924404562	ENSG00000138135	5	CH25H
SAT	Heart_Failure	ENSG00000184887.9	0.000860458	0.765699499	0.002887395	0.076055479	0.000965892	0.737962553	0.002887112	0.076035323	-0.000105434	1.12E-05	1	0.971109414	1	0.960499534	ENSG00000138135	5	CH25H
SAT	Heart_Failure	ENSG00000184983.5	0.000526317	0.477822995	0.000741491	0.076055414	0.000514466	0.487744949	0.000741412	0.076035265	1.19E-05	0.025458428	1	0.896874856	1	0.894608312	ENSG00000138135	5	CH25H
SAT	Heart_Failure	ENSG00000185043.6	-0.00113256	0.491266728	0.001645455	0.076055419	-0.001170356	0.476872076	0.001645276	0.076035261	3.78E-05	0.000845891	1	0.898909993	1	0.894608312	ENSG00000138172	6	CALHM2
SAT	Heart_Failure	ENSG00000185163.5	0.00012424	0.934040817	0.001501178	0.076055492	0.000148888	0.920986474	0.001501021	0.076035339	-2.46E-05	0.029230958	1	0.989642217	1	0.984731983	ENSG00000138175	8	ARL3
SAT	Heart_Failure	ENSG00000185245.6	-0.000745422	0.834036866	0.00355764	0.076055486	-0.000794193	0.823332688	0.003557244	0.076035332	4.88E-05	0.035064042	1	0.976669362	1	0.972791643	ENSG00000138175	8	ARL3
SAT	Heart_Failure	ENSG00000185344.9	0.002213795	0.494315228	0.003239089	0.07605542	0.002376278	0.463131227	0.003238755	0.076035256	-0.000162484	6.03E-11	1	0.898909993	1	0.892844363	ENSG00000138175	8	ARL3
SAT	Heart_Failure	ENSG00000185404.12	0.002094883	0.55218713	0.003523854	0.076055438	0.002164082	0.539094798	0.003523515	0.076035281	-6.92E-05	0.021097086	1	0.920018143	1	0.91051702	ENSG00000138175	8	ARL3
SAT	Heart_Failure	ENSG00000185499.12	0.011733361	4.37E-05	0.002870906	0.076052886	0.011829975	3.77E-05	0.002870621	0.07603269	-9.66E-05	4.22E-05	0.143404981	0.005736199	0.123763084	0.009269033	ENSG00000138175	8	ARL3
SAT	Heart_Failure	ENSG00000185619.13	0.000613807	0.831718164	0.002888523	0.076055486	0.000534774	0.853105804	0.002888216	0.076035335	7.90E-05	0.000164471	1	0.976669362	1	0.972791643	ENSG00000138193	10	PLCE1
SAT	Heart_Failure	ENSG00000185684.8	0.0004591	0.649539159	0.001010338	0.076055461	0.000426856	0.672635659	0.00101023	0.076035312	3.22E-05	7.85E-06	1	0.962760695	1	0.956878334	ENSG00000138193	10	PLCE1
SAT	Heart_Failure	ENSG00000185697.12	-0.007981874	0.004218584	0.002789548	0.076054215	-0.005884944	0.046441211	0.00295523	0.076034721	-0.002096929	0.031784313	1	0.1521008	1	0.467403724	ENSG00000138286	10	FAM149B1
SAT	Heart_Failure	ENSG00000185710.5	-0.006356555	0.089105862	0.003738849	0.076055042	-0.006277248	0.0931347	0.003738477	0.0760349	-7.93E-05	0.00890211	1	0.597756036	1	0.599166569	ENSG00000138286	10	FAM149B1
SAT	Heart_Failure	ENSG00000185774.10	0.004188563	0.190280741	0.003197984	0.076055225	0.004109615	0.198723648	0.003197651	0.076035082	7.89E-05	0.000993664	1	0.722912434	1	0.725655076	ENSG00000138286	10	FAM149B1
SAT	Heart_Failure	ENSG00000185864.12	-0.000416948	0.881400541	0.002794669	0.07605549	-0.000351456	0.899911707	0.002794367	0.076035338	-6.55E-05	0.00079266	1	0.979223495	1	0.980373283	ENSG00000138303	13	ASCC1
SAT	Heart_Failure	ENSG00000185904.7	-0.00121376	0.750679388	0.003819933	0.076055477	-0.001288541	0.735849223	0.003819538	0.076035322	7.48E-05	0.010181683	1	0.966658386	1	0.960001834	ENSG00000138303	13	ASCC1
SAT	Heart_Failure	ENSG00000185988.7	-0.006354802	0.090688303	0.003756279	0.076055046	-0.006262099	0.095458783	0.003755871	0.076034906	-9.27E-05	0.00034927	1	0.599895813	1	0.603495	ENSG00000138326	14	RPS24
SAT	Heart_Failure	ENSG00000186230.6	0.001857708	0.410436665	0.002256893	0.076055387	0.001905063	0.398562274	0.002256669	0.076035229	-4.74E-05	0.009718617	1	0.870429337	1	0.864869591	ENSG00000138346	10	DNA2
SAT	Heart_Failure	ENSG00000186448.10	-0.00150515	0.498062092	0.002221484	0.076055421	-0.001552498	0.484596785	0.002221253	0.076035264	4.73E-05	0.004782481	1	0.899263643	1	0.894608312	ENSG00000138346	10	DNA2
SAT	Heart_Failure	ENSG00000186468.8	0.000950182	0.595301105	0.001788847	0.076055449	0.000916086	0.608534678	0.00178865	0.076035299	3.41E-05	0.003923896	1	0.943566631	1	0.94401999	ENSG00000138346	10	DNA2
SAT	Heart_Failure	ENSG00000186532.7	-0.004344201	0.131369218	0.002879382	0.076055138	-0.004436786	0.123308503	0.002879091	0.07603497	9.26E-05	5.36E-05	1	0.653539914	1	0.647747917	ENSG00000138363	10	ATIC
SAT	Heart_Failure	ENSG00000186684.8	-0.000203764	0.946352399	0.00302824	0.076055492	-0.000144584	0.961915406	0.003027932	0.07603534	-5.92E-05	0.012921456	1	0.992414898	1	0.99592587	ENSG00000138363	10	ATIC
SAT	Heart_Failure	ENSG00000186715.6	0.002404121	0.129765309	0.001586847	0.076055135	0.002428699	0.125847944	0.001586674	0.076034975	-2.46E-05	0.025105036	1	0.649888404	1	0.647874406	ENSG00000138380	13	CARF
SAT	Heart_Failure	ENSG00000186766.7	-0.003546019	0.081784158	0.002037438	0.07605502	-0.003467988	0.088698952	0.002037232	0.076034888	-7.80E-05	1.12E-06	1	0.592308253	1	0.596493272	ENSG00000138380	13	CARF
SAT	Heart_Failure	ENSG00000186792.11	-0.000931422	0.461621734	0.00126521	0.076055408	-0.000909813	0.472036719	0.001265089	0.076035259	-2.16E-05	0.044669969	1	0.890157253	1	0.894608312	ENSG00000138385	11	SSB
SAT	Heart_Failure	ENSG00000186854.6	0.000211954	0.86914922	0.001286602	0.076055489	0.002233514	0.133211329	0.001487462	0.076034988	-0.00202156	0.006786929	1	0.979223495	1	0.649215182	ENSG00000138385	11	SSB
SAT	Heart_Failure	ENSG00000186867.6	0.0006911	0.860280253	0.003926316	0.076055488	0.000610625	0.876396976	0.00392588	0.076035336	8.05E-05	0.001774418	1	0.976669362	1	0.974665278	ENSG00000138448	7	ITGAV
SAT	Heart_Failure	ENSG00000187048.8	0.000771859	0.840462297	0.003834327	0.076055487	0.000678389	0.859552303	0.003833916	0.076035335	9.35E-05	0.000592994	1	0.976669362	1	0.972791643	ENSG00000138459	4	SLC35A5
SAT	Heart_Failure	ENSG00000187068.2	0.000143482	0.949033225	0.002244676	0.076055492	0.000188512	0.933064253	0.002244453	0.076035339	-4.50E-05	0.013442038	1	0.992913907	1	0.988742522	ENSG00000138459	4	SLC35A5
SAT	Heart_Failure	ENSG00000187145.10	-0.010963452	0.001747416	0.003502559	0.076053964	-0.011069905	0.001574271	0.003502414	0.076033781	0.000106453	0.024439328	1	0.088204203	1	0.09221864	ENSG00000138468	11	SENP7
SAT	Heart_Failure	ENSG00000187166.1	-0.001008337	0.510152677	0.001531029	0.076055425	-0.000985391	0.519783077	0.00153087	0.076035275	-2.29E-05	0.04647717	1	0.903297859	1	0.904369708	ENSG00000138468	11	SENP7
SAT	Heart_Failure	ENSG00000187630.10	-0.000155561	0.901030583	0.001250896	0.076055491	-0.000130842	0.916685577	0.001250762	0.076035338	-2.47E-05	0.005132397	1	0.980849815	1	0.984094962	ENSG00000138468	11	SENP7
SAT	Heart_Failure	ENSG00000187741.10	-0.001143401	0.602825162	0.0021974	0.076055451	-0.001238035	0.573118353	0.002197181	0.076035291	9.46E-05	1.08E-07	1	0.945538861	1	0.925849984	ENSG00000138472	6	GUCA1C
SAT	Heart_Failure	ENSG00000187778.9	0.012475282	6.06E-05	0.00311079	0.076052983	0.012376657	6.92E-05	0.003110541	0.07603287	9.86E-05	0.001938011	0.198972651	0.007106166	0.227154091	0.011955478	ENSG00000138592	9	USP8
SAT	Heart_Failure	ENSG00000187862.7	6.48E-05	0.98021267	0.002614234	0.076055493	2.82E-05	0.991383098	0.002613941	0.07603534	3.66E-05	0.028799397	1	0.998920999	1	0.999768899	ENSG00000138592	9	USP8
SAT	Heart_Failure	ENSG00000187942.7	-0.003989537	0.086460333	0.002327107	0.076055034	-0.005168717	0.026683045	0.002332317	0.076034574	0.00117918	1.96E-13	1	0.592308253	1	0.360276017	ENSG00000138592	9	USP8
SAT	Heart_Failure	ENSG00000187953.6	0.003205736	0.23612871	0.002705901	0.076055274	0.003267192	0.227223792	0.002705653	0.076035113	-6.15E-05	0.011997447	1	0.755348658	1	0.747837725	ENSG00000138600	5	SPPL2A
SAT	Heart_Failure	ENSG00000187987.5	0.000500102	0.594014395	0.000938228	0.076055449	0.000755674	0.424794361	0.000946804	0.076035241	-0.000255572	0.045958659	1	0.943350063	1	0.873390936	ENSG00000138613	9	APH1B
SAT	Heart_Failure	ENSG00000188056.7	0.001344601	0.639528217	0.002870878	0.076055459	0.001419985	0.620834922	0.002870583	0.076035302	-7.54E-05	0.000647904	1	0.959428651	1	0.94798204	ENSG00000138613	9	APH1B
SAT	Heart_Failure	ENSG00000188086.8	0.0020941	0.418217272	0.002586846	0.076055391	0.002137721	0.408541756	0.002586589	0.076035234	-4.36E-05	0.038366175	1	0.873994184	1	0.867451005	ENSG00000138613	9	APH1B
SAT	Heart_Failure	ENSG00000188312.9	-0.001553401	0.155791	0.001094428	0.076055179	-0.001524222	0.163661749	0.001094308	0.076035037	-2.92E-05	8.64E-05	1	0.677064618	1	0.679583582	ENSG00000138613	9	APH1B
SAT	Heart_Failure	ENSG00000188372.10	-0.005547217	0.019014528	0.002365295	0.076054635	-0.005593395	0.018028918	0.00236504	0.076034467	4.62E-05	0.005651411	1	0.302151987	1	0.300690097	ENSG00000138621	7	PPCDC
SAT	Heart_Failure	ENSG00000188383.7	0.004253215	0.126303432	0.002781978	0.076055128	0.004167849	0.134054035	0.002781705	0.07603499	8.54E-05	0.000200945	1	0.64967372	1	0.649215182	ENSG00000138621	7	PPCDC
SAT	Heart_Failure	ENSG00000188599.13	-0.000825123	0.296117743	0.000789746	0.076055323	-0.00081156	0.304074334	0.000789657	0.076035175	-1.36E-05	0.007468886	1	0.813063328	1	0.811772083	ENSG00000138621	7	PPCDC
SAT	Heart_Failure	ENSG00000188672.12	-0.001722151	0.56658568	0.003005041	0.076055442	-0.001780244	0.553526613	0.00300471	0.076035285	5.81E-05	0.004004391	1	0.927165893	1	0.915531921	ENSG00000138621	7	PPCDC
SAT	Heart_Failure	ENSG00000188820.8	-0.002397733	0.133522697	0.001598109	0.076055142	-0.002362435	0.13930418	0.001597979	0.076034999	-3.53E-05	0.029025043	1	0.659800431	1	0.653383835	ENSG00000138621	7	PPCDC
SAT	Heart_Failure	ENSG00000188868.9	0.000407095	0.908950658	0.003559696	0.076055491	0.00035032	0.921596122	0.003559312	0.076035339	5.68E-05	0.023164572	1	0.983702811	1	0.984731983	ENSG00000138660	7	AP1AR
SAT	Heart_Failure	ENSG00000188878.12	0.001322509	0.301875634	0.001280979	0.076055327	0.001288985	0.314247788	0.001280852	0.076035182	3.35E-05	0.00137229	1	0.815859931	1	0.823487262	ENSG00000138669	5	PRKG2
SAT	Heart_Failure	ENSG00000189014.6	0.011078993	0.00193532	0.003573869	0.076053993	0.011142765	0.001819776	0.003573475	0.076033823	-6.38E-05	0.007456515	1	0.09524259	1	0.101198063	ENSG00000138669	5	PRKG2
SAT	Heart_Failure	ENSG00000189046.6	-0.00131401	0.690798368	0.003303423	0.076055468	-0.001373987	0.677432623	0.003303097	0.076035313	6.00E-05	0.027498637	1	0.966658386	1	0.956878334	ENSG00000138686	5	BBS7
SAT	Heart_Failure	ENSG00000189157.9	0.009109544	0.015354992	0.00375821	0.076054576	0.009451556	0.01190603	0.003758183	0.076034353	-0.000342011	9.51E-09	1	0.273107546	1	0.246278972	ENSG00000138686	5	BBS7
SAT	Heart_Failure	ENSG00000189180.11	0.001518382	0.613386423	0.003005234	0.076055453	0.001593752	0.595849964	0.003004935	0.076035296	-7.54E-05	0.00203074	1	0.954216727	1	0.937611754	ENSG00000138744	10	NAAA
SAT	Heart_Failure	ENSG00000189190.7	-5.90E-05	0.975757198	0.001940261	0.076055493	-2.15E-05	0.991153364	0.001940048	0.07603534	-3.75E-05	0.004073164	1	0.998920999	1	0.999768899	ENSG00000138744	10	NAAA
SAT	Heart_Failure	ENSG00000189339.7	-0.00081757	0.531289087	0.001305939	0.076055432	-0.000850702	0.5147437	0.00130582	0.076035274	3.31E-05	0.005325995	1	0.908320477	1	0.903395669	ENSG00000138750	10	NUP54
SAT	Heart_Failure	ENSG00000189362.7	-0.000163938	0.947223436	0.002476627	0.076055492	-0.000229455	0.926175774	0.002476379	0.076035339	6.55E-05	0.00099989	1	0.992414898	1	0.986938199	ENSG00000138756	13	BMP2K
SAT	Heart_Failure	ENSG00000189398.5	-0.00103058	0.784147484	0.003762362	0.076055481	-0.001126514	0.764597826	0.003761959	0.076035326	9.59E-05	0.000340385	1	0.972730696	1	0.965679451	ENSG00000138756	13	BMP2K
SAT	Heart_Failure	ENSG00000196126.6	-0.014418107	1.33E-11	0.002131234	0.076048351	0.000333289	0.909033454	0.002916992	0.076035338	-0.014751396	1.30E-13	4.37E-08	6.25E-09	1	0.982067422	ENSG00000138756	13	BMP2K
SAT	Heart_Failure	ENSG00000196151.6	-0.002436196	0.228959953	0.002025027	0.076055267	-0.002509384	0.215227492	0.002024807	0.0760351	7.32E-05	1.69E-07	1	0.751217604	1	0.736362287	ENSG00000138757	10	G3BP2
SAT	Heart_Failure	ENSG00000196196.2	0.00254431	0.496430559	0.003740987	0.076055421	0.002456596	0.511350951	0.003740613	0.076035273	8.77E-05	0.003679383	1	0.898994301	1	0.902750226	ENSG00000138757	10	G3BP2
SAT	Heart_Failure	ENSG00000196220.11	-0.000370999	0.858673716	0.002083529	0.076055488	-0.000333462	0.872832748	0.002083338	0.076035336	-3.75E-05	0.045676794	1	0.976669362	1	0.974665278	ENSG00000138757	10	G3BP2
SAT	Heart_Failure	ENSG00000196301.3	0.007891862	1.13E-09	0.001295805	0.076049704	0.002414639	0.106311676	0.001495134	0.076034933	0.005477222	2.12E-13	3.70E-06	4.63E-07	1	0.625105036	ENSG00000138760	4	SCARB2
SAT	Heart_Failure	ENSG00000196405.8	0.001024819	0.472897628	0.001427776	0.076055413	0.000999585	0.483818296	0.001427621	0.076035264	2.52E-05	0.010536019	1	0.895715287	1	0.894608312	ENSG00000138767	8	CNOT6L
SAT	Heart_Failure	ENSG00000196456.4	0.002482917	0.364213885	0.002736402	0.076055365	0.002520957	0.356859508	0.002736106	0.076035208	-3.80E-05	0.047172658	1	0.85040303	1	0.845383425	ENSG00000138767	8	CNOT6L
SAT	Heart_Failure	ENSG00000196511.9	-0.002051388	0.406070124	0.002469082	0.076055385	-0.002012747	0.414918116	0.00246881	0.076035236	-3.86E-05	0.01859136	1	0.870429337	1	0.867451005	ENSG00000138767	8	CNOT6L
SAT	Heart_Failure	ENSG00000196535.10	0.000106814	0.968846304	0.002734931	0.076055493	1.89E-05	0.994477058	0.002734632	0.07603534	8.79E-05	2.28E-06	1	0.996609664	1	0.999768899	ENSG00000138777	15	PPA2
SAT	Heart_Failure	ENSG00000196693.10	-0.001717152	0.23149588	0.001435127	0.07605527	-0.001691181	0.23857895	0.00143497	0.076035123	-2.60E-05	0.008519662	1	0.752763116	1	0.752162449	ENSG00000138777	15	PPA2
SAT	Heart_Failure	ENSG00000196712.12	0.002314383	0.456357993	0.003107147	0.076055406	0.002262072	0.466551549	0.003106806	0.076035257	5.23E-05	0.012766572	1	0.88796436	1	0.892844363	ENSG00000138777	15	PPA2
SAT	Heart_Failure	ENSG00000196735.7	-0.021431787	8.15E-21	0.002290178	0.076041827	-0.004269906	0.323756806	0.004327162	0.076035188	-0.017161881	2.95E-06	2.67E-17	8.91E-18	1	0.828598718	ENSG00000138777	15	PPA2
SAT	Heart_Failure	ENSG00000196793.9	0.003859521	0.146462917	0.002657813	0.076055164	0.003819361	0.15066716	0.002657536	0.076035018	4.02E-05	0.044402583	1	0.673975917	1	0.665121824	ENSG00000138792	5	ENPEP
SAT	Heart_Failure	ENSG00000196821.5	0.000872641	0.643659938	0.001886432	0.07605546	0.001136073	0.547019706	0.001886435	0.076035284	-0.000263432	1.61E-17	1	0.960732494	1	0.912597963	ENSG00000138801	4	PAPSS1
SAT	Heart_Failure	ENSG00000196872.6	0.003814364	0.114784786	0.002418671	0.076055105	0.00375333	0.120669575	0.002418427	0.076034964	6.10E-05	0.001442809	1	0.641850091	1	0.64481576	ENSG00000138801	4	PAPSS1
SAT	Heart_Failure	ENSG00000196912.8	0.000899381	0.683920847	0.002209135	0.076055467	0.000939466	0.670610552	0.002208894	0.076035312	-4.01E-05	0.007655858	1	0.966658386	1	0.956878334	ENSG00000138821	8	SLC39A8
SAT	Heart_Failure	ENSG00000196961.8	9.43E-05	0.971968758	0.002684371	0.076055493	3.71E-05	0.988969908	0.002684117	0.07603534	5.72E-05	0.014305874	1	0.99766005	1	0.999768899	ENSG00000138821	8	SLC39A8
SAT	Heart_Failure	ENSG00000196966.3	0.00177879	0.45258363	0.002368198	0.076055405	0.001375054	0.561576735	0.002368735	0.076035288	0.000403736	1.98E-10	1	0.887019168	1	0.918876404	ENSG00000138821	8	SLC39A8
SAT	Heart_Failure	ENSG00000196981.2	0.001551332	0.663831569	0.003569321	0.076055464	0.00147581	0.679228218	0.003568925	0.076035313	7.55E-05	0.001220155	1	0.962994646	1	0.956878334	ENSG00000138829	6	FBN2
SAT	Heart_Failure	ENSG00000197134.7	-0.002278409	0.500914817	0.003385193	0.076055422	-0.002390048	0.480145633	0.003385007	0.076035262	0.000111639	0.008145724	1	0.900549813	1	0.894608312	ENSG00000138834	8	MAPK8IP3
SAT	Heart_Failure	ENSG00000197165.6	0.001304845	0.364734698	0.001439621	0.076055365	0.001266484	0.378954435	0.001439473	0.076035219	3.84E-05	0.000551843	1	0.85040303	1	0.854967426	ENSG00000138834	8	MAPK8IP3
SAT	Heart_Failure	ENSG00000197181.7	-0.001252969	0.748820936	0.003913148	0.076055477	-0.001142519	0.770288948	0.003912773	0.076035327	-0.00011045	0.000987517	1	0.966658386	1	0.965963214	ENSG00000138834	8	MAPK8IP3
SAT	Heart_Failure	ENSG00000197217.8	-0.002288242	0.453965588	0.003055794	0.076055406	-0.002432851	0.425911331	0.003055546	0.076035241	0.000144609	2.94E-06	1	0.887019168	1	0.873465455	ENSG00000139133	2	ALG10

SAT	Heart_Failure	ENSG00000197226.8	-0.000406008	0.776477632	0.001430048	0.07605548	-0.000367154	0.797357188	0.001429901	0.07603533	-3.89E-05	0.000413142	1	0.972664863	1	0.971094631	ENSG00000139133	2	ALG10
SAT	Heart_Failure	ENSG00000197410.8	0.00135215	0.653574709	0.003012799	0.076055462	0.001263766	0.674844413	0.003012489	0.076035313	8.84E-05	0.000137238	1	0.962760695	1	0.956878334	ENSG00000139168	3	ZCRB1
SAT	Heart_Failure	ENSG00000197442.8	-0.002956296	0.320777154	0.002977546	0.076055339	-0.00286978	0.335091784	0.00297723	0.076035195	-8.65E-05	6.08E-05	1	0.828692762	1	0.836830236	ENSG00000139178	6	C1RL
SAT	Heart_Failure	ENSG00000197563.5	0.001550145	0.187302244	0.001175596	0.076055222	0.001512225	0.198279409	0.001175483	0.076035082	3.79E-05	0.000167338	1	0.72128951	1	0.725655076	ENSG00000139178	6	C1RL
SAT	Heart_Failure	ENSG00000197629.5	0.003167305	0.120857266	0.002041857	0.076055117	0.003499025	0.087050883	0.002044818	0.076034883	-0.00033172	0.003896035	1	0.646585619	1	0.596493272	ENSG00000139187	5	KLRG1
SAT	Heart_Failure	ENSG00000197734.4	-0.000481756	0.819576081	0.002112128	0.076055485	-0.000523243	0.804323615	0.002111933	0.076035331	4.15E-05	0.02866064	1	0.976669362	1	0.972791643	ENSG00000139190	12	VAMP1
SAT	Heart_Failure	ENSG00000197782.10	-0.000836508	0.309275568	0.000822732	0.076055332	-0.000820147	0.318782331	0.000822644	0.076035185	-1.64E-05	0.004821405	1	0.81701541	1	0.823562856	ENSG00000139190	12	VAMP1
SAT	Heart_Failure	ENSG00000197915.5	0.001905185	0.024704036	0.000848254	0.076054706	0.001889665	0.02588594	0.000848172	0.076034566	1.55E-05	0.030663166	1	0.336323411	1	0.355059412	ENSG00000139192	7	TAPBPL
SAT	Heart_Failure	ENSG00000197935.6	-0.000696709	0.772147697	0.002406036	0.07605548	-0.000385252	0.872952622	0.002409193	0.076035336	-0.000311456	0.016047446	1	0.971467105	1	0.974665278	ENSG00000139192	7	TAPBPL
SAT	Heart_Failure	ENSG00000198015.8	0.00123626	0.403632258	0.00148028	0.076055384	0.001274447	0.389216452	0.001480125	0.076035224	-3.82E-05	0.000556052	1	0.870429337	1	0.858921514	ENSG00000139197	6	PEX5
SAT	Heart_Failure	ENSG00000198026.6	-0.003707016	0.131442007	0.002457513	0.076055138	-0.003655423	0.136855092	0.00245725	0.076034995	-5.16E-05	0.003257495	1	0.653539914	1	0.649215182	ENSG00000139200	9	PIANP
SAT	Heart_Failure	ENSG00000198099.4	-0.00218691	0.283976406	0.002041112	0.076055314	-0.002219585	0.2767927	0.002040897	0.076035156	3.27E-05	0.029330371	1	0.798394677	1	0.786282985	ENSG00000139200	9	PIANP
SAT	Heart_Failure	ENSG00000198105.7	-0.004753598	0.153245994	0.003328474	0.076055175	-0.004864476	0.143842742	0.003328113	0.076035007	0.000110878	1.71E-06	1	0.677064618	1	0.659101398	ENSG00000139220	12	PPFIA2
SAT	Heart_Failure	ENSG00000198169.4	0.003188614	0.393997621	0.003740788	0.07605538	0.002991653	0.423817356	0.003740423	0.07603524	0.00019696	2.82E-10	1	0.864419838	1	0.873390936	ENSG00000139343	6	SNRPF
SAT	Heart_Failure	ENSG00000198185.7	0.002334196	0.413895518	0.002856829	0.076055389	0.002274135	0.425960409	0.002856509	0.07603524	6.01E-05	0.00098504	1	0.873306234	1	0.873465455	ENSG00000139343	6	SNRPF
SAT	Heart_Failure	ENSG00000198208.7	0.005005094	0.188027484	0.003801995	0.076055223	0.005064336	0.182808787	0.003801603	0.076035063	-5.92E-05	0.041510195	1	0.722191019	1	0.715224819	ENSG00000139343	6	SNRPF
SAT	Heart_Failure	ENSG00000198218.6	0.002199043	0.17347188	0.001615596	0.076055204	0.002151213	0.182970536	0.00161543	0.076035063	4.78E-05	0.000117807	1	0.704583343	1	0.715224819	ENSG00000139343	6	SNRPF
SAT	Heart_Failure	ENSG00000198315.6	0.001955953	0.569874403	0.003442143	0.076055443	0.001106429	0.747930471	0.003442856	0.076035324	0.000849523	2.78E-21	1	0.92942386	1	0.962415493	ENSG00000139344	3	AMDHD1
SAT	Heart_Failure	ENSG00000198466.8	0.008480051	0.048286051	0.004294029	0.076054884	0.008553597	0.046351601	0.004293581	0.076034721	-7.35E-05	0.022804887	1	0.478630006	1	0.467403724	ENSG00000139344	3	AMDHD1
SAT	Heart_Failure	ENSG00000198502.5	-0.001371275	0.03722127	0.000658211	0.076054816	0.000778606	0.311675473	0.000769591	0.07603518	-0.002149881	7.08E-08	1	0.436153525	1	0.821155716	ENSG00000139344	3	AMDHD1
SAT	Heart_Failure	ENSG00000198646.9	-0.001225039	0.718691839	0.003400922	0.076055473	-0.001038722	0.760029341	0.003400714	0.076035326	-0.000186317	4.43E-06	1	0.966658386	1	0.963545699	ENSG00000139344	3	AMDHD1
SAT	Heart_Failure	ENSG00000198874.8	0.00167842	0.305041048	0.001636391	0.076055329	0.001730961	0.290097803	0.001636211	0.076035166	-5.25E-05	1.73E-06	1	0.816225947	1	0.803578953	ENSG00000139405	11	RITA1
SAT	Heart_Failure	ENSG00000198959.7	0.000438977	0.811259894	0.001838255	0.076055484	0.000405253	0.825498479	0.001838059	0.076035333	3.37E-05	0.011190389	1	0.976669362	1	0.972791643	ENSG00000139405	11	RITA1
SAT	Heart_Failure	ENSG00000203301.2	0.0007781	0.796224898	0.003013134	0.076055483	0.00085523	0.77651512	0.003012822	0.076035328	-7.71E-05	0.000772631	1	0.976456935	1	0.965963214	ENSG00000139505	10	MTMR6
SAT	Heart_Failure	ENSG00000203668.1	-0.000264992	0.897851765	0.00206419	0.07605549	-0.000230401	0.911117363	0.002063983	0.076035338	-3.46E-05	0.036567096	1	0.979990566	1	0.982887891	ENSG00000139508	10	SLC46A3
SAT	Heart_Failure	ENSG00000203705.6	-0.002256615	0.137358901	0.001518889	0.076055149	-0.002302081	0.129573636	0.001518736	0.076034982	4.55E-05	0.000165816	1	0.663104757	1	0.649215182	ENSG00000139531	8	SUOX
SAT	Heart_Failure	ENSG00000203734.7	0.006946922	0.064423318	0.00375663	0.076054959	0.00699848	0.062438539	0.003756216	0.076034798	-5.16E-05	0.040389695	1	0.523200263	1	0.509604095	ENSG00000139537	6	CCDC65
SAT	Heart_Failure	ENSG00000203907.5	0.003728359	0.202741932	0.002927004	0.07605524	0.003819298	0.191897432	0.002926691	0.076035074	-9.09E-05	1.55E-05	1	0.735350411	1	0.721677191	ENSG00000139537	6	CCDC65
SAT	Heart_Failure	ENSG00000204147.5	0.001364554	0.67472288	0.003251452	0.076055466	0.00141371	0.663677371	0.003251089	0.076035311	-4.92E-05	0.019456375	1	0.966658386	1	0.956878334	ENSG00000139620	8	KANSL2
SAT	Heart_Failure	ENSG00000204161.9	0.006723585	0.05953664	0.003568377	0.076054939	0.006623641	0.063396974	0.003568	0.076034802	9.99E-05	0.000134133	1	0.507375885	1	0.511069956	ENSG00000139620	8	KANSL2
SAT	Heart_Failure	ENSG00000204177.6	0.001666853	0.489186368	0.002410126	0.076055418	0.001731705	0.472395209	0.002409872	0.07603526	-6.49E-05	0.000245057	1	0.898909993	1	0.894608312	ENSG00000139620	8	KANSL2
SAT	Heart_Failure	ENSG00000204227.4	-0.000472823	0.880862637	0.003154761	0.07605549	-0.003274895	0.301135141	0.003167199	0.076035173	0.002802072	8.54E-23	1	0.979223495	1	0.810520426	ENSG00000139624	8	CERS5
SAT	Heart_Failure	ENSG00000204228.3	0.002952715	0.261150543	0.002627725	0.076055296	-0.000112926	0.965964435	0.002646471	0.07603534	0.00306564	4.39E-22	1	0.774632399	1	0.996957946	ENSG00000139624	8	CERS5
SAT	Heart_Failure	ENSG00000204237.4	0.001488676	0.684166508	0.003659619	0.076055467	0.001426236	0.696710515	0.003659221	0.076035316	6.24E-05	0.012882517	1	0.966658386	1	0.956878334	ENSG00000139624	8	CERS5
SAT	Heart_Failure	ENSG00000204252.8	-0.00525209	0.035283043	0.002494935	0.076054801	-0.004240879	0.091559494	0.002513513	0.076034896	-0.001011211	0.001014793	1	0.425601709	1	0.599166569	ENSG00000139624	8	CERS5
SAT	Heart_Failure	ENSG00000204267.9	-0.001238152	0.266639494	0.001114613	0.0760553	-9.02E-05	0.936097232	0.001124678	0.076035339	-0.00114798	3.15E-14	1	0.779024202	1	0.988983601	ENSG00000139636	11	LMBR1L
SAT	Heart_Failure	ENSG00000204287.9	0.010722186	8.85E-07	0.002181173	0.076051722	0.003055095	0.204270454	0.002406572	0.076035089	0.007667091	4.85E-14	0.002902194	0.000233648	1	0.729840368	ENSG00000139651	9	ZNF740
SAT	Heart_Failure	ENSG00000204305.9	0.002784295	0.288140657	0.002621228	0.076055317	-0.000177403	0.952860436	0.003000967	0.07603534	0.002961698	0.04275421	1	0.801524014	1	0.991228627	ENSG00000139651	9	ZNF740
SAT	Heart_Failure	ENSG00000204314.6	0.007967833	0.000718812	0.002355728	0.076053708	0.002764885	0.253936918	0.002423551	0.076035137	0.005202948	7.54E-20	1	0.046243566	1	0.766534574	ENSG00000139835	9	GRTP1
SAT	Heart_Failure	ENSG00000204316.8	-0.004507962	0.141663802	0.003067422	0.076055156	-0.004360044	0.155159238	0.003067118	0.076035025	-0.000147918	3.37E-09	1	0.669181587	1	0.669469928	ENSG00000139899	6	CBLN3
SAT	Heart_Failure	ENSG00000204338.4	-0.000437595	0.739338878	0.001315167	0.076055476	-0.003298254	0.0293683	0.001513993	0.0760346	0.00286066	0.000137528	1	0.966658386	1	0.383605048	ENSG00000139899	6	CBLN3
SAT	Heart_Failure	ENSG00000204344.10	-0.000271979	0.870759425	0.001671721	0.076055489	0.001517976	0.366455833	0.001680789	0.076035213	-0.001789955	3.59E-24	1	0.979223495	1	0.849290727	ENSG00000139899	6	CBLN3
SAT	Heart_Failure	ENSG00000204348.5	0.00314953	0.08630531	0.001836224	0.076055034	0.001941983	0.298755713	0.001868897	0.076035172	0.001207547	0.00054406	1	0.592308253	1	0.810520426	ENSG00000139910	15	NOVA1
SAT	Heart_Failure	ENSG00000204351.7	-0.000633865	0.338616426	0.000662413	0.07605535	-0.00165883	0.015469487	0.000685126	0.076034425	0.001024965	5.00E-09	1	0.84294423	1	0.276579386	ENSG00000139970	12	RTN1
SAT	Heart_Failure	ENSG00000204387.8	-0.000634405	0.745057479	0.001951032	0.076055477	-0.001851504	0.345545122	0.001962865	0.076035201	0.0012171	2.21E-08	1	0.966658386	1	0.839802625	ENSG00000139970	12	RTN1
SAT	Heart_Failure	ENSG00000204410.10	-0.001733802	0.524850069	0.002726587	0.07605543	-0.009117455	0.001236007	0.002822342	0.076033712	0.007383652	5.03E-24	1	0.906724424	1	0.081106807	ENSG00000139971	11	C14orf37
SAT	Heart_Failure	ENSG00000204427.7	0.001314377	0.538051848	0.002134553	0.076055434	-0.001512885	0.483158352	0.002157466	0.076035263	0.002827262	3.22E-19	1	0.912011429	1	0.894608312	ENSG00000139998	10	RAB15
SAT	Heart_Failure	ENSG00000204428.8	0.001714331	0.122193054	0.001109143	0.07605512	0.002374532	0.033834975	0.001118986	0.076034638	-0.0006602	9.66E-06	1	0.646585619	1	0.412686066	ENSG00000140009	14	ESR2
SAT	Heart_Failure	ENSG00000204438.6	-0.005083757	0.020866482	0.00220038	0.07605466	-0.009692416	3.15E-05	0.002328415	0.076032636	0.004608659	1.49E-09	1	0.31549736	0.103222284	0.008601857	ENSG00000140025	11	EFCAB11
SAT	Heart_Failure	ENSG00000204463.8	0.006679661	2.63E-06	0.001421958	0.076052049	0.003001342	0.042092563	0.001476595	0.076034695	0.003678319	2.72E-20	0.00864292	0.000576195	1	0.45628996	ENSG00000140025	11	EFCAB11
SAT	Heart_Failure	ENSG00000204516.5	0.002748059	0.000682295	0.000809057	0.076053692	0.00338079	3.96E-05	0.000822552	0.076032704	-0.000632731	2.17E-05	1	0.044772199	0.129766464	0.009269033	ENSG00000140043	7	PTGR2
SAT	Heart_Failure	ENSG00000204520.8	0.00380571	1.41E-05	0.000876446	0.07605255	0.002861829	0.001166519	0.000881387	0.076033695	0.000943881	1.26E-23	0.046290748	0.002204321	1	0.078109189	ENSG00000140105	13	WARS
SAT	Heart_Failure	ENSG00000204525.10	-0.003579571	0.002615442	0.001189356	0.076054079	-0.001840203	0.140557129	0.001248677	0.076035001	-0.001739368	4.92E-06	1	0.113128813	1	0.655068096	ENSG00000140254	8	DUOXA1
SAT	Heart_Failure	ENSG00000204538.3	0.000971806	0.223992142	0.000799194	0.076055262	-0.000477697	0.564098611	0.000828236	0.076035288	0.001449503	2.82E-11	1	0.751217604	1	0.92032393	ENSG00000140264	15	SERF2
SAT	Heart_Failure	ENSG00000204540.6	-0.000237146	0.699850986	0.000615131	0.07605547	-0.001006447	0.106231975	0.000623046	0.076034933	0.0007693	1.07E-14	1	0.966658386	1	0.625105036	ENSG00000140264	15	SERF2
SAT	Heart_Failure	ENSG00000204622.6	-0.001178725	0.376808261	0.001333711	0.076055371	-0.003312899	0.014208298	0.001351122	0.076034402	0.002134174	9.25E-23	1	0.85759885	1	0.266385282	ENSG00000140265	8	ZSCAN29
SAT	Heart_Failure	ENSG00000204632.7	-0.002833692	0.165583598	0.002043719	0.076055193	-0.004843553	0.020420942	0.002089062	0.076034501	0.002009861	3.68E-06	1	0.689582559	1	0.319052908	ENSG00000140319	6	SRP14
SAT	Heart_Failure	ENSG00000204650.9	0.001465097	0.671142924	0.003450695	0.076055465	0.001540833	0.6551806	0.003450318	0.076035309	-7.57E-05	0.00124337	1	0.965985276	1	0.95563262	ENSG00000140319	6	SRP14
SAT	Heart_Failure	ENSG00000204709.4	0.001427852	0.257714747	0.001261564	0.076055293	0.001081693	0.395108353	0.001271995	0.076035227	0.000346159	0.034631131	1	0.773173321	1	0.862334842	ENSG00000140319	6	SRP14
SAT	Heart_Failure	ENSG00000204713.6	3.05E-05	0.986225857	0.001764607	0.076055493	-0.000764876	0.666214748	0.001773217	0.076035311	0.00079534	6.91E-06	1	0.999322741	1	0.956878334	ENSG00000140323	4	DISP2
SAT	Heart_Failure	ENSG00000204740.5	-0.002618145	0.276290445	0.002404855	0.076055308	-0.002670242	0.266794425	0.002404592	0.076035148	5.21E-05	0.001536023	1	0.792402928	1	0.775062994	ENSG00000140323	4	DISP2
SAT	Heart_Failure	ENSG00000204778.4	-0.000579393	0.878333672	0.003784852	0.076055489	-0.000516219	0.891500975	0.00378445	0.076035337	-6.32E-05	0.021330606	1	0.979223495	1	0.977805317	ENSG00000140323	4	DISP2
SAT	Heart_Failure	ENSG00000204839.4	-0.000766809	0.742192199	0.002331069	0.076055476	-0.000809507	0.728363302	0.002330834	0.076035321	4.27E-05	0.02153504	1	0.966658386	1	0.958028875	ENSG00000140323	4	DISP2
SAT	Heart_Failure	ENSG00000204933.2	-0.000330048	0.898888146	0.002597451	0.07605549	-0.000364875	0.888272763	0.002597156	0.076035337	3.48E-05	0.028938033	1	0.980097109	1	0.977226037	ENSG00000140386	8	SCAPER
SAT	Heart_Failure	ENSG00000204946.5	-0.000788705	0.77986375	0.002821875	0.076055481	-0.000831066	0.768345001	0.002821568	0.076035327	4.24E-05	0.029479429	1	0.972664863	1	0.965963214	ENSG00000140386	8	SCAPER
SAT	Heart_Failure	ENSG00000204947.4	-0.001325698	0.586407553	0.002436722	0.076055447	-0.001379091	0.571378376	0.002436458	0.07603529	5.34E-05	0.001527552	1	0.940373012	1	0.924404562	ENSG00000140400	10	MAN2C1
SAT	Heart_Failure	ENSG00000204959.3	0.003188289	0.225062278	0.002628047	0.076055263	0.003227593	0.219346125	0.002627754	0.076035105	-3.93E-05	0.021990524	1	0.751217604	1	0.742028061	ENSG00000140463	9	BBS4
SAT	Heart_Failure	ENSG00000204991.6	-0.000214528	0.89111965	0.001567179	0.07605549	-0.000268253	0.864077138	0.001567019	0.076035336	5.37E-05	9.52E-06	1	0.979270459	1	0.973904875	ENSG00000140470	9	ADAMTS17
SAT	Heart_Failure	ENSG00000205047.3	-0.000290324	0.880207503	0.001926424	0.076055489	-0.00034595	0.857466394	0.001926223	0.076035335	5.56E-05	0.000110422	1	0.979223495	1	0.972791643	ENSG00000140471	12	LINS
SAT	Heart_Failure	ENSG00000205057.3	0.009263663	0.019523383	0.003966642	0.076054642	0.009203849	0.020310231	0.003966194	0.0760345	5.98E-05	0.015746482	1	0.302151987	1	0.318841471	ENSG00000140471	12	LINS
SAT	Heart_Failure	ENSG00000205078.5	-0.000754006	0.757178405	0.002438663	0.076055478	-0.000706642	0.771972119	0.00243841	0.076035327	-4.74E-05	0.01023946	1	0.967625883	1	0.965963214	ENSG00000140471	12	LINS
SAT	Heart_Failure	ENSG00000205084.6	0.001643578	0.492165111	0.002392852	0.076055419	0.001596178	0.504689311	0.002392604	0.076035271	4.74E-05	0.008848735	1	0.898909993	1	0.900571724	ENSG00000140471	12	LINS
SAT	Heart_Failure	ENSG00000205108.5	-0.010547559	0.006090788	0.003845453	0.076054319	-0.010620657	0.005742141	0.003845046	0.07603415	7.31E-05	0.009638519	1	0.172274799	1	0.179428249	ENSG00000140471	12	LINS
SAT	Heart_Failure	ENSG00000205269.4	-0.000172345	0.928293812	0.001915123	0.076055492	-0.000133029	0.944616183	0.001914936	0.076035339	-3.93E-05	0.014018973	1	0.988739396	1	0.990503578	ENSG00000140471	12	LINS
SAT	Heart_Failure	ENSG00000205309.9	0.002237617	0.364872074	0.002469446	0.076055365	0.002159538	0.381799335	0.00246921	0.076035221	7.81E-05	0.000237233	1	0.85040303	1	0.854967426	ENSG00000140497	12	SCAMP2
SAT	Heart_Failure	ENSG00000205464.7	-0.002843848	0.460683296	0.003854901	0.076055408	-0.002795291	0.468324691	0.003854468	0.076035258	-4.86E-05	0.047071459	1	0.890157253	1	0.892844363	ENSG00000140553	12	UNC45A
SAT	Heart_Failure	ENSG00000205485.9	-0.003274599	0.011841547	0.001301075	0.076054504	-0.003307168	0.011018062	0.001300941	0.076034332	3.26E-05	0.001095707	1	0.24440108	1	0.242619208	ENSG00000140575	8	IQGAP1
SAT	Heart_Failure	ENSG00000205571.8	-0.000796118	0.580828425	0.001441784	0.076055445	-0.000818055	0.570407555	0.001441629	0.07603529	2.19E-05	0.032253863	1	0.936460965	1	0.924404562	ENSG00000140575	8	IQGAP1
SAT	Heart_Failure	ENSG00000205710.3	-0.001924677	0.156334748	0.001357788	0.076055179	-0.001961421	0.148535253	0.001357641	0.076035014	3.67E-05	0.000102687	1	0.677064618	1	0.665121824	ENSG00000140575	8	IQGAP1
SAT	Heart_Failure	ENSG00000205937.7	0.001016114	0.719051225	0.00282468	0.076055473	0.001060269	0.707362643	0.002824367	0.076035318	-4.42E-05	0.017650502	1	0.966658386	1	0.958028875	ENSG00000140575	8	IQGAP1
SAT	Heart_Failure	ENSG00000206077.6	0.001785735	0.580680515	0.003232736	0.076055445	0.001851869	0.56671061	0.003232431	0.076035289	-6.61E-05	0.019014614	1	0.936460965	1	0.922318901	ENSG00000140577	11	CRTC3
SAT	Heart_Failure	ENSG00000206341.6	-0.001629602	0.309870775	0.001604738	0.076055332	-0.003063949	0.057813604	0.001615059	0.076034779	0.001434347	6.86E-15	1	0.817271716	1	0.503576399	ENSG00000140839	7	CLEC18B
SAT	Heart_Failure	ENSG00000206344.6	0.000191185	0.818790488	0.000834501	0.076055485	-0.001192652	0.160775029	0.000850393	0.076035033	0.001383837	3.53E-17	1	0.976669362	1	0.676259259	ENSG00000140876	7	NUDT7
SAT	Heart_Failure	ENSG00000206562.7	-0.000197477	0.956528545	0.003622735	0.076055493	-0.000270871	0.9403914	0.003622331	0.076035339	7.34E-05	0.001799542	1	0.994657967	1	0.988983601	ENSG00000140876	7	NUDT7
SAT	Heart_Failure	ENSG00000212125.2	0.00128717	0.564092486	0.002231671	0.076055441	0.001358259	0.542731709	0.002231454	0.076035282	-7.11E-05	0.000142196	1	0.925856651	1	0.912245255	ENSG00000140876	7	NUDT7
SAT	Heart_Failure	ENSG00000212916.3	0.001917388	0.62396644	0.003911145	0.076055456	0.001974248	0.613678481	0.003910719	0.0760353	-5.69E-05	0.034266995	1	0.955683491	1	0.945647463	ENSG00000140876	7	NUDT7
SAT	Heart_Failure	ENSG00000213123.6	0.00178015	0.604195714	0.003434085	0.076055451	0.001862717	0.587488066	0.003433705	0.076035294	-8.26E-05	0.000279083	1	0.9466887	1	0.933194299	ENSG00000140955	6	ADAD2
SAT	Heart_Failure	ENSG00000213132.2	0.001335556	0.698625352	0.003449472	0.07605547	0.001428701	0.678713959	0.003449144	0.076035313	-9.31E-05	0.001857448	1	0.966658386	1	0.956878334	ENSG00000140955	6	ADAD2
SAT	Heart_Failure	ENSG00000213277.3	-0.002867917	0.318699891	0.00287616	0.076055338	-0.002807071	0.329023117	0.002875848	0.076035191	-6.08E-05	0.00238916	1	0.827258183	1	0.833382337	ENSG00000140955	6	ADAD2
SAT	Heart_Failure	ENSG00000213366.8	0.001288708	0.650979786	0.002848591	0.076055461	0.001428366	0.616036138	0.002848321	0.076035301	-0.000139658	1.65E-08	1	0.962760695	1	0.94759239	ENSG00000140955	6	ADAD2
SAT	Heart_Failure	ENSG00000213443.2	-0.000385231	0.750751689	0.001212757	0.076055477	-0.000417168	0.730834279	0.00121264	0.076035322	3.19E-05	0.001910807	1	0.966658386	1	0.958028875	ENSG00000140993	6	TIGD7
SAT	Heart_Failure	ENSG00000213462.4	0.002371463	0.156957982	0.001675496	0.07605518	0.002406053	0.150951669	0.001675314	0.076035018	-3.46E-05	0.002637134	1	0.678274948	1	0.665121824	ENSG00000140995	12	DEF8
SAT	Heart_Failure	ENSG00000213523.5	-0.000840465	0.701215687	0.002190535	0.07605547	-0.000873364	0.690081874	0.002190286	0.076035315	3.29E-05	0.014191811	1	0.966658386	1	0.956878334	ENSG00000140995	12	DEF8
SAT	Heart_Failure	ENSG00000213676.6	0.005698643	4.43E-06	0.001241521	0.076052205	0.003446098	0.007058878	0.001279144	0.076034208	0.002252544	2.87E-13	0.014543183	0.000908949	1	0.194766089	ENSG00000141002	14	TCF25
SAT	Heart_Failure	ENSG00000213689.5	0.00028345	0.856899097	0.001571901	0.076055488	0.000244434	0.876413825	0.00157175	0.076035336	3.90E-05	0.003538191	1	0.976669362	1	0.974665278	ENSG00000141002	14	TCF25
SAT	Heart_Failure	ENSG00000213719.4	-0.007298771	0.047733228	0.003686731	0.076054881	-0.014485839	0.001101869	0.004439213	0.076033679	0.007187068	0.003665782	1	0.478075483	1	0.076874944	ENSG00000141012	8	GALNS
SAT	Heart_Failure	ENSG00000213760.6	0.002843771	0.023236082	0.001253018	0.076054689	-3.11E-05	0.980808833	0.001291882	0.07603534	0.002874847	7.32E-20	1	0.327199936	1	0.999768899	ENSG00000141012	8	GALNS
SAT	Heart_Failure	ENSG00000214076.3	0.000618425	0.795355936	0.002384406	0.076055483	0.00058797	0.80520408	0.002384132	0.076035331	3.05E-05	0.031379335	1	0.976267425	1	0.972791643	ENSG00000141013	10	GAS8
SAT	Heart_Failure	ENSG00000214078.7	-0.002448255	0.000382831	0.000689325	0.076053524	-0.002434439	0.000412456	0.000689251	0.076033394	-1.38E-05	0.004421867	1	0.031401733	1	0.038968793	ENSG00000141013	10	GAS8
SAT	Heart_Failure	ENSG00000214087.4	-0.000123414	0.845066762	0.000631542	0.076055487	-0.00010058	0.873450793	0.00063148	0.076035336	-2.28E-05	1.03E-05	1	0.976669362	1	0.974665278	ENSG00000141013	10	GAS8
SAT	Heart_Failure	ENSG00000214279.8	-0.003598709	0.186005599	0.002721161	0.07605522	-0.003658967	0.178696708	0.002720865	0.076035058	6.03E-05	0.001335254	1	0.720656885	1	0.706126577	ENSG00000141076	13	CIRH1A
SAT	Heart_Failure	ENSG00000214376.5	-0.001949619	0.428670553	0.002463291	0.076055395	-0.001892934	0.442170734	0.002463047	0.076035248	-5.67E-05	0.004947752	1	0.878482333	1	0.880611614	ENSG00000141084	6	RANBP10
SAT	Heart_Failure	ENSG00000214425.2	-0.000366343	0.808696062	0.001513137	0.076055484	-0.000399485	0.791749095	0.00151297	0.076035329	3.31E-05	0.001156489	1	0.976669362	1	0.970635778	ENSG00000141098	8	GFOD2
SAT	Heart_Failure	ENSG00000214435.3	1.52E-05	0.981791685	0.000664834	0.076055493	-5.26E-06	0.993684723	0.000664762	0.07603534	2.04E-05	1.07E-05	1	0.998920999	1	0.999768899	ENSG00000141127	10	PRPSAP2
SAT	Heart_Failure	ENSG00000214517.4	-0.001137044	0.507255883	0.001714696	0.076055424	-0.001103081	0.519984846	0.001714538	0.076035276	-3.40E-05	0.027489558	1	0.901211406	1	0.904369708	ENSG00000141127	10	PRPSAP2
SAT	Heart_Failure	ENSG00000214597.4	-0.003315462	0.085509834	0.001928067	0.076055032	-0.003260182	0.090820215	0.001927858	0.076034894	-5.53E-05	3.50E-05	1	0.592308253	1	0.598355674	ENSG00000141127	10	PRPSAP2
SAT	Heart_Failure	ENSG00000214914.3	-0.001098879	0.499377738	0.001626836	0.076055422	-0.001158391	0.476390135	0.001626677	0.076035261	5.95E-05	1.10E-05	1	0.899263643	1	0.894608312	ENSG00000141140	12	MYO19
SAT	Heart_Failure	ENSG00000215452.3	-0.001168465	0.767602713	0.003954031	0.076055479	-0.001221104	0.757428892	0.003953596	0.076035325	5.26E-05	0.047066373	1	0.971467105	1	0.962699472	ENSG00000141198	9	TOM1L1
SAT	Heart_Failure	ENSG00000215527.3	0.000525796	0.844142487	0.002674476	0.076055487	0.000577726	0.828958917	0.002674198	0.076035333	-5.19E-05	0.010533366	1	0.976669362	1	0.972791643	ENSG00000141219	11	C17orf80
SAT	Heart_Failure	ENSG00000217930.3	0.004451657	0.119918506	0.002862584	0.076055116	0.004366346	0.127144099	0.002862307	0.076034977	8.53E-05	0.000411745	1	0.646585619	1	0.647874406	ENSG00000141252	15	VPS53
SAT	Heart_Failure	ENSG00000219481.6	-0.000681359	0.773432712	0.00236675	0.07605548	-0.000626115	0.791339565	0.002366525	0.076035329	-5.52E-05	0.006854216	1	0.972273076	1	0.970635778	ENSG00000141252	15	VPS53
SAT	Heart_Failure	ENSG00000219755.1	0.001018321	0.400053874	0.001210089	0.076055382	0.000999738	0.408658056	0.001209958	0.076035234	1.86E-05	0.026540417	1	0.870429337	1	0.867451005	ENSG00000141258	8	SGSM2
SAT	Heart_Failure	ENSG00000221823.6	0.002066788	0.563131542	0.003574541	0.076055441	0.002132551	0.550741503	0.003574209	0.076035285	-6.58E-05	0.038956588	1	0.925856651	1	0.914465015	ENSG00000141258	8	SGSM2
SAT	Heart_Failure	ENSG00000221923.4	-0.001072649	0.232316468	0.000898055	0.07605527	-0.001055581	0.239783054	0.000897961	0.076035125	-1.71E-05	0.011861417	1	0.752934502	1	0.752162449	ENSG00000141295	9	SCRN2
SAT	Heart_Failure	ENSG00000222009.4	0.002940692	0.417340453	0.003625808	0.07605539	0.003006714	0.406911144	0.00362542	0.076035233	-6.60E-05	0.01062055	1	0.87398914	1	0.867451005	ENSG00000141295	9	SCRN2
SAT	Heart_Failure	ENSG00000222019.3	0.001581721	0.305022311	0.001542054	0.076055329	0.001538644	0.318329213	0.001541884	0.076035185	4.31E-05	3.17E-05	1	0.816225947	1	0.823562856	ENSG00000141314	8	RHBDL3
SAT	Heart_Failure	ENSG00000223396.2	-0.001230335	0.724264049	0.00348768	0.076055474	-0.001307435	0.707730269	0.003487363	0.076035318	7.71E-05	0.01518971	1	0.966658386	1	0.958028875	ENSG00000141391	9	SLMO1
SAT	Heart_Failure	ENSG00000223496.1	0.000902527	0.74267882	0.002749024	0.076055476	0.000969492	0.724312552	0.002748762	0.076035321	-6.70E-05	0.004756913	1	0.966658386	1	0.958028875	ENSG00000141391	9	SLMO1
SAT	Heart_Failure	ENSG00000223756.2	0.000295404	0.905456655	0.002487167	0.076055491	0.000227267	0.927186168	0.002486897	0.076035339	6.81E-05	6.74E-05	1	0.981697482	1	0.987053153	ENSG00000141391	9	SLMO1
SAT	Heart_Failure	ENSG00000223865.6	-0.002244793	0.374026784	0.002525199	0.07605537	0.001652948	0.519059604	0.00256352	0.076035275	-0.00389774	1.51E-18	1	0.85759885	1	0.903946158	ENSG00000141391	9	SLMO1
SAT	Heart_Failure	ENSG00000224316.1	-0.002348284	0.092660964	0.001396517	0.076055052	-0.00231528	0.097302744	0.001396365	0.076034911	-3.30E-05	0.000551558	1	0.604414756	1	0.60854036	ENSG00000141425	13	RPRD1A
SAT	Heart_Failure	ENSG00000224389.4	0.000484243	0.784708774	0.001772564	0.076055481	0.004639166	0.024351262	0.002060433	0.076034549	-0.004154922	7.69E-05	1	0.972730696	1	0.342903392	ENSG00000141425	13	RPRD1A
SAT	Heart_Failure	ENSG00000224429.3	-0.004444568	0.242712342	0.003804514	0.07605528	-0.004543625	0.23232135	0.003804101	0.076035118	9.91E-05	0.000167756	1	0.759865642	1	0.750242469	ENSG00000141452	5	C18orf8
SAT	Heart_Failure	ENSG00000224497.1	0.002086699	0.008142554	0.000788591	0.0760544	0.002067097	0.00875462	0.000788517	0.076034268	1.96E-05	0.004400447	1	0.203936797	1	0.215969227	ENSG00000141452	5	C18orf8
SAT	Heart_Failure	ENSG00000224557.3	-0.001195913	0.070302237	0.000660739	0.076054982	-0.001776354	0.007637995	0.00066588	0.07603423	0.000580441	3.21E-12	1	0.540191194	1	0.202724069	ENSG00000141452	5	C18orf8
SAT	Heart_Failure	ENSG00000224635.1	-0.000746298	0.639105904	0.001591425	0.076055459	-0.000707213	0.656728515	0.001591265	0.076035309	-3.91E-05	0.002020015	1	0.959428651	1	0.956286421	ENSG00000141452	5	C18orf8
SAT	Heart_Failure	ENSG00000224796.1	0.000559977	0.464062472	0.000764813	0.076055409	0.002254325	0.004272628	0.000788967	0.076034066	-0.001694349	2.61E-18	1	0.890605014	1	0.161355485	ENSG00000141458	8	NPC1
SAT	Heart_Failure	ENSG00000224818.1	0.000531839	0.857690548	0.00296595	0.076055488	0.000461038	0.876460048	0.002965667	0.076035336	7.08E-05	0.005371629	1	0.976669362	1	0.974665278	ENSG00000141458	8	NPC1
SAT	Heart_Failure	ENSG00000224997.1	-0.002592813	0.092491251	0.001541136	0.076055051	-0.002630777	0.087782804	0.001540976	0.076034885	3.80E-05	0.001149212	1	0.604414756	1	0.596493272	ENSG00000141519	10	CCDC40
SAT	Heart_Failure	ENSG00000225492.2	-0.005008863	0.111484994	0.003147147	0.076055098	-0.005114138	0.104129008	0.003146851	0.076034928	0.000105275	0.000138083	1	0.640599412	1	0.620049499	ENSG00000141519	10	CCDC40
SAT	Heart_Failure	ENSG00000225498.1	0.000255685	0.923649901	0.002667912	0.076055492	0.000212093	0.936630304	0.002667639	0.076035339	4.36E-05	0.034606809	1	0.988739396	1	0.988983601	ENSG00000141519	10	CCDC40
SAT	Heart_Failure	ENSG00000225684.3	0.004095641	0.12960752	0.002702227	0.076055134	0.004031371	0.135692797	0.002701943	0.076034993	6.43E-05	0.001306846	1	0.649888404	1	0.649215182	ENSG00000141556	16	TBCD
SAT	Heart_Failure	ENSG00000225726.1	-0.000472744	0.880822188	0.003153156	0.07605549	-0.000521182	0.868702806	0.003152822	0.076035336	4.84E-05	0.035303499	1	0.979223495	1	0.974432105	ENSG00000141556	16	TBCD
SAT	Heart_Failure	ENSG00000225851.1	-0.002418101	0.082302347	0.001391726	0.076055022	-0.005296477	0.000246642	0.001444856	0.076033243	0.002878376	1.35E-13	1	0.592308253	0.809233072	0.027247586	ENSG00000141556	16	TBCD
SAT	Heart_Failure	ENSG00000226002.1	-0.000647266	0.523034893	0.001013452	0.076055429	-0.000617405	0.542341255	0.001013341	0.076035282	-2.99E-05	1.24E-05	1	0.906724424	1	0.912245255	ENSG00000141560	10	FN3KRP
SAT	Heart_Failure	ENSG00000226180.2	-0.000349285	0.875294627	0.002225642	0.076055489	-0.000400196	0.857286088	0.002225413	0.076035335	5.09E-05	0.003024539	1	0.979223495	1	0.972791643	ENSG00000141560	10	FN3KRP
SAT	Heart_Failure	ENSG00000226232.4	-0.001804946	0.129992816	0.001192066	0.076055135	-0.001769302	0.137711302	0.001191954	0.076034996	-3.56E-05	0.000722645	1	0.649888404	1	0.649215182	ENSG00000141560	10	FN3KRP

SAT	Heart_Failure	ENSG00000226259.6	-0.000136473	0.969272002	0.003542797	0.076055493	-0.00023765	0.946512299	0.003542405	0.076035339	0.000101177	1.55E-05	1	0.996609664	1	0.990755659	ENSG00000141560	10	FN3KRP
SAT	Heart_Failure	ENSG00000226314.3	0.000710364	0.466497224	0.000975519	0.07605541	0.000256246	0.793799834	0.000980358	0.076035329	0.000454118	4.09E-06	1	0.892464951	1	0.970635778	ENSG00000141560	10	FN3KRP
SAT	Heart_Failure	ENSG00000226479.3	-0.005373987	0.077530537	0.003044405	0.076055007	-0.005428633	0.07453292	0.003044096	0.076034844	5.46E-05	0.023099124	1	0.575515143	1	0.557044443	ENSG00000141562	13	NARF
SAT	Heart_Failure	ENSG00000227113.2	-0.000457614	0.681441925	0.001114783	0.076055467	-0.000426428	0.702043939	0.001114661	0.076035317	-3.12E-05	3.76E-05	1	0.966658386	1	0.956878334	ENSG00000141569	6	TRIM65
SAT	Heart_Failure	ENSG00000227766.1	-0.001956688	0.236793365	0.001653947	0.076055275	-0.003807954	0.02208954	0.001663711	0.076034523	0.001851266	2.71E-24	1	0.755348658	1	0.330737054	ENSG00000141569	6	TRIM65
SAT	Heart_Failure	ENSG00000228049.5	-0.001499309	0.096002804	0.000900728	0.076055061	-0.001525886	0.090222706	0.000900639	0.076034892	2.66E-05	0.000296785	1	0.612811673	1	0.598355674	ENSG00000141569	6	TRIM65
SAT	Heart_Failure	ENSG00000228157.2	0.001105749	0.774809728	0.003865046	0.07605548	0.001044791	0.786893763	0.003864637	0.076035329	6.10E-05	0.030347635	1	0.972664863	1	0.969773699	ENSG00000141569	6	TRIM65
SAT	Heart_Failure	ENSG00000228653.2	-0.001348988	0.421552261	0.001678406	0.076055392	-0.001380302	0.410807901	0.001678233	0.076035235	3.13E-05	0.014855744	1	0.876497446	1	0.867451005	ENSG00000141569	6	TRIM65
SAT	Heart_Failure	ENSG00000228696.4	-0.001018181	0.751899748	0.00322071	0.076055477	-0.000925914	0.773716961	0.003220385	0.076035327	-9.23E-05	0.000315959	1	0.966658386	1	0.965963214	ENSG00000141569	6	TRIM65
SAT	Heart_Failure	ENSG00000228716.2	0.001130886	0.433422393	0.001443655	0.076055397	0.001095592	0.447862933	0.0014435	0.07603525	3.53E-05	0.000580868	1	0.879986926	1	0.882462767	ENSG00000141574	3	SECTM1
SAT	Heart_Failure	ENSG00000228960.4	-0.002265596	0.516752632	0.00349435	0.076055427	-0.002182721	0.532161065	0.00349397	0.076035279	-8.29E-05	0.000562269	1	0.906069117	1	0.908441675	ENSG00000141574	3	SECTM1
SAT	Heart_Failure	ENSG00000229186.3	0.000107234	0.904860973	0.000897179	0.076055491	0.000130576	0.884272417	0.000897087	0.076035337	-2.33E-05	0.000659075	1	0.981517198	1	0.974898455	ENSG00000141576	10	RNF157
SAT	Heart_Failure	ENSG00000229212.3	0.00168951	0.640306585	0.0036157	0.076055459	0.001842397	0.610372347	0.003615787	0.0760353	-0.000152887	0.016934694	1	0.959428651	1	0.945083374	ENSG00000141576	10	RNF157
SAT	Heart_Failure	ENSG00000229291.1	0.002543975	0.21814866	0.002065808	0.076055256	0.002573908	0.212729411	0.002065576	0.076035098	-2.99E-05	0.022313753	1	0.747815981	1	0.733958243	ENSG00000141577	9	AZI1
SAT	Heart_Failure	ENSG00000229390.1	-0.001288074	0.557957597	0.002198535	0.076055439	-0.002928115	0.184162374	0.002204823	0.076035065	0.001640041	5.79E-22	1	0.922459771	1	0.715224819	ENSG00000141577	9	AZI1
SAT	Heart_Failure	ENSG00000229391.3	0.005667797	5.57E-12	0.000822568	0.076048084	0.00130563	0.19531984	0.001008206	0.076035078	0.004362167	7.36E-14	1.83E-08	3.66E-09	1	0.7243687	ENSG00000141580	11	WDR45B
SAT	Heart_Failure	ENSG00000229694.2	4.90E-05	0.98907484	0.003579347	0.076055493	-3.93E-05	0.99123883	0.003578973	0.07603534	8.83E-05	0.000939567	1	0.999440735	1	0.999768899	ENSG00000141580	11	WDR45B
SAT	Heart_Failure	ENSG00000230521.1	0.000672631	0.499260573	0.000995526	0.076055422	0.000942332	0.344014283	0.000995845	0.0760352	-0.000269701	2.23E-19	1	0.899263643	1	0.839547455	ENSG00000141580	11	WDR45B
SAT	Heart_Failure	ENSG00000230596.2	-0.003264257	0.056670074	0.001712755	0.076054926	-0.003222605	0.05987415	0.001712581	0.076034788	-4.17E-05	0.001950781	1	0.503931718	1	0.504627254	ENSG00000141580	11	WDR45B
SAT	Heart_Failure	ENSG00000230795.2	-0.000429385	0.6426711	0.000925464	0.076055459	-0.001796503	0.057594068	0.000946136	0.076034778	0.001367118	4.22E-12	1	0.960639581	1	0.503576399	ENSG00000141580	11	WDR45B
SAT	Heart_Failure	ENSG00000230953.2	-0.003147212	0.376612085	0.003559564	0.076055371	-0.003208214	0.367382339	0.003559192	0.076035213	6.10E-05	0.021824184	1	0.85759885	1	0.849290727	ENSG00000141639	7	MAPK4
SAT	Heart_Failure	ENSG00000231106.2	-0.000535485	0.508894055	0.000810651	0.076055425	-0.000565214	0.485613857	0.000810572	0.076035264	2.97E-05	1.08E-05	1	0.901555828	1	0.894608312	ENSG00000141639	7	MAPK4
SAT	Heart_Failure	ENSG00000231130.1	-5.20E-05	0.967983923	0.001295241	0.076055493	-0.001981248	0.138985129	0.001339058	0.076034999	0.001929261	1.45E-08	1	0.996534437	1	0.653383835	ENSG00000141664	5	ZCCHC2
SAT	Heart_Failure	ENSG00000231412.2	0.002188631	0.311292039	0.002161577	0.076055333	0.002129914	0.324399018	0.002161342	0.076035189	5.87E-05	7.06E-05	1	0.819702392	1	0.828598718	ENSG00000141698	12	NT5C3B
SAT	Heart_Failure	ENSG00000231500.2	0.00359843	0.010188326	0.001400505	0.076054463	0.002118763	0.133368214	0.001411612	0.076034989	0.001479667	9.91E-17	1	0.230796464	1	0.649215182	ENSG00000141753	5	IGFBP4
SAT	Heart_Failure	ENSG00000231784.4	0.002475531	0.356790895	0.00268642	0.07605536	0.002428082	0.366029168	0.002686119	0.076035213	4.74E-05	0.005357011	1	0.848363544	1	0.849290727	ENSG00000141753	5	IGFBP4
SAT	Heart_Failure	ENSG00000231925.7	-0.002016237	0.242272518	0.001724272	0.07605528	-0.00072268	0.676043041	0.00172944	0.076035313	-0.001293558	2.62E-21	1	0.759865642	1	0.956878334	ENSG00000141756	14	FKBP10
SAT	Heart_Failure	ENSG00000232491.1	0.013354322	8.26E-06	0.002995372	0.076052391	0.0133915	7.78E-06	0.002995025	0.076032221	-3.72E-05	0.032835772	0.027108694	0.001355435	0.025520246	0.00258496	ENSG00000141759	10	TXNL4A
SAT	Heart_Failure	ENSG00000232629.4	0.006390158	2.01E-14	0.000835259	0.076046359	0.002273927	0.05387884	0.001179537	0.07603476	0.004116231	7.74E-07	6.58E-11	1.64E-11	1	0.491574717	ENSG00000141759	10	TXNL4A
SAT	Heart_Failure	ENSG00000232637.3	-0.0017013	0.581814385	0.003089134	0.076055446	-0.001765179	0.567681831	0.003088842	0.076035289	6.39E-05	0.017918811	1	0.937265914	1	0.923432865	ENSG00000141759	10	TXNL4A
SAT	Heart_Failure	ENSG00000232677.2	0.000412518	0.840919285	0.002055215	0.076055487	0.00037722	0.854357064	0.002055001	0.076035335	3.53E-05	0.021700992	1	0.976669362	1	0.972791643	ENSG00000141933	8	TPGS1
SAT	Heart_Failure	ENSG00000232926.1	0.000482088	0.856545194	0.002666801	0.076055488	0.000429795	0.871949747	0.00266651	0.076035336	5.23E-05	0.004217541	1	0.976669362	1	0.974665278	ENSG00000141933	8	TPGS1
SAT	Heart_Failure	ENSG00000233005.1	0.002587547	0.20985088	0.002063472	0.076055248	0.002519148	0.222102886	0.002063257	0.076035108	6.84E-05	9.02E-06	1	0.739879495	1	0.746638902	ENSG00000141934	5	PPAP2C
SAT	Heart_Failure	ENSG00000233265.1	-0.000747052	0.490369839	0.001083121	0.076055419	-0.001631064	0.133263574	0.001086392	0.076034988	0.000884012	9.80E-25	1	0.898909993	1	0.649215182	ENSG00000141956	9	PRDM15
SAT	Heart_Failure	ENSG00000233396.3	-0.006570948	0.04009057	0.003200937	0.076054835	-0.006511901	0.041892548	0.003200594	0.076034694	-5.90E-05	0.009443684	1	0.450042677	1	0.45628996	ENSG00000141956	9	PRDM15
SAT	Heart_Failure	ENSG00000233448.2	-0.000676491	0.741351993	0.002049577	0.076055476	-0.000746414	0.715694934	0.002049357	0.076035319	6.99E-05	1.60E-06	1	0.966658386	1	0.958028875	ENSG00000141959	12	PFKL
SAT	Heart_Failure	ENSG00000233836.3	-0.006572973	0.086416688	0.003833501	0.076055034	-0.006437832	0.093049089	0.003833111	0.0760349	-0.000135141	6.93E-06	1	0.592308253	1	0.599166569	ENSG00000141959	12	PFKL
SAT	Heart_Failure	ENSG00000233892.1	-0.005819382	0.05602968	0.003045506	0.076054923	-0.008191073	0.007326211	0.003054488	0.076034218	0.002371691	3.89E-23	1	0.503931718	1	0.198655363	ENSG00000141971	8	MVB12A
SAT	Heart_Failure	ENSG00000233916.1	-0.002654782	0.126764363	0.001738573	0.076055129	-0.003643763	0.03685313	0.001745604	0.07603466	0.000988981	5.10E-10	1	0.649888404	1	0.429320811	ENSG00000141971	8	MVB12A
SAT	Heart_Failure	ENSG00000234127.4	0.000852403	0.685785453	0.002106877	0.076055467	-0.00149376	0.482783709	0.00212837	0.076035263	0.002346164	1.11E-14	1	0.966658386	1	0.894608312	ENSG00000141971	8	MVB12A
SAT	Heart_Failure	ENSG00000234444.5	0.003198455	0.008769532	0.001220355	0.076054421	0.003158134	0.009649597	0.001220229	0.076034295	4.03E-05	1.15E-05	1	0.210020691	1	0.22454133	ENSG00000141994	11	DUS3L
SAT	Heart_Failure	ENSG00000234585.2	-0.000474704	0.609616097	0.000929659	0.076055452	-0.000448669	0.629330277	0.000929556	0.076035304	-2.60E-05	2.61E-05	1	0.950190221	1	0.94798204	ENSG00000141994	11	DUS3L
SAT	Heart_Failure	ENSG00000234745.5	-0.001638855	0.289182061	0.001546208	0.076055318	-0.004158745	0.008037085	0.001569031	0.076034244	0.002519891	5.12E-21	1	0.802034101	1	0.209225934	ENSG00000141994	11	DUS3L
SAT	Heart_Failure	ENSG00000235231.3	0.002743383	0.136597263	0.001842947	0.076055147	0.002703607	0.142332352	0.001842745	0.076035004	3.98E-05	0.001422678	1	0.663104757	1	0.658003792	ENSG00000141994	11	DUS3L
SAT	Heart_Failure	ENSG00000235374.1	-0.002746129	0.06778555	0.001503547	0.076054972	-0.002724969	0.06990072	0.001503384	0.076034828	-2.12E-05	0.042228672	1	0.535424809	1	0.529994954	ENSG00000141994	11	DUS3L
SAT	Heart_Failure	ENSG00000235421.1	-0.00063344	0.719559529	0.001764224	0.076055473	-0.000577094	0.743558578	0.001764034	0.076035323	-5.63E-05	5.85E-06	1	0.966658386	1	0.962349048	ENSG00000142002	12	DPP9
SAT	Heart_Failure	ENSG00000235477.2	-0.000317684	0.842377353	0.001597575	0.076055487	-0.000270301	0.865630009	0.001597408	0.076035336	-4.74E-05	6.98E-05	1	0.976669362	1	0.974123167	ENSG00000142002	12	DPP9
SAT	Heart_Failure	ENSG00000235641.3	-0.000422006	0.739157117	0.001267399	0.076055476	-0.000450498	0.722223775	0.001267264	0.07603532	2.85E-05	0.001720275	1	0.966658386	1	0.958028875	ENSG00000142046	10	TMEM91
SAT	Heart_Failure	ENSG00000235821.1	-0.000762339	0.554738186	0.001290622	0.076055439	-0.001643287	0.215730942	0.001327414	0.076035101	0.000880948	0.004626416	1	0.921542737	1	0.736538211	ENSG00000142046	10	TMEM91
SAT	Heart_Failure	ENSG00000235944.3	0.000620568	0.8751668	0.003950171	0.076055489	0.000553925	0.888468537	0.003949766	0.076035337	6.66E-05	0.029133215	1	0.979223495	1	0.977226037	ENSG00000142102	11	ATHL1
SAT	Heart_Failure	ENSG00000235963.1	0.002411603	0.246261163	0.0020799	0.076055283	0.00078426	0.713089455	0.002132822	0.076035319	0.001627342	0.000586502	1	0.761161032	1	0.958028875	ENSG00000142102	11	ATHL1
SAT	Heart_Failure	ENSG00000236104.2	0.008639788	0.000533674	0.002494682	0.076053621	0.007932823	0.001500173	0.002498788	0.076033768	0.000706964	2.01E-06	1	0.037254953	1	0.09221864	ENSG00000142102	11	ATHL1
SAT	Heart_Failure	ENSG00000236532.1	0.002077288	0.38344941	0.002383417	0.076055374	0.002161953	0.364312171	0.002383158	0.076035212	-8.47E-05	3.14E-07	1	0.85759885	1	0.849290727	ENSG00000142156	10	COL6A1
SAT	Heart_Failure	ENSG00000236682.1	-0.001645768	0.447531991	0.002166805	0.076055403	-0.001604404	0.45898486	0.002166591	0.076035255	-4.14E-05	0.019462792	1	0.884975994	1	0.890430886	ENSG00000142156	10	COL6A1
SAT	Heart_Failure	ENSG00000236710.1	0.006820391	0.048233597	0.003452822	0.076054884	0.006765758	0.050032457	0.003452461	0.076034741	5.46E-05	0.034581524	1	0.478630006	1	0.480888199	ENSG00000142166	8	IFNAR1
SAT	Heart_Failure	ENSG00000237135.1	0.002027555	0.553983645	0.003426071	0.076055438	0.001939102	0.571365329	0.00342572	0.07603529	8.85E-05	0.000870858	1	0.921261889	1	0.924404562	ENSG00000142178	7	SIK1
SAT	Heart_Failure	ENSG00000237310.1	0.000356035	0.71420151	0.000972192	0.076055472	0.000397326	0.682736958	0.000972096	0.076035314	-4.13E-05	3.06E-07	1	0.966658386	1	0.956878334	ENSG00000142185	12	TRPM2
SAT	Heart_Failure	ENSG00000237541.3	0.013315141	1.47E-22	0.001362376	0.076040588	0.006147784	0.002608409	0.002042122	0.076033926	0.007167356	2.46E-06	4.82E-19	2.41E-19	1	0.122791633	ENSG00000142185	12	TRPM2
SAT	Heart_Failure	ENSG00000237669.1	-0.000672213	0.41866741	0.00083119	0.076055391	-6.34E-05	0.941802048	0.000868797	0.076035339	-0.000608787	0.016203516	1	0.874377957	1	0.988983601	ENSG00000142185	12	TRPM2
SAT	Heart_Failure	ENSG00000237984.2	-0.001785016	0.635060687	0.003760967	0.076055458	-0.001699851	0.651255599	0.003760575	0.076035308	-8.52E-05	0.00260346	1	0.959428651	1	0.954583686	ENSG00000142185	12	TRPM2
SAT	Heart_Failure	ENSG00000238083.3	0.004957063	0.115587213	0.003150199	0.076055107	0.005043021	0.109372262	0.003149872	0.07603494	-8.60E-05	0.000335835	1	0.641850091	1	0.631382451	ENSG00000142188	12	TMEM50B
SAT	Heart_Failure	ENSG00000238243.2	0.000471379	0.819253307	0.002062878	0.076055485	0.000414908	0.840580331	0.002062668	0.076035334	5.65E-05	0.000463357	1	0.976669362	1	0.972791643	ENSG00000142197	8	DOPEY2
SAT	Heart_Failure	ENSG00000239388.4	0.001593208	0.527150532	0.002519455	0.076055431	0.001634349	0.516490329	0.002519167	0.076035274	-4.11E-05	0.007153046	1	0.906724424	1	0.903395669	ENSG00000142207	5	URB1
SAT	Heart_Failure	ENSG00000239556.2	0.003039576	0.082766693	0.001752061	0.076055023	0.003064793	0.080215072	0.001751868	0.076034863	-2.52E-05	0.031303946	1	0.592308253	1	0.57843	ENSG00000142207	5	URB1
SAT	Heart_Failure	ENSG00000239617.1	0.000192079	0.928518406	0.002141127	0.076055492	0.000144424	0.946216031	0.002140907	0.076035339	4.77E-05	0.003811016	1	0.988739396	1	0.990755659	ENSG00000142230	7	SAE1
SAT	Heart_Failure	ENSG00000240053.8	-0.001015929	0.222127812	0.000832121	0.07605526	0.000255046	0.763314327	0.00084696	0.076035326	-0.001270976	1.03E-15	1	0.751090303	1	0.965635578	ENSG00000142273	6	CBLC
SAT	Heart_Failure	ENSG00000240065.3	0.006211607	0.00160865	0.001969218	0.07605394	0.004737196	0.020034034	0.002036875	0.076034496	0.001474411	0.004700152	1	0.083777454	1	0.318841471	ENSG00000142273	6	CBLC
SAT	Heart_Failure	ENSG00000240522.1	0.001443798	0.702452453	0.003779455	0.07605547	0.00153072	0.685439921	0.003779077	0.076035315	-8.69E-05	0.004392338	1	0.966658386	1	0.956878334	ENSG00000142273	6	CBLC
SAT	Heart_Failure	ENSG00000241057.1	0.005472601	0.13212713	0.003634423	0.076055139	0.005537476	0.127562978	0.003634026	0.076034978	-6.49E-05	0.00904416	1	0.655838294	1	0.647874406	ENSG00000142319	14	SLC6A3
SAT	Heart_Failure	ENSG00000241106.2	0.001252847	0.144389321	0.000858325	0.07605516	0.000490458	0.570075094	0.000863572	0.07603529	0.000762389	2.07E-15	1	0.669181587	1	0.924404562	ENSG00000142330	15	CAPN10
SAT	Heart_Failure	ENSG00000241186.3	-0.00429973	0.055439352	0.002244796	0.07605492	-0.004377924	0.051120996	0.002244551	0.076034747	7.82E-05	4.53E-07	1	0.503306524	1	0.485739162	ENSG00000142330	15	CAPN10
SAT	Heart_Failure	ENSG00000241549.4	0.000228038	0.82560358	0.001034918	0.076055485	0.000457141	0.658747494	0.001035091	0.07603531	-0.000229103	1.50E-19	1	0.976669362	1	0.956791995	ENSG00000142599	13	RERE
SAT	Heart_Failure	ENSG00000241679.2	-0.00133815	0.644421664	0.002899405	0.07605546	-0.001402837	0.628465665	0.002899095	0.076035304	6.47E-05	0.001837052	1	0.960776086	1	0.94798204	ENSG00000142621	15	FHAD1
SAT	Heart_Failure	ENSG00000241973.6	0.002529966	0.381479531	0.002890815	0.076055373	0.002585077	0.371144848	0.002890512	0.076035215	-5.51E-05	0.01071886	1	0.85759885	1	0.849768918	ENSG00000142632	12	ARHGEF19
SAT	Heart_Failure	ENSG00000242372.2	-0.008626285	0.004980029	0.003071672	0.076054262	-0.008685238	0.004687418	0.0030714	0.076034092	5.90E-05	0.041041627	1	0.160190922	1	0.163610845	ENSG00000142632	12	ARHGEF19
SAT	Heart_Failure	ENSG00000242435.1	0.000210186	0.93441342	0.002554099	0.076055492	0.000112737	0.964789573	0.002553847	0.07603534	9.74E-05	3.36E-06	1	0.989642217	1	0.996058713	ENSG00000142655	8	PEX14
SAT	Heart_Failure	ENSG00000242441.3	4.39E-05	0.973273321	0.001310454	0.076055493	2.39E-05	0.985442107	0.001310305	0.07603534	2.00E-05	0.013168694	1	0.99766005	1	0.999768899	ENSG00000142655	8	PEX14
SAT	Heart_Failure	ENSG00000242516.1	-0.000794815	0.501109352	0.001181449	0.076055422	-0.000820798	0.487174146	0.001181326	0.076035265	2.60E-05	0.003480215	1	0.900549813	1	0.894608312	ENSG00000142675	13	CNKSR1
SAT	Heart_Failure	ENSG00000242779.2	0.001216367	0.709239382	0.003262105	0.076055471	0.001349122	0.679156705	0.003261786	0.076035313	-0.000132754	1.03E-06	1	0.966658386	1	0.956878334	ENSG00000142675	13	CNKSR1
SAT	Heart_Failure	ENSG00000243244.1	0.004519909	0.179777727	0.003369441	0.076055212	0.00444264	0.187284677	0.003369066	0.076035069	7.73E-05	0.000416146	1	0.714462461	1	0.7171788	ENSG00000142675	13	CNKSR1
SAT	Heart_Failure	ENSG00000243478.3	0.001378772	0.392758722	0.001613304	0.076055379	0.001404535	0.383924767	0.001613132	0.076035222	-2.58E-05	0.025287364	1	0.863144683	1	0.854967426	ENSG00000142677	3	IL22RA1
SAT	Heart_Failure	ENSG00000243753.1	-0.001853235	0.021162824	0.000803981	0.076054664	-0.002967725	0.000261247	0.000812852	0.07603326	0.00111449	2.31E-20	1	0.315614662	0.857152676	0.027247586	ENSG00000142677	3	IL22RA1
SAT	Heart_Failure	ENSG00000243961.2	-0.001875207	0.451430009	0.002490204	0.076055405	-0.001822676	0.464157194	0.002489929	0.076035257	-5.25E-05	0.001535051	1	0.887019168	1	0.892844363	ENSG00000142677	3	IL22RA1
SAT	Heart_Failure	ENSG00000244026.2	0.003986225	0.113478809	0.002518542	0.076055102	0.003911645	0.120352367	0.002518282	0.076034964	7.46E-05	0.000108508	1	0.641850091	1	0.64481576	ENSG00000142677	3	IL22RA1
SAT	Heart_Failure	ENSG00000244560.2	0.000345619	0.829490031	0.001604876	0.076055486	0.000321075	0.841415094	0.001604705	0.076035334	2.45E-05	0.033731175	1	0.976669362	1	0.972791643	ENSG00000142684	7	ZNF593
SAT	Heart_Failure	ENSG00000244731.3	-0.001565464	0.046675809	0.000786968	0.076054875	-0.003205945	0.004626258	0.001132051	0.076034089	0.001640481	0.043836891	1	0.475002578	1	0.163212403	ENSG00000142684	7	ZNF593
SAT	Heart_Failure	ENSG00000245958.2	-0.001513117	0.312805325	0.001499094	0.076055334	-0.00155408	0.299833002	0.001498931	0.076035172	4.10E-05	6.81E-05	1	0.822137009	1	0.810520426	ENSG00000142686	7	C1orf216
SAT	Heart_Failure	ENSG00000246523.3	0.001090304	0.65970917	0.002476191	0.076055463	0.001218726	0.62255967	0.002475947	0.076035302	-0.000128422	3.39E-10	1	0.962994646	1	0.94798204	ENSG00000142686	7	C1orf216
SAT	Heart_Failure	ENSG00000246575.2	-0.00344137	0.248862409	0.002984403	0.076055285	-0.003295367	0.269510632	0.002984417	0.07603515	-0.000146003	0.0031006	1	0.765246076	1	0.780780192	ENSG00000142694	6	EVA1B
SAT	Heart_Failure	ENSG00000248202.1	-0.000629946	0.700472048	0.001637568	0.07605547	-0.00068782	0.674433755	0.00163739	0.076035313	5.79E-05	2.73E-07	1	0.966658386	1	0.956878334	ENSG00000142794	14	NBPF3
SAT	Heart_Failure	ENSG00000248375.1	-0.002654684	0.3430565	0.002799873	0.076055353	-0.002748662	0.32619484	0.002799592	0.07603519	9.40E-05	2.81E-05	1	0.847011103	1	0.830490914	ENSG00000142794	14	NBPF3
SAT	Heart_Failure	ENSG00000248408.1	-0.002980225	0.427512908	0.003756008	0.076055395	-0.003442397	0.359872874	0.003759683	0.076035209	0.000462172	0.009061574	1	0.878482333	1	0.848283315	ENSG00000142798	12	HSPG2
SAT	Heart_Failure	ENSG00000248794.1	-0.00141833	0.718366379	0.00393278	0.076055473	-0.001500068	0.702857905	0.003932381	0.076035317	8.17E-05	0.008441446	1	0.966658386	1	0.956878334	ENSG00000142798	12	HSPG2
SAT	Heart_Failure	ENSG00000249087.3	-0.00207271	0.422516949	0.002584224	0.076055393	-0.002019994	0.434366231	0.002583963	0.076035245	-5.27E-05	0.010227458	1	0.876565321	1	0.877927942	ENSG00000142798	12	HSPG2
SAT	Heart_Failure	ENSG00000249476.1	0.001126033	0.476072058	0.001580098	0.076055414	0.001095862	0.487922578	0.001579923	0.076035265	3.02E-05	0.003707439	1	0.895715287	1	0.894608312	ENSG00000142798	12	HSPG2
SAT	Heart_Failure	ENSG00000249740.1	-0.00227641	0.50875931	0.00344508	0.076055425	-0.00239931	0.486105142	0.003444724	0.076035264	0.0001229	2.88E-06	1	0.901555828	1	0.894608312	ENSG00000142798	12	HSPG2
SAT	Heart_Failure	ENSG00000249839.1	-0.001438506	0.541468274	0.00235591	0.076055435	-0.001386178	0.556235914	0.002355674	0.076035286	-5.23E-05	0.005635171	1	0.913266518	1	0.915531921	ENSG00000142937	7	RPS8
SAT	Heart_Failure	ENSG00000250075.1	0.000608554	0.738582959	0.001823488	0.076055476	0.000631038	0.729267522	0.001823282	0.076035321	-2.25E-05	0.049044484	1	0.966658386	1	0.958028875	ENSG00000142937	7	RPS8
SAT	Heart_Failure	ENSG00000250138.3	0.002767674	0.122189081	0.001790618	0.07605512	0.002795213	0.1184778	0.001790429	0.07603496	-2.75E-05	0.035989811	1	0.646585619	1	0.64481576	ENSG00000142973	8	CYP4B1
SAT	Heart_Failure	ENSG00000250378.1	-0.002318301	0.214471098	0.00186754	0.076055253	-0.002368576	0.20464893	0.001867345	0.076035089	5.03E-05	0.000343545	1	0.745086527	1	0.729840368	ENSG00000142973	8	CYP4B1
SAT	Heart_Failure	ENSG00000250412.1	0.004584433	0.114689734	0.002906207	0.076055105	0.004662234	0.108626861	0.002905907	0.076034938	-7.78E-05	0.000498898	1	0.641850091	1	0.631382451	ENSG00000143028	7	SYPL2
SAT	Heart_Failure	ENSG00000250506.2	-8.17E-06	0.995285604	0.001382698	0.076055493	-4.55E-05	0.973724033	0.001382564	0.07603534	3.74E-05	0.001354209	1	0.999479422	1	0.998213938	ENSG00000143067	4	ZNF697
SAT	Heart_Failure	ENSG00000250519.2	0.002618241	0.329711	0.002686209	0.076055345	0.002570673	0.338522016	0.002685924	0.076035197	4.76E-05	0.014528012	1	0.83656615	1	0.8376162	ENSG00000143119	8	CD53
SAT	Heart_Failure	ENSG00000250535.1	-0.000232255	0.80539819	0.000942717	0.076055484	-0.00139937	0.142545781	0.000954303	0.076035005	0.001167115	4.88E-15	1	0.976669362	1	0.658003792	ENSG00000143155	8	TIPRL
SAT	Heart_Failure	ENSG00000250656.2	-0.002852163	0.453565557	0.003805499	0.076055405	-0.002751888	0.469549921	0.003805091	0.076035259	-0.000100275	0.000209605	1	0.887019168	1	0.894229386	ENSG00000143156	9	NME7
SAT	Heart_Failure	ENSG00000250770.2	-0.000864618	0.772092443	0.002985156	0.07605548	-0.000804952	0.787405405	0.002984823	0.076035329	-5.97E-05	0.002015511	1	0.971467105	1	0.969773699	ENSG00000143185	3	XCL2
SAT	Heart_Failure	ENSG00000251383.1	-0.004027074	0.188507265	0.003062391	0.076055223	-0.003507457	0.252328275	0.003064047	0.076035136	-0.000519617	3.74E-06	1	0.722191019	1	0.765423527	ENSG00000143185	3	XCL2
SAT	Heart_Failure	ENSG00000251417.1	-0.001389039	0.280395952	0.001286824	0.076055311	-0.001354247	0.292568189	0.001286689	0.076035167	-3.48E-05	0.000233756	1	0.795640478	1	0.806652294	ENSG00000143185	3	XCL2
SAT	Heart_Failure	ENSG00000251580.1	-0.003076884	0.436560312	0.003954786	0.076055399	-0.003155941	0.424821856	0.003954395	0.076035241	7.91E-05	0.014893807	1	0.880339889	1	0.873390936	ENSG00000143303	7	RRNAD1
SAT	Heart_Failure	ENSG00000251593.1	0.00329502	0.18691832	0.002496695	0.076055221	0.003238617	0.194529252	0.002496432	0.076035078	5.64E-05	0.002115744	1	0.720656885	1	0.723506187	ENSG00000143367	11	TUFT1
SAT	Heart_Failure	ENSG00000251600.1	0.00393107	0.123982085	0.002555506	0.076055124	0.00387731	0.129170772	0.002555262	0.076034981	5.38E-05	0.014592736	1	0.647388543	1	0.649215182	ENSG00000143376	8	SNX27
SAT	Heart_Failure	ENSG00000251615.3	-0.002269033	0.273061473	0.002070209	0.076055306	-0.002301885	0.266126748	0.002069989	0.076035147	3.29E-05	0.028696365	1	0.786649253	1	0.774729353	ENSG00000143376	8	SNX27
SAT	Heart_Failure	ENSG00000253304.1	0.009201957	0.006831396	0.003401877	0.076054351	0.008967328	0.008417502	0.003403343	0.076034257	0.000234629	0.039901767	1	0.181474985	1	0.209225934	ENSG00000143418	15	CERS2
SAT	Heart_Failure	ENSG00000253326.2	0.004530616	0.210340601	0.00361688	0.076055248	0.004452267	0.218288077	0.003616511	0.076035104	7.83E-05	0.005422262	1	0.739879495	1	0.742028061	ENSG00000143437	16	ARNT
SAT	Heart_Failure	ENSG00000253333.1	0.004257711	0.278947264	0.003932536	0.07605531	0.004311156	0.272903693	0.003932097	0.076035153	-5.34E-05	0.035530561	1	0.793905695	1	0.783093703	ENSG00000143437	16	ARNT
SAT	Heart_Failure	ENSG00000253485.1	-0.007673449	0.037753571	0.003693551	0.076054819	-0.007620272	0.039080182	0.003693161	0.076034676	-5.32E-05	0.048356133	1	0.439253432	1	0.443745602	ENSG00000143442	17	POGZ
SAT	Heart_Failure	ENSG00000253669.3	0.003642286	0.152434648	0.002545297	0.076055173	0.003601727	0.157009062	0.002545021	0.076035028	4.06E-05	0.022084029	1	0.677064618	1	0.669469928	ENSG00000143442	17	POGZ
SAT	Heart_Failure	ENSG00000253731.1	0.002142539	0.326005424	0.002181383	0.076055342	0.00220462	0.312134671	0.002181164	0.076035181	-6.21E-05	0.000381647	1	0.833010855	1	0.821155716	ENSG00000143442	17	POGZ
SAT	Heart_Failure	ENSG00000254352.1	0.00018571	0.890560904	0.00134969	0.07605549	0.000133215	0.921367966	0.001349546	0.076035339	5.25E-05	7.76E-08	1	0.979270459	1	0.984731983	ENSG00000143442	17	POGZ
SAT	Heart_Failure	ENSG00000254521.2	-0.001082446	0.316591085	0.001080837	0.076055336	-0.001113392	0.302903568	0.001080724	0.076035175	3.09E-05	0.000134781	1	0.824393135	1	0.810625291	ENSG00000143442	17	POGZ
SAT	Heart_Failure	ENSG00000254681.2	-0.007140698	0.038949814	0.00345843	0.076054828	-0.007020958	0.042326153	0.003458082	0.076034697	-0.000119739	1.33E-05	1	0.445276442	1	0.45628996	ENSG00000143452	11	HORMAD1
SAT	Heart_Failure	ENSG00000254731.1	-0.003829553	0.249725213	0.0033271	0.076055286	-0.00417007	0.210144957	0.003327621	0.076035095	0.000340516	2.09E-05	1	0.766457153	1	0.733958243	ENSG00000143458	7	GABPB2
SAT	Heart_Failure	ENSG00000255328.1	-0.001244206	0.262942751	0.00111143	0.076055297	-0.001215437	0.274090946	0.001111316	0.076035153	-2.88E-05	0.000771081	1	0.775544307	1	0.784122993	ENSG00000143486	11	EIF2D
SAT	Heart_Failure	ENSG00000255374.1	-0.001294344	0.222636488	0.00106133	0.076055261	-0.001271747	0.230766744	0.001061215	0.076035116	-2.26E-05	0.002385439	1	0.751090303	1	0.750242469	ENSG00000143486	11	EIF2D
SAT	Heart_Failure	ENSG00000256061.3	0.00107234	0.762035422	0.003541307	0.076055479	0.001135954	0.748357426	0.003540936	0.076035324	-6.36E-05	0.015601295	1	0.969898847	1	0.962415493	ENSG00000143486	11	EIF2D
SAT	Heart_Failure	ENSG00000256087.2	0.000494204	0.864407886	0.002894044	0.076055488	0.000588569	0.838828146	0.002893754	0.076035334	-9.44E-05	4.87E-05	1	0.978183841	1	0.972791643	ENSG00000143603	14	KCNN3
SAT	Heart_Failure	ENSG00000256092.2	-1.07E-05	0.997524715	0.003442131	0.076055493	0.000127526	0.970443443	0.003441788	0.07603534	-0.000138204	7.64E-07	1	0.999479422	1	0.99755193	ENSG00000143622	6	RIT1
SAT	Heart_Failure	ENSG00000256223.1	0.002212032	0.308727949	0.002173142	0.076055331	0.00217144	0.317636796	0.002172902	0.076035184	4.06E-05	0.005029594	1	0.816225947	1	0.823487262	ENSG00000143631	10	FLG
SAT	Heart_Failure	ENSG00000256274.1	-0.001657162	0.112726522	0.00104483	0.0760551	-0.00164246	0.115913725	0.001044714	0.076034954	-1.47E-05	0.034043526	1	0.641850091	1	0.64481576	ENSG00000143631	10	FLG
SAT	Heart_Failure	ENSG00000256433.1	0.004997109	0.056934786	0.002624786	0.076054927	0.004956829	0.058937317	0.002624514	0.076034784	4.03E-05	0.043265726	1	0.503931718	1	0.503576399	ENSG00000143702	11	CEP170
SAT	Heart_Failure	ENSG00000256885.1	-0.001130667	0.422418621	0.0014094	0.076055393	-0.001149115	0.414836473	0.001409244	0.076035236	1.84E-05	0.049015254	1	0.876565321	1	0.867451005	ENSG00000143740	10	SNAP47
SAT	Heart_Failure	ENSG00000257218.1	-0.00383433	0.155127452	0.002697092	0.076055178	-0.003920117	0.146055471	0.002696817	0.07603501	8.58E-05	5.10E-05	1	0.677064618	1	0.663722991	ENSG00000143786	3	CNIH3
SAT	Heart_Failure	ENSG00000257534.1	0.001991757	0.429048942	0.002518594	0.076055395	0.002049782	0.415676603	0.002518328	0.076035237	-5.80E-05	0.001714866	1	0.878482333	1	0.867451005	ENSG00000143811	12	PYCR2
SAT	Heart_Failure	ENSG00000258230.2	0.003819625	0.166170777	0.002758618	0.076055194	0.003869391	0.160677532	0.002758337	0.076035033	-4.98E-05	0.020873122	1	0.690134582	1	0.676259259	ENSG00000143811	12	PYCR2
SAT	Heart_Failure	ENSG00000258713.2	0.000350448	0.917508206	0.003383582	0.076055491	0.000259307	0.938906367	0.003383244	0.076035339	9.11E-05	0.000873432	1	0.986998171	1	0.988983601	ENSG00000143811	12	PYCR2
SAT	Heart_Failure	ENSG00000258819.1	-0.006940037	0.057596585	0.003655036	0.07605493	-0.006874091	0.059983124	0.003654639	0.076034788	-6.59E-05	0.009016661	1	0.503931718	1	0.504627254	ENSG00000143811	12	PYCR2
SAT	Heart_Failure	ENSG00000258873.2	-0.00148456	0.344828038	0.001571512	0.076055354	-0.001459225	0.353072688	0.001571346	0.076035206	-2.53E-05	0.027462989	1	0.847011103	1	0.843366096	ENSG00000143891	12	GALM

SAT	Heart_Failure	ENSG00000259056.1	0.000364244	0.904791432	0.003045237	0.076055491	0.002784787	0.385853245	0.003211378	0.076035223	-0.002420544	0.017724763	1	0.981517198	1	0.857132361	ENSG00000143951	11	WDPCP
SAT	Heart_Failure	ENSG00000259205.2	0.001729474	0.162817204	0.001239179	0.076055189	0.001691901	0.172100999	0.001239047	0.076035049	3.76E-05	2.47E-05	1	0.686431329	1	0.697976981	ENSG00000143951	11	WDPCP
SAT	Heart_Failure	ENSG00000259207.3	0.00157004	0.584426551	0.002870638	0.076055446	0.001611659	0.574463235	0.002870321	0.076035291	-4.16E-05	0.029241196	1	0.93857245	1	0.927160988	ENSG00000143954	8	REG3G
SAT	Heart_Failure	ENSG00000259295.2	-0.004094938	0.111816075	0.002575293	0.076055098	-0.003968468	0.123331965	0.002575355	0.07603497	-0.00012647	0.005457426	1	0.641378571	1	0.647747917	ENSG00000144021	2	CIAO1
SAT	Heart_Failure	ENSG00000259433.2	-0.001981883	0.443935308	0.002588783	0.076055402	-0.002033238	0.432169548	0.002588513	0.076035244	5.14E-05	0.008060496	1	0.882758633	1	0.876305924	ENSG00000144026	7	ZNF514
SAT	Heart_Failure	ENSG00000259648.1	-0.003424988	0.233778363	0.002876488	0.076055272	-0.003494623	0.224357954	0.002876179	0.07603511	6.96E-05	0.000587779	1	0.754244409	1	0.747837725	ENSG00000144026	7	ZNF514
SAT	Heart_Failure	ENSG00000259658.3	0.000196091	0.943820305	0.002782656	0.076055492	0.000245243	0.929763879	0.002782368	0.076035339	-4.92E-05	0.02021672	1	0.992414898	1	0.988475359	ENSG00000144118	9	RALB
SAT	Heart_Failure	ENSG00000259673.1	0.002088279	0.195874201	0.001614569	0.076055232	0.002061534	0.201611111	0.001614387	0.076035086	2.67E-05	0.008627773	1	0.726878652	1	0.728906397	ENSG00000144152	8	FBLN7
SAT	Heart_Failure	ENSG00000259715.1	-0.000357462	0.756260987	0.001151638	0.076055478	-0.000380844	0.740847002	0.001151517	0.076035323	2.34E-05	0.006084875	1	0.967625883	1	0.960758504	ENSG00000144152	8	FBLN7
SAT	Heart_Failure	ENSG00000259758.1	0.00222517	0.569948432	0.003916672	0.076055443	0.002143094	0.584222232	0.003916272	0.076035293	8.21E-05	0.007178363	1	0.92942386	1	0.933194299	ENSG00000144152	8	FBLN7
SAT	Heart_Failure	ENSG00000259982.1	0.002716093	0.093590794	0.001619849	0.076055054	0.002666978	0.099639159	0.001619679	0.076034917	4.91E-05	4.20E-05	1	0.604759516	1	0.613289124	ENSG00000144152	8	FBLN7
SAT	Heart_Failure	ENSG00000260065.1	-0.000262777	0.799698096	0.001035622	0.076055483	-0.000297599	0.773813455	0.001035523	0.076035327	3.48E-05	9.28E-05	1	0.976669362	1	0.965963214	ENSG00000144152	8	FBLN7
SAT	Heart_Failure	ENSG00000260075.1	0.003382233	0.132575355	0.002248787	0.07605514	0.003323418	0.139399172	0.002248539	0.076034999	5.88E-05	8.38E-05	1	0.657069095	1	0.653383835	ENSG00000144152	8	FBLN7
SAT	Heart_Failure	ENSG00000260265.1	0.000686745	0.722637804	0.001934842	0.076055473	0.000831547	0.667347811	0.00193477	0.076035311	-0.000144802	6.05E-08	1	0.966658386	1	0.956878334	ENSG00000144182	12	LIPT1
SAT	Heart_Failure	ENSG00000260302.1	-0.000244534	0.929638882	0.002769378	0.076055492	-0.000197397	0.94317001	0.002769082	0.076035339	-4.71E-05	0.017577168	1	0.988739396	1	0.989303326	ENSG00000144182	12	LIPT1
SAT	Heart_Failure	ENSG00000260388.1	-0.003440023	0.109568761	0.00214983	0.076055093	-0.003388496	0.11494756	0.002149594	0.076034952	-5.15E-05	0.000351024	1	0.640599412	1	0.64481576	ENSG00000144182	12	LIPT1
SAT	Heart_Failure	ENSG00000260778.2	0.002934696	0.351137684	0.003147513	0.076055357	0.002831126	0.368345231	0.003147171	0.076035214	0.00010357	1.90E-06	1	0.848363544	1	0.849290727	ENSG00000144182	12	LIPT1
SAT	Heart_Failure	ENSG00000260947.1	-0.001049023	0.227286924	0.000868844	0.076055266	-0.001025402	0.237876498	0.000868755	0.076035123	-2.36E-05	0.000474724	1	0.751217604	1	0.752162449	ENSG00000144199	7	FAHD2B
SAT	Heart_Failure	ENSG00000261143.1	0.003778109	0.086481857	0.002203932	0.076055034	0.003822315	0.082831211	0.002203707	0.076034871	-4.42E-05	0.010362772	1	0.592308253	1	0.5844499	ENSG00000144214	5	LYG1
SAT	Heart_Failure	ENSG00000261455.1	0.003606566	0.196219739	0.002790598	0.076055232	0.003554367	0.202727757	0.002790321	0.076035087	5.22E-05	0.02151707	1	0.726878652	1	0.729735526	ENSG00000144214	5	LYG1
SAT	Heart_Failure	ENSG00000261556.4	0.001311639	0.572276253	0.002322708	0.076055443	0.001357789	0.558797251	0.002322479	0.076035287	-4.61E-05	0.015839081	1	0.931368247	1	0.916248766	ENSG00000144214	5	LYG1
SAT	Heart_Failure	ENSG00000261584.1	0.000128449	0.91177299	0.001159261	0.076055491	0.000293376	0.800557093	0.001161305	0.07603533	-0.000164927	0.020927977	1	0.984491733	1	0.972791643	ENSG00000144214	5	LYG1
SAT	Heart_Failure	ENSG00000261586.2	0.00136642	0.588716795	0.002527146	0.076055447	0.001542896	0.541558831	0.002527441	0.076035282	-0.000176476	0.001763099	1	0.941136084	1	0.91214298	ENSG00000144214	5	LYG1
SAT	Heart_Failure	ENSG00000261652.2	-0.005332236	0.044146358	0.00264929	0.076054861	-0.005413152	0.041006731	0.002649003	0.076034689	8.09E-05	1.23E-05	1	0.464244235	1	0.453733817	ENSG00000144214	5	LYG1
SAT	Heart_Failure	ENSG00000261794.1	-0.002718781	0.18426754	0.002047689	0.076055218	-0.002778231	0.174814646	0.002047489	0.076035053	5.94E-05	0.000488214	1	0.720580417	1	0.700325828	ENSG00000144218	14	AFF3
SAT	Heart_Failure	ENSG00000262500.1	0.001247156	0.513409145	0.001908311	0.076055426	0.001294743	0.497423708	0.001908105	0.076035268	-4.76E-05	0.000354731	1	0.905155107	1	0.894608312	ENSG00000144230	12	GPR17
SAT	Heart_Failure	ENSG00000262539.1	0.00056926	0.786873048	0.002105458	0.076055482	0.000612161	0.771218419	0.002105227	0.076035327	-4.29E-05	0.002683679	1	0.974020771	1	0.965963214	ENSG00000144230	12	GPR17
SAT	Heart_Failure	ENSG00000262855.1	-0.000936827	0.496813648	0.001378676	0.076055421	-0.000917012	0.505914993	0.001378525	0.076035271	-1.98E-05	0.033968826	1	0.899087467	1	0.900571724	ENSG00000144230	12	GPR17
SAT	Heart_Failure	ENSG00000263627.1	-0.002189257	0.40491411	0.002628546	0.076055385	-0.002226741	0.396868519	0.002628267	0.076035228	3.75E-05	0.049443045	1	0.870429337	1	0.862334842	ENSG00000144230	12	GPR17
SAT	Heart_Failure	ENSG00000263874.1	0.000113049	0.964780783	0.002560259	0.076055493	5.34E-05	0.983368814	0.002559983	0.07603534	5.97E-05	0.000902	1	0.996432082	1	0.997768899	ENSG00000144230	12	GPR17
SAT	Heart_Failure	ENSG00000264469.1	-0.002348828	0.033777649	0.001106517	0.07605479	-0.002374377	0.03187018	0.001106399	0.076034622	2.55E-05	0.001273811	1	0.411793489	1	0.401948008	ENSG00000144362	7	PHOSPHO2
SAT	Heart_Failure	ENSG00000264538.2	0.001795019	0.144724998	0.001230796	0.076055161	0.001817202	0.139782826	0.001230664	0.076035	-2.22E-05	0.011894628	1	0.669735853	1	0.654247435	ENSG00000144407	5	PTH2R
SAT	Heart_Failure	ENSG00000265798.2	-0.006172742	0.00646516	0.002266708	0.076054336	-0.006098786	0.007126764	0.002266471	0.07603421	-7.40E-05	1.03E-05	1	0.179416801	1	0.194857595	ENSG00000144407	5	PTH2R
SAT	Heart_Failure	ENSG00000266904.1	0.000483484	0.662490825	0.001107695	0.076055463	0.000527504	0.633884159	0.001107581	0.076035305	-4.40E-05	1.71E-07	1	0.962994646	1	0.94798204	ENSG00000144426	14	NBEAL1
SAT	Heart_Failure	ENSG00000267058.1	-0.00212321	0.191580284	0.001625834	0.076055227	-0.00214995	0.186001384	0.001625667	0.076035067	2.67E-05	0.033551617	1	0.722912434	1	0.715224819	ENSG00000144426	14	NBEAL1
SAT	Heart_Failure	ENSG00000267108.1	-0.000561369	0.804692864	0.00227019	0.076055483	-0.000508321	0.822806662	0.002269933	0.076035332	-5.30E-05	0.000186946	1	0.976669362	1	0.972791643	ENSG00000144476	5	ACKR3
SAT	Heart_Failure	ENSG00000267152.1	-0.002842571	0.453867078	0.003795235	0.076055405	-0.002968036	0.434140418	0.003794827	0.076035245	0.000125466	3.29E-06	1	0.887019168	1	0.877927942	ENSG00000144476	5	ACKR3
SAT	Heart_Failure	ENSG00000267405.1	0.003070984	0.191970063	0.00235365	0.076055227	0.003027624	0.198276503	0.002353419	0.076035082	4.34E-05	0.025553806	1	0.722912434	1	0.725655076	ENSG00000144504	11	ANKMY1
SAT	Heart_Failure	ENSG00000267505.1	0.012192873	0.002002968	0.003946158	0.076054003	0.012108484	0.00214975	0.00394575	0.076033871	8.44E-05	0.005037603	1	0.09524259	1	0.111957631	ENSG00000144559	6	TAMM41
SAT	Heart_Failure	ENSG00000267594.5	-0.004212223	0.255777843	0.003706545	0.076055291	-0.004119443	0.26635011	0.003706174	0.076035147	-9.28E-05	0.001825662	1	0.770621765	1	0.774729353	ENSG00000144580	9	RQCD1
SAT	Heart_Failure	ENSG00000267653.1	0.00180614	0.590728025	0.003358501	0.076055448	0.001873632	0.576890666	0.003358168	0.076035292	-6.75E-05	0.014074178	1	0.941376309	1	0.927910133	ENSG00000144580	9	RQCD1
SAT	Heart_Failure	ENSG00000268340.1	0.008077257	0.010353342	0.003150491	0.076054467	0.00799544	0.011146781	0.003150201	0.076034335	8.18E-05	0.003842215	1	0.230796464	1	0.243469768	ENSG00000144580	9	RQCD1
SAT	Heart_Failure	ENSG00000269086.2	0.000100621	0.969653633	0.002644938	0.076055493	4.15E-05	0.987477463	0.002644657	0.07603534	5.91E-05	0.001941495	1	0.996689715	1	0.997768899	ENSG00000144677	10	CTDSPL
SAT	Heart_Failure	ENSG00000269202.1	-0.001028199	0.64383596	0.002223891	0.07605546	-0.000941747	0.671922842	0.002223668	0.076035312	-8.65E-05	1.30E-06	1	0.960732494	1	0.956878334	ENSG00000144677	10	CTDSPL
SAT	Heart_Failure	ENSG00000269430.1	0.004085797	0.043086138	0.002019793	0.076054854	0.004154062	0.039698366	0.002019594	0.07603468	-6.83E-05	4.39E-05	1	0.463882417	1	0.447595661	ENSG00000144677	10	CTDSPL
SAT	Heart_Failure	ENSG00000269486.1	0.003663808	0.330401116	0.003764287	0.076055345	0.003798799	0.312845061	0.003763902	0.076035181	-0.000134991	4.34E-06	1	0.837101206	1	0.821155716	ENSG00000144792	5	ZNF660
SAT	Heart_Failure	ENSG00000269558.1	-0.000788342	0.631496275	0.001643672	0.076055457	-0.000738325	0.653258009	0.001643494	0.076035309	-5.00E-05	1.11E-05	1	0.959428651	1	0.954716939	ENSG00000144834	8	TAGLN3
SAT	Heart_Failure	ENSG00000269893.2	0.001284697	0.62208765	0.002606435	0.076055455	0.001228677	0.637319434	0.002606165	0.076035305	5.60E-05	0.004730994	1	0.954216727	1	0.94798204	ENSG00000144834	8	TAGLN3
SAT	Heart_Failure	ENSG00000269928.1	0.000867285	0.72464175	0.002462044	0.076055474	0.000818728	0.73945457	0.002461778	0.076035323	4.86E-05	0.004696492	1	0.966658386	1	0.960499534	ENSG00000144837	4	PLA1A
SAT	Heart_Failure	ENSG00000269963.1	-0.00541483	0.071107874	0.003000252	0.076054985	-0.005539077	0.064836906	0.002999957	0.076034808	0.000124247	6.20E-07	1	0.545104986	1	0.514983161	ENSG00000144843	7	ADPRH
SAT	Heart_Failure	ENSG00000269983.1	-0.000413948	0.845822042	0.002128795	0.076055487	-0.000474427	0.823623969	0.002128561	0.076035332	6.05E-05	2.33E-05	1	0.976669362	1	0.972791643	ENSG00000144843	7	ADPRH
SAT	Heart_Failure	ENSG00000270020.1	-0.003175928	0.010640662	0.001243369	0.076054475	-0.003199352	0.010070605	0.001243237	0.076034307	2.34E-05	0.009358768	1	0.232334342	1	0.230558242	ENSG00000144843	7	ADPRH
SAT	Heart_Failure	ENSG00000270030.1	-0.002441227	0.527530556	0.003864042	0.076055431	-0.002363277	0.540758646	0.003863668	0.076035282	-7.80E-05	0.016865746	1	0.906724424	1	0.911336308	ENSG00000144847	8	IGSF11
SAT	Heart_Failure	ENSG00000270093.1	0.0019837	0.498099779	0.002928044	0.076055421	0.002041687	0.48557433	0.002927713	0.076035264	-5.80E-05	0.001557332	1	0.899263643	1	0.894608312	ENSG00000144847	8	IGSF11
SAT	Heart_Failure	ENSG00000270127.1	0.001807825	0.142788718	0.0012336	0.076055158	0.00177061	0.151152762	0.001233468	0.076035019	3.72E-05	2.10E-05	1	0.669181587	1	0.665121824	ENSG00000144847	8	IGSF11
SAT	Heart_Failure	ENSG00000270182.1	-0.000239854	0.940369299	0.003206362	0.076055492	-4.27E-05	0.989383146	0.003206121	0.07603534	-0.000197192	9.30E-09	1	0.992074492	1	0.997768899	ENSG00000145020	10	AMT
SAT	Heart_Failure	ENSG00000270672.1	0.0061009	0.118857041	0.003911857	0.076055113	0.005997261	0.125214813	0.003911469	0.076034973	0.000103639	0.001135611	1	0.646149939	1	0.647874406	ENSG00000145020	10	AMT
SAT	Heart_Failure	ENSG00000270777.1	0.000678947	0.835415647	0.003267924	0.076055486	0.000732119	0.822714304	0.003267581	0.076035332	-5.32E-05	0.028556977	1	0.976669362	1	0.972791643	ENSG00000145029	7	NICN1
SAT	Heart_Failure	ENSG00000270948.1	0.001768638	0.478506653	0.002495584	0.076055415	0.001698646	0.496039753	0.002495313	0.076035268	7.00E-05	4.55E-05	1	0.897131616	1	0.894608312	ENSG00000145029	7	NICN1
SAT	Heart_Failure	ENSG00000270964.1	0.003064279	0.116479387	0.00195211	0.076055108	0.003030361	0.120536648	0.001951888	0.076034964	3.39E-05	0.004818869	1	0.642397685	1	0.64481576	ENSG00000145103	8	ILDR1
SAT	Heart_Failure	ENSG00000270972.1	-0.001789572	0.253574571	0.001567447	0.07605529	-0.001749109	0.26441753	0.001567285	0.076035146	-4.05E-05	0.000659329	1	0.768323136	1	0.774729353	ENSG00000145103	8	ILDR1
SAT	Heart_Failure	ENSG00000271095.1	-0.00192066	0.536518573	0.003107428	0.076055433	-0.001970461	0.525964877	0.003107109	0.076035277	4.98E-05	0.037683793	1	0.911137391	1	0.905399141	ENSG00000145113	17	MUC4
SAT	Heart_Failure	ENSG00000271538.1	0.001756534	0.483648152	0.002507731	0.076055416	0.001721672	0.49232161	0.002507455	0.076035267	3.49E-05	0.038137262	1	0.897349195	1	0.894608312	ENSG00000145113	17	MUC4
SAT	Heart_Failure	ENSG00000271581.1	0.00153247	0.143462064	0.001047469	0.076055159	-0.000365478	0.739248592	0.001098031	0.076035323	0.001897948	8.67E-09	1	0.669181587	1	0.960499534	ENSG00000145113	17	MUC4
SAT	Heart_Failure	ENSG00000271623.1	-0.003981308	0.180638149	0.0029738	0.076055213	-0.003910563	0.188462202	0.002973487	0.07603507	-7.07E-05	0.001231891	1	0.714462461	1	0.717336988	ENSG00000145217	9	SLC26A1
SAT	Heart_Failure	ENSG00000272003.1	0.001163121	0.761453848	0.003831446	0.076055479	0.001071649	0.779686635	0.003831046	0.076035328	9.15E-05	0.0014457	1	0.969898847	1	0.966957854	ENSG00000145217	9	SLC26A1
SAT	Heart_Failure	ENSG00000272005.1	0.003480565	0.371351531	0.003893487	0.076055368	0.003594266	0.355876992	0.003893055	0.076035207	-0.000113701	9.23E-06	1	0.856926425	1	0.844885969	ENSG00000145217	9	SLC26A1
SAT	Heart_Failure	ENSG00000272010.1	-0.001210442	0.434958657	0.001550388	0.076055398	-0.001292131	0.40466903	0.001550597	0.076035232	8.17E-05	0.022654285	1	0.880339889	1	0.867451005	ENSG00000145241	6	CENPC
SAT	Heart_Failure	ENSG00000272047.1	0.00669432	0.01111805	0.002636622	0.076054487	0.006633634	0.01186242	0.002636349	0.076034352	6.07E-05	0.002415645	1	0.236383009	1	0.246278972	ENSG00000145241	6	CENPC
SAT	Heart_Failure	ENSG00000272156.1	-0.000349409	0.904934326	0.002925628	0.076055491	-0.000403484	0.890297563	0.002925326	0.076035337	5.41E-05	0.015680415	1	0.981517198	1	0.977805317	ENSG00000145358	2	DDIT4L
SAT	Heart_Failure	ENSG00000272168.1	0.005218725	0.039691827	0.002537122	0.076054833	0.006040518	0.018621154	0.002567115	0.076034476	-0.000821793	0.03674464	1	0.448303633	1	0.304660546	ENSG00000145390	7	USP53
SAT	Heart_Failure	ENSG00000272236.1	-0.001712896	0.211360385	0.001370497	0.076055249	-0.002554394	0.069227371	0.001405879	0.076034825	0.000841498	0.007406069	1	0.740890409	1	0.529994954	ENSG00000145390	7	USP53
SAT	Heart_Failure	ENSG00000272462.2	-0.000532939	0.386069511	0.000614858	0.076055376	-0.00066188	0.282876975	0.000616344	0.07603516	0.00012894	0.003338373	1	0.859117766	1	0.795303647	ENSG00000145569	5	FAM105A
SAT	Heart_Failure	ENSG00000272636.1	0.003597382	0.235721529	0.003033845	0.076055274	0.003671959	0.226106808	0.003033549	0.076035112	-7.46E-05	0.003258345	1	0.755348658	1	0.747837725	ENSG00000145569	5	FAM105A
SAT	Heart_Failure	ENSG00000272720.1	0.000218128	0.886733276	0.001531377	0.07605549	0.000243652	0.873571276	0.001531206	0.076035336	-2.55E-05	0.010168368	1	0.979270459	1	0.974665278	ENSG00000145692	10	BHMT
SAT	Heart_Failure	ENSG00000272733.1	-0.002020918	0.346518355	0.002146796	0.076055355	-0.00196181	0.36075937	0.002146588	0.07603521	-5.91E-05	0.00115111	1	0.847011103	1	0.848283315	ENSG00000145715	10	RASA1
SAT	Heart_Failure	ENSG00000272810.1	-0.000710968	0.673371411	0.001686654	0.076055465	-0.001293475	0.443461927	0.001687814	0.076035248	0.000582507	1.53E-17	1	0.966658386	1	0.882352081	ENSG00000145736	10	GTF2H2
SAT	Heart_Failure	ENSG00000272849.1	-0.003403278	0.114161931	0.002154298	0.076055104	-0.003372135	0.117472448	0.002154066	0.076034958	-3.11E-05	0.041583023	1	0.641850091	1	0.64481576	ENSG00000145777	10	TSLP
SAT	Heart_Failure	ENSG00000273448.1	0.004113872	0.078682738	0.002339575	0.07605501	0.004174866	0.074321595	0.002339342	0.076034843	-6.10E-05	0.001360556	1	0.580653917	1	0.55673323	ENSG00000145781	4	COMMD10
SAT	HTN	ENSG00000001561.6	-0.000429865	0.882755228	0.002914785	0.240335803	-0.0004705	0.871760622	0.002914708	0.240312213	4.06E-05	0.037520217	1	0.957468	1	0.955645373	ENSG00000145781	4	COMMD10
SAT	HTN	ENSG00000005243.5	0.017101783	6.11E-05	0.004266163	0.240327888	0.017019524	6.62E-05	0.004266076	0.240304376	8.23E-05	0.010881207	0.200344429	0.001500008	0.21727313	0.001621441	ENSG00000145781	4	COMMD10
SAT	HTN	ENSG00000008853.12	-0.003063163	0.585452442	0.005615967	0.240335667	-0.002897073	0.605949012	0.005615953	0.240312094	-0.00016609	0.002167501	1	0.813238553	1	0.83080598	ENSG00000145781	4	COMMD10
SAT	HTN	ENSG00000009830.7	-0.003681659	0.598414582	0.006990286	0.240335677	-0.003812987	0.585424312	0.006990162	0.240312079	0.000131329	0.017792548	1	0.822882751	1	0.814247906	ENSG00000145979	13	TBC1D7
SAT	HTN	ENSG00000009950.11	-0.010343565	0.047148722	0.005210951	0.24033387	-0.013659519	0.011266158	0.005389768	0.240309058	0.003315954	0.016098864	1	0.205985296	1	0.083456744	ENSG00000145990	6	GFOD1
SAT	HTN	ENSG00000011523.9	0.014871827	5.87E-08	0.002742418	0.24032131	0.014929688	5.21E-08	0.002742348	0.24029761	-5.79E-05	0.002200884	0.000192501	4.70E-06	0.000170892	4.07E-06	ENSG00000145990	6	GFOD1
SAT	HTN	ENSG00000016864.12	-0.003531031	0.480340558	0.005003193	0.240335568	-0.003614007	0.470077196	0.005003096	0.240311968	8.30E-05	0.031742696	1	0.746918185	1	0.736442251	ENSG00000145990	6	GFOD1
SAT	HTN	ENSG00000020922.8	-0.002915274	0.012985012	0.001173531	0.24033277	-0.002896502	0.013577924	0.001173505	0.240309221	-1.88E-05	0.027464015	1	0.091228745	1	0.094584223	ENSG00000146001	4	PCDHB18
SAT	HTN	ENSG00000030110.8	0.001813124	0.571457821	0.00320393	0.240335655	0.001138442	0.723714048	0.003220489	0.240312164	0.000674682	0.039509659	1	0.805147909	1	0.88865024	ENSG00000146007	6	ZMAT2
SAT	HTN	ENSG00000034533.7	0.000193631	0.952436681	0.003246279	0.240335812	0.000251912	0.938145044	0.003246218	0.240312222	-5.83E-05	0.020566696	1	0.982687029	1	0.978139775	ENSG00000146007	6	ZMAT2
SAT	HTN	ENSG00000035115.17	-0.006111531	0.092820431	0.003636285	0.24033442	-0.006179345	0.089246071	0.00363621	0.240310801	6.78E-05	0.013513363	1	0.311394513	1	0.304120181	ENSG00000146007	6	ZMAT2
SAT	HTN	ENSG00000043514.11	0.002766751	0.307283837	0.002710007	0.240335299	0.002716825	0.316083961	0.002709946	0.24031173	4.99E-05	0.01141056	1	0.601695266	1	0.604641264	ENSG00000146094	9	DOK3
SAT	HTN	ENSG00000044090.4	0.004185375	0.372332265	0.004691525	0.240335421	0.004244203	0.365635274	0.004691385	0.240311822	-5.88E-05	0.042933271	1	0.659719927	1	0.649864212	ENSG00000146109	3	ABT1
SAT	HTN	ENSG00000048140.13	-0.000586686	0.866494451	0.003489833	0.240335799	-0.000521494	0.881209974	0.003489756	0.240312214	-6.52E-05	0.010959546	1	0.952737363	1	0.957809595	ENSG00000146109	3	ABT1
SAT	HTN	ENSG00000050393.7	-0.001238202	0.826343669	0.00564375	0.24033579	-0.001346534	0.81142182	0.005643661	0.240312197	0.000108332	0.018623609	1	0.935230623	1	0.926770342	ENSG00000146109	3	ABT1
SAT	HTN	ENSG00000054938.11	0.00844675	0.221660243	0.006911516	0.240335077	0.008346648	0.227172488	0.00691133	0.240311506	0.000100102	0.02944585	1	0.515379933	1	0.517461433	ENSG00000146109	3	ABT1
SAT	HTN	ENSG00000065057.3	0.005837984	0.367915923	0.006483876	0.240335414	0.005714833	0.378102039	0.006483816	0.240311842	0.000123151	0.033405439	1	0.655337755	1	0.66057124	ENSG00000146205	9	ANO7
SAT	HTN	ENSG00000065060.12	0.010235483	7.98E-07	0.002073684	0.240323798	0.010163766	9.52E-07	0.002073662	0.240300379	7.17E-05	7.21E-05	0.002618615	3.91E-05	0.003123699	4.53E-05	ENSG00000146205	9	ANO7
SAT	HTN	ENSG00000066379.10	0.007144086	0.045684113	0.003575072	0.240333844	0.00893268	0.014446493	0.003651993	0.240309275	-0.001788595	0.016572303	1	0.203102407	1	0.099073573	ENSG00000146205	9	ANO7
SAT	HTN	ENSG00000068654.11	-0.006958328	0.000109572	0.001798767	0.240328433	-0.006997956	0.000100095	0.001798778	0.240304762	3.96E-05	0.036470282	0.359505355	0.002462365	0.328412008	0.002296587	ENSG00000146205	9	ANO7
SAT	HTN	ENSG00000072864.8	0.004831697	0.437295324	0.006220248	0.240335516	0.004964574	0.424791564	0.006220205	0.240311911	-0.000132877	0.020141242	1	0.710984122	1	0.697917436	ENSG00000146243	9	IRAK1BP1
SAT	HTN	ENSG00000073146.11	6.13E-05	0.988614397	0.004296836	0.240335813	-8.80E-08	0.999983652	0.004296716	0.240312225	6.14E-05	0.028062472	1	0.99730223	1	0.999983652	ENSG00000146243	9	IRAK1BP1
SAT	HTN	ENSG00000075151.15	0.016237927	0.008585138	0.006178444	0.240332407	0.016352953	0.008125345	0.006178323	0.240308771	-0.000115026	0.015296663	1	0.069550213	1	0.067346705	ENSG00000146263	7	MMS22L
SAT	HTN	ENSG00000076344.11	-0.013783774	0.016762904	0.005762747	0.240332992	-0.013702147	0.017417704	0.005762585	0.240309437	-8.16E-05	0.028562707	1	0.109125177	1	0.111398611	ENSG00000146263	7	MMS22L
SAT	HTN	ENSG00000076685.14	0.019655101	0.003524734	0.006736166	0.240331614	0.019531652	0.003737122	0.006736091	0.240308079	0.000123449	0.035221289	1	0.035150923	1	0.037382609	ENSG00000146263	7	MMS22L
SAT	HTN	ENSG00000079150.13	-0.003254426	0.269592317	0.002947841	0.240335212	-0.003297043	0.263358811	0.002947765	0.240311608	4.26E-05	0.034100105	1	0.563396428	1	0.553542766	ENSG00000146263	7	MMS22L
SAT	HTN	ENSG00000079257.3	0.002980051	0.651478731	0.006597278	0.240335713	0.002869697	0.663568982	0.006597138	0.240312132	0.000110354	0.024593433	1	0.847542314	1	0.859631339	ENSG00000146386	7	ABRACL
SAT	HTN	ENSG00000082516.8	-0.003969209	0.453614738	0.005296495	0.240335536	-0.00387109	0.464844907	0.005296384	0.240311962	-9.81E-05	0.013321908	1	0.726359177	1	0.734100068	ENSG00000146386	7	ABRACL
SAT	HTN	ENSG00000083097.10	0.000354391	0.864206757	0.002072197	0.240335799	0.000320873	0.876939204	0.002072149	0.240312214	3.35E-05	0.023953185	1	0.952456288	1	0.956781838	ENSG00000146386	7	ABRACL
SAT	HTN	ENSG00000083720.8	-0.01102256	0.091733938	0.00653644	0.240334411	-0.011553932	0.077362923	0.00654169	0.240310687	0.000531372	0.049012885	1	0.308696461	1	0.282344552	ENSG00000146409	6	SLC18B1
SAT	HTN	ENSG00000084092.6	-0.010541156	0.017016241	0.004417244	0.240333005	-0.01012442	0.021979213	0.004419727	0.240309638	-0.000416736	0.006949305	1	0.109471152	1	0.129468218	ENSG00000146409	6	SLC18B1
SAT	HTN	ENSG00000085117.7	0.008589795	0.103341983	0.005273481	0.240334505	0.008497633	0.107087047	0.005273355	0.240310945	9.22E-05	0.013543861	1	0.332597644	1	0.34237775	ENSG00000146425	6	DYNLT1
SAT	HTN	ENSG00000085365.13	0.015799944	0.00369282	0.005442084	0.240331656	0.015609337	0.00412717	0.005442039	0.240308168	0.000190607	0.000101144	1	0.036384812	1	0.039725318	ENSG00000146425	6	DYNLT1
SAT	HTN	ENSG00000087301.4	-0.006466559	0.267610529	0.005833174	0.240335207	-0.006624428	0.256100507	0.005833122	0.240311589	0.000157869	0.002541464	1	0.561046104	1	0.548573946	ENSG00000146425	6	DYNLT1
SAT	HTN	ENSG00000087884.10	-0.011161106	2.21E-05	0.002630919	0.240326938	-0.011124874	2.35E-05	0.002630854	0.240303407	-3.62E-05	0.047550759	0.072607824	0.00066164	0.077161004	0.000682841	ENSG00000146425	6	DYNLT1
SAT	HTN	ENSG00000088325.11	0.001243983	0.735912845	0.003688378	0.240335757	0.001340835	0.716205772	0.003688328	0.24031216	-9.69E-05	0.001839383	1	0.893569851	1	0.88553283	ENSG00000146425	6	DYNLT1
SAT	HTN	ENSG00000089335.16	-0.001269923	0.637037008	0.002691393	0.240335704	-0.001227367	0.648358289	0.002691332	0.240312123	-4.26E-05	0.029250455	1	0.840834873	1	0.849876055	ENSG00000146453	8	PNLDC1
SAT	HTN	ENSG00000090238.7	0.005184095	9.29E-05	0.001326367	0.240328279	0.005156261	0.000101286	0.001326362	0.240304773	2.78E-05	0.027850052	0.304759692	0.002131187	0.332320906	0.002307784	ENSG00000146453	8	PNLDC1
SAT	HTN	ENSG00000092295.7	0.01937856	0.000198023	0.005207116	0.240328983	0.019496423	0.000180944	0.00520701	0.240305312	-0.000117864	0.002874605	0.649715088	0.00389051	0.593676249	0.003619977	ENSG00000146453	8	PNLDC1
SAT	HTN	ENSG00000092931.7	-0.004705891	0.162947364	0.003372844	0.240334853	-0.004770151	0.157269899	0.003372763	0.240311239	6.43E-05	0.006917792	1	0.433572431	1	0.427154418	ENSG00000146457	10	WTAP
SAT	HTN	ENSG00000093000.14	0.007839287	0.014465619	0.003205601	0.240332864	0.00776559	0.015412382	0.003205544	0.240309331	7.37E-05	0.003655263	1	0.098878534	1	0.102989871	ENSG00000146457	10	WTAP
SAT	HTN	ENSG00000094963.9	0.015358875	0.008718704	0.00585568	0.24033242	0.01525799	0.009168717	0.005855601	0.240308877	0.000100885	0.040767126	1	0.070125913	1	0.073193579	ENSG00000146469	8	VIP
SAT	HTN	ENSG00000095485.12	-0.002223917	0.228932246	0.001848465	0.240335099	-0.002257061	0.22205892	0.001848424	0.24031149	3.31E-05	0.013943144	1	0.520170844	1	0.513081208	ENSG00000146535	9	GNA12
SAT	HTN	ENSG00000099330.4	-0.001701201	0.789428112	0.006370265	0.240335778	-0.001817502	0.77540169	0.006370121	0.240312185	0.0001163	0.012301635	1	0.918290338	1	0.911861271	ENSG00000146535	9	GNA12
SAT	HTN	ENSG00000100014.15	-0.005423029	0.113419325	0.003425766	0.240334577	-0.005361028	0.117595269	0.003425686	0.240311018	-6.20E-05	0.011645649	1	0.355763675	1	0.365369392	ENSG00000146535	9	GNA12
SAT	HTN	ENSG00000100024.10	-0.001447527	0.692731768	0.003663226	0.240335736	-0.001377234	0.706942028	0.003663183	0.240312156	-7.03E-05	0.02640529	1	0.869846415	1	0.880236222	ENSG00000146555	14	SDK1
SAT	HTN	ENSG00000100106.15	-0.008515344	0.004765005	0.003016932	0.240331884	-0.008560302	0.004547328	0.003016869	0.240308255	4.50E-05	0.048606944	1	0.043792664	1	0.042219581	ENSG00000146555	14	SDK1
SAT	HTN	ENSG00000100300.13	-0.000116216	0.980071619	0.004652511	0.240335813	-0.00020474	0.96489853	0.004652392	0.240312224	8.85E-05	0.005439012	1	0.99356442	1	0.988156741	ENSG00000146555	14	SDK1
SAT	HTN	ENSG00000100505.9	0.004880635	0.430320343	0.00618864	0.240335507	0.004965177	0.42236486	0.006188479	0.240311908	-8.45E-05	0.044297364	1	0.705948972	1	0.696721522	ENSG00000146556	10	WASH2P
SAT	HTN	ENSG00000100997.14	0.004255445	0.393332418	0.004985348	0.240335454	0.004180015	0.401763545	0.00498525	0.240311879	7.54E-05	0.04798446	1	0.676956485	1	0.681743181	ENSG00000146556	10	WASH2P
SAT	HTN	ENSG00000103196.7	0.01639055	0.003220515	0.005563887	0.240331533	0.021845417	0.000180824	0.005834109	0.240305311	-0.005454868	0.001893153	1	0.032917472	0.593283879	0.003619977	ENSG00000146556	10	WASH2P
SAT	HTN	ENSG00000103269.9	0.008590265	0.128500536	0.005651326	0.240334674	0.008693481	0.123968106	0.005651239	0.240311058	-0.000103216	0.025895021	1	0.382239581	1	0.374529795	ENSG00000146556	10	WASH2P
SAT	HTN	ENSG00000103550.9	0.006863974	0.124633079	0.004469848	0.24033465	0.006779161	0.129353793	0.004469803	0.240311091	8.48E-05	0.031562667	1	0.374127295	1	0.384081261	ENSG00000146556	10	WASH2P
SAT	HTN	ENSG00000104442.5	-0.003577687	0.157611782	0.002531708	0.240334828	-0.003371984	0.183001775	0.002532335	0.240311351	-0.000205703	0.000850277	1	0.427022509	1	0.463293845	ENSG00000146556	10	WASH2P
SAT	HTN	ENSG00000105088.4	0.001422427	0.74329366	0.004343354	0.24033576	0.001561518	0.719203923	0.004343305	0.240312162	-0.000139092	0.00022607	1	0.896532567	1	0.886108926	ENSG00000146574	11	CCZ1B
SAT	HTN	ENSG00000105146.8	-0.000251383	0.951541513	0.004136556	0.240335812	-0.00019241	0.962899086	0.004136444	0.240312224	-5.90E-05	0.032212432	1	0.982072256	1	0.988156741	ENSG00000146574	11	CCZ1B
SAT	HTN	ENSG00000105185.7	0.001867621	0.703999397	0.00491573	0.240335742	0.001962538	0.689713184	0.004915629	0.240312147	-9.49E-05	0.010647664	1	0.876281552	1	0.870030357	ENSG00000146707	10	POMZP3
SAT	HTN	ENSG00000105258.4	-0.001675418	0.789094132	0.006263543	0.240335778	-0.001577147	0.801192815	0.006263394	0.240312194	-9.83E-05	0.027453626	1	0.918290338	1	0.923833778	ENSG00000146707	10	POMZP3

SAT	HTN	ENSG00000105329.5	-0.012526614	0.050953761	0.006417738	0.240333934	-0.012411435	0.05311781	0.006417606	0.240310381	-0.000115179	0.017279185	1	0.219395395	1	0.227405237	ENSG00000146733	9	PSPH
SAT	HTN	ENSG00000105447.8	-0.006394369	0.069284981	0.003520041	0.240334186	-0.00645936	0.06649913	0.003519994	0.240310565	6.50E-05	0.029351589	1	0.266812233	1	0.262480731	ENSG00000146733	9	PSPH
SAT	HTN	ENSG00000105618.9	-0.005465605	0.332065346	0.005634858	0.240335349	-0.00536108	0.341385078	0.005634743	0.240311779	-0.000104524	0.014091454	1	0.62016399	1	0.627146943	ENSG00000146733	9	PSPH
SAT	HTN	ENSG00000105699.12	0.007501647	0.11633597	0.004777076	0.240334597	0.007571142	0.11298269	0.004776954	0.240310987	-6.95E-05	0.034546914	1	0.362335772	1	0.355413428	ENSG00000146733	9	PSPH
SAT	HTN	ENSG00000105968.14	-0.004109052	0.54887457	0.006854728	0.240335636	-0.003942554	0.565180019	0.00685465	0.240312062	-0.000166498	0.005110295	1	0.791241216	1	0.803251455	ENSG00000146733	9	PSPH
SAT	HTN	ENSG00000105982.12	0.002564077	0.540281101	0.004187011	0.240335628	0.002627646	0.530275394	0.00418691	0.240312031	-6.36E-05	0.031217871	1	0.786663493	1	0.779696379	ENSG00000146830	9	GIGYF1
SAT	HTN	ENSG00000106009.11	0.002821638	0.485722739	0.004047516	0.240335574	0.002923603	0.470093142	0.004047472	0.240311968	-0.000101965	0.004040779	1	0.74889864	1	0.736442251	ENSG00000146833	11	TRIM4
SAT	HTN	ENSG00000106133.13	-0.006555082	0.036317939	0.003131375	0.240333652	-0.006268303	0.045456804	0.003133513	0.240310252	-0.000286779	0.016645311	1	0.176751156	1	0.203193151	ENSG00000146833	11	TRIM4
SAT	HTN	ENSG00000106263.13	-0.006690325	0.276525806	0.006148302	0.240335229	-0.006569667	0.285275333	0.006148237	0.240311662	-0.000120658	0.025279438	1	0.571695759	1	0.577147307	ENSG00000146833	11	TRIM4
SAT	HTN	ENSG00000106603.13	0.001868131	0.467039959	0.002568569	0.240335552	0.001915914	0.455712934	0.002568507	0.240311951	-4.78E-05	0.008525323	1	0.738333035	1	0.727942617	ENSG00000146833	11	TRIM4
SAT	HTN	ENSG00000106733.16	-0.000552971	0.927425188	0.006070947	0.240335809	-0.000658604	0.913609187	0.00607079	0.24031222	0.000105632	0.010701658	1	0.975926571	1	0.972372648	ENSG00000146918	15	NCAPG2
SAT	HTN	ENSG00000107099.11	0.004273889	0.348710571	0.004560789	0.24033538	0.005231865	0.252507408	0.004572183	0.24031158	-0.000957977	0.003275046	1	0.636328912	1	0.544334301	ENSG00000147419	12	CCDC25
SAT	HTN	ENSG00000107443.11	-0.010645615	0.133704085	0.007098703	0.240334704	-0.010516437	0.138478888	0.007098594	0.240311143	-0.000129178	0.02651812	1	0.386164703	1	0.393375959	ENSG00000147457	9	CHMP7
SAT	HTN	ENSG00000107890.12	0.000354225	0.938421127	0.004585174	0.24033581	0.000459369	0.920195839	0.004585108	0.24031222	-0.000105143	0.005830213	1	0.978294984	1	0.974491801	ENSG00000147457	9	CHMP7
SAT	HTN	ENSG00000108272.9	-0.007609289	0.146649617	0.00524247	0.240334774	-0.007511155	0.15192388	0.005242397	0.240311213	-9.81E-05	0.025495028	1	0.407415235	1	0.417123222	ENSG00000147457	9	CHMP7
SAT	HTN	ENSG00000108384.10	-0.007340308	8.21E-05	0.001863879	0.240328164	-0.007310663	8.77E-05	0.001863834	0.240304638	-2.96E-05	0.023637343	0.26940511	0.00191236	0.287714717	0.002055105	ENSG00000147509	9	RGS20
SAT	HTN	ENSG00000108465.10	-0.009752059	0.09778707	0.005890059	0.240334461	-0.009658601	0.101038519	0.005889956	0.240310899	-9.35E-05	0.045534435	1	0.321804792	1	0.329208389	ENSG00000147509	9	RGS20
SAT	HTN	ENSG00000109171.10	0.009830934	0.067195249	0.005371009	0.240334161	0.009681463	0.071460006	0.00537099	0.240310623	0.000149471	0.003514154	1	0.262909169	1	0.272311592	ENSG00000147576	11	ADHFE1
SAT	HTN	ENSG00000109265.8	0.001503255	0.608329579	0.002933411	0.240335684	0.001400731	0.632997785	0.002933394	0.240312113	0.000102524	0.000169867	1	0.829908253	1	0.840496048	ENSG00000147576	11	ADHFE1
SAT	HTN	ENSG00000109854.9	-0.000251472	0.909681166	0.002216773	0.240335807	-0.000214279	0.92299254	0.002216719	0.240312221	-3.72E-05	0.017469118	1	0.968235044	1	0.974491801	ENSG00000147576	11	ADHFE1
SAT	HTN	ENSG00000110025.8	-0.007765033	0.009776174	0.003005442	0.240332521	-0.005391799	0.079807647	0.00307785	0.240310712	-0.002373234	0.000353559	1	0.0752949	1	0.285677612	ENSG00000147576	11	ADHFE1
SAT	HTN	ENSG00000110455.9	0.003125422	0.122475723	0.002023624	0.240334637	0.003159698	0.118420145	0.002023577	0.240311023	-3.43E-05	0.018587332	1	0.372076709	1	0.366889987	ENSG00000147576	11	ADHFE1
SAT	HTN	ENSG00000110700.2	0.002643322	0.496185548	0.003884358	0.240335585	0.002553533	0.510922753	0.003884279	0.240312012	8.98E-05	0.002327583	1	0.757316081	1	0.763012086	ENSG00000147576	11	ADHFE1
SAT	HTN	ENSG00000110711.5	-0.007524017	0.23505601	0.006336353	0.240335118	-0.007949924	0.209635708	0.006336775	0.240311449	0.000425906	1.01E-05	1	0.526060442	1	0.499064033	ENSG00000147576	11	ADHFE1
SAT	HTN	ENSG00000111341.5	-0.003200202	0.603180075	0.006156209	0.24033568	-0.00310661	0.613811916	0.006156092	0.2403121	-9.36E-05	0.049760203	1	0.826982828	1	0.834690078	ENSG00000147576	11	ADHFE1
SAT	HTN	ENSG00000111615.8	0.000409416	0.947782133	0.006251368	0.240335811	0.000302902	0.961353965	0.00625125	0.240312224	0.000106514	0.028654173	1	0.981389706	1	0.98809363	ENSG00000147576	11	ADHFE1
SAT	HTN	ENSG00000111801.11	-0.003306349	0.037269651	0.001587451	0.240333674	-0.003051833	0.054907307	0.001589817	0.240310408	-0.000254516	0.003859375	1	0.178482982	1	0.23245274	ENSG00000147804	5	SLC39A4
SAT	HTN	ENSG00000111879.14	0.007157672	0.264317617	0.006412274	0.240335199	0.007043087	0.272031406	0.006412147	0.24031163	0.000114585	0.019268231	1	0.556219915	1	0.564538294	ENSG00000147813	11	NAPRT1
SAT	HTN	ENSG00000112053.9	0.002088999	0.513752202	0.003199046	0.240335603	0.002174818	0.496627686	0.003199174	0.240311998	-8.58E-05	0.004732497	1	0.765147969	1	0.755559631	ENSG00000147813	11	NAPRT1
SAT	HTN	ENSG00000112110.5	0.001644154	0.602493845	0.00315687	0.24033568	0.001692165	0.591930673	0.003156784	0.240312084	-4.80E-05	0.021858179	1	0.826759642	1	0.81946183	ENSG00000147853	12	AK3
SAT	HTN	ENSG00000112308.8	-0.002071786	0.764279649	0.006909044	0.240335769	-0.002655274	0.700917515	0.006913291	0.240312153	0.000583487	0.020472279	1	0.905600735	1	0.87708252	ENSG00000147853	12	AK3
SAT	HTN	ENSG00000112378.11	-0.00064314	0.835403678	0.003095348	0.240335792	-0.000697314	0.821760868	0.003095311	0.2403122	5.42E-05	0.042721007	1	0.940381331	1	0.934090633	ENSG00000147894	10	C9orf72
SAT	HTN	ENSG00000112667.8	0.001796483	0.554290433	0.003037967	0.240335641	0.00187437	0.537237461	0.003037903	0.240312038	-7.79E-05	0.000608516	1	0.795829275	1	0.782997282	ENSG00000147894	10	C9orf72
SAT	HTN	ENSG00000112763.11	0.005079039	0.035208683	0.00241175	0.240333626	0.005557821	0.021623074	0.002419672	0.240309624	-0.000478782	0.015143752	1	0.174237842	1	0.128291693	ENSG00000147912	8	FBXO10
SAT	HTN	ENSG00000112964.9	0.004690239	0.423843411	0.005864471	0.240335498	0.002481564	0.677173876	0.005960672	0.24031214	0.002208674	0.038661337	1	0.700922495	1	0.865678672	ENSG00000147912	8	FBXO10
SAT	HTN	ENSG00000112977.11	0.001476657	0.823064333	0.00660385	0.240335789	0.001588764	0.80987514	0.006603692	0.240312197	-0.000112107	0.016915922	1	0.933773386	1	0.925531291	ENSG00000147955	12	SIGMAR1
SAT	HTN	ENSG00000113597.13	0.011060152	0.074718493	0.006205925	0.240334247	0.01117678	0.071700489	0.006205809	0.240310626	-0.000116628	0.015932305	1	0.276027247	1	0.27259479	ENSG00000147955	12	SIGMAR1
SAT	HTN	ENSG00000113971.14	0.003747708	0.003345313	0.001277293	0.240331568	0.00372186	0.003569222	0.001277263	0.240308038	2.58E-05	0.004342903	1	0.033793366	1	0.036032666	ENSG00000147955	12	SIGMAR1
SAT	HTN	ENSG00000114742.9	0.000521401	0.888360526	0.003714232	0.240335804	0.000365524	0.92160918	0.003714409	0.240312221	0.000155877	0.002561085	1	0.958471189	1	0.974491801	ENSG00000147955	12	SIGMAR1
SAT	HTN	ENSG00000114988.7	0.00379255	0.546731258	0.006292955	0.240335634	0.003888314	0.536644505	0.006292834	0.240312037	-9.58E-05	0.048885031	1	0.789827969	1	0.782991949	ENSG00000147955	12	SIGMAR1
SAT	HTN	ENSG00000115221.6	0.002342778	0.597672313	0.004439172	0.240335676	0.002438643	0.582759032	0.004439066	0.240312077	-9.59E-05	0.00239686	1	0.822882751	1	0.812928219	ENSG00000148110	11	HIATL1
SAT	HTN	ENSG00000115423.14	0.00095575	0.63061031	0.001987551	0.240335699	3.38E-05	0.986647087	0.002016936	0.240312225	0.000921995	0.007287743	1	0.837963781	1	0.994833771	ENSG00000148110	11	HIATL1
SAT	HTN	ENSG00000115486.7	0.00112311	0.691243501	0.002827808	0.24033573	0.001048644	0.710768242	0.002827869	0.240312158	7.45E-05	0.026622258	1	0.869622269	1	0.882008549	ENSG00000148110	11	HIATL1
SAT	HTN	ENSG00000115568.11	-0.018078878	0.000366391	0.005073771	0.240329552	-0.018198099	0.000334844	0.005073688	0.240305881	0.000119221	0.00366228	1	0.006327001	1	0.005782235	ENSG00000148218	11	ALAD
SAT	HTN	ENSG00000116157.5	0.001844147	0.362543947	0.002025357	0.240335404	0.001882845	0.352554038	0.002025332	0.240311799	-3.87E-05	0.025106386	1	0.650299517	1	0.636902944	ENSG00000148225	11	WDR31
SAT	HTN	ENSG00000116221.11	0.004767022	0.467135467	0.006555777	0.240335553	0.004570517	0.485688991	0.006555698	0.240311986	0.000196505	0.000491664	1	0.738333035	1	0.747907685	ENSG00000148225	11	WDR31
SAT	HTN	ENSG00000116874.7	-0.003564614	0.362426417	0.003913923	0.240335404	-0.00365401	0.350503087	0.003913831	0.240311796	8.94E-05	0.001403435	1	0.650299517	1	0.635605707	ENSG00000148225	11	WDR31
SAT	HTN	ENSG00000117480.11	0.00465855	0.103398231	0.002860462	0.240334505	0.00461348	0.106770257	0.00286039	0.240310943	4.51E-05	0.023338029	1	0.332597644	1	0.34176899	ENSG00000148225	11	WDR31
SAT	HTN	ENSG00000118307.14	-0.009754564	0.10438276	0.006006614	0.240334513	-0.009617107	0.109351548	0.006006497	0.240310961	-0.000137457	0.002861268	1	0.334126667	1	0.347994595	ENSG00000148229	8	POLE3
SAT	HTN	ENSG00000118507.11	-0.005632765	0.14350401	0.003850493	0.240334758	-0.005540945	0.150132774	0.003850567	0.240311204	-9.18E-05	0.04141884	1	0.401395273	1	0.414759254	ENSG00000148229	8	POLE3
SAT	HTN	ENSG00000118579.7	-0.005623192	0.138679178	0.003797581	0.240334732	-0.005541881	0.144470066	0.003797506	0.240311175	-8.13E-05	0.005053763	1	0.391877031	1	0.404787607	ENSG00000148229	8	POLE3
SAT	HTN	ENSG00000118640.6	0.001804769	0.771302364	0.006208955	0.240335772	0.001513423	0.807437308	0.006209335	0.240312196	0.000291346	0.001577151	1	0.909321975	1	0.925323719	ENSG00000148229	8	POLE3
SAT	HTN	ENSG00000118900.10	0.002547944	0.705014148	0.006730606	0.240335743	0.002419993	0.719176819	0.006730442	0.240312162	0.000127951	0.006685459	1	0.876817876	1	0.886108926	ENSG00000148290	5	SURF1
SAT	HTN	ENSG00000118961.10	-0.001797153	0.515468792	0.002763377	0.240335605	-0.001866406	0.499410635	0.002763335	0.2403012	6.93E-05	0.002256125	1	0.765896967	1	0.756843553	ENSG00000148357	12	HMCN2
SAT	HTN	ENSG00000119333.7	-0.003129282	0.526325068	0.004938688	0.240335615	-0.003212405	0.51538975	0.0049386	0.240312017	8.31E-05	0.033026259	1	0.776122494	1	0.76520719	ENSG00000148386	5	LCN9
SAT	HTN	ENSG00000119878.5	-0.013093964	0.05975797	0.006955327	0.240334065	-0.013192626	0.057852322	0.006955129	0.240310451	9.87E-05	0.027565247	1	0.245081663	1	0.240880037	ENSG00000148396	14	SEC16A
SAT	HTN	ENSG00000120055.5	0.003934062	0.420897455	0.004887847	0.240335494	0.004017599	0.411095407	0.004887779	0.240311892	-8.35E-05	0.041712215	1	0.698776883	1	0.687814395	ENSG00000148396	14	SEC16A
SAT	HTN	ENSG00000121101.11	0.042946388	3.76E-10	0.006856271	0.240316464	0.042853366	4.10E-10	0.006856083	0.24029296	9.30E-05	0.038628102	1.23E-06	7.71E-08	1.34E-06	8.40E-08	ENSG00000148484	13	RSU1
SAT	HTN	ENSG00000121691.4	-0.002890231	0.428576853	0.003650987	0.240335504	-0.002956913	0.417992947	0.00365092	0.240311902	6.67E-05	0.019826293	1	0.705195915	1	0.692294225	ENSG00000148655	10	C10orf11
SAT	HTN	ENSG00000122376.7	-0.00906254	0.037193541	0.004349381	0.240333672	-0.009334701	0.031876755	0.004349898	0.240309954	0.000272161	0.000643215	1	0.178436214	1	0.164445963	ENSG00000148655	10	C10orf11
SAT	HTN	ENSG00000122696.8	-0.006618897	0.019665138	0.00283746	0.24033313	-0.006675551	0.018637887	0.002837396	0.240309496	5.67E-05	0.006173289	1	0.119705596	1	0.114982479	ENSG00000148655	10	C10orf11
SAT	HTN	ENSG00000122912.10	0.012505724	0.062730045	0.006719722	0.240334105	0.012690886	0.058904505	0.006719585	0.240310466	-0.000185163	0.000282141	1	0.251407587	1	0.243426139	ENSG00000148655	10	C10orf11
SAT	HTN	ENSG00000123454.6	-0.006381552	0.208471698	0.005073661	0.240335033	-0.006457941	0.203063251	0.00507351	0.240311426	7.64E-05	0.015740731	1	0.499334127	1	0.489890094	ENSG00000148814	13	LRRC27
SAT	HTN	ENSG00000123815.7	0.001094304	0.857971928	0.006114914	0.240335798	0.001223326	0.841435165	0.006114849	0.240312206	-0.000129022	0.016202322	1	0.951893842	1	0.94371963	ENSG00000148824	14	MTG1
SAT	HTN	ENSG00000124508.12	0.006986376	0.002591739	0.002319179	0.240331338	0.007426189	0.001391382	0.002323261	0.240307187	-0.000439813	0.001631017	1	0.028064341	1	0.017448468	ENSG00000148824	14	MTG1
SAT	HTN	ENSG00000124549.10	-0.005109932	0.058226859	0.002697971	0.240334044	-0.004413582	0.104049623	0.00271516	0.240310922	-0.00069635	0.022945075	1	0.240304811	1	0.334692955	ENSG00000148834	8	GSTO1
SAT	HTN	ENSG00000124562.5	-0.013316794	2.92E-07	0.002596565	0.240322842	-0.013184049	3.83E-07	0.002596601	0.240299512	-0.000132745	4.99E-06	0.000958002	1.71E-05	0.00125572	2.13E-05	ENSG00000148843	9	PDCD11
SAT	HTN	ENSG00000124575.5	0.02943827	2.95E-07	0.005742404	0.240322853	0.029707316	2.31E-07	0.005743393	0.240299032	-0.000269046	0.026066505	0.000968997	1.71E-05	0.000758423	1.38E-05	ENSG00000148843	9	PDCD11
SAT	HTN	ENSG00000124613.4	-0.009647156	3.92E-05	0.00234605	0.240327474	-0.010722599	6.15E-06	0.002371534	0.240302144	0.001075443	0.001973279	0.128670306	0.001037664	0.020162759	0.000224031	ENSG00000148843	9	PDCD11
SAT	HTN	ENSG00000124713.5	-0.002952819	0.311499833	0.002917572	0.240335308	-0.002910785	0.318425726	0.002917498	0.240311735	-4.20E-05	0.035800167	1	0.60449332	1	0.606006269	ENSG00000149054	10	ZNF215
SAT	HTN	ENSG00000124787.9	0.000286088	0.961759366	0.005966886	0.240335812	0.000180547	0.975860694	0.005966781	0.240312225	0.00010554	0.025869885	1	0.986748469	1	0.990655611	ENSG00000149054	10	ZNF215
SAT	HTN	ENSG00000124920.9	0.001075404	0.824813798	0.004858212	0.240335789	0.001293526	0.790050914	0.004858411	0.24031219	-0.000218122	0.000819718	1	0.934466185	1	0.919969807	ENSG00000149100	8	EIF3M
SAT	HTN	ENSG00000124939.4	-0.001365835	0.773253789	0.00474048	0.240335772	-0.000962243	0.839223796	0.004742765	0.240312205	-0.000403591	0.009000775	1	0.909987691	1	0.942977149	ENSG00000149100	8	EIF3M
SAT	HTN	ENSG00000125388.15	0.006033382	0.00041035	0.001707548	0.240329656	0.006005631	0.000436206	0.001707516	0.240306125	2.78E-05	0.036686616	1	0.006903399	1	0.006981431	ENSG00000149177	8	PTPRJ
SAT	HTN	ENSG00000125450.6	-0.008357478	0.076785784	0.004722668	0.240334269	-0.008432068	0.074181714	0.004722539	0.240310653	7.46E-05	0.016793724	1	0.278996851	1	0.276576499	ENSG00000149179	9	C11orf49
SAT	HTN	ENSG00000125611.11	-0.014496243	0.02227038	0.006342058	0.240333237	-0.014323455	0.023914285	0.006341991	0.24030971	-0.000172788	0.001903905	1	0.130247978	1	0.135984003	ENSG00000149179	9	C11orf49
SAT	HTN	ENSG00000125656.4	0.004096104	0.446810462	0.005384341	0.240335528	0.003977377	0.460088092	0.005384275	0.240311956	0.000118727	0.01031363	1	0.7196446	1	0.731635082	ENSG00000149179	9	C11orf49
SAT	HTN	ENSG00000125966.8	0.00800784	0.236885345	0.006770188	0.240335123	0.008094137	0.231856781	0.006769988	0.24031152	-8.63E-05	0.041877111	1	0.52728685	1	0.519577173	ENSG00000149231	7	CCDC82
SAT	HTN	ENSG00000126247.6	-0.000416638	0.931282814	0.004831645	0.24033581	-0.000269617	0.955499125	0.004831616	0.240312224	-0.000147021	0.001043833	1	0.976204842	1	0.985694958	ENSG00000149260	10	CAPN5
SAT	HTN	ENSG00000126500.3	-0.01020323	0.034398877	0.004823377	0.240333606	-0.009996909	0.038237715	0.004824057	0.240310108	-0.000206321	0.028196862	1	0.172046825	1	0.181823106	ENSG00000149273	10	RPS3
SAT	HTN	ENSG00000126870.11	0.002687657	0.619761602	0.005416604	0.240335691	0.002609786	0.629931309	0.005416473	0.240312111	7.79E-05	0.041809053	1	0.833771344	1	0.839506103	ENSG00000149273	10	RPS3
SAT	HTN	ENSG00000127337.2	0.005609413	0.01173242	0.002225868	0.240332681	0.005641633	0.011256826	0.002225819	0.240309057	-3.22E-05	0.048436893	1	0.084864703	1	0.083456744	ENSG00000149292	12	TTC12
SAT	HTN	ENSG00000127903.12	0.001703197	0.758900113	0.005549216	0.240335767	0.001574001	0.77679073	0.005549105	0.240312186	0.000129195	0.002287971	1	0.902979276	1	0.912575524	ENSG00000149308	12	NPAT
SAT	HTN	ENSG00000129003.11	0.004397454	0.301533569	0.00425635	0.240335287	0.004472006	0.293401187	0.004256254	0.240311681	-7.46E-05	0.016185797	1	0.595982915	1	0.58377762	ENSG00000149308	12	NPAT
SAT	HTN	ENSG00000129055.8	-0.002809326	0.083341821	0.001622368	0.240334335	-0.002782248	0.086352224	0.001622335	0.240310775	-2.71E-05	0.027154161	1	0.293903036	1	0.299568267	ENSG00000149328	10	GLB1L2
SAT	HTN	ENSG00000129562.6	-0.007719446	0.103766876	0.004744989	0.240334508	-0.007592624	0.109563696	0.004744917	0.240310963	-0.000126822	0.001182169	1	0.332804614	1	0.348332534	ENSG00000149328	10	GLB1L2
SAT	HTN	ENSG00000129657.10	0.002369733	0.603455953	0.004562115	0.24033568	0.002433243	0.593776184	0.004561998	0.240312085	-6.35E-05	0.042585673	1	0.826982828	1	0.820640533	ENSG00000149328	10	GLB1L2
SAT	HTN	ENSG00000129667.8	0.008578797	0.070392094	0.004741282	0.240334199	0.008502706	0.07291431	0.004741187	0.240310639	7.61E-05	0.035151525	1	0.268554024	1	0.27407569	ENSG00000149346	10	SLX4IP
SAT	HTN	ENSG00000130518.12	-0.002870406	0.137126746	0.001930885	0.240334723	-0.0028401	0.141316928	0.001930849	0.240311158	-3.03E-05	0.044863916	1	0.390551916	1	0.398676562	ENSG00000149380	7	P4HA3
SAT	HTN	ENSG00000130520.6	0.003581509	0.104042069	0.002203234	0.24033451	0.003629242	0.099502776	0.002203183	0.240310887	-4.77E-05	0.002578906	1	0.333361356	1	0.325816974	ENSG00000149380	7	P4HA3
SAT	HTN	ENSG00000131437.11	-0.001935301	0.685058088	0.004771795	0.240335732	-0.002036647	0.669514752	0.004771739	0.240312136	0.000101345	0.013998702	1	0.866100648	1	0.86204999	ENSG00000149403	7	GRIK4
SAT	HTN	ENSG00000131797.8	-0.003774573	0.518709303	0.005848998	0.240335608	-0.003646484	0.532998722	0.005849016	0.240312034	-0.000128089	0.032073296	1	0.768692512	1	0.780736132	ENSG00000149480	2	MTA2
SAT	HTN	ENSG00000131849.10	0.004585077	0.434413862	0.005865808	0.240335512	0.00448189	0.444805393	0.005865676	0.240311937	0.000103088	0.015906183	1	0.709110389	1	0.717505651	ENSG00000149480	2	MTA2
SAT	HTN	ENSG00000132205.6	-0.007684772	0.207426029	0.006095753	0.240335023	-0.007566479	0.214497917	0.006095614	0.240311466	-0.000118294	0.012946494	1	0.498217277	1	0.504492951	ENSG00000149485	12	FADS1
SAT	HTN	ENSG00000132305.16	-0.009415516	0.037133033	0.004517343	0.240333671	-0.009527999	0.03492994	0.004517405	0.240310032	0.000112483	0.026358706	1	0.178436214	1	0.173459202	ENSG00000149489	4	ROM1
SAT	HTN	ENSG00000132313.10	0.006291679	0.015455327	0.002598215	0.240332921	0.006424487	0.013424883	0.002598583	0.240309211	-0.000132808	0.00889516	1	0.103207817	1	0.09371711	ENSG00000149489	4	ROM1
SAT	HTN	ENSG00000132432.9	-0.000615883	0.851111494	0.003281202	0.240335796	-0.000548374	0.867270478	0.00328119	0.240312212	-6.75E-05	0.031163956	1	0.948538319	1	0.954550298	ENSG00000149489	4	ROM1
SAT	HTN	ENSG00000133816.9	-0.00445807	0.235759569	0.00376001	0.24033512	-0.00451493	0.229826251	0.003759909	0.240311514	5.69E-05	0.024219062	1	0.526207273	1	0.518610682	ENSG00000149489	4	ROM1
SAT	HTN	ENSG00000133884.5	-0.000665014	0.914849788	0.006219536	0.240335808	-0.001580976	0.799492724	0.006224221	0.240312194	0.000915962	0.000237393	1	0.969141477	1	0.923639306	ENSG00000149499	7	EML3
SAT	HTN	ENSG00000133983.10	0.006301742	0.196133441	0.004875064	0.240334989	0.00638796	0.190013907	0.004874935	0.240311378	-8.71E-05	0.00785647	1	0.48072934	1	0.472131871	ENSG00000149499	7	EML3
SAT	HTN	ENSG00000133997.7	0.00327657	0.545993581	0.00542682	0.240335634	0.003379043	0.533499058	0.005426664	0.240312034	-0.000102465	0.003140329	1	0.789731191	1	0.780736132	ENSG00000149499	7	EML3
SAT	HTN	ENSG00000134201.6	-0.009267884	0.085359663	0.005387039	0.240334354	-0.009155626	0.08921203	0.005387017	0.240310801	-0.000112259	0.02785383	1	0.296748329	1	0.304120181	ENSG00000149548	10	CCDC15
SAT	HTN	ENSG00000134809.4	-0.005656992	0.330389827	0.005811998	0.240335346	-0.005266781	0.364951577	0.005813409	0.240311821	-0.000390211	0.005443216	1	0.62016399	1	0.64900061	ENSG00000149575	5	SCN2B
SAT	HTN	ENSG00000134874.13	-0.007146052	0.28854189	0.00673313	0.240335258	-0.007236498	0.28246962	0.006732946	0.240311656	9.04E-05	0.041925854	1	0.581565115	1	0.575181454	ENSG00000149577	11	SIDT2
SAT	HTN	ENSG00000135074.11	0.003303097	0.131708045	0.00219125	0.240334693	0.003266079	0.136083137	0.002191206	0.24031113	3.70E-05	0.026877119	1	0.384460941	1	0.389946525	ENSG00000149577	11	SIDT2
SAT	HTN	ENSG00000135211.5	0.011305697	0.010257947	0.00440421	0.240332563	0.011391714	0.009693056	0.004404125	0.240308926	-8.60E-05	0.011533321	1	0.077370859	1	0.075362365	ENSG00000149577	11	SIDT2
SAT	HTN	ENSG00000135747.7	0.003271525	0.362980243	0.003596261	0.240335405	0.003216559	0.371088332	0.003596182	0.240311831	5.50E-05	0.037306465	1	0.650299517	1	0.653687263	ENSG00000149577	11	SIDT2
SAT	HTN	ENSG00000135917.9	-0.001960915	0.768652648	0.006666652	0.240335771	-0.0102114	0.157427028	0.007222762	0.24031124	0.008250485	0.002999739	1	0.907788321	1	0.427227526	ENSG00000149591	12	TAGLN
SAT	HTN	ENSG00000136146.10	-0.004099039	0.450104196	0.005427452	0.240335532	-0.004019602	0.458923395	0.005427337	0.240311955	-7.94E-05	0.049935675	1	0.722523685	1	0.730581106	ENSG00000149591	12	TAGLN
SAT	HTN	ENSG00000136213.7	-0.015547844	0.005807158	0.005636364	0.240332061	-0.015433861	0.006175575	0.005636247	0.240308528	-0.000113983	0.007046903	1	0.051081192	1	0.05390513	ENSG00000149591	12	TAGLN
SAT	HTN	ENSG00000136247.10	0.002137309	0.756156003	0.006882716	0.240335766	0.002009944	0.770260596	0.006882562	0.240312183	0.000127365	0.011404293	1	0.901979395	1	0.91001726	ENSG00000149679	7	CABLES2
SAT	HTN	ENSG00000136279.14	0.003450045	0.575458058	0.006160446	0.240335659	0.003348471	0.586749848	0.006160348	0.240312068	0.000101574	0.042832872	1	0.808945111	1	0.815726353	ENSG00000149679	7	CABLES2
SAT	HTN	ENSG00000136371.5	0.002452337	0.342345397	0.002582652	0.240335372	0.002406007	0.351527424	0.002582574	0.240311797	4.63E-05	0.009246801	1	0.629901559	1	0.636161875	ENSG00000149743	9	TRPT1
SAT	HTN	ENSG00000136715.13	-0.006055751	0.342500573	0.006379599	0.240335369	-0.005951613	0.350858027	0.006379504	0.240311796	-0.000104138	0.0485929	1	0.629901559	1	0.635651677	ENSG00000149743	9	TRPT1
SAT	HTN	ENSG00000136816.11	0.006101329	0.036391032	0.002915757	0.240333654	0.006052388	0.037914126	0.002915707	0.2403101	4.89E-05	0.035456566	1	0.176751156	1	0.180808499	ENSG00000149922	6	TBX6
SAT	HTN	ENSG00000136856.13	0.016017911	0.002574615	0.005313715	0.240331332	0.015911073	0.00274977	0.005313566	0.240307804	0.000106839	0.001912395	1	0.027977934	1	0.029292189	ENSG00000149922	6	TBX6
SAT	HTN	ENSG00000137310.7	-0.001972178	0.750694238	0.006207199	0.240335764	-0.009012139	0.178841364	0.0067038	0.240311334	0.007039961	0.005445612	1	0.899394965	1	0.458067006	ENSG00000150054	14	MPP7
SAT	HTN	ENSG00000137411.12	0.00307253	0.010665728	0.001203275	0.240332598	0.00372299	0.002599218	0.001236233	0.240307753	-0.00065046	0.021914997	1	0.079172522	1	0.028332336	ENSG00000150076	18	C10ORF68
SAT	HTN	ENSG00000137574.6	4.68E-05	0.989533833	0.003564792	0.240335812	-6.54E-06	0.998536639	0.003564706	0.240312225	5.33E-05	0.034191185	1	0.99730223	1	0.999787914	ENSG00000150316	7	CWC15
SAT	HTN	ENSG00000138135.5	-0.001893386	0.702000694	0.004948459	0.240335741	-0.001964875	0.691309764	0.00494833	0.240312148	7.15E-05	0.033597497	1	0.875941799	1	0.871374312	ENSG00000150316	7	CWC15
SAT	HTN	ENSG00000138175.8	0.021826371	6.98E-07	0.004398758	0.240323671	0.021719004	7.91E-07	0.004398679	0.240300203	0.000107366	0.001887866	0.002290671	3.52E-05	0.002595531	3.82E-05	ENSG00000150316	7	CWC15
SAT	HTN	ENSG00000138621.7	0.003022618	0.093892374	0.001804315	0.240334429	0.00299854	0.096527711	0.001804268	0.240310863	2.41E-05	0.049546175	1	0.313707617	1	0.319658873	ENSG00000150337	9	FCGR1A
SAT	HTN	ENSG00000138756.13	0.00023744	0.955331764	0.004239042	0.240335812	-0.000662838	0.876366422	0.00426051	0.240312213	0.000900278	0.035952713	1	0.983048498	1	0.956781838	ENSG00000150656	10	CNDP1
SAT	HTN	ENSG00000138767.8	-0.003155067	0.229602039	0.002626189	0.240335102	-0.003349884	0.20238383	0.002627791	0.240311424	0.000194817	0.041071298	1	0.52039215	1	0.488970062	ENSG00000150656	10	CNDP1
SAT	HTN	ENSG00000138821.8	0.008149541	0.175058602	0.006009387	0.240334906	0.007972455	0.184612169	0.006009287	0.240311357	0.000177086	0.000242675	1	0.450479504	1	0.465896151	ENSG00000150656	10	CNDP1
SAT	HTN	ENSG00000139343.6	-0.002471422	0.435595541	0.003170916	0.240335514	-0.002524308	0.42582833	0.003169843	0.240311913	5.29E-05	0.034099864	1	0.710685714	1	0.699140398	ENSG00000150893	9	FREM2
SAT	HTN	ENSG00000139344.3	0.000394575	0.948112896	0.006063223	0.240335811	0.00025661	0.966241123	0.006063107	0.240312225	0.000137965	0.003236353	1	0.981389706	1	0.988536677	ENSG00000151006	7	PRSS53
SAT	HTN	ENSG00000139531.8	0.00431473	0.432801259	0.005500621	0.24033551	0.00392811	0.475383066	0.005503494	0.240311974	0.00038662	0.03758682	1	0.708593279	1	0.74149608	ENSG00000151006	7	PRSS53
SAT	HTN	ENSG00000139620.8	0.00278857	0.382817522	0.003195272	0.240335438	0.002723554	0.394002257	0.003195225	0.240311867	6.50E-05	0.013973407	1	0.668098026	1	0.673994476	ENSG00000151006	7	PRSS53
SAT	HTN	ENSG00000139624.8	0.014863412	0.007461169	0.005555296	0.240332283	0.014703729	0.008128404	0.005555495	0.240308771	0.000159682	0.027258915	1	0.062290319	1	0.067346705	ENSG00000151117	4	TMEM86A
SAT	HTN	ENSG00000140319.6	0.006385491	0.215499343	0.005155467	0.240335057	0.006457672	0.210345174	0.005155338	0.240311452	-7.22E-05	0.044034966	1	0.507557983	1	0.499379533	ENSG00000151117	4	TMEM86A
SAT	HTN	ENSG00000140400.10	-8.32E-05	0.980449665	0.003398511	0.240335811	-0.000106175	0.975094407	0.003398559	0.240312221	2.30E-05	0.024876473	1	0.978294984	1	0.974491801	ENSG00000151148	9	UBE3B
SAT	HTN	ENSG00000140471.12	0.004895145	0.117157907	0.003124259	0.240334603	0.004968202	0.111789071	0.003124251	0.240310978	-7.31E-05	0.015380892	1	0.363550738	1	0.352908944	ENSG00000151148	9	UBE3B
SAT	HTN	ENSG00000140876.7	0.009598789	0.167345225	0.006951682	0.240334873	0.00972055	0.162017632	0.006951592	0.240311261	-0.000121762	0.037865555	1	0.439214192	1	0.434297263	ENSG00000151148	9	UBE3B
SAT	HTN	ENSG00000141569.6	-0.012583847	0.00027754	0.003461442	0.240329295	-0.012649129	0.000257874	0.003461402	0.24030564	6.53E-05	0.030013277	0.910609609	0.005091434	0.846086136	0.004726738	ENSG00000151148	9	UBE3B
SAT	HTN	ENSG00000141933.8	0.009351153	0.164740108	0.006730805	0.240334861	0.009224884	0.170511941	0.006730728	0.240311299	0.000126269	0.030367877	1	0.435897688	1	0.443378165	ENSG00000151176	3	PLBD2
SAT	HTN	ENSG00000142002.12	-0.000492168	0.935281185	0.006061023	0.24033581	-0.000399528	0.947442337	0.006060887	0.240312223	-9.26E-05	0.036264939	1	0.97727948	1	0.981538823	ENSG00000151176	3	PLBD2

SAT	HTN	ENSG00000142599.13	0.024014294	8.90E-05	0.006128116	0.24032824	0.024103573	8.38E-05	0.006127982	0.240304596	-8.93E-05	0.049211472	0.292130826	0.002057259	0.274874119	0.001977512	ENSG00000151176	3	PLBD2
SAT	HTN	ENSG00000142677.3	-0.002020186	0.766652939	0.006807568	0.24033577	-0.001921194	0.777774227	0.006807411	0.240312186	-9.90E-05	0.04431138	1	0.906772997	1	0.91334189	ENSG00000151208	12	DLG5
SAT	HTN	ENSG00000143442.17	-0.00725319	0.074054473	0.004060499	0.24033424	-0.007131329	0.079040448	0.004060463	0.240310704	-0.000121861	0.000803533	1	0.276027247	1	0.285677612	ENSG00000151208	12	DLG5
SAT	HTN	ENSG00000143486.11	-0.00320216	0.482705575	0.004561753	0.24033557	-0.003299075	0.469550703	0.004561706	0.240311967	9.69E-05	0.015936813	1	0.747733474	1	0.736442251	ENSG00000151208	12	DLG5
SAT	HTN	ENSG00000144152.8	0.001498048	0.72664361	0.004285109	0.240335753	0.00155429	0.716807578	0.004284992	0.240312161	-5.62E-05	0.046098375	1	0.886948544	1	0.88553283	ENSG00000151276	19	MAGI1
SAT	HTN	ENSG00000144214.5	0.000378991	0.91242541	0.003446	0.240335807	0.000316005	0.926933401	0.003445934	0.240312221	6.30E-05	0.018098521	1	0.969071845	1	0.975149159	ENSG00000151276	19	MAGI1
SAT	HTN	ENSG00000144580.9	0.003479129	0.610465556	0.006829732	0.240335685	0.003334726	0.625352804	0.006829558	0.240312108	0.000144403	0.002082056	1	0.830870282	1	0.837966728	ENSG00000151348	9	EXT2
SAT	HTN	ENSG00000144677.10	-0.021509455	0.001223545	0.006652369	0.240330657	-0.021331105	0.001343634	0.006652433	0.240307155	-0.00017835	0.013168597	1	0.015930364	1	0.016955623	ENSG00000151348	9	EXT2
SAT	HTN	ENSG00000144837.4	-0.005791209	0.318079372	0.005800412	0.240335322	-0.005670907	0.328230212	0.005800337	0.240311754	-0.000120302	0.014724879	1	0.610360453	1	0.615105129	ENSG00000151348	9	EXT2
SAT	HTN	ENSG00000145990.6	0.010519818	0.012418749	0.004207884	0.240332731	0.01182034	0.005283333	0.00423783	0.240308389	-0.001300522	0.00996956	1	0.088578075	1	0.048151705	ENSG00000151491	8	EPS8
SAT	HTN	ENSG00000146007.6	0.011553027	0.01120433	0.004555124	0.240332641	0.011626737	0.010694974	0.004555003	0.240309012	-7.37E-05	0.015936943	1	0.082725873	1	0.08048213	ENSG00000151532	9	VTI1A
SAT	HTN	ENSG00000146109.3	0.013438086	0.000692174	0.003960903	0.240330137	0.014008239	0.000411778	0.003965596	0.240306072	-0.000570153	0.003780788	1	0.010414304	1	0.006721602	ENSG00000151532	9	VTI1A
SAT	HTN	ENSG00000146205.9	-0.004690429	0.136386862	0.00314924	0.240334719	-0.004635958	0.140989087	0.003149171	0.240311157	-5.45E-05	0.018964357	1	0.389587189	1	0.398436861	ENSG00000151532	9	VTI1A
SAT	HTN	ENSG00000146263.7	-0.014562731	0.024787128	0.006487575	0.240333328	-0.014455956	0.025861491	0.00648746	0.240309777	-0.000106775	0.037623859	1	0.139829894	1	0.142607652	ENSG00000151552	7	QDPR
SAT	HTN	ENSG00000146425.6	-0.010945811	0.076191625	0.006172818	0.240334263	-0.011052662	0.073361246	0.00617267	0.240310644	0.000106851	0.014594412	1	0.278675024	1	0.274769689	ENSG00000151552	7	QDPR
SAT	HTN	ENSG00000146556.10	-0.02868272	8.67E-06	0.006448388	0.240326056	-0.028580901	9.32E-06	0.006448241	0.240302537	-0.000101819	0.029352455	0.028437867	0.00030253	0.030588524	0.000321984	ENSG00000151640	8	DPYSL4
SAT	HTN	ENSG00000147576.11	-0.010778136	0.001552548	0.003405742	0.240330874	-0.008896289	0.010833411	0.003491429	0.240309024	-0.001881847	0.014456946	1	0.019007131	1	0.081337348	ENSG00000151640	8	DPYSL4
SAT	HTN	ENSG00000147853.12	-0.008884965	0.156883324	0.006276315	0.240334825	-0.009007304	0.151241245	0.006276153	0.24031121	0.00012234	0.004415502	1	0.425752014	1	0.415945118	ENSG00000151640	8	DPYSL4
SAT	HTN	ENSG00000147955.12	-0.006703334	0.163334657	0.004808892	0.240334855	-0.006619943	0.168627669	0.004808808	0.240311291	-8.34E-05	0.028954153	1	0.433927943	1	0.442141749	ENSG00000151640	8	DPYSL4
SAT	HTN	ENSG00000148843.9	0.06581016	1.37E-22	0.006728725	0.240288645	0.065679101	1.66E-22	0.006728585	0.240265246	0.000131059	0.008974326	4.50E-19	2.25E-19	5.45E-19	2.72E-19	ENSG00000151640	8	DPYSL4
SAT	HTN	ENSG00000149179.9	-0.021246354	0.001485414	0.006686424	0.240330834	-0.021343736	0.001412147	0.006686267	0.2403072	9.74E-05	0.042409743	1	0.018460765	1	0.017483974	ENSG00000151657	7	KIN
SAT	HTN	ENSG00000149403.7	0.007779319	0.074786932	0.004366056	0.240334248	0.007719771	0.077031378	0.004365941	0.240310684	5.95E-05	0.042373001	1	0.276027247	1	0.282212646	ENSG00000151657	7	KIN
SAT	HTN	ENSG00000150656.10	0.000295479	0.955004169	0.005236776	0.240335812	0.000428245	0.934823312	0.005236678	0.240312222	-0.000132766	0.001153421	1	0.983048498	1	0.977239819	ENSG00000151725	7	CENPU
SAT	HTN	ENSG00000151006.7	-0.012223548	0.028704634	0.005587842	0.240333453	-0.01249598	0.025345126	0.005588271	0.24030976	0.000272432	0.002146352	1	0.154393286	1	0.140973783	ENSG00000151725	7	CENPU
SAT	HTN	ENSG00000151532.9	0.006719179	0.259130502	0.005954344	0.240335185	0.00662027	0.266197008	0.005954206	0.240311616	9.89E-05	0.020957768	1	0.550295907	1	0.55733039	ENSG00000151725	7	CENPU
SAT	HTN	ENSG00000151657.7	-0.005750539	0.267715534	0.005188425	0.240335208	-0.005611326	0.2794646	0.005188351	0.240311649	-0.000139212	0.001305522	1	0.561046104	1	0.572417553	ENSG00000151725	7	CENPU
SAT	HTN	ENSG00000151849.10	0.006555744	0.257305456	0.005787284	0.240335181	0.006459539	0.264346364	0.005787193	0.240311611	9.62E-05	0.041486655	1	0.549440772	1	0.554552698	ENSG00000151849	10	CENPJ
SAT	HTN	ENSG00000151883.12	0.00352052	0.484765584	0.005038969	0.240335573	0.003621472	0.472324291	0.005038899	0.240311971	-0.000100952	0.017074248	1	0.748376275	1	0.738432275	ENSG00000151849	10	CENPJ
SAT	HTN	ENSG00000152219.4	-0.023354061	6.13E-05	0.005826962	0.240327891	-0.023451201	5.71E-05	0.005826808	0.240304237	9.71E-05	0.013484311	0.201001009	0.001500008	0.1871961	0.001428978	ENSG00000151883	12	PARP8
SAT	HTN	ENSG00000152253.4	0.003468851	0.346083145	0.003681592	0.240335376	0.004042309	0.273638027	0.003692536	0.240311634	-0.000573458	0.045274705	1	0.633986113	1	0.566796949	ENSG00000151883	12	PARP8
SAT	HTN	ENSG00000152291.9	-0.009322672	0.093036593	0.005550544	0.240334422	-0.009697234	0.080725376	0.005552393	0.240310721	0.000374562	0.014684542	1	0.311800881	1	0.287266767	ENSG00000151883	12	PARP8
SAT	HTN	ENSG00000152465.13	-0.005011517	0.471634494	0.006962154	0.240335558	-0.0048807	0.483273937	0.006962014	0.240311983	-0.000130817	0.013552308	1	0.740044368	1	0.747074465	ENSG00000151883	12	PARP8
SAT	HTN	ENSG00000152689.13	0.004121935	0.297231411	0.003954312	0.240335277	0.007050446	0.09086856	0.004169796	0.240310816	-0.002928512	0.026942648	1	0.590682168	1	0.308633276	ENSG00000151883	12	PARP8
SAT	HTN	ENSG00000152942.14	-0.006084276	0.019850194	0.002612202	0.240333138	-0.006023758	0.021108088	0.002612151	0.240309603	-6.05E-05	0.002527771	1	0.120385374	1	0.125919337	ENSG00000152061	17	RABGAP1L
SAT	HTN	ENSG00000153714.5	-0.000686789	0.8939951	0.005154109	0.240335805	-0.000550838	0.91488789	0.005154021	0.24031222	-0.000135951	0.000977916	1	0.960795963	1	0.972383274	ENSG00000152061	17	RABGAP1L
SAT	HTN	ENSG00000154240.12	0.007035023	0.249660496	0.006111163	0.24033516	0.006001694	0.326529827	0.006117133	0.240311751	0.001033329	0.000189558	1	0.541398603	1	0.615105129	ENSG00000152078	5	TMEM56
SAT	HTN	ENSG00000155085.11	0.021930058	0.001019806	0.006675766	0.240330491	0.021832095	0.001073954	0.006675625	0.240306951	9.80E-05	0.048994719	1	0.013826371	1	0.014323756	ENSG00000152219	4	ARL14EP
SAT	HTN	ENSG00000156172.5	-0.011587303	0.017082589	0.004858544	0.240333008	-0.011484611	0.018087774	0.004858487	0.24030947	-0.000102692	0.01452011	1	0.109682924	1	0.113472249	ENSG00000152219	4	ARL14EP
SAT	HTN	ENSG00000156603.10	0.00524905	0.020606568	0.002267287	0.24033317	0.005532728	0.014782197	0.002269669	0.240309295	-0.000283678	0.007670508	1	0.122927543	1	0.10024166	ENSG00000152219	4	ARL14EP
SAT	HTN	ENSG00000156886.11	0.003218186	0.557079153	0.005480694	0.240335643	0.002736563	0.617721545	0.005483188	0.240312103	0.000481623	0.005649956	1	0.797697485	1	0.835426376	ENSG00000152223	8	EPG5
SAT	HTN	ENSG00000157045.4	-0.009659788	0.126209885	0.006316796	0.24033466	-0.009801179	0.120750838	0.006316688	0.240311038	0.000141391	0.00522347	1	0.376792204	1	0.369887656	ENSG00000152240	8	HAUS1
SAT	HTN	ENSG00000157540.15	-0.006263749	0.407145293	0.007556441	0.240335474	-0.006570211	0.384643521	0.007557412	0.240311853	0.000306462	0.03142072	1	0.687516061	1	0.667766115	ENSG00000152240	8	HAUS1
SAT	HTN	ENSG00000158201.5	-0.000657069	0.897377671	0.005094556	0.240335805	-0.000524275	0.918033996	0.005094472	0.24031222	-0.000132794	0.001226221	1	0.962469823	1	0.974136703	ENSG00000152240	8	HAUS1
SAT	HTN	ENSG00000158552.8	-0.009461575	0.099790804	0.005748671	0.240334477	-0.009553008	0.096550425	0.005748537	0.240310863	9.14E-05	0.026980044	1	0.325460864	1	0.319658873	ENSG00000152253	4	SPC25
SAT	HTN	ENSG00000158805.7	0.026162674	1.46E-05	0.006035764	0.240326547	0.026253204	1.36E-05	0.006035606	0.240302895	-9.05E-05	0.027472726	0.047918603	0.000460756	0.044732652	0.000430122	ENSG00000152253	4	SPC25
SAT	HTN	ENSG00000158850.10	0.005517006	0.264384405	0.004943158	0.240335199	0.005609926	0.256412203	0.004943043	0.24031159	-9.29E-05	0.009040161	1	0.556219915	1	0.548573946	ENSG00000152253	4	SPC25
SAT	HTN	ENSG00000158869.6	0.009334868	0.029912592	0.004299302	0.240333488	0.009408738	0.028634282	0.004299189	0.240309863	-7.39E-05	0.011003618	1	0.158807793	1	0.153011528	ENSG00000152270	4	PDE3B
SAT	HTN	ENSG00000159579.9	0.002464679	0.70102588	0.006419501	0.240335741	0.00260989	0.684328253	0.006419379	0.240312144	-0.000145211	0.003518716	1	0.875548501	1	0.867573802	ENSG00000152291	9	TGOLN2
SAT	HTN	ENSG00000159588.10	-0.016115729	0.015014578	0.006626436	0.240332896	-0.01595016	0.016081033	0.006626353	0.240309368	-0.000165569	0.003456911	1	0.101155706	1	0.106374736	ENSG00000152291	9	TGOLN2
SAT	HTN	ENSG00000159733.9	-0.002360542	0.583278314	0.004302825	0.240335665	-0.002424691	0.57307615	0.004302704	0.240312069	6.41E-05	0.021139912	1	0.812626814	1	0.806980304	ENSG00000152291	9	TGOLN2
SAT	HTN	ENSG00000159904.7	0.00405236	0.25471873	0.003557935	0.240335174	0.004105487	0.248532557	0.003557853	0.240311569	-5.31E-05	0.039188389	1	0.548740745	1	0.537886095	ENSG00000152348	11	ATG10
SAT	HTN	ENSG00000160049.7	0.004957215	0.33737532	0.005167183	0.240335359	0.005111914	0.322509293	0.005167122	0.240311743	-0.000154699	0.000538625	1	0.625030166	1	0.611295778	ENSG00000152348	11	ATG10
SAT	HTN	ENSG00000160179.14	0.003240746	0.476194516	0.004548817	0.240335563	0.003312147	0.46651882	0.004548682	0.240311964	-7.14E-05	0.01160616	1	0.743997242	1	0.734476126	ENSG00000152348	11	ATG10
SAT	HTN	ENSG00000160194.13	0.001889022	0.174965878	0.001392651	0.240334906	0.001908542	0.170540458	0.001392617	0.240311299	-1.95E-05	0.045191396	1	0.450479504	1	0.443378165	ENSG00000152348	11	ATG10
SAT	HTN	ENSG00000160321.10	-0.005024778	0.472687505	0.006997189	0.240335559	-0.005153907	0.461381403	0.006997128	0.240311958	0.00012913	0.040127141	1	0.740564567	1	0.732029465	ENSG00000152348	11	ATG10
SAT	HTN	ENSG00000160752.10	0.014865409	0.000868645	0.004464138	0.240330345	0.014940592	0.000817375	0.004464057	0.240306701	-7.52E-05	0.032512136	1	0.01203601	1	0.011411947	ENSG00000152359	10	POC5
SAT	HTN	ENSG00000161243.4	-0.00413586	0.4341675	0.005288278	0.240335512	-0.004047469	0.444043987	0.005288163	0.240311937	-8.84E-05	0.02363982	1	0.709110389	1	0.716629769	ENSG00000152439	8	ZNF773
SAT	HTN	ENSG00000161249.16	0.006282195	0.202839842	0.004932999	0.240335014	0.006204206	0.208493779	0.0049329	0.240311445	7.80E-05	0.037920898	1	0.490794632	1	0.497295906	ENSG00000152439	8	ZNF773
SAT	HTN	ENSG00000161912.13	0.001719831	0.642310133	0.00370277	0.240335707	0.001610227	0.66365321	0.003702734	0.240312132	0.000109604	0.000832185	1	0.844302676	1	0.859631339	ENSG00000152439	8	ZNF773
SAT	HTN	ENSG00000161996.13	0.005930031	0.041574184	0.002910069	0.240333765	0.005981646	0.039828101	0.002910022	0.240310142	-5.16E-05	0.029009833	1	0.191311216	1	0.185366123	ENSG00000152439	8	ZNF773
SAT	HTN	ENSG00000161999.7	0.002021525	0.569155815	0.003550923	0.240335654	0.001952975	0.582320183	0.003550869	0.240312076	6.85E-05	0.018780317	1	0.804220599	1	0.812928219	ENSG00000152465	13	NMT2
SAT	HTN	ENSG00000162244.6	-0.002490603	0.624590389	0.00508956	0.240335695	-0.00258386	0.611670793	0.005089441	0.240312098	9.33E-05	0.01051008	1	0.834397828	1	0.833930915	ENSG00000152465	13	NMT2
SAT	HTN	ENSG00000162341.11	-0.007003154	0.285326333	0.006554611	0.24033525	-0.006742522	0.303653883	0.006554853	0.240311704	-0.000260633	0.002429728	1	0.57930427	1	0.595866261	ENSG00000152465	13	NMT2
SAT	HTN	ENSG00000162383.7	0.00474276	0.124089336	0.003084047	0.240334647	0.004699333	0.127560451	0.003083966	0.24031108	4.34E-05	0.03584692	1	0.374127295	1	0.381499044	ENSG00000152465	13	NMT2
SAT	HTN	ENSG00000162384.9	-0.004204446	0.436973483	0.005408936	0.240335515	-0.00406013	0.452863639	0.005408818	0.240311948	-0.000144316	0.000298163	1	0.710902942	1	0.725510546	ENSG00000152465	13	NMT2
SAT	HTN	ENSG00000162704.11	-0.004149132	0.163068692	0.002974657	0.240334854	-0.004105791	0.167498563	0.00297459	0.240311286	-4.33E-05	0.044645618	1	0.433572431	1	0.440923826	ENSG00000152465	13	NMT2
SAT	HTN	ENSG00000162885.8	-0.007019909	0.068110028	0.003848039	0.240334172	-0.007077271	0.065882473	0.00384796	0.240310557	5.74E-05	0.049014818	1	0.264460356	1	0.262366273	ENSG00000152492	9	CCDC50
SAT	HTN	ENSG00000163138.14	0.00220197	0.529708766	0.003503805	0.240335619	0.002256641	0.519530488	0.003503721	0.240312021	-5.47E-05	0.027563595	1	0.778612574	1	0.769056242	ENSG00000152492	9	CCDC50
SAT	HTN	ENSG00000163453.7	-0.014838463	0.008480565	0.00563702	0.240332396	-0.014061956	0.012744898	0.005645494	0.240309166	-0.000776508	0.013435599	1	0.069215759	1	0.089955233	ENSG00000152492	9	CCDC50
SAT	HTN	ENSG00000163554.7	0.000375629	0.886121121	0.002622848	0.240335803	0.000631314	0.809856932	0.002623798	0.240312197	-0.000255685	0.000675904	1	0.958390933	1	0.925531291	ENSG00000152492	9	CCDC50
SAT	HTN	ENSG00000164031.12	0.009159019	0.030896712	0.004243417	0.240333516	0.009232905	0.029565935	0.004243329	0.240309891	-7.39E-05	0.020878548	1	0.16193628	1	0.156286405	ENSG00000152527	9	PLEKHH2
SAT	HTN	ENSG00000164144.10	0.00202574	0.734833008	0.005980859	0.240335757	0.001908133	0.749690301	0.00598071	0.240312175	0.000117606	0.004716249	1	0.893035719	1	0.898697069	ENSG00000152527	9	PLEKHH2
SAT	HTN	ENSG00000164465.14	-0.006159994	0.274860758	0.005641339	0.240335225	-0.007806623	0.168852061	0.005673815	0.240311292	0.001646629	0.006836306	1	0.570049397	1	0.442141749	ENSG00000152527	9	PLEKHH2
SAT	HTN	ENSG00000164850.10	0.007313905	0.022711195	0.003210295	0.240333253	0.007243168	0.024055416	0.003210268	0.240309715	7.07E-05	0.014381242	1	0.13136175	1	0.136079	ENSG00000152683	10	SLC30A6
SAT	HTN	ENSG00000164924.13	-0.004863305	0.13060005	0.00321702	0.240334686	-0.004807803	0.135041159	0.003216961	0.240311124	-5.55E-05	0.027894164	1	0.382588181	1	0.388719394	ENSG00000152689	13	RASGRP3
SAT	HTN	ENSG00000165178.8	0.014513033	0.000407774	0.004105496	0.24032965	0.014610602	0.000372526	0.004105435	0.24030598	-9.76E-05	0.004062189	1	0.00689643	1	0.006267988	ENSG00000152689	13	RASGRP3
SAT	HTN	ENSG00000165244.6	0.020202921	0.002375352	0.006648322	0.240331259	0.020100828	0.002498579	0.006648134	0.240307717	0.000102093	0.016988116	1	0.02652693	1	0.027695394	ENSG00000152689	13	RASGRP3
SAT	HTN	ENSG00000165650.7	-0.001210715	0.657936615	0.002734446	0.240335717	-0.00230091	0.405770124	0.002767643	0.240311885	0.001090196	0.010904111	1	0.852573121	1	0.683130653	ENSG00000152689	13	RASGRP3
SAT	HTN	ENSG00000165684.3	-0.001539162	0.553770565	0.002599409	0.24033564	-0.001498308	0.564335894	0.002599362	0.240312062	-4.09E-05	0.045293019	1	0.79549966	1	0.803251455	ENSG00000152689	13	RASGRP3
SAT	HTN	ENSG00000165806.15	-0.00604255	0.010372334	0.002357449	0.240332573	-0.006076051	0.009953665	0.002357393	0.240308949	3.35E-05	0.045491311	1	0.077784342	1	0.076584716	ENSG00000152689	13	RASGRP3
SAT	HTN	ENSG00000166148.2	0.005934216	0.385587075	0.006839422	0.240335442	0.005775967	0.398388912	0.006839489	0.240311874	0.000158249	0.032839239	1	0.669725354	1	0.678667716	ENSG00000152749	7	GPR180
SAT	HTN	ENSG00000166548.11	0.002044439	0.720499893	0.005714059	0.24033575	0.001941262	0.734053472	0.005713986	0.240312168	0.000103176	0.034464527	1	0.885985829	1	0.892066631	ENSG00000152749	7	GPR180
SAT	HTN	ENSG00000166669.9	-0.002065653	0.752869286	0.006560578	0.240335764	-0.001930737	0.768530005	0.006560473	0.240312183	-0.000134916	0.011475747	1	0.900432066	1	0.908993132	ENSG00000152749	7	GPR180
SAT	HTN	ENSG00000166801.11	-0.003338856	0.156623457	0.002357081	0.240334824	-0.002903189	0.219337594	0.002363596	0.240311481	-0.000435667	0.013791021	1	0.425751089	1	0.509427202	ENSG00000152749	7	GPR180
SAT	HTN	ENSG00000166881.5	0.001333673	0.749584114	0.004178332	0.240335763	0.001049454	0.801746634	0.004179638	0.240312194	0.000284219	0.011440342	1	0.899241283	1	0.923833778	ENSG00000152749	7	GPR180
SAT	HTN	ENSG00000167395.10	0.023878767	0.000731681	0.007070066	0.240330188	0.024064028	0.00066502	0.007070106	0.240306512	-0.000185262	0.012354556	1	0.010622319	1	0.009917863	ENSG00000152749	7	GPR180
SAT	HTN	ENSG00000167528.8	0.007109728	0.002888918	0.00238635	0.240331436	0.007155712	0.002712043	0.002386322	0.240307791	-4.60E-05	0.0262235	1	0.030282878	1	0.028984413	ENSG00000152778	7	IFIT5
SAT	HTN	ENSG00000167595.10	6.42E-05	0.981195796	0.002723406	0.240335813	0.00011905	0.965131773	0.002723342	0.240312224	-5.49E-05	0.004906177	1	0.993612162	1	0.988156741	ENSG00000152784	11	PRDM8
SAT	HTN	ENSG00000167711.9	0.008922424	0.119075841	0.005724394	0.240334615	0.009035527	0.114464553	0.005724329	0.240310997	-0.000113102	0.023168684	1	0.366076286	1	0.358450758	ENSG00000152784	11	PRDM8
SAT	HTN	ENSG00000167799.5	0.00653602	0.217330285	0.005298073	0.240335063	0.007982753	0.132615193	0.005308136	0.24031111	-0.001446733	1.23E-05	1	0.509681988	1	0.386764842	ENSG00000152926	10	ZNF117
SAT	HTN	ENSG00000167972.9	-2.18E-05	0.997009858	0.005809287	0.240335813	-0.000147867	0.97969272	0.005809165	0.240312225	0.000126097	0.003788634	1	0.998836441	1	0.992702845	ENSG00000152942	14	RAD17
SAT	HTN	ENSG00000167987.6	0.001382316	0.822306771	0.00615514	0.240335789	0.001603014	0.794534024	0.006155313	0.240312192	-0.000220698	0.003912839	1	0.93323712	1	0.922042681	ENSG00000152942	14	RAD17
SAT	HTN	ENSG00000168016.9	0.002911188	0.658200506	0.006580453	0.240335717	0.002755644	0.675384837	0.006580337	0.240312139	0.000155544	0.002868488	1	0.852573121	1	0.864587456	ENSG00000152942	14	RAD17
SAT	HTN	ENSG00000168028.9	0.002757947	0.110078907	0.001726046	0.240334554	0.001800269	0.303977221	0.001751332	0.240311704	0.000957678	0.001262215	1	0.348282442	1	0.596144208	ENSG00000152990	9	GPR125
SAT	HTN	ENSG00000168038.6	0.002476842	0.21497655	0.001997455	0.240335055	0.002415856	0.226480383	0.001997439	0.240311504	6.10E-05	0.000750024	1	0.507575983	1	0.517461433	ENSG00000153044	5	CENPH
SAT	HTN	ENSG00000168056.10	0.010281047	0.040480518	0.00501804	0.240333743	0.006843354	0.181847219	0.005125759	0.240311346	0.003437693	0.001019304	1	0.188077921	1	0.461796228	ENSG00000153044	5	CENPH
SAT	HTN	ENSG00000168329.9	-0.007390188	0.289323768	0.006974458	0.24033526	-0.001249196	0.862708448	0.00722381	0.240312211	-0.006140992	0.001107909	1	0.581565115	1	0.952725149	ENSG00000153048	6	CARHSP1
SAT	HTN	ENSG00000168356.7	-0.000348982	0.896296	0.002677433	0.240335805	-0.000196255	0.941574026	0.002677719	0.240312223	-0.000152727	0.001228349	1	0.961971599	1	0.9791773	ENSG00000153048	6	CARHSP1
SAT	HTN	ENSG00000168522.8	0.009925984	0.141865735	0.006757514	0.240334749	0.009834675	0.14555736	0.006757338	0.240311181	9.13E-05	0.046214573	1	0.39809709	1	0.406791907	ENSG00000153157	8	SYCP2L
SAT	HTN	ENSG00000168679.13	0.001280693	0.784369945	0.004680403	0.240335776	0.001361039	0.771205636	0.004680353	0.240312184	-8.03E-05	0.049972045	1	0.916412446	1	0.91001726	ENSG00000153201	11	RANBP2
SAT	HTN	ENSG00000168702.12	-0.015084309	0.014305902	0.006158123	0.240332854	-0.022536887	0.000402906	0.006369596	0.240306052	0.007452579	4.76E-06	1	0.098401814	1	0.00660968	ENSG00000153208	12	MERTK
SAT	HTN	ENSG00000168813.12	-0.002819446	0.682088873	0.006883167	0.240335731	-0.002676654	0.697368268	0.006883058	0.240312151	-0.000142792	0.01083944	1	0.865403555	1	0.875982116	ENSG00000153246	7	PLA2R1
SAT	HTN	ENSG00000168936.6	0.010411117	0.003745229	0.003591436	0.240331669	0.010356987	0.003928514	0.003591363	0.240308124	5.41E-05	0.047198652	1	0.036790708	1	0.038361473	ENSG00000153246	7	PLA2R1
SAT	HTN	ENSG00000168944.11	-0.013842068	0.015161731	0.005699839	0.240332905	-0.013940616	0.014451455	0.005699702	0.240309275	9.85E-05	0.014418942	1	0.101937783	1	0.099073573	ENSG00000153246	7	PLA2R1
SAT	HTN	ENSG00000169047.5	-0.020539792	3.56E-09	0.003479268	0.240318626	-0.020587958	3.27E-09	0.00347917	0.240294958	4.82E-05	0.032999576	1.17E-05	3.83E-07	1.07E-05	3.83E-07	ENSG00000153291	11	SLC25A27
SAT	HTN	ENSG00000169085.7	0.004915038	0.000794335	0.001465084	0.240330263	0.004106623	0.006290732	0.001503024	0.240308544	0.000808415	0.01608431	1	0.011331359	1	0.054602888	ENSG00000153406	9	NMRAL1
SAT	HTN	ENSG00000169609.9	0.000460617	0.883026948	0.003130612	0.240335803	0.000605183	0.846746146	0.003131253	0.240312207	-0.000144566	0.040382344	1	0.957468	1	0.946566986	ENSG00000153406	9	NMRAL1
SAT	HTN	ENSG00000169862.14	-0.006418413	0.13493413	0.004293462	0.240334711	-0.006351041	0.139069071	0.004293364	0.240311146	-6.74E-05	0.030049926	1	0.386992028	1	0.394186122	ENSG00000153406	9	NMRAL1
SAT	HTN	ENSG00000170322.10	0.007053393	0.225315805	0.005817158	0.240335088	0.007154732	0.218711845	0.005817034	0.240311479	-0.000101339	0.01925266	1	0.516604581	1	0.509292807	ENSG00000153446	11	C16orf89
SAT	HTN	ENSG00000170509.7	-0.002269536	0.485155848	0.003251323	0.240335573	-0.00217005	0.504498907	0.003251361	0.240312006	-9.95E-05	0.005455818	1	0.748376275	1	0.758328059	ENSG00000153531	8	ADPRHL1
SAT	HTN	ENSG00000170745.7	0.008012193	0.032332016	0.003743512	0.240333554	0.007948973	0.033717122	0.003743433	0.240310002	6.32E-05	0.024067799	1	0.1664956	1	0.170718945	ENSG00000153714	5	LURAP1L
SAT	HTN	ENSG00000170776.15	-0.007950932	0.224828075	0.006550494	0.240335087	-0.007601052	0.245930186	0.006550978	0.240311561	-0.00034988	0.000652704	1	0.51644005	1	0.536079363	ENSG00000153714	5	LURAP1L
SAT	HTN	ENSG00000170837.2	-0.004632468	0.437028721	0.005960297	0.240335515	-0.004741706	0.426287374	0.005960201	0.240311913	0.000109238	0.02436939	1	0.710902942	1	0.699305573	ENSG00000153714	5	LURAP1L
SAT	HTN	ENSG00000170955.9	0.017460223	6.48E-05	0.004370858	0.240327943	0.01733567	7.30E-05	0.004370792	0.240304468	0.000124552	0.000526317	0.212571166	0.001574601	0.239584078	0.001774697	ENSG00000153714	5	LURAP1L
SAT	HTN	ENSG00000171530.9	0.01006824	0.113091921	0.006354405	0.240334575	0.009980769	0.116246326	0.006354229	0.240311009	8.75E-05	0.034520165	1	0.355076165	1	0.362072419	ENSG00000153714	5	LURAP1L
SAT	HTN	ENSG00000171574.13	0.004057021	0.173875342	0.002983414	0.240334901	0.004006202	0.179319863	0.002983348	0.240311336	5.08E-05	0.020387207	1	0.449200786	1	0.458229807	ENSG00000153774	4	CFDP1
SAT	HTN	ENSG00000172247.3	-0.000206373	0.899976452	0.001641902	0.240335806	-0.000176038	0.914615842	0.001641863	0.24031222	-3.03E-05	0.009725393	1	0.963714993	1	0.972383274	ENSG00000153814	7	JAZF1
SAT	HTN	ENSG00000172349.12	0.005648262	0.388121638	0.006544692	0.240335446	0.005463863	0.403799437	0.00654468	0.240311882	0.000184399	0.003769443	1	0.672703167	1	0.682217278	ENSG00000153814	7	JAZF1
SAT	HTN	ENSG00000172613.3	0.00311272	0.033917814	0.001467535	0.240333595	0.003416288	0.020048434	0.001469088	0.240309559	-0.000303568	1.12E-05	1	0.170681517	1	0.121363303	ENSG00000153814	7	JAZF1
SAT	HTN	ENSG00000172638.8	0.014522648	0.018542064	0.006167726	0.240333079	-0.003896544	0.645408689	0.008467984	0.240312121	0.018419192	0.001501758	1	0.11478587	1	0.847979199	ENSG00000153822	9	KCNJ16
SAT	HTN	ENSG00000172803.13	0.012305424	0.003611276	0.004228269	0.240331636	-0.007174849	0.389672448	0.008340788	0.24031186	0.019480272	0.006739185	1	0.035796367	1	0.670742545	ENSG00000153822	9	KCNJ16
SAT	HTN	ENSG00000172922.4	-0.007517662	0.029436959	0.003452284	0.240333475	-0.003724397	0.309732324	0.003666522	0.240311717	-0.003793265	0.002137077	1	0.157300752	1	0.602376784	ENSG00000154079	5	C6orf57
SAT	HTN	ENSG00000172927.3	-0.015934442	0.002389868	0.005246833	0.240331265	-0.016180647	0.002043549	0.005246905	0.240307535	0.000246205	2.98E-05	1	0.026580189	1	0.023608743	ENSG00000154134	10	ROBO3
SAT	HTN	ENSG00000172932.10	-0.001333793	0.81249953	0.005623007	0.240335786	-0.000511812	0.927513927	0.005625963	0.240312221	-0.000821981	1.62E-05	1	0.928207158	1	0.975149159	ENSG00000154222	10	CC2D1B
SAT	HTN	ENSG00000173156.2	-0.006665486	0.046955705	0.003355047	0.240333867	-0.006877271	0.040404068	0.003355422	0.240310154	0.000211784	0.000438195	1	0.205689811	1	0.18777018	ENSG00000154222	10	CC2D1B
SAT	HTN	ENSG00000173200.8	0.00903478	0.084109268	0.005230492	0.240334342	0.008918972	0.088152262	0.005230364	0.240310791	0.000115808	0.001573014	1	0.294571399	1	0.301592879	ENSG00000154222	10	CC2D1B
SAT	HTN	ENSG00000173226.12	0.00140228	0.522067682	0.002190508	0.240335611	0.001432367	0.513165962	0.002190444	0.240312015	-3.01E-05	0.028600918	1	0.771578407	1	0.76520719	ENSG00000154240	12	CEP112
SAT	HTN	ENSG00000173511.5	-0.023209806	3.33E-09	0.00392405	0.24031856	-0.0233707	2.59E-09	0.00392423	0.240294735	0.000160894	0.003002944	1.09E-05	3.76E-07	8.51E-06	3.41E-07	ENSG00000154240	12	CEP112
SAT	HTN	ENSG00000174080.6	-0.001615994	0.614494306	0.003208444	0.240335688	-0.003379812	0.296077721	0.003234633	0.240311687	0.001763818	1.86E-05	1	0.832284161	1	0.586298316	ENSG00000154240	12	CEP112
SAT	HTN	ENSG00000174226.4	-0.027022839	2.85E-05	0.006456298	0.240327174	-0.026903752	3.09E-05	0.006456219	0.240303662	-0.000119087	0.031252231	0.093373483	0.000819066	0.101233222	0.00084361	ENSG00000154240	12	CEP112
SAT	HTN	ENSG00000174353.13	-0.005584898	0.080354943	0.003193863	0.240334305	-0.005397298	0.091102212	0.003194395	0.240310818	-0.0001876	0.004636537	1	0.287194519	1	0.309106886	ENSG00000154240	12	CEP112
SAT	HTN	ENSG00000174652.13	9.77E-05	0.939889305	0.001295785	0.240335811	0.000122976	0.924388954	0.001295752	0.240312221	-2.53E-05	0.00471477	1	0.978294984	1	0.974588739	ENSG00000154263	13	ABCA10
SAT	HTN	ENSG00000175334.3	0.005420809	0.120998393	0.003495945	0.240334628	0.010312241	0.004814434	0.003657851	0.240308306	-0.004891432	5.56E-06	1	0.369642206	1	0.044371228	ENSG00000154265	11	ABCA5
SAT	HTN	ENSG00000175387.11	-0.000202624	0.956016854	0.003673868	0.240335812	-0.000122421	0.973417275	0.003673807	0.240312225	-8.02E-05	0.006740002	1	0.983048498	1	0.990013044	ENSG00000154265	11	ABCA5
SAT	HTN	ENSG00000175416.8	0.009060645	0.062579707	0.004865637	0.240334103	0.009187759	0.05898317	0.004865565	0.240310467	-0.000127115	0.001618117	1	0.251314587	1	0.243426139	ENSG00000154265	11	ABCA5
SAT	HTN	ENSG00000175445.10	-0.008422893	0.082883956	0.004856944	0.24033433	-0.011454066	0.020663183	0.004949706	0.240309585	0.003031173	0.001504269	1	0.293674147	1	0.124168322	ENSG00000154309	7	DISP1
SAT	HTN	ENSG00000175463.7	0.002460508	0.68455287	0.00605651	0.240335732	0.002820025	0.641518656	0.006057056	0.240312119	-0.000359516	0.000375048	1	0.866100648	1	0.845498554	ENSG00000154316	10	TDH

SAT	HTN	ENSG00000176024.12	-0.000723156	0.877572219	0.004694367	0.240335802	-0.000650214	0.88983504	0.004694244	0.240312216	-7.29E-05	0.021204485	1	0.957442803	1	0.960935133	ENSG00000154518	5	ATP5G3
SAT	HTN	ENSG00000176390.10	0.023842265	0.000190109	0.006388878	0.240328945	0.023940506	0.000178765	0.006388725	0.2403053	-9.82E-05	0.030498067	0.623746086	0.003780279	0.586527662	0.003619977	ENSG00000154518	5	ATP5G3
SAT	HTN	ENSG00000176428.5	-0.004137508	0.204524101	0.003261041	0.240335019	-0.004770856	0.145336355	0.003276224	0.24031118	0.000633348	0.045524636	1	0.493414393	1	0.40652053	ENSG00000154518	5	ATP5G3
SAT	HTN	ENSG00000176933.4	0.002386153	0.611831234	0.004702136	0.240335686	0.002916869	0.535411147	0.004706393	0.240312036	-0.000530716	0.009805137	1	0.830870282	1	0.782451496	ENSG00000154710	11	RABGEF1
SAT	HTN	ENSG00000176998.3	0.001131402	0.655557351	0.00253646	0.240335715	0.002991646	0.256123894	0.002634415	0.240311589	-0.001860244	0.008997723	1	0.850824236	1	0.548573946	ENSG00000154710	11	RABGEF1
SAT	HTN	ENSG00000177590.6	-0.005582247	0.30636778	0.005457413	0.240335297	-0.00548622	0.314751325	0.005457279	0.240311727	-9.60E-05	0.011834762	1	0.601393141	1	0.604641264	ENSG00000154710	11	RABGEF1
SAT	HTN	ENSG00000177663.9	-0.003519872	0.220740801	0.002874392	0.240335074	-0.003592887	0.211313119	0.002874391	0.240311455	7.30E-05	0.01007029	1	0.51391887	1	0.50095256	ENSG00000154710	11	RABGEF1
SAT	HTN	ENSG00000177990.7	-0.003202432	0.447812798	0.004218906	0.240335529	-0.01425261	0.011306139	0.005626546	0.240309061	0.011050178	0.002996709	1	0.720446806	1	0.083456744	ENSG00000154719	9	MRPL39
SAT	HTN	ENSG00000178381.7	-0.01408143	0.037413877	0.006765935	0.240333677	-0.014217325	0.035610713	0.006765803	0.240310048	0.000135896	0.009230929	1	0.178682575	1	0.175170538	ENSG00000154719	9	MRPL39
SAT	HTN	ENSG00000178971.9	0.010515428	0.000243919	0.002866341	0.240329176	0.010463225	0.000261805	0.002866281	0.240305654	5.22E-05	0.014707737	0.800297117	0.004573126	0.858981653	0.004769464	ENSG00000154719	9	MRPL39
SAT	HTN	ENSG00000179163.11	0.003764961	0.504954401	0.00564703	0.240335594	0.003850094	0.495360297	0.005646884	0.240311996	-8.51E-05	0.026742144	1	0.762947945	1	0.754680724	ENSG00000154743	13	TSEN2
SAT	HTN	ENSG00000180089.4	0.003850685	0.370488347	0.004299751	0.240335418	0.003734258	0.385122289	0.004299674	0.240311853	0.000116427	0.000589748	1	0.6577772	1	0.668210592	ENSG00000154743	13	TSEN2
SAT	HTN	ENSG00000180855.11	0.019970138	0.000126722	0.005210361	0.240328568	0.01984662	0.000139471	0.005210269	0.24030507	0.000123518	0.002742251	0.415774597	0.002809288	0.457603234	0.002990871	ENSG00000154760	9	SLFN13
SAT	HTN	ENSG00000181061.9	-0.010281919	0.057548947	0.005414036	0.240334035	-0.01019966	0.05956959	0.005413918	0.240310475	-8.23E-05	0.039165575	1	0.238708085	1	0.245399526	ENSG00000154768	4	C17orf50
SAT	HTN	ENSG00000181092.5	0.000475335	0.903667001	0.003927401	0.240335806	-0.002786674	0.5001712	0.004133177	0.240312001	0.003262009	0.011352423	1	0.965816745	1	0.757052571	ENSG00000154803	8	FLCN
SAT	HTN	ENSG00000181781.5	0.000710357	0.913591382	0.006546484	0.240335808	0.00057955	0.929455403	0.006546363	0.240312222	0.000130807	0.010698025	1	0.969071845	1	0.975149159	ENSG00000154803	8	FLCN
SAT	HTN	ENSG00000182134.11	0.010252302	0.057524319	0.005397909	0.240334034	0.01040243	0.053974151	0.005398115	0.240310394	-0.000150128	0.035364169	1	0.238708085	1	0.22939014	ENSG00000154803	8	FLCN
SAT	HTN	ENSG00000182150.11	0.00398885	0.224662463	0.0032851	0.240335086	0.003911511	0.233766741	0.003285015	0.240311526	7.73E-05	0.000522072	1	0.51644005	1	0.522471851	ENSG00000154803	8	FLCN
SAT	HTN	ENSG00000182240.11	-0.000607095	0.865617113	0.003587429	0.240335799	-0.001723715	0.634202975	0.003622621	0.240312114	0.00111662	0.027021556	1	0.952485811	1	0.841755648	ENSG00000154856	8	APCDD1
SAT	HTN	ENSG00000182796.8	0.010837473	0.08307772	0.006253214	0.240334332	0.011549694	0.06511646	0.006261823	0.240310548	-0.000712221	0.033023175	1	0.293726291	1	0.260227901	ENSG00000154864	7	PIEZO2
SAT	HTN	ENSG00000182871.10	0.011510396	0.000824124	0.003441504	0.240330296	0.010930603	0.001548288	0.003453047	0.240307284	0.000579793	0.041280238	1	0.011654964	1	0.018884508	ENSG00000154864	7	PIEZO2
SAT	HTN	ENSG00000182902.9	0.010766084	0.111585677	0.006766402	0.240334565	0.01063684	0.115943457	0.006766294	0.240311007	0.000129244	0.018666839	1	0.352031353	1	0.361606923	ENSG00000154864	7	PIEZO2
SAT	HTN	ENSG00000182919.10	-0.000641814	0.683130136	0.001572318	0.240335731	-0.000681851	0.664530631	0.001572292	0.240312133	4.00E-05	0.001529201	1	0.865648254	1	0.86030249	ENSG00000154889	12	MPPE1
SAT	HTN	ENSG00000182923.12	-0.004136307	0.099735145	0.002512728	0.240334477	-0.004095589	0.103108602	0.002512678	0.240310915	-4.07E-05	0.03469501	1	0.325460864	1	0.33261627	ENSG00000155016	13	CYP2U1
SAT	HTN	ENSG00000182952.4	-0.01650057	0.003432699	0.005639123	0.240331591	-0.01546564	0.006209685	0.005651592	0.240308532	-0.00103493	0.006354059	1	0.034345697	1	0.054042377	ENSG00000155085	11	AK9
SAT	HTN	ENSG00000183208.8	-0.003731058	0.565094351	0.006485507	0.24033565	-0.003874298	0.550247272	0.006485374	0.240312049	0.00014324	0.003487958	1	0.801993972	1	0.793216739	ENSG00000155085	11	AK9
SAT	HTN	ENSG00000183426.11	0.003449726	0.025881345	0.001548355	0.240333365	0.003479254	0.024634352	0.001548334	0.240309735	-2.95E-05	0.023695583	1	0.144416143	1	0.138583634	ENSG00000155085	11	AK9
SAT	HTN	ENSG00000183458.9	-0.003954619	0.45347119	0.005275349	0.240335536	-0.004061222	0.441389417	0.005275344	0.240311933	0.000106603	0.039276652	1	0.726359177	1	0.713749964	ENSG00000155085	11	AK9
SAT	HTN	ENSG00000183506.12	0.002404878	0.329301256	0.002465218	0.240335344	0.002368277	0.336701743	0.002465148	0.24031177	3.66E-05	0.020990764	1	0.62016399	1	0.620977189	ENSG00000155085	11	AK9
SAT	HTN	ENSG00000183604.10	-0.013340544	0.000964788	0.004041831	0.240330441	-0.013430969	0.000890578	0.004041806	0.24030678	9.04E-05	0.015927738	1	0.013134726	1	0.012122569	ENSG00000155115	6	GTF3C6
SAT	HTN	ENSG00000183628.8	-0.001427789	0.457689112	0.001922538	0.240335541	-0.001383373	0.471789494	0.001922497	0.24031197	-4.44E-05	0.001848641	1	0.730033046	1	0.738169447	ENSG00000155189	7	AGPAT5
SAT	HTN	ENSG00000183828.10	0.006112771	0.317721334	0.006117956	0.240335321	0.006209765	0.310092773	0.006117823	0.240311717	-9.70E-05	0.032409401	1	0.610330034	1	0.602376784	ENSG00000155393	8	HEATR3
SAT	HTN	ENSG00000184110.10	0.018018177	6.70E-07	0.003625383	0.240323632	0.017946603	7.41E-07	0.003625314	0.240300141	7.16E-05	0.010337784	0.002197402	3.43E-05	0.002431272	3.63E-05	ENSG00000155393	8	HEATR3
SAT	HTN	ENSG00000184115.12	0.003962589	0.190961453	0.0030301	0.24033497	0.004011897	0.185489286	0.003030034	0.240311361	-4.93E-05	0.028312402	1	0.47377744	1	0.466710388	ENSG00000155393	8	HEATR3
SAT	HTN	ENSG00000184371.9	-0.003868529	0.341181657	0.00406429	0.240335367	-0.006315775	0.126649989	0.004134851	0.240311075	0.002447246	0.001314449	1	0.629532484	1	0.380181715	ENSG00000155542	7	SETD9
SAT	HTN	ENSG00000184385.2	-0.009981811	0.000364449	0.002800263	0.240329547	-0.009934233	0.000388661	0.002800197	0.240306019	-4.76E-05	0.017504874	1	0.006327001	1	0.006408027	ENSG00000155542	7	SETD9
SAT	HTN	ENSG00000184402.10	-0.005696622	0.399401979	0.006760041	0.240335463	-0.00583391	0.38813128	0.00675994	0.240311858	0.000137287	0.014073747	1	0.681058492	1	0.670742545	ENSG00000155542	7	SETD9
SAT	HTN	ENSG00000184743.8	-0.011995923	0.016751603	0.00501476	0.240332991	-0.011374388	0.023425543	0.005018638	0.240309692	-0.000621535	0.002237527	1	0.109125177	1	0.134604566	ENSG00000155542	7	SETD9
SAT	HTN	ENSG00000184967.2	0.001536101	0.69717462	0.003947451	0.240335739	0.00147314	0.709003123	0.003947368	0.240312157	6.30E-05	0.032750082	1	0.873398216	1	0.880526418	ENSG00000155542	7	SETD9
SAT	HTN	ENSG00000184990.8	-0.000397035	0.94141522	0.00540248	0.240335811	-0.000505623	0.925433502	0.005402433	0.240312221	0.000108588	0.025377171	1	0.978480835	1	0.975038588	ENSG00000155542	7	SETD9
SAT	HTN	ENSG00000185298.8	0.002585399	0.391384871	0.003016417	0.240335451	0.002539823	0.399776869	0.003016343	0.240311876	4.56E-05	0.030867725	1	0.675184407	1	0.679972995	ENSG00000155542	7	SETD9
SAT	HTN	ENSG00000185404.12	-0.002873175	0.646471471	0.006264147	0.24033571	-0.002682756	0.66844955	0.006264067	0.240312135	-0.000190419	0.000357368	1	0.845722845	1	0.86204999	ENSG00000155542	7	SETD9
SAT	HTN	ENSG00000185664.10	-0.00226427	0.536646144	0.003664502	0.240335625	-0.001489507	0.685400031	0.003676836	0.240312144	-0.000774763	0.010581463	1	0.783266351	1	0.86859695	ENSG00000155592	11	ZKSCAN2
SAT	HTN	ENSG00000185684.8	-0.00326126	0.069396477	0.001796012	0.240334187	-0.00329425	0.066618177	0.00179597	0.240310566	3.30E-05	0.010111504	1	0.266928303	1	0.262480731	ENSG00000155957	12	TMBIM4
SAT	HTN	ENSG00000185697.12	-0.017735104	0.000348261	0.00495879	0.240329505	-0.014282968	0.006555713	0.005253756	0.240308581	-0.003452136	0.046779369	1	0.00614325	1	0.056160037	ENSG00000155957	12	TMBIM4
SAT	HTN	ENSG00000185739.9	0.006970309	0.074461567	0.003907626	0.240334244	0.007051959	0.071123358	0.003907567	0.240310619	-8.16E-05	0.011253359	1	0.276027247	1	0.271659766	ENSG00000155957	12	TMBIM4
SAT	HTN	ENSG00000186132.10	0.001253167	0.831241264	0.005880379	0.240335791	0.001153906	0.844427039	0.005880254	0.240312206	9.93E-05	0.023847583	1	0.938268032	1	0.944940352	ENSG00000156042	13	TTC18
SAT	HTN	ENSG00000186470.9	0.000616753	0.586640861	0.00113434	0.240335668	0.001310847	0.266349909	0.00117934	0.240311616	-0.000694095	0.031574131	1	0.813855672	1	0.55733039	ENSG00000156042	13	TTC18
SAT	HTN	ENSG00000186665.8	-0.006035265	0.364345986	0.006653246	0.240335408	-0.005873879	0.377306419	0.006653157	0.240311841	-0.000161386	0.004046684	1	0.651454594	1	0.66057124	ENSG00000156042	13	TTC18
SAT	HTN	ENSG00000186854.6	0.003335238	0.144765289	0.002287111	0.240334765	0.000433289	0.869848016	0.0026444	0.240312212	0.002901949	0.02882313	1	0.403890231	1	0.955333968	ENSG00000156096	8	UGT2B4
SAT	HTN	ENSG00000187166.1	0.004125007	0.129609317	0.002721615	0.24033468	0.004173821	0.125125171	0.002721558	0.240311066	-4.88E-05	0.017193529	1	0.382482133	1	0.376984101	ENSG00000156096	8	UGT2B4
SAT	HTN	ENSG00000187987.5	-0.003138688	0.059849873	0.001667827	0.240334067	-0.003672648	0.029115064	0.00168321	0.240309878	0.000533961	0.019003478	1	0.24515285	1	0.154824192	ENSG00000156113	16	KCNMA1
SAT	HTN	ENSG00000188321.9	-0.003631858	0.002190255	0.001185658	0.240331186	-0.003648849	0.002087143	0.00118563	0.240307555	1.70E-05	0.047887935	1	0.025126664	1	0.024027772	ENSG00000156136	5	DCK
SAT	HTN	ENSG00000188878.12	-0.011511595	4.30E-07	0.002277063	0.240323209	-0.011550304	3.93E-07	0.002277027	0.240299537	3.87E-05	0.037640718	0.00140924	2.43E-05	0.001288415	2.15E-05	ENSG00000156136	5	DCK
SAT	HTN	ENSG00000189007.11	0.003791837	0.475553322	0.005314617	0.240335562	0.003712509	0.4848261	0.005314501	0.240311985	7.93E-05	0.043373692	1	0.743349428	1	0.747867623	ENSG00000156162	12	DPY19L4
SAT	HTN	ENSG00000189045.9	-0.000189206	0.958941734	0.00367521	0.240335812	-0.000132821	0.971170332	0.003675129	0.240312225	-5.64E-05	0.036876615	1	0.985679145	1	0.990013044	ENSG00000156171	10	DRAM2
SAT	HTN	ENSG00000189143.8	-0.0058714	0.174979644	0.004328735	0.240334906	-0.005997755	0.165895674	0.004328899	0.240311279	0.000126355	0.026944393	1	0.450479504	1	0.439073216	ENSG00000156171	10	DRAM2
SAT	HTN	ENSG00000189190.7	0.003402047	0.323955561	0.003449083	0.240335334	0.003452728	0.316787057	0.003448991	0.240311731	-5.07E-05	0.028779213	1	0.616849723	1	0.604641264	ENSG00000156172	5	C8orf37
SAT	HTN	ENSG00000189306.6	-0.001079129	0.826904484	0.004934887	0.24033579	-0.000974161	0.843508302	0.00493476	0.240312206	-0.000104968	0.001848449	1	0.935512501	1	0.944556566	ENSG00000156172	5	C8orf37
SAT	HTN	ENSG00000196126.6	-0.022573475	2.55E-09	0.003788609	0.240318306	-0.031825735	8.40E-10	0.005185596	0.240293651	0.009252259	0.008976919	8.37E-06	3.32E-07	2.76E-06	1.31E-07	ENSG00000156172	5	C8orf37
SAT	HTN	ENSG00000196152.6	0.00358361	0.269425296	0.003244883	0.240335212	0.003527274	0.277014966	0.003244811	0.240311643	5.63E-05	0.017988234	1	0.563396428	1	0.570293485	ENSG00000156172	5	C8orf37
SAT	HTN	ENSG00000196535.10	-0.00266115	0.584126355	0.004861722	0.240335666	-0.002762517	0.569877309	0.004861596	0.240312066	0.000101367	0.002165698	1	0.812965812	1	0.804893436	ENSG00000156172	5	C8orf37
SAT	HTN	ENSG00000196730.8	-0.001666844	0.786720068	0.006160437	0.240335777	-0.001825369	0.766994381	0.00616036	0.240312182	0.000158525	0.002629187	1	0.917660669	1	0.908486846	ENSG00000156219	12	ART3
SAT	HTN	ENSG00000196793.9	0.005052539	0.284889245	0.004724641	0.240335249	0.004954951	0.294285234	0.004724543	0.240311683	9.76E-05	0.006000425	1	0.578774993	1	0.58518173	ENSG00000156219	12	ART3
SAT	HTN	ENSG00000197062.7	0.006863732	0.001119889	0.002106373	0.240330577	0.008927332	4.77E-05	0.002195311	0.24030407	-0.002063599	0.000855582	1	0.014936405	0.156577668	0.001223263	ENSG00000156219	12	ART3
SAT	HTN	ENSG00000197165.6	0.010999228	1.72E-05	0.002559082	0.240326703	0.010937042	1.92E-05	0.002559033	0.240303218	6.22E-05	0.001633234	0.056528272	0.000538364	0.063035388	0.000572975	ENSG00000156219	12	ART3
SAT	HTN	ENSG00000197279.3	0.014406169	0.0048163	0.005110232	0.240331894	0.017284393	0.000894136	0.005203166	0.240306783	-0.002878224	0.00332746	1	0.044140448	1	0.012122569	ENSG00000156219	12	ART3
SAT	HTN	ENSG00000197302.6	-0.002673948	0.595755339	0.005040289	0.240335675	-0.002532493	0.615353285	0.005040318	0.240312101	-0.000141455	0.00727466	1	0.820946353	1	0.834690078	ENSG00000156232	6	WHAMM
SAT	HTN	ENSG00000197345.8	0.001512789	0.293076241	0.001438835	0.240335268	0.001535715	0.285811051	0.001438801	0.240311664	-2.29E-05	0.02594745	1	0.58529001	1	0.577147307	ENSG00000156253	2	RWDD2B
SAT	HTN	ENSG00000197629.5	0.003954048	0.275995058	0.003629693	0.240335228	0.004360355	0.230349024	0.00363526	0.240311516	-0.000406307	0.046735318	1	0.571318478	1	0.519406797	ENSG00000156253	2	RWDD2B
SAT	HTN	ENSG00000197728.5	-0.006615008	3.81E-06	0.00143139	0.240325281	-0.006573244	4.39E-06	0.001431432	0.240301826	-4.18E-05	0.019720192	0.012509804	0.000157075	0.014402644	0.000167473	ENSG00000156261	8	CCT8
SAT	HTN	ENSG00000197847.8	-0.006498948	0.294009833	0.006193209	0.24033527	-0.007887975	0.203525627	0.006203323	0.240311428	0.001389027	0.000111218	1	0.586767799	1	0.490644807	ENSG00000156411	5	C14orf2
SAT	HTN	ENSG00000197903.6	-0.003925886	0.158126203	0.002781547	0.240334831	-0.005284	0.062408052	0.002835692	0.240310513	0.001358114	0.013915771	1	0.427709869	1	0.251548918	ENSG00000156411	5	C14orf2
SAT	HTN	ENSG00000197935.6	0.003190965	0.4556291	0.004277066	0.240335539	0.003998582	0.350517592	0.004283032	0.240311796	-0.000807617	0.000444739	1	0.728518069	1	0.635605707	ENSG00000156413	9	FUT6
SAT	HTN	ENSG00000197958.8	-0.002209279	0.287519094	0.002077208	0.240335255	-0.002173154	0.295463298	0.002077162	0.240311686	-3.61E-05	0.017658918	1	0.581565115	1	0.586298316	ENSG00000156414	14	TDRD9
SAT	HTN	ENSG00000197969.7	3.50E-05	0.991681433	0.003355749	0.240335813	9.20E-05	0.97813641	0.003355689	0.240312225	-5.70E-05	0.031659625	1	0.99730223	1	0.991752513	ENSG00000156414	14	TDRD9
SAT	HTN	ENSG00000198026.6	0.008741565	0.045391362	0.004368566	0.240333839	0.008805087	0.043842046	0.004368463	0.240310222	-6.35E-05	0.041577649	1	0.202074707	1	0.19786211	ENSG00000156463	13	SH3RF2
SAT	HTN	ENSG00000198131.9	0.00392762	0.283834615	0.003664693	0.240335247	0.003863963	0.291701573	0.003664618	0.240311677	6.37E-05	0.021826591	1	0.578137942	1	0.582047821	ENSG00000156521	9	TYSND1
SAT	HTN	ENSG00000198315.6	0.01337501	0.028825993	0.006118865	0.240333457	0.012934802	0.034574993	0.006120645	0.240310023	0.000440208	0.005770502	1	0.15453935	1	0.173191684	ENSG00000156603	10	MED19
SAT	HTN	ENSG00000198515.9	-0.005393714	0.303558226	0.005242551	0.240335291	-0.005819062	0.26733183	0.005246041	0.240311619	0.000425348	0.031931503	1	0.598182906	1	0.559028511	ENSG00000156603	10	MED19
SAT	HTN	ENSG00000198874.8	0.00066005	0.820497282	0.002908918	0.240335788	0.000706887	0.807995123	0.002908841	0.240312196	-4.68E-05	0.016470004	1	0.932182301	1	0.925531291	ENSG00000156603	10	MED19
SAT	HTN	ENSG00000198920.5	0.001634316	0.770090265	0.005592051	0.240335771	0.001725101	0.757703357	0.005591931	0.240312178	-9.08E-05	0.028657182	1	0.908545905	1	0.904156036	ENSG00000156603	10	MED19
SAT	HTN	ENSG00000198945.3	0.008947716	0.082681535	0.00515618	0.240334328	0.009031324	0.079843453	0.005156046	0.240310712	-8.36E-05	0.017507172	1	0.293273641	1	0.285677612	ENSG00000156711	12	MAPK13
SAT	HTN	ENSG00000203668.1	0.003445203	0.347780078	0.003669385	0.240335379	0.003506772	0.339224187	0.003669322	0.240311775	-6.16E-05	0.036340888	1	0.63533766	1	0.624576071	ENSG00000156711	12	MAPK13
SAT	HTN	ENSG00000204060.4	0.008478151	0.179801478	0.006320524	0.240334926	0.008565544	0.175343852	0.006320347	0.24031132	-8.74E-05	0.033312784	1	0.457488848	1	0.452759918	ENSG00000156711	12	MAPK13
SAT	HTN	ENSG00000204237.4	0.013911042	0.032487658	0.006505458	0.240333558	0.013774435	0.03422475	0.006505293	0.240310014	0.000136607	0.00220894	1	0.1664956	1	0.172226081	ENSG00000156795	2	WDYHV1
SAT	HTN	ENSG00000204305.9	0.005824058	0.211334668	0.004659598	0.240335043	0.000464853	0.930567143	0.005335086	0.240312222	0.005359205	0.039184111	1	0.502455829	1	0.975149159	ENSG00000156795	2	WDYHV1
SAT	HTN	ENSG00000204316.8	0.017293243	0.001516718	0.005452729	0.240330853	0.017387823	0.001428342	0.005452643	0.240307211	-9.46E-05	0.03346605	1	0.018699145	1	0.017618005	ENSG00000156860	11	FBRS
SAT	HTN	ENSG00000204338.4	0.014085648	1.69E-09	0.002337808	0.24031791	0.020866772	8.98E-15	0.002691407	0.240282585	-0.006781124	3.70E-07	5.55E-06	2.47E-07	2.95E-11	5.90E-12	ENSG00000156886	11	ITGAD
SAT	HTN	ENSG00000204348.5	-0.000486975	0.881404835	0.003264156	0.240335802	0.001153819	0.728386037	0.003322508	0.240312166	-0.001640794	0.008216629	1	0.957468	1	0.889755957	ENSG00000156886	11	ITGAD
SAT	HTN	ENSG00000204356.7	0.005662249	0.021949776	0.002471256	0.240333224	0.004348718	0.084083831	0.002517391	0.240310754	0.001313531	0.006245134	1	0.129527365	1	0.296007563	ENSG00000156931	11	VPS8
SAT	HTN	ENSG00000204516.5	-0.003590575	0.012541504	0.001438221	0.240332739	-0.004195103	0.004120911	0.001462336	0.240308167	0.000604528	0.022470465	1	0.089025167	1	0.039725318	ENSG00000156931	11	VPS8
SAT	HTN	ENSG00000204525.10	-0.006677261	0.001587437	0.002114246	0.240330894	-0.008179527	0.000228991	0.002219859	0.24030553	0.001502266	0.026466432	1	0.019362006	0.751320646	0.004297476	ENSG00000156968	8	MPV17L
SAT	HTN	ENSG00000204531.11	-0.003428321	0.02096531	0.001485011	0.240333185	-0.004663311	0.002803926	0.001560435	0.240307821	0.00123499	0.010008865	1	0.12461446	1	0.029676388	ENSG00000156968	8	MPV17L
SAT	HTN	ENSG00000204536.9	-0.004139406	0.002223461	0.001353342	0.240331199	-0.005751687	8.04E-05	0.001458584	0.240304557	0.001612281	0.003048923	1	0.025330473	0.263683982	0.001918893	ENSG00000157045	4	NTAN1
SAT	HTN	ENSG00000204538.3	0.004931397	0.000518198	0.001420664	0.240329871	0.00381193	0.009628859	0.001472418	0.24030892	0.001119467	0.003835826	1	0.008213566	1	0.075041061	ENSG00000157045	4	NTAN1
SAT	HTN	ENSG00000204540.6	0.002988309	0.006278938	0.001093473	0.24033213	0.002513065	0.023278166	0.00110764	0.240309687	0.000475244	0.00722137	1	0.054031376	1	0.134059059	ENSG00000157045	4	NTAN1
SAT	HTN	ENSG00000204574.8	-0.002556129	0.514223251	0.003918782	0.240335604	-0.005735899	0.15612423	0.004044407	0.240311233	0.00317977	0.001489555	1	0.765198628	1	0.425451494	ENSG00000157107	9	FCHO2
SAT	HTN	ENSG00000204592.5	-0.003313971	0.41290837	0.004047415	0.240335483	-0.006692269	0.118250142	0.004283975	0.240311022	0.003378298	0.016155327	1	0.693677605	1	0.366889987	ENSG00000157107	9	FCHO2
SAT	HTN	ENSG00000204625.6	-0.005423039	0.079498626	0.003092507	0.240334297	-0.006396764	0.03943187	0.003105743	0.240310133	0.000973725	0.000725258	1	0.285290525	1	0.18508722	ENSG00000157150	4	TIMP4
SAT	HTN	ENSG00000204642.9	0.006245832	0.062537776	0.003353525	0.240334103	0.007242234	0.032169469	0.003380581	0.240309962	-0.000996402	0.019958994	1	0.251314587	1	0.165401029	ENSG00000157150	4	TIMP4
SAT	HTN	ENSG00000204644.5	-0.002164738	0.079301765	0.001233641	0.240334295	-0.00353013	0.007382663	0.001317664	0.240308686	0.001365392	0.003199364	1	0.285290525	1	0.061950171	ENSG00000157150	4	TIMP4
SAT	HTN	ENSG00000204709.4	-0.004115208	0.066504252	0.002242603	0.240334153	-0.005083944	0.024563186	0.002261329	0.240309733	0.000968737	0.000882072	1	0.26155018	1	0.138473904	ENSG00000157214	9	STEAP2
SAT	HTN	ENSG00000204778.4	0.003361238	0.617369831	0.006728104	0.24033569	0.00322686	0.631497816	0.006727951	0.240312112	0.000134378	0.00587947	1	0.833285913	1	0.840204515	ENSG00000157214	9	STEAP2
SAT	HTN	ENSG00000205269.4	0.006800668	0.045758461	0.003404386	0.240333845	0.006887228	0.043065998	0.003404337	0.240310207	-8.66E-05	0.002346016	1	0.203157659	1	0.195435048	ENSG00000157214	9	STEAP2
SAT	HTN	ENSG00000205485.9	-0.004987029	0.031066302	0.002312848	0.24033352	-0.005024783	0.029811305	0.002312802	0.240309898	3.78E-05	0.033275051	1	0.16204855	1	0.156999826	ENSG00000157214	9	STEAP2
SAT	HTN	ENSG00000205571.8	-0.006764557	0.008306592	0.002562956	0.240332378	-0.006728469	0.008656533	0.002562895	0.240308826	-3.61E-05	0.047548693	1	0.068649697	1	0.069955871	ENSG00000157214	9	STEAP2
SAT	HTN	ENSG00000205643.6	-0.00025982	0.955747357	0.004682208	0.240335812	-0.000370873	0.936867598	0.0046823	0.240312222	0.000111053	0.043051949	1	0.983048498	1	0.978139775	ENSG00000157214	9	STEAP2
SAT	HTN	ENSG00000205771.2	-0.005755518	0.299700915	0.005549755	0.240335283	-0.005854305	0.291478234	0.005549707	0.240311677	9.88E-05	0.047584261	1	0.59311359	1	0.582047821	ENSG00000157214	9	STEAP2
SAT	HTN	ENSG00000206341.6	-0.000779793	0.784578653	0.002852649	0.240335777	-0.001966449	0.493421688	0.002871243	0.240311994	0.001186656	0.000290296	1	0.916412446	1	0.753334834	ENSG00000157214	9	STEAP2
SAT	HTN	ENSG00000206344.6	0.002279651	0.124359195	0.001483439	0.240334649	0.00148092	0.327303594	0.001511821	0.240311752	0.000798731	0.006217556	1	0.374127295	1	0.615105129	ENSG00000157322	12	CLEC18A
SAT	HTN	ENSG00000206503.7	0.002731237	0.30918606	0.00268576	0.240335303	0.004110303	0.133926602	0.002742399	0.240311118	-0.001379066	0.01298375	1	0.602619487	1	0.387527756	ENSG00000157322	12	CLEC18A
SAT	HTN	ENSG00000212694.4	-0.004417235	0.156257143	0.003115611	0.240334822	-0.004371914	0.160539165	0.003115535	0.240311254	-4.53E-05	0.038985245	1	0.425460321	1	0.432099261	ENSG00000157445	10	CACNA2D3
SAT	HTN	ENSG00000213090.2	0.007836285	0.11644039	0.004991601	0.240334598	0.007748828	0.120569219	0.004991543	0.240311037	8.75E-05	0.042463927	1	0.362335772	1	0.369887656	ENSG00000157540	15	DYRK1A
SAT	HTN	ENSG00000213398.3	-0.002817568	0.401163232	0.00335606	0.240335466	-0.002893362	0.388618552	0.003356078	0.240311859	7.58E-05	0.030233882	1	0.681924009	1	0.670742545	ENSG00000157540	15	DYRK1A
SAT	HTN	ENSG00000213402.2	0.000270573	0.929177366	0.003044252	0.240335809	-0.000385371	0.899378249	0.003047694	0.240312218	0.000655944	9.22E-06	1	0.976204842	1	0.965911631	ENSG00000157601	9	MX1
SAT	HTN	ENSG00000213443.2	-0.001176106	0.585380511	0.002155846	0.240335667	-0.001227719	0.569023083	0.002155817	0.240312065	5.16E-05	0.004780984	1	0.813238553	1	0.804544406	ENSG00000157601	9	MX1
SAT	HTN	ENSG00000213445.4	0.029362665	2.63E-09	0.004932121	0.240318335	0.027571394	2.75E-08	0.004961769	0.240296999	0.001791271	0.000983486	8.63E-06	3.32E-07	9.02E-05	2.65E-06	ENSG00000157653	7	C9orf43
SAT	HTN	ENSG00000213462.4	0.005146118	0.084025224	0.002978427	0.240334341	0.005188932	0.081471222	0.002978351	0.240310729	-4.28E-05	0.036289286	1	0.294571399	1	0.289293376	ENSG00000157833	8	GAREML
SAT	HTN	ENSG00000213719.4	0.023083505	0.000427939	0.006553625	0.240329695	0.04350156	3.54E-08	0.007891823	0.240297242	-0.020418055	3.43E-06	1	0.007020346	0.000116308	2.92E-06	ENSG00000157833	8	GAREML
SAT	HTN	ENSG00000214078.7	-0.001371438	0.263060354	0.001225386	0.240335196	-0.00134436	0.272590526	0.001225357	0.240311632	-2.71E-05	0.001701067	1	0.555406063	1	0.565119463	ENSG00000157837	11	SPPL3
SAT	HTN	ENSG00000214087.4	0.002035825	0.069769537	0.001122651	0.240334192	0.002063362	0.06606818	0.001122634	0.240310559	-2.75E-05	0.002776776	1	0.267422724	1	0.262433049	ENSG00000157837	11	SPPL3
SAT	HTN	ENSG00000214226.4	-0.003028335	0.528912194	0.004809416	0.240335618	-0.003097818	0.519491524	0.004809307	0.240312021	6.95E-05	0.046718587	1	0.778537869	1	0.769056242	ENSG00000157870	10	FAM213B
SAT	HTN	ENSG00000214376.5	-0.002512173	0.566166627	0.004378847	0.240335651	-0.002389299	0.585303936	0.004378779	0.240312079	-0.000122874	0.000610856	1	0.802415854	1	0.814247906	ENSG00000157870	10	FAM213B
SAT	HTN	ENSG00000214517.4	-0.009127995	0.00274761	0.003048086	0.24033139	-0.009065405	0.002938107	0.00304806	0.240307863	-6.26E-05	0.02229834	1	0.029080345	1	0.030700413	ENSG00000157895	7	C12orf43
SAT	HTN	ENSG00000214659.4	0.00069658	0.916320114	0.006629659	0.240335808	0.003098055	0.642581486	0.006675508	0.240312119	-0.002401475	0.002185217	1	0.969508641	1	0.845691879	ENSG00000157895	7	C12orf43
SAT	HTN	ENSG00000214872.4	-0.001988673	0.253242752	0.001740618	0.24033517	-0.001922287	0.269496949	0.00174085	0.240311624	-6.64E-05	0.045844309	1	0.54654986	1	0.560697202	ENSG00000157911	5	PEX10
SAT	HTN	ENSG00000215908.5	0.006744047	0.132810923	0.004486724	0.240334699	0.006856076	0.126489086	0.004486676	0.240311074	-0.000112029	0.004347113	1	0.385638593	1	0.380046421	ENSG00000157911	5	PEX10
SAT	HTN	ENSG00000216901.1	-0.004849954	0.00601754	0.001765651	0.240332092	-0.005755527	0.001304625	0.001790222	0.240307128	0.000905573	0.002228425	1	0.05264946	1	0.016590986	ENSG00000157911	5	PEX10
SAT	HTN	ENSG00000219392.1	0.004702185	0.008422006	0.001784729	0.24033239	0.006301656	0.000737134	0.001866934	0.240306607	-0.001599472	0.003526739	1	0.069215759	1	0.010515378	ENSG00000157978	7	LDLRAP1
SAT	HTN	ENSG00000219481.6	-0.000822749	0.844957771	0.004207232	0.240335795	-0.000737042	0.860933133	0.004207182	0.24031221	-8.57E-05	0.018293391	1	0.944567784	1	0.952725149	ENSG00000157978	7	LDLRAP1
SAT	HTN	ENSG00000219891.2	-0.007145537	0.047063557	0.003598438	0.240333869	-0.006670515	0.064236131	0.003604629	0.240310537	-0.000475021	0.026523176	1	0.205887374	1	0.257651278	ENSG00000157992	8	KRTCAP3
SAT	HTN	ENSG00000221829.5	-0.002851968	0.39958476	0.003385672	0.240335463	-0.002902198	0.391323812	0.003385593	0.240311863	5.02E-05	0.03848866	1	0.681058492	1	0.671162273	ENSG00000158006	9	PAFAH2
SAT	HTN	ENSG00000221947.3	-0.001099288	0.820855511	0.004854548	0.240335788	-0.001175721	0.808629769	0.004854457	0.240312196	7.64E-05	0.042976447	1	0.932182301	1	0.925531291	ENSG00000158006	9	PAFAH2
SAT	HTN	ENSG00000224389.4	-0.005420599	0.085379586	0.003150972	0.240334354	-0.010576109	0.003886077	0.003663003	0.240308114	0.005155511	0.005786158	1	0.296748329	1	0.038072244	ENSG00000158022	6	TRIM63
SAT	HTN	ENSG00000224497.1	0.002963755	0.03449891	0.001401835	0.240333609	0.002920192	0.037238646	0.00140182	0.240310085	4.36E-05	0.000370714	1	0.172091642	1	0.179701644	ENSG00000158023	5	WDR66
SAT	HTN	ENSG00000224930.2	0.000587105	0.909804523	0.005182539	0.240335807	0.000476897	0.926680886	0.005182446	0.240312221	0.000110208	0.006992322	1	0.968235044	1	0.975149159	ENSG00000158104	7	HPD

SAT	HTN	ENSG00000225880.4	-0.005521485	0.272344872	0.005030141	0.240335219	-0.005366502	0.286023018	0.005030053	0.240311664	-0.000154983	9.97E-05	1	0.567410704	1	0.577147307	ENSG00000158158	7	CNNM4
SAT	HTN	ENSG00000226015.2	-0.003494188	0.403562492	0.004183271	0.240335469	-0.003390832	0.417606712	0.004183212	0.240311901	-0.000103356	0.00309129	1	0.683930028	1	0.692294225	ENSG00000158158	7	CNNM4
SAT	HTN	ENSG00000226479.3	-0.001762975	0.744605682	0.005411874	0.240335761	-0.001883128	0.727863943	0.005411777	0.240312166	0.000120153	0.004958318	1	0.896532567	1	0.889755957	ENSG00000158161	11	EYA3
SAT	HTN	ENSG00000227124.4	0.003076085	0.006198272	0.001123842	0.240332119	0.003060231	0.006467988	0.001123815	0.240308569	1.59E-05	0.045307749	1	0.053800349	1	0.055699396	ENSG00000158161	11	EYA3
SAT	HTN	ENSG00000228078.1	-0.000842481	0.711555738	0.002278406	0.240335746	-0.002120793	0.361289106	0.002323107	0.240311814	0.001278313	0.004877598	1	0.880322163	1	0.645706614	ENSG00000158161	11	EYA3
SAT	HTN	ENSG00000228789.2	-0.003992493	0.028777999	0.001825957	0.240333455	-0.003355961	0.068875893	0.001844708	0.240310593	-0.000636532	0.015492186	1	0.154534558	1	0.266192155	ENSG00000158161	11	EYA3
SAT	HTN	ENSG00000229390.1	-0.008107921	0.038024334	0.003908189	0.240333691	-0.008990035	0.021816553	0.003919696	0.240309631	0.000882114	0.003562649	1	0.179766342	1	0.128741203	ENSG00000158161	11	EYA3
SAT	HTN	ENSG00000229759.1	-0.01403672	1.24E-11	0.002071658	0.240313173	-0.014069908	1.11E-11	0.002071616	0.240289479	3.32E-05	0.035426547	4.07E-08	4.99E-09	3.64E-08	4.04E-09	ENSG00000158186	8	MRAS
SAT	HTN	ENSG00000230092.3	-0.002590474	0.615507875	0.005157972	0.240335689	-0.002507601	0.626846469	0.005157863	0.240312109	-8.29E-05	0.031353605	1	0.832284161	1	0.837966728	ENSG00000158186	8	MRAS
SAT	HTN	ENSG00000230224.1	-0.012114377	0.040057657	0.005900359	0.240333734	-0.012208655	0.038528839	0.005900211	0.240310114	9.43E-05	0.021485351	1	0.186954726	1	0.182677922	ENSG00000158201	5	ABHD3
SAT	HTN	ENSG00000230267.3	0.006437259	0.222976601	0.005282275	0.240335081	0.007696184	0.14641832	0.005299301	0.240311185	-0.001258925	0.003245619	1	0.515564644	1	0.40780858	ENSG00000158201	5	ABHD3
SAT	HTN	ENSG00000230387.1	0.007278392	0.052512685	0.003753854	0.240333959	0.007353141	0.050129881	0.003753788	0.240310333	-7.47E-05	0.012092717	1	0.223758598	1	0.218427807	ENSG00000158201	5	ABHD3
SAT	HTN	ENSG00000230521.1	0.002160921	0.222057877	0.001769686	0.240335078	0.002307092	0.192525264	0.0017704	0.240311388	-0.000146171	0.00606597	1	0.515379933	1	0.474949011	ENSG00000158201	5	ABHD3
SAT	HTN	ENSG00000230596.2	0.007155254	0.018768697	0.003044658	0.240333089	0.007210643	0.017868645	0.003044602	0.240309459	-5.54E-05	0.020502392	1	0.115418861	1	0.112744278	ENSG00000158411	6	MITD1
SAT	HTN	ENSG00000231130.1	0.005439027	0.018163934	0.00230246	0.240333061	0.006676282	0.005039457	0.002380549	0.240308347	-0.001237255	0.040907822	1	0.113515937	1	0.046258571	ENSG00000158411	6	MITD1
SAT	HTN	ENSG00000231784.4	0.003934965	0.409944591	0.004775491	0.240335479	0.003859902	0.418920169	0.004775352	0.240311903	7.51E-05	0.013210676	1	0.690214399	1	0.692780784	ENSG00000158411	6	MITD1
SAT	HTN	ENSG00000231793.4	0.004714631	0.484880429	0.006749891	0.240335573	0.00221503	0.743704287	0.006774786	0.240312173	0.002499601	1.87E-05	1	0.748376275	1	0.896382412	ENSG00000158411	6	MITD1
SAT	HTN	ENSG00000231925.7	0.004579998	0.13511748	0.003065133	0.240334712	0.005397338	0.079178316	0.00307457	0.240310706	-0.000817341	0.000756361	1	0.387179433	1	0.285677612	ENSG00000158457	4	TSPAN33
SAT	HTN	ENSG00000232629.4	0.000754433	0.611399184	0.001484878	0.240335686	-0.00322223	0.124388886	0.00209697	0.240311061	0.003976663	0.007240861	1	0.830870282	1	0.375110233	ENSG00000158457	4	TSPAN33
SAT	HTN	ENSG00000233265.1	-0.001996705	0.299720309	0.001925399	0.240335283	-0.002357116	0.222301539	0.001931377	0.240311491	0.000360411	0.018532547	1	0.59311359	1	0.513280331	ENSG00000158552	8	ZFAND2B
SAT	HTN	ENSG00000233754.1	-0.005535627	0.361468386	0.006065965	0.240335403	-0.005439513	0.369853781	0.00606582	0.240311829	-9.61E-05	0.025815955	1	0.650299517	1	0.653687263	ENSG00000158552	8	ZFAND2B
SAT	HTN	ENSG00000233916.1	-0.001274115	0.680149365	0.003090564	0.24033573	-0.001974823	0.524544359	0.003103329	0.240312026	0.000700709	0.013237124	1	0.864026219	1	0.773149166	ENSG00000158552	8	ZFAND2B
SAT	HTN	ENSG00000234127.4	0.002650244	0.479178761	0.003745269	0.240335566	0.001565287	0.679107292	0.003783795	0.240312141	0.001084957	0.044455946	1	0.746582289	1	0.865678672	ENSG00000158623	10	COPG2
SAT	HTN	ENSG00000234147.1	-0.002422902	0.49882704	0.003582389	0.240335588	-0.00253941	0.478407209	0.003582349	0.240311978	0.000116508	0.000191788	1	0.759114805	1	0.743207411	ENSG00000158623	10	COPG2
SAT	HTN	ENSG00000234745.5	0.008323166	0.002460521	0.002748579	0.240331291	0.007018203	0.011869186	0.002789407	0.240309104	0.001304963	0.006141287	1	0.027273547	1	0.085400878	ENSG00000158717	6	RNF166
SAT	HTN	ENSG00000235109.3	-0.001449521	0.542625223	0.002380759	0.240335631	-0.002393477	0.32133982	0.002413486	0.24031174	0.000943955	0.017368335	1	0.788531091	1	0.610136545	ENSG00000158769	13	F11R
SAT	HTN	ENSG00000235290.1	-0.001953954	0.506747511	0.002943096	0.240335596	-0.002186937	0.457760108	0.002945208	0.240311954	0.000232983	0.043271253	1	0.763819444	1	0.729437064	ENSG00000158793	9	NIT1
SAT	HTN	ENSG00000235477.2	-0.000239099	0.932903556	0.002839915	0.24033581	-0.000187413	0.947382612	0.002839855	0.240312223	-5.17E-05	0.014679116	1	0.976204842	1	0.981538823	ENSG00000158793	9	NIT1
SAT	HTN	ENSG00000235677.1	0.00236603	0.590181434	0.004393143	0.24033567	0.002300757	0.600467399	0.004393031	0.24031209	6.53E-05	0.030876861	1	0.816351301	1	0.825747189	ENSG00000158793	9	NIT1
SAT	HTN	ENSG00000235821.1	-0.001218568	0.595324195	0.002294263	0.240335674	-0.000122265	0.95867994	0.002359866	0.240312224	-0.001096302	0.047437403	1	0.820696926	1	0.98710697	ENSG00000158793	9	NIT1
SAT	HTN	ENSG00000235963.1	0.004976981	0.178268486	0.003697313	0.24033492	0.00679545	0.073102614	0.003791695	0.240310641	-0.001818469	0.030706214	1	0.455174244	1	0.274427548	ENSG00000158805	7	ZNF276
SAT	HTN	ENSG00000237491.4	-0.005642965	0.222944658	0.004630175	0.240335081	-0.005532936	0.232088699	0.004630083	0.240311521	-0.000110029	0.001857568	1	0.515564644	1	0.519577173	ENSG00000158805	7	ZNF276
SAT	HTN	ENSG00000237510.3	0.004002437	0.143039125	0.002732842	0.240334755	0.004055807	0.137774739	0.002732782	0.240311139	-5.34E-05	0.008009935	1	0.400778284	1	0.392735811	ENSG00000158825	5	CDA
SAT	HTN	ENSG00000237541.3	0.009977545	3.80E-05	0.002422011	0.240327444	0.018590358	3.04E-07	0.003630397	0.240299294	-0.008612813	0.001449444	0.124582329	0.001029606	0.00099886	1.72E-05	ENSG00000158825	5	CDA
SAT	HTN	ENSG00000237669.1	-0.001677163	0.256337072	0.001477556	0.240335178	-0.002650451	0.086158886	0.001544533	0.240310773	0.000973288	0.030606516	1	0.54929286	1	0.299456891	ENSG00000158825	5	CDA
SAT	HTN	ENSG00000237753.1	0.006275009	0.022571888	0.002751459	0.240333248	0.006224391	0.023682333	0.002751415	0.240309702	5.06E-05	0.023003063	1	0.130898125	1	0.135133452	ENSG00000158850	10	B4GALT3
SAT	HTN	ENSG00000237927.1	0.013133775	0.04140651	0.006439888	0.240333762	0.01323697	0.03983027	0.006439748	0.240310142	-0.000103194	0.030682581	1	0.191175896	1	0.185366123	ENSG00000158850	10	B4GALT3
SAT	HTN	ENSG00000238024.1	-0.019703982	0.002549568	0.006530095	0.240331323	-0.024601528	0.000234573	0.006687802	0.240305552	0.004897546	0.000703005	1	0.027883779	0.769635591	0.004323795	ENSG00000158864	8	NDUFS2
SAT	HTN	ENSG00000238083.3	0.008582762	0.125360913	0.005599922	0.240334655	0.00868645	0.120853266	0.005599809	0.240311039	-0.000103688	0.014968621	1	0.3756248	1	0.369887656	ENSG00000158869	6	FCER1G
SAT	HTN	ENSG00000239257.1	-0.000359579	0.791742931	0.001361795	0.240335779	-0.001309623	0.352014838	0.001407154	0.240311798	0.000950044	0.007389526	1	0.918363088	1	0.636570061	ENSG00000158869	6	FCER1G
SAT	HTN	ENSG00000239557.1	0.027192557	0.000104662	0.007009199	0.240328391	0.027326064	9.67E-05	0.007009026	0.24030473	-0.000133507	0.006673134	0.343397295	0.002368257	0.317354766	0.002234893	ENSG00000158869	6	FCER1G
SAT	HTN	ENSG00000240065.3	-0.002184171	0.532665686	0.003500597	0.240335621	-0.00452317	0.211629688	0.003621148	0.240311456	0.002338999	0.011650786	1	0.78125888	1	0.500979081	ENSG00000159055	3	MIS18A
SAT	HTN	ENSG00000240399.1	-0.024920525	8.07E-05	0.006321471	0.240328149	-0.025047249	7.43E-05	0.006321447	0.240304483	0.000126724	0.035519106	0.264933018	0.001905993	0.243622707	0.001791343	ENSG00000159055	3	MIS18A
SAT	HTN	ENSG00000241549.4	-0.000863668	0.638742473	0.001839713	0.240335705	-0.000761203	0.679124601	0.001840174	0.240312141	-0.000102465	0.022883036	1	0.84165223	1	0.865678672	ENSG00000159055	3	MIS18A
SAT	HTN	ENSG00000241837.2	-0.012339235	0.046050236	0.006185273	0.240333851	-0.012239999	0.047824284	0.006185156	0.240310294	-9.92E-05	0.038230578	1	0.203626447	1	0.21061943	ENSG00000159055	3	MIS18A
SAT	HTN	ENSG00000242110.3	-0.000699841	0.686203097	0.001732223	0.240335733	-0.000668506	0.699547781	0.001732189	0.240312152	-3.13E-05	0.017620172	1	0.866935834	1	0.876659689	ENSG00000159110	15	IFNAR2
SAT	HTN	ENSG00000242435.1	0.000307121	0.946069224	0.00454027	0.240335811	0.000229251	0.959729117	0.004540201	0.240312224	7.79E-05	0.036711859	1	0.980743483	1	0.98710697	ENSG00000159111	8	MRPL10
SAT	HTN	ENSG00000242779.2	0.005644768	0.330340305	0.005798846	0.240335346	0.005752159	0.321215948	0.005798762	0.24031174	-0.000107391	0.026184929	1	0.62016399	1	0.610136545	ENSG00000159111	8	MRPL10
SAT	HTN	ENSG00000243819.3	0.006562627	0.322758802	0.006636924	0.240335331	0.006660716	0.31556717	0.006636758	0.240311729	-9.81E-05	0.032927904	1	0.61568118	1	0.604641264	ENSG00000159128	10	IFNGR2
SAT	HTN	ENSG00000244301.1	-0.003282583	0.496721794	0.004829761	0.240335586	-0.003352257	0.487617621	0.004829622	0.240311988	6.97E-05	0.023110814	1	0.757316081	1	0.75005786	ENSG00000159128	10	IFNGR2
SAT	HTN	ENSG00000244607.1	-0.005101392	0.11899525	0.003272205	0.240334615	-0.005029457	0.124280199	0.003272136	0.24031106	-7.19E-05	0.003236465	1	0.366076286	1	0.375110233	ENSG00000159128	10	IFNGR2
SAT	HTN	ENSG00000244731.3	0.000189488	0.892256133	0.001398952	0.240335804	0.005429881	0.006975728	0.002012549	0.240308636	-0.005240393	0.000292541	1	0.959833565	1	0.05914047	ENSG00000159173	14	TNNI1
SAT	HTN	ENSG00000246523.3	-0.002555417	0.561549388	0.004401777	0.240335647	-0.002432618	0.580501444	0.004401711	0.240312075	-0.000122799	0.000730864	1	0.799324746	1	0.811169181	ENSG00000159228	8	CBR1
SAT	HTN	ENSG00000246575.2	0.001558965	0.76886822	0.005305203	0.240335771	0.001318693	0.803713582	0.005305669	0.240312195	0.000240272	0.006185482	1	0.907788321	1	0.923833778	ENSG00000159231	5	CBR3
SAT	HTN	ENSG00000248540.2	-0.002599757	0.482218798	0.003699463	0.24033557	-0.002548486	0.490887832	0.003699367	0.240311991	-5.13E-05	0.040994357	1	0.747733474	1	0.75109625	ENSG00000159259	7	CHAF1B
SAT	HTN	ENSG00000248994.1	0.001707872	0.722783906	0.004814421	0.240335751	0.00162158	0.736250554	0.00481434	0.240312169	8.63E-05	0.025558546	1	0.885985829	1	0.893357273	ENSG00000159263	11	SIM2
SAT	HTN	ENSG00000250599.1	-0.000768616	0.874112346	0.004851236	0.240335801	-0.001240404	0.798311822	0.00485419	0.240312193	0.000471788	0.007354069	1	0.956418386	1	0.922924978	ENSG00000159267	10	HLCS
SAT	HTN	ENSG00000253326.2	0.007916776	0.2182045	0.006429513	0.240335066	0.007807838	0.224596602	0.006429393	0.240311498	0.000108938	0.029639078	1	0.509681988	1	0.516432023	ENSG00000159267	10	HLCS
SAT	HTN	ENSG00000253813.1	0.00670309	0.133695282	0.00446965	0.240334704	0.006792711	0.128569664	0.004469568	0.240311086	-8.96E-05	0.010592386	1	0.386164703	1	0.382792259	ENSG00000159445	8	THEM4
SAT	HTN	ENSG00000254152.1	0.003783112	0.539078306	0.006159336	0.240335627	0.003899869	0.526620948	0.006159246	0.240312028	-0.000116756	0.022370341	1	0.786082216	1	0.775861396	ENSG00000159445	8	THEM4
SAT	HTN	ENSG00000254614.1	-0.000626947	0.828542083	0.002894866	0.24033579	0.000447542	0.877656526	0.002907235	0.240312214	-0.001074488	6.66E-05	1	0.935875598	1	0.956781838	ENSG00000159479	12	MED8
SAT	HTN	ENSG00000254681.2	-0.012234964	0.046578105	0.006147848	0.24033386	-0.012127942	0.048524854	0.006147741	0.240310306	-0.000107022	0.028527946	1	0.205131225	1	0.212847655	ENSG00000159579	9	RSPRY1
SAT	HTN	ENSG00000254986.3	0.00048128	0.861217603	0.002752935	0.240335798	-0.000297997	0.913978082	0.002758661	0.24031222	0.000779277	1.45E-05	1	0.952246908	1	0.972372648	ENSG00000159579	9	RSPRY1
SAT	HTN	ENSG00000255147.1	0.009897276	0.08597394	0.005764177	0.240334359	0.009422248	0.102207427	0.005765495	0.240310908	0.000475028	0.000468243	1	0.298497879	1	0.330732247	ENSG00000159579	9	RSPRY1
SAT	HTN	ENSG00000255200.1	-0.001602726	0.467211237	0.002204501	0.240335553	-0.000753126	0.733834481	0.002214884	0.240312168	-0.000849601	7.95E-05	1	0.738333035	1	0.892066631	ENSG00000159588	10	CCDC17
SAT	HTN	ENSG00000255298.2	-0.009302845	0.042957804	0.004595983	0.240333793	-0.009366394	0.041549869	0.004595862	0.240310177	6.35E-05	0.040509429	1	0.194632687	1	0.192216555	ENSG00000159588	10	CCDC17
SAT	HTN	ENSG00000256436.1	-0.005557434	0.228890473	0.004618789	0.240335099	-0.005485528	0.234960607	0.004618697	0.24031153	-7.19E-05	0.041774051	1	0.520170844	1	0.524037868	ENSG00000159588	10	CCDC17
SAT	HTN	ENSG00000257431.1	-0.008053	0.08729884	0.004709838	0.240334372	-0.008248508	0.07993503	0.004710562	0.240310713	0.000195508	0.039294169	1	0.300939215	1	0.28569372	ENSG00000159588	10	CCDC17
SAT	HTN	ENSG00000258100.1	0.009267912	0.075512426	0.005214475	0.240334255	0.00915376	0.079177791	0.00521439	0.240310706	0.000114152	0.006762228	1	0.277442632	1	0.285677612	ENSG00000159720	7	ATP6V0D1
SAT	HTN	ENSG00000259056.1	0.006829683	0.207078229	0.005413329	0.240335028	0.002346387	0.681082465	0.005709155	0.240312142	0.004483296	0.013490498	1	0.497746278	1	0.865857127	ENSG00000159733	9	ZFYVE28
SAT	HTN	ENSG00000260082.1	0.007010052	0.274201681	0.006411014	0.240335224	0.007401907	0.248368918	0.006412344	0.240311568	-0.000391855	0.006984958	1	0.569402351	1	0.537886095	ENSG00000159733	9	ZFYVE28
SAT	HTN	ENSG00000260176.1	-0.012482545	0.033785494	0.005880702	0.240333591	-0.012351898	0.035690143	0.005880608	0.24031005	-0.000130647	0.006210233	1	0.17053878	1	0.17529844	ENSG00000159761	10	C16orf86
SAT	HTN	ENSG00000260257.1	-0.001628289	0.78987768	0.006110598	0.240335778	-0.001517386	0.803884619	0.006110535	0.240312195	-0.000110902	0.039233597	1	0.918290338	1	0.923833778	ENSG00000159904	7	ZNF890P
SAT	HTN	ENSG00000260274.1	2.20E-05	0.992111392	0.002221164	0.240335813	-2.75E-05	0.990131063	0.002221157	0.240312225	4.94E-05	0.020499234	1	0.99730223	1	0.996509208	ENSG00000159904	7	ZNF890P
SAT	HTN	ENSG00000260302.1	-0.000173414	0.97189989	0.004922958	0.240335813	-0.000101016	0.98362874	0.004922843	0.240312225	-7.24E-05	0.040230453	1	0.988749111	1	0.994404109	ENSG00000159904	7	ZNF890P
SAT	HTN	ENSG00000260388.1	-0.002785889	0.466015027	0.003821633	0.240335551	-0.002730648	0.474891967	0.003821531	0.240311974	-5.52E-05	0.031122756	1	0.737932097	1	0.74149608	ENSG00000159904	7	ZNF890P
SAT	HTN	ENSG00000260518.1	-0.002587604	0.399491951	0.003071232	0.240335463	-0.002009653	0.51383727	0.00307816	0.240312015	-0.000577951	0.005599596	1	0.681058492	1	0.76520719	ENSG00000159917	10	ZNF235
SAT	HTN	ENSG00000260539.1	0.003708001	0.29649383	0.003551784	0.240335276	0.003767809	0.28875978	0.003551697	0.24031167	-5.98E-05	0.016843728	1	0.59028899	1	0.579178516	ENSG00000160049	7	DFFA
SAT	HTN	ENSG00000260787.1	0.005893124	0.301195139	0.005700035	0.240335286	0.005753698	0.31276576	0.00569991	0.240311723	0.000139426	0.000874918	1	0.595672845	1	0.603663737	ENSG00000160049	7	DFFA
SAT	HTN	ENSG00000260877.1	-0.021329137	1.43E-06	0.004424036	0.24032435	-0.021502211	1.17E-06	0.004424075	0.240300576	0.000173074	0.000289536	0.004683446	6.60E-05	0.003847323	5.42E-05	ENSG00000160049	7	DFFA
SAT	HTN	ENSG00000260911.1	0.009465266	0.006311998	0.003465697	0.240332135	0.009549892	0.00586003	0.003465726	0.240308481	-8.46E-05	0.022832943	1	0.05407223	1	0.051964215	ENSG00000160049	7	DFFA
SAT	HTN	ENSG00000261143.1	0.004803437	0.220179253	0.003917804	0.240335072	0.004877399	0.213149	0.003917732	0.240311461	-7.40E-05	0.015841141	1	0.513073955	1	0.503485868	ENSG00000160072	15	ATAD3B
SAT	HTN	ENSG00000261584.1	-0.002454343	0.233655203	0.002060746	0.240335114	-0.002079122	0.313908045	0.002064553	0.240311725	-0.00037522	0.003124367	1	0.524724656	1	0.603711779	ENSG00000160072	15	ATAD3B
SAT	HTN	ENSG00000262370.1	0.003043721	0.247788532	0.002633572	0.240335155	0.002982789	0.257370864	0.00263351	0.240311593	6.09E-05	0.001238047	1	0.539579113	1	0.548573946	ENSG00000160072	15	ATAD3B
SAT	HTN	ENSG00000262663.1	0.002748242	0.590447299	0.005106469	0.240335671	0.00285258	0.576412986	0.005106365	0.240312072	-0.000104338	0.00701969	1	0.816374879	1	0.808901201	ENSG00000160072	15	ATAD3B
SAT	HTN	ENSG00000264538.2	0.008718424	6.75E-05	0.002187881	0.240327982	0.008750856	6.34E-05	0.00218783	0.240304336	-3.24E-05	0.038592064	0.221555214	0.001629082	0.208043723	0.001564239	ENSG00000160172	6	FAM86C2P
SAT	HTN	ENSG00000267651.1	0.007907418	0.105835289	0.004889567	0.240334524	0.007980777	0.102628203	0.004889443	0.240310912	-7.34E-05	0.029813748	1	0.338116439	1	0.331746931	ENSG00000160179	14	ABCG1
SAT	HTN	ENSG00000268040.1	0.002317095	0.578874291	0.004174718	0.240335661	0.002423794	0.561527448	0.004174819	0.240312059	-0.000106699	0.034605919	1	0.81131968	1	0.801717772	ENSG00000160179	14	ABCG1
SAT	HTN	ENSG00000268340.1	0.00865063	0.122437106	0.005600464	0.240334637	0.008440828	0.131765518	0.005600416	0.240311105	0.000209802	3.05E-05	1	0.372076709	1	0.386347332	ENSG00000160179	14	ABCG1
SAT	HTN	ENSG00000269921.1	0.001787377	0.692375289	0.004517754	0.240335736	0.001693451	0.707772651	0.004517685	0.240312156	9.39E-05	0.01138122	1	0.869846415	1	0.880273494	ENSG00000160179	14	ABCG1
SAT	HTN	ENSG00000269990.1	0.017764284	0.002812303	0.005946089	0.240331411	0.015147871	0.011277879	0.005977901	0.240309059	0.002616413	2.34E-05	1	0.029669341	1	0.083456744	ENSG00000160194	13	NDUFV3
SAT	HTN	ENSG00000270547.1	0.012630248	0.060773888	0.006735625	0.240334079	0.012533503	0.062769724	0.006735461	0.240310518	9.67E-05	0.040879505	1	0.247326914	1	0.252696275	ENSG00000160194	13	NDUFV3
SAT	HTN	ENSG00000270775.1	0.000287264	0.887856598	0.002037087	0.240335804	8.37E-05	0.967256736	0.002037822	0.240312225	0.000203613	0.000483675	1	0.958390933	1	0.989225546	ENSG00000160194	13	NDUFV3
SAT	HTN	ENSG00000271581.1	0.009753943	1.62E-07	0.001861975	0.24032228	0.01143513	4.68E-09	0.001951999	0.240295302	-0.001681187	0.00413752	0.000531387	1.06E-05	1.54E-05	5.12E-07	ENSG00000160194	13	NDUFV3
SAT	HTN	ENSG00000271623.1	-0.023785837	6.81E-06	0.005286248	0.240325828	-0.023676658	7.50E-06	0.005286133	0.240302332	-0.000109178	0.005029115	0.022345974	0.000251078	0.024609466	0.000261923	ENSG00000160194	13	NDUFV3
SAT	HTN	ENSG00000272010.1	0.003730155	0.175911693	0.002756029	0.24033491	0.003518198	0.201861573	0.002756631	0.240311422	0.000211956	0.000879404	1	0.451616796	1	0.488427597	ENSG00000160200	13	CBS
SAT	HTN	ENSG00000272047.1	-0.001519162	0.74584533	0.004686996	0.240335762	-0.001606093	0.731841618	0.004686901	0.240312168	8.69E-05	0.014506975	1	0.89736653	1	0.891634737	ENSG00000160200	13	CBS
SAT	HTN	ENSG00000272221.1	-0.002164845	0.072259974	0.001204378	0.24033422	-0.001752659	0.148825126	0.001214008	0.240311198	-0.000412186	0.007084583	1	0.272198594	1	0.412411518	ENSG00000160200	13	CBS
SAT	HTN	ENSG00000272236.1	-0.000703628	0.772722753	0.002436255	0.240335772	0.000433514	0.862297899	0.002499364	0.240312211	-0.001137143	0.041790449	1	0.90968904	1	0.952725149	ENSG00000160201	7	U2AF1
SAT	HTN	ENSG00000272462.2	0.001058233	0.332947513	0.001092996	0.240335351	0.000872334	0.425962102	0.00109573	0.240311913	0.000185899	0.017312787	1	0.620682267	1	0.699140398	ENSG00000160201	7	U2AF1
SAT	HTN	ENSG00000272541.1	0.005616097	0.138787294	0.003793828	0.240334733	0.005691857	0.133530043	0.003793741	0.240311115	-7.58E-05	0.005708418	1	0.391877031	1	0.387527756	ENSG00000160201	7	U2AF1
SAT	HTN	ENSG00000272849.1	-0.005382102	0.159900996	0.003829569	0.240334839	-0.005327049	0.164206583	0.003829478	0.240311271	-5.51E-05	0.042750861	1	0.430734949	1	0.436688665	ENSG00000160209	14	PDXK
SAT	HTN	ENSG00000273340.1	0.001449574	0.330986626	0.001491133	0.240335347	0.00190655	0.205060079	0.001504457	0.240311433	-0.000456976	0.022545551	1	0.62016399	1	0.493618576	ENSG00000160209	14	PDXK
SAT	Obesity	ENSG00000001460.13	0.004697743	0.257426386	0.004148131	0.183453989	0.004359361	0.293523485	0.004150094	0.183420853	0.000338382	0.015116507	1	0.550238419	1	0.59812588	ENSG00000160213	5	CSTB
SAT	Obesity	ENSG00000001461.12	0.008336993	0.129229894	0.005495173	0.183453604	0.007930819	0.149124234	0.005497458	0.183420484	0.000406174	0.020154919	1	0.37959112	1	0.414396534	ENSG00000160221	12	C21orf33
SAT	Obesity	ENSG00000001561.6	0.00130929	0.607878062	0.002551699	0.183454375	0.00127098	0.618395829	0.002551526	0.183421176	3.83E-05	0.02599928	1	0.823471479	1	0.828495448	ENSG00000160221	12	C21orf33
SAT	Obesity	ENSG00000004809.9	-0.014566224	0.011529505	0.005765978	0.183452062	-0.014439096	0.012269998	0.00576574	0.1834189	-0.000127128	0.025969505	1	0.085007432	1	0.094213678	ENSG00000160223	12	ICOSLG
SAT	Obesity	ENSG00000005156.7	0.000654453	0.741108822	0.001980878	0.183454433	0.000696385	0.725159277	0.001980775	0.183421223	-4.19E-05	0.015622728	1	0.894949594	1	0.889105974	ENSG00000160223	12	ICOSLG
SAT	Obesity	ENSG00000005189.15	0.002661207	0.483786135	0.003800494	0.183454289	0.002721818	0.473858258	0.003800275	0.183421076	-6.06E-05	0.049358916	1	0.744513279	1	0.742320719	ENSG00000160226	11	C21orf2
SAT	Obesity	ENSG00000005243.5	0.01049366	0.004979989	0.003736608	0.183451493	0.010393316	0.005408519	0.003736383	0.183418346	0.000100344	0.000540925	1	0.049363573	1	0.055454223	ENSG00000160226	11	C21orf2
SAT	Obesity	ENSG00000005955.8	-0.025436996	5.70E-11	0.003882597	0.183438251	-0.025349744	6.61E-11	0.003882366	0.183405159	-8.73E-05	0.004121427	1.87E-07	5.20E-09	2.17E-07	6.57E-09	ENSG00000160226	11	C21orf2
SAT	Obesity	ENSG00000006715.11	-0.001181687	0.728721313	0.003407136	0.183454429	-0.001129487	0.740247203	0.003406932	0.183421229	-5.22E-05	0.049652889	1	0.888823282	1	0.897054301	ENSG00000160229	7	ZNF66
SAT	Obesity	ENSG00000008324.6	-0.003139919	0.585492692	0.005757308	0.183454362	-0.0030587	0.595204759	0.00575691	0.183421164	-8.12E-05	0.031036368	1	0.80802013	1	0.817137678	ENSG00000160229	7	ZNF66
SAT	Obesity	ENSG00000008441.12	-0.008986622	0.113765504	0.005682358	0.183453529	-0.009090177	0.109640068	0.005682014	0.183420303	0.000103555	0.018672947	1	0.350483211	1	0.347900449	ENSG00000160229	7	ZNF66
SAT	Obesity	ENSG00000008853.12	-0.006206776	0.206831625	0.004916931	0.183453872	-0.006083183	0.215996611	0.004916715	0.183420692	-0.000123593	0.009214537	1	0.493814259	1	0.508175311	ENSG00000160233	6	LRRC3
SAT	Obesity	ENSG00000010292.8	0.000621668	0.746414558	0.001922462	0.183454435	0.000688399	0.720291531	0.001922528	0.183421222	-6.67E-05	0.02827519	1	0.89640782	1	0.885797449	ENSG00000160256	8	FAM207A
SAT	Obesity	ENSG00000010704.14	-0.018755012	3.11E-09	0.003164967	0.183441201	-0.016780294	1.51E-07	0.003195462	0.183410849	-0.001974718	8.02E-06	1.02E-05	2.22E-07	0.000495766	8.70E-06	ENSG00000160282	9	FTCD
SAT	Obesity	ENSG00000010803.12	0.007969577	0.155269606	0.00560778	0.183453711	0.007864009	0.160792165	0.005607479	0.183420527	0.000105568	0.028244923	1	0.417573423	1	0.428934236	ENSG00000160284	10	SPATC1L
SAT	Obesity	ENSG00000011523.9	0.001425657	0.552660862	0.002400993	0.183454341	0.001478056	0.538130639	0.002400834	0.183421127	-5.24E-05	0.001624676	1	0.793558114	1	0.786461749	ENSG00000160285	10	LSS
SAT	Obesity	ENSG00000013810.14	-0.001204153	0.839248369	0.005936026	0.183454459	-0.001107051	0.852046422	0.005935675	0.183421257	-9.71E-05	0.038782505	1	0.940107169	1	0.94476658	ENSG00000160285	10	LSS
SAT	Obesity	ENSG00000016864.12	-0.005189273	0.236132923	0.004380207	0.183453944	-0.005284476	0.227618621	0.004379941	0.18342072	9.52E-05	0.004973449	1	0.525379039	1	0.522317542	ENSG00000160285	10	LSS
SAT	Obesity	ENSG00000018699.7	-0.001209971	0.763277071	0.00401743	0.18345444	-0.001274531	0.751037531	0.00401716	0.183421232	6.46E-05	0.018550253	1	0.904538582	1	0.901114525	ENSG00000160299	12	PCNT
SAT	Obesity	ENSG00000020922.8	-0.002494183	0.015193314	0.001027366	0.183452246	-0.0024749	0.015990869	0.001027299	0.183419077	-1.93E-05	0.008946622	1	0.102994344	1	0.112106928	ENSG00000160299	12	PCNT
SAT	Obesity	ENSG00000023041.7	0.004473692	0.367921546	0.004968702	0.183454168	0.004554025	0.359351547	0.004968377	0.183420953	-8.03E-05	0.022213273	1	0.664730502	1	0.660152534	ENSG00000160307	5	S100B
SAT	Obesity	ENSG00000025800.9	0.009860906	0.074546529	0.005529744	0.183453272	0.009749107	0.07787684	0.005529388	0.183420095	0.000111798	0.005165775	1	0.274343367	1	0.287417225	ENSG00000160321	10	ZNF208
SAT	Obesity	ENSG00000030110.8	0.005724676	0.041239296	0.002804672	0.183452899	0.004221479	0.134269426	0.002819058	0.183420423	0.001503197	1.61E-07	1	0.192979684	1	0.392603841	ENSG00000160321	10	ZNF208
SAT	Obesity	ENSG00000032742.13	0.002698844	0.565721913	0.004698841	0.183454349	0.002788113	0.552914382	0.004698547	0.183421137	-8.93E-05	0.010911606	1	0.797234594	1	0.793924694	ENSG00000160321	10	ZNF208
SAT	Obesity	ENSG00000033178.8	-0.008149944	0.01675209	0.003407007	0.183452311	-0.008223077	0.015791569	0.003406826	0.183419068	7.31E-05	0.013249172	1	0.10970782	1	0.111772978	ENSG00000160321	10	ZNF208
SAT	Obesity	ENSG00000034533.7	-0.005373112	0.058658788	0.002841794	0.183453123	-0.005319809	0.061192955	0.002841622	0.183419946	-5.33E-05	0.015106772	1	0.24059281	1	0.250029993	ENSG00000160321	10	ZNF208
SAT	Obesity	ENSG00000035115.17	-0.013765809	1.53E-05	0.003183298	0.183447406	-0.01384339	1.37E-05	0.003183101	0.183414122	7.76E-05	0.001317823	0.050195442	0.000418295	0.044874705	0.000439948	ENSG00000160321	10	ZNF208
SAT	Obesity	ENSG00000037757.9	0.002266411	0.55690386	0.00385807	0.183454344	0.002190793	0.570114877	0.003857827	0.183421148	7.56E-05	0.008138809	1	0.794232732	1	0.801777501	ENSG00000160323	14	ADAMTS13
SAT	Obesity	ENSG00000038219.8	0.002050599	0.526700345	0.00323923	0.183454323	0.002005957	0.535710681	0.003239005	0.183421125	4.46E-05	0.033975179	1	0.778775949	1	0.78622818	ENSG00000160323	14	ADAMTS13
SAT	Obesity	ENSG00000040199.14	0.001102984	0.843104727	0.00557277	0.183454459	0.001180397	0.832240346	0.005572399	0.183421253	-7.74E-05	0.04455791	1	0.941066406	1	0.936326075	ENSG00000160339	11	FCN2
SAT	Obesity	ENSG00000040531.10	-0.000930003	0.639872215	0.001987705	0.183454391	-0.00089368	0.652974281	0.001987571	0.183421194	-3.63E-05	0.007444062	1	0.842174268	1	0.849151255	ENSG00000160339	11	FCN2
SAT	Obesity	ENSG00000041353.5	0.001680481	0.774710539	0.005871312	0.183454443	-0.007590912	0.273259663	0.00692862	0.183420817	0.009271393	0.011747354	1	0.909817544	1	0.576940124	ENSG00000160439	11	RDH13
SAT	Obesity	ENSG00000041802.6	0.003223347	0.002720929	0.001075295	0.183451078	0.003202284	0.002899481	0.001075236	0.183417918	2.11E-05	0.020514544	1	0.030997801	1	0.035691396	ENSG00000160695	10	VPS11
SAT	Obesity	ENSG00000042429.6	0.005029471	0.347117149	0.005349381	0.18345414	0.004948468	0.354908779	0.005349055	0.183420947	8.10E-05	0.048249256	1	0.644898849	1	0.656771407	ENSG00000160695	10	VPS11

SAT	Obesity	ENSG00000043514.11	0.000584358	0.80547717	0.002372881	0.183454451	0.000547895	0.81738205	0.002372728	0.18342125	3.65E-05	0.033531092	1	0.924045663	1	0.930870102	ENSG00000160752	10	FDPS
SAT	Obesity	ENSG00000044090.4	-0.005375265	0.190803523	0.004108878	0.183453827	-0.005307031	0.196463981	0.004108584	0.18342064	-6.82E-05	0.007211556	1	0.467532754	1	0.48140278	ENSG00000160752	10	FDPS
SAT	Obesity	ENSG00000048140.13	0.00753509	0.013654681	0.003055306	0.183452175	0.007621112	0.012612244	0.003055112	0.183418918	-8.60E-05	0.000133526	1	0.096553901	1	0.096011077	ENSG00000160766	10	GBAP1
SAT	Obesity	ENSG00000048544.5	-0.001688723	0.140847933	0.001146729	0.183453654	-0.001717591	0.134156933	0.001146657	0.183420422	2.89E-05	0.000748728	1	0.396214067	1	0.392603841	ENSG00000160766	10	GBAP1
SAT	Obesity	ENSG00000050130.13	0.01029324	0.004466849	0.003620334	0.183451419	0.010533917	0.003623772	0.003620904	0.183418072	-0.000240677	0.002865163	1	0.045656481	1	0.042161685	ENSG00000160766	10	GBAP1
SAT	Obesity	ENSG00000050393.7	-0.000161468	0.973930535	0.004941034	0.183454474	-0.000295004	0.952387969	0.004940755	0.183421269	0.000133536	0.001066626	1	0.993452012	1	0.987557383	ENSG00000160783	15	PMF1
SAT	Obesity	ENSG00000051382.4	-0.010315334	0.081167781	0.005914913	0.183453324	-0.010161838	0.0857756	0.005914514	0.183420155	-0.000153496	0.000133796	1	0.287792178	1	0.300351913	ENSG00000160783	15	PMF1
SAT	Obesity	ENSG00000054938.11	-0.004214786	0.486052539	0.006050498	0.183454291	-0.004333515	0.473823052	0.006050087	0.183421076	0.000118729	0.003520714	1	0.746950062	1	0.742320719	ENSG00000160828	13	STAG3L2
SAT	Obesity	ENSG00000057608.12	-0.003953537	0.136285295	0.002653789	0.183453635	-0.003904854	0.141151715	0.002653623	0.183420452	-4.87E-05	0.013896427	1	0.389540014	1	0.401725489	ENSG00000160828	13	STAG3L2
SAT	Obesity	ENSG00000058085.10	-0.000129936	0.942856327	0.001812713	0.183454472	-0.000181206	0.920369634	0.001812633	0.183421266	5.13E-05	0.003386069	1	0.981335116	1	0.974203713	ENSG00000160828	13	STAG3L2
SAT	Obesity	ENSG00000058453.12	0.003256158	0.054626144	0.001694289	0.183453078	0.003227529	0.056771481	0.001694178	0.183419899	2.86E-05	0.016494871	1	0.229192299	1	0.239958924	ENSG00000160856	16	FCRL3
SAT	Obesity	ENSG00000063046.13	0.004971341	0.05762742	0.002618527	0.183453112	0.004918031	0.060344302	0.002618378	0.183419937	5.33E-05	0.012858439	1	0.237234083	1	0.24841864	ENSG00000160856	16	FCRL3
SAT	Obesity	ENSG00000064607.12	-0.006290747	0.185287513	0.004748984	0.183453811	-0.006192174	0.192244565	0.00474871	0.183420628	-9.86E-05	0.010399574	1	0.460203128	1	0.475323599	ENSG00000160856	16	FCRL3
SAT	Obesity	ENSG00000064995.12	-0.008781332	0.002221421	0.002870721	0.183450937	-0.008993872	0.001731465	0.002870856	0.183417561	0.000212539	8.18E-06	1	0.026030297	1	0.022999738	ENSG00000160886	9	LY6K
SAT	Obesity	ENSG00000065060.12	0.017974582	4.15E-23	0.001815486	0.183417429	0.017877663	7.04E-23	0.001815392	0.183384625	9.69E-05	8.09E-10	1.36E-19	1.36E-20	2.31E-19	2.31E-20	ENSG00000160917	10	CPSF4
SAT	Obesity	ENSG00000066379.10	-0.007240234	0.020716082	0.003130063	0.183452452	-0.003814689	0.232847341	0.003197405	0.183420732	-0.003425545	1.63E-07	1	0.127045729	1	0.525427871	ENSG00000160932	6	LY6E
SAT	Obesity	ENSG00000069493.10	-0.002926992	0.356727223	0.00317592	0.183454153	-0.003000454	0.344771469	0.003175822	0.183420933	7.35E-05	0.034123421	1	0.654232542	1	0.648774721	ENSG00000160932	6	LY6E
SAT	Obesity	ENSG00000070501.7	-0.005124899	0.292688294	0.004870449	0.183454056	-0.005014205	0.303213812	0.004870208	0.18342087	-0.000110693	0.012049829	1	0.592053202	1	0.60366779	ENSG00000160948	9	VPS28
SAT	Obesity	ENSG00000072958.4	-0.002303308	0.433397283	0.002940172	0.183454242	-0.00225563	0.442947454	0.002939981	0.183421048	-4.77E-05	0.023127011	1	0.714561047	1	0.722357979	ENSG00000160948	9	VPS28
SAT	Obesity	ENSG00000073578.12	0.008653131	0.022541473	0.003793361	0.183452507	0.008724229	0.021448654	0.003793143	0.183419271	-7.11E-05	0.02159153	1	0.133740638	1	0.134299683	ENSG00000160948	9	VPS28
SAT	Obesity	ENSG00000075643.5	0.00426925	0.290031455	0.004035003	0.183454051	0.004207293	0.297055925	0.004034733	0.183420859	6.20E-05	0.02519226	1	0.588792914	1	0.601026164	ENSG00000160948	9	VPS28
SAT	Obesity	ENSG00000076344.11	-0.002084639	0.679417463	0.005044395	0.18345441	-0.001998264	0.691984758	0.005044041	0.183421211	-8.64E-05	0.007089299	1	0.858646871	1	0.868937924	ENSG00000160953	10	MUM1
SAT	Obesity	ENSG00000076554.11	-0.003880111	0.498716804	0.005735476	0.183454301	-0.004062743	0.478703519	0.00573518	0.183421081	0.000182632	0.000303682	1	0.75749331	1	0.743208721	ENSG00000161010	10	C5orf45
SAT	Obesity	ENSG00000076685.14	0.027520054	3.05E-06	0.005896393	0.18344624	0.027327284	3.57E-06	0.005896086	0.183413152	0.00019277	0.000177994	0.010017246	0.00011008	0.011724467	0.000160609	ENSG00000161021	7	MAML1
SAT	Obesity	ENSG00000077616.6	0.006230368	0.310199767	0.006139479	0.183454085	0.00635	0.300971042	0.006139095	0.183420866	-0.000119632	0.009183529	1	0.607417315	1	0.601026164	ENSG00000161021	7	MAML1
SAT	Obesity	ENSG00000079150.13	-0.001900161	0.461575717	0.002580845	0.183454269	-0.001970048	0.445234036	0.002580674	0.18342105	6.99E-05	9.63E-05	1	0.731904575	1	0.724809903	ENSG00000161021	7	MAML1
SAT	Obesity	ENSG00000079257.3	-0.004103865	0.477343472	0.005775355	0.183454283	-0.00420611	0.466410932	0.005774992	0.18342107	0.000102244	0.017211379	1	0.739803464	1	0.737265369	ENSG00000161036	6	LRWD1
SAT	Obesity	ENSG00000079950.9	-0.001114761	0.803547539	0.004481295	0.183454451	-0.000981953	0.826549372	0.004481158	0.183421252	-0.000132808	0.006725095	1	0.922904531	1	0.935786229	ENSG00000161036	6	LRWD1
SAT	Obesity	ENSG00000080815.14	-0.000665144	0.849533985	0.003506048	0.183454461	-0.000761068	0.828141785	0.003505842	0.183421252	9.59E-05	0.000579002	1	0.94293674	1	0.936326075	ENSG00000161036	6	LRWD1
SAT	Obesity	ENSG00000082516.8	-0.004728431	0.307789147	0.004636294	0.183454081	-0.004600503	0.32103103	0.004636004	0.183420898	-0.000127928	0.00022402	1	0.606520234	1	0.6232561	ENSG00000161036	6	LRWD1
SAT	Obesity	ENSG00000083097.10	0.005210585	0.004069354	0.001813798	0.183451355	0.005179793	0.00429089	0.001813679	0.183418188	3.08E-05	0.016284193	1	0.042520858	1	0.047531447	ENSG00000161180	6	CCDC116
SAT	Obesity	ENSG00000083937.4	0.001393233	0.704766369	0.0036771	0.18345442	0.00131444	0.720727568	0.00367689	0.183421222	7.88E-05	0.008963767	1	0.874485947	1	0.885797449	ENSG00000161180	6	CCDC116
SAT	Obesity	ENSG00000084092.6	-0.006167398	0.110724772	0.003866836	0.183453513	-0.005785448	0.134812952	0.003868852	0.183420425	-0.00038195	0.004738515	1	0.343697234	1	0.39281238	ENSG00000161202	13	DVL3
SAT	Obesity	ENSG00000085117.7	0.009650409	0.036558572	0.004615951	0.183452822	0.009584407	0.03784809	0.004615651	0.183419641	6.60E-05	0.044996789	1	0.1809418	1	0.189433997	ENSG00000161204	7	ABCF3
SAT	Obesity	ENSG00000085365.13	0.017678615	0.000206583	0.004764027	0.183449269	0.017474778	0.000244244	0.004763794	0.183416185	0.000203837	2.45E-06	0.677799119	0.003786587	0.80136467	0.00471391	ENSG00000161217	7	PCYT1A
SAT	Obesity	ENSG00000085644.9	-0.009596373	0.026314773	0.004319691	0.183452609	-0.009523696	0.027463747	0.004319393	0.183419433	-7.27E-05	0.010006983	1	0.146997736	1	0.155047129	ENSG00000161243	4	FBXO27
SAT	Obesity	ENSG00000087301.4	-0.001712141	0.737437562	0.005107076	0.183454432	-0.001875839	0.71338062	0.005106826	0.183421219	0.000163697	0.000439161	1	0.892158054	1	0.882946041	ENSG00000161243	4	FBXO27
SAT	Obesity	ENSG00000088205.8	0.005848349	0.311721724	0.005781189	0.183454087	0.005996527	0.299596808	0.005780902	0.183420864	-0.000148178	0.004591228	1	0.608768261	1	0.601026164	ENSG00000161243	4	FBXO27
SAT	Obesity	ENSG00000088325.11	-0.004352818	0.177606724	0.003228704	0.183453787	-0.004282141	0.184726144	0.003228527	0.183420605	-7.07E-05	0.009642076	1	0.451027601	1	0.462308526	ENSG00000161243	4	FBXO27
SAT	Obesity	ENSG00000089916.13	-0.001850587	0.646835149	0.004039141	0.183454395	-0.001955815	0.628213674	0.004038912	0.183421182	0.000105228	0.001580753	1	0.844125734	1	0.833806256	ENSG00000161243	4	FBXO27
SAT	Obesity	ENSG00000090432.5	-0.00101829	0.843139807	0.005146026	0.183454459	-0.001093155	0.83176206	0.005145657	0.183421253	7.49E-05	0.017858272	1	0.941066406	1	0.936326075	ENSG00000161249	16	DMKN
SAT	Obesity	ENSG00000091542.8	0.006949071	0.028559622	0.003173789	0.183452662	0.006993878	0.027539791	0.003173573	0.183419435	-4.48E-05	0.034699205	1	0.154014794	1	0.155047129	ENSG00000161249	16	DMKN
SAT	Obesity	ENSG00000092295.7	0.002157476	0.636000535	0.004558384	0.183454389	0.002342059	0.607375933	0.004558104	0.18342117	-0.000184583	1.02E-07	1	0.840744539	1	0.822933669	ENSG00000161249	16	DMKN
SAT	Obesity	ENSG00000093000.14	-0.00077829	0.781517757	0.00280625	0.183454445	-0.000878685	0.754177817	0.002806084	0.183421233	0.000100394	6.35E-06	1	0.913071213	1	0.901441682	ENSG00000161281	6	COX7A1
SAT	Obesity	ENSG00000095485.12	-0.001150472	0.477051109	0.001617981	0.183454283	-0.001196148	0.459706309	0.001617878	0.183421064	4.57E-05	0.0001195	1	0.739803464	1	0.732714066	ENSG00000161328	10	LRRC56
SAT	Obesity	ENSG00000095787.17	0.006271329	0.246476992	0.005411214	0.183453966	0.006137621	0.256679874	0.005411053	0.183420784	0.000133708	0.024853559	1	0.534848552	1	0.55551891	ENSG00000161542	12	PRPSAP1
SAT	Obesity	ENSG00000097046.8	-0.002926775	0.32448049	0.002970461	0.183454107	-0.002859693	0.335664292	0.002970282	0.18342092	-6.71E-05	0.003628206	1	0.622949379	1	0.638443212	ENSG00000161542	12	PRPSAP1
SAT	Obesity	ENSG00000099956.13	0.007618727	0.001399586	0.002384761	0.183450616	0.00767829	0.001282303	0.002384614	0.183417352	-5.96E-05	0.001046471	1	0.018078898	1	0.018134645	ENSG00000161551	8	ZNF577
SAT	Obesity	ENSG00000099974.7	-0.003269452	0.227199967	0.002707386	0.183453923	-0.00331673	0.220520541	0.00270721	0.183420703	4.73E-05	0.014771354	1	0.514945339	1	0.512776681	ENSG00000161609	5	CCDC155
SAT	Obesity	ENSG00000100014.15	-0.001991969	0.50650129	0.002998617	0.183454307	-0.001922279	0.52146022	0.002998423	0.183421115	-6.97E-05	0.001234051	1	0.763007683	1	0.775353375	ENSG00000161609	5	CCDC155
SAT	Obesity	ENSG00000100024.10	-0.00432434	0.177544326	0.003207118	0.183453787	-0.004255334	0.184538797	0.003206948	0.183420605	-6.90E-05	0.012581077	1	0.451027601	1	0.462308526	ENSG00000161618	5	ALDH16A1
SAT	Obesity	ENSG00000100033.12	0.001657064	0.770544773	0.005681442	0.183454442	0.001769058	0.755501214	0.005681091	0.183421234	-0.000111994	0.009478954	1	0.90745061	1	0.90236603	ENSG00000161640	11	SIGLEC11
SAT	Obesity	ENSG00000100129.13	0.00285951	0.520159122	0.004446443	0.183454318	0.002929762	0.509933887	0.004446167	0.183421106	-7.03E-05	0.036034922	1	0.772585821	1	0.767826106	ENSG00000161664	2	ASB16
SAT	Obesity	ENSG00000100196.6	-0.003398098	0.331843635	0.003501721	0.183454118	-0.003516268	0.315282182	0.003501557	0.183420889	0.00011817	0.000313109	1	0.630089167	1	0.615584597	ENSG00000161664	2	ASB16
SAT	Obesity	ENSG00000100197.16	-0.006332564	0.125960004	0.004138304	0.183453589	-0.006274563	0.129437615	0.004138004	0.183420401	-5.80E-05	0.01948704	1	0.374273307	1	0.383362075	ENSG00000161692	13	DBF4B
SAT	Obesity	ENSG00000100201.14	-0.000294294	0.947469108	0.004466765	0.183454472	-0.000383665	0.931547029	0.004466488	0.183421267	8.94E-05	0.008163865	1	0.983182517	1	0.979468354	ENSG00000161692	13	DBF4B
SAT	Obesity	ENSG00000100211.6	-0.002520378	0.133472304	0.001679634	0.183453623	-0.002489899	0.138206158	0.001679519	0.18342044	-3.05E-05	0.00623008	1	0.386967743	1	0.398466084	ENSG00000161813	16	LARP4
SAT	Obesity	ENSG00000100225.13	0.013028985	0.000605855	0.003799643	0.18345003	0.013094019	0.00056832	0.003799418	0.183416781	-6.50E-05	0.030395222	1	0.009245634	1	0.009661437	ENSG00000161813	16	LARP4
SAT	Obesity	ENSG00000100300.13	0.008676641	0.033147066	0.00407296	0.183452759	0.008537038	0.036067858	0.004072689	0.18341961	0.000139603	6.73E-07	1	0.168954102	1	0.184615666	ENSG00000161888	7	SPC24
SAT	Obesity	ENSG00000100302.6	-0.012313883	0.017646533	0.005189249	0.183452346	-0.012226143	0.018462611	0.005188891	0.183419172	-8.77E-05	0.009792174	1	0.113720642	1	0.123121596	ENSG00000161888	7	SPC24
SAT	Obesity	ENSG00000100336.13	0.005080731	0.105387923	0.003137651	0.183453483	0.004998411	0.111127443	0.003137441	0.183420311	8.23E-05	0.00013872	1	0.333987547	1	0.350248935	ENSG00000161896	6	IP6K3
SAT	Obesity	ENSG00000100413.12	-0.004955565	0.108365063	0.003086449	0.1834535	-0.004997438	0.105390648	0.003086236	0.183420279	4.19E-05	0.039661044	1	0.337971267	1	0.340341256	ENSG00000161896	6	IP6K3
SAT	Obesity	ENSG00000100429.13	0.000702231	0.87701023	0.004537536	0.183454465	0.000626828	0.890120567	0.004537246	0.183421263	7.54E-05	0.022877315	1	0.956287991	1	0.962898024	ENSG00000161896	6	IP6K3
SAT	Obesity	ENSG00000100445.12	-0.001034493	0.634711758	0.002177396	0.183454389	-0.001078965	0.620205009	0.002177264	0.183421177	4.45E-05	0.008393502	1	0.840052149	1	0.830073312	ENSG00000161896	6	IP6K3
SAT	Obesity	ENSG00000100505.9	0.002940278	0.587381962	0.005418528	0.183454363	0.003053648	0.573029886	0.005418159	0.18342115	-0.00011337	0.001768017	1	0.808728584	1	0.802779912	ENSG00000161904	7	LEMD2
SAT	Obesity	ENSG00000100554.7	-0.001716935	0.609006252	0.003356713	0.183454375	-0.001762469	0.59951715	0.003356473	0.183421166	4.55E-05	0.028083867	1	0.824225687	1	0.81924855	ENSG00000161904	7	LEMD2
SAT	Obesity	ENSG00000100564.4	0.005541483	0.144345326	0.003796053	0.183453669	0.005604443	0.139815822	0.003795814	0.183420446	-6.30E-05	0.02595296	1	0.401443831	1	0.400118009	ENSG00000161904	7	LEMD2
SAT	Obesity	ENSG00000100614.13	-0.00358362	0.444314666	0.004684911	0.183454253	-0.003477128	0.457950292	0.004684724	0.183421062	-0.000106493	0.023822247	1	0.720967567	1	0.731563013	ENSG00000161904	7	LEMD2
SAT	Obesity	ENSG00000100979.10	0.007241431	0.024498316	0.003219516	0.183452562	0.007190854	0.025505945	0.003219315	0.183419385	5.06E-05	0.036674761	1	0.141512278	1	0.14864122	ENSG00000161904	7	LEMD2
SAT	Obesity	ENSG00000100994.7	0.007615504	0.12764081	0.004998775	0.183453597	0.007739358	0.121539973	0.004998472	0.183420364	-0.000123854	0.001376607	1	0.376521527	1	0.369554001	ENSG00000161904	7	LEMD2
SAT	Obesity	ENSG00000101197.8	-0.000408682	0.914252558	0.003795467	0.18345447	-0.000325257	0.931703972	0.003795248	0.183421267	-8.34E-05	0.006690992	1	0.969823034	1	0.979468354	ENSG00000161912	13	ADCY10P1
SAT	Obesity	ENSG00000101310.10	-0.001222937	0.654635769	0.002733848	0.183454398	-0.001160797	0.671109384	0.002733691	0.183421202	-6.21E-05	0.005056063	1	0.849292194	1	0.859449605	ENSG00000161912	13	ADCY10P1
SAT	Obesity	ENSG00000101417.7	0.01056273	0.035717915	0.00502956	0.183452807	0.010653447	0.034150917	0.005029264	0.183419574	-9.07E-05	0.023859331	1	0.177830774	1	0.178269465	ENSG00000161912	13	ADCY10P1
SAT	Obesity	ENSG00000102760.12	-0.000567682	0.772048755	0.001959576	0.183454442	-0.000538579	0.783421235	0.001959441	0.183421242	-2.91E-05	0.023932253	1	0.908895574	1	0.91311015	ENSG00000161912	13	ADCY10P1
SAT	Obesity	ENSG00000102967.7	0.002478032	0.330242807	0.002545158	0.183454116	0.002517887	0.322491956	0.002544989	0.1834209	-3.99E-05	0.02371252	1	0.628809457	1	0.62535231	ENSG00000161912	13	ADCY10P1
SAT	Obesity	ENSG00000103168.12	-0.002273077	0.108906433	0.001417898	0.183453503	-0.002222578	0.116993606	0.001417894	0.183420342	-5.05E-05	0.006919655	1	0.339015187	1	0.36350002	ENSG00000161912	13	ADCY10P1
SAT	Obesity	ENSG00000103196.7	0.003275384	0.501354022	0.004871464	0.183454303	0.00913971	0.073552254	0.005107717	0.18342006	-0.005864326	0.000135796	1	0.758937763	1	0.276431783	ENSG00000161929	10	SCIMP
SAT	Obesity	ENSG00000103269.9	-0.011104089	0.024786241	0.004946748	0.183452569	-0.010996952	0.026203286	0.004946469	0.183419402	-0.000107138	0.008533045	1	0.142182373	1	0.15056564	ENSG00000161970	8	RPL26
SAT	Obesity	ENSG00000103356.11	-0.000732759	0.76366104	0.002437031	0.18345444	-0.000678142	0.780793808	0.002436863	0.183421241	-5.46E-05	0.000686659	1	0.904538582	1	0.91293214	ENSG00000161996	13	WDR90
SAT	Obesity	ENSG00000103472.5	0.000547969	0.836353118	0.002652826	0.183454458	0.000623199	0.814260135	0.002652657	0.183421249	-7.52E-05	0.000108469	1	0.938145156	1	0.928924722	ENSG00000161996	13	WDR90
SAT	Obesity	ENSG00000104231.6	0.002516501	0.467266565	0.0034618	0.183454274	0.002588536	0.454587669	0.003461585	0.183421059	-7.20E-05	0.005639775	1	0.73389258	1	0.730054891	ENSG00000161996	13	WDR90
SAT	Obesity	ENSG00000104442.5	0.000312826	0.88776237	0.002216482	0.183454467	0.000551566	0.803518989	0.002216942	0.183421247	-0.00023874	1.03E-05	1	0.960985924	1	0.923968271	ENSG00000161996	13	WDR90
SAT	Obesity	ENSG00000104689.5	0.00494367	0.142207831	0.003368492	0.18345366	0.004883668	0.147084733	0.00336826	0.183420476	6.00E-05	0.006586431	1	0.398449097	1	0.412113586	ENSG00000161999	7	JMJD8
SAT	Obesity	ENSG00000104852.10	-0.023935343	5.28E-05	0.005920641	0.183448297	-0.024042722	4.89E-05	0.005920308	0.183415037	0.000107379	0.028777244	0.173396153	0.00120414	0.16030374	0.001292772	ENSG00000162040	5	HS3ST6
SAT	Obesity	ENSG00000104953.14	-0.007108877	0.161504344	0.005077653	0.183453733	-0.007025212	0.16646676	0.00507731	0.183420547	-8.37E-05	0.014923519	1	0.425835921	1	0.436829777	ENSG00000162066	10	AMDHD2
SAT	Obesity	ENSG00000104983.4	-0.00481988	0.307686416	0.004724957	0.183454081	-0.004908487	0.298850161	0.004724681	0.183420862	8.86E-05	0.019263367	1	0.606520234	1	0.601026164	ENSG00000162194	8	C11orf48
SAT	Obesity	ENSG00000105088.4	0.007528426	0.047703087	0.003802218	0.183452992	0.007639676	0.044498003	0.003802018	0.183419744	-0.00011125	0.000766859	1	0.210507537	1	0.207089289	ENSG00000162194	8	C11orf48
SAT	Obesity	ENSG00000105146.8	0.003147943	0.384692982	0.003621312	0.183454188	0.003211913	0.375074931	0.003621063	0.183420973	-6.40E-05	0.00758623	1	0.677497409	1	0.670638065	ENSG00000162194	8	C11orf48
SAT	Obesity	ENSG00000105185.7	0.002696312	0.530927013	0.004303139	0.183454326	0.002784791	0.517507076	0.004302873	0.183421112	-8.85E-05	0.006704467	1	0.781854367	1	0.773197048	ENSG00000162222	9	TTC9C
SAT	Obesity	ENSG00000105193.4	-0.007701804	0.061375808	0.004116889	0.183453151	-0.007778001	0.058836708	0.004116617	0.183419921	7.62E-05	0.008151385	1	0.246479835	1	0.245276282	ENSG00000162244	6	RPL29
SAT	Obesity	ENSG00000105258.4	-0.000943125	0.863432106	0.005483063	0.183454463	-0.000816379	0.881632074	0.005482708	0.183421262	-0.000126746	0.001283487	1	0.950006955	1	0.959414539	ENSG00000162244	6	RPL29
SAT	Obesity	ENSG00000105270.10	0.003459458	0.098130027	0.002091582	0.18345344	0.00341215	0.102791437	0.002091455	0.183420264	4.73E-05	0.003266446	1	0.319058996	1	0.334582049	ENSG00000162244	6	RPL29
SAT	Obesity	ENSG00000105287.8	-0.003446127	0.267652878	0.003108861	0.18345401	-0.003387071	0.275915268	0.00310871	0.183420822	-5.91E-05	0.037455434	1	0.563881016	1	0.579934654	ENSG00000162244	6	RPL29
SAT	Obesity	ENSG00000105447.8	-0.008061433	0.008896072	0.003081549	0.183451887	-0.008156608	0.008119631	0.003081378	0.183418622	9.52E-05	0.000229218	1	0.072606992	1	0.072001372	ENSG00000162300	7	ZFPL1
SAT	Obesity	ENSG00000105519.8	0.004264535	0.357533058	0.004634977	0.183454154	0.00434721	0.348263012	0.004634726	0.183420938	-8.27E-05	0.036990786	1	0.655187068	1	0.650436499	ENSG00000162341	11	TPCN2
SAT	Obesity	ENSG00000105640.8	0.000642912	0.85840754	0.003603729	0.183454462	0.000758809	0.833219958	0.003603529	0.183421253	-0.000115897	0.000118953	1	0.947247673	1	0.936326075	ENSG00000162341	11	TPCN2
SAT	Obesity	ENSG00000105656.8	-0.020462231	0.000771691	0.006084923	0.1834502	-0.020595188	0.000712265	0.006084546	0.18341694	0.000132958	0.003896118	1	0.011203176	1	0.01139972	ENSG00000162341	11	TPCN2
SAT	Obesity	ENSG00000105699.12	-0.004441571	0.288210637	0.004182049	0.183454048	-0.004354993	0.297679412	0.004181768	0.18342086	-8.66E-05	0.002449324	1	0.587064948	1	0.601026164	ENSG00000162341	11	TPCN2
SAT	Obesity	ENSG00000105750.10	0.0019267	0.698258853	0.00496992	0.183454417	0.001815926	0.71481233	0.004969683	0.18342122	0.000110773	0.015795859	1	0.870105316	1	0.884017812	ENSG00000162341	11	TPCN2
SAT	Obesity	ENSG00000105755.3	0.002344826	0.562421311	0.004048043	0.183454347	0.002283815	0.572609077	0.004047784	0.18342115	6.10E-05	0.03856076	1	0.797231826	1	0.802779912	ENSG00000162341	11	TPCN2
SAT	Obesity	ENSG00000105771.9	-0.004888694	0.375555041	0.005516986	0.183454177	-0.00499314	0.365411517	0.005516657	0.183420961	0.000104446	0.015915428	1	0.670275034	1	0.665460489	ENSG00000162365	7	CYP4A22
SAT	Obesity	ENSG00000105880.4	0.003368802	0.568396931	0.005905885	0.183454351	0.003256211	0.581371198	0.00590553	0.183421155	0.000112591	0.014374251	1	0.797993295	1	0.807911436	ENSG00000162365	7	CYP4A22
SAT	Obesity	ENSG00000105928.9	0.004618086	0.360933685	0.005054888	0.183454159	0.004719764	0.350426609	0.005054562	0.183420941	-0.000101677	0.005424454	1	0.65717171	1	0.65249005	ENSG00000162368	9	CMPK1
SAT	Obesity	ENSG00000105968.14	-0.00968961	0.106525636	0.006003434	0.183453489	-0.009565348	0.111071909	0.006003118	0.183420311	-0.000124262	0.017301951	1	0.335175838	1	0.350248935	ENSG00000162368	9	CMPK1
SAT	Obesity	ENSG00000105982.12	-0.003430053	0.34943359	0.003665803	0.183454143	-0.003373983	0.357335686	0.003665562	0.18342095	-5.61E-05	0.029249186	1	0.647953427	1	0.658105737	ENSG00000162383	7	SLC1A7
SAT	Obesity	ENSG00000106009.11	-0.007755863	0.028587324	0.003542885	0.183452663	-0.007637826	0.031089335	0.0035427	0.183419514	-0.000118037	0.000134358	1	0.154014794	1	0.166130468	ENSG00000162383	7	SLC1A7
SAT	Obesity	ENSG00000106263.13	-0.010986428	0.041211661	0.005381811	0.183452899	-0.010857107	0.043646446	0.005381531	0.183419732	-0.000129321	0.005986407	1	0.192979684	1	0.204577127	ENSG00000162383	7	SLC1A7
SAT	Obesity	ENSG00000106591.3	0.004004074	0.294975205	0.003823342	0.18345406	0.003951498	0.301329061	0.003823086	0.183420866	5.26E-05	0.045333607	1	0.593750704	1	0.601253497	ENSG00000162384	9	C1orf123
SAT	Obesity	ENSG00000106603.13	0.000672399	0.764903804	0.002248467	0.18345444	0.00070481	0.75391393	0.00224832	0.183421233	-3.24E-05	0.042902946	1	0.904704175	1	0.901441682	ENSG00000162384	9	C1orf123
SAT	Obesity	ENSG00000106733.16	0.005846298	0.27135466	0.00531506	0.183454017	0.005706747	0.282928473	0.005314706	0.183420834	0.000139551	0.000144609	1	0.567681645	1	0.587152637	ENSG00000162384	9	C1orf123
SAT	Obesity	ENSG00000107099.11	0.00018105	0.963830114	0.003992467	0.183454473	0.001072812	0.788658515	0.004002235	0.183421243	-0.000891762	0.001726095	1	0.990703823	1	0.917518993	ENSG00000162384	9	C1orf123
SAT	Obesity	ENSG00000107890.12	0.005150484	0.19942671	0.004013817	0.183453852	0.005225666	0.192919278	0.004013589	0.18342063	-7.52E-05	0.022396327	1	0.481470961	1	0.476274003	ENSG00000162402	8	USP24
SAT	Obesity	ENSG00000108107.8	0.002311427	0.306905653	0.002262251	0.183454079	0.002367106	0.295368462	0.002262103	0.183420856	-5.57E-05	0.000506421	1	0.606475433	1	0.599693023	ENSG00000162402	8	USP24
SAT	Obesity	ENSG00000108187.11	0.001791442	0.40123763	0.002134158	0.183454208	0.001836136	0.389568319	0.002134046	0.18342099	-4.47E-05	0.016010894	1	0.691650463	1	0.683150002	ENSG00000162402	8	USP24
SAT	Obesity	ENSG00000108272.9	-0.007808063	0.088952916	0.004590411	0.18345338	-0.007706404	0.093172482	0.004590155	0.183420205	-0.000101659	0.007703539	1	0.30212683	1	0.316069472	ENSG00000162415	6	ZSWIM5
SAT	Obesity	ENSG00000108384.10	-0.00580268	0.00037613	0.001631655	0.183449694	-0.005753469	0.000421317	0.00163155	0.18341657	-4.92E-05	2.42E-05	1	0.006201415	1	0.007477175	ENSG00000162415	6	ZSWIM5
SAT	Obesity	ENSG00000108439.5	-0.001664701	0.673259366	0.003947789	0.183454407	-0.001571697	0.690522104	0.003947532	0.18342121	-9.30E-05	0.000927342	1	0.855855862	1	0.868937924	ENSG00000162461	7	SLC25A34
SAT	Obesity	ENSG00000108465.10	0.006143666	0.233391798	0.005155515	0.183453937	0.006247719	0.225542801	0.005155215	0.183420715	-0.000104053	0.012019644	1	0.522565341	1	0.518492259	ENSG00000162520	10	SYNC
SAT	Obesity	ENSG00000108556.7	0.007894562	0.00104283	0.002407812	0.183450411	0.00785795	0.001099647	0.002407661	0.183417244	3.66E-05	0.042288609	1	0.01443682	1	0.016106882	ENSG00000162600	7	OMA1
SAT	Obesity	ENSG00000108602.13	0.000673915	0.871228551	0.00415745	0.183454464	0.000535187	0.897565373	0.004157191	0.183421264	0.000138729	9.12E-06	1	0.955362338	1	0.963222823	ENSG00000162600	7	OMA1
SAT	Obesity	ENSG00000108639.3	-0.008198759	0.04687267	0.004125257	0.183452981	-0.008114683	0.049163816	0.004125048	0.183419808	-8.41E-05	0.022033066	1	0.208104508	1	0.221531933	ENSG00000162600	7	OMA1
SAT	Obesity	ENSG00000108771.8	0.003184155	0.236508638	0.002689865	0.183453944	0.003220261	0.231202985	0.002689676	0.183420728	-3.61E-05	0.03496254	1	0.525379039	1	0.524775447	ENSG00000162631	14	NTNG1
SAT	Obesity	ENSG00000108823.11	-0.002209326	0.461680484	0.003001462	0.183454269	-0.002272657	0.448912238	0.003001276	0.183421053	6.33E-05	0.005195499	1	0.731904575	1	0.726631007	ENSG00000162694	9	EXTL2
SAT	Obesity	ENSG00000109084.9	0.006536232	0.149895577	0.004539357	0.18345369	0.006612537	0.145171011	0.00453907	0.183420468	-7.63E-05	0.022795275	1	0.409839489	1	0.40919767	ENSG00000162704	11	ARPC5
SAT	Obesity	ENSG00000109265.8	0.005441395	0.034117716	0.00256829	0.183452777	0.005334367	0.037792042	0.002568169	0.18341964	0.000107028	7.27E-06	1	0.172215733	1	0.189433997	ENSG00000162722	8	TRIM58
SAT	Obesity	ENSG00000109771.11	0.004098258	0.332561974	0.004229511	0.183454119	0.004026444	0.34107147	0.00422923	0.183420928	7.18E-05	0.014721322	1	0.630349991	1	0.64461722	ENSG00000162734	8	PEA15
SAT	Obesity	ENSG00000110025.8	-0.006061025	0.021260828	0.002631421	0.183452469	-0.002908339	0.28047356	0.002694759	0.18342083	-0.003152686	6.01E-08	1	0.129189682	1	0.584646601	ENSG00000162734	8	PEA15
SAT	Obesity	ENSG00000110169.6	0.005064743	0.237693235	0.004289343	0.183453947	0.004993599	0.244317588	0.004289061	0.183420758	7.11E-05	0.018189256	1	0.526060746	1	0.539991654	ENSG00000162817	6	C1orf115
SAT	Obesity	ENSG00000110455.9	0.001499683	0.397244275	0.001771515	0.183454203	0.001532397	0.38699722	0.0017714	0.183420987	-3.27E-05	0.009996582	1	0.688878681	1	0.680459742	ENSG00000162817	6	C1orf115
SAT	Obesity	ENSG00000110660.10	-0.005201175	0.106085352	0.00321846	0.183453487	-0.005157117	0.109054133	0.003218246	0.1834203	-4.41E-05	0.047877805	1	0.334678885	1	0.346730908	ENSG00000162817	6	C1orf115
SAT	Obesity	ENSG00000110700.2	-0.004492789	0.186845029	0.0034037	0.183453816	-0.004599247	0.176589209	0.003403488	0.18342058	0.000106459	3.00E-05	1	0.461882921	1	0.452214424	ENSG00000162836	7	ACP6
SAT	Obesity	ENSG00000110711.5	0.004563011	0.410858666	0.005548514	0.183454218	0.004130831	0.456590887	0.005548666	0.183421061	0.00043218	3.89E-07	1	0.698355005	1	0.731541927	ENSG00000162877	8	PM20D1
SAT	Obesity	ENSG00000111058.3	0.001842816	0.617110699	0.003686003	0.18345438	0.002105717	0.567944119	0.003687239	0.183421147	-0.000262901	0.014536923	1	0.829471612	1	0.800784122	ENSG00000162877	8	PM20D1
SAT	Obesity	ENSG00000111341.5	-0.000851372	0.874482205	0.005389527	0.183454465	-0.000734402	0.89160566	0.005389202	0.183421263	-0.00011697	0.005248512	1	0.955754868	1	0.962922373	ENSG00000162877	8	PM20D1
SAT	Obesity	ENSG00000111639.3	0.000813373	0.549756009	0.001359868	0.183454339	0.000859171	0.527531111	0.001359922	0.183421119	-4.58E-05	0.036408398	1	0.793070817	1	0.779574758	ENSG00000162885	8	B3GALNT2
SAT	Obesity	ENSG00000111666.6	-0.000151639	0.93307592	0.001805749	0.183454471	-0.0001263	0.944234957	0.001805625	0.183421268	-2.53E-05	0.035543449	1	0.978715503	1	0.983503141	ENSG00000162885	8	B3GALNT2
SAT	Obesity	ENSG00000111801.11	0.003409344	0.014239553	0.001390904	0.183452203	0.003787027	0.006552622	0.001392916	0.183418477	-0.000377683	9.91E-07	1	0.099045337	1	0.063606962	ENSG00000162885	8	B3GALNT2

SAT	Obesity	ENSG00000111879.14	0.008505501	0.129721484	0.005613437	0.183453606	0.008403143	0.134377968	0.005613092	0.183420423	0.000102358	0.016591086	1	0.380265236	1	0.392603841	ENSG00000162885	8	B3GALNT2
SAT	Obesity	ENSG00000112053.9	-0.008650478	0.002011295	0.002800798	0.183450868	-0.008512259	0.002371971	0.002800795	0.183417779	-0.00013822	0.000219142	1	0.023996583	1	0.030048015	ENSG00000162885	8	B3GALNT2
SAT	Obesity	ENSG00000112110.5	0.001220878	0.658634437	0.00276342	0.1834544	0.001270745	0.645605272	0.002763232	0.18342119	-4.99E-05	0.006948736	1	0.852457431	1	0.846276827	ENSG00000162910	14	MRPL55
SAT	Obesity	ENSG00000112130.12	0.003703626	0.310280835	0.003650209	0.183454085	0.003613666	0.32215113	0.003649991	0.1834209	9.00E-05	0.001660531	1	0.607417315	1	0.625060826	ENSG00000162913	9	C1orf145
SAT	Obesity	ENSG00000112290.8	0.002919108	0.375036698	0.003290696	0.183454177	0.00302815	0.357435057	0.003290522	0.18342095	-0.000109042	0.000130185	1	0.670275034	1	0.658105737	ENSG00000162994	11	CLHC1
SAT	Obesity	ENSG00000112308.8	0.005459574	0.367122489	0.006053566	0.183454167	0.004244637	0.483443594	0.006057059	0.183421085	0.001214937	3.93E-08	1	0.664384383	1	0.747492193	ENSG00000163041	5	H3F3A
SAT	Obesity	ENSG00000112339.10	0.001306971	0.645512157	0.002841204	0.183454394	0.001391987	0.624170352	0.002841086	0.183421179	-8.50E-05	0.002502384	1	0.844125734	1	0.831978382	ENSG00000163041	5	H3F3A
SAT	Obesity	ENSG00000112378.11	-0.000796638	0.768790034	0.002710038	0.183454441	-0.000868125	0.748700366	0.002709897	0.183421231	7.15E-05	0.00267955	1	0.907300451	1	0.900789844	ENSG00000163041	5	H3F3A
SAT	Obesity	ENSG00000112763.11	-0.005736365	0.006596863	0.002111636	0.183451685	-0.004788321	0.02380765	0.002118514	0.183419339	-0.000948044	4.15E-08	1	0.05946238	1	0.143326422	ENSG00000163041	5	H3F3A
SAT	Obesity	ENSG00000112812.11	0.020884891	1.09E-08	0.00365393	0.183442126	0.017307355	3.78E-06	0.003743771	0.183413193	0.003577535	1.18E-05	3.59E-05	6.64E-07	0.012414489	0.000167763	ENSG00000163125	11	RPRD2
SAT	Obesity	ENSG00000112852.4	-0.004627868	0.124274912	0.003010822	0.183453581	-0.004694371	0.118934634	0.003010633	0.183420351	6.65E-05	0.003023648	1	0.370341494	1	0.367443065	ENSG00000163131	6	CTSS
SAT	Obesity	ENSG00000112964.9	0.003950692	0.441928672	0.005137834	0.183454251	0.001989036	0.703268182	0.005221764	0.183421215	0.001961656	0.003902753	1	0.720521812	1	0.876674335	ENSG00000163131	6	CTSS
SAT	Obesity	ENSG00000112977.11	0.008914544	0.122958926	0.005779378	0.183453575	0.009066424	0.116682042	0.005779002	0.18342034	-0.00015188	0.000232282	1	0.367513287	1	0.363219906	ENSG00000163131	6	CTSS
SAT	Obesity	ENSG00000112992.12	0.002284977	0.49978935	0.003386045	0.183454302	0.002217572	0.512495058	0.003385823	0.183421108	6.74E-05	0.004879313	1	0.757767494	1	0.769450316	ENSG00000163138	14	PACRGL
SAT	Obesity	ENSG00000113141.11	-0.011862083	0.000212246	0.003202502	0.183449288	-0.011799974	0.000228853	0.003202286	0.183416139	-6.21E-05	0.004014116	0.696379361	0.0038474	0.750867837	0.004550714	ENSG00000163138	14	PACRGL
SAT	Obesity	ENSG00000113249.8	-0.004314926	0.350232011	0.004619141	0.183454144	-0.004236593	0.359015878	0.004618835	0.183420952	-7.83E-05	0.014777589	1	0.648116881	1	0.660152534	ENSG00000163138	14	PACRGL
SAT	Obesity	ENSG00000113273.11	-0.0044882	0.330126843	0.004608674	0.183454116	-0.004609462	0.317197799	0.004608384	0.183420892	0.000121261	0.000393097	1	0.628809457	1	0.617275195	ENSG00000163138	14	PACRGL
SAT	Obesity	ENSG00000113593.7	-0.000381735	0.842369271	0.001919578	0.183454459	-0.000332616	0.862427119	0.001919468	0.183421259	-4.91E-05	0.001707755	1	0.941066406	1	0.949538046	ENSG00000163138	14	PACRGL
SAT	Obesity	ENSG00000113597.13	-0.008889738	0.101800416	0.005433193	0.183453462	-0.00872734	0.108187533	0.005432869	0.183420295	-0.000162399	0.000134426	1	0.325294064	1	0.345600181	ENSG00000163155	7	LYSMD1
SAT	Obesity	ENSG00000113971.14	-0.000668287	0.550082447	0.001118216	0.183454339	-0.000695252	0.534079433	0.001118143	0.183421124	2.70E-05	0.000670711	1	0.793070817	1	0.784735611	ENSG00000163155	7	LYSMD1
SAT	Obesity	ENSG00000114125.9	0.000232479	0.927581384	0.002557857	0.183454471	0.000158182	0.950686188	0.002557707	0.183421269	7.43E-05	0.000257084	1	0.976869566	1	0.987089045	ENSG00000163161	8	ERCC3
SAT	Obesity	ENSG00000114735.5	0.007299605	0.1305093	0.004827465	0.18345361	0.007206225	0.135480362	0.0048272	0.183420428	9.34E-05	0.021695231	1	0.381300991	1	0.394070094	ENSG00000163161	8	ERCC3
SAT	Obesity	ENSG00000114742.9	0.003604394	0.267657511	0.003251671	0.18345401	0.00340115	0.295579101	0.003251696	0.183420857	0.000203244	8.10E-06	1	0.563881016	1	0.599749554	ENSG00000163281	7	GNPDA2
SAT	Obesity	ENSG00000114796.11	0.018662524	0.000684476	0.005495856	0.183450115	0.018763661	0.000639387	0.005495523	0.183416864	-0.000101137	0.017863371	1	0.010208026	1	0.010446545	ENSG00000163281	7	GNPDA2
SAT	Obesity	ENSG00000114988.7	-0.002005706	0.715753633	0.005508062	0.183454424	-0.001847556	0.737287408	0.005507729	0.183421228	-0.00015815	0.000210123	1	0.882189207	1	0.896583401	ENSG00000163281	7	GNPDA2
SAT	Obesity	ENSG00000115084.8	-0.001817356	0.585133879	0.003329091	0.183454361	-0.001763856	0.596205392	0.003328874	0.183421164	-5.35E-05	0.023629612	1	0.808006842	1	0.817137678	ENSG00000163281	7	GNPDA2
SAT	Obesity	ENSG00000115221.6	-0.01036429	0.007651139	0.003885977	0.183451785	-0.010251088	0.008336543	0.003885729	0.1834186	-0.000113202	6.42E-05	1	0.065715675	1	0.07313422	ENSG00000163281	7	GNPDA2
SAT	Obesity	ENSG00000115310.13	-0.013581579	0.006416845	0.004982794	0.183451666	-0.014383428	0.003917062	0.004985973	0.183418125	0.000801849	2.52E-05	1	0.058320407	1	0.044436554	ENSG00000163281	7	GNPDA2
SAT	Obesity	ENSG00000115423.14	0.00218821	0.208529702	0.001739961	0.183453876	0.00128826	0.465621506	0.001765653	0.183421069	0.00089995	0.00279215	1	0.496146448	1	0.736953286	ENSG00000163297	12	ANTXR2
SAT	Obesity	ENSG00000115486.7	0.005863627	0.017849419	0.002475425	0.18345253	0.00579988	0.01912816	0.002475374	0.183419195	6.37E-05	0.029254258	1	0.114606541	1	0.125770525	ENSG00000163297	12	ANTXR2
SAT	Obesity	ENSG00000115561.10	-0.012578491	1.71E-07	0.002405883	0.183444142	-0.012536593	1.88E-07	0.002405729	0.183411007	-4.19E-05	0.016293763	0.000561766	8.14E-06	0.000616035	1.06E-05	ENSG00000163297	12	ANTXR2
SAT	Obesity	ENSG00000115568.11	0.006889473	0.120956479	0.004442602	0.183453565	0.006760695	0.128040605	0.004442341	0.183420395	0.000128777	0.000263307	1	0.364377321	1	0.381612058	ENSG00000163297	12	ANTXR2
SAT	Obesity	ENSG00000116138.8	-0.015379779	0.000886847	0.004626635	0.183450297	-0.015460011	0.00083258	0.00462632	0.18341705	8.02E-05	0.009954404	1	0.012434808	1	0.012764933	ENSG00000163297	12	ANTXR2
SAT	Obesity	ENSG00000116157.5	-0.000323015	0.855445102	0.001773098	0.183454462	-0.000276487	0.876078182	0.001773003	0.183421261	-4.65E-05	0.002386303	1	0.945022013	1	0.958314845	ENSG00000163354	10	DCST2
SAT	Obesity	ENSG00000116221.11	-0.005310146	0.355059894	0.005741814	0.183454151	-0.005579786	0.331133809	0.005741503	0.183420913	0.00026964	3.78E-08	1	0.652999726	1	0.632292088	ENSG00000163389	6	POGLUT1
SAT	Obesity	ENSG00000116667.8	0.007431693	0.065547857	0.004035657	0.183453192	0.00749234	0.063339238	0.004035387	0.183419967	-6.12E-05	0.026695312	1	0.257251818	1	0.256563012	ENSG00000163389	6	POGLUT1
SAT	Obesity	ENSG00000116785.9	-0.004756642	0.305608411	0.004642975	0.183454077	-0.004832469	0.297930453	0.004642664	0.183420861	7.58E-05	0.017277938	1	0.605495891	1	0.601026164	ENSG00000163389	6	POGLUT1
SAT	Obesity	ENSG00000116791.9	0.002356296	0.3773798	0.002669312	0.18345418	0.002412412	0.366095745	0.002669154	0.183420961	-5.61E-05	0.007824232	1	0.671085989	1	0.665462682	ENSG00000163389	6	POGLUT1
SAT	Obesity	ENSG00000116857.12	-0.003244444	0.282323215	0.003017756	0.183454037	-0.003318935	0.271389438	0.003017571	0.183420813	7.45E-05	0.001303288	1	0.579661774	1	0.574631165	ENSG00000163393	8	SLC22A15
SAT	Obesity	ENSG00000116874.7	0.007367155	0.031510264	0.003425668	0.183452726	0.007289022	0.033345497	0.003425446	0.183419559	7.81E-05	0.001443792	1	0.163843387	1	0.175894815	ENSG00000163395	12	IGFN1
SAT	Obesity	ENSG00000116977.14	-0.001245383	0.64613365	0.002712424	0.183454394	-0.001197452	0.658853846	0.002712258	0.183421197	-4.79E-05	0.021232371	1	0.844125734	1	0.852666776	ENSG00000163395	12	IGFN1
SAT	Obesity	ENSG00000117174.6	-0.000301187	0.912535361	0.002742021	0.18345447	-0.00023877	0.930604981	0.002741852	0.183421267	-6.24E-05	0.002777145	1	0.968941268	1	0.979254311	ENSG00000164331	11	LMOD1
SAT	Obesity	ENSG00000117222.5	-0.003467996	0.470550951	0.004806092	0.183454277	-0.003894195	0.417934709	0.004807587	0.183421022	0.000426198	0.000159534	1	0.735593387	1	0.703737593	ENSG00000163444	7	TMEM183A
SAT	Obesity	ENSG00000117481.6	-0.010668015	0.041668388	0.005237577	0.183452906	-0.010761027	0.039907182	0.005237238	0.183419675	9.30E-05	0.013526459	1	0.19344648	1	0.195087821	ENSG00000163453	7	IGFBP7
SAT	Obesity	ENSG00000118307.14	-0.006559788	0.212222694	0.005258438	0.183453886	-0.00640691	0.223041945	0.005258113	0.183420709	-0.000152879	0.000124291	1	0.501426545	1	0.516186585	ENSG00000163453	7	IGFBP7
SAT	Obesity	ENSG00000118407.10	0.001331696	0.79841986	0.005214307	0.183454449	0.001438385	0.78264647	0.005213957	0.183421241	-0.000106689	0.002732245	1	0.919633097	1	0.91293214	ENSG00000163453	7	IGFBP7
SAT	Obesity	ENSG00000118507.11	3.23E-05	0.992300006	0.003371891	0.183454474	0.000111077	0.973720333	0.003371819	0.18342127	-7.88E-05	0.046777729	1	0.996612544	1	0.992266083	ENSG00000163468	10	CCT3
SAT	Obesity	ENSG00000118514.9	0.000413135	0.797748493	0.001612156	0.183454449	0.000458408	0.776137423	0.001612091	0.18342124	-4.53E-05	0.005122237	1	0.919633097	1	0.911862059	ENSG00000163486	8	SRGAP2
SAT	Obesity	ENSG00000118579.7	-0.002709461	0.415049797	0.003324329	0.183454223	-0.002610992	0.432179649	0.003324124	0.183421037	-9.85E-05	9.41E-05	1	0.700759618	1	0.715518881	ENSG00000163492	9	CCDC141
SAT	Obesity	ENSG00000118640.6	0.013042436	0.016433067	0.005436249	0.183452298	0.012717123	0.019321355	0.005436341	0.183419202	0.000325313	4.33E-05	1	0.108703412	1	0.126415995	ENSG00000163521	11	GLB1L
SAT	Obesity	ENSG00000118900.10	-0.013262197	0.022406976	0.005892559	0.183452559	-0.013418796	0.022763247	0.005892176	0.18341931	0.000156599	0.000210112	1	0.141482842	1	0.139600399	ENSG00000163521	11	GLB1L
SAT	Obesity	ENSG00000118961.10	0.001566711	0.517247348	0.002419276	0.183454316	0.001458763	0.546502864	0.002419139	0.183421133	0.000107947	6.01E-08	1	0.771001245	1	0.788841592	ENSG00000163521	11	GLB1L
SAT	Obesity	ENSG00000119471.10	-0.001459054	0.788921443	0.005450097	0.183454447	-0.001346889	0.804794862	0.005449758	0.183421247	-0.000112165	0.006095172	1	0.914498218	1	0.923968271	ENSG00000163521	11	GLB1L
SAT	Obesity	ENSG00000119711.8	-0.000278438	0.942853609	0.003884259	0.183454472	-0.00020218	0.958485261	0.003884005	0.183421269	-7.63E-05	0.005644587	1	0.981335116	1	0.990485083	ENSG00000163545	7	NUAK2
SAT	Obesity	ENSG00000119718.6	-0.00464785	0.033149546	0.002181809	0.183452759	-0.004719516	0.030523638	0.002181694	0.183419502	7.17E-05	0.000168532	1	0.168954102	1	0.164446725	ENSG00000163545	7	NUAK2
SAT	Obesity	ENSG00000119946.6	0.003525746	0.056919944	0.001851826	0.183453104	0.003495443	0.059068339	0.001851705	0.183419923	3.03E-05	0.021567415	1	0.235503577	1	0.245320532	ENSG00000163545	7	NUAK2
SAT	Obesity	ENSG00000120055.5	0.005907306	0.167406861	0.004278839	0.183453754	0.00603249	0.158564175	0.004278607	0.183420519	-0.000125184	0.000603885	1	0.433856169	1	0.426084405	ENSG00000163545	7	NUAK2
SAT	Obesity	ENSG00000120314.14	-0.021539141	0.000159867	0.005705356	0.183449087	-0.021424112	0.000173122	0.005704962	0.18341594	-0.000115029	0.002037209	0.524522634	0.002997272	0.568012556	0.003641106	ENSG00000163545	7	NUAK2
SAT	Obesity	ENSG00000120539.10	-0.004647975	0.006946425	0.001721846	0.18345172	-0.004567826	0.007986883	0.001721996	0.183418611	-8.01E-05	0.013353246	1	0.061597897	1	0.071403169	ENSG00000163545	7	NUAK2
SAT	Obesity	ENSG00000120784.11	0.002460161	0.252269576	0.002148884	0.183453979	0.00250416	0.243859211	0.002148762	0.183420757	-4.40E-05	0.013228657	1	0.542731309	1	0.539515895	ENSG00000163554	7	SPTA1
SAT	Obesity	ENSG00000120805.9	0.001140865	0.78781633	0.004238809	0.183454447	0.00107315	0.800121796	0.004238522	0.183421246	6.77E-05	0.018011038	1	0.914498218	1	0.92223015	ENSG00000163554	7	SPTA1
SAT	Obesity	ENSG00000120860.6	-0.001646049	0.705902456	0.004361954	0.18345442	-0.001559235	0.720729334	0.004361686	0.183421222	-8.68E-05	0.009084514	1	0.875173806	1	0.885797449	ENSG00000163554	7	SPTA1
SAT	Obesity	ENSG00000120907.13	0.008208412	7.43E-05	0.002071681	0.183448854	0.00823833	6.98E-05	0.002071544	0.183415293	-2.99E-05	0.038796461	0.243690948	0.001562121	0.229108206	0.001660204	ENSG00000163554	7	SPTA1
SAT	Obesity	ENSG00000121101.11	0.019184731	0.001392009	0.006002128	0.183450612	0.019050009	0.001503146	0.006001716	0.183417463	0.000134721	0.000655871	1	0.018078898	1	0.020513962	ENSG00000163558	8	PRKCI
SAT	Obesity	ENSG00000121350.11	0.007297807	0.022783163	0.003204927	0.183452514	0.007468327	0.019808004	0.003205328	0.183419218	-0.00017052	0.010433487	1	0.134930607	1	0.127967724	ENSG00000163576	13	EFHB
SAT	Obesity	ENSG00000121552.3	0.007959234	0.163206458	0.005708121	0.183453739	0.007866337	0.168147936	0.005707766	0.183420552	9.29E-05	0.030586068	1	0.428041877	1	0.437851886	ENSG00000163576	13	EFHB
SAT	Obesity	ENSG00000121691.4	0.001778195	0.578038773	0.003196742	0.183454357	0.00171855	0.590834902	0.00319655	0.183421161	5.96E-05	0.016440285	1	0.804985236	1	0.815193151	ENSG00000163576	13	EFHB
SAT	Obesity	ENSG00000122367.15	0.003554745	0.553416844	0.005998069	0.183454341	0.020816038	0.007169301	0.007741506	0.183418538	-0.017261293	0.0004214	1	0.793948695	1	0.067399648	ENSG00000163576	13	EFHB
SAT	Obesity	ENSG00000122376.7	0.000436351	0.908750033	0.003807088	0.183454469	8.41E-05	0.982381909	0.00380739	0.18342127	0.000352273	5.11E-07	1	0.967797137	1	0.995364986	ENSG00000163576	13	EFHB
SAT	Obesity	ENSG00000122507.16	-0.004765217	0.43304436	0.006078134	0.183454242	-0.005238539	0.389077718	0.006082188	0.18342099	0.000473322	0.04546734	1	0.714441885	1	0.682866142	ENSG00000163576	13	EFHB
SAT	Obesity	ENSG00000122696.8	0.006115728	0.01379911	0.002483585	0.183452182	0.006059563	0.014687708	0.00248343	0.18341902	5.62E-05	0.002424856	1	0.097156393	1	0.107328215	ENSG00000163596	12	ICA1L
SAT	Obesity	ENSG00000122912.10	0.010103861	0.08604269	0.005885781	0.1834533	0.010274203	0.080863248	0.005885425	0.183420118	-0.000170343	0.000198255	1	0.294990664	1	0.293162781	ENSG00000163596	12	ICA1L
SAT	Obesity	ENSG00000122952.12	0.004506026	0.245434753	0.003879452	0.183453964	0.004442884	0.25207923	0.003879181	0.183420774	6.31E-05	0.011305211	1	0.534004366	1	0.549184565	ENSG00000163596	12	ICA1L
SAT	Obesity	ENSG00000123009.3	0.009672778	0.016920488	0.004049832	0.183452318	0.009773183	0.015806968	0.004049632	0.183419069	-0.000100406	0.006396529	1	0.110151033	1	0.111772978	ENSG00000163596	12	ICA1L
SAT	Obesity	ENSG00000123213.18	-0.002112582	0.556820223	0.003595447	0.183454344	-0.002185666	0.543229226	0.003595216	0.183421131	7.31E-05	0.005039547	1	0.79423732	1	0.788643845	ENSG00000163611	7	SPICE1
SAT	Obesity	ENSG00000123454.6	-0.003701295	0.40464972	0.004441483	0.183454212	-0.003811373	0.390787304	0.004441169	0.183420992	0.000110078	8.43E-05	1	0.694017632	1	0.684921551	ENSG00000163611	7	SPICE1
SAT	Obesity	ENSG00000123815.7	-0.003627952	0.497926389	0.005352884	0.1834543	-0.003443775	0.519976169	0.005352607	0.183421114	-0.000184177	9.54E-05	1	0.756691284	1	0.774417527	ENSG00000163618	13	CADPS
SAT	Obesity	ENSG00000123989.9	-0.003792049	0.3753422	0.004277493	0.183454177	-0.003871553	0.365381458	0.004277207	0.183420961	7.95E-05	0.00673703	1	0.670275034	1	0.665460489	ENSG00000163625	11	WDFY3
SAT	Obesity	ENSG00000124116.14	-0.000431946	0.76190854	0.00142568	0.183454439	-0.000408761	0.774318389	0.001425589	0.183421239	-2.32E-05	0.026643784	1	0.904421823	1	0.911862059	ENSG00000163629	8	PTPN13
SAT	Obesity	ENSG00000124164.11	0.000987218	0.463129136	0.001345525	0.183454271	0.001017561	0.449468251	0.001345442	0.183421054	-3.03E-05	0.002903381	1	0.733008537	1	0.727172255	ENSG00000163682	11	RPL9
SAT	Obesity	ENSG00000124191.13	-0.002014189	0.293131247	0.001915951	0.183454056	-0.001970599	0.303677892	0.001915845	0.18342087	-4.36E-05	0.006782926	1	0.592218978	1	0.604225085	ENSG00000163682	11	RPL9
SAT	Obesity	ENSG00000124508.12	0.002455639	0.226496086	0.002030401	0.183453921	0.003147436	0.121745278	0.002033895	0.183420365	-0.000691797	1.54E-08	1	0.514945339	1	0.369554001	ENSG00000163682	11	RPL9
SAT	Obesity	ENSG00000124532.10	0.000499373	0.911637811	0.004499955	0.183454469	0.000586281	0.896333769	0.004499672	0.183421264	-8.69E-05	0.009202248	1	0.968941268	1	0.963222823	ENSG00000163728	6	TTC14
SAT	Obesity	ENSG00000124549.10	0.002786548	0.238098204	0.002361972	0.183453948	0.004328916	0.068574193	0.002376933	0.183420017	-0.001542368	8.89E-09	1	0.526060746	1	0.265839301	ENSG00000163728	6	TTC14
SAT	Obesity	ENSG00000124562.5	-0.022949773	5.75E-24	0.002273026	0.183415949	-0.02280671	1.09E-23	0.002272965	0.183383227	-0.000143062	1.88E-08	1.89E-20	2.36E-21	3.57E-20	3.96E-21	ENSG00000163743	9	RCHY1
SAT	Obesity	ENSG00000124575.5	0.004009335	0.425356298	0.005029499	0.183454234	0.004562894	0.364350436	0.005030158	0.183420959	-0.000553559	1.73E-07	1	0.708423357	1	0.664705409	ENSG00000163743	9	RCHY1
SAT	Obesity	ENSG00000124613.4	0.002366725	0.249153883	0.002053721	0.183453972	0.000665508	0.748531376	0.00207597	0.183421231	0.001701217	2.28E-08	1	0.538484566	1	0.900879844	ENSG00000163743	9	RCHY1
SAT	Obesity	ENSG00000124635.7	0.033750509	5.61E-10	0.005442512	0.183439939	0.027951843	6.71E-07	0.005624569	0.183411937	0.005798666	4.49E-05	1.84E-06	4.60E-08	0.002202038	3.34E-05	ENSG00000163743	9	RCHY1
SAT	Obesity	ENSG00000124641.10	0.00401678	0.416483162	0.004943496	0.183454225	0.003936861	0.425788212	0.004943195	0.18342103	7.99E-05	0.035775776	1	0.700759618	1	0.710947136	ENSG00000163743	9	RCHY1
SAT	Obesity	ENSG00000124678.13	-0.00044387	0.919183444	0.00437472	0.18345447	-0.000273304	0.950183562	0.004374521	0.183421269	-0.000170566	4.00E-05	1	0.972851897	1	0.986879476	ENSG00000163788	9	SNRK
SAT	Obesity	ENSG00000124713.5	-0.004692293	0.066192003	0.00255415	0.183453198	-0.004650885	0.068602303	0.00255398	0.183420017	-4.14E-05	0.019422246	1	0.25885097	1	0.265839301	ENSG00000163788	9	SNRK
SAT	Obesity	ENSG00000124784.4	0.000483224	0.887391659	0.003412468	0.183454467	0.000434061	0.89877629	0.003412227	0.183421264	4.92E-05	0.022654506	1	0.960985924	1	0.963222823	ENSG00000163788	9	SNRK
SAT	Obesity	ENSG00000124787.9	0.003143771	0.547263953	0.005223378	0.183454337	0.003024483	0.562547539	0.005223072	0.183421143	0.000119288	0.004166924	1	0.792029236	1	0.797062812	ENSG00000163807	4	KIAA1143
SAT	Obesity	ENSG00000124920.9	-0.004215514	0.321655183	0.00425353	0.183454103	-0.004011529	0.345627769	0.004253515	0.183420934	-0.000203985	0.000394337	1	0.619974849	1	0.648774721	ENSG00000163807	4	KIAA1143
SAT	Obesity	ENSG00000124939.4	-0.0011946	0.772713536	0.004152861	0.183454443	-0.000869959	0.834142417	0.004154695	0.183421254	-0.000329501	0.015012341	1	0.909351905	1	0.936326075	ENSG00000163864	10	NMNAT3
SAT	Obesity	ENSG00000125107.12	0.007465631	0.177892275	0.005541282	0.183453788	0.007567645	0.172012529	0.005540946	0.183420565	-0.000102014	0.017335025	1	0.451403367	1	0.443997285	ENSG00000163864	10	NMNAT3
SAT	Obesity	ENSG00000125409.8	0.006171326	0.055304854	0.003220139	0.183453086	0.006220942	0.053358051	0.003219922	0.183419859	-4.96E-05	0.023434998	1	0.231153156	1	0.230862672	ENSG00000163870	10	TPRA1
SAT	Obesity	ENSG00000125450.6	-0.001627973	0.693748435	0.004134287	0.183454415	-0.001689765	0.682724889	0.004134002	0.183421207	6.18E-05	0.023082109	1	0.867449929	1	0.86636807	ENSG00000163870	10	TPRA1
SAT	Obesity	ENSG00000125611.11	-0.008175587	0.1409271	0.005552748	0.183453655	-0.007977204	0.150804865	0.005552463	0.18342049	-0.000198382	5.30E-05	1	0.396214067	1	0.414511276	ENSG00000163870	10	TPRA1
SAT	Obesity	ENSG00000125821.7	-0.009093099	0.098411782	0.00550228	0.183453442	-0.008874087	0.106775352	0.005502078	0.183420287	-0.000219011	0.000123193	1	0.319375923	1	0.343124319	ENSG00000163904	8	SENP2
SAT	Obesity	ENSG00000125844.11	-0.001097704	0.812426876	0.00462585	0.183454453	-0.001167069	0.800802712	0.004625564	0.183421246	6.94E-05	0.047810737	1	0.927799965	1	0.92223015	ENSG00000163904	8	SENP2
SAT	Obesity	ENSG00000125901.5	-0.002091337	0.222104997	0.001712875	0.183453911	-0.002051069	0.231107366	0.001712774	0.183420728	-4.03E-05	0.003142738	1	0.509598947	1	0.524775447	ENSG00000163904	8	SENP2
SAT	Obesity	ENSG00000125965.4	-0.004190461	0.370392758	0.004678218	0.183454171	-0.004324667	0.355242884	0.004678004	0.183420947	0.000134207	0.002462916	1	0.666625694	1	0.657019111	ENSG00000163913	7	IFT122
SAT	Obesity	ENSG00000126226.17	0.000198165	0.973848942	0.006045063	0.183454474	0.000118547	0.984352883	0.006044628	0.18342127	7.96E-05	0.030674107	1	0.993452012	1	0.995364986	ENSG00000163913	7	IFT122
SAT	Obesity	ENSG00000126247.6	-0.003442981	0.41563258	0.004229591	0.183454224	-0.003259495	0.440898783	0.004229392	0.183421046	-0.000183486	3.21E-06	1	0.700759618	1	0.721131061	ENSG00000163938	12	GNL3
SAT	Obesity	ENSG00000126251.5	0.001619688	0.55687886	0.002756991	0.183454344	0.001685505	0.540939738	0.002756827	0.183421129	-6.58E-05	0.002481973	1	0.79423732	1	0.788109805	ENSG00000163945	11	UVSSA
SAT	Obesity	ENSG00000126500.3	0.006431554	0.127725834	0.004222579	0.183453597	0.006767379	0.109045181	0.004223007	0.1834203	-0.000335826	4.96E-05	1	0.376521527	1	0.346730908	ENSG00000163945	11	UVSSA
SAT	Obesity	ENSG00000126790.7	-0.003244688	0.305309706	0.003165196	0.183454077	-0.003430437	0.278519574	0.003165626	0.183420826	0.000185749	0.005814934	1	0.605495891	1	0.581682192	ENSG00000163945	11	UVSSA
SAT	Obesity	ENSG00000127220.5	0.00320619	0.335871911	0.003331609	0.183454124	0.003258227	0.328053276	0.003331372	0.183420909	-5.20E-05	0.01223909	1	0.633626214	1	0.630177282	ENSG00000163945	11	UVSSA
SAT	Obesity	ENSG00000127337.2	0.006059552	0.001872435	0.001948553	0.183450819	0.006090088	0.0017743	0.00194843	0.183417578	-3.05E-05	0.035391409	1	0.022586247	1	0.023473705	ENSG00000163947	7	ARHGEF3
SAT	Obesity	ENSG00000127616.13	-0.004043762	0.394441049	0.004748474	0.1834542	-0.004193924	0.377099299	0.004748254	0.183420975	0.000150162	0.000761497	1	0.687020457	1	0.671441012	ENSG00000163956	6	LRPAP1
SAT	Obesity	ENSG00000127903.12	-0.000843344	0.862189626	0.004858318	0.183454463	-0.000986099	0.839147878	0.004858024	0.183421255	0.000142756	0.000148108	1	0.949276565	1	0.937115108	ENSG00000164002	7	EXO5
SAT	Obesity	ENSG00000128039.6	0.005010247	0.388618696	0.005811503	0.183454193	0.004923121	0.396884847	0.005811105	0.183420999	8.71E-05	0.023753011	1	0.679668413	1	0.691351433	ENSG00000164024	7	METAP1
SAT	Obesity	ENSG00000128262.4	-0.004944155	0.374011821	0.005561576	0.183454175	-0.004814259	0.386672422	0.00556132	0.183420987	-0.000129896	0.013211367	1	0.669832306	1	0.680459742	ENSG00000164024	7	METAP1
SAT	Obesity	ENSG00000128463.8	0.012217475	0.013516465	0.004946601	0.183452168	0.012316742	0.012770815	0.004946271	0.183418927	-9.93E-05	0.00362326	1	0.095990306	1	0.096541971	ENSG00000164031	12	DNAJB14
SAT	Obesity	ENSG00000128604.14	-0.001247292	0.760654691	0.004094564	0.183454439	-0.0011665	0.77755913	0.004094315	0.18342124	-9.06E-05	0.003966951	1	0.904420994	1	0.912114423	ENSG00000164031	12	DNAJB14
SAT	Obesity	ENSG00000128655.11	-0.005412648	0.280463979	0.00501506	0.183454034	-0.0053218	0.288586877	0.005014757	0.183420845	-9.08E-05	0.019557905	1	0.579036375	1	0.594383895	ENSG00000164031	12	DNAJB14
SAT	Obesity	ENSG00000128829.7	0.004712836	0.044510898	0.002345567	0.183452948	0.004761829	0.042329051	0.00234541	0.183419712	-4.90E-05	0.002422248	1	0.202271825	1	0.200986421	ENSG00000164039	10	BDH2
SAT	Obesity	ENSG00000129055.6	-0.001603255	0.259010356	0.0014204	0.183453994	-0.001577906	0.266586991	0.001420311	0.183420804	-2.53E-05	0.016780211	1	0.552544198	1	0.569447863	ENSG00000164045	7	CDC25A
SAT	Obesity	ENSG00000129187.10	-0.000487694	0.828281903	0.002248414	0.183454456	-0.000452792	0.84038897	0.002248266	0.183421255	-3.49E-05	0.027168094	1	0.936525064	1	0.937310206	ENSG00000164048	9	ZNF589
SAT	Obesity	ENSG00000129219.9	0.010670227	0.03618517	0.005105377	0.183452823	0.010792259	0.034511786	0.005105024	0.183419581	-0.000122032	0.000255463	1	0.1809418	1	0.179450348	ENSG00000164053	13	ATRIP
SAT	Obesity	ENSG00000129347.15	0.000454782	0.791729304	0.001722229	0.183454448	0.00049047	0.77579584	0.001722134	0.18342124	-3.57E-05	0.013827267	1	0.915154651	1	0.911862059	ENSG00000164062	8	APEH
SAT	Obesity	ENSG00000129484.9	-0.000804122	0.881120242	0.005376969	0.183454466	-0.000700823	0.896293348	0.005376665	0.183421264	-0.0001033	0.019577574	1	0.958494761	1	0.963222823	ENSG00000164062	8	APEH
SAT	Obesity	ENSG00000129562.6	-0.00387452	0.35098977	0.004154215	0.183454145	-0.003740582	0.367864744	0.00415398	0.183420964	-0.000133938	9.85E-05	1	0.648421979	1	0.66613537	ENSG00000164068	11	RNF123
SAT	Obesity	ENSG00000130035.2	0.005669705	0.126375998	0.003709198	0.183453591	0.005727923	0.122502167	0.003708934	0.183420369	-5.82E-05	0.011529163	1	0.374273307	1	0.371126141	ENSG00000164070	7	HSPA4L
SAT	Obesity	ENSG00000130158.9	-0.005095573	0.391347132	0.005944594	0.183454196	-0.005200496	0.381635372	0.005944186	0.183420981	0.000104922	0.007456784	1	0.683347493	1	0.676469843	ENSG00000164070	7	HSPA4L
SAT	Obesity	ENSG00000130270.12	-0.013963459	1.62E-08	0.002472035	0.183442414	-0.01400295	1.47E-08	0.002471887	0.183409143	3.95E-05	0.04085933	5.31E-05	9.66E-07	4.83E-05	1.03E-06	ENSG00000164074	10	C4orf29
SAT	Obesity	ENSG00000130312.2	0.001275406	0.337521219	0.001329829	0.183454126	0.001249712	0.347311855	0.001329739	0.183420936	2.57E-05	0.00437624	1	0.6350426	1	0.650045748	ENSG00000164078	8	MST1R
SAT	Obesity	ENSG00000130348.7	-0.001166038	0.45738621	0.00156903	0.183454265	-0.001190287	0.448053983	0.001568925	0.183421053	2.42E-05	0.025559263	1	0.731029138	1	0.726019131	ENSG00000164080	9	RAD54L2
SAT	Obesity	ENSG00000130413.11	0.006757143	0.25072304	0.005882957	0.183453975	0.006660299	0.257548389	0.005882566	0.183420786	9.68E-05	0.025945876	1	0.540132826	1	0.556825651	ENSG00000164080	9	RAD54L2
SAT	Obesity	ENSG00000130517.9	0.009715726	0.001797798	0.003112252	0.18345079	0.009807601	0.001624761	0.003112099	0.183417517	-9.19E-05	0.001187549	1	0.021927791	1	0.021937612	ENSG00000164081	8	TEX264
SAT	Obesity	ENSG00000130520.6	0.005081272	0.008422551	0.001928629	0.183451845	0.005131015	0.007799945	0.00192853	0.183418595	-4.97E-05	0.000296791	1	0.069783813	1	0.071403169	ENSG00000164081	8	TEX264
SAT	Obesity	ENSG00000130640.9	0.008710218	0.141741598	0.005927985	0.183453658	0.008603551	0.146658954	0.005927611	0.183420474	0.000106667	0.014799016	1	0.39770483	1	0.411271818	ENSG00000164081	8	TEX264
SAT	Obesity	ENSG00000130733.6	-0.008417416	0.126506887	0.005508688	0.183453592	-0.00852174	0.121851792	0.005508379	0.183420366	0.000104324	0.02261741	1	0.374273307	1	0.369554001	ENSG00000164082	10	GRM2
SAT	Obesity	ENSG00000130803.10	-0.009091868	0.045003909	0.004499533	0.183452955	-0.008913599	0.047581543	0.004499337	0.183419787	-0.000106268	0.01465771	1	0.203947272	1	0.216826648	ENSG00000164099	3	PRSS12
SAT	Obesity	ENSG00000131437.11	0.008158012	0.05099271	0.004180282	0.183453034	0.008078926	0.053270005	0.004180058	0.183419858	7.91E-05	0.027794692	1	0.219563101	1	0.230862672	ENSG00000164111	10	ANXA5
SAT	Obesity	ENSG00000131473.12	-0.004526301	0.435407171	0.005803152	0.183454244	-0.004626068	0.425327182	0.005802794	0.18342103	9.98E-05	0.023799422	1	0.716072224	1	0.710538943	ENSG00000164111	10	ANXA5

SAT	Obesity	ENSG00000131845.10	-0.007738949	0.121502498	0.004997705	0.183453568	-0.007624125	0.127107533	0.004997425	0.183420391	-0.000114824	0.005603207	1	0.364910204	1	0.379817682	ENSG00000164111	10	ANXA5
SAT	Obesity	ENSG00000131849.10	-0.000392735	0.939031308	0.00513463	0.183454472	-0.000471773	0.926788268	0.005134303	0.183421267	7.90E-05	0.036082422	1	0.981045593	1	0.976804467	ENSG00000164118	8	CEP44
SAT	Obesity	ENSG00000131943.13	-0.010023303	0.076277198	0.005654222	0.183453286	-0.009837426	0.081871882	0.00565392	0.183420126	-0.000185877	0.000129222	1	0.277764135	1	0.295121602	ENSG00000164118	8	CEP44
SAT	Obesity	ENSG00000132205.6	0.026691431	5.69E-07	0.005336648	0.183445019	0.026821135	5.01E-07	0.005336327	0.183411723	-0.000129704	0.001940128	0.001866738	2.49E-05	0.001642551	2.65E-05	ENSG00000164118	8	CEP44
SAT	Obesity	ENSG00000132305.16	0.005666708	0.15186374	0.003954488	0.183453698	0.005498496	0.16438303	0.00395438	0.183420539	0.000168212	0.000153287	1	0.411789199	1	0.433205398	ENSG00000164144	10	ARFIP1
SAT	Obesity	ENSG00000132313.10	-0.000777107	0.732691749	0.002275253	0.18345443	-0.000642815	0.777562951	0.002275478	0.18342124	-0.000134292	0.002427315	1	0.889784069	1	0.912114423	ENSG00000164144	10	ARFIP1
SAT	Obesity	ENSG00000132906.13	-0.003620583	0.093221922	0.002156849	0.183453409	-0.003657499	0.089910563	0.002156706	0.183420183	3.69E-05	0.013936293	1	0.310854834	1	0.309870332	ENSG00000164144	10	ARFIP1
SAT	Obesity	ENSG00000133059.12	-0.003293232	0.482423621	0.004688473	0.183454288	-0.00283866	0.545091157	0.004690971	0.183421132	-0.000454572	0.006031573	1	0.743462612	1	0.788841592	ENSG00000164167	5	LSM6
SAT	Obesity	ENSG00000133069.10	-0.003848396	0.457984145	0.005185322	0.183454266	-0.003363234	0.516816055	0.005188081	0.183421111	-0.000485161	0.008011604	1	0.731214589	1	0.772996237	ENSG00000164167	5	LSM6
SAT	Obesity	ENSG00000133597.5	-0.001614389	0.430549878	0.002048063	0.183454239	-0.001642817	0.422444087	0.002047918	0.183421027	2.84E-05	0.02796653	1	0.71201318	1	0.708004523	ENSG00000164171	6	ITGA2
SAT	Obesity	ENSG00000133816.9	0.000432426	0.895499168	0.003292184	0.183454468	0.000382823	0.907422552	0.003291962	0.183421265	4.96E-05	0.026475437	1	0.962747126	1	0.966723909	ENSG00000164171	6	ITGA2
SAT	Obesity	ENSG00000133884.5	0.003744454	0.491651445	0.005445012	0.183454295	0.002595136	0.633885256	0.005448924	0.183421184	0.001149318	1.54E-07	1	0.752031883	1	0.837394946	ENSG00000164171	6	ITGA2
SAT	Obesity	ENSG00000133983.10	-0.001642354	0.700413339	0.004268483	0.183454418	-0.001581459	0.710993973	0.004268196	0.183421218	-6.09E-05	0.035635504	1	0.871446033	1	0.882288663	ENSG00000164171	6	ITGA2
SAT	Obesity	ENSG00000133997.7	0.006604472	0.164446202	0.004750481	0.183453743	0.006681755	0.159534289	0.004750147	0.183420522	-7.73E-05	0.010829695	1	0.429576424	1	0.427990191	ENSG00000164181	9	ELOVL7
SAT	Obesity	ENSG00000134222.12	0.00012995	0.978615331	0.004847991	0.183454474	0.00025716	0.957693616	0.004847673	0.183421269	-0.00012721	0.000205165	1	0.993452012	1	0.990485083	ENSG00000164187	6	LMBRD2
SAT	Obesity	ENSG00000134283.13	0.007651469	0.081943418	0.004398601	0.18345333	0.007731883	0.078764936	0.00439836	0.183420102	-8.04E-05	0.030690754	1	0.288332309	1	0.289069078	ENSG00000164187	6	LMBRD2
SAT	Obesity	ENSG00000134531.5	-0.003119939	0.50242266	0.004651882	0.183454304	-0.00322494	0.48812412	0.004651606	0.183421089	0.000105001	0.004240476	1	0.760004033	1	0.750604943	ENSG00000164253	8	WDR41
SAT	Obesity	ENSG00000134744.9	0.007452941	0.14042533	0.005055544	0.183453653	0.007526927	0.136500955	0.005055188	0.183420432	-7.40E-05	0.02121526	1	0.396084299	1	0.395635718	ENSG00000164253	8	WDR41
SAT	Obesity	ENSG00000134809.4	0.004399074	0.387226253	0.005087638	0.183454191	0.004696378	0.356054209	0.005088659	0.183420948	-0.000297304	0.015465315	1	0.67818082	1	0.657778074	ENSG00000164305	13	CASP3
SAT	Obesity	ENSG00000134851.8	-0.015294821	0.004917379	0.005438333	0.183451484	-0.015204635	0.005173944	0.005437971	0.183418316	-9.02E-05	0.016330597	1	0.049039272	1	0.053857262	ENSG00000164307	8	ERAP1
SAT	Obesity	ENSG00000134874.13	0.011334339	0.054543275	0.00589562	0.183453077	0.011226074	0.056875803	0.005895216	0.1834199	0.000108264	0.005435928	1	0.229137624	1	0.239958924	ENSG00000164307	8	ERAP1
SAT	Obesity	ENSG00000134905.12	-0.00084797	0.806094279	0.003454505	0.183454451	-0.000780832	0.821164115	0.003454275	0.183421251	-6.71E-05	0.00505132	1	0.924430385	1	0.932801998	ENSG00000164308	12	ERAP2
SAT	Obesity	ENSG00000135074.11	-0.003134952	0.102196636	0.001918223	0.183453464	-0.003168003	0.098610321	0.001918105	0.183420239	3.31E-05	0.023707816	1	0.325540933	1	0.327348022	ENSG00000164323	8	KIAA1430
SAT	Obesity	ENSG00000135314.8	0.000187695	0.943180287	0.002633461	0.183454472	0.000137271	0.958425563	0.00263328	0.183421269	5.04E-05	0.003780128	1	0.981335116	1	0.990485083	ENSG00000164332	6	UBLCP1
SAT	Obesity	ENSG00000135480.10	-0.007857268	0.158843582	0.00557659	0.183453724	-0.007981043	0.15235933	0.005576276	0.183420496	0.000123775	0.007442463	1	0.421996593	1	0.415882664	ENSG00000164334	11	FAM170A
SAT	Obesity	ENSG00000135968.15	-0.002952073	0.30168057	0.002858217	0.183454071	-0.002994017	0.294832195	0.002858029	0.183420855	4.19E-05	0.037253254	1	0.601710607	1	0.599223798	ENSG00000164344	11	KLKB1
SAT	Obesity	ENSG00000135972.4	0.006587331	0.003875183	0.002280805	0.183451321	0.006534016	0.004170656	0.002280657	0.183418168	5.33E-05	0.001104738	1	0.040751521	1	0.046543954	ENSG00000164346	5	NSA2
SAT	Obesity	ENSG00000136014.7	0.004869484	0.274746227	0.004458422	0.183454023	0.004941123	0.267713678	0.004458111	0.183420806	-7.16E-05	0.012857791	1	0.570894471	1	0.569985864	ENSG00000164465	14	DCBLD1
SAT	Obesity	ENSG00000136146.10	0.005469789	0.249606013	0.004750934	0.183453973	0.005546664	0.24298375	0.004750635	0.183420755	-7.69E-05	0.029359423	1	0.538787717	1	0.53813209	ENSG00000164465	14	DCBLD1
SAT	Obesity	ENSG00000136169.12	0.000560704	0.88283181	0.003804468	0.183454466	0.000459201	0.903922033	0.00380422	0.183421265	0.000101503	0.000171217	1	0.958494761	1	0.96423859	ENSG00000164535	10	DAGLB
SAT	Obesity	ENSG00000136213.7	-0.005305936	0.282322297	0.004935211	0.183454037	-0.005168401	0.294954719	0.004934905	0.183420856	-0.000137535	0.000212004	1	0.579661774	1	0.599223798	ENSG00000164535	10	DAGLB
SAT	Obesity	ENSG00000136247.10	-0.010763156	0.074122679	0.006026878	0.183453269	-0.010856033	0.071643237	0.006026501	0.183420044	9.29E-05	0.039236223	1	0.273561877	1	0.273327281	ENSG00000164543	5	STK17A
SAT	Obesity	ENSG00000136279.14	0.003732043	0.488882977	0.005392447	0.183454293	0.003630464	0.500764238	0.005392138	0.183421099	0.000101579	0.020857486	1	0.749894833	1	0.761367956	ENSG00000164556	7	FAM183B
SAT	Obesity	ENSG00000136371.5	-0.000647998	0.77440562	0.002260847	0.183454443	-0.000682233	0.762818781	0.002260685	0.183421236	3.42E-05	0.013809689	1	0.909817544	1	0.904912194	ENSG00000164556	7	FAM183B
SAT	Obesity	ENSG00000136715.13	-0.004589138	0.41100991	0.005582088	0.183454219	-0.004438498	0.426511026	0.005581774	0.183421031	-0.00015064	0.001102506	1	0.698355005	1	0.711073464	ENSG00000164574	11	GALNT10
SAT	Obesity	ENSG00000136816.11	-0.002133603	0.403201484	0.002552407	0.18345421	-0.002214779	0.385518991	0.002552256	0.183420986	8.12E-05	5.94E-05	1	0.692257493	1	0.680080079	ENSG00000164574	11	GALNT10
SAT	Obesity	ENSG00000136856.13	0.007563408	0.103966401	0.004651759	0.183453475	0.007475789	0.108011191	0.004651437	0.183420294	8.76E-05	0.003878564	1	0.330216614	1	0.345600181	ENSG00000164609	5	SLU7
SAT	Obesity	ENSG00000136866.9	-0.004674275	0.35951488	0.005101301	0.183454157	-0.004593274	0.36786923	0.005100959	0.183420964	-8.10E-05	0.020430736	1	0.655680001	1	0.66613537	ENSG00000164609	5	SLU7
SAT	Obesity	ENSG00000136932.9	0.006464235	0.126981096	0.004235737	0.183453594	0.006557352	0.121577812	0.004235503	0.183420364	-9.31E-05	0.008863576	1	0.374999979	1	0.369554001	ENSG00000164615	3	CAMLG
SAT	Obesity	ENSG00000136982.5	0.003457947	0.003448426	0.001182339	0.183451241	0.003482088	0.003226864	0.001182264	0.183417992	-2.41E-05	0.005083897	1	0.037340878	1	0.038781468	ENSG00000164615	3	CAMLG
SAT	Obesity	ENSG00000137185.7	0.011806211	1.67E-06	0.00246512	0.183445804	0.009808909	8.76E-05	0.002500657	0.183415455	0.001997302	2.13E-06	0.005492674	6.24E-05	0.287531101	0.001973489	ENSG00000164620	4	RELL2
SAT	Obesity	ENSG00000137216.14	-0.012268533	0.003566133	0.004209909	0.183451264	-0.012363997	0.003313348	0.004209623	0.18341801	9.55E-05	0.000751389	1	0.038236873	1	0.039245833	ENSG00000164659	10	KIAA1324L
SAT	Obesity	ENSG00000137310.7	0.02246155	3.57E-05	0.005433677	0.183448015	0.01361106	0.020379116	0.005868604	0.183419237	0.00885049	6.61E-05	0.117107381	0.000867462	1	0.13047531	ENSG00000164669	8	INTS4L1
SAT	Obesity	ENSG00000137312.10	0.009315128	0.008038518	0.003514534	0.183451819	0.006185409	0.084918119	0.00359024	0.183420148	0.003129719	2.05E-05	1	0.067813996	1	0.298677775	ENSG00000164669	8	INTS4L1
SAT	Obesity	ENSG00000137411.12	-0.001579698	0.133653898	0.001053237	0.183453624	-0.000273326	0.800578372	0.001082054	0.183421246	-0.001306372	1.46E-07	1	0.386967743	1	0.92223015	ENSG00000164733	16	CTSB
SAT	Obesity	ENSG00000137474.15	-0.003478086	0.473861043	0.004856226	0.18345428	-0.003389603	0.485156075	0.004855926	0.183421086	-8.85E-05	0.016198832	1	0.737541785	1	0.748543071	ENSG00000164733	16	CTSB
SAT	Obesity	ENSG00000137574.6	-0.0006054	0.846197078	0.003121053	0.18345446	-0.000651126	0.834731414	0.003120851	0.183421254	4.57E-05	0.040531258	1	0.942344807	1	0.936326075	ENSG00000164733	16	CTSB
SAT	Obesity	ENSG00000137675.4	0.005556998	0.129807091	0.003668311	0.183453607	0.005614368	0.125868974	0.003668083	0.183420385	-5.74E-05	0.038012269	1	0.380265236	1	0.378183246	ENSG00000164733	16	CTSB
SAT	Obesity	ENSG00000137692.7	0.006518386	0.217681869	0.005287819	0.1834539	0.006432441	0.223782632	0.005287519	0.183420711	8.59E-05	0.048097895	1	0.505890452	1	0.516186585	ENSG00000164825	3	DEFB1
SAT	Obesity	ENSG00000137709.5	-0.006674535	0.106212699	0.004131673	0.183453488	-0.00659788	0.110264926	0.004131405	0.183420306	-7.67E-05	0.009409686	1	0.334758757	1	0.34859618	ENSG00000164825	3	DEFB1
SAT	Obesity	ENSG00000137806.4	0.004194911	0.261448433	0.003735532	0.183453997	0.004280686	0.251792105	0.0037353	0.183420774	-8.58E-05	0.002360754	1	0.555218322	1	0.548923519	ENSG00000164825	3	DEFB1
SAT	Obesity	ENSG00000137876.5	-0.004648717	0.07977452	0.002653378	0.183453314	-0.004607981	0.082429919	0.002653203	0.18342013	-4.07E-05	0.028057924	1	0.285743367	1	0.295578034	ENSG00000164828	13	SUN1
SAT	Obesity	ENSG00000137992.10	5.45E-05	0.992720505	0.005976614	0.183454474	-0.000100516	0.986581119	0.005976369	0.18342127	0.000155044	0.009161302	1	0.996669516	1	0.995364986	ENSG00000164828	13	SUN1
SAT	Obesity	ENSG00000137996.8	0.005400361	0.062527326	0.002899457	0.183453163	0.005451873	0.060051727	0.002899288	0.183419934	-5.15E-05	0.02680978	1	0.250185557	1	0.24752477	ENSG00000164830	13	OXR1
SAT	Obesity	ENSG00000138030.8	-0.002942232	0.047734717	0.00148618	0.183452993	-0.002964096	0.046090384	0.001486083	0.183419767	2.19E-05	0.037452511	1	0.210507537	1	0.211796289	ENSG00000164830	13	OXR1
SAT	Obesity	ENSG00000138085.12	-0.000336922	0.951531892	0.005543005	0.183454473	-0.00022827	0.967149067	0.005542653	0.18342127	-0.000108652	0.00769603	1	0.985117699	1	0.992119797	ENSG00000164850	10	GPER1
SAT	Obesity	ENSG00000138135.5	0.004075177	0.346870264	0.004332169	0.18345414	0.004016618	0.353811115	0.004331879	0.183420945	5.86E-05	0.048037908	1	0.644805289	1	0.656220616	ENSG00000164850	10	GPER1
SAT	Obesity	ENSG00000138175.8	0.002143637	0.577792234	0.003851218	0.183454357	0.002065394	0.591731969	0.003850989	0.183421161	7.82E-05	0.009793004	1	0.804985236	1	0.81608768	ENSG00000164850	10	GPER1
SAT	Obesity	ENSG00000138592.9	0.001265443	0.750831838	0.003985107	0.183454436	0.001200016	0.76330409	0.003984844	0.183421236	6.54E-05	0.018660739	1	0.899291443	1	0.904912194	ENSG00000164904	11	ALDH7A1
SAT	Obesity	ENSG00000138621.7	-0.003720143	0.018565872	0.001580254	0.183452379	-0.00375242	0.017562245	0.001580147	0.183419139	3.23E-05	0.002517579	1	0.117823264	1	0.118319765	ENSG00000164924	13	YWHAZ
SAT	Obesity	ENSG00000138756.13	-0.007602705	0.040525861	0.003711617	0.183452888	-0.008774396	0.018662881	0.003730285	0.183419179	0.001171691	0.001837796	1	0.192172349	1	0.123670158	ENSG00000164924	13	YWHAZ
SAT	Obesity	ENSG00000138757.10	0.004533994	0.189948381	0.003459127	0.183453825	0.004609457	0.182665875	0.003459012	0.183420599	-7.55E-05	0.041819124	1	0.46712282	1	0.459606393	ENSG00000164924	13	YWHAZ
SAT	Obesity	ENSG00000138767.8	-0.004785503	0.0374498	0.002299803	0.183452837	-0.005019696	0.029152041	0.002301103	0.183419472	0.000234193	0.004943402	1	0.183666357	1	0.161406237	ENSG00000164924	13	YWHAZ
SAT	Obesity	ENSG00000138777.15	-0.003650896	0.305999719	0.003566537	0.183454078	-0.003883089	0.276308851	0.003566885	0.183420822	0.000232193	0.000791345	1	0.605655297	1	0.580018771	ENSG00000164924	13	YWHAZ
SAT	Obesity	ENSG00000138801.4	-0.005042806	0.219416157	0.00410624	0.183453904	-0.005148764	0.209860202	0.004106031	0.183420676	0.000105957	0.003720129	1	0.506545751	1	0.501023309	ENSG00000164941	9	INTS8
SAT	Obesity	ENSG00000138821.8	0.001901064	0.717797684	0.005260219	0.183454425	0.00172823	0.74248382	0.005259915	0.183421229	0.000172834	4.72E-05	1	0.883049944	1	0.897931963	ENSG00000164941	9	INTS8
SAT	Obesity	ENSG00000138829.6	-0.000202758	0.879592023	0.001338454	0.183454465	-0.000176311	0.895193275	0.001338371	0.183421264	-2.64E-05	0.008389228	1	0.957347365	1	0.963222823	ENSG00000164941	9	INTS8
SAT	Obesity	ENSG00000139190.12	-0.001249541	0.647051576	0.002729077	0.183454395	-0.001329093	0.626244588	0.002729033	0.183421181	7.96E-05	0.017040428	1	0.844125734	1	0.832877378	ENSG00000164967	5	RPP25L
SAT	Obesity	ENSG00000139200.9	0.002908681	0.625552286	0.00596045	0.183454384	0.003154202	0.596697964	0.005960824	0.183421164	-0.000245521	0.018636347	1	0.833307775	1	0.817137678	ENSG00000164967	5	RPP25L
SAT	Obesity	ENSG00000139220.12	0.001386338	0.729903751	0.004015418	0.183454429	0.002830266	0.485196869	0.004055002	0.183421086	-0.001443928	0.010988908	1	0.8889108	1	0.748543071	ENSG00000164967	5	RPP25L
SAT	Obesity	ENSG00000139343.6	-0.005679888	0.040648464	0.002774598	0.18345289	-0.005735832	0.038698075	0.002774433	0.183419655	5.59E-05	0.010810773	1	0.192172349	1	0.191795143	ENSG00000164978	13	NUDT2
SAT	Obesity	ENSG00000139344.3	-0.008834946	0.096019568	0.00530797	0.183453427	-0.0090333	0.088767106	0.005307646	0.183420175	0.000198353	1.23E-06	1	0.315355558	1	0.307220331	ENSG00000164978	13	NUDT2
SAT	Obesity	ENSG00000139405.11	-0.007204281	0.002893817	0.002418507	0.18345112	-0.007247464	0.002727848	0.002418348	0.183417876	4.32E-05	0.011224809	1	0.032627535	1	0.034160566	ENSG00000164978	13	NUDT2
SAT	Obesity	ENSG00000139620.8	-0.003864395	0.167114301	0.002797169	0.183453753	-0.003979824	0.154770374	0.002797012	0.183420505	0.000115429	6.73E-07	1	0.433440333	1	0.418287971	ENSG00000164989	11	CCDC171
SAT	Obesity	ENSG00000140323.4	-5.98E-05	0.977729618	0.002140789	0.183454474	-1.73E-05	0.993561087	0.002140641	0.18342127	-4.25E-05	0.00245062	1	0.993452012	1	0.997818772	ENSG00000164989	11	CCDC171
SAT	Obesity	ENSG00000140471.12	0.001348097	0.622146287	0.002735526	0.183454382	0.001441713	0.598155357	0.00273541	0.183421165	-9.36E-05	0.000506259	1	0.832018977	1	0.817909964	ENSG00000165055	11	METTL2B
SAT	Obesity	ENSG00000140497.12	0.00672156	0.072705319	0.003745269	0.183453257	0.006795693	0.069588842	0.003745047	0.183420026	-7.41E-05	0.012217483	1	0.269848588	1	0.268612932	ENSG00000165055	11	METTL2B
SAT	Obesity	ENSG00000140876.7	-0.000955459	0.875255008	0.00608622	0.183454465	-0.000791351	0.896542359	0.00608589	0.183421264	-0.000164107	0.001539633	1	0.955789527	1	0.963222823	ENSG00000165055	11	METTL2B
SAT	Obesity	ENSG00000140995.12	-0.002429442	0.505644189	0.003649806	0.183454307	-0.002364054	0.517137074	0.003649553	0.183421112	-6.54E-05	0.005928178	1	0.762495381	1	0.772996237	ENSG00000165055	11	METTL2B
SAT	Obesity	ENSG00000141013.10	0.001903053	0.304850877	0.001854669	0.183454076	0.001929169	0.29822879	0.001854541	0.183420861	-2.61E-05	0.032262203	1	0.605495891	1	0.601026164	ENSG00000165055	11	METTL2B
SAT	Obesity	ENSG00000141576.10	0.006021814	0.091304649	0.003566245	0.183453396	0.006101516	0.087081271	0.003566047	0.183420164	-7.97E-05	0.007765053	1	0.30630936	1	0.302983724	ENSG00000165118	10	C9orf64
SAT	Obesity	ENSG00000141580.11	-0.003631617	0.361154341	0.00397694	0.183454159	-0.003713701	0.350375397	0.003976712	0.183420941	8.21E-05	0.011226847	1	0.657208759	1	0.65249005	ENSG00000165171	6	WBSCR27
SAT	Obesity	ENSG00000141933.8	0.008552864	0.146669558	0.005892843	0.183453678	0.008394379	0.154279315	0.005892529	0.183420503	0.000158485	0.001809493	1	0.405752798	1	0.417993751	ENSG00000165171	6	WBSCR27
SAT	Obesity	ENSG00000141971.8	0.006599171	0.063235459	0.003552647	0.18345317	0.006661608	0.060760868	0.00355241	0.183419941	-6.24E-05	0.011247861	1	0.250993039	1	0.249243536	ENSG00000165171	6	WBSCR27
SAT	Obesity	ENSG00000142002.12	-0.002518178	0.635077825	0.005305982	0.183454389	-0.002424086	0.647751535	0.005305642	0.183421191	-9.41E-05	0.014789055	1	0.840197719	1	0.846421941	ENSG00000165171	6	WBSCR27
SAT	Obesity	ENSG00000142046.10	0.002705394	0.424291136	0.003385977	0.183454233	0.002641645	0.43526054	0.003385761	0.18342104	6.37E-05	0.00984779	1	0.707008237	1	0.717633082	ENSG00000165171	6	WBSCR27
SAT	Obesity	ENSG00000142166.8	-0.002639347	0.078528172	0.001500231	0.183453304	-0.002612215	0.081629281	0.00150014	0.183420124	-2.71E-05	0.019838314	1	0.282822099	1	0.294809859	ENSG00000165171	6	WBSCR27
SAT	Obesity	ENSG00000142207.5	-0.001071012	0.685724018	0.002646664	0.183454412	-0.001019162	0.700165415	0.002646504	0.183421214	-5.19E-05	0.011293197	1	0.862676574	1	0.876141391	ENSG00000165178	8	NCF1C
SAT	Obesity	ENSG00000142230.7	0.001566454	0.679198843	0.00378776	0.183454409	0.001894448	0.617099016	0.003789152	0.183421176	-0.000327994	0.004227974	1	0.858646871	1	0.828495448	ENSG00000165202	2	OR1Q1
SAT	Obesity	ENSG00000142632.12	-0.001302952	0.207110328	0.001032816	0.183453873	-0.001322602	0.200317073	0.001032758	0.18342065	1.97E-05	0.022470045	1	0.493814259	1	0.487105125	ENSG00000165202	2	OR1Q1
SAT	Obesity	ENSG00000142677.3	0.003841006	0.519291506	0.005960238	0.183454317	0.003986897	0.50352215	0.005959856	0.183421101	-0.000145891	0.000755024	1	0.772402624	1	0.763780016	ENSG00000165233	13	C9orf89
SAT	Obesity	ENSG00000142794.14	0.002103381	0.309294596	0.002068823	0.183454083	0.002149054	0.298876463	0.002068692	0.183420862	-4.57E-05	0.002727251	1	0.60688449	1	0.601026164	ENSG00000165233	13	C9orf89
SAT	Obesity	ENSG00000142798.12	-0.000564804	0.756504952	0.001821516	0.183454438	-0.000100573	0.956069377	0.001825718	0.183421269	-0.000464232	0.000234652	1	0.901627438	1	0.989546885	ENSG00000165244	6	ZNF367
SAT	Obesity	ENSG00000142937.7	0.0056392	0.068099543	0.003091071	0.183453216	0.005698773	0.065222371	0.003090885	0.183419985	-5.96E-05	0.013516019	1	0.261549209	1	0.260334062	ENSG00000165244	6	ZNF367
SAT	Obesity	ENSG00000143442.17	0.007849306	0.027222515	0.003554426	0.183452631	0.007951255	0.025279334	0.003554247	0.183419379	-0.000101949	0.001379404	1	0.149609837	1	0.147845799	ENSG00000165244	6	ZNF367
SAT	Obesity	ENSG00000143486.11	-0.004309996	0.280538184	0.00399402	0.183454034	-0.004399206	0.270677054	0.00399381	0.183420812	8.92E-05	0.010431695	1	0.579036375	1	0.574073313	ENSG00000165271	12	NOL6
SAT	Obesity	ENSG00000143811.12	-0.00258457	0.616609134	0.005162303	0.183454379	-0.00267942	0.603710829	0.005161944	0.183421168	9.49E-05	0.004525275	1	0.829137118	1	0.821898436	ENSG00000165271	12	NOL6
SAT	Obesity	ENSG00000144026.7	0.000244325	0.852926329	0.001317924	0.183454461	0.000276697	0.833700938	0.00131787	0.183421254	-3.24E-05	0.01298772	1	0.943648754	1	0.936326075	ENSG00000165271	12	NOL6
SAT	Obesity	ENSG00000144152.8	-0.009859154	0.00859581	0.003751959	0.183451864	-0.009798696	0.009006959	0.003751702	0.183418692	-6.05E-05	0.01496342	1	0.070683838	1	0.077624322	ENSG00000165280	11	VCP
SAT	Obesity	ENSG00000144214.5	0.003951432	0.190262885	0.003016812	0.183453826	0.003866542	0.199933729	0.00301663	0.183420649	8.49E-05	0.00028158	1	0.46712282	1	0.48663395	ENSG00000165280	11	VCP
SAT	Obesity	ENSG00000144218.14	-0.020248974	0.000761508	0.00601495	0.18345019	-0.020145986	0.000809544	0.006014568	0.18341703	-0.000102988	0.019377351	1	0.011104481	1	0.012532499	ENSG00000165410	10	CFL2
SAT	Obesity	ENSG00000144230.12	0.001007295	0.680766107	0.002448348	0.18345441	0.000964594	0.693580334	0.002448201	0.183421212	4.27E-05	0.024808384	1	0.859735796	1	0.869649775	ENSG00000165410	10	CFL2
SAT	Obesity	ENSG00000144407.5	-0.001937023	0.733914796	0.005698419	0.18345443	-0.002041376	0.720148979	0.00569803	0.183421222	0.000104353	0.005990139	1	0.88986491	1	0.885797449	ENSG00000165475	9	CRYL1
SAT	Obesity	ENSG00000144580.9	0.008553206	0.152609595	0.005979688	0.183453701	0.008449717	0.157608122	0.005979289	0.183420515	0.000103489	0.012178732	1	0.412938483	1	0.424210212	ENSG00000165476	8	REEP3
SAT	Obesity	ENSG00000144792.5	-0.001234583	0.54740986	0.002052008	0.183454337	-0.001191601	0.56141664	0.00205187	0.183421143	-4.30E-05	0.002028137	1	0.792029636	1	0.796027656	ENSG00000165478	6	HEPACAM
SAT	Obesity	ENSG00000145113.17	-0.001946989	0.431436529	0.002474767	0.18345424	-0.002024755	0.413235893	0.002474606	0.183421017	7.78E-05	1.04E-05	1	0.71240224	1	0.701318401	ENSG00000165502	6	RPL36AL
SAT	Obesity	ENSG00000145241.6	-0.014669883	9.49E-06	0.003312596	0.183447061	-0.014759239	8.36E-06	0.003312402	0.183413767	8.94E-05	0.000764915	0.031138176	0.000275559	0.027435231	0.00029764	ENSG00000165502	6	RPL36AL
SAT	Obesity	ENSG00000145715.10	-0.003842539	0.31535314	0.003827025	0.183454093	-0.003779063	0.323381035	0.003826764	0.183420902	-6.35E-05	0.013359918	1	0.613321667	1	0.626705952	ENSG00000165568	13	AKR1E2
SAT	Obesity	ENSG00000145990.6	0.0075942	0.03926252	0.003683947	0.183452868	0.00951102	0.010359296	0.003710011	0.183418786	-0.00191682	1.44E-05	1	0.189487109	1	0.083928965	ENSG00000165629	15	ATP5C1
SAT	Obesity	ENSG00000146007.6	-0.010087278	0.011415652	0.003987507	0.183452055	-0.009979617	0.012318704	0.003987237	0.183418903	-0.000107661	6.33E-05	1	0.084739265	1	0.094213678	ENSG00000165632	7	TAF3
SAT	Obesity	ENSG00000146109.3	0.011640342	0.000788459	0.003467653	0.183450215	0.012643625	0.000270544	0.003471605	0.183416257	-0.001003283	5.66E-09	1	0.011353235	0.887656266	0.005101473	ENSG00000165632	7	TAF3
SAT	Obesity	ENSG00000146425.6	0.005128378	0.342582713	0.005403552	0.183454134	0.004953537	0.35925916	0.005403199	0.183420953	0.000174842	4.97E-06	1	0.641194456	1	0.660152534	ENSG00000165650	7	PDZD8
SAT	Obesity	ENSG00000146453.8	0.008070042	0.166235933	0.00582926	0.18345375	0.008179773	0.160522934	0.005828884	0.183420526	-0.000109732	0.009116725	1	0.43218708	1	0.428934236	ENSG00000165650	7	PDZD8
SAT	Obesity	ENSG00000146535.9	-0.004347358	0.504326952	0.006510969	0.183454306	-0.004449484	0.494334479	0.006510504	0.183421094	0.000102126	0.011248586	1	0.761480317	1	0.755382565	ENSG00000165650	7	PDZD8
SAT	Obesity	ENSG00000146556.10	-0.002780939	0.622302521	0.005645545	0.183454382	-0.002653163	0.638364542	0.005645184	0.183421187	-0.000127776	0.001927595	1	0.832018977	1	0.840143029	ENSG00000165650	7	PDZD8
SAT	Obesity	ENSG00000146733.9	-0.003894974	0.467026201	0.005355192	0.183454274	-0.00401592	0.453277198	0.005354822	0.183421058	0.000120946	0.000582249	1	0.733866362	1	0.728306801	ENSG00000165682	10	CLEC1B
SAT	Obesity	ENSG00000147419.12	9.24E-05	0.986706117	0.005546247	0.183454474	0.000229374	0.967010165	0.00554601	0.18342127	-0.000136962	0.011637802	1	0.994152558	1	0.992119797	ENSG00000165684	3	SNAPC4
SAT	Obesity	ENSG00000147576.11	-0.009376989	0.001660852	0.002981535	0.183450735	-0.007454924	0.014723654	0.003056406	0.183419022	-0.001922065	0.004319295	1	0.020719605	1	0.107351796	ENSG00000165684	3	SNAPC4
SAT	Obesity	ENSG00000147853.12	-0.003343788	0.542861323	0.005495202	0.183454334	-0.003460772	0.528811033	0.005494837	0.18342112	0.000116984	0.002181429	1	0.790819813	1	0.780841134	ENSG00000165684	3	SNAPC4
SAT	Obesity	ENSG00000147955.12	-0.001660666	0.693254609	0.00421016	0.183454415	-0.001550628	0.712629036	0.004209914	0.183421219	-0.000110038	0.001105952	1	0.867162933	1	0.882648497	ENSG00000165684	3	SNAPC4
SAT	Obesity	ENSG00000148229.8	-0.004721624	0.360804447	0.005166828	0.183454158	-0.004606319	0.372620103	0.005166485	0.18342097	-0.000115304	0.001308371	1	0.65717171	1	0.669643631	ENSG00000165684	3	SNAPC4
SAT	Obesity	ENSG00000148814.13	-0.001276022	0.507168523	0.001923882	0.183454308	-0.00131762	0.493398033	0.001923772	0.183421093	4.16E-05	0.007837695	1	0.763662195	1	0.755129058	ENSG00000165684	3	SNAPC4
SAT	Obesity	ENSG00000149179.9	-0.000325492	0.955653728	0.005853278	0.183454473	-0.00047698	0.935048672	0.005852903	0.183421268	0.000151488	0.000376596	1	0.986058193	1	0.979844999	ENSG00000165684	3	SNAPC4
SAT	Obesity	ENSG00000150054.14	-0.001727267	0.447907501	0.002275987	0.183454256	-0.00035815	0.87892448	0.002351099	0.183421261	-0.001369117	0.02038019	1	0.723220724	1	0.958406391	ENSG00000165730	10	STOX1
SAT	Obesity	ENSG00000150316.7	0.00269348	0.339870969	0.002822108	0.18345413	0.002747344	0.330269856	0.002821923	0.183420912	-5.39E-05	0.006975767	1	0.638034522	1	0.632292088	ENSG00000165730	10	STOX1
SAT	Obesity	ENSG00000151006.7	-0.023527874	1.50E-06	0.004890781	0.183445727	-0.023778637	1.16E-06	0.004890954	0.183412337	0.000250763	0.001252572	0.004937235	5.67E-05	0.003818547	5.62E-05	ENSG00000165730	10	STOX1
SAT	Obesity	ENSG00000151148.9	0.008436753	0.01641345	0.003515902	0.183452298	0.008361935	0.017385482	0.003515692	0.183419132	7.48E-05	0.006566792	1	0.108703412	1	0.118319765	ENSG00000165733	7	BMS1
SAT	Obesity	ENSG00000151208.12	-0.001661362	0.674541626	0.003956346	0.183454407	-0.001571894	0.691125867	0.003956162	0.183421211	-8.95E-05	0.016043125	1	0.856857118	1	0.868937924	ENSG00000165794	5	SLC39A2
SAT	Obesity	ENSG00000151348.9	0.00112608	0.551752403	0.001892137	0.18345434	0.001093986	0.563118439	0.001892001	0.183421144	3.21E-05	0.005158104	1	0.793295194	1	0.797062812	ENSG00000165806	15	CASP7
SAT	Obesity	ENSG00000151532.9	0.006993095	0.179747067	0.005212751	0.183453794	0.006882203	0.186719473	0.005212416	0.183420611	0.000110891	0.003250322	1	0.452624689	1	0.466081875	ENSG00000165806	15	CASP7
SAT	Obesity	ENSG00000151640.8	0.005976272	0.000142848	0.001571374	0.183449007	0.005947517	0.000153636	0.001571277	0.183415855	2.88E-05	0.016516826	0.468683054	0.002709151	0.504078468	0.003273237	ENSG00000165832	4	TRUB1
SAT	Obesity	ENSG00000151657.7	0.002899045	0.523320603	0.00454228	0.18345432	0.002995448	0.509577699	0.004542026	0.183421106	-9.64E-05	0.010764878	1	0.775526151	1	0.767790037	ENSG00000165868	8	HSPA12A
SAT	Obesity	ENSG00000151725.7	0.002130938	0.535023064	0.003435018	0.183454329	0.0020731	0.54613995	0.003434816	0.183421133	5.78E-05	0.034102871	1	0.784632613	1	0.788841592	ENSG00000165868	8	HSPA12A
SAT	Obesity	ENSG00000151883.12	-0.005933669	0.178525652	0.004410632	0.18345379	-0.005792587	0.189049669	0.004410384	0.183420618	-0.000141082	0.000109748	1	0.45217888	1	0.469903003	ENSG00000165868	8	HSPA12A
SAT	Obesity	ENSG00000152061.17	0.010005882	0.001587738	0.003168255	0.183450704	0.00993509	0.001712666	0.003168055	0.183417553	7.08E-05	0.002440989	1	0.019959267	1	0.022842502	ENSG00000165912	11	PACSIN3
SAT	Obesity	ENSG00000152219.4	-0.022319951	1.21E-05	0.005100491	0.183447236	-0.022440645	1.08E-05	0.005100145	0.183413954	0.000120694	0.00048148	0.039660702	0.000336108	0.035524439	0.000355244	ENSG00000165912	11	PACSIN3
SAT	Obesity	ENSG00000152223.8	0.000841108	0.813327121	0.003561961	0.183454453	0.000784707	0.825625575	0.003561744	0.183421252	5.64E-05	0.039219293	1	0.927860321	1	0.935559334	ENSG00000165912	11	PACSIN3

SAT	Obesity	ENSG00000152253.4	0.000705455	0.826728483	0.003222737	0.183454456	0.001235901	0.702188417	0.003232227	0.183421215	-0.000530446	0.034767449	1	0.9363121	1	0.876674335	ENSG00000165916	4	PSMC3
SAT	Obesity	ENSG00000152291.9	-0.006183971	0.203150148	0.004859207	0.183453862	-0.006621082	0.173138957	0.00486062	0.183420569	0.000437111	0.001127777	1	0.487947024	1	0.445195076	ENSG00000165917	5	RAPSN
SAT	Obesity	ENSG00000152348.11	-0.000554765	0.621977447	0.00112517	0.183454382	-0.000571669	0.611380398	0.001125101	0.183421173	1.69E-05	0.048496138	1	0.832018977	1	0.82447147	ENSG00000165917	5	RAPSN
SAT	Obesity	ENSG00000152527.9	-7.95E-05	0.968095754	0.001988486	0.183454474	-3.81E-05	0.984719192	0.001988381	0.18342127	-4.14E-05	0.016143758	1	0.992600678	1	0.995364986	ENSG00000165917	5	RAPSN
SAT	Obesity	ENSG00000152689.13	0.005168608	0.135404292	0.003461597	0.183453631	0.00755332	0.038520216	0.003650214	0.183419652	-0.002384711	0.039657799	1	0.38936151	1	0.191782742	ENSG00000165929	8	TC2N
SAT	Obesity	ENSG00000152942.14	-0.00344271	0.132314526	0.002287455	0.183453618	-0.003388049	0.13854494	0.002287316	0.183420441	-5.47E-05	0.001931022	1	0.384861667	1	0.398742061	ENSG00000165934	8	CPSF2
SAT	Obesity	ENSG00000153044.5	0.008939065	0.026540407	0.00402984	0.183452614	0.008874345	0.027644804	0.004029586	0.183419437	6.47E-05	0.029942143	1	0.14734192	1	0.155261977	ENSG00000165934	8	CPSF2
SAT	Obesity	ENSG00000153714.5	0.006660376	0.14010927	0.004514326	0.183453651	0.006792319	0.132401223	0.004514061	0.183420415	-0.000131943	0.000252847	1	0.396084299	1	0.389207047	ENSG00000165934	8	CPSF2
SAT	Obesity	ENSG00000153814.7	0.020296208	3.37E-05	0.004894456	0.183447974	0.020498389	2.82E-05	0.004894936	0.183414643	-0.000202181	0.03355512	0.110651336	0.000831965	0.092484676	0.000818448	ENSG00000165934	8	CPSF2
SAT	Obesity	ENSG00000154222.10	0.00513515	0.028582514	0.00234567	0.183452663	0.005186885	0.027007793	0.002345509	0.183419422	-5.17E-05	0.00078685	1	0.154014794	1	0.153308944	ENSG00000165983	10	PTER
SAT	Obesity	ENSG00000154240.12	0.01592239	0.002916291	0.005349472	0.183451125	0.015441913	0.003927644	0.00535447	0.183418127	0.000480477	0.047259533	1	0.032768325	1	0.044436554	ENSG00000165985	8	C1QL3
SAT	Obesity	ENSG00000154309.7	0.000267185	0.930737937	0.003074042	0.183454471	0.000215974	0.943984739	0.003073821	0.183421268	5.12E-05	0.00607147	1	0.977387556	1	0.983503141	ENSG00000166037	6	CEP57
SAT	Obesity	ENSG00000154743.13	-0.011276601	0.040156339	0.005495038	0.183452882	-0.01140813	0.037875252	0.005494696	0.183419641	0.00013153	0.001484163	1	0.191780129	1	0.189433997	ENSG00000166037	6	CEP57
SAT	Obesity	ENSG00000155085.11	0.009461962	0.105459036	0.005844512	0.183453483	0.009332624	0.110284314	0.005844142	0.183420306	0.000129338	0.002683228	1	0.333987547	1	0.34859618	ENSG00000166037	6	CEP57
SAT	Obesity	ENSG00000155115.6	0.003422508	0.426293008	0.004302055	0.183454235	0.003364329	0.434167212	0.004301764	0.183421039	5.82E-05	0.045127801	1	0.708903881	1	0.717272217	ENSG00000166037	6	CEP57
SAT	Obesity	ENSG00000155393.8	0.00048565	0.774600367	0.001695925	0.183454443	0.000518044	0.75999883	0.001695821	0.183421235	-3.24E-05	0.011747079	1	0.909817544	1	0.904912194	ENSG00000166037	6	CEP57
SAT	Obesity	ENSG00000155542.7	-0.006318458	0.018973393	0.00269322	0.183452394	-0.006351998	0.018340415	0.00269303	0.183419168	3.35E-05	0.049134431	1	0.119714811	1	0.122805922	ENSG00000166037	6	CEP57
SAT	Obesity	ENSG00000155592.11	0.008361886	0.011741537	0.003318435	0.183452074	0.008419267	0.01117183	0.003318216	0.183418837	-5.74E-05	0.013331985	1	0.085991032	1	0.087690846	ENSG00000166037	6	CEP57
SAT	Obesity	ENSG00000156414.14	-0.0096398	0.014248521	0.003933095	0.183452203	-0.009580518	0.014849637	0.003932834	0.183419027	-5.93E-05	0.029463762	1	0.099045337	1	0.107919055	ENSG00000166140	13	ZFYVE19
SAT	Obesity	ENSG00000156795.2	-0.001039659	0.757841364	0.003372046	0.183454438	-0.001093918	0.745612654	0.003371816	0.18342123	5.43E-05	0.015907675	1	0.902205195	1	0.898760093	ENSG00000166148	2	AVPR1A
SAT	Obesity	ENSG00000156886.11	-0.003200233	0.504752629	0.004797727	0.183454306	-0.003678892	0.44338913	0.004799702	0.183421048	0.000478659	0.00164774	1	0.761772481	1	0.722682432	ENSG00000166166	8	TRMT61A
SAT	Obesity	ENSG00000157045.4	0.000934622	0.865784309	0.005529792	0.183454463	0.000780787	0.887708285	0.005529467	0.183421263	0.000153835	0.000477649	1	0.951956541	1	0.962898024	ENSG00000166166	8	TRMT61A
SAT	Obesity	ENSG00000157214.9	-0.011559894	0.00021588	0.00312455	0.1834493	-0.011628705	0.000197743	0.00312439	0.183416035	6.88E-05	0.013238182	0.70830134	0.003891766	0.648793464	0.004054959	ENSG00000166206	9	GABRB3
SAT	Obesity	ENSG00000157870.10	-0.005257976	0.269931681	0.004766022	0.183454014	-0.00517504	0.277527178	0.004765703	0.183420825	-8.29E-05	0.011191826	1	0.566269722	1	0.580588871	ENSG00000166220	8	TBATA
SAT	Obesity	ENSG00000158022.6	0.005384886	0.19589504	0.004163559	0.183453842	0.005323749	0.200989736	0.004163282	0.183420652	6.11E-05	0.033481901	1	0.476192171	1	0.48811793	ENSG00000166224	12	SGPL1
SAT	Obesity	ENSG00000158161.11	0.002776322	0.439055073	0.003587951	0.183454248	0.002707683	0.450427417	0.003587745	0.183421055	6.86E-05	0.018741821	1	0.717400247	1	0.727311113	ENSG00000166224	12	SGPL1
SAT	Obesity	ENSG00000158201.5	0.001557874	0.726803615	0.004458958	0.183454428	0.001680881	0.706181576	0.004458695	0.183421216	-0.000123007	0.000525851	1	0.887859509	1	0.879167935	ENSG00000166228	4	PCBD1
SAT	Obesity	ENSG00000158552.8	-0.007592652	0.13139448	0.005032819	0.183453614	-0.007757367	0.123206483	0.005032493	0.183420372	0.000164715	5.56E-06	1	0.382864378	1	0.372571864	ENSG00000166228	4	PCBD1
SAT	Obesity	ENSG00000158805.7	-0.011182676	0.034321151	0.005284109	0.183452781	-0.011083747	0.035931853	0.005283753	0.183419607	-9.89E-05	0.005845221	1	0.172711189	1	0.184495162	ENSG00000166228	4	PCBD1
SAT	Obesity	ENSG00000158850.10	0.005961845	0.168232727	0.004326741	0.183453756	0.006044212	0.162403999	0.00432646	0.183420533	-8.24E-05	0.008002389	1	0.434800182	1	0.430422843	ENSG00000166260	6	COX11
SAT	Obesity	ENSG00000158864.8	0.002979316	0.352737934	0.003206004	0.183454148	0.002927948	0.361067965	0.003205784	0.183420955	5.14E-05	0.015287923	1	0.650001169	1	0.6621934	ENSG00000166260	6	COX11
SAT	Obesity	ENSG00000158869.6	0.008650149	0.021544632	0.003763701	0.183452477	0.008721382	0.020482828	0.003763448	0.183419241	-7.12E-05	0.005436472	1	0.129941061	1	0.13047531	ENSG00000166262	11	FAM227B
SAT	Obesity	ENSG00000159111.8	-0.00260914	0.581455962	0.004733052	0.183454359	-0.002710283	0.566871565	0.004732752	0.183421146	0.000101142	0.003691237	1	0.806390405	1	0.800506675	ENSG00000166262	11	FAM227B
SAT	Obesity	ENSG00000159267.10	-0.000496816	0.847564622	0.002584531	0.18345446	-0.00046325	0.85773952	0.002584345	0.183421258	-3.36E-05	0.033362568	1	0.942344807	1	0.947556688	ENSG00000166268	6	MYRFL
SAT	Obesity	ENSG00000159479.12	0.004839115	0.323392777	0.004900316	0.183454105	0.004914055	0.31592491	0.0049	0.18342089	-7.49E-05	0.033835249	1	0.622290867	1	0.615584597	ENSG00000166268	6	MYRFL
SAT	Obesity	ENSG00000159588.10	0.005118838	0.377850538	0.005804563	0.18345418	0.005218739	0.368586876	0.005804246	0.183420965	-9.99E-05	0.042329881	1	0.671085989	1	0.667026933	ENSG00000166275	11	C10orf32
SAT	Obesity	ENSG00000159720.7	-0.012106076	0.012935407	0.004870579	0.183452139	-0.01217431	0.012429271	0.004870258	0.183418909	6.82E-05	0.046234913	1	0.09286886	1	0.094838226	ENSG00000166275	11	C10orf32
SAT	Obesity	ENSG00000159904.7	0.001025495	0.741958551	0.003114535	0.183454433	0.001078673	0.729074176	0.003114333	0.183421225	-5.32E-05	0.01655326	1	0.895159095	1	0.891559411	ENSG00000166278	10	C2
SAT	Obesity	ENSG00000160049.7	0.01275909	0.00479807	0.004524018	0.183451468	0.012898403	0.00435504	0.004523781	0.183418198	-0.000139314	0.000431394	1	0.047995329	1	0.047949285	ENSG00000166278	10	C2
SAT	Obesity	ENSG00000160072.15	0.001925734	0.74531773	0.005928625	0.183454434	0.001841726	0.756051421	0.005928233	0.183421234	8.40E-05	0.04256594	1	0.895746327	1	0.902694582	ENSG00000166278	10	C2
SAT	Obesity	ENSG00000160179.14	0.001452151	0.715428258	0.003983125	0.183454424	0.001523171	0.702140009	0.00398284	0.183421215	-7.10E-05	0.003874576	1	0.882123626	1	0.876674335	ENSG00000166321	9	NUDT13
SAT	Obesity	ENSG00000160194.13	0.001505744	0.216879581	0.001219354	0.183453898	0.001524472	0.211186256	0.001219273	0.183420679	-1.87E-05	0.027140202	1	0.505890452	1	0.502449821	ENSG00000166321	9	NUDT13
SAT	Obesity	ENSG00000160201.7	-0.004858183	0.286750528	0.004560483	0.183454045	-0.004784594	0.294082006	0.004560188	0.183420854	-7.36E-05	0.024564332	1	0.585820974	1	0.598191606	ENSG00000166321	9	NUDT13
SAT	Obesity	ENSG00000160213.5	0.000550338	0.845283278	0.002820221	0.18345446	0.000608873	0.829058791	0.002820043	0.183421253	-5.85E-05	0.005102947	1	0.941763438	1	0.936326075	ENSG00000166321	9	NUDT13
SAT	Obesity	ENSG00000160226.11	-0.000448597	0.809391679	0.001859772	0.183454452	-0.000488564	0.792767255	0.001859646	0.183421244	4.00E-05	0.001593135	1	0.925742147	1	0.919712977	ENSG00000166348	13	USP54
SAT	Obesity	ENSG00000160229.7	0.004045638	0.316253237	0.004036805	0.183454094	0.003951773	0.32759628	0.004036672	0.183420908	9.39E-05	0.030162089	1	0.614343914	1	0.630037159	ENSG00000166394	10	CYB5R2
SAT	Obesity	ENSG00000160233.6	-0.002343865	0.69320219	0.005941154	0.183454415	-0.00242909	0.682623112	0.005940743	0.183421207	8.52E-05	0.0279661	1	0.867162933	1	0.86636807	ENSG00000166394	10	CYB5R2
SAT	Obesity	ENSG00000160321.10	-0.011712806	0.055881837	0.006126071	0.183453092	-0.011836238	0.053334702	0.006125765	0.183419859	0.000123433	0.025524977	1	0.232463336	1	0.230862672	ENSG00000166402	4	TUB
SAT	Obesity	ENSG00000160752.10	0.002162016	0.580100763	0.003907932	0.183454358	0.002225423	0.569019487	0.003907703	0.183421148	-6.34E-05	0.042380114	1	0.806390405	1	0.801026154	ENSG00000166435	11	XRRA1
SAT	Obesity	ENSG00000160783.15	0.012047544	0.005734184	0.004360914	0.183451589	0.012131913	0.005400709	0.004360672	0.183418345	-8.44E-05	0.020680062	1	0.054062808	1	0.055454223	ENSG00000166435	11	XRRA1
SAT	Obesity	ENSG00000160948.9	0.001091163	0.55561042	0.001851392	0.183454343	0.001126626	0.542813489	0.001851285	0.18342113	-3.55E-05	0.017801829	1	0.794232732	1	0.788389136	ENSG00000166436	11	TRIM66
SAT	Obesity	ENSG00000161243.4	0.010461365	0.023849703	0.004629847	0.183452544	0.010580811	0.022284497	0.004629557	0.183419296	-0.000119445	0.000579469	1	0.140084245	1	0.137177177	ENSG00000166436	11	TRIM66
SAT	Obesity	ENSG00000161904.7	-0.01103483	0.006718127	0.00407112	0.183451697	-0.01129126	0.005563891	0.004072671	0.183418365	0.00025643	0.040349074	1	0.060224517	1	0.056517422	ENSG00000166436	11	TRIM66
SAT	Obesity	ENSG00000161912.13	0.004336278	0.180872304	0.003240682	0.183453797	0.004194709	0.195508057	0.003240515	0.183420637	0.00014157	7.24E-07	1	0.453456712	1	0.479418486	ENSG00000166446	10	CDYL2
SAT	Obesity	ENSG00000161929.10	-0.010176793	0.032532918	0.004760389	0.183452747	-0.010264827	0.031050422	0.004760097	0.183419513	8.80E-05	0.015485459	1	0.16809528	1	0.166130468	ENSG00000166446	10	CDYL2
SAT	Obesity	ENSG00000161996.13	-0.003718024	0.144414275	0.002547376	0.183453669	-0.003665595	0.150136606	0.00254723	0.183420488	-5.24E-05	0.011476037	1	0.401443831	1	0.414396534	ENSG00000166446	10	CDYL2
SAT	Obesity	ENSG00000162244.6	0.000132786	0.976264773	0.004463088	0.183454474	5.04E-05	0.990983953	0.004462799	0.18342127	8.24E-05	0.010275817	1	0.993452012	1	0.996450613	ENSG00000166446	10	CDYL2
SAT	Obesity	ENSG00000162341.11	0.016030447	0.005200773	0.005736755	0.183451523	0.016427474	0.004189299	0.005736735	0.183418171	-0.000397027	1.64E-07	1	0.05087712	1	0.046593526	ENSG00000166529	10	ZSCAN21
SAT	Obesity	ENSG00000162383.7	-0.000649727	0.809829963	0.002699932	0.183454452	-0.000710306	0.792473006	0.002699748	0.183421244	6.06E-05	0.000799772	1	0.925742147	1	0.919712977	ENSG00000166529	10	ZSCAN21
SAT	Obesity	ENSG00000162384.9	-0.007494974	0.113425585	0.00473471	0.183453527	-0.007365911	0.119750961	0.004734416	0.183420355	-0.000129064	0.000285244	1	0.350423113	1	0.368420209	ENSG00000166532	11	RIMKLB
SAT	Obesity	ENSG00000162402.8	-3.51E-05	0.992195832	0.00359313	0.183454474	-0.000114359	0.97460829	0.003592911	0.18342127	7.92E-05	0.004099402	1	0.996612544	1	0.992763055	ENSG00000166532	11	RIMKLB
SAT	Obesity	ENSG00000162415.6	0.004593176	0.2967648	0.004402135	0.183454063	0.004506794	0.30591159	0.004401856	0.183420874	8.64E-05	0.007585142	1	0.596760583	1	0.606176583	ENSG00000166548	11	TK2
SAT	Obesity	ENSG00000162600.7	0.001563518	0.563480831	0.002706549	0.183454348	0.001514649	0.57571359	0.002706388	0.183421152	4.89E-05	0.022218939	1	0.797231826	1	0.80345227	ENSG00000166557	8	TMED3
SAT	Obesity	ENSG00000162694.9	0.000290494	0.945520542	0.004251145	0.183454472	0.000357138	0.933044757	0.004250894	0.183421268	-6.66E-05	0.047812876	1	0.982191461	1	0.979844999	ENSG00000166557	8	TMED3
SAT	Obesity	ENSG00000163041.5	0.002388564	0.359257272	0.002605378	0.183454156	0.002315267	0.374164451	0.002605227	0.183420972	7.33E-05	0.000472694	1	0.655574588	1	0.669643631	ENSG00000166557	8	TMED3
SAT	Obesity	ENSG00000163138.14	0.003637757	0.23565102	0.003067433	0.183453942	0.003691953	0.228715425	0.003067231	0.183420723	-5.42E-05	0.011978898	1	0.525001456	1	0.523302168	ENSG00000166573	4	GALR1
SAT	Obesity	ENSG00000163297.12	0.023477296	6.77E-05	0.005892422	0.183448474	0.024608233	3.11E-05	0.005907977	0.183414713	-0.001130938	0.009461325	0.222069572	0.00144201	0.102058163	0.000895247	ENSG00000166595	7	FAM96B
SAT	Obesity	ENSG00000163453.7	-0.009384744	0.057243893	0.004935576	0.183453107	-0.008749175	0.076713737	0.004942801	0.183420086	-0.000635569	0.0209356	1	0.236545607	1	0.284725986	ENSG00000166664	9	CHRFAM7A
SAT	Obesity	ENSG00000163521.11	-0.008042336	0.05597258	0.004207885	0.183453093	-0.008127533	0.053405903	0.004207606	0.18341986	8.52E-05	0.003630987	1	0.232463336	1	0.230862672	ENSG00000166667	8	SPDYE6
SAT	Obesity	ENSG00000163545.7	0.000779337	0.82704928	0.003566963	0.183454456	0.000591607	0.868293646	0.003567626	0.18342126	0.000187731	0.025194097	1	0.936352204	1	0.954321831	ENSG00000166669	9	ATF7IP2
SAT	Obesity	ENSG00000163554.7	0.000167582	0.941804557	0.002295575	0.183454472	0.000414806	0.856649745	0.002296307	0.183421258	-0.000247225	0.000168493	1	0.981335116	1	0.947314554	ENSG00000166669	9	ATF7IP2
SAT	Obesity	ENSG00000163576.13	0.001215968	0.201420157	0.000951821	0.183453857	0.001198306	0.208019943	0.000951769	0.183420671	1.77E-05	0.029395043	1	0.485212581	1	0.497821614	ENSG00000166669	9	ATF7IP2
SAT	Obesity	ENSG00000163611.7	0.002172785	0.641923384	0.004672541	0.183454392	0.002250892	0.629979498	0.004672266	0.183421182	-7.81E-05	0.035973905	1	0.843249571	1	0.834462145	ENSG00000166669	9	ATF7IP2
SAT	Obesity	ENSG00000163728.6	0.000751188	0.844463459	0.003828929	0.18345446	0.000804373	0.833596044	0.00382867	0.183421254	-5.32E-05	0.039787446	1	0.941763438	1	0.936326075	ENSG00000166716	5	ZNF592
SAT	Obesity	ENSG00000164031.12	0.006967433	0.060758087	0.003715456	0.183453145	0.007057464	0.05748532	0.003715223	0.183419906	-9.00E-05	0.001183097	1	0.244597894	1	0.24057313	ENSG00000166762	12	CATSPER2
SAT	Obesity	ENSG00000164045.7	-0.000500265	0.89105048	0.003652228	0.183454467	-0.000571128	0.875727662	0.003652007	0.183421261	7.09E-05	0.012350405	1	0.962747126	1	0.958314845	ENSG00000166762	12	CATSPER2
SAT	Obesity	ENSG00000164048.9	-0.000291498	0.833609827	0.001387597	0.183454457	-0.000318783	0.818284947	0.00138751	0.18342125	2.73E-05	0.00903385	1	0.936669124	1	0.931572835	ENSG00000166763	7	STRCP1
SAT	Obesity	ENSG00000164062.8	-0.022469708	1.10E-12	0.003157126	0.183435329	-0.022516413	9.88E-13	0.003156923	0.183402046	4.67E-05	0.040786092	3.62E-09	1.29E-10	3.24E-09	1.20E-10	ENSG00000166770	6	ZNF667-AS1
SAT	Obesity	ENSG00000164144.10	0.002069338	0.692650761	0.005235377	0.183454415	0.001942402	0.710608242	0.005235028	0.183421218	0.000126936	0.00042805	1	0.867162933	1	0.882143641	ENSG00000166787	3	SAA3P
SAT	Obesity	ENSG00000164465.14	0.007691907	0.119351352	0.004938609	0.183453557	0.006276508	0.206350645	0.004966906	0.183420667	0.001415399	0.007981441	1	0.361914776	1	0.495716632	ENSG00000166796	7	LDHC
SAT	Obesity	ENSG00000164556.7	0.000718703	0.861674006	0.004124701	0.183454463	0.000639338	0.876811046	0.004124411	0.183421261	7.94E-05	0.002478913	1	0.94902733	1	0.958314845	ENSG00000166796	7	LDHC
SAT	Obesity	ENSG00000164574.11	-0.012159702	0.029200535	0.005575863	0.183452676	-0.012075294	0.030328709	0.005575502	0.183419497	-8.44E-05	0.034725846	1	0.156547313	1	0.163665286	ENSG00000166796	7	LDHC
SAT	Obesity	ENSG00000164609.5	0.007353221	0.222009604	0.006021291	0.18345391	0.007202548	0.231598974	0.006020931	0.183420729	0.000150673	0.001393261	1	0.509598947	1	0.524775447	ENSG00000166801	11	FAM111A
SAT	Obesity	ENSG00000164825.3	-0.007618896	0.159402851	0.005414667	0.183453726	-0.007521128	0.164801955	0.005414376	0.183420541	-9.78E-05	0.03503692	1	0.423139768	1	0.433943657	ENSG00000166801	11	FAM111A
SAT	Obesity	ENSG00000164850.10	0.005114922	0.068773861	0.002810541	0.183453222	0.005034277	0.07324507	0.002810397	0.183420057	8.06E-05	0.001188733	1	0.262380278	1	0.275592976	ENSG00000166801	11	FAM111A
SAT	Obesity	ENSG00000164924.13	-0.010931669	0.000103656	0.00281606	0.183448778	-0.010821797	0.000121501	0.002815896	0.183415688	-0.000109873	1.06E-06	0.340096136	0.002024382	0.398643746	0.002622656	ENSG00000166801	11	FAM111A
SAT	Obesity	ENSG00000165055.11	0.003592767	0.041485567	0.001762327	0.183452903	0.003621919	0.039849499	0.001762224	0.183419674	-2.92E-05	0.040014133	1	0.193338752	1	0.195087821	ENSG00000166821	4	PEX11A
SAT	Obesity	ENSG00000165233.13	0.003781989	0.381450098	0.004321146	0.183454185	0.003865205	0.37103377	0.004320888	0.183420968	-8.32E-05	0.014295096	1	0.674683434	1	0.668682157	ENSG00000166839	12	ANKDD1A
SAT	Obesity	ENSG00000165244.6	0.007065398	0.224908779	0.005821947	0.183453917	0.006932063	0.233748074	0.005821541	0.183420734	0.000133335	0.000370702	1	0.513161129	1	0.527090757	ENSG00000166839	12	ANKDD1A
SAT	Obesity	ENSG00000165502.6	-0.000897997	0.713855482	0.002448971	0.183454423	-0.000950326	0.697960794	0.00244882	0.183421213	5.23E-05	0.004835085	1	0.881706793	1	0.8747171	ENSG00000166847	5	DCTN5
SAT	Obesity	ENSG00000165632.7	0.012341178	0.024214558	0.005475927	0.183452554	0.012431014	0.023192772	0.005475611	0.183419322	-8.98E-05	0.043069598	1	0.141366488	1	0.141441422	ENSG00000166847	5	DCTN5
SAT	Obesity	ENSG00000165650.7	0.001029718	0.667065617	0.002393696	0.183454404	-0.000367291	0.879498172	0.002422673	0.183421262	0.001397008	0.000194773	1	0.854600118	1	0.958406391	ENSG00000166881	5	TMEM194A
SAT	Obesity	ENSG00000165684.3	-0.001927989	0.396744705	0.002275047	0.183454203	-0.001882031	0.408068407	0.002274912	0.183421012	-4.60E-05	0.010772229	1	0.688610432	1	0.698420679	ENSG00000166881	5	TMEM194A
SAT	Obesity	ENSG00000165832.4	0.003764042	0.290143562	0.003558342	0.183454051	0.003811674	0.284050024	0.0035581	0.183420836	-4.76E-05	0.046189464	1	0.588792914	1	0.588363718	ENSG00000166881	5	TMEM194A
SAT	Obesity	ENSG00000165868.8	-0.007232765	0.155793205	0.005095774	0.183453713	-0.007327503	0.150425563	0.005095507	0.183420489	9.47E-05	0.033124952	1	0.417953806	1	0.414396534	ENSG00000166902	4	MRPL16
SAT	Obesity	ENSG00000166037.6	-0.002465424	0.499430311	0.003650388	0.183454302	-0.002389786	0.512654188	0.003650139	0.183421108	-7.56E-05	0.001888188	1	0.7576285	1	0.769450316	ENSG00000166902	4	MRPL16
SAT	Obesity	ENSG00000166206.9	-0.00864468	0.116802554	0.005511995	0.183453544	-0.00854447	0.121081096	0.005511656	0.183420362	-0.00010021	0.016966604	1	0.357489907	1	0.369554001	ENSG00000166902	4	MRPL16
SAT	Obesity	ENSG00000166260.6	0.002891858	0.581290479	0.005243613	0.183454359	0.00277936	0.596054867	0.005243258	0.183421164	0.000112498	0.00148478	1	0.806390405	1	0.817137678	ENSG00000166912	12	MTMR10
SAT	Obesity	ENSG00000166796.7	0.00097147	0.789724514	0.003642986	0.183454447	0.000905203	0.803752784	0.003642766	0.183421247	6.63E-05	0.019206907	1	0.914498218	1	0.923968271	ENSG00000166912	12	MTMR10
SAT	Obesity	ENSG00000166881.5	0.0049791	0.173404831	0.003657481	0.183453774	0.00478217	0.191164424	0.003658487	0.183420624	0.00019693	0.046408698	1	0.442843988	1	0.473366395	ENSG00000166913	8	YWHAB
SAT	Obesity	ENSG00000166912.12	0.014173675	0.01588945	0.005877648	0.183452276	0.01427255	0.015164156	0.005877242	0.183419041	-9.89E-05	0.010329659	1	0.106394462	1	0.108395637	ENSG00000166913	8	YWHAB
SAT	Obesity	ENSG00000166913.8	-0.014866709	0.002099463	0.004833436	0.183450898	-0.014732008	0.002302946	0.004833155	0.183417759	-0.000134701	0.000511312	1	0.024689388	1	0.029515494	ENSG00000166924	4	NYAP1
SAT	Obesity	ENSG00000167136.6	0.00621506	0.119909638	0.003996423	0.18345356	0.006138784	0.124496298	0.003996151	0.183420378	7.63E-05	0.004289427	1	0.362555014	1	0.375654748	ENSG00000166928	6	MS4A14
SAT	Obesity	ENSG00000167216.12	0.001590586	0.665587453	0.003680105	0.183454403	0.001657066	0.652490265	0.003679869	0.183421194	-6.65E-05	0.012544425	1	0.854600118	1	0.849060146	ENSG00000166928	6	MS4A14
SAT	Obesity	ENSG00000167306.14	0.002521412	0.547036828	0.004186955	0.183454337	0.002464432	0.556104637	0.004186671	0.183421139	5.70E-05	0.043418797	1	0.792029636	1	0.795394048	ENSG00000166938	8	DIS3L
SAT	Obesity	ENSG00000167325.10	0.000280366	0.896662496	0.002158678	0.183454468	0.000331944	0.877781663	0.002158534	0.183421261	-5.16E-05	0.000574102	1	0.962747126	1	0.958406391	ENSG00000166946	9	CCNDBP1
SAT	Obesity	ENSG00000167333.8	-0.007715502	0.158585531	0.005472581	0.183453723	-0.007855935	0.151118009	0.005472247	0.183420491	0.000140434	0.00083947	1	0.421652454	1	0.414563702	ENSG00000166946	9	CCNDBP1
SAT	Obesity	ENSG00000167395.10	0.036794767	2.77E-09	0.006189141	0.183441115	0.036937091	2.40E-09	0.006188923	0.183407809	-0.000142324	0.029269954	9.07E-06	2.02E-07	7.87E-06	1.92E-07	ENSG00000166997	3	CNPY4
SAT	Obesity	ENSG00000167528.8	-0.000580291	0.781185093	0.002089067	0.183454445	-0.000543603	0.794688693	0.002088957	0.183421245	-3.67E-05	0.043340705	1	0.913071213	1	0.920289292	ENSG00000167083	2	GNGT2
SAT	Obesity	ENSG00000167595.10	0.003566291	0.134697859	0.002384151	0.183453628	0.00362468	0.128405748	0.002383997	0.183420397	-5.84E-05	0.000683772	1	0.388010251	1	0.382304228	ENSG00000167103	7	PIP5KL1
SAT	Obesity	ENSG00000167711.9	0.007914714	0.114255858	0.005011366	0.183453531	0.008028279	0.109134536	0.005011103	0.1834203	-0.000113565	0.009336659	1	0.351663667	1	0.346730908	ENSG00000167103	7	PIP5KL1
SAT	Obesity	ENSG00000167720.8	0.005848491	9.98E-06	0.001323863	0.183447097	0.005885123	8.76E-06	0.001323782	0.183413801	-3.66E-05	0.000359714	0.032730066	0.000282156	0.028751567	0.000299495	ENSG00000167103	7	PIP5KL1
SAT	Obesity	ENSG00000167799.5	-0.012771176	0.005898114	0.004638322	0.183451608	-0.011141303	0.01650507	0.004646935	0.183419098	-0.001629874	1.79E-08	1	0.055219818	1	0.114488656	ENSG00000167103	7	PIP5KL1
SAT	Obesity	ENSG00000167972.9	-0.009446345	0.06324206	0.005085542	0.18345317	-0.009523403	0.061102346	0.005085228	0.183419945	7.71E-05	0.04539636	1	0.250993039	1	0.250029993	ENSG00000167123	14	CERCAM
SAT	Obesity	ENSG00000167987.6	-0.010420449	0.053094943	0.005387604	0.18345306	-0.010251272	0.057070022	0.005387527	0.183419902	-0.000169178	0.010877869	1	0.225653508	1	0.240368091	ENSG00000167123	14	CERCAM
SAT	Obesity	ENSG00000168004.5	0.001801587	0.570065526	0.003172057	0.183454352	0.001968033	0.535006334	0.00317229	0.183421125	-0.000166446	0.003751301	1	0.799651556	1	0.78574565	ENSG00000167136	6	ENDOG
SAT	Obesity	ENSG00000168016.9	0.011907041	0.038725569	0.005760273	0.183452859	0.011777784	0.040877357	0.005759935	0.18341969	0.000129257	0.004869859	1	0.188234951	1	0.196654851	ENSG00000167136	6	ENDOG
SAT	Obesity	ENSG00000168028.9	0.001460962	0.333624406	0.001511069	0.183454121	5.83E-05	0.969652081	0.001533169	0.18342127	0.001402633	6.99E-08	1	0.631217486	1	0.992266083	ENSG00000167136	6	ENDOG
SAT	Obesity	ENSG00000168038.6	0.002111555	0.227260241	0.001748775	0.183453923	0.002067665	0.237043956	0.001748688	0.183420742	4.39E-05	0.005325143	1	0.514945339	1	0.530519249	ENSG00000167216	12	KATNAL2
SAT	Obesity	ENSG00000168056.10	0.005143829	0.241690996	0.004393536	0.183453956	7.39E-05	0.986870558	0.004487895	0.18342127	0.005069976	3.26E-08	1	0.530497704	1	0.995364986	ENSG00000167220	7	HDHD2
SAT	Obesity	ENSG00000168246.5	0.009741332	0.083669447	0.00563153	0.183453343	0.010666039	0.058908171	0.005646746	0.183419922	-0.000924707	0.028123921	1	0.291421926	1	0.245276282	ENSG00000167220	7	HDHD2
SAT	Obesity	ENSG00000168255.14	-0.000286186	0.934704933	0.003493188	0.183454472	-0.000335295	0.923527167	0.003492958	0.183421267	4.91E-05	0.045193123	1	0.979129279	1	0.975006702	ENSG00000167236	2	CCL23
SAT	Obesity	ENSG00000168329.9	-0.004641878	0.447025523	0.006104651	0.183454256	0.004319216	0.494532688	0.006322797	0.183421094	-0.008961094	5.46E-08	1	0.722507754	1	0.755382565	ENSG00000167236	2	CCL23
SAT	Obesity	ENSG00000168356.7	-0.001580709	0.500036971	0.002343763	0.183454302	-0.001378841	0.55635592	0.002343918	0.183421139	-0.000201868	1.14E-06	1	0.75779275	1	0.795394048	ENSG00000167257	6	RNF214
SAT	Obesity	ENSG00000168394.9	0.009017789	0.136534903	0.006056996	0.183453636	0.001617069	0.796196427	0.006261085	0.183421245	0.007400721	3.14E-06	1	0.389540014	1	0.921453431	ENSG00000167257	6	RNF214
SAT	Obesity	ENSG00000168405.10	0.02517499	9.75E-06	0.005692367	0.183447081	0.023295629	4.43E-05	0.005704671	0.183414968	0.001879361	8.65E-07	0.032005176	0.000278795	0.145510061	0.001202562	ENSG00000167257	6	RNF214
SAT	Obesity	ENSG00000168653.6	0.007636315	0.149348126	0.005296229	0.183453688	0.007715089	0.145170379	0.00529589	0.183420468	-7.88E-05	0.041705197	1	0.409366084	1	0.40919767	ENSG00000167306	14	MYO5B
SAT	Obesity	ENSG00000168661.10	0.002046201	0.583572058	0.003732752	0.183454361	0.001933613	0.604426349	0.003732511	0.183421169	0.000112588	2.83E-05	1	0.806765832	1	0.822274447	ENSG00000167306	14	MYO5B
SAT	Obesity	ENSG00000168702.12	-0.001589831	0.768122292	0.005392327	0.183454441	-0.006924513	0.214396951	0.005577237	0.183420688	0.005334682	0.000183451	1	0.90720275	1	0.506069351	ENSG00000167311	9	ART5
SAT	Obesity	ENSG00000168813.12	-0.014699691	0.014718471	0.006026336	0.183452225	-0.014473403	0.016313689	0.006025991	0.18341909	-0.000226288	3.93E-06	1	0.101452318	1	0.113641644	ENSG00000167311	9	ART5
SAT	Obesity	ENSG00000168827.10	0.003575394	0.170518215	0.002608743	0.183453764	0.003621377	0.165059946	0.002608581	0.183420542	-4.60E-05	0.019525648	1	0.437770159	1	0.433943657	ENSG00000167311	9	ART5
SAT	Obesity	ENSG00000168944.11	0.000782438	0.875387848	0.004989445	0.183454465	0.000685417	0.890728642	0.004989118	0.183421263	9.70E-05	0.005873244	1	0.955789527	1	0.962898024	ENSG00000167325	10	RRM1
SAT	Obesity	ENSG00000169047.5	0.005636284	0.064240636	0.0030458	0.18345318	0.00556658	0.067587357	0.003045588	0.183420008	6.97E-05	0.000380883	1	0.253944006	1	0.265533656	ENSG00000167325	10	RRM1
SAT	Obesity	ENSG00000169085.7	0.004589355	0.000346132	0.001282623	0.183449635	0.003778689	0.004081271	0.00131578	0.183418153	0.000810666	0.005827625	1	0.005794173	1	0.04585839	ENSG00000167325	10	RRM1
SAT	Obesity	ENSG00000169203.12	0.016386935	1.78E-13	0.002225174	0.183433976	0.016424187	1.57E-13	0.002225037	0.18340068	-3.73E-05	0.029353052	5.85E-10	2.34E-11	5.14E-10	2.14E-11	ENSG00000167325	10	RRM1
SAT	Obesity	ENSG00000169220.13	-0.000309324	0.895999776	0.002366375	0.183454468	-0.000357594	0.879877658	0.002366222	0.183421262	4.83E-05	0.004603858	1	0.962747126	1	0.958459029	ENSG00000167325	10	RRM1
SAT	Obesity	ENSG00000169313.9	0.004163771	0.387564979	0.004818943	0.183454192	0.004285268	0.373837649	0.004818651	0.183420971	-0.000121497	0.001136794	1	0.678187037	1	0.669643631	ENSG00000167332	7	OR51E2

SAT	Obesity	ENSG00000169862.14	-0.00897906	0.016878672	0.003757953	0.183452316	-0.008924112	0.017555097	0.003757712	0.183419139	-5.49E-05	0.043750329	1	0.110097259	1	0.118319765	ENSG00000167332	7	OR51E2
SAT	Obesity	ENSG00000169914.5	0.00333197	0.153183283	0.002332692	0.183453703	0.003367635	0.14880656	0.002332542	0.183420482	-3.57E-05	0.033897997	1	0.413998642	1	0.414396534	ENSG00000167333	8	TRIM68
SAT	Obesity	ENSG00000170175.6	-0.002989454	0.369660558	0.003332315	0.18345417	-0.002915997	0.381511201	0.003332127	0.183420981	-7.35E-05	0.007389141	1	0.665673046	1	0.676469831	ENSG00000167395	10	ZNF646
SAT	Obesity	ENSG00000170209.4	-0.001768126	0.554151364	0.002988964	0.183454342	-0.001719259	0.565129848	0.002988772	0.183421145	-4.89E-05	0.023716296	1	0.794232732	1	0.798875928	ENSG00000167395	10	ZNF646
SAT	Obesity	ENSG00000170248.9	-0.001102006	0.785586048	0.004050794	0.183454446	-0.001157657	0.775027269	0.004050506	0.183421239	5.57E-05	0.02779951	1	0.914334098	1	0.911862059	ENSG00000167395	10	ZNF646
SAT	Obesity	ENSG00000170264.8	-0.004893307	0.426990688	0.006160121	0.183454236	-0.004983791	0.418460891	0.00615971	0.183421023	9.05E-05	0.033246911	1	0.70970438	1	0.703737593	ENSG00000167434	5	CA4
SAT	Obesity	ENSG00000170322.10	-0.008026528	0.114832405	0.005090253	0.183453534	-0.007921248	0.119647473	0.005089933	0.183420355	-0.00010528	0.005339533	1	0.353106955	1	0.368420209	ENSG00000167461	7	RAB8A
SAT	Obesity	ENSG00000170356.8	-0.003610785	0.157448827	0.002554127	0.183453719	-0.003564533	0.162812135	0.00255398	0.183420534	-4.63E-05	0.025195918	1	0.420045334	1	0.430795656	ENSG00000167476	6	JSRP1
SAT	Obesity	ENSG00000170509.7	-0.002618294	0.357687043	0.002846645	0.183454154	-0.002519652	0.376072982	0.002846563	0.183420974	-9.86E-05	0.001792487	1	0.655187068	1	0.671325056	ENSG00000167525	9	PROCA1
SAT	Obesity	ENSG00000170745.7	-0.000532834	0.870861778	0.003277685	0.183454464	-0.000626636	0.848373355	0.003277483	0.183421256	9.38E-05	0.000154068	1	0.955298393	1	0.942286045	ENSG00000167528	8	ZNF641
SAT	Obesity	ENSG00000170776.15	0.005593634	0.329403193	0.005735192	0.183454115	0.005850517	0.307693615	0.005735382	0.183420877	-0.000256884	0.004418673	1	0.628809457	1	0.607792143	ENSG00000167528	8	ZNF641
SAT	Obesity	ENSG00000170837.2	0.009124035	0.080307627	0.005216991	0.183453318	0.009037197	0.083209827	0.005216692	0.183420136	8.68E-05	0.040869247	1	0.286713084	1	0.29736676	ENSG00000167528	8	ZNF641
SAT	Obesity	ENSG00000170955.9	0.000580738	0.879376402	0.003826692	0.183454465	0.000474973	0.901213886	0.003826476	0.183421264	0.000105765	0.000838971	1	0.957347365	1	0.963222823	ENSG00000167528	8	ZNF641
SAT	Obesity	ENSG00000171310.6	0.000399596	0.935015895	0.004900863	0.183454472	0.000470412	0.923527085	0.004900542	0.183421267	-7.08E-05	0.041161114	1	0.979129279	1	0.975006702	ENSG00000167535	3	CACNB3
SAT	Obesity	ENSG00000171421.8	0.00938636	0.119004083	0.006020872	0.183453555	0.00951018	0.114192607	0.006020517	0.183420327	-0.00012382	0.009617074	1	0.361529998	1	0.357164865	ENSG00000167595	10	C19orf55
SAT	Obesity	ENSG00000171503.7	-0.000667168	0.718346585	0.001849805	0.183454425	-0.000706146	0.702636894	0.001849695	0.183421215	3.90E-05	0.007427362	1	0.883393983	1	0.876674335	ENSG00000167595	10	C19orf55
SAT	Obesity	ENSG00000171530.9	-0.007506251	0.177212499	0.005562701	0.183453786	-0.007592323	0.172267157	0.005562316	0.183420566	8.61E-05	0.017942156	1	0.450724192	1	0.443997285	ENSG00000167595	10	C19orf55
SAT	Obesity	ENSG00000171574.13	-0.000170149	0.948051291	0.002611481	0.183454473	-0.000214265	0.934604938	0.002611316	0.183421268	4.41E-05	0.022687499	1	0.983182517	1	0.979844999	ENSG00000167595	10	C19orf55
SAT	Obesity	ENSG00000171763.13	0.00367347	0.157673687	0.002599875	0.183453719	0.003724109	0.151999928	0.002599718	0.183420495	-5.06E-05	0.012122153	1	0.420249688	1	0.415593137	ENSG00000167644	7	C19orf33
SAT	Obesity	ENSG00000172113.4	-0.000393722	0.87665025	0.002536589	0.183454465	-0.000338124	0.893950892	0.002536435	0.183421263	-5.56E-05	0.004600575	1	0.956287991	1	0.963222823	ENSG00000167644	7	C19orf33
SAT	Obesity	ENSG00000172247.3	-0.011362341	2.67E-15	0.001437265	0.183430853	-0.011342034	2.98E-15	0.001437172	0.183397734	-2.03E-05	0.048425759	8.77E-12	4.18E-13	9.78E-12	4.89E-13	ENSG00000167644	7	C19orf33
SAT	Obesity	ENSG00000172349.12	0.003088926	0.589788586	0.005729329	0.183454364	0.002882232	0.614902596	0.00572908	0.183421175	0.000206694	0.000200894	1	0.811021102	1	0.827520679	ENSG00000167644	7	C19orf33
SAT	Obesity	ENSG00000172590.14	0.004611824	0.120004494	0.002966266	0.18345356	0.004696401	0.11334076	0.002966104	0.183420323	-8.46E-05	0.000761468	1	0.362555014	1	0.354838771	ENSG00000167646	9	DNAAF3
SAT	Obesity	ENSG00000172613.3	0.001726681	0.178882722	0.001284537	0.183453791	0.002069567	0.10750849	0.001285848	0.183420291	-0.000342886	1.52E-08	1	0.452218367	1	0.345142226	ENSG00000167646	9	DNAAF3
SAT	Obesity	ENSG00000172638.8	0.026720038	7.47E-07	0.005399254	0.183445217	0.010674639	0.149844836	0.007412522	0.183420486	0.016045398	0.001582624	0.002450336	3.10E-05	1	0.414396534	ENSG00000167646	9	DNAAF3
SAT	Obesity	ENSG00000172661.13	0.003256139	0.170431961	0.002375324	0.183453764	0.003304152	0.164194102	0.002375199	0.183420539	-4.80E-05	0.020319108	1	0.437770159	1	0.433055345	ENSG00000167670	11	CHAF1A
SAT	Obesity	ENSG00000172803.13	0.01794911	1.24E-06	0.003700967	0.183445584	-0.000715321	0.921949518	0.007300811	0.183421267	0.018664432	0.003019659	0.004055228	4.77E-05	1	0.974898873	ENSG00000167671	7	UBXN6
SAT	Obesity	ENSG00000172890.7	0.009722045	0.067246242	0.005312509	0.183453208	0.009844504	0.063858083	0.005312241	0.183419972	-0.000122459	0.009994687	1	0.26053725	1	0.257054802	ENSG00000167695	10	FAM57A
SAT	Obesity	ENSG00000172922.4	0.000142568	0.962371826	0.003021962	0.183454473	0.006659987	0.037980283	0.003209519	0.183419643	-0.006517418	1.70E-09	1	0.989966035	1	0.18967018	ENSG00000167695	10	FAM57A
SAT	Obesity	ENSG00000172927.3	-0.000558456	0.903237594	0.004593605	0.183454469	-0.000797621	0.862147028	0.004593484	0.183421259	0.000239165	4.42E-06	1	0.964060685	1	0.949538046	ENSG00000167695	10	FAM57A
SAT	Obesity	ENSG00000172932.10	-0.004795514	0.329973336	0.004922674	0.183454115	-0.00391271	0.426934342	0.004925059	0.183421032	-0.000882805	1.23E-07	1	0.628809457	1	0.711073464	ENSG00000167700	4	MFSD3
SAT	Obesity	ENSG00000172955.13	-0.003908162	0.306405489	0.003821062	0.183454079	-0.003963437	0.299583138	0.00382081	0.183420864	5.53E-05	0.039080795	1	0.605977341	1	0.601026164	ENSG00000167711	9	SERPINF2
SAT	Obesity	ENSG00000173113.2	0.000603298	0.869894474	0.003683305	0.183454464	0.000662583	0.85723161	0.003683077	0.183421258	-5.93E-05	0.033349209	1	0.954875801	1	0.947314554	ENSG00000167711	9	SERPINF2
SAT	Obesity	ENSG00000173156.2	-0.004048221	0.168196559	0.0029377	0.183453756	-0.004280143	0.145153394	0.002937906	0.183420468	0.000231921	1.07E-05	1	0.434800182	1	0.40919767	ENSG00000167711	9	SERPINF2
SAT	Obesity	ENSG00000173193.9	0.001853491	0.217902897	0.001504304	0.1834539	0.001873524	0.212938326	0.0015042	0.183420684	-2.00E-05	0.042103575	1	0.505890452	1	0.503707333	ENSG00000167711	9	SERPINF2
SAT	Obesity	ENSG00000173200.8	0.002006929	0.661180801	0.004579064	0.183454401	0.001868012	0.683293489	0.004578762	0.183421207	0.000138917	1.39E-05	1	0.852929209	1	0.86636807	ENSG00000167711	9	SERPINF2
SAT	Obesity	ENSG00000173226.12	-0.00642587	0.000805341	0.001917613	0.18345023	-0.006389418	0.000861686	0.001917477	0.183417074	-3.65E-05	0.002321311	1	0.011499836	1	0.013149728	ENSG00000167720	8	SRR
SAT	Obesity	ENSG00000173511.5	0.000634061	0.853582205	0.003435716	0.183454461	0.00042552	0.901433042	0.003435735	0.183421264	0.00020854	1.21E-05	1	0.943648754	1	0.963222823	ENSG00000167720	8	SRR
SAT	Obesity	ENSG00000173917.9	0.010953294	0.003078692	0.003700707	0.183451163	0.010888814	0.003255678	0.003700506	0.183417998	6.45E-05	0.040448546	1	0.034241321	1	0.038984968	ENSG00000167720	8	SRR
SAT	Obesity	ENSG00000174007.7	-0.005005078	0.187060903	0.003793664	0.183453816	-0.005056944	0.18250282	0.0037934	0.183420599	5.19E-05	0.034972997	1	0.461882921	1	0.459606393	ENSG00000167720	8	SRR
SAT	Obesity	ENSG00000174080.6	-0.004592283	0.10199488	0.002808287	0.183453463	-0.006723147	0.017561695	0.002831108	0.183419139	0.002130864	3.51E-09	1	0.325530352	1	0.118319765	ENSG00000167747	9	C19orf48
SAT	Obesity	ENSG00000174194.11	0.001658471	0.312891872	0.001643395	0.183454089	0.001685225	0.305114813	0.001643276	0.183420873	-2.68E-05	0.006766376	1	0.609761023	1	0.605981659	ENSG00000167747	9	C19orf48
SAT	Obesity	ENSG00000174226.4	-0.000549702	0.922520011	0.005651887	0.183454471	-0.000339193	0.952141808	0.005651581	0.183421269	-0.000210509	1.23E-05	1	0.97398945	1	0.987557383	ENSG00000167757	9	KLK11
SAT	Obesity	ENSG00000174405.9	0.000981705	0.873287526	0.006155503	0.183454464	0.000888388	0.885236791	0.006155082	0.183421262	9.33E-05	0.022281308	1	0.95575129	1	0.961975704	ENSG00000167766	14	ZNF83
SAT	Obesity	ENSG00000174652.13	0.001340823	0.237210995	0.00113438	0.183453946	0.001363739	0.229259666	0.001134304	0.183420724	-2.29E-05	0.003490241	1	0.526060746	1	0.523816827	ENSG00000167780	7	SOAT2
SAT	Obesity	ENSG00000174705.7	0.006738117	0.210691845	0.005383315	0.183453882	0.00899886	0.101660132	0.005497632	0.183420258	-0.002260743	0.043101616	1	0.499840884	1	0.333213679	ENSG00000167785	4	ZNF558
SAT	Obesity	ENSG00000175334.3	-0.001459692	0.633405833	0.003060542	0.183454388	0.004078363	0.202804791	0.003202225	0.183420657	-0.005538054	4.27E-09	1	0.839339474	1	0.491434652	ENSG00000167799	5	NUDT8
SAT	Obesity	ENSG00000175390.8	-0.006210363	0.223542272	0.00510232	0.183453914	-0.0061062	0.231377173	0.005102019	0.183420729	-0.000104163	0.009890662	1	0.511109543	1	0.524775447	ENSG00000167799	5	NUDT8
SAT	Obesity	ENSG00000175416.8	0.002448807	0.565359389	0.004259537	0.183454349	0.002606322	0.540595212	0.004259297	0.183421129	-0.000157515	7.78E-06	1	0.797234594	1	0.787957747	ENSG00000167799	5	NUDT8
SAT	Obesity	ENSG00000175445.10	-0.002781901	0.512981867	0.004252357	0.183454312	-0.004996392	0.24891635	0.004333434	0.183420768	0.002214491	0.008098685	1	0.767484499	1	0.545189948	ENSG00000167799	5	NUDT8
SAT	Obesity	ENSG00000175463.7	0.004004393	0.450061882	0.005301638	0.183454258	0.004462329	0.39998457	0.005301885	0.183421002	-0.000457936	1.85E-07	1	0.725628027	1	0.692729827	ENSG00000167799	5	NUDT8
SAT	Obesity	ENSG00000175575.8	-0.000909287	0.489766172	0.001316507	0.183454294	-0.000889716	0.499130951	0.001316424	0.183421098	-1.96E-05	0.046757284	1	0.75041911	1	0.759577297	ENSG00000167799	5	NUDT8
SAT	Obesity	ENSG00000175582.15	-0.003565141	0.430418324	0.004521556	0.183454239	-0.003468653	0.442969904	0.004521253	0.183421048	-9.65E-05	0.001896409	1	0.71201318	1	0.722357979	ENSG00000167800	9	TBX10
SAT	Obesity	ENSG00000176024.12	0.00576352	0.160750534	0.004109303	0.183453731	0.005848224	0.154660586	0.004109026	0.183420505	-8.47E-05	0.002392725	1	0.424997988	1	0.418287971	ENSG00000167800	9	TBX10
SAT	Obesity	ENSG00000176393.6	-0.005892854	0.134544437	0.003937963	0.183453628	-0.005783524	0.141913529	0.00393784	0.183420455	-0.000109329	0.010696252	1	0.388010251	1	0.40243586	ENSG00000167840	9	ZNF232
SAT	Obesity	ENSG00000176422.10	0.000848717	0.806660784	0.003467885	0.183454451	0.000754878	0.827675584	0.003467771	0.183421252	9.38E-05	0.01180312	1	0.924756825	1	0.936326075	ENSG00000167840	9	ZNF232
SAT	Obesity	ENSG00000176472.6	0.001157963	0.407561417	0.00139818	0.183454215	0.001132053	0.418106026	0.001398094	0.183421022	2.59E-05	0.015076898	1	0.695625456	1	0.703737593	ENSG00000167840	9	ZNF232
SAT	Obesity	ENSG00000176715.11	-0.006581045	0.076889032	0.003720138	0.183453291	-0.006524521	0.07943833	0.003719884	0.183420108	-5.65E-05	0.023163588	1	0.279062958	1	0.290241828	ENSG00000167840	9	ZNF232
SAT	Obesity	ENSG00000176998.3	-0.008538986	0.000120495	0.002220717	0.183448885	-0.005616912	0.014883452	0.00230654	0.183419029	-0.002922074	2.83E-06	0.39534337	0.00231195	1	0.107919055	ENSG00000167895	10	TMC8
SAT	Obesity	ENSG00000177590.6	0.009081307	0.057320178	0.004777458	0.183453108	0.009168118	0.054964261	0.004777143	0.183419878	-8.68E-05	0.00958842	1	0.236562896	1	0.235120913	ENSG00000167920	4	TMEM99
SAT	Obesity	ENSG00000177663.9	-0.002709946	0.281473417	0.00251616	0.183454036	-0.002803818	0.265119699	0.00251605	0.183420801	9.39E-05	0.000109383	1	0.579661774	1	0.567932065	ENSG00000167920	4	TMEM99
SAT	Obesity	ENSG00000177990.7	-0.001676563	0.649865537	0.003693283	0.183454396	-0.011044634	0.024939948	0.004925498	0.18341937	0.009368071	0.004049351	1	0.846029897	1	0.146382772	ENSG00000167930	11	ITFG3
SAT	Obesity	ENSG00000178295.10	-0.000241958	0.890095177	0.001750983	0.183454467	-0.000212418	0.903437291	0.001750881	0.183421265	-2.95E-05	0.03625148	1	0.962241277	1	0.96423859	ENSG00000167972	9	ABCA3
SAT	Obesity	ENSG00000178381.7	0.003860629	0.514556356	0.005923391	0.183454314	0.003683957	0.533960625	0.005923028	0.183421124	0.000176672	9.57E-05	1	0.769062939	1	0.784735611	ENSG00000167972	9	ABCA3
SAT	Obesity	ENSG00000178397.8	-0.004929761	0.17272245	0.003615493	0.183453771	-0.004987166	0.16774808	0.003615256	0.183420551	5.74E-05	0.024800812	1	0.442045521	1	0.437505128	ENSG00000167972	9	ABCA3
SAT	Obesity	ENSG00000178665.10	-0.002000881	0.667843555	0.004662865	0.183454405	-0.00211868	0.649540304	0.004662575	0.183421192	0.000117799	0.000817229	1	0.854600118	1	0.847588987	ENSG00000167972	9	ABCA3
SAT	Obesity	ENSG00000178922.12	-0.005298858	0.038325714	0.002558154	0.183452852	-0.005345081	0.036658194	0.002557998	0.18341962	4.62E-05	0.01894154	1	0.187401887	1	0.186062873	ENSG00000167972	9	ABCA3
SAT	Obesity	ENSG00000179242.11	-3.40E-06	0.999414195	0.004629844	0.183454474	9.37E-05	0.983853444	0.004629583	0.18342127	-9.71E-05	0.011101271	1	0.99968511	1	0.995364986	ENSG00000167972	9	ABCA3
SAT	Obesity	ENSG00000179409.6	-0.004941426	0.18528491	0.003730339	0.183453811	-0.004999288	0.180159048	0.003730067	0.183420591	5.79E-05	0.009380035	1	0.460203128	1	0.457509161	ENSG00000167987	6	VPS37C
SAT	Obesity	ENSG00000179698.9	-0.004747379	0.042503892	0.002340273	0.183452919	-0.004708019	0.044235158	0.002340131	0.18341974	-3.94E-05	0.029696018	1	0.195589441	1	0.206451713	ENSG00000167987	6	VPS37C
SAT	Obesity	ENSG00000180089.4	0.003506382	0.351616965	0.0037644	0.183454146	0.00338162	0.368989297	0.003764177	0.183420965	0.000124762	2.74E-05	1	0.649215117	1	0.667026933	ENSG00000168000	10	BSCL2
SAT	Obesity	ENSG00000180537.8	-0.008857993	0.142757087	0.006043924	0.183453662	-0.009110869	0.131684996	0.006043722	0.183420411	0.000252876	9.36E-05	1	0.39896593	1	0.388541792	ENSG00000168000	10	BSCL2
SAT	Obesity	ENSG00000180610.9	0.003908739	0.512887	0.005973473	0.183454312	0.004002962	0.502752124	0.005973088	0.1834211	-9.42E-05	0.02886737	1	0.767484499	1	0.763656037	ENSG00000168000	10	BSCL2
SAT	Obesity	ENSG00000180822.7	-0.003352212	0.498915892	0.004957449	0.183454301	-0.003445028	0.487076195	0.004957109	0.183421088	9.28E-05	0.004748875	1	0.75749331	1	0.750011464	ENSG00000168004	5	HRASLS5
SAT	Obesity	ENSG00000180855.11	0.004644349	0.308584714	0.004561345	0.183454082	0.004499602	0.323877727	0.004561078	0.183420902	0.000144748	6.92E-05	1	0.60688449	1	0.626706567	ENSG00000168016	9	TRANK1
SAT	Obesity	ENSG00000181027.6	0.006392619	0.008578046	0.002432097	0.183451863	0.006348159	0.009046891	0.002431977	0.183418695	4.45E-05	0.043355611	1	0.070683838	1	0.07770379	ENSG00000168016	9	TRANK1
SAT	Obesity	ENSG00000181061.9	-0.00257571	0.586837054	0.004739765	0.183454362	-0.002497053	0.5982883	0.004739464	0.183421165	-7.87E-05	0.023798229	1	0.808391759	1	0.817909964	ENSG00000168016	9	TRANK1
SAT	Obesity	ENSG00000181092.5	-5.59E-05	0.987031634	0.003437833	0.183454474	-0.002685179	0.457979405	0.003617968	0.183421062	0.0026293	0.019793606	1	0.994152558	1	0.731563013	ENSG00000168016	9	TRANK1
SAT	Obesity	ENSG00000181126.9	-0.002501733	0.231281259	0.002089887	0.183453932	-0.002010013	0.339106575	0.002102671	0.183420925	-0.00049172	0.034986251	1	0.520105422	1	0.641968257	ENSG00000168026	12	TTC21A
SAT	Obesity	ENSG00000181315.6	-0.019185218	9.98E-09	0.003347546	0.183442059	-0.01832876	4.55E-08	0.003351943	0.18340997	-0.000856458	1.35E-06	3.28E-05	6.30E-07	0.000149321	2.93E-06	ENSG00000168028	9	RPSA
SAT	Obesity	ENSG00000181619.11	0.008307364	0.137276507	0.005590374	0.183453639	0.008768275	0.116921965	0.005592626	0.183420341	-0.000460911	0.008704532	1	0.390599089	1	0.36350002	ENSG00000168028	9	RPSA
SAT	Obesity	ENSG00000181744.4	0.000107756	0.977675609	0.003850742	0.183454474	4.84E-05	0.989970046	0.003850473	0.18342127	5.94E-05	0.016131811	1	0.993452012	1	0.996265811	ENSG00000168028	9	RPSA
SAT	Obesity	ENSG00000181781.5	-0.005872505	0.305491942	0.005730792	0.183454077	-0.006019889	0.293484858	0.005730451	0.183420853	0.000147384	0.001105519	1	0.605495891	1	0.59812588	ENSG00000168028	9	RPSA
SAT	Obesity	ENSG00000181885.14	-0.015922684	0.004619446	0.00562152	0.183451442	-0.015828684	0.004864296	0.005621184	0.183418273	-9.40E-05	0.032396593	1	0.046951916	1	0.051649693	ENSG00000168028	9	RPSA
SAT	Obesity	ENSG00000182013.13	0.003897432	0.027373919	0.001766621	0.183452634	0.00397366	0.02449853	0.001766678	0.183419358	-7.62E-05	0.005968693	1	0.149689712	1	0.14496253	ENSG00000168028	9	RPSA
SAT	Obesity	ENSG00000182117.4	-0.010646833	0.008386093	0.004038816	0.183451847	-0.010781962	0.007591105	0.004038571	0.183418576	0.000135128	1.59E-05	1	0.069748174	1	0.070158914	ENSG00000168028	9	RPSA
SAT	Obesity	ENSG00000182134.11	-0.00684383	0.147542502	0.004725524	0.183453681	-0.00670679	0.155819491	0.004725504	0.183420509	-0.00013704	0.027144216	1	0.406454198	1	0.420430716	ENSG00000168038	6	ULK4
SAT	Obesity	ENSG00000182141.5	-0.004290041	0.415214471	0.005265454	0.183454223	-0.004068384	0.439701429	0.005265166	0.183421045	-0.000221658	6.16E-07	1	0.700759618	1	0.719531366	ENSG00000168038	6	ULK4
SAT	Obesity	ENSG00000182150.11	0.002235522	0.436956518	0.002875848	0.183454246	0.002159547	0.452667072	0.002875652	0.183421057	7.60E-05	7.65E-05	1	0.717185761	1	0.727870316	ENSG00000168038	6	ULK4
SAT	Obesity	ENSG00000182902.9	0.000170593	0.977023795	0.005923281	0.183454474	1.78E-05	0.997601554	0.005922933	0.18342127	0.000152788	0.001225821	1	0.993452012	1	0.998819255	ENSG00000168038	6	ULK4
SAT	Obesity	ENSG00000182919.10	0.00026854	0.845319514	0.001376464	0.18345446	0.000223427	0.871046986	0.001376385	0.18342126	4.51E-05	6.08E-05	1	0.941763438	1	0.955501559	ENSG00000168038	6	ULK4
SAT	Obesity	ENSG00000182923.12	-0.002126243	0.333789192	0.002199917	0.183454121	-0.002091136	0.341802296	0.002199781	0.183420929	-3.51E-05	0.035070714	1	0.631217486	1	0.645626559	ENSG00000168038	6	ULK4
SAT	Obesity	ENSG00000182952.4	-0.006190688	0.20988234	0.004937183	0.18345388	-0.004475784	0.365688894	0.004947919	0.183420961	-0.001714904	2.48E-07	1	0.498280722	1	0.665460489	ENSG00000168056	10	LTBP3
SAT	Obesity	ENSG00000182986.8	0.000849684	0.435225397	0.001088944	0.183454244	0.000826028	0.448091661	0.001088884	0.183421053	2.37E-05	0.00909537	1	0.716072224	1	0.726019131	ENSG00000168056	10	LTBP3
SAT	Obesity	ENSG00000183208.8	0.009998839	0.078234675	0.005677849	0.183453302	0.009894691	0.081370615	0.005677495	0.183420122	0.000104147	0.014194038	1	0.282822099	1	0.294351696	ENSG00000168056	10	LTBP3
SAT	Obesity	ENSG00000183458.9	0.006102996	0.186373901	0.004618638	0.183453814	0.005961406	0.196779819	0.004618444	0.183420641	0.00014159	0.001840031	1	0.461852544	1	0.481816855	ENSG00000168056	10	LTBP3
SAT	Obesity	ENSG00000183527.7	0.006567255	3.63E-05	0.001590154	0.183448027	0.006590148	3.40E-05	0.001590042	0.183414778	-2.29E-05	0.021744578	0.119069587	0.000875512	0.111687929	0.000954598	ENSG00000168056	10	LTBP3
SAT	Obesity	ENSG00000183604.10	-0.027019379	2.23E-14	0.00353793	0.18343243	-0.027129336	1.74E-14	0.003537764	0.183399048	0.000109958	0.000918754	7.30E-11	3.32E-12	5.72E-11	2.60E-12	ENSG00000168056	10	LTBP3
SAT	Obesity	ENSG00000183628.8	0.001465773	0.383730748	0.001682778	0.183454187	0.001512041	0.368869053	0.001682673	0.183420965	-4.63E-05	0.000269925	1	0.67678409	1	0.667026933	ENSG00000168060	10	NAALADL1
SAT	Obesity	ENSG00000183807.6	0.004403796	0.396086921	0.005189293	0.183454202	0.004276162	0.409893091	0.005188997	0.183421014	0.000127634	0.002690022	1	0.6883269	1	0.700812523	ENSG00000168060	10	NAALADL1
SAT	Obesity	ENSG00000183828.10	-0.005759383	0.282258554	0.005356266	0.183454037	-0.005577293	0.297722936	0.005355932	0.18342086	-0.00018209	5.62E-06	1	0.579661774	1	0.601026164	ENSG00000168060	10	NAALADL1
SAT	Obesity	ENSG00000183833.12	0.01068395	0.015755874	0.004424856	0.18345227	0.010760146	0.0150207	0.004424606	0.183419035	-7.62E-05	0.036674568	1	0.105715792	1	0.108076574	ENSG00000168116	9	KIAA1586
SAT	Obesity	ENSG00000183891.5	0.00020241	0.966626224	0.004837701	0.183454473	0.000108095	0.982172042	0.004837371	0.18342127	9.43E-05	0.00358309	1	0.991713771	1	0.995364986	ENSG00000168116	9	KIAA1586
SAT	Obesity	ENSG00000183977.9	0.00722149	0.208968383	0.005747723	0.183453877	0.007313359	0.203204474	0.005747343	0.183420658	-9.19E-05	0.022235712	1	0.49647014	1	0.491652405	ENSG00000168116	9	KIAA1586
SAT	Obesity	ENSG00000184371.9	0.003368676	0.343813805	0.003558495	0.183454135	0.00104653	0.772514221	0.003620111	0.183421239	0.002322146	0.000496469	1	0.642232408	1	0.911785759	ENSG00000168159	9	RNF187
SAT	Obesity	ENSG00000184385.2	0.004293473	0.079861831	0.002451323	0.183453315	0.004332721	0.077125421	0.002451164	0.183420089	-3.92E-05	0.024806888	1	0.285743367	1	0.285607794	ENSG00000168159	9	RNF187
SAT	Obesity	ENSG00000184402.10	-0.005885374	0.32005083	0.005918788	0.1834541	-0.006071688	0.304943676	0.005918456	0.183420872	0.000186314	0.000145148	1	0.618425661	1	0.605981659	ENSG00000168246	5	UBTD2
SAT	Obesity	ENSG00000184508.6	0.006801274	0.269646511	0.006161254	0.183454013	0.006892332	0.263255473	0.006160848	0.183420797	-9.11E-05	0.035778292	1	0.5660334	1	0.566387677	ENSG00000168255	14	POLR2J3
SAT	Obesity	ENSG00000184743.8	0.005108586	0.244552352	0.004390008	0.183453962	0.005641413	0.199098386	0.004393191	0.183420647	-0.000532827	0.002663827	1	0.533140377	1	0.485479182	ENSG00000168255	14	POLR2J3
SAT	Obesity	ENSG00000184923.8	-0.010086798	0.082880577	0.005816348	0.183453337	-0.011087992	0.057206723	0.005830469	0.183419903	0.001001195	0.015360997	1	0.290088201	1	0.24057313	ENSG00000168291	8	PDHB
SAT	Obesity	ENSG00000184967.2	-0.008961543	0.009524419	0.003456527	0.183451933	-0.009049532	0.008838122	0.003456311	0.183418679	8.80E-05	0.000687242	1	0.075158099	1	0.077122017	ENSG00000168291	8	PDHB
SAT	Obesity	ENSG00000184990.8	0.004454268	0.346314681	0.004729714	0.183454139	0.004328314	0.3600982	0.004729473	0.183420954	0.000125954	0.002707356	1	0.644591556	1	0.660784225	ENSG00000168297	11	PXK
SAT	Obesity	ENSG00000185070.6	0.000168395	0.973355794	0.005041794	0.183454474	0.001327714	0.793205326	0.005064698	0.183421244	-0.00115932	0.017032286	1	0.993452012	1	0.919712977	ENSG00000168329	9	CX3CR1
SAT	Obesity	ENSG00000185271.6	0.010203665	0.012293542	0.004075575	0.183452105	0.010148191	0.012768507	0.004075299	0.183418927	5.55E-05	0.044085853	1	0.08923697	1	0.096541971	ENSG00000168329	9	CX3CR1
SAT	Obesity	ENSG00000185290.3	-0.001206764	0.669522193	0.002827442	0.183454405	-0.001254483	0.657251451	0.002827251	0.183421196	4.77E-05	0.01242219	1	0.855746909	1	0.852011857	ENSG00000168329	9	CX3CR1
SAT	Obesity	ENSG00000185298.8	0.00147324	0.576858894	0.002640314	0.183454356	0.001428057	0.588575484	0.002640141	0.18342116	4.52E-05	0.015141329	1	0.804708347	1	0.813760607	ENSG00000168356	7	SCN11A
SAT	Obesity	ENSG00000185324.17	-8.91E-05	0.958789734	0.001724891	0.183454473	-0.000126028	0.941751407	0.001724786	0.183421268	3.69E-05	0.005658839	1	0.987453658	1	0.982135449	ENSG00000168356	7	SCN11A
SAT	Obesity	ENSG00000185404.12	-0.002113023	0.700020909	0.005484206	0.183454418	-0.001953662	0.721650893	0.005483909	0.183421222	-0.000159362	0.000647478	1	0.871308271	1	0.885797449	ENSG00000168356	7	SCN11A
SAT	Obesity	ENSG00000185619.13	0.011497658	0.01053333	0.004495099	0.183452001	0.011588893	0.009929558	0.00449481	0.183418758	-9.12E-05	0.005280183	1	0.07998762	1	0.081872378	ENSG00000168356	7	SCN11A
SAT	Obesity	ENSG00000185664.10	0.006550838	0.041170946	0.003208349	0.183452898	0.00714347	0.026477216	0.003219017	0.183419409	-0.000592631	0.025557467	1	0.192979684	1	0.151209505	ENSG00000168385	13	SEPT2
SAT	Obesity	ENSG00000185697.12	-0.011155564	0.010183381	0.004341447	0.183451978	-0.006925359	0.132143603	0.004599413	0.183420413	-0.004230205	0.005378197	1	0.078431158	1	0.388845886	ENSG00000168385	13	SEPT2
SAT	Obesity	ENSG00000185739.9	0.007625298	0.025821797	0.003421122	0.183452596	0.007704946	0.024303782	0.003420924	0.183419353	-7.96E-05	0.003979322	1	0.145319582	1	0.144457803	ENSG00000168394	9	TAP1
SAT	Obesity	ENSG00000185829.11	-0.002484973	0.460479932	0.003366911	0.183454268	-0.002433818	0.469736589	0.003366704	0.183421073	-5.12E-05	0.046266934	1	0.731904575	1	0.738833053	ENSG00000168394	9	TAP1
SAT	Obesity	ENSG00000186132.10	0.00896584	0.081568778	0.005147874	0.183453327	0.008881725	0.084451228	0.005147553	0.183420145	8.41E-05	0.029251168	1	0.287792178	1	0.298677775	ENSG00000168404	8	MLKL
SAT	Obesity	ENSG00000186230.6	-0.003641524	0.299789394	0.00351198	0.183454068	-0.003575874	0.308559013	0.003511779	0.183420878	-6.56E-05	0.021858725	1	0.599396101	1	0.609083345	ENSG00000168404	8	MLKL
SAT	Obesity	ENSG00000186453.8	0.000909071	0.641288339	0.00195122	0.183454392	0.000871451	0.655131432	0.001951103	0.183421195	3.76E-05	0.013710359	1	0.843249571	1	0.850607926	ENSG00000168405	10	CMAHP
SAT	Obesity	ENSG00000186470.9	-0.006685308	1.66E-11	0.000992885	0.183437339	-0.005600123	5.80E-08	0.001032274	0.183410148	-0.001085185	0.000123658	5.45E-08	1.65E-09	0.000190203	3.66E-06	ENSG00000168405	10	CMAHP
SAT	Obesity	ENSG00000186522.10	-0.002800573	0.42772562	0.003531216	0.183454236	-0.002748035	0.436413578	0.003530979	0.183421041	-5.25E-05	0.028683946	1	0.709992059	1	0.717711418	ENSG00000168405	10	CMAHP
SAT	Obesity	ENSG00000186575.13	-0.004582519	0.294617467	0.004372436	0.183454059	-0.004679644	0.284473374	0.004372169	0.183420837	9.71E-05	0.003763416	1	0.593750704	1	0.588868858	ENSG00000168405	10	CMAHP
SAT	Obesity	ENSG00000186577.7	-0.000469455	0.929182949	0.005282319	0.183454471	-0.000994736	0.85064725	0.005282921	0.183421257	0.000525281	8.71E-07	1	0.977131172	1	0.943853103	ENSG00000168405	10	CMAHP
SAT	Obesity	ENSG00000186665.8	-0.002331443	0.689011428	0.005825735	0.183454414	-0.002154286	0.711525576	0.005825415	0.183421219	-0.000177157	0.000317085	1	0.864822684	1	0.882614523	ENSG00000168405	10	CMAHP
SAT	Obesity	ENSG00000186792.11	-0.008896385	6.22E-06	0.001968676	0.183446755	-0.008932908	5.69E-06	0.001968567	0.183413488	3.65E-05	0.027358486	0.020393468	0.00020165	0.018657521	0.000222113	ENSG00000168522	8	FNTA
SAT	Obesity	ENSG00000186854.6	0.005375562	0.007261374	0.002002351	0.18345175	0.002969232	0.19963753	0.002315035	0.183420648	0.002406329	0.038402083	1	0.063194226	1	0.486273745	ENSG00000168522	8	FNTA
SAT	Obesity	ENSG00000186867.6	-0.008286044	0.175135377	0.006111156	0.183453779	-0.008191039	0.180104942	0.006110737	0.183420591	-9.50E-05	0.018571663	1	0.446826727	1	0.457509161	ENSG00000168569	7	TMEM223
SAT	Obesity	ENSG00000187049.5	0.001720602	0.700662796	0.004475768	0.183454418	0.001960613	0.661365296	0.004475987	0.183421198	-0.000240011	0.00132145	1	0.871446033	1	0.854643378	ENSG00000168612	4	ZSWIM1
SAT	Obesity	ENSG00000187091.9	0.001850096	0.671607271	0.004364017	0.183454406	0.001726974	0.692290074	0.004363807	0.183421211	0.000123122	0.002216069	1	0.855855862	1	0.868937924	ENSG00000168653	6	NDUFS5
SAT	Obesity	ENSG00000187166.1	9.40E-05	0.968531392	0.002382471	0.183454474	0.000136475	0.954316933	0.002382325	0.183421269	-4.25E-05	0.018852939	1	0.992734289	1	0.988489312	ENSG00000168653	6	NDUFS5

SAT	Obesity	ENSG00000187741.10	-0.008411486	0.013903545	0.003419644	0.183452187	-0.008349448	0.014616508	0.003419449	0.183419017	-6.20E-05	0.02620649	1	0.097265525	1	0.107046342	ENSG00000168653	6	NDUFS5
SAT	Obesity	ENSG00000187762.5	0.011410306	0.005756579	0.004132154	0.183451592	0.010660933	0.009917109	0.004134202	0.183418757	0.000749373	1.15E-07	1	0.054118445	1	0.081872378	ENSG00000168653	6	NDUFS5
SAT	Obesity	ENSG00000187942.7	-0.001520275	0.674569319	0.00362069	0.183454407	-0.000598685	0.868964409	0.00362896	0.18342126	-0.000921591	0.000222847	1	0.856857118	1	0.954321831	ENSG00000168661	10	ZNF30
SAT	Obesity	ENSG00000187987.5	0.004389296	0.002644468	0.001460024	0.183451058	0.003336434	0.023552678	0.001473458	0.183419332	0.001052862	1.30E-07	1	0.030231706	1	0.142476911	ENSG00000168661	10	ZNF30
SAT	Obesity	ENSG00000188321.9	-7.46E-05	0.942709838	0.001038341	0.183454472	-9.68E-05	0.925719092	0.001038275	0.183421267	2.22E-05	0.003182193	1	0.981335116	1	0.976304835	ENSG00000168661	10	ZNF30
SAT	Obesity	ENSG00000188629.7	-0.000247234	0.894821475	0.001870069	0.183454467	-0.000286203	0.878355891	0.001869948	0.183421261	3.90E-05	0.003837363	1	0.962747126	1	0.958406391	ENSG00000168661	10	ZNF30
SAT	Obesity	ENSG00000188677.10	-0.001926454	0.487835624	0.002776846	0.183454292	-0.001972407	0.4774843	0.002776649	0.183421079	4.60E-05	0.007931827	1	0.748926431	1	0.742751548	ENSG00000168661	10	ZNF30
SAT	Obesity	ENSG00000188738.9	-0.004551263	0.13162776	0.003018647	0.183453615	-0.004593488	0.128056957	0.003018434	0.183420395	4.22E-05	0.027655709	1	0.383203799	1	0.381612058	ENSG00000168672	3	FAM84B
SAT	Obesity	ENSG00000189007.11	0.009022316	0.052457061	0.004652193	0.183453052	0.008906109	0.055555471	0.004651897	0.183419885	0.000116207	0.000661999	1	0.224103668	1	0.236724026	ENSG00000168679	13	SLC16A4
SAT	Obesity	ENSG00000189045.9	-0.00376663	0.241723276	0.003217439	0.183453956	-0.00368772	0.251696643	0.003217234	0.183420774	-7.89E-05	0.000748509	1	0.530497704	1	0.548923519	ENSG00000168679	13	SLC16A4
SAT	Obesity	ENSG00000189068.5	0.005641784	0.321250424	0.005687898	0.183454102	0.005712516	0.315186859	0.005687497	0.183420889	-7.07E-05	0.049534288	1	0.619648819	1	0.615584597	ENSG00000168679	13	SLC16A4
SAT	Obesity	ENSG00000189180.11	0.000863995	0.853402152	0.004675817	0.183454461	0.000962956	0.836825557	0.004675549	0.183421254	-9.90E-05	0.009330774	1	0.943648754	1	0.937115108	ENSG00000168679	13	SLC16A4
SAT	Obesity	ENSG00000189182.5	-0.011239987	0.046000682	0.005632971	0.183452969	-0.011411623	0.042767435	0.005632645	0.183419719	0.000171636	0.00016425	1	0.206468178	1	0.201899213	ENSG00000168679	13	SLC16A4
SAT	Obesity	ENSG00000189190.7	-0.004999586	0.097701957	0.003018889	0.183453437	-0.004948875	0.101127502	0.003018683	0.183420254	-5.07E-05	0.011964934	1	0.318015993	1	0.331799333	ENSG00000168702	12	LRP1B
SAT	Obesity	ENSG00000189306.6	-0.006788105	0.116067575	0.004319511	0.183453541	-0.006686918	0.121580326	0.004319221	0.183420364	-0.000101186	0.000583172	1	0.356237338	1	0.369554001	ENSG00000168702	12	LRP1B
SAT	Obesity	ENSG00000196151.6	-0.002540475	0.42026734	0.003152118	0.183454229	-0.002485779	0.430312192	0.003151909	0.183421035	-5.47E-05	0.01278529	1	0.703340943	1	0.714674593	ENSG00000168702	12	LRP1B
SAT	Obesity	ENSG00000196152.6	0.00143817	0.612653325	0.002840606	0.183454377	0.001374283	0.628506935	0.002840427	0.183421182	6.39E-05	0.002435118	1	0.826188064	1	0.83385817	ENSG00000168702	12	LRP1B
SAT	Obesity	ENSG00000196236.8	-0.00136156	0.576261929	0.002436345	0.183454356	-0.00132726	0.585882449	0.002436176	0.183421158	-3.43E-05	0.030825304	1	0.804708347	1	0.812070604	ENSG00000168702	12	LRP1B
SAT	Obesity	ENSG00000196263.3	-0.004015268	0.445503836	0.005262938	0.183454254	-0.004113524	0.434418074	0.005262586	0.183421039	9.83E-05	0.006699369	1	0.722137485	1	0.717325467	ENSG00000168743	8	NPNT
SAT	Obesity	ENSG00000196535.10	-0.004556881	0.284593311	0.004258532	0.183454041	-0.004637183	0.276158904	0.004258243	0.183420822	8.03E-05	0.004999735	1	0.582865575	1	0.580018771	ENSG00000168765	11	GSTM4
SAT	Obesity	ENSG00000196730.8	-0.00159981	0.766715171	0.005392476	0.183454441	-0.001782706	0.740939928	0.005392187	0.183421229	0.000182896	8.01E-05	1	0.906519811	1	0.897054301	ENSG00000168765	11	GSTM4
SAT	Obesity	ENSG00000196743.4	0.004978364	0.071802267	0.002765184	0.183453249	0.00503683	0.068512525	0.00276502	0.183420016	-5.85E-05	0.007661246	1	0.268623988	1	0.265839301	ENSG00000168778	7	TCTN2
SAT	Obesity	ENSG00000196793.9	0.00777086	0.060293721	0.00413642	0.18345314	0.007666727	0.0637994	0.004136165	0.183419972	0.000104133	0.000920503	1	0.243144411	1	0.257054802	ENSG00000168778	7	TCTN2
SAT	Obesity	ENSG00000196966.3	0.003348394	0.36368424	0.003686165	0.183454162	0.003802931	0.302352838	0.003687147	0.183420868	-0.000454537	3.98E-06	1	0.660713174	1	0.602319163	ENSG00000168778	7	TCTN2
SAT	Obesity	ENSG00000197062.7	-0.009707507	1.40E-07	0.001843861	0.183443997	-0.007421931	0.000112433	0.001921736	0.183415633	-0.002285576	2.48E-05	0.000460727	6.88E-06	0.368891926	0.002475785	ENSG00000168778	7	TCTN2
SAT	Obesity	ENSG00000197165.6	0.025093694	3.96E-29	0.002239842	0.183407042	0.025056674	4.73E-29	0.002239707	0.183373979	3.70E-05	0.032057273	1.30E-25	3.25E-26	1.55E-25	3.88E-26	ENSG00000168778	7	TCTN2
SAT	Obesity	ENSG00000197168.7	-0.003446251	0.475551692	0.004830228	0.183454282	-0.003305122	0.493784126	0.00482991	0.183421093	-0.000141129	2.93E-05	1	0.738771355	1	0.755129058	ENSG00000168778	7	TCTN2
SAT	Obesity	ENSG00000197223.7	0.001342356	0.774508069	0.004685633	0.183454443	0.001234967	0.792103327	0.004685352	0.183421244	0.000107389	0.003267634	1	0.909817544	1	0.919712977	ENSG00000168778	7	TCTN2
SAT	Obesity	ENSG00000197279.3	-0.017494393	9.20E-05	0.004473433	0.183448693	-0.013316878	0.003458675	0.004554738	0.18341804	-0.004177515	1.15E-06	0.301950166	0.00184116	1	0.040673517	ENSG00000168778	7	TCTN2
SAT	Obesity	ENSG00000197302.6	-0.002587983	0.557668002	0.004414024	0.183454344	-0.002487582	0.573038099	0.004413868	0.18342115	-0.000100401	0.030260791	1	0.794232732	1	0.802779912	ENSG00000168778	7	TCTN2
SAT	Obesity	ENSG00000197345.8	-0.004619649	0.000244758	0.001259546	0.183449389	-0.00459975	0.000260086	0.001259465	0.183416229	-1.99E-05	0.03042095	0.803050333	0.004279083	0.853342504	0.004934546	ENSG00000168778	7	TCTN2
SAT	Obesity	ENSG00000197728.5	-0.004999392	6.61E-05	0.001253025	0.183448457	-0.004951639	7.76E-05	0.00125301	0.183415368	-4.78E-05	0.002334033	0.216946595	0.00142728	0.254532644	0.00181809	ENSG00000168781	17	PPIP5K1
SAT	Obesity	ENSG00000197822.6	-0.007926497	0.011852073	0.003149775	0.18345208	-0.007883171	0.012316671	0.003149554	0.183418903	-4.33E-05	0.030012306	1	0.086607243	1	0.094213678	ENSG00000168781	17	PPIP5K1
SAT	Obesity	ENSG00000197847.8	0.010813001	0.046145572	0.005422594	0.183452971	0.009107576	0.093565028	0.005431237	0.183420207	0.001705425	6.01E-08	1	0.20655337	1	0.316481295	ENSG00000168803	10	ADAL
SAT	Obesity	ENSG00000197903.6	0.006975662	0.004177575	0.002435257	0.183451373	0.004522051	0.068541378	0.002482686	0.183420016	0.00245361	3.99E-07	1	0.043375388	1	0.265839301	ENSG00000168803	10	ADAL
SAT	Obesity	ENSG00000197935.6	0.003571896	0.340157401	0.003744694	0.18345413	0.004632561	0.21667539	0.003749791	0.183420693	-0.001060665	1.49E-07	1	0.638111167	1	0.508520712	ENSG00000168813	12	ZNF507
SAT	Obesity	ENSG00000197958.8	-0.001001194	0.581914925	0.001818401	0.18345436	-0.000960952	0.597157189	0.001818286	0.183421165	-4.02E-05	0.002825685	1	0.806390405	1	0.817137678	ENSG00000168813	12	ZNF507
SAT	Obesity	ENSG00000197969.7	-0.00459892	0.117480898	0.002937785	0.183453548	-0.004546697	0.121682611	0.002937613	0.183420365	-5.22E-05	0.025885684	1	0.358562629	1	0.369554001	ENSG00000168813	12	ZNF507
SAT	Obesity	ENSG00000198015.8	-0.005643355	0.014295474	0.002303635	0.183452206	-0.005704107	0.013275962	0.002303491	0.183418953	6.08E-05	0.000400141	1	0.099161625	1	0.09922194	ENSG00000168827	10	GFM1
SAT	Obesity	ENSG00000198026.6	0.003083179	0.42037537	0.003826372	0.183454229	0.003142829	0.411410689	0.003826119	0.183421015	-5.97E-05	0.025695756	1	0.703340943	1	0.701318401	ENSG00000168827	10	GFM1
SAT	Obesity	ENSG00000198093.6	-0.010987172	0.004712029	0.003887749	0.183451455	-0.011036791	0.004524819	0.003887477	0.183418224	4.96E-05	0.047792054	1	0.047569744	1	0.049651945	ENSG00000168827	10	GFM1
SAT	Obesity	ENSG00000198099.4	-0.012718738	6.23E-05	0.003176619	0.183448415	-0.012664397	6.69E-05	0.003176418	0.183415263	-5.43E-05	0.020687286	0.204492663	0.001354256	0.219571995	0.001602715	ENSG00000168827	10	GFM1
SAT	Obesity	ENSG00000198131.9	-0.000960846	0.764550408	0.003208051	0.18345444	-0.001050672	0.743266117	0.00320785	0.18342123	8.98E-05	0.000187156	1	0.904612293	1	0.898125261	ENSG00000168874	8	ATOH8
SAT	Obesity	ENSG00000198203.5	0.00103775	0.780553966	0.00372491	0.183454445	0.00097439	0.793627301	0.003724682	0.183421244	6.34E-05	0.025907612	1	0.913071213	1	0.919712977	ENSG00000168884	10	TNIP2
SAT	Obesity	ENSG00000198208.7	-0.001522833	0.796866521	0.005916106	0.183454449	-0.001687333	0.77546989	0.005915742	0.183421239	0.0001645	0.000261585	1	0.919310497	1	0.911862059	ENSG00000168936	6	TMEM129
SAT	Obesity	ENSG00000198336.5	0.007745693	0.149679386	0.005376462	0.18345369	0.007659955	0.154210901	0.005376099	0.183420503	8.57E-05	0.018400718	1	0.409839489	1	0.417993751	ENSG00000168936	6	TMEM129
SAT	Obesity	ENSG00000198502.5	-0.001580005	0.122989817	0.001024416	0.183453575	0.000758679	0.526497447	0.00119786	0.183421119	-0.002338684	0.00016584	1	0.367513287	1	0.778826927	ENSG00000168944	11	CEP120
SAT	Obesity	ENSG00000198515.9	0.000124149	0.978423742	0.004590422	0.183454474	-0.000377518	0.934496715	0.004593313	0.183421268	0.000501667	0.00399056	1	0.993452012	1	0.979844999	ENSG00000168944	11	CEP120
SAT	Obesity	ENSG00000198755.6	0.003617949	0.110704867	0.002268256	0.183453512	0.003337317	0.141311642	0.002268854	0.183420452	0.000280632	3.31E-06	1	0.343697234	1	0.401725489	ENSG00000168961	12	LGALS9
SAT	Obesity	ENSG00000198794.7	-0.005627431	0.038589098	0.002720475	0.183452857	-0.00568549	0.036615417	0.002720288	0.183419619	5.81E-05	0.001301049	1	0.188234951	1	0.186062873	ENSG00000168961	12	LGALS9
SAT	Obesity	ENSG00000198912.6	0.001922947	0.667775827	0.004480276	0.183454404	0.00199297	0.656419492	0.004479971	0.183421195	-7.00E-05	0.019471032	1	0.854600118	1	0.851269705	ENSG00000168961	12	LGALS9
SAT	Obesity	ENSG00000198920.5	8.47E-05	0.98619511	0.004895479	0.183454474	0.000166115	0.972929496	0.004895171	0.18342127	-8.14E-05	0.025056669	1	0.994152558	1	0.992266083	ENSG00000168961	12	LGALS9
SAT	Obesity	ENSG00000198945.3	0.006035288	0.181189195	0.0045137	0.183453798	0.006124978	0.174760779	0.004513396	0.183420574	-8.97E-05	0.003638936	1	0.453456712	1	0.448311272	ENSG00000168992	3	OR7E102P
SAT	Obesity	ENSG00000203778.3	0.002516112	0.415357905	0.003089142	0.183454223	0.002589832	0.401801062	0.003088982	0.183421005	-7.37E-05	0.006601977	1	0.700759618	1	0.692729827	ENSG00000168992	3	OR7E102P
SAT	Obesity	ENSG00000204060.4	-0.009165158	0.097658673	0.005533463	0.183453437	-0.009003294	0.103700414	0.00553308	0.18342027	-0.000161865	6.82E-06	1	0.318015993	1	0.336539128	ENSG00000168993	10	CPLX1
SAT	Obesity	ENSG00000204169.6	0.003032414	0.21775636	0.002460341	0.1834539	0.003072628	0.211683369	0.002460166	0.183420681	-4.02E-05	0.008262393	1	0.505890452	1	0.502914061	ENSG00000168993	10	CPLX1
SAT	Obesity	ENSG00000204237.4	0.002458604	0.665910403	0.005694275	0.183454404	0.002300909	0.686138681	0.005693897	0.183421208	0.000157695	6.78E-05	1	0.854600118	1	0.86636807	ENSG00000168993	10	CPLX1
SAT	Obesity	ENSG00000204252.8	7.17E-05	0.985256887	0.003882101	0.183454474	-0.002500374	0.522634459	0.003911172	0.183421116	0.002572111	7.81E-08	1	0.994152558	1	0.776262408	ENSG00000168993	10	CPLX1
SAT	Obesity	ENSG00000204267.9	-0.000598377	0.730147701	0.001734782	0.183454429	-0.001652774	0.345087629	0.00175052	0.183420933	0.001054397	7.48E-06	1	0.8889108	1	0.648774721	ENSG00000169047	5	IRS1
SAT	Obesity	ENSG00000204287.9	-0.010017838	0.003165553	0.003394495	0.183451182	-0.005832897	0.11941829	0.003745706	0.183420354	-0.00418494	0.008249264	1	0.034736391	1	0.368420209	ENSG00000169047	5	IRS1
SAT	Obesity	ENSG00000204305.9	0.01380219	0.000714009	0.004078463	0.183450145	0.005015805	0.282762654	0.004669622	0.183420834	0.008786384	0.000112145	1	0.010600293	1	0.587152637	ENSG00000169047	5	IRS1
SAT	Obesity	ENSG00000204314.6	0.000765944	0.834465484	0.003665188	0.183454458	0.002766467	0.46318396	0.003771008	0.183421067	-0.002000523	0.024340891	1	0.936988793	1	0.735223306	ENSG00000169047	5	IRS1
SAT	Obesity	ENSG00000204338.4	-0.004178097	0.041152679	0.002046087	0.183452898	0.001854319	0.431195463	0.002355743	0.183421036	-0.006032415	2.40E-07	1	0.192979684	1	0.714882423	ENSG00000169047	5	IRS1
SAT	Obesity	ENSG00000204348.5	-0.002891559	0.311609271	0.002857689	0.183454087	-7.44E-05	0.979601763	0.002908737	0.18342127	-0.002817188	2.22E-07	1	0.608768261	1	0.994983894	ENSG00000169062	10	UPF3A
SAT	Obesity	ENSG00000204351.7	-0.005350322	2.10E-07	0.001030888	0.183444293	-0.004266343	6.31E-05	0.001066332	0.18341522	-0.001083979	7.15E-05	0.000690205	9.86E-06	0.207024255	0.001544957	ENSG00000169085	7	C8orf46
SAT	Obesity	ENSG00000204356.7	0.010126793	2.86E-06	0.002163634	0.183446194	0.008249118	0.000181971	0.002203971	0.183415976	0.001877675	8.05E-06	0.009394045	0.000104378	0.597045615	0.00377877	ENSG00000169085	7	C8orf46
SAT	Obesity	ENSG00000204387.8	-0.002309367	0.446993564	0.003036893	0.183454256	-0.000657986	0.829496979	0.003055472	0.183421253	-0.001651381	1.10E-06	1	0.722507754	1	0.936326075	ENSG00000169085	7	C8orf46
SAT	Obesity	ENSG00000204410.10	-0.001354793	0.749456476	0.004242262	0.183454436	0.001386245	0.752263407	0.004391625	0.183421233	-0.002741038	0.015922188	1	0.898416769	1	0.901441682	ENSG00000169085	7	C8orf46
SAT	Obesity	ENSG00000204428.8	-0.000535192	0.756531648	0.001726209	0.183454438	-0.00161319	0.354314707	0.001741629	0.183420946	0.001077998	3.51E-06	1	0.901627438	1	0.656608804	ENSG00000169085	7	C8orf46
SAT	Obesity	ENSG00000204438.6	-0.011442876	0.00083082	0.003423614	0.183450251	-0.006523699	0.071773772	0.003623162	0.183420045	-0.004919177	3.39E-05	1	0.011749661	1	0.27339388	ENSG00000169085	7	C8orf46
SAT	Obesity	ENSG00000204498.6	0.008923711	0.060153375	0.004747483	0.183453139	0.00290362	0.55456837	0.004913654	0.183421138	0.006020092	2.08E-06	1	0.243144411	1	0.794558438	ENSG00000169085	7	C8orf46
SAT	Obesity	ENSG00000204516.5	0.001391575	0.269047937	0.001259049	0.183454012	0.000101478	0.936815494	0.001280112	0.183421268	0.001290096	2.64E-08	1	0.5655005	1	0.980364798	ENSG00000169085	7	C8orf46
SAT	Obesity	ENSG00000204525.10	0.004798001	0.009529341	0.001850748	0.183451934	0.002050477	0.291344937	0.001943254	0.183420849	0.002747524	3.59E-06	1	0.075158099	1	0.597439211	ENSG00000169116	7	PARM1
SAT	Obesity	ENSG00000204531.11	0.00635109	1.04E-06	0.001300143	0.183445455	0.004670615	0.000629435	0.001366226	0.183416853	0.001680475	6.32E-05	0.003396026	4.14E-05	1	0.010377772	ENSG00000169116	7	PARM1
SAT	Obesity	ENSG00000204536.9	0.006442758	5.37E-08	0.001184642	0.183443294	0.004687221	0.000241626	0.001276823	0.183416177	0.001755537	0.00022979	0.000176309	2.84E-06	0.792776436	0.004690985	ENSG00000169129	10	AFAP1L2
SAT	Obesity	ENSG00000204538.3	0.00043641	0.725665429	0.001243696	0.183454428	-0.000641986	0.618449814	0.001289002	0.183421176	0.001078396	0.001474517	1	0.887405245	1	0.828495448	ENSG00000169129	10	AFAP1L2
SAT	Obesity	ENSG00000204574.8	0.014218964	3.40E-05	0.003430609	0.183447981	0.010231213	0.003855244	0.003540477	0.183418114	0.003987752	5.34E-06	0.111641196	0.000833143	1	0.043920336	ENSG00000169203	12	RP11-231C14.4
SAT	Obesity	ENSG00000204592.5	0.026707702	4.78E-14	0.003543013	0.183432997	0.022714046	1.39E-09	0.003750173	0.183407407	0.003993657	0.001165501	1.57E-10	6.81E-12	4.56E-06	1.17E-07	ENSG00000169203	12	RP11-231C14.4
SAT	Obesity	ENSG00000204622.6	-0.006463708	0.001842252	0.002075314	0.183450807	-0.005585899	0.007889514	0.002102513	0.183418603	-0.000877809	0.009446654	1	0.022304168	1	0.071403169	ENSG00000169203	12	RP11-231C14.4
SAT	Obesity	ENSG00000204625.6	0.000898058	0.740123366	0.00270752	0.183454433	-2.93E-05	0.991389299	0.002719042	0.18342127	0.000927402	0.000242421	1	0.894088647	1	0.996552785	ENSG00000169220	13	RGS14
SAT	Obesity	ENSG00000204632.7	-0.008159872	0.010289628	0.003180057	0.183451985	-0.005103234	0.116449416	0.003250767	0.183420339	-0.003056638	6.06E-06	1	0.07900256	1	0.362840014	ENSG00000169220	13	RGS14
SAT	Obesity	ENSG00000204642.9	0.000621544	0.832381203	0.00293668	0.183454457	0.002689765	0.363546525	0.002960246	0.183420958	-0.002068221	3.45E-08	1	0.936669124	1	0.664705409	ENSG00000169220	13	RGS14
SAT	Obesity	ENSG00000204644.5	0.00603426	2.31E-08	0.001080036	0.183442675	0.004463343	0.000109397	0.001153683	0.183415614	0.001570918	0.000108348	7.58E-05	1.35E-06	0.358933116	0.002425224	ENSG00000169220	13	RGS14
SAT	Obesity	ENSG00000204709.4	0.003254238	0.097398344	0.00196322	0.183453436	0.001998297	0.312751281	0.001979557	0.183420885	0.001255941	8.54E-07	1	0.31765802	1	0.613351437	ENSG00000169231	9	THBS3
SAT	Obesity	ENSG00000204713.6	-0.004775887	0.081991479	0.002745947	0.183453331	-0.003405212	0.217197386	0.002759454	0.183420695	-0.001370676	6.30E-07	1	0.288332309	1	0.508767414	ENSG00000169231	9	THBS3
SAT	Obesity	ENSG00000204718.3	-0.002805463	0.600661764	0.005359568	0.183454371	-0.002667593	0.61865707	0.00535925	0.183421177	-0.000137871	0.001090785	1	0.818086861	1	0.828495448	ENSG00000169231	9	THBS3
SAT	Obesity	ENSG00000204778.4	-0.003995121	0.497586104	0.005889961	0.1834543	-0.004209071	0.474817443	0.005889585	0.183421077	0.00021395	6.67E-07	1	0.756691284	1	0.742537751	ENSG00000169247	7	SH3TC2
SAT	Obesity	ENSG00000204789.3	0.014353267	2.14E-07	0.002767244	0.183444305	0.012733339	4.93E-06	0.002787582	0.183413385	0.001619928	1.65E-06	0.000702043	9.89E-06	0.016168856	0.000199916	ENSG00000169248	8	CXCL11
SAT	Obesity	ENSG00000204792.2	-0.003815533	0.431075978	0.004846029	0.18345424	-0.003720666	0.442593429	0.004845742	0.183421047	-9.49E-05	0.013240757	1	0.712165298	1	0.722357979	ENSG00000169302	10	STK32A
SAT	Obesity	ENSG00000204933.2	-0.003609887	0.37185999	0.004042447	0.183454173	-0.003561055	0.378329436	0.004042155	0.183420977	-4.88E-05	0.045861799	1	0.666706353	1	0.673155574	ENSG00000169313	9	P2RY12
SAT	Obesity	ENSG00000205269.4	-0.000794984	0.789653108	0.002980133	0.183454447	-0.000705267	0.812913275	0.002979967	0.183421249	-8.97E-05	0.00030527	1	0.914498218	1	0.928356581	ENSG00000169313	9	P2RY12
SAT	Obesity	ENSG00000205309.9	-0.003160914	0.410759466	0.003842783	0.183454218	-0.003239275	0.399230894	0.003842572	0.183421002	7.84E-05	0.015856972	1	0.698355005	1	0.692729827	ENSG00000169330	4	KIAA1024
SAT	Obesity	ENSG00000205485.9	0.000253408	0.900400101	0.002024738	0.183454468	0.000216045	0.915019832	0.002024616	0.183421266	3.74E-05	0.016843733	1	0.963816005	1	0.970950863	ENSG00000169385	2	RNASE2
SAT	Obesity	ENSG00000205571.8	-0.006625826	0.003139993	0.002243225	0.183451176	-0.006592112	0.003294321	0.002243081	0.183418006	-3.37E-05	0.036797944	1	0.034571535	1	0.039204918	ENSG00000169442	4	CD52
SAT	Obesity	ENSG00000205643.6	-0.002656576	0.516919351	0.004099009	0.183454315	-0.002787612	0.496450727	0.004098915	0.183421095	0.000131037	0.005960253	1	0.771001245	1	0.756387058	ENSG00000169442	4	CD52
SAT	Obesity	ENSG00000205771.2	-0.009839294	0.042761131	0.004856414	0.183452922	-0.009975532	0.03995703	0.004856168	0.183419675	0.000136238	0.001686578	1	0.195948701	1	0.195087821	ENSG00000169442	4	CD52
SAT	Obesity	ENSG00000206344.6	0.001427796	0.271524835	0.001298517	0.183454017	0.00085163	0.519876486	0.00132336	0.183421114	0.000576166	0.02430067	1	0.567681645	1	0.774417527	ENSG00000169442	4	CD52
SAT	Obesity	ENSG00000206503.7	-0.003299237	0.160553345	0.002351198	0.18345373	-0.000696514	0.771722828	0.002400759	0.183421238	-0.002602723	8.70E-08	1	0.424818971	1	0.911785759	ENSG00000169442	4	CD52
SAT	Obesity	ENSG00000212694.4	-0.010235616	0.000174913	0.002727484	0.183449151	-0.010187849	0.000187365	0.002727306	0.183415997	-4.78E-05	0.013387633	0.573888544	0.00326073	0.614743135	0.003866309	ENSG00000169519	15	METTL15
SAT	Obesity	ENSG00000213090.2	0.001877062	0.667491024	0.004369385	0.183454404	0.001763682	0.686457944	0.004369152	0.183421209	0.000113381	0.002659549	1	0.854600118	1	0.86636807	ENSG00000169519	15	METTL15
SAT	Obesity	ENSG00000213246.2	0.0098064	0.070193843	0.00541592	0.183453235	0.00971026	0.072969777	0.005415579	0.183420055	9.61E-05	0.01644907	1	0.26532949	1	0.275592976	ENSG00000169519	15	METTL15
SAT	Obesity	ENSG00000213402.2	0.005236968	0.049413063	0.002665103	0.183453015	0.004546446	0.088371491	0.002668016	0.183420173	0.000690522	1.00E-07	1	0.21559077	1	0.306497741	ENSG00000169592	10	INO80E
SAT	Obesity	ENSG00000213443.2	0.001008728	0.592960915	0.001887059	0.183454366	0.000968459	0.607784946	0.001886958	0.183421171	4.03E-05	0.013034632	1	0.812973298	1	0.823005533	ENSG00000169592	10	INO80E
SAT	Obesity	ENSG00000213445.4	0.012919381	0.002777038	0.004318823	0.183451092	0.010280986	0.017963181	0.004344597	0.183419154	0.002638395	2.97E-08	1	0.031527553	1	0.120772946	ENSG00000169609	9	C15orf40
SAT	Obesity	ENSG00000213523.5	-0.002785733	0.413758806	0.003408469	0.183454222	-0.002830636	0.406239363	0.003408227	0.18342101	4.49E-05	0.034415496	1	0.7004864	1	0.696744041	ENSG00000169609	9	C15orf40
SAT	Obesity	ENSG00000213676.6	0.001726794	0.371420056	0.001931931	0.183454172	0.000688745	0.729334184	0.001990525	0.183421225	0.001038049	0.030609161	1	0.666706353	1	0.891559411	ENSG00000169609	9	C15orf40
SAT	Obesity	ENSG00000213719.4	-0.028043223	1.02E-06	0.005736602	0.183445441	-0.014992298	0.030007583	0.006908904	0.183419491	-0.013050925	0.000701207	0.003335249	4.12E-05	1	0.162878683	ENSG00000169609	9	C15orf40
SAT	Obesity	ENSG00000213760.6	-0.004803573	0.013760014	0.001949916	0.18345218	-0.003410436	0.089835142	0.002010549	0.183420183	-0.001393137	0.004525288	1	0.097089474	1	0.309870332	ENSG00000169609	9	C15orf40
SAT	Obesity	ENSG00000213839.4	0.01032299	0.066484018	0.005625147	0.183453201	0.010420851	0.063930728	0.005624784	0.183419973	-9.79E-05	0.016008144	1	0.259683409	1	0.257054802	ENSG00000169609	9	C15orf40
SAT	Obesity	ENSG00000213903.4	-7.28E-05	0.978388152	0.002688813	0.183454474	-0.000130537	0.961276774	0.002688639	0.183421269	5.77E-05	0.002865989	1	0.993452012	1	0.9914961	ENSG00000169609	9	C15orf40
SAT	Obesity	ENSG00000214078.7	-0.002688489	0.012228296	0.001073036	0.183452101	-0.002664263	0.013025433	0.001072966	0.18341894	-2.42E-05	0.001380902	1	0.089157865	1	0.097795071	ENSG00000169612	3	FAM103A1
SAT	Obesity	ENSG00000214087.4	-0.001695085	0.084584457	0.000982836	0.18345335	-0.001677227	0.087894684	0.000982781	0.183420169	-1.79E-05	0.028224658	1	0.29243583	1	0.305166621	ENSG00000169629	7	RGPD8
SAT	Obesity	ENSG00000214102.3	0.009673476	0.049864908	0.004932613	0.18345302	0.009755405	0.047944978	0.004932301	0.183419792	-8.19E-05	0.024561409	1	0.216697698	1	0.218179573	ENSG00000169683	3	LRRC45
SAT	Obesity	ENSG00000214135.4	-0.007584673	0.024494284	0.003372025	0.183452562	-0.007633458	0.02357969	0.003371793	0.183419333	4.88E-05	0.028864797	1	0.141512278	1	0.142476911	ENSG00000169683	3	LRRC45
SAT	Obesity	ENSG00000214376.5	-0.006291536	0.100696292	0.003832819	0.183453456	-0.00612898	0.109783688	0.003832603	0.183420304	-0.000162556	2.63E-07	1	0.323906407	1	0.348019594	ENSG00000169683	3	LRRC45
SAT	Obesity	ENSG00000214517.4	0.002145725	0.421315315	0.002668341	0.18345423	0.00220889	0.407752412	0.002668206	0.183421011	-6.32E-05	0.007905186	1	0.704195389	1	0.698244083	ENSG00000169813	12	HNRNPF
SAT	Obesity	ENSG00000214562.9	0.008126751	0.067073856	0.004437979	0.183453207	0.008539886	0.054425264	0.0044399	0.183419872	-0.000413135	0.0040273	1	0.26053725	1	0.234035768	ENSG00000169862	14	CTNND2
SAT	Obesity	ENSG00000214659.4	0.018539281	0.001399453	0.005802987	0.183450616	0.02253179	0.000115136	0.005842867	0.18341565	-0.003992509	5.94E-09	1	0.018078898	0.377760746	0.002501727	ENSG00000169862	14	CTNND2
SAT	Obesity	ENSG00000214765.4	-0.002037596	0.254614858	0.0017886	0.183453984	-0.002003272	0.262675513	0.00178849	0.183420796	-3.43E-05	0.011912981	1	0.546364519	1	0.565510733	ENSG00000169894	13	MUC3A
SAT	Obesity	ENSG00000214982.6	0.003271343	0.581484861	0.005934759	0.183454359	0.003153694	0.595120623	0.005934347	0.183421163	0.000117649	0.002236165	1	0.806390405	1	0.817137678	ENSG00000169902	9	TPST1
SAT	Obesity	ENSG00000215908.5	0.002448342	0.53311077	0.003928253	0.183454327	0.002546239	0.516843534	0.003928052	0.183421111	-9.79E-05	0.005035672	1	0.784366115	1	0.772996237	ENSG00000169902	9	TPST1
SAT	Obesity	ENSG00000216901.1	0.005590088	0.000298355	0.001545611	0.18344953	0.004326528	0.005765072	0.001567092	0.183418389	0.00126356	1.11E-06	0.978902294	0.005121427	1	0.058022089	ENSG00000169914	5	OTUD3
SAT	Obesity	ENSG00000217930.3	0.001029218	0.817309663	0.004455358	0.183454454	0.001124155	0.800786854	0.004455114	0.183421246	-9.49E-05	0.011618438	1	0.930695292	1	0.92223015	ENSG00000169914	5	OTUD3
SAT	Obesity	ENSG00000218537.1	0.002061424	0.165191903	0.001485366	0.183453746	0.002087931	0.159796351	0.00148527	0.183420523	-2.65E-05	0.012577231	1	0.430496136	1	0.428342998	ENSG00000169914	5	OTUD3
SAT	Obesity	ENSG00000219392.1	-0.006499315	3.18E-05	0.001562245	0.183447932	-0.004336629	0.007961925	0.001634188	0.183418609	-0.002162685	6.60E-06	0.10432488	0.00079034	1	0.071403169	ENSG00000169946	9	ZFPM2
SAT	Obesity	ENSG00000219891.2	0.002070762	0.511050292	0.003150868	0.183454311	0.003172072	0.314874421	0.003156141	0.183420888	-0.00110131	4.07E-09	1	0.766692277	1	0.615584597	ENSG00000169967	12	MAP3K2
SAT	Obesity	ENSG00000221829.5	-0.000650057	0.826438758	0.002964624	0.183454456	-0.000694327	0.814814841	0.00296443	0.183421249	4.43E-05	0.033931411	1	0.9363121	1	0.929234443	ENSG00000169972	7	PUSL1
SAT	Obesity	ENSG00000221944.3	-0.00015987	0.954252922	0.002786799	0.183454473	-0.000109999	0.968512447	0.002786613	0.18342127	-4.99E-05	0.009527564	1	0.985800956	1	0.992119797	ENSG00000170035	11	UBE2E3
SAT	Obesity	ENSG00000221947.3	0.008997311	0.034249493	0.004249772	0.18345278	0.008907832	0.036065097	0.004249517	0.18341961	8.95E-05	0.006953198	1	0.172615341	1	0.184615666	ENSG00000170092	10	AC018720.10
SAT	Obesity	ENSG00000222019.3	-7.60E-05	0.974736329	0.002399598	0.183454474	-2.85E-05	0.990519996	0.002399435	0.18342127	-4.75E-05	0.003212942	1	0.993452012	1	0.996289426	ENSG00000170144	14	HNRNPA3
SAT	Obesity	ENSG00000223473.1	-0.007080993	0.041289771	0.003470031	0.1834529	-0.00712321	0.040079821	0.003469776	0.183419677	4.22E-05	0.037858111	1	0.192979684	1	0.195229009	ENSG00000170144	14	HNRNPA3
SAT	Obesity	ENSG00000223718.2	0.014512026	0.007233012	0.005402975	0.183451747	0.014623306	0.006795891	0.005402641	0.183418501	-0.00011128	0.006790253	1	0.063115725	1	0.06519684	ENSG00000170175	6	CHRNB1
SAT	Obesity	ENSG00000223865.6	0.003556655	0.365403331	0.003929494	0.183454164	0.001485878	0.709554352	0.003989418	0.183421218	0.002070777	0.002723549	1	0.663101952	1	0.881168747	ENSG00000170175	6	CHRNB1
SAT	Obesity	ENSG00000224389.4	0.015904982	8.10E-09	0.00275817	0.183441906	0.011035607	0.000578937	0.003206797	0.183416794	0.004869375	0.002919755	2.66E-05	5.54E-07	1	0.009691288	ENSG00000170175	6	CHRNB1
SAT	Obesity	ENSG00000224429.3	-0.000586354	0.921116949	0.005921162	0.18345447	-0.000479658	0.935431789	0.005920767	0.183421268	-0.000106696	0.00888414	1	0.973380029	1	0.979933493	ENSG00000170175	6	CHRNB1
SAT	Obesity	ENSG00000224497.1	0.002315376	0.059271311	0.001227548	0.183453129	0.002274424	0.06389517	0.001227485	0.183419973	4.10E-05	0.00015416	1	0.241576612	1	0.257054802	ENSG00000170209	4	ANKK1
SAT	Obesity	ENSG00000224557.3	0.000384383	0.708551225	0.001028304	0.183454421	1.33E-05	0.989776543	0.00103634	0.18342127	0.000371104	0.004175906	1	0.87726663	1	0.996265811	ENSG00000170209	4	ANKK1

SAT	Obesity	ENSG00000224635.1	0.000154659	0.950203504	0.002476481	0.183454473	9.82E-05	0.96835344	0.002476336	0.18342127	5.64E-05	0.004227805	1	0.98440723	1	0.992119797	ENSG00000170209	4	ANKK1
SAT	Obesity	ENSG00000224796.1	-0.000229337	0.847215991	0.001190297	0.18345446	0.000469882	0.701966114	0.001227911	0.183421215	-0.000699219	0.020604111	1	0.942344807	1	0.876674335	ENSG00000170209	4	ANKK1
SAT	Obesity	ENSG00000224818.1	0.001296345	0.778794176	0.004615119	0.183454444	0.001379002	0.765079828	0.004614871	0.183421236	-8.27E-05	0.037073343	1	0.913071213	1	0.906138181	ENSG00000170248	9	PDCD6IP
SAT	Obesity	ENSG00000224843.2	0.031905543	1.41E-09	0.00527	0.18344062	0.027332415	3.56E-07	0.005368377	0.183411474	0.004573127	8.16E-06	4.63E-06	1.08E-07	0.001166758	1.91E-05	ENSG00000170264	8	FAM161A
SAT	Obesity	ENSG00000224930.2	-0.000848921	0.851604988	0.004537959	0.183454461	-0.000936473	0.836496957	0.004537695	0.183421254	8.76E-05	0.016229958	1	0.943639299	1	0.937115108	ENSG00000170264	8	FAM161A
SAT	Obesity	ENSG00000224997.1	-0.010734664	7.61E-06	0.00239835	0.183446902	-0.01067946	8.47E-06	0.002398202	0.183413776	-5.52E-05	0.002389275	0.024976216	0.000233423	0.027775013	0.00029764	ENSG00000170275	10	CRTAP
SAT	Obesity	ENSG00000225194.2	-0.003211694	0.288015005	0.003022807	0.183454047	-0.003146391	0.297900353	0.003022621	0.183420861	-6.53E-05	0.004593069	1	0.587064948	1	0.601026164	ENSG00000170275	10	CRTAP
SAT	Obesity	ENSG00000225398.2	0.000646591	0.833460965	0.003075126	0.183454457	0.000582217	0.849824066	0.003074932	0.18342157	6.44E-05	0.004710042	1	0.936669124	1	0.943577922	ENSG00000170289	8	CNGB3
SAT	Obesity	ENSG00000225498.1	-0.01096889	0.008244574	0.004151899	0.183451836	-0.011046356	0.007797741	0.004151649	0.183418595	7.75E-05	0.016681289	1	0.068830653	1	0.071403169	ENSG00000170322	10	NFRKB
SAT	Obesity	ENSG00000225684.3	-0.001917584	0.648459025	0.004206111	0.183454396	-0.001822365	0.664801971	0.004205847	0.183421199	-9.52E-05	0.0023763	1	0.845625621	1	0.856049947	ENSG00000170322	10	NFRKB
SAT	Obesity	ENSG00000225726.1	-0.01476772	0.002629017	0.004909321	0.183451054	-0.014695957	0.002756575	0.00490901	0.183417883	-7.18E-05	0.04678054	1	0.030160152	1	0.034389064	ENSG00000170356	8	OR2A20P
SAT	Obesity	ENSG00000225784.5	0.001388772	0.543574756	0.002286358	0.183454335	0.001423221	0.533594301	0.002286192	0.183421124	-3.44E-05	0.011678423	1	0.790819813	1	0.784725639	ENSG00000170356	8	OR2A20P
SAT	Obesity	ENSG00000225851.1	-0.001244486	0.565564974	0.002165846	0.183454349	0.001706192	0.448003788	0.002248695	0.183421053	-0.002950678	1.10E-06	1	0.797234594	1	0.726019131	ENSG00000170356	8	OR2A20P
SAT	Obesity	ENSG00000225880.4	-0.004117264	0.349837658	0.004403937	0.183454144	-0.003996192	0.364160371	0.004403678	0.183420959	-0.000121071	0.000536149	1	0.647953427	1	0.664705409	ENSG00000170430	9	MGMT
SAT	Obesity	ENSG00000226015.2	-0.002228931	0.542786916	0.003662364	0.183454334	-0.002142711	0.558484061	0.003662159	0.183421141	-8.62E-05	0.004687439	1	0.790819813	1	0.795455172	ENSG00000170439	5	METTL7B
SAT	Obesity	ENSG00000226278.1	-0.001212765	0.633777408	0.002545595	0.183454388	-0.001260008	0.620594281	0.002545426	0.183421178	4.72E-05	0.00754048	1	0.839492804	1	0.830073312	ENSG00000170439	5	METTL7B
SAT	Obesity	ENSG00000226314.3	0.000773861	0.610292471	0.001518397	0.183454376	0.001583737	0.299346082	0.001525997	0.183420863	-0.000809877	1.32E-07	1	0.824707644	1	0.601026164	ENSG00000170454	5	KRT75
SAT	Obesity	ENSG00000226479.3	-0.002800921	0.554356983	0.004737333	0.183454342	-0.002890775	0.541697821	0.004737049	0.183421129	8.99E-05	0.015340484	1	0.794232732	1	0.788255443	ENSG00000170454	5	KRT75
SAT	Obesity	ENSG00000227124.4	0.001813521	0.065300516	0.000983899	0.18345319	0.001793111	0.0683685	0.000983834	0.183420015	2.04E-05	0.003250551	1	0.256588015	1	0.265839301	ENSG00000170469	6	SPATA24
SAT	Obesity	ENSG00000227766.1	-0.005590447	0.029881713	0.002574272	0.183452691	-0.004944163	0.056231485	0.002589595	0.183419893	-0.000646284	0.022574366	1	0.157820771	1	0.238744104	ENSG00000170486	6	KRT72
SAT	Obesity	ENSG00000227827.3	-0.001354586	0.480528294	0.001920163	0.183454286	-0.001313514	0.493906418	0.001920035	0.183421093	-4.11E-05	0.001979048	1	0.741872665	1	0.755129058	ENSG00000170509	7	HSD17B13
SAT	Obesity	ENSG00000228065.6	0.000214626	0.928949834	0.002407037	0.183454471	0.000117577	0.961044675	0.002407249	0.183421269	9.70E-05	0.033009519	1	0.977131172	1	0.9914961	ENSG00000170509	7	HSD17B13
SAT	Obesity	ENSG00000228078.1	0.006181456	0.001940812	0.001994558	0.183450844	0.004232268	0.037428194	0.0020337	0.183419633	0.001949188	9.67E-07	1	0.023240164	1	0.188635799	ENSG00000170540	10	ARL6IP1
SAT	Obesity	ENSG00000228157.2	0.002593278	0.66645316	0.00601659	0.183454404	0.002477764	0.680450797	0.006016206	0.183421206	0.000115514	0.00842259	1	0.854600118	1	0.86636807	ENSG00000170540	10	ARL6IP1
SAT	Obesity	ENSG00000228446.2	0.000176028	0.936216843	0.002199645	0.183454472	0.000224158	0.918825575	0.002199503	0.183421266	-4.81E-05	0.002316467	1	0.979192687	1	0.974044172	ENSG00000170545	12	SMAGP
SAT	Obesity	ENSG00000228789.2	-0.000846349	0.59646873	0.001598436	0.183454368	0.00041628	0.796568408	0.001614797	0.183421245	-0.001262629	4.18E-08	1	0.81491093	1	0.921558867	ENSG00000170545	12	SMAGP
SAT	Obesity	ENSG00000229425.1	-0.007579189	0.038682976	0.003665784	0.183452858	-0.007486428	0.041117038	0.003665589	0.183419694	-9.28E-05	0.003323235	1	0.188234951	1	0.197229533	ENSG00000170571	7	EMB
SAT	Obesity	ENSG00000229759.1	-0.000577797	0.750060057	0.001813776	0.183454436	-0.000617308	0.733581655	0.001813663	0.183421226	3.95E-05	0.003853362	1	0.898811924	1	0.893959582	ENSG00000170571	7	EMB
SAT	Obesity	ENSG00000230189.2	-0.012737482	0.016795632	0.005326906	0.183452313	-0.012800981	0.016250241	0.005326519	0.183419087	6.35E-05	0.046185214	1	0.109773844	1	0.113440509	ENSG00000170571	7	EMB
SAT	Obesity	ENSG00000230224.1	-0.014412691	0.005264515	0.0051651	0.183451531	-0.014526974	0.004912768	0.005164758	0.18341828	0.000114283	0.001531206	1	0.050941439	1	0.051828914	ENSG00000170632	9	ARMC10
SAT	Obesity	ENSG00000230267.3	0.002683012	0.561723409	0.004623619	0.183454347	0.0038129	0.41105582	0.004638351	0.183421015	-0.001129888	0.002559772	1	0.797151602	1	0.701318401	ENSG00000170632	9	ARMC10
SAT	Obesity	ENSG00000230295.1	0.003437945	0.185659514	0.002597555	0.183453812	0.003364065	0.195261101	0.002597386	0.183420636	7.39E-05	6.55E-05	1	0.460778263	1	0.479171034	ENSG00000170632	9	ARMC10
SAT	Obesity	ENSG00000230387.1	0.005929717	0.071174869	0.003286319	0.183453243	0.00599853	0.067939508	0.003286124	0.183420011	-6.88E-05	0.007990662	1	0.268419248	1	0.265839301	ENSG00000170632	9	ARMC10
SAT	Obesity	ENSG00000230438.5	-0.001220674	0.661237743	0.002785623	0.183454402	-0.001272521	0.647779791	0.002785431	0.183421191	5.18E-05	0.004570002	1	0.852929209	1	0.846421941	ENSG00000170632	9	ARMC10
SAT	Obesity	ENSG00000230795.2	-0.00319263	0.026649206	0.001440311	0.183452617	-0.00181986	0.216517721	0.001472566	0.183420693	-0.00137277	7.82E-06	1	0.147637149	1	0.508514418	ENSG00000170632	9	ARMC10
SAT	Obesity	ENSG00000230911.1	0.004727982	0.237470758	0.004002241	0.183453947	0.004802734	0.230112908	0.004002047	0.183420726	-7.48E-05	0.041775968	1	0.526060746	1	0.523921586	ENSG00000170632	9	ARMC10
SAT	Obesity	ENSG00000231130.1	-0.004866469	0.015747722	0.002015335	0.18345227	-0.002373792	0.254593308	0.002083618	0.18342078	-0.002492677	2.53E-06	1	0.105715792	1	0.55282637	ENSG00000170633	12	RNF34
SAT	Obesity	ENSG00000231500.2	0.00683004	0.001725553	0.002179459	0.183450762	0.008023035	0.000260188	0.002196861	0.18341623	-0.001192995	1.72E-05	1	0.02144523	0.853676482	0.004934546	ENSG00000170633	12	RNF34
SAT	Obesity	ENSG00000231784.4	0.00103678	0.804280355	0.004183745	0.183454451	0.000973357	0.816018884	0.004183448	0.18342125	6.34E-05	0.015098201	1	0.92331835	1	0.929961083	ENSG00000170647	2	TMEM133
SAT	Obesity	ENSG00000231793.4	0.012327609	0.036949923	0.005908774	0.183452829	0.009476136	0.110066302	0.005930375	0.183420305	0.002851473	2.53E-08	1	0.181758169	1	0.348578702	ENSG00000170667	10	RASA4B
SAT	Obesity	ENSG00000231852.2	0.01076893	9.32E-10	0.001759432	0.183440314	0.008473329	5.63E-05	0.002103628	0.183415139	0.002295602	0.046537445	3.06E-06	7.46E-08	0.184626332	0.001429286	ENSG00000170667	10	RASA4B
SAT	Obesity	ENSG00000231943.3	0.000539471	0.899531647	0.004272934	0.183454468	0.000465714	0.913203268	0.004272637	0.183421266	7.38E-05	0.007772631	1	0.963553161	1	0.969964365	ENSG00000170667	10	RASA4B
SAT	Obesity	ENSG00000232388.2	-0.002973469	0.25485478	0.002611427	0.183453984	-0.002867689	0.272127313	0.002611316	0.183420814	-0.00010578	3.47E-05	1	0.546521917	1	0.575660681	ENSG00000170745	7	KCNS3
SAT	Obesity	ENSG00000232677.2	0.000444175	0.889547612	0.003198337	0.183454467	0.000505736	0.874350904	0.003198138	0.183421261	-6.16E-05	0.010237554	1	0.962025309	1	0.957844846	ENSG00000170745	7	KCNS3
SAT	Obesity	ENSG00000232926.1	0.003480651	0.401686352	0.004150481	0.183454208	0.003545773	0.392904975	0.0041502	0.183420994	-6.51E-05	0.020587802	1	0.691650463	1	0.68753132	ENSG00000170745	7	KCNS3
SAT	Obesity	ENSG00000233754.1	0.00376453	0.478395573	0.005310487	0.183454284	0.003890871	0.463727047	0.005310144	0.183421067	-0.000126341	0.000953228	1	0.74073425	1	0.735373824	ENSG00000170745	7	KCNS3
SAT	Obesity	ENSG00000233916.1	0.003307483	0.22160137	0.002705989	0.183453909	0.00241407	0.374273206	0.002717022	0.183420972	0.000893414	0.000302745	1	0.50915553	1	0.669643631	ENSG00000170745	7	KCNS3
SAT	Obesity	ENSG00000234127.4	-0.000876284	0.789257243	0.003278582	0.183454447	-0.002254009	0.496160156	0.003312067	0.183421095	0.001377724	0.003502404	1	0.914498218	1	0.756387058	ENSG00000170776	15	AKAP13
SAT	Obesity	ENSG00000234147.1	-0.008754032	0.005245661	0.003135892	0.183451528	-0.008864824	0.004698227	0.003135726	0.18341825	0.000110792	4.59E-05	1	0.050941439	1	0.050822526	ENSG00000170776	15	AKAP13
SAT	Obesity	ENSG00000234353.2	-0.00146309	0.521985282	0.002285047	0.183454319	-0.001499821	0.511566057	0.002284914	0.183421107	3.67E-05	0.045589511	1	0.774246705	1	0.768872301	ENSG00000170776	15	AKAP13
SAT	Obesity	ENSG00000234409.4	-0.008284761	0.019372291	0.003543076	0.183452407	-0.008212353	0.020448403	0.003542824	0.183419239	-7.24E-05	0.001008226	1	0.121067591	1	0.13047531	ENSG00000170776	15	AKAP13
SAT	Obesity	ENSG00000234465.6	-0.001170759	0.688785409	0.002923212	0.183454413	-0.001113714	0.703193067	0.002923026	0.183421215	-5.70E-05	0.007615711	1	0.864822684	1	0.876674335	ENSG00000170837	2	GPR27
SAT	Obesity	ENSG00000234882.1	0.000515479	0.82197508	0.00229096	0.183454455	0.000470087	0.837411166	0.002290805	0.183421254	4.54E-05	0.002611854	1	0.933051052	1	0.937115108	ENSG00000170837	2	GPR27
SAT	Obesity	ENSG00000235109.3	0.008033854	0.000116029	0.002084334	0.183448859	0.00630703	0.002836556	0.002112956	0.183417903	0.001726824	6.80E-07	0.38069216	0.002239366	1	0.035252799	ENSG00000170837	2	GPR27
SAT	Obesity	ENSG00000235241.1	0.00908808	0.017676784	0.00383087	0.183452347	0.009027217	0.018443979	0.003830627	0.183419171	6.09E-05	0.030316254	1	0.113720642	1	0.123121596	ENSG00000170837	2	GPR27
SAT	Obesity	ENSG00000235821.1	-0.008368769	3.08E-05	0.002008254	0.18344791	-0.006112398	0.003086069	0.002065664	0.183417961	-0.002256372	3.18E-06	0.10119758	0.000772501	1	0.037501449	ENSG00000170837	2	GPR27
SAT	Obesity	ENSG00000235963.1	-0.000471533	0.884192547	0.003237294	0.183454466	0.003698993	0.265211987	0.00331999	0.183420801	-0.004170527	1.57E-08	1	0.959020082	1	0.567932065	ENSG00000170837	2	GPR27
SAT	Obesity	ENSG00000236104.2	0.006277797	0.105852915	0.003882083	0.183453486	0.007264532	0.061742252	0.003888631	0.183419951	-0.000986736	2.04E-05	1	0.334504825	1	0.251335397	ENSG00000170866	7	LILRA3
SAT	Obesity	ENSG00000236397.2	-0.000175444	0.930389515	0.002008409	0.183454471	-0.000224237	0.911096159	0.00200828	0.183421265	4.88E-05	0.00084612	1	0.977387556	1	0.968667044	ENSG00000170866	7	LILRA3
SAT	Obesity	ENSG00000237276.4	-0.007241151	0.126208994	0.004735173	0.18345359	-0.00734123	0.121035782	0.004734923	0.183420362	0.000100079	0.015128926	1	0.374273307	1	0.369554001	ENSG00000170866	7	LILRA3
SAT	Obesity	ENSG00000237310.1	0.003249719	0.031711818	0.001512883	0.18345273	0.003215342	0.033551467	0.001512798	0.183419563	3.44E-05	0.005900939	1	0.164370418	1	0.176545003	ENSG00000170866	7	LILRA3
SAT	Obesity	ENSG00000237491.4	0.000598491	0.88264581	0.004054374	0.183454466	0.000668494	0.869028796	0.004054124	0.18342126	-7.00E-05	0.022946507	1	0.958494761	1	0.954321831	ENSG00000170915	8	PAQR8
SAT	Obesity	ENSG00000237510.3	0.000222704	0.925824765	0.002392115	0.183454471	0.000307695	0.897645021	0.002391964	0.183421264	-8.50E-05	1.80E-06	1	0.976102523	1	0.963222823	ENSG00000170915	8	PAQR8
SAT	Obesity	ENSG00000237669.1	0.005025548	0.000102329	0.001293566	0.183448769	0.003227087	0.017007339	0.001352193	0.183419118	0.001798461	5.06E-06	0.335743054	0.002010437	1	0.11722916	ENSG00000170915	8	PAQR8
SAT	Obesity	ENSG00000237693.4	0.000862337	0.819787747	0.003785202	0.183454454	0.000917998	0.808361294	0.003784923	0.183421248	-5.57E-05	0.011113377	1	0.932313205	1	0.924767575	ENSG00000170915	8	PAQR8
SAT	Obesity	ENSG00000237753.1	0.004525005	0.060322935	0.002408929	0.1834531	0.004443585	0.065076256	0.002408789	0.183419984	8.14E-05	2.64E-05	1	0.243144411	1	0.260067232	ENSG00000170955	9	PRKCDBP
SAT	Obesity	ENSG00000237927.1	0.008934761	0.113003202	0.00563764	0.183453525	0.009100503	0.106454023	0.005637285	0.183420285	-0.000165742	7.56E-05	1	0.349776893	1	0.342763151	ENSG00000170955	9	PRKCDBP
SAT	Obesity	ENSG00000238024.1	0.006955223	0.22376162	0.005716991	0.183453915	9.19E-05	0.987482117	0.005855007	0.18342127	0.006863361	5.93E-08	1	0.511254788	1	0.99567573	ENSG00000170955	9	PRKCDBP
SAT	Obesity	ENSG00000238286.1	0.001231728	0.681608533	0.003002242	0.18345441	0.001291019	0.667163132	0.003002055	0.1834212	-5.93E-05	0.008856384	1	0.860468487	1	0.857743823	ENSG00000170955	9	PRKCDBP
SAT	Obesity	ENSG00000239257.1	0.004648721	9.63E-05	0.001192045	0.183448726	0.003205245	0.009262706	0.001231738	0.183418711	0.001443476	3.35E-06	0.315930883	0.001914733	1	0.078523369	ENSG00000171045	10	TSNARE1
SAT	Obesity	ENSG00000239557.1	0.035129419	1.03E-08	0.006135616	0.183442084	0.03522512	9.39E-09	0.006135211	0.183408812	-9.57E-05	0.026660096	3.39E-05	6.39E-07	3.08E-05	6.85E-07	ENSG00000171045	10	TSNARE1
SAT	Obesity	ENSG00000239697.6	0.001749813	0.550146047	0.002928353	0.183454339	0.001808309	0.536869097	0.002928175	0.183421126	-5.85E-05	0.009737847	1	0.793070817	1	0.78622818	ENSG00000171084	11	FAM86JP
SAT	Obesity	ENSG00000240053.8	-0.002353124	0.069226008	0.001295098	0.183453226	-0.00340294	0.009839979	0.001318247	0.183418752	0.001049816	2.07E-05	1	0.262882561	1	0.081872378	ENSG00000171084	11	FAM86JP
SAT	Obesity	ENSG00000240065.3	0.011318795	0.000221051	0.003064363	0.183449317	0.008075804	0.01084637	0.003169941	0.183418817	0.003242992	6.54E-05	0.72526703	0.003941669	1	0.086166924	ENSG00000171084	11	FAM86JP
SAT	Obesity	ENSG00000240522.1	0.006854391	0.243931719	0.005882502	0.183453961	0.006649733	0.258269869	0.005882162	0.183420787	0.000204659	1.70E-05	1	0.532140939	1	0.557489106	ENSG00000171103	6	TRMT61B
SAT	Obesity	ENSG00000240972.1	0.001623785	0.220372546	0.00132495	0.183453906	0.001649426	0.213142027	0.001324868	0.183420684	-2.56E-05	0.005923836	1	0.507398121	1	0.503707333	ENSG00000171109	14	MFN1
SAT	Obesity	ENSG00000241106.2	0.001437145	0.282039282	0.001335946	0.183454037	0.002054613	0.1263783	0.001344164	0.183420387	-0.000617468	3.63E-05	1	0.579661774	1	0.37867367	ENSG00000171109	14	MFN1
SAT	Obesity	ENSG00000241679.2	0.004638032	0.303971848	0.004511905	0.183454075	0.00473754	0.293683207	0.004511611	0.183420853	-9.95E-05	0.002048191	1	0.604809967	1	0.59812588	ENSG00000171109	14	MFN1
SAT	Obesity	ENSG00000241837.2	0.004093814	0.449636474	0.005414942	0.183454258	0.004214783	0.436328425	0.005414613	0.183421041	-0.000120968	0.003824301	1	0.72529856	1	0.717711418	ENSG00000171130	13	ATP6V0E2
SAT	Obesity	ENSG00000242086.4	-0.002030405	0.451488868	0.002696651	0.18345426	-0.00211688	0.432421945	0.002696475	0.183421037	8.65E-05	6.23E-06	1	0.726500723	1	0.715518881	ENSG00000171298	8	GAA
SAT	Obesity	ENSG00000242516.1	-0.003734415	0.042287245	0.00183899	0.183452915	-0.003697195	0.044371018	0.001838875	0.183419742	-3.72E-05	0.006540349	1	0.194865803	1	0.206791634	ENSG00000171298	8	GAA
SAT	Obesity	ENSG00000243478.3	0.001504016	0.549261611	0.002511435	0.183454339	0.001463236	0.560117729	0.002511273	0.183421142	4.08E-05	0.024186752	1	0.792840892	1	0.795863471	ENSG00000171298	8	GAA
SAT	Obesity	ENSG00000243753.1	0.001617965	0.19593387	0.001251109	0.183453842	0.002225676	0.078505417	0.001265	0.1834201	-0.000607711	0.001206477	1	0.476192171	1	0.288439275	ENSG00000171298	8	GAA
SAT	Obesity	ENSG00000243819.3	0.01054527	0.069495018	0.005809451	0.183453229	0.010673383	0.066156329	0.005809064	0.183419994	-0.000128113	0.00140404	1	0.262990952	1	0.26214845	ENSG00000171298	8	GAA
SAT	Obesity	ENSG00000244301.1	-0.003265448	0.43994049	0.004228243	0.183454249	-0.003337377	0.429901205	0.004227945	0.183421035	7.19E-05	0.007264561	1	0.718489173	1	0.714541973	ENSG00000171310	6	CHST11
SAT	Obesity	ENSG00000244607.1	-0.00046331	0.871502432	0.00286435	0.183454464	-0.000399848	0.888973403	0.002864173	0.183421263	-6.35E-05	0.003267445	1	0.955362338	1	0.962898024	ENSG00000171310	6	CHST11
SAT	Obesity	ENSG00000244731.3	-0.006635662	5.97E-08	0.001224332	0.183443371	-0.00366357	0.037545095	0.001761508	0.183419635	-0.002972092	0.018944066	0.000195832	3.11E-06	1	0.18893475	ENSG00000171310	6	CHST11
SAT	Obesity	ENSG00000246375.2	-0.000261202	0.926338614	0.002825258	0.183454471	-0.000191488	0.945960314	0.002825111	0.183421268	-6.97E-05	0.004923554	1	0.976330547	1	0.984050663	ENSG00000171314	8	PGAM1
SAT	Obesity	ENSG00000246523.3	-0.005962868	0.121778522	0.00385359	0.183453569	-0.005846407	0.129212269	0.003853373	0.1834204	-0.000116461	0.00025378	1	0.36522425	1	0.383362075	ENSG00000171314	8	PGAM1
SAT	Obesity	ENSG00000246575.2	-0.004705773	0.310972203	0.004644534	0.183454086	-0.004959838	0.285594838	0.004644754	0.183420839	0.000254065	0.000998651	1	0.608407751	1	0.589702117	ENSG00000171357	5	LURAP1
SAT	Obesity	ENSG00000247121.2	0.010265439	0.021262551	0.004456845	0.183452469	0.010201512	0.022073874	0.004456557	0.18341929	6.39E-05	0.045480534	1	0.129189682	1	0.136607693	ENSG00000171357	5	LURAP1
SAT	Obesity	ENSG00000248202.1	0.001362019	0.59303452	0.002548477	0.183454366	0.001401078	0.582451751	0.002548307	0.183421156	-3.91E-05	0.026100588	1	0.812973298	1	0.809070362	ENSG00000171357	5	LURAP1
SAT	Obesity	ENSG00000248290.1	-0.006998812	4.55E-06	0.001526614	0.18344653	-0.006363871	3.32E-05	0.001533448	0.183414761	-0.000634941	1.37E-05	0.014932386	0.000153942	0.109084671	0.000940385	ENSG00000171357	5	LURAP1
SAT	Obesity	ENSG00000248375.1	0.002645087	0.544004973	0.004359303	0.183454335	0.00275335	0.527622622	0.004359051	0.183421119	-0.000108263	0.002174066	1	0.790819813	1	0.779574758	ENSG00000171357	5	LURAP1
SAT	Obesity	ENSG00000248408.1	-0.025916451	9.27E-06	0.005845588	0.183447044	-0.026588763	5.52E-06	0.005851536	0.183413467	0.000672312	0.014624395	0.030425754	0.000271659	0.01812343	0.000218355	ENSG00000171357	5	LURAP1
SAT	Obesity	ENSG00000248508.2	0.003197604	0.018310471	0.001355326	0.18345237	0.003227211	0.017252331	0.001355234	0.183419127	-2.96E-05	0.001236225	1	0.116653699	1	0.118173061	ENSG00000171357	5	LURAP1
SAT	Obesity	ENSG00000248787.1	-0.006390405	0.180226917	0.00476875	0.183453795	-0.006285175	0.187480428	0.004768464	0.183420614	-0.00010523	0.004648292	1	0.452624689	1	0.46706856	ENSG00000171421	8	MRPL36
SAT	Obesity	ENSG00000248794.1	-9.99E-05	0.986971229	0.006119282	0.183454474	1.95E-05	0.99745519	0.006118914	0.18342127	-0.000119443	0.012319232	1	0.994152558	1	0.998819255	ENSG00000171421	8	MRPL36
SAT	Obesity	ENSG00000248905.4	-0.004611922	0.455174938	0.006175445	0.183454263	-0.008414068	0.180981178	0.006289754	0.183420594	0.003802146	0.001485893	1	0.730067807	1	0.458532235	ENSG00000171421	8	MRPL36
SAT	Obesity	ENSG00000248994.1	0.006605857	0.117109094	0.004215535	0.183453546	0.006462966	0.125222103	0.004215287	0.183420382	0.00014289	1.99E-05	1	0.35809407	1	0.376584526	ENSG00000171421	8	MRPL36
SAT	Obesity	ENSG00000250535.1	-0.000253165	0.863000817	0.001467152	0.183454463	0.00074081	0.617936947	0.001485254	0.183421176	-0.000993975	1.84E-05	1	0.94985095	1	0.828495448	ENSG00000171421	8	MRPL36
SAT	Obesity	ENSG00000251615.3	-0.002710597	0.400103733	0.003221391	0.183454206	-0.002636374	0.413101237	0.003221183	0.183421017	-7.42E-05	0.00140843	1	0.691279804	1	0.701318401	ENSG00000171421	8	MRPL36
SAT	Obesity	ENSG00000253326.2	0.004768924	0.396879709	0.00562899	0.183454203	0.004676982	0.406016971	0.005628652	0.183421009	9.19E-05	0.036309025	1	0.688610432	1	0.696726821	ENSG00000171446	6	KRT27
SAT	Obesity	ENSG00000253731.1	0.007946618	0.019286537	0.003396058	0.183452404	0.007862159	0.020601335	0.003395859	0.183419244	8.45E-05	0.001804213	1	0.120761697	1	0.130740771	ENSG00000171446	6	KRT27
SAT	Obesity	ENSG00000253813.1	0.007897194	0.04357116	0.003912992	0.183452934	0.008020935	0.040370441	0.003912758	0.183419682	-0.000123741	5.67E-05	1	0.199104425	1	0.195361972	ENSG00000171450	4	CDK5R2
SAT	Obesity	ENSG00000254126.2	-0.001528794	0.687122484	0.003795761	0.183454413	-0.001466018	0.699311454	0.003795513	0.183421214	-6.28E-05	0.019588988	1	0.863266708	1	0.87540667	ENSG00000171469	6	ZNF561
SAT	Obesity	ENSG00000254152.1	-0.015379927	0.004342341	0.005392356	0.183451399	-0.015222672	0.004755415	0.00539206	0.183418258	-0.000157255	0.000546724	1	0.04466213	1	0.050822526	ENSG00000171469	6	ZNF561
SAT	Obesity	ENSG00000254352.1	-0.000338704	0.871902563	0.00210059	0.183454464	-0.000294996	0.888309257	0.002100456	0.183421263	-4.37E-05	0.004543097	1	0.955463635	1	0.962898024	ENSG00000171502	10	COL24A1
SAT	Obesity	ENSG00000254614.1	-0.003662781	0.14834496	0.002534087	0.183453684	-0.002347092	0.356374728	0.002544836	0.183420949	-0.001315689	2.51E-08	1	0.407296916	1	0.657825701	ENSG00000171502	10	COL24A1
SAT	Obesity	ENSG00000254986.3	0.007193198	0.002840258	0.002410159	0.183451107	0.006309343	0.008989069	0.002415078	0.183418691	0.000883855	1.95E-08	1	0.03213409	1	0.077624322	ENSG00000171502	10	COL24A1
SAT	Obesity	ENSG00000255147.1	-0.000548242	0.913477328	0.005045785	0.1834544	-0.001147509	0.820131128	0.005046736	0.183421251	0.000599267	4.99E-07	1	0.96957309	1	0.932801998	ENSG00000171503	7	ETFDH
SAT	Obesity	ENSG00000255200.1	-0.003215559	0.095654373	0.00192976	0.183453425	-0.002163177	0.264536518	0.001938792	0.1834208	-0.001052382	2.44E-08	1	0.315355558	1	0.567283867	ENSG00000171503	7	ETFDH
SAT	Obesity	ENSG00000256061.3	0.008183389	0.137501356	0.005510096	0.18345364	0.008014121	0.145798847	0.00550975	0.183420471	0.000169268	3.62E-05	1	0.390599089	1	0.410262452	ENSG00000171530	9	TBCA
SAT	Obesity	ENSG00000256073.2	-0.001903928	0.557409672	0.003245182	0.183454344	-0.001965398	0.54473455	0.003245	0.183421132	6.15E-05	0.022790795	1	0.794232732	1	0.788841592	ENSG00000171530	9	TBCA
SAT	Obesity	ENSG00000256092.2	-0.011894359	0.026406404	0.005357364	0.183452611	-0.011979992	0.02533243	0.005357054	0.18341938	8.56E-05	0.048332164	1	0.147095776	1	0.147892708	ENSG00000171530	9	TBCA
SAT	Obesity	ENSG00000256269.2	0.02056763	2.56E-11	0.003083638	0.183437659	0.020655418	2.11E-11	0.003083541	0.183404313	-8.78E-05	0.008955652	8.41E-08	2.47E-09	6.91E-08	2.16E-09	ENSG00000171552	8	BCL2L1
SAT	Obesity	ENSG00000256576.1	-0.009723021	0.00927465	0.003737077	0.183451915	-0.009633677	0.00993703	0.003736847	0.183418758	-8.93E-05	0.001594597	1	0.074401287	1	0.081872378	ENSG00000171574	13	ZNF584
SAT	Obesity	ENSG00000256885.1	-0.001604377	0.464425117	0.002193029	0.183454272	-0.001559648	0.476940107	0.002192879	0.183421079	-4.47E-05	0.00226843	1	0.733866362	1	0.742751548	ENSG00000171574	13	ZNF584
SAT	Obesity	ENSG00000257093.2	0.011154741	0.030506376	0.005155975	0.183452705	0.011071927	0.031751552	0.005155655	0.183419527	8.28E-05	0.032863994	1	0.159635439	1	0.168844153	ENSG00000171574	13	ZNF584
SAT	Obesity	ENSG00000257431.1	-0.005353329	0.194212538	0.004123593	0.183453837	-0.005683871	0.168135026	0.004124057	0.183420552	0.000330542	7.02E-05	1	0.474115579	1	0.437851886	ENSG00000171574	13	ZNF584
SAT	Obesity	ENSG00000257790.1	-0.001915256	0.721048554	0.005363992	0.183454426	-0.001793537	0.738089536	0.005363696	0.183421228	-0.000121719	0.00680437	1	0.884077949	1	0.896583401	ENSG00000171700	9	RGS19
SAT	Obesity	ENSG00000258100.1	-8.10E-05	0.985838179	0.004565659	0.183454474	-0.000207849	0.963687234	0.004565394	0.183421269	0.000126808	0.000565874	1	0.994152558	1	0.991742114	ENSG00000171700	9	RGS19
SAT	Obesity	ENSG00000258586.1	-0.008849992	0.0179408	0.00373915	0.183452357	-0.008789395	0.018733334	0.00373889	0.183419182	-6.06E-05	0.011984527	1	0.114968288	1	0.123670158	ENSG00000171723	11	GPHN
SAT	Obesity	ENSG00000258819.1	-0.004659789	0.412733204	0.005688955	0.183454221	-0.004549258	0.423874605	0.005688578	0.183421028	-0.000110531	0.005142837	1	0.699592222	1	0.709195604	ENSG00000171723	11	GPHN
SAT	Obesity	ENSG00000259056.1	0.014021695	0.003089194	0.004739084	0.183451165	0.010104495	0.043205136	0.004997953	0.183419725	0.0039172	0.01368519	1	0.034242044	1	0.2030889	ENSG00000171735	14	CAMTA1
SAT	Obesity	ENSG00000259539.1	0.000707612	0.876926449	0.004569168	0.183454465	0.000563373	0.901865441	0.004568929	0.183421264	0.000144239	0.000306052	1	0.956287991	1	0.963222823	ENSG00000171747	4	LGALS4
SAT	Obesity	ENSG00000260065.1	0.003172937	0.048977258	0.001611614	0.183453009	0.00313974	0.051379614	0.001611528	0.183419836	3.32E-05	0.016770327	1	0.21425918	1	0.226581335	ENSG00000171757	11	LRRC34
SAT	Obesity	ENSG00000260082.1	0.014668504	0.008949547	0.005611547	0.183451891	0.015013648	0.007472084	0.005612476	0.183418566	-0.000345144	0.006610506	1	0.07286219	1	0.069450161	ENSG00000171763	13	SPATA5L1
SAT	Obesity	ENSG00000260176.1	0.010507702	0.041257584	0.005148469	0.1834529	0.010620581	0.039114978	0.005148172	0.183419662	-0.000112879	0.007011441	1	0.192979684	1	0.192986833	ENSG00000171763	13	SPATA5L1
SAT	Obesity	ENSG00000260257.1	0.000387389	0.942268725	0.00534929	0.183454472	0.000483843	0.927926052	0.005349014	0.183421267	-9.65E-05	0.041046839	1	0.981335116	1	0.977375723	ENSG00000171763	13	SPATA5L1
SAT	Obesity	ENSG00000260302.1	-0.009247684	0.031906376	0.004310093	0.183452734	-0.0091778	0.033212315	0.004309811	0.183419556	-6.99E-05	0.021565602	1	0.165118011	1	0.175474405	ENSG00000171772	11	SYCE1
SAT	Obesity	ENSG00000260518.1	0.003119815	0.245903035	0.002688662	0.183453965	0.003679196	0.172136031	0.002694642	0.183420566	-0.000559381	0.002244045	1	0.534004366	1	0.443997285	ENSG00000171806	7	METTL18
SAT	Obesity	ENSG00000260539.1	-0.007960941	0.010441302	0.003108685	0.183451995	-0.007888912	0.01115328	0.003108479	0.183418836	-7.20E-05	0.000854979	1	0.079855276	1	0.087690846	ENSG00000171806	7	METTL18
SAT	Obesity	ENSG00000260787.1	0.007297029	0.143644428	0.004989914	0.183453666	0.007182152	0.150030579	0.004989595	0.183420487	0.000114877	0.001585131	1	0.400717148	1	0.414396534	ENSG00000171806	7	METTL18
SAT	Obesity	ENSG00000260877.1	0.003361227	0.385469544	0.003872991	0.183454189	0.003173384	0.412565847	0.003872874	0.183421016	0.000187843	9.98E-06	1	0.677819359	1	0.701318401	ENSG00000171860	4	C3AR1
SAT	Obesity	ENSG00000260907.1	-0.005747356	0.157219944	0.004063212	0.183453718	-0.005797174	0.153624228	0.004062922	0.183420501	4.98E-05	0.048052336	1	0.420045334	1	0.417598255	ENSG00000171860	4	C3AR1
SAT	Obesity	ENSG00000261143.1	0.000662116	0.84694154	0.003430254	0.18345446	0.0007326	0.830872266	0.003430048	0.183421253	-7.05E-05	0.00835294	1	0.942344807	1	0.936326075	ENSG00000171862	5	PTEN

SAT	Obesity	ENSG00000261341.1	0.002828509	0.466113989	0.003880959	0.183454273	0.002884207	0.457350346	0.003880704	0.183421061	-5.57E-05	0.041045778	1	0.733866362	1	0.731563013	ENSG00000171862	5	PTEN
SAT	Obesity	ENSG00000261584.1	0.000266048	0.882788533	0.001804507	0.183454466	0.000854814	0.636316437	0.001807769	0.183421186	-0.000588766	1.21E-07	1	0.958494761	1	0.838966415	ENSG00000171928	9	TVP23B
SAT	Obesity	ENSG00000262165.1	-0.011348618	0.022279313	0.004965314	0.1834525	-0.011435671	0.021264587	0.004964991	0.183419265	8.71E-05	0.014448492	1	0.132906229	1	0.133679732	ENSG00000171928	9	TVP23B
SAT	Obesity	ENSG00000262370.1	0.00175893	0.445497142	0.002305451	0.183454254	0.001700572	0.460709881	0.002305301	0.183421065	5.84E-05	0.000358301	1	0.722137485	1	0.732714066	ENSG00000171953	11	ATPAF2
SAT	Obesity	ENSG00000262663.1	-0.001592587	0.721739324	0.004471859	0.183454426	-0.001476739	0.74121128	0.004471581	0.183421229	-0.000115848	0.000581686	1	0.884077949	1	0.897054301	ENSG00000171953	11	ATPAF2
SAT	Obesity	ENSG00000266930.2	0.00225433	0.493162957	0.003289614	0.183454297	0.002188446	0.505862858	0.003289443	0.183421103	6.59E-05	0.022545292	1	0.752324516	1	0.765560903	ENSG00000171962	13	LRRC48
SAT	Obesity	ENSG00000266992.1	0.002617535	0.218807038	0.002128583	0.183453903	0.002583796	0.224772331	0.002128443	0.183420713	3.37E-05	0.024798877	1	0.506281943	1	0.517165511	ENSG00000171984	10	C20orf196
SAT	Obesity	ENSG00000268225.1	0.001255333	0.685161306	0.003096296	0.183454412	0.00119936	0.69847974	0.003096132	0.183421213	5.60E-05	0.037029299	1	0.86231772	1	0.875033229	ENSG00000172000	3	ZNF556
SAT	Obesity	ENSG00000268340.1	0.00877536	0.073519172	0.004903537	0.183453264	0.008612459	0.079009584	0.004903289	0.183420104	0.000162902	0.000199345	1	0.271914102	1	0.289319694	ENSG00000172000	3	ZNF556
SAT	Obesity	ENSG00000269202.1	0.001848876	0.593204555	0.003461029	0.183454366	0.001783076	0.606401311	0.003460823	0.18342117	6.58E-05	0.015677195	1	0.812973298	1	0.822933669	ENSG00000172000	3	ZNF556
SAT	Obesity	ENSG00000269446.1	-0.001119796	0.779100098	0.003992255	0.183454444	-0.001195277	0.764620284	0.003991984	0.183421236	7.55E-05	0.004996313	1	0.913071213	1	0.906001861	ENSG00000172071	7	EIF2AK3
SAT	Obesity	ENSG00000269921.1	0.011309931	0.004236423	0.003954504	0.183451382	0.011209713	0.004585172	0.003954278	0.183418233	0.000100218	0.001874525	1	0.043847645	1	0.050146494	ENSG00000172071	7	EIF2AK3
SAT	Obesity	ENSG00000269963.1	-0.026870697	8.66E-09	0.004668921	0.183441955	-0.026754741	1.00E-08	0.00466866	0.183408859	-0.000115956	0.002704859	2.84E-05	5.57E-07	3.28E-05	7.14E-07	ENSG00000172113	4	NME6
SAT	Obesity	ENSG00000269990.1	0.026243341	4.63E-07	0.005206023	0.183444869	0.023598735	6.52E-06	0.005233744	0.183413587	0.002644606	1.08E-06	0.001520332	2.05E-05	0.021379575	0.000245156	ENSG00000172113	4	NME6
SAT	Obesity	ENSG00000270228.1	0.004438871	0.386107437	0.005121583	0.18345419	0.004522792	0.377161166	0.005121264	0.183420975	-8.39E-05	0.02877519	1	0.677819359	1	0.671441012	ENSG00000172113	4	NME6
SAT	Obesity	ENSG00000270547.1	-0.003446188	0.558924411	0.005896561	0.183454345	-0.003568992	0.544975545	0.005896173	0.183421132	0.000122805	0.003050799	1	0.794898566	1	0.788841592	ENSG00000172116	17	CD8B
SAT	Obesity	ENSG00000270775.1	0.000352958	0.843113143	0.001783401	0.183454459	7.13E-05	0.968098725	0.00178397	0.18342127	0.000281612	3.45E-08	1	0.941066406	1	0.992119797	ENSG00000172123	8	SLFN12
SAT	Obesity	ENSG00000271040.1	-0.011018512	0.067757329	0.006032182	0.183453213	-0.010912288	0.070431015	0.006031779	0.183420033	-0.000106224	0.01050169	1	0.261235952	1	0.270590351	ENSG00000172123	8	SLFN12
SAT	Obesity	ENSG00000271324.1	-0.001014464	0.760914484	0.003333977	0.183454439	-0.001065758	0.749206504	0.003333766	0.183421232	5.13E-05	0.036911885	1	0.904420994	1	0.900889371	ENSG00000172167	3	MTBP
SAT	Obesity	ENSG00000271581.1	-0.003321477	0.041543377	0.001629716	0.183452904	-0.000920065	0.590213026	0.001708483	0.183421161	-0.002401411	2.88E-06	1	0.193338752	1	0.815020596	ENSG00000172171	6	TEFM
SAT	Obesity	ENSG00000271584.1	0.006489497	0.01506005	0.002669542	0.18345224	0.006432889	0.015957665	0.00266937	0.183419075	5.66E-05	0.003234065	1	0.102604462	1	0.112106928	ENSG00000172171	6	TEFM
SAT	Obesity	ENSG00000271623.1	-0.051206482	1.83E-28	0.004626909	0.183408188	-0.051129969	2.18E-28	0.004626618	0.183375124	-7.65E-05	0.024636876	5.99E-25	1.20E-25	7.15E-25	1.43E-25	ENSG00000172183	10	ISG20
SAT	Obesity	ENSG00000271751.1	-0.031143475	1.90E-09	0.005185445	0.18344084	-0.031308692	1.56E-09	0.005185331	0.183407493	0.000165216	0.006620156	6.25E-06	1.42E-07	5.13E-06	1.28E-07	ENSG00000172247	3	C1QTNF4
SAT	Obesity	ENSG00000271811.1	-0.002659732	0.438956191	0.003436534	0.183454248	-0.002708175	0.430634462	0.003436303	0.183421035	4.84E-05	0.039097197	1	0.717400247	1	0.714674593	ENSG00000172247	3	C1QTNF4
SAT	Obesity	ENSG00000272010.1	-0.001135985	0.63777103	0.00241279	0.18345439	-0.001381456	0.567015395	0.002413221	0.183421146	0.000245471	1.17E-05	1	0.841561068	1	0.800506675	ENSG00000172247	3	C1QTNF4
SAT	Obesity	ENSG00000272168.1	0.003649226	0.355335961	0.003948141	0.183454151	0.002436128	0.542000695	0.003995023	0.18342113	0.001213098	0.047651216	1	0.653141338	1	0.788255443	ENSG00000172247	3	C1QTNF4
SAT	Obesity	ENSG00000272221.1	-0.002346445	0.026061464	0.001054439	0.183452602	-0.001721055	0.105381325	0.001062833	0.183420279	-0.000625389	3.08E-06	1	0.146166945	1	0.340341256	ENSG00000172247	3	C1QTNF4
SAT	Obesity	ENSG00000272236.1	-0.005036719	0.018186356	0.002132568	0.183452366	-0.002518386	0.249684347	0.002187774	0.183420769	-0.002518334	2.63E-07	1	0.116221806	1	0.546117316	ENSG00000172247	3	C1QTNF4
SAT	Obesity	ENSG00000272462.2	0.00630067	4.51E-11	0.000956608	0.183438077	0.006008114	3.73E-10	0.000958971	0.183406436	0.000292556	1.92E-05	1.48E-07	4.23E-09	1.22E-06	3.40E-08	ENSG00000172322	9	CLEC12A
SAT	Obesity	ENSG00000272541.1	0.000969001	0.770460924	0.003321086	0.183454442	0.001029666	0.75651613	0.003320871	0.183421234	-6.07E-05	0.01095314	1	0.90745061	1	0.902920853	ENSG00000172322	9	CLEC12A
SAT	Obesity	ENSG00000272556.1	0.001797596	0.479204547	0.002540473	0.183454285	0.001751938	0.490414288	0.002540327	0.18342109	4.57E-05	0.027062214	1	0.741636848	1	0.752696629	ENSG00000172322	9	CLEC12A
SAT	Obesity	ENSG00000272720.1	-0.002972877	0.212277193	0.002383393	0.183453886	-0.003016778	0.205572077	0.002383228	0.183420665	4.39E-05	0.004680667	1	0.501426545	1	0.494851053	ENSG00000172322	9	CLEC12A
SAT	Obesity	ENSG00000272787.1	-0.002314162	0.592810825	0.004327416	0.183454366	-0.002254976	0.60227903	0.004327121	0.183421168	-5.92E-05	0.040543218	1	0.812973298	1	0.820971125	ENSG00000172322	9	CLEC12A
SAT	Obesity	ENSG00000272849.1	-0.013221074	8.03E-05	0.003352544	0.183448596	-0.013163843	8.61E-05	0.003352327	0.183415443	-5.72E-05	0.016751441	0.263401104	0.001656611	0.282504754	0.001973489	ENSG00000172340	10	SUCLG2
SAT	Obesity	ENSG00000273073.1	-0.001004835	0.873313735	0.006301844	0.183454464	-0.001112078	0.859917242	0.006301451	0.183421258	0.000107243	0.023237506	1	0.95575129	1	0.948758084	ENSG00000172340	10	SUCLG2
SAT	Obesity	ENSG00000273142.1	0.001546645	0.308887073	0.001519953	0.183454083	0.001526637	0.31515294	0.001519844	0.183420889	2.00E-05	0.032762744	1	0.60688449	1	0.615584597	ENSG00000172349	12	IL16
SAT	Obesity	ENSG00000273295.1	0.001498465	0.241663895	0.001279821	0.183453956	0.001522912	0.234039031	0.001279736	0.183420735	-2.44E-05	0.006157816	1	0.530497704	1	0.527090757	ENSG00000172349	12	IL16
SAT	Obesity	ENSG00000273340.1	-0.000248774	0.848911209	0.001305848	0.18345446	0.000735457	0.576684025	0.001317468	0.183421152	-0.000984232	2.04E-08	1	0.942883439	1	0.804464408	ENSG00000172432	14	GTPBP2
SAT	Stroke	ENSG00000026950.12	0.001136542	0.307639929	0.001114052	0.057490515	0.001180344	0.289452676	0.001114242	0.057488926	-4.38E-05	0.040660719	1	0.871327204	1	0.865823277	ENSG00000172432	14	GTPBP2
SAT	Stroke	ENSG00000041353.5	-0.000563737	0.863511055	0.003279322	0.057490634	-0.004697905	0.224818157	0.003870357	0.057488885	0.004134169	0.044320871	1	0.985453834	1	0.846254871	ENSG00000172493	16	AFF1
SAT	Stroke	ENSG00000090054.9	-0.007254096	0.001328798	0.002260051	0.057489422	-0.007212832	0.001416155	0.002260115	0.057487857	-4.13E-05	0.046433953	1	0.36331564	1	0.38845011	ENSG00000172493	16	AFF1
SAT	Stroke	ENSG00000121552.3	0.001572011	0.621755984	0.003186314	0.057490609	0.001520956	0.633125282	0.003186363	0.057489032	5.11E-05	0.035366279	1	0.94794674	1	0.950783463	ENSG00000172493	16	AFF1
SAT	Stroke	ENSG00000163545.7	0.001403052	0.481390475	0.001992777	0.057490579	0.001303719	0.513078128	0.001993293	0.057489008	9.93E-05	0.032695256	1	0.923200221	1	0.930997953	ENSG00000172548	10	NIPAL4
SAT	Stroke	ENSG00000163743.9	-0.00347589	0.119491588	0.002232551	0.057490352	-0.00355089	0.111767395	0.00223284	0.05748876	7.50E-05	0.047135356	1	0.771457664	1	0.769465514	ENSG00000172572	6	PDE3A
SAT	Stroke	ENSG00000166278.10	-6.07E-05	0.984773951	0.003183168	0.057490638	-0.003306201	0.342315294	0.003481672	0.057488952	0.003245453	0.021404895	1	0.998676328	1	0.892510712	ENSG00000172590	14	MRPL52
SAT	Stroke	ENSG00000176933.4	0.00232425	0.312186851	0.002299769	0.057490517	0.00253826	0.270173122	0.002301932	0.057488915	-0.00021401	0.033232244	1	0.875457313	1	0.858951562	ENSG00000172590	14	MRPL52
SAT	Stroke	ENSG00000182240.11	0.001314012	0.453914615	0.001754577	0.057490572	0.000810071	0.647535471	0.001771853	0.057489034	0.000503941	0.041318426	1	0.909770222	1	0.955863681	ENSG00000172594	8	SMPDL3A
SAT	Stroke	ENSG00000184371.9	-0.000795924	0.688859858	0.001987807	0.057490619	-0.001707334	0.398549516	0.002022392	0.057488975	0.00091141	0.014431837	1	0.955101857	1	0.907572399	ENSG00000172594	8	SMPDL3A
SAT	Stroke	ENSG00000185344.9	-0.001528854	0.58720764	0.002816153	0.057490603	-0.001482619	0.598567968	0.002816197	0.057489026	-4.62E-05	0.032261515	1	0.943546012	1	0.946914901	ENSG00000172613	3	RAD9A
SAT	Stroke	ENSG00000189157.9	0.001885006	0.564011525	0.003267509	0.057490598	0.00200874	0.538757705	0.003267875	0.057489014	-0.000123735	0.016944184	1	0.937689169	1	0.93449293	ENSG00000172613	3	RAD9A
SAT	Stroke	ENSG00000198315.6	0.000442505	0.882452002	0.002992694	0.057490635	0.00026682	0.92898013	0.002993669	0.057489058	0.000175685	0.024291091	1	0.986976745	1	0.995096555	ENSG00000172613	3	RAD9A
SAT	Stroke	ENSG00000204228.3	-0.002240176	0.326816277	0.002284617	0.057490524	-0.00294047	0.201317925	0.002301184	0.057488866	0.000700294	0.011143515	1	0.882615609	1	0.833541007	ENSG00000172613	3	RAD9A
SAT	Stroke	ENSG00000204287.9	-0.002023265	0.286022787	0.001896416	0.057490503	-0.003818911	0.068005591	0.002092584	0.057488666	0.001795646	0.04238155	1	0.864235017	1	0.732839136	ENSG00000172613	3	RAD9A
SAT	Stroke	ENSG00000204344.10	0.000519108	0.720973427	0.001453439	0.057490623	0.000836023	0.567301024	0.001461498	0.05748902	-0.000316915	0.03890885	1	0.960236691	1	0.938376016	ENSG00000172613	3	RAD9A
SAT	Stroke	ENSG00000204427.7	-0.00027768	0.881060401	0.00185584	0.057490635	-0.000894895	0.633343004	0.001875983	0.057489032	0.000617214	0.024465266	1	0.986976745	1	0.950783463	ENSG00000172613	3	RAD9A
SAT	Stroke	ENSG00000204520.8	-0.000271102	0.722015516	0.000762021	0.057490623	-0.000464798	0.544203488	0.000766401	0.057489015	0.000193697	0.01805239	1	0.960236691	1	0.937286953	ENSG00000172638	8	EFEMP2
SAT	Stroke	ENSG00000206344.6	-8.55E-05	0.906239289	0.000725538	0.057490636	-0.000379235	0.608045792	0.000739444	0.057489028	0.000293779	0.039636211	1	0.992541054	1	0.94961806	ENSG00000172638	8	EFEMP2
SAT	Stroke	ENSG00000229390.1	-0.001025638	0.591564046	0.001911466	0.057490604	-0.00135228	0.480590022	0.001917164	0.057489	0.000326642	0.027349051	1	0.943546012	1	0.92815772	ENSG00000172638	8	EFEMP2
SAT	Stroke	ENSG00000229391.3	0.000377981	0.597154205	0.000715198	0.057490605	-0.000788221	0.368593977	0.000876667	0.057488963	0.001166201	0.021437365	1	0.943916331	1	0.905937727	ENSG00000172650	8	AGAP5
SAT	Stroke	ENSG00000231925.7	-0.001655302	0.269517534	0.001499129	0.057490494	-0.00140462	0.350279963	0.0015038	0.057488956	-0.000250681	0.034674415	1	0.858043425	1	0.898540262	ENSG00000172650	8	AGAP5
SAT	Stroke	ENSG00000233265.1	-0.000396111	0.674021298	0.000941695	0.057490617	-0.000544922	0.564041566	0.000944653	0.057489019	0.000148812	0.046825824	1	0.955009236	1	0.938376016	ENSG00000172661	13	FAM21C
SAT	Stroke	ENSG00000233892.1	-0.001238417	0.639995212	0.002647856	0.057490612	-0.001660871	0.531754498	0.002655989	0.057489013	0.000422453	0.042418219	1	0.951392057	1	0.933410978	ENSG00000172661	13	FAM21C
SAT	Stroke	ENSG00000234127.4	-0.000776175	0.671764511	0.001831776	0.057490617	-0.001366615	0.460248715	0.001850683	0.057488994	0.00059044	0.025340838	1	0.955009236	1	0.92077807	ENSG00000172671	15	ZFAND4
SAT	Stroke	ENSG00000234745.5	-0.00064658	0.630535788	0.001344316	0.05749061	-0.001146293	0.400803257	0.001364329	0.057488975	0.000499713	0.031928826	1	0.949661187	1	0.907572399	ENSG00000172687	9	ZNF738
SAT	Stroke	ENSG00000242372.2	-0.004322936	0.105510862	0.00267061	0.057490329	-0.004381778	0.100862248	0.002670691	0.057488741	5.88E-05	0.01901498	1	0.749315451	1	0.758998867	ENSG00000172687	9	ZNF738
SAT	Stroke	ENSG00000272810.1	-5.21E-05	0.971646174	0.001466424	0.057490638	-0.000179777	0.902505633	0.001467607	0.057489057	0.000127655	0.031676526	1	0.997892448	1	0.991426711	ENSG00000172728	11	FUT10
SAT	T2DM	ENSG00000000457.9	-0.003902753	0.216073418	0.003154916	0.081436703	-0.003950083	0.210515677	0.003154639	0.081418869	4.73E-05	0.022963663	1	0.580276647	1	0.579977552	ENSG00000172728	11	FUT10
SAT	T2DM	ENSG00000001561.6	0.001191819	0.482411864	0.001696711	0.081436876	0.001150779	0.497581862	0.001696564	0.081419054	4.10E-05	0.000307082	1	0.800198851	1	0.81497549	ENSG00000172728	11	FUT10
SAT	T2DM	ENSG00000005156.7	0.000173968	0.894931117	0.001317264	0.081436956	0.000198723	0.880077342	0.001317168	0.081419127	-2.48E-05	0.028115552	1	0.970180416	1	0.966700287	ENSG00000172746	6	RP11-344H11.4
SAT	T2DM	ENSG00000005243.5	0.003510866	0.157439326	0.002483392	0.081436624	0.003437026	0.166323429	0.002483191	0.081418811	7.38E-05	8.60E-05	1	0.507474184	1	0.526744374	ENSG00000172775	12	FAM192A
SAT	T2DM	ENSG00000006715.11	-0.001513243	0.504082897	0.002265063	0.081436884	-0.001463594	0.518142194	0.002264881	0.081419061	-4.96E-05	0.00419252	1	0.810314363	1	0.826056628	ENSG00000172780	12	RAB43
SAT	T2DM	ENSG00000006744.14	0.002165345	0.486449019	0.003111269	0.081436877	0.002098326	0.500003962	0.003111009	0.081419055	6.70E-05	0.002604218	1	0.801645999	1	0.815984012	ENSG00000172780	12	RAB43
SAT	T2DM	ENSG00000007541.10	-0.002054442	0.550436902	0.003440666	0.081436899	-0.001984608	0.564038326	0.003440399	0.081419075	-6.98E-05	0.010945292	1	0.83552458	1	0.84223981	ENSG00000172780	12	RAB43
SAT	T2DM	ENSG00000008853.12	-0.002546775	0.435951408	0.003269083	0.081436857	-0.002447608	0.454001667	0.003268877	0.081419037	-9.92E-05	0.001659278	1	0.772274669	1	0.794600418	ENSG00000172780	12	RAB43
SAT	T2DM	ENSG00000010292.8	-0.000430207	0.736436775	0.001278187	0.081436939	-0.000375966	0.768654505	0.001278206	0.081419116	-5.42E-05	0.007171937	1	0.923995816	1	0.937978714	ENSG00000172789	3	HOXC5
SAT	T2DM	ENSG00000010704.14	-0.002785577	0.185686937	0.002104787	0.081436666	-0.001007282	0.635491566	0.002125013	0.081419093	-0.001778295	1.48E-09	1	0.542563333	1	0.884482576	ENSG00000172789	3	HOXC5
SAT	T2DM	ENSG00000011523.9	0.009656686	1.46E-09	0.001596364	0.081430844	0.009709795	1.18E-09	0.001596227	0.081412949	-5.31E-05	1.38E-06	4.78E-06	3.68E-07	3.87E-06	6.46E-07	ENSG00000172803	13	SNX32
SAT	T2DM	ENSG00000014164.6	0.001836644	0.419497065	0.00227506	0.08143685	0.00188506	0.407308424	0.002274884	0.081419016	-4.84E-05	0.007985757	1	0.76380126	1	0.761035842	ENSG00000172803	13	SNX32
SAT	T2DM	ENSG00000016864.12	-0.000562756	0.846780158	0.002912386	0.081436952	-0.000670296	0.817958209	0.002912154	0.081419122	0.000107541	1.74E-06	1	0.963201596	1	0.957271954	ENSG00000172803	13	SNX32
SAT	T2DM	ENSG00000018189.8	-0.001264725	0.578153932	0.002274343	0.081436907	-0.001231475	0.588155284	0.002274144	0.081419082	-3.32E-05	0.02722525	1	0.842939695	1	0.853229568	ENSG00000172803	13	SNX32
SAT	T2DM	ENSG00000018699.7	-0.001511634	0.571437099	0.002671031	0.081436905	-0.001555286	0.560345277	0.002670803	0.081419074	4.37E-05	0.018661049	1	0.840736467	1	0.84223981	ENSG00000172803	13	SNX32
SAT	T2DM	ENSG00000022556.11	-0.000590485	0.865845096	0.003495263	0.081436954	-0.000669018	0.848195094	0.003494993	0.081419125	7.85E-05	0.005057962	1	0.965942795	1	0.965561588	ENSG00000172803	13	SNX32
SAT	T2DM	ENSG00000023041.7	-0.003593474	0.276784049	0.003304121	0.081436761	-0.003513708	0.28754581	0.003303841	0.081418942	-7.98E-05	0.000612986	1	0.63926506	1	0.653237213	ENSG00000172803	13	SNX32
SAT	T2DM	ENSG00000023191.12	0.000277684	0.888506642	0.00198071	0.081436955	0.000310143	0.875563914	0.00198054	0.081419127	-3.25E-05	0.01768525	1	0.969612316	1	0.966700287	ENSG00000172890	7	NADSYN1
SAT	T2DM	ENSG00000023572.4	-0.001362482	0.590610265	0.002532719	0.08143691	-0.001429604	0.572421681	0.002532563	0.081419078	6.71E-05	0.00677407	1	0.846620707	1	0.847525062	ENSG00000172890	7	NADSYN1
SAT	T2DM	ENSG00000025800.9	0.006322987	0.085385282	0.003675593	0.081436464	0.006237623	0.089663176	0.003675286	0.08141865	8.54E-05	0.001192388	1	0.402357222	1	0.421804746	ENSG00000172890	7	NADSYN1
SAT	T2DM	ENSG00000026950.12	-0.001289955	0.330616076	0.001325921	0.0814368	-0.001166077	0.379195503	0.001326021	0.081419002	-0.000123878	1.15E-06	1	0.693131851	1	0.739239717	ENSG00000172890	7	NADSYN1
SAT	T2DM	ENSG00000030110.8	0.00532108	0.004329525	0.001865009	0.081435598	0.00443273	0.018044911	0.00187454	0.081418197	0.000888349	3.20E-06	1	0.069633188	1	0.185291928	ENSG00000172922	4	RNASEH2C
SAT	T2DM	ENSG00000032742.13	-0.001399527	0.65419715	0.003124366	0.081436925	-0.001336764	0.668734263	0.003124112	0.0814191	-6.28E-05	0.007106508	1	0.882995924	1	0.896403813	ENSG00000172922	4	RNASEH2C
SAT	T2DM	ENSG00000033178.8	-0.006508455	0.004070223	0.002265638	0.081435579	-0.006550995	0.003832212	0.002265474	0.081417734	4.25E-05	0.029618303	1	0.068134697	1	0.072792717	ENSG00000172922	4	RNASEH2C
SAT	T2DM	ENSG00000034533.7	0.00036665	0.846154288	0.001889676	0.081436952	0.000402664	0.831246552	0.001889526	0.081419123	-3.60E-05	0.013947082	1	0.963201596	1	0.961544037	ENSG00000172922	4	RNASEH2C
SAT	T2DM	ENSG00000035115.17	-0.005031337	0.01745549	0.002116695	0.081436014	-0.005069136	0.016619393	0.002116523	0.081418172	3.78E-05	0.018021267	1	0.172324494	1	0.178781076	ENSG00000172922	4	RNASEH2C
SAT	T2DM	ENSG00000037757.9	-0.002898628	0.258506441	0.002565318	0.081436745	-0.002964451	0.247811479	0.002565107	0.081418908	6.58E-05	0.000543359	1	0.622274126	1	0.620663712	ENSG00000172922	4	RNASEH2C
SAT	T2DM	ENSG00000040199.14	0.002762004	0.456102639	0.003705993	0.081436866	0.00283617	0.444058624	0.003705677	0.081419033	-7.42E-05	0.003942381	1	0.786411516	1	0.790106478	ENSG00000172927	3	MYEOV
SAT	T2DM	ENSG00000041353.5	0.011311338	0.003753828	0.003902946	0.081435555	0.001403515	0.760582798	0.004605982	0.081419115	0.009907823	5.12E-05	1	0.064822685	1	0.932999239	ENSG00000172927	3	MYEOV
SAT	T2DM	ENSG00000041802.6	-0.000115304	0.871875969	0.000714949	0.081436954	-0.000127932	0.857976087	0.000714897	0.081419126	1.26E-05	0.039795933	1	0.966468088	1	0.965566132	ENSG00000172927	3	MYEOV
SAT	T2DM	ENSG00000042429.6	0.009583775	0.007045391	0.003556524	0.081435745	0.009493453	0.007596231	0.00355624	0.08141794	9.03E-05	0.000939287	1	0.09396718	1	0.108835078	ENSG00000172932	10	ANKRD13D
SAT	T2DM	ENSG00000042493.11	-0.004194961	0.19966623	0.003270913	0.081436684	-0.004116466	0.20818605	0.003270747	0.081418866	-7.85E-05	0.026670145	1	0.56039769	1	0.579977552	ENSG00000172932	10	ANKRD13D
SAT	T2DM	ENSG00000044090.4	0.000816965	0.76482662	0.002730963	0.081436943	0.000871466	0.749624543	0.002730717	0.081419114	-5.45E-05	0.001272464	1	0.93459819	1	0.928470414	ENSG00000172932	10	ANKRD13D
SAT	T2DM	ENSG00000048140.13	0.005145588	0.01131007	0.002031437	0.081435886	0.005175572	0.010836103	0.002031269	0.081418046	-3.00E-05	0.044409669	1	0.131589853	1	0.138879898	ENSG00000172935	8	MRGPRF
SAT	T2DM	ENSG00000048544.5	-0.000967364	0.204596149	0.000762564	0.081436689	-0.000980657	0.198407109	0.000762502	0.081418855	1.33E-05	0.019366256	1	0.565931149	1	0.566556767	ENSG00000172935	8	MRGPRF
SAT	T2DM	ENSG00000049192.10	-0.001935766	0.42054271	0.002403243	0.08143685	-0.001996602	0.40605333	0.002403056	0.081419016	6.08E-05	0.001404621	1	0.764009208	1	0.759884747	ENSG00000172935	8	MRGPRF
SAT	T2DM	ENSG00000049449.4	-0.007751281	0.029867639	0.00356898	0.08143617	-0.007821157	0.028408598	0.003568691	0.081418328	6.99E-05	0.009565673	1	0.240185597	1	0.247237694	ENSG00000172955	13	ADH6
SAT	T2DM	ENSG00000050130.13	6.74E-05	0.977656149	0.002407602	0.081436958	-7.78E-05	0.974209217	0.002407937	0.081419131	0.000145279	0.006788347	1	0.995203291	1	0.996688631	ENSG00000172955	13	ADH6
SAT	T2DM	ENSG00000050393.7	-0.001327052	0.686255826	0.003285256	0.081436931	-0.001403992	0.669092636	0.003285006	0.0814191	7.69E-05	0.004091552	1	0.895709047	1	0.896403813	ENSG00000172992	7	DCAKD
SAT	T2DM	ENSG00000053900.6	0.007144346	0.023867609	0.003162249	0.081436105	0.007205193	0.022685602	0.003161981	0.081418263	-6.08E-05	0.006246606	1	0.212275376	1	0.215743361	ENSG00000173113	2	TRMT112
SAT	T2DM	ENSG00000054938.11	0.006687534	0.096466831	0.004023225	0.081436497	0.006622092	0.099741775	0.004022873	0.081418678	6.54E-05	0.014459357	1	0.420328914	1	0.440431278	ENSG00000173113	2	TRMT112
SAT	T2DM	ENSG00000055955.11	0.005097369	7.22E-06	0.001135978	0.081433594	0.005059899	8.41E-06	0.001135894	0.081415816	3.75E-05	8.53E-05	0.02368204	0.000526268	0.027589044	0.000689726	ENSG00000173113	2	TRMT112
SAT	T2DM	ENSG00000057608.12	0.001767891	0.316431837	0.001764682	0.081436791	0.00181757	0.302985334	0.00176454	0.081418954	-4.97E-05	0.000210649	1	0.677242569	1	0.671232195	ENSG00000173113	2	TRMT112
SAT	T2DM	ENSG00000058085.10	-2.47E-05	0.983651481	0.001205416	0.081436958	-5.24E-05	0.965326623	0.001205341	0.081419131	2.77E-05	0.017901725	1	0.995937959	1	0.99427016	ENSG00000173113	2	TRMT112
SAT	T2DM	ENSG00000061938.12	0.000858738	0.738670166	0.00257404	0.08143694	0.000917281	0.721550973	0.002573835	0.08141911	-5.85E-05	0.003284289	1	0.924676388	1	0.920812547	ENSG00000173156	2	RHOD
SAT	T2DM	ENSG00000063046.13	-0.001186839	0.495363794	0.001740736	0.081436881	-0.001224014	0.481923591	0.001740602	0.081419048	3.72E-05	0.008415053	1	0.806594843	1	0.80426821	ENSG00000173156	2	RHOD
SAT	T2DM	ENSG00000064313.7	0.003311768	0.223465512	0.002720437	0.081436711	0.003265421	0.229974852	0.00272022	0.08141889	4.63E-05	0.027309797	1	0.587734143	1	0.605344964	ENSG00000173156	2	RHOD
SAT	T2DM	ENSG00000064995.12	-0.002973907	0.119262075	0.001908943	0.081436553	-0.003165027	0.097327132	0.001908994	0.081418672	0.00019112	1.36E-09	1	0.454470229	1	0.435054932	ENSG00000173193	9	PARP14
SAT	T2DM	ENSG00000065060.12	0.00035905	0.766130631	0.001207133	0.081436944	0.00029587	0.806364621	0.001207046	0.081419121	6.32E-05	1.89E-09	1	0.934630346	1	0.952410029	ENSG00000173193	9	PARP14
SAT	T2DM	ENSG00000066185.8	0.002547712	0.334681633	0.002640861	0.081436803	0.00249414	0.344903863	0.00264064	0.081418982	5.36E-05	0.00476236	1	0.695221474	1	0.713247438	ENSG00000173193	9	PARP14
SAT	T2DM	ENSG00000066379.10	-0.001631263	0.433125126	0.002081075	0.081436856	-1.82E-05	0.993185805	0.002125729	0.081419131	-0.001613108	0.000205187	1	0.772274669	1	0.998405122	ENSG00000173200	8	PARP15
SAT	T2DM	ENSG00000067533.5	0.000463622	0.697165454	0.001191372	0.081436933	0.000430409	0.717873496	0.001191272	0.081419109	3.32E-05	9.53E-05	1	0.900770568	1	0.918559531	ENSG00000173200	8	PARP15
SAT	T2DM	ENSG00000068028.13	0.001469721	0.636411206	0.003109055	0.081436921	0.001415475	0.648886133	0.003108813	0.081419096	5.42E-05	0.028232128	1	0.873655621	1	0.891013292	ENSG00000173200	8	PARP15
SAT	T2DM	ENSG00000068078.13	0.002289087	0.100778477	0.001394854	0.081436508	0.002272803	0.103193137	0.001394726	0.081418687	1.63E-05	0.049227379	1	0.425727224	1	0.440431278	ENSG00000173200	8	PARP15
SAT	T2DM	ENSG00000069998.8	0.001844892	0.449417498	0.002439085	0.081436863	0.001796276	0.46141712	0.002438883	0.08141904	4.86E-05	0.006315974	1	0.78183394	1	0.797039301	ENSG00000173200	8	PARP15
SAT	T2DM	ENSG00000072864.8	-0.005804917	0.108891186	0.003620832	0.081436529	-0.005719037	0.114202003	0.003620589	0.081418714	-8.59E-05	0.009874925	1	0.436210685	1	0.455762334	ENSG00000173209	18	AHSA2
SAT	T2DM	ENSG00000073350.9	0.002435843	0.398675936	0.002886107	0.081436839	0.002395005	0.406587925	0.002885848	0.081419016	4.08E-05	0.024181955	1	0.750003279	1	0.760122496	ENSG00000173209	18	AHSA2
SAT	T2DM	ENSG00000073670.9	-0.000463602	0.81135765	0.0019424	0.081436949	-0.000497953	0.797656064	0.001942232	0.08141912	3.44E-05	0.009070043	1	0.951539016	1	0.952160336	ENSG00000173214	5	KIAA1919
SAT	T2DM	ENSG00000075643.5	0.001632947	0.542620369	0.002681993	0.081436896	0.001593752	0.552316442	0.002681762	0.081419072	3.92E-05	0.031988678	1	0.832700048	1	0.842050121	ENSG00000173226	12	IQCB1
SAT	T2DM	ENSG00000076242.10	-0.000871989	0.74888351	0.002724013	0.081436941	-0.000793533	0.770795649	0.002723787	0.081419117	-7.85E-05	7.49E-05	1	0.928253417	1	0.938518235	ENSG00000173226	12	IQCB1
SAT	T2DM	ENSG00000076344.11	-0.00244185	0.46666095	0.003354543	0.08143687	-0.002373286	0.479227157	0.003354247	0.081419047	-6.86E-05	0.001580089	1	0.79334291	1	0.80363893	ENSG00000173226	12	IQCB1
SAT	T2DM	ENSG00000076554.11	0.002453045	0.520058684	0.003813485	0.081436889	0.00231449	0.543873916	0.003813213	0.081419069	0.000138555	3.20E-05	1	0.819948363	1	0.840136685	ENSG00000173258	8	ZNF483
SAT	T2DM	ENSG00000079257.3	0.002164617	0.57298794	0.00384031	0.081436905	0.002043727	0.594573127	0.003839996	0.081419084	0.00012089	2.33E-05	1	0.840775238	1	0.854809759	ENSG00000173258	8	ZNF483
SAT	T2DM	ENSG00000079335.13	0.000700951	0.772267791	0.002421999	0.081436944	0.001302053	0.591497196	0.002426179	0.081419083	-0.000601102	4.23E-05	1	0.936021656	1	0.854809759	ENSG00000173272	9	MZT2A
SAT	T2DM	ENSG00000082516.8	-0.001158887	0.707005154	0.003083119	0.081436935	-0.001077237	0.7267689	0.003082868	0.08141911	-8.17E-05	0.000402543	1	0.906221055	1	0.921455409	ENSG00000173442	7	EHBP1L1
SAT	T2DM	ENSG00000083097.10	0.001928844	0.109807138	0.001206234	0.081436531	0.001908109	0.11364895	0.001206133	0.081418713	2.07E-05	0.016403969	1	0.436210685	1	0.455762334	ENSG00000173442	7	EHBP1L1
SAT	T2DM	ENSG00000083099.6	0.001372751	0.394610843	0.001612561	0.081436837	0.001345214	0.404122141	0.00161242	0.081419015	2.75E-05	0.010908966	1	0.74451879	1	0.758538184	ENSG00000173442	7	EHBP1L1
SAT	T2DM	ENSG00000084092.6	-0.001967353	0.444202956	0.002571315	0.081436861	-0.001702387	0.508140045	0.002572604	0.081419058	-0.000264966	0.003193497	1	0.779791277	1	0.817738235	ENSG00000173511	5	VEGFB
SAT	T2DM	ENSG00000085117.7	0.008066249	0.008596882	0.003069708	0.081435805	0.00799963	0.009155298	0.003069449	0.081417996	6.66E-05	0.0021672	1	0.109327018	1	0.124126174	ENSG00000173511	5	VEGFB
SAT	T2DM	ENSG00000085365.13	0.005328774	0.092545396	0.003167884	0.081436486	0.005191806	0.101213829	0.003167666	0.081418682	0.000136968	1.59E-06	1	0.414244809	1	0.440431278	ENSG00000173511	5	VEGFB
SAT	T2DM	ENSG00000086205.12	0.001496241	0.350235243	0.001601742	0.081436813	0.001577953	0.324539087	0.0016017	0.081418969	-8.17E-05	8.02E-05	1	0.708896874	1	0.693504571	ENSG00000173530	5	TNFRSF10D

SAT	T2DM	ENSG00000086475.10	0.004095361	0.333326273	0.004233208	0.081436802	0.003962451	0.349216344	0.004232885	0.081418984	0.000132909	0.000113022	1	0.695221474	1	0.717008026	ENSG00000173531	11	MST1
SAT	T2DM	ENSG00000087263.12	-0.004269882	0.124463802	0.002779316	0.081436564	-0.004320128	0.120066987	0.002779119	0.081418727	5.02E-05	0.039976227	1	0.463429598	1	0.467258033	ENSG00000173531	11	MST1
SAT	T2DM	ENSG00000087301.4	0.000249047	0.941531054	0.003395525	0.081436958	0.000161752	0.962003197	0.00339529	0.081419131	8.73E-05	0.004138365	1	0.982870948	1	0.993672317	ENSG00000173535	9	TNFRSF10C
SAT	T2DM	ENSG00000087448.5	0.004906895	0.135805023	0.003289689	0.081436587	0.004812729	0.143442459	0.00328942	0.081418773	9.42E-05	0.000125815	1	0.478599658	1	0.501004535	ENSG00000173540	8	GMPPB
SAT	T2DM	ENSG00000088298.8	-0.004189974	0.021985308	0.001829181	0.081436082	-0.004126184	0.0240762	0.001829048	0.081418281	-6.38E-05	5.08E-05	1	0.20149105	1	0.223974689	ENSG00000173727	7	AP000769.1
SAT	T2DM	ENSG00000088325.11	-0.000798991	0.709789807	0.002147024	0.081436935	-0.000700733	0.744123363	0.002146866	0.081419113	-9.83E-05	5.66E-08	1	0.907710061	1	0.927432137	ENSG00000173727	7	AP000769.1
SAT	T2DM	ENSG00000088538.12	-0.002277442	0.548894448	0.003799422	0.081436898	-0.002218057	0.5593271	0.003799073	0.081419074	-5.94E-05	0.008198562	1	0.83552458	1	0.842199272	ENSG00000173727	7	AP000769.1
SAT	T2DM	ENSG00000089063.10	0.004565758	0.203465232	0.00359016	0.081436688	0.004639165	0.196259987	0.003589901	0.081418852	-7.34E-05	0.018123938	1	0.565257769	1	0.564850016	ENSG00000173805	11	HAP1
SAT	T2DM	ENSG00000090054.9	-0.00110648	0.680818057	0.002689892	0.08143693	-0.00103697	0.699843277	0.002689711	0.081419106	-6.95E-05	0.004820659	1	0.892786811	1	0.909380512	ENSG00000173821	15	RNF213
SAT	T2DM	ENSG00000090238.7	0.001859299	0.016034849	0.000772093	0.081435989	0.00184462	0.016882279	0.000772043	0.081418177	1.47E-05	0.046281474	1	0.162066172	1	0.179453096	ENSG00000173821	15	RNF213
SAT	T2DM	ENSG00000090316.11	-0.001856982	0.216955299	0.001504036	0.081436704	-0.001897844	0.20697585	0.001503926	0.081418865	4.09E-05	0.001508697	1	0.581494104	1	0.579921232	ENSG00000173905	4	GOLIM4
SAT	T2DM	ENSG00000090621.9	-0.010191109	4.98E-09	0.001742702	0.081431244	-0.010218366	4.52E-09	0.001742547	0.081413386	2.73E-05	0.014226059	1.63E-05	1.17E-06	1.48E-05	1.85E-06	ENSG00000173917	9	HOXB2
SAT	T2DM	ENSG00000091640.3	0.000186728	0.900358484	0.001491337	0.081436956	0.000229713	0.877575629	0.001491225	0.081419127	-4.30E-05	0.000506731	1	0.972055342	1	0.966700287	ENSG00000173917	9	HOXB2
SAT	T2DM	ENSG00000092108.16	0.004221912	0.146062534	0.002904486	0.081436605	0.004156064	0.152424152	0.00290426	0.081418789	6.58E-05	0.004350513	1	0.492023791	1	0.512250482	ENSG00000173917	9	HOXB2
SAT	T2DM	ENSG00000092295.7	0.002208713	0.466200182	0.003031131	0.08143687	0.00230518	0.446918645	0.003030887	0.081419034	-9.65E-05	2.77E-05	1	0.79334291	1	0.791227469	ENSG00000174004	5	NRROS
SAT	T2DM	ENSG00000093000.14	0.002503272	0.17975424	0.001866005	0.081436658	0.002447611	0.189592497	0.001865859	0.081418843	5.57E-05	0.000162396	1	0.533249243	1	0.559400165	ENSG00000174007	7	CEP19
SAT	T2DM	ENSG00000094963.9	0.004087484	0.230468858	0.003408641	0.081436718	0.004026824	0.237426619	0.003408389	0.081418898	6.07E-05	0.034566716	1	0.5943147	1	0.613383258	ENSG00000174007	7	CEP19
SAT	T2DM	ENSG00000095485.12	-0.004128795	0.000124452	0.001075987	0.081434498	-0.00414476	0.000116993	0.001075898	0.081416651	1.60E-05	0.041875036	0.408327732	0.00600482	0.383854135	0.006618175	ENSG00000174007	7	CEP19
SAT	T2DM	ENSG00000097046.8	-0.001413146	0.474366507	0.001975342	0.081436873	-0.001373952	0.486675374	0.001975185	0.08141905	-3.92E-05	0.010193968	1	0.796607042	1	0.807270931	ENSG00000174007	7	CEP19
SAT	T2DM	ENSG00000100014.15	-0.000523022	0.79310828	0.001994161	0.081436947	-0.000470645	0.813408564	0.001993994	0.081419122	-5.24E-05	0.000250998	1	0.94681813	1	0.955415085	ENSG00000174080	6	CTSF
SAT	T2DM	ENSG00000100033.12	-0.006618986	0.079641563	0.003776294	0.081436445	-0.006552584	0.082683551	0.003775994	0.081418628	-6.64E-05	0.02331988	1	0.394965061	1	0.412914353	ENSG00000174080	6	CTSF
SAT	T2DM	ENSG00000100109.12	0.002767069	0.109341172	0.001728161	0.08143653	0.002720292	0.115434761	0.001728014	0.081418717	4.68E-05	9.97E-05	1	0.436210685	1	0.456640392	ENSG00000174080	6	CTSF
SAT	T2DM	ENSG00000100129.13	-0.0063078	0.032856623	0.002956083	0.081436197	-0.006248751	0.034512961	0.002955844	0.081418384	-5.90E-05	0.007979325	1	0.252465059	1	0.272172417	ENSG00000174080	6	CTSF
SAT	T2DM	ENSG00000100196.6	0.001562925	0.5020967	0.002328571	0.081436883	0.001480339	0.524926264	0.002328418	0.081419063	8.26E-05	0.000145728	1	0.810314363	1	0.827311811	ENSG00000174080	6	CTSF
SAT	T2DM	ENSG00000100225.13	0.000765623	0.761861352	0.002526494	0.081436943	0.000845671	0.737815619	0.002526294	0.081419112	-8.00E-05	5.08E-05	1	0.93459819	1	0.925041991	ENSG00000174080	6	CTSF
SAT	T2DM	ENSG00000100296.9	-0.002852674	0.29427786	0.002719982	0.081436775	-0.002781535	0.306442454	0.002719752	0.081418956	-7.11E-05	0.000222667	1	0.651501794	1	0.674696375	ENSG00000174125	3	TLR1
SAT	T2DM	ENSG00000100300.13	0.00092278	0.733307547	0.002708251	0.081436939	0.000876361	0.746228125	0.002708018	0.081419113	4.64E-05	0.012270425	1	0.922893004	1	0.927766001	ENSG00000174177	8	CTU2
SAT	T2DM	ENSG00000100302.6	-0.003496582	0.310871884	0.003450363	0.081436787	-0.003423875	0.320998023	0.00345006	0.081418966	-7.27E-05	0.001322424	1	0.671033323	1	0.691873008	ENSG00000174194	11	AGAP8
SAT	T2DM	ENSG00000100350.10	0.00339697	0.125861596	0.002219328	0.081436567	0.003357329	0.130306254	0.002219138	0.081418748	3.96E-05	0.009241096	1	0.464512818	1	0.480064491	ENSG00000174194	11	AGAP8
SAT	T2DM	ENSG00000100429.13	-0.001588768	0.598544604	0.003017631	0.081436912	-0.001661938	0.581778854	0.003017379	0.08141908	7.32E-05	0.000768178	1	0.850140626	1	0.851796934	ENSG00000174226	4	SNX31
SAT	T2DM	ENSG00000100580.7	-0.007528337	0.006070312	0.002743592	0.0814357	-0.007568377	0.005801349	0.002743343	0.081417859	4.00E-05	0.017423724	1	0.085847824	1	0.091952785	ENSG00000174226	4	SNX31
SAT	T2DM	ENSG00000100731.11	0.00321104	0.014791751	0.001317379	0.081435966	0.003187387	0.015533534	0.001317263	0.081418153	2.37E-05	0.006868315	1	0.15455967	1	0.171731497	ENSG00000174226	4	SNX31
SAT	T2DM	ENSG00000100994.7	-0.001742374	0.600190474	0.003324335	0.081436913	-0.001660042	0.617497663	0.003324071	0.081419089	-8.23E-05	0.001372817	1	0.851004731	1	0.869905467	ENSG00000174226	4	SNX31
SAT	T2DM	ENSG00000101000.4	-0.003612632	0.069506876	0.001990305	0.081436408	-0.003557653	0.073838535	0.001990166	0.081418597	-5.50E-05	0.001817539	1	0.366054671	1	0.395413696	ENSG00000174226	4	SNX31
SAT	T2DM	ENSG00000101052.8	0.005852071	0.084875162	0.00339629	0.081436462	0.005790824	0.08816031	0.003396007	0.081418645	6.12E-05	0.012113422	1	0.401115612	1	0.421383296	ENSG00000174226	4	SNX31
SAT	T2DM	ENSG00000101181.13	-0.000447448	0.912792246	0.004085639	0.081436956	-0.000549699	0.892963548	0.0040853	0.081419128	0.000102251	0.000570904	1	0.976164068	1	0.972100523	ENSG00000174353	13	STAG3L3
SAT	T2DM	ENSG00000101191.12	0.00568754	0.026653688	0.002565932	0.081436137	0.005646308	0.027759904	0.002565723	0.081418322	4.12E-05	0.031788339	1	0.225970935	1	0.242234694	ENSG00000174353	13	STAG3L3
SAT	T2DM	ENSG00000101197.8	-0.005422578	0.031648798	0.002523509	0.081436187	-0.005375363	0.033149247	0.002523316	0.081418373	-4.72E-05	0.021364391	1	0.248186538	1	0.267755401	ENSG00000174353	13	STAG3L3
SAT	T2DM	ENSG00000101335.5	0.003709085	0.363441036	0.00408117	0.08143682	0.002866122	0.483547871	0.004090906	0.081419049	0.000842962	0.003471375	1	0.721005279	1	0.805749399	ENSG00000174353	13	STAG3L3
SAT	T2DM	ENSG00000101363.8	0.001881927	0.103880525	0.001157163	0.081436516	0.001849736	0.10991478	0.001157113	0.081418704	3.22E-05	0.01534278	1	0.429259448	1	0.452205306	ENSG00000174353	13	STAG3L3
SAT	T2DM	ENSG00000101464.6	0.002314448	0.343086375	0.00244118	0.081436808	0.002369632	0.331663372	0.002440983	0.081418973	-5.52E-05	0.002742976	1	0.703910998	1	0.700003408	ENSG00000174405	9	LIG4
SAT	T2DM	ENSG00000101751.6	0.00084371	0.362022864	0.00092561	0.08143682	0.000814999	0.378568664	0.00092557	0.081419001	2.87E-05	0.007104311	1	0.720313533	1	0.738661557	ENSG00000174446	8	SNAPC5
SAT	T2DM	ENSG00000103269.9	-0.004275815	0.193680168	0.003289669	0.081436676	-0.004207026	0.200912787	0.00328942	0.081418858	-6.88E-05	0.001074874	1	0.553257876	1	0.570731475	ENSG00000174529	1	TMEM81
SAT	T2DM	ENSG00000103275.14	0.004204164	0.23983732	0.00357681	0.081436728	0.004154326	0.245414671	0.003576505	0.081418905	4.98E-05	0.044703556	1	0.60584911	1	0.620663712	ENSG00000174652	13	ZNF266
SAT	T2DM	ENSG00000103415.7	0.002979638	0.119488651	0.001913795	0.081436553	0.003015025	0.115133491	0.001913651	0.081418716	-3.54E-05	0.024893237	1	0.45480541	1	0.456639917	ENSG00000174652	13	ZNF266
SAT	T2DM	ENSG00000103550.9	-0.001425573	0.583766914	0.002601928	0.081436908	-0.001524194	0.55798635	0.002601744	0.081419074	9.86E-05	1.75E-05	1	0.845251212	1	0.842050121	ENSG00000174652	13	ZNF266
SAT	T2DM	ENSG00000103742.7	0.002817609	0.024997846	0.001257051	0.081436119	0.0028405	0.023831578	0.001256947	0.081418278	-2.29E-05	0.012343964	1	0.216409969	1	0.223866198	ENSG00000174652	13	ZNF266
SAT	T2DM	ENSG00000103811.11	0.001362185	0.082026278	0.000783292	0.081436453	0.00134752	0.085348308	0.000783229	0.081418636	1.47E-05	0.01486962	1	0.395495266	1	0.415329589	ENSG00000174652	13	ZNF266
SAT	T2DM	ENSG00000104388.10	0.005471884	0.055000378	0.002851606	0.081436343	0.005421621	0.0572498	0.002851379	0.081418527	5.03E-05	0.021891621	1	0.325754403	1	0.345923743	ENSG00000174652	13	ZNF266
SAT	T2DM	ENSG00000104442.5	-0.002331742	0.113601067	0.00147372	0.08143654	-0.002150986	0.144462689	0.001473996	0.081418775	-0.000180756	4.75E-07	1	0.443192746	1	0.501004535	ENSG00000174705	7	SH3PXD2B
SAT	T2DM	ENSG00000104689.5	0.003249445	0.146864588	0.002239918	0.081436607	0.003195329	0.153677204	0.002239719	0.081418791	5.41E-05	0.000166222	1	0.492391981	1	0.513457135	ENSG00000174718	7	KIAA1551
SAT	T2DM	ENSG00000104774.8	0.002378202	0.297778571	0.002284075	0.081436777	0.002427146	0.287909598	0.0022839	0.081418942	-4.89E-05	0.008203362	1	0.654394836	1	0.653237213	ENSG00000174775	12	HRAS
SAT	T2DM	ENSG00000104835.10	-0.004422857	0.128268729	0.002907924	0.081436572	-0.004377354	0.132212223	0.002907695	0.081418752	-4.55E-05	0.045549739	1	0.468889004	1	0.485221815	ENSG00000174780	11	SRP72
SAT	T2DM	ENSG00000104866.6	-0.005284583	0.169311862	0.003844954	0.081436643	-0.005378621	0.161819277	0.00384467	0.081418804	9.40E-05	0.003790121	1	0.523170556	1	0.520626624	ENSG00000174808	7	BTC
SAT	T2DM	ENSG00000105088.4	-0.00168391	0.505393656	0.002528288	0.081436884	-0.001593079	0.528598819	0.002528107	0.081419065	-9.08E-05	3.51E-05	1	0.810314363	1	0.82995678	ENSG00000174808	7	BTC
SAT	T2DM	ENSG00000105193.4	-0.00462803	0.090897367	0.002737367	0.081436481	-0.004668201	0.088100572	0.002737134	0.081418645	4.02E-05	0.035003359	1	0.413867045	1	0.421383296	ENSG00000174808	7	BTC
SAT	T2DM	ENSG00000105258.4	0.005508281	0.130850279	0.003646033	0.081436577	0.005570517	0.126523543	0.003645726	0.081418741	-6.22E-05	0.016433704	1	0.47229897	1	0.47645145	ENSG00000174851	10	YIF1A
SAT	T2DM	ENSG00000105479.11	0.000765019	0.743204578	0.00233513	0.08143694	0.00081546	0.726908407	0.002334947	0.081419111	-5.04E-05	0.00592017	1	0.927272852	1	0.921455409	ENSG00000174891	8	RSRC1
SAT	T2DM	ENSG00000105497.3	-0.004187725	0.030714194	0.001938077	0.081436178	-0.004160643	0.031796168	0.001937914	0.081418361	-2.71E-05	0.047984651	1	0.244489871	1	0.263779676	ENSG00000174891	8	RSRC1
SAT	T2DM	ENSG00000105501.7	-0.003924806	0.035726848	0.00186893	0.081436221	-0.003957898	0.034181784	0.001868761	0.081418381	3.31E-05	0.004170815	1	0.263415251	1	0.271550686	ENSG00000174915	7	PTDSS2
SAT	T2DM	ENSG00000105640.8	0.002672621	0.264415786	0.002394787	0.08143675	0.002755733	0.249811487	0.002394606	0.08141891	-8.31E-05	2.40E-05	1	0.627688499	1	0.621386978	ENSG00000174915	7	PTDSS2
SAT	T2DM	ENSG00000105656.8	0.008719664	0.031160282	0.004046199	0.081436175	0.008638351	0.032753128	0.004045872	0.081418369	8.13E-05	0.007799825	1	0.245761744	1	0.267755401	ENSG00000174989	8	FBXW8
SAT	T2DM	ENSG00000105968.14	0.00317049	0.426865773	0.003990172	0.081436853	0.003260053	0.41388345	0.003989855	0.081419019	-8.96E-05	0.009638767	1	0.771499322	1	0.766308941	ENSG00000175115	7	PACS1
SAT	T2DM	ENSG00000106009.11	-0.001014835	0.666664892	0.002356081	0.081436927	-0.000959584	0.683781448	0.002355914	0.081419103	-5.53E-05	0.00744263	1	0.888630991	1	0.901372745	ENSG00000175115	7	PACS1
SAT	T2DM	ENSG00000106263.13	-0.002008067	0.574746527	0.003578962	0.081436906	-0.001897606	0.59593963	0.003578708	0.081419084	-0.000110461	0.000433923	1	0.841779887	1	0.854809759	ENSG00000175115	7	PACS1
SAT	T2DM	ENSG00000106603.13	0.000591495	0.692398841	0.001495178	0.081436932	0.000617115	0.679774276	0.001495052	0.081419102	-2.56E-05	0.01539197	1	0.900268043	1	0.90041962	ENSG00000175130	6	MARCKSL1
SAT	T2DM	ENSG00000106610.10	-0.007731214	0.019057002	0.003297706	0.081436054	-0.007810095	0.017859152	0.003297439	0.081418194	7.89E-05	0.001565284	1	0.181761693	1	0.184263769	ENSG00000175213	2	ZNF408
SAT	T2DM	ENSG00000106733.16	0.005347363	0.130241569	0.003533922	0.081436576	0.005297411	0.133836193	0.003533617	0.081418755	5.00E-05	0.038123582	1	0.472179657	1	0.486825442	ENSG00000175220	7	ARHGAP1
SAT	T2DM	ENSG00000107020.5	-0.001347058	0.422130484	0.001678093	0.081436851	-0.001377143	0.411799768	0.001677946	0.081419018	3.01E-05	0.006325256	1	0.766045419	1	0.765504236	ENSG00000175220	7	ARHGAP1
SAT	T2DM	ENSG00000107099.11	0.003696298	0.163838832	0.002654856	0.081436634	0.004361966	0.101209925	0.002661327	0.081418682	-0.000665667	0.000447192	1	0.515393296	1	0.440431278	ENSG00000175220	7	ARHGAP1
SAT	T2DM	ENSG00000107443.11	0.004437406	0.282885469	0.004132197	0.081436766	0.004571569	0.268548387	0.004131883	0.081418926	-0.000134163	7.54E-05	1	0.641542051	1	0.639410201	ENSG00000175283	7	DOLK
SAT	T2DM	ENSG00000107537.9	-0.001152922	0.39918291	0.001367508	0.08143684	-0.001177152	0.389308102	0.001367393	0.081419007	2.42E-05	0.011878918	1	0.750125503	1	0.749514821	ENSG00000175305	12	CCNE2
SAT	T2DM	ENSG00000107779.7	0.001115034	0.642871421	0.002404706	0.081436922	0.000205306	0.932847643	0.002436502	0.08141913	0.000909727	0.020929364	1	0.877329554	1	0.983506786	ENSG00000175334	3	BANF1
SAT	T2DM	ENSG00000107890.12	0.002572068	0.335214987	0.002669051	0.081436803	0.002626601	0.32503298	0.002668851	0.081418969	-5.45E-05	0.014019711	1	0.695221474	1	0.693840734	ENSG00000175334	3	BANF1
SAT	T2DM	ENSG00000108264.12	0.000614063	0.477427052	0.000864332	0.081436874	0.000639448	0.459379233	0.00086427	0.081419039	-2.54E-05	0.000745648	1	0.79660704	1	0.795972372	ENSG00000175334	3	BANF1
SAT	T2DM	ENSG00000108272.9	-0.009646259	0.001572363	0.003051645	0.081435289	-0.009583119	0.001686433	0.003051418	0.081417483	-6.31E-05	0.013540319	1	0.035825853	1	0.043914172	ENSG00000175344	12	CHRNA7
SAT	T2DM	ENSG00000108439.5	0.00307485	0.241425055	0.002624844	0.081436729	0.003121092	0.234378419	0.002624626	0.081418895	-4.62E-05	0.015538495	1	0.607040297	1	0.610313962	ENSG00000175356	8	SCUBE2
SAT	T2DM	ENSG00000108468.10	0.001938109	0.388611205	0.002248023	0.081436834	0.001894994	0.399210895	0.00224783	0.081419012	4.31E-05	0.005531182	1	0.739604086	1	0.756241886	ENSG00000175387	11	SMAD2
SAT	T2DM	ENSG00000108509.16	0.005341395	0.098385777	0.003231855	0.081436502	0.00538593	0.095583039	0.003231575	0.081418667	-4.45E-05	0.040642342	1	0.421965667	1	0.432044519	ENSG00000175387	11	SMAD2
SAT	T2DM	ENSG00000108556.7	0.00113986	0.476533195	0.001601175	0.081436874	0.001092808	0.494885609	0.001601045	0.081419053	4.71E-05	8.90E-05	1	0.79660704	1	0.813894578	ENSG00000175387	11	SMAD2
SAT	T2DM	ENSG00000108602.13	-0.000898037	0.745278243	0.002764282	0.081436941	-0.000957665	0.728988931	0.002764055	0.081419111	5.96E-05	0.003479054	1	0.927287795	1	0.922641931	ENSG00000175390	8	EIF3F
SAT	T2DM	ENSG00000108639.3	-0.006696499	0.014632067	0.002742928	0.081435962	-0.006595824	0.016180166	0.002742735	0.081418165	-0.000100675	3.12E-05	1	0.153379588	1	0.176369183	ENSG00000175390	8	EIF3F
SAT	T2DM	ENSG00000108654.7	0.001571814	0.48829834	0.00226807	0.081436878	0.001341945	0.554357686	0.0022697	0.081419072	0.000229869	0.012762135	1	0.80306108	1	0.842050121	ENSG00000175390	8	EIF3F
SAT	T2DM	ENSG00000108669.12	0.011228556	0.003015359	0.003785524	0.081435488	0.01117446	0.003155794	0.003785187	0.081417675	5.41E-05	0.025276807	1	0.054659637	1	0.064643525	ENSG00000175390	8	EIF3F
SAT	T2DM	ENSG00000108733.5	-0.004284727	0.180610167	0.003200231	0.081436659	-0.004178519	0.191643138	0.003200127	0.081418846	-0.000106208	0.007405457	1	0.534339005	1	0.560852203	ENSG00000175390	8	EIF3F
SAT	T2DM	ENSG00000109265.8	0.002207998	0.195984618	0.001707552	0.081436679	0.002144773	0.209067627	0.001707439	0.081418867	6.32E-05	6.79E-05	1	0.55576969	1	0.579977552	ENSG00000175390	8	EIF3F
SAT	T2DM	ENSG00000109321.6	0.001822364	0.576964502	0.003266913	0.081436906	0.001590591	0.62635686	0.00326703	0.081419091	0.000231773	3.16E-05	1	0.842502133	1	0.876644981	ENSG00000175395	11	ZNF25
SAT	T2DM	ENSG00000109854.9	0.00064898	0.615012552	0.001290395	0.081436916	0.00066697	0.605214636	0.001290286	0.081419086	-1.80E-05	0.04827489	1	0.859969008	1	0.858870727	ENSG00000175395	11	ZNF25
SAT	T2DM	ENSG00000110025.8	-0.002075057	0.235587094	0.001749493	0.081436723	-0.000570747	0.750044753	0.00179153	0.081419114	-0.001504311	0.000100075	1	0.600737047	1	0.928640315	ENSG00000175416	8	CLTB
SAT	T2DM	ENSG00000110169.6	0.001530319	0.591559521	0.002851999	0.08143691	0.001477512	0.604384804	0.002851759	0.081419086	5.28E-05	0.009099436	1	0.846620707	1	0.858807511	ENSG00000175416	8	CLTB
SAT	T2DM	ENSG00000110700.2	0.002304389	0.308135397	0.002261104	0.081436785	0.002250251	0.319600384	0.002260922	0.081418965	5.41E-05	0.001609863	1	0.66864566	1	0.690783176	ENSG00000175416	8	CLTB
SAT	T2DM	ENSG00000110711.5	-0.000363877	0.921413397	0.00368843	0.081436957	-0.000586691	0.873620364	0.003688453	0.081419127	0.000222815	7.22E-05	1	0.978648294	1	0.966700287	ENSG00000175445	10	LPL
SAT	T2DM	ENSG00000110768.7	-0.001907014	0.564387266	0.003308849	0.081436903	-0.001845963	0.576890148	0.003308573	0.081419079	-6.11E-05	0.010274263	1	0.837959912	1	0.849979671	ENSG00000175445	10	LPL
SAT	T2DM	ENSG00000111341.5	0.00734804	0.040316832	0.003583547	0.081436256	0.007406397	0.038740109	0.003583262	0.081418417	-5.84E-05	0.035574423	1	0.276735412	1	0.283087523	ENSG00000175445	10	LPL
SAT	T2DM	ENSG00000111530.8	-0.002438966	0.226180923	0.002015246	0.081436714	-0.002481968	0.218063403	0.002015082	0.081418877	4.30E-05	0.004294011	1	0.591314429	1	0.591783312	ENSG00000175445	10	LPL
SAT	T2DM	ENSG00000111639.3	-0.000333169	0.712506874	0.000904144	0.081436936	-0.000290829	0.747713421	0.000904162	0.081419114	-4.23E-05	0.003597898	1	0.908107919	1	0.927854665	ENSG00000175463	7	TBC1D10C
SAT	T2DM	ENSG00000111725.6	4.90E-05	0.978711033	0.001836357	0.081436958	7.80E-05	0.966129844	0.001836201	0.081419131	-2.90E-05	0.023752474	1	0.995203291	1	0.994313682	ENSG00000175463	7	TBC1D10C
SAT	T2DM	ENSG00000111801.11	-0.000445981	0.629360142	0.000924068	0.081436919	-0.000119032	0.897650951	0.000925388	0.081419128	-0.000326949	1.81E-10	1	0.868347614	1	0.972878089	ENSG00000175463	7	TBC1D10C
SAT	T2DM	ENSG00000111879.14	0.004988234	0.181422233	0.003732617	0.08143666	0.004919268	0.187497649	0.003732317	0.081418841	6.90E-05	0.015522537	1	0.535187534	1	0.557313872	ENSG00000175489	9	LRRC25
SAT	T2DM	ENSG00000112031.11	0.003953718	0.041597075	0.001940442	0.081436265	0.003909584	0.043911346	0.001940297	0.081418453	4.41E-05	0.006093114	1	0.28140207	1	0.303311846	ENSG00000175575	8	PAAF1
SAT	T2DM	ENSG00000112053.9	0.00166483	0.371310548	0.001862181	0.081436825	0.001845278	0.321713667	0.001862143	0.081418967	-0.000180448	4.15E-13	1	0.725886698	1	0.691873008	ENSG00000175575	8	PAAF1
SAT	T2DM	ENSG00000112110.5	0.000294276	0.872771889	0.001837631	0.081436954	0.000333508	0.855972237	0.00183747	0.081419125	-3.92E-05	0.001287083	1	0.966468088	1	0.965566132	ENSG00000175575	8	PAAF1
SAT	T2DM	ENSG00000112130.12	-0.001431011	0.555477546	0.002427201	0.0814369	-0.00151448	0.532620637	0.002427009	0.081419066	8.35E-05	1.01E-05	1	0.835948344	1	0.83186484	ENSG00000175575	8	PAAF1
SAT	T2DM	ENSG00000112293.10	0.005236458	0.161489437	0.003740108	0.081436631	0.005390557	0.14948782	0.003739949	0.081418784	-0.0001541	0.000369587	1	0.512811064	1	0.509843595	ENSG00000175575	8	PAAF1
SAT	T2DM	ENSG00000112308.8	0.004798402	0.232829823	0.004021786	0.081436721	0.004276004	0.28795438	0.004024016	0.081418942	0.000522399	0.000364194	1	0.598143699	1	0.653237213	ENSG00000175581	9	MRPL48
SAT	T2DM	ENSG00000112339.10	-0.000779482	0.6799084	0.001889249	0.08143693	-0.000705362	0.708866858	0.001889133	0.081419108	-7.41E-05	5.78E-05	1	0.892786811	1	0.912941362	ENSG00000175581	9	MRPL48
SAT	T2DM	ENSG00000112378.11	0.000121882	0.946069253	0.001801818	0.081436958	7.78E-05	0.96557705	0.001801688	0.081419131	4.41E-05	0.00457127	1	0.984788457	1	0.99427016	ENSG00000175581	9	MRPL48
SAT	T2DM	ENSG00000112619.6	-0.005417464	0.103612652	0.00332852	0.081436516	-0.005349376	0.107995516	0.003328235	0.081418699	-6.81E-05	0.002897655	1	0.429233725	1	0.450218948	ENSG00000175582	15	RAB6A
SAT	T2DM	ENSG00000112667.8	0.001860454	0.292778978	0.001768415	0.081436773	0.00191549	0.278695358	0.001768271	0.081418935	-5.50E-05	3.17E-05	1	0.649937638	1	0.644397089	ENSG00000175582	15	RAB6A
SAT	T2DM	ENSG00000112763.11	-0.001894249	0.177247647	0.001403896	0.081436654	-0.001258962	0.371386951	0.001408425	0.081418997	-0.000635287	3.08E-08	1	0.530526907	1	0.734641671	ENSG00000175643	7	RMI2
SAT	T2DM	ENSG00000112812.11	-0.000752645	0.756767055	0.00243001	0.081436942	-0.003386193	0.173785069	0.002489585	0.081418822	0.002633548	1.21E-06	1	0.93284139	1	0.532948952	ENSG00000175643	7	RMI2
SAT	T2DM	ENSG00000112977.11	-0.000272382	0.943512019	0.003844136	0.081436958	-0.000189474	0.960685639	0.003843812	0.08141913	-8.29E-05	0.00240749	1	0.98331034	1	0.993672317	ENSG00000175643	7	RMI2
SAT	T2DM	ENSG00000113068.5	0.00239346	0.359918194	0.002614313	0.081436818	0.002353182	0.368019589	0.002614094	0.081418995	4.03E-05	0.032287418	1	0.71830389	1	0.732878736	ENSG00000175643	7	RMI2
SAT	T2DM	ENSG00000113141.11	-0.001685822	0.428518586	0.002129289	0.081436854	-0.001645094	0.439718378	0.002129104	0.081419031	-4.07E-05	0.004577104	1	0.771792681	1	0.786806474	ENSG00000175662	13	TOM1L2
SAT	T2DM	ENSG00000113593.7	-0.000881313	0.489829728	0.001276192	0.081436879	-0.000830866	0.514981752	0.001276094	0.081419066	-5.04E-05	9.88E-07	1	0.80382562	1	0.824105482	ENSG00000175730	7	BAK1P1
SAT	T2DM	ENSG00000113597.13	0.00256976	0.532124743	0.003612512	0.081436893	0.0023932	0.507633338	0.003612226	0.081419058	-0.000136224	1.32E-06	1	0.827049399	1	0.817738235	ENSG00000175764	10	TTLL11
SAT	T2DM	ENSG00000113734.13	0.000637359	0.5707192	0.001124103	0.081436905	0.000619383	0.581601281	0.001124011	0.081419078	1.80E-05	0.02974585	1	0.840736467	1	0.851796934	ENSG00000175826	7	CTDNEP1
SAT	T2DM	ENSG00000113971.14	0.002052223	0.005777786	0.00074352	0.081435685	0.002036605	0.006155928	0.000743457	0.081417877	1.56E-05	0.003069502	1	0.083144374	1	0.095234728	ENSG00000175826	7	CTDNEP1
SAT	T2DM	ENSG00000114200.5	-0.002079713	0.547936226	0.003461268	0.081436895	-0.002001033	0.563150989	0.003461024	0.081419075	-7.87E-05	0.001120177	1	0.835027823	1	0.84223981	ENSG00000175832	8	ETV4
SAT	T2DM	ENSG00000114302.11	-0.004533103	0.126526242	0.002966793	0.081436568	-0.004595007	0.121397835	0.002966558	0.08141873	6.19E-05	0.007328224	1	0.464874211	1	0.469700823	ENSG00000175832	8	ETV4
SAT	T2DM	ENSG00000114544.11	-0.002039971	0.607884206	0.003975805	0.081436914	-0.002110275	0.595541988	0.003975481	0.081419084	7.03E-05	0.018225932	1	0.855260755	1	0.854809759	ENSG00000175854	7	SWI5
SAT	T2DM	ENSG00000114742.9	0.00161285	0.455683358	0.002162073	0.081436865	0.001526809	0.480072083	0.002162045	0.081419048	8.60E-05	0.000243481	1	0.786411516	1	0.80363893	ENSG00000175879	7	HOXD8
SAT	T2DM	ENSG00000114796.11	0.003052195	0.403595472	0.00365437	0.081436842	0.00311837	0.393441055	0.003654078	0.081419009	-6.62E-05	0.018700037	1	0.753240469	1	0.753672849	ENSG00000175911	4	AC127496.1
SAT	T2DM	ENSG00000115084.8	0.003624923	0.101508492	0.002213576	0.08143651	0.003686567	0.095798647	0.002213389	0.081418667	-6.16E-05	8.77E-05	1	0.425727224	1	0.432044519	ENSG00000175938	6	ORAI3
SAT	T2DM	ENSG00000115221.6	-0.005761825	0.025763638	0.002584053	0.081436128	-0.005687153	0.027732923	0.002583836	0.081418321	-7.47E-05	4.85E-05	1	0.220131498	1	0.242234694	ENSG00000176024	12	ZNF613
SAT	T2DM	ENSG00000115486.7	-0.000858878	0.601830072	0.001646082	0.081436913	-0.00092936	0.572338621	0.001646018	0.081419078	7.05E-05	0.00031209	1	0.851489636	1	0.847525062	ENSG00000176024	12	ZNF613
SAT	T2DM	ENSG00000115561.10	-0.007122278	8.48E-06	0.001599492	0.081433645	-0.007094507	9.17E-06	0.0015936	0.081415843	-2.78E-05	0.017239456	0.027810273	0.000596732	0.030097647	0.000734089	ENSG00000176024	12	ZNF613
SAT	T2DM	ENSG00000116151.9	0.002906816	0.418489148	0.003592894	0.081436849	0.00296399	0.409357615	0.003592605	0.081419017	-5.72E-05	0.003739799	1	0.763661232	1	0.761827756	ENSG00000176024	12	ZNF613
SAT	T2DM	ENSG00000116157.5	-0.004135879	0.000451391	0.001178957	0.081434902	-0.004077206	0.000543185	0.001178872	0.081417132	-5.87E-05	5.40E-09	1	0.014727922	1	0.019368778	ENSG00000176142	8	TMEM39A
SAT	T2DM	ENSG00000116198.8	0.001126722	0.55318148	0.001900037	0.0814369	0.001093243	0.56500147	0.001899875	0.081419076	3.35E-05	0.011001557	1	0.835948344	1	0.84223981	ENSG00000176148	11	TCP11L1
SAT	T2DM	ENSG00000116221.11	0.00842396	0.0272825	0.003816135	0.081436144	0.008266659	0.030293482	0.003815859	0.081418347	0.000157901	1.50E-06	1	0.227073047	1	0.256527252	ENSG00000176148	11	TCP11L1
SAT	T2DM	ENSG00000116747.7	-0.002026699	0.373702515	0.002278315	0.081436826	-0.002090552	0.358795367	0.002278124	0.08141899	6.39E-05	8.88E-05	1	0.72766644	1	0.725902637	ENSG00000176148	11	TCP11L1
SAT	T2DM	ENSG00000117222.9	0.006004917	0.060226932	0.003195578	0.081436368	0.005649331	0.077171053	0.003196507	0.081418609	0.000355586	8.27E-05	1	0.342468915	1	0.404470007	ENSG00000176148	11	TCP11L1
SAT	T2DM	ENSG00000117450.9	0.00172283	0.619161971	0.003466188	0.081436917	0.001654229	0.633157232	0.003465892	0.081419093	6.86E-05	0.004198373	1	0.862986588	1	0.883243571	ENSG00000176155	14	CCDC57
SAT	T2DM	ENSG00000117480.11	0.003577017	0.031694654	0.001665087	0.081436187	0.003538532	0.033560849	0.001664945	0.081418376	3.85E-05	0.000878125	1	0.248186538	1	0.267915193	ENSG00000176155	14	CCDC57
SAT	T2DM	ENSG00000117595.6	0.003861002	0.118603045	0.002473944	0.081436551	0.003775092	0.12699646	0.002473757	0.081418742	8.59E-05	2.29E-05	1	0.453748792	1	0.476745292	ENSG00000176171	7	BNIP3
SAT	T2DM	ENSG00000117697.10	-0.000283539	0.92462137	0.002996795	0.081436957	-0.00022248	0.940815166	0.002996551	0.08141913	-6.11E-05	0.006554908	1	0.978648294	1	0.986202735	ENSG00000176171	7	BNIP3

SAT	T2DM	ENSG00000117868.11	-0.003228104	0.424856546	0.004045117	0.081436852	-0.003172519	0.432833623	0.004044762	0.081419028	-5.56E-05	0.036308435	1	0.770140513	1	0.781577939	ENSG00000176194	13	CIDEA
SAT	T2DM	ENSG00000118017.3	-0.002892106	0.097722537	0.001746441	0.0814365	-0.002842486	0.103584479	0.001746297	0.081418688	-4.96E-05	0.000103642	1	0.421965667	1	0.441377502	ENSG00000176268	5	CYCSP34
SAT	T2DM	ENSG00000118197.9	-0.001980045	0.281571683	0.001838829	0.081436765	-0.00195039	0.288801379	0.00183868	0.081418943	-2.97E-05	0.031321321	1	0.641415693	1	0.653487811	ENSG00000176293	15	ZNF135
SAT	T2DM	ENSG00000118307.14	-0.005817061	0.096175056	0.003496481	0.081436496	-0.005758522	0.099542364	0.003496202	0.081418678	-5.85E-05	0.029112458	1	0.420328914	1	0.440431278	ENSG00000176293	15	ZNF135
SAT	T2DM	ENSG00000118402.5	0.00887519	0.001737748	0.002833935	0.081435319	0.008916566	0.001651815	0.0028337	0.081417477	-4.14E-05	0.045914723	1	0.038414593	1	0.04335685	ENSG00000176386	4	CDC26
SAT	T2DM	ENSG00000118507.11	-0.002998212	0.181009326	0.002241393	0.081436659	-0.002916714	0.193139722	0.002241301	0.081418848	-8.15E-05	0.001872883	1	0.535037478	1	0.561285588	ENSG00000176386	4	CDC26
SAT	T2DM	ENSG00000118514.9	-0.000806841	0.451635855	0.001071942	0.081436864	-0.000772135	0.471305457	0.001071877	0.081419044	-3.47E-05	0.001063928	1	0.783398902	1	0.80363893	ENSG00000176390	10	CRLF3
SAT	T2DM	ENSG00000118579.7	-0.001507919	0.495155499	0.002210596	0.081436881	-0.001470843	0.505786195	0.002210418	0.081419057	-3.71E-05	0.028072159	1	0.806594843	1	0.817738235	ENSG00000176390	10	CRLF3
SAT	T2DM	ENSG00000118900.10	0.000981992	0.802091683	0.003917922	0.081436948	0.000863495	0.825548173	0.003917589	0.081419123	0.000118498	1.59E-05	1	0.948256967	1	0.959754419	ENSG00000176390	10	CRLF3
SAT	T2DM	ENSG00000118961.10	-0.002403295	0.135162333	0.001608573	0.081436585	-0.002469554	0.124695809	0.001608451	0.081418737	6.63E-05	5.16E-07	1	0.477360189	1	0.474800718	ENSG00000176390	10	CRLF3
SAT	T2DM	ENSG00000119431.5	-0.002932871	0.017685111	0.001236375	0.081436018	-0.002915851	0.018344446	0.001236264	0.081418201	-1.70E-05	0.028226976	1	0.172693001	1	0.187482528	ENSG00000176393	6	RNPEP
SAT	T2DM	ENSG00000119471.10	0.000745712	0.836958196	0.003623727	0.081436951	0.000804915	0.824203722	0.003623434	0.081419123	-5.92E-05	0.030419774	1	0.959823323	1	0.959280742	ENSG00000176393	6	RNPEP
SAT	T2DM	ENSG00000119599.12	0.005890171	0.036475986	0.002816125	0.081436227	0.005841101	0.038049497	0.002815902	0.081418412	4.91E-05	0.02501078	1	0.266450175	1	0.281806774	ENSG00000176422	10	SPRYD4
SAT	T2DM	ENSG00000119878.5	-0.005677109	0.16085853	0.004048739	0.08143663	-0.005738381	0.156351634	0.004048379	0.081418795	6.13E-05	0.018729246	1	0.512133793	1	0.51775889	ENSG00000176422	10	SPRYD4
SAT	T2DM	ENSG00000119943.6	0.001487186	0.227070508	0.001231174	0.081436715	0.001466617	0.233522853	0.00123107	0.081418894	2.06E-05	0.017992328	1	0.591955891	1	0.610313962	ENSG00000176422	10	SPRYD4
SAT	T2DM	ENSG00000120071.8	0.002737168	0.303786377	0.002661713	0.081436782	0.002813182	0.290516027	0.002661499	0.081418944	-7.60E-05	0.000192882	1	0.66271483	1	0.655417523	ENSG00000176422	10	SPRYD4
SAT	T2DM	ENSG00000120314.14	-0.003113364	0.411802341	0.003793423	0.081436846	-0.003050976	0.421194568	0.00379309	0.081419023	-6.24E-05	0.012129469	1	0.757945834	1	0.770311805	ENSG00000176422	10	SPRYD4
SAT	T2DM	ENSG00000120328.4	-0.001580255	0.43560328	0.002026903	0.081436857	-0.001606484	0.427980941	0.00202672	0.081419026	2.62E-05	0.036149631	1	0.772274669	1	0.777953168	ENSG00000176428	5	VPS37D
SAT	T2DM	ENSG00000120539.10	-0.002802619	0.014364199	0.001144845	0.081435957	-0.002760072	0.015921946	0.001144922	0.08141816	-4.25E-05	0.047651654	1	0.151551916	1	0.174133018	ENSG00000176428	5	VPS37D
SAT	T2DM	ENSG00000120805.9	0.001332922	0.636211218	0.002818	0.081436921	0.001289671	0.647172155	0.002817754	0.081419096	4.33E-05	0.021836832	1	0.873655621	1	0.890302658	ENSG00000176428	5	VPS37D
SAT	T2DM	ENSG00000120963.7	-0.001043703	0.747263525	0.003238801	0.081436941	-0.000985513	0.760892151	0.00323852	0.081419115	-5.82E-05	0.007696742	1	0.927647229	1	0.932999239	ENSG00000176428	5	VPS37D
SAT	T2DM	ENSG00000121350.11	-0.003581239	0.092856478	0.002131028	0.081436486	-0.003494157	0.101112888	0.002131252	0.081418682	-8.71E-05	0.048476268	1	0.414991889	1	0.440431278	ENSG00000176472	6	ZNF575
SAT	T2DM	ENSG00000121552.3	-0.001270942	0.737520511	0.003792284	0.08143694	-0.001419433	0.708162671	0.003791979	0.081419107	0.000148491	2.71E-07	1	0.924295185	1	0.912941362	ENSG00000176472	6	ZNF575
SAT	T2DM	ENSG00000121957.8	0.007920118	0.049325853	0.004029018	0.081436313	0.008019387	0.046531298	0.004028736	0.081418469	-9.93E-05	0.005652669	1	0.30826309	1	0.310935212	ENSG00000176485	6	PLA2G16
SAT	T2DM	ENSG00000122376.7	0.000706114	0.780324044	0.002531809	0.081436945	0.000594454	0.814379259	0.002531957	0.081419122	0.00011166	0.016153765	1	0.940919952	1	0.955647609	ENSG00000176485	6	PLA2G16
SAT	T2DM	ENSG00000122417.11	0.004633477	0.077676925	0.002626195	0.081436438	0.004681773	0.074608118	0.002625976	0.0814186	-4.83E-05	0.010911646	1	0.389096167	1	0.395620568	ENSG00000176531	6	PHLDB3
SAT	T2DM	ENSG00000122477.8	0.000432086	0.811005302	0.001806914	0.081436949	0.000480594	0.790244161	0.001806786	0.081419119	-4.85E-05	0.002306069	1	0.951539016	1	0.947957612	ENSG00000176715	11	ACSF3
SAT	T2DM	ENSG00000122696.8	0.003466706	0.035828958	0.001651702	0.081436222	0.003435122	0.037533502	0.001651565	0.081418408	3.16E-05	0.008719313	1	0.263575813	1	0.280518039	ENSG00000176783	10	RUFY1
SAT	T2DM	ENSG00000122873.7	-0.001486253	0.286870204	0.001395524	0.081436769	-0.001516795	0.277043985	0.001395415	0.081418933	3.05E-05	0.005482056	1	0.644230759	1	0.64327119	ENSG00000176783	10	RUFY1
SAT	T2DM	ENSG00000122912.10	0.00526685	0.178150935	0.003911592	0.081436655	0.005405066	0.166997335	0.003911276	0.081418812	-0.000138216	3.21E-06	1	0.530526907	1	0.527859591	ENSG00000176909	7	MAMSTR
SAT	T2DM	ENSG00000123096.7	0.007993065	0.012798624	0.003210926	0.081435923	0.007928236	0.013536519	0.003210667	0.081418112	6.48E-05	0.007867527	1	0.143532419	1	0.157494043	ENSG00000176915	10	ANKLE2
SAT	T2DM	ENSG00000123143.8	-0.000876374	0.797572088	0.003416788	0.081436947	-0.000826337	0.808883856	0.003416504	0.081419121	-5.00E-05	0.043154338	1	0.94681813	1	0.953285895	ENSG00000176933	4	TOB2P1
SAT	T2DM	ENSG00000123191.9	-0.000774774	0.44468803	0.001013703	0.081436861	-0.000759124	0.453904536	0.001013622	0.081419037	-1.57E-05	0.046114548	1	0.780081459	1	0.794600418	ENSG00000176933	4	TOB2P1
SAT	T2DM	ENSG00000123213.18	0.004791799	0.045051524	0.002390904	0.081436287	0.00471993	0.048350237	0.002390707	0.08141848	7.19E-05	4.08E-05	1	0.292287338	1	0.319189392	ENSG00000176933	4	TOB2P1
SAT	T2DM	ENSG00000123427.11	-0.002177231	0.257446997	0.001922589	0.081436744	-0.00222216	0.247725573	0.001922462	0.081418908	4.49E-05	0.012016424	1	0.622005594	1	0.620663712	ENSG00000176933	4	TOB2P1
SAT	T2DM	ENSG00000123454.6	-0.00494541	0.094037011	0.002953401	0.08143649	-0.004988154	0.091199999	0.002953135	0.081418654	4.27E-05	0.020259311	1	0.417503971	1	0.424593011	ENSG00000176933	4	TOB2P1
SAT	T2DM	ENSG00000123545.5	0.001041597	0.49200482	0.001515878	0.08143688	0.00101952	0.501192044	0.001515754	0.081419055	2.21E-05	0.049869732	1	0.803918235	1	0.816084912	ENSG00000176946	7	THAP4
SAT	T2DM	ENSG00000123815.7	0.004049777	0.25523309	0.003559519	0.081436742	0.004130389	0.245861842	0.003559266	0.081418906	-8.06E-05	0.009857967	1	0.620408004	1	0.620663712	ENSG00000176974	13	SHMT1
SAT	T2DM	ENSG00000123870.9	-0.005201603	0.099769933	0.003160199	0.081436506	-0.005158089	0.102607197	0.003159923	0.081418686	-4.35E-05	0.038196975	1	0.425727224	1	0.440431278	ENSG00000176974	13	SHMT1
SAT	T2DM	ENSG00000124191.13	-0.001104756	0.38580675	0.001273864	0.081436833	-0.001066794	0.402306748	0.001273769	0.081419014	-3.80E-05	0.000372207	1	0.736376933	1	0.757591271	ENSG00000176974	13	SHMT1
SAT	T2DM	ENSG00000124207.12	-0.00282912	0.233964978	0.002376995	0.081436722	-0.002872696	0.22679979	0.00237679	0.081418887	4.36E-05	0.007242907	1	0.598969104	1	0.601281476	ENSG00000176998	3	HCG4
SAT	T2DM	ENSG00000124226.7	-0.002958386	0.446271953	0.003884203	0.081436861	-0.002900554	0.455171199	0.00388386	0.081419038	-5.78E-05	0.022197446	1	0.780081459	1	0.794600418	ENSG00000176998	3	HCG4
SAT	T2DM	ENSG00000124257.6	0.004561033	0.039346314	0.0022135	0.081436249	0.004524676	0.040925603	0.002213326	0.081418433	3.64E-05	0.035569603	1	0.274087595	1	0.289545565	ENSG00000176998	3	HCG4
SAT	T2DM	ENSG00000124508.12	0.001087728	0.420408181	0.001350018	0.08143685	0.001661	0.21934769	0.001352313	0.081418879	-0.000573272	1.73E-12	1	0.764009208	1	0.592816944	ENSG00000176998	3	HCG4
SAT	T2DM	ENSG00000124532.10	0.001659229	0.579207211	0.002992063	0.081436907	0.001759919	0.556368836	0.002991819	0.081419073	-0.00010069	6.66E-06	1	0.842939695	1	0.842050121	ENSG00000176998	3	HCG4
SAT	T2DM	ENSG00000124549.10	0.000257654	0.8696856	0.001570509	0.081436954	0.001227113	0.437484074	0.001580417	0.08141903	-0.000969459	5.33E-08	1	0.966468088	1	0.786081735	ENSG00000176998	3	HCG4
SAT	T2DM	ENSG00000124562.5	0.000173242	0.908750619	0.001511515	0.081436956	0.000286279	0.84977336	0.001511444	0.081419125	-0.000113037	2.42E-11	1	0.974472349	1	0.965561588	ENSG00000177042	10	TMEM80
SAT	T2DM	ENSG00000124575.5	-0.00080717	0.809193293	0.003342774	0.081436949	-0.000470673	0.888037989	0.003343149	0.081419128	-0.000336496	1.74E-06	1	0.951539016	1	0.971217547	ENSG00000177042	10	TMEM80
SAT	T2DM	ENSG00000124587.9	-0.000932806	0.410227117	0.001132741	0.081436845	-0.000949962	0.401631542	0.001132644	0.081419013	1.72E-05	0.026831831	1	0.757820042	1	0.757591271	ENSG00000177156	6	TALDO1
SAT	T2DM	ENSG00000124613.4	0.0016677	0.222026326	0.00136567	0.081436709	0.000622486	0.652035211	0.001380428	0.081419097	0.001045215	2.39E-07	1	0.587001107	1	0.891013292	ENSG00000177202	1	SPACA4
SAT	T2DM	ENSG00000124635.7	0.007316809	0.043213081	0.003619227	0.081436275	0.002532316	0.49836327	0.003740122	0.081419054	0.004784493	4.09E-07	1	0.286428522	1	0.81497549	ENSG00000177294	6	FBXO39
SAT	T2DM	ENSG00000124659.5	-0.004095362	0.124430671	0.002665485	0.081436564	-0.004052283	0.12841004	0.002665267	0.081418745	-4.31E-05	0.029656214	1	0.463429598	1	0.478221727	ENSG00000177311	6	ZBTB38
SAT	T2DM	ENSG00000124713.5	-0.001097482	0.518143333	0.001698335	0.081436889	-0.001061095	0.532077055	0.001698189	0.081419066	-3.64E-05	0.001796671	1	0.818675014	1	0.831703104	ENSG00000177311	6	ZBTB38
SAT	T2DM	ENSG00000124733.3	-0.000666667	0.796970828	0.002591316	0.081436947	-0.000633908	0.806727499	0.002591081	0.081419121	-3.28E-05	0.039748042	1	0.94681813	1	0.952410029	ENSG00000177370	4	TIMM22
SAT	T2DM	ENSG00000124920.9	-0.001646213	0.560489963	0.00282799	0.081436902	-0.001492783	0.597589586	0.002827935	0.081419084	-0.00015343	5.26E-05	1	0.837416925	1	0.855769266	ENSG00000177409	7	SAMD9L
SAT	T2DM	ENSG00000124939.4	-0.002106677	0.445202003	0.002759457	0.081436861	-0.001745723	0.527147884	0.002760621	0.081419064	-0.000360954	5.99E-05	1	0.780081459	1	0.828775497	ENSG00000177409	7	SAMD9L
SAT	T2DM	ENSG00000125107.12	0.005212753	0.157073039	0.003683959	0.081436624	0.005290724	0.150927174	0.003683664	0.081418786	-7.80E-05	0.00586716	1	0.507474184	1	0.5109822	ENSG00000177409	7	SAMD9L
SAT	T2DM	ENSG00000125388.15	0.003459211	0.000501109	0.000993973	0.081434934	0.003432358	0.000553525	0.000993894	0.081417138	2.69E-05	0.000514398	1	0.015422559	1	0.019528141	ENSG00000177479	15	ARIH2
SAT	T2DM	ENSG00000125450.6	0.000995294	0.717319368	0.002749098	0.081436937	0.000939716	0.732458626	0.002748857	0.081419111	5.56E-05	0.002206355	1	0.911865496	1	0.923670747	ENSG00000177570	9	SAMD12
SAT	T2DM	ENSG00000125510.11	-0.014155629	8.55E-06	0.003180309	0.081433648	-0.014209361	7.89E-06	0.003180062	0.081415795	5.37E-05	0.034136514	0.02804641	0.000596732	0.025878571	0.000663553	ENSG00000177590	6	RP5-1051J4.6
SAT	T2DM	ENSG00000125611.11	0.00038549	0.916837006	0.003691765	0.081436957	0.000453702	0.902182891	0.003691503	0.081419128	-6.82E-05	0.035226446	1	0.977304164	1	0.972878089	ENSG00000177590	6	RP5-1051J4.6
SAT	T2DM	ENSG00000125648.10	0.002344123	0.384815122	0.002697311	0.081436832	0.002404458	0.372657633	0.002697071	0.081418998	-6.03E-05	0.000519058	1	0.734911767	1	0.735400932	ENSG00000177595	13	PIDD
SAT	T2DM	ENSG00000125821.7	-0.001731248	0.636055244	0.00365843	0.081436921	-0.001577214	0.666364905	0.00365822	0.0814191	-0.000154034	3.72E-05	1	0.873655621	1	0.896403813	ENSG00000177628	11	GBA
SAT	T2DM	ENSG00000125966.8	0.00472836	0.230218154	0.003940962	0.081436718	0.004783715	0.224765946	0.003940607	0.081418885	-5.54E-05	0.024940281	1	0.594292496	1	0.599071543	ENSG00000177628	11	GBA
SAT	T2DM	ENSG00000126091.15	0.000780117	0.808435218	0.003217702	0.081436949	0.000721111	0.822659957	0.003217446	0.081419123	5.90E-05	0.017922323	1	0.951539016	1	0.958398582	ENSG00000177628	11	GBA
SAT	T2DM	ENSG00000126247.6	0.003769325	0.180182803	0.002812521	0.081436658	0.003860193	0.169879139	0.002812334	0.081418816	-9.09E-05	0.000499183	1	0.533555754	1	0.531756789	ENSG00000177628	11	GBA
SAT	T2DM	ENSG00000126602.6	0.00025728	0.70489417	0.000679336	0.081436934	0.000269245	0.691831763	0.000679275	0.081419105	-1.20E-05	0.005480855	1	0.906221055	1	0.904075912	ENSG00000177646	13	ACAD9
SAT	T2DM	ENSG00000126698.6	-0.005237655	0.092020593	0.003108701	0.081436484	-0.005309026	0.087650217	0.003108459	0.081418644	7.14E-05	0.003956707	1	0.414244809	1	0.420439126	ENSG00000177663	9	IL17RA
SAT	T2DM	ENSG00000126790.7	0.001005989	0.632696464	0.002104865	0.08143692	0.00112834	0.591958705	0.002105112	0.081419083	-0.000122351	0.00631771	1	0.871484928	1	0.854809759	ENSG00000177663	9	IL17RA
SAT	T2DM	ENSG00000126858.12	0.003014821	0.375731476	0.003403542	0.081436827	0.002936861	0.388164375	0.003403274	0.081419006	7.80E-05	0.003488351	1	0.728082648	1	0.748716823	ENSG00000177663	9	IL17RA
SAT	T2DM	ENSG00000126870.11	-0.000807887	0.79777712	0.003153034	0.081436947	-0.000870496	0.782467401	0.003152768	0.081419118	6.26E-05	0.004931082	1	0.94681813	1	0.944893464	ENSG00000177674	11	AGTRAP
SAT	T2DM	ENSG00000127220.5	0.000291347	0.895363044	0.002215206	0.081436956	0.000319281	0.885386411	0.002215006	0.081419127	-2.79E-05	0.041405673	1	0.970180416	1	0.968806494	ENSG00000177674	11	AGTRAP
SAT	T2DM	ENSG00000127337.2	0.001959545	0.130445268	0.001295695	0.081436576	0.001979163	0.126608117	0.001295588	0.081418741	-1.96E-05	0.038983996	1	0.47229897	1	0.47645145	ENSG00000177674	11	AGTRAP
SAT	T2DM	ENSG00000127903.12	-0.002705144	0.402341577	0.003230226	0.081436841	-0.002801928	0.385679985	0.003229966	0.081419005	9.68E-05	8.66E-05	1	0.752518444	1	0.746964998	ENSG00000177685	12	EFCAB4A
SAT	T2DM	ENSG00000127952.12	-0.000734029	0.593227981	0.001374162	0.081436911	-0.000709119	0.605799663	0.001374052	0.081419086	-2.49E-05	0.017104239	1	0.846620707	1	0.85932931	ENSG00000177694	10	NAALADL2
SAT	T2DM	ENSG00000128262.4	-0.000171007	0.963092066	0.003695552	0.081436958	-9.23E-05	0.980066457	0.003695312	0.081419131	-7.87E-05	0.023966843	1	0.9918095	1	0.997085517	ENSG00000177697	13	CD151
SAT	T2DM	ENSG00000128408.7	0.007084352	0.004013598	0.00246232	0.081435575	0.006993672	0.004506719	0.002462265	0.081417783	9.07E-05	0.005219207	1	0.068072202	1	0.079766896	ENSG00000177839	4	PCDHB9
SAT	T2DM	ENSG00000128463.8	0.008865954	0.007025839	0.003289012	0.081435744	0.008933702	0.006598677	0.003288731	0.081417898	-6.77E-05	0.003055815	1	0.09396718	1	0.099770777	ENSG00000177989	9	ODF3B
SAT	T2DM	ENSG00000128699.9	0.002497582	0.216122543	0.002019217	0.081436703	0.002457257	0.223597195	0.002019077	0.081418883	4.03E-05	0.026139345	1	0.580276647	1	0.597775996	ENSG00000177990	7	DPY19L2
SAT	T2DM	ENSG00000129003.11	-0.001361794	0.582571862	0.002477646	0.081436908	-0.00131982	0.594217058	0.00247744	0.081419083	-4.20E-05	0.020019925	1	0.845212395	1	0.854809759	ENSG00000177990	7	DPY19L2
SAT	T2DM	ENSG00000129204.12	0.000152131	0.916592924	0.001452648	0.081436957	0.000180502	0.901103048	0.001452522	0.081419128	-2.84E-05	0.003567418	1	0.977304164	1	0.972878089	ENSG00000177990	7	DPY19L2
SAT	T2DM	ENSG00000129219.9	0.006337841	0.061910584	0.003394775	0.081436376	0.006420696	0.058556392	0.003394475	0.081418533	-8.29E-05	0.000170474	1	0.345456849	1	0.349315498	ENSG00000177990	7	DPY19L2
SAT	T2DM	ENSG00000129317.10	0.000544588	0.839070593	0.002681611	0.081436952	0.000604643	0.821598074	0.002681463	0.081419122	-6.01E-05	0.031604788	1	0.959889764	1	0.958012298	ENSG00000177990	7	DPY19L2
SAT	T2DM	ENSG00000129484.9	-0.00040825	0.909078587	0.003574844	0.081436956	-0.000314477	0.929895644	0.003574574	0.08141913	-9.38E-05	0.001479331	1	0.974472349	1	0.982921265	ENSG00000178015	4	GPR150
SAT	T2DM	ENSG00000129562.6	0.000336368	0.903073237	0.00276209	0.081436956	0.000433313	0.875331339	0.002761882	0.081419127	-9.69E-05	2.05E-05	1	0.97231278	1	0.966700287	ENSG00000178078	7	STAP2
SAT	T2DM	ENSG00000129657.10	-0.001191502	0.653670059	0.002655631	0.081436925	-0.001152869	0.66417278	0.002655403	0.081419099	-3.86E-05	0.03408026	1	0.882995924	1	0.895996588	ENSG00000178149	12	DALRD3
SAT	T2DM	ENSG00000130035.2	-0.004444119	0.071508065	0.002465885	0.081436416	-0.004407257	0.073864717	0.002465662	0.081418597	-3.69E-05	0.015077233	1	0.373553186	1	0.395413696	ENSG00000178149	12	DALRD3
SAT	T2DM	ENSG00000130193.7	-0.003880062	0.238443594	0.003291291	0.081436726	-0.00392701	0.232770148	0.003291007	0.081418893	4.69E-05	0.035906551	1	0.60584911	1	0.610313962	ENSG00000178188	10	SH2B1
SAT	T2DM	ENSG00000130312.2	-0.000967055	0.274098294	0.000884226	0.081436759	-0.000980817	0.26728617	0.000884149	0.081418925	1.38E-05	0.020719095	1	0.6356488	1	0.638593145	ENSG00000178209	10	PLEC
SAT	T2DM	ENSG00000130363.7	4.68E-05	0.974234288	0.001448905	0.081436958	2.40E-05	0.986790099	0.001448778	0.081419131	2.28E-05	0.016801211	1	0.995031844	1	0.997162029	ENSG00000178248	10	AP000345.1
SAT	T2DM	ENSG00000130520.6	5.38E-06	0.996650523	0.001282518	0.081436958	3.34E-05	0.979232997	0.001282411	0.081419131	-2.80E-05	0.002388432	1	0.998171663	1	0.997085517	ENSG00000178252	13	WDR6
SAT	T2DM	ENSG00000130714.11	0.000844881	0.82571304	0.003836824	0.08143695	0.000753267	0.844342541	0.003836506	0.081419124	9.16E-05	0.001027634	1	0.955051074	1	0.96477102	ENSG00000178295	10	GEN1
SAT	T2DM	ENSG00000130733.6	-0.005536535	0.130679649	0.003663111	0.081436577	-0.005606182	0.125879327	0.003662835	0.081418739	6.96E-05	0.021547517	1	0.47229897	1	0.47645145	ENSG00000178295	10	GEN1
SAT	T2DM	ENSG00000130770.13	-0.002318901	0.192759909	0.001780401	0.081436675	-0.002361994	0.184588018	0.001780269	0.081418837	4.31E-05	0.004042511	1	0.552241504	1	0.550575716	ENSG00000178295	10	GEN1
SAT	T2DM	ENSG00000130856.11	-0.006775837	0.065498954	0.003678838	0.081436391	-0.006709994	0.068137785	0.003678526	0.081418575	-6.58E-05	0.010540232	1	0.357574154	1	0.380219613	ENSG00000178295	10	GEN1
SAT	T2DM	ENSG00000131165.10	0.002220091	0.390382134	0.002584731	0.081436835	0.002163458	0.402551801	0.00258455	0.081419014	5.66E-05	0.013753029	1	0.741307668	1	0.757591271	ENSG00000178295	10	GEN1
SAT	T2DM	ENSG00000131401.7	0.002523547	0.53397372	0.004057463	0.081436894	0.002432878	0.548738836	0.004057154	0.081419071	9.07E-05	0.00613802	1	0.828750699	1	0.841179809	ENSG00000178381	7	ZFAND2A
SAT	T2DM	ENSG00000131437.11	0.00018911	0.945720578	0.002777687	0.081436958	6.99E-05	0.979928621	0.002777487	0.081419131	0.000119233	6.81E-07	1	0.984737929	1	0.997085517	ENSG00000178381	7	ZFAND2A
SAT	T2DM	ENSG00000131626.11	-0.003027735	0.436189907	0.003888472	0.081436857	-0.002970883	0.444814205	0.003888134	0.081419033	-5.69E-05	0.029447956	1	0.772274669	1	0.790845546	ENSG00000178381	7	ZFAND2A
SAT	T2DM	ENSG00000131711.10	0.00184828	0.200973134	0.00144534	0.081436685	0.001822241	0.207351776	0.001445211	0.081418865	2.60E-05	0.004464298	1	0.561663418	1	0.579977552	ENSG00000178386	8	ZNF223
SAT	T2DM	ENSG00000131845.10	-0.003387597	0.308016242	0.003323146	0.081436785	-0.003326376	0.316804585	0.003322897	0.081418963	-6.12E-05	0.026353298	1	0.66864566	1	0.68742915	ENSG00000178386	8	ZNF223
SAT	T2DM	ENSG00000131849.10	0.006085935	0.074688731	0.003414509	0.081436428	0.005983027	0.079708822	0.003414226	0.081418618	0.000102908	3.55E-05	1	0.382299104	1	0.409271745	ENSG00000178397	8	FAM220A
SAT	T2DM	ENSG00000132139.8	0.000674141	0.627194772	0.001388038	0.081436919	0.000630784	0.649486334	0.001387937	0.081419096	4.34E-05	0.000256454	1	0.866453072	1	0.891013292	ENSG00000178397	8	FAM220A
SAT	T2DM	ENSG00000132205.6	0.002553511	0.471754096	0.003548374	0.081436872	0.002690874	0.448211982	0.003548093	0.081419035	-0.000137363	7.13E-07	1	0.79607871	1	0.791227469	ENSG00000178467	13	P4HTM
SAT	T2DM	ENSG00000132305.16	0.004248608	0.106159392	0.002629574	0.081436522	0.004164504	0.113241698	0.002629451	0.081418712	8.41E-05	0.00433314	1	0.433220108	1	0.455325994	ENSG00000178467	13	P4HTM
SAT	T2DM	ENSG00000132382.10	0.000967501	0.748580513	0.003018613	0.081436941	0.001011302	0.73758533	0.003018336	0.081419112	-4.38E-05	0.014718704	1	0.92822852	1	0.925041991	ENSG00000178538	5	CA8
SAT	T2DM	ENSG00000132432.9	0.001245196	0.514444171	0.001910004	0.081436887	0.001289415	0.499594578	0.001909882	0.081419055	-4.42E-05	0.015307618	1	0.816039871	1	0.815958247	ENSG00000178623	7	GPR35
SAT	T2DM	ENSG00000132563.11	-0.002846764	0.132058072	0.001890234	0.081436579	-0.00281436	0.136479367	0.001890059	0.08141876	-3.24E-05	0.003422569	1	0.472458584	1	0.492616944	ENSG00000178623	7	GPR35
SAT	T2DM	ENSG00000132824.9	-0.001635401	0.121457193	0.001055991	0.081436558	-0.001653068	0.117455314	0.001055905	0.081418721	1.77E-05	0.025142601	1	0.459102591	1	0.462451956	ENSG00000178623	7	GPR35
SAT	T2DM	ENSG00000132840.5	-0.001408887	0.434687856	0.0018035	0.081436856	-0.00137229	0.44667462	0.001803341	0.081419034	-3.66E-05	0.001692439	1	0.772274669	1	0.791227469	ENSG00000178665	10	ZNF713
SAT	T2DM	ENSG00000133048.8	0.002101841	0.185705158	0.001588221	0.081436666	0.002064941	0.193512861	0.0015881	0.081418848	3.69E-05	0.00444526	1	0.542563333	1	0.561375505	ENSG00000178665	10	ZNF713
SAT	T2DM	ENSG00000133059.12	-0.010796959	0.00053458	0.003117962	0.081434955	-0.010468085	0.000791863	0.003119545	0.081417249	-0.000328874	0.002669781	1	0.015863016	1	0.024351274	ENSG00000178665	10	ZNF713
SAT	T2DM	ENSG00000133069.10	-0.010873286	0.001614993	0.003448331	0.081435297	-0.010512396	0.002311502	0.003450081	0.08141758	-0.00036089	0.002878277	1	0.036293105	1	0.051945464	ENSG00000178685	9	PARP10
SAT	T2DM	ENSG00000133597.5	-0.002056192	0.131068337	0.001361802	0.081436577	-0.002084321	0.125844883	0.001361681	0.081418739	2.81E-05	0.0011499	1	0.472458584	1	0.47645145	ENSG00000178695	4	KCTD12
SAT	T2DM	ENSG00000133731.5	-0.000867262	0.40910458	0.001050627	0.081436845	-0.00088992	0.396933788	0.001050536	0.081419011	2.27E-05	0.001371603	1	0.756926483	1	0.756180206	ENSG00000178695	4	KCTD12
SAT	T2DM	ENSG00000133816.9	0.002297928	0.293767012	0.002188722	0.081436774	0.002237976	0.306501032	0.002188532	0.081418956	6.00E-05	4.46E-05	1	0.651249707	1	0.674696375	ENSG00000178718	5	RPP25
SAT	T2DM	ENSG00000133884.5	-0.000116038	0.974431431	0.003620425	0.081436958	-0.00061593	0.86500336	0.003622933	0.081419126	0.000499892	0.000568556	1	0.995031844	1	0.966241215	ENSG00000178718	5	RPP25
SAT	T2DM	ENSG00000133983.10	-3.70E-05	0.989591989	0.002837805	0.081436958	2.99E-05	0.991606033	0.002837559	0.081419131	-6.69E-05	0.000451497	1	0.996461007	1	0.998267105	ENSG00000178750	2	STX19
SAT	T2DM	ENSG00000133997.7	0.002752511	0.38357471	0.003158979	0.081436832	0.002811374	0.373443642	0.003158698	0.081418999	-5.89E-05	0.003556003	1	0.734252407	1	0.735400932	ENSG00000178802	13	MPI
SAT	T2DM	ENSG00000134184.8	0.000977835	0.475122512	0.001369192	0.081436873	0.001022064	0.455346363	0.001369083	0.081419038	-4.42E-05	3.01E-05	1	0.796607042	1	0.794600418	ENSG00000178809	7	TRIM73
SAT	T2DM	ENSG00000134201.6	0.00132051	0.673679841	0.003135833	0.081436929	0.00147789	0.637411296	0.00313563	0.081419094	-0.000157381	1.18E-07	1	0.891627092	1	0.884719251	ENSG00000178809	7	TRIM73
SAT	T2DM	ENSG00000134202.6	0.001487511	0.249974547	0.001293024	0.081436737	0.001516307	0.240883963	0.001292915	0.081418901	-2.88E-05	0.001737024	1	0.615333003	1	0.618903903	ENSG00000178852	11	EFCAB13
SAT	T2DM	ENSG00000134222.12	-0.000321088	0.920658703	0.003223641	0.081436957	-0.00022929	0.943291443	0.003223368	0.08141913	-9.18E-05	5.44E-05	1	0.978648294	1	0.98735976	ENSG00000178852	11	EFCAB13
SAT	T2DM	ENSG00000134262.8	0.007795714	0.038332478	0.003763705	0.081436241	0.007897047	0.03587364	0.003763433	0.081418395	-0.000101333	0.00182526	1	0.271246148	1	0.27605566	ENSG00000178922	12	HYI
SAT	T2DM	ENSG00000134531.5	-0.002787794	0.367415308	0.003092991	0.081436823	-0.002840673	0.358360313	0.003092748	0.08141899	5.29E-05	0.029497334	1	0.723275188	1	0.725902637	ENSG00000178922	12	HYI
SAT	T2DM	ENSG00000134809.4	-0.006206917	0.066560168	0.003383186	0.081436396	-0.005918488	0.080280691	0.003383804	0.08141862	-0.00028843	0.000415968	1	0.358786109	1	0.409385619	ENSG00000178952	4	TUFM
SAT	T2DM	ENSG00000134851.8	0.006129203	0.090105321	0.003616378	0.081436478	0.006184749	0.08720134	0.003616067	0.081418642	-5.55E-05	0.024871188	1	0.41272218	1	0.419512605	ENSG00000178952	4	TUFM
SAT	T2DM	ENSG00000135052.12	-0.003273355	0.355933895	0.003545886	0.081436816	-0.003212125	0.36496138	0.003545577	0.081418994	-6.12E-05	0.009779098	1	0.715575434	1	0.730228601	ENSG00000178952	4	TUFM
SAT	T2DM	ENSG00000135269.13	-0.001431279	0.564597586	0.002484746	0.081436903	-0.001384166	0.577452132	0.002484544	0.081419079	-4.71E-05	0.011467007	1	0.837959912	1	0.849979671	ENSG00000178966	11	RMI1
SAT	T2DM	ENSG00000135424.11	-0.005792992	0.101437398	0.003536781	0.08143651	-0.007753441	0.033223927	0.003641185	0.081418373	0.001960449	0.02379487	1	0.425727224	1	0.267755401	ENSG00000178966	11	RMI1
SAT	T2DM	ENSG00000135587.4	-0.000623391	0.793051095	0.002376172	0.081436947	-0.000543199	0.819163912	0.002375986	0.081419122	-8.02E-05	1.99E-05	1	0.94681813	1	0.957271954	ENSG00000178971	9	CTC1
SAT	T2DM	ENSG00000135747.7	-0.000542243	0.795616401	0.002093404	0.081436947	-0.000576442	0.783021571	0.002093231	0.081419118	3.42E-05	0.026021047	1	0.94681813	1	0.945214781	ENSG00000178971	9	CTC1
SAT	T2DM	ENSG00000135845.5	0.000962369	0.7558501	0.00309508	0.081436942	0.00087222	0.778072492	0.003094824	0.081419118	9.01E-05	6.85E-05	1	0.932309841	1	0.941773638	ENSG00000178971	9	CTC1
SAT	T2DM	ENSG00000135917.9	0.001027259	0.791232849	0.003880693	0.081436947	-0.002690595	0.522183363	0.004204161	0.081419062	0.003717854	0.021586999	1	0.94681813	1	0.826644476	ENSG00000179021	5	C3orf38
SAT	T2DM	ENSG00000135926.8	-0.008621575	0.001092398	0.00264012	0.081435176	-0.008560775	0.001183505	0.002639896	0.081417374	-6.08E-05	0.001082603	1	0.027029242	1	0.033188714	ENSG00000179021	5	C3orf38
SAT	T2DM	ENSG00000136014.7	-0.003624344	0.221518038	0.002964691	0.081436709	-0.003585516	0.226465925	0.002964427	0.081418886	-3.88E-05	0.039410407	1	0.586129584	1	0.601160762	ENSG00000179023	7	KLHDC7A
SAT	T2DM	ENSG00000136048.9	-0.000509223	0.860016577	0.002887519	0.081436953	-0.000560949	0.845954494	0.002887281	0.081419125	5.17E-05	0.015058379	1	0.965007451	1	0.96477102	ENSG00000179163	11	FUCA1

SAT	T2DM	ENSG00000136104.14	0.001361311	0.607101876	0.002647358	0.081436914	0.00142593	0.590115565	0.002647137	0.081419082	-6.46E-05	0.000694919	1	0.854526494	1	0.854809759	ENSG00000179163	11	FUCA1
SAT	T2DM	ENSG00000136143.9	-0.001606946	0.529093492	0.002553177	0.081436892	-0.001667538	0.513643909	0.002552973	0.08141906	6.06E-05	0.002155631	1	0.823749329	1	0.82328562	ENSG00000179163	11	FUCA1
SAT	T2DM	ENSG00000136146.10	-0.004882571	0.122240781	0.003159342	0.081436559	-0.00483395	0.125974268	0.003159085	0.08141874	-4.86E-05	0.039251513	1	0.459418101	1	0.47645145	ENSG00000179163	11	FUCA1
SAT	T2DM	ENSG00000136169.12	0.000219607	0.930806173	0.002529138	0.081436957	0.000159235	0.949793948	0.002528924	0.08141913	6.04E-05	0.000749565	1	0.980452059	1	0.989619036	ENSG00000179163	11	FUCA1
SAT	T2DM	ENSG00000136197.8	-0.001893965	0.330950389	0.001948118	0.0814368	-0.001858907	0.339939511	0.001947957	0.081418979	-3.51E-05	0.013220015	1	0.693389672	1	0.707255253	ENSG00000179163	11	FUCA1
SAT	T2DM	ENSG00000136213.7	-0.001344769	0.681903002	0.003280982	0.08143693	-0.001224435	0.708983417	0.003280716	0.081419108	-0.000120334	1.02E-06	1	0.892786811	1	0.912941362	ENSG00000179163	11	FUCA1
SAT	T2DM	ENSG00000136247.10	0.001006544	0.801636484	0.004006465	0.081436948	0.00093213	0.816013311	0.004006134	0.081419122	7.44E-05	0.01109924	1	0.948256967	1	0.95619274	ENSG00000179242	11	CDH4
SAT	T2DM	ENSG00000136279.14	-0.000986436	0.783256838	0.003586029	0.081436946	-0.001059039	0.767729485	0.003585755	0.081419116	7.26E-05	0.012877893	1	0.942378322	1	0.937978714	ENSG00000179296	9	CTGLF12P
SAT	T2DM	ENSG00000136371.5	-1.91E-05	0.989872977	0.001503377	0.081436958	-4.10E-05	0.978245395	0.00150324	0.081419131	2.19E-05	0.01678826	1	0.996461007	1	0.99701704	ENSG00000179299	12	NSUN7
SAT	T2DM	ENSG00000136448.7	0.002128049	0.150415917	0.001479799	0.081436613	0.002094016	0.157012947	0.00147967	0.081418796	3.40E-05	0.000522074	1	0.496972349	1	0.51775889	ENSG00000179299	12	NSUN7
SAT	T2DM	ENSG00000136715.13	-0.005020529	0.176397552	0.003713594	0.081436653	-0.004842435	0.192209261	0.003713316	0.081418847	-0.000178094	6.86E-09	1	0.530526907	1	0.560852203	ENSG00000179344	12	HLA-DQB1
SAT	T2DM	ENSG00000136783.9	-0.006853433	0.070996351	0.003795854	0.081436414	-0.006773878	0.074310343	0.003795524	0.081418599	-7.96E-05	0.001767733	1	0.373189908	1	0.395620568	ENSG00000179344	12	HLA-DQB1
SAT	T2DM	ENSG00000136816.11	-3.86E-05	0.9818333	0.001697285	0.081436958	-8.75E-05	0.958865745	0.001697153	0.08141913	4.89E-05	0.000307274	1	0.995935491	1	0.993672317	ENSG00000179361	13	ARID3B
SAT	T2DM	ENSG00000136856.13	-0.000180595	0.953442013	0.00309317	0.081436958	-0.000249443	0.935720184	0.003092897	0.08141913	6.88E-05	0.000590639	1	0.988996775	1	0.984952815	ENSG00000179361	13	ARID3B
SAT	T2DM	ENSG00000136875.8	0.003938397	0.008570613	0.001498211	0.081435804	0.003964673	0.008133198	0.001498081	0.081417961	-2.63E-05	0.009710353	1	0.109327018	1	0.115021654	ENSG00000179406	6	LINC00174
SAT	T2DM	ENSG00000137054.11	0.001772997	0.349328074	0.001894441	0.081436812	0.001743405	0.35739188	0.00189429	0.081418989	2.96E-05	0.043258213	1	0.708371701	1	0.725119681	ENSG00000179406	6	LINC00174
SAT	T2DM	ENSG00000137106.13	-0.002867512	0.157065145	0.002026491	0.081436624	-0.002818444	0.164251564	0.002026322	0.081418808	-4.91E-05	0.000832784	1	0.507474184	1	0.523492072	ENSG00000179406	6	LINC00174
SAT	T2DM	ENSG00000137185.7	0.001743186	0.287587672	0.001639212	0.081436769	0.000644013	0.698528281	0.001662793	0.081419106	0.001099173	8.69E-05	1	0.644566475	1	0.908954249	ENSG00000179409	6	GEMIN4
SAT	T2DM	ENSG00000137274.8	0.001553902	0.50124565	0.002310526	0.081436883	0.001516549	0.511556029	0.002310342	0.081419059	3.74E-05	0.036720583	1	0.810314363	1	0.821142531	ENSG00000179409	6	GEMIN4
SAT	T2DM	ENSG00000137285.9	0.001919888	0.468203953	0.002646643	0.08143687	0.001847279	0.485164569	0.002646453	0.081419049	7.26E-05	0.001609611	1	0.79334291	1	0.806395618	ENSG00000179528	11	LBX2
SAT	T2DM	ENSG00000137310.7	0.006017389	0.095838551	0.003613233	0.081436495	-0.003313914	0.395732901	0.003902086	0.08141901	0.009331304	2.46E-10	1	0.420328914	1	0.755715818	ENSG00000179528	11	LBX2
SAT	T2DM	ENSG00000137312.10	0.006087638	0.00918323	0.002336756	0.081435824	0.00499607	0.036343647	0.002386961	0.081418399	0.001091568	0.025353363	1	0.114563415	1	0.276092282	ENSG00000179639	6	FCER1A
SAT	T2DM	ENSG00000137404.10	-0.001420962	0.674922021	0.003388061	0.081436929	-0.000466584	0.890718955	0.00339594	0.081419128	-0.000954378	5.47E-05	1	0.892191439	1	0.971822875	ENSG00000179639	6	FCER1A
SAT	T2DM	ENSG00000137411.12	0.001254617	0.073262163	0.000700435	0.081436422	0.001860896	0.009707149	0.000719576	0.081418013	-0.000606278	0.000242606	1	0.37912505	1	0.129468108	ENSG00000179639	6	FCER1A
SAT	T2DM	ENSG00000137509.6	0.007147416	0.016053492	0.002968559	0.08143599	0.007094266	0.016848213	0.002968298	0.081418176	5.31E-05	0.006170277	1	0.162066172	1	0.179453096	ENSG00000179698	9	KIAA1875
SAT	T2DM	ENSG00000137561.4	-0.004110668	0.057903821	0.002167582	0.081436357	-0.00415061	0.055490504	0.002167397	0.081418518	3.99E-05	0.008164628	1	0.333873157	1	0.341997131	ENSG00000179698	9	KIAA1875
SAT	T2DM	ENSG00000137709.5	0.000445042	0.871279855	0.002746611	0.081436954	0.000499131	0.855785771	0.002746379	0.081419125	-5.41E-05	0.005265412	1	0.966468088	1	0.965566132	ENSG00000179698	9	KIAA1875
SAT	T2DM	ENSG00000137806.4	-0.006876048	0.005646764	0.002484453	0.081435678	-0.006794835	0.006235207	0.002484254	0.081417881	-8.12E-05	1.90E-05	1	0.082656666	1	0.095436475	ENSG00000179774	7	ATOH7
SAT	T2DM	ENSG00000137841.7	0.001244482	0.48589762	0.00178587	0.081436877	0.001270077	0.476934427	0.001785716	0.081419046	-2.56E-05	0.035425146	1	0.801645999	1	0.80363893	ENSG00000179913	6	B3GNT3
SAT	T2DM	ENSG00000137992.10	-0.000411882	0.917451636	0.003973993	0.081436957	-0.000519358	0.89601489	0.003973752	0.081419128	0.000107476	0.006160403	1	0.977324291	1	0.9725607	ENSG00000179954	10	SSC5D
SAT	T2DM	ENSG00000138030.8	-0.001765711	0.073972253	0.000988203	0.081436425	-0.00177958	0.071707144	0.000988119	0.081418589	1.39E-05	0.044296531	1	0.379223381	1	0.390815844	ENSG00000179978	10	RP11-1319K7.1
SAT	T2DM	ENSG00000138085.12	0.001431988	0.697578568	0.003685073	0.081436933	0.001488366	0.686268759	0.003684766	0.081419104	-5.64E-05	0.034514614	1	0.900770568	1	0.902825425	ENSG00000179978	10	RP11-1319K7.1
SAT	T2DM	ENSG00000138135.5	0.001378984	0.632133142	0.002880524	0.08143692	0.00133196	0.643764698	0.002880275	0.081419095	4.70E-05	0.016338723	1	0.871484928	1	0.888950368	ENSG00000179979	7	CRIPAK
SAT	T2DM	ENSG00000138175.8	0.001688984	0.509508456	0.002560604	0.081436886	0.001638196	0.522290155	0.002560403	0.081419062	5.08E-05	0.011563607	1	0.812130745	1	0.826644476	ENSG00000179979	7	CRIPAK
SAT	T2DM	ENSG00000138380.13	0.007750453	0.00067346	0.002279422	0.081435026	0.007705973	0.00072242	0.002279235	0.081417221	4.45E-05	0.008182761	1	0.018725617	1	0.023237829	ENSG00000179979	7	CRIPAK
SAT	T2DM	ENSG00000138459.4	0.002055401	0.060898897	0.001096662	0.081436371	0.002033288	0.063707847	0.001096571	0.081418556	2.21E-05	0.005323779	1	0.34323074	1	0.366069085	ENSG00000179979	7	CRIPAK
SAT	T2DM	ENSG00000138621.7	0.000551151	0.59975465	0.001050304	0.081436912	0.000519876	0.620586391	0.001050213	0.08141909	3.13E-05	1.15E-05	1	0.850754434	1	0.87257824	ENSG00000180089	4	TMEM86B
SAT	T2DM	ENSG00000138744.10	0.003170958	0.148828592	0.002196435	0.08143661	0.003220947	0.142496702	0.002196262	0.081418772	-5.00E-05	0.003477451	1	0.493635361	1	0.500269293	ENSG00000180089	4	TMEM86B
SAT	T2DM	ENSG00000138750.10	0.005214399	0.050529547	0.002666586	0.081436319	0.005157969	0.053059068	0.002666382	0.081418506	5.64E-05	0.009040917	1	0.311148486	1	0.332861953	ENSG00000180089	4	TMEM86B
SAT	T2DM	ENSG00000138756.13	-0.002874049	0.24412855	0.002467565	0.081436732	-0.003810349	0.124419573	0.002479909	0.081418736	0.0009363	0.000178503	1	0.61016229	1	0.474800718	ENSG00000180113	11	TDRD6
SAT	T2DM	ENSG00000138757.10	-0.004805735	0.03667407	0.002300077	0.081436229	-0.004855835	0.034749639	0.002299959	0.081418386	5.01E-05	0.043588674	1	0.266450175	1	0.272172417	ENSG00000180155	14	LYNX1
SAT	T2DM	ENSG00000138767.8	-0.002460708	0.107473648	0.001528717	0.081436525	-0.002642638	0.08403993	0.001529556	0.081418632	0.00018193	0.001047647	1	0.435334617	1	0.414575198	ENSG00000180185	7	FAHD1
SAT	T2DM	ENSG00000138821.8	-0.00077277	0.825161575	0.003498102	0.08143695	-0.000882898	0.800722112	0.003497832	0.08141912	0.000110127	8.82E-05	1	0.955051074	1	0.952160336	ENSG00000180185	7	FAHD1
SAT	T2DM	ENSG00000139190.12	0.002443029	0.178189775	0.001814555	0.081436655	0.002359415	0.193492646	0.001814491	0.081418848	8.36E-05	0.000155509	1	0.530526907	1	0.561375505	ENSG00000180185	7	FAHD1
SAT	T2DM	ENSG00000139192.7	-0.001066353	0.219303927	0.000868095	0.081436706	-0.001030769	0.235076024	0.0008681	0.081418895	-3.56E-05	0.006842311	1	0.584513552	1	0.611160407	ENSG00000180185	7	FAHD1
SAT	T2DM	ENSG00000139200.9	0.002330904	0.556427527	0.003963067	0.081436901	0.002544108	0.520921628	0.003963243	0.081419062	-0.000213205	0.002171355	1	0.835948344	1	0.826644476	ENSG00000180211	5	RP1-278E11.3
SAT	T2DM	ENSG00000139343.6	-0.001928975	0.295841774	0.001845215	0.081436776	-0.001987793	0.28132279	0.00184507	0.081418937	5.88E-05	5.15E-05	1	0.653201117	1	0.647765649	ENSG00000180211	5	RP1-278E11.3
SAT	T2DM	ENSG00000139344.3	0.00309971	0.379810511	0.003529432	0.08143683	0.003006197	0.394315043	0.003529151	0.08141901	9.35E-05	0.000606725	1	0.732172907	1	0.754765468	ENSG00000180353	6	HCLS1
SAT	T2DM	ENSG00000139405.11	-0.000924979	0.565130977	0.001607995	0.081436903	-0.000958949	0.55089925	0.001607858	0.081419071	3.40E-05	0.002453302	1	0.837959912	1	0.842050121	ENSG00000180398	7	MCFD2
SAT	T2DM	ENSG00000139624.8	-0.005747444	0.075517186	0.00323378	0.081436431	-0.005854133	0.070241947	0.0032337	0.081418583	0.000106689	0.011269687	1	0.382364025	1	0.386976108	ENSG00000180398	7	MCFD2
SAT	T2DM	ENSG00000139971.11	-0.001530852	0.432022564	0.001948304	0.081436855	-0.001570519	0.420151044	0.001948148	0.081419022	3.97E-05	0.008068386	1	0.772274669	1	0.769260924	ENSG00000180481	6	GLIPR1L2
SAT	T2DM	ENSG00000140319.6	0.007254543	0.015633641	0.003001011	0.081435982	0.007303191	0.014942245	0.003000755	0.081418141	-4.86E-05	0.019717312	1	0.159794321	1	0.166753416	ENSG00000180535	3	BHLHA15
SAT	T2DM	ENSG00000140386.8	-0.000667803	0.769898875	0.002283031	0.081436944	-0.000701781	0.758525855	0.002282827	0.081419115	3.40E-05	0.019224328	1	0.935400227	1	0.932894921	ENSG00000180537	8	RNF182
SAT	T2DM	ENSG00000140471.12	0.001189239	0.513168433	0.001818651	0.081436887	0.001234175	0.497351376	0.001818537	0.081419054	-4.49E-05	0.010446215	1	0.81574885	1	0.81497549	ENSG00000180537	8	RNF182
SAT	T2DM	ENSG00000140876.7	-0.007800035	0.053911549	0.004046603	0.081436338	-0.007695781	0.057181299	0.004046307	0.081418526	-0.000104254	0.002478655	1	0.322192699	1	0.345923743	ENSG00000180543	3	TSPYL5
SAT	T2DM	ENSG00000140993.6	0.007213607	0.026060408	0.003241608	0.081436131	0.007161191	0.027152614	0.00324135	0.081418315	5.24E-05	0.036110204	1	0.221604234	1	0.240777641	ENSG00000180543	3	TSPYL5
SAT	T2DM	ENSG00000141012.8	-0.004951388	0.166066208	0.00357512	0.081436638	-0.005007663	0.161272195	0.003574839	0.081418803	5.63E-05	0.046044114	1	0.518423625	1	0.520626624	ENSG00000180610	9	ZBTB12P1
SAT	T2DM	ENSG00000141258.8	-0.003262402	0.222345476	0.002673406	0.08143671	-0.003309948	0.215640428	0.002673181	0.081418875	4.75E-05	0.012389255	1	0.587161187	1	0.587638077	ENSG00000180610	9	ZBTB12P1
SAT	T2DM	ENSG00000141391.9	0.000181965	0.924244678	0.001913645	0.081436957	0.000226136	0.905924139	0.001913473	0.081419129	-4.42E-05	0.000225824	1	0.978648294	1	0.975470314	ENSG00000180773	10	SLC36A4
SAT	T2DM	ENSG00000141452.5	-0.010326129	0.000128161	0.002696119	0.081434507	-0.010261089	0.000141143	0.002695897	0.08141671	-6.50E-05	0.00104193	0.420494863	0.006094128	0.463090023	0.007718167	ENSG00000180785	8	OR51E1
SAT	T2DM	ENSG00000141458.8	-0.016496259	6.72E-06	0.003663866	0.081433571	-0.016433383	7.27E-06	0.003663559	0.081415769	-6.29E-05	0.016413767	0.022048263	0.000501097	0.023857506	0.000627829	ENSG00000180822	7	PSMG4
SAT	T2DM	ENSG00000141519.10	0.003923258	0.289670348	0.00370522	0.081436771	0.003975843	0.283213393	0.003704904	0.081418938	-5.26E-05	0.041688795	1	0.646536335	1	0.649244222	ENSG00000180822	7	PSMG4
SAT	T2DM	ENSG00000141569.6	-0.000616948	0.75946359	0.00201495	0.081436943	-0.000708828	0.724981231	0.002014806	0.08141911	9.19E-05	1.55E-07	1	0.934086217	1	0.921192527	ENSG00000180828	1	BHLHE22
SAT	T2DM	ENSG00000141577.9	0.006836584	0.078487563	0.003885453	0.081436441	0.006759602	0.081882853	0.003885124	0.081418625	7.70E-05	0.004859156	1	0.391645528	1	0.411433676	ENSG00000180828	1	BHLHE22
SAT	T2DM	ENSG00000141580.11	0.004662432	0.077842448	0.002644079	0.081436439	0.004579259	0.083269874	0.002643877	0.08141863	8.32E-05	0.000104948	1	0.389330903	1	0.414575198	ENSG00000180828	1	BHLHE22
SAT	T2DM	ENSG00000141639.7	0.005172921	0.146882367	0.003565972	0.081436607	0.005230425	0.142406975	0.003565659	0.081418771	-5.75E-05	0.014673835	1	0.492391981	1	0.500269293	ENSG00000180834	3	MAP6D1
SAT	T2DM	ENSG00000141756.14	-0.003196841	0.255273028	0.002810073	0.081436742	-0.003141186	0.263598491	0.002809829	0.081418922	-5.57E-05	0.003156272	1	0.620408004	1	0.63733725	ENSG00000180834	3	MAP6D1
SAT	T2DM	ENSG00000141759.10	-0.001673275	0.222623748	0.001372007	0.08143671	-0.001653496	0.228101495	0.001371893	0.081418888	-1.98E-05	0.049840799	1	0.587161187	1	0.602834606	ENSG00000180855	11	ZNF443
SAT	T2DM	ENSG00000141956.9	0.00185597	0.234220469	0.001560217	0.081436722	0.001905931	0.221834482	0.001560107	0.081418882	-5.00E-05	0.000310033	1	0.598969104	1	0.596100684	ENSG00000180855	11	ZNF443
SAT	T2DM	ENSG00000141971.8	0.000445449	0.850427955	0.002362218	0.081436952	0.000498904	0.832715925	0.002362016	0.081419123	-5.35E-05	0.001071594	1	0.963534946	1	0.961544037	ENSG00000180855	11	ZNF443
SAT	T2DM	ENSG00000142185.12	0.005743679	0.027806956	0.002610756	0.08143615	0.005795099	0.026427623	0.002610551	0.081418308	-5.14E-05	0.011976399	1	0.230185673	1	0.236264392	ENSG00000180867	9	PDIA3P1
SAT	T2DM	ENSG00000142330.15	0.001680209	0.390620019	0.001957159	0.081436835	0.001715754	0.380635693	0.001957001	0.081419002	-3.55E-05	0.016735151	1	0.741307668	1	0.740726993	ENSG00000180901	6	KCTD2
SAT	T2DM	ENSG00000142675.13	-0.001556934	0.138996865	0.00105231	0.081436593	-0.001540397	0.143206747	0.001052216	0.081418773	-1.65E-05	0.01273825	1	0.483935715	1	0.501004535	ENSG00000180953	7	ST20
SAT	T2DM	ENSG00000142677.3	-0.00303742	0.443379793	0.003962719	0.08143686	-0.002907586	0.463073302	0.003962389	0.081419041	-0.000129834	5.85E-06	1	0.779180021	1	0.797402301	ENSG00000180953	7	ST20
SAT	T2DM	ENSG00000142684.7	-0.004669277	0.152171868	0.003260887	0.081436616	-0.004616195	0.156848553	0.003260594	0.081418796	-5.31E-05	0.009707257	1	0.499775674	1	0.51775889	ENSG00000180953	7	ST20
SAT	T2DM	ENSG00000142794.14	-0.002525723	0.066361443	0.001375682	0.081436395	-0.002501371	0.068999615	0.001375569	0.081418578	-2.44E-05	0.016122604	1	0.358786109	1	0.38370803	ENSG00000180979	5	LRRC57
SAT	T2DM	ENSG00000142973.8	0.004396616	0.089510328	0.002589306	0.081436477	0.004345553	0.093267161	0.002589085	0.08141866	5.11E-05	0.004238583	1	0.411321268	1	0.428129221	ENSG00000180979	5	LRRC57
SAT	T2DM	ENSG00000143442.17	-0.000304667	0.897439062	0.002363646	0.081436956	-0.000218223	0.926435047	0.002363482	0.081419129	-8.64E-05	4.42E-05	1	0.970180416	1	0.981878481	ENSG00000180979	5	LRRC57
SAT	T2DM	ENSG00000143786.3	0.004667496	0.252218047	0.004076487	0.081436739	0.004737239	0.245161473	0.004076154	0.081418905	-6.97E-05	0.021502891	1	0.617791928	1	0.620663712	ENSG00000180992	5	MRPL14
SAT	T2DM	ENSG00000144026.7	-0.001444373	0.099312329	0.000876334	0.081436504	-0.001425387	0.103815782	0.000876281	0.081418689	-1.90E-05	0.028259417	1	0.424828882	1	0.441789338	ENSG00000181027	6	FKRP
SAT	T2DM	ENSG00000144152.8	0.004076864	0.10217109	0.002494378	0.081436512	0.0041172	0.098793	0.002494159	0.081418676	-4.03E-05	0.013993427	1	0.425727224	1	0.439213865	ENSG00000181027	6	FKRP
SAT	T2DM	ENSG00000144182.12	0.001147181	0.585266849	0.002102188	0.081436909	0.001087473	0.604917498	0.002102038	0.081419086	5.97E-05	0.00117083	1	0.845534946	1	0.858820558	ENSG00000181027	6	FKRP
SAT	T2DM	ENSG00000144214.5	-0.002159733	0.281627047	0.002005932	0.081436765	-0.002201876	0.272305781	0.002005773	0.08141893	4.21E-05	0.006588118	1	0.641415693	1	0.64192524	ENSG00000181027	6	FKRP
SAT	T2DM	ENSG00000144230.12	0.001634986	0.315359087	0.001628405	0.08143679	0.001599092	0.326062427	0.001628276	0.08141897	3.59E-05	0.004520353	1	0.675828325	1	0.695122755	ENSG00000181061	9	HIGD1A
SAT	T2DM	ENSG00000144426.14	0.006445889	0.00052482	0.001858794	0.081434949	0.006406391	0.000567287	0.001858641	0.081417146	3.95E-05	0.004053583	1	0.015797562	1	0.019800723	ENSG00000181061	9	HIGD1A
SAT	T2DM	ENSG00000144580.9	-0.00274143	0.49047126	0.003975622	0.081436879	-0.002816611	0.478615629	0.003975281	0.081419047	7.52E-05	0.005898374	1	0.80382562	1	0.80363893	ENSG00000181092	5	ADIPOQ
SAT	T2DM	ENSG00000144677.10	-0.009889657	0.010653092	0.003872395	0.081435868	-0.009806296	0.011325915	0.003872198	0.081418059	-8.34E-05	0.046507488	1	0.127894796	1	0.141833307	ENSG00000181092	5	ADIPOQ
SAT	T2DM	ENSG00000144843.7	6.83E-05	0.969881927	0.001808694	0.081436958	1.48E-05	0.993449141	0.001808569	0.081419131	5.34E-05	0.000944816	1	0.9941737	1	0.998405122	ENSG00000181092	5	ADIPOQ
SAT	T2DM	ENSG00000145020.10	0.000897334	0.348790459	0.000957729	0.081436812	0.000920139	0.336639764	0.000957653	0.081418977	-2.28E-05	0.002062651	1	0.708371701	1	0.704365099	ENSG00000181092	5	ADIPOQ
SAT	T2DM	ENSG00000145029.7	0.001538162	0.336196299	0.001599405	0.081436804	0.001575159	0.324664133	0.001599277	0.081418969	-3.70E-05	0.00268406	1	0.696147596	1	0.693504571	ENSG00000181092	5	ADIPOQ
SAT	T2DM	ENSG00000145103.8	-0.00314076	0.217107674	0.002544656	0.081436704	-0.003034706	0.233004889	0.002544495	0.081418893	-0.000106054	1.45E-05	1	0.581494104	1	0.610313962	ENSG00000181092	5	ADIPOQ
SAT	T2DM	ENSG00000145113.17	-0.001378077	0.402283613	0.001645366	0.081436841	-0.001405224	0.393037887	0.001645227	0.081419009	2.71E-05	0.019155489	1	0.752518444	1	0.753672849	ENSG00000181092	5	ADIPOQ
SAT	T2DM	ENSG00000145781.4	-0.001916392	0.467694332	0.0026388	0.08143687	-0.001864689	0.479759972	0.002638626	0.081419047	-5.17E-05	0.036255771	1	0.79334291	1	0.80363893	ENSG00000181092	5	ADIPOQ
SAT	T2DM	ENSG00000146007.6	0.000118529	0.964345458	0.002651579	0.081436958	0.00019878	0.94023598	0.002651349	0.08141913	-8.03E-05	6.53E-06	1	0.992060403	1	0.986202735	ENSG00000181104	6	F2R
SAT	T2DM	ENSG00000146109.3	0.001470929	0.523502347	0.002305689	0.08143689	0.001973878	0.392480374	0.002308282	0.081419009	-0.000502949	1.14E-05	1	0.821144329	1	0.753498015	ENSG00000181104	6	F2R
SAT	T2DM	ENSG00000146205.9	-0.002160376	0.23860656	0.001833191	0.081436726	-0.0021075	0.250255905	0.00183304	0.08141891	-5.29E-05	9.12E-05	1	0.60584911	1	0.621566711	ENSG00000181104	6	F2R
SAT	T2DM	ENSG00000146263.7	-0.005384117	0.153954568	0.003776463	0.081436619	-0.005310233	0.15965079	0.003776168	0.0814188	-7.39E-05	0.013458689	1	0.504116703	1	0.519656987	ENSG00000181104	6	F2R
SAT	T2DM	ENSG00000146425.6	-0.003800043	0.290259255	0.003593237	0.081436772	-0.003879598	0.280238227	0.003592934	0.081418936	7.96E-05	0.001784204	1	0.646746051	1	0.646143093	ENSG00000181126	9	HLA-V
SAT	T2DM	ENSG00000146453.8	0.001314965	0.734326849	0.003874659	0.081436939	0.001426838	0.712665032	0.003874334	0.081419108	-0.000111873	5.70E-05	1	0.923192618	1	0.915526221	ENSG00000181126	9	HLA-V
SAT	T2DM	ENSG00000146535.9	-0.00040688	0.925028395	0.004323833	0.081436957	-0.000482405	0.911157371	0.00432344	0.081419129	7.55E-05	0.004196027	1	0.978648294	1	0.978562139	ENSG00000181126	9	HLA-V
SAT	T2DM	ENSG00000146555.14	-0.001385686	0.466726853	0.001903898	0.08143687	-7.79E-05	0.967871723	0.001933552	0.081419131	-0.001307807	0.0001118	1	0.79334291	1	0.995169891	ENSG00000181126	9	HLA-V
SAT	T2DM	ENSG00000146556.10	-0.000138418	0.97058475	0.003753717	0.081436958	-6.99E-05	0.985142734	0.003753404	0.081419131	-6.85E-05	0.011770028	1	0.994419011	1	0.997162029	ENSG00000181126	9	HLA-V
SAT	T2DM	ENSG00000146733.9	0.007359933	0.038668907	0.003559481	0.081436244	0.007303247	0.040174712	0.003559168	0.081418427	5.67E-05	0.015083981	1	0.272844483	1	0.2890641	ENSG00000181126	9	HLA-V
SAT	T2DM	ENSG00000147457.9	0.002906456	0.359876055	0.003174365	0.081436818	0.003062242	0.334800294	0.003174982	0.081418975	-0.000155786	0.046522885	1	0.71830389	1	0.702779133	ENSG00000181126	9	HLA-V
SAT	T2DM	ENSG00000147509.9	-0.000694282	0.81648996	0.002991796	0.081436949	-0.000654124	0.826916236	0.002991532	0.081419123	-4.02E-05	0.038697129	1	0.95232974	1	0.960053847	ENSG00000181126	9	HLA-V
SAT	T2DM	ENSG00000147576.11	-0.005456844	0.005914443	0.001982505	0.081435692	-0.003510539	0.084095897	0.002032264	0.081418632	-0.001946305	1.39E-05	1	0.084370819	1	0.414575198	ENSG00000181126	9	HLA-V
SAT	T2DM	ENSG00000147894.10	0.002398935	0.529248273	0.003812951	0.081436892	0.002499882	0.512032163	0.003812673	0.081419059	-0.000100947	0.001911564	1	0.823749329	1	0.821219305	ENSG00000181218	4	HIST3H2A
SAT	T2DM	ENSG00000148229.8	-0.001612845	0.638724182	0.003435358	0.081436922	-0.001552586	0.651282609	0.003435066	0.081419097	-6.03E-05	0.012117551	1	0.874326778	1	0.891013292	ENSG00000181218	4	HIST3H2A
SAT	T2DM	ENSG00000148396.14	0.006888877	0.009463648	0.002654832	0.081435833	0.006932081	0.009019349	0.002654617	0.081417992	-4.32E-05	0.031133029	1	0.116626425	1	0.123302012	ENSG00000181222	10	POLR2A
SAT	T2DM	ENSG00000148843.9	0.00104136	0.790360808	0.003917211	0.081436947	0.000959983	0.806388288	0.003916891	0.081419121	8.14E-05	0.005317451	1	0.94681813	1	0.952410029	ENSG00000181291	5	TMEM132E
SAT	T2DM	ENSG00000149054.10	0.001005512	0.496946514	0.001480212	0.081436881	0.001034021	0.484792179	0.001480098	0.081419049	-2.85E-05	0.016946764	1	0.807969034	1	0.806185068	ENSG00000181291	5	TMEM132E
SAT	T2DM	ENSG00000149100.8	-0.01067829	0.000562712	0.003096053	0.08143497	-0.010746473	0.000518027	0.003095824	0.081417118	6.82E-05	0.009006468	1	0.016484453	1	0.018677439	ENSG00000181315	6	ZNF322
SAT	T2DM	ENSG00000149179.9	0.004403461	0.257910725	0.003892238	0.081436745	0.004294704	0.269813006	0.003891912	0.081418927	0.000108758	9.86E-05	1	0.622209623	1	0.640098679	ENSG00000181315	6	ZNF322
SAT	T2DM	ENSG00000149308.12	0.001113835	0.65448267	0.002488773	0.081436925	0.001178285	0.635872714	0.002488575	0.081419093	-6.44E-05	0.000848033	1	0.882995924	1	0.884482576	ENSG00000181315	6	ZNF322
SAT	T2DM	ENSG00000149380.7	0.007409183	0.020303209	0.003192644	0.081436058	0.007504038	0.018743342	0.003192386	0.081418208	-9.49E-05	9.44E-05	1	0.189785835	1	0.18980526	ENSG00000181315	6	ZNF322
SAT	T2DM	ENSG00000149480.2	-0.000702921	0.820654614	0.003100623	0.08143695	-0.000638269	0.83689684	0.003100434	0.081419124	-6.47E-05	0.034569943	1	0.953693013	1	0.963362294	ENSG00000181315	6	ZNF322
SAT	T2DM	ENSG00000150316.7	-0.000696668	0.710453958	0.001876564	0.081436935	-0.000660018	0.725028952	0.001876405	0.08141911	-3.67E-05	0.005491126	1	0.907710061	1	0.921192527	ENSG00000181396	8	OGFOD3
SAT	T2DM	ENSG00000150656.10	0.001870133	0.539552887	0.003048354	0.081436896	0.00195053	0.52222871	0.003048113	0.081419062	-8.04E-05	0.000721469	1	0.831736309	1	0.826644476	ENSG00000181619	11	GPR135
SAT	T2DM	ENSG00000151006.7	-0.008404719	0.009768844	0.003252706	0.081435843	-0.008535582	0.008687971	0.003252759	0.08141798	0.000130863	0.011313822	1	0.11916337	1	0.121125172	ENSG00000181619	11	GPR135
SAT	T2DM	ENSG00000151117.4	-0.002150846	0.333901714	0.002225891	0.081436802	-0.002188784	0.325409196	0.002225723	0.081418969	3.79E-05	0.038827213	1	0.695221474	1	0.694192179	ENSG00000181619	11	GPR135
SAT	T2DM	ENSG00000151208.12	-0.003234376	0.218892877	0.002630688	0.081436706	-0.003177576	0.227061185	0.002630519	0.081418887	-5.68E-05	0.023047398	1	0.583892301	1	0.601281476	ENSG00000181625	13	SLX1B
SAT	T2DM	ENSG00000151348.9	-0.000718105	0.568146695	0.001258103	0.081436904	-0.000740162	0.556284491	0.001257989	0.081419073	2.21E-05	0.003994074	1	0.840032807	1	0.842050121	ENSG00000181626	10	ANKRD62
SAT	T2DM	ENSG00000151552.7	-0.002495521	0.089249924	0.001468497	0.081436476	-0.002529208	0.084986474	0.001468368	0.081418635	3.37E-05	0.000481435	1	0.410699859	1	0.414941399	ENSG00000181744	4	C3orf58
SAT	T2DM	ENSG00000152219.4	-0.017421228	2.80E-07	0.003391869	0.08143255	-0.017520121	2.40E-07	0.003391574	0.081414672	9.89E-05	1.55E-05	0.00092031	3.54E-05	0.000785911	4.37E-05	ENSG00000181744	4	C3orf58
SAT	T2DM	ENSG00000152240.8	-0.001203241	0.245341095	0.001035722	0.081436733	-0.001188136	0.251278293	0.001035637	0.081418911	-1.51E-05	0.049446608	1	0.61016229	1	0.622908325	ENSG00000181754	6	AMIGO1
SAT	T2DM	ENSG00000152465.13	-0.004809679	0.235313745	0.004052703	0.081436723	-0.004734687	0.242657004	0.004052377	0.081418903	-7.50E-05	0.015033319	1	0.600737047	1	0.620663712	ENSG00000181754	6	AMIGO1
SAT	T2DM	ENSG00000152492.9	0.000305893	0.850839832	0.001626685	0.081436953	0.000262093	0.871988581	0.001626561	0.081419127	4.38E-05	0.000949664	1	0.963534946	1	0.966383771	ENSG00000181773	6	GPR3
SAT	T2DM	ENSG00000152527.9	0.001659821	0.209360644	0.001322222	0.081436695	0.001686164	0.20218906	0.001322128	0.081418859	-2.63E-05	0.022502293	1	0.571950268	1	0.573364137	ENSG00000181781	5	ODF3L2
SAT	T2DM	ENSG00000152784.11	-0.002416095	0.55504431	0.00409356	0.0814369	-0.002276297	0.578144141	0.004093339	0.081419079	-0.000139798	0.001189443	1	0.835948344	1	0.849979671	ENSG00000181781	5	ODF3L2
SAT	T2DM	ENSG00000152942.14	-0.001686774	0.267303931	0.001520583	0.081436753	-0.00165878	0.275285806	0.001520461	0.081418932	-2.80E-05	0.016398213	1	0.630625715	1	0.64327119	ENSG00000181781	5	ODF3L2
SAT	T2DM	ENSG00000153406.9	0.001994787	0.092965266	0.0011874	0.081436487	0.002018148	0.089176329	0.001187312	0.081418648	-2.34E-05	0.019994617	1	0.414991889	1	0.421804746	ENSG00000181826	5	RELL1
SAT	T2DM	ENSG00000153714.5	0.003051969	0.309037474	0.003000231	0.081436785	0.003163848	0.291601221	0.002999998	0.081418945	-0.000111879	3.15E-06	1	0.669717274	1	0.657104124	ENSG00000181826	5	RELL1
SAT	T2DM	ENSG00000154240.12	0.001221659	0.731283657	0.003557345	0.081436939	0.000856263	0.809956209	0.003560604	0.081419121	0.000365395	0.02335785	1	0.92246416	1	0.953864437	ENSG00000181826	5	RELL1
SAT	T2DM	ENSG00000154518.5	0.005872931	0.033693748	0.002765393	0.081436205	0.00581314	0.035535155	0.002765248	0.081418392	5.98E-05	0.042637663	1	0.256395884	1	0.274331399	ENSG00000181885	14	CLDN7
SAT	T2DM	ENSG00000154719.9	0.000714081	0.565619941	0.001242929	0.081436903	0.000734599	0.554470961	0.001242821	0.081419073	-2.05E-05	0.013617143	1	0.837959912	1	0.842050121	ENSG00000181915	3	ADO
SAT	T2DM	ENSG00000154760.9	-0.00281361	0.141009547	0.001911362	0.081436596	-0.002129838	0.267636889	0.001921331	0.081418926	-0.000683772	0.000535394	1	0.485469384	1	0.638630278	ENSG00000181915	3	ADO
SAT	T2DM	ENSG00000154803.8	0.000523052	0.875554117	0.00333989	0.081436954	0.000572239	0.863949438	0.003339612	0.081419126	-4.92E-05	0.042519759	1	0.967171747	1	0.966241215	ENSG00000182013	13	PNMAL1

SAT	T2DM	ENSG00000154889.12	-0.000300651	0.781748834	0.001085224	0.081436946	-0.00032187	0.766760421	0.001085144	0.081419116	2.12E-05	0.020598694	1	0.941848527	1	0.937659687	ENSG00000182013	13	PNMAL1
SAT	T2DM	ENSG00000155085.11	0.000497294	0.898173359	0.003886041	0.081436956	0.000419987	0.913928605	0.003885724	0.081419129	7.73E-05	0.007608618	1	0.970287017	1	0.979654273	ENSG00000182048	7	TRPC2
SAT	T2DM	ENSG00000155542.7	-0.003923804	0.028450422	0.001790853	0.081436156	-0.003949124	0.027429245	0.001790693	0.081418318	2.53E-05	0.026322033	1	0.233364583	1	0.241274407	ENSG00000182048	7	TRPC2
SAT	T2DM	ENSG00000156136.5	-0.002346407	0.550565115	0.003930897	0.081436899	-0.002422453	0.537687596	0.00393055	0.081419067	7.60E-05	0.003078061	1	0.83552458	1	0.836091471	ENSG00000182077	6	PTCHD3
SAT	T2DM	ENSG00000156172.5	-0.002537984	0.369513701	0.002828198	0.081436824	-0.002463162	0.383758556	0.002827994	0.081419004	-7.48E-05	0.002217915	1	0.723805643	1	0.745584853	ENSG00000182117	4	NOP10
SAT	T2DM	ENSG00000156411.5	-0.003073835	0.08547485	0.001787352	0.081436464	-0.003046699	0.088242513	0.001787187	0.081418645	-2.71E-05	0.009396773	1	0.402357222	1	0.421383296	ENSG00000182117	4	NOP10
SAT	T2DM	ENSG00000156463.13	0.004135665	0.255921413	0.003640276	0.081436743	0.004066794	0.263884432	0.003639976	0.081418922	6.89E-05	0.010030618	1	0.62099686	1	0.637558779	ENSG00000182134	11	TDRKH
SAT	T2DM	ENSG00000156795.2	-0.002633368	0.24020905	0.002242184	0.081436728	-0.00266611	0.234372374	0.002241988	0.081418895	3.27E-05	0.027911955	1	0.60584911	1	0.610313962	ENSG00000182134	11	TDRKH
SAT	T2DM	ENSG00000156968.8	0.001612682	0.48021816	0.002284406	0.081436875	0.00158388	0.488054285	0.002284199	0.081419051	2.88E-05	0.040351307	1	0.798174155	1	0.807924374	ENSG00000182134	11	TDRKH
SAT	T2DM	ENSG00000157045.4	-0.000167341	0.963701085	0.003677049	0.081436958	-0.000257097	0.944253648	0.003676765	0.08141913	8.98E-05	0.002319576	1	0.992060403	1	0.987598412	ENSG00000182134	11	TDRKH
SAT	T2DM	ENSG00000157214.9	0.004341759	0.036637498	0.002077609	0.081436229	0.00425384	0.04059844	0.002077463	0.08141843	8.79E-05	1.68E-06	1	0.266450175	1	0.289075371	ENSG00000182141	5	ZNF708
SAT	T2DM	ENSG00000157833.8	0.004170214	0.018036729	0.0017634	0.081436024	0.004198059	0.017272827	0.001763256	0.081418184	-2.78E-05	0.034498967	1	0.175084346	1	0.179453096	ENSG00000182141	5	ZNF708
SAT	T2DM	ENSG00000157837.11	-0.003999511	0.169725934	0.002912788	0.081436643	-0.004050202	0.164342834	0.002912525	0.081418808	5.07E-05	0.005319719	1	0.523374802	1	0.523492072	ENSG00000182141	5	ZNF708
SAT	T2DM	ENSG00000157895.7	0.008297923	0.002240692	0.002714987	0.081435397	0.008349819	0.002100165	0.002714765	0.08141755	-5.19E-05	0.010379881	1	0.044750749	1	0.049971446	ENSG00000182141	5	ZNF708
SAT	T2DM	ENSG00000158186.8	-0.007925252	0.043131303	0.003918656	0.081436275	-0.007866415	0.044687235	0.003918334	0.081418457	-5.88E-05	0.042036055	1	0.286428522	1	0.306542588	ENSG00000182141	5	ZNF708
SAT	T2DM	ENSG00000158201.5	0.00490351	0.098232781	0.00296556	0.081436502	0.005001287	0.091683571	0.002965331	0.081418656	-9.78E-05	4.33E-05	1	0.421965667	1	0.424878243	ENSG00000182150	11	ERCC6L2
SAT	T2DM	ENSG00000158411.6	-0.00123438	0.603613902	0.002377416	0.081436913	-0.001160573	0.625408796	0.002377251	0.081419091	-7.38E-05	0.000548614	1	0.852910082	1	0.876644981	ENSG00000182150	11	ERCC6L2
SAT	T2DM	ENSG00000158457.4	-0.004386691	0.045038569	0.002188641	0.081436287	-0.004437358	0.042601724	0.002188479	0.081418444	5.07E-05	0.006041824	1	0.292287338	1	0.296764878	ENSG00000182150	11	ERCC6L2
SAT	T2DM	ENSG00000158552.8	-0.000478642	0.886263066	0.003346341	0.081436955	-0.000598296	0.858090107	0.003346061	0.081419126	0.000119654	6.60E-07	1	0.969599573	1	0.965566132	ENSG00000182154	7	MRPL41
SAT	T2DM	ENSG00000158623.10	-0.001657899	0.151985124	0.0011573	0.081436615	-0.00167599	0.147530462	0.001157202	0.08141878	1.81E-05	0.025503576	1	0.499700102	1	0.506773649	ENSG00000182230	7	FAM153B
SAT	T2DM	ENSG00000158869.6	0.003719904	0.137178047	0.002502653	0.081436589	0.003754187	0.133559858	0.002502436	0.081418755	-3.43E-05	0.042639473	1	0.480267343	1	0.486825442	ENSG00000182240	11	BACE2
SAT	T2DM	ENSG00000159111.8	-0.001559723	0.620048765	0.003145989	0.081436917	-0.001636363	0.602934252	0.003145727	0.081419086	7.66E-05	0.000774243	1	0.863241392	1	0.858233093	ENSG00000182240	11	BACE2
SAT	T2DM	ENSG00000159579.9	-0.00498071	0.182573693	0.003736819	0.081436662	-0.004868015	0.192637005	0.003736523	0.081418847	-0.000112695	9.98E-05	1	0.537241514	1	0.560852203	ENSG00000182240	11	BACE2
SAT	T2DM	ENSG00000159588.10	-0.004302662	0.264654294	0.003857302	0.08143675	-0.004118526	0.28561134	0.003857021	0.08141894	-0.000184136	2.32E-08	1	0.627688499	1	0.651854486	ENSG00000182240	11	BACE2
SAT	T2DM	ENSG00000159904.7	-0.002542482	0.219596047	0.002071092	0.081436707	-0.002512626	0.225019639	0.00207092	0.081418885	-2.99E-05	0.046477809	1	0.58481707	1	0.599260906	ENSG00000182240	11	BACE2
SAT	T2DM	ENSG00000160049.7	0.004731339	0.115718894	0.003007839	0.081436545	0.004846522	0.10709036	0.003007622	0.081418697	-0.000115183	9.56E-06	1	0.447728409	1	0.448503105	ENSG00000182240	11	BACE2
SAT	T2DM	ENSG00000160179.14	0.001090884	0.680352575	0.002647891	0.08143693	0.001155859	0.662430873	0.002647653	0.081419099	-6.50E-05	7.95E-05	1	0.892786811	1	0.895728881	ENSG00000182362	9	YBEY
SAT	T2DM	ENSG00000160194.13	0.000193255	0.81157954	0.000810671	0.081436949	0.000213868	0.791904685	0.000810602	0.081419119	-2.06E-05	0.000279552	1	0.951539016	1	0.948608715	ENSG00000182362	9	YBEY
SAT	T2DM	ENSG00000160201.7	-0.003766091	0.214301917	0.003032708	0.081436701	-0.003683731	0.224454614	0.003032453	0.081418884	-8.24E-05	0.000134861	1	0.576804422	1	0.598728122	ENSG00000182389	14	CACNB4
SAT	T2DM	ENSG00000160226.11	0.00063652	0.606728611	0.001236563	0.081436914	0.000616063	0.618309323	0.001236457	0.081419089	2.05E-05	0.015858551	1	0.854526494	1	0.87067506	ENSG00000182446	9	NPLOC4
SAT	T2DM	ENSG00000160299.12	0.000516054	0.736768077	0.00153525	0.08143694	0.000553909	0.718233773	0.001535137	0.081419109	-3.79E-05	0.003736177	1	0.924058127	1	0.918559531	ENSG00000182446	9	NPLOC4
SAT	T2DM	ENSG00000160321.10	-0.000558737	0.890890724	0.004073103	0.081436955	-0.000688613	0.86573754	0.004072821	0.081419126	0.000129876	0.000390422	1	0.970180416	1	0.966241215	ENSG00000182472	4	CAPN12
SAT	T2DM	ENSG00000160323.14	-0.000765085	0.824969223	0.003459443	0.08143695	-0.000872719	0.800814939	0.003459151	0.08141912	0.000107634	1.04E-05	1	0.955051074	1	0.952160336	ENSG00000182511	7	FES
SAT	T2DM	ENSG00000160339.11	0.003598193	0.147447466	0.002483896	0.081436608	0.003910715	0.115888524	0.002487305	0.081418718	-0.000312522	0.02088501	1	0.492642703	1	0.457557457	ENSG00000182511	7	FES
SAT	T2DM	ENSG00000160783.15	0.001856793	0.521976466	0.00289987	0.08143689	0.001922878	0.50724066	0.002899653	0.081419057	-6.61E-05	0.006155199	1	0.821144329	1	0.817738235	ENSG00000182511	7	FES
SAT	T2DM	ENSG00000160856.16	-0.003169906	0.266342437	0.00285185	0.081436752	-0.003232383	0.256994885	0.002851623	0.081418916	6.25E-05	0.004834734	1	0.630350538	1	0.629723837	ENSG00000182512	4	GLRX5
SAT	T2DM	ENSG00000160948.9	0.003612786	0.003342325	0.001231193	0.081435519	0.003632437	0.003172084	0.001231099	0.081417676	-1.97E-05	0.049294353	1	0.059276589	1	0.064643525	ENSG00000182533	6	CAV3
SAT	T2DM	ENSG00000160953.10	0.004470581	0.25656993	0.003940446	0.081436743	0.004412831	0.262722394	0.003940094	0.081418921	5.78E-05	0.020913932	1	0.62099686	1	0.636156586	ENSG00000182534	9	MXRA7
SAT	T2DM	ENSG00000161010.10	-0.002800125	0.389352187	0.003252957	0.081436835	-0.002749986	0.397856731	0.003252666	0.081419011	-5.01E-05	0.014784164	1	0.740559146	1	0.756180206	ENSG00000182534	9	MXRA7
SAT	T2DM	ENSG00000161036.6	0.001096569	0.582076703	0.001992479	0.081436908	0.001048894	0.598566016	0.001992337	0.081419085	4.77E-05	0.006153649	1	0.845212395	1	0.855952281	ENSG00000182534	9	MXRA7
SAT	T2DM	ENSG00000161243.4	-4.89E-05	0.987324677	0.003078336	0.081436958	5.15E-05	0.986645136	0.003078083	0.081419131	-0.000100428	1.00E-05	1	0.996461007	1	0.997162029	ENSG00000182544	8	MFSD5
SAT	T2DM	ENSG00000161249.16	0.001688814	0.556449614	0.002871529	0.081436901	0.00163296	0.569547521	0.002871298	0.081419077	5.59E-05	0.010651034	1	0.835948344	1	0.845822679	ENSG00000182606	10	TRAK1
SAT	T2DM	ENSG00000161328.10	-0.001968208	0.426343201	0.002474285	0.081436853	-0.00200615	0.417439534	0.002474065	0.081419021	3.79E-05	0.016945467	1	0.77145714	1	0.768154297	ENSG00000182670	9	TTC3
SAT	T2DM	ENSG00000161664.2	0.000200645	0.949561777	0.003171894	0.081436958	0.000146275	0.963214666	0.003171617	0.081419131	5.44E-05	0.009790298	1	0.987171163	1	0.993672317	ENSG00000182685	3	BRICD5
SAT	T2DM	ENSG00000161904.7	0.000465029	0.86361175	0.002707145	0.081436953	-0.000105722	0.96885971	0.002708145	0.081419131	0.000570751	9.54E-12	1	0.965214867	1	0.995561763	ENSG00000182771	13	GRID1
SAT	T2DM	ENSG00000161912.13	-0.001269927	0.555738488	0.002155402	0.0814369	-0.001330354	0.537062157	0.00215525	0.081419067	6.04E-05	0.001549101	1	0.835948344	1	0.835911261	ENSG00000182796	8	TMEM198B
SAT	T2DM	ENSG00000161996.13	-0.001136311	0.502350747	0.001693973	0.081436883	-0.001101317	0.515570848	0.001693844	0.08141906	-3.50E-05	0.010989074	1	0.810314363	1	0.824105482	ENSG00000182796	8	TMEM198B
SAT	T2DM	ENSG00000162244.6	0.000210333	0.943402079	0.002962661	0.081436958	0.000141456	0.961915298	0.002962412	0.081419131	6.89E-05	0.001168224	1	0.98331034	1	0.993672317	ENSG00000182827	8	ACBD3
SAT	T2DM	ENSG00000162341.11	0.002228667	0.559145025	0.003815477	0.081436901	0.002457855	0.519449008	0.003815387	0.081419062	-0.000229188	4.64E-06	1	0.837033211	1	0.826644476	ENSG00000182841	8	RRP7B
SAT	T2DM	ENSG00000162383.7	0.001535651	0.392330133	0.001795243	0.081436836	0.001507728	0.400954296	0.001795087	0.081419013	2.79E-05	0.020429552	1	0.742778516	1	0.757591271	ENSG00000182851	2	GPIHBP1
SAT	T2DM	ENSG00000162384.9	-0.008997015	0.004269912	0.003148546	0.081435594	-0.008913031	0.004639404	0.003148287	0.081417792	-8.40E-05	0.000299019	1	0.06901272	1	0.0804112	ENSG00000182858	9	ALG12
SAT	T2DM	ENSG00000162402.8	-0.000987712	0.679309982	0.00238921	0.08143693	-0.00106667	0.655244267	0.002389018	0.081419098	7.90E-05	1.54E-05	1	0.892786811	1	0.891871358	ENSG00000182858	9	ALG12
SAT	T2DM	ENSG00000162885.8	-0.001587348	0.478543909	0.00223997	0.081436874	-0.001645159	0.462635094	0.002239789	0.081419041	5.78E-05	0.000653631	1	0.796855602	1	0.797402301	ENSG00000182858	9	ALG12
SAT	T2DM	ENSG00000162994.11	0.001469042	0.03255195	0.000687248	0.081436195	0.001481192	0.031129127	0.000687193	0.081418355	-1.22E-05	0.021374064	1	0.252459754	1	0.262336831	ENSG00000182871	10	COL18A1
SAT	T2DM	ENSG00000163041.5	0.002844008	0.100675937	0.001732473	0.081436508	0.002780936	0.108427365	0.001732341	0.0814187	6.31E-05	7.84E-06	1	0.425727224	1	0.450887431	ENSG00000182871	10	COL18A1
SAT	T2DM	ENSG00000163138.14	0.001740398	0.393487229	0.002039582	0.081436837	0.001808268	0.375261242	0.00203941	0.081419	-6.79E-05	2.61E-06	1	0.743169868	1	0.736823541	ENSG00000182902	9	SLC25A18
SAT	T2DM	ENSG00000163161.8	0.002907597	0.056788469	0.001526346	0.081436352	0.002880653	0.059100947	0.001526218	0.081418536	2.69E-05	0.013626404	1	0.330946654	1	0.351641584	ENSG00000182902	9	SLC25A18
SAT	T2DM	ENSG00000163281.7	0.000319103	0.777559343	0.001129563	0.081436945	0.000356892	0.752018989	0.001129481	0.081419114	-3.78E-05	8.39E-05	1	0.939432466	1	0.929871654	ENSG00000182919	10	C11orf54
SAT	T2DM	ENSG00000163297.12	0.002252423	0.565411593	0.003918466	0.081436903	0.003267524	0.405580107	0.003928741	0.081419015	-0.001015102	0.000461137	1	0.837959912	1	0.759868776	ENSG00000182919	10	C11orf54
SAT	T2DM	ENSG00000163389.6	-0.000419484	0.625778302	0.000860166	0.081436919	-0.000373721	0.663958674	0.000860207	0.081419099	-4.58E-05	0.002674368	1	0.866359035	1	0.895996588	ENSG00000182919	10	C11orf54
SAT	T2DM	ENSG00000163453.7	-0.001694331	0.605610251	0.003281362	0.081436914	-0.001167462	0.722384921	0.003286094	0.08141911	-0.000526869	0.003956989	1	0.853892236	1	0.920812547	ENSG00000182919	10	C11orf54
SAT	T2DM	ENSG00000163521.11	-0.00022821	0.934981515	0.002797407	0.081436957	-0.000327509	0.906791971	0.002797167	0.081419129	9.93E-05	2.54E-07	1	0.981839134	1	0.975470314	ENSG00000182919	10	C11orf54
SAT	T2DM	ENSG00000163545.7	0.001111966	0.639187577	0.002371762	0.081436922	0.000911881	0.700673535	0.002372148	0.081419106	0.000200084	0.00030033	1	0.874326778	1	0.909587128	ENSG00000182919	10	C11orf54
SAT	T2DM	ENSG00000163554.7	-0.004818244	0.001600321	0.001526758	0.081435294	-0.004697565	0.002098839	0.001527219	0.08141755	-0.000120678	0.005845953	1	0.036211407	1	0.049971446	ENSG00000182919	10	C11orf54
SAT	T2DM	ENSG00000163576.13	-0.000269512	0.670197108	0.000632839	0.081436928	-0.00029024	0.646475171	0.000632793	0.081419096	2.07E-05	0.000129756	1	0.889125844	1	0.890183286	ENSG00000182923	12	CEP63
SAT	T2DM	ENSG00000163596.12	-0.00808681	0.000625091	0.002364212	0.081435003	-0.008032222	0.000679613	0.00236402	0.081417202	-5.46E-05	0.002039068	1	0.017680364	1	0.022298088	ENSG00000182923	12	CEP63
SAT	T2DM	ENSG00000163611.7	0.002070852	0.505177963	0.003107683	0.081436884	0.002151111	0.488783828	0.003107442	0.081419051	-8.03E-05	0.001270742	1	0.810314363	1	0.808724024	ENSG00000182923	12	CEP63
SAT	T2DM	ENSG00000163743.9	-0.004079907	0.124673184	0.002657135	0.081436564	-0.00419662	0.114262258	0.002657224	0.081418714	0.000116713	0.009437878	1	0.463429598	1	0.455762334	ENSG00000182923	12	CEP63
SAT	T2DM	ENSG00000163807.4	4.26E-05	0.973147314	0.001264565	0.081436958	6.85E-05	0.956818689	0.001264459	0.08141913	-2.59E-05	0.004606789	1	0.995031844	1	0.993142081	ENSG00000182923	12	CEP63
SAT	T2DM	ENSG00000163945.11	0.002112884	0.117570734	0.001350038	0.081436549	0.002153399	0.110673837	0.001349946	0.081418706	-4.05E-05	0.000909048	1	0.451697397	1	0.452205306	ENSG00000182950	2	ODF3L1
SAT	T2DM	ENSG00000164031.12	0.004530854	0.066615132	0.002470118	0.081436396	0.004598991	0.062603472	0.002469918	0.081418552	-6.81E-05	0.000252133	1	0.358786109	1	0.362901045	ENSG00000182952	4	HMGN4
SAT	T2DM	ENSG00000164039.10	-0.007833929	0.00024875	0.002138331	0.081434715	-0.007877137	0.000229539	0.002138146	0.081416863	4.32E-05	0.002742951	0.816147259	0.009170194	0.753118457	0.009909453	ENSG00000182952	4	HMGN4
SAT	T2DM	ENSG00000164080.9	-0.001439396	0.652270206	0.003194321	0.081436924	-0.001493271	0.640133055	0.003194073	0.081419094	5.39E-05	0.035155628	1	0.882995924	1	0.885818875	ENSG00000182952	4	HMGN4
SAT	T2DM	ENSG00000164144.10	-0.000488259	0.888466997	0.00348149	0.081436955	-0.000572212	0.869438106	0.003481193	0.081419126	8.40E-05	0.000529197	1	0.969612316	1	0.966336865	ENSG00000182952	4	HMGN4
SAT	T2DM	ENSG00000164253.8	-0.007129774	0.029181936	0.003268999	0.081436163	-0.007081261	0.030282614	0.003268706	0.081418347	-4.85E-05	0.018245245	1	0.236994883	1	0.256527252	ENSG00000182952	4	HMGN4
SAT	T2DM	ENSG00000164332.6	0.002781477	0.237725217	0.0023558	0.081436725	0.002818107	0.231562065	0.002355595	0.081418892	-3.66E-05	0.020463651	1	0.604632897	1	0.608290741	ENSG00000182952	4	HMGN4
SAT	T2DM	ENSG00000164556.7	-0.003470609	0.205760531	0.002742891	0.081436691	-0.003529483	0.198133168	0.002742645	0.081418854	5.89E-05	0.000684305	1	0.568086176	1	0.566556767	ENSG00000182952	4	HMGN4
SAT	T2DM	ENSG00000164609.5	-0.000854452	0.830998839	0.004003603	0.081436951	-0.00091948	0.818339805	0.004003291	0.081419122	6.50E-05	0.040856735	1	0.95668325	1	0.957271954	ENSG00000182952	4	HMGN4
SAT	T2DM	ENSG00000164615.3	0.006972839	0.004228602	0.002437546	0.081435591	0.00692952	0.00446827	0.002437334	0.08141778	4.33E-05	0.008356084	1	0.068881061	1	0.079676052	ENSG00000182986	8	ZNF320
SAT	T2DM	ENSG00000164825.3	-0.004391514	0.222530053	0.003600104	0.08143671	-0.004285101	0.23390622	0.003599842	0.081418894	-0.000106413	0.000537109	1	0.587161187	1	0.610313962	ENSG00000182986	8	ZNF320
SAT	T2DM	ENSG00000164830.13	0.004848067	0.187881413	0.003681496	0.081436669	0.004990351	0.175218653	0.003681213	0.081418824	-0.000142284	1.89E-06	1	0.54503883	1	0.535281564	ENSG00000182986	8	ZNF320
SAT	T2DM	ENSG00000164850.10	0.000845283	0.65103309	0.001868739	0.081436924	0.000810297	0.664552806	0.001868611	0.081419099	3.50E-05	0.037552773	1	0.882256298	1	0.895996588	ENSG00000182986	8	ZNF320
SAT	T2DM	ENSG00000164904.11	0.001165529	0.663376838	0.002677801	0.081436927	0.001210189	0.65129043	0.002677583	0.081419097	-4.47E-05	0.026012984	1	0.887596281	1	0.891013292	ENSG00000183060	11	LYSMD4
SAT	T2DM	ENSG00000164924.13	-0.000810675	0.66508543	0.001872648	0.081436927	-0.000759862	0.68488944	0.001872501	0.081419103	-5.08E-05	0.0005434	1	0.888056281	1	0.901372745	ENSG00000183060	11	LYSMD4
SAT	T2DM	ENSG00000165202.2	-0.005956156	0.006715353	0.002197315	0.081435731	-0.006007386	0.006253838	0.002197145	0.081417882	5.12E-05	0.003592929	1	0.090914049	1	0.095436475	ENSG00000183060	11	LYSMD4
SAT	T2DM	ENSG00000165244.6	0.005733924	0.138443568	0.003870051	0.081436592	0.005630564	0.145660017	0.003869708	0.081418777	0.00010336	3.30E-05	1	0.483226964	1	0.503061344	ENSG00000183172	8	SMDT1
SAT	T2DM	ENSG00000165475.9	-0.001090103	0.605930655	0.002113049	0.081436914	-0.001045503	0.620724661	0.002112878	0.08141909	-4.46E-05	0.00525494	1	0.85397701	1	0.87257824	ENSG00000183172	8	SMDT1
SAT	T2DM	ENSG00000165476.8	-0.010033452	0.000986441	0.003045621	0.081435145	-0.009984603	0.001043225	0.003045366	0.081417335	-4.88E-05	0.024218892	1	0.024896248	1	0.029763665	ENSG00000183208	8	GDPGP1
SAT	T2DM	ENSG00000165478.6	0.002688211	0.122431179	0.001740335	0.08143656	0.002775003	0.110863593	0.001740549	0.081418706	-8.68E-05	0.020783506	1	0.459607206	1	0.452417225	ENSG00000183208	8	GDPGP1
SAT	T2DM	ENSG00000165568.13	-0.006303164	0.05887613	0.003336558	0.081436362	-0.006372416	0.056130478	0.003336298	0.081418521	6.93E-05	0.009205317	1	0.337124925	1	0.342949902	ENSG00000183208	8	GDPGP1
SAT	T2DM	ENSG00000165629.15	-0.003963342	0.328367687	0.004054951	0.081436799	-0.003907235	0.335217721	0.004054586	0.081418976	-5.61E-05	0.02566798	1	0.692334629	1	0.702779133	ENSG00000183250	7	C21orf67
SAT	T2DM	ENSG00000165650.7	0.003498932	0.027935528	0.001591728	0.081436151	0.002177445	0.176489796	0.00161096	0.081418826	0.001321487	1.15E-07	1	0.230185673	1	0.537662971	ENSG00000183255	7	PTTG1IP
SAT	T2DM	ENSG00000165684.3	0.000455385	0.763448687	0.00151313	0.081436943	0.000501205	0.740445942	0.001513011	0.081419113	-4.58E-05	0.000114621	1	0.93459819	1	0.925595787	ENSG00000183281	10	PLGLB1
SAT	T2DM	ENSG00000165868.8	0.002233736	0.509727268	0.003388232	0.081436886	0.002151641	0.525377061	0.003387989	0.081419064	8.21E-05	0.005344007	1	0.812130745	1	0.827311811	ENSG00000183309	7	ZNF623
SAT	T2DM	ENSG00000165912.11	-0.000307447	0.851966748	0.001647538	0.081436953	-0.000360743	0.826672942	0.001647451	0.081419123	5.33E-05	0.002268386	1	0.963534946	1	0.960053847	ENSG00000183309	7	ZNF623
SAT	T2DM	ENSG00000166166.8	-0.018953919	1.17E-06	0.003900012	0.081433011	-0.019052313	1.03E-06	0.003899687	0.081415143	9.84E-05	0.000512455	0.003853313	9.88E-05	0.003384238	0.000120866	ENSG00000183346	6	C10orf107
SAT	T2DM	ENSG00000166260.6	-0.000870261	0.802882613	0.003486367	0.081436948	-0.000928	0.790083338	0.003486067	0.081419119	5.77E-05	0.015118091	1	0.948256967	1	0.947957612	ENSG00000183386	5	FHL3
SAT	T2DM	ENSG00000166262.11	0.003989177	0.187799294	0.003028714	0.081436669	0.004056652	0.180404724	0.003028455	0.081418831	-6.75E-05	0.001347256	1	0.54503883	1	0.543907656	ENSG00000183426	11	NPIPA1
SAT	T2DM	ENSG00000166278.10	0.026394479	3.24E-12	0.00378835	0.081428847	0.01966209	2.08E-06	0.004143324	0.081415368	0.006732389	6.06E-05	1.06E-08	1.18E-09	0.006826591	0.000206866	ENSG00000183426	11	NPIPA1
SAT	T2DM	ENSG00000166394.10	0.001114276	0.765927585	0.003742871	0.081436944	0.001172609	0.754038019	0.003742535	0.081419114	-5.83E-05	0.013099727	1	0.934630346	1	0.930424498	ENSG00000183426	11	NPIPA1
SAT	T2DM	ENSG00000166762.12	0.001863236	0.488820182	0.002691811	0.081436878	0.001913657	0.477096895	0.002691576	0.081419046	-5.04E-05	0.004947296	1	0.803516541	1	0.80363893	ENSG00000183426	11	NPIPA1
SAT	T2DM	ENSG00000166796.7	0.002329028	0.336298061	0.00242227	0.081436804	0.002267714	0.349135665	0.002422078	0.081418984	6.13E-05	0.001143966	1	0.696147596	1	0.717008026	ENSG00000183426	11	NPIPA1
SAT	T2DM	ENSG00000166801.11	0.000904182	0.509902266	0.001372071	0.081436886	0.001153331	0.401856573	0.00137578	0.081419013	-0.000249149	0.015539948	1	0.812130745	1	0.757591271	ENSG00000183431	7	SF3A3
SAT	T2DM	ENSG00000166839.12	-0.002754738	0.49518675	0.004038713	0.081436881	-0.002821303	0.484785407	0.004038353	0.081419049	6.66E-05	0.009634717	1	0.806594843	1	0.806185068	ENSG00000183431	7	SF3A3
SAT	T2DM	ENSG00000166912.12	0.005457378	0.162582496	0.003908063	0.081436633	0.005522289	0.157605097	0.003907719	0.081418797	-6.49E-05	0.011165285	1	0.513507272	1	0.51775889	ENSG00000183431	7	SF3A3
SAT	T2DM	ENSG00000166913.8	0.000509558	0.874014925	0.003213643	0.081436954	0.000569329	0.859371984	0.003213393	0.081419126	-5.98E-05	0.019649158	1	0.966670474	1	0.965566132	ENSG00000183444	10	OR7E38P
SAT	T2DM	ENSG00000167103.7	0.001432303	0.36695901	0.001587594	0.081436822	0.001404838	0.376177667	0.001587456	0.081419	2.75E-05	0.010311603	1	0.723118627	1	0.737979921	ENSG00000183444	10	OR7E38P
SAT	T2DM	ENSG00000167123.14	0.001504715	0.548042944	0.002504954	0.081436898	0.001468529	0.557674211	0.002504741	0.081419073	3.62E-05	0.038330744	1	0.835027823	1	0.842050121	ENSG00000183458	9	RP11-958N24.1
SAT	T2DM	ENSG00000167306.14	0.003099357	0.265511526	0.002783538	0.081436751	0.003047704	0.273517673	0.002783294	0.081418931	5.17E-05	0.005133206	1	0.628984344	1	0.64192524	ENSG00000183458	9	RP11-958N24.1
SAT	T2DM	ENSG00000167311.9	-0.001036046	0.704760144	0.002734331	0.081436934	-0.000961002	0.725223807	0.002734111	0.08141911	-7.50E-05	0.000316868	1	0.906221055	1	0.921192527	ENSG00000183458	9	RP11-958N24.1
SAT	T2DM	ENSG00000167332.7	-0.000495332	0.895502171	0.003771218	0.081436956	-0.000370286	0.921777268	0.003770913	0.081419129	-0.000125046	1.11E-05	1	0.970180416	1	0.979654273	ENSG00000183486	8	MX2
SAT	T2DM	ENSG00000167395.10	0.00607931	0.139635569	0.004115562	0.081436594	0.00617057	0.133768363	0.004115337	0.081418755	-9.13E-05	0.034235626	1	0.484402728	1	0.486825442	ENSG00000183486	8	MX2
SAT	T2DM	ENSG00000167528.8	0.004255612	0.002187311	0.001389106	0.08143539	0.004292774	0.001998061	0.001389006	0.081417535	-3.72E-05	0.002026596	1	0.044524014	1	0.049345351	ENSG00000183486	8	MX2
SAT	T2DM	ENSG00000167595.10	-0.002659965	0.09336939	0.001585304	0.081436488	-0.002636296	0.096293356	0.001585171	0.081418669	-2.37E-05	0.037060007	1	0.416229576	1	0.432792465	ENSG00000183506	12	PI4KAP2
SAT	T2DM	ENSG00000167644.7	0.001664867	0.330192436	0.001709787	0.0814368	0.001639581	0.337548231	0.001709639	0.081418977	2.53E-05	0.027972853	1	0.692789976	1	0.704513834	ENSG00000183506	12	PI4KAP2
SAT	T2DM	ENSG00000167695.10	-0.002053957	0.391379518	0.00239635	0.081436836	-0.002015094	0.400364834	0.002396153	0.081419013	-3.89E-05	0.027927352	1	0.741834893	1	0.757591271	ENSG00000183506	12	PI4KAP2
SAT	T2DM	ENSG00000167711.9	0.00261298	0.43294711	0.003332206	0.081436856	0.002711557	0.415759516	0.003331967	0.08141902	-9.86E-05	0.000673861	1	0.772274669	1	0.766754833	ENSG00000183506	12	PI4KAP2
SAT	T2DM	ENSG00000167720.8	0.000629243	0.474772451	0.000880385	0.081436873	0.000648161	0.461558876	0.000880315	0.08141904	-1.89E-05	0.005649685	1	0.796607042	1	0.797039301	ENSG00000183506	12	PI4KAP2
SAT	T2DM	ENSG00000167785.4	0.001425511	0.445000256	0.001866396	0.081436861	0.001389158	0.456661903	0.001866257	0.081419038	3.64E-05	0.019729425	1	0.780081459	1	0.794600418	ENSG00000183527	7	PSMG1
SAT	T2DM	ENSG00000167799.5	3.55E-05	0.990820269	0.003084041	0.081436958	0.000790952	0.797954584	0.003089714	0.08141912	-0.000755469	8.79E-05	1	0.996461007	1	0.952160336	ENSG00000183527	7	PSMG1
SAT	T2DM	ENSG00000167840.9	-0.000200904	0.86030076	0.001141557	0.081436953	-0.000175952	0.877493798	0.001141457	0.081419127	-2.50E-05	0.001138882	1	0.965007451	1	0.966700287	ENSG00000183527	7	PSMG1
SAT	T2DM	ENSG00000167972.9	0.001454301	0.667151557	0.003381616	0.081436928	0.001378635	0.683480621	0.003381341	0.081419103	7.57E-05	0.002838185	1	0.888630991	1	0.901372745	ENSG00000183527	7	PSMG1
SAT	T2DM	ENSG00000168000.10	-0.002068669	0.133321502	0.00137807	0.081436582	-0.001969196	0.153156832	0.001378534	0.08141879	-9.95E-05	0.015606922	1	0.474948803	1	0.512250482	ENSG00000183562	2	AC131971.1
SAT	T2DM	ENSG00000168016.9	-0.003507149	0.359886688	0.003830514	0.081436818	-0.003617047	0.344993503	0.003830215	0.081418982	0.000109898	0.000295688	1	0.71830389	1	0.713247438	ENSG00000183562	2	AC131971.1
SAT	T2DM	ENSG00000168028.9	0.001420842	0.15732216	0.001004741	0.081436624	0.000724662	0.477163958	0.001019398	0.081419046	0.00069618	5.65E-05	1	0.507474184	1	0.80363893	ENSG00000183570	12	PCBP3
SAT	T2DM	ENSG00000168038.6	-0.001492819	0.199180125	0.001162729	0.081436683	-0.001526773	0.189121485	0.001162649	0.081418843	3.40E-05	0.001264203	1	0.559511977	1	0.55901585	ENSG00000183604	10	RP11-347C12.2
SAT	T2DM	ENSG00000168056.10	0.012743118	1.29E-05	0.002920983	0.081433778	0.011064621	0.000208443	0.002983514	0.081416833	0.001678497	0.005855984	0.04217545	0.00082697	0.68390041	0.009368499	ENSG00000183604	10	RP11-347C12.2
SAT	T2DM	ENSG00000168159.9	0.002175362	0.459971985	0.002944081	0.081436867	0.002235317	0.447665096	0.002943863	0.081419035	-6.00E-05	0.01601185	1	0.789726889	1	0.791227469	ENSG00000183604	10	RP11-347C12.2
SAT	T2DM	ENSG00000168291.8	-0.009186963	0.012914705	0.003695299	0.081435926	-0.009128977	0.013487487	0.003694985	0.081418111	-5.80E-05	0.024556454	1	0.143739766	1	0.157494043	ENSG00000183617	4	MRPL54
SAT	T2DM	ENSG00000168329.9	-0.002747407	0.498582351	0.004059872	0.081436882	0.001711667	0.683950816	0.004204763	0.081419103	-0.004459074	4.73E-05	1	0.809826086	1	0.901372745	ENSG00000183624	9	HMCES
SAT	T2DM	ENSG00000168356.7	0.000168626	0.913841408	0.001558548	0.081436956	0.000251425	0.871847222	0.00155862	0.081419127	-8.28E-05	0.002608551	1	0.976649401	1	0.966383771	ENSG00000183628	8	DGCR6
SAT	T2DM	ENSG00000168404.8	0.001324104	0.303505306	0.001286854	0.081436781	0.001348681	0.294581587	0.001286756	0.081418947	-2.46E-05	0.019094661	1	0.662542189	1	0.661548383	ENSG00000183628	8	DGCR6
SAT	T2DM	ENSG00000168405.10	0.001639097	0.664984959	0.003785082	0.081436927	0.000371277	0.922027551	0.003793184	0.081419129	0.00126782	6.01E-07	1	0.888056281	1	0.979654273	ENSG00000183628	8	DGCR6
SAT	T2DM	ENSG00000168653.6	-0.005649253	0.108676958	0.003521602	0.081436528	-0.005592353	0.112254204	0.003521311	0.081418709	-5.69E-05	0.027131898	1	0.436210685	1	0.453578872	ENSG00000183628	8	DGCR6
SAT	T2DM	ENSG00000168661.10	0.002093878	0.398889278	0.002482052	0.081436839	0.002029165	0.413583956	0.002481844	0.081419019	6.47E-05	0.000270956	1	0.750003279	1	0.766308941	ENSG00000183628	8	DGCR6
SAT	T2DM	ENSG00000168679.13	0.004913811	0.071297999	0.002724478	0.081436415	0.004987915	0.067114852	0.002724284	0.081418571	-7.41E-05	0.001896244	1	0.373189908	1	0.377708111	ENSG00000183648	5	NDUFB1
SAT	T2DM	ENSG00000168702.12	-0.00256251	0.474703833	0.003584696	0.081436873	-0.007718131	0.037369012	0.003707583	0.081418407	0.005155622	5.41E-08	1	0.796607042	1	0.280518039	ENSG00000183684	6	ALYREF

SAT	T2DM	ENSG00000168765.11	0.003557657	0.084689577	0.002063481	0.081436462	0.00360978	0.080204281	0.002063314	0.081418619	-5.21E-05	0.000881775	1	0.401115612	1	0.409385619	ENSG00000183684	6	ALYREF
SAT	T2DM	ENSG00000168813.12	-0.010857239	0.006733186	0.004006698	0.081435731	-0.010752354	0.007279334	0.004006393	0.081417927	-0.000104885	0.001303515	1	0.090914049	1	0.10521364	ENSG00000183684	6	ALYREF
SAT	T2DM	ENSG00000168827.10	-0.000228236	0.895314865	0.001734553	0.081436956	-0.000176133	0.919112196	0.001734413	0.081419129	-5.21E-05	8.13E-05	1	0.970180416	1	0.979654273	ENSG00000183688	4	FAM101B
SAT	T2DM	ENSG00000168884.10	-0.002228312	0.197647282	0.001729673	0.081436681	-0.002252327	0.192819654	0.00172952	0.081418848	2.40E-05	0.032275566	1	0.558130589	1	0.560852203	ENSG00000183762	8	KREMEN1
SAT	T2DM	ENSG00000169085.7	0.002322095	0.006473546	0.000852837	0.081435719	0.001470023	0.092903917	0.000874869	0.081418659	0.000852072	1.31E-05	1	0.088498767	1	0.428114821	ENSG00000183779	5	ZNF703
SAT	T2DM	ENSG00000169116.7	-0.000559944	0.889642011	0.004035418	0.081436955	0.003226321	0.431954286	0.004105505	0.081419028	-0.002666378	0.000433365	1	0.969612316	1	0.781358298	ENSG00000183779	5	ZNF703
SAT	T2DM	ENSG00000169203.12	0.002363501	0.110253568	0.00147991	0.081436532	0.002397776	0.105158128	0.00147979	0.081418692	-3.43E-05	0.002276897	1	0.436277088	1	0.444046099	ENSG00000183793	9	NPIPA5
SAT	T2DM	ENSG00000169247.7	-0.001952978	0.62340504	0.003977305	0.081436918	-0.001887282	0.635108258	0.003976996	0.081419093	-6.57E-05	0.038083856	1	0.864876358	1	0.884482576	ENSG00000183793	9	NPIPA5
SAT	T2DM	ENSG00000169302.10	-0.004995362	0.131843727	0.00331505	0.081436579	-0.00493928	0.136205948	0.003314793	0.08141876	-5.61E-05	0.033561644	1	0.472458584	1	0.492171492	ENSG00000183807	6	FAM162B
SAT	T2DM	ENSG00000169442.4	0.002317418	0.534460197	0.003730476	0.081436894	0.00239747	0.520408205	0.003730212	0.081419062	-8.01E-05	0.014936239	1	0.82870699	1	0.826644476	ENSG00000183807	6	FAM162B
SAT	T2DM	ENSG00000169519.15	-0.003160193	0.229989075	0.002632642	0.081436718	-0.00311041	0.237372406	0.002632413	0.081418898	-4.98E-05	0.004757015	1	0.594168627	1	0.613383258	ENSG00000183828	10	NUDT14
SAT	T2DM	ENSG00000169609.9	0.000252487	0.889805331	0.001822346	0.081436955	0.000333205	0.85494108	0.001822609	0.081419125	-8.07E-05	0.049266777	1	0.969612316	1	0.965566132	ENSG00000183828	10	NUDT14
SAT	T2DM	ENSG00000169683.3	-0.004762479	0.01764823	0.002007008	0.081436017	-0.004791944	0.016949456	0.002006836	0.081418178	2.95E-05	0.03408761	1	0.172693001	1	0.179453096	ENSG00000183828	10	NUDT14
SAT	T2DM	ENSG00000170175.6	0.001249167	0.572894263	0.002215642	0.081436905	0.001300551	0.557183896	0.002215476	0.081419073	-5.14E-05	0.005241065	1	0.840775238	1	0.842050121	ENSG00000183833	12	MAATS1
SAT	T2DM	ENSG00000170209.4	-0.003432039	0.084179945	0.001987358	0.08143646	-0.003379275	0.089032463	0.001987193	0.081418619	-5.28E-05	0.000246465	1	0.401115612	1	0.421804746	ENSG00000183833	12	MAATS1
SAT	T2DM	ENSG00000170264.8	0.000199242	0.961203247	0.004095946	0.081436958	0.00013496	0.973712432	0.004095595	0.081419131	6.43E-05	0.022796977	1	0.99110869	1	0.996491107	ENSG00000183833	12	MAATS1
SAT	T2DM	ENSG00000170454.5	0.005439587	0.045602754	0.002721077	0.081436291	0.005393357	0.047456706	0.002720861	0.081418474	4.62E-05	0.028795229	1	0.293843465	1	0.314543215	ENSG00000183844	12	FAM3B
SAT	T2DM	ENSG00000170540.10	-0.00420796	0.078543967	0.002391972	0.081436441	-0.00416631	0.081520742	0.002391783	0.081418624	-4.16E-05	0.001683456	1	0.391645528	1	0.411433676	ENSG00000183844	12	FAM3B
SAT	T2DM	ENSG00000170571.7	-0.011110069	0.000242929	0.003027573	0.081434708	-0.011055356	0.000260389	0.003027333	0.081416903	-5.47E-05	0.020238047	0.797048645	0.009057371	0.854336845	0.011095284	ENSG00000183844	12	FAM3B
SAT	T2DM	ENSG00000170632.9	0.000994479	0.68278898	0.00243351	0.081436931	0.000941016	0.698961161	0.002433306	0.081419106	5.35E-05	0.002130407	1	0.893590205	1	0.908954249	ENSG00000183844	12	FAM3B
SAT	T2DM	ENSG00000170633.12	-0.003794697	0.164305223	0.002728547	0.081436635	-0.003744919	0.169875827	0.00272833	0.081418816	-4.98E-05	0.018085574	1	0.516146667	1	0.531756789	ENSG00000183889	8	NPIPA7
SAT	T2DM	ENSG00000170667.10	-0.001948807	0.571917394	0.00344781	0.081436905	-0.002026227	0.556712825	0.003447538	0.081419073	7.74E-05	0.004065669	1	0.840736467	1	0.842050121	ENSG00000183889	8	NPIPA7
SAT	T2DM	ENSG00000170745.7	0.0048086	0.027337353	0.002179117	0.081436145	0.004761981	0.028855798	0.00217894	0.081418333	4.66E-05	0.004260912	1	0.227073047	1	0.249804413	ENSG00000183891	5	TTC32
SAT	T2DM	ENSG00000170776.15	0.005860018	0.124337411	0.003813074	0.081436564	0.006091248	0.110161618	0.003813124	0.081418705	-0.00023123	0.000108713	1	0.463429598	1	0.452205306	ENSG00000183891	5	TTC32
SAT	T2DM	ENSG00000170837.2	-0.000827332	0.811526693	0.003469522	0.081436949	-0.000951194	0.783947867	0.003469257	0.081419118	0.000123862	1.16E-05	1	0.951539016	1	0.945615795	ENSG00000183977	9	PP2D1
SAT	T2DM	ENSG00000170866.7	-0.00118498	0.655634067	0.002657207	0.081436925	-0.001127842	0.671214778	0.002656986	0.081419101	-5.71E-05	0.003090087	1	0.883423151	1	0.898343777	ENSG00000183977	9	PP2D1
SAT	T2DM	ENSG00000170955.9	-0.001547842	0.542957533	0.00254434	0.081436897	-0.001615312	0.525485466	0.002544148	0.081419064	6.75E-05	0.001253169	1	0.832700048	1	0.827311811	ENSG00000184106	4	TREML3P
SAT	T2DM	ENSG00000171045.10	0.001508782	0.297192846	0.001447311	0.081436777	0.001545045	0.285695444	0.001447195	0.08141894	-3.63E-05	0.00115466	1	0.654394836	1	0.651854486	ENSG00000184110	10	EIF3C
SAT	T2DM	ENSG00000171084.11	-0.00278566	0.145385388	0.00191319	0.081436604	-0.002813105	0.141425737	0.00191302	0.0814187	2.74E-05	0.024583206	1	0.491255879	1	0.499917672	ENSG00000184110	10	EIF3C
SAT	T2DM	ENSG00000171109.14	0.006758461	0.038312717	0.003262594	0.081436241	0.006831282	0.036261336	0.003262327	0.081418398	-7.28E-05	0.002685691	1	0.271246148	1	0.276092282	ENSG00000184110	10	EIF3C
SAT	T2DM	ENSG00000171298.8	-0.000478419	0.668672778	0.001117878	0.081436928	-0.000454493	0.684301884	0.001117788	0.081419103	-2.39E-05	0.004608752	1	0.888630991	1	0.901372745	ENSG00000184110	10	EIF3C
SAT	T2DM	ENSG00000171310.6	-0.005035626	0.122208977	0.003258102	0.081436559	-0.004936221	0.129725151	0.003257825	0.081418747	-9.94E-05	1.22E-05	1	0.459418101	1	0.479851431	ENSG00000184110	10	EIF3C
SAT	T2DM	ENSG00000171502.10	-0.002703211	0.120155474	0.001739379	0.081436555	-0.002740871	0.115049616	0.001739242	0.081418716	3.77E-05	0.005697345	1	0.456565492	1	0.456639917	ENSG00000184115	12	RP13-1039J1.2
SAT	T2DM	ENSG00000171530.9	-0.001624456	0.660540106	0.003698942	0.081436926	-0.001680432	0.649583695	0.003698615	0.081419096	5.60E-05	0.020120085	1	0.885563559	1	0.891013292	ENSG00000184115	12	RP13-1039J1.2
SAT	T2DM	ENSG00000171574.13	0.003530079	0.042084761	0.001736657	0.081436268	0.003471316	0.045607358	0.001736514	0.081418463	5.88E-05	4.08E-06	1	0.28326473	1	0.308531429	ENSG00000184117	7	NIPSNAP1
SAT	T2DM	ENSG00000171723.11	-0.008481236	0.001509733	0.002673087	0.081435276	-0.008564429	0.001354533	0.002672887	0.081417415	8.32E-05	0.000188244	1	0.034843648	1	0.037035189	ENSG00000184117	7	NIPSNAP1
SAT	T2DM	ENSG00000171763.13	-0.002675896	0.121658647	0.001728781	0.081436558	-0.002621509	0.129390747	0.001728644	0.081418747	-5.44E-05	5.46E-05	1	0.459236339	1	0.479366489	ENSG00000184164	10	CRELD2
SAT	T2DM	ENSG00000172123.8	8.02E-05	0.931743225	0.000935797	0.081436957	0.000147208	0.875011044	0.000935864	0.081419127	-6.71E-05	0.000171372	1	0.980452059	1	0.966700287	ENSG00000184164	10	CRELD2
SAT	T2DM	ENSG00000172247.3	-0.003588133	0.000173865	0.000955746	0.081434603	-0.00357062	0.000186796	0.000955666	0.081416798	-1.75E-05	0.010338429	0.570449786	0.007408439	0.612876884	0.008632069	ENSG00000184164	10	CRELD2
SAT	T2DM	ENSG00000172340.10	0.00379343	0.133489698	0.002528137	0.081436582	0.003836658	0.129069643	0.002527936	0.081418746	-4.32E-05	0.026661186	1	0.475032212	1	0.479121176	ENSG00000184164	10	CRELD2
SAT	T2DM	ENSG00000172590.14	0.0035121	0.07494876	0.001972224	0.081436428	0.003545845	0.072173874	0.001972079	0.081418591	-3.37E-05	0.043765174	1	0.382364025	1	0.39204956	ENSG00000184206	7	GOLGA6L4
SAT	T2DM	ENSG00000172613.7	0.000413672	0.628212651	0.000854264	0.081436919	0.000581776	0.496285332	0.000855117	0.081419054	-0.000168103	2.93E-05	1	0.867129031	1	0.814437198	ENSG00000184232	4	OAF
SAT	T2DM	ENSG00000172638.8	0.002498658	0.486468877	0.003590284	0.081436877	-0.013376006	0.00665231	0.004928924	0.08141779	0.015874664	2.60E-06	1	0.801645999	1	0.100085232	ENSG00000184232	4	OAF
SAT	T2DM	ENSG00000172661.13	-0.000656245	0.67779975	0.001579533	0.08143693	-0.000694765	0.660019766	0.00157942	0.081419099	-3.85E-05	0.005124663	1	0.892786811	1	0.894350146	ENSG00000184277	8	TM2D3
SAT	T2DM	ENSG00000172728.11	-0.001765719	0.206271153	0.001397054	0.081436691	-0.00174599	0.211345883	0.001396932	0.08141887	-1.97E-05	0.0338845	1	0.568086176	1	0.58173309	ENSG00000184277	8	TM2D3
SAT	T2DM	ENSG00000172789.3	-0.000326442	0.934418838	0.003967132	0.081436957	-0.000264058	0.9469266	0.003966809	0.08141913	-6.24E-05	0.035423832	1	0.981839134	1	0.988503397	ENSG00000184292	5	TACSTD2
SAT	T2DM	ENSG00000172803.13	0.008143767	0.000937244	0.002461291	0.081435129	-0.000108485	0.982172551	0.004854927	0.081419131	0.008252252	0.048620796	1	0.023837978	1	0.997162029	ENSG00000184292	5	TACSTD2
SAT	T2DM	ENSG00000172922.4	-0.007181474	0.000352105	0.002009576	0.081434824	-0.005343799	0.012282422	0.002134161	0.081418083	-0.001837675	0.010600941	1	0.012289954	1	0.150368007	ENSG00000184313	15	MROH7
SAT	T2DM	ENSG00000172927.3	-0.007416551	0.015170295	0.003054222	0.081435973	-0.007509924	0.013933481	0.003054078	0.081418121	9.34E-05	0.006519173	1	0.156521191	1	0.160406141	ENSG00000184371	9	CSF1
SAT	T2DM	ENSG00000172932.10	0.000242162	0.941023309	0.003273182	0.081436958	0.000688287	0.833524958	0.003274705	0.081419124	-0.000446125	5.82E-05	1	0.982870948	1	0.961601754	ENSG00000184371	9	CSF1
SAT	T2DM	ENSG00000173113.2	0.002031157	0.406845221	0.002448773	0.081436843	0.002073445	0.397108734	0.002448574	0.081419011	-4.23E-05	0.021501236	1	0.756247299	1	0.756180206	ENSG00000184371	9	CSF1
SAT	T2DM	ENSG00000173156.2	-0.000470371	0.8096747	0.001952998	0.081436949	-0.000561417	0.77376826	0.001953099	0.081419117	9.10E-05	0.009410815	1	0.951539016	1	0.939923606	ENSG00000184371	9	CSF1
SAT	T2DM	ENSG00000173200.8	-0.001405962	0.644244633	0.003044706	0.081436923	-0.001461552	0.631176741	0.003044447	0.081419092	5.56E-05	0.009141526	1	0.877766855	1	0.881139272	ENSG00000184371	9	CSF1
SAT	T2DM	ENSG00000173511.5	-0.010139549	9.04E-06	0.002284246	0.081433666	-0.010267639	6.96E-06	0.002284213	0.081415755	0.00012809	4.94E-05	0.029672588	0.000618179	0.022826296	0.000616927	ENSG00000184381	14	PLA2G6
SAT	T2DM	ENSG00000173535.9	0.00815589	0.359676919	0.001982124	0.081436818	0.00753832	0.008850615	0.002879659	0.081417986	-0.005722731	0.006157627	1	0.71830389	1	0.12252687	ENSG00000184381	14	PLA2G6
SAT	T2DM	ENSG00000174007.7	0.000862784	0.732392645	0.002523168	0.081436939	0.000819074	0.745446084	0.002522944	0.081419113	4.37E-05	0.006963399	1	0.922803482	1	0.927496626	ENSG00000184385	2	C21orf128
SAT	T2DM	ENSG00000174080.6	-0.000545515	0.770220902	0.001867653	0.081436944	-0.00155108	0.410040894	0.001882784	0.081419017	0.001005564	2.75E-05	1	0.935400227	1	0.762667765	ENSG00000184385	2	C21orf128
SAT	T2DM	ENSG00000174194.11	-0.000861806	0.43031405	0.001092754	0.081436854	-0.00083687	0.443733005	0.001092655	0.081419033	-2.49E-05	0.000159287	1	0.772274669	1	0.789955502	ENSG00000184385	2	C21orf128
SAT	T2DM	ENSG00000174226.4	0.004697195	0.211367036	0.003758307	0.081436697	0.004869364	0.195072047	0.003758033	0.08141885	-0.000172169	8.81E-08	1	0.575258443	1	0.563293473	ENSG00000184385	2	C21orf128
SAT	T2DM	ENSG00000174353.13	-0.001760782	0.343598243	0.001859169	0.081436809	-0.001673395	0.368130489	0.001859366	0.081418996	-8.74E-05	0.023463802	1	0.704151053	1	0.732878736	ENSG00000184385	2	C21orf128
SAT	T2DM	ENSG00000174652.13	-0.001686045	0.025397744	0.00075428	0.081436123	-0.001674315	0.026423022	0.000754215	0.081418307	-1.17E-05	0.024172387	1	0.217571794	1	0.236264392	ENSG00000184389	8	A3GALT2
SAT	T2DM	ENSG00000174915.7	-0.002245685	0.393609184	0.00263241	0.081436837	-0.002200334	0.403194454	0.002632197	0.081419014	-4.54E-05	0.023091499	1	0.743169968	1	0.757663806	ENSG00000184389	8	A3GALT2
SAT	T2DM	ENSG00000175334.3	0.004434607	0.02931988	0.002035003	0.081436165	0.007415485	0.000496071	0.002129117	0.081417104	-0.002980878	1.98E-06	1	0.237037613	1	0.018287726	ENSG00000184389	8	A3GALT2
SAT	T2DM	ENSG00000175387.11	0.000674759	0.752368862	0.002138578	0.081436942	0.000726461	0.734067849	0.002138413	0.081419112	-5.17E-05	0.002693688	1	0.929413492	1	0.924370544	ENSG00000184389	8	A3GALT2
SAT	T2DM	ENSG00000175390.8	-0.002983876	0.379129247	0.003392686	0.081436829	-0.002907178	0.3914661	0.003392422	0.081419008	-7.67E-05	0.004594088	1	0.731289276	1	0.752431326	ENSG00000184389	8	A3GALT2
SAT	T2DM	ENSG00000175395.11	-0.002313752	0.091352547	0.001370455	0.081436482	-0.002345088	0.08702247	0.001370338	0.081418642	3.13E-05	0.00101075	1	0.413867045	1	0.419266847	ENSG00000184402	10	SS18L1
SAT	T2DM	ENSG00000175416.8	0.002748417	0.331859328	0.00283232	0.081436801	0.002796241	0.323478572	0.002832108	0.081418968	-4.78E-05	0.041578661	1	0.693964599	1	0.691873008	ENSG00000184402	10	SS18L1
SAT	T2DM	ENSG00000175445.10	-0.006328478	0.025196162	0.002827247	0.081436121	-0.008048614	0.005212355	0.002881068	0.081417827	0.001720136	0.00197264	1	0.216409969	1	0.085938379	ENSG00000184402	10	SS18L1
SAT	T2DM	ENSG00000175463.7	0.000252976	0.942796552	0.003525527	0.081436958	0.000444027	0.899774482	0.003525632	0.081419128	-0.000191051	0.001164342	1	0.983253492	1	0.972878089	ENSG00000184428	8	TOP1MT
SAT	T2DM	ENSG00000175582.15	-0.00090448	0.76347823	0.00300575	0.081436943	-0.0008194	0.785763868	0.003005491	0.081419119	-8.75E-05	2.14E-05	1	0.93459819	1	0.946500713	ENSG00000184465	11	WDR27
SAT	T2DM	ENSG00000175643.7	-0.001001657	0.652488836	0.002224384	0.081436925	-0.000954137	0.667938935	0.002224206	0.0814191	-4.75E-05	0.005237015	1	0.882995924	1	0.896403813	ENSG00000184470	15	TXNRD2
SAT	T2DM	ENSG00000175832.8	0.007593656	0.044122567	0.00377244	0.081436281	0.007526779	0.046002113	0.003772105	0.081418466	6.69E-05	0.005850532	1	0.290112507	1	0.309334779	ENSG00000184470	15	TXNRD2
SAT	T2DM	ENSG00000176024.12	0.004146753	0.129139449	0.002732609	0.081436574	0.004199147	0.12433963	0.002732372	0.081418736	-5.24E-05	0.004460899	1	0.470262523	1	0.474800718	ENSG00000184508	6	HDDC3
SAT	T2DM	ENSG00000176148.11	0.00274338	0.080244848	0.001568299	0.081436447	0.002714565	0.083444703	0.001568167	0.08141863	2.88E-05	0.009827981	1	0.395495266	1	0.414575198	ENSG00000184508	6	HDDC3
SAT	T2DM	ENSG00000176386.4	-0.005052534	0.013232886	0.002039233	0.081435933	-0.005089781	0.012555533	0.002039058	0.081418091	3.72E-05	0.000582451	1	0.145107	1	0.150896353	ENSG00000184508	6	HDDC3
SAT	T2DM	ENSG00000176390.10	0.006077134	0.102247197	0.003719043	0.081436512	0.006154388	0.097931081	0.003718729	0.081418673	-7.73E-05	0.003467349	1	0.425727224	1	0.436565051	ENSG00000184574	5	LPAR5
SAT	T2DM	ENSG00000176422.10	-0.000558847	0.808551151	0.002306491	0.081436949	-0.000626746	0.785817721	0.00230637	0.081419119	6.79E-05	0.005904999	1	0.951539016	1	0.946500713	ENSG00000184574	5	LPAR5
SAT	T2DM	ENSG00000176485.6	0.002395613	0.313738351	0.002377993	0.081436789	0.000337664	0.890447327	0.002451489	0.081419128	0.00205795	0.000564144	1	0.674437649	1	0.971822875	ENSG00000184608	4	C8orf12
SAT	T2DM	ENSG00000176783.10	-0.000166914	0.935089271	0.00204945	0.081436957	-0.000122473	0.952343693	0.002049279	0.08141913	-4.44E-05	0.002575011	1	0.981839134	1	0.99107822	ENSG00000184608	4	C8orf12
SAT	T2DM	ENSG00000176933.4	-0.001946319	0.47703614	0.002737137	0.081436874	-0.001226209	0.654433946	0.002739451	0.081419097	-0.000720109	1.74E-09	1	0.796607042	1	0.891871358	ENSG00000184608	4	C8orf12
SAT	T2DM	ENSG00000176974.13	0.00435947	0.177928991	0.003236043	0.081436655	0.004414738	0.172461931	0.003235811	0.081418821	-5.53E-05	0.049244619	1	0.530526907	1	0.532948952	ENSG00000184608	4	C8orf12
SAT	T2DM	ENSG00000176998.3	-0.001700894	0.24932633	0.001476485	0.081436737	0.000417683	0.785324372	0.001533416	0.081419118	-0.002118577	3.21E-07	1	0.615333003	1	0.946500713	ENSG00000184674	8	GSTT1
SAT	T2DM	ENSG00000177042.10	-0.000572669	0.788782817	0.002137688	0.081436946	-0.000540028	0.800542819	0.0021375	0.08141912	-3.26E-05	0.020201763	1	0.946250977	1	0.952160336	ENSG00000184674	8	GSTT1
SAT	T2DM	ENSG00000177311.6	-0.007518737	0.024832541	0.003350589	0.081436117	-0.007472663	0.025718441	0.003350303	0.0814183	-4.61E-05	0.047676976	1	0.21611556	1	0.233772911	ENSG00000184674	8	GSTT1
SAT	T2DM	ENSG00000177479.15	0.001630088	0.521162378	0.002540839	0.08143689	0.001684601	0.507292538	0.002540647	0.081419057	-5.45E-05	0.009503794	1	0.82089955	1	0.817738235	ENSG00000184674	8	GSTT1
SAT	T2DM	ENSG00000177595.13	-0.000863206	0.679668323	0.002090512	0.08143693	-0.000896716	0.66793946	0.002090349	0.0814191	3.35E-05	0.043608176	1	0.892786811	1	0.896403813	ENSG00000184743	8	ATL3
SAT	T2DM	ENSG00000177663.9	0.001746498	0.296574905	0.001673199	0.081436776	0.001686349	0.313493268	0.001673098	0.081418961	6.01E-05	0.000270413	1	0.654379465	1	0.682529139	ENSG00000184743	8	ATL3
SAT	T2DM	ENSG00000177839.4	0.003738576	0.165584441	0.002696346	0.081436637	0.003780858	0.160816142	0.002696118	0.081418802	-4.23E-05	0.026583206	1	0.518060238	1	0.520626624	ENSG00000184743	8	ATL3
SAT	T2DM	ENSG00000178149.12	0.004660296	0.135432718	0.00312139	0.081436586	0.004721225	0.130368016	0.003121152	0.081418749	-6.09E-05	0.016812267	1	0.477800803	1	0.480064491	ENSG00000184743	8	ATL3
SAT	T2DM	ENSG00000178252.13	-0.000267604	0.715329623	0.000733749	0.081436936	-0.000281881	0.700833971	0.000733693	0.081419106	1.43E-05	0.018917995	1	0.910394295	1	0.909587128	ENSG00000184887	9	BTBD6
SAT	T2DM	ENSG00000178381.7	-0.000869378	0.825296506	0.003938504	0.08143695	-0.000935613	0.812211334	0.00393819	0.081419121	6.62E-05	0.02926178	1	0.955051074	1	0.95480666	ENSG00000184887	9	BTBD6
SAT	T2DM	ENSG00000178386.8	-0.002091234	0.051818993	0.001075384	0.081436326	-0.002063652	0.05496573	0.001075294	0.081418515	-2.76E-05	0.00032921	1	0.315548759	1	0.341997131	ENSG00000184923	8	NUTM2A
SAT	T2DM	ENSG00000178397.8	-0.00073621	0.759428854	0.0024041	0.081436943	-0.000774196	0.747408214	0.002403897	0.081419114	3.80E-05	0.025094018	1	0.934086217	1	0.927854665	ENSG00000184923	8	NUTM2A
SAT	T2DM	ENSG00000178467.13	0.002365867	0.551312677	0.003970944	0.081436899	0.002437052	0.539366304	0.003970615	0.081419068	-7.12E-05	0.014063621	1	0.83588581	1	0.836431746	ENSG00000184945	9	AQP12A
SAT	T2DM	ENSG00000178665.10	0.001512163	0.625806435	0.003100995	0.081436919	0.001461275	0.637450872	0.003100742	0.081419094	5.09E-05	0.02837002	1	0.866359035	1	0.884719251	ENSG00000184945	9	AQP12A
SAT	T2DM	ENSG00000178695.4	-0.002442089	0.342448859	0.002572411	0.081436808	-0.002373257	0.356185843	0.002572196	0.081418989	-6.88E-05	0.000193171	1	0.703113082	1	0.724067999	ENSG00000184945	9	AQP12A
SAT	T2DM	ENSG00000178718.5	-0.001423365	0.546319024	0.002359356	0.081436898	-0.001467068	0.534033183	0.002359154	0.081419066	4.37E-05	0.007688419	1	0.835027823	1	0.832950936	ENSG00000184949	11	FAM227A
SAT	T2DM	ENSG00000178809.7	-0.010410695	0.003614541	0.003577568	0.081435543	-0.010472327	0.003417695	0.003577286	0.081417699	6.16E-05	0.028182767	1	0.062747674	1	0.066764938	ENSG00000184949	11	FAM227A
SAT	T2DM	ENSG00000178852.11	0.000629071	0.744590871	0.001930968	0.081436941	0.000668501	0.729171145	0.001930808	0.081419111	-3.94E-05	0.005259662	1	0.927287795	1	0.922641931	ENSG00000184967	2	NOC4L
SAT	T2DM	ENSG00000178966.11	0.004892086	0.064139219	0.002642635	0.081436386	0.004849187	0.066486401	0.002642415	0.081418568	4.29E-05	0.024802603	1	0.35132016	1	0.375381853	ENSG00000184967	2	NOC4L
SAT	T2DM	ENSG00000178971.9	0.002481779	0.136909017	0.001668532	0.081436589	0.002452718	0.141533679	0.001668395	0.08141877	2.91E-05	0.019642706	1	0.479912916	1	0.499917672	ENSG00000184967	2	NOC4L
SAT	T2DM	ENSG00000179163.11	0.00345427	0.293334473	0.003287164	0.081436774	0.003539577	0.281534232	0.00328688	0.081418937	-8.53E-05	0.000136963	1	0.650730497	1	0.647765649	ENSG00000184967	2	NOC4L
SAT	T2DM	ENSG00000179296.9	-0.0012093	0.664141686	0.002784486	0.081436927	-0.001160816	0.676736397	0.002784258	0.081419102	-4.82E-05	0.019882308	1	0.887596281	1	0.898778511	ENSG00000184983	5	NDUFA6
SAT	T2DM	ENSG00000179299.12	-0.001113864	0.56245426	0.001923106	0.081436902	-0.001170849	0.54261365	0.001923001	0.081419069	5.70E-05	0.004629377	1	0.837959912	1	0.839771408	ENSG00000184983	5	NDUFA6
SAT	T2DM	ENSG00000179361.13	-0.011076775	0.000424072	0.003873422	0.081435592	-0.011013463	0.00446131	0.003873112	0.081417778	-6.33E-05	0.03264365	1	0.068881061	1	0.079676052	ENSG00000184983	5	NDUFA6
SAT	T2DM	ENSG00000179409.6	-0.001697264	0.493765258	0.002480173	0.081436883	-0.001726732	0.486255111	0.002479946	0.081419051	2.95E-05	0.001760351	1	0.805591154	1	0.807270931	ENSG00000184983	5	NDUFA6
SAT	T2DM	ENSG00000179797.7	0.001456626	0.202875434	0.001143884	0.081436687	0.001484617	0.194296944	0.001143796	0.081418849	-2.80E-05	0.002497228	1	0.565054583	1	0.562655141	ENSG00000184988	4	TMEM106A
SAT	T2DM	ENSG00000180089.4	9.99E-05	0.968173492	0.00250291	0.081436958	3.92E-05	0.987516514	0.002502714	0.081419131	6.07E-05	0.002082894	1	0.993613755	1	0.997162029	ENSG00000184988	4	TMEM106A
SAT	T2DM	ENSG00000180610.9	0.00713194	0.072447198	0.003970347	0.081436419	0.007194753	0.069944529	0.003970015	0.081418582	-6.28E-05	0.027877401	1	0.376702465	1	0.38680588	ENSG00000184990	8	SIVA1
SAT	T2DM	ENSG00000180773.10	-0.007015696	0.006021496	0.002554301	0.081435698	-0.007058294	0.005718218	0.002554087	0.081417855	4.26E-05	0.020374412	1	0.085526101	1	0.091112401	ENSG00000184990	8	SIVA1
SAT	T2DM	ENSG00000180822.7	-0.00148967	0.651272403	0.003295763	0.081436924	-0.001542945	0.639640922	0.003295473	0.081419094	5.33E-05	0.013851652	1	0.882256298	1	0.885701439	ENSG00000184990	8	SIVA1
SAT	T2DM	ENSG00000180855.11	-0.00243983	0.421153644	0.003033022	0.081436853	-0.00253665	0.402924952	0.003032785	0.081419014	9.68E-05	5.49E-05	1	0.764695687	1	0.757591271	ENSG00000185022	7	MAFF
SAT	T2DM	ENSG00000180953.7	-0.000180202	0.844147918	0.000916636	0.081436951	-0.000157006	0.863989132	0.000916563	0.081419126	-2.32E-05	0.000988265	1	0.962017825	1	0.966241215	ENSG00000185043	6	CIB1
SAT	T2DM	ENSG00000181092.5	-0.002593919	0.256535415	0.002286158	0.081436743	-0.004560743	0.057995663	0.002405791	0.08141853	0.001966824	0.008729333	1	0.62099686	1	0.347397154	ENSG00000185043	6	CIB1
SAT	T2DM	ENSG00000181104.6	0.000181324	0.94051202	0.002429753	0.081436957	0.000224078	0.926515019	0.002429541	0.081419129	-4.28E-05	0.008366668	1	0.982870948	1	0.981878481	ENSG00000185043	6	CIB1
SAT	T2DM	ENSG00000181126.9	-0.000308092	0.824445885	0.001388861	0.08143695	-0.000939891	0.501183585	0.001397339	0.081419055	0.000631799	4.62E-05	1	0.955051074	1	0.816084912	ENSG00000185070	6	FLRT2
SAT	T2DM	ENSG00000181218.4	-0.000949592	0.81563181	0.004072583	0.081436949	-0.001038755	0.798662341	0.004072292	0.081419112	-8.92E-05	0.011990564	1	0.952005681	1	0.952160336	ENSG00000185101	8	ANO9
SAT	T2DM	ENSG00000181315.6	-0.001691886	0.447221342	0.002226001	0.081436862	-0.001192112	0.592751894	0.00222886	0.081419083	-0.000499773	2.13E-05	1	0.780081459	1	0.854809759	ENSG00000185101	8	ANO9
SAT	T2DM	ENSG00000181619.11	-0.000293595	0.937047373	0.003717259	0.081436957	-0.000657673	0.859621661	0.003718696	0.081419126	0.000364079	0.001875084	1	0.982560792	1	0.965566132	ENSG00000185101	8	ANO9
SAT	T2DM	ENSG00000181744.4	0.000423866	0.868527038	0.002560664	0.081436954	0.000390283	0.878849294	0.002560436	0.081419127	3.36E-05	0.040294552	1	0.966468088	1	0.966700287	ENSG00000185163	5	DDX51
SAT	T2DM	ENSG00000181754.6	-0.002219859	0.186847363	0.001681756	0.081436667	-0.002265639	0.17788352	0.001681612	0.081418828	4.58E-05	8.19E-05	1	0.544446003	1	0.539691192	ENSG00000185245	6	GP1BA
SAT	T2DM	ENSG00000181781.5	0.002527509	0.507164899	0.003810741	0.081436885	0.002465891	0.517541228	0.003810441	0.081419061	6.16E-05	0.038864524	1	0.810627063	1	0.825499645	ENSG00000185271	6	KLHL33
SAT	T2DM	ENSG00000182048.7	-0.002407427	0.519204523	0.00373492	0.081436889	-0.002478612	0.506890443	0.003734605	0.081419057	7.12E-05	0.006931088	1	0.819389148	1	0.817738235	ENSG00000185290	3	NUPR1L
SAT	T2DM	ENSG00000182117.4	-0.006142052	0.022089299	0.002683483	0.081436083	-0.006196772	0.020921064	0.002683267	0.081418264	5.47E-05	0.007404145	1	0.201880195	1	0.204291699	ENSG00000185290	3	NUPR1L
SAT	T2DM	ENSG00000182141.5	-0.003551929	0.310413577	0.003501661	0.081436786	-0.003478999	0.320417592	0.003501404	0.081418966	-7.29E-05	0.014182865	1	0.670485151	1	0.691183511	ENSG00000185290	3	NUPR1L
SAT	T2DM	ENSG00000182150.11	0.00041429	0.828483316	0.001912277	0.081436951	0.000371532	0.845937448	0.001912112	0.081419125	4.28E-05	0.000984087	1	0.956121618	1	0.96477102	ENSG00000185290	3	NUPR1L
SAT	T2DM	ENSG00000182240.11	0.000382188	0.85478465	0.002088261	0.081436953	-0.001162609	0.581386976	0.002108619	0.081419081	0.001544797	1.48E-07	1	0.963534946	1	0.851769934	ENSG00000185290	3	NUPR1L
SAT	T2DM	ENSG00000182362.9	0.000160055	0.839354994	0.000789544	0.081436952	0.000135253	0.863973965	0.000789486	0.081419126	2.48E-05	0.000197333	1	0.959889764	1	0.966241215	ENSG00000185298	8	CCDC137
SAT	T2DM	ENSG00000182533.6	-0.000103256	0.959250484	0.002020905	0.081436958	-7.21E-05	0.971543574	0.002020728	0.081419131	-3.12E-05	0.019726981	1	0.990651822	1	0.996397533	ENSG00000185298	8	CCDC137
SAT	T2DM	ENSG00000182796.8	0.001095162	0.763517145	0.003640039	0.081436943	0.001485665	0.683561255	0.003644832	0.081419103	-0.000390502	0.044635917	1	0.93459819	1	0.901372745	ENSG00000185298	8	CCDC137
SAT	T2DM	ENSG00000182919.10	-0.000516729	0.572363778	0.000915255	0.081436905	-0.000541157	0.554313332	0.000915184	0.081419072	2.44E-05	0.000894414	1	0.840736467	1	0.842050121	ENSG00000185298	8	CCDC137
SAT	T2DM	ENSG00000182952.4	-0.00506215	0.12304442	0.003282584	0.08143656	-0.003702278	0.260405557	0.003289644	0.081418919	-0.001359873	7.26E-10	1	0.460484574	1	0.633351098	ENSG00000185298	8	CCDC137
SAT	T2DM	ENSG00000183060.11	0.000265558	0.8511417	0.001415088	0.081436953	0.0002481	0.860812087	0.00141496	0.081419126	1.75E-05	0.046507572	1	0.963534946	1	0.965732208	ENSG00000185324	17	CDK10
SAT	T2DM	ENSG00000183172.8	-7.67E-05	0.923218609	0.000796135	0.081436957	-8.90E-05	0.910937362	0.000796069	0.081419129	1.23E-05	0.035608906	1	0.978648294	1	0.978562139	ENSG00000185324	17	CDK10
SAT	T2DM	ENSG00000183309.7	-0.001438201	0.713138433	0.003911934	0.081436936	-0.001587951	0.684777158	0.003911659	0.081419103	0.000149749	1.56E-05	1	0.908310248	1	0.901372745	ENSG00000185324	17	CDK10
SAT	T2DM	ENSG00000183426.11	0.001278638	0.156002503	0.000901308	0.081436622	0.001295059	0.150726501	0.000901242	0.081418786	-1.64E-05	0.003068842	1	0.506776449	1	0.51088187	ENSG00000185324	17	CDK10
SAT	T2DM	ENSG00000183458.9	0.002540702	0.40802635	0.003070808	0.081436844	0.002479227	0.419434857	0.00307062	0.081419022	6.15E-05	0.041133496	1	0.756247299	1	0.768824902	ENSG00000185340	11	GAS2L1

SAT	T2DM	ENSG00000183486.8	-0.00265109	0.440432255	0.003436439	0.081436859	-0.001508033	0.662223143	0.003452092	0.081419099	-0.001143057	0.000581214	1	0.776495555	1	0.895728881	ENSG00000185340	11	GAS2L1
SAT	T2DM	ENSG00000183506.12	4.98E-05	0.972341156	0.001435018	0.081436958	2.30E-05	0.987212445	0.001434891	0.081419131	2.68E-05	0.003744225	1	0.995031844	1	0.997162029	ENSG00000185340	11	GAS2L1
SAT	T2DM	ENSG00000183527.7	0.00118919	0.26077697	0.001057473	0.081436747	0.001203209	0.255155673	0.001057378	0.081418915	-1.40E-05	0.034905589	1	0.625445349	1	0.62709046	ENSG00000185344	9	ATP6V0A2
SAT	T2DM	ENSG00000183604.10	-0.002829171	0.229181031	0.002352794	0.081436717	-0.002885285	0.220047078	0.002352638	0.08141888	5.61E-05	0.010170348	1	0.593149171	1	0.593241137	ENSG00000185344	9	ATP6V0A2
SAT	T2DM	ENSG00000183628.8	-0.001964812	0.079143594	0.001119117	0.081436443	-0.00193378	0.083972196	0.001119025	0.081418632	-3.10E-05	0.000186019	1	0.393439592	1	0.414575198	ENSG00000185344	9	ATP6V0A2
SAT	T2DM	ENSG00000183779.5	-0.013872951	7.45E-05	0.003502045	0.081434336	-0.013951765	6.77E-05	0.003501798	0.081416479	7.88E-05	0.010910868	0.244521641	0.003943897	0.22220762	0.004114956	ENSG00000185344	9	ATP6V0A2
SAT	T2DM	ENSG00000183807.6	0.00195811	0.570439158	0.003450991	0.081436905	0.001895131	0.582870301	0.003450729	0.08141908	6.30E-05	0.026248128	1	0.840736467	1	0.85184742	ENSG00000185344	9	ATP6V0A2
SAT	T2DM	ENSG00000183828.10	8.06E-06	0.998194947	0.003561298	0.081436958	8.18E-05	0.981669797	0.003561006	0.081419131	-7.38E-05	0.005190955	1	0.998511991	1	0.997162029	ENSG00000185344	9	ATP6V0A2
SAT	T2DM	ENSG00000183844.12	-0.000681321	0.53425762	0.001096217	0.081436894	-0.000731982	0.504334837	0.001096298	0.081419056	5.07E-05	0.015834902	1	0.828750699	1	0.817738235	ENSG00000185404	12	SP140L
SAT	T2DM	ENSG00000183977.9	0.007733516	0.043038125	0.003822144	0.081436274	0.007831105	0.040457497	0.003821817	0.081418429	-9.76E-05	0.000237095	1	0.286428522	1	0.289075371	ENSG00000185404	12	SP140L
SAT	T2DM	ENSG00000184110.10	0.00740093	0.000453374	0.002110382	0.081434903	0.007364308	0.000483352	0.002110213	0.081417096	3.66E-05	0.024188753	1	0.014727922	1	0.018228475	ENSG00000185404	12	SP140L
SAT	T2DM	ENSG00000184277.8	0.000345533	0.913537701	0.003182367	0.081436956	0.000395138	0.901176633	0.003182099	0.081419128	-4.96E-05	0.028778981	1	0.976642944	1	0.972878089	ENSG00000185404	12	SP140L
SAT	T2DM	ENSG00000184292.5	-0.001347844	0.448839787	0.001779681	0.081436863	-0.001316919	0.459280856	0.001779543	0.081419039	-3.09E-05	0.029418485	1	0.78183394	1	0.795972372	ENSG00000185418	11	TARSL2
SAT	T2DM	ENSG00000184371.9	0.004573745	0.053206528	0.002365838	0.081436334	0.001791297	0.456712247	0.002406778	0.081419038	0.002782448	3.48E-10	1	0.320901873	1	0.794600418	ENSG00000185437	9	SH3BGR
SAT	T2DM	ENSG00000184381.14	0.006959711	0.068559877	0.00382126	0.081436404	0.006876286	0.071919203	0.003820937	0.08141859	8.34E-05	0.001998821	1	0.363988605	1	0.391321567	ENSG00000185437	9	SH3BGR
SAT	T2DM	ENSG00000184385.2	-0.000833897	0.60895105	0.001630069	0.081436915	-0.000792094	0.626990418	0.001629932	0.081419091	-4.18E-05	0.000335321	1	0.855383565	1	0.876877903	ENSG00000185499	12	MUC1
SAT	T2DM	ENSG00000184402.10	0.002707245	0.491464854	0.003935056	0.081436879	0.002589622	0.510448623	0.00393476	0.081419059	0.000117623	0.000301356	1	0.80382562	1	0.819765996	ENSG00000185499	12	MUC1
SAT	T2DM	ENSG00000184508.6	-0.001089585	0.790253953	0.004096475	0.081436947	-0.001019042	0.803529644	0.004096127	0.081419121	-7.05E-05	0.013487173	1	0.94681813	1	0.952314593	ENSG00000185499	12	MUC1
SAT	T2DM	ENSG00000184743.8	-0.003109937	0.286711871	0.002919132	0.081436769	-0.002575564	0.377952126	0.002921212	0.081419001	-0.000534373	6.33E-06	1	0.644230759	1	0.738661557	ENSG00000185591	5	SP1
SAT	T2DM	ENSG00000184967.2	-0.000525756	0.819020551	0.002297832	0.08143695	-0.000598156	0.794606032	0.002297645	0.08141912	7.24E-05	2.46E-05	1	0.953693013	1	0.950456578	ENSG00000185619	13	PCGF3
SAT	T2DM	ENSG00000184990.8	-0.001756158	0.576550836	0.003144811	0.081436906	-0.001845822	0.557215703	0.003144594	0.081419073	8.97E-05	0.001516843	1	0.842502133	1	0.842050121	ENSG00000185619	13	PCGF3
SAT	T2DM	ENSG00000185043.6	-0.001024891	0.54722092	0.001702674	0.081436898	-0.00106579	0.531312396	0.001702528	0.081419065	4.09E-05	0.000483132	1	0.835027823	1	0.831380066	ENSG00000185619	13	PCGF3
SAT	T2DM	ENSG00000185290.3	0.003211859	0.087541823	0.001879915	0.081436471	0.00318025	0.090676102	0.001879752	0.081418653	3.16E-05	0.012840513	1	0.407026915	1	0.423536093	ENSG00000185619	13	PCGF3
SAT	T2DM	ENSG00000185298.8	0.002215943	0.206942423	0.001755871	0.081436692	0.002191827	0.21188847	0.001755722	0.08141887	2.41E-05	0.049714557	1	0.568658367	1	0.582737696	ENSG00000185624	10	P4HB
SAT	T2DM	ENSG00000185324.17	0.000436445	0.703589495	0.001147091	0.081436934	0.000414435	0.717859839	0.001147	0.081419109	2.20E-05	0.013425262	1	0.905285151	1	0.918559531	ENSG00000185627	13	PSMD13
SAT	T2DM	ENSG00000185344.9	-0.000615551	0.85428653	0.003351726	0.081436953	-0.000502714	0.880765719	0.003351458	0.081419127	-0.000112837	1.13E-05	1	0.963534946	1	0.967132638	ENSG00000185627	13	PSMD13
SAT	T2DM	ENSG00000185404.12	-0.003228703	0.375913443	0.003646391	0.081436827	-0.00309416	0.396095337	0.003646124	0.081419011	-0.000134543	1.47E-05	1	0.728082648	1	0.755715818	ENSG00000185664	10	PMEL
SAT	T2DM	ENSG00000185499.12	0.005560851	0.061227984	0.002970779	0.081436373	0.005641715	0.057537008	0.002970553	0.081418528	-8.09E-05	0.00092598	1	0.343400026	1	0.346763912	ENSG00000185664	10	PMEL
SAT	T2DM	ENSG00000185591.5	0.001129336	0.770949315	0.003879099	0.081436944	0.001200858	0.756866511	0.003878762	0.081419115	-7.15E-05	0.005894258	1	0.935460319	1	0.932511837	ENSG00000185664	10	PMEL
SAT	T2DM	ENSG00000185684.8	3.64E-05	0.972257872	0.001045472	0.081436958	1.50E-05	0.988541083	0.001045384	0.081419131	2.13E-05	0.004251667	1	0.995031844	1	0.997162029	ENSG00000185684	8	EP400NL
SAT	T2DM	ENSG00000185697.12	-0.006383812	0.026997319	0.002886561	0.081436141	-0.001953203	0.523016321	0.003058076	0.081419063	-0.004430609	1.16E-05	1	0.226852733	1	0.82668068	ENSG00000185684	8	EP400NL
SAT	T2DM	ENSG00000185739.9	-0.000175014	0.938670723	0.002274657	0.081436957	-0.000116853	0.959026365	0.002274486	0.08141913	-5.82E-05	0.001922585	1	0.982870948	1	0.993672317	ENSG00000185684	8	EP400NL
SAT	T2DM	ENSG00000185774.10	-0.003228708	0.329224353	0.003309193	0.081436799	-0.003345222	0.312031415	0.003308924	0.08141896	0.000116514	2.66E-06	1	0.692334629	1	0.68070151	ENSG00000185684	8	EP400NL
SAT	T2DM	ENSG00000186153.12	0.002269078	0.450282909	0.003005624	0.081436863	0.002212866	0.461544279	0.003005358	0.08141904	5.62E-05	0.00363898	1	0.782095408	1	0.797039301	ENSG00000185684	8	EP400NL
SAT	T2DM	ENSG00000186230.6	-0.003929252	0.092473129	0.002335369	0.081436485	-0.003874801	0.097054339	0.002335191	0.081418671	-5.45E-05	0.004063689	1	0.414244809	1	0.434658078	ENSG00000185684	8	EP400NL
SAT	T2DM	ENSG00000186448.10	-0.001581583	0.491438272	0.002298734	0.081436879	-0.00162812	0.478743456	0.002298548	0.081419047	4.65E-05	0.007367498	1	0.80382562	1	0.80363893	ENSG00000185684	8	EP400NL
SAT	T2DM	ENSG00000186453.8	-0.001941847	0.134353952	0.001297026	0.081436584	-0.001966503	0.129447946	0.001296922	0.081418747	2.47E-05	0.012945689	1	0.475922014	1	0.479366489	ENSG00000185697	12	MYBL1
SAT	T2DM	ENSG00000186468.8	-0.002268929	0.220292551	0.00185105	0.081436707	-0.002293645	0.215267143	0.001850888	0.081418874	2.47E-05	0.043336139	1	0.585721118	1	0.587596919	ENSG00000185697	12	MYBL1
SAT	T2DM	ENSG00000186470.9	-0.000139011	0.833258237	0.000660305	0.081436951	0.000816314	0.234374662	0.000686459	0.081418895	-0.000955324	3.71E-07	1	0.957925815	1	0.610313962	ENSG00000185697	12	MYBL1
SAT	T2DM	ENSG00000186501.10	0.000340317	0.868636464	0.002057652	0.081436954	0.000311807	0.879542976	0.002057471	0.081419127	2.85E-05	0.034030352	1	0.966468088	1	0.966700287	ENSG00000185697	12	MYBL1
SAT	T2DM	ENSG00000186522.10	0.001228485	0.600826153	0.002347962	0.081436913	0.001261706	0.590985703	0.002347759	0.081419083	-3.32E-05	0.036495765	1	0.851317044	1	0.854809759	ENSG00000185710	5	RP11-645C24.2
SAT	T2DM	ENSG00000186532.7	-0.000392715	0.895138625	0.002979516	0.081436956	-0.000445015	0.881262011	0.002979285	0.081419127	5.23E-05	0.027453582	1	0.970180416	1	0.96735385	ENSG00000185739	9	SRL
SAT	T2DM	ENSG00000186575.13	0.002281178	0.432698813	0.002907506	0.081436856	0.00220428	0.448334397	0.002907271	0.081419035	7.69E-05	0.000495722	1	0.772274669	1	0.791227469	ENSG00000185739	9	SRL
SAT	T2DM	ENSG00000186577.7	-0.003151645	0.369282768	0.003510336	0.081436824	-0.003481566	0.321339851	0.003510672	0.081418967	0.000329922	3.53E-06	1	0.723805643	1	0.691873008	ENSG00000185739	9	SRL
SAT	T2DM	ENSG00000186665.8	0.004446271	0.250948267	0.003872888	0.081436738	0.004530738	0.242022909	0.003872602	0.081418902	-8.45E-05	0.009746385	1	0.615827421	1	0.620663712	ENSG00000185742	6	C11orf87
SAT	T2DM	ENSG00000186766.7	-0.002513231	0.233234371	0.002108291	0.081436721	-0.00244555	0.246025446	0.002108126	0.081418906	-6.77E-05	4.48E-05	1	0.598312722	1	0.620663712	ENSG00000185742	6	C11orf87
SAT	T2DM	ENSG00000187048.8	0.003411874	0.389832893	0.003967659	0.081436835	0.00332537	0.401924707	0.003967325	0.081419014	8.65E-05	0.002126723	1	0.741043872	1	0.757591271	ENSG00000185774	10	KCNIP4
SAT	T2DM	ENSG00000187049.5	0.006174338	0.038009589	0.002975933	0.081436239	0.006272252	0.035066155	0.002976017	0.081418389	-9.79E-05	0.047416766	1	0.27051944	1	0.272172417	ENSG00000185774	10	KCNIP4
SAT	T2DM	ENSG00000187091.9	0.000573025	0.843456774	0.002901777	0.081436952	0.000505729	0.861634377	0.002901581	0.081419126	6.73E-05	0.011339102	1	0.961656125	1	0.965732208	ENSG00000185800	7	DMWD
SAT	T2DM	ENSG00000187105.4	0.002714211	0.183030678	0.002038487	0.081436662	0.002745629	0.177978016	0.002038315	0.081418828	-3.14E-05	0.030174672	1	0.537621892	1	0.539691192	ENSG00000185829	11	ARL17A
SAT	T2DM	ENSG00000187166.1	0.005281354	0.000857185	0.001584252	0.081435101	0.005316135	0.000791175	0.001584123	0.081417249	-3.48E-05	0.003538979	1	0.022145067	1	0.024351274	ENSG00000185864	12	NPIPB4
SAT	T2DM	ENSG00000187741.10	-0.00115148	0.612569457	0.002273812	0.081436916	-0.001194768	0.599244861	0.002273638	0.081419085	4.33E-05	0.018858968	1	0.857440439	1	0.855952281	ENSG00000185864	12	NPIPB4
SAT	T2DM	ENSG00000187762.5	-0.00143688	0.600929678	0.002747042	0.081436913	-0.001861146	0.498288974	0.002748357	0.081419054	0.000424266	6.73E-06	1	0.851317044	1	0.81497549	ENSG00000185869	9	ZNF829
SAT	T2DM	ENSG00000187862.7	-0.003075474	0.255580193	0.002705137	0.081436742	-0.003116388	0.249268876	0.002704897	0.081418909	4.09E-05	0.018215602	1	0.620694754	1	0.621386978	ENSG00000185869	9	ZNF829
SAT	T2DM	ENSG00000187987.5	0.000907988	0.349661534	0.000970854	0.081436812	0.000302262	0.757695324	0.000979751	0.081419115	0.000605726	4.85E-06	1	0.708609939	1	0.932832405	ENSG00000185869	9	ZNF829
SAT	T2DM	ENSG00000188051.6	0.000683556	0.602355772	0.001311968	0.081436913	0.000700948	0.593119775	0.001311849	0.081419083	-1.74E-05	0.030548228	1	0.851866072	1	0.854809759	ENSG00000185880	8	TRIM69
SAT	T2DM	ENSG00000188056.7	0.000402784	0.892149273	0.002970711	0.081436955	0.000457929	0.877483362	0.002970473	0.081419127	-5.51E-05	0.01590327	1	0.970180416	1	0.966700287	ENSG00000185904	7	LINC00839
SAT	T2DM	ENSG00000188312.9	-5.55E-05	0.960931671	0.001132488	0.081436958	-3.43E-05	0.97584007	0.00113239	0.081419131	-2.12E-05	0.005889096	1	0.99110869	1	0.99701704	ENSG00000185917	9	SETD4
SAT	T2DM	ENSG00000188493.10	0.001407153	0.673510345	0.003339745	0.081436929	0.001455923	0.66285407	0.003339454	0.081419099	-4.88E-05	0.029924657	1	0.891627092	1	0.895728881	ENSG00000185986	10	SDHAP3
SAT	T2DM	ENSG00000188878.12	-0.000321117	0.808581758	0.001325525	0.081436949	-0.000354221	0.789275921	0.001325424	0.081419119	3.31E-05	0.002257226	1	0.951539016	1	0.947957612	ENSG00000185988	7	PLK5
SAT	T2DM	ENSG00000188906.9	0.005548166	0.0443861	0.002759689	0.081436283	0.005503108	0.046123722	0.002759467	0.081418466	4.51E-05	0.031993564	1	0.29068023	1	0.309334779	ENSG00000186049	4	KRT73
SAT	T2DM	ENSG00000188958.5	-9.87E-05	0.978509884	0.00366589	0.081436958	-0.000152346	0.966848534	0.003665578	0.081419131	5.36E-05	0.0359364	1	0.995203291	1	0.994741311	ENSG00000186081	7	KRT5
SAT	T2DM	ENSG00000189007.11	0.006035353	0.051071845	0.003093656	0.081436322	0.005985071	0.053017128	0.003093402	0.081418505	5.03E-05	0.0278216	1	0.312624484	1	0.332861953	ENSG00000186132	10	C2orf76
SAT	T2DM	ENSG00000189045.9	-0.002957395	0.166856601	0.002139355	0.081436639	-0.00291053	0.173646173	0.002139179	0.081418822	-4.69E-05	0.002879509	1	0.519037734	1	0.532948952	ENSG00000186132	10	C2orf76
SAT	T2DM	ENSG00000189046.6	0.009591563	0.005016725	0.003418269	0.081435643	0.009508518	0.005404608	0.003418011	0.081417838	8.30E-05	0.003182614	1	0.074817614	1	0.088387036	ENSG00000186153	12	WWOX
SAT	T2DM	ENSG00000189157.9	0.003501074	0.367977333	0.003888918	0.081436823	0.003701161	0.341247293	0.003888981	0.08141898	-0.000200087	0.001175698	1	0.723275188	1	0.708179865	ENSG00000186160	4	CYP4Z1
SAT	T2DM	ENSG00000189180.11	0.000405387	0.896281308	0.003109738	0.081436956	0.00050781	0.870275187	0.003109501	0.081419126	-0.000102423	5.07E-05	1	0.970180416	1	0.966383771	ENSG00000186230	6	ZNF749
SAT	T2DM	ENSG00000189182.5	0.004718668	0.207763107	0.003745742	0.081436693	0.004591059	0.220286804	0.003745457	0.08141888	0.000127609	2.94E-05	1	0.569276333	1	0.593399838	ENSG00000186230	6	ZNF749
SAT	T2DM	ENSG00000189190.7	0.000151379	0.939897938	0.002007731	0.081436957	0.000204195	0.918984184	0.002007557	0.081419129	-5.28E-05	9.05E-05	1	0.982870948	1	0.979654273	ENSG00000186230	6	ZNF749
SAT	T2DM	ENSG00000189306.6	-0.004054939	0.158073336	0.002872618	0.081436625	-0.003992144	0.164577271	0.002872371	0.081418808	-6.28E-05	0.001373969	1	0.507474184	1	0.523492072	ENSG00000186230	6	ZNF749
SAT	T2DM	ENSG00000189339.7	0.000720643	0.593842865	0.001351351	0.081436911	0.000676876	0.616426193	0.001351259	0.081419089	4.38E-05	0.000374651	1	0.8467616	1	0.869142389	ENSG00000186230	6	ZNF749
SAT	T2DM	ENSG00000196072.7	-0.002284396	0.014092932	0.000930547	0.081435951	-0.002300122	0.013436131	0.000930468	0.08141811	1.57E-05	0.016138231	1	0.151493517	1	0.157494043	ENSG00000186230	6	ZNF749
SAT	T2DM	ENSG00000196152.6	-0.002018477	0.285241538	0.001888863	0.081436768	-0.00206853	0.273425374	0.001888707	0.08141893	5.01E-05	0.000304716	1	0.643214767	1	0.64192524	ENSG00000186364	7	NUDT17
SAT	T2DM	ENSG00000196196.2	-0.003914818	0.311873278	0.003871074	0.081436788	-0.003979869	0.303863868	0.003870776	0.081418954	6.51E-05	0.037380571	1	0.67252715	1	0.671707395	ENSG00000186409	10	CCDC30
SAT	T2DM	ENSG00000196236.8	-0.003036662	0.060850272	0.00161991	0.081436371	-0.003007224	0.063371851	0.001619767	0.081418555	-2.94E-05	0.005152701	1	0.34323074	1	0.365339457	ENSG00000186417	9	GLDN
SAT	T2DM	ENSG00000196301.3	0.007061771	1.39E-07	0.001340879	0.081432323	0.002446034	0.113882837	0.001547162	0.081418713	0.004615737	2.25E-09	0.00045635	1.90E-05	1	0.455762334	ENSG00000186417	9	GLDN
SAT	T2DM	ENSG00000196511.9	0.004861411	0.057073266	0.002554934	0.081436353	0.004899223	0.055146992	0.002554711	0.081418516	-3.78E-05	0.026034814	1	0.331288318	1	0.341997131	ENSG00000186448	10	ZNF197
SAT	T2DM	ENSG00000196535.10	-0.002988554	0.290962205	0.002830032	0.081436772	-0.00308148	0.276179156	0.002829787	0.081418933	9.29E-05	1.37E-06	1	0.647523626	1	0.64327119	ENSG00000186448	10	ZNF197
SAT	T2DM	ENSG00000196653.7	0.000971289	0.645722217	0.002112815	0.081436923	0.000935558	0.657881794	0.002112632	0.081419098	3.57E-05	0.012566334	1	0.878338086	1	0.892315075	ENSG00000186453	8	FAM228A
SAT	T2DM	ENSG00000196712.12	0.002653023	0.409285912	0.003215194	0.081436845	0.002596866	0.419231751	0.003214916	0.081419022	5.62E-05	0.009783611	1	0.756926483	1	0.768824902	ENSG00000186453	8	FAM228A
SAT	T2DM	ENSG00000196730.8	-0.000431499	0.90422326	0.003586023	0.081436956	-0.000541449	0.879975924	0.003585762	0.081419127	0.00010995	0.000337938	1	0.973026079	1	0.966700287	ENSG00000186453	8	FAM228A
SAT	T2DM	ENSG00000196735.7	-0.013411484	1.52E-08	0.002369952	0.081431607	0.000612058	0.891276763	0.004477742	0.081419128	-0.014023541	0.000223263	5.00E-05	2.76E-06	1	0.971822875	ENSG00000186453	8	FAM228A
SAT	T2DM	ENSG00000196743.4	-0.000546453	0.766277242	0.001838375	0.081436944	-0.000515393	0.779190095	0.001838229	0.081419118	-3.11E-05	0.029980513	1	0.934630346	1	0.941975941	ENSG00000186468	8	RPS23
SAT	T2DM	ENSG00000196793.9	0.002149266	0.434518877	0.00275024	0.081436856	0.002082965	0.448788272	0.002750017	0.081419035	6.63E-05	0.001340404	1	0.772274669	1	0.791227469	ENSG00000186468	8	RPS23
SAT	T2DM	ENSG00000196821.5	0.004887867	0.012279908	0.001952019	0.081435911	0.005103516	0.008938257	0.001952066	0.081417989	-0.000215648	1.59E-11	1	0.138932334	1	0.1231999	ENSG00000186468	8	RPS23
SAT	T2DM	ENSG00000196872.6	0.001797213	0.472704561	0.002502783	0.081436872	0.001757397	0.482534797	0.002502587	0.081419048	3.98E-05	0.044588361	1	0.796581235	1	0.804878835	ENSG00000186468	8	RPS23
SAT	T2DM	ENSG00000196961.8	-0.001957989	0.480877941	0.002777716	0.081436875	-0.00202048	0.466956053	0.002777517	0.081419042	6.25E-05	0.009732265	1	0.798461803	1	0.800114089	ENSG00000186470	9	BTN3A2
SAT	T2DM	ENSG00000196963.2	0.001187177	0.554253656	0.002007405	0.0814369	0.00121552	0.544799872	0.002007228	0.08141907	-2.83E-05	0.030899743	1	0.835948344	1	0.840498821	ENSG00000186470	9	BTN3A2
SAT	T2DM	ENSG00000196967.6	0.00395216	0.060988577	0.002109413	0.081436372	0.0039155	0.063403623	0.002109241	0.081418555	3.67E-05	0.019707556	1	0.34323074	1	0.365339457	ENSG00000186470	9	BTN3A2
SAT	T2DM	ENSG00000197062.7	-0.00335057	0.006283357	0.001226135	0.081435711	-0.00146661	0.251084042	0.001277845	0.081418911	-0.00188396	1.70E-07	1	0.086986048	1	0.622908325	ENSG00000186470	9	BTN3A2
SAT	T2DM	ENSG00000197165.6	0.002446279	0.10055924	0.001489679	0.081436508	0.002421589	0.104012536	0.00148956	0.081418689	2.47E-05	0.031678259	1	0.425727224	1	0.442053277	ENSG00000186470	9	BTN3A2
SAT	T2DM	ENSG00000197168.7	-0.002491755	0.437791144	0.003211318	0.081436858	-0.002405316	0.453811698	0.003211043	0.081419037	-8.64E-05	9.92E-05	1	0.77433571	1	0.794600418	ENSG00000186470	9	BTN3A2
SAT	T2DM	ENSG00000197181.7	0.004307661	0.287408469	0.004049219	0.081436769	0.004405531	0.276562874	0.004048925	0.081418933	-9.79E-05	0.00479201	1	0.644566475	1	0.64327119	ENSG00000186501	10	TMEM222
SAT	T2DM	ENSG00000197217.8	0.00012468	0.96854754	0.003162058	0.081436958	4.64E-05	0.988280126	0.003161874	0.081419131	7.82E-05	0.014525177	1	0.993613755	1	0.997162029	ENSG00000186522	10	SEPT10
SAT	T2DM	ENSG00000197223.7	-0.007386936	0.017752542	0.003115868	0.081436019	-0.007483515	0.016308975	0.003115619	0.081418167	9.66E-05	5.92E-05	1	0.172837064	1	0.177184592	ENSG00000186522	10	SEPT10
SAT	T2DM	ENSG00000197226.8	-0.004204542	0.004492361	0.001479765	0.081435609	-0.004173472	0.004793786	0.001479647	0.081417802	-3.11E-05	0.006349906	1	0.071190619	1	0.081103506	ENSG00000186532	7	SMYD4
SAT	T2DM	ENSG00000197279.3	-0.004669885	0.116447756	0.00297471	0.081436547	-0.001399485	0.644021648	0.003028641	0.081419095	-0.0032704	1.00E-08	1	0.4484332	1	0.888950368	ENSG00000186532	7	SMYD4
SAT	T2DM	ENSG00000197498.8	0.001820316	0.588967498	0.003368877	0.08143691	0.001770403	0.599192638	0.003368589	0.081419085	4.99E-05	0.033010396	1	0.846620707	1	0.855952281	ENSG00000186532	7	SMYD4
SAT	T2DM	ENSG00000197535.10	0.00258206	0.487957873	0.003722903	0.081436878	0.002244658	0.546890907	0.003726031	0.08141907	0.000337402	0.037599247	1	0.802903602	1	0.841095699	ENSG00000186532	7	SMYD4
SAT	T2DM	ENSG00000197563.5	0.000499041	0.681633973	0.001216478	0.08143693	0.000466139	0.70156019	0.001216389	0.081419106	3.29E-05	0.001599812	1	0.892786811	1	0.909657964	ENSG00000186567	8	CEACAM19
SAT	T2DM	ENSG00000197728.5	-0.001893909	0.023028815	0.000833234	0.081436095	-0.001872118	0.024648155	0.000833208	0.081418287	-2.18E-05	0.036606843	1	0.20700696	1	0.227804495	ENSG00000186567	8	CEACAM19
SAT	T2DM	ENSG00000197822.6	-1.30E-05	0.995044486	0.002094728	0.081436958	1.51E-05	0.994259478	0.00209454	0.081419131	-2.81E-05	0.033770903	1	0.99758396	1	0.998405122	ENSG00000186567	8	CEACAM19
SAT	T2DM	ENSG00000197847.8	-0.006908254	0.055333876	0.00360509	0.081436345	-0.007748233	0.031883475	0.003610758	0.081418362	0.000839979	5.94E-05	1	0.326529581	1	0.263779676	ENSG00000186567	8	CEACAM19
SAT	T2DM	ENSG00000197903.6	0.001440851	0.373531663	0.001619155	0.081436826	-0.000321647	0.845495427	0.001650578	0.081419125	0.001762498	4.17E-08	1	0.72766644	1	0.96477102	ENSG00000186575	13	NF2
SAT	T2DM	ENSG00000197935.6	-0.003003838	0.22762303	0.0024897	0.081436715	-0.002160647	0.386119647	0.002493025	0.081419005	-0.000843191	2.99E-10	1	0.591955891	1	0.746964998	ENSG00000186575	13	NF2
SAT	T2DM	ENSG00000197958.8	0.001328858	0.271769382	0.001209154	0.081436757	0.001361317	0.260193363	0.001209054	0.081418919	-3.25E-05	0.000249697	1	0.634354473	1	0.633304468	ENSG00000186577	7	C6orf1
SAT	T2DM	ENSG00000197969.7	0.002358014	0.227380475	0.001953396	0.081436715	0.002401053	0.218973104	0.001953243	0.081418878	-4.30E-05	0.00529635	1	0.591955891	1	0.592332986	ENSG00000186577	7	C6orf1
SAT	T2DM	ENSG00000198026.6	0.000218902	0.931402173	0.002542976	0.081436957	0.000271181	0.915068189	0.002542762	0.081419129	-5.23E-05	0.003961607	1	0.980452059	1	0.979654273	ENSG00000186665	8	C17orf58
SAT	T2DM	ENSG00000198093.6	-0.002217791	0.390875424	0.002584743	0.081436835	-0.002275997	0.378519387	0.002584514	0.081419001	5.82E-05	0.000483851	1	0.741307668	1	0.738661557	ENSG00000186665	8	C17orf58
SAT	T2DM	ENSG00000198105.7	-0.007517364	0.029065012	0.003444208	0.081436162	-0.00758277	0.027680739	0.003443914	0.081418321	6.54E-05	0.006376426	1	0.236875227	1	0.242234694	ENSG00000186665	8	C17orf58
SAT	T2DM	ENSG00000198131.9	0.00226256	0.288861749	0.002133237	0.08143677	0.002197486	0.302916131	0.002133065	0.081418954	6.51E-05	5.63E-05	1	0.645569425	1	0.671232195	ENSG00000186684	8	CYP27C1
SAT	T2DM	ENSG00000198169.4	0.005084311	0.18902139	0.003870866	0.08143667	0.005010243	0.195512964	0.003870578	0.081418851	7.41E-05	0.021870324	1	0.547377916	1	0.563318267	ENSG00000186715	6	MST1L
SAT	T2DM	ENSG00000198203.5	0.004093778	0.09827155	0.002476134	0.081436502	0.004040203	0.102724518	0.002475934	0.081418686	5.36E-05	0.00441697	1	0.421965667	1	0.440431278	ENSG00000186715	6	MST1L
SAT	T2DM	ENSG00000198208.7	0.00417232	0.288907089	0.003934208	0.08143677	0.004102424	0.297022523	0.003933893	0.081418949	6.99E-05	0.020122367	1	0.645569425	1	0.66437884	ENSG00000186715	6	MST1L
SAT	T2DM	ENSG00000198218.6	-0.002642833	0.113912066	0.001671776	0.081436541	-0.002671501	0.110014683	0.001671642	0.081418704	2.87E-05	0.025723022	1	0.443351708	1	0.452205306	ENSG00000186715	6	MST1L
SAT	T2DM	ENSG00000198315.6	0.004563166	0.200149419	0.003561835	0.081436684	0.003847549	0.280157731	0.003562656	0.081418936	0.000715617	1.26E-14	1	0.561024613	1	0.646143093	ENSG00000186715	6	MST1L
SAT	T2DM	ENSG00000198336.5	0.002134817	0.550155579	0.003572751	0.081436899	0.002074305	0.561482896	0.003572442	0.081419075	6.05E-05	0.012408345	1	0.83552458	1	0.84223981	ENSG00000186766	7	FOXI2
SAT	T2DM	ENSG00000198453.8	0.01172958	0.000536664	0.00338831	0.081434956	0.011664832	0.000575495	0.003388053	0.08141715	6.47E-05	0.019573323	1	0.015863016	1	0.019875797	ENSG00000186766	7	FOXI2
SAT	T2DM	ENSG00000198502.5	-0.004801532	1.79E-12	0.000681068	0.081428653	-0.002858768	0.00033098	0.000796362	0.081416978	-0.001942763	2.52E-06	5.88E-09	7.35E-10	1	0.013406744	ENSG00000186766	7	FOXI2
SAT	T2DM	ENSG00000198646.9	-0.001926087	0.584165728	0.003519185	0.081436908	-0.001791618	0.61066862	0.003519051	0.081419088	-0.000134469	0.001367534	1	0.845302694	1	0.863994715	ENSG00000186766	7	FOXI2
SAT	T2DM	ENSG00000198755.6	-0.002357641	0.117985272	0.001508128	0.08143655	-0.0026075	0.083891624	0.001508496	0.081418632	0.000249859	4.76E-10	1	0.452230931	1	0.414575198	ENSG00000186792	11	HYAL3
SAT	T2DM	ENSG00000198794.7	0.00102311	0.571488137	0.001808057	0.081436905	0.000983383	0.586485	0.001807898	0.081419081	3.97E-05	0.00092437	1	0.840736467	1	0.853229568	ENSG00000186792	11	HYAL3
SAT	T2DM	ENSG00000198837.5	-0.008559116	0.034593647	0.004050523	0.081436212	-0.008483407	0.036211183	0.004050221	0.081418398	-7.57E-05	0.025292101	1	0.257958536	1	0.276092282	ENSG00000186854	6	TRABD2A
SAT	T2DM	ENSG00000198874.8	0.002085105	0.21817771	0.001693294	0.081436705	0.002120059	0.210518845	0.001693147	0.081418869	-3.50E-05	0.002105074	1	0.582458151	1	0.579977552	ENSG00000186854	6	TRABD2A
SAT	T2DM	ENSG00000198945.3	-0.002435052	0.417197858	0.003001447	0.081436848	-0.002389288	0.425966209	0.003001188	0.081419025	-4.58E-05	0.025467427	1	0.763620456	1	0.776441739	ENSG00000186854	6	TRABD2A
SAT	T2DM	ENSG00000203301.2	-0.00036472	0.906879267	0.003117913	0.081436956	-0.000293131	0.925091158	0.003117662	0.081419129	-7.16E-05	0.002562097	1	0.974472349	1	0.981878481	ENSG00000186854	6	TRABD2A
SAT	T2DM	ENSG00000203652.3	0.000552485	0.855140497	0.00302626	0.081436953	0.000605127	0.841499926	0.003026008	0.081419124	-5.26E-05	0.015766408	1	0.963534946	1	0.96477102	ENSG00000186854	6	TRABD2A
SAT	T2DM	ENSG00000203705.6	-0.000512641	0.744297707	0.00157171	0.081436941	-0.00054974	0.726489673	0.001571588	0.08141911	3.71E-05	0.002981239	1	0.927287795	1	0.921455409	ENSG00000186867	6	QRFPR
SAT	T2DM	ENSG00000203778.3	-0.006265299	0.002288322	0.002054177	0.081435404	-0.006208468	0.002506473	0.002054033	0.081417604	-5.68E-05	0.001730483	1	0.045228813	1	0.054078961	ENSG00000186867	6	QRFPR
SAT	T2DM	ENSG00000203907.5	-0.003560809	0.239733445	0.003028788	0.081436727	-0.003486783	0.249605201	0.003028535	0.081418909	-7.40E-05	0.000676157	1	0.60584911	1	0.621386978	ENSG00000186889	5	TMEM17
SAT	T2DM	ENSG00000204060.4	-0.006261722	0.088770589	0.003679206	0.081436474	-0.00617543	0.093227474	0.003678881	0.08141866	-8.63E-05	0.000305785	1	0.410282965	1	0.428129221	ENSG00000187010	14	RHD
SAT	T2DM	ENSG00000204161.9	0.003499474	0.343266564	0.003692473	0.081436808	0.003409898	0.355721717	0.003692169	0.081418988	8.96E-05	0.000941446	1	0.703910998	1	0.724067999	ENSG00000187010	14	RHD
SAT	T2DM	ENSG00000204169.6	-0.000432973	0.791252974	0.001635812	0.081436947	-0.000399068	0.807247224	0.001635664	0.081419121	-3.39E-05	0.000847164	1	0.94681813	1	0.952410029	ENSG00000187013	2	C17orf82
SAT	T2DM	ENSG00000204177.6	0.001314617	0.59810692	0.002493936	0.081436912	0.001360483	0.58536762	0.00249373	0.081419081	-4.59E-05	0.012191499	1	0.84988688	1	0.853216864	ENSG00000187048	8	CYP4A11
SAT	T2DM	ENSG00000204227.4	-0.001586412	0.626993022	0.003264464	0.081436919	-0.003048117	0.352351894	0.003277412	0.081418986	0.001461706	7.26E-07	1	0.866453072	1	0.720881299	ENSG00000187048	8	CYP4A11
SAT	T2DM	ENSG00000204228.3	-0.00216211	0.426523393	0.002719103	0.081436853	-0.004358074	0.111525408	0.002738555	0.081418708	0.002195964	2.26E-11	1	0.77145714	1	0.45286493	ENSG00000187049	5	TMEM216
SAT	T2DM	ENSG00000204237.4	0.011874764	0.001713981	0.003786841	0.081435315	0.011762554	0.001893709	0.003786516	0.081417519	0.00011221	1.57E-05	1	0.038255586	1	0.047650044	ENSG00000187049	5	TMEM216

SAT	T2DM	ENSG00000204252.8	-0.001519933	0.556041193	0.002581705	0.0814369	-0.002684697	0.30198569	0.002600983	0.081418953	0.001164764	0.00025406	1	0.835948344	1	0.670829416	ENSG00000187049	5	TMEM216
SAT	T2DM	ENSG00000204305.9	0.008647889	0.001431037	0.002712353	0.08143526	-0.000576269	0.852781729	0.003105394	0.081419125	0.009224159	1.07E-09	1	0.03377865	1	0.965561588	ENSG00000187049	5	TMEM216
SAT	T2DM	ENSG00000204314.6	0.00867025	0.00037541	0.002437643	0.081434844	0.006909595	0.005866451	0.002507869	0.081417863	0.001760655	0.002861264	1	0.012965483	1	0.092094861	ENSG00000187049	5	TMEM216
SAT	T2DM	ENSG00000204316.8	0.001222942	0.70002394	0.003174095	0.081436934	0.001363262	0.667538459	0.003173853	0.0814191	-0.00014032	5.96E-08	1	0.902046837	1	0.896403813	ENSG00000187049	5	TMEM216
SAT	T2DM	ENSG00000204338.4	-0.000301164	0.824861155	0.001360901	0.08143695	0.003075668	0.049626088	0.001566678	0.081418487	-0.003376831	1.37E-05	1	0.955051074	1	0.324677987	ENSG00000187068	2	C3orf70
SAT	T2DM	ENSG00000204344.10	0.003060123	0.076892251	0.001729847	0.081436435	0.004050044	0.019881184	0.001739268	0.081418225	-0.00098992	5.94E-08	1	0.387125475	1	0.195966491	ENSG00000187091	9	PLCD1
SAT	T2DM	ENSG00000204348.5	-0.005686142	0.002766223	0.001900065	0.081435462	-0.004966238	0.010230045	0.00193392	0.081418029	-0.000719904	0.046339867	1	0.050988644	1	0.133193565	ENSG00000187091	9	PLCD1
SAT	T2DM	ENSG00000204356.7	0.005372573	0.000187889	0.001438518	0.081434627	0.003748757	0.010516772	0.001465292	0.081418037	0.001623815	6.33E-09	0.616462728	0.007553524	1	0.136385495	ENSG00000187105	4	HEATR4
SAT	T2DM	ENSG00000204410.10	0.005454754	0.053192903	0.002821391	0.081436334	0.003295674	0.259138543	0.002920581	0.081418918	0.00215908	0.004281412	1	0.320901873	1	0.631674265	ENSG00000187145	10	MRPS21
SAT	T2DM	ENSG00000204427.7	0.000900405	0.683532146	0.002208781	0.081436931	-0.00163063	0.465150872	0.002232541	0.081419042	0.002531035	9.03E-15	1	0.89384973	1	0.799014258	ENSG00000187145	10	MRPS21
SAT	T2DM	ENSG00000204428.8	-5.14E-05	0.964247975	0.001147715	0.081436958	-0.00047079	0.684317479	0.001157929	0.081419103	0.000419345	0.00660857	1	0.992060403	1	0.901372745	ENSG00000187145	10	MRPS21
SAT	T2DM	ENSG00000204438.6	-0.002576224	0.257862817	0.002276906	0.081436744	-0.000868633	0.71846866	0.002409481	0.081419109	-0.00170759	0.030432367	1	0.622209623	1	0.918559531	ENSG00000187166	1	H1FNT
SAT	T2DM	ENSG00000204463.8	0.004708289	0.001375216	0.001471423	0.081435247	0.002008444	0.188697938	0.001527981	0.081418842	0.002699846	5.89E-11	1	0.032934918	1	0.558266847	ENSG00000187166	1	H1FNT
SAT	T2DM	ENSG00000204498.6	0.009607239	0.002337094	0.003156446	0.08143541	0.004337524	0.184264656	0.003266848	0.081418836	0.005269715	4.13E-10	1	0.045916193	1	0.550111317	ENSG00000187166	1	H1FNT
SAT	T2DM	ENSG00000204516.5	0.000623056	0.456747651	0.000837201	0.081436866	8.37E-05	0.9216273	0.000851189	0.081419129	0.000539313	0.000468734	1	0.786668754	1	0.979654273	ENSG00000187166	1	H1FNT
SAT	T2DM	ENSG00000204520.8	-0.000949876	0.294942822	0.00090694	0.081436775	-0.001647376	0.070886672	0.000912064	0.081418586	0.0006975	8.38E-13	1	0.652072776	1	0.387631954	ENSG00000187260	11	WDR86
SAT	T2DM	ENSG00000204525.10	-0.000627864	0.609941474	0.001230726	0.081436915	-0.001540394	0.233208513	0.001292129	0.081418893	0.00091253	0.020559185	1	0.855586993	1	0.610313962	ENSG00000187595	10	ZNF385C
SAT	T2DM	ENSG00000204531.11	0.000592815	0.492851432	0.000864437	0.08143688	-0.000955323	0.292899835	0.00090829	0.081418946	0.001548138	2.91E-08	1	0.80490072	1	0.658673309	ENSG00000187630	10	DHRS4L2
SAT	T2DM	ENSG00000204536.9	0.000852857	0.278991834	0.000787794	0.081436763	-0.000427969	0.614205556	0.000849011	0.081419088	0.001280826	5.26E-05	1	0.63926506	1	0.867485217	ENSG00000187630	10	DHRS4L2
SAT	T2DM	ENSG00000204538.3	0.003098635	0.000179015	0.000826974	0.081434612	0.001675333	0.050612035	0.000857053	0.081418492	0.001423302	2.69E-10	0.58734888	0.007434796	1	0.326243786	ENSG00000187688	10	TRPV2
SAT	T2DM	ENSG00000204540.6	0.001516835	0.017171609	0.000636518	0.081436009	0.001198233	0.063096336	0.000644726	0.081418554	0.000318602	0.001974024	1	0.171768447	1	0.365113014	ENSG00000187688	10	TRPV2
SAT	T2DM	ENSG00000204574.8	0.001244035	0.585509112	0.002281144	0.081436909	-0.001973678	0.40181267	0.002354131	0.081419013	0.003217713	3.34E-08	1	0.845534946	1	0.757591271	ENSG00000187715	9	KBTBD12
SAT	T2DM	ENSG00000204592.5	0.003273725	0.164676557	0.002356019	0.081436636	-0.000245013	0.921727794	0.00249358	0.081419129	0.003518738	1.68E-05	1	0.516146667	1	0.979654273	ENSG00000187715	9	KBTBD12
SAT	T2DM	ENSG00000204622.6	0.004830308	0.000465236	0.001380074	0.081434911	0.004299143	0.002105722	0.001398133	0.081417551	0.000531165	0.018184564	1	0.014911936	1	0.049971446	ENSG00000187741	10	FANCA
SAT	T2DM	ENSG00000204632.7	0.00284377	0.17872044	0.002114787	0.081436656	0.004309662	0.046197513	0.00216176	0.081418467	-0.001465892	0.001104181	1	0.530902204	1	0.309334779	ENSG00000187741	10	FANCA
SAT	T2DM	ENSG00000204642.9	-0.000550549	0.777922388	0.001952111	0.081436945	0.000926027	0.637924135	0.001967744	0.081419094	-0.001476577	3.13E-09	1	0.939432466	1	0.884889906	ENSG00000187741	10	FANCA
SAT	T2DM	ENSG00000204644.5	0.001174122	0.102045629	0.000718109	0.081436512	-3.77E-06	0.996079761	0.000766978	0.081419131	0.001177891	1.25E-05	1	0.425727224	1	0.998405122	ENSG00000187741	10	FANCA
SAT	T2DM	ENSG00000204650.9	0.010244766	0.004115918	0.00357066	0.081435583	0.010322513	0.003838202	0.003570351	0.081417734	-7.77E-05	0.0013598	1	0.068549877	1	0.072792717	ENSG00000187741	10	FANCA
SAT	T2DM	ENSG00000204709.4	0.003423686	0.008724968	0.001305426	0.081435809	0.002354833	0.073608893	0.001316254	0.081418596	0.001068853	2.90E-10	1	0.11010239	1	0.395413696	ENSG00000187741	10	FANCA
SAT	T2DM	ENSG00000204713.6	-0.00178135	0.329280621	0.001825968	0.081436799	-0.001283938	0.484100055	0.001834921	0.081419049	-0.000497412	0.006576872	1	0.692334629	1	0.806185068	ENSG00000187741	10	FANCA
SAT	T2DM	ENSG00000204740.5	2.64E-05	0.991535653	0.002488484	0.081436958	-1.74E-05	0.994418751	0.00248827	0.081419131	4.38E-05	0.010052448	1	0.996461007	1	0.998405122	ENSG00000187762	5	HSPE1P11
SAT	T2DM	ENSG00000204778.4	0.003376442	0.38862464	0.003916463	0.081436834	0.00331346	0.397495033	0.003916137	0.081419011	6.30E-05	0.026562218	1	0.739604086	1	0.756180206	ENSG00000187762	5	HSPE1P11
SAT	T2DM	ENSG00000204789.3	0.002474154	0.17880126	0.001840263	0.081436656	0.001176047	0.525801959	0.001853713	0.081419064	0.001298108	7.41E-09	1	0.530902204	1	0.827413059	ENSG00000187775	12	DNAH17
SAT	T2DM	ENSG00000204792.2	-0.004015163	0.212753954	0.003222364	0.081436699	-0.003960701	0.218988087	0.003222112	0.081418878	-5.45E-05	0.032207396	1	0.575921597	1	0.592332986	ENSG00000187778	9	MCRS1
SAT	T2DM	ENSG00000204839.4	-0.000452065	0.851336847	0.002412129	0.081436953	-0.000512044	0.831876859	0.002411942	0.081419123	6.00E-05	0.001807536	1	0.963534946	1	0.961544037	ENSG00000187790	6	FANCM
SAT	T2DM	ENSG00000204946.5	0.001677212	0.565705945	0.002920002	0.081436903	0.001634978	0.575499084	0.002919752	0.081419079	4.22E-05	0.035949601	1	0.837959912	1	0.84981421	ENSG00000187790	6	FANCM
SAT	T2DM	ENSG00000204947.4	-0.002279113	0.366055394	0.002521455	0.081436822	-0.002314891	0.358537658	0.00252124	0.08141899	3.58E-05	0.040131813	1	0.721771482	1	0.725902637	ENSG00000187790	6	FANCM
SAT	T2DM	ENSG00000204954.5	-0.004962314	0.075495073	0.002791819	0.08143643	-0.004902749	0.079046212	0.0027916	0.081418616	-5.96E-05	0.006670282	1	0.382364025	1	0.407783994	ENSG00000187815	5	ZFP69
SAT	T2DM	ENSG00000204959.3	-0.001988983	0.464538195	0.002719437	0.081436869	-0.001934179	0.476895597	0.002719197	0.081419046	-5.48E-05	0.002025457	1	0.792954767	1	0.80363893	ENSG00000187824	4	TMEM220
SAT	T2DM	ENSG00000204991.6	0.003449187	0.033425819	0.001621669	0.081436202	0.003417118	0.035089586	0.00162154	0.081418389	3.21E-05	0.010644288	1	0.25564129	1	0.272172417	ENSG00000187837	2	HIST1H1C
SAT	T2DM	ENSG00000205078.5	0.004402675	0.081037792	0.002523457	0.08143645	0.004446486	0.078035545	0.002523253	0.081418612	-4.38E-05	0.0217247	1	0.395495266	1	0.406404163	ENSG00000187848	8	P2RX2
SAT	T2DM	ENSG00000205269.4	0.000578551	0.770329599	0.001981719	0.081436944	0.000655379	0.740843185	0.001981571	0.081419113	-7.68E-05	3.49E-06	1	0.935400227	1	0.925595787	ENSG00000187862	7	TTC24
SAT	T2DM	ENSG00000205309.9	0.000590573	0.817225551	0.00255532	0.081436949	0.00049539	0.846269733	0.002555135	0.081419125	9.52E-05	1.49E-05	1	0.952848981	1	0.96477102	ENSG00000187862	7	TTC24
SAT	T2DM	ENSG00000205485.9	-0.002028183	0.131949943	0.001346324	0.081436579	-0.002058829	0.126179715	0.001346216	0.08141874	3.06E-05	0.002990723	1	0.472458584	1	0.47645145	ENSG00000187942	7	LDLRAD2
SAT	T2DM	ENSG00000205710.3	0.002341195	0.095648826	0.001405003	0.081436494	0.002302577	0.101217854	0.001404883	0.081418682	3.86E-05	7.98E-05	1	0.420328914	1	0.440431278	ENSG00000187942	7	LDLRAD2
SAT	T2DM	ENSG00000205771.2	-0.004256393	0.187655445	0.003230539	0.081436668	-0.004390354	0.174112438	0.003230316	0.081418822	0.000133961	3.92E-06	1	0.54503883	1	0.532948952	ENSG00000187942	7	LDLRAD2
SAT	T2DM	ENSG00000206341.6	0.002237008	0.177930204	0.001660539	0.081436655	0.001116341	0.504158239	0.001671265	0.081419056	0.001120667	4.11E-09	1	0.530526907	1	0.817738235	ENSG00000187942	7	LDLRAD2
SAT	T2DM	ENSG00000206344.6	0.00550573	1.82E-10	0.000863484	0.081430165	0.004371075	6.79E-07	0.000879964	0.081415008	0.001134655	2.43E-11	5.96E-07	5.42E-08	0.002227894	0.000101268	ENSG00000187953	6	PMS2CL
SAT	T2DM	ENSG00000206503.7	-0.000774331	0.620397197	0.001563396	0.081436917	0.000946645	0.553157765	0.001596272	0.081419072	-0.001720977	1.00E-07	1	0.863241392	1	0.842050121	ENSG00000187987	5	ZSCAN23
SAT	T2DM	ENSG00000212694.4	-0.003329401	0.066389997	0.001813611	0.081436395	-0.003285499	0.070028251	0.001813458	0.081418582	-4.39E-05	0.000591347	1	0.358786109	1	0.38680588	ENSG00000187987	5	ZSCAN23
SAT	T2DM	ENSG00000213132.2	0.00057414	0.872211951	0.003569424	0.081436954	0.00066352	0.852520731	0.003569167	0.081419125	-8.94E-05	0.00390302	1	0.966468088	1	0.965561588	ENSG00000187987	5	ZSCAN23
SAT	T2DM	ENSG00000213277.3	0.004390328	0.1401703	0.002976171	0.081436595	0.004436417	0.13602182	0.002975918	0.08141876	-4.61E-05	0.026207892	1	0.484614073	1	0.492171492	ENSG00000187987	5	ZSCAN23
SAT	T2DM	ENSG00000213366.8	0.001263607	0.668153955	0.002947648	0.081436928	0.001393071	0.636471613	0.002947437	0.081419094	-0.000129463	4.26E-07	1	0.888630991	1	0.884482576	ENSG00000187987	5	ZSCAN23
SAT	T2DM	ENSG00000213402.2	0.001054228	0.551902499	0.001772074	0.081436899	0.00070615	0.690584919	0.001773971	0.081419104	0.000348079	5.28E-05	1	0.835918602	1	0.904075912	ENSG00000187987	5	ZSCAN23
SAT	T2DM	ENSG00000213443.2	-0.001459857	0.244708422	0.001254928	0.081436732	-0.001499141	0.232208405	0.001254836	0.081418892	3.93E-05	0.000224888	1	0.61016229	1	0.609500622	ENSG00000187987	5	ZSCAN23
SAT	T2DM	ENSG00000213445.4	0.008889284	0.001960715	0.00287109	0.081435356	0.007720366	0.007515601	0.002888168	0.081417937	0.001168918	0.000220585	1	0.041237855	1	0.108152135	ENSG00000187987	5	ZSCAN23
SAT	T2DM	ENSG00000213462.4	0.002398678	0.166508568	0.00173376	0.081436639	0.002429986	0.161008297	0.001733611	0.081418803	-3.13E-05	0.00853016	1	0.519037734	1	0.520626624	ENSG00000187987	5	ZSCAN23
SAT	T2DM	ENSG00000213523.5	-0.002174522	0.337392398	0.002266706	0.081436805	-0.002209477	0.329639921	0.002266501	0.081418972	3.50E-05	0.011803494	1	0.696216641	1	0.698223745	ENSG00000187987	5	ZSCAN23
SAT	T2DM	ENSG00000213676.6	0.004997626	0.000100214	0.001284702	0.08143443	0.002928322	0.026946831	0.001323659	0.081418313	0.002069303	9.10E-11	0.328801711	0.005087718	1	0.2402515	ENSG00000188051	6	TMEM221
SAT	T2DM	ENSG00000213689.5	-0.00181391	0.264773204	0.00162656	0.081436751	-0.001846454	0.256261461	0.001626441	0.081418916	3.25E-05	0.018718935	1	0.627688499	1	0.628866009	ENSG00000188056	7	TREML4
SAT	T2DM	ENSG00000213719.4	-0.013289074	0.000495011	0.003814901	0.081434931	-0.003607597	0.432260642	0.004593735	0.081419028	-0.009681477	0.000155352	1	0.015422559	1	0.781403397	ENSG00000188056	7	TREML4
SAT	T2DM	ENSG00000213760.6	0.00226023	0.081298129	0.001296593	0.081436451	0.001553301	0.245266586	0.001336835	0.081418905	0.000706928	0.030167642	1	0.395495266	1	0.620663712	ENSG00000188056	7	TREML4
SAT	T2DM	ENSG00000213839.4	0.002888057	0.439986035	0.003739953	0.081436859	0.002949653	0.430256875	0.003739642	0.081419027	-6.16E-05	0.023224664	1	0.776495555	1	0.780817802	ENSG00000188056	7	TREML4
SAT	T2DM	ENSG00000214078.7	-0.000889673	0.212303957	0.000713304	0.081436698	-0.000869873	0.222616135	0.000713243	0.081418882	-1.98E-05	8.08E-05	1	0.575921597	1	0.596734918	ENSG00000188086	8	PRSS45
SAT	T2DM	ENSG00000214087.4	-0.000518818	0.427252402	0.000653503	0.081436853	-0.000500692	0.443543005	0.000653453	0.081419033	-1.81E-05	0.000717382	1	0.771499796	1	0.789955502	ENSG00000188186	6	LAMTOR4
SAT	T2DM	ENSG00000214376.5	0.000758981	0.76588483	0.00254895	0.081436944	0.000899395	0.724181465	0.002548757	0.08141911	-0.000140414	1.73E-11	1	0.934630346	1	0.921192527	ENSG00000188227	8	ZNF793
SAT	T2DM	ENSG00000214425.2	-0.004514889	0.003932405	0.001565741	0.081435569	-0.004545639	0.003691053	0.001565605	0.081417722	3.08E-05	0.003572988	1	0.067550897	1	0.071237315	ENSG00000188234	9	AGAP4
SAT	T2DM	ENSG00000214435.3	-0.000499869	0.467468513	0.000687952	0.08143687	-0.000513604	0.455285334	0.000687894	0.081419038	1.37E-05	0.004241054	1	0.79334291	1	0.794600418	ENSG00000188234	9	AGAP4
SAT	T2DM	ENSG00000214517.4	-0.002950401	0.096345911	0.001774318	0.081436496	-0.002902283	0.101875849	0.001774196	0.081418684	-4.81E-05	0.002542572	1	0.420328914	1	0.440431278	ENSG00000188312	9	CENPP
SAT	T2DM	ENSG00000214659.4	-0.010280909	0.007720923	0.003859131	0.081435772	-0.009070516	0.01957536	0.003885593	0.08141822	-0.001210393	0.007977549	1	0.100525186	1	0.195966491	ENSG00000188312	9	CENPP
SAT	T2DM	ENSG00000214982.6	0.004435368	0.260994775	0.003945901	0.081436747	0.004375656	0.267426693	0.003945554	0.081418925	5.97E-05	0.020259644	1	0.625510487	1	0.638593145	ENSG00000188312	9	CENPP
SAT	T2DM	ENSG00000216901.1	0.001649287	0.108565572	0.0010278	0.081436528	0.000564156	0.588236904	0.001042046	0.081419082	0.001085131	3.07E-10	1	0.436210685	1	0.853229568	ENSG00000188321	9	ZNF559
SAT	T2DM	ENSG00000219392.1	-0.00254718	0.014214627	0.001038901	0.081435954	-0.00105198	0.333019269	0.001086698	0.081418974	-0.0014952	2.79E-06	1	0.151551916	1	0.701740984	ENSG00000188321	9	ZNF559
SAT	T2DM	ENSG00000219481.6	-0.000397369	0.871105763	0.002449052	0.081436954	-0.000353541	0.885209233	0.002448875	0.081419127	-4.38E-05	0.038174586	1	0.966468088	1	0.968806494	ENSG00000188321	9	ZNF559
SAT	T2DM	ENSG00000219755.1	0.000988111	0.430042674	0.001252169	0.081436854	0.000959859	0.443306396	0.001252062	0.081419033	2.83E-05	0.001118646	1	0.772274669	1	0.789955502	ENSG00000188321	9	ZNF559
SAT	T2DM	ENSG00000219891.2	0.003335252	0.111328172	0.002094673	0.081436535	0.004067576	0.05254423	0.002098146	0.081418503	-0.000732324	4.21E-09	1	0.437446384	1	0.332371916	ENSG00000188321	9	ZNF559
SAT	T2DM	ENSG00000220267.1	0.000454798	0.778488232	0.001616829	0.081436945	0.000495874	0.759058948	0.001616715	0.081419115	-4.11E-05	0.003932652	1	0.939432466	1	0.932998601	ENSG00000188372	10	ZP3
SAT	T2DM	ENSG00000221823.6	0.001424743	0.70009969	0.003698843	0.081436934	0.001503845	0.68430185	0.003698585	0.081419103	-7.91E-05	0.01639872	1	0.902046837	1	0.901372745	ENSG00000188372	10	ZP3
SAT	T2DM	ENSG00000221829.5	-0.003282082	0.095844827	0.001970814	0.081436495	-0.003320897	0.091954553	0.001970648	0.081418656	3.88E-05	0.00600424	1	0.420328914	1	0.425532987	ENSG00000188383	7	AC018892.9
SAT	T2DM	ENSG00000221944.3	-0.000901619	0.626601516	0.001853215	0.081436919	-0.000867247	0.639778242	0.001853057	0.081419094	-3.44E-05	0.007475566	1	0.866453072	1	0.885701439	ENSG00000188404	4	SELL
SAT	T2DM	ENSG00000221995.4	0.005887497	0.08201443	0.003385333	0.081436453	0.005827608	0.085146407	0.003385036	0.081418636	5.99E-05	0.007544119	1	0.395495266	1	0.415104547	ENSG00000188404	4	SELL
SAT	T2DM	ENSG00000222009.4	0.002904687	0.438817279	0.003751892	0.081436858	0.002977323	0.427417615	0.003751577	0.081419026	-7.26E-05	0.006600372	1	0.775314751	1	0.777953168	ENSG00000188404	4	SELL
SAT	T2DM	ENSG00000223509.4	0.003986465	0.307270262	0.003904594	0.081436784	0.004063265	0.298010478	0.003904318	0.08141895	-7.68E-05	0.025453179	1	0.667651477	1	0.664773075	ENSG00000188488	9	SERPINA5
SAT	T2DM	ENSG00000223756.2	-0.008984814	0.000481053	0.002573624	0.081434922	-0.009024297	0.000453644	0.002573403	0.081417076	3.95E-05	0.025640364	1	0.015176308	1	0.017307053	ENSG00000188488	9	SERPINA5
SAT	T2DM	ENSG00000223865.6	0.001618368	0.535686189	0.002613011	0.081436894	0.003080055	0.245604952	0.002652722	0.081418906	-0.001461687	0.001447602	1	0.828930489	1	0.620663712	ENSG00000188493	10	C19orf54
SAT	T2DM	ENSG00000224389.4	0.004647019	0.011291585	0.001834191	0.081435886	0.001527378	0.473770219	0.002132142	0.081419045	0.00311964	0.004119519	1	0.131589853	1	0.80363893	ENSG00000188529	10	SRSF10
SAT	T2DM	ENSG00000224497.1	0.001194064	0.143390595	0.000816017	0.081436601	0.001162292	0.154317138	0.000815959	0.081418792	3.18E-05	8.17E-06	1	0.488540542	1	0.514547286	ENSG00000188529	10	SRSF10
SAT	T2DM	ENSG00000224557.3	0.00180599	0.00825561	0.000683713	0.081435792	0.001146993	0.095995262	0.000689054	0.081418668	0.000658997	2.09E-14	1	0.105807246	1	0.432044519	ENSG00000188599	13	NPIPP1
SAT	T2DM	ENSG00000224796.1	-0.00245562	0.001916615	0.000791401	0.081435349	-0.000914995	0.262402834	0.000816427	0.081418921	-0.001540625	1.74E-14	1	0.04093133	1	0.636156586	ENSG00000188611	10	ASAH2
SAT	T2DM	ENSG00000224843.2	0.007278909	0.037726636	0.00350315	0.081436237	0.004057193	0.255536174	0.003568312	0.081418915	0.003221716	2.22E-06	1	0.269351866	1	0.62755553	ENSG00000188629	7	ZNF177
SAT	T2DM	ENSG00000224930.2	-0.005621615	0.062399123	0.003016773	0.081436378	-0.005668567	0.060222628	0.003016536	0.081418541	4.70E-05	0.048360112	1	0.346391409	1	0.35334636	ENSG00000188629	7	ZNF177
SAT	T2DM	ENSG00000225492.2	-0.000580233	0.858588204	0.003256594	0.081436953	-0.000637936	0.844685018	0.003256363	0.081419124	5.77E-05	0.043493726	1	0.964974089	1	0.96477102	ENSG00000188629	7	ZNF177
SAT	T2DM	ENSG00000225498.1	0.007537057	0.00633059	0.002760665	0.081435713	0.007435898	0.007065963	0.002760446	0.081417919	0.000101159	2.15E-06	1	0.087271699	1	0.103497438	ENSG00000188629	7	ZNF177
SAT	T2DM	ENSG00000225784.5	-0.001258728	0.407705085	0.001520314	0.081436844	-0.001228284	0.419097565	0.001520175	0.081419022	-3.04E-05	0.000852847	1	0.756247299	1	0.768824902	ENSG00000188659	5	FAM154B
SAT	T2DM	ENSG00000225880.4	0.001946445	0.506208932	0.002928074	0.081436885	0.001994306	0.495776053	0.002927845	0.081419053	-4.79E-05	0.038965496	1	0.810627063	1	0.814419437	ENSG00000188662	5	HILS1
SAT	T2DM	ENSG00000226015.2	-0.005456986	0.025028065	0.002435093	0.081436119	-0.005415484	0.026142267	0.002434912	0.081418304	-4.15E-05	0.041254361	1	0.216409969	1	0.235639496	ENSG00000188662	5	HILS1
SAT	T2DM	ENSG00000226194.1	0.002362471	0.221447279	0.001932191	0.081436709	0.00232839	0.228146649	0.001932036	0.081418888	3.41E-05	0.021588454	1	0.586129584	1	0.602834606	ENSG00000188672	12	RHCE
SAT	T2DM	ENSG00000226259.6	0.005369819	0.142984936	0.003665986	0.0814366	0.005276595	0.15001959	0.003665665	0.081418785	9.32E-05	0.00011916	1	0.488540542	1	0.51082416	ENSG00000188677	10	PARVB
SAT	T2DM	ENSG00000226266.2	-0.007104829	0.024728686	0.003163858	0.081436116	-0.007051059	0.025827639	0.003163611	0.081418301	-5.38E-05	0.031805552	1	0.21611556	1	0.234089731	ENSG00000188677	10	PARVB
SAT	T2DM	ENSG00000226278.1	0.00270996	0.109346375	0.001692518	0.08143653	0.002681437	0.113098016	0.001692374	0.081418711	2.85E-05	0.015271243	1	0.436210685	1	0.455306244	ENSG00000188707	4	ZBED6CL
SAT	T2DM	ENSG00000226314.3	-0.000475896	0.637323024	0.001009442	0.081436921	-0.000225539	0.82406391	0.001014473	0.081419123	-0.000250358	0.014114501	1	0.874187643	1	0.959280742	ENSG00000188738	9	FSIP2
SAT	T2DM	ENSG00000226328.2	0.00389933	0.134194769	0.002603431	0.081436584	0.003860173	0.138114874	0.002603212	0.081418764	3.92E-05	0.034250811	1	0.475922014	1	0.49579311	ENSG00000188760	6	TMEM198
SAT	T2DM	ENSG00000226479.3	-0.003422737	0.277263741	0.003150277	0.081436761	-0.003498345	0.266751797	0.00315003	0.081418925	7.56E-05	0.002385289	1	0.63926506	1	0.638375379	ENSG00000188786	9	MTF1
SAT	T2DM	ENSG00000227124.4	-9.60E-05	0.883274556	0.000654199	0.081436955	-0.000105889	0.871405546	0.000654144	0.081419127	9.84E-06	0.032821941	1	0.969020186	1	0.966383771	ENSG00000188786	9	MTF1
SAT	T2DM	ENSG00000227766.1	0.001334259	0.435625784	0.001711463	0.081436857	0.000581225	0.735661272	0.001721613	0.081419112	0.000753035	6.40E-05	1	0.772274669	1	0.92442683	ENSG00000188818	8	ZDHHC11
SAT	T2DM	ENSG00000227827.3	0.003127206	0.014324407	0.001276915	0.081435956	0.003161393	0.013286035	0.001276806	0.081418107	-3.42E-05	0.000112973	1	0.151551916	1	0.157369966	ENSG00000188820	8	FAM26F
SAT	T2DM	ENSG00000227877.2	-0.001722781	0.656749097	0.00387659	0.081436925	-0.002023072	0.601925271	0.003878334	0.081419085	0.000300291	0.020595442	1	0.884093538	1	0.858221553	ENSG00000188820	8	FAM26F
SAT	T2DM	ENSG00000228049.5	-0.000504792	0.588100195	0.000932052	0.081436909	-0.000531025	0.568826683	0.000931981	0.081419077	2.62E-05	0.000558332	1	0.846296816	1	0.845271624	ENSG00000188868	9	ZNF563
SAT	T2DM	ENSG00000228078.1	0.000781882	0.555503335	0.001326272	0.0814369	-0.00045773	0.734982957	0.001352212	0.081419112	0.001239613	2.73E-06	1	0.835948344	1	0.92442683	ENSG00000188878	12	FBF1
SAT	T2DM	ENSG00000228106.1	0.002527176	0.227551921	0.002094303	0.081436715	0.002559974	0.221536131	0.002094125	0.081418881	-3.28E-05	0.025313334	1	0.591955891	1	0.595786923	ENSG00000188878	12	FBF1
SAT	T2DM	ENSG00000228157.2	0.000486537	0.903175431	0.003999449	0.081436956	0.00039606	0.921109019	0.003999118	0.081419129	9.05E-05	0.001895663	1	0.97231278	1	0.979654273	ENSG00000188878	12	FBF1
SAT	T2DM	ENSG00000228300.9	-0.005303212	0.091441358	0.003141999	0.081436482	-0.005238422	0.0954423	0.003141735	0.081418666	-6.48E-05	0.003734136	1	0.413867045	1	0.432044519	ENSG00000188878	12	FBF1
SAT	T2DM	ENSG00000228446.2	0.000311064	0.831554522	0.001462396	0.081436951	0.000347564	0.812123483	0.001462273	0.081419121	-3.65E-05	0.000497552	1	0.956754735	1	0.95480666	ENSG00000188895	7	MSL1
SAT	T2DM	ENSG00000228653.2	5.85E-05	0.973121966	0.001736772	0.081436958	3.20E-05	0.985278878	0.001736633	0.081419131	2.65E-05	0.046573849	1	0.995031844	1	0.997162029	ENSG00000188906	9	LRRK2
SAT	T2DM	ENSG00000228696.4	-0.005316958	0.110625407	0.003332698	0.081436533	-0.005252317	0.114999267	0.003332439	0.081418716	-6.46E-05	0.014746415	1	0.436277088	1	0.456639917	ENSG00000188906	9	LRRK2
SAT	T2DM	ENSG00000228789.2	-0.001746334	0.10038607	0.001062901	0.081436507	-0.00079551	0.458773502	0.001073753	0.081419039	-0.000950825	5.23E-10	1	0.425727224	1	0.795972372	ENSG00000188921	12	PTPLAD2
SAT	T2DM	ENSG00000228914.1	-0.003995416	0.013285089	0.001613629	0.081435934	-0.004022657	0.012662692	0.001613499	0.081418092	2.72E-05	0.025721566	1	0.145107	1	0.151628804	ENSG00000188921	12	PTPLAD2
SAT	T2DM	ENSG00000228960.4	-0.00033087	0.927091353	0.003615864	0.081436957	-0.000265705	0.941416625	0.003615554	0.08141913	-6.52E-05	0.008769647	1	0.978695859	1	0.986380169	ENSG00000188921	12	PTPLAD2
SAT	T2DM	ENSG00000229291.1	0.000859345	0.68768034	0.002137647	0.081436931	0.000895557	0.675228851	0.002137456	0.081419102	-3.62E-05	0.007553126	1	0.896416049	1	0.898778511	ENSG00000188958	5	UTS2B
SAT	T2DM	ENSG00000229390.1	4.18E-05	0.985337155	0.002274988	0.081436958	-0.001317125	0.563741193	0.00228155	0.081419075	0.001358935	1.22E-14	1	0.996461007	1	0.84223981	ENSG00000189007	11	ADAT2
SAT	T2DM	ENSG00000229425.1	-0.000961312	0.693262707	0.002437209	0.081436932	-0.000910677	0.708640701	0.002437034	0.081419108	-5.06E-05	0.016505543	1	0.900268043	1	0.912941362	ENSG00000189007	11	ADAT2
SAT	T2DM	ENSG00000230224.1	-0.009995794	0.003610869	0.003434615	0.081435543	-0.010080736	0.003332494	0.003434321	0.081417691	8.49E-05	0.000372067	1	0.062747674	1	0.065866952	ENSG00000189007	11	ADAT2
SAT	T2DM	ENSG00000230295.1	-0.001145357	0.507228698	0.001727121	0.081436885	-0.001188077	0.491483089	0.001726976	0.081419052	4.27E-05	0.000502991	1	0.810627063	1	0.811177221	ENSG00000189011	9	AC138783.12
SAT	T2DM	ENSG00000230387.1	0.002715431	0.213986949	0.002185143	0.0814367	0.002754633	0.207411364	0.002184973	0.081418865	-3.92E-05	0.023759113	1	0.576804422	1	0.579977552	ENSG00000189011	9	AC138783.12
SAT	T2DM	ENSG00000230438.5	-0.000554413	0.764664305	0.001851984	0.081436943	-0.000596253	0.747466452	0.001851821	0.081419114	4.18E-05	0.000574614	1	0.93459819	1	0.927854665	ENSG00000189011	9	AC138783.12
SAT	T2DM	ENSG00000230521.1	-0.000438118	0.670619838	0.001030145	0.081436928	-0.000318222	0.757470961	0.001030499	0.081419115	-0.000119896	0.000110138	1	0.889125844	1	0.932832405	ENSG00000189014	6	FAM35DP
SAT	T2DM	ENSG00000230555.2	0.000403881	0.915125665	0.003789618	0.081436957	0.000472561	0.900753338	0.003789285	0.081419128	-6.87E-05	0.005827824	1	0.977304164	1	0.972878089	ENSG00000189045	9	ANKDD1B
SAT	T2DM	ENSG00000230596.2	0.00075796	0.668895219	0.001772321	0.081436928	0.000789464	0.655976039	0.001772182	0.081419098	-3.15E-05	0.023564864	1	0.888630991	1	0.891942554	ENSG00000189045	9	ANKDD1B
SAT	T2DM	ENSG00000231130.1	0.001468482	0.27323122	0.00134028	0.081436758	0.002197974	0.112686458	0.001385654	0.08141871	-0.000729492	0.038366465	1	0.634986223	1	0.4547654	ENSG00000189045	9	ANKDD1B
SAT	T2DM	ENSG00000231412.2	-0.000683935	0.75977819	0.002236746	0.081436943	-0.000713919	0.749571094	0.002236553	0.081419114	3.00E-05	0.049849077	1	0.934086217	1	0.928470414	ENSG00000189046	6	ALKBH2
SAT	T2DM	ENSG00000231784.4	-0.004191415	0.131608034	0.002779834	0.081436578	-0.004242207	0.126960348	0.002779585	0.081418742	5.08E-05	0.003967916	1	0.472458584	1	0.476745292	ENSG00000189046	6	ALKBH2
SAT	T2DM	ENSG00000231793.4	0.001785213	0.649576422	0.003929148	0.081436924	0.000401761	0.918850397	0.003943401	0.081419129	0.001383451	4.71E-05	1	0.881625728	1	0.979654273	ENSG00000189046	6	ALKBH2
SAT	T2DM	ENSG00000231852.2	0.009025723	1.22E-14	0.001170015	0.081427015	0.006404531	4.68E-06	0.001398775	0.081415628	0.002621192	0.000628984	4.00E-11	5.72E-12	0.015356883	0.000430718	ENSG00000189046	6	ALKBH2
SAT	T2DM	ENSG00000231925.7	0.003348955	0.060521735	0.001784227	0.08143637	0.004356753	0.014913948	0.00178961	0.081418141	-0.001007798	9.67E-13	1	0.342956501	1	0.166753416	ENSG00000189068	5	VSTM1
SAT	T2DM	ENSG00000232388.2	6.80E-05	0.968780982	0.001736283	0.081436958	0.00012058	0.944630363	0.001736174	0.08141913	-5.26E-05	0.001719646	1	0.993613755	1	0.987677572	ENSG00000189143	8	CLDN4
SAT	T2DM	ENSG00000232629.4	0.004746812	3.98E-08	0.000864329	0.081431918	-0.000561416	0.645547911	0.001220586	0.081419096	0.005308228	7.33E-10	0.000130525	6.53E-06	1	0.889933905	ENSG00000189144	9	ZNF573
SAT	T2DM	ENSG00000233005.1	0.001594217	0.455289866	0.00213523	0.081436865	0.001529931	0.473635671	0.002135055	0.081419045	6.43E-05	5.53E-05	1	0.786213711	1	0.80363893	ENSG00000189144	9	ZNF573

SAT	T2DM	ENSG00000233265.1	0.000289591	0.796112129	0.001120786	0.081436947	-0.000216147	0.847532449	0.001124199	0.081419125	0.000505738	1.37E-08	1	0.94681813	1	0.965539571	ENSG00000189157	9	FAM47E
SAT	T2DM	ENSG00000233448.2	0.002904765	0.170804089	0.002120844	0.081436645	0.002848382	0.179222573	0.002120666	0.081418829	5.64E-05	0.000184635	1	0.524235936	1	0.541962453	ENSG00000189157	9	FAM47E
SAT	T2DM	ENSG00000233836.3	-0.008949515	0.024064466	0.003966798	0.081436108	-0.008871912	0.025305211	0.003966486	0.081418295	-7.76E-05	0.012601856	1	0.212818095	1	0.232403611	ENSG00000189157	9	FAM47E
SAT	T2DM	ENSG00000233892.1	0.006887001	0.028861959	0.003151407	0.08143616	0.005655124	0.073591457	0.00316079	0.081418596	0.001231876	6.61E-07	1	0.236149844	1	0.395413696	ENSG00000189180	11	ZNF33A
SAT	T2DM	ENSG00000233916.1	0.002055456	0.253232447	0.001799032	0.08143674	0.000735335	0.683947652	0.001806355	0.081419103	0.00132012	1.07E-15	1	0.618206663	1	0.901372745	ENSG00000189180	11	ZNF33A
SAT	T2DM	ENSG00000234127.4	0.00186506	0.392287505	0.00218014	0.081436836	-0.000748342	0.734022583	0.002202434	0.081419112	0.002613402	9.03E-17	1	0.742778516	1	0.924370544	ENSG00000189180	11	ZNF33A
SAT	T2DM	ENSG00000234147.1	0.000973403	0.640652714	0.002085328	0.081436922	0.00091666	0.660221847	0.002085179	0.081419099	5.67E-05	0.00180437	1	0.875315948	1	0.894350146	ENSG00000189182	5	KRT77
SAT	T2DM	ENSG00000234409.4	-0.003408205	0.148005535	0.002355987	0.081436609	-0.003378653	0.151515558	0.002355774	0.081418787	-2.96E-05	0.041980625	1	0.493001178	1	0.511442948	ENSG00000189182	5	KRT77
SAT	T2DM	ENSG00000234444.5	0.000870118	0.490799434	0.0012628	0.081436879	0.000843226	0.504263183	0.001262698	0.081419056	2.69E-05	0.004697495	1	0.80382562	1	0.817738235	ENSG00000189190	7	ZNF600
SAT	T2DM	ENSG00000234745.5	0.00623999	9.62E-05	0.001599952	0.081434416	0.004396489	0.006772984	0.001623629	0.081417906	0.001843501	2.92E-11	0.315506995	0.005008048	1	0.100552768	ENSG00000189190	7	ZNF600
SAT	T2DM	ENSG00000235081.1	0.013959332	0.000159101	0.00369642	0.081434575	0.013909923	0.000167623	0.003696083	0.081416764	4.94E-05	0.027055031	0.522008791	0.006960117	0.549971522	0.00820853	ENSG00000189190	7	ZNF600
SAT	T2DM	ENSG00000235109.3	0.002269822	0.101452258	0.001385849	0.08143651	0.00102071	0.467485129	0.001404819	0.081419043	0.001249113	6.37E-08	1	0.425727224	1	0.800114089	ENSG00000189190	7	ZNF600
SAT	T2DM	ENSG00000235290.1	0.001569904	0.359477273	0.001713191	0.081436818	0.001755709	0.305767191	0.001714317	0.081418956	-0.000185805	0.005620646	1	0.71830389	1	0.674208436	ENSG00000189190	7	ZNF600
SAT	T2DM	ENSG00000235370.5	-0.003989875	0.269392172	0.003612497	0.081436755	-0.003913502	0.278638569	0.003612294	0.081418935	-7.64E-05	0.040516972	1	0.632695575	1	0.644397089	ENSG00000189190	7	ZNF600
SAT	T2DM	ENSG00000235477.2	-0.0036547	0.027228761	0.001653121	0.081436144	-0.003603497	0.029258549	0.001652986	0.081418337	-4.70E-05	0.000138903	1	0.227073047	1	0.251961415	ENSG00000189269	8	C22orf43
SAT	T2DM	ENSG00000235641.3	-0.000746144	0.569399276	0.001311471	0.081436904	-0.00076857	0.557818147	0.001311362	0.081419074	2.24E-05	0.017109251	1	0.840736467	1	0.842050121	ENSG00000189298	9	ZKSCAN3
SAT	T2DM	ENSG00000235821.1	-0.000868223	0.515621483	0.001335502	0.081436888	0.000400005	0.764892731	0.001373607	0.081419117	-0.001268228	8.15E-05	1	0.816877879	1	0.938518235	ENSG00000189298	9	ZKSCAN3
SAT	T2DM	ENSG00000235878.4	-0.001114199	0.278930864	0.001029069	0.081436763	-0.001092177	0.288516869	0.001029014	0.081418943	-2.20E-05	0.042673446	1	0.63926506	1	0.65329458	ENSG00000189298	9	ZKSCAN3
SAT	T2DM	ENSG00000235963.1	-0.001682537	0.434352861	0.002152228	0.081436856	-6.84E-05	0.975259098	0.002207039	0.081419131	-0.00161409	0.000983787	1	0.772274669	1	0.99701704	ENSG00000189298	9	ZKSCAN3
SAT	T2DM	ENSG00000236104.2	0.000772309	0.764806833	0.002581463	0.081436943	0.001091493	0.672940808	0.002585767	0.081419101	-0.000319184	0.03813576	1	0.93459819	1	0.898778511	ENSG00000189306	6	RRP7A
SAT	T2DM	ENSG00000236209.1	-0.002128142	0.271838583	0.001936717	0.081436757	-0.002091752	0.280080991	0.001936559	0.081418936	-3.64E-05	0.011361075	1	0.634354473	1	0.646143093	ENSG00000189306	6	RRP7A
SAT	T2DM	ENSG00000236682.1	-0.001988162	0.375230157	0.002242153	0.081436827	-0.00194215	0.386345505	0.002241983	0.081419006	-4.60E-05	0.012012602	1	0.728082648	1	0.746964998	ENSG00000189306	6	RRP7A
SAT	T2DM	ENSG00000236710.1	0.002845351	0.425817474	0.003572902	0.081436852	0.002780775	0.436357613	0.003572611	0.08141903	6.46E-05	0.015786499	1	0.771328212	1	0.784487303	ENSG00000189306	6	RRP7A
SAT	T2DM	ENSG00000237510.3	-0.001426905	0.369734914	0.001590804	0.081436824	-0.001391849	0.381570907	0.001590673	0.081419003	-3.51E-05	0.002769309	1	0.723806834	1	0.742106784	ENSG00000189339	7	SLC35E2B
SAT	T2DM	ENSG00000237669.1	0.000445067	0.604833355	0.000860094	0.081436914	-0.000667057	0.458102908	0.000899029	0.081419039	0.001112124	2.19E-05	1	0.83560529	1	0.795256953	ENSG00000189339	7	SLC35E2B
SAT	T2DM	ENSG00000237927.1	0.004187635	0.263956971	0.003748704	0.08143675	0.004253354	0.256496119	0.003748396	0.081418916	-6.57E-05	0.018050807	1	0.627688499	1	0.628971425	ENSG00000189362	7	TMEM194B
SAT	T2DM	ENSG00000238024.1	0.004934119	0.194277099	0.003801231	0.081436677	0.000520736	0.893585851	0.003892824	0.081419128	0.004413382	1.56E-07	1	0.553799446	1	0.972100523	ENSG00000189362	7	TMEM194B
SAT	T2DM	ENSG00000238083.3	0.004392815	0.177789492	0.003259746	0.081436655	0.004461645	0.171056251	0.003259483	0.081418818	-6.88E-05	0.005522802	1	0.530526907	1	0.531976833	ENSG00000189367	10	KIAA0408
SAT	T2DM	ENSG00000238286.1	-0.001157049	0.562177221	0.00199625	0.081436902	-0.001117686	0.575521846	0.001996089	0.081419079	-3.94E-05	0.008968519	1	0.837959912	1	0.84981421	ENSG00000189398	5	OR7E12P
SAT	T2DM	ENSG00000239257.1	0.00146518	0.064555474	0.000792705	0.081436387	0.000399178	0.626003752	0.000819063	0.081419091	0.001066002	2.42E-07	1	0.353010849	1	0.876644981	ENSG00000196072	7	BLOC1S2
SAT	T2DM	ENSG00000239388.4	0.006043307	0.020446433	0.002607053	0.081435606	0.006075187	0.019780066	0.002606815	0.081418223	-3.19E-05	0.043994014	1	0.190581663	1	0.195966491	ENSG00000196072	7	BLOC1S2
SAT	T2DM	ENSG00000239557.1	0.013320553	0.001095669	0.00408011	0.081435177	0.013394316	0.001026754	0.004079763	0.0814173	-7.38E-05	0.010026356	1	0.027029242	1	0.029550704	ENSG00000196072	7	BLOC1S2
SAT	T2DM	ENSG00000239697.6	-0.000962413	0.621198317	0.001947602	0.081436918	-0.000920848	0.636320842	0.001947445	0.081419094	-4.16E-05	0.005033029	1	0.863989689	1	0.884482576	ENSG00000196126	6	HLA-DRB1
SAT	T2DM	ENSG00000240065.3	0.000431847	0.832164297	0.002037717	0.081436951	-0.003765401	0.074027384	0.002107759	0.081418598	0.004197248	7.44E-15	1	0.957003525	1	0.395576296	ENSG00000196126	6	HLA-DRB1
SAT	T2DM	ENSG00000240399.1	-0.00767506	0.037004009	0.003679804	0.081436231	-0.007759487	0.034962023	0.003679568	0.081418388	8.44E-05	0.016114346	1	0.266835505	1	0.272172417	ENSG00000196126	6	HLA-DRB1
SAT	T2DM	ENSG00000240522.1	-0.000238853	0.951300393	0.003910882	0.081436958	-0.000304694	0.937895404	0.003910581	0.0814193	6.58E-05	0.037057914	1	0.988605339	1	0.985647128	ENSG00000196126	6	HLA-DRB1
SAT	T2DM	ENSG00000240972.6	-0.000639106	0.468188339	0.000881003	0.081436873	-0.000626184	0.477193904	0.000880927	0.081419046	-1.29E-05	0.034087623	1	0.79334291	1	0.80363893	ENSG00000196126	6	HLA-DRB1
SAT	T2DM	ENSG00000241186.3	-0.00227038	0.328368501	0.002322862	0.081436799	-0.0023141	0.319098592	0.002322663	0.081418965	4.37E-05	0.006409963	1	0.692334629	1	0.690153251	ENSG00000196151	6	WDSUB1
SAT	T2DM	ENSG00000241549.4	-0.002038586	0.056960991	0.001070903	0.081436353	-0.001937899	0.070412904	0.001071107	0.081418584	-0.000100687	0.000122383	1	0.331288318	1	0.386976108	ENSG00000196151	6	WDSUB1
SAT	T2DM	ENSG00000241837.2	0.000517469	0.88572031	0.003600495	0.081436955	0.000596432	0.86841992	0.003600209	0.081419126	-7.90E-05	0.004611415	1	0.969439538	1	0.966241215	ENSG00000196151	6	WDSUB1
SAT	T2DM	ENSG00000242086.4	-0.001926909	0.282488777	0.001792896	0.081436765	-0.001964804	0.273089832	0.001792744	0.08141893	3.79E-05	0.00248837	1	0.641415693	1	0.64192524	ENSG00000196151	6	WDSUB1
SAT	T2DM	ENSG00000242110.3	0.000569306	0.572346372	0.001008336	0.081436905	0.000585829	0.561218956	0.001008255	0.081419074	-1.65E-05	0.031549695	1	0.840736467	1	0.84223981	ENSG00000196151	6	WDSUB1
SAT	T2DM	ENSG00000242372.1	-0.000425702	0.893456972	0.003178512	0.081436956	-0.000509178	0.872720242	0.003178304	0.081419127	8.35E-05	0.005178962	1	0.970180416	1	0.966383771	ENSG00000196151	6	WDSUB1
SAT	T2DM	ENSG00000242435.1	0.004311019	0.102856204	0.002642908	0.081436514	0.004224966	0.10988218	0.002642708	0.081418704	8.61E-05	7.31E-05	1	0.42676853	1	0.452205306	ENSG00000196151	6	WDSUB1
SAT	T2DM	ENSG00000242779.2	-0.001242972	0.712702802	0.003375542	0.081436936	-0.001147742	0.73382465	0.003375289	0.081419112	-9.52E-05	0.000705727	1	0.908107919	1	0.924370544	ENSG00000196152	6	ZNF79
SAT	T2DM	ENSG00000243819.3	0.000703925	0.855422774	0.003863392	0.081436953	0.000759858	0.844063588	0.003863062	0.081419124	-5.59E-05	0.036655809	1	0.963534946	1	0.96477102	ENSG00000196152	6	ZNF79
SAT	T2DM	ENSG00000243961.2	0.000417464	0.87129915	0.002576799	0.081436954	0.000460257	0.858227092	0.002576574	0.081419126	-4.28E-05	0.012633087	1	0.966468088	1	0.965566132	ENSG00000196152	6	ZNF79
SAT	T2DM	ENSG00000244026.2	0.000259531	0.920673767	0.002606128	0.081436957	0.000212707	0.934945197	0.002605919	0.08141913	4.68E-05	0.01885137	1	0.978648294	1	0.984768922	ENSG00000196152	6	ZNF79
SAT	T2DM	ENSG00000244607.1	-0.004299459	0.023995071	0.001904763	0.081436107	-0.004258526	0.025358273	0.001904607	0.081418296	-4.09E-05	0.003997663	1	0.212777913	1	0.232403611	ENSG00000196152	6	ZNF79
SAT	T2DM	ENSG00000244731.3	-0.004152367	3.41E-07	0.000814315	0.081432613	-0.001597309	0.172716075	0.001171451	0.08141882	-0.002555058	0.002415015	0.001119471	4.15E-05	1	0.532948952	ENSG00000196152	6	ZNF79
SAT	T2DM	ENSG00000246375.2	-0.003623393	0.053710496	0.001878214	0.081436336	-0.003591416	0.055840729	0.00187808	0.08141852	-3.20E-05	0.049802168	1	0.321968593	1	0.342399143	ENSG00000196196	2	HRCT1
SAT	T2DM	ENSG00000246523.3	-0.009442157	0.000228651	0.002562263	0.081434689	-0.009302668	0.000282444	0.002562071	0.081416928	-0.000139489	4.35E-11	0.750203982	0.008723302	0.926699671	0.011730376	ENSG00000196196	2	HRCT1
SAT	T2DM	ENSG00000247121.2	-0.003654863	0.216426715	0.0029568	0.081436703	-0.003705614	0.210076727	0.002956553	0.081418868	5.08E-05	0.017145591	1	0.580618195	1	0.579977552	ENSG00000196196	2	HRCT1
SAT	T2DM	ENSG00000248290.1	-0.003633813	0.000342139	0.00101471	0.081434815	-0.003004014	0.003205274	0.001019226	0.081417679	-0.000629798	8.30E-11	1	0.012070532	1	0.064916684	ENSG00000196204	7	RNF216P1
SAT	T2DM	ENSG00000248408.1	-0.001935473	0.618496	0.003886621	0.081436917	-0.002576407	0.507825414	0.003890513	0.081419058	0.000640934	0.000469764	1	0.862424724	1	0.817738235	ENSG00000196204	7	RNF216P1
SAT	T2DM	ENSG00000248905.4	0.006253936	0.127693256	0.004105614	0.081436571	0.004195252	0.315741639	0.004181669	0.081418963	0.002058684	0.009719749	1	0.467990294	1	0.685604445	ENSG00000196204	7	RNF216P1
SAT	T2DM	ENSG00000250075.1	0.003963733	0.03567044	0.00188689	0.081436221	0.003989643	0.034465235	0.00188672	0.081418384	-2.59E-05	0.02839214	1	0.263415251	1	0.272172417	ENSG00000196209	8	SIRPB2
SAT	T2DM	ENSG00000250506.2	-0.001293595	0.365932405	0.001430779	0.081436822	-0.001322669	0.355222373	0.001430673	0.081418988	2.91E-05	0.015992378	1	0.721771482	1	0.724067999	ENSG00000196209	8	SIRPB2
SAT	T2DM	ENSG00000250519.2	0.006598195	0.017607048	0.002779606	0.081436017	0.00655233	0.018399687	0.002779375	0.081418202	4.59E-05	0.02277168	1	0.172693001	1	0.187482528	ENSG00000196220	11	SRGAP3
SAT	T2DM	ENSG00000250599.1	-0.001401253	0.619748769	0.00282393	0.081436917	-0.001606162	0.569725121	0.002825478	0.081419077	0.000204909	0.045497907	1	0.863241392	1	0.845822679	ENSG00000196220	11	SRGAP3
SAT	T2DM	ENSG00000250656.2	0.008966145	0.022790501	0.003937812	0.081436092	0.009042289	0.021649681	0.00393748	0.08141825	-7.61E-05	0.006520465	1	0.206365356	1	0.208306757	ENSG00000196236	8	XPNPEP3
SAT	T2DM	ENSG00000251383.1	-0.00322185	0.309289887	0.003168885	0.081436786	-0.002835727	0.371128755	0.003170671	0.081418997	-0.000386123	0.000896035	1	0.669821861	1	0.734641671	ENSG00000196236	8	XPNPEP3
SAT	T2DM	ENSG00000251532.1	-0.000593121	0.778750268	0.002111142	0.081436945	-0.000565202	0.788893879	0.002110954	0.081419119	-2.79E-05	0.039432093	1	0.939432466	1	0.947957612	ENSG00000196247	7	ZNF107
SAT	T2DM	ENSG00000251580.1	0.005928468	0.147425524	0.004092303	0.081436608	0.005850432	0.152796139	0.004091993	0.081418789	7.80E-05	0.020195381	1	0.492642703	1	0.512250482	ENSG00000196247	7	ZNF107
SAT	T2DM	ENSG00000251593.1	-0.003733459	0.148428034	0.002583514	0.081436609	-0.003793541	0.141972968	0.002583301	0.081418771	6.01E-05	0.001556816	1	0.493635361	1	0.499917672	ENSG00000196247	7	ZNF107
SAT	T2DM	ENSG00000253305.1	0.00389033	0.172950831	0.002854691	0.081436648	0.003939692	0.167527235	0.002854446	0.081418813	-4.94E-05	0.01212853	1	0.526970477	1	0.529024888	ENSG00000196260	3	SFTA2
SAT	T2DM	ENSG00000253326.2	-0.00310093	0.40284439	0.003742657	0.081436841	-0.003201933	0.392224027	0.00374236	0.081419009	7.10E-05	0.014878461	1	0.752695013	1	0.75344674	ENSG00000196260	3	SFTA2
SAT	T2DM	ENSG00000253813.1	0.009751325	0.000178306	0.002601775	0.081434611	0.009791969	0.000167324	0.00260157	0.081416764	-4.06E-05	0.046437954	0.585021353	0.007434796	0.548990588	0.00820853	ENSG00000196260	3	SFTA2
SAT	T2DM	ENSG00000254126.2	-0.000283016	0.910715539	0.002523863	0.081436956	-0.000218602	0.930972531	0.00252365	0.08141913	-6.44E-05	0.000287591	1	0.975126969	1	0.983109389	ENSG00000196260	3	SFTA2
SAT	T2DM	ENSG00000254521.2	-0.000973687	0.383978581	0.001118422	0.081436832	-0.000998669	0.37185808	0.001118331	0.081418998	2.50E-05	0.002899835	1	0.734596925	1	0.734979735	ENSG00000196263	3	ZNF471
SAT	T2DM	ENSG00000254614.1	0.001142691	0.497703569	0.001685116	0.081436882	0.001769135	0.295812373	0.001692213	0.081418948	-0.000626445	6.48E-05	1	0.808799113	1	0.662977391	ENSG00000196301	3	HLA-DRB9
SAT	T2DM	ENSG00000254731.1	-0.003963979	0.249575038	0.003442796	0.081436737	-0.004166146	0.226322477	0.003443415	0.081418886	0.000202167	0.014631232	1	0.615333003	1	0.601160762	ENSG00000196301	3	HLA-DRB9
SAT	T2DM	ENSG00000254986.3	0.00084767	0.596827909	0.001602497	0.081436912	0.000401027	0.802783052	0.001605734	0.08141912	0.000446642	1.96E-05	1	0.848804669	1	0.952314593	ENSG00000196301	3	HLA-DRB9
SAT	T2DM	ENSG00000255147.1	-0.000943373	0.778592751	0.003355367	0.081436945	-0.001255165	0.708394	0.003355931	0.081419108	0.000311792	7.99E-05	1	0.939432466	1	0.912941362	ENSG00000196357	7	ZNF565
SAT	T2DM	ENSG00000255200.1	0.000656615	0.608874958	0.001283252	0.081436915	0.001159612	0.368402777	0.001289217	0.081418996	-0.000502997	5.98E-05	1	0.855383565	1	0.732878736	ENSG00000196357	7	ZNF565
SAT	T2DM	ENSG00000256061.3	0.000541725	0.882474954	0.003664452	0.081436955	0.000488212	0.894003654	0.003664153	0.081419128	5.35E-05	0.049322988	1	0.969020186	1	0.972150687	ENSG00000196357	7	ZNF565
SAT	T2DM	ENSG00000256269.2	0.008952537	1.27E-05	0.00205055	0.081433773	0.009006153	1.12E-05	0.002050451	0.081415908	-5.36E-05	0.015920674	0.041544541	0.00082697	0.036810281	0.000856053	ENSG00000196381	6	ZNF781
SAT	T2DM	ENSG00000256576.1	-0.002321657	0.35010984	0.002484711	0.081436813	-0.002256578	0.363742165	0.002484511	0.081418993	-6.51E-05	0.000593755	1	0.708896874	1	0.729039733	ENSG00000196405	8	EVL
SAT	T2DM	ENSG00000257093.2	0.004082613	0.233653528	0.003427882	0.081436721	0.004023224	0.240487277	0.003427605	0.081418901	5.94E-05	0.021850775	1	0.598919708	1	0.618368932	ENSG00000196456	4	ZNF775
SAT	T2DM	ENSG00000257534.1	-0.000976451	0.707907517	0.002606177	0.081436935	-0.000926217	0.722273522	0.002605962	0.08141911	-5.02E-05	0.008707082	1	0.906221055	1	0.920812547	ENSG00000196458	6	ZNF605
SAT	T2DM	ENSG00000258100.1	0.001028255	0.734793611	0.003035383	0.081436939	0.000934017	0.758285433	0.00303515	0.081419115	9.42E-05	0.000122429	1	0.923192618	1	0.932894921	ENSG00000196458	6	ZNF605
SAT	T2DM	ENSG00000258289.3	0.00035943	0.76042191	0.001178742	0.081436943	0.000342704	0.771233344	0.001178641	0.081419117	1.67E-05	0.037837353	1	0.934086217	1	0.938518235	ENSG00000196458	6	ZNF605
SAT	T2DM	ENSG00000258405.5	0.000499416	0.812471914	0.002105123	0.081436949	0.000462541	0.826073294	0.002104941	0.081419123	3.69E-05	0.009560102	1	0.951539016	1	0.959754419	ENSG00000196460	8	RFX8
SAT	T2DM	ENSG00000258586.1	-0.003390265	0.172809515	0.002486928	0.081436648	-0.00335494	0.177289478	0.002486707	0.081418827	-3.53E-05	0.026255508	1	0.526970477	1	0.539255458	ENSG00000196511	9	TPK1
SAT	T2DM	ENSG00000258713.2	5.71E-06	0.998699494	0.003501242	0.081436958	-6.29E-05	0.985675577	0.003500974	0.081419131	6.86E-05	0.015533724	1	0.998699494	1	0.997162029	ENSG00000196511	9	TPK1
SAT	T2DM	ENSG00000258819.1	-0.006820931	0.071316694	0.003782137	0.081436415	-0.006742119	0.074623725	0.003781814	0.0814186	-7.88E-05	0.00256164	1	0.373189908	1	0.395620568	ENSG00000196535	10	MYO18A
SAT	T2DM	ENSG00000258873.2	-0.001113494	0.493509615	0.001626161	0.08143688	-0.001086835	0.503879171	0.001626026	0.081419056	-2.67E-05	0.024951295	1	0.805574651	1	0.817738235	ENSG00000196535	10	MYO18A
SAT	T2DM	ENSG00000259005.1	0.000616545	0.655483268	0.001381898	0.081436925	0.000640265	0.643106762	0.001381788	0.081419095	-2.37E-05	0.026550662	1	0.883423151	1	0.888435068	ENSG00000196535	10	MYO18A
SAT	T2DM	ENSG00000259205.2	-0.000365115	0.77584294	0.001282273	0.081436945	-0.000388627	0.761811936	0.001282165	0.081419116	2.35E-05	0.010759166	1	0.939125277	1	0.932999239	ENSG00000196535	10	MYO18A
SAT	T2DM	ENSG00000259433.2	0.00411271	0.124715571	0.0026788	0.081436565	0.004068525	0.128785869	0.002678582	0.081418745	4.42E-05	0.027598091	1	0.463429598	1	0.478535034	ENSG00000196535	10	MYO18A
SAT	T2DM	ENSG00000259539.1	-0.001755762	0.563285201	0.003037813	0.081436903	-0.001839126	0.544876865	0.003037593	0.08141907	8.34E-05	0.001441917	1	0.837959912	1	0.840498821	ENSG00000196535	10	MYO18A
SAT	T2DM	ENSG00000259758.1	0.00385587	0.341405375	0.004052867	0.081436807	0.003753271	0.354367885	0.004052547	0.081418988	0.000102599	0.001163511	1	0.702729632	1	0.723060342	ENSG00000196562	10	SULF2
SAT	T2DM	ENSG00000259869.1	0.005978443	0.003484514	0.002046414	0.081435532	0.005925845	0.003780377	0.002046257	0.08141773	5.26E-05	0.001478665	1	0.06146607	1	0.072534597	ENSG00000196653	7	ZNF502
SAT	T2DM	ENSG00000260065.1	0.001929843	0.071727677	0.001071631	0.081436416	0.001883336	0.078820371	0.001071553	0.081418615	4.65E-05	4.55E-07	1	0.373553186	1	0.407783994	ENSG00000196655	5	TRAPPC4
SAT	T2DM	ENSG00000260075.1	0.00231793	0.319198609	0.002326989	0.081436788	0.002272771	0.328675633	0.002326786	0.081418971	4.52E-05	0.003519136	1	0.680500738	1	0.696630976	ENSG00000196666	3	FAM180B
SAT	T2DM	ENSG00000260265.1	-0.00399058	0.046242196	0.002002116	0.081436295	-0.003897697	0.051557452	0.002002089	0.081418498	-9.29E-05	0.000784941	1	0.295751746	1	0.328466017	ENSG00000196693	10	ZNF33B
SAT	T2DM	ENSG00000260302.1	0.005898798	0.039548641	0.002865667	0.08143625	0.005975805	0.037025827	0.002865427	0.081418404	-7.70E-05	0.000177825	1	0.274913329	1	0.279268363	ENSG00000196693	10	ZNF33B
SAT	T2DM	ENSG00000260388.1	0.001818544	0.413658552	0.002224592	0.081436847	0.001865005	0.401789067	0.002224399	0.081419013	-4.65E-05	0.001842338	1	0.760343415	1	0.757591271	ENSG00000196693	10	ZNF33B
SAT	T2DM	ENSG00000260778.2	-0.001445291	0.657220937	0.003256967	0.081436926	-0.001531746	0.638113233	0.003256688	0.081419094	8.65E-05	0.000121453	1	0.884109018	1	0.884889906	ENSG00000196693	10	ZNF33B
SAT	T2DM	ENSG00000260787.1	0.008615086	0.009419035	0.003318001	0.081435832	0.008549713	0.009967116	0.003317728	0.081418021	6.54E-05	0.007345328	1	0.116618318	1	0.131863332	ENSG00000196712	12	NF1
SAT	T2DM	ENSG00000260877.1	-0.007276278	0.004722129	0.002575295	0.081435624	-0.007333316	0.004403692	0.002575163	0.081417776	5.70E-05	0.040160364	1	0.073427987	1	0.079676052	ENSG00000196712	12	NF1
SAT	T2DM	ENSG00000261143.1	5.90E-05	0.979355131	0.002280578	0.081436958	9.82E-05	0.965642443	0.002280398	0.081419131	-3.92E-05	0.027996293	1	0.995203291	1	0.99427016	ENSG00000196730	8	DAPK1
SAT	T2DM	ENSG00000261584.1	-0.00236122	0.049023471	0.001199568	0.081436311	-0.001869711	0.119739381	0.001201713	0.081418726	-0.00049151	2.92E-11	1	0.306958031	1	0.466585403	ENSG00000196730	8	DAPK1
SAT	T2DM	ENSG00000262165.1	-0.00711624	0.031125102	0.003301471	0.081436182	-0.007197024	0.029248448	0.003301193	0.081418337	8.08E-05	0.000617769	1	0.245761744	1	0.251961415	ENSG00000196730	8	DAPK1
SAT	T2DM	ENSG00000262370.1	9.10E-05	0.952684927	0.001533018	0.081436958	5.20E-05	0.972913989	0.00153289	0.081419131	3.89E-05	0.000394179	1	0.988645274	1	0.996397533	ENSG00000196735	7	HLA-DQA1
SAT	T2DM	ENSG00000262500.1	0.005548827	0.004953996	0.001974656	0.081435639	0.005586931	0.004661389	0.001974487	0.081417793	-3.81E-05	0.005714515	1	0.074555423	1	0.0804112	ENSG00000196735	7	HLA-DQA1
SAT	T2DM	ENSG00000262539.1	0.005413489	0.012963479	0.002178659	0.081435927	0.005446393	0.012416175	0.00217847	0.081418087	-3.29E-05	0.026088756	1	0.143739766	1	0.150879531	ENSG00000196735	7	HLA-DQA1
SAT	T2DM	ENSG00000262855.1	-1.06E-05	0.994083803	0.001426618	0.081436958	1.45E-05	0.991877709	0.001426495	0.081419131	-2.51E-05	0.009442139	1	0.99758396	1	0.998267105	ENSG00000196735	7	HLA-DQA1
SAT	T2DM	ENSG00000263627.1	-0.004731968	0.081907159	0.002719945	0.081436453	-0.004781002	0.078764576	0.002719718	0.081418615	4.90E-05	0.01300018	1	0.395495266	1	0.407783994	ENSG00000196735	7	HLA-DQA1
SAT	T2DM	ENSG00000264176.1	0.002591628	0.162411356	0.001855126	0.081436632	0.002616141	0.15843643	0.001854956	0.081418799	-2.45E-05	0.027587218	1	0.513507272	1	0.51775889	ENSG00000196735	7	HLA-DQA1
SAT	T2DM	ENSG00000264538.2	0.002220074	0.08130692	0.001273594	0.081436451	0.00224817	0.077503037	0.001273487	0.08141861	-2.81E-05	0.002079455	1	0.395495266	1	0.405562145	ENSG00000196735	7	HLA-DQA1
SAT	T2DM	ENSG00000265798.2	-0.000914295	0.69668465	0.002345548	0.081436933	-0.000870383	0.710556328	0.002345356	0.081419108	-4.39E-05	0.011387352	1	0.900770568	1	0.913890753	ENSG00000196735	7	HLA-DQA1
SAT	T2DM	ENSG00000266904.1	0.000218637	0.848723892	0.001146215	0.081436952	0.000251966	0.825994398	0.001146122	0.081419123	-3.33E-05	0.000130503	1	0.963201596	1	0.959754419	ENSG00000196743	4	GM2A
SAT	T2DM	ENSG00000267102.1	-0.000387843	0.799823274	0.001529496	0.081436948	-0.000290534	0.849348818	0.001529533	0.081419125	-9.73E-05	0.000101782	1	0.948056417	1	0.965561588	ENSG00000196743	4	GM2A
SAT	T2DM	ENSG00000267108.1	-0.000690073	0.768944012	0.002349133	0.081436944	-0.000643196	0.784218403	0.002348922	0.081419118	-4.69E-05	0.001420492	1	0.935400227	1	0.945615795	ENSG00000196743	4	GM2A
SAT	T2DM	ENSG00000267369.1	-0.001094315	0.764563675	0.003653887	0.081436943	0.000696531	0.850205807	0.003688159	0.081419125	-0.001790846	0.000385826	1	0.93459819	1	0.965561588	ENSG00000196743	4	GM2A
SAT	T2DM	ENSG00000267547.1	0.000179027	0.880451926	0.001190366	0.081436955	0.00030092	0.800468105	0.001190625	0.08141912	-0.000121894	6.29E-05	1	0.968084038	1	0.952160336	ENSG00000196743	4	GM2A
SAT	T2DM	ENSG00000267592.1	0.004704605	0.198348146	0.003657545	0.081436682	0.005091175	0.164093049	0.003658929	0.081418807	-0.00038657	0.000716803	1	0.558130589	1	0.523492072	ENSG00000196743	4	GM2A
SAT	T2DM	ENSG00000267648.1	-2.48E-05	0.983424905	0.001193379	0.081436958	5.52E-05	0.96308001	0.001193437	0.081419131	-8.00E-05	0.000166145	1	0.995937959	1	0.993672317	ENSG00000196787	2	HIST1H2AG
SAT	T2DM	ENSG00000268340.1	0.005177476	0.112252218	0.003260059	0.081436537	0.005062717	0.120409553	0.003259834	0.081418728	0.000114759	8.91E-05	1	0.44002333	1	0.467258033	ENSG00000196793	9	ZNF239
SAT	T2DM	ENSG00000268362.1	0.000447482	0.877663687	0.00290702	0.081436954	0.000500643	0.863254259	0.002906775	0.081419126	-5.32E-05	0.009833282	1	0.96728739	1	0.966241215	ENSG00000196793	9	ZNF239
SAT	T2DM	ENSG00000268734.1	-0.001255597	0.642011524	0.002700857	0.081436922	-0.001211735	0.653659446	0.00270064	0.081419097	-4.39E-05	0.032764931	1	0.87658752	1	0.891871358	ENSG00000196793	9	ZNF239
SAT	T2DM	ENSG00000269430.1	0.002925368	0.161611289	0.002090034	0.081436631	0.002959562	0.15673459	0.002089876	0.081418796	-3.42E-05	0.047945964	1	0.512811064	1	0.51775889	ENSG00000196793	9	ZNF239
SAT	T2DM	ENSG00000269486.1	-0.00541202	0.164707577	0.003895182	0.081436636	-0.005351804	0.169422648	0.003894874	0.081418815	-6.02E-05	0.047648494	1	0.516146667	1	0.531756789	ENSG00000196821	5	C6orf106
SAT	T2DM	ENSG00000269549.1	-0.00170707	0.523075708	0.002673094	0.08143689	-0.001661701	0.534144703	0.002672868	0.081419066	-4.54E-05	0.015925651	1	0.821144329	1	0.832950936	ENSG00000196821	5	C6orf106
SAT	T2DM	ENSG00000269921.1	0.000320194	0.903092585	0.002629809	0.081436956	0.000260941	0.920954289	0.00262961	0.081419129	5.93E-05	0.006090278	1	0.97231278	1	0.979654273	ENSG00000196821	5	C6orf106
SAT	T2DM	ENSG00000269983.1	0.002678099	0.22407679	0.002202818	0.081436711	0.002621336	0.234009488	0.002202627	0.081418894	5.68E-05	0.000124437	1	0.588156759	1	0.610313962	ENSG00000196821	5	C6orf106
SAT	T2DM	ENSG00000269990.1	0.009734587	0.004916828	0.003461253	0.081435637	0.008164478	0.018955608	0.003479561	0.081418211	0.001570109	1.30E-05	1	0.07434153	1	0.191364154	ENSG00000196839	8	ADA
SAT	T2DM	ENSG00000270093.1	-0.00012111	0.968115373	0.003029865	0.081436958	-7.34E-05	0.980675088	0.003029593	0.081419131	-4.77E-05	0.011856462	1	0.993613755	1	0.997085517	ENSG00000196859	3	KRT39
SAT	T2DM	ENSG00000270127.1	-0.000523521	0.681716526	0.0012765	0.08143693	-0.000547239	0.668113583	0.001276392	0.0814191	2.37E-05	0.008788106	1	0.892786811	1	0.896403813	ENSG00000196872	6	KIAA1211L
SAT	T2DM	ENSG00000270182.1	0.009364953	0.004763613	0.003317832	0.081435627	0.009548328	0.004001812	0.003317658	0.081417747	-0.000183376	2.46E-07	1	0.073507661	1	0.075459453	ENSG00000196872	6	KIAA1211L
SAT	T2DM	ENSG00000270672.1	0.002242403	0.579601327	0.004047896	0.081436908	0.002109437	0.602256213	0.004047788	0.081419086	0.000132966	5.46E-05	1	0.842939695	1	0.858221553	ENSG00000196912	8	ANKRD36B
SAT	T2DM	ENSG00000270775.1	-0.000982337	0.40743396	0.001185798	0.081436844	-0.001106163	0.351046255	0.001186154	0.081418986	0.000123826	0.000266385	1	0.756247299	1	0.71896552	ENSG00000196912	8	ANKRD36B
SAT	T2DM	ENSG00000271040.1	0.004569461	0.254667495	0.004011512	0.081436742	0.004637083	0.247663902	0.004011166	0.081418908	-6.76E-05	0.013477546	1	0.620408004	1	0.620663712	ENSG00000196912	8	ANKRD36B
SAT	T2DM	ENSG00000271324.1	-0.00161947	0.465104382	0.00221703	0.081436869	-0.001658205	0.454459684	0.002216848	0.081419037	3.87E-05	0.018348154	1	0.792954767	1	0.794600418	ENSG00000196912	8	ANKRD36B
SAT	T2DM	ENSG00000271538.1	0.002266882	0.382347403	0.002594933	0.081436831	0.002226393	0.39086435	0.002594708	0.081419008	4.05E-05	0.019911126	1	0.732758077	1	0.751715083	ENSG00000196912	8	ANKRD36B
SAT	T2DM	ENSG00000271550.1	0.001481391	0.507142845	0.002233386	0.081436885	0.001516506	0.497089894	0.00223319	0.081419054	-3.51E-05	0.016250733	1	0.810627063	1	0.81497549	ENSG00000196912	8	ANKRD36B

SAT	T2DM	ENSG00000271553.1	0.001433733	0.091578017	0.000849804	0.081436483	0.001420959	0.094477196	0.000849732	0.081418664	1.28E-05	0.036300289	1	0.413867045	1	0.429887299	ENSG00000196912	8	ANKRD36B
SAT	T2DM	ENSG00000271584.1	0.004734072	0.007658772	0.001775212	0.08143577	0.004707035	0.008077716	0.001775064	0.081417956	2.70E-05	0.035559055	1	0.100113265	1	0.114231808	ENSG00000196961	8	AP2A1
SAT	T2DM	ENSG00000271623.1	-0.004107636	0.181923857	0.003077211	0.081436661	-0.004055202	0.187529082	0.003076958	0.081418841	-5.24E-05	0.020642634	1	0.535809853	1	0.557313872	ENSG00000196961	8	AP2A1
SAT	T2DM	ENSG00000271751.1	-0.01280102	0.000205406	0.003448271	0.081434655	-0.012907761	0.00018156	0.003448129	0.08141679	0.000106741	0.008105044	0.673938506	0.008023077	0.595698457	0.008583862	ENSG00000196963	2	PCDHB16
SAT	T2DM	ENSG00000271811.1	-0.002458421	0.282124118	0.002285711	0.081436765	-0.002495028	0.27497851	0.002285514	0.081418932	3.66E-05	0.019444958	1	0.641415693	1	0.64327119	ENSG00000196963	2	PCDHB16
SAT	T2DM	ENSG00000272003.1	0.008612197	0.029838037	0.003964662	0.08143617	0.008502415	0.031975399	0.00396434	0.081418362	0.000109782	0.000220357	1	0.240185597	1	0.263779676	ENSG00000196963	2	PCDHB16
SAT	T2DM	ENSG00000272010.1	0.002592251	0.106134644	0.001604298	0.081436522	0.002408323	0.133373623	0.001604551	0.081418754	0.000183927	7.08E-07	1	0.433220108	1	0.486825442	ENSG00000196966	3	HIST1H3E
SAT	T2DM	ENSG00000272047.1	0.001631005	0.549970486	0.002728325	0.081436899	0.001580461	0.562369716	0.002728105	0.081419075	5.05E-05	0.01461579	1	0.83552458	1	0.84223981	ENSG00000196966	3	HIST1H3E
SAT	T2DM	ENSG00000272156.1	-0.002452444	0.417887209	0.003027361	0.081436849	-0.002514168	0.406229151	0.003027118	0.081419016	6.17E-05	0.007690153	1	0.763620456	1	0.759884747	ENSG00000196966	3	HIST1H3E
SAT	T2DM	ENSG00000272221.1	-0.003490018	6.42E-07	0.000701059	0.081432817	-0.003233973	4.73E-06	0.000706624	0.081415631	-0.000256045	0.004054741	0.002106314	6.58E-05	0.01550583	0.000430718	ENSG00000196966	3	HIST1H3E
SAT	T2DM	ENSG00000272236.1	-0.0008708	0.539191676	0.001418157	0.081436895	0.000498255	0.731983125	0.001454805	0.081419111	-0.001369054	2.55E-05	1	0.831736309	1	0.923670747	ENSG00000196966	3	HIST1H3E
SAT	T2DM	ENSG00000272369.1	0.009633511	0.005668318	0.003482343	0.081435679	0.009735531	0.005175601	0.003482063	0.081417825	-0.00010202	0.000129231	1	0.082656666	1	0.08576336	ENSG00000196967	6	ZNF585A
SAT	T2DM	ENSG00000272462.2	7.00E-05	0.912442136	0.000636239	0.081436956	-0.000249064	0.696160393	0.000637792	0.081419105	0.000319024	2.27E-12	1	0.976107808	1	0.907470103	ENSG00000196968	6	FUT11
SAT	T2DM	ENSG00000272541.1	0.003551298	0.107818005	0.002208407	0.081436526	0.003598086	0.103228178	0.002208222	0.081418687	-4.68E-05	0.003359428	1	0.435653784	1	0.440431278	ENSG00000196968	6	FUT11
SAT	T2DM	ENSG00000272733.1	-0.003255238	0.142820798	0.002221445	0.0814366	-0.003210213	0.148400363	0.002221282	0.081418782	-4.50E-05	0.016712812	1	0.488540542	1	0.507874308	ENSG00000196968	6	FUT11
SAT	T2DM	ENSG00000272810.1	-0.001137416	0.514595091	0.001745306	0.081436887	-0.001698046	0.330936091	0.001746546	0.081418973	0.00056063	2.22E-15	1	0.816039871	1	0.700003408	ENSG00000196981	2	WDR5B
SAT	T2DM	ENSG00000272849.1	-0.00415791	0.062154623	0.002229209	0.081436377	-0.004103507	0.065630124	0.002229021	0.081418565	-5.44E-05	0.00058205	1	0.346229741	1	0.37355836	ENSG00000197008	5	ZNF138
SAT	T2DM	ENSG00000272871.1	0.003744004	0.347572759	0.003985914	0.081436811	0.003794991	0.341003711	0.003985561	0.081418979	-5.10E-05	0.047619586	1	0.708371701	1	0.708179865	ENSG00000197020	6	ZNF100
SAT	T2DM	ENSG00000273073.1	-0.004588709	0.273464046	0.004190137	0.081436758	-0.004656705	0.266379832	0.004189796	0.081418924	6.80E-05	0.03044191	1	0.634986223	1	0.638375379	ENSG00000197054	7	ZNF763
SAT	T2DM	ENSG00000273142.1	-0.000574018	0.570034138	0.001010592	0.081436905	-0.00058803	0.560621319	0.001010501	0.081419074	1.40E-05	0.023662764	1	0.840736467	1	0.84223981	ENSG00000197054	7	ZNF763
SAT	T2DM	ENSG00000273340.1	-0.000580507	0.503630151	0.000867997	0.081436884	2.44E-05	0.977795164	0.000875701	0.081419131	-0.000604881	2.13E-07	1	0.810314363	1	0.99701704	ENSG00000197054	7	ZNF763
SAT	T2DM	ENSG00000273448.1	0.005992552	0.013312163	0.002420924	0.081435934	0.006043067	0.012547379	0.002420739	0.08141809	-5.05E-05	0.010363148	1	0.145107	1	0.150896353	ENSG00000197062	7	RP5-874C20.3
VAT	CAD	ENSG00000058453.12	0.000842682	0.543536932	0.00138719	0.138947791	0.000865581	0.532646462	0.001387212	0.138943761	-2.29E-05	0.033550653	1	0.84421043	1	0.841020729	ENSG00000197062	7	RP5-874C20.3
VAT	CAD	ENSG00000077420.11	-0.007947745	0.116224314	0.005059604	0.138947193	-0.008571477	0.090862625	0.005069276	0.138943057	0.000623732	0.047133318	1	0.535009893	1	0.519153608	ENSG00000197062	7	RP5-874C20.3
VAT	CAD	ENSG00000096433.6	0.019464309	6.84E-05	0.004888315	0.138943376	0.020058784	4.21E-05	0.004897574	0.138939089	-0.000594475	0.049184682	0.188795807	0.017163255	0.116207024	0.0124879	ENSG00000197062	7	RP5-874C20.3
VAT	CAD	ENSG00000106638.11	0.005695742	0.090105006	0.003360622	0.138947077	0.006322662	0.061038047	0.003375284	0.138942872	-0.000626921	0.046429039	1	0.513821934	1	0.434398716	ENSG00000197062	7	RP5-874C20.3
VAT	CAD	ENSG00000120555.9	-0.007697391	0.104965746	0.004747827	0.138947147	-0.007610605	0.108952653	0.004747959	0.138943139	-8.68E-05	0.046586359	1	0.526589031	1	0.535978172	ENSG00000197062	7	RP5-874C20.3
VAT	CAD	ENSG00000124116.14	-0.004654821	0.001615201	0.001476238	0.138945062	-0.00467946	0.001525524	0.001476263	0.138941007	2.46E-05	0.035876406	1	0.080942347	1	0.081258362	ENSG00000197062	7	RP5-874C20.3
VAT	CAD	ENSG00000127337.2	0.003432641	0.068168941	0.001882041	0.138946948	0.00358708	0.056843375	0.001883458	0.138942838	-0.000154439	0.036281088	1	0.461578079	1	0.422852497	ENSG00000197062	7	RP5-874C20.3
VAT	CAD	ENSG00000134440.7	0.001556356	0.761091544	0.005118786	0.13894787	0.001638886	0.748843068	0.005118873	0.138943843	-8.25E-05	0.041720942	1	0.918627635	1	0.916133253	ENSG00000197081	8	IGF2R
VAT	CAD	ENSG00000144152.8	0.005631779	0.164963179	0.004055802	0.138947347	0.006491574	0.111463306	0.004078511	0.13894315	-0.000859796	0.045735161	1	0.616100643	1	0.535978172	ENSG00000197119	8	SLC25A29
VAT	CAD	ENSG00000181092.5	-0.009254553	0.003120185	0.003131132	0.138945406	-0.011737578	0.000338054	0.003274746	0.138940209	0.002483025	0.009646373	1	0.10764638	0.933027817	0.038876159	ENSG00000197128	7	ZNF772
VAT	CAD	ENSG00000186715.6	0.00288358	0.462314356	0.003923014	0.138947742	0.002962195	0.450209344	0.003923091	0.138943709	-7.86E-05	0.015409428	1	0.811043869	1	0.799828966	ENSG00000197134	7	ZNF257
VAT	CAD	ENSG00000187049.5	-0.004319557	0.270632518	0.003921137	0.13894755	-0.004397839	0.26206154	0.003921274	0.138943513	7.83E-05	0.044884596	1	0.697428338	1	0.696035802	ENSG00000197134	7	ZNF257
VAT	CAD	ENSG00000204792.2	-0.002285126	0.352785665	0.002459239	0.13894765	-0.002239823	0.36242283	0.002459293	0.138943636	-4.53E-05	0.03124847	1	0.749857359	1	0.756460842	ENSG00000197134	7	ZNF257
VAT	CAD	ENSG00000230836.1	-0.003083312	0.332250414	0.00318001	0.138947628	-0.003024469	0.34156971	0.003180073	0.138943614	-5.88E-05	0.025249008	1	0.743723555	1	0.747632686	ENSG00000197146	2	AL133458.1
VAT	CAD	ENSG00000236209.1	-0.003060177	0.295511794	0.002925298	0.138947584	-0.003007317	0.30394165	0.002925353	0.138943571	-5.29E-05	0.026425113	1	0.712469718	1	0.724807082	ENSG00000197146	2	AL133458.1
VAT	CAD	ENSG00000251417.1	-0.003185601	0.220334954	0.002599134	0.138947468	-0.003137626	0.227374271	0.002599198	0.138943457	-4.80E-05	0.036178862	1	0.675304527	1	0.678432654	ENSG00000197157	6	SND1
VAT	CAD	ENSG00000261824.2	-0.000670176	0.840320627	0.003326209	0.138947885	-0.000614394	0.853457225	0.003326277	0.138943862	-5.58E-05	0.044580493	1	0.949931696	1	0.959970096	ENSG00000197157	6	SND1
VAT	Heart_Failure	ENSG00000001561.6	-0.004283975	0.106751299	0.002655952	0.076055087	-0.004393073	0.098123341	0.002655991	0.07604845	0.000109097	0.000145843	1	0.637447692	1	0.624888429	ENSG00000197157	6	SND1
VAT	Heart_Failure	ENSG00000003249.9	-0.003282875	0.114022649	0.002077281	0.076055103	-0.003326311	0.109311877	0.002077261	0.076048477	4.34E-05	0.010763657	1	0.655403408	1	0.628112053	ENSG00000197165	6	SULT1A2
VAT	Heart_Failure	ENSG00000006432.11	0.003055538	0.199853015	0.002383466	0.076055237	0.003094109	0.194226771	0.002383425	0.076048614	-3.86E-05	0.024758821	1	0.756191037	1	0.738152304	ENSG00000197165	6	SULT1A2
VAT	Heart_Failure	ENSG00000008394.8	0.004378696	0.13547362	0.002933086	0.076055145	0.003583264	0.22502103	0.002953354	0.076048647	0.000795432	0.021688884	1	0.688213472	1	0.76157815	ENSG00000197165	6	SULT1A2
VAT	Heart_Failure	ENSG00000009950.11	0.002386657	0.50427979	0.003574063	0.076055423	0.000331232	0.928895076	0.003711905	0.076048876	0.002055425	0.04037544	1	0.904941123	1	0.990517646	ENSG00000197165	6	SULT1A2
VAT	Heart_Failure	ENSG00000011523.9	-0.000258509	0.91794659	0.002509296	0.076055491	-0.000201336	0.9360489	0.002509266	0.076048876	-5.72E-05	0.00423219	1	0.984985763	1	0.990517646	ENSG00000197165	6	SULT1A2
VAT	Heart_Failure	ENSG00000013573.12	0.000129369	0.950804221	0.00209685	0.076055492	9.26E-05	0.96476985	0.002096817	0.076048877	3.68E-05	0.018088972	1	0.992630426	1	0.993760632	ENSG00000197165	6	SULT1A2
VAT	Heart_Failure	ENSG00000018280.12	-0.002133741	0.423235704	0.002664442	0.076055393	-0.005908731	0.062587744	0.003173132	0.076048336	0.00377499	0.02849475	1	0.87173921	1	0.543610282	ENSG00000197165	6	SULT1A2
VAT	Heart_Failure	ENSG00000020922.8	0.000351197	0.58777763	0.000647894	0.076055447	0.000362775	0.575523756	0.000647888	0.076048828	-1.16E-05	0.03282663	1	0.929665478	1	0.92458997	ENSG00000197168	7	NEK5
VAT	Heart_Failure	ENSG00000030110.8	-0.001730713	0.542871458	0.002844336	0.076055435	-0.000917079	0.747992104	0.00285438	0.076048861	-0.000813634	0.00072423	1	0.921324909	1	0.960126407	ENSG00000197168	7	NEK5
VAT	Heart_Failure	ENSG00000040531.10	0.000267104	0.828124342	0.00123028	0.076055486	0.000242013	0.844049865	0.001230265	0.076048871	2.51E-05	0.010277241	1	0.972185844	1	0.969484287	ENSG00000197168	7	NEK5
VAT	Heart_Failure	ENSG00000050130.13	0.002620645	0.368279248	0.002912792	0.076055367	0.00273684	0.34743619	0.00291285	0.076048739	-0.000116195	0.000393092	1	0.834524405	1	0.821053473	ENSG00000197168	7	NEK5
VAT	Heart_Failure	ENSG00000050555.13	-0.0029879	0.420904974	0.003712355	0.076055392	-0.005924749	0.135356517	0.003967526	0.076048529	0.002936849	0.035970945	1	0.870783317	1	0.670707336	ENSG00000197181	7	PIWIL2
VAT	Heart_Failure	ENSG00000054277.8	-0.002795182	0.251616732	0.002438156	0.076055288	-0.002842037	0.243751557	0.00243813	0.076048665	4.69E-05	0.01783083	1	0.790959203	1	0.775956515	ENSG00000197181	7	PIWIL2
VAT	Heart_Failure	ENSG00000055211.8	0.00056881	0.836977381	0.002764414	0.076055486	0.000520335	0.850696286	0.002764357	0.076048871	4.85E-05	0.00921759	1	0.972185844	1	0.972627071	ENSG00000197181	7	PIWIL2
VAT	Heart_Failure	ENSG00000058453.12	0.002463484	0.016379215	0.001026297	0.076054594	0.002492713	0.01514613	0.001026283	0.076047956	-2.92E-05	0.000245513	1	0.282541459	1	0.274406299	ENSG00000197181	7	PIWIL2
VAT	Heart_Failure	ENSG00000059573.4	0.003394117	0.354235376	0.003663746	0.076055359	0.003467761	0.343883518	0.003663693	0.076048737	-7.36E-05	0.008435496	1	0.826718867	1	0.81750087	ENSG00000197181	7	PIWIL2
VAT	Heart_Failure	ENSG00000064703.7	-0.000144778	0.961415367	0.00299267	0.076055493	-8.63E-05	0.976995826	0.002992618	0.076048877	-5.85E-05	0.006810982	1	0.993060803	1	0.995646729	ENSG00000197217	8	ENTPD4
VAT	Heart_Failure	ENSG00000065060.12	-0.002845245	0.094127135	0.001699647	0.076055056	-0.002917448	0.086087687	0.001699739	0.076048417	7.22E-05	0.002331743	1	0.635185556	1	0.607677788	ENSG00000197217	8	ENTPD4
VAT	Heart_Failure	ENSG00000066855.11	-0.005261417	0.111467328	0.003305668	0.076055098	-0.005339709	0.106237578	0.003305624	0.07604847	7.83E-05	0.002358473	1	0.64371215	1	0.628112053	ENSG00000197217	8	ENTPD4
VAT	Heart_Failure	ENSG00000066926.6	0.000333038	0.758602183	0.001083696	0.076055478	0.0003141	0.771933977	0.001083678	0.076048864	1.89E-05	0.016779635	1	0.964199078	1	0.965780945	ENSG00000197223	7	C1D
VAT	Heart_Failure	ENSG00000068654.11	-0.00266542	0.08465042	0.001545779	0.076055029	-0.002702799	0.080380223	0.001545792	0.0760484	3.74E-05	0.018200657	1	0.623489698	1	0.600796054	ENSG00000197223	7	C1D
VAT	Heart_Failure	ENSG00000070190.8	0.002865728	0.2906763	0.002712113	0.076055319	0.007128524	0.01460563	0.002919108	0.076047946	-0.004262796	7.91E-05	1	0.795899392	1	0.274406299	ENSG00000197226	8	TBC1D9B
VAT	Heart_Failure	ENSG00000075826.12	-0.003745111	0.126704654	0.002452226	0.076055129	-0.003789082	0.122302191	0.002452187	0.076048504	4.40E-05	0.016160134	1	0.673805094	1	0.652670563	ENSG00000197226	8	TBC1D9B
VAT	Heart_Failure	ENSG00000079263.14	-0.004507583	0.233630408	0.003784512	0.076055272	-0.004418739	0.242969093	0.003784466	0.076048664	-8.88E-05	0.0030417	1	0.779051326	1	0.774790476	ENSG00000197226	8	TBC1D9B
VAT	Heart_Failure	ENSG00000083099.6	-5.44E-05	0.977808252	0.001955512	0.076055493	-8.40E-05	0.965716444	0.001955479	0.076048877	2.97E-05	0.037062363	1	0.994659842	1	0.993760632	ENSG00000197261	7	C6orf141
VAT	Heart_Failure	ENSG00000085117.7	0.004526803	0.059890851	0.002405826	0.07605494	0.004491504	0.061908115	0.002405788	0.076048333	3.53E-05	0.048703225	1	0.546800541	1	0.54177348	ENSG00000197261	7	C6orf141
VAT	Heart_Failure	ENSG00000085514.11	0.000722575	0.42911941	0.000913843	0.076055395	-0.000644745	0.542115414	0.001057623	0.076048819	0.001367321	0.010232842	1	0.872618112	1	0.913136234	ENSG00000197261	7	C6orf141
VAT	Heart_Failure	ENSG00000086015.16	0.011513686	8.62E-05	0.002932242	0.076053087	0.011575077	7.90E-05	0.002932205	0.076046445	-6.14E-05	0.007869365	0.237839757	0.011325703	0.217930737	0.011470039	ENSG00000197279	3	ZNF165
VAT	Heart_Failure	ENSG00000086300.11	0.000481076	0.868431371	0.002904147	0.076055489	0.000434798	0.880987342	0.002904116	0.076048873	4.63E-05	0.049017606	1	0.982255997	1	0.982211765	ENSG00000197279	3	ZNF165
VAT	Heart_Failure	ENSG00000086598.6	0.00319082	0.121169147	0.002058743	0.076055118	0.003224474	0.117287917	0.002058707	0.076048494	-3.37E-05	0.023501174	1	0.669281111	1	0.64556656	ENSG00000197279	3	ZNF165
VAT	Heart_Failure	ENSG00000088876.7	0.003546614	0.245366437	0.003053006	0.076055282	0.003488009	0.253248059	0.00305297	0.076048673	5.86E-05	0.015725493	1	0.782288072	1	0.784151852	ENSG00000197279	3	ZNF165
VAT	Heart_Failure	ENSG00000090054.9	0.001853942	0.510136343	0.002814863	0.076055425	0.00190648	0.498219454	0.002814846	0.076048805	-5.25E-05	0.031129929	1	0.906664036	1	0.899741745	ENSG00000197279	3	ZNF165
VAT	Heart_Failure	ENSG00000090238.7	0.000508077	0.746857202	0.001574035	0.076055477	0.00046257	0.768852868	0.001574032	0.076048863	4.55E-05	0.001421894	1	0.963913289	1	0.965438542	ENSG00000197302	6	ZNF720
VAT	Heart_Failure	ENSG00000095485.12	0.000627294	0.42149114	0.000780374	0.076055392	0.000600395	0.441693872	0.000780406	0.076048784	2.69E-05	0.008421544	1	0.870783317	1	0.875281919	ENSG00000197302	6	ZNF720
VAT	Heart_Failure	ENSG00000096433.6	0.003768421	0.29742702	0.003616638	0.076055324	0.004700609	0.194529268	0.003623384	0.076048614	-0.000932188	3.06E-05	1	0.802442401	1	0.738152304	ENSG00000197345	8	MRPL21
VAT	Heart_Failure	ENSG00000096996.11	-0.002826674	0.339558353	0.002959747	0.076055351	-0.002777796	0.34796908	0.002959709	0.076048739	-4.89E-05	0.035504472	1	0.815649307	1	0.821053473	ENSG00000197345	8	MRPL21
VAT	Heart_Failure	ENSG00000099949.14	0.003500782	0.198696321	0.002723758	0.076055235	0.003438497	0.206796068	0.002723721	0.076048628	6.23E-05	0.003090576	1	0.756191037	1	0.748456354	ENSG00000197363	5	ZNF517
VAT	Heart_Failure	ENSG00000100151.11	-0.001715207	0.471646042	0.00238288	0.076055412	-0.001674195	0.482304133	0.00238285	0.0760488	-4.10E-05	0.027720015	1	0.884782233	1	0.895800408	ENSG00000197363	5	ZNF517
VAT	Heart_Failure	ENSG00000100284.16	-0.001117515	0.673561653	0.00265276	0.076055465	-0.001056576	0.690415902	0.002652774	0.076048852	-6.09E-05	0.02000967	1	0.963913289	1	0.960065682	ENSG00000197410	8	DCHS2
VAT	Heart_Failure	ENSG00000100731.11	-0.000291912	0.910274223	0.00259034	0.076055491	-0.000345924	0.893761594	0.002590297	0.076048874	5.40E-05	0.004265211	1	0.984985763	1	0.98412197	ENSG00000197415	7	VEPH1
VAT	Heart_Failure	ENSG00000101052.8	-0.002517341	0.186895512	0.001907336	0.076055221	-0.002549218	0.181371342	0.001907317	0.076048598	3.19E-05	0.042032156	1	0.756191037	1	0.732920798	ENSG00000197415	7	VEPH1
VAT	Heart_Failure	ENSG00000101224.13	0.000488432	0.894177371	0.003671858	0.07605549	0.000549797	0.880974115	0.003671814	0.076048873	-6.14E-05	0.035588827	1	0.984985763	1	0.982211765	ENSG00000197442	8	MAP3K5
VAT	Heart_Failure	ENSG00000103248.13	0.001839993	0.091461075	0.001090209	0.076055048	0.001855169	0.088812664	0.001090187	0.076048425	-1.52E-05	0.042023012	1	0.623489698	1	0.608245538	ENSG00000197457	5	STMN3
VAT	Heart_Failure	ENSG00000103472.5	-0.003985895	0.288145197	0.003752488	0.076055317	-0.00390205	0.298399944	0.003752434	0.076048708	-8.38E-05	0.003281005	1	0.795233108	1	0.800488833	ENSG00000197479	4	PCDHB11
VAT	Heart_Failure	ENSG00000104219.8	-0.004437696	0.2203561	0.003620885	0.076055259	-0.004494107	0.214536845	0.003620811	0.076048636	5.64E-05	0.021773026	1	0.772901704	1	0.756570334	ENSG00000197479	4	PCDHB11
VAT	Heart_Failure	ENSG00000104835.10	0.00169236	0.318545309	0.001696682	0.076055338	0.001725689	0.309099003	0.00169665	0.076048715	-3.33E-05	0.005041728	1	0.811008798	1	0.807108087	ENSG00000197498	8	RPF2
VAT	Heart_Failure	ENSG00000104967.6	0.00056559	0.848389329	0.002958509	0.076055487	0.000653523	0.8251769	0.002958567	0.076048869	-8.79E-05	0.008123251	1	0.977595833	1	0.96872144	ENSG00000197535	10	MYO5A
VAT	Heart_Failure	ENSG00000105258.4	0.001002551	0.793361528	0.003827287	0.076055482	0.000919861	0.810063169	0.003827257	0.076048868	8.27E-05	0.010470969	1	0.972185844	1	0.96824489	ENSG00000197563	5	PIGN
VAT	Heart_Failure	ENSG00000105364.9	0.001249969	0.703990996	0.003289923	0.07605547	0.001201224	0.715015422	0.003289865	0.076048856	4.87E-05	0.039116043	1	0.963913289	1	0.960065682	ENSG00000197563	5	PIGN
VAT	Heart_Failure	ENSG00000106049.4	0.005530453	0.117435213	0.003532407	0.07605511	0.005603451	0.112675194	0.003532435	0.076048484	-7.30E-05	0.041800898	1	0.661746357	1	0.637261341	ENSG00000197563	5	PIGN
VAT	Heart_Failure	ENSG00000106125.14	-0.002332136	0.343363831	0.002461252	0.076055353	-0.00227792	0.35469636	0.002461235	0.076048743	-5.42E-05	0.010032391	1	0.818147112	1	0.822657104	ENSG00000197568	9	HHLA3
VAT	Heart_Failure	ENSG00000106133.13	0.000440091	0.765091116	0.001472851	0.076055479	0.000199491	0.892619631	0.001477817	0.076048874	0.0002406	0.04828452	1	0.967686555	1	0.98412197	ENSG00000197568	9	HHLA3
VAT	Heart_Failure	ENSG00000106236.3	0.003034656	0.240210964	0.002583871	0.076055278	0.002897393	0.262223089	0.002584296	0.076048681	0.000137264	0.009176403	1	0.779051326	1	0.791877836	ENSG00000197568	9	HHLA3
VAT	Heart_Failure	ENSG00000106290.10	0.000934149	0.607879284	0.001820586	0.076055452	0.001227783	0.501005477	0.001824589	0.076048806	-0.000293634	0.016084579	1	0.938337149	1	0.901880257	ENSG00000197580	7	BCO2
VAT	Heart_Failure	ENSG00000106330.7	-0.000547178	0.858671506	0.003072899	0.076055488	-0.001068688	0.728509749	0.003078827	0.076048858	0.00052151	0.006920138	1	0.979476883	1	0.960065682	ENSG00000197580	7	BCO2
VAT	Heart_Failure	ENSG00000106565.13	-0.002647424	0.099243188	0.001605924	0.076055069	-0.002613148	0.103695254	0.001605917	0.076048464	-3.43E-05	0.016029741	1	0.637447692	1	0.624888429	ENSG00000197586	8	ENTPD6
VAT	Heart_Failure	ENSG00000106635.3	-0.002655572	0.381558933	0.003034842	0.076055374	-0.002538086	0.403016181	0.00303509	0.076048768	-0.000117486	0.014473262	1	0.837245697	1	0.852871973	ENSG00000197629	5	MPEG1
VAT	Heart_Failure	ENSG00000106638.11	-0.001549939	0.533033623	0.002486338	0.076055432	-0.001071049	0.667985626	0.002497114	0.076048848	-0.00047889	0.039761517	1	0.9158182	1	0.957156395	ENSG00000197629	5	MPEG1
VAT	Heart_Failure	ENSG00000107099.11	-0.000191957	0.889864466	0.001386215	0.07605549	0.000386659	0.784696261	0.001415275	0.076048865	-0.000578616	0.042784455	1	0.984985763	1	0.965780945	ENSG00000197653	10	DNAH10
VAT	Heart_Failure	ENSG00000107554.11	0.00252431	0.346847464	0.002683373	0.076055355	0.002162909	0.420677422	0.002686018	0.076048776	0.000361401	0.002996362	1	0.820677452	1	0.871425441	ENSG00000197728	5	RPS26
VAT	Heart_Failure	ENSG00000107593.15	0.000466686	0.903069459	0.003832054	0.076055491	0.000336029	0.930125814	0.003832161	0.076048876	0.000130657	0.004353568	1	0.984985763	1	0.990517646	ENSG00000197728	5	RPS26
VAT	Heart_Failure	ENSG00000108264.12	-0.001881687	0.064209841	0.00101673	0.076054958	-0.001863696	0.066795835	0.001016719	0.076048352	-1.80E-05	0.027329389	1	0.564941346	1	0.560354119	ENSG00000197728	5	RPS26
VAT	Heart_Failure	ENSG00000108666.5	-0.002072653	0.284424316	0.001936272	0.076055314	-0.002042477	0.291486613	0.001936241	0.076048703	-3.02E-05	0.035237518	1	0.795233108	1	0.79496349	ENSG00000197728	5	RPS26
VAT	Heart_Failure	ENSG00000108774.10	0.009931808	0.006380826	0.003641289	0.076054332	0.009994086	0.006056989	0.003641241	0.076047701	-6.23E-05	0.028669851	1	0.195678662	1	0.192152742	ENSG00000197734	4	C14orf178
VAT	Heart_Failure	ENSG00000108924.9	-0.001795962	0.54184263	0.002944057	0.076055435	-0.001855427	0.528540522	0.00294402	0.076048815	5.95E-05	0.009940097	1	0.920870181	1	0.908819144	ENSG00000197782	10	ZNF780A
VAT	Heart_Failure	ENSG00000110628.9	-0.000443071	0.705062607	0.001170611	0.07605547	-0.000424585	0.716821654	0.001170591	0.076048856	-1.85E-05	0.028974617	1	0.963913289	1	0.960065682	ENSG00000197782	10	ZNF780A
VAT	Heart_Failure	ENSG00000110768.7	-0.001498733	0.663254001	0.003441999	0.076055463	-0.001433159	0.67713474	0.00344198	0.07604885	-6.56E-05	0.02892263	1	0.963584692	1	0.958291901	ENSG00000197816	9	CCDC180
VAT	Heart_Failure	ENSG00000111144.5	-0.004009361	0.201535813	0.003139205	0.076055238	-0.003951325	0.208130504	0.00313915	0.07604863	-5.80E-05	0.010291215	1	0.756841892	1	0.749921919	ENSG00000197816	9	CCDC180
VAT	Heart_Failure	ENSG00000111215.7	0.001485798	0.346599451	0.00157861	0.076055355	0.001453424	0.357206943	0.001578606	0.076048745	3.24E-05	0.023009848	1	0.820677452	1	0.826396615	ENSG00000197816	9	CCDC180
VAT	Heart_Failure	ENSG00000112294.8	-0.002825045	0.39874248	0.003347726	0.076055382	-0.002915272	0.383860974	0.00334779	0.076048758	9.02E-05	0.0159753	1	0.850486278	1	0.838615487	ENSG00000197816	9	CCDC180
VAT	Heart_Failure	ENSG00000113119.8	-0.00831038	0.009032593	0.003183043	0.076054429	-0.008258113	0.009475041	0.003183015	0.076047826	-5.23E-05	0.047919758	1	0.232990241	1	0.239368781	ENSG00000197822	6	OCLN
VAT	Heart_Failure	ENSG00000113971.14	-0.000572764	0.526753373	0.000904883	0.07605543	-0.000592092	0.512893972	0.000904871	0.07604881	1.93E-05	0.00681293	1	0.913789635	1	0.904747387	ENSG00000197822	6	OCLN
VAT	Heart_Failure	ENSG00000115163.10	-0.005390113	0.148973332	0.003734913	0.076055168	-0.005321876	0.154180939	0.003734859	0.07604856	-6.82E-05	0.016494543	1	0.721315241	1	0.704981382	ENSG00000197822	6	OCLN
VAT	Heart_Failure	ENSG00000115275.7	0.004043443	0.03380545	0.001905136	0.07605479	0.004007995	0.035396087	0.001905118	0.076048186	3.54E-05	0.023908318	1	0.438042449	1	0.430366522	ENSG00000197847	8	SLC22A20
VAT	Heart_Failure	ENSG00000115687.9	-0.003871178	0.056148887	0.002026919	0.076054924	-0.003825211	0.059132116	0.002026909	0.076048321	-4.60E-05	0.009714032	1	0.533809522	1	0.532804147	ENSG00000197847	8	SLC22A20
VAT	Heart_Failure	ENSG00000115750.12	0.007659161	0.01365409	0.003105595	0.076054544	0.007717379	0.012954894	0.003105565	0.076047913	-5.82E-05	0.023049122	1	0.266586081	1	0.264855602	ENSG00000197847	8	SLC22A20
VAT	Heart_Failure	ENSG00000116198.8	0.000525542	0.857501719	0.002926905	0.076055488	0.000474652	0.871171019	0.002926854	0.076048873	5.09E-05	0.016107457	1	0.978786081	1	0.980677945	ENSG00000197903	6	HIST1H2BK
VAT	Heart_Failure	ENSG00000117115.8	0.000719262	0.834048674	0.003433036	0.076055486	0.000648263	0.850224702	0.00343302	0.076048871	7.10E-05	0.018775124	1	0.972185844	1	0.97249075	ENSG00000197903	6	HIST1H2BK
VAT	Heart_Failure	ENSG00000117122.9	-0.0027164	0.073386648	0.001517184	0.076054993	-0.002738281	0.071094582	0.001517157	0.076048368	2.19E-05	0.043770991	1	0.603545069	1	0.580042752	ENSG00000197903	6	HIST1H2BK
VAT	Heart_Failure	ENSG00000117226.7	-0.000631129	0.529851034	0.001004609	0.076055431	-0.000612666	0.541952612	0.001004595	0.076048819	-1.85E-05	0.017426579	1	0.915695819	1	0.913136234	ENSG00000197903	6	HIST1H2BK
VAT	Heart_Failure	ENSG00000118600.7	-0.004829173	0.177741745	0.003583156	0.07605521	-0.004954064	0.166822946	0.003583444	0.076048579	0.000124891	0.026917407	1	0.74593461	1	0.71714634	ENSG00000197903	6	HIST1H2BK
VAT	Heart_Failure	ENSG00000119865.4	0.002880365	0.321392993	0.002904764	0.07605534	0.00283267	0.329464635	0.002904725	0.076048728	4.77E-05	0.034593985	1	0.811008798	1	0.812752882	ENSG00000197903	6	HIST1H2BK
VAT	Heart_Failure	ENSG00000120915.9	-0.005478442	0.142787352	0.003738295	0.076055158	-0.005569585	0.136260782	0.003738319	0.07604853	9.11E-05	0.01478487	1	0.707715645	1	0.672705156	ENSG00000197903	6	HIST1H2BK
VAT	Heart_Failure	ENSG00000121594.7	0.004431789	0.124904008	0.002888074	0.076055126	0.004496057	0.119523374	0.002888049	0.076048499	-6.43E-05	0.007612436	1	0.67041525	1	0.64556656	ENSG00000197903	6	HIST1H2BK
VAT	Heart_Failure	ENSG00000121716.14	0.00090999	0.30874787	0.000894028	0.076055331	-0.000335332	0.744908808	0.001030651	0.07604886	0.001245322	0.015173847	1	0.811008798	1	0.960065682	ENSG00000197903	6	HIST1H2BK
VAT	Heart_Failure	ENSG00000123427.11	-0.001006299	0.557436159	0.001715319	0.076055439	-0.000980409	0.567614569	0.001715295	0.076048826	-2.59E-05	0.049692879	1	0.92326913	1	0.921911445	ENSG00000197914	2	HIST1H4K
VAT	Heart_Failure	ENSG00000123545.5	0.002093042	0.341355945	0.002199751	0.076055352	0.002047643	0.351926733	0.002199734	0.076048742	4.54E-05	0.014967998	1	0.818052084	1	0.822453669	ENSG00000197915	5	HRNR
VAT	Heart_Failure	ENSG00000124116.14	-0.001298761	0.234388608	0.001092193	0.076055272	-0.001316688	0.22798776	0.00109218	0.07604865	1.79E-05	0.039046225	1	0.779051326	1	0.763693205	ENSG00000197915	5	HRNR
VAT	Heart_Failure	ENSG00000124191.13	0.000642057	0.756039393	0.002066578	0.076055478	-0.000828602	0.700746762	0.002156061	0.076048854	0.001470659	0.016788868	1	0.964199078	1	0.960065682	ENSG00000197915	5	HRNR
VAT	Heart_Failure	ENSG00000124587.9	-0.000377848	0.677159542	0.000907542	0.076055466	-0.000393052	0.664941945	0.000907531	0.076048848	1.52E-05	0.034841866	1	0.963913289	1	0.957156395	ENSG00000197935	6	ZNF311
VAT	Heart_Failure	ENSG00000124641.10	-0.005189563	0.088421915	0.003045902	0.07605504	-0.00513379	0.091897717	0.003045902	0.076048433	-5.58E-05	0.048866352	1	0.623489698	1	0.616607764	ENSG00000197935	6	ZNF311
VAT	Heart_Failure	ENSG00000124678.13	0.002692817	0.165155147	0.00194015	0.076055192	0.002757257	0.15528422	0.001940208	0.076048562	-6.44E-05	0.006147637	1	0.729325131	1	0.704981382	ENSG00000197935	6	ZNF311
VAT	Heart_Failure	ENSG00000124713.5	-0.002415003	0.456469801	0.003243039	0.076055406	-0.002364156	0.465999109	0.003242991	0.076048794	-5.08E-05	0.03869371	1	0.881202785	1	0.884473968	ENSG00000197935	6	ZNF311
VAT	Heart_Failure	ENSG00000125124.7	-0.000363621	0.720741017	0.00101721	0.076055473	-0.000384628	0.705336551	0.001017194	0.076048855	2.10E-05	0.005439213	1	0.963913289	1	0.960065682	ENSG00000197935	6	ZNF311
VAT	Heart_Failure	ENSG00000125388.15	0.001973233	0.021197605	0.000856269	0.076054664	0.001954263	0.022471415	0.000856265	0.076048064	1.90E-05	0.011453374	1	0.33431651	1	0.351455777	ENSG00000197935	6	ZNF311
VAT	Heart_Failure	ENSG00000125534.5	0.005444101	0.046304648	0.002732145	0.076054873	0.005490687	0.044463972	0.002732099	0.076048246	-4.66E-05	0.019764189	1	0.497279488	1	0.477344364	ENSG00000197935	6	ZNF311
VAT	Heart_Failure	ENSG00000126746.13	-0.008903907	0.011704046	0.00353196	0.076054501	-0.008839572	0.012323117	0.003531925	0.076047899	-6.43E-05	0.026157866	1	0.256374348	1	0.258531674	ENSG00000197935	6	ZNF311

VAT	Heart_Failure	ENSG00000127337.2	-0.001567752	0.260199316	0.001392417	0.076055295	-0.001411633	0.311029061	0.001393426	0.076048717	-0.00015612	0.004224574	1	0.79317431	1	0.809810347	ENSG00000197935	6	ZNF311
VAT	Heart_Failure	ENSG00000128218.7	-0.003369043	0.087773276	0.001973356	0.076055038	-0.00331216	0.093282816	0.001973482	0.076048437	-5.69E-05	0.049345969	1	0.623489698	1	0.617411444	ENSG00000197935	6	ZNF311
VAT	Heart_Failure	ENSG00000128617.2	-0.000546208	0.811865225	0.002294793	0.076055484	-0.000592798	0.796154776	0.00229476	0.076048866	4.66E-05	0.007848029	1	0.972185844	1	0.965780945	ENSG00000197935	6	ZNF311
VAT	Heart_Failure	ENSG00000128815.13	-0.000383794	0.891362157	0.002810011	0.07605549	0.004542758	0.13026796	0.003002387	0.07604852	-0.004926551	3.20E-06	1	0.984985763	1	0.665314041	ENSG00000197951	4	ZNF71
VAT	Heart_Failure	ENSG00000128891.11	0.000673555	0.550141423	0.001127198	0.076055437	0.000692236	0.539128165	0.001127178	0.076048818	-1.87E-05	0.020892138	1	0.92326913	1	0.911760867	ENSG00000197951	4	ZNF71
VAT	Heart_Failure	ENSG00000129055.8	0.002031161	0.055964522	0.001062703	0.076054923	0.002053798	0.053281189	0.001062691	0.076048294	-2.26E-05	0.007951464	1	0.533809522	1	0.508844578	ENSG00000197958	8	RPL12
VAT	Heart_Failure	ENSG00000129317.10	-0.003255749	0.252545405	0.002845462	0.076055289	-0.003214487	0.258599237	0.00284541	0.076048678	-4.13E-05	0.042027249	1	0.791175162	1	0.786050543	ENSG00000197958	8	RPL12
VAT	Heart_Failure	ENSG00000129354.7	-0.000850048	0.794385669	0.003261638	0.076055482	-0.000919344	0.778043675	0.003261593	0.076048864	6.93E-05	0.005768306	1	0.972185844	1	0.965780945	ENSG00000197958	8	RPL12
VAT	Heart_Failure	ENSG00000129465.11	-0.00365105	0.164631652	0.002627291	0.076055192	-0.003614363	0.168906934	0.002627239	0.076048581	-3.67E-05	0.043843908	1	0.729325131	1	0.71714634	ENSG00000197958	8	RPL12
VAT	Heart_Failure	ENSG00000130758.3	0.003326197	0.380485651	0.003792687	0.076055373	0.003242996	0.392514107	0.003792678	0.076048763	8.32E-05	0.015268594	1	0.837245697	1	0.842989349	ENSG00000197958	8	RPL12
VAT	Heart_Failure	ENSG00000130764.5	-0.001234678	0.713680528	0.003365	0.076055472	-0.001171674	0.727688922	0.00336493	0.076048858	-6.30E-05	0.005655131	1	0.963913289	1	0.960065682	ENSG00000197958	8	RPL12
VAT	Heart_Failure	ENSG00000131495.4	0.00402012	0.068153136	0.002204017	0.076054974	0.003984191	0.070650844	0.002203988	0.076048367	3.59E-05	0.036084939	1	0.586322251	1	0.580042752	ENSG00000197969	7	VPS13A
VAT	Heart_Failure	ENSG00000132141.9	0.00186371	0.525541646	0.002935778	0.07605543	0.001802991	0.539121545	0.002935786	0.076048818	6.07E-05	0.03197183	1	0.91226097	1	0.911760867	ENSG00000197969	7	VPS13A
VAT	Heart_Failure	ENSG00000132824.9	0.000564953	0.703350347	0.001483588	0.07605547	0.000531925	0.719944488	0.001483616	0.076048857	3.30E-05	0.046296509	1	0.963913289	1	0.960065682	ENSG00000197969	7	VPS13A
VAT	Heart_Failure	ENSG00000132837.10	-0.009805122	0.012945566	0.003945291	0.076054529	-0.009733096	0.013623142	0.003945223	0.076047927	-7.20E-05	0.011547335	1	0.266586081	1	0.267212404	ENSG00000197969	7	VPS13A
VAT	Heart_Failure	ENSG00000132840.5	-0.002661964	0.126439888	0.001741786	0.076055128	-0.002609898	0.134047541	0.001741868	0.076048526	-5.21E-05	0.026316321	1	0.673695158	1	0.6690257	ENSG00000198015	8	MRPL42
VAT	Heart_Failure	ENSG00000133661.11	-0.000408484	0.739698359	0.001229436	0.076055476	-0.000385156	0.754064688	0.001229414	0.076048862	-2.33E-05	0.007541337	1	0.963913289	1	0.960414647	ENSG00000198015	8	MRPL42
VAT	Heart_Failure	ENSG00000134440.7	-0.003477286	0.358517856	0.003787093	0.076055361	-0.003410274	0.367849369	0.003787047	0.07604875	-6.70E-05	0.025425483	1	0.82873678	1	0.830566839	ENSG00000198026	6	ZNF335
VAT	Heart_Failure	ENSG00000135525.14	0.002668523	0.321153964	0.002689798	0.076055339	0.002596021	0.334476045	0.002689792	0.076048731	7.25E-05	0.002932067	1	0.811008798	1	0.812752882	ENSG00000198026	6	ZNF335
VAT	Heart_Failure	ENSG00000135596.13	0.004543061	0.054520364	0.002362875	0.076054916	0.0044935	0.057207276	0.002362851	0.076048312	4.96E-05	0.010085067	1	0.529845795	1	0.526306936	ENSG00000198026	6	ZNF335
VAT	Heart_Failure	ENSG00000135968.15	-0.001864354	0.505154964	0.002797645	0.076055424	-0.001906155	0.495647987	0.002797599	0.076048804	4.18E-05	0.04166447	1	0.904963929	1	0.898557334	ENSG00000198026	6	ZNF335
VAT	Heart_Failure	ENSG00000136448.7	0.00439196	0.000893267	0.001322015	0.07605377	0.004411477	0.000846964	0.001321992	0.076047139	-1.95E-05	0.039910828	1	0.0632158	1	0.058440502	ENSG00000198028	3	ZNF560
VAT	Heart_Failure	ENSG00000136932.9	-5.37E-05	0.987837901	0.003521621	0.076055493	-0.000105043	0.976203808	0.003521545	0.076048877	5.14E-05	0.0283649	1	0.996216992	1	0.995646729	ENSG00000198039	7	ZNF273
VAT	Heart_Failure	ENSG00000137070.13	-0.004634107	0.069339311	0.002551531	0.076054978	-0.00451383	0.076944282	0.002552059	0.076048389	-0.000120277	0.035061161	1	0.592493737	1	0.597746085	ENSG00000198039	7	ZNF273
VAT	Heart_Failure	ENSG00000137312.10	0.003113204	0.082775733	0.001794554	0.076055023	0.004194915	0.024473449	0.001864721	0.076048087	-0.001081711	0.032880719	1	0.619367186	1	0.371135825	ENSG00000198046	7	ZNF667
VAT	Heart_Failure	ENSG00000139178.6	-0.001332963	0.387958692	0.001543986	0.076055377	-0.001290479	0.403260118	0.001543979	0.076048768	-4.25E-05	0.001885728	1	0.840475658	1	0.852871973	ENSG00000198046	7	ZNF667
VAT	Heart_Failure	ENSG00000139537.6	0.001260433	0.730161839	0.003654378	0.076055474	0.001334407	0.714992762	0.003654317	0.076048856	-7.40E-05	0.005689938	1	0.963913289	1	0.960065682	ENSG00000198053	7	SIRPA
VAT	Heart_Failure	ENSG00000139970.12	-0.002082288	0.552790746	0.003507993	0.076055438	-0.001140478	0.745628526	0.003515555	0.07604886	-0.00094181	5.20E-05	1	0.92326913	1	0.960126407	ENSG00000198093	6	ZNF649
VAT	Heart_Failure	ENSG00000140025.11	0.001430842	0.690829499	0.003597522	0.076055468	0.001276884	0.722642082	0.003597565	0.076048857	0.000153958	4.88E-05	1	0.963913289	1	0.960065682	ENSG00000198093	6	ZNF649
VAT	Heart_Failure	ENSG00000140577.11	0.005042632	0.173251203	0.003702827	0.076055204	0.004960896	0.180319658	0.003702787	0.076048597	8.17E-05	0.006262165	1	0.73906232	1	0.732359213	ENSG00000198093	6	ZNF649
VAT	Heart_Failure	ENSG00000141013.10	-0.004801644	0.133006664	0.003196083	0.076055141	-0.004860992	0.128274691	0.003196038	0.076048516	5.93E-05	0.015393348	1	0.68290901	1	0.662992784	ENSG00000198099	4	ADH4
VAT	Heart_Failure	ENSG00000141556.16	0.002378367	0.545783072	0.003937098	0.076055436	0.00245534	0.532857842	0.003937048	0.076048816	-7.70E-05	0.013082381	1	0.921324909	1	0.908819144	ENSG00000198099	4	ADH4
VAT	Heart_Failure	ENSG00000141753.5	-0.006445935	0.076003002	0.003632807	0.076055002	-0.006541727	0.071740743	0.003632752	0.076048371	9.58E-05	0.000466029	1	0.612031969	1	0.580042752	ENSG00000198099	4	ADH4
VAT	Heart_Failure	ENSG00000141971.8	0.001261504	0.567588824	0.002206944	0.076055442	0.001225589	0.57865964	0.0022069	0.076048829	3.59E-05	0.017929929	1	0.92639479	1	0.92586219	ENSG00000198105	7	ZNF248
VAT	Heart_Failure	ENSG00000142185.12	-0.00012532	0.920598727	0.00125723	0.076055491	-0.000107551	0.931826477	0.001257208	0.076048876	-1.78E-05	0.048520403	1	0.984985763	1	0.990517646	ENSG00000198105	7	ZNF248
VAT	Heart_Failure	ENSG00000142798.12	-0.002896456	0.108028675	0.001802265	0.07605509	-0.003262021	0.070960601	0.00180648	0.076048368	0.000365566	0.003320703	1	0.639208475	1	0.580042752	ENSG00000198131	9	ZNF544
VAT	Heart_Failure	ENSG00000144152.8	0.006005852	0.045337157	0.003000649	0.076054868	0.006632701	0.027936802	0.003017361	0.076048123	-0.000626848	0.048976941	1	0.49654981	1	0.387205367	ENSG00000198131	9	ZNF544
VAT	Heart_Failure	ENSG00000144476.5	0.005283993	0.080776818	0.003025995	0.076055017	0.005328429	0.078253114	0.00302594	0.076048393	-4.44E-05	0.039952539	1	0.619367186	1	0.597746085	ENSG00000198131	9	ZNF544
VAT	Heart_Failure	ENSG00000145390.7	0.00763122	0.010300266	0.002974447	0.076054466	0.007581163	0.010810133	0.00297442	0.076047863	5.01E-05	0.041868456	1	0.240921465	1	0.25004753	ENSG00000198169	4	ZNF251
VAT	Heart_Failure	ENSG00000146243.9	0.0035739	0.353174736	0.003849319	0.076055358	0.003515527	0.361085113	0.003849256	0.076048747	5.84E-05	0.039623089	1	0.826718867	1	0.829804255	ENSG00000198169	4	ZNF251
VAT	Heart_Failure	ENSG00000146830.9	0.003914233	0.212093188	0.003136827	0.07605525	0.004435444	0.158172954	0.003142929	0.076048566	-0.000521211	0.008463065	1	0.766257518	1	0.704981382	ENSG00000198171	8	DDRGK1
VAT	Heart_Failure	ENSG00000146833.11	0.000201189	0.774025462	0.000700728	0.07605548	0.000440301	0.533155806	0.00070652	0.076048816	-0.000239112	0.008253724	1	0.969984771	1	0.908819144	ENSG00000198185	7	ZNF334
VAT	Heart_Failure	ENSG00000147576.11	-0.003599189	0.088610285	0.002113716	0.07605504	-0.00323655	0.126594512	0.002118615	0.076048513	-0.000362639	0.012577881	1	0.623489698	1	0.658005371	ENSG00000198203	5	SULT1C2
VAT	Heart_Failure	ENSG00000147894.10	0.000763752	0.694794844	0.001946576	0.076055469	0.000136044	0.944588474	0.001957357	0.076048876	0.000627708	0.002305549	1	0.963913289	1	0.991197882	ENSG00000198203	5	SULT1C2
VAT	Heart_Failure	ENSG00000148225.11	-0.000128233	0.954150816	0.002230333	0.076055493	-6.78E-05	0.975737525	0.002230303	0.076048877	-6.04E-05	0.000472087	1	0.993060803	1	0.995646729	ENSG00000198203	5	SULT1C2
VAT	Heart_Failure	ENSG00000149346.10	0.001740086	0.493191857	0.002539376	0.07605542	0.001780384	0.48322681	0.002539334	0.0760488	-4.03E-05	0.030237419	1	0.896935503	1	0.896227848	ENSG00000198208	7	RPS6KL1
VAT	Heart_Failure	ENSG00000149679.7	-0.00346258	0.21245516	0.002777074	0.07605525	-0.003419871	0.218142181	0.00277703	0.07604864	-4.27E-05	0.038188897	1	0.766257518	1	0.761153501	ENSG00000198208	7	RPS6KL1
VAT	Heart_Failure	ENSG00000149922.6	0.002732058	0.035146205	0.001296857	0.0760548	0.00269561	0.037658258	0.001296864	0.076048203	3.64E-05	0.004236213	1	0.442938475	1	0.442284224	ENSG00000198208	7	RPS6KL1
VAT	Heart_Failure	ENSG00000150076.18	0.002331606	0.349469667	0.002492046	0.076055356	0.002376188	0.340331246	0.002492038	0.076048735	-4.46E-05	0.0461725	1	0.825801611	1	0.812752882	ENSG00000198218	6	QRICH1
VAT	Heart_Failure	ENSG00000151176.3	-0.003701168	0.260112549	0.003286636	0.076055295	-0.00377591	0.250607456	0.003286611	0.076048671	7.47E-05	0.007127241	1	0.79317431	1	0.781555457	ENSG00000198218	6	QRICH1
VAT	Heart_Failure	ENSG00000152240.8	-0.00135783	0.257078876	0.001198096	0.076055293	-0.001381248	0.248957725	0.001198078	0.076048669	2.34E-05	0.010472047	1	0.79317431	1	0.780278252	ENSG00000198237	7	RP11-98J23.2
VAT	Heart_Failure	ENSG00000152749.7	-0.003138572	0.276428046	0.002883711	0.076055308	-0.003185919	0.269240747	0.00288367	0.076048686	4.73E-05	0.032173747	1	0.795233108	1	0.791877836	ENSG00000198270	8	TMEM116
VAT	Heart_Failure	ENSG00000153157.8	-0.005058141	0.055751587	0.00264412	0.076054922	-0.005096715	0.053905926	0.002644078	0.076048297	3.86E-05	0.049750838	1	0.533809522	1	0.512041201	ENSG00000198315	6	ZKSCAN8
VAT	Heart_Failure	ENSG00000155393.8	0.002104822	0.138077333	0.001419308	0.07605515	0.002071654	0.144391284	0.001419297	0.076048544	3.32E-05	0.005412736	1	0.694159268	1	0.696882186	ENSG00000198315	6	ZKSCAN8
VAT	Heart_Failure	ENSG00000156171.10	0.001717925	0.442985997	0.002239327	0.076055401	0.001748982	0.43477597	0.002239283	0.076048782	-3.11E-05	0.043128626	1	0.873500102	1	0.875281919	ENSG00000198315	6	ZKSCAN8
VAT	Heart_Failure	ENSG00000156172.5	0.001914622	0.32150221	0.001931276	0.07605534	0.001944372	0.314033033	0.001931246	0.076048719	-2.97E-05	0.038365881	1	0.811008798	1	0.812343568	ENSG00000198315	6	ZKSCAN8
VAT	Heart_Failure	ENSG00000157150.4	-0.000866006	0.681516662	0.002110178	0.076055467	-0.000935953	0.657373408	0.002110176	0.076048846	6.99E-05	0.00034965	1	0.963913289	1	0.956411867	ENSG00000198315	6	ZKSCAN8
VAT	Heart_Failure	ENSG00000159904.7	-0.002361426	0.258316929	0.002089056	0.076055294	-0.002319014	0.266964635	0.002089049	0.076048685	-4.24E-05	0.023348512	1	0.79317431	1	0.791877836	ENSG00000198336	5	MYL4
VAT	Heart_Failure	ENSG00000160285.10	0.000247167	0.849614103	0.001303548	0.076055487	0.000292154	0.822667017	0.001303584	0.076048869	-4.50E-05	0.003708286	1	0.977595833	1	0.968476262	ENSG00000198336	5	MYL4
VAT	Heart_Failure	ENSG00000160828.13	-0.003138925	0.268264934	0.002835348	0.076055302	-0.003095005	0.275011836	0.002835306	0.076048691	-4.39E-05	0.040213529	1	0.795233108	1	0.791877836	ENSG00000198453	8	ZNF568
VAT	Heart_Failure	ENSG00000160856.16	-0.007766197	0.003248761	0.002638711	0.076054141	-0.007817183	0.003051361	0.002638684	0.076047507	5.10E-05	0.018379231	1	0.122857446	1	0.126139004	ENSG00000198453	8	ZNF568
VAT	Heart_Failure	ENSG00000160917.10	-0.003863932	0.309900026	0.003805207	0.076055332	-0.002934975	0.442483805	0.003821551	0.076048785	-0.000928957	0.008841797	1	0.811008798	1	0.875576038	ENSG00000198466	8	ZNF587
VAT	Heart_Failure	ENSG00000161180.6	-0.000634154	0.729680932	0.001835201	0.076055474	-0.000686378	0.708399125	0.001835199	0.076048855	5.22E-05	0.002023527	1	0.963913289	1	0.960065682	ENSG00000198466	8	ZNF587
VAT	Heart_Failure	ENSG00000161551.8	0.001030365	0.748130743	0.003208806	0.076055477	0.001428624	0.656787915	0.00321507	0.076048846	-0.00039826	0.049565777	1	0.963913289	1	0.956411867	ENSG00000198466	8	ZNF587
VAT	Heart_Failure	ENSG00000161640.11	-0.000423241	0.881054145	0.002828528	0.07605549	-0.00047463	0.866738895	0.002828508	0.076048872	5.14E-05	0.033335558	1	0.984137328	1	0.980029484	ENSG00000198466	8	ZNF587
VAT	Heart_Failure	ENSG00000162365.7	0.001082667	0.743708718	0.003311452	0.076055476	0.001148806	0.728647856	0.003311397	0.076048858	-6.61E-05	0.006506445	1	0.963913289	1	0.960065682	ENSG00000198468	3	FLVCR1-AS1
VAT	Heart_Failure	ENSG00000163297.12	0.003105304	0.327153103	0.003169113	0.076055343	0.00338262	0.285997343	0.00317038	0.076048699	-0.000277317	0.003309335	1	0.811008798	1	0.794840503	ENSG00000198468	3	FLVCR1-AS1
VAT	Heart_Failure	ENSG00000163393.8	-0.000920899	0.784393459	0.003365881	0.076055481	-0.000861725	0.797934861	0.003365839	0.076048867	-5.92E-05	0.025634954	1	0.972185844	1	0.965780945	ENSG00000198478	6	SH3BGRL2
VAT	Heart_Failure	ENSG00000163468.10	0.000399348	0.862415591	0.00230437	0.076055488	0.000369267	0.87268427	0.00230432	0.076048873	3.01E-05	0.048508059	1	0.980340622	1	0.980677945	ENSG00000198478	6	SH3BGRL2
VAT	Heart_Failure	ENSG00000163558.8	0.000326055	0.876236547	0.002093558	0.076055489	0.000282986	0.892477553	0.002093559	0.076048874	4.31E-05	0.028584552	1	0.983439565	1	0.98412197	ENSG00000198478	6	SH3BGRL2
VAT	Heart_Failure	ENSG00000163629.8	0.002599518	0.28654724	0.002439195	0.076055316	0.002664707	0.274628294	0.002439164	0.076048691	-6.52E-05	0.000696378	1	0.795233108	1	0.791877836	ENSG00000198478	6	SH3BGRL2
VAT	Heart_Failure	ENSG00000163682.11	0.000708833	0.194525197	0.000546387	0.07605523	0.000719552	0.187859255	0.000546382	0.076048606	-1.07E-05	0.017942213	1	0.756191037	1	0.738152304	ENSG00000198502	5	HLA-DRB5
VAT	Heart_Failure	ENSG00000163904.8	-0.002063212	0.334364968	0.002137246	0.076055348	-0.001907132	0.372355301	0.002137871	0.076048753	-0.00015608	0.004818291	1	0.813847772	1	0.834172589	ENSG00000198502	5	HLA-DRB5
VAT	Heart_Failure	ENSG00000163945.11	0.000879646	0.57612314	0.001573445	0.076055444	0.000849439	0.589289543	0.001573424	0.076048831	3.02E-05	0.013328313	1	0.92639479	1	0.927008193	ENSG00000198502	5	HLA-DRB5
VAT	Heart_Failure	ENSG00000164070.7	0.004652333	0.208914971	0.003702447	0.076055247	0.00472431	0.201956709	0.003702442	0.076048623	-7.20E-05	0.034024401	1	0.765744116	1	0.74232065	ENSG00000198502	5	HLA-DRB5
VAT	Heart_Failure	ENSG00000164334.11	0.00191214	0.498633322	0.002825925	0.076055422	0.0018633	0.509661463	0.002825899	0.076048809	4.88E-05	0.036369604	1	0.900611917	1	0.904026759	ENSG00000198502	5	HLA-DRB5
VAT	Heart_Failure	ENSG00000164535.10	-0.000384662	0.738921632	0.001154164	0.076055476	-0.000400936	0.728299283	0.001154142	0.076048858	1.63E-05	0.044213061	1	0.963913289	1	0.960065682	ENSG00000198502	5	HLA-DRB5
VAT	Heart_Failure	ENSG00000164828.13	0.001783223	0.445019072	0.002334839	0.076055402	0.001752742	0.452828982	0.002334787	0.076048789	3.05E-05	0.044842936	1	0.873500102	1	0.879776385	ENSG00000198515	9	CNGA1
VAT	Heart_Failure	ENSG00000164967.5	-0.006772479	0.050148983	0.003457647	0.076054894	-0.006603392	0.056216098	0.00345843	0.076048308	-0.000169086	0.035287955	1	0.51300547	1	0.520659166	ENSG00000198515	9	CNGA1
VAT	Heart_Failure	ENSG00000164978.13	0.001390843	0.088417382	0.000816314	0.07605504	0.001476896	0.070714252	0.000817178	0.076048367	-8.61E-05	0.024813048	1	0.623489698	1	0.580042752	ENSG00000198569	5	SLC34A3
VAT	Heart_Failure	ENSG00000165271.12	0.001957556	0.552071027	0.003291891	0.076055438	0.002115926	0.520467835	0.003292631	0.076048812	-0.00015837	0.037775967	1	0.92326913	1	0.90770989	ENSG00000198569	5	SLC34A3
VAT	Heart_Failure	ENSG00000165983.10	-0.003154034	0.416418905	0.003881169	0.07605539	-0.003230442	0.405213823	0.003881126	0.076048769	7.64E-05	0.014523502	1	0.869982193	1	0.854385142	ENSG00000198624	8	CCDC69
VAT	Heart_Failure	ENSG00000166140.13	-0.004784463	0.027161939	0.00216571	0.076054731	-0.004821595	0.025990079	0.002165679	0.076048103	3.71E-05	0.024717519	1	0.390557318	1	0.383743978	ENSG00000198624	8	CCDC69
VAT	Heart_Failure	ENSG00000166435.11	-0.002854963	0.191139729	0.002184001	0.076055226	-0.002904663	0.18352475	0.002183986	0.076048601	4.97E-05	0.007736116	1	0.756191037	1	0.733556367	ENSG00000198624	8	CCDC69
VAT	Heart_Failure	ENSG00000166446.10	-0.000279181	0.856177938	0.001540377	0.076055488	-0.000306625	0.842215285	0.00154036	0.076048871	2.74E-05	0.02786895	1	0.978786081	1	0.969450344	ENSG00000198624	8	CCDC69
VAT	Heart_Failure	ENSG00000166529.10	-0.000540386	0.727838691	0.001552824	0.076055474	-4.40E-05	0.977592311	0.001565566	0.076048877	-0.000496413	0.012997909	1	0.963913289	1	0.995646729	ENSG00000198633	6	ZNF534
VAT	Heart_Failure	ENSG00000166664.9	-0.00216943	0.151888885	0.001514021	0.076055173	-0.002196151	0.146899924	0.00151399	0.076048548	2.67E-05	0.01024431	1	0.721315241	1	0.701115705	ENSG00000198646	9	NCOA6
VAT	Heart_Failure	ENSG00000166716.5	0.001270389	0.715676948	0.003487753	0.076055472	0.001212467	0.728111211	0.003487714	0.076048858	5.79E-05	0.038978503	1	0.963913289	1	0.960065682	ENSG00000198646	9	NCOA6
VAT	Heart_Failure	ENSG00000166847.5	-0.001171298	0.327487019	0.001196191	0.076055343	-0.00119564	0.317530702	0.001196183	0.076048721	2.43E-05	0.01771503	1	0.811008798	1	0.812752882	ENSG00000198646	9	NCOA6
VAT	Heart_Failure	ENSG00000166924.4	0.00223421	0.352714121	0.002404087	0.076055358	0.003373528	0.166303856	0.002437203	0.076048578	-0.001139318	0.004499322	1	0.826718867	1	0.71714634	ENSG00000198677	6	TTC37
VAT	Heart_Failure	ENSG00000166997.3	0.004855461	0.14408407	0.003323946	0.07605516	0.005433324	0.102768578	0.003330099	0.076048461	-0.000577862	0.004765187	1	0.709748879	1	0.624888429	ENSG00000198677	6	TTC37
VAT	Heart_Failure	ENSG00000167236.2	-0.001849164	0.098142893	0.001118044	0.076055066	-0.001824682	0.102673177	0.001118042	0.076048461	-2.45E-05	0.016656203	1	0.637447692	1	0.624888429	ENSG00000198755	6	RPL10A
VAT	Heart_Failure	ENSG00000167311.9	-4.43E-05	0.98721423	0.002765977	0.076055493	-4.72E-06	0.998637732	0.002765925	0.076048877	-3.96E-05	0.041990423	1	0.996216992	1	0.999361908	ENSG00000198755	6	RPL10A
VAT	Heart_Failure	ENSG00000167525.9	0.001549936	0.532500201	0.002483097	0.076055432	0.00158265	0.52387478	0.002483038	0.076048813	-3.27E-05	0.035261875	1	0.9158182	1	0.908734352	ENSG00000198756	6	COLGALT2
VAT	Heart_Failure	ENSG00000168661.10	0.002410424	0.200703049	0.001883802	0.076055237	0.002437098	0.19575676	0.001883766	0.076048616	-2.67E-05	0.04303569	1	0.756749202	1	0.738152304	ENSG00000198756	6	COLGALT2
VAT	Heart_Failure	ENSG00000168778.7	0.000415632	0.65223467	0.000922274	0.076055461	0.00043176	0.639675632	0.000922264	0.076048843	-1.61E-05	0.030354581	1	0.96297588	1	0.953349011	ENSG00000198756	6	COLGALT2
VAT	Heart_Failure	ENSG00000168961.12	0.005336788	0.095861273	0.003204775	0.07605506	0.005391476	0.092501657	0.003204729	0.076048435	-5.47E-05	0.025616072	1	0.637447692	1	0.616677715	ENSG00000198756	6	COLGALT2
VAT	Heart_Failure	ENSG00000168993.10	0.000852053	0.790070935	0.003200577	0.076055482	0.000901727	0.778140931	0.003200534	0.076048864	-4.97E-05	0.046227939	1	0.972185844	1	0.965780945	ENSG00000198785	4	GRIN3A
VAT	Heart_Failure	ENSG00000169085.7	0.002120555	0.103508755	0.001302489	0.076055079	0.00190051	0.145391888	0.001305291	0.076048546	0.000220045	0.01080457	1	0.637447692	1	0.697881062	ENSG00000198794	7	SCAMP5
VAT	Heart_Failure	ENSG00000169129.10	-0.000852794	0.589054911	0.001578645	0.076055447	-0.001161683	0.463864187	0.001585917	0.076048793	0.000308889	0.04269435	1	0.930570288	1	0.881725315	ENSG00000198794	7	SCAMP5
VAT	Heart_Failure	ENSG00000169894.13	-0.002455367	0.43935366	0.003175239	0.0760554	-0.0029488	0.353920272	0.00318097	0.076048743	0.000493433	0.010633646	1	0.873500102	1	0.822657104	ENSG00000198794	7	SCAMP5
VAT	Heart_Failure	ENSG00000169914.5	0.001384358	0.431636676	0.001760386	0.076055397	0.001348989	0.443498298	0.001760393	0.076048785	3.54E-05	0.039893657	1	0.872618112	1	0.875576038	ENSG00000198837	5	DENND4B
VAT	Heart_Failure	ENSG00000170356.8	0.001686312	0.438537551	0.002176826	0.076055399	0.001735885	0.425196772	0.002176825	0.076048778	-4.96E-05	0.014631501	1	0.872855704	1	0.875281919	ENSG00000198874	8	TYW1
VAT	Heart_Failure	ENSG00000170632.9	0.001097521	0.648917122	0.002410718	0.076055461	0.001142623	0.635514669	0.002410702	0.076048842	-4.51E-05	0.029153448	1	0.961874179	1	0.950579326	ENSG00000198874	8	TYW1
VAT	Heart_Failure	ENSG00000170837.2	0.002253506	0.321093376	0.002271188	0.076055339	0.002178294	0.337534193	0.002271304	0.076048733	7.52E-05	0.016116527	1	0.811008798	1	0.812752882	ENSG00000198874	8	TYW1
VAT	Heart_Failure	ENSG00000170866.7	-0.000908543	0.656913051	0.002045442	0.076055462	-0.000865113	0.672331463	0.002045422	0.076048849	-4.34E-05	0.01003947	1	0.963584692	1	0.957156395	ENSG00000198885	5	ITPRIPL1
VAT	Heart_Failure	ENSG00000171421.8	-0.002169846	0.125246126	0.001415311	0.076055126	-0.00219744	0.120512224	0.001415302	0.076048501	2.76E-05	0.024036768	1	0.67041525	1	0.648517413	ENSG00000198885	5	ITPRIPL1
VAT	Heart_Failure	ENSG00000171446.6	-0.001107857	0.462244951	0.001506969	0.076055409	-0.001140594	0.449117698	0.001506953	0.076048787	3.27E-05	0.007430459	1	0.88303338	1	0.877588101	ENSG00000198885	5	ITPRIPL1
VAT	Heart_Failure	ENSG00000171806.7	0.0015911	0.07095052	0.000881106	0.076054984	0.001553202	0.077968452	0.000881199	0.076048392	3.79E-05	0.01250337	1	0.597905872	1	0.597746085	ENSG00000198885	5	ITPRIPL1
VAT	Heart_Failure	ENSG00000171860.4	0.002522865	0.481246207	0.003582087	0.076055416	0.002460923	0.492073954	0.003582054	0.076048803	6.19E-05	0.036481728	1	0.892788619	1	0.897302672	ENSG00000198885	5	ITPRIPL1
VAT	Heart_Failure	ENSG00000171928.9	0.001266704	0.57057415	0.002233233	0.076055443	0.001214786	0.586468929	0.002233225	0.076048831	5.19E-05	0.009087508	1	0.92639479	1	0.927008193	ENSG00000198912	6	C1orf174
VAT	Heart_Failure	ENSG00000172116.17	0.001300163	0.581010646	0.002355755	0.076055445	0.00133657	0.570461736	0.002355721	0.076048827	-3.64E-05	0.043153639	1	0.92639479	1	0.922350742	ENSG00000198912	6	C1orf174
VAT	Heart_Failure	ENSG00000174177.8	-0.00246055	0.315039372	0.002449027	0.076055335	-0.002426327	0.321806297	0.002448971	0.076048724	-3.42E-05	0.029490186	1	0.811008798	1	0.812752882	ENSG00000198917	7	C9orf114
VAT	Heart_Failure	ENSG00000174353.13	-0.001472471	0.314434778	0.001463745	0.076055335	-0.001392815	0.341438865	0.001464076	0.076048736	-7.97E-05	0.019027579	1	0.811008798	1	0.814313543	ENSG00000198920	5	KIAA0753
VAT	Heart_Failure	ENSG00000175489.9	-0.002233662	0.569085941	0.003922844	0.076055442	-0.002285639	0.560121749	0.003922759	0.076048824	5.20E-05	0.045514117	1	0.92639479	1	0.921646963	ENSG00000198920	5	KIAA0753
VAT	Heart_Failure	ENSG00000175826.7	0.007512204	0.026188892	0.003378692	0.076054721	0.00744421	0.027574682	0.003378677	0.076048119	6.80E-05	0.022564296	1	0.383927688	1	0.387205367	ENSG00000198920	5	KIAA0753
VAT	Heart_Failure	ENSG00000175938.6	0.002480854	0.360706037	0.002714219	0.076055363	0.002528763	0.351502951	0.002714201	0.076048741	-4.79E-05	0.039520082	1	0.829623884	1	0.822453669	ENSG00000198925	6	ATG9A
VAT	Heart_Failure	ENSG00000176148.11	0.002385224	0.114220666	0.001510108	0.076055104	0.002361108	0.117923261	0.001510091	0.076048495	2.41E-05	0.048700548	1	0.655403408	1	0.64556656	ENSG00000198945	3	L3MBTL3
VAT	Heart_Failure	ENSG00000176386.4	-0.002439513	0.194895154	0.001881999	0.076055231	-0.002409401	0.200457253	0.001881974	0.076048621	-3.01E-05	0.040647804	1	0.756191037	1	0.74232065	ENSG00000198945	3	L3MBTL3
VAT	Heart_Failure	ENSG00000176428.5	0.001032605	0.657603917	0.002329758	0.076055462	0.000601514	0.796908204	0.002337333	0.076048866	0.000431091	0.022747866	1	0.963584692	1	0.965780945	ENSG00000198945	3	L3MBTL3
VAT	Heart_Failure	ENSG00000177674.11	-0.00095117	0.789785826	0.003567928	0.076055482	-0.000891132	0.802768661	0.003567872	0.076048867	-6.00E-05	0.023521093	1	0.972185844	1	0.966005801	ENSG00000198945	3	L3MBTL3
VAT	Heart_Failure	ENSG00000177697.13	0.005097956	0.081011266	0.002921713	0.076055018	0.00515886	0.077447442	0.002921714	0.07604839	-6.09E-05	0.026533015	1	0.619367186	1	0.597746085	ENSG00000198945	3	L3MBTL3
VAT	Heart_Failure	ENSG00000178295.10	-0.002068464	0.068962831	0.001137352	0.076054977	-0.002035534	0.073502371	0.001137359	0.076048377	-3.29E-05	0.003635574	1	0.591109981	1	0.584037962	ENSG00000198959	7	TGM2
VAT	Heart_Failure	ENSG00000178718.5	-0.003902768	0.090552279	0.002305926	0.076055046	-0.003934967	0.087917474	0.002305883	0.076048422	3.22E-05	0.048167577	1	0.623489698	1	0.608245538	ENSG00000198959	7	TGM2
VAT	Heart_Failure	ENSG00000178952.4	-0.000301106	0.885944964	0.002099217	0.07605549	-0.000356658	0.865087025	0.002099195	0.076048872	5.56E-05	0.001104066	1	0.98475518	1	0.980029484	ENSG00000198961	5	PJA2
VAT	Heart_Failure	ENSG00000179344.12	-0.015162325	4.21E-25	0.001464968	0.076038778	-0.017737781	1.19E-22	0.001810986	0.076033908	0.002575457	0.015569498	1.16E-21	1.16E-21	3.30E-19	3.30E-19	ENSG00000203301	2	AL590822.1
VAT	Heart_Failure	ENSG00000179913.6	-2.21E-06	0.999310141	0.002555273	0.076055493	-5.71E-05	0.982158767	0.002555247	0.076048877	5.49E-05	0.008317687	1	0.999535911	1	0.995646729	ENSG00000203301	2	AL590822.1
VAT	Heart_Failure	ENSG00000180543.3	0.005174543	0.108804191	0.003226841	0.076055092	0.005231276	0.104978198	0.003226819	0.076048467	-5.67E-05	0.040272245	1	0.64079958	1	0.626491505	ENSG00000203403	2	AC020907.2
VAT	Heart_Failure	ENSG00000180834.3	0.004017421	0.197624928	0.003118269	0.076055234	0.004081812	0.190530321	0.003118235	0.076048609	-6.44E-05	0.010457508	1	0.756191037	1	0.738152304	ENSG00000203652	3	AC099048.1
VAT	Heart_Failure	ENSG00000180953.7	-0.000430478	0.678087354	0.001037111	0.076055466	-0.00041504	0.689013465	0.001037095	0.076048852	-1.54E-05	0.049901513	1	0.963913289	1	0.960065682	ENSG00000203666	8	EFCAB2
VAT	Heart_Failure	ENSG00000180979.5	-0.004342252	0.052096433	0.002235577	0.076054904	-0.004387337	0.049700392	0.002235542	0.076048276	4.51E-05	0.006947846	1	0.518983358	1	0.500631689	ENSG00000203668	1	CHML
VAT	Heart_Failure	ENSG00000181092.5	-0.002519946	0.276686111	0.002316563	0.076055308	-0.005100968	0.035252822	0.002422747	0.076048185	0.002581023	0.000276334	1	0.795233108	1	0.430366522	ENSG00000203668	1	CHML
VAT	Heart_Failure	ENSG00000181126.9	6.53E-05	0.950049784	0.001042537	0.076055492	-0.000477792	0.648263807	0.001047386	0.076048844	0.000543101	7.86E-08	1	0.992630426	1	0.955604921	ENSG00000203668	1	CHML
VAT	Heart_Failure	ENSG00000181291.5	-0.000519587	0.727153126	0.001489149	0.076055474	-0.000546348	0.713702792	0.001489142	0.076048856	2.68E-05	0.041244034	1	0.963913289	1	0.960065682	ENSG00000203705	6	TATDN3
VAT	Heart_Failure	ENSG00000181826.5	0.00635959	0.081698976	0.003653014	0.07605502	0.006143149	0.092760269	0.003654424	0.076048436	0.000216442	0.04314758	1	0.619367186	1	0.616911666	ENSG00000203705	6	TATDN3
VAT	Heart_Failure	ENSG00000182240.11	0.001043072	0.436024546	0.001339119	0.076055398	0.001018494	0.44690745	0.001339099	0.076048787	2.46E-05	0.016282075	1	0.872618112	1	0.877588101	ENSG00000203705	6	TATDN3
VAT	Heart_Failure	ENSG00000182606.10	-0.002417488	0.327583578	0.00246936	0.076055343	-0.002368652	0.337442727	0.002469324	0.076048733	-4.88E-05	0.009114527	1	0.811008798	1	0.812752882	ENSG00000203722	3	RAET1G

VAT	Heart_Failure	ENSG00000182851.2	-0.000563458	0.792056896	0.002137221	0.076055482	-0.000604257	0.777381437	0.002137199	0.076048864	4.08E-05	0.019597587	1	0.972185844	1	0.965780945	ENSG00000203734	7	ECT2L
VAT	Heart_Failure	ENSG00000182923.12	0.00390685	0.052650486	0.002016146	0.076054907	0.003944862	0.050389175	0.00201613	0.076048279	-3.80E-05	0.025649096	1	0.518983358	1	0.502072644	ENSG00000203756	3	TMEM244
VAT	Heart_Failure	ENSG00000183684.6	0.000677097	0.850491302	0.003592187	0.076055487	0.000610576	0.865030014	0.003592158	0.076048872	6.65E-05	0.028076577	1	0.977595833	1	0.980029484	ENSG00000203778	3	FAM229B
VAT	Heart_Failure	ENSG00000184389.8	-0.005000802	0.012934037	0.002011918	0.076054529	-0.005043302	0.012185806	0.002011907	0.076047896	4.25E-05	0.015831174	1	0.266586081	1	0.258531674	ENSG00000203778	3	FAM229B
VAT	Heart_Failure	ENSG00000185298.8	-0.000289612	0.844102343	0.001472738	0.076055487	-0.000318588	0.828732505	0.001472713	0.07604887	2.90E-05	0.007128909	1	0.975350458	1	0.96872144	ENSG00000203791	9	METTL10
VAT	Heart_Failure	ENSG00000185344.9	0.005776716	0.119419336	0.003709638	0.076055115	0.005849407	0.114833864	0.003709585	0.076048489	-7.27E-05	0.010367535	1	0.663876855	1	0.641581914	ENSG00000203791	9	METTL10
VAT	Heart_Failure	ENSG00000185437.9	0.00249531	0.063867153	0.001346553	0.076054957	0.002518174	0.061467119	0.001346527	0.076048331	-2.29E-05	0.015872043	1	0.564941346	1	0.540284228	ENSG00000203791	9	METTL10
VAT	Heart_Failure	ENSG00000185684.8	0.000768773	0.493696353	0.001123212	0.07605542	0.000747045	0.505982144	0.001123194	0.076048808	2.17E-05	0.009891709	1	0.897038797	1	0.902059208	ENSG00000203871	4	C6orf164
VAT	Heart_Failure	ENSG00000185742.6	-0.003337608	0.347003836	0.00354907	0.076055355	-0.003419191	0.335338458	0.003549023	0.076048732	8.16E-05	0.003171177	1	0.820677452	1	0.812752882	ENSG00000203871	4	C6orf164
VAT	Heart_Failure	ENSG00000186417.9	0.000404582	0.697471975	0.001040765	0.076055469	0.003934038	0.043085275	0.001944763	0.076048238	-0.003529455	0.031685967	1	0.963913289	1	0.477344364	ENSG00000203872	6	C6orf163
VAT	Heart_Failure	ENSG00000186532.7	-0.005686669	0.038180915	0.002743323	0.076054822	-0.005774646	0.03529232	0.002743307	0.076048185	8.80E-05	0.000231712	1	0.462190028	1	0.430366522	ENSG00000203872	6	C6orf163
VAT	Heart_Failure	ENSG00000186567.8	-0.001180206	0.383434261	0.001354088	0.076055374	-0.001157	0.392849697	0.001354069	0.076048763	-2.32E-05	0.024858885	1	0.837245697	1	0.842989349	ENSG00000203907	5	OOEP
VAT	Heart_Failure	ENSG00000186715.6	0.003910861	0.177834805	0.002902409	0.07605521	0.003997706	0.168392933	0.002902382	0.076048581	-8.68E-05	0.000297648	1	0.74593461	1	0.71714634	ENSG00000203907	5	OOEP
VAT	Heart_Failure	ENSG00000186889.5	0.005350857	0.04375745	0.002653653	0.076054858	0.005292922	0.046091399	0.002653678	0.076048256	5.79E-05	0.03410465	1	0.491334625	1	0.483462092	ENSG00000204060	4	FOXO6
VAT	Heart_Failure	ENSG00000187049.5	-0.00104584	0.718468363	0.002901027	0.076055473	-0.001135316	0.695540685	0.002901044	0.076048853	8.95E-05	0.001943095	1	0.963913289	1	0.960065682	ENSG00000204060	4	FOXO6
VAT	Heart_Failure	ENSG00000187741.10	-0.000444401	0.787468957	0.001648378	0.076055482	-0.000484972	0.768592515	0.001648351	0.076048863	4.06E-05	0.000799235	1	0.972185844	1	0.965438542	ENSG00000204060	4	FOXO6
VAT	Heart_Failure	ENSG00000187775.12	-0.002699907	0.436020441	0.003466167	0.076055398	-0.002750646	0.42743784	0.003466104	0.076048779	5.07E-05	0.039724603	1	0.872618112	1	0.875281919	ENSG00000204084	8	INPP5B
VAT	Heart_Failure	ENSG00000187942.7	-0.00511139	0.124402211	0.003326519	0.076055125	-0.005774388	0.08328539	0.003334063	0.076048409	0.000662998	0.003396207	1	0.67041525	1	0.604015714	ENSG00000204140	8	CLPSL1
VAT	Heart_Failure	ENSG00000188056.7	0.001017882	0.444262602	0.001330537	0.076055402	0.001041639	0.433698346	0.001330524	0.076048781	-2.38E-05	0.028455437	1	0.873500102	1	0.875281919	ENSG00000204140	8	CLPSL1
VAT	Heart_Failure	ENSG00000188234.9	0.001750148	0.504235804	0.002620609	0.076055423	0.001610804	0.538874265	0.002621254	0.076048818	0.000139344	0.027140757	1	0.904941123	1	0.911760867	ENSG00000204140	8	CLPSL1
VAT	Heart_Failure	ENSG00000188404.4	-0.003676366	0.290400592	0.00347731	0.076055319	-0.003538996	0.308824868	0.003477473	0.076048715	-0.00013737	0.003268244	1	0.795899392	1	0.807108087	ENSG00000204140	8	CLPSL1
VAT	Heart_Failure	ENSG00000188895.7	-0.005844981	0.000308823	0.001620091	0.076053462	-0.005811648	0.000334166	0.001620067	0.076046869	-3.33E-05	0.006225832	0.852352241	0.032782778	0.922297947	0.036010503	ENSG00000204147	5	ASAH2B
VAT	Heart_Failure	ENSG00000189362.7	-0.00340501	0.239553495	0.002895158	0.076055277	-0.003448097	0.233651055	0.002895108	0.076048655	4.31E-05	0.039997109	1	0.779051326	1	0.76771061	ENSG00000204147	5	ASAH2B
VAT	Heart_Failure	ENSG00000196072.7	0.00059036	0.644080728	0.001277833	0.07605546	0.000614185	0.630767223	0.001277829	0.076048841	-2.38E-05	0.037514777	1	0.960091406	1	0.950282497	ENSG00000204147	5	ASAH2B
VAT	Heart_Failure	ENSG00000196126.6	-0.009921092	1.39E-07	0.00188383	0.076051164	-0.006717242	0.002291595	0.002202664	0.076047425	-0.00320385	0.005006287	0.000384045	4.27E-05	1	0.110580144	ENSG00000204149	5	AGAP6
VAT	Heart_Failure	ENSG00000196151.6	-0.001477068	0.609920777	0.002895151	0.076055452	-0.001544559	0.593688801	0.002895151	0.076048832	6.75E-05	0.01241343	1	0.93938691	1	0.929058336	ENSG00000204149	5	AGAP6
VAT	Heart_Failure	ENSG00000196204.7	0.002767743	0.37820113	0.003140821	0.076055372	0.00281674	0.369811494	0.003140783	0.076048751	-4.90E-05	0.049532981	1	0.837245697	1	0.830566839	ENSG00000204161	9	C10orf128
VAT	Heart_Failure	ENSG00000196381.6	-0.000237887	0.850924921	0.001265768	0.076055488	-0.000214759	0.86527049	0.001265754	0.076048872	-2.31E-05	0.023251937	1	0.977595833	1	0.980029484	ENSG00000204161	9	C10orf128
VAT	Heart_Failure	ENSG00000196458.6	-0.000285491	0.899274956	0.002255464	0.076055491	-0.000320627	0.886955374	0.002255431	0.076048874	3.51E-05	0.040285597	1	0.984985763	1	0.98412197	ENSG00000204169	6	AGAP7
VAT	Heart_Failure	ENSG00000196912.8	0.000994932	0.67824014	0.002398201	0.076055466	0.001044864	0.66305832	0.002398154	0.076048847	-4.99E-05	0.002690723	1	0.963913289	1	0.957156395	ENSG00000204169	6	AGAP7
VAT	Heart_Failure	ENSG00000196966.3	0.002080813	0.260863021	0.001850672	0.076055296	0.001977661	0.285277866	0.001850808	0.076048699	0.000103152	0.000262941	1	0.79317431	1	0.794840503	ENSG00000204169	6	AGAP7
VAT	Heart_Failure	ENSG00000197165.6	0.003966641	0.273870701	0.003625174	0.076055306	0.003871903	0.285490468	0.003625147	0.076048699	9.47E-05	0.002108197	1	0.795233108	1	0.794840503	ENSG00000204169	6	AGAP7
VAT	Heart_Failure	ENSG00000197935.6	0.001118879	0.644871864	0.002427605	0.07605546	0.000689582	0.776688648	0.002431211	0.076048864	0.000429297	0.001388128	1	0.960521503	1	0.965780945	ENSG00000204169	6	AGAP7
VAT	Heart_Failure	ENSG00000198453.8	0.002949307	0.417024073	0.003633961	0.07605539	0.003008548	0.407722903	0.003633916	0.07604877	-5.92E-05	0.038814507	1	0.869982193	1	0.855404372	ENSG00000204169	6	AGAP7
VAT	Heart_Failure	ENSG00000198502.5	-0.001839618	0.00474411	0.00065144	0.076054248	2.07E-05	0.982219161	0.000930664	0.076048877	-0.001860359	0.005127668	1	0.1596798	1	0.995646729	ENSG00000204173	6	LRRC37A5P
VAT	Heart_Failure	ENSG00000203666.8	0.002174044	0.367355939	0.002411751	0.076055366	0.002137575	0.375440307	0.002411715	0.076048754	3.65E-05	0.046568517	1	0.833801308	1	0.834984084	ENSG00000204177	6	BMS1P1
VAT	Heart_Failure	ENSG00000204149.5	0.00149338	0.454680353	0.00199747	0.076055406	0.001545876	0.438981934	0.001997477	0.076048783	-5.25E-05	0.006696255	1	0.879409792	1	0.875281919	ENSG00000204177	6	BMS1P1
VAT	Heart_Failure	ENSG00000204173.6	0.000503333	0.890587169	0.003658969	0.07605549	0.000560996	0.878143127	0.003658904	0.076048873	-5.77E-05	0.028131012	1	0.984985763	1	0.982211765	ENSG00000204209	6	DAXX
VAT	Heart_Failure	ENSG00000204237.4	-0.000101894	0.96300525	0.002196806	0.076055493	-0.000145645	0.94713998	0.002196792	0.076048876	4.38E-05	0.020366641	1	0.993060803	1	0.991197882	ENSG00000204209	6	DAXX
VAT	Heart_Failure	ENSG00000204314.6	0.00617409	0.001048165	0.001883902	0.076053817	0.002929597	0.162006056	0.002095025	0.076048572	0.003244494	0.000401	1	0.070559418	1	0.70956483	ENSG00000204227	4	RING1
VAT	Heart_Failure	ENSG00000204338.4	-0.000460584	0.683776689	0.00113078	0.076055467	-0.001589416	0.191003119	0.001215504	0.07604861	0.001128832	0.011370312	1	0.963913289	1	0.738152304	ENSG00000204227	4	RING1
VAT	Heart_Failure	ENSG00000204344.10	-0.008783169	0.001683277	0.002796213	0.076053953	-0.005909516	0.04015136	0.002879609	0.07604822	-0.002873653	2.99E-05	1	0.089726808	1	0.461626679	ENSG00000204227	4	RING1
VAT	Heart_Failure	ENSG00000204351.7	-0.001628628	0.110495393	0.001020461	0.076055095	-0.002299337	0.026864179	0.001038782	0.076048112	0.000670709	0.000563409	1	0.643497633	1	0.385375739	ENSG00000204228	3	HSD17B8
VAT	Heart_Failure	ENSG00000204428.8	0.00190086	0.118436055	0.001217428	0.076055113	0.003147682	0.011645211	0.00124773	0.076047884	-0.001246822	5.18E-06	1	0.663876855	1	0.257959522	ENSG00000204228	3	HSD17B8
VAT	Heart_Failure	ENSG00000204463.8	0.005381983	0.014244082	0.00219578	0.076054555	0.004405685	0.0462442	0.002210399	0.076048257	0.000976297	0.000125643	1	0.271128728	1	0.483462092	ENSG00000204228	3	HSD17B8
VAT	Heart_Failure	ENSG00000204516.5	0.002451008	0.002310812	0.000804377	0.076054044	0.002793396	0.000577761	0.000811593	0.076047028	-0.000342389	0.001564142	1	0.11189196	1	0.04577203	ENSG00000204228	3	HSD17B8
VAT	Heart_Failure	ENSG00000204525.10	-0.002250566	0.030322233	0.001039109	0.076054761	-0.001381774	0.217358975	0.00112013	0.076048639	-0.000868792	0.037836783	1	0.412262874	1	0.759489439	ENSG00000204228	3	HSD17B8
VAT	Heart_Failure	ENSG00000204538.3	-0.000173323	0.830964101	0.000811947	0.076055486	-0.001000913	0.22504943	0.000825011	0.076048647	0.000827591	1.59E-08	1	0.972185844	1	0.76157815	ENSG00000204228	3	HSD17B8
VAT	Heart_Failure	ENSG00000204540.6	-0.000765731	0.206817501	0.000606583	0.076055244	-0.0013053	0.034123178	0.000616109	0.076048176	0.000539569	5.96E-07	1	0.762104543	1	0.425960325	ENSG00000204228	3	HSD17B8
VAT	Heart_Failure	ENSG00000204560.5	0.000461683	0.869093651	0.002801304	0.076055489	0.00092835	0.740858866	0.002807088	0.07604886	-0.000466667	0.010346567	1	0.982255997	1	0.960065682	ENSG00000204228	3	HSD17B8
VAT	Heart_Failure	ENSG00000204574.8	-0.0023776	0.235473808	0.002004084	0.076055273	-0.003346961	0.103264376	0.002054317	0.076048463	0.000969361	0.031951599	1	0.779051326	1	0.624888429	ENSG00000204228	3	HSD17B8
VAT	Heart_Failure	ENSG00000204592.5	-0.004475214	0.177405961	0.003317954	0.076055209	-0.005347961	0.10932579	0.0033399	0.076048477	0.000872747	0.022869298	1	0.74593461	1	0.628112053	ENSG00000204237	4	OXLD1
VAT	Heart_Failure	ENSG00000204622.6	-0.001395307	0.195195901	0.001077156	0.076055231	-0.002248258	0.039506148	0.001091981	0.076048215	0.000852951	2.05E-06	1	0.756191037	1	0.460071599	ENSG00000204237	4	OXLD1
VAT	Heart_Failure	ENSG00000204625.6	0.001344123	0.68605477	0.003325266	0.076055468	-4.13E-05	0.990168043	0.003350981	0.076048877	0.001385416	0.000854894	1	0.963913289	1	0.998073832	ENSG00000204237	4	OXLD1
VAT	Heart_Failure	ENSG00000204632.7	-0.005175355	0.009653969	0.001999756	0.076054448	-0.006276493	0.001924175	0.002023559	0.076047375	0.001101138	0.000383293	1	0.237475638	1	0.098346714	ENSG00000204237	4	OXLD1
VAT	Heart_Failure	ENSG00000204677.6	-0.002486578	0.389183651	0.002887678	0.076055377	-0.002437323	0.398640158	0.002887642	0.076048766	-4.93E-05	0.029571503	1	0.840717801	1	0.848955891	ENSG00000204237	4	OXLD1
VAT	Heart_Failure	ENSG00000204713.6	-3.23E-05	0.987915184	0.002131049	0.076055493	-0.000570006	0.789873796	0.002139064	0.076048866	0.000537728	0.003852156	1	0.996216992	1	0.965780945	ENSG00000204237	4	OXLD1
VAT	Heart_Failure	ENSG00000204792.2	-0.002143199	0.238821558	0.001819448	0.076055276	-0.002111046	0.245937358	0.001819436	0.076048667	-3.22E-05	0.038800136	1	0.779051326	1	0.778245128	ENSG00000204237	4	OXLD1
VAT	Heart_Failure	ENSG00000204920.6	-0.007405707	0.00947721	0.002854549	0.076054443	-0.007336463	0.010166593	0.00285452	0.076047846	-6.92E-05	0.002919402	1	0.237475638	1	0.241894809	ENSG00000204248	6	COL11A2
VAT	Heart_Failure	ENSG00000204991.6	5.67E-06	0.997040468	0.001529569	0.076055493	-3.24E-05	0.983118548	0.001529545	0.076048877	3.80E-05	0.000882072	1	0.998849979	1	0.995646729	ENSG00000204252	8	HLA-DOA
VAT	Heart_Failure	ENSG00000205578.4	-0.001885697	0.243501125	0.001616845	0.076055281	-0.002094427	0.196038275	0.001619916	0.076048616	0.00020873	0.038453361	1	0.779655573	1	0.738152304	ENSG00000204252	8	HLA-DOA
VAT	Heart_Failure	ENSG00000205837.3	0.004543518	0.183115513	0.003413035	0.076055216	0.00447625	0.189679735	0.003413002	0.076048608	6.73E-05	0.01636313	1	0.754326591	1	0.738152304	ENSG00000204252	8	HLA-DOA
VAT	Heart_Failure	ENSG00000206341.6	-0.003743964	0.037343946	0.001798263	0.076054816	-0.004751607	0.008869418	0.00181563	0.076047808	0.001007642	6.00E-05	1	0.458085743	1	0.236280118	ENSG00000204256	8	BRD2
VAT	Heart_Failure	ENSG00000206344.6	0.000272435	0.821212652	0.001205539	0.076055485	-0.00112566	0.366855994	0.001247436	0.07604875	0.001398095	1.31E-05	1	0.972185844	1	0.830566839	ENSG00000204257	10	HLA-DMA
VAT	Heart_Failure	ENSG00000212127.5	0.00214345	0.262074782	0.001911232	0.076055297	0.00211326	0.268846747	0.001911201	0.076048686	3.02E-05	0.032086639	1	0.79317431	1	0.791877836	ENSG00000204257	10	HLA-DMA
VAT	Heart_Failure	ENSG00000213199.3	-0.006106929	0.056282091	0.003199272	0.076054924	-0.00623541	0.051304446	0.003199403	0.076048284	0.000128481	0.002007785	1	0.533809522	1	0.50391556	ENSG00000204267	9	TAP2
VAT	Heart_Failure	ENSG00000213221.4	-0.000385397	0.659714866	0.000875291	0.076055463	-0.000400777	0.64703653	0.000875282	0.076048844	1.54E-05	0.032081517	1	0.963584692	1	0.955604921	ENSG00000204267	9	TAP2
VAT	Heart_Failure	ENSG00000213494.5	0.002010939	0.106756667	0.001246748	0.076055087	0.001974865	0.113194152	0.001246757	0.076048485	3.61E-05	0.0040912	1	0.637447692	1	0.638887238	ENSG00000204267	9	TAP2
VAT	Heart_Failure	ENSG00000213676.6	0.008801328	8.49E-06	0.001976725	0.076052399	0.007146307	0.000376644	0.002009673	0.076046903	0.001655022	5.07E-06	0.02343312	0.00146457	1	0.036010503	ENSG00000204267	9	TAP2
VAT	Heart_Failure	ENSG00000213760.6	0.000273187	0.832755016	0.001293683	0.076055486	-0.000833629	0.525100161	0.00131176	0.076048814	0.001106815	3.54E-07	1	0.972185844	1	0.908819144	ENSG00000204267	9	TAP2
VAT	Heart_Failure	ENSG00000214253.4	-0.000146823	0.894203848	0.00110404	0.07605549	-2.36E-05	0.982939174	0.001104852	0.076048877	-0.000123196	0.004718322	1	0.984985763	1	0.995646729	ENSG00000204287	9	HLA-DRA
VAT	Heart_Failure	ENSG00000214309.3	8.47E-05	0.965715651	0.001970372	0.076055493	0.000486717	0.805486193	0.001976488	0.076048867	-0.000402026	0.010176729	1	0.993060803	1	0.967412756	ENSG00000204287	9	HLA-DRA
VAT	Heart_Failure	ENSG00000214659.4	0.000872839	0.754017678	0.002785541	0.076055478	0.000766624	0.783165626	0.002785735	0.076048865	0.000106215	0.011332097	1	0.963913289	1	0.965780945	ENSG00000204287	9	HLA-DRA
VAT	Heart_Failure	ENSG00000215790.2	-0.001581244	0.592084796	0.002951086	0.076055448	-0.00154158	0.601400507	0.002951026	0.076048834	-3.97E-05	0.047055919	1	0.930611638	1	0.931462064	ENSG00000204301	5	NOTCH4
VAT	Heart_Failure	ENSG00000215908.5	-0.009995388	0.002740951	0.003336908	0.076054093	-0.010049561	0.002598041	0.003336842	0.076047461	5.42E-05	0.018917656	1	0.122016507	1	0.117595502	ENSG00000204301	5	NOTCH4
VAT	Heart_Failure	ENSG00000219755.1	0.000717099	0.579223534	0.001293189	0.076055445	0.000692517	0.592293875	0.001293179	0.076048832	2.46E-05	0.025714316	1	0.92639479	1	0.928824486	ENSG00000204301	5	NOTCH4
VAT	Heart_Failure	ENSG00000220583.1	-0.001094977	0.774230598	0.003817302	0.07605548	-0.001380601	0.717682762	0.00381848	0.076048856	0.000285624	0.004806155	1	0.969984771	1	0.960065682	ENSG00000204305	9	AGER
VAT	Heart_Failure	ENSG00000221838.5	0.000246765	0.866580329	0.001468805	0.076055489	0.000371078	0.800678963	0.001469799	0.076048867	-0.000124313	0.025797257	1	0.982255997	1	0.965780945	ENSG00000204305	9	AGER
VAT	Heart_Failure	ENSG00000224557.3	-0.001723113	0.07105548	0.000954566	0.076054984	-0.002103758	0.029541774	0.000966718	0.076048138	0.000380645	0.012888366	1	0.597905872	1	0.398269521	ENSG00000204305	9	AGER
VAT	Heart_Failure	ENSG00000224796.1	0.000977323	0.313315797	0.000969288	0.076055334	0.001400761	0.154241565	0.00098319	0.07604856	-0.000423438	0.010282099	1	0.811008798	1	0.704981382	ENSG00000204305	9	AGER
VAT	Heart_Failure	ENSG00000225492.2	-0.001936046	0.463121409	0.002638681	0.076055409	-0.001991531	0.450396794	0.002638649	0.076048788	5.55E-05	0.007669361	1	0.88303338	1	0.877892056	ENSG00000204305	9	AGER
VAT	Heart_Failure	ENSG00000225851.1	-0.001272984	0.230414728	0.001061446	0.076055269	-0.002316404	0.033606226	0.00109019	0.076048172	0.00104342	2.76E-05	1	0.777367927	1	0.423530522	ENSG00000204305	9	AGER
VAT	Heart_Failure	ENSG00000226314.3	0.002897904	0.192316423	0.00222273	0.076055228	0.002388427	0.283870578	0.002228705	0.076048698	0.000509477	0.001939133	1	0.756191037	1	0.794840503	ENSG00000204305	9	AGER
VAT	Heart_Failure	ENSG00000227262.3	-0.00212994	0.573275921	0.003781627	0.076055444	-0.003780198	0.326128479	0.003849713	0.076048726	0.001650258	0.022217298	1	0.92639479	1	0.812752882	ENSG00000204305	9	AGER
VAT	Heart_Failure	ENSG00000227766.1	-0.001448271	0.289613276	0.00136762	0.076055318	-0.002189582	0.114199169	0.001386163	0.076048487	0.000741311	0.00105582	1	0.795899392	1	0.640561135	ENSG00000204305	9	AGER
VAT	Heart_Failure	ENSG00000228106.1	0.001698068	0.353788591	0.001831262	0.076055359	0.001669096	0.362055069	0.001831239	0.076048747	2.90E-05	0.04466479	1	0.826718867	1	0.830566839	ENSG00000204305	9	AGER
VAT	Heart_Failure	ENSG00000228696.4	-0.005362764	0.069768284	0.002957272	0.07605498	-0.005421175	0.066772984	0.002957215	0.076048352	5.84E-05	0.004708581	1	0.592493737	1	0.560354119	ENSG00000204308	6	RNF5
VAT	Heart_Failure	ENSG00000230224.1	0.001811253	0.408528396	0.002191508	0.076055386	0.00173637	0.428196038	0.002191604	0.076048779	7.49E-05	0.009551307	1	0.861373852	1	0.875281919	ENSG00000204314	6	PRRT1
VAT	Heart_Failure	ENSG00000230521.1	0.00067001	0.675680804	0.001601499	0.076055466	0.000992515	0.537218903	0.00160855	0.076048817	-0.000322504	0.032914649	1	0.963913289	1	0.911760867	ENSG00000204314	6	PRRT1
VAT	Heart_Failure	ENSG00000230606.6	0.001882338	0.154085019	0.001320706	0.076055176	0.001912061	0.147679278	0.001320685	0.07604855	-2.97E-05	0.002681129	1	0.721315241	1	0.701115705	ENSG00000204314	6	PRRT1
VAT	Heart_Failure	ENSG00000230795.2	-0.000821653	0.461018081	0.001114601	0.076055408	-0.001331462	0.238533064	0.001129638	0.07604866	0.000509809	0.005593581	1	0.88303338	1	0.771971926	ENSG00000204314	6	PRRT1
VAT	Heart_Failure	ENSG00000230836.1	-0.004119819	0.079929042	0.002352701	0.076055014	-0.00407838	0.083006706	0.00235268	0.076048408	-4.14E-05	0.033177004	1	0.619367186	1	0.604015714	ENSG00000204314	6	PRRT1
VAT	Heart_Failure	ENSG00000230939.1	0.001403965	0.525293053	0.002210245	0.07605543	0.001449511	0.511938886	0.002210219	0.07604881	-4.55E-05	0.009844868	1	0.91226097	1	0.904578313	ENSG00000204316	8	MRPL38
VAT	Heart_Failure	ENSG00000231925.7	-0.003568317	0.148097759	0.002467229	0.076055167	-0.002945814	0.2331994	0.00247099	0.076048655	-0.000622503	6.65E-06	1	0.719389945	1	0.76771061	ENSG00000204316	8	MRPL38
VAT	Heart_Failure	ENSG00000232112.3	-0.003063208	0.239293108	0.002603093	0.076055277	-0.003151982	0.225975388	0.002603238	0.076048648	8.88E-05	0.015399667	1	0.779051326	1	0.76157815	ENSG00000204316	8	MRPL38
VAT	Heart_Failure	ENSG00000233265.1	-0.001010901	0.279603792	0.000934971	0.076055311	-0.001358223	0.147230132	0.000937102	0.076048549	0.000347322	5.13E-08	1	0.795233108	1	0.701115705	ENSG00000204316	8	MRPL38
VAT	Heart_Failure	ENSG00000233448.2	-0.00056757	0.801088118	0.002252805	0.076055483	-0.000605589	0.788069548	0.002252782	0.076048866	3.80E-05	0.038282934	1	0.972185844	1	0.965780945	ENSG00000204338	4	CYP21A1P
VAT	Heart_Failure	ENSG00000233836.3	-0.006679438	0.089309807	0.003931271	0.076055042	-0.006760776	0.085475187	0.003931213	0.076048415	8.13E-05	0.006523962	1	0.623489698	1	0.607677788	ENSG00000204338	4	CYP21A1P
VAT	Heart_Failure	ENSG00000234127.4	0.002839716	0.231442338	0.002373048	0.07605527	0.002317326	0.331116556	0.002384403	0.076048729	0.00052239	0.025245851	1	0.777647581	1	0.812752882	ENSG00000204338	4	CYP21A1P
VAT	Heart_Failure	ENSG00000234585.2	-0.000530275	0.566506382	0.000925106	0.076055442	-0.000423825	0.647350195	0.0009265	0.076048844	-0.00010645	0.038896577	1	0.92639479	1	0.955604921	ENSG00000204338	4	CYP21A1P
VAT	Heart_Failure	ENSG00000235241.1	0.004450919	0.034600015	0.002106431	0.076054796	0.004513059	0.032150278	0.002106403	0.07604816	-6.21E-05	0.000154604	1	0.440073928	1	0.414648442	ENSG00000204338	4	CYP21A1P
VAT	Heart_Failure	ENSG00000235290.1	-0.000788104	0.627648812	0.001624825	0.076055456	-0.001077847	0.507497086	0.001626349	0.076048808	0.000289744	5.73E-05	1	0.952138605	1	0.903672231	ENSG00000204338	4	CYP21A1P
VAT	Heart_Failure	ENSG00000235878.4	-0.00195317	0.096088593	0.001173694	0.076055061	-0.001926893	0.10064377	0.001173687	0.076048456	-2.63E-05	0.009881258	1	0.637447692	1	0.624888429	ENSG00000204338	4	CYP21A1P
VAT	Heart_Failure	ENSG00000236104.2	0.012914392	0.000409686	0.003654545	0.076053544	0.011893973	0.001178173	0.003666304	0.076047234	0.001020419	0.000548825	1	0.035335395	1	0.073737866	ENSG00000204338	4	CYP21A1P
VAT	Heart_Failure	ENSG00000236209.1	-0.003512705	0.104578567	0.002164257	0.076055082	-0.003475912	0.108259148	0.002164234	0.076048474	-3.68E-05	0.036750567	1	0.637447692	1	0.628112053	ENSG00000204338	4	CYP21A1P
VAT	Heart_Failure	ENSG00000236624.4	-0.001470334	0.695053553	0.00375079	0.076055469	-0.001549152	0.679588391	0.003750745	0.07604885	7.88E-05	0.008132989	1	0.963913289	1	0.960065682	ENSG00000204344	10	STK19
VAT	Heart_Failure	ENSG00000237988.2	0.002577026	0.324437361	0.002615258	0.076055341	0.002110264	0.420873229	0.002621746	0.076048776	0.000466762	0.012058354	1	0.811008798	1	0.871425441	ENSG00000204344	10	STK19
VAT	Heart_Failure	ENSG00000238142.1	0.005091028	0.060575302	0.002712919	0.076054943	0.0051344	0.058410486	0.002712869	0.076048318	-4.34E-05	0.024561517	1	0.546800541	1	0.532055913	ENSG00000204344	10	STK19
VAT	Heart_Failure	ENSG00000239672.3	0.001792013	0.281379403	0.001663543	0.076055312	0.001817268	0.274645615	0.001663513	0.076048691	-2.53E-05	0.033275244	1	0.795233108	1	0.791877836	ENSG00000204344	10	STK19
VAT	Heart_Failure	ENSG00000240053.8	0.001659515	0.233526614	0.001392999	0.076055272	0.002974086	0.036931232	0.001425375	0.076048197	-0.001314571	1.38E-05	1	0.779051326	1	0.441256279	ENSG00000204344	10	STK19
VAT	Heart_Failure	ENSG00000240522.1	-0.003381354	0.383111058	0.003876903	0.076055374	-0.003315157	0.392486949	0.003876847	0.076048763	-6.62E-05	0.025177905	1	0.837245697	1	0.842989349	ENSG00000204348	5	DXO
VAT	Heart_Failure	ENSG00000241186.3	-0.002165234	0.492905683	0.003157721	0.07605542	-0.002228189	0.480415823	0.00315771	0.076048799	6.30E-05	0.02634287	1	0.896935503	1	0.892894055	ENSG00000204348	5	DXO
VAT	Heart_Failure	ENSG00000242294.2	0.001239584	0.295738764	0.001185505	0.076055322	-0.000398938	0.770278936	0.001366178	0.076048864	0.001638522	0.015826767	1	0.799450526	1	0.965780945	ENSG00000204348	5	DXO
VAT	Heart_Failure	ENSG00000242516.1	-0.000497325	0.578193495	0.000894428	0.076055445	-0.000512403	0.566717743	0.000894415	0.076048826	1.51E-05	0.024719704	1	0.92639479	1	0.92172808	ENSG00000204348	5	DXO
VAT	Heart_Failure	ENSG00000243753.1	-0.000988	0.1546881	0.000694226	0.076055177	-0.001536657	0.028338944	0.000700848	0.076048127	0.000548657	1.24E-08	1	0.721315241	1	0.387205367	ENSG00000204351	7	SKIV2L
VAT	Heart_Failure	ENSG00000244219.2	0.001598375	0.600340844	0.003050853	0.07605545	0.001903071	0.532959813	0.003052266	0.076048816	-0.000304697	0.001707657	1	0.93401394	1	0.908819144	ENSG00000204351	7	SKIV2L
VAT	Heart_Failure	ENSG00000244291.1	-0.003818327	0.326022568	0.003887692	0.076055342	-0.003765094	0.332803068	0.003887615	0.07604873	-5.32E-05	0.047663801	1	0.811008798	1	0.812752882	ENSG00000204351	7	SKIV2L
VAT	Heart_Failure	ENSG00000244482.5	0.007709962	0.003343134	0.002627528	0.076054149	0.00775741	0.003153274	0.002627491	0.076047516	-4.74E-05	0.019384552	1	0.124689861	1	0.126139004	ENSG00000204351	7	SKIV2L
VAT	Heart_Failure	ENSG00000244623.1	-0.000576769	0.566536211	0.001006296	0.076055442	-0.00085948	0.395626027	0.001011797	0.076048764	0.000282711	0.007525991	1	0.92639479	1	0.845800026	ENSG00000204351	7	SKIV2L
VAT	Heart_Failure	ENSG00000244731.3	-0.002651827	0.007596889	0.000993383	0.076054381	-0.004493592	0.000752122	0.001333466	0.076047105	0.001841765	0.038424729	1	0.21841057	1	0.05462779	ENSG00000204351	7	SKIV2L
VAT	Heart_Failure	ENSG00000245958.2	-0.001673226	0.381139559	0.001910513	0.076055373	-0.00171219	0.370143917	0.00191049	0.076048751	3.90E-05	0.010194068	1	0.837245697	1	0.830566839	ENSG00000204351	7	SKIV2L
VAT	Heart_Failure	ENSG00000248290.1	-0.004029981	0.00153618	0.001272179	0.076053927	-0.0045749	0.000346877	0.001278784	0.076046879	0.000544918	2.92E-05	1	0.085321306	0.957379669	0.036010503	ENSG00000204351	7	SKIV2L
VAT	Heart_Failure	ENSG00000250535.1	-0.001683947	0.085286813	0.000978581	0.076055031	-0.002297812	0.024817065	0.001023868	0.076048091	0.000613865	0.041601692	1	0.623489698	1	0.372255978	ENSG00000204351	7	SKIV2L
VAT	Heart_Failure	ENSG00000251417.1	-0.002542299	0.186141149	0.00192295	0.07605522	-0.002488574	0.195614091	0.001922941	0.076048615	-5.37E-05	0.001519364	1	0.756191037	1	0.738152304	ENSG00000204356	7	NELFE
VAT	Heart_Failure	ENSG00000251580.1	-0.00215019	0.401664087	0.002563859	0.076055383	-0.002193725	0.392196523	0.002563837	0.076048763	4.35E-05	0.042031438	1	0.85407772	1	0.842989349	ENSG00000204356	7	NELFE
VAT	Heart_Failure	ENSG00000254999.2	-0.00048608	0.762726021	0.001610054	0.076055479	-0.000459112	0.775524899	0.001610038	0.076048864	-2.70E-05	0.041638675	1	0.966718282	1	0.965780945	ENSG00000204356	7	NELFE
VAT	Heart_Failure	ENSG00000256087.2	0.001068123	0.616355724	0.002131888	0.076055454	0.001159776	0.586498803	0.002132267	0.076048831	-9.17E-05	0.041061496	1	0.945604112	1	0.927008193	ENSG00000204371	7	EHMT2
VAT	Heart_Failure	ENSG00000257595.2	-0.002445204	0.395163121	0.002875723	0.07605538	-0.002386203	0.406659347	0.00287568	0.076048769	-5.90E-05	0.006690927	1	0.846538141	1	0.855404372	ENSG00000204371	7	EHMT2
VAT	Heart_Failure	ENSG00000257742.1	0.002011623	0.59602464	0.003794612	0.076055449	0.001911578	0.614427564	0.003794592	0.076048837	0.000100045	0.002570181	1	0.931133617	1	0.942122265	ENSG00000204371	7	EHMT2
VAT	Heart_Failure	ENSG00000257877.1	-0.005739071	0.110333986	0.00359434	0.076055095	-0.005674149	0.114419072	0.003594325	0.076048488	-6.49E-05	0.041342636	1	0.643497633	1	0.640561135	ENSG00000204371	7	EHMT2
VAT	Heart_Failure	ENSG00000258289.3	9.61E-05	0.939890866	0.001275062	0.076055492	6.85E-05	0.957154058	0.001275054	0.076048876	2.76E-05	0.01189036	1	0.990975896	1	0.992070179	ENSG00000204387	8	C6orf48
VAT	Heart_Failure	ENSG00000259134.1	-0.001967861	0.422359639	0.002452668	0.076055393	-0.004764632	0.08086156	0.002729334	0.076048401	0.002796771	0.019526646	1	0.871415448	1	0.600796054	ENSG00000204387	8	C6orf48
VAT	Heart_Failure	ENSG00000259205.2	0.002016204	0.090631016	0.001191552	0.076055046	0.001994685	0.094124084	0.001191542	0.076048439	2.15E-05	0.030284149	1	0.623489698	1	0.620005898	ENSG00000204387	8	C6orf48
VAT	Heart_Failure	ENSG00000259915.1	-0.00086613	0.624875049	0.001771397	0.076055456	-0.000896807	0.612660558	0.001771368	0.076048837	3.07E-05	0.018785643	1	0.952138605	1	0.940457808	ENSG00000204387	8	C6orf48
VAT	Heart_Failure	ENSG00000259982.1	0.001071026	0.527792768	0.001696327	0.076055431	0.00102866	0.544242429	0.00169631	0.076048819	4.24E-05	0.00235111	1	0.915017613	1	0.913136234	ENSG00000204410	10	MSH5
VAT	Heart_Failure	ENSG00000260077.1	0.000120071	0.972232984	0.003449528	0.076055493	3.84E-05	0.991115311	0.003449506	0.076048877	8.17E-05	0.006117612	1	0.994659842	1	0.998073832	ENSG00000204410	10	MSH5
VAT	Heart_Failure	ENSG00000260302.1	0.002899172	0.423906643	0.003625493	0.076055393	0.003020762	0.404736113	0.003625523	0.076048768	-0.00012159	0.000993985	1	0.871819921	1	0.85403033	ENSG00000204410	10	MSH5

VAT	Heart_Failure	ENSG00000260382.1	-0.001129695	0.71858697	0.003135011	0.076055473	-0.001179059	0.706844009	0.003134985	0.076048855	4.94E-05	0.049189596	1	0.963913289	1	0.960065682	ENSG00000204410	10	MSH5
VAT	Heart_Failure	ENSG00000260482.1	-0.007211105	0.03374685	0.003396515	0.07605479	-0.007302345	0.031558052	0.003396486	0.076048155	9.12E-05	0.001392214	1	0.438042449	1	0.409322718	ENSG00000204427	7	ABHD16A
VAT	Heart_Failure	ENSG00000260761.1	0.001419462	0.6127765	0.002804627	0.076055453	0.001368937	0.62547514	0.00280459	0.07604884	5.05E-05	0.020515992	1	0.942733077	1	0.947481552	ENSG00000204427	7	ABHD16A
VAT	Heart_Failure	ENSG00000261037.1	-0.000340474	0.875051609	0.002165244	0.076055489	-0.000378007	0.861409089	0.002165226	0.076048872	3.75E-05	0.039319966	1	0.983439565	1	0.979196493	ENSG00000204427	7	ABHD16A
VAT	Heart_Failure	ENSG00000261353.1	-0.005018428	0.169085998	0.003649371	0.076055398	-0.005296466	0.1469277	0.003651547	0.076048548	0.000278038	0.033236825	1	0.733769426	1	0.701115705	ENSG00000204427	7	ABHD16A
VAT	Heart_Failure	ENSG00000261794.1	-0.001262491	0.381077186	0.001441341	0.076055373	-0.001298976	0.367462852	0.001441326	0.07604875	3.65E-05	0.001852337	1	0.837245697	1	0.830566839	ENSG00000204428	8	LY6G5C
VAT	Heart_Failure	ENSG00000261824.2	-0.001878576	0.445237966	0.00246087	0.076055402	-0.00183195	0.456611842	0.002460849	0.07604879	-4.66E-05	0.023248351	1	0.873500102	1	0.880469391	ENSG00000204428	8	LY6G5C
VAT	Heart_Failure	ENSG00000268069.1	0.0035269	0.249324562	0.003061564	0.076055286	0.003464816	0.25774906	0.003061525	0.076048677	6.21E-05	0.00990027	1	0.789536793	1	0.786050543	ENSG00000204428	8	LY6G5C
VAT	Heart_Failure	ENSG00000269486.1	0.003333746	0.309266746	0.003278784	0.076055332	0.00339146	0.300955793	0.003278715	0.07604871	-5.77E-05	0.00850064	1	0.811008798	1	0.801314322	ENSG00000204428	8	LY6G5C
VAT	Heart_Failure	ENSG00000270020.1	-0.002499892	0.134314203	0.001669593	0.076055143	-0.002524764	0.130475142	0.00166956	0.07604852	2.49E-05	0.031044979	1	0.686494815	1	0.665314041	ENSG00000204428	8	LY6G5C
VAT	Heart_Failure	ENSG00000270127.1	0.001709834	0.122095181	0.001105943	0.07605512	0.001690534	0.126363331	0.001105933	0.076048512	1.93E-05	0.035531773	1	0.669945723	1	0.658005371	ENSG00000204428	8	LY6G5C
VAT	Heart_Failure	ENSG00000270228.1	-0.001797159	0.549783387	0.003004855	0.076055437	-0.001861826	0.535513649	0.003004826	0.076048817	6.47E-05	0.00862114	1	0.92326913	1	0.910109404	ENSG00000204438	6	GPANK1
VAT	Heart_Failure	ENSG00000270604.1	0.003788891	0.257962993	0.003349385	0.076055293	0.006234355	0.065761245	0.003388141	0.076048348	-0.002445464	1.79E-06	1	0.79317431	1	0.560188382	ENSG00000204438	6	GPANK1
VAT	Heart_Failure	ENSG00000270773.1	0.000124339	0.970483362	0.00336033	0.076055493	0.000197261	0.953188649	0.003360315	0.076048876	-7.29E-05	0.014014867	1	0.994628325	1	0.992070179	ENSG00000204438	6	GPANK1
VAT	Heart_Failure	ENSG00000271581.1	0.003105033	0.436239216	0.003988174	0.076055398	-0.003111695	0.478960569	0.004395199	0.07604879	0.006216728	0.000766102	1	0.872618112	1	0.891945927	ENSG00000204438	6	GPANK1
VAT	Heart_Failure	ENSG00000271840.1	-0.004307985	0.220872207	0.003518981	0.076055259	-0.004487848	0.202255481	0.003519457	0.076048623	0.000179863	0.006878619	1	0.772901704	1	0.74232065	ENSG00000204438	6	GPANK1
VAT	Heart_Failure	ENSG00000272221.1	0.002824148	0.000350861	0.00079007	0.076053499	0.003118532	8.58E-05	0.000794011	0.076046469	-0.000294384	0.000208211	0.968377101	0.034584896	0.236867022	0.011843351	ENSG00000204439	3	C6orf47
VAT	Heart_Failure	ENSG00000273038.1	0.003204933	0.320406901	0.003225502	0.076055339	0.003263762	0.311600938	0.003225477	0.07604871	-5.88E-05	0.030648398	1	0.811008798	1	0.809810347	ENSG00000204463	8	BAG6
VAT	Heart_Failure	ENSG00000273142.1	-0.000768684	0.545223811	0.001270692	0.076055436	-0.000999321	0.433513731	0.001275956	0.076048781	0.000230637	0.04753125	1	0.921324909	1	0.875281919	ENSG00000204463	8	BAG6
VAT	Heart_Failure	ENSG00000273172.1	0.00440934	0.195708218	0.003407849	0.076055232	0.00446598	0.19002317	0.003407812	0.076048609	-5.66E-05	0.039788271	1	0.756191037	1	0.738152304	ENSG00000204463	8	BAG6
VAT	HTN	ENSG00000002919.10	0.000427465	0.872139307	0.002656024	0.240335801	0.000498242	0.851194099	0.002655944	0.240302367	-7.08E-05	0.002705902	1	0.953686405	1	0.945771221	ENSG00000204463	8	BAG6
VAT	HTN	ENSG00000003056.3	-0.014374984	0.000647766	0.004214543	0.240330076	-0.014919899	0.000411253	0.004223274	0.24029623	0.000544915	0.048344661	1	0.01103602	1	0.00822505	ENSG00000204463	8	BAG6
VAT	HTN	ENSG00000005156.7	-0.000338164	0.931163534	0.003914792	0.24033581	-0.000504928	0.897377879	0.003914946	0.240302377	0.000166764	0.00392035	1	0.973508158	1	0.964471552	ENSG00000204463	8	BAG6
VAT	HTN	ENSG00000006530.11	0.00939445	0.050140359	0.004796098	0.240333921	0.00930933	0.05224934	0.004795958	0.240300527	8.51E-05	0.047884744	1	0.226864576	1	0.229630856	ENSG00000204498	6	NFKBIL1
VAT	HTN	ENSG00000008394.8	0.007681419	0.140687673	0.005213972	0.240334743	0.005391271	0.304451734	0.005249868	0.240301865	0.002290147	0.000200486	1	0.408304917	1	0.604088272	ENSG00000204498	6	NFKBIL1
VAT	HTN	ENSG00000009830.7	0.009399045	0.07642743	0.005304783	0.240334265	0.00948333	0.073812892	0.005304542	0.240300809	-8.43E-05	0.022471877	1	0.292971817	1	0.285327146	ENSG00000204516	5	MICB
VAT	HTN	ENSG00000009950.11	-0.012514191	0.048874336	0.006353377	0.2403339	-0.019846191	0.00263143	0.006598199	0.240297923	0.007332	3.90E-05	1	0.222963912	1	0.030644506	ENSG00000204516	5	MICB
VAT	HTN	ENSG00000011523.9	0.025914169	6.26E-09	0.004460472	0.240319168	0.026018476	5.44E-09	0.004460302	0.240285605	-0.000104307	0.003327953	1.73E-05	8.23E-07	1.50E-05	6.52E-07	ENSG00000204516	5	MICB
VAT	HTN	ENSG00000012061.11	-0.000313988	0.945481985	0.004591704	0.240335811	-0.000206112	0.964195288	0.004591516	0.240302384	-0.000107876	0.001925112	1	0.976968793	1	0.985245375	ENSG00000204516	5	MICB
VAT	HTN	ENSG00000018510.8	0.005079416	0.239547286	0.004318789	0.240335131	0.005316382	0.218318715	0.004318706	0.240301637	-0.000236966	4.95E-08	1	0.549661061	1	0.517020606	ENSG00000204516	5	MICB
VAT	HTN	ENSG00000031691.6	-0.003550229	0.021691389	0.001546444	0.240333214	-0.003655697	0.018110111	0.001546817	0.24029963	0.000105469	0.006248271	1	0.133634452	1	0.116241644	ENSG00000204516	5	MICB
VAT	HTN	ENSG00000035115.17	-0.017882023	0.004921881	0.006358922	0.240331913	-0.018036684	0.004561216	0.006358771	0.240298417	0.000154661	0.011071013	1	0.049397792	1	0.046861116	ENSG00000204516	5	MICB
VAT	HTN	ENSG00000037474.10	0.005403638	0.437224999	0.006955488	0.240335516	0.005283831	0.447441304	0.006955262	0.2403021	0.000119807	0.045497108	1	0.716423851	1	0.721401667	ENSG00000204516	5	MICB
VAT	HTN	ENSG00000038219.8	0.00053913	0.850902759	0.002868218	0.240335796	0.000463049	0.871741004	0.002868106	0.240302372	7.61E-05	0.000696798	1	0.942297189	1	0.956043422	ENSG00000204516	5	MICB
VAT	HTN	ENSG00000041802.6	-0.001315552	0.326405241	0.001340511	0.240335338	-0.0013405	0.317241544	0.001340457	0.240301891	2.51E-05	0.015464539	1	0.627494074	1	0.616610324	ENSG00000204516	5	MICB
VAT	HTN	ENSG00000050555.13	-0.005421198	0.41136818	0.006599233	0.240335481	-0.018949936	0.007211288	0.007052615	0.240298825	0.013528738	5.48E-08	1	0.700167128	1	0.065470903	ENSG00000204516	5	MICB
VAT	HTN	ENSG00000055211.8	0.001358538	0.782198603	0.004914135	0.240335776	0.00128752	0.79330947	0.004913904	0.240302351	7.10E-05	0.031867244	1	0.904048637	1	0.918168712	ENSG00000204520	8	MICA
VAT	HTN	ENSG00000056661.9	0.004369065	0.287663266	0.004109111	0.240335256	0.004292235	0.296207605	0.004108968	0.240301847	7.68E-05	0.024631393	1	0.59321241	1	0.596304149	ENSG00000204520	8	MICA
VAT	HTN	ENSG00000058063.11	0.003719797	0.563713137	0.006443043	0.240335649	0.003625162	0.57365836	0.006442759	0.240302229	9.46E-05	0.03971606	1	0.792605307	1	0.801328599	ENSG00000204520	8	MICA
VAT	HTN	ENSG00000058453.12	-2.52E-06	0.998897878	0.001824398	0.240335813	2.73E-05	0.988053784	0.001824326	0.240302385	-2.98E-05	0.035231162	1	0.999622242	1	0.997085354	ENSG00000204520	8	MICA
VAT	HTN	ENSG00000059573.4	-0.001473662	0.820991038	0.006512828	0.240335788	-0.001343536	0.836557397	0.006512565	0.240302364	-0.000130126	0.008835937	1	0.926718801	1	0.939635856	ENSG00000204520	8	MICA
VAT	HTN	ENSG00000063046.13	0.007498404	0.009074973	0.002873803	0.240332456	0.007287529	0.011223149	0.002873991	0.240299214	0.000210876	7.76E-06	1	0.075215989	1	0.086044142	ENSG00000204520	8	MICA
VAT	HTN	ENSG00000063854.8	0.012686668	0.004467781	0.004484313	0.240331866	0.012754888	0.004484515	0.004484115	0.240298395	-6.82E-05	0.032886065	1	0.047539021	1	0.046161039	ENSG00000204525	10	HLA-C
VAT	HTN	ENSG00000065060.12	0.015153473	5.29E-07	0.003021293	0.240323407	0.015011965	6.75E-07	0.003021078	0.240290211	0.000141507	0.000789119	0.001459982	3.29E-05	0.001862089	3.88E-05	ENSG00000204525	10	HLA-C
VAT	HTN	ENSG00000065665.16	-0.003513066	0.546485956	0.005825646	0.240335634	-0.003667228	0.529006102	0.005825394	0.240302189	0.000154161	0.000259053	1	0.783126292	1	0.771700234	ENSG00000204525	10	HLA-C
VAT	HTN	ENSG00000066933.11	-0.003813862	0.459550252	0.005156737	0.240335544	-0.003938403	0.445004	0.005156522	0.240302097	0.000124541	0.001237824	1	0.733862963	1	0.720405231	ENSG00000204525	10	HLA-C
VAT	HTN	ENSG00000069869.11	0.008157435	0.042384649	0.004018977	0.240333782	0.008238171	0.040375324	0.004018828	0.240300313	-8.07E-05	0.012707189	1	0.204075679	1	0.197548996	ENSG00000204525	10	HLA-C
VAT	HTN	ENSG00000070540.8	-0.001665559	0.721233376	0.004667901	0.240335751	-0.001568986	0.736769345	0.004667728	0.240302329	-9.66E-05	0.010078054	1	0.869815229	1	0.880677087	ENSG00000204525	10	HLA-C
VAT	HTN	ENSG00000071462.7	-0.001734661	0.641480128	0.003725402	0.240335706	-0.001251283	0.737142494	0.003728051	0.240302329	-0.000483378	0.001024608	1	0.830433937	1	0.880741681	ENSG00000204525	10	HLA-C
VAT	HTN	ENSG00000071575.7	0.002163427	0.67023116	0.005080481	0.240335724	0.002078583	0.682432261	0.005080288	0.240302302	8.48E-05	0.03269405	1	0.843623709	1	0.856249354	ENSG00000204525	10	HLA-C
VAT	HTN	ENSG00000071967.7	0.001395311	0.829377658	0.006474764	0.24033579	0.00159263	0.805694478	0.00647452	0.240302355	-0.000197319	0.000137526	1	0.931521652	1	0.924385659	ENSG00000204525	10	HLA-C
VAT	HTN	ENSG00000072736.14	-0.004152978	0.484542026	0.005941173	0.240335572	-0.004288266	0.470407029	0.005940928	0.240302128	0.000135288	0.00247593	1	0.748369329	1	0.73755282	ENSG00000204525	10	HLA-C
VAT	HTN	ENSG00000073331.13	-0.001689015	0.770768416	0.005796802	0.240335772	-0.001799048	0.756282728	0.005796536	0.240302337	0.000110033	0.005669728	1	0.899121229	1	0.892265229	ENSG00000204525	10	HLA-C
VAT	HTN	ENSG00000075461.5	0.005561026	0.374079145	0.006256362	0.240335424	0.005689183	0.363145936	0.006256058	0.240301977	-0.000128157	0.001593395	1	0.671507939	1	0.662009765	ENSG00000204525	10	HLA-C
VAT	HTN	ENSG00000075975.11	0.001583707	0.679049465	0.003827591	0.240335729	0.001511161	0.692972212	0.003827421	0.240302308	7.25E-05	0.007433587	1	0.849581379	1	0.861145117	ENSG00000204525	10	HLA-C
VAT	HTN	ENSG00000076258.5	0.012253474	0.076230395	0.006911178	0.240334263	0.012635207	0.067554381	0.006912145	0.240300737	-0.000381733	0.00699368	1	0.292622035	1	0.269826471	ENSG00000204531	11	POU5F1
VAT	HTN	ENSG00000078070.7	-0.004550387	0.493700142	0.006648373	0.240335582	-0.004417742	0.506363928	0.006648115	0.240302167	-0.000132645	0.010798206	1	0.752792925	1	0.76099237	ENSG00000204531	11	POU5F1
VAT	HTN	ENSG00000079263.14	-0.007352586	0.274431916	0.006727504	0.240335224	-0.007241158	0.281752302	0.006727247	0.240301813	-0.000111428	0.036534633	1	0.583987732	1	0.58432047	ENSG00000204531	11	POU5F1
VAT	HTN	ENSG00000083828.11	0.003198166	0.548908087	0.005335635	0.240335636	0.003119667	0.558743273	0.005335413	0.240302216	7.85E-05	0.049075898	1	0.783206863	1	0.792055179	ENSG00000204531	11	POU5F1
VAT	HTN	ENSG00000084207.11	-0.001126477	0.759297724	0.003676451	0.240335767	-0.001453715	0.692555815	0.003676668	0.240302308	0.000327238	2.85E-08	1	0.89291083	1	0.861126259	ENSG00000204531	11	POU5F1
VAT	HTN	ENSG00000087263.12	-0.009666993	0.06738625	0.005285129	0.240334163	-0.009578157	0.069932114	0.005284926	0.240300765	-8.88E-05	0.033073979	1	0.269986697	1	0.275732335	ENSG00000204531	11	POU5F1
VAT	HTN	ENSG00000087301.4	-0.005927143	0.246443817	0.005113874	0.240335151	-0.0060194	0.239150454	0.005113692	0.240301701	9.23E-05	0.028525972	1	0.555714288	1	0.545951408	ENSG00000204531	11	POU5F1
VAT	HTN	ENSG00000087365.10	-0.004793272	0.264934177	0.004299648	0.2403352	-0.006623009	0.124835602	0.004315249	0.240301223	0.001829737	7.69E-07	1	0.572277862	1	0.382404287	ENSG00000204531	11	POU5F1
VAT	HTN	ENSG00000087495.12	-0.004944914	0.372170835	0.005541046	0.240335421	-0.004845217	0.381867786	0.005540805	0.240302008	-9.97E-05	0.012424636	1	0.670600511	1	0.673023578	ENSG00000204531	11	POU5F1
VAT	HTN	ENSG00000088833.13	0.010581729	0.033014035	0.004963477	0.240333572	0.010688519	0.031277585	0.004963256	0.240300098	-0.00010679	0.002457411	1	0.17572191	1	0.171622533	ENSG00000204531	11	POU5F1
VAT	HTN	ENSG00000088876.7	0.003260487	0.54799156	0.005427154	0.240335635	0.00313902	0.562962556	0.005426948	0.24030222	0.000121284	0.004924983	1	0.783206863	1	0.794767593	ENSG00000204536	9	CCHCR1
VAT	HTN	ENSG00000089335.16	-0.001569605	0.635397372	0.003310397	0.240335703	-0.0014912	0.652368048	0.003310283	0.240302285	-7.84E-05	0.004818715	1	0.827629557	1	0.843737494	ENSG00000204536	9	CCHCR1
VAT	HTN	ENSG00000090054.9	-0.009455071	0.058814937	0.005003799	0.240334052	-0.00956276	0.05598442	0.005003638	0.240300584	0.000107689	0.01294064	1	0.251992588	1	0.242158595	ENSG00000204536	9	CCHCR1
VAT	HTN	ENSG00000090238.7	0.011175921	6.49E-05	0.002798024	0.240327945	0.011233713	5.95E-05	0.002797944	0.240294436	-5.78E-05	0.022657891	0.179171607	0.001794776	0.164102622	0.001676064	ENSG00000204536	9	CCHCR1
VAT	HTN	ENSG00000090263.11	0.002179864	0.74323268	0.006654537	0.24033576	0.002285829	0.731212015	0.006654254	0.240302327	-0.000105965	0.030295058	1	0.880557529	1	0.878114224	ENSG00000204536	9	CCHCR1
VAT	HTN	ENSG00000091640.3	-0.000718078	0.751770371	0.002270201	0.240335764	-0.000677688	0.765303975	0.002270136	0.240302341	-4.04E-05	0.049158227	1	0.887082609	1	0.898827402	ENSG00000204536	9	CCHCR1
VAT	HTN	ENSG00000092094.6	0.002486031	0.665718663	0.0057428	0.240335721	0.002578737	0.654037133	0.00575404	0.240302286	-9.27E-05	0.031053106	1	0.842665958	1	0.843796725	ENSG00000204536	9	CCHCR1
VAT	HTN	ENSG00000093000.14	0.00279801	0.508498527	0.004231857	0.240335598	0.002694224	0.524334532	0.004231687	0.240302185	0.000103786	0.001373846	1	0.765243148	1	0.769652481	ENSG00000204538	3	PSORS1C2
VAT	HTN	ENSG00000094963.9	0.009862716	0.016793209	0.004124567	0.240332993	0.004435554	0.321145576	0.00447084	0.240301899	0.005427161	0.001662983	1	0.110883392	1	0.619471487	ENSG00000204538	3	PSORS1C2
VAT	HTN	ENSG00000095485.12	-0.001550549	0.263680437	0.001387223	0.240335197	-0.001596866	0.249680088	0.001387245	0.240301731	4.63E-05	0.010703792	1	0.571238623	1	0.556098166	ENSG00000204538	3	PSORS1C2
VAT	HTN	ENSG00000095713.9	0.00154668	0.733263118	0.004538535	0.240335756	0.001623825	0.720494658	0.004538385	0.240302322	-7.71E-05	0.046747179	1	0.875348705	1	0.872943483	ENSG00000204538	3	PSORS1C2
VAT	HTN	ENSG00000096433.6	0.020932359	0.001130343	0.00642902	0.240330585	0.021988685	0.000640344	0.006440841	0.240296637	-0.001056326	0.007867766	1	0.016835	1	0.01096636	ENSG00000204538	3	PSORS1C2
VAT	HTN	ENSG00000099810.14	-0.021241701	0.001200846	0.006558716	0.24033064	-0.021152367	0.00125882	0.006558416	0.240297255	-8.93E-05	0.048166383	1	0.01768665	1	0.018581684	ENSG00000204538	3	PSORS1C2
VAT	HTN	ENSG00000099949.14	0.005478246	0.257873393	0.004841865	0.240335185	0.005399397	0.264768533	0.004841673	0.240301771	7.88E-05	0.035117179	1	0.567568234	1	0.569128623	ENSG00000204538	3	PSORS1C2
VAT	HTN	ENSG00000099956.13	0.001340323	0.530145962	0.002135008	0.240335619	0.000791546	0.71161417	0.002141113	0.240302317	0.000548777	0.000789965	1	0.775412218	1	0.869767456	ENSG00000204540	6	PSORS1C1
VAT	HTN	ENSG00000099974.7	-0.00276412	0.506155237	0.004157984	0.240335598	-0.003733733	0.370144228	0.004166164	0.240301989	0.000969613	0.000260767	1	0.763441997	1	0.665877875	ENSG00000204540	6	PSORS1C1
VAT	HTN	ENSG00000099977.9	-0.004945973	0.449776485	0.006544124	0.240335532	-0.004531079	0.488763721	0.006545182	0.240302148	-0.000414894	0.003188993	1	0.72510695	1	0.748605922	ENSG00000204540	6	PSORS1C1
VAT	HTN	ENSG00000099984.6	-0.003017625	0.261001848	0.002684653	0.24033519	-0.0064426	0.021970359	0.002812273	0.240299797	0.003424975	4.39E-05	1	0.570001694	1	0.135655909	ENSG00000204540	6	PSORS1C1
VAT	HTN	ENSG00000099985.3	-0.00300472	0.511598736	0.004577918	0.240335601	-0.002922472	0.523205614	0.004577719	0.240302184	-8.22E-05	0.012672818	1	0.767398104	1	0.769652481	ENSG00000204540	6	PSORS1C1
VAT	HTN	ENSG00000099994.10	-0.002295333	0.404957901	0.002756164	0.240335471	-0.002451574	0.373771472	0.002756337	0.240301995	0.00015624	0.000493889	1	0.695156362	1	0.668408979	ENSG00000204540	6	PSORS1C1
VAT	HTN	ENSG00000100014.15	-0.006014901	0.122337621	0.003893043	0.240334636	-0.006137636	0.114896227	0.003893042	0.240301159	0.000122735	0.007496077	1	0.378109558	1	0.366605303	ENSG00000204540	6	PSORS1C1
VAT	HTN	ENSG00000100023.13	-0.000960148	0.79423704	0.00368137	0.24033578	-0.001018099	0.782113932	0.003681224	0.240302347	5.80E-05	0.041911372	1	0.911852841	1	0.912852444	ENSG00000204560	5	DHX16
VAT	HTN	ENSG00000100068.7	-0.000983698	0.869490161	0.00598698	0.2403358	-0.001113094	0.852503465	0.005986785	0.240302368	0.000129396	0.011886871	1	0.95263744	1	0.94611263	ENSG00000204560	5	DHX16
VAT	HTN	ENSG00000100129.13	-0.012992474	0.05205007	0.00668776	0.240333952	-0.012856625	0.054542834	0.006687434	0.240300562	-0.000135849	0.001587154	1	0.229853111	1	0.238570877	ENSG00000204560	5	DHX16
VAT	HTN	ENSG00000100147.9	0.0076875	0.219970833	0.006266676	0.240335071	0.007588526	0.225900549	0.006266392	0.240301662	9.82E-05	0.024328223	1	0.523830457	1	0.526616698	ENSG00000204560	5	DHX16
VAT	HTN	ENSG00000100211.6	-7.24E-05	0.976882858	0.002498505	0.240335813	-2.30E-05	0.992661261	0.002498409	0.240302385	-4.94E-05	0.012294079	1	0.98866831	1	0.997393679	ENSG00000204560	5	DHX16
VAT	HTN	ENSG00000100284.16	-0.005290788	0.261878214	0.004715648	0.240335193	-0.005141511	0.275567863	0.00471555	0.240301799	-0.000149277	0.001348023	1	0.570001694	1	0.580170048	ENSG00000204560	5	DHX16
VAT	HTN	ENSG00000100330.11	0.003102247	0.299601911	0.002990727	0.240335283	0.003026091	0.311602331	0.002990603	0.24030188	7.62E-05	0.000661311	1	0.601382744	1	0.610862846	ENSG00000204560	5	DHX16
VAT	HTN	ENSG00000100441.5	-0.000131312	0.967813266	0.00325769	0.240335813	-0.000289394	0.929216094	0.003257794	0.240302381	0.000158081	0.000662684	1	0.985704227	1	0.973666067	ENSG00000204574	8	ABCF1
VAT	HTN	ENSG00000100445.12	-0.003048017	0.126182067	0.001993034	0.24033466	-0.003075262	0.122812703	0.001992941	0.240301211	2.72E-05	0.042660468	1	0.388252513	1	0.379153311	ENSG00000204574	8	ABCF1
VAT	HTN	ENSG00000100478.10	-0.009852233	0.108331967	0.006135639	0.240334542	-0.009989088	0.103504322	0.006135426	0.240301078	0.000136855	0.007722973	1	0.353658534	1	0.344320158	ENSG00000204574	8	ABCF1
VAT	HTN	ENSG00000100580.7	0.004729315	0.406637955	0.005699169	0.240335474	0.004598555	0.419716791	0.005698945	0.240302064	0.00013076	0.003150389	1	0.696665428	1	0.703433516	ENSG00000204574	8	ABCF1
VAT	HTN	ENSG00000101198.10	-0.007629276	0.20628294	0.006036516	0.240335026	-0.007525019	0.212531219	0.006036255	0.240301618	-0.000104257	0.017358183	1	0.5053674	1	0.51251931	ENSG00000204574	8	ABCF1
VAT	HTN	ENSG00000101343.10	-0.005478805	0.138572224	0.003699068	0.240334731	-0.005544339	0.133895764	0.003698896	0.240301277	6.55E-05	0.009189635	1	0.406439256	1	0.400381698	ENSG00000204574	8	ABCF1
VAT	HTN	ENSG00000103148.11	0.005436448	0.303266233	0.005280897	0.240335292	0.005555148	0.292811749	0.005280687	0.240301878	-0.000118699	0.003517113	1	0.605216777	1	0.592989502	ENSG00000204574	8	ABCF1
VAT	HTN	ENSG00000103168.12	0.001895595	0.242634718	0.001622347	0.24033514	0.001868487	0.249420983	0.001622291	0.240301731	2.71E-05	0.044664695	1	0.552590633	1	0.556098166	ENSG00000204592	5	HLA-E
VAT	HTN	ENSG00000103174.7	-0.000328464	0.937596548	0.004195418	0.24033581	-0.000392379	0.925482885	0.004195241	0.24030238	6.39E-05	0.039638041	1	0.976192895	1	0.973079147	ENSG00000204592	5	HLA-E
VAT	HTN	ENSG00000103199.9	-0.010509289	0.013081437	0.004234963	0.240332776	-0.010598966	0.012321052	0.004234805	0.240299384	8.97E-05	0.008397951	1	0.094022827	1	0.090201866	ENSG00000204592	5	HLA-E
VAT	HTN	ENSG00000103356.11	9.25E-05	0.980174018	0.003722267	0.240335813	0.000165471	0.964540943	0.003722125	0.240302384	-7.30E-05	0.013334509	1	0.990945161	1	0.985245375	ENSG00000204592	5	HLA-E
VAT	HTN	ENSG00000103811.11	0.002689386	0.173870393	0.001977673	0.240334901	0.002637424	0.182318979	0.001977597	0.240301508	5.20E-05	0.000906274	1	0.458916465	1	0.472932691	ENSG00000204592	5	HLA-E
VAT	HTN	ENSG00000104442.5	-0.005743279	0.193706036	0.004418943	0.24033498	-0.005674093	0.199109155	0.004418747	0.240301572	-6.92E-05	0.027558906	1	0.489138755	1	0.493820082	ENSG00000204592	5	HLA-E
VAT	HTN	ENSG00000104450.8	-0.00138845	0.783301896	0.005048563	0.240335776	-0.001473981	0.770306548	0.005048327	0.240302343	8.55E-05	0.012120644	1	0.904415117	1	0.903546992	ENSG00000204592	5	HLA-E
VAT	HTN	ENSG00000104522.11	0.014299976	0.038323943	0.006903924	0.240333697	0.014443919	0.036419101	0.006903623	0.240300226	-0.000143943	0.003908486	1	0.19180587	1	0.186988481	ENSG00000204616	6	TRIM31
VAT	HTN	ENSG00000104714.9	0.000826608	0.896123139	0.006331234	0.240335805	0.000728385	0.908404598	0.006330964	0.240302378	9.82E-05	0.034114541	1	0.959290439	1	0.96802961	ENSG00000204616	6	TRIM31
VAT	HTN	ENSG00000104731.9	-0.002712129	0.557214123	0.004620441	0.240335643	-0.002576392	0.577100119	0.004620288	0.240302231	-0.000135737	0.00056661	1	0.788268056	1	0.80282073	ENSG00000204616	6	TRIM31
VAT	HTN	ENSG00000104866.6	-0.003514401	0.460173531	0.004758439	0.240335544	-0.00344348	0.469257723	0.004758239	0.240302127	-7.09E-05	0.044279733	1	0.733862963	1	0.73755282	ENSG00000204618	4	RNF39
VAT	HTN	ENSG00000104870.8	0.004270556	0.536113996	0.006902461	0.240335625	0.004732043	0.49315907	0.006905134	0.240302153	-0.000461487	0.026981985	1	0.777236628	1	0.749901432	ENSG00000204618	4	RNF39
VAT	HTN	ENSG00000104901.2	-0.002094741	0.590255472	0.003890199	0.240335667	-0.002186462	0.574078181	0.003890114	0.240302229	9.17E-05	0.031867256	1	0.807604154	1	0.801328599	ENSG00000204618	4	RNF39
VAT	HTN	ENSG00000104953.14	0.003909603	0.540488292	0.006387455	0.240335629	0.003997912	0.531361078	0.006387154	0.240302192	-8.83E-05	0.039440253	1	0.778579499	1	0.773365062	ENSG00000204618	4	RNF39
VAT	HTN	ENSG00000105088.4	0.002025062	0.666949471	0.004705672	0.240335722	0.001927909	0.682013211	0.004705458	0.240302302	9.72E-05	0.00287391	1	0.842887455	1	0.856249354	ENSG00000204618	4	RNF39
VAT	HTN	ENSG00000105136.15	0.001715488	0.475814554	0.002405842	0.240335563	0.001675176	0.486223116	0.002405725	0.240302146	4.03E-05	0.009829773	1	0.741910088	1	0.746371412	ENSG00000204618	4	RNF39
VAT	HTN	ENSG00000105695.10	-0.003842988	0.023223415	0.001693135	0.240333273	-0.003882329	0.021845433	0.001693084	0.240299792	3.93E-05	0.008712286	1	0.139902718	1	0.135186986	ENSG00000204618	4	RNF39
VAT	HTN	ENSG00000105699.12	0.005456065	0.194559937	0.004206004	0.240334983	0.005560455	0.186141611	0.004205834	0.240301523	-0.00010439	0.001154556	1	0.489950206	1	0.477318114	ENSG00000204622	6	HLA-J
VAT	HTN	ENSG00000106077.14	0.004869576	0.276155766	0.004471616	0.240335228	0.004730079	0.290139991	0.004471553	0.240301833	0.000139497	0.003058341	1	0.584870208	1	0.59182601	ENSG00000204622	6	HLA-J
VAT	HTN	ENSG00000106133.13	-0.007001726	0.00748941	0.00261818	0.240332266	-0.007756566	0.00315018	0.002626936	0.240298085	0.00075484	0.00049121	1	0.066465499	1	0.035927668	ENSG00000204622	6	HLA-J
VAT	HTN	ENSG00000106153.12	0.001629219	0.661766686	0.003724137	0.240335719	0.001567394	0.673834997	0.003723992	0.240302297	6.18E-05	0.034067941	1	0.840330084	1	0.850381614	ENSG00000204622	6	HLA-J
VAT	HTN	ENSG00000106635.3	0.003078085	0.56829902	0.00539486	0.240335653	0.00336029	0.533393018	0.00539516	0.240302193	-0.000282212	0.000951944	1	0.794096482	1	0.774008796	ENSG00000204622	6	HLA-J
VAT	HTN	ENSG00000106638.11	0.006355327	0.150457492	0.004419807	0.240334794	0.007843448	0.077228372	0.004438843	0.240300845	-0.001488121	0.000324997	1	0.423737427	1	0.293595464	ENSG00000204622	6	HLA-J
VAT	HTN	ENSG00000106733.16	0.004388593	0.496552546	0.00645453	0.240335585	0.004189371	0.516288239	0.006454434	0.240302177	0.000199223	0.000568913	1	0.754348741	1	0.768034541	ENSG00000204622	6	HLA-J
VAT	HTN	ENSG00000107130.6	0.006082695	0.319719806	0.006113047	0.240335325	0.005076983	0.406510797	0.006116477	0.240302045	0.001005712	3.60E-06	1	0.622518701	1	0.690868103	ENSG00000204622	6	HLA-J
VAT	HTN	ENSG00000107554.11	0.002717014	0.568925277	0.004770076	0.240335653	0.00210114	0.659892867	0.004774653	0.240302289	0.000615874	0.004434994	1	0.794096482	1	0.846845836	ENSG00000204622	6	HLA-J
VAT	HTN	ENSG00000107593.15	-0.004051397	0.552015464	0.006812012	0.240335639	-0.004275005	0.530287587	0.006812025	0.240302191	0.000223608	0.000647697	1	0.785341587	1	0.772344982	ENSG00000204625	6	HCG9
VAT	HTN	ENSG00000108556.7	0.001752896	0.319323796	0.001760201	0.240335324	0.001725354	0.326966061	0.001760127	0.240301911	2.75E-05	0.03312299	1	0.622410789	1	0.624860924	ENSG00000204625	6	HCG9
VAT	HTN	ENSG00000108846.11	0.00020064	0.973878611	0.006127492	0.240335813	0.000313247	0.959227101	0.006127252	0.240302383	-0.000112607	0.018195734	1	0.988563798	1	0.983460383	ENSG00000204625	6	HCG9
VAT	HTN	ENSG00000109854.9	-0.000350983	0.925762948	0.003766844	0.240335809	-0.0002733	0.942151965	0.003766675	0.240302382	-7.77E-05	0.003457333	1	0.971153834	1	0.978587755	ENSG00000204625	6	HCG9
VAT	HTN	ENSG00000109861.11	-0.006903108	0.199721401	0.00538318	0.240335002	-0.006999128	0.193158155	0.005382937	0.240301551	9.60E-05	0.010563929	1	0.49587241	1	0.485554643	ENSG00000204625	6	HCG9
VAT	HTN	ENSG00000110011.9	0.00402744	0.340038748	0.004221243	0.240335364	0.004998984	0.236927985	0.00422675	0.240301695	-0.00097154	1.14E-05	1	0.642812975	1	0.542174105	ENSG00000204625	6	HCG9
VAT	HTN	ENSG00000110025.8	-0.011584044	0.022776773	0.005087045	0.240333256	-0.007966754	0.123012267	0.005165653	0.240301212	-0.00361729	5.80E-05	1	0.138162403	1	0.379345092	ENSG00000204625	6	HCG9
VAT	HTN	ENSG00000110060.4	0.001367014	0.705030854	0.003611296	0.240335743	0.001445158	0.689019913	0.003611218	0.240302306	-7.81E-05	0.027087465	1	0.861817357	1	0.860491307	ENSG00000204625	6	HCG9
VAT	HTN	ENSG00000110768.7	-0.006503306	0.287841691	0.006118631	0.240335256	-0.006385105	0.296678392	0.006118438	0.240301848	-0.000118201	0.026743843	1	0.59321241	1	0.59681659	ENSG00000204625	6	HCG9
VAT	HTN	ENSG00000110888.13	-1.66E-05	0.994198379	0.00228339	0.240335813	1.57E-05	0.994507306	0.00228329	0.240302385	-3.23E-05	0.047235928	1	0.997230388	1	0.997760874	ENSG00000204632	7	HLA-G
VAT	HTN	ENSG00000111144.5	0.005775761	0.300663894	0.005580381	0.240335285	0.005859381	0.293699176	0.005580138	0.240301841	-8.36E-05	0.037545981	1	0.603075834	1	0.592989502	ENSG00000204632	7	HLA-G

VAT	HTN	ENSG00000111450.9	0.002597335	0.535210216	0.004188759	0.240335624	0.0026675	0.524222518	0.004188582	0.240302185	-7.02E-05	0.023547409	1	0.777236628	1	0.769652481	ENSG00000204632	7	HLA-G
VAT	HTN	ENSG00000111666.6	-0.002850723	0.510387901	0.004330863	0.2403356	-0.002771028	0.522265574	0.004330696	0.240302185	-7.97E-05	0.019373197	1	0.767061751	1	0.769595828	ENSG00000204632	7	HLA-G
VAT	HTN	ENSG00000111850.6	0.005453946	0.281298252	0.005062095	0.240335241	0.005358734	0.289763101	0.005061892	0.240301832	9.52E-05	0.013926794	1	0.586749061	1	0.59182601	ENSG00000204632	7	HLA-G
VAT	HTN	ENSG00000112159.7	-0.001082302	0.87284843	0.006762635	0.240335801	-0.000934943	0.890038048	0.006762396	0.240302375	-0.00014736	0.008471544	1	0.954083829	1	0.961073949	ENSG00000204632	7	HLA-G
VAT	HTN	ENSG00000112357.8	-0.00118001	0.620014004	0.002379863	0.240335692	-0.001126951	0.635816519	0.002379761	0.240302274	-5.31E-05	0.002163839	1	0.821428835	1	0.832512881	ENSG00000204632	7	HLA-G
VAT	HTN	ENSG00000112619.6	0.000567756	0.768852663	0.001931956	0.240335771	0.000527538	0.784802116	0.001931902	0.240302348	4.02E-05	0.022604337	1	0.898405313	1	0.913561299	ENSG00000204632	7	HLA-G
VAT	HTN	ENSG00000113248.3	-0.003886918	0.366405524	0.004303365	0.240335411	-0.003797154	0.377558408	0.004303189	0.240302001	-8.98E-05	0.005767942	1	0.667014755	1	0.669772956	ENSG00000204642	9	HLA-F
VAT	HTN	ENSG00000113360.12	-0.003095734	0.450498207	0.004102563	0.240335533	-0.00332347	0.41790453	0.004102732	0.240302061	0.000227736	0.000191538	1	0.725846499	1	0.702429993	ENSG00000204642	9	HLA-F
VAT	HTN	ENSG00000114054.9	0.027687316	3.01E-08	0.00499663	0.240320671	0.027607456	3.29E-08	0.004996429	0.24028733	7.99E-05	0.035688624	8.30E-05	3.19E-06	9.08E-05	3.24E-06	ENSG00000204642	9	HLA-F
VAT	HTN	ENSG00000114405.6	-0.002192902	0.718916171	0.006092954	0.240335749	-0.002083917	0.732326179	0.006092737	0.240302327	-0.000108985	0.029578225	1	0.86836264	1	0.878114224	ENSG00000204642	9	HLA-F
VAT	HTN	ENSG00000114503.6	0.004321756	0.536108878	0.006985128	0.240335625	0.004528502	0.516772548	0.006984883	0.240302177	-0.000206746	0.00037212	1	0.777236628	1	0.768034541	ENSG00000204642	9	HLA-F
VAT	HTN	ENSG00000115274.10	0.005477176	0.29012408	0.005177646	0.240335261	0.00540039	0.296918374	0.005177421	0.240301848	7.68E-05	0.039684028	1	0.59321241	1	0.596864321	ENSG00000204642	9	HLA-F
VAT	HTN	ENSG00000115677.12	0.003209815	0.635920999	0.006780203	0.240335703	0.003346741	0.62157425	0.006779985	0.240302265	-0.000136926	0.01939499	1	0.827897149	1	0.826686941	ENSG00000204642	9	HLA-F
VAT	HTN	ENSG00000115750.12	0.004845368	0.380118401	0.005520661	0.240335433	0.004963973	0.368549346	0.005520465	0.240301986	-0.000118605	0.009198381	1	0.673481092	1	0.664239406	ENSG00000204644	5	ZFP57
VAT	HTN	ENSG00000115825.5	0.0134173	0.039075949	0.006502546	0.240333714	0.013542726	0.037274195	0.006502317	0.240300246	-0.000125426	0.020125598	1	0.192933127	1	0.189809556	ENSG00000204644	5	ZFP57
VAT	HTN	ENSG00000115840.9	-0.006455239	0.326478801	0.006578708	0.240335339	-0.006563626	0.318401278	0.00657843	0.240301894	0.000108387	0.025858613	1	0.627494074	1	0.616693002	ENSG00000204644	5	ZFP57
VAT	HTN	ENSG00000115947.9	0.010754131	0.030034439	0.004956638	0.240333492	0.010835223	0.028808133	0.004956397	0.240300028	-8.11E-05	0.011703822	1	0.167127118	1	0.164277785	ENSG00000204644	5	ZFP57
VAT	HTN	ENSG00000116044.11	0.002954879	0.539874907	0.004820336	0.240335628	0.003119169	0.517565822	0.004820207	0.240302178	-0.00016429	0.000226788	1	0.778497687	1	0.768034541	ENSG00000204644	5	ZFP57
VAT	HTN	ENSG00000116151.9	-0.0147576	0.003376498	0.005034628	0.240331576	-0.014851418	0.003178075	0.00503441	0.240298093	9.38E-05	0.010154132	1	0.037128021	1	0.03594872	ENSG00000204644	5	ZFP57
VAT	HTN	ENSG00000116539.6	0.00489329	0.422031643	0.006094503	0.240335495	0.004780334	0.432808079	0.006094286	0.240302081	0.000112956	0.024196686	1	0.706371944	1	0.711889332	ENSG00000204650	9	CRHR1-IT1
VAT	HTN	ENSG00000116679.11	-0.00012453	0.977205489	0.004358375	0.240335813	-4.95E-05	0.990936923	0.004358213	0.240302385	-7.50E-05	0.032676404	1	0.98866831	1	0.997393679	ENSG00000204650	9	CRHR1-IT1
VAT	HTN	ENSG00000116701.10	0.006022248	0.362796936	0.006617496	0.240335405	0.0091636	0.17482335	0.006753488	0.240301477	-0.003141352	0.020037731	1	0.665980483	1	0.461734397	ENSG00000204657	2	OR2H2
VAT	HTN	ENSG00000116857.12	-0.007907868	0.051628001	0.004063178	0.240333945	-0.007969232	0.049831793	0.004063012	0.240300488	6.14E-05	0.046738959	1	0.229793907	1	0.224365004	ENSG00000204677	6	FAM153C
VAT	HTN	ENSG00000117115.8	-0.004837662	0.427948172	0.006102701	0.240335503	-0.005056039	0.407377815	0.006102512	0.240302046	0.000218377	4.78E-05	1	0.710672055	1	0.69191555	ENSG00000204677	6	FAM153C
VAT	HTN	ENSG00000117122.9	-0.000137799	0.959251316	0.002697017	0.240335812	-0.000182304	0.946105974	0.002696898	0.240302383	4.45E-05	0.021050607	1	0.98228229	1	0.9791881	ENSG00000204677	6	FAM153C
VAT	HTN	ENSG00000117450.9	-0.004416687	0.434077262	0.005646245	0.240335512	-0.004319	0.444292202	0.005646003	0.240302096	-9.77E-05	0.017789713	1	0.713724511	1	0.720050779	ENSG00000204677	6	FAM153C
VAT	HTN	ENSG00000117477.8	-0.009278381	0.129085254	0.006113362	0.240334677	-0.009423927	0.123177287	0.006113177	0.240301213	0.000145546	0.007292169	1	0.391942027	1	0.379430035	ENSG00000204681	6	GABBR1
VAT	HTN	ENSG00000117481.6	0.003669897	0.485138274	0.005257259	0.240335573	0.00376297	0.474118807	0.005257056	0.240302132	-9.31E-05	0.024595489	1	0.748439173	1	0.740140219	ENSG00000204709	4	C6orf100
VAT	HTN	ENSG00000117523.11	0.011374051	0.037713602	0.005473663	0.240333684	0.011291417	0.039117338	0.005473416	0.240300286	8.26E-05	0.030616059	1	0.189598436	1	0.193604012	ENSG00000204709	4	C6orf100
VAT	HTN	ENSG00000117533.10	-0.000299247	0.913907159	0.00276794	0.240335808	2.68E-05	0.992279471	0.002769441	0.240302385	-0.000326045	0.000760369	1	0.966800981	1	0.997393679	ENSG00000204709	4	C6orf100
VAT	HTN	ENSG00000118096.3	-0.006022483	0.186058351	0.004554442	0.240334951	-0.006126829	0.178534878	0.004554309	0.240301492	0.000104346	0.010769649	1	0.477250044	1	0.46743134	ENSG00000204709	4	C6orf100
VAT	HTN	ENSG00000118197.9	-0.008744725	0.007808064	0.003287157	0.240332323	-0.008702276	0.008109568	0.003286998	0.240298928	-4.24E-05	0.046028598	1	0.067768104	1	0.070885362	ENSG00000204709	4	C6orf100
VAT	HTN	ENSG00000118961.10	-0.003568326	0.390155493	0.004152422	0.240335449	-0.003666171	0.377275116	0.004152283	0.240302	9.78E-05	0.005565291	1	0.682680345	1	0.669772956	ENSG00000204709	4	C6orf100
VAT	HTN	ENSG00000119203.9	-0.007659344	0.159089796	0.005439328	0.240334835	-0.007548449	0.165192211	0.005439065	0.240301435	-0.000110895	0.001720057	1	0.43652375	1	0.449635604	ENSG00000204709	4	C6orf100
VAT	HTN	ENSG00000119431.5	-0.004730355	0.060158099	0.002516631	0.240334071	-0.004677109	0.063092103	0.002516542	0.240300681	-5.32E-05	0.010159904	1	0.252880519	1	0.260935385	ENSG00000204713	6	TRIM27
VAT	HTN	ENSG00000119471.10	0.008307396	0.154398073	0.005833152	0.240334813	0.008395044	0.150079187	0.005832913	0.240301363	-8.76E-05	0.047181202	1	0.430829979	1	0.423969862	ENSG00000204713	6	TRIM27
VAT	HTN	ENSG00000119715.10	0.003704994	0.523764923	0.005811271	0.240335613	0.00380251	0.512878783	0.005811017	0.240302174	-9.75E-05	0.019733152	1	0.772678586	1	0.76521159	ENSG00000204713	6	TRIM27
VAT	HTN	ENSG00000119899.11	0.000420526	0.949516605	0.006641933	0.240335811	0.000573488	0.931190799	0.00664169	0.240302381	-0.000152962	0.004615603	1	0.979347123	1	0.974255726	ENSG00000204713	6	TRIM27
VAT	HTN	ENSG00000120159.7	0.000490433	0.889828467	0.003540486	0.240335804	0.000418683	0.905862614	0.003540396	0.240302378	7.17E-05	0.030682481	1	0.958223397	1	0.967584999	ENSG00000204713	6	TRIM27
VAT	HTN	ENSG00000120328.4	-0.00924399	0.036175059	0.00441249	0.240333649	-0.009155899	0.037981726	0.004412359	0.240300261	-8.81E-05	0.025396762	1	0.185702856	1	0.191644541	ENSG00000204713	6	TRIM27
VAT	HTN	ENSG00000120437.7	0.002771032	0.613329739	0.005483646	0.240335687	0.002676321	0.625495837	0.005483403	0.240302267	9.47E-05	0.015070341	1	0.819907808	1	0.827116213	ENSG00000204718	3	CNN2P12
VAT	HTN	ENSG00000120784.11	0.013321105	5.21E-05	0.003292517	0.24032774	0.013377832	4.84E-05	0.003292379	0.240294243	-5.67E-05	0.020541572	0.143896838	0.001530817	0.133575816	0.001451911	ENSG00000204740	5	MALRD1
VAT	HTN	ENSG00000120805.9	-0.010590046	0.094686396	0.00633686	0.240334436	-0.010693906	0.091481236	0.006336608	0.24030098	0.00010386	0.034016844	1	0.329967743	1	0.324534976	ENSG00000204740	5	MALRD1
VAT	HTN	ENSG00000120915.9	-0.012563888	0.058673988	0.006645335	0.24033405	-0.012705759	0.055874316	0.006645205	0.240300582	0.000141872	0.032799649	1	0.251851022	1	0.242158595	ENSG00000204778	4	RP11-15J10.1
VAT	HTN	ENSG00000121064.8	0.001722663	0.604789677	0.003328654	0.240335681	0.001667465	0.616399556	0.00332854	0.240302261	5.52E-05	0.048498374	1	0.81487424	1	0.824255221	ENSG00000204778	4	RP11-15J10.1
VAT	HTN	ENSG00000121350.11	-0.000676433	0.851192621	0.003605777	0.240335796	-0.000622264	0.862980315	0.003605623	0.24030237	-5.42E-05	0.03971432	1	0.942297189	1	0.949828955	ENSG00000204778	4	RP11-15J10.1
VAT	HTN	ENSG00000121413.8	-0.006430709	0.330662179	0.006610638	0.240335347	-0.006326447	0.338542648	0.006610364	0.240301933	-0.000104262	0.035281734	1	0.630703258	1	0.637283706	ENSG00000204778	4	RP11-15J10.1
VAT	HTN	ENSG00000122085.12	-0.005301729	0.28405763	0.004949106	0.240335247	-0.005393497	0.275784843	0.004948896	0.240301799	9.18E-05	0.011871724	1	0.589701944	1	0.580170048	ENSG00000204778	4	RP11-15J10.1
VAT	HTN	ENSG00000122674.8	0.002696957	0.32772721	0.002755644	0.240335341	0.002752825	0.317789324	0.002755547	0.240301893	-5.59E-05	0.014655093	1	0.627864285	1	0.616693002	ENSG00000204789	3	ZNF204P
VAT	HTN	ENSG00000122696.8	-0.006050898	0.036774325	0.00289757	0.240333663	-0.00609776	0.035333162	0.002897451	0.240300201	4.69E-05	0.03292148	1	0.18691922	1	0.183949871	ENSG00000204789	3	ZNF204P
VAT	HTN	ENSG00000122733.11	0.014454048	0.012155542	0.005764083	0.240332712	0.014550091	0.011590379	0.005763814	0.240299242	-9.60E-05	0.013932582	1	0.089779421	1	0.08740286	ENSG00000204789	3	ZNF204P
VAT	HTN	ENSG00000122783.12	-0.007122097	0.016552971	0.002971877	0.240332981	-0.00718892	0.015560983	0.002971781	0.240299499	6.68E-05	0.009085121	1	0.110087228	1	0.105524112	ENSG00000204789	3	ZNF204P
VAT	HTN	ENSG00000123374.6	-0.019225149	0.003412163	0.006566069	0.240331585	-0.019071512	0.00367685	0.006565864	0.240298224	-0.000153637	0.007395161	1	0.037371304	1	0.039953174	ENSG00000204789	3	ZNF204P
VAT	HTN	ENSG00000123415.10	-0.000394876	0.871613095	0.002443387	0.2403358	-0.000488748	0.84145359	0.002443315	0.240302365	9.39E-05	1.79E-05	1	0.953488761	1	0.940295692	ENSG00000204789	3	ZNF204P
VAT	HTN	ENSG00000124532.10	-0.000737064	0.835881581	0.003557869	0.240335792	-0.000842185	0.812875504	0.003557752	0.240302357	0.000105121	0.000566303	1	0.934399823	1	0.928203638	ENSG00000204792	2	AC104135.3
VAT	HTN	ENSG00000124562.5	-0.017504869	1.62E-05	0.004059497	0.240326643	-0.017368984	1.88E-05	0.004059756	0.240293359	-0.000135885	0.040102766	0.04464555	0.00055118	0.051980452	0.000641734	ENSG00000204792	2	AC104135.3
VAT	HTN	ENSG00000124713.5	0.005294769	0.358388734	0.005764956	0.240335397	0.005381761	0.350526972	0.00576472	0.240301955	-8.70E-05	0.046612275	1	0.662083605	1	0.650170996	ENSG00000204792	2	AC104135.3
VAT	HTN	ENSG00000124733.3	0.006681546	0.163400512	0.004794011	0.240334855	0.006792434	0.156512614	0.004793868	0.240301395	-0.000110888	0.009370476	1	0.441724348	1	0.433565668	ENSG00000204792	2	AC104135.3
VAT	HTN	ENSG00000125450.6	-0.006614355	0.199786275	0.005158749	0.240335003	-0.006708684	0.193434968	0.005158597	0.240301551	9.43E-05	0.040900412	1	0.49587241	1	0.485554643	ENSG00000204792	2	AC104135.3
VAT	HTN	ENSG00000125498.15	0.004935495	0.474048648	0.006894038	0.240335561	0.00480517	0.48578452	0.006893781	0.240302145	0.000130325	0.018689189	1	0.741580968	1	0.746371412	ENSG00000204792	2	AC104135.3
VAT	HTN	ENSG00000125534.5	0.003812377	0.432478309	0.004856788	0.240335509	0.003902956	0.421603632	0.00485658	0.240302066	-9.06E-05	0.010789813	1	0.713571923	1	0.704374107	ENSG00000204792	2	AC104135.3
VAT	HTN	ENSG00000125630.11	0.001217019	0.649975831	0.002681863	0.240335712	0.001111282	0.67861266	0.002681941	0.2403023	0.000105737	0.005000299	1	0.835367368	1	0.853678642	ENSG00000204839	4	MROH6
VAT	HTN	ENSG00000125901.5	0.004275023	0.161248238	0.003051652	0.240334845	0.004333829	0.155552393	0.003051579	0.24030139	-5.88E-05	0.043630622	1	0.439333798	1	0.432828844	ENSG00000204839	4	MROH6
VAT	HTN	ENSG00000126215.9	-0.009374133	0.060356479	0.00499106	0.240334074	-0.009452707	0.058224141	0.004990841	0.240300616	7.86E-05	0.028255045	1	0.252880519	1	0.248759487	ENSG00000204920	6	ZNF155
VAT	HTN	ENSG00000126251.5	0.000984699	0.630473605	0.002046934	0.240335699	0.001018224	0.618864793	0.002046845	0.240302263	-3.35E-05	0.023940323	1	0.825823207	1	0.825554802	ENSG00000204920	6	ZNF155
VAT	HTN	ENSG00000127337.2	0.004830661	0.050984366	0.002475211	0.240333935	0.005068328	0.04073654	0.002476938	0.24030032	-0.000237666	0.014282636	1	0.229180536	1	0.198159466	ENSG00000204933	2	CD177P1
VAT	HTN	ENSG00000128185.5	0.007079439	0.06037091	0.00376951	0.240334074	0.007135162	0.058364979	0.003769333	0.240300618	-5.57E-05	0.027818783	1	0.252880519	1	0.2489758	ENSG00000204933	2	CD177P1
VAT	HTN	ENSG00000128218.7	-0.004006774	0.253368491	0.003507923	0.24033517	-0.003826116	0.275420693	0.003508056	0.240301798	-0.000180659	0.000445014	1	0.563738197	1	0.580170048	ENSG00000204946	5	ZNF783
VAT	HTN	ENSG00000128262.4	-0.001942375	0.637604116	0.004123487	0.240335704	-0.001071148	0.79540375	0.004130917	0.240302352	-0.000871227	0.000556148	1	0.828194673	1	0.918168712	ENSG00000204946	5	ZNF783
VAT	HTN	ENSG00000128271.15	0.006109848	0.217211425	0.004951339	0.240335062	0.005679163	0.25147728	0.004952312	0.240301736	0.000430685	0.000161682	1	0.519951026	1	0.558388812	ENSG00000204947	4	ZNF425
VAT	HTN	ENSG00000128274.11	-0.010996396	0.030326225	0.005077268	0.2403335	-0.011085218	0.02900733	0.00507706	0.240300034	8.88E-05	0.020694249	1	0.168253907	1	0.164732983	ENSG00000204947	4	ZNF425
VAT	HTN	ENSG00000128604.14	-0.007644489	0.082135603	0.004397351	0.240334323	-0.007548917	0.08602806	0.00439725	0.240300931	-9.56E-05	0.024330063	1	0.303472909	1	0.314034154	ENSG00000204947	4	ZNF425
VAT	HTN	ENSG00000128645.11	-0.007225401	0.239343732	0.006140758	0.240335131	-0.008416144	0.171617776	0.006156536	0.240301463	0.001190744	0.007648572	1	0.549661061	1	0.456168919	ENSG00000204947	4	ZNF425
VAT	HTN	ENSG00000128652.7	-0.000809048	0.857145933	0.004494495	0.240335797	-0.001902828	0.673557443	0.004516874	0.240302297	0.00109378	0.015570232	1	0.945910746	1	0.850381614	ENSG00000204954	5	C12orf73
VAT	HTN	ENSG00000128655.12	0.001360259	0.742627474	0.004142381	0.24033576	0.0012929	0.754943968	0.004142221	0.240302337	6.74E-05	0.038549873	1	0.880434634	1	0.892265229	ENSG00000204959	3	ARHGEF34P
VAT	HTN	ENSG00000128709.10	-0.001208953	0.756109562	0.003892394	0.240335766	-0.004456721	0.276750075	0.004097568	0.240301801	0.003247767	0.011246662	1	0.890491808	1	0.581087867	ENSG00000204959	3	ARHGEF34P
VAT	HTN	ENSG00000128944.9	-0.003325096	0.03401232	0.001568492	0.240333597	-0.003295706	0.035617605	0.001568434	0.240300207	-2.94E-05	0.020071473	1	0.178807628	1	0.184436381	ENSG00000204959	3	ARHGEF34P
VAT	HTN	ENSG00000129055.8	-0.003177324	0.092584961	0.001889105	0.240334418	-0.003143724	0.096073888	0.001889034	0.240301019	-3.36E-05	0.026678076	1	0.32589131	1	0.333838771	ENSG00000204977	5	TRIM13
VAT	HTN	ENSG00000129187.10	-0.003864671	0.388975412	0.004486095	0.240335447	-0.003784392	0.39888398	0.004485914	0.240302034	-8.03E-05	0.019205252	1	0.682680345	1	0.68668204	ENSG00000204991	6	SPIRE2
VAT	HTN	ENSG00000129204.12	-0.001201657	0.717037952	0.003315642	0.240335749	-0.001107712	0.738303637	0.003315502	0.24030233	-9.39E-05	0.000124054	1	0.867520033	1	0.881179801	ENSG00000204991	6	SPIRE2
VAT	HTN	ENSG00000130177.10	0.009643055	0.000229056	0.002617099	0.240329118	0.009688663	0.000213751	0.002616992	0.240295626	-4.56E-05	0.021787159	0.632193253	0.005057546	0.589951873	0.004835671	ENSG00000204991	6	SPIRE2
VAT	HTN	ENSG00000130300.4	0.004378075	0.369356605	0.0048771	0.240335416	0.004538191	0.352089672	0.00487692	0.240301958	-0.000160116	4.85E-05	1	0.668640123	1	0.651755529	ENSG00000204991	6	SPIRE2
VAT	HTN	ENSG00000130332.10	0.007031341	0.033405599	0.003305475	0.240333582	0.006925391	0.036153028	0.003305348	0.24030022	0.00010595	4.74E-05	1	0.176610754	1	0.186465689	ENSG00000204991	6	SPIRE2
VAT	HTN	ENSG00000130511.11	0.006896631	0.315019086	0.006864046	0.240335315	0.007036241	0.305304342	0.006863783	0.240301867	-0.00013961	0.010196752	1	0.618387395	1	0.604212978	ENSG00000204991	6	SPIRE2
VAT	HTN	ENSG00000130529.11	-0.022965247	0.000165273	0.006096507	0.240328815	-0.023118435	0.000149318	0.006096261	0.240295293	0.000153188	0.001051882	0.456152124	0.003801268	0.412116568	0.003552729	ENSG00000205038	7	PKHD1L1
VAT	HTN	ENSG00000130724.4	0.005883347	0.262272038	0.005248123	0.240335194	0.005964783	0.255705811	0.005247915	0.240301748	-8.14E-05	0.045026568	1	0.570001694	1	0.562797479	ENSG00000205038	7	PKHD1L1
VAT	HTN	ENSG00000130943.5	-0.003726059	0.31983026	0.003745507	0.240335325	-0.003432981	0.359613507	0.003747376	0.240301971	-0.000293078	0.020329204	1	0.622518701	1	0.657742398	ENSG00000205045	4	SLFN12L
VAT	HTN	ENSG00000131558.10	0.02497382	1.05E-05	0.005668137	0.240326239	0.024846442	1.17E-05	0.005667903	0.240292908	0.000127377	0.002699129	0.029067997	0.000398192	0.032209103	0.000435258	ENSG00000205045	4	SLFN12L
VAT	HTN	ENSG00000131845.10	0.000691692	0.874576243	0.004382002	0.240335801	0.000772065	0.860138427	0.004381803	0.240302369	-8.04E-05	0.008385419	1	0.954460432	1	0.949732956	ENSG00000205047	3	FLJ00104
VAT	HTN	ENSG00000132141.9	-0.009288399	0.075106793	0.005218743	0.240334251	-0.009416857	0.071157968	0.005218622	0.240300779	0.000128458	0.010692665	1	0.290259707	1	0.278181293	ENSG00000205047	3	FLJ00104
VAT	HTN	ENSG00000132286.7	0.010619409	0.04129088	0.005204055	0.2403376	0.010730292	0.039210036	0.005203874	0.240300288	-0.000110883	0.010700553	1	0.200638783	1	0.193604012	ENSG00000205057	3	CLLU1OS
VAT	HTN	ENSG00000132382.10	0.004650136	0.252019166	0.004059623	0.240335166	0.004713891	0.245556368	0.004059458	0.24030172	-6.38E-05	0.039077566	1	0.562761245	1	0.551175422	ENSG00000205078	5	SYCE1L
VAT	HTN	ENSG00000132383.7	-0.005117979	0.205707311	0.004044365	0.240335024	-0.005188554	0.199507889	0.004044216	0.240301573	7.06E-05	0.031402284	1	0.505148194	1	0.494292435	ENSG00000205078	5	SYCE1L
VAT	HTN	ENSG00000132600.12	-0.005348295	0.10076015	0.003258805	0.240334485	-0.005297145	0.104044569	0.003258665	0.240301082	-5.11E-05	0.030660116	1	0.33914392	1	0.344320158	ENSG00000205084	6	TMEM231
VAT	HTN	ENSG00000132837.10	-0.00394057	0.574206945	0.007013351	0.240335658	-0.003785345	0.589364451	0.007013047	0.240302241	-0.000155225	0.00219823	1	0.797991524	1	0.809098734	ENSG00000205108	5	FAM205A
VAT	HTN	ENSG00000133433.6	-0.001208647	0.566223037	0.00210704	0.240335651	-0.002882164	0.180706145	0.002153141	0.240301501	0.001673517	0.000163125	1	0.79306004	1	0.469962518	ENSG00000205269	4	TMEM170B
VAT	HTN	ENSG00000133460.15	0.015147241	0.007077869	0.005624318	0.240332236	0.014889446	0.00811586	0.005624555	0.240298929	0.000257795	0.002137501	1	0.064259602	1	0.070885362	ENSG00000205269	4	TMEM170B
VAT	HTN	ENSG00000133661.11	0.002230812	0.307380113	0.002185493	0.2403353	0.002263062	0.300418035	0.002185397	0.240301856	-3.23E-05	0.037702368	1	0.609460569	1	0.599966554	ENSG00000205269	4	TMEM170B
VAT	HTN	ENSG00000133706.13	-0.002666156	0.135614788	0.001786579	0.240334715	-0.002622114	0.142179601	0.001786516	0.240301322	-4.40E-05	0.00285392	1	0.402902923	1	0.412928922	ENSG00000205269	4	TMEM170B
VAT	HTN	ENSG00000133739.11	-0.003705231	0.542594527	0.006085176	0.240335631	-0.003868102	0.524983046	0.006084949	0.240302186	0.00016287	0.000871609	1	0.780738853	1	0.769900748	ENSG00000205269	4	TMEM170B
VAT	HTN	ENSG00000134440.7	-0.000246006	0.970850051	0.006732096	0.240335813	-4.83E-05	0.994278036	0.006731839	0.240302385	-0.000197728	0.000207508	1	0.987235058	1	0.997760874	ENSG00000205269	4	TMEM170B
VAT	HTN	ENSG00000134825.9	-0.001947678	0.765766763	0.006537657	0.24033577	-0.001595243	0.807226266	0.00653772	0.240302355	-0.000352435	1.83E-05	1	0.896317331	1	0.924888243	ENSG00000205269	4	TMEM170B
VAT	HTN	ENSG00000134905.12	-0.002115131	0.544537063	0.003490507	0.240335632	-0.002053291	0.556348087	0.00349036	0.240302214	-6.18E-05	0.0166438	1	0.780738853	1	0.791790437	ENSG00000205309	9	NT5M
VAT	HTN	ENSG00000135297.11	0.000380644	0.908284367	0.003304115	0.240335807	0.000293829	0.929136338	0.003303986	0.240302381	8.68E-05	0.000792584	1	0.964197726	1	0.973666067	ENSG00000205309	9	NT5M
VAT	HTN	ENSG00000135480.10	0.009407759	0.146045193	0.006471838	0.240334771	0.009593395	0.138241412	0.006471642	0.240301301	-0.000185635	0.001227144	1	0.417271979	1	0.407127621	ENSG00000205309	9	NT5M
VAT	HTN	ENSG00000135541.16	0.000459626	0.820790656	0.002028999	0.240335788	0.000502259	0.804481967	0.002028912	0.240302355	-4.26E-05	0.004138857	1	0.926718801	1	0.924383942	ENSG00000205356	5	TECPR1
VAT	HTN	ENSG00000136371.5	0.001741713	0.772446437	0.006023012	0.240335772	0.001572813	0.793980717	0.006022754	0.240302351	0.000168901	0.000128406	1	0.900698	1	0.918168712	ENSG00000205464	7	ATP6AP1L
VAT	HTN	ENSG00000136449.9	0.003031462	0.36673902	0.003358591	0.240335412	0.00307924	0.359213894	0.003358445	0.24030197	-4.78E-05	0.048688433	1	0.667014755	1	0.65744718	ENSG00000205464	7	ATP6AP1L
VAT	HTN	ENSG00000137070.13	-0.012250573	0.006914821	0.004535686	0.240332216	-0.01180958	0.009235234	0.004536508	0.240299043	-0.000440993	1.38E-05	1	0.062986484	1	0.076483154	ENSG00000205464	7	ATP6AP1L
VAT	HTN	ENSG00000137225.8	0.006507568	0.147226884	0.00448984	0.240334777	0.006401791	0.153902901	0.004489705	0.240301382	0.000105777	0.007983692	1	0.418982277	1	0.429931182	ENSG00000205476	4	CCDC85C
VAT	HTN	ENSG00000137285.9	-0.004287108	0.308725263	0.004211712	0.240335302	-0.004191371	0.319634972	0.004211547	0.240301896	-9.57E-05	0.003542618	1	0.611674165	1	0.617799211	ENSG00000205485	9	AC004980.7
VAT	HTN	ENSG00000137563.7	-0.005203102	0.082027762	0.002991933	0.240334322	-0.005134439	0.086131831	0.002991804	0.240300932	-6.87E-05	0.001478231	1	0.303472909	1	0.314034154	ENSG00000205485	9	AC004980.7
VAT	HTN	ENSG00000137709.5	-0.005903846	0.337769739	0.006158935	0.24033536	-0.005658905	0.358226914	0.006159353	0.240301969	-0.000244941	0.016417308	1	0.640277802	1	0.656234077	ENSG00000205485	9	AC004980.7
VAT	HTN	ENSG00000137767.9	0.006494722	0.11437372	0.004113607	0.240334584	0.006408136	0.11926796	0.004113432	0.240301188	8.66E-05	0.00414006	1	0.36551286	1	0.374067693	ENSG00000205485	9	AC004980.7
VAT	HTN	ENSG00000137996.8	0.008488347	0.15453684	0.005962215	0.240334814	0.008586278	0.149819412	0.005961981	0.240301362	-9.79E-05	0.034998525	1	0.430829979	1	0.42366965	ENSG00000205517	8	RGL3
VAT	HTN	ENSG00000138119.12	-0.00371599	0.237597276	0.003146437	0.240335125	-0.002999913	0.3413978	0.00315313	0.240301938	-0.000716077	0.00059899	1	0.549204878	1	0.639686306	ENSG00000205517	8	RGL3
VAT	HTN	ENSG00000138175.8	0.009662708	0.002245386	0.003162179	0.240331208	0.009614262	0.002361738	0.003162034	0.240297826	4.84E-05	0.025927094	1	0.027915609	1	0.02884247	ENSG00000205517	8	RGL3
VAT	HTN	ENSG00000138193.10	-0.004971532	0.473831103	0.006940959	0.24033556	-0.00508631	0.463665018	0.006940681	0.24030212	0.000114778	0.031223773	1	0.741580968	1	0.733361289	ENSG00000205531	8	NAP1L4
VAT	HTN	ENSG00000138346.10	-0.000784073	0.700105316	0.002035609	0.2403574	-0.000835699	0.681398891	0.002035534	0.240302302	5.16E-05	0.001658964	1	0.860778235	1	0.856249354	ENSG00000205560	8	CPT1B
VAT	HTN	ENSG00000138363.10	0.003401973	0.34059866	0.003569811	0.240335365	0.003457406	0.332769165	0.003569664	0.240301922	-5.54E-05	0.039177246	1	0.642908166	1	0.631637966	ENSG00000205560	8	CPT1B
VAT	HTN	ENSG00000138385.11	-0.004238005	0.361021543	0.004639706	0.240335402	-0.004127473	0.3736625	0.004639514	0.240301994	-0.000110533	0.00149603	1	0.665223012	1	0.668408979	ENSG00000205560	8	CPT1B
VAT	HTN	ENSG00000138448.7	-0.002320357	0.693883896	0.005895365	0.240335737	-0.002175705	0.712079761	0.005895187	0.240302318	-0.000144652	0.005655797	1	0.858028474	1	0.869767456	ENSG00000205560	8	CPT1B
VAT	HTN	ENSG00000138468.11	-0.010423136	0.078768456	0.005929376	0.240334289	-0.010291125	0.08262202	0.005929179	0.240300899	-0.000132011	0.008845076	1	0.297402103	1	0.306698747	ENSG00000205565	6	RP11-497H16.6
VAT	HTN	ENSG00000139133.2	-0.010124069	0.108532166	0.006308504	0.240334543	-0.010019492	0.11221449	0.00630823	0.240301141	-0.000104577	0.021365655	1	0.353658534	1	0.362660414	ENSG00000205565	6	RP11-497H16.6
VAT	HTN	ENSG00000139178.6	0.00037064	0.892579674	0.002744654	0.240335804	0.000442707	0.871854895	0.002744571	0.240302372	-7.21E-05	0.003020984	1	0.959290439	1	0.956043422	ENSG00000205571	8	SMN2
VAT	HTN	ENSG00000139636.11	-0.009114302	0.0874382	0.005332895	0.240334373	-0.009035271	0.090203831	0.005332661	0.240300969	-7.90E-05	0.038688236	1	0.315463309	1	0.32217795	ENSG00000205571	8	SMN2
VAT	HTN	ENSG00000139899.6	0.00016439	0.906135688	0.001394148	0.240335807	0.00019406	0.889291472	0.001394097	0.240302375	-2.97E-05	0.008936233	1	0.963884879	1	0.960643625	ENSG00000205571	8	SMN2
VAT	HTN	ENSG00000139910.15	0.010971407	0.015239733	0.004521234	0.240332909	0.007206738	0.128305718	0.00473872	0.240301244	0.003764669	0.008024307	1	0.105417704	1	0.390434159	ENSG00000205571	8	SMN2
VAT	HTN	ENSG00000140043.7	0.003138911	0.114706338	0.001989939	0.240334586	0.003089364	0.120531771	0.001989867	0.240301196	4.95E-05	0.002213235	1	0.365576781	1	0.376532767	ENSG00000205572	5	SERF1B
VAT	HTN	ENSG00000140323.4	0.000988229	0.598214639	0.001875303	0.240335676	0.001021718	0.585858665	0.001875237	0.240302238	-3.35E-05	0.031032934	1	0.81054119	1	0.807829017	ENSG00000205572	5	SERF1B
VAT	HTN	ENSG00000140471.12	0.008287685	0.051833652	0.004262078	0.240333949	0.008394324	0.048885549	0.004261958	0.240300472	-0.000106639	0.005978118	1	0.229793907	1	0.221550272	ENSG00000205578	4	POM121B
VAT	HTN	ENSG00000140955.6	1.74E-05	0.994918358	0.002734858	0.240335813	-4.82E-05	0.9859397	0.002734766	0.240302385	6.56E-05	0.004620682	1	0.997448117	1	0.996044499	ENSG00000205578	4	POM121B
VAT	HTN	ENSG00000141098.8	-0.003737885	0.415323613	0.004588829	0.240335486	-0.00367006	0.423818148	0.004588634	0.240302069	-6.78E-05	0.044436139	1	0.701684662	1	0.704889231	ENSG00000205578	4	POM121B
VAT	HTN	ENSG00000141252.15	-0.010864962	0.000605586	0.003168438	0.240330014	-0.010920708	0.000567152	0.00316829	0.240296526	5.57E-05	0.009214846	1	0.010578596	1	0.010098967	ENSG00000205578	4	POM121B
VAT	HTN	ENSG00000141391.9	-0.002808355	0.324814807	0.002852243	0.240335335	-0.002743317	0.336127103	0.002852136	0.240301929	-6.50E-05	0.004267197	1	0.626300732	1	0.635876512	ENSG00000205578	4	POM121B
VAT	HTN	ENSG00000141425.13	0.012178569	0.034783237	0.005769431	0.240333616	0.012294314	0.033086614	0.00576917	0.240300145	-0.000115745	0.004188466	1	0.18113535	1	0.176632601	ENSG00000205583	9	STAG3L1
VAT	HTN	ENSG00000141452.5	-0.026710432	2.14E-07	0.005149977	0.240322547	-0.02660741	2.38E-07	0.00514983	0.240289221	-0.000103022	0.026886975	0.000591608	1.64E-05	0.000658012	1.88E-05	ENSG00000205583	9	STAG3L1

VAT	HTN	ENSG00000141556.16	0.004241125	0.544525813	0.006998744	0.240335632	0.004456652	0.524253229	0.006998474	0.240302185	-0.000215527	9.27E-05	1	0.780738853	1	0.769652481	ENSG00000205643	6	CDPF1
VAT	HTN	ENSG00000141580.11	-0.006549573	0.351206661	0.007025538	0.240335385	-0.006664361	0.342811718	0.007025284	0.240301941	0.000114788	0.045504677	1	0.655395798	1	0.641030042	ENSG00000205643	6	CDPF1
VAT	HTN	ENSG00000141753.5	0.013156465	0.04162127	0.006457818	0.240335366	0.013003722	0.044039184	0.006457553	0.240300385	0.000152743	0.001694539	1	0.20153457	1	0.209205076	ENSG00000205659	6	LIN52
VAT	HTN	ENSG00000141759.10	-0.009802887	0.031018806	0.004545028	0.240333519	-0.009914163	0.029153321	0.004544834	0.240300038	0.000111276	0.000881549	1	0.169864891	1	0.165136315	ENSG00000205704	5	LINC00634
VAT	HTN	ENSG00000142102.11	-0.004110806	0.195936609	0.003178742	0.240334989	-0.004161319	0.190480478	0.003178615	0.24030124	5.05E-05	0.038814327	1	0.491622256	1	0.482167409	ENSG00000205710	3	C17orf107
VAT	HTN	ENSG00000142185.12	0.001935969	0.386357292	0.002234902	0.240335443	0.001980536	0.375497429	0.002234803	0.240301997	-4.46E-05	0.005378083	1	0.680067683	1	0.668627681	ENSG00000205710	3	C17orf107
VAT	HTN	ENSG00000142675.13	-0.00087303	0.737731322	0.002607153	0.240335758	-0.000808444	0.756485738	0.00260705	0.240302337	-6.46E-05	0.001357132	1	0.878176701	1	0.892265229	ENSG00000205710	3	C17orf107
VAT	HTN	ENSG00000142798.12	0.002588395	0.419138356	0.003203785	0.240335491	0.002061217	0.520948699	0.003211198	0.240302182	0.000527178	0.017211757	1	0.704520013	1	0.768793407	ENSG00000205710	3	C17orf107
VAT	HTN	ENSG00000143185.3	4.52E-05	0.99417445	0.006195834	0.240335813	0.000201988	0.973992102	0.006195595	0.240302384	-0.00015675	0.001287689	1	0.997230388	1	0.989761439	ENSG00000205771	2	CATSPER2P1
VAT	HTN	ENSG00000143376.8	0.024144966	3.91E-05	0.005870643	0.240327471	0.023751049	5.24E-05	0.005872228	0.240294318	0.000393917	0.009972471	0.107888249	0.001198758	0.144656132	0.001530313	ENSG00000205771	2	CATSPER2P1
VAT	HTN	ENSG00000143811.12	0.002249956	0.693834757	0.005715529	0.240335737	0.002085045	0.715247554	0.005715305	0.240302319	0.000164911	0.00021101	1	0.858028474	1	0.870791023	ENSG00000205771	2	CATSPER2P1
VAT	HTN	ENSG00000143951.11	-0.003829536	0.573285871	0.006799371	0.240335657	-0.005060156	0.457679007	0.006813422	0.240302113	0.00123062	0.005648931	1	0.797624595	1	0.728905978	ENSG00000205771	2	CATSPER2P1
VAT	HTN	ENSG00000144214.5	-0.002465639	0.534939457	0.003973735	0.240335623	-0.002519936	0.525965627	0.003973553	0.240302186	5.43E-05	0.047350043	1	0.777236628	1	0.770114128	ENSG00000205771	2	CATSPER2P1
VAT	HTN	ENSG00000144843.7	0.002541749	0.705079799	0.006715809	0.240335743	0.002663959	0.691601969	0.006715599	0.240302307	-0.00012221	0.037763131	1	0.861817357	1	0.861126259	ENSG00000205771	2	CATSPER2P1
VAT	HTN	ENSG00000144847.8	0.004579621	0.464621533	0.006262656	0.24033555	0.0047298	0.450088238	0.006262409	0.240302103	-0.000150179	0.001986285	1	0.737028926	1	0.723286064	ENSG00000205822	6	TPTE2P6
VAT	HTN	ENSG00000145217.9	0.005440835	0.096542095	0.003273949	0.240334451	0.005386497	0.099903794	0.003273823	0.24030105	5.43E-05	0.035111025	1	0.333487087	1	0.33915679	ENSG00000205837	3	LINC00487
VAT	HTN	ENSG00000145390.7	0.004738387	0.370176398	0.00528753	0.240335417	0.004835646	0.360417794	0.005287343	0.240301972	-9.73E-05	0.026142706	1	0.66884126	1	0.658776895	ENSG00000205918	5	PDPK2
VAT	HTN	ENSG00000145569.5	0.008389141	0.170407501	0.006119451	0.240334886	0.008480814	0.165766951	0.006119194	0.240301438	-9.17E-05	0.04385992	1	0.454626907	1	0.450561028	ENSG00000205937	7	RNPS1
VAT	HTN	ENSG00000145781.4	0.000966462	0.823264941	0.00432716	0.240335789	0.000876646	0.839447462	0.004326969	0.240302365	8.98E-05	0.00354412	1	0.928190865	1	0.94007326	ENSG00000205937	7	RNPS1
VAT	HTN	ENSG00000146094.9	-0.012677882	0.027273612	0.005742871	0.24033341	-0.012757982	0.026308351	0.005742606	0.240299951	8.01E-05	0.041393603	1	0.15617255	1	0.15378118	ENSG00000205978	5	NYNRIN
VAT	HTN	ENSG00000146205.9	-0.003944218	0.444063233	0.00515348	0.240335524	-0.003838582	0.456343778	0.005153279	0.240302111	-0.000105637	0.008768552	1	0.721374057	1	0.728497638	ENSG00000205978	5	NYNRIN
VAT	HTN	ENSG00000146386.7	0.000407247	0.417416355	0.004941652	0.240335489	0.003877929	0.432590594	0.004941504	0.240302081	0.000129318	0.003233546	1	0.702909786	1	0.711889332	ENSG00000205978	5	NYNRIN
VAT	HTN	ENSG00000146409.6	-0.016325341	0.000485476	0.004679534	0.240329811	-0.016244236	0.000517647	0.004679343	0.240296442	-8.11E-05	0.021847921	1	0.009115068	1	0.009771271	ENSG00000206013	2	IFITM5
VAT	HTN	ENSG00000146556.10	-0.019194977	5.32E-06	0.004217048	0.240325596	-0.019096743	5.94E-06	0.004216946	0.240292272	-9.82E-05	0.014180058	0.014686681	0.000225949	0.016393759	0.000252212	ENSG00000206013	2	IFITM5
VAT	HTN	ENSG00000146574.11	-0.003231092	0.239452676	0.002746693	0.240335131	-0.003286192	0.231521597	0.002746619	0.240301679	5.51E-05	0.030008282	1	0.549661061	1	0.534280609	ENSG00000206013	2	IFITM5
VAT	HTN	ENSG00000146733.9	0.004369627	0.271480488	0.003973613	0.240335217	0.004433165	0.264552793	0.003973452	0.240301771	-6.35E-05	0.035651443	1	0.579737555	1	0.569128623	ENSG00000206028	1	CTA-373H7.7
VAT	HTN	ENSG00000147576.11	-0.013078995	0.00049983	0.003757392	0.240329838	-0.01213279	0.001274589	0.003766006	0.240297267	-0.000946205	0.000249255	1	0.009321154	1	0.018581684	ENSG00000206077	6	ZDHHC11B
VAT	HTN	ENSG00000148110.11	0.006704518	0.184606514	0.005053506	0.240334945	0.006594582	0.191894341	0.00505333	0.240301545	0.000109936	0.008868708	1	0.474407803	1	0.484121007	ENSG00000206113	3	RP11-503N18.1
VAT	HTN	ENSG00000148655.10	0.005941854	0.133956995	0.003964722	0.240334706	0.006049	0.127068659	0.003964565	0.240301237	-0.000107146	0.000485215	1	0.400743043	1	0.387952984	ENSG00000206145	4	P2RX6P
VAT	HTN	ENSG00000149273.10	-0.002412541	0.59338885	0.004518443	0.240335673	-0.00255847	0.571260696	0.004518698	0.240302227	0.000145928	0.041950834	1	0.808777562	1	0.801076492	ENSG00000206145	4	P2RX6P
VAT	HTN	ENSG00000149292.12	0.000736061	0.893189836	0.005481983	0.240335804	0.000818158	0.881335557	0.005481747	0.240302374	-8.21E-05	0.040224543	1	0.959290439	1	0.959100799	ENSG00000206172	4	HBA1
VAT	HTN	ENSG00000149489.4	0.010829674	0.001366551	0.003382537	0.240330758	0.010890964	0.001282493	0.003382405	0.240297272	-6.13E-05	0.020932534	1	0.019243274	1	0.018581684	ENSG00000206172	4	HBA1
VAT	HTN	ENSG00000149499.7	0.001494034	0.688051805	0.003721126	0.240335734	0.001410767	0.70458886	0.003721029	0.240302314	8.33E-05	0.016346621	1	0.855718693	1	0.868203529	ENSG00000206341	6	HLA-H
VAT	HTN	ENSG00000149548.10	0.012778606	0.036964434	0.006125413	0.240333667	0.012624727	0.03929477	0.006125261	0.24030029	0.000153878	0.007836077	1	0.187472424	1	0.193604012	ENSG00000206341	6	HLA-H
VAT	HTN	ENSG00000149743.9	-0.0033317	0.431117522	0.004231904	0.240335508	-0.003259348	0.44117098	0.004231721	0.240302092	-7.24E-05	0.018142294	1	0.713571923	1	0.717128893	ENSG00000206341	6	HLA-H
VAT	HTN	ENSG00000149922.6	-0.013990739	0.001968088	0.004520399	0.240331089	-0.014109572	0.001800158	0.004520299	0.24029758	0.000118833	0.006999627	1	0.0258663	1	0.024236277	ENSG00000206341	6	HLA-H
VAT	HTN	ENSG00000151640.8	0.007490868	0.001156702	0.002305332	0.240330606	0.007556028	0.001046632	0.002305282	0.240297087	-6.52E-05	0.00402222	1	0.01716397	1	0.016048355	ENSG00000206341	6	HLA-H
VAT	HTN	ENSG00000151883.12	0.001864831	0.213729101	0.00149981	0.240335051	0.001826495	0.223276027	0.001499752	0.240301653	3.83E-05	0.001106708	1	0.513843482	1	0.523569953	ENSG00000206341	6	HLA-H
VAT	HTN	ENSG00000152465.13	-0.006214606	0.077555382	0.003520916	0.240334277	-0.005059896	0.152412565	0.003535758	0.240301375	-0.0011547	0.000401024	1	0.294028647	1	0.427064649	ENSG00000206341	6	HLA-H
VAT	HTN	ENSG00000152492.9	-0.009534494	0.111821846	0.00599631	0.240334566	-0.009453522	0.114881502	0.005996034	0.240301159	-8.10E-05	0.048779958	1	0.361931749	1	0.366605303	ENSG00000206341	6	HLA-H
VAT	HTN	ENSG00000153246.7	-0.002620547	0.400705817	0.003118355	0.240335465	-0.002666792	0.392425812	0.003118227	0.240302024	4.62E-05	0.049092311	1	0.69221916	1	0.681708076	ENSG00000206341	6	HLA-H
VAT	HTN	ENSG00000153291.11	0.009254341	0.005510473	0.003334196	0.240332014	0.008523796	0.010852081	0.003346029	0.240299185	0.000730545	0.010061598	1	0.053552481	1	0.084874033	ENSG00000206341	6	HLA-H
VAT	HTN	ENSG00000153814.7	-0.002629079	0.348098766	0.002802007	0.240335379	-0.002688104	0.337367187	0.002801914	0.240301931	-5.90E-05	0.013666929	1	0.650475689	1	0.636028046	ENSG00000206341	6	HLA-H
VAT	HTN	ENSG00000154518.5	-0.008757991	0.010306691	0.00341392	0.240332568	-0.00914269	0.007446369	0.0034169	0.240298853	0.000384699	0.003953178	1	0.081742724	1	0.066988633	ENSG00000206341	6	HLA-H
VAT	HTN	ENSG00000154803.8	0.00421815	0.133695673	0.002812683	0.240334704	0.005180326	0.067918199	0.002837675	0.240300741	-0.000962176	0.010759592	1	0.400651528	1	0.270496723	ENSG00000206344	6	HCG27
VAT	HTN	ENSG00000155016.13	-0.00445862	0.407568876	0.005383636	0.240335475	-0.004633844	0.389373261	0.005383463	0.240302019	0.000175224	0.000171174	1	0.696936974	1	0.679740797	ENSG00000206344	6	HCG27
VAT	HTN	ENSG00000155085.11	-0.00122492	0.803947625	0.004934389	0.240335783	-0.001314535	0.789922525	0.004934231	0.24030235	8.96E-05	0.03603581	1	0.918797285	1	0.915481504	ENSG00000206344	6	HCG27
VAT	HTN	ENSG00000156096.8	0.010410386	0.117730602	0.006654669	0.240334606	0.010244518	0.123687883	0.006654533	0.240301216	0.000165869	0.011743179	1	0.369484015	1	0.379887847	ENSG00000206344	6	HCG27
VAT	HTN	ENSG00000156219.12	-0.000236551	0.958628746	0.004560069	0.240335812	-0.00039629	0.930745142	0.004559909	0.24030381	0.000159739	2.46E-05	1	0.98228229	1	0.974255726	ENSG00000206344	6	HCG27
VAT	HTN	ENSG00000156521.9	-0.015298048	0.002611524	0.005082195	0.240331345	-0.015385284	0.002466677	0.005081987	0.240297865	8.72E-05	0.023582377	1	0.030626438	1	0.029600124	ENSG00000206344	6	HCG27
VAT	HTN	ENSG00000156603.10	0.0037668	0.524096558	0.005912936	0.240335613	0.003663602	0.535510556	0.005912692	0.240302195	0.000103198	0.020225575	1	0.772678586	1	0.776265302	ENSG00000206344	6	HCG27
VAT	HTN	ENSG00000157911.5	0.012397515	0.002214721	0.004051696	0.240331196	0.012466744	0.00209083	0.004051546	0.240297716	-6.92E-05	0.034152496	1	0.02785549	1	0.026978653	ENSG00000206344	6	HCG27
VAT	HTN	ENSG00000157992.8	-0.00565304	0.353319398	0.006090516	0.240335388	-0.005850021	0.336776925	0.006090257	0.24030193	0.000196981	1.10E-05	1	0.657128126	1	0.636028046	ENSG00000206344	6	HCG27
VAT	HTN	ENSG00000158006.9	-0.003535733	0.316930431	0.003532954	0.240335319	-0.003583764	0.310376584	0.003532775	0.240301877	4.80E-05	0.027362596	1	0.620374461	1	0.60927411	ENSG00000206344	6	HCG27
VAT	HTN	ENSG00000158161.11	-0.005362307	0.181661176	0.004014726	0.240334934	-0.005301709	0.186628578	0.004014559	0.240301525	-6.06E-05	0.043948465	1	0.470059822	1	0.477318114	ENSG00000206344	6	HCG27
VAT	HTN	ENSG00000158769.13	-0.008589604	0.148096386	0.005939059	0.240334782	-0.008698946	0.142989213	0.005938852	0.240301327	0.000109342	0.027344849	1	0.418982277	1	0.412957063	ENSG00000206344	6	HCG27
VAT	HTN	ENSG00000158825.5	-0.007424358	0.038890442	0.003594722	0.24033371	-0.007324669	0.041585913	0.003594672	0.240300337	-9.97E-05	0.008577025	1	0.192789179	1	0.201363369	ENSG00000206344	6	HCG27
VAT	HTN	ENSG00000159228.8	0.000555598	0.886287849	0.003885218	0.240335803	0.000471615	0.903381223	0.003885081	0.240302378	8.40E-05	0.009080453	1	0.957399007	1	0.967157554	ENSG00000206503	7	HLA-A
VAT	HTN	ENSG00000159445.8	0.000492382	0.870134722	0.00301174	0.2403358	0.000443499	0.882924441	0.003011611	0.240302374	4.89E-05	0.026240405	1	0.95263744	1	0.959100799	ENSG00000206503	7	HLA-A
VAT	HTN	ENSG00000159761.10	0.005002016	0.230572598	0.004172217	0.240335104	0.004895842	0.240623352	0.004172243	0.240301706	0.000106174	0.038465633	1	0.53748342	1	0.54683908	ENSG00000206503	7	HLA-A
VAT	HTN	ENSG00000160221.12	0.009816742	0.137588849	0.006611347	0.240334726	0.009680271	0.143128578	0.006611116	0.240301327	0.000136472	0.012999254	1	0.404531314	1	0.412957063	ENSG00000206503	7	HLA-A
VAT	HTN	ENSG00000160321.10	-0.009179167	0.081780108	0.00527401	0.240334319	-0.00932359	0.07707632	0.005273787	0.240300843	0.000144423	0.000222186	1	0.3029706	1	0.293421577	ENSG00000206503	7	HLA-A
VAT	HTN	ENSG00000161036.6	-0.027058115	5.52E-05	0.006709757	0.240327793	-0.026928126	5.98E-05	0.006709484	0.240294442	-0.000129988	0.010648576	0.152239551	0.001602522	0.165182137	0.001676064	ENSG00000206503	7	HLA-A
VAT	HTN	ENSG00000161609.5	-0.002531469	0.591365216	0.004715333	0.240335671	-0.002639652	0.575605635	0.004715219	0.24030223	0.000108183	0.0159615	1	0.807604154	1	0.802143866	ENSG00000206503	7	HLA-A
VAT	HTN	ENSG00000161912.13	-0.002175207	0.614334062	0.004316769	0.240335688	-0.002262002	0.600262827	0.004316609	0.240302249	8.68E-05	0.012486258	1	0.819907808	1	0.814916578	ENSG00000206503	7	HLA-A
VAT	HTN	ENSG00000162194.8	0.00288508	0.585027304	0.005283482	0.240335666	0.002793168	0.597024294	0.005283237	0.240302247	9.19E-05	0.010582881	1	0.804121195	1	0.814290189	ENSG00000206503	7	HLA-A
VAT	HTN	ENSG00000162885.8	-0.002764332	0.647921408	0.006053476	0.240335711	-0.002592522	0.668445039	0.006053287	0.240302294	-0.00017181	0.00117605	1	0.835218069	1	0.849405298	ENSG00000206503	7	HLA-A
VAT	HTN	ENSG00000163131.6	-0.005169061	0.081138226	0.002963702	0.240334313	-0.005108908	0.084732709	0.002963635	0.240300919	-6.02E-05	0.035977967	1	0.301756109	1	0.311050019	ENSG00000206535	3	LNP1
VAT	HTN	ENSG00000163155.7	-0.003736103	0.286513305	0.003505435	0.240335253	-0.002435218	0.493413406	0.003555631	0.240302153	-0.001300885	0.029265981	1	0.592342114	1	0.749901432	ENSG00000206562	7	METTL6
VAT	HTN	ENSG00000163554.7	-0.013734132	2.85E-05	0.003281437	0.240327174	-0.01359099	3.45E-05	0.003281518	0.240293926	-0.000143142	0.00143558	0.078580679	0.000913729	0.095173587	0.001119689	ENSG00000206562	7	METTL6
VAT	HTN	ENSG00000163576.13	0.000186476	0.953248746	0.003180678	0.240335812	0.0001377	0.965466743	0.003180538	0.240302384	4.88E-05	0.031523803	1	0.981080063	1	0.985461617	ENSG00000212125	2	TAS2R15
VAT	HTN	ENSG00000163682.11	0.000536465	0.6834937	0.001315831	0.240335731	0.000558055	0.671473953	0.001315776	0.240302296	-2.16E-05	0.027647681	1	0.852822158	1	0.850122986	ENSG00000212127	5	TAS2R14
VAT	HTN	ENSG00000163743.9	0.000933932	0.336277241	0.00097128	0.240335357	0.000961821	0.322029978	0.000971246	0.240301901	-2.79E-05	0.000531952	1	0.638628342	1	0.620239176	ENSG00000212127	5	TAS2R14
VAT	HTN	ENSG00000163870.10	-0.002758205	0.289617929	0.002604633	0.24033526	-0.002707732	0.298522456	0.002604569	0.240301852	-5.05E-05	0.041565546	1	0.59321241	1	0.597477868	ENSG00000212127	5	TAS2R14
VAT	HTN	ENSG00000163904.8	-0.015662133	0.011542947	0.006200794	0.240332667	-0.01554734	0.012161671	0.006200514	0.240299284	-0.000114793	0.007848089	1	0.087283652	1	0.089985214	ENSG00000212127	5	TAS2R14
VAT	HTN	ENSG00000163913.7	0.003921936	0.301936462	0.003799255	0.240335288	0.004245928	0.263878767	0.003800265	0.240301769	-0.000323992	0.000995275	1	0.60414959	1	0.569128623	ENSG00000212694	4	AC084018.1
VAT	HTN	ENSG00000164053.13	0.002450425	0.613303423	0.004848831	0.240335687	0.002358428	0.626677363	0.004848652	0.240302268	9.20E-05	0.018606524	1	0.819907808	1	0.827573934	ENSG00000212694	4	AC084018.1
VAT	HTN	ENSG00000164118.8	-0.006447706	0.047327809	0.003250901	0.240333873	-0.006564458	0.043471303	0.003251086	0.240300374	0.000116752	0.024049038	1	0.21807219	1	0.207579233	ENSG00000212694	4	AC084018.1
VAT	HTN	ENSG00000164167.5	-0.012839956	0.016518027	0.005356069	0.240332979	-0.01266191	0.018073572	0.005355876	0.240299629	-0.000178046	4.68E-05	1	0.110087128	1	0.116241644	ENSG00000212694	4	AC084018.1
VAT	HTN	ENSG00000164574.11	0.004198837	0.547497431	0.006980438	0.240335635	0.004310011	0.536926064	0.006980132	0.240302197	-0.000111174	0.026358195	1	0.783206863	1	0.776342689	ENSG00000212694	4	AC084018.1
VAT	HTN	ENSG00000164733.16	0.003820767	0.566348311	0.00666291	0.240335651	0.003985736	0.549695264	0.006662689	0.240302208	-0.000164969	0.003770942	1	0.79306004	1	0.786610816	ENSG00000212694	4	AC084018.1
VAT	HTN	ENSG00000164924.13	-0.006470111	0.089010989	0.003804513	0.240334387	-0.005699355	0.136014769	0.003823019	0.240301289	-0.000770757	0.041635273	1	0.317403528	1	0.403615733	ENSG00000212694	4	AC084018.1
VAT	HTN	ENSG00000164967.5	-0.007289179	0.107807858	0.004532712	0.240334538	-0.007194528	0.112445035	0.004532553	0.240301142	-9.47E-05	0.011846842	1	0.35296523	1	0.362980465	ENSG00000212807	1	OR2A42
VAT	HTN	ENSG00000164978.13	-0.022137558	0.000316158	0.006146396	0.240329416	-0.021529835	0.000461586	0.006147631	0.240296337	-0.000607723	2.08E-05	0.872594828	0.006610567	1	0.008847074	ENSG00000212916	3	MAP10
VAT	HTN	ENSG00000164989.11	-0.003864076	0.007748506	0.001451107	0.240332316	-0.003580656	0.013702203	0.001452607	0.240299388	-0.00028342	3.21E-05	1	0.067627177	1	0.096721436	ENSG00000213024	6	NUP62
VAT	HTN	ENSG00000165171.6	0.002662181	0.690740388	0.006691406	0.240335735	0.002569022	0.701018794	0.006691107	0.240302312	9.32E-05	0.047955411	1	0.857708065	1	0.866075144	ENSG00000213077	5	FAM106A
VAT	HTN	ENSG00000165271.12	-0.0003443	0.783322884	0.001252037	0.240335776	-0.000320317	0.798067605	0.001251977	0.240302353	-2.40E-05	0.003569189	1	0.904415117	1	0.92007794	ENSG00000213088	5	DARC
VAT	HTN	ENSG00000165280.11	-0.003440965	0.556520343	0.0058518	0.240335643	-0.003046315	0.602733475	0.005852962	0.240302251	-0.00039465	0.003590541	1	0.788086792	1	0.816595742	ENSG00000213088	5	DARC
VAT	HTN	ENSG00000165682.10	-0.001079953	0.781263102	0.00388929	0.240335775	-0.000935753	0.809871593	0.003889381	0.240302356	-0.0001442	0.006491937	1	0.903724293	1	0.92710311	ENSG00000213090	2	AC007256.5
VAT	HTN	ENSG00000165684.3	-0.001562712	0.761476193	0.00514824	0.240335768	0.009359907	0.185891458	0.007075651	0.240301522	-0.010922619	0.024443169	1	0.893569002	1	0.47726551	ENSG00000213090	2	AC007256.5
VAT	HTN	ENSG00000165730.10	-0.000730696	0.81964567	0.003204794	0.240335788	-0.000664244	0.835795787	0.003204661	0.240302364	-6.65E-05	0.005514049	1	0.926718801	1	0.939506392	ENSG00000213123	6	TCTEX1D2
VAT	HTN	ENSG00000165912.11	0.013874298	0.022256386	0.006069324	0.240333236	0.013755022	0.023426857	0.006069085	0.240299852	0.000119276	0.01127014	1	0.135901828	1	0.142418781	ENSG00000213132	2	AC022498.1
VAT	HTN	ENSG00000165917.5	-0.007830078	0.029776779	0.003603261	0.240333484	-0.007770574	0.03103513	0.003603112	0.240300091	-5.95E-05	0.028381517	1	0.166662296	1	0.171622533	ENSG00000213132	2	AC022498.1
VAT	HTN	ENSG00000166037.6	0.003020404	0.596796142	0.005709501	0.240335675	0.003118399	0.584929308	0.005709271	0.240302238	-9.80E-05	0.022154251	1	0.810316927	1	0.807829017	ENSG00000213171	2	LINGO4
VAT	HTN	ENSG00000166532.11	-0.01729796	0.00041986	0.004904023	0.240329677	-0.017208976	0.000449318	0.004903812	0.240296312	-8.90E-05	0.012194668	1	0.00816066	1	0.008733218	ENSG00000213171	2	LINGO4
VAT	HTN	ENSG00000166557.8	-0.015595237	9.68E-09	0.002718634	0.240319585	-0.018036485	9.29E-11	0.002784199	0.240281691	0.002441248	4.96E-05	2.67E-05	1.11E-06	2.57E-07	3.66E-08	ENSG00000213199	3	ASIC3
VAT	HTN	ENSG00000166595.7	-0.004316321	0.117626516	0.002758356	0.240334606	-0.004368366	0.113251538	0.002758244	0.240301148	5.20E-05	0.013658266	1	0.369484015	1	0.364132457	ENSG00000213221	4	DNLZ
VAT	HTN	ENSG00000166667.8	0.001469499	0.69803842	0.00378766	0.240335739	0.001521509	0.687888709	0.003787466	0.240302305	-5.20E-05	0.023014288	1	0.860778235	1	0.859471632	ENSG00000213221	4	DNLZ
VAT	HTN	ENSG00000166881.5	0.003067231	0.418697877	0.003792872	0.240335491	0.003132279	0.408884276	0.003792751	0.240302048	-6.50E-05	0.0481588	1	0.704520013	1	0.692521557	ENSG00000213221	4	DNLZ
VAT	HTN	ENSG00000166928.6	-0.004058123	0.143150083	0.002771634	0.240334756	-0.004010399	0.147896855	0.002771522	0.240301359	-4.77E-05	0.024127969	1	0.411985639	1	0.420521528	ENSG00000213221	4	DNLZ
VAT	HTN	ENSG00000167103.7	-0.008913668	0.03237582	0.00416576	0.240333555	-0.007720602	0.065040316	0.004184642	0.240300706	-0.001193066	0.00286117	1	0.174179193	1	0.263213011	ENSG00000213221	4	DNLZ
VAT	HTN	ENSG00000167220.7	0.001838647	0.686229963	0.004551307	0.240335733	0.001748788	0.700789644	0.004551113	0.240302312	8.99E-05	0.007232644	1	0.854300251	1	0.866075144	ENSG00000213221	4	DNLZ
VAT	HTN	ENSG00000167325.10	-0.00144721	0.574181223	0.002575543	0.240335658	-0.001366403	0.595732591	0.002575459	0.240302246	-8.08E-05	0.000244644	1	0.797991524	1	0.814290189	ENSG00000213221	4	DNLZ
VAT	HTN	ENSG00000167644.7	-0.001305167	0.458254328	0.001759638	0.240335542	-0.001270269	0.470342289	0.001759564	0.240302128	-3.49E-05	0.007317487	1	0.733206925	1	0.73755282	ENSG00000213240	7	NOTCH2NL
VAT	HTN	ENSG00000167646.9	0.009744755	0.106581352	0.006038561	0.240334529	0.009646456	0.110146161	0.006038321	0.240301126	9.83E-05	0.03476774	1	0.350613267	1	0.358494581	ENSG00000213246	2	SUPT4H1
VAT	HTN	ENSG00000167780.7	-0.002220957	0.657418725	0.005008021	0.240335716	-0.002298176	0.646293859	0.005007823	0.240302281	7.72E-05	0.046379806	1	0.837990221	1	0.839816879	ENSG00000213277	3	MARCKSL1P1
VAT	HTN	ENSG00000167799.5	0.02005027	0.002059877	0.006506707	0.24033113	0.009136039	0.236004636	0.007709517	0.240301692	0.010914232	0.008315492	1	0.026691367	1	0.541007306	ENSG00000213277	3	MARCKSL1P1
VAT	HTN	ENSG00000167840.9	-0.004656698	0.21662735	0.003768934	0.24033506	-0.003975267	0.291891783	0.003771666	0.240301837	-0.000681431	5.72E-06	1	0.519003026	1	0.592804504	ENSG00000213315	4	CTD-3035D6.1
VAT	HTN	ENSG00000168038.6	-0.000664195	0.739947897	0.002001054	0.240335759	-0.000605416	0.762224514	0.002000971	0.24030234	-5.88E-05	8.87E-05	1	0.879146016	1	0.896852841	ENSG00000213347	6	MXD3
VAT	HTN	ENSG00000168056.10	0.003283147	0.3425071	0.003458769	0.240335369	0.003155676	0.361560877	0.00345867	0.240301914	0.000127471	4.56E-05	1	0.643512318	1	0.660428868	ENSG00000213366	8	GSTM2
VAT	HTN	ENSG00000168060.10	-0.002085842	0.736540081	0.006199766	0.240335758	-0.006214181	0.321182499	0.006264094	0.240301899	0.004128339	4.33E-06	1	0.877742066	1	0.619471487	ENSG00000213366	8	GSTM2
VAT	HTN	ENSG00000168116.9	0.002712599	0.606167626	0.005261545	0.240335682	0.003238817	0.538316433	0.005263277	0.240302198	-0.000526218	0.000399581	1	0.815710701	1	0.777474283	ENSG00000213398	3	LCAT
VAT	HTN	ENSG00000168679.13	-0.005918012	0.181641212	0.004430583	0.240334933	-0.005820373	0.188934544	0.004430386	0.240301534	-9.76E-05	0.001839397	1	0.470059822	1	0.48016514	ENSG00000213402	2	PTPRCAP
VAT	HTN	ENSG00000168778.7	0.005556469	0.19078067	0.004247172	0.240334969	0.005644744	0.183809402	0.004246982	0.240301514	-8.83E-05	0.003204568	1	0.483521258	1	0.475904267	ENSG00000213402	2	PTPRCAP
VAT	HTN	ENSG00000168781.17	-0.003669316	0.025213724	0.001639464	0.240333343	-0.003615204	0.027440908	0.001639403	0.240299987	-5.41E-05	4.37E-05	1	0.147535207	1	0.158114629	ENSG00000213402	2	PTPRCAP
VAT	HTN	ENSG00000168936.6	-0.002626178	0.65725503	0.005918729	0.240335716	-0.00279467	0.636790466	0.005918513	0.240302275	0.000168492	0.000465494	1	0.837990221	1	0.833353099	ENSG00000213402	2	PTPRCAP
VAT	HTN	ENSG00000168961.12	0.016646259	0.006548415	0.006122224	0.240332167	0.0167672	0.006167932	0.006122079	0.240298686	-0.00012046	0.004133879	1	0.06063534	1	0.058100659	ENSG00000213402	2	PTPRCAP
VAT	HTN	ENSG00000168993.10	0.001264499	0.048009169	0.005696932	0.240333885	0.01139965	0.045382533	0.005696702	0.24030041	-0.000135151	0.001915366	1	0.220474719	1	0.213136987	ENSG00000213402	2	PTPRCAP
VAT	HTN	ENSG00000169047.5	0.00481416	0.39746812	0.005689471	0.240335458	0.004942494	0.384988176	0.005689247	0.240302013	-0.000128334	0.003767096	1	0.69081361	1	0.675933439	ENSG00000213433	2	RP11-54C4.1
VAT	HTN	ENSG00000169085.7	-0.008274233	0.052702551	0.004270892	0.240333962	-0.008412297	0.048867693	0.004270746	0.240300467	0.000138064	0.000122488	1	0.232362685	1	0.221550272	ENSG00000213443	2	RP11-75L1.2
VAT	HTN	ENSG00000169220.13	0.007767576	0.000794181	0.002315337	0.240330263	0.007202816	0.001907316	0.00232026	0.240297633	0.00056476	0.000232943	1	0.01289377	1	0.025341822	ENSG00000213443	2	RP11-75L1.2
VAT	HTN	ENSG00000169442.4	-0.006666493	0.051203283	0.003419105	0.240333938	-0.006742432	0.048602321	0.003418964	0.240300467	7.59E-05	0.0313021	1	0.229790343	1	0.221550272	ENSG00000213443	2	RP11-75L1.2
VAT	HTN	ENSG00000169592.10	-0.003885747	0.305789362	0.003794311	0.240335296	-0.003823384	0.313597448	0.003794157	0.240301884	-6.24E-05	0.030571288	1	0.607969384	1	0.612903088	ENSG00000213443	2	RP11-75L1.2
VAT	HTN	ENSG00000169609.9	-0.017418799	5.34E-10	0.002805443	0.240316802	-0.017507831	4.35E-10	0.002805345	0.240283179	8.90E-05	0.000148554	1.47E-06	1.23E-07	1.20E-06	1.00E-07	ENSG00000213445	4	SIPA1
VAT	HTN	ENSG00000169683.5	-0.002395254	0.457230195	0.003221956	0.240335541	-0.002458177	0.445474742	0.003221803	0.240302098	6.29E-05	0.003406147	1	0.73258513	1	0.720517323	ENSG00000213445	4	SIPA1
VAT	HTN	ENSG00000169902.9	-0.006018178	0.171907481	0.004405364	0.240334893	-0.005908186	0.179867473	0.004405264	0.240301498	-0.000109992	0.009806566	1	0.45577776	1	0.468776417	ENSG00000213445	4	SIPA1
VAT	HTN	ENSG00000169946.9	0.004132237	0.279150468	0.003818258	0.240335236	0.005867585	0.113989777	0.003895352	0.240301266	-0.001735348	0.02467544	1	0.58589756	1	0.395969331	ENSG00000213462	4	ERV3-1
VAT	HTN	ENSG00000170035.11	-0.007927521	0.241841368	0.006773354	0.240335138	-0.008531538	0.208081349	0.006777191	0.240301603	0.000604017	0.01243452	1	0.552094438	1	0.5074681	ENSG00000213462	4	ERV3-1
VAT	HTN	ENSG00000170144.14	-0.01344031	0.042817426	0.006635579	0.24033379	-0.013320848	0.044691132	0.006635358	0.240300397	-0.000119462	0.034556256	1	0.204346659	1	0.211215515	ENSG00000213462	4	ERV3-1
VAT	HTN	ENSG00000170275.10	0.011592837	0.068298924	0.006359091	0.240334174	0.011461539	0.071475301	0.006358868	0.240300783	0.000131297	0.012706193	1	0.271760451	1	0.278635048	ENSG00000213462	4	ERV3-1
VAT	HTN	ENSG00000170469.6	0.005786894	0.29380511	0.005512315	0.24033527	0.00587843	0.286219139	0.005512129	0.240301824	-9.15E-05	0.049775321	1	0.594939182	1	0.590747324	ENSG00000213465	3	ARL2
VAT	HTN	ENSG00000170486.6	-0.007609429	0.074415732	0.004265249	0.240334244	-0.007544332	0.076916821	0.004265066	0.240300842	-6.51E-05	0.036552122	1	0.288060897	1	0.293421577	ENSG00000213494	5	CCL14
VAT	HTN	ENSG00000170571.7	0.005053555	0.41379378	0.006183713	0.240335484	0.000918024	0.883693236	0.006275412	0.240302374	0.00413553	0.000113309	1	0.70100582	1	0.959100799	ENSG00000213494	5	CCL14
VAT	HTN	ENSG00000170647.2	-0.014835669	0.013840784	0.006027383	0.240332825	-0.015135541	0.012037471	0.006027567	0.240299275	0.000299872	0.000441696	1	0.098709469	1	0.089310271	ENSG00000213494	5	CCL14
VAT	HTN	ENSG00000170915.8	-0.004633802	0.476058973	0.006502158	0.240335563	-0.004476791	0.491131149	0.006502131	0.240302151	-0.000157011	0.034552226	1	0.741910088	1	0.749321156	ENSG00000213494	5	CCL14
VAT	HTN	ENSG00000171084.11	-0.022055487	0.000712534	0.006516169	0.240330163	-0.021779112	0.000831422	0.006516522	0.240296877	-0.000276375	0.006095045	1	0.011705911	1	0.013419446	ENSG00000213523	5	SRA1
VAT	HTN	ENSG00000171084.11	4.02E-05	0.989933694	0.003184621	0.240335813	9.15E-05	0.977087166	0.003184472	0.240302384	-5.13E-05	0.017000679	1	0.996068901	1	0.991091723	ENSG00000213523	5	SRA1

VAT	HTN	ENSG00000171298.8	0.000472997	0.813852852	0.002008834	0.240335786	0.000522978	0.794594905	0.00200806	0.240302351	-5.00E-05	0.002111272	1	0.923869473	1	0.918168712	ENSG00000213523	5	SRA1
VAT	HTN	ENSG00000171357.5	-1.82E-05	0.993335826	0.00218413	0.240335813	4.41E-05	0.983880349	0.00218405	0.240302385	-6.24E-05	0.000454757	1	0.997230388	1	0.995421467	ENSG00000213523	5	SRA1
VAT	HTN	ENSG00000171446.6	0.004003969	0.135003116	0.002678844	0.240334712	0.003956517	0.1396754	0.002678746	0.240301309	4.75E-05	0.029048613	1	0.40220992	1	0.410547502	ENSG00000213588	4	ZBTB9
VAT	HTN	ENSG00000171700.9	-0.01916446	7.92E-09	0.00332121	0.240319393	-0.019110622	8.70E-09	0.003321062	0.240286057	-5.38E-05	0.021818334	2.18E-05	9.93E-07	2.40E-05	1.00E-06	ENSG00000213588	4	ZBTB9
VAT	HTN	ENSG00000171806.7	0.00766827	0.624431221	0.001566295	0.240335695	0.000660508	0.673267769	0.001566314	0.240302297	0.000106319	8.09E-05	1	0.825002475	1	0.850381614	ENSG00000213588	4	ZBTB9
VAT	HTN	ENSG00000172171.6	0.013379949	0.017855387	0.005648858	0.240333046	0.013458818	0.017187301	0.005648595	0.240299585	-7.89E-05	0.039790877	1	0.114606667	1	0.1118796	ENSG00000213676	6	ATF6B
VAT	HTN	ENSG00000172247.3	-0.000994657	0.718138623	0.00275568	0.240335749	-0.000952611	0.729564775	0.002755562	0.240302326	-4.20E-05	0.036689386	1	0.86780324	1	0.878043729	ENSG00000213676	6	ATF6B
VAT	HTN	ENSG00000172322.9	-0.000605746	0.711591755	0.001638394	0.240335746	0.002778239	0.21749675	0.002252846	0.240301635	-0.003383985	0.028648091	1	0.864869003	1	0.517020606	ENSG00000213676	6	ATF6B
VAT	HTN	ENSG00000172432.14	-0.006400211	0.09572193	0.003841755	0.240334445	-0.006295833	0.101243916	0.003841603	0.24030106	-0.000104378	0.000444013	1	0.332536044	1	0.342746569	ENSG00000213676	6	ATF6B
VAT	HTN	ENSG00000172613.3	0.004538348	0.070370302	0.002508035	0.240334198	0.005042081	0.044581296	0.00251026	0.240300395	-0.000503733	4.48E-06	1	0.27741042	1	0.211053818	ENSG00000213676	6	ATF6B
VAT	HTN	ENSG00000172746.6	-0.018706185	0.002761551	0.006249728	0.240331395	-0.018830743	0.002585544	0.006249496	0.240297908	0.000124558	0.01354134	1	0.031626067	1	0.030627042	ENSG00000213676	6	ATF6B
VAT	HTN	ENSG00000172775.12	-0.001948201	0.65335348	0.004337928	0.240335714	-0.001885826	0.663743928	0.004337722	0.240302292	-6.24E-05	0.030087326	1	0.837554855	1	0.847332674	ENSG00000213676	6	ATF6B
VAT	HTN	ENSG00000172780.12	0.00263213	0.626328092	0.005405874	0.240335696	0.002744414	0.611669736	0.00540567	0.240302258	-0.000112284	0.00926801	1	0.825696865	1	0.821992369	ENSG00000213676	6	ATF6B
VAT	HTN	ENSG00000172803.13	0.005016913	0.062860578	0.002697003	0.240334107	-0.008504894	0.032410428	0.003975517	0.240300128	0.013521807	3.67E-06	1	0.25933512	1	0.175054366	ENSG00000213676	6	ATF6B
VAT	HTN	ENSG00000172890.7	0.002573561	0.563726166	0.004457802	0.240335649	0.002677758	0.548031106	0.004457628	0.240302207	-0.000104196	0.002825937	1	0.792605307	1	0.785748494	ENSG00000213689	5	TREX1
VAT	HTN	ENSG00000172922.4	-0.00413982	0.071974018	0.002300812	0.240334217	-0.001522515	0.523983486	0.002389317	0.240302185	-0.002617305	4.93E-05	1	0.282170866	1	0.769652481	ENSG00000213689	5	TREX1
VAT	HTN	ENSG00000172935.8	-0.008477907	0.139312576	0.005734677	0.240334735	-0.008063446	0.159725602	0.005735037	0.24030141	-0.000414461	8.74E-06	1	0.40653371	1	0.439962736	ENSG00000213689	5	TREX1
VAT	HTN	ENSG00000173113.2	-0.000395297	0.918085948	0.003843613	0.240335808	-0.00030403	0.936950593	0.003843468	0.240302382	-9.13E-05	0.002836757	1	0.968477978	1	0.975842882	ENSG00000213719	4	CLIC1
VAT	HTN	ENSG00000173193.9	-0.001740014	0.51683373	0.002684237	0.240335606	-0.001657307	0.53697036	0.002684327	0.240302197	-8.27E-05	0.031922478	1	0.769926105	1	0.776342689	ENSG00000213719	4	CLIC1
VAT	HTN	ENSG00000173214.5	0.006090919	0.289069325	0.005745247	0.240335259	0.006190407	0.281249901	0.005745066	0.240301812	-9.95E-05	0.047601182	1	0.59321241	1	0.584169217	ENSG00000213719	4	CLIC1
VAT	HTN	ENSG00000173442.7	-0.002405998	0.539222312	0.003918629	0.240335627	-0.002929684	0.454940612	0.003920861	0.240302109	0.000523686	0.000186359	1	0.778154916	1	0.727062009	ENSG00000213719	4	CLIC1
VAT	HTN	ENSG00000173727.7	0.008546549	0.014581547	0.003498931	0.240332871	0.006689157	0.057899247	0.003527172	0.240300611	0.001857392	3.29E-05	1	0.102351368	1	0.247754918	ENSG00000213760	6	ATP6V1G2
VAT	HTN	ENSG00000173821.15	-0.00075831	0.909162752	0.00664632	0.240335807	-0.000654093	0.92159992	0.006646033	0.24030238	-0.000104217	0.030944679	1	0.964369407	1	0.972406143	ENSG00000213760	6	ATP6V1G2
VAT	HTN	ENSG00000174080.6	0.000781837	0.811739974	0.003282519	0.240335785	-5.87E-05	0.985761253	0.003287648	0.240302385	0.000840511	7.45E-06	1	0.923115916	1	0.996044499	ENSG00000213760	6	ATP6V1G2
VAT	HTN	ENSG00000174353.13	0.00080549	0.756892811	0.002602015	0.240335766	0.001028699	0.692645053	0.002602534	0.240302308	-0.00022321	0.000218498	1	0.890841859	1	0.861126259	ENSG00000213760	6	ATP6V1G2
VAT	HTN	ENSG00000174446.8	0.012299711	0.062657864	0.006607008	0.240334104	0.012405685	0.060417451	0.006606714	0.240300646	-0.000105974	0.023439592	1	0.259273918	1	0.253180669	ENSG00000213760	6	ATP6V1G2
VAT	HTN	ENSG00000175115.7	-0.000433596	0.924345382	0.004566013	0.240335809	-0.000885392	0.846266771	0.004566607	0.240302366	0.000451796	7.34E-07	1	0.970404433	1	0.942573159	ENSG00000213760	6	ATP6V1G2
VAT	HTN	ENSG00000175575.8	-0.000231161	0.879204412	0.00152102	0.240335802	-0.000181702	0.90490676	0.001520963	0.240302378	-4.95E-05	5.14E-05	1	0.955786877	1	0.967289953	ENSG00000213760	6	ATP6V1G2
VAT	HTN	ENSG00000175643.7	-0.000579453	0.899826106	0.004603181	0.240335806	-0.000711303	0.87719696	0.004603199	0.240302373	0.000131849	0.018023928	1	0.959290439	1	0.9580782	ENSG00000213760	6	ATP6V1G2
VAT	HTN	ENSG00000175826.7	0.023924886	6.79E-05	0.006006033	0.240327988	0.023690121	8.00E-05	0.006005851	0.240294712	0.000234765	9.42E-06	0.187471523	0.001837956	0.220732361	0.002143033	ENSG00000213760	6	ATP6V1G2
VAT	HTN	ENSG00000175879.7	-0.006418707	0.147748138	0.004434236	0.24033478	-0.008463843	0.059998534	0.004500108	0.24030064	0.002045136	0.007823842	1	0.418982277	1	0.252944336	ENSG00000213760	6	ATP6V1G2
VAT	HTN	ENSG00000176155.14	-0.001361826	0.695866371	0.003483762	0.240335738	-0.00129848	0.709343294	0.003483622	0.240302316	-6.33E-05	0.017726044	1	0.859324915	1	0.869462276	ENSG00000213761	4	MT1P1
VAT	HTN	ENSG00000176422.10	-0.001764264	0.65979111	0.004007852	0.240335718	-0.001828184	0.648266056	0.004007658	0.240302282	6.39E-05	0.014604492	1	0.838794779	1	0.840256636	ENSG00000213839	4	RP11-3J10.4
VAT	HTN	ENSG00000176428.5	-0.005929446	0.152222611	0.004141464	0.240334802	-0.007157675	0.084935941	0.004154817	0.240300921	0.001228229	0.000261621	1	0.426965859	1	0.311318987	ENSG00000213839	4	RP11-3J10.4
VAT	HTN	ENSG00000177674.11	0.003171009	0.617101642	0.006342493	0.24033569	0.00328156	0.60486712	0.006342227	0.240302253	-0.00011055	0.018972032	1	0.821428835	1	0.817356612	ENSG00000213901	6	SLC23A3
VAT	HTN	ENSG00000177694.10	0.002428223	0.655996761	0.005451202	0.240335716	0.001896104	0.728060191	0.005453162	0.240302325	0.000532119	0.000860824	1	0.837990221	1	0.877487391	ENSG00000213901	6	SLC23A3
VAT	HTN	ENSG00000178295.10	-0.006435552	0.001457171	0.002021788	0.240330816	-0.006371754	0.001623851	0.002021748	0.240297487	-6.38E-05	0.001526866	1	0.020312084	1	0.022409137	ENSG00000213901	6	SLC23A3
VAT	HTN	ENSG00000178952.4	0.006097784	0.102243874	0.003731644	0.240334496	0.005990542	0.108407625	0.003731507	0.240301114	0.000107242	0.000395373	1	0.342052233	1	0.354088812	ENSG00000213901	6	SLC23A3
VAT	HTN	ENSG00000179528.11	-0.00585013	0.210129989	0.004668967	0.240335039	-0.0057769	0.215959092	0.004668781	0.24030163	-7.32E-05	0.041914948	1	0.508766538	1	0.515497803	ENSG00000213901	6	SLC23A3
VAT	HTN	ENSG00000180113.11	-8.57E-05	0.963481191	0.001870894	0.240335812	-0.000131018	0.944168619	0.001870848	0.240302382	4.54E-05	0.010820104	1	0.983071382	1	0.978965976	ENSG00000213903	4	LTB4R
VAT	HTN	ENSG00000180398.7	0.006821626	0.277451683	0.00628106	0.240335232	0.006664304	0.288663224	0.0062808	0.24030183	0.000157322	0.00082313	1	0.585762318	1	0.59182601	ENSG00000214063	6	TSPAN4
VAT	HTN	ENSG00000180828.1	-0.001572151	0.805245247	0.006376222	0.240335783	-0.001395754	0.826721755	0.006375982	0.240302361	-0.000176397	0.000532716	1	0.918978716	1	0.936295463	ENSG00000214076	3	CPSF1P1
VAT	HTN	ENSG00000180834.3	0.009147231	0.09890578	0.005543156	0.24033447	0.009251129	0.095119962	0.00554295	0.240301011	-0.000103898	0.020124048	1	0.336272805	1	0.332830936	ENSG00000214078	7	CPNE1
VAT	HTN	ENSG00000180979.5	-0.000234365	0.952973161	0.003974068	0.240335812	-0.000317324	0.936355063	0.003973902	0.240302382	8.30E-05	0.005203164	1	0.981080063	1	0.975590779	ENSG00000214078	7	CPNE1
VAT	HTN	ENSG00000181027.6	0.000209659	0.93868973	0.002725788	0.24033581	0.000163248	0.952241153	0.00272568	0.240302383	4.64E-05	0.026885729	1	0.976192895	1	0.98139865	ENSG00000214078	7	CPNE1
VAT	HTN	ENSG00000181092.5	-0.004960634	0.228351489	0.004118015	0.240335098	-0.009114116	0.0343212	0.004306661	0.240300176	0.004153482	0.000994363	1	0.534110263	1	0.180088428	ENSG00000214078	7	CPNE1
VAT	HTN	ENSG00000181773.6	0.005823806	0.109711614	0.003641031	0.240334552	0.005889836	0.105733485	0.003640929	0.240301094	-6.60E-05	0.04662793	1	0.356659663	1	0.348224683	ENSG00000214078	7	CPNE1
VAT	HTN	ENSG00000181826.5	0.005431294	0.402937984	0.006493762	0.240335468	0.004907611	0.449967074	0.006496098	0.240302103	0.000523684	0.005913346	1	0.693767209	1	0.723286064	ENSG00000214087	4	ARL16
VAT	HTN	ENSG00000181915.3	0.026665426	1.17E-09	0.004382104	0.240317553	0.026755312	1.02E-09	0.004381897	0.240284001	-8.99E-05	0.0023042	3.22E-06	1.89E-07	2.82E-06	1.76E-07	ENSG00000214087	4	ARL16
VAT	HTN	ENSG00000182134.11	0.003377409	0.573364927	0.005997852	0.240335657	0.003686567	0.538803069	0.005998081	0.240302198	-0.000309158	0.000449573	1	0.797624595	1	0.777770121	ENSG00000214087	4	ARL16
VAT	HTN	ENSG00000182141.5	-0.007427708	0.021982968	0.003242593	0.240333226	-0.007349713	0.023409675	0.003242489	0.240299851	-7.80E-05	0.005407018	1	0.13482887	1	0.142418781	ENSG00000214087	4	ARL16
VAT	HTN	ENSG00000182511.7	-0.057540774	2.09E-19	0.00638713	0.240295792	-0.057726927	1.60E-19	0.006386854	0.240262105	0.000186153	7.08E-05	5.77E-16	5.77E-16	4.40E-16	4.40E-16	ENSG00000214087	4	ARL16
VAT	HTN	ENSG00000182534.9	-0.004221085	0.065131199	0.002288644	0.240334136	-0.00428129	0.061384494	0.002288575	0.240300659	6.02E-05	0.003683121	1	0.263580805	1	0.255210403	ENSG00000214102	3	WEE2
VAT	HTN	ENSG00000182544.8	-0.004660711	0.390172684	0.005423815	0.240335449	-0.004243724	0.434018602	0.005424439	0.240302083	-0.000416987	6.29E-05	1	0.682680345	1	0.712632987	ENSG00000214135	4	AC024560.3
VAT	HTN	ENSG00000182919.10	-0.000354336	0.827702074	0.00162801	0.24033579	-0.0003843	0.813382154	0.001627942	0.240302357	3.00E-05	0.012851533	1	0.930911869	1	0.928203638	ENSG00000214135	4	AC024560.3
VAT	HTN	ENSG00000182923.12	-0.005919972	0.098579265	0.003583987	0.240334468	-0.005845735	0.102864502	0.003583866	0.240301073	-7.42E-05	0.014218356	1	0.336272805	1	0.344320158	ENSG00000214135	4	AC024560.3
VAT	HTN	ENSG00000182986.8	0.004894115	0.000164036	0.001298578	0.240328808	0.00485425	0.000185348	0.001298545	0.240295494	3.99E-05	0.001109221	0.452738877	0.003801268	0.511559856	0.004298822	ENSG00000214226	4	C17orf67
VAT	HTN	ENSG00000183386.5	-0.000598513	0.895528726	0.004557955	0.240335805	-0.000498019	0.912990065	0.004557778	0.240302379	-0.000100494	0.005023952	1	0.959290439	1	0.968801453	ENSG00000214253	4	FIS1
VAT	HTN	ENSG00000183431.7	-0.001257845	0.661912175	0.002876553	0.240335719	-0.001211554	0.673609318	0.002876435	0.240302297	-4.63E-05	0.033275451	1	0.840330084	1	0.850381614	ENSG00000214279	8	RP11-108K14.4
VAT	HTN	ENSG00000183506.12	-0.00144663	0.635451591	0.003051199	0.240335703	-0.001493876	0.624399173	0.003051058	0.240302267	4.72E-05	0.023593944	1	0.827629557	1	0.826939404	ENSG00000214309	3	MBLAC1
VAT	HTN	ENSG00000183527.7	0.002812206	0.579409088	0.005073908	0.240335662	0.002736756	0.58961033	0.005073693	0.240302241	7.54E-05	0.043845893	1	0.800563876	1	0.809098734	ENSG00000214338	6	SOGA3
VAT	HTN	ENSG00000183628.8	-0.001343136	0.566214866	0.002341445	0.240335651	-0.001299093	0.578997877	0.002341343	0.240302233	-4.40E-05	0.009211404	1	0.79306004	1	0.805054983	ENSG00000214338	6	SOGA3
VAT	HTN	ENSG00000183684.6	0.005809986	0.362898989	0.006385612	0.240335405	0.005644017	0.376753444	0.006385395	0.240302	0.000165969	0.002052332	1	0.665980483	1	0.669772956	ENSG00000214376	5	VSTM5
VAT	HTN	ENSG00000183793.9	-0.002366975	0.160477455	0.001686517	0.240334842	-0.002336766	0.165868376	0.001686459	0.240301438	-3.02E-05	0.033382288	1	0.438197474	1	0.450561028	ENSG00000214376	5	VSTM5
VAT	HTN	ENSG00000184313.15	0.003115626	0.547378574	0.005178096	0.240335635	0.003017489	0.560058845	0.005177976	0.240302217	9.81E-05	0.048961777	1	0.783206863	1	0.793105393	ENSG00000214376	5	VSTM5
VAT	HTN	ENSG00000184428.8	-0.005520617	0.03540402	0.002624226	0.240333631	-0.005579089	0.033500013	0.002624163	0.240300156	5.85E-05	0.01984352	1	0.183674992	1	0.177661645	ENSG00000214376	5	VSTM5
VAT	HTN	ENSG00000184470.15	0.006323494	0.365974146	0.006994699	0.24033541	0.006510823	0.351924986	0.006994396	0.240301957	-0.00018733	0.00022602	1	0.667014755	1	0.651755529	ENSG00000214376	5	VSTM5
VAT	HTN	ENSG00000184674.8	-0.005672047	0.407549316	0.006848522	0.240335475	-0.012849776	0.070070255	0.007093601	0.240300767	0.00717773	0.000104811	1	0.696936974	1	0.275849438	ENSG00000214376	5	VSTM5
VAT	HTN	ENSG00000184949.11	0.006020036	0.157023781	0.004253977	0.240334826	0.005950792	0.161830701	0.004253777	0.24030142	6.92E-05	0.015161346	1	0.435044054	1	0.4435479	ENSG00000214376	5	VSTM5
VAT	HTN	ENSG00000185290.3	0.005549037	0.102498141	0.003398345	0.240334498	0.005605844	0.09901519	0.003398202	0.240301043	-5.68E-05	0.024296637	1	0.342135622	1	0.337802132	ENSG00000214425	2	LRRC37A4P
VAT	HTN	ENSG00000185344.9	-0.001455502	0.825312675	0.006594419	0.240335789	-0.001356058	0.837067945	0.006594153	0.240302364	-9.94E-05	0.048527852	1	0.930119634	1	0.939635856	ENSG00000214425	2	LRRC37A4P
VAT	HTN	ENSG00000185437.9	-0.004080793	0.088229706	0.002393688	0.24033438	-0.004045971	0.090963095	0.002393581	0.240300976	-3.48E-05	0.038783635	1	0.316251933	1	0.323528535	ENSG00000214435	3	AS3MT
VAT	HTN	ENSG00000185627.13	-0.009471652	0.045868679	0.004743879	0.240333847	-0.009394831	0.04764671	0.00474364	0.24030045	-7.68E-05	0.008885855	1	0.214201949	1	0.218810182	ENSG00000214435	3	AS3MT
VAT	HTN	ENSG00000185664.10	-0.001617219	0.630078355	0.003357894	0.240335699	-0.001695632	0.613569386	0.003357785	0.240302259	7.84E-05	0.006724205	1	0.825823207	1	0.823173728	ENSG00000214491	4	SEC14L6
VAT	HTN	ENSG00000185864.12	-0.003030532	0.441785567	0.003939941	0.240335522	-0.002958081	0.452759422	0.003939783	0.240302107	-7.25E-05	0.01664264	1	0.720643123	1	0.724217571	ENSG00000214517	4	PPME1
VAT	HTN	ENSG00000185869.9	0.012426135	0.011649359	0.004925921	0.240332675	0.012511484	0.011084863	0.004925739	0.240299203	-8.53E-05	0.031990576	1	0.087847622	1	0.085698103	ENSG00000214517	4	PPME1
VAT	HTN	ENSG00000186049.4	0.008450482	0.017374739	0.00355258	0.240333023	0.00756262	0.033653066	0.003560203	0.24030016	0.000887862	0.000174169	1	0.112568733	1	0.17793575	ENSG00000214517	4	PPME1
VAT	HTN	ENSG00000186081.7	0.001429409	0.571867313	0.002528567	0.240335656	0.00133013	0.598864565	0.002528599	0.240302248	9.93E-05	0.002182857	1	0.796745978	1	0.814290189	ENSG00000214517	4	PPME1
VAT	HTN	ENSG00000186230.6	-0.001441357	0.424429104	0.00180449	0.240335499	-0.001388946	0.441449996	0.001804418	0.240302093	-5.24E-05	0.000145157	1	0.708665655	1	0.717128893	ENSG00000214530	3	STARD10
VAT	HTN	ENSG00000186417.9	0.00135825	0.462859334	0.001850106	0.240335548	0.011377274	0.000998015	0.003456976	0.240297043	-0.010019024	0.000601844	1	0.735882351	1	0.015650693	ENSG00000214562	9	NUTM2D
VAT	HTN	ENSG00000186715.6	0.00252072	0.625150655	0.005159448	0.240335696	0.002658608	0.606338458	0.005159266	0.240302254	-0.000137887	0.001233547	1	0.825288349	1	0.817934576	ENSG00000214597	4	TMEM249
VAT	HTN	ENSG00000187049.5	0.000371754	0.942532256	0.005156984	0.240335811	0.000179469	0.972237682	0.005156881	0.240302384	0.000192285	0.000179491	1	0.976968793	1	0.989080723	ENSG00000214659	4	KRT8P26
VAT	HTN	ENSG00000187145.10	-0.003299616	0.159109744	0.002343354	0.240334836	-0.003219193	0.169526521	0.002343397	0.240301454	-8.04E-05	0.009707582	1	0.43652375	1	0.454706703	ENSG00000214659	4	KRT8P26
VAT	HTN	ENSG00000187260.11	0.002847698	0.688295471	0.00709849	0.240335734	-0.00249882	0.728593159	0.007201257	0.240302325	0.005346518	1.08E-05	1	0.855718693	1	0.87774645	ENSG00000214659	4	KRT8P26
VAT	HTN	ENSG00000187790.6	-0.009906347	0.096727978	0.005964334	0.240334453	-0.010006367	0.0933927	0.005964088	0.240300997	0.00010002	0.025966556	1	0.333711525	1	0.329200323	ENSG00000214659	4	KRT8P26
VAT	HTN	ENSG00000187942.7	0.003895354	0.51006494	0.005913367	0.240335599	0.002917714	0.622503306	0.005926628	0.240302265	0.00097764	0.015097635	1	0.767061751	1	0.826686941	ENSG00000214694	6	ARHGEF33
VAT	HTN	ENSG00000188404.4	0.000197694	0.974486427	0.006181414	0.240335813	0.000609247	0.921488403	0.006181542	0.24030238	-0.000411553	7.15E-07	1	0.98866831	1	0.972406143	ENSG00000214706	6	IFRD2
VAT	HTN	ENSG00000188529.10	0.009245964	0.031412859	0.004296832	0.240333353	0.009168276	0.032858985	0.004296673	0.240300139	7.77E-05	0.02475004	1	0.171134257	1	0.17644124	ENSG00000214765	4	SEPT7P2
VAT	HTN	ENSG00000188818.8	-0.001856699	0.766651496	0.006256614	0.240335769	-0.001739401	0.780998026	0.006256413	0.240302347	-0.000117299	0.030300362	1	0.896662855	1	0.912597186	ENSG00000214765	4	SEPT7P2
VAT	HTN	ENSG00000188921.12	-0.00870358	0.11402302	0.005513082	0.240334584	-0.008604915	0.118551035	0.005512836	0.240301183	-9.87E-05	0.011236197	1	0.36551286	1	0.372666123	ENSG00000214826	4	DDX12P
VAT	HTN	ENSG00000189144.9	-0.00047557	0.908627747	0.004143461	0.240335807	-0.000544833	0.895381684	0.004143291	0.240302376	6.93E-05	0.026583819	1	0.964197726	1	0.962700992	ENSG00000214872	4	SMTNL1
VAT	HTN	ENSG00000189269.8	0.000165189	0.954359026	0.002886223	0.240335812	-4.94E-06	0.998634087	0.002886523	0.240302385	0.000170131	0.001545377	1	0.981382605	1	0.998848822	ENSG00000214872	4	SMTNL1
VAT	HTN	ENSG00000189367.10	-0.002457661	0.711636777	0.006648455	0.240335746	-0.002273247	0.732400188	0.006648192	0.240302327	-0.000184413	0.000343129	1	0.864869003	1	0.878114224	ENSG00000214914	3	RPL23AP3
VAT	HTN	ENSG00000196072.7	-0.002253794	0.321103442	0.002271526	0.240335265	-0.002199132	0.33296584	0.002271459	0.240301923	-5.47E-05	0.007257627	1	0.624075039	1	0.631637966	ENSG00000214982	6	PARGP1
VAT	HTN	ENSG00000196152.6	0.002099858	0.26149278	0.00187008	0.240335192	0.002045239	0.27408512	0.00187001	0.240301795	5.46E-05	0.000285217	1	0.570001694	1	0.579436835	ENSG00000214982	6	PARGP1
VAT	HTN	ENSG00000196209.8	-0.003127332	0.640054763	0.006687728	0.240335706	-0.003224927	0.629636597	0.006687408	0.24030227	9.76E-05	0.026535127	1	0.829366735	1	0.829101626	ENSG00000215114	3	UBXN2B
VAT	HTN	ENSG00000196220.11	0.009046637	0.140238963	0.006133712	0.240334741	0.01011916	0.09930162	0.006139328	0.240301045	-0.001072523	8.24E-05	1	0.408123093	1	0.338361074	ENSG00000215146	4	RP11-313J2.1
VAT	HTN	ENSG00000196247.7	0.001045309	0.653346821	0.002327471	0.240335714	0.0017432	0.45878199	0.002349072	0.240302114	-0.000695011	0.029360385	1	0.837554855	1	0.728992166	ENSG00000215237	5	RP11-54D18.2
VAT	HTN	ENSG00000196357.7	-0.00533901	0.176857777	0.003953372	0.240334914	-0.005442241	0.168620419	0.003953243	0.24030145	0.000103231	0.002413729	1	0.462241918	1	0.45395764	ENSG00000215305	5	VPS16
VAT	HTN	ENSG00000196693.10	-0.000189113	0.958692025	0.00365118	0.240335812	-0.000275747	0.939766815	0.003651062	0.240302382	8.66E-05	0.006023663	1	0.98228229	1	0.977875185	ENSG00000215399	2	HMGB3P7
VAT	HTN	ENSG00000196912.8	-0.010883558	0.01068144	0.004263111	0.240332599	-0.010820128	0.011142815	0.004262918	0.240299208	-6.34E-05	0.031977592	1	0.083044439	1	0.085905504	ENSG00000215399	2	HMGB3P7
VAT	HTN	ENSG00000196966.3	0.017659445	7.96E-08	0.003289737	0.240321602	0.017547845	9.62E-08	0.003289893	0.240288356	0.0001116	0.026340207	0.000219786	6.66E-06	0.000265476	8.30E-06	ENSG00000215452	3	ZNF663P
VAT	HTN	ENSG00000197062.7	0.004644433	0.028490525	0.002120289	0.240333447	0.006047241	0.006704325	0.00223047	0.24029876	-0.001402808	0.042893902	1	0.16246663	1	0.061679788	ENSG00000215452	3	ZNF663P
VAT	HTN	ENSG00000197128.7	-0.007346752	0.037069266	0.003523612	0.240333669	-0.007284567	0.038691987	0.003523449	0.240300277	-6.22E-05	0.009830012	1	0.187472424	1	0.193570362	ENSG00000215452	3	ZNF663P
VAT	HTN	ENSG00000197165.6	0.027863242	1.53E-05	0.006444141	0.240326961	0.027713692	1.70E-05	0.006443927	0.240293264	0.00014955	0.006326786	0.042334961	0.000549805	0.046990952	0.000594822	ENSG00000215464	4	AP000354.2
VAT	HTN	ENSG00000197168.7	0.002544655	0.499046409	0.003764331	0.240335588	0.002641906	0.482770441	0.003764181	0.240302142	-9.73E-05	0.000817392	1	0.755550241	1	0.74386019	ENSG00000215527	3	AP005482.1
VAT	HTN	ENSG00000197181.7	-0.003421343	0.593849942	0.006415831	0.240335673	-0.003295591	0.607471408	0.006415569	0.240302255	-0.000125751	0.009595994	1	0.808777562	1	0.818845598	ENSG00000215790	2	SLC35E2
VAT	HTN	ENSG00000197479.4	-0.00815336	0.004352562	0.002859399	0.240331803	-0.008101643	0.004605358	0.00285938	0.240298426	-5.17E-05	0.037144118	1	0.045161918	1	0.047053283	ENSG00000215908	5	CROCCP2
VAT	HTN	ENSG00000197816.9	0.001881919	0.70518472	0.004974264	0.240335743	0.001802549	0.717061351	0.00497407	0.24030232	7.94E-05	0.041203891	1	0.861817357	1	0.872614342	ENSG00000215908	5	CROCCP2
VAT	HTN	ENSG00000197951.4	0.00304547	0.528903433	0.004836527	0.240335618	0.002923091	0.545578216	0.004836354	0.240302205	0.000122379	0.002096408	1	0.774633714	1	0.78353797	ENSG00000215908	5	CROCCP2
VAT	HTN	ENSG00000197958.8	-0.002649272	0.279871088	0.002451645	0.240335237	-0.00257432	0.293683608	0.002451556	0.240301841	-7.50E-05	0.000168678	1	0.586749061	1	0.592989502	ENSG00000215915	5	ATAD3C
VAT	HTN	ENSG00000198039.7	-0.007737309	0.253565126	0.006776804	0.240335177	-0.006031581	0.378136058	0.006823251	0.240302002	-0.001723727	0.030926826	1	0.563738197	1	0.669868754	ENSG00000216775	2	RP1-152L7.5
VAT	HTN	ENSG00000198466.8	-0.001277925	0.813000816	0.005402194	0.240335786	-0.00135789	0.801527662	0.005401944	0.240302354	8.00E-05	0.030023692	1	0.923788494	1	0.923295638	ENSG00000216901	1	AL022393.7
VAT	HTN	ENSG00000198478.6	0.001859277	0.631036646	0.003871327	0.2403357	0.001942929	0.61573738	0.003871141	0.240302261	-8.37E-05	0.000955662	1	0.825823207	1	0.823768865	ENSG00000216901	1	AL022393.7
VAT	HTN	ENSG00000198569.5	0.004886912	0.281597719	0.004538626	0.240335242	0.004804547	0.289765362	0.004538424	0.240301832	8.24E-05	0.01052396	1	0.586749061	1	0.59182601	ENSG00000216901	1	AL022393.7
VAT	HTN	ENSG00000198646.9	-0.010290886	0.003587202	0.003533516	0.24033163	-0.010396593	0.003259267	0.003533637	0.240298116	0.000105707	0.037997993	1	0.03843057	1	0.036419338	ENSG00000216901	1	AL022393.7
VAT	HTN	ENSG00000198677.6	-0.004389109	0.395388272	0.005164343	0.240335457	-0.004295693	0.405505733	0.005164153	0.240302044	-9.34E-05	0.024981871	1	0.688065342	1	0.690868103	ENSG00000216901	1	AL022393.7
VAT	HTN	ENSG00000198885.5	-0.006022139	0.035506578	0.002864222	0.240333633	-0.005943596	0.037969209	0.002864118	0.240300261	-7.85E-05	0.000742213	1	0.183861456	1	0.191644541	ENSG00000216901	1	AL022393.7
VAT	HTN	ENSG00000203791.9	-0.00615576	0.284128533	0.005747181	0.240335248	-0.00633377	0.270416033	0.005746968	0.240301786	0.000178009	0.000125485	1	0.589701944	1	0.575885996	ENSG00000216906	2	RP11-350J20.9
VAT	HTN	ENSG00000203871.4	-0.006279243	0.117241341	0.004008553	0.240334603	-0.006185729	0.122784924	0.004008399	0.24030121	-9.35E-05	0.003091358	1	0.369484015	1	0.379153311	ENSG00000217930	3	PAM16
VAT	HTN	ENSG00000203872.6	-0.004823825	0.139340902	0.003263195	0.240334736	-0.004751378	0.145363712	0.00326307	0.240301339	-7.24E-05	0.004939647	1	0.40653371	1	0.41661874	ENSG00000217930	3	PAM16
VAT	HTN	ENSG00000204169.6	-0.000255506	0.923156692	0.002648876	0.240335809	-0.00018895	0.943131677	0.002648799	0.240302382	-6.66E-05	0.005236667	1	0.970079269	1	0.978587755	ENSG00000218422	2	AC016773.1
VAT	HTN	ENSG00000204428.8	0.005001029	0.020841019	0.002164142	0.24033318	0.006046108	0.006411267	0.002217956	0.24029872	-0.001045079	0.031630859	1	0.129552281	1	0.05937952	ENSG00000218537	1	AP000350.4
VAT	HTN	ENSG00000204525.10	-0.010028822	5.66E-08	0.001847115	0.240321275	-0.01185149	2.65E-09	0.001991068	0.240284914	0.001822668	0.014248766	0.000156089	5.34E-06	7.30E-06	4.06E-07	ENSG00000218537	1	AP000350.4
VAT	HTN	ENSG00000204531.11	-0.003601571	0.000931258	0.001087913	0.240330408	-0.00413257	0.000224101	0.001119876	0.24029567	0.000530998	0.045881742	1	0.014521318	0.618518387	0.005028605	ENSG00000218537	1	AP000350.4
VAT	HTN	ENSG00000204536.9	-0.002542766	0.072780324	0.001417207	0.240334226	-0.004114244	0.00847294	0.001562789	0.240298967	0.001571478	0.017076411	1	0.283719908	1	0.072176896	ENSG00000219159	3	AC011298.2
VAT	HTN	ENSG00000204538.3	0.004332237	0.002686284	0.001443337	0.24033137	0.003735061	0.010869582	0.001466528	0.240299186	0.000597176	0.021786046	1	0.031021521	1	0.084874033	ENSG00000219159	3	AC011298.2
VAT	HTN	ENSG00000204574.8	0.000250821	0.943871352	0.003562546	0.240335811	-0.001695903	0.642355561	0.003651751	0.240302278	0.001946724	0.015378186	1	0.976968793	1	0.837994779	ENSG00000219159	3	AC011298.2
VAT	HTN	ENSG00000204592.5	-0.009132938	0.121515264	0.005898126	0.240334631	-0.011021815	0.063387544	0.005936982	0.240300685	0.001888877	0.005594544	1	0.376410917	1	0.261118836	ENSG00000219201	3	RP11-181C21.4
VAT	HTN	ENSG00000204622.6	0.003473645	0.069661425	0.001914793	0.24033419	0.002677528	0.167776795	0.001941103	0.240301446	0.000796117	0.012653765	1	0.276243583	1	0.452652937	ENSG00000219392	1	RP1-265C24.5
VAT	HTN	ENSG00000204632.7	0.002204295	0.535207092	0.00355487	0.240335624	0.000580154	0.871870034	0.003597102	0.240302372	0.001624141	0.003211848	1	0.777236628	1	0.956043422	ENSG00000219392	1	RP1-265C24.5
VAT	HTN	ENSG00000204644.5	-0.001869038	0.076221151	0.001054137	0.240334263	-0.00276715	0.014347312	0.001130161	0.240299428	0.000898113	0.02760349	1	0.292622935	1	0.100249572	ENSG00000219392	1	RP1-265C24.5
VAT	HTN	ENSG00000204677.6	0.002615879	0.610335882	0.005133257	0.240335685	0.002727113	0.595221985	0.005133057	0.240302246	-0.000111234	0.005706794	1	0.819230676	1	0.814079622	ENSG00000219392	1	RP1-265C24.5
VAT	HTN	ENSG00000204709.4	-0.004503023	0.131139267	0.002982871	0.240334689	-0.006514285	0.038714072	0.003151232	0.240300277	0.002011262	0.047939319	1	0.397304475	1	0.193570362	ENSG00000219392	1	RP1-265C24.5
VAT	HTN	ENSG00000204792.2	0.001468889	0.649717037	0.003234329	0.240335712	0.001532506	0.635613697	0.003234222	0.240302274	-6.36E-05	0.021450448	1	0.835367368	1	0.832512881	ENSG00000219392	1	RP1-265C24.5
VAT	HTN	ENSG00000204920.6	-0.008368532	0.099113023	0.005074383	0.240334472	-0.008230915	0.104780221	0.0050742	0.240301087	-0.000137617	0.000876758	1	0.336272805	1	0.345925131	ENSG00000219481	6	NBPF1
VAT	HTN	ENSG00000204947.4	-0.007739447	0.11269989	0.004879309	0.240334573	-0.007664409	0.116213047	0.004879079	0.240301168	-7.50E-05	0.021349466	1	0.363803153	1	0.368868078	ENSG00000219481	6	NBPF1

VAT	HTN	ENSG00000205038.7	0.004083121	0.520287618	0.006351072	0.24033561	0.008029803	0.213333156	0.006452471	0.240301621	-0.003946682	0.00054785	1	0.77245499	1	0.513786658	ENSG00000219481	6	NBPF1
VAT	HTN	ENSG00000205047.3	0.003526801	0.529333038	0.005606775	0.240335618	0.003353297	0.54977256	0.00560657	0.240302208	0.000173504	0.00013398	1	0.774633714	1	0.786610816	ENSG00000219481	6	NBPF1
VAT	HTN	ENSG00000205269.4	0.017365668	0.004838097	0.006163211	0.240331898	0.017507615	0.004500833	0.006163009	0.240298405	-0.000141947	0.007305415	1	0.048912633	1	0.046525467	ENSG00000219481	6	NBPF1
VAT	HTN	ENSG00000205531.8	0.005691825	0.350282548	0.006093755	0.240335383	0.005782446	0.342644232	0.006093491	0.240301941	-9.06E-05	0.039909109	1	0.654113554	1	0.641030042	ENSG00000219626	4	FAM228B
VAT	HTN	ENSG00000205565.6	-0.004717385	0.005422116	0.001696391	0.240332	-0.004684917	0.005748297	0.001696317	0.240298623	-3.25E-05	0.008084191	1	0.053256368	1	0.055667722	ENSG00000219755	1	RP1-199J3.5
VAT	HTN	ENSG00000205572.5	0.004686333	0.321308199	0.004725201	0.240335328	0.004776426	0.312077554	0.004725049	0.240301881	-9.01E-05	0.0274413	1	0.624075039	1	0.610875211	ENSG00000219755	1	RP1-199J3.5
VAT	HTN	ENSG00000205578.4	-0.001451551	0.61353613	0.002874173	0.240335688	-0.002081175	0.469839095	0.002879556	0.240302127	0.000629624	0.000443699	1	0.819907808	1	0.73755282	ENSG00000219755	1	RP1-199J3.5
VAT	HTN	ENSG00000205710.3	0.003588121	0.310646819	0.003539046	0.240335306	0.003533592	0.318036889	0.003538896	0.240301893	5.45E-05	0.036681037	1	0.613294148	1	0.616693002	ENSG00000219755	1	RP1-199J3.5
VAT	HTN	ENSG00000205771.2	-0.00347257	0.310026532	0.003420687	0.240335305	-0.003546982	0.299757307	0.003420574	0.240301855	7.44E-05	0.010997481	1	0.612581159	1	0.599080498	ENSG00000219891	2	ZSCAN12P1
VAT	HTN	ENSG00000206013.2	0.001871417	0.588003688	0.003454509	0.240335669	0.001938712	0.574641971	0.003454406	0.240302229	-6.73E-05	0.029127825	1	0.806605456	1	0.801420839	ENSG00000219891	2	ZSCAN12P1
VAT	HTN	ENSG00000206113.3	0.011588071	0.085625026	0.006741386	0.240334356	0.01169174	0.082847466	0.00674108	0.240300901	-0.000103669	0.027145441	1	0.31229706	1	0.306924839	ENSG00000219891	2	ZSCAN12P1
VAT	HTN	ENSG00000212694.4	-0.004641565	0.173922689	0.003413646	0.240334902	-0.004587892	0.178935871	0.003413507	0.240301494	-5.37E-05	0.037499779	1	0.458916465	1	0.46743134	ENSG00000219891	2	ZSCAN12P1
VAT	HTN	ENSG00000213171.2	-0.001707907	0.721056445	0.004783418	0.240335751	-0.00203083	0.671295962	0.004785513	0.240302296	0.000322923	0.034139207	1	0.869815229	1	0.850122986	ENSG00000219891	2	ZSCAN12P1
VAT	HTN	ENSG00000213221.4	0.000284484	0.854926947	0.001555953	0.240335797	0.000259357	0.867611877	0.001555897	0.240302371	2.51E-05	0.048847385	1	0.944984744	1	0.953267827	ENSG00000220267	1	ACTBP8
VAT	HTN	ENSG00000213402.2	-0.000385183	0.883261301	0.002623216	0.240335803	-0.001220661	0.642465241	0.00262929	0.240302278	0.000835478	4.05E-06	1	0.956145095	1	0.837994779	ENSG00000220583	1	RPL35P2
VAT	HTN	ENSG00000213494.5	-0.001997634	0.36740385	0.002216274	0.240335413	-0.002050604	0.354828166	0.002216232	0.240301963	5.30E-05	0.017710741	1	0.667567233	1	0.6537555	ENSG00000220583	1	RPL35P2
VAT	HTN	ENSG00000214826.4	-0.004958106	0.415417659	0.006088066	0.240335486	-0.005292302	0.384799144	0.006089486	0.240302012	0.000334197	0.025748006	1	0.701684662	1	0.675933439	ENSG00000220583	1	RPL35P2
VAT	HTN	ENSG00000214872.4	-0.00287536	0.242659365	0.002461008	0.24033514	-0.002830038	0.250143769	0.002460899	0.240301733	-4.53E-05	0.009523232	1	0.552590633	1	0.556098166	ENSG00000220583	1	RPL35P2
VAT	HTN	ENSG00000215399.2	-0.002233607	0.745028741	0.006868378	0.240335761	-0.001969414	0.774306587	0.006868136	0.240302344	-0.000264193	3.34E-06	1	0.881766434	1	0.907082419	ENSG00000221823	6	PPP3R1
VAT	HTN	ENSG00000215464.4	-0.021562122	0.000904797	0.006497374	0.240330382	-0.021754908	0.000813093	0.006497261	0.240296856	0.000192786	0.003753613	1	0.01418886	1	0.013200804	ENSG00000221823	6	PPP3R1
VAT	HTN	ENSG00000218422.2	0.01707219	0.000311983	0.004735478	0.240329403	0.017156351	0.000291116	0.00473526	0.240295912	-8.42E-05	0.009791397	0.86107294	0.006573076	0.803478988	0.006228519	ENSG00000221829	5	FANCG
VAT	HTN	ENSG00000218537.1	0.0025189	0.2738068	0.002301755	0.240335223	0.003121343	0.176113631	0.002307285	0.240301482	-0.000602442	0.000199644	1	0.583904713	1	0.463882578	ENSG00000221829	5	FANCG
VAT	HTN	ENSG00000219481.6	0.001108461	0.732893569	0.003247958	0.240335756	0.001164168	0.720010025	0.003247825	0.240302321	-5.57E-05	0.022728013	1	0.875286132	1	0.872943483	ENSG00000221829	5	FANCG
VAT	HTN	ENSG00000219755.1	0.000532998	0.816649465	0.002298825	0.240335787	0.000483997	0.833240059	0.002298749	0.240302363	4.90E-05	0.012373129	1	0.925513978	1	0.939506392	ENSG00000221838	5	AP4M1
VAT	HTN	ENSG00000220583.1	-0.012732544	0.060606419	0.006785767	0.240334077	-0.013232556	0.051236827	0.006787682	0.240300511	0.000500013	0.005487428	1	0.252880519	1	0.227450279	ENSG00000221923	4	ZNF880
VAT	HTN	ENSG00000221944.3	0.000751682	0.878460173	0.00491547	0.240335802	0.00087736	0.85833472	0.004915333	0.240302369	-0.000125678	0.005120393	1	0.955786877	1	0.949120123	ENSG00000221944	3	TIGD1
VAT	HTN	ENSG00000221983.3	0.004597802	0.117778725	0.002939455	0.240334607	0.004539018	0.12253127	0.002939325	0.240301209	5.88E-05	0.004926472	1	0.369484015	1	0.379153311	ENSG00000221944	3	TIGD1
VAT	HTN	ENSG00000223861.1	-0.007343489	0.267270402	0.006619503	0.240335206	-0.007142141	0.280605369	0.006619458	0.240301811	-0.000201349	0.006579369	1	0.573612994	1	0.584169217	ENSG00000221944	3	TIGD1
VAT	HTN	ENSG00000224287.2	-0.002543077	0.461560368	0.003453952	0.240335546	-0.00247901	0.472903132	0.003453795	0.240302131	-6.41E-05	0.007628621	1	0.734663562	1	0.739078508	ENSG00000221944	3	TIGD1
VAT	HTN	ENSG00000224316.1	0.003126207	0.171811682	0.002287902	0.240334893	0.002645503	0.250205792	0.002300736	0.240301733	0.000480703	0.048970577	1	0.45577776	1	0.556098166	ENSG00000221947	3	XKR9
VAT	HTN	ENSG00000224769.1	4.75E-05	0.988476015	0.003286995	0.240335813	0.000149583	0.963701956	0.003286922	0.240302384	-0.000102107	0.001374072	1	0.995476191	1	0.985245375	ENSG00000221947	3	XKR9
VAT	HTN	ENSG00000225241.3	-0.001114555	0.865820699	0.006596191	0.240335799	-0.001228709	0.852223003	0.006595932	0.240302368	0.000114154	0.026235555	1	0.950651672	1	0.94611263	ENSG00000221947	3	XKR9
VAT	HTN	ENSG00000225282.1	0.003162116	0.291232904	0.002996068	0.240335264	0.003978951	0.185288948	0.003003782	0.240301519	-0.000816835	0.000179621	1	0.59321241	1	0.477049902	ENSG00000221955	6	SLC12A8
VAT	HTN	ENSG00000226002.1	0.001664884	0.256911553	0.001468511	0.240335179	0.001506968	0.305345484	0.001470157	0.240301867	0.000157916	0.02755063	1	0.566122845	1	0.604212978	ENSG00000221955	6	SLC12A8
VAT	HTN	ENSG00000226180.2	0.00439329	0.318975857	0.00440843	0.240335324	0.004267775	0.332983058	0.004408295	0.240301923	0.000125516	0.001219847	1	0.622410789	1	0.631637966	ENSG00000221983	3	UBA52
VAT	HTN	ENSG00000226278.1	0.005245407	0.05392571	0.002721441	0.240333981	0.005302861	0.051341132	0.002721339	0.240300512	-5.75E-05	0.008445705	1	0.236976525	1	0.227450279	ENSG00000221983	3	UBA52
VAT	HTN	ENSG00000226328.2	0.010382196	0.084373828	0.006015677	0.240334344	0.010222129	0.0892613	0.006015456	0.240300961	0.000160067	0.001011233	1	0.309258654	1	0.321201027	ENSG00000221988	8	PPT2
VAT	HTN	ENSG00000226383.1	-0.010457598	0.055029183	0.005450497	0.240333998	-0.010651246	0.05067879	0.005450453	0.240300502	0.000193647	0.001370539	1	0.239559218	1	0.226332458	ENSG00000221988	8	PPT2
VAT	HTN	ENSG00000227113.2	0.002615101	0.200498839	0.002042835	0.240335005	0.002508335	0.219546431	0.002043057	0.240301641	0.000106766	0.005594471	1	0.497193887	1	0.517020606	ENSG00000221988	8	PPT2
VAT	HTN	ENSG00000228022.1	0.004782018	0.327808853	0.004886901	0.240335341	0.009675759	0.07202152	0.005378452	0.240300789	-0.004893742	0.029419384	1	0.627864285	1	0.27997098	ENSG00000221995	4	TIAF1
VAT	HTN	ENSG00000228049.5	-0.000682676	0.72625818	0.001949905	0.240335753	-0.000717995	0.712698728	0.001949832	0.240302318	3.53E-05	0.023815894	1	0.873302353	1	0.869990486	ENSG00000222009	4	BTBD19
VAT	HTN	ENSG00000228050.1	-0.007529021	0.288648083	0.007095533	0.240335258	-0.007379259	0.298329346	0.007095279	0.240301851	-0.000149762	0.010179021	1	0.59321241	1	0.597477868	ENSG00000222009	4	BTBD19
VAT	HTN	ENSG00000228065.6	-0.000738872	0.684660984	0.001819382	0.240335732	-0.00079155	0.663502869	0.001819312	0.240302291	5.27E-05	0.000234959	1	0.853121587	1	0.847332674	ENSG00000222019	3	URAHP
VAT	HTN	ENSG00000228075.4	0.004393562	0.469880674	0.006079592	0.240335556	0.00428714	0.480685626	0.006079319	0.24030214	0.000106421	0.012636454	1	0.740158428	1	0.743661619	ENSG00000222019	3	URAHP
VAT	HTN	ENSG00000228914.1	-0.006376123	0.054092468	0.00331038	0.240333984	-0.006429018	0.052116881	0.003310221	0.240300525	5.29E-05	0.015126787	1	0.236976525	1	0.229414021	ENSG00000222019	3	URAHP
VAT	HTN	ENSG00000230224.1	-0.004585717	0.239148421	0.003895711	0.24033513	-0.00471889	0.225787575	0.003895779	0.240301661	0.000133173	0.009517643	1	0.549661061	1	0.526616698	ENSG00000222019	3	URAHP
VAT	HTN	ENSG00000230438.5	0.006730569	0.079011877	0.003831911	0.240334292	0.006651911	0.082564498	0.003831746	0.240300899	7.87E-05	0.004713849	1	0.297599038	1	0.306698747	ENSG00000222019	3	URAHP
VAT	HTN	ENSG00000230712.2	-0.002181707	0.656119633	0.004899661	0.240335716	-0.00104919	0.830858768	0.004911929	0.240302362	-0.001132517	0.001281953	1	0.837990221	1	0.939438836	ENSG00000223396	2	RPS10P7
VAT	HTN	ENSG00000230836.1	0.003410427	0.414815607	0.004182268	0.240335485	0.003479807	0.405371172	0.00418212	0.240302043	-6.94E-05	0.044843908	1	0.701684662	1	0.690868103	ENSG00000223473	1	GS1-124K5.3
VAT	HTN	ENSG00000230939.1	0.003062774	0.435670316	0.003929021	0.240335514	0.003154366	0.422051474	0.003928872	0.240302067	-9.16E-05	0.003498443	1	0.715318306	1	0.704580121	ENSG00000223496	1	EXOSC6
VAT	HTN	ENSG00000231236.2	-0.005293765	0.153339724	0.003707544	0.240334808	-0.005228297	0.158471817	0.003707402	0.240301404	-6.55E-05	0.024853407	1	0.429662578	1	0.437382215	ENSG00000223496	1	EXOSC6
VAT	HTN	ENSG00000231698.2	-0.000815568	0.880263996	0.005414216	0.240335802	-0.001053469	0.845721874	0.005414065	0.240302366	0.0002379	1.54E-06	1	0.955786877	1	0.942573159	ENSG00000223496	1	EXOSC6
VAT	HTN	ENSG00000231784.4	0.005763311	0.400336113	0.006852749	0.240335465	0.005636578	0.41075917	0.00685249	0.240302051	0.000126734	0.02025869	1	0.692212916	1	0.69398935	ENSG00000223509	4	RP11-632K20.7
VAT	HTN	ENSG00000231793.4	0.002251613	0.664420259	0.005190217	0.240335721	0.00039005	0.940303675	0.005208428	0.240302382	0.001861564	2.27E-05	1	0.842351822	1	0.977875185	ENSG00000223572	5	CKMT1A
VAT	HTN	ENSG00000231925.7	0.007344656	0.094007816	0.004385843	0.24033443	0.008063247	0.066399267	0.00439241	0.240300723	-0.000718591	0.003442236	1	0.328017159	1	0.266756879	ENSG00000223718	2	AC093107.7
VAT	HTN	ENSG00000232075.1	0.027443794	4.55E-06	0.005986222	0.240325448	0.027344422	4.92E-06	0.005985969	0.240292095	9.94E-05	0.02419142	0.012563585	0.000199422	0.01358799	0.000219161	ENSG00000223756	2	TSSC2
VAT	HTN	ENSG00000232546.1	0.01055131	0.066215086	0.005743869	0.240334149	0.009497868	0.099441383	0.00576477	0.240301046	0.001053441	0.03334604	1	0.26718368	1	0.338419502	ENSG00000223756	2	TSSC2
VAT	HTN	ENSG00000232615.4	0.002267275	0.642503177	0.004884241	0.240335707	0.002148061	0.660072454	0.004884028	0.240302289	0.000119214	0.000705358	1	0.830978804	1	0.846845836	ENSG00000223756	2	TSSC2
VAT	HTN	ENSG00000232653.4	0.005175886	0.138799763	0.003496564	0.240334733	0.005121339	0.142993487	0.003496424	0.240301327	5.45E-05	0.041466148	1	0.40653371	1	0.412957063	ENSG00000223756	2	TSSC2
VAT	HTN	ENSG00000232926.1	0.005312546	0.228958019	0.004415903	0.2403351	0.005435135	0.218375147	0.004415715	0.240301638	-0.000122589	0.000168455	1	0.534622786	1	0.517020606	ENSG00000223823	1	RP11-465B22.5
VAT	HTN	ENSG00000233265.1	-0.002360722	0.155499061	0.001662041	0.240334818	-0.002618481	0.115969944	0.001665786	0.240301166	0.000257758	0.022966865	1	0.431768017	1	0.368752358	ENSG00000223861	1	RP11-74C1.2
VAT	HTN	ENSG00000233297.4	0.004110256	0.522366394	0.006425263	0.240335612	0.00434875	0.498507771	0.006425077	0.240302159	-0.000238493	3.89E-05	1	0.772678586	1	0.754734749	ENSG00000223861	1	RP11-74C1.2
VAT	HTN	ENSG00000233448.2	-0.007946409	0.047224078	0.004004661	0.240333871	-0.00787698	0.049180974	0.004004516	0.240300477	-6.94E-05	0.033301338	1	0.21807219	1	0.222523752	ENSG00000223865	6	HLA-DPB1
VAT	HTN	ENSG00000233569.1	-0.011394144	0.104281574	0.007014177	0.240334512	-0.011295114	0.107311227	0.007013856	0.240301106	-9.90E-05	0.040377097	1	0.346052657	1	0.351756515	ENSG00000223865	6	HLA-DPB1
VAT	HTN	ENSG00000234232.2	-0.005351621	0.376358427	0.006049581	0.240335427	-0.005789419	0.338756124	0.006051909	0.240301933	0.000437798	0.016396846	1	0.672329618	1	0.637283706	ENSG00000223865	6	HLA-DPB1
VAT	HTN	ENSG00000234444.5	0.003159374	0.086384816	0.001842429	0.240334363	0.002911905	0.114800874	0.001846508	0.240301158	0.000247469	0.046959232	1	0.314113273	1	0.366605303	ENSG00000223865	6	HLA-DPB1
VAT	HTN	ENSG00000235231.3	-0.002830051	0.540153286	0.004619876	0.240335628	-0.007841236	0.102379388	0.004800475	0.240301069	0.005011185	0.000123805	1	0.778497687	1	0.344320158	ENSG00000223865	6	HLA-DPB1
VAT	HTN	ENSG00000235421.1	0.00270712	0.372837117	0.003037706	0.240335422	0.002460968	0.418105976	0.003039312	0.240302061	0.000246152	0.019141443	1	0.670600511	1	0.702429993	ENSG00000224132	2	AC112715.2
VAT	HTN	ENSG00000236209.1	0.003394712	0.377578613	0.003847278	0.240335429	0.003457367	0.368820555	0.003847137	0.240301987	-6.27E-05	0.045415481	1	0.673387034	1	0.664239406	ENSG00000224183	1	SDHDP6
VAT	HTN	ENSG00000236397.2	0.009007688	5.90E-05	0.002242488	0.240327856	0.008955716	6.50E-05	0.002242438	0.240294519	5.20E-05	0.016895311	0.162827818	0.001690495	0.179533751	0.001777562	ENSG00000224287	2	MSL3P1
VAT	HTN	ENSG00000236611.1	0.005201828	0.390315067	0.006055348	0.240335449	0.005083667	0.401149707	0.006055081	0.240302037	0.000118161	0.006268026	1	0.682680345	1	0.687928367	ENSG00000224287	2	MSL3P1
VAT	HTN	ENSG00000237190.3	-0.00379238	0.364427887	0.004181414	0.240335408	-0.003869335	0.354756377	0.00418124	0.240301962	7.70E-05	0.013781169	1	0.667014755	1	0.6537555	ENSG00000224316	1	RP11-479O9.2
VAT	HTN	ENSG00000237310.1	0.001186644	0.543785539	0.001954614	0.240335632	0.000570236	0.773208007	0.00197874	0.240302344	0.000616408	0.045996696	1	0.780738853	1	0.906180085	ENSG00000224316	1	RP11-479O9.2
VAT	HTN	ENSG00000237510.3	0.005838158	0.011991111	0.002323723	0.2403327	0.005798236	0.01258385	0.002323622	0.240299314	3.99E-05	0.017244781	1	0.089447203	1	0.090807843	ENSG00000224316	1	RP11-479O9.2
VAT	HTN	ENSG00000237753.1	0.00724784	0.016881635	0.003033478	0.240332998	0.007151856	0.018391535	0.003033471	0.240299644	9.60E-05	0.006355731	1	0.11097825	1	0.11723011	ENSG00000224389	4	C4B
VAT	HTN	ENSG00000237988.2	0.001133382	0.807392891	0.004648993	0.240335784	0.000414136	0.929191071	0.004660404	0.240302381	0.000719246	0.029540705	1	0.920447905	1	0.973666067	ENSG00000224389	4	C4B
VAT	HTN	ENSG00000238243.2	-0.010907096	0.107271091	0.006772138	0.240334534	-0.011091849	0.101441386	0.006771958	0.240301062	0.000184753	0.00329264	1	0.351624954	1	0.342746569	ENSG00000224389	4	C4B
VAT	HTN	ENSG00000239257.1	-0.000333073	0.780921476	0.001197592	0.240335775	-0.000938372	0.447739513	0.001236018	0.240302101	0.000605299	0.048010277	1	0.903724293	1	0.721401667	ENSG00000224389	4	C4B
VAT	HTN	ENSG00000239713.3	-0.001410657	0.383459861	0.00161858	0.240335439	-0.001386637	0.391591095	0.001618511	0.240302023	-2.40E-05	0.042329491	1	0.67626148	1	0.68145739	ENSG00000224389	4	C4B
VAT	HTN	ENSG00000240356.2	0.011265955	0.011045019	0.00443318	0.240332628	0.011147013	0.011919533	0.004433045	0.240299267	0.000118943	0.002417147	1	0.084345828	1	0.088913277	ENSG00000224389	4	C4B
VAT	HTN	ENSG00000240399.1	-0.012079303	0.000356163	0.003382965	0.240329526	-0.012143521	0.000331028	0.003382832	0.24029603	6.42E-05	0.014859072	0.983010846	0.007252285	0.91363702	0.006869451	ENSG00000224389	4	C4B
VAT	HTN	ENSG00000240563.1	-0.006194362	0.195572146	0.004785982	0.240334987	-0.006315628	0.186949594	0.004785804	0.240301526	0.000121266	0.001642443	1	0.491154798	1	0.477318114	ENSG00000224429	3	LINC00422
VAT	HTN	ENSG00000240771.2	0.00263214	0.626404357	0.00540709	0.240335697	0.002776372	0.607607168	0.005406842	0.240302255	-0.000144232	0.000104148	1	0.825696865	1	0.818845598	ENSG00000224429	3	LINC00422
VAT	HTN	ENSG00000240972.1	0.003287095	0.319134215	0.003299502	0.240335324	0.004164795	0.207982238	0.003307664	0.240301603	-0.0008777	0.000193649	1	0.622410789	1	0.5074681	ENSG00000224497	1	RPL36P4
VAT	HTN	ENSG00000241186.3	-0.013557508	0.015724176	0.005613261	0.240332936	-0.013680827	0.014797557	0.005613096	0.240299455	0.000123319	0.014381652	1	0.107157347	1	0.102874703	ENSG00000224497	1	RPL36P4
VAT	HTN	ENSG00000241644.2	-0.004298271	0.492230272	0.006258708	0.240335581	-0.004207472	0.501399331	0.006258417	0.240302162	-9.08E-05	0.03227046	1	0.75257754	1	0.756622282	ENSG00000224497	1	RPL36P4
VAT	HTN	ENSG00000241839.5	-0.010022706	0.097345629	0.006045555	0.240334458	-0.010124574	0.093976708	0.006045291	0.240301002	0.000101868	0.019469877	1	0.334303787	1	0.330414924	ENSG00000224497	1	RPL36P4
VAT	HTN	ENSG00000242110.3	-0.000880226	0.642158324	0.001894248	0.240335707	-0.000829732	0.661353468	0.001894169	0.24030229	-5.05E-05	0.000363914	1	0.830922164	1	0.847332674	ENSG00000224497	1	RPL36P4
VAT	HTN	ENSG00000243753.1	0.001027784	0.404941161	0.001234086	0.240335471	0.000657662	0.597575229	0.001245829	0.240302247	0.000370122	0.030688922	1	0.695156362	1	0.814290189	ENSG00000224497	1	RPL36P4
VAT	HTN	ENSG00000244165.1	0.001424705	0.626642989	0.002928734	0.240335697	0.00134824	0.645252279	0.002928613	0.24030228	7.65E-05	0.000506179	1	0.825696865	1	0.839493846	ENSG00000224557	3	HLA-DPB2
VAT	HTN	ENSG00000244482.5	0.005576873	0.23248718	0.004670834	0.24033511	0.005648223	0.226547556	0.004670648	0.240301664	-7.14E-05	0.047946958	1	0.541032561	1	0.526616698	ENSG00000224557	3	HLA-DPB2
VAT	HTN	ENSG00000244625.1	0.004335963	0.42064181	0.005384223	0.240335494	0.004419945	0.411680024	0.005383995	0.240302052	-8.40E-05	0.03460002	1	0.705324046	1	0.694521313	ENSG00000224557	3	HLA-DPB2
VAT	HTN	ENSG00000244723.3	-0.010342759	0.060551014	0.005510953	0.240334076	-0.01053076	0.056014985	0.005510822	0.240300584	0.000188001	0.000372032	1	0.252880519	1	0.242158595	ENSG00000224557	3	HLA-DPB2
VAT	HTN	ENSG00000244731.3	0.000659006	0.709010277	0.001765891	0.240335745	0.004227084	0.074539171	0.002370381	0.240300817	-0.003568079	0.024052089	1	0.863959543	1	0.287731625	ENSG00000224557	3	HLA-DPB2
VAT	HTN	ENSG00000245958.2	0.006885142	0.042631154	0.003396194	0.240333786	0.00679507	0.045407445	0.003396065	0.240300411	9.01E-05	0.000834468	1	0.204284466	1	0.213136987	ENSG00000224557	3	HLA-DPB2
VAT	HTN	ENSG00000246067.3	-0.014298673	0.042868375	0.007061089	0.240333791	-0.014444705	0.040780644	0.007060805	0.240300321	0.000146032	0.007070562	1	0.204346659	1	0.198159466	ENSG00000224557	3	HLA-DPB2
VAT	HTN	ENSG00000247157.2	0.003042501	0.02473298	0.001354899	0.240333326	0.003067757	0.023557212	0.001354848	0.240299856	-2.53E-05	0.01887388	1	0.146802202	1	0.142896495	ENSG00000224635	1	RP4-564F22.5
VAT	HTN	ENSG00000248265.1	0.000815537	0.839576458	0.00402863	0.240335793	0.000957639	0.812101541	0.004028502	0.240302357	-0.000142102	5.08E-05	1	0.937501866	1	0.928116047	ENSG00000224635	1	RP4-564F22.5
VAT	HTN	ENSG00000248290.1	0.001564729	0.48900096	0.002261499	0.240335577	0.000968364	0.670112594	0.002273188	0.240302295	0.000596366	0.010076301	1	0.75021826	1	0.850122986	ENSG00000224635	1	RP4-564F22.5
VAT	HTN	ENSG00000248794.1	-0.01151489	0.044695028	0.005735882	0.240333826	-0.011382807	0.047194975	0.005735708	0.240300443	-0.000132083	0.00923444	1	0.210868848	1	0.217096886	ENSG00000224635	1	RP4-564F22.5
VAT	HTN	ENSG00000249731.1	-0.002313508	0.602456379	0.004441608	0.24033568	-0.002425142	0.585050091	0.004441461	0.240302238	0.000111633	0.003211431	1	0.814289719	1	0.807829017	ENSG00000224635	1	RP4-564F22.5
VAT	HTN	ENSG00000249839.1	-0.004124209	0.284910705	0.00385673	0.240335249	-0.004047743	0.293916536	0.003856574	0.240301842	-7.65E-05	0.009288009	1	0.589912638	1	0.592989502	ENSG00000224635	1	RP4-564F22.5
VAT	HTN	ENSG00000250476.1	0.008736098	0.176635232	0.006465485	0.240334913	0.00861217	0.182835145	0.006465214	0.24030151	0.000123927	0.009998627	1	0.462097857	1	0.473826292	ENSG00000224687	1	RASAL2-AS1
VAT	HTN	ENSG00000250506.2	-0.002079757	0.515221157	0.003196038	0.240335605	-0.002150985	0.500920425	0.003195912	0.240302161	7.12E-05	0.004150408	1	0.769486144	1	0.756313114	ENSG00000224769	1	AC069213.1
VAT	HTN	ENSG00000251417.1	-0.014745981	1.60E-05	0.003418254	0.240326635	-0.01466861	1.78E-05	0.00341815	0.240293303	-7.74E-05	0.010198147	0.044280439	0.00055118	0.04901568	0.000612696	ENSG00000224769	1	AC069213.1
VAT	HTN	ENSG00000253540.1	0.001378567	0.780527244	0.004947624	0.240335775	0.001090908	0.825539895	0.004949102	0.240302361	0.000287659	0.032157171	1	0.903724293	1	0.935340768	ENSG00000224796	1	RPL32P1
VAT	HTN	ENSG00000253771.1	-0.002002052	0.437381953	0.002577897	0.240335516	-0.001958471	0.44740199	0.00257777	0.2403021	-4.36E-05	0.008042042	1	0.716423851	1	0.721401667	ENSG00000224796	1	RPL32P1
VAT	HTN	ENSG00000253816.2	0.004360874	0.327122922	0.004450206	0.24033534	0.00444432	0.317936716	0.00445007	0.240301893	-8.34E-05	0.033987954	1	0.627864285	1	0.616693002	ENSG00000224796	1	RPL32P1
VAT	HTN	ENSG00000254126.2	-0.006339371	0.18304446	0.004761284	0.240334939	-0.006412765	0.178009145	0.004761086	0.24030149	7.34E-05	0.039251093	1	0.472152066	1	0.466576677	ENSG00000224796	1	RPL32P1
VAT	HTN	ENSG00000254352.1	-0.002578739	0.575440302	0.004604415	0.240335659	-0.002496622	0.587649233	0.004604222	0.24030224	-8.21E-05	0.016323709	1	0.798499363	1	0.808584778	ENSG00000224796	1	RPL32P1
VAT	HTN	ENSG00000254614.1	-0.000842318	0.69149008	0.002122596	0.240335736	0.000438643	0.837706928	0.002141525	0.240302364	-0.001280962	7.07E-06	1	0.857708065	1	0.939635856	ENSG00000224796	1	RPL32P1
VAT	HTN	ENSG00000254634.3	-0.010877268	0.004803311	0.003857256	0.240331891	-0.010939008	0.004567261	0.003857084	0.240298418	6.17E-05	0.024231017	1	0.048739475	1	0.046861116	ENSG00000224796	1	RPL32P1
VAT	HTN	ENSG00000254667.1	0.007583604	0.113865259	0.004796535	0.240334581	0.0074165	0.122038507	0.004796362	0.240301206	0.000167104	1.92E-05	1	0.36506767	1	0.379153311	ENSG00000224796	1	RPL32P1
VAT	HTN	ENSG00000254912.2	0.002896912	0.46010396	0.003921762	0.240335544	0.002788974	0.476973842	0.003921622	0.240302135	0.000107938	0.000830801	1	0.733862963	1	0.740826001	ENSG00000224796	1	RPL32P1
VAT	HTN	ENSG00000254986.3	0.000581121	0.814033628	0.002470484	0.240335786	-0.000274064	0.911929086	0.002477848	0.240302379	0.000855186	9.47E-06	1	0.923869473	1	0.968393726	ENSG00000224796	1	RPL32P1
VAT	HTN	ENSG00000255147.1	-0.000382033	0.940478905	0.005116423	0.240335811	0.000524633	0.918391751	0.005120374	0.24030238	-0.000906666	1.57E-05	1	0.976202248	1	0.971917651	ENSG00000224818	1	RP11-134G8.8
VAT	HTN	ENSG00000255200.1	-0.002446705	0.495494561	0.003589669	0.240335584	-0.000427814	0.905883927	0.003618431	0.240302378	-0.002018891	1.01E-05	1	0.753479333	1	0.967584999	ENSG00000224818	1	RP11-134G8.8
VAT	HTN	ENSG00000255837.1	0.008829996	0.060317738	0.004700637	0.240334073	0.008927607	0.057525664	0.004700473	0.240300606	-9.76E-05	0.01276557	1	0.252880519	1	0.246538561	ENSG00000224843	2	LINC00240
VAT	HTN	ENSG00000256274.1	0.00040574	0.804883525	0.00164246	0.240335783	0.000345914	0.83319096	0.001642433	0.240302363	5.98E-05	0.000401868	1	0.918978716	1	0.939506392	ENSG00000224843	2	LINC00240
VAT	HTN	ENSG00000256612.3	-0.00682086	0.289108363	0.006434283	0.240335259	-0.006676251	0.299434373	0.006434021	0.240301854	-0.00014461	0.003043665	1	0.59321241	1	0.598868745	ENSG00000224843	2	LINC00240
VAT	HTN	ENSG00000256660.1	-0.000863048	0.737350677	0.002573465	0.240335758	0.004518945	0.201384255	0.003537004	0.24030158	-0.005381992	0.026563554	1	0.878176701	1	0.497601204	ENSG00000224843	2	LINC00240
VAT	HTN	ENSG00000257742.1	0.007757967	0.250101677	0.006745449	0.240335161	0.007524254	0.264640571	0.006745238	0.240301771	0.000233713	7.43E-05	1	0.56074787	1	0.569128623	ENSG00000224870	3	RP4-758J18.2
VAT	HTN	ENSG00000257877.1	-0.023437635	0.00024427	0.006389373	0.240329177	-0.023318786	0.000262543	0.00638918	0.240295816	-0.000118849	0.035642981	0.674184667	0.005267068	0.724618892	0.005750944	ENSG00000224930	2	LINC00162
VAT	HTN	ENSG00000257949.2	-0.001587059	0.700162006	0.004121139	0.24033574	-0.001678208	0.683834547	0.004120967	0.240302303	9.11E-05	0.002940624	1	0.860778235	1	0.856733249	ENSG00000224930	2	LINC00162
VAT	HTN	ENSG00000258819.1	-0.02148579	0.00124762	0.006656517	0.240330675	-0.021289961	0.001381589	0.006656257	0.24029734	-0.000195829	0.000156425	1	0.018219212	1	0.019554794	ENSG00000224930	2	LINC00162
VAT	HTN	ENSG00000259205.2	-0.003348739	0.113885241	0.002118153	0.240334581	-0.003407082	0.107710628	0.002118079	0.240301109	5.83E-05	0.000952668	1	0.36506767	1	0.352646896	ENSG00000224997	1	AL049840.1
VAT	HTN	ENSG00000259803.2	-0.010068362	0.060901451	0.005372043	0.240334081	-0.009932978	0.064447329	0.005371858	0.240300699	-0.000135384	0.002587326	1	0.253144583	1	0.26197345	ENSG00000224997	1	AL049840.1
VAT	HTN	ENSG00000259982.1	0.011430503	0.000150248	0.003015416	0.240328727	0.011369371	0.000162907	0.003015308	0.240295374	6.11E-05	0.013538506	0.41468463	0.003519708	0.449623483	0.003842936	ENSG00000224997	1	AL049840.1
VAT	HTN	ENSG00000260077.1	-0.01975808	0.001272407	0.006131955	0.240330693	-0.019889877	0.001179756	0.006131756	0.240297196	0.000131797	0.012804985	1	0.018405959	1	0.017696346	ENSG00000225194	2	LINC00092
VAT	HTN	ENSG00000260302.1	0.006151996	0.339798637	0.006444822	0.240335364	0.006317255	0.326976237	0.006444708	0.240301911	-0.000165259	0.011828163	1	0.64279934	1	0.624860924	ENSG00000225194	2	LINC00092
VAT	HTN	ENSG00000260693.1	0.002345985	0.614547213	0.004658489	0.240335688	0.002271452	0.625823285	0.004658297	0.240302268	7.45E-05	0.033559458	1	0.819907808	1	0.827116213	ENSG00000225241	3	RP11-640M9.2
VAT	HTN	ENSG00000260911.1	0.008910713	0.000101634	0.002292623	0.240328363	0.008962444	9.25E-05	0.002292541	0.240294848	-5.17E-05	0.006400555	0.280510026	0.002597315	0.255413501	0.00243251	ENSG00000225282	1	AP000350.6
VAT	HTN	ENSG00000260947.1	-0.003874721	0.004470225	0.001362931	0.240331827	-0.003800907	0.005295542	0.001363067	0.24029855	-7.38E-05	0.003244148	1	0.046036649	1	0.052198915	ENSG00000225282	1	AP000350.6
VAT	HTN	ENSG00000261064.1	0.000291304	0.958693826	0.005624418	0.240335812	0.000180897	0.974341243	0.005624198	0.240302384	0.000110407	0.011703554	1	0.98228229	1	0.989761439	ENSG00000225294	1	OSTCP2
VAT	HTN	ENSG00000261556.4	-0.01017361	9.36E-05	0.002604213	0.240328287	-0.010232066	8.53E-05	0.002604155	0.240294772	5.85E-05	0.020871239	0.258385761	0.002437602	0.235331206	0.0022628	ENSG00000225398	2	RP11-395L14.5
VAT	HTN	ENSG00000261589.1	0.008163651	0.157696218	0.005778085	0.240334829	0.008311586	0.1502868	0.005777868	0.240301364	-0.000147936	0.001385681	1	0.435044054	1	0.424122257	ENSG00000225492	2	GBP1P1
VAT	HTN	ENSG00000261925.1	0.000131907	0.976866731	0.004548941	0.240335813	0.000203235	0.964362987	0.004548764	0.240302384	-7.13E-05	0.045140435	1	0.98866831	1	0.985245375	ENSG00000225492	2	GBP1P1

VAT	HTN	ENSG00000262497.1	0.006022034	0.137225481	0.004051953	0.240334724	0.00608275	0.133289891	0.004051773	0.240301273	-6.07E-05	0.034440728	1	0.404207393	1	0.399435503	ENSG00000225492	2	GBP1P1
VAT	HTN	ENSG00000262855.1	-0.001640863	0.558056842	0.002801395	0.240335644	-0.001575491	0.573829977	0.002801271	0.240302229	-6.54E-05	0.000984172	1	0.788352234	1	0.801328599	ENSG00000225498	1	AC002064.5
VAT	HTN	ENSG00000263004.1	0.010134529	0.031150182	0.004702459	0.240333523	0.010045806	0.032649665	0.004702275	0.240300134	8.87E-05	0.015501713	1	0.170246542	1	0.176002101	ENSG00000225498	1	AC002064.5
VAT	HTN	ENSG00000266173.1	0.003466214	0.471368639	0.004812485	0.240335558	0.003351608	0.48614159	0.004812353	0.240302146	0.000114606	0.009501462	1	0.741297688	1	0.746371412	ENSG00000225498	1	AC002064.5
VAT	HTN	ENSG00000266714.2	0.005509053	0.147925775	0.003807489	0.240334781	0.005586675	0.142280944	0.003807317	0.240301323	-7.76E-05	0.003534071	1	0.418982277	1	0.412928922	ENSG00000225498	1	AC002064.5
VAT	HTN	ENSG00000266992.1	-0.017211435	0.000817018	0.00514237	0.240330289	-0.017329672	0.000751499	0.005142201	0.240296784	0.000118237	0.007329852	1	0.013186962	1	0.012346048	ENSG00000225498	1	AC002064.5
VAT	HTN	ENSG00000267058.1	-0.001108831	0.80006679	0.004378218	0.240335782	-0.001026449	0.814635021	0.004378099	0.240302358	-8.24E-05	0.040662054	1	0.916259063	1	0.928320668	ENSG00000225684	3	FAM225B
VAT	HTN	ENSG00000267102.1	0.000397199	0.945358304	0.005795411	0.240335811	0.000210519	0.971022369	0.005795261	0.240302384	0.00018668	0.000557188	1	0.976968793	1	0.988573124	ENSG00000225684	3	FAM225B
VAT	HTN	ENSG00000267108.1	-0.001725987	0.559348114	0.002956417	0.240335645	-0.001671572	0.571782858	0.002956295	0.240302227	-5.44E-05	0.014352648	1	0.788860907	1	0.801076492	ENSG00000225684	3	FAM225B
VAT	HTN	ENSG00000267302.1	-0.002559951	0.486515388	0.003678819	0.240335575	-0.002631258	0.474438183	0.003678656	0.240302132	7.13E-05	0.006444524	1	0.749738957	1	0.740220116	ENSG00000225726	1	AC007000.10
VAT	HTN	ENSG00000267346.1	0.003646384	0.390065857	0.004242454	0.240335449	0.003728905	0.379408425	0.004242273	0.240302004	-8.25E-05	0.007971207	1	0.682680345	1	0.671045748	ENSG00000225726	1	AC007000.10
VAT	HTN	ENSG00000267508.1	-0.008826049	0.023161095	0.003886802	0.24033327	-0.0087699	0.024043853	0.003886618	0.240299874	-5.61E-05	0.029767978	1	0.139878824	1	0.144666325	ENSG00000225784	5	RP11-592B15.4
VAT	HTN	ENSG00000267939.1	0.011376911	0.099663462	0.006909794	0.240334476	0.011238119	0.103849981	0.006909508	0.24030108	0.000138792	0.007245228	1	0.336272805	1	0.344320158	ENSG00000225784	5	RP11-592B15.4
VAT	HTN	ENSG00000268225.1	0.014323374	0.000131702	0.003746353	0.240328604	0.014209688	0.000148836	0.003746259	0.24029529	0.000113686	0.001261807	0.363496717	0.003188568	0.410786292	0.003552729	ENSG00000225784	5	RP11-592B15.4
VAT	HTN	ENSG00000268945.1	0.00330664	0.602857839	0.00635531	0.24033568	0.003578631	0.573385456	0.006355536	0.240302228	-0.000271991	0.003165026	1	0.814433497	1	0.801328599	ENSG00000225849	1	MKRN7P
VAT	HTN	ENSG00000269558.1	0.002985966	0.202686765	0.002343891	0.240335013	0.00261204	0.266508841	0.002350776	0.240301776	0.000373926	0.039808654	1	0.501009921	1	0.572019261	ENSG00000225851	1	HLA-S
VAT	HTN	ENSG00000270071.1	0.005210723	0.070458226	0.002880512	0.240334199	0.005265322	0.067552371	0.002880397	0.240300737	-5.46E-05	0.013835907	1	0.27741042	1	0.269826471	ENSG00000225851	1	HLA-S
VAT	HTN	ENSG00000270127.1	-0.003049467	0.120872186	0.00196597	0.240334627	-0.003103491	0.114414477	0.001965901	0.240301156	5.40E-05	0.000931766	1	0.37568382	1	0.366605303	ENSG00000225851	1	HLA-S
VAT	HTN	ENSG00000271553.1	0.000782695	0.792260677	0.002971776	0.240335779	0.000889933	0.764579303	0.002971665	0.240302341	-0.000107238	6.92E-06	1	0.909962326	1	0.898738874	ENSG00000225851	1	HLA-S
VAT	HTN	ENSG00000272010.1	0.005368727	0.210327036	0.004285833	0.240335039	0.005301224	0.216098173	0.004285647	0.24030163	6.75E-05	0.029020826	1	0.508766538	1	0.515497803	ENSG00000225872	2	AC002398.5
VAT	HTN	ENSG00000272129.1	-0.003725867	0.161803549	0.002663172	0.240334848	-0.003772334	0.156617743	0.00266306	0.240301395	4.65E-05	0.018433582	1	0.440412026	1	0.433565668	ENSG00000225880	4	LINC00115
VAT	HTN	ENSG00000272221.1	-0.002844496	0.042835951	0.001404474	0.24033379	-0.002299526	0.103272066	0.001411448	0.240301076	-0.00054497	0.000112273	1	0.204346659	1	0.344320158	ENSG00000225880	4	LINC00115
VAT	HTN	ENSG00000272732.1	-0.003305445	0.63067222	0.006875154	0.240335699	-0.003489768	0.611729103	0.006874938	0.240302258	0.000184323	0.00213303	1	0.825823207	1	0.821992369	ENSG00000225880	4	LINC00115
VAT	HTN	ENSG00000272733.1	-0.00010307	0.969639383	0.002708057	0.240335813	-0.000314306	0.907619543	0.002708558	0.240302378	0.000211235	0.000550252	1	0.987165141	1	0.96802961	ENSG00000225892	3	RP11-384K6.2
VAT	HTN	ENSG00000272787.1	-0.000269569	0.937565448	0.003441443	0.24033581	-0.001120851	0.745222593	0.003449352	0.240302333	0.000851282	0.000327376	1	0.976192895	1	0.884511252	ENSG00000225892	3	RP11-384K6.2
VAT	HTN	ENSG00000272849.1	-0.006429603	0.179319382	0.004788008	0.240334924	-0.006350047	0.184742945	0.004787812	0.240301517	-7.96E-05	0.02807208	1	0.467789692	1	0.476781711	ENSG00000226002	1	RP11-460N20.5
VAT	HTN	ENSG00000272902.1	0.012272877	0.045849153	0.00614632	0.240333847	0.012426078	0.043199098	0.006146092	0.240300369	-0.000153201	0.002008523	1	0.214201949	1	0.20699568	ENSG00000226002	1	RP11-460N20.5
VAT	HTN	ENSG00000273142.1	0.000641707	0.776343417	0.002258835	0.240335774	0.000186905	0.934325035	0.002268134	0.240302381	0.000454802	0.027936873	1	0.902839726	1	0.974609668	ENSG00000226002	1	RP11-460N20.5
VAT	HTN	ENSG00000273172.1	0.006788488	0.262460967	0.006057933	0.240335194	0.006914072	0.253717662	0.00605771	0.240301742	-0.000125584	0.010334262	1	0.570001694	1	0.559962186	ENSG00000226015	2	CCT8P1
VAT	HTN	ENSG00000273270.1	-0.001010066	0.657431618	0.002277683	0.240335716	-0.000970654	0.669979342	0.002277587	0.240302295	-3.94E-05	0.019624319	1	0.837990221	1	0.850122986	ENSG00000226015	2	CCT8P1
VAT	HTN	ENSG00000273295.1	0.002246199	0.332948505	0.002319992	0.240335351	0.002864551	0.218091587	0.00232584	0.240301637	-0.000618352	0.000215367	1	0.634625603	1	0.517020606	ENSG00000226015	2	CCT8P1
VAT	Obesity	ENSG00000002919.10	-0.001594678	0.49287072	0.002325448	0.183454296	-0.001545043	0.506404442	0.002325306	0.183417379	-4.96E-05	0.016112667	1	0.766182814	1	0.783011911	ENSG00000226180	2	AC010536.1
VAT	Obesity	ENSG00000004809.9	0.000708704	0.729413134	0.002048832	0.183454429	0.000768348	0.70762726	0.002048683	0.183417493	-5.96E-05	0.000103856	1	0.883749012	1	0.876422561	ENSG00000226180	2	AC010536.1
VAT	Obesity	ENSG00000005156.7	-0.000176369	0.958954928	0.003426964	0.183454473	-0.000339547	0.921074824	0.003426997	0.183417542	0.000163178	0.001341853	1	0.98546132	1	0.973721371	ENSG00000226194	1	RP1-137D17.1
VAT	Obesity	ENSG00000005801.12	0.002765582	0.421207102	0.003438369	0.18345423	0.002839188	0.408920157	0.003438122	0.183417288	-7.36E-05	0.004835485	1	0.71233554	1	0.72254778	ENSG00000226232	4	RP11-419C5.2
VAT	Obesity	ENSG00000006071.7	0.005035704	0.356841113	0.005465263	0.183454153	0.004840221	0.375783039	0.005464892	0.18341725	0.000195483	9.57E-06	1	0.668015266	1	0.699838858	ENSG00000226232	4	RP11-419C5.2
VAT	Obesity	ENSG00000007341.14	-0.001450766	0.455920643	0.001945818	0.183454264	-0.00142153	0.465018214	0.001945677	0.183417344	-2.92E-05	0.044463116	1	0.739580277	1	0.76239704	ENSG00000226232	4	RP11-419C5.2
VAT	Obesity	ENSG00000007372.16	-0.004473224	0.217639789	0.003628419	0.1834539	-0.00440946	0.224240572	0.003628197	0.183416988	-6.38E-05	0.047897885	1	0.521961016	1	0.533537912	ENSG00000226259	6	GTF2H2B
VAT	Obesity	ENSG00000008394.8	-0.004705423	0.30259372	0.004564435	0.183454072	-0.007272907	0.113528799	0.004595741	0.1834166	0.002567484	1.94E-06	1	0.611389947	1	0.36994036	ENSG00000226259	6	GTF2H2B
VAT	Obesity	ENSG00000009950.11	0.011407537	0.040235531	0.005561049	0.183452884	0.003620645	0.530717659	0.00577537	0.183417398	0.007786892	6.05E-07	1	0.193804653	1	0.786996417	ENSG00000226266	2	AC009961.3
VAT	Obesity	ENSG00000010219.9	-0.001149732	0.80714216	0.004709805	0.183454452	-0.001221347	0.795372801	0.004709437	0.183417521	7.16E-05	0.022904179	1	0.921269427	1	0.918573915	ENSG00000226278	1	PSPHP1
VAT	Obesity	ENSG00000010539.7	0.001427245	0.616769817	0.002852015	0.183454379	0.001379669	0.628536763	0.002851806	0.183417458	4.76E-05	0.024497353	1	0.831570896	1	0.842487235	ENSG00000226278	1	PSPHP1
VAT	Obesity	ENSG00000011105.7	0.002018167	0.590057941	0.003746001	0.183454364	0.002078055	0.579046958	0.003745743	0.18341743	-5.99E-05	0.04513677	1	0.817872626	1	0.816782844	ENSG00000226278	1	PSPHP1
VAT	Obesity	ENSG00000011485.10	0.001207149	0.769506206	0.004119666	0.183454442	0.00112213	0.785314863	0.004119425	0.183417518	8.50E-05	0.02438535	1	0.902991976	1	0.913850934	ENSG00000226314	3	ZNF192P1
VAT	Obesity	ENSG00000011523.9	0.003153053	0.419433109	0.003905166	0.183454228	0.003239871	0.406712353	0.0039049	0.183417286	-8.68E-05	0.005872346	1	0.712286006	1	0.721556426	ENSG00000226314	3	ZNF192P1
VAT	Obesity	ENSG00000012061.11	-0.00350155	0.383834192	0.004020822	0.183454187	-0.003401439	0.397543892	0.004020535	0.183417276	-0.000100111	0.00109913	1	0.691502852	1	0.715434904	ENSG00000226314	3	ZNF192P1
VAT	Obesity	ENSG00000013306.11	-0.001716139	0.618640033	0.003447592	0.18345438	-0.001648248	0.63256495	0.003447351	0.18341746	-6.79E-05	0.012158	1	0.831754083	1	0.84382758	ENSG00000226314	3	ZNF192P1
VAT	Obesity	ENSG00000014914.15	0.006989675	0.034527444	0.003306588	0.183452785	0.006833962	0.038774682	0.003306901	0.183415932	0.000155713	0.017166657	1	0.174534333	1	0.194932828	ENSG00000226314	3	ZNF192P1
VAT	Obesity	ENSG00000015133.14	-0.000567478	0.907906421	0.004905595	0.183454469	0.00323329	0.51752932	0.004996128	0.183417388	-0.003800767	6.24E-05	1	0.967125327	1	0.784425829	ENSG00000226314	3	ZNF192P1
VAT	Obesity	ENSG00000018510.8	0.003076039	0.415769575	0.003779924	0.183454224	0.003263047	0.387972841	0.003779736	0.183417264	-0.000187008	9.45E-07	1	0.710460915	1	0.708846095	ENSG00000226314	3	ZNF192P1
VAT	Obesity	ENSG00000020922.8	-0.002464749	0.014505407	0.001008281	0.183452215	-0.00243779	0.015609554	0.001008215	0.183415337	-2.70E-05	0.001422334	1	0.098365905	1	0.108519823	ENSG00000226328	2	CTA-217C2.1
VAT	Obesity	ENSG00000026297.11	-0.003278104	0.418588206	0.004052678	0.183454227	-0.003379845	0.404259762	0.004052385	0.183417283	0.00010174	0.000883043	1	0.712286006	1	0.720669275	ENSG00000226328	2	CTA-217C2.1
VAT	Obesity	ENSG00000026950.12	-0.003520957	0.059575746	0.001868948	0.183453133	-0.00440635	0.018875314	0.001876646	0.183415462	0.000885392	2.60E-07	1	0.248007631	1	0.120957701	ENSG00000226328	2	CTA-217C2.1
VAT	Obesity	ENSG00000030110.8	0.003701988	0.402943185	0.004426218	0.18345421	0.002465896	0.578774775	0.004441651	0.18341743	0.001236091	0.000986316	1	0.706963646	1	0.816782844	ENSG00000226328	2	CTA-217C2.1
VAT	Obesity	ENSG00000031691.6	-0.003800752	0.004997916	0.001353939	0.183451495	-0.00386726	0.004294372	0.001354224	0.183414464	6.65E-05	0.049128678	1	0.044071084	1	0.043575249	ENSG00000226383	1	AC093375.1
VAT	Obesity	ENSG00000033178.8	-0.006783735	0.011829289	0.002694946	0.183452079	-0.006850515	0.011017352	0.002694764	0.183415104	6.68E-05	0.002297039	1	0.084112755	1	0.084466367	ENSG00000226383	1	AC093375.1
VAT	Obesity	ENSG00000033800.9	0.034124219	9.38E-08	0.006392272	0.183443702	0.033988019	1.05E-07	0.00639182	0.183406861	0.0001362	0.005605716	0.000258986	4.89E-06	0.000290608	6.46E-06	ENSG00000226479	3	TMEM185B
VAT	Obesity	ENSG00000035115.17	-0.02607395	2.82E-06	0.005567445	0.183446184	-0.026225758	2.47E-06	0.005567143	0.183409159	0.000151808	0.004787049	0.007793827	0.000108248	0.006811713	0.000111667	ENSG00000226479	3	TMEM185B
VAT	Obesity	ENSG00000037474.10	0.01496502	0.013987275	0.006089285	0.183452191	0.014799805	0.015073352	0.0060889	0.183415313	0.000165215	0.001685018	1	0.095320688	1	0.107059606	ENSG00000226479	3	TMEM185B
VAT	Obesity	ENSG00000037749.7	-0.001364668	0.54389782	0.002248478	0.183454335	-0.001325163	0.555594673	0.002248334	0.183417415	-3.95E-05	0.040273163	1	0.79046238	1	0.805802048	ENSG00000226479	3	TMEM185B
VAT	Obesity	ENSG00000040531.10	-0.000789085	0.680185367	0.001914278	0.18345441	-0.000745169	0.697057637	0.001914147	0.183417489	-4.39E-05	0.004235479	1	0.862732905	1	0.870280156	ENSG00000226686	3	AC012309.5
VAT	Obesity	ENSG00000048544.5	-0.00164174	0.172512427	0.001203465	0.183453771	-0.001667401	0.165869197	0.001203376	0.18341682	2.57E-05	0.003722598	1	0.459620793	1	0.456484191	ENSG00000226913	1	BSN-AS2
VAT	Obesity	ENSG00000049247.9	-0.016280483	0.012621234	0.006527099	0.183452122	-0.01613937	0.013404387	0.006526628	0.183415235	-0.000141113	0.004122831	1	0.0877446	1	0.098393908	ENSG00000227057	3	WDR46
VAT	Obesity	ENSG00000050555.13	0.003067899	0.595354659	0.005776576	0.183454367	-0.002077068	0.736524364	0.006173305	0.183417503	0.005144967	0.018212016	1	0.818796889	1	0.891658005	ENSG00000227057	3	WDR46
VAT	Obesity	ENSG00000055147.13	-0.002278066	0.663169211	0.005230416	0.183454402	-0.002113189	0.686181207	0.005230108	0.183417484	-0.000164877	0.000556155	1	0.854105003	1	0.869882588	ENSG00000227113	2	RP11-460N20.4
VAT	Obesity	ENSG00000057608.12	-0.004774725	0.366465432	0.005286953	0.183454166	-0.004664988	0.377547542	0.005286556	0.183417252	-0.000109737	0.003694234	1	0.674746226	1	0.700657728	ENSG00000227113	2	RP11-460N20.4
VAT	Obesity	ENSG00000058453.12	0.003272499	0.040446169	0.001596988	0.183452887	0.003305304	0.038466315	0.001596876	0.183415927	-3.28E-05	0.008231604	1	0.194479835	1	0.193735457	ENSG00000227113	2	RP11-460N20.4
VAT	Obesity	ENSG00000060558.3	-0.003564464	0.545188986	0.005891819	0.183454336	-0.003453347	0.557765358	0.005891431	0.183417416	-0.000111118	0.023752192	1	0.791125973	1	0.808055518	ENSG00000227124	4	ZNF717
VAT	Obesity	ENSG00000060642.6	-0.002795656	0.298663802	0.00268993	0.183454066	-0.002741241	0.308131487	0.002689728	0.183417153	-5.44E-05	0.004744087	1	0.608797706	1	0.629957707	ENSG00000227124	4	ZNF717
VAT	Obesity	ENSG00000064309.10	-0.001753248	0.737380877	0.005228519	0.183454432	-0.000497266	0.924775502	0.005266533	0.183417543	-0.001255981	0.048276975	1	0.887171413	1	0.974118595	ENSG00000227124	4	ZNF717
VAT	Obesity	ENSG00000065060.12	0.02639805	1.87E-23	0.00264497	0.183416829	0.026202429	3.92E-23	0.002644965	0.183380463	0.000195621	1.16E-07	5.15E-20	8.58E-21	1.08E-19	1.80E-20	ENSG00000227262	3	HCG4B
VAT	Obesity	ENSG00000065665.16	-0.008645774	0.090016951	0.005099814	0.183453388	-0.0087782	0.085177571	0.005099432	0.183416426	0.000132426	0.000307738	1	0.314092016	1	0.311377611	ENSG00000227262	3	HCG4B
VAT	Obesity	ENSG00000066185.8	-0.001902951	0.412670653	0.002322928	0.18345422	-0.001860423	0.423161796	0.002322775	0.183417304	-4.25E-05	0.027777932	1	0.708533979	1	0.728768041	ENSG00000227262	3	HCG4B
VAT	Obesity	ENSG00000066379.10	-0.007666781	0.005714364	0.002774051	0.183451587	-0.004886431	0.083993926	0.002827842	0.183416418	-0.00278035	4.36E-07	1	0.048828617	1	0.308446194	ENSG00000227262	3	HCG4B
VAT	Obesity	ENSG00000066427.17	0.003569633	0.177177842	0.002645157	0.183453786	0.003464488	0.190272657	0.002645102	0.183416898	0.000105146	0.001628445	1	0.4663773	1	0.500365103	ENSG00000227262	3	HCG4B
VAT	Obesity	ENSG00000066933.11	-0.001775042	0.694474035	0.00451905	0.183454416	-0.001882569	0.676959735	0.004518718	0.18341748	0.000107526	0.001212288	1	0.866149932	1	0.865004105	ENSG00000227262	3	HCG4B
VAT	Obesity	ENSG00000067167.3	0.004316396	0.473090917	0.006016221	0.18345428	0.004219981	0.482999459	0.006015764	0.18341736	9.64E-05	0.022603185	1	0.755994642	1	0.775358255	ENSG00000227262	3	HCG4B
VAT	Obesity	ENSG00000069966.14	-0.001462995	0.686776852	0.00362816	0.183454413	-0.00176335	0.627178194	0.003630514	0.183417457	0.000300356	0.032534397	1	0.863333733	1	0.842487235	ENSG00000227531	1	RP11-202G18.1
VAT	Obesity	ENSG00000070540.8	-0.012203573	0.002824998	0.004086683	0.183451103	-0.012133575	0.00298535	0.0040864	0.183414214	-7.00E-05	0.030746761	1	0.029202227	1	0.033090625	ENSG00000227531	1	RP11-202G18.1
VAT	Obesity	ENSG00000071462.7	0.002436998	0.454963812	0.003261656	0.183454263	0.003179336	0.330010104	0.003263889	0.183417187	-0.000742338	9.44E-09	1	0.739081884	1	0.650677127	ENSG00000227578	1	RPS3AP53
VAT	Obesity	ENSG00000071575.7	-0.000343974	0.938350964	0.004447406	0.183454472	-0.000449479	0.919492962	0.004447099	0.183417542	0.000105505	0.002869694	1	0.979148832	1	0.973721371	ENSG00000227694	1	AC005884.1
VAT	Obesity	ENSG00000072134.11	-0.001016183	0.726295483	0.0029029	0.183454428	-0.001071612	0.711995508	0.002902699	0.183417495	5.54E-05	0.016490523	1	0.882200818	1	0.877671998	ENSG00000227755	1	AP000344.4
VAT	Obesity	ENSG00000075461.5	0.009663071	0.077838135	0.005479877	0.183453299	0.009766315	0.074691947	0.005479443	0.183416345	-0.000103244	0.004106909	1	0.286642319	1	0.291599575	ENSG00000227766	1	HCG4P5
VAT	Obesity	ENSG00000076242.10	-0.00189825	0.676798656	0.00455395	0.183454408	-0.001996042	0.661138395	0.004553616	0.183417473	9.78E-05	0.003662308	1	0.860416532	1	0.855080586	ENSG00000227766	1	HCG4P5
VAT	Obesity	ENSG00000076685.14	0.009780495	0.000141643	0.002570222	0.183449001	0.009746773	0.000149162	0.002570019	0.18341211	3.37E-05	0.046399732	0.390934604	0.002874519	0.41168648	0.003293492	ENSG00000227766	1	HCG4P5
VAT	Obesity	ENSG00000077585.9	0.000107122	0.983124662	0.005064459	0.183454474	1.34E-05	0.997882508	0.005064097	0.183417546	9.37E-05	0.015650315	1	0.99502166	1	0.999330814	ENSG00000227766	1	HCG4P5
VAT	Obesity	ENSG00000079150.13	-0.001221342	0.802083179	0.004872658	0.18345445	-0.00131277	0.787595119	0.004872307	0.183417519	9.14E-05	0.013056043	1	0.918961541	1	0.915268433	ENSG00000227766	1	HCG4P5
VAT	Obesity	ENSG00000081665.9	0.012321992	0.028111249	0.00561178	0.183452652	0.012404202	0.027067548	0.005611367	0.183415699	-8.22E-05	0.046974784	1	0.152430349	1	0.155231297	ENSG00000227766	1	HCG4P5
VAT	Obesity	ENSG00000082684.10	-0.003479031	0.425549634	0.004366084	0.183454234	-0.003564359	0.414259395	0.004365831	0.183417294	8.53E-05	0.034572117	1	0.714174021	1	0.72386593	ENSG00000227766	1	HCG4P5
VAT	Obesity	ENSG00000083097.10	0.006341945	0.004068008	0.002207542	0.183451354	0.006290028	0.004378496	0.002207387	0.183414477	5.19E-05	0.002451653	1	0.037931426	1	0.0439491	ENSG00000227825	3	SLC9A7P1
VAT	Obesity	ENSG00000083845.4	0.000858329	0.84304168	0.004334897	0.183454459	0.00093005	0.830105568	0.004334557	0.183417529	-7.17E-05	0.012930433	1	0.938891035	1	0.936897537	ENSG00000227825	3	SLC9A7P1
VAT	Obesity	ENSG00000084072.12	0.006747231	0.013782104	0.002739545	0.183452181	0.006696175	0.014507531	0.002739334	0.183415288	5.11E-05	0.006577721	1	0.094623402	1	0.104818812	ENSG00000227827	3	RP11-958N24.2
VAT	Obesity	ENSG00000084207.11	0.013859775	1.66E-05	0.003218386	0.183447464	0.013535305	2.61E-05	0.003218472	0.183410862	0.00032447	2.34E-10	0.045798762	0.000487221	0.071910214	0.00080798	ENSG00000227827	3	RP11-958N24.2
VAT	Obesity	ENSG00000085117.7	0.008955991	0.016749783	0.003743888	0.183452311	0.008859399	0.017955869	0.003743616	0.183415429	9.66E-05	0.000552363	1	0.108012621	1	0.119020171	ENSG00000227827	3	RP11-958N24.2
VAT	Obesity	ENSG00000086015.16	0.014924507	0.001073733	0.004563402	0.183450431	0.014842102	0.001143441	0.004563087	0.183413548	8.24E-05	0.022976729	1	0.014179435	1	0.016436964	ENSG00000227827	3	RP11-958N24.2
VAT	Obesity	ENSG00000086598.6	-0.004845865	0.130401265	0.003203827	0.183453609	-0.004794795	0.134473352	0.003203589	0.183416699	-5.11E-05	0.028726353	1	0.387831349	1	0.405181715	ENSG00000227877	2	LINC00948
VAT	Obesity	ENSG00000087365.10	-0.004121519	0.273444553	0.003763374	0.183454021	-0.006010332	0.111536658	0.003776931	0.183416589	0.001888813	5.68E-09	1	0.578319515	1	0.366914394	ENSG00000227921	1	AL353791.1
VAT	Obesity	ENSG00000088053.7	-0.00606974	0.038204594	0.002928481	0.18345285	-0.006159966	0.03541163	0.002928262	0.183415874	9.02E-05	1.95E-05	1	0.186351781	1	0.184211409	ENSG00000227921	1	AL353791.1
VAT	Obesity	ENSG00000088833.13	0.012266138	0.004760622	0.004345359	0.183451462	0.012381224	0.004378799	0.004345031	0.183414477	-0.000115086	0.000191069	1	0.042384891	1	0.0439491	ENSG00000227958	1	RP11-399H11.3
VAT	Obesity	ENSG00000089048.10	-0.003478253	0.521341186	0.005423924	0.183454319	-0.003565962	0.510862362	0.00542355	0.183417383	8.77E-05	0.041822516	1	0.778563922	1	0.784425829	ENSG00000227986	1	TRIM60P18
VAT	Obesity	ENSG00000090054.9	-0.000333004	0.93941657	0.004381445	0.183454472	-0.000511416	0.907073694	0.00438117	0.183417541	0.000178412	2.79E-06	1	0.979890299	1	0.970734158	ENSG00000228022	1	HCG20
VAT	Obesity	ENSG00000090263.11	-0.003469921	0.551396781	0.005825251	0.18345434	-0.003368907	0.563013961	0.00582482	0.18341742	-0.000101014	0.017222544	1	0.793904838	1	0.809555852	ENSG00000228022	1	HCG20
VAT	Obesity	ENSG00000090316.11	-0.001447096	0.670389314	0.00340002	0.183454406	-0.001527076	0.653311431	0.003399797	0.18341747	8.00E-05	0.005022909	1	0.857647266	1	0.852951538	ENSG00000228022	1	HCG20
VAT	Obesity	ENSG00000090621.9	-0.029530608	6.03E-11	0.004513151	0.183438292	-0.029430877	6.96E-11	0.004512839	0.183401473	-9.97E-05	0.005154988	1.66E-07	4.89E-09	1.92E-07	5.82E-09	ENSG00000228022	1	HCG20
VAT	Obesity	ENSG00000091640.3	0.003411091	0.086093404	0.001987377	0.183453361	0.003454021	0.082196949	0.00198726	0.183416404	-4.29E-05	0.018160715	1	0.303083921	1	0.305457316	ENSG00000228049	5	POLR2J2
VAT	Obesity	ENSG00000093000.14	0.000359167	0.922769297	0.003704819	0.183454471	0.000223074	0.951983434	0.003704554	0.183417545	0.000136093	1.56E-06	1	0.974681691	1	0.984072763	ENSG00000228049	5	POLR2J2
VAT	Obesity	ENSG00000093072.11	0.000978568	0.272404275	0.000891598	0.183454019	0.000959805	0.281668867	0.000891532	0.183417108	1.88E-05	0.003989629	1	0.576561196	1	0.600777491	ENSG00000228049	5	POLR2J2
VAT	Obesity	ENSG00000095713.9	-0.004727052	0.234374034	0.003975091	0.18345394	-0.004657176	0.241331996	0.003974834	0.183417027	-6.99E-05	0.038746562	1	0.529788971	1	0.547310032	ENSG00000228049	5	POLR2J2
VAT	Obesity	ENSG00000095917.9	-0.010555982	0.052646512	0.005447366	0.183453055	-0.010646559	0.050632257	0.005446957	0.183416102	9.06E-05	0.020196689	1	0.231376391	1	0.236113017	ENSG00000228050	1	TOP3BP1
VAT	Obesity	ENSG00000096433.6	0.024937978	9.41E-06	0.005628991	0.183447055	0.026003821	4.00E-06	0.005639176	0.18340951	-0.001065843	0.002206137	0.025980893	0.000309296	0.011048227	0.000172629	ENSG00000228050	1	TOP3BP1
VAT	Obesity	ENSG00000096872.11	0.010907708	0.046085147	0.005468571	0.18345297	0.010788666	0.048504135	0.005468348	0.183416075	0.000119043	0.046461811	1	0.212642286	1	0.229164841	ENSG00000228050	1	TOP3BP1
VAT	Obesity	ENSG00000096996.11	-0.012467464	0.006789148	0.004605596	0.183451704	-0.012391257	0.007131169	0.004605272	0.18341481	-7.62E-05	0.033573221	1	0.055111911	1	0.062273965	ENSG00000228065	6	RP11-222A11.1
VAT	Obesity	ENSG00000099256.14	8.07E-05	0.988718483	0.005704963	0.183454474	-0.000170436	0.976167361	0.005705126	0.183417546	0.000251103	0.006167864	1	0.997756129	1	0.993659205	ENSG00000228065	6	RP11-222A11.1
VAT	Obesity	ENSG00000099330.4	-0.007458388	0.048875457	0.003786596	0.183453008	-0.007518217	0.047075176	0.003786317	0.183416056	5.98E-05	0.031160501	1	0.219637062	1	0.223627344	ENSG00000228065	6	RP11-222A11.1
VAT	Obesity	ENSG00000099338.18	-0.00257737	0.238532841	0.002186689	0.183453949	-0.002531225	0.247010008	0.002186532	0.18341704	-4.61E-05	0.005278907	1	0.533077442	1	0.554266359	ENSG00000228065	6	RP11-222A11.1
VAT	Obesity	ENSG00000099810.14	-0.007338634	0.201181159	0.005741403	0.183453857	-0.007229178	0.207948919	0.005740964	0.183416947	-0.000109456	0.006189079	1	0.501589882	1	0.520051841	ENSG00000228065	6	RP11-222A11.1
VAT	Obesity	ENSG00000099949.14	-0.00813013	0.055117023	0.004238953	0.183453084	-0.00826893	0.051076795	0.004238653	0.183416108	0.000138799	2.47E-05	1	0.236951687	1	0.237726734	ENSG00000228065	6	RP11-222A11.1
VAT	Obesity	ENSG00000099991.12	0.01122281	0.049708992	0.005718732	0.183453018	0.011140203	0.051395219	0.005718291	0.183416112	8.26E-05	0.035120938	1	0.222001323	1	0.238404712	ENSG00000228065	6	RP11-222A11.1
VAT	Obesity	ENSG00000100023.13	-0.001830931	0.56995105	0.003222767	0.183454352	-0.001906768	0.554053812	0.003222542	0.183417414	7.58E-05	0.002813871	1	0.806905296	1	0.805615291	ENSG00000228075	4	BOD1L2
VAT	Obesity	ENSG00000100068.7	5.67E-05	0.991365587	0.005240826	0.183454474	-7.37E-05	0.988780569	0.005240489	0.183417546	0.000130407	0.003574943	1	0.997766076	1	0.996361581	ENSG00000228075	4	BOD1L2
VAT	Obesity	ENSG00000100079.5	-0.001057642	0.799813848	0.004170708	0.18345445	-0.000968735	0.816313429	0.004170384	0.183417526	-8.89E-05	0.001647911	1	0.918637629	1	0.929465786	ENSG00000228078	1	HLA-U
VAT	Obesity	ENSG00000100211.6	-0.003233157	0.139347989	0.002187187	0.183453648	-0.00318074	0.145846381	0.002187034	0.183416747	-5.24E-05	0.002081187	1	0.401503968	1	0.421062772	ENSG00000228078	1	HLA-U
VAT	Obesity	ENSG00000100461.13	-0.002078695	0.573629114	0.003694044	0.183454354	-0.001996784	0.588796547	0.003693773	0.183417436	-8.19E-05	0.002699349	1	0.806905296	1	0.823637411	ENSG00000228078	1	HLA-U
VAT	Obesity	ENSG00000100580.7	0.011299341	0.023518711	0.004988872	0.183452535	0.011191464	0.024868794	0.004988525	0.183415644	0.000107876	0.005949108	1	0.134392636	1	0.146976171	ENSG00000228078	1	HLA-U
VAT	Obesity	ENSG00000100908.9	-0.001137421	0.776853752	0.004013178	0.183454444	-0.001240845	0.757158529	0.004012894	0.18341751	0.000103424	0.000892638	1	0.905454542	1	0.900448937	ENSG00000228078	1	HLA-U
VAT	Obesity	ENSG00000101019.17	0.001186333	0.795818043	0.004584629	0.183454449	0.00108585	0.812764544	0.004584331	0.183417525	0.000100483	0.009329516	1	0.915954045	1	0.928683673	ENSG00000228078	1	HLA-U
VAT	Obesity	ENSG00000101052.8	0.001000042	0.7362316	0.002968821	0.183454431	0.001088427	0.713885085	0.002968624	0.183417495	-8.84E-05	0.00031413	1	0.886561612	1	0.878823744	ENSG00000228106	1	RP11-452F19.3
VAT	Obesity	ENSG00000101282.4	-3.49E-05	0.992910346	0.003932787	0.183454474	3.41E-05	0.993087731	0.003932511	0.183417546	-6.90E-05	0.024478588	1	0.997766076	1	0.997787455	ENSG00000228106	1	RP11-452F19.3
VAT	Obesity	ENSG00000101310.10	-0.001077554	0.720972142	0.003017005	0.183454426	-0.001003403	0.739430312	0.003016774	0.183417504	-7.42E-05	0.000390223	1	0.880089833	1	0.892750508	ENSG00000228106	1	RP11-452F19.3
VAT	Obesity	ENSG00000101353.10	-0.000983319	0.814092016	0.004181659	0.183454453	-0.001111301	0.790415075	0.004181412	0.183417519	0.000127982	0.000789635	1	0.92437246	1	0.917009082	ENSG00000228106	1	RP11-452F19.3
VAT	Obesity	ENSG00000101363.8	0.008637711	0.005740178	0.003127025	0.18345159	0.00821851	0.008681617	0.003131631	0.183414943	0.000419202	0.01688208	1	0.048897816	1	0.071955746	ENSG00000228106	1	RP11-452F19.3
VAT	Obesity	ENSG00000101425.8	-0.001382862	0.642630838	0.002980152	0.183454393	-0.001436528	0.629761182	0.002979958	0.183417458	5.37E-05	0.032618479	1	0.84339568	1	0.842487235	ENSG00000228157	2	AC007952.5
VAT	Obesity	ENSG00000101546.8	-0.003135405	0.569694924	0.005515212	0.183454352	-0.003043903	0.580981395	0.005514795	0.183417431	-9.15E-05	0.019402989	1	0.806905296	1	0.816866353	ENSG00000228157	2	AC007952.5
VAT	Obesity	ENSG00000101639.14	-0.00497622	0.011053124	0.001958352	0.183452033	-0.004913942	0.012094349	0.001958222	0.183415166	-6.23E-05	0.000130459	1	0.079444329	1	0.090106525	ENSG00000228157	2	AC007952.5
VAT	Obesity	ENSG00000101751.6	0.002908693	0.072897844	0.001621821	0.183453258	0.002951695	0.068743757	0.001621718	0.183416294	-4.30E-05	0.002048278	1	0.279186061	1	0.277387091	ENSG00000228262	4	AC073218.2
VAT	Obesity	ENSG00000102699.5	0.005919491	0.294477472	0.005646478	0.183454059	0.00584182	0.300820905	0.00564604	0.183417141	7.77E-05	0.04250389	1	0.603383685	1	0.623322597	ENSG00000228262	4	AC073218.2
VAT	Obesity	ENSG00000102780.12	0.008426757	0.131150593	0.005582176	0.183453613	0.008269939	0.138449801	0.005581796	0.183416716	0.000156818	0.000483206	1	0.387974943	1	0.412910374	ENSG00000228300	9	C19orf24

VAT	Obesity	ENSG00000103148.11	-0.004683941	0.311079333	0.00462401	0.183454086	-0.004541726	0.325965555	0.004623687	0.183417181	-0.000142215	8.84E-05	1	0.619458427	1	0.646775652	ENSG00000228409	1	CCT6P1
VAT	Obesity	ENSG00000103174.7	-0.002325169	0.526684324	0.003672811	0.183454323	-0.002393761	0.514530868	0.003672543	0.183417385	6.86E-05	0.011969298	1	0.779823873	1	0.784425829	ENSG00000228418	2	CTSLP3
VAT	Obesity	ENSG00000103226.13	-0.001316349	0.753001529	0.00418308	0.183454437	-0.001401796	0.737524132	0.004182792	0.183417504	8.54E-05	0.010283407	1	0.893787648	1	0.892400966	ENSG00000228446	2	AC073052.1
VAT	Obesity	ENSG00000103351.8	0.005782985	0.001339036	0.001802958	0.183450585	0.00581963	0.001246349	0.001802818	0.183413608	-3.66E-05	0.00291629	1	0.01687521	1	0.01764063	ENSG00000228446	2	AC073052.1
VAT	Obesity	ENSG00000103356.11	-0.000723393	0.82431215	0.003258491	0.183454455	-0.00065723	0.840142232	0.003258264	0.183417531	-6.62E-05	0.009571067	1	0.927365584	1	0.939531177	ENSG00000228653	2	HNRNPCP7
VAT	Obesity	ENSG00000103811.11	0.001418548	0.412667665	0.001731607	0.18345422	0.00138915	0.42238555	0.001731487	0.183417303	2.94E-05	0.03229319	1	0.708533979	1	0.728159973	ENSG00000228653	2	HNRNPCP7
VAT	Obesity	ENSG00000103932.7	-0.018991067	0.001625312	0.00602634	0.18345072	-0.01914295	0.001489429	0.006025948	0.183413732	0.000151883	0.002844318	1	0.01958891	1	0.020451858	ENSG00000228672	2	PROB1
VAT	Obesity	ENSG00000104442.5	-0.000250605	0.948350671	0.003868664	0.183454473	-0.000195438	0.959706385	0.003868373	0.183417545	-5.52E-05	0.045279631	1	0.982080274	1	0.98754495	ENSG00000228672	2	PROB1
VAT	Obesity	ENSG00000104679.6	0.005406987	0.101858249	0.003305178	0.183453463	0.005488419	0.09677903	0.003304929	0.183416504	-8.14E-05	0.000541467	1	0.342005798	1	0.339834761	ENSG00000228672	2	PROB1
VAT	Obesity	ENSG00000104774.8	-0.010487607	0.022859797	0.004608363	0.183452516	-0.010386515	0.024199704	0.004608137	0.183415626	-0.000101092	0.030633182	1	0.132827449	1	0.143946518	ENSG00000228696	4	ARL17B
VAT	Obesity	ENSG00000104866.6	-0.003552364	0.393791628	0.004165715	0.183454199	-0.003443601	0.408399828	0.00416541	0.183417288	-0.000108763	0.00042946	1	0.701655839	1	0.72254778	ENSG00000228696	4	ARL17B
VAT	Obesity	ENSG00000104953.14	-0.002618851	0.639556306	0.005591941	0.183454334	-0.002512925	0.653130143	0.005591705	0.18341747	-0.00010589	0.005320154	1	0.841849822	1	0.852951536	ENSG00000228696	4	ARL17B
VAT	Obesity	ENSG00000104967.6	0.01044811	0.023211563	0.00460281	0.183452526	0.010550471	0.021890686	0.004602637	0.18341556	-0.00010236	0.047688758	1	0.133602008	1	0.13459386	ENSG00000228716	2	DHFR
VAT	Obesity	ENSG00000105088.4	-0.004423486	0.282920189	0.004119532	0.183454038	-0.004501273	0.274504312	0.004119217	0.183417095	7.78E-05	0.006830894	1	0.588439881	1	0.593290447	ENSG00000228782	3	MRPL45P2
VAT	Obesity	ENSG00000105146.8	0.002532648	0.588758366	0.004684566	0.183454364	0.002631383	0.574284754	0.004684238	0.183417427	-9.87E-05	0.007007251	1	0.816569392	1	0.814504585	ENSG00000228789	2	HCG22
VAT	Obesity	ENSG00000105383.10	-0.004244041	0.275942651	0.003895473	0.183454025	-0.004308717	0.268659919	0.003895219	0.183417084	6.47E-05	0.048987925	1	0.580931898	1	0.584363319	ENSG00000228789	2	HCG22
VAT	Obesity	ENSG00000106077.14	-0.000525359	0.893235876	0.003914426	0.183454467	-0.000745711	0.848908168	0.003914253	0.183417532	0.000220353	1.17E-07	1	0.961627151	1	0.944752639	ENSG00000228789	2	HCG22
VAT	Obesity	ENSG00000106125.14	0.003863016	0.313224995	0.003830536	0.183454094	0.003930186	0.30485441	0.003830292	0.183417148	6.72E-05	0.04121847	1	0.619765744	1	0.626973302	ENSG00000228789	2	HCG22
VAT	Obesity	ENSG00000106133.13	-0.004069356	0.075815584	0.002291946	0.183453283	-0.005094811	0.026721063	0.002299543	0.183415691	0.001025454	6.38E-08	1	0.283034564	1	0.154937256	ENSG00000228789	2	HCG22
VAT	Obesity	ENSG00000106178.2	-0.009516413	0.087171641	0.005563484	0.183453368	-0.009690941	0.081510106	0.005563131	0.183416399	0.000174528	0.000277662	1	0.306098991	1	0.304422046	ENSG00000228789	2	HCG22
VAT	Obesity	ENSG00000106344.4	-0.004802738	0.408638306	0.005812391	0.183454216	-0.004949302	0.394459271	0.005812048	0.183417272	0.000146564	0.005682342	1	0.708044263	1	0.714375058	ENSG00000228914	1	OR1H1P
VAT	Obesity	ENSG00000106610.10	-0.001982913	0.258306593	0.001754163	0.18345399	-0.001523042	0.385967813	0.001756774	0.183417262	-0.000459872	3.32E-06	1	0.557845225	1	0.707997923	ENSG00000228914	1	OR1H1P
VAT	Obesity	ENSG00000106635.3	-0.005575859	0.237704314	0.00472232	0.183453947	-0.00522538	0.268509872	0.004722432	0.183417083	-0.00035048	2.47E-06	1	0.532768171	1	0.584363319	ENSG00000228960	4	OR2A9P
VAT	Obesity	ENSG00000106638.11	0.009993365	0.009802877	0.003869324	0.183451953	0.012038339	0.001948608	0.003885219	0.183413919	-0.002044974	1.70E-08	1	0.072731024	1	0.023938382	ENSG00000228960	4	OR2A9P
VAT	Obesity	ENSG00000106688.7	0.002366986	0.131499311	0.001569392	0.183453614	0.002314372	0.140272509	0.0015693	0.183416724	5.26E-05	0.000246898	1	0.387974943	1	0.416292606	ENSG00000228998	3	RP11-697E2.7
VAT	Obesity	ENSG00000107099.11	0.000263813	0.902663956	0.00215715	0.183454468	0.001568143	0.476426064	0.002202253	0.183417354	-0.00130433	0.003340946	1	0.965567705	1	0.772080125	ENSG00000228998	3	RP11-697E2.7
VAT	Obesity	ENSG00000107130.6	-0.00798379	0.135825918	0.005352791	0.183453633	-0.008412366	0.11624057	0.005355624	0.183416614	0.000428576	0.024136819	1	0.395299075	1	0.375232716	ENSG00000229089	2	ANKRD20A8P
VAT	Obesity	ENSG00000107742.8	-0.000757167	0.794720965	0.002910108	0.183454449	0.003850171	0.229511396	0.003204151	0.183417	-0.004607339	0.000593639	1	0.915072951	1	0.539107619	ENSG00000229089	2	ANKRD20A8P
VAT	Obesity	ENSG00000108384.10	-0.011532065	4.85E-05	0.002838393	0.183448235	-0.011455808	5.43E-05	0.002838205	0.183411389	-7.63E-05	0.001135891	0.133797875	0.001205386	0.149901575	0.001441361	ENSG00000229091	2	HSPA8P8
VAT	Obesity	ENSG00000108666.5	0.002392757	0.427058737	0.003012655	0.183454236	0.002442487	0.417480146	0.003012436	0.183417298	-4.97E-05	0.027404761	1	0.715653985	1	0.725782338	ENSG00000229186	3	ADAM1A
VAT	Obesity	ENSG00000108846.11	0.01039639	0.052604148	0.005364051	0.183453054	0.010516266	0.049920941	0.005363675	0.183416093	-0.00011987	0.004262775	1	0.231376391	1	0.234322784	ENSG00000229212	3	RP11-561C5.4
VAT	Obesity	ENSG00000108924.9	-0.00891714	0.051613983	0.004581481	0.183453042	-0.008800413	0.054732223	0.004581161	0.18341615	-0.000116727	0.001140458	1	0.22939548	1	0.245627538	ENSG00000229212	3	RP11-561C5.4
VAT	Obesity	ENSG00000109917.6	-0.004428452	0.441894619	0.005758727	0.183454251	-0.004562679	0.428149802	0.005758323	0.183417309	0.000134226	0.002850105	1	0.727515266	1	0.732605984	ENSG00000229212	3	RP11-561C5.4
VAT	Obesity	ENSG00000110011.9	-0.001638989	0.657352355	0.003694979	0.1834544	-0.000538105	0.884358706	0.003699663	0.183417538	-0.001100884	1.33E-08	1	0.852180602	1	0.960056703	ENSG00000229212	3	RP11-561C5.4
VAT	Obesity	ENSG00000110025.8	-0.01061548	0.017142106	0.004453449	0.183452326	-0.006340499	0.160881772	0.004522098	0.183416803	-0.004274981	5.66E-08	1	0.108763707	1	0.449882159	ENSG00000229212	3	RP11-561C5.4
VAT	Obesity	ENSG00000110455.9	0.000938425	0.576746237	0.001681331	0.183454356	0.000975992	0.561557485	0.001681208	0.183417419	-3.76E-05	0.002672632	1	0.806947626	1	0.809555852	ENSG00000229291	1	RP4-580N22.2
VAT	Obesity	ENSG00000110906.8	-0.015723627	0.001134082	0.004830637	0.183450469	-0.015803936	0.001068617	0.004830307	0.1834135	8.03E-05	0.038546761	1	0.014764466	1	0.015746258	ENSG00000229291	1	RP4-580N22.2
VAT	Obesity	ENSG00000111664.6	0.001906222	0.571833244	0.003371732	0.183454353	0.001988534	0.555322386	0.003371518	0.183417415	-8.23E-05	0.004539902	1	0.806905296	1	0.805802048	ENSG00000229390	1	MICD
VAT	Obesity	ENSG00000111666.6	-0.000590017	0.876320886	0.003791031	0.183454465	-0.000454298	0.904607251	0.003790766	0.183417541	-0.000135718	4.77E-06	1	0.951543054	1	0.970729398	ENSG00000229390	1	MICD
VAT	Obesity	ENSG00000111676.10	-0.003501305	0.31040811	0.003451714	0.183454085	-0.003555844	0.30289773	0.00345147	0.183417145	5.45E-05	0.041424256	1	0.618575006	1	0.624344835	ENSG00000229390	1	MICD
VAT	Obesity	ENSG00000111801.11	0.019542063	1.51E-10	0.003051066	0.183438968	0.016884885	6.22E-08	0.003119694	0.183406476	0.002657178	4.60E-05	4.15E-07	1.12E-08	0.000171758	3.90E-06	ENSG00000229390	1	MICD
VAT	Obesity	ENSG00000112031.11	0.004518367	0.075121775	0.002538802	0.183453277	0.004475424	0.077910617	0.002538608	0.183416371	4.29E-05	0.015286847	1	0.282737093	1	0.297007324	ENSG00000229390	1	MICD
VAT	Obesity	ENSG00000112041.8	-0.017011823	0.00032436	0.004732011	0.183449589	-0.016852345	0.000368778	0.004731812	0.183412752	-0.000159478	0.001805306	0.895232629	0.005526127	1	0.006696239	ENSG00000229390	1	MICD
VAT	Obesity	ENSG00000112053.9	-0.009030464	0.005477992	0.003251285	0.183451558	-0.008932531	0.006005828	0.003251178	0.183414693	-9.79E-05	0.009603069	1	0.047544835	1	0.054526597	ENSG00000229390	1	MICD
VAT	Obesity	ENSG00000112339.10	0.000425708	0.929924979	0.004840927	0.183454471	0.00052281	0.913991692	0.004840604	0.183417542	-9.71E-05	0.01491921	1	0.976170888	1	0.972419923	ENSG00000229390	1	MICD
VAT	Obesity	ENSG00000112357.8	0.000330534	0.873924815	0.002083087	0.183454465	0.000364709	0.861005977	0.002082933	0.183417534	-3.42E-05	0.024896422	1	0.951116912	1	0.950288579	ENSG00000229390	1	MICD
VAT	Obesity	ENSG00000112514.11	-0.009872461	0.000463978	0.002820092	0.183449842	-0.01119687	7.77E-05	0.002833565	0.183411645	0.00132441	2.06E-06	1	0.00731759	0.214367329	0.001981293	ENSG00000229390	1	MICD
VAT	Obesity	ENSG00000112619.6	-0.003572071	0.034676652	0.001691225	0.183452788	-0.003634061	0.031642968	0.001691128	0.183415801	6.20E-05	7.78E-05	1	0.17455266	1	0.172598009	ENSG00000229390	1	MICD
VAT	Obesity	ENSG00000112977.11	0.005273545	0.168299968	0.003827819	0.183453757	0.005327005	0.163994266	0.003827518	0.183416814	-5.35E-05	0.034956084	1	0.450948512	1	0.455295507	ENSG00000229291	3	HLA-DRB6
VAT	Obesity	ENSG00000113119.8	-0.018109156	0.000256284	0.004953373	0.183449422	-0.017969029	0.000285796	0.004953049	0.183412572	-0.000140128	0.000703086	0.707344985	0.004684404	0.788795859	0.005533516	ENSG00000229391	3	HLA-DRB6
VAT	Obesity	ENSG00000113141.11	-0.010945354	0.000283795	0.00301551	0.183449494	-0.010831715	0.000327891	0.003015316	0.183412669	-0.00011364	9.45E-06	0.783274966	0.005022832	0.904978408	0.006073681	ENSG00000229391	3	HLA-DRB6
VAT	Obesity	ENSG00000114503.6	-0.005495108	0.368899894	0.006115618	0.183454169	-0.005329887	0.383438883	0.006115213	0.183417259	-0.000165221	0.001104598	1	0.676520736	1	0.707013865	ENSG00000229391	3	HLA-DRB6
VAT	Obesity	ENSG00000114841.13	0.010988886	0.060493906	0.0085393	0.183453142	0.01113751	0.057085482	0.005853652	0.183416178	-0.000148625	0.013825785	1	0.25037186	1	0.251902356	ENSG00000229425	1	AJ006998.2
VAT	Obesity	ENSG00000115274.10	-0.004533318	0.31729381	0.004533157	0.183454096	-0.004637556	0.306258422	0.004532822	0.18341715	0.000104238	0.001696926	1	0.623838113	1	0.628923546	ENSG00000229425	1	AJ006998.2
VAT	Obesity	ENSG00000115275.7	0.002073587	0.484299282	0.002964787	0.183454289	0.0021393	0.470529956	0.002964589	0.183417349	-6.57E-05	0.007091169	1	0.764245865	1	0.76685518	ENSG00000229694	2	RP11-305L7.6
VAT	Obesity	ENSG00000115423.14	0.002399533	0.292448299	0.002279263	0.183454055	0.002537527	0.265594164	0.00227935	0.183417078	-0.000137993	0.000282492	1	0.601010652	1	0.579936623	ENSG00000229694	2	RP11-305L7.6
VAT	Obesity	ENSG00000115457.5	3.68E-05	0.994863009	0.005712069	0.183454474	0.000669243	0.906742828	0.005712797	0.183417541	-0.000632467	2.18E-07	1	0.997766076	1	0.970734158	ENSG00000229759	1	MRPS18AP1
VAT	Obesity	ENSG00000115486.7	0.009837497	0.015584117	0.004067568	0.183452263	0.009634957	0.017858473	0.004067876	0.183415426	0.000202541	0.007968358	1	0.10325657	1	0.119020171	ENSG00000229759	1	MRPS18AP1
VAT	Obesity	ENSG00000115687.9	0.001507045	0.632794424	0.003154148	0.183454388	0.001582329	0.615880439	0.00315395	0.183417451	-7.53E-05	0.005979287	1	0.840477676	1	0.839253732	ENSG00000229759	1	MRPS18AP1
VAT	Obesity	ENSG00000115947.9	0.004964015	0.252620196	0.004339138	0.183453979	0.005055899	0.24390711	0.004338792	0.183417033	-9.19E-05	0.001140291	1	0.549867303	1	0.550886762	ENSG00000229827	1	AC093899.3
VAT	Obesity	ENSG00000116157.5	-0.000108746	0.945337394	0.001586078	0.183454472	-6.07E-05	0.969454674	0.001585991	0.183417545	-4.80E-05	0.001526511	1	0.981611028	1	0.990920808	ENSG00000229836	1	XXbac-BPG248L24.10
VAT	Obesity	ENSG00000116539.6	0.011112002	0.037220345	0.005333732	0.183452833	0.01100033	0.039156212	0.005333372	0.183415938	0.000111672	0.00990284	1	0.182790008	1	0.196136378	ENSG00000230092	3	RP11-206L10.8
VAT	Obesity	ENSG00000116663.6	-0.003388882	0.516335864	0.005221662	0.183454315	-0.003281467	0.529690509	0.005221284	0.183417397	-0.000107414	0.00614392	1	0.776929212	1	0.786996417	ENSG00000230189	2	GS1-124K5.2
VAT	Obesity	ENSG00000116688.12	0.005887536	0.284425526	0.005500151	0.183454041	0.00578469	0.292886817	0.005499743	0.183417128	0.000102847	0.010054801	1	0.59068055	1	0.615229003	ENSG00000230189	2	GS1-124K5.2
VAT	Obesity	ENSG00000116857.12	-0.004325505	0.223930379	0.003556735	0.183453915	-0.004410063	0.214973021	0.00355648	0.183416965	8.46E-05	0.001753774	1	0.52199987	1	0.526433789	ENSG00000230224	1	PHBP9
VAT	Obesity	ENSG00000116906.7	0.008741162	0.125525755	0.005701684	0.183453586	0.00865386	0.129042804	0.005701285	0.183416675	8.73E-05	0.025178229	1	0.379063468	1	0.395731266	ENSG00000230224	1	PHBP9
VAT	Obesity	ENSG00000117115.8	-0.010753172	0.04412347	0.005342073	0.183452943	-0.010946494	0.040439396	0.005341739	0.183415959	0.000193322	3.59E-05	1	0.206758534	1	0.201467029	ENSG00000230224	1	PHBP9
VAT	Obesity	ENSG00000117122.9	-0.005926226	0.012065138	0.002360818	0.183452092	-0.005970751	0.01142585	0.002360641	0.183415128	4.45E-05	0.008478976	1	0.085384052	1	0.087142608	ENSG00000230224	1	PHBP9
VAT	Obesity	ENSG00000117153.11	-0.00999564	0.063290562	0.00538225	0.18345317	-0.010119867	0.060061253	0.005381912	0.18341621	0.000124227	0.000012798	1	0.258023563	1	0.25801405	ENSG00000230224	1	PHBP9
VAT	Obesity	ENSG00000117174.6	-0.003739994	0.548689246	0.006236173	0.183454338	-0.003617885	0.561789947	0.00623574	0.183417419	-0.00012211	0.013058701	1	0.793647904	1	0.809555852	ENSG00000230267	3	HERC2P4
VAT	Obesity	ENSG00000117450.9	-0.003294267	0.505026167	0.004941874	0.183454306	-0.003211722	0.515726799	0.004941514	0.183417386	-8.25E-05	0.024894074	1	0.770249732	1	0.784425829	ENSG00000230267	3	HERC2P4
VAT	Obesity	ENSG00000117523.11	0.016749774	0.000474739	0.004792986	0.183449858	0.01663217	0.000519791	0.004792624	0.183412995	0.000117604	0.00052198	1	0.007444768	1	0.0087477	ENSG00000230267	3	HERC2P4
VAT	Obesity	ENSG00000117569.14	0.006049173	0.10662206	0.003748945	0.18345349	0.006133798	0.10178955	0.00374871	0.183416534	-8.46E-05	0.0098324	1	0.351165734	1	0.351173964	ENSG00000230295	1	RP11-458F8.2
VAT	Obesity	ENSG00000117595.6	0.005119082	0.114783792	0.003245978	0.183453534	0.004941884	0.127902234	0.003246052	0.18341667	0.000177198	0.000507231	1	0.365551911	1	0.395731266	ENSG00000230295	1	RP11-458F8.2
VAT	Obesity	ENSG00000118197.9	-0.00012711	0.964769197	0.002877777	0.183454473	-7.37E-05	0.97956526	0.00287755	0.183417546	-5.34E-05	0.004738359	1	0.985729121	1	0.994336196	ENSG00000230310	1	CTD-2192J16.11
VAT	Obesity	ENSG00000118307.14	-0.005195086	0.350503012	0.00555929	0.183454144	-0.005097805	0.359113088	0.005558873	0.183417228	-9.73E-05	0.014716978	1	0.661582235	1	0.683932408	ENSG00000230387	1	RP4-737E23.2
VAT	Obesity	ENSG00000118514.9	-0.000150608	0.935018976	0.001847231	0.183454472	-0.000105715	0.954359914	0.00184711	0.183417545	-4.49E-05	0.00378911	1	0.977307539	1	0.984913318	ENSG00000230387	1	RP4-737E23.2
VAT	Obesity	ENSG00000118961.10	0.001839852	0.612779993	0.003635285	0.183454377	0.001713999	0.637269752	0.003635051	0.183417462	0.000125854	4.79E-05	1	0.83120188	1	0.845380113	ENSG00000230387	1	RP4-737E23.2
VAT	Obesity	ENSG00000119203.9	-0.010853869	0.022643061	0.004761698	0.18345251	-0.01076847	0.02371921	0.004761325	0.183415613	-8.54E-05	0.006908361	1	0.132124418	1	0.141699174	ENSG00000230387	1	RP4-737E23.2
VAT	Obesity	ENSG00000119431.5	-0.004362245	0.047720506	0.002203317	0.183452992	-0.0042924	0.051381444	0.002203168	0.183416111	-6.98E-05	0.000100302	1	0.216270272	1	0.238404712	ENSG00000230387	1	RP4-737E23.2
VAT	Obesity	ENSG00000119471.10	-0.002700313	0.597015939	0.005107488	0.183454368	-0.002578684	0.613615595	0.00510712	0.18341745	-0.000121629	0.001613477	1	0.819937013	1	0.839253732	ENSG00000230438	5	RP11-420G6.4
VAT	Obesity	ENSG00000119509.8	0.011322848	7.00E-07	0.002282117	0.183445169	0.011394059	5.94E-07	0.002281946	0.183408124	-7.12E-05	1.70E-05	0.001930744	2.93E-05	0.001640224	2.98E-05	ENSG00000230438	5	RP11-420G6.4
VAT	Obesity	ENSG00000119715.10	0.004203309	0.408682101	0.005087422	0.183454216	0.004317057	0.396083263	0.005087043	0.183417274	-0.000113748	0.00197143	1	0.708044263	1	0.715434904	ENSG00000230438	5	RP11-420G6.4
VAT	Obesity	ENSG00000119718.6	-0.004076222	0.028359531	0.00185935	0.183452657	-0.004114527	0.026895115	0.001859216	0.183415695	3.83E-05	0.006646339	1	0.153475108	1	0.155231297	ENSG00000230438	5	RP11-420G6.4
VAT	Obesity	ENSG00000119772.12	-0.000740291	0.882480255	0.005007855	0.183454466	-0.000666664	0.89408785	0.005007488	0.183417539	-7.36E-05	0.046750583	1	0.95522585	1	0.968028006	ENSG00000230438	5	RP11-420G6.4
VAT	Obesity	ENSG00000119977.16	-0.005825781	0.128016002	0.003827777	0.183453599	-0.005767163	0.131870553	0.003827503	0.183416688	-5.86E-05	0.04463034	1	0.383214931	1	0.399591076	ENSG00000230438	5	RP11-420G6.4
VAT	Obesity	ENSG00000120437.7	0.004068801	0.396631636	0.004800078	0.183454203	0.003977707	0.40725308	0.004799716	0.183417286	9.11E-05	0.007511774	1	0.701928581	1	0.721556426	ENSG00000230454	1	U73166.2
VAT	Obesity	ENSG00000120555.9	-0.008903871	0.103296066	0.005466095	0.183453471	-0.008773409	0.108461189	0.005465783	0.183416572	-0.000130463	0.010514865	1	0.344846701	1	0.365064489	ENSG00000230454	1	U73166.2
VAT	Obesity	ENSG00000120669.11	-0.000923629	0.616367556	0.001843552	0.183454379	-0.000889629	0.627455637	0.001843412	0.183417457	-2.90E-05	0.026096865	1	0.831570896	1	0.842487235	ENSG00000230454	1	U73166.2
VAT	Obesity	ENSG00000120784.11	0.004181378	0.146985415	0.002883183	0.183453679	0.004234835	0.141857304	0.002882974	0.183416731	-5.35E-05	0.013536574	1	0.413958925	1	0.417278146	ENSG00000230521	1	HCG4P7
VAT	Obesity	ENSG00000120907.13	0.005857508	0.001473888	0.001842103	0.183450652	0.005940957	0.001259209	0.001842079	0.183413615	-8.34E-05	0.000650606	1	0.018160406	1	0.017731721	ENSG00000230521	1	HCG4P7
VAT	Obesity	ENSG00000121064.8	0.004286197	0.141283475	0.002913737	0.183453656	0.004204949	0.148953419	0.002913547	0.183416759	8.12E-05	0.000908523	1	0.405767317	1	0.426597721	ENSG00000230521	1	HCG4P7
VAT	Obesity	ENSG00000121350.11	0.007166296	0.023193541	0.003156626	0.183452526	0.007218639	0.022198608	0.003156392	0.183415569	-5.23E-05	0.022933629	1	0.133602008	1	0.135971568	ENSG00000230521	1	HCG4P7
VAT	Obesity	ENSG00000121577.9	-0.001934433	0.695549177	0.004943153	0.183454416	-0.001848369	0.70844157	0.004942822	0.183417493	-8.61E-05	0.033556986	1	0.866149932	1	0.876422561	ENSG00000230521	1	HCG4P7
VAT	Obesity	ENSG00000122085.12	-0.007769832	0.072961343	0.004333241	0.183453259	-0.007882273	0.068887828	0.004332922	0.183416295	0.000112442	0.00043245	1	0.279186061	1	0.277410173	ENSG00000230521	1	HCG4P7
VAT	Obesity	ENSG00000122376.7	0.001848246	0.642189593	0.00397776	0.183454392	0.001720504	0.665336126	0.00397751	0.183417475	0.000127742	0.000222091	1	0.843374123	1	0.856496133	ENSG00000230521	1	HCG4P7
VAT	Obesity	ENSG00000122678.10	-0.001137517	0.568630572	0.001995399	0.183454351	-0.001201369	0.547103657	0.001995278	0.183417409	6.39E-05	0.0003372	1	0.806905296	1	0.799791363	ENSG00000230528	3	NOS2P3
VAT	Obesity	ENSG00000122696.8	0.007588087	0.002774936	0.002536427	0.183451091	0.007546505	0.002925596	0.002536244	0.18341442	4.16E-05	0.029855662	1	0.029101822	1	0.032690866	ENSG00000230528	3	NOS2P3
VAT	Obesity	ENSG00000122733.11	0.007946237	0.115283421	0.005045595	0.183453537	0.008044282	0.110837624	0.005045201	0.183416585	-9.80E-05	0.003771209	1	0.366186259	1	0.366800769	ENSG00000230555	2	RP11-517P14.2
VAT	Obesity	ENSG00000122783.12	0.005902935	0.023282538	0.002601836	0.18345253	0.005830119	0.025032172	0.002601672	0.183415648	7.28E-05	0.001168548	1	0.133602008	1	0.147625628	ENSG00000230596	2	AC112229.4
VAT	Obesity	ENSG00000122873.7	-0.00071676	0.759365663	0.002339951	0.183454439	-0.000753561	0.74740237	0.002339767	0.183417507	3.68E-05	0.017406526	1	0.897964531	1	0.89626095	ENSG00000230596	2	AC112229.4
VAT	Obesity	ENSG00000123096.7	0.00354889	0.389399529	0.004123223	0.183454194	0.003479787	0.398664623	0.004122925	0.183417277	6.91E-05	0.025861016	1	0.696527997	1	0.716012529	ENSG00000230596	2	AC112229.4
VAT	Obesity	ENSG00000123243.10	0.01401068	0.004364492	0.004915072	0.183451403	0.013920304	0.004620509	0.004914705	0.183414514	9.04E-05	0.010695075	1	0.040287616	1	0.044966202	ENSG00000230606	6	AC159540.1
VAT	Obesity	ENSG00000123415.10	0.005131385	0.016427912	0.002138722	0.183452298	0.00507873	0.017558846	0.002138593	0.183415415	5.27E-05	0.00591379	1	0.106434359	1	0.117913417	ENSG00000230606	6	AC159540.1
VAT	Obesity	ENSG00000123643.8	0.003476615	0.162038787	0.002486405	0.183453735	0.003436244	0.166935432	0.002486212	0.183416824	4.04E-05	0.016087417	1	0.441391512	1	0.457993828	ENSG00000230712	2	AP000354.4
VAT	Obesity	ENSG00000124164.11	0.001047666	0.533896721	0.001684163	0.183454328	0.001075973	0.522870969	0.001684032	0.183417392	-2.83E-05	0.012630371	1	0.783986954	1	0.784841324	ENSG00000230795	2	HLA-K
VAT	Obesity	ENSG00000124532.10	0.003723603	0.231901659	0.003114745	0.183453934	0.003662893	0.239571419	0.003114495	0.183417023	6.07E-05	0.022484094	1	0.52715189	1	0.546616798	ENSG00000230795	2	HLA-K
VAT	Obesity	ENSG00000124562.5	-0.036341463	1.53E-24	0.003553866	0.183414956	-0.036021943	3.86E-24	0.003553989	0.183378729	-0.000319521	3.71E-08	4.21E-21	8.43E-22	1.07E-20	2.13E-21	ENSG00000230795	2	HLA-K
VAT	Obesity	ENSG00000124613.4	0.001681958	0.246202583	0.001450434	0.183453966	0.00083714	0.566887709	0.001461891	0.183417422	0.000844818	4.30E-06	1	0.543180758	1	0.811940881	ENSG00000230795	2	HLA-K
VAT	Obesity	ENSG00000124678.13	0.008710428	0.00391321	0.00301912	0.183451328	0.008563181	0.004562759	0.003019035	0.183414506	0.000147247	4.91E-05	1	0.036733053	1	0.044966202	ENSG00000230795	2	HLA-K
VAT	Obesity	ENSG00000124733.3	-0.001338301	0.74976842	0.004196025	0.183454436	-0.001171921	0.780006683	0.004195763	0.183417517	-0.00016638	5.77E-06	1	0.892799597	1	0.911107144	ENSG00000230795	2	HLA-K
VAT	Obesity	ENSG00000124767.6	0.016017362	0.008777804	0.006112098	0.183451878	0.015835343	0.009570391	0.006111703	0.183415009	0.000182018	0.000430687	1	0.06692469	1	0.076785697	ENSG00000230795	2	HLA-K
VAT	Obesity	ENSG00000125409.8	-0.005020226	0.094263768	0.003000148	0.183453416	-0.005138433	0.086743851	0.002999961	0.183416437	0.000118207	6.87E-06	1	0.323190063	1	0.316168943	ENSG00000230795	2	HLA-K
VAT	Obesity	ENSG00000125450.6	-0.003897016	0.388166031	0.00451593	0.183454193	-0.004048102	0.370008307	0.004515659	0.183417242	0.000151086	0.000215602	1	0.695743688	1	0.692818993	ENSG00000230795	2	HLA-K
VAT	Obesity	ENSG00000125485.13	-0.007868745	0.01774991	0.003319022	0.18345235	-0.007795892	0.018823674	0.003318796	0.18341546	-7.29E-05	0.006398864	1	0.110802893	1	0.120957701	ENSG00000230795	2	HLA-K
VAT	Obesity	ENSG00000125821.7	-0.005452302	0.222267442	0.004467187	0.183453911	-0.005323479	0.233356504	0.004466911	0.183417009	-0.000128823	0.001059089	1	0.52199987	1	0.540524906	ENSG00000230836	1	AC104135.4
VAT	Obesity	ENSG00000125863.13	-0.010511407	0.080559233	0.006015269	0.18345332	-0.010664596	0.076221978	0.00601485	0.183416358	0.000153189	0.001227498	1	0.291025502	1	0.293816565	ENSG00000230836	1	AC104135.4
VAT	Obesity	ENSG00000125885.9	0.001489668	0.504398662	0.002231427	0.183454306	0.001522804	0.494930923	0.002231255	0.1834137	-3.31E-05	0.02979099	1	0.770249732	1	0.775358255	ENSG00000230836	1	AC104135.4
VAT	Obesity	ENSG00000125912.6	0.000216697	0.970943897	0.0059492	0.183454474	0.002636031	0.659609135	0.005984816	0.183417473	-0.002419335	0.000233116	1	0.988370418	1	0.854766662	ENSG00000230836	1	AC104135.4
VAT	Obesity	ENSG00000126246.5	0.005189585	0.218263134	0.004215195	0.183453901	0.00525749	0.212268553	0.004214907	0.183416958	-6.79E-05	0.045506162	1	0.521961016	1	0.524026123	ENSG00000230911	1	PPIHP1
VAT	Obesity	ENSG00000127616.13	-0.002068059	0.671163929	0.004871163	0.183454406	-0.002263879	0.64208813	0.004870846	0.183417464	0.00019582	1.90E-06	1	0.857647266	1	0.848972395	ENSG00000230939	1	RP11-314C16.1
VAT	Obesity	ENSG00000127952.12	0.005381446	0.005810689	0.001951223	0.183451599	0.005431133	0.005375979	0.001951112	0.183414618	-4.97E-05	0.000512404	1	0.049240505	1	0.05044591	ENSG00000230939	1	RP11-314C16.1
VAT	Obesity	ENSG00000128604.14	0.000875894	0.820011835	0.003849578	0.183454455	0.001086107	0.777827159	0.00384937	0.183417516	-0.000210213	1.62E-08	1	0.927365584	1	0.910459344	ENSG00000230939	1	RP11-314C16.1
VAT	Obesity	ENSG00000128655.12	-0.004414388	0.222530238	0.003626686	0.183453914	-0.004472189	0.217494093	0.003626431	0.183416971	5.78E-05	0.004648538	1	0.52199987	1	0.529248865	ENSG00000230939	1	RP11-314C16.1
VAT	Obesity	ENSG00000128928.4	0.004280894	0.228246247	0.003552933	0.183453925	0.004339465	0.221921451	0.003552686	0.183416982	-5.86E-05	0.037174176	1	0.524101338	1	0.532716487	ENSG00000230953	2	RP11-631H13.6
VAT	Obesity	ENSG00000129055.8	-0.001971874	0.231648899	0.001653913	0.183453937	-0.001936454	0.241634794	0.00165838	0.183417028	-3.54E-05	0.007723255	1	0.527920526	1	0.547564824	ENSG00000231007	4	CDC20P1
VAT	Obesity	ENSG00000129197.10	-0.020349422	1.68E-05	0.004728849	0.183447475	-0.020264101	1.82E-05	0.004728568	0.183410605	-8.53E-05	0.046947536	0.046462655	0.000480981	0.050340929	0.000606517	ENSG00000231043	2	AC007238.1
VAT	Obesity	ENSG00000129473.5	0.002788405	0.608453413	0.005443098	0.183454375	0.00268976	0.621171921	0.005442765	0.183417454	9.86E-05	0.041280945	1	0.828767095	1	0.840409069	ENSG00000231066	2	AP000688.8
VAT	Obesity	ENSG00000129480.8	-0.00090599	0.750469226	0.002848837	0.183454436	-0.000853775	0.764394664	0.002848622	0.183417512	-5.22E-05	0.009633996	1	0.892799597	1	0.904298874	ENSG00000231130	1	HLA-T
VAT	Obesity	ENSG00000130300.4	0.005004423	0.241113184	0.00426922	0.183453955	0.005088836	0.233236483	0.004268926	0.183417009	-8.44E-05	0.01342419	1	0.535635762	1	0.540524906	ENSG00000231130	1	HLA-T
VAT	Obesity	ENSG00000130414.7	0.000501048	0.747379539	0.001555583	0.183454435	0.000528733	0.733916856	0.001555462	0.183417502	-2.77E-05	0.008714259	1	0.892799597	1	0.890236789	ENSG00000231130	1	HLA-T
VAT	Obesity	ENSG00000130511.11	0.006055934	0.313502022	0.006008456	0.183454095	0.00620419	0.301768807	0.006008035	0.183417143	-0.000148256	0.001652689	1	0.619877551	1	0.623730655	ENSG00000231130	1	HLA-T
VAT	Obesity	ENSG00000130517.9	0.008752042	0.001207561	0.002703661	0.183450513	0.008814609	0.001112362	0.00270348	0.183413528	-6.26E-05	0.004787193	1	0.015501716	1	0.016244016	ENSG00000231130	1	HLA-T
VAT	Obesity	ENSG00000130733.6	0.005469992	0.231872985	0.004575295	0.183453934	0.005368762	0.240589175	0.004574934	0.183417026	0.00010123	0.000749821	1	0.52715189	1	0.546973742	ENSG00000231130	1	HLA-T
VAT	Obesity	ENSG00000130943.5	-0.009894041	0.002549521	0.003278977	0.183451033	-0.009607804	0.003403407	0.00328051	0.183414304	-0.000286237	0.009583504	1	0.027812961	1	0.036161216	ENSG00000231205	7	ZNF826P
VAT	Obesity	ENSG00000131351.10	0.002804536	0.551423744	0.004708533	0.1834534	0.002693495	0.567264742	0.004708209	0.183417422	0.00011041	0.003114253	1	0.793908438	1	0.81202149	ENSG00000231205	7	ZNF826P
VAT	Obesity	ENSG00000131495.4	0.013248535	0.000112175	0.003429903	0.183448834	0.013137927	0.000127808	0.003429662	0.183412	0.000110608	3.52E-05	0.309603817	0.00240003	0.352748922	0.00291528	ENSG00000231205	7	ZNF826P

VAT	Obesity	ENSG00000131558.10	0.019366818	9.50E-05	0.004961938	0.183448716	0.019289287	0.000101199	0.004961579	0.183411834	7.75E-05	0.037341893	0.262175373	0.002112498	0.279309184	0.002428776	ENSG00000231205	7	ZNF826P
VAT	Obesity	ENSG00000131697.13	-0.003365396	0.463671466	0.004592421	0.183454271	-0.003475553	0.44913706	0.004592098	0.18341733	0.000110157	0.00210723	1	0.746635499	1	0.747200025	ENSG00000231236	2	AP001604.3
VAT	Obesity	ENSG00000131941.3	0.001277897	0.800503487	0.005057059	0.18345445	0.00118459	0.814782415	0.005056709	0.183417525	9.33E-05	0.019819908	1	0.918665124	1	0.928683673	ENSG00000231402	1	WASF5P
VAT	Obesity	ENSG00000131944.5	0.001870959	0.506650155	0.002817441	0.183454307	0.001938535	0.491395771	0.002817266	0.183417367	-6.76E-05	0.006285105	1	0.771793347	1	0.775358255	ENSG00000231402	1	WASF5P
VAT	Obesity	ENSG00000132139.8	-0.001536728	0.661624952	0.003511149	0.183454402	-0.001654872	0.637404353	0.003511059	0.183417462	0.000118144	0.005995617	1	0.854099711	1	0.845380113	ENSG00000231402	1	WASF5P
VAT	Obesity	ENSG00000132141.9	-0.000562777	0.901969192	0.004568948	0.183454468	-0.000663254	0.884573984	0.0045687	0.183417538	0.000100477	0.022360129	1	0.965567705	1	0.960056703	ENSG00000231412	2	CTC-490G23.2
VAT	Obesity	ENSG00000132286.7	0.001571905	0.730067519	0.004555773	0.183454429	0.001690376	0.710589284	0.004555473	0.183417494	-0.000118471	0.001835667	1	0.884153731	1	0.876721691	ENSG00000231412	2	CTC-490G23.2
VAT	Obesity	ENSG00000132406.7	0.002161746	0.523576317	0.003389151	0.18345432	0.002222665	0.511910147	0.003388897	0.183417383	-6.09E-05	0.011916828	1	0.779013819	1	0.784425829	ENSG00000231443	2	AC024937.6
VAT	Obesity	ENSG00000132837.10	-0.014512505	0.018086609	0.006139355	0.183452362	-0.014413209	0.018882579	0.0061389	0.183415463	-9.93E-05	0.025856832	1	0.111926101	1	0.120957701	ENSG00000231464	1	AC024937.4
VAT	Obesity	ENSG00000132906.13	-0.003649861	0.119150025	0.002342126	0.183453556	-0.003696275	0.114497875	0.002341937	0.183416605	4.64E-05	0.001907523	1	0.372684435	1	0.371781334	ENSG00000231500	2	RPS18
VAT	Obesity	ENSG00000133661.11	0.003788917	0.047678747	0.001913377	0.183452992	0.003816042	0.046092236	0.001913232	0.183416043	-2.71E-05	0.045478187	1	0.216270272	1	0.220094413	ENSG00000231500	2	RPS18
VAT	Obesity	ENSG00000134201.6	0.016095568	0.001780487	0.005151217	0.183450784	0.016020355	0.001869425	0.005150832	0.18341389	7.52E-05	0.042291767	1	0.020647668	1	0.023559873	ENSG00000231500	2	RPS18
VAT	Obesity	ENSG00000134440.7	0.011312132	0.05496147	0.005894236	0.183453082	0.011414305	0.052788085	0.005893824	0.183416129	-0.000102173	0.027176735	1	0.236651571	1	0.241837823	ENSG00000231500	2	RPS18
VAT	Obesity	ENSG00000134490.9	-0.005687921	0.200676297	0.004444977	0.183453855	-0.00562547	0.205630338	0.004444643	0.183416941	-6.25E-05	0.049173058	1	0.501589882	1	0.517356182	ENSG00000231500	2	RPS18
VAT	Obesity	ENSG00000134825.9	-0.003977838	0.487014649	0.00572297	0.183454291	-0.003703503	0.517539465	0.005722847	0.183417388	-0.000274336	0.00013889	1	0.765031548	1	0.784425829	ENSG00000231500	2	RPS18
VAT	Obesity	ENSG00000134905.12	7.32E-05	0.980894616	0.003055914	0.183454474	0.000146995	0.961632389	0.003055691	0.183417545	-7.38E-05	0.001123279	1	0.99502166	1	0.98754495	ENSG00000231500	2	RPS18
VAT	Obesity	ENSG00000135052.12	-0.003831759	0.107503798	0.002380687	0.183453495	-0.003789743	0.111389487	0.002380519	0.183416588	-4.20E-05	0.022393784	1	0.353035267	1	0.366914394	ENSG00000231576	1	Z95114.3
VAT	Obesity	ENSG00000135093.8	0.003959891	0.330941246	0.004073037	0.183454117	0.003897228	0.338615844	0.004072744	0.1834172	6.27E-05	0.042313294	1	0.640950731	1	0.660896608	ENSG00000231698	2	AP002856.5
VAT	Obesity	ENSG00000135213.8	-0.036547248	1.25E-17	0.004275348	0.183426856	-0.03665955	9.92E-18	0.004275084	0.183389759	0.000112302	0.003075767	3.46E-14	2.66E-15	2.74E-14	2.11E-15	ENSG00000231698	2	AP002856.5
VAT	Obesity	ENSG00000135318.7	0.00042483	0.933618513	0.005100426	0.183454471	0.000507074	0.920801026	0.005100074	0.183417542	-8.22E-05	0.042579869	1	0.977307539	1	0.973721371	ENSG00000231768	1	RP5-855F14.1
VAT	Obesity	ENSG00000135480.10	0.005180458	0.360501599	0.005665352	0.183454158	0.005319266	0.347746423	0.005664999	0.183417213	-0.000138808	0.005161692	1	0.670499496	1	0.670656147	ENSG00000231768	1	RP5-855F14.1
VAT	Obesity	ENSG00000135951.10	-0.003829269	0.324951742	0.003890213	0.183454108	-0.003931763	0.312138873	0.003889964	0.18341716	0.000102494	0.002129471	1	0.633061352	1	0.634391229	ENSG00000231784	4	DBIL5P
VAT	Obesity	ENSG00000136371.5	0.000957202	0.855936316	0.0052724	0.183454462	0.000851036	0.871758511	0.00527201	0.183417536	0.000106166	0.005817521	1	0.944116239	1	0.955921132	ENSG00000231784	4	DBIL5P
VAT	Obesity	ENSG00000136875.8	0.002151237	0.181146555	0.00160872	0.183453798	0.002186664	0.174035495	0.001608607	0.183416848	-3.54E-05	0.004529744	1	0.473899992	1	0.470919574	ENSG00000231784	4	DBIL5P
VAT	Obesity	ENSG00000137070.13	-0.011004602	0.00558056	0.00397067	0.183451571	-0.010681376	0.007152481	0.003971256	0.183414812	-0.000323226	0.000255719	1	0.048283214	1	0.062273965	ENSG00000231784	4	DBIL5P
VAT	Obesity	ENSG00000137225.8	-0.003856014	0.32657802	0.003930574	0.18345411	-0.003976321	0.311681461	0.003930332	0.183417159	0.000120307	0.000547326	1	0.634310581	1	0.633928395	ENSG00000231784	4	DBIL5P
VAT	Obesity	ENSG00000137275.9	0.000817266	0.861373728	0.004680098	0.183454463	0.000734683	0.875252198	0.004679785	0.183417537	8.26E-05	0.031848307	1	0.944533766	1	0.956332568	ENSG00000231784	4	DBIL5P
VAT	Obesity	ENSG00000137285.9	-0.003725005	0.312422511	0.003687565	0.183454088	-0.003668658	0.319764549	0.003687305	0.183417172	-5.63E-05	0.049112525	1	0.619458427	1	0.641269508	ENSG00000231793	4	DOC2GP
VAT	Obesity	ENSG00000137310.7	0.016127751	0.003126383	0.0054577	0.183451173	0.008345427	0.149229867	0.005786357	0.18341676	0.007782324	5.23E-05	1	0.031840649	1	0.426812885	ENSG00000231793	4	DOC2GP
VAT	Obesity	ENSG00000137312.10	0.006194203	0.026562002	0.002792821	0.183452615	0.002436847	0.401075493	0.002902035	0.18341728	0.003757356	1.95E-06	1	0.147671738	1	0.718344166	ENSG00000231793	4	DOC2GP
VAT	Obesity	ENSG00000137411.12	-0.002843931	0.048940867	0.001444272	0.183453008	-0.001103273	0.457327937	0.001484381	0.183417337	-0.001740659	3.98E-07	1	0.219637062	1	0.755285959	ENSG00000231793	4	DOC2GP
VAT	Obesity	ENSG00000137563.7	-0.002013618	0.442070259	0.002619502	0.183454251	-0.001966031	0.452898453	0.002619306	0.183417333	-4.76E-05	0.011606719	1	0.727515266	1	0.750751641	ENSG00000231793	4	DOC2GP
VAT	Obesity	ENSG00000137700.12	0.024469577	6.96E-08	0.004537951	0.183443484	0.024583661	6.05E-08	0.004537804	0.183406454	-0.000114084	0.031684423	0.000192178	3.70E-06	0.000166866	3.88E-06	ENSG00000231793	4	DOC2GP
VAT	Obesity	ENSG00000137876.5	-0.021287286	0.000100368	0.005472687	0.183448755	-0.021077465	0.000117355	0.005472366	0.18341194	-0.000209821	2.70E-05	0.277016792	0.002198546	0.32389977	0.002744913	ENSG00000231799	2	RP13-93L13.2
VAT	Obesity	ENSG00000137877.8	0.001460895	0.49545392	0.002143142	0.183454298	0.001493785	0.485766857	0.002142985	0.183417362	-3.29E-05	0.038281884	1	0.766551816	1	0.775358255	ENSG00000231816	1	RP4-782L23.1
VAT	Obesity	ENSG00000138111.10	0.001442475	0.28907056	0.001360615	0.183454049	0.001462632	0.282349404	0.001360515	0.183417109	-2.02E-05	0.044446889	1	0.59807702	1	0.601299657	ENSG00000231852	2	CYP21A2
VAT	Obesity	ENSG00000138193.10	-0.00194799	0.748511413	0.006076019	0.183454435	-0.002043685	0.736587048	0.006075586	0.183417503	9.57E-05	0.03987224	1	0.892799597	1	0.891658005	ENSG00000231852	2	CYP21A2
VAT	Obesity	ENSG00000138303.13	-0.004225546	0.277235467	0.00388895	0.183454028	-0.007530535	0.059540541	0.003996706	0.183416204	0.003304988	0.000345202	1	0.582299881	1	0.257361325	ENSG00000231925	7	TAPBP
VAT	Obesity	ENSG00000138363.10	-3.61E-05	0.990779578	0.003125289	0.183454474	3.80E-05	0.990292539	0.003125064	0.183417546	-7.41E-05	0.001732599	1	0.997766076	1	0.99714778	ENSG00000231925	7	TAPBP
VAT	Obesity	ENSG00000138385.11	0.00537429	0.18588029	0.004062609	0.183453813	0.005464522	0.178569679	0.004062316	0.183416862	-9.02E-05	0.003324074	1	0.481717936	1	0.480362878	ENSG00000231925	7	TAPBP
VAT	Obesity	ENSG00000138468.11	-0.006999591	0.177500868	0.005190681	0.183453787	-0.006819332	0.188897837	0.005190349	0.183416894	-0.000180259	4.97E-05	1	0.466573709	1	0.497954184	ENSG00000231925	7	TAPBP
VAT	Obesity	ENSG00000138600.5	-0.00099827	0.808113508	0.004110466	0.183454452	-0.000313664	0.939351998	0.004122577	0.183417544	-0.000684606	0.033001942	1	0.921269427	1	0.980488158	ENSG00000231925	7	TAPBP
VAT	Obesity	ENSG00000138621.7	-0.005225461	0.015674044	0.002162474	0.183452267	-0.005254252	0.015101812	0.0021623	0.183415315	2.88E-05	0.035311518	1	0.103365207	1	0.107059606	ENSG00000231925	7	TAPBP
VAT	Obesity	ENSG00000138669.5	0.003181976	0.514169022	0.004877636	0.183454313	0.003061757	0.530160811	0.004877265	0.183417397	0.00012022	0.000441463	1	0.776929212	1	0.786996417	ENSG00000231925	7	TAPBP
VAT	Obesity	ENSG00000139133.2	-0.000227466	0.967157413	0.005524533	0.183454473	-0.000145195	0.979031025	0.005524121	0.183417546	-8.23E-05	0.038970576	1	0.985729121	1	0.994336196	ENSG00000231925	7	TAPBP
VAT	Obesity	ENSG00000139344.3	-0.00543617	0.08520846	0.003158293	0.183453354	-0.005360963	0.089599222	0.003158117	0.183416457	-7.52E-05	0.011986007	1	0.301506858	1	0.322837926	ENSG00000231925	7	TAPBP
VAT	Obesity	ENSG00000139899.6	-0.00168163	0.168268153	0.001220525	0.183453757	-0.001652267	0.175792555	0.001220443	0.183416853	-2.94E-05	0.00316227	1	0.450948512	1	0.474743104	ENSG00000231943	3	RP11-395L14.4
VAT	Obesity	ENSG00000140254.8	-0.011387919	0.022808393	0.005002081	0.183452515	-0.011523942	0.021223989	0.005001747	0.18341554	0.000136023	0.000960731	1	0.132808366	1	0.132256412	ENSG00000232040	2	SCAND3
VAT	Obesity	ENSG00000140323.4	-0.001338628	0.414800111	0.001641529	0.183454223	-0.001266744	0.44027109	0.001641421	0.183417321	-7.19E-05	1.56E-07	1	0.709763365	1	0.742750586	ENSG00000232040	2	SCAND3
VAT	Obesity	ENSG00000140463.9	0.003303608	0.536777271	0.00534829	0.18345433	0.00339236	0.525860818	0.005347878	0.183417394	-8.88E-05	0.015607071	1	0.78620379	1	0.785965627	ENSG00000232040	2	SCAND3
VAT	Obesity	ENSG00000140471.12	0.000944135	0.800237595	0.003731171	0.18345445	0.001048185	0.778754109	0.003730952	0.183417516	-0.000104051	0.002267505	1	0.918665124	1	0.910529706	ENSG00000232040	2	SCAND3
VAT	Obesity	ENSG00000141002.14	-0.009122855	0.071238146	0.005057126	0.183453244	-0.008996477	0.075224238	0.005056762	0.18341635	-0.000126378	0.000922361	1	0.274916222	1	0.291599575	ENSG00000232053	2	AC009784.3
VAT	Obesity	ENSG00000141252.15	0.001527903	0.581731303	0.002773678	0.183454359	0.001483049	0.59283787	0.002773463	0.183417438	4.49E-05	0.016967413	1	0.810488842	1	0.825129864	ENSG00000232063	1	RP11-307E17.8
VAT	Obesity	ENSG00000141391.9	-0.000754103	0.762642007	0.002496918	0.18345444	-0.000708407	0.776615967	0.002496747	0.183417516	-4.57E-05	0.021870136	1	0.899221841	1	0.909787806	ENSG00000232075	1	MRPL35P2
VAT	Obesity	ENSG00000141425.13	-0.004649934	0.357194312	0.005050295	0.183454154	-0.004575934	0.36486108	0.005049908	0.183417236	-7.40E-05	0.034581094	1	0.668015266	1	0.688411445	ENSG00000232075	1	MRPL35P2
VAT	Obesity	ENSG00000141452.5	-0.037212439	1.54E-16	0.004508587	0.183428727	-0.037094171	1.91E-16	0.004508325	0.183391964	-0.000118268	0.004264177	4.25E-13	3.04E-14	5.27E-13	3.76E-14	ENSG00000232075	1	MRPL35P2
VAT	Obesity	ENSG00000141519.10	-0.004519018	0.37293082	0.005071862	0.183454174	-0.004397279	0.385914418	0.005071533	0.183417262	-0.000121739	0.004541638	1	0.68300535	1	0.707997923	ENSG00000232112	3	TMA7
VAT	Obesity	ENSG00000141556.16	-0.002566894	0.67532553	0.006128423	0.183454408	-0.002450868	0.689196378	0.006127997	0.183417486	-0.000116025	0.016188677	1	0.860416532	1	0.869882588	ENSG00000232210	1	PHBP15
VAT	Obesity	ENSG00000141560.10	0.001417706	0.246818305	0.001224152	0.183453967	0.001390544	0.255959865	0.001224077	0.183417058	2.72E-05	0.013088206	1	0.543398386	1	0.566137648	ENSG00000232388	2	LINC00493
VAT	Obesity	ENSG00000141574.3	0.005386238	0.350361261	0.005767531	0.183454144	0.007436393	0.198620171	0.005784851	0.183416922	-0.002050156	6.55E-06	1	0.661582235	1	0.51015583	ENSG00000232388	2	LINC00493
VAT	Obesity	ENSG00000141580.11	-0.000450969	0.941562512	0.006151858	0.183454472	-0.000574098	0.9256435	0.006151443	0.183417543	0.000123129	0.014003747	1	0.981016434	1	0.974118595	ENSG00000232388	2	LINC00493
VAT	Obesity	ENSG00000141934.5	-0.002570406	0.164673984	0.001849846	0.183453744	-0.002542607	0.169257116	0.001849711	0.183416832	-2.78E-05	0.042803476	1	0.445152005	1	0.463442103	ENSG00000232388	2	LINC00493
VAT	Obesity	ENSG00000141994.11	-0.000760524	0.737319089	0.002267473	0.183454432	-0.000822449	0.716798243	0.002267316	0.183417496	6.19E-05	0.000565463	1	0.887171413	1	0.880054782	ENSG00000232450	1	RP4-730K3.3
VAT	Obesity	ENSG00000142046.10	0.001481703	0.515392917	0.002277917	0.183454314	0.001426902	0.53101807	0.002277748	0.183417398	5.48E-05	0.000867379	1	0.776929212	1	0.786996417	ENSG00000232491	1	RP4-756H11.1
VAT	Obesity	ENSG00000142102.11	0.000479156	0.863294031	0.002782835	0.183454463	0.000420885	0.879775237	0.002782634	0.183417537	5.83E-05	0.005345829	1	0.94551251	1	0.958352832	ENSG00000232546	1	RP11-458F8.1
VAT	Obesity	ENSG00000142156.10	0.00832192	0.001119516	0.002553794	0.18345046	0.008914991	0.000524442	0.002570662	0.183413001	-0.000593071	0.045299527	1	0.014643911	1	0.008772485	ENSG00000232546	1	RP11-458F8.1
VAT	Obesity	ENSG00000142632.12	-0.002190221	0.120944287	0.001412294	0.183453565	-0.00221664	0.116499969	0.001412198	0.183416615	2.64E-05	0.020642362	1	0.373803171	1	0.37563074	ENSG00000232559	2	GS1-124K5.12
VAT	Obesity	ENSG00000142655.8	0.002793007	0.621609441	0.005658774	0.183454382	0.002905514	0.607611066	0.0056584	0.183417446	-0.000112507	0.016739602	1	0.833933632	1	0.834474842	ENSG00000232561	2	GTF2IP1
VAT	Obesity	ENSG00000142686.7	0.005366383	0.13816419	0.003619418	0.183453643	0.005306171	0.142614533	0.003619177	0.183416734	6.02E-05	0.044243695	1	0.399279871	1	0.418027797	ENSG00000232561	2	GTF2IP1
VAT	Obesity	ENSG00000142694.6	0.002709921	0.526530976	0.004278974	0.183454322	0.002784302	0.515213139	0.004278655	0.183417386	-7.44E-05	0.015819364	1	0.779823873	1	0.784425829	ENSG00000232561	2	GTF2IP1
VAT	Obesity	ENSG00000143156.9	-0.009898538	0.056820429	0.005196917	0.183453103	-0.010007393	0.05413257	0.005196543	0.183416144	0.000108855	0.005682233	1	0.24109463	1	0.24496821	ENSG00000232615	4	CTD-2012J19.1
VAT	Obesity	ENSG00000143185.3	-0.005322079	0.326494144	0.005424046	0.18345411	-0.005203612	0.337344104	0.005423666	0.183417198	-0.000118467	0.005163116	1	0.634310581	1	0.659397824	ENSG00000232615	4	CTD-2012J19.1
VAT	Obesity	ENSG00000143376.8	0.007904673	0.123983135	0.005138675	0.18345358	0.007513225	0.143811154	0.005139894	0.183416739	0.000391448	0.003367741	1	0.37657343	1	0.419132824	ENSG00000232615	4	CTD-2012J19.1
VAT	Obesity	ENSG00000143437.16	-0.002728778	0.576238575	0.004882515	0.183454356	-0.001607514	0.742662741	0.004896039	0.183417505	-0.001121264	0.002454073	1	0.806947626	1	0.893915902	ENSG00000232615	4	CTD-2012J19.1
VAT	Obesity	ENSG00000143458.7	-0.003745104	0.398192343	0.004432837	0.183454204	-0.004025596	0.363853694	0.004433245	0.183417234	0.000280492	0.001269576	1	0.703592104	1	0.687833011	ENSG00000232615	4	CTD-2012J19.1
VAT	Obesity	ENSG00000143603.14	-0.008434712	0.132300678	0.005604111	0.183453618	-0.008518767	0.128460822	0.005603707	0.183416673	8.41E-05	0.046996467	1	0.388871002	1	0.395731266	ENSG00000232629	4	HLA-DQB2
VAT	Obesity	ENSG00000143631.10	-0.003119733	0.183470604	0.002345406	0.183453805	-0.003033672	0.195840911	0.002345331	0.183416914	-8.61E-05	0.001770315	1	0.477265662	1	0.508008379	ENSG00000232629	4	HLA-DQB2
VAT	Obesity	ENSG00000143811.12	-0.003177412	0.525274896	0.005001941	0.183454322	-0.00332708	0.505919812	0.005001587	0.183417379	0.000149667	0.000108619	1	0.779823873	1	0.782701055	ENSG00000232629	4	HLA-DQB2
VAT	Obesity	ENSG00000143951.11	0.005609101	0.346006214	0.005952153	0.183454138	0.004099303	0.491888798	0.005964286	0.183417368	0.001509798	0.000105952	1	0.657697762	1	0.775358255	ENSG00000232629	4	HLA-DQB2
VAT	Obesity	ENSG00000143954.8	-0.001779193	0.77196279	0.006139198	0.183454442	-0.001918014	0.754705337	0.006138799	0.183417509	0.000138822	0.007366051	1	0.904559068	1	0.898976285	ENSG00000232629	4	HLA-DQB2
VAT	Obesity	ENSG00000144182.12	-0.001480604	0.513435554	0.002265659	0.183454313	-0.001518565	0.50266798	0.002265507	0.183417376	3.80E-05	0.040742958	1	0.776692113	1	0.778980138	ENSG00000232637	3	WI2-3658N16.1
VAT	Obesity	ENSG00000144199.7	-0.015262743	0.001632529	0.00484524	0.183450723	-0.015170688	0.001740586	0.004844889	0.18341384	-9.21E-05	0.011445396	1	0.019590343	1	0.022554068	ENSG00000232653	4	GOLGA8N
VAT	Obesity	ENSG00000144214.5	0.002901396	0.404276868	0.003478859	0.183454211	0.002814375	0.418483462	0.003478593	0.183417299	8.70E-05	0.000310791	1	0.707119945	1	0.725782338	ENSG00000232653	4	GOLGA8N
VAT	Obesity	ENSG00000144230.12	0.001110876	0.597128042	0.002101802	0.183454369	0.001043718	0.619460783	0.002101664	0.183417453	6.72E-05	0.000135703	1	0.819937013	1	0.8401532	ENSG00000232653	4	GOLGA8N
VAT	Obesity	ENSG00000144476.5	0.001235689	0.793162115	0.004712652	0.183454448	0.00131591	0.780052964	0.004712297	0.183417517	-8.02E-05	0.016653457	1	0.914040683	1	0.911107144	ENSG00000232677	2	LINC00665
VAT	Obesity	ENSG00000144847.8	0.005446036	0.320776365	0.005485174	0.183454101	0.005581823	0.308825426	0.005484793	0.183417155	-0.000135787	0.001724604	1	0.628794579	1	0.630590092	ENSG00000232677	2	LINC00665
VAT	Obesity	ENSG00000145569.5	-0.002964363	0.580111615	0.00535836	0.183454358	-0.002877707	0.591206254	0.005357969	0.183417437	-8.67E-05	0.029428811	1	0.809049044	1	0.824941855	ENSG00000232677	2	LINC00665
VAT	Obesity	ENSG00000145781.4	-0.004915284	0.194452122	0.003788207	0.183453838	-0.005032532	0.183989614	0.003787924	0.183416879	0.000117248	1.71E-05	1	0.492826316	1	0.489908029	ENSG00000232859	5	LYRM9
VAT	Obesity	ENSG00000146409.6	-0.006176842	0.131574111	0.00409625	0.183453615	-0.006073754	0.138110647	0.004095956	0.183416715	-0.000103088	0.000919601	1	0.387974943	1	0.412910374	ENSG00000232926	1	AC000078.5
VAT	Obesity	ENSG00000146556.10	-0.00172958	0.639430653	0.003691778	0.183454391	-0.001637764	0.657296328	0.003691573	0.183417472	-9.18E-05	0.008814613	1	0.841849822	1	0.854766662	ENSG00000232926	1	AC000078.5
VAT	Obesity	ENSG00000147576.11	-0.012271954	0.000191001	0.003289486	0.183449213	-0.010850738	0.00099774	0.003296911	0.183413452	-0.001421215	3.12E-10	0.527162594	0.003738742	1	0.015047886	ENSG00000232926	1	AC000078.5
VAT	Obesity	ENSG00000147813.11	0.001219264	0.574588471	0.002172187	0.183454355	0.001186404	0.584915019	0.002172026	0.183417433	3.29E-05	0.037555099	1	0.806905296	1	0.820551558	ENSG00000232926	1	AC000078.5
VAT	Obesity	ENSG00000147912.8	0.005169868	0.364913739	0.005705988	0.183454164	0.005090123	0.372321168	0.005705554	0.183417245	7.97E-05	0.046094969	1	0.674137831	1	0.695586169	ENSG00000233005	1	AC067959.1
VAT	Obesity	ENSG00000148110.11	-0.007365997	0.095928295	0.004424221	0.183453426	-0.007479686	0.090888033	0.004423925	0.183416466	0.000113688	0.001798702	1	0.326866783	1	0.32624827	ENSG00000233005	1	AC067959.1
VAT	Obesity	ENSG00000148225.11	0.006961019	0.04496242	0.003471811	0.183452955	0.007065824	0.041817167	0.003471569	0.18341598	-0.000104805	0.000128084	1	0.209268597	1	0.205736949	ENSG00000233265	1	MICF
VAT	Obesity	ENSG00000148229.8	-0.002921315	0.378645712	0.003318186	0.183454181	-0.002862728	0.388246864	0.003317944	0.183417265	-5.86E-05	0.017548774	1	0.687088866	1	0.708846095	ENSG00000233265	1	MICF
VAT	Obesity	ENSG00000148824.14	0.007275592	0.000440618	0.002070163	0.183449805	0.007243889	0.000466278	0.002070014	0.183412918	3.17E-05	0.043250346	1	0.007102966	1	0.00819516	ENSG00000233265	1	MICF
VAT	Obesity	ENSG00000149231.7	-0.001738987	0.146346795	0.001197189	0.183453677	-0.001763202	0.140779954	0.001197099	0.183416726	2.42E-05	0.005073206	1	0.413425951	1	0.417278146	ENSG00000233265	1	MICF
VAT	Obesity	ENSG00000149273.10	-0.003539303	0.460942682	0.004800382	0.183454269	-0.00345566	0.47157152	0.004800028	0.18341735	-8.36E-05	0.01747579	1	0.744619218	1	0.766963697	ENSG00000233265	1	MICF
VAT	Obesity	ENSG00000149489.4	0.001529172	0.638711333	0.003257011	0.183454391	0.001454417	0.655181392	0.003256819	0.183417471	7.48E-05	0.012151615	1	0.841849822	1	0.854180748	ENSG00000233265	1	MICF
VAT	Obesity	ENSG00000149499.7	0.000493479	0.926687547	0.005363125	0.183454471	0.000353092	0.947504776	0.005362826	0.183417544	0.000140387	0.005748913	1	0.976170888	1	0.981754039	ENSG00000233265	1	MICF
VAT	Obesity	ENSG00000149743.9	-0.010071655	0.01093075	0.003957561	0.183452026	-0.01019455	0.009992262	0.003957347	0.183415038	0.000122895	0.001320375	1	0.079183388	1	0.079124756	ENSG00000233265	1	MICF
VAT	Obesity	ENSG00000150337.9	-0.003465602	0.444097181	0.004528461	0.183454253	-0.004073334	0.368756504	0.00453194	0.183417241	0.000607733	0.001288949	1	0.729116989	1	0.69094905	ENSG00000233297	4	RASA4DP
VAT	Obesity	ENSG00000151148.9	0.005315696	0.028050238	0.002419977	0.18345265	0.005271185	0.029381521	0.002419822	0.183415753	4.45E-05	0.031531697	1	0.152430349	1	0.163493946	ENSG00000233297	4	RASA4DP
VAT	Obesity	ENSG00000151348.9	0.001468017	0.574461927	0.00261449	0.183454355	0.001420708	0.586828471	0.002614297	0.183417434	4.73E-05	0.013349137	1	0.806905296	1	0.822155624	ENSG00000233327	6	USP32P2
VAT	Obesity	ENSG00000151491.8	0.00188829	0.681662846	0.004603393	0.183454411	0.001348609	0.769747785	0.0046074	0.183417514	0.000539682	0.007816451	1	0.863333733	1	0.907738428	ENSG00000233396	3	RP11-458D21.1
VAT	Obesity	ENSG00000151640.8	0.004078411	0.00189479	0.001312964	0.183450827	0.004053569	0.002018203	0.001312873	0.183413943	2.48E-05	0.016289057	1	0.021521074	1	0.024430883	ENSG00000233448	2	PMS2P9
VAT	Obesity	ENSG00000151725.7	0.002557211	0.671270974	0.006025408	0.183454406	0.002414686	0.688585874	0.006025039	0.183417485	0.000142525	0.007608845	1	0.857647266	1	0.869882588	ENSG00000233448	2	PMS2P9
VAT	Obesity	ENSG00000151883.12	-0.003870217	0.209207486	0.003081998	0.183453878	-0.002850086	0.357105742	0.003094909	0.183417226	-0.001020131	0.000356735	1	0.509182242	1	0.683029694	ENSG00000233448	2	PMS2P9
VAT	Obesity	ENSG00000152240.8	-0.000247452	0.894405482	0.001864299	0.183454467	-0.00020775	0.911264313	0.001864165	0.183417541	-3.97E-05	0.004896964	1	0.962401221	1	0.971531422	ENSG00000233448	2	PMS2P9
VAT	Obesity	ENSG00000152348.11	-0.000683719	0.522124261	0.001068187	0.183454319	-0.000729764	0.494475415	0.001068142	0.18341737	4.60E-05	7.29E-05	1	0.778563922	1	0.775358255	ENSG00000233448	2	PMS2P9
VAT	Obesity	ENSG00000152439.8	0.000404495	0.875309555	0.002577751	0.183454465	0.000353774	0.890831405	0.002577545	0.183417539	5.07E-05	0.002243048	1	0.951543054	1	0.965708829	ENSG00000233569	1	RP11-500B12.1
VAT	Obesity	ENSG00000152465.13	-0.014364828	0.006213033	0.005249663	0.183451644	-0.014222156	0.006741471	0.00524926	0.183414772	-0.000142672	7.93E-05	1	0.051797881	1	0.059827845	ENSG00000233569	1	RP11-500B12.1
VAT	Obesity	ENSG00000152683.10	-0.002530832	0.674220245	0.006020582	0.183454407	-0.002428625	0.686642106	0.006020143	0.183417485	-0.000102207	0.022584317	1	0.859911219	1	0.869882588	ENSG00000233590	1	RP11-153K11.3
VAT	Obesity	ENSG00000152926.10	0.004793221	0.023901107	0.002122094	0.183452546	-0.00082623	0.742655609	0.002516395	0.183417505	0.005619451	3.26E-05	1	0.136014549	1	0.893915902	ENSG00000233609	3	RP11-62H7.2
VAT	Obesity	ENSG00000153048.6	-0.001474515	0.708196648	0.00393961	0.183454421	-0.001587469	0.686962588	0.003939318	0.183417485	0.000112954	8.15E-05	1	0.872628294	1	0.869882588	ENSG00000233609	3	RP11-62H7.2
VAT	Obesity	ENSG00000153246.7	0.009450462	0.001202623	0.002918362	0.18345051	0.008905953	0.002358135	0.002928635	0.183414051	0.00054451	0.028448951	1	0.015501716	1	0.027933277	ENSG00000233609	3	RP11-62H7.2
VAT	Obesity	ENSG00000154222.10	0.007955296	0.062435412	0.004269706	0.183453162	0.008022346	0.060238688	0.00426937	0.183416212	-6.71E-05	0.017780972	1	0.255670231	1	0.25801405	ENSG00000233690	1	EBAG9P1
VAT	Obesity	ENSG00000154710.11	-0.002967422	0.108892891	0.001850946	0.183453503	1.95E-05	0.992089784	0.001969938	0.183417546	-0.002986952	9.57E-06	1	0.355343638	1	0.99714778	ENSG00000233754	1	AP001628.7
VAT	Obesity	ENSG00000155085.11	0.010834643	0.062904	0.005825473	0.183453167	0.010701631	0.06619059	0.005825174	0.18341627	0.000133012	0.021535162	1	0.257207467	1	0.27337482	ENSG00000233754	1	AP001628.7
VAT	Obesity	ENSG00000155542.7	-0.003155568	0.208656183	0.002509855	0.183453877	-0.003230401	0.198034715	0.002509687	0.18341692	7.48E-05	0.000287325	1	0.508805001	1	0.509865497	ENSG00000233820	2	RP11-535M15.2
VAT	Obesity	ENSG00000155957.12	0.003289751	0.231078224	0.002746982	0.183453932	0.003243993	0.237595634	0.002746774	0.183417019	4.58E-05	0.018001946	1	0.52708752	1	0.544132965	ENSG00000233836	3	RP11-255H23.2
VAT	Obesity	ENSG00000156042.13	0.000152358	0.943372205	0.002144913	0.183454472	-0.000178764	0.933639291	0.002146873	0.183417543	0.000331121	0.000611648	1	0.981243858	1	0.977187882	ENSG00000233836	3	RP11-255H23.2
VAT	Obesity	ENSG00000156096.8	0.006899516	0.083938167	0.003992122	0.183453345	0.006745851	0.091047535	0.003991862	0.183416467	0.000153664	4.70E-06	1	0.298928183	1	0.326336482	ENSG00000233836	3	RP11-255H23.2
VAT	Obesity	ENSG00000156219.12	0.008166974	0.066386415	0.004448704	0.1834532	0.008085203	0.069131994	0.004448383	0.183416298	8.18E-05	0.014685892	1	0.264946219	1	0.277735522	ENSG00000233892	1	PAIP1P1
VAT	Obesity	ENSG00000156261.8	-0.002350817	0.060506533	0.001252374	0.183453142	-0.0023308	0.062711208	0.001252285	0.183416237	-2.00E-05	0.037576711	1	0.25037186	1	0.263845936	ENSG00000233892	1	PAIP1P1
VAT	Obesity	ENSG00000156603.10	-0.006976181	0.049216215	0.003547117	0.183453012	-0.00690583	0.05153339	0.003546879	0.183416113	-7.04E-05	0.015386357	1	0.22051421	1	0.238644559	ENSG00000233892	1	PAIP1P1
VAT	Obesity	ENSG00000156860.11	0.003362976	0.491800349	0.004891966	0.183454295	0.00346174	0.479140041	0.004891627	0.183417357	-9.88E-05	0.010658139	1	0.766182814	1	0.774198351	ENSG00000233892	1	PAIP1P1
VAT	Obesity	ENSG00000157107.9	0.010759815	0.050381404	0.005498914	0.183453027	0.010339075	0.060191252	0.00550128	0.183416211	0.00042074	0.018845449	1	0.224640832	1	0.25801405	ENSG00000233916	1	ZDHHC20P1
VAT	Obesity	ENSG00000157150.4	-0.004473759	0.173127332	0.003284154	0.183453773	-0.004554998	0.165429517	0.003283965	0.183416819	8.12E-05	0.007691291	1	0.460092631	1	0.456129337	ENSG00000233916	1	ZDHHC20P1
VAT	Obesity	ENSG00000157214.9	-0.014329603	0.000448894	0.004083023	0.183449818	-0.014480599	0.000390016	0.004082752	0.183412792	0.000150996	8.83E-06	1	0.007148695	1	0.007035575	ENSG00000233916	1	ZDHHC20P1
VAT	Obesity	ENSG00000157601.9	0.002485334	0.44397803	0.003246708	0.183454253	0.002549036	0.432363281	0.00324654	0.183417313	-6.37E-05	0.047741035	1	0.729116989	1	0.737081891	ENSG00000233916	1	ZDHHC20P1
VAT	Obesity	ENSG00000157911.5	0.005464432	0.305387359	0.005331414	0.183454077	0.005267237	0.323136203	0.005331025	0.183417177	0.000197196	5.96E-07	1	0.613888646	1	0.643939293	ENSG00000233916	1	ZDHHC20P1
VAT	Obesity	ENSG00000157978.7	0.002751706	0.368792855	0.003061746	0.183454169	0.002853313	0.351340381	0.003061516	0.183417218	-0.000101606	3.50E-06	1	0.676520736	1	0.674808247	ENSG00000233922	1	AL133493.2
VAT	Obesity	ENSG00000158006.9	-0.001185807	0.735848657	0.003515	0.183454431	-0.001132079	0.747381433	0.003514746	0.183417507	-5.37E-05	0.042002543	1	0.886561612	1	0.89626095	ENSG00000234127	4	TRIM26
VAT	Obesity	ENSG00000158161.11	-0.000346327	0.946922587	0.00520231	0.183454472	-0.000482867	0.926043453	0.005201968	0.183417543	0.00013654	0.001669454	1	0.981782998	1	0.974118595	ENSG00000234127	4	TRIM26
VAT	Obesity	ENSG00000158411.6	0.007752502	0.157212667	0.005480695	0.183453718	0.007883865	0.150272453	0.005480341	0.183416764	-0.000131363	0.004748116	1	0.432609133	1	0.42846278	ENSG00000234127	4	TRIM26

VAT	Obesity	ENSG00000158717.6	-0.000700923	0.86733301	0.004195956	0.183454464	-0.000772305	0.853955581	0.004195625	0.183417533	7.14E-05	0.009885258	1	0.948025973	1	0.947822399	ENSG00000234127	4	TRIM26
VAT	Obesity	ENSG00000158825.5	-0.001827447	0.591039824	0.003400976	0.183454365	-0.00197501	0.561404393	0.00340075	0.183417419	0.000147563	1.58E-07	1	0.818499706	1	0.809555852	ENSG00000234127	4	TRIM26
VAT	Obesity	ENSG00000159263.11	0.00054481	0.849842529	0.002877729	0.183454461	0.000497486	0.862740367	0.002877522	0.183417535	4.73E-05	0.030547482	1	0.941875299	1	0.950564236	ENSG00000234127	4	TRIM26
VAT	Obesity	ENSG00000159445.8	-0.000571305	0.875701118	0.003652353	0.183454465	-0.000690829	0.849974372	0.003652259	0.183417533	0.000119524	0.007492431	1	0.951543054	1	0.945557947	ENSG00000234127	4	TRIM26
VAT	Obesity	ENSG00000160221.12	-0.004557413	0.323827036	0.004619195	0.183454106	-0.0046505	0.314006885	0.004618856	0.183417163	9.31E-05	0.006419931	1	0.633061352	1	0.636904895	ENSG00000234127	4	TRIM26
VAT	Obesity	ENSG00000160223.12	0.000507825	0.867662328	0.003047648	0.183454464	0.000451326	0.882262548	0.003047407	0.183417538	5.65E-05	0.004764056	1	0.948025973	1	0.959812626	ENSG00000234127	4	TRIM26
VAT	Obesity	ENSG00000160226.11	-0.004749696	0.030430221	0.002194411	0.183452703	-0.004788738	0.029081619	0.002194268	0.183415746	3.90E-05	0.034717749	1	0.160281317	1	0.162152058	ENSG00000234127	4	TRIM26
VAT	Obesity	ENSG00000160282.9	-0.001857314	0.518445238	0.002876237	0.183454316	-0.001950559	0.497677763	0.002876301	0.183417372	9.32E-05	0.038492184	1	0.778044231	1	0.776039901	ENSG00000234127	4	TRIM26
VAT	Obesity	ENSG00000160284.10	0.000583089	0.608293144	0.001137708	0.183454375	0.000553623	0.626509026	0.001137628	0.183417457	2.95E-05	0.00082346	1	0.828767095	1	0.842487235	ENSG00000234127	4	TRIM26
VAT	Obesity	ENSG00000160307.5	-0.00302522	0.068090022	0.001658186	0.183453216	-0.003001117	0.070293261	0.001658057	0.183416308	-2.41E-05	0.030767519	1	0.26846923	1	0.281173044	ENSG00000234147	1	RP3-460G2.2
VAT	Obesity	ENSG00000160321.10	0.007416922	0.206563529	0.005872125	0.183453871	0.007532895	0.199522475	0.005871702	0.183416924	-0.000115972	0.009192209	1	0.507220054	1	0.510412237	ENSG00000234147	1	RP3-460G2.2
VAT	Obesity	ENSG00000160439.11	-0.001742817	0.607142692	0.003389661	0.183454374	-0.001804095	0.594535092	0.003389398	0.183417439	6.13E-05	0.007940157	1	0.827969162	1	0.825553026	ENSG00000234147	1	RP3-460G2.2
VAT	Obesity	ENSG00000160695.10	-0.006412625	0.1268456	0.004200421	0.183453593	-0.006513276	0.12097426	0.004200217	0.183416637	0.000100651	0.018988011	1	0.381781739	1	0.382826475	ENSG00000234147	1	RP3-460G2.2
VAT	Obesity	ENSG00000160828.13	-0.00809562	0.066541042	0.004412346	0.183453202	-0.008012505	0.069361851	0.004412028	0.1834163	-8.31E-05	0.012594739	1	0.264946219	1	0.278253938	ENSG00000234232	2	RP11-353N4.5
VAT	Obesity	ENSG00000161036.6	-0.012409902	0.00264616	0.004128209	0.183451058	-0.012490064	0.002480624	0.004127977	0.183414086	8.02E-05	0.039604275	1	0.028230105	1	0.029010686	ENSG00000234232	2	RP11-353N4.5
VAT	Obesity	ENSG00000161202.13	0.001441229	0.733127207	0.004226864	0.18345443	0.001362772	0.747128651	0.004226595	0.183417507	7.85E-05	0.030904245	1	0.884914569	1	0.89626095	ENSG00000234338	1	RP11-797H7.1
VAT	Obesity	ENSG00000161609.5	-0.005375797	0.154875485	0.003779061	0.183453709	-0.005480286	0.146984259	0.003778808	0.183416751	0.000104489	0.000735359	1	0.428742567	1	0.423904445	ENSG00000234353	2	AP000347.4
VAT	Obesity	ENSG00000161692.13	-0.001023507	0.444504124	0.0013386	0.183454253	-0.001046833	0.434159219	0.001338499	0.183417315	2.33E-05	0.014564022	1	0.729116989	1	0.737402735	ENSG00000234409	4	AC006547.14
VAT	Obesity	ENSG00000161912.13	0.007377056	0.110611157	0.004623794	0.183453512	0.007243394	0.11719295	0.004623439	0.183416618	0.000133662	2.64E-05	1	0.357478681	1	0.376596898	ENSG00000234409	4	AC006547.14
VAT	Obesity	ENSG00000162040.5	0.014745951	0.011004346	0.005799623	0.18345203	0.014839909	0.010499081	0.00579921	0.183415071	-9.40E-05	0.035240638	1	0.079444329	1	0.081397369	ENSG00000234444	5	ZNF736
VAT	Obesity	ENSG00000162194.8	-0.005492982	0.299948102	0.005299314	0.183454068	-0.005267769	0.320170066	0.005298982	0.183417173	-0.000225213	1.09E-06	1	0.608848729	1	0.641269508	ENSG00000234444	5	ZNF736
VAT	Obesity	ENSG00000162365.7	-0.004067997	0.429831303	0.005152748	0.183454239	-0.004156135	0.419871032	0.005152368	0.1834173	8.81E-05	0.019571121	1	0.718120094	1	0.726092763	ENSG00000234444	5	ZNF736
VAT	Obesity	ENSG00000162368.9	-0.016643241	0.006053411	0.006063363	0.183451626	-0.016533407	0.006392063	0.006062916	0.183414736	-0.000109834	0.013578423	1	0.050937236	1	0.057279527	ENSG00000234444	5	ZNF736
VAT	Obesity	ENSG00000162520.10	-0.008750567	0.106324283	0.005418508	0.183453488	-0.00884309	0.102651277	0.005418105	0.183416539	9.25E-05	0.018531122	1	0.35060337	1	0.353263747	ENSG00000234465	6	PINLYP
VAT	Obesity	ENSG00000162877.8	0.010363641	0.017784667	0.004372698	0.183452351	0.010460442	0.016741948	0.004372483	0.183415383	-9.68E-05	0.029196115	1	0.110802893	1	0.113642834	ENSG00000234585	2	CCT6P3
VAT	Obesity	ENSG00000162910.14	0.007059964	0.119435871	0.004533905	0.183453558	0.007128645	0.115856107	0.004533578	0.183416612	-6.87E-05	0.045381016	1	0.372684435	1	0.37486853	ENSG00000234585	2	CCT6P3
VAT	Obesity	ENSG00000162913.9	0.007351956	0.225286689	0.006063012	0.183453918	0.007441192	0.219671048	0.006062541	0.183416977	-8.92E-05	0.030040518	1	0.52272452	1	0.531115411	ENSG00000234585	2	CCT6P3
VAT	Obesity	ENSG00000163041.5	0.000511639	0.843951838	0.002599247	0.183454459	0.000470962	0.856207315	0.002599068	0.183417534	4.07E-05	0.048931983	1	0.938891035	1	0.948422961	ENSG00000234745	5	HLA-B
VAT	Obesity	ENSG00000163131.6	-0.004843115	0.114472757	0.003068356	0.183453532	-0.003271044	0.293244355	0.003112221	0.183417129	-0.001572071	0.002622003	1	0.365254115	1	0.615229003	ENSG00000234745	5	HLA-B
VAT	Obesity	ENSG00000163155.7	0.005705534	0.04702461	0.002872756	0.183452983	0.005859642	0.041375915	0.002872728	0.183415974	-0.000154108	6.92E-05	1	0.21488067	1	0.204683628	ENSG00000234745	5	HLA-B
VAT	Obesity	ENSG00000163297.12	0.021204101	1.71E-05	0.004931925	0.183447487	0.021509946	1.30E-05	0.004933608	0.183410362	-0.000305845	0.037106966	0.04728667	0.000492569	0.035924051	0.000466546	ENSG00000234745	5	HLA-B
VAT	Obesity	ENSG00000163682.11	-0.001314533	0.122134295	0.000850346	0.183453571	-0.001294475	0.127911134	0.000850289	0.18341667	-2.01E-05	0.004161552	1	0.374700593	1	0.395731266	ENSG00000234745	5	HLA-B
VAT	Obesity	ENSG00000163728.6	-0.014344982	0.003400937	0.004897606	0.183451231	-0.014450928	0.003169532	0.004897269	0.183414255	0.000105946	0.006823023	1	0.033404223	1	0.034440579	ENSG00000234745	5	HLA-B
VAT	Obesity	ENSG00000163864.10	-0.000405848	0.930280836	0.004638708	0.183454471	-0.000285838	0.95086337	0.004638519	0.183417545	-0.00012001	0.018011067	1	0.976170888	1	0.983283215	ENSG00000234745	5	HLA-B
VAT	Obesity	ENSG00000163870.10	-0.01312716	0.015600721	0.005428633	0.183452264	-0.013042303	0.016275658	0.005428224	0.183415364	-8.49E-05	0.027715651	1	0.10325657	1	0.111743324	ENSG00000234745	5	HLA-B
VAT	Obesity	ENSG00000163904.8	-0.003958288	0.23398755	0.00332587	0.183453939	-0.003611254	0.27767733	0.003326649	0.183417101	-0.000347035	5.49E-05	1	0.529348883	1	0.597807668	ENSG00000234745	5	HLA-B
VAT	Obesity	ENSG00000164024.7	-0.001724398	0.710881854	0.004652084	0.183454422	-0.001829714	0.694073593	0.004651817	0.183417488	0.000105316	0.014921987	1	0.873568084	1	0.870280156	ENSG00000234882	1	EIF3EP1
VAT	Obesity	ENSG00000164070.7	-0.001590026	0.782682784	0.005764625	0.183454445	-0.001693689	0.768891937	0.005764281	0.183417513	0.000103662	0.047235045	1	0.909245983	1	0.907673971	ENSG00000234882	1	EIF3EP1
VAT	Obesity	ENSG00000164099.3	0.007034236	0.226137001	0.005811635	0.18345392	0.00694366	0.232135412	0.005811189	0.183417006	9.06E-05	0.024232608	1	0.52272452	1	0.540524906	ENSG00000234882	1	EIF3EP1
VAT	Obesity	ENSG00000164118.8	-0.008849461	0.059141187	0.004689334	0.183453128	-0.008759453	0.061751889	0.004689021	0.183416227	-9.00E-05	0.019536891	1	0.246943534	1	0.2614037	ENSG00000234882	1	EIF3EP1
VAT	Obesity	ENSG00000164167.5	-0.010736458	0.078956306	0.006111449	0.183453308	-0.010571505	0.083645909	0.006110993	0.183416415	-0.000164953	0.000182557	1	0.289017777	1	0.308446194	ENSG00000234981	1	RP11-534L20.4
VAT	Obesity	ENSG00000164171.6	0.004408539	0.271222177	0.004006839	0.183454017	0.004504691	0.260872722	0.004006548	0.183417068	-9.62E-05	0.001373782	1	0.575086695	1	0.574169626	ENSG00000235021	1	RP11-439E19.7
VAT	Obesity	ENSG00000164187.6	-0.010800795	0.065420819	0.005862428	0.183453191	-0.010916456	0.062568975	0.005861983	0.183416235	0.000115661	0.005026397	1	0.264360266	1	0.263845936	ENSG00000235081	1	AC010492.2
VAT	Obesity	ENSG00000164307.8	-0.018529837	5.76E-06	0.004085926	0.1834467	-0.018613439	5.22E-06	0.004085645	0.183409702	8.36E-05	0.010697325	0.015899387	0.000201258	0.014406061	0.000218274	ENSG00000235109	3	ZSCAN31
VAT	Obesity	ENSG00000164344.11	-0.001551054	0.453832972	0.00207072	0.183454262	-0.001511332	0.465448918	0.002070588	0.183417345	-3.97E-05	0.025493763	1	0.738859748	1	0.76239704	ENSG00000235109	3	ZSCAN31
VAT	Obesity	ENSG00000164543.5	0.000535298	0.924006795	0.005611801	0.183454471	0.00090707	0.8716425	0.005614008	0.183417536	-0.000371772	0.035069902	1	0.975615438	1	0.955921132	ENSG00000235109	3	ZSCAN31
VAT	Obesity	ENSG00000164669.8	-0.000982919	0.751771061	0.003107503	0.183454436	-0.002310166	0.459100503	0.003120457	0.183417339	0.001327247	3.89E-06	1	0.892809005	1	0.756487993	ENSG00000235109	3	ZSCAN31
VAT	Obesity	ENSG00000164828.13	0.001161492	0.749204877	0.003633203	0.183454435	0.001225079	0.735953845	0.003632915	0.183417503	-6.36E-05	0.007452253	1	0.892799597	1	0.891658005	ENSG00000235109	3	ZSCAN31
VAT	Obesity	ENSG00000164924.13	-0.015779778	6.98E-05	0.003967809	0.183448496	-0.015663421	7.89E-05	0.003967549	0.183411656	-0.000116357	0.000452062	0.192689484	0.001632962	0.217643783	0.001981293	ENSG00000235109	3	ZSCAN31
VAT	Obesity	ENSG00000164967.5	-0.019492159	0.000291702	0.00538073	0.183449514	-0.019022999	0.000408121	0.005381633	0.183412824	-0.00046916	0.00016504	0.805097424	0.005063506	1	0.007220609	ENSG00000235109	3	ZSCAN31
VAT	Obesity	ENSG00000164978.13	-0.000524385	0.679790783	0.001270471	0.18345441	-0.000264902	0.834995898	0.001271741	0.18341753	-0.000259484	1.34E-05	1	0.862732905	1	0.938350439	ENSG00000235109	3	ZSCAN31
VAT	Obesity	ENSG00000165055.11	0.002839975	0.125595641	0.001854125	0.183453587	0.002812026	0.129331823	0.001853988	0.183416677	2.79E-05	0.038316805	1	0.37967576	1	0.396177394	ENSG00000235109	3	ZSCAN31
VAT	Obesity	ENSG00000165171.6	-5.58E-05	0.959396582	0.001096281	0.183454473	-2.87E-05	0.979082738	0.001096195	0.183417546	-2.71E-05	0.000206081	1	0.98546132	1	0.994336196	ENSG00000235231	3	AP000351.10
VAT	Obesity	ENSG00000165271.12	0.007895591	0.123184287	0.005121861	0.183453576	0.008271184	0.106396092	0.005122717	0.183416561	-0.000375593	0.001540757	1	0.37609362	1	0.361633427	ENSG00000235231	3	AP000351.10
VAT	Obesity	ENSG00000165280.11	0.001422238	0.676274038	0.003406124	0.183454408	0.001557526	0.64747228	0.003406091	0.183417467	-0.000135288	0.003204259	1	0.860416532	1	0.851774782	ENSG00000235241	1	RP11-108M9.5
VAT	Obesity	ENSG00000165502.6	-0.000858287	0.661858114	0.001962473	0.183454402	-0.000907078	0.643909286	0.001962352	0.183417465	4.88E-05	0.004754403	1	0.854099711	1	0.849655254	ENSG00000235241	1	RP11-108M9.5
VAT	Obesity	ENSG00000165684.3	-0.002115305	0.450882156	0.002805646	0.183454259	-0.002059575	0.462866755	0.002805444	0.183417342	-5.57E-05	0.00867852	1	0.735047106	1	0.76223881	ENSG00000235241	1	RP11-108M9.5
VAT	Obesity	ENSG00000165912.11	-0.007620553	0.015692037	0.003154191	0.183452268	-0.00755229	0.016641578	0.003153962	0.183415379	-6.83E-05	0.003812318	1	0.103365207	1	0.113409271	ENSG00000235290	1	HLA-W
VAT	Obesity	ENSG00000165917.5	-0.040707936	3.80E-16	0.004997839	0.1834294	-0.040591581	4.58E-16	0.004997485	0.183392616	-0.000116355	0.002453135	1.05E-12	6.55E-14	1.26E-12	7.90E-14	ENSG00000235290	1	HLA-W
VAT	Obesity	ENSG00000165929.8	0.000349264	0.928854855	0.003911758	0.183454471	0.003710105	0.358129064	0.004037389	0.183417227	-0.003360841	0.00078543	1	0.976170888	1	0.683932408	ENSG00000235290	1	HLA-W
VAT	Obesity	ENSG00000165934.8	-0.000600796	0.724748841	0.001706228	0.183454427	-0.000694482	0.684008255	0.001706344	0.183417483	9.37E-05	0.002794144	1	0.881580785	1	0.869882588	ENSG00000235290	1	HLA-W
VAT	Obesity	ENSG00000166037.6	-0.002748702	0.521961746	0.004292668	0.183454319	-0.002655877	0.536083583	0.004292349	0.183417401	-9.28E-05	0.002913821	1	0.778563922	1	0.791224967	ENSG00000235290	1	HLA-W
VAT	Obesity	ENSG00000166321.9	-0.002162401	0.35469415	0.002336409	0.18345415	-0.002443955	0.295779234	0.00233753	0.183417133	0.000281553	0.000406946	1	0.66550364	1	0.617860951	ENSG00000235290	1	HLA-W
VAT	Obesity	ENSG00000166436.11	0.002396313	0.10405532	0.001474195	0.183453475	0.002352781	0.110472824	0.001474105	0.183416583	4.35E-05	0.000908266	1	0.346851066	1	0.366472348	ENSG00000235290	1	HLA-W
VAT	Obesity	ENSG00000166446.10	-0.003692595	0.123395524	0.002396732	0.183453577	-0.003638626	0.128948169	0.002396568	0.183416675	-5.40E-05	0.005006085	1	0.376322261	1	0.395731266	ENSG00000235290	1	HLA-W
VAT	Obesity	ENSG00000166557.8	-0.000366574	0.879324064	0.002414431	0.183454465	-0.000415133	0.863476458	0.00241426	0.183417535	4.86E-05	0.009360772	1	0.953234256	1	0.95099562	ENSG00000235290	1	HLA-W
VAT	Obesity	ENSG00000166573.4	7.29E-05	0.981223477	0.003098267	0.183454474	0.000147056	0.962140714	0.003098044	0.183417545	-7.41E-05	0.001579714	1	0.99502166	1	0.98754495	ENSG00000235328	1	AC006946.12
VAT	Obesity	ENSG00000166787.3	-0.001354987	0.78798245	0.005038409	0.183454447	-0.001520092	0.762866594	0.005038108	0.183417512	0.000165105	0.000297172	1	0.911115024	1	0.903653133	ENSG00000235370	5	DNM1P51
VAT	Obesity	ENSG00000166938.8	-0.000811082	0.818314996	0.003530853	0.183454454	-0.000864841	0.806489273	0.003530572	0.183417523	5.38E-05	0.019667479	1	0.926394336	1	0.925919465	ENSG00000235370	5	DNM1P51
VAT	Obesity	ENSG00000166946.9	0.011991797	0.033022884	0.005625169	0.183452756	0.01215746	0.030663431	0.005624757	0.18341578	-0.000165663	7.39E-05	1	0.16909677	1	0.168587788	ENSG00000235374	1	SSR4P1
VAT	Obesity	ENSG00000167103.7	0.008829659	0.026749336	0.003986009	0.183452619	0.008723553	0.028618763	0.003985714	0.183415736	0.000106106	0.000271352	1	0.147671738	1	0.160694428	ENSG00000235374	1	SSR4P1
VAT	Obesity	ENSG00000167136.6	0.007407744	0.031284782	0.003439962	0.183452721	0.007348787	0.032642496	0.003439704	0.183415821	5.90E-05	0.016431887	1	0.163240261	1	0.17527877	ENSG00000235374	1	SSR4P1
VAT	Obesity	ENSG00000167220.7	0.002961998	0.188951339	0.002254717	0.183453822	0.003017047	0.180835503	0.002254575	0.183416869	-5.50E-05	0.00479081	1	0.485121578	1	0.483914845	ENSG00000235421	1	RP11-667F9.1
VAT	Obesity	ENSG00000167325.10	0.001404958	0.361765958	0.001540513	0.18345416	0.001454573	0.345025926	0.0015404	0.183417209	-4.96E-05	1.28E-05	1	0.671468759	1	0.667792116	ENSG00000235421	1	RP11-667F9.1
VAT	Obesity	ENSG00000167461.7	0.001647674	0.723822961	0.004662952	0.183454427	0.001746602	0.707960627	0.004662622	0.183417493	-9.89E-05	0.005975644	1	0.881231307	1	0.876422561	ENSG00000235421	1	RP11-667F9.1
VAT	Obesity	ENSG00000167476.6	-0.002157757	0.682162111	0.005269058	0.183454411	-0.002044459	0.697987193	0.005268691	0.183417489	-0.000113299	0.006253453	1	0.863333733	1	0.870280156	ENSG00000235477	2	RP11-122G18.5
VAT	Obesity	ENSG00000167646.9	-0.000360513	0.934471144	0.004384688	0.183454472	-0.000469805	0.914666506	0.004384376	0.183417542	0.000109293	0.001170339	1	0.977307539	1	0.972419923	ENSG00000235477	2	RP11-122G18.5
VAT	Obesity	ENSG00000167720.8	0.006163013	2.26E-05	0.001454423	0.183447687	0.006216213	1.92E-05	0.001454327	0.183410642	-5.32E-05	1.39E-05	0.062413418	0.000630439	0.052932954	0.000626206	ENSG00000235477	2	RP11-122G18.5
VAT	Obesity	ENSG00000167747.9	0.003637454	0.04614954	0.001824174	0.183452971	0.003675399	0.043908994	0.001824052	0.183416012	-3.79E-05	0.011590442	1	0.212642286	1	0.212985632	ENSG00000235477	2	RP11-122G18.5
VAT	Obesity	ENSG00000167766.14	0.008821622	0.047139402	0.004444029	0.183452985	0.00888875	0.045467973	0.004443699	0.183416034	-6.71E-05	0.038284348	1	0.215024497	1	0.218780414	ENSG00000235477	2	RP11-122G18.5
VAT	Obesity	ENSG00000167799.5	-0.009024749	0.00623076	0.003299248	0.183451646	-0.008288947	0.012051762	0.003301535	0.183415164	-0.000735802	2.13E-08	1	0.051797881	1	0.090106525	ENSG00000235602	4	POU5F1P3
VAT	Obesity	ENSG00000168038.6	0.002191569	0.469167393	0.003027719	0.183454276	0.002043417	0.499712269	0.00302754	0.183417374	0.000148152	7.04E-08	1	0.751667156	1	0.776858792	ENSG00000235641	3	LINC00484
VAT	Obesity	ENSG00000168056.10	0.000680317	0.900267719	0.005428502	0.183454468	-0.00397789	0.468293433	0.005484784	0.183417347	0.004658207	3.32E-09	1	0.965087305	1	0.76491497	ENSG00000235641	3	LINC00484
VAT	Obesity	ENSG00000168060.10	-0.000330273	0.942856332	0.004607577	0.183454472	0.000341085	0.941006599	0.004608957	0.183417544	-0.000671358	2.60E-07	1	0.981243858	1	0.980488158	ENSG00000235641	3	LINC00484
VAT	Obesity	ENSG00000168116.9	-0.012755508	0.001004704	0.003877966	0.183450385	-0.012692844	0.00106302	0.003877675	0.183413497	-6.27E-05	0.023437973	1	0.013660016	1	0.015746258	ENSG00000235677	1	NPM1P26
VAT	Obesity	ENSG00000168356.7	-0.000571564	0.873725058	0.003596355	0.183454465	-0.000661422	0.854070401	0.003596101	0.183417533	8.99E-05	0.001315534	1	0.951116912	1	0.947822399	ENSG00000235677	1	NPM1P26
VAT	Obesity	ENSG00000168405.10	0.010834544	0.000666363	0.003183747	0.183450097	0.010253168	0.001290601	0.003186113	0.183413632	0.000581376	8.68E-06	1	0.009679794	1	0.017990194	ENSG00000235821	1	IFITM4P
VAT	Obesity	ENSG00000168661.10	0.001885573	0.520093971	0.002931546	0.183454318	0.001834148	0.531507725	0.002931324	0.183417398	5.14E-05	0.012951141	1	0.778563922	1	0.786996417	ENSG00000235821	1	IFITM4P
VAT	Obesity	ENSG00000168679.13	0.005548237	0.135744115	0.003719079	0.183453633	0.005604952	0.131761334	0.003718797	0.183416688	-5.67E-05	0.030599515	1	0.395299075	1	0.399591076	ENSG00000235821	1	IFITM4P
VAT	Obesity	ENSG00000168743.8	0.016333557	0.003917953	0.00566212	0.183451329	0.018508377	0.001310122	0.005759082	0.183413643	-0.002174819	0.039347935	1	0.036733053	1	0.018170536	ENSG00000235821	1	IFITM4P
VAT	Obesity	ENSG00000168778.7	-0.002557857	0.074838477	0.001435823	0.183453274	-0.002518818	0.07936529	0.001435728	0.183416383	-3.90E-05	0.000936016	1	0.282737093	1	0.298885799	ENSG00000235821	1	IFITM4P
VAT	Obesity	ENSG00000168781.17	-0.008126163	0.116664775	0.005179428	0.183453544	-0.008210752	0.112883342	0.005179082	0.183416596	8.46E-05	0.047062715	1	0.367994033	1	0.368271896	ENSG00000235821	1	IFITM4P
VAT	Obesity	ENSG00000168874.8	0.000777563	0.856386516	0.0042965	0.183454462	0.000874233	0.838756975	0.004296326	0.18341753	-9.67E-05	0.04021504	1	0.944116239	1	0.939543177	ENSG00000235821	1	IFITM4P
VAT	Obesity	ENSG00000168961.12	0.006203682	0.213556145	0.004987492	0.183453889	0.006278946	0.208020736	0.004987136	0.183416947	-7.53E-05	0.048914184	1	0.516511799	1	0.520051841	ENSG00000235821	1	IFITM4P
VAT	Obesity	ENSG00000168992.3	-0.002658522	0.424428083	0.003328297	0.183454233	-0.002596563	0.43527175	0.00332806	0.183417316	-6.20E-05	0.015087773	1	0.714174021	1	0.738383547	ENSG00000235821	1	IFITM4P
VAT	Obesity	ENSG00000168993.10	0.012991767	0.00909089	0.004980307	0.183451902	0.013112841	0.008460695	0.004979962	0.183414926	-0.000121074	0.002171881	1	0.067950356	1	0.070977262	ENSG00000235821	1	IFITM4P
VAT	Obesity	ENSG00000169047.5	0.010514241	0.004916799	0.003738466	0.183451484	0.010381771	0.005483251	0.003738223	0.183414631	0.00013247	2.48E-05	1	0.043494762	1	0.05044591	ENSG00000235863	2	B3GALT4
VAT	Obesity	ENSG00000169062.10	-0.006590576	0.074902545	0.003700358	0.183453275	-0.006518465	0.078124176	0.003700144	0.183416373	-7.21E-05	0.034796787	1	0.282737093	1	0.297410657	ENSG00000235863	2	B3GALT4
VAT	Obesity	ENSG00000169085.7	0.007252847	0.000346074	0.002026985	0.183449635	0.006408825	0.001604268	0.002031228	0.183413784	0.000844023	3.24E-10	0.955165045	0.005824177	1	0.021287397	ENSG00000235863	2	B3GALT4
VAT	Obesity	ENSG00000169220.13	0.000552322	0.853600045	0.002993177	0.183454461	0.000480669	0.872408599	0.00299296	0.183417536	7.17E-05	0.001464557	1	0.943884665	1	0.956254064	ENSG00000235878	4	AP001468.1
VAT	Obesity	ENSG00000169442.4	0.002347803	0.479702913	0.003321826	0.183454285	0.002417449	0.466736869	0.003321588	0.183417346	-6.96E-05	0.005864357	1	0.762547611	1	0.763147961	ENSG00000235878	4	AP001468.1
VAT	Obesity	ENSG00000169592.10	-0.019513676	1.93E-15	0.002455737	0.18343061	-0.019555013	1.68E-15	0.002455575	0.183393581	4.13E-05	0.044136993	5.32E-12	3.01E-13	4.63E-12	2.72E-13	ENSG00000235944	3	ZNF815P
VAT	Obesity	ENSG00000169609.9	0.001397652	0.620245426	0.002820673	0.183454381	0.001337236	0.635413955	0.002820453	0.183417461	6.04E-05	0.001412668	1	0.833030354	1	0.844363271	ENSG00000235963	1	MCCD1P1
VAT	Obesity	ENSG00000169902.9	-0.00275965	0.408970411	0.003342164	0.183454216	0.000413015	0.90358392	0.003409539	0.18341754	-0.003172666	2.70E-06	1	0.708044263	1	0.970385844	ENSG00000235963	1	MCCD1P1
VAT	Obesity	ENSG00000170092.10	0.041569515	4.76E-19	0.004661227	0.183424415	0.041701703	3.66E-19	0.004660919	0.183387297	-0.000132189	0.000716823	1.31E-15	1.19E-16	1.01E-15	9.19E-17	ENSG00000235963	1	MCCD1P1
VAT	Obesity	ENSG00000170175.6	-0.002868605	0.130823724	0.001898652	0.183453611	-0.002819951	0.137459691	0.001898547	0.183416712	-4.87E-05	0.00731291	1	0.387974943	1	0.412354078	ENSG00000235963	1	MCCD1P1
VAT	Obesity	ENSG00000170289.8	0.019927458	0.000641584	0.005837975	0.18345007	0.019824786	0.000683629	0.005837543	0.183413187	0.000102673	0.015779413	1	0.00936916	1	0.010986495	ENSG00000235994	4	RP3-470B24.5
VAT	Obesity	ENSG00000170356.8	-0.006230573	0.065855172	0.003387263	0.183453195	-0.006160226	0.068950499	0.003387069	0.183416296	-7.03E-05	0.025988671	1	0.264689498	1	0.277410173	ENSG00000235994	4	RP3-470B24.5
VAT	Obesity	ENSG00000170540.10	-0.000855393	0.86014088	0.004854813	0.183454462	-0.0007659	0.874635284	0.004854426	0.183417537	-8.95E-05	0.004441027	1	0.944533766	1	0.956332568	ENSG00000236104	2	ZBTB22
VAT	Obesity	ENSG00000170571.7	0.002936518	0.57788226	0.00527694	0.183454357	0.00278631	0.597488432	0.005276941	0.183417441	0.000150208	0.044911111	1	0.80716348	1	0.827015081	ENSG00000236104	2	ZBTB22
VAT	Obesity	ENSG00000170632.9	0.002347496	0.531514516	0.003751814	0.183454326	0.002417895	0.519250934	0.003751577	0.183417389	-7.04E-05	0.029905003	1	0.783174974	1	0.784425829	ENSG00000236104	2	ZBTB22
VAT	Obesity	ENSG00000170745.7	0.000523041	0.903252893	0.004302975	0.183454469	0.00041962	0.922309096	0.004302666	0.183417542	0.000103421	0.00156358	1	0.965567705	1	0.974118595	ENSG00000236104	2	ZBTB22
VAT	Obesity	ENSG00000170915.8	-0.00969804	0.089129513	0.005704693	0.183453382	-0.00938138	0.100080555	0.005704817	0.183416524	-0.000316661	0.000384305	1	0.311783847	1	0.347887069	ENSG00000236104	2	ZBTB22
VAT	Obesity	ENSG00000171109.14	0.004723106	0.162884267	0.003384675	0.183453738	0.004792132	0.156795362	0.003384431	0.183416788	-6.90E-05	0.00702394	1	0.441873282	1	0.441136798	ENSG00000236155	2	RP11-231P20.2
VAT	Obesity	ENSG00000171298.8	-0.000983429	0.576021919	0.001758618	0.183454356	-0.000942184	0.592105005	0.0017585	0.183417438	-4.12E-05	0.003925141	1	0.806947626	1	0.824941855	ENSG00000236209	1	AC104135.2
VAT	Obesity	ENSG00000171314.8	-0.002050868	0.324733346	0.002082568	0.183454108	-0.002090754	0.315377216	0.002082416	0.183417165	3.99E-05	0.010392891	1	0.633061352	1	0.637753261	ENSG00000236209	1	AC104135.2
VAT	Obesity	ENSG00000171357.5	-0.004710296	0.01376668	0.001912187	0.18345218	-0.004664234	0.014712826	0.001912057	0.183415297	-4.61E-05	0.002903867	1	0.094623402	1	0.105748437	ENSG00000236209	1	AC104135.2
VAT	Obesity	ENSG00000171421.8	0.002592094	0.239202574	0.002202319	0.18345395	0.002646312	0.22948896	0.002202181	0.183417	-5.42E-05	0.004723148	1	0.533709866	1	0.539107619	ENSG00000236209	1	AC104135.2
VAT	Obesity	ENSG00000171469.6	0.00351516	0.07397229	0.001967306	0.183453267	0.003545307	0.071505508	0.001967148	0.183416318	-3.01E-05	0.016029261	1	0.281216969	1	0.283556326	ENSG00000236397	2	DDX11L2
VAT	Obesity	ENSG00000171700.9	0.008753089	0.002608842	0.002907579	0.183451048	0.008799469	0.002473166	0.002907357	0.183414084	-4.64E-05	0.022152655	1	0.028230105	1	0.029010686	ENSG00000236397	2	DDX11L2
VAT	Obesity	ENSG00000171735.14	0.017589656	0.001873237	0.005656485	0.183450819	0.017679161	0.001774017	0.005656091	0.183413854	-8.95E-05	0.04452364	1	0.021380487	1	0.02256353	ENSG00000236397	2	DDX11L2
VAT	Obesity	ENSG00000171757.11	-0.003498706	0.22824993	0.002903778	0.183453925	-0.003456699	0.233848918	0.002903559	0.18341701	-4.20E-05	0.042575421	1	0.524101338	1	0.540555288	ENSG00000236411	1	NDUFAF4P3
VAT	Obesity	ENSG00000171860.4	-0.006448905	0.247344574	0.005574661	0.183453968	-0.006323804	0.256603094	0.005574296	0.18341706	-0.000125101	0.007450393	1	0.543960975	1	0.566579631	ENSG00000236417	2	CTSLP1
VAT	Obesity	ENSG00000171953.11	-0.009801708	0.031471058	0.004556663	0.183452725	-0.009712213	0.033041644	0.004556338	0.183415829	-8.95E-05	0.01020918	1	0.163578381	1	0.176392531	ENSG00000236417	2	CTSLP1
VAT	Obesity	ENSG00000172071.7	0.0028874	0.637428151	0.006126485	0.18345439	0.003006359	0.623602044	0.006126024	0.183417455	-0.000118959	0.006493659	1	0.841638558	1	0.841508821	ENSG00000236485	1	RP1-156L9.1
VAT	Obesity	ENSG00000172167.3	0.000869241	0.870179958	0.005318739	0.183454464	0.000964046	0.856158598	0.005318397	0.183417534	-9.48E-05	0.036644715	1	0.948684727	1	0.948422961	ENSG00000236532	1	AL035610.2
VAT	Obesity	ENSG00000172247.3	-0.019162525	1.96E-15	0.002412239	0.183430623	-0.019118297	2.27E-15	0.002412061	0.183393806	-4.42E-05	0.012162849	5.42E-12	3.01E-13	6.25E-12	3.47E-13	ENSG00000236603	1	RANP1
VAT	Obesity	ENSG00000172432.14	-0.001885957	0.574901771	0.003362691	0.183454355	-0.001805074	0.591383697	0.003362454	0.183417437	-8.09E-05	0.001962932	1	0.806905296	1	0.824941855	ENSG00000236611	1	AP000343.2
VAT	Obesity	ENSG00000172594.8	0.00271995	0.44733443	0.003579508	0.183454256	0.002582031	0.470671259	0.003579255	0.183417349	0.000137918	6.98E-07	1	0.731423594	1	0.76685518	ENSG00000236611	1	AP000343.2
VAT	Obesity	ENSG00000172613.3	0.002910228	0.184941909	0.00219525	0.18345381	0.003462898	0.115003644	0.002197132	0.183416607	-0.000552669	9.02E-09	1	0.480635362	1	0.37298479	ENSG00000236624	4	CCDC163P
VAT	Obesity	ENSG00000172650.8	-0.01350837	0.001062118	0.004126516	0.183450423	-0.013083155	0.001526428	0.004127661	0.183413749	-0.000425214	0.000176251	1	0.014093491	1	0.020753408	ENSG00000236624	4	CCDC163P
VAT	Obesity	ENSG00000172687.9	0.002401574	0.167055662	0.001738094	0.183453752	0.002445929	0.1593251	0.001737972	0.183416797	-4.44E-05	0.001066368	1	0.449389499	1	0.446887475	ENSG00000236624	4	CCDC163P
VAT	Obesity	ENSG00000172803.13	0.009233168	9.17E-05	0.002360494	0.183448691	-0.00206327	0.553260256	0.003480067	0.183417413	0.011296438	9.99E-06	0.253151054	0.002075009	1	0.805509891	ENSG00000236624	4	CCDC163P
VAT	Obesity	ENSG00000172890.7	0.007224752	0.064122306	0.00390246	0.183453179	0.007313744	0.060894234	0.003902189	0.183416218	-8.90E-05	0.003921276	1	0.259497897	1	0.258964693	ENSG00000236624	4	CCDC163P
VAT	Obesity	ENSG00000172922.4	0.001137912	0.572078676	0.002014028	0.183454353	0.004614818	0.027349022	0.002091459	0.183415706	-0.003476905	7.35E-10	1	0.806905296	1	0.155231297	ENSG00000236624	4	CCDC163P
VAT	Obesity	ENSG00000172935.8	0.020374379	4.93E-05	0.005019835	0.183448247	0.020836133	3.32E-05	0.00501999	0.183411035	-0.000461754	1.57E-08	0.13617451	0.001215844	0.091525667	0.000984147	ENSG00000236682	1	AC068282.3
VAT	Obesity	ENSG00000173113.2	-0.004139871	0.218638864	0.003365319	0.183453902	-0.004028108	0.231296098	0.00336509	0.183417005	-0.000111763	3.80E-05	1	0.521961016	1	0.540524906	ENSG00000236682	1	AC068282.3

VAT	Obesity	ENSG00000173193.9	0.001436482	0.541038006	0.002350094	0.183454333	0.001514691	0.519236535	0.002350096	0.183417389	-7.82E-05	0.019463744	1	0.788540277	1	0.784425829	ENSG00000236682	1	AC068282.3
VAT	Obesity	ENSG00000173200.8	0.001695326	0.644250179	0.003671405	0.183454394	0.001631656	0.65671605	0.003671165	0.183417471	6.37E-05	0.038446083	1	0.844163628	1	0.854766662	ENSG00000236682	1	AC068282.3
VAT	Obesity	ENSG00000173226.12	-0.01109916	0.001646544	0.003526285	0.183450729	-0.010749192	0.002321423	0.003529287	0.18341404	-0.000349969	0.022914586	1	0.019672995	1	0.027736481	ENSG00000236699	4	ARHGEF38
VAT	Obesity	ENSG00000173258.8	0.000635563	0.900398482	0.005078086	0.183454468	0.000746966	0.883048119	0.005077728	0.183417538	-0.000111403	0.00480771	1	0.965087305	1	0.960056703	ENSG00000236710	1	AC108448.2
VAT	Obesity	ENSG00000173442.7	-0.000951314	0.781543294	0.003430524	0.183454445	-0.001577393	0.645830855	0.003432384	0.183417466	0.00062608	3.61E-07	1	0.90900105	1	0.850570266	ENSG00000236710	1	AC108448.2
VAT	Obesity	ENSG00000173531.11	-0.011036681	0.001730967	0.003522829	0.183450764	-0.01511439	0.000208654	0.004075795	0.183412349	0.004077709	0.04673641	1	0.0201581	0.575885908	0.004290423	ENSG00000236710	1	AC108448.2
VAT	Obesity	ENSG00000173727.7	0.000271785	0.929317262	0.003063958	0.183454471	-0.002048044	0.507277459	0.003088667	0.18341738	0.002319829	3.36E-09	1	0.976170888	1	0.783244156	ENSG00000236834	1	LINC00421
VAT	Obesity	ENSG00000173821.15	-0.006972022	0.230850149	0.005818876	0.183453931	-0.006877063	0.237229935	0.005818452	0.183417018	-9.50E-05	0.028327219	1	0.52708752	1	0.544132965	ENSG00000237135	1	DDX10P1
VAT	Obesity	ENSG00000173917.9	0.013336825	0.000552538	0.003861353	0.183449965	0.013230121	0.00061136	0.003861066	0.183413109	0.000106704	0.000123983	1	0.008472254	1	0.009984341	ENSG00000237190	3	CDKN2AIPNL
VAT	Obesity	ENSG00000174080.6	0.001872141	0.514723837	0.002873584	0.183454314	0.00949958	0.741340734	0.002877974	0.183417505	0.000922183	1.89E-08	1	0.776929212	1	0.893915902	ENSG00000237276	4	ANO7P1
VAT	Obesity	ENSG00000174226.4	-0.001195434	0.667261421	0.002780662	0.183454404	-0.001108319	0.690180946	0.002780461	0.183417486	-8.71E-05	3.36E-05	1	0.857608118	1	0.869882588	ENSG00000237310	1	GS1-124K5.4
VAT	Obesity	ENSG00000174353.13	6.61E-05	0.976854278	0.002277958	0.183454474	0.000371732	0.870393226	0.002278339	0.183417536	-0.000305642	6.98E-09	1	0.992659365	1	0.955921132	ENSG00000237310	1	GS1-124K5.4
VAT	Obesity	ENSG00000174652.13	0.001353332	0.23164131	0.001131413	0.183453933	0.001370469	0.22574989	0.001131328	0.183416991	-1.71E-05	0.034325484	1	0.52715189	1	0.535743505	ENSG00000237310	1	GS1-124K5.4
VAT	Obesity	ENSG00000174775.12	0.007126894	0.122184976	0.004610877	0.183453571	0.007058934	0.125759684	0.004610543	0.18341666	6.80E-05	0.049018624	1	0.374700593	1	0.391460914	ENSG00000237310	1	GS1-124K5.4
VAT	Obesity	ENSG00000174989.8	0.006844987	0.190061221	0.005223586	0.183453825	0.006663656	0.202038379	0.005223253	0.183416931	0.000181331	5.24E-05	1	0.487064968	1	0.512836579	ENSG00000237491	4	RP11-206L10.9
VAT	Obesity	ENSG00000175115.7	-0.014823072	0.000208853	0.003997496	0.183449277	-0.015369721	0.000120833	0.003997888	0.18341196	0.000546649	7.64E-12	0.576433352	0.004003009	0.333499937	0.00280252	ENSG00000237491	4	RP11-206L10.9
VAT	Obesity	ENSG00000175390.8	-0.006581493	0.063048748	0.003540623	0.183453168	-0.006498544	0.066427452	0.003540414	0.183416273	-8.29E-05	0.010150775	1	0.257417965	1	0.27337482	ENSG00000237510	3	AC008268.2
VAT	Obesity	ENSG00000175575.8	-0.000477063	0.720209656	0.001331914	0.183454426	-0.00044409	0.738798573	0.001331823	0.183417504	-3.30E-05	0.002118256	1	0.879937428	1	0.892750508	ENSG00000237510	3	AC008268.2
VAT	Obesity	ENSG00000175643.7	0.001256748	0.755131214	0.004029578	0.183454437	0.001144895	0.776310305	0.004029465	0.183417516	0.000111853	0.021286123	1	0.894874259	1	0.909787806	ENSG00000237510	3	AC008268.2
VAT	Obesity	ENSG00000176024.12	0.007328578	0.161529182	0.005234889	0.183453733	0.007579606	0.147673785	0.00523526	0.183416754	-0.000251028	0.009659893	1	0.441056458	1	0.424562132	ENSG00000237510	3	AC008268.2
VAT	Obesity	ENSG00000176155.14	-0.010017561	0.001020217	0.003049568	0.183450395	-0.009931938	0.001125781	0.003049351	0.183413537	-8.56E-05	0.000247907	1	0.013782824	1	0.016353444	ENSG00000237541	3	HLA-DQA2
VAT	Obesity	ENSG00000176293.15	-0.002832006	0.173201538	0.002079315	0.183453773	-0.002891813	0.164273748	0.002079181	0.183416815	5.98E-05	0.000742223	1	0.460092631	1	0.455295507	ENSG00000237541	3	HLA-DQA2
VAT	Obesity	ENSG00000176390.10	0.000956637	0.78554272	0.003515714	0.183454446	0.001007131	0.774504963	0.003515446	0.183417515	-5.05E-05	0.040366754	1	0.909319831	1	0.909523293	ENSG00000237541	3	HLA-DQA2
VAT	Obesity	ENSG00000176422.10	-0.010917876	0.001859885	0.003508589	0.183450814	-0.010975319	0.001757854	0.003508312	0.183413847	5.74E-05	0.012673928	1	0.021380487	1	0.02256353	ENSG00000237669	1	HCG4P3
VAT	Obesity	ENSG00000176428.5	0.006541163	0.071190923	0.003625396	0.183453244	0.005039155	0.165889001	0.003636969	0.183416821	0.001502009	3.35E-07	1	0.274916222	1	0.456484191	ENSG00000237669	1	HCG4P3
VAT	Obesity	ENSG00000176998.3	-0.009913193	5.48E-05	0.002457409	0.183448323	-0.007038922	0.005446824	0.002532569	0.183414627	-0.002874271	2.78E-06	0.151367782	0.001339538	1	0.05044591	ENSG00000237669	1	HCG4P3
VAT	Obesity	ENSG00000177409.7	0.003349393	0.206088352	0.002649005	0.18345387	0.003396773	0.199715973	0.002648839	0.183416925	-4.74E-05	0.039713964	1	0.507220054	1	0.510412237	ENSG00000237669	1	HCG4P3
VAT	Obesity	ENSG00000178209.10	-0.00231595	0.407080221	0.00279352	0.183454214	-0.002368244	0.396535068	0.002793312	0.183417274	5.23E-05	0.009822572	1	0.708044263	1	0.715434904	ENSG00000237669	1	HCG4P3
VAT	Obesity	ENSG00000178295.10	-0.000291541	0.869161241	0.001769877	0.183454464	-0.000218743	0.901633174	0.001769786	0.18341754	-7.28E-05	3.34E-05	1	0.948684727	1	0.969573834	ENSG00000237669	1	HCG4P3
VAT	Obesity	ENSG00000178809.7	-0.040223622	1.98E-13	0.005472469	0.183434055	-0.04038699	1.58E-13	0.005472089	0.183396961	0.000163369	0.000177277	5.47E-10	2.38E-11	4.36E-10	1.98E-11	ENSG00000237669	1	HCG4P3
VAT	Obesity	ENSG00000179406.6	-0.004866876	0.02914508	0.002230951	0.183452675	-0.00128908	0.5890798	0.002386432	0.183417436	-0.003577797	2.45E-05	1	0.155892287	1	0.823637411	ENSG00000237669	1	HCG4P3
VAT	Obesity	ENSG00000179528.11	0.004957814	0.225192113	0.004087777	0.183453918	0.005082944	0.213670734	0.00408749	0.183416962	-0.00012513	8.77E-05	1	0.52272452	1	0.525139114	ENSG00000237669	1	HCG4P3
VAT	Obesity	ENSG00000179639.6	-0.004447144	0.47091429	0.006168084	0.183454278	-0.004220808	0.493771779	0.006167863	0.183417369	-0.000226336	0.001256315	1	0.753462863	1	0.775358255	ENSG00000237669	1	HCG4P3
VAT	Obesity	ENSG00000179698.9	-0.012719442	0.02098737	0.0055105	0.18345246	-0.012584454	0.022378533	0.005510101	0.183415575	-0.000134987	0.001082373	1	0.125108299	1	0.136056182	ENSG00000237669	1	HCG4P3
VAT	Obesity	ENSG00000179978.10	0.003399296	0.333368281	0.003514019	0.18345412	0.003458076	0.325040286	0.003513755	0.18341718	-5.88E-05	0.018833151	1	0.642540807	1	0.64633371	ENSG00000237693	4	IRGM
VAT	Obesity	ENSG00000179979.7	-0.000735893	0.82652082	0.003357698	0.183454456	-0.000681317	0.839192802	0.003357464	0.18341753	-5.46E-05	0.038438677	1	0.928070571	1	0.939543177	ENSG00000237753	1	AC079922.3
VAT	Obesity	ENSG00000180185.7	-0.001427318	0.206294748	0.001129367	0.18345387	-0.001406211	0.21305171	0.001129287	0.18341696	-2.11E-05	0.01641728	1	0.507220054	1	0.525074761	ENSG00000237753	1	AC079922.3
VAT	Obesity	ENSG00000180353.6	-0.003081979	0.606401831	0.005981909	0.183454374	-0.00184202	0.759298032	0.006011759	0.183417511	-0.001239959	0.040185533	1	0.827729503	1	0.90093259	ENSG00000237753	1	AC079922.3
VAT	Obesity	ENSG00000180398.7	0.008918028	0.104834198	0.005498646	0.18345348	0.008801494	0.109425925	0.005498247	0.183416578	0.000116534	0.00472263	1	0.347941332	1	0.365676336	ENSG00000237854	3	LINC00674
VAT	Obesity	ENSG00000180828.1	0.001980662	0.722796437	0.00558367	0.183454427	0.002136108	0.702023967	0.005583283	0.183417491	-0.000155446	0.000442321	1	0.88098326	1	0.872786554	ENSG00000237887	1	RPL23AP32
VAT	Obesity	ENSG00000181092.5	0.002899808	0.42119178	0.00360513	0.18345423	-0.000806833	0.830542633	0.003770145	0.183417529	0.003706641	0.000789798	1	0.71233554	1	0.936897537	ENSG00000237927	1	RP3-393E18.2
VAT	Obesity	ENSG00000181104.6	9.49E-06	0.998354567	0.004604146	0.183454474	0.000123741	0.978557302	0.004603869	0.183417546	-0.000114246	0.005778378	1	0.999228023	1	0.994336196	ENSG00000237927	1	RP3-393E18.2
VAT	Obesity	ENSG00000181291.5	-0.001438156	0.534926891	0.002317726	0.183454329	-0.00149448	0.519028804	0.002317585	0.183417389	5.63E-05	0.006414356	1	0.784524453	1	0.784425829	ENSG00000237927	1	RP3-393E18.2
VAT	Obesity	ENSG00000181315.6	-0.011559534	0.00115808	0.003557844	0.183450484	-0.012943813	0.000294877	0.00357585	0.183412594	0.001384279	0.000131487	1	0.015006104	0.813859235	0.005574378	ENSG00000237984	2	PTENP1
VAT	Obesity	ENSG00000181826.5	0.011531291	0.042586673	0.005686752	0.18345292	0.0111183	0.050644531	0.00568861	0.183416102	0.000412991	0.012970795	1	0.201957418	1	0.236113017	ENSG00000237984	2	PTENP1
VAT	Obesity	ENSG00000181915.3	0.002667916	0.486797561	0.003836455	0.183454291	0.002719473	0.478383459	0.003836157	0.183417356	-5.16E-05	0.047298357	1	0.765031548	1	0.77393807	ENSG00000237988	2	OR2I1P
VAT	Obesity	ENSG00000182013.13	0.003577587	0.076091176	0.002016867	0.183453285	0.003635077	0.071473047	0.002016726	0.183416318	-5.75E-05	0.000307844	1	0.283034564	1	0.283556326	ENSG00000237988	2	OR2I1P
VAT	Obesity	ENSG00000182134.11	-0.014303135	0.006447745	0.005250573	0.183451669	-0.013954465	0.007868248	0.005250613	0.183414877	-0.000348671	6.20E-06	1	0.052963619	1	0.066819583	ENSG00000237988	2	OR2I1P
VAT	Obesity	ENSG00000182141.5	-0.003697732	0.192637506	0.002838257	0.183453833	-0.003603128	0.20423992	0.002838079	0.183416937	-9.46E-05	0.000130192	1	0.490311549	1	0.515267074	ENSG00000237988	2	OR2I1P
VAT	Obesity	ENSG00000182446.9	-0.00587553	0.131364118	0.003894311	0.183453614	-0.006005957	0.122989842	0.003894036	0.183416647	0.000130427	1.65E-05	1	0.387974943	1	0.385025715	ENSG00000238024	1	DDX39BP2
VAT	Obesity	ENSG00000182511.7	0.00939725	0.092997879	0.005594285	0.183453408	0.009269741	0.097495324	0.005593877	0.183416508	0.000127509	0.002241433	1	0.319981445	1	0.341914986	ENSG00000238024	1	DDX39BP2
VAT	Obesity	ENSG00000182871.10	0.020499536	0.000249891	0.005597298	0.183449404	0.020748871	0.000209858	0.005597406	0.183412353	-0.000249335	0.004077566	0.689699497	0.004597997	0.57920706	0.004290423	ENSG00000238024	1	DDX39BP2
VAT	Obesity	ENSG00000182919.10	0.000573067	0.687611313	0.00142519	0.183454413	0.000523688	0.713262628	0.001425086	0.183417495	4.94E-05	3.23E-06	1	0.863613457	1	0.878449288	ENSG00000238034	1	RP5-1185K9.1
VAT	Obesity	ENSG00000182923.12	-0.003470315	0.268742376	0.003137817	0.183454012	-0.003393004	0.279521364	0.003137614	0.183417104	-7.73E-05	0.003589176	1	0.57160411	1	0.59920191	ENSG00000238034	1	RP5-1185K9.1
VAT	Obesity	ENSG00000182952.4	-0.0034168	0.219190624	0.00278086	0.183453903	-0.001412303	0.615361698	0.002810916	0.183417451	-0.002004497	1.13E-06	1	0.521961016	1	0.839253732	ENSG00000238034	1	RP5-1185K9.1
VAT	Obesity	ENSG00000182986.8	0.00062332	0.58348755	0.001136827	0.18345436	0.000593218	0.601776353	0.001136764	0.183417443	3.01E-05	0.00528383	1	0.810955212	1	0.829207556	ENSG00000238034	1	RP5-1185K9.1
VAT	Obesity	ENSG00000183060.11	0.012641372	0.002703049	0.004214284	0.183451073	0.01255758	0.002882733	0.004213969	0.18341419	8.38E-05	0.005558763	1	0.0286939	1	0.032474874	ENSG00000238083	3	LRRC37A2
VAT	Obesity	ENSG00000183250.7	-0.012264232	0.004349358	0.004300736	0.1834514	-0.012185771	0.00460266	0.004300432	0.183414512	-7.85E-05	0.018367712	1	0.040282648	1	0.044966202	ENSG00000238083	3	LRRC37A2
VAT	Obesity	ENSG00000183255.7	-0.004757576	0.126219861	0.003111189	0.18345359	-0.004703555	0.1305531	0.003110967	0.183416682	-5.40E-05	0.023358835	1	0.381145314	1	0.398993348	ENSG00000238083	3	LRRC37A2
VAT	Obesity	ENSG00000183281.10	0.012445078	0.000967381	0.00377139	0.183450358	0.01237416	0.001033511	0.003771159	0.183413477	7.09E-05	0.034276884	1	0.013283447	1	0.015502666	ENSG00000238105	3	GOLGA2P5
VAT	Obesity	ENSG00000183527.7	0.016113282	0.000286	0.00444175	0.1834495	0.016017532	0.000310513	0.004441426	0.183412631	9.58E-05	0.003752329	0.789359804	0.005022832	0.85701644	0.005830044	ENSG00000238142	1	RP11-108M9.4
VAT	Obesity	ENSG00000183648.5	0.001087396	0.853152522	0.005874715	0.183454461	0.000470516	0.936189918	0.005877075	0.183417544	0.00061688	0.000924093	1	0.943767921	1	0.979114882	ENSG00000238142	1	RP11-108M9.4
VAT	Obesity	ENSG00000183684.6	0.000417338	0.940495364	0.005590801	0.183454472	0.000323866	0.953802622	0.005590435	0.183417545	9.35E-05	0.046396255	1	0.980274624	1	0.984913318	ENSG00000238243	2	OR2W3
VAT	Obesity	ENSG00000183844.12	-0.003261686	0.090513357	0.001926914	0.183453391	-0.003210506	0.095663269	0.001926779	0.183416497	-5.12E-05	0.00068267	1	0.314235052	1	0.337231331	ENSG00000238243	2	OR2W3
VAT	Obesity	ENSG00000183889.8	0.005747992	0.134336245	0.003839106	0.183453627	0.005686353	0.138534423	0.003838828	0.183416717	6.16E-05	0.032529207	1	0.392762749	1	0.412910374	ENSG00000238286	1	SLC35E1P1
VAT	Obesity	ENSG00000184117.7	-0.003477994	0.449672986	0.004600759	0.183454258	-0.003555538	0.439602012	0.004600457	0.18341732	7.75E-05	0.044018086	1	0.733942898	1	0.74253461	ENSG00000238286	1	SLC35E1P1
VAT	Obesity	ENSG00000184206.7	-0.005242692	0.326984798	0.00534856	0.183454111	-0.005318738	0.319979281	0.005348145	0.183417172	7.60E-05	0.036775214	1	0.634654038	1	0.641269508	ENSG00000239257	1	RPL23AP1
VAT	Obesity	ENSG00000184470.15	0.004402548	0.472183029	0.006123732	0.183454279	0.004594176	0.453086086	0.006123277	0.183417333	-0.000191628	1.64E-05	1	0.755055133	1	0.750751641	ENSG00000239257	1	RPL23AP1
VAT	Obesity	ENSG00000184887.9	-0.006572683	0.221433984	0.005375437	0.183453909	-0.006672352	0.214474824	0.005375051	0.183416964	9.97E-05	0.014805122	1	0.52199987	1	0.526178235	ENSG00000239257	1	RPL23AP1
VAT	Obesity	ENSG00000184949.11	-0.001636071	0.660388533	0.003723615	0.183454401	-0.001686807	0.650521706	0.003723324	0.183417468	5.07E-05	0.04178097	1	0.853311026	1	0.85253557	ENSG00000239257	1	RPL23AP1
VAT	Obesity	ENSG00000184988.4	-0.005512988	0.271005322	0.005008385	0.183454016	-0.005605566	0.263007486	0.005008044	0.183417073	9.26E-05	0.022318278	1	0.575086695	1	0.577946387	ENSG00000239257	1	RPL23AP1
VAT	Obesity	ENSG00000185022.7	-0.014569039	0.000270665	0.004000399	0.183449461	-0.014628714	0.000255125	0.0040001	0.183412492	5.97E-05	0.039285853	0.747034477	0.004882578	0.704144699	0.004995804	ENSG00000239257	1	RPL23AP1
VAT	Obesity	ENSG00000185298.8	0.002035438	0.374527036	0.002292092	0.183454176	0.001999205	0.383054647	0.002291923	0.183417258	3.62E-05	0.031401165	1	0.684277319	1	0.707013865	ENSG00000239257	1	RPL23AP1
VAT	Obesity	ENSG00000185324.17	0.000181132	0.894911945	0.001371265	0.183454468	0.000156443	0.90916269	0.001371165	0.183417541	2.47E-05	0.016452257	1	0.962570916	1	0.971175886	ENSG00000239257	1	RPL23AP1
VAT	Obesity	ENSG00000185619.13	0.00614909	0.04163268	0.003018434	0.183452905	0.006207132	0.039732068	0.003018256	0.183415948	-5.80E-05	0.035254226	1	0.199144188	1	0.198660338	ENSG00000239257	1	RPL23AP1
VAT	Obesity	ENSG00000185742.6	-0.003733826	0.499056019	0.005523604	0.183454301	-0.003842672	0.486597778	0.005523218	0.183417363	0.000108847	0.011814941	1	0.768206699	1	0.775358255	ENSG00000239272	1	RPL21P10
VAT	Obesity	ENSG00000185800.7	-0.003348359	0.466264653	0.004595791	0.183454273	-0.00324316	0.480355833	0.004595463	0.183417358	-0.000105198	0.002778882	1	0.749063121	1	0.774858036	ENSG00000239388	4	ASB14
VAT	Obesity	ENSG00000185869.9	-0.000209475	0.961256459	0.004312228	0.183454473	-0.000129355	0.97606763	0.004311933	0.183417546	-8.01E-05	0.020724201	1	0.985729121	1	0.993659205	ENSG00000239388	4	ASB14
VAT	Obesity	ENSG00000185880.8	-0.002014858	0.282533641	0.001874904	0.183454038	-0.00195317	0.297494374	0.001874762	0.183417136	-6.17E-05	3.42E-06	1	0.588079072	1	0.619387123	ENSG00000239556	2	AC004951.5
VAT	Obesity	ENSG00000186160.4	-0.0028017	0.192435069	0.002149514	0.183453832	-0.002873131	0.18130832	0.002149358	0.183416871	7.14E-05	9.55E-06	1	0.490311549	1	0.48442494	ENSG00000239556	2	AC004951.5
VAT	Obesity	ENSG00000186409.10	0.002894936	0.406003192	0.003483894	0.183454213	0.002799486	0.421654051	0.003483873	0.183417302	9.54E-05	0.046577886	1	0.708044263	1	0.727353238	ENSG00000239557	1	RP11-168J18.6
VAT	Obesity	ENSG00000186453.8	0.00195577	0.341662887	0.002056788	0.183454132	0.001872486	0.362584107	0.002056653	0.183417233	8.33E-05	1.31E-06	1	0.651685949	1	0.686859082	ENSG00000239557	1	RP11-168J18.6
VAT	Obesity	ENSG00000186468.8	0.002365581	0.512683145	0.003613416	0.183454312	0.002502982	0.488510897	0.003613476	0.183417365	-0.000137401	0.013005181	1	0.776196094	1	0.775358255	ENSG00000239557	1	RP11-168J18.6
VAT	Obesity	ENSG00000186470.9	-0.005843218	7.70E-11	0.00089805	0.183438473	-0.004828131	2.76E-07	0.000939412	0.183407564	-0.001015087	0.000234339	2.12E-07	5.90E-09	0.000760503	1.52E-05	ENSG00000239617	1	RP11-302B13.1
VAT	Obesity	ENSG00000186532.7	0.005322825	0.212440906	0.004268904	0.183453886	0.005238348	0.219758623	0.004268635	0.183416977	8.45E-05	0.022489196	1	0.515160001	1	0.531115411	ENSG00000239672	3	NME1
VAT	Obesity	ENSG00000186567.8	-0.003312957	0.11577571	0.002106465	0.183453539	-0.003345015	0.112266959	0.002106315	0.183416593	3.21E-05	0.049110542	1	0.367288459	1	0.367944097	ENSG00000239697	6	TNFSF12
VAT	Obesity	ENSG00000186715.6	0.015111198	0.00081952	0.004516012	0.183450242	0.015239998	0.000738525	0.00451571	0.183413242	-0.0001288	0.000526472	1	0.011599357	1	0.011600152	ENSG00000239697	6	TNFSF12
VAT	Obesity	ENSG00000186766.7	-0.003599588	0.300062521	0.003473498	0.183454068	-0.003526284	0.309981167	0.003473273	0.183417156	-7.33E-05	0.012577931	1	0.608848729	1	0.631867076	ENSG00000239697	6	TNFSF12
VAT	Obesity	ENSG00000186854.6	0.006671717	0.016870452	0.002792066	0.183452316	0.006751908	0.015588571	0.002791871	0.183415336	-8.02E-05	0.000267316	1	0.108150968	1	0.108519823	ENSG00000239713	3	APOBEC3G
VAT	Obesity	ENSG00000187049.5	0.005937849	0.188451914	0.004514876	0.18345382	0.005751485	0.202677284	0.004514642	0.183416933	0.000186364	2.95E-05	1	0.484397268	1	0.512836579	ENSG00000240053	8	LY6G5B
VAT	Obesity	ENSG00000187145.10	0.002744828	0.180932199	0.002051606	0.183453798	0.002837234	0.166680822	0.002051581	0.183416823	-9.24E-05	0.000713836	1	0.4737883	1	0.457993828	ENSG00000240053	8	LY6G5B
VAT	Obesity	ENSG00000187630.10	-0.001479233	0.486603626	0.002126185	0.183454291	-0.001550356	0.465863817	0.00212603	0.183417345	7.11E-05	6.53E-06	1	0.765031548	1	0.762624042	ENSG00000240053	8	LY6G5B
VAT	Obesity	ENSG00000187688.10	-0.000676351	0.904131055	0.005615453	0.183454469	-0.000580493	0.917659981	0.005615043	0.183417542	-9.59E-05	0.021493831	1	0.965567705	1	0.973721371	ENSG00000240053	8	LY6G5B
VAT	Obesity	ENSG00000187715.9	0.005003325	0.412819224	0.00610949	0.183454221	0.005141316	0.400019511	0.006109071	0.183417278	-0.000137991	0.004863672	1	0.708533979	1	0.716918084	ENSG00000240053	8	LY6G5B
VAT	Obesity	ENSG00000187790.6	-0.007498893	0.150971983	0.005221674	0.183453695	-0.00770167	0.140199862	0.005221296	0.183416724	0.000202778	2.56E-07	1	0.420095411	1	0.416292606	ENSG00000240053	8	LY6G5B
VAT	Obesity	ENSG00000187824.4	0.001043067	0.741065001	0.003156567	0.183454433	0.001090043	0.729830676	0.003156334	0.183417501	-4.70E-05	0.04212248	1	0.889278002	1	0.887801393	ENSG00000240053	8	LY6G5B
VAT	Obesity	ENSG00000187848.8	-0.002152214	0.434479136	0.002753775	0.183454243	-0.002103765	0.444861451	0.002753581	0.183417325	-4.84E-05	0.024001606	1	0.722822432	1	0.745369224	ENSG00000240053	8	LY6G5B
VAT	Obesity	ENSG00000187987.5	0.004450056	0.000986268	0.00135078	0.183450372	0.003533143	0.009633654	0.001364823	0.183415013	0.000916913	2.96E-06	1	0.013475737	1	0.077028846	ENSG00000240053	8	LY6G5B
VAT	Obesity	ENSG00000188186.6	0.005795366	0.252836631	0.005068148	0.18345398	0.005586585	0.270375632	0.005068581	0.183417087	0.000208781	0.032641023	1	0.54990473	1	0.586203255	ENSG00000240065	3	PSMB9
VAT	Obesity	ENSG00000188662.5	0.001408292	0.754114204	0.004496185	0.183454437	0.001496046	0.739316327	0.004495884	0.183417504	-8.78E-05	0.0175122	1	0.89443713	1	0.892750508	ENSG00000240065	3	PSMB9
VAT	Obesity	ENSG00000188906.9	-0.00391977	0.244954202	0.003371283	0.183453963	-0.003326568	0.324872268	0.003378956	0.18341718	-0.000593203	0.010742166	1	0.541424131	1	0.64633371	ENSG00000240065	3	PSMB9
VAT	Obesity	ENSG00000188921.12	0.002163831	0.653929815	0.004826642	0.183454398	0.002321568	0.630497901	0.004826278	0.183417459	-0.000157737	4.40E-06	1	0.849804294	1	0.842487235	ENSG00000240065	3	PSMB9
VAT	Obesity	ENSG00000189046.6	0.00370651	0.228122349	0.003075403	0.183453925	0.003657061	0.234354599	0.003075182	0.183417012	4.94E-05	0.03385797	1	0.524101338	1	0.541270873	ENSG00000240065	3	PSMB9
VAT	Obesity	ENSG00000189190.7	-0.005254167	0.017123791	0.002203886	0.183452326	-0.005213049	0.018004067	0.002203744	0.183415431	-4.11E-05	0.02800946	1	0.108763707	1	0.119020171	ENSG00000240065	3	PSMB9
VAT	Obesity	ENSG00000189298.9	-0.019016201	1.16E-08	0.00333331	0.183442173	-0.015262222	1.03E-05	0.003459473	0.18341019	-0.003753979	5.11E-05	3.21E-05	6.99E-07	0.02831071	0.000403836	ENSG00000240065	3	PSMB9
VAT	Obesity	ENSG00000196152.6	0.000869113	0.595495668	0.001637089	0.183454368	0.000812975	0.619449352	0.001636979	0.183417453	5.61E-05	2.61E-05	1	0.818796889	1	0.8401532	ENSG00000240065	3	PSMB9
VAT	Obesity	ENSG00000196196.2	-0.006699489	0.111350044	0.004207806	0.183453516	-0.006586089	0.117513602	0.004207561	0.18341662	-0.000113401	0.003362521	1	0.359025843	1	0.377136675	ENSG00000240065	3	PSMB9
VAT	Obesity	ENSG00000196204.7	0.001201558	0.805950959	0.00489128	0.183454451	0.001374226	0.778730604	0.004890939	0.183417516	-0.000172668	6.81E-06	1	0.921063356	1	0.910529706	ENSG00000240280	2	TCAM1P
VAT	Obesity	ENSG00000196209.8	-0.004963034	0.396742241	0.005856401	0.183454203	-0.005049143	0.388564113	0.005855946	0.183417265	8.61E-05	0.030167861	1	0.701928581	1	0.708846095	ENSG00000240344	4	PPIL3
VAT	Obesity	ENSG00000196247.7	-0.001672503	0.411771013	0.002037695	0.183454219	-0.000332221	0.87166831	0.002056587	0.183417536	-0.001340282	1.64E-06	1	0.708533979	1	0.955921132	ENSG00000240344	4	PPIL3
VAT	Obesity	ENSG00000196357.7	0.003913178	0.258242042	0.003461283	0.183453991	0.003843771	0.266751612	0.003461062	0.18341708	6.94E-05	0.019432123	1	0.557845225	1	0.582003517	ENSG00000240356	2	RPL23AP7
VAT	Obesity	ENSG00000196458.6	0.012959803	0.000221918	0.003509583	0.18344932	0.013017935	0.000207675	0.00350933	0.183412346	-5.81E-05	0.02932811	0.612494646	0.004195169	0.57318292	0.004290423	ENSG00000240399	1	RP1-228P16.1
VAT	Obesity	ENSG00000196693.10	0.003925196	0.219535805	0.003197025	0.183453904	0.003838895	0.22981093	0.003196821	0.183417001	8.63E-05	0.00166081	1	0.521961016	1	0.539352182	ENSG00000240399	1	RP1-228P16.1
VAT	Obesity	ENSG00000196735.7	-0.00499442	0.226013974	0.00412526	0.18345392	0.002139408	0.674686674	0.005097168	0.183417479	-0.007133828	0.017202557	1	0.52272452	1	0.862964608	ENSG00000240399	1	RP1-228P16.1
VAT	Obesity	ENSG00000196963.2	0.000160996	0.95770016	0.003035381	0.183454473	0.00022515	0.940867353	0.003035192	0.183417544	-6.42E-05	0.015538038	1	0.985127657	1	0.980488158	ENSG00000240399	1	RP1-228P16.1
VAT	Obesity	ENSG00000196968.6	0.004115572	0.400347822	0.004893659	0.183454207	0.003700724	0.449673639	0.004895397	0.18341733	0.000414848	0.00498776	1	0.705593862	1	0.747200025	ENSG00000240522	1	RPL7AP10
VAT	Obesity	ENSG00000197008.5	0.010408986	0.039294531	0.005050222	0.183452868	-0.001835538	0.752099836	0.005811018	0.183417508	0.012244523	2.06E-05	1	0.190602646	1	0.89822395	ENSG00000240522	1	RPL7AP10
VAT	Obesity	ENSG00000197020.6	-0.00542324	0.045225655	0.002708161	0.183452958	-0.005476254	0.043148153	0.002707962	0.183416	5.30E-05	0.00836982	1	0.209588906	1	0.210230467	ENSG00000240522	1	RPL7AP10
VAT	Obesity	ENSG00000197062.7	-0.010324302	2.66E-08	0.00185593	0.183442777	-0.007990855	4.26E-05	0.001952418	0.183411215	-0.002333446	0.000119915	7.33E-05	1.53E-06	0.117648929	0.001170502	ENSG00000240522	1	RPL7AP10
VAT	Obesity	ENSG00000197119.8	0.009428414	0.077085792	0.005333251	0.183453293	0.009308697	0.080894514	0.005332899	0.183416395	0.000119717	0.006893134	1	0.284920982	1	0.303354427	ENSG00000240522	1	RPL7AP10
VAT	Obesity	ENSG00000197134.7	-0.005358032	0.300041422	0.00517012	0.183454068	-0.005474982	0.289579096	0.005169727	0.183417122	0.00011695	0.001274401	1	0.608848729	1	0.611488195	ENSG00000240563	1	L1TD1
VAT	Obesity	ENSG00000197165.6	0.06296627	6.18E-29	0.005640208	0.183407376	0.062849037	7.74E-29	0.005639844	0.183370625	0.000117233	0.013833941	1.71E-25	8.53E-26	2.14E-25	1.07E-25	ENSG00000240771	2	ARHGEF25
VAT	Obesity	ENSG00000197168.7	-0.004796852	0.145589168	0.003296145	0.183453674	-0.004734021	0.150909054	0.003295914	0.183416766	-6.28E-05	0.014801183	1	0.412455621	1	0.42939071	ENSG00000240875	1	LINC00886
VAT	Obesity	ENSG00000197181.7	-2.78E-05	0.996049969	0.005616404	0.183454474	9.52E-05	0.986475474	0.005615997	0.183417546	-0.000123003	0.003527849	1	0.998582606	1	0.996361581	ENSG00000240929	2	HIST2H2BB
VAT	Obesity	ENSG00000197261.7	-0.002408331	0.394817478	0.002830287	0.1834542	0.011248937	0.035503456	0.005350076	0.183415875	-0.013657267	0.002629737	1	0.701928581	1	0.184211409	ENSG00000240972	1	MIF
VAT	Obesity	ENSG00000197279.3	-0.012127725	0.000280042	0.003338101	0.183449485	-0.008700823	0.010999238	0.003421839	0.183415103	-0.003426902	5.46E-06	0.772915908	0.005018934	1	0.084466367	ENSG00000240972	1	MIF
VAT	Obesity	ENSG00000197563.5	-0.005196729	0.016794935	0.002173295	0.183452313	-0.005286805	0.014992832	0.00217335	0.18341531	9.01E-05	0.008984204	1	0.108051331	1	0.107059606	ENSG00000240972	1	MIF
VAT	Obesity	ENSG00000197568.9	-0.001924835	0.492533824	0.002804718	0.183454296	-0.00187216	0.504423715	0.002804541	0.183417378	-5.27E-05	0.030599067	1	0.766182814	1	0.78126232	ENSG00000240972	1	MIF
VAT	Obesity	ENSG00000197653.10	0.038906975	7.15E-13	0.005421214	0.183435007	0.038775569	8.50E-13	0.005420844	0.183398211	0.000131406	0.002444105	1.97E-09	8.22E-11	2.35E-09	9.77E-11	ENSG00000240975	1	RP11-313M3.1
VAT	Obesity	ENSG00000197782.10	-0.004419385	4.52E-05	0.001083433	0.183448185	-0.004433373	4.27E-05	0.001083347	0.183411217	1.40E-05	0.046986894	0.12482401	0.001145174	0.117906757	0.001170502	ENSG00000240975	1	RP11-313M3.1
VAT	Obesity	ENSG00000197903.6	0.014004748	0.000394452	0.003951909	0.183449727	0.009871607	0.015238396	0.004067962	0.183415321	0.00413314	1.89E-05	1	0.006519084	1	0.107453986	ENSG00000241057	1	AC004985.12
VAT	Obesity	ENSG00000197915.5	0.001897307	0.10014979	0.001153988	0.183453452	0.001869574	0.10519499	0.001153932	0.183416554	2.77E-05	0.01927757	1	0.337913718	1	0.359264719	ENSG00000241057	1	AC004985.12
VAT	Obesity	ENSG00000197935.6	0.002604284	0.490574267	0.00377763	0.183454294	0.002127962	0.573778417	0.00378307	0.183417426	0.000476322	0.023158132	1	0.766182814	1	0.814504585	ENSG00000241106	2	HLA-DOB
VAT	Obesity	ENSG00000197951.4	0.003853234	0.362792738	0.004234057	0.183454161	0.003773762	0.372743458	0.004233774	0.183417246	7.95E-05	0.022633463	1	0.672794162	1	0.695586169	ENSG00000241106	2	HLA-DOB
VAT	Obesity	ENSG00000197958.8	-0.000604239	0.778300556	0.002146235	0.183454444	-0.000530757	0.804665932	0.002146091	0.183417523	-7.35E-05	3.05E-05	1	0.90675793	1	0.924210558	ENSG00000241106	2	HLA-DOB
VAT	Obesity	ENSG00000198028.3	0.009787347	0.023400297	0.004317613	0.183452532	0.009868379	0.022267808	0.0043173	0.183415571	-8.10E-05	0.012544791	1	0.133993401	1	0.135971568	ENSG00000241106	2	HLA-DOB
VAT	Obesity	ENSG00000198039.7	-0.00930964	0.116596899	0.005932647	0.183453543	-0.006002162	0.314979262	0.005973312	0.183417164	-0.003307478	2.36E-06	1	0.367994033	1	0.637753261	ENSG00000241106	2	HLA-DOB

VAT	Obesity	ENSG00000198046.7	-0.006347935	0.304427033	0.006181124	0.183454075	-0.006131415	0.321185081	0.006180697	0.183417174	-0.00021652	9.82E-06	1	0.613298257	1	0.641712662	ENSG00000241106	2	HLA-DOB
VAT	Obesity	ENSG00000198093.6	-0.002327404	0.42549021	0.00292045	0.183454234	-0.00248738	0.394457359	0.00292096	0.183417272	0.000159976	0.019565543	1	0.714174021	1	0.714375058	ENSG00000241186	3	TDGF1
VAT	Obesity	ENSG00000198203.5	0.001610482	0.713518485	0.00438662	0.183454423	0.001514638	0.729862812	0.004386336	0.183417501	9.58E-05	0.010091317	1	0.875776046	1	0.887801393	ENSG00000241186	3	TDGF1
VAT	Obesity	ENSG00000198237.7	0.0015757	0.759162083	0.005139581	0.183454439	0.001401302	0.785109645	0.005139251	0.183417518	0.000174398	6.91E-05	1	0.897964537	1	0.913850934	ENSG00000241186	3	TDGF1
VAT	Obesity	ENSG00000198466.8	-0.000384793	0.935170004	0.004730553	0.183454472	-0.000468192	0.921154529	0.004730185	0.183417542	8.34E-05	0.009369588	1	0.977307539	1	0.973721371	ENSG00000241186	3	TDGF1
VAT	Obesity	ENSG00000198468.3	0.001904865	0.246760947	0.001644601	0.183453967	0.001931221	0.24024648	0.001644471	0.183417025	-2.64E-05	0.01551751	1	0.543398386	1	0.546644917	ENSG00000241258	2	CRCP
VAT	Obesity	ENSG00000198478.6	0.00868444	0.01039125	0.003388996	0.183451992	0.008749844	0.009822039	0.00338873	0.183415026	-6.54E-05	0.003632478	1	0.075863144	1	0.078123421	ENSG00000241258	2	CRCP
VAT	Obesity	ENSG00000198502.5	-0.001071417	0.290640509	0.001013909	0.183454052	0.001059174	0.464612119	0.001448395	0.183417344	-0.002130591	0.039429287	1	0.599975921	1	0.76239704	ENSG00000241484	5	ARHGAP8
VAT	Obesity	ENSG00000198569.5	0.003635418	0.360439292	0.00397518	0.183454158	0.003579261	0.367870961	0.003974883	0.18341724	5.62E-05	0.049767141	1	0.670499496	1	0.690690966	ENSG00000241484	5	ARHGAP8
VAT	Obesity	ENSG00000198633.6	-0.003992511	0.183356506	0.003000776	0.183453805	-0.004122769	0.169467793	0.003000739	0.183416833	0.000130257	0.001095397	1	0.477265662	1	0.463559076	ENSG00000241549	4	GUSBP2
VAT	Obesity	ENSG00000198677.6	-0.007510073	0.096731479	0.004521652	0.183453431	-0.007437549	0.099973481	0.004521347	0.183416523	-7.25E-05	0.048521881	1	0.328791727	1	0.347887069	ENSG00000241549	4	GUSBP2
VAT	Obesity	ENSG00000198785.4	-0.006223499	0.263023132	0.005560292	0.183454001	-0.006030116	0.278112427	0.005559911	0.183417101	-0.000193383	1.44E-05	1	0.564497547	1	0.59781176	ENSG00000241549	4	GUSBP2
VAT	Obesity	ENSG00000198885.5	-0.003784013	0.131231039	0.002507185	0.183453613	-0.003686807	0.14140095	0.002507015	0.183416729	-9.72E-05	1.64E-06	1	0.387974943	1	0.417278146	ENSG00000241549	4	GUSBP2
VAT	Obesity	ENSG00000198912.6	0.002136507	0.684678417	0.005261198	0.183454412	0.002278414	0.664951589	0.005260866	0.183417475	-0.000141907	0.001879556	1	0.863333733	1	0.856400553	ENSG00000241549	4	GUSBP2
VAT	Obesity	ENSG00000198917.7	0.005406454	0.039719469	0.002628754	0.183452875	0.005348045	0.041892813	0.002628563	0.183415982	5.84E-05	0.002848644	1	0.191653384	1	0.205736949	ENSG00000241549	4	GUSBP2
VAT	Obesity	ENSG00000198920.5	0.00049458	0.927182193	0.005411708	0.183454471	0.000594976	0.912448503	0.005411299	0.183417541	-0.000100397	0.0089835	1	0.976170888	1	0.971531422	ENSG00000241549	4	GUSBP2
VAT	Obesity	ENSG00000198945.3	0.004634456	0.068754188	0.002546354	0.183453222	0.004685874	0.065715303	0.002546166	0.183416266	-5.14E-05	0.005724496	1	0.269094893	1	0.272854254	ENSG00000241549	4	GUSBP2
VAT	Obesity	ENSG00000198959.7	0.00101243	0.789410359	0.003790785	0.183454447	-0.001129507	0.770853572	0.003878021	0.183417514	0.002141937	0.008971998	1	0.911620331	1	0.908047742	ENSG00000241634	1	RP11-381E24.1
VAT	Obesity	ENSG00000203668.1	0.005086854	0.371235968	0.005688966	0.183454172	0.004928001	0.386331092	0.005688622	0.183417262	0.000158853	0.001829361	1	0.68035277	1	0.707997923	ENSG00000241644	2	INMT
VAT	Obesity	ENSG00000203791.9	-0.012019696	0.016888792	0.005030997	0.183452317	-0.012182603	0.015449646	0.005030658	0.18341533	0.000162907	7.40E-05	1	0.108150968	1	0.108225945	ENSG00000241644	2	INMT
VAT	Obesity	ENSG00000203871.4	-0.00908989	0.009590089	0.003509231	0.183451938	-0.009014391	0.010201175	0.003508986	0.183415052	-7.55E-05	0.006033237	1	0.071344059	1	0.080066313	ENSG00000241644	2	INMT
VAT	Obesity	ENSG00000203872.6	-0.007503849	0.008622901	0.002856798	0.183451866	-0.007456128	0.009050733	0.002856598	0.183414971	-4.77E-05	0.033221443	1	0.066293054	1	0.073687385	ENSG00000241679	2	RP11-80H8.4
VAT	Obesity	ENSG00000204209.6	0.002879959	0.394829178	0.003384631	0.1834542	0.001876034	0.580251072	0.003392354	0.18341743	0.001003925	1.75E-05	1	0.701928581	1	0.816782844	ENSG00000241679	2	RP11-80H8.4
VAT	Obesity	ENSG00000204228.3	-0.000441046	0.915997379	0.004181445	0.18345447	-0.005192952	0.223831255	0.004269098	0.183416987	0.004751906	3.63E-08	1	0.972156914	1	0.533483819	ENSG00000241697	3	TMEFF1
VAT	Obesity	ENSG00000204237.4	-0.002082575	0.542372825	0.003418378	0.183454334	-0.002196983	0.520394308	0.003418162	0.18341739	0.000114408	0.00011539	1	0.789547633	1	0.784425829	ENSG00000241837	2	ATP5O
VAT	Obesity	ENSG00000204267.9	0.000423464	0.857734858	0.002362312	0.183454462	-0.003175874	0.194398887	0.002447352	0.18341691	0.003599338	1.91E-08	1	0.944116239	1	0.505693617	ENSG00000241837	2	ATP5O
VAT	Obesity	ENSG00000204305.9	0.005638796	0.204235654	0.004441473	0.183453865	-0.001709064	0.71572942	0.004693008	0.183417496	0.00734786	1.28E-06	1	0.505551932	1	0.879525022	ENSG00000241837	2	ATP5O
VAT	Obesity	ENSG00000204338.4	-0.004680699	0.007803568	0.001759354	0.183451799	-0.001599766	0.397607963	0.001891196	0.183417276	-0.003080933	9.07E-06	1	0.06153671	1	0.715434904	ENSG00000241837	2	ATP5O
VAT	Obesity	ENSG00000204351.7	-0.007917177	6.21E-07	0.001588327	0.183445083	-0.006943576	1.75E-05	0.001616797	0.183410576	-0.000973602	0.001305824	0.001714273	2.68E-05	0.048301174	0.000589039	ENSG00000241839	5	PLEKHO2
VAT	Obesity	ENSG00000204387.8	0.00279926	0.368424197	0.003112261	0.183454168	-0.002489137	0.446973028	0.003273148	0.183417327	0.005288397	1.86E-07	1	0.676520736	1	0.74540517	ENSG00000241839	5	PLEKHO2
VAT	Obesity	ENSG00000204428.8	-0.001133205	0.549784019	0.001894725	0.183454339	-0.002389779	0.218423663	0.001941754	0.183416974	0.001256574	0.003154294	1	0.793904838	1	0.529803765	ENSG00000241839	5	PLEKHO2
VAT	Obesity	ENSG00000204438.6	-0.023851173	1.69E-12	0.003379184	0.183435644	-0.019733244	1.37E-07	0.00374496	0.183407053	-0.004117928	0.010769324	4.66E-09	1.86E-10	0.00037817	7.88E-06	ENSG00000241839	5	PLEKHO2
VAT	Obesity	ENSG00000204463.8	-0.000174156	0.9593533	0.00341716	0.183454473	0.001520556	0.658448389	0.00343973	0.183417472	-0.001694712	1.91E-05	1	0.98546132	1	0.854766662	ENSG00000241839	5	PLEKHO2
VAT	Obesity	ENSG00000204516.5	0.000224928	0.857389484	0.0012517	0.183454462	-0.000599046	0.635246406	0.001262862	0.183417461	0.000823974	1.00E-06	1	0.944116239	1	0.844363271	ENSG00000241973	6	PI4KA
VAT	Obesity	ENSG00000204520.8	-0.002984265	0.157705406	0.002112256	0.18345372	-0.003215766	0.128398807	0.002115011	0.183416672	0.000231501	0.038761966	1	0.433532788	1	0.395731266	ENSG00000242086	4	LINC00969
VAT	Obesity	ENSG00000204525.10	0.004396101	0.006554414	0.001616995	0.18345168	0.00173033	0.320872782	0.001743112	0.183417174	0.00266577	4.26E-05	1	0.053526038	1	0.641712662	ENSG00000242086	4	LINC00969
VAT	Obesity	ENSG00000204531.11	0.003372572	0.000399013	0.000952499	0.183449735	0.002241651	0.022242512	0.00098051	0.183415571	0.001130921	1.22E-06	1	0.006555209	1	0.135971568	ENSG00000242086	4	LINC00969
VAT	Obesity	ENSG00000204536.9	0.005729579	3.87E-06	0.001240664	0.183446413	0.003457349	0.011508964	0.001368238	0.183415133	0.00227223	8.25E-05	0.010688541	0.000140639	1	0.087265774	ENSG00000242110	3	AMACR
VAT	Obesity	ENSG00000204540.6	-0.001275589	0.176517637	0.000943792	0.183453784	-0.000797827	0.40524168	0.000958584	0.183417284	-0.000477762	0.004523263	1	0.465763555	1	0.720669275	ENSG00000242110	3	AMACR
VAT	Obesity	ENSG00000204560.5	0.001044658	0.810735086	0.00436223	0.183454452	0.000176006	0.967881123	0.004371079	0.183417545	0.000868652	0.002287783	1	0.923114207	1	0.990920808	ENSG00000242110	3	AMACR
VAT	Obesity	ENSG00000204574.8	0.009781839	0.001708198	0.003118425	0.183450755	0.00686076	0.031840969	0.003196392	0.183415805	0.002921079	3.25E-05	1	0.020002655	1	0.172994243	ENSG00000242110	3	AMACR
VAT	Obesity	ENSG00000204592.5	0.011197748	0.030095309	0.005163012	0.183452696	0.008583677	0.098593633	0.005196832	0.183416515	0.002614071	1.17E-05	1	0.159430046	1	0.344453707	ENSG00000242110	3	AMACR
VAT	Obesity	ENSG00000204618.4	-0.026497967	4.38E-06	0.005769663	0.183446502	-0.018908854	0.001756312	0.006043805	0.183413847	-0.007589113	2.52E-05	0.012082985	0.000156922	1	0.02256353	ENSG00000242163	1	RP11-299L17.1
VAT	Obesity	ENSG00000204625.6	-0.008897104	0.085669031	0.005176637	0.183453358	-0.006341706	0.224096108	0.005216464	0.183416987	-0.002555398	7.93E-05	1	0.301975129	1	0.533537912	ENSG00000242193	5	RP11-568K15.1
VAT	Obesity	ENSG00000204632.7	0.004931849	0.113098862	0.003112716	0.183453525	0.0030823	0.327767604	0.003149635	0.183417184	0.001849549	0.000127787	1	0.362126285	1	0.648715527	ENSG00000242252	1	BGLAP
VAT	Obesity	ENSG00000204644.5	0.005374648	5.75E-09	0.000922861	0.183441654	0.004078199	3.77E-05	0.000989502	0.183411126	0.001296449	0.00028386	1.59E-05	3.69E-07	0.103922864	0.001105562	ENSG00000242252	1	BGLAP
VAT	Obesity	ENSG00000204677.6	-0.007483449	0.095907975	0.004494491	0.183453426	-0.007382236	0.100461842	0.004494179	0.183416526	-0.000101213	0.004316032	1	0.326866783	1	0.348773187	ENSG00000242294	2	STAG3L5P
VAT	Obesity	ENSG00000204709.4	0.014907423	1.14E-08	0.002611382	0.183442156	0.011580548	2.70E-05	0.002758823	0.183410887	0.003326875	0.000186927	3.15E-05	6.99E-07	0.074453131	0.000827257	ENSG00000242372	2	EIF6
VAT	Obesity	ENSG00000204713.6	-0.011417757	0.000575485	0.003316285	0.183449993	-0.010341312	0.001891382	0.003328611	0.183413898	-0.001076444	0.000205117	1	0.008727132	1	0.023620883	ENSG00000242372	2	EIF6
VAT	Obesity	ENSG00000204792.2	-0.002569119	0.36421688	0.002831422	0.183454163	-0.002517046	0.373989703	0.002831241	0.183417247	-5.21E-05	0.031786662	1	0.673301132	1	0.697440257	ENSG00000242372	2	EIF6
VAT	Obesity	ENSG00000204977.5	0.005732036	0.22631644	0.004737597	0.183453921	0.005644583	0.23344409	0.004737235	0.183417009	8.75E-05	0.007898097	1	0.52272452	1	0.540524906	ENSG00000242435	1	UPK3BP1
VAT	Obesity	ENSG00000205038.7	0.003922609	0.480436037	0.005559237	0.183454286	0.006103429	0.279863972	0.005648049	0.183417105	-0.002180821	0.029325911	1	0.762547611	1	0.59924326	ENSG00000242435	1	UPK3BP1
VAT	Obesity	ENSG00000205045.4	-0.016155067	0.004685479	0.005712722	0.183451451	-0.01367359	0.017902638	0.005775213	0.183415427	-0.002481477	0.003548413	1	0.042123525	1	0.119020171	ENSG00000242435	1	UPK3BP1
VAT	Obesity	ENSG00000205269.4	-0.003755734	0.486357886	0.005395288	0.183454291	-0.003545549	0.51105318	0.005394938	0.183417383	-0.000210185	4.17E-06	1	0.765031548	1	0.784425829	ENSG00000242441	3	GTF2A1L
VAT	Obesity	ENSG00000205464.7	-0.002078223	0.54423783	0.003427049	0.183454335	-0.002204681	0.520017121	0.003427039	0.18341739	0.000126458	0.008252021	1	0.79046238	1	0.784425829	ENSG00000242516	1	LINC00960
VAT	Obesity	ENSG00000205560.8	0.000425228	0.824139495	0.001913511	0.183454455	0.000386882	0.83976398	0.001913406	0.183417531	3.83E-05	0.036123091	1	0.927365584	1	0.939543177	ENSG00000242516	1	LINC00960
VAT	Obesity	ENSG00000205571.8	-0.007091874	0.006074731	0.002584755	0.183451629	-0.007179246	0.005474262	0.002584575	0.18341463	8.74E-05	2.07E-05	1	0.05096127	1	0.05044591	ENSG00000242516	1	LINC00960
VAT	Obesity	ENSG00000205572.5	-0.006617691	0.109640183	0.004136534	0.183453507	-0.006535571	0.114092795	0.004136269	0.183416602	-8.21E-05	0.02009526	1	0.355833781	1	0.370902372	ENSG00000242600	2	MBL1P
VAT	Obesity	ENSG00000205578.4	-0.002543769	0.312034919	0.002516185	0.183454088	-0.003434504	0.173056338	0.002520828	0.183416844	0.000890736	1.42E-08	1	0.619458427	1	0.470490411	ENSG00000242616	3	GNG10
VAT	Obesity	ENSG00000205583.9	-0.018798565	3.87E-20	0.002045077	0.183422541	-0.018842512	3.15E-20	0.002044932	0.183385464	4.39E-05	0.005765058	1.07E-16	1.19E-17	8.68E-17	9.65E-18	ENSG00000242779	2	ZNF702P
VAT	Obesity	ENSG00000205771.2	-0.002700906	0.366933405	0.002993579	0.183454166	-0.002806603	0.348449607	0.002993386	0.183417214	0.000105697	3.37E-05	1	0.675157466	1	0.670656147	ENSG00000242779	2	ZNF702P
VAT	Obesity	ENSG00000206013.2	-0.000490094	0.871255769	0.003024084	0.183454464	-0.000390556	0.897233834	0.003023895	0.18341754	-9.95E-05	0.000168095	1	0.949335145	1	0.968028006	ENSG00000242779	2	ZNF702P
VAT	Obesity	ENSG00000206145.4	0.009369088	0.052132317	0.004824343	0.183453048	0.009287123	0.054205519	0.00482399	0.183416145	8.20E-05	0.021689438	1	0.229848555	1	0.24496821	ENSG00000243056	1	EIF4EBP3
VAT	Obesity	ENSG00000206172.4	-0.01002136	0.047450334	0.005055514	0.183452989	-0.009894323	0.05031703	0.005055181	0.183416098	-0.000127036	0.002591761	1	0.215754403	1	0.23538136	ENSG00000243056	1	EIF4EBP3
VAT	Obesity	ENSG00000206341.6	-0.000765298	0.784484859	0.002798376	0.183454446	0.000831184	0.768606325	0.002825249	0.183417513	-0.001596482	4.40E-05	1	0.909319831	1	0.907673971	ENSG00000243056	1	EIF4EBP3
VAT	Obesity	ENSG00000206344.6	-0.002268564	0.226513958	0.001875793	0.183453921	-0.001131956	0.559764142	0.001940965	0.183417417	-0.001136608	0.022861248	1	0.52272452	1	0.809555852	ENSG00000243244	1	STON1
VAT	Obesity	ENSG00000206503.7	-0.009078523	0.039477611	0.004408807	0.183452871	-0.007051307	0.111138452	0.004426155	0.183416587	-0.002027215	3.15E-07	1	0.191154746	1	0.366914394	ENSG00000243444	3	PALM2
VAT	Obesity	ENSG00000206535.3	-0.000238701	0.965102883	0.005455903	0.183454473	-8.12E-05	0.988119418	0.005455514	0.183417546	-0.000157465	0.000164573	1	0.985729121	1	0.996361581	ENSG00000243444	3	PALM2
VAT	Obesity	ENSG00000212694.4	-0.011835421	7.48E-05	0.002988435	0.183448545	-0.011770043	8.19E-05	0.002988221	0.183411683	-6.54E-05	0.0040622	0.206537765	0.001721148	0.226036704	0.002018185	ENSG00000243478	3	AOX2P
VAT	Obesity	ENSG00000212807.1	-0.007433418	0.055522083	0.003882141	0.183453088	-0.007367787	0.057697205	0.003881877	0.183416185	-6.56E-05	0.036147161	1	0.237582866	1	0.252768707	ENSG00000243478	3	AOX2P
VAT	Obesity	ENSG00000213024.6	-0.005252988	0.081431782	0.003014728	0.183453326	-0.005195438	0.084805824	0.003014537	0.183416423	-5.76E-05	0.027234439	1	0.293409555	1	0.310429805	ENSG00000243646	4	IL10RB
VAT	Obesity	ENSG00000213077.5	0.000330963	0.948410326	0.005115089	0.183454473	0.000251231	0.96082436	0.005114719	0.183417545	7.97E-05	0.038432291	1	0.982080274	1	0.98754495	ENSG00000243646	4	IL10RB
VAT	Obesity	ENSG00000213088.5	0.005236009	0.293546583	0.004984899	0.183454057	0.005367348	0.281592103	0.004984763	0.183417108	-0.000131339	0.029775961	1	0.602370683	1	0.600777491	ENSG00000243646	4	IL10RB
VAT	Obesity	ENSG00000213171.2	0.00331496	0.42858028	0.004187543	0.183454237	0.002927342	0.484691796	0.004189237	0.183417361	0.000387617	0.003593713	1	0.71733267	1	0.775358255	ENSG00000243725	2	TTC4
VAT	Obesity	ENSG00000213221.4	0.000402739	0.767504915	0.00136226	0.183454441	0.000358576	0.792366515	0.001362169	0.18341752	4.42E-05	8.38E-05	1	0.901410028	1	0.918062321	ENSG00000243753	1	HLA-L
VAT	Obesity	ENSG00000213402.2	0.008193612	0.00035941	0.002296256	0.183449661	0.007284576	0.00155021	0.002301506	0.18341376	0.000909036	1.01E-08	0.991971782	0.005975734	1	0.020868421	ENSG00000243753	1	HLA-L
VAT	Obesity	ENSG00000213433.5	0.002973158	0.439500479	0.003846072	0.183454248	0.003032046	0.43045901	0.003845787	0.183417311	-5.89E-05	0.035797359	1	0.726033105	1	0.735057673	ENSG00000243753	1	HLA-L
VAT	Obesity	ENSG00000213462.4	0.008572563	0.012385967	0.003427706	0.18345211	-8.44E-05	0.983378242	0.004050701	0.183417546	0.008656954	6.08E-05	1	0.086985415	1	0.996238962	ENSG00000243753	1	HLA-L
VAT	Obesity	ENSG00000213494.5	-0.000512017	0.791902051	0.001940623	0.183454448	-0.00056414	0.771269497	0.001940527	0.183417514	5.21E-05	0.007876598	1	0.913008119	1	0.908150091	ENSG00000243753	1	HLA-L
VAT	Obesity	ENSG00000213689.5	0.005252967	0.002783653	0.001756442	0.183451093	0.005209813	0.00301453	0.001756353	0.183414221	4.32E-05	0.013927948	1	0.029101822	1	0.033147822	ENSG00000243753	1	HLA-L
VAT	Obesity	ENSG00000213760.6	-0.004680351	0.020070182	0.002013019	0.183452431	-0.003895155	0.056339424	0.002041052	0.18341617	-0.000785196	0.020288104	1	0.120683447	1	0.24999487	ENSG00000243753	1	HLA-L
VAT	Obesity	ENSG00000213901.6	0.009722559	0.004841748	0.003450909	0.183451474	0.009568048	0.005564308	0.003451152	0.183414641	0.000154511	0.015479844	1	0.04296857	1	0.051021564	ENSG00000243753	1	HLA-L
VAT	Obesity	ENSG00000214063.6	0.014886186	0.003764004	0.005137942	0.183451301	0.014773845	0.004032406	0.005137604	0.183414421	0.000112342	0.008675888	1	0.036051294	1	0.041068046	ENSG00000243759	1	ST13P15
VAT	Obesity	ENSG00000214135.4	-0.008956801	0.028768807	0.004096135	0.183452667	-0.009025438	0.027554953	0.004095822	0.183415711	6.86E-05	0.016324546	1	0.154178459	1	0.15580997	ENSG00000243789	6	JMJD7
VAT	Obesity	ENSG00000214338.6	-0.001404261	0.721660041	0.003941883	0.183454426	-0.001472168	0.708779198	0.003941585	0.183417493	6.79E-05	0.015298619	1	0.880540103	1	0.876447308	ENSG00000243789	6	JMJD7
VAT	Obesity	ENSG00000214530.3	-0.003364759	0.123868793	0.0021867	0.183453579	-0.003413865	0.118454217	0.002186556	0.183416625	4.91E-05	0.007308581	1	0.37657343	1	0.37883388	ENSG00000243819	3	RN7SL832P
VAT	Obesity	ENSG00000214694.6	-0.009069252	0.104228913	0.005582137	0.183453476	-0.008973971	0.10789248	0.005581725	0.183416569	-9.53E-05	0.019898525	1	0.347010616	1	0.363593705	ENSG00000243819	3	RN7SL832P
VAT	Obesity	ENSG00000214706.6	-0.004623271	0.221783643	0.003783981	0.18345391	-0.004472481	0.237248695	0.003784167	0.183417018	-0.00015079	0.02124728	1	0.52199987	1	0.544132965	ENSG00000243819	3	RN7SL832P
VAT	Obesity	ENSG00000215114.3	0.001049872	0.827503176	0.004818027	0.183454456	0.001198095	0.803604385	0.004817716	0.183417523	-0.000148222	0.000303767	1	0.928795757	1	0.92407402	ENSG00000243961	2	RP5-839B4.8
VAT	Obesity	ENSG00000215399.2	-0.011884746	0.048076582	0.00601243	0.183452997	-0.011631896	0.053019071	0.00601203	0.183416131	-0.00025285	3.15E-07	1	0.216635313	1	0.242272577	ENSG00000243961	2	RP5-839B4.8
VAT	Obesity	ENSG00000215452.3	0.001156462	0.622523918	0.002349211	0.183454383	0.001113859	0.63537574	0.002349048	0.183417461	4.26E-05	0.022107016	1	0.833933632	1	0.844363271	ENSG00000244026	2	FAM86DP
VAT	Obesity	ENSG00000216901.1	0.00609358	3.22E-06	0.001308694	0.183446279	0.004908492	0.00023558	0.001334745	0.183412435	0.001185088	6.62E-06	0.008890984	0.000120148	0.650200703	0.004745991	ENSG00000244026	2	FAM86DP
VAT	Obesity	ENSG00000219392.1	-0.001778373	0.397425836	0.002101529	0.183454203	0.000453291	0.833012594	0.002149935	0.183417529	-0.002231664	9.16E-07	1	0.702687577	1	0.93758435	ENSG00000244045	6	TMEM199
VAT	Obesity	ENSG00000219481.6	0.00492702	0.083075198	0.002842863	0.183453339	0.0049904	0.079167577	0.002842658	0.183416381	-6.34E-05	0.003136426	1	0.297357955	1	0.298885799	ENSG00000244045	6	TMEM199
VAT	Obesity	ENSG00000219626.4	-0.001658929	0.351137717	0.001779231	0.183454145	-0.001599442	0.368647554	0.001779114	0.183417241	-5.95E-05	6.02E-05	1	0.661582235	1	0.69094905	ENSG00000244119	1	PDCL3P4
VAT	Obesity	ENSG00000219891.2	-0.012048954	0.001346548	0.003758382	0.183450589	-0.008793149	0.021147769	0.003814247	0.183415537	-0.003255805	6.04E-07	1	0.01689306	1	0.132256412	ENSG00000244165	1	P2RY11
VAT	Obesity	ENSG00000220583.1	-0.007847039	0.186624892	0.005941884	0.183453815	-0.008647526	0.145672693	0.005943358	0.183416746	0.000800487	3.43E-07	1	0.482741052	1	0.421062772	ENSG00000244165	1	P2RY11
VAT	Obesity	ENSG00000221947.3	0.003875688	0.068824242	0.002129995	0.183453223	0.003834302	0.071820465	0.002129862	0.183416321	4.14E-05	0.02595129	1	0.269094893	1	0.283582953	ENSG00000244165	1	P2RY11
VAT	Obesity	ENSG00000221955.6	-0.006620182	0.221893957	0.005419675	0.18345391	-0.006493544	0.230827018	0.005419268	0.183417003	-0.000126638	0.00106084	1	0.52199987	1	0.540207072	ENSG00000244165	1	P2RY11
VAT	Obesity	ENSG00000223756.2	0.000218478	0.949622701	0.003457988	0.183454473	0.000304828	0.92975104	0.003457739	0.183417543	-8.63E-05	0.000965017	1	0.982080274	1	0.976079449	ENSG00000244165	1	P2RY11
VAT	Obesity	ENSG00000223861.1	-0.022249633	0.000125171	0.005800516	0.183448913	-0.022070677	0.000141769	0.005800302	0.183412074	-0.000178956	0.006188773	0.345471582	0.002637188	0.39128379	0.003155514	ENSG00000244165	1	P2RY11
VAT	Obesity	ENSG00000224132.2	-0.001500551	0.726241582	0.004285702	0.183454428	-0.001414205	0.741400152	0.004285472	0.183417505	-8.63E-05	0.037344505	1	0.882200818	1	0.893915902	ENSG00000244219	2	GS1-259H13.2
VAT	Obesity	ENSG00000224183.1	-0.000403688	0.869649636	0.002459961	0.183454464	-0.000357651	0.88439654	0.002459804	0.183417538	-4.60E-05	0.029566676	1	0.948684727	1	0.960056703	ENSG00000244219	2	GS1-259H13.2
VAT	Obesity	ENSG00000224287.2	-0.002781876	0.357484981	0.003023224	0.183454154	-0.002738995	0.364907951	0.003022994	0.183417236	-4.29E-05	0.043748823	1	0.668015266	1	0.688411445	ENSG00000244219	2	GS1-259H13.2
VAT	Obesity	ENSG00000224316.1	0.002299169	0.250977122	0.002002795	0.183453976	0.001291349	0.521399569	0.002013989	0.183417391	0.00100782	2.46E-06	1	0.549323438	1	0.784425829	ENSG00000244219	2	GS1-259H13.2
VAT	Obesity	ENSG00000224389.4	0.014273928	0.000128122	0.003726804	0.183448929	0.002444052	0.614001843	0.004845756	0.18341745	0.011829876	0.000133919	0.353616871	0.002678916	1	0.839253732	ENSG00000244291	1	C7orf13
VAT	Obesity	ENSG00000224497.1	0.001931546	0.054092077	0.001002826	0.183453072	0.001907719	0.057113505	0.001002772	0.183416178	2.38E-05	0.014090989	1	0.236224894	1	0.251902356	ENSG00000244301	1	AOX3P
VAT	Obesity	ENSG00000224557.3	0.000739088	0.61883744	0.001485606	0.183454381	-0.000323913	0.829530804	0.001504449	0.183417529	0.001063001	8.16E-06	1	0.831754083	1	0.936897537	ENSG00000244301	1	AOX3P
VAT	Obesity	ENSG00000224635.1	0.00032938	0.893337686	0.002456557	0.183454467	0.000236042	0.923447531	0.002456421	0.183417543	9.33E-05	6.72E-05	1	0.961627151	1	0.974118595	ENSG00000244414	2	CFHR1
VAT	Obesity	ENSG00000224687.1	-0.004866417	0.334732609	0.005044874	0.183454122	-0.004624698	0.359312316	0.005045067	0.183417228	-0.00024172	0.004026963	1	0.64291023	1	0.683932408	ENSG00000244414	2	CFHR1
VAT	Obesity	ENSG00000224796.1	-0.000429154	0.776016082	0.001508372	0.183454444	0.000709543	0.642809086	0.00152993	0.183417465	-0.001138697	9.26E-06	1	0.904860323	1	0.849283426	ENSG00000244414	2	CFHR1
VAT	Obesity	ENSG00000224843.2	0.00734849	0.000401411	0.002076326	0.183449739	0.006041627	0.003900377	0.002093337	0.183414398	0.001306863	1.07E-06	1	0.006555585	1	0.040018734	ENSG00000244482	5	LILRA6
VAT	Obesity	ENSG00000224997.1	-0.028945432	3.98E-08	0.005270455	0.183443073	-0.028787917	4.70E-08	0.005270076	0.183406269	-0.000157514	7.42E-05	0.000109728	2.24E-06	0.000129621	3.09E-06	ENSG00000244482	5	LILRA6
VAT	Obesity	ENSG00000225194.2	-0.001537211	0.485628365	0.002204584	0.18345429	-0.001504016	0.495067821	0.002204425	0.18341737	-3.32E-05	0.046473865	1	0.765003071	1	0.775358255	ENSG00000244482	5	LILRA6
VAT	Obesity	ENSG00000225498.1	-0.019149433	0.001863007	0.006154877	0.183450815	-0.019430182	0.001593722	0.006154496	0.183413779	0.000280749	2.39E-07	1	0.021380487	1	0.021249624	ENSG00000244560	2	RP4-800G7.2
VAT	Obesity	ENSG00000225684.3	-0.000941007	0.833588914	0.00447883	0.183454457	-0.000861483	0.847460661	0.004478505	0.183417532	-7.95E-05	0.018217698	1	0.933349047	1	0.944145183	ENSG00000244607	1	CCDC13
VAT	Obesity	ENSG00000225851.1	0.000481394	0.77070857	0.00165173	0.183454442	0.002038434	0.229510966	0.001696403	0.183417	-0.00155704	5.86E-05	1	0.904018552	1	0.539107619	ENSG00000244607	1	CCDC13
VAT	Obesity	ENSG00000225892.3	0.000269125	0.963640039	0.005903632	0.183454473	0.000186142	0.97484494	0.005903186	0.183417546	8.30E-05	0.046894855	1	0.985729121	1	0.993586204	ENSG00000244607	1	CCDC13
VAT	Obesity	ENSG00000226002.1	0.001157831	0.367758307	0.001285506	0.183454167	0.000853667	0.507109764	0.001286912	0.18341738	0.000304164	1.29E-06	1	0.676224469	1	0.783244156	ENSG00000244607	1	CCDC13
VAT	Obesity	ENSG00000226314.3	0.005226518	0.130882315	0.003459817	0.183453612	0.006316933	0.068606738	0.003468927	0.183416293	-0.001090415	2.06E-05	1	0.387974943	1	0.277239528	ENSG00000244623	1	OR2AE1
VAT	Obesity	ENSG00000226328.2	-0.002378677	0.651484674	0.005266045	0.183454397	-0.002605958	0.620674874	0.005265686	0.183417453	0.000227281	8.24E-08	1	0.848236169	1	0.840409069	ENSG00000244625	1	CTA-211A9.5
VAT	Obesity	ENSG00000226383.1	-0.007427982	0.119550477	0.004771725	0.183453558	-0.007569554	0.112649551	0.004771533	0.183416595	0.000141571	0.006962282	1	0.372684435	1	0.367944097	ENSG00000244625	1	CTA-211A9.5
VAT	Obesity	ENSG00000227113.2	0.001520923	0.395052746	0.001788288	0.183454201	0.001355336	0.448549725	0.00178843	0.183417329	0.000165586	1.06E-06	1	0.701928581	1	0.747131709	ENSG00000244693	1	CTAGE8
VAT	Obesity	ENSG00000227262.3	-0.017157582	0.00355177	0.005885027	0.183451261	-0.011915489	0.046704336	0.005990764	0.183416051	-0.005242093	3.08E-06	1	0.034455189	1	0.222632069	ENSG00000244723	3	ASLP1
VAT	Obesity	ENSG00000227531.1	0.002570862	0.439828751	0.00332805	0.183454249	0.002516804	0.449473568	0.003327814	0.18341733	5.41E-05	0.034901133	1	0.726033105	1	0.747200025	ENSG00000244731	3	C4A
VAT	Obesity	ENSG00000227694.1	-0.014698894	0.017686187	0.006196492	0.183452347	-0.014558631	0.018790783	0.006196049	0.18341546	-0.000140263	0.002978122	1	0.110689061	1	0.120957701	ENSG00000244731	3	C4A
VAT	Obesity	ENSG00000227766.1	-0.005852545	0.005960705	0.002128239	0.183451616	-0.00433856	0.044289832	0.002157047	0.183416017	-0.001513985	1.76E-05	1	0.050310539	1	0.214456031	ENSG00000244731	3	C4A
VAT	Obesity	ENSG00000227827.3	-0.001515923	0.319045968	0.001521368	0.183454099	-0.001483757	0.329389458	0.001521263	0.183417187	-3.22E-05	0.008373212	1	0.626292228	1	0.650296785	ENSG00000244731	3	C4A
VAT	Obesity	ENSG00000227921.1	0.006576188	0.04354673	0.003258066	0.183452934	0.006660618	0.040905055	0.003257828	0.183415966	-8.44E-05	0.000506537	1	0.205215702	1	0.203053868	ENSG00000244731	3	C4A
VAT	Obesity	ENSG00000227986.1	0.003339077	0.145853872	0.002295946	0.183453675	9.13E-05	0.96973808	0.002405317	0.183417545	0.003247826	6.05E-06	1	0.412455621	1	0.990920808	ENSG00000244731	3	C4A
VAT	Obesity	ENSG00000228022.1	-0.021249736	6.79E-07	0.004277871	0.183445148	-0.013910012	0.003132945	0.004708239	0.183414247	-0.007339724	0.000190878	0.001873842	2.88E-05	1	0.03417758	ENSG00000244731	3	C4A
VAT	Obesity	ENSG00000228050.1	-0.001032652	0.867993346	0.006213009	0.183454464	-0.0008268	0.894126624	0.006212598	0.183417539	-0.000205851	6.58E-05	1	0.948025973	1	0.968028006	ENSG00000244731	3	C4A
VAT	Obesity	ENSG00000228065.6	0.000536887	0.73604818	0.001592703	0.183454431	0.000505346	0.751008197	0.001592592	0.183417508	3.15E-05	0.011230133	1	0.886561612	1	0.897653804	ENSG00000244731	3	C4A
VAT	Obesity	ENSG00000228075.4	-0.008234403	0.121893724	0.005323246	0.18345357	-0.00831483	0.118264223	0.005322841	0.183416624	8.04E-05	0.031703144	1	0.374639953	1	0.37866503	ENSG00000244731	3	C4A
VAT	Obesity	ENSG00000228078.1	0.007474284	6.80E-05	0.001876402	0.183448477	0.005851993	0.002134658	0.001905662	0.183413982	0.001622292	1.16E-06	0.187604572	0.001603458	1	0.025615891	ENSG00000244752	2	CRYBB2
VAT	Obesity	ENSG00000228106.1	-0.001503597	0.597719346	0.00284943	0.183454369	-0.001426014	0.616730065	0.002849233	0.183417451	-7.76E-05	0.000610253	1	0.820340823	1	0.839253732	ENSG00000245958	2	RP11-33B1.1
VAT	Obesity	ENSG00000228409.1	-0.00537163	0.357283822	0.005835215	0.183454154	-0.005736906	0.325557738	0.005835515	0.183417181	0.000365276	0.000332089	1	0.668015266	1	0.646431191	ENSG00000245958	2	RP11-33B1.1

VAT	Obesity	ENSG00000228789.2	-0.002400239	0.135150375	0.001606479	0.18345363	-0.000345096	0.835723755	0.001664182	0.18341753	-0.002055143	2.31E-06	1	0.394724904	1	0.938551038	ENSG00000245958	2	RP11-33B1.1
VAT	Obesity	ENSG00000228998.3	0.005190599	0.376841483	0.005873501	0.183454179	0.005054623	0.389437675	0.005873109	0.183417266	0.000135976	0.004889258	1	0.685170491	1	0.709470616	ENSG00000245958	2	RP11-33B1.1
VAT	Obesity	ENSG00000229390.1	-0.005313194	0.067464481	0.002905653	0.1834321	-0.003159058	0.280234199	0.002936577	0.183417109	-0.002154136	4.54E-07	1	0.266383359	1	0.601092193	ENSG00000246067	3	RAB30-AS1
VAT	Obesity	ENSG00000230189.2	-0.006373163	0.024812283	0.002839666	0.18345257	-0.002377923	0.426733716	0.002991874	0.18341307	-0.00399524	2.27E-05	1	0.13753347	1	0.73108942	ENSG00000246067	3	RAB30-AS1
VAT	Obesity	ENSG00000230267.3	0.002026367	0.554902369	0.003432016	0.183454342	0.001952198	0.569455213	0.003431805	0.183417424	7.42E-05	0.014640442	1	0.795474689	1	0.812246196	ENSG00000246375	2	RP11-10L7.1
VAT	Obesity	ENSG00000230387.4	0.008774748	0.066620535	0.004783887	0.183453202	0.008954583	0.061214812	0.004783557	0.183416222	-0.000179835	6.58E-06	1	0.264946219	1	0.259777168	ENSG00000246375	2	RP11-10L7.1
VAT	Obesity	ENSG00000230438.5	0.001071664	0.749380662	0.00335465	0.183454436	0.001005778	0.764300813	0.003354404	0.183417512	6.59E-05	0.007889972	1	0.892799597	1	0.904298874	ENSG00000246523	3	RP11-736K20.6
VAT	Obesity	ENSG00000230521.1	0.007566621	0.00239821	0.002492371	0.1834509	0.006426216	0.010253329	0.002503223	0.18341505	0.001140406	1.27E-06	1	0.026266109	1	0.080167672	ENSG00000246523	3	RP11-736K20.6
VAT	Obesity	ENSG00000230528.3	-0.004048155	0.27386216	0.003699604	0.183454022	-0.004121018	0.26528756	0.003699359	0.183417077	7.29E-05	0.017324169	1	0.578759235	1	0.579936623	ENSG00000246523	3	RP11-736K20.6
VAT	Obesity	ENSG00000230795.2	-0.003862541	0.025965391	0.001734632	0.1834526	-0.002565024	0.144541989	0.001757966	0.183416741	-0.001297517	5.93E-06	1	0.145075383	1	0.420375017	ENSG00000246523	3	RP11-736K20.6
VAT	Obesity	ENSG00000230836.1	-0.003107076	0.396101183	0.00366139	0.183454202	-0.003047146	0.405244037	0.003661146	0.183417284	-5.99E-05	0.047093499	1	0.701928581	1	0.720669275	ENSG00000246575	2	AC093162.5
VAT	Obesity	ENSG00000230939.1	0.007469301	0.029872823	0.003439296	0.183452463	0.007592465	0.027262455	0.003439059	0.183415704	-0.000123074	7.31E-06	1	0.158861254	1	0.155231297	ENSG00000246575	2	AC093162.5
VAT	Obesity	ENSG00000231130.1	-0.005799475	0.008420245	0.00220115	0.18345185	-0.002864044	0.210150085	0.002285467	0.183416953	-0.002935431	1.87E-06	1	0.065097695	1	0.521595534	ENSG00000246575	2	AC093162.5
VAT	Obesity	ENSG00000231576.1	0.006644786	0.102533083	0.004069819	0.183453466	0.006584236	0.105675607	0.004069511	0.183416557	6.06E-05	0.035876612	1	0.343436055	1	0.360079845	ENSG00000247121	2	CTD-2260A17.2
VAT	Obesity	ENSG00000231698.2	-0.002794014	0.555519462	0.004739553	0.183454343	-0.002949527	0.533706391	0.004739272	0.1834174	0.000155513	0.000313356	1	0.795474689	1	0.788982131	ENSG00000247121	2	CTD-2260A17.2
VAT	Obesity	ENSG00000231768.1	0.004022962	0.348080129	0.004287406	0.183454141	0.003896832	0.363367891	0.004287099	0.183417234	0.00012613	0.00010696	1	0.660100296	1	0.687385454	ENSG00000247157	2	RP11-434C1.1
VAT	Obesity	ENSG00000231784.4	-0.002456446	0.682427299	0.00600373	0.183454411	-0.002641306	0.659955572	0.006003313	0.183417473	0.00018486	9.90E-05	1	0.863333733	1	0.854766662	ENSG00000247157	2	RP11-434C1.1
VAT	Obesity	ENSG00000231793.4	0.005114993	0.260303964	0.004543937	0.183453999	0.002853358	0.531465106	0.004559744	0.183417398	0.002261635	4.14E-09	1	0.561280421	1	0.786996417	ENSG00000247157	2	RP11-434C1.1
VAT	Obesity	ENSG00000231816.1	0.00098432	0.772152596	0.003399359	0.183454442	0.000881267	0.795431759	0.003399112	0.183417521	0.000103053	4.86E-05	1	0.904559068	1	0.918573915	ENSG00000247596	4	TWF2
VAT	Obesity	ENSG00000231925.7	0.013159031	0.000608689	0.003838987	0.183450033	0.013618858	0.000396691	0.003844637	0.183412804	-0.000459827	0.032791561	1	0.009032155	1	0.007109524	ENSG00000247596	4	TWF2
VAT	Obesity	ENSG00000232075.1	0.018410362	0.000442649	0.005240218	0.183449809	0.018270716	0.000488716	0.005239832	0.183412951	0.000139646	0.000262429	1	0.007102966	1	0.008430344	ENSG00000248098	6	BCKDHA
VAT	Obesity	ENSG00000232388.2	-0.007823816	0.145345522	0.005372862	0.183453673	-0.007649272	0.154512043	0.005372526	0.1834178	-0.000174544	0.000189663	1	0.412285344	1	0.436939795	ENSG00000248098	6	BCKDHA
VAT	Obesity	ENSG00000232450.1	0.003080289	0.617671951	0.006171034	0.18345438	0.002970997	0.630178092	0.006170595	0.183417458	0.000109292	0.021299329	1	0.831570896	1	0.842487235	ENSG00000248161	1	RP11-499E18.1
VAT	Obesity	ENSG00000232546.1	0.00602959	0.23509491	0.005029291	0.18345391	0.003950284	0.433850462	0.00504751	0.183417315	0.002079306	1.69E-06	1	0.52708752	1	0.737402735	ENSG00000248202	1	RP11-455B3.1
VAT	Obesity	ENSG00000232559.2	-0.005071942	0.012579291	0.002032458	0.1834521	-0.003316696	0.109598089	0.002072929	0.183416579	-0.001755246	1.74E-05	1	0.0877446	1	0.365676336	ENSG00000248202	1	RP11-455B3.1
VAT	Obesity	ENSG00000232561.2	-0.01741927	4.53E-08	0.003185141	0.183443169	-0.01750575	3.88E-08	0.003184915	0.183406129	8.65E-05	0.000465278	0.000125033	2.45E-06	0.000107003	2.63E-06	ENSG00000248202	1	RP11-455B3.1
VAT	Obesity	ENSG00000232615.4	0.002039848	0.633392371	0.00427679	0.183454388	0.00194474	0.649286779	0.004276481	0.183417468	9.51E-05	0.002113914	1	0.840565826	1	0.852367106	ENSG00000248202	1	RP11-455B3.1
VAT	Obesity	ENSG00000232926.1	0.001558216	0.686913361	0.003866085	0.183454413	0.001680213	0.663827405	0.003865801	0.183417475	-0.000121997	2.00E-05	1	0.863333733	1	0.855751349	ENSG00000248202	1	RP11-455B3.1
VAT	Obesity	ENSG00000233297.4	-0.007805979	0.165267946	0.005625638	0.183453746	-0.007655655	0.173535956	0.005625305	0.183416846	-0.000150324	0.003327906	1	0.446320479	1	0.470490411	ENSG00000248265	1	RP11-834C11.3
VAT	Obesity	ENSG00000233327.6	-0.000545902	0.903003014	0.004479426	0.183454468	-0.000617295	0.890385214	0.004479094	0.183417539	7.14E-05	0.028621005	1	0.965567705	1	0.965604397	ENSG00000248265	1	RP11-834C11.3
VAT	Obesity	ENSG00000233448.2	-0.002881975	0.411025858	0.003505667	0.183454219	-0.002817764	0.421496477	0.00350543	0.183417302	-6.42E-05	0.024624122	1	0.708533979	1	0.727353238	ENSG00000248265	1	RP11-834C11.3
VAT	Obesity	ENSG00000233569.1	-0.025205882	4.04E-05	0.006140368	0.183448105	-0.025122472	4.28E-05	0.006139898	0.183411219	-8.34E-05	0.049858233	0.111634994	0.001043318	0.118220734	0.001170502	ENSG00000248290	1	TNXA
VAT	Obesity	ENSG00000234232.2	0.009330705	0.078087442	0.005295831	0.183453301	0.00893113	0.091826269	0.005297717	0.183416472	0.000399575	0.012524771	1	0.286642319	1	0.327866111	ENSG00000248290	1	TNXA
VAT	Obesity	ENSG00000234338.1	-0.003505281	0.297839489	0.0033697	0.183454064	0.00368412	0.319111031	0.003697848	0.183417171	-0.00718940	2.60E-06	1	0.608015553	1	0.641269508	ENSG00000248290	1	TNXA
VAT	Obesity	ENSG00000234444.5	0.000766425	0.63475925	0.001613392	0.183454389	0.000255415	0.874485204	0.001616916	0.183417537	0.00051101	2.83E-06	1	0.840660043	1	0.956332568	ENSG00000248290	1	TNXA
VAT	Obesity	ENSG00000234585.2	0.000490688	0.733216304	0.001439599	0.183454409	0.000854084	0.55356847	0.001441682	0.183417413	-0.000363396	5.75E-06	1	0.884914569	1	0.805509891	ENSG00000248375	1	RP11-177B4.1
VAT	Obesity	ENSG00000235109.3	0.008014927	2.53E-07	0.001554647	0.18344428	0.006747713	1.93E-05	0.001579257	0.183410647	0.001267214	5.37E-06	0.000698563	1.20E-05	0.053308014	0.000626206	ENSG00000248375	1	RP11-177B4.1
VAT	Obesity	ENSG00000235421.1	0.002068475	0.436636072	0.002659095	0.183454245	0.001618063	0.543056747	0.002660424	0.183417406	0.000450412	1.02E-06	1	0.725099615	1	0.797218136	ENSG00000248408	1	RP11-452C8.1
VAT	Obesity	ENSG00000235821.1	-0.004093119	0.001390058	0.00128041	0.183450611	-0.002759237	0.034895089	0.001307956	0.183415864	-0.001333881	6.21E-07	1	0.017360006	1	0.183099707	ENSG00000248408	1	RP11-452C8.1
VAT	Obesity	ENSG00000235994.4	-0.003080006	0.552357461	0.005183173	0.183454341	-0.003158571	0.542235921	0.005182779	0.183417406	7.86E-05	0.030436386	1	0.793904838	1	0.796907844	ENSG00000248408	1	RP11-452C8.1
VAT	Obesity	ENSG00000236104.2	0.00721647	0.174548396	0.005687153	0.183453777	0.009424962	0.098531763	0.005705125	0.183416515	-0.001703315	0.000211035	1	0.46322459	1	0.344453707	ENSG00000248508	2	SRP14-AS1
VAT	Obesity	ENSG00000236611.1	-0.009823086	0.063861239	0.005300746	0.183453176	-0.009919712	0.061273528	0.005300348	0.183416222	9.66E-05	0.010943256	1	0.2592015	1	0.259777168	ENSG00000248540	2	RP11-247C2.2
VAT	Obesity	ENSG00000236682.1	0.001703677	0.562016435	0.002938136	0.183454326	0.001750773	0.551228287	0.002937929	0.183417412	-4.71E-05	0.003918898	1	0.802465266	1	0.803268253	ENSG00000248778	1	RP11-311D14.1
VAT	Obesity	ENSG00000237310.1	0.004732189	0.005678275	0.001710956	0.183451583	0.003527459	0.04169598	0.001732078	0.183415979	0.00120473	8.59E-06	1	0.048670928	1	0.205736949	ENSG00000248787	1	RP11-666A20.4
VAT	Obesity	ENSG00000237669.1	0.004628896	0.00075157	0.001373534	0.183450181	0.00260112	0.071125433	0.00144132	0.183416315	0.002027776	3.52E-06	1	0.010692438	1	0.283270125	ENSG00000248794	1	CTD-2248H3.1
VAT	Obesity	ENSG00000237854.3	0.016457482	3.47E-05	0.003975163	0.183447995	0.016517246	3.25E-05	0.003974862	0.18341102	-5.98E-05	0.033529241	0.095840376	0.00092542	0.089627377	0.000974211	ENSG00000248794	1	CTD-2248H3.1
VAT	Obesity	ENSG00000237988.2	0.006603415	0.104912619	0.004072429	0.1834548	0.005396353	0.186205966	0.004082306	0.183416886	0.001207062	3.07E-05	1	0.347941332	1	0.493687287	ENSG00000248794	1	CTD-2248H3.1
VAT	Obesity	ENSG00000239257.1	0.004061363	0.000107112	0.001048386	0.183448802	0.00284328	0.008595219	0.001082017	0.183414936	0.001218083	5.51E-06	0.295630061	0.00230961	1	0.071670101	ENSG00000248905	4	FMN1
VAT	Obesity	ENSG00000239556.2	-0.001682479	0.702842662	0.004410327	0.183454419	-0.001788678	0.685043716	0.004410059	0.183417484	0.000106199	0.00697231	1	0.871233847	1	0.869882588	ENSG00000248905	4	FMN1
VAT	Obesity	ENSG00000240053.8	-0.001355807	0.531764457	0.002168197	0.183454326	-0.00285365	0.198326784	0.002218435	0.183416921	0.001497843	0.001453611	1	0.783174974	1	0.510141588	ENSG00000248905	4	FMN1
VAT	Obesity	ENSG00000240065.3	0.00769467	1.20E-06	0.001584496	0.18344556	0.005532217	0.00190308	0.001781728	0.183413902	0.002162453	0.007976922	0.003303224	4.79E-05	1	0.023659912	ENSG00000248905	4	FMN1
VAT	Obesity	ENSG00000240344.4	-0.000729253	0.611900819	0.001437341	0.183454377	-0.000757442	0.598186572	0.001437242	0.183417441	2.82E-05	0.013939462	1	0.83120188	1	0.827151773	ENSG00000248927	1	CTD-2334D19.1
VAT	Obesity	ENSG00000240399.1	0.000948228	0.748819467	0.002961392	0.183454435	0.000876836	0.76714643	0.002961186	0.183417513	7.14E-05	0.002243639	1	0.892799597	1	0.906388761	ENSG00000248927	1	CTD-2334D19.1
VAT	Obesity	ENSG00000240522.1	0.011488922	0.056954239	0.006035155	0.183453104	0.011378891	0.059353864	0.00603473	0.183416202	0.000110031	0.019005106	1	0.24109463	1	0.257169016	ENSG00000248927	1	CTD-2334D19.1
VAT	Obesity	ENSG00000240875.1	-0.001867333	0.375954474	0.002109085	0.183454178	-0.001900325	0.367545792	0.002108939	0.183417239	3.30E-05	0.048661508	1	0.685170491	1	0.690555366	ENSG00000248994	1	RP11-259O2.1
VAT	Obesity	ENSG00000240975.1	0.003116542	0.579581492	0.005625563	0.183454358	0.003320252	0.555025996	0.005625201	0.183417414	-0.00020371	2.13E-05	1	0.808718361	1	0.805802048	ENSG00000248994	1	RP11-259O2.1
VAT	Obesity	ENSG00000241057.1	-0.001724279	0.712065584	0.004671784	0.183454423	-0.001863526	0.689960071	0.004671551	0.183417486	0.000139247	0.003086943	1	0.874633294	1	0.869882588	ENSG00000249087	3	C1orf213
VAT	Obesity	ENSG00000241106.2	0.000877603	0.530659365	0.001399685	0.183454326	0.001813214	0.199726527	0.001413996	0.183416925	-0.000935611	3.49E-06	1	0.78238297	1	0.510412237	ENSG00000249109	1	RP11-1026M7.2
VAT	Obesity	ENSG00000241549.4	0.003714074	0.042140206	0.001827668	0.183452913	0.003136788	0.086826544	0.001831825	0.183416438	0.000577287	4.61E-06	1	0.200529258	1	0.316168943	ENSG00000249242	3	TMEM150C
VAT	Obesity	ENSG00000241644.2	-0.008704279	0.11225813	0.005480844	0.183453521	-0.008617485	0.115855178	0.005480417	0.183416612	-8.68E-05	0.018817887	1	0.361110068	1	0.37486853	ENSG00000249242	3	TMEM150C
VAT	Obesity	ENSG00000241839.5	0.007658149	0.147898593	0.005292446	0.183453683	0.007499091	0.15646953	0.005292052	0.183416787	0.000159058	3.24E-05	1	0.415682399	1	0.440669287	ENSG00000249476	1	CTD-2587M2.1
VAT	Obesity	ENSG00000242110.3	-0.001348699	0.415980014	0.001658068	0.183454224	-0.001309297	0.429697556	0.001657947	0.1834131	-3.94E-05	0.001417932	1	0.710460915	1	0.734343811	ENSG00000249565	2	SERBP1P5
VAT	Obesity	ENSG00000242163.1	0.003023415	0.547837206	0.005030602	0.183454338	0.002908738	0.563096401	0.005030252	0.183417442	0.000114677	0.003886356	1	0.793647904	1	0.809555852	ENSG00000249626	1	RP11-496H1.1
VAT	Obesity	ENSG00000242193.5	-0.016851306	9.54E-05	0.004318705	0.183448719	-0.016776811	0.00010237	0.004318426	0.183411842	-7.45E-05	0.041368108	0.263413849	0.002112498	0.282541352	0.002435701	ENSG00000249731	1	RP11-259O2.3
VAT	Obesity	ENSG00000242600.2	0.003647492	0.544445284	0.006017921	0.183454335	0.003798856	0.527843729	0.0060175	0.183417395	-0.000151364	0.00132643	1	0.79046238	1	0.786996417	ENSG00000249731	1	RP11-259O2.3
VAT	Obesity	ENSG00000243646.4	0.002476306	0.643298191	0.005347324	0.183454393	0.002389797	0.654912607	0.005346921	0.183417471	8.65E-05	0.022859916	1	0.843870251	1	0.854180748	ENSG00000249740	1	CTD-2127H9.1
VAT	Obesity	ENSG00000243789.6	0.000748415	0.773348082	0.002598679	0.183454443	0.000798806	0.758530354	0.002598492	0.18341751	-5.04E-05	0.010673541	1	0.904681739	1	0.900448937	ENSG00000249780	1	RP11-352E6.2
VAT	Obesity	ENSG00000244045.6	0.00467412	0.146953702	0.00322269	0.183453679	0.004600789	0.153371889	0.003222464	0.183416776	7.33E-05	0.003472205	1	0.413958925	1	0.434702786	ENSG00000249839	1	AC011330.5
VAT	Obesity	ENSG00000244165.1	-0.000575709	0.822350216	0.002564137	0.183454455	-0.000648519	0.800317108	0.002563955	0.183417522	7.28E-05	0.00022869	1	0.927365584	1	0.921516563	ENSG00000249839	1	AC011330.5
VAT	Obesity	ENSG00000244607.1	0.004519757	0.338064454	0.004717921	0.183454127	0.00444271	0.3463346	0.004717595	0.183417211	7.71E-05	0.039602438	1	0.646762125	1	0.669386202	ENSG00000249839	1	AC011330.5
VAT	Obesity	ENSG00000244731.3	-0.008055735	1.87E-07	0.001545514	0.183444205	-0.004633435	0.025525384	0.002074644	0.183415661	-0.0034223	0.013418272	0.000514893	9.19E-06	1	0.149575496	ENSG00000249863	2	RP11-177C12.1
VAT	Obesity	ENSG00000245958.2	-0.009187066	0.002000069	0.002972931	0.183450864	-0.009238838	0.001884532	0.002972725	0.183413896	5.18E-05	0.028614783	1	0.022348948	1	0.023620883	ENSG00000249863	2	RP11-177C12.1
VAT	Obesity	ENSG00000246067.3	-0.010468346	0.090417649	0.006182573	0.18345339	-0.010565329	0.087449792	0.006182135	0.183416442	9.70E-05	0.042381986	1	0.314235052	1	0.317028233	ENSG00000249911	1	RP11-122C5.1
VAT	Obesity	ENSG00000248098.6	-0.000821431	0.881691052	0.005519408	0.183454466	-0.000716964	0.89663939	0.005519023	0.18341754	-0.000104467	0.015994078	1	0.95522585	1	0.968028006	ENSG00000250075	1	RP11-584P21.2
VAT	Obesity	ENSG00000248265.1	-0.008466759	0.016349574	0.00352631	0.183452295	-0.008382363	0.017443057	0.003526088	0.18341541	-8.44E-05	0.005898904	1	0.106176055	1	0.117421555	ENSG00000250075	1	RP11-584P21.2
VAT	Obesity	ENSG00000249242.3	0.002881922	0.504032014	0.004313224	0.183454305	0.002973665	0.490522751	0.004312921	0.183417366	-9.17E-05	0.00650003	1	0.770249732	1	0.775358255	ENSG00000250138	3	RP11-848G14.5
VAT	Obesity	ENSG00000249565.2	-0.007644773	0.066007257	0.004158432	0.183453197	-0.007731868	0.062966661	0.00415819	0.183416239	8.71E-05	0.023062076	1	0.264689498	1	0.264517478	ENSG00000250327	1	RP11-173M11.2
VAT	Obesity	ENSG00000249731.1	0.003296222	0.396710558	0.003889295	0.183454203	0.003193849	0.411509003	0.003889048	0.183417291	0.000102374	0.00223037	1	0.701928581	1	0.722625422	ENSG00000250376	1	CTD-2005D20.1
VAT	Obesity	ENSG00000249839.1	0.007395383	0.028506343	0.003376497	0.183452661	0.007447733	0.02739045	0.003376256	0.183415707	-5.23E-05	0.043588147	1	0.153667004	1	0.155231297	ENSG00000250376	1	CTD-2005D20.1
VAT	Obesity	ENSG00000249863.2	-0.00321612	0.363130639	0.003536464	0.183454161	-0.00327552	0.354298042	0.003536188	0.183417222	5.94E-05	0.012223512	1	0.672794162	1	0.679543152	ENSG00000250376	1	CTD-2005D20.1
VAT	Obesity	ENSG00000249911.1	-0.004272091	0.473318385	0.005957531	0.183454258	-0.004153759	0.485628662	0.005957099	0.183417362	-0.000118332	0.008047146	1	0.755994642	1	0.775358255	ENSG00000250378	1	RP11-119J18.1
VAT	Obesity	ENSG00000250506.2	0.000434	0.876729705	0.002797899	0.183454465	0.000362484	0.896910853	0.002797704	0.183417754	7.15E-05	0.001156551	1	0.951543054	1	0.968028006	ENSG00000250412	1	KLHL2P1
VAT	Obesity	ENSG00000250535.1	-0.003297128	0.030384614	0.00152289	0.183452702	-0.001027777	0.518901843	0.001593355	0.183417389	-0.002269351	1.31E-06	1	0.160281317	1	0.784425829	ENSG00000250476	1	ENPP7P9
VAT	Obesity	ENSG00000251136.4	-0.004983929	0.044563194	0.002481098	0.183452949	-0.005016102	0.04318865	0.002480902	0.183416001	3.22E-05	0.049822255	1	0.208112376	1	0.210230467	ENSG00000250506	2	CDK3
VAT	Obesity	ENSG00000251169.2	0.00192376	0.692685567	0.004867648	0.183454415	0.001832599	0.706536269	0.004867302	0.183417492	9.12E-05	0.014849397	1	0.866149932	1	0.876422518	ENSG00000250506	2	CDK3
VAT	Obesity	ENSG00000251580.1	0.001301825	0.744207844	0.003989823	0.183454434	0.00121667	0.760394159	0.003989562	0.183417511	8.52E-05	0.010980484	1	0.892204507	1	0.901498229	ENSG00000250506	2	CDK3
VAT	Obesity	ENSG00000253203.2	-0.001237157	0.768016584	0.004194172	0.183454441	-0.001296233	0.757261531	0.004193855	0.18341751	5.91E-05	0.046875769	1	0.901627295	1	0.900448937	ENSG00000250506	2	CDK3
VAT	Obesity	ENSG00000253704.1	-0.023056799	4.83E-09	0.003939343	0.183441526	-0.022984484	5.38E-09	0.003939007	0.183404679	-7.23E-05	0.020108523	1.33E-05	3.18E-07	1.49E-05	3.91E-07	ENSG00000250506	2	CDK3
VAT	Obesity	ENSG00000253731.1	0.004539381	0.173117329	0.003332249	0.183453773	0.004470277	0.179723085	0.003332024	0.183416866	6.91E-05	0.010629946	1	0.460092631	1	0.482525015	ENSG00000250506	2	CDK3
VAT	Obesity	ENSG00000253771.1	0.000854237	0.705056055	0.002256875	0.18345442	0.000900754	0.689784646	0.002256695	0.183417486	-4.65E-05	0.00137197	1	0.871233847	1	0.869882588	ENSG00000250519	2	RP11-680H20.2
VAT	Obesity	ENSG00000253813.1	0.007991683	0.003200161	0.002711028	0.183451189	0.008117002	0.002752625	0.002710992	0.183414158	-0.000125319	0.000471787	1	0.032235198	1	0.031393572	ENSG00000250519	2	RP11-680H20.2
VAT	Obesity	ENSG00000253816.2	-0.002597656	0.505127542	0.003897784	0.183454306	-0.002465267	0.527048674	0.003897543	0.183417395	-0.000132389	0.000115252	1	0.770249732	1	0.7867249	ENSG00000250535	1	STK19P
VAT	Obesity	ENSG00000254352.1	-0.000347326	0.931337419	0.004031064	0.183454471	-0.000276886	0.945234081	0.004030772	0.183417544	-7.04E-05	0.019904455	1	0.976259505	1	0.981754039	ENSG00000250535	1	STK19P
VAT	Obesity	ENSG00000254614.1	-0.002811535	0.130239027	0.001858052	0.183453609	-0.001398662	0.455599476	0.001874598	0.183417336	-0.001412873	1.57E-08	1	0.387831349	1	0.752966798	ENSG00000250535	1	STK19P
VAT	Obesity	ENSG00000254667.1	-0.0017939	0.669267138	0.004199654	0.183454405	-0.001934252	0.645081863	0.004199369	0.183417466	0.000140352	3.64E-05	1	0.857647266	1	0.850251166	ENSG00000250535	1	STK19P
VAT	Obesity	ENSG00000254726.2	-0.003522924	0.524155836	0.005530902	0.183454321	-0.003622797	0.512429369	0.005530481	0.183417384	9.99E-05	0.009860957	1	0.779212983	1	0.784425829	ENSG00000250535	1	STK19P
VAT	Obesity	ENSG00000254851.1	0.001648237	0.188022468	0.001252028	0.183453819	0.001631003	0.192647893	0.001251933	0.183416905	1.72E-05	0.048294936	1	0.484270267	1	0.50208516	ENSG00000250535	1	STK19P
VAT	Obesity	ENSG00000254912.2	0.000660776	0.847470948	0.003435343	0.18345446	0.000529293	0.877544342	0.003435113	0.183417537	0.000131483	3.16E-06	1	0.940876837	1	0.957699638	ENSG00000250535	1	STK19P
VAT	Obesity	ENSG00000254986.3	0.007094833	0.00103713	0.002162877	0.183450407	0.006169819	0.004452802	0.00216928	0.183414489	0.000925014	4.64E-08	1	0.013828397	1	0.044318348	ENSG00000250535	1	STK19P
VAT	Obesity	ENSG00000255147.1	0.00890558	0.046771787	0.00447847	0.18345298	0.009912568	0.026997748	0.004482167	0.183415698	-0.00100698	4.37E-08	1	0.214079822	1	0.155231297	ENSG00000250535	1	STK19P
VAT	Obesity	ENSG00000255200.1	-0.004462977	0.155522286	0.003142287	0.183453712	-0.002200127	0.487299535	0.003167424	0.183417364	-0.00226285	1.63E-08	1	0.429671181	1	0.775358255	ENSG00000250599	1	AC064853.3
VAT	Obesity	ENSG00000255328.1	-0.007258162	0.218139756	0.005893799	0.183453901	-0.007369022	0.21115926	0.005893399	0.183416955	0.00011086	0.020267004	1	0.521961016	1	0.522690187	ENSG00000250599	1	AC064853.3
VAT	Obesity	ENSG00000256087.2	0.00051542	0.876558794	0.003318162	0.183454465	0.000378281	0.90924583	0.00331855	0.183417541	0.000137139	0.047440485	1	0.951543054	1	0.971175886	ENSG00000250656	2	ST3GAL1P1
VAT	Obesity	ENSG00000256612.3	0.006511484	0.247565561	0.005631388	0.183453969	0.006672329	0.236045186	0.005630984	0.183417015	-0.000160845	0.000173898	1	0.544013495	1	0.543810279	ENSG00000250656	2	ST3GAL1P1
VAT	Obesity	ENSG00000256885.1	-0.002172791	0.496497994	0.00319523	0.183454299	-0.002096613	0.51168349	0.003194996	0.183417383	-7.62E-05	0.001216431	1	0.766551816	1	0.784425829	ENSG00000250656	2	ST3GAL1P1
VAT	Obesity	ENSG00000257512.1	0.001508672	0.689390346	0.003774673	0.183454414	0.001583954	0.67473827	0.003774427	0.183417479	-7.53E-05	0.018174415	1	0.86447858	1	0.862964608	ENSG00000250656	2	ST3GAL1P1
VAT	Obesity	ENSG00000257742.1	0.011020701	0.062033635	0.005905867	0.183453158	0.010838109	0.066468434	0.005905504	0.183416273	0.000182592	0.000458603	1	0.254402428	1	0.27337482	ENSG00000250656	2	ST3GAL1P1
VAT	Obesity	ENSG00000257949.2	-0.008148553	0.023899593	0.003607555	0.183452546	-0.008221934	0.022652388	0.003607291	0.183415583	7.34E-05	0.006173274	1	0.136014549	1	0.136806542	ENSG00000250770	2	RP5-1063M23.1
VAT	Obesity	ENSG00000258230.2	0.001234921	0.749910331	0.003874167	0.183454436	0.001359257	0.725688344	0.003873995	0.18341175	-0.000124336	0.002511643	1	0.892799597	1	0.885846895	ENSG00000250974	1	RP11-122F24.1
VAT	Obesity	ENSG00000258512.1	0.012985928	0.004520732	0.004573555	0.183451427	0.012873274	0.004879288	0.004573246	0.183414451	0.000112653	0.002357482	1	0.041315295	1	0.046598047	ENSG00000250986	1	AC141928.1
VAT	Obesity	ENSG00000258572.1	0.000581717	0.70904724	0.001558993	0.183454421	0.000559476	0.719672296	0.001558875	0.183417497	2.22E-05	0.0416578	1	0.872868145	1	0.881622519	ENSG00000250986	1	AC141928.1
VAT	Obesity	ENSG00000258819.1	-0.001339403	0.818199976	0.005827017	0.183454454	-0.001163777	0.841688024	0.005826607	0.18341753	-0.000175627	0.000107708	1	0.926394336	1	0.940035744	ENSG00000250986	1	AC141928.1
VAT	Obesity	ENSG00000259207.3	-0.006153667	0.12302276	0.003990163	0.183453575	-0.006250631	0.117203962	0.003989935	0.183416618	9.70E-05	0.009284871	1	0.37609362	1	0.376956898	ENSG00000251008	1	ORAOV1P1
VAT	Obesity	ENSG00000259363.1	0.003832891	0.529468808	0.006095394	0.183454325	0.003935841	0.518437445	0.006094932	0.183417388	-0.000102951	0.016829314	1	0.781416001	1	0.784425829	ENSG00000251008	1	ORAOV1P1
VAT	Obesity	ENSG00000259715.1	0.003172045	0.206222312	0.002509483	0.1834587	0.003378217	0.178465032	0.002510756	0.183416862	-0.000206172	0.01847197	1	0.507220054	1	0.480362878	ENSG00000251136	4	RP11-37B2.1
VAT	Obesity	ENSG00000260302.1	-0.005300633	0.347483713	0.005642079	0.1834514	-0.005134566	0.36277408	0.005641805	0.183417233	-0.000166067	0.00391675	1	0.660045114	1	0.686859902	ENSG00000251169	2	AC005355.2
VAT	Obesity	ENSG00000260613.1	-0.00150547	0.775815068	0.005286486	0.183454443	-0.001373611	0.794977929	0.005286127	0.183417521	-0.000131859	0.002039665	1	0.904860323	1	0.918573915	ENSG00000251169	2	AC005355.2
VAT	Obesity	ENSG00000260947.1	-0.005307225	8.74E-06	0.001193642	0.183447002	-0.005234709	1.16E-05	0.001193725	0.183410279	-7.25E-05	0.000956916	0.024123505	0.000294189	0.031990896	0.000420933	ENSG00000251226	1	RP11-1469N6.1
VAT	Obesity	ENSG00000260973.5	-0.005769493	0.000363927	0.00161542	0.183449528	-0.005737526	0.000434588	0.001615458	0.183412958	-3.20E-05	0.021541507	1	0.004896306	1	0.005044591	ENSG00000251350	1	RP11-1328N19.1
VAT	Obesity	ENSG00000261064.1	-0.000634133	0.897508614	0.004923035	0.183454468	-0.000715363	0.884378523	0.004922692	0.183417538	8.17E-05	0.034646667	1	0.963797845	1	0.960056703	ENSG00000251365	1	RP11-332J15.3
VAT	Obesity	ENSG00000261584.1	0.000840935	0.666381571	0.001950583	0.183454404	0.001273369	0.51434102	0.00195274	0.183417385	-0.000432435	6.43E-06	1	0.857042468	1	0.784425829	ENSG00000251383	1	RP11-542G1.3
VAT	Obesity	ENSG00000261589.1	0.005164812	0.307153365	0.005057519	0.183454108	0.005304505	0.294221285	0.005057173	0.18341713	-0.000139694	0.000573405	1	0.615029354	1	0.616533352	ENSG00000251383	1	RP11-542G1.3
VAT	Obesity	ENSG00000261672.1	0.004901052	0.143970658	0.003354207	0.183453667	0.004957498	0.139383782	0.003353985	0.18341672	-5.64E-05	0.042404161	1	0.410919356	1	0.414546593	ENSG00000251417	1	RP11-1348G14.4
VAT	Obesity	ENSG00000261754.2	-0.002627144	0.440179006	0.003403515	0.183454249	-0.002682045	0.430647464	0.003403248	0.183417311	5.49E-05	0.015580944	1	0.726069693	1	0.735057673	ENSG00000251417	1	RP11-1348G14.4
VAT	Obesity	ENSG00000262118.1	0.000903265	0.80275952	0.003616277	0.183454451	0.000799269	0.825064992	0.003616025	0.183417528	0.000103995	0.000249459	1	0.918961541	1	0.934468454	ENSG00000251417	1	RP11-1348G14.4
VAT	Obesity	ENSG00000262468.1	0.001565559	0.727034781	0.004484906	0.183454428	0.001628767	0.716459879	0.00448456	0.183417496	-6.32E-05	0.009388589	1	0.882348289	1	0.880030826	ENSG00000251417	1	RP11-1348G14.4
VAT	Obesity	ENSG00000262877.3	-0.007586081	0.162980798	0.00543759	0.183453738	-0.007516667	0.166829752	0.005437515	0.183416824	-6.94E-05	0.04246985	1	0.441873282	1	0.457993828	ENSG00000251417	1	RP11-1348G14.4
VAT	Obesity	ENSG00000266173.1	-0.012165229	0.003883482	0.004213083	0.183451323	-0.012274283	0.003573702	0.004212839	0.183414338	0.000109053	0.005118287	1	0.036721461	1	0.037380051	ENSG00000251417	1	RP11-1348G14.4
VAT	Obesity	ENSG00000266714.2	0.003099854	0.3523702	0.003333167	0.183454147	0.003178385	0.340268726	0.003332915	0.183417202	-7.85E-05	0.000856992	1	0.663398193	1	0.661367382	ENSG00000251417	1	RP11-1348G14.4
VAT	Obesity	ENSG00000266992.1	-0.006323731	0.160165421	0.004502422	0.183453728	-0.006424809	0.153563484	0.004502132	0.183416776	0.000101079	0.008560405	1	0.438548177	1	0.434702786	ENSG00000251468	2	RP11-369K16.1
VAT	Obesity	ENSG00000267056.2	0.000799948	0.892324312	0.005909616	0.183454467	0.000901856	0.878699037	0.00590921	0.183417537	-0.000101908	0.030852141	1	0.961627151	1	0.958352832	ENSG00000251532	1	CTD-2245E15.3
VAT	Obesity	ENSG00000267102.1	-0.00639077	0.207978459	0.005075483	0.183453875	-0.006535234	0.197857529	0.005075195	0.183416919	0.000144464	0.002420433	1	0.508805001	1	0.509865497	ENSG00000251532	1	CTD-2245E15.3
VAT	Obesity	ENSG00000267302.1	0.00251852	0.434238423	0.003220778	0.183454243	0.002466495	0.443757062	0.003220535	0.183417324	5.20E-05	0.022840918	1	0.722822432	1	0.744875937	ENSG00000251580	1	RP11-539L10.3
VAT	Obesity	ENSG00000267346.1	0.008672078	0.01958134	0.003715093	0.183452414	0.008785919	0.018025519	0.003714817	0.183415432	-0.000113841	2.41E-05	1	0.118518637	1	0.119020171	ENSG00000251580	1	RP11-539L10.3
VAT	Obesity	ENSG00000267508.1	0.000680365	0.84154312	0.003403182	0.183454459	0.000731524	0.82979104	0.003402916	0.183417529	-5.12E-05	0.024726788	1	0.938448085	1	0.936897537	ENSG00000251580	1	RP11-539L10.3
VAT	Obesity	ENSG00000267534.1	0.007150479	0.121427946	0.004616755	0.183453567	0.007073037	0.125487717	0.004616433	0.183416659	7.74E-05	0.0325302	1	0.374041441	1	0.391351525	ENSG00000251580	1	RP11-539L10.3
VAT	Obesity	ENSG00000267594.5	-0.000857022	0.848976927	0.004500596	0.18345446	-0.000937727	0.834940803	0.004500314	0.18341753	8.07E-05	0.03957968	1	0.941412743	1	0.938350439	ENSG00000251580	1	RP11-539L10.3

VAT	Obesity	ENSG00000267648.1	0.003688551	0.488558663	0.00532563	0.183454293	0.003495346	0.511593154	0.005325354	0.183417383	0.000193204	0.00023685	1	0.76550139	1	0.784425829	ENSG00000251593	1	MSNP1
VAT	Obesity	ENSG00000268087.1	-0.00185185	0.408307761	0.002239573	0.183454216	-0.001809522	0.419070436	0.002239408	0.183417299	-4.23E-05	0.010022167	1	0.708044263	1	0.725782338	ENSG00000251593	1	MSNP1
VAT	Obesity	ENSG00000268117.1	-0.003236236	0.459077715	0.004371124	0.183454267	-0.003349866	0.44342745	0.004370803	0.183417324	0.00011363	0.000415296	1	0.742704861	1	0.744875937	ENSG00000251600	1	RP11-673E1.1
VAT	Obesity	ENSG00000268225.1	0.001742326	0.595248997	0.003279701	0.183454367	0.001657162	0.613343879	0.003279519	0.18341745	8.52E-05	0.006206908	1	0.818796889	1	0.839253732	ENSG00000251600	1	RP11-673E1.1
VAT	Obesity	ENSG00000268518.1	-0.002675741	0.528219451	0.004242314	0.183454324	-0.00276822	0.514033192	0.00424202	0.183417385	9.25E-05	0.006103254	1	0.780452723	1	0.784425829	ENSG00000251615	3	RP11-774O3.3
VAT	Obesity	ENSG00000268658.1	-0.002168796	0.333008901	0.002240325	0.18345412	-0.002123444	0.343184216	0.002240172	0.183417206	-4.54E-05	0.011534262	1	0.642540807	1	0.665851594	ENSG00000251615	3	RP11-774O3.3
VAT	Obesity	ENSG00000269430.1	0.002231364	0.455905627	0.002992684	0.183454264	0.002162362	0.469931735	0.002992513	0.183417349	6.90E-05	0.013312895	1	0.739580277	1	0.766555312	ENSG00000251639	2	RP11-20I20.1
VAT	Obesity	ENSG00000269558.1	0.002075553	0.311745021	0.002051817	0.183454087	0.001315953	0.522500412	0.002057796	0.183417391	0.0007596	1.89E-06	1	0.619458427	1	0.784841324	ENSG00000251639	2	RP11-20I20.1
VAT	Obesity	ENSG00000269963.1	-0.027562862	9.16E-06	0.006213181	0.183447035	-0.027372986	1.05E-05	0.006212746	0.18341021	-0.000189876	8.81E-05	0.02527726	0.000304545	0.029076197	0.000403836	ENSG00000253203	2	GUSBP3
VAT	Obesity	ENSG00000270346.1	-0.007569272	0.127121643	0.004961655	0.183453594	-0.007475048	0.131894011	0.00496128	0.183416688	-9.42E-05	0.007263759	1	0.381801917	1	0.399591076	ENSG00000253304	1	TMEM200B
VAT	Obesity	ENSG00000270547.1	-0.003822401	0.422516241	0.004765704	0.183454231	-0.003899403	0.413198528	0.004765373	0.183417293	7.70E-05	0.040862377	1	0.712802461	1	0.72386593	ENSG00000253304	1	TMEM200B
VAT	Obesity	ENSG00000270775.1	0.002231735	0.624150692	0.004554779	0.183454383	0.002409314	0.596820541	0.004554653	0.18341744	-0.000177579	0.001248435	1	0.835148305	1	0.827015081	ENSG00000253305	1	PCDHGB6
VAT	Obesity	ENSG00000270810.1	-0.003649338	0.311928222	0.003608971	0.183454088	-0.003590896	0.319705268	0.003608706	0.183417172	-5.84E-05	0.028148664	1	0.619458427	1	0.641269508	ENSG00000253326	2	RP11-261C10.7
VAT	Obesity	ENSG00000270876.1	0.001691692	0.62702604	0.00348144	0.183454385	0.00174593	0.61599453	0.003481168	0.183417451	-5.42E-05	0.020328875	1	0.836438797	1	0.839253732	ENSG00000253326	2	RP11-261C10.7
VAT	Obesity	ENSG00000270972.1	-0.004475845	0.253641809	0.00392085	0.183453982	-0.004555614	0.245246669	0.003920583	0.183417036	7.98E-05	0.011745557	1	0.549998458	1	0.552555761	ENSG00000253326	2	RP11-261C10.7
VAT	Obesity	ENSG00000271303.1	0.000378149	0.946045411	0.005587823	0.183454472	0.000478344	0.931775961	0.005587432	0.183417543	-0.000100195	0.022093966	1	0.981611028	1	0.976719199	ENSG00000253326	2	RP11-261C10.7
VAT	Obesity	ENSG00000271550.1	0.000703047	0.886013888	0.00490441	0.183454466	-0.000279188	0.954649519	0.004909307	0.183417545	0.000982235	1.95E-05	1	0.957634941	1	0.984913318	ENSG00000253333	1	RP11-497H16.8
VAT	Obesity	ENSG00000271551.1	0.007038933	0.253676825	0.006166575	0.183453982	0.007138915	0.246962465	0.006166143	0.183417039	-1.00E-04	0.038159579	1	0.549998458	1	0.554266359	ENSG00000253395	1	KB-1460A1.1
VAT	Obesity	ENSG00000271553.1	-0.000974052	0.708120979	0.002601767	0.183454421	-0.000908334	0.726980211	0.002601591	0.1834175	-6.57E-05	0.001866501	1	0.872628294	1	0.886980127	ENSG00000253444	1	AC133633.2
VAT	Obesity	ENSG00000271581.1	-0.011675575	0.059926101	0.006205985	0.183453136	-0.001091628	0.873194997	0.006839704	0.183417536	-0.010583948	0.000233842	1	0.24909042	1	0.956332568	ENSG00000253485	1	PCDHGA5
VAT	Obesity	ENSG00000271736.1	-0.002897972	0.605559267	0.005611625	0.183454373	-0.003008768	0.591819993	0.005611271	0.183417437	0.000110796	0.022789579	1	0.827343939	1	0.824941855	ENSG00000253540	1	FAM86HP
VAT	Obesity	ENSG00000271755.1	0.000939457	0.708707825	0.002514659	0.183454421	6.16E-05	0.980497064	0.002521458	0.183417546	0.000877818	3.19E-06	1	0.872839624	1	0.99462398	ENSG00000253540	1	FAM86HP
VAT	Obesity	ENSG00000271811.1	-0.002463566	0.500613705	0.003657721	0.183454303	-0.002517797	0.491199875	0.003657451	0.183417367	5.42E-05	0.043155256	1	0.768636987	1	0.775358255	ENSG00000253669	3	KB-1732A1.1
VAT	Obesity	ENSG00000271912.1	-0.000748453	0.882624098	0.005069324	0.183454466	-0.000659596	0.896468306	0.005068979	0.18341754	-8.89E-05	0.029748195	1	0.95522585	1	0.968028006	ENSG00000253704	1	RP11-267M23.4
VAT	Obesity	ENSG00000272010.1	0.00089686	0.811081351	0.003752058	0.183454453	0.000839753	0.822891194	0.003751778	0.183417527	5.71E-05	0.035132232	1	0.923127641	1	0.933105873	ENSG00000253731	1	PCDHGA6
VAT	Obesity	ENSG00000272221.1	-0.002032557	0.098331107	0.001229616	0.183453441	-0.001635348	0.185693542	0.00123569	0.183416884	-0.000397209	0.001310479	1	0.333396805	1	0.492802092	ENSG00000253731	1	PCDHGA6
VAT	Obesity	ENSG00000272236.1	-0.015225219	0.000624548	0.004450848	0.183450051	-0.010471587	0.021645912	0.00455974	0.183415553	-0.004753632	1.67E-06	1	0.00916889	1	0.13365261	ENSG00000253731	1	PCDHGA6
VAT	Obesity	ENSG00000272282.1	0.00082394	0.891657765	0.006049177	0.183454467	0.000950678	0.875110415	0.006048702	0.183417537	-0.000126739	0.001624163	1	0.961627151	1	0.956332568	ENSG00000253731	1	PCDHGA6
VAT	Obesity	ENSG00000272338.1	-0.020774282	0.000195936	0.005578145	0.183449231	-0.020686037	0.000208345	0.005577697	0.183412348	-8.82E-05	0.013088252	0.540783532	0.003808335	0.575030975	0.004290423	ENSG00000253745	1	RP11-350F16.1
VAT	Obesity	ENSG00000272462.2	0.007044907	1.08E-11	0.001036676	0.183437019	0.00669793	1.25E-10	0.001041098	0.183401905	0.000346977	0.000343545	2.98E-08	1.03E-09	3.44E-07	1.01E-08	ENSG00000253771	1	TPTE2P1
VAT	Obesity	ENSG00000272636.1	0.000287906	0.924799305	0.003050175	0.183454471	0.000374634	0.902244839	0.003050115	0.18341754	-8.67E-05	0.02540391	1	0.975705689	1	0.969573834	ENSG00000253771	1	TPTE2P1
VAT	Obesity	ENSG00000272732.1	0.00783017	0.193295363	0.006019069	0.183453834	0.00771467	0.199918921	0.006018696	0.183416925	0.0001155	0.029054225	1	0.490645638	1	0.510431287	ENSG00000253813	1	COX6B1P4
VAT	Obesity	ENSG00000272849.1	-0.016615508	7.37E-05	0.004191653	0.183448535	-0.01652107	8.09E-05	0.004191353	0.183411674	-9.44E-05	0.00311467	0.203494252	0.001710036	0.223329467	0.002011977	ENSG00000253813	1	COX6B1P4
VAT	Obesity	ENSG00000272902.1	-0.006041863	0.261418717	0.005379892	0.183453997	-0.005941203	0.269415494	0.005379522	0.183417085	-0.000100661	0.019174472	1	0.563244075	1	0.585237719	ENSG00000253813	1	COX6B1P4
VAT	Obesity	ENSG00000273009.1	0.001431053	0.738800673	0.004291757	0.183454432	0.001351753	0.752770888	0.004291443	0.183417509	7.93E-05	0.01240157	1	0.888105338	1	0.898390751	ENSG00000253813	1	COX6B1P4
VAT	Obesity	ENSG00000273038.1	-0.006869525	0.171087653	0.005018943	0.183453766	-0.006963	0.165310056	0.005018616	0.183416819	9.35E-05	0.027279158	1	0.457560003	1	0.456129337	ENSG00000253816	2	RP11-1415C14.3
VAT	Obesity	ENSG00000273142.1	0.002207319	0.264343012	0.001977554	0.183454003	0.001337658	0.500531499	0.001985674	0.183417375	0.000869661	1.67E-06	1	0.565471432	1	0.776886587	ENSG00000253816	2	RP11-1415C14.3
VAT	Obesity	ENSG00000273340.1	-0.001865609	0.223177159	0.001531542	0.183453913	-0.000270961	0.86214587	0.001560444	0.183417535	-0.001594649	1.03E-07	1	0.52199987	1	0.950288579	ENSG00000253816	2	RP11-1415C14.3
VAT	Stroke	ENSG00000197261.7	0.002822017	0.074174397	0.001580484	0.057490262	0.008307609	0.005442403	0.002988753	0.057488797	-0.005485592	0.030579464	1	0.73651102	1	0.551358311	ENSG00000254126	2	CD8BP
VAT	T2DM	ENSG00000001561.6	0.003011447	0.273191547	0.002748314	0.081436758	0.002925737	0.287068745	0.002748267	0.081424463	8.57E-05	0.003930626	1	0.652447775	1	0.663927047	ENSG00000254126	2	CD8BP
VAT	T2DM	ENSG00000003056.3	0.00323482	0.187323195	0.002453332	0.081436668	0.003568463	0.14663084	0.002458402	0.0814243	-0.000333643	0.037831661	1	0.570024276	1	0.514898084	ENSG00000254126	2	CD8BP
VAT	T2DM	ENSG00000003249.9	-0.005362978	0.012596792	0.002149508	0.081435918	-0.005418758	0.011701337	0.002149418	0.08142359	5.58E-05	0.00155036	1	0.14522599	1	0.148828067	ENSG00000254152	1	CTD-3107M8.2
VAT	T2DM	ENSG00000004809.9	0.001407328	0.301450946	0.001361938	0.08143678	0.001430793	0.293440056	0.001361873	0.081424468	-2.35E-05	0.022319663	1	0.675668349	1	0.66767894	ENSG00000254152	1	CTD-3107M8.2
VAT	T2DM	ENSG00000006071.7	0.001194897	0.742302189	0.003634043	0.08143694	0.001113779	0.759225721	0.003633888	0.081424637	8.11E-05	0.005963098	1	0.916157236	1	0.915051408	ENSG00000254352	1	RP11-578O24.2
VAT	T2DM	ENSG00000006282.15	0.001398367	0.60360006	0.002693152	0.081436913	0.001329389	0.621559169	0.002693021	0.081424612	6.90E-05	0.000505236	1	0.857656828	1	0.868609269	ENSG00000254352	1	RP11-578O24.2
[truncated: 165,598 more chars]
