# Supplementary material for: CausalCellInfer: Prioritizing Cell‐Type‐Specific Disease Associations From Genetically Predicted Transcriptomes in Biobank Cohorts
Source: Adv Sci (Weinh). 2026 Oct 14:e78197. Online ahead of print. doi: 10.1002/advs.78197 (PMC13669230; doi:10.1002/advs.78197)
Supplement: Supplementary file 2 [file ADVS-9999-e78197-s002.zip › advs78197-sup-00011-S10.pdf]

Table S10 Cell-type common and unique associated genes identified from TWAS analysis, based on estimated CTS expression profiles of all cell types in the target tissue

		Summary of trait-associated genes (p(bonferroni)<0.05)				*Summary of trait-associated genes (FDR<=0.05)*					
Tissue	Disease/Disorder	Cell type specific TWAS gene number	Cell type common TWAS gene number	Total TWAS gene number	Proportion of cell type common TWAS gene	Cell type specific TWAS gene number	Cell type common TWAS gene number	Total TWAS gene number	Proportion of cell type common TWAS gene	Case size	Sample size
Frontal cortex	Anxiety	0	0	0		0	0	0	0.000	7163	479906
Frontal cortex	Bipolar	1	0	1	0.000	186	0	186	0.000	1603	479906
Frontal cortex	Depression	0	2	2	1.000	0	0	0	0.000	27875	479906
Frontal cortex	Psychosis	0	0	0		0	2	2	0.000	1070	479906
Pancreas	Prediabetes	5	0	5	0.000	1367	772	2139	0.361	1039	479906
Pancreas	T1DM	3153	29	3182	0.009	3576	70	3646	0.019	3965	479906
Pancreas	T2DM	16	27	43	0.628	2970	235	3205	0.073	43371	479962
Pancreas	T2DM related hospitalization	46	43	89	0.483	3123	235	3358	0.070	38553	479906
Pancreas	T2DM requires insulin	2	10	12	0.833	3128	20	3148	0.006	5627	479906
Subcutaneous adipose	Obesity	1857	2457	4314	0.570	106	6145	6251	0.983	122160	479906
Subcutaneous adipose	CAD	6	11	17	0.647	605	46	651	0.071	80164	479962
Subcutaneous adipose	T2DM	2323	988	3311	0.298	104	6148	6252	0.983	43371	479962
Subcutaneous adipose	Hypertension	69	138	207	0.667	2160	3567	5727	0.623	194236	479962
Subcutaneous adipose	Heart failure	4732	46	4778	0.010	1524	4552	6076	0.749	39744	479962
Subcutaneous adipose	Stroke	1	2	3	0.667	2	2	4	0.500	29348	479962
Visceral adipose	Obesity	1856	2207	4063	0.543	125	4694	4819	0.974	122160	479962
Visceral adipose	CAD	20	11	31	0.355	781	870	1651	0.527	80164	479962
Visceral adipose	T2DM	2559	851	3410	0.250	90	4738	4828	0.981	43371	479962
Visceral adipose	Hypertension	1560	280	1840	0.152	391	4371	4762	0.918	194236	479962
Visceral adipose	Heart failure	1796	56	1852	0.030	1982	2258	4240	0.533	39744	479962
Visceral adipose	Stroke	3	0	3	0.000	806	0	806	0.000	29348	479962
