# Supplementary material for: CausalCellInfer: Prioritizing Cell‐Type‐Specific Disease Associations From Genetically Predicted Transcriptomes in Biobank Cohorts
Source: Adv Sci (Weinh). 2026 Oct 14:e78197. Online ahead of print. doi: 10.1002/advs.78197 (PMC13669230; doi:10.1002/advs.78197)
Supplement: Supplementary file 2 [file ADVS-9999-e78197-s002.zip › advs78197-sup-00012-S11.pdf]

Table S11 Identified trait-associated genes from CTS TWAS analysis

a. Identified trait-associated genes from CTS TWAS analysis in frontal cortex

disorder	celltype	gene	Estimates	Pvalues	Pvalues_BH	Pvalue_Bonferroni	Absolute Estimates
Bipolar	Ex	*SCYL3*	0.027141259	0.001944181	0.036916749	1	0.027141259
Bipolar	Ex	*HS3ST1*	0.100104921	0.000349965	0.017785524	0.999850749	0.100104921
Bipolar	Ex	*LRRC23*	0.023913402	9.74E-05	0.017785524	0.278253741	0.023913402
Bipolar	Ex	*TTC27*	0.096481997	0.000254131	0.017785524	0.726052582	0.096481997
Bipolar	Ex	*MIPEP*	0.096386215	0.000484428	0.019268993	1	0.096386215
Bipolar	Ex	*DAPK2*	0.109473131	0.001524137	0.032255251	1	0.109473131
Bipolar	Ex	*CNTLN*	0.067101237	0.000881344	0.022684674	1	0.067101237
Bipolar	Ex	*HSPA5*	0.08921208	0.000252358	0.017785524	0.720986834	0.08921208
Bipolar	Ex	*SEC61A1*	0.028233956	0.001544632	0.032448629	1	0.028233956
Bipolar	Ex	*POLR3E*	0.068081267	0.002891056	0.046403074	1	0.068081267
Bipolar	Ex	*TM7SF3*	0.074207345	0.003004127	0.047267494	1	0.074207345
Bipolar	Ex	*CNN2*	0.51795813	0.000830444	0.022173639	1	0.51795813
Bipolar	Ex	*THUMPD1*	0.068140106	0.001015879	0.024863087	1	0.068140106
Bipolar	Ex	*ZXDC*	0.083688634	0.000533128	0.019627556	1	0.083688634
Bipolar	Ex	*SIRT6*	0.101995073	0.001198255	0.027608192	1	0.101995073
Bipolar	Ex	*SAR1A*	0.065256008	0.000757159	0.021374262	1	0.065256008
Bipolar	Ex	*BZW1*	0.077678083	0.002798199	0.045423038	1	0.077678083
Bipolar	Ex	*PIBF1*	0.07365563	0.000268893	0.017785524	0.768226125	0.07365563
Bipolar	Ex	*TDRD3*	0.069803176	8.57E-05	0.017785524	0.244783206	0.069803176
Bipolar	Ex	*AGBL5*	0.055402727	0.001649448	0.034148349	1	0.055402727
Bipolar	Ex	*CD59*	0.089714863	0.000385965	0.017785524	1	0.089714863
Bipolar	Ex	*COQ9*	0.061369046	0.002110635	0.03816509	1	0.061369046
Bipolar	Ex	*ANAPC5*	0.069349178	0.000294314	0.017785524	0.840855155	0.069349178
Bipolar	Ex	*NOS1*	0.075982365	0.000591722	0.019814124	1	0.075982365
Bipolar	Ex	*TOX4*	0.090542364	0.000850148	0.022340816	1	0.090542364
Bipolar	Ex	*NUP50*	0.095825454	0.001074835	0.025590029	1	0.095825454
Bipolar	Ex	*LRRFIP2*	0.029712671	0.002818444	0.045493189	1	0.029712671
Bipolar	Ex	*IFT74*	0.003835244	0.002143996	0.038524515	1	0.003835244
Bipolar	Ex	*SH3GLB1*	0.092761915	0.000314031	0.017785524	0.897187562	0.092761915
Bipolar	Ex	*MAPK1*	0.068642355	0.000519466	0.019627556	1	0.068642355
Bipolar	Ex	*TXN2*	0.104890464	0.000359287	0.017785524	1	0.104890464
Bipolar	Ex	*COCH*	0.03222192	0.001964069	0.036916749	1	0.03222192
Bipolar	Ex	*GSTZ1*	0.168281563	0.00062136	0.019946344	1	0.168281563
Bipolar	Ex	*PLTP*	0.160106249	0.000744799	0.021374262	1	0.160106249
Bipolar	Ex	*MIB1*	0.072834656	0.000283396	0.017785524	0.809662824	0.072834656
Bipolar	Ex	*GGA2*	0.026268798	0.002783907	0.045423038	1	0.026268798
Bipolar	Ex	*C16orf62*	0.078618965	0.000282072	0.017785524	0.805879132	0.078618965
Bipolar	Ex	*AKAP8*	0.089840368	0.002071635	0.037977054	1	0.089840368
Bipolar	Ex	*PLA2G4C*	0.00798525	0.002492841	0.042220698	1	0.00798525
Bipolar	Ex	*RPA3*	0.101612217	0.001266052	0.028936885	1	0.101612217
Bipolar	Ex	*CDK5RAP3*	0.04789584	0.001354497	0.029712702	1	0.04789584
Bipolar	Ex	*EZH1*	0.088657948	0.000314276	0.017785524	0.897885754	0.088657948
Bipolar	Ex	*SLAIN2*	0.084587842	0.000771069	0.021374262	1	0.084587842
Bipolar	Ex	*LAMTOR3*	0.08507558	0.000100579	0.017785524	0.287354044	0.08507558
Bipolar	Ex	*MTCH2*	0.088978841	0.000369774	0.017785524	1	0.088978841
Bipolar	Ex	*TSPAN11*	0.025285227	0.003131499	0.048889038	1	0.025285227
Bipolar	Ex	*ALDH2*	0.111958062	0.000273746	0.017785524	0.782092437	0.111958062
Bipolar	Ex	*DDX55*	0.006418404	0.001033249	0.025016877	1	0.006418404
Bipolar	Ex	*SPSB2*	0.015717161	0.001065481	0.025580492	1	0.015717161
Bipolar	Ex	*NUDT12*	0.041237775	0.002500812	0.042220698	1	0.041237775
Bipolar	Ex	*ARSB*	0.046080327	0.001834948	0.035907173	1	0.046080327
Bipolar	Ex	*SLC27A6*	0.092439543	0.00060615	0.019814124	1	0.092439543
Bipolar	Ex	*ACTR8*	0.175078832	0.00030816	0.017785524	0.880414456	0.175078832
Bipolar	Ex	*TFG*	0.034998372	0.000304446	0.017785524	0.86980309	0.034998372
Bipolar	Ex	*ABCC5*	0.017175059	0.000151509	0.017785524	0.432859798	0.017175059
Bipolar	Ex	*NKTR*	0.039812138	0.001196746	0.027608192	1	0.039812138
Bipolar	Ex	*EPB41L5*	0.083281089	0.000288846	0.017785524	0.825232363	0.083281089
Bipolar	Ex	*TTC31*	0.175935511	0.001091151	0.025763787	1	0.175935511
Bipolar	Ex	*COX7A2L*	0.073856939	0.000871573	0.02263713	1	0.073856939
Bipolar	Ex	*THADA*	0.067236538	0.000473523	0.019268993	1	0.067236538
Bipolar	Ex	*ARMC2*	0.048790082	0.00199552	0.037262758	1	0.048790082
Bipolar	Ex	*DNAL1*	0.006796564	0.000542976	0.019627556	1	0.006796564
Bipolar	Ex	*CRIPT*	0.110097355	0.001792646	0.035588271	1	0.110097355
Bipolar	Ex	*CRHR1*	0.026704615	0.001916577	0.036916749	1	0.026704615
Bipolar	Ex	*ZSCAN18*	0.089169923	0.000603034	0.019814124	1	0.089169923

Bipolar	Ex	*FLT3*	0.10508716	0.00023961	0.017785524	0.684566052	0.10508716
Bipolar	Ex	*NQO2*	0.07944372	0.000562472	0.019627556	1	0.07944372
Bipolar	Ex	*GLO1*	0.084905566	0.001310929	0.029260342	1	0.084905566
Bipolar	Ex	*BBS2*	0.072794748	0.00039399	0.017867153	1	0.072794748
Bipolar	Ex	*MIF4GD*	0.09284618	0.002336129	0.040450422	1	0.09284618
Bipolar	Ex	*PCNXL4*	0.055244581	0.000435895	0.018048587	1	0.055244581
Bipolar	Ex	*POT1*	0.045588472	0.000316037	0.017785524	0.902917046	0.045588472
Bipolar	Ex	*ORMDL1*	0.055217599	0.000673753	0.020593961	1	0.055217599
Bipolar	Ex	*NDUFA10*	0.07823106	0.000374433	0.017785524	1	0.07823106
Bipolar	Ex	*PNPLA7*	0.091927315	0.000434478	0.018048587	1	0.091927315
Bipolar	Ex	*ATPIF1*	0.049497135	0.00059642	0.019814124	1	0.049497135
Bipolar	Ex	*PPFIA1*	0.042877336	0.00301109	0.047267494	1	0.042877336
Bipolar	Ex	*SNRPA1*	0.095458655	0.000125363	0.017785524	0.358162572	0.095458655
Bipolar	Ex	*C1QTNF6*	0.201866358	0.000417104	0.018048587	1	0.201866358
Bipolar	Ex	*LARS*	0.084731397	0.000280605	0.017785524	0.801689144	0.084731397
Bipolar	Ex	*WDR36*	0.055361015	0.00171756	0.034815981	1	0.055361015
Bipolar	Ex	*CCDC64*	0.011232023	0.000750787	0.021374262	1	0.011232023
Bipolar	Ex	*PWP1*	0.066378203	0.001950972	0.036916749	1	0.066378203
Bipolar	Ex	*NUPL2*	0.060666103	0.002949858	0.047082369	1	0.060666103
Bipolar	Ex	*XPA*	0.091935539	0.000261206	0.017785524	0.746266786	0.091935539
Bipolar	Ex	*CPEB2*	0.064782168	0.000492347	0.019268993	1	0.064782168
Bipolar	Ex	*NARS2*	0.084885456	0.000762955	0.021374262	1	0.084885456
Bipolar	Ex	*PPP2R1B*	0.106139082	0.000289573	0.017785524	0.82731083	0.106139082
Bipolar	Ex	*MAPKBP1*	0.061315766	0.002395418	0.040980292	1	0.061315766
Bipolar	Ex	*RPS24*	0.039562745	0.000408819	0.017969172	1	0.039562745
Bipolar	Ex	*DISP2*	0.08258394	0.001018194	0.024863087	1	0.08258394
Bipolar	Ex	*SCAPER*	0.054086865	0.000577386	0.019814124	1	0.054086865
Bipolar	Ex	*DEF8*	0.084094877	0.000383374	0.017785524	1	0.084094877
Bipolar	Ex	*FBXO15*	0.150368226	0.001953981	0.036916749	1	0.150368226
Bipolar	Ex	*ZNF385B*	0.055008804	0.001685745	0.034648726	1	0.055008804
Bipolar	Ex	*SCD5*	0.030230211	0.002793724	0.045423038	1	0.030230211
Bipolar	Ex	*GTF2H2*	0.095484945	0.00029895	0.017785524	0.854101009	0.095484945
Bipolar	Ex	*C7orf50*	0.013379487	0.001457322	0.031459942	1	0.013379487
Bipolar	Ex	*GBAS*	0.081642271	9.15E-05	0.017785524	0.26142673	0.081642271
Bipolar	Ex	*NTRK2*	0.045855516	0.000186156	0.017785524	0.531846302	0.045855516
Bipolar	Ex	*UGCG*	0.086479669	0.000773263	0.021374262	1	0.086479669
Bipolar	Ex	*CACNA1B*	0.061681866	0.000900376	0.022967637	1	0.061681866
Bipolar	Ex	*ADD3*	0.054544478	0.000174752	0.017785524	0.499265881	0.054544478
Bipolar	Ex	*PPP1R1C*	0.126958766	0.001464534	0.031459942	1	0.126958766
Bipolar	Ex	*NBAS*	0.032031615	0.002533954	0.042336302	1	0.032031615
Bipolar	Ex	*GRID2*	0.051183446	0.000302675	0.017785524	0.864743807	0.051183446
Bipolar	Ex	*NMRAL1*	0.072871299	0.000684783	0.020593961	1	0.072871299
Bipolar	Ex	*RPIA*	0.106106099	0.00096705	0.024024889	1	0.106106099
Bipolar	Ex	*ANKH*	0.056180455	6.88E-05	0.017785524	0.196485395	0.056180455
Bipolar	Ex	*AGPAT5*	0.082089537	0.000275737	0.017785524	0.787779563	0.082089537
Bipolar	Ex	*FAM126B*	0.072355301	0.000333375	0.017785524	0.952453598	0.072355301
Bipolar	Ex	*NMNAT2*	0.070866523	0.000372573	0.017785524	1	0.070866523
Bipolar	Ex	*DYRK1A*	0.069469653	0.000334181	0.017785524	0.954756487	0.069469653
Bipolar	Ex	*RADIL*	0.134966366	0.001353803	0.029712702	1	0.134966366
Bipolar	Ex	*CDA*	0.325855322	0.000674062	0.020593961	1	0.325855322
Bipolar	Ex	*JAK1*	0.081417443	0.000259488	0.017785524	0.74135734	0.081417443
Bipolar	Ex	*ZNF496*	0.113499486	0.000227774	0.017785524	0.650748975	0.113499486
Bipolar	Ex	*PPP1R21*	0.008303401	0.00274856	0.045390957	1	0.008303401
Bipolar	Ex	*GABRB1*	0.059323896	0.000337325	0.017785524	0.963738874	0.059323896
Bipolar	Ex	*ADORA1*	0.03059968	0.002333218	0.040450422	1	0.03059968
Bipolar	Ex	*HPS3*	0.120526185	0.000273919	0.017785524	0.782585362	0.120526185
Bipolar	Ex	*LRPAP1*	0.046350286	0.001514412	0.032255251	1	0.046350286
Bipolar	Ex	*WDR41*	0.082374125	0.000610306	0.019814124	1	0.082374125
Bipolar	Ex	*INTS1*	0.078919481	0.001279412	0.029010159	1	0.078919481
Bipolar	Ex	*YWHAZ*	0.027268685	2.33E-05	0.017785524	0.066694858	0.027268685
Bipolar	Ex	*CCDC171*	0.016986638	0.002236563	0.039408386	1	0.016986638
Bipolar	Ex	*GABRB3*	0.054555523	0.002083059	0.037977054	1	0.054555523
Bipolar	Ex	*DCHS1*	0.081688452	0.001784529	0.035588271	1	0.081688452
Bipolar	Ex	*CRK*	0.097178487	0.000402305	0.01795913	1	0.097178487
Bipolar	Ex	*ING5*	0.095483126	0.000542557	0.019627556	1	0.095483126
Bipolar	Ex	*PTPN9*	0.086552303	0.000232363	0.017785524	0.66386013	0.086552303
Bipolar	Ex	*CNTROB*	0.131029358	0.000759637	0.021374262	1	0.131029358
Bipolar	Ex	*MGMT*	0.102037103	0.000758561	0.021374262	1	0.102037103
Bipolar	Ex	*EIF2AK3*	0.097631548	0.000322153	0.017785524	0.920389816	0.097631548

Bipolar	Ex	*FUT9*	0.062582622	0.000295001	0.017785524	0.842818135	0.062582622
Bipolar	Ex	*GXYLT2*	0.082007876	0.000192866	0.017785524	0.55101816	0.082007876
Bipolar	Ex	*PACS1*	0.026509846	0.001834214	0.035907173	1	0.026509846
Bipolar	Ex	*LRRC37A3*	0.071894219	0.000258223	0.017785524	0.737743364	0.071894219
Bipolar	Ex	*PLEC*	0.065986922	0.002641062	0.043869266	1	0.065986922
Bipolar	Ex	*EFCAB13*	0.117430785	0.000193719	0.017785524	0.553454873	0.117430785
Bipolar	Ex	*LRRC3B*	0.065131992	0.000956915	0.024024889	1	0.065131992
Bipolar	Ex	*CCDC66*	0.066485001	0.000485877	0.019268993	1	0.066485001
Bipolar	Ex	*NME9*	0.087499911	6.65E-05	0.017785524	0.189982597	0.087499911
Bipolar	Ex	*ERCC6L2*	0.041414669	0.003185609	0.049463506	1	0.041414669
Bipolar	Ex	*C12orf60*	0.073863876	0.000959891	0.024024889	1	0.073863876
Bipolar	Ex	*SMDT1*	0.095007563	0.000353988	0.017785524	1	0.095007563
Bipolar	Ex	*ZNF623*	0.010667589	0.000132344	0.017785524	0.378106927	0.010667589
Bipolar	Ex	*ACSM5*	0.076415806	0.002080731	0.037977054	1	0.076415806
Bipolar	Ex	*KCTD16*	0.058667499	0.000340094	0.017785524	0.971647275	0.058667499
Bipolar	Ex	*TM2D3*	0.061862318	0.003009502	0.047267494	1	0.061862318
Bipolar	Ex	*TXNRD2*	0.106776798	0.001362396	0.029712702	1	0.106776798
Bipolar	Ex	*CHRM5*	0.08461714	0.000679139	0.020593961	1	0.08461714
Bipolar	Ex	*GSAP*	0.083861341	0.000299412	0.017785524	0.855419845	0.083861341
Bipolar	Ex	*NBPF12*	0.092534241	0.000385157	0.017785524	1	0.092534241
Bipolar	Ex	*ISG15*	0.113102001	0.000348952	0.017785524	0.996956921	0.113102001
Bipolar	Ex	*TMEM220*	0.15028626	0.000324361	0.017785524	0.926699434	0.15028626
Bipolar	Ex	*UROS*	0.056937689	0.000793	0.021573372	1	0.056937689
Bipolar	Ex	*APOD*	0.051244742	0.002086943	0.037977054	1	0.051244742
Bipolar	Ex	*FAM47E*	0.361722939	0.001646514	0.034148349	1	0.361722939
Bipolar	Ex	*OR2L13*	0.076867029	0.001793739	0.035588271	1	0.076867029
Bipolar	Ex	*GTF2IRD2*	0.100104685	0.00055076	0.019627556	1	0.100104685
Bipolar	Ex	*ZNF124*	0.079406911	0.000203051	0.017785524	0.580118082	0.079406911
Bipolar	Ex	*MYO18A*	0.090569031	0.002512257	0.042220698	1	0.090569031
Bipolar	Ex	*MYO6*	0.048803713	0.000563339	0.019627556	1	0.048803713
Bipolar	Ex	*SLC39A10*	0.055583284	0.001718255	0.034815981	1	0.055583284
Bipolar	Ex	*PARVA*	0.06918228	0.000778062	0.021374262	1	0.06918228
Bipolar	Ex	*PRMT6*	0.141695773	0.00187245	0.036391759	1	0.141695773
Bipolar	Ex	*ZNF814*	0.093145595	0.000431188	0.018048587	1	0.093145595
Bipolar	Ex	*DPF3*	0.071020405	0.001141723	0.026736912	1	0.071020405
Bipolar	Ex	*CSNK1E*	0.051574822	0.000642662	0.020400957	1	0.051574822
Bipolar	Ex	*PGM5P2*	0.107340433	0.000318251	0.017785524	0.909241805	0.107340433
Bipolar	Ex	*LINC01103*	0.109029541	0.000800412	0.021573372	1	0.109029541
Bipolar	Ex	*RP4-669L17.10*	0.131358897	0.002230447	0.039408386	1	0.131358897
Bipolar	Ex	*FAM200B*	0.092173628	0.000512969	0.019627556	1	0.092173628
Bipolar	Ex	*LRRC37A2*	0.023790525	0.000852345	0.022340816	1	0.023790525
Bipolar	Ex	*AC007405.6*	0.123102328	0.001295014	0.029132721	1	0.123102328
Bipolar	Ex	*MRPL20*	0.093141622	0.000655639	0.02058419	1	0.093141622
Bipolar	Ex	*NEAT1*	0.027171008	0.002236689	0.039408386	1	0.027171008
Bipolar	Ex	*LINC00290*	0.128420856	0.000286072	0.017785524	0.817308685	0.128420856
Bipolar	Ex	*AP006621.5*	0.11497268	0.000539088	0.019627556	1	0.11497268
Bipolar	Ex	*HMBS*	0.032824608	0.003230179	0.04988444	1	0.032824608
Bipolar	Ex	*PWAR6*	0.061004539	0.000239215	0.017785524	0.683438221	0.061004539
Bipolar	Ex	*LINC01003*	0.150607602	0.000302919	0.017785524	0.865438351	0.150607602
Bipolar	Ex	*CTC-498M16.4*	0.054336635	0.002394209	0.040980292	1	0.054336635
Bipolar	Ex	*RP11-301G7.1*	0.165188597	0.002248361	0.039408386	1	0.165188597
Bipolar	Inhib	*TSSC1*	-0.058200782	1.51E-05	0.043049224	0.043049224	0.058200782
Depression	Astros	*ZSCAN9*	-1.544015402	2.16E-07	0.000617702	0.000617702	1.544015402
Depression	Astros	*BTN3A2*	0.904951231	1.04E-05	0.014797886	0.029595772	0.904951231
Depression	Ex	*ZSCAN9*	-0.050278755	1.10E-07	0.000314214	0.000314214	0.050278755
Depression	Ex	*BTN3A2*	0.028083903	1.26E-05	0.017941799	0.035883599	0.028083903
Depression	Inhib	*ZSCAN9*	-0.067792998	5.92E-08	0.000169066	0.000169066	0.067792998
Depression	Inhib	*BTN3A2*	0.035683921	2.52E-05	0.036023585	0.07204717	0.035683921
Depression	Micro_Macro	*ZSCAN9*	-2.028384466	2.88E-07	0.000823229	0.000823229	2.028384466
Depression	Micro_Macro	*BTN3A2*	1.191006204	2.07E-05	0.029509561	0.059019123	1.191006204
Depression	Oligos	*ZSCAN9*	-0.112933231	1.33E-07	0.000378634	0.000378634	0.112933231
Depression	OPCs	*ZSCAN9*	-0.425614173	3.19E-08	9.12E-05	9.12E-05	0.425614173

b. Identified trait-associated genes from CTS TWAS analysis in pancreas

disorder	celltype	gene	Estimates	Pvalues	Pvalues_BH	Pvalue_Bonferroni
Prediabetes	duct_epithelial_cell	*STPG1*	-0.164458875	7.44E-06	0.008963481	0.029218456
Prediabetes	duct_epithelial_cell	*M6PR*	-0.162374367	0.002265684	0.013326143	1
Prediabetes	duct_epithelial_cell	*NDUFAF7*	-0.234139996	0.002443751	0.013915212	1
Prediabetes	duct_epithelial_cell	*PLXND1*	-0.11676646	0.004978932	0.021832837	1
Prediabetes	duct_epithelial_cell	*KDM1A*	-0.130241815	0.009347995	0.033088535	1
Prediabetes	duct_epithelial_cell	*RECQL*	-0.092816137	0.01369914	0.044117967	1
Prediabetes	duct_epithelial_cell	*SLC25A13*	-0.26018646	0.001380988	0.010271223	1
Prediabetes	duct_epithelial_cell	*KMT2E*	-0.140269826	0.00058333	0.008963481	1
Prediabetes	duct_epithelial_cell	*IBTK*	-0.107859807	0.000842593	0.008963481	1
Prediabetes	duct_epithelial_cell	*GGNBP2*	-0.082247068	0.006086741	0.024776105	1
Prediabetes	duct_epithelial_cell	*SYNRG*	-0.136021584	0.002066413	0.012568013	1
Prediabetes	duct_epithelial_cell	*VPS41*	-0.071307492	0.012878112	0.042129976	1
Prediabetes	duct_epithelial_cell	*CCDC124*	-0.134728403	0.004266027	0.019612585	1
Prediabetes	duct_epithelial_cell	*BAIAP3*	-0.064871685	0.007128577	0.027648747	1
Prediabetes	duct_epithelial_cell	*CYB561*	-0.091110785	0.004242887	0.019551648	1
Prediabetes	duct_epithelial_cell	*MED24*	-0.200540851	0.005435313	0.023196885	1
Prediabetes	duct_epithelial_cell	*RPS20*	-0.082678623	0.000891397	0.00905104	1
Prediabetes	duct_epithelial_cell	*REV3L*	-0.13111087	0.004712464	0.020968597	1
Prediabetes	duct_epithelial_cell	*ETV7*	-0.361470073	0.005971416	0.024567217	1
Prediabetes	duct_epithelial_cell	*FUZ*	-0.24765069	0.006176398	0.024966119	1
Prediabetes	duct_epithelial_cell	*LRRC23*	-0.068835012	0.007142713	0.027676253	1
Prediabetes	duct_epithelial_cell	*RABGAP1*	-0.193667678	0.000202707	0.008963481	0.796435181
Prediabetes	duct_epithelial_cell	*PPP5C*	-0.264869766	0.000715204	0.008963481	1
Prediabetes	duct_epithelial_cell	*ERCC1*	-0.165407848	0.000627605	0.008963481	1
Prediabetes	duct_epithelial_cell	*EXTL3*	-0.104751838	0.005046085	0.02202293	1
Prediabetes	duct_epithelial_cell	*MAN2B2*	-0.220189247	0.009889357	0.034568759	1
Prediabetes	duct_epithelial_cell	*UFL1*	-0.145404715	0.001047051	0.009336398	1
Prediabetes	duct_epithelial_cell	*MDH1*	-0.126607466	0.002110454	0.012737289	1
Prediabetes	duct_epithelial_cell	*NUDCD3*	-0.111108683	0.009473597	0.033412712	1
Prediabetes	duct_epithelial_cell	*RNH1*	-0.097655502	0.003746436	0.018194991	1
Prediabetes	duct_epithelial_cell	*NDUFS1*	-0.101281622	0.004095261	0.019223751	1
Prediabetes	duct_epithelial_cell	*RNASET2*	-0.085633389	0.0046819	0.02090362	1
Prediabetes	duct_epithelial_cell	*AGPAT4*	-0.08036806	0.007345992	0.028324243	1
Prediabetes	duct_epithelial_cell	*VEZT*	-0.104723807	0.006088516	0.024776105	1
Prediabetes	duct_epithelial_cell	*RABEP1*	-0.091026879	0.005188664	0.022377892	1
Prediabetes	duct_epithelial_cell	*ARID4A*	-0.108047355	0.00541767	0.02316216	1
Prediabetes	duct_epithelial_cell	*ABCF2*	-0.269688271	0.000365826	0.008963481	1
Prediabetes	duct_epithelial_cell	*UBA6*	-0.185232982	0.000469162	0.008963481	1
Prediabetes	duct_epithelial_cell	*FAM136A*	-0.194861329	0.000364529	0.008963481	1
Prediabetes	duct_epithelial_cell	*DAPK2*	-0.069331892	0.015344246	0.04807619	1
Prediabetes	duct_epithelial_cell	*RPL26L1*	-0.106098795	0.003546532	0.017534673	1
Prediabetes	duct_epithelial_cell	*NSUN2*	-0.147770331	0.008430261	0.030897851	1
Prediabetes	duct_epithelial_cell	*AGA*	-0.146276957	0.004528701	0.020499155	1
Prediabetes	duct_epithelial_cell	*DNAH5*	-0.168907936	0.00118627	0.009719245	1
Prediabetes	duct_epithelial_cell	*PHLPP2*	-0.206399117	0.004242991	0.019551648	1
Prediabetes	duct_epithelial_cell	*INPP4A*	-0.282310192	0.000857473	0.008963481	1
Prediabetes	duct_epithelial_cell	*PSMA4*	-0.170337862	0.000344864	0.008963481	1
Prediabetes	duct_epithelial_cell	*CNTLN*	-0.310803401	0.000950248	0.009132479	1
Prediabetes	duct_epithelial_cell	*WDR37*	-0.107116305	0.002331512	0.013496739	1
Prediabetes	duct_epithelial_cell	*YTHDC2*	-0.1222248	0.001399497	0.010278575	1
Prediabetes	duct_epithelial_cell	*DTNBP1*	-0.22472068	0.0032736	0.016769199	1
Prediabetes	duct_epithelial_cell	*GOPC*	-0.186129871	0.000793832	0.008963481	1
Prediabetes	duct_epithelial_cell	*ZNF800*	-0.156231503	0.00065039	0.008963481	1
Prediabetes	duct_epithelial_cell	*H6PD*	-0.244342683	0.00616527	0.024966119	1
Prediabetes	duct_epithelial_cell	*VAMP3*	-0.122819369	0.002777039	0.01511602	1
Prediabetes	duct_epithelial_cell	*RCN1*	-0.0806333	0.005355881	0.022972989	1
Prediabetes	duct_epithelial_cell	*JKAMP*	-0.087350648	0.012438877	0.041069201	1
Prediabetes	duct_epithelial_cell	*BCAR1*	-0.128404414	0.005238708	0.022568952	1
Prediabetes	duct_epithelial_cell	*HEBP2*	-0.154416261	0.000581163	0.008963481	1
Prediabetes	duct_epithelial_cell	*RRP12*	-0.174505075	0.000986317	0.009283441	1
Prediabetes	duct_epithelial_cell	*FNIP2*	-0.098793072	0.001572717	0.010878879	1
Prediabetes	duct_epithelial_cell	*MCF2L2*	-0.089162453	0.00899366	0.032207801	1

Prediabetes	duct_epithelial_cell	*LY75*	-0.223537603	0.00128572	0.010042933	1
Prediabetes	duct_epithelial_cell	*KIF1B*	-0.165598061	0.000617782	0.008963481	1
Prediabetes	duct_epithelial_cell	*TBC1D22A*	-0.139957163	0.001635012	0.011037642	1
Prediabetes	duct_epithelial_cell	*SYNE2*	-0.193843821	0.002912767	0.015486146	1
Prediabetes	duct_epithelial_cell	*ATP9A*	-0.19453204	0.001366217	0.010271223	1
Prediabetes	duct_epithelial_cell	*USP36*	-0.167385445	0.000751437	0.008963481	1
Prediabetes	duct_epithelial_cell	*MCOLN3*	-0.075975211	0.007552374	0.028893162	1
Prediabetes	duct_epithelial_cell	*GDI2*	-0.115252629	0.000361547	0.008963481	1
Prediabetes	duct_epithelial_cell	*RIMBP2*	-0.053537323	0.016146299	0.049755927	1
Prediabetes	duct_epithelial_cell	*MPC1*	-0.16272113	0.000447158	0.008963481	1
Prediabetes	duct_epithelial_cell	*EIF4B*	-0.088954365	0.000963846	0.009147222	1
Prediabetes	duct_epithelial_cell	*HAGH*	-0.085028847	0.004163819	0.019406459	1
Prediabetes	duct_epithelial_cell	*RNF4*	-0.24012995	0.000126159	0.008963481	0.495676775
Prediabetes	duct_epithelial_cell	*ST3GAL6*	-0.086481589	0.000365966	0.008963481	1
Prediabetes	duct_epithelial_cell	*HIPK2*	-0.140475053	0.001105045	0.009564575	1
Prediabetes	duct_epithelial_cell	*PMS1*	-0.158265121	0.006378496	0.02552048	1
Prediabetes	duct_epithelial_cell	*IPO5*	-0.132641939	0.003283817	0.016799632	1
Prediabetes	duct_epithelial_cell	*OAT*	-0.087474003	0.010969958	0.037414245	1
Prediabetes	duct_epithelial_cell	*GSTO2*	-0.135042175	0.001007982	0.009336398	1
Prediabetes	duct_epithelial_cell	*ATXN3*	-0.164324015	0.000206372	0.008963481	0.810836149
Prediabetes	duct_epithelial_cell	*EML1*	-0.305050405	0.000639555	0.008963481	1
Prediabetes	duct_epithelial_cell	*STOML1*	-0.264961023	0.00022401	0.008963481	0.880136968
Prediabetes	duct_epithelial_cell	*REEP1*	-0.062988974	0.013869891	0.044485554	1
Prediabetes	duct_epithelial_cell	*POLR1A*	-0.05592608	0.002300459	0.013430167	1
Prediabetes	duct_epithelial_cell	*LAPTM4A*	-0.126826638	0.000450758	0.008963481	1
Prediabetes	duct_epithelial_cell	*KIF2A*	-0.128941797	0.011449081	0.038546222	1
Prediabetes	duct_epithelial_cell	*MAST4*	-0.159706485	0.000614616	0.008963481	1
Prediabetes	duct_epithelial_cell	*RAB27A*	-0.068266437	0.005966214	0.024567217	1
Prediabetes	duct_epithelial_cell	*PTPN3*	-0.201621016	0.0054376	0.023196885	1
Prediabetes	duct_epithelial_cell	*FGF10*	-0.260384319	0.001584846	0.010911694	1
Prediabetes	duct_epithelial_cell	*DGCR2*	-0.175237706	0.000868203	0.009000449	1
Prediabetes	duct_epithelial_cell	*ZXDC*	-0.212925266	0.000584427	0.008963481	1
Prediabetes	duct_epithelial_cell	*WIPI1*	-0.217414107	0.004092969	0.019223751	1
Prediabetes	duct_epithelial_cell	*ASNS*	-0.116844339	0.006335171	0.025438743	1
Prediabetes	duct_epithelial_cell	*RPL31*	-0.081781741	0.001111068	0.009564575	1
Prediabetes	duct_epithelial_cell	*SEL1L*	-0.128277264	0.000153946	0.008963481	0.604852939
Prediabetes	duct_epithelial_cell	*CYBRD1*	-0.141352674	0.004616346	0.020788435	1
Prediabetes	duct_epithelial_cell	*TRNT1*	-0.225478528	0.000621891	0.008963481	1
Prediabetes	duct_epithelial_cell	*CRMP1*	-0.054384437	0.004768806	0.021123605	1
Prediabetes	duct_epithelial_cell	*DERL2*	-0.170819322	0.000340585	0.008963481	1
Prediabetes	duct_epithelial_cell	*NDE1*	-0.195809056	0.002605065	0.014586734	1
Prediabetes	duct_epithelial_cell	*SMARCE1*	-0.110385989	0.001246296	0.009849499	1
Prediabetes	duct_epithelial_cell	*MRPS34*	-0.077548227	0.013290539	0.043155809	1
Prediabetes	duct_epithelial_cell	*ZZEF1*	-0.151108079	0.000903609	0.009057947	1
Prediabetes	duct_epithelial_cell	*ANO8*	-0.133126966	0.011581419	0.038858577	1
Prediabetes	duct_epithelial_cell	*GRAMD4*	-0.055853377	0.011001638	0.037424619	1
Prediabetes	duct_epithelial_cell	*CACNG5*	-1.570507843	0.001915189	0.012108933	1
Prediabetes	duct_epithelial_cell	*DLG1*	-0.172336987	0.001085616	0.009519336	1
Prediabetes	duct_epithelial_cell	*MKRN2*	-0.152360163	0.007018059	0.027368761	1
Prediabetes	duct_epithelial_cell	*TPD52*	-0.090423779	0.00086624	0.009000449	1
Prediabetes	duct_epithelial_cell	*ACACB*	-0.062167899	0.006159876	0.024966119	1
Prediabetes	duct_epithelial_cell	*GPATCH1*	-0.11665869	0.003861838	0.018571801	1
Prediabetes	duct_epithelial_cell	*XAB2*	-0.193627554	0.002105953	0.012729677	1
Prediabetes	duct_epithelial_cell	*APBB1IP*	0.053622004	0.008648281	0.031435931	1
Prediabetes	duct_epithelial_cell	*N4BP2*	-0.118378118	0.001845517	0.011790302	1
Prediabetes	duct_epithelial_cell	*MLLT10*	-0.148618985	0.001684224	0.011177897	1
Prediabetes	duct_epithelial_cell	*PCM1*	-0.093461398	0.002769476	0.01511602	1
Prediabetes	duct_epithelial_cell	*TP73*	-1.490527174	0.006966218	0.027335463	1
Prediabetes	duct_epithelial_cell	*TOLLIP*	-0.091782514	0.002192467	0.012987238	1
Prediabetes	duct_epithelial_cell	*FKBP7*	-0.083420566	0.013830565	0.044431963	1
Prediabetes	duct_epithelial_cell	*LXN*	-0.048530363	0.006172969	0.024966119	1
Prediabetes	duct_epithelial_cell	*SAR1A*	-0.107489185	0.0012267	0.009805258	1
Prediabetes	duct_epithelial_cell	*FDFT1*	-0.155768917	4.25E-05	0.008963481	0.166857771
Prediabetes	duct_epithelial_cell	*DDX1*	-0.081890501	0.01040756	0.035995866	1

Prediabetes	duct_epithelial_cell	*RABL2B*	-0.275458599	0.009493529	0.033452982	1
Prediabetes	duct_epithelial_cell	*RIF1*	-0.146864888	0.004244331	0.019551648	1
Prediabetes	duct_epithelial_cell	*COL5A3*	-0.181774095	0.003232258	0.016709924	1
Prediabetes	duct_epithelial_cell	*IGSF9B*	-0.165361571	0.008047815	0.030163992	1
Prediabetes	duct_epithelial_cell	*ZNF506*	-0.173530921	0.000662868	0.008963481	1
Prediabetes	duct_epithelial_cell	*ME2*	-0.184551431	0.00068626	0.008963481	1
Prediabetes	duct_epithelial_cell	*BCKDHB*	-0.170625034	0.01161572	0.038940413	1
Prediabetes	duct_epithelial_cell	*PIBF1*	-0.149861283	0.006860949	0.027010691	1
Prediabetes	duct_epithelial_cell	*CHMP2B*	-0.157538202	0.00053169	0.008963481	1
Prediabetes	duct_epithelial_cell	*NCOA1*	-0.188866175	0.000445589	0.008963481	1
Prediabetes	duct_epithelial_cell	*SLC25A24*	-0.14042076	0.004476117	0.020307924	1
Prediabetes	duct_epithelial_cell	*CPNE3*	-0.097504302	0.001730522	0.011369938	1
Prediabetes	duct_epithelial_cell	*RRN3*	-0.135098676	0.000281907	0.008963481	1
Prediabetes	duct_epithelial_cell	*EPDR1*	-0.233292159	0.001419028	0.010342775	1
Prediabetes	duct_epithelial_cell	*TMED2*	-0.092568383	0.00144746	0.010415881	1
Prediabetes	duct_epithelial_cell	*PGS1*	-0.19131308	0.001668738	0.01113209	1
Prediabetes	duct_epithelial_cell	*PSMC5*	-0.06758318	0.006215766	0.025099428	1
Prediabetes	duct_epithelial_cell	*UIMC1*	-0.135915766	0.005555777	0.023430359	1
Prediabetes	duct_epithelial_cell	*SF3B2*	-0.08355709	0.003984934	0.018886376	1
Prediabetes	duct_epithelial_cell	*ZNF343*	-0.150041072	0.013865715	0.044485554	1
Prediabetes	duct_epithelial_cell	*MAVS*	-0.15824973	0.009298589	0.032973065	1
Prediabetes	duct_epithelial_cell	*SLC23A2*	-0.113955874	0.009020709	0.03224965	1
Prediabetes	duct_epithelial_cell	*TMEM230*	-0.096568265	0.002771139	0.01511602	1
Prediabetes	duct_epithelial_cell	*GRAMD1A*	-0.141360827	0.000504704	0.008963481	1
Prediabetes	duct_epithelial_cell	*DDX24*	-0.087216868	0.001350942	0.010256616	1
Prediabetes	duct_epithelial_cell	*GPATCH2L*	-0.147930189	0.000610286	0.008963481	1
Prediabetes	duct_epithelial_cell	*STRN4*	-0.158215128	0.001502966	0.010620779	1
Prediabetes	duct_epithelial_cell	*MUL1*	-0.213072598	0.000189546	0.008963481	0.744727995
Prediabetes	duct_epithelial_cell	*SPG21*	-0.177621691	0.000537844	0.008963481	1
Prediabetes	duct_epithelial_cell	*GLG1*	-0.1586374	0.000421181	0.008963481	1
Prediabetes	duct_epithelial_cell	*NRCAM*	-0.054828482	0.009214671	0.032764203	1
Prediabetes	duct_epithelial_cell	*TMEM101*	-0.107513116	0.014697362	0.046474444	1
Prediabetes	duct_epithelial_cell	*SCFD1*	-0.10968843	0.002141718	0.012886383	1
Prediabetes	duct_epithelial_cell	*NUP50*	-0.146004979	0.000265869	0.008963481	1
Prediabetes	duct_epithelial_cell	*COMT*	-0.158048846	0.006971274	0.027335463	1
Prediabetes	duct_epithelial_cell	*SUCO*	-0.072853694	0.003956007	0.018817373	1
Prediabetes	duct_epithelial_cell	*MSH2*	-0.411825349	0.001347345	0.010256616	1
Prediabetes	duct_epithelial_cell	*TMEM38B*	-0.067969129	0.014384978	0.045690039	1
Prediabetes	duct_epithelial_cell	*PSMD5*	-0.243318537	0.000558668	0.008963481	1
Prediabetes	duct_epithelial_cell	*CRAT*	-0.157428029	0.005080291	0.022048127	1
Prediabetes	duct_epithelial_cell	*IKZF5*	-0.206582954	2.46E-05	0.008963481	0.096573773
Prediabetes	duct_epithelial_cell	*SRPK1*	-0.140181168	0.003778997	0.018285321	1
Prediabetes	duct_epithelial_cell	*EFHC1*	-0.071337772	0.004045816	0.019105782	1
Prediabetes	duct_epithelial_cell	*ABL1*	-0.21585135	0.000378841	0.008963481	1
Prediabetes	duct_epithelial_cell	*SH3GLB1*	-0.11650065	0.000306923	0.008963481	1
Prediabetes	duct_epithelial_cell	*ABLIM1*	-0.09967574	0.003517105	0.017534673	1
Prediabetes	duct_epithelial_cell	*HNRNPM*	-0.089585044	0.001726967	0.011365586	1
Prediabetes	duct_epithelial_cell	*TIMM13*	-0.118308666	0.00092166	0.009057947	1
Prediabetes	duct_epithelial_cell	*POLR2E*	-0.191801406	0.000444909	0.008963481	1
Prediabetes	duct_epithelial_cell	*ARVCF*	-0.169136747	0.000444135	0.008963481	1
Prediabetes	duct_epithelial_cell	*TRMT2A*	-0.106678145	0.001376619	0.010271223	1
Prediabetes	duct_epithelial_cell	*MED15*	-0.244447595	0.002404615	0.013772204	1
Prediabetes	duct_epithelial_cell	*CABIN1*	-0.101106483	0.011066599	0.037577086	1
Prediabetes	duct_epithelial_cell	*SPECC1L*	-0.126971762	0.001424865	0.010342775	1
Prediabetes	duct_epithelial_cell	*SLC25A1*	-0.191077659	0.000835983	0.008963481	1
Prediabetes	duct_epithelial_cell	*HPS4*	-0.099834592	0.003249361	0.016734297	1
Prediabetes	duct_epithelial_cell	*CBY1*	-0.160606658	0.002796245	0.015172844	1
Prediabetes	duct_epithelial_cell	*XBP1*	-0.076627491	0.00089887	0.009057947	1
Prediabetes	duct_epithelial_cell	*TIMP3*	-0.068954928	0.000449074	0.008963481	1
Prediabetes	duct_epithelial_cell	*MIOX*	-0.130748461	0.013065457	0.042604802	1
Prediabetes	duct_epithelial_cell	*TOM1*	-0.166106802	0.008738289	0.031543827	1
Prediabetes	duct_epithelial_cell	*TTLL12*	-0.330541617	0.005337434	0.022918885	1
Prediabetes	duct_epithelial_cell	*RBFOX2*	-0.120260998	0.002333918	0.013496739	1
Prediabetes	duct_epithelial_cell	*SAMM50*	-0.223997728	0.003392811	0.017190742	1

Prediabetes	duct_epithelial_cell	*SGSM3*	-0.184422713	0.002883339	0.015391907	1
Prediabetes	duct_epithelial_cell	*IFT27*	-0.095034928	0.002968044	0.01569508	1
Prediabetes	duct_epithelial_cell	*ST13*	-0.097621489	0.000915212	0.009057947	1
Prediabetes	duct_epithelial_cell	*POLR3H*	-0.15512586	0.002643769	0.014712986	1
Prediabetes	duct_epithelial_cell	*PMM1*	-0.195340569	0.001416299	0.010342775	1
Prediabetes	duct_epithelial_cell	*PRMT5*	-0.25336405	0.000480426	0.008963481	1
Prediabetes	duct_epithelial_cell	*VCPKMT*	-0.258287756	0.000199508	0.008963481	0.783867646
Prediabetes	duct_epithelial_cell	*SOS2*	-0.109031558	0.015872917	0.049339155	1
Prediabetes	duct_epithelial_cell	*CDKL1*	-0.122507496	0.005532339	0.023421841	1
Prediabetes	duct_epithelial_cell	*NIN*	-0.271533375	0.00104677	0.009336398	1
Prediabetes	duct_epithelial_cell	*TRIM9*	-0.057688366	0.014149619	0.045198253	1
Prediabetes	duct_epithelial_cell	*GSTZ1*	-0.112790312	0.012348	0.040906653	1
Prediabetes	duct_epithelial_cell	*TMED8*	-0.083616202	0.011575621	0.038858577	1
Prediabetes	duct_epithelial_cell	*DAAM1*	-0.097229095	0.001970717	0.01235437	1
Prediabetes	duct_epithelial_cell	*DHRS7*	-0.166855972	0.001044915	0.009336398	1
Prediabetes	duct_epithelial_cell	*GSKIP*	-0.245284434	0.00066521	0.008963481	1
Prediabetes	duct_epithelial_cell	*TRIP11*	-0.193966711	0.000411487	0.008963481	1
Prediabetes	duct_epithelial_cell	*NELFCD*	-0.159672569	0.000878478	0.009011853	1
Prediabetes	duct_epithelial_cell	*MTG2*	-0.242883035	5.48E-05	0.008963481	0.215365873
Prediabetes	duct_epithelial_cell	*POFUT1*	-0.28011742	0.000832973	0.008963481	1
Prediabetes	duct_epithelial_cell	*IDH3B*	-0.193293238	0.000655778	0.008963481	1
Prediabetes	duct_epithelial_cell	*MAPRE1*	-0.09530991	0.00749572	0.028732375	1
Prediabetes	duct_epithelial_cell	*TTI1*	-0.331958544	0.000565488	0.008963481	1
Prediabetes	duct_epithelial_cell	*CHMP4B*	-0.1372382	0.000433459	0.008963481	1
Prediabetes	duct_epithelial_cell	*DNTTIP1*	-0.160701424	0.002541624	0.014327176	1
Prediabetes	duct_epithelial_cell	*VAPA*	-0.114607585	0.000621925	0.008963481	1
Prediabetes	duct_epithelial_cell	*LIPG*	-0.332084603	0.000654721	0.008963481	1
Prediabetes	duct_epithelial_cell	*RIOK3*	-0.111227811	0.001593448	0.010911694	1
Prediabetes	duct_epithelial_cell	*NALCN*	-0.076613837	0.010109162	0.035211789	1
Prediabetes	duct_epithelial_cell	*FGF14*	-0.050433785	0.004073315	0.019166532	1
Prediabetes	duct_epithelial_cell	*FNDC3A*	-0.087969239	0.001637807	0.011037642	1
Prediabetes	duct_epithelial_cell	*DNAJC3*	-0.117830284	0.000834139	0.008963481	1
Prediabetes	duct_epithelial_cell	*UGGT2*	-0.141312993	0.002315183	0.013483149	1
Prediabetes	duct_epithelial_cell	*ARHGEF7*	-0.116417247	0.001872424	0.011928428	1
Prediabetes	duct_epithelial_cell	*CLN5*	-0.174107709	0.000364759	0.008963481	1
Prediabetes	duct_epithelial_cell	*MGRN1*	-0.125443297	0.000286915	0.008963481	1
Prediabetes	duct_epithelial_cell	*LONP2*	-0.111642562	0.002128091	0.012824032	1
Prediabetes	duct_epithelial_cell	*USB1*	-0.162646285	0.006849417	0.026992335	1
Prediabetes	duct_epithelial_cell	*CYB5B*	-0.195690772	0.000801056	0.008963481	1
Prediabetes	duct_epithelial_cell	*NME3*	-0.112567537	0.000315507	0.008963481	1
Prediabetes	duct_epithelial_cell	*NPRL3*	-0.107165788	0.002669544	0.014746113	1
Prediabetes	duct_epithelial_cell	*NAGPA*	-0.185205291	0.006791376	0.026844381	1
Prediabetes	duct_epithelial_cell	*NME4*	-0.124759247	0.001187206	0.009719245	1
Prediabetes	duct_epithelial_cell	*NOMO3*	-0.111845941	0.000961836	0.009147222	1
Prediabetes	duct_epithelial_cell	*LMF1*	-0.229140116	0.010324925	0.035836244	1
Prediabetes	duct_epithelial_cell	*HAGHL*	-0.076437516	0.01113412	0.037693769	1
Prediabetes	duct_epithelial_cell	*METRN*	-0.082378936	0.002874033	0.015391043	1
Prediabetes	duct_epithelial_cell	*HMOX2*	-0.202333873	0.000452245	0.008963481	1
Prediabetes	duct_epithelial_cell	*RBL2*	-0.080386147	0.015975802	0.049463299	1
Prediabetes	duct_epithelial_cell	*KAT8*	-0.150123335	0.006444824	0.02571346	1
Prediabetes	duct_epithelial_cell	*RNF40*	-0.118806742	0.0072169	0.027881219	1
Prediabetes	duct_epithelial_cell	*AP3B2*	-0.086043789	0.00965663	0.033906075	1
Prediabetes	duct_epithelial_cell	*RPAP1*	-0.224221797	0.001048637	0.009336398	1
Prediabetes	duct_epithelial_cell	*DMXL2*	-0.088927341	0.00431652	0.019789508	1
Prediabetes	duct_epithelial_cell	*DNAJC17*	-0.189360626	0.012604367	0.04147618	1
Prediabetes	duct_epithelial_cell	*ZDHHC2*	-0.21165749	0.000391329	0.008963481	1
Prediabetes	duct_epithelial_cell	*NBN*	-0.126828828	0.006446363	0.02571346	1
Prediabetes	duct_epithelial_cell	*RAB2A*	-0.056812572	0.011218154	0.037931263	1
Prediabetes	duct_epithelial_cell	*EIF3E*	-0.085497064	0.001601177	0.01094091	1
Prediabetes	duct_epithelial_cell	*ESRP1*	-0.222827131	0.000899696	0.009057947	1
Prediabetes	duct_epithelial_cell	*ARMC1*	-0.118371804	0.001290794	0.010044933	1
Prediabetes	duct_epithelial_cell	*CHRAC1*	-0.164503012	0.001386851	0.010271223	1
Prediabetes	duct_epithelial_cell	*INTS10*	-0.116519051	0.001242493	0.009849499	1
Prediabetes	duct_epithelial_cell	*LEPROTL1*	-0.172074824	0.001668704	0.01113209	1

Prediabetes	duct_epithelial_cell	*KLHDC4*	-0.105122124	0.004630558	0.020792527	1
Prediabetes	duct_epithelial_cell	*ASAH1*	-0.099424083	0.001311151	0.010116188	1
Prediabetes	duct_epithelial_cell	*MAN2B1*	-0.096305189	0.008704182	0.031543827	1
Prediabetes	duct_epithelial_cell	*HNRNPL*	-0.108216198	0.001920047	0.012108933	1
Prediabetes	duct_epithelial_cell	*LIN7B*	-0.080640554	0.00129109	0.010044933	1
Prediabetes	duct_epithelial_cell	*ERCC2*	-0.144387207	0.010796063	0.036981459	1
Prediabetes	duct_epithelial_cell	*NUMBL*	-0.104816808	0.004268491	0.019612585	1
Prediabetes	duct_epithelial_cell	*PLIN3*	-0.045181725	0.012199694	0.040552114	1
Prediabetes	duct_epithelial_cell	*ZNF175*	-0.141808458	0.014039268	0.044918798	1
Prediabetes	duct_epithelial_cell	*PLA2G4C*	-0.073031667	0.016047468	0.049607005	1
Prediabetes	duct_epithelial_cell	*GCDH*	-0.187481739	0.001122237	0.009564575	1
Prediabetes	duct_epithelial_cell	*PIK3R2*	-0.238318037	0.001017636	0.009336398	1
Prediabetes	duct_epithelial_cell	*UPK1A*	-0.722949664	0.002875293	0.015391043	1
Prediabetes	duct_epithelial_cell	*COPE*	-0.106853279	0.000764238	0.008963481	1
Prediabetes	duct_epithelial_cell	*SIPA1L3*	-0.211269271	0.000544795	0.008963481	1
Prediabetes	duct_epithelial_cell	*PMPCB*	-0.202506536	0.000690321	0.008963481	1
Prediabetes	duct_epithelial_cell	*TSPAN12*	-0.124529689	0.003493719	0.017450459	1
Prediabetes	duct_epithelial_cell	*CHCHD2*	-0.071513831	0.002887207	0.015391907	1
Prediabetes	duct_epithelial_cell	*EIF3B*	-0.115584871	0.001109091	0.009564575	1
Prediabetes	duct_epithelial_cell	*C1GALT1*	-0.151708782	0.000615999	0.008963481	1
Prediabetes	duct_epithelial_cell	*RPA3*	-0.121464597	0.000444789	0.008963481	1
Prediabetes	duct_epithelial_cell	*TMEM106B*	-0.110913799	0.005078551	0.022048127	1
Prediabetes	duct_epithelial_cell	*ACTR3C*	-0.241691309	0.011826672	0.03954638	1
Prediabetes	duct_epithelial_cell	*TSPAN13*	-0.07349659	0.002818512	0.015228566	1
Prediabetes	duct_epithelial_cell	*COA1*	-0.192862989	0.000493702	0.008963481	1
Prediabetes	duct_epithelial_cell	*BLVRA*	-0.171432337	0.000349637	0.008963481	1
Prediabetes	duct_epithelial_cell	*TMEM248*	-0.104887499	0.002339343	0.013496739	1
Prediabetes	duct_epithelial_cell	*RHEB*	-0.102118637	0.000681382	0.008963481	1
Prediabetes	duct_epithelial_cell	*SPIN1*	-0.198275344	0.000281449	0.008963481	1
Prediabetes	duct_epithelial_cell	*NMRK1*	-0.135740724	0.000793494	0.008963481	1
Prediabetes	duct_epithelial_cell	*SUSD1*	-0.116012425	0.002200136	0.012987238	1
Prediabetes	duct_epithelial_cell	*DNM1*	-0.081468799	0.005467628	0.023274443	1
Prediabetes	duct_epithelial_cell	*NPDC1*	-0.050959021	0.005864987	0.02428191	1
Prediabetes	duct_epithelial_cell	*HPS1*	-0.189705927	0.001776158	0.01151572	1
Prediabetes	duct_epithelial_cell	*ATE1*	-0.158562958	0.003745175	0.018194991	1
Prediabetes	duct_epithelial_cell	*PLEKHA1*	-0.118522765	0.000118976	0.008963481	0.467457682
Prediabetes	duct_epithelial_cell	*SPOCK2*	-0.65536316	0.000698866	0.008963481	1
Prediabetes	duct_epithelial_cell	*BMPR1A*	-0.293794992	0.001184207	0.009719245	1
Prediabetes	duct_epithelial_cell	*LIPA*	-0.206818286	0.000838229	0.008963481	1
Prediabetes	duct_epithelial_cell	*TNKS2*	-0.127714501	0.000674747	0.008963481	1
Prediabetes	duct_epithelial_cell	*ACBD5*	-0.195915416	0.000742108	0.008963481	1
Prediabetes	duct_epithelial_cell	*GTPBP4*	-0.162780104	0.000844938	0.008963481	1
Prediabetes	duct_epithelial_cell	*XPNPEP1*	-0.20477485	0.003887107	0.01867047	1
Prediabetes	duct_epithelial_cell	*SMC3*	-0.126954288	0.00113691	0.00963464	1
Prediabetes	duct_epithelial_cell	*CWC25*	-0.160392519	0.004039963	0.019101102	1
Prediabetes	duct_epithelial_cell	*TRIM37*	-0.107022542	0.002562474	0.014403376	1
Prediabetes	duct_epithelial_cell	*MED13*	-0.08618318	0.00187321	0.011928428	1
Prediabetes	duct_epithelial_cell	*CCDC47*	-0.144444913	0.000482087	0.008963481	1
Prediabetes	duct_epithelial_cell	*DRG2*	-0.184193579	0.000518696	0.008963481	1
Prediabetes	duct_epithelial_cell	*SMARCD2*	-0.198927944	0.004680611	0.02090362	1
Prediabetes	duct_epithelial_cell	*C17orf75*	-0.072273951	0.014930271	0.046928828	1
Prediabetes	duct_epithelial_cell	*NAGLU*	-0.107981825	1.51E-05	0.008963481	0.059205576
Prediabetes	duct_epithelial_cell	*RND2*	-0.419276645	0.000368551	0.008963481	1
Prediabetes	duct_epithelial_cell	*MPP2*	-0.070562521	0.007275707	0.028080799	1
Prediabetes	duct_epithelial_cell	*HLF*	-0.06249945	0.009874087	0.034546116	1
Prediabetes	duct_epithelial_cell	*EFNB3*	-0.100512923	0.003418556	0.017241986	1
Prediabetes	duct_epithelial_cell	*RANGRF*	-0.175348894	0.001352234	0.010256616	1
Prediabetes	duct_epithelial_cell	*DPH1*	-0.247810079	0.001120451	0.009564575	1
Prediabetes	duct_epithelial_cell	*OCIAD1*	-0.100749352	0.000874323	0.009011853	1
Prediabetes	duct_epithelial_cell	*UBE2D3*	-0.078236769	0.00529699	0.022770103	1
Prediabetes	duct_epithelial_cell	*TRIM2*	-0.104343162	0.002249011	0.01324792	1
Prediabetes	duct_epithelial_cell	*ST3GAL4*	-0.100671419	0.009320625	0.033021401	1
Prediabetes	duct_epithelial_cell	*PRPF19*	-0.119585103	0.004116851	0.019282694	1
Prediabetes	duct_epithelial_cell	*MDK*	-0.160028007	0.002898229	0.01542973	1

Prediabetes	duct_epithelial_cell	*MADD*	-0.091906058	0.006039165	0.02471654	1
Prediabetes	duct_epithelial_cell	*NAA40*	-0.183999389	0.008675447	0.031502618	1
Prediabetes	duct_epithelial_cell	*NUP98*	-0.167143219	0.000705297	0.008963481	1
Prediabetes	duct_epithelial_cell	*GTF2H1*	-0.123133921	0.001591647	0.010911694	1
Prediabetes	duct_epithelial_cell	*METAP2*	-0.10371939	0.000639856	0.008963481	1
Prediabetes	duct_epithelial_cell	*ITFG2*	-0.2595012	0.00119087	0.009719245	1
Prediabetes	duct_epithelial_cell	*CREBL2*	-0.149128842	0.004972854	0.021830553	1
Prediabetes	duct_epithelial_cell	*LTBR*	-0.103694089	0.015007454	0.047096075	1
Prediabetes	duct_epithelial_cell	*CAND1*	-0.103332583	0.001916969	0.012108933	1
Prediabetes	duct_epithelial_cell	*KRR1*	-0.124381751	9.13E-05	0.008963481	0.358668134
Prediabetes	duct_epithelial_cell	*ATN1*	-0.121778877	0.011959987	0.039890314	1
Prediabetes	duct_epithelial_cell	*GOLT1B*	-0.11488142	0.001743014	0.011413834	1
Prediabetes	duct_epithelial_cell	*C2CD5*	-0.269758313	0.000253938	0.008963481	0.997723735
Prediabetes	duct_epithelial_cell	*SRSF9*	-0.087856859	0.008120756	0.030300524	1
Prediabetes	duct_epithelial_cell	*RSPH4A*	-0.052072841	0.010418972	0.036003641	1
Prediabetes	duct_epithelial_cell	*SMIM8*	-0.202385421	0.003477246	0.017403948	1
Prediabetes	duct_epithelial_cell	*NEDD9*	-0.127419771	0.001030973	0.009336398	1
Prediabetes	duct_epithelial_cell	*CEP85L*	-0.08671947	0.012359217	0.04090932	1
Prediabetes	duct_epithelial_cell	*RNF8*	-0.151405584	0.002864374	0.015391043	1
Prediabetes	duct_epithelial_cell	*GLP1R*	-0.048447344	0.011822573	0.03954638	1
Prediabetes	duct_epithelial_cell	*FBXL4*	-0.217031287	0.000658973	0.008963481	1
Prediabetes	duct_epithelial_cell	*ASCC3*	-0.093229828	0.015418314	0.048231336	1
Prediabetes	duct_epithelial_cell	*C6orf62*	-0.170259197	0.000484421	0.008963481	1
Prediabetes	duct_epithelial_cell	*B3GAT2*	-0.354106693	0.000581964	0.008963481	1
Prediabetes	duct_epithelial_cell	*HBS1L*	-0.143145962	0.00101661	0.009336398	1
Prediabetes	duct_epithelial_cell	*PEX7*	-0.21485091	0.003532712	0.017534673	1
Prediabetes	duct_epithelial_cell	*PHACTR2*	-0.117421093	0.000925919	0.009057947	1
Prediabetes	duct_epithelial_cell	*TFEB*	-0.173281403	0.001192933	0.009719245	1
Prediabetes	duct_epithelial_cell	*DUSP22*	-0.250273745	0.004342217	0.019814833	1
Prediabetes	duct_epithelial_cell	*FBRSL1*	-0.248106112	0.000195644	0.008963481	0.768687046
Prediabetes	duct_epithelial_cell	*SEMA5A*	-0.099719233	0.002992631	0.015740359	1
Prediabetes	duct_epithelial_cell	*GHR*	-1.014259622	0.0005803	0.008963481	1
Prediabetes	duct_epithelial_cell	*BRD8*	-0.207830839	0.000646606	0.008963481	1
Prediabetes	duct_epithelial_cell	*NNT*	-0.092916216	0.000784445	0.008963481	1
Prediabetes	duct_epithelial_cell	*IK*	-0.138143581	0.000601177	0.008963481	1
Prediabetes	duct_epithelial_cell	*PDE8B*	-0.082596462	0.010721337	0.036757533	1
Prediabetes	duct_epithelial_cell	*GOLPH3*	-0.163401718	0.000392002	0.008963481	1
Prediabetes	duct_epithelial_cell	*RAD50*	-0.182629289	0.000447926	0.008963481	1
Prediabetes	duct_epithelial_cell	*SMAD5*	-0.110391943	0.001994551	0.012380078	1
Prediabetes	duct_epithelial_cell	*HMGXB3*	-0.120639871	0.002039034	0.012486647	1
Prediabetes	duct_epithelial_cell	*STC2*	-0.129480977	0.00322394	0.016688879	1
Prediabetes	duct_epithelial_cell	*ACTR8*	-0.307787274	0.000913432	0.009057947	1
Prediabetes	duct_epithelial_cell	*TIMMDC1*	-0.117125874	0.004136965	0.019327151	1
Prediabetes	duct_epithelial_cell	*RBP1*	-0.154546837	0.001248422	0.009849499	1
Prediabetes	duct_epithelial_cell	*RNF7*	-0.080372355	0.003542376	0.017534673	1
Prediabetes	duct_epithelial_cell	*WNT5A*	-0.087320811	0.016121831	0.049752675	1
Prediabetes	duct_epithelial_cell	*CBLB*	-0.141219456	0.003310076	0.016870817	1
Prediabetes	duct_epithelial_cell	*NCBP2*	-0.131144214	0.004823325	0.021317033	1
Prediabetes	duct_epithelial_cell	*ATP6V1A*	-0.168462236	0.000535618	0.008963481	1
Prediabetes	duct_epithelial_cell	*PODXL2*	-0.054768955	0.008629967	0.031424597	1
Prediabetes	duct_epithelial_cell	*PLSCR4*	-0.268129764	0.008375313	0.030753836	1
Prediabetes	duct_epithelial_cell	*COMMD2*	-0.196629601	0.000263706	0.008963481	1
Prediabetes	duct_epithelial_cell	*ARHGEF26*	-0.294436456	0.005084144	0.022048127	1
Prediabetes	duct_epithelial_cell	*PLCH1*	-0.280575543	0.00105043	0.009336398	1
Prediabetes	duct_epithelial_cell	*NKTR*	-0.100074748	0.000832859	0.008963481	1
Prediabetes	duct_epithelial_cell	*NEK4*	-0.186633014	0.00549884	0.023344611	1
Prediabetes	duct_epithelial_cell	*ACTR3*	-0.130137059	0.000448402	0.008963481	1
Prediabetes	duct_epithelial_cell	*POMC*	-0.108930966	0.002928534	0.015548931	1
Prediabetes	duct_epithelial_cell	*RTN4*	-0.12992592	0.000286964	0.008963481	1
Prediabetes	duct_epithelial_cell	*HTRA2*	-0.142161501	0.014541765	0.046039156	1
Prediabetes	duct_epithelial_cell	*WDR75*	-0.212354078	0.000672205	0.008963481	1
Prediabetes	duct_epithelial_cell	*OTX1*	-0.155963914	0.000200663	0.008963481	0.788404783
Prediabetes	duct_epithelial_cell	*SF3B1*	-0.119880018	0.000706886	0.008963481	1
Prediabetes	duct_epithelial_cell	*CNPPD1*	-0.078083175	0.007582956	0.028953774	1

Prediabetes	duct_epithelial_cell	*HDLBP*	-0.086972235	0.002384312	0.013695852	1
Prediabetes	duct_epithelial_cell	*CEBPZ*	-0.096346095	0.00144422	0.010411636	1
Prediabetes	duct_epithelial_cell	*WIPF1*	-0.18287194	0.002011457	0.012406615	1
Prediabetes	duct_epithelial_cell	*COX7A2L*	-0.094899996	0.001048316	0.009336398	1
Prediabetes	duct_epithelial_cell	*PNO1*	-0.112240749	0.011084714	0.037577086	1
Prediabetes	duct_epithelial_cell	*ARID3A*	-0.144939642	0.001594124	0.010911694	1
Prediabetes	duct_epithelial_cell	*ALMS1*	-0.112861521	0.001616946	0.011022922	1
Prediabetes	duct_epithelial_cell	*MARK1*	-0.13807993	0.00343633	0.017287247	1
Prediabetes	duct_epithelial_cell	*SCP2*	-0.124981087	0.000740503	0.008963481	1
Prediabetes	duct_epithelial_cell	*FAM20B*	-0.12803453	0.007817989	0.029507089	1
Prediabetes	duct_epithelial_cell	*WRAP73*	-0.304373543	0.001053248	0.009336398	1
Prediabetes	duct_epithelial_cell	*PARK7*	-0.087910162	0.001985305	0.012369325	1
Prediabetes	duct_epithelial_cell	*EDEM3*	-0.096950478	0.003547993	0.017534673	1
Prediabetes	duct_epithelial_cell	*FBXO6*	-0.250885965	0.013550783	0.043806482	1
Prediabetes	duct_epithelial_cell	*SMG7*	-0.16966424	0.001142719	0.00963464	1
Prediabetes	duct_epithelial_cell	*BCAS2*	-0.107558989	0.005022583	0.021950756	1
Prediabetes	duct_epithelial_cell	*PLEKHM2*	-0.119554036	0.00340418	0.017213674	1
Prediabetes	duct_epithelial_cell	*UAP1*	-0.119576022	0.000100104	0.008963481	0.393307251
Prediabetes	duct_epithelial_cell	*CTBS*	-0.071601187	0.004117633	0.019282694	1
Prediabetes	duct_epithelial_cell	*CD46*	-0.118236156	0.000688703	0.008963481	1
Prediabetes	duct_epithelial_cell	*CCDC181*	-0.061393609	0.013620451	0.043936578	1
Prediabetes	duct_epithelial_cell	*NSUN4*	-0.130128999	0.002037273	0.012486647	1
Prediabetes	duct_epithelial_cell	*TMED5*	-0.176022849	0.000507513	0.008963481	1
Prediabetes	duct_epithelial_cell	*DR1*	-0.120358487	0.00308209	0.01610824	1
Prediabetes	duct_epithelial_cell	*PTBP2*	-0.093341381	0.009149229	0.032620074	1
Prediabetes	duct_epithelial_cell	*NSL1*	-0.129039203	0.001986534	0.012369325	1
Prediabetes	duct_epithelial_cell	*ESYT2*	-0.125722257	0.007034123	0.027390555	1
Prediabetes	duct_epithelial_cell	*TTR*	-0.02676366	0.006306624	0.025388037	1
Prediabetes	duct_epithelial_cell	*SPCS2*	-0.115608782	0.000852545	0.008963481	1
Prediabetes	duct_epithelial_cell	*ELOVL4*	-0.078079708	0.010453606	0.036028262	1
Prediabetes	duct_epithelial_cell	*FILIP1*	-0.26639268	3.39E-05	0.008963481	0.133315594
Prediabetes	duct_epithelial_cell	*RNF146*	-0.120244883	0.002396768	0.013747304	1
Prediabetes	duct_epithelial_cell	*MED28*	-0.095654541	0.011308542	0.038171186	1
Prediabetes	duct_epithelial_cell	*ARMC2*	-0.072757969	0.002705713	0.014880018	1
Prediabetes	duct_epithelial_cell	*PKD2*	-0.250635749	0.000679972	0.008963481	1
Prediabetes	duct_epithelial_cell	*CCDC92*	-0.199547415	0.001323328	0.010174863	1
Prediabetes	duct_epithelial_cell	*RBM18*	-0.242813993	0.000388079	0.008963481	1
Prediabetes	duct_epithelial_cell	*DCAF4*	-0.076574054	0.008275733	0.030617094	1
Prediabetes	duct_epithelial_cell	*IFI27L2*	-0.082540793	0.004625128	0.020791908	1
Prediabetes	duct_epithelial_cell	*ZC2HC1C*	-0.161587115	0.000173404	0.008963481	0.681303087
Prediabetes	duct_epithelial_cell	*AFTPH*	-0.097004478	0.008002729	0.030059963	1
Prediabetes	duct_epithelial_cell	*EPCAM*	-0.128007344	0.002691328	0.014830613	1
Prediabetes	duct_epithelial_cell	*SLC17A5*	-0.185098432	3.87E-06	0.008963481	0.015204514
Prediabetes	duct_epithelial_cell	*HELLS*	-0.191029292	0.014239606	0.045368317	1
Prediabetes	duct_epithelial_cell	*RCL1*	-0.158181529	0.001215219	0.009784005	1
Prediabetes	duct_epithelial_cell	*CD274*	-0.28743252	0.002546017	0.014331379	1
Prediabetes	duct_epithelial_cell	*SLC10A7*	-0.08948795	0.003720799	0.01813774	1
Prediabetes	duct_epithelial_cell	*IQSEC3*	-0.060341458	0.012511135	0.041203899	1
Prediabetes	duct_epithelial_cell	*DNAJC15*	-0.125672476	0.001194807	0.009719245	1
Prediabetes	duct_epithelial_cell	*PROSER1*	-0.23513474	0.002653217	0.014744682	1
Prediabetes	duct_epithelial_cell	*WBP4*	-0.178617954	0.000464167	0.008963481	1
Prediabetes	duct_epithelial_cell	*KBTBD7*	-0.146397585	0.002750025	0.015090572	1
Prediabetes	duct_epithelial_cell	*FAM53C*	-0.094417924	0.010344159	0.035853926	1
Prediabetes	duct_epithelial_cell	*SIL1*	-0.060613187	0.014021417	0.044898247	1
Prediabetes	duct_epithelial_cell	*UTP20*	-0.10724264	0.00609786	0.024776105	1
Prediabetes	duct_epithelial_cell	*PPP3CC*	-0.16026733	0.000855341	0.008963481	1
Prediabetes	duct_epithelial_cell	*EPHX2*	-0.219092994	0.000619483	0.008963481	1
Prediabetes	duct_epithelial_cell	*IAPP*	-0.026628457	0.008337048	0.030749039	1
Prediabetes	duct_epithelial_cell	*ZNF211*	-0.045002347	0.010448593	0.036028262	1
Prediabetes	duct_epithelial_cell	*POLK*	-0.092868652	0.011079887	0.037577086	1
Prediabetes	duct_epithelial_cell	*UBL3*	-0.098355002	0.001372276	0.010271223	1
Prediabetes	duct_epithelial_cell	*NAA60*	-0.220047845	0.000192148	0.008963481	0.754949697
Prediabetes	duct_epithelial_cell	*KLHL7*	-0.2467194	0.002764132	0.01511602	1
Prediabetes	duct_epithelial_cell	*CBX3*	-0.188284312	0.000451995	0.008963481	1

Prediabetes	duct_epithelial_cell	*INHBA*	-0.062222592	0.006734108	0.026644824	1
Prediabetes	duct_epithelial_cell	*BHLHE41*	-0.184640755	0.000492884	0.008963481	1
Prediabetes	duct_epithelial_cell	*USP45*	-0.17532423	0.001541362	0.010718604	1
Prediabetes	duct_epithelial_cell	*ARFGEF2*	-0.149032086	0.001168334	0.009719245	1
Prediabetes	duct_epithelial_cell	*ZNFX1*	-0.274161595	0.001466662	0.01048303	1
Prediabetes	duct_epithelial_cell	*BCAS4*	-0.140446551	0.010583647	0.036348905	1
Prediabetes	duct_epithelial_cell	*PEPD*	-0.129463033	0.002050211	0.012527651	1
Prediabetes	duct_epithelial_cell	*SNRNP27*	-0.094273429	0.006093513	0.024776105	1
Prediabetes	duct_epithelial_cell	*USP22*	-0.129182657	0.000743592	0.008963481	1
Prediabetes	duct_epithelial_cell	*MRS2*	-0.249102474	0.000477541	0.008963481	1
Prediabetes	duct_epithelial_cell	*SNRPC*	-0.162046574	0.000199209	0.008963481	0.78269333
Prediabetes	duct_epithelial_cell	*SERPINB6*	-0.115965993	0.001028465	0.009336398	1
Prediabetes	duct_epithelial_cell	*GLO1*	-0.165354504	0.000783274	0.008963481	1
Prediabetes	duct_epithelial_cell	*ATXN1*	-0.203531134	0.000296402	0.008963481	1
Prediabetes	duct_epithelial_cell	*LRRFIP1*	-0.12550133	0.002079562	0.012608949	1
Prediabetes	duct_epithelial_cell	*SH3TC1*	-0.166447795	0.00198967	0.012369325	1
Prediabetes	duct_epithelial_cell	*MT1G*	-0.022136699	0.011323344	0.038188343	1
Prediabetes	duct_epithelial_cell	*TM9SF2*	-0.100439392	0.012796104	0.041896577	1
Prediabetes	duct_epithelial_cell	*MRPS7*	-0.119489427	0.003599122	0.017765011	1
Prediabetes	duct_epithelial_cell	*PPDPF*	-0.144802707	0.000526025	0.008963481	1
Prediabetes	duct_epithelial_cell	*CHCHD5*	-0.110970536	0.003045754	0.015976995	1
Prediabetes	duct_epithelial_cell	*NAPB*	-0.070813719	0.00597835	0.024570017	1
Prediabetes	duct_epithelial_cell	*RBCK1*	-0.083124111	0.015466624	0.048305536	1
Prediabetes	duct_epithelial_cell	*TUBGCP3*	-0.193240371	0.000917366	0.009057947	1
Prediabetes	duct_epithelial_cell	*MCF2L*	-0.095117888	0.000954055	0.009132479	1
Prediabetes	duct_epithelial_cell	*NR1D1*	-0.087574846	0.006005665	0.024630748	1
Prediabetes	duct_epithelial_cell	*PRMT1*	-0.08865452	0.0024385	0.013905466	1
Prediabetes	duct_epithelial_cell	*BECN1*	-0.128977006	0.005884928	0.024287691	1
Prediabetes	duct_epithelial_cell	*ZNF384*	-0.294094964	0.000389421	0.008963481	1
Prediabetes	duct_epithelial_cell	*HSPA2*	-0.096833387	0.00935706	0.03309081	1
Prediabetes	duct_epithelial_cell	*ABHD8*	-0.093228832	0.009933762	0.034693111	1
Prediabetes	duct_epithelial_cell	*YEATS4*	-0.105904353	0.007648475	0.029024613	1
Prediabetes	duct_epithelial_cell	*IDUA*	-0.290135448	0.000588465	0.008963481	1
Prediabetes	duct_epithelial_cell	*UBR4*	-0.138896284	0.001402985	0.010284193	1
Prediabetes	duct_epithelial_cell	*WDR24*	-0.472765615	0.000531211	0.008963481	1
Prediabetes	duct_epithelial_cell	*KDM4B*	-0.18488678	0.007201356	0.027848551	1
Prediabetes	duct_epithelial_cell	*METTL25*	-0.30310594	0.001025825	0.009336398	1
Prediabetes	duct_epithelial_cell	*POR*	-0.206938688	0.001521971	0.010704482	1
Prediabetes	duct_epithelial_cell	*RBM48*	-0.257488573	0.000320137	0.008963481	1
Prediabetes	duct_epithelial_cell	*A4GALT*	0.042714311	0.003671633	0.017966065	1
Prediabetes	duct_epithelial_cell	*NAA38*	-0.102924548	0.00067299	0.008963481	1
Prediabetes	duct_epithelial_cell	*PRKRIP1*	-0.159710061	0.001753254	0.011442746	1
Prediabetes	duct_epithelial_cell	*VGF*	-0.037232182	0.005486065	0.02332765	1
Prediabetes	duct_epithelial_cell	*MTX2*	-0.183102013	0.000517805	0.008963481	1
Prediabetes	duct_epithelial_cell	*PSMG2*	-0.127688187	0.002272092	0.013326143	1
Prediabetes	duct_epithelial_cell	*TWSG1*	-0.114004901	0.009625142	0.033856026	1
Prediabetes	duct_epithelial_cell	*TMOD2*	-0.12288885	0.002489217	0.014112749	1
Prediabetes	duct_epithelial_cell	*ELL3*	-0.082245558	0.00828732	0.03063112	1
Prediabetes	duct_epithelial_cell	*SUMF2*	-0.120628635	0.005451489	0.023230913	1
Prediabetes	duct_epithelial_cell	*DCTD*	-0.134059596	0.011023952	0.037468087	1
Prediabetes	duct_epithelial_cell	*MTUS1*	-0.096895839	0.008069381	0.030194855	1
Prediabetes	duct_epithelial_cell	*HEATR5A*	-0.219317982	0.00339527	0.017190742	1
Prediabetes	duct_epithelial_cell	*MIS18BP1*	-0.067613258	0.003064682	0.016054848	1
Prediabetes	duct_epithelial_cell	*RNASE1*	-0.081754656	0.005502944	0.023344611	1
Prediabetes	duct_epithelial_cell	*TEP1*	-0.122495008	0.003729525	0.018157748	1
Prediabetes	duct_epithelial_cell	*REEP5*	-0.102923082	0.000959773	0.009147222	1
Prediabetes	duct_epithelial_cell	*MAU2*	-0.112773158	0.004236888	0.019551648	1
Prediabetes	duct_epithelial_cell	*SHC2*	-0.226686607	0.000624333	0.008963481	1
Prediabetes	duct_epithelial_cell	*CBFA2T3*	-0.089366442	0.010143777	0.035301061	1
Prediabetes	duct_epithelial_cell	*PHF10*	-0.1920159	0.001819057	0.011681295	1
Prediabetes	duct_epithelial_cell	*ECSIT*	-0.135040705	0.005603603	0.023493587	1
Prediabetes	duct_epithelial_cell	*CDC16*	-0.137968737	0.002980367	0.015696866	1
Prediabetes	duct_epithelial_cell	*DPP6*	-0.046239447	0.01069353	0.036694217	1
Prediabetes	duct_epithelial_cell	*TULP4*	-0.149023921	9.82E-05	0.008963481	0.385941856

Prediabetes	duct_epithelial_cell	*NDUFA10*	-0.097902729	0.001339897	0.010242129	1
Prediabetes	duct_epithelial_cell	*UBAC1*	-0.164900678	0.000407138	0.008963481	1
Prediabetes	duct_epithelial_cell	*ATXN10*	-0.130084444	0.001368768	0.010271223	1
Prediabetes	duct_epithelial_cell	*PAK4*	-0.264187352	0.001011945	0.009336398	1
Prediabetes	duct_epithelial_cell	*ADRM1*	-0.162040327	0.000419224	0.008963481	1
Prediabetes	duct_epithelial_cell	*UCK1*	-0.225727602	0.000365321	0.008963481	1
Prediabetes	duct_epithelial_cell	*MAP3K10*	-0.196908053	0.000198603	0.008963481	0.780311971
Prediabetes	duct_epithelial_cell	*DNMT1*	-0.209815417	0.001528433	0.010707166	1
Prediabetes	duct_epithelial_cell	*HSD17B3*	-0.946365795	0.00267224	0.014746113	1
Prediabetes	duct_epithelial_cell	*SYNE1*	-0.19621394	0.000556383	0.008963481	1
Prediabetes	duct_epithelial_cell	*PDLIM4*	-0.11287976	0.001470673	0.01048303	1
Prediabetes	duct_epithelial_cell	*MGAT1*	-0.144171763	0.000721303	0.008963481	1
Prediabetes	duct_epithelial_cell	*DIAPH1*	-0.137347286	0.013795947	0.044357017	1
Prediabetes	duct_epithelial_cell	*PPFIA1*	-0.141004895	0.008374299	0.030753836	1
Prediabetes	duct_epithelial_cell	*TRAF7*	-0.113576685	0.012682412	0.041663208	1
Prediabetes	duct_epithelial_cell	*CHD1L*	-0.103753551	0.004383035	0.019977896	1
Prediabetes	duct_epithelial_cell	*PIAS3*	-0.114208649	0.003317779	0.016885431	1
Prediabetes	duct_epithelial_cell	*ZNF304*	-0.351583002	0.00097082	0.00916911	1
Prediabetes	duct_epithelial_cell	*GCH1*	-0.091783632	0.005608421	0.023493587	1
Prediabetes	duct_epithelial_cell	*ENOSF1*	-0.227894872	0.000737412	0.008963481	1
Prediabetes	duct_epithelial_cell	*IMMT*	-0.111869616	0.000787538	0.008963481	1
Prediabetes	duct_epithelial_cell	*TMEM128*	-0.154818237	0.00122696	0.009805258	1
Prediabetes	duct_epithelial_cell	*SEC61G*	-0.075844807	0.001642054	0.011047314	1
Prediabetes	duct_epithelial_cell	*FIGNL1*	-0.145058724	0.00480373	0.021254343	1
Prediabetes	duct_epithelial_cell	*EIF5A*	-0.075638628	0.007442076	0.028573059	1
Prediabetes	duct_epithelial_cell	*ANKEF1*	-0.318085255	0.000373891	0.008963481	1
Prediabetes	duct_epithelial_cell	*BTBD3*	-0.094600875	0.002606232	0.014586734	1
Prediabetes	duct_epithelial_cell	*SERINC3*	-0.0679166	0.012423876	0.041069201	1
Prediabetes	duct_epithelial_cell	*TRPC4*	-0.178246749	0.008389602	0.030777541	1
Prediabetes	duct_epithelial_cell	*RFC3*	-0.136773454	0.005154454	0.022303801	1
Prediabetes	duct_epithelial_cell	*CNDP2*	-0.191974445	0.00624151	0.02517751	1
Prediabetes	duct_epithelial_cell	*SLC2A11*	-0.115113094	0.004244733	0.019551648	1
Prediabetes	duct_epithelial_cell	*FAM83F*	-0.262707264	0.000464301	0.008963481	1
Prediabetes	duct_epithelial_cell	*AMPD3*	-0.06346267	0.005880761	0.024287691	1
Prediabetes	duct_epithelial_cell	*HSD17B4*	-0.190597673	0.001373348	0.010271223	1
Prediabetes	duct_epithelial_cell	*COX16*	-0.116552112	0.001117119	0.009564575	1
Prediabetes	duct_epithelial_cell	*ELP3*	-0.166332806	0.001987419	0.012369325	1
Prediabetes	duct_epithelial_cell	*MRPS36*	-0.08611608	0.00578419	0.024023343	1
Prediabetes	duct_epithelial_cell	*THUMPD3*	-0.102703325	0.00698305	0.027354339	1
Prediabetes	duct_epithelial_cell	*SORT1*	-0.229934628	0.000838807	0.008963481	1
Prediabetes	duct_epithelial_cell	*CEPT1*	-0.153273453	0.0014728	0.01048303	1
Prediabetes	duct_epithelial_cell	*NAPG*	-0.213030694	0.001001065	0.009336398	1
Prediabetes	duct_epithelial_cell	*SPIRE1*	-0.127863303	0.001160705	0.009685284	1
Prediabetes	duct_epithelial_cell	*FKBP11*	-0.045759637	0.015791623	0.049125325	1
Prediabetes	duct_epithelial_cell	*KIDINS220*	-0.160671919	0.000495376	0.008963481	1
Prediabetes	duct_epithelial_cell	*LPIN1*	-0.067115746	0.003529514	0.017534673	1
Prediabetes	duct_epithelial_cell	*IAH1*	-0.149518099	0.001747589	0.011424754	1
Prediabetes	duct_epithelial_cell	*KLRC1*	-2.519034006	0.012458399	0.041099117	1
Prediabetes	duct_epithelial_cell	*PRPF38A*	-0.198391661	0.003720401	0.01813774	1
Prediabetes	duct_epithelial_cell	*TIMM10*	-0.142239198	0.001734479	0.011376911	1
Prediabetes	duct_epithelial_cell	*TMEM165*	-0.172927456	0.000543098	0.008963481	1
Prediabetes	duct_epithelial_cell	*TMED7*	-0.130622728	0.000639974	0.008963481	1
Prediabetes	duct_epithelial_cell	*NREP*	-0.297461669	0.000918949	0.009057947	1
Prediabetes	duct_epithelial_cell	*WDR36*	-0.308290061	0.000754225	0.008963481	1
Prediabetes	duct_epithelial_cell	*OSTF1*	-0.192212289	0.002197431	0.012987238	1
Prediabetes	duct_epithelial_cell	*MSI1*	-0.086147691	0.00980452	0.034333298	1
Prediabetes	duct_epithelial_cell	*PNPLA8*	-0.090774193	0.000835417	0.008963481	1
Prediabetes	duct_epithelial_cell	*MTO1*	-0.242091058	0.000421409	0.008963481	1
Prediabetes	duct_epithelial_cell	*LCA5*	-0.287859243	0.000298641	0.008963481	1
Prediabetes	duct_epithelial_cell	*LMO2*	-0.100333563	0.005064372	0.022038603	1
Prediabetes	duct_epithelial_cell	*TFCP2*	-0.244216691	0.000457235	0.008963481	1
Prediabetes	duct_epithelial_cell	*PKIB*	-0.053316925	0.008250103	0.030579862	1
Prediabetes	duct_epithelial_cell	*GNS*	-0.168843916	0.000515597	0.008963481	1
Prediabetes	duct_epithelial_cell	*MPHOSPH6*	-0.169520192	0.007653219	0.029024613	1

Prediabetes	duct_epithelial_cell	*NTPCR*	-0.111611056	0.008906456	0.031928346	1
Prediabetes	duct_epithelial_cell	*GLUL*	-0.175995498	0.001424925	0.010342775	1
Prediabetes	duct_epithelial_cell	*USP37*	-0.115725068	0.005736309	0.023925645	1
Prediabetes	duct_epithelial_cell	*ARMC9*	-0.264403627	0.000681585	0.008963481	1
Prediabetes	duct_epithelial_cell	*COX5B*	-0.108604305	0.000779692	0.008963481	1
Prediabetes	duct_epithelial_cell	*REV1*	-0.098851544	6.54E-05	0.008963481	0.257104008
Prediabetes	duct_epithelial_cell	*TSGA10*	-0.055336696	5.49E-05	0.008963481	0.215687485
Prediabetes	duct_epithelial_cell	*TGFBRAP1*	-0.105225866	0.003253705	0.016734297	1
Prediabetes	duct_epithelial_cell	*MRPS9*	-0.168158819	0.000337709	0.008963481	1
Prediabetes	duct_epithelial_cell	*ANKRD36*	-0.360400764	0.001496041	0.010590892	1
Prediabetes	duct_epithelial_cell	*DRAM1*	-0.288015862	0.000371721	0.008963481	1
Prediabetes	duct_epithelial_cell	*VPS36*	-0.272479498	0.00010542	0.008963481	0.414195525
Prediabetes	duct_epithelial_cell	*RNASEH2B*	-0.122899881	0.013006794	0.042515553	1
Prediabetes	duct_epithelial_cell	*SUCLA2*	-0.143814672	0.0010983	0.00956008	1
Prediabetes	duct_epithelial_cell	*KDELR2*	-0.089811616	0.00147133	0.01048303	1
Prediabetes	duct_epithelial_cell	*ALPK3*	-0.073490838	0.004672466	0.02090362	1
Prediabetes	duct_epithelial_cell	*CIB2*	-0.049087877	0.003984021	0.018886376	1
Prediabetes	duct_epithelial_cell	*TEX2*	-0.193549235	0.007385182	0.028419569	1
Prediabetes	duct_epithelial_cell	*MRPL47*	-0.129907848	0.00136096	0.010271223	1
Prediabetes	duct_epithelial_cell	*SMPD4*	-0.20104017	0.000602906	0.008963481	1
Prediabetes	duct_epithelial_cell	*CCDC115*	-0.120081894	0.005184733	0.022377892	1
Prediabetes	duct_epithelial_cell	*NIPSNAP3A*	-0.129616948	0.001999885	0.012393611	1
Prediabetes	duct_epithelial_cell	*CDK9*	-0.245990833	0.000294426	0.008963481	1
Prediabetes	duct_epithelial_cell	*TXN*	-0.141158932	0.000756596	0.008963481	1
Prediabetes	duct_epithelial_cell	*TOR1A*	-0.158761342	0.003762956	0.018230151	1
Prediabetes	duct_epithelial_cell	*STX17*	-0.09893476	0.008736081	0.031543827	1
Prediabetes	duct_epithelial_cell	*ATP6V1G1*	-0.058864788	0.011397686	0.038406096	1
Prediabetes	duct_epithelial_cell	*ARPC5L*	-0.133397864	0.00071638	0.008963481	1
Prediabetes	duct_epithelial_cell	*APTX*	-0.160011829	0.001348162	0.010256616	1
Prediabetes	duct_epithelial_cell	*TMEM8B*	-0.199748107	0.001212594	0.009782919	1
Prediabetes	duct_epithelial_cell	*PPIL1*	-0.148516613	0.002057412	0.012532667	1
Prediabetes	duct_epithelial_cell	*KIAA0319*	-0.118563161	0.01470291	0.046474444	1
Prediabetes	duct_epithelial_cell	*RIPK1*	-0.193615976	0.001787304	0.011538034	1
Prediabetes	duct_epithelial_cell	*MTCH1*	-0.12612543	0.000803092	0.008963481	1
Prediabetes	duct_epithelial_cell	*VARS2*	-0.026393901	0.01305541	0.042604802	1
Prediabetes	duct_epithelial_cell	*ANKRD42*	-0.129181347	0.002167093	0.012920349	1
Prediabetes	duct_epithelial_cell	*CREBZF*	-0.18178001	0.000712659	0.008963481	1
Prediabetes	duct_epithelial_cell	*NARS2*	-0.265102806	0.008903916	0.031928346	1
Prediabetes	duct_epithelial_cell	*GGH*	-0.058017474	0.005010184	0.02192095	1
Prediabetes	duct_epithelial_cell	*FDX1*	-0.139764556	0.002017659	0.012425367	1
Prediabetes	duct_epithelial_cell	*C11orf1*	-0.079753966	0.010946098	0.037397582	1
Prediabetes	duct_epithelial_cell	*ALKBH8*	-0.462941761	0.00036501	0.008963481	1
Prediabetes	duct_epithelial_cell	*TUBGCP4*	-0.153861066	0.000585026	0.008963481	1
Prediabetes	duct_epithelial_cell	*ADAM10*	-0.186685308	0.00094626	0.009132479	1
Prediabetes	duct_epithelial_cell	*ZNF280D*	-0.119627058	0.001534267	0.010707166	1
Prediabetes	duct_epithelial_cell	*RSL24D1*	-0.09296825	0.002272465	0.013326143	1
Prediabetes	duct_epithelial_cell	*SPTBN5*	0.069400509	0.008138526	0.030336239	1
Prediabetes	duct_epithelial_cell	*RTCA*	-0.159426119	0.00044396	0.008963481	1
Prediabetes	duct_epithelial_cell	*ACTR2*	-0.139826548	0.000105159	0.008963481	0.413169196
Prediabetes	duct_epithelial_cell	*ATRAID*	-0.092753994	0.002944703	0.015613682	1
Prediabetes	duct_epithelial_cell	*LRPPRC*	-0.145170784	0.000256043	0.008963481	1
Prediabetes	duct_epithelial_cell	*NCOA4*	-0.137741042	0.001650168	0.011064011	1
Prediabetes	duct_epithelial_cell	*ASCC1*	-0.168158637	0.008362578	0.030753836	1
Prediabetes	duct_epithelial_cell	*RPS24*	-0.067231703	0.001175238	0.009719245	1
Prediabetes	duct_epithelial_cell	*USP8*	-0.119886812	0.001189828	0.009719245	1
Prediabetes	duct_epithelial_cell	*SPPL2A*	-0.1648329	0.000302668	0.008963481	1
Prediabetes	duct_epithelial_cell	*RAP1GDS1*	-0.214295626	0.001001728	0.009336398	1
Prediabetes	duct_epithelial_cell	*G3BP2*	-0.109028753	0.001227871	0.009805258	1
Prediabetes	duct_epithelial_cell	*SCARB2*	-0.066039481	0.00762018	0.028976426	1
Prediabetes	duct_epithelial_cell	*CNOT6L*	-0.129539463	0.002072397	0.012584925	1
Prediabetes	duct_epithelial_cell	*USO1*	-0.125622942	0.00201066	0.012406615	1
Prediabetes	duct_epithelial_cell	*ZCRB1*	-0.116338748	0.000837853	0.008963481	1
Prediabetes	duct_epithelial_cell	*PEX5*	-0.126681235	0.001230337	0.009805258	1
Prediabetes	duct_epithelial_cell	*SNRPF*	-0.074260064	0.010357395	0.035853926	1

Prediabetes	duct_epithelial_cell	*GLTP*	-0.211007215	0.000436422	0.008963481	1
Prediabetes	duct_epithelial_cell	*MTMR6*	-0.110851495	0.002661964	0.014746113	1
Prediabetes	duct_epithelial_cell	*KANSL2*	-0.309491233	0.000109234	0.008963481	0.429179406
Prediabetes	duct_epithelial_cell	*WDFY2*	-0.137974265	0.003623943	0.017842699	1
Prediabetes	duct_epithelial_cell	*TMX1*	-0.150377878	0.00072821	0.008963481	1
Prediabetes	duct_epithelial_cell	*NIPA2*	-0.206677394	0.000416407	0.008963481	1
Prediabetes	duct_epithelial_cell	*SERF2*	-0.077048268	0.000579687	0.008963481	1
Prediabetes	duct_epithelial_cell	*BNIP2*	-0.196080806	0.000482247	0.008963481	1
Prediabetes	duct_epithelial_cell	*GTF2A2*	-0.129986746	0.000426728	0.008963481	1
Prediabetes	duct_epithelial_cell	*SRP14*	-0.097196157	0.000627007	0.008963481	1
Prediabetes	duct_epithelial_cell	*TLE3*	-0.184647696	0.000637982	0.008963481	1
Prediabetes	duct_epithelial_cell	*SCAPER*	-0.081061745	0.002458614	0.013979585	1
Prediabetes	duct_epithelial_cell	*TSPAN3*	-0.175546807	0.000101853	0.008963481	0.400180227
Prediabetes	duct_epithelial_cell	*DNAJA4*	-0.049405829	0.013557822	0.043806482	1
Prediabetes	duct_epithelial_cell	*IGF1R*	-0.079427489	0.01048557	0.036075136	1
Prediabetes	duct_epithelial_cell	*CELF6*	-0.090086065	0.008613512	0.031393773	1
Prediabetes	duct_epithelial_cell	*POLG*	-0.291401793	0.000701664	0.008963481	1
Prediabetes	duct_epithelial_cell	*PMM2*	-0.189301452	0.000234422	0.008963481	0.921043976
Prediabetes	duct_epithelial_cell	*UQCRC2*	-0.210070113	3.10E-05	0.008963481	0.121653861
Prediabetes	duct_epithelial_cell	*ARHGAP17*	-0.240732424	0.00046718	0.008963481	1
Prediabetes	duct_epithelial_cell	*KIFC3*	-0.123346633	0.014053429	0.044927521	1
Prediabetes	duct_epithelial_cell	*NUDT7*	-0.112265132	0.006892725	0.027108625	1
Prediabetes	duct_epithelial_cell	*GCSH*	-0.164423071	0.000430816	0.008963481	1
Prediabetes	duct_epithelial_cell	*MAP1LC3B*	-0.114345137	0.000901125	0.009057947	1
Prediabetes	duct_epithelial_cell	*RPS2*	-0.05612199	0.00322193	0.016688879	1
Prediabetes	duct_epithelial_cell	*PDPK1*	-0.106198924	0.01613258	0.049752675	1
Prediabetes	duct_epithelial_cell	*DEF8*	-0.119442539	0.001082456	0.009514476	1
Prediabetes	duct_epithelial_cell	*NPEPPS*	-0.148193654	0.002412653	0.013798128	1
Prediabetes	duct_epithelial_cell	*PTRH2*	-0.227946226	0.000805243	0.008963481	1
Prediabetes	duct_epithelial_cell	*GNAL*	-0.079684266	0.006342491	0.025438743	1
Prediabetes	duct_epithelial_cell	*RPRD1A*	-0.158051661	0.000798318	0.008963481	1
Prediabetes	duct_epithelial_cell	*SAT2*	-0.167974119	0.000414794	0.008963481	1
Prediabetes	duct_epithelial_cell	*ARHGDIA*	-0.119849738	0.000108516	0.008963481	0.426360458
Prediabetes	duct_epithelial_cell	*TTYH2*	-0.74231099	0.000747452	0.008963481	1
Prediabetes	duct_epithelial_cell	*NARF*	-0.127255107	0.000954448	0.009132479	1
Prediabetes	duct_epithelial_cell	*WDR45B*	-0.082203435	0.005601787	0.023493587	1
Prediabetes	duct_epithelial_cell	*PMAIP1*	-0.234069167	0.000545651	0.008963481	1
Prediabetes	duct_epithelial_cell	*TXNL4A*	-0.077223005	0.009549759	0.033620969	1
Prediabetes	duct_epithelial_cell	*SH3GL1*	-0.096164714	0.015667478	0.048855176	1
Prediabetes	duct_epithelial_cell	*SIRT3*	-0.274788895	0.006021091	0.024668265	1
Prediabetes	duct_epithelial_cell	*COL6A1*	-0.062592997	0.004389817	0.019985619	1
Prediabetes	duct_epithelial_cell	*AKT1*	-0.108826516	0.006071159	0.024776105	1
Prediabetes	duct_epithelial_cell	*RERE*	-0.194743587	0.000425484	0.008963481	1
Prediabetes	duct_epithelial_cell	*ZNF593*	-0.187042777	0.000272561	0.008963481	1
Prediabetes	duct_epithelial_cell	*CELA3A*	-0.021126512	0.007667907	0.029052273	1
Prediabetes	duct_epithelial_cell	*PRKACB*	-0.142054552	0.000861872	0.00898221	1
Prediabetes	duct_epithelial_cell	*PIGK*	-0.162655812	0.000921004	0.009057947	1
Prediabetes	duct_epithelial_cell	*RPS8*	-0.063507356	0.001030226	0.009336398	1
Prediabetes	duct_epithelial_cell	*ALDH9A1*	-0.179408	0.00098765	0.009283441	1
Prediabetes	duct_epithelial_cell	*MGST3*	-0.139450995	0.000536555	0.008963481	1
Prediabetes	duct_epithelial_cell	*LYPLAL1*	-0.172263257	0.003135698	0.016318092	1
Prediabetes	duct_epithelial_cell	*SNX27*	-0.165524071	0.000336547	0.008963481	1
Prediabetes	duct_epithelial_cell	*CTSK*	-0.129729817	0.00916723	0.03265462	1
Prediabetes	duct_epithelial_cell	*TAF1A*	-0.253987928	0.009241052	0.032821135	1
Prediabetes	duct_epithelial_cell	*KCNN3*	-0.325682697	0.00216364	0.012919364	1
Prediabetes	duct_epithelial_cell	*SRP9*	-0.129534998	0.000184375	0.008963481	0.724410987
Prediabetes	duct_epithelial_cell	*ARL8A*	-0.145182598	0.001770309	0.011496768	1
Prediabetes	duct_epithelial_cell	*GALM*	-0.388820836	0.007045832	0.027408984	1
Prediabetes	duct_epithelial_cell	*EML4*	-0.170631341	0.001095252	0.00956008	1
Prediabetes	duct_epithelial_cell	*WDPCP*	-0.222308166	0.003083074	0.01610824	1
Prediabetes	duct_epithelial_cell	*CIAO1*	-0.116586745	0.008158468	0.030354755	1
Prediabetes	duct_epithelial_cell	*TEX261*	-0.248262295	0.001121215	0.009564575	1
Prediabetes	duct_epithelial_cell	*LIPT1*	-0.239909816	0.000186944	0.008963481	0.734501109
Prediabetes	duct_epithelial_cell	*FAM171B*	-0.074692951	0.003528474	0.017534673	1

Prediabetes	duct_epithelial_cell	*METTL21A*	-0.154860802	0.007197914	0.027848551	1
Prediabetes	duct_epithelial_cell	*HES6*	-0.143148077	0.001392393	0.010271223	1
Prediabetes	duct_epithelial_cell	*RAB5A*	-0.182355308	3.81E-05	0.008963481	0.149882245
Prediabetes	duct_epithelial_cell	*UBA3*	-0.149155146	0.001532543	0.010707166	1
Prediabetes	duct_epithelial_cell	*ARL6IP5*	-0.16750958	0.000409312	0.008963481	1
Prediabetes	duct_epithelial_cell	*TAGLN3*	-0.032748112	0.013571003	0.043813042	1
Prediabetes	duct_epithelial_cell	*EIF2A*	-0.090771106	0.003446252	0.017314992	1
Prediabetes	duct_epithelial_cell	*TRPC1*	-0.113198538	0.004559141	0.020613192	1
Prediabetes	duct_epithelial_cell	*PYURF*	-0.090732767	0.002286299	0.013387283	1
Prediabetes	duct_epithelial_cell	*TIFA*	-0.155589208	0.003683872	0.018002406	1
Prediabetes	duct_epithelial_cell	*SSBP2*	-0.126840682	0.001713762	0.011297601	1
Prediabetes	duct_epithelial_cell	*GTF2H2*	-0.283294475	0.000375491	0.008963481	1
Prediabetes	duct_epithelial_cell	*BTF3*	-0.080968435	0.002329872	0.013496739	1
Prediabetes	duct_epithelial_cell	*COMMD10*	-0.306404129	0.000831504	0.008963481	1
Prediabetes	duct_epithelial_cell	*ATG12*	-0.146026038	0.000322557	0.008963481	1
Prediabetes	duct_epithelial_cell	*PCYOX1L*	-0.273354857	0.001082456	0.009514476	1
Prediabetes	duct_epithelial_cell	*TBC1D7*	-0.321830108	0.00050891	0.008963481	1
Prediabetes	duct_epithelial_cell	*ZMAT2*	-0.178456727	0.000553373	0.008963481	1
Prediabetes	duct_epithelial_cell	*SLC17A4*	0.056017371	0.010315937	0.035836244	1
Prediabetes	duct_epithelial_cell	*TRIM7*	-0.180386043	3.39E-05	0.008963481	0.133280029
Prediabetes	duct_epithelial_cell	*RPL7L1*	-0.105350859	0.001038685	0.009336398	1
Prediabetes	duct_epithelial_cell	*IRAK1BP1*	-0.193707439	0.000776559	0.008963481	1
Prediabetes	duct_epithelial_cell	*FAXC*	-0.149368289	0.007625763	0.028976426	1
Prediabetes	duct_epithelial_cell	*DYNLT1*	-0.113630004	0.00506512	0.022038603	1
Prediabetes	duct_epithelial_cell	*GIGYF1*	-0.147092628	0.006315443	0.02539752	1
Prediabetes	duct_epithelial_cell	*TMEM209*	-0.098666868	0.003272818	0.016769199	1
Prediabetes	duct_epithelial_cell	*CCDC25*	-0.130302257	0.003100228	0.016176354	1
Prediabetes	duct_epithelial_cell	*LACTB2*	-0.181925343	0.001159285	0.009685284	1
Prediabetes	duct_epithelial_cell	*TERF1*	-0.17172447	0.00047312	0.008963481	1
Prediabetes	duct_epithelial_cell	*LRP12*	-0.10847017	0.005763637	0.023988697	1
Prediabetes	duct_epithelial_cell	*ZNF7*	-0.270953351	0.000391569	0.008963481	1
Prediabetes	duct_epithelial_cell	*AK3*	-0.171320279	0.0003998	0.008963481	1
Prediabetes	duct_epithelial_cell	*C9orf72*	-0.142808306	0.000313285	0.008963481	1
Prediabetes	duct_epithelial_cell	*FBXO10*	-0.087693237	0.012748265	0.041783516	1
Prediabetes	duct_epithelial_cell	*ZNF462*	-0.074457408	0.005644776	0.023593961	1
Prediabetes	duct_epithelial_cell	*SURF6*	-0.214690984	0.000452619	0.008963481	1
Prediabetes	duct_epithelial_cell	*GPR107*	-0.162547162	0.002617285	0.014627755	1
Prediabetes	duct_epithelial_cell	*GLUD1*	-0.163881823	0.000485686	0.008963481	1
Prediabetes	duct_epithelial_cell	*DNAJB12*	-0.182836181	0.000633833	0.008963481	1
Prediabetes	duct_epithelial_cell	*PTPRJ*	-0.100979401	0.004605872	0.02077666	1
Prediabetes	duct_epithelial_cell	*CAPN5*	0.029597336	0.005579115	0.023475689	1
Prediabetes	duct_epithelial_cell	*RPS3*	-0.060998165	0.001522984	0.010704482	1
Prediabetes	duct_epithelial_cell	*AASDHPPT*	-0.144472043	0.000852528	0.008963481	1
Prediabetes	duct_epithelial_cell	*DUSP15*	-0.29415639	3.13E-05	0.008963481	0.123054651
Prediabetes	duct_epithelial_cell	*YTHDF1*	-0.203834667	0.000445791	0.008963481	1
Prediabetes	duct_epithelial_cell	*TMEM218*	-0.249010295	0.000710159	0.008963481	1
Prediabetes	duct_epithelial_cell	*MIA2*	-0.087825044	0.007021561	0.027368761	1
Prediabetes	duct_epithelial_cell	*DLG5*	-0.131205561	0.00138142	0.010271223	1
Prediabetes	duct_epithelial_cell	*SLC2A13*	-0.081351214	0.000541399	0.008963481	1
Prediabetes	duct_epithelial_cell	*MAGI1*	-0.089013954	0.014871882	0.046782724	1
Prediabetes	duct_epithelial_cell	*CSNK1G3*	-0.106838798	0.001369922	0.010271223	1
Prediabetes	duct_epithelial_cell	*AKAP6*	-0.139383094	0.00846561	0.030969628	1
Prediabetes	duct_epithelial_cell	*TMEM18*	-0.172831927	0.000629404	0.008963481	1
Prediabetes	duct_epithelial_cell	*NDUFC2*	-0.089735063	0.00121008	0.009782723	1
Prediabetes	duct_epithelial_cell	*QDPR*	-0.058277782	0.002799774	0.015172844	1
Prediabetes	duct_epithelial_cell	*ZNF827*	-0.120285376	0.001031711	0.009336398	1
Prediabetes	duct_epithelial_cell	*KIN*	-0.215469291	0.000815151	0.008963481	1
Prediabetes	duct_epithelial_cell	*BICD1*	-0.104327935	0.008759347	0.031544891	1
Prediabetes	duct_epithelial_cell	*GUF1*	-0.114590481	0.004326807	0.019802187	1
Prediabetes	duct_epithelial_cell	*CACUL1*	-0.136445603	0.000843516	0.008963481	1
Prediabetes	duct_epithelial_cell	*RABGAP1L*	-0.132485617	0.001227156	0.009805258	1
Prediabetes	duct_epithelial_cell	*ARL14EP*	-0.166919752	0.000524243	0.008963481	1
Prediabetes	duct_epithelial_cell	*EPG5*	-0.254858506	9.59E-05	0.008963481	0.376712789
Prediabetes	duct_epithelial_cell	*PDK1*	-0.145616719	0.001630428	0.011037642	1

Prediabetes	duct_epithelial_cell	*PDE3B*	-0.044442537	0.000756898	0.008963481	1
Prediabetes	duct_epithelial_cell	*TGOLN2*	-0.147700476	0.000114902	0.008963481	0.451449662
Prediabetes	duct_epithelial_cell	*USP12*	-0.078550035	0.006942265	0.027276159	1
Prediabetes	duct_epithelial_cell	*IGSF10*	-0.308015012	0.002532526	0.014316973	1
Prediabetes	duct_epithelial_cell	*GPR180*	-0.092873286	0.006480487	0.025823362	1
Prediabetes	duct_epithelial_cell	*IFIT5*	-0.16497803	0.000100703	0.008963481	0.395661704
Prediabetes	duct_epithelial_cell	*MED21*	-0.147497615	0.002813473	0.015226082	1
Prediabetes	duct_epithelial_cell	*SRP19*	-0.110040747	0.000815016	0.008963481	1
Prediabetes	duct_epithelial_cell	*CAST*	-0.135807213	0.00346965	0.017388079	1
Prediabetes	duct_epithelial_cell	*PLA2R1*	-0.911524241	0.002707868	0.014880018	1
Prediabetes	duct_epithelial_cell	*RPIA*	-0.176744819	0.003412686	0.017234503	1
Prediabetes	duct_epithelial_cell	*GTF2E1*	-0.291184747	0.000518724	0.008963481	1
Prediabetes	duct_epithelial_cell	*JAZF1*	-0.089684011	0.000924598	0.009057947	1
Prediabetes	duct_epithelial_cell	*CMIP*	-0.142020976	0.00087653	0.009011853	1
Prediabetes	duct_epithelial_cell	*NUS1*	-0.135445457	0.000669901	0.008963481	1
Prediabetes	duct_epithelial_cell	*PITPNC1*	-0.176329857	0.001382705	0.010271223	1
Prediabetes	duct_epithelial_cell	*CEP112*	-0.195443182	0.010986253	0.037414245	1
Prediabetes	duct_epithelial_cell	*TSEN2*	-0.139458002	0.000944767	0.009132479	1
Prediabetes	duct_epithelial_cell	*FLCN*	-0.175685394	0.001647928	0.011064011	1
Prediabetes	duct_epithelial_cell	*RAB6B*	-0.213711569	0.001782585	0.011538034	1
Prediabetes	duct_epithelial_cell	*ANKRD40*	-0.129122182	0.004225348	0.019551648	1
Prediabetes	duct_epithelial_cell	*ATP6V1C1*	-0.156088773	0.00084114	0.008963481	1
Prediabetes	duct_epithelial_cell	*MARVELD1*	-0.154381251	0.009067511	0.0323875	1
Prediabetes	duct_epithelial_cell	*DBI*	-0.087865585	0.008697543	0.031543827	1
Prediabetes	duct_epithelial_cell	*LRGUK*	-0.127134631	0.001890349	0.011998678	1
Prediabetes	duct_epithelial_cell	*MIER3*	-0.150570086	0.003615199	0.017821977	1
Prediabetes	duct_epithelial_cell	*RMND1*	-0.081758818	0.015937062	0.049382268	1
Prediabetes	duct_epithelial_cell	*PSD3*	-0.147561569	0.003341513	0.016984223	1
Prediabetes	duct_epithelial_cell	*CCT8*	-0.053367215	0.007995756	0.030059963	1
Prediabetes	duct_epithelial_cell	*ZDHHC5*	-0.156433876	0.014186996	0.045268501	1
Prediabetes	duct_epithelial_cell	*MED19*	-0.17717621	0.002367143	0.013617139	1
Prediabetes	duct_epithelial_cell	*MAPK13*	-0.065530849	0.006720995	0.026639575	1
Prediabetes	duct_epithelial_cell	*EXOG*	-0.152309923	5.97E-05	0.008963481	0.234492697
Prediabetes	duct_epithelial_cell	*ZFYVE9*	-0.191988088	0.002055671	0.012532667	1
Prediabetes	duct_epithelial_cell	*GATAD1*	-0.162160476	0.000767435	0.008963481	1
Prediabetes	duct_epithelial_cell	*DHRS4*	-0.127722182	0.004704391	0.020956406	1
Prediabetes	duct_epithelial_cell	*SLC38A10*	-0.133825572	0.000381616	0.008963481	1
Prediabetes	duct_epithelial_cell	*SLC37A3*	-0.186839085	0.002321381	0.013492166	1
Prediabetes	duct_epithelial_cell	*SPPL3*	-0.183026451	0.00033123	0.008963481	1
Prediabetes	duct_epithelial_cell	*TNFRSF14*	-0.3649251	0.001257353	0.009900082	1
Prediabetes	duct_epithelial_cell	*PEX10*	-0.167704139	0.010103431	0.035211789	1
Prediabetes	duct_epithelial_cell	*LDLRAP1*	-0.114515345	0.001245236	0.009849499	1
Prediabetes	duct_epithelial_cell	*XKR8*	-0.148169753	0.014481187	0.045921375	1
Prediabetes	duct_epithelial_cell	*FAIM*	-0.171216144	0.01048424	0.036075136	1
Prediabetes	duct_epithelial_cell	*PINK1*	-0.137537024	0.000855375	0.008963481	1
Prediabetes	duct_epithelial_cell	*CCAR2*	-0.269580265	0.000436691	0.008963481	1
Prediabetes	duct_epithelial_cell	*EPB41*	-0.122738064	0.006537581	0.025971847	1
Prediabetes	duct_epithelial_cell	*ADPGK*	-0.144967692	0.004181316	0.019464918	1
Prediabetes	duct_epithelial_cell	*THEM4*	-0.128069246	0.007885631	0.029705317	1
Prediabetes	duct_epithelial_cell	*UBR1*	-0.261397202	0.000473534	0.008963481	1
Prediabetes	duct_epithelial_cell	*TEPP*	-2.666297899	0.000746848	0.008963481	1
Prediabetes	duct_epithelial_cell	*ZDHHC1*	-0.156271372	0.008347905	0.030749039	1
Prediabetes	duct_epithelial_cell	*PDXK*	-0.176541048	3.85E-05	0.008963481	0.151459877
Prediabetes	duct_epithelial_cell	*FTCD*	-0.045147495	0.013066629	0.042604802	1
Prediabetes	duct_epithelial_cell	*LSS*	-0.060246412	0.008527303	0.031108424	1
Prediabetes	duct_epithelial_cell	*SLC25A44*	-0.132883869	0.005797474	0.024053088	1
Prediabetes	duct_epithelial_cell	*VPS28*	-0.08612628	0.005862665	0.02428191	1
Prediabetes	duct_epithelial_cell	*TONSL*	-0.590473693	0.000363393	0.008963481	1
Prediabetes	duct_epithelial_cell	*ZNF333*	-0.225965268	0.000255804	0.008963481	1
Prediabetes	duct_epithelial_cell	*RPL8*	-0.062706068	0.001620845	0.011022922	1
Prediabetes	duct_epithelial_cell	*MAML1*	-0.111587023	0.013204202	0.042946447	1
Prediabetes	duct_epithelial_cell	*RPL26*	-0.07282838	0.000423271	0.008963481	1
Prediabetes	duct_epithelial_cell	*ZNF75A*	-0.165516343	0.001276278	0.010008972	1
Prediabetes	duct_epithelial_cell	*ZFPL1*	-0.109537513	0.003121707	0.016266825	1

Prediabetes	duct_epithelial_cell	*CTRC*	-0.024651373	0.011238161	0.037966239	1
Prediabetes	duct_epithelial_cell	*TM2D1*	-0.210755893	0.001010207	0.009336398	1
Prediabetes	duct_epithelial_cell	*ZNF496*	-0.268674385	0.000564219	0.008963481	1
Prediabetes	duct_epithelial_cell	*FLVCR1*	-0.106647452	0.001985901	0.012369325	1
Prediabetes	duct_epithelial_cell	*ACP6*	-0.068125732	0.013161123	0.042877323	1
Prediabetes	duct_epithelial_cell	*SMC6*	-0.268419434	0.000470125	0.008963481	1
Prediabetes	duct_epithelial_cell	*MSX1*	-0.088784909	0.010505241	0.036111192	1
Prediabetes	duct_epithelial_cell	*PACRGL*	-0.11509856	0.008776383	0.031577296	1
Prediabetes	duct_epithelial_cell	*DCAF16*	-0.121021175	0.002635355	0.014686963	1
Prediabetes	duct_epithelial_cell	*NPPC*	-0.807586342	0.000529857	0.008963481	1
Prediabetes	duct_epithelial_cell	*EOGT*	-0.210808379	0.000592195	0.008963481	1
Prediabetes	duct_epithelial_cell	*ARPC2*	-0.162060637	0.000754906	0.008963481	1
Prediabetes	duct_epithelial_cell	*CWC22*	-0.207313159	0.000705984	0.008963481	1
Prediabetes	duct_epithelial_cell	*HDAC11*	-0.119523229	0.013752872	0.044254737	1
Prediabetes	duct_epithelial_cell	*SUCLG1*	-0.208861733	0.000389337	0.008963481	1
Prediabetes	duct_epithelial_cell	*ATXN7*	-0.155373713	0.000163277	0.008963481	0.641513494
Prediabetes	duct_epithelial_cell	*PSMD6*	-0.106606381	0.001313122	0.010116188	1
Prediabetes	duct_epithelial_cell	*IL17RC*	-0.11279427	0.000780268	0.008963481	1
Prediabetes	duct_epithelial_cell	*TTC14*	-0.238563814	0.000955319	0.009132479	1
Prediabetes	duct_epithelial_cell	*NMNAT3*	-0.092616384	0.014309074	0.045485722	1
Prediabetes	duct_epithelial_cell	*SMIM12*	-0.168127	0.001260913	0.009908255	1
Prediabetes	duct_epithelial_cell	*TPRA1*	-0.230970703	0.008487885	0.031022233	1
Prediabetes	duct_epithelial_cell	*MEAF6*	-0.136981516	0.0004609	0.008963481	1
Prediabetes	duct_epithelial_cell	*DNALI1*	-0.0845404	0.003967294	0.018848246	1
Prediabetes	duct_epithelial_cell	*RPN1*	-0.137739996	0.001632351	0.011037642	1
Prediabetes	duct_epithelial_cell	*RFC4*	-0.148145338	0.001494047	0.010590892	1
Prediabetes	duct_epithelial_cell	*TKT*	-0.128975155	0.000578756	0.008963481	1
Prediabetes	duct_epithelial_cell	*LRPAP1*	-0.055069928	0.008649082	0.031435931	1
Prediabetes	duct_epithelial_cell	*PIGX*	-0.147252343	0.001560935	0.010816429	1
Prediabetes	duct_epithelial_cell	*SAP30*	-0.126239913	0.000735611	0.008963481	1
Prediabetes	duct_epithelial_cell	*ARFIP1*	-0.095633201	0.00558063	0.023475689	1
Prediabetes	duct_epithelial_cell	*STARD4*	-0.12179822	0.001940316	0.012217151	1
Prediabetes	duct_epithelial_cell	*CMBL*	-0.125906315	0.006403342	0.025593825	1
Prediabetes	duct_epithelial_cell	*PRRC1*	-0.166869126	0.000745085	0.008963481	1
Prediabetes	duct_epithelial_cell	*AGGF1*	-0.182726473	0.000297872	0.008963481	1
Prediabetes	duct_epithelial_cell	*RICTOR*	-0.153434528	0.000694731	0.008963481	1
Prediabetes	duct_epithelial_cell	*UTP15*	-0.310663861	0.000684237	0.008963481	1
Prediabetes	duct_epithelial_cell	*NSA2*	-0.072750475	0.004864296	0.021463151	1
Prediabetes	duct_epithelial_cell	*PDSS2*	-0.179010791	0.005748243	0.023949997	1
Prediabetes	duct_epithelial_cell	*SAP30L*	-0.124888315	0.008741784	0.031543827	1
Prediabetes	duct_epithelial_cell	*COG5*	-0.204202388	0.000816802	0.008963481	1
Prediabetes	duct_epithelial_cell	*SLU7*	-0.138554056	0.000588539	0.008963481	1
Prediabetes	duct_epithelial_cell	*CAMLG*	-0.08093184	0.003800493	0.018364621	1
Prediabetes	duct_epithelial_cell	*BRI3*	-0.109918667	0.000683199	0.008963481	1
Prediabetes	duct_epithelial_cell	*LMTK2*	-0.228287191	2.09E-05	0.008963481	0.082073335
Prediabetes	duct_epithelial_cell	*PEX2*	-0.162366651	0.000582088	0.008963481	1
Prediabetes	duct_epithelial_cell	*SUN1*	-0.190062919	0.000131961	0.008963481	0.518474291
Prediabetes	duct_epithelial_cell	*INTS1*	-0.267241769	0.001133512	0.00963464	1
Prediabetes	duct_epithelial_cell	*YWHAZ*	-0.141373177	0.000818388	0.008963481	1
Prediabetes	duct_epithelial_cell	*DCAF13*	-0.125798439	0.002632425	0.014686963	1
Prediabetes	duct_epithelial_cell	*GEM*	-0.082561519	0.002201451	0.012987238	1
Prediabetes	duct_epithelial_cell	*RPP25L*	-0.246161	0.003671863	0.017966065	1
Prediabetes	duct_epithelial_cell	*FAM219A*	-0.179258977	0.00170255	0.011242555	1
Prediabetes	duct_epithelial_cell	*NUDT2*	-0.068607724	0.004573735	0.020655409	1
Prediabetes	duct_epithelial_cell	*NOL6*	-0.220414953	0.001885738	0.01198878	1
Prediabetes	duct_epithelial_cell	*WRN*	-0.20106498	0.000608382	0.008963481	1
Prediabetes	duct_epithelial_cell	*CFL2*	-0.116659965	0.007604553	0.028976426	1
Prediabetes	duct_epithelial_cell	*PGM2L1*	-0.082745331	0.01185783	0.03961685	1
Prediabetes	duct_epithelial_cell	*PHYHIPL*	-0.058623592	0.011952227	0.039890314	1
Prediabetes	duct_epithelial_cell	*INPPL1*	-0.156320714	0.003755917	0.018218516	1
Prediabetes	duct_epithelial_cell	*OXGR1*	-0.168361994	0.009409001	0.033244573	1
Prediabetes	duct_epithelial_cell	*VDAC2*	-0.128847762	0.000602711	0.008963481	1
Prediabetes	duct_epithelial_cell	*TSC1*	-0.185714005	0.000917899	0.009057947	1
Prediabetes	duct_epithelial_cell	*ZMYND19*	-0.222218255	0.014297858	0.045485722	1

Prediabetes	duct_epithelial_cell	*METTL3*	-0.175318055	0.013446583	0.043518636	1
Prediabetes	duct_epithelial_cell	*TRUB1*	-0.179089329	0.000437193	0.008963481	1
Prediabetes	duct_epithelial_cell	*IFI27L1*	-0.114281239	0.000620101	0.008963481	1
Prediabetes	duct_epithelial_cell	*HTRA1*	-0.104921447	0.00541091	0.023158459	1
Prediabetes	duct_epithelial_cell	*CEP57*	-0.108565508	0.002201116	0.012987238	1
Prediabetes	duct_epithelial_cell	*BAG5*	-0.123620824	0.000937571	0.009132479	1
Prediabetes	duct_epithelial_cell	*LARP6*	-0.120713266	0.00975896	0.034204242	1
Prediabetes	duct_epithelial_cell	*ZNF319*	-0.348617431	0.001434034	0.010357204	1
Prediabetes	duct_epithelial_cell	*FRS2*	-0.071157748	0.005608802	0.023493587	1
Prediabetes	duct_epithelial_cell	*PCBD1*	-0.081176109	0.015775277	0.049113362	1
Prediabetes	duct_epithelial_cell	*COX11*	-0.149958224	0.000244982	0.008963481	0.962534711
Prediabetes	duct_epithelial_cell	*RIC3*	-0.147876157	0.000552689	0.008963481	1
Prediabetes	duct_epithelial_cell	*ZNF143*	-0.27512056	0.000442446	0.008963481	1
Prediabetes	duct_epithelial_cell	*COG1*	-0.172713956	0.000473164	0.008963481	1
Prediabetes	duct_epithelial_cell	*B2M*	-0.083576763	0.000450933	0.008963481	1
Prediabetes	duct_epithelial_cell	*SAAL1*	-0.257790314	0.007880787	0.029705317	1
Prediabetes	duct_epithelial_cell	*GREM1*	-0.087485057	0.004941443	0.021716922	1
Prediabetes	duct_epithelial_cell	*TSC22D4*	-0.078299879	0.003258592	0.016735957	1
Prediabetes	duct_epithelial_cell	*NAT16*	-0.046510287	0.012432312	0.041069201	1
Prediabetes	duct_epithelial_cell	*ENDOG*	-0.150185973	0.000184413	0.008963481	0.72455921
Prediabetes	duct_epithelial_cell	*ZNF23*	-0.26352581	0.000432757	0.008963481	1
Prediabetes	duct_epithelial_cell	*ZNF180*	-0.354151904	0.00875497	0.031544891	1
Prediabetes	duct_epithelial_cell	*GPX4*	-0.083222928	0.00393959	0.018783824	1
Prediabetes	duct_epithelial_cell	*ZNF641*	-0.195396018	0.005166129	0.022329724	1
Prediabetes	duct_epithelial_cell	*RAB4B*	-0.087101122	0.008222247	0.03050539	1
Prediabetes	duct_epithelial_cell	*UBXN6*	-0.168155859	0.000121302	0.008963481	0.476594996
Prediabetes	duct_epithelial_cell	*NDUFV1*	-0.152101374	0.00700786	0.027368761	1
Prediabetes	duct_epithelial_cell	*HID1*	-0.160893277	0.006074633	0.024776105	1
Prediabetes	duct_epithelial_cell	*SRP68*	-0.166636226	0.000751921	0.008963481	1
Prediabetes	duct_epithelial_cell	*TMEM68*	-0.185692168	0.006248145	0.025178422	1
Prediabetes	duct_epithelial_cell	*MLST8*	-0.087547677	0.00528809	0.022756741	1
Prediabetes	duct_epithelial_cell	*C2CD3*	-0.228783758	0.000641601	0.008963481	1
Prediabetes	duct_epithelial_cell	*RPSA*	-0.050909815	0.009191503	0.032711427	1
Prediabetes	duct_epithelial_cell	*ENTPD3*	-0.049232213	0.002316397	0.013483149	1
Prediabetes	duct_epithelial_cell	*LTBP3*	-0.060240753	0.002835799	0.015283753	1
Prediabetes	duct_epithelial_cell	*SAC3D1*	-0.257561408	0.00706191	0.027444355	1
Prediabetes	duct_epithelial_cell	*LMBRD1*	-0.141208374	0.002845871	0.015317022	1
Prediabetes	duct_epithelial_cell	*TTC39C*	-0.107592707	0.005557927	0.023430359	1
Prediabetes	duct_epithelial_cell	*POLR2J3*	-0.110225931	0.004192052	0.019468837	1
Prediabetes	duct_epithelial_cell	*NT5DC2*	-0.084928779	0.015511651	0.048407686	1
Prediabetes	duct_epithelial_cell	*COA6*	-0.23628923	0.002336969	0.013496739	1
Prediabetes	duct_epithelial_cell	*PXK*	-0.126308174	0.001041199	0.009336398	1
Prediabetes	duct_epithelial_cell	*ING5*	-0.15759742	0.001161051	0.009685284	1
Prediabetes	duct_epithelial_cell	*HEXIM2*	-0.348886295	0.00083581	0.008963481	1
Prediabetes	duct_epithelial_cell	*TRAPPC11*	-0.177506894	0.00924739	0.032821135	1
Prediabetes	duct_epithelial_cell	*COL3A1*	-0.061215889	0.004619064	0.020788435	1
Prediabetes	duct_epithelial_cell	*ING2*	-0.073910496	0.008040879	0.030163992	1
Prediabetes	duct_epithelial_cell	*DNAJC21*	-0.167875611	0.000472711	0.008963481	1
Prediabetes	duct_epithelial_cell	*CNNM3*	-0.193373796	0.000857793	0.008963481	1
Prediabetes	duct_epithelial_cell	*PPIP5K1*	-0.231313243	7.31E-05	0.008963481	0.287196018
Prediabetes	duct_epithelial_cell	*ZNF507*	-0.195843431	0.001099811	0.00956008	1
Prediabetes	duct_epithelial_cell	*FSTL5*	-0.036560605	0.008053456	0.030163992	1
Prediabetes	duct_epithelial_cell	*CTRB1*	-0.023284854	0.012065744	0.040194196	1
Prediabetes	duct_epithelial_cell	*MFF*	-0.172909339	0.000828517	0.008963481	1
Prediabetes	duct_epithelial_cell	*FAM110B*	-0.228949776	0.005557863	0.023430359	1
Prediabetes	duct_epithelial_cell	*PRELID1*	-0.113493205	0.000521613	0.008963481	1
Prediabetes	duct_epithelial_cell	*NMD3*	-0.129903275	0.001422801	0.010342775	1
Prediabetes	duct_epithelial_cell	*SDC2*	-0.087570558	0.000918384	0.009057947	1
Prediabetes	duct_epithelial_cell	*HINT1*	-0.073624894	0.002297606	0.013430167	1
Prediabetes	duct_epithelial_cell	*MT1E*	-0.033594698	0.007574673	0.028950283	1
Prediabetes	duct_epithelial_cell	*LIMS1*	-0.114485216	0.001507201	0.010631588	1
Prediabetes	duct_epithelial_cell	*AVEN*	-0.093164295	0.006821378	0.026908829	1
Prediabetes	duct_epithelial_cell	*TM4SF4*	-0.09556379	0.001668822	0.01113209	1
Prediabetes	duct_epithelial_cell	*OTUD3*	-0.123718459	0.000608381	0.008963481	1

Prediabetes	duct_epithelial_cell	*MAP3K2*	-0.08467784	0.002767489	0.01511602	1
Prediabetes	duct_epithelial_cell	*UBE2E1*	-0.219647965	0.000417126	0.008963481	1
Prediabetes	duct_epithelial_cell	*USP47*	-0.139944764	0.000701718	0.008963481	1
Prediabetes	duct_epithelial_cell	*CRTAP*	-0.180894839	0.000603333	0.008963481	1
Prediabetes	duct_epithelial_cell	*NFRKB*	-0.220607952	0.014743286	0.046564606	1
Prediabetes	duct_epithelial_cell	*MGMT*	-0.219606191	0.000314296	0.008963481	1
Prediabetes	duct_epithelial_cell	*NUDT9*	-0.04584725	0.014835108	0.046765612	1
Prediabetes	duct_epithelial_cell	*IRX1*	-0.104834469	0.014816513	0.046758297	1
Prediabetes	duct_epithelial_cell	*HSPA4*	-0.106219591	0.005880378	0.024287691	1
Prediabetes	duct_epithelial_cell	*ACYP2*	-0.110791022	0.00364911	0.01792169	1
Prediabetes	duct_epithelial_cell	*FOXN2*	-0.114596171	0.001808249	0.011646905	1
Prediabetes	duct_epithelial_cell	*MTSS1*	-0.100597245	0.001832078	0.011742633	1
Prediabetes	duct_epithelial_cell	*RPS9*	-0.068128483	0.000501439	0.008963481	1
Prediabetes	duct_epithelial_cell	*NDUFA3*	-0.121627704	0.001203577	0.009750216	1
Prediabetes	duct_epithelial_cell	*ZNF160*	-0.122997604	0.013440285	0.043518636	1
Prediabetes	duct_epithelial_cell	*TSNARE1*	-0.108454232	0.007723929	0.029236335	1
Prediabetes	duct_epithelial_cell	*TRMT61B*	-0.052964683	0.008119489	0.030300524	1
Prediabetes	duct_epithelial_cell	*MFN1*	-0.087706012	0.010156574	0.035314317	1
Prediabetes	duct_epithelial_cell	*JAGN1*	-0.193770374	0.000369284	0.008963481	1
Prediabetes	duct_epithelial_cell	*TRIM8*	-0.141131263	0.010807308	0.036987729	1
Prediabetes	duct_epithelial_cell	*ZNF440*	-0.138038706	0.013179916	0.042902975	1
Prediabetes	duct_epithelial_cell	*EXOSC1*	-0.132014291	0.003495421	0.017450459	1
Prediabetes	duct_epithelial_cell	*MRPL36*	-0.138213612	0.000110908	0.008963481	0.435759048
Prediabetes	duct_epithelial_cell	*PTGER4*	-0.11194702	0.008270571	0.030617094	1
Prediabetes	duct_epithelial_cell	*VAT1L*	-0.086136201	0.008091894	0.030250286	1
Prediabetes	duct_epithelial_cell	*PWWP2B*	-0.225853417	0.008215471	0.03050539	1
Prediabetes	duct_epithelial_cell	*PTEN*	-0.065101982	0.01333212	0.043255079	1
Prediabetes	duct_epithelial_cell	*TVP23B*	-0.088735651	0.004335579	0.019807548	1
Prediabetes	duct_epithelial_cell	*SRGAP2C*	-0.213257492	0.000280726	0.008963481	1
Prediabetes	duct_epithelial_cell	*ZNF554*	-0.116119081	0.016017696	0.049553959	1
Prediabetes	duct_epithelial_cell	*REG1B*	-0.022233531	0.0153736	0.048129782	1
Prediabetes	duct_epithelial_cell	*NME6*	-0.14183716	0.010853144	0.037112275	1
Prediabetes	duct_epithelial_cell	*TEFM*	-0.174245321	0.003944173	0.018783824	1
Prediabetes	duct_epithelial_cell	*ZNF131*	-0.222152084	0.000284682	0.008963481	1
Prediabetes	duct_epithelial_cell	*BSG*	-0.093402152	0.001158534	0.009685284	1
Prediabetes	duct_epithelial_cell	*HINFP*	-0.364686052	0.000134235	0.008963481	0.527408959
Prediabetes	duct_epithelial_cell	*CERS6*	-0.076423316	0.007737566	0.029259765	1
Prediabetes	duct_epithelial_cell	*SUCLG2*	-0.225368258	0.002538645	0.014327176	1
Prediabetes	duct_epithelial_cell	*ZNF24*	-0.101927445	0.00064402	0.008963481	1
Prediabetes	duct_epithelial_cell	*AFF1*	-0.126792907	0.003372574	0.01711995	1
Prediabetes	duct_epithelial_cell	*DCP2*	-0.185226625	0.002028995	0.012475623	1
Prediabetes	duct_epithelial_cell	*DMXL1*	-0.133559714	0.00178841	0.011538034	1
Prediabetes	duct_epithelial_cell	*LCLAT1*	-0.20314786	0.000109949	0.008963481	0.43198872
Prediabetes	duct_epithelial_cell	*TADA2B*	-0.207267083	0.00038188	0.008963481	1
Prediabetes	duct_epithelial_cell	*IQCB1*	-0.123704163	0.003424296	0.017248792	1
Prediabetes	duct_epithelial_cell	*ZNF483*	-0.057332834	0.015092026	0.04732368	1
Prediabetes	duct_epithelial_cell	*NDNF*	-0.981340384	0.01364685	0.043985621	1
Prediabetes	duct_epithelial_cell	*ARV1*	-0.207251323	0.004004415	0.018955838	1
Prediabetes	duct_epithelial_cell	*PPP1R14B*	-0.106738254	0.005050308	0.02202293	1
Prediabetes	duct_epithelial_cell	*NUDT18*	-0.307950246	0.000520668	0.008963481	1
Prediabetes	duct_epithelial_cell	*UQCRH*	-0.121635038	0.000159599	0.008963481	0.627064688
Prediabetes	duct_epithelial_cell	*DPY19L1*	-0.281800646	0.000681131	0.008963481	1
Prediabetes	duct_epithelial_cell	*RALGAPA1*	-0.079822667	0.003809449	0.018364621	1
Prediabetes	duct_epithelial_cell	*GOLT1A*	-0.208139746	0.001055068	0.009336398	1
Prediabetes	duct_epithelial_cell	*ANGEL2*	-0.171700631	0.000663102	0.008963481	1
Prediabetes	duct_epithelial_cell	*BRSK2*	-0.062102753	0.012304973	0.04084842	1
Prediabetes	duct_epithelial_cell	*BRMS1*	-0.168680399	0.000621164	0.008963481	1
Prediabetes	duct_epithelial_cell	*HRAS*	-0.159515369	0.000580988	0.008963481	1
Prediabetes	duct_epithelial_cell	*SRP72*	-0.089909186	0.008350528	0.030749039	1
Prediabetes	duct_epithelial_cell	*DENND6A*	-0.10023822	0.006367417	0.025502123	1
Prediabetes	duct_epithelial_cell	*NDUFA11*	-0.111835039	8.36E-05	0.008963481	0.328420668
Prediabetes	duct_epithelial_cell	*DHX36*	-0.115401647	0.000815484	0.008963481	1
Prediabetes	duct_epithelial_cell	*ZNF519*	-0.146658252	0.003461492	0.017369351	1
Prediabetes	duct_epithelial_cell	*TMEM9B*	-0.113130191	0.001768875	0.011496768	1

Prediabetes	duct_epithelial_cell	*EIF3F*	-0.101095721	0.001193479	0.009719245	1
Prediabetes	duct_epithelial_cell	*ZNF25*	-0.092353868	0.009126644	0.032569106	1
Prediabetes	duct_epithelial_cell	*PPP2R2D*	-0.145598735	0.001312901	0.010116188	1
Prediabetes	duct_epithelial_cell	*UCP2*	-0.079365879	0.014249046	0.045368317	1
Prediabetes	duct_epithelial_cell	*SWI5*	-0.054609484	0.012193819	0.040552114	1
Prediabetes	duct_epithelial_cell	*ZDHHC21*	-0.12114135	0.006151944	0.024966119	1
Prediabetes	duct_epithelial_cell	*BNIP3*	-0.058046686	0.011560438	0.038854544	1
Prediabetes	duct_epithelial_cell	*SMIM19*	-0.120468558	0.003639838	0.017898526	1
Prediabetes	duct_epithelial_cell	*SLC35G1*	-0.117483007	0.006510299	0.02591587	1
Prediabetes	duct_epithelial_cell	*COX8A*	-0.078282582	0.001835732	0.011746889	1
Prediabetes	duct_epithelial_cell	*WDR25*	-0.123885848	0.002962269	0.015685656	1
Prediabetes	duct_epithelial_cell	*KBTBD11*	-0.167558431	0.003045015	0.015976995	1
Prediabetes	duct_epithelial_cell	*ANKLE2*	-0.10251994	0.003667146	0.017966065	1
Prediabetes	duct_epithelial_cell	*DPP7*	-0.089810906	0.001491365	0.010590892	1
Prediabetes	duct_epithelial_cell	*DEAF1*	-0.190621236	0.000554927	0.008963481	1
Prediabetes	duct_epithelial_cell	*MAN1B1*	-0.173941181	0.000891513	0.00905104	1
Prediabetes	duct_epithelial_cell	*PPFIA3*	-0.09689483	0.00750322	0.028733091	1
Prediabetes	duct_epithelial_cell	*PAWR*	-0.134882025	0.001399602	0.010278575	1
Prediabetes	duct_epithelial_cell	*AGTRAP*	-0.241511837	0.0003732	0.008963481	1
Prediabetes	duct_epithelial_cell	*THAP5*	-0.101355693	0.002040323	0.012486647	1
Prediabetes	duct_epithelial_cell	*CHID1*	-0.067101953	0.001902429	0.012055874	1
Prediabetes	duct_epithelial_cell	*ZBTB41*	-0.110240362	0.002777747	0.01511602	1
Prediabetes	duct_epithelial_cell	*BET1L*	-0.164499957	0.001533212	0.010707166	1
Prediabetes	duct_epithelial_cell	*IMP3*	-0.119714047	0.012750938	0.041783516	1
Prediabetes	duct_epithelial_cell	*NT5DC1*	-0.120199479	0.007625156	0.028976426	1
Prediabetes	duct_epithelial_cell	*ZNF713*	-0.289358536	0.0158944	0.049366875	1
Prediabetes	duct_epithelial_cell	*RPP25*	-0.155112296	0.000873917	0.009011853	1
Prediabetes	duct_epithelial_cell	*SPTY2D1*	-0.16587181	0.000498814	0.008963481	1
Prediabetes	duct_epithelial_cell	*CALR*	-0.084051316	0.003922717	0.018769071	1
Prediabetes	duct_epithelial_cell	*ELMOD2*	-0.192133204	0.000758186	0.008963481	1
Prediabetes	duct_epithelial_cell	*RPRML*	-0.054122682	0.004272938	0.019612585	1
Prediabetes	duct_epithelial_cell	*CCDC66*	-0.135488381	0.002093546	0.012674181	1
Prediabetes	duct_epithelial_cell	*MCFD2*	-0.131896568	0.000428794	0.008963481	1
Prediabetes	duct_epithelial_cell	*PPA1*	-0.092658636	0.0006387	0.008963481	1
Prediabetes	duct_epithelial_cell	*MAP6D1*	-0.099034887	0.00973565	0.034153006	1
Prediabetes	duct_epithelial_cell	*D2HGDH*	-0.167248949	0.000255908	0.008963481	1
Prediabetes	duct_epithelial_cell	*LRRC57*	-0.110736551	0.010351001	0.035853926	1
Prediabetes	duct_epithelial_cell	*ZFP82*	-0.165851748	0.001140393	0.00963464	1
Prediabetes	duct_epithelial_cell	*RPH3AL*	-0.05356274	0.008854455	0.031799959	1
Prediabetes	duct_epithelial_cell	*HIGD1A*	-0.141886668	0.000675387	0.008963481	1
Prediabetes	duct_epithelial_cell	*ZNF707*	-0.391152384	0.012344831	0.040906653	1
Prediabetes	duct_epithelial_cell	*OGFOD3*	-0.062220368	0.001020121	0.009336398	1
Prediabetes	duct_epithelial_cell	*ZNF467*	-0.168478756	0.01166782	0.039081727	1
Prediabetes	duct_epithelial_cell	*TMEM45A*	-0.066276288	0.014194653	0.045268501	1
Prediabetes	duct_epithelial_cell	*GPR135*	-0.162590161	0.004868675	0.021463151	1
Prediabetes	duct_epithelial_cell	*RFX7*	-0.174807969	0.001590742	0.010911694	1
Prediabetes	duct_epithelial_cell	*CCDC149*	-0.164495084	0.001311631	0.010116188	1
Prediabetes	duct_epithelial_cell	*ZNF708*	-0.177840771	0.001624401	0.011022922	1
Prediabetes	duct_epithelial_cell	*IST1*	-0.175188755	0.00046551	0.008963481	1
Prediabetes	duct_epithelial_cell	*ERCC6L2*	-0.176727979	0.000496899	0.008963481	1
Prediabetes	duct_epithelial_cell	*MRPL41*	-0.138758995	0.000483238	0.008963481	1
Prediabetes	duct_epithelial_cell	*TP53TG1*	-0.081972063	0.001188086	0.009719245	1
Prediabetes	duct_epithelial_cell	*ARL6IP4*	-0.075640282	0.002604268	0.014586734	1
Prediabetes	duct_epithelial_cell	*FIGN*	-0.097822575	0.008834133	0.031756002	1
Prediabetes	duct_epithelial_cell	*NPLOC4*	-0.138911845	0.001188496	0.009719245	1
Prediabetes	duct_epithelial_cell	*SATB1*	-0.192115572	0.00214785	0.012899732	1
Prediabetes	duct_epithelial_cell	*C11orf54*	-0.051761733	0.015915127	0.049373666	1
Prediabetes	duct_epithelial_cell	*CEP63*	-0.096187912	0.000756684	0.008963481	1
Prediabetes	duct_epithelial_cell	*GPC6*	-0.071548359	0.013235277	0.043011913	1
Prediabetes	duct_epithelial_cell	*FANCF*	-0.300807458	0.000112048	0.008963481	0.440236997
Prediabetes	duct_epithelial_cell	*CALN1*	-0.18888279	0.003207596	0.016648141	1
Prediabetes	duct_epithelial_cell	*BOLA2*	-0.12211825	0.004648867	0.02085091	1
Prediabetes	duct_epithelial_cell	*MRPL54*	-0.083433327	0.002786604	0.015143249	1
Prediabetes	duct_epithelial_cell	*TRMT12*	-0.265775403	0.000286227	0.008963481	1

Prediabetes	duct_epithelial_cell	*CBX6*	-0.095864343	0.003154143	0.016392366	1
Prediabetes	duct_epithelial_cell	*UQCR10*	-0.10146397	0.000219713	0.008963481	0.863252581
Prediabetes	duct_epithelial_cell	*CRELD2*	-0.058024167	0.002979804	0.015696866	1
Prediabetes	duct_epithelial_cell	*UBE2F*	-0.135925873	0.000202302	0.008963481	0.794844999
Prediabetes	duct_epithelial_cell	*TM2D3*	-0.124221803	0.001054425	0.009336398	1
Prediabetes	duct_epithelial_cell	*COPB2*	-0.125759586	0.003254009	0.016734297	1
Prediabetes	duct_epithelial_cell	*CCR10*	-0.058904138	0.005636673	0.023585185	1
Prediabetes	duct_epithelial_cell	*ZFP1*	-0.264804864	0.000813536	0.008963481	1
Prediabetes	duct_epithelial_cell	*XPOT*	-0.08509942	0.00406066	0.019152863	1
Prediabetes	duct_epithelial_cell	*ZBTB40*	-0.107314748	0.015455539	0.048305536	1
Prediabetes	duct_epithelial_cell	*UBE2G2*	-0.060157651	0.012071558	0.040194196	1
Prediabetes	duct_epithelial_cell	*TMED9*	-0.103331766	0.004933777	0.021707514	1
Prediabetes	duct_epithelial_cell	*BTBD6*	-0.231473828	0.000993433	0.009315508	1
Prediabetes	duct_epithelial_cell	*LCN12*	-0.079522196	0.010099724	0.035211789	1
Prediabetes	duct_epithelial_cell	*SIVA1*	-0.093388398	0.001329578	0.010202955	1
Prediabetes	duct_epithelial_cell	*BRI3BP*	-0.208459967	0.001463452	0.01048303	1
Prediabetes	duct_epithelial_cell	*CIB1*	-0.155511083	0.000340913	0.008963481	1
Prediabetes	duct_epithelial_cell	*ZNF445*	-0.257009492	0.007765693	0.029337893	1
Prediabetes	duct_epithelial_cell	*RBM11*	-0.085418793	0.00742177	0.02853242	1
Prediabetes	duct_epithelial_cell	*ATP6V0A2*	-0.142867627	0.001142302	0.00963464	1
Prediabetes	duct_epithelial_cell	*RAD51D*	-0.162509714	0.002506838	0.014192172	1
Prediabetes	duct_epithelial_cell	*GPRIN3*	-0.073499791	0.008221605	0.03050539	1
Prediabetes	duct_epithelial_cell	*P4HB*	-0.074890008	0.001690262	0.011199058	1
Prediabetes	duct_epithelial_cell	*DRG1*	-0.223091209	1.21E-05	0.008963481	0.047710491
Prediabetes	duct_epithelial_cell	*MORF4L1*	-0.089749355	0.000890195	0.00905104	1
Prediabetes	duct_epithelial_cell	*DMWD*	-0.115974165	0.000371853	0.008963481	1
Prediabetes	duct_epithelial_cell	*ZNF529*	-0.16396428	0.000474032	0.008963481	1
Prediabetes	duct_epithelial_cell	*AIDA*	-0.084791539	0.014509093	0.045972762	1
Prediabetes	duct_epithelial_cell	*WWOX*	-0.223476664	0.000232543	0.008963481	0.913662547
Prediabetes	duct_epithelial_cell	*POLR1D*	-0.108784167	0.000723912	0.008963481	1
Prediabetes	duct_epithelial_cell	*TOR3A*	-0.173020805	0.000267127	0.008963481	1
Prediabetes	duct_epithelial_cell	*ZNF555*	-0.086519477	0.004329366	0.019802187	1
Prediabetes	duct_epithelial_cell	*ZNF197*	-0.175925496	0.001087855	0.009519336	1
Prediabetes	duct_epithelial_cell	*RPS23*	-0.065564808	0.001393373	0.010271223	1
Prediabetes	duct_epithelial_cell	*ZNF396*	-0.114832741	0.004519013	0.020478893	1
Prediabetes	duct_epithelial_cell	*FAM86B1*	-0.845942662	0.000386516	0.008963481	1
Prediabetes	duct_epithelial_cell	*RTN4RL2*	-0.135798555	0.005396469	0.023121839	1
Prediabetes	duct_epithelial_cell	*TMEM232*	-0.128785419	0.004768783	0.021123605	1
Prediabetes	duct_epithelial_cell	*FAM183A*	-0.0491613	0.015717205	0.048971372	1
Prediabetes	duct_epithelial_cell	*BCAM*	-0.065841093	0.004688817	0.020910738	1
Prediabetes	duct_epithelial_cell	*CHP1*	-0.165807034	0.003895868	0.018689701	1
Prediabetes	duct_epithelial_cell	*DHRS4L2*	-0.167106057	0.000704352	0.008963481	1
Prediabetes	duct_epithelial_cell	*KBTBD12*	-0.095333655	0.001116307	0.009564575	1
Prediabetes	duct_epithelial_cell	*KLHL17*	-0.129079284	0.010437066	0.036028262	1
Prediabetes	duct_epithelial_cell	*RINL*	-0.177033969	0.001338608	0.010242129	1
Prediabetes	duct_epithelial_cell	*ZNF490*	-0.122346001	0.005538022	0.023421841	1
Prediabetes	duct_epithelial_cell	*FAM166A*	-0.238735576	0.000232479	0.008963481	0.913410247
Prediabetes	duct_epithelial_cell	*LAMTOR4*	-0.069420079	0.00814577	0.030336239	1
Prediabetes	duct_epithelial_cell	*ZNF559*	-0.029144201	0.006522925	0.025939852	1
Prediabetes	duct_epithelial_cell	*FOCAD*	-0.095304909	0.006802162	0.026859993	1
Prediabetes	duct_epithelial_cell	*FBLL1*	-0.081557978	0.009651274	0.033906075	1
Prediabetes	duct_epithelial_cell	*UROS*	-0.211604115	0.000440334	0.008963481	1
Prediabetes	duct_epithelial_cell	*FAM221A*	-0.079112087	0.008460389	0.030969628	1
Prediabetes	duct_epithelial_cell	*MTF1*	-0.100743472	0.002883203	0.015391907	1
Prediabetes	duct_epithelial_cell	*RPL14*	-0.070414022	0.000542522	0.008963481	1
Prediabetes	duct_epithelial_cell	*ZNF567*	-0.164606362	0.000926774	0.009057947	1
Prediabetes	duct_epithelial_cell	*ARID2*	-0.127183932	0.00063895	0.008963481	1
Prediabetes	duct_epithelial_cell	*PNRC2*	-0.119324592	0.001944038	0.012221002	1
Prediabetes	duct_epithelial_cell	*SPATA41*	-0.181947269	0.007379223	0.028419569	1
Prediabetes	duct_epithelial_cell	*ZNF681*	-0.221684816	0.002464574	0.013993224	1
Prediabetes	duct_epithelial_cell	*MPHOSPH8*	-0.095873125	0.001201183	0.009750216	1
Prediabetes	duct_epithelial_cell	*LCOR*	-0.142174782	0.000264682	0.008963481	1
Prediabetes	duct_epithelial_cell	*XPNPEP3*	-0.14187082	0.001111016	0.009564575	1
Prediabetes	duct_epithelial_cell	*ZNF471*	-0.324026754	0.001388528	0.010271223	1

Prediabetes	duct_epithelial_cell	*ZNF493*	-0.212402748	0.000492332	0.008963481	1
Prediabetes	duct_epithelial_cell	*GTF2IRD2*	-0.168105658	0.004656867	0.020862975	1
Prediabetes	duct_epithelial_cell	*LONP1*	-0.121219682	0.00499096	0.021861184	1
Prediabetes	duct_epithelial_cell	*ZNF34*	-0.223533372	0.004223145	0.019551648	1
Prediabetes	duct_epithelial_cell	*ZNF124*	-0.352449188	0.002175049	0.012948135	1
Prediabetes	duct_epithelial_cell	*XRCC6*	-0.093031318	0.002422972	0.013837002	1
Prediabetes	duct_epithelial_cell	*ZNF502*	-0.29594826	0.000447311	0.008963481	1
Prediabetes	duct_epithelial_cell	*ZNF33B*	-0.15118933	0.014432504	0.045803964	1
Prediabetes	duct_epithelial_cell	*AMZ2*	-0.11771932	0.002153786	0.012899732	1
Prediabetes	duct_epithelial_cell	*ZNF431*	-0.175048916	0.002669524	0.014746113	1
Prediabetes	duct_epithelial_cell	*FAM3C*	-0.140059529	0.000706042	0.008963481	1
Prediabetes	duct_epithelial_cell	*AP2A1*	-0.145618433	0.000885005	0.00905104	1
Prediabetes	duct_epithelial_cell	*ZNF429*	-0.246461849	0.000442139	0.008963481	1
Prediabetes	duct_epithelial_cell	*ZNF682*	-0.208893105	0.001151958	0.009685284	1
Prediabetes	duct_epithelial_cell	*SND1*	-0.074418783	0.011138322	0.037693769	1
Prediabetes	duct_epithelial_cell	*PIWIL2*	-0.063667119	0.001759013	0.011461296	1
Prediabetes	duct_epithelial_cell	*GTF2E2*	-0.136639352	0.004068263	0.019165715	1
Prediabetes	duct_epithelial_cell	*OGDHL*	-0.037402247	0.003536487	0.017534673	1
Prediabetes	duct_epithelial_cell	*RPS26*	-0.044933798	0.013396309	0.043427474	1
Prediabetes	duct_epithelial_cell	*LHFPL5*	-0.119905092	0.008524026	0.031108424	1
Prediabetes	duct_epithelial_cell	*ATAD3A*	-0.12787455	0.005142936	0.022278498	1
Prediabetes	duct_epithelial_cell	*SLC9A8*	-0.262842784	0.003248872	0.016734297	1
Prediabetes	duct_epithelial_cell	*RPL12*	-0.054493815	0.002003084	0.012393885	1
Prediabetes	duct_epithelial_cell	*ENTPD7*	-0.091558492	0.008742995	0.031543827	1
Prediabetes	duct_epithelial_cell	*ZNF273*	-0.194000289	0.012309628	0.04084842	1
Prediabetes	duct_epithelial_cell	*ZNF84*	-0.159275135	0.001385709	0.010271223	1
Prediabetes	duct_epithelial_cell	*ZNF667*	-0.160782503	0.002153655	0.012899732	1
Prediabetes	duct_epithelial_cell	*CD2AP*	-0.216567958	0.00071169	0.008963481	1
Prediabetes	duct_epithelial_cell	*SFI1*	-0.078957448	0.007008366	0.027368761	1
Prediabetes	duct_epithelial_cell	*SVIP*	-0.09084218	0.001295536	0.010059608	1
Prediabetes	duct_epithelial_cell	*DDRGK1*	-0.115019782	0.010989066	0.037414245	1
Prediabetes	duct_epithelial_cell	*HSD17B11*	-0.106129459	0.00799701	0.030059963	1
Prediabetes	duct_epithelial_cell	*HELZ*	-0.15448286	0.000840524	0.008963481	1
Prediabetes	duct_epithelial_cell	*ZNF583*	-0.152153939	0.002198765	0.012987238	1
Prediabetes	duct_epithelial_cell	*ZNF587*	-0.215482759	0.000623038	0.008963481	1
Prediabetes	duct_epithelial_cell	*ZNF627*	-0.169840243	0.001698885	0.01123724	1
Prediabetes	duct_epithelial_cell	*NCOA6*	-0.212762132	0.003310613	0.016870817	1
Prediabetes	duct_epithelial_cell	*PAPSS2*	-0.062678376	0.002869966	0.015391043	1
Prediabetes	duct_epithelial_cell	*CEP290*	-0.159797365	0.001068597	0.009434874	1
Prediabetes	duct_epithelial_cell	*ECI2*	-0.102126597	0.005995852	0.024616198	1
Prediabetes	duct_epithelial_cell	*DENND4B*	-0.10180204	0.004722025	0.020987373	1
Prediabetes	duct_epithelial_cell	*TSEN15*	-0.184651935	0.000465799	0.008963481	1
Prediabetes	duct_epithelial_cell	*CCDC152*	-0.109685723	0.001623866	0.011022922	1
Prediabetes	duct_epithelial_cell	*MAP3K3*	-0.201602828	0.001541343	0.010718604	1
Prediabetes	duct_epithelial_cell	*C1orf174*	-0.127139443	0.002821684	0.015228566	1
Prediabetes	duct_epithelial_cell	*TBKBP1*	-0.049382619	0.010987813	0.037414245	1
Prediabetes	duct_epithelial_cell	*CCDC167*	-0.138051792	0.016090905	0.049702174	1
Prediabetes	duct_epithelial_cell	*SOWAHA*	-0.115329767	0.004126077	0.019299233	1
Prediabetes	duct_epithelial_cell	*PJA2*	-0.061691875	0.00759873	0.028976426	1
Prediabetes	duct_epithelial_cell	*EFCAB2*	-0.081707432	0.005507879	0.023344611	1
Prediabetes	duct_epithelial_cell	*SAMD5*	-0.07731194	0.004412237	0.020064443	1
Prediabetes	duct_epithelial_cell	*PCMTD2*	-0.153903808	0.00095146	0.009132479	1
Prediabetes	duct_epithelial_cell	*NOTCH4*	-0.031744308	0.007005839	0.027368761	1
Prediabetes	duct_epithelial_cell	*MRPL38*	-0.117660115	0.006345118	0.025438743	1
Prediabetes	duct_epithelial_cell	*ABCF1*	-0.105180199	0.003926744	0.018769071	1
Prediabetes	duct_epithelial_cell	*ASPDH*	-0.185896287	0.004741392	0.021049638	1
Prediabetes	duct_epithelial_cell	*SARNP*	-0.228017037	2.29E-05	0.008963481	0.089950414
Prediabetes	duct_epithelial_cell	*LCMT1*	-0.19422821	0.000387537	0.008963481	1
Prediabetes	duct_epithelial_cell	*LIN52*	-0.180276067	0.00216242	0.012919364	1
Prediabetes	duct_epithelial_cell	*ZNF254*	-0.174111134	0.000313569	0.008963481	1
Prediabetes	duct_epithelial_cell	*SUPT4H1*	-0.198859505	0.000789137	0.008963481	1
Prediabetes	duct_epithelial_cell	*C8orf82*	-0.104390078	0.004872774	0.021463151	1
Prediabetes	duct_epithelial_cell	*LEPROT*	-0.158370173	0.000674577	0.008963481	1
Prediabetes	duct_epithelial_cell	*PPP1CB*	-0.109359547	0.000835863	0.008963481	1

Prediabetes	duct_epithelial_cell	*NUDT19*	-0.103879393	0.002663968	0.014746113	1
Prediabetes	duct_epithelial_cell	*ZNF90*	-0.50979216	0.000262568	0.008963481	1
Prediabetes	duct_epithelial_cell	*REPIN1*	-0.169830974	0.000692775	0.008963481	1
Prediabetes	duct_epithelial_cell	*MEG3*	-0.060536668	0.003900812	0.018690598	1
Prediabetes	duct_epithelial_cell	*EML6*	-0.127754589	0.005778837	0.024023343	1
Prediabetes	duct_epithelial_cell	*IFRD2*	-0.23584343	0.000334034	0.008963481	1
Prediabetes	duct_epithelial_cell	*C5orf49*	-0.102297948	0.004192068	0.019468837	1
Prediabetes	duct_epithelial_cell	*VPS16*	-0.10601946	0.009000806	0.032207801	1
Prediabetes	duct_epithelial_cell	*ATAD3C*	-0.079969868	0.009455938	0.033380395	1
Prediabetes	duct_epithelial_cell	*PAM16*	-0.142555843	0.000514842	0.008963481	1
Prediabetes	duct_epithelial_cell	*CELA3B*	-0.024184468	0.012744221	0.041783516	1
Prediabetes	duct_epithelial_cell	*VAMP2*	-0.074823997	0.001431675	0.010357204	1
Prediabetes	duct_epithelial_cell	*PPP3R1*	-0.139969392	0.000943564	0.009132479	1
Prediabetes	duct_epithelial_cell	*CEBPD*	-0.121383927	0.000553481	0.008963481	1
Prediabetes	duct_epithelial_cell	*UBA52*	-0.063406228	0.006073164	0.024776105	1
Prediabetes	duct_epithelial_cell	*ARL17B*	-0.076538514	0.015921719	0.049373666	1
Prediabetes	duct_epithelial_cell	*HSBP1*	-0.108109729	0.001578445	0.010899314	1
Prediabetes	duct_epithelial_cell	*RPS18*	-0.059257462	0.000966218	0.009147642	1
Prediabetes	duct_epithelial_cell	*SNHG15*	-0.112690205	0.003293789	0.016828736	1
Prediabetes	duct_epithelial_cell	*RPS28*	-0.061265523	0.001819535	0.011681295	1
Prediabetes	duct_epithelial_cell	*ZNF879*	-0.398628588	7.42E-05	0.008963481	0.291378099
Prediabetes	duct_epithelial_cell	*PINLYP*	0.104427179	0.008308274	0.030679707	1
Prediabetes	duct_epithelial_cell	*ANKRD65*	-0.081665187	0.005712848	0.023853116	1
Prediabetes	duct_epithelial_cell	*ZNF737*	-0.298947187	2.12E-05	0.008963481	0.083265099
Prediabetes	duct_epithelial_cell	*TXNDC5*	-0.089590881	0.005946999	0.024518112	1
Prediabetes	duct_epithelial_cell	*NME1*	-0.067449697	0.008742093	0.031543827	1
Prediabetes	duct_epithelial_cell	*CDRT4*	-0.087503633	0.007083016	0.027499182	1
Prediabetes	duct_epithelial_cell	*SMKR1*	-0.07463146	0.006726001	0.026639575	1
Prediabetes	duct_epithelial_cell	*LINC00886*	-0.058633573	0.014854539	0.046765612	1
Prediabetes	duct_epithelial_cell	*MIF*	-0.063283251	0.001460945	0.01048303	1
Prediabetes	duct_epithelial_cell	*NSUN6*	-0.192380817	0.000662442	0.008963481	1
Prediabetes	duct_epithelial_cell	*CRCP*	-0.091151813	0.007446885	0.028573059	1
Prediabetes	duct_epithelial_cell	*PI4KA*	-0.13563189	0.0010099	0.009336398	1
Prediabetes	duct_epithelial_cell	*DECR2*	-0.181681809	0.014956726	0.046974403	1
Prediabetes	duct_epithelial_cell	*GNG10*	-0.048210212	0.012477539	0.041127729	1
Prediabetes	duct_epithelial_cell	*PSG4*	0.20815524	0.002345215	0.013510775	1
Prediabetes	duct_epithelial_cell	*MICAL3*	-0.199460315	0.006570216	0.026075129	1
Prediabetes	duct_epithelial_cell	*VSIG8*	-0.467056533	0.000528266	0.008963481	1
Prediabetes	duct_epithelial_cell	*C4orf48*	-0.082691657	0.001426771	0.010342775	1
Prediabetes	duct_epithelial_cell	*IL10RB*	-0.123917794	0.001283013	0.010041748	1
Prediabetes	duct_epithelial_cell	*TMEM141*	-0.166034899	5.69E-05	0.008963481	0.223444659
Prediabetes	duct_epithelial_cell	*NEAT1*	-0.054627875	0.003812854	0.018364621	1
Prediabetes	duct_epithelial_cell	*LINC01091*	-0.048004845	0.011504318	0.038699029	1
Prediabetes	duct_epithelial_cell	*PDCD6*	-0.126003121	0.004161554	0.019406459	1
Prediabetes	duct_epithelial_cell	*ATP6V1E2*	-0.097437558	0.012636188	0.041546093	1
Prediabetes	duct_epithelial_cell	*ZNF550*	-0.161837938	0.001241003	0.009849499	1
Prediabetes	duct_epithelial_cell	*ALG11*	-0.370308369	0.000187071	0.008963481	0.735003271
Prediabetes	duct_epithelial_cell	*SNHG9*	-0.094512024	0.000391153	0.008963481	1
Prediabetes	duct_epithelial_cell	*ZNF432*	-0.159142287	0.000676356	0.008963481	1
Prediabetes	duct_epithelial_cell	*ZNF10*	-0.166713698	0.001681537	0.011177897	1
Prediabetes	duct_epithelial_cell	*GATC*	-0.150631505	0.004437547	0.020156211	1
Prediabetes	duct_epithelial_cell	*TEN1*	-0.095114243	0.00394324	0.018783824	1
Prediabetes	duct_epithelial_cell	*CHURC1*	-0.062963335	0.01484694	0.046765612	1
Prediabetes	duct_epithelial_cell	*TMEM179*	-0.04699401	0.013882688	0.044490278	1
Prediabetes	duct_epithelial_cell	*TMEM178B*	-0.076751892	0.001971542	0.01235437	1
Prediabetes	duct_epithelial_cell	*LINC01003*	-0.186899669	0.000410644	0.008963481	1
Prediabetes	duct_epithelial_cell	*MRPL12*	-0.186844707	0.000579441	0.008963481	1
Prediabetes	duct_epithelial_cell	*LINC00667*	-0.119642634	0.001680254	0.011177897	1
Prediabetes	duct_epithelial_cell	*FBXO17*	-0.103000842	0.003814083	0.018364621	1
Prediabetes	duct_epithelial_cell	*SNHG8*	-0.062797446	0.00297207	0.015695248	1
Prediabetes	duct_epithelial_cell	*GTF2H5*	-0.138211901	0.001544681	0.010722704	1
Prediabetes	PP_cell	*GCLC*	-0.310359378	0.002550512	0.01537007	1
Prediabetes	PP_cell	*LASP1*	-0.307808579	0.001461864	0.011646409	1
Prediabetes	PP_cell	*SLC25A13*	-0.411728505	0.00307958	0.016741022	1

Prediabetes	PP_cell	*PON1*	-0.084080162	0.01463689	0.039524633	1
Prediabetes	PP_cell	*IBTK*	-0.309129654	0.001280359	0.011111559	1
Prediabetes	PP_cell	*SYNRG*	-0.361715777	0.001077152	0.010267243	1
Prediabetes	PP_cell	*IL32*	-0.176244993	0.000839134	0.009444968	1
Prediabetes	PP_cell	*MED24*	-0.443513327	0.001989727	0.013597804	1
Prediabetes	PP_cell	*ETV7*	-0.156593721	0.006028735	0.024356489	1
Prediabetes	PP_cell	*GIPR*	-0.147509042	0.011824748	0.035704358	1
Prediabetes	PP_cell	*TTC19*	-0.223754562	0.016563731	0.04234151	1
Prediabetes	PP_cell	*LARS2*	-0.248445647	0.015084481	0.040072295	1
Prediabetes	PP_cell	*PPP5C*	-0.420817798	0.001783552	0.012957043	1
Prediabetes	PP_cell	*ERCC1*	-0.299060671	0.000220517	0.008519859	0.866412642
Prediabetes	PP_cell	*EXTL3*	-0.292673616	0.003553067	0.018224541	1
Prediabetes	PP_cell	*ALOX5*	-0.132694136	0.009036808	0.030982214	1
Prediabetes	PP_cell	*MAN2B2*	-0.448612726	0.000327958	0.008519859	1
Prediabetes	PP_cell	*DDX11*	-0.243064383	0.00691085	0.026361874	1
Prediabetes	PP_cell	*MDH1*	-0.271411813	0.000737584	0.009084543	1
Prediabetes	PP_cell	*DPEP1*	-0.084119265	0.010387016	0.033588961	1
Prediabetes	PP_cell	*TTC27*	-0.268240799	0.01460159	0.039474478	1
Prediabetes	PP_cell	*RNASET2*	-0.255099557	0.000406965	0.008519859	1
Prediabetes	PP_cell	*CD44*	-0.109458713	0.001115776	0.010487757	1
Prediabetes	PP_cell	*AGPAT4*	-0.37446771	0.002095823	0.01395676	1
Prediabetes	PP_cell	*SH2D2A*	-0.249213869	0.015781821	0.04125534	1
Prediabetes	PP_cell	*VEZT*	-0.284942902	0.005936981	0.024153447	1
Prediabetes	PP_cell	*BAK1*	-0.137668222	0.014265308	0.039085352	1
Prediabetes	PP_cell	*ABCF2*	-0.448939198	0.00404678	0.019357575	1
Prediabetes	PP_cell	*APBA2*	-0.174459108	0.012949847	0.037800852	1
Prediabetes	PP_cell	*ASTE1*	-0.187613116	0.009973314	0.032763505	1
Prediabetes	PP_cell	*SH3YL1*	-0.128795719	0.003154772	0.017002881	1
Prediabetes	PP_cell	*FAM136A*	-0.282815814	0.000306634	0.008519859	1
Prediabetes	PP_cell	*MYOM2*	-0.383366355	0.00434788	0.020119092	1
Prediabetes	PP_cell	*RPL26L1*	-0.268461711	0.004437193	0.020248234	1
Prediabetes	PP_cell	*NSUN2*	-0.412329336	0.000357579	0.008519859	1
Prediabetes	PP_cell	*AGA*	-0.385339396	0.000273321	0.008519859	1
Prediabetes	PP_cell	*SPATA7*	-0.266154052	0.0068627	0.026305899	1
Prediabetes	PP_cell	*CAPG*	-0.077468341	0.003986957	0.019154283	1
Prediabetes	PP_cell	*ADRB1*	-0.07591933	0.014391632	0.039235459	1
Prediabetes	PP_cell	*PREX2*	-0.250074336	0.017354859	0.043822135	1
Prediabetes	PP_cell	*WDR37*	-0.379193475	0.001291416	0.011111559	1
Prediabetes	PP_cell	*DTNBP1*	-0.4024696	0.002044997	0.01385309	1
Prediabetes	PP_cell	*GOPC*	-0.364496815	0.002787585	0.0160564	1
Prediabetes	PP_cell	*MRPS10*	-0.18061212	0.007662043	0.027998993	1
Prediabetes	PP_cell	*ERCC8*	-0.436224737	0.000552092	0.008519859	1
Prediabetes	PP_cell	*CYBA*	-0.299113375	0.001684573	0.012559178	1
Prediabetes	PP_cell	*THOC3*	-0.185376532	0.00155739	0.011998012	1
Prediabetes	PP_cell	*HEBP2*	-0.264225639	0.001590245	0.012147101	1
Prediabetes	PP_cell	*FNIP2*	-0.298704297	0.000438465	0.008519859	1
Prediabetes	PP_cell	*MRTO4*	-0.221284652	0.013426633	0.03803406	1
Prediabetes	PP_cell	*MCF2L2*	-0.226007096	0.001996486	0.013597804	1
Prediabetes	PP_cell	*LAMA3*	-0.104958868	0.011785733	0.035704358	1
Prediabetes	PP_cell	*ANAPC4*	-0.336764711	0.001468982	0.011646409	1
Prediabetes	PP_cell	*LY75*	-0.244884609	0.002674002	0.015685035	1
Prediabetes	PP_cell	*OPN3*	-0.192135395	0.017028308	0.04320283	1
Prediabetes	PP_cell	*TBC1D22A*	-0.326875084	0.000415136	0.008519859	1
Prediabetes	PP_cell	*SYNE2*	-0.290185791	0.008400742	0.029688131	1
Prediabetes	PP_cell	*ATP9A*	-0.157708789	0.001616795	0.012263296	1
Prediabetes	PP_cell	*CYFIP2*	-0.238549108	0.016512417	0.042268461	1
Prediabetes	PP_cell	*MCOLN3*	-0.044675175	0.012125513	0.036255474	1
Prediabetes	PP_cell	*ITIH4*	-0.080301654	0.014854281	0.039783551	1
Prediabetes	PP_cell	*GDI2*	-0.324186778	0.000365435	0.008519859	1
Prediabetes	PP_cell	*LAMC2*	-0.068818347	0.018809749	0.046626816	1
Prediabetes	PP_cell	*RASGRF1*	-0.127372816	0.016153869	0.041700755	1
Prediabetes	PP_cell	*CROCC*	-0.236071812	0.01056552	0.033914974	1
Prediabetes	PP_cell	*GNA15*	-0.353067775	0.01951053	0.047834311	1
Prediabetes	PP_cell	*RIMBP2*	-0.146077979	0.003735878	0.018556591	1

Prediabetes	PP_cell	*MPC1*	-0.293368281	0.001752767	0.012855972	1
Prediabetes	PP_cell	*BCAT1*	-0.06999088	0.009895836	0.032720527	1
Prediabetes	PP_cell	*EIF4B*	-0.191751446	0.009598874	0.032069706	1
Prediabetes	PP_cell	*SPHK2*	-0.344827305	0.002073459	0.013902083	1
Prediabetes	PP_cell	*RNF4*	-0.342645052	0.003770429	0.018659548	1
Prediabetes	PP_cell	*TM7SF3*	-0.229298926	0.002379587	0.014840315	1
Prediabetes	PP_cell	*ST3GAL6*	-0.29339817	7.45E-05	0.008519859	0.29254693
Prediabetes	PP_cell	*TMEM161A*	-0.476991096	0.001744811	0.012855972	1
Prediabetes	PP_cell	*SNX24*	-0.322963858	0.003632551	0.01839612	1
Prediabetes	PP_cell	*PMS1*	-0.185645027	0.004085492	0.019452089	1
Prediabetes	PP_cell	*IPO5*	-0.266884985	0.00941047	0.031819051	1
Prediabetes	PP_cell	*OAT*	-0.242410285	0.014299957	0.039125719	1
Prediabetes	PP_cell	*GSTO2*	-0.281038498	0.000197392	0.008519859	0.775554376
Prediabetes	PP_cell	*CTNNA2*	-0.166281946	0.009487346	0.031941544	1
Prediabetes	PP_cell	*STOML1*	-0.188219376	0.001820141	0.013073736	1
Prediabetes	PP_cell	*HEATR6*	-0.326245823	0.019092341	0.047089646	1
Prediabetes	PP_cell	*REEP1*	-0.234526692	0.003383886	0.017633008	1
Prediabetes	PP_cell	*POLR1A*	-0.194939492	0.000677728	0.008780065	1
Prediabetes	PP_cell	*MAST4*	-0.355698335	0.000335739	0.008519859	1
Prediabetes	PP_cell	*CLEC2D*	-0.527568422	0.001624262	0.012296195	1
Prediabetes	PP_cell	*PLA2G10*	-0.297950135	0.004989624	0.021710114	1
Prediabetes	PP_cell	*RAB27A*	-0.251793687	0.000674282	0.008780065	1
Prediabetes	PP_cell	*PTPN3*	-0.291322523	0.000451904	0.008519859	1
Prediabetes	PP_cell	*FGF10*	-0.235557421	0.003197257	0.017184708	1
Prediabetes	PP_cell	*DGCR2*	-0.397603139	0.000294413	0.008519859	1
Prediabetes	PP_cell	*ASNS*	-0.168962699	0.004119429	0.019523809	1
Prediabetes	PP_cell	*RPL31*	-0.224284809	0.000991738	0.009839747	1
Prediabetes	PP_cell	*SEL1L*	-0.277307253	0.002238474	0.014348148	1
Prediabetes	PP_cell	*CPSF1*	-0.18108201	0.011857614	0.035727429	1
Prediabetes	PP_cell	*ALDH3A2*	-0.165234225	0.00535022	0.022774663	1
Prediabetes	PP_cell	*P4HA2*	-0.191693543	0.012266119	0.036372141	1
Prediabetes	PP_cell	*TRNT1*	-0.456140227	0.002338926	0.014690752	1
Prediabetes	PP_cell	*CRMP1*	-0.306656127	0.000208254	0.008519859	0.818228967
Prediabetes	PP_cell	*DERL2*	-0.233224915	0.017093826	0.043330092	1
Prediabetes	PP_cell	*NDE1*	-0.288730809	0.000179717	0.008519859	0.70610884
Prediabetes	PP_cell	*SCARB1*	-0.21814856	0.005594518	0.023253761	1
Prediabetes	PP_cell	*NLE1*	-0.068772371	0.01860953	0.046247213	1
Prediabetes	PP_cell	*MRPS34*	-0.232265107	0.002476991	0.015206402	1
Prediabetes	PP_cell	*IPCEF1*	-0.137634446	0.007610695	0.027900502	1
Prediabetes	PP_cell	*ANO8*	-0.177808691	0.003596562	0.018323322	1
Prediabetes	PP_cell	*GRAMD4*	-0.157466037	0.001129151	0.010537852	1
Prediabetes	PP_cell	*TIMM21*	-0.359339612	0.007359007	0.027276923	1
Prediabetes	PP_cell	*CACNG5*	-2.297660509	0.003086167	0.016741022	1
Prediabetes	PP_cell	*EXOSC7*	-0.317867499	0.009918592	0.032720527	1
Prediabetes	PP_cell	*MKRN2*	-0.258456177	0.001458715	0.011646409	1
Prediabetes	PP_cell	*TPD52*	-0.285799682	0.00018572	0.008519859	0.729693382
Prediabetes	PP_cell	*ACACB*	-0.266007197	0.000444686	0.008519859	1
Prediabetes	PP_cell	*GPATCH1*	-0.284550211	0.020586376	0.049760752	1
Prediabetes	PP_cell	*ICAM3*	-0.10276832	0.017564789	0.044153587	1
Prediabetes	PP_cell	*ACTL6B*	-0.168967643	0.002651394	0.01561651	1
Prediabetes	PP_cell	*APBB1IP*	0.105745311	0.00583552	0.023907984	1
Prediabetes	PP_cell	*NAALAD2*	-0.191712784	0.010044151	0.032886224	1
Prediabetes	PP_cell	*MCCC1*	-0.177702535	0.002084353	0.013904889	1
Prediabetes	PP_cell	*N4BP2*	-0.317923778	0.008904829	0.030690414	1
Prediabetes	PP_cell	*VDAC3*	-0.212593639	0.015151172	0.040167986	1
Prediabetes	PP_cell	*TP73*	-0.790487395	0.013474365	0.038114313	1
Prediabetes	PP_cell	*TOLLIP*	-0.28145024	0.001952558	0.013500582	1
Prediabetes	PP_cell	*CLUL1*	-0.398687505	0.009589116	0.032064372	1
Prediabetes	PP_cell	*FKBP7*	-0.36173626	0.001996929	0.013597804	1
Prediabetes	PP_cell	*LXN*	-0.188149204	0.000509639	0.008519859	1
Prediabetes	PP_cell	*FDFT1*	-0.322151141	1.37E-05	0.008519859	0.05389867
Prediabetes	PP_cell	*SCGN*	-0.141184438	0.006298117	0.024961783	1
Prediabetes	PP_cell	*SCTR*	-0.063648034	0.015914497	0.041381906	1
Prediabetes	PP_cell	*ZNF506*	-0.28986405	0.014653138	0.039533432	1

Prediabetes	PP_cell	*CADPS2*	-0.276327265	0.001790714	0.012963792	1
Prediabetes	PP_cell	*ME2*	-0.396750669	0.000397697	0.008519859	1
Prediabetes	PP_cell	*CCNT2*	-0.19736883	0.011344067	0.035126529	1
Prediabetes	PP_cell	*PIBF1*	-0.386475421	0.001341174	0.011283666	1
Prediabetes	PP_cell	*CHMP2B*	-0.236848796	0.00650413	0.02547829	1
Prediabetes	PP_cell	*PPIE*	-0.238507941	0.001017928	0.009973668	1
Prediabetes	PP_cell	*CD82*	-0.124755258	0.013187659	0.037909761	1
Prediabetes	PP_cell	*SLC25A24*	-0.29932633	0.003916228	0.019030442	1
Prediabetes	PP_cell	*CPNE3*	-0.285913008	0.000980242	0.009817609	1
Prediabetes	PP_cell	*DDHD2*	-0.193868528	0.001271548	0.011102024	1
Prediabetes	PP_cell	*TTC39A*	-0.194885217	0.002956015	0.016546556	1
Prediabetes	PP_cell	*EPDR1*	-0.304301477	0.001231681	0.010954577	1
Prediabetes	PP_cell	*ITPKC*	-0.385471051	0.015220091	0.040296319	1
Prediabetes	PP_cell	*TMED2*	-0.213329086	0.007975892	0.028855691	1
Prediabetes	PP_cell	*ACOX3*	-0.350538184	0.000274513	0.008519859	1
Prediabetes	PP_cell	*TRIP6*	-0.087666525	0.008205125	0.029383885	1
Prediabetes	PP_cell	*PGS1*	-0.312563009	0.018295563	0.045524551	1
Prediabetes	PP_cell	*UIMC1*	-0.25979263	0.017032625	0.04320283	1
Prediabetes	PP_cell	*METTL2A*	-0.226464234	0.001256623	0.011020698	1
Prediabetes	PP_cell	*SLC15A1*	-0.130517283	0.015844621	0.041316373	1
Prediabetes	PP_cell	*TGDS*	-0.167121861	0.00590921	0.024134393	1
Prediabetes	PP_cell	*DOCK3*	-0.209066765	0.003687713	0.018410449	1
Prediabetes	PP_cell	*ARHGAP28*	-0.167322302	0.013411814	0.03803406	1
Prediabetes	PP_cell	*PPP1R13B*	-0.361066171	0.010912777	0.034411155	1
Prediabetes	PP_cell	*ZNF343*	-0.281759904	0.002165435	0.014132881	1
Prediabetes	PP_cell	*DZANK1*	-0.128672742	0.011735607	0.035704358	1
Prediabetes	PP_cell	*GRAMD1A*	-0.357679412	0.003326051	0.017447335	1
Prediabetes	PP_cell	*CDIP1*	-0.242474182	0.01090254	0.03440649	1
Prediabetes	PP_cell	*DDX24*	-0.194064007	0.011776749	0.035704358	1
Prediabetes	PP_cell	*SPTLC1*	-0.177523666	0.011351509	0.035126529	1
Prediabetes	PP_cell	*LYZ*	-0.068657907	0.014264203	0.039085352	1
Prediabetes	PP_cell	*NRCAM*	-0.158484166	0.001514742	0.011846392	1
Prediabetes	PP_cell	*PITPNM3*	-0.18093891	0.013606499	0.038249975	1
Prediabetes	PP_cell	*SCFD1*	-0.33244441	0.000544593	0.008519859	1
Prediabetes	PP_cell	*EZR*	-0.295557335	0.000674484	0.008780065	1
Prediabetes	PP_cell	*NUP50*	-0.336172076	0.002969031	0.016546556	1
Prediabetes	PP_cell	*COMT*	-0.321052577	0.000412019	0.008519859	1
Prediabetes	PP_cell	*SUCO*	-0.280149653	0.000536111	0.008519859	1
Prediabetes	PP_cell	*TMEM38B*	-0.233764614	0.006308743	0.024961783	1
Prediabetes	PP_cell	*CRAT*	-0.366032805	0.00507661	0.02194376	1
Prediabetes	PP_cell	*CWF19L1*	-0.123808478	0.003248605	0.017225057	1
Prediabetes	PP_cell	*SEMA4G*	-0.197843779	0.008627452	0.030190258	1
Prediabetes	PP_cell	*TLL2*	-0.264595681	0.013342421	0.037957281	1
Prediabetes	PP_cell	*SRPK1*	-0.253864562	0.001001166	0.009851066	1
Prediabetes	PP_cell	*ITPR3*	-0.103932585	0.006065396	0.024391958	1
Prediabetes	PP_cell	*IFT74*	-0.248011756	0.005009553	0.021772712	1
Prediabetes	PP_cell	*SYDE2*	-0.064145737	0.010932982	0.034447222	1
Prediabetes	PP_cell	*PCSK5*	-0.094761427	0.018171393	0.045272926	1
Prediabetes	PP_cell	*ABLIM1*	-0.267012467	0.000282458	0.008519859	1
Prediabetes	PP_cell	*MZF1*	-0.165905183	0.007109768	0.026721477	1
Prediabetes	PP_cell	*HSD3B7*	-0.121775134	0.004679686	0.020822749	1
Prediabetes	PP_cell	*TIMM13*	-0.233751647	0.011259602	0.034971522	1
Prediabetes	PP_cell	*CEP170B*	-0.114728497	0.00447544	0.020304855	1
Prediabetes	PP_cell	*RASSF7*	-0.359168877	0.002739909	0.015948298	1
Prediabetes	PP_cell	*ARVCF*	-0.44323773	0.000107581	0.008519859	0.422685569
Prediabetes	PP_cell	*MED15*	-0.339688636	0.011791438	0.035704358	1
Prediabetes	PP_cell	*SPECC1L*	-0.334261875	0.002441793	0.015060919	1
Prediabetes	PP_cell	*SLC25A1*	-0.306548181	0.007202874	0.02687568	1
Prediabetes	PP_cell	*GCAT*	-0.092866132	0.009799171	0.032572709	1
Prediabetes	PP_cell	*HSCB*	-0.200083773	0.003014218	0.016609903	1
Prediabetes	PP_cell	*CBY1*	-0.14443253	0.003176641	0.017097291	1
Prediabetes	PP_cell	*XBP1*	-0.152652828	0.000400662	0.008519859	1
Prediabetes	PP_cell	*MIOX*	-0.159518422	0.010579896	0.033933396	1
Prediabetes	PP_cell	*LMF2*	-0.138991987	0.009097421	0.031136291	1

Prediabetes	PP_cell	*TOM1*	-0.36855483	0.003377895	0.017625163	1
Prediabetes	PP_cell	*CHKB*	-0.228556845	0.002948763	0.016546556	1
Prediabetes	PP_cell	*BIK*	-0.086221471	0.014558649	0.03942173	1
Prediabetes	PP_cell	*ARSA*	-0.095245384	0.014524667	0.039384002	1
Prediabetes	PP_cell	*RBFOX2*	-0.311298539	0.001415966	0.011542178	1
Prediabetes	PP_cell	*SYNGR1*	-0.106497989	0.002966058	0.016546556	1
Prediabetes	PP_cell	*SGSM3*	-0.146331145	0.002252957	0.014369919	1
Prediabetes	PP_cell	*ST13*	-0.242494127	0.002573629	0.015428508	1
Prediabetes	PP_cell	*PMM1*	-0.204820024	0.001295264	0.011111559	1
Prediabetes	PP_cell	*PRMT5*	-0.514178486	0.000524167	0.008519859	1
Prediabetes	PP_cell	*VCPKMT*	-0.402524411	0.008980998	0.030871689	1
Prediabetes	PP_cell	*TRIM9*	-0.309900005	0.000764815	0.009244451	1
Prediabetes	PP_cell	*ATP6V1D*	-0.270710946	0.002502245	0.015266026	1
Prediabetes	PP_cell	*GSTZ1*	-0.146848184	0.002944614	0.016546556	1
Prediabetes	PP_cell	*TMED8*	-0.281398712	0.005683363	0.02351138	1
Prediabetes	PP_cell	*DAAM1*	-0.340563602	0.000488959	0.008519859	1
Prediabetes	PP_cell	*DHRS7*	-0.301288969	0.0008769	0.009444968	1
Prediabetes	PP_cell	*MTHFD1*	-0.400081564	0.007342337	0.027240832	1
Prediabetes	PP_cell	*ABHD12*	-0.238170784	0.009537989	0.032025881	1
Prediabetes	PP_cell	*CD40*	-0.468945074	0.001661943	0.012440588	1
Prediabetes	PP_cell	*SALL4*	-0.173240307	0.001915713	0.01336916	1
Prediabetes	PP_cell	*NELFCD*	-0.282015766	0.000448823	0.008519859	1
Prediabetes	PP_cell	*MTG2*	-0.362027201	0.004570974	0.020408359	1
Prediabetes	PP_cell	*ARFRP1*	-0.233185053	0.002053898	0.013865574	1
Prediabetes	PP_cell	*POFUT1*	-0.383791922	0.007455143	0.027529377	1
Prediabetes	PP_cell	*MROH8*	-0.102567708	0.011153749	0.034835517	1
Prediabetes	PP_cell	*NOP56*	-0.230439719	0.012862018	0.03760809	1
Prediabetes	PP_cell	*MANBAL*	-0.245674698	0.007189393	0.026854533	1
Prediabetes	PP_cell	*IDH3B*	-0.277367105	0.01179104	0.035704358	1
Prediabetes	PP_cell	*MAPRE1*	-0.23190292	0.010848986	0.034320181	1
Prediabetes	PP_cell	*TTI1*	-0.434075326	0.000407387	0.008519859	1
Prediabetes	PP_cell	*DNTTIP1*	-0.361922951	0.002137477	0.014043725	1
Prediabetes	PP_cell	*APMAP*	-0.117152447	0.016430437	0.04220961	1
Prediabetes	PP_cell	*MYOM1*	-0.116575722	0.010776116	0.034287057	1
Prediabetes	PP_cell	*RIOK3*	-0.257412695	0.003233517	0.017219313	1
Prediabetes	PP_cell	*FNDC3A*	-0.323588052	0.000277993	0.008519859	1
Prediabetes	PP_cell	*KLF5*	-0.073695551	0.007545863	0.027760015	1
Prediabetes	PP_cell	*DNAJC3*	-0.222329671	0.008757114	0.030367193	1
Prediabetes	PP_cell	*UGGT2*	-0.199911203	0.000982536	0.009817609	1
Prediabetes	PP_cell	*PARP4*	-0.25867269	0.008442889	0.029750772	1
Prediabetes	PP_cell	*CLN5*	-0.266369906	0.006841064	0.026292348	1
Prediabetes	PP_cell	*OLFM4*	-0.056047132	0.015463535	0.040748644	1
Prediabetes	PP_cell	*DHODH*	-0.194259005	0.004359301	0.020119092	1
Prediabetes	PP_cell	*USB1*	-0.41656957	0.001133438	0.010552788	1
Prediabetes	PP_cell	*CYB5B*	-0.308015023	0.010703721	0.03417498	1
Prediabetes	PP_cell	*NME3*	-0.342628326	0.000203613	0.008519859	0.799993617
Prediabetes	PP_cell	*VAC14*	-0.296090572	0.001521564	0.011846392	1
Prediabetes	PP_cell	*SMPD3*	-0.226082527	0.006636268	0.025799843	1
Prediabetes	PP_cell	*HAGHL*	-0.10435504	0.006811317	0.026269771	1
Prediabetes	PP_cell	*METRN*	-0.251347379	0.001651477	0.012440588	1
Prediabetes	PP_cell	*RHBDL1*	-0.154255959	0.005508753	0.023090348	1
Prediabetes	PP_cell	*PIEZO1*	-0.140417157	0.005730147	0.023599316	1
Prediabetes	PP_cell	*AQP8*	-0.063669948	0.017388681	0.043823045	1
Prediabetes	PP_cell	*HMOX2*	-0.36077933	0.000123217	0.008519859	0.48412085
Prediabetes	PP_cell	*KNOP1*	-0.356964594	0.008512244	0.029941457	1
Prediabetes	PP_cell	*TRIP4*	-0.355025479	0.002759598	0.015968275	1
Prediabetes	PP_cell	*AP3B2*	-0.183828207	0.003212524	0.017212803	1
Prediabetes	PP_cell	*RPAP1*	-0.185950461	0.003151182	0.017002881	1
Prediabetes	PP_cell	*HOMER2*	-0.170719819	0.001752987	0.012855972	1
Prediabetes	PP_cell	*DMXL2*	-0.308817223	0.000431449	0.008519859	1
Prediabetes	PP_cell	*ZDHHC2*	-0.310680991	0.000344216	0.008519859	1
Prediabetes	PP_cell	*TRIM35*	-0.173827189	0.003857941	0.018923659	1
Prediabetes	PP_cell	*RIPK2*	-0.245968617	0.004089444	0.019452089	1
Prediabetes	PP_cell	*LAPTM4B*	-0.300481618	0.001582029	0.012117789	1

Prediabetes	PP_cell	*EIF3E*	-0.258385461	0.000692565	0.008780065	1
Prediabetes	PP_cell	*ESRP1*	-0.373616014	0.000454995	0.008519859	1
Prediabetes	PP_cell	*ARMC1*	-0.322638589	0.000483752	0.008519859	1
Prediabetes	PP_cell	*SNX16*	-0.447724455	0.000379186	0.008519859	1
Prediabetes	PP_cell	*SH2D4A*	-0.16039282	0.011407667	0.035181102	1
Prediabetes	PP_cell	*INTS10*	-0.322627476	0.001765808	0.012915503	1
Prediabetes	PP_cell	*LEPROTL1*	-0.321185981	0.002959937	0.016546556	1
Prediabetes	PP_cell	*KLHDC4*	-0.280849655	0.000462674	0.008519859	1
Prediabetes	PP_cell	*MCM4*	-0.51086888	0.001791636	0.012963792	1
Prediabetes	PP_cell	*FGL1*	-0.065254341	0.013609943	0.038249975	1
Prediabetes	PP_cell	*ECH1*	-0.188681179	0.005334837	0.022733811	1
Prediabetes	PP_cell	*HNRNPL*	-0.222626991	0.011070906	0.034770255	1
Prediabetes	PP_cell	*SARS2*	-0.085921164	0.006848418	0.026292348	1
Prediabetes	PP_cell	*LIN7B*	-0.391077269	5.08E-05	0.008519859	0.199679214
Prediabetes	PP_cell	*PPP1R13L*	-0.082622123	0.009563991	0.032025881	1
Prediabetes	PP_cell	*ERCC2*	-0.414515046	0.00793464	0.028742703	1
Prediabetes	PP_cell	*KLC3*	-0.066491919	0.008857513	0.030634833	1
Prediabetes	PP_cell	*AMH*	-0.575951526	0.00068866	0.008780065	1
Prediabetes	PP_cell	*NUMBL*	-0.467065587	0.00024079	0.008519859	0.946063805
Prediabetes	PP_cell	*MYH14*	-0.158284479	0.004818082	0.021151112	1
Prediabetes	PP_cell	*CAPS*	-0.155128695	0.007033827	0.026624187	1
Prediabetes	PP_cell	*PPP2R1A*	-0.233404856	0.009916788	0.032720527	1
Prediabetes	PP_cell	*GCDH*	-0.17379527	0.002110496	0.013980215	1
Prediabetes	PP_cell	*PIK3R2*	-0.207362199	0.001538719	0.011877462	1
Prediabetes	PP_cell	*UPK1A*	-1.185796777	0.003064594	0.016741022	1
Prediabetes	PP_cell	*COPE*	-0.220884486	0.005804137	0.023804231	1
Prediabetes	PP_cell	*SIPA1L3*	-0.451097791	0.00039351	0.008519859	1
Prediabetes	PP_cell	*ETHE1*	-0.253605618	0.002283056	0.014499386	1
Prediabetes	PP_cell	*SMG9*	-0.217055774	0.019162768	0.047174509	1
Prediabetes	PP_cell	*ITGB8*	-0.33584716	0.00013013	0.008519859	0.51127946
Prediabetes	PP_cell	*DNAH11*	-0.37201856	0.001313156	0.011188791	1
Prediabetes	PP_cell	*ANKRD7*	-0.127165284	0.007667833	0.027998993	1
Prediabetes	PP_cell	*TSPAN12*	-0.478800411	0.000526694	0.008519859	1
Prediabetes	PP_cell	*ABHD11*	-0.182627179	0.002211798	0.014319626	1
Prediabetes	PP_cell	*CHCHD2*	-0.172733171	0.006794409	0.026255163	1
Prediabetes	PP_cell	*AGFG2*	-0.312322628	0.002228365	0.014336949	1
Prediabetes	PP_cell	*C1GALT1*	-0.266127371	0.013870059	0.038431214	1
Prediabetes	PP_cell	*RPA3*	-0.364143587	0.000226449	0.008519859	0.889719406
Prediabetes	PP_cell	*CLDN15*	-0.095388926	0.002611521	0.015570055	1
Prediabetes	PP_cell	*TMEM106B*	-0.254841269	0.002229543	0.014336949	1
Prediabetes	PP_cell	*TSPAN13*	-0.297059492	0.000295504	0.008519859	1
Prediabetes	PP_cell	*AGR2*	-0.047701311	0.015756623	0.04125534	1
Prediabetes	PP_cell	*COA1*	-0.369280137	6.84E-05	0.008519859	0.268893334
Prediabetes	PP_cell	*BLVRA*	-0.401359023	0.000122988	0.008519859	0.483220597
Prediabetes	PP_cell	*TMEM248*	-0.237427349	0.005438664	0.022930133	1
Prediabetes	PP_cell	*RHEB*	-0.252455844	0.001463371	0.011646409	1
Prediabetes	PP_cell	*LIMK1*	-0.251119037	0.002858251	0.016251909	1
Prediabetes	PP_cell	*SLC1A1*	-0.074524886	0.010221436	0.033269422	1
Prediabetes	PP_cell	*NMRK1*	-0.336524663	0.003325573	0.017447335	1
Prediabetes	PP_cell	*SUSD1*	-0.344670386	0.004467023	0.020304368	1
Prediabetes	PP_cell	*AK1*	-0.171097017	0.007068186	0.026651539	1
Prediabetes	PP_cell	*PLGRKT*	-0.165309724	0.008135956	0.029272821	1
Prediabetes	PP_cell	*NPDC1*	-0.228747749	0.00528597	0.022550027	1
Prediabetes	PP_cell	*HPS1*	-0.326108717	0.000448262	0.008519859	1
Prediabetes	PP_cell	*CXCL12*	-0.096651629	0.014151843	0.03888293	1
Prediabetes	PP_cell	*PLEKHA1*	-0.3177807	0.00027081	0.008519859	1
Prediabetes	PP_cell	*PALD1*	-0.33209836	0.003664374	0.01839612	1
Prediabetes	PP_cell	*SPOCK2*	-0.114480672	0.011685278	0.035704358	1
Prediabetes	PP_cell	*BMPR1A*	-0.353669726	0.000453175	0.008519859	1
Prediabetes	PP_cell	*LIPA*	-0.447307775	0.000766557	0.009244451	1
Prediabetes	PP_cell	*NPM3*	-0.141022387	0.005078946	0.02194376	1
Prediabetes	PP_cell	*NEURL1*	-0.137321209	0.002643768	0.01561651	1
Prediabetes	PP_cell	*SORCS1*	-0.244162706	0.011903217	0.035755154	1
Prediabetes	PP_cell	*XPNPEP1*	-0.328404977	0.011307839	0.0350936	1

Prediabetes	PP_cell	*SMC3*	-0.208823223	0.017858991	0.044721463	1
Prediabetes	PP_cell	*PBLD*	-0.155837529	0.003320447	0.017447335	1
Prediabetes	PP_cell	*DHRS11*	-0.377673774	0.000319617	0.008519859	1
Prediabetes	PP_cell	*TRIM37*	-0.405759511	0.000422199	0.008519859	1
Prediabetes	PP_cell	*TRIM16L*	-0.099267943	0.017483984	0.044034984	1
Prediabetes	PP_cell	*CDK5RAP3*	-0.1521252	0.014745575	0.039627472	1
Prediabetes	PP_cell	*MED13*	-0.221379956	0.004557361	0.020393929	1
Prediabetes	PP_cell	*RAI1*	-0.442751762	0.00391846	0.019030442	1
Prediabetes	PP_cell	*DRG2*	-0.440335564	0.000427172	0.008519859	1
Prediabetes	PP_cell	*SMARCD2*	-0.435987055	0.000507614	0.008519859	1
Prediabetes	PP_cell	*SYNGR2*	-0.249735942	0.003224389	0.017212803	1
Prediabetes	PP_cell	*C17orf75*	-0.126916483	0.002238589	0.014348148	1
Prediabetes	PP_cell	*NAGLU*	-0.27979974	5.40E-06	0.008519859	0.021207864
Prediabetes	PP_cell	*CNTNAP1*	0.089855303	0.006255484	0.024851158	1
Prediabetes	PP_cell	*RND2*	-0.749490895	0.00092272	0.009590919	1
Prediabetes	PP_cell	*ABCC3*	-0.066716891	0.003023629	0.016638427	1
Prediabetes	PP_cell	*MPP2*	-0.296082608	0.000487017	0.008519859	1
Prediabetes	PP_cell	*EFNB3*	-0.392524314	0.000260677	0.008519859	1
Prediabetes	PP_cell	*RANGRF*	-0.397813118	0.000303274	0.008519859	1
Prediabetes	PP_cell	*AADAT*	-0.350372903	0.005411891	0.022930133	1
Prediabetes	PP_cell	*TRIM2*	-0.333897613	0.003716545	0.01850736	1
Prediabetes	PP_cell	*SH3D19*	-0.503987442	0.000551443	0.008519859	1
Prediabetes	PP_cell	*GLRB*	-0.43525092	0.001656584	0.012440588	1
Prediabetes	PP_cell	*FAM149A*	-0.241984065	0.008764672	0.030367193	1
Prediabetes	PP_cell	*HTATIP2*	-0.160506945	0.007061896	0.026651539	1
Prediabetes	PP_cell	*B3GAT1*	-0.156913475	0.016062956	0.041521635	1
Prediabetes	PP_cell	*P2RX3*	-1.2737616	0.013163535	0.037909761	1
Prediabetes	PP_cell	*VWA5A*	-0.227470519	0.018794243	0.046626816	1
Prediabetes	PP_cell	*PRPF19*	-0.316919067	0.001208127	0.010887001	1
Prediabetes	PP_cell	*CEP164*	-0.247336284	0.004692494	0.020832552	1
Prediabetes	PP_cell	*MDK*	-0.311496476	0.001213937	0.010904418	1
Prediabetes	PP_cell	*MADD*	-0.260473001	0.000872098	0.009444968	1
Prediabetes	PP_cell	*GTF2H1*	-0.248045241	0.00054315	0.008519859	1
Prediabetes	PP_cell	*CORO1C*	-0.436583708	0.000562584	0.008521817	1
Prediabetes	PP_cell	*KRT18*	-0.058084582	0.004384554	0.02017203	1
Prediabetes	PP_cell	*ACSS3*	-0.154540449	0.01331179	0.0379275	1
Prediabetes	PP_cell	*WNT5B*	-0.144517721	0.013299896	0.0379275	1
Prediabetes	PP_cell	*ITFG2*	-0.219596888	0.001732089	0.012837572	1
Prediabetes	PP_cell	*GSG1*	-0.453136907	0.020606534	0.049760752	1
Prediabetes	PP_cell	*RASAL1*	-0.450887432	0.009953037	0.032751739	1
Prediabetes	PP_cell	*MDM1*	-0.186168201	0.002575999	0.015428508	1
Prediabetes	PP_cell	*NUP107*	-0.380535396	0.002806354	0.01609659	1
Prediabetes	PP_cell	*GNB3*	-0.21983803	0.00572842	0.023599316	1
Prediabetes	PP_cell	*CDCA3*	-0.169194397	0.002065873	0.013874899	1
Prediabetes	PP_cell	*SPSB2*	-0.292903565	0.011890804	0.035745194	1
Prediabetes	PP_cell	*NT5DC3*	-0.118235082	0.015248858	0.040345295	1
Prediabetes	PP_cell	*GOLT1B*	-0.325642111	0.003413954	0.017742628	1
Prediabetes	PP_cell	*C2CD5*	-0.385196147	0.000330946	0.008519859	1
Prediabetes	PP_cell	*FRK*	-0.464978896	0.00365639	0.01839612	1
Prediabetes	PP_cell	*RSPH4A*	-0.216014335	0.003563403	0.018246003	1
Prediabetes	PP_cell	*NEDD9*	-0.307647661	0.000151421	0.008519859	0.594932644
Prediabetes	PP_cell	*CEP85L*	-0.286918981	0.001346929	0.01130353	1
Prediabetes	PP_cell	*TPD52L1*	-0.155614269	0.000632307	0.008645299	1
Prediabetes	PP_cell	*RNF8*	-0.376731091	0.001470252	0.011646409	1
Prediabetes	PP_cell	*GLP1R*	-0.061191289	0.008413229	0.029699528	1
Prediabetes	PP_cell	*BACH2*	-0.270976555	0.000633488	0.008645299	1
Prediabetes	PP_cell	*WASF1*	-0.356474975	0.013796302	0.038362117	1
Prediabetes	PP_cell	*ALDH5A1*	-0.3559123	0.002932687	0.016546556	1
Prediabetes	PP_cell	*C6orf62*	-0.306848467	0.002084495	0.013904889	1
Prediabetes	PP_cell	*HBS1L*	-0.30591142	0.006979969	0.026522533	1
Prediabetes	PP_cell	*PEX7*	-0.38988709	0.000213475	0.008519859	0.838741516
Prediabetes	PP_cell	*PHACTR2*	-0.241436825	0.01054762	0.033892939	1
Prediabetes	PP_cell	*EPM2A*	-0.227163789	0.00601026	0.024326947	1
Prediabetes	PP_cell	*DNPH1*	-0.15973435	0.006796004	0.026255163	1

Prediabetes	PP_cell	*FBRSL1*	-0.295627519	0.000342918	0.008519859	1
Prediabetes	PP_cell	*NUDT12*	-0.128428856	0.004283333	0.019996719	1
Prediabetes	PP_cell	*SEMA5A*	-0.194259471	0.000974321	0.009804971	1
Prediabetes	PP_cell	*DAP*	-0.187761349	0.003341229	0.017480279	1
Prediabetes	PP_cell	*BRD8*	-0.349897233	0.015683741	0.041136895	1
Prediabetes	PP_cell	*NNT*	-0.22665756	0.00619662	0.024667192	1
Prediabetes	PP_cell	*TMCO6*	-0.122091573	0.012284508	0.036372141	1
Prediabetes	PP_cell	*PDE8B*	-0.103220049	0.005445979	0.022930133	1
Prediabetes	PP_cell	*CCNG1*	-0.227717063	0.002129579	0.014043725	1
Prediabetes	PP_cell	*GOLPH3*	-0.336913283	0.001383221	0.011450342	1
Prediabetes	PP_cell	*RAD50*	-0.353195508	0.000392589	0.008519859	1
Prediabetes	PP_cell	*PPWD1*	-0.339609205	0.010716105	0.03417498	1
Prediabetes	PP_cell	*WWC1*	-0.086937355	0.010307769	0.033442795	1
Prediabetes	PP_cell	*STC2*	-0.142849469	0.003707229	0.018484393	1
Prediabetes	PP_cell	*EHHADH*	-0.203933258	0.011254861	0.034971522	1
Prediabetes	PP_cell	*ACTR8*	-0.480678292	0.000327662	0.008519859	1
Prediabetes	PP_cell	*CLDN16*	-0.887894319	0.01577841	0.04125534	1
Prediabetes	PP_cell	*FAM162A*	-0.194763134	0.01083973	0.034318532	1
Prediabetes	PP_cell	*RBP1*	-0.169731152	0.000606946	0.008645299	1
Prediabetes	PP_cell	*RNF7*	-0.163863191	0.016582227	0.042361229	1
Prediabetes	PP_cell	*SERPINI2*	-0.087936952	0.00618356	0.024659802	1
Prediabetes	PP_cell	*WNT5A*	-0.374705933	0.001387041	0.011450342	1
Prediabetes	PP_cell	*TUSC2*	-0.233457578	0.002182733	0.014198607	1
Prediabetes	PP_cell	*C3orf14*	-0.151535275	0.01113663	0.034835517	1
Prediabetes	PP_cell	*NCBP2*	-0.271502852	0.011185062	0.034877863	1
Prediabetes	PP_cell	*C3orf52*	-0.057103388	0.012239315	0.036372141	1
Prediabetes	PP_cell	*SLC41A3*	-0.2226307	0.002956249	0.016546556	1
Prediabetes	PP_cell	*ATP6V1A*	-0.329781466	0.000868156	0.009444968	1
Prediabetes	PP_cell	*GORASP1*	-0.206807264	0.016250549	0.041846259	1
Prediabetes	PP_cell	*PLCH1*	-0.248492516	0.002791174	0.0160564	1
Prediabetes	PP_cell	*NEK4*	-0.428852987	0.002588015	0.015476883	1
Prediabetes	PP_cell	*KANSL3*	-0.202481095	0.005156078	0.022091856	1
Prediabetes	PP_cell	*ACTR1B*	-0.229496628	0.000778307	0.009253454	1
Prediabetes	PP_cell	*TFCP2L1*	-0.069212572	0.012973952	0.037843101	1
Prediabetes	PP_cell	*TANC1*	-0.155024948	0.020643936	0.049760752	1
Prediabetes	PP_cell	*REEP6*	-0.102686463	0.010894327	0.03440649	1
Prediabetes	PP_cell	*INO80B*	-0.0766001	0.009871076	0.032714383	1
Prediabetes	PP_cell	*MOGS*	-0.220841454	0.014736229	0.039627472	1
Prediabetes	PP_cell	*GALNT3*	-0.062576002	0.009953061	0.032751739	1
Prediabetes	PP_cell	*MRPL19*	-0.202335696	0.017646696	0.044302791	1
Prediabetes	PP_cell	*OTX1*	-0.559118173	5.86E-05	0.008519859	0.230154106
Prediabetes	PP_cell	*IL1R2*	-0.093361489	0.010474255	0.033815404	1
Prediabetes	PP_cell	*IL1RL2*	-0.195282627	0.004769279	0.021054489	1
Prediabetes	PP_cell	*CNPPD1*	-0.273560158	0.000842227	0.009444968	1
Prediabetes	PP_cell	*HDLBP*	-0.257194415	0.000609843	0.008645299	1
Prediabetes	PP_cell	*TAF1B*	-0.445116684	0.000542934	0.008519859	1
Prediabetes	PP_cell	*COX7A2L*	-0.258760452	0.000655318	0.008752256	1
Prediabetes	PP_cell	*PNO1*	-0.276825389	0.016436931	0.04220961	1
Prediabetes	PP_cell	*ARID3A*	-0.305872463	0.000482599	0.008519859	1
Prediabetes	PP_cell	*ALMS1*	-0.31840867	0.000153071	0.008519859	0.601414632
Prediabetes	PP_cell	*MARK1*	-0.413206314	0.000707302	0.008816433	1
Prediabetes	PP_cell	*MORN1*	-0.351578841	0.010144394	0.033119439	1
Prediabetes	PP_cell	*RALGPS2*	-0.269460845	0.000660846	0.008757086	1
Prediabetes	PP_cell	*PARK7*	-0.183161558	0.012110092	0.036238044	1
Prediabetes	PP_cell	*MECR*	-0.188994706	0.004470165	0.020304368	1
Prediabetes	PP_cell	*FBXO6*	-0.12921114	0.005146543	0.022075073	1
Prediabetes	PP_cell	*SMG7*	-0.312212383	0.009408117	0.031819051	1
Prediabetes	PP_cell	*TMEM9*	-0.131622849	0.001592201	0.012147101	1
Prediabetes	PP_cell	*WARS2*	-0.288996003	0.00933574	0.031675407	1
Prediabetes	PP_cell	*HPCAL4*	-0.187535738	0.008783918	0.03040706	1
Prediabetes	PP_cell	*BMP8B*	-0.143773441	0.006116917	0.024498846	1
Prediabetes	PP_cell	*UAP1*	-0.32923226	2.24E-05	0.008519859	0.087859594
Prediabetes	PP_cell	*CTBS*	-0.273684998	0.000898395	0.009499809	1
Prediabetes	PP_cell	*CD46*	-0.276929818	0.000865497	0.009444968	1

Prediabetes	PP_cell	*TMED5*	-0.328761004	0.001390128	0.011450342	1
Prediabetes	PP_cell	*DR1*	-0.234394815	0.020720655	0.049888972	1
Prediabetes	PP_cell	*PTBP2*	-0.233003318	0.00129272	0.011111559	1
Prediabetes	PP_cell	*NSL1*	-0.339369538	0.000436914	0.008519859	1
Prediabetes	PP_cell	*RPA2*	-0.170693176	0.016476398	0.042228159	1
Prediabetes	PP_cell	*DDX59*	-0.385354543	0.00109452	0.010322435	1
Prediabetes	PP_cell	*TTR*	-0.152619639	0.000432983	0.008519859	1
Prediabetes	PP_cell	*SPCS2*	-0.187706286	0.018041629	0.045006706	1
Prediabetes	PP_cell	*ELOVL4*	-0.562676716	0.003930044	0.019039633	1
Prediabetes	PP_cell	*FILIP1*	-0.597271781	1.59E-05	0.008519859	0.0624835
Prediabetes	PP_cell	*RNF146*	-0.468450371	0.000471809	0.008519859	1
Prediabetes	PP_cell	*VAMP8*	-0.145685492	0.002132742	0.014043725	1
Prediabetes	PP_cell	*SPP1*	-0.058060458	0.006390517	0.025183893	1
Prediabetes	PP_cell	*DNAH7*	-0.103234328	0.00603178	0.024356489	1
Prediabetes	PP_cell	*PIGZ*	-0.055753688	0.0112369	0.03495628	1
Prediabetes	PP_cell	*NEK6*	-0.193363932	0.010548621	0.033892939	1
Prediabetes	PP_cell	*RBM18*	-0.385584814	0.000321473	0.008519859	1
Prediabetes	PP_cell	*DCAF4*	-0.15179742	0.001120235	0.01050454	1
Prediabetes	PP_cell	*IFI27L2*	-0.257136221	0.000845896	0.009444968	1
Prediabetes	PP_cell	*KLHL29*	-0.083800621	0.016779871	0.042754938	1
Prediabetes	PP_cell	*BCL11A*	-0.087818692	0.013759062	0.038295695	1
Prediabetes	PP_cell	*CRIPT*	-0.269816249	0.017508437	0.044068322	1
Prediabetes	PP_cell	*EPCAM*	-0.168583341	0.000434379	0.008519859	1
Prediabetes	PP_cell	*PRLHR*	-0.089567443	0.013065597	0.037909761	1
Prediabetes	PP_cell	*HOXB3*	-0.262003406	0.013232145	0.037909761	1
Prediabetes	PP_cell	*RCL1*	-0.4641047	0.000795176	0.009253454	1
Prediabetes	PP_cell	*CD274*	-0.088400578	0.012361645	0.036545451	1
Prediabetes	PP_cell	*PLEKHG1*	-0.138342582	0.002295479	0.014546671	1
Prediabetes	PP_cell	*PCDHB10*	-0.119717573	0.010292223	0.033419952	1
Prediabetes	PP_cell	*SLC10A7*	-0.14765659	0.001733549	0.012837572	1
Prediabetes	PP_cell	*MRC1*	-0.074072004	0.008711748	0.030237155	1
Prediabetes	PP_cell	*IQSEC3*	-0.107917533	0.005444708	0.022930133	1
Prediabetes	PP_cell	*CCDC77*	-0.111698114	0.017106978	0.043335473	1
Prediabetes	PP_cell	*MTRF1*	-0.154576282	0.003820991	0.018812875	1
Prediabetes	PP_cell	*KBTBD7*	-0.454834477	0.003404494	0.017716895	1
Prediabetes	PP_cell	*SIL1*	-0.182079965	0.00148573	0.01172175	1
Prediabetes	PP_cell	*PLS1*	-0.281972785	0.001671687	0.012486802	1
Prediabetes	PP_cell	*UTP20*	-0.384069236	0.000543573	0.008519859	1
Prediabetes	PP_cell	*APAF1*	-0.112497449	0.011597521	0.035654664	1
Prediabetes	PP_cell	*PPP3CC*	-0.34199254	0.00645507	0.025367051	1
Prediabetes	PP_cell	*EPHX2*	-0.205305355	0.001779892	0.012957043	1
Prediabetes	PP_cell	*CRISPLD1*	-0.343408441	0.020602334	0.049760752	1
Prediabetes	PP_cell	*TRIM6*	-0.172744032	0.003282689	0.017358932	1
Prediabetes	PP_cell	*IAPP*	-0.05831874	0.003045342	0.016712144	1
Prediabetes	PP_cell	*ZNF211*	-0.17696113	0.001023076	0.00999917	1
Prediabetes	PP_cell	*CAT*	-0.37881541	0.001218386	0.010904418	1
Prediabetes	PP_cell	*CSMD2*	-0.349004462	0.009153502	0.031273138	1
Prediabetes	PP_cell	*UBL3*	-0.211810842	0.014359859	0.039207704	1
Prediabetes	PP_cell	*NBPF14*	-0.247829932	0.010615485	0.033992045	1
Prediabetes	PP_cell	*PMS2*	-0.265150945	0.002429438	0.015031909	1
Prediabetes	PP_cell	*EEPD1*	-0.335480292	0.001250266	0.010991254	1
Prediabetes	PP_cell	*NPY*	-0.066599985	0.009026382	0.030973499	1
Prediabetes	PP_cell	*INHBA*	-0.068214581	0.006358509	0.025082914	1
Prediabetes	PP_cell	*BHLHE41*	-0.357552003	0.000391784	0.008519859	1
Prediabetes	PP_cell	*EBPL*	-0.242097199	0.008143343	0.029272821	1
Prediabetes	PP_cell	*IKZF4*	-0.292121916	0.003247201	0.017225057	1
Prediabetes	PP_cell	*SARDH*	-0.118259188	0.019137249	0.047170798	1
Prediabetes	PP_cell	*USP45*	-0.481806499	0.000364619	0.008519859	1
Prediabetes	PP_cell	*C4BPA*	-0.136442783	0.00594395	0.024153447	1
Prediabetes	PP_cell	*ARHGAP40*	-3.331089861	0.014686139	0.039551271	1
Prediabetes	PP_cell	*MATN4*	-0.149820255	0.018805821	0.046626816	1
Prediabetes	PP_cell	*PARD6B*	-0.0448849	0.013630612	0.038253338	1
Prediabetes	PP_cell	*ARFGEF2*	-0.33445742	0.004202649	0.019870287	1
Prediabetes	PP_cell	*BCAS4*	-0.389115343	0.000953619	0.009706653	1

Prediabetes	PP_cell	*PEPD*	-0.263146712	0.012157923	0.036270675	1
Prediabetes	PP_cell	*SNRNP27*	-0.239088945	0.013543274	0.038233958	1
Prediabetes	PP_cell	*USP22*	-0.209586685	0.014432815	0.039243274	1
Prediabetes	PP_cell	*BTN2A2*	-0.142513863	0.015430817	0.040717045	1
Prediabetes	PP_cell	*MRS2*	-0.334430786	0.000404512	0.008519859	1
Prediabetes	PP_cell	*ABCC10*	-0.380086894	0.000882177	0.009444968	1
Prediabetes	PP_cell	*MOCS1*	-0.106549901	0.014972805	0.0399258	1
Prediabetes	PP_cell	*KCNK17*	-0.053352014	0.015206813	0.040288313	1
Prediabetes	PP_cell	*ATXN1*	-0.369203794	0.000383366	0.008519859	1
Prediabetes	PP_cell	*LRRFIP1*	-0.262683756	0.000219944	0.008519859	0.864158377
Prediabetes	PP_cell	*MT1G*	-0.085210155	0.007184007	0.026854533	1
Prediabetes	PP_cell	*CLYBL*	-0.271210977	0.002344571	0.014691894	1
Prediabetes	PP_cell	*TM9SF2*	-0.23046581	0.012202292	0.036320307	1
Prediabetes	PP_cell	*GRK4*	-0.198510596	0.00251123	0.015273409	1
Prediabetes	PP_cell	*HS3ST3B1*	-0.20885188	0.009819355	0.032612212	1
Prediabetes	PP_cell	*PPDPF*	-0.210856808	0.004775886	0.021059995	1
Prediabetes	PP_cell	*CHCHD5*	-0.267202481	0.002690497	0.0157471	1
Prediabetes	PP_cell	*INSIG2*	-0.488152457	7.08E-06	0.008519859	0.027830768
Prediabetes	PP_cell	*PSD4*	-0.226361874	0.009767791	0.032495895	1
Prediabetes	PP_cell	*CLPP*	-0.225811564	0.016252836	0.041846259	1
Prediabetes	PP_cell	*SH2D3A*	-0.110237056	0.000148586	0.008519859	0.583792537
Prediabetes	PP_cell	*GPR108*	-0.249560356	0.020638304	0.049760752	1
Prediabetes	PP_cell	*SYMPK*	-0.235175896	0.002554506	0.01537007	1
Prediabetes	PP_cell	*NAPB*	-0.305051973	0.001951316	0.013500582	1
Prediabetes	PP_cell	*PSMF1*	-0.187053995	0.018222588	0.045371704	1
Prediabetes	PP_cell	*RBCK1*	-0.207869112	0.001185696	0.010758887	1
Prediabetes	PP_cell	*MGME1*	-0.158019594	0.005417527	0.022930133	1
Prediabetes	PP_cell	*ITPA*	-0.292531724	0.010808186	0.034287057	1
Prediabetes	PP_cell	*BANF2*	-0.899269185	0.013232421	0.037909761	1
Prediabetes	PP_cell	*MCF2L*	-0.321930099	0.00017152	0.008519859	0.673900154
Prediabetes	PP_cell	*PROZ*	-0.989144219	0.008901993	0.030690414	1
Prediabetes	PP_cell	*ASL*	-0.386659509	0.000480029	0.008519859	1
Prediabetes	PP_cell	*TRAP1*	-0.136715399	0.005121164	0.021990223	1
Prediabetes	PP_cell	*ZNF384*	-0.52518175	0.000836953	0.009444968	1
Prediabetes	PP_cell	*RHOJ*	-0.113635562	0.011146018	0.034835517	1
Prediabetes	PP_cell	*HSPA2*	-0.441736366	0.002306066	0.014586494	1
Prediabetes	PP_cell	*PLEKHG3*	-0.423242231	0.000529916	0.008519859	1
Prediabetes	PP_cell	*FGD3*	-0.388706495	0.01400367	0.038637936	1
Prediabetes	PP_cell	*HELB*	-0.358462077	0.003896857	0.019001519	1
Prediabetes	PP_cell	*YEATS4*	-0.278941842	0.002552976	0.01537007	1
Prediabetes	PP_cell	*IDUA*	-0.619283644	0.000225196	0.008519859	0.884794244
Prediabetes	PP_cell	*EMC1*	-0.300684986	0.020392828	0.049458901	1
Prediabetes	PP_cell	*SYNGR3*	-0.134279625	0.009966074	0.032763505	1
Prediabetes	PP_cell	*KDM4B*	-0.386097008	0.001145207	0.010597251	1
Prediabetes	PP_cell	*METTL25*	-0.436352252	0.000868525	0.009444968	1
Prediabetes	PP_cell	*POR*	-0.368745887	0.000513368	0.008519859	1
Prediabetes	PP_cell	*RBM48*	-0.430697239	0.000273154	0.008519859	1
Prediabetes	PP_cell	*SRD5A3*	-0.174627433	0.01003712	0.032886224	1
Prediabetes	PP_cell	*GALR3*	-0.620859684	0.005548935	0.023167203	1
Prediabetes	PP_cell	*NAA38*	-0.247874094	0.004981721	0.021699757	1
Prediabetes	PP_cell	*PRKRIP1*	-0.346291777	0.005882747	0.024051313	1
Prediabetes	PP_cell	*VGF*	-0.216642868	0.001956775	0.013500582	1
Prediabetes	PP_cell	*MTX2*	-0.374100835	0.0003961	0.008519859	1
Prediabetes	PP_cell	*GAD1*	-0.179348613	0.005944613	0.024153447	1
Prediabetes	PP_cell	*TWSG1*	-0.271396161	0.020523778	0.049653894	1
Prediabetes	PP_cell	*EIF2AK4*	-0.256169797	0.008476512	0.029842488	1
Prediabetes	PP_cell	*MYO5C*	-0.236751273	0.001216425	0.010904418	1
Prediabetes	PP_cell	*CGNL1*	-0.075908317	0.011757254	0.035704358	1
Prediabetes	PP_cell	*ANAPC13*	-0.078665962	0.011761286	0.035704358	1
Prediabetes	PP_cell	*SUMF2*	-0.297621482	0.001871098	0.013293931	1
Prediabetes	PP_cell	*DCTD*	-0.262085737	0.020319825	0.049330421	1
Prediabetes	PP_cell	*ATP1B2*	-0.200169995	0.008872583	0.030659964	1
Prediabetes	PP_cell	*AP1M2*	-0.089137914	0.006832429	0.026292348	1
Prediabetes	PP_cell	*DTD2*	-0.328988204	0.013966602	0.03856274	1

Prediabetes	PP_cell	*MIS18BP1*	-0.160830995	0.017945004	0.044810578	1
Prediabetes	PP_cell	*RNASE1*	-0.080819374	0.006187355	0.024659802	1
Prediabetes	PP_cell	*EPB41L4A*	-0.762465209	0.002340649	0.014690752	1
Prediabetes	PP_cell	*REEP5*	-0.214299465	0.00694077	0.026424694	1
Prediabetes	PP_cell	*SHC2*	-0.387841768	0.00045849	0.008519859	1
Prediabetes	PP_cell	*SYT5*	-0.144149583	0.008157601	0.029297271	1
Prediabetes	PP_cell	*CBFA2T3*	-0.204383492	0.002786767	0.0160564	1
Prediabetes	PP_cell	*ERMARD*	-0.172997914	0.016452976	0.042210376	1
Prediabetes	PP_cell	*ECSIT*	-0.335625185	0.002004042	0.013622633	1
Prediabetes	PP_cell	*CDC16*	-0.345756722	0.00052801	0.008519859	1
Prediabetes	PP_cell	*DPP6*	-0.288698471	0.000338156	0.008519859	1
Prediabetes	PP_cell	*TULP4*	-0.233262722	0.009875097	0.032714383	1
Prediabetes	PP_cell	*RSPH3*	-0.367293097	0.001232355	0.010954577	1
Prediabetes	PP_cell	*NDUFA10*	-0.192031423	0.011803725	0.035704358	1
Prediabetes	PP_cell	*SCO2*	-0.310397023	0.00109556	0.010322435	1
Prediabetes	PP_cell	*ATXN10*	-0.264104968	0.00733195	0.027228007	1
Prediabetes	PP_cell	*PNPLA7*	-0.357464676	0.001958598	0.013500582	1
Prediabetes	PP_cell	*PAK4*	-0.343391394	0.000493822	0.008519859	1
Prediabetes	PP_cell	*LAMA5*	-0.19966642	0.020327297	0.049330421	1
Prediabetes	PP_cell	*UCK1*	-0.418520684	0.000997532	0.009851066	1
Prediabetes	PP_cell	*MAP3K10*	-0.260907284	0.00021518	0.008519859	0.845444122
Prediabetes	PP_cell	*SMPDL3B*	-0.062299962	0.017951604	0.044810578	1
Prediabetes	PP_cell	*CLIP1*	-0.208732887	0.013606112	0.038249975	1
Prediabetes	PP_cell	*ZNF317*	-0.210737847	0.009371859	0.031770521	1
Prediabetes	PP_cell	*LRP3*	-0.31362986	0.003093403	0.016741022	1
Prediabetes	PP_cell	*HSD17B3*	-0.956499823	0.003794583	0.018729796	1
Prediabetes	PP_cell	*C1QL1*	-0.089005845	0.009935449	0.03274864	1
Prediabetes	PP_cell	*F12*	-0.09843622	0.014179314	0.038931185	1
Prediabetes	PP_cell	*NFATC1*	-0.297962519	0.007688077	0.028046848	1
Prediabetes	PP_cell	*PDLIM4*	-0.32972699	0.000341733	0.008519859	1
Prediabetes	PP_cell	*MGAT1*	-0.278291153	0.007445349	0.027521593	1
Prediabetes	PP_cell	*DIAPH1*	-0.298209865	0.000907618	0.009499809	1
Prediabetes	PP_cell	*EXOC4*	-0.27896846	0.015442428	0.040720336	1
Prediabetes	PP_cell	*ANO1*	-0.193536576	0.005494079	0.023062216	1
Prediabetes	PP_cell	*CHD1L*	-0.422657426	0.0005118	0.008519859	1
Prediabetes	PP_cell	*PIAS3*	-0.372153898	0.002216704	0.014319626	1
Prediabetes	PP_cell	*ZNF304*	-0.364558411	0.00117995	0.010731532	1
Prediabetes	PP_cell	*RHPN2*	-0.101642126	0.012555383	0.037006826	1
Prediabetes	PP_cell	*GCH1*	-0.28699103	0.00062764	0.008645299	1
Prediabetes	PP_cell	*CCT6B*	-0.217739613	0.00267472	0.015685035	1
Prediabetes	PP_cell	*PPARG*	-0.483943604	0.002031877	0.01378799	1
Prediabetes	PP_cell	*ENOSF1*	-0.360507494	0.00039087	0.008519859	1
Prediabetes	PP_cell	*IMMT*	-0.244181402	0.005712199	0.02357482	1
Prediabetes	PP_cell	*TMEM128*	-0.282535799	0.000555124	0.008519859	1
Prediabetes	PP_cell	*SEC61G*	-0.185143511	0.007369158	0.027288805	1
Prediabetes	PP_cell	*FIGNL1*	-0.23912218	0.001836165	0.013116896	1
Prediabetes	PP_cell	*EIF5A*	-0.192333583	0.011085221	0.034787408	1
Prediabetes	PP_cell	*RBM38*	-0.312418988	0.007937346	0.028742703	1
Prediabetes	PP_cell	*POSTN*	-0.322576992	0.01360328	0.038249975	1
Prediabetes	PP_cell	*EPHB2*	-0.127890515	0.004525217	0.020366067	1
Prediabetes	PP_cell	*BTBD2*	-0.111070654	0.005058121	0.02194376	1
Prediabetes	PP_cell	*CNDP2*	-0.352745396	0.000325842	0.008519859	1
Prediabetes	PP_cell	*SLC2A11*	-0.416527486	0.003333856	0.017464961	1
Prediabetes	PP_cell	*FAM83F*	-0.374379498	0.000325751	0.008519859	1
Prediabetes	PP_cell	*GIMAP6*	-0.11661617	0.019157307	0.047174509	1
Prediabetes	PP_cell	*AGAP3*	-0.349827352	0.004359349	0.020119092	1
Prediabetes	PP_cell	*SFTPD*	-0.1111293	0.006306808	0.024961783	1
Prediabetes	PP_cell	*IMPA1*	-0.17680396	0.004506623	0.020366067	1
Prediabetes	PP_cell	*RRAS2*	-0.229966979	0.006006046	0.024326947	1
Prediabetes	PP_cell	*HSD17B4*	-0.385705249	0.000580681	0.008616393	1
Prediabetes	PP_cell	*COX16*	-0.261328751	0.003004335	0.016581525	1
Prediabetes	PP_cell	*ELP3*	-0.276097657	0.001089453	0.010314364	1
Prediabetes	PP_cell	*PEBP4*	-0.098726502	0.011017839	0.034659	1
Prediabetes	PP_cell	*MRO*	-0.526458532	0.013752427	0.038295695	1

Prediabetes	PP_cell	*MRPS36*	-0.249864687	0.001250468	0.010991254	1
Prediabetes	PP_cell	*THUMPD3*	-0.235607971	0.014978526	0.0399258	1
Prediabetes	PP_cell	*REG4*	-0.079606943	0.016007494	0.041435884	1
Prediabetes	PP_cell	*SORT1*	-0.261347776	0.00048077	0.008519859	1
Prediabetes	PP_cell	*NOTCH2*	-0.085821515	0.003234373	0.017219313	1
Prediabetes	PP_cell	*CEPT1*	-0.43136282	0.003256774	0.017245099	1
Prediabetes	PP_cell	*NAPG*	-0.25990262	0.018109604	0.045147611	1
Prediabetes	PP_cell	*FKBP11*	-0.107314963	0.002644318	0.01561651	1
Prediabetes	PP_cell	*IAH1*	-0.322089517	0.000718094	0.008872296	1
Prediabetes	PP_cell	*ECHDC3*	-0.256264416	0.003871046	0.018940647	1
Prediabetes	PP_cell	*KLRC1*	-2.036010899	0.013148518	0.037909761	1
Prediabetes	PP_cell	*DSC2*	-0.186966466	0.001399383	0.011478447	1
Prediabetes	PP_cell	*TPGS2*	-0.254052268	0.005118055	0.021990223	1
Prediabetes	PP_cell	*DAGLA*	-0.161910591	0.004491878	0.020343144	1
Prediabetes	PP_cell	*TIMM10*	-0.279586755	0.00427037	0.019996719	1
Prediabetes	PP_cell	*CLOCK*	-0.22381984	0.00683702	0.026292348	1
Prediabetes	PP_cell	*GGACT*	-0.180692985	0.014019703	0.038655027	1
Prediabetes	PP_cell	*CLDN10*	-0.033197074	0.009535225	0.032025881	1
Prediabetes	PP_cell	*CARS2*	-0.230272285	0.001174871	0.010731532	1
Prediabetes	PP_cell	*TMED7*	-0.29205269	0.001239937	0.010970459	1
Prediabetes	PP_cell	*OSTF1*	-0.404893675	0.001281635	0.011111559	1
Prediabetes	PP_cell	*GOLM1*	-0.174341109	0.00079156	0.009253454	1
Prediabetes	PP_cell	*MSI1*	-0.162172815	0.003866296	0.018940647	1
Prediabetes	PP_cell	*PNPLA8*	-0.211319594	0.008991996	0.030882477	1
Prediabetes	PP_cell	*TES*	-0.18217552	0.002986413	0.016581525	1
Prediabetes	PP_cell	*LCA5*	-0.450702175	0.000288409	0.008519859	1
Prediabetes	PP_cell	*TFCP2*	-0.359720199	0.000437654	0.008519859	1
Prediabetes	PP_cell	*KRT7*	-0.034494	0.008704881	0.030237155	1
Prediabetes	PP_cell	*PKIB*	-0.314337192	0.001824128	0.013078464	1
Prediabetes	PP_cell	*GNS*	-0.35620279	0.000624192	0.008645299	1
Prediabetes	PP_cell	*MPHOSPH6*	-0.354546438	0.010168019	0.03312616	1
Prediabetes	PP_cell	*NTPCR*	-0.238140602	0.000974889	0.009804971	1
Prediabetes	PP_cell	*GLUL*	-0.304679958	0.006050416	0.024381623	1
Prediabetes	PP_cell	*USP37*	-0.317943032	0.000386056	0.008519859	1
Prediabetes	PP_cell	*ARMC9*	-0.610322997	0.00048935	0.008519859	1
Prediabetes	PP_cell	*COX5B*	-0.211540397	0.004494235	0.020343144	1
Prediabetes	PP_cell	*REV1*	-0.259789458	0.000331745	0.008519859	1
Prediabetes	PP_cell	*TSGA10*	-0.102626104	8.35E-05	0.008519859	0.32811416
Prediabetes	PP_cell	*MRPS9*	-0.411680737	0.000136406	0.008519859	0.53594097
Prediabetes	PP_cell	*ANKRD36*	-0.434328033	0.020761	0.049920422	1
Prediabetes	PP_cell	*USP44*	-0.099462587	0.013443808	0.038055275	1
Prediabetes	PP_cell	*DRAM1*	-0.480655922	0.001659595	0.012440588	1
Prediabetes	PP_cell	*VPS36*	-0.34145479	0.000195196	0.008519859	0.766926611
Prediabetes	PP_cell	*RNASEH2B*	-0.301291745	0.000430402	0.008519859	1
Prediabetes	PP_cell	*SUCLA2*	-0.310354246	0.003596191	0.018323322	1
Prediabetes	PP_cell	*LMO7*	-0.210705725	0.000847891	0.009444968	1
Prediabetes	PP_cell	*C7orf25*	-0.18715331	0.008305017	0.029556532	1
Prediabetes	PP_cell	*RAPGEF5*	-0.296967453	0.000688468	0.008780065	1
Prediabetes	PP_cell	*KDELR2*	-0.271391625	0.00108821	0.010314364	1
Prediabetes	PP_cell	*TBRG4*	-0.213390992	0.009253677	0.031505802	1
Prediabetes	PP_cell	*ALPK3*	-0.324532525	0.003819784	0.018812875	1
Prediabetes	PP_cell	*CIB2*	-0.171157778	0.00188078	0.013314564	1
Prediabetes	PP_cell	*TEX2*	-0.389082684	0.000930365	0.009619487	1
Prediabetes	PP_cell	*BLK*	-0.995188266	0.010370858	0.033564334	1
Prediabetes	PP_cell	*SMPD4*	-0.373054933	0.006549752	0.025605945	1
Prediabetes	PP_cell	*CCDC115*	-0.292953871	0.000250602	0.008519859	0.984616854
Prediabetes	PP_cell	*HS6ST1*	-0.42920877	0.002109916	0.013980215	1
Prediabetes	PP_cell	*NIPSNAP3A*	-0.229480753	0.00045224	0.008519859	1
Prediabetes	PP_cell	*TXN*	-0.163606356	0.01926451	0.047346586	1
Prediabetes	PP_cell	*SLC2A8*	-0.43328336	0.000329395	0.008519859	1
Prediabetes	PP_cell	*STX17*	-0.252353166	0.010228929	0.033269422	1
Prediabetes	PP_cell	*FPGS*	-0.506400174	0.001533683	0.011877462	1
Prediabetes	PP_cell	*POLR1E*	-0.183405355	0.004533917	0.020368512	1
Prediabetes	PP_cell	*TMEM8B*	-0.179009062	0.001836025	0.013116896	1

Prediabetes	PP_cell	*ARHGEF39*	-0.272211858	0.005527183	0.023127052	1
Prediabetes	PP_cell	*PPIL1*	-0.142262164	0.00363576	0.01839612	1
Prediabetes	PP_cell	*GMPR*	-0.214939456	0.002065566	0.013874899	1
Prediabetes	PP_cell	*KIAA0319*	-0.588450243	0.002061703	0.013874899	1
Prediabetes	PP_cell	*LRRC1*	-0.400654304	0.000909119	0.009499809	1
Prediabetes	PP_cell	*UQCC2*	-0.182504479	0.008181022	0.029327769	1
Prediabetes	PP_cell	*HMGA1*	-0.169049237	0.000843082	0.009444968	1
Prediabetes	PP_cell	*VARS2*	-0.095951919	0.001232245	0.010954577	1
Prediabetes	PP_cell	*ANKRD42*	-0.296048997	0.000301256	0.008519859	1
Prediabetes	PP_cell	*GGH*	-0.200720004	0.00049582	0.008519859	1
Prediabetes	PP_cell	*SDCBP*	-0.200982614	0.015884512	0.041331289	1
Prediabetes	PP_cell	*NEK1*	-0.153367746	0.012247018	0.036372141	1
Prediabetes	PP_cell	*MMP7*	-0.038886499	0.015951835	0.041414773	1
Prediabetes	PP_cell	*FDX1*	-0.296213433	0.000788268	0.009253454	1
Prediabetes	PP_cell	*C11orf1*	-0.25247705	0.00128533	0.011111559	1
Prediabetes	PP_cell	*ALKBH8*	-0.511253277	0.000694986	0.008780065	1
Prediabetes	PP_cell	*TUBGCP4*	-0.388055417	0.000143528	0.008519859	0.563921771
Prediabetes	PP_cell	*ADAM10*	-0.267284245	0.011205889	0.034915098	1
Prediabetes	PP_cell	*RSL24D1*	-0.237608838	0.002886258	0.01636379	1
Prediabetes	PP_cell	*SPTBN5*	0.218967228	0.014961464	0.0399258	1
Prediabetes	PP_cell	*TTLL7*	-0.239414745	0.007008471	0.026553791	1
Prediabetes	PP_cell	*SLC44A5*	-0.152446985	0.018759897	0.046591426	1
Prediabetes	PP_cell	*RTCA*	-0.396522183	0.000338414	0.008519859	1
Prediabetes	PP_cell	*DYNC2LI1*	-0.222904203	0.008236265	0.029391721	1
Prediabetes	PP_cell	*DUSP5*	-0.31275067	0.000187241	0.008519859	0.735670836
Prediabetes	PP_cell	*DNAJC13*	-0.438756548	0.014687021	0.039551271	1
Prediabetes	PP_cell	*ATIC*	-0.136629144	0.00836638	0.029640674	1
Prediabetes	PP_cell	*SMARCAL1*	-0.574994939	0.000622996	0.008645299	1
Prediabetes	PP_cell	*BARD1*	-0.187251421	0.003986479	0.019154283	1
Prediabetes	PP_cell	*FASTKD1*	-0.150526295	0.015626215	0.041056337	1
Prediabetes	PP_cell	*WDR12*	-0.181001756	0.01049818	0.03386482	1
Prediabetes	PP_cell	*SLC35A5*	-0.185586887	0.004033538	0.019326551	1
Prediabetes	PP_cell	*HCN4*	-0.112117044	0.00274871	0.015953783	1
Prediabetes	PP_cell	*SEMA7A*	-0.155311667	0.013081038	0.037909761	1
Prediabetes	PP_cell	*AP1AR*	-0.226164809	0.019274108	0.047346586	1
Prediabetes	PP_cell	*RAP1GDS1*	-0.437340783	0.000479041	0.008519859	1
Prediabetes	PP_cell	*G3BP2*	-0.257035523	0.003928767	0.019039633	1
Prediabetes	PP_cell	*SCARB2*	-0.183161389	0.00705153	0.026639866	1
Prediabetes	PP_cell	*USO1*	-0.318493621	0.001520488	0.011846392	1
Prediabetes	PP_cell	*SHROOM3*	-0.12105196	0.00432586	0.020113968	1
Prediabetes	PP_cell	*EGF*	-0.186938821	0.011855443	0.035727429	1
Prediabetes	PP_cell	*RGS3*	-0.340189816	0.000103923	0.008519859	0.408313501
Prediabetes	PP_cell	*ETV6*	-0.407977323	0.000193221	0.008519859	0.759164103
Prediabetes	PP_cell	*ALG10*	-0.933591803	0.003937237	0.019050989	1
Prediabetes	PP_cell	*ZCRB1*	-0.264224854	0.00151762	0.011846392	1
Prediabetes	PP_cell	*KLRG1*	-0.446491932	0.009231118	0.031483562	1
Prediabetes	PP_cell	*PEX5*	-0.39726327	0.000188792	0.008519859	0.741762592
Prediabetes	PP_cell	*SLC38A4*	-0.072476051	0.012263606	0.036372141	1
Prediabetes	PP_cell	*LRIG3*	-0.339823661	0.004435743	0.020248234	1
Prediabetes	PP_cell	*GLIPR1*	-0.491838776	0.001449614	0.011646409	1
Prediabetes	PP_cell	*ASCL1*	-0.101464113	0.014328544	0.039167161	1
Prediabetes	PP_cell	*PDX1*	-0.164006468	0.002456293	0.015103998	1
Prediabetes	PP_cell	*KANSL2*	-0.499952812	0.000131917	0.008519859	0.518300599
Prediabetes	PP_cell	*GALNT6*	-0.124013646	0.016756366	0.042722754	1
Prediabetes	PP_cell	*RAB20*	-0.226804016	0.006603977	0.0257511	1
Prediabetes	PP_cell	*GRTP1*	-0.217646128	0.008656233	0.030204563	1
Prediabetes	PP_cell	*SERF2*	-0.17976053	0.004285375	0.019996719	1
Prediabetes	PP_cell	*GTF2A2*	-0.20334701	0.016073914	0.041521635	1
Prediabetes	PP_cell	*SCAPER*	-0.259132937	0.000773885	0.009253454	1
Prediabetes	PP_cell	*DNAJA4*	-0.239889075	0.000969322	0.009804971	1
Prediabetes	PP_cell	*TPM1*	-0.140876543	0.000946976	0.009678612	1
Prediabetes	PP_cell	*IGF1R*	-0.264099427	0.004839423	0.021221086	1
Prediabetes	PP_cell	*ULK3*	-0.235084022	0.019031552	0.047015043	1
Prediabetes	PP_cell	*CELF6*	-0.519821334	0.000363775	0.008519859	1

Prediabetes	PP_cell	*WDR93*	-0.279365799	0.010357984	0.033559662	1
Prediabetes	PP_cell	*PMM2*	-0.406586306	0.001041662	0.010105406	1
Prediabetes	PP_cell	*SLC5A2*	-0.262115674	0.010805915	0.034287057	1
Prediabetes	PP_cell	*TXNL4B*	-0.205943778	0.002048746	0.013854598	1
Prediabetes	PP_cell	*MARVELD3*	-0.133904233	0.006076494	0.024411598	1
Prediabetes	PP_cell	*KIFC3*	-0.338456759	0.001945111	0.013500582	1
Prediabetes	PP_cell	*NUDT7*	-0.179389721	0.002754074	0.015959817	1
Prediabetes	PP_cell	*GCSH*	-0.325088915	0.000682152	0.008780065	1
Prediabetes	PP_cell	*OSGIN1*	-0.305420493	0.002280827	0.014499386	1
Prediabetes	PP_cell	*PDPK1*	-0.2111952	0.001299485	0.011123481	1
Prediabetes	PP_cell	*DEF8*	-0.236504613	0.011979388	0.035956468	1
Prediabetes	PP_cell	*GALNS*	-0.260756624	0.009097598	0.031136291	1
Prediabetes	PP_cell	*GAS8*	-0.279080447	0.000165131	0.008519859	0.648801445
Prediabetes	PP_cell	*TOM1L1*	-0.118849663	0.006084096	0.024417172	1
Prediabetes	PP_cell	*NPEPPS*	-0.290089685	0.000238225	0.008519859	0.935985278
Prediabetes	PP_cell	*SPACA3*	-0.075995408	0.003606784	0.018332544	1
Prediabetes	PP_cell	*PTRH2*	-0.409788845	0.001156989	0.010645921	1
Prediabetes	PP_cell	*RPRD1A*	-0.353888359	0.000614561	0.008645299	1
Prediabetes	PP_cell	*NPC1*	-0.305719803	0.008348965	0.029619945	1
Prediabetes	PP_cell	*SLC14A1*	-0.10449589	0.015958759	0.041414773	1
Prediabetes	PP_cell	*ZMYND15*	-0.241334916	0.001242518	0.010970459	1
Prediabetes	PP_cell	*TMC6*	-0.120058438	0.012081085	0.036178797	1
Prediabetes	PP_cell	*TTYH2*	-0.725635234	0.000679185	0.008780065	1
Prediabetes	PP_cell	*TBCD*	-0.290839761	0.006732848	0.026062425	1
Prediabetes	PP_cell	*NARF*	-0.284280331	0.003223195	0.017212803	1
Prediabetes	PP_cell	*WDR45B*	-0.193692343	0.014505071	0.039384002	1
Prediabetes	PP_cell	*TXNL4A*	-0.201133919	0.015130226	0.040139539	1
Prediabetes	PP_cell	*AKT1*	-0.224979661	0.016524397	0.042268461	1
Prediabetes	PP_cell	*CBLC*	-0.069573313	0.009302328	0.031644023	1
Prediabetes	PP_cell	*CTU1*	-0.276649924	0.006703993	0.025976319	1
Prediabetes	PP_cell	*CNKSR1*	-0.140490129	0.010687112	0.034165713	1
Prediabetes	PP_cell	*ZNF593*	-0.266485801	0.000175343	0.008519859	0.688920821
Prediabetes	PP_cell	*CELA3A*	-0.061872008	0.001311786	0.011188791	1
Prediabetes	PP_cell	*PIGK*	-0.289270914	0.000483821	0.008519859	1
Prediabetes	PP_cell	*SLC44A3*	-0.104359352	0.012862586	0.03760809	1
Prediabetes	PP_cell	*CELSR2*	-0.360002925	0.001878072	0.013314564	1
Prediabetes	PP_cell	*ALDH9A1*	-0.25712671	0.016249609	0.041846259	1
Prediabetes	PP_cell	*ATP1B1*	-0.105043585	0.001177494	0.010731532	1
Prediabetes	PP_cell	*NME7*	-0.067992954	0.007574306	0.027812568	1
Prediabetes	PP_cell	*LYPLAL1*	-0.289295559	0.000884639	0.009444968	1
Prediabetes	PP_cell	*CTSK*	-0.211253633	0.003426203	0.017782763	1
Prediabetes	PP_cell	*TAF1A*	-0.106027334	0.015064858	0.040047244	1
Prediabetes	PP_cell	*SLC27A3*	-0.311486931	0.001388543	0.011450342	1
Prediabetes	PP_cell	*PSEN2*	-0.329490744	0.002629683	0.015607286	1
Prediabetes	PP_cell	*LBR*	-0.192939516	0.001001009	0.009851066	1
Prediabetes	PP_cell	*ARL8A*	-0.310199605	0.00086872	0.009444968	1
Prediabetes	PP_cell	*GALM*	-0.240097097	0.003045532	0.016712144	1
Prediabetes	PP_cell	*EML4*	-0.35863383	0.000789191	0.009253454	1
Prediabetes	PP_cell	*WDPCP*	-0.560782527	0.001032266	0.010051656	1
Prediabetes	PP_cell	*REG3G*	-0.04819581	0.013880751	0.038433735	1
Prediabetes	PP_cell	*CIAO1*	-0.273035719	0.006904831	0.026361874	1
Prediabetes	PP_cell	*THNSL2*	-0.062091569	0.015853746	0.041316373	1
Prediabetes	PP_cell	*RALB*	-0.247232557	0.018833202	0.046655517	1
Prediabetes	PP_cell	*LIPT1*	-0.186022711	0.001995667	0.013597804	1
Prediabetes	PP_cell	*FAM171B*	-0.281435087	0.000277372	0.008519859	1
Prediabetes	PP_cell	*SUMF1*	-0.340810275	0.001768527	0.012915503	1
Prediabetes	PP_cell	*HES6*	-0.425624453	0.000455695	0.008519859	1
Prediabetes	PP_cell	*ESPNL*	-0.99887796	0.007167599	0.02684604	1
Prediabetes	PP_cell	*RAB5A*	-0.224240319	0.016596614	0.042370433	1
Prediabetes	PP_cell	*CNTN4*	-0.084894457	0.011749662	0.035704358	1
Prediabetes	PP_cell	*POMGNT2*	-0.291489712	0.01407532	0.038726844	1
Prediabetes	PP_cell	*IL17RD*	-0.27877186	0.011056746	0.034753564	1
Prediabetes	PP_cell	*UBA3*	-0.392517841	0.000505737	0.008519859	1
Prediabetes	PP_cell	*ARL6IP5*	-0.294555028	0.000581152	0.008616393	1

Prediabetes	PP_cell	*TAGLN3*	-0.160482654	0.002102517	0.013977644	1
Prediabetes	PP_cell	*EIF2A*	-0.278105009	0.001146305	0.010597251	1
Prediabetes	PP_cell	*ALDH1L1*	-0.090343043	0.014031775	0.038661181	1
Prediabetes	PP_cell	*NCEH1*	-0.167322195	0.001428433	0.011619697	1
Prediabetes	PP_cell	*DGKQ*	-0.105477348	0.008580926	0.030075341	1
Prediabetes	PP_cell	*SCD5*	-0.06575034	0.007987197	0.028870005	1
Prediabetes	PP_cell	*TBCK*	-0.126456628	0.015632548	0.041056337	1
Prediabetes	PP_cell	*TIFA*	-0.35975142	0.000704358	0.008813447	1
Prediabetes	PP_cell	*RNF175*	-3.862678103	0.016958059	0.043069304	1
Prediabetes	PP_cell	*IQGAP2*	-0.206179484	0.000273488	0.008519859	1
Prediabetes	PP_cell	*GTF2H2*	-0.152424224	0.002962743	0.016546556	1
Prediabetes	PP_cell	*SPATA9*	-0.47485228	0.019532231	0.047834311	1
Prediabetes	PP_cell	*COMMD10*	-0.347487822	0.00048687	0.008519859	1
Prediabetes	PP_cell	*PCYOX1L*	-0.737934691	0.001438344	0.011628097	1
Prediabetes	PP_cell	*TBC1D7*	-0.536792689	0.000101822	0.008519859	0.400056853
Prediabetes	PP_cell	*ZMAT2*	-0.350819238	0.001045602	0.01010918	1
Prediabetes	PP_cell	*DCDC2*	-0.408223141	0.000896013	0.009499809	1
Prediabetes	PP_cell	*TRIM7*	-0.270197613	0.000126089	0.008519859	0.495403559
Prediabetes	PP_cell	*ANO7*	-0.213763091	0.009615895	0.032072031	1
Prediabetes	PP_cell	*IRAK1BP1*	-0.404714968	0.000302141	0.008519859	1
Prediabetes	PP_cell	*FAXC*	-0.062877977	0.013545856	0.038233958	1
Prediabetes	PP_cell	*ABRACL*	-0.189033192	0.001710053	0.012724999	1
Prediabetes	PP_cell	*DYNLT1*	-0.222168027	0.005088461	0.02194376	1
Prediabetes	PP_cell	*TMEM209*	-0.353541486	0.007105466	0.026721477	1
Prediabetes	PP_cell	*CSGALNACT1*	-0.299333163	0.014893342	0.039860996	1
Prediabetes	PP_cell	*BIN3*	-0.459132641	0.00250145	0.015266026	1
Prediabetes	PP_cell	*CHMP7*	-0.246193173	0.014660272	0.039533432	1
Prediabetes	PP_cell	*LACTB2*	-0.372593974	0.000416591	0.008519859	1
Prediabetes	PP_cell	*AK3*	-0.250518757	0.008049518	0.029041834	1
Prediabetes	PP_cell	*C9orf72*	-0.292228477	0.00754487	0.027760015	1
Prediabetes	PP_cell	*FBXO10*	-0.199046959	0.002951589	0.016546556	1
Prediabetes	PP_cell	*ZNF462*	-0.415670125	0.000293799	0.008519859	1
Prediabetes	PP_cell	*SURF4*	-0.19998983	0.002501929	0.015266026	1
Prediabetes	PP_cell	*SURF6*	-0.424684247	0.000390544	0.008519859	1
Prediabetes	PP_cell	*SH3GLB2*	-0.307153756	0.001434733	0.011622813	1
Prediabetes	PP_cell	*LCN2*	-0.030665584	0.01122134	0.034935536	1
Prediabetes	PP_cell	*GPR107*	-0.367557482	0.002204352	0.014315539	1
Prediabetes	PP_cell	*SEC16A*	-0.211527797	0.013852733	0.038410295	1
Prediabetes	PP_cell	*MSRB2*	-0.222825492	0.001842975	0.01314165	1
Prediabetes	PP_cell	*FRA10AC1*	-0.313357357	0.002434937	0.015042244	1
Prediabetes	PP_cell	*DNAJB12*	-0.374483801	0.000522352	0.008519859	1
Prediabetes	PP_cell	*PAOX*	-0.285546073	0.019235389	0.047323634	1
Prediabetes	PP_cell	*APIP*	-0.119454102	0.010783468	0.034287057	1
Prediabetes	PP_cell	*PTPRJ*	-0.155109098	0.000431342	0.008519859	1
Prediabetes	PP_cell	*CCDC82*	-0.127970832	0.017373572	0.043823045	1
Prediabetes	PP_cell	*AASDHPPT*	-0.356523541	0.001340456	0.011283666	1
Prediabetes	PP_cell	*EML3*	-0.493359688	0.004704302	0.020861403	1
Prediabetes	PP_cell	*CHEK1*	-0.184943468	0.01181261	0.035704358	1
Prediabetes	PP_cell	*SIDT2*	-0.247758505	0.004856462	0.021272062	1
Prediabetes	PP_cell	*DUSP15*	-0.123103171	0.003668888	0.01839612	1
Prediabetes	PP_cell	*YTHDF1*	-0.25997527	0.020621253	0.049760752	1
Prediabetes	PP_cell	*TRPT1*	-0.338505805	0.001054731	0.01015696	1
Prediabetes	PP_cell	*TBX6*	-0.190696479	0.017236256	0.043597099	1
Prediabetes	PP_cell	*MPP7*	-0.318660246	0.000987008	0.009817609	1
Prediabetes	PP_cell	*MIA2*	-0.257545009	0.00117673	0.010731532	1
Prediabetes	PP_cell	*LYPD1*	-0.104409732	0.013817565	0.038387898	1
Prediabetes	PP_cell	*MTMR12*	-0.328132852	0.003971587	0.019146462	1
Prediabetes	PP_cell	*IL18*	-0.104126402	0.013926864	0.038507143	1
Prediabetes	PP_cell	*DLG5*	-0.367969635	0.000374609	0.008519859	1
Prediabetes	PP_cell	*AKAP6*	-0.140833989	0.005556339	0.023167203	1
Prediabetes	PP_cell	*NDUFC2*	-0.171102689	0.017193608	0.043526859	1
Prediabetes	PP_cell	*C4orf33*	-0.291553649	0.006342776	0.025045996	1
Prediabetes	PP_cell	*QDPR*	-0.219380519	0.002748972	0.015953783	1
Prediabetes	PP_cell	*TEX9*	-0.359937031	0.001450779	0.011646409	1

Prediabetes	PP_cell	*KIN*	-0.343922135	0.006186513	0.024659802	1
Prediabetes	PP_cell	*CENPU*	-0.188423329	0.01961114	0.047918016	1
Prediabetes	PP_cell	*BICD1*	-0.375919609	0.00117086	0.010731532	1
Prediabetes	PP_cell	*GUF1*	-0.365794573	0.000507523	0.008519859	1
Prediabetes	PP_cell	*CCL28*	-0.415091535	0.002352817	0.014710984	1
Prediabetes	PP_cell	*RABGAP1L*	-0.380893852	0.001062539	0.010207131	1
Prediabetes	PP_cell	*ARL14EP*	-0.360570845	0.001374207	0.011450342	1
Prediabetes	PP_cell	*EPG5*	-0.408282468	0.003216578	0.017212803	1
Prediabetes	PP_cell	*PDE3B*	-0.169156288	9.33E-05	0.008519859	0.366636485
Prediabetes	PP_cell	*POC5*	-0.061504661	0.0131064	0.037909761	1
Prediabetes	PP_cell	*GUCY1A2*	-0.502741247	0.000479283	0.008519859	1
Prediabetes	PP_cell	*DCLRE1C*	-0.210934378	0.004106289	0.019508595	1
Prediabetes	PP_cell	*ZSCAN1*	-0.210972561	0.004447152	0.020270141	1
Prediabetes	PP_cell	*GRIA4*	-0.059831002	0.014771914	0.039644024	1
Prediabetes	PP_cell	*IGSF10*	-0.583258352	0.000358665	0.008519859	1
Prediabetes	PP_cell	*GPD1L*	-0.565802736	0.001465288	0.011646409	1
Prediabetes	PP_cell	*CCNO*	-0.080736213	0.011868275	0.035732147	1
Prediabetes	PP_cell	*CATSPER3*	-0.527749895	0.009452663	0.031889469	1
Prediabetes	PP_cell	*GPR180*	-0.270531572	0.013963479	0.03856274	1
Prediabetes	PP_cell	*MED21*	-0.263816175	0.017893685	0.044751296	1
Prediabetes	PP_cell	*ACMSD*	-0.090615338	0.013762668	0.038295695	1
Prediabetes	PP_cell	*TMEM87B*	-0.238862106	0.000975756	0.009804971	1
Prediabetes	PP_cell	*PLA2R1*	-0.539307759	0.004517298	0.020366067	1
Prediabetes	PP_cell	*CMTM7*	-0.106692683	0.006930605	0.026411589	1
Prediabetes	PP_cell	*PTPRD*	-0.075495885	0.008218136	0.029383885	1
Prediabetes	PP_cell	*ZDHHC7*	-0.276175894	0.003770853	0.018659548	1
Prediabetes	PP_cell	*JAZF1*	-0.219994578	0.000149033	0.008519859	0.585551619
Prediabetes	PP_cell	*CMIP*	-0.351533685	0.000354964	0.008519859	1
Prediabetes	PP_cell	*PID1*	-0.140902876	0.004431305	0.020248234	1
Prediabetes	PP_cell	*KCTD15*	-0.666437728	0.002720523	0.015858955	1
Prediabetes	PP_cell	*DDAH1*	-0.309675597	6.94E-05	0.008519859	0.272530487
Prediabetes	PP_cell	*NUS1*	-0.346735218	0.000937887	0.009671806	1
Prediabetes	PP_cell	*CC2D1B*	-0.117188846	0.009465088	0.031893938	1
Prediabetes	PP_cell	*MRPL39*	-0.211639291	0.01254574	0.037006165	1
Prediabetes	PP_cell	*TSEN2*	-0.534920306	0.001047197	0.01010918	1
Prediabetes	PP_cell	*FLCN*	-0.309139233	0.000629628	0.008645299	1
Prediabetes	PP_cell	*MPPE1*	-0.164889163	0.011534459	0.035488558	1
Prediabetes	PP_cell	*RAB6B*	-0.511676383	0.000455536	0.008519859	1
Prediabetes	PP_cell	*TRMT44*	-0.234556895	0.002784569	0.0160564	1
Prediabetes	PP_cell	*PPM1J*	-0.172692997	0.011825637	0.035704358	1
Prediabetes	PP_cell	*HEATR3*	-0.072633152	0.011753122	0.035704358	1
Prediabetes	PP_cell	*LRGUK*	-0.520137979	0.000791182	0.009253454	1
Prediabetes	PP_cell	*MIER3*	-0.375007597	0.000766562	0.009244451	1
Prediabetes	PP_cell	*TTN*	-0.498062171	0.006532535	0.025564074	1
Prediabetes	PP_cell	*RMND1*	-0.288329219	0.003316746	0.017447335	1
Prediabetes	PP_cell	*PSD3*	-0.153167535	0.002954978	0.016546556	1
Prediabetes	PP_cell	*MMP16*	-0.105538858	0.012716241	0.03734089	1
Prediabetes	PP_cell	*ADK*	-0.266034979	0.000796047	0.009253454	1
Prediabetes	PP_cell	*BATF*	-0.972956858	0.013489719	0.038130291	1
Prediabetes	PP_cell	*DRAM2*	-0.243644746	0.002319217	0.014602893	1
Prediabetes	PP_cell	*CCT8*	-0.154500585	0.011125559	0.034830535	1
Prediabetes	PP_cell	*MAPK13*	-0.196616504	0.000221736	0.008519859	0.87120178
Prediabetes	PP_cell	*TBC1D31*	-0.125779826	0.013409756	0.03803406	1
Prediabetes	PP_cell	*MPV17L*	-0.194616532	0.000757876	0.009244451	1
Prediabetes	PP_cell	*EXOG*	-0.291099061	0.005791194	0.023775968	1
Prediabetes	PP_cell	*ZFYVE9*	-0.130975297	0.004237115	0.019985143	1
Prediabetes	PP_cell	*SLC6A1*	-1.613361554	0.001858509	0.013228408	1
Prediabetes	PP_cell	*STEAP2*	-0.337583067	0.009563151	0.032025881	1
Prediabetes	PP_cell	*GATAD1*	-0.295788786	0.004301182	0.020046669	1
Prediabetes	PP_cell	*SLC38A10*	-0.247237672	0.015857748	0.041316373	1
Prediabetes	PP_cell	*AP3S2*	-0.308857626	0.003004847	0.016581525	1
Prediabetes	PP_cell	*TNFRSF14*	-0.279252126	0.001395449	0.011470127	1
Prediabetes	PP_cell	*PEX10*	-0.30088293	0.000857935	0.009444968	1
Prediabetes	PP_cell	*LDLRAP1*	-0.381630874	0.000253836	0.008519859	0.997322368

Prediabetes	PP_cell	*KRTCAP3*	-0.046766981	0.012907031	0.037703885	1
Prediabetes	PP_cell	*PAFAH2*	-0.211415269	0.003434021	0.017799828	1
Prediabetes	PP_cell	*EXTL1*	-0.340215285	0.019521622	0.047834311	1
Prediabetes	PP_cell	*TRIM63*	-1.280646068	0.00107618	0.010267243	1
Prediabetes	PP_cell	*XKR8*	-0.129205977	0.007163195	0.02684604	1
Prediabetes	PP_cell	*CNNM4*	-0.229262963	0.000134786	0.008519859	0.529573471
Prediabetes	PP_cell	*ESYT3*	-0.138213229	0.013753474	0.038295695	1
Prediabetes	PP_cell	*FAIM*	-0.317807693	0.014535931	0.039387361	1
Prediabetes	PP_cell	*COPG2*	-0.081465041	0.015834263	0.041316373	1
Prediabetes	PP_cell	*NPM2*	-0.097453425	0.00744603	0.027521593	1
Prediabetes	PP_cell	*CDA*	-0.792218731	0.019967792	0.048728853	1
Prediabetes	PP_cell	*EPB41*	-0.281890343	0.000907231	0.009499809	1
Prediabetes	PP_cell	*MIS18A*	-0.154795982	0.011123797	0.034830535	1
Prediabetes	PP_cell	*ALG8*	-0.584012133	0.0018963	0.013352263	1
Prediabetes	PP_cell	*GJD2*	-0.177844821	0.011765844	0.035704358	1
Prediabetes	PP_cell	*CLDN14*	-0.326176842	0.001483955	0.01172175	1
Prediabetes	PP_cell	*ADPGK*	-0.375532532	0.004391203	0.020178992	1
Prediabetes	PP_cell	*UBR1*	-0.40511296	0.000366305	0.008519859	1
Prediabetes	PP_cell	*TEPP*	-0.577057245	0.010550029	0.033892939	1
Prediabetes	PP_cell	*NPR2*	-0.256872709	0.016845717	0.042867114	1
Prediabetes	PP_cell	*TMPRSS3*	-0.20872093	0.014498569	0.039384002	1
Prediabetes	PP_cell	*SLC37A1*	-0.181535588	0.010157527	0.033119439	1
Prediabetes	PP_cell	*NDUFV3*	-0.110299167	0.013834885	0.038387898	1
Prediabetes	PP_cell	*PDXK*	-0.30789584	3.10E-05	0.008519859	0.121938146
Prediabetes	PP_cell	*ZNF66*	-0.329129594	0.006101038	0.024460181	1
Prediabetes	PP_cell	*LSS*	-0.220312242	0.001945669	0.013500582	1
Prediabetes	PP_cell	*VAV2*	-0.456537987	0.000964809	0.009795175	1
Prediabetes	PP_cell	*RDH13*	-0.094185674	0.003724614	0.018524062	1
Prediabetes	PP_cell	*MPZL3*	-0.104895773	0.008440712	0.029750772	1
Prediabetes	PP_cell	*S100A1*	-0.151637094	0.014070044	0.038726844	1
Prediabetes	PP_cell	*SLC25A44*	-0.356895751	0.000877867	0.009444968	1
Prediabetes	PP_cell	*CCR5*	-9.022961597	0.016390775	0.042146174	1
Prediabetes	PP_cell	*TONSL*	-0.17788178	0.006400585	0.025198295	1
Prediabetes	PP_cell	*PGAP3*	-0.618969884	0.002156857	0.0141015	1
Prediabetes	PP_cell	*PRPSAP1*	-0.30359402	0.002410339	0.014960855	1
Prediabetes	PP_cell	*SIGLEC11*	-1.329819892	0.006188487	0.024659802	1
Prediabetes	PP_cell	*SCIMP*	-0.147090444	0.014422992	0.039243274	1
Prediabetes	PP_cell	*AMDHD2*	-0.210147196	0.013912168	0.038493597	1
Prediabetes	PP_cell	*ZFPL1*	-0.3010667	0.002568318	0.015428508	1
Prediabetes	PP_cell	*TPCN2*	-0.167148687	0.00518166	0.022153146	1
Prediabetes	PP_cell	*PDZK1IP1*	-0.046409204	0.015684161	0.041136895	1
Prediabetes	PP_cell	*CTRC*	-0.073549283	0.001904738	0.01336916	1
Prediabetes	PP_cell	*OMA1*	-0.13040884	0.01320191	0.037909761	1
Prediabetes	PP_cell	*TM2D1*	-0.28473267	0.013629498	0.038253338	1
Prediabetes	PP_cell	*FUBP1*	-0.241503782	0.005942614	0.024153447	1
Prediabetes	PP_cell	*VCAM1*	-0.118164699	0.002143678	0.014060951	1
Prediabetes	PP_cell	*ZNF496*	-0.490267354	0.000781362	0.009253454	1
Prediabetes	PP_cell	*ACP6*	-0.194651299	0.001349289	0.01130353	1
Prediabetes	PP_cell	*MSX1*	-0.285315577	0.000847244	0.009444968	1
Prediabetes	PP_cell	*PACRGL*	-0.340697929	0.004405183	0.020219583	1
Prediabetes	PP_cell	*TDRD10*	-1.26222529	0.00856096	0.030058993	1
Prediabetes	PP_cell	*NPPC*	-0.154195465	0.013264094	0.037909824	1
Prediabetes	PP_cell	*NIPAL1*	-0.150469557	0.003794061	0.018729796	1
Prediabetes	PP_cell	*DAPL1*	-0.102930129	0.008284771	0.029511212	1
Prediabetes	PP_cell	*ATP1A1*	-0.236638661	0.000506557	0.008519859	1
Prediabetes	PP_cell	*SRGAP2*	-0.309264559	0.004467048	0.020304368	1
Prediabetes	PP_cell	*EOMES*	-0.455604047	0.006459421	0.025367051	1
Prediabetes	PP_cell	*HDAC11*	-0.15812647	0.002485587	0.015235369	1
Prediabetes	PP_cell	*NUAK2*	-0.186235272	0.003082635	0.016741022	1
Prediabetes	PP_cell	*CADPS*	-0.153608526	0.003464121	0.017885059	1
Prediabetes	PP_cell	*ATXN7*	-0.283611523	0.007265138	0.027056614	1
Prediabetes	PP_cell	*PSMD6*	-0.251308327	0.005630342	0.023335038	1
Prediabetes	PP_cell	*IL17RC*	-0.23030658	0.000183305	0.008519859	0.720205294
Prediabetes	PP_cell	*CRELD1*	-0.137865727	0.010534855	0.033892939	1

Prediabetes	PP_cell	*CXCL1*	-0.221771946	0.004337518	0.020119092	1
Prediabetes	PP_cell	*CCDC158*	-0.203022522	0.004901525	0.02139788	1
Prediabetes	PP_cell	*TM4SF18*	-0.09794602	0.012700337	0.03734089	1
Prediabetes	PP_cell	*NMNAT3*	-0.348317376	0.002407321	0.014960855	1
Prediabetes	PP_cell	*TPRA1*	-0.372691547	0.013699561	0.038295695	1
Prediabetes	PP_cell	*MEAF6*	-0.26328916	0.003854313	0.018923659	1
Prediabetes	PP_cell	*RPN1*	-0.271597938	0.003657198	0.01839612	1
Prediabetes	PP_cell	*RFC4*	-0.214867017	0.000917452	0.009561452	1
Prediabetes	PP_cell	*TKT*	-0.279544447	0.000486055	0.008519859	1
Prediabetes	PP_cell	*UVSSA*	-0.18290714	0.020494107	0.04961266	1
Prediabetes	PP_cell	*ARHGEF3*	-0.293718433	0.015767817	0.04125534	1
Prediabetes	PP_cell	*LRPAP1*	-0.171049701	0.00835299	0.029619945	1
Prediabetes	PP_cell	*PIGX*	-0.360191215	0.001265876	0.011077124	1
Prediabetes	PP_cell	*EXO5*	-0.258864672	0.016860222	0.042870225	1
Prediabetes	PP_cell	*ERMAP*	-0.205523871	0.006996912	0.026553791	1
Prediabetes	PP_cell	*CEP44*	-0.286665771	0.012713146	0.03734089	1
Prediabetes	PP_cell	*ARFIP1*	-0.337688285	0.001748197	0.012855972	1
Prediabetes	PP_cell	*ITGA2*	-0.149877493	0.003077722	0.016741022	1
Prediabetes	PP_cell	*STARD4*	-0.330214984	0.010360873	0.033559662	1
Prediabetes	PP_cell	*PRRC1*	-0.269180389	0.012407859	0.036654496	1
Prediabetes	PP_cell	*WDR41*	-0.202203401	0.002903362	0.016437045	1
Prediabetes	PP_cell	*SERINC5*	-0.449747069	0.000331056	0.008519859	1
Prediabetes	PP_cell	*UTP15*	-0.483716884	0.00032872	0.008519859	1
Prediabetes	PP_cell	*NSA2*	-0.167302686	0.016235772	0.041846259	1
Prediabetes	PP_cell	*DACT2*	-0.200616588	0.010057319	0.03290192	1
Prediabetes	PP_cell	*PDSS2*	-0.403758484	0.001138454	0.01057443	1
Prediabetes	PP_cell	*STK17A*	-0.360150283	6.23E-05	0.008519859	0.244854228
Prediabetes	PP_cell	*GALNT10*	-0.267793385	0.013247672	0.037909761	1
Prediabetes	PP_cell	*COG5*	-0.358450935	0.016009079	0.041435884	1
Prediabetes	PP_cell	*SLU7*	-0.233126552	0.015560117	0.040920818	1
Prediabetes	PP_cell	*STEAP1*	-0.29221506	0.020442655	0.049518615	1
Prediabetes	PP_cell	*CHMP4C*	-0.091079561	0.004896852	0.02139788	1
Prediabetes	PP_cell	*LMTK2*	-0.348728802	4.43E-05	0.008519859	0.174119503
Prediabetes	PP_cell	*PEX2*	-0.341241313	0.001662334	0.012440588	1
Prediabetes	PP_cell	*CSMD3*	-4.605035106	0.013038594	0.037891004	1
Prediabetes	PP_cell	*OSGIN2*	-0.359510925	0.000364308	0.008519859	1
Prediabetes	PP_cell	*DEFB1*	-0.057381196	0.004796994	0.0211057	1
Prediabetes	PP_cell	*INTS1*	-0.417212517	0.002880069	0.016352298	1
Prediabetes	PP_cell	*YWHAZ*	-0.184220581	0.015877222	0.041331289	1
Prediabetes	PP_cell	*GEM*	-0.415870445	0.000168679	0.008519859	0.662738319
Prediabetes	PP_cell	*RPP25L*	-0.539517802	0.000274721	0.008519859	1
Prediabetes	PP_cell	*FAM219A*	-0.341915373	0.016791907	0.042757876	1
Prediabetes	PP_cell	*NUDT2*	-0.195598348	0.008328022	0.029611582	1
Prediabetes	PP_cell	*METTL2B*	-0.153641149	0.017699065	0.044405892	1
Prediabetes	PP_cell	*MAMDC2*	-0.365499288	0.005100566	0.021949753	1
Prediabetes	PP_cell	*PRSS37*	-37.8743355	0.01316267	0.037909761	1
Prediabetes	PP_cell	*C9orf64*	-0.436639778	0.000488876	0.008519859	1
Prediabetes	PP_cell	*CLDN3*	-0.089804988	0.003752864	0.018617428	1
Prediabetes	PP_cell	*WNK2*	-0.084836682	0.00975793	0.032490598	1
Prediabetes	PP_cell	*NOL6*	-0.50169353	0.000478078	0.008519859	1
Prediabetes	PP_cell	*ARHGAP12*	-0.37020719	0.001126194	0.010535274	1
Prediabetes	PP_cell	*WRN*	-0.400546003	0.003668699	0.01839612	1
Prediabetes	PP_cell	*CFL2*	-0.273901896	0.006333101	0.025032951	1
Prediabetes	PP_cell	*INPPL1*	-0.475530472	0.000601927	0.008645299	1
Prediabetes	PP_cell	*NGB*	0.787414796	0.01928087	0.047346586	1
Prediabetes	PP_cell	*VDAC2*	-0.26008519	0.001292528	0.011111559	1
Prediabetes	PP_cell	*SNAPC4*	-0.19971427	0.00349204	0.018005545	1
Prediabetes	PP_cell	*TSC1*	-0.268670285	0.000605958	0.008645299	1
Prediabetes	PP_cell	*ZMYND19*	-0.444326973	0.005562669	0.023167203	1
Prediabetes	PP_cell	*METTL3*	-0.456932798	0.004367927	0.020119092	1
Prediabetes	PP_cell	*SLC39A13*	-0.433827166	0.000461916	0.008519859	1
Prediabetes	PP_cell	*IFI27L1*	-0.285779778	0.00013096	0.008519859	0.514542325
Prediabetes	PP_cell	*LIPC*	-0.094870198	0.013647396	0.038273105	1
Prediabetes	PP_cell	*CEP57*	-0.306417807	0.000643907	0.008645299	1

Prediabetes	PP_cell	*BTRC*	-0.23508889	0.002814977	0.016099046	1
Prediabetes	PP_cell	*ZNF319*	-0.164338941	0.005093588	0.02194376	1
Prediabetes	PP_cell	*SGPL1*	-0.159809436	0.007296213	0.027120926	1
Prediabetes	PP_cell	*FRS2*	-0.260415618	0.000482711	0.008519859	1
Prediabetes	PP_cell	*PCBD1*	-0.158147072	0.001894882	0.013352263	1
Prediabetes	PP_cell	*COX11*	-0.35653798	0.000277124	0.008519859	1
Prediabetes	PP_cell	*ZNF202*	-0.637329125	0.000709084	0.008816433	1
Prediabetes	PP_cell	*WBP1L*	-0.173361979	0.006613098	0.0257511	1
Prediabetes	PP_cell	*TUB*	-0.16215936	0.01889641	0.046769771	1
Prediabetes	PP_cell	*RIC3*	-0.315314883	0.000384659	0.008519859	1
Prediabetes	PP_cell	*XRRA1*	-0.179568741	0.007006991	0.026553791	1
Prediabetes	PP_cell	*TRIM66*	-0.126975138	0.010792866	0.034287057	1
Prediabetes	PP_cell	*ZNF143*	-0.417111425	0.000317448	0.008519859	1
Prediabetes	PP_cell	*SPDYE6*	-0.219822995	0.014425703	0.039243274	1
Prediabetes	PP_cell	*ATF7IP2*	-0.264305748	0.00070006	0.008813447	1
Prediabetes	PP_cell	*COG1*	-0.404596174	0.000287696	0.008519859	1
Prediabetes	PP_cell	*AP1G1*	-0.262501941	0.004319847	0.020109809	1
Prediabetes	PP_cell	*SLFN5*	-0.343706423	0.004363902	0.020119092	1
Prediabetes	PP_cell	*SCNN1G*	-0.586121492	0.014601478	0.039474478	1
Prediabetes	PP_cell	*STAT6*	-0.372197198	0.004344272	0.020119092	1
Prediabetes	PP_cell	*TSC22D4*	-0.262348898	0.00054131	0.008519859	1
Prediabetes	PP_cell	*URM1*	-0.263560283	0.00284568	0.016227394	1
Prediabetes	PP_cell	*CERCAM*	-0.119223131	0.015360873	0.040614315	1
Prediabetes	PP_cell	*DOLPP1*	-0.273008735	0.012643981	0.037212134	1
Prediabetes	PP_cell	*ENDOG*	-0.292355447	0.005089073	0.02194376	1
Prediabetes	PP_cell	*PRR15L*	-0.105693211	0.007045085	0.026639866	1
Prediabetes	PP_cell	*ACAA2*	-0.273830278	0.000524944	0.008519859	1
Prediabetes	PP_cell	*ZNF229*	-0.102310396	0.008178491	0.029327769	1
Prediabetes	PP_cell	*PROCA1*	-0.393074975	0.011786213	0.035704358	1
Prediabetes	PP_cell	*TP53I13*	-0.153200371	0.003963576	0.019131314	1
Prediabetes	PP_cell	*ZNF528*	-0.390569661	0.00033076	0.008519859	1
Prediabetes	PP_cell	*TMC4*	-0.127254241	0.002136966	0.014043725	1
Prediabetes	PP_cell	*C19orf33*	-0.130776385	0.005566261	0.023167203	1
Prediabetes	PP_cell	*UBXN6*	-0.248215259	0.010749299	0.034253037	1
Prediabetes	PP_cell	*KIFC2*	-0.160300493	0.020030538	0.048760833	1
Prediabetes	PP_cell	*TRPV3*	-0.309097212	0.018939122	0.046829333	1
Prediabetes	PP_cell	*NDUFV1*	-0.289941953	0.005600866	0.023253761	1
Prediabetes	PP_cell	*HID1*	-0.364442654	0.002284327	0.014499386	1
Prediabetes	PP_cell	*TMEM68*	-0.35673063	0.000536699	0.008519859	1
Prediabetes	PP_cell	*MLST8*	-0.286633832	0.000483953	0.008519859	1
Prediabetes	PP_cell	*ECI1*	-0.338697643	0.000877231	0.009444968	1
Prediabetes	PP_cell	*ABCA3*	-0.193294244	0.009504488	0.031971859	1
Prediabetes	PP_cell	*RPSA*	-0.125459286	0.019339298	0.047460401	1
Prediabetes	PP_cell	*ENTPD3*	-0.118431387	0.000607852	0.008645299	1
Prediabetes	PP_cell	*LTBP3*	-0.200222739	0.00107925	0.010267243	1
Prediabetes	PP_cell	*SAC3D1*	-0.402900251	0.0165193	0.042268461	1
Prediabetes	PP_cell	*CCDC88B*	-0.072426581	0.01851558	0.046042858	1
Prediabetes	PP_cell	*PBK*	-0.145464863	0.01597193	0.041421592	1
Prediabetes	PP_cell	*FAM83B*	-0.079298279	0.009863997	0.032714383	1
Prediabetes	PP_cell	*LMBRD1*	-0.228701962	0.017380805	0.043823045	1
Prediabetes	PP_cell	*TTC39C*	-0.119787564	0.001991362	0.013597804	1
Prediabetes	PP_cell	*NT5DC2*	-0.128230722	0.00401655	0.019268653	1
Prediabetes	PP_cell	*COA6*	-0.423803049	0.000471311	0.008519859	1
Prediabetes	PP_cell	*ING5*	-0.432807523	0.001433935	0.011622813	1
Prediabetes	PP_cell	*HEXIM2*	-0.218026761	0.004243163	0.019989673	1
Prediabetes	PP_cell	*SERINC2*	-0.067814196	0.005755578	0.023679231	1
Prediabetes	PP_cell	*TRAPPC11*	-0.252763817	0.001408848	0.011508037	1
Prediabetes	PP_cell	*COL3A1*	-0.216284184	0.020013435	0.048749401	1
Prediabetes	PP_cell	*ING2*	-0.205323839	0.000908126	0.009499809	1
Prediabetes	PP_cell	*ZSWIM1*	-0.171875728	0.014287272	0.039118253	1
Prediabetes	PP_cell	*ZNF30*	-0.150556245	0.005604813	0.023253761	1
Prediabetes	PP_cell	*SLC16A4*	-0.219516354	0.003202462	0.01718917	1
Prediabetes	PP_cell	*PPIP5K1*	-0.076699727	0.006497716	0.02547829	1
Prediabetes	PP_cell	*NSG1*	-0.050641351	0.01313256	0.037909761	1

Prediabetes	PP_cell	*FSTL5*	-0.170634554	0.001497323	0.011789544	1
Prediabetes	PP_cell	*CTRB1*	-0.071501293	0.001910056	0.01336916	1
Prediabetes	PP_cell	*CTRB2*	-0.063450389	0.013570538	0.038249975	1
Prediabetes	PP_cell	*MFF*	-0.273768845	0.004073197	0.019421835	1
Prediabetes	PP_cell	*XPO6*	-0.319600997	0.003442558	0.017820568	1
Prediabetes	PP_cell	*PRELID1*	-0.175858792	0.020724239	0.049888972	1
Prediabetes	PP_cell	*CXCL10*	-11.68687263	0.013196877	0.037909761	1
Prediabetes	PP_cell	*NMD3*	-0.329751793	0.000366803	0.008519859	1
Prediabetes	PP_cell	*SNUPN*	-0.492024992	0.000386875	0.008519859	1
Prediabetes	PP_cell	*CRADD*	-0.293434534	0.017282497	0.043667479	1
Prediabetes	PP_cell	*SDC2*	-0.171363683	0.000456459	0.008519859	1
Prediabetes	PP_cell	*CLIC4*	-0.320547605	0.007109625	0.026721477	1
Prediabetes	PP_cell	*AGPAT2*	-0.205501339	0.002693373	0.0157471	1
Prediabetes	PP_cell	*MT1E*	-0.077974438	0.001806423	0.013030484	1
Prediabetes	PP_cell	*LDB2*	-0.179985895	0.002797347	0.016068387	1
Prediabetes	PP_cell	*RAC3*	-0.295080514	0.002655085	0.01561651	1
Prediabetes	PP_cell	*AVEN*	-0.461623465	0.000386561	0.008519859	1
Prediabetes	PP_cell	*CTNND2*	-0.063311151	0.009193363	0.031382034	1
Prediabetes	PP_cell	*WNT10B*	-4.015588291	0.013833006	0.038387898	1
Prediabetes	PP_cell	*OTUD3*	-0.315658055	0.000119774	0.008519859	0.470590606
Prediabetes	PP_cell	*PUSL1*	-0.263004343	0.005512534	0.023090348	1
Prediabetes	PP_cell	*MYRIP*	-0.186058609	0.009328488	0.031675407	1
Prediabetes	PP_cell	*CNTROB*	-0.184636575	0.003987835	0.019154283	1
Prediabetes	PP_cell	*KCNAB3*	0.096110556	0.019084687	0.047089646	1
Prediabetes	PP_cell	*FAM153A*	-0.187697283	0.019552533	0.047834311	1
Prediabetes	PP_cell	*ZNF778*	-0.288916333	0.002518858	0.015295049	1
Prediabetes	PP_cell	*CST2*	-0.240898465	0.006889779	0.026358267	1
Prediabetes	PP_cell	*KRT8*	-0.062509399	0.005443143	0.022930133	1
Prediabetes	PP_cell	*MGMT*	-0.328520434	0.007218334	0.026907811	1
Prediabetes	PP_cell	*NPAS2*	-0.391847029	0.000159895	0.008519859	0.628228698
Prediabetes	PP_cell	*NUDT9*	-0.147212533	0.019624586	0.047921067	1
Prediabetes	PP_cell	*SMAGP*	-0.179589961	0.010898286	0.03440649	1
Prediabetes	PP_cell	*IRX1*	-0.098017876	0.013026637	0.037884276	1
Prediabetes	PP_cell	*CDH2*	-0.071633646	0.010255096	0.033326942	1
Prediabetes	PP_cell	*ACYP2*	-0.298513281	0.000811023	0.009399738	1
Prediabetes	PP_cell	*FOXN2*	-0.450304209	0.000282936	0.008519859	1
Prediabetes	PP_cell	*MTSS1*	-0.290899007	0.000904106	0.009499809	1
Prediabetes	PP_cell	*NDUFA3*	-0.293381719	0.0023158	0.014602893	1
Prediabetes	PP_cell	*TSNARE1*	-0.124466854	0.004524633	0.020366067	1
Prediabetes	PP_cell	*SOX7*	-0.150071677	0.007859387	0.02853931	1
Prediabetes	PP_cell	*TRMT61B*	-0.188475982	0.002605176	0.01555583	1
Prediabetes	PP_cell	*MFN1*	-0.262204811	0.000430193	0.008519859	1
Prediabetes	PP_cell	*KCNMB3*	-0.371294607	0.020427738	0.049513007	1
Prediabetes	PP_cell	*ATP6V0E2*	-0.110230108	0.00806951	0.029087251	1
Prediabetes	PP_cell	*JAGN1*	-0.306334231	0.000317842	0.008519859	1
Prediabetes	PP_cell	*MORN4*	-0.126817217	0.00496816	0.021664708	1
Prediabetes	PP_cell	*TRIM8*	-0.250832799	0.012316123	0.036438288	1
Prediabetes	PP_cell	*EXOSC1*	-0.289141454	0.009726676	0.032414002	1
Prediabetes	PP_cell	*ZNF561*	-0.360720586	0.000742939	0.009121901	1
Prediabetes	PP_cell	*PTGER4*	-0.183978045	0.004055674	0.019361778	1
Prediabetes	PP_cell	*NMUR1*	-0.738748245	0.013148248	0.037909761	1
Prediabetes	PP_cell	*PIK3CD*	-0.439054917	0.002213942	0.014319626	1
Prediabetes	PP_cell	*ENC1*	-0.130333789	0.006612558	0.0257511	1
Prediabetes	PP_cell	*RGS19*	-0.201869107	0.003677966	0.018402303	1
Prediabetes	PP_cell	*ANO5*	-0.141121718	0.010521928	0.033892939	1
Prediabetes	PP_cell	*GPHN*	-0.391237954	0.003520403	0.018080605	1
Prediabetes	PP_cell	*VAT1L*	-0.113435176	0.005684859	0.02351138	1
Prediabetes	PP_cell	*LGALS4*	-0.040610148	0.00822659	0.029383885	1
Prediabetes	PP_cell	*SPATA5L1*	-0.26055312	0.000415994	0.008519859	1
Prediabetes	PP_cell	*CTPS1*	-0.182683602	0.008022575	0.028971229	1
Prediabetes	PP_cell	*PWWP2B*	-0.191875441	0.001981505	0.013597804	1
Prediabetes	PP_cell	*TVP23B*	-0.218860082	0.000878229	0.009444968	1
Prediabetes	PP_cell	*SRGAP2C*	-0.356095137	0.01007632	0.032936658	1
Prediabetes	PP_cell	*ZNF556*	-0.108404035	0.01550937	0.040814678	1

Prediabetes	PP_cell	*GAP43*	-0.050858358	0.020098499	0.0488355	1
Prediabetes	PP_cell	*REG1B*	-0.071398039	0.002395532	0.014916081	1
Prediabetes	PP_cell	*NME6*	-0.367550701	0.000233004	0.008519859	0.915474431
Prediabetes	PP_cell	*SNTB1*	-0.170842698	0.007278849	0.027082005	1
Prediabetes	PP_cell	*BSG*	-0.213695118	0.008402444	0.029688131	1
Prediabetes	PP_cell	*CERS6*	-0.267488654	0.000817689	0.009434491	1
Prediabetes	PP_cell	*SPTLC3*	-0.206741395	0.014399779	0.039235459	1
Prediabetes	PP_cell	*SUCLG2*	-0.319253647	0.000456015	0.008519859	1
Prediabetes	PP_cell	*CSDC2*	-0.230242075	0.004767041	0.021054489	1
Prediabetes	PP_cell	*AFF1*	-0.297820549	0.006672937	0.025881509	1
Prediabetes	PP_cell	*SMPDL3A*	-0.235342473	0.006303815	0.024961783	1
Prediabetes	PP_cell	*EFEMP2*	-0.522640654	0.002625461	0.015605802	1
Prediabetes	PP_cell	*AGAP5*	-1.352947627	0.000369745	0.008519859	1
Prediabetes	PP_cell	*FUT10*	-0.176240984	0.001381014	0.011450342	1
Prediabetes	PP_cell	*NAA16*	-0.241689542	0.009110295	0.031152611	1
Prediabetes	PP_cell	*DMXL1*	-0.345988444	0.000566349	0.008521817	1
Prediabetes	PP_cell	*NADSYN1*	-0.240339329	0.014956299	0.0399258	1
Prediabetes	PP_cell	*TADA2B*	-0.388126826	0.000458619	0.008519859	1
Prediabetes	PP_cell	*NOC3L*	-0.164121882	0.020051332	0.048781228	1
Prediabetes	PP_cell	*RHOD*	-0.153819341	0.012587613	0.037074012	1
Prediabetes	PP_cell	*VANGL1*	-0.374279332	0.000929091	0.009619487	1
Prediabetes	PP_cell	*IQCB1*	-0.303649518	0.000553102	0.008519859	1
Prediabetes	PP_cell	*ARV1*	-0.357433982	0.000260851	0.008519859	1
Prediabetes	PP_cell	*PPP1R14B*	-0.207718308	0.007646146	0.027971794	1
Prediabetes	PP_cell	*TNFRSF10D*	-0.174301203	0.013135207	0.037909761	1
Prediabetes	PP_cell	*GMPPB*	-0.243468503	0.013418014	0.03803406	1
Prediabetes	PP_cell	*NUDT18*	-0.556404892	0.000293757	0.008519859	1
Prediabetes	PP_cell	*PC*	-0.098910189	0.014978431	0.0399258	1
Prediabetes	PP_cell	*UQCRH*	-0.18471351	0.011620578	0.035697617	1
Prediabetes	PP_cell	*HEG1*	-0.360624051	0.000771684	0.009253454	1
Prediabetes	PP_cell	*DPY19L1*	-0.444172832	0.004806888	0.021125572	1
Prediabetes	PP_cell	*GPR160*	-0.187271611	0.007113939	0.026721477	1
Prediabetes	PP_cell	*C9orf131*	-0.148639134	0.01495222	0.0399258	1
Prediabetes	PP_cell	*CTU2*	-0.315047317	0.002974218	0.016551989	1
Prediabetes	PP_cell	*ADCY6*	-0.103400906	0.006203734	0.024670519	1
Prediabetes	PP_cell	*RALGAPA1*	-0.265836952	0.001807486	0.013030484	1
Prediabetes	PP_cell	*GOLT1A*	-0.120735275	0.003451399	0.017842824	1
Prediabetes	PP_cell	*BRSK2*	-0.343341303	0.000513594	0.008519859	1
Prediabetes	PP_cell	*BRMS1*	-0.304120603	0.00455465	0.020393929	1
Prediabetes	PP_cell	*HRAS*	-0.290533708	0.002624223	0.015605802	1
Prediabetes	PP_cell	*YIF1A*	-0.151542013	0.008635328	0.030190258	1
Prediabetes	PP_cell	*NDUFA11*	-0.24359127	0.001561556	0.012006559	1
Prediabetes	PP_cell	*PPM1E*	-0.051328568	0.015800094	0.041275644	1
Prediabetes	PP_cell	*ZNF408*	-0.089997387	0.01890313	0.046769771	1
Prediabetes	PP_cell	*MED16*	-0.214022498	0.008636765	0.030190258	1
Prediabetes	PP_cell	*CHST1*	-0.135087139	0.013210361	0.037909761	1
Prediabetes	PP_cell	*DOLK*	-0.324293339	6.74E-05	0.008519859	0.264886874
Prediabetes	PP_cell	*ZNF519*	-0.351954981	0.000420381	0.008519859	1
Prediabetes	PP_cell	*CLCF1*	-0.256706861	0.000704045	0.008813447	1
Prediabetes	PP_cell	*UCP2*	-0.134880975	0.005559512	0.023167203	1
Prediabetes	PP_cell	*PAAF1*	-0.116057516	0.007190371	0.026854533	1
Prediabetes	PP_cell	*TMEM70*	-0.193432202	0.006638748	0.025799843	1
Prediabetes	PP_cell	*RPS6KB2*	-0.264852417	0.003077266	0.016741022	1
Prediabetes	PP_cell	*RMI2*	-0.24928134	0.007788232	0.028333299	1
Prediabetes	PP_cell	*SWI5*	-0.197454512	0.002506648	0.015269176	1
Prediabetes	PP_cell	*ZDHHC21*	-0.349355081	0.00083736	0.009444968	1
Prediabetes	PP_cell	*CHMP6*	-0.318742194	0.00098574	0.009817609	1
Prediabetes	PP_cell	*DLEU1*	-0.299139103	0.005194847	0.022185385	1
Prediabetes	PP_cell	*CCDC57*	-0.138064722	0.00221956	0.014319626	1
Prediabetes	PP_cell	*BNIP3*	-0.221024262	0.001522634	0.011846392	1
Prediabetes	PP_cell	*SMIM19*	-0.26674229	0.001468116	0.011646409	1
Prediabetes	PP_cell	*SLC35G1*	-0.431421079	0.000564821	0.008521817	1
Prediabetes	PP_cell	*COX8A*	-0.173861628	0.01204574	0.036128026	1
Prediabetes	PP_cell	*CRLF3*	-0.205244173	0.00443697	0.020248234	1

Prediabetes	PP_cell	*RNPEP*	-0.36158207	0.00364278	0.01839612	1
Prediabetes	PP_cell	*DMRTA1*	-0.0594169	0.017837935	0.044697223	1
Prediabetes	PP_cell	*WDR25*	-0.091224602	0.006153538	0.024620417	1
Prediabetes	PP_cell	*PHLDB3*	-0.07517216	0.020083603	0.048829502	1
Prediabetes	PP_cell	*PRR15*	-0.146474463	0.020007668	0.048749401	1
Prediabetes	PP_cell	*RMDN1*	-0.211150297	0.008693136	0.030237155	1
Prediabetes	PP_cell	*LRRC37A*	-0.245304333	0.013270031	0.037909824	1
Prediabetes	PP_cell	*ACSF3*	-0.456122792	0.002998143	0.016581525	1
Prediabetes	PP_cell	*PFN4*	0.134334978	0.013583527	0.038249975	1
Prediabetes	PP_cell	*VSIG10*	-0.269855445	0.001193745	0.01078212	1
Prediabetes	PP_cell	*WSB2*	-0.192174364	0.006903254	0.026361874	1
Prediabetes	PP_cell	*MUC20*	-0.122044027	0.004900667	0.02139788	1
Prediabetes	PP_cell	*SHMT1*	-0.062133028	0.019544975	0.047834311	1
Prediabetes	PP_cell	*DPP7*	-0.275929502	0.0004134	0.008519859	1
Prediabetes	PP_cell	*DEAF1*	-0.351310413	0.000339618	0.008519859	1
Prediabetes	PP_cell	*DSCAML1*	-0.244888081	0.003243018	0.017225057	1
Prediabetes	PP_cell	*KCNA2*	-0.439138774	0.013178963	0.037909761	1
Prediabetes	PP_cell	*PPFIA3*	-0.311104396	0.000767038	0.009244451	1
Prediabetes	PP_cell	*PAWR*	-0.327859823	0.000402328	0.008519859	1
Prediabetes	PP_cell	*ACOT4*	-0.322118078	0.00307745	0.016741022	1
Prediabetes	PP_cell	*RBM44*	-0.164445325	0.013302963	0.0379275	1
Prediabetes	PP_cell	*IL17RA*	-0.216634485	0.004715687	0.020888312	1
Prediabetes	PP_cell	*AGTRAP*	-0.161338583	0.00158219	0.012117789	1
Prediabetes	PP_cell	*THAP5*	-0.357862331	0.00064102	0.008645299	1
Prediabetes	PP_cell	*CD151*	-0.292466464	0.000526236	0.008519859	1
Prediabetes	PP_cell	*CHID1*	-0.240878205	0.000420914	0.008519859	1
Prediabetes	PP_cell	*ZBTB41*	-0.370163284	0.000557841	0.008521817	1
Prediabetes	PP_cell	*BET1L*	-0.225269502	0.000906624	0.009499809	1
Prediabetes	PP_cell	*GPR150*	-0.081567086	0.009569454	0.032025881	1
Prediabetes	PP_cell	*ALS2CL*	-0.119380449	0.01135421	0.035126529	1
Prediabetes	PP_cell	*STAP2*	-0.138332858	0.011522819	0.035480529	1
Prediabetes	PP_cell	*DALRD3*	-0.254700523	0.005785771	0.023775968	1
Prediabetes	PP_cell	*ZNF114*	-0.179168624	0.014729203	0.039627472	1
Prediabetes	PP_cell	*WDR6*	-0.181002824	0.00646282	0.025367051	1
Prediabetes	PP_cell	*NT5DC1*	-0.265656263	0.000866474	0.009444968	1
Prediabetes	PP_cell	*P4HTM*	-0.164799882	0.003619864	0.018375251	1
Prediabetes	PP_cell	*ERBB4*	-0.231897798	0.016868763	0.042870225	1
Prediabetes	PP_cell	*ERN1*	-0.239032914	0.013201016	0.037909761	1
Prediabetes	PP_cell	*RPP25*	-0.267667257	0.000524685	0.008519859	1
Prediabetes	PP_cell	*GAK*	-0.255043595	0.005460648	0.022946401	1
Prediabetes	PP_cell	*IGSF22*	-0.126734175	0.014200578	0.03896234	1
Prediabetes	PP_cell	*FARSA*	-0.155300604	0.003353594	0.017521636	1
Prediabetes	PP_cell	*SPTY2D1*	-0.349864234	0.001537386	0.011877462	1
Prediabetes	PP_cell	*FUCA1*	-0.230628762	0.000640853	0.008645299	1
Prediabetes	PP_cell	*HLA-DQB1*	-0.071420427	0.014784146	0.039649766	1
Prediabetes	PP_cell	*ELMOD2*	-0.345621146	0.000284041	0.008519859	1
Prediabetes	PP_cell	*DNHD1*	-0.138093488	0.005426013	0.022930133	1
Prediabetes	PP_cell	*ZBTB42*	-0.488388859	0.005400502	0.022930133	1
Prediabetes	PP_cell	*RPRML*	-0.195358505	0.001405397	0.011503757	1
Prediabetes	PP_cell	*SYCN*	-0.047537571	0.015120751	0.040139539	1
Prediabetes	PP_cell	*CITED4*	-0.210016555	0.001651687	0.012440588	1
Prediabetes	PP_cell	*SSC5D*	-0.088384791	0.01227556	0.036372141	1
Prediabetes	PP_cell	*ANKRD18A*	-0.0659255	0.004541312	0.020368512	1
Prediabetes	PP_cell	*TH*	-0.345294388	0.016673652	0.042539466	1
Prediabetes	PP_cell	*ZNF816*	-0.386000238	0.002649621	0.01561651	1
Prediabetes	PP_cell	*MCFD2*	-0.261127072	0.002355105	0.014710984	1
Prediabetes	PP_cell	*TSPYL5*	-0.164471733	0.002178426	0.014194086	1
Prediabetes	PP_cell	*CHST13*	-5.572960075	0.013018676	0.037884276	1
Prediabetes	PP_cell	*PPA1*	-0.253761425	0.000257345	0.008519859	1
Prediabetes	PP_cell	*MAP6D1*	-0.357448059	0.005410479	0.022930133	1
Prediabetes	PP_cell	*D2HGDH*	-0.123087411	0.002554351	0.01537007	1
Prediabetes	PP_cell	*ZFP82*	-0.191469917	0.00188739	0.013337331	1
Prediabetes	PP_cell	*RPH3AL*	-0.277364043	0.000384165	0.008519859	1
Prediabetes	PP_cell	*HIGD1A*	-0.275909789	0.000299729	0.008519859	1

Prediabetes	PP_cell	*OGFOD3*	-0.213439009	0.000661963	0.008757086	1
Prediabetes	PP_cell	*ZNF467*	-0.408175768	0.001454921	0.011646409	1
Prediabetes	PP_cell	*ZNF678*	-0.124871061	0.011378254	0.035145567	1
Prediabetes	PP_cell	*TMEM45A*	-0.533494159	0.002077887	0.013904889	1
Prediabetes	PP_cell	*ACBD4*	-0.856876819	0.008579359	0.030075341	1
Prediabetes	PP_cell	*SGSH*	-0.248819769	0.013709599	0.038295695	1
Prediabetes	PP_cell	*GPR135*	-0.192250421	0.003897996	0.019001519	1
Prediabetes	PP_cell	*GPR88*	-1.714165873	0.001378662	0.011450342	1
Prediabetes	PP_cell	*ZNF329*	-0.2859448	0.008272779	0.029495235	1
Prediabetes	PP_cell	*IDH2*	-0.29872328	0.000630091	0.008645299	1
Prediabetes	PP_cell	*ERCC6L2*	-0.432303062	0.0009484	0.009678612	1
Prediabetes	PP_cell	*TP53TG1*	-0.313419883	0.000205044	0.008519859	0.805618528
Prediabetes	PP_cell	*ARL6IP4*	-0.168764453	0.012758333	0.037436514	1
Prediabetes	PP_cell	*FAM153B*	-0.169921046	0.01774465	0.04449185	1
Prediabetes	PP_cell	*BACE2*	-0.36226316	0.001002908	0.009851066	1
Prediabetes	PP_cell	*ZNF804B*	-4.933063687	0.011812815	0.035704358	1
Prediabetes	PP_cell	*NPLOC4*	-0.316691278	0.002113578	0.013980215	1
Prediabetes	PP_cell	*SATB1*	-0.374587456	0.001241372	0.010970459	1
Prediabetes	PP_cell	*ACTL10*	-0.121210327	0.00990717	0.032720527	1
Prediabetes	PP_cell	*SKA2*	-0.270106281	0.008100505	0.029172212	1
Prediabetes	PP_cell	*ANXA2*	-0.256035159	0.000220395	0.008519859	0.86593054
Prediabetes	PP_cell	*GPIHBP1*	-0.602707173	0.013425666	0.03803406	1
Prediabetes	PP_cell	*ALG12*	-0.164218941	0.009546314	0.032025881	1
Prediabetes	PP_cell	*SPATA13*	-0.412994517	0.001598629	0.012172507	1
Prediabetes	PP_cell	*SLC25A21*	-0.281562994	0.008541674	0.0300181	1
Prediabetes	PP_cell	*LYSMD4*	-0.30448249	0.004536495	0.020368512	1
Prediabetes	PP_cell	*BEGAIN*	-0.274996781	0.000752448	0.009209872	1
Prediabetes	PP_cell	*GPC6*	-0.342337475	0.000947968	0.009678612	1
Prediabetes	PP_cell	*FANCF*	-0.12152023	0.003509454	0.018071617	1
Prediabetes	PP_cell	*CALN1*	-0.47891947	0.000844758	0.009444968	1
Prediabetes	PP_cell	*MX2*	-0.163292999	0.017627527	0.044282962	1
Prediabetes	PP_cell	*SFXN4*	-0.204578156	0.002857963	0.016251909	1
Prediabetes	PP_cell	*MRPL54*	-0.255500176	0.000684671	0.008780065	1
Prediabetes	PP_cell	*MACC1*	-0.054556201	0.010812335	0.034287057	1
Prediabetes	PP_cell	*KREMEN1*	-0.16584698	0.004049867	0.019357575	1
Prediabetes	PP_cell	*NUDT14*	-0.214168861	0.002157038	0.0141015	1
Prediabetes	PP_cell	*TMEM236*	-0.092816174	0.013351225	0.037957281	1
Prediabetes	PP_cell	*UQCR10*	-0.191410944	0.005061405	0.02194376	1
Prediabetes	PP_cell	*CRELD2*	-0.19159081	0.000366723	0.008519859	1
Prediabetes	PP_cell	*UBE2F*	-0.355172659	0.000178739	0.008519859	0.702263708
Prediabetes	PP_cell	*OAF*	-0.2652699	0.015036635	0.039999281	1
Prediabetes	PP_cell	*TM2D3*	-0.341436114	0.000620978	0.008645299	1
Prediabetes	PP_cell	*COPB2*	-0.279256213	0.004566755	0.020408359	1
Prediabetes	PP_cell	*LRRC19*	0.113067768	0.013707441	0.038295695	1
Prediabetes	PP_cell	*CCR10*	-0.279505051	0.000657143	0.008752256	1
Prediabetes	PP_cell	*NCMAP*	-0.158087084	0.004784829	0.021075778	1
Prediabetes	PP_cell	*WDR27*	-0.209693003	0.004606387	0.020543126	1
Prediabetes	PP_cell	*ZFP1*	-0.419712727	0.008647702	0.030201619	1
Prediabetes	PP_cell	*XPOT*	-0.306241166	0.000714306	0.008853338	1
Prediabetes	PP_cell	*C14orf180*	-6.923210078	0.013247502	0.037909761	1
Prediabetes	PP_cell	*ZBTB40*	-0.258973739	0.001939602	0.013500582	1
Prediabetes	PP_cell	*LCN12*	-0.16416116	0.00423522	0.019985143	1
Prediabetes	PP_cell	*MUC6*	-0.043352977	0.011363761	0.035128417	1
Prediabetes	PP_cell	*BRI3BP*	-0.376111411	0.000425792	0.008519859	1
Prediabetes	PP_cell	*UBOX5*	-0.351931069	0.016069157	0.041521635	1
Prediabetes	PP_cell	*CIB1*	-0.274908828	0.000478996	0.008519859	1
Prediabetes	PP_cell	*INPP5J*	-0.135873718	0.010156611	0.033119439	1
Prediabetes	PP_cell	*MFSD6L*	-0.109969973	0.01071127	0.03417498	1
Prediabetes	PP_cell	*ZNF445*	-0.298529505	0.00181706	0.013073736	1
Prediabetes	PP_cell	*ZNF74*	-0.102351057	0.012536746	0.037006165	1
Prediabetes	PP_cell	*RBM11*	-0.174098218	0.001734984	0.012837572	1
Prediabetes	PP_cell	*ATP6V0A2*	-0.365894472	0.003517251	0.018080605	1
Prediabetes	PP_cell	*RAD51D*	-0.153624858	0.00411127	0.019508673	1
Prediabetes	PP_cell	*GPRIN3*	-0.310648509	0.000552992	0.008519859	1

Prediabetes	PP_cell	*MUC1*	-0.044783848	0.0121436	0.036255474	1
Prediabetes	PP_cell	*IRF7*	-0.368515236	0.000670063	0.008780065	1
Prediabetes	PP_cell	*FAM131C*	-0.240365054	0.017536222	0.044109998	1
Prediabetes	PP_cell	*LSAMP*	-0.107736753	0.00433322	0.020119092	1
Prediabetes	PP_cell	*PDIA2*	-0.044771549	0.012068011	0.036167213	1
Prediabetes	PP_cell	*P4HB*	-0.226266767	0.000531491	0.008519859	1
Prediabetes	PP_cell	*DRG1*	-0.452411573	6.26E-05	0.008519859	0.245894704
Prediabetes	PP_cell	*DMWD*	-0.257258252	0.004425708	0.020248234	1
Prediabetes	PP_cell	*TRIM69*	-0.118929909	0.017946385	0.044810578	1
Prediabetes	PP_cell	*ZNF529*	-0.467498586	0.000846766	0.009444968	1
Prediabetes	PP_cell	*WWOX*	-0.22722799	0.000644712	0.008645299	1
Prediabetes	PP_cell	*POLR1D*	-0.25437502	0.001192171	0.01078212	1
Prediabetes	PP_cell	*ZNF749*	-0.081339055	0.019590277	0.047896825	1
Prediabetes	PP_cell	*TOR3A*	-0.303901719	0.000223052	0.008519859	0.876370609
Prediabetes	PP_cell	*ZNF555*	-0.37590253	0.001616129	0.012263296	1
Prediabetes	PP_cell	*FAM86B1*	-0.142729488	0.01327664	0.037909824	1
Prediabetes	PP_cell	*BCR*	-0.363475072	0.001913211	0.01336916	1
Prediabetes	PP_cell	*RTN4RL2*	-0.098920378	0.009319059	0.031673516	1
Prediabetes	PP_cell	*TMEM232*	-0.108117799	0.008953715	0.030831853	1
Prediabetes	PP_cell	*ESPN*	-1.444212284	0.005079544	0.02194376	1
Prediabetes	PP_cell	*TMEM216*	-0.121883265	0.016000807	0.041435884	1
Prediabetes	PP_cell	*BCAM*	-0.240198137	0.001519585	0.011846392	1
Prediabetes	PP_cell	*CHP1*	-0.251142402	0.014399983	0.039235459	1
Prediabetes	PP_cell	*RGPD1*	-7.445059191	0.012850262	0.03760809	1
Prediabetes	PP_cell	*KBTBD12*	-0.296052579	0.000289883	0.008519859	1
Prediabetes	PP_cell	*PALM3*	-0.082681691	0.013660847	0.0382835	1
Prediabetes	PP_cell	*KLHL17*	-0.152463551	0.007745748	0.028204861	1
Prediabetes	PP_cell	*ZNF490*	-0.289095379	0.001152687	0.010631239	1
Prediabetes	PP_cell	*NRN1L*	-0.177541082	0.013002879	0.037884276	1
Prediabetes	PP_cell	*FAM166A*	-0.709812695	7.55E-05	0.008519859	0.296662989
Prediabetes	PP_cell	*ZNF559*	-0.113562292	0.00129381	0.011111559	1
Prediabetes	PP_cell	*FOCAD*	-0.399747432	0.007151599	0.026837281	1
Prediabetes	PP_cell	*SERPINA5*	-0.060892448	0.004687528	0.020832552	1
Prediabetes	PP_cell	*FBLL1*	-0.391839069	0.000508309	0.008519859	1
Prediabetes	PP_cell	*UROS*	-0.350112935	0.001325157	0.011220999	1
Prediabetes	PP_cell	*FAM221A*	-0.325237538	6.83E-05	0.008519859	0.268484702
Prediabetes	PP_cell	*TMEM198*	-0.088191063	0.008889756	0.030690414	1
Prediabetes	PP_cell	*RPL14*	-0.165302293	0.004271221	0.019996719	1
Prediabetes	PP_cell	*ZNF567*	-0.421996115	0.00057238	0.008550883	1
Prediabetes	PP_cell	*ARID2*	-0.350311722	0.000615805	0.008645299	1
Prediabetes	PP_cell	*CLDN4*	-0.062250096	0.003670796	0.01839612	1
Prediabetes	PP_cell	*FAM47E*	-0.127123674	0.011709312	0.035704358	1
Prediabetes	PP_cell	*S100A14*	-0.060073418	0.009455646	0.031889469	1
Prediabetes	PP_cell	*CXCL17*	-0.057192794	0.011681833	0.035704358	1
Prediabetes	PP_cell	*SPATA41*	-0.348867005	0.003566539	0.018246003	1
Prediabetes	PP_cell	*PTPRT*	-0.087824998	0.011166279	0.034846952	1
Prediabetes	PP_cell	*ZNF676*	-0.189903571	0.014519698	0.039384002	1
Prediabetes	PP_cell	*HLA-DRB1*	-0.060957105	0.003303562	0.017445616	1
Prediabetes	PP_cell	*ZNF681*	-0.068323681	0.012828353	0.03760809	1
Prediabetes	PP_cell	*HRCT1*	-0.429167325	0.015419017	0.04071325	1
Prediabetes	PP_cell	*XPNPEP3*	-0.294321215	0.000245896	0.008519859	0.966124909
Prediabetes	PP_cell	*ZNF471*	-0.647708062	0.000504666	0.008519859	1
Prediabetes	PP_cell	*ZNF493*	-0.439581745	0.000685426	0.008780065	1
Prediabetes	PP_cell	*SUPT3H*	-0.604789145	8.70E-05	0.008519859	0.34177395
Prediabetes	PP_cell	*LONP1*	-0.321572535	0.002810832	0.016098772	1
Prediabetes	PP_cell	*EVL*	-0.170599806	0.004280279	0.019996719	1
Prediabetes	PP_cell	*ZNF124*	-0.387521297	0.000619129	0.008645299	1
Prediabetes	PP_cell	*PIK3R4*	-0.259950141	0.002416086	0.014972873	1
Prediabetes	PP_cell	*C20orf96*	-0.289378016	0.003909832	0.019030442	1
Prediabetes	PP_cell	*SULT1A1*	-0.182957949	0.006661717	0.025863524	1
Prediabetes	PP_cell	*MYO18A*	-0.226521112	0.003600307	0.018323322	1
Prediabetes	PP_cell	*XRCC2*	-0.187205949	0.013346672	0.037957281	1
Prediabetes	PP_cell	*MYO6*	-0.308048209	0.003307962	0.017445616	1
Prediabetes	PP_cell	*MMP1*	-0.102672766	0.00867729	0.030231024	1

Prediabetes	PP_cell	*TRAPPC4*	-0.181458406	0.003661631	0.01839612	1
Prediabetes	PP_cell	*ZNF33B*	-0.129156208	0.004256294	0.019996719	1
Prediabetes	PP_cell	*AMZ2*	-0.31094535	0.011889119	0.035745194	1
Prediabetes	PP_cell	*MVB12B*	-0.253341533	0.004646164	0.020697029	1
Prediabetes	PP_cell	*ANKRD36B*	-0.127798036	0.01313877	0.037909761	1
Prediabetes	PP_cell	*FAM3C*	-0.221571319	0.013741899	0.038295695	1
Prediabetes	PP_cell	*AP2A1*	-0.302175683	0.00822521	0.029383885	1
Prediabetes	PP_cell	*ZNF100*	-0.241346073	0.00307228	0.016741022	1
Prediabetes	PP_cell	*ARRDC1*	-0.132935723	0.008699043	0.030237155	1
Prediabetes	PP_cell	*ACSL5*	-0.182773217	0.002456466	0.015103998	1
Prediabetes	PP_cell	*SND1*	-0.250834075	0.00197736	0.013597804	1
Prediabetes	PP_cell	*PIWIL2*	-0.17114521	0.000182282	0.008519859	0.716185733
Prediabetes	PP_cell	*C6orf141*	-0.118933249	0.020807903	0.049972037	1
Prediabetes	PP_cell	*ZNF165*	-0.11532065	0.010592047	0.033944658	1
Prediabetes	PP_cell	*SVIL*	-0.432113804	0.001069798	0.010251798	1
Prediabetes	PP_cell	*ZNF98*	-0.109986339	0.010628311	0.034005404	1
Prediabetes	PP_cell	*OGDHL*	-0.087141662	0.000865244	0.009444968	1
Prediabetes	PP_cell	*ATG7*	-0.405740728	0.015394833	0.040676731	1
Prediabetes	PP_cell	*HHLA3*	-0.152321703	0.007554297	0.027765046	1
Prediabetes	PP_cell	*ENTPD6*	-0.229231553	0.00133483	0.011278593	1
Prediabetes	PP_cell	*DPP4*	-0.053929453	0.01301277	0.037884276	1
Prediabetes	PP_cell	*NMB*	-0.193613007	0.000434908	0.008519859	1
Prediabetes	PP_cell	*LHFPL5*	-0.132409929	0.005881061	0.024051313	1
Prediabetes	PP_cell	*FAM118B*	-0.20317648	0.011831783	0.035704358	1
Prediabetes	PP_cell	*SLC9A8*	-0.364478549	0.000449813	0.008519859	1
Prediabetes	PP_cell	*ZNF181*	-0.33189706	0.016458716	0.042210376	1
Prediabetes	PP_cell	*TEAD4*	-0.225139083	0.001908512	0.01336916	1
Prediabetes	PP_cell	*RPL12*	-0.138260711	0.008976711	0.030871689	1
Prediabetes	PP_cell	*LEKR1*	-0.113130343	0.013747469	0.038295695	1
Prediabetes	PP_cell	*ENTPD7*	-0.342917828	0.000641537	0.008645299	1
Prediabetes	PP_cell	*ZNF84*	-0.393450578	0.00426035	0.019996719	1
Prediabetes	PP_cell	*ZNF667*	-0.404897418	0.01083096	0.034318421	1
Prediabetes	PP_cell	*CD2AP*	-0.324510859	0.002988872	0.016581525	1
Prediabetes	PP_cell	*SFI1*	-0.294498796	9.55E-05	0.008519859	0.375243518
Prediabetes	PP_cell	*SOWAHC*	-0.466748411	0.000170713	0.008519859	0.670730702
Prediabetes	PP_cell	*DDRGK1*	-0.249714675	0.015727444	0.041222901	1
Prediabetes	PP_cell	*ZNF607*	-0.108249041	0.014608269	0.039474478	1
Prediabetes	PP_cell	*ZNF587*	-0.362761388	0.000406213	0.008519859	1
Prediabetes	PP_cell	*HLA-DRB5*	-0.062072966	0.019998896	0.048749401	1
Prediabetes	PP_cell	*CNGA1*	-0.136845787	0.008373945	0.029640748	1
Prediabetes	PP_cell	*ZNF789*	-0.151240036	0.010466629	0.033815404	1
Prediabetes	PP_cell	*NCOA6*	-0.417563974	0.000945544	0.009678612	1
Prediabetes	PP_cell	*STK39*	-0.104887294	0.006952629	0.026444221	1
Prediabetes	PP_cell	*PAPSS2*	-0.181512185	0.000500478	0.008519859	1
Prediabetes	PP_cell	*ECI2*	-0.249377433	0.014819076	0.039716338	1
Prediabetes	PP_cell	*MSRB1*	-0.310308578	0.014996084	0.039945502	1
Prediabetes	PP_cell	*COLGALT2*	-0.07281752	0.007910789	0.028699438	1
Prediabetes	PP_cell	*DENND4B*	-0.287488249	0.000606775	0.008645299	1
Prediabetes	PP_cell	*TSEN15*	-0.235459414	0.000586859	0.008635842	1
Prediabetes	PP_cell	*CCDC152*	-0.47339769	0.000692814	0.008780065	1
Prediabetes	PP_cell	*TYW1*	-0.170945324	0.006012081	0.024326947	1
Prediabetes	PP_cell	*ITPRIPL1*	-0.117904558	0.007540472	0.027760015	1
Prediabetes	PP_cell	*C1orf174*	-0.294234582	0.000371572	0.008519859	1
Prediabetes	PP_cell	*TBKBP1*	-0.152773023	0.000619401	0.008645299	1
Prediabetes	PP_cell	*CCDC167*	-0.367446457	0.000293892	0.008519859	1
Prediabetes	PP_cell	*INF2*	-0.162496537	0.003681397	0.018402303	1
Prediabetes	PP_cell	*CHML*	-0.263835129	0.000641208	0.008645299	1
Prediabetes	PP_cell	*RAET1G*	-0.395899469	0.011017824	0.034659	1
Prediabetes	PP_cell	*PCMTD2*	-0.290314721	0.019038161	0.047015043	1
Prediabetes	PP_cell	*HSD17B8*	-0.101811398	0.012260785	0.036372141	1
Prediabetes	PP_cell	*NOTCH4*	-0.136880051	0.000124953	0.008519859	0.490939425
Prediabetes	PP_cell	*RNF5*	-0.119910628	0.009419766	0.031823094	1
Prediabetes	PP_cell	*MRPL38*	-0.301881699	0.001316875	0.011188791	1
Prediabetes	PP_cell	*LAYN*	-0.557090453	0.006040087	0.024364991	1

Prediabetes	PP_cell	*PSORS1C1*	-0.059719248	0.015948725	0.041414773	1
Prediabetes	PP_cell	*DHX16*	-0.315007647	0.006888534	0.026358267	1
Prediabetes	PP_cell	*LILRB3*	-0.176627494	0.005984293	0.024289554	1
Prediabetes	PP_cell	*ZNF783*	-0.684750635	0.000594922	0.008645299	1
Prediabetes	PP_cell	*ZNF425*	-0.167063765	0.012190208	0.036311846	1
Prediabetes	PP_cell	*PABPN1L*	-6.033700647	0.012864666	0.03760809	1
Prediabetes	PP_cell	*SLC35B4*	-0.52662041	0.009381198	0.031774765	1
Prediabetes	PP_cell	*SARNP*	-0.268195297	0.006813158	0.026269771	1
Prediabetes	PP_cell	*MT1A*	-0.038513302	0.011407129	0.035181102	1
Prediabetes	PP_cell	*ATP6AP1L*	-0.219091041	0.011678681	0.035704358	1
Prediabetes	PP_cell	*RGL3*	-0.149744404	0.007636647	0.027963081	1
Prediabetes	PP_cell	*CPT1B*	-0.091415287	0.005449417	0.022930133	1
Prediabetes	PP_cell	*LCMT1*	-0.337613982	0.000266334	0.008519859	1
Prediabetes	PP_cell	*LIN52*	-0.637132408	0.000448384	0.008519859	1
Prediabetes	PP_cell	*GOLGA8O*	-0.480015042	0.00088266	0.009444968	1
Prediabetes	PP_cell	*MAP10*	-0.14299727	0.012189319	0.036311846	1
Prediabetes	PP_cell	*CCL14*	-0.138939073	0.00300287	0.016581525	1
Prediabetes	PP_cell	*ZBTB9*	-0.170110144	0.005694609	0.02352694	1
Prediabetes	PP_cell	*PPP1CB*	-0.205816893	0.012138719	0.036255474	1
Prediabetes	PP_cell	*NUDT19*	-0.427393565	0.00016569	0.008519859	0.650997891
Prediabetes	PP_cell	*AP1G2*	-0.192061496	0.002522574	0.015295049	1
Prediabetes	PP_cell	*ZNF90*	-0.766230902	0.000268245	0.008519859	1
Prediabetes	PP_cell	*MBLAC1*	-0.193829479	0.020797291	0.049972037	1
Prediabetes	PP_cell	*SEC14L6*	-0.041212018	0.017878324	0.04474136	1
Prediabetes	PP_cell	*MEG3*	-0.113466272	0.00153561	0.011877462	1
Prediabetes	PP_cell	*IFRD2*	-0.446496766	0.00027966	0.008519859	1
Prediabetes	PP_cell	*UBXN2B*	-0.110827294	0.002640466	0.01561651	1
Prediabetes	PP_cell	*C5orf49*	-0.214909364	0.001318506	0.011188791	1
Prediabetes	PP_cell	*ATAD3C*	-0.352885794	0.000681617	0.008780065	1
Prediabetes	PP_cell	*PAM16*	-0.250664248	0.000355091	0.008519859	1
Prediabetes	PP_cell	*CELA3B*	-0.06486107	0.002697327	0.0157471	1
Prediabetes	PP_cell	*PPP3R1*	-0.326220175	0.004273627	0.019996719	1
Prediabetes	PP_cell	*TRIM16*	-0.570983367	0.003826009	0.018814004	1
Prediabetes	PP_cell	*SLC12A8*	-0.458526644	0.002822577	0.016119051	1
Prediabetes	PP_cell	*LINC00365*	-0.187417408	0.013190305	0.037909761	1
Prediabetes	PP_cell	*LINC00937*	-0.098222817	0.007612456	0.027900502	1
Prediabetes	PP_cell	*CYP4F26P*	-3.288813334	0.015030876	0.039999281	1
Prediabetes	PP_cell	*EPPK1*	-0.159428801	0.007541211	0.027760015	1
Prediabetes	PP_cell	*ARL17B*	-0.251713217	0.00054477	0.008519859	1
Prediabetes	PP_cell	*PSMG3-AS1*	-0.2052873	0.014118264	0.038817816	1
Prediabetes	PP_cell	*HSBP1*	-0.210643389	0.011770858	0.035704358	1
Prediabetes	PP_cell	*RPS18*	-0.126684111	0.013704449	0.038295695	1
Prediabetes	PP_cell	*LYRM9*	-0.183034221	0.016358397	0.042090466	1
Prediabetes	PP_cell	*ANKRD65*	-0.266799519	0.008679205	0.030231024	1
Prediabetes	PP_cell	*C12orf75*	-0.20705527	0.000398645	0.008519859	1
Prediabetes	PP_cell	*ZNF737*	-0.387625001	0.003092271	0.016741022	1
Prediabetes	PP_cell	*SHISA9*	-0.497211049	0.014335041	0.039167161	1
Prediabetes	PP_cell	*LRRC37A2*	-0.215781001	0.000568266	0.008521817	1
Prediabetes	PP_cell	*TXNDC5*	-0.258555654	0.010029828	0.032886224	1
Prediabetes	PP_cell	*RBM14*	-0.175074005	0.005175754	0.022152003	1
Prediabetes	PP_cell	*NME1*	-0.194104127	0.007736093	0.028195833	1
Prediabetes	PP_cell	*CDRT4*	-0.108049849	0.008336241	0.029614005	1
Prediabetes	PP_cell	*AMY2B*	-0.046764078	0.01146595	0.035333113	1
Prediabetes	PP_cell	*SMKR1*	-0.289298138	0.000777907	0.009253454	1
Prediabetes	PP_cell	*LINC00886*	-0.116715917	0.003949085	0.019084817	1
Prediabetes	PP_cell	*MIF*	-0.16074376	0.005932165	0.024153447	1
Prediabetes	PP_cell	*PI4KA*	-0.3792914	0.000450719	0.008519859	1
Prediabetes	PP_cell	*EIF4EBP3*	-0.208789862	0.006062176	0.024391958	1
Prediabetes	PP_cell	*MICAL3*	-0.298766761	0.000586279	0.008635842	1
Prediabetes	PP_cell	*VSIG8*	-0.17975039	0.009607354	0.032070769	1
Prediabetes	PP_cell	*C4orf48*	-0.25357959	0.000621435	0.008645299	1
Prediabetes	PP_cell	*IL10RB*	-0.34012149	0.000339996	0.008519859	1
Prediabetes	PP_cell	*RN7SL832P*	-0.30342327	0.007848541	0.02852629	1
Prediabetes	PP_cell	*GSTA1*	-0.043817022	0.015477337	0.040757678	1

Prediabetes	PP_cell	*GSTA2*	-0.063601218	0.006573107	0.025671708	1
Prediabetes	PP_cell	*TMEM141*	-0.209941149	0.000137547	0.008519859	0.540421437
Prediabetes	PP_cell	*UBAP1L*	-0.315375029	0.002243737	0.014351084	1
Prediabetes	PP_cell	*BCKDHA*	-0.350912841	0.000879476	0.009444968	1
Prediabetes	PP_cell	*PCDHAC1*	-0.123907945	0.020735223	0.049888972	1
Prediabetes	PP_cell	*FMN1*	-0.099383935	0.004522907	0.020366067	1
Prediabetes	PP_cell	*PDCD6*	-0.264957419	0.002246352	0.014351084	1
Prediabetes	PP_cell	*PCDHA10*	-0.200184246	0.004189246	0.01983078	1
Prediabetes	PP_cell	*ATP6V1E2*	-0.151462661	0.002551777	0.01537007	1
Prediabetes	PP_cell	*SNHG18*	-0.179021821	0.002309188	0.014586494	1
Prediabetes	PP_cell	*ZNF550*	-0.466705094	0.000208444	0.008519859	0.818975429
Prediabetes	PP_cell	*LINC01099*	-0.233971357	0.003575527	0.018268201	1
Prediabetes	PP_cell	*LYN*	-0.35923479	0.001359517	0.011364982	1
Prediabetes	PP_cell	*AP5B1*	-0.328967384	0.009250355	0.031505802	1
Prediabetes	PP_cell	*PABPC4L*	-1.046294151	0.00545094	0.022930133	1
Prediabetes	PP_cell	*NPIPA2*	-0.749586497	0.010007339	0.032847816	1
Prediabetes	PP_cell	*EID3*	-0.300707455	0.001033563	0.010051656	1
Prediabetes	PP_cell	*SNHG9*	-0.287264474	0.000863944	0.009444968	1
Prediabetes	PP_cell	*ZNF10*	-0.378669196	0.017243546	0.043597099	1
Prediabetes	PP_cell	*LIMS3*	-1.323072494	0.006852472	0.026292348	1
Prediabetes	PP_cell	*GATC*	-0.358596656	0.001753831	0.012855972	1
Prediabetes	PP_cell	*TEN1*	-0.238555467	0.011743224	0.035704358	1
Prediabetes	PP_cell	*LINC00638*	-0.575327495	0.004731078	0.020932887	1
Prediabetes	PP_cell	*TMEM179*	-0.17042538	0.001784108	0.012957043	1
Prediabetes	PP_cell	*SH3RF3-AS1*	-0.345715596	0.011321368	0.035107856	1
Prediabetes	PP_cell	*TMEM178B*	-0.339257613	0.003112631	0.016821908	1
Prediabetes	PP_cell	*MRPL12*	-0.367908002	0.003885811	0.018989244	1
Prediabetes	PP_cell	*LINC00667*	-0.252446741	0.014760542	0.03964058	1
Prediabetes	PP_cell	*FSBP*	-0.117408563	0.011114787	0.034830535	1
Prediabetes	PP_cell	*ZNF285*	-0.394216429	0.009891861	0.032720527	1
Prediabetes	PP_cell	*FBXO17*	-0.293593331	0.000562949	0.008521817	1
Prediabetes	PP_cell	*SNHG8*	-0.235732351	0.000790385	0.009253454	1
Prediabetes	PP_cell	*GTF2H5*	-0.305227523	0.000818824	0.009434491	1
T1DM	acinar_cell	*CEP68*	0.2918121	0.000375486	0.046102602	1
T1DM	acinar_cell	*UQCC2*	0.420967841	3.74E-05	0.005069852	0.147025694
T1DM	acinar_cell	*HLA-DQB1*	-0.313913978	1.25E-28	8.21E-26	4.92E-25
T1DM	acinar_cell	*BTN3A2*	-0.073687797	2.38E-05	0.003335122	0.093383413
T1DM	acinar_cell	*ZKSCAN3*	-0.467317287	2.51E-09	5.58E-07	9.88E-06
T1DM	acinar_cell	*HLA-DRB1*	-0.113206073	1.05E-08	2.07E-06	4.13E-05
T1DM	acinar_cell	*SFTA2*	-0.206287569	2.56E-09	5.58E-07	1.00E-05
T1DM	acinar_cell	*RPS26*	0.230137041	0.000410187	0.048388528	1
T1DM	acinar_cell	*HLA-DRB5*	-0.204406187	1.05E-23	5.17E-21	4.13E-20
T1DM	acinar_cell	*HSD17B8*	0.64987386	5.90E-28	3.31E-25	2.32E-24
T1DM	acinar_cell	*HLA-DMA*	0.212329234	1.96E-29	1.92E-26	7.69E-26
T1DM	acinar_cell	*NOTCH4*	-0.423133901	5.86E-29	4.61E-26	2.30E-25
T1DM	acinar_cell	*AGER*	0.37811136	4.65E-06	0.000676734	0.018271809
T1DM	acinar_cell	*RNF5*	-0.347499166	1.81E-14	5.93E-12	7.11E-11
T1DM	acinar_cell	*EHMT2*	0.457659235	0.000151076	0.019147691	0.593578434
T1DM	acinar_cell	*MICA*	0.102564971	1.91E-07	3.41E-05	0.000749455
T1DM	acinar_cell	*POU5F1*	0.147460858	0.000418735	0.048388528	1
T1DM	acinar_cell	*CCHCR1*	0.369408365	9.26E-09	1.91E-06	3.64E-05
T1DM	acinar_cell	*PSORS1C1*	-0.153212321	4.44E-11	1.09E-08	1.74E-07
T1DM	acinar_cell	*RNF39*	0.303782609	1.50E-13	4.54E-11	5.90E-10
T1DM	acinar_cell	*ZFP57*	0.239327096	3.28E-07	5.39E-05	0.001288796
T1DM	acinar_cell	*HCG27*	0.229720492	2.42E-08	4.52E-06	9.49E-05
T1DM	acinar_cell	*ATF6B*	0.342432947	4.43E-11	1.09E-08	1.74E-07
T1DM	acinar_cell	*PPT2*	0.537468961	2.28E-12	6.40E-10	8.96E-09
T1DM	acinar_cell	*C4B*	1.82037135	6.46E-07	0.000101488	0.002537198
T1DM	acinar_cell	*HLA-DQB2*	0.950641717	1.53E-52	6.02E-49	6.02E-49
T1DM	acinar_cell	*HLA-B*	0.136705996	4.35E-05	0.005695233	0.170856997
T1DM	acinar_cell	*ZSCAN31*	0.144010099	3.29E-07	5.39E-05	0.001292888
T1DM	acinar_cell	*B3GALT4*	-0.248056779	1.59E-06	0.000239598	0.00622954
T1DM	acinar_cell	*HLA-DQA2*	0.371724601	8.15E-33	1.07E-29	3.20E-29
T1DM	acinar_cell	*PSMB9*	0.528297116	7.87E-21	3.09E-18	3.09E-17

T1DM	acinar_cell	*HLA-DOB*	0.605244643	4.49E-20	1.61E-17	1.77E-16
T1DM	acinar_cell	*HLA-DMB*	1.01527798	6.03E-35	1.18E-31	2.37E-31
T1DM	acinar_cell	*C4A*	-2.28398029	1.72E-23	7.50E-21	6.75E-20
T1DM	alpha_cell	*UQCC2*	0.072238164	9.31E-05	0.011795073	0.365647278
T1DM	alpha_cell	*HLA-DQB1*	-0.051911614	1.40E-29	9.14E-27	5.49E-26
T1DM	alpha_cell	*CAMK1D*	0.113129848	0.000202874	0.024154352	0.797093606
T1DM	alpha_cell	*BTN3A2*	-0.01215429	1.54E-05	0.002165613	0.060637152
T1DM	alpha_cell	*ZKSCAN3*	-0.078897783	7.59E-10	1.75E-07	2.98E-06
T1DM	alpha_cell	*HLA-DRB1*	-0.01957382	1.93E-09	4.20E-07	7.56E-06
T1DM	alpha_cell	*SFTA2*	-0.032123119	7.97E-09	1.57E-06	3.13E-05
T1DM	alpha_cell	*HLA-DRB5*	-0.036470321	9.33E-27	4.58E-24	3.66E-23
T1DM	alpha_cell	*HSD17B8*	0.114337254	2.12E-30	2.08E-27	8.32E-27
T1DM	alpha_cell	*HLA-DMA*	0.034550439	7.84E-30	6.16E-27	3.08E-26
T1DM	alpha_cell	*NOTCH4*	-0.068553376	3.69E-29	2.07E-26	1.45E-25
T1DM	alpha_cell	*AGER*	0.065009243	3.62E-06	0.00052681	0.014223861
T1DM	alpha_cell	*RNF5*	-0.058890951	1.39E-15	4.57E-13	5.48E-12
T1DM	alpha_cell	*EHMT2*	0.076807654	0.000391922	0.043996045	1
T1DM	alpha_cell	*SLC44A4*	-0.021575313	0.000186426	0.022889677	0.732469659
T1DM	alpha_cell	*MICA*	0.016782553	1.55E-07	2.65E-05	0.000608818
T1DM	alpha_cell	*POU5F1*	0.024848145	0.000227018	0.02623394	0.891953945
T1DM	alpha_cell	*CCHCR1*	0.060980108	9.99E-09	1.87E-06	3.93E-05
T1DM	alpha_cell	*PSORS1C1*	-0.02455395	5.67E-11	1.48E-08	2.23E-07
T1DM	alpha_cell	*TRIM31*	-0.124949522	5.81E-05	0.00761292	0.228387612
T1DM	alpha_cell	*RNF39*	0.051477346	2.71E-14	8.18E-12	1.06E-10
T1DM	alpha_cell	*ZFP57*	0.039163627	2.44E-07	3.83E-05	0.000957722
T1DM	alpha_cell	*HCG27*	0.039072963	6.87E-09	1.42E-06	2.70E-05
T1DM	alpha_cell	*ATF6B*	0.055703081	8.02E-11	1.97E-08	3.15E-07
T1DM	alpha_cell	*PPT2*	0.097887353	1.03E-13	2.90E-11	4.06E-10
T1DM	alpha_cell	*C4B*	0.302846823	2.83E-07	4.28E-05	0.001111755
T1DM	alpha_cell	*HLA-DQB2*	0.162495579	4.13E-56	1.62E-52	1.62E-52
T1DM	alpha_cell	*HLA-B*	0.023038146	4.03E-05	0.005458968	0.158310069
T1DM	alpha_cell	*ZSCAN31*	0.023627342	2.23E-07	3.66E-05	0.000877697
T1DM	alpha_cell	*B3GALT4*	-0.046647473	1.09E-07	1.95E-05	0.000429029
T1DM	alpha_cell	*HLA-DQA2*	0.062491959	1.45E-34	1.90E-31	5.70E-31
T1DM	alpha_cell	*PSMB9*	0.089432151	9.27E-22	3.64E-19	3.64E-18
T1DM	alpha_cell	*HLA-DOB*	0.097766656	3.48E-20	1.24E-17	1.37E-16
T1DM	alpha_cell	*HLA-DMB*	0.177258168	3.35E-38	6.58E-35	1.32E-34
T1DM	alpha_cell	*C4A*	-0.375616261	3.57E-24	1.56E-21	1.40E-20
T1DM	beta_cell	*BTN2A2*	-0.019729908	0.000272089	0.031442283	1
T1DM	beta_cell	*RHPN2*	-0.080181934	0.000140952	0.017306283	0.55380106
T1DM	beta_cell	*UQCC2*	0.051425045	2.46E-05	0.003335101	0.096717937
T1DM	beta_cell	*HLA-DQB1*	-0.031759006	3.84E-30	3.77E-27	1.51E-26
T1DM	beta_cell	*BTN3A2*	-0.007339744	1.56E-05	0.002194721	0.061452201
T1DM	beta_cell	*ZKSCAN3*	-0.04808706	5.44E-10	1.26E-07	2.14E-06
T1DM	beta_cell	*HLA-DRB1*	-0.011980257	1.24E-09	2.70E-07	4.86E-06
T1DM	beta_cell	*SFTA2*	-0.020345715	1.70E-09	3.52E-07	6.68E-06
T1DM	beta_cell	*HLA-DRB5*	-0.021873579	3.90E-26	1.92E-23	1.53E-22
T1DM	beta_cell	*HSD17B8*	0.069411637	7.71E-30	6.06E-27	3.03E-26
T1DM	beta_cell	*HLA-DMA*	0.020712566	3.16E-29	1.78E-26	1.24E-25
T1DM	beta_cell	*NOTCH4*	-0.041715914	1.67E-29	1.09E-26	6.54E-26
T1DM	beta_cell	*AGER*	0.039850728	2.78E-06	0.000404766	0.010928678
T1DM	beta_cell	*RNF5*	-0.036373338	5.73E-16	1.88E-13	2.25E-12
T1DM	beta_cell	*EHMT2*	0.052458389	0.000129578	0.016423026	0.50911381
T1DM	beta_cell	*SLC44A4*	-0.013015399	0.000195645	0.023293644	0.768690247
T1DM	beta_cell	*MICA*	0.009983383	2.42E-07	4.14E-05	0.000952655
T1DM	beta_cell	*POU5F1*	0.014645442	0.000327521	0.035745305	1
T1DM	beta_cell	*CCHCR1*	0.036442046	1.77E-08	3.31E-06	6.95E-05
T1DM	beta_cell	*PSORS1C1*	-0.015064586	2.78E-11	7.29E-09	1.09E-07
T1DM	beta_cell	*TRIM31*	-0.068930038	0.000293186	0.032912229	1
T1DM	beta_cell	*RNF39*	0.030459535	1.13E-13	3.43E-11	4.46E-10
T1DM	beta_cell	*ZFP57*	0.023571544	2.74E-07	4.48E-05	0.001075696
T1DM	beta_cell	*HCG27*	0.023467723	8.84E-09	1.74E-06	3.48E-05
T1DM	beta_cell	*ATF6B*	0.033593953	1.27E-10	3.11E-08	4.97E-07
T1DM	beta_cell	*PPT2*	0.059239994	1.41E-13	3.96E-11	5.54E-10

T1DM	beta_cell	*C4B*	0.180788363	4.07E-07	6.14E-05	0.00159749
T1DM	beta_cell	*HLA-DQB2*	0.098617133	3.02E-56	1.19E-52	1.19E-52
T1DM	beta_cell	*HLA-B*	0.013841291	6.91E-05	0.009044847	0.271345401
T1DM	beta_cell	*ZSCAN31*	0.01409498	3.25E-07	5.11E-05	0.001278307
T1DM	beta_cell	*B3GALT4*	-0.028884486	6.38E-08	1.14E-05	0.000250539
T1DM	beta_cell	*HLA-DQA2*	0.038117607	1.29E-34	1.69E-31	5.08E-31
T1DM	beta_cell	*PSMB9*	0.053596119	5.13E-21	2.01E-18	2.01E-17
T1DM	beta_cell	*HLA-DOB*	0.058757729	4.12E-20	1.47E-17	1.62E-16
T1DM	beta_cell	*HLA-DMB*	0.105824216	6.07E-37	1.19E-33	2.39E-33
T1DM	beta_cell	*C4A*	-0.225552654	4.36E-23	1.90E-20	1.71E-19
T1DM	delta_cell	*HLA-DQB1*	-0.267478713	1.67E-28	1.31E-25	6.57E-25
T1DM	delta_cell	*BTN3A2*	-0.063536436	1.22E-05	0.001841323	0.047874394
T1DM	delta_cell	*ZKSCAN3*	-0.207486451	8.84E-05	0.01253115	0.347463168
T1DM	delta_cell	*HLA-DRB1*	-0.100954905	4.52E-09	1.04E-06	1.77E-05
T1DM	delta_cell	*SFTA2*	-0.167027428	1.23E-08	2.55E-06	4.84E-05
T1DM	delta_cell	*HLA-DRB5*	-0.178335213	2.53E-25	1.24E-22	9.94E-22
T1DM	delta_cell	*HSD17B8*	0.538616806	2.43E-27	1.59E-24	9.56E-24
T1DM	delta_cell	*HLA-DMA*	0.176725873	4.12E-29	4.04E-26	1.62E-25
T1DM	delta_cell	*NOTCH4*	-0.335777135	2.72E-26	1.52E-23	1.07E-22
T1DM	delta_cell	*RNF5*	-0.272389816	9.34E-14	3.06E-11	3.67E-10
T1DM	delta_cell	*MICA*	0.087189029	2.02E-07	3.97E-05	0.000793131
T1DM	delta_cell	*POU5F1*	0.129020996	0.000264049	0.035774095	1
T1DM	delta_cell	*CCHCR1*	0.309306516	5.54E-09	1.21E-06	2.18E-05
T1DM	delta_cell	*PSORS1C1*	-0.125618447	1.80E-10	4.71E-08	7.06E-07
T1DM	delta_cell	*RNF39*	0.233257689	4.41E-13	1.33E-10	1.73E-09
T1DM	delta_cell	*ZFP57*	0.202255811	4.01E-07	6.86E-05	0.001577147
T1DM	delta_cell	*HCG27*	0.132785248	4.96E-07	8.13E-05	0.001950029
T1DM	delta_cell	*ATF6B*	0.254831973	2.49E-09	6.11E-07	9.77E-06
T1DM	delta_cell	*PPT2*	0.464504911	3.90E-12	1.10E-09	1.53E-08
T1DM	delta_cell	*C4B*	1.561576925	2.24E-07	4.19E-05	0.000879877
T1DM	delta_cell	*HLA-DQB2*	0.786938286	3.38E-52	1.33E-48	1.33E-48
T1DM	delta_cell	*HLA-B*	0.095617957	8.93E-05	0.01253115	0.350872208
T1DM	delta_cell	*ZSCAN31*	0.121859034	3.68E-07	6.57E-05	0.001444568
T1DM	delta_cell	*B3GALT4*	-0.213409569	6.06E-07	9.53E-05	0.002381902
T1DM	delta_cell	*HLA-DQA2*	0.297316379	8.51E-32	1.67E-28	3.35E-28
T1DM	delta_cell	*PSMB9*	0.428161294	1.52E-20	5.45E-18	5.99E-17
T1DM	delta_cell	*HLA-DOB*	0.533061398	1.08E-20	4.25E-18	4.25E-17
T1DM	delta_cell	*HLA-DMB*	0.684494405	1.20E-29	1.57E-26	4.72E-26
T1DM	delta_cell	*C4A*	-1.955120837	2.45E-21	1.07E-18	9.64E-18
T1DM	duct_epithelial_cell	*BTN2A2*	-0.113589964	0.000372411	0.045725078	1
T1DM	duct_epithelial_cell	*UQCC2*	0.210846591	0.000208686	0.026449273	0.819927461
T1DM	duct_epithelial_cell	*HLA-DQB1*	-0.200854382	2.25E-30	2.21E-27	8.86E-27
T1DM	duct_epithelial_cell	*BTN3A2*	-0.046017969	1.67E-05	0.002342122	0.065579414
T1DM	duct_epithelial_cell	*ZKSCAN3*	-0.291259675	8.85E-10	2.17E-07	3.48E-06
T1DM	duct_epithelial_cell	*HLA-DRB1*	-0.080194801	2.30E-10	6.02E-08	9.03E-07
T1DM	duct_epithelial_cell	*SFTA2*	-0.100032476	7.05E-08	1.54E-05	0.000277113
T1DM	duct_epithelial_cell	*HLA-DRB5*	-0.127785976	4.91E-25	2.41E-22	1.93E-21
T1DM	duct_epithelial_cell	*HSD17B8*	0.419646829	1.54E-28	8.66E-26	6.06E-25
T1DM	duct_epithelial_cell	*HLA-DMA*	0.130533846	3.57E-29	2.81E-26	1.40E-25
T1DM	duct_epithelial_cell	*NOTCH4*	-0.255098961	6.37E-29	4.17E-26	2.50E-25
T1DM	duct_epithelial_cell	*AGER*	0.243132997	1.75E-06	0.000264795	0.006884663
T1DM	duct_epithelial_cell	*RNF5*	-0.207990136	1.14E-14	3.72E-12	4.47E-11
T1DM	duct_epithelial_cell	*EHMT2*	0.285117566	0.000204997	0.026449273	0.805431376
T1DM	duct_epithelial_cell	*MICA*	0.059005349	9.03E-07	0.000141931	0.003548279
T1DM	duct_epithelial_cell	*CCHCR1*	0.206334126	1.23E-07	2.53E-05	0.000481572
T1DM	duct_epithelial_cell	*PSORS1C1*	-0.072213488	2.63E-09	6.09E-07	1.03E-05
T1DM	duct_epithelial_cell	*RNF39*	0.075513781	1.01E-05	0.00147337	0.039780979
T1DM	duct_epithelial_cell	*ZFP57*	0.150083998	2.23E-07	4.18E-05	0.000877126
T1DM	duct_epithelial_cell	*HCG27*	0.128880372	1.41E-07	2.77E-05	0.000553319
T1DM	duct_epithelial_cell	*ATF6B*	0.210857879	1.01E-10	2.83E-08	3.96E-07
T1DM	duct_epithelial_cell	*PPT2*	0.373847219	5.92E-14	1.79E-11	2.33E-10
T1DM	duct_epithelial_cell	*C4B*	1.116200311	6.93E-07	0.00011351	0.002724241
T1DM	duct_epithelial_cell	*HLA-DQB2*	0.62342939	1.75E-56	6.90E-53	6.90E-53
T1DM	duct_epithelial_cell	*HLA-B*	0.086893685	3.08E-05	0.00417461	0.121063692

T1DM	duct_epithelial_cell	*ZSCAN31*	0.088912833	3.52E-07	6.28E-05	0.001382052
T1DM	duct_epithelial_cell	*B3GALT4*	-0.162350135	6.89E-07	0.00011351	0.002707219
T1DM	duct_epithelial_cell	*HLA-DQA2*	0.248218403	9.42E-36	1.23E-32	3.70E-32
T1DM	duct_epithelial_cell	*PSMB9*	0.259386952	2.94E-16	1.05E-13	1.15E-12
T1DM	duct_epithelial_cell	*HLA-DOB*	0.334105561	7.19E-18	2.82E-15	2.82E-14
T1DM	duct_epithelial_cell	*HLA-DMB*	0.660749828	2.48E-36	4.87E-33	9.74E-33
T1DM	duct_epithelial_cell	*C4A*	-1.446395005	3.63E-22	1.58E-19	1.43E-18
T1DM	endothelial_cell	*USH1C*	-0.172630568	0.00075327	0.047033508	1
T1DM	endothelial_cell	*BAIAP3*	-0.181622087	0.000780852	0.047033508	1
T1DM	endothelial_cell	*CEP68*	0.250335056	0.000245964	0.025431367	0.96639196
T1DM	endothelial_cell	*RNASET2*	-0.206332283	0.000774936	0.047033508	1
T1DM	endothelial_cell	*GPATCH1*	0.428399869	8.59E-05	0.009926625	0.337505243
T1DM	endothelial_cell	*CTSH*	0.089434422	0.000338777	0.032464756	1
T1DM	endothelial_cell	*VIPR1*	0.351193879	4.78E-05	0.007217939	0.187666416
T1DM	endothelial_cell	*PCDHB10*	0.274161266	0.000459568	0.03611288	1
T1DM	endothelial_cell	*EPS8L1*	0.182685937	0.000625917	0.043721785	1
T1DM	endothelial_cell	*PIAS3*	0.338420983	5.51E-05	0.00746945	0.216614055
T1DM	endothelial_cell	*RHPN2*	-0.495529888	8.32E-05	0.009910504	0.327046637
T1DM	endothelial_cell	*NHSL1*	0.397635268	0.000433064	0.03585838	1
T1DM	endothelial_cell	*SPTBN5*	0.113622459	0.000551055	0.040850829	1
T1DM	endothelial_cell	*IFITM3*	0.149336654	0.000449672	0.036056347	1
T1DM	endothelial_cell	*ARHGEF19*	0.129014431	0.000294134	0.028891357	1
T1DM	endothelial_cell	*SPC25*	-0.120056268	0.000767467	0.047033508	1
T1DM	endothelial_cell	*SLC25A27*	-0.13383973	0.000597635	0.042692883	1
T1DM	endothelial_cell	*CDK20*	-0.244311821	0.000485509	0.037403226	1
T1DM	endothelial_cell	*DIP2A*	0.241843072	0.000140466	0.015330349	0.551892563
T1DM	endothelial_cell	*SRGAP2*	0.240968143	6.33E-05	0.008295095	0.248852851
T1DM	endothelial_cell	*EXO5*	0.212426875	0.000539531	0.04076569	1
T1DM	endothelial_cell	*ZNF589*	-0.113172074	0.000276542	0.027859872	1
T1DM	endothelial_cell	*LMBRD2*	-0.276999619	0.000721504	0.047033508	1
T1DM	endothelial_cell	*WBP1L*	0.465327683	0.000634294	0.043721785	1
T1DM	endothelial_cell	*ENGASE*	0.134862876	0.000802048	0.047033508	1
T1DM	endothelial_cell	*CACNB3*	0.181607691	0.000425801	0.03585838	1
T1DM	endothelial_cell	*SMAGP*	0.137568624	0.000666213	0.045130171	1
T1DM	endothelial_cell	*UBE2U*	-0.478011335	0.000879764	0.049379895	1
T1DM	endothelial_cell	*HLA-DQB1*	-0.241813964	1.98E-24	1.56E-21	7.78E-21
T1DM	endothelial_cell	*LBX2*	0.595874436	0.000429591	0.03585838	1
T1DM	endothelial_cell	*TH*	1.030000948	0.000118262	0.013275776	0.464652152
T1DM	endothelial_cell	*ZBTB40*	0.263963309	0.000570063	0.041477338	1
T1DM	endothelial_cell	*RBM11*	-0.21008307	0.000687841	0.045805555	1
T1DM	endothelial_cell	*C19orf54*	0.309388256	0.00085407	0.048632506	1
T1DM	endothelial_cell	*ZKSCAN3*	-0.287880507	2.82E-06	0.000502975	0.011065445
T1DM	endothelial_cell	*HLA-DRB1*	-0.067970205	5.14E-05	0.00746945	0.201923325
T1DM	endothelial_cell	*MVB12B*	0.455301854	8.19E-05	0.009910504	0.321735518
T1DM	endothelial_cell	*HLA-DRB5*	-0.084895944	7.04E-10	1.84E-07	2.77E-06
T1DM	endothelial_cell	*HSD17B8*	0.399795302	2.32E-18	1.14E-15	9.11E-15
T1DM	endothelial_cell	*HLA-DMA*	0.185961368	3.12E-32	3.07E-29	1.23E-28
T1DM	endothelial_cell	*AGER*	0.256243939	1.06E-07	2.31E-05	0.000415277
T1DM	endothelial_cell	*RNF5*	-0.294741057	7.07E-15	3.09E-12	2.78E-11
T1DM	endothelial_cell	*EHMT2*	0.360194048	0.000763076	0.047033508	1
T1DM	endothelial_cell	*SLC44A4*	-0.119507823	4.20E-05	0.006606389	0.165159727
T1DM	endothelial_cell	*MICB*	0.070487359	0.000438076	0.03585838	1
T1DM	endothelial_cell	*MICA*	0.090838821	3.55E-09	8.72E-07	1.40E-05
T1DM	endothelial_cell	*HLA-C*	0.101833135	3.45E-05	0.005652281	0.135654736
T1DM	endothelial_cell	*POU5F1*	0.137368208	6.95E-05	0.008805333	0.272965308
T1DM	endothelial_cell	*CCHCR1*	0.352108132	1.04E-10	3.14E-08	4.08E-07
T1DM	endothelial_cell	*PSORS1C1*	-0.093096795	2.61E-07	5.13E-05	0.0010252
T1DM	endothelial_cell	*TRIM31*	-0.675751789	0.000399223	0.035648823	1
T1DM	endothelial_cell	*RNF39*	0.224850273	3.54E-11	1.39E-08	1.39E-07
T1DM	endothelial_cell	*HCG9*	0.206906724	0.000828831	0.047889343	1
T1DM	endothelial_cell	*ZFP57*	0.180152183	3.17E-06	0.000541389	0.012451952
T1DM	endothelial_cell	*HCG27*	0.131800372	5.33E-05	0.00746945	0.209476479
T1DM	endothelial_cell	*ATF6B*	0.289033505	5.65E-11	1.85E-08	2.22E-07
T1DM	endothelial_cell	*LTB4R*	0.376217735	0.000794406	0.047033508	1

T1DM	endothelial_cell	*PPT2*	0.207613287	5.39E-10	1.51E-07	2.12E-06
T1DM	endothelial_cell	*C4B*	1.615558904	1.47E-07	3.05E-05	0.000579043
T1DM	endothelial_cell	*HLA-DQB2*	0.85179145	1.66E-55	6.53E-52	6.53E-52
T1DM	endothelial_cell	*HLA-B*	0.132562651	3.19E-07	5.96E-05	0.001252
T1DM	endothelial_cell	*ZSCAN31*	0.083430313	0.000154124	0.016366333	0.605554324
T1DM	endothelial_cell	*B3GALT4*	-0.224353038	3.35E-08	7.75E-06	0.000131812
T1DM	endothelial_cell	*HLA-DQA2*	0.341567382	1.09E-35	2.14E-32	4.27E-32
T1DM	endothelial_cell	*PSMB9*	0.133193866	5.27E-11	1.85E-08	2.07E-07
T1DM	endothelial_cell	*PCDHGC3*	0.228195793	0.000353669	0.033084936	1
T1DM	endothelial_cell	*HLA-DOB*	0.510281778	8.91E-20	5.00E-17	3.50E-16
T1DM	endothelial_cell	*HLA-DMB*	0.725085828	3.61E-35	4.72E-32	1.42E-31
T1DM	endothelial_cell	*C4A*	-2.003959178	4.82E-23	3.16E-20	1.90E-19
T1DM	endothelial_cell	*GOLGA8K*	-1.782772651	0.000393075	0.035648823	1
T1DM	epsilon_cell	*HLA-DQB1*	-2.321486487	1.21E-22	1.19E-19	4.74E-19
T1DM	epsilon_cell	*BTN3A2*	-0.792895094	1.80E-05	0.004413869	0.070621911
T1DM	epsilon_cell	*HLA-DRB1*	-1.133798914	1.73E-09	6.18E-07	6.80E-06
T1DM	epsilon_cell	*SFTA2*	-2.018488114	3.40E-09	1.03E-06	1.34E-05
T1DM	epsilon_cell	*HLA-DRB5*	-2.224245973	3.13E-17	1.54E-14	1.23E-13
T1DM	epsilon_cell	*HLA-DMA*	1.458547128	3.99E-18	2.24E-15	1.57E-14
T1DM	epsilon_cell	*NOTCH4*	-3.262658665	3.35E-23	4.39E-20	1.32E-19
T1DM	epsilon_cell	*RNF5*	-0.926517314	4.78E-06	0.001251774	0.018776606
T1DM	epsilon_cell	*PSORS1C1*	-1.654965124	8.46E-12	3.32E-09	3.32E-08
T1DM	epsilon_cell	*RNF39*	2.387380195	2.62E-09	8.59E-07	1.03E-05
T1DM	epsilon_cell	*C4B*	17.7296358	3.58E-06	0.001004827	0.014067581
T1DM	epsilon_cell	*HLA-DQB2*	8.558347809	2.24E-45	8.79E-42	8.79E-42
T1DM	epsilon_cell	*HLA-DQA2*	3.660692057	1.19E-29	2.33E-26	4.66E-26
T1DM	epsilon_cell	*HLA-DOB*	6.547150285	4.28E-20	2.80E-17	1.68E-16
T1DM	epsilon_cell	*HLA-DMB*	5.239849096	6.46E-16	2.82E-13	2.54E-12
T1DM	epsilon_cell	*C4A*	-24.84005838	3.52E-22	2.77E-19	1.38E-18
T1DM	leukocyte	*GCLC*	-0.927374905	9.06E-09	1.72E-08	3.56E-05
T1DM	leukocyte	*NFYA*	-0.773456522	2.29E-09	8.21E-09	8.98E-06
T1DM	leukocyte	*STPG1*	-0.665720209	8.04E-10	8.14E-09	3.16E-06
T1DM	leukocyte	*LASP1*	-0.626746433	4.20E-09	9.96E-09	1.65E-05
T1DM	leukocyte	*SNX11*	-0.680640514	2.26E-08	3.79E-08	8.89E-05
T1DM	leukocyte	*TMEM176A*	-0.395563141	2.02E-06	2.62E-06	0.007919863
T1DM	leukocyte	*M6PR*	-1.079925561	1.66E-06	2.17E-06	0.006533214
T1DM	leukocyte	*DBNDD1*	-0.434971516	2.78E-07	3.90E-07	0.001093499
T1DM	leukocyte	*CASP10*	-1.537979902	1.27E-07	1.85E-07	0.000500249
T1DM	leukocyte	*NDUFAF7*	-1.358263824	4.48E-09	1.03E-08	1.76E-05
T1DM	leukocyte	*POLDIP2*	-0.514870385	2.64E-08	4.36E-08	0.000103735
T1DM	leukocyte	*PLXND1*	-1.354957685	5.23E-09	1.13E-08	2.05E-05
T1DM	leukocyte	*CD38*	-3.071299585	0.000581045	0.000674026	1
T1DM	leukocyte	*KDM1A*	-0.701262181	1.25E-09	8.14E-09	4.93E-06
T1DM	leukocyte	*RBM6*	-0.736118875	1.46E-09	8.14E-09	5.72E-06
T1DM	leukocyte	*RECQL*	-0.690037	2.43E-09	8.21E-09	9.55E-06
T1DM	leukocyte	*SLC25A13*	-0.686111315	1.51E-09	8.14E-09	5.93E-06
T1DM	leukocyte	*MEOX1*	1.182917444	0.000161103	0.000191	0.632974828
T1DM	leukocyte	*PON1*	-0.797918548	2.92E-09	8.49E-09	1.15E-05
T1DM	leukocyte	*KMT2E*	-0.684586557	3.56E-09	9.10E-09	1.40E-05
T1DM	leukocyte	*SOX8*	-0.79200015	0.001089193	0.00125203	1
T1DM	leukocyte	*IBTK*	-0.669977221	3.66E-09	9.22E-09	1.44E-05
T1DM	leukocyte	*ZNF195*	-0.943699462	8.46E-09	1.62E-08	3.33E-05
T1DM	leukocyte	*GGNBP2*	-0.703491442	2.83E-09	8.43E-09	1.11E-05
T1DM	leukocyte	*ASB4*	0.88290214	0.006648967	0.007434204	1
T1DM	leukocyte	*TMEM98*	-0.520262093	4.67E-09	1.06E-08	1.83E-05
T1DM	leukocyte	*SYNRG*	-0.925506413	8.39E-09	1.61E-08	3.30E-05
T1DM	leukocyte	*SPATA20*	-0.710685758	6.96E-09	1.39E-08	2.73E-05
T1DM	leukocyte	*FARP2*	-1.383043417	1.71E-05	2.11E-05	0.067180039
T1DM	leukocyte	*USH1C*	-0.733328085	1.19E-10	8.14E-09	4.67E-07
T1DM	leukocyte	*VPS41*	-0.852656818	1.37E-08	2.45E-08	5.40E-05
T1DM	leukocyte	*ELAC2*	-0.733118436	2.05E-09	8.14E-09	8.06E-06
T1DM	leukocyte	*CCDC124*	-0.590330845	3.07E-09	8.60E-09	1.20E-05
T1DM	leukocyte	*NOS2*	-11.19298429	1.61E-09	8.14E-09	6.32E-06
T1DM	leukocyte	*CD79B*	-2.252071228	0.000113337	0.000135145	0.445302712

T1DM	leukocyte	*ST7L*	-0.561630236	0.000788689	0.000911132	1
T1DM	leukocyte	*BAIAP3*	-0.702259964	1.41E-10	8.14E-09	5.54E-07
T1DM	leukocyte	*FMO3*	-1.625708588	0.000589916	0.000684114	1
T1DM	leukocyte	*E2F2*	-0.883931499	0.03878369	0.042082606	1
T1DM	leukocyte	*CYB561*	-0.606468371	1.88E-09	8.14E-09	7.37E-06
T1DM	leukocyte	*MGST1*	-0.455874056	1.63E-08	2.83E-08	6.40E-05
T1DM	leukocyte	*ST3GAL1*	-1.240292852	1.59E-07	2.29E-07	0.000625842
T1DM	leukocyte	*IL32*	-0.318669351	1.40E-09	8.14E-09	5.49E-06
T1DM	leukocyte	*MED24*	-0.934243164	6.86E-10	8.14E-09	2.70E-06
T1DM	leukocyte	*RPS20*	-0.447213483	3.28E-09	8.79E-09	1.29E-05
T1DM	leukocyte	*REV3L*	-0.580394713	1.86E-09	8.14E-09	7.32E-06
T1DM	leukocyte	*MLXIPL*	-0.51305447	3.14E-06	4.03E-06	0.012349103
T1DM	leukocyte	*ETV7*	-1.256673436	1.93E-09	8.14E-09	7.57E-06
T1DM	leukocyte	*NCAPD2*	-1.072929221	1.06E-07	1.57E-07	0.000416722
T1DM	leukocyte	*GIPR*	-0.55459422	3.42E-09	8.93E-09	1.35E-05
T1DM	leukocyte	*FUZ*	-1.043838607	1.15E-09	8.14E-09	4.53E-06
T1DM	leukocyte	*LRRC23*	-0.513799381	1.83E-09	8.14E-09	7.19E-06
T1DM	leukocyte	*TSPAN9*	-0.589524909	1.41E-09	8.14E-09	5.53E-06
T1DM	leukocyte	*BTBD7*	-0.728493421	2.23E-09	8.21E-09	8.75E-06
T1DM	leukocyte	*APBA3*	-1.273925654	1.66E-09	8.14E-09	6.50E-06
T1DM	leukocyte	*TTC19*	-0.553413586	1.95E-09	8.14E-09	7.64E-06
T1DM	leukocyte	*DPF1*	-0.682879364	4.19E-07	5.76E-07	0.001646206
T1DM	leukocyte	*LARS2*	0.908336856	0.042465715	0.046001597	1
T1DM	leukocyte	*RABGAP1*	-0.66326967	2.53E-09	8.21E-09	9.93E-06
T1DM	leukocyte	*PPP5C*	-0.669151542	1.51E-09	8.14E-09	5.92E-06
T1DM	leukocyte	*CEP68*	-0.615539774	4.43E-05	5.39E-05	0.173995424
T1DM	leukocyte	*ERCC1*	-0.806015113	9.44E-10	8.14E-09	3.71E-06
T1DM	leukocyte	*CD22*	0.302020225	1.62E-06	2.12E-06	0.006373764
T1DM	leukocyte	*LTF*	-1.74310081	2.57E-09	8.21E-09	1.01E-05
T1DM	leukocyte	*EXTL3*	-0.606097227	1.87E-09	8.14E-09	7.34E-06
T1DM	leukocyte	*ALOX5*	0.34536668	2.03E-08	3.43E-08	7.97E-05
T1DM	leukocyte	*CALCOCO1*	-0.927024019	0.009819041	0.010894948	1
T1DM	leukocyte	*UBR7*	-0.690614476	3.15E-09	8.69E-09	1.24E-05
T1DM	leukocyte	*MAN2B2*	-0.950769348	2.26E-09	8.21E-09	8.89E-06
T1DM	leukocyte	*ANGEL1*	-0.881050057	1.96E-08	3.34E-08	7.71E-05
T1DM	leukocyte	*DDX11*	-0.727110128	2.74E-09	8.30E-09	1.08E-05
T1DM	leukocyte	*GPRC5A*	-0.778651836	7.89E-08	1.19E-07	0.000309996
T1DM	leukocyte	*TACC3*	0.342134298	0.007225339	0.008069459	1
T1DM	leukocyte	*UFL1*	-0.640424517	2.49E-09	8.21E-09	9.77E-06
T1DM	leukocyte	*POLA2*	-1.204070075	1.23E-09	8.14E-09	4.85E-06
T1DM	leukocyte	*ZC3H3*	-0.613796709	1.92E-08	3.28E-08	7.55E-05
T1DM	leukocyte	*MDH1*	-0.637692425	2.62E-09	8.21E-09	1.03E-05
T1DM	leukocyte	*MTMR11*	-2.252858438	2.60E-08	4.30E-08	0.000102058
T1DM	leukocyte	*DPEP1*	-0.420480754	9.15E-10	8.14E-09	3.60E-06
T1DM	leukocyte	*NUDCD3*	-0.594744968	2.87E-09	8.44E-09	1.13E-05
T1DM	leukocyte	*TTC27*	-1.359723432	2.51E-08	4.16E-08	9.86E-05
T1DM	leukocyte	*SNAI2*	-1.547682763	5.15E-07	7.02E-07	0.002023666
T1DM	leukocyte	*PLEKHB1*	-0.623559488	4.42E-08	7.00E-08	0.000173745
T1DM	leukocyte	*OSBPL5*	-1.190554777	2.76E-06	3.56E-06	0.010862841
T1DM	leukocyte	*NLRP2*	-1.264823029	6.62E-08	1.01E-07	0.00026001
T1DM	leukocyte	*ZDHHC6*	-0.892234582	1.78E-10	8.14E-09	6.98E-07
T1DM	leukocyte	*RNH1*	-0.615865074	4.54E-09	1.04E-08	1.78E-05
T1DM	leukocyte	*NDUFS1*	-0.583612667	3.74E-09	9.32E-09	1.47E-05
T1DM	leukocyte	*TYMP*	0.323316881	9.90E-08	1.47E-07	0.000389164
T1DM	leukocyte	*RNASET2*	0.527378254	0.001049919	0.001207591	1
T1DM	leukocyte	*CD44*	-0.35213849	3.05E-09	8.59E-09	1.20E-05
T1DM	leukocyte	*KCNG1*	-2.037269288	4.38E-07	6.02E-07	0.001722741
T1DM	leukocyte	*AGPAT4*	-1.247318837	0.001485535	0.001697199	1
T1DM	leukocyte	*SH2D2A*	-1.581271871	0.00414375	0.004671677	1
T1DM	leukocyte	*VEZT*	-0.67871326	3.51E-09	9.03E-09	1.38E-05
T1DM	leukocyte	*RABEP1*	-1.0371908	2.89E-08	4.73E-08	0.000113385
T1DM	leukocyte	*BAK1*	-1.237623002	2.16E-09	8.21E-09	8.48E-06
T1DM	leukocyte	*ARID4A*	-0.857597467	4.37E-09	1.02E-08	1.72E-05
T1DM	leukocyte	*PNPLA6*	-0.858228002	6.18E-10	8.14E-09	2.43E-06

T1DM	leukocyte	*ABCF2*	-0.914201335	1.86E-09	8.14E-09	7.31E-06
T1DM	leukocyte	*LRRC7*	-0.727579054	1.18E-05	1.47E-05	0.046399933
T1DM	leukocyte	*UBA6*	-0.982919712	7.57E-09	1.49E-08	2.97E-05
T1DM	leukocyte	*APBA2*	-1.788097264	1.39E-09	8.14E-09	5.48E-06
T1DM	leukocyte	*ASTE1*	-0.672487845	5.65E-06	7.14E-06	0.022198804
T1DM	leukocyte	*RNF19A*	-0.595270137	4.02E-10	8.14E-09	1.58E-06
T1DM	leukocyte	*SH3YL1*	-0.448910206	1.42E-09	8.14E-09	5.59E-06
T1DM	leukocyte	*FAM136A*	-0.635630978	2.21E-09	8.21E-09	8.67E-06
T1DM	leukocyte	*DAPK2*	-0.716596692	1.03E-09	8.14E-09	4.06E-06
T1DM	leukocyte	*TIMP2*	-0.998971121	7.47E-07	1.00E-06	0.002933049
T1DM	leukocyte	*MYOM2*	0.88667154	9.80E-09	1.83E-08	3.85E-05
T1DM	leukocyte	*RPL26L1*	-0.586537164	2.65E-09	8.21E-09	1.04E-05
T1DM	leukocyte	*NSUN2*	-0.953113529	8.41E-10	8.14E-09	3.30E-06
T1DM	leukocyte	*MRI1*	-0.784185231	2.70E-09	8.26E-09	1.06E-05
T1DM	leukocyte	*AGA*	-0.765492121	1.12E-09	8.14E-09	4.41E-06
T1DM	leukocyte	*BOD1L1*	-0.546728348	6.30E-08	9.69E-08	0.000247627
T1DM	leukocyte	*DNAH5*	-0.981592531	3.58E-09	9.11E-09	1.41E-05
T1DM	leukocyte	*PNKP*	-0.818471808	0.000596629	0.000691695	1
T1DM	leukocyte	*PHLPP2*	-0.614632444	1.68E-09	8.14E-09	6.61E-06
T1DM	leukocyte	*SPDL1*	0.940076503	5.47E-07	7.44E-07	0.002149098
T1DM	leukocyte	*CTNS*	-0.859676041	1.03E-09	8.14E-09	4.06E-06
T1DM	leukocyte	*INPP4A*	-0.647111331	1.05E-09	8.14E-09	4.14E-06
T1DM	leukocyte	*PSMA4*	-0.607646465	1.59E-09	8.14E-09	6.25E-06
T1DM	leukocyte	*LSG1*	-0.399028695	5.13E-06	6.49E-06	0.020139911
T1DM	leukocyte	*SPATA7*	-0.871674909	3.98E-09	9.64E-09	1.56E-05
T1DM	leukocyte	*CAPG*	0.603492157	1.68E-06	2.19E-06	0.006585852
T1DM	leukocyte	*TRIT1*	-1.413306361	5.51E-09	1.17E-08	2.17E-05
T1DM	leukocyte	*CNTLN*	-0.929348908	2.43E-09	8.21E-09	9.53E-06
T1DM	leukocyte	*PREX2*	-1.320230183	1.11E-09	8.14E-09	4.36E-06
T1DM	leukocyte	*WDR37*	-0.903083807	4.58E-09	1.04E-08	1.80E-05
T1DM	leukocyte	*YTHDC2*	-0.956262894	9.08E-09	1.72E-08	3.57E-05
T1DM	leukocyte	*DTNBP1*	-0.744840923	2.06E-09	8.14E-09	8.09E-06
T1DM	leukocyte	*GOPC*	-0.747896372	1.99E-09	8.14E-09	7.81E-06
T1DM	leukocyte	*ZNF800*	-0.730736503	2.82E-09	8.41E-09	1.11E-05
T1DM	leukocyte	*LMO3*	-0.635317888	0.000130188	0.000154815	0.511507278
T1DM	leukocyte	*MRPS10*	-0.740774219	1.15E-07	1.68E-07	0.000450107
T1DM	leukocyte	*COL9A2*	-0.716383658	2.01E-09	8.14E-09	7.88E-06
T1DM	leukocyte	*ERCC8*	-1.926550126	2.13E-06	2.76E-06	0.008366492
T1DM	leukocyte	*H6PD*	-1.486829908	6.21E-08	9.56E-08	0.000244057
T1DM	leukocyte	*VAMP3*	-0.73594355	6.60E-09	1.34E-08	2.59E-05
T1DM	leukocyte	*RCN1*	-0.532930935	2.16E-09	8.21E-09	8.47E-06
T1DM	leukocyte	*RFC2*	-0.711308552	8.88E-09	1.69E-08	3.49E-05
T1DM	leukocyte	*JKAMP*	-0.632230686	9.78E-10	8.14E-09	3.84E-06
T1DM	leukocyte	*PTGER3*	-0.577535758	3.18E-08	5.17E-08	0.000124969
T1DM	leukocyte	*BCAR1*	-0.535164167	2.02E-09	8.14E-09	7.94E-06
T1DM	leukocyte	*CYBA*	0.696573967	1.27E-05	1.58E-05	0.05009404
T1DM	leukocyte	*THOC3*	-0.537157294	1.87E-09	8.14E-09	7.35E-06
T1DM	leukocyte	*HEBP2*	-0.569110159	4.53E-09	1.04E-08	1.78E-05
T1DM	leukocyte	*RRP12*	-0.486708328	1.38E-09	8.14E-09	5.42E-06
T1DM	leukocyte	*FNIP2*	-0.916188855	0.000103136	0.000123242	0.405221163
T1DM	leukocyte	*MRTO4*	-0.77785074	1.44E-09	8.14E-09	5.68E-06
T1DM	leukocyte	*MCF2L2*	-1.071172841	1.89E-09	8.14E-09	7.44E-06
T1DM	leukocyte	*LAMA3*	-1.062836493	2.97E-09	8.50E-09	1.17E-05
T1DM	leukocyte	*ANAPC4*	-1.379328203	0.00470011	0.005286783	1
T1DM	leukocyte	*LY75*	-2.370949294	1.33E-08	2.38E-08	5.21E-05
T1DM	leukocyte	*ARID4B*	-0.55412006	5.84E-09	1.22E-08	2.30E-05
T1DM	leukocyte	*OPN3*	-0.83614891	0.002057833	0.002340152	1
T1DM	leukocyte	*SDCCAG8*	-0.645622053	2.41E-09	8.21E-09	9.46E-06
T1DM	leukocyte	*KIF1B*	-0.634113478	1.41E-09	8.14E-09	5.56E-06
T1DM	leukocyte	*TBC1D22A*	-0.685293706	1.71E-09	8.14E-09	6.73E-06
T1DM	leukocyte	*SYNE2*	-0.619475749	1.81E-09	8.14E-09	7.11E-06
T1DM	leukocyte	*ATP9A*	-0.673405421	2.55E-09	8.21E-09	1.00E-05
T1DM	leukocyte	*CHRDL2*	-2.499543449	9.66E-10	8.14E-09	3.79E-06
T1DM	leukocyte	*GALC*	-0.871416712	2.00E-08	3.39E-08	7.86E-05

T1DM	leukocyte	*CYFIP2*	-0.53348876	4.47E-09	1.03E-08	1.76E-05
T1DM	leukocyte	*USP36*	-0.955645916	4.07E-09	9.78E-09	1.60E-05
T1DM	leukocyte	*MCOLN3*	-0.652388665	8.86E-10	8.14E-09	3.48E-06
T1DM	leukocyte	*ITIH4*	-1.334087608	3.61E-09	9.13E-09	1.42E-05
T1DM	leukocyte	*PCGF2*	-0.641579923	2.53E-09	8.21E-09	9.92E-06
T1DM	leukocyte	*GDI2*	-0.667777814	2.32E-09	8.21E-09	9.12E-06
T1DM	leukocyte	*ATP11B*	-1.034514159	5.79E-09	1.21E-08	2.28E-05
T1DM	leukocyte	*LAMC2*	-0.466295278	7.35E-09	1.46E-08	2.89E-05
T1DM	leukocyte	*RASGRF1*	-0.512765309	1.92E-09	8.14E-09	7.54E-06
T1DM	leukocyte	*CROCC*	-1.022594374	2.22E-09	8.21E-09	8.73E-06
T1DM	leukocyte	*FLYWCH1*	-0.435396508	8.13E-08	1.22E-07	0.000319528
T1DM	leukocyte	*OGFR*	-1.158052046	0.000108994	0.000130084	0.428237175
T1DM	leukocyte	*GNA15*	1.155596148	5.61E-09	1.18E-08	2.20E-05
T1DM	leukocyte	*PIGV*	0.794424204	7.70E-08	1.16E-07	0.000302621
T1DM	leukocyte	*RIMBP2*	-0.555139539	1.31E-09	8.14E-09	5.15E-06
T1DM	leukocyte	*MPC1*	-0.5985703	2.25E-09	8.21E-09	8.84E-06
T1DM	leukocyte	*BCAT1*	0.269838821	2.38E-08	3.97E-08	9.34E-05
T1DM	leukocyte	*MRPS35*	-0.685808187	1.74E-09	8.14E-09	6.84E-06
T1DM	leukocyte	*ZNF112*	-0.936409927	1.08E-09	8.14E-09	4.23E-06
T1DM	leukocyte	*EIF4B*	-0.573110968	5.98E-09	1.24E-08	2.35E-05
T1DM	leukocyte	*SLC6A16*	-1.818980434	1.26E-09	8.14E-09	4.96E-06
T1DM	leukocyte	*SPHK2*	-0.807084115	2.48E-09	8.21E-09	9.75E-06
T1DM	leukocyte	*HAGH*	-0.736099141	4.59E-09	1.04E-08	1.80E-05
T1DM	leukocyte	*RNF4*	-0.757152822	2.77E-09	8.34E-09	1.09E-05
T1DM	leukocyte	*TM7SF3*	-1.083279335	2.61E-08	4.31E-08	0.000102402
T1DM	leukocyte	*ATP2C2*	-0.790700904	0.00036977	0.000431618	1
T1DM	leukocyte	*HIPK2*	-0.545520434	1.93E-09	8.14E-09	7.57E-06
T1DM	leukocyte	*TMEM161A*	-0.741686913	3.98E-09	9.64E-09	1.56E-05
T1DM	leukocyte	*SNX24*	-1.303483613	5.17E-08	8.09E-08	0.000203207
T1DM	leukocyte	*ABCA7*	-0.948720739	3.73E-09	9.32E-09	1.47E-05
T1DM	leukocyte	*BCAS1*	3.931557661	7.09E-08	1.08E-07	0.000278616
T1DM	leukocyte	*PMS1*	-0.656819404	2.33E-09	8.21E-09	9.17E-06
T1DM	leukocyte	*IPO5*	-0.576756928	1.52E-09	8.14E-09	5.99E-06
T1DM	leukocyte	*OAT*	-0.596864927	2.86E-09	8.43E-09	1.12E-05
T1DM	leukocyte	*WDR18*	-0.710535685	8.90E-09	1.69E-08	3.50E-05
T1DM	leukocyte	*MCM10*	1.389628116	1.04E-08	1.93E-08	4.10E-05
T1DM	leukocyte	*ERBB3*	-0.582269977	1.75E-09	8.14E-09	6.86E-06
T1DM	leukocyte	*ANKRD44*	-1.316323861	0.004654678	0.00523868	1
T1DM	leukocyte	*ADAT1*	-0.644803223	6.98E-09	1.40E-08	2.74E-05
T1DM	leukocyte	*MYLK*	-0.610009064	1.51E-09	8.14E-09	5.92E-06
T1DM	leukocyte	*GSTO2*	-0.455748464	1.98E-09	8.14E-09	7.80E-06
T1DM	leukocyte	*SEC61A2*	-1.876336464	1.56E-09	8.14E-09	6.13E-06
T1DM	leukocyte	*PRKCQ*	-1.415597888	2.42E-07	3.43E-07	0.000952174
T1DM	leukocyte	*TBC1D1*	-0.69419579	1.27E-09	8.14E-09	5.01E-06
T1DM	leukocyte	*CTNNA2*	-0.851261432	2.21E-09	8.21E-09	8.67E-06
T1DM	leukocyte	*CD84*	0.108545375	6.19E-10	8.14E-09	2.43E-06
T1DM	leukocyte	*ATXN3*	-0.901760398	1.32E-07	1.91E-07	0.000518363
T1DM	leukocyte	*EML1*	-1.006440537	2.34E-09	8.21E-09	9.20E-06
T1DM	leukocyte	*STOML1*	-0.613266387	1.32E-09	8.14E-09	5.18E-06
T1DM	leukocyte	*DNTTIP2*	-0.591096461	2.38E-09	8.21E-09	9.36E-06
T1DM	leukocyte	*RRP15*	-0.703433129	7.44E-10	8.14E-09	2.92E-06
T1DM	leukocyte	*HEATR6*	1.427658153	0.000101765	0.000121678	0.399832964
T1DM	leukocyte	*REEP1*	-0.610712681	4.00E-09	9.65E-09	1.57E-05
T1DM	leukocyte	*POLR1A*	-0.53538599	1.02E-07	1.51E-07	0.000400884
T1DM	leukocyte	*LAPTM4A*	-0.555888006	3.00E-09	8.53E-09	1.18E-05
T1DM	leukocyte	*KIF2A*	-0.62519762	1.70E-09	8.14E-09	6.67E-06
T1DM	leukocyte	*MAST4*	-0.700310057	2.39E-09	8.21E-09	9.38E-06
T1DM	leukocyte	*ADAM7*	-5.083552021	0.00920897	0.010235373	1
T1DM	leukocyte	*CLEC2D*	1.689000718	1.19E-07	1.74E-07	0.000466203
T1DM	leukocyte	*DRD4*	-2.204135485	2.39E-09	8.21E-09	9.41E-06
T1DM	leukocyte	*PLA2G10*	-2.468363517	2.60E-09	8.21E-09	1.02E-05
T1DM	leukocyte	*PIGB*	-0.89651222	0.030311755	0.033026868	1
T1DM	leukocyte	*GNB5*	-0.776624579	1.08E-08	1.99E-08	4.25E-05
T1DM	leukocyte	*RAB27A*	-0.568538632	1.86E-08	3.19E-08	7.31E-05

T1DM	leukocyte	*PTPN3*	-1.018879496	2.33E-09	8.21E-09	9.17E-06
T1DM	leukocyte	*SPTB*	-0.506243485	2.19E-07	3.11E-07	0.000859957
T1DM	leukocyte	*FGF10*	-2.146455158	2.80E-10	8.14E-09	1.10E-06
T1DM	leukocyte	*DGCR2*	-0.848006573	2.49E-09	8.21E-09	9.78E-06
T1DM	leukocyte	*ZXDC*	-1.390547799	4.94E-08	7.74E-08	0.000194012
T1DM	leukocyte	*WIPI1*	-1.316016045	1.31E-07	1.91E-07	0.00051642
T1DM	leukocyte	*GBA2*	-0.657063032	4.23E-09	9.97E-09	1.66E-05
T1DM	leukocyte	*ASNS*	-0.671312611	1.39E-09	8.14E-09	5.47E-06
T1DM	leukocyte	*AP3M2*	-0.895722731	4.16E-07	5.72E-07	0.00163287
T1DM	leukocyte	*MAP4K4*	-0.576036938	1.68E-09	8.14E-09	6.61E-06
T1DM	leukocyte	*RPL31*	-0.492262133	3.27E-09	8.79E-09	1.28E-05
T1DM	leukocyte	*SEL1L*	-0.557082142	4.29E-09	1.00E-08	1.68E-05
T1DM	leukocyte	*TCF3*	-0.638045291	5.46E-10	8.14E-09	2.15E-06
T1DM	leukocyte	*CPSF1*	-0.594840829	2.83E-09	8.42E-09	1.11E-05
T1DM	leukocyte	*CYBRD1*	0.513187876	2.23E-08	3.74E-08	8.77E-05
T1DM	leukocyte	*LNX1*	-0.52444978	1.37E-09	8.14E-09	5.37E-06
T1DM	leukocyte	*ALDH3A2*	-0.46484141	8.04E-10	8.14E-09	3.16E-06
T1DM	leukocyte	*P4HA2*	-0.500451663	4.96E-09	1.09E-08	1.95E-05
T1DM	leukocyte	*TRNT1*	-1.819032179	9.69E-08	1.44E-07	0.000380555
T1DM	leukocyte	*CRMP1*	-0.553432649	2.12E-09	8.16E-09	8.32E-06
T1DM	leukocyte	*DERL2*	-0.662776429	1.89E-09	8.14E-09	7.42E-06
T1DM	leukocyte	*SIDT1*	-0.440628894	0.017561574	0.019322157	1
T1DM	leukocyte	*NDE1*	1.164178375	0.00040435	0.000471003	1
T1DM	leukocyte	*XRCC1*	-0.875678145	1.59E-08	2.77E-08	6.23E-05
T1DM	leukocyte	*SCARB1*	-1.613197577	1.31E-09	8.14E-09	5.13E-06
T1DM	leukocyte	*NLE1*	-0.674307647	7.16E-08	1.09E-07	0.000281384
T1DM	leukocyte	*SMARCE1*	-0.571352381	2.66E-09	8.24E-09	1.05E-05
T1DM	leukocyte	*MRPS34*	-0.524321566	5.91E-09	1.23E-08	2.32E-05
T1DM	leukocyte	*TEAD2*	-0.879439731	1.63E-08	2.83E-08	6.39E-05
T1DM	leukocyte	*IPCEF1*	-1.070613667	6.29E-07	8.48E-07	0.002469791
T1DM	leukocyte	*ZZEF1*	-0.904294449	4.23E-09	9.97E-09	1.66E-05
T1DM	leukocyte	*ANO8*	1.548397214	3.86E-05	4.71E-05	0.151657607
T1DM	leukocyte	*WSCD2*	-0.729130984	1.57E-09	8.14E-09	6.17E-06
T1DM	leukocyte	*TIPIN*	-0.780404735	2.13E-07	3.03E-07	0.000837209
T1DM	leukocyte	*SEMA3A*	-1.927702441	1.64E-09	8.14E-09	6.45E-06
T1DM	leukocyte	*TTC38*	-1.179653789	7.49E-09	1.48E-08	2.94E-05
T1DM	leukocyte	*GRAMD4*	-0.695358897	1.43E-05	1.77E-05	0.056250949
T1DM	leukocyte	*CELSR1*	-3.304622979	8.45E-09	1.62E-08	3.32E-05
T1DM	leukocyte	*TIMM21*	-0.988297443	8.80E-10	8.14E-09	3.46E-06
T1DM	leukocyte	*VPS9D1*	-0.646829014	9.59E-09	1.80E-08	3.77E-05
T1DM	leukocyte	*ZNF37A*	-0.583852226	1.18E-07	1.72E-07	0.000462308
T1DM	leukocyte	*MARK3*	-0.712061021	3.56E-10	8.14E-09	1.40E-06
T1DM	leukocyte	*CACNG5*	-20.6763649	5.15E-10	8.14E-09	2.02E-06
T1DM	leukocyte	*MOCOS*	-1.572165404	4.02E-06	5.12E-06	0.015811256
T1DM	leukocyte	*ATP12A*	1.89464192	7.13E-08	1.08E-07	0.000280307
T1DM	leukocyte	*DLG1*	-0.640622539	1.53E-09	8.14E-09	6.01E-06
T1DM	leukocyte	*SEC31B*	0.892084117	1.92E-05	2.36E-05	0.075366733
T1DM	leukocyte	*ARHGAP15*	-2.944814635	0.004756255	0.005346874	1
T1DM	leukocyte	*EXOSC7*	-0.565755362	2.09E-09	8.15E-09	8.20E-06
T1DM	leukocyte	*MKRN2*	-0.816166597	1.38E-09	8.14E-09	5.43E-06
T1DM	leukocyte	*MLH1*	-0.865680593	7.86E-09	1.54E-08	3.09E-05
T1DM	leukocyte	*TPD52*	-0.513365199	3.53E-09	9.07E-09	1.39E-05
T1DM	leukocyte	*ACACB*	-0.803429081	1.41E-09	8.14E-09	5.52E-06
T1DM	leukocyte	*GPATCH1*	-0.593146505	9.27E-08	1.38E-07	0.000364206
T1DM	leukocyte	*ICAM3*	-0.7119138	4.25E-10	8.14E-09	1.67E-06
T1DM	leukocyte	*XAB2*	-0.688441891	6.36E-10	8.14E-09	2.50E-06
T1DM	leukocyte	*ACTL6B*	-0.526110001	1.33E-07	1.93E-07	0.000524518
T1DM	leukocyte	*IL4R*	-0.574452738	3.39E-08	5.49E-08	0.000133129
T1DM	leukocyte	*APBB1IP*	0.288128782	9.55E-09	1.80E-08	3.75E-05
T1DM	leukocyte	*GPR137B*	-0.809422006	3.54E-06	4.52E-06	0.013906469
T1DM	leukocyte	*NAALAD2*	-1.712327053	4.84E-08	7.59E-08	0.000190085
T1DM	leukocyte	*MCCC1*	-0.694394411	2.32E-09	8.21E-09	9.12E-06
T1DM	leukocyte	*N4BP2*	-0.914154088	4.21E-09	9.96E-09	1.65E-05
T1DM	leukocyte	*MLLT10*	-0.853472969	2.11E-09	8.16E-09	8.28E-06

T1DM	leukocyte	*VDAC3*	-0.659481716	2.34E-08	3.91E-08	9.18E-05
T1DM	leukocyte	*PCM1*	-0.605586664	1.58E-09	8.14E-09	6.22E-06
T1DM	leukocyte	*MYH7B*	-0.977502542	0.001687633	0.001923618	1
T1DM	leukocyte	*TP73*	2.067906618	4.84E-09	1.08E-08	1.90E-05
T1DM	leukocyte	*TOLLIP*	-0.673705683	3.42E-09	8.93E-09	1.34E-05
T1DM	leukocyte	*CLUL1*	-1.744684504	3.18E-09	8.70E-09	1.25E-05
T1DM	leukocyte	*FKBP7*	-0.889709405	1.75E-09	8.14E-09	6.87E-06
T1DM	leukocyte	*LXN*	-0.47661009	5.75E-08	8.91E-08	0.000225975
T1DM	leukocyte	*SAR1A*	-0.53026602	1.59E-09	8.14E-09	6.25E-06
T1DM	leukocyte	*CDC14A*	-0.956621315	1.39E-08	2.48E-08	5.48E-05
T1DM	leukocyte	*FDFT1*	-0.731788048	6.53E-10	8.14E-09	2.57E-06
T1DM	leukocyte	*SCGN*	-0.308483665	9.90E-09	1.84E-08	3.89E-05
T1DM	leukocyte	*DDX1*	-0.583474625	4.89E-09	1.08E-08	1.92E-05
T1DM	leukocyte	*RABL2B*	-1.005044638	1.38E-09	8.14E-09	5.44E-06
T1DM	leukocyte	*DCT*	-4.02948659	3.55E-09	9.10E-09	1.40E-05
T1DM	leukocyte	*SCTR*	-0.697291801	8.42E-10	8.14E-09	3.31E-06
T1DM	leukocyte	*RIF1*	-1.072423175	1.69E-08	2.92E-08	6.62E-05
T1DM	leukocyte	*IGSF9B*	-1.223029995	1.26E-08	2.28E-08	4.95E-05
T1DM	leukocyte	*COL4A4*	-1.52248225	1.65E-09	8.14E-09	6.48E-06
T1DM	leukocyte	*ZNF506*	-0.665073647	1.08E-09	8.14E-09	4.26E-06
T1DM	leukocyte	*CADPS2*	-0.716883951	1.26E-09	8.14E-09	4.94E-06
T1DM	leukocyte	*C1QTNF3*	2.14450914	2.20E-07	3.11E-07	0.000863126
T1DM	leukocyte	*ME2*	-1.246474767	1.61E-07	2.31E-07	0.000633005
T1DM	leukocyte	*CCNT2*	-0.748460823	6.47E-08	9.92E-08	0.000254033
T1DM	leukocyte	*COL19A1*	-10.50840369	0.015954406	0.017573553	1
T1DM	leukocyte	*SEMA5B*	1.133292316	2.67E-09	8.25E-09	1.05E-05
T1DM	leukocyte	*BCKDHB*	-1.67438068	1.25E-08	2.27E-08	4.93E-05
T1DM	leukocyte	*DIS3*	-0.641193418	3.60E-09	9.12E-09	1.41E-05
T1DM	leukocyte	*PIBF1*	-0.976768688	1.12E-08	2.05E-08	4.39E-05
T1DM	leukocyte	*CHMP2B*	-0.571318825	2.41E-09	8.21E-09	9.45E-06
T1DM	leukocyte	*PPIE*	-0.715669786	1.02E-08	1.90E-08	4.02E-05
T1DM	leukocyte	*NCOA1*	-0.745805304	2.90E-09	8.49E-09	1.14E-05
T1DM	leukocyte	*AGBL5*	-1.121491887	4.48E-08	7.08E-08	0.00017606
T1DM	leukocyte	*CAD*	-0.697345087	1.86E-07	2.66E-07	0.000730976
T1DM	leukocyte	*CD59*	-0.415745001	3.17E-09	8.70E-09	1.25E-05
T1DM	leukocyte	*CD82*	-0.94260626	9.78E-07	1.30E-06	0.003842378
T1DM	leukocyte	*MECOM*	-0.473439676	8.08E-09	1.57E-08	3.18E-05
T1DM	leukocyte	*SLC25A24*	-0.646848963	4.16E-09	9.92E-09	1.64E-05
T1DM	leukocyte	*PILRA*	0.244282295	5.72E-09	1.20E-08	2.25E-05
T1DM	leukocyte	*ZNF213*	-0.806103535	8.74E-11	8.14E-09	3.43E-07
T1DM	leukocyte	*CPNE3*	-0.508718346	2.02E-09	8.14E-09	7.94E-06
T1DM	leukocyte	*RRN3*	-0.586753478	1.83E-09	8.14E-09	7.19E-06
T1DM	leukocyte	*WNT11*	-1.28999027	4.08E-05	4.97E-05	0.160289651
T1DM	leukocyte	*DDHD2*	-0.68580844	1.55E-07	2.22E-07	0.00060706
T1DM	leukocyte	*TTC39A*	-0.668389863	1.40E-08	2.48E-08	5.49E-05
T1DM	leukocyte	*EPS15*	-0.738837944	7.94E-07	1.06E-06	0.003120277
T1DM	leukocyte	*ORC1*	-1.944754465	9.77E-07	1.30E-06	0.003839021
T1DM	leukocyte	*MGST2*	-0.47676153	3.79E-09	9.41E-09	1.49E-05
T1DM	leukocyte	*USP40*	-1.005666304	3.31E-09	8.82E-09	1.30E-05
T1DM	leukocyte	*POMGNT1*	-0.850807468	1.51E-09	8.14E-09	5.91E-06
T1DM	leukocyte	*NFX1*	-1.011687087	5.16E-09	1.12E-08	2.03E-05
T1DM	leukocyte	*EIF2AK1*	-0.626379942	3.06E-08	4.99E-08	0.000120148
T1DM	leukocyte	*EPDR1*	1.347674301	0.001878883	0.002138508	1
T1DM	leukocyte	*ITPKC*	-0.83783512	1.61E-09	8.14E-09	6.32E-06
T1DM	leukocyte	*TMED2*	-0.492591038	2.33E-09	8.21E-09	9.17E-06
T1DM	leukocyte	*ACOX3*	-1.243475797	2.34E-07	3.32E-07	0.000920741
T1DM	leukocyte	*TRIP6*	-0.492791848	8.42E-08	1.26E-07	0.000330628
T1DM	leukocyte	*ACHE*	0.596116956	0.003069403	0.003473412	1
T1DM	leukocyte	*PGS1*	-1.030300057	1.41E-09	8.14E-09	5.53E-06
T1DM	leukocyte	*PSMC5*	-0.550932229	3.83E-10	8.14E-09	1.50E-06
T1DM	leukocyte	*UIMC1*	-0.718225765	3.22E-09	8.75E-09	1.27E-05
T1DM	leukocyte	*SH3BP2*	0.869407851	1.73E-07	2.48E-07	0.000680678
T1DM	leukocyte	*SF3B2*	-0.545925027	2.62E-09	8.21E-09	1.03E-05
T1DM	leukocyte	*KLHL42*	-0.56829096	1.81E-09	8.14E-09	7.13E-06

T1DM	leukocyte	*TFAP2C*	-1.517947867	1.35E-06	1.77E-06	0.005285234
T1DM	leukocyte	*METTL2A*	-1.001350976	2.40E-09	8.21E-09	9.44E-06
T1DM	leukocyte	*GP6*	1.091319928	1.66E-08	2.88E-08	6.52E-05
T1DM	leukocyte	*EDEM2*	-0.71792082	4.13E-08	6.55E-08	0.000162163
T1DM	leukocyte	*SLC15A1*	-1.24987474	6.68E-08	1.02E-07	0.000262419
T1DM	leukocyte	*TGDS*	-0.690034774	2.69E-09	8.26E-09	1.06E-05
T1DM	leukocyte	*DOCK3*	-0.782571042	1.53E-09	8.14E-09	6.02E-06
T1DM	leukocyte	*ARHGAP28*	-1.76620239	1.32E-09	8.14E-09	5.19E-06
T1DM	leukocyte	*PPP1R13B*	-0.495043444	5.60E-09	1.18E-08	2.20E-05
T1DM	leukocyte	*ZNF343*	-0.956905714	5.55E-10	8.14E-09	2.18E-06
T1DM	leukocyte	*MAVS*	-1.134622602	9.24E-08	1.38E-07	0.00036295
T1DM	leukocyte	*DYNLL1*	-0.509084571	1.95E-08	3.32E-08	7.68E-05
T1DM	leukocyte	*RBBP9*	-0.628547831	6.91E-10	8.14E-09	2.72E-06
T1DM	leukocyte	*SLC23A2*	-1.011765722	1.38E-08	2.46E-08	5.43E-05
T1DM	leukocyte	*SLC8B1*	0.223448568	4.67E-09	1.06E-08	1.84E-05
T1DM	leukocyte	*TMEM230*	-0.624482831	5.62E-10	8.14E-09	2.21E-06
T1DM	leukocyte	*KIF16B*	-0.785365716	1.40E-06	1.84E-06	0.005500812
T1DM	leukocyte	*ZNF302*	-0.641518179	6.41E-10	8.14E-09	2.52E-06
T1DM	leukocyte	*GRAMD1A*	-0.987316991	5.61E-09	1.18E-08	2.20E-05
T1DM	leukocyte	*CDIP1*	-0.704694112	1.71E-09	8.14E-09	6.73E-06
T1DM	leukocyte	*MLF2*	-0.661460517	2.83E-09	8.42E-09	1.11E-05
T1DM	leukocyte	*DDX24*	-0.53446751	2.44E-09	8.21E-09	9.60E-06
T1DM	leukocyte	*ANKRD24*	-4.893161637	8.48E-08	1.27E-07	0.000333256
T1DM	leukocyte	*GPATCH2L*	-0.751758237	3.90E-09	9.52E-09	1.53E-05
T1DM	leukocyte	*SLC9A1*	-0.587822794	6.25E-09	1.28E-08	2.46E-05
T1DM	leukocyte	*SPTLC1*	-0.625976504	5.50E-09	1.17E-08	2.16E-05
T1DM	leukocyte	*YPEL3*	-0.664915067	1.05E-07	1.55E-07	0.00041294
T1DM	leukocyte	*NDUFB2*	-0.481081963	1.32E-08	2.38E-08	5.20E-05
T1DM	leukocyte	*MAEA*	-0.528920716	1.24E-07	1.81E-07	0.000486929
T1DM	leukocyte	*STRN4*	-0.641139247	1.82E-09	8.14E-09	7.17E-06
T1DM	leukocyte	*LYZ*	0.169911896	3.34E-09	8.82E-09	1.31E-05
T1DM	leukocyte	*MUL1*	-0.704758251	9.57E-10	8.14E-09	3.76E-06
T1DM	leukocyte	*SPG21*	-0.844142401	2.48E-09	8.21E-09	9.75E-06
T1DM	leukocyte	*USP48*	-0.640119386	7.27E-09	1.44E-08	2.86E-05
T1DM	leukocyte	*GLG1*	-0.644947553	1.46E-09	8.14E-09	5.73E-06
T1DM	leukocyte	*NRCAM*	-0.444685531	1.46E-09	8.14E-09	5.72E-06
T1DM	leukocyte	*TF*	-0.905756859	8.03E-07	1.07E-06	0.003155084
T1DM	leukocyte	*MYO15A*	-1.418241946	2.92E-07	4.08E-07	0.00114923
T1DM	leukocyte	*PITPNM3*	-1.916509921	1.29E-09	8.14E-09	5.08E-06
T1DM	leukocyte	*SPAG7*	-0.650063004	6.76E-08	1.03E-07	0.000265533
T1DM	leukocyte	*TMEM101*	-0.803442045	3.83E-09	9.46E-09	1.51E-05
T1DM	leukocyte	*PPP2R3C*	-0.771780849	5.51E-06	6.96E-06	0.021649383
T1DM	leukocyte	*OSGEP*	-0.918432991	5.78E-07	7.83E-07	0.002272517
T1DM	leukocyte	*SCFD1*	-0.653324243	3.90E-09	9.52E-09	1.53E-05
T1DM	leukocyte	*RPGRIP1*	0.953375489	2.97E-09	8.50E-09	1.17E-05
T1DM	leukocyte	*EZR*	-0.45822211	1.51E-09	8.14E-09	5.95E-06
T1DM	leukocyte	*NUP50*	-1.049142434	1.17E-08	2.13E-08	4.60E-05
T1DM	leukocyte	*COMT*	-0.580815103	1.84E-09	8.14E-09	7.22E-06
T1DM	leukocyte	*ECHDC1*	-0.547156036	2.16E-09	8.21E-09	8.50E-06
T1DM	leukocyte	*CBX5*	-0.547851627	1.60E-09	8.14E-09	6.27E-06
T1DM	leukocyte	*SUCO*	-0.458109599	2.39E-09	8.21E-09	9.40E-06
T1DM	leukocyte	*MSH2*	-1.363584967	2.21E-09	8.21E-09	8.67E-06
T1DM	leukocyte	*TMEM38B*	-0.831221567	4.57E-08	7.21E-08	0.000179498
T1DM	leukocyte	*PSMD5*	-0.628956999	1.59E-09	8.14E-09	6.26E-06
T1DM	leukocyte	*CRAT*	-1.050276335	2.55E-09	8.21E-09	1.00E-05
T1DM	leukocyte	*SH2D3C*	-0.968204611	2.13E-09	8.18E-09	8.37E-06
T1DM	leukocyte	*CWF19L1*	-0.632198764	5.53E-09	1.17E-08	2.17E-05
T1DM	leukocyte	*SEMA4G*	-2.307657455	0.034205871	0.037177004	1
T1DM	leukocyte	*IKZF5*	-0.957517447	5.01E-09	1.10E-08	1.97E-05
T1DM	leukocyte	*TLL2*	-2.810672794	1.06E-09	8.14E-09	4.16E-06
T1DM	leukocyte	*TPSD1*	-22.0801647	4.12E-07	5.68E-07	0.001619679
T1DM	leukocyte	*SRPK1*	-0.755353107	3.20E-09	8.73E-09	1.26E-05
T1DM	leukocyte	*PGC*	-0.345549303	0.000794818	0.000917942	1
T1DM	leukocyte	*EFHC1*	-0.575006458	7.74E-09	1.52E-08	3.04E-05

T1DM	leukocyte	*ITPR3*	-0.70337988	3.60E-09	9.12E-09	1.41E-05
T1DM	leukocyte	*IFT74*	-0.863860505	6.39E-09	1.30E-08	2.51E-05
T1DM	leukocyte	*ABL1*	-0.679101515	2.58E-09	8.21E-09	1.01E-05
T1DM	leukocyte	*SH3GLB1*	-0.662018426	3.39E-09	8.89E-09	1.33E-05
T1DM	leukocyte	*CDC7*	-0.89524395	1.22E-08	2.20E-08	4.78E-05
T1DM	leukocyte	*SYDE2*	-0.734775719	1.93E-08	3.28E-08	7.57E-05
T1DM	leukocyte	*ABLIM1*	-0.536328405	9.73E-09	1.82E-08	3.82E-05
T1DM	leukocyte	*ERMP1*	-0.661897182	1.93E-09	8.14E-09	7.57E-06
T1DM	leukocyte	*PALMD*	-0.460804726	1.86E-06	2.42E-06	0.007297552
T1DM	leukocyte	*MZF1*	-0.637876342	1.39E-07	2.02E-07	0.00054783
T1DM	leukocyte	*OCEL1*	-0.851655685	5.45E-09	1.16E-08	2.14E-05
T1DM	leukocyte	*CATSPERG*	-0.803631596	0.00019733	0.000233246	0.775308517
T1DM	leukocyte	*STX1B*	0.663187839	2.08E-06	2.70E-06	0.008175093
T1DM	leukocyte	*HSD3B7*	-1.682672944	1.07E-06	1.42E-06	0.004212056
T1DM	leukocyte	*HNRNPM*	-0.518121843	4.22E-09	9.97E-09	1.66E-05
T1DM	leukocyte	*TIMM13*	-0.6377243	2.78E-09	8.34E-09	1.09E-05
T1DM	leukocyte	*CEP170B*	-0.961531477	2.76E-09	8.33E-09	1.08E-05
T1DM	leukocyte	*POLR2E*	-0.849208626	3.81E-09	9.44E-09	1.50E-05
T1DM	leukocyte	*RASSF7*	-0.592841065	1.62E-09	8.14E-09	6.36E-06
T1DM	leukocyte	*ARVCF*	-1.271759784	7.70E-10	8.14E-09	3.03E-06
T1DM	leukocyte	*TRMT2A*	-0.657680352	4.86E-10	8.14E-09	1.91E-06
T1DM	leukocyte	*MED15*	-1.409247926	3.34E-07	4.64E-07	0.001312828
T1DM	leukocyte	*LZTR1*	-0.935307256	7.21E-07	9.69E-07	0.002832351
T1DM	leukocyte	*SMARCB1*	-0.687667018	2.91E-09	8.49E-09	1.14E-05
T1DM	leukocyte	*DDT*	-0.632730005	2.84E-08	4.66E-08	0.000111459
T1DM	leukocyte	*CABIN1*	-0.857906927	3.15E-09	8.69E-09	1.24E-05
T1DM	leukocyte	*SPECC1L*	-0.97016319	6.11E-09	1.26E-08	2.40E-05
T1DM	leukocyte	*PES1*	-0.694740314	5.09E-10	8.14E-09	2.00E-06
T1DM	leukocyte	*TOP3B*	-1.028489185	8.91E-09	1.69E-08	3.50E-05
T1DM	leukocyte	*CARD10*	-0.466794497	2.77E-08	4.55E-08	0.000108714
T1DM	leukocyte	*SLC25A1*	-0.725579103	2.97E-09	8.50E-09	1.17E-05
T1DM	leukocyte	*HPS4*	-0.745772327	2.71E-09	8.26E-09	1.07E-05
T1DM	leukocyte	*GCAT*	-0.953404026	1.32E-09	8.14E-09	5.19E-06
T1DM	leukocyte	*CCDC134*	-1.637123669	3.14E-06	4.02E-06	0.012331363
T1DM	leukocyte	*CYP2D6*	1.906830427	8.28E-09	1.60E-08	3.25E-05
T1DM	leukocyte	*DDX17*	-0.622117339	1.32E-08	2.37E-08	5.18E-05
T1DM	leukocyte	*HSCB*	-0.980893114	2.66E-07	3.73E-07	0.00104535
T1DM	leukocyte	*CBY1*	-0.725612589	2.41E-09	8.21E-09	9.48E-06
T1DM	leukocyte	*XBP1*	-0.462362858	2.71E-09	8.26E-09	1.06E-05
T1DM	leukocyte	*TIMP3*	-0.736016955	4.78E-09	1.07E-08	1.88E-05
T1DM	leukocyte	*CYB5R3*	-0.606682764	6.90E-09	1.39E-08	2.71E-05
T1DM	leukocyte	*MIOX*	-1.631469137	1.27E-09	8.14E-09	4.99E-06
T1DM	leukocyte	*LMF2*	-1.111054484	8.48E-08	1.27E-07	0.000333232
T1DM	leukocyte	*RHBDD3*	-0.741556592	6.53E-09	1.33E-08	2.56E-05
T1DM	leukocyte	*AP1B1*	0.704526993	0.009530711	0.010583992	1
T1DM	leukocyte	*TOM1*	-0.983204501	1.74E-08	3.01E-08	6.85E-05
T1DM	leukocyte	*CHKB*	-1.512887726	6.43E-08	9.87E-08	0.00025247
T1DM	leukocyte	*BIK*	-1.03398048	2.43E-06	3.13E-06	0.009530081
T1DM	leukocyte	*ARSA*	-0.450234341	0.00022882	0.000269494	0.89903246
T1DM	leukocyte	*TTLL12*	-1.02127702	1.40E-09	8.14E-09	5.51E-06
T1DM	leukocyte	*RBFOX2*	-0.620788922	5.89E-09	1.22E-08	2.31E-05
T1DM	leukocyte	*SYNGR1*	-1.316019501	4.13E-06	5.25E-06	0.016226648
T1DM	leukocyte	*MTMR3*	-0.764247034	7.14E-08	1.08E-07	0.000280393
T1DM	leukocyte	*APOL4*	-1.205763427	0.002378622	0.002700262	1
T1DM	leukocyte	*SAMM50*	-0.641295376	8.03E-10	8.14E-09	3.15E-06
T1DM	leukocyte	*FOXRED2*	-0.773662834	3.13E-09	8.69E-09	1.23E-05
T1DM	leukocyte	*SGSM3*	-0.94042904	4.88E-09	1.08E-08	1.92E-05
T1DM	leukocyte	*IFT27*	-0.828425249	1.03E-09	8.14E-09	4.05E-06
T1DM	leukocyte	*FAM118A*	-0.608920777	9.20E-05	0.00011017	0.361577428
T1DM	leukocyte	*ST13*	-0.558036615	3.03E-09	8.56E-09	1.19E-05
T1DM	leukocyte	*POLR3H*	-1.295607135	5.39E-08	8.40E-08	0.000211704
T1DM	leukocyte	*PMM1*	-0.857077329	3.43E-09	8.93E-09	1.35E-05
T1DM	leukocyte	*DESI1*	-0.871382994	2.95E-09	8.50E-09	1.16E-05
T1DM	leukocyte	*HDAC10*	1.064769873	2.83E-05	3.47E-05	0.111145414

T1DM	leukocyte	*KHNYN*	-0.996924136	5.94E-11	8.14E-09	2.33E-07
T1DM	leukocyte	*RBM23*	-0.599101883	3.28E-08	5.33E-08	0.000128976
T1DM	leukocyte	*PRMT5*	-0.648975846	1.46E-09	8.14E-09	5.75E-06
T1DM	leukocyte	*VCPKMT*	-0.948726615	1.71E-09	8.14E-09	6.70E-06
T1DM	leukocyte	*SOS2*	-0.822187331	4.59E-09	1.04E-08	1.80E-05
T1DM	leukocyte	*CDKL1*	-1.147942641	3.63E-08	5.84E-08	0.000142731
T1DM	leukocyte	*NIN*	-1.361746387	1.73E-08	2.98E-08	6.78E-05
T1DM	leukocyte	*PYGL*	1.394869731	1.27E-05	1.57E-05	0.049834837
T1DM	leukocyte	*TRIM9*	-0.537890642	1.19E-09	8.14E-09	4.67E-06
T1DM	leukocyte	*ATP6V1D*	-0.603233626	1.66E-09	8.14E-09	6.51E-06
T1DM	leukocyte	*PLEK2*	-0.965235226	5.00E-08	7.83E-08	0.000196554
T1DM	leukocyte	*GSTZ1*	-0.931646096	1.54E-08	2.70E-08	6.03E-05
T1DM	leukocyte	*TMED8*	-0.743805592	3.33E-09	8.82E-09	1.31E-05
T1DM	leukocyte	*DAAM1*	-0.598454395	1.54E-09	8.14E-09	6.07E-06
T1DM	leukocyte	*DHRS7*	-0.676668248	5.38E-09	1.15E-08	2.11E-05
T1DM	leukocyte	*MTHFD1*	-1.858983857	8.93E-07	1.19E-06	0.003509091
T1DM	leukocyte	*TELO2*	-0.652359393	1.15E-05	1.43E-05	0.045110413
T1DM	leukocyte	*GSKIP*	-0.671662322	1.28E-09	8.14E-09	5.03E-06
T1DM	leukocyte	*TRIP11*	-0.574886656	1.59E-09	8.14E-09	6.23E-06
T1DM	leukocyte	*DCAF11*	-0.814046113	1.35E-08	2.41E-08	5.29E-05
T1DM	leukocyte	*EMC9*	-0.673526909	2.02E-08	3.41E-08	7.92E-05
T1DM	leukocyte	*NFATC4*	-0.958380738	1.51E-09	8.14E-09	5.94E-06
T1DM	leukocyte	*PYGB*	-0.603901223	5.79E-07	7.83E-07	0.002274174
T1DM	leukocyte	*ABHD12*	-0.976698521	8.38E-07	1.12E-06	0.003291963
T1DM	leukocyte	*PROCR*	-0.622388395	4.98E-10	8.14E-09	1.96E-06
T1DM	leukocyte	*CD40*	-0.747134759	1.48E-09	8.14E-09	5.83E-06
T1DM	leukocyte	*IFT52*	-0.658772985	1.02E-09	8.14E-09	4.00E-06
T1DM	leukocyte	*MYBL2*	-1.494628864	4.49E-07	6.16E-07	0.001765925
T1DM	leukocyte	*PABPC1L*	-0.320616062	0.019056689	0.020926141	1
T1DM	leukocyte	*SALL4*	0.93719024	1.71E-06	2.24E-06	0.006737105
T1DM	leukocyte	*NELFCD*	-0.622943545	1.66E-09	8.14E-09	6.52E-06
T1DM	leukocyte	*MTG2*	-0.741937959	3.27E-09	8.79E-09	1.29E-05
T1DM	leukocyte	*DIDO1*	-0.730434328	6.83E-08	1.04E-07	0.000268518
T1DM	leukocyte	*SLC17A9*	0.805488738	5.51E-08	8.56E-08	0.000216335
T1DM	leukocyte	*CDC25B*	-1.097201622	2.20E-07	3.11E-07	0.00086258
T1DM	leukocyte	*ISM1*	1.094897809	5.86E-09	1.22E-08	2.30E-05
T1DM	leukocyte	*ARFRP1*	-1.006970173	4.23E-09	9.97E-09	1.66E-05
T1DM	leukocyte	*SLC52A3*	-1.336041956	2.98E-06	3.82E-06	0.01169006
T1DM	leukocyte	*RSPO4*	-5.784273787	0.0034132	0.003858015	1
T1DM	leukocyte	*SIRPB1*	0.506153589	3.74E-09	9.32E-09	1.47E-05
T1DM	leukocyte	*PLCB4*	-0.541107573	9.39E-08	1.40E-07	0.000368831
T1DM	leukocyte	*POFUT1*	-0.658076556	1.42E-09	8.14E-09	5.59E-06
T1DM	leukocyte	*NOP56*	-0.872732658	6.05E-09	1.25E-08	2.38E-05
T1DM	leukocyte	*MANBAL*	-0.613413908	1.91E-09	8.14E-09	7.51E-06
T1DM	leukocyte	*IDH3B*	-0.797774362	1.96E-09	8.14E-09	7.72E-06
T1DM	leukocyte	*MAPRE1*	-0.53320056	5.47E-09	1.17E-08	2.15E-05
T1DM	leukocyte	*OXT*	0.660475684	7.39E-09	1.46E-08	2.90E-05
T1DM	leukocyte	*TTI1*	-1.012991175	2.04E-09	8.14E-09	8.03E-06
T1DM	leukocyte	*CHMP4B*	-0.546487965	2.07E-09	8.14E-09	8.14E-06
T1DM	leukocyte	*DHX35*	-1.070558405	1.63E-08	2.83E-08	6.39E-05
T1DM	leukocyte	*DNTTIP1*	-1.28803477	2.33E-08	3.90E-08	9.15E-05
T1DM	leukocyte	*MAP1LC3A*	-0.420588827	5.93E-08	9.15E-08	0.00023296
T1DM	leukocyte	*APMAP*	-0.592920563	2.09E-06	2.71E-06	0.008202977
T1DM	leukocyte	*VAPA*	-0.526991405	2.61E-09	8.21E-09	1.02E-05
T1DM	leukocyte	*MYOM1*	-1.59815577	7.16E-09	1.43E-08	2.82E-05
T1DM	leukocyte	*CEP192*	-0.52684932	1.30E-07	1.89E-07	0.00051236
T1DM	leukocyte	*LIPG*	-0.753937058	1.35E-09	8.14E-09	5.32E-06
T1DM	leukocyte	*LAMA1*	-2.723414237	2.87E-05	3.52E-05	0.112955646
T1DM	leukocyte	*POLI*	-0.713920354	0.000266553	0.000313091	1
T1DM	leukocyte	*RIOK3*	-0.581863725	3.16E-09	8.69E-09	1.24E-05
T1DM	leukocyte	*NALCN*	-0.652017529	1.38E-09	8.14E-09	5.40E-06
T1DM	leukocyte	*FGF14*	-0.573586638	2.46E-07	3.47E-07	0.000967184
T1DM	leukocyte	*FNDC3A*	-0.512434637	1.75E-09	8.14E-09	6.88E-06
T1DM	leukocyte	*KLF5*	-0.497520655	1.20E-09	8.14E-09	4.71E-06

T1DM	leukocyte	*ACP5*	0.046549814	4.28E-09	1.00E-08	1.68E-05
T1DM	leukocyte	*DNAJC3*	-0.564942904	2.81E-09	8.40E-09	1.10E-05
T1DM	leukocyte	*UGGT2*	-0.614862736	3.16E-09	8.69E-09	1.24E-05
T1DM	leukocyte	*ARHGEF7*	-0.847141508	3.28E-09	8.79E-09	1.29E-05
T1DM	leukocyte	*PARP4*	-1.396030326	8.01E-07	1.07E-06	0.00314753
T1DM	leukocyte	*MEDAG*	-1.023376395	1.75E-08	3.01E-08	6.86E-05
T1DM	leukocyte	*CLN5*	-0.775091892	4.76E-09	1.07E-08	1.87E-05
T1DM	leukocyte	*OLFM4*	-0.330828356	2.26E-09	8.21E-09	8.86E-06
T1DM	leukocyte	*MGRN1*	-0.745156904	7.46E-08	1.13E-07	0.000293043
T1DM	leukocyte	*LONP2*	-0.639135394	2.02E-09	8.14E-09	7.93E-06
T1DM	leukocyte	*CBLN1*	-1.84355106	6.00E-07	8.11E-07	0.002359136
T1DM	leukocyte	*DHODH*	-1.787013066	6.65E-09	1.35E-08	2.61E-05
T1DM	leukocyte	*PARD6A*	-0.48961139	4.14E-09	9.90E-09	1.63E-05
T1DM	leukocyte	*MMP15*	-1.150215044	1.18E-06	1.55E-06	0.004622287
T1DM	leukocyte	*USB1*	-0.942119322	3.01E-09	8.54E-09	1.18E-05
T1DM	leukocyte	*CYB5B*	-1.110285405	1.92E-08	3.28E-08	7.55E-05
T1DM	leukocyte	*CCDC113*	-0.774750697	3.33E-07	4.62E-07	0.001307933
T1DM	leukocyte	*NME3*	-0.681421843	8.49E-09	1.63E-08	3.34E-05
T1DM	leukocyte	*VAC14*	-0.733572248	8.96E-10	8.14E-09	3.52E-06
T1DM	leukocyte	*SMPD3*	1.479924953	3.32E-06	4.24E-06	0.013044792
T1DM	leukocyte	*NPRL3*	-0.930093219	3.47E-08	5.61E-08	0.000136284
T1DM	leukocyte	*TAF1C*	0.323187141	0.018146902	0.019949407	1
T1DM	leukocyte	*NAGPA*	-1.006969774	0.045098712	0.048826905	1
T1DM	leukocyte	*ZNF500*	-1.296024152	9.58E-09	1.80E-08	3.76E-05
T1DM	leukocyte	*NME4*	-0.694982849	3.24E-09	8.77E-09	1.27E-05
T1DM	leukocyte	*NOMO3*	-1.016937697	1.08E-09	8.14E-09	4.24E-06
T1DM	leukocyte	*LMF1*	-0.692826023	1.55E-09	8.14E-09	6.08E-06
T1DM	leukocyte	*MTHFSD*	-0.333791104	0.015503556	0.017096119	1
T1DM	leukocyte	*HAGHL*	-1.08981238	1.97E-09	8.14E-09	7.75E-06
T1DM	leukocyte	*METRN*	-0.538915191	1.19E-09	8.14E-09	4.67E-06
T1DM	leukocyte	*PIEZO1*	-0.801642049	4.11E-09	9.85E-09	1.62E-05
T1DM	leukocyte	*CLUAP1*	-0.663694807	5.43E-07	7.39E-07	0.002133504
T1DM	leukocyte	*UBFD1*	-0.787397272	3.39E-07	4.70E-07	0.001331638
T1DM	leukocyte	*AQP8*	-1.121701432	1.42E-09	8.14E-09	5.59E-06
T1DM	leukocyte	*USP31*	-0.499402352	8.35E-09	1.61E-08	3.28E-05
T1DM	leukocyte	*HMOX2*	-0.602632662	3.80E-09	9.42E-09	1.49E-05
T1DM	leukocyte	*RBL2*	-0.833825284	3.82E-09	9.44E-09	1.50E-05
T1DM	leukocyte	*KAT8*	-0.786804307	2.02E-09	8.14E-09	7.93E-06
T1DM	leukocyte	*NOMO1*	-0.909693401	5.23E-07	7.13E-07	0.002054376
T1DM	leukocyte	*RNF40*	-1.106087019	1.73E-08	2.98E-08	6.78E-05
T1DM	leukocyte	*KNOP1*	-0.832863319	1.91E-09	8.14E-09	7.51E-06
T1DM	leukocyte	*TRIP4*	-0.731836379	1.99E-09	8.14E-09	7.81E-06
T1DM	leukocyte	*AP3B2*	-0.752209404	1.41E-09	8.14E-09	5.55E-06
T1DM	leukocyte	*IGDCC4*	1.824997136	2.75E-08	4.53E-08	0.000108084
T1DM	leukocyte	*RPAP1*	-1.040681207	1.24E-09	8.14E-09	4.88E-06
T1DM	leukocyte	*HOMER2*	-0.52526979	6.00E-09	1.24E-08	2.36E-05
T1DM	leukocyte	*CEP152*	0.750621704	0.005347699	0.005998032	1
T1DM	leukocyte	*ATP8B4*	0.402129818	3.40E-09	8.90E-09	1.33E-05
T1DM	leukocyte	*DNAJC17*	-0.679636441	2.23E-09	8.21E-09	8.75E-06
T1DM	leukocyte	*OIP5*	-2.500260067	1.84E-06	2.40E-06	0.007235422
T1DM	leukocyte	*SLC30A4*	-1.074729918	0.000842491	0.000972429	1
T1DM	leukocyte	*ZDHHC2*	-0.467952473	1.40E-09	8.14E-09	5.49E-06
T1DM	leukocyte	*TRIM35*	-1.549263575	3.13E-09	8.69E-09	1.23E-05
T1DM	leukocyte	*RIPK2*	-0.82637981	8.41E-10	8.14E-09	3.30E-06
T1DM	leukocyte	*NBN*	-0.947791158	1.85E-09	8.14E-09	7.28E-06
T1DM	leukocyte	*LAPTM4B*	-0.435144531	1.34E-09	8.14E-09	5.28E-06
T1DM	leukocyte	*RAB2A*	-0.481547505	1.29E-09	8.14E-09	5.06E-06
T1DM	leukocyte	*EIF3E*	-0.528421688	2.55E-09	8.21E-09	1.00E-05
T1DM	leukocyte	*ESRP1*	-0.559223147	1.97E-09	8.14E-09	7.75E-06
T1DM	leukocyte	*ARMC1*	-0.726712453	5.58E-09	1.18E-08	2.19E-05
T1DM	leukocyte	*SPAG1*	-0.621616281	3.35E-09	8.83E-09	1.31E-05
T1DM	leukocyte	*CHRAC1*	-0.562017686	9.98E-10	8.14E-09	3.92E-06
T1DM	leukocyte	*SNX16*	-1.798535432	1.40E-08	2.49E-08	5.52E-05
T1DM	leukocyte	*GSDMD*	-0.541998437	4.27E-09	1.00E-08	1.68E-05

T1DM	leukocyte	*SH2D4A*	-0.776786483	8.81E-10	8.14E-09	3.46E-06
T1DM	leukocyte	*INTS10*	-0.821335537	5.78E-09	1.21E-08	2.27E-05
T1DM	leukocyte	*ERI1*	-1.464292355	2.93E-08	4.79E-08	0.000114953
T1DM	leukocyte	*LEPROTL1*	-0.770645142	9.98E-10	8.14E-09	3.92E-06
T1DM	leukocyte	*ERICH1*	-0.942419758	7.14E-10	8.14E-09	2.81E-06
T1DM	leukocyte	*ARHGEF10*	-0.713021657	5.51E-09	1.17E-08	2.17E-05
T1DM	leukocyte	*KLHDC4*	-0.763176153	8.28E-08	1.25E-07	0.000325399
T1DM	leukocyte	*MCM4*	-0.845170504	1.55E-09	8.14E-09	6.09E-06
T1DM	leukocyte	*FGL1*	-1.143059221	2.18E-08	3.66E-08	8.56E-05
T1DM	leukocyte	*ASAH1*	0.504089888	2.03E-06	2.64E-06	0.007980047
T1DM	leukocyte	*MAN2B1*	0.726118819	7.74E-06	9.70E-06	0.030399322
T1DM	leukocyte	*ECH1*	-0.645171145	1.77E-10	8.14E-09	6.94E-07
T1DM	leukocyte	*HNRNPL*	-0.660369377	2.48E-09	8.21E-09	9.76E-06
T1DM	leukocyte	*SARS2*	-0.79866916	1.75E-09	8.14E-09	6.88E-06
T1DM	leukocyte	*LIN7B*	-0.847152116	4.41E-09	1.02E-08	1.73E-05
T1DM	leukocyte	*PPP1R13L*	-0.710656797	3.05E-08	4.97E-08	0.000119802
T1DM	leukocyte	*ERCC2*	-0.751517836	1.20E-09	8.14E-09	4.71E-06
T1DM	leukocyte	*KLC3*	-0.702009498	1.15E-10	8.14E-09	4.51E-07
T1DM	leukocyte	*AMH*	-0.76450466	2.11E-09	8.16E-09	8.31E-06
T1DM	leukocyte	*TLE6*	-0.965295216	2.89E-08	4.73E-08	0.000113438
T1DM	leukocyte	*IL27RA*	-0.860420225	6.59E-08	1.01E-07	0.000258955
T1DM	leukocyte	*C19orf44*	-0.584277628	1.27E-05	1.57E-05	0.049733767
T1DM	leukocyte	*CASP14*	1.601993487	0.000204312	0.000241136	0.802742263
T1DM	leukocyte	*PDCD5*	-0.515902839	3.62E-09	9.14E-09	1.42E-05
T1DM	leukocyte	*NUMBL*	-0.583743707	1.42E-09	8.14E-09	5.57E-06
T1DM	leukocyte	*SLC1A5*	-0.832478809	9.89E-09	1.84E-08	3.88E-05
T1DM	leukocyte	*PRKD2*	-0.807555845	2.40E-10	8.14E-09	9.43E-07
T1DM	leukocyte	*APLP1*	-0.307418489	4.79E-08	7.52E-08	0.000188068
T1DM	leukocyte	*PLIN3*	-0.690089049	2.39E-09	8.21E-09	9.38E-06
T1DM	leukocyte	*MYH14*	-0.567776205	9.29E-10	8.14E-09	3.65E-06
T1DM	leukocyte	*CARD8*	-0.926011284	0.006591519	0.007372069	1
T1DM	leukocyte	*ZNF175*	-0.652488009	8.80E-10	8.14E-09	3.46E-06
T1DM	leukocyte	*PLA2G4C*	-0.865846861	4.99E-09	1.10E-08	1.96E-05
T1DM	leukocyte	*CAPS*	-0.893852658	2.17E-09	8.21E-09	8.51E-06
T1DM	leukocyte	*RASIP1*	-0.721816361	4.41E-07	6.04E-07	0.001731537
T1DM	leukocyte	*PPP2R1A*	-0.564491656	3.37E-09	8.87E-09	1.33E-05
T1DM	leukocyte	*GCDH*	-0.827247337	3.53E-10	8.14E-09	1.39E-06
T1DM	leukocyte	*PIK3R2*	-1.504096988	1.00E-06	1.33E-06	0.003936409
T1DM	leukocyte	*UPK1A*	-10.04180692	3.44E-09	8.94E-09	1.35E-05
T1DM	leukocyte	*COPE*	-0.556682567	3.05E-09	8.59E-09	1.20E-05
T1DM	leukocyte	*ETV2*	-0.757282392	5.70E-07	7.72E-07	0.002238957
T1DM	leukocyte	*PBX4*	-2.587525782	1.95E-08	3.32E-08	7.67E-05
T1DM	leukocyte	*SIPA1L3*	-1.424773018	1.20E-07	1.75E-07	0.000470527
T1DM	leukocyte	*ETHE1*	-0.896895225	1.04E-07	1.54E-07	0.000409743
T1DM	leukocyte	*SMG9*	-0.633971269	2.28E-09	8.21E-09	8.94E-06
T1DM	leukocyte	*RUNDC3B*	-3.014558977	4.55E-08	7.18E-08	0.000178792
T1DM	leukocyte	*PMPCB*	-0.755720537	3.18E-09	8.70E-09	1.25E-05
T1DM	leukocyte	*PON2*	-0.575262351	2.61E-09	8.21E-09	1.03E-05
T1DM	leukocyte	*ITGB8*	-0.607092518	1.87E-09	8.14E-09	7.37E-06
T1DM	leukocyte	*DNAH11*	-2.124811876	1.13E-05	1.40E-05	0.044236549
T1DM	leukocyte	*ADAP1*	1.901232543	4.22E-08	6.70E-08	0.000165809
T1DM	leukocyte	*CAV2*	-0.414823529	4.42E-09	1.02E-08	1.74E-05
T1DM	leukocyte	*CAV1*	-0.505585846	1.37E-09	8.14E-09	5.40E-06
T1DM	leukocyte	*ANKRD7*	-1.200085952	3.82E-09	9.44E-09	1.50E-05
T1DM	leukocyte	*VIPR2*	-0.550032819	0.000668316	0.00077298	1
T1DM	leukocyte	*TSPAN12*	-0.99970462	1.85E-09	8.14E-09	7.28E-06
T1DM	leukocyte	*CPVL*	0.098668279	3.93E-09	9.56E-09	1.55E-05
T1DM	leukocyte	*ABHD11*	-0.892822075	4.69E-09	1.06E-08	1.84E-05
T1DM	leukocyte	*CHCHD2*	-0.446912014	1.87E-09	8.14E-09	7.34E-06
T1DM	leukocyte	*EIF3B*	-0.645266648	4.30E-09	1.01E-08	1.69E-05
T1DM	leukocyte	*WASL*	-0.538715357	5.11E-09	1.12E-08	2.01E-05
T1DM	leukocyte	*TFR2*	1.052623175	0.0002011	0.00023756	0.790122977
T1DM	leukocyte	*AGFG2*	-0.668321072	1.04E-09	8.14E-09	4.10E-06
T1DM	leukocyte	*C1GALT1*	-0.576157369	1.58E-09	8.14E-09	6.21E-06

T1DM	leukocyte	*RPA3*	-0.705082142	2.06E-09	8.14E-09	8.10E-06
T1DM	leukocyte	*ZNHIT1*	-0.571849553	1.81E-09	8.14E-09	7.12E-06
T1DM	leukocyte	*CLDN15*	-0.464073616	1.51E-06	1.98E-06	0.005913984
T1DM	leukocyte	*TMEM106B*	-0.570887999	4.88E-09	1.08E-08	1.92E-05
T1DM	leukocyte	*ACTR3C*	-1.071140935	4.28E-09	1.00E-08	1.68E-05
T1DM	leukocyte	*TSPAN13*	-0.446747263	1.86E-09	8.14E-09	7.31E-06
T1DM	leukocyte	*RARRES2*	-0.327490985	7.06E-09	1.41E-08	2.77E-05
T1DM	leukocyte	*AGR2*	-0.475765692	1.99E-08	3.38E-08	7.84E-05
T1DM	leukocyte	*TMEM176B*	-0.356469628	0.000256859	0.000301975	1
T1DM	leukocyte	*COA1*	-0.804213028	5.86E-09	1.22E-08	2.30E-05
T1DM	leukocyte	*BLVRA*	-1.009782247	4.34E-08	6.88E-08	0.000170619
T1DM	leukocyte	*TMEM248*	-0.744739266	8.10E-09	1.57E-08	3.18E-05
T1DM	leukocyte	*RHEB*	-0.554217614	2.36E-09	8.21E-09	9.26E-06
T1DM	leukocyte	*TBL2*	-0.610328656	3.57E-08	5.75E-08	0.000140101
T1DM	leukocyte	*LIMK1*	-1.616101388	3.04E-05	3.73E-05	0.119566792
T1DM	leukocyte	*SLC1A1*	-0.724167693	0.000490233	0.000569691	1
T1DM	leukocyte	*SPIN1*	-0.916276091	8.07E-09	1.57E-08	3.17E-05
T1DM	leukocyte	*NMRK1*	-0.702352504	3.09E-09	8.63E-09	1.21E-05
T1DM	leukocyte	*C5*	-0.634023015	4.84E-05	5.88E-05	0.190141959
T1DM	leukocyte	*OGN*	-1.497367014	2.71E-09	8.26E-09	1.06E-05
T1DM	leukocyte	*SUSD1*	-1.067679506	3.56E-09	9.10E-09	1.40E-05
T1DM	leukocyte	*DNM1*	-0.494760625	1.30E-09	8.14E-09	5.10E-06
T1DM	leukocyte	*AK1*	-0.494128441	8.50E-09	1.63E-08	3.34E-05
T1DM	leukocyte	*PLGRKT*	-0.57454277	3.46E-09	8.98E-09	1.36E-05
T1DM	leukocyte	*DOCK8*	0.340402651	9.38E-09	1.77E-08	3.68E-05
T1DM	leukocyte	*MPDZ*	-0.560063027	4.30E-09	1.01E-08	1.69E-05
T1DM	leukocyte	*RAPGEF1*	-0.518694811	0.010249171	0.011368999	1
T1DM	leukocyte	*NPDC1*	-0.46270205	2.54E-09	8.21E-09	9.98E-06
T1DM	leukocyte	*HPS1*	-0.769256814	2.40E-09	8.21E-09	9.42E-06
T1DM	leukocyte	*CXCL12*	-0.657954184	2.52E-09	8.21E-09	9.90E-06
T1DM	leukocyte	*TRDMT1*	-1.332864491	3.46E-08	5.59E-08	0.000135907
T1DM	leukocyte	*ATE1*	-1.012982674	4.80E-09	1.07E-08	1.89E-05
T1DM	leukocyte	*NSMCE4A*	-0.537966957	2.21E-07	3.13E-07	0.000868838
T1DM	leukocyte	*PLEKHA1*	-0.50438864	3.09E-09	8.64E-09	1.21E-05
T1DM	leukocyte	*PALD1*	-3.616821685	4.64E-08	7.29E-08	0.000182124
T1DM	leukocyte	*SPOCK2*	-1.052046433	1.41E-09	8.14E-09	5.54E-06
T1DM	leukocyte	*BMPR1A*	-1.316675005	9.67E-09	1.81E-08	3.80E-05
T1DM	leukocyte	*ACTA2*	-0.446157626	0.033139444	0.036057844	1
T1DM	leukocyte	*LIPA*	0.241962025	9.56E-09	1.80E-08	3.76E-05
T1DM	leukocyte	*SFXN3*	-1.028391216	6.06E-10	8.14E-09	2.38E-06
T1DM	leukocyte	*NPM3*	-0.751158683	2.06E-09	8.14E-09	8.08E-06
T1DM	leukocyte	*TNKS2*	-0.701356741	3.55E-09	9.10E-09	1.40E-05
T1DM	leukocyte	*ACBD5*	-0.586547641	2.21E-09	8.21E-09	8.70E-06
T1DM	leukocyte	*LHPP*	-1.042708475	9.13E-08	1.37E-07	0.000358709
T1DM	leukocyte	*GTPBP4*	-0.556186131	1.79E-09	8.14E-09	7.03E-06
T1DM	leukocyte	*NEURL1*	-0.772837906	2.55E-09	8.21E-09	1.00E-05
T1DM	leukocyte	*SORCS1*	-2.151901355	0.009396455	0.010440801	1
T1DM	leukocyte	*XPNPEP1*	-0.643410458	1.36E-09	8.14E-09	5.33E-06
T1DM	leukocyte	*SMC3*	-0.543903127	1.54E-09	8.14E-09	6.03E-06
T1DM	leukocyte	*RPL28*	-0.377146787	7.78E-08	1.17E-07	0.000305539
T1DM	leukocyte	*PBLD*	-0.543717517	2.78E-08	4.57E-08	0.000109063
T1DM	leukocyte	*KRT23*	-0.259216299	0.000335413	0.000392214	1
T1DM	leukocyte	*DHRS11*	-0.597680805	2.57E-09	8.21E-09	1.01E-05
T1DM	leukocyte	*CWC25*	-0.770375243	2.84E-09	8.43E-09	1.11E-05
T1DM	leukocyte	*WNT3*	-0.8470709	2.52E-05	3.10E-05	0.098976032
T1DM	leukocyte	*RAD51C*	-0.70724229	2.57E-09	8.21E-09	1.01E-05
T1DM	leukocyte	*TRIM37*	-0.640323086	1.59E-09	8.14E-09	6.23E-06
T1DM	leukocyte	*RPS6KB1*	-0.719045272	9.43E-09	1.78E-08	3.70E-05
T1DM	leukocyte	*TRIM16L*	-0.98343874	3.03E-08	4.95E-08	0.000119244
T1DM	leukocyte	*CDK5RAP3*	-0.889495034	2.43E-07	3.43E-07	0.000955056
T1DM	leukocyte	*CAMTA2*	-0.968209091	1.86E-08	3.18E-08	7.29E-05
T1DM	leukocyte	*MED13*	-0.631606293	2.05E-09	8.14E-09	8.04E-06
T1DM	leukocyte	*RAI1*	-0.55496067	1.32E-09	8.14E-09	5.18E-06
T1DM	leukocyte	*CCDC47*	-0.605444907	1.18E-09	8.14E-09	4.65E-06

T1DM	leukocyte	*DRG2*	-0.713596904	9.90E-10	8.14E-09	3.89E-06
T1DM	leukocyte	*FTSJ3*	-0.637022935	1.96E-09	8.14E-09	7.69E-06
T1DM	leukocyte	*SMARCD2*	-1.314581104	8.37E-09	1.61E-08	3.29E-05
T1DM	leukocyte	*SYNGR2*	-0.577531726	3.08E-09	8.62E-09	1.21E-05
T1DM	leukocyte	*C17orf75*	-0.87537938	1.18E-08	2.15E-08	4.64E-05
T1DM	leukocyte	*DHX58*	-1.292281247	4.45E-09	1.03E-08	1.75E-05
T1DM	leukocyte	*NAGLU*	-0.697819221	2.85E-09	8.43E-09	1.12E-05
T1DM	leukocyte	*HSD17B1*	-0.780805514	1.49E-09	8.14E-09	5.84E-06
T1DM	leukocyte	*CNTNAP1*	-0.411461213	0.030911367	0.03366152	1
T1DM	leukocyte	*RND2*	-1.600406216	2.80E-09	8.39E-09	1.10E-05
T1DM	leukocyte	*ABCC3*	-0.494772321	7.48E-08	1.13E-07	0.000293864
T1DM	leukocyte	*MPP2*	-0.491433441	2.09E-09	8.15E-09	8.21E-06
T1DM	leukocyte	*HLF*	-0.442584126	1.79E-09	8.14E-09	7.02E-06
T1DM	leukocyte	*EFNB3*	-0.638283312	8.65E-10	8.14E-09	3.40E-06
T1DM	leukocyte	*RANGRF*	-0.813115218	2.96E-09	8.50E-09	1.16E-05
T1DM	leukocyte	*DPH1*	-1.56220974	4.89E-10	8.14E-09	1.92E-06
T1DM	leukocyte	*MYH3*	-1.341431393	0.00060557	0.000701647	1
T1DM	leukocyte	*VTN*	-0.39431369	3.21E-06	4.11E-06	0.012607169
T1DM	leukocyte	*OCIAD1*	-0.555012517	1.74E-09	8.14E-09	6.84E-06
T1DM	leukocyte	*UBE2D3*	-0.509360781	1.53E-08	2.69E-08	6.01E-05
T1DM	leukocyte	*MAPK10*	-0.51959695	1.57E-08	2.74E-08	6.15E-05
T1DM	leukocyte	*ANXA10*	-0.55502603	3.53E-05	4.31E-05	0.138821653
T1DM	leukocyte	*AADAT*	-1.91931467	2.00E-09	8.14E-09	7.86E-06
T1DM	leukocyte	*TRIM2*	-0.619166452	2.66E-09	8.23E-09	1.04E-05
T1DM	leukocyte	*CLNK*	-1.133241468	0.008458065	0.00942477	1
T1DM	leukocyte	*SH3D19*	-1.263167164	4.10E-09	9.83E-09	1.61E-05
T1DM	leukocyte	*GLRB*	-0.639181819	4.60E-09	1.04E-08	1.81E-05
T1DM	leukocyte	*LRP2BP*	-0.770080947	6.82E-10	8.14E-09	2.68E-06
T1DM	leukocyte	*UFSP2*	-0.768639783	3.94E-08	6.29E-08	0.000154982
T1DM	leukocyte	*KLHL5*	-0.800973703	2.27E-09	8.21E-09	8.91E-06
T1DM	leukocyte	*FAM149A*	-0.636351379	4.64E-09	1.05E-08	1.82E-05
T1DM	leukocyte	*DDX25*	-0.589373772	3.72E-07	5.14E-07	0.001462713
T1DM	leukocyte	*HTATIP2*	-0.764337363	9.91E-08	1.47E-07	0.000389351
T1DM	leukocyte	*B3GAT1*	-1.679347149	2.05E-09	8.14E-09	8.06E-06
T1DM	leukocyte	*P2RX3*	-13.35316837	1.18E-09	8.14E-09	4.62E-06
T1DM	leukocyte	*VWA5A*	-0.708407094	3.44E-08	5.56E-08	0.000135027
T1DM	leukocyte	*DTX4*	-0.611376873	1.66E-06	2.17E-06	0.006538647
T1DM	leukocyte	*FOXRED1*	-1.008059788	1.31E-07	1.91E-07	0.000516061
T1DM	leukocyte	*ST3GAL4*	-1.105854473	2.45E-09	8.21E-09	9.63E-06
T1DM	leukocyte	*PRPF19*	-0.589000502	2.57E-09	8.21E-09	1.01E-05
T1DM	leukocyte	*APOA5*	-3.570242902	0.023134044	0.025325622	1
T1DM	leukocyte	*CEP164*	-1.074750825	2.02E-07	2.88E-07	0.000795459
T1DM	leukocyte	*PDHX*	-0.729669367	1.14E-07	1.67E-07	0.000447474
T1DM	leukocyte	*SLC1A2*	-0.717970782	2.28E-09	8.21E-09	8.97E-06
T1DM	leukocyte	*CD5*	-2.45329741	1.95E-06	2.53E-06	0.007661835
T1DM	leukocyte	*ACCS*	0.37712244	6.15E-09	1.26E-08	2.42E-05
T1DM	leukocyte	*MDK*	-0.636792558	1.96E-09	8.14E-09	7.71E-06
T1DM	leukocyte	*MADD*	-0.843806746	6.81E-09	1.37E-08	2.68E-05
T1DM	leukocyte	*NAA40*	-0.806538312	3.70E-09	9.29E-09	1.46E-05
T1DM	leukocyte	*CD81*	-0.393006464	1.32E-07	1.92E-07	0.000520134
T1DM	leukocyte	*SLC35F2*	-0.687886223	8.34E-09	1.61E-08	3.28E-05
T1DM	leukocyte	*NUP98*	-0.689252247	1.79E-09	8.14E-09	7.04E-06
T1DM	leukocyte	*GTF2H1*	-1.073910245	5.51E-09	1.17E-08	2.16E-05
T1DM	leukocyte	*PTPN5*	1.479697915	3.06E-09	8.60E-09	1.20E-05
T1DM	leukocyte	*PRDM4*	-0.531799646	4.89E-09	1.08E-08	1.92E-05
T1DM	leukocyte	*COQ5*	-0.276771972	0.020999363	0.023014364	1
T1DM	leukocyte	*CORO1C*	-0.772563721	1.84E-09	8.14E-09	7.24E-06
T1DM	leukocyte	*LIN7A*	-1.553239362	1.27E-09	8.14E-09	5.00E-06
T1DM	leukocyte	*KRT18*	-0.243224699	2.18E-09	8.21E-09	8.56E-06
T1DM	leukocyte	*ACSS3*	0.862504996	0.02133864	0.023379675	1
T1DM	leukocyte	*METAP2*	-0.597121252	5.56E-09	1.18E-08	2.18E-05
T1DM	leukocyte	*SLC6A12*	-3.526540134	3.38E-07	4.69E-07	0.001327357
T1DM	leukocyte	*WNT5B*	-1.599497404	1.33E-09	8.14E-09	5.22E-06
T1DM	leukocyte	*ITFG2*	-1.482914059	0.001871846	0.002131116	1

T1DM	leukocyte	*PRR4*	-0.509972772	0.00093659	0.001079772	1
T1DM	leukocyte	*VPS29*	-0.593175854	6.33E-10	8.14E-09	2.49E-06
T1DM	leukocyte	*AKAP3*	-0.484465235	0.003320015	0.003754847	1
T1DM	leukocyte	*CREBL2*	-0.815768471	2.44E-09	8.21E-09	9.58E-06
T1DM	leukocyte	*GPRC5D*	-11.43902562	0.005248469	0.005890099	1
T1DM	leukocyte	*GSG1*	-3.976299261	1.16E-06	1.53E-06	0.004548296
T1DM	leukocyte	*LTBR*	-0.733798229	2.03E-09	8.14E-09	7.97E-06
T1DM	leukocyte	*CDK2AP1*	-0.623172947	4.19E-09	9.96E-09	1.65E-05
T1DM	leukocyte	*OAS3*	3.142036771	1.87E-05	2.31E-05	0.073660165
T1DM	leukocyte	*RASAL1*	2.186982905	0.001018669	0.001172678	1
T1DM	leukocyte	*DDX55*	-0.948220032	1.14E-08	2.09E-08	4.49E-05
T1DM	leukocyte	*STX2*	-0.853927584	1.03E-08	1.90E-08	4.04E-05
T1DM	leukocyte	*CAND1*	-0.721338537	3.59E-09	9.11E-09	1.41E-05
T1DM	leukocyte	*NUP107*	-0.862448479	1.96E-09	8.14E-09	7.69E-06
T1DM	leukocyte	*KRR1*	-0.632795939	4.33E-09	1.01E-08	1.70E-05
T1DM	leukocyte	*GNB3*	-0.824981494	3.03E-09	8.56E-09	1.19E-05
T1DM	leukocyte	*CDCA3*	-1.305192209	5.35E-09	1.15E-08	2.10E-05
T1DM	leukocyte	*CHPT1*	-0.893406223	1.38E-07	2.00E-07	0.000543388
T1DM	leukocyte	*SPSB2*	-0.707131151	9.62E-10	8.14E-09	3.78E-06
T1DM	leukocyte	*ATN1*	-0.679386203	4.57E-09	1.04E-08	1.80E-05
T1DM	leukocyte	*NT5DC3*	-0.796433961	1.13E-08	2.06E-08	4.43E-05
T1DM	leukocyte	*GOLT1B*	-0.627815444	1.88E-09	8.14E-09	7.40E-06
T1DM	leukocyte	*PRKAB1*	-0.597838642	9.00E-09	1.70E-08	3.53E-05
T1DM	leukocyte	*C2CD5*	-0.652112905	1.04E-09	8.14E-09	4.08E-06
T1DM	leukocyte	*SRSF9*	-0.561289117	1.78E-09	8.14E-09	6.98E-06
T1DM	leukocyte	*BTN3A3*	-0.514180224	0.000249537	0.000293454	0.980430364
T1DM	leukocyte	*FRK*	-0.702817349	1.76E-09	8.14E-09	6.92E-06
T1DM	leukocyte	*RSPH4A*	-0.642683802	2.11E-07	3.00E-07	0.000829207
T1DM	leukocyte	*SMIM8*	-0.788594961	1.57E-09	8.14E-09	6.16E-06
T1DM	leukocyte	*NEDD9*	-0.38600162	1.33E-09	8.14E-09	5.23E-06
T1DM	leukocyte	*CEP85L*	-0.683764163	6.75E-09	1.36E-08	2.65E-05
T1DM	leukocyte	*GABRR2*	-0.921128151	0.002045286	0.002326558	1
T1DM	leukocyte	*HDDC2*	-0.535045868	1.79E-09	8.14E-09	7.05E-06
T1DM	leukocyte	*TPD52L1*	-0.568494305	4.57E-09	1.04E-08	1.80E-05
T1DM	leukocyte	*MTRF1L*	-0.755284948	3.90E-09	9.52E-09	1.53E-05
T1DM	leukocyte	*MRPL18*	-0.874849123	4.38E-09	1.02E-08	1.72E-05
T1DM	leukocyte	*RNF8*	-0.673880125	1.50E-09	8.14E-09	5.91E-06
T1DM	leukocyte	*MDGA1*	1.216754477	1.68E-09	8.14E-09	6.60E-06
T1DM	leukocyte	*CD83*	0.148730574	2.32E-09	8.21E-09	9.12E-06
T1DM	leukocyte	*GLP1R*	-0.641893212	1.16E-08	2.11E-08	4.55E-05
T1DM	leukocyte	*BACH2*	-0.636003639	2.43E-10	8.14E-09	9.56E-07
T1DM	leukocyte	*FBXL4*	-0.798844116	1.61E-09	8.14E-09	6.34E-06
T1DM	leukocyte	*ASCC3*	-0.560036129	1.40E-09	8.14E-09	5.51E-06
T1DM	leukocyte	*WASF1*	-0.611788502	1.86E-09	8.14E-09	7.33E-06
T1DM	leukocyte	*ALDH5A1*	-1.547208068	1.35E-06	1.78E-06	0.005317884
T1DM	leukocyte	*C6orf62*	-0.728741224	3.44E-09	8.93E-09	1.35E-05
T1DM	leukocyte	*B3GAT2*	-0.992691058	1.61E-09	8.14E-09	6.34E-06
T1DM	leukocyte	*HBS1L*	-0.670750003	1.75E-09	8.14E-09	6.86E-06
T1DM	leukocyte	*TRIM38*	0.6938188	0.001669903	0.001904513	1
T1DM	leukocyte	*PEX7*	-0.641834223	9.92E-10	8.14E-09	3.90E-06
T1DM	leukocyte	*PHACTR2*	-0.516804717	1.69E-09	8.14E-09	6.64E-06
T1DM	leukocyte	*EPM2A*	-1.10607995	8.43E-10	8.14E-09	3.31E-06
T1DM	leukocyte	*CCR6*	2.31035677	4.43E-07	6.07E-07	0.001738994
T1DM	leukocyte	*TFEB*	-0.827274615	8.44E-10	8.14E-09	3.31E-06
T1DM	leukocyte	*PRPH2*	-0.715184913	2.88E-06	3.70E-06	0.011327224
T1DM	leukocyte	*DNPH1*	-0.590806997	3.33E-07	4.62E-07	0.001307445
T1DM	leukocyte	*DUSP22*	-0.853706894	9.49E-10	8.14E-09	3.73E-06
T1DM	leukocyte	*LAMA4*	-0.548091851	1.47E-09	8.14E-09	5.79E-06
T1DM	leukocyte	*FBRSL1*	-0.794132808	1.91E-09	8.14E-09	7.51E-06
T1DM	leukocyte	*ENPP5*	-0.569215549	8.89E-09	1.69E-08	3.49E-05
T1DM	leukocyte	*NUDT12*	-0.84041499	4.27E-09	1.00E-08	1.68E-05
T1DM	leukocyte	*CEP72*	-2.034120754	1.03E-08	1.91E-08	4.06E-05
T1DM	leukocyte	*SEMA5A*	-0.783204708	6.45E-09	1.31E-08	2.54E-05
T1DM	leukocyte	*GHR*	-1.833599424	1.28E-09	8.14E-09	5.05E-06

T1DM	leukocyte	*DAP*	-0.52718907	3.89E-09	9.52E-09	1.53E-05
T1DM	leukocyte	*NME5*	-0.84421973	1.28E-06	1.68E-06	0.005010624
T1DM	leukocyte	*BRD8*	-0.780347998	1.62E-09	8.14E-09	6.37E-06
T1DM	leukocyte	*NNT*	-0.849634978	1.38E-07	2.00E-07	0.000542562
T1DM	leukocyte	*TMCO6*	1.206315221	3.26E-07	4.53E-07	0.001280997
T1DM	leukocyte	*IK*	-0.634246678	2.94E-09	8.50E-09	1.16E-05
T1DM	leukocyte	*PDE8B*	-0.454353162	2.42E-09	8.21E-09	9.50E-06
T1DM	leukocyte	*ITK*	1.004559926	7.01E-05	8.44E-05	0.275420013
T1DM	leukocyte	*BTNL8*	5.89928522	2.51E-07	3.54E-07	0.00098807
T1DM	leukocyte	*MSH3*	-0.730651347	1.25E-09	8.14E-09	4.92E-06
T1DM	leukocyte	*CCNG1*	-0.98853739	2.85E-07	3.98E-07	0.00111893
T1DM	leukocyte	*DROSHA*	-0.590306282	1.42E-09	8.14E-09	5.57E-06
T1DM	leukocyte	*GOLPH3*	-0.6458861	2.64E-09	8.21E-09	1.04E-05
T1DM	leukocyte	*NPR3*	-1.992184209	4.67E-09	1.06E-08	1.83E-05
T1DM	leukocyte	*RAD50*	-0.798745457	3.44E-09	8.93E-09	1.35E-05
T1DM	leukocyte	*PPWD1*	1.174385548	5.11E-06	6.47E-06	0.020060186
T1DM	leukocyte	*WWC1*	-0.395653657	2.70E-09	8.26E-09	1.06E-05
T1DM	leukocyte	*SMAD5*	-0.58020335	5.95E-09	1.23E-08	2.34E-05
T1DM	leukocyte	*HMGXB3*	-0.829782326	1.47E-09	8.14E-09	5.78E-06
T1DM	leukocyte	*BNIP1*	-0.499714839	2.33E-06	3.00E-06	0.009135434
T1DM	leukocyte	*STC2*	-0.527886887	1.31E-09	8.14E-09	5.16E-06
T1DM	leukocyte	*EHHADH*	-0.910323527	2.34E-09	8.21E-09	9.21E-06
T1DM	leukocyte	*ACTR8*	-0.662496608	1.27E-09	8.14E-09	5.00E-06
T1DM	leukocyte	*TIMMDC1*	-0.721269161	3.89E-09	9.52E-09	1.53E-05
T1DM	leukocyte	*CLDN16*	3.29830364	4.80E-08	7.53E-08	0.000188583
T1DM	leukocyte	*NPHP3*	-0.705472937	3.25E-07	4.52E-07	0.001278001
T1DM	leukocyte	*FAM162A*	-0.756754563	1.04E-09	8.14E-09	4.09E-06
T1DM	leukocyte	*RBP1*	-0.555716931	2.65E-09	8.23E-09	1.04E-05
T1DM	leukocyte	*RNF7*	-0.579022188	9.46E-09	1.78E-08	3.72E-05
T1DM	leukocyte	*SERPINI2*	-0.452117383	2.60E-09	8.21E-09	1.02E-05
T1DM	leukocyte	*WNT5A*	1.704700511	9.01E-06	1.13E-05	0.035417694
T1DM	leukocyte	*HYAL1*	-0.453962128	0.009004991	0.010014324	1
T1DM	leukocyte	*TUSC2*	-0.820548727	2.42E-09	8.21E-09	9.50E-06
T1DM	leukocyte	*CYB561D2*	-0.571201421	1.47E-08	2.60E-08	5.78E-05
T1DM	leukocyte	*C3orf14*	-0.518012842	1.89E-08	3.23E-08	7.42E-05
T1DM	leukocyte	*CBLB*	-1.033075737	2.69E-09	8.26E-09	1.06E-05
T1DM	leukocyte	*IFT57*	-0.570087019	7.27E-09	1.44E-08	2.86E-05
T1DM	leukocyte	*NCBP2*	-0.60541879	1.19E-09	8.14E-09	4.67E-06
T1DM	leukocyte	*C3orf52*	-0.576565594	4.02E-09	9.69E-09	1.58E-05
T1DM	leukocyte	*SLC41A3*	-0.563331735	1.38E-07	2.00E-07	0.000541439
T1DM	leukocyte	*ATP6V1A*	-0.724241227	3.67E-09	9.23E-09	1.44E-05
T1DM	leukocyte	*PODXL2*	-0.517361049	1.45E-09	8.14E-09	5.71E-06
T1DM	leukocyte	*UPK1B*	-0.866056658	8.54E-07	1.14E-06	0.003356346
T1DM	leukocyte	*NEK11*	-0.559062124	2.20E-09	8.21E-09	8.64E-06
T1DM	leukocyte	*PLSCR4*	-1.062091189	2.02E-09	8.14E-09	7.95E-06
T1DM	leukocyte	*COMMD2*	-0.625253808	7.60E-10	8.14E-09	2.98E-06
T1DM	leukocyte	*GORASP1*	-1.170277154	1.78E-06	2.32E-06	0.006985055
T1DM	leukocyte	*AADAC*	-0.439331209	5.42E-06	6.85E-06	0.021296182
T1DM	leukocyte	*ARHGEF26*	-1.538407283	1.48E-08	2.61E-08	5.82E-05
T1DM	leukocyte	*PLCH1*	-2.503537383	5.82E-08	9.01E-08	0.000228673
T1DM	leukocyte	*VIPR1*	-0.881272999	1.63E-06	2.14E-06	0.006418135
T1DM	leukocyte	*NKTR*	-0.608122022	4.33E-09	1.01E-08	1.70E-05
T1DM	leukocyte	*NEK4*	-1.518778104	1.23E-07	1.80E-07	0.000484212
T1DM	leukocyte	*KANSL3*	-1.254388699	7.66E-09	1.50E-08	3.01E-05
T1DM	leukocyte	*ACTR1B*	-1.006840624	1.62E-08	2.82E-08	6.35E-05
T1DM	leukocyte	*SLC35F5*	-0.611447774	1.09E-08	2.00E-08	4.28E-05
T1DM	leukocyte	*ACTR3*	-0.690101965	2.53E-09	8.21E-09	9.94E-06
T1DM	leukocyte	*POMC*	-1.01060992	4.91E-09	1.09E-08	1.93E-05
T1DM	leukocyte	*TANC1*	-0.528431662	6.68E-08	1.02E-07	0.000262392
T1DM	leukocyte	*REEP6*	-0.845192923	1.25E-09	8.14E-09	4.89E-06
T1DM	leukocyte	*INO80B*	-1.936983668	2.99E-08	4.88E-08	0.000117467
T1DM	leukocyte	*MOGS*	-1.0751636	9.54E-10	8.14E-09	3.75E-06
T1DM	leukocyte	*RTN4*	-0.516838167	2.58E-09	8.21E-09	1.01E-05
T1DM	leukocyte	*HTRA2*	-1.007463293	5.29E-09	1.14E-08	2.08E-05

T1DM	leukocyte	*GALNT3*	-0.735300174	1.49E-09	8.14E-09	5.86E-06
T1DM	leukocyte	*MRPL19*	-0.662929421	7.45E-09	1.47E-08	2.93E-05
T1DM	leukocyte	*WDR75*	-1.110845587	3.21E-09	8.73E-09	1.26E-05
T1DM	leukocyte	*FANCL*	-1.440552434	1.97E-10	8.14E-09	7.74E-07
T1DM	leukocyte	*DNAH6*	-0.376799356	0.000101709	0.000121649	0.399616577
T1DM	leukocyte	*GGCX*	-0.794523163	6.28E-09	1.29E-08	2.47E-05
T1DM	leukocyte	*OTX1*	-1.098775655	0.042427963	0.045973377	1
T1DM	leukocyte	*SF3B1*	-0.679370163	5.26E-09	1.14E-08	2.07E-05
T1DM	leukocyte	*CHMP3*	-0.56806522	1.56E-09	8.14E-09	6.15E-06
T1DM	leukocyte	*IL1R2*	-0.702171571	1.25E-09	8.14E-09	4.90E-06
T1DM	leukocyte	*IL1R1*	-0.611242503	2.29E-09	8.21E-09	9.00E-06
T1DM	leukocyte	*IL1RL2*	0.708433985	5.07E-09	1.11E-08	1.99E-05
T1DM	leukocyte	*IL18R1*	0.621524238	3.32E-08	5.39E-08	0.000130445
T1DM	leukocyte	*MLPH*	-0.958596158	4.41E-08	6.98E-08	0.000173214
T1DM	leukocyte	*CNPPD1*	-0.559659797	2.91E-09	8.49E-09	1.14E-05
T1DM	leukocyte	*HDLBP*	-0.492686737	1.27E-09	8.14E-09	5.01E-06
T1DM	leukocyte	*PROC*	0.901904809	1.60E-08	2.79E-08	6.27E-05
T1DM	leukocyte	*TAF1B*	-0.628685733	2.85E-09	8.43E-09	1.12E-05
T1DM	leukocyte	*STRN*	-1.031685376	5.75E-08	8.91E-08	0.000225774
T1DM	leukocyte	*CEBPZ*	-0.59261582	8.36E-09	1.61E-08	3.28E-05
T1DM	leukocyte	*WIPF1*	0.462125261	1.62E-08	2.82E-08	6.35E-05
T1DM	leukocyte	*COX7A2L*	-0.520095522	2.50E-09	8.21E-09	9.84E-06
T1DM	leukocyte	*PNO1*	-0.679576287	1.92E-09	8.14E-09	7.54E-06
T1DM	leukocyte	*ORC4*	-0.679154909	2.42E-09	8.21E-09	9.51E-06
T1DM	leukocyte	*C2orf42*	1.138214995	2.44E-07	3.44E-07	0.000956956
T1DM	leukocyte	*ARID3A*	0.837209323	0.020254008	0.022216079	1
T1DM	leukocyte	*SUMO1*	-0.55833012	1.42E-08	2.52E-08	5.59E-05
T1DM	leukocyte	*GRIN3B*	1.10065407	2.93E-06	3.76E-06	0.011498683
T1DM	leukocyte	*ALMS1*	-0.81708416	1.93E-09	8.14E-09	7.58E-06
T1DM	leukocyte	*MARK1*	-1.048681391	2.22E-09	8.21E-09	8.73E-06
T1DM	leukocyte	*MORN1*	-1.192449614	1.12E-08	2.05E-08	4.41E-05
T1DM	leukocyte	*GPX7*	-0.610405357	3.56E-09	9.10E-09	1.40E-05
T1DM	leukocyte	*SCP2*	-0.604057624	5.05E-09	1.11E-08	1.99E-05
T1DM	leukocyte	*RALGPS2*	-0.638920947	1.44E-09	8.14E-09	5.64E-06
T1DM	leukocyte	*ANGPTL1*	-1.204362974	8.79E-10	8.14E-09	3.45E-06
T1DM	leukocyte	*CEP104*	-0.496019919	0.005662924	0.006344348	1
T1DM	leukocyte	*FAM20B*	-0.713961659	9.25E-09	1.75E-08	3.63E-05
T1DM	leukocyte	*WRAP73*	-1.737036368	2.69E-06	3.47E-06	0.010576488
T1DM	leukocyte	*ICMT*	-0.814761825	6.71E-09	1.36E-08	2.64E-05
T1DM	leukocyte	*PARK7*	-0.5041593	4.53E-09	1.04E-08	1.78E-05
T1DM	leukocyte	*MECR*	-1.631615071	2.05E-08	3.46E-08	8.04E-05
T1DM	leukocyte	*EDEM3*	-0.73303053	1.86E-09	8.14E-09	7.29E-06
T1DM	leukocyte	*FBXO6*	-1.712537276	0.00034521	0.00040343	1
T1DM	leukocyte	*SMG7*	-0.655327324	1.70E-09	8.14E-09	6.70E-06
T1DM	leukocyte	*PDC*	-6.449235299	2.96E-08	4.83E-08	0.000116179
T1DM	leukocyte	*RGS2*	0.423766453	1.03E-08	1.90E-08	4.03E-05
T1DM	leukocyte	*BCAS2*	-0.580869436	2.03E-09	8.14E-09	7.97E-06
T1DM	leukocyte	*AGMAT*	-0.649062783	0.000148908	0.000176808	0.585058755
T1DM	leukocyte	*TNNI3K*	-1.671158808	6.44E-08	9.89E-08	0.000252964
T1DM	leukocyte	*PLEKHM2*	-1.131056638	9.02E-06	1.13E-05	0.035435275
T1DM	leukocyte	*CRYZ*	-0.474765393	1.81E-09	8.14E-09	7.10E-06
T1DM	leukocyte	*TMEM9*	-0.570126473	4.07E-08	6.47E-08	0.000159874
T1DM	leukocyte	*WARS2*	-0.688317099	1.69E-09	8.14E-09	6.65E-06
T1DM	leukocyte	*C1orf109*	-0.45291492	2.72E-07	3.81E-07	0.001067663
T1DM	leukocyte	*LGALS8*	-0.806019732	1.68E-09	8.14E-09	6.61E-06
T1DM	leukocyte	*HPCAL4*	-1.78242467	3.55E-09	9.10E-09	1.40E-05
T1DM	leukocyte	*BMP8B*	-0.625720872	8.31E-10	8.14E-09	3.27E-06
T1DM	leukocyte	*MYCL*	0.370225504	3.99E-09	9.64E-09	1.57E-05
T1DM	leukocyte	*MFAP2*	-0.392854849	5.21E-05	6.32E-05	0.204808367
T1DM	leukocyte	*UAP1*	-0.490866594	1.24E-09	8.14E-09	4.85E-06
T1DM	leukocyte	*CTBS*	-0.752486173	2.88E-09	8.46E-09	1.13E-05
T1DM	leukocyte	*ZNHIT6*	-0.461768325	2.91E-08	4.77E-08	0.00011441
T1DM	leukocyte	*GBP3*	-0.213580652	0.003519083	0.003974268	1
T1DM	leukocyte	*CD46*	-0.513733058	2.00E-09	8.14E-09	7.86E-06

T1DM	leukocyte	*SLC2A1*	-0.934975668	1.06E-09	8.14E-09	4.16E-06
T1DM	leukocyte	*PRDX1*	-0.532114846	4.53E-09	1.04E-08	1.78E-05
T1DM	leukocyte	*CCDC181*	-0.540143079	8.59E-10	8.14E-09	3.37E-06
T1DM	leukocyte	*NSUN4*	-1.351791362	6.89E-05	8.31E-05	0.270847301
T1DM	leukocyte	*TMED5*	-0.836309479	6.07E-09	1.25E-08	2.39E-05
T1DM	leukocyte	*DR1*	-0.814370542	8.84E-09	1.68E-08	3.47E-05
T1DM	leukocyte	*PTBP2*	-0.660167877	1.16E-08	2.11E-08	4.55E-05
T1DM	leukocyte	*NSL1*	-0.961826265	6.28E-09	1.29E-08	2.47E-05
T1DM	leukocyte	*RPA2*	-0.825335269	4.48E-09	1.03E-08	1.76E-05
T1DM	leukocyte	*ESYT2*	-0.707297034	4.87E-09	1.08E-08	1.91E-05
T1DM	leukocyte	*DDX59*	-0.599699301	5.59E-10	8.14E-09	2.20E-06
T1DM	leukocyte	*TTR*	-0.25671243	1.71E-09	8.14E-09	6.74E-06
T1DM	leukocyte	*SPCS2*	-0.572979966	2.97E-09	8.50E-09	1.17E-05
T1DM	leukocyte	*ELOVL4*	-0.955144576	1.57E-09	8.14E-09	6.18E-06
T1DM	leukocyte	*FILIP1*	-0.734647049	2.13E-09	8.18E-09	8.37E-06
T1DM	leukocyte	*AKAP7*	-1.107431399	4.22E-07	5.79E-07	0.001656558
T1DM	leukocyte	*ALDH8A1*	-0.51581511	0.005586636	0.006260665	1
T1DM	leukocyte	*RNF146*	-0.737268098	1.46E-09	8.14E-09	5.74E-06
T1DM	leukocyte	*MED28*	-0.864877725	5.19E-08	8.11E-08	0.000203891
T1DM	leukocyte	*VAMP8*	-0.631521013	1.36E-08	2.43E-08	5.34E-05
T1DM	leukocyte	*ARMC2*	-0.581251445	9.64E-09	1.81E-08	3.79E-05
T1DM	leukocyte	*PKD2*	-0.550758043	1.40E-09	8.14E-09	5.50E-06
T1DM	leukocyte	*SPP1*	-0.281276084	2.98E-08	4.86E-08	0.000116957
T1DM	leukocyte	*WDR35*	-0.613151278	2.00E-09	8.14E-09	7.86E-06
T1DM	leukocyte	*DNAH7*	-0.62522741	3.84E-08	6.14E-08	0.000150875
T1DM	leukocyte	*NDUFB3*	-0.606870537	1.54E-09	8.14E-09	6.04E-06
T1DM	leukocyte	*GTF3C3*	-0.884378086	5.18E-08	8.10E-08	0.000203557
T1DM	leukocyte	*CPSF3*	-0.945941277	3.61E-09	9.13E-09	1.42E-05
T1DM	leukocyte	*PIGZ*	0.409517754	0.039395367	0.042734511	1
T1DM	leukocyte	*CCDC92*	-0.691539267	1.73E-09	8.14E-09	6.82E-06
T1DM	leukocyte	*CTNNAL1*	-0.489063694	5.41E-08	8.43E-08	0.000212535
T1DM	leukocyte	*NEK6*	-0.724728739	3.94E-08	6.28E-08	0.00015478
T1DM	leukocyte	*BSPRY*	-0.48445832	3.90E-07	5.38E-07	0.001532588
T1DM	leukocyte	*RBM18*	-0.834372292	1.94E-09	8.14E-09	7.61E-06
T1DM	leukocyte	*INVS*	-0.520997191	9.18E-07	1.22E-06	0.003608494
T1DM	leukocyte	*DCAF4*	-0.830119211	3.24E-09	8.77E-09	1.27E-05
T1DM	leukocyte	*IFI27L2*	-0.550325733	5.84E-09	1.22E-08	2.30E-05
T1DM	leukocyte	*ACOT2*	-0.9229397	1.94E-07	2.77E-07	0.00076177
T1DM	leukocyte	*ZC2HC1C*	-1.613262069	3.82E-06	4.86E-06	0.015000174
T1DM	leukocyte	*ESRRB*	-1.258340636	0.026707819	0.029156716	1
T1DM	leukocyte	*KLHL29*	-1.800765483	3.94E-06	5.02E-06	0.015493189
T1DM	leukocyte	*AFTPH*	-0.666001609	8.24E-10	8.14E-09	3.24E-06
T1DM	leukocyte	*BCL11A*	-1.135131523	1.03E-09	8.14E-09	4.04E-06
T1DM	leukocyte	*CRIPT*	-0.760586907	2.00E-09	8.14E-09	7.86E-06
T1DM	leukocyte	*EPCAM*	-0.411802715	1.79E-09	8.14E-09	7.04E-06
T1DM	leukocyte	*SLC17A5*	-1.051496825	0.001477508	0.00168852	1
T1DM	leukocyte	*OGFRL1*	0.791645121	0.000193634	0.000229222	0.760786893
T1DM	leukocyte	*CUTC*	-0.714377603	6.30E-08	9.69E-08	0.000247651
T1DM	leukocyte	*PYROXD2*	-1.136283961	1.48E-10	8.14E-09	5.81E-07
T1DM	leukocyte	*HELLS*	-0.826200347	3.75E-10	8.14E-09	1.47E-06
T1DM	leukocyte	*PRLHR*	-0.943197366	1.41E-09	8.14E-09	5.54E-06
T1DM	leukocyte	*WDR11*	-0.985135042	9.96E-09	1.85E-08	3.91E-05
T1DM	leukocyte	*KANSL1*	-0.690661849	1.44E-09	8.14E-09	5.65E-06
T1DM	leukocyte	*HOXB3*	-0.881963993	1.35E-08	2.41E-08	5.30E-05
T1DM	leukocyte	*RCL1*	-1.098769448	3.63E-09	9.16E-09	1.43E-05
T1DM	leukocyte	*CD274*	-1.82906526	5.08E-09	1.11E-08	1.99E-05
T1DM	leukocyte	*PLEKHG1*	-0.650865924	6.88E-09	1.38E-08	2.70E-05
T1DM	leukocyte	*PCDHB10*	-1.092562999	4.71E-08	7.41E-08	0.000185165
T1DM	leukocyte	*MRPS14*	-0.975433928	5.61E-08	8.71E-08	0.000220314
T1DM	leukocyte	*GORAB*	-0.686051943	1.40E-09	8.14E-09	5.48E-06
T1DM	leukocyte	*SNX19*	-0.623381116	6.29E-10	8.14E-09	2.47E-06
T1DM	leukocyte	*KCNJ5*	0.241209169	1.41E-09	8.14E-09	5.53E-06
T1DM	leukocyte	*SLC10A7*	-1.180855238	9.58E-09	1.80E-08	3.77E-05
T1DM	leukocyte	*MASTL*	-0.502945982	5.49E-06	6.94E-06	0.021560807

T1DM	leukocyte	*MRC1*	0.218737631	7.11E-09	1.42E-08	2.79E-05
T1DM	leukocyte	*IQSEC3*	-0.726006765	1.43E-09	8.14E-09	5.61E-06
T1DM	leukocyte	*MTRF1*	-1.172339474	6.83E-06	8.59E-06	0.026849332
T1DM	leukocyte	*DNAJC15*	-0.636859472	3.05E-09	8.59E-09	1.20E-05
T1DM	leukocyte	*PROSER1*	-0.859945719	1.87E-09	8.14E-09	7.35E-06
T1DM	leukocyte	*WBP4*	-0.648510791	1.92E-09	8.14E-09	7.56E-06
T1DM	leukocyte	*KBTBD7*	-1.292545291	2.62E-09	8.21E-09	1.03E-05
T1DM	leukocyte	*FAM53C*	-1.160109504	4.55E-08	7.18E-08	0.00017867
T1DM	leukocyte	*SIL1*	-0.628103203	5.45E-09	1.16E-08	2.14E-05
T1DM	leukocyte	*PLS1*	-0.564630345	1.13E-09	8.14E-09	4.43E-06
T1DM	leukocyte	*ZFP30*	-0.865036274	5.97E-09	1.24E-08	2.35E-05
T1DM	leukocyte	*UTP20*	-1.211081444	8.75E-08	1.31E-07	0.000343923
T1DM	leukocyte	*TMPO*	-0.844396453	9.80E-08	1.45E-07	0.000384857
T1DM	leukocyte	*APAF1*	-0.746082671	0.045459882	0.049177279	1
T1DM	leukocyte	*PPP3CC*	-0.793892345	2.81E-09	8.40E-09	1.10E-05
T1DM	leukocyte	*EPHX2*	-0.771355943	2.15E-09	8.19E-09	8.44E-06
T1DM	leukocyte	*CRISPLD1*	-3.599097146	1.22E-08	2.20E-08	4.78E-05
T1DM	leukocyte	*TEX14*	-1.234900324	7.00E-05	8.43E-05	0.275141804
T1DM	leukocyte	*TRIM6*	0.870049843	1.41E-07	2.04E-07	0.000552916
T1DM	leukocyte	*PYROXD1*	-0.553064941	1.12E-09	8.14E-09	4.39E-06
T1DM	leukocyte	*IAPP*	-0.233266936	2.91E-09	8.49E-09	1.14E-05
T1DM	leukocyte	*ZSCAN18*	-0.537009282	1.33E-08	2.39E-08	5.23E-05
T1DM	leukocyte	*ZNF211*	-0.428465581	0.015521656	0.017111276	1
T1DM	leukocyte	*LHX4*	-1.049519594	0.001597407	0.001822361	1
T1DM	leukocyte	*TRMT1L*	-0.688685685	3.48E-09	9.00E-09	1.37E-05
T1DM	leukocyte	*CAT*	-1.223846817	6.21E-08	9.56E-08	0.000243869
T1DM	leukocyte	*PILRB*	-0.358942167	0.000602483	0.000698276	1
T1DM	leukocyte	*CPXM2*	-0.606065473	0.032091602	0.034927397	1
T1DM	leukocyte	*CSMD2*	0.944822679	4.81E-09	1.07E-08	1.89E-05
T1DM	leukocyte	*ACVR2A*	-0.62011291	1.70E-09	8.14E-09	6.70E-06
T1DM	leukocyte	*POLK*	-0.877763258	2.42E-09	8.21E-09	9.49E-06
T1DM	leukocyte	*FLT3*	-0.631904514	0.033253122	0.036161504	1
T1DM	leukocyte	*UBL3*	-0.563160827	2.30E-09	8.21E-09	9.04E-06
T1DM	leukocyte	*FMOD*	-0.774870418	2.25E-06	2.92E-06	0.008859288
T1DM	leukocyte	*LDB3*	-0.936194747	2.01E-07	2.86E-07	0.000788444
T1DM	leukocyte	*NAA60*	-0.837210901	1.68E-09	8.14E-09	6.59E-06
T1DM	leukocyte	*TRMT13*	-1.428911071	3.85E-09	9.49E-09	1.51E-05
T1DM	leukocyte	*LRRC39*	-0.572150568	0.000138203	0.000164247	0.542999299
T1DM	leukocyte	*RWDD3*	-0.66649946	1.15E-09	8.14E-09	4.53E-06
T1DM	leukocyte	*CCDC18*	-0.681701103	4.25E-09	9.99E-09	1.67E-05
T1DM	leukocyte	*NBPF14*	-1.871906215	1.72E-09	8.14E-09	6.78E-06
T1DM	leukocyte	*PMS2*	-1.159530754	1.68E-07	2.41E-07	0.00066009
T1DM	leukocyte	*EEPD1*	0.411612184	1.35E-08	2.41E-08	5.29E-05
T1DM	leukocyte	*KLHL7*	-0.920524022	1.99E-09	8.14E-09	7.82E-06
T1DM	leukocyte	*CBX3*	-0.678392654	2.91E-09	8.49E-09	1.14E-05
T1DM	leukocyte	*NPY*	-0.316919136	1.89E-09	8.14E-09	7.43E-06
T1DM	leukocyte	*INHBA*	-0.594577152	1.63E-09	8.14E-09	6.39E-06
T1DM	leukocyte	*CCZ1*	-0.898349552	1.19E-08	2.17E-08	4.69E-05
T1DM	leukocyte	*POLM*	-0.541593436	7.41E-07	9.95E-07	0.002911234
T1DM	leukocyte	*SMU1*	-0.733805027	1.47E-09	8.14E-09	5.76E-06
T1DM	leukocyte	*SLC25A51*	-0.775629041	1.56E-08	2.74E-08	6.13E-05
T1DM	leukocyte	*ACO1*	-0.649105572	1.10E-09	8.14E-09	4.32E-06
T1DM	leukocyte	*PLAU*	-0.988290197	1.29E-09	8.14E-09	5.08E-06
T1DM	leukocyte	*CHST3*	0.73815687	1.13E-05	1.41E-05	0.044564189
T1DM	leukocyte	*RBM19*	-0.605377558	1.56E-09	8.14E-09	6.13E-06
T1DM	leukocyte	*BHLHE41*	-0.975437198	1.94E-07	2.76E-07	0.000761133
T1DM	leukocyte	*ITPR2*	-1.566899116	5.43E-08	8.45E-08	0.000213342
T1DM	leukocyte	*EBPL*	-0.576674473	2.53E-09	8.21E-09	9.95E-06
T1DM	leukocyte	*ATP7B*	-0.535614334	0.006235313	0.006977655	1
T1DM	leukocyte	*TSFM*	-0.829938819	8.00E-09	1.56E-08	3.14E-05
T1DM	leukocyte	*CDK2*	-1.073078812	1.51E-06	1.98E-06	0.005937154
T1DM	leukocyte	*IKZF4*	-0.918223597	2.20E-08	3.69E-08	8.63E-05
T1DM	leukocyte	*SARDH*	0.995668371	0.001494802	0.00170729	1
T1DM	leukocyte	*USP45*	-0.56935339	2.19E-09	8.21E-09	8.60E-06

T1DM	leukocyte	*SLC36A1*	0.392456995	0.033236058	0.036152955	1
T1DM	leukocyte	*C4BPA*	-0.977680196	2.14E-06	2.77E-06	0.008400061
T1DM	leukocyte	*C4BPB*	-1.593092677	2.58E-07	3.63E-07	0.001014913
T1DM	leukocyte	*RAB38*	-1.892221234	2.75E-08	4.54E-08	0.000108242
T1DM	leukocyte	*CKS2*	-0.613370138	4.97E-09	1.10E-08	1.95E-05
T1DM	leukocyte	*SNX21*	-0.754438163	4.07E-10	8.14E-09	1.60E-06
T1DM	leukocyte	*WFDC3*	-0.441408184	0.040216903	0.043601604	1
T1DM	leukocyte	*ARHGAP40*	-36.03515973	7.47E-10	8.14E-09	2.93E-06
T1DM	leukocyte	*NCOA3*	-0.683429649	4.98E-09	1.10E-08	1.96E-05
T1DM	leukocyte	*MATN4*	-3.944183973	5.52E-07	7.49E-07	0.00216865
T1DM	leukocyte	*VAPB*	-0.563986534	6.36E-09	1.30E-08	2.50E-05
T1DM	leukocyte	*PARD6B*	-0.847223667	1.49E-09	8.14E-09	5.87E-06
T1DM	leukocyte	*ARFGEF2*	-0.799855582	3.56E-09	9.10E-09	1.40E-05
T1DM	leukocyte	*ZNFX1*	-1.520572174	1.18E-05	1.46E-05	0.04627059
T1DM	leukocyte	*CSE1L*	-0.773301893	2.36E-09	8.21E-09	9.28E-06
T1DM	leukocyte	*BCAS4*	-0.510033085	1.04E-09	8.14E-09	4.09E-06
T1DM	leukocyte	*KCNK15*	-0.640028276	8.94E-09	1.70E-08	3.51E-05
T1DM	leukocyte	*PEPD*	-0.963375637	4.73E-09	1.06E-08	1.86E-05
T1DM	leukocyte	*PAIP2B*	-0.454848653	2.09E-07	2.98E-07	0.000822436
T1DM	leukocyte	*SNRNP27*	-0.621392376	7.03E-09	1.40E-08	2.76E-05
T1DM	leukocyte	*USP22*	-0.743128366	6.94E-09	1.39E-08	2.73E-05
T1DM	leukocyte	*CRISP2*	-20.01033639	0.003956941	0.004464911	1
T1DM	leukocyte	*BTN2A2*	-1.239075816	5.95E-10	8.14E-09	2.34E-06
T1DM	leukocyte	*SIRT5*	0.959444492	9.48E-08	1.41E-07	0.00037261
T1DM	leukocyte	*MRS2*	-1.043799258	3.66E-09	9.22E-09	1.44E-05
T1DM	leukocyte	*SNRPC*	-0.678800175	2.68E-09	8.26E-09	1.05E-05
T1DM	leukocyte	*SERPINB6*	-0.595073248	2.77E-09	8.34E-09	1.09E-05
T1DM	leukocyte	*ABCC10*	-0.876237264	8.58E-10	8.14E-09	3.37E-06
T1DM	leukocyte	*PEX6*	-0.607630705	8.63E-08	1.30E-07	0.000339194
T1DM	leukocyte	*NQO2*	-1.216838396	6.59E-07	8.87E-07	0.002590568
T1DM	leukocyte	*UNC5CL*	-0.626507158	2.93E-09	8.50E-09	1.15E-05
T1DM	leukocyte	*ZNF391*	-0.365307379	0.000652851	0.00075576	1
T1DM	leukocyte	*MOCS1*	0.757293033	7.35E-06	9.23E-06	0.028888596
T1DM	leukocyte	*MED20*	-0.962504305	2.70E-08	4.46E-08	0.000106226
T1DM	leukocyte	*TREM1*	0.728997569	7.92E-08	1.19E-07	0.000311064
T1DM	leukocyte	*GLO1*	-0.675611719	4.00E-09	9.65E-09	1.57E-05
T1DM	leukocyte	*KCNK17*	-0.52794038	3.49E-09	9.01E-09	1.37E-05
T1DM	leukocyte	*ATXN1*	-0.836973672	5.25E-09	1.14E-08	2.06E-05
T1DM	leukocyte	*LRRFIP1*	-0.534386455	1.86E-09	8.14E-09	7.30E-06
T1DM	leukocyte	*CXCL6*	-1.022669494	8.91E-08	1.33E-07	0.000350053
T1DM	leukocyte	*SH3TC1*	-0.720502428	3.33E-09	8.82E-09	1.31E-05
T1DM	leukocyte	*MT1G*	-0.278996329	0.002210192	0.002512686	1
T1DM	leukocyte	*DOK4*	-0.506826053	1.08E-09	8.14E-09	4.25E-06
T1DM	leukocyte	*CLYBL*	-1.490151154	2.36E-08	3.95E-08	9.29E-05
T1DM	leukocyte	*ABCC4*	0.865691151	1.86E-05	2.30E-05	0.073165438
T1DM	leukocyte	*TM9SF2*	-0.583897192	2.07E-09	8.14E-09	8.13E-06
T1DM	leukocyte	*KIF25*	-9.472310768	1.02E-09	8.14E-09	4.02E-06
T1DM	leukocyte	*BMP4*	-1.201930839	7.46E-07	1.00E-06	0.002932216
T1DM	leukocyte	*GRK4*	-0.785974194	1.43E-07	2.07E-07	0.000562377
T1DM	leukocyte	*HS3ST3B1*	-1.600486044	1.36E-09	8.14E-09	5.35E-06
T1DM	leukocyte	*MRPS7*	-0.552440517	2.34E-09	8.21E-09	9.18E-06
T1DM	leukocyte	*GGA3*	-1.29090899	2.11E-07	3.01E-07	0.000830937
T1DM	leukocyte	*DDX31*	-0.820940122	3.28E-05	4.02E-05	0.129040062
T1DM	leukocyte	*OPRL1*	0.945424035	6.29E-05	7.60E-05	0.24705324
T1DM	leukocyte	*PPDPF*	-0.48723985	2.46E-09	8.21E-09	9.67E-06
T1DM	leukocyte	*CHCHD5*	-0.588821843	2.53E-09	8.21E-09	9.95E-06
T1DM	leukocyte	*PAX8*	0.298013581	0.037124884	0.040316106	1
T1DM	leukocyte	*INSIG2*	-0.58107561	1.12E-09	8.14E-09	4.40E-06
T1DM	leukocyte	*POLR1B*	-0.556895539	1.65E-05	2.04E-05	0.064913147
T1DM	leukocyte	*PSD4*	-0.865587317	2.41E-08	4.02E-08	9.47E-05
T1DM	leukocyte	*CLPP*	-0.63452978	5.56E-09	1.18E-08	2.19E-05
T1DM	leukocyte	*SH2D3A*	-0.268998303	0.005664612	0.00634443	1
T1DM	leukocyte	*GPR108*	-0.888032686	9.75E-09	1.82E-08	3.83E-05
T1DM	leukocyte	*TNFSF14*	-0.957262966	0.012164034	0.013462674	1

T1DM	leukocyte	*FOSB*	-0.452104963	0.008536846	0.009504468	1
T1DM	leukocyte	*SYMPK*	-0.955517809	6.89E-06	8.66E-06	0.027069433
T1DM	leukocyte	*NAPB*	-0.586862085	1.89E-08	3.24E-08	7.44E-05
T1DM	leukocyte	*PSMF1*	-0.68894771	1.58E-08	2.76E-08	6.20E-05
T1DM	leukocyte	*RBCK1*	-0.586531581	1.93E-08	3.28E-08	7.57E-05
T1DM	leukocyte	*FLRT3*	-0.589869775	1.31E-09	8.14E-09	5.15E-06
T1DM	leukocyte	*MGME1*	-2.284597473	1.28E-07	1.86E-07	0.000502853
T1DM	leukocyte	*ITPA*	-0.873275505	1.93E-09	8.14E-09	7.58E-06
T1DM	leukocyte	*BANF2*	-9.46542881	1.44E-09	8.14E-09	5.64E-06
T1DM	leukocyte	*MRPS26*	-0.57415212	9.42E-10	8.14E-09	3.70E-06
T1DM	leukocyte	*ZNF436*	-1.613626435	4.42E-10	8.14E-09	1.74E-06
T1DM	leukocyte	*TUBGCP3*	-0.830250803	8.44E-10	8.14E-09	3.32E-06
T1DM	leukocyte	*MCF2L*	-0.50779849	2.82E-09	8.42E-09	1.11E-05
T1DM	leukocyte	*PROZ*	-4.812132715	0.001470191	0.001681125	1
T1DM	leukocyte	*IGFLR1*	0.258494843	5.67E-09	1.19E-08	2.23E-05
T1DM	leukocyte	*NR1D1*	-0.576062672	1.70E-09	8.14E-09	6.66E-06
T1DM	leukocyte	*PRMT1*	-0.55705311	7.32E-10	8.14E-09	2.88E-06
T1DM	leukocyte	*PRR12*	-0.696473648	3.71E-09	9.29E-09	1.46E-05
T1DM	leukocyte	*FLRT1*	-1.104946955	0.000411026	0.000478637	1
T1DM	leukocyte	*ASL*	-0.710549021	2.11E-09	8.16E-09	8.28E-06
T1DM	leukocyte	*WNK4*	-0.763297164	5.44E-07	7.40E-07	0.002139052
T1DM	leukocyte	*BECN1*	-0.690025215	4.67E-09	1.06E-08	1.83E-05
T1DM	leukocyte	*PRKCG*	1.986046823	1.28E-07	1.86E-07	0.000501412
T1DM	leukocyte	*TRAP1*	-0.503489151	1.25E-07	1.82E-07	0.000490411
T1DM	leukocyte	*ZNF384*	-0.846942468	1.61E-09	8.14E-09	6.33E-06
T1DM	leukocyte	*RHOJ*	-1.161755122	1.65E-09	8.14E-09	6.46E-06
T1DM	leukocyte	*L3HYPDH*	-0.7116166	6.59E-07	8.87E-07	0.002589708
T1DM	leukocyte	*HSPA2*	-1.091703721	4.68E-09	1.06E-08	1.84E-05
T1DM	leukocyte	*ZBTB1*	-0.881659255	2.35E-09	8.21E-09	9.21E-06
T1DM	leukocyte	*PLEKHG3*	-0.939214843	3.49E-09	9.01E-09	1.37E-05
T1DM	leukocyte	*FGD3*	2.181371269	9.93E-09	1.85E-08	3.90E-05
T1DM	leukocyte	*BCL11B*	1.328893616	3.71E-09	9.29E-09	1.46E-05
T1DM	leukocyte	*ABHD8*	-1.168441893	4.25E-06	5.40E-06	0.016708109
T1DM	leukocyte	*BEST3*	-1.489169563	5.11E-07	6.98E-07	0.002009576
T1DM	leukocyte	*YEATS4*	-0.582904519	2.58E-08	4.27E-08	0.000101363
T1DM	leukocyte	*TAS2R5*	-2.414345568	0.002622687	0.002973893	1
T1DM	leukocyte	*LRRC61*	-0.982167943	4.47E-09	1.03E-08	1.76E-05
T1DM	leukocyte	*IDUA*	-0.698805534	1.07E-09	8.14E-09	4.20E-06
T1DM	leukocyte	*FGFRL1*	-0.587530208	1.08E-07	1.59E-07	0.000423097
T1DM	leukocyte	*EMC1*	-0.766418705	2.95E-09	8.50E-09	1.16E-05
T1DM	leukocyte	*UBR4*	-0.710379476	2.95E-09	8.50E-09	1.16E-05
T1DM	leukocyte	*SYNGR3*	-0.832959109	2.21E-09	8.21E-09	8.69E-06
T1DM	leukocyte	*WFIKKN1*	-0.718637824	4.54E-09	1.04E-08	1.78E-05
T1DM	leukocyte	*WDR24*	1.588911896	1.17E-05	1.45E-05	0.045968708
T1DM	leukocyte	*KDM4B*	-1.344575974	4.64E-08	7.29E-08	0.00018219
T1DM	leukocyte	*METTL25*	1.557754185	0.004662759	0.005246272	1
T1DM	leukocyte	*PNKD*	-0.835059953	1.14E-09	8.14E-09	4.47E-06
T1DM	leukocyte	*PTPN12*	-0.647194345	7.56E-09	1.49E-08	2.97E-05
T1DM	leukocyte	*POR*	-0.702774868	3.34E-09	8.82E-09	1.31E-05
T1DM	leukocyte	*STYXL1*	-0.827980516	0.013308711	0.014712978	1
T1DM	leukocyte	*RBM48*	-1.18598941	4.79E-09	1.07E-08	1.88E-05
T1DM	leukocyte	*SRD5A3*	-0.788069235	4.65E-08	7.32E-08	0.00018281
T1DM	leukocyte	*PPAT*	-0.760198749	5.72E-09	1.20E-08	2.25E-05
T1DM	leukocyte	*A4GALT*	-0.585666718	1.50E-08	2.65E-08	5.91E-05
T1DM	leukocyte	*GALR3*	-1.900307548	0.033837636	0.03678696	1
T1DM	leukocyte	*NAA38*	-0.545271979	2.00E-09	8.14E-09	7.85E-06
T1DM	leukocyte	*PRKRIP1*	-0.65325984	1.70E-09	8.14E-09	6.69E-06
T1DM	leukocyte	*VGF*	-0.347271125	1.87E-09	8.14E-09	7.35E-06
T1DM	leukocyte	*IRF5*	0.316567353	5.23E-09	1.13E-08	2.06E-05
T1DM	leukocyte	*MTX2*	-0.898231771	4.65E-09	1.05E-08	1.83E-05
T1DM	leukocyte	*GAD1*	-2.398425376	3.47E-09	8.99E-09	1.36E-05
T1DM	leukocyte	*ORMDL1*	-0.593671414	2.45E-08	4.07E-08	9.61E-05
T1DM	leukocyte	*PSMG2*	-0.722383735	4.59E-09	1.04E-08	1.80E-05
T1DM	leukocyte	*TWSG1*	-0.966959666	2.98E-09	8.50E-09	1.17E-05

T1DM	leukocyte	*ARHGAP22*	0.646698674	0.000988235	0.001138644	1
T1DM	leukocyte	*EIF2AK4*	-0.881546187	1.36E-08	2.43E-08	5.36E-05
T1DM	leukocyte	*MYO5C*	-0.485255904	2.00E-09	8.14E-09	7.84E-06
T1DM	leukocyte	*CGNL1*	-0.751293582	2.28E-09	8.21E-09	8.97E-06
T1DM	leukocyte	*TMOD2*	-0.754508717	1.68E-09	8.14E-09	6.62E-06
T1DM	leukocyte	*ELL3*	-1.246784691	0.00020467	0.000241486	0.804148848
T1DM	leukocyte	*IVD*	-0.706004592	7.57E-06	9.49E-06	0.029729596
T1DM	leukocyte	*KNSTRN*	-0.356348705	0.00051965	0.000603163	1
T1DM	leukocyte	*CHAC1*	-0.762053101	8.60E-09	1.64E-08	3.38E-05
T1DM	leukocyte	*ACKR4*	-1.236081227	1.10E-07	1.62E-07	0.000432416
T1DM	leukocyte	*ANAPC13*	-0.448931838	7.56E-09	1.49E-08	2.97E-05
T1DM	leukocyte	*SUMF2*	-0.721987837	8.54E-09	1.63E-08	3.35E-05
T1DM	leukocyte	*TPH1*	-1.558964233	0.000107885	0.000128799	0.423878899
T1DM	leukocyte	*DCTD*	-0.780731023	3.40E-09	8.90E-09	1.34E-05
T1DM	leukocyte	*PLD2*	1.259177385	0.001003228	0.00115558	1
T1DM	leukocyte	*ATP1B2*	-0.852134324	0.035722058	0.038803419	1
T1DM	leukocyte	*KRI1*	-0.576279489	2.49E-09	8.21E-09	9.79E-06
T1DM	leukocyte	*AP1M2*	-0.598086078	2.99E-09	8.52E-09	1.18E-05
T1DM	leukocyte	*CDKN2D*	-0.580398741	1.75E-08	3.02E-08	6.89E-05
T1DM	leukocyte	*MTUS1*	-0.557948027	2.69E-09	8.26E-09	1.06E-05
T1DM	leukocyte	*ADCY4*	-0.735275681	5.22E-09	1.13E-08	2.05E-05
T1DM	leukocyte	*DTD2*	-0.594260782	3.17E-09	8.69E-09	1.24E-05
T1DM	leukocyte	*HEATR5A*	-1.225300799	1.98E-08	3.36E-08	7.78E-05
T1DM	leukocyte	*MIS18BP1*	-0.904902665	4.11E-05	5.01E-05	0.161526263
T1DM	leukocyte	*RNASE1*	0.12947153	5.60E-09	1.18E-08	2.20E-05
T1DM	leukocyte	*TEP1*	1.046267808	4.42E-05	5.39E-05	0.173815789
T1DM	leukocyte	*EPB41L4A*	-0.798150283	1.37E-09	8.14E-09	5.37E-06
T1DM	leukocyte	*REEP5*	-0.540459313	3.19E-09	8.72E-09	1.26E-05
T1DM	leukocyte	*MAU2*	-1.373793109	3.33E-09	8.82E-09	1.31E-05
T1DM	leukocyte	*SHC2*	-0.741959573	1.94E-09	8.14E-09	7.61E-06
T1DM	leukocyte	*SYT5*	-0.391796189	5.59E-07	7.59E-07	0.002197627
T1DM	leukocyte	*CBFA2T3*	-0.678338071	1.40E-09	8.14E-09	5.52E-06
T1DM	leukocyte	*ERMARD*	-1.202562165	2.10E-08	3.54E-08	8.25E-05
T1DM	leukocyte	*PHF10*	-0.688211532	1.19E-09	8.14E-09	4.69E-06
T1DM	leukocyte	*SH3BP4*	-0.420374959	2.85E-09	8.43E-09	1.12E-05
T1DM	leukocyte	*DOCK6*	-0.528306097	3.34E-09	8.82E-09	1.31E-05
T1DM	leukocyte	*ECSIT*	-0.749429189	8.78E-09	1.67E-08	3.45E-05
T1DM	leukocyte	*CDC16*	-0.88038442	3.10E-09	8.64E-09	1.22E-05
T1DM	leukocyte	*DPP6*	-0.374856309	1.45E-09	8.14E-09	5.68E-06
T1DM	leukocyte	*GTPBP3*	-0.70297643	1.98E-09	8.14E-09	7.77E-06
T1DM	leukocyte	*PLVAP*	-0.348130185	2.95E-09	8.50E-09	1.16E-05
T1DM	leukocyte	*MRPL34*	-0.467895598	2.99E-07	4.16E-07	0.001174044
T1DM	leukocyte	*LSM7*	-0.459881047	1.62E-08	2.83E-08	6.38E-05
T1DM	leukocyte	*TULP4*	-0.668378228	4.68E-09	1.06E-08	1.84E-05
T1DM	leukocyte	*QRSL1*	-0.788137119	4.24E-10	8.14E-09	1.67E-06
T1DM	leukocyte	*RSPH3*	-0.741801306	1.04E-08	1.92E-08	4.08E-05
T1DM	leukocyte	*STK33*	-0.576590636	1.15E-09	8.14E-09	4.51E-06
T1DM	leukocyte	*NDUFA10*	-0.653116369	1.32E-09	8.14E-09	5.19E-06
T1DM	leukocyte	*EPO*	-0.818115128	0.013822302	0.015263582	1
T1DM	leukocyte	*SCO2*	0.764377927	3.98E-08	6.34E-08	0.000156452
T1DM	leukocyte	*TRPM4*	-1.886318447	7.99E-05	9.59E-05	0.313985478
T1DM	leukocyte	*UBAC1*	-0.650252905	2.06E-09	8.14E-09	8.10E-06
T1DM	leukocyte	*ATXN10*	-0.56341057	1.44E-09	8.14E-09	5.67E-06
T1DM	leukocyte	*PNPLA7*	-1.355531389	1.35E-09	8.14E-09	5.32E-06
T1DM	leukocyte	*PAK4*	-0.84356921	1.46E-09	8.14E-09	5.72E-06
T1DM	leukocyte	*CEP85*	0.890197927	4.13E-06	5.26E-06	0.016244863
T1DM	leukocyte	*LAMA5*	-0.464097611	5.29E-09	1.14E-08	2.08E-05
T1DM	leukocyte	*ADRM1*	-0.61982949	2.49E-09	8.21E-09	9.80E-06
T1DM	leukocyte	*UCK1*	-1.067398991	1.21E-08	2.20E-08	4.77E-05
T1DM	leukocyte	*MAP3K10*	-1.324589608	9.50E-08	1.41E-07	0.0003734
T1DM	leukocyte	*SMPDL3B*	-0.583940003	3.77E-09	9.36E-09	1.48E-05
T1DM	leukocyte	*CLIP1*	-0.723237593	1.52E-08	2.67E-08	5.97E-05
T1DM	leukocyte	*ZNF317*	-0.82601278	4.08E-08	6.49E-08	0.000160437
T1DM	leukocyte	*DNMT1*	-1.051454029	2.33E-09	8.21E-09	9.14E-06

T1DM	leukocyte	*ZNF426*	-0.925874773	2.19E-09	8.21E-09	8.60E-06
T1DM	leukocyte	*LRP3*	-1.087407927	9.23E-10	8.14E-09	3.63E-06
T1DM	leukocyte	*PKDREJ*	0.751750568	0.000295487	0.000346145	1
T1DM	leukocyte	*HSD17B3*	-8.666678651	8.91E-10	8.14E-09	3.50E-06
T1DM	leukocyte	*FBP2*	-1.563339218	0.000102808	0.000122888	0.403931948
T1DM	leukocyte	*SYNE1*	-0.596208988	1.52E-09	8.14E-09	5.96E-06
T1DM	leukocyte	*EPS8L1*	-1.374327981	0.000124253	0.000147892	0.488190807
T1DM	leukocyte	*C1QL1*	-0.3042386	7.83E-09	1.53E-08	3.08E-05
T1DM	leukocyte	*F12*	-0.773438658	2.03E-08	3.43E-08	7.96E-05
T1DM	leukocyte	*TRAF3*	-0.554690211	8.04E-09	1.56E-08	3.16E-05
T1DM	leukocyte	*GALNT15*	-0.84980347	0.000129987	0.000154623	0.510718435
T1DM	leukocyte	*NAPSA*	-1.592612199	6.69E-05	8.07E-05	0.262689916
T1DM	leukocyte	*PDLIM4*	-0.737333307	2.53E-09	8.21E-09	9.94E-06
T1DM	leukocyte	*DIAPH1*	-0.516301896	3.24E-09	8.77E-09	1.27E-05
T1DM	leukocyte	*EXOC4*	-1.185268711	9.15E-08	1.37E-07	0.000359435
T1DM	leukocyte	*C1orf159*	-0.954029597	0.025348556	0.027711318	1
T1DM	leukocyte	*ANO1*	-1.697120807	8.43E-10	8.14E-09	3.31E-06
T1DM	leukocyte	*PPFIA1*	-0.603381932	1.78E-09	8.14E-09	6.98E-06
T1DM	leukocyte	*TRAF7*	-0.883187499	1.52E-09	8.14E-09	5.96E-06
T1DM	leukocyte	*MAP1B*	-0.426821125	3.75E-09	9.33E-09	1.47E-05
T1DM	leukocyte	*CHD1L*	-1.37720164	8.05E-08	1.21E-07	0.000316378
T1DM	leukocyte	*PIAS3*	-0.512067195	2.21E-08	3.72E-08	8.70E-05
T1DM	leukocyte	*RBM8A*	-0.459532185	3.27E-09	8.79E-09	1.28E-05
T1DM	leukocyte	*ZNF304*	-2.316041831	3.65E-07	5.04E-07	0.001433609
T1DM	leukocyte	*ZNF132*	0.600559194	0.000397339	0.000462972	1
T1DM	leukocyte	*RHPN2*	-0.709318416	1.22E-10	8.14E-09	4.79E-07
T1DM	leukocyte	*GCH1*	-0.497618745	1.15E-09	8.14E-09	4.51E-06
T1DM	leukocyte	*CC2D1A*	-0.894923933	5.73E-09	1.20E-08	2.25E-05
T1DM	leukocyte	*CCT6B*	-1.065896884	1.64E-09	8.14E-09	6.46E-06
T1DM	leukocyte	*PPARG*	-1.086628061	4.16E-09	9.92E-09	1.63E-05
T1DM	leukocyte	*ENOSF1*	-1.338259605	3.78E-07	5.22E-07	0.001485939
T1DM	leukocyte	*TRIM22*	0.850383894	3.08E-06	3.95E-06	0.012095119
T1DM	leukocyte	*IMMT*	-0.719143521	1.51E-09	8.14E-09	5.93E-06
T1DM	leukocyte	*MRPL35*	-0.721634995	4.13E-09	9.87E-09	1.62E-05
T1DM	leukocyte	*SCLY*	-1.038469436	1.60E-07	2.30E-07	0.000628831
T1DM	leukocyte	*CARD6*	-1.259340986	1.43E-08	2.53E-08	5.61E-05
T1DM	leukocyte	*INPP5K*	-0.741845561	4.42E-09	1.02E-08	1.73E-05
T1DM	leukocyte	*RPA1*	-0.811029053	5.45E-09	1.16E-08	2.14E-05
T1DM	leukocyte	*TMEM128*	-0.969506499	3.94E-09	9.57E-09	1.55E-05
T1DM	leukocyte	*SEC61G*	-0.507926723	9.36E-10	8.14E-09	3.68E-06
T1DM	leukocyte	*FIGNL1*	-1.938001846	8.28E-08	1.25E-07	0.000325453
T1DM	leukocyte	*ITGB4*	-1.338945162	1.10E-07	1.62E-07	0.000432443
T1DM	leukocyte	*TRIM47*	-0.67761824	1.44E-09	8.14E-09	5.67E-06
T1DM	leukocyte	*EIF5A*	-0.582905374	3.51E-10	8.14E-09	1.38E-06
T1DM	leukocyte	*PRMT7*	-0.934696497	0.000368915	0.000430748	1
T1DM	leukocyte	*ANKEF1*	-0.60144983	1.38E-09	8.14E-09	5.43E-06
T1DM	leukocyte	*BTBD3*	-0.56113884	1.71E-09	8.14E-09	6.72E-06
T1DM	leukocyte	*IGHMBP2*	-0.395069438	0.004652627	0.005237872	1
T1DM	leukocyte	*LPIN3*	-0.697575246	7.93E-05	9.53E-05	0.311745004
T1DM	leukocyte	*RBM38*	-1.065062713	3.48E-10	8.14E-09	1.37E-06
T1DM	leukocyte	*VSTM2L*	-0.57955753	2.77E-09	8.34E-09	1.09E-05
T1DM	leukocyte	*SERINC3*	-0.621242634	1.08E-08	1.99E-08	4.26E-05
T1DM	leukocyte	*BHMT2*	-0.787207509	4.88E-09	1.08E-08	1.92E-05
T1DM	leukocyte	*WASF3*	-0.708286586	4.02E-09	9.69E-09	1.58E-05
T1DM	leukocyte	*SCO1*	-0.988857515	2.82E-07	3.95E-07	0.001108414
T1DM	leukocyte	*CHI3L1*	-0.608038592	3.74E-08	5.99E-08	0.000146891
T1DM	leukocyte	*SLC41A1*	-0.7769999	1.68E-09	8.14E-09	6.61E-06
T1DM	leukocyte	*TRPC4*	-2.095153645	2.52E-10	8.14E-09	9.90E-07
T1DM	leukocyte	*POSTN*	0.719376509	4.84E-09	1.08E-08	1.90E-05
T1DM	leukocyte	*RFC3*	-0.584760397	1.27E-09	8.14E-09	5.00E-06
T1DM	leukocyte	*EPHB2*	-1.187576366	0.00708222	0.007911869	1
T1DM	leukocyte	*BTBD2*	-1.390142408	1.49E-08	2.63E-08	5.85E-05
T1DM	leukocyte	*CNDP2*	-0.684341274	1.82E-09	8.14E-09	7.15E-06
T1DM	leukocyte	*WDR74*	-0.89342377	1.02E-07	1.50E-07	0.000398973

T1DM	leukocyte	*GSTT2B*	-0.595880347	3.60E-08	5.80E-08	0.000141414
T1DM	leukocyte	*SLC2A11*	-1.045601703	2.64E-09	8.21E-09	1.04E-05
T1DM	leukocyte	*C1QTNF6*	-1.166761132	6.17E-10	8.14E-09	2.43E-06
T1DM	leukocyte	*FAM83F*	-0.737084642	1.67E-09	8.14E-09	6.56E-06
T1DM	leukocyte	*GIMAP6*	-1.341337865	3.24E-10	8.14E-09	1.27E-06
T1DM	leukocyte	*AGAP3*	-1.379805846	4.86E-05	5.90E-05	0.190992013
T1DM	leukocyte	*SFTPD*	-0.590381162	1.40E-06	1.84E-06	0.005487494
T1DM	leukocyte	*IMPA1*	-0.602292807	1.52E-08	2.67E-08	5.96E-05
T1DM	leukocyte	*LRRCC1*	-1.061561412	2.11E-09	8.16E-09	8.27E-06
T1DM	leukocyte	*RRAS2*	-0.562342739	9.46E-09	1.78E-08	3.72E-05
T1DM	leukocyte	*HSD17B4*	-1.101084264	5.56E-09	1.18E-08	2.19E-05
T1DM	leukocyte	*RNF122*	-0.979154978	5.86E-05	7.09E-05	0.230244976
T1DM	leukocyte	*COX16*	-0.625837899	3.30E-09	8.81E-09	1.30E-05
T1DM	leukocyte	*ELP3*	-1.315959763	4.41E-09	1.02E-08	1.73E-05
T1DM	leukocyte	*PEBP4*	-0.954607686	3.59E-09	9.11E-09	1.41E-05
T1DM	leukocyte	*MRO*	-1.663946805	9.25E-08	1.38E-07	0.000363337
T1DM	leukocyte	*MRPS36*	-0.709075995	5.34E-09	1.15E-08	2.10E-05
T1DM	leukocyte	*THUMPD3*	-0.730917595	1.70E-09	8.14E-09	6.67E-06
T1DM	leukocyte	*CHL1*	-0.743198746	5.81E-08	9.00E-08	0.000228369
T1DM	leukocyte	*GSTM1*	1.308198236	4.59E-07	6.29E-07	0.0018046
T1DM	leukocyte	*REG4*	-0.785915645	1.90E-09	8.14E-09	7.48E-06
T1DM	leukocyte	*GSTM5*	-0.978704005	6.95E-05	8.37E-05	0.273003003
T1DM	leukocyte	*GSTM3*	-0.460414953	4.55E-08	7.18E-08	0.000178856
T1DM	leukocyte	*PTPN22*	-1.79196484	0.007006343	0.00782933	1
T1DM	leukocyte	*SORT1*	-0.819764923	3.28E-09	8.79E-09	1.29E-05
T1DM	leukocyte	*NOTCH2*	-0.654511773	1.84E-05	2.27E-05	0.072306569
T1DM	leukocyte	*CEPT1*	-1.087916481	1.54E-08	2.71E-08	6.07E-05
T1DM	leukocyte	*NAPG*	-0.679881247	2.07E-09	8.14E-09	8.11E-06
T1DM	leukocyte	*SPIRE1*	-0.702099558	1.27E-09	8.14E-09	4.98E-06
T1DM	leukocyte	*FKBP11*	-0.413857136	2.95E-09	8.50E-09	1.16E-05
T1DM	leukocyte	*KIDINS220*	-0.701699657	2.89E-09	8.48E-09	1.13E-05
T1DM	leukocyte	*LPIN1*	-0.497353773	4.28E-09	1.00E-08	1.68E-05
T1DM	leukocyte	*IAH1*	-0.798327191	1.85E-08	3.18E-08	7.29E-05
T1DM	leukocyte	*ANO3*	1.240171272	1.52E-09	8.14E-09	5.97E-06
T1DM	leukocyte	*IL2RA*	0.240264345	4.36E-09	1.02E-08	1.71E-05
T1DM	leukocyte	*ECHDC3*	-0.676967617	1.54E-09	8.14E-09	6.05E-06
T1DM	leukocyte	*TMEM241*	-1.226291403	9.38E-10	8.14E-09	3.69E-06
T1DM	leukocyte	*KLRC1*	37.32803231	7.20E-07	9.68E-07	0.002830109
T1DM	leukocyte	*LRP4*	-0.527966138	0.00579062	0.006481865	1
T1DM	leukocyte	*AGO4*	-1.139571516	8.66E-09	1.65E-08	3.40E-05
T1DM	leukocyte	*PRPF38A*	-0.676160749	1.75E-09	8.14E-09	6.86E-06
T1DM	leukocyte	*TPGS2*	-0.791118163	5.35E-09	1.15E-08	2.10E-05
T1DM	leukocyte	*DAGLA*	-0.795549027	5.88E-09	1.22E-08	2.31E-05
T1DM	leukocyte	*TIMM10*	-0.676008412	3.14E-09	8.69E-09	1.23E-05
T1DM	leukocyte	*FADS2*	-0.762218488	5.33E-09	1.15E-08	2.09E-05
T1DM	leukocyte	*TMEM165*	-0.561061562	1.48E-09	8.14E-09	5.80E-06
T1DM	leukocyte	*CLOCK*	-0.776030933	3.30E-09	8.81E-09	1.30E-05
T1DM	leukocyte	*GGACT*	-1.124663036	1.70E-09	8.14E-09	6.67E-06
T1DM	leukocyte	*CLDN10*	-0.301213768	3.64E-08	5.85E-08	0.000142948
T1DM	leukocyte	*CARS2*	-0.670875935	9.00E-10	8.14E-09	3.54E-06
T1DM	leukocyte	*TMED7*	-0.59423691	2.42E-09	8.21E-09	9.49E-06
T1DM	leukocyte	*NREP*	-2.319521774	7.36E-05	8.85E-05	0.28921711
T1DM	leukocyte	*WDR36*	-0.729362535	1.36E-09	8.14E-09	5.33E-06
T1DM	leukocyte	*OSTF1*	-0.927172409	6.12E-09	1.26E-08	2.40E-05
T1DM	leukocyte	*GOLM1*	-0.533467301	8.33E-10	8.14E-09	3.27E-06
T1DM	leukocyte	*ADAM19*	-1.974730924	0.001100042	0.001264131	1
T1DM	leukocyte	*SDS*	0.051401582	3.05E-09	8.59E-09	1.20E-05
T1DM	leukocyte	*MSI1*	-1.167529479	2.05E-09	8.14E-09	8.07E-06
T1DM	leukocyte	*DTX1*	-1.737276781	1.30E-09	8.14E-09	5.12E-06
T1DM	leukocyte	*PNPLA8*	-0.537012313	2.40E-09	8.21E-09	9.43E-06
T1DM	leukocyte	*TES*	-0.454744793	3.16E-09	8.69E-09	1.24E-05
T1DM	leukocyte	*MDFIC*	-0.992419129	0.000464355	0.000540098	1
T1DM	leukocyte	*MTO1*	-0.664060128	1.11E-09	8.14E-09	4.37E-06
T1DM	leukocyte	*LCA5*	-1.564853454	3.62E-08	5.83E-08	0.000142152

T1DM	leukocyte	*LMO2*	-0.604658456	2.63E-09	8.21E-09	1.03E-05
T1DM	leukocyte	*RDH5*	-0.613254535	1.52E-09	8.14E-09	5.95E-06
T1DM	leukocyte	*TFCP2*	-1.725426222	4.53E-08	7.15E-08	0.000177814
T1DM	leukocyte	*KRT7*	-0.330671506	3.59E-09	9.12E-09	1.41E-05
T1DM	leukocyte	*NHSL1*	-0.856288863	3.88E-08	6.19E-08	0.000152379
T1DM	leukocyte	*AHI1*	-0.497953911	6.42E-10	8.14E-09	2.52E-06
T1DM	leukocyte	*HEY2*	-0.995244458	2.32E-09	8.21E-09	9.12E-06
T1DM	leukocyte	*PKIB*	-0.483604902	1.66E-09	8.14E-09	6.50E-06
T1DM	leukocyte	*SMPD2*	-0.898589237	0.001184971	0.001359344	1
T1DM	leukocyte	*SEMA4F*	-0.985019473	7.64E-10	8.14E-09	3.00E-06
T1DM	leukocyte	*GNS*	-1.070127489	6.28E-08	9.66E-08	0.000246739
T1DM	leukocyte	*MPHOSPH6*	-0.618808816	1.73E-09	8.14E-09	6.79E-06
T1DM	leukocyte	*AGT*	-0.464037998	3.02E-10	8.14E-09	1.19E-06
T1DM	leukocyte	*NTPCR*	-0.853850522	1.19E-08	2.17E-08	4.69E-05
T1DM	leukocyte	*USP37*	-0.881611045	1.55E-09	8.14E-09	6.10E-06
T1DM	leukocyte	*ARMC9*	-1.24412781	0.01925393	0.021136823	1
T1DM	leukocyte	*COX5B*	-0.509389415	5.19E-09	1.13E-08	2.04E-05
T1DM	leukocyte	*REV1*	-0.630928171	2.51E-09	8.21E-09	9.88E-06
T1DM	leukocyte	*TSGA10*	-0.581545787	3.69E-06	4.70E-06	0.014480895
T1DM	leukocyte	*TGFBRAP1*	-0.779082225	1.89E-09	8.14E-09	7.45E-06
T1DM	leukocyte	*GCC2*	-0.69427473	7.05E-09	1.41E-08	2.77E-05
T1DM	leukocyte	*MRPS9*	-0.596570062	2.38E-09	8.21E-09	9.34E-06
T1DM	leukocyte	*ANKRD36*	-0.907109209	1.45E-09	8.14E-09	5.71E-06
T1DM	leukocyte	*USP44*	-0.856235299	3.67E-08	5.90E-08	0.000144312
T1DM	leukocyte	*PLXNC1*	1.281717249	3.14E-07	4.37E-07	0.001232249
T1DM	leukocyte	*DRAM1*	-1.229875603	4.21E-09	9.96E-09	1.65E-05
T1DM	leukocyte	*VILL*	1.012333141	1.76E-08	3.03E-08	6.91E-05
T1DM	leukocyte	*FLNB*	-0.550006176	1.37E-08	2.45E-08	5.39E-05
T1DM	leukocyte	*NEK3*	-0.457167293	1.12E-07	1.65E-07	0.00044129
T1DM	leukocyte	*VPS36*	-0.971138702	4.37E-09	1.02E-08	1.72E-05
T1DM	leukocyte	*RNASEH2B*	-1.12069331	5.73E-08	8.89E-08	0.000225279
T1DM	leukocyte	*CKAP2*	-0.502792408	1.50E-05	1.86E-05	0.058922129
T1DM	leukocyte	*TBC1D4*	-0.720038652	1.50E-09	8.14E-09	5.89E-06
T1DM	leukocyte	*SUCLA2*	-0.764691158	1.77E-09	8.14E-09	6.96E-06
T1DM	leukocyte	*LMO7*	-0.400166239	1.21E-09	8.14E-09	4.76E-06
T1DM	leukocyte	*SETDB2*	0.963440934	7.84E-06	9.82E-06	0.030811664
T1DM	leukocyte	*C7orf25*	-0.573715046	1.02E-08	1.90E-08	4.02E-05
T1DM	leukocyte	*RAPGEF5*	-0.495418221	1.43E-09	8.14E-09	5.63E-06
T1DM	leukocyte	*KDELR2*	-0.585463501	3.01E-09	8.53E-09	1.18E-05
T1DM	leukocyte	*TBRG4*	-1.403911539	5.74E-10	8.14E-09	2.25E-06
T1DM	leukocyte	*DBNL*	-1.449670569	2.21E-06	2.85E-06	0.008668853
T1DM	leukocyte	*ALPK3*	-0.50318591	1.52E-09	8.14E-09	5.96E-06
T1DM	leukocyte	*CIB2*	-0.481052013	0.00042863	0.000498989	1
T1DM	leukocyte	*NMT1*	-0.583557346	1.14E-09	8.14E-09	4.46E-06
T1DM	leukocyte	*CHAD*	-2.323760126	0.001143115	0.001313246	1
T1DM	leukocyte	*TEX2*	-0.496783673	2.12E-09	8.16E-09	8.33E-06
T1DM	leukocyte	*LIMD2*	0.621995849	6.71E-10	8.14E-09	2.64E-06
T1DM	leukocyte	*MRPL47*	-0.607790082	5.29E-09	1.14E-08	2.08E-05
T1DM	leukocyte	*SMPD4*	-1.343481288	6.51E-08	9.97E-08	0.000255654
T1DM	leukocyte	*CCDC115*	-0.719397729	4.49E-09	1.03E-08	1.76E-05
T1DM	leukocyte	*BIN1*	-1.148304324	5.52E-06	6.98E-06	0.021699287
T1DM	leukocyte	*HS6ST1*	-0.947777999	2.73E-09	8.29E-09	1.07E-05
T1DM	leukocyte	*GYPC*	-0.61387902	1.20E-07	1.76E-07	0.000472057
T1DM	leukocyte	*NIPSNAP3A*	-0.9120156	6.88E-09	1.38E-08	2.70E-05
T1DM	leukocyte	*CDK9*	-0.795613623	1.49E-09	8.14E-09	5.84E-06
T1DM	leukocyte	*TXN*	-0.468830454	3.14E-09	8.69E-09	1.23E-05
T1DM	leukocyte	*TOR1B*	-1.050126363	5.32E-09	1.15E-08	2.09E-05
T1DM	leukocyte	*TOR1A*	-0.638357944	1.89E-09	8.14E-09	7.44E-06
T1DM	leukocyte	*SLC2A8*	-0.731484353	1.44E-09	8.14E-09	5.66E-06
T1DM	leukocyte	*ZFP37*	-0.643855079	1.82E-09	8.14E-09	7.14E-06
T1DM	leukocyte	*STX17*	-0.624122925	5.52E-09	1.17E-08	2.17E-05
T1DM	leukocyte	*PRPF4*	-0.835826949	2.87E-08	4.71E-08	0.000112724
T1DM	leukocyte	*FPGS*	-0.856349297	1.47E-09	8.14E-09	5.78E-06
T1DM	leukocyte	*ATP6V1G1*	-0.484641414	6.94E-09	1.39E-08	2.73E-05

T1DM	leukocyte	*ARPC5L*	-0.509683016	2.30E-09	8.21E-09	9.04E-06
T1DM	leukocyte	*DSCC1*	-0.167432513	0.029968735	0.032680311	1
T1DM	leukocyte	*POLR1E*	-0.459301192	2.03E-08	3.43E-08	7.96E-05
T1DM	leukocyte	*APTX*	-0.577426665	1.21E-09	8.14E-09	4.76E-06
T1DM	leukocyte	*CD72*	-1.166367156	5.97E-08	9.21E-08	0.000234726
T1DM	leukocyte	*TMEM8B*	-0.893065859	8.96E-10	8.14E-09	3.52E-06
T1DM	leukocyte	*GRHPR*	-0.636870768	3.18E-09	8.70E-09	1.25E-05
T1DM	leukocyte	*ALDH1B1*	-1.10951239	4.33E-07	5.94E-07	0.001700759
T1DM	leukocyte	*ARHGEF39*	-2.496882617	2.48E-09	8.21E-09	9.75E-06
T1DM	leukocyte	*PPIL1*	-1.420271484	1.10E-08	2.02E-08	4.32E-05
T1DM	leukocyte	*GMPR*	-1.147365269	1.18E-09	8.14E-09	4.64E-06
T1DM	leukocyte	*KIAA0319*	-0.723745213	2.24E-09	8.21E-09	8.79E-06
T1DM	leukocyte	*LRRC1*	-0.629838126	1.79E-09	8.14E-09	7.03E-06
T1DM	leukocyte	*RIPK1*	-0.697382316	1.26E-09	8.14E-09	4.93E-06
T1DM	leukocyte	*TUBB2B*	-0.56029622	3.62E-09	9.14E-09	1.42E-05
T1DM	leukocyte	*UQCC2*	-0.552409732	2.40E-07	3.39E-07	0.000941292
T1DM	leukocyte	*HMGA1*	-0.486977004	1.18E-09	8.14E-09	4.63E-06
T1DM	leukocyte	*TCF19*	0.580734345	3.70E-08	5.94E-08	0.000145333
T1DM	leukocyte	*CLPS*	-0.24406181	1.01E-07	1.49E-07	0.000396515
T1DM	leukocyte	*MTCH1*	-0.576582108	2.39E-09	8.21E-09	9.37E-06
T1DM	leukocyte	*FCHSD2*	0.854942589	4.97E-05	6.03E-05	0.195302162
T1DM	leukocyte	*ANKRD42*	-0.95763258	1.35E-09	8.14E-09	5.29E-06
T1DM	leukocyte	*CREBZF*	-0.841673558	3.17E-09	8.70E-09	1.25E-05
T1DM	leukocyte	*PRCP*	-0.749419895	8.61E-07	1.15E-06	0.003381146
T1DM	leukocyte	*NARS2*	-0.809835399	2.12E-09	8.17E-09	8.34E-06
T1DM	leukocyte	*TTPA*	-4.666326987	3.69E-07	5.10E-07	0.001451555
T1DM	leukocyte	*GGH*	-0.703079957	6.84E-07	9.20E-07	0.002687285
T1DM	leukocyte	*SDCBP*	-0.791337907	3.54E-06	4.52E-06	0.013919224
T1DM	leukocyte	*NEK1*	-0.893257388	4.63E-05	5.63E-05	0.181744486
T1DM	leukocyte	*TRPC6*	-0.946142828	0.002660333	0.00301484	1
T1DM	leukocyte	*MMP7*	-0.289457293	4.95E-09	1.09E-08	1.95E-05
T1DM	leukocyte	*TRIM29*	-1.862124833	6.68E-06	8.41E-06	0.026256455
T1DM	leukocyte	*POU2F3*	-1.022959854	0.002397366	0.002719968	1
T1DM	leukocyte	*FDX1*	-0.650777065	2.94E-09	8.50E-09	1.16E-05
T1DM	leukocyte	*C11orf1*	-0.659799793	1.37E-09	8.14E-09	5.40E-06
T1DM	leukocyte	*FXYD2*	-0.365377674	2.26E-09	8.21E-09	8.88E-06
T1DM	leukocyte	*CASP1*	0.287798907	3.25E-09	8.77E-09	1.28E-05
T1DM	leukocyte	*ALKBH8*	-0.952762933	1.37E-09	8.14E-09	5.37E-06
T1DM	leukocyte	*NDUFAF1*	-0.629926609	3.04E-06	3.90E-06	0.011953436
T1DM	leukocyte	*TUBGCP4*	-1.000056904	5.65E-09	1.19E-08	2.22E-05
T1DM	leukocyte	*UACA*	-0.567279977	1.38E-08	2.46E-08	5.43E-05
T1DM	leukocyte	*PLCB2*	0.440362336	1.22E-08	2.21E-08	4.80E-05
T1DM	leukocyte	*PAK6*	-0.382050088	8.13E-09	1.57E-08	3.19E-05
T1DM	leukocyte	*ADAM10*	-0.784236708	5.85E-09	1.22E-08	2.30E-05
T1DM	leukocyte	*DUOX1*	-0.77908962	5.88E-08	9.08E-08	0.000230866
T1DM	leukocyte	*CYP19A1*	-5.397194598	5.86E-08	9.06E-08	0.000230252
T1DM	leukocyte	*ZNF280D*	-0.7871168	2.99E-09	8.51E-09	1.17E-05
T1DM	leukocyte	*RSL24D1*	-0.588881819	4.83E-09	1.08E-08	1.90E-05
T1DM	leukocyte	*TTLL7*	-0.649688295	1.35E-09	8.14E-09	5.32E-06
T1DM	leukocyte	*GIPC2*	-0.493127901	8.99E-05	0.000107744	0.353293538
T1DM	leukocyte	*SLC44A5*	-0.720745658	5.20E-05	6.31E-05	0.20429396
T1DM	leukocyte	*RTCA*	-1.180108035	4.50E-06	5.70E-06	0.01766502
T1DM	leukocyte	*KHK*	-1.385902411	0.000606428	0.000702433	1
T1DM	leukocyte	*DYNC2LI1*	-0.769022413	4.76E-10	8.14E-09	1.87E-06
T1DM	leukocyte	*ACTR2*	-0.581383329	1.91E-09	8.14E-09	7.51E-06
T1DM	leukocyte	*ATRAID*	-0.591341374	9.77E-09	1.83E-08	3.84E-05
T1DM	leukocyte	*LRPPRC*	-0.697204764	7.34E-09	1.46E-08	2.88E-05
T1DM	leukocyte	*MYOF*	-0.36302747	1.69E-06	2.21E-06	0.006647999
T1DM	leukocyte	*BTBD16*	-4.355941166	0.000274456	0.000322181	1
T1DM	leukocyte	*DUSP5*	-1.064242071	3.07E-08	5.01E-08	0.000120712
T1DM	leukocyte	*CALHM2*	0.583464455	6.26E-05	7.56E-05	0.245802524
T1DM	leukocyte	*ARL3*	-0.523975147	8.34E-08	1.25E-07	0.000327828
T1DM	leukocyte	*PLCE1*	-0.772692122	1.09E-08	2.00E-08	4.29E-05
T1DM	leukocyte	*DNAJC13*	-0.614188829	1.80E-09	8.14E-09	7.07E-06

T1DM	leukocyte	*FAM149B1*	-0.716440693	1.96E-09	8.14E-09	7.70E-06
T1DM	leukocyte	*NCOA4*	-1.069992648	3.87E-07	5.33E-07	0.001519829
T1DM	leukocyte	*ASCC1*	-0.790974751	1.97E-09	8.14E-09	7.75E-06
T1DM	leukocyte	*RPS24*	-0.432504436	3.96E-09	9.61E-09	1.55E-05
T1DM	leukocyte	*DNA2*	-0.749896745	5.33E-05	6.45E-05	0.209223332
T1DM	leukocyte	*MYPN*	2.950083814	3.62E-08	5.83E-08	0.000142351
T1DM	leukocyte	*ATIC*	-0.982374999	3.22E-08	5.23E-08	0.000126478
T1DM	leukocyte	*SMARCAL1*	-0.925686346	1.37E-09	8.14E-09	5.40E-06
T1DM	leukocyte	*BARD1*	-0.735656872	3.20E-09	8.73E-09	1.26E-05
T1DM	leukocyte	*STAT4*	0.939409742	9.43E-08	1.41E-07	0.00037043
T1DM	leukocyte	*FASTKD1*	-0.781770053	8.06E-09	1.56E-08	3.17E-05
T1DM	leukocyte	*FAM117B*	-0.835416809	1.07E-09	8.14E-09	4.21E-06
T1DM	leukocyte	*WDR12*	-0.962181167	5.71E-10	8.14E-09	2.24E-06
T1DM	leukocyte	*SLC35A5*	-0.925768277	1.65E-09	8.14E-09	6.49E-06
T1DM	leukocyte	*USP8*	-0.762780551	5.58E-09	1.18E-08	2.19E-05
T1DM	leukocyte	*SPPL2A*	-0.68547792	2.69E-09	8.26E-09	1.06E-05
T1DM	leukocyte	*APH1B*	-0.939589932	2.97E-08	4.85E-08	0.000116566
T1DM	leukocyte	*HCN4*	-0.927354092	1.52E-09	8.14E-09	5.95E-06
T1DM	leukocyte	*SEMA7A*	-1.606622367	1.48E-09	8.14E-09	5.82E-06
T1DM	leukocyte	*AP1AR*	-0.566887097	2.44E-09	8.21E-09	9.59E-06
T1DM	leukocyte	*BBS7*	-0.788517893	4.20E-09	9.96E-09	1.65E-05
T1DM	leukocyte	*RAP1GDS1*	-0.78882026	2.51E-09	8.21E-09	9.86E-06
T1DM	leukocyte	*PRDM5*	-0.682155332	1.39E-09	8.14E-09	5.46E-06
T1DM	leukocyte	*NUP54*	-0.541132668	9.52E-08	1.42E-07	0.000373948
T1DM	leukocyte	*G3BP2*	-0.543708172	9.82E-10	8.14E-09	3.86E-06
T1DM	leukocyte	*SCARB2*	-0.609728003	1.52E-09	8.14E-09	5.98E-06
T1DM	leukocyte	*CNOT6L*	-0.785567223	3.30E-09	8.82E-09	1.30E-05
T1DM	leukocyte	*USO1*	-0.546444731	1.19E-09	8.14E-09	4.69E-06
T1DM	leukocyte	*SHROOM3*	-1.142444571	4.51E-09	1.04E-08	1.77E-05
T1DM	leukocyte	*ANXA3*	-0.490314085	3.05E-10	8.14E-09	1.20E-06
T1DM	leukocyte	*PPA2*	-0.647086612	1.86E-09	8.14E-09	7.31E-06
T1DM	leukocyte	*EGF*	-1.385168812	5.32E-06	6.73E-06	0.020894676
T1DM	leukocyte	*MAPK8IP3*	-0.700669425	3.31E-09	8.82E-09	1.30E-05
T1DM	leukocyte	*RGS3*	-0.617610913	6.98E-10	8.14E-09	2.74E-06
T1DM	leukocyte	*ETV6*	-1.180902557	1.47E-08	2.60E-08	5.78E-05
T1DM	leukocyte	*ALG10*	-1.22162568	1.35E-09	8.14E-09	5.31E-06
T1DM	leukocyte	*PIK3C2G*	1.361772072	0.001008109	0.001160861	1
T1DM	leukocyte	*SLCO1C1*	-10.71024755	1.86E-09	8.14E-09	7.31E-06
T1DM	leukocyte	*ZCRB1*	-0.574283826	2.94E-09	8.50E-09	1.16E-05
T1DM	leukocyte	*C1RL*	-0.739715045	2.50E-07	3.53E-07	0.000983231
T1DM	leukocyte	*CLSTN3*	-0.867209092	4.72E-07	6.46E-07	0.001855819
T1DM	leukocyte	*KLRG1*	-4.478588817	8.73E-10	8.14E-09	3.43E-06
T1DM	leukocyte	*VAMP1*	0.480687233	1.63E-06	2.13E-06	0.006396006
T1DM	leukocyte	*TAPBPL*	-0.443629401	3.01E-05	3.68E-05	0.118120781
T1DM	leukocyte	*PEX5*	-0.663666851	2.13E-09	8.18E-09	8.37E-06
T1DM	leukocyte	*SLC38A4*	-0.945158837	4.12E-09	9.86E-09	1.62E-05
T1DM	leukocyte	*LRIG3*	-0.692012879	8.91E-10	8.14E-09	3.50E-06
T1DM	leukocyte	*GLIPR1*	-0.782811415	1.48E-09	8.14E-09	5.83E-06
T1DM	leukocyte	*SNRPF*	-0.51158247	5.91E-09	1.23E-08	2.32E-05
T1DM	leukocyte	*AMDHD1*	-0.720552652	0.000617096	0.000714581	1
T1DM	leukocyte	*ASCL1*	-1.085065552	1.05E-09	8.14E-09	4.13E-06
T1DM	leukocyte	*RITA1*	-0.615119626	0.000121898	0.000145264	0.478935404
T1DM	leukocyte	*GLTP*	-0.539652205	1.51E-09	8.14E-09	5.94E-06
T1DM	leukocyte	*MTMR6*	-0.791016707	1.05E-08	1.94E-08	4.13E-05
T1DM	leukocyte	*PDX1*	-0.894394124	1.96E-09	8.14E-09	7.68E-06
T1DM	leukocyte	*SUOX*	-0.82519582	6.87E-10	8.14E-09	2.70E-06
T1DM	leukocyte	*CCDC65*	-0.885606273	1.21E-06	1.59E-06	0.004738131
T1DM	leukocyte	*SLC39A5*	-0.200495872	0.007677996	0.008567693	1
T1DM	leukocyte	*KANSL2*	-1.378641917	2.09E-08	3.53E-08	8.23E-05
T1DM	leukocyte	*CERS5*	-0.722327252	2.46E-07	3.47E-07	0.000967512
T1DM	leukocyte	*GALNT6*	2.258404025	0.002284606	0.002596534	1
T1DM	leukocyte	*ZNF740*	-1.314172886	1.85E-08	3.17E-08	7.26E-05
T1DM	leukocyte	*WDFY2*	-0.755611035	1.53E-09	8.14E-09	5.99E-06
T1DM	leukocyte	*GRTP1*	-1.447074471	9.52E-09	1.79E-08	3.74E-05

T1DM	leukocyte	*TSSK4*	-5.153966865	2.26E-06	2.92E-06	0.008886397
T1DM	leukocyte	*TMX1*	-0.877700392	6.02E-09	1.24E-08	2.37E-05
T1DM	leukocyte	*RAB15*	-0.88880528	2.08E-08	3.51E-08	8.18E-05
T1DM	leukocyte	*ESR2*	-0.893370576	3.33E-06	4.26E-06	0.01309602
T1DM	leukocyte	*PTGR2*	-0.44702253	9.09E-07	1.21E-06	0.003573035
T1DM	leukocyte	*NIPA2*	-0.559669356	1.47E-09	8.14E-09	5.76E-06
T1DM	leukocyte	*SERF2*	-0.474038101	3.56E-09	9.10E-09	1.40E-05
T1DM	leukocyte	*ZSCAN29*	-0.739603113	0.008503333	0.009469839	1
T1DM	leukocyte	*BNIP2*	-0.97755697	9.28E-09	1.75E-08	3.65E-05
T1DM	leukocyte	*GTF2A2*	-0.581574052	2.27E-09	8.21E-09	8.93E-06
T1DM	leukocyte	*SRP14*	-0.465596116	4.24E-09	9.99E-09	1.67E-05
T1DM	leukocyte	*BAHD1*	-0.746989678	5.13E-09	1.12E-08	2.02E-05
T1DM	leukocyte	*DISP2*	-0.48418537	2.28E-07	3.23E-07	0.000897
T1DM	leukocyte	*TLE3*	-1.211267414	1.09E-07	1.60E-07	0.000426643
T1DM	leukocyte	*SCAPER*	-0.557551166	2.33E-08	3.89E-08	9.14E-05
T1DM	leukocyte	*TSPAN3*	-0.507274455	2.64E-09	8.21E-09	1.04E-05
T1DM	leukocyte	*MAN2C1*	-0.584963964	6.99E-08	1.06E-07	0.000274464
T1DM	leukocyte	*DNAJA4*	-0.576013451	4.07E-09	9.78E-09	1.60E-05
T1DM	leukocyte	*TPM1*	-0.379773142	1.10E-09	8.14E-09	4.33E-06
T1DM	leukocyte	*IGF1R*	-0.561414642	1.09E-09	8.14E-09	4.27E-06
T1DM	leukocyte	*ADAMTS17*	-1.188571287	1.44E-05	1.79E-05	0.056678004
T1DM	leukocyte	*ULK3*	-0.741631808	4.74E-11	8.14E-09	1.86E-07
T1DM	leukocyte	*CELF6*	-0.809140264	1.32E-09	8.14E-09	5.18E-06
T1DM	leukocyte	*POLG*	-1.035934166	1.41E-09	8.14E-09	5.54E-06
T1DM	leukocyte	*WDR93*	-1.842823229	3.32E-05	4.06E-05	0.130312011
T1DM	leukocyte	*UNC45A*	-0.748807753	5.24E-10	8.14E-09	2.06E-06
T1DM	leukocyte	*IQGAP1*	-0.613015109	2.11E-08	3.56E-08	8.30E-05
T1DM	leukocyte	*PMM2*	-0.663026326	1.54E-09	8.14E-09	6.07E-06
T1DM	leukocyte	*SLC5A2*	-2.665152477	1.73E-09	8.14E-09	6.82E-06
T1DM	leukocyte	*UQCRC2*	-0.684020244	1.91E-09	8.14E-09	7.50E-06
T1DM	leukocyte	*ARHGAP17*	-1.409142371	4.40E-08	6.97E-08	0.000172991
T1DM	leukocyte	*TXNL4B*	-1.270708595	2.62E-07	3.68E-07	0.001028656
T1DM	leukocyte	*MARVELD3*	-0.552886766	1.17E-08	2.14E-08	4.61E-05
T1DM	leukocyte	*KIFC3*	-0.720751776	4.19E-09	9.96E-09	1.65E-05
T1DM	leukocyte	*NUDT7*	-0.618310575	1.45E-09	8.14E-09	5.68E-06
T1DM	leukocyte	*GCSH*	-0.615430927	1.65E-09	8.14E-09	6.49E-06
T1DM	leukocyte	*MAP1LC3B*	-0.526418832	2.53E-09	8.21E-09	9.95E-06
T1DM	leukocyte	*OSGIN1*	-0.535434029	1.12E-09	8.14E-09	4.40E-06
T1DM	leukocyte	*RPL3L*	-1.219570169	3.05E-06	3.91E-06	0.011965528
T1DM	leukocyte	*RPS2*	-0.4031318	2.43E-09	8.21E-09	9.57E-06
T1DM	leukocyte	*PDPK1*	-0.773181239	1.86E-08	3.19E-08	7.32E-05
T1DM	leukocyte	*DEF8*	-0.706542051	4.59E-09	1.04E-08	1.80E-05
T1DM	leukocyte	*GALNS*	0.777246823	0.013573751	0.014997544	1
T1DM	leukocyte	*GAS8*	-1.089809496	1.08E-08	1.99E-08	4.24E-05
T1DM	leukocyte	*RANBP10*	-0.607672421	5.30E-08	8.28E-08	0.000208421
T1DM	leukocyte	*PRPSAP2*	-0.796332379	5.42E-09	1.16E-08	2.13E-05
T1DM	leukocyte	*TOM1L1*	-0.638198232	2.28E-09	8.21E-09	8.96E-06
T1DM	leukocyte	*C17orf80*	-1.000983961	6.43E-08	9.87E-08	0.000252592
T1DM	leukocyte	*VPS53*	-0.618202305	5.87E-08	9.08E-08	0.000230787
T1DM	leukocyte	*NPEPPS*	-0.799491303	3.80E-08	6.09E-08	0.000149376
T1DM	leukocyte	*LRRC46*	0.577738847	6.45E-05	7.78E-05	0.253283212
T1DM	leukocyte	*SCRN2*	-0.981594675	1.12E-06	1.49E-06	0.004414723
T1DM	leukocyte	*RHBDL3*	-0.746776699	0.00147061	0.001681125	1
T1DM	leukocyte	*SPACA3*	-0.415592024	7.98E-05	9.58E-05	0.313549333
T1DM	leukocyte	*ARSG*	-1.191098259	0.00325506	0.003682444	1
T1DM	leukocyte	*PTRH2*	-0.739526319	1.85E-09	8.14E-09	7.27E-06
T1DM	leukocyte	*GNAL*	-0.985627922	2.42E-09	8.21E-09	9.51E-06
T1DM	leukocyte	*RPRD1A*	-0.649724298	4.06E-09	9.77E-09	1.60E-05
T1DM	leukocyte	*NPC1*	-1.196236497	2.36E-08	3.94E-08	9.26E-05
T1DM	leukocyte	*SLC14A1*	-0.789672065	1.03E-05	1.28E-05	0.040446031
T1DM	leukocyte	*ZMYND15*	0.170168935	2.95E-09	8.50E-09	1.16E-05
T1DM	leukocyte	*SAT2*	-0.728611579	6.62E-09	1.34E-08	2.60E-05
T1DM	leukocyte	*CCDC40*	-0.700106284	6.91E-09	1.39E-08	2.71E-05
T1DM	leukocyte	*ARHGDIA*	-0.570525028	1.82E-08	3.13E-08	7.16E-05

T1DM	leukocyte	*TMC6*	-1.099279993	1.28E-09	8.14E-09	5.05E-06
T1DM	leukocyte	*TTYH2*	-1.218247218	1.47E-09	8.14E-09	5.79E-06
T1DM	leukocyte	*EIF4A3*	-0.718286495	2.41E-09	8.21E-09	9.45E-06
T1DM	leukocyte	*TBCD*	-1.051088746	1.94E-08	3.30E-08	7.61E-05
T1DM	leukocyte	*FN3KRP*	-0.531665376	0.0002781	0.000326361	1
T1DM	leukocyte	*NARF*	-0.866793478	5.97E-09	1.24E-08	2.35E-05
T1DM	leukocyte	*TRIM65*	-0.909335216	9.35E-08	1.40E-07	0.000367416
T1DM	leukocyte	*RNF157*	-0.800920192	5.95E-10	8.14E-09	2.34E-06
T1DM	leukocyte	*WDR45B*	-0.528610169	1.01E-08	1.88E-08	3.99E-05
T1DM	leukocyte	*TNFRSF11A*	-0.632255411	9.23E-10	8.14E-09	3.63E-06
T1DM	leukocyte	*ZCCHC2*	-0.852550911	1.18E-08	2.15E-08	4.64E-05
T1DM	leukocyte	*PMAIP1*	-0.632711655	1.39E-09	8.14E-09	5.48E-06
T1DM	leukocyte	*NT5C3B*	-0.614898114	9.56E-08	1.42E-07	0.000375701
T1DM	leukocyte	*PIP4K2B*	-0.65591991	3.43E-09	8.93E-09	1.35E-05
T1DM	leukocyte	*TXNL4A*	-0.549625164	9.66E-09	1.81E-08	3.79E-05
T1DM	leukocyte	*PRDM15*	-0.854106099	1.26E-07	1.84E-07	0.000496493
T1DM	leukocyte	*MVB12A*	-0.74679532	4.03E-08	6.42E-08	0.000158411
T1DM	leukocyte	*SH3GL1*	-0.616197479	1.97E-09	8.14E-09	7.75E-06
T1DM	leukocyte	*DUS3L*	-0.485098131	4.60E-08	7.24E-08	0.000180574
T1DM	leukocyte	*SIRT3*	-0.936200136	1.41E-09	8.14E-09	5.53E-06
T1DM	leukocyte	*IFITM3*	-0.413195606	7.95E-09	1.55E-08	3.12E-05
T1DM	leukocyte	*COL6A1*	-0.894062224	7.85E-10	8.14E-09	3.08E-06
T1DM	leukocyte	*IFNAR1*	-0.622906832	9.34E-10	8.14E-09	3.67E-06
T1DM	leukocyte	*SIK1*	-0.861216324	0.001404816	0.001608253	1
T1DM	leukocyte	*TMEM50B*	-0.56751356	2.94E-07	4.10E-07	0.00115609
T1DM	leukocyte	*URB1*	-0.432621194	0.006296624	0.007044258	1
T1DM	leukocyte	*AKT1*	-0.893325572	1.62E-08	2.82E-08	6.36E-05
T1DM	leukocyte	*CBLC*	-0.647233538	1.41E-08	2.51E-08	5.55E-05
T1DM	leukocyte	*EVI5L*	-0.744166685	2.48E-07	3.49E-07	0.000972683
T1DM	leukocyte	*CTU1*	-1.04843821	2.84E-09	8.43E-09	1.12E-05
T1DM	leukocyte	*RERE*	-0.559371294	1.57E-09	8.14E-09	6.18E-06
T1DM	leukocyte	*FHAD1*	-0.687031908	5.82E-09	1.21E-08	2.29E-05
T1DM	leukocyte	*PGD*	-0.843644383	6.78E-10	8.14E-09	2.66E-06
T1DM	leukocyte	*CNKSR1*	-0.973202385	3.90E-06	4.97E-06	0.015333898
T1DM	leukocyte	*ZNF593*	-0.613226057	3.09E-09	8.63E-09	1.21E-05
T1DM	leukocyte	*FCN3*	-0.322762979	6.26E-07	8.46E-07	0.002461126
T1DM	leukocyte	*CELA3A*	-0.297923361	1.46E-09	8.14E-09	5.74E-06
T1DM	leukocyte	*NBPF3*	-0.606086381	7.89E-06	9.88E-06	0.031009085
T1DM	leukocyte	*HSPG2*	-0.356265678	1.51E-08	2.65E-08	5.92E-05
T1DM	leukocyte	*ITGB3BP*	-0.565323403	6.48E-07	8.73E-07	0.002545484
T1DM	leukocyte	*PRKACB*	-0.729288967	4.97E-09	1.10E-08	1.95E-05
T1DM	leukocyte	*PIGK*	-1.038648961	5.85E-09	1.22E-08	2.30E-05
T1DM	leukocyte	*RPS8*	-0.389782115	2.41E-09	8.21E-09	9.47E-06
T1DM	leukocyte	*CYP4B1*	3.758109142	5.28E-06	6.69E-06	0.020750349
T1DM	leukocyte	*SYPL2*	-2.011757738	2.52E-09	8.21E-09	9.90E-06
T1DM	leukocyte	*SLC44A3*	-0.660134193	2.64E-09	8.21E-09	1.04E-05
T1DM	leukocyte	*ZNF697*	-0.830164415	2.79E-06	3.60E-06	0.010981546
T1DM	leukocyte	*CELSR2*	-0.772377673	1.25E-08	2.26E-08	4.90E-05
T1DM	leukocyte	*ALDH9A1*	-0.586377611	1.60E-09	8.14E-09	6.27E-06
T1DM	leukocyte	*ATP1B1*	-0.402180467	1.41E-09	8.14E-09	5.53E-06
T1DM	leukocyte	*TIPRL*	-0.566310217	3.84E-09	9.48E-09	1.51E-05
T1DM	leukocyte	*NME7*	-0.925910185	8.06E-09	1.56E-08	3.17E-05
T1DM	leukocyte	*CREG1*	0.514509891	6.66E-08	1.02E-07	0.000261715
T1DM	leukocyte	*XCL1*	-11.3570334	0.009130189	0.010150682	1
T1DM	leukocyte	*MGST3*	-0.588455364	1.32E-08	2.37E-08	5.18E-05
T1DM	leukocyte	*LYPLAL1*	-0.793953041	1.09E-09	8.14E-09	4.29E-06
T1DM	leukocyte	*SNX27*	-0.979481144	8.66E-09	1.65E-08	3.40E-05
T1DM	leukocyte	*CTSK*	-1.871375766	6.67E-08	1.02E-07	0.000262165
T1DM	leukocyte	*SELENBP1*	-0.804144714	1.35E-09	8.14E-09	5.31E-06
T1DM	leukocyte	*POGZ*	-0.696166397	9.79E-09	1.83E-08	3.85E-05
T1DM	leukocyte	*GABPB2*	-1.121707437	4.20E-09	9.96E-09	1.65E-05
T1DM	leukocyte	*TAF1A*	1.53448456	1.08E-07	1.59E-07	0.000424527
T1DM	leukocyte	*SLC27A3*	-0.917548617	2.58E-09	8.21E-09	1.01E-05
T1DM	leukocyte	*KCNN3*	-1.225344227	1.05E-09	8.14E-09	4.12E-06

T1DM	leukocyte	*RIT1*	-0.671294931	0.001286449	0.001474032	1
T1DM	leukocyte	*SRP9*	-0.607771246	4.31E-09	1.01E-08	1.69E-05
T1DM	leukocyte	*CNIH4*	-0.645374166	2.37E-09	8.21E-09	9.31E-06
T1DM	leukocyte	*PSEN2*	-1.22978948	1.37E-08	2.45E-08	5.38E-05
T1DM	leukocyte	*PYCR2*	-0.727672405	2.69E-09	8.26E-09	1.06E-05
T1DM	leukocyte	*LBR*	-0.729962662	4.33E-08	6.86E-08	0.000169938
T1DM	leukocyte	*ARL8A*	-0.940378164	1.53E-08	2.68E-08	5.99E-05
T1DM	leukocyte	*GALM*	1.634784665	0.00117488	0.001348555	1
T1DM	leukocyte	*EML4*	-0.977502272	1.72E-08	2.97E-08	6.74E-05
T1DM	leukocyte	*WDPCP*	-1.569003483	1.46E-08	2.58E-08	5.74E-05
T1DM	leukocyte	*REG3G*	-0.641340351	3.72E-08	5.96E-08	0.000146077
T1DM	leukocyte	*CIAO1*	-0.842449792	7.57E-09	1.49E-08	2.97E-05
T1DM	leukocyte	*ZNF514*	-0.749498629	2.94E-10	8.14E-09	1.15E-06
T1DM	leukocyte	*TPRKB*	-0.573952464	2.15E-09	8.19E-09	8.44E-06
T1DM	leukocyte	*TEX261*	-0.751166184	1.86E-09	8.14E-09	7.31E-06
T1DM	leukocyte	*THNSL2*	-0.325500288	0.030096127	0.032801021	1
T1DM	leukocyte	*RALB*	-0.724738588	3.07E-10	8.14E-09	1.21E-06
T1DM	leukocyte	*RABL2A*	-1.009076812	2.54E-09	8.21E-09	9.98E-06
T1DM	leukocyte	*SLC20A1*	-0.98662473	3.88E-09	9.52E-09	1.53E-05
T1DM	leukocyte	*LIPT1*	1.235732788	0.003525153	0.003979979	1
T1DM	leukocyte	*LYG1*	-2.653342919	3.40E-05	4.15E-05	0.133518648
T1DM	leukocyte	*AFF3*	-0.796351623	0.019814172	0.021739705	1
T1DM	leukocyte	*GULP1*	-0.647822389	2.77E-09	8.34E-09	1.09E-05
T1DM	leukocyte	*FAM171B*	-0.599387037	1.48E-09	8.14E-09	5.83E-06
T1DM	leukocyte	*METTL21A*	-0.688082562	2.09E-09	8.15E-09	8.20E-06
T1DM	leukocyte	*SUMF1*	-0.919411015	1.17E-09	8.14E-09	4.60E-06
T1DM	leukocyte	*HES6*	-1.341376355	1.72E-07	2.47E-07	0.000677566
T1DM	leukocyte	*ESPNL*	-5.046093011	0.00070443	0.00081451	1
T1DM	leukocyte	*TAMM41*	-0.781903276	2.27E-08	3.80E-08	8.91E-05
T1DM	leukocyte	*RAB5A*	-0.614142007	2.57E-09	8.21E-09	1.01E-05
T1DM	leukocyte	*CNTN4*	-0.628976017	2.51E-08	4.16E-08	9.86E-05
T1DM	leukocyte	*POMGNT2*	-1.113722036	3.72E-09	9.31E-09	1.46E-05
T1DM	leukocyte	*CTDSPL*	-0.626101541	1.33E-09	8.14E-09	5.21E-06
T1DM	leukocyte	*IL17RD*	-2.763403848	4.54E-09	1.04E-08	1.78E-05
T1DM	leukocyte	*UBA3*	-0.952327253	3.02E-09	8.56E-09	1.19E-05
T1DM	leukocyte	*ARL6IP5*	-0.593489411	2.48E-09	8.21E-09	9.76E-06
T1DM	leukocyte	*LIMD1*	-1.051414641	6.79E-09	1.37E-08	2.67E-05
T1DM	leukocyte	*ZNF660*	-0.610106013	6.07E-05	7.35E-05	0.2385573
T1DM	leukocyte	*NXPE3*	-0.602279254	2.13E-08	3.58E-08	8.36E-05
T1DM	leukocyte	*PHLDB2*	-0.503789202	1.38E-09	8.14E-09	5.42E-06
T1DM	leukocyte	*ABHD10*	-0.721756634	9.02E-09	1.71E-08	3.54E-05
T1DM	leukocyte	*TAGLN3*	-0.426555323	5.48E-09	1.17E-08	2.15E-05
T1DM	leukocyte	*EIF2A*	-0.556844577	1.33E-09	8.14E-09	5.21E-06
T1DM	leukocyte	*ALDH1L1*	-0.958080028	1.43E-09	8.14E-09	5.61E-06
T1DM	leukocyte	*TRPC1*	-0.809035288	1.43E-09	8.14E-09	5.61E-06
T1DM	leukocyte	*NCEH1*	-0.420263921	2.25E-09	8.21E-09	8.85E-06
T1DM	leukocyte	*AMT*	-0.76781445	4.78E-05	5.81E-05	0.187887389
T1DM	leukocyte	*DGKQ*	-0.711394001	1.34E-08	2.40E-08	5.26E-05
T1DM	leukocyte	*SCD5*	-0.569193533	1.40E-09	8.14E-09	5.52E-06
T1DM	leukocyte	*PYURF*	-0.527277137	2.58E-09	8.21E-09	1.01E-05
T1DM	leukocyte	*TBCK*	-0.760867176	4.64E-06	5.89E-06	0.018238745
T1DM	leukocyte	*DDIT4L*	-1.124318584	9.83E-05	0.000117615	0.386131126
T1DM	leukocyte	*TIFA*	-0.820768259	1.83E-09	8.14E-09	7.21E-06
T1DM	leukocyte	*RNF175*	-12.51515212	0.027666121	0.030194497	1
T1DM	leukocyte	*CYP4V2*	-0.709038711	0.002529874	0.002869479	1
T1DM	leukocyte	*SSBP2*	-0.554743006	1.42E-09	8.14E-09	5.58E-06
T1DM	leukocyte	*IQGAP2*	0.39995143	2.65E-07	3.72E-07	0.001040754
T1DM	leukocyte	*GTF2H2*	-1.505626254	4.75E-08	7.47E-08	0.000186794
T1DM	leukocyte	*BTF3*	-0.476399934	4.40E-09	1.02E-08	1.73E-05
T1DM	leukocyte	*SPATA9*	-4.563052175	1.14E-07	1.68E-07	0.000448466
T1DM	leukocyte	*COMMD10*	-0.778840172	1.52E-09	8.14E-09	5.98E-06
T1DM	leukocyte	*ATG12*	-0.597846265	4.93E-09	1.09E-08	1.94E-05
T1DM	leukocyte	*PCYOX1L*	-2.444439697	7.00E-06	8.80E-06	0.027505667
T1DM	leukocyte	*MYLK4*	1.584193815	5.83E-05	7.06E-05	0.229144098

T1DM	leukocyte	*ZMAT2*	-0.783047019	2.37E-09	8.21E-09	9.31E-06
T1DM	leukocyte	*KLHL3*	-1.454709438	0.001167897	0.001340931	1
T1DM	leukocyte	*DCDC2*	-0.515719302	1.48E-09	8.14E-09	5.80E-06
T1DM	leukocyte	*TRIM7*	-1.757058732	2.17E-07	3.08E-07	0.000850946
T1DM	leukocyte	*ANO7*	-1.827546284	8.24E-09	1.59E-08	3.24E-05
T1DM	leukocyte	*RPL7L1*	-0.597107366	1.59E-09	8.14E-09	6.24E-06
T1DM	leukocyte	*IRAK1BP1*	-0.588296162	1.07E-09	8.14E-09	4.21E-06
T1DM	leukocyte	*MMS22L*	-0.941067828	4.75E-09	1.07E-08	1.87E-05
T1DM	leukocyte	*FAXC*	-1.023799346	1.48E-09	8.14E-09	5.81E-06
T1DM	leukocyte	*SCML4*	-1.702353085	4.70E-05	5.71E-05	0.184529378
T1DM	leukocyte	*TBC1D32*	-0.908594986	0.010944048	0.012129524	1
T1DM	leukocyte	*ABRACL*	-0.620484218	4.80E-08	7.54E-08	0.00018872
T1DM	leukocyte	*DYNLT1*	-0.527779679	2.90E-09	8.49E-09	1.14E-05
T1DM	leukocyte	*WTAP*	-0.547921589	1.06E-09	8.14E-09	4.18E-06
T1DM	leukocyte	*SLC22A3*	-4.263483528	5.40E-08	8.42E-08	0.00021226
T1DM	leukocyte	*VWDE*	-0.614584537	2.12E-08	3.57E-08	8.33E-05
T1DM	leukocyte	*GNA12*	-0.952734721	4.18E-08	6.64E-08	0.000164249
T1DM	leukocyte	*SDK1*	-0.806820453	3.75E-09	9.33E-09	1.47E-05
T1DM	leukocyte	*CCZ1B*	-0.893015149	3.16E-07	4.40E-07	0.001243227
T1DM	leukocyte	*RBAK*	-1.314319011	3.43E-08	5.55E-08	0.000134795
T1DM	leukocyte	*POMZP3*	-0.721343859	7.52E-09	1.48E-08	2.95E-05
T1DM	leukocyte	*PSPH*	-0.753050091	4.21E-06	5.34E-06	0.016526507
T1DM	leukocyte	*GIGYF1*	-1.322954824	7.99E-10	8.14E-09	3.14E-06
T1DM	leukocyte	*TRIM4*	-0.529694492	3.52E-08	5.68E-08	0.000138272
T1DM	leukocyte	*TMEM209*	-0.980673428	1.88E-09	8.14E-09	7.38E-06
T1DM	leukocyte	*NCAPG2*	0.68785511	3.28E-08	5.33E-08	0.000128967
T1DM	leukocyte	*RAB19*	-3.641333855	2.78E-05	3.41E-05	0.109195222
T1DM	leukocyte	*CSGALNACT1*	-0.610475074	3.04E-09	8.59E-09	1.20E-05
T1DM	leukocyte	*CCDC25*	-0.542358735	1.36E-09	8.14E-09	5.35E-06
T1DM	leukocyte	*BIN3*	-1.54116065	2.13E-07	3.02E-07	0.000835224
T1DM	leukocyte	*CHMP7*	-0.970945923	2.50E-09	8.21E-09	9.83E-06
T1DM	leukocyte	*STAR*	0.524800681	7.43E-09	1.47E-08	2.92E-05
T1DM	leukocyte	*ADHFE1*	1.300599674	0.004154724	0.004682706	1
T1DM	leukocyte	*LACTB2*	-0.604565823	1.18E-09	8.14E-09	4.65E-06
T1DM	leukocyte	*TERF1*	-1.125603841	2.88E-08	4.73E-08	0.000113251
T1DM	leukocyte	*LRP12*	-0.673255672	1.37E-09	8.14E-09	5.39E-06
T1DM	leukocyte	*ZNF7*	-0.591124446	1.38E-09	8.14E-09	5.41E-06
T1DM	leukocyte	*AK3*	-0.580907422	2.08E-09	8.15E-09	8.19E-06
T1DM	leukocyte	*CER1*	2.773120846	1.83E-09	8.14E-09	7.20E-06
T1DM	leukocyte	*C9orf72*	-0.925918801	2.55E-09	8.21E-09	1.00E-05
T1DM	leukocyte	*FBXO10*	-0.960358051	8.96E-09	1.70E-08	3.52E-05
T1DM	leukocyte	*SIGMAR1*	-0.761513957	1.75E-09	8.14E-09	6.88E-06
T1DM	leukocyte	*ZNF462*	-0.593076358	1.95E-09	8.14E-09	7.67E-06
T1DM	leukocyte	*UGCG*	-0.638647299	5.91E-09	1.23E-08	2.32E-05
T1DM	leukocyte	*ALAD*	-0.692975784	5.41E-08	8.43E-08	0.000212568
T1DM	leukocyte	*SURF4*	-0.561393086	6.46E-10	8.14E-09	2.54E-06
T1DM	leukocyte	*SURF1*	-0.586783476	7.30E-07	9.81E-07	0.002868269
T1DM	leukocyte	*SURF6*	-0.925294652	2.63E-09	8.21E-09	1.03E-05
T1DM	leukocyte	*SH3GLB2*	-1.00583602	2.25E-09	8.21E-09	8.85E-06
T1DM	leukocyte	*LCN2*	-0.289450069	1.33E-09	8.14E-09	5.24E-06
T1DM	leukocyte	*GPR107*	-0.868809261	4.66E-09	1.06E-08	1.83E-05
T1DM	leukocyte	*INPP5E*	-1.125715176	1.49E-07	2.15E-07	0.000585428
T1DM	leukocyte	*SEC16A*	-0.790262855	1.43E-07	2.06E-07	0.000561464
T1DM	leukocyte	*MSRB2*	-0.985840416	8.09E-10	8.14E-09	3.18E-06
T1DM	leukocyte	*RSU1*	-0.856776778	7.30E-10	8.14E-09	2.87E-06
T1DM	leukocyte	*GLUD1*	-0.749435595	2.78E-09	8.34E-09	1.09E-05
T1DM	leukocyte	*FRA10AC1*	-0.511993911	2.79E-09	8.35E-09	1.10E-05
T1DM	leukocyte	*DNAJB12*	-0.809140683	2.72E-09	8.27E-09	1.07E-05
T1DM	leukocyte	*FUOM*	0.74325801	2.92E-07	4.07E-07	0.001145381
T1DM	leukocyte	*LRRC27*	-0.723996116	0.039475101	0.042809184	1
T1DM	leukocyte	*MTG1*	-0.563273814	8.37E-09	1.61E-08	3.29E-05
T1DM	leukocyte	*PAOX*	-1.162609314	0.025843155	0.028226272	1
T1DM	leukocyte	*GSTO1*	-0.683677111	5.85E-07	7.91E-07	0.002297808
T1DM	leukocyte	*ZNF215*	-0.929561629	0.000126676	0.00015073	0.497710493

T1DM	leukocyte	*HSD17B12*	-0.344278028	1.08E-06	1.42E-06	0.004225724
T1DM	leukocyte	*APIP*	-0.582091556	3.65E-08	5.87E-08	0.000143557
T1DM	leukocyte	*PTPRJ*	-0.890252596	1.52E-09	8.14E-09	5.96E-06
T1DM	leukocyte	*CCDC82*	-0.664438417	9.42E-10	8.14E-09	3.70E-06
T1DM	leukocyte	*KLHL35*	-0.476663335	0.000550798	0.000639127	1
T1DM	leukocyte	*CAPN5*	-0.419213584	0.001396948	0.001599711	1
T1DM	leukocyte	*RPS3*	-0.41057529	3.12E-09	8.69E-09	1.23E-05
T1DM	leukocyte	*TTC12*	-0.532879361	6.08E-05	7.36E-05	0.238983342
T1DM	leukocyte	*AASDHPPT*	-0.682969127	2.07E-09	8.14E-09	8.14E-06
T1DM	leukocyte	*GLB1L2*	-0.648801228	3.95E-08	6.30E-08	0.000155326
T1DM	leukocyte	*FADS1*	-0.411345041	1.53E-05	1.89E-05	0.059964595
T1DM	leukocyte	*TMC2*	-46.43375462	4.42E-07	6.06E-07	0.001735715
T1DM	leukocyte	*EML3*	-1.751450819	0.000159987	0.000189734	0.628587344
T1DM	leukocyte	*CHEK1*	-0.84148752	2.48E-09	8.21E-09	9.74E-06
T1DM	leukocyte	*SIDT2*	-0.855391711	4.96E-10	8.14E-09	1.95E-06
T1DM	leukocyte	*TAGLN*	-0.550703059	3.37E-09	8.87E-09	1.32E-05
T1DM	leukocyte	*DUSP15*	-0.864298174	1.58E-09	8.14E-09	6.20E-06
T1DM	leukocyte	*DSN1*	-0.791957188	2.99E-09	8.52E-09	1.18E-05
T1DM	leukocyte	*YTHDF1*	-0.717539985	2.47E-09	8.21E-09	9.72E-06
T1DM	leukocyte	*CABLES2*	-1.02074819	1.12E-11	3.16E-09	4.42E-08
T1DM	leukocyte	*TRPT1*	-0.672597065	1.67E-09	8.14E-09	6.56E-06
T1DM	leukocyte	*TBX6*	-1.048338404	0.000112064	0.000133667	0.440298584
T1DM	leukocyte	*DOC2A*	1.082460219	2.88E-06	3.70E-06	0.011296507
T1DM	leukocyte	*MPP7*	-0.558236363	1.75E-09	8.14E-09	6.86E-06
T1DM	leukocyte	*DCUN1D2*	-0.772082445	5.36E-08	8.37E-08	0.000210592
T1DM	leukocyte	*TMCO3*	-0.729939836	8.30E-09	1.60E-08	3.26E-05
T1DM	leukocyte	*TMEM218*	-1.020038184	8.80E-10	8.14E-09	3.46E-06
T1DM	leukocyte	*MIA2*	-0.619865958	3.14E-09	8.69E-09	1.23E-05
T1DM	leukocyte	*LYPD1*	-1.109811009	1.19E-09	8.14E-09	4.66E-06
T1DM	leukocyte	*LYPD6B*	-1.811628182	1.22E-09	8.14E-09	4.81E-06
T1DM	leukocyte	*WDR17*	-0.552105355	1.05E-08	1.94E-08	4.14E-05
T1DM	leukocyte	*CNDP1*	-2.055968084	0.000292501	0.000342851	1
T1DM	leukocyte	*PRSS23*	-0.529082651	7.94E-10	8.14E-09	3.12E-06
T1DM	leukocyte	*MTMR12*	-0.552900882	2.56E-09	8.21E-09	1.01E-05
T1DM	leukocyte	*DOCK1*	-0.690548616	1.88E-09	8.14E-09	7.40E-06
T1DM	leukocyte	*IL18*	0.427424727	6.23E-09	1.28E-08	2.45E-05
T1DM	leukocyte	*PRSS53*	0.45707225	4.29E-06	5.45E-06	0.016854687
T1DM	leukocyte	*TMEM86A*	-0.855322592	1.39E-09	8.14E-09	5.48E-06
T1DM	leukocyte	*DLG5*	-0.66103288	6.19E-10	8.14E-09	2.43E-06
T1DM	leukocyte	*SLC2A13*	-0.510879391	3.15E-09	8.69E-09	1.24E-05
T1DM	leukocyte	*MAGI1*	-0.680299708	1.39E-09	8.14E-09	5.48E-06
T1DM	leukocyte	*CSNK1G3*	-0.745066515	3.91E-09	9.54E-09	1.54E-05
T1DM	leukocyte	*AKAP6*	-0.829035396	1.25E-09	8.14E-09	4.93E-06
T1DM	leukocyte	*MBIP*	-0.711508309	8.54E-09	1.63E-08	3.36E-05
T1DM	leukocyte	*MIPOL1*	-0.639861172	1.91E-09	8.14E-09	7.49E-06
T1DM	leukocyte	*TMEM18*	-0.701611854	1.91E-09	8.14E-09	7.50E-06
T1DM	leukocyte	*NDUFC2*	-0.512088293	2.85E-09	8.43E-09	1.12E-05
T1DM	leukocyte	*VIPAS39*	-1.045855644	9.59E-09	1.80E-08	3.77E-05
T1DM	leukocyte	*C4orf33*	-1.188035768	4.17E-07	5.74E-07	0.001639496
T1DM	leukocyte	*QDPR*	-0.503905358	1.62E-09	8.14E-09	6.36E-06
T1DM	leukocyte	*TEX9*	-1.14082712	3.58E-09	9.11E-09	1.41E-05
T1DM	leukocyte	*ZNF827*	-0.633002822	8.36E-10	8.14E-09	3.28E-06
T1DM	leukocyte	*AKR1C2*	-1.230719935	2.34E-06	3.02E-06	0.009195026
T1DM	leukocyte	*DPYSL4*	-0.487101518	0.001864117	0.002122932	1
T1DM	leukocyte	*KIN*	-1.054246707	1.78E-08	3.07E-08	7.01E-05
T1DM	leukocyte	*TMEM45B*	-1.065272997	6.48E-09	1.32E-08	2.55E-05
T1DM	leukocyte	*CENPU*	-1.127114686	0.000243536	0.000286483	0.956854293
T1DM	leukocyte	*BICD1*	-0.59585445	1.37E-09	8.14E-09	5.40E-06
T1DM	leukocyte	*GUF1*	-1.452225165	1.98E-08	3.36E-08	7.77E-05
T1DM	leukocyte	*CCL28*	-0.869155054	2.55E-10	8.14E-09	1.00E-06
T1DM	leukocyte	*CACUL1*	-0.872629659	7.68E-09	1.51E-08	3.02E-05
T1DM	leukocyte	*KCNE4*	-0.772770658	8.75E-08	1.31E-07	0.000343932
T1DM	leukocyte	*RABGAP1L*	-0.739138133	2.25E-09	8.21E-09	8.84E-06
T1DM	leukocyte	*PTPN14*	-0.568091956	3.86E-08	6.17E-08	0.00015158

T1DM	leukocyte	*ARL14EP*	-0.852253053	3.13E-09	8.69E-09	1.23E-05
T1DM	leukocyte	*EPG5*	-1.044351721	3.47E-09	8.98E-09	1.36E-05
T1DM	leukocyte	*HAUS1*	-0.90000936	1.17E-09	8.14E-09	4.60E-06
T1DM	leukocyte	*SPC25*	-0.819264842	2.76E-09	8.33E-09	1.08E-05
T1DM	leukocyte	*PDK1*	-0.825315709	2.14E-09	8.18E-09	8.39E-06
T1DM	leukocyte	*PDE3B*	-0.43252596	6.18E-10	8.14E-09	2.43E-06
T1DM	leukocyte	*TGOLN2*	-0.661446708	1.07E-09	8.14E-09	4.19E-06
T1DM	leukocyte	*ATG10*	-0.519205873	2.12E-05	2.61E-05	0.083193278
T1DM	leukocyte	*POC5*	-0.500430305	0.000512472	0.000595007	1
T1DM	leukocyte	*GUCY1A2*	-0.699954962	1.35E-09	8.14E-09	5.32E-06
T1DM	leukocyte	*ZNF773*	-0.52142521	1.65E-06	2.16E-06	0.006490617
T1DM	leukocyte	*DCLRE1C*	-1.227188835	0.000155218	0.000184134	0.609852055
T1DM	leukocyte	*ZSCAN1*	-0.720656834	4.41E-10	8.14E-09	1.73E-06
T1DM	leukocyte	*USP12*	-0.604577631	5.04E-09	1.11E-08	1.98E-05
T1DM	leukocyte	*CCDC50*	-0.514867413	5.60E-09	1.18E-08	2.20E-05
T1DM	leukocyte	*PLEKHH2*	-0.571471721	2.13E-06	2.76E-06	0.008362166
T1DM	leukocyte	*GRIA4*	-0.782479905	1.04E-07	1.54E-07	0.000408029
T1DM	leukocyte	*IGSF10*	-1.674252986	2.30E-09	8.21E-09	9.05E-06
T1DM	leukocyte	*SPEF2*	-0.688567635	5.50E-07	7.47E-07	0.002161564
T1DM	leukocyte	*GPD1L*	-0.791975897	1.65E-09	8.14E-09	6.50E-06
T1DM	leukocyte	*CCNO*	-0.741593816	4.45E-08	7.03E-08	0.000174664
T1DM	leukocyte	*CLEC4F*	-81.76471542	4.32E-05	5.26E-05	0.169725237
T1DM	leukocyte	*RASGRP3*	-0.713110521	1.55E-07	2.23E-07	0.000608136
T1DM	leukocyte	*CATSPER3*	-3.856485521	1.29E-09	8.14E-09	5.07E-06
T1DM	leukocyte	*GPR180*	-0.603127042	4.77E-09	1.07E-08	1.88E-05
T1DM	leukocyte	*IFIT5*	-0.738765896	2.23E-09	8.21E-09	8.76E-06
T1DM	leukocyte	*PRDM8*	0.759777106	4.75E-07	6.49E-07	0.001865387
T1DM	leukocyte	*ZNF117*	-0.485231178	1.19E-05	1.48E-05	0.046798156
T1DM	leukocyte	*MED21*	-0.983512115	2.30E-09	8.21E-09	9.04E-06
T1DM	leukocyte	*LGI2*	-1.076678675	2.72E-09	8.27E-09	1.07E-05
T1DM	leukocyte	*SRP19*	-0.548475199	2.53E-09	8.21E-09	9.95E-06
T1DM	leukocyte	*DAB2*	-0.861937572	2.69E-09	8.26E-09	1.06E-05
T1DM	leukocyte	*ACMSD*	-0.952265113	2.04E-09	8.14E-09	8.03E-06
T1DM	leukocyte	*CAST*	-0.562592345	4.51E-09	1.04E-08	1.77E-05
T1DM	leukocyte	*SYCP2L*	-1.588910002	3.91E-06	4.98E-06	0.015360016
T1DM	leukocyte	*MERTK*	0.380107993	6.36E-06	8.01E-06	0.024992883
T1DM	leukocyte	*TMEM87B*	-0.731866867	4.88E-09	1.08E-08	1.92E-05
T1DM	leukocyte	*PLA2R1*	-2.413418646	1.15E-09	8.14E-09	4.53E-06
T1DM	leukocyte	*SLC25A27*	-0.956074487	1.19E-09	8.14E-09	4.68E-06
T1DM	leukocyte	*ASAP1*	-0.608447379	4.88E-09	1.08E-08	1.92E-05
T1DM	leukocyte	*NMRAL1*	-0.74260346	1.14E-07	1.68E-07	0.000448387
T1DM	leukocyte	*TEX29*	-0.860354812	0.000902661	0.001040961	1
T1DM	leukocyte	*CMTM7*	-0.561031926	1.72E-08	2.98E-08	6.77E-05
T1DM	leukocyte	*RPIA*	-0.698418332	1.77E-09	8.14E-09	6.95E-06
T1DM	leukocyte	*PTPRD*	-0.552974687	5.38E-06	6.81E-06	0.021155457
T1DM	leukocyte	*GTF2E1*	-1.518177404	7.02E-09	1.40E-08	2.76E-05
T1DM	leukocyte	*CFDP1*	-0.604960008	6.89E-10	8.14E-09	2.71E-06
T1DM	leukocyte	*ZDHHC7*	-1.059033307	4.25E-08	6.75E-08	0.000167133
T1DM	leukocyte	*JAZF1*	-0.796086048	2.92E-09	8.49E-09	1.15E-05
T1DM	leukocyte	*CMIP*	-0.687480505	2.11E-09	8.16E-09	8.29E-06
T1DM	leukocyte	*KCNJ16*	-0.330377763	0.030826058	0.033577927	1
T1DM	leukocyte	*KCTD15*	-0.984831483	1.71E-09	8.14E-09	6.72E-06
T1DM	leukocyte	*MCOLN2*	-2.833796373	0.001567334	0.001788573	1
T1DM	leukocyte	*LGI4*	0.8538153	1.16E-07	1.70E-07	0.000456365
T1DM	leukocyte	*DDAH1*	-1.369716575	6.59E-09	1.34E-08	2.59E-05
T1DM	leukocyte	*NUS1*	-0.743691058	1.26E-09	8.14E-09	4.95E-06
T1DM	leukocyte	*PPP2R5E*	-0.74909321	2.05E-09	8.14E-09	8.04E-06
T1DM	leukocyte	*THY1*	0.599094724	3.17E-05	3.88E-05	0.124416555
T1DM	leukocyte	*ROBO3*	-2.082277038	1.36E-09	8.14E-09	5.34E-06
T1DM	leukocyte	*PITPNC1*	-0.752468226	1.86E-09	8.14E-09	7.33E-06
T1DM	leukocyte	*LRRK1*	1.187462216	1.16E-07	1.71E-07	0.000457497
T1DM	leukocyte	*CEP112*	-0.581396439	1.79E-09	8.14E-09	7.04E-06
T1DM	leukocyte	*ABCA5*	-0.884447747	4.05E-08	6.44E-08	0.000159153
T1DM	leukocyte	*GBP5*	-14.24338052	5.95E-07	8.04E-07	0.002337231

T1DM	leukocyte	*SRSF12*	-1.188681441	2.66E-08	4.40E-08	0.00010465
T1DM	leukocyte	*PDLIM3*	-0.452909735	1.34E-08	2.40E-08	5.26E-05
T1DM	leukocyte	*PDE1C*	-0.74056367	1.58E-06	2.07E-06	0.006202244
T1DM	leukocyte	*RABGEF1*	-0.724142461	1.06E-09	8.14E-09	4.18E-06
T1DM	leukocyte	*MRPL39*	-0.667018364	4.86E-07	6.63E-07	0.001908643
T1DM	leukocyte	*TSEN2*	-1.593871377	5.75E-10	8.14E-09	2.26E-06
T1DM	leukocyte	*C17orf50*	-0.949671583	0.008487781	0.009455201	1
T1DM	leukocyte	*FLCN*	-1.019836835	3.96E-09	9.62E-09	1.56E-05
T1DM	leukocyte	*MPPE1*	-0.735995006	8.92E-08	1.34E-07	0.00035058
T1DM	leukocyte	*RAB6B*	-0.852822197	1.31E-09	8.14E-09	5.17E-06
T1DM	leukocyte	*ACSS1*	-0.939320448	3.36E-07	4.66E-07	0.001319184
T1DM	leukocyte	*ANKRD40*	-0.846649936	2.68E-08	4.42E-08	0.000105275
T1DM	leukocyte	*ATP6V1C1*	-0.635287602	1.63E-09	8.14E-09	6.40E-06
T1DM	leukocyte	*MARVELD1*	-1.670505435	8.58E-06	1.07E-05	0.033706739
T1DM	leukocyte	*TRMT44*	-0.631465102	8.50E-09	1.63E-08	3.34E-05
T1DM	leukocyte	*PPM1J*	-1.780148419	1.56E-09	8.14E-09	6.15E-06
T1DM	leukocyte	*DBI*	-0.598727931	1.01E-08	1.87E-08	3.96E-05
T1DM	leukocyte	*HEATR3*	-0.531319223	4.66E-07	6.38E-07	0.001831214
T1DM	leukocyte	*LRGUK*	-1.759495878	3.32E-06	4.24E-06	0.013034446
T1DM	leukocyte	*SETD9*	-0.761680346	8.01E-10	8.14E-09	3.15E-06
T1DM	leukocyte	*MIER3*	-1.294891159	6.40E-09	1.31E-08	2.52E-05
T1DM	leukocyte	*TTN*	-1.576993496	4.92E-09	1.09E-08	1.93E-05
T1DM	leukocyte	*DEPTOR*	-0.758803796	1.35E-08	2.41E-08	5.30E-05
T1DM	leukocyte	*RMND1*	-1.059969643	2.62E-08	4.32E-08	0.00010277
T1DM	leukocyte	*TMBIM4*	-0.557572434	1.67E-08	2.90E-08	6.56E-05
T1DM	leukocyte	*PSD3*	-0.611964476	1.19E-09	8.14E-09	4.69E-06
T1DM	leukocyte	*FAM161B*	-1.010579862	1.44E-07	2.07E-07	0.000564766
T1DM	leukocyte	*MMP16*	-0.58106943	5.87E-09	1.22E-08	2.31E-05
T1DM	leukocyte	*ADK*	-0.720284114	8.52E-09	1.63E-08	3.35E-05
T1DM	leukocyte	*BATF*	-10.27075069	1.21E-09	8.14E-09	4.73E-06
T1DM	leukocyte	*DRAM2*	-0.903798167	1.02E-06	1.35E-06	0.003989805
T1DM	leukocyte	*CCT8*	-0.574899931	2.15E-10	8.14E-09	8.44E-07
T1DM	leukocyte	*CDK20*	-0.725131754	5.87E-11	8.14E-09	2.31E-07
T1DM	leukocyte	*SFXN2*	-0.330641445	0.003615941	0.004081308	1
T1DM	leukocyte	*FUT6*	-0.922528662	0.00030969	0.000362566	1
T1DM	leukocyte	*TDRD9*	-0.456925652	2.11E-09	8.16E-09	8.28E-06
T1DM	leukocyte	*HKDC1*	-1.499349781	2.73E-07	3.83E-07	0.001073225
T1DM	leukocyte	*ZDHHC5*	-0.790595621	2.12E-09	8.16E-09	8.31E-06
T1DM	leukocyte	*MED19*	-0.648216687	2.07E-09	8.14E-09	8.14E-06
T1DM	leukocyte	*MAPK13*	-0.782749944	3.49E-09	9.01E-09	1.37E-05
T1DM	leukocyte	*TBC1D31*	1.824311664	1.41E-07	2.04E-07	0.000553824
T1DM	leukocyte	*COX6A2*	-0.73006683	1.20E-06	1.58E-06	0.004704103
T1DM	leukocyte	*MPV17L*	-0.669969345	2.92E-09	8.50E-09	1.15E-05
T1DM	leukocyte	*TATDN2*	-1.088754645	1.53E-08	2.68E-08	6.00E-05
T1DM	leukocyte	*EXOG*	-0.821526714	4.68E-09	1.06E-08	1.84E-05
T1DM	leukocyte	*ZFYVE9*	-0.74942969	1.24E-09	8.14E-09	4.89E-06
T1DM	leukocyte	*SLC6A1*	-4.08395471	1.32E-09	8.14E-09	5.17E-06
T1DM	leukocyte	*NECAP2*	-0.858115032	2.01E-09	8.14E-09	7.91E-06
T1DM	leukocyte	*STEAP2*	-1.020131705	3.50E-09	9.02E-09	1.38E-05
T1DM	leukocyte	*GATAD1*	-0.831064479	4.76E-09	1.07E-08	1.87E-05
T1DM	leukocyte	*TMED6*	-0.295688815	8.10E-05	9.71E-05	0.318126164
T1DM	leukocyte	*CLEC18A*	-2.232110515	2.21E-10	8.14E-09	8.69E-07
T1DM	leukocyte	*DHRS4*	-0.793079699	1.73E-09	8.14E-09	6.79E-06
T1DM	leukocyte	*DHRS1*	-0.799311279	3.57E-08	5.75E-08	0.00014029
T1DM	leukocyte	*AASDH*	-0.535215698	1.92E-08	3.28E-08	7.56E-05
T1DM	leukocyte	*SLC38A10*	-0.765823435	2.55E-09	8.21E-09	1.00E-05
T1DM	leukocyte	*SLC37A3*	-0.562742194	6.90E-10	8.14E-09	2.71E-06
T1DM	leukocyte	*AP3S2*	-0.820367673	4.40E-09	1.02E-08	1.73E-05
T1DM	leukocyte	*SPPL3*	-0.747870526	2.43E-09	8.21E-09	9.57E-06
T1DM	leukocyte	*TNFRSF14*	0.55789313	2.51E-08	4.16E-08	9.86E-05
T1DM	leukocyte	*CIB4*	-10.11900979	2.54E-07	3.58E-07	0.000998619
T1DM	leukocyte	*C12orf43*	-0.804237185	1.16E-08	2.11E-08	4.55E-05
T1DM	leukocyte	*PEX10*	-1.03359249	8.44E-09	1.62E-08	3.31E-05
T1DM	leukocyte	*LDLRAP1*	-0.558677279	2.69E-09	8.26E-09	1.06E-05

T1DM	leukocyte	*KRTCAP3*	-0.687047654	1.41E-09	8.14E-09	5.54E-06
T1DM	leukocyte	*PAFAH2*	-1.791147431	3.70E-09	9.28E-09	1.45E-05
T1DM	leukocyte	*EXTL1*	-2.672320296	3.32E-06	4.24E-06	0.013054008
T1DM	leukocyte	*TRIM63*	-8.648809509	7.29E-08	1.11E-07	0.000286255
T1DM	leukocyte	*XKR8*	-1.215858774	1.26E-09	8.14E-09	4.97E-06
T1DM	leukocyte	*CNNM4*	-0.68330354	0.000654844	0.000757844	1
T1DM	leukocyte	*ESYT3*	-1.229037717	2.14E-07	3.03E-07	0.000839185
T1DM	leukocyte	*FAIM*	-0.719311741	6.78E-10	8.14E-09	2.66E-06
T1DM	leukocyte	*AHCYL2*	-0.740140099	1.94E-07	2.77E-07	0.000762157
T1DM	leukocyte	*COPG2*	-0.574799968	5.54E-07	7.52E-07	0.002176624
T1DM	leukocyte	*RNF166*	-0.873122276	1.38E-08	2.46E-08	5.42E-05
T1DM	leukocyte	*NIT1*	-0.940991301	4.54E-08	7.17E-08	0.00017832
T1DM	leukocyte	*NPM2*	-0.813999783	1.83E-09	8.14E-09	7.17E-06
T1DM	leukocyte	*FGF17*	-1.495772938	5.48E-07	7.44E-07	0.002151799
T1DM	leukocyte	*VWA5B1*	2.119991762	4.72E-09	1.06E-08	1.86E-05
T1DM	leukocyte	*CDA*	-8.562375664	4.17E-09	9.92E-09	1.64E-05
T1DM	leukocyte	*PINK1*	-0.773072725	1.12E-08	2.04E-08	4.38E-05
T1DM	leukocyte	*DMTN*	-0.689020697	1.98E-07	2.82E-07	0.00077636
T1DM	leukocyte	*SLC5A11*	1.196644645	3.71E-05	4.53E-05	0.145915766
T1DM	leukocyte	*CCAR2*	-0.953578732	2.74E-09	8.30E-09	1.08E-05
T1DM	leukocyte	*EPB41*	-0.698544675	7.71E-10	8.14E-09	3.03E-06
T1DM	leukocyte	*MIS18A*	-0.723834314	1.46E-07	2.10E-07	0.00057316
T1DM	leukocyte	*ALG8*	-0.675549303	1.32E-09	8.14E-09	5.20E-06
T1DM	leukocyte	*IFNAR2*	-1.072887036	1.19E-06	1.57E-06	0.004655888
T1DM	leukocyte	*IFNGR2*	-0.645535793	1.28E-08	2.31E-08	5.03E-05
T1DM	leukocyte	*C1QC*	0.045239889	3.33E-09	8.82E-09	1.31E-05
T1DM	leukocyte	*GJD2*	-0.470053784	3.18E-10	8.14E-09	1.25E-06
T1DM	leukocyte	*CLDN14*	-1.763723683	1.00E-09	8.14E-09	3.94E-06
T1DM	leukocyte	*HLCS*	-0.645306452	2.98E-09	8.50E-09	1.17E-05
T1DM	leukocyte	*ADPGK*	0.815061513	3.32E-06	4.24E-06	0.013057619
T1DM	leukocyte	*THEM4*	-0.585383977	1.01E-09	8.14E-09	3.95E-06
T1DM	leukocyte	*UBR1*	-0.874529564	1.99E-09	8.14E-09	7.80E-06
T1DM	leukocyte	*TEPP*	2.915476562	1.11E-08	2.03E-08	4.36E-05
T1DM	leukocyte	*ZDHHC1*	-0.705123741	3.26E-09	8.79E-09	1.28E-05
T1DM	leukocyte	*ZFYVE28*	-0.947459523	3.28E-09	8.79E-09	1.29E-05
T1DM	leukocyte	*NPR2*	-0.88268598	2.56E-09	8.21E-09	1.01E-05
T1DM	leukocyte	*ATAD3B*	-1.419476669	2.73E-07	3.82E-07	0.001072052
T1DM	leukocyte	*ANKLE1*	0.414735895	1.42E-06	1.87E-06	0.005593481
T1DM	leukocyte	*KALRN*	-0.995817468	2.77E-09	8.34E-09	1.09E-05
T1DM	leukocyte	*CILP2*	-0.741750896	0.009761882	0.010837647	1
T1DM	leukocyte	*TMPRSS3*	-2.071764332	1.07E-08	1.97E-08	4.20E-05
T1DM	leukocyte	*RSPH1*	-0.469535847	8.65E-09	1.65E-08	3.40E-05
T1DM	leukocyte	*SLC37A1*	-0.753261409	2.58E-09	8.21E-09	1.01E-05
T1DM	leukocyte	*NDUFV3*	-0.504957484	1.06E-08	1.95E-08	4.16E-05
T1DM	leukocyte	*HSF2BP*	-1.629055801	6.04E-06	7.62E-06	0.023736372
T1DM	leukocyte	*PDXK*	-0.773565003	3.58E-09	9.11E-09	1.41E-05
T1DM	leukocyte	*CSTB*	-0.653431393	1.57E-07	2.25E-07	0.000614985
T1DM	leukocyte	*ICOSLG*	0.369878109	2.22E-08	3.73E-08	8.72E-05
T1DM	leukocyte	*ZNF66*	-1.630134204	4.19E-07	5.76E-07	0.001646004
T1DM	leukocyte	*FTCD*	-0.989646899	8.03E-10	8.14E-09	3.15E-06
T1DM	leukocyte	*SPATC1L*	-0.356111404	0.000122695	0.00014617	0.482067789
T1DM	leukocyte	*LSS*	-0.814166642	1.04E-09	8.14E-09	4.07E-06
T1DM	leukocyte	*VAV2*	-1.035940451	6.11E-09	1.26E-08	2.40E-05
T1DM	leukocyte	*MCM3AP*	-0.762359215	4.62E-05	5.62E-05	0.181558392
T1DM	leukocyte	*DIP2A*	-0.513367754	5.25E-07	7.16E-07	0.002064421
T1DM	leukocyte	*ZNF208*	-0.707787666	4.94E-11	8.14E-09	1.94E-07
T1DM	leukocyte	*ADAMTS13*	-0.503099195	0.011942686	0.013221418	1
T1DM	leukocyte	*SLC2A6*	-1.191517424	3.31E-09	8.82E-09	1.30E-05
T1DM	leukocyte	*RDH13*	-0.831356515	2.74E-09	8.31E-09	1.08E-05
T1DM	leukocyte	*MPZL3*	-0.747797127	6.03E-09	1.25E-08	2.37E-05
T1DM	leukocyte	*S100A1*	-1.466238511	2.41E-08	4.03E-08	9.48E-05
T1DM	leukocyte	*VPS11*	-1.395283933	9.19E-11	8.14E-09	3.61E-07
T1DM	leukocyte	*PAQR6*	-0.824767703	3.01E-09	8.54E-09	1.18E-05
T1DM	leukocyte	*SLC25A44*	-1.077770711	3.40E-09	8.90E-09	1.33E-05

T1DM	leukocyte	*CCR5*	-89.25643941	2.40E-08	4.01E-08	9.43E-05
T1DM	leukocyte	*NBEAL2*	-0.505312791	2.46E-08	4.09E-08	9.65E-05
T1DM	leukocyte	*LY6K*	-1.12227913	3.88E-07	5.36E-07	0.001526357
T1DM	leukocyte	*LY6E*	-0.717262565	8.45E-09	1.62E-08	3.32E-05
T1DM	leukocyte	*VPS28*	-0.581805394	5.73E-09	1.20E-08	2.25E-05
T1DM	leukocyte	*TONSL*	-2.90794089	1.45E-07	2.10E-07	0.000570999
T1DM	leukocyte	*ZNF333*	-1.575251348	6.71E-05	8.09E-05	0.263517336
T1DM	leukocyte	*RPL8*	-0.402167434	2.64E-09	8.21E-09	1.04E-05
T1DM	leukocyte	*MAML1*	-1.132947695	6.62E-08	1.01E-07	0.000260042
T1DM	leukocyte	*LRWD1*	-1.013345096	2.75E-09	8.32E-09	1.08E-05
T1DM	leukocyte	*CCDC116*	-5.122986348	1.33E-05	1.64E-05	0.052128281
T1DM	leukocyte	*U2AF1L4*	-0.531151498	3.54E-08	5.71E-08	0.000139195
T1DM	leukocyte	*ZNF382*	-0.697691483	8.34E-09	1.61E-08	3.28E-05
T1DM	leukocyte	*DUSP14*	-0.873841473	1.79E-09	8.14E-09	7.02E-06
T1DM	leukocyte	*PGAP3*	-0.766499302	1.81E-09	8.14E-09	7.10E-06
T1DM	leukocyte	*PRPSAP1*	-0.687123072	7.97E-10	8.14E-09	3.13E-06
T1DM	leukocyte	*SIGLEC11*	-11.92426086	6.92E-09	1.39E-08	2.72E-05
T1DM	leukocyte	*DBF4B*	-0.761765543	1.03E-05	1.28E-05	0.040402545
T1DM	leukocyte	*SPC24*	-1.799175563	1.24E-07	1.81E-07	0.000486997
T1DM	leukocyte	*IP6K3*	1.25835801	7.20E-05	8.66E-05	0.282698564
T1DM	leukocyte	*LEMD2*	-1.212984895	1.14E-07	1.68E-07	0.000448995
T1DM	leukocyte	*CXCL16*	0.290504583	8.18E-09	1.58E-08	3.21E-05
T1DM	leukocyte	*RPL26*	-0.427390069	2.60E-09	8.21E-09	1.02E-05
T1DM	leukocyte	*SNRNP25*	-0.616440611	9.65E-10	8.14E-09	3.79E-06
T1DM	leukocyte	*WDR90*	-0.995887823	3.10E-08	5.04E-08	0.00012163
T1DM	leukocyte	*CCDC78*	-2.174435368	1.77E-09	8.14E-09	6.96E-06
T1DM	leukocyte	*AMDHD2*	0.764447916	1.86E-08	3.19E-08	7.32E-05
T1DM	leukocyte	*ZNF75A*	-1.162471298	1.92E-08	3.28E-08	7.55E-05
T1DM	leukocyte	*ASRGL1*	-0.573572452	1.74E-09	8.14E-09	6.85E-06
T1DM	leukocyte	*TAF6L*	-0.87434192	4.62E-08	7.27E-08	0.000181406
T1DM	leukocyte	*ZFPL1*	-0.748410614	6.37E-09	1.30E-08	2.50E-05
T1DM	leukocyte	*LRP5*	-1.25838486	1.59E-09	8.14E-09	6.24E-06
T1DM	leukocyte	*TPCN2*	-0.826789137	2.76E-07	3.86E-07	0.001082744
T1DM	leukocyte	*PDZK1IP1*	-0.498382729	1.66E-09	8.14E-09	6.52E-06
T1DM	leukocyte	*CTRC*	-0.352534165	1.61E-09	8.14E-09	6.34E-06
T1DM	leukocyte	*SDC3*	-1.349565559	1.28E-09	8.14E-09	5.03E-06
T1DM	leukocyte	*OMA1*	-0.962281013	1.79E-08	3.07E-08	7.03E-05
T1DM	leukocyte	*TM2D1*	-0.911150899	6.70E-09	1.36E-08	2.63E-05
T1DM	leukocyte	*FUBP1*	-0.573336037	1.29E-09	8.14E-09	5.05E-06
T1DM	leukocyte	*SNX7*	-0.569200106	1.93E-09	8.14E-09	7.59E-06
T1DM	leukocyte	*NTNG1*	0.963389686	7.62E-07	1.02E-06	0.002994184
T1DM	leukocyte	*HFM1*	-1.312321443	7.79E-09	1.53E-08	3.06E-05
T1DM	leukocyte	*VCAM1*	-0.39411309	2.28E-09	8.21E-09	8.97E-06
T1DM	leukocyte	*ZNF496*	-0.694005209	1.84E-09	8.14E-09	7.24E-06
T1DM	leukocyte	*TRIM58*	-2.465359864	6.53E-07	8.80E-07	0.002565506
T1DM	leukocyte	*PEA15*	-0.621640792	3.93E-09	9.56E-09	1.54E-05
T1DM	leukocyte	*FLVCR1*	-0.566026402	6.72E-10	8.14E-09	2.64E-06
T1DM	leukocyte	*TDRD5*	-2.349813364	6.22E-09	1.28E-08	2.44E-05
T1DM	leukocyte	*C1orf115*	-0.435470873	1.59E-06	2.09E-06	0.006256849
T1DM	leukocyte	*ACP6*	-0.892743622	5.52E-09	1.17E-08	2.17E-05
T1DM	leukocyte	*OXER1*	-1.180044209	0.011204851	0.012411576	1
T1DM	leukocyte	*HAAO*	-0.80793726	2.64E-09	8.21E-09	1.04E-05
T1DM	leukocyte	*RFTN2*	-1.779208917	1.77E-11	4.65E-09	6.97E-08
T1DM	leukocyte	*KCNJ3*	-0.645448985	2.27E-09	8.21E-09	8.93E-06
T1DM	leukocyte	*CLHC1*	-0.520582678	4.73E-07	6.47E-07	0.001860335
T1DM	leukocyte	*DUSP19*	-1.024821638	4.08E-09	9.80E-09	1.60E-05
T1DM	leukocyte	*SMC6*	-1.096008518	5.41E-09	1.16E-08	2.12E-05
T1DM	leukocyte	*NOSTRIN*	-0.628055197	9.87E-07	1.31E-06	0.003876199
T1DM	leukocyte	*CTSS*	0.17857312	1.21E-08	2.20E-08	4.76E-05
T1DM	leukocyte	*MSX1*	-0.783103574	1.47E-09	8.14E-09	5.78E-06
T1DM	leukocyte	*PACRGL*	-0.790546711	2.05E-09	8.14E-09	8.05E-06
T1DM	leukocyte	*ERCC3*	-0.868723032	1.40E-08	2.49E-08	5.51E-05
T1DM	leukocyte	*TDRD10*	-11.08304818	6.88E-08	1.05E-07	0.000270272
T1DM	leukocyte	*CCNYL1*	-1.494672888	1.47E-08	2.60E-08	5.77E-05

T1DM	leukocyte	*DCAF16*	-0.84321827	3.74E-09	9.32E-09	1.47E-05
T1DM	leukocyte	*NPPC*	-5.333696453	1.05E-06	1.39E-06	0.004119592
T1DM	leukocyte	*GNPDA2*	-0.449677743	1.08E-05	1.35E-05	0.042495826
T1DM	leukocyte	*NIPAL1*	-0.607572916	1.65E-08	2.87E-08	6.49E-05
T1DM	leukocyte	*CGGBP1*	-0.612800819	1.68E-08	2.91E-08	6.59E-05
T1DM	leukocyte	*DAPL1*	-1.103579632	0.001042562	0.00119948	1
T1DM	leukocyte	*EOGT*	-0.70936071	1.56E-09	8.14E-09	6.15E-06
T1DM	leukocyte	*POGLUT1*	-0.575309602	2.85E-09	8.43E-09	1.12E-05
T1DM	leukocyte	*IGFN1*	-0.784712747	1.21E-07	1.77E-07	0.00047654
T1DM	leukocyte	*ATP1A1*	-0.459225589	1.48E-09	8.14E-09	5.83E-06
T1DM	leukocyte	*ARPC2*	-0.648425735	5.16E-09	1.12E-08	2.03E-05
T1DM	leukocyte	*CCT3*	-0.509141673	1.64E-09	8.14E-09	6.45E-06
T1DM	leukocyte	*SRGAP2*	-0.691213924	0.031677071	0.034485788	1
T1DM	leukocyte	*CCDC141*	-0.799478513	0.001983565	0.002257002	1
T1DM	leukocyte	*EOMES*	-3.822267386	2.93E-08	4.79E-08	0.000114927
T1DM	leukocyte	*CWC22*	-0.741634637	2.52E-09	8.21E-09	9.89E-06
T1DM	leukocyte	*HDAC11*	-1.439135286	7.53E-06	9.45E-06	0.029602888
T1DM	leukocyte	*SUCLG1*	-0.647928866	2.45E-09	8.21E-09	9.61E-06
T1DM	leukocyte	*NUAK2*	0.796900948	4.64E-05	5.64E-05	0.182325697
T1DM	leukocyte	*EFHB*	-0.352931055	4.05E-05	4.95E-05	0.159284404
T1DM	leukocyte	*ICA1L*	-0.554429653	3.45E-07	4.78E-07	0.001355533
T1DM	leukocyte	*RYBP*	-0.529229851	7.21E-10	8.14E-09	2.83E-06
T1DM	leukocyte	*GTPBP8*	-1.096294442	2.48E-09	8.21E-09	9.75E-06
T1DM	leukocyte	*CADPS*	-0.354962722	7.62E-09	1.50E-08	2.99E-05
T1DM	leukocyte	*ATXN7*	-0.735831549	5.69E-09	1.20E-08	2.24E-05
T1DM	leukocyte	*PSMD6*	-0.582725196	2.62E-09	8.21E-09	1.03E-05
T1DM	leukocyte	*PRICKLE2*	-0.743854229	1.79E-09	8.14E-09	7.04E-06
T1DM	leukocyte	*RPL9*	-0.42441974	1.36E-09	8.14E-09	5.34E-06
T1DM	leukocyte	*IL17RC*	-0.76141085	9.30E-10	8.14E-09	3.65E-06
T1DM	leukocyte	*CRELD1*	-0.508643347	2.18E-08	3.66E-08	8.56E-05
T1DM	leukocyte	*TTC14*	-1.017565753	7.46E-09	1.47E-08	2.93E-05
T1DM	leukocyte	*CXCL3*	0.659388382	1.59E-07	2.28E-07	0.000623909
T1DM	leukocyte	*CXCL1*	-0.48312949	8.17E-10	8.14E-09	3.21E-06
T1DM	leukocyte	*CCDC158*	-1.720073939	2.54E-08	4.21E-08	9.98E-05
T1DM	leukocyte	*TM4SF18*	-0.725268565	2.47E-09	8.21E-09	9.70E-06
T1DM	leukocyte	*SNRK*	-0.487456897	3.47E-09	8.99E-09	1.36E-05
T1DM	leukocyte	*KIAA1143*	-0.561247864	3.40E-10	8.14E-09	1.34E-06
T1DM	leukocyte	*CCR1*	0.629531192	1.14E-07	1.68E-07	0.000448812
T1DM	leukocyte	*NMNAT3*	-0.645349	8.40E-10	8.14E-09	3.30E-06
T1DM	leukocyte	*SMIM12*	-0.647377929	2.10E-09	8.16E-09	8.24E-06
T1DM	leukocyte	*TPRA1*	-1.149066718	4.75E-09	1.07E-08	1.87E-05
T1DM	leukocyte	*MEAF6*	-0.581410379	2.33E-09	8.21E-09	9.14E-06
T1DM	leukocyte	*DNALI1*	-0.718075568	3.29E-09	8.81E-09	1.29E-05
T1DM	leukocyte	*CAMK2N2*	-0.4567714	1.10E-09	8.14E-09	4.33E-06
T1DM	leukocyte	*RPN1*	-0.70044653	2.08E-09	8.15E-09	8.18E-06
T1DM	leukocyte	*IFT122*	-0.762874712	5.42E-07	7.37E-07	0.002128143
T1DM	leukocyte	*RFC4*	-0.861613242	1.69E-09	8.14E-09	6.64E-06
T1DM	leukocyte	*TKT*	-0.664289852	4.78E-09	1.07E-08	1.88E-05
T1DM	leukocyte	*RFT1*	-1.168249153	1.91E-09	8.14E-09	7.50E-06
T1DM	leukocyte	*UVSSA*	-0.918525299	1.42E-06	1.87E-06	0.005581262
T1DM	leukocyte	*ARHGEF3*	-0.672614199	4.35E-09	1.01E-08	1.71E-05
T1DM	leukocyte	*LRPAP1*	-0.54961834	7.96E-09	1.55E-08	3.13E-05
T1DM	leukocyte	*SLC51A*	1.22567284	2.55E-05	3.13E-05	0.100243754
T1DM	leukocyte	*PIGX*	-0.878678282	3.89E-09	9.52E-09	1.53E-05
T1DM	leukocyte	*EXO5*	-0.93746713	1.48E-06	1.94E-06	0.005811201
T1DM	leukocyte	*ERMAP*	-1.612611416	2.07E-06	2.69E-06	0.008151201
T1DM	leukocyte	*BDH2*	-0.655618981	2.84E-09	8.43E-09	1.12E-05
T1DM	leukocyte	*CDC25A*	-1.006622252	1.90E-07	2.72E-07	0.000748046
T1DM	leukocyte	*TEX264*	-0.759026318	7.55E-09	1.49E-08	2.97E-05
T1DM	leukocyte	*PPM1M*	-0.433873734	0.005305081	0.005951931	1
T1DM	leukocyte	*SAP30*	-0.66635873	2.64E-09	8.21E-09	1.04E-05
T1DM	leukocyte	*ANXA5*	-0.431119914	2.97E-09	8.50E-09	1.17E-05
T1DM	leukocyte	*CEP44*	-0.759600131	1.96E-09	8.14E-09	7.69E-06
T1DM	leukocyte	*HPGD*	-1.028313552	8.55E-07	1.14E-06	0.003361096

T1DM	leukocyte	*NPY1R*	1.565019911	6.41E-06	8.08E-06	0.025203078
T1DM	leukocyte	*ARFIP1*	-0.93458899	5.37E-09	1.15E-08	2.11E-05
T1DM	leukocyte	*ITGA2*	-0.47589852	7.62E-09	1.50E-08	2.99E-05
T1DM	leukocyte	*ELOVL7*	-0.663408608	5.32E-09	1.15E-08	2.09E-05
T1DM	leukocyte	*LMBRD2*	-0.823179118	9.24E-11	8.14E-09	3.63E-07
T1DM	leukocyte	*STARD4*	-0.920098998	4.34E-10	8.14E-09	1.71E-06
T1DM	leukocyte	*CMBL*	-0.7207857	1.76E-09	8.14E-09	6.90E-06
T1DM	leukocyte	*PRRC1*	-0.545620757	1.36E-09	8.14E-09	5.34E-06
T1DM	leukocyte	*AGGF1*	-0.842890282	5.76E-09	1.20E-08	2.26E-05
T1DM	leukocyte	*WDR41*	-1.025422719	3.11E-09	8.66E-09	1.22E-05
T1DM	leukocyte	*SERINC5*	-1.373122146	0.0009853	0.001135596	1
T1DM	leukocyte	*RICTOR*	-0.888361773	6.41E-09	1.31E-08	2.52E-05
T1DM	leukocyte	*UTP15*	-0.632342686	1.38E-09	8.14E-09	5.42E-06
T1DM	leukocyte	*KLKB1*	-0.848114071	0.000139139	0.00016526	0.546678474
T1DM	leukocyte	*NSA2*	-0.461610935	2.69E-09	8.26E-09	1.06E-05
T1DM	leukocyte	*LEAP2*	-1.107522584	2.01E-09	8.14E-09	7.88E-06
T1DM	leukocyte	*DACT2*	-3.022567926	1.70E-09	8.14E-09	6.67E-06
T1DM	leukocyte	*PDSS2*	-0.705429903	1.01E-09	8.14E-09	3.98E-06
T1DM	leukocyte	*STK17A*	-0.59352169	3.24E-09	8.77E-09	1.27E-05
T1DM	leukocyte	*GALNT10*	-1.237170078	3.56E-07	4.92E-07	0.001398542
T1DM	leukocyte	*SAP30L*	-0.88817672	3.23E-09	8.77E-09	1.27E-05
T1DM	leukocyte	*HCN1*	-1.645323891	3.21E-06	4.11E-06	0.01260495
T1DM	leukocyte	*COG5*	-1.134841567	1.34E-09	8.14E-09	5.26E-06
T1DM	leukocyte	*SLU7*	-0.678723514	3.44E-09	8.93E-09	1.35E-05
T1DM	leukocyte	*CAMLG*	-0.535953308	4.17E-09	9.93E-09	1.64E-05
T1DM	leukocyte	*RELL2*	-0.919263882	3.03E-06	3.89E-06	0.011914213
T1DM	leukocyte	*KIF6*	-1.184552513	1.69E-08	2.94E-08	6.66E-05
T1DM	leukocyte	*STEAP1*	-0.944292908	4.50E-09	1.04E-08	1.77E-05
T1DM	leukocyte	*FNDC1*	-3.009288302	0.000200623	0.000237067	0.788247653
T1DM	leukocyte	*CHMP4C*	-0.661658812	1.59E-09	8.14E-09	6.25E-06
T1DM	leukocyte	*BRI3*	-0.690602197	5.36E-08	8.37E-08	0.000210707
T1DM	leukocyte	*LMTK2*	-0.680725818	3.67E-09	9.23E-09	1.44E-05
T1DM	leukocyte	*CTSB*	0.381353011	3.09E-08	5.03E-08	0.000121287
T1DM	leukocyte	*DLC1*	-0.666017276	2.60E-07	3.66E-07	0.001021785
T1DM	leukocyte	*PEX2*	-0.589318997	1.69E-09	8.14E-09	6.64E-06
T1DM	leukocyte	*MED30*	-0.687593215	1.52E-09	8.14E-09	5.96E-06
T1DM	leukocyte	*TNFRSF11B*	-1.949109847	1.64E-07	2.35E-07	0.000644984
T1DM	leukocyte	*CSMD3*	-48.52474452	1.38E-09	8.14E-09	5.44E-06
T1DM	leukocyte	*OSGIN2*	-0.883606786	4.63E-09	1.05E-08	1.82E-05
T1DM	leukocyte	*DEFB1*	-0.322569237	1.63E-09	8.14E-09	6.41E-06
T1DM	leukocyte	*SUN1*	-0.539186428	2.23E-09	8.21E-09	8.75E-06
T1DM	leukocyte	*TMEM184A*	-0.723734865	2.64E-09	8.21E-09	1.04E-05
T1DM	leukocyte	*INTS1*	-0.716549585	1.57E-09	8.14E-09	6.17E-06
T1DM	leukocyte	*YWHAZ*	-0.565040566	3.39E-09	8.89E-09	1.33E-05
T1DM	leukocyte	*DCAF13*	-0.695331153	2.29E-09	8.21E-09	9.00E-06
T1DM	leukocyte	*INTS8*	-0.797664466	1.45E-06	1.91E-06	0.005703822
T1DM	leukocyte	*GEM*	-0.706424102	9.95E-09	1.85E-08	3.91E-05
T1DM	leukocyte	*RPP25L*	-1.04322884	3.06E-09	8.60E-09	1.20E-05
T1DM	leukocyte	*FAM219A*	-1.03670416	2.30E-09	8.21E-09	9.04E-06
T1DM	leukocyte	*NUDT2*	-0.737289681	4.21E-09	9.96E-09	1.65E-05
T1DM	leukocyte	*NIPSNAP3B*	1.104981851	4.34E-05	5.29E-05	0.170551216
T1DM	leukocyte	*METTL2B*	-0.614299874	1.62E-06	2.12E-06	0.006375688
T1DM	leukocyte	*FXN*	-1.289655315	9.14E-09	1.73E-08	3.59E-05
T1DM	leukocyte	*MAMDC2*	-1.513500838	1.52E-08	2.67E-08	5.97E-05
T1DM	leukocyte	*PRSS37*	-396.6281638	1.71E-09	8.14E-09	6.73E-06
T1DM	leukocyte	*C9orf64*	-0.892561661	2.94E-09	8.50E-09	1.15E-05
T1DM	leukocyte	*ANKS6*	-0.627417629	5.47E-08	8.50E-08	0.000214784
T1DM	leukocyte	*CLDN3*	-0.691309701	1.22E-09	8.14E-09	4.78E-06
T1DM	leukocyte	*WNK2*	-1.049188584	3.33E-09	8.82E-09	1.31E-05
T1DM	leukocyte	*NOL6*	-1.313882796	1.91E-09	8.14E-09	7.50E-06
T1DM	leukocyte	*PIGO*	-1.111332039	1.86E-07	2.66E-07	0.000732407
T1DM	leukocyte	*ARHGAP12*	-0.720483342	3.91E-09	9.54E-09	1.54E-05
T1DM	leukocyte	*WRN*	-0.772178342	1.79E-09	8.14E-09	7.02E-06
T1DM	leukocyte	*CFL2*	-0.673418684	3.82E-09	9.44E-09	1.50E-05

T1DM	leukocyte	*PGM2L1*	-1.050318347	3.37E-08	5.47E-08	0.000132491
T1DM	leukocyte	*PHYHIPL*	-0.707168084	1.63E-09	8.14E-09	6.41E-06
T1DM	leukocyte	*PKNOX2*	2.867712586	9.23E-08	1.38E-07	0.000362584
T1DM	leukocyte	*RPL36AL*	-0.464064741	1.82E-08	3.12E-08	7.14E-05
T1DM	leukocyte	*TMEM63C*	-0.614442898	4.11E-09	9.85E-09	1.62E-05
T1DM	leukocyte	*NGB*	-3.07104838	0.012668885	0.014009583	1
T1DM	leukocyte	*NOXRED1*	-2.515159685	4.93E-05	5.98E-05	0.193598901
T1DM	leukocyte	*AKR1E2*	-1.954012379	0.000316292	0.000369965	1
T1DM	leukocyte	*OXGR1*	-1.590820129	2.64E-09	8.21E-09	1.04E-05
T1DM	leukocyte	*VDAC2*	-0.573940755	2.96E-09	8.50E-09	1.16E-05
T1DM	leukocyte	*SNAPC4*	-0.721681337	2.24E-09	8.21E-09	8.81E-06
T1DM	leukocyte	*TSC1*	-0.954052037	1.98E-09	8.14E-09	7.79E-06
T1DM	leukocyte	*ZMYND19*	-0.695750782	1.41E-09	8.14E-09	5.53E-06
T1DM	leukocyte	*METTL17*	-0.909159954	8.02E-09	1.56E-08	3.15E-05
T1DM	leukocyte	*CASP7*	-0.416373449	5.26E-07	7.17E-07	0.002067777
T1DM	leukocyte	*VWA2*	-1.864339142	9.00E-10	8.14E-09	3.54E-06
T1DM	leukocyte	*METTL3*	-0.757929515	1.95E-09	8.14E-09	7.68E-06
T1DM	leukocyte	*TRUB1*	-0.929191417	3.44E-09	8.93E-09	1.35E-05
T1DM	leukocyte	*ARHGAP42*	-1.093724027	2.56E-09	8.21E-09	1.01E-05
T1DM	leukocyte	*SLC39A13*	-0.678799849	1.55E-09	8.14E-09	6.08E-06
T1DM	leukocyte	*PSMC3*	-0.521821634	1.08E-08	1.99E-08	4.24E-05
T1DM	leukocyte	*CPSF2*	-0.550907257	5.03E-08	7.87E-08	0.000197551
T1DM	leukocyte	*IFI27L1*	-0.710495904	2.98E-09	8.50E-09	1.17E-05
T1DM	leukocyte	*C1QL3*	-1.078440123	0.0165531	0.018227895	1
T1DM	leukocyte	*SMCO4*	-0.722737286	3.34E-09	8.82E-09	1.31E-05
T1DM	leukocyte	*ABTB2*	-1.038430084	3.25E-09	8.77E-09	1.28E-05
T1DM	leukocyte	*HTRA1*	0.827705755	0.002331227	0.002647988	1
T1DM	leukocyte	*CEP57*	-0.591801406	2.26E-09	8.21E-09	8.89E-06
T1DM	leukocyte	*JAM3*	-0.559941983	6.15E-09	1.26E-08	2.42E-05
T1DM	leukocyte	*IKBIP*	-0.785058713	8.93E-09	1.70E-08	3.51E-05
T1DM	leukocyte	*ZFYVE19*	-0.641672935	4.91E-08	7.69E-08	0.000192769
T1DM	leukocyte	*CKB*	-0.518800986	1.95E-10	8.14E-09	7.68E-07
T1DM	leukocyte	*BTRC*	-1.142965808	6.71E-08	1.02E-07	0.000263763
T1DM	leukocyte	*BAG5*	-0.692613358	3.85E-09	9.49E-09	1.51E-05
T1DM	leukocyte	*LARP6*	-0.593057135	2.04E-09	8.14E-09	8.03E-06
T1DM	leukocyte	*ZNF319*	-1.838386054	4.74E-09	1.06E-08	1.86E-05
T1DM	leukocyte	*SGPL1*	-1.084262402	1.53E-09	8.14E-09	6.02E-06
T1DM	leukocyte	*FRS2*	-0.676261411	4.63E-08	7.29E-08	0.000181927
T1DM	leukocyte	*PCBD1*	-0.643575652	1.37E-09	8.14E-09	5.40E-06
T1DM	leukocyte	*COX11*	-0.746525829	3.97E-09	9.63E-09	1.56E-05
T1DM	leukocyte	*ZNF202*	0.958675946	8.16E-08	1.23E-07	0.000320541
T1DM	leukocyte	*MYRFL*	-0.425427561	0.001239019	0.001420929	1
T1DM	leukocyte	*WBP1L*	-1.269873911	9.50E-08	1.41E-07	0.000373104
T1DM	leukocyte	*NUDT13*	-0.496843218	0.008884142	0.009882728	1
T1DM	leukocyte	*CYB5R2*	-0.622498989	6.30E-07	8.50E-07	0.002474613
T1DM	leukocyte	*TUB*	-0.900489879	8.05E-09	1.56E-08	3.16E-05
T1DM	leukocyte	*RIC3*	-0.537385881	1.68E-09	8.14E-09	6.61E-06
T1DM	leukocyte	*XRRA1*	-0.721151441	3.88E-09	9.51E-09	1.52E-05
T1DM	leukocyte	*TRIM66*	-0.762488626	3.10E-06	3.98E-06	0.012197734
T1DM	leukocyte	*CDYL2*	-0.732967295	2.85E-09	8.43E-09	1.12E-05
T1DM	leukocyte	*ZNF143*	-0.695760131	1.46E-09	8.14E-09	5.74E-06
T1DM	leukocyte	*CCDC68*	-0.685383115	2.04E-08	3.45E-08	8.02E-05
T1DM	leukocyte	*ZSCAN21*	-1.039760528	6.40E-09	1.31E-08	2.51E-05
T1DM	leukocyte	*RIMKLB*	-0.674487148	1.63E-09	8.14E-09	6.39E-06
T1DM	leukocyte	*GALR1*	0.919502382	1.30E-09	8.14E-09	5.10E-06
T1DM	leukocyte	*SPDYE6*	1.224827713	2.51E-06	3.24E-06	0.009867738
T1DM	leukocyte	*ATF7IP2*	-0.894147729	2.11E-09	8.16E-09	8.28E-06
T1DM	leukocyte	*COG1*	-0.669025937	2.17E-09	8.21E-09	8.54E-06
T1DM	leukocyte	*B2M*	-0.427054752	2.18E-09	8.21E-09	8.55E-06
T1DM	leukocyte	*ACSM1*	-1.409971703	0.00033983	0.000397261	1
T1DM	leukocyte	*AP1G1*	-0.513592066	3.74E-09	9.32E-09	1.47E-05
T1DM	leukocyte	*SLFN5*	-0.571267353	1.69E-09	8.14E-09	6.65E-06
T1DM	leukocyte	*SAAL1*	-0.683155967	1.61E-09	8.14E-09	6.34E-06
T1DM	leukocyte	*SCNN1G*	-6.301304667	9.82E-10	8.14E-09	3.86E-06

T1DM	leukocyte	*GLYATL1*	-4.691372152	0.013736934	0.015173577	1
T1DM	leukocyte	*DCTN5*	-0.767151976	4.61E-10	8.14E-09	1.81E-06
T1DM	leukocyte	*STAT6*	-1.104643981	1.06E-08	1.95E-08	4.16E-05
T1DM	leukocyte	*PATL1*	-0.607789489	1.44E-09	8.14E-09	5.65E-06
T1DM	leukocyte	*MRPL16*	-0.871886556	6.31E-06	7.96E-06	0.024805887
T1DM	leukocyte	*GREM1*	-1.012289981	1.95E-08	3.32E-08	7.66E-05
T1DM	leukocyte	*TSC22D4*	-0.699361285	3.72E-06	4.74E-06	0.014619097
T1DM	leukocyte	*MS4A14*	0.168992939	1.36E-08	2.42E-08	5.33E-05
T1DM	leukocyte	*DIS3L*	-1.049271799	4.17E-09	9.93E-09	1.64E-05
T1DM	leukocyte	*MS4A8*	-1.329159585	0.000176926	0.00020957	0.695143636
T1DM	leukocyte	*RCCD1*	-1.476200507	6.74E-08	1.03E-07	0.000264976
T1DM	leukocyte	*MAPRE2*	-0.633531663	6.86E-09	1.38E-08	2.70E-05
T1DM	leukocyte	*NAT16*	-0.468790641	2.07E-09	8.14E-09	8.13E-06
T1DM	leukocyte	*GNGT2*	0.334639325	7.38E-09	1.46E-08	2.90E-05
T1DM	leukocyte	*SLC27A4*	-0.762271639	6.30E-09	1.29E-08	2.47E-05
T1DM	leukocyte	*URM1*	-0.833714102	3.51E-08	5.67E-08	0.000137904
T1DM	leukocyte	*CERCAM*	-0.606898574	2.11E-08	3.55E-08	8.28E-05
T1DM	leukocyte	*DOLPP1*	-0.796480783	1.08E-08	1.99E-08	4.26E-05
T1DM	leukocyte	*ENDOG*	-0.773987055	6.54E-09	1.33E-08	2.57E-05
T1DM	leukocyte	*PRR15L*	-0.96526895	7.80E-09	1.53E-08	3.07E-05
T1DM	leukocyte	*RNF214*	-1.027882083	9.73E-08	1.45E-07	0.000382428
T1DM	leukocyte	*POP5*	-0.762523275	4.54E-10	8.14E-09	1.79E-06
T1DM	leukocyte	*ENGASE*	-0.36898788	0.000766759	0.000886058	1
T1DM	leukocyte	*ACAA2*	-0.71418931	1.98E-09	8.14E-09	7.77E-06
T1DM	leukocyte	*FN3K*	-0.906718286	3.88E-09	9.51E-09	1.52E-05
T1DM	leukocyte	*ZNF23*	-0.672798122	1.55E-09	8.14E-09	6.10E-06
T1DM	leukocyte	*ZNF229*	-0.95066565	1.23E-09	8.14E-09	4.83E-06
T1DM	leukocyte	*ZNF180*	-0.746235706	1.38E-09	8.14E-09	5.44E-06
T1DM	leukocyte	*CA4*	-0.44520753	1.11E-06	1.47E-06	0.004370751
T1DM	leukocyte	*GPX4*	-0.624062389	1.31E-08	2.35E-08	5.13E-05
T1DM	leukocyte	*ZNF641*	-1.069085779	3.26E-09	8.78E-09	1.28E-05
T1DM	leukocyte	*CACNB3*	-0.709436575	1.09E-05	1.35E-05	0.042686513
T1DM	leukocyte	*TP53I13*	-0.640656689	1.84E-08	3.17E-08	7.24E-05
T1DM	leukocyte	*TUBA1C*	-0.450440317	3.07E-09	8.60E-09	1.21E-05
T1DM	leukocyte	*ZNF528*	-1.933351434	9.91E-07	1.31E-06	0.003892904
T1DM	leukocyte	*RAB4B*	-0.801427843	4.13E-09	9.87E-09	1.62E-05
T1DM	leukocyte	*TMC4*	-0.701865811	1.46E-09	8.14E-09	5.73E-06
T1DM	leukocyte	*PPP1R14A*	-0.414069883	0.000218351	0.000257473	0.857900361
T1DM	leukocyte	*C19orf33*	-0.616282379	1.32E-09	8.14E-09	5.19E-06
T1DM	leukocyte	*DNAAF3*	-2.507018219	1.19E-09	8.14E-09	4.66E-06
T1DM	leukocyte	*CHAF1A*	-0.817293204	1.79E-06	2.32E-06	0.007015048
T1DM	leukocyte	*UBXN6*	-0.845997845	6.43E-09	1.31E-08	2.53E-05
T1DM	leukocyte	*NXN*	-0.502512618	4.25E-09	9.99E-09	1.67E-05
T1DM	leukocyte	*KIFC2*	-2.128173952	6.53E-08	1.00E-07	0.000256442
T1DM	leukocyte	*SLC43A2*	0.506035206	2.13E-05	2.62E-05	0.083594317
T1DM	leukocyte	*SERPINF2*	0.32581462	4.81E-07	6.57E-07	0.001889709
T1DM	leukocyte	*SRR*	-0.674335012	2.94E-07	4.10E-07	0.001154103
T1DM	leukocyte	*TRPV3*	-3.559118859	2.92E-10	8.14E-09	1.15E-06
T1DM	leukocyte	*C19orf48*	-0.965640788	1.60E-06	2.10E-06	0.006288348
T1DM	leukocyte	*KLK11*	-0.384817738	0.007375825	0.008235185	1
T1DM	leukocyte	*ZNF83*	-0.867148932	2.23E-09	8.21E-09	8.74E-06
T1DM	leukocyte	*ZNF558*	-0.781691653	2.51E-07	3.54E-07	0.000987885
T1DM	leukocyte	*NDUFV1*	-0.629427693	1.34E-09	8.14E-09	5.26E-06
T1DM	leukocyte	*ZNF232*	-0.530348375	8.36E-07	1.12E-06	0.003285342
T1DM	leukocyte	*MIS12*	-0.881090032	3.37E-10	8.14E-09	1.32E-06
T1DM	leukocyte	*HID1*	-0.748209795	1.96E-09	8.14E-09	7.69E-06
T1DM	leukocyte	*EVPL*	-0.771078605	0.03519023	0.038236287	1
T1DM	leukocyte	*SRP68*	-0.58213484	1.49E-09	8.14E-09	5.85E-06
T1DM	leukocyte	*TMC8*	0.695268572	7.39E-07	9.92E-07	0.002901859
T1DM	leukocyte	*TMEM68*	-0.894326359	4.38E-09	1.02E-08	1.72E-05
T1DM	leukocyte	*GSDMA*	-2.161612623	0.008561189	0.00952887	1
T1DM	leukocyte	*MLST8*	-0.567185197	2.31E-09	8.21E-09	9.09E-06
T1DM	leukocyte	*ECI1*	-0.548213519	1.91E-09	8.14E-09	7.52E-06
T1DM	leukocyte	*ABCA3*	-0.587219187	1.12E-07	1.65E-07	0.000440455

T1DM	leukocyte	*BSCL2*	-0.505740238	1.54E-07	2.21E-07	0.000604571
T1DM	leukocyte	*C2CD3*	-1.266670291	1.01E-09	8.14E-09	3.96E-06
T1DM	leukocyte	*TRANK1*	-1.66235315	1.18E-08	2.14E-08	4.62E-05
T1DM	leukocyte	*RPSA*	-0.42757119	5.87E-09	1.22E-08	2.31E-05
T1DM	leukocyte	*ENTPD3*	-0.434627609	2.19E-08	3.68E-08	8.61E-05
T1DM	leukocyte	*ULK4*	-0.716421144	1.59E-06	2.08E-06	0.006241768
T1DM	leukocyte	*LTBP3*	-0.579745733	1.77E-09	8.14E-09	6.96E-06
T1DM	leukocyte	*SAC3D1*	-0.815079802	1.19E-09	8.14E-09	4.67E-06
T1DM	leukocyte	*PBK*	-1.563549547	1.96E-09	8.14E-09	7.70E-06
T1DM	leukocyte	*KIAA1586*	-0.881148439	7.29E-10	8.14E-09	2.86E-06
T1DM	leukocyte	*FAM83B*	-0.670346218	2.65E-07	3.72E-07	0.001040654
T1DM	leukocyte	*LMBRD1*	-0.779751529	2.27E-09	8.21E-09	8.91E-06
T1DM	leukocyte	*TTC39C*	-0.635174559	3.34E-09	8.82E-09	1.31E-05
T1DM	leukocyte	*POLR2J3*	-0.728663486	3.79E-09	9.41E-09	1.49E-05
T1DM	leukocyte	*FOXI1*	2.164820032	6.35E-06	8.00E-06	0.024933702
T1DM	leukocyte	*COA6*	-0.745017419	1.83E-09	8.14E-09	7.20E-06
T1DM	leukocyte	*PXK*	-0.884235251	3.57E-09	9.10E-09	1.40E-05
T1DM	leukocyte	*ING5*	-1.120564878	7.23E-09	1.44E-08	2.84E-05
T1DM	leukocyte	*RFWD3*	-0.581012094	0.00019707	0.000233009	0.774289412
T1DM	leukocyte	*BMP1*	-0.823457254	9.78E-10	8.14E-09	3.84E-06
T1DM	leukocyte	*HEXIM2*	-0.877874243	1.40E-09	8.14E-09	5.50E-06
T1DM	leukocyte	*SERINC2*	-0.47267808	2.29E-09	8.21E-09	8.99E-06
T1DM	leukocyte	*TRAPPC11*	-0.586180322	1.23E-09	8.14E-09	4.83E-06
T1DM	leukocyte	*COL3A1*	0.791397591	0.0023181	0.002633838	1
T1DM	leukocyte	*ING2*	-0.498374237	8.65E-09	1.65E-08	3.40E-05
T1DM	leukocyte	*ADAM9*	-1.143011285	7.52E-08	1.14E-07	0.000295585
T1DM	leukocyte	*NDUFS5*	-0.510940929	1.87E-09	8.14E-09	7.35E-06
T1DM	leukocyte	*ZNF30*	-0.608586418	0.027826941	0.030361581	1
T1DM	leukocyte	*DNAJC21*	-0.725140111	1.90E-09	8.14E-09	7.46E-06
T1DM	leukocyte	*CNNM3*	-0.675487628	2.08E-09	8.15E-09	8.17E-06
T1DM	leukocyte	*GSTM4*	-0.797522665	2.88E-09	8.46E-09	1.13E-05
T1DM	leukocyte	*TCTN2*	-0.461847428	0.003373858	0.003814645	1
T1DM	leukocyte	*SHOX2*	-5.704497485	1.06E-07	1.57E-07	0.000416846
T1DM	leukocyte	*PPIP5K1*	-1.750911227	1.49E-09	8.14E-09	5.85E-06
T1DM	leukocyte	*ADAL*	-1.514981811	2.75E-06	3.54E-06	0.010810415
T1DM	leukocyte	*LCMT2*	-1.091310049	5.46E-10	8.14E-09	2.15E-06
T1DM	leukocyte	*ZNF507*	-0.837270453	2.47E-09	8.21E-09	9.70E-06
T1DM	leukocyte	*NSG1*	-0.532583863	1.42E-09	8.14E-09	5.59E-06
T1DM	leukocyte	*ZBTB49*	-1.838842574	2.93E-09	8.50E-09	1.15E-05
T1DM	leukocyte	*GFM1*	-0.587539081	9.50E-10	8.14E-09	3.73E-06
T1DM	leukocyte	*FSTL5*	-0.472525209	4.26E-09	1.00E-08	1.67E-05
T1DM	leukocyte	*CTRB1*	-0.303120947	1.64E-09	8.14E-09	6.45E-06
T1DM	leukocyte	*CTRB2*	-0.280536625	3.51E-09	9.03E-09	1.38E-05
T1DM	leukocyte	*TMEM129*	-0.803392362	6.67E-07	8.98E-07	0.002621954
T1DM	leukocyte	*PPIC*	-0.345901305	1.27E-07	1.85E-07	0.000499948
T1DM	leukocyte	*STXBP6*	-0.747997377	6.56E-09	1.33E-08	2.58E-05
T1DM	leukocyte	*MFF*	-0.646742639	3.07E-09	8.60E-09	1.21E-05
T1DM	leukocyte	*CPLX1*	-0.709022912	5.53E-09	1.17E-08	2.17E-05
T1DM	leukocyte	*ROR2*	-1.767695888	4.71E-07	6.45E-07	0.001852357
T1DM	leukocyte	*HSPBAP1*	1.272507284	0.00020351	0.000240262	0.799590298
T1DM	leukocyte	*FAM110B*	-0.736690329	1.19E-09	8.14E-09	4.67E-06
T1DM	leukocyte	*ATF5*	-0.7958717	4.63E-09	1.05E-08	1.82E-05
T1DM	leukocyte	*XPO6*	-1.08408043	1.33E-08	2.39E-08	5.24E-05
T1DM	leukocyte	*RGS14*	-0.905406796	6.57E-08	1.01E-07	0.000258253
T1DM	leukocyte	*TBC1D10B*	-0.877690271	8.18E-10	8.14E-09	3.21E-06
T1DM	leukocyte	*PRELID1*	-0.505897002	4.09E-09	9.81E-09	1.61E-05
T1DM	leukocyte	*CXCL10*	-122.9291475	1.48E-09	8.14E-09	5.80E-06
T1DM	leukocyte	*NMD3*	-0.657343849	2.02E-09	8.14E-09	7.92E-06
T1DM	leukocyte	*STK32A*	-0.768463822	1.42E-08	2.52E-08	5.57E-05
T1DM	leukocyte	*P2RY12*	-25.03357988	0.003043583	0.003445186	1
T1DM	leukocyte	*SNUPN*	-1.689914474	2.35E-09	8.21E-09	9.25E-06
T1DM	leukocyte	*CRADD*	-0.750630548	1.70E-09	8.14E-09	6.67E-06
T1DM	leukocyte	*COL22A1*	-7.422228919	5.78E-10	8.14E-09	2.27E-06
T1DM	leukocyte	*SDC2*	-0.913520992	0.00039496	0.000460337	1

T1DM	leukocyte	*CLIC4*	-0.415261625	1.72E-09	8.14E-09	6.75E-06
T1DM	leukocyte	*SLC38A11*	-6.145753406	3.83E-11	8.14E-09	1.51E-07
T1DM	leukocyte	*CCDC8*	-0.876555736	1.05E-05	1.31E-05	0.041366154
T1DM	leukocyte	*HINT1*	-0.462850219	1.00E-09	8.14E-09	3.95E-06
T1DM	leukocyte	*DTWD2*	-1.373437412	0.000289116	0.000339086	1
T1DM	leukocyte	*INO80E*	-0.826576177	1.09E-08	2.00E-08	4.28E-05
T1DM	leukocyte	*DFFB*	0.969243852	3.08E-07	4.28E-07	0.00120909
T1DM	leukocyte	*C15orf40*	-0.630825006	3.28E-08	5.33E-08	0.000128876
T1DM	leukocyte	*HIC2*	-1.012016256	0.030049866	0.03275969	1
T1DM	leukocyte	*AGPAT2*	-0.689757084	1.84E-09	8.14E-09	7.23E-06
T1DM	leukocyte	*FASN*	-1.093853008	4.20E-09	9.96E-09	1.65E-05
T1DM	leukocyte	*MT1E*	-0.391478586	9.46E-09	1.78E-08	3.72E-05
T1DM	leukocyte	*LDB2*	-0.738203982	2.68E-08	4.42E-08	0.000105187
T1DM	leukocyte	*RAC3*	-0.971972804	7.36E-10	8.14E-09	2.89E-06
T1DM	leukocyte	*LIMS1*	-0.759933762	2.04E-09	8.14E-09	8.02E-06
T1DM	leukocyte	*CSGALNACT2*	-1.144613342	9.02E-05	0.000108037	0.354360083
T1DM	leukocyte	*AVEN*	-1.630302496	7.30E-08	1.11E-07	0.000286677
T1DM	leukocyte	*CTNND2*	-0.735170865	3.91E-09	9.52E-09	1.53E-05
T1DM	leukocyte	*WNT10B*	-42.6415921	1.33E-09	8.14E-09	5.23E-06
T1DM	leukocyte	*CALML6*	0.844566147	2.64E-09	8.21E-09	1.04E-05
T1DM	leukocyte	*TM4SF4*	-0.638216158	9.77E-10	8.14E-09	3.84E-06
T1DM	leukocyte	*OTUD3*	-0.527165678	1.08E-09	8.14E-09	4.24E-06
T1DM	leukocyte	*MAP3K2*	-0.776354618	1.46E-08	2.59E-08	5.75E-05
T1DM	leukocyte	*PUSL1*	-1.277245482	5.88E-08	9.09E-08	0.000231202
T1DM	leukocyte	*IFFO2*	-0.667449827	6.27E-10	8.14E-09	2.46E-06
T1DM	leukocyte	*MYRIP*	-0.936456158	1.83E-09	8.14E-09	7.19E-06
T1DM	leukocyte	*KCNAB3*	0.677647003	8.13E-07	1.09E-06	0.003193507
T1DM	leukocyte	*FAM153A*	-1.460881044	1.47E-08	2.60E-08	5.78E-05
T1DM	leukocyte	*ZNF778*	-0.908444075	1.45E-09	8.14E-09	5.70E-06
T1DM	leukocyte	*NIPA1*	-0.85117852	6.56E-10	8.14E-09	2.58E-06
T1DM	leukocyte	*FOXD4*	-6.755974902	8.21E-05	9.85E-05	0.322623513
T1DM	leukocyte	*UBE2E1*	-1.059536232	9.47E-09	1.78E-08	3.72E-05
T1DM	leukocyte	*ANKK1*	-19.92259845	2.13E-10	8.14E-09	8.39E-07
T1DM	leukocyte	*USP47*	-0.752124221	5.05E-09	1.11E-08	1.99E-05
T1DM	leukocyte	*PDCD6IP*	-0.651962696	2.40E-09	8.21E-09	9.44E-06
T1DM	leukocyte	*ZNF212*	0.517088953	0.020359557	0.022325621	1
T1DM	leukocyte	*CRTAP*	-1.095990346	1.72E-08	2.97E-08	6.75E-05
T1DM	leukocyte	*STX8*	-0.790830897	1.44E-07	2.07E-07	0.000564097
T1DM	leukocyte	*NFRKB*	-1.143751657	4.51E-09	1.04E-08	1.77E-05
T1DM	leukocyte	*SETMAR*	-0.690132802	7.86E-10	8.14E-09	3.09E-06
T1DM	leukocyte	*CST5*	-2.325251065	0.002938181	0.003326833	1
T1DM	leukocyte	*CST2*	-1.079776095	9.19E-06	1.15E-05	0.036103032
T1DM	leukocyte	*KRT8*	-0.297138583	1.88E-09	8.14E-09	7.37E-06
T1DM	leukocyte	*ADORA2B*	1.318827078	0.000195346	0.000231109	0.767513896
T1DM	leukocyte	*MGMT*	-0.862295323	2.54E-09	8.21E-09	9.99E-06
T1DM	leukocyte	*NFXL1*	-1.148489884	2.71E-10	8.14E-09	1.06E-06
T1DM	leukocyte	*DNAJC18*	-0.581877299	0.000110963	0.000132394	0.435973995
T1DM	leukocyte	*KRT4*	-25.04554711	0.000225478	0.000265638	0.885902504
T1DM	leukocyte	*SLC23A1*	-5.620035958	3.63E-08	5.84E-08	0.000142714
T1DM	leukocyte	*NPAS2*	-0.559053705	5.23E-09	1.13E-08	2.06E-05
T1DM	leukocyte	*NUDT9*	-0.564079981	1.14E-08	2.09E-08	4.50E-05
T1DM	leukocyte	*SMAGP*	-0.483008924	9.70E-08	1.44E-07	0.000380948
T1DM	leukocyte	*IRX1*	-1.033267945	1.39E-09	8.14E-09	5.47E-06
T1DM	leukocyte	*CDH2*	-0.573405836	1.45E-09	8.14E-09	5.71E-06
T1DM	leukocyte	*HSPA4*	-0.682225002	4.38E-09	1.02E-08	1.72E-05
T1DM	leukocyte	*ARMC10*	-0.885008992	1.29E-08	2.32E-08	5.07E-05
T1DM	leukocyte	*ACYP2*	-0.618496112	2.34E-09	8.21E-09	9.18E-06
T1DM	leukocyte	*ATF7*	-0.72739853	1.60E-09	8.14E-09	6.29E-06
T1DM	leukocyte	*RASA4B*	-0.576394115	0.01234354	0.013657497	1
T1DM	leukocyte	*KCNS3*	-0.933989221	3.74E-09	9.32E-09	1.47E-05
T1DM	leukocyte	*FOXN2*	-1.400046775	9.46E-06	1.18E-05	0.037163016
T1DM	leukocyte	*GPR27*	-0.715908341	6.90E-10	8.14E-09	2.71E-06
T1DM	leukocyte	*MTSS1*	-0.753418915	1.49E-06	1.96E-06	0.005863211
T1DM	leukocyte	*TMEM43*	-0.692273725	2.03E-09	8.14E-09	7.97E-06

T1DM	leukocyte	*RPS9*	-0.417033205	2.64E-09	8.21E-09	1.04E-05
T1DM	leukocyte	*NDUFA3*	-0.690243464	5.07E-09	1.11E-08	1.99E-05
T1DM	leukocyte	*ZNF160*	-0.693424901	2.30E-09	8.21E-09	9.02E-06
T1DM	leukocyte	*ZNF415*	-0.67282962	9.51E-09	1.79E-08	3.74E-05
T1DM	leukocyte	*TSNARE1*	0.739375099	7.51E-09	1.48E-08	2.95E-05
T1DM	leukocyte	*SOX7*	-0.933509105	1.48E-09	8.14E-09	5.81E-06
T1DM	leukocyte	*TRMT61B*	-0.585563048	8.67E-09	1.65E-08	3.41E-05
T1DM	leukocyte	*INSR*	-0.492587079	2.71E-09	8.26E-09	1.06E-05
T1DM	leukocyte	*MFN1*	-0.714173758	5.73E-09	1.20E-08	2.25E-05
T1DM	leukocyte	*KCNMB3*	-1.247697692	2.35E-10	8.14E-09	9.24E-07
T1DM	leukocyte	*ATP6V0E2*	-0.537928814	2.90E-08	4.74E-08	0.00011375
T1DM	leukocyte	*JAGN1*	-0.736008556	2.22E-09	8.21E-09	8.73E-06
T1DM	leukocyte	*MORN4*	-0.557626756	4.84E-11	8.14E-09	1.90E-07
T1DM	leukocyte	*TRIM8*	-0.757618912	1.90E-09	8.14E-09	7.47E-06
T1DM	leukocyte	*CDC42BPG*	-0.504663656	3.70E-09	9.29E-09	1.46E-05
T1DM	leukocyte	*ZNF440*	-2.001839024	1.73E-08	2.98E-08	6.78E-05
T1DM	leukocyte	*GAA*	-0.505976087	0.000354181	0.000413667	1
T1DM	leukocyte	*EXOSC1*	-0.864383037	3.15E-09	8.69E-09	1.24E-05
T1DM	leukocyte	*PGAM1*	-0.553547296	3.02E-08	4.94E-08	0.00011884
T1DM	leukocyte	*ESCO2*	-2.359286127	2.62E-07	3.69E-07	0.001031068
T1DM	leukocyte	*MRPL36*	-0.67562254	2.14E-09	8.19E-09	8.43E-06
T1DM	leukocyte	*MCC*	-0.843156656	2.07E-08	3.49E-08	8.12E-05
T1DM	leukocyte	*ZNF561*	-0.834568495	4.82E-10	8.14E-09	1.89E-06
T1DM	leukocyte	*PPID*	-0.785508994	4.19E-06	5.33E-06	0.016468726
T1DM	leukocyte	*RXFP1*	-11.34765297	6.79E-05	8.19E-05	0.266875675
T1DM	leukocyte	*PTGER4*	0.483787195	1.26E-08	2.28E-08	4.95E-05
T1DM	leukocyte	*MAP6*	-0.482619959	9.78E-09	1.83E-08	3.84E-05
T1DM	leukocyte	*ZNF584*	-0.709354483	0.000154925	0.000183842	0.608701012
T1DM	leukocyte	*NMUR1*	-7.76197343	1.25E-09	8.14E-09	4.90E-06
T1DM	leukocyte	*CLSTN1*	-0.61408651	9.41E-11	8.14E-09	3.70E-07
T1DM	leukocyte	*PIK3CD*	0.690353044	1.71E-08	2.95E-08	6.70E-05
T1DM	leukocyte	*ENC1*	-0.578892953	1.62E-07	2.33E-07	0.000638018
T1DM	leukocyte	*RGS19*	-1.039160853	1.33E-08	2.39E-08	5.22E-05
T1DM	leukocyte	*ANO5*	-0.876461076	1.91E-09	8.14E-09	7.51E-06
T1DM	leukocyte	*GPHN*	-1.062459968	3.72E-08	5.96E-08	0.000146063
T1DM	leukocyte	*VAT1L*	-0.868673176	5.23E-09	1.13E-08	2.06E-05
T1DM	leukocyte	*LGALS4*	-0.398435253	1.60E-09	8.14E-09	6.27E-06
T1DM	leukocyte	*LRRC34*	-0.857803664	3.42E-09	8.93E-09	1.34E-05
T1DM	leukocyte	*SPATA5L1*	-1.291268643	2.51E-09	8.21E-09	9.88E-06
T1DM	leukocyte	*CTPS1*	-0.689888869	3.61E-10	8.14E-09	1.42E-06
T1DM	leukocyte	*METTL18*	-0.897050696	2.77E-09	8.34E-09	1.09E-05
T1DM	leukocyte	*PWWP2B*	-0.855137219	1.26E-09	8.14E-09	4.96E-06
T1DM	leukocyte	*PCDHB1*	0.990678601	0.015787227	0.017399162	1
T1DM	leukocyte	*FAM90A1*	-1.327110692	0.001856462	0.002114827	1
T1DM	leukocyte	*PTEN*	-0.79915139	4.35E-10	8.14E-09	1.71E-06
T1DM	leukocyte	*CYP4F11*	-1.672965796	7.08E-08	1.08E-07	0.00027829
T1DM	leukocyte	*TLN2*	-1.398666915	1.36E-09	8.14E-09	5.34E-06
T1DM	leukocyte	*TVP23B*	-1.008981936	1.16E-07	1.71E-07	0.000457128
T1DM	leukocyte	*SRGAP2C*	-1.290148068	2.52E-09	8.21E-09	9.88E-06
T1DM	leukocyte	*ATPAF2*	-0.843503905	8.95E-09	1.70E-08	3.52E-05
T1DM	leukocyte	*ZNF556*	0.600651235	2.59E-07	3.65E-07	0.001018517
T1DM	leukocyte	*ZNF554*	-0.790954525	1.40E-09	8.14E-09	5.49E-06
T1DM	leukocyte	*GAP43*	-0.560410475	1.49E-09	8.14E-09	5.86E-06
T1DM	leukocyte	*REG1B*	-0.32925581	6.24E-10	8.14E-09	2.45E-06
T1DM	leukocyte	*NME6*	-1.074218374	6.76E-08	1.03E-07	0.000265521
T1DM	leukocyte	*SLFN12*	0.758943634	1.33E-08	2.39E-08	5.23E-05
T1DM	leukocyte	*SLC9C1*	-2.230276823	0.00266198	0.003015836	1
T1DM	leukocyte	*TEFM*	-0.897970333	1.83E-09	8.14E-09	7.20E-06
T1DM	leukocyte	*MBOAT1*	-1.160841082	1.48E-06	1.94E-06	0.005800816
T1DM	leukocyte	*ZNF131*	-0.711825047	1.52E-09	8.14E-09	5.99E-06
T1DM	leukocyte	*BSG*	-0.568012018	3.07E-09	8.60E-09	1.21E-05
T1DM	leukocyte	*HINFP*	-1.756323128	5.53E-08	8.59E-08	0.000217163
T1DM	leukocyte	*CERS6*	-0.660160656	1.45E-09	8.14E-09	5.71E-06
T1DM	leukocyte	*SPTLC3*	0.898714027	1.38E-08	2.45E-08	5.41E-05

T1DM	leukocyte	*COPRS*	-0.470179237	3.81E-08	6.10E-08	0.000149782
T1DM	leukocyte	*B3GALT1*	-1.131862076	0.005782391	0.006474498	1
T1DM	leukocyte	*SUCLG2*	-1.123946559	6.82E-09	1.37E-08	2.68E-05
T1DM	leukocyte	*CSDC2*	-1.60854965	2.34E-06	3.02E-06	0.009178155
T1DM	leukocyte	*ARNT2*	-0.570303351	1.55E-08	2.72E-08	6.11E-05
T1DM	leukocyte	*FUT9*	-2.027081283	0.000203084	0.000239831	0.797916492
T1DM	leukocyte	*ZNF24*	-0.73247111	5.28E-10	8.14E-09	2.07E-06
T1DM	leukocyte	*MANEA*	-0.774504381	4.54E-09	1.04E-08	1.78E-05
T1DM	leukocyte	*AFF1*	-0.761706805	9.95E-10	8.14E-09	3.91E-06
T1DM	leukocyte	*PDE3A*	-0.576581993	0.000897292	0.001035074	1
T1DM	leukocyte	*SMPDL3A*	-0.847146548	0.005538362	0.006208338	1
T1DM	leukocyte	*RAD9A*	-1.150689014	3.10E-08	5.05E-08	0.000121967
T1DM	leukocyte	*EFEMP2*	-0.764641964	6.41E-10	8.14E-09	2.52E-06
T1DM	leukocyte	*AGAP5*	3.246831587	2.17E-06	2.81E-06	0.008525882
T1DM	leukocyte	*ZNF738*	-0.803698492	1.12E-07	1.65E-07	0.000439045
T1DM	leukocyte	*NAA16*	-0.690067569	2.32E-09	8.21E-09	9.11E-06
T1DM	leukocyte	*RAB43*	0.617674825	0.02368405	0.025913293	1
T1DM	leukocyte	*SNX32*	-0.985425099	0.004235826	0.004772745	1
T1DM	leukocyte	*OVOL1*	-0.695334887	0.023865377	0.026104417	1
T1DM	leukocyte	*DMXL1*	-0.995697493	1.85E-08	3.17E-08	7.25E-05
T1DM	leukocyte	*NADSYN1*	-1.083623089	3.21E-07	4.47E-07	0.001262474
T1DM	leukocyte	*RNASEH2C*	-0.67718411	1.11E-08	2.03E-08	4.36E-05
T1DM	leukocyte	*LCLAT1*	-0.719452245	8.02E-10	8.14E-09	3.15E-06
T1DM	leukocyte	*SH3RF3*	-1.172219171	1.82E-10	8.14E-09	7.13E-07
T1DM	leukocyte	*DCAKD*	-0.528189285	1.13E-08	2.06E-08	4.43E-05
T1DM	leukocyte	*TADA2B*	-0.789399874	2.40E-09	8.21E-09	9.42E-06
T1DM	leukocyte	*EVC2*	-1.349114615	0.000475066	0.000552393	1
T1DM	leukocyte	*TRMT112*	-0.587820695	3.35E-09	8.83E-09	1.32E-05
T1DM	leukocyte	*NOC3L*	-0.701131673	1.33E-09	8.14E-09	5.22E-06
T1DM	leukocyte	*RHOD*	-0.494518417	5.27E-10	8.14E-09	2.07E-06
T1DM	leukocyte	*PARP14*	-0.679190587	1.34E-08	2.40E-08	5.26E-05
T1DM	leukocyte	*VANGL1*	-0.723996941	9.46E-10	8.14E-09	3.72E-06
T1DM	leukocyte	*IQCB1*	-1.441001698	4.09E-08	6.50E-08	0.00016078
T1DM	leukocyte	*ZNF483*	-0.570238967	4.49E-09	1.03E-08	1.76E-05
T1DM	leukocyte	*SLC2A14*	-2.810553878	1.95E-09	8.14E-09	7.65E-06
T1DM	leukocyte	*MZT2A*	-0.495112628	6.34E-08	9.75E-08	0.000249255
T1DM	leukocyte	*PPP1R3B*	-1.378559534	1.85E-07	2.65E-07	0.000727837
T1DM	leukocyte	*MAP3K11*	-0.654164333	1.16E-09	8.14E-09	4.57E-06
T1DM	leukocyte	*NDNF*	0.80067785	3.89E-09	9.52E-09	1.53E-05
T1DM	leukocyte	*ARV1*	-0.636110881	2.00E-09	8.14E-09	7.85E-06
T1DM	leukocyte	*EHBP1L1*	-0.677766187	5.40E-08	8.41E-08	0.000212018
T1DM	leukocyte	*PPP1R14B*	-0.540073468	6.78E-09	1.37E-08	2.66E-05
T1DM	leukocyte	*ZNF417*	0.53012987	8.28E-09	1.60E-08	3.25E-05
T1DM	leukocyte	*PEAK1*	-0.536617321	1.16E-07	1.70E-07	0.000454404
T1DM	leukocyte	*TNFRSF10D*	-0.859537904	2.10E-09	8.16E-09	8.23E-06
T1DM	leukocyte	*GMPPB*	-1.0119745	7.84E-09	1.53E-08	3.08E-05
T1DM	leukocyte	*NUDT18*	-0.722448247	1.45E-09	8.14E-09	5.69E-06
T1DM	leukocyte	*NUDT4*	-0.62131381	8.33E-09	1.61E-08	3.27E-05
T1DM	leukocyte	*PC*	-1.084589343	2.64E-09	8.21E-09	1.04E-05
T1DM	leukocyte	*LRFN4*	1.402505848	1.97E-05	2.43E-05	0.077542188
T1DM	leukocyte	*UQCRH*	-0.573668738	1.32E-09	8.14E-09	5.20E-06
T1DM	leukocyte	*HEG1*	-0.708180492	1.26E-09	8.14E-09	4.97E-06
T1DM	leukocyte	*HAP1*	-0.865615007	2.36E-07	3.34E-07	0.000927065
T1DM	leukocyte	*ENDOV*	-1.233259343	7.39E-09	1.46E-08	2.90E-05
T1DM	leukocyte	*DPY19L1*	-0.849306362	1.03E-09	8.14E-09	4.04E-06
T1DM	leukocyte	*TUBB8*	-12.79308011	0.002758072	0.003123801	1
T1DM	leukocyte	*GPR160*	-0.993342065	8.28E-09	1.60E-08	3.25E-05
T1DM	leukocyte	*XXYLT1*	-1.052349992	1.47E-08	2.60E-08	5.77E-05
T1DM	leukocyte	*GNG5*	-0.573031916	2.87E-09	8.44E-09	1.13E-05
T1DM	leukocyte	*C9orf131*	-1.386089586	6.17E-08	9.51E-08	0.000242416
T1DM	leukocyte	*FAM53A*	-2.592278997	2.12E-06	2.75E-06	0.008330439
T1DM	leukocyte	*SELP*	-4.286617657	1.30E-09	8.14E-09	5.12E-06
T1DM	leukocyte	*CTU2*	-0.658595993	7.19E-09	1.43E-08	2.83E-05
T1DM	leukocyte	*SNX31*	-3.603707568	0.000506145	0.000587834	1

T1DM	leukocyte	*ADCY6*	-0.790447093	8.81E-10	8.14E-09	3.46E-06
T1DM	leukocyte	*PODN*	-0.684368165	9.70E-08	1.44E-07	0.000381017
T1DM	leukocyte	*SLC6A19*	0.629354223	0.025848339	0.028226272	1
T1DM	leukocyte	*RALGAPA1*	-0.630245875	3.14E-09	8.69E-09	1.23E-05
T1DM	leukocyte	*CNTNAP2*	1.508055202	6.84E-09	1.38E-08	2.69E-05
T1DM	leukocyte	*GCSAM*	-2.377815436	0.045163453	0.048883528	1
T1DM	leukocyte	*GOLT1A*	-0.667972163	2.47E-09	8.21E-09	9.70E-06
T1DM	leukocyte	*NPAS4*	-1.310787295	4.57E-08	7.21E-08	0.000179596
T1DM	leukocyte	*ANGEL2*	-0.903447529	2.64E-09	8.21E-09	1.04E-05
T1DM	leukocyte	*ZNF266*	-0.461389353	7.75E-07	1.04E-06	0.003046597
T1DM	leukocyte	*BRSK2*	-0.658692389	1.38E-09	8.14E-09	5.43E-06
T1DM	leukocyte	*BRMS1*	-0.626924627	1.94E-09	8.14E-09	7.63E-06
T1DM	leukocyte	*HRAS*	-0.622118226	1.69E-09	8.14E-09	6.63E-06
T1DM	leukocyte	*SRP72*	-0.520086171	2.15E-09	8.19E-09	8.43E-06
T1DM	leukocyte	*PCP2*	-1.96939054	1.60E-05	1.98E-05	0.062925806
T1DM	leukocyte	*BTC*	-1.044408645	8.13E-09	1.57E-08	3.20E-05
T1DM	leukocyte	*DENND6A*	-0.668998159	9.40E-10	8.14E-09	3.69E-06
T1DM	leukocyte	*YIF1A*	-0.53723309	6.85E-08	1.04E-07	0.000269116
T1DM	leukocyte	*NDUFA11*	-0.593632121	3.88E-09	9.51E-09	1.52E-05
T1DM	leukocyte	*RSRC1*	-1.087379267	3.71E-07	5.13E-07	0.001459606
T1DM	leukocyte	*FUT1*	-1.223450591	1.44E-09	8.14E-09	5.66E-06
T1DM	leukocyte	*DHX36*	-0.670110715	2.35E-09	8.21E-09	9.24E-06
T1DM	leukocyte	*PSMD2*	-0.554991858	3.35E-09	8.83E-09	1.32E-05
T1DM	leukocyte	*FAM182B*	-0.608807902	0.040936851	0.0443699	1
T1DM	leukocyte	*PPM1E*	-0.567127017	5.14E-10	8.14E-09	2.02E-06
T1DM	leukocyte	*ZNF408*	-0.899067093	0.008692758	0.009672571	1
T1DM	leukocyte	*ARHGAP1*	-0.625261441	1.20E-08	2.18E-08	4.71E-05
T1DM	leukocyte	*MED16*	-0.632732209	2.41E-09	8.21E-09	9.49E-06
T1DM	leukocyte	*GAL3ST3*	24.33800015	2.30E-07	3.26E-07	0.000904726
T1DM	leukocyte	*CHST1*	-1.419265816	1.45E-09	8.14E-09	5.71E-06
T1DM	leukocyte	*DOLK*	-1.171882198	7.05E-08	1.07E-07	0.000276882
T1DM	leukocyte	*PHYHD1*	-0.377691429	0.000346944	0.000405336	1
T1DM	leukocyte	*CCNE2*	-0.745227248	3.39E-07	4.70E-07	0.001331371
T1DM	leukocyte	*ZNF519*	-1.315055264	3.40E-10	8.14E-09	1.34E-06
T1DM	leukocyte	*CHRNA7*	-1.140754055	5.43E-08	8.45E-08	0.000213333
T1DM	leukocyte	*TMEM9B*	-0.637873421	5.19E-09	1.13E-08	2.04E-05
T1DM	leukocyte	*SCUBE2*	-0.372341225	2.57E-05	3.16E-05	0.101042448
T1DM	leukocyte	*EIF3F*	-0.631204476	8.00E-09	1.56E-08	3.14E-05
T1DM	leukocyte	*ZNF25*	-0.649268823	2.79E-09	8.35E-09	1.10E-05
T1DM	leukocyte	*PPP2R2D*	-0.640625544	1.39E-09	8.14E-09	5.48E-06
T1DM	leukocyte	*CLCF1*	-0.983503738	1.61E-09	8.14E-09	6.31E-06
T1DM	leukocyte	*UCP2*	0.607246114	1.56E-08	2.74E-08	6.14E-05
T1DM	leukocyte	*PAAF1*	-0.440755935	0.006667553	0.007452864	1
T1DM	leukocyte	*MRPL48*	-0.840197536	3.77E-09	9.36E-09	1.48E-05
T1DM	leukocyte	*TMEM70*	-0.634647162	3.07E-09	8.60E-09	1.21E-05
T1DM	leukocyte	*RPS6KB2*	-0.949389196	3.33E-09	8.82E-09	1.31E-05
T1DM	leukocyte	*RMI2*	-2.166222728	3.51E-08	5.67E-08	0.000137868
T1DM	leukocyte	*SFN*	-1.035184309	6.32E-05	7.64E-05	0.248484115
T1DM	leukocyte	*SWI5*	-0.817809421	6.28E-07	8.48E-07	0.002467485
T1DM	leukocyte	*ZDHHC21*	-1.080639887	8.67E-08	1.30E-07	0.000340586
T1DM	leukocyte	*LRRN1*	-0.477755678	0.007648054	0.008536706	1
T1DM	leukocyte	*TUBB6*	-0.367486946	3.43E-09	8.93E-09	1.35E-05
T1DM	leukocyte	*CHMP6*	-0.694987979	4.15E-09	9.91E-09	1.63E-05
T1DM	leukocyte	*DLEU1*	-0.790750147	1.21E-08	2.20E-08	4.76E-05
T1DM	leukocyte	*TCP11L1*	0.965026962	2.34E-05	2.87E-05	0.091860748
T1DM	leukocyte	*CCDC57*	-0.76263322	7.05E-08	1.07E-07	0.000277128
T1DM	leukocyte	*BNIP3*	-0.505426054	1.31E-09	8.14E-09	5.14E-06
T1DM	leukocyte	*SMIM19*	-0.561630508	4.60E-09	1.04E-08	1.81E-05
T1DM	leukocyte	*SLC35G1*	-0.970975636	1.44E-09	8.14E-09	5.65E-06
T1DM	leukocyte	*COX8A*	-0.469191012	3.99E-09	9.64E-09	1.57E-05
T1DM	leukocyte	*CDC26*	-0.707626759	1.89E-09	8.14E-09	7.41E-06
T1DM	leukocyte	*RNPEP*	-1.106364105	2.04E-08	3.44E-08	8.00E-05
T1DM	leukocyte	*DMRTA1*	-0.674280912	3.83E-10	8.14E-09	1.51E-06
T1DM	leukocyte	*CLEC14A*	-0.44412676	1.50E-09	8.14E-09	5.88E-06

T1DM	leukocyte	*WDR25*	-1.449681932	4.35E-08	6.89E-08	0.000170917
T1DM	leukocyte	*DIRAS1*	-0.832568618	2.33E-08	3.90E-08	9.15E-05
T1DM	leukocyte	*PHLDB3*	-0.606836231	6.77E-09	1.37E-08	2.66E-05
T1DM	leukocyte	*PRR15*	1.080321791	9.41E-09	1.78E-08	3.70E-05
T1DM	leukocyte	*KBTBD11*	-0.614094237	2.05E-09	8.14E-09	8.07E-06
T1DM	leukocyte	*RMDN1*	-0.754627152	2.51E-08	4.17E-08	9.88E-05
T1DM	leukocyte	*LRRC37A*	-3.755328202	1.48E-09	8.14E-09	5.83E-06
T1DM	leukocyte	*ACSF3*	-0.918029423	1.43E-09	8.14E-09	5.63E-06
T1DM	leukocyte	*PFN4*	1.294524206	0.000796152	0.000919213	1
T1DM	leukocyte	*RUFY1*	-1.396832306	6.46E-08	9.91E-08	0.000253791
T1DM	leukocyte	*VSIG10*	-0.890108912	1.12E-07	1.65E-07	0.000441939
T1DM	leukocyte	*WSB2*	-0.573325102	1.72E-08	2.98E-08	6.78E-05
T1DM	leukocyte	*ANKLE2*	-0.525980131	3.50E-08	5.66E-08	0.000137634
T1DM	leukocyte	*FUT2*	-0.21573348	0.004884006	0.005487349	1
T1DM	leukocyte	*MUC20*	-0.715349096	2.31E-09	8.21E-09	9.07E-06
T1DM	leukocyte	*NFATC2IP*	-0.957603721	3.74E-09	9.32E-09	1.47E-05
T1DM	leukocyte	*DPP7*	-0.607403776	3.22E-09	8.74E-09	1.26E-05
T1DM	leukocyte	*MTHFR*	-0.753136937	7.19E-10	8.14E-09	2.82E-06
T1DM	leukocyte	*DEAF1*	-0.866810797	3.14E-09	8.69E-09	1.24E-05
T1DM	leukocyte	*TMEM80*	-0.592743657	7.08E-10	8.14E-09	2.78E-06
T1DM	leukocyte	*DSCAML1*	-0.855253313	9.86E-09	1.84E-08	3.87E-05
T1DM	leukocyte	*PUS1*	-1.364684572	7.92E-09	1.55E-08	3.11E-05
T1DM	leukocyte	*MAN1B1*	-1.073225896	8.05E-09	1.56E-08	3.16E-05
T1DM	leukocyte	*KCNA2*	-2.66718292	0.000235968	0.000277747	0.927118888
T1DM	leukocyte	*PPFIA3*	-0.573913873	1.07E-09	8.14E-09	4.20E-06
T1DM	leukocyte	*UBE2U*	-3.373557583	9.96E-09	1.85E-08	3.91E-05
T1DM	leukocyte	*PAWR*	-0.651535133	1.31E-09	8.14E-09	5.14E-06
T1DM	leukocyte	*ACOT4*	-0.906380632	6.77E-09	1.37E-08	2.66E-05
T1DM	leukocyte	*ARIH2*	-0.583247074	3.93E-08	6.27E-08	0.000154216
T1DM	leukocyte	*RBM44*	3.156918282	1.68E-06	2.19E-06	0.006594077
T1DM	leukocyte	*IRX3*	-0.830163696	0.001264533	0.001449344	1
T1DM	leukocyte	*SAMD12*	-0.832517145	1.82E-07	2.60E-07	0.000713753
T1DM	leukocyte	*CD163*	0.074771034	2.27E-09	8.21E-09	8.92E-06
T1DM	leukocyte	*ACAD9*	-0.642508048	7.54E-06	9.46E-06	0.029622156
T1DM	leukocyte	*IL17RA*	-0.730011552	0.001182944	0.001357415	1
T1DM	leukocyte	*AGTRAP*	-0.733614478	2.59E-09	8.21E-09	1.02E-05
T1DM	leukocyte	*THAP5*	-0.692580041	2.18E-09	8.21E-09	8.56E-06
T1DM	leukocyte	*CD151*	-0.549370006	1.58E-09	8.14E-09	6.21E-06
T1DM	leukocyte	*CHID1*	-0.551988032	5.29E-09	1.14E-08	2.08E-05
T1DM	leukocyte	*ZBTB41*	-0.851291396	2.98E-09	8.50E-09	1.17E-05
T1DM	leukocyte	*BET1L*	-0.932469077	9.25E-09	1.75E-08	3.63E-05
T1DM	leukocyte	*RIC8A*	-0.855173953	3.81E-09	9.43E-09	1.50E-05
T1DM	leukocyte	*IMP3*	-0.721292338	5.70E-09	1.20E-08	2.24E-05
T1DM	leukocyte	*DPY19L2*	-0.59345691	1.37E-06	1.80E-06	0.005364474
T1DM	leukocyte	*GPR150*	-0.634472188	5.89E-07	7.96E-07	0.002313535
T1DM	leukocyte	*ADAMTSL1*	-2.344221238	7.69E-05	9.24E-05	0.302301797
T1DM	leukocyte	*ALS2CL*	-0.977716919	2.07E-09	8.14E-09	8.12E-06
T1DM	leukocyte	*STAP2*	-0.553205679	3.87E-10	8.14E-09	1.52E-06
T1DM	leukocyte	*DALRD3*	-0.696963633	7.30E-08	1.11E-07	0.000286912
T1DM	leukocyte	*ZNF114*	-1.890274542	2.30E-09	8.21E-09	9.03E-06
T1DM	leukocyte	*PLEC*	-1.0977152	1.62E-08	2.83E-08	6.37E-05
T1DM	leukocyte	*WDR6*	-0.724924584	1.15E-09	8.14E-09	4.51E-06
T1DM	leukocyte	*AQP11*	-0.502517447	0.000749763	0.000866673	1
T1DM	leukocyte	*ZFAND2A*	-0.820985369	8.13E-09	1.57E-08	3.19E-05
T1DM	leukocyte	*DNAJC22*	-0.288460124	0.000292202	0.000342603	1
T1DM	leukocyte	*NT5DC1*	-0.650034421	3.71E-09	9.29E-09	1.46E-05
T1DM	leukocyte	*COX14*	-0.648515859	1.69E-08	2.94E-08	6.66E-05
T1DM	leukocyte	*P4HTM*	-0.479149414	7.25E-09	1.44E-08	2.85E-05
T1DM	leukocyte	*ERBB4*	-5.240389496	3.10E-08	5.05E-08	0.00012184
T1DM	leukocyte	*ERN1*	-0.815511239	2.50E-08	4.15E-08	9.82E-05
T1DM	leukocyte	*GPR35*	0.615219076	0.012453283	0.013775042	1
T1DM	leukocyte	*PARP10*	-0.827767999	1.77E-08	3.06E-08	6.97E-05
T1DM	leukocyte	*RPP25*	-1.055324878	3.34E-09	8.82E-09	1.31E-05
T1DM	leukocyte	*ZHX2*	-0.784123203	8.90E-10	8.14E-09	3.50E-06

T1DM	leukocyte	*TRIM73*	0.961854668	9.98E-06	1.25E-05	0.039203325
T1DM	leukocyte	*EFCAB13*	-0.360096781	0.010599358	0.011750812	1
T1DM	leukocyte	*MSC*	0.767782305	2.20E-05	2.70E-05	0.086247876
T1DM	leukocyte	*HYI*	-0.7751287	3.87E-09	9.51E-09	1.52E-05
T1DM	leukocyte	*GAK*	-0.784482872	4.70E-09	1.06E-08	1.84E-05
T1DM	leukocyte	*TUFM*	-0.50519858	4.02E-09	9.69E-09	1.58E-05
T1DM	leukocyte	*RMI1*	0.74072301	0.000163302	0.000193548	0.6416118
T1DM	leukocyte	*C3orf38*	-0.935367327	7.33E-08	1.11E-07	0.000288079
T1DM	leukocyte	*KLHDC7A*	1.072429554	5.94E-07	8.03E-07	0.002333029
T1DM	leukocyte	*IGSF22*	-0.540452053	1.51E-09	8.14E-09	5.93E-06
T1DM	leukocyte	*HTR1F*	-1.848151406	6.72E-09	1.36E-08	2.64E-05
T1DM	leukocyte	*TMTC2*	-1.351268197	1.42E-10	8.14E-09	5.59E-07
T1DM	leukocyte	*FARSA*	-0.723486535	4.73E-10	8.14E-09	1.86E-06
T1DM	leukocyte	*SPTY2D1*	-0.696342556	3.86E-09	9.50E-09	1.52E-05
T1DM	leukocyte	*SAMD4B*	-0.571609464	1.60E-08	2.80E-08	6.29E-05
T1DM	leukocyte	*TCAIM*	-0.938526134	2.23E-09	8.21E-09	8.75E-06
T1DM	leukocyte	*FUCA1*	0.411585484	5.10E-08	7.98E-08	0.000200316
T1DM	leukocyte	*CALR*	-0.491718943	1.25E-08	2.26E-08	4.90E-05
T1DM	leukocyte	*CDH4*	-0.910645019	0.003468351	0.0039181	1
T1DM	leukocyte	*SMCO3*	-15.50853889	3.62E-07	5.00E-07	0.0014216
T1DM	leukocyte	*NSUN7*	-0.851195974	0.00013493	0.000160405	0.530139973
T1DM	leukocyte	*HLA-DQB1*	0.082700401	1.56E-05	1.93E-05	0.061228186
T1DM	leukocyte	*ELMOD2*	-1.091453562	1.05E-08	1.93E-08	4.11E-05
T1DM	leukocyte	*LINC00174*	-0.6893889	7.22E-10	8.14E-09	2.84E-06
T1DM	leukocyte	*GEMIN4*	-0.720289916	1.57E-09	8.14E-09	6.19E-06
T1DM	leukocyte	*LBX2*	0.838261477	1.06E-09	8.14E-09	4.17E-06
T1DM	leukocyte	*CDC42EP4*	-0.576076041	3.57E-09	9.10E-09	1.40E-05
T1DM	leukocyte	*ZBTB42*	-1.544128279	5.56E-09	1.18E-08	2.18E-05
T1DM	leukocyte	*SYCN*	-0.354662117	2.80E-08	4.60E-08	0.000110101
T1DM	leukocyte	*ATOH7*	-0.426221027	0.008345461	0.009304573	1
T1DM	leukocyte	*CITED4*	-0.497293392	4.89E-10	8.14E-09	1.92E-06
T1DM	leukocyte	*ABCA13*	-13.8842351	7.96E-08	1.20E-07	0.00031278
T1DM	leukocyte	*ZNF154*	-0.61807159	7.69E-07	1.03E-06	0.003019591
T1DM	leukocyte	*B3GNT3*	-1.508152706	5.71E-08	8.86E-08	0.000224438
T1DM	leukocyte	*LINC00652*	-10.59420479	0.001035693	0.001191927	1
T1DM	leukocyte	*PUF60*	-0.630338533	1.54E-09	8.14E-09	6.05E-06
T1DM	leukocyte	*SSC5D*	1.107877515	0.000274352	0.000322155	1
T1DM	leukocyte	*TSHZ1*	-0.570770072	6.79E-09	1.37E-08	2.67E-05
T1DM	leukocyte	*ANKRD18A*	-0.408417489	1.26E-05	1.56E-05	0.049340963
T1DM	leukocyte	*EXOC3*	-0.858387811	9.14E-06	1.14E-05	0.035897352
T1DM	leukocyte	*TH*	-3.170028182	2.83E-07	3.96E-07	0.001113246
T1DM	leukocyte	*FAHD1*	-0.57809615	4.30E-07	5.91E-07	0.001690496
T1DM	leukocyte	*ZNF816*	-1.027698138	9.34E-10	8.14E-09	3.67E-06
T1DM	leukocyte	*CCDC66*	-1.349086329	2.65E-07	3.73E-07	0.001043099
T1DM	leukocyte	*MCFD2*	-0.518313508	1.84E-09	8.14E-09	7.21E-06
T1DM	leukocyte	*SERTM1*	0.605145721	1.55E-08	2.72E-08	6.10E-05
T1DM	leukocyte	*GLIPR1L2*	-1.049875251	8.33E-07	1.11E-06	0.003273277
T1DM	leukocyte	*TSPYL5*	-0.522215965	5.30E-09	1.14E-08	2.08E-05
T1DM	leukocyte	*SHISA2*	-0.973104384	1.39E-05	1.72E-05	0.054694721
T1DM	leukocyte	*CHST13*	-58.75171988	1.38E-09	8.14E-09	5.41E-06
T1DM	leukocyte	*PPA1*	-0.488018883	2.02E-09	8.14E-09	7.95E-06
T1DM	leukocyte	*PSMG4*	-0.584972298	4.62E-10	8.14E-09	1.82E-06
T1DM	leukocyte	*MAP6D1*	-1.329905443	3.39E-08	5.48E-08	0.000133005
T1DM	leukocyte	*ZNF792*	-1.523925143	5.67E-09	1.19E-08	2.23E-05
T1DM	leukocyte	*D2HGDH*	-1.870419055	7.44E-07	9.98E-07	0.002922171
T1DM	leukocyte	*FAM83H*	-1.035245339	3.27E-09	8.79E-09	1.29E-05
T1DM	leukocyte	*LRRC57*	-1.037650779	1.65E-08	2.86E-08	6.47E-05
T1DM	leukocyte	*MRPL14*	-0.658541198	4.79E-09	1.07E-08	1.88E-05
T1DM	leukocyte	*ZFP82*	-0.661833675	1.65E-09	8.14E-09	6.48E-06
T1DM	leukocyte	*LSMEM1*	-0.680200155	0.00048699	0.00056609	1
T1DM	leukocyte	*FKRP*	-0.684918346	2.02E-10	8.14E-09	7.93E-07
T1DM	leukocyte	*RPH3AL*	-0.524884144	1.29E-09	8.14E-09	5.08E-06
T1DM	leukocyte	*HIGD1A*	-0.532720802	1.54E-09	8.14E-09	6.05E-06
T1DM	leukocyte	*F2R*	-0.628529172	3.86E-11	8.14E-09	1.52E-07

T1DM	leukocyte	*ZNF707*	-0.888049193	1.38E-09	8.14E-09	5.44E-06
T1DM	leukocyte	*SLC25A41*	-1.719019506	0.010417218	0.011552144	1
T1DM	leukocyte	*OGFOD3*	-0.568522986	4.22E-09	9.96E-09	1.66E-05
T1DM	leukocyte	*ZNF467*	-0.881931557	5.12E-09	1.12E-08	2.01E-05
T1DM	leukocyte	*ZNF678*	-0.577690949	0.020369071	0.022329821	1
T1DM	leukocyte	*TMEM45A*	-0.571222032	1.34E-09	8.14E-09	5.26E-06
T1DM	leukocyte	*ACBD4*	1.85384378	8.84E-07	1.18E-06	0.003472215
T1DM	leukocyte	*SGSH*	-0.816236468	0.017203842	0.018939169	1
T1DM	leukocyte	*GPR135*	-0.817519663	1.45E-09	8.14E-09	5.71E-06
T1DM	leukocyte	*ANKRD62*	-0.824902595	1.24E-06	1.64E-06	0.00487992
T1DM	leukocyte	*GPR88*	-5.938755547	0.004328806	0.004876112	1
T1DM	leukocyte	*RFX7*	-1.503175493	3.18E-09	8.70E-09	1.25E-05
T1DM	leukocyte	*ZNF329*	-0.855633759	2.84E-08	4.67E-08	0.000111689
T1DM	leukocyte	*CCDC149*	-1.042342377	2.50E-09	8.21E-09	9.84E-06
T1DM	leukocyte	*IDH2*	-0.536311326	2.12E-09	8.16E-09	8.32E-06
T1DM	leukocyte	*TNRC18*	-0.822636389	2.95E-09	8.50E-09	1.16E-05
T1DM	leukocyte	*TDRKH*	-0.753431006	3.59E-09	9.11E-09	1.41E-05
T1DM	leukocyte	*ZNF708*	-0.544717717	1.53E-09	8.14E-09	6.03E-06
T1DM	leukocyte	*IST1*	-0.913090794	9.69E-09	1.81E-08	3.81E-05
T1DM	leukocyte	*ERCC6L2*	-1.415661296	4.44E-06	5.64E-06	0.017456073
T1DM	leukocyte	*MRPL41*	-0.60941996	3.16E-09	8.69E-09	1.24E-05
T1DM	leukocyte	*TP53TG1*	-0.690204607	2.25E-09	8.21E-09	8.84E-06
T1DM	leukocyte	*ARL6IP4*	-0.479853388	1.27E-08	2.29E-08	4.98E-05
T1DM	leukocyte	*FAM153B*	-0.980025022	7.92E-09	1.55E-08	3.11E-05
T1DM	leukocyte	*BACE2*	-0.421411951	7.96E-10	8.14E-09	3.13E-06
T1DM	leukocyte	*SYNM*	-0.557260053	1.62E-06	2.12E-06	0.006354192
T1DM	leukocyte	*FIGN*	-0.679610462	2.35E-09	8.21E-09	9.24E-06
T1DM	leukocyte	*IZUMO1*	-2.058129123	0.038382992	0.041659331	1
T1DM	leukocyte	*B4GALNT4*	-0.998553261	1.08E-05	1.35E-05	0.042524662
T1DM	leukocyte	*ZNF804B*	-50.9666607	1.46E-09	8.14E-09	5.74E-06
T1DM	leukocyte	*YBEY*	-0.515999995	0.000103369	0.000123484	0.406137371
T1DM	leukocyte	*NPLOC4*	-0.681954049	2.61E-09	8.21E-09	1.02E-05
T1DM	leukocyte	*CAPN12*	-1.234070078	5.12E-09	1.12E-08	2.01E-05
T1DM	leukocyte	*GLRX5*	-0.523628126	1.98E-07	2.83E-07	0.000779881
T1DM	leukocyte	*MXRA7*	-0.553698805	5.16E-09	1.12E-08	2.03E-05
T1DM	leukocyte	*ADI1*	-1.217268568	2.80E-08	4.60E-08	0.000109997
T1DM	leukocyte	*SATB1*	-0.973479559	3.24E-09	8.77E-09	1.27E-05
T1DM	leukocyte	*EPHB3*	-1.441082421	1.71E-05	2.11E-05	0.066995366
T1DM	leukocyte	*ACTL10*	2.176655969	2.09E-05	2.57E-05	0.082119722
T1DM	leukocyte	*SKA2*	-1.011616305	1.63E-08	2.84E-08	6.41E-05
T1DM	leukocyte	*TTC3*	-0.392327144	2.46E-08	4.09E-08	9.66E-05
T1DM	leukocyte	*BRICD5*	-1.13651574	2.73E-05	3.35E-05	0.107129737
T1DM	leukocyte	*TSKU*	-0.775042881	1.10E-09	8.14E-09	4.34E-06
T1DM	leukocyte	*ANXA2*	-0.402627092	1.08E-09	8.14E-09	4.26E-06
T1DM	leukocyte	*SLC35D3*	-1.107809762	7.43E-08	1.13E-07	0.000292096
T1DM	leukocyte	*GPIHBP1*	-6.34063553	1.51E-09	8.14E-09	5.93E-06
T1DM	leukocyte	*VMO1*	0.085625462	2.88E-09	8.46E-09	1.13E-05
T1DM	leukocyte	*ALG12*	-1.078237486	1.48E-07	2.13E-07	0.000580843
T1DM	leukocyte	*COL18A1*	-0.298960731	2.83E-07	3.96E-07	0.001112662
T1DM	leukocyte	*C11orf54*	-0.860494952	5.16E-08	8.08E-08	0.000202852
T1DM	leukocyte	*CEP63*	-0.806966932	4.08E-10	8.14E-09	1.60E-06
T1DM	leukocyte	*HMGN4*	-0.794088329	7.98E-08	1.20E-07	0.000313669
T1DM	leukocyte	*SPATA13*	-0.638372081	2.26E-09	8.21E-09	8.89E-06
T1DM	leukocyte	*ZNF320*	0.244607973	0.015239962	0.016814886	1
T1DM	leukocyte	*SLC25A21*	-1.756076367	1.36E-09	8.14E-09	5.34E-06
T1DM	leukocyte	*CAMK1D*	-1.321623506	6.14E-06	7.74E-06	0.024124546
T1DM	leukocyte	*LYSMD4*	-0.763048284	1.59E-08	2.77E-08	6.23E-05
T1DM	leukocyte	*BEGAIN*	-0.984384401	1.39E-09	8.14E-09	5.48E-06
T1DM	leukocyte	*GPC6*	-0.774357202	4.77E-09	1.07E-08	1.88E-05
T1DM	leukocyte	*FAM43B*	-3.113533839	0.000216961	0.000255911	0.85243981
T1DM	leukocyte	*FANCF*	-1.713590854	1.91E-06	2.48E-06	0.007496668
T1DM	leukocyte	*CALN1*	-2.707136037	4.61E-06	5.85E-06	0.018130366
T1DM	leukocyte	*SMDT1*	-0.42744348	1.21E-07	1.76E-07	0.000474448
T1DM	leukocyte	*POTEC*	-10.62557719	0.02280018	0.024967087	1

T1DM	leukocyte	*PTTG1IP*	-0.503168411	3.04E-07	4.23E-07	0.001194564
T1DM	leukocyte	*ZNF623*	-0.621345841	2.35E-09	8.21E-09	9.24E-06
T1DM	leukocyte	*CCDC125*	-1.204638178	5.78E-06	7.30E-06	0.022706929
T1DM	leukocyte	*BOLA2*	-0.635312119	4.84E-09	1.08E-08	1.90E-05
T1DM	leukocyte	*FHL3*	-0.663138977	3.67E-08	5.90E-08	0.000144226
T1DM	leukocyte	*SF3A3*	-0.555643825	2.05E-08	3.47E-08	8.06E-05
T1DM	leukocyte	*MX2*	0.92707197	9.44E-08	1.41E-07	0.00037104
T1DM	leukocyte	*PCBP3*	-0.867530282	1.12E-06	1.48E-06	0.004397173
T1DM	leukocyte	*SFXN4*	-0.780496069	1.83E-10	8.14E-09	7.17E-07
T1DM	leukocyte	*MRPL54*	-0.627029261	1.20E-09	8.14E-09	4.73E-06
T1DM	leukocyte	*DGCR6*	-0.572105213	2.33E-06	3.01E-06	0.009164482
T1DM	leukocyte	*NDUFB1*	-0.551201066	1.51E-07	2.17E-07	0.000592489
T1DM	leukocyte	*TRMT12*	-0.773155395	1.06E-09	8.14E-09	4.15E-06
T1DM	leukocyte	*FIGLA*	-12.27429656	3.21E-06	4.11E-06	0.012610721
T1DM	leukocyte	*CBX6*	-0.539612781	2.42E-08	4.03E-08	9.49E-05
T1DM	leukocyte	*MACC1*	-0.510713874	1.06E-09	8.14E-09	4.17E-06
T1DM	leukocyte	*NPIPA5*	0.437513684	0.000232943	0.000274268	0.915231297
T1DM	leukocyte	*NUDT14*	-0.641265694	1.29E-09	8.14E-09	5.09E-06
T1DM	leukocyte	*FAM3B*	-0.606676213	1.00E-09	8.14E-09	3.93E-06
T1DM	leukocyte	*TOB2*	-0.740111397	2.95E-09	8.50E-09	1.16E-05
T1DM	leukocyte	*TTC32*	-0.669722211	8.20E-10	8.14E-09	3.22E-06
T1DM	leukocyte	*LRRC55*	-5.105664391	0.00025827	0.000303452	1
T1DM	leukocyte	*TMEM236*	-0.527017375	4.63E-09	1.05E-08	1.82E-05
T1DM	leukocyte	*UQCR10*	-0.553553402	8.90E-10	8.14E-09	3.50E-06
T1DM	leukocyte	*EIF3C*	-0.632886614	5.36E-09	1.15E-08	2.11E-05
T1DM	leukocyte	*NIPSNAP1*	-0.642291159	3.17E-09	8.69E-09	1.24E-05
T1DM	leukocyte	*CRELD2*	-0.538032612	1.43E-09	8.14E-09	5.63E-06
T1DM	leukocyte	*UBE2F*	-0.907062102	7.45E-08	1.13E-07	0.00029253
T1DM	leukocyte	*GOLGA6L4*	-0.739917262	1.15E-07	1.69E-07	0.000453247
T1DM	leukocyte	*PGP*	-0.907612421	8.91E-10	8.14E-09	3.50E-06
T1DM	leukocyte	*ACOT1*	-3.700992955	1.72E-07	2.46E-07	0.000675416
T1DM	leukocyte	*OAF*	-0.797125262	5.18E-09	1.13E-08	2.04E-05
T1DM	leukocyte	*TM2D3*	-0.635689335	2.06E-09	8.14E-09	8.08E-06
T1DM	leukocyte	*MROH7*	-0.660758396	0.001523489	0.001739044	1
T1DM	leukocyte	*PLA2G6*	0.659895741	6.55E-07	8.82E-07	0.002572059
T1DM	leukocyte	*TOP1MT*	-1.473341942	9.87E-09	1.84E-08	3.88E-05
T1DM	leukocyte	*COPB2*	-0.713114298	2.16E-09	8.21E-09	8.50E-06
T1DM	leukocyte	*CCR10*	-0.897557657	0.000377873	0.000440945	1
T1DM	leukocyte	*NCMAP*	-0.677963777	1.66E-09	8.14E-09	6.52E-06
T1DM	leukocyte	*WDR27*	-1.199717158	2.60E-06	3.35E-06	0.010203169
T1DM	leukocyte	*TXNRD2*	-0.698110894	1.36E-09	8.14E-09	5.32E-06
T1DM	leukocyte	*ZFP1*	-0.888336858	7.81E-10	8.14E-09	3.07E-06
T1DM	leukocyte	*XPOT*	-0.646628443	5.44E-10	8.14E-09	2.14E-06
T1DM	leukocyte	*C14orf180*	-72.83102587	1.45E-09	8.14E-09	5.71E-06
T1DM	leukocyte	*ZBTB40*	-1.006481625	3.46E-08	5.59E-08	0.000135907
T1DM	leukocyte	*UBE2G2*	-0.632619957	2.78E-08	4.58E-08	0.00010932
T1DM	leukocyte	*OSBP2*	-0.803728277	1.37E-09	8.14E-09	5.37E-06
T1DM	leukocyte	*TMED9*	-0.547628764	3.70E-09	9.28E-09	1.45E-05
T1DM	leukocyte	*BTBD6*	-0.83227309	2.48E-09	8.21E-09	9.73E-06
T1DM	leukocyte	*CLCNKB*	2.377364749	0.014628436	0.016144698	1
T1DM	leukocyte	*JAG2*	-0.645578096	9.68E-10	8.14E-09	3.80E-06
T1DM	leukocyte	*LCN12*	-1.974809814	8.10E-08	1.22E-07	0.000318361
T1DM	leukocyte	*FAM227A*	-0.712374698	2.41E-08	4.03E-08	9.49E-05
T1DM	leukocyte	*MUC6*	-0.443581733	1.73E-09	8.14E-09	6.78E-06
T1DM	leukocyte	*NOC4L*	-1.024167308	2.07E-09	8.14E-09	8.14E-06
T1DM	leukocyte	*NDUFA6*	-0.425447512	5.76E-07	7.80E-07	0.002261901
T1DM	leukocyte	*SIVA1*	-0.624141215	4.03E-09	9.70E-09	1.58E-05
T1DM	leukocyte	*BRI3BP*	-0.916851669	1.40E-09	8.14E-09	5.50E-06
T1DM	leukocyte	*UBOX5*	-0.690724211	1.31E-09	8.14E-09	5.14E-06
T1DM	leukocyte	*MAFF*	-0.454098402	9.02E-10	8.14E-09	3.55E-06
T1DM	leukocyte	*SEMA4B*	-0.681970215	1.66E-09	8.14E-09	6.53E-06
T1DM	leukocyte	*CIB1*	-0.599196489	2.98E-09	8.50E-09	1.17E-05
T1DM	leukocyte	*ANO9*	-0.563041214	0.001673224	0.001907747	1
T1DM	leukocyte	*MFSD6L*	-1.964648896	1.26E-08	2.28E-08	4.96E-05

T1DM	leukocyte	*AQP12B*	-1.553240922	0.043601042	0.047218438	1
T1DM	leukocyte	*ZNF445*	-1.894316708	3.27E-05	4.00E-05	0.12851162
T1DM	leukocyte	*ZNF74*	-1.332987256	2.08E-07	2.96E-07	0.000817694
T1DM	leukocyte	*RBM11*	-0.626429523	1.70E-10	8.14E-09	6.69E-07
T1DM	leukocyte	*RGPD2*	-2.04451463	6.86E-09	1.38E-08	2.70E-05
T1DM	leukocyte	*GAS2L1*	-1.047187135	0.000258157	0.000303409	1
T1DM	leukocyte	*ATP6V0A2*	-1.003754368	6.39E-09	1.30E-08	2.51E-05
T1DM	leukocyte	*RAD51D*	-1.564674038	7.82E-09	1.53E-08	3.07E-05
T1DM	leukocyte	*SH3BGR*	-0.335347502	7.57E-06	9.49E-06	0.029742252
T1DM	leukocyte	*GPRIN3*	0.931537779	0.000308574	0.000361367	1
T1DM	leukocyte	*MUC1*	-0.468351725	1.11E-09	8.14E-09	4.38E-06
T1DM	leukocyte	*IRF7*	-1.08804812	1.24E-08	2.25E-08	4.89E-05
T1DM	leukocyte	*SV2B*	-0.525538516	3.18E-05	3.89E-05	0.125008666
T1DM	leukocyte	*FAM131C*	-0.499682436	2.40E-09	8.21E-09	9.42E-06
T1DM	leukocyte	*LSAMP*	-0.394272916	2.55E-09	8.21E-09	1.00E-05
T1DM	leukocyte	*OLFML2A*	-1.984198594	1.04E-09	8.14E-09	4.10E-06
T1DM	leukocyte	*PDIA2*	-0.365379017	1.41E-09	8.14E-09	5.54E-06
T1DM	leukocyte	*PCGF3*	-0.708773103	1.27E-09	8.14E-09	4.99E-06
T1DM	leukocyte	*P4HB*	-0.447280852	2.07E-09	8.14E-09	8.14E-06
T1DM	leukocyte	*PSMD13*	-0.603124096	1.42E-07	2.05E-07	0.000557093
T1DM	leukocyte	*BRWD1*	-0.712299421	9.65E-10	8.14E-09	3.79E-06
T1DM	leukocyte	*DRG1*	-0.626989497	1.96E-09	8.14E-09	7.71E-06
T1DM	leukocyte	*ADAMTSL5*	-3.977743348	2.04E-05	2.51E-05	0.080207453
T1DM	leukocyte	*MORF4L1*	-0.490173444	2.51E-09	8.21E-09	9.86E-06
T1DM	leukocyte	*DMWD*	-1.064976658	4.42E-09	1.02E-08	1.74E-05
T1DM	leukocyte	*PIGP*	-0.511943405	6.14E-09	1.26E-08	2.41E-05
T1DM	leukocyte	*NPAP1*	-13.83152141	0.000459629	0.000534759	1
T1DM	leukocyte	*ARL17A*	-0.5427096	3.73E-08	5.98E-08	0.000146675
T1DM	leukocyte	*TRIM69*	-0.747549685	5.01E-10	8.14E-09	1.97E-06
T1DM	leukocyte	*IFITM1*	-1.273218022	9.53E-07	1.27E-06	0.003743714
T1DM	leukocyte	*SETD4*	-1.42208464	0.000454655	0.000529129	1
T1DM	leukocyte	*ZNF529*	-1.09787128	1.62E-08	2.83E-08	6.38E-05
T1DM	leukocyte	*AIDA*	-0.606345482	2.20E-09	8.21E-09	8.62E-06
T1DM	leukocyte	*GSAP*	0.856388949	1.22E-07	1.77E-07	0.000478077
T1DM	leukocyte	*WWOX*	-0.671161924	2.03E-09	8.14E-09	7.99E-06
T1DM	leukocyte	*POLR1D*	-0.562597964	3.31E-09	8.82E-09	1.30E-05
T1DM	leukocyte	*FFAR4*	-0.416139563	0.000659167	0.000762623	1
T1DM	leukocyte	*EDARADD*	-0.724965936	9.68E-10	8.14E-09	3.80E-06
T1DM	leukocyte	*ZNF749*	-0.478209179	1.71E-08	2.96E-08	6.72E-05
T1DM	leukocyte	*TOR3A*	-0.702890916	1.59E-09	8.14E-09	6.26E-06
T1DM	leukocyte	*GABRA5*	12.39546516	8.86E-07	1.18E-06	0.003481185
T1DM	leukocyte	*ZNF555*	-0.572060488	1.39E-09	8.14E-09	5.46E-06
T1DM	leukocyte	*NUDT17*	-0.993290942	7.33E-10	8.14E-09	2.88E-06
T1DM	leukocyte	*CYP4X1*	1.071628124	2.40E-09	8.21E-09	9.41E-06
T1DM	leukocyte	*ZNF197*	-0.626993145	1.93E-09	8.14E-09	7.57E-06
T1DM	leukocyte	*FAM228A*	-0.632363739	1.14E-05	1.42E-05	0.044962409
T1DM	leukocyte	*RPS23*	-0.42841383	3.87E-09	9.51E-09	1.52E-05
T1DM	leukocyte	*BTN3A2*	-0.413113184	6.75E-09	1.36E-08	2.65E-05
T1DM	leukocyte	*ZNF396*	-1.429196622	1.74E-06	2.26E-06	0.006822687
T1DM	leukocyte	*FAM86B1*	2.376441206	3.38E-07	4.69E-07	0.001329008
T1DM	leukocyte	*SMYD4*	-0.412465434	0.002364884	0.002685442	1
T1DM	leukocyte	*CEACAM19*	-0.438637282	4.90E-06	6.21E-06	0.019269621
T1DM	leukocyte	*ZFP91*	-0.679661964	9.03E-08	1.35E-07	0.000354735
T1DM	leukocyte	*BCR*	-0.630127133	2.61E-09	8.21E-09	1.03E-05
T1DM	leukocyte	*FSCN2*	-1.165849039	7.86E-08	1.19E-07	0.000309006
T1DM	leukocyte	*RTN4RL2*	-0.720836942	1.42E-09	8.14E-09	5.60E-06
T1DM	leukocyte	*TMEM232*	-1.088240631	5.71E-10	8.14E-09	2.24E-06
T1DM	leukocyte	*FAM183A*	-0.578721721	2.02E-09	8.14E-09	7.95E-06
T1DM	leukocyte	*EMID1*	-0.789457194	5.97E-08	9.21E-08	0.000234722
T1DM	leukocyte	*RHD*	-0.607963956	0.01165322	0.012904595	1
T1DM	leukocyte	*ESPN*	-11.98657955	5.90E-08	9.11E-08	0.000231764
T1DM	leukocyte	*LYPD6*	-2.13923146	5.24E-05	6.35E-05	0.205784489
T1DM	leukocyte	*AKR1C1*	-0.529949005	1.09E-09	8.14E-09	4.29E-06
T1DM	leukocyte	*BCAM*	-0.470106931	2.46E-08	4.09E-08	9.67E-05

T1DM	leukocyte	*CHP1*	-0.564994701	1.25E-09	8.14E-09	4.91E-06
T1DM	leukocyte	*KCNJ11*	-1.996301841	7.62E-09	1.50E-08	2.99E-05
T1DM	leukocyte	*ZNF385C*	-2.288150544	9.95E-10	8.14E-09	3.91E-06
T1DM	leukocyte	*EXD3*	-0.74349333	1.29E-09	8.14E-09	5.08E-06
T1DM	leukocyte	*C17orf97*	-0.477625245	2.94E-06	3.77E-06	0.011545865
T1DM	leukocyte	*RGPD1*	-78.53317337	1.36E-09	8.14E-09	5.36E-06
T1DM	leukocyte	*DHRS4L2*	-0.686534613	1.56E-09	8.14E-09	6.14E-06
T1DM	leukocyte	*KBTBD12*	-1.417296573	1.50E-05	1.86E-05	0.058901902
T1DM	leukocyte	*FANCA*	-0.677694132	0.000313482	0.000366787	1
T1DM	leukocyte	*MCRS1*	-0.749310907	5.41E-05	6.55E-05	0.21263357
T1DM	leukocyte	*ZFP69*	1.576637873	0.000310662	0.000363595	1
T1DM	leukocyte	*TMEM220*	-0.511189056	0.00039087	0.000455976	1
T1DM	leukocyte	*PALM3*	-0.876139188	1.28E-09	8.14E-09	5.02E-06
T1DM	leukocyte	*KLHL17*	-1.231708206	3.23E-10	8.14E-09	1.27E-06
T1DM	leukocyte	*ZSCAN23*	-1.187679123	0.000391483	0.000456555	1
T1DM	leukocyte	*RINL*	-0.852696488	2.00E-09	8.14E-09	7.86E-06
T1DM	leukocyte	*OR7D2*	-8.351267673	0.000149189	0.000177089	0.586163118
T1DM	leukocyte	*ZNF490*	-1.211856408	1.73E-09	8.14E-09	6.79E-06
T1DM	leukocyte	*NRN1L*	-1.863541544	1.48E-09	8.14E-09	5.82E-06
T1DM	leukocyte	*NWD1*	-0.985787677	0.008457286	0.00942477	1
T1DM	leukocyte	*RAB42*	0.391547138	1.94E-08	3.30E-08	7.61E-05
T1DM	leukocyte	*MAPK12*	-0.840162055	2.63E-09	8.21E-09	1.03E-05
T1DM	leukocyte	*FAM166A*	-2.830568238	8.86E-05	0.000106256	0.348305591
T1DM	leukocyte	*LINC00265*	-1.47314832	1.31E-09	8.14E-09	5.14E-06
T1DM	leukocyte	*LAMTOR4*	-0.567480844	2.08E-08	3.51E-08	8.17E-05
T1DM	leukocyte	*PRKAR1B*	-0.814942673	5.12E-09	1.12E-08	2.01E-05
T1DM	leukocyte	*NUTM2B*	-6.985929966	0.001507488	0.001721279	1
T1DM	leukocyte	*AGAP4*	-1.660556848	1.36E-09	8.14E-09	5.33E-06
T1DM	leukocyte	*CENPP*	-0.602653566	8.02E-10	8.14E-09	3.15E-06
T1DM	leukocyte	*ZNF559*	-0.453302368	7.97E-06	9.97E-06	0.031303278
T1DM	leukocyte	*FOCAD*	-0.722901216	2.18E-09	8.21E-09	8.55E-06
T1DM	leukocyte	*ZP3*	-0.911934845	1.37E-08	2.45E-08	5.39E-05
T1DM	leukocyte	*INSC*	-2.033824642	0.005039797	0.005659149	1
T1DM	leukocyte	*SERPINA5*	-0.353876933	7.66E-05	9.20E-05	0.300842763
T1DM	leukocyte	*SRSF10*	-0.611048416	5.36E-09	1.15E-08	2.11E-05
T1DM	leukocyte	*RALGAPA2*	-0.72405841	8.63E-10	8.14E-09	3.39E-06
T1DM	leukocyte	*NDOR1*	-2.016893127	1.37E-09	8.14E-09	5.38E-06
T1DM	leukocyte	*FBLL1*	-1.404326351	2.29E-09	8.21E-09	9.01E-06
T1DM	leukocyte	*ASAH2*	-1.34006143	3.47E-07	4.80E-07	0.001362326
T1DM	leukocyte	*ZNF177*	-0.441170011	0.004727881	0.005316498	1
T1DM	leukocyte	*S100A16*	-0.376906997	1.35E-09	8.14E-09	5.29E-06
T1DM	leukocyte	*PARVB*	-0.456452927	1.45E-08	2.56E-08	5.68E-05
T1DM	leukocyte	*UROS*	-0.782771363	3.24E-09	8.77E-09	1.27E-05
T1DM	leukocyte	*QRFP*	-5.359095261	0.011195809	0.012405058	1
T1DM	leukocyte	*FAM221A*	-0.88679146	6.69E-09	1.36E-08	2.63E-05
T1DM	leukocyte	*NOXA1*	-1.280522265	1.30E-09	8.14E-09	5.11E-06
T1DM	leukocyte	*TMEM198*	-0.574005784	4.85E-09	1.08E-08	1.91E-05
T1DM	leukocyte	*ZNF548*	-0.763810189	1.24E-07	1.80E-07	0.000486079
T1DM	leukocyte	*MTF1*	-0.651957436	7.22E-09	1.44E-08	2.84E-05
T1DM	leukocyte	*ZDHHC11*	-1.712496679	4.62E-08	7.27E-08	0.000181326
T1DM	leukocyte	*ENTPD8*	-2.925042622	8.67E-05	0.000103909	0.340509138
T1DM	leukocyte	*RPL14*	-0.420295564	2.56E-09	8.21E-09	1.01E-05
T1DM	leukocyte	*FBF1*	0.686963598	4.73E-09	1.06E-08	1.86E-05
T1DM	leukocyte	*MSL1*	-0.593641456	5.53E-07	7.51E-07	0.002173692
T1DM	leukocyte	*NOC2L*	-0.72227313	7.46E-08	1.13E-07	0.000293147
T1DM	leukocyte	*ZNF567*	-0.664126885	5.70E-09	1.20E-08	2.24E-05
T1DM	leukocyte	*ARID2*	-0.860123479	2.35E-09	8.21E-09	9.25E-06
T1DM	leukocyte	*PLAC9*	-0.682448963	4.46E-09	1.03E-08	1.75E-05
T1DM	leukocyte	*CLDN4*	-0.391143834	1.74E-09	8.14E-09	6.84E-06
T1DM	leukocyte	*FAM47E*	-0.552985072	0.000224525	0.000264674	0.882158797
T1DM	leukocyte	*ZNF527*	-0.904523433	1.05E-08	1.93E-08	4.11E-05
T1DM	leukocyte	*ZNF600*	-0.646961809	1.69E-07	2.43E-07	0.00066591
T1DM	leukocyte	*PNRC2*	-0.562242193	4.70E-09	1.06E-08	1.85E-05
T1DM	leukocyte	*ZKSCAN3*	-2.032888682	1.44E-17	8.08E-15	5.66E-14

T1DM	leukocyte	*RRP7A*	-0.721669286	4.34E-09	1.01E-08	1.70E-05
T1DM	leukocyte	*S100A14*	-0.587987547	3.28E-09	8.79E-09	1.29E-05
T1DM	leukocyte	*SLC35E2B*	-0.750128818	0.002632645	0.002984323	1
T1DM	leukocyte	*CXCL17*	-0.572019519	4.21E-09	9.96E-09	1.65E-05
T1DM	leukocyte	*SPATA41*	-2.701499303	9.75E-08	1.45E-07	0.000383189
T1DM	leukocyte	*BLOC1S2*	-0.586971139	2.49E-07	3.51E-07	0.000977993
T1DM	leukocyte	*PTPRT*	1.473675143	0.018105907	0.01990991	1
T1DM	leukocyte	*ZNF676*	-0.945315761	1.49E-10	8.14E-09	5.85E-07
T1DM	leukocyte	*HLA-DRB1*	0.090465349	8.32E-07	1.11E-06	0.00326993
T1DM	leukocyte	*WDSUB1*	-0.880815255	2.20E-09	8.21E-09	8.64E-06
T1DM	leukocyte	*ZNF681*	-2.118738592	7.80E-05	9.37E-05	0.306489302
T1DM	leukocyte	*TMEM63A*	1.020533223	1.31E-06	1.73E-06	0.005143407
T1DM	leukocyte	*HRCT1*	0.867981143	7.46E-09	1.47E-08	2.93E-05
T1DM	leukocyte	*MPHOSPH8*	-0.644145983	1.99E-09	8.14E-09	7.82E-06
T1DM	leukocyte	*SIRPB2*	1.343616783	3.68E-09	9.24E-09	1.44E-05
T1DM	leukocyte	*LCOR*	-0.597636871	2.33E-09	8.21E-09	9.15E-06
T1DM	leukocyte	*SUPT5H*	-0.572477287	2.87E-09	8.44E-09	1.13E-05
T1DM	leukocyte	*XPNPEP3*	-0.937898242	2.91E-09	8.49E-09	1.14E-05
T1DM	leukocyte	*ZNF107*	-0.739166785	3.58E-09	9.11E-09	1.41E-05
T1DM	leukocyte	*SFTA2*	-1.059829121	3.69E-15	1.61E-12	1.45E-11
T1DM	leukocyte	*PPIA*	-0.511733151	5.54E-09	1.18E-08	2.18E-05
T1DM	leukocyte	*ZNF471*	-0.99735032	1.40E-09	8.14E-09	5.48E-06
T1DM	leukocyte	*ZNF493*	-0.871218591	2.39E-09	8.21E-09	9.38E-06
T1DM	leukocyte	*GTF2IRD2*	-0.887192467	5.29E-09	1.14E-08	2.08E-05
T1DM	leukocyte	*SUPT3H*	-0.603478329	1.96E-09	8.14E-09	7.71E-06
T1DM	leukocyte	*POM121*	-0.696603481	3.53E-09	9.06E-09	1.39E-05
T1DM	leukocyte	*LONP1*	-0.930742609	2.48E-09	8.21E-09	9.75E-06
T1DM	leukocyte	*ZNF34*	-0.9633072	1.95E-09	8.14E-09	7.64E-06
T1DM	leukocyte	*ZNF781*	-0.68927756	6.77E-05	8.16E-05	0.265948515
T1DM	leukocyte	*EVL*	-1.028015559	2.20E-05	2.70E-05	0.086330477
T1DM	leukocyte	*ZNF124*	-1.106737065	1.80E-09	8.14E-09	7.09E-06
T1DM	leukocyte	*XRCC6*	-0.588866821	5.17E-09	1.13E-08	2.03E-05
T1DM	leukocyte	*PIK3R4*	-1.096823497	1.34E-09	8.14E-09	5.26E-06
T1DM	leukocyte	*ZNF605*	-0.994502678	0.000394867	0.000460337	1
T1DM	leukocyte	*C20orf96*	-1.151063713	1.17E-07	1.72E-07	0.000461238
T1DM	leukocyte	*SULT1A1*	-1.303398129	2.05E-09	8.14E-09	8.04E-06
T1DM	leukocyte	*ARL9*	0.885813211	2.52E-08	4.17E-08	9.89E-05
T1DM	leukocyte	*TPK1*	-1.458929996	5.62E-07	7.62E-07	0.002208594
T1DM	leukocyte	*AFAP1*	-0.779075276	1.63E-09	8.14E-09	6.42E-06
T1DM	leukocyte	*MYO18A*	-0.565432848	1.96E-08	3.34E-08	7.71E-05
T1DM	leukocyte	*XRCC2*	-2.572000865	1.40E-09	8.14E-09	5.51E-06
T1DM	leukocyte	*MYO6*	-0.500099002	1.61E-09	8.14E-09	6.34E-06
T1DM	leukocyte	*MMP1*	0.684384505	4.05E-07	5.57E-07	0.001589503
T1DM	leukocyte	*ZNF502*	-1.760512374	3.36E-05	4.10E-05	0.131847338
T1DM	leukocyte	*TRAPPC4*	-0.614631592	3.12E-09	8.69E-09	1.23E-05
T1DM	leukocyte	*ZNF33B*	-1.170005865	8.88E-10	8.14E-09	3.49E-06
T1DM	leukocyte	*AMZ2*	-0.799664974	2.21E-09	8.21E-09	8.69E-06
T1DM	leukocyte	*ZNF431*	-0.679243883	2.04E-09	8.14E-09	8.01E-06
T1DM	leukocyte	*DAPK1*	-1.024961354	3.38E-08	5.48E-08	0.000132938
T1DM	leukocyte	*HLA-DQA1*	0.066555411	3.41E-09	8.93E-09	1.34E-05
T1DM	leukocyte	*GM2A*	0.588839158	4.06E-07	5.59E-07	0.001593459
T1DM	leukocyte	*CLPSL2*	-0.817894446	2.48E-08	4.12E-08	9.75E-05
T1DM	leukocyte	*ADA*	0.354788722	5.68E-08	8.81E-08	0.000223011
T1DM	leukocyte	*PPTC7*	-0.974597009	1.63E-09	8.14E-09	6.41E-06
T1DM	leukocyte	*ZFP28*	-0.965190186	1.68E-09	8.14E-09	6.59E-06
T1DM	leukocyte	*ANKRD36B*	-0.752462115	2.75E-08	4.53E-08	0.000108104
T1DM	leukocyte	*SRGAP1*	-0.782501467	1.94E-09	8.14E-09	7.64E-06
T1DM	leukocyte	*FAM3C*	-0.544909016	1.41E-09	8.14E-09	5.54E-06
T1DM	leukocyte	*AP2A1*	-0.810042374	3.22E-09	8.75E-09	1.27E-05
T1DM	leukocyte	*WDR5B*	-1.7785553	1.01E-07	1.50E-07	0.000397066
T1DM	leukocyte	*ZNF138*	-0.524272306	3.97E-08	6.32E-08	0.000155995
T1DM	leukocyte	*ZNF429*	-0.656236307	1.50E-09	8.14E-09	5.91E-06
T1DM	leukocyte	*ZNF100*	-0.792125026	9.68E-08	1.44E-07	0.000380476
T1DM	leukocyte	*ZNF763*	-1.336592122	3.82E-08	6.11E-08	0.000150014

T1DM	leukocyte	*ARRDC1*	-0.641118996	1.98E-09	8.14E-09	7.79E-06
T1DM	leukocyte	*ZNF682*	-1.215309605	2.92E-09	8.49E-09	1.15E-05
T1DM	leukocyte	*ZNF772*	0.519054911	0.019291008	0.021171612	1
T1DM	leukocyte	*ZNF257*	-1.1587458	4.13E-11	8.14E-09	1.62E-07
T1DM	leukocyte	*ADAM32*	0.865413492	5.67E-08	8.80E-08	0.000222836
T1DM	leukocyte	*ACSL5*	-1.151734907	1.11E-09	8.14E-09	4.38E-06
T1DM	leukocyte	*SND1*	-0.644477214	2.71E-09	8.26E-09	1.06E-05
T1DM	leukocyte	*SULT1A2*	-1.737077099	3.44E-05	4.21E-05	0.135291031
T1DM	leukocyte	*PIWIL2*	0.837416271	9.67E-07	1.28E-06	0.003801023
T1DM	leukocyte	*TBC1D9B*	-0.921232162	2.04E-06	2.64E-06	0.00800583
T1DM	leukocyte	*C6orf141*	-1.218636895	2.80E-08	4.60E-08	0.000109828
T1DM	leukocyte	*GTF2E2*	-0.653468292	1.19E-09	8.14E-09	4.69E-06
T1DM	leukocyte	*GUCA2A*	-0.691253013	0.015884495	0.017501453	1
T1DM	leukocyte	*ZNF165*	-0.574226905	2.14E-06	2.77E-06	0.008406379
T1DM	leukocyte	*SVIL*	-0.632121165	1.12E-09	8.14E-09	4.40E-06
T1DM	leukocyte	*MRPL21*	-0.629659655	6.03E-09	1.25E-08	2.37E-05
T1DM	leukocyte	*ZNF98*	-1.000851381	8.12E-10	8.14E-09	3.19E-06
T1DM	leukocyte	*ZNF786*	0.354571741	0.025409533	0.027770252	1
T1DM	leukocyte	*ZNF517*	-1.455490872	0.001145532	0.001315637	1
T1DM	leukocyte	*SLC22A5*	-1.013724306	1.27E-09	8.14E-09	4.98E-06
T1DM	leukocyte	*ADARB1*	0.601438514	0.045288686	0.049005577	1
T1DM	leukocyte	*C5AR1*	0.097478513	3.64E-09	9.18E-09	1.43E-05
T1DM	leukocyte	*VEPH1*	-1.120411731	1.85E-06	2.41E-06	0.007268021
T1DM	leukocyte	*SHPK*	-0.863260437	7.81E-08	1.18E-07	0.000307039
T1DM	leukocyte	*OGDHL*	-0.550736228	5.33E-05	6.46E-05	0.209551304
T1DM	leukocyte	*PCDHB11*	-1.012138485	2.52E-06	3.25E-06	0.009909304
T1DM	leukocyte	*MIB2*	-0.630535171	1.97E-08	3.34E-08	7.72E-05
T1DM	leukocyte	*ATG7*	-1.219554546	6.33E-09	1.30E-08	2.49E-05
T1DM	leukocyte	*PIGN*	-0.609458884	7.05E-09	1.41E-08	2.77E-05
T1DM	leukocyte	*HHLA3*	-0.557446489	2.25E-07	3.19E-07	0.000884883
T1DM	leukocyte	*BCO2*	-0.736739402	5.56E-09	1.18E-08	2.19E-05
T1DM	leukocyte	*ENTPD6*	-0.776730802	1.20E-09	8.14E-09	4.70E-06
T1DM	leukocyte	*ENPP1*	-0.572093196	3.82E-09	9.44E-09	1.50E-05
T1DM	leukocyte	*CCDC154*	2.197481734	4.82E-05	5.86E-05	0.189421123
T1DM	leukocyte	*DPP4*	-0.484015014	4.96E-10	8.14E-09	1.95E-06
T1DM	leukocyte	*NMB*	-0.651353035	2.86E-09	8.44E-09	1.13E-05
T1DM	leukocyte	*HSPB9*	-0.325810114	0.013376501	0.014783762	1
T1DM	leukocyte	*RPS26*	-0.389328738	7.53E-07	1.01E-06	0.002956857
T1DM	leukocyte	*LHFPL5*	-1.527784443	1.00E-07	1.49E-07	0.000394583
T1DM	leukocyte	*ZNF780A*	-0.54883114	6.47E-08	9.92E-08	0.000254092
T1DM	leukocyte	*ATAD3A*	-1.123658081	1.60E-09	8.14E-09	6.30E-06
T1DM	leukocyte	*FAM118B*	-1.317811485	1.51E-07	2.17E-07	0.000591345
T1DM	leukocyte	*CCDC180*	-1.146173353	3.39E-05	4.15E-05	0.133380022
T1DM	leukocyte	*SLC9A8*	-1.808409902	2.84E-07	3.97E-07	0.00111488
T1DM	leukocyte	*OCLN*	-0.482623609	9.42E-10	8.14E-09	3.70E-06
T1DM	leukocyte	*ZNF181*	-0.93881104	2.68E-09	8.26E-09	1.05E-05
T1DM	leukocyte	*TEAD4*	-0.593513655	1.76E-09	8.14E-09	6.92E-06
T1DM	leukocyte	*C2orf27A*	-1.105729492	2.04E-10	8.14E-09	8.01E-07
T1DM	leukocyte	*PLCG2*	-0.733875405	3.99E-09	9.64E-09	1.57E-05
T1DM	leukocyte	*RPL12*	-0.414863622	1.06E-09	8.14E-09	4.15E-06
T1DM	leukocyte	*VPS13A*	-0.626678419	5.21E-09	1.13E-08	2.05E-05
T1DM	leukocyte	*LEKR1*	-1.188586611	1.82E-09	8.14E-09	7.14E-06
T1DM	leukocyte	*ENTPD7*	-1.08455962	6.65E-06	8.38E-06	0.026143031
T1DM	leukocyte	*AGAP9*	-1.279393087	4.94E-08	7.74E-08	0.000194136
T1DM	leukocyte	*ZNF273*	-1.275323418	1.82E-09	8.14E-09	7.16E-06
T1DM	leukocyte	*ZNF84*	-1.240840118	5.86E-08	9.06E-08	0.000230055
T1DM	leukocyte	*ZNF667*	-0.519486508	1.16E-09	8.14E-09	4.57E-06
T1DM	leukocyte	*SIRPA*	0.600991712	9.14E-05	0.000109403	0.358951
T1DM	leukocyte	*CD2AP*	-0.651301966	2.00E-09	8.14E-09	7.85E-06
T1DM	leukocyte	*SFI1*	-1.209173814	2.47E-07	3.49E-07	0.000971886
T1DM	leukocyte	*HIBCH*	-0.629349438	1.08E-09	8.14E-09	4.24E-06
T1DM	leukocyte	*TMEM229B*	-3.022229271	5.49E-09	1.17E-08	2.16E-05
T1DM	leukocyte	*SOWAHC*	-1.100027751	4.41E-09	1.02E-08	1.73E-05
T1DM	leukocyte	*SVIP*	-0.571551129	1.69E-09	8.14E-09	6.64E-06

T1DM	leukocyte	*DDRGK1*	-0.680595772	7.35E-09	1.46E-08	2.89E-05
T1DM	leukocyte	*ZNF607*	-0.874741251	7.22E-10	8.14E-09	2.83E-06
T1DM	leukocyte	*HSD17B11*	-0.916680034	1.74E-08	3.01E-08	6.85E-05
T1DM	leukocyte	*RPS6KL1*	-0.835735618	2.44E-08	4.06E-08	9.58E-05
T1DM	leukocyte	*QRICH1*	-1.048950941	3.49E-09	9.01E-09	1.37E-05
T1DM	leukocyte	*DDX42*	-0.558001586	1.01E-08	1.88E-08	3.98E-05
T1DM	leukocyte	*HELZ*	-0.700441318	2.24E-09	8.21E-09	8.79E-06
T1DM	leukocyte	*CARD11*	-1.260518257	0.001793794	0.00204403	1
T1DM	leukocyte	*ZNF583*	-0.618922022	1.42E-09	8.14E-09	5.60E-06
T1DM	leukocyte	*ZNF568*	-0.6674488	3.48E-07	4.82E-07	0.001368876
T1DM	leukocyte	*ZNF587*	-0.778994969	1.56E-09	8.14E-09	6.14E-06
T1DM	leukocyte	*TPM2*	-0.756835058	1.13E-09	8.14E-09	4.43E-06
T1DM	leukocyte	*HLA-DRB5*	0.063134155	1.89E-05	2.33E-05	0.074197122
T1DM	leukocyte	*CNGA1*	-0.922110221	2.07E-06	2.68E-06	0.008128414
T1DM	leukocyte	*ZNF789*	-1.228139976	1.76E-05	2.17E-05	0.06910549
T1DM	leukocyte	*CCDC69*	-0.565579042	9.86E-05	0.000118003	0.387521615
T1DM	leukocyte	*NCOA6*	-0.625755977	1.08E-09	8.14E-09	4.24E-06
T1DM	leukocyte	*STK39*	-0.611942892	3.40E-09	8.90E-09	1.33E-05
T1DM	leukocyte	*PAPSS2*	-0.562652278	1.72E-09	8.14E-09	6.74E-06
T1DM	leukocyte	*CEP290*	-0.752997725	1.49E-09	8.14E-09	5.86E-06
T1DM	leukocyte	*ECI2*	-0.699298008	7.54E-09	1.48E-08	2.96E-05
T1DM	leukocyte	*MSRB1*	-0.889008608	1.03E-09	8.14E-09	4.05E-06
T1DM	leukocyte	*OXCT2*	-0.697940999	3.29E-07	4.57E-07	0.001292963
T1DM	leukocyte	*COLGALT2*	-0.675969533	0.000238797	0.000280992	0.938233401
T1DM	leukocyte	*PNP*	-0.397380367	2.87E-07	4.01E-07	0.001128985
T1DM	leukocyte	*DENND4B*	-0.70564589	5.27E-09	1.14E-08	2.07E-05
T1DM	leukocyte	*CES1*	-1.196417657	2.36E-06	3.05E-06	0.009281499
T1DM	leukocyte	*TSEN15*	-0.80315977	2.50E-09	8.21E-09	9.81E-06
T1DM	leukocyte	*LTN1*	-0.700878543	4.94E-10	8.14E-09	1.94E-06
T1DM	leukocyte	*CCDC152*	0.817456262	8.47E-08	1.27E-07	0.000332897
T1DM	leukocyte	*TYW1*	-1.005061034	6.07E-09	1.25E-08	2.38E-05
T1DM	leukocyte	*ITPRIPL1*	-0.664971783	0.000194789	0.00023052	0.765327105
T1DM	leukocyte	*MAP3K3*	-0.743372568	8.43E-10	8.14E-09	3.31E-06
T1DM	leukocyte	*C1orf174*	-0.795905279	1.52E-08	2.67E-08	5.96E-05
T1DM	leukocyte	*KIAA0753*	-0.520975933	0.023437323	0.025650485	1
T1DM	leukocyte	*ATG9A*	-0.796896791	2.57E-08	4.25E-08	0.000100861
T1DM	leukocyte	*TBKBP1*	-0.686406995	3.86E-08	6.17E-08	0.000151697
T1DM	leukocyte	*CCDC167*	-0.546747783	2.63E-09	8.21E-09	1.03E-05
T1DM	leukocyte	*SOWAHA*	-0.623058162	1.42E-09	8.14E-09	5.56E-06
T1DM	leukocyte	*L3MBTL3*	-0.705622217	0.000124033	0.000147675	0.487326252
T1DM	leukocyte	*MFAP3L*	-1.126848735	1.42E-09	8.14E-09	5.59E-06
T1DM	leukocyte	*PJA2*	-0.554612815	7.41E-09	1.47E-08	2.91E-05
T1DM	leukocyte	*INF2*	-0.973203546	3.32E-09	8.82E-09	1.31E-05
T1DM	leukocyte	*EFCAB2*	-0.6419391	5.86E-08	9.06E-08	0.000230255
T1DM	leukocyte	*RAET1G*	-1.734853908	0.00548356	0.00614866	1
T1DM	leukocyte	*SAMD5*	-0.799033804	3.03E-09	8.57E-09	1.19E-05
T1DM	leukocyte	*SPRN*	-3.291866967	1.01E-05	1.26E-05	0.039567091
T1DM	leukocyte	*DDO*	-1.12525612	0.000105153	0.000125576	0.413144243
T1DM	leukocyte	*C6orf163*	-0.809883349	0.000295087	0.000345779	1
T1DM	leukocyte	*PCMTD2*	-0.653308548	1.58E-09	8.14E-09	6.22E-06
T1DM	leukocyte	*LIME1*	-0.596969615	0.001452966	0.001661923	1
T1DM	leukocyte	*EFCAB7*	1.133616264	0.001412727	0.001616838	1
T1DM	leukocyte	*FOXO6*	-1.33765777	2.79E-09	8.35E-09	1.10E-05
T1DM	leukocyte	*SYS1*	-0.672522851	2.72E-09	8.27E-09	1.07E-05
T1DM	leukocyte	*INPP5B*	-0.822666445	6.27E-06	7.90E-06	0.02462985
T1DM	leukocyte	*TRAF3IP1*	-1.464068978	5.36E-07	7.29E-07	0.002104783
T1DM	leukocyte	*C2orf72*	-1.913781764	3.22E-06	4.11E-06	0.012637873
T1DM	leukocyte	*CLPSL1*	-0.264189692	2.04E-05	2.51E-05	0.079966564
T1DM	leukocyte	*HLA-DOA*	0.128276863	1.69E-09	8.14E-09	6.62E-06
T1DM	leukocyte	*HLA-DMA*	0.234652493	3.98E-20	2.61E-17	1.56E-16
T1DM	leukocyte	*NOTCH4*	-1.333638859	1.58E-32	3.10E-29	6.21E-29
T1DM	leukocyte	*RNF5*	-0.989259939	2.63E-21	2.06E-18	1.03E-17
T1DM	leukocyte	*MRPL38*	-0.676787839	2.39E-09	8.21E-09	9.41E-06
T1DM	leukocyte	*SMIM5*	-0.598914548	3.38E-10	8.14E-09	1.33E-06

T1DM	leukocyte	*LAYN*	-0.660974048	1.47E-09	8.14E-09	5.79E-06
T1DM	leukocyte	*SLC44A4*	-0.597451968	4.78E-12	1.45E-09	1.88E-08
T1DM	leukocyte	*VWA7*	-0.607326628	7.44E-05	8.95E-05	0.292467776
T1DM	leukocyte	*MICB*	0.288071595	0.018749904	0.020600776	1
T1DM	leukocyte	*HLA-C*	-0.215660404	0.001060976	0.001219951	1
T1DM	leukocyte	*POU5F1*	0.554593827	0.001447103	0.001655698	1
T1DM	leukocyte	*CCHCR1*	1.356489238	2.76E-14	1.08E-11	1.08E-10
T1DM	leukocyte	*PSORS1C1*	-0.761517967	6.49E-16	3.19E-13	2.55E-12
T1DM	leukocyte	*DHX16*	-0.749017444	1.98E-08	3.36E-08	7.76E-05
T1DM	leukocyte	*ABCF1*	-0.643072746	1.83E-08	3.14E-08	7.18E-05
T1DM	leukocyte	*LILRB3*	0.635032853	1.51E-06	1.98E-06	0.005934214
T1DM	leukocyte	*LINC01123*	-1.856080657	3.00E-05	3.68E-05	0.118065667
T1DM	leukocyte	*ZNF468*	-1.332778737	3.69E-07	5.10E-07	0.001451664
T1DM	leukocyte	*TRIM31*	-2.273443894	0.004584763	0.005162951	1
T1DM	leukocyte	*RNF39*	0.449727935	0.002389428	0.002711746	1
T1DM	leukocyte	*HLA-G*	-0.486325436	2.75E-05	3.37E-05	0.107891271
T1DM	leukocyte	*HLA-F*	-0.37152315	5.12E-09	1.12E-08	2.01E-05
T1DM	leukocyte	*ZFP57*	0.627399061	0.000882199	0.001017962	1
T1DM	leukocyte	*ASPDH*	-1.23526857	1.40E-09	8.14E-09	5.48E-06
T1DM	leukocyte	*KRT40*	-0.706507492	0.037626172	0.040849193	1
T1DM	leukocyte	*ZNF425*	-1.72698858	7.42E-09	1.47E-08	2.92E-05
T1DM	leukocyte	*PCDHA5*	-0.967710197	6.84E-07	9.20E-07	0.002688533
T1DM	leukocyte	*PCDHA4*	-0.329033865	0.003444216	0.003891954	1
T1DM	leukocyte	*SPIRE2*	-0.558970597	6.56E-08	1.01E-07	0.00025779
T1DM	leukocyte	*PABPN1L*	-95.11083259	1.40E-09	8.14E-09	5.49E-06
T1DM	leukocyte	*PKHD1L1*	-2.89010175	9.96E-10	8.14E-09	3.91E-06
T1DM	leukocyte	*SYCE1L*	-0.997117381	1.42E-08	2.52E-08	5.60E-05
T1DM	leukocyte	*TMEM231*	-0.70861642	5.15E-09	1.12E-08	2.02E-05
T1DM	leukocyte	*LINC00654*	-2.000630407	0.000167291	0.000198216	0.657285575
T1DM	leukocyte	*SAP25*	-0.347609148	0.001323448	0.001515985	1
T1DM	leukocyte	*SARNP*	-0.632866573	2.43E-09	8.21E-09	9.54E-06
T1DM	leukocyte	*MT1A*	-0.298857455	6.26E-05	7.57E-05	0.245984459
T1DM	leukocyte	*ATP6AP1L*	1.338999748	1.01E-07	1.50E-07	0.000396925
T1DM	leukocyte	*CCDC85C*	-0.719593501	2.27E-09	8.21E-09	8.90E-06
T1DM	leukocyte	*RGL3*	-0.926287314	6.91E-06	8.68E-06	0.027131039
T1DM	leukocyte	*CPT1B*	-0.763841108	2.48E-08	4.11E-08	9.73E-05
T1DM	leukocyte	*SMN2*	-0.863927166	3.01E-09	8.54E-09	1.18E-05
T1DM	leukocyte	*SERF1B*	-5.55327236	1.43E-06	1.88E-06	0.005618315
T1DM	leukocyte	*HMGN1*	-0.508393542	2.24E-09	8.21E-09	8.81E-06
T1DM	leukocyte	*LCMT1*	-0.885782855	2.81E-09	8.40E-09	1.10E-05
T1DM	leukocyte	*LIN52*	-1.409451648	3.38E-09	8.89E-09	1.33E-05
T1DM	leukocyte	*DENND1C*	0.297837365	3.00E-09	8.53E-09	1.18E-05
T1DM	leukocyte	*GMNC*	-1.017993599	0.009813548	0.010891929	1
T1DM	leukocyte	*LINC00487*	-10.18061188	0.018348931	0.02016586	1
T1DM	leukocyte	*DEFB134*	-77.72504757	1.76E-06	2.30E-06	0.006922123
T1DM	leukocyte	*RNPS1*	-0.629089016	1.35E-09	8.14E-09	5.29E-06
T1DM	leukocyte	*ZDHHC11B*	-1.178164263	2.29E-09	8.21E-09	8.99E-06
T1DM	leukocyte	*GOLGA8O*	-1.968240629	3.93E-08	6.27E-08	0.000154407
T1DM	leukocyte	*HLA-A*	-0.328136203	3.47E-06	4.43E-06	0.013625702
T1DM	leukocyte	*STK38L*	-1.063194235	1.21E-07	1.76E-07	0.000473907
T1DM	leukocyte	*TAS2R14*	-0.402496643	0.017745705	0.019519282	1
T1DM	leukocyte	*MAP10*	-0.834277038	7.28E-10	8.14E-09	2.86E-06
T1DM	leukocyte	*NUP62*	-0.739097872	1.58E-09	8.14E-09	6.20E-06
T1DM	leukocyte	*FAM106A*	-0.826104565	5.05E-07	6.88E-07	0.001982285
T1DM	leukocyte	*ZNF254*	-0.801225668	1.87E-09	8.14E-09	7.37E-06
T1DM	leukocyte	*LINGO4*	-0.791579811	4.76E-06	6.03E-06	0.018689744
T1DM	leukocyte	*ASIC3*	-0.882974382	1.18E-07	1.73E-07	0.00046402
T1DM	leukocyte	*ARHGEF35*	-0.48680877	2.01E-09	8.14E-09	7.90E-06
T1DM	leukocyte	*SUPT4H1*	-0.763242679	3.21E-09	8.74E-09	1.26E-05
T1DM	leukocyte	*MXD3*	-0.670567517	5.99E-08	9.24E-08	0.000235429
T1DM	leukocyte	*COG8*	-0.990863675	1.49E-09	8.14E-09	5.85E-06
T1DM	leukocyte	*ERV3-1*	-0.697061691	0.000191499	0.000226763	0.752398222
T1DM	leukocyte	*CCL14*	-0.538940142	0.004050904	0.004568313	1
T1DM	leukocyte	*RBMXL1*	-0.608497066	4.72E-07	6.46E-07	0.001856105

T1DM	leukocyte	*SRA1*	-0.526504404	2.29E-09	8.21E-09	9.01E-06
T1DM	leukocyte	*C8orf82*	-1.079889668	1.33E-07	1.93E-07	0.000523521
T1DM	leukocyte	*ZBTB9*	1.06867395	7.25E-06	9.11E-06	0.028493584
T1DM	leukocyte	*LEPROT*	-0.640355938	3.36E-09	8.85E-09	1.32E-05
T1DM	leukocyte	*PPP1CB*	-0.524365627	2.40E-09	8.21E-09	9.42E-06
T1DM	leukocyte	*NCKIPSD*	-0.800585386	1.02E-06	1.35E-06	0.004010674
T1DM	leukocyte	*TREX1*	4.69327075	7.79E-06	9.76E-06	0.030593115
T1DM	leukocyte	*ATP6V1G2*	-1.108211799	8.47E-07	1.13E-06	0.003329313
T1DM	leukocyte	*ZNF888*	-1.020077127	1.34E-09	8.14E-09	5.28E-06
T1DM	leukocyte	*EMP2*	-0.735804474	9.33E-10	8.14E-09	3.67E-06
T1DM	leukocyte	*LTB4R*	-0.919608367	5.82E-09	1.21E-08	2.29E-05
T1DM	leukocyte	*DNASE1*	-0.661463501	1.17E-09	8.14E-09	4.58E-06
T1DM	leukocyte	*NUDT19*	-0.885155324	4.22E-09	9.96E-09	1.66E-05
T1DM	leukocyte	*AP1G2*	-1.90022981	2.88E-06	3.70E-06	0.01132152
T1DM	leukocyte	*ZNF90*	-0.873466273	1.33E-09	8.14E-09	5.24E-06
T1DM	leukocyte	*REPIN1*	-0.724084025	2.49E-09	8.21E-09	9.79E-06
T1DM	leukocyte	*TSPAN4*	-0.601881198	6.07E-06	7.65E-06	0.023835033
T1DM	leukocyte	*CPNE1*	-0.372613384	0.017482004	0.019239999	1
T1DM	leukocyte	*ARL16*	-0.408844598	6.15E-05	7.44E-05	0.241729253
T1DM	leukocyte	*WEE2*	-2.016395981	0.004832691	0.005431248	1
T1DM	leukocyte	*FIS1*	-0.530846869	2.54E-09	8.21E-09	9.99E-06
T1DM	leukocyte	*MBLAC1*	-0.715062642	4.47E-05	5.45E-05	0.175804705
T1DM	leukocyte	*VSTM5*	-1.02291089	0.015390532	0.016976249	1
T1DM	leukocyte	*SEC14L6*	-0.28134513	7.23E-05	8.70E-05	0.284047221
T1DM	leukocyte	*MEG3*	-0.430016815	1.62E-09	8.14E-09	6.35E-06
T1DM	leukocyte	*EML6*	-0.870737087	2.73E-09	8.29E-09	1.07E-05
T1DM	leukocyte	*C12orf71*	-8.679765491	8.01E-06	1.00E-05	0.031455634
T1DM	leukocyte	*IFRD2*	-0.724088873	1.59E-09	8.14E-09	6.26E-06
T1DM	leukocyte	*ARHGEF28*	-0.88417464	7.14E-09	1.42E-08	2.81E-05
T1DM	leukocyte	*COL28A1*	-3.112359637	2.91E-07	4.07E-07	0.001144887
T1DM	leukocyte	*UBXN2B*	-1.033143683	1.08E-08	1.99E-08	4.25E-05
T1DM	leukocyte	*C5orf49*	-0.592158463	1.38E-09	8.14E-09	5.41E-06
T1DM	leukocyte	*GOLGA8B*	-0.946090804	1.92E-09	8.14E-09	7.54E-06
T1DM	leukocyte	*VPS16*	-1.025922777	9.08E-08	1.36E-07	0.000356877
T1DM	leukocyte	*LINC01139*	-1.635635619	1.96E-06	2.54E-06	0.007691201
T1DM	leukocyte	*ATAD3C*	-0.647508087	1.28E-09	8.14E-09	5.04E-06
T1DM	leukocyte	*SYCE3*	-1.30788106	1.16E-08	2.11E-08	4.55E-05
T1DM	leukocyte	*PAM16*	-0.618698371	1.62E-09	8.14E-09	6.35E-06
T1DM	leukocyte	*LINC00339*	-0.747168288	2.44E-08	4.06E-08	9.58E-05
T1DM	leukocyte	*CELA3B*	-0.33972163	1.72E-09	8.14E-09	6.77E-06
T1DM	leukocyte	*FAM228B*	-0.502706966	6.30E-05	7.60E-05	0.247374843
T1DM	leukocyte	*VAMP2*	-0.477619859	3.16E-09	8.69E-09	1.24E-05
T1DM	leukocyte	*PPP3R1*	-0.947243741	8.21E-09	1.59E-08	3.22E-05
T1DM	leukocyte	*FANCG*	-1.019518634	7.76E-07	1.04E-06	0.003047815
T1DM	leukocyte	*AP4M1*	-0.590674286	1.45E-09	8.14E-09	5.70E-06
T1DM	leukocyte	*ZNF880*	-0.530525715	5.42E-08	8.43E-08	0.000212884
T1DM	leukocyte	*TRIM16*	-0.977420052	1.69E-09	8.14E-09	6.65E-06
T1DM	leukocyte	*TIGD1*	-1.040616616	1.41E-08	2.51E-08	5.54E-05
T1DM	leukocyte	*XKR9*	-0.427325722	0.005234812	0.00587645	1
T1DM	leukocyte	*SLC12A8*	-1.073975356	2.73E-10	8.14E-09	1.07E-06
T1DM	leukocyte	*UBA52*	-0.478806925	4.80E-09	1.07E-08	1.89E-05
T1DM	leukocyte	*PPT2*	-0.350511678	0.024053994	0.026303408	1
T1DM	leukocyte	*EXOSC6*	-0.718543287	3.53E-09	9.06E-09	1.39E-05
T1DM	leukocyte	*CKMT1A*	-0.68767838	8.32E-09	1.60E-08	3.27E-05
T1DM	leukocyte	*HLA-DPB1*	0.083092708	9.83E-09	1.83E-08	3.86E-05
T1DM	leukocyte	*C4B*	5.464021612	0.000283458	0.000332549	1
T1DM	leukocyte	*LINC00365*	3.237859776	4.30E-07	5.90E-07	0.001688046
T1DM	leukocyte	*LINC00115*	-1.864911899	1.85E-09	8.14E-09	7.25E-06
T1DM	leukocyte	*LINC00937*	-0.83245945	1.24E-09	8.14E-09	4.86E-06
T1DM	leukocyte	*TMEM185B*	-0.752009813	1.54E-09	8.14E-09	6.03E-06
T1DM	leukocyte	*CYP4F26P*	3.824068683	3.99E-09	9.64E-09	1.57E-05
T1DM	leukocyte	*WDR46*	-0.640881331	6.68E-09	1.35E-08	2.62E-05
T1DM	leukocyte	*ZNF717*	-0.423927112	5.47E-07	7.44E-07	0.002151104
T1DM	leukocyte	*EPPK1*	-1.124833322	7.01E-06	8.80E-06	0.027536177

T1DM	leukocyte	*ZNF883*	0.784576259	0.000493561	0.000573389	1
T1DM	leukocyte	*PROB1*	-1.324825358	1.98E-09	8.14E-09	7.80E-06
T1DM	leukocyte	*ARL17B*	-1.5140467	4.18E-07	5.75E-07	0.001643118
T1DM	leukocyte	*DHFR*	-0.864718329	4.84E-07	6.60E-07	0.001899678
T1DM	leukocyte	*PSMG3-AS1*	-0.854199739	3.21E-09	8.74E-09	1.26E-05
T1DM	leukocyte	*HSBP1*	-0.573520977	2.61E-09	8.21E-09	1.02E-05
T1DM	leukocyte	*RPS18*	-0.386509452	3.15E-09	8.69E-09	1.24E-05
T1DM	leukocyte	*DIRC3*	-1.97684783	2.71E-05	3.32E-05	0.106324467
T1DM	leukocyte	*LINC00865*	-1.730354084	2.58E-09	8.21E-09	1.01E-05
T1DM	leukocyte	*HLA-DQB2*	1.716026932	2.08E-35	8.18E-32	8.18E-32
T1DM	leukocyte	*GOLGA8N*	-0.756112353	1.21E-07	1.76E-07	0.000474311
T1DM	leukocyte	*LINC00665*	-0.527902673	3.18E-09	8.70E-09	1.25E-05
T1DM	leukocyte	*LYRM9*	-0.811470903	1.39E-09	8.14E-09	5.46E-06
T1DM	leukocyte	*SNHG15*	-0.634235964	4.80E-09	1.07E-08	1.89E-05
T1DM	leukocyte	*RPS28*	-0.414003762	2.92E-09	8.49E-09	1.15E-05
T1DM	leukocyte	*TRIM26*	-0.897179124	4.94E-11	8.14E-09	1.94E-07
T1DM	leukocyte	*ZNF879*	-0.912690472	2.14E-09	8.18E-09	8.39E-06
T1DM	leukocyte	*PINLYP*	-1.409252819	5.36E-09	1.15E-08	2.11E-05
T1DM	leukocyte	*MCIDAS*	25.90276846	2.20E-07	3.12E-07	0.000864304
T1DM	leukocyte	*HLA-B*	-0.250661303	0.000138256	0.00016426	0.543207933
T1DM	leukocyte	*ANKRD65*	-0.630906757	1.51E-09	8.14E-09	5.92E-06
T1DM	leukocyte	*C12orf75*	-0.389604348	1.37E-09	8.14E-09	5.38E-06
T1DM	leukocyte	*LINC00957*	-1.049249006	3.43E-07	4.75E-07	0.001346664
T1DM	leukocyte	*B3GALT4*	-0.677907326	7.99E-13	2.85E-10	3.14E-09
T1DM	leukocyte	*ARHGEF38*	-0.734394458	0.000224919	0.000265059	0.8837071
T1DM	leukocyte	*ZNF737*	-0.9345784	2.73E-09	8.28E-09	1.07E-05
T1DM	leukocyte	*SHISA9*	-5.323278378	9.79E-10	8.14E-09	3.85E-06
T1DM	leukocyte	*HLA-DQA2*	0.093856908	4.45E-12	1.45E-09	1.75E-08
T1DM	leukocyte	*LRRC37A2*	-0.815458758	8.49E-07	1.13E-06	0.003335952
T1DM	leukocyte	*TXNDC5*	-0.686357625	1.21E-09	8.14E-09	4.76E-06
T1DM	leukocyte	*RBM14*	-0.868122161	3.93E-09	9.56E-09	1.54E-05
T1DM	leukocyte	*PCDHA13*	-1.478878509	0.000196799	0.000232758	0.773223412
T1DM	leukocyte	*NME1*	-0.502153882	2.50E-09	8.21E-09	9.82E-06
T1DM	leukocyte	*CDRT4*	-1.518388513	1.13E-09	8.14E-09	4.42E-06
T1DM	leukocyte	*APOBEC3G*	1.227270374	6.21E-07	8.39E-07	0.002441087
T1DM	leukocyte	*AMY2B*	-0.570969792	1.47E-09	8.14E-09	5.78E-06
T1DM	leukocyte	*LY6G5B*	-0.675800741	1.21E-06	1.60E-06	0.0047729
T1DM	leukocyte	*PSMB9*	-0.197028437	0.029269253	0.0319264	1
T1DM	leukocyte	*PCDHGC3*	-0.746483963	1.20E-07	1.75E-07	0.000470891
T1DM	leukocyte	*SMKR1*	-0.846184709	7.24E-09	1.44E-08	2.85E-05
T1DM	leukocyte	*C1QTNF9*	-4.678587569	0.018908449	0.020769162	1
T1DM	leukocyte	*LINC00886*	-0.890499914	1.70E-07	2.44E-07	0.000669636
T1DM	leukocyte	*MIF*	-0.401870237	2.91E-09	8.49E-09	1.14E-05
T1DM	leukocyte	*NSUN6*	-0.668871976	1.48E-09	8.14E-09	5.83E-06
T1DM	leukocyte	*HLA-DOB*	2.408624987	4.88E-27	6.40E-24	1.92E-23
T1DM	leukocyte	*CRCP*	-0.779509521	7.53E-10	8.14E-09	2.96E-06
T1DM	leukocyte	*ARHGAP8*	-0.573359258	0.000123091	0.000146598	0.483625865
T1DM	leukocyte	*PLEKHO2*	0.497156347	0.001243431	0.001425573	1
T1DM	leukocyte	*C8orf58*	-1.007322632	3.95E-08	6.30E-08	0.000155337
T1DM	leukocyte	*PWP2*	2.118421309	0.008132501	0.009069712	1
T1DM	leukocyte	*PI4KA*	-0.948399907	1.88E-07	2.69E-07	0.000738799
T1DM	leukocyte	*AMACR*	-0.57576344	4.97E-09	1.10E-08	1.95E-05
T1DM	leukocyte	*ARHGDIG*	-0.682917462	8.19E-06	1.03E-05	0.032195689
T1DM	leukocyte	*BGLAP*	-0.87990552	6.69E-06	8.42E-06	0.026296636
T1DM	leukocyte	*MRPL20*	-0.526207694	2.47E-08	4.10E-08	9.70E-05
T1DM	leukocyte	*LINC00960*	-0.394394112	6.85E-05	8.26E-05	0.269248223
T1DM	leukocyte	*HLA-DMB*	0.087393404	1.62E-10	8.14E-09	6.36E-07
T1DM	leukocyte	*DECR2*	-0.713280751	1.64E-09	8.14E-09	6.46E-06
T1DM	leukocyte	*GNG10*	-0.554651578	1.31E-08	2.35E-08	5.13E-05
T1DM	leukocyte	*EIF4EBP3*	-0.927076379	2.77E-10	8.14E-09	1.09E-06
T1DM	leukocyte	*PSG4*	2.246323025	2.98E-07	4.15E-07	0.001169801
T1DM	leukocyte	*MICAL3*	-0.662489524	1.48E-09	8.14E-09	5.82E-06
T1DM	leukocyte	*STON1*	-0.823933974	7.55E-07	1.01E-06	0.002968301
T1DM	leukocyte	*VSIG8*	-1.809625604	1.45E-09	8.14E-09	5.69E-06

T1DM	leukocyte	*C4orf48*	-0.486452423	2.21E-09	8.21E-09	8.68E-06
T1DM	leukocyte	*UPK3B*	1.63660568	0.004891843	0.005494583	1
T1DM	leukocyte	*IL10RB*	-1.200653286	8.81E-07	1.17E-06	0.003461453
T1DM	leukocyte	*PLA2G4B*	-0.901513519	6.34E-07	8.55E-07	0.002491107
T1DM	leukocyte	*LEFTY1*	-5.532421282	1.97E-08	3.34E-08	7.73E-05
T1DM	leukocyte	*TTC4*	-0.718982967	2.08E-09	8.15E-09	8.17E-06
T1DM	leukocyte	*JMJD7*	0.834121246	0.009493112	0.010545218	1
T1DM	leukocyte	*APOBEC3D*	-2.640945823	2.23E-08	3.74E-08	8.76E-05
T1DM	leukocyte	*GSTA1*	-0.400657475	3.46E-09	8.97E-09	1.36E-05
T1DM	leukocyte	*GSTA2*	-0.532044246	1.44E-09	8.14E-09	5.66E-06
T1DM	leukocyte	*TMEM141*	-0.773814782	1.26E-08	2.27E-08	4.94E-05
T1DM	leukocyte	*CFHR1*	5.160101915	2.38E-06	3.07E-06	0.009345148
T1DM	leukocyte	*APOBEC3C*	-1.303985633	1.32E-07	1.91E-07	0.000517796
T1DM	leukocyte	*C4A*	-9.995810018	3.04E-24	2.99E-21	1.19E-20
T1DM	leukocyte	*NEAT1*	-0.440835924	5.83E-09	1.22E-08	2.29E-05
T1DM	leukocyte	*LINC00920*	-5.142378259	5.63E-07	7.63E-07	0.002212141
T1DM	leukocyte	*UBAP1L*	-0.735392418	2.70E-09	8.26E-09	1.06E-05
T1DM	leukocyte	*TWF2*	-0.718693236	1.55E-06	2.03E-06	0.006088762
T1DM	leukocyte	*LINC00926*	3.995444818	1.05E-06	1.39E-06	0.004137932
T1DM	leukocyte	*BCKDHA*	-0.684956018	5.42E-09	1.16E-08	2.13E-05
T1DM	leukocyte	*PCDHAC1*	-1.079607217	1.46E-09	8.14E-09	5.72E-06
T1DM	leukocyte	*TMEM150C*	-0.80810391	3.50E-09	9.01E-09	1.37E-05
T1DM	leukocyte	*LINC01091*	-0.512391689	7.77E-10	8.14E-09	3.05E-06
T1DM	leukocyte	*PDCD6*	-0.636605294	2.50E-09	8.21E-09	9.82E-06
T1DM	leukocyte	*GOLGA8K*	-12.15079308	2.45E-07	3.46E-07	0.000962329
T1DM	leukocyte	*YJEFN3*	-1.231494442	9.66E-10	8.14E-09	3.79E-06
T1DM	leukocyte	*PCDHA10*	-0.657722436	1.29E-09	8.14E-09	5.07E-06
T1DM	leukocyte	*ZNF718*	-0.852271998	3.82E-08	6.11E-08	0.000150064
T1DM	leukocyte	*CDK3*	0.333717758	3.34E-06	4.27E-06	0.013128511
T1DM	leukocyte	*SNHG18*	-0.702454902	1.82E-09	8.14E-09	7.15E-06
T1DM	leukocyte	*ZNF550*	-0.779353491	5.33E-09	1.15E-08	2.09E-05
T1DM	leukocyte	*LINC01099*	-0.850706855	1.52E-09	8.14E-09	5.97E-06
T1DM	leukocyte	*PCDHA12*	-0.85827359	1.02E-05	1.27E-05	0.039904843
T1DM	leukocyte	*ALG11*	-1.195140809	2.51E-09	8.21E-09	9.88E-06
T1DM	leukocyte	*ZNF260*	-0.994361513	2.84E-06	3.66E-06	0.011171098
T1DM	leukocyte	*LYN*	0.68091902	0.021641119	0.023704477	1
T1DM	leukocyte	*AP5B1*	-1.074731554	9.85E-08	1.46E-07	0.000386968
T1DM	leukocyte	*SIGLEC12*	1.517241578	3.64E-09	9.18E-09	1.43E-05
T1DM	leukocyte	*PABPC4L*	-1.987353578	1.63E-09	8.14E-09	6.42E-06
T1DM	leukocyte	*FPGT*	0.693762549	0.014001887	0.015457549	1
T1DM	leukocyte	*NPIPA2*	-7.612300136	1.01E-09	8.14E-09	3.98E-06
T1DM	leukocyte	*DPP3*	-0.500085456	1.12E-05	1.40E-05	0.044042279
T1DM	leukocyte	*BRK1*	-0.502381703	4.98E-09	1.10E-08	1.96E-05
T1DM	leukocyte	*EID3*	-1.35176857	3.86E-09	9.50E-09	1.52E-05
T1DM	leukocyte	*SNHG9*	-0.595375295	9.60E-09	1.80E-08	3.77E-05
T1DM	leukocyte	*PCDHA3*	-0.894943833	4.34E-06	5.50E-06	0.017036123
T1DM	leukocyte	*ZNF432*	-1.291980043	1.96E-09	8.14E-09	7.72E-06
T1DM	leukocyte	*LINC01152*	-3.38676528	1.50E-09	8.14E-09	5.91E-06
T1DM	leukocyte	*SMLR1*	2.800222641	5.75E-09	1.20E-08	2.26E-05
T1DM	leukocyte	*ZNF10*	-0.871952583	8.66E-10	8.14E-09	3.40E-06
T1DM	leukocyte	*HMBS*	-0.310210226	0.025695217	0.028074668	1
T1DM	leukocyte	*TAS2R31*	-2.549361225	6.55E-09	1.33E-08	2.58E-05
T1DM	leukocyte	*LIMS3*	-4.870709803	0.008080956	0.009014786	1
T1DM	leukocyte	*GATC*	-0.893469997	9.57E-10	8.14E-09	3.76E-06
T1DM	leukocyte	*TEN1*	-1.143410848	0.000115213	0.00013734	0.45267212
T1DM	leukocyte	*CHURC1*	-0.533078785	4.03E-09	9.70E-09	1.58E-05
T1DM	leukocyte	*LINC00638*	-4.302582634	2.74E-07	3.84E-07	0.001076133
T1DM	leukocyte	*SLC25A21-AS1*	-0.702478052	1.61E-09	8.14E-09	6.32E-06
T1DM	leukocyte	*TMEM179*	-0.509779288	1.78E-09	8.14E-09	6.98E-06
T1DM	leukocyte	*IQCH-AS1*	-1.033819116	4.47E-07	6.12E-07	0.001755341
T1DM	leukocyte	*SLC22A31*	-0.526064224	0.003963945	0.004471531	1
T1DM	leukocyte	*SH3RF3-AS1*	1.546382496	1.73E-06	2.25E-06	0.006780538
T1DM	leukocyte	*PWRN1*	-0.874586076	7.01E-08	1.07E-07	0.000275253
T1DM	leukocyte	*TMEM178B*	-0.636623485	3.56E-09	9.10E-09	1.40E-05

T1DM	leukocyte	*LINC01003*	-0.779717304	4.21E-09	9.96E-09	1.65E-05
T1DM	leukocyte	*GOLGA8H*	-10.52581079	1.74E-07	2.49E-07	0.000682581
T1DM	leukocyte	*MRPL12*	-0.83122724	2.42E-09	8.21E-09	9.52E-06
T1DM	leukocyte	*LINC00667*	-0.718762068	2.23E-09	8.21E-09	8.76E-06
T1DM	leukocyte	*FSBP*	-1.011489174	1.35E-09	8.14E-09	5.31E-06
T1DM	leukocyte	*MYO15B*	-1.315655666	2.19E-07	3.11E-07	0.000859956
T1DM	leukocyte	*LINC00663*	-0.924021006	8.01E-07	1.07E-06	0.00314802
T1DM	leukocyte	*ZNF285*	-1.637516309	4.52E-09	1.04E-08	1.78E-05
T1DM	leukocyte	*FBXO17*	-0.581498697	1.13E-09	8.14E-09	4.45E-06
T1DM	leukocyte	*LINC00528*	0.420810732	3.38E-08	5.48E-08	0.000132725
T1DM	leukocyte	*SNHG8*	-0.489792752	1.80E-09	8.14E-09	7.07E-06
T1DM	leukocyte	*MILR1*	0.657393354	1.79E-08	3.07E-08	7.02E-05
T1DM	leukocyte	*GTF2H5*	-0.618552173	4.60E-09	1.04E-08	1.81E-05
T1DM	mesenchymal_cell	*CEP68*	0.348351975	0.000159927	0.019041039	0.628354292
T1DM	mesenchymal_cell	*GPATCH1*	0.558100378	0.000208894	0.024139529	0.820743997
T1DM	mesenchymal_cell	*BTN2A2*	-0.212060841	0.000391173	0.042692206	1
T1DM	mesenchymal_cell	*UQCC2*	0.452736717	1.15E-05	0.001677589	0.045294908
T1DM	mesenchymal_cell	*ENGASE*	0.194384966	0.00043889	0.046605381	1
T1DM	mesenchymal_cell	*HLA-DQB1*	-0.288176327	2.56E-23	2.01E-20	1.01E-19
T1DM	mesenchymal_cell	*BTN3A2*	-0.085453142	1.27E-05	0.001784055	0.049953552
T1DM	mesenchymal_cell	*ZKSCAN3*	-0.525284169	9.45E-09	2.18E-06	3.71E-05
T1DM	mesenchymal_cell	*HLA-DRB1*	-0.098962803	2.84E-07	5.07E-05	0.001114982
T1DM	mesenchymal_cell	*SFTA2*	-0.234955038	1.02E-09	2.50E-07	4.00E-06
T1DM	mesenchymal_cell	*RPS26*	0.242443253	0.000159785	0.019041039	0.627796376
T1DM	mesenchymal_cell	*HLA-DRB5*	-0.140271059	7.97E-16	3.48E-13	3.13E-12
T1DM	mesenchymal_cell	*HSD17B8*	0.603100824	4.32E-24	4.24E-21	1.70E-20
T1DM	mesenchymal_cell	*HLA-DMA*	0.242350085	1.60E-28	2.09E-25	6.27E-25
T1DM	mesenchymal_cell	*NOTCH4*	-0.260066779	2.85E-17	1.60E-14	1.12E-13
T1DM	mesenchymal_cell	*AGER*	0.476404239	2.14E-06	0.00033678	0.008419508
T1DM	mesenchymal_cell	*RNF5*	-0.386473182	1.95E-13	6.37E-11	7.64E-10
T1DM	mesenchymal_cell	*EHMT2*	0.608159436	2.59E-05	0.003280272	0.101688444
T1DM	mesenchymal_cell	*MICA*	0.093123479	1.14E-05	0.001677589	0.044758602
T1DM	mesenchymal_cell	*HLA-C*	0.125137352	0.000283464	0.031820855	1
T1DM	mesenchymal_cell	*CCHCR1*	0.387088224	1.12E-07	2.33E-05	0.000441929
T1DM	mesenchymal_cell	*PSORS1C1*	-0.177825285	3.54E-11	9.94E-09	1.39E-07
T1DM	mesenchymal_cell	*RNF39*	0.351438706	5.49E-14	1.96E-11	2.16E-10
T1DM	mesenchymal_cell	*ZFP57*	0.268512124	8.13E-07	0.000133152	0.003195642
T1DM	mesenchymal_cell	*HCG27*	0.256802562	1.36E-08	2.97E-06	5.35E-05
T1DM	mesenchymal_cell	*ATF6B*	0.407481973	1.29E-11	3.89E-09	5.06E-08
T1DM	mesenchymal_cell	*PPT2*	0.384973498	2.41E-07	4.74E-05	0.000947363
T1DM	mesenchymal_cell	*C4B*	2.164702984	2.59E-07	4.84E-05	0.001015811
T1DM	mesenchymal_cell	*HLA-DQB2*	1.081329738	1.25E-51	4.93E-48	4.93E-48
T1DM	mesenchymal_cell	*HLA-B*	0.160946451	2.41E-05	0.003158508	0.094755236
T1DM	mesenchymal_cell	*ZSCAN31*	0.15202471	3.22E-07	5.50E-05	0.001264232
T1DM	mesenchymal_cell	*B3GALT4*	-0.254859134	2.27E-05	0.003076309	0.089212952
T1DM	mesenchymal_cell	*HLA-DQA2*	0.448564337	6.98E-33	1.37E-29	2.74E-29
T1DM	mesenchymal_cell	*PSMB9*	0.293126454	1.02E-09	2.50E-07	3.99E-06
T1DM	mesenchymal_cell	*HLA-DOB*	0.596012192	1.60E-16	7.86E-14	6.29E-13
T1DM	mesenchymal_cell	*HLA-DMB*	0.490643988	5.23E-14	1.96E-11	2.06E-10
T1DM	mesenchymal_cell	*C4A*	-2.679420158	1.84E-21	1.20E-18	7.23E-18
T1DM	PP_cell	*BTN2A2*	-0.417583381	0.000257227	0.037431237	1
T1DM	PP_cell	*ENGASE*	0.324638666	0.000222071	0.03355832	0.872516314
T1DM	PP_cell	*HLA-DQB1*	-0.631844149	1.17E-28	1.84E-25	4.58E-25
T1DM	PP_cell	*BTN3A2*	-0.142114363	8.75E-05	0.014318254	0.343638107
T1DM	PP_cell	*ZKSCAN3*	-0.731676744	1.34E-06	0.000262646	0.00525292
T1DM	PP_cell	*HLA-DRB1*	-0.248757107	6.63E-10	1.74E-07	2.61E-06
T1DM	PP_cell	*SFTA2*	-0.370112356	4.77E-09	1.17E-06	1.87E-05
T1DM	PP_cell	*HLA-DRB5*	-0.429361523	1.16E-16	5.69E-14	4.55E-13
T1DM	PP_cell	*HSD17B8*	0.438575183	2.76E-08	6.37E-06	0.000108281
T1DM	PP_cell	*HLA-DMA*	0.419682567	2.28E-27	2.24E-24	8.95E-24
T1DM	PP_cell	*NOTCH4*	-0.726933956	9.35E-26	7.35E-23	3.68E-22
T1DM	PP_cell	*RNF5*	-0.638819902	1.05E-12	4.13E-10	4.13E-09
T1DM	PP_cell	*SLC44A4*	-0.205907889	0.000319121	0.043525559	1
T1DM	PP_cell	*MICA*	0.209373819	4.06E-07	8.40E-05	0.001595457

T1DM	PP_cell	*PSORS1C1*	-0.322838073	1.95E-11	6.38E-09	7.65E-08
T1DM	PP_cell	*TRIM31*	-1.716230849	0.000321263	0.043525559	1
T1DM	PP_cell	*RNF39*	0.378700201	2.04E-07	4.45E-05	0.00080189
T1DM	PP_cell	*ZFP57*	0.461264161	2.23E-06	0.000416437	0.008745173
T1DM	PP_cell	*ATF6B*	0.586626748	3.85E-10	1.08E-07	1.51E-06
T1DM	PP_cell	*PPT2*	1.14728567	9.13E-13	3.99E-10	3.59E-09
T1DM	PP_cell	*C4B*	3.131498624	1.99E-05	0.003555647	0.078224229
T1DM	PP_cell	*HLA-DQB2*	1.793953017	9.15E-46	3.60E-42	3.60E-42
T1DM	PP_cell	*HLA-B*	0.243435054	0.000167764	0.026365863	0.659146581
T1DM	PP_cell	*B3GALT4*	-0.397828616	8.10E-05	0.013842616	0.318380163
T1DM	PP_cell	*HLA-DQA2*	0.701517073	1.41E-28	1.84E-25	5.53E-25
T1DM	PP_cell	*PSMB9*	0.669560985	6.66E-12	2.38E-09	2.62E-08
T1DM	PP_cell	*HLA-DOB*	1.240414239	5.06E-19	2.84E-16	1.99E-15
T1DM	PP_cell	*HLA-DMB*	0.693262541	1.29E-10	3.91E-08	5.09E-07
T1DM	PP_cell	*C4A*	-4.851704286	2.48E-22	1.63E-19	9.76E-19
T2DM	acinar_cell	*RBM6*	-0.754793139	1.80E-07	6.44E-05	0.000708573
T2DM	acinar_cell	*MLXIPL*	0.535189476	0.00010249	0.011185597	0.402681487
T2DM	acinar_cell	*CEP68*	0.964270143	6.24E-06	0.001226297	0.024525945
T2DM	acinar_cell	*LTF*	-0.914212248	0.000686743	0.049966895	1
T2DM	acinar_cell	*BAK1*	1.280540908	4.27E-06	0.000922451	0.016794411
T2DM	acinar_cell	*ITIH4*	-0.7115692	9.74E-05	0.011185597	0.382739294
T2DM	acinar_cell	*TCF3*	-1.425565827	2.98E-06	0.000700712	0.011697388
T2DM	acinar_cell	*ST6GAL1*	0.669957685	0.000389308	0.032544479	1
T2DM	acinar_cell	*MARK3*	-1.620499992	4.46E-06	0.000922451	0.017526562
T2DM	acinar_cell	*CWF19L1*	-0.496144474	7.08E-05	0.009592382	0.278179092
T2DM	acinar_cell	*CCDC134*	1.164240546	0.000204076	0.020045325	0.801813018
T2DM	acinar_cell	*OIP5*	2.012782462	0.000100362	0.011185597	0.394323735
T2DM	acinar_cell	*HSD17B1*	-0.625785989	0.000339439	0.02899253	1
T2DM	acinar_cell	*CNTNAP1*	0.622289993	4.11E-05	0.006451962	0.161299052
T2DM	acinar_cell	*PRR4*	0.449767647	0.000539124	0.041533688	1
T2DM	acinar_cell	*DNAH7*	-0.672412341	0.000178542	0.017986968	0.701491756
T2DM	acinar_cell	*FLT3*	0.775955002	0.000505201	0.039698698	1
T2DM	acinar_cell	*OPRL1*	-0.856803103	0.000480377	0.038518359	1
T2DM	acinar_cell	*PTPN12*	-0.610007861	0.000297198	0.026538452	1
T2DM	acinar_cell	*MAU2*	-1.495475188	3.03E-06	0.000700712	0.011912098
T2DM	acinar_cell	*NOTCH2*	-0.6194768	2.12E-09	1.67E-06	8.33E-06
T2DM	acinar_cell	*MAN2C1*	-0.468478519	1.79E-05	0.003050403	0.070159278
T2DM	acinar_cell	*NPC1*	-1.842344081	2.79E-05	0.004571552	0.109717244
T2DM	acinar_cell	*GIGYF1*	-1.64654733	7.59E-05	0.00994128	0.298238411
T2DM	acinar_cell	*HSD17B12*	-0.285134048	0.000105609	0.011214586	0.414939684
T2DM	acinar_cell	*APIP*	-0.407448892	0.000553641	0.041831821	1
T2DM	acinar_cell	*PTPN14*	-0.513777922	4.62E-05	0.006981613	0.18152193
T2DM	acinar_cell	*JAZF1*	-1.600126564	3.25E-12	4.26E-09	1.28E-08
T2DM	acinar_cell	*CLEC18A*	-2.220775946	3.04E-09	1.99E-06	1.19E-05
T2DM	acinar_cell	*CILP2*	-0.723637829	9.55E-05	0.011185597	0.375337955
T2DM	acinar_cell	*SGPL1*	-0.999672114	0.000668356	0.049546606	1
T2DM	acinar_cell	*INO80E*	-0.744666203	0.000240927	0.022013989	0.946601511
T2DM	acinar_cell	*IRX3*	2.653787077	3.34E-23	1.31E-19	1.31E-19
T2DM	acinar_cell	*FARSA*	-1.332008435	5.62E-05	0.008181197	0.220892326
T2DM	acinar_cell	*HLA-DQB1*	-0.42004321	1.13E-08	5.41E-06	4.46E-05
T2DM	acinar_cell	*UBA7*	-0.593708176	1.41E-06	0.000462888	0.005554653
T2DM	acinar_cell	*VMO1*	0.584895189	0.000337597	0.02899253	1
T2DM	acinar_cell	*CAMK1D*	1.80842789	0.000227406	0.021273249	0.893476444
T2DM	acinar_cell	*FAM43B*	2.943995455	0.000165969	0.01716033	0.652092525
T2DM	acinar_cell	*HLA-DRB1*	-0.329684647	1.49E-10	1.47E-07	5.87E-07
T2DM	acinar_cell	*HLA-DQA1*	-0.208280171	0.000401532	0.032867099	1
T2DM	acinar_cell	*PIWIL2*	-0.593794866	0.000218863	0.020973448	0.859911383
T2DM	acinar_cell	*HLA-DRB5*	-0.229387518	1.49E-05	0.002711248	0.058700589
T2DM	acinar_cell	*NOTCH4*	-0.5612611	1.24E-08	5.41E-06	4.87E-05
T2DM	acinar_cell	*RNF5*	-0.55849212	2.20E-06	0.000575108	0.008626619
T2DM	acinar_cell	*HCG27*	0.514807343	1.54E-06	0.000464581	0.00603955
T2DM	acinar_cell	*DNLZ*	0.314544719	8.21E-05	0.010078575	0.322514391
T2DM	acinar_cell	*ATF6B*	0.537276606	7.07E-05	0.009592382	0.277749948
T2DM	acinar_cell	*SLC12A8*	-2.535840194	1.43E-07	5.61E-05	0.000561213

T2DM	acinar_cell	*PPT2*	0.944873489	2.12E-06	0.000575108	0.008336986
T2DM	acinar_cell	*HLA-DQB2*	0.953737898	4.04E-09	2.27E-06	1.59E-05
T2DM	acinar_cell	*HLA-B*	0.376317742	1.52E-05	0.002711248	0.059647459
T2DM	acinar_cell	*HLA-DQA2*	0.595067619	2.11E-13	4.14E-10	8.28E-10
T2DM	acinar_cell	*JMJD7*	1.027246108	8.19E-05	0.010078575	0.321590816
T2DM	alpha_cell	*RBM6*	-0.127977665	7.62E-08	2.72E-05	0.000299239
T2DM	alpha_cell	*BAIAP3*	-0.185238573	0.000636709	0.041010293	1
T2DM	alpha_cell	*MLXIPL*	0.085565517	0.000273378	0.022377128	1
T2DM	alpha_cell	*GIPR*	-0.150925617	0.000516749	0.035005266	1
T2DM	alpha_cell	*CEP68*	0.179209104	4.84E-07	0.000146417	0.00190342
T2DM	alpha_cell	*BAK1*	0.219087846	2.33E-06	0.00057226	0.009156166
T2DM	alpha_cell	*ITIH4*	-0.170227305	0.000197303	0.018457222	0.775203311
T2DM	alpha_cell	*TCF3*	-0.225101587	1.11E-05	0.001985953	0.04369096
T2DM	alpha_cell	*MARK3*	-0.366412534	6.21E-08	2.44E-05	0.000244148
T2DM	alpha_cell	*CWF19L1*	-0.083146528	4.32E-05	0.005853332	0.169746627
T2DM	alpha_cell	*CCDC134*	0.179308131	0.000616429	0.040365829	1
T2DM	alpha_cell	*AP1B1*	-0.294874627	0.000228264	0.020382933	0.896849032
T2DM	alpha_cell	*OIP5*	0.318902279	0.000156486	0.015845631	0.61483363
T2DM	alpha_cell	*GCDH*	-0.363032588	0.000121373	0.014025759	0.476875803
T2DM	alpha_cell	*HSD17B1*	-0.103610128	0.000264989	0.022151985	1
T2DM	alpha_cell	*CNTNAP1*	0.095472266	0.00011457	0.013640744	0.450144552
T2DM	alpha_cell	*NNT*	-0.155930566	0.000161884	0.015901092	0.636043678
T2DM	alpha_cell	*DNAH7*	-0.124894315	2.88E-05	0.004192955	0.113209793
T2DM	alpha_cell	*FLT3*	0.127152456	0.000395166	0.028229227	1
T2DM	alpha_cell	*OPRL1*	-0.142829009	0.000322327	0.02389476	1
T2DM	alpha_cell	*PTPN12*	-0.093286377	0.000812371	0.047685791	1
T2DM	alpha_cell	*MAU2*	-0.261600723	2.50E-06	0.00057883	0.009840118
T2DM	alpha_cell	*PPARG*	-0.359325626	0.000157287	0.015845631	0.617979617
T2DM	alpha_cell	*NOTCH2*	-0.096931937	1.21E-08	5.29E-06	4.76E-05
T2DM	alpha_cell	*FXYD2*	-0.16098264	0.000320388	0.02389476	1
T2DM	alpha_cell	*MAN2C1*	-0.080040997	6.86E-06	0.001347115	0.026942305
T2DM	alpha_cell	*NPC1*	-0.364045405	1.03E-05	0.001919713	0.040313977
T2DM	alpha_cell	*CNTN4*	-0.078155116	0.000457277	0.032082913	1
T2DM	alpha_cell	*GIGYF1*	-0.308013921	0.000135135	0.015169824	0.530943843
T2DM	alpha_cell	*HSD17B12*	-0.047549048	7.55E-05	0.009569469	0.296653549
T2DM	alpha_cell	*APIP*	-0.071289964	0.000226538	0.020382933	0.890066031
T2DM	alpha_cell	*PTPN14*	-0.076779464	0.000234608	0.020483902	0.921775592
T2DM	alpha_cell	*JAZF1*	-0.345268477	7.65E-17	1.50E-13	3.00E-13
T2DM	alpha_cell	*NUS1*	-0.31179408	0.000861979	0.049740491	1
T2DM	alpha_cell	*CC2D1B*	-0.086156546	0.000813171	0.047685791	1
T2DM	alpha_cell	*CLEC18A*	-0.34917244	7.75E-09	5.08E-06	3.05E-05
T2DM	alpha_cell	*AP3S2*	0.288153372	0.000914793	0.049919746	1
T2DM	alpha_cell	*EXTL1*	-0.207631412	0.000898848	0.049740491	1
T2DM	alpha_cell	*CILP2*	-0.118780579	7.10E-05	0.00930407	0.279122105
T2DM	alpha_cell	*ICA1L*	-0.067856921	0.000892756	0.049740491	1
T2DM	alpha_cell	*CASP7*	0.065229004	0.000305925	0.023568214	1
T2DM	alpha_cell	*CKB*	-0.111437658	0.000255607	0.021832143	1
T2DM	alpha_cell	*PCBD1*	-0.201531273	0.000394839	0.028229227	1
T2DM	alpha_cell	*DNAJC21*	-0.292096367	0.000799849	0.047685791	1
T2DM	alpha_cell	*INO80E*	-0.151417015	1.41E-05	0.002410525	0.055442065
T2DM	alpha_cell	*PPP1R3B*	-0.151157566	0.000882254	0.049740491	1
T2DM	alpha_cell	*IRX3*	0.426096048	2.07E-23	8.13E-20	8.13E-20
T2DM	alpha_cell	*FARSA*	-0.238267161	2.23E-05	0.003365322	0.087498378
T2DM	alpha_cell	*HLA-DQB1*	-0.068420431	1.05E-08	5.16E-06	4.13E-05
T2DM	alpha_cell	*UBA7*	-0.092152265	4.79E-06	0.000990221	0.018814201
T2DM	alpha_cell	*VMO1*	0.09171573	0.00046602	0.032122687	1
T2DM	alpha_cell	*ZNF320*	0.039540615	0.000702122	0.044494123	1
T2DM	alpha_cell	*CAMK1D*	0.340727812	1.70E-05	0.002775206	0.066604953
T2DM	alpha_cell	*FAM43B*	0.433126065	0.000537115	0.035768194	1
T2DM	alpha_cell	*DMWD*	-0.158910531	0.000772949	0.047685791	1
T2DM	alpha_cell	*NUTM2B*	-0.954190279	0.000151651	0.015845631	0.595838548
T2DM	alpha_cell	*HLA-DRB1*	-0.05361681	2.58E-10	2.53E-07	1.01E-06
T2DM	alpha_cell	*XRCC6*	-0.226310491	0.000297967	0.023414237	1
T2DM	alpha_cell	*HLA-DQA1*	-0.034680007	0.00028432	0.02279782	1

T2DM	alpha_cell	*PIWIL2*	-0.089785581	0.000792935	0.047685791	1
T2DM	alpha_cell	*HLA-DRB5*	-0.036766336	3.33E-05	0.004674874	0.130896462
T2DM	alpha_cell	*NOTCH4*	-0.092005969	7.35E-09	5.08E-06	2.89E-05
T2DM	alpha_cell	*RNF5*	-0.091256173	1.97E-06	0.000551556	0.007721781
T2DM	alpha_cell	*HCG27*	0.082946359	2.27E-06	0.00057226	0.008899946
T2DM	alpha_cell	*DNLZ*	0.048814151	0.000176385	0.016902873	0.693017812
T2DM	alpha_cell	*ATF6B*	0.084600094	0.000147658	0.015845631	0.580149086
T2DM	alpha_cell	*SLC12A8*	-0.522598855	2.31E-07	7.58E-05	0.00090948
T2DM	alpha_cell	*PPT2*	0.159753729	3.08E-06	0.000672542	0.012105754
T2DM	alpha_cell	*HLA-DQB2*	0.153646367	9.70E-09	5.16E-06	3.81E-05
T2DM	alpha_cell	*HLA-B*	0.062434979	1.89E-05	0.002972209	0.07430522
T2DM	alpha_cell	*HLA-DQA2*	0.095677146	5.34E-13	7.00E-10	2.10E-09
T2DM	alpha_cell	*JMJD7*	0.16592955	9.16E-05	0.011252725	0.360087211
T2DM	beta_cell	*RBM6*	-0.073336338	5.02E-07	0.0001408	0.001971204
T2DM	beta_cell	*MLXIPL*	0.059033694	3.90E-05	0.004929304	0.153319364
T2DM	beta_cell	*CEP68*	0.126274583	1.11E-08	5.45E-06	4.36E-05
T2DM	beta_cell	*BAK1*	0.152283218	1.26E-07	3.80E-05	0.000494197
T2DM	beta_cell	*ANAPC4*	0.528951373	3.99E-05	0.004929304	0.156820791
T2DM	beta_cell	*ITIH4*	-0.099962396	0.000388247	0.023884252	1
T2DM	beta_cell	*TCF3*	-0.126373996	6.63E-05	0.007657555	0.260356855
T2DM	beta_cell	*ST6GAL1*	0.065692652	0.000675566	0.033598695	1
T2DM	beta_cell	*MARK3*	-0.241527256	7.12E-07	0.000186514	0.002797703
T2DM	beta_cell	*CD59*	0.173250668	0.000389449	0.023884252	1
T2DM	beta_cell	*MGST2*	0.187469391	0.000210526	0.01621875	0.827156231
T2DM	beta_cell	*UIMC1*	0.208035336	0.000769076	0.036611569	1
T2DM	beta_cell	*CWF19L1*	-0.047557424	0.000117364	0.01152805	0.461122014
T2DM	beta_cell	*CCDC134*	0.114003257	0.000335673	0.023137856	1
T2DM	beta_cell	*TOM1*	0.31823681	5.32E-05	0.006338095	0.209157139
T2DM	beta_cell	*RBL2*	0.126759431	0.000363618	0.023420566	1
T2DM	beta_cell	*NOMO1*	0.114546712	0.000628535	0.032071586	1
T2DM	beta_cell	*OIP5*	0.200153358	8.69E-05	0.008981758	0.341306789
T2DM	beta_cell	*ACBD5*	0.385867982	0.000596166	0.031084775	1
T2DM	beta_cell	*DHRS11*	0.153928941	0.000238365	0.018010316	0.936536429
T2DM	beta_cell	*RAI1*	0.576488723	0.000687705	0.033774888	1
T2DM	beta_cell	*HSD17B1*	-0.057466036	0.000849606	0.039406797	1
T2DM	beta_cell	*CNTNAP1*	0.059062163	8.35E-05	0.008868126	0.328120666
T2DM	beta_cell	*PDHX*	0.080199146	0.001061096	0.046843227	1
T2DM	beta_cell	*PRR4*	0.044526584	0.000913507	0.041616947	1
T2DM	beta_cell	*CDK2AP1*	0.080902947	4.01E-05	0.004929304	0.157737732
T2DM	beta_cell	*SEMA5A*	0.249219582	0.00017513	0.0146401	0.688084682
T2DM	beta_cell	*NNT*	-0.085707213	0.000974102	0.043491454	1
T2DM	beta_cell	*DNAH7*	-0.070473906	0.000111835	0.011266642	0.439399039
T2DM	beta_cell	*BCL11A*	0.268334069	0.000450811	0.025804744	1
T2DM	beta_cell	*IAPP*	0.125601849	6.27E-06	0.001071367	0.024641433
T2DM	beta_cell	*FLT3*	0.077767186	0.000347695	0.023420566	1
T2DM	beta_cell	*BHLHE41*	0.244659956	0.000245007	0.018162877	0.962632464
T2DM	beta_cell	*OPRL1*	-0.085537254	0.000358434	0.023420566	1
T2DM	beta_cell	*BECN1*	0.60394207	1.94E-06	0.000362411	0.007610634
T2DM	beta_cell	*PLEKHG3*	0.569098148	0.000458816	0.025804744	1
T2DM	beta_cell	*MAU2*	-0.147090622	3.80E-05	0.004929304	0.149321066
T2DM	beta_cell	*SYT5*	0.059729639	0.000400953	0.023884252	1
T2DM	beta_cell	*TRAF3*	0.119851979	0.001116036	0.048185758	1
T2DM	beta_cell	*PPARG*	-0.202164404	0.000664537	0.033473921	1
T2DM	beta_cell	*NOTCH2*	-0.057509769	2.65E-08	8.68E-06	0.000104114
T2DM	beta_cell	*MAN2C1*	-0.045870579	2.17E-05	0.003417891	0.085447265
T2DM	beta_cell	*NPC1*	-0.218059335	0.00015067	0.012869143	0.591980599
T2DM	beta_cell	*POGZ*	0.105791846	0.000192497	0.015126395	0.756319738
T2DM	beta_cell	*CNTN4*	-0.045495729	0.000852527	0.039406797	1
T2DM	beta_cell	*SEC16A*	0.102123649	0.000407032	0.023884252	1
T2DM	beta_cell	*HSD17B12*	-0.027255742	0.000190758	0.015126395	0.749488625
T2DM	beta_cell	*APIP*	-0.040607943	0.000556977	0.030393921	1
T2DM	beta_cell	*PTPN14*	-0.04531086	0.000383729	0.023884252	1
T2DM	beta_cell	*ACMSD*	0.722682214	0.000148217	0.012869143	0.582345149
T2DM	beta_cell	*JAZF1*	-0.206359772	2.11E-15	4.15E-12	8.29E-12

T2DM	beta_cell	*CLEC18A*	-0.205441803	2.16E-08	8.29E-06	8.47E-05
T2DM	beta_cell	*AP3S2*	0.429731809	5.26E-10	3.45E-07	2.07E-06
T2DM	beta_cell	*EXTL1*	-0.123099829	0.001111691	0.048185758	1
T2DM	beta_cell	*CILP2*	-0.071584956	7.40E-05	0.008311021	0.290885748
T2DM	beta_cell	*CASP7*	0.041769701	0.000135761	0.012122878	0.533406618
T2DM	beta_cell	*POLR2J3*	0.194452781	0.000584015	0.031008035	1
T2DM	beta_cell	*INO80E*	-0.085646542	7.65E-05	0.008347736	0.300518486
T2DM	beta_cell	*PHYHD1*	0.031071775	0.000704769	0.034185655	1
T2DM	beta_cell	*IRX3*	0.268091009	1.28E-24	5.05E-21	5.05E-21
T2DM	beta_cell	*IMP3*	0.290767858	1.70E-06	0.000334427	0.006688542
T2DM	beta_cell	*FARSA*	-0.132118327	0.000190447	0.015126395	0.74826477
T2DM	beta_cell	*HLA-DQB1*	-0.040459271	2.32E-08	8.29E-06	9.12E-05
T2DM	beta_cell	*UBA7*	-0.055557383	4.98E-06	0.000889012	0.019558267
T2DM	beta_cell	*ARL6IP4*	0.119681523	0.000268719	0.019551793	1
T2DM	beta_cell	*VMO1*	0.057591949	0.000303409	0.02128739	1
T2DM	beta_cell	*ZNF320*	0.024276237	0.000568042	0.030573089	1
T2DM	beta_cell	*CAMK1D*	0.372391357	2.08E-10	2.04E-07	8.15E-07
T2DM	beta_cell	*FAM43B*	0.277842445	0.000298788	0.02128739	1
T2DM	beta_cell	*ZBTB40*	0.200950607	0.000121167	0.011611322	0.47606421
T2DM	beta_cell	*NUTM2B*	-0.518862928	0.000601284	0.031084775	1
T2DM	beta_cell	*ZNF567*	0.189864267	0.000459743	0.025804744	1
T2DM	beta_cell	*HLA-DRB1*	-0.032165101	3.60E-10	2.83E-07	1.41E-06
T2DM	beta_cell	*HLA-DQA1*	-0.020614873	0.000362043	0.023420566	1
T2DM	beta_cell	*DDX42*	0.146854886	0.000407291	0.023884252	1
T2DM	beta_cell	*HLA-DRB5*	-0.022358449	3.25E-05	0.004565161	0.127824511
T2DM	beta_cell	*NOTCH4*	-0.054303094	1.66E-08	7.26E-06	6.54E-05
T2DM	beta_cell	*RNF5*	-0.052157336	8.12E-06	0.001329303	0.03190328
T2DM	beta_cell	*HCG27*	0.051717086	1.10E-06	0.000260467	0.004332086
T2DM	beta_cell	*TAS2R14*	0.042655791	0.000921526	0.041616947	1
T2DM	beta_cell	*DNLZ*	0.030037997	0.000133023	0.012122878	0.522647812
T2DM	beta_cell	*ATF6B*	0.056804703	2.91E-05	0.004236584	0.114387778
T2DM	beta_cell	*SLC12A8*	-0.307916471	1.31E-06	0.000285318	0.005135727
T2DM	beta_cell	*PPT2*	0.101443639	1.13E-06	0.000260467	0.004427935
T2DM	beta_cell	*HLA-DQB2*	0.09374773	7.75E-09	4.35E-06	3.04E-05
T2DM	beta_cell	*HLA-B*	0.043358	1.65E-06	0.000334427	0.006486625
T2DM	beta_cell	*HLA-DQA2*	0.059239238	2.29E-13	3.00E-10	8.99E-10
T2DM	beta_cell	*JMJD7*	0.108769846	2.44E-05	0.003683469	0.095770183
T2DM	beta_cell	*NEAT1*	0.181013908	0.000125359	0.011727034	0.492535408
T2DM	beta_cell	*BCKDHA*	0.184039446	0.000773418	0.036611569	1
T2DM	beta_cell	*TAS2R31*	0.939670117	0.000519751	0.028761995	1
T2DM	delta_cell	*RBM6*	-0.589289967	3.28E-07	0.00014299	0.001286913
T2DM	delta_cell	*CEP68*	0.612190801	6.47E-05	0.012098448	0.254067404
T2DM	delta_cell	*BAK1*	0.868954619	7.60E-05	0.013569574	0.298530623
T2DM	delta_cell	*TCF3*	-0.79741734	5.02E-05	0.010514442	0.197330373
T2DM	delta_cell	*MARK3*	-0.739955226	0.000378489	0.046735952	1
T2DM	delta_cell	*CWF19L1*	-0.360869172	0.000235152	0.032996855	0.923911944
T2DM	delta_cell	*HSD17B1*	-0.53563126	0.000144275	0.022674288	0.566857199
T2DM	delta_cell	*CNTNAP1*	0.455305661	0.00041015	0.04883275	1
T2DM	delta_cell	*MAU2*	-0.954947839	1.92E-05	0.005029363	0.075440439
T2DM	delta_cell	*NOTCH2*	-0.451759965	1.34E-07	6.58E-05	0.000526797
T2DM	delta_cell	*MAN2C1*	-0.410393508	6.23E-06	0.002038418	0.024461017
T2DM	delta_cell	*HSD17B12*	-0.243635288	6.44E-05	0.012098448	0.253191129
T2DM	delta_cell	*APIP*	-0.37375048	0.000133109	0.021791062	0.522985488
T2DM	delta_cell	*JAZF1*	-1.34058846	1.26E-13	2.48E-10	4.97E-10
T2DM	delta_cell	*CC2D1B*	-0.454690478	0.000266231	0.036069708	1
T2DM	delta_cell	*CLEC18A*	-1.351171726	1.68E-06	0.00059883	0.006587126
T2DM	delta_cell	*CILP2*	-0.576932895	0.000193255	0.029203819	0.759299297
T2DM	delta_cell	*INO80E*	-0.693548723	1.72E-05	0.004815893	0.067422498
T2DM	delta_cell	*IRX3*	1.436886734	2.43E-14	9.54E-11	9.54E-11
T2DM	delta_cell	*FARSA*	-0.986281883	5.08E-05	0.010514442	0.199774403
T2DM	delta_cell	*HLA-DQB1*	-0.369178488	4.22E-09	3.31E-06	1.66E-05
T2DM	delta_cell	*UBA7*	-0.462951059	1.42E-05	0.004278471	0.055620128
T2DM	delta_cell	*FAM43B*	2.353355859	0.000380644	0.046735952	1
T2DM	delta_cell	*HLA-DRB1*	-0.28851223	1.18E-10	1.16E-07	4.63E-07

T2DM	delta_cell	*HLA-DQA1*	-0.185795562	0.000228339	0.032996855	0.897143811
T2DM	delta_cell	*HLA-DRB5*	-0.181741181	4.63E-05	0.010514442	0.182102274
T2DM	delta_cell	*NOTCH4*	-0.461254576	2.12E-08	1.19E-05	8.33E-05
T2DM	delta_cell	*RNF5*	-0.47227765	6.86E-07	0.000269479	0.00269479
T2DM	delta_cell	*HCG27*	0.264525114	0.000118014	0.020159919	0.463678142
T2DM	delta_cell	*PPT2*	0.737696359	2.26E-05	0.005557908	0.088926522
T2DM	delta_cell	*HLA-DQB2*	0.774841324	8.73E-09	5.71E-06	3.43E-05
T2DM	delta_cell	*HLA-B*	0.22642686	0.000361743	0.046735952	1
T2DM	delta_cell	*HLA-DQA2*	0.466021723	1.55E-12	2.03E-09	6.09E-09
T2DM	duct_epithelial_cell	*RBM6*	-0.396360465	5.41E-06	0.001516945	0.021237225
T2DM	duct_epithelial_cell	*MLXIPL*	0.315263694	5.88E-05	0.009569986	0.230889356
T2DM	duct_epithelial_cell	*CEP68*	0.689445793	1.65E-07	8.11E-05	0.000648466
T2DM	duct_epithelial_cell	*BAK1*	0.648257522	8.31E-05	0.011666462	0.326660924
T2DM	duct_epithelial_cell	*TCF3*	-0.633451251	2.02E-05	0.004175602	0.079336445
T2DM	duct_epithelial_cell	*CWF19L1*	-0.289998526	0.000168284	0.020036034	0.661189115
T2DM	duct_epithelial_cell	*OIP5*	1.150965603	5.13E-05	0.009170606	0.201753337
T2DM	duct_epithelial_cell	*HSD17B1*	-0.402373928	0.000190602	0.021396452	0.748875821
T2DM	duct_epithelial_cell	*CNTNAP1*	0.379721894	2.40E-05	0.004716974	0.094339474
T2DM	duct_epithelial_cell	*CDK2AP1*	0.408049473	0.000324159	0.033516316	1
T2DM	duct_epithelial_cell	*MAU2*	-0.796360824	6.38E-05	0.009644654	0.250761002
T2DM	duct_epithelial_cell	*PPARG*	-1.228072302	0.00060309	0.049594302	1
T2DM	duct_epithelial_cell	*NOTCH2*	-0.30598468	1.89E-08	1.06E-05	7.42E-05
T2DM	duct_epithelial_cell	*MAN2C1*	-0.276497979	4.20E-05	0.007859605	0.165051704
T2DM	duct_epithelial_cell	*NPC1*	-1.006442432	0.000349693	0.034348625	1
T2DM	duct_epithelial_cell	*HSD17B12*	-0.155194742	0.000529534	0.047284978	1
T2DM	duct_epithelial_cell	*APIP*	-0.261115217	0.000381595	0.036567956	1
T2DM	duct_epithelial_cell	*PTPN14*	-0.294873672	7.10E-05	0.010332015	0.278964393
T2DM	duct_epithelial_cell	*JAZF1*	-0.859560004	3.27E-10	4.29E-07	1.29E-06
T2DM	duct_epithelial_cell	*CLEC18A*	-0.936561864	8.23E-06	0.002072492	0.032330103
T2DM	duct_epithelial_cell	*AP3S2*	0.942323587	0.000340759	0.034329243	1
T2DM	duct_epithelial_cell	*EXTL1*	-0.773307442	0.00023386	0.024833409	0.918836119
T2DM	duct_epithelial_cell	*CILP2*	-0.462547936	1.85E-05	0.004028615	0.072515074
T2DM	duct_epithelial_cell	*TMC8*	-0.325015952	0.000442683	0.041411944	1
T2DM	duct_epithelial_cell	*INO80E*	-0.474856647	0.000206493	0.022536401	0.811310454
T2DM	duct_epithelial_cell	*RGS19*	-0.494641433	0.000605886	0.049594302	1
T2DM	duct_epithelial_cell	*DSCAML1*	-0.580855511	0.000476747	0.043561353	1
T2DM	duct_epithelial_cell	*FARSA*	-0.83738955	5.42E-05	0.009250605	0.212763919
T2DM	duct_epithelial_cell	*HLA-DQB1*	-0.266646872	5.07E-09	3.32E-06	1.99E-05
T2DM	duct_epithelial_cell	*UBA7*	-0.333804121	2.03E-06	0.000663067	0.007956801
T2DM	duct_epithelial_cell	*ZNF320*	0.154363537	0.000567373	0.049537962	1
T2DM	duct_epithelial_cell	*CAMK1D*	1.588267309	6.85E-07	0.000299006	0.002691052
T2DM	duct_epithelial_cell	*FAM43B*	1.716695352	9.65E-05	0.013078654	0.379280955
T2DM	duct_epithelial_cell	*HLA-DRB1*	-0.220196697	2.20E-11	4.33E-08	8.66E-08
T2DM	duct_epithelial_cell	*HLA-DQA1*	-0.137572365	0.000189892	0.021396452	0.746086703
T2DM	duct_epithelial_cell	*HLA-DRB5*	-0.143260641	8.44E-06	0.002072492	0.033159867
T2DM	duct_epithelial_cell	*NOTCH4*	-0.3483444	4.68E-09	3.32E-06	1.84E-05
T2DM	duct_epithelial_cell	*RNF5*	-0.329101887	2.63E-06	0.000796259	0.010351371
T2DM	duct_epithelial_cell	*HCG27*	0.255348226	6.09E-05	0.009569986	0.239249657
T2DM	duct_epithelial_cell	*TAS2R14*	0.278498928	0.000583378	0.049594302	1
T2DM	duct_epithelial_cell	*DNLZ*	0.1880497	0.000161511	0.01983057	0.634578248
T2DM	duct_epithelial_cell	*ATF6B*	0.329209106	0.000104229	0.013650573	0.4095172
T2DM	duct_epithelial_cell	*SLC12A8*	-1.512075929	1.26E-06	0.00049485	0.0049485
T2DM	duct_epithelial_cell	*PPT2*	0.623624387	1.47E-06	0.000524197	0.005766172
T2DM	duct_epithelial_cell	*HLA-DQB2*	0.605560482	3.37E-09	3.31E-06	1.32E-05
T2DM	duct_epithelial_cell	*HLA-B*	0.23817545	1.13E-05	0.002606935	0.044317892
T2DM	duct_epithelial_cell	*HLA-DQA2*	0.389402711	5.20E-14	2.04E-10	2.04E-10
T2DM	duct_epithelial_cell	*JMJD7*	0.621408661	0.000121573	0.015408452	0.477662013
T2DM	endothelial_cell	*RBM6*	-0.678296428	1.79E-08	1.41E-05	7.05E-05
T2DM	endothelial_cell	*ZNF195*	-0.763597996	0.001207648	0.040554277	1
T2DM	endothelial_cell	*GGNBP2*	-0.820058565	0.00045448	0.027055351	1
T2DM	endothelial_cell	*BAIAP3*	-0.495360063	0.000427841	0.026682363	1
T2DM	endothelial_cell	*GIPR*	-0.569743244	0.000126884	0.012085623	0.4985256
T2DM	endothelial_cell	*CEP68*	0.844275635	2.00E-06	0.000561312	0.007858364
T2DM	endothelial_cell	*LTF*	-0.874550696	0.000445329	0.026918395	1

T2DM	endothelial_cell	*SH2D2A*	-1.013734713	0.000444149	0.026918395	1
T2DM	endothelial_cell	*BAK1*	1.041957899	9.60E-06	0.001886494	0.037729887
T2DM	endothelial_cell	*ARID4A*	-0.838090342	0.00179515	0.04551528	1
T2DM	endothelial_cell	*GOPC*	-1.136448764	0.001481766	0.043247251	1
T2DM	endothelial_cell	*HEBP2*	-0.563922837	0.001965166	0.047080096	1
T2DM	endothelial_cell	*USP36*	-1.247026171	0.001031844	0.037888925	1
T2DM	endothelial_cell	*ITIH4*	-1.001288235	1.85E-05	0.003155572	0.072578147
T2DM	endothelial_cell	*HAGH*	-0.789619084	0.00030358	0.021299366	1
T2DM	endothelial_cell	*SEC61A2*	-0.83795835	0.00144949	0.043247251	1
T2DM	endothelial_cell	*ST6GAL1*	0.412951834	0.00049378	0.027313218	1
T2DM	endothelial_cell	*MARK3*	-1.567766718	2.85E-08	1.80E-05	0.00011184
T2DM	endothelial_cell	*XAB2*	-1.393587676	0.001114765	0.038551247	1
T2DM	endothelial_cell	*FDFT1*	-0.556197863	0.001486358	0.043247251	1
T2DM	endothelial_cell	*POMGNT1*	-0.948738885	0.000580994	0.028754401	1
T2DM	endothelial_cell	*KLHL42*	-0.490032923	0.001706068	0.045291503	1
T2DM	endothelial_cell	*SUCO*	-0.585306732	0.001309611	0.042953994	1
T2DM	endothelial_cell	*CWF19L1*	-0.443023717	2.52E-05	0.003937403	0.098937669
T2DM	endothelial_cell	*IKZF5*	-1.146130644	0.00083447	0.034123028	1
T2DM	endothelial_cell	*AP1B1*	-1.311736521	1.58E-05	0.002928447	0.06211393
T2DM	endothelial_cell	*RBFOX2*	-0.981455883	0.001405291	0.043242585	1
T2DM	endothelial_cell	*SYNGR1*	-0.465335764	0.001733041	0.045394121	1
T2DM	endothelial_cell	*POLR3H*	-1.315319212	0.001052149	0.038019184	1
T2DM	endothelial_cell	*RPAP1*	-2.0653448	0.000713829	0.032580379	1
T2DM	endothelial_cell	*LEPROTL1*	-1.034850306	0.002027102	0.04797882	1
T2DM	endothelial_cell	*TMEM248*	-0.713037551	0.00196345	0.047080096	1
T2DM	endothelial_cell	*SLC1A1*	0.441373635	0.001857496	0.046190526	1
T2DM	endothelial_cell	*SMARCD2*	-1.139007104	0.001645038	0.044731778	1
T2DM	endothelial_cell	*HSD17B1*	-0.575276589	2.61E-05	0.003937403	0.102372485
T2DM	endothelial_cell	*CNTNAP1*	0.492789211	0.000141202	0.012112199	0.554782247
T2DM	endothelial_cell	*SRSF9*	-0.715631559	0.001311906	0.042953994	1
T2DM	endothelial_cell	*PEX7*	-1.03462421	0.0020112	0.047890929	1
T2DM	endothelial_cell	*NNT*	-0.701015243	1.64E-05	0.002928447	0.064425842
T2DM	endothelial_cell	*C3orf14*	-0.352109898	0.001812273	0.04551528	1
T2DM	endothelial_cell	*NEK11*	-0.52615063	0.001491663	0.043247251	1
T2DM	endothelial_cell	*CHMP3*	-0.677514135	0.000269511	0.020363641	1
T2DM	endothelial_cell	*ANGPTL1*	-2.421681898	6.88E-05	0.00900829	0.270248704
T2DM	endothelial_cell	*CCDC181*	-0.593307843	0.001363895	0.043242585	1
T2DM	endothelial_cell	*DNAH7*	-0.574681073	5.27E-06	0.001380539	0.02070808
T2DM	endothelial_cell	*SLC10A7*	-0.670810343	0.001137425	0.038860382	1
T2DM	endothelial_cell	*DNAJC15*	-0.835666815	0.000844873	0.034123028	1
T2DM	endothelial_cell	*FAM53C*	-0.913479004	0.000864049	0.034123028	1
T2DM	endothelial_cell	*POLK*	-0.874059588	0.000425333	0.026682363	1
T2DM	endothelial_cell	*PEPD*	-1.177789104	0.000239374	0.018809989	0.940499461
T2DM	endothelial_cell	*PRMT1*	-0.842250404	0.000133896	0.012085623	0.526076359
T2DM	endothelial_cell	*EIF2AK4*	-1.019689646	0.000141807	0.012112199	0.557161166
T2DM	endothelial_cell	*MAU2*	-1.360412005	2.58E-07	0.000126905	0.001015237
T2DM	endothelial_cell	*CDC16*	-1.030555668	0.000549184	0.028754401	1
T2DM	endothelial_cell	*GTPBP3*	-0.459666922	0.001354569	0.043242585	1
T2DM	endothelial_cell	*UCK1*	-1.212178165	0.001749248	0.045515201	1
T2DM	endothelial_cell	*BHMT2*	-0.631466388	0.000625418	0.030336639	1
T2DM	endothelial_cell	*HSD17B4*	-1.070193902	0.001650829	0.044731778	1
T2DM	endothelial_cell	*THUMPD3*	-0.608367596	0.001818758	0.04551528	1
T2DM	endothelial_cell	*NOTCH2*	-0.52519944	2.19E-09	2.15E-06	8.59E-06
T2DM	endothelial_cell	*LRP4*	0.376565228	0.000868491	0.034123028	1
T2DM	endothelial_cell	*GCC2*	-0.665937393	0.001107964	0.038551247	1
T2DM	endothelial_cell	*FXYD2*	-0.378299304	0.000328678	0.022655706	1
T2DM	endothelial_cell	*TUBGCP4*	-0.97356872	0.000578812	0.028754401	1
T2DM	endothelial_cell	*MYPN*	1.699969761	0.00138856	0.043242585	1
T2DM	endothelial_cell	*G3BP2*	-0.794657155	0.001019926	0.037804605	1
T2DM	endothelial_cell	*USO1*	-0.65251791	0.001589967	0.044731778	1
T2DM	endothelial_cell	*CERS5*	-0.579273434	0.001424752	0.043242585	1
T2DM	endothelial_cell	*MAN2C1*	-0.407617552	9.26E-06	0.001886494	0.036372822
T2DM	endothelial_cell	*OSGIN1*	-0.885890765	0.001677449	0.045141762	1
T2DM	endothelial_cell	*VPS53*	-0.591885792	0.000507474	0.027313218	1

T2DM	endothelial_cell	*NPC1*	-1.645400178	8.16E-06	0.001886494	0.032043317
T2DM	endothelial_cell	*ZNF593*	-0.615236204	0.001639707	0.044731778	1
T2DM	endothelial_cell	*NME7*	-0.562275133	0.00058548	0.028754401	1
T2DM	endothelial_cell	*CIAO1*	-0.978169863	0.000758724	0.032882406	1
T2DM	endothelial_cell	*ZNF514*	-0.592843332	0.00029552	0.021299366	1
T2DM	endothelial_cell	*CNTN4*	-0.444949704	3.69E-05	0.005171633	0.144805734
T2DM	endothelial_cell	*TBC1D7*	-1.928773453	0.000250179	0.019273585	0.982952844
T2DM	endothelial_cell	*RAB19*	-1.783145754	0.000930445	0.036113394	1
T2DM	endothelial_cell	*ADHFE1*	-0.698742184	0.00101686	0.037804605	1
T2DM	endothelial_cell	*LRP12*	-1.497101513	0.000734209	0.032780767	1
T2DM	endothelial_cell	*HSD17B12*	-0.266595156	8.69E-06	0.001886494	0.034149658
T2DM	endothelial_cell	*APIP*	-0.380689437	0.000133459	0.012085623	0.524359446
T2DM	endothelial_cell	*DOC2A*	-0.549272337	0.00181148	0.04551528	1
T2DM	endothelial_cell	*DLG5*	-0.769613808	0.001885967	0.046603561	1
T2DM	endothelial_cell	*ARL14EP*	-1.143387574	0.001370842	0.043242585	1
T2DM	endothelial_cell	*POC5*	-0.309748087	0.000167218	0.013687476	0.656998842
T2DM	endothelial_cell	*JAZF1*	-1.047186529	6.21E-13	8.14E-10	2.44E-09
T2DM	endothelial_cell	*NUS1*	-1.282717263	0.000133222	0.012085623	0.523428286
T2DM	endothelial_cell	*CC2D1B*	-0.494962221	0.000146886	0.012279069	0.57711626
T2DM	endothelial_cell	*DHRS4*	-1.396708293	0.000577566	0.028754401	1
T2DM	endothelial_cell	*EXTL1*	-1.183738343	0.000209047	0.016762191	0.82134734
T2DM	endothelial_cell	*FAIM*	-1.001047891	0.001629338	0.044731778	1
T2DM	endothelial_cell	*CILP2*	-0.654900656	2.42E-05	0.003937403	0.095111087
T2DM	endothelial_cell	*PDXK*	-0.568388564	0.000756027	0.032882406	1
T2DM	endothelial_cell	*PAQR6*	-0.781780418	0.000288242	0.021299366	1
T2DM	endothelial_cell	*ZNF75A*	-1.297106184	0.000767148	0.032882406	1
T2DM	endothelial_cell	*DCAF16*	-1.047799596	0.000367648	0.0244048	1
T2DM	endothelial_cell	*ICA1L*	-0.389793875	8.48E-05	0.010586276	0.333187321
T2DM	endothelial_cell	*LMBRD2*	-0.668983455	0.001694287	0.045284719	1
T2DM	endothelial_cell	*BRI3*	-0.67177911	0.001118565	0.038551247	1
T2DM	endothelial_cell	*INPPL1*	-1.234384321	0.000705008	0.032580379	1
T2DM	endothelial_cell	*VDAC2*	-0.673231507	0.000853178	0.034123028	1
T2DM	endothelial_cell	*CASP7*	0.281091409	0.000107316	0.012085623	0.4216434
T2DM	endothelial_cell	*CEP57*	-0.923005722	0.000111024	0.012085623	0.436213554
T2DM	endothelial_cell	*CKB*	-0.396060598	8.62E-05	0.010586276	0.338760836
T2DM	endothelial_cell	*SGPL1*	-0.70122196	0.00010966	0.012085623	0.430852905
T2DM	endothelial_cell	*PCBD1*	-0.434525043	0.000505719	0.027313218	1
T2DM	endothelial_cell	*POP5*	-0.956966561	0.000658013	0.031528461	1
T2DM	endothelial_cell	*ACAA2*	-0.634950482	0.0008245	0.034123028	1
T2DM	endothelial_cell	*TP53I13*	-0.445200113	0.000847139	0.034123028	1
T2DM	endothelial_cell	*TRANK1*	-1.025714141	0.000769962	0.032882406	1
T2DM	endothelial_cell	*SAC3D1*	-1.714360834	0.00129798	0.042953994	1
T2DM	endothelial_cell	*KIAA1586*	-0.730159087	0.000685035	0.032066829	1
T2DM	endothelial_cell	*NDUFS5*	-0.576798043	0.001199363	0.040554277	1
T2DM	endothelial_cell	*DNAJC21*	-1.094528042	0.00161708	0.044731778	1
T2DM	endothelial_cell	*PPIP5K1*	-0.725439668	0.001794981	0.04551528	1
T2DM	endothelial_cell	*TMEM129*	-0.454649596	0.001950753	0.047080096	1
T2DM	endothelial_cell	*INO80E*	-0.825529505	1.24E-06	0.000444446	0.004888908
T2DM	endothelial_cell	*NIPA1*	-1.101228315	0.000860929	0.034123028	1
T2DM	endothelial_cell	*ZNF440*	-1.414143834	0.000937533	0.036113394	1
T2DM	endothelial_cell	*CLSTN1*	-0.608395187	0.001484903	0.043247251	1
T2DM	endothelial_cell	*CTSW*	-0.231812544	0.00150295	0.043247251	1
T2DM	endothelial_cell	*LCLAT1*	-0.709260704	0.001507985	0.043247251	1
T2DM	endothelial_cell	*YIF1A*	-0.494986967	0.000122243	0.012085623	0.480294439
T2DM	endothelial_cell	*CCDC57*	-0.400671791	0.001073163	0.038019184	1
T2DM	endothelial_cell	*COX8A*	-0.451589369	0.001604588	0.044731778	1
T2DM	endothelial_cell	*DSCAML1*	-0.471742581	0.000503772	0.027313218	1
T2DM	endothelial_cell	*ARIH2*	-0.576977038	0.00192149	0.047080096	1
T2DM	endothelial_cell	*IRX3*	1.426540934	1.07E-13	4.20E-10	4.20E-10
T2DM	endothelial_cell	*TRIM73*	-0.720049302	0.001002393	0.037804605	1
T2DM	endothelial_cell	*FARSA*	-1.200373149	1.62E-06	0.000488429	0.006349583
T2DM	endothelial_cell	*HLA-DQB1*	-0.303729551	8.44E-07	0.000368647	0.003317825
T2DM	endothelial_cell	*D2HGDH*	-1.104221046	0.000466473	0.027313218	1
T2DM	endothelial_cell	*VMO1*	0.502322966	9.69E-05	0.011541365	0.380865054

T2DM	endothelial_cell	*ZNF320*	0.206563464	0.000685572	0.032066829	1
T2DM	endothelial_cell	*FAM43B*	2.309801041	0.000496952	0.027313218	1
T2DM	endothelial_cell	*TRMT12*	-1.503835653	0.001019519	0.037804605	1
T2DM	endothelial_cell	*MACC1*	-0.462137717	0.001381592	0.043242585	1
T2DM	endothelial_cell	*PGP*	-0.835468015	0.000299756	0.021299366	1
T2DM	endothelial_cell	*BRWD1*	-0.828077635	0.00211222	0.049694078	1
T2DM	endothelial_cell	*DMWD*	-0.807232046	0.000502215	0.027313218	1
T2DM	endothelial_cell	*NUTM2B*	-4.122132213	3.04E-05	0.004423456	0.1194333
T2DM	endothelial_cell	*ASAH2*	-0.651951848	0.000558203	0.028754401	1
T2DM	endothelial_cell	*HLA-DRB1*	-0.21373167	9.85E-07	0.000386982	0.003869822
T2DM	endothelial_cell	*XPNPEP3*	-0.789762315	0.00143078	0.043242585	1
T2DM	endothelial_cell	*SUPT3H*	-1.577804661	0.001074098	0.038019184	1
T2DM	endothelial_cell	*XRCC6*	-0.843828692	4.65E-05	0.006300362	0.182710508
T2DM	endothelial_cell	*HLA-DQA1*	-0.164031853	0.001336483	0.043242585	1
T2DM	endothelial_cell	*PIWIL2*	-0.483050537	0.00053352	0.028327055	1
T2DM	endothelial_cell	*KIAA0753*	-0.372273262	0.001546607	0.044033456	1
T2DM	endothelial_cell	*RNF5*	-0.475844257	1.36E-06	0.000446462	0.005357548
T2DM	endothelial_cell	*HMGN1*	-0.655627662	0.001790445	0.04551528	1
T2DM	endothelial_cell	*HCG27*	0.286918296	0.000721429	0.032580379	1
T2DM	endothelial_cell	*DNLZ*	0.257567697	0.000135344	0.012085623	0.531767416
T2DM	endothelial_cell	*ATF6B*	0.408338871	0.000372687	0.0244048	1
T2DM	endothelial_cell	*IFRD2*	-1.164047888	0.001422404	0.043242585	1
T2DM	endothelial_cell	*SLC12A8*	-0.606147151	0.000370283	0.0244048	1
T2DM	endothelial_cell	*PPT2*	0.333804544	0.000124781	0.012085623	0.49026372
T2DM	endothelial_cell	*HLA-DQB2*	0.780829438	3.21E-08	1.80E-05	0.000125992
T2DM	endothelial_cell	*HLA-B*	0.299083867	9.26E-06	0.001886494	0.036396578
T2DM	endothelial_cell	*B3GALT4*	-0.372696383	0.000421652	0.026682363	1
T2DM	endothelial_cell	*HLA-DQA2*	0.515409689	4.72E-13	8.14E-10	1.86E-09
T2DM	endothelial_cell	*JMJD7*	0.840481171	0.000127324	0.012085623	0.50025792
T2DM	endothelial_cell	*LINC01091*	-0.528013709	0.001949944	0.047080096	1
T2DM	endothelial_cell	*TAS2R20*	0.953515631	0.001729901	0.045394121	1
T2DM	endothelial_cell	*ZNF432*	-1.2127724	0.001058698	0.038019184	1
T2DM	epsilon_cell	*IRX3*	14.2725765	1.07E-13	4.21E-10	4.21E-10
T2DM	epsilon_cell	*UBA7*	-5.898332582	9.01E-06	0.007080607	0.035403033
T2DM	epsilon_cell	*HLA-DRB1*	-2.484785461	3.92E-07	0.000513569	0.001540708
T2DM	epsilon_cell	*HLA-DQB2*	7.369618614	2.88E-06	0.002827766	0.011311064
T2DM	epsilon_cell	*HLA-DQA2*	6.105339943	4.16E-13	8.18E-10	1.64E-09
T2DM	leukocyte	*GCLC*	-1.428864345	0.000663897	0.004990048	1
T2DM	leukocyte	*NFYA*	-0.964070269	0.004191306	0.007244893	1
T2DM	leukocyte	*STPG1*	-0.863510803	0.002186771	0.005356498	1
T2DM	leukocyte	*LASP1*	-0.899031832	0.001194904	0.004990048	1
T2DM	leukocyte	*SNX11*	-0.757163971	0.016818342	0.022627392	1
T2DM	leukocyte	*TMEM176A*	-0.577384489	0.007669579	0.011409987	1
T2DM	leukocyte	*M6PR*	-2.027633553	0.000544902	0.004990048	1
T2DM	leukocyte	*DBNDD1*	-0.635397361	0.003914914	0.007085756	1
T2DM	leukocyte	*CASP10*	-2.642188976	0.000485422	0.004990048	1
T2DM	leukocyte	*NDUFAF7*	-2.066377509	0.000603261	0.004990048	1
T2DM	leukocyte	*POLDIP2*	-0.690227958	0.004141384	0.007238288	1
T2DM	leukocyte	*PLXND1*	-1.974513092	0.001070398	0.004990048	1
T2DM	leukocyte	*KDM1A*	-0.906675734	0.002541208	0.00567203	1
T2DM	leukocyte	*RBM6*	-1.735769141	4.18E-08	3.28E-05	0.000164088
T2DM	leukocyte	*RECQL*	-0.963409157	0.001364834	0.004990048	1
T2DM	leukocyte	*SLC25A13*	-0.907358777	0.002124278	0.005299229	1
T2DM	leukocyte	*MEOX1*	1.986469378	0.01485814	0.020234881	1
T2DM	leukocyte	*PON1*	-0.900627702	0.010006588	0.014296686	1
T2DM	leukocyte	*KMT2E*	-1.002928621	0.000885577	0.004990048	1
T2DM	leukocyte	*IBTK*	-0.981412908	0.000894939	0.004990048	1
T2DM	leukocyte	*ZNF195*	-1.599118618	0.000175909	0.004990048	0.691147926
T2DM	leukocyte	*GGNBP2*	-1.119045011	0.000280361	0.004990048	1
T2DM	leukocyte	*TMEM98*	-0.57278085	0.013160217	0.018219343	1
T2DM	leukocyte	*SYNRG*	-1.403074411	0.000787915	0.004990048	1
T2DM	leukocyte	*SPATA20*	-0.897547164	0.004925232	0.007976602	1
T2DM	leukocyte	*FARP2*	-2.937105307	0.000447871	0.004990048	1
T2DM	leukocyte	*USH1C*	-1.028856737	0.000513416	0.004990048	1

T2DM	leukocyte	*VPS41*	-1.291093741	0.000952718	0.004990048	1
T2DM	leukocyte	*ELAC2*	-0.976412912	0.002150484	0.005327398	1
T2DM	leukocyte	*CCDC124*	-0.827813723	0.001395912	0.004990048	1
T2DM	leukocyte	*NOS2*	-13.7593998	0.004358629	0.007368783	1
T2DM	leukocyte	*CD79B*	-5.294546636	0.000485669	0.004990048	1
T2DM	leukocyte	*ST7L*	-1.517689988	0.000488531	0.004990048	1
T2DM	leukocyte	*BAIAP3*	-1.033623076	0.000284037	0.004990048	1
T2DM	leukocyte	*CYB561*	-0.842283897	0.001338136	0.004990048	1
T2DM	leukocyte	*MGST1*	-0.581520965	0.005618136	0.008825931	1
T2DM	leukocyte	*ST3GAL1*	-2.027859106	0.000986689	0.004990048	1
T2DM	leukocyte	*IL32*	-0.392222555	0.004167721	0.007242343	1
T2DM	leukocyte	*MED24*	-1.264584471	0.001327147	0.004990048	1
T2DM	leukocyte	*RPS20*	-0.65462262	0.000870422	0.004990048	1
T2DM	leukocyte	*REV3L*	-0.794515164	0.001565541	0.005009694	1
T2DM	leukocyte	*ETV7*	-1.7246826	0.001537539	0.005006447	1
T2DM	leukocyte	*NCAPD2*	-1.436717188	0.006211483	0.009563054	1
T2DM	leukocyte	*GIPR*	-0.83944537	0.000584675	0.004990048	1
T2DM	leukocyte	*FUZ*	-1.348331585	0.002508792	0.005648735	1
T2DM	leukocyte	*LRRC23*	-0.630652651	0.004557734	0.007575016	1
T2DM	leukocyte	*TSPAN9*	-0.725310539	0.004189302	0.007244617	1
T2DM	leukocyte	*BTBD7*	-0.981798428	0.001946762	0.005192687	1
T2DM	leukocyte	*APBA3*	-1.696335977	0.002030112	0.005251028	1
T2DM	leukocyte	*TTC19*	-0.720075661	0.002680552	0.005854302	1
T2DM	leukocyte	*RABGAP1*	-0.866435341	0.00276533	0.005956822	1
T2DM	leukocyte	*QPCTL*	-3.575408647	0.000632971	0.004990048	1
T2DM	leukocyte	*PPP5C*	-0.886272926	0.002090959	0.005283012	1
T2DM	leukocyte	*ERCC1*	-1.180881082	0.000569107	0.004990048	1
T2DM	leukocyte	*CD22*	0.390616145	0.017115067	0.022950546	1
T2DM	leukocyte	*LTF*	-2.888581529	0.000147724	0.004990048	0.580406627
T2DM	leukocyte	*EXTL3*	-0.774945939	0.003143764	0.006311945	1
T2DM	leukocyte	*CALCOCO1*	-2.811432088	0.002609605	0.005766669	1
T2DM	leukocyte	*UBR7*	-0.800612329	0.008299371	0.012180885	1
T2DM	leukocyte	*MAN2B2*	-1.341079903	0.001190253	0.004990048	1
T2DM	leukocyte	*ANGEL1*	-0.906429592	0.026367296	0.033744986	1
T2DM	leukocyte	*DDX11*	-0.908110519	0.004304863	0.007328649	1
T2DM	leukocyte	*GPRC5A*	-0.897405994	0.017362675	0.023234996	1
T2DM	leukocyte	*UFL1*	-0.91299115	0.00108512	0.004990048	1
T2DM	leukocyte	*POLA2*	-1.631148846	0.001556294	0.005009694	1
T2DM	leukocyte	*ZC3H3*	-0.654098938	0.021345628	0.027937033	1
T2DM	leukocyte	*MDH1*	-0.922675571	0.000928197	0.004990048	1
T2DM	leukocyte	*MTMR11*	-3.277860881	0.001849217	0.005146173	1
T2DM	leukocyte	*DPEP1*	-0.50049721	0.005080944	0.008170331	1
T2DM	leukocyte	*NUDCD3*	-0.79984365	0.002138543	0.005317933	1
T2DM	leukocyte	*TTC27*	-1.933217691	0.002321902	0.005502263	1
T2DM	leukocyte	*SNAI2*	-2.060835242	0.010173454	0.014519251	1
T2DM	leukocyte	*PLEKHB1*	-0.608203496	0.040160452	0.049776157	1
T2DM	leukocyte	*NLRP2*	-1.629578988	0.007471603	0.011166196	1
T2DM	leukocyte	*ZDHHC6*	-1.177976221	0.001205175	0.004990048	1
T2DM	leukocyte	*RNH1*	-0.906211326	0.000911619	0.004990048	1
T2DM	leukocyte	*NDUFS1*	-0.815597943	0.001539514	0.005006447	1
T2DM	leukocyte	*TYMP*	0.325878486	0.038967225	0.048496113	1
T2DM	leukocyte	*CD44*	-0.477497987	0.001998713	0.005227473	1
T2DM	leukocyte	*KCNG1*	-3.024046638	0.0039469	0.007116737	1
T2DM	leukocyte	*AGPAT4*	-2.428707703	0.017389282	0.023249955	1
T2DM	leukocyte	*SH2D2A*	-5.830663208	4.83E-05	0.004515102	0.189634264
T2DM	leukocyte	*VEZT*	-0.914562293	0.002220076	0.005407737	1
T2DM	leukocyte	*RABEP1*	-1.529581678	0.001659187	0.005041098	1
T2DM	leukocyte	*ARID4A*	-1.342002685	0.000414299	0.004990048	1
T2DM	leukocyte	*PNPLA6*	-1.110893246	0.002084413	0.005283012	1
T2DM	leukocyte	*ABCF2*	-1.258873035	0.00146754	0.004990048	1
T2DM	leukocyte	*UBA6*	-1.48229157	0.000809698	0.004990048	1
T2DM	leukocyte	*APBA2*	-2.201225413	0.004157835	0.007242343	1
T2DM	leukocyte	*ASTE1*	-0.908913894	0.018358311	0.024409047	1
T2DM	leukocyte	*RNF19A*	-0.811799058	0.001044556	0.004990048	1

T2DM	leukocyte	*SH3YL1*	-0.52717157	0.006287747	0.009657763	1
T2DM	leukocyte	*FAM136A*	-0.872402786	0.001599255	0.005024491	1
T2DM	leukocyte	*DAPK2*	-0.916625957	0.002687299	0.00586252	1
T2DM	leukocyte	*TIMP2*	-1.979172603	0.000163873	0.004990048	0.643858015
T2DM	leukocyte	*MYOM2*	0.842142322	0.036301617	0.045437736	1
T2DM	leukocyte	*RPL26L1*	-0.822486111	0.001334675	0.004990048	1
T2DM	leukocyte	*NSUN2*	-1.405192125	0.000505035	0.004990048	1
T2DM	leukocyte	*MRI1*	-1.023675552	0.002833601	0.006014705	1
T2DM	leukocyte	*AGA*	-1.097216294	0.000790787	0.004990048	1
T2DM	leukocyte	*BOD1L1*	-0.768082995	0.003481899	0.006621675	1
T2DM	leukocyte	*DNAH5*	-1.381050145	0.001411192	0.004990048	1
T2DM	leukocyte	*PNKP*	-1.609491749	0.009453093	0.013599854	1
T2DM	leukocyte	*PHLPP2*	-0.786186158	0.003047568	0.006220403	1
T2DM	leukocyte	*CTNS*	-1.045711424	0.004314018	0.007328649	1
T2DM	leukocyte	*INPP4A*	-0.793301756	0.004039779	0.007166394	1
T2DM	leukocyte	*PSMA4*	-0.855107094	0.001096181	0.004990048	1
T2DM	leukocyte	*LSG1*	-0.591103462	0.009419611	0.013556649	1
T2DM	leukocyte	*SPATA7*	-1.296864011	0.000763211	0.004990048	1
T2DM	leukocyte	*TRIT1*	-1.939378471	0.002099339	0.005283012	1
T2DM	leukocyte	*CNTLN*	-1.286391758	0.00150012	0.004993935	1
T2DM	leukocyte	*PREX2*	-1.461421727	0.009525598	0.013691804	1
T2DM	leukocyte	*WDR37*	-1.347846799	0.000771024	0.004990048	1
T2DM	leukocyte	*YTHDC2*	-1.486186564	0.000595861	0.004990048	1
T2DM	leukocyte	*DTNBP1*	-1.015539499	0.001683376	0.005060368	1
T2DM	leukocyte	*GOPC*	-1.071883033	0.000950009	0.004990048	1
T2DM	leukocyte	*ZNF800*	-1.053739543	0.000988956	0.004990048	1
T2DM	leukocyte	*MRPS10*	-1.308011858	0.000319688	0.004990048	1
T2DM	leukocyte	*COL9A2*	-0.755239211	0.015078656	0.02047841	1
T2DM	leukocyte	*ERCC8*	-2.591480017	0.014231571	0.019503259	1
T2DM	leukocyte	*H6PD*	-2.574485568	0.000314915	0.004990048	1
T2DM	leukocyte	*VAMP3*	-1.054202498	0.001402173	0.004990048	1
T2DM	leukocyte	*RCN1*	-0.703674016	0.002380949	0.005560194	1
T2DM	leukocyte	*RFC2*	-0.937235402	0.003581496	0.006727272	1
T2DM	leukocyte	*JKAMP*	-0.827307045	0.002105315	0.005283012	1
T2DM	leukocyte	*PTGER3*	-0.788448184	0.003699307	0.006868344	1
T2DM	leukocyte	*BCAR1*	-0.688844764	0.003008944	0.006176667	1
T2DM	leukocyte	*THOC3*	-0.67236824	0.003838189	0.007020598	1
T2DM	leukocyte	*HEBP2*	-0.867835986	0.000588059	0.004990048	1
T2DM	leukocyte	*RRP12*	-0.603402461	0.003889026	0.00705773	1
T2DM	leukocyte	*FNIP2*	-2.07409232	0.000726881	0.004990048	1
T2DM	leukocyte	*MRTO4*	-1.042568387	0.001824929	0.00513989	1
T2DM	leukocyte	*MCF2L2*	-1.45995675	0.001649119	0.005035955	1
T2DM	leukocyte	*LAMA3*	-1.37221642	0.003233692	0.006381841	1
T2DM	leukocyte	*ANAPC4*	-3.101703136	0.014539148	0.01985302	1
T2DM	leukocyte	*LY75*	-3.824525302	0.000425533	0.004990048	1
T2DM	leukocyte	*ARID4B*	-0.721477862	0.003573448	0.006719453	1
T2DM	leukocyte	*SDCCAG8*	-0.859408714	0.002261036	0.005456717	1
T2DM	leukocyte	*KIF1B*	-0.879113167	0.001254139	0.004990048	1
T2DM	leukocyte	*TBC1D22A*	-0.915593036	0.001979341	0.005222499	1
T2DM	leukocyte	*SYNE2*	-0.844320674	0.001627093	0.005035955	1
T2DM	leukocyte	*ATP9A*	-0.955157451	0.001160088	0.004990048	1
T2DM	leukocyte	*CHRDL2*	-3.006135237	0.004698026	0.007719684	1
T2DM	leukocyte	*GALC*	-1.250445961	0.001963946	0.005212073	1
T2DM	leukocyte	*CYFIP2*	-0.722231983	0.002268219	0.005457772	1
T2DM	leukocyte	*USP36*	-1.507700651	0.000361411	0.004990048	1
T2DM	leukocyte	*MCOLN3*	-0.8098272	0.003450744	0.006578347	1
T2DM	leukocyte	*ITIH4*	-2.659462704	6.12E-06	0.001302453	0.024052853
T2DM	leukocyte	*PCGF2*	-0.849195549	0.002426379	0.005602682	1
T2DM	leukocyte	*GDI2*	-0.923378838	0.001497309	0.004993935	1
T2DM	leukocyte	*ATP11B*	-1.592267959	0.000571046	0.004990048	1
T2DM	leukocyte	*LAMC2*	-0.563657716	0.007208645	0.01083503	1
T2DM	leukocyte	*RASGRF1*	-0.61529224	0.005610721	0.008817809	1
T2DM	leukocyte	*CROCC*	-1.305218734	0.003341635	0.006470236	1
T2DM	leukocyte	*FLYWCH1*	-0.671761805	0.001465354	0.004990048	1

T2DM	leukocyte	*OGFR*	-1.94469777	0.012489835	0.017401617	1
T2DM	leukocyte	*GNA15*	1.170007623	0.023310489	0.030176906	1
T2DM	leukocyte	*RIMBP2*	-0.682471857	0.004147027	0.007238372	1
T2DM	leukocyte	*MPC1*	-0.842231849	0.001221599	0.004990048	1
T2DM	leukocyte	*BCAT1*	0.261437359	0.03762152	0.046955195	1
T2DM	leukocyte	*MRPS35*	-0.921280107	0.0018778	0.005146173	1
T2DM	leukocyte	*ZNF112*	-1.058948305	0.008032106	0.011844895	1
T2DM	leukocyte	*EIF4B*	-0.838832289	0.001063673	0.004990048	1
T2DM	leukocyte	*SLC6A16*	-2.294580416	0.003235589	0.006381841	1
T2DM	leukocyte	*SPHK2*	-0.947441266	0.007130834	0.0107345	1
T2DM	leukocyte	*HAGH*	-1.229489552	0.000167544	0.004990048	0.658280623
T2DM	leukocyte	*RNF4*	-1.085039713	0.001057778	0.004990048	1
T2DM	leukocyte	*TM7SF3*	-1.656627173	0.001067639	0.004990048	1
T2DM	leukocyte	*HIPK2*	-0.727977565	0.002070719	0.005273744	1
T2DM	leukocyte	*TMEM161A*	-0.999429138	0.002300463	0.005478715	1
T2DM	leukocyte	*SNX24*	-2.180253528	0.000463166	0.004990048	1
T2DM	leukocyte	*ABCA7*	-1.278163851	0.002262755	0.005456717	1
T2DM	leukocyte	*PMS1*	-0.802627667	0.005021811	0.008092984	1
T2DM	leukocyte	*IPO5*	-0.787793958	0.001512426	0.004993935	1
T2DM	leukocyte	*OAT*	-0.809392091	0.00195994	0.00520663	1
T2DM	leukocyte	*WDR18*	-1.054961702	0.001030636	0.004990048	1
T2DM	leukocyte	*MCM10*	1.425823234	0.023984173	0.030997966	1
T2DM	leukocyte	*ERBB3*	-0.807094439	0.001338586	0.004990048	1
T2DM	leukocyte	*ANKRD44*	-4.130194584	0.000641443	0.004990048	1
T2DM	leukocyte	*ADAT1*	-0.832762068	0.004038082	0.007166394	1
T2DM	leukocyte	*MYLK*	-0.579213538	0.027388899	0.034870702	1
T2DM	leukocyte	*GSTO2*	-0.610169977	0.002018644	0.005244224	1
T2DM	leukocyte	*SEC61A2*	-3.228544742	6.50E-05	0.004990048	0.255424531
T2DM	leukocyte	*PRKCQ*	-2.068288013	0.00373057	0.006898954	1
T2DM	leukocyte	*TBC1D1*	-0.911154064	0.002192234	0.005366534	1
T2DM	leukocyte	*CTNNA2*	-0.93867699	0.011225196	0.015824828	1
T2DM	leukocyte	*CD84*	0.123392197	0.006872428	0.010409317	1
T2DM	leukocyte	*ATXN3*	-1.373795761	0.002002379	0.005227473	1
T2DM	leukocyte	*EML1*	-1.410668218	0.001291656	0.004990048	1
T2DM	leukocyte	*STOML1*	-0.780414586	0.003008449	0.006176667	1
T2DM	leukocyte	*DNTTIP2*	-0.808486394	0.00169768	0.005060368	1
T2DM	leukocyte	*RRP15*	-0.837489855	0.004837896	0.007890449	1
T2DM	leukocyte	*REEP1*	-0.647360586	0.016499807	0.022224114	1
T2DM	leukocyte	*POLR1A*	-0.770414732	0.003234647	0.006381841	1
T2DM	leukocyte	*LAPTM4A*	-0.801519632	0.001010268	0.004990048	1
T2DM	leukocyte	*KIF2A*	-0.83194653	0.002058678	0.00526598	1
T2DM	leukocyte	*MAST4*	-1.012979253	0.000903416	0.004990048	1
T2DM	leukocyte	*DRD4*	-2.547539146	0.00800689	0.011817833	1
T2DM	leukocyte	*PLA2G10*	-3.259409977	0.002503886	0.005644159	1
T2DM	leukocyte	*GNB5*	-0.999810855	0.004663319	0.007686961	1
T2DM	leukocyte	*RAB27A*	-0.837076688	0.001455844	0.004990048	1
T2DM	leukocyte	*PTPN3*	-1.535327	0.000540717	0.004990048	1
T2DM	leukocyte	*FGF10*	-2.309687671	0.009057444	0.013102613	1
T2DM	leukocyte	*DGCR2*	-1.249814808	0.000730411	0.004990048	1
T2DM	leukocyte	*ZXDC*	-2.355824837	0.000382836	0.004990048	1
T2DM	leukocyte	*WIPI1*	-2.324799474	0.000339184	0.004990048	1
T2DM	leukocyte	*GBA2*	-0.79693132	0.006162139	0.009503098	1
T2DM	leukocyte	*ASNS*	-0.824540099	0.004243066	0.007289465	1
T2DM	leukocyte	*AP3M2*	-0.978438268	0.033550533	0.042317832	1
T2DM	leukocyte	*TESK2*	-2.534124281	0.034142903	0.042913457	1
T2DM	leukocyte	*MAP4K4*	-0.821614138	0.000953654	0.004990048	1
T2DM	leukocyte	*RPL31*	-0.716314947	0.0009325	0.004990048	1
T2DM	leukocyte	*SEL1L*	-0.800918545	0.001171869	0.004990048	1
T2DM	leukocyte	*TCF3*	-0.985863748	0.000228119	0.004990048	0.896277925
T2DM	leukocyte	*CPSF1*	-0.736122028	0.00470944	0.007722616	1
T2DM	leukocyte	*LNX1*	-0.657083151	0.003518764	0.006649019	1
T2DM	leukocyte	*ALDH3A2*	-0.584892532	0.002960675	0.006135605	1
T2DM	leukocyte	*P4HA2*	-0.594831277	0.007535128	0.011246286	1
T2DM	leukocyte	*TRNT1*	-2.975358518	0.000799578	0.004990048	1

T2DM	leukocyte	*CRMP1*	-0.66756489	0.005490296	0.008659724	1
T2DM	leukocyte	*DERL2*	-0.935326373	0.001118832	0.004990048	1
T2DM	leukocyte	*XRCC1*	-1.205130269	0.002788538	0.005983705	1
T2DM	leukocyte	*SCARB1*	-2.671600322	0.000112436	0.004990048	0.441759334
T2DM	leukocyte	*NLE1*	-0.675992035	0.037893478	0.047264596	1
T2DM	leukocyte	*SMARCE1*	-0.805967623	0.001251031	0.004990048	1
T2DM	leukocyte	*ST6GAL1*	2.253133459	0.002885684	0.00605886	1
T2DM	leukocyte	*MRPS34*	-0.733441036	0.00175288	0.005084476	1
T2DM	leukocyte	*TEAD2*	-0.98054012	0.015495981	0.02102338	1
T2DM	leukocyte	*IPCEF1*	-1.737480256	0.001882517	0.005146173	1
T2DM	leukocyte	*ZZEF1*	-1.342967564	0.000796946	0.004990048	1
T2DM	leukocyte	*WSCD2*	-0.863923929	0.005965606	0.009262768	1
T2DM	leukocyte	*TIPIN*	-0.80452621	0.039804351	0.049412732	1
T2DM	leukocyte	*SEMA3A*	-2.347129754	0.004768588	0.007800075	1
T2DM	leukocyte	*TTC38*	-1.93335306	0.000271357	0.004990048	1
T2DM	leukocyte	*GRAMD4*	-1.26952199	0.002325499	0.005507466	1
T2DM	leukocyte	*CELSR1*	-4.250503538	0.004405707	0.007420205	1
T2DM	leukocyte	*TIMM21*	-1.320732039	0.001638521	0.005035955	1
T2DM	leukocyte	*VPS9D1*	-0.73731257	0.011929128	0.016697379	1
T2DM	leukocyte	*ZNF37A*	-0.902478244	0.001646766	0.005035955	1
T2DM	leukocyte	*MARK3*	-1.17565035	6.87E-05	0.004990048	0.269803645
T2DM	leukocyte	*CACNG5*	-24.06846005	0.005421995	0.008576094	1
T2DM	leukocyte	*MOCOS*	-2.509446885	0.004673663	0.007686961	1
T2DM	leukocyte	*DLG1*	-0.874575404	0.001522712	0.004993935	1
T2DM	leukocyte	*SEC31B*	1.339144188	0.013646007	0.018785971	1
T2DM	leukocyte	*EXOSC7*	-0.707117989	0.003997637	0.007143456	1
T2DM	leukocyte	*MKRN2*	-1.109779235	0.00154399	0.00500936	1
T2DM	leukocyte	*MLH1*	-1.256127082	0.001285483	0.004990048	1
T2DM	leukocyte	*TPD52*	-0.714739555	0.00157652	0.005009694	1
T2DM	leukocyte	*ACACB*	-1.103228677	0.00139338	0.004990048	1
T2DM	leukocyte	*GPATCH1*	-0.722664328	0.01237726	0.017257011	1
T2DM	leukocyte	*ICAM3*	-0.817927666	0.005817506	0.009073831	1
T2DM	leukocyte	*XAB2*	-0.939217425	0.001188586	0.004990048	1
T2DM	leukocyte	*ACTL6B*	-0.78145194	0.002601088	0.005751212	1
T2DM	leukocyte	*IL4R*	-0.751011122	0.005534512	0.008722462	1
T2DM	leukocyte	*APBB1IP*	0.327282912	0.012220785	0.017063064	1
T2DM	leukocyte	*GPR137B*	-1.373578728	0.002488538	0.005622792	1
T2DM	leukocyte	*NAALAD2*	-2.450580082	0.002680171	0.005854302	1
T2DM	leukocyte	*MCCC1*	-0.920115552	0.002344725	0.005519727	1
T2DM	leukocyte	*N4BP2*	-1.357439826	0.000796836	0.004990048	1
T2DM	leukocyte	*MLLT10*	-1.184303823	0.001399123	0.004990048	1
T2DM	leukocyte	*VDAC3*	-1.000366351	0.001127289	0.004990048	1
T2DM	leukocyte	*PCM1*	-0.842120568	0.001253974	0.004990048	1
T2DM	leukocyte	*TP73*	2.10003024	0.022326102	0.029074993	1
T2DM	leukocyte	*TOLLIP*	-0.935875261	0.001599806	0.005024491	1
T2DM	leukocyte	*CLUL1*	-2.277951571	0.002957007	0.006135605	1
T2DM	leukocyte	*FKBP7*	-1.263604899	0.001014747	0.004990048	1
T2DM	leukocyte	*LXN*	-0.611461741	0.007447677	0.011134674	1
T2DM	leukocyte	*SAR1A*	-0.717903864	0.001685283	0.005060368	1
T2DM	leukocyte	*CDC14A*	-1.329658085	0.002431457	0.005603046	1
T2DM	leukocyte	*FDFT1*	-1.107362204	0.000326658	0.004990048	1
T2DM	leukocyte	*SCGN*	-0.428558923	0.0022035	0.005384049	1
T2DM	leukocyte	*DDX1*	-0.814521391	0.00169052	0.005060368	1
T2DM	leukocyte	*RABL2B*	-1.317442782	0.002271178	0.005457772	1
T2DM	leukocyte	*DCT*	-6.024250747	0.000691469	0.004990048	1
T2DM	leukocyte	*SCTR*	-0.821125101	0.005469853	0.008631969	1
T2DM	leukocyte	*RIF1*	-1.732123176	0.000460513	0.004990048	1
T2DM	leukocyte	*COL5A3*	-4.359093491	0.008061957	0.011881256	1
T2DM	leukocyte	*IGSF9B*	-1.601393195	0.004175593	0.007242343	1
T2DM	leukocyte	*COL4A4*	-2.019336863	0.002112848	0.005283012	1
T2DM	leukocyte	*ZNF506*	-0.8605112	0.002427835	0.005602682	1
T2DM	leukocyte	*CADPS2*	-0.837108413	0.006412633	0.009838046	1
T2DM	leukocyte	*ME2*	-2.167812806	0.00046042	0.004990048	1
T2DM	leukocyte	*CCNT2*	-0.83660621	0.020200632	0.026544577	1

T2DM	leukocyte	*SEMA5B*	1.245841676	0.01191212	0.016679515	1
T2DM	leukocyte	*BCKDHB*	-2.215515924	0.003788255	0.006955165	1
T2DM	leukocyte	*DIS3*	-0.898143697	0.001483936	0.004990048	1
T2DM	leukocyte	*PIBF1*	-1.334325207	0.002704389	0.005880214	1
T2DM	leukocyte	*CHMP2B*	-0.807108602	0.001191797	0.004990048	1
T2DM	leukocyte	*PPIE*	-1.014824571	0.001798923	0.005114303	1
T2DM	leukocyte	*NCOA1*	-1.076397317	0.000988202	0.004990048	1
T2DM	leukocyte	*AGBL5*	-1.43828591	0.00699554	0.010563211	1
T2DM	leukocyte	*CAD*	-0.79388996	0.022533317	0.029296295	1
T2DM	leukocyte	*CD59*	-0.532029465	0.003574359	0.006719453	1
T2DM	leukocyte	*CD82*	-1.690263877	0.000738381	0.004990048	1
T2DM	leukocyte	*MECOM*	-0.483524205	0.023575265	0.030489537	1
T2DM	leukocyte	*SLC25A24*	-0.864467986	0.002532104	0.005660135	1
T2DM	leukocyte	*PILRA*	0.293835388	0.007075778	0.010655705	1
T2DM	leukocyte	*ZNF213*	-1.007703976	0.001824291	0.00513989	1
T2DM	leukocyte	*CPNE3*	-0.67828095	0.00211764	0.005286028	1
T2DM	leukocyte	*RRN3*	-0.751115207	0.003090994	0.00625753	1
T2DM	leukocyte	*WNT11*	-1.728972109	0.034521426	0.043375338	1
T2DM	leukocyte	*DDHD2*	-0.81056631	0.017127507	0.022959391	1
T2DM	leukocyte	*TTC39A*	-0.794503373	0.009527461	0.013691804	1
T2DM	leukocyte	*EPS15*	-1.145499735	0.003257937	0.006405338	1
T2DM	leukocyte	*ORC1*	-3.3018759	0.001395497	0.004990048	1
T2DM	leukocyte	*MGST2*	-0.559290427	0.007872737	0.011663643	1
T2DM	leukocyte	*USP40*	-1.45858206	0.000973614	0.004990048	1
T2DM	leukocyte	*POMGNT1*	-1.286394022	0.00044349	0.004990048	1
T2DM	leukocyte	*NFX1*	-1.418167157	0.001643622	0.005035955	1
T2DM	leukocyte	*EIF2AK1*	-0.887321839	0.002563393	0.00569337	1
T2DM	leukocyte	*ITPKC*	-1.079267221	0.002812805	0.006001505	1
T2DM	leukocyte	*TMED2*	-0.686055135	0.001385412	0.004990048	1
T2DM	leukocyte	*ACOX3*	-2.007729062	0.00133313	0.004990048	1
T2DM	leukocyte	*TRIP6*	-0.872984924	0.000263553	0.004990048	1
T2DM	leukocyte	*PGS1*	-1.422810208	0.00130873	0.004990048	1
T2DM	leukocyte	*PSMC5*	-0.781002202	0.000645362	0.004990048	1
T2DM	leukocyte	*UIMC1*	-0.901849492	0.004270075	0.007310294	1
T2DM	leukocyte	*SF3B2*	-0.7464054	0.001754	0.005084476	1
T2DM	leukocyte	*KLHL42*	-0.854920577	0.000505813	0.004990048	1
T2DM	leukocyte	*METTL2A*	-1.421627303	0.001126078	0.004990048	1
T2DM	leukocyte	*GP6*	1.115609996	0.026560182	0.033969712	1
T2DM	leukocyte	*SLC15A1*	-1.488618627	0.013431038	0.018535492	1
T2DM	leukocyte	*TGDS*	-0.853908664	0.004651116	0.007681478	1
T2DM	leukocyte	*DOCK3*	-0.963926851	0.004231832	0.007276528	1
T2DM	leukocyte	*ARHGAP28*	-2.178406119	0.00403488	0.007166394	1
T2DM	leukocyte	*PPP1R13B*	-0.608925727	0.005853882	0.009116093	1
T2DM	leukocyte	*ZNF343*	-1.018307115	0.011168098	0.015766962	1
T2DM	leukocyte	*MAVS*	-1.750093288	0.001540684	0.005006447	1
T2DM	leukocyte	*DYNLL1*	-0.805122577	0.000639536	0.004990048	1
T2DM	leukocyte	*RBBP9*	-0.698488263	0.008417042	0.012330558	1
T2DM	leukocyte	*SLC23A2*	-1.542464132	0.000880416	0.004990048	1
T2DM	leukocyte	*SLC8B1*	0.221868287	0.025347457	0.032577742	1
T2DM	leukocyte	*TMEM230*	-0.841180015	0.001324296	0.004990048	1
T2DM	leukocyte	*KIF16B*	-1.477410698	0.000484524	0.004990048	1
T2DM	leukocyte	*ZNF302*	-0.89566518	0.000910515	0.004990048	1
T2DM	leukocyte	*GRAMD1A*	-1.473779863	0.000825167	0.004990048	1
T2DM	leukocyte	*CDIP1*	-0.89386001	0.003316455	0.006450669	1
T2DM	leukocyte	*MLF2*	-0.951400261	0.001020114	0.004990048	1
T2DM	leukocyte	*DDX24*	-0.769227876	0.00096598	0.004990048	1
T2DM	leukocyte	*ANKRD24*	-6.103997059	0.010209665	0.014560353	1
T2DM	leukocyte	*GPATCH2L*	-1.109621066	0.000834266	0.004990048	1
T2DM	leukocyte	*SLC9A1*	-0.73744306	0.00508082	0.008170331	1
T2DM	leukocyte	*SPTLC1*	-0.858314389	0.002114445	0.005283012	1
T2DM	leukocyte	*NDUFB2*	-0.816682028	0.000208512	0.004990048	0.819242871
T2DM	leukocyte	*MAEA*	-0.752236808	0.003842766	0.007022431	1
T2DM	leukocyte	*STRN4*	-0.873035187	0.00164783	0.005035955	1
T2DM	leukocyte	*LYZ*	0.183052526	0.014319122	0.019616398	1

T2DM	leukocyte	*MUL1*	-0.968334402	0.001235475	0.004990048	1
T2DM	leukocyte	*SPG21*	-1.22953384	0.000842094	0.004990048	1
T2DM	leukocyte	*USP48*	-0.805321708	0.005150195	0.008249407	1
T2DM	leukocyte	*GLG1*	-0.881332168	0.001485487	0.004990048	1
T2DM	leukocyte	*NRCAM*	-0.559561274	0.003432414	0.006559316	1
T2DM	leukocyte	*TF*	-1.212887593	0.01108079	0.015660584	1
T2DM	leukocyte	*MYO15A*	-1.940178764	0.007000542	0.010565863	1
T2DM	leukocyte	*PITPNM3*	-2.367334508	0.00395964	0.007124893	1
T2DM	leukocyte	*SPAG7*	-0.838760254	0.007425941	0.011106403	1
T2DM	leukocyte	*TMEM101*	-1.176792055	0.000909852	0.004990048	1
T2DM	leukocyte	*PPP2R3C*	-1.64909572	0.000189448	0.004990048	0.744340782
T2DM	leukocyte	*OSGEP*	-1.595421824	0.000844803	0.004990048	1
T2DM	leukocyte	*SCFD1*	-0.911386611	0.001591088	0.005021192	1
T2DM	leukocyte	*RPGRIP1*	0.920280361	0.027685941	0.035237467	1
T2DM	leukocyte	*EZR*	-0.609380714	0.002007035	0.005232069	1
T2DM	leukocyte	*NUP50*	-1.695391933	0.000395241	0.004990048	1
T2DM	leukocyte	*COMT*	-0.795921942	0.001541818	0.005006447	1
T2DM	leukocyte	*ECHDC1*	-0.680667344	0.004208896	0.007263735	1
T2DM	leukocyte	*CBX5*	-0.784322335	0.000897318	0.004990048	1
T2DM	leukocyte	*SUCO*	-0.644923524	0.001237583	0.004990048	1
T2DM	leukocyte	*MSH2*	-1.911124644	0.001269603	0.004990048	1
T2DM	leukocyte	*TMEM38B*	-1.457310677	0.000228947	0.004990048	0.899531627
T2DM	leukocyte	*PSMD5*	-0.810240428	0.002804573	0.006001505	1
T2DM	leukocyte	*CRAT*	-1.507741632	0.001009443	0.004990048	1
T2DM	leukocyte	*SH2D3C*	-1.166309154	0.005561602	0.008754621	1
T2DM	leukocyte	*CWF19L1*	-1.305422578	3.69E-06	0.0010358	0.014501198
T2DM	leukocyte	*SEMA4G*	-8.311742455	0.003367338	0.006494978	1
T2DM	leukocyte	*IKZF5*	-1.521312302	0.000355785	0.004990048	1
T2DM	leukocyte	*TLL2*	-3.427002804	0.004246876	0.007292822	1
T2DM	leukocyte	*TPSD1*	-28.03606079	0.013460208	0.018569226	1
T2DM	leukocyte	*SRPK1*	-1.106118874	0.000859466	0.004990048	1
T2DM	leukocyte	*PGC*	-0.82129192	0.002176975	0.005349178	1
T2DM	leukocyte	*EFHC1*	-0.735006668	0.004551083	0.007568852	1
T2DM	leukocyte	*ITPR3*	-0.881294966	0.004476954	0.007504245	1
T2DM	leukocyte	*IFT74*	-1.255052406	0.001184121	0.004990048	1
T2DM	leukocyte	*ABL1*	-0.930310984	0.001708996	0.005068796	1
T2DM	leukocyte	*SH3GLB1*	-0.936354294	0.001308215	0.004990048	1
T2DM	leukocyte	*CDC7*	-1.008301261	0.013634382	0.018776546	1
T2DM	leukocyte	*SYDE2*	-0.770986322	0.023435673	0.030318986	1
T2DM	leukocyte	*ABLIM1*	-0.742553082	0.002269837	0.005457772	1
T2DM	leukocyte	*ERMP1*	-0.832094529	0.003715448	0.006879357	1
T2DM	leukocyte	*PALMD*	-0.829827341	0.000963684	0.004990048	1
T2DM	leukocyte	*MZF1*	-0.815188332	0.009681744	0.013893197	1
T2DM	leukocyte	*OCEL1*	-1.234973747	0.001148804	0.004990048	1
T2DM	leukocyte	*STX1B*	1.087933705	0.002766913	0.005956822	1
T2DM	leukocyte	*HSD3B7*	-2.619124389	0.003515142	0.006649019	1
T2DM	leukocyte	*HNRNPM*	-0.776272236	0.000715041	0.004990048	1
T2DM	leukocyte	*TIMM13*	-0.920075878	0.00097808	0.004990048	1
T2DM	leukocyte	*CEP170B*	-1.338101746	0.001469987	0.004990048	1
T2DM	leukocyte	*POLR2E*	-1.251776141	0.000841893	0.004990048	1
T2DM	leukocyte	*RASSF7*	-0.787740367	0.002062188	0.00526847	1
T2DM	leukocyte	*ARVCF*	-1.505541902	0.005124583	0.008214804	1
T2DM	leukocyte	*TRMT2A*	-0.781928245	0.004451098	0.007480054	1
T2DM	leukocyte	*MED15*	-2.588917052	0.000313606	0.004990048	1
T2DM	leukocyte	*SMARCB1*	-0.990768073	0.001009547	0.004990048	1
T2DM	leukocyte	*DDT*	-1.029761866	0.000514737	0.004990048	1
T2DM	leukocyte	*CABIN1*	-1.163573517	0.002012008	0.005238686	1
T2DM	leukocyte	*SPECC1L*	-1.416315132	0.001104015	0.004990048	1
T2DM	leukocyte	*PES1*	-0.887691843	0.002263794	0.005456717	1
T2DM	leukocyte	*GGT1*	-5.648239162	0.024265207	0.031330265	1
T2DM	leukocyte	*TOP3B*	-1.275302502	0.006126893	0.009458767	1
T2DM	leukocyte	*CARD10*	-0.645628964	0.00313897	0.006311945	1
T2DM	leukocyte	*SLC25A1*	-0.987447908	0.001909686	0.00515389	1
T2DM	leukocyte	*HPS4*	-1.016862796	0.001822697	0.00513989	1

T2DM	leukocyte	*GCAT*	-1.283583921	0.001696778	0.005060368	1
T2DM	leukocyte	*CYP2D6*	1.845368875	0.032049824	0.04052905	1
T2DM	leukocyte	*DDX17*	-0.977827296	0.000594533	0.004990048	1
T2DM	leukocyte	*HSCB*	-1.563124227	0.00161946	0.005035955	1
T2DM	leukocyte	*CBY1*	-1.000383837	0.001564068	0.005009694	1
T2DM	leukocyte	*XBP1*	-0.661677334	0.001066792	0.004990048	1
T2DM	leukocyte	*TIMP3*	-0.944070795	0.003891906	0.007059695	1
T2DM	leukocyte	*CYB5R3*	-0.897784427	0.000982302	0.004990048	1
T2DM	leukocyte	*MIOX*	-2.019339682	0.003868699	0.007040351	1
T2DM	leukocyte	*LMF2*	-1.432956349	0.007914503	0.011703456	1
T2DM	leukocyte	*RHBDD3*	-1.063084529	0.001385142	0.004990048	1
T2DM	leukocyte	*TOM1*	-1.329489193	0.003395208	0.006510382	1
T2DM	leukocyte	*CHKB*	-2.371893901	0.001120523	0.004990048	1
T2DM	leukocyte	*TTLL12*	-1.327714846	0.002477926	0.005622792	1
T2DM	leukocyte	*RBFOX2*	-0.931900436	0.000783894	0.004990048	1
T2DM	leukocyte	*SYNGR1*	-3.06136173	3.83E-05	0.004122285	0.150356863
T2DM	leukocyte	*MTMR3*	-1.199448758	0.001152168	0.004990048	1
T2DM	leukocyte	*SAMM50*	-0.847071557	0.001809637	0.005122825	1
T2DM	leukocyte	*FOXRED2*	-0.932878159	0.006028208	0.009335763	1
T2DM	leukocyte	*SGSM3*	-1.350916927	0.001233325	0.004990048	1
T2DM	leukocyte	*IFT27*	-1.19696262	0.000697676	0.004990048	1
T2DM	leukocyte	*FAM118A*	-0.953811058	0.018533122	0.024600215	1
T2DM	leukocyte	*ST13*	-0.807887552	0.000966174	0.004990048	1
T2DM	leukocyte	*POLR3H*	-2.374418316	0.000127597	0.004990048	0.501330534
T2DM	leukocyte	*PMM1*	-1.202020107	0.001443368	0.004990048	1
T2DM	leukocyte	*DESI1*	-1.156733315	0.002458988	0.005622792	1
T2DM	leukocyte	*KHNYN*	-1.325661705	0.000820625	0.004990048	1
T2DM	leukocyte	*RBM23*	-1.008462902	0.000349558	0.004990048	1
T2DM	leukocyte	*PRMT5*	-0.828868473	0.002982913	0.006152298	1
T2DM	leukocyte	*VCPKMT*	-1.348729718	0.000995518	0.004990048	1
T2DM	leukocyte	*SOS2*	-1.204871604	0.000960511	0.004990048	1
T2DM	leukocyte	*CDKL1*	-1.486364166	0.006117025	0.00945096	1
T2DM	leukocyte	*NIN*	-2.178566962	0.000526347	0.004990048	1
T2DM	leukocyte	*TRIM9*	-0.650883898	0.004669876	0.007686961	1
T2DM	leukocyte	*ATP6V1D*	-0.852266202	0.001060582	0.004990048	1
T2DM	leukocyte	*PLEK2*	-1.055198599	0.021974983	0.028655728	1
T2DM	leukocyte	*GSTZ1*	-1.252702994	0.003452844	0.006579158	1
T2DM	leukocyte	*TMED8*	-1.100705876	0.000766617	0.004990048	1
T2DM	leukocyte	*DAAM1*	-0.804173566	0.001809704	0.005122825	1
T2DM	leukocyte	*DHRS7*	-1.009936421	0.00081463	0.004990048	1
T2DM	leukocyte	*MTHFD1*	-3.821538098	0.000103131	0.004990048	0.405201463
T2DM	leukocyte	*GSKIP*	-0.880610951	0.002219013	0.005407737	1
T2DM	leukocyte	*TRIP11*	-0.768176929	0.001934848	0.005184924	1
T2DM	leukocyte	*DCAF11*	-1.173244246	0.001649599	0.005035955	1
T2DM	leukocyte	*EMC9*	-0.735235216	0.018552916	0.02460986	1
T2DM	leukocyte	*NFATC4*	-1.188050934	0.003981018	0.007132431	1
T2DM	leukocyte	*PYGB*	-1.149033212	0.00025647	0.004990048	1
T2DM	leukocyte	*ABHD12*	-1.811240049	0.000445013	0.004990048	1
T2DM	leukocyte	*PROCR*	-0.685447853	0.008458567	0.012372937	1
T2DM	leukocyte	*CD40*	-0.991659145	0.002036196	0.005253355	1
T2DM	leukocyte	*IFT52*	-0.885145072	0.00160976	0.005033031	1
T2DM	leukocyte	*MYBL2*	-2.280296329	0.003078443	0.006253982	1
T2DM	leukocyte	*NELFCD*	-0.837769679	0.001831261	0.005146173	1
T2DM	leukocyte	*MTG2*	-1.041518829	0.001407246	0.004990048	1
T2DM	leukocyte	*DIDO1*	-0.936364311	0.007841821	0.011622224	1
T2DM	leukocyte	*CDC25B*	-1.531088657	0.005440307	0.008601596	1
T2DM	leukocyte	*ISM1*	1.136723054	0.020179977	0.026526307	1
T2DM	leukocyte	*ARFRP1*	-1.046028802	0.018974629	0.025084163	1
T2DM	leukocyte	*SLC52A3*	-1.532614462	0.039357467	0.048919799	1
T2DM	leukocyte	*SIRPB1*	0.563584189	0.011627869	0.016328055	1
T2DM	leukocyte	*PLCB4*	-0.742780749	0.004849549	0.00790211	1
T2DM	leukocyte	*POFUT1*	-0.85826567	0.002408692	0.005586019	1
T2DM	leukocyte	*NOP56*	-1.168510695	0.002761778	0.005955558	1
T2DM	leukocyte	*MANBAL*	-0.822415792	0.001968347	0.005212073	1

T2DM	leukocyte	*IDH3B*	-1.092230987	0.001587967	0.005020048	1
T2DM	leukocyte	*MAPRE1*	-0.733782233	0.002033326	0.005252426	1
T2DM	leukocyte	*OXT*	0.678069564	0.022503191	0.029266811	1
T2DM	leukocyte	*TTI1*	-1.389543498	0.001573379	0.005009694	1
T2DM	leukocyte	*CHMP4B*	-0.759980447	0.001358605	0.004990048	1
T2DM	leukocyte	*DHX35*	-1.577364077	0.001379777	0.004990048	1
T2DM	leukocyte	*DNTTIP1*	-2.138792409	0.000363349	0.004990048	1
T2DM	leukocyte	*MAP1LC3A*	-0.549608523	0.006467424	0.009910495	1
T2DM	leukocyte	*APMAP*	-0.973762183	0.002740319	0.005930931	1
T2DM	leukocyte	*VAPA*	-0.751651405	0.001095586	0.004990048	1
T2DM	leukocyte	*MYOM1*	-1.99431643	0.005502975	0.008676239	1
T2DM	leukocyte	*CEP192*	-0.72682958	0.005123138	0.008214804	1
T2DM	leukocyte	*LIPG*	-0.926882076	0.00417942	0.007242343	1
T2DM	leukocyte	*LAMA1*	-3.597953766	0.033634777	0.042410475	1
T2DM	leukocyte	*POLI*	-1.326382944	0.009219333	0.013317191	1
T2DM	leukocyte	*RIOK3*	-0.838465147	0.001034663	0.004990048	1
T2DM	leukocyte	*NALCN*	-0.768392453	0.006059599	0.009380679	1
T2DM	leukocyte	*FGF14*	-0.874561493	0.002488621	0.005622792	1
T2DM	leukocyte	*FNDC3A*	-0.691821539	0.001784519	0.005100156	1
T2DM	leukocyte	*KLF5*	-0.613112772	0.003972148	0.007124893	1
T2DM	leukocyte	*ACP5*	0.048580304	0.018464941	0.024526286	1
T2DM	leukocyte	*DNAJC3*	-0.804873852	0.001136533	0.004990048	1
T2DM	leukocyte	*UGGT2*	-0.840088947	0.001864415	0.005146173	1
T2DM	leukocyte	*ARHGEF7*	-1.231573281	0.000944394	0.004990048	1
T2DM	leukocyte	*PARP4*	-2.662817183	0.000296226	0.004990048	1
T2DM	leukocyte	*MEDAG*	-1.443317845	0.002248678	0.005439981	1
T2DM	leukocyte	*CLN5*	-1.159614357	0.000758285	0.004990048	1
T2DM	leukocyte	*OLFM4*	-0.319149465	0.026626808	0.034038245	1
T2DM	leukocyte	*MGRN1*	-1.16989571	0.001167074	0.004990048	1
T2DM	leukocyte	*LONP2*	-0.854324292	0.002062333	0.00526847	1
T2DM	leukocyte	*DHODH*	-2.663319001	0.000891827	0.004990048	1
T2DM	leukocyte	*PARD6A*	-0.680701169	0.001680196	0.005060368	1
T2DM	leukocyte	*MMP15*	-1.752532608	0.004423208	0.007442734	1
T2DM	leukocyte	*USB1*	-1.28697494	0.001843279	0.005146173	1
T2DM	leukocyte	*CYB5B*	-1.879024208	0.000256767	0.004990048	1
T2DM	leukocyte	*CCDC113*	-1.204512358	0.002286386	0.005462378	1
T2DM	leukocyte	*NME3*	-0.984715661	0.001379933	0.004990048	1
T2DM	leukocyte	*VAC14*	-0.969824632	0.001847524	0.005146173	1
T2DM	leukocyte	*NPRL3*	-1.415555235	0.00125041	0.004990048	1
T2DM	leukocyte	*NAGPA*	-4.843850528	0.000211205	0.004990048	0.829822961
T2DM	leukocyte	*ZNF500*	-1.731083842	0.003217148	0.006372159	1
T2DM	leukocyte	*NME4*	-0.971664431	0.001466394	0.004990048	1
T2DM	leukocyte	*NOMO3*	-1.451540925	0.000821367	0.004990048	1
T2DM	leukocyte	*LMF1*	-0.888241036	0.002917117	0.006099708	1
T2DM	leukocyte	*MTHFSD*	-0.933025977	0.009301146	0.0134061	1
T2DM	leukocyte	*CLCN7*	-2.005887427	0.008989989	0.013019413	1
T2DM	leukocyte	*HAGHL*	-1.514489875	0.001349271	0.004990048	1
T2DM	leukocyte	*METRN*	-0.773004761	0.000798073	0.004990048	1
T2DM	leukocyte	*PIEZO1*	-1.106405545	0.001812368	0.005123568	1
T2DM	leukocyte	*CLUAP1*	-0.805895302	0.019349111	0.025536667	1
T2DM	leukocyte	*UBFD1*	-1.152935275	0.004093742	0.007206818	1
T2DM	leukocyte	*AQP8*	-1.357640453	0.004859226	0.007912101	1
T2DM	leukocyte	*USP31*	-0.657686882	0.003538686	0.006674746	1
T2DM	leukocyte	*HMOX2*	-0.808623239	0.002369974	0.005551753	1
T2DM	leukocyte	*RBL2*	-0.872177374	0.017833174	0.02375137	1
T2DM	leukocyte	*KAT8*	-1.112933475	0.001112092	0.004990048	1
T2DM	leukocyte	*NOMO1*	-0.972998349	0.039104503	0.048651549	1
T2DM	leukocyte	*RNF40*	-1.751356857	0.000600558	0.004990048	1
T2DM	leukocyte	*KNOP1*	-1.160861839	0.001292894	0.004990048	1
T2DM	leukocyte	*TRIP4*	-0.926295945	0.003514315	0.006649019	1
T2DM	leukocyte	*AP3B2*	-0.944357074	0.003480899	0.006621675	1
T2DM	leukocyte	*IGDCC4*	2.163151469	0.011348517	0.01597003	1
T2DM	leukocyte	*RPAP1*	-1.417071741	0.001474379	0.004990048	1
T2DM	leukocyte	*HOMER2*	-0.693018952	0.003177194	0.006330221	1

T2DM	leukocyte	*ATP8B4*	0.427771244	0.015641602	0.021198984	1
T2DM	leukocyte	*DMXL2*	-2.244638205	0.026631093	0.034038245	1
T2DM	leukocyte	*DNAJC17*	-0.815231554	0.005823452	0.009077881	1
T2DM	leukocyte	*ZDHHC2*	-0.595082707	0.003073293	0.006249984	1
T2DM	leukocyte	*TRIM35*	-2.25948884	0.000895372	0.004990048	1
T2DM	leukocyte	*RIPK2*	-1.168931471	0.000846769	0.004990048	1
T2DM	leukocyte	*NBN*	-1.316944618	0.00132638	0.004990048	1
T2DM	leukocyte	*LAPTM4B*	-0.557320061	0.002839593	0.006020917	1
T2DM	leukocyte	*RAB2A*	-0.64543304	0.001766551	0.00509228	1
T2DM	leukocyte	*EIF3E*	-0.746330763	0.001217165	0.004990048	1
T2DM	leukocyte	*ESRP1*	-0.711980386	0.003319861	0.006454098	1
T2DM	leukocyte	*ARMC1*	-1.048053903	0.001231818	0.004990048	1
T2DM	leukocyte	*SPAG1*	-0.822209255	0.002640106	0.005811191	1
T2DM	leukocyte	*CHRAC1*	-0.715287367	0.002798108	0.005997385	1
T2DM	leukocyte	*SNX16*	-2.377323377	0.003945538	0.007116737	1
T2DM	leukocyte	*SH2D4A*	-0.986722598	0.002761634	0.005955558	1
T2DM	leukocyte	*INTS10*	-1.245510633	0.000687899	0.004990048	1
T2DM	leukocyte	*ERI1*	-2.639718052	0.000121452	0.004990048	0.47718579
T2DM	leukocyte	*LEPROTL1*	-1.119639958	0.000644292	0.004990048	1
T2DM	leukocyte	*ERICH1*	-1.28675167	0.001218018	0.004990048	1
T2DM	leukocyte	*ARHGEF10*	-0.93470781	0.003299747	0.006432819	1
T2DM	leukocyte	*KLHDC4*	-1.103957053	0.002873421	0.006055188	1
T2DM	leukocyte	*MCM4*	-1.129700256	0.001915379	0.005158002	1
T2DM	leukocyte	*FGL1*	-1.259245609	0.017772732	0.023686929	1
T2DM	leukocyte	*ECH1*	-0.814696664	0.001953924	0.005198138	1
T2DM	leukocyte	*HNRNPL*	-0.924037187	0.00134024	0.004990048	1
T2DM	leukocyte	*SARS2*	-1.150842262	0.000855283	0.004990048	1
T2DM	leukocyte	*LIN7B*	-1.238645159	0.00097322	0.004990048	1
T2DM	leukocyte	*PPP1R13L*	-0.868007503	0.009309012	0.013407297	1
T2DM	leukocyte	*ERCC2*	-0.878432779	0.006294231	0.009663944	1
T2DM	leukocyte	*KLC3*	-0.869928485	0.002136361	0.005317933	1
T2DM	leukocyte	*AMH*	-1.008613128	0.002387666	0.005560842	1
T2DM	leukocyte	*IL27RA*	-1.042331559	0.011883016	0.016650632	1
T2DM	leukocyte	*PDCD5*	-0.673137756	0.003080361	0.006254644	1
T2DM	leukocyte	*NUMBL*	-0.730333235	0.003599021	0.006745301	1
T2DM	leukocyte	*SLC1A5*	-1.106708971	0.003393934	0.006510382	1
T2DM	leukocyte	*PRKD2*	-1.159435189	0.000473446	0.004990048	1
T2DM	leukocyte	*APLP1*	-0.390809449	0.007635994	0.011372941	1
T2DM	leukocyte	*PLIN3*	-0.989206159	0.001005173	0.004990048	1
T2DM	leukocyte	*MYH14*	-0.737324656	0.002245331	0.005438799	1
T2DM	leukocyte	*ZNF175*	-0.826278276	0.00284597	0.006031185	1
T2DM	leukocyte	*PLA2G4C*	-1.303546266	0.00071404	0.004990048	1
T2DM	leukocyte	*CAPS*	-1.111885347	0.004212033	0.007263735	1
T2DM	leukocyte	*PPP2R1A*	-0.752882343	0.002435569	0.005609232	1
T2DM	leukocyte	*GCDH*	-1.148542946	0.000813417	0.004990048	1
T2DM	leukocyte	*PIK3R2*	-2.883335097	0.00031284	0.004990048	1
T2DM	leukocyte	*UPK1A*	-11.79217218	0.007644581	0.011381417	1
T2DM	leukocyte	*COPE*	-0.805361992	0.000976309	0.004990048	1
T2DM	leukocyte	*ETV2*	-0.999084487	0.01120143	0.015796991	1
T2DM	leukocyte	*PBX4*	-3.321504638	0.005583095	0.008784934	1
T2DM	leukocyte	*SIPA1L3*	-2.504933568	0.000346475	0.004990048	1
T2DM	leukocyte	*ETHE1*	-1.251064428	0.004342757	0.007358292	1
T2DM	leukocyte	*SMG9*	-0.73200505	0.00798289	0.011786837	1
T2DM	leukocyte	*RUNDC3B*	-5.103683682	0.000372764	0.004990048	1
T2DM	leukocyte	*PMPCB*	-1.080776613	0.001131443	0.004990048	1
T2DM	leukocyte	*PON2*	-0.803891462	0.001381541	0.004990048	1
T2DM	leukocyte	*ITGB8*	-0.809541994	0.002071109	0.005273744	1
T2DM	leukocyte	*DNAH11*	-3.46069706	0.005969287	0.009262768	1
T2DM	leukocyte	*CAV2*	-0.524476333	0.004347841	0.007358292	1
T2DM	leukocyte	*CAV1*	-0.622282977	0.004150688	0.007238372	1
T2DM	leukocyte	*ANKRD7*	-1.390313441	0.0086922	0.012653447	1
T2DM	leukocyte	*TSPAN12*	-1.405591416	0.001160405	0.004990048	1
T2DM	leukocyte	*CPVL*	0.102469514	0.018763998	0.024856287	1
T2DM	leukocyte	*ABHD11*	-1.340376918	0.000723128	0.004990048	1

T2DM	leukocyte	*CHCHD2*	-0.627479515	0.00118224	0.004990048	1
T2DM	leukocyte	*EIF3B*	-0.911392929	0.001431545	0.004990048	1
T2DM	leukocyte	*WASL*	-0.700144733	0.003505857	0.006641519	1
T2DM	leukocyte	*AGFG2*	-0.81619394	0.004171571	0.007242343	1
T2DM	leukocyte	*C1GALT1*	-0.771098119	0.001900508	0.005146173	1
T2DM	leukocyte	*RPA3*	-0.996796541	0.001126555	0.004990048	1
T2DM	leukocyte	*ZNHIT1*	-0.746850053	0.002534023	0.005660135	1
T2DM	leukocyte	*CLDN15*	-0.712096512	0.004546375	0.007568852	1
T2DM	leukocyte	*TMEM106B*	-0.783578924	0.002020371	0.005244224	1
T2DM	leukocyte	*ACTR3C*	-1.41468613	0.002865776	0.006053568	1
T2DM	leukocyte	*TSPAN13*	-0.592571061	0.002182467	0.005356498	1
T2DM	leukocyte	*RARRES2*	-0.392961095	0.007575108	0.01129082	1
T2DM	leukocyte	*AGR2*	-0.549001184	0.012789122	0.017761916	1
T2DM	leukocyte	*TMEM176B*	-0.651848956	0.010184863	0.014530256	1
T2DM	leukocyte	*COA1*	-1.183300943	0.000993881	0.004990048	1
T2DM	leukocyte	*BLVRA*	-1.62724446	0.000692811	0.004990048	1
T2DM	leukocyte	*TMEM248*	-1.231084166	0.000248086	0.004990048	0.974728953
T2DM	leukocyte	*RHEB*	-0.795830285	0.000980608	0.004990048	1
T2DM	leukocyte	*TBL2*	-0.599710545	0.037373799	0.04667567	1
T2DM	leukocyte	*LIMK1*	-3.250791671	0.001261513	0.004990048	1
T2DM	leukocyte	*SPIN1*	-1.382556247	0.000822662	0.004990048	1
T2DM	leukocyte	*NMRK1*	-0.933822389	0.002452375	0.005621365	1
T2DM	leukocyte	*C5*	-0.998018187	0.01394475	0.019143579	1
T2DM	leukocyte	*OGN*	-2.030230147	0.001932457	0.005184924	1
T2DM	leukocyte	*SUSD1*	-1.632709036	0.000520089	0.004990048	1
T2DM	leukocyte	*DNM1*	-0.622175352	0.003356232	0.006483106	1
T2DM	leukocyte	*AK1*	-0.706294981	0.001556677	0.005009694	1
T2DM	leukocyte	*PLGRKT*	-0.726277552	0.004094074	0.007206818	1
T2DM	leukocyte	*DOCK8*	0.321740783	0.036966521	0.046211091	1
T2DM	leukocyte	*MPDZ*	-0.686109972	0.00568568	0.008916678	1
T2DM	leukocyte	*NPDC1*	-0.634682254	0.001678026	0.005059834	1
T2DM	leukocyte	*HPS1*	-1.08494635	0.001213158	0.004990048	1
T2DM	leukocyte	*CXCL12*	-0.906581388	0.0015945	0.005024491	1
T2DM	leukocyte	*TRDMT1*	-2.001709351	0.001449036	0.004990048	1
T2DM	leukocyte	*ATE1*	-1.510168703	0.000794129	0.004990048	1
T2DM	leukocyte	*NSMCE4A*	-0.754608911	0.005214688	0.008330015	1
T2DM	leukocyte	*PLEKHA1*	-0.679105723	0.002159233	0.005342335	1
T2DM	leukocyte	*PALD1*	-6.174050286	0.000335809	0.004990048	1
T2DM	leukocyte	*SPOCK2*	-1.2902233	0.004313562	0.007328649	1
T2DM	leukocyte	*BMPR1A*	-2.064594499	0.000544659	0.004990048	1
T2DM	leukocyte	*LIPA*	0.234137531	0.032800875	0.041412158	1
T2DM	leukocyte	*SFXN3*	-1.446403968	0.000819737	0.004990048	1
T2DM	leukocyte	*NPM3*	-0.977596726	0.00271421	0.005895041	1
T2DM	leukocyte	*TNKS2*	-1.016189559	0.001008124	0.004990048	1
T2DM	leukocyte	*ACBD5*	-0.747992784	0.00336638	0.006494978	1
T2DM	leukocyte	*LHPP*	-1.510993814	0.002916069	0.006099708	1
T2DM	leukocyte	*GTPBP4*	-0.76064206	0.001563704	0.005009694	1
T2DM	leukocyte	*NEURL1*	-0.899452818	0.007682424	0.011424771	1
T2DM	leukocyte	*XPNPEP1*	-0.837816415	0.002416187	0.005594105	1
T2DM	leukocyte	*SMC3*	-0.755052908	0.001266291	0.004990048	1
T2DM	leukocyte	*RPL28*	-0.630827897	0.000551884	0.004990048	1
T2DM	leukocyte	*PBLD*	-0.730385491	0.004124022	0.007233608	1
T2DM	leukocyte	*KRT23*	-0.452358535	0.016136307	0.021794276	1
T2DM	leukocyte	*DHRS11*	-0.646239203	0.013292343	0.018363438	1
T2DM	leukocyte	*CWC25*	-1.095721975	0.001161716	0.004990048	1
T2DM	leukocyte	*RAD51C*	-0.986355215	0.00140434	0.004990048	1
T2DM	leukocyte	*TRIM37*	-0.852803569	0.002001952	0.005227473	1
T2DM	leukocyte	*RPS6KB1*	-0.870836706	0.007525589	0.011238327	1
T2DM	leukocyte	*TRIM16L*	-1.284495355	0.005414834	0.008574413	1
T2DM	leukocyte	*CDK5RAP3*	-1.139410877	0.011012152	0.015569178	1
T2DM	leukocyte	*CAMTA2*	-1.141095156	0.010822618	0.015317747	1
T2DM	leukocyte	*MED13*	-0.89776687	0.001055936	0.004990048	1
T2DM	leukocyte	*RAI1*	-0.673441459	0.00466499	0.007686961	1
T2DM	leukocyte	*CCDC47*	-0.857149549	0.000932454	0.004990048	1

T2DM	leukocyte	*DRG2*	-0.897549129	0.003129915	0.006306376	1
T2DM	leukocyte	*FTSJ3*	-1.004562575	0.00027474	0.004990048	1
T2DM	leukocyte	*SMARCD2*	-2.197206397	0.000214415	0.004990048	0.84243803
T2DM	leukocyte	*SYNGR2*	-0.77126975	0.002343644	0.005519727	1
T2DM	leukocyte	*C17orf75*	-0.996923915	0.01254373	0.017458135	1
T2DM	leukocyte	*DHX58*	-2.082019844	0.000279796	0.004990048	1
T2DM	leukocyte	*NAGLU*	-0.866847586	0.004563039	0.007578894	1
T2DM	leukocyte	*HSD17B1*	-1.432142816	2.02E-05	0.0030497	0.0792922
T2DM	leukocyte	*RND2*	-2.311093896	0.000970824	0.004990048	1
T2DM	leukocyte	*ABCC3*	-0.645245677	0.007002674	0.010565863	1
T2DM	leukocyte	*MPP2*	-0.610599331	0.004219249	0.007267615	1
T2DM	leukocyte	*HLF*	-0.519399701	0.006646919	0.010135575	1
T2DM	leukocyte	*EFNB3*	-0.778147755	0.00405348	0.007180398	1
T2DM	leukocyte	*RANGRF*	-1.176667098	0.000963712	0.004990048	1
T2DM	leukocyte	*DPH1*	-1.611745784	0.013592994	0.01873268	1
T2DM	leukocyte	*OCIAD1*	-0.779920545	0.001145778	0.004990048	1
T2DM	leukocyte	*UBE2D3*	-0.721965831	0.002049674	0.00526208	1
T2DM	leukocyte	*MAPK10*	-0.731914873	0.002200904	0.005384049	1
T2DM	leukocyte	*ANXA10*	-0.7786026	0.025720346	0.032981475	1
T2DM	leukocyte	*AADAT*	-2.53606174	0.002315938	0.005496836	1
T2DM	leukocyte	*TRIM2*	-0.825917888	0.00227529	0.005460974	1
T2DM	leukocyte	*SH3D19*	-1.832928053	0.001038413	0.004990048	1
T2DM	leukocyte	*GLRB*	-0.846957621	0.002831418	0.006013319	1
T2DM	leukocyte	*LRP2BP*	-0.9357478	0.003950458	0.007119885	1
T2DM	leukocyte	*UFSP2*	-1.078475429	0.003047665	0.006220403	1
T2DM	leukocyte	*KLHL5*	-1.124791563	0.001252398	0.004990048	1
T2DM	leukocyte	*FAM149A*	-0.65599437	0.020236575	0.026582917	1
T2DM	leukocyte	*DDX25*	-0.709936412	0.018599032	0.024662706	1
T2DM	leukocyte	*HTATIP2*	-1.234129714	0.000942231	0.004990048	1
T2DM	leukocyte	*B3GAT1*	-2.021826529	0.005539175	0.00872631	1
T2DM	leukocyte	*P2RX3*	-16.43355389	0.004002769	0.007145551	1
T2DM	leukocyte	*VWA5A*	-1.019029046	0.00228055	0.005462378	1
T2DM	leukocyte	*DTX4*	-1.04890237	0.001581273	0.005009694	1
T2DM	leukocyte	*FOXRED1*	-1.435888694	0.003859436	0.007033267	1
T2DM	leukocyte	*ST3GAL4*	-1.709027579	0.000394789	0.004990048	1
T2DM	leukocyte	*PRPF19*	-0.807035309	0.001704158	0.00506478	1
T2DM	leukocyte	*CEP164*	-1.627101402	0.00249012	0.005622805	1
T2DM	leukocyte	*PDHX*	-0.745663909	0.037231348	0.046512549	1
T2DM	leukocyte	*SLC1A2*	-0.830109075	0.00790346	0.011693207	1
T2DM	leukocyte	*CD5*	-3.516844017	0.008735701	0.012702654	1
T2DM	leukocyte	*ACCS*	0.44399644	0.008521753	0.012446828	1
T2DM	leukocyte	*MDK*	-0.922701958	0.000830143	0.004990048	1
T2DM	leukocyte	*MADD*	-1.291676607	0.000648825	0.004990048	1
T2DM	leukocyte	*NAA40*	-1.080923308	0.002381727	0.005560194	1
T2DM	leukocyte	*CD81*	-0.569421778	0.00330039	0.006432819	1
T2DM	leukocyte	*SLC35F2*	-1.046449453	0.000753892	0.004990048	1
T2DM	leukocyte	*NUP98*	-0.941659985	0.001581764	0.005009694	1
T2DM	leukocyte	*GTF2H1*	-1.655769513	0.000548363	0.004990048	1
T2DM	leukocyte	*PTPN5*	1.602081725	0.013609571	0.01874895	1
T2DM	leukocyte	*PRDM4*	-0.717566536	0.002407395	0.005586019	1
T2DM	leukocyte	*CORO1C*	-1.034788674	0.001968304	0.005212073	1
T2DM	leukocyte	*LIN7A*	-1.972931545	0.003034164	0.006212209	1
T2DM	leukocyte	*KRT18*	-0.297937015	0.004836763	0.007890449	1
T2DM	leukocyte	*METAP2*	-0.861296138	0.001227902	0.004990048	1
T2DM	leukocyte	*WNT5B*	-1.982947344	0.003853753	0.007029432	1
T2DM	leukocyte	*ITFG2*	-4.188652684	0.00073354	0.004990048	1
T2DM	leukocyte	*VPS29*	-0.833489826	0.000840502	0.004990048	1
T2DM	leukocyte	*CREBL2*	-1.13469325	0.001424012	0.004990048	1
T2DM	leukocyte	*GSG1*	-7.338813638	0.000560491	0.004990048	1
T2DM	leukocyte	*LTBR*	-1.045131533	0.001028823	0.004990048	1
T2DM	leukocyte	*DDX55*	-1.51665827	0.000448839	0.004990048	1
T2DM	leukocyte	*STX2*	-1.11899745	0.00392177	0.007087688	1
T2DM	leukocyte	*CAND1*	-1.067632478	0.000784804	0.004990048	1
T2DM	leukocyte	*NUP107*	-1.179529768	0.001604631	0.005033031	1

T2DM	leukocyte	*KRR1*	-0.922604249	0.001000259	0.004990048	1
T2DM	leukocyte	*GNB3*	-1.019141462	0.004862399	0.007913988	1
T2DM	leukocyte	*CDCA3*	-1.742801826	0.002739857	0.005930931	1
T2DM	leukocyte	*CHPT1*	-1.578564733	0.000346543	0.004990048	1
T2DM	leukocyte	*SPSB2*	-0.871624677	0.003759026	0.00692001	1
T2DM	leukocyte	*ATN1*	-0.966515606	0.001347036	0.004990048	1
T2DM	leukocyte	*NT5DC3*	-0.958372884	0.008256709	0.012133749	1
T2DM	leukocyte	*GOLT1B*	-0.864454269	0.001473834	0.004990048	1
T2DM	leukocyte	*PRKAB1*	-0.778373615	0.004012829	0.00714972	1
T2DM	leukocyte	*C2CD5*	-0.811710026	0.003496398	0.006633196	1
T2DM	leukocyte	*SRSF9*	-0.848976769	0.000467949	0.004990048	1
T2DM	leukocyte	*FRK*	-0.911257458	0.002703108	0.005880214	1
T2DM	leukocyte	*RSPH4A*	-0.661315635	0.04010597	0.049736615	1
T2DM	leukocyte	*SMIM8*	-1.061769993	0.001780242	0.005098084	1
T2DM	leukocyte	*NEDD9*	-0.486536804	0.003304003	0.006432819	1
T2DM	leukocyte	*CEP85L*	-0.91681058	0.002809057	0.006001505	1
T2DM	leukocyte	*HDDC2*	-0.724177086	0.001749249	0.005084476	1
T2DM	leukocyte	*TPD52L1*	-0.738023286	0.003439566	0.006566174	1
T2DM	leukocyte	*MTRF1L*	-0.866799342	0.009384808	0.01351151	1
T2DM	leukocyte	*MRPL18*	-1.271877932	0.001037941	0.004990048	1
T2DM	leukocyte	*RNF8*	-0.901076187	0.001892403	0.005146173	1
T2DM	leukocyte	*MDGA1*	1.17096093	0.025797579	0.033069719	1
T2DM	leukocyte	*CD83*	0.136086019	0.035637372	0.044677484	1
T2DM	leukocyte	*GLP1R*	-0.833613595	0.004394328	0.007406827	1
T2DM	leukocyte	*BACH2*	-0.785022087	0.002664499	0.005838039	1
T2DM	leukocyte	*FBXL4*	-1.070077762	0.001894398	0.005146173	1
T2DM	leukocyte	*ASCC3*	-0.729039031	0.002444699	0.005621365	1
T2DM	leukocyte	*WASF1*	-0.763925161	0.0039214	0.007087688	1
T2DM	leukocyte	*ALDH5A1*	-2.202861647	0.008182292	0.012040533	1
T2DM	leukocyte	*C6orf62*	-1.067534508	0.000876046	0.004990048	1
T2DM	leukocyte	*B3GAT2*	-1.327199492	0.001932464	0.005184924	1
T2DM	leukocyte	*HBS1L*	-0.927027637	0.001382657	0.004990048	1
T2DM	leukocyte	*PEX7*	-0.840922653	0.002086737	0.005283012	1
T2DM	leukocyte	*PHACTR2*	-0.698534007	0.001745108	0.005084476	1
T2DM	leukocyte	*EPM2A*	-1.504844345	0.001330063	0.004990048	1
T2DM	leukocyte	*TFEB*	-1.07878497	0.002097374	0.005283012	1
T2DM	leukocyte	*PRPH2*	-1.168868063	0.003285945	0.006432725	1
T2DM	leukocyte	*DNPH1*	-0.847029644	0.004910399	0.007970068	1
T2DM	leukocyte	*DUSP22*	-1.138478052	0.001711853	0.005068796	1
T2DM	leukocyte	*LAMA4*	-0.704320587	0.002815476	0.006001505	1
T2DM	leukocyte	*FBRSL1*	-1.074035991	0.001795612	0.005114303	1
T2DM	leukocyte	*ENPP5*	-0.672309966	0.009029068	0.013066375	1
T2DM	leukocyte	*NUDT12*	-1.157991938	0.001864407	0.005146173	1
T2DM	leukocyte	*CEP72*	-2.608847502	0.004762599	0.007793525	1
T2DM	leukocyte	*SEMA5A*	-0.990927519	0.004756064	0.007788402	1
T2DM	leukocyte	*GHR*	-2.257407784	0.004073993	0.007189	1
T2DM	leukocyte	*DAP*	-0.687008885	0.003179167	0.006330941	1
T2DM	leukocyte	*NME5*	-1.114920426	0.013934801	0.019136607	1
T2DM	leukocyte	*BRD8*	-1.049182023	0.001824361	0.00513989	1
T2DM	leukocyte	*NNT*	-1.871587808	8.17E-06	0.001605004	0.032100082
T2DM	leukocyte	*TMCO6*	1.610879378	0.008742766	0.012708224	1
T2DM	leukocyte	*IK*	-0.910931217	0.001050855	0.004990048	1
T2DM	leukocyte	*PDE8B*	-0.567481191	0.004172836	0.007242343	1
T2DM	leukocyte	*MSH3*	-1.003351346	0.001345646	0.004990048	1
T2DM	leukocyte	*CCNG1*	-1.753229959	0.000465753	0.004990048	1
T2DM	leukocyte	*DROSHA*	-0.64144394	0.011452523	0.016104854	1
T2DM	leukocyte	*GOLPH3*	-0.913993133	0.001203633	0.004990048	1
T2DM	leukocyte	*NPR3*	-2.624992609	0.003002353	0.006172668	1
T2DM	leukocyte	*RAD50*	-1.167960489	0.000894965	0.004990048	1
T2DM	leukocyte	*WWC1*	-0.478427729	0.005687247	0.008916678	1
T2DM	leukocyte	*SMAD5*	-0.80655399	0.001877098	0.005146173	1
T2DM	leukocyte	*HMGXB3*	-1.105425812	0.001955421	0.005198138	1
T2DM	leukocyte	*BNIP1*	-0.738044579	0.007331258	0.010998287	1
T2DM	leukocyte	*STC2*	-0.64487368	0.004393914	0.007406827	1

T2DM	leukocyte	*EHHADH*	-1.123335117	0.004613972	0.007636182	1
T2DM	leukocyte	*ACTR8*	-0.843640323	0.002960832	0.006135605	1
T2DM	leukocyte	*TIMMDC1*	-0.970706152	0.002314086	0.005496836	1
T2DM	leukocyte	*CLDN16*	3.276938594	0.037094069	0.046355787	1
T2DM	leukocyte	*FAM162A*	-1.126067624	0.000481431	0.004990048	1
T2DM	leukocyte	*RBP1*	-0.797175863	0.001031001	0.004990048	1
T2DM	leukocyte	*RNF7*	-0.868655848	0.00093204	0.004990048	1
T2DM	leukocyte	*SERPINI2*	-0.585890448	0.00301345	0.006182688	1
T2DM	leukocyte	*TUSC2*	-1.201253543	0.000785245	0.004990048	1
T2DM	leukocyte	*CYB561D2*	-0.846308109	0.001253719	0.004990048	1
T2DM	leukocyte	*C3orf14*	-0.869213823	0.000287586	0.004990048	1
T2DM	leukocyte	*CBLB*	-1.50840419	0.000839729	0.004990048	1
T2DM	leukocyte	*IFT57*	-0.746376206	0.003600127	0.006745301	1
T2DM	leukocyte	*NCBP2*	-0.82745132	0.001397066	0.004990048	1
T2DM	leukocyte	*C3orf52*	-0.693997901	0.006484624	0.009925239	1
T2DM	leukocyte	*SLC41A3*	-0.829307975	0.002870404	0.006055188	1
T2DM	leukocyte	*ATP6V1A*	-1.057259765	0.000932952	0.004990048	1
T2DM	leukocyte	*PODXL2*	-0.67307846	0.002482733	0.005622792	1
T2DM	leukocyte	*UPK1B*	-0.973521936	0.033413736	0.042158821	1
T2DM	leukocyte	*NEK11*	-0.809661593	0.000866896	0.004990048	1
T2DM	leukocyte	*PLSCR4*	-1.481048581	0.001308535	0.004990048	1
T2DM	leukocyte	*COMMD2*	-0.864278953	0.001077225	0.004990048	1
T2DM	leukocyte	*GORASP1*	-1.976914	0.001921118	0.005162839	1
T2DM	leukocyte	*AADAC*	-0.876054103	0.000490129	0.004990048	1
T2DM	leukocyte	*ARHGEF26*	-2.406945393	0.000658516	0.004990048	1
T2DM	leukocyte	*PLCH1*	-4.196732888	0.000473442	0.004990048	1
T2DM	leukocyte	*VIPR1*	-1.253984169	0.00873279	0.012702654	1
T2DM	leukocyte	*NKTR*	-0.85753222	0.00146002	0.004990048	1
T2DM	leukocyte	*NEK4*	-2.754307016	0.000227046	0.004990048	0.892064599
T2DM	leukocyte	*KANSL3*	-1.987352679	0.000435573	0.004990048	1
T2DM	leukocyte	*ACTR1B*	-1.565052313	0.000737354	0.004990048	1
T2DM	leukocyte	*SLC35F5*	-0.828700315	0.002901074	0.006075864	1
T2DM	leukocyte	*ACTR3*	-0.976944453	0.001182554	0.004990048	1
T2DM	leukocyte	*POMC*	-1.516408885	0.000739904	0.004990048	1
T2DM	leukocyte	*TANC1*	-0.619520301	0.014955657	0.020332448	1
T2DM	leukocyte	*REEP6*	-1.048407312	0.003774706	0.006933529	1
T2DM	leukocyte	*INO80B*	-2.887676554	0.001493022	0.004993935	1
T2DM	leukocyte	*MOGS*	-1.500180137	0.001034635	0.004990048	1
T2DM	leukocyte	*RTN4*	-0.753279846	0.000846671	0.004990048	1
T2DM	leukocyte	*HTRA2*	-1.466010308	0.001092999	0.004990048	1
T2DM	leukocyte	*GALNT3*	-0.974417278	0.002072829	0.005274707	1
T2DM	leukocyte	*MRPL19*	-0.902719296	0.00247953	0.005622792	1
T2DM	leukocyte	*WDR75*	-1.611380536	0.000961704	0.004990048	1
T2DM	leukocyte	*FANCL*	-2.014975199	0.000622265	0.004990048	1
T2DM	leukocyte	*GGCX*	-1.144228054	0.001300074	0.004990048	1
T2DM	leukocyte	*SF3B1*	-1.024110795	0.000715886	0.004990048	1
T2DM	leukocyte	*CHMP3*	-0.875364306	0.000348323	0.004990048	1
T2DM	leukocyte	*IL1R2*	-0.850111275	0.004699792	0.007719684	1
T2DM	leukocyte	*IL1R1*	-0.845027587	0.001494588	0.004993935	1
T2DM	leukocyte	*IL1RL2*	0.696973551	0.027076492	0.034528898	1
T2DM	leukocyte	*IL18R1*	0.744841791	0.010939137	0.015477086	1
T2DM	leukocyte	*MLPH*	-1.185059039	0.009289744	0.013402558	1
T2DM	leukocyte	*CNPPD1*	-0.696603954	0.00450429	0.007539852	1
T2DM	leukocyte	*HDLBP*	-0.663921114	0.001662831	0.005041098	1
T2DM	leukocyte	*PROC*	1.026782579	0.013398735	0.01849741	1
T2DM	leukocyte	*TAF1B*	-0.748408042	0.006565392	0.010030007	1
T2DM	leukocyte	*STRN*	-1.326161539	0.00733119	0.010998287	1
T2DM	leukocyte	*CEBPZ*	-0.853359866	0.001427745	0.004990048	1
T2DM	leukocyte	*COX7A2L*	-0.738429924	0.001139888	0.004990048	1
T2DM	leukocyte	*PNO1*	-0.951777508	0.001226047	0.004990048	1
T2DM	leukocyte	*ORC4*	-0.984685929	0.000882593	0.004990048	1
T2DM	leukocyte	*SUMO1*	-0.838552898	0.001060825	0.004990048	1
T2DM	leukocyte	*ALMS1*	-1.091699212	0.002045556	0.00526208	1
T2DM	leukocyte	*MARK1*	-1.556251007	0.000645406	0.004990048	1

T2DM	leukocyte	*MORN1*	-1.373884879	0.01142549	0.016072593	1
T2DM	leukocyte	*GPX7*	-1.029454696	0.000129536	0.004990048	0.50894764
T2DM	leukocyte	*SCP2*	-0.890350897	0.000926339	0.004990048	1
T2DM	leukocyte	*RALGPS2*	-0.834786808	0.002371046	0.005551753	1
T2DM	leukocyte	*ANGPTL1*	-1.650382203	0.001242094	0.004990048	1
T2DM	leukocyte	*FAM20B*	-1.01535571	0.001688775	0.005060368	1
T2DM	leukocyte	*WRAP73*	-3.533604644	0.000242611	0.004990048	0.953220402
T2DM	leukocyte	*ICMT*	-1.209451046	0.000938061	0.004990048	1
T2DM	leukocyte	*PARK7*	-0.73092898	0.001083626	0.004990048	1
T2DM	leukocyte	*MECR*	-2.680948649	0.000396808	0.004990048	1
T2DM	leukocyte	*EDEM3*	-1.046203159	0.0009762	0.004990048	1
T2DM	leukocyte	*FBXO6*	-4.339072959	0.000490864	0.004990048	1
T2DM	leukocyte	*SMG7*	-0.891951793	0.001623272	0.005035955	1
T2DM	leukocyte	*PDC*	-8.959678821	0.003069158	0.006249984	1
T2DM	leukocyte	*BCAS2*	-0.792603545	0.001662029	0.005041098	1
T2DM	leukocyte	*AGMAT*	-1.365308538	0.002162346	0.005345956	1
T2DM	leukocyte	*PLEKHM2*	-2.292911953	0.000540935	0.004990048	1
T2DM	leukocyte	*CRYZ*	-0.60711091	0.00311239	0.006284318	1
T2DM	leukocyte	*TMEM9*	-0.89793189	0.000892108	0.004990048	1
T2DM	leukocyte	*WARS2*	-0.860916476	0.003770231	0.00692855	1
T2DM	leukocyte	*C1orf109*	-0.651447594	0.004467511	0.007498014	1
T2DM	leukocyte	*LGALS8*	-1.186041806	0.000653266	0.004990048	1
T2DM	leukocyte	*HPCAL4*	-2.227629641	0.004563926	0.007578894	1
T2DM	leukocyte	*BMP8B*	-0.769707258	0.003698137	0.006868344	1
T2DM	leukocyte	*MYCL*	0.415569513	0.011111624	0.015698515	1
T2DM	leukocyte	*UAP1*	-0.639057774	0.002361332	0.005542217	1
T2DM	leukocyte	*CTBS*	-1.145170821	0.000512605	0.004990048	1
T2DM	leukocyte	*ZNHIT6*	-0.614051995	0.004577715	0.007595372	1
T2DM	leukocyte	*CD46*	-0.712731187	0.001380579	0.004990048	1
T2DM	leukocyte	*SLC2A1*	-1.200655775	0.002600525	0.005751212	1
T2DM	leukocyte	*PRDX1*	-0.773853691	0.001045663	0.004990048	1
T2DM	leukocyte	*CCDC181*	-0.726387925	0.001520184	0.004993935	1
T2DM	leukocyte	*NSUN4*	-3.429375454	0.000103893	0.004990048	0.408197123
T2DM	leukocyte	*TMED5*	-1.279690131	0.000625652	0.004990048	1
T2DM	leukocyte	*DR1*	-1.158686626	0.001656334	0.005041098	1
T2DM	leukocyte	*PTBP2*	-0.874613972	0.00366228	0.006825948	1
T2DM	leukocyte	*NSL1*	-1.428785581	0.000909259	0.004990048	1
T2DM	leukocyte	*RPA2*	-1.076506942	0.003272188	0.006415383	1
T2DM	leukocyte	*ESYT2*	-1.026722293	0.001093365	0.004990048	1
T2DM	leukocyte	*DDX59*	-0.7226067	0.004072711	0.007189	1
T2DM	leukocyte	*TTR*	-0.34692105	0.001755445	0.005084476	1
T2DM	leukocyte	*SPCS2*	-0.833878785	0.000902731	0.004990048	1
T2DM	leukocyte	*ELOVL4*	-1.261566084	0.002176114	0.005349178	1
T2DM	leukocyte	*FILIP1*	-0.847776018	0.007904596	0.011693207	1
T2DM	leukocyte	*AKAP7*	-1.919868958	0.000747918	0.004990048	1
T2DM	leukocyte	*RNF146*	-0.984718562	0.001899295	0.005146173	1
T2DM	leukocyte	*MED28*	-1.412864746	0.000628253	0.004990048	1
T2DM	leukocyte	*VAMP8*	-0.945889821	0.00107764	0.004990048	1
T2DM	leukocyte	*ARMC2*	-0.82899864	0.001659286	0.005041098	1
T2DM	leukocyte	*PKD2*	-0.710036634	0.002691383	0.005864439	1
T2DM	leukocyte	*SPP1*	-0.441876644	0.000816109	0.004990048	1
T2DM	leukocyte	*WDR35*	-0.759989532	0.004266699	0.007310294	1
T2DM	leukocyte	*DNAH7*	-1.262699733	1.97E-05	0.0030497	0.077349156
T2DM	leukocyte	*NDUFB3*	-0.818044079	0.001748308	0.005084476	1
T2DM	leukocyte	*GTF3C3*	-1.176224123	0.005372161	0.008521283	1
T2DM	leukocyte	*CPSF3*	-1.247910774	0.00276556	0.005956822	1
T2DM	leukocyte	*CCDC92*	-0.966432871	0.001218572	0.004990048	1
T2DM	leukocyte	*CTNNAL1*	-0.583932805	0.012574682	0.017495017	1
T2DM	leukocyte	*NEK6*	-0.797455508	0.020142633	0.026486122	1
T2DM	leukocyte	*BSPRY*	-0.789235901	0.001485965	0.004990048	1
T2DM	leukocyte	*RBM18*	-1.152019839	0.001442124	0.004990048	1
T2DM	leukocyte	*INVS*	-0.574699626	0.037406129	0.046701202	1
T2DM	leukocyte	*DCAF4*	-0.924598134	0.011268375	0.015873701	1
T2DM	leukocyte	*IFI27L2*	-0.79136496	0.001291634	0.004990048	1

T2DM	leukocyte	*ACOT2*	-1.033635609	0.02503435	0.032217478	1
T2DM	leukocyte	*ZC2HC1C*	-2.70632553	0.002882742	0.006056841	1
T2DM	leukocyte	*KLHL29*	-2.421204571	0.017081397	0.022921042	1
T2DM	leukocyte	*AFTPH*	-0.865661324	0.00215522	0.005335765	1
T2DM	leukocyte	*BCL11A*	-1.264367904	0.008947625	0.012962839	1
T2DM	leukocyte	*CRIPT*	-1.089102983	0.000961858	0.004990048	1
T2DM	leukocyte	*EPCAM*	-0.56357884	0.001551914	0.005009694	1
T2DM	leukocyte	*SLC17A5*	-3.034610195	0.000420457	0.004990048	1
T2DM	leukocyte	*CUTC*	-0.936213694	0.00642164	0.009848019	1
T2DM	leukocyte	*PYROXD2*	-1.289813449	0.005175203	0.00827906	1
T2DM	leukocyte	*HELLS*	-1.04033651	0.002429243	0.005602682	1
T2DM	leukocyte	*PRLHR*	-1.156808491	0.004311172	0.007328649	1
T2DM	leukocyte	*WDR11*	-1.556305086	0.000500258	0.004990048	1
T2DM	leukocyte	*KANSL1*	-0.990045178	0.000854623	0.004990048	1
T2DM	leukocyte	*HOXB3*	-1.033989148	0.010478804	0.014891758	1
T2DM	leukocyte	*RCL1*	-1.637957472	0.000721894	0.004990048	1
T2DM	leukocyte	*CD274*	-2.697980417	0.000919482	0.004990048	1
T2DM	leukocyte	*PLEKHG1*	-0.80073283	0.006144448	0.009482144	1
T2DM	leukocyte	*PCDHB10*	-1.53093968	0.00326102	0.006405338	1
T2DM	leukocyte	*MRPS14*	-1.450548144	0.001905672	0.00515389	1
T2DM	leukocyte	*GORAB*	-0.847963938	0.004009756	0.00714972	1
T2DM	leukocyte	*SNX19*	-0.830698915	0.001538711	0.005006447	1
T2DM	leukocyte	*KCNJ5*	0.296473505	0.004227063	0.007272975	1
T2DM	leukocyte	*SLC10A7*	-2.042170425	0.000136422	0.004990048	0.536001278
T2DM	leukocyte	*MASTL*	-0.993572169	0.000557119	0.004990048	1
T2DM	leukocyte	*MRC1*	0.208506418	0.033924699	0.042693832	1
T2DM	leukocyte	*IQSEC3*	-0.942332055	0.002529955	0.005660135	1
T2DM	leukocyte	*DNAJC15*	-0.977503071	0.000468307	0.004990048	1
T2DM	leukocyte	*PROSER1*	-1.208701955	0.001168108	0.004990048	1
T2DM	leukocyte	*WBP4*	-0.893978193	0.001464603	0.004990048	1
T2DM	leukocyte	*KBTBD7*	-2.004375322	0.000386433	0.004990048	1
T2DM	leukocyte	*FAM53C*	-2.154167297	9.49E-05	0.004990048	0.373042938
T2DM	leukocyte	*SIL1*	-0.940659034	0.000785516	0.004990048	1
T2DM	leukocyte	*PLS1*	-0.691652117	0.004132712	0.007236049	1
T2DM	leukocyte	*ZFP30*	-1.058651863	0.006202516	0.009552993	1
T2DM	leukocyte	*UTP20*	-1.486452565	0.011582551	0.016276053	1
T2DM	leukocyte	*TMPO*	-1.332560289	0.001221936	0.004990048	1
T2DM	leukocyte	*PPP3CC*	-1.086139131	0.001776792	0.005098084	1
T2DM	leukocyte	*EPHX2*	-1.06737385	0.001451368	0.004990048	1
T2DM	leukocyte	*CRISPLD1*	-5.100608949	0.001910196	0.00515389	1
T2DM	leukocyte	*PYROXD1*	-0.777352521	0.00099709	0.004990048	1
T2DM	leukocyte	*IAPP*	-0.265826773	0.009304768	0.0134061	1
T2DM	leukocyte	*ZSCAN18*	-0.758922152	0.002023478	0.005244224	1
T2DM	leukocyte	*LHX4*	-2.408571281	0.005360115	0.008509048	1
T2DM	leukocyte	*TRMT1L*	-0.887683123	0.003423179	0.006544851	1
T2DM	leukocyte	*CAT*	-2.190583055	0.000195861	0.004990048	0.769536992
T2DM	leukocyte	*CSMD2*	0.934624549	0.02602211	0.033324925	1
T2DM	leukocyte	*ACVR2A*	-0.650387936	0.015156188	0.020576594	1
T2DM	leukocyte	*POLK*	-1.442726322	0.000163167	0.004990048	0.641082067
T2DM	leukocyte	*UBL3*	-0.788960724	0.001291827	0.004990048	1
T2DM	leukocyte	*FMOD*	-1.129843569	0.008031848	0.011844895	1
T2DM	leukocyte	*LDB3*	-1.306488411	0.005289143	0.008423609	1
T2DM	leukocyte	*NAA60*	-1.209107528	0.000820878	0.004990048	1
T2DM	leukocyte	*TRMT13*	-2.122958744	0.000767605	0.004990048	1
T2DM	leukocyte	*RWDD3*	-0.899510598	0.001589447	0.005020048	1
T2DM	leukocyte	*CCDC18*	-0.846493156	0.005048523	0.008126033	1
T2DM	leukocyte	*NBPF14*	-2.24890083	0.00541875	0.008574413	1
T2DM	leukocyte	*PMS2*	-1.686696829	0.003439983	0.006566174	1
T2DM	leukocyte	*EEPD1*	0.400009786	0.033846163	0.0426223	1
T2DM	leukocyte	*KLHL7*	-1.264267097	0.001540418	0.005006447	1
T2DM	leukocyte	*CBX3*	-0.978039582	0.001001635	0.004990048	1
T2DM	leukocyte	*NPY*	-0.414439469	0.002531198	0.005660135	1
T2DM	leukocyte	*INHBA*	-0.797520836	0.001872302	0.005146173	1
T2DM	leukocyte	*CCZ1*	-1.265238774	0.002026753	0.00524925	1

T2DM	leukocyte	*POLM*	-0.948792279	0.000857022	0.004990048	1
T2DM	leukocyte	*SMU1*	-1.048839558	0.00089029	0.004990048	1
T2DM	leukocyte	*SLC25A51*	-0.873494985	0.014366028	0.019667292	1
T2DM	leukocyte	*ACO1*	-0.805991482	0.003627857	0.006781089	1
T2DM	leukocyte	*RBM19*	-0.743449585	0.004365155	0.007376642	1
T2DM	leukocyte	*BHLHE41*	-1.483682604	0.002338147	0.005515664	1
T2DM	leukocyte	*ITPR2*	-2.146251146	0.004201666	0.007256417	1
T2DM	leukocyte	*EBPL*	-0.748593909	0.002941595	0.006124816	1
T2DM	leukocyte	*ATP7B*	-1.345465746	0.008261696	0.012134656	1
T2DM	leukocyte	*TSFM*	-1.241350625	0.000911168	0.004990048	1
T2DM	leukocyte	*CDK2*	-1.295681194	0.02558443	0.032828617	1
T2DM	leukocyte	*IKZF4*	-1.1953966	0.00510499	0.00819008	1
T2DM	leukocyte	*USP45*	-0.679118147	0.006080823	0.009402422	1
T2DM	leukocyte	*C4BPB*	-1.966620721	0.01450015	0.019816031	1
T2DM	leukocyte	*RAB38*	-2.033486542	0.021712615	0.028370092	1
T2DM	leukocyte	*CKS2*	-0.783117235	0.004101137	0.00720956	1
T2DM	leukocyte	*SNX21*	-0.841656004	0.007343511	0.011008262	1
T2DM	leukocyte	*ARHGAP40*	-45.22893927	0.002976973	0.006152298	1
T2DM	leukocyte	*NCOA3*	-0.892280669	0.003335795	0.00646588	1
T2DM	leukocyte	*MATN4*	-6.229855173	0.002363236	0.005543376	1
T2DM	leukocyte	*VAPB*	-0.856475562	0.000699098	0.004990048	1
T2DM	leukocyte	*PARD6B*	-1.14516177	0.001684261	0.005060368	1
T2DM	leukocyte	*ARFGEF2*	-1.146598651	0.00114194	0.004990048	1
T2DM	leukocyte	*ZNFX1*	-3.222537919	0.000357426	0.004990048	1
T2DM	leukocyte	*CSE1L*	-1.110427386	0.000981576	0.004990048	1
T2DM	leukocyte	*BCAS4*	-0.617023099	0.004536403	0.007568852	1
T2DM	leukocyte	*KCNK15*	-0.60815035	0.035713862	0.044744823	1
T2DM	leukocyte	*PEPD*	-1.621867208	0.000150642	0.004990048	0.591873793
T2DM	leukocyte	*SNRNP27*	-0.86825024	0.001870566	0.005146173	1
T2DM	leukocyte	*USP22*	-1.071050255	0.001331166	0.004990048	1
T2DM	leukocyte	*BTN2A2*	-1.221388101	0.018969815	0.025084163	1
T2DM	leukocyte	*MRS2*	-1.539724453	0.00082295	0.004990048	1
T2DM	leukocyte	*SNRPC*	-0.99940405	0.000758611	0.004990048	1
T2DM	leukocyte	*SERPINB6*	-0.860944243	0.000946095	0.004990048	1
T2DM	leukocyte	*ABCC10*	-1.055061271	0.004523861	0.007557079	1
T2DM	leukocyte	*PEX6*	-0.976882427	0.000938151	0.004990048	1
T2DM	leukocyte	*NQO2*	-2.432109595	0.000132983	0.004990048	0.522489091
T2DM	leukocyte	*UNC5CL*	-0.824756544	0.002667187	0.005838092	1
T2DM	leukocyte	*ZNF391*	-0.615314774	0.027308493	0.0347796	1
T2DM	leukocyte	*MED20*	-1.336386682	0.003003854	0.006172668	1
T2DM	leukocyte	*GLO1*	-0.952531712	0.00142711	0.004990048	1
T2DM	leukocyte	*KCNK17*	-0.579502191	0.012688018	0.017627731	1
T2DM	leukocyte	*ATXN1*	-1.250136209	0.000800501	0.004990048	1
T2DM	leukocyte	*LRRFIP1*	-0.746028632	0.001258937	0.004990048	1
T2DM	leukocyte	*CXCL6*	-1.119661013	0.024407497	0.031503632	1
T2DM	leukocyte	*SH3TC1*	-0.965412437	0.002316814	0.005496836	1
T2DM	leukocyte	*MT1G*	-0.59465237	0.012158125	0.016987651	1
T2DM	leukocyte	*DOK4*	-0.584527085	0.00687167	0.010409317	1
T2DM	leukocyte	*CLYBL*	-2.266341215	0.001098395	0.004990048	1
T2DM	leukocyte	*TM9SF2*	-0.799730691	0.001605125	0.005033031	1
T2DM	leukocyte	*KIF25*	-12.68423923	0.001672962	0.005048438	1
T2DM	leukocyte	*BMP4*	-2.226521724	0.000424868	0.004990048	1
T2DM	leukocyte	*HS3ST3B1*	-1.963708204	0.00426155	0.007310294	1
T2DM	leukocyte	*MRPS7*	-0.745186356	0.001955363	0.005198138	1
T2DM	leukocyte	*GGA3*	-2.148854203	0.000899017	0.004990048	1
T2DM	leukocyte	*DDX31*	-1.189914371	0.020674288	0.027094489	1
T2DM	leukocyte	*PPDPF*	-0.691774601	0.001133692	0.004990048	1
T2DM	leukocyte	*CHCHD5*	-0.818519396	0.001450118	0.004990048	1
T2DM	leukocyte	*INSIG2*	-0.777463422	0.001730301	0.005070552	1
T2DM	leukocyte	*POLR1B*	-0.693133205	0.039316172	0.048883937	1
T2DM	leukocyte	*PSD4*	-1.007041247	0.012582047	0.017499066	1
T2DM	leukocyte	*CLPP*	-0.900413638	0.001473385	0.004990048	1
T2DM	leukocyte	*GPR108*	-1.217037268	0.002516231	0.005655212	1
T2DM	leukocyte	*FOSB*	-1.448504369	0.001198396	0.004990048	1

T2DM	leukocyte	*SYMPK*	-1.888559673	0.000633651	0.004990048	1
T2DM	leukocyte	*NAPB*	-0.820153322	0.002527902	0.005660135	1
T2DM	leukocyte	*PSMF1*	-1.081737118	0.000645153	0.004990048	1
T2DM	leukocyte	*RBCK1*	-0.905505922	0.000854918	0.004990048	1
T2DM	leukocyte	*FLRT3*	-0.734050793	0.003710576	0.006873574	1
T2DM	leukocyte	*MGME1*	-3.710314628	0.000975892	0.004990048	1
T2DM	leukocyte	*ITPA*	-1.254728547	0.000913313	0.004990048	1
T2DM	leukocyte	*MCM8*	-0.898902588	0.012917338	0.017908335	1
T2DM	leukocyte	*BANF2*	-11.65446282	0.004181065	0.007242343	1
T2DM	leukocyte	*MRPS26*	-0.727724644	0.002869773	0.006055188	1
T2DM	leukocyte	*ZNF436*	-2.250096463	0.000826037	0.004990048	1
T2DM	leukocyte	*TUBGCP3*	-1.094031514	0.001881303	0.005146173	1
T2DM	leukocyte	*MCF2L*	-0.578315523	0.009292076	0.013402558	1
T2DM	leukocyte	*IGFLR1*	0.262185413	0.02311258	0.029979969	1
T2DM	leukocyte	*NR1D1*	-0.782971473	0.001645607	0.005035955	1
T2DM	leukocyte	*PRMT1*	-0.848327863	0.00031152	0.004990048	1
T2DM	leukocyte	*PRR12*	-0.716975215	0.019627313	0.025877756	1
T2DM	leukocyte	*FLRT1*	-1.980850408	0.014905758	0.020278644	1
T2DM	leukocyte	*ASL*	-0.965246185	0.001762549	0.005084476	1
T2DM	leukocyte	*WNK4*	-0.875712044	0.027139467	0.034597977	1
T2DM	leukocyte	*BECN1*	-0.935306197	0.002268478	0.005457772	1
T2DM	leukocyte	*TRAP1*	-0.681526921	0.005947416	0.009239777	1
T2DM	leukocyte	*ZNF384*	-1.135812384	0.001870865	0.005146173	1
T2DM	leukocyte	*RHOJ*	-1.473004702	0.003295613	0.006432819	1
T2DM	leukocyte	*L3HYPDH*	-1.053127367	0.004667674	0.007686961	1
T2DM	leukocyte	*HSPA2*	-1.537663813	0.001514806	0.004993935	1
T2DM	leukocyte	*ZBTB1*	-1.304525483	0.000682057	0.004990048	1
T2DM	leukocyte	*PLEKHG3*	-1.275057014	0.002052331	0.00526208	1
T2DM	leukocyte	*FGD3*	2.075541962	0.036040564	0.045139743	1
T2DM	leukocyte	*BCL11B*	1.330647702	0.023227893	0.030105511	1
T2DM	leukocyte	*ABHD8*	-1.658620976	0.01209469	0.016905029	1
T2DM	leukocyte	*HELB*	-3.906509739	0.033995775	0.042769367	1
T2DM	leukocyte	*BEST3*	-2.291057948	0.002977793	0.006152298	1
T2DM	leukocyte	*YEATS4*	-0.835943767	0.002144765	0.005323324	1
T2DM	leukocyte	*LRRC61*	-1.32482192	0.002354548	0.005532907	1
T2DM	leukocyte	*IDUA*	-0.874452106	0.003350186	0.006474609	1
T2DM	leukocyte	*FGFRL1*	-0.973047533	0.000717867	0.004990048	1
T2DM	leukocyte	*EMC1*	-0.991759325	0.003158747	0.006314657	1
T2DM	leukocyte	*UBR4*	-1.012972077	0.001142279	0.004990048	1
T2DM	leukocyte	*SYNGR3*	-0.962305975	0.007898016	0.011692277	1
T2DM	leukocyte	*WFIKKN1*	-0.709916094	0.025982465	0.033285003	1
T2DM	leukocyte	*KDM4B*	-2.319017504	0.000291106	0.004990048	1
T2DM	leukocyte	*PNKD*	-1.223828948	0.000602943	0.004990048	1
T2DM	leukocyte	*PTPN12*	-1.223507971	2.68E-05	0.003310187	0.105377589
T2DM	leukocyte	*POR*	-0.984161892	0.001454273	0.004990048	1
T2DM	leukocyte	*RBM48*	-1.771055964	0.000777522	0.004990048	1
T2DM	leukocyte	*SRD5A3*	-1.14948588	0.002185945	0.005356498	1
T2DM	leukocyte	*PPAT*	-1.170179192	0.000567724	0.004990048	1
T2DM	leukocyte	*A4GALT*	-0.70650296	0.008661015	0.0126174	1
T2DM	leukocyte	*RIBC2*	2.659439609	0.034727086	0.043598559	1
T2DM	leukocyte	*NAA38*	-0.752993581	0.001452697	0.004990048	1
T2DM	leukocyte	*PRKRIP1*	-0.871733031	0.002000993	0.005227473	1
T2DM	leukocyte	*VGF*	-0.465996176	0.001938867	0.00518571	1
T2DM	leukocyte	*IRF5*	0.335550847	0.01734104	0.023213951	1
T2DM	leukocyte	*MTX2*	-1.343321612	0.000755784	0.004990048	1
T2DM	leukocyte	*GAD1*	-3.428085776	0.001171242	0.004990048	1
T2DM	leukocyte	*ORMDL1*	-0.868970527	0.001700593	0.005060368	1
T2DM	leukocyte	*PSMG2*	-1.024881086	0.001389751	0.004990048	1
T2DM	leukocyte	*TWSG1*	-1.430569749	0.000741055	0.004990048	1
T2DM	leukocyte	*EIF2AK4*	-1.573692332	9.76E-05	0.004990048	0.383612134
T2DM	leukocyte	*MYO5C*	-0.628550098	0.002821055	0.006006579	1
T2DM	leukocyte	*CGNL1*	-1.095275434	0.000810496	0.004990048	1
T2DM	leukocyte	*TMOD2*	-1.015493414	0.0018238	0.00513989	1
T2DM	leukocyte	*ELL3*	-2.81601822	0.001264379	0.004990048	1

T2DM	leukocyte	*IVD*	-1.046808278	0.010716074	0.015172416	1
T2DM	leukocyte	*CHAC1*	-1.113805837	0.001220085	0.004990048	1
T2DM	leukocyte	*ACKR4*	-1.307945957	0.030804333	0.039016835	1
T2DM	leukocyte	*ANAPC13*	-0.547320981	0.006772187	0.010281269	1
T2DM	leukocyte	*SUMF2*	-1.069810028	0.001039941	0.004990048	1
T2DM	leukocyte	*TPH1*	-2.343811566	0.025230044	0.032437448	1
T2DM	leukocyte	*DCTD*	-1.101993809	0.001340066	0.004990048	1
T2DM	leukocyte	*AP1M2*	-0.824401575	0.001671321	0.005048289	1
T2DM	leukocyte	*CDKN2D*	-0.89028868	0.000891847	0.004990048	1
T2DM	leukocyte	*MTUS1*	-0.716235428	0.003326555	0.006456709	1
T2DM	leukocyte	*ADCY4*	-0.904755721	0.005737628	0.008970609	1
T2DM	leukocyte	*DTD2*	-0.751874345	0.003968918	0.007124893	1
T2DM	leukocyte	*HEATR5A*	-1.966354495	0.00053408	0.004990048	1
T2DM	leukocyte	*MIS18BP1*	-1.553994303	0.006781737	0.010291791	1
T2DM	leukocyte	*RNASE1*	0.129733825	0.024768804	0.031917557	1
T2DM	leukocyte	*EPB41L4A*	-0.983329166	0.004108547	0.007219356	1
T2DM	leukocyte	*REEP5*	-0.765738578	0.001259869	0.004990048	1
T2DM	leukocyte	*MAU2*	-3.027396751	5.44E-07	0.000266998	0.002135982
T2DM	leukocyte	*SHC2*	-1.031123466	0.001341645	0.004990048	1
T2DM	leukocyte	*CBFA2T3*	-0.838963967	0.003993181	0.007143456	1
T2DM	leukocyte	*ERMARD*	-1.846198983	0.000943306	0.004990048	1
T2DM	leukocyte	*PHF10*	-0.936832173	0.001462558	0.004990048	1
T2DM	leukocyte	*SH3BP4*	-0.449167085	0.014696635	0.020042722	1
T2DM	leukocyte	*DOCK6*	-0.653380058	0.004929245	0.007979813	1
T2DM	leukocyte	*ECSIT*	-1.042456943	0.002097211	0.005283012	1
T2DM	leukocyte	*CDC16*	-1.392999077	0.000312624	0.004990048	1
T2DM	leukocyte	*DPP6*	-0.467088476	0.003755807	0.00692001	1
T2DM	leukocyte	*GTPBP3*	-1.115431243	0.000252745	0.004990048	0.993033826
T2DM	leukocyte	*PLVAP*	-0.428051888	0.005031832	0.008102486	1
T2DM	leukocyte	*MRPL34*	-0.772557423	0.001144409	0.004990048	1
T2DM	leukocyte	*LSM7*	-0.584959825	0.005750862	0.008987724	1
T2DM	leukocyte	*TULP4*	-0.842454982	0.004533875	0.007568852	1
T2DM	leukocyte	*QRSL1*	-0.977074485	0.00291991	0.006102301	1
T2DM	leukocyte	*RSPH3*	-1.016353108	0.002571103	0.005704045	1
T2DM	leukocyte	*STK33*	-0.790004127	0.001345613	0.004990048	1
T2DM	leukocyte	*NDUFA10*	-0.887465792	0.001535089	0.005006447	1
T2DM	leukocyte	*TRPM4*	-3.386663174	0.00647968	0.009921536	1
T2DM	leukocyte	*UBAC1*	-0.922543249	0.001081721	0.004990048	1
T2DM	leukocyte	*ATXN10*	-0.769132726	0.001497407	0.004993935	1
T2DM	leukocyte	*PNPLA7*	-1.747112239	0.002673981	0.005846451	1
T2DM	leukocyte	*PAK4*	-1.157499827	0.001419162	0.004990048	1
T2DM	leukocyte	*LAMA5*	-0.601543333	0.003630087	0.006782032	1
T2DM	leukocyte	*ADRM1*	-0.881125053	0.001122456	0.004990048	1
T2DM	leukocyte	*UCK1*	-1.747940376	0.000334786	0.004990048	1
T2DM	leukocyte	*MAP3K10*	-2.188931332	0.000699751	0.004990048	1
T2DM	leukocyte	*SMPDL3B*	-0.725029338	0.00490524	0.007970068	1
T2DM	leukocyte	*CLIP1*	-1.015893425	0.002244165	0.005438799	1
T2DM	leukocyte	*ZNF317*	-1.173737904	0.002723893	0.005904436	1
T2DM	leukocyte	*DNMT1*	-1.510384722	0.000972553	0.004990048	1
T2DM	leukocyte	*ZNF426*	-1.373758625	0.000643667	0.004990048	1
T2DM	leukocyte	*LRP3*	-1.339976727	0.0037313	0.006898954	1
T2DM	leukocyte	*HSD17B3*	-11.99808734	0.00110997	0.004990048	1
T2DM	leukocyte	*SYNE1*	-0.804256832	0.001728099	0.005070552	1
T2DM	leukocyte	*EPS8L1*	-2.782216789	0.002822399	0.006006579	1
T2DM	leukocyte	*C1QL1*	-0.378338922	0.00579348	0.009041044	1
T2DM	leukocyte	*F12*	-0.916714042	0.010590793	0.015022103	1
T2DM	leukocyte	*TRAF3*	-0.628902095	0.011944409	0.016712814	1
T2DM	leukocyte	*PDLIM4*	-1.027610012	0.001409141	0.004990048	1
T2DM	leukocyte	*MGAT1*	-2.059578283	0.007920298	0.011707619	1
T2DM	leukocyte	*DIAPH1*	-0.680986298	0.002691605	0.005864439	1
T2DM	leukocyte	*EXOC4*	-1.791501352	0.001907032	0.00515389	1
T2DM	leukocyte	*C1orf159*	-3.056494605	0.005889697	0.009160973	1
T2DM	leukocyte	*ANO1*	-2.18536571	0.002384977	0.005560842	1
T2DM	leukocyte	*PPFIA1*	-0.799900571	0.002166685	0.005345956	1

T2DM	leukocyte	*TRAF7*	-1.208890208	0.001475579	0.004990048	1
T2DM	leukocyte	*MAP1B*	-0.512050286	0.006557046	0.010024372	1
T2DM	leukocyte	*CHD1L*	-2.11815861	0.001511325	0.004993935	1
T2DM	leukocyte	*PIAS3*	-0.673943954	0.004649839	0.007681478	1
T2DM	leukocyte	*RBM8A*	-0.71410442	0.000407956	0.004990048	1
T2DM	leukocyte	*ZNF304*	-4.227554986	0.000358301	0.004990048	1
T2DM	leukocyte	*ZNF132*	1.013038418	0.021638323	0.028282425	1
T2DM	leukocyte	*RHPN2*	-0.825114866	0.003997105	0.007143456	1
T2DM	leukocyte	*GCH1*	-0.623415801	0.003372014	0.006497619	1
T2DM	leukocyte	*CC2D1A*	-1.321419673	0.000946291	0.004990048	1
T2DM	leukocyte	*CCT6B*	-1.459495126	0.001505307	0.004993935	1
T2DM	leukocyte	*PPARG*	-1.672888635	0.000504141	0.004990048	1
T2DM	leukocyte	*ENOSF1*	-2.449250356	0.000351962	0.004990048	1
T2DM	leukocyte	*IMMT*	-0.984839075	0.00146531	0.004990048	1
T2DM	leukocyte	*MRPL35*	-0.906918892	0.004508757	0.007541467	1
T2DM	leukocyte	*CARD6*	-1.765061217	0.002251852	0.005441283	1
T2DM	leukocyte	*INPP5K*	-0.814435384	0.013273188	0.018344791	1
T2DM	leukocyte	*RPA1*	-1.050092607	0.003695203	0.006868344	1
T2DM	leukocyte	*TMEM128*	-1.428920906	0.000852356	0.004990048	1
T2DM	leukocyte	*SEC61G*	-0.711411651	0.000984117	0.004990048	1
T2DM	leukocyte	*FIGNL1*	-2.774323139	0.003177194	0.006330221	1
T2DM	leukocyte	*TRIM47*	-0.71189886	0.014542375	0.01985302	1
T2DM	leukocyte	*EIF5A*	-0.789092685	0.001094465	0.004990048	1
T2DM	leukocyte	*PRMT7*	-1.589310554	0.019920748	0.026220642	1
T2DM	leukocyte	*ANKEF1*	-0.743934858	0.003973184	0.007124893	1
T2DM	leukocyte	*BTBD3*	-0.759724872	0.001719721	0.005070552	1
T2DM	leukocyte	*IGHMBP2*	-1.209758547	0.000863944	0.004990048	1
T2DM	leukocyte	*LPIN3*	-0.998878311	0.029834786	0.03786204	1
T2DM	leukocyte	*RBM38*	-1.589105724	0.000318796	0.004990048	1
T2DM	leukocyte	*VSTM2L*	-0.684458925	0.00695867	0.010520217	1
T2DM	leukocyte	*SERINC3*	-0.897198632	0.001504468	0.004993935	1
T2DM	leukocyte	*BHMT2*	-1.29160758	0.000223793	0.004990048	0.879281151
T2DM	leukocyte	*CASP9*	-1.589706182	0.011188432	0.015789997	1
T2DM	leukocyte	*WASF3*	-0.934118777	0.002857656	0.006048134	1
T2DM	leukocyte	*SCO1*	-1.649567734	0.000991734	0.004990048	1
T2DM	leukocyte	*CHI3L1*	-0.63671119	0.026755976	0.03416452	1
T2DM	leukocyte	*SLC41A1*	-1.010113367	0.002600826	0.005751212	1
T2DM	leukocyte	*TRPC4*	-2.602454065	0.002524154	0.005660135	1
T2DM	leukocyte	*POSTN*	0.734504327	0.021614705	0.028260957	1
T2DM	leukocyte	*RFC3*	-0.718013495	0.004164904	0.007242343	1
T2DM	leukocyte	*EPHB2*	-3.370558711	0.003302229	0.006432819	1
T2DM	leukocyte	*BTBD2*	-2.229076483	0.000482028	0.004990048	1
T2DM	leukocyte	*CNDP2*	-0.932737821	0.00162999	0.005035955	1
T2DM	leukocyte	*WDR74*	-1.541436778	0.000414018	0.004990048	1
T2DM	leukocyte	*GSTT2B*	-0.791792034	0.004889897	0.007948864	1
T2DM	leukocyte	*SLC2A11*	-1.527510218	0.000829714	0.004990048	1
T2DM	leukocyte	*C1QTNF6*	-1.405697128	0.004171706	0.007242343	1
T2DM	leukocyte	*FAM83F*	-0.998346927	0.001700374	0.005060368	1
T2DM	leukocyte	*GIMAP6*	-1.805320176	0.001143526	0.004990048	1
T2DM	leukocyte	*AGAP3*	-3.171062937	0.000332234	0.004990048	1
T2DM	leukocyte	*SFTPD*	-0.725344748	0.022666346	0.029430296	1
T2DM	leukocyte	*IMPA1*	-0.917338521	0.000920869	0.004990048	1
T2DM	leukocyte	*LRRCC1*	-1.404715895	0.002313982	0.005496836	1
T2DM	leukocyte	*RRAS2*	-0.755546159	0.003029863	0.006206637	1
T2DM	leukocyte	*HSD17B4*	-1.774697131	0.000304118	0.004990048	1
T2DM	leukocyte	*COX16*	-0.911715604	0.000922951	0.004990048	1
T2DM	leukocyte	*ELP3*	-1.956163395	0.000798895	0.004990048	1
T2DM	leukocyte	*PEBP4*	-1.111314292	0.00825799	0.012133749	1
T2DM	leukocyte	*MRO*	-2.477923163	0.002232339	0.005430873	1
T2DM	leukocyte	*MRPS36*	-0.881140393	0.005304275	0.008434033	1
T2DM	leukocyte	*THUMPD3*	-1.102785745	0.00047569	0.004990048	1
T2DM	leukocyte	*CHL1*	-0.828646959	0.020074138	0.026413694	1
T2DM	leukocyte	*REG4*	-0.963245662	0.004660367	0.007686961	1
T2DM	leukocyte	*GSTM5*	-1.428407496	0.02562446	0.032869248	1

T2DM	leukocyte	*GSTM3*	-0.593227703	0.006763593	0.010278358	1
T2DM	leukocyte	*PTPN22*	-4.267293523	0.013569125	0.018706349	1
T2DM	leukocyte	*SORT1*	-1.185799922	0.001001274	0.004990048	1
T2DM	leukocyte	*NOTCH2*	-2.710476667	9.16E-12	3.60E-08	3.60E-08
T2DM	leukocyte	*CEPT1*	-1.617346856	0.001226489	0.004990048	1
T2DM	leukocyte	*NAPG*	-0.931443162	0.001599781	0.005024491	1
T2DM	leukocyte	*SPIRE1*	-0.989911403	0.000999628	0.004990048	1
T2DM	leukocyte	*FKBP11*	-0.546332889	0.002601146	0.005751212	1
T2DM	leukocyte	*KIDINS220*	-1.010559837	0.00101178	0.004990048	1
T2DM	leukocyte	*LPIN1*	-0.653818534	0.002997957	0.006172668	1
T2DM	leukocyte	*IAH1*	-1.207787165	0.001070232	0.004990048	1
T2DM	leukocyte	*ANO3*	1.187725357	0.026116407	0.033434787	1
T2DM	leukocyte	*IL2RA*	0.247966468	0.019869571	0.026163627	1
T2DM	leukocyte	*ECHDC3*	-0.853509333	0.003418695	0.006539461	1
T2DM	leukocyte	*TMEM241*	-1.381755542	0.00803427	0.011844895	1
T2DM	leukocyte	*AGO4*	-1.772710587	0.000578616	0.004990048	1
T2DM	leukocyte	*PRPF38A*	-0.925608505	0.001536142	0.005006447	1
T2DM	leukocyte	*DSC2*	-2.022227678	0.021230554	0.027795683	1
T2DM	leukocyte	*TPGS2*	-0.985079357	0.005215535	0.008330015	1
T2DM	leukocyte	*DAGLA*	-0.991581963	0.005294578	0.008425434	1
T2DM	leukocyte	*TIMM10*	-0.967744977	0.00111352	0.004990048	1
T2DM	leukocyte	*FADS2*	-1.1187484	0.000990807	0.004990048	1
T2DM	leukocyte	*TMEM165*	-0.754228989	0.001779103	0.005098084	1
T2DM	leukocyte	*CLOCK*	-1.041416009	0.002273932	0.005460974	1
T2DM	leukocyte	*GGACT*	-1.569794147	0.001227297	0.004990048	1
T2DM	leukocyte	*CLDN10*	-0.371967617	0.008936713	0.012951806	1
T2DM	leukocyte	*CARS2*	-0.979255087	0.000587232	0.004990048	1
T2DM	leukocyte	*TMED7*	-0.84132643	0.001164116	0.004990048	1
T2DM	leukocyte	*NREP*	-5.341076361	0.000449643	0.004990048	1
T2DM	leukocyte	*WDR36*	-0.944908165	0.002542236	0.00567203	1
T2DM	leukocyte	*OSTF1*	-1.232401548	0.002973934	0.006152298	1
T2DM	leukocyte	*GOLM1*	-0.7170405	0.001515358	0.004993935	1
T2DM	leukocyte	*SDS*	0.053511191	0.017662348	0.023570678	1
T2DM	leukocyte	*MSI1*	-1.61833251	0.001405623	0.004990048	1
T2DM	leukocyte	*TBX3*	-1.460168199	0.039205779	0.04876211	1
T2DM	leukocyte	*DTX1*	-2.276345209	0.002243494	0.005438799	1
T2DM	leukocyte	*PNPLA8*	-0.739466483	0.001582344	0.005009694	1
T2DM	leukocyte	*TES*	-0.594086705	0.002935031	0.006117633	1
T2DM	leukocyte	*MDFIC*	-2.177790998	0.003147899	0.006311945	1
T2DM	leukocyte	*MTO1*	-0.836939685	0.003160385	0.006314657	1
T2DM	leukocyte	*LCA5*	-2.567617914	0.000511871	0.004990048	1
T2DM	leukocyte	*LMO2*	-0.797758097	0.002533137	0.005660135	1
T2DM	leukocyte	*RDH5*	-0.788380817	0.002824443	0.006006579	1
T2DM	leukocyte	*TFCP2*	-3.002257977	0.000254102	0.004990048	0.998365013
T2DM	leukocyte	*KRT7*	-0.409514011	0.004959246	0.00801517	1
T2DM	leukocyte	*NHSL1*	-0.960719801	0.017764039	0.023686929	1
T2DM	leukocyte	*AHI1*	-0.586367821	0.005152685	0.008249756	1
T2DM	leukocyte	*HEY2*	-1.235253918	0.004371059	0.007383444	1
T2DM	leukocyte	*PKIB*	-0.639767566	0.002171698	0.005349178	1
T2DM	leukocyte	*SMPD2*	-2.229035862	0.001988573	0.005227473	1
T2DM	leukocyte	*SEMA4F*	-1.098483353	0.008354601	0.012252792	1
T2DM	leukocyte	*GNS*	-1.831341491	0.0003717	0.004990048	1
T2DM	leukocyte	*MPHOSPH6*	-0.74998749	0.005025718	0.00809596	1
T2DM	leukocyte	*AGT*	-0.576199827	0.002647241	0.005823633	1
T2DM	leukocyte	*NTPCR*	-1.271041861	0.001106157	0.004990048	1
T2DM	leukocyte	*USP37*	-1.057785027	0.005348156	0.008493495	1
T2DM	leukocyte	*ARMC9*	-4.244227778	0.002144775	0.005323324	1
T2DM	leukocyte	*COX5B*	-0.735357422	0.001189838	0.004990048	1
T2DM	leukocyte	*REV1*	-0.863614497	0.001711098	0.005068796	1
T2DM	leukocyte	*TSGA10*	-0.921754812	0.004801867	0.007847977	1
T2DM	leukocyte	*TGFBRAP1*	-1.143072579	0.000704725	0.004990048	1
T2DM	leukocyte	*GCC2*	-1.126216549	0.000305852	0.004990048	1
T2DM	leukocyte	*MRPS9*	-0.763189653	0.003327777	0.006456709	1
T2DM	leukocyte	*ANKRD36*	-1.191542424	0.002253378	0.005441623	1

T2DM	leukocyte	*USP44*	-1.092577742	0.006918882	0.010467574	1
T2DM	leukocyte	*DRAM1*	-1.837430328	0.000739617	0.004990048	1
T2DM	leukocyte	*VILL*	0.971094927	0.037747	0.047096845	1
T2DM	leukocyte	*FLNB*	-0.684028107	0.00664647	0.010135575	1
T2DM	leukocyte	*NEK3*	-0.54007957	0.015978862	0.021618784	1
T2DM	leukocyte	*VPS36*	-1.46274435	0.000677815	0.004990048	1
T2DM	leukocyte	*RNASEH2B*	-1.858247699	0.000541972	0.004990048	1
T2DM	leukocyte	*TBC1D4*	-0.944921279	0.002293916	0.005475575	1
T2DM	leukocyte	*SUCLA2*	-1.124175981	0.000672471	0.004990048	1
T2DM	leukocyte	*RCBTB1*	-1.056839353	0.022564493	0.029327123	1
T2DM	leukocyte	*LMO7*	-0.506278274	0.003113969	0.006284318	1
T2DM	leukocyte	*C7orf25*	-0.816632593	0.001726535	0.005070552	1
T2DM	leukocyte	*RAPGEF5*	-0.609336852	0.004219122	0.007267615	1
T2DM	leukocyte	*KDELR2*	-0.84563028	0.000990467	0.004990048	1
T2DM	leukocyte	*TBRG4*	-2.108607999	0.000345928	0.004990048	1
T2DM	leukocyte	*DBNL*	-2.788918408	0.000464129	0.004990048	1
T2DM	leukocyte	*ALPK3*	-0.654655048	0.002510527	0.005649404	1
T2DM	leukocyte	*CIB2*	-0.855905247	0.016006514	0.021640595	1
T2DM	leukocyte	*NMT1*	-0.676316224	0.006673527	0.010162902	1
T2DM	leukocyte	*CHAD*	-3.814557871	0.04011589	0.049736615	1
T2DM	leukocyte	*TEX2*	-0.606998347	0.004913083	0.007970068	1
T2DM	leukocyte	*LIMD2*	0.687116169	0.008760919	0.0127299	1
T2DM	leukocyte	*MRPL47*	-0.884538255	0.001091032	0.004990048	1
T2DM	leukocyte	*SMPD4*	-2.303556546	0.000367973	0.004990048	1
T2DM	leukocyte	*CCDC115*	-0.965893036	0.00246891	0.005622792	1
T2DM	leukocyte	*BIN1*	-2.071793618	0.001624558	0.005035955	1
T2DM	leukocyte	*HS6ST1*	-1.360128053	0.001035322	0.004990048	1
T2DM	leukocyte	*GYPC*	-0.803361406	0.007748307	0.011505329	1
T2DM	leukocyte	*NIPSNAP3A*	-1.206190941	0.003223727	0.006377655	1
T2DM	leukocyte	*CDK9*	-1.078675544	0.001627877	0.005035955	1
T2DM	leukocyte	*TXN*	-0.662740294	0.001285968	0.004990048	1
T2DM	leukocyte	*TOR1B*	-1.096777685	0.019106901	0.025242439	1
T2DM	leukocyte	*TOR1A*	-0.816664318	0.003137538	0.006311945	1
T2DM	leukocyte	*SLC2A8*	-0.958638602	0.002301546	0.005478715	1
T2DM	leukocyte	*ZFP37*	-0.625234876	0.024786215	0.031929522	1
T2DM	leukocyte	*STX17*	-0.892823881	0.001344716	0.004990048	1
T2DM	leukocyte	*PRPF4*	-0.867330893	0.026857781	0.034280595	1
T2DM	leukocyte	*FPGS*	-1.155054258	0.001715357	0.005070552	1
T2DM	leukocyte	*ATP6V1G1*	-0.676894416	0.001871604	0.005146173	1
T2DM	leukocyte	*ARPC5L*	-0.698435761	0.001647279	0.005035955	1
T2DM	leukocyte	*POLR1E*	-0.681973725	0.001365429	0.004990048	1
T2DM	leukocyte	*APTX*	-0.779404644	0.00161061	0.005033031	1
T2DM	leukocyte	*CD72*	-1.73992406	0.001884824	0.005146173	1
T2DM	leukocyte	*TMEM8B*	-1.165879881	0.002107021	0.005283012	1
T2DM	leukocyte	*GRHPR*	-0.890597769	0.001454914	0.004990048	1
T2DM	leukocyte	*ALDH1B1*	-1.810152032	0.00152649	0.004997983	1
T2DM	leukocyte	*ARHGEF39*	-3.200933671	0.003298828	0.006432819	1
T2DM	leukocyte	*PPIL1*	-2.232730071	0.000553626	0.004990048	1
T2DM	leukocyte	*GMPR*	-1.456185774	0.003001342	0.006172668	1
T2DM	leukocyte	*KIAA0319*	-0.918845829	0.003519969	0.006649019	1
T2DM	leukocyte	*LRRC1*	-0.822862055	0.002517426	0.005655212	1
T2DM	leukocyte	*RIPK1*	-0.918797445	0.00210036	0.005283012	1
T2DM	leukocyte	*TUBB2B*	-0.715860087	0.003753749	0.00692001	1
T2DM	leukocyte	*UQCC2*	-0.84858173	0.002285542	0.005462378	1
T2DM	leukocyte	*HMGA1*	-0.638663966	0.002163924	0.005345956	1
T2DM	leukocyte	*CLPS*	-0.392786902	0.000986333	0.004990048	1
T2DM	leukocyte	*MTCH1*	-0.808826799	0.001287233	0.004990048	1
T2DM	leukocyte	*ANKRD42*	-1.426701156	0.000517533	0.004990048	1
T2DM	leukocyte	*CREBZF*	-1.213894718	0.001025649	0.004990048	1
T2DM	leukocyte	*PRCP*	-1.003874379	0.011276024	0.015873701	1
T2DM	leukocyte	*NARS2*	-1.038891667	0.00314737	0.006311945	1
T2DM	leukocyte	*TTPA*	-5.418775892	0.023237858	0.030105511	1
T2DM	leukocyte	*GGH*	-1.270156427	0.000563505	0.004990048	1
T2DM	leukocyte	*SDCBP*	-1.443574974	0.001148447	0.004990048	1

T2DM	leukocyte	*TRPC6*	-1.924232077	0.01882808	0.024919309	1
T2DM	leukocyte	*MMP7*	-0.340146686	0.008240403	0.012116971	1
T2DM	leukocyte	*FDX1*	-0.929154267	0.001124645	0.004990048	1
T2DM	leukocyte	*C11orf1*	-0.844368439	0.002878873	0.006055188	1
T2DM	leukocyte	*FXYD2*	-0.559969971	0.000428542	0.004990048	1
T2DM	leukocyte	*CASP1*	0.279956488	0.026892061	0.03430484	1
T2DM	leukocyte	*ALKBH8*	-1.18616802	0.003733981	0.006900664	1
T2DM	leukocyte	*TUBGCP4*	-1.643039384	0.000233239	0.004990048	0.916396285
T2DM	leukocyte	*UACA*	-0.751670476	0.003841655	0.007022431	1
T2DM	leukocyte	*PLCB2*	0.479160051	0.017181718	0.023024205	1
T2DM	leukocyte	*PAK6*	-0.5321167	0.002021956	0.005244224	1
T2DM	leukocyte	*ADAM10*	-1.136251511	0.001186895	0.004990048	1
T2DM	leukocyte	*DUOX1*	-0.820875929	0.028072811	0.035695169	1
T2DM	leukocyte	*CYP19A1*	-6.005219016	0.020368026	0.026746649	1
T2DM	leukocyte	*ZNF280D*	-1.135448806	0.001003102	0.004990048	1
T2DM	leukocyte	*RSL24D1*	-0.899854216	0.000586022	0.004990048	1
T2DM	leukocyte	*TTLL7*	-0.850177583	0.002297736	0.005478037	1
T2DM	leukocyte	*SLC44A5*	-1.206666074	0.009208455	0.013306369	1
T2DM	leukocyte	*RTCA*	-1.975335406	0.003162148	0.006314657	1
T2DM	leukocyte	*KHK*	-4.30195504	4.29E-05	0.004212864	0.168514541
T2DM	leukocyte	*DYNC2LI1*	-1.007764817	0.001708839	0.005068796	1
T2DM	leukocyte	*ACTR2*	-0.854100069	0.000695749	0.004990048	1
T2DM	leukocyte	*ATRAID*	-0.857796269	0.00138488	0.004990048	1
T2DM	leukocyte	*LRPPRC*	-0.966612939	0.002055773	0.00526208	1
T2DM	leukocyte	*DUSP5*	-1.703847167	0.000654789	0.004990048	1
T2DM	leukocyte	*ARL3*	-0.849949957	0.000831833	0.004990048	1
T2DM	leukocyte	*PLCE1*	-0.779004353	0.026752145	0.03416452	1
T2DM	leukocyte	*DNAJC13*	-0.729646384	0.006016895	0.009321917	1
T2DM	leukocyte	*FAM149B1*	-1.010070139	0.001144547	0.004990048	1
T2DM	leukocyte	*NCOA4*	-1.93072748	0.000430795	0.004990048	1
T2DM	leukocyte	*ASCC1*	-1.012227484	0.003160944	0.006314657	1
T2DM	leukocyte	*RPS24*	-0.638709612	0.000833662	0.004990048	1
T2DM	leukocyte	*DNA2*	-1.369646682	0.004552099	0.007568852	1
T2DM	leukocyte	*MYPN*	4.469546344	0.0013364	0.004990048	1
T2DM	leukocyte	*ATIC*	-1.364886865	0.003146858	0.006311945	1
T2DM	leukocyte	*SMARCAL1*	-1.13857457	0.004175878	0.007242343	1
T2DM	leukocyte	*BARD1*	-0.992189794	0.002141902	0.005322917	1
T2DM	leukocyte	*FASTKD1*	-1.059251973	0.002665676	0.005838039	1
T2DM	leukocyte	*FAM117B*	-1.095547922	0.002110311	0.005283012	1
T2DM	leukocyte	*WDR12*	-1.173139318	0.003672266	0.006838073	1
T2DM	leukocyte	*SLC35A5*	-1.172170672	0.00334219	0.006470236	1
T2DM	leukocyte	*USP8*	-1.139430094	0.000816848	0.004990048	1
T2DM	leukocyte	*SPPL2A*	-0.996064517	0.00089018	0.004990048	1
T2DM	leukocyte	*APH1B*	-1.462187359	0.000912687	0.004990048	1
T2DM	leukocyte	*HCN4*	-1.138478824	0.004348678	0.007358292	1
T2DM	leukocyte	*SEMA7A*	-1.972573053	0.004322038	0.007335329	1
T2DM	leukocyte	*AP1AR*	-0.760045876	0.002108818	0.005283012	1
T2DM	leukocyte	*BBS7*	-1.178201981	0.000737534	0.004990048	1
T2DM	leukocyte	*RAP1GDS1*	-1.082927469	0.001656864	0.005041098	1
T2DM	leukocyte	*PRDM5*	-0.79245136	0.006838025	0.010369201	1
T2DM	leukocyte	*G3BP2*	-0.765223659	0.000943288	0.004990048	1
T2DM	leukocyte	*SCARB2*	-0.862315763	0.001020436	0.004990048	1
T2DM	leukocyte	*CNOT6L*	-1.175537516	0.000666398	0.004990048	1
T2DM	leukocyte	*USO1*	-0.754494891	0.001248093	0.004990048	1
T2DM	leukocyte	*SHROOM3*	-1.686777976	0.000873548	0.004990048	1
T2DM	leukocyte	*ANXA3*	-0.634422968	0.001738681	0.005075243	1
T2DM	leukocyte	*PPA2*	-0.894361274	0.001408623	0.004990048	1
T2DM	leukocyte	*MAPK8IP3*	-0.968534904	0.001670049	0.005048289	1
T2DM	leukocyte	*RGS3*	-0.806351762	0.001968622	0.005212073	1
T2DM	leukocyte	*ETV6*	-1.7140537	0.001573507	0.005009694	1
T2DM	leukocyte	*ALG10*	-1.542369259	0.003263814	0.006405357	1
T2DM	leukocyte	*SLCO1C1*	-13.76724257	0.00298259	0.006152298	1
T2DM	leukocyte	*ZCRB1*	-0.823312164	0.001072709	0.004990048	1
T2DM	leukocyte	*CLSTN3*	-1.520515455	0.000686025	0.004990048	1

T2DM	leukocyte	*KLRG1*	-5.445018118	0.004165337	0.007242343	1
T2DM	leukocyte	*TAPBPL*	-0.658268412	0.01730033	0.023167347	1
T2DM	leukocyte	*PEX5*	-0.869588519	0.002562937	0.00569337	1
T2DM	leukocyte	*SLC38A4*	-1.245590469	0.002897649	0.006071927	1
T2DM	leukocyte	*LRIG3*	-0.876071543	0.002862937	0.006050822	1
T2DM	leukocyte	*GLIPR1*	-1.041787783	0.001980535	0.005222499	1
T2DM	leukocyte	*SNRPF*	-0.728228443	0.001451389	0.004990048	1
T2DM	leukocyte	*AMDHD1*	-1.294098	0.018083375	0.024060135	1
T2DM	leukocyte	*ASCL1*	-1.183105978	0.010544675	0.014967495	1
T2DM	leukocyte	*RITA1*	-1.30717619	0.001696547	0.005060368	1
T2DM	leukocyte	*GLTP*	-0.712897724	0.002149531	0.005327398	1
T2DM	leukocyte	*MTMR6*	-1.21421201	0.000734291	0.004990048	1
T2DM	leukocyte	*PDX1*	-1.237441824	0.00141349	0.004990048	1
T2DM	leukocyte	*CCDC65*	-1.255133382	0.008172511	0.012030646	1
T2DM	leukocyte	*KANSL2*	-2.239402189	0.000466573	0.004990048	1
T2DM	leukocyte	*CERS5*	-1.382439286	0.000146657	0.004990048	0.57621634
T2DM	leukocyte	*ZNF740*	-1.877781647	0.002001809	0.005227473	1
T2DM	leukocyte	*WDFY2*	-1.031803084	0.001517262	0.004993935	1
T2DM	leukocyte	*GRTP1*	-1.841729045	0.004988086	0.008055154	1
T2DM	leukocyte	*TSSK4*	-6.087221262	0.031807513	0.040235582	1
T2DM	leukocyte	*TMX1*	-1.334074983	0.000678144	0.004990048	1
T2DM	leukocyte	*RAB15*	-1.212970354	0.003277362	0.006422321	1
T2DM	leukocyte	*NIPA2*	-0.732662531	0.002338531	0.005515664	1
T2DM	leukocyte	*SERF2*	-0.721970854	0.000547941	0.004990048	1
T2DM	leukocyte	*BNIP2*	-1.539422107	0.000507537	0.004990048	1
T2DM	leukocyte	*GTF2A2*	-0.825723089	0.001105674	0.004990048	1
T2DM	leukocyte	*SRP14*	-0.667955548	0.001196941	0.004990048	1
T2DM	leukocyte	*BAHD1*	-1.033879605	0.001879714	0.005146173	1
T2DM	leukocyte	*DISP2*	-0.555764793	0.022414807	0.029180839	1
T2DM	leukocyte	*TLE3*	-2.251800618	0.000147076	0.004990048	0.577860509
T2DM	leukocyte	*SCAPER*	-0.788482589	0.002392465	0.005565421	1
T2DM	leukocyte	*TSPAN3*	-0.705982768	0.001450395	0.004990048	1
T2DM	leukocyte	*MAN2C1*	-1.426190477	4.35E-07	0.000266998	0.001707834
T2DM	leukocyte	*DNAJA4*	-0.802589262	0.00163242	0.005035955	1
T2DM	leukocyte	*TPM1*	-0.494309567	0.002296295	0.005477925	1
T2DM	leukocyte	*IGF1R*	-0.754629313	0.001633629	0.005035955	1
T2DM	leukocyte	*ADAMTS17*	-1.552761068	0.029393271	0.037313785	1
T2DM	leukocyte	*ULK3*	-0.852837726	0.003634748	0.006787511	1
T2DM	leukocyte	*CELF6*	-1.046934241	0.002555128	0.005681968	1
T2DM	leukocyte	*HAPLN3*	-5.349458718	0.014403674	0.019702973	1
T2DM	leukocyte	*POLG*	-1.425621479	0.001361005	0.004990048	1
T2DM	leukocyte	*WDR93*	-2.770882172	0.016438398	0.022152044	1
T2DM	leukocyte	*UNC45A*	-0.98619815	0.001661583	0.005041098	1
T2DM	leukocyte	*IQGAP1*	-0.935496147	0.001013865	0.004990048	1
T2DM	leukocyte	*PMM2*	-0.888409208	0.001864925	0.005146173	1
T2DM	leukocyte	*SLC5A2*	-3.061964399	0.007835788	0.01162205	1
T2DM	leukocyte	*UQCRC2*	-0.996215272	0.000773466	0.004990048	1
T2DM	leukocyte	*ARHGAP17*	-2.37579736	0.000388647	0.004990048	1
T2DM	leukocyte	*TXNL4B*	-2.317075921	0.000307089	0.004990048	1
T2DM	leukocyte	*MARVELD3*	-0.697111882	0.005702893	0.008935705	1
T2DM	leukocyte	*KIFC3*	-1.043384224	0.001074925	0.004990048	1
T2DM	leukocyte	*NUDT7*	-0.763573058	0.004076503	0.007189	1
T2DM	leukocyte	*GCSH*	-0.854628837	0.001289991	0.004990048	1
T2DM	leukocyte	*MAP1LC3B*	-0.750524154	0.001090405	0.004990048	1
T2DM	leukocyte	*OSGIN1*	-0.681431267	0.002881691	0.006056841	1
T2DM	leukocyte	*RPL3L*	-1.471998998	0.030333122	0.038457024	1
T2DM	leukocyte	*RPS2*	-0.58838256	0.000816556	0.004990048	1
T2DM	leukocyte	*PDPK1*	-1.172069039	0.001047532	0.004990048	1
T2DM	leukocyte	*DEF8*	-1.046401037	0.000847008	0.004990048	1
T2DM	leukocyte	*GAS8*	-1.736912505	0.000459988	0.004990048	1
T2DM	leukocyte	*RANBP10*	-0.765457286	0.008424966	0.012337567	1
T2DM	leukocyte	*PRPSAP2*	-0.994445752	0.005103685	0.00819008	1
T2DM	leukocyte	*TOM1L1*	-0.828491217	0.002859829	0.006048134	1
T2DM	leukocyte	*C17orf80*	-1.418560725	0.003226671	0.006380267	1

T2DM	leukocyte	*VPS53*	-1.14048515	0.00012027	0.004990048	0.472541883
T2DM	leukocyte	*NPEPPS*	-1.298291899	0.000596473	0.004990048	1
T2DM	leukocyte	*SCRN2*	-2.148150358	4.21E-05	0.004212864	0.165304763
T2DM	leukocyte	*RHBDL3*	-1.254712467	0.039959885	0.049590142	1
T2DM	leukocyte	*SPACA3*	-0.957906609	0.00047332	0.004990048	1
T2DM	leukocyte	*ARSG*	-2.727397664	0.009593467	0.013781621	1
T2DM	leukocyte	*PTRH2*	-0.994561076	0.001888631	0.005146173	1
T2DM	leukocyte	*GNAL*	-1.196901025	0.005346999	0.008493495	1
T2DM	leukocyte	*RPRD1A*	-0.871424083	0.002425993	0.005602682	1
T2DM	leukocyte	*NPC1*	-2.414904964	1.47E-05	0.00262752	0.057805445
T2DM	leukocyte	*SLC14A1*	-1.28262906	0.005885695	0.009158375	1
T2DM	leukocyte	*ZMYND15*	0.174371915	0.019414795	0.025614751	1
T2DM	leukocyte	*SAT2*	-1.125884968	0.000570278	0.004990048	1
T2DM	leukocyte	*CCDC40*	-0.742166846	0.018240344	0.024260769	1
T2DM	leukocyte	*ARHGDIA*	-0.842196319	0.001404932	0.004990048	1
T2DM	leukocyte	*TMC6*	-1.356730498	0.003983392	0.00713343	1
T2DM	leukocyte	*TTYH2*	-1.496983794	0.004284001	0.007324561	1
T2DM	leukocyte	*EIF4A3*	-0.965535846	0.002044525	0.00526208	1
T2DM	leukocyte	*TBCD*	-1.516543627	0.001834774	0.005146173	1
T2DM	leukocyte	*FN3KRP*	-0.945011036	0.013005221	0.018017459	1
T2DM	leukocyte	*NARF*	-1.342749495	0.000531592	0.004990048	1
T2DM	leukocyte	*TRIM65*	-1.433280828	0.001217105	0.004990048	1
T2DM	leukocyte	*RNF157*	-0.995705956	0.003086135	0.00625753	1
T2DM	leukocyte	*WDR45B*	-0.749896535	0.001785532	0.005100156	1
T2DM	leukocyte	*TNFRSF11A*	-0.768294039	0.004239657	0.007286795	1
T2DM	leukocyte	*ZCCHC2*	-1.258016922	0.001217796	0.004990048	1
T2DM	leukocyte	*PMAIP1*	-0.808048112	0.002947794	0.006131224	1
T2DM	leukocyte	*NT5C3B*	-0.887908077	0.003063531	0.006246297	1
T2DM	leukocyte	*PIP4K2B*	-0.818679406	0.004577379	0.007595372	1
T2DM	leukocyte	*TXNL4A*	-0.843461695	0.000714026	0.004990048	1
T2DM	leukocyte	*PRDM15*	-0.944396723	0.024706665	0.031847929	1
T2DM	leukocyte	*MVB12A*	-0.795020846	0.02467465	0.031817099	1
T2DM	leukocyte	*SH3GL1*	-0.886206709	0.000909402	0.004990048	1
T2DM	leukocyte	*SIRT3*	-1.21518775	0.00251948	0.005656594	1
T2DM	leukocyte	*IFITM3*	-0.549721569	0.00317108	0.006327666	1
T2DM	leukocyte	*COL6A1*	-1.379267586	0.000266417	0.004990048	1
T2DM	leukocyte	*IFNAR1*	-0.930332214	0.000441628	0.004990048	1
T2DM	leukocyte	*TMEM50B*	-0.656406803	0.022630461	0.029393415	1
T2DM	leukocyte	*AKT1*	-1.21007756	0.003269208	0.00641274	1
T2DM	leukocyte	*CBLC*	-0.926335015	0.001805178	0.005122825	1
T2DM	leukocyte	*EVI5L*	-0.79531459	0.034032158	0.042787952	1
T2DM	leukocyte	*CTU1*	-1.586681584	0.000548467	0.004990048	1
T2DM	leukocyte	*RERE*	-0.757751322	0.001669466	0.005048289	1
T2DM	leukocyte	*FHAD1*	-0.91770182	0.002795454	0.005995273	1
T2DM	leukocyte	*ARHGEF19*	-1.545105159	0.007033761	0.010600555	1
T2DM	leukocyte	*PGD*	-1.209033083	0.000674695	0.004990048	1
T2DM	leukocyte	*CNKSR1*	-1.24409479	0.023292875	0.030164042	1
T2DM	leukocyte	*ZNF593*	-0.90619315	0.000760243	0.004990048	1
T2DM	leukocyte	*FCN3*	-0.505174115	0.002717448	0.005898814	1
T2DM	leukocyte	*CELA3A*	-0.397489181	0.001920892	0.005162839	1
T2DM	leukocyte	*NBPF3*	-1.136674833	0.001276507	0.004990048	1
T2DM	leukocyte	*HSPG2*	-0.550198388	0.000777748	0.004990048	1
T2DM	leukocyte	*ITGB3BP*	-0.787621787	0.007694534	0.011438451	1
T2DM	leukocyte	*PRKACB*	-1.060824304	0.001075229	0.004990048	1
T2DM	leukocyte	*PIGK*	-1.576796271	0.000681396	0.004990048	1
T2DM	leukocyte	*RPS8*	-0.545337419	0.001330287	0.004990048	1
T2DM	leukocyte	*SYPL2*	-2.603806348	0.003022693	0.006198413	1
T2DM	leukocyte	*SLC44A3*	-0.817706378	0.004592102	0.007609603	1
T2DM	leukocyte	*ZNF697*	-1.064675106	0.020895139	0.027374792	1
T2DM	leukocyte	*CELSR2*	-0.942718615	0.007560095	0.011272719	1
T2DM	leukocyte	*ALDH9A1*	-0.792071414	0.001728309	0.005070552	1
T2DM	leukocyte	*ATP1B1*	-0.549150898	0.001482837	0.004990048	1
T2DM	leukocyte	*TIPRL*	-0.783498861	0.001730916	0.005070552	1
T2DM	leukocyte	*NME7*	-1.657684858	7.21E-05	0.004990048	0.28346605

T2DM	leukocyte	*MGST3*	-0.905964594	0.00076898	0.004990048	1
T2DM	leukocyte	*LYPLAL1*	-1.089846502	0.001298726	0.004990048	1
T2DM	leukocyte	*SNX27*	-1.394721884	0.001631488	0.005035955	1
T2DM	leukocyte	*CTSK*	-3.219258878	0.0003559	0.004990048	1
T2DM	leukocyte	*SELENBP1*	-0.989722676	0.004134701	0.007236049	1
T2DM	leukocyte	*POGZ*	-0.70840513	0.024891902	0.032055156	1
T2DM	leukocyte	*GABPB2*	-1.335479306	0.007160688	0.010771187	1
T2DM	leukocyte	*SLC27A3*	-1.299766937	0.001180989	0.004990048	1
T2DM	leukocyte	*KCNN3*	-1.509202552	0.00386553	0.007037843	1
T2DM	leukocyte	*RIT1*	-1.534666646	0.00467085	0.007686961	1
T2DM	leukocyte	*SRP9*	-0.891877916	0.000925113	0.004990048	1
T2DM	leukocyte	*CNIH4*	-0.910263542	0.001208296	0.004990048	1
T2DM	leukocyte	*PSEN2*	-1.753676851	0.001858231	0.005146173	1
T2DM	leukocyte	*PYCR2*	-0.973628835	0.002212844	0.005400163	1
T2DM	leukocyte	*LBR*	-1.122068468	0.00121062	0.004990048	1
T2DM	leukocyte	*ARL8A*	-1.564033833	0.000297034	0.004990048	1
T2DM	leukocyte	*EML4*	-1.523579062	0.000729394	0.004990048	1
T2DM	leukocyte	*WDPCP*	-2.380285747	0.000951969	0.004990048	1
T2DM	leukocyte	*REG3G*	-1.073734239	0.000396949	0.004990048	1
T2DM	leukocyte	*CIAO1*	-1.385726126	0.000258864	0.004990048	1
T2DM	leukocyte	*ZNF514*	-1.175210602	0.000145504	0.004990048	0.571683877
T2DM	leukocyte	*TPRKB*	-0.793616232	0.001463243	0.004990048	1
T2DM	leukocyte	*TEX261*	-1.031877823	0.001506851	0.004993935	1
T2DM	leukocyte	*RALB*	-0.903470931	0.002555367	0.005681968	1
T2DM	leukocyte	*RABL2A*	-1.092681957	0.013124248	0.018175949	1
T2DM	leukocyte	*SLC20A1*	-1.408868034	0.001226435	0.004990048	1
T2DM	leukocyte	*LYG1*	-4.029828205	0.015521245	0.021050387	1
T2DM	leukocyte	*GULP1*	-0.841281203	0.003001481	0.006172668	1
T2DM	leukocyte	*FAM171B*	-0.788424901	0.002234511	0.005432793	1
T2DM	leukocyte	*METTL21A*	-0.901420654	0.002553282	0.005681968	1
T2DM	leukocyte	*SUMF1*	-1.304557824	0.000905121	0.004990048	1
T2DM	leukocyte	*HES6*	-2.396732778	0.000330607	0.004990048	1
T2DM	leukocyte	*TAMM41*	-0.800657429	0.027772355	0.035327696	1
T2DM	leukocyte	*RAB5A*	-0.845166056	0.001625514	0.005035955	1
T2DM	leukocyte	*CNTN4*	-1.263988585	1.67E-05	0.002853237	0.065624451
T2DM	leukocyte	*POMGNT2*	-1.720136249	0.000464141	0.004990048	1
T2DM	leukocyte	*CTDSPL*	-0.823197167	0.002176637	0.005349178	1
T2DM	leukocyte	*IL17RD*	-3.3046154	0.007031666	0.010600555	1
T2DM	leukocyte	*UBA3*	-1.377551495	0.000975349	0.004990048	1
T2DM	leukocyte	*ARL6IP5*	-0.843135471	0.001129177	0.004990048	1
T2DM	leukocyte	*LIMD1*	-1.560390213	0.000944177	0.004990048	1
T2DM	leukocyte	*NXPE3*	-0.791227933	0.004673983	0.007686961	1
T2DM	leukocyte	*PHLDB2*	-0.644840148	0.002876416	0.006055188	1
T2DM	leukocyte	*ABHD10*	-0.944087622	0.003848664	0.007027251	1
T2DM	leukocyte	*TAGLN3*	-0.40338361	0.034006491	0.042769367	1
T2DM	leukocyte	*EIF2A*	-0.762690333	0.001408741	0.004990048	1
T2DM	leukocyte	*ALDH1L1*	-1.183939715	0.004040102	0.007166394	1
T2DM	leukocyte	*TRPC1*	-1.055960787	0.00239187	0.005565421	1
T2DM	leukocyte	*NCEH1*	-0.529028179	0.003817061	0.006991718	1
T2DM	leukocyte	*AMT*	-1.602409667	0.001106268	0.004990048	1
T2DM	leukocyte	*DGKQ*	-0.882183178	0.006767631	0.010278358	1
T2DM	leukocyte	*SCD5*	-0.738847567	0.002516666	0.005655212	1
T2DM	leukocyte	*PYURF*	-0.736786418	0.00137726	0.004990048	1
T2DM	leukocyte	*DDIT4L*	-2.499698304	0.000872363	0.004990048	1
T2DM	leukocyte	*TIFA*	-1.128962477	0.001479176	0.004990048	1
T2DM	leukocyte	*CYP4V2*	-1.599650439	0.008820729	0.012807333	1
T2DM	leukocyte	*SSBP2*	-0.716495827	0.002652338	0.005824744	1
T2DM	leukocyte	*GTF2H2*	-2.546809452	0.000384526	0.004990048	1
T2DM	leukocyte	*BTF3*	-0.711440816	0.000754577	0.004990048	1
T2DM	leukocyte	*COMMD10*	-1.031730163	0.002092974	0.005283012	1
T2DM	leukocyte	*ATG12*	-0.88842369	0.00083294	0.004990048	1
T2DM	leukocyte	*PCYOX1L*	-5.052444085	0.000356405	0.004990048	1
T2DM	leukocyte	*TBC1D7*	-3.126644702	0.01438781	0.019689901	1
T2DM	leukocyte	*ZMAT2*	-1.128557407	0.000940779	0.004990048	1

T2DM	leukocyte	*KLHL3*	-3.90558463	0.000806026	0.004990048	1
T2DM	leukocyte	*DCDC2*	-0.651026498	0.003342983	0.006470236	1
T2DM	leukocyte	*TRIM7*	-3.243516302	0.000234126	0.004990048	0.919881559
T2DM	leukocyte	*ANO7*	-2.472903418	0.002718957	0.005898831	1
T2DM	leukocyte	*RPL7L1*	-0.835586685	0.001168068	0.004990048	1
T2DM	leukocyte	*IRAK1BP1*	-0.7271178	0.003762062	0.00692001	1
T2DM	leukocyte	*MMS22L*	-1.242061669	0.002969118	0.006149534	1
T2DM	leukocyte	*FAXC*	-1.383257021	0.001686702	0.005060368	1
T2DM	leukocyte	*SCML4*	-3.563017319	0.001057376	0.004990048	1
T2DM	leukocyte	*ABRACL*	-0.866651642	0.003381645	0.006503419	1
T2DM	leukocyte	*DYNLT1*	-0.769966651	0.000870207	0.004990048	1
T2DM	leukocyte	*WTAP*	-0.789915565	0.000723976	0.004990048	1
T2DM	leukocyte	*SLC22A3*	-4.417705238	0.030323351	0.038457024	1
T2DM	leukocyte	*VWDE*	-0.879637952	0.002055628	0.00526208	1
T2DM	leukocyte	*GNA12*	-1.350301607	0.002814787	0.006001505	1
T2DM	leukocyte	*SDK1*	-0.964895534	0.006728394	0.010230596	1
T2DM	leukocyte	*CCZ1B*	-1.311942237	0.00388011	0.007054583	1
T2DM	leukocyte	*RBAK*	-1.795200806	0.003765042	0.00692225	1
T2DM	leukocyte	*POMZP3*	-1.012892079	0.001812614	0.005123568	1
T2DM	leukocyte	*GIGYF1*	-2.350878155	2.70E-05	0.003310187	0.105925972
T2DM	leukocyte	*TRIM4*	-0.942661241	0.00016209	0.004990048	0.636852299
T2DM	leukocyte	*TMEM209*	-1.444850695	0.000667218	0.004990048	1
T2DM	leukocyte	*RAB19*	-8.873280475	8.65E-05	0.004990048	0.339700603
T2DM	leukocyte	*CSGALNACT1*	-0.782310511	0.003491269	0.006628411	1
T2DM	leukocyte	*CCDC25*	-0.751768737	0.001242625	0.004990048	1
T2DM	leukocyte	*BIN3*	-2.584440495	0.000824746	0.004990048	1
T2DM	leukocyte	*CHMP7*	-1.203086066	0.004518603	0.007554719	1
T2DM	leukocyte	*STAR*	0.541441768	0.021863505	0.028548259	1
T2DM	leukocyte	*LACTB2*	-0.79529436	0.002099301	0.005283012	1
T2DM	leukocyte	*TERF1*	-1.889498701	0.000343134	0.004990048	1
T2DM	leukocyte	*LRP12*	-0.922881051	0.001409967	0.004990048	1
T2DM	leukocyte	*ZNF7*	-0.752307689	0.003038546	0.006214704	1
T2DM	leukocyte	*AK3*	-0.809659037	0.001327528	0.004990048	1
T2DM	leukocyte	*CER1*	2.880374493	0.016377537	0.022082136	1
T2DM	leukocyte	*C9orf72*	-1.393477069	0.000567222	0.004990048	1
T2DM	leukocyte	*FBXO10*	-1.203194681	0.005624773	0.008832827	1
T2DM	leukocyte	*SIGMAR1*	-1.172743868	0.000366016	0.004990048	1
T2DM	leukocyte	*ZNF462*	-0.778879754	0.002445925	0.005621365	1
T2DM	leukocyte	*UGCG*	-0.94813811	0.00089681	0.004990048	1
T2DM	leukocyte	*ALAD*	-0.93353982	0.004866832	0.007914645	1
T2DM	leukocyte	*SURF4*	-0.806888815	0.000640631	0.004990048	1
T2DM	leukocyte	*SURF1*	-0.936589508	0.002372469	0.005551774	1
T2DM	leukocyte	*SURF6*	-1.330926103	0.000996049	0.004990048	1
T2DM	leukocyte	*SH3GLB2*	-1.461869036	0.000837461	0.004990048	1
T2DM	leukocyte	*LCN2*	-0.360432103	0.003704245	0.006868344	1
T2DM	leukocyte	*GPR107*	-1.167222199	0.00247985	0.005622792	1
T2DM	leukocyte	*INPP5E*	-1.182369919	0.033901346	0.042678112	1
T2DM	leukocyte	*SEC16A*	-0.853898289	0.02885791	0.03665785	1
T2DM	leukocyte	*MSRB2*	-1.446712349	0.000529369	0.004990048	1
T2DM	leukocyte	*RSU1*	-1.134782079	0.001712566	0.005068796	1
T2DM	leukocyte	*GLUD1*	-1.070389926	0.001100103	0.004990048	1
T2DM	leukocyte	*FRA10AC1*	-0.660584526	0.003200066	0.006353934	1
T2DM	leukocyte	*DNAJB12*	-1.169007371	0.000955882	0.004990048	1
T2DM	leukocyte	*LRRC27*	-1.892392806	0.038541074	0.048011376	1
T2DM	leukocyte	*MTG1*	-0.769453972	0.002485025	0.005622792	1
T2DM	leukocyte	*PAOX*	-5.372634293	7.53E-05	0.004990048	0.295792562
T2DM	leukocyte	*GSTO1*	-1.117179882	0.001698944	0.005060368	1
T2DM	leukocyte	*HSD17B12*	-0.880015286	1.65E-06	0.00053944	0.006473279
T2DM	leukocyte	*APIP*	-1.241822897	6.30E-06	0.001302453	0.0247466
T2DM	leukocyte	*PTPRJ*	-1.191776503	0.001873741	0.005146173	1
T2DM	leukocyte	*CCDC82*	-0.9648036	0.000636617	0.004990048	1
T2DM	leukocyte	*RPS3*	-0.596076518	0.000944203	0.004990048	1
T2DM	leukocyte	*AASDHPPT*	-0.949857415	0.00135745	0.004990048	1
T2DM	leukocyte	*GLB1L2*	-0.719089678	0.019269342	0.025439934	1

T2DM	leukocyte	*TMC2*	-54.75723831	0.022064521	0.028762941	1
T2DM	leukocyte	*EML3*	-4.043515278	0.000807338	0.004990048	1
T2DM	leukocyte	*CCDC15*	-1.007571235	0.003390606	0.006510382	1
T2DM	leukocyte	*CHEK1*	-1.102993945	0.002660561	0.00583507	1
T2DM	leukocyte	*SIDT2*	-1.217430247	0.000666119	0.004990048	1
T2DM	leukocyte	*TAGLN*	-0.872778267	0.000315791	0.004990048	1
T2DM	leukocyte	*DUSP15*	-1.052205185	0.004730582	0.007750816	1
T2DM	leukocyte	*DSN1*	-1.079967704	0.001873827	0.005146173	1
T2DM	leukocyte	*YTHDF1*	-0.999975943	0.001400539	0.004990048	1
T2DM	leukocyte	*CABLES2*	-1.130083362	0.003858247	0.007033267	1
T2DM	leukocyte	*TRPT1*	-0.852957579	0.003301766	0.006432819	1
T2DM	leukocyte	*MPP7*	-0.700200571	0.00370091	0.006868344	1
T2DM	leukocyte	*DCUN1D2*	-1.021715528	0.005661404	0.008886798	1
T2DM	leukocyte	*TMCO3*	-0.999393951	0.002423604	0.005602682	1
T2DM	leukocyte	*TMEM218*	-1.413596789	0.001092705	0.004990048	1
T2DM	leukocyte	*MIA2*	-0.898289579	0.000966553	0.004990048	1
T2DM	leukocyte	*LYPD1*	-1.373967214	0.003798098	0.006966727	1
T2DM	leukocyte	*LYPD6B*	-2.243744379	0.003812987	0.006987512	1
T2DM	leukocyte	*WDR17*	-0.724679723	0.003888566	0.00705773	1
T2DM	leukocyte	*CNDP1*	-3.249385655	0.027774816	0.035327696	1
T2DM	leukocyte	*PRSS23*	-0.697770963	0.001833795	0.005146173	1
T2DM	leukocyte	*MTMR12*	-0.754714678	0.001772148	0.005097196	1
T2DM	leukocyte	*DOCK1*	-0.93556329	0.001755779	0.005084476	1
T2DM	leukocyte	*IL18*	0.409843259	0.0322199	0.040731013	1
T2DM	leukocyte	*TMEM86A*	-1.05318579	0.004149033	0.007238372	1
T2DM	leukocyte	*PLBD2*	-2.168347339	0.008295045	0.012179085	1
T2DM	leukocyte	*DLG5*	-0.898135253	0.001235026	0.004990048	1
T2DM	leukocyte	*SLC2A13*	-0.736371047	0.001030178	0.004990048	1
T2DM	leukocyte	*MAGI1*	-0.740701909	0.011254469	0.015860404	1
T2DM	leukocyte	*CSNK1G3*	-0.946903382	0.004021403	0.00715584	1
T2DM	leukocyte	*AKAP6*	-1.075517887	0.002456575	0.005621365	1
T2DM	leukocyte	*MBIP*	-1.014165191	0.001606988	0.005033031	1
T2DM	leukocyte	*MIPOL1*	-0.776550055	0.005082283	0.008170331	1
T2DM	leukocyte	*TMEM18*	-0.96561974	0.001487295	0.004990249	1
T2DM	leukocyte	*NDUFC2*	-0.740200384	0.000965435	0.004990048	1
T2DM	leukocyte	*VIPAS39*	-1.659873532	0.000464176	0.004990048	1
T2DM	leukocyte	*C4orf33*	-1.91961594	0.001669659	0.005048289	1
T2DM	leukocyte	*QDPR*	-0.652275838	0.002686021	0.00586252	1
T2DM	leukocyte	*TEX9*	-1.717461053	0.000635785	0.004990048	1
T2DM	leukocyte	*ZNF827*	-0.81829939	0.002286997	0.005462378	1
T2DM	leukocyte	*AKR1C2*	-2.619840941	0.000111701	0.004990048	0.438874434
T2DM	leukocyte	*KIN*	-1.59008076	0.001093862	0.004990048	1
T2DM	leukocyte	*TMEM45B*	-1.198750869	0.012054251	0.016860503	1
T2DM	leukocyte	*BICD1*	-0.736773478	0.003980738	0.007132431	1
T2DM	leukocyte	*GUF1*	-2.366468819	0.000437128	0.004990048	1
T2DM	leukocyte	*CCL28*	-1.109334786	0.001917435	0.005160002	1
T2DM	leukocyte	*CACUL1*	-1.346086059	0.000615783	0.004990048	1
T2DM	leukocyte	*KCNE4*	-1.112068928	0.00307593	0.00625211	1
T2DM	leukocyte	*RABGAP1L*	-1.036246187	0.001272821	0.004990048	1
T2DM	leukocyte	*PTPN14*	-1.147239902	1.98E-05	0.0030497	0.077661022
T2DM	leukocyte	*ARL14EP*	-1.298332702	0.00052144	0.004990048	1
T2DM	leukocyte	*EPG5*	-1.530313673	0.000875404	0.004990048	1
T2DM	leukocyte	*HAUS1*	-1.2609468	0.001050086	0.004990048	1
T2DM	leukocyte	*SPC25*	-1.10275084	0.002096052	0.005283012	1
T2DM	leukocyte	*PDK1*	-1.10054597	0.002147644	0.005327078	1
T2DM	leukocyte	*PDE3B*	-0.53249322	0.003417578	0.006539461	1
T2DM	leukocyte	*TGOLN2*	-0.941013385	0.000851156	0.004990048	1
T2DM	leukocyte	*ATG10*	-0.703094281	0.02686433	0.034280595	1
T2DM	leukocyte	*POC5*	-1.711392318	4.94E-06	0.001213482	0.019415718
T2DM	leukocyte	*GUCY1A2*	-0.862683639	0.004085351	0.007197912	1
T2DM	leukocyte	*ZNF773*	-0.659199775	0.019870772	0.026163627	1
T2DM	leukocyte	*DCLRE1C*	-2.63974418	0.001761396	0.005084476	1
T2DM	leukocyte	*ZSCAN1*	-0.990409847	0.00098127	0.004990048	1
T2DM	leukocyte	*USP12*	-0.83632393	0.001880055	0.005146173	1

T2DM	leukocyte	*CCDC50*	-0.625148441	0.006521126	0.009977222	1
T2DM	leukocyte	*PLEKHH2*	-0.745902593	0.017374433	0.023242815	1
T2DM	leukocyte	*GRIA4*	-1.216432932	0.001477159	0.004990048	1
T2DM	leukocyte	*IGSF10*	-1.951943102	0.007412897	0.011095342	1
T2DM	leukocyte	*SPEF2*	-0.885807228	0.013266205	0.018344791	1
T2DM	leukocyte	*GPD1L*	-0.927526873	0.006646088	0.010135575	1
T2DM	leukocyte	*CCNO*	-0.98058684	0.005411485	0.008573276	1
T2DM	leukocyte	*RASGRP3*	-0.851618378	0.016011507	0.021640595	1
T2DM	leukocyte	*CATSPER3*	-4.751227344	0.004052228	0.007180398	1
T2DM	leukocyte	*GPR180*	-0.847371852	0.001566306	0.005009694	1
T2DM	leukocyte	*IFIT5*	-0.935678144	0.003596161	0.006744304	1
T2DM	leukocyte	*MED21*	-1.554554185	0.000282974	0.004990048	1
T2DM	leukocyte	*LGI2*	-1.203180185	0.01061915	0.015056889	1
T2DM	leukocyte	*SRP19*	-0.779821318	0.001125607	0.004990048	1
T2DM	leukocyte	*DAB2*	-1.23548529	0.001044733	0.004990048	1
T2DM	leukocyte	*ACMSD*	-1.121232887	0.006665751	0.010154996	1
T2DM	leukocyte	*CAST*	-0.818066281	0.001045649	0.004990048	1
T2DM	leukocyte	*SYCP2L*	-2.135205088	0.017098061	0.022935569	1
T2DM	leukocyte	*MERTK*	0.457245146	0.036854538	0.046100439	1
T2DM	leukocyte	*TMEM87B*	-0.965997047	0.002989958	0.006163456	1
T2DM	leukocyte	*PLA2R1*	-2.975885562	0.003914054	0.007085756	1
T2DM	leukocyte	*SLC25A27*	-1.399777192	0.000620196	0.004990048	1
T2DM	leukocyte	*ASAP1*	-0.833816058	0.002053993	0.00526208	1
T2DM	leukocyte	*NMRAL1*	-0.934259025	0.010333262	0.014709923	1
T2DM	leukocyte	*CMTM7*	-0.798854794	0.002029984	0.005251028	1
T2DM	leukocyte	*RPIA*	-0.958360986	0.001502434	0.004993935	1
T2DM	leukocyte	*PTPRD*	-0.692030572	0.028631256	0.036393466	1
T2DM	leukocyte	*GTF2E1*	-2.332693775	0.000625992	0.004990048	1
T2DM	leukocyte	*CFDP1*	-0.7378749	0.003824536	0.007002145	1
T2DM	leukocyte	*ZDHHC7*	-1.191314215	0.017803909	0.023720433	1
T2DM	leukocyte	*JAZF1*	-1.976653238	1.46E-08	1.91E-05	5.74E-05
T2DM	leukocyte	*CMIP*	-0.95771465	0.001340415	0.004990048	1
T2DM	leukocyte	*PID1*	-1.659783995	0.009224111	0.013319197	1
T2DM	leukocyte	*KCTD15*	-1.220925024	0.004100073	0.00720956	1
T2DM	leukocyte	*LGI4*	1.13749046	0.006647843	0.010135575	1
T2DM	leukocyte	*DDAH1*	-2.145097492	0.000478761	0.004990048	1
T2DM	leukocyte	*NUS1*	-1.127427492	0.000402033	0.004990048	1
T2DM	leukocyte	*PPP2R5E*	-0.990913768	0.002303598	0.005478715	1
T2DM	leukocyte	*ROBO3*	-2.562603018	0.00414361	0.007238335	1
T2DM	leukocyte	*PITPNC1*	-1.034860508	0.001488698	0.004990695	1
T2DM	leukocyte	*CC2D1B*	-2.243991756	0.000138459	0.004990048	0.544006304
T2DM	leukocyte	*CEP112*	-0.738183732	0.003324367	0.006456709	1
T2DM	leukocyte	*ABCA5*	-1.35912768	0.001185932	0.004990048	1
T2DM	leukocyte	*SRSF12*	-1.466104343	0.008359669	0.01225565	1
T2DM	leukocyte	*PDLIM3*	-0.572246802	0.005794184	0.009041044	1
T2DM	leukocyte	*PDE1C*	-0.922566096	0.021497729	0.028126732	1
T2DM	leukocyte	*RABGEF1*	-1.042776215	0.0007342	0.004990048	1
T2DM	leukocyte	*MRPL39*	-0.983440696	0.00434609	0.007358292	1
T2DM	leukocyte	*TSEN2*	-1.991582393	0.002914453	0.006099708	1
T2DM	leukocyte	*FLCN*	-1.507357092	0.000825618	0.004990048	1
T2DM	leukocyte	*MPPE1*	-1.103742451	0.002051425	0.00526208	1
T2DM	leukocyte	*RAB6B*	-1.110918904	0.002387127	0.005560842	1
T2DM	leukocyte	*ANKRD40*	-1.167458568	0.003200416	0.006353934	1
T2DM	leukocyte	*ATP6V1C1*	-0.852452566	0.001864989	0.005146173	1
T2DM	leukocyte	*MARVELD1*	-3.312087207	0.000694358	0.004990048	1
T2DM	leukocyte	*TRMT44*	-0.7958612	0.005275088	0.00840804	1
T2DM	leukocyte	*PPM1J*	-2.176716639	0.004541069	0.007568852	1
T2DM	leukocyte	*DBI*	-0.936689713	0.00056956	0.004990048	1
T2DM	leukocyte	*LRGUK*	-3.13770258	0.001433896	0.004990048	1
T2DM	leukocyte	*SETD9*	-1.161256733	0.000316666	0.004990048	1
T2DM	leukocyte	*MIER3*	-2.106195334	0.000283205	0.004990048	1
T2DM	leukocyte	*TTN*	-2.352383882	0.000795301	0.004990048	1
T2DM	leukocyte	*DEPTOR*	-0.854788794	0.013911948	0.019114815	1
T2DM	leukocyte	*RMND1*	-1.738436074	0.000450186	0.004990048	1

T2DM	leukocyte	*TMBIM4*	-0.848409282	0.000964263	0.004990048	1
T2DM	leukocyte	*PSD3*	-0.788888475	0.002583503	0.00572832	1
T2DM	leukocyte	*MMP16*	-0.730042125	0.00493838	0.007991307	1
T2DM	leukocyte	*ADK*	-0.970553546	0.002860126	0.006048134	1
T2DM	leukocyte	*BATF*	-12.66145167	0.003963995	0.007124893	1
T2DM	leukocyte	*DRAM2*	-1.328562029	0.005737341	0.008970609	1
T2DM	leukocyte	*ART3*	-3.595878446	0.013914067	0.019114815	1
T2DM	leukocyte	*CCT8*	-0.643752121	0.006266913	0.009633295	1
T2DM	leukocyte	*CDK20*	-0.860710073	0.002814742	0.006001505	1
T2DM	leukocyte	*TDRD9*	-0.644582269	0.001162778	0.004990048	1
T2DM	leukocyte	*HKDC1*	-1.9475662	0.010256161	0.014621356	1
T2DM	leukocyte	*ZDHHC5*	-1.103137618	0.001317447	0.004990048	1
T2DM	leukocyte	*MED19*	-0.907293665	0.001264206	0.004990048	1
T2DM	leukocyte	*MAPK13*	-0.90636517	0.008558595	0.012491352	1
T2DM	leukocyte	*COX6A2*	-0.910984703	0.019842139	0.026143448	1
T2DM	leukocyte	*MPV17L*	-0.847735259	0.003886712	0.00705773	1
T2DM	leukocyte	*TATDN2*	-1.599882392	0.001391579	0.004990048	1
T2DM	leukocyte	*EXOG*	-1.21448821	0.000871577	0.004990048	1
T2DM	leukocyte	*ZFYVE9*	-0.988715594	0.002065792	0.005273744	1
T2DM	leukocyte	*SLC6A1*	-5.032831268	0.004062338	0.007184034	1
T2DM	leukocyte	*NECAP2*	-1.204455244	0.001213775	0.004990048	1
T2DM	leukocyte	*STEAP2*	-1.121767354	0.012539299	0.017458135	1
T2DM	leukocyte	*GATAD1*	-1.220404868	0.000948787	0.004990048	1
T2DM	leukocyte	*CLEC18A*	-5.129525168	2.07E-08	2.03E-05	8.13E-05
T2DM	leukocyte	*DHRS4*	-1.120878679	0.001070188	0.004990048	1
T2DM	leukocyte	*DHRS1*	-1.017160373	0.007022112	0.010591123	1
T2DM	leukocyte	*AASDH*	-0.665446764	0.007242914	0.010874058	1
T2DM	leukocyte	*SLC38A10*	-1.083640923	0.001191495	0.004990048	1
T2DM	leukocyte	*SLC37A3*	-0.709642384	0.002788176	0.005983705	1
T2DM	leukocyte	*AP3S2*	-0.930391748	0.01051333	0.014928397	1
T2DM	leukocyte	*SPPL3*	-1.090585659	0.00082517	0.004990048	1
T2DM	leukocyte	*C12orf43*	-1.109460268	0.002480005	0.005622792	1
T2DM	leukocyte	*PEX10*	-1.538053467	0.000985696	0.004990048	1
T2DM	leukocyte	*LDLRAP1*	-0.756927214	0.001945242	0.005192687	1
T2DM	leukocyte	*KRTCAP3*	-0.898779573	0.002330683	0.005513096	1
T2DM	leukocyte	*PAFAH2*	-2.697328128	0.000640599	0.004990048	1
T2DM	leukocyte	*EXTL1*	-6.151233395	3.88E-05	0.004122285	0.152524531
T2DM	leukocyte	*TRIM63*	-14.28003871	0.000632682	0.004990048	1
T2DM	leukocyte	*XKR8*	-1.740664029	0.00083304	0.004990048	1
T2DM	leukocyte	*CNNM4*	-1.523492394	0.003491433	0.006628411	1
T2DM	leukocyte	*ESYT3*	-1.746787929	0.00459748	0.007615304	1
T2DM	leukocyte	*FAIM*	-0.973632464	0.001322913	0.004990048	1
T2DM	leukocyte	*AHCYL2*	-0.996301166	0.007073861	0.010655705	1
T2DM	leukocyte	*COPG2*	-0.915066436	0.002184763	0.005356498	1
T2DM	leukocyte	*RNF166*	-1.183964634	0.003089404	0.00625753	1
T2DM	leukocyte	*NIT1*	-1.098430275	0.014133573	0.019389249	1
T2DM	leukocyte	*NPM2*	-1.025776466	0.003584391	0.006728653	1
T2DM	leukocyte	*VWA5B1*	2.151589599	0.022317913	0.029073965	1
T2DM	leukocyte	*CDA*	-10.47558898	0.005706297	0.008935847	1
T2DM	leukocyte	*PINK1*	-1.207355754	0.00060505	0.004990048	1
T2DM	leukocyte	*DMTN*	-0.853318667	0.013274177	0.018344791	1
T2DM	leukocyte	*CCAR2*	-1.368456026	0.001036622	0.004990048	1
T2DM	leukocyte	*EPB41*	-0.896401283	0.002412243	0.005588268	1
T2DM	leukocyte	*MIS18A*	-1.276735999	0.000363949	0.004990048	1
T2DM	leukocyte	*ALG8*	-0.844068754	0.003581943	0.006727272	1
T2DM	leukocyte	*IFNAR2*	-1.752741924	0.002281568	0.005462378	1
T2DM	leukocyte	*IFNGR2*	-0.964232459	0.001089632	0.004990048	1
T2DM	leukocyte	*C1QC*	0.047879092	0.01612283	0.021783562	1
T2DM	leukocyte	*GJD2*	-0.602116492	0.001953954	0.005198138	1
T2DM	leukocyte	*CLDN14*	-1.890399695	0.01183766	0.016598917	1
T2DM	leukocyte	*HLCS*	-0.797894934	0.004804649	0.007849258	1
T2DM	leukocyte	*THEM4*	-0.775893743	0.001855881	0.005146173	1
T2DM	leukocyte	*UBR1*	-1.200380999	0.001549396	0.005009694	1
T2DM	leukocyte	*TEPP*	2.74706872	0.038520977	0.04800156	1

T2DM	leukocyte	*ZDHHC1*	-0.985247514	0.001480231	0.004990048	1
T2DM	leukocyte	*ZFYVE28*	-1.25696638	0.002548529	0.005679621	1
T2DM	leukocyte	*LYPD5*	-9.337893481	0.022422233	0.029180839	1
T2DM	leukocyte	*NPR2*	-0.798291537	0.038338723	0.047789607	1
T2DM	leukocyte	*ATAD3B*	-2.37850639	0.000927511	0.004990048	1
T2DM	leukocyte	*ANKLE1*	0.750289931	0.000799038	0.004990048	1
T2DM	leukocyte	*KALRN*	-1.166919992	0.007407499	0.011091488	1
T2DM	leukocyte	*CILP2*	-3.410474918	4.94E-06	0.001213482	0.019394961
T2DM	leukocyte	*TMPRSS3*	-2.376034051	0.01168416	0.016401238	1
T2DM	leukocyte	*RSPH1*	-0.624588386	0.003250574	0.006400784	1
T2DM	leukocyte	*SLC37A1*	-0.916496102	0.005337682	0.008483718	1
T2DM	leukocyte	*NDUFV3*	-0.538649	0.018980712	0.025084163	1
T2DM	leukocyte	*HSF2BP*	-3.124326463	0.000849859	0.004990048	1
T2DM	leukocyte	*PDXK*	-1.27498898	0.000184241	0.004990048	0.723882111
T2DM	leukocyte	*CSTB*	-1.116346326	0.000572296	0.004990048	1
T2DM	leukocyte	*ICOSLG*	0.356871198	0.038012188	0.047397615	1
T2DM	leukocyte	*ZNF66*	-2.123637664	0.0112755	0.015873701	1
T2DM	leukocyte	*FTCD*	-1.46745813	0.000461234	0.004990048	1
T2DM	leukocyte	*SPATC1L*	-0.76277273	0.001564842	0.005009694	1
T2DM	leukocyte	*LSS*	-0.945511562	0.006432806	0.009861293	1
T2DM	leukocyte	*VAV2*	-1.40543938	0.002429399	0.005602682	1
T2DM	leukocyte	*MCM3AP*	-1.228872994	0.011588159	0.016278112	1
T2DM	leukocyte	*DIP2A*	-0.739488406	0.005470502	0.008631969	1
T2DM	leukocyte	*ZNF208*	-0.941311289	0.00077862	0.004990048	1
T2DM	leukocyte	*SLC2A6*	-1.711907136	0.001087114	0.004990048	1
T2DM	leukocyte	*RDH13*	-1.088254597	0.00277077	0.005961859	1
T2DM	leukocyte	*MPZL3*	-1.000640533	0.002775549	0.005965608	1
T2DM	leukocyte	*S100A1*	-1.612139786	0.018364252	0.024409047	1
T2DM	leukocyte	*VPS11*	-2.231250736	6.80E-05	0.004990048	0.267158568
T2DM	leukocyte	*PAQR6*	-1.264047314	0.000475363	0.004990048	1
T2DM	leukocyte	*SLC25A44*	-1.556597483	0.001031227	0.004990048	1
T2DM	leukocyte	*CCR5*	-108.9403803	0.008838241	0.012823283	1
T2DM	leukocyte	*NBEAL2*	-0.653732898	0.005550278	0.008740298	1
T2DM	leukocyte	*LY6E*	-1.111046757	0.000605008	0.004990048	1
T2DM	leukocyte	*VPS28*	-0.745318804	0.004127745	0.00723368	1
T2DM	leukocyte	*TONSL*	-4.7331673	0.00100142	0.004990048	1
T2DM	leukocyte	*ZNF333*	-3.417668516	0.000884875	0.004990048	1
T2DM	leukocyte	*RPL8*	-0.583391682	0.000902726	0.004990048	1
T2DM	leukocyte	*MAML1*	-1.651152697	0.002478418	0.005622792	1
T2DM	leukocyte	*LRWD1*	-1.266227308	0.004290068	0.00732537	1
T2DM	leukocyte	*U2AF1L4*	-0.758062865	0.002492997	0.005623364	1
T2DM	leukocyte	*ZNF382*	-0.828378842	0.008548665	0.012481496	1
T2DM	leukocyte	*DUSP14*	-1.192484656	0.001599612	0.005024491	1
T2DM	leukocyte	*PGAP3*	-0.955431924	0.003953822	0.00712268	1
T2DM	leukocyte	*PRPSAP1*	-1.000339248	0.000583417	0.004990048	1
T2DM	leukocyte	*SIGLEC11*	-12.59257378	0.018690336	0.024775415	1
T2DM	leukocyte	*SPC24*	-2.539594273	0.00411682	0.007227428	1
T2DM	leukocyte	*IP6K3*	2.196481105	0.007729212	0.011485655	1
T2DM	leukocyte	*LEMD2*	-1.829184097	0.002113782	0.005283012	1
T2DM	leukocyte	*RPL26*	-0.625789956	0.000802861	0.004990048	1
T2DM	leukocyte	*SNRNP25*	-0.819828595	0.001770435	0.005097196	1
T2DM	leukocyte	*WDR90*	-1.436846809	0.002138249	0.005317933	1
T2DM	leukocyte	*CCDC78*	-2.567996882	0.006298776	0.009667145	1
T2DM	leukocyte	*ZNF75A*	-2.024259783	0.000169122	0.004990048	0.664481785
T2DM	leukocyte	*ASRGL1*	-0.753391375	0.00236826	0.005551753	1
T2DM	leukocyte	*TAF6L*	-1.044558049	0.012073688	0.01688168	1
T2DM	leukocyte	*ZFPL1*	-1.025157016	0.002231945	0.005430873	1
T2DM	leukocyte	*LRP5*	-1.816578183	0.00081092	0.004990048	1
T2DM	leukocyte	*PDZK1IP1*	-0.671379358	0.001798597	0.005114303	1
T2DM	leukocyte	*CTRC*	-0.467761302	0.002091084	0.005283012	1
T2DM	leukocyte	*SDC3*	-1.746466573	0.00253143	0.005660135	1
T2DM	leukocyte	*OMA1*	-1.187570129	0.007550307	0.011262397	1
T2DM	leukocyte	*TM2D1*	-1.386490251	0.000694724	0.004990048	1
T2DM	leukocyte	*FUBP1*	-0.785221387	0.001397041	0.004990048	1

T2DM	leukocyte	*SNX7*	-0.722102423	0.00341469	0.006538167	1
T2DM	leukocyte	*HFM1*	-1.739733908	0.003262174	0.006405338	1
T2DM	leukocyte	*VCAM1*	-0.486720708	0.004551879	0.007568852	1
T2DM	leukocyte	*ZNF496*	-0.903593596	0.002624958	0.005790825	1
T2DM	leukocyte	*TRIM58*	-3.421142457	0.007959497	0.011761137	1
T2DM	leukocyte	*PEA15*	-0.844893531	0.002098687	0.005283012	1
T2DM	leukocyte	*FLVCR1*	-0.722712963	0.002448664	0.005621365	1
T2DM	leukocyte	*TDRD5*	-2.735929873	0.009303471	0.0134061	1
T2DM	leukocyte	*ACP6*	-1.279648192	0.001315027	0.004990048	1
T2DM	leukocyte	*OXER1*	-2.969244011	0.014158311	0.019416406	1
T2DM	leukocyte	*HAAO*	-1.019457339	0.003884999	0.00705773	1
T2DM	leukocyte	*RFTN2*	-1.551451334	0.024216655	0.031277856	1
T2DM	leukocyte	*KCNJ3*	-0.732769114	0.009096599	0.013154413	1
T2DM	leukocyte	*DUSP19*	-1.434182084	0.001558346	0.005009694	1
T2DM	leukocyte	*SMC6*	-1.658072028	0.000691871	0.004990048	1
T2DM	leukocyte	*NOSTRIN*	-0.932815105	0.005200197	0.008312275	1
T2DM	leukocyte	*CTSS*	0.176684482	0.030218638	0.038336786	1
T2DM	leukocyte	*MSX1*	-0.988326092	0.003346871	0.006472193	1
T2DM	leukocyte	*PACRGL*	-0.982335575	0.004195601	0.007249127	1
T2DM	leukocyte	*ERCC3*	-1.142917186	0.004115426	0.007227428	1
T2DM	leukocyte	*TDRD10*	-15.36832206	0.004035328	0.007166394	1
T2DM	leukocyte	*CCNYL1*	-2.394711403	0.000484715	0.004990048	1
T2DM	leukocyte	*DCAF16*	-1.32999112	0.000350925	0.004990048	1
T2DM	leukocyte	*NPPC*	-10.25229789	0.000309087	0.004990048	1
T2DM	leukocyte	*GNPDA2*	-0.727081657	0.006239131	0.009598099	1
T2DM	leukocyte	*NIPAL1*	-0.698264979	0.012635689	0.017567454	1
T2DM	leukocyte	*CGGBP1*	-0.793235969	0.004990163	0.008055197	1
T2DM	leukocyte	*DAPL1*	-3.36453001	0.000121706	0.004990048	0.478184689
T2DM	leukocyte	*EOGT*	-0.940741473	0.002084342	0.005283012	1
T2DM	leukocyte	*POGLUT1*	-0.809901099	0.001306644	0.004990048	1
T2DM	leukocyte	*ATP1A1*	-0.620402266	0.001689882	0.005060368	1
T2DM	leukocyte	*ARPC2*	-0.977026803	0.000715118	0.004990048	1
T2DM	leukocyte	*CCT3*	-0.632119156	0.00400471	0.007145551	1
T2DM	leukocyte	*EOMES*	-3.787935813	0.034624333	0.04349073	1
T2DM	leukocyte	*CWC22*	-1.002914776	0.001945836	0.005192687	1
T2DM	leukocyte	*HDAC11*	-2.397552889	0.004133574	0.007236049	1
T2DM	leukocyte	*SUCLG1*	-0.9229957	0.00108893	0.004990048	1
T2DM	leukocyte	*ICA1L*	-1.249492095	1.01E-05	0.00188355	0.039554547
T2DM	leukocyte	*RYBP*	-0.664636478	0.002934868	0.006117633	1
T2DM	leukocyte	*GTPBP8*	-1.819736877	0.000142028	0.004990048	0.558027549
T2DM	leukocyte	*CADPS*	-0.470678279	0.003234412	0.006381841	1
T2DM	leukocyte	*ATXN7*	-1.099725226	0.000817462	0.004990048	1
T2DM	leukocyte	*PSMD6*	-0.816347354	0.001345509	0.004990048	1
T2DM	leukocyte	*PRICKLE2*	-0.888700014	0.005731819	0.008968665	1
T2DM	leukocyte	*RPL9*	-0.658193072	0.000302985	0.004990048	1
T2DM	leukocyte	*IL17RC*	-1.052284127	0.001146534	0.004990048	1
T2DM	leukocyte	*CRELD1*	-0.611940538	0.009638017	0.013835502	1
T2DM	leukocyte	*TTC14*	-1.569107604	0.000611825	0.004990048	1
T2DM	leukocyte	*CXCL1*	-0.617622623	0.002544095	0.005672956	1
T2DM	leukocyte	*CCDC158*	-2.008920993	0.01238746	0.017265105	1
T2DM	leukocyte	*TM4SF18*	-0.80979119	0.010479947	0.014891758	1
T2DM	leukocyte	*SNRK*	-0.662765603	0.002017787	0.005244224	1
T2DM	leukocyte	*KIAA1143*	-0.69118531	0.00295314	0.006135605	1
T2DM	leukocyte	*NMNAT3*	-0.707575885	0.009690188	0.013900237	1
T2DM	leukocyte	*SMIM12*	-0.898930649	0.001386777	0.004990048	1
T2DM	leukocyte	*TPRA1*	-1.717022647	0.000769184	0.004990048	1
T2DM	leukocyte	*MEAF6*	-0.812802515	0.001327575	0.004990048	1
T2DM	leukocyte	*DNALI1*	-0.939478016	0.002925031	0.006103547	1
T2DM	leukocyte	*CAMK2N2*	-0.602340459	0.002008134	0.005232069	1
T2DM	leukocyte	*RPN1*	-1.027985142	0.000724771	0.004990048	1
T2DM	leukocyte	*IFT122*	-1.139365502	0.004016145	0.00714972	1
T2DM	leukocyte	*RFC4*	-1.095212723	0.003240057	0.006387448	1
T2DM	leukocyte	*TKT*	-0.995687853	0.000742366	0.004990048	1
T2DM	leukocyte	*RFT1*	-1.753064676	0.000531944	0.004990048	1

T2DM	leukocyte	*UVSSA*	-1.114070088	0.024557839	0.031676871	1
T2DM	leukocyte	*ARHGEF3*	-0.864052959	0.003744284	0.006913201	1
T2DM	leukocyte	*LRPAP1*	-0.815145343	0.001004742	0.004990048	1
T2DM	leukocyte	*PIGX*	-1.351806499	0.000496713	0.004990048	1
T2DM	leukocyte	*EXO5*	-1.476760745	0.003555312	0.006699674	1
T2DM	leukocyte	*ERMAP*	-3.289868963	0.000197665	0.004990048	0.776626095
T2DM	leukocyte	*BDH2*	-1.040276259	0.000290675	0.004990048	1
T2DM	leukocyte	*CDC25A*	-1.691258432	0.000768359	0.004990048	1
T2DM	leukocyte	*TEX264*	-1.158829773	0.000696011	0.004990048	1
T2DM	leukocyte	*PPM1M*	-1.536865809	0.000146929	0.004990048	0.57728302
T2DM	leukocyte	*SAP30*	-0.870280723	0.002803912	0.006001505	1
T2DM	leukocyte	*ANXA5*	-0.575800566	0.002318302	0.005497049	1
T2DM	leukocyte	*CEP44*	-1.033443325	0.001697631	0.005060368	1
T2DM	leukocyte	*HPGD*	-1.321237692	0.01505024	0.020446886	1
T2DM	leukocyte	*ARFIP1*	-1.456814569	0.000471202	0.004990048	1
T2DM	leukocyte	*ITGA2*	-0.597902199	0.005273148	0.00840804	1
T2DM	leukocyte	*ELOVL7*	-0.939998663	0.001477123	0.004990048	1
T2DM	leukocyte	*LMBRD2*	-1.143379761	0.000541593	0.004990048	1
T2DM	leukocyte	*STARD4*	-1.272366881	0.000907668	0.004990048	1
T2DM	leukocyte	*CMBL*	-0.984274313	0.00158071	0.005009694	1
T2DM	leukocyte	*PRRC1*	-0.726042465	0.001935934	0.005184924	1
T2DM	leukocyte	*AGGF1*	-1.281690447	0.000663804	0.004990048	1
T2DM	leukocyte	*WDR41*	-1.518961263	0.000739852	0.004990048	1
T2DM	leukocyte	*SERINC5*	-3.645017206	0.000773679	0.004990048	1
T2DM	leukocyte	*RICTOR*	-1.356352724	0.000655658	0.004990048	1
T2DM	leukocyte	*UTP15*	-0.80458784	0.003045956	0.006220403	1
T2DM	leukocyte	*NSA2*	-0.57084823	0.004679217	0.00769232	1
T2DM	leukocyte	*LEAP2*	-1.336050919	0.005417388	0.008574413	1
T2DM	leukocyte	*DACT2*	-3.787985255	0.003704256	0.006868344	1
T2DM	leukocyte	*PDSS2*	-0.929056159	0.001988352	0.005227473	1
T2DM	leukocyte	*STK17A*	-0.798551455	0.002202992	0.005384049	1
T2DM	leukocyte	*GALNT10*	-1.607683334	0.010984642	0.015535874	1
T2DM	leukocyte	*SAP30L*	-1.27770722	0.001062853	0.004990048	1
T2DM	leukocyte	*COG5*	-1.684647275	0.000540851	0.004990048	1
T2DM	leukocyte	*SLU7*	-0.986920075	0.000956317	0.004990048	1
T2DM	leukocyte	*CAMLG*	-0.763675203	0.001285527	0.004990048	1
T2DM	leukocyte	*KIF6*	-1.642968787	0.002634482	0.005802062	1
T2DM	leukocyte	*STEAP1*	-0.938425383	0.025066813	0.032248693	1
T2DM	leukocyte	*FNDC1*	-4.330914903	0.039679886	0.049289368	1
T2DM	leukocyte	*CHMP4C*	-0.890327877	0.001798609	0.005114303	1
T2DM	leukocyte	*BRI3*	-1.237931406	0.000178434	0.004990048	0.701068301
T2DM	leukocyte	*LMTK2*	-0.983778045	0.00104967	0.004990048	1
T2DM	leukocyte	*PEX2*	-0.795842076	0.001761112	0.005084476	1
T2DM	leukocyte	*MED30*	-0.939108521	0.001511626	0.004993935	1
T2DM	leukocyte	*TNFRSF11B*	-2.809203028	0.003720809	0.006886038	1
T2DM	leukocyte	*CSMD3*	-59.50096086	0.004301858	0.007328649	1
T2DM	leukocyte	*OSGIN2*	-1.323287981	0.000742161	0.004990048	1
T2DM	leukocyte	*DEFB1*	-0.409924668	0.003218576	0.006372159	1
T2DM	leukocyte	*SUN1*	-0.73365236	0.001759714	0.005084476	1
T2DM	leukocyte	*TMEM184A*	-1.007903516	0.00143947	0.004990048	1
T2DM	leukocyte	*INTS1*	-0.956859144	0.00194218	0.005191039	1
T2DM	leukocyte	*YWHAZ*	-0.832921544	0.000808463	0.004990048	1
T2DM	leukocyte	*DCAF13*	-0.933487823	0.002042933	0.00526208	1
T2DM	leukocyte	*INTS8*	-1.157185747	0.00721561	0.010841351	1
T2DM	leukocyte	*GEM*	-0.975402921	0.002348487	0.005521966	1
T2DM	leukocyte	*RPP25L*	-1.548117303	0.000719816	0.004990048	1
T2DM	leukocyte	*FAM219A*	-1.51139296	0.00081244	0.004990048	1
T2DM	leukocyte	*NUDT2*	-1.076301198	0.000976088	0.004990048	1
T2DM	leukocyte	*METTL2B*	-0.726064231	0.029344703	0.037264168	1
T2DM	leukocyte	*FXN*	-1.803633372	0.002007521	0.005232069	1
T2DM	leukocyte	*MAMDC2*	-2.108154744	0.002442916	0.005621365	1
T2DM	leukocyte	*PRSS37*	-488.4734141	0.004352311	0.007361269	1
T2DM	leukocyte	*C9orf64*	-1.276092682	0.001106838	0.004990048	1
T2DM	leukocyte	*ANKS6*	-0.677910371	0.023972325	0.030992848	1

T2DM	leukocyte	*CLDN3*	-0.989977434	0.000820193	0.004990048	1
T2DM	leukocyte	*WNK2*	-1.538562703	0.00085634	0.004990048	1
T2DM	leukocyte	*NOL6*	-1.83337211	0.001276297	0.004990048	1
T2DM	leukocyte	*PIGO*	-2.186848831	8.05E-05	0.004990048	0.316381002
T2DM	leukocyte	*ARHGAP12*	-1.016298104	0.001410139	0.004990048	1
T2DM	leukocyte	*WRN*	-1.053011922	0.001612365	0.005033031	1
T2DM	leukocyte	*CFL2*	-0.940221827	0.001565904	0.005009694	1
T2DM	leukocyte	*PGM2L1*	-1.711984664	0.000541632	0.004990048	1
T2DM	leukocyte	*PHYHIPL*	-0.918165446	0.002612801	0.005770487	1
T2DM	leukocyte	*PKNOX2*	3.201250027	0.021902887	0.028590181	1
T2DM	leukocyte	*RPL36AL*	-0.710869845	0.000918301	0.004990048	1
T2DM	leukocyte	*TMEM63C*	-0.812273896	0.002809746	0.006001505	1
T2DM	leukocyte	*OXGR1*	-1.974735624	0.004505873	0.007539852	1
T2DM	leukocyte	*VDAC2*	-0.872915457	0.000522061	0.004990048	1
T2DM	leukocyte	*SNAPC4*	-0.912658998	0.003653109	0.006815321	1
T2DM	leukocyte	*TSC1*	-1.333404783	0.001267956	0.004990048	1
T2DM	leukocyte	*ZMYND19*	-0.89911052	0.002631911	0.005799651	1
T2DM	leukocyte	*METTL17*	-1.326516733	0.00121593	0.004990048	1
T2DM	leukocyte	*VWA2*	-2.473823449	0.001779024	0.005098084	1
T2DM	leukocyte	*METTL3*	-0.940914285	0.004182954	0.007242343	1
T2DM	leukocyte	*TRUB1*	-1.407986169	0.000577218	0.004990048	1
T2DM	leukocyte	*ARHGAP42*	-1.366853391	0.004209721	0.007263735	1
T2DM	leukocyte	*SLC39A13*	-0.903641063	0.001997654	0.005227473	1
T2DM	leukocyte	*PSMC3*	-0.759394488	0.001380183	0.004990048	1
T2DM	leukocyte	*CPSF2*	-0.789745512	0.002669158	0.005839155	1
T2DM	leukocyte	*IFI27L1*	-1.005784671	0.00124447	0.004990048	1
T2DM	leukocyte	*SMCO4*	-0.969864809	0.002283013	0.005462378	1
T2DM	leukocyte	*ABTB2*	-1.236011644	0.006767654	0.010278358	1
T2DM	leukocyte	*CEP57*	-0.903689932	0.000450011	0.004990048	1
T2DM	leukocyte	*JAM3*	-0.712316176	0.004476914	0.007504245	1
T2DM	leukocyte	*IKBIP*	-0.982282154	0.005682775	0.008916678	1
T2DM	leukocyte	*ZFYVE19*	-0.731219261	0.016874261	0.022685009	1
T2DM	leukocyte	*CKB*	-0.830102412	9.05E-05	0.004990048	0.35540689
T2DM	leukocyte	*BTRC*	-1.647282273	0.002780672	0.005973352	1
T2DM	leukocyte	*BAG5*	-0.914247685	0.002799492	0.005997385	1
T2DM	leukocyte	*LARP6*	-0.82523617	0.001344363	0.004990048	1
T2DM	leukocyte	*ZNF319*	-2.792984664	0.000626149	0.004990048	1
T2DM	leukocyte	*SGPL1*	-1.955795675	2.81E-05	0.003340136	0.110224481
T2DM	leukocyte	*FRS2*	-1.112522672	0.00054824	0.004990048	1
T2DM	leukocyte	*PCBD1*	-1.032508398	0.000186684	0.004990048	0.733481118
T2DM	leukocyte	*COX11*	-1.113097137	0.000742702	0.004990048	1
T2DM	leukocyte	*WBP1L*	-1.999895717	0.00123587	0.004990048	1
T2DM	leukocyte	*CYB5R2*	-1.219119528	0.000176459	0.004990048	0.693308306
T2DM	leukocyte	*TUB*	-1.351931353	0.000873117	0.004990048	1
T2DM	leukocyte	*RIC3*	-0.726112857	0.001748635	0.005084476	1
T2DM	leukocyte	*XRRA1*	-1.057135699	0.000904486	0.004990048	1
T2DM	leukocyte	*CDYL2*	-0.853823715	0.007815845	0.011596849	1
T2DM	leukocyte	*ZNF143*	-0.910379092	0.00234775	0.005521966	1
T2DM	leukocyte	*CCDC68*	-0.852320408	0.007339284	0.011006125	1
T2DM	leukocyte	*ZSCAN21*	-1.412478465	0.002429873	0.005602682	1
T2DM	leukocyte	*RIMKLB*	-0.911048312	0.001738445	0.005075243	1
T2DM	leukocyte	*GALR1*	0.976284375	0.013267077	0.018344791	1
T2DM	leukocyte	*ATF7IP2*	-1.355521819	0.000482346	0.004990048	1
T2DM	leukocyte	*COG1*	-0.952702921	0.001053455	0.004990048	1
T2DM	leukocyte	*B2M*	-0.61546798	0.00091579	0.004990048	1
T2DM	leukocyte	*AP1G1*	-0.670156843	0.003105764	0.00627703	1
T2DM	leukocyte	*SLFN5*	-0.745534751	0.002507225	0.005648444	1
T2DM	leukocyte	*SAAL1*	-0.866666229	0.003261763	0.006405338	1
T2DM	leukocyte	*SCNN1G*	-7.859895386	0.00338113	0.006503419	1
T2DM	leukocyte	*DCTN5*	-1.074583005	0.00079179	0.004990048	1
T2DM	leukocyte	*STAT6*	-1.67791014	0.000836455	0.004990048	1
T2DM	leukocyte	*PATL1*	-0.862198362	0.000967732	0.004990048	1
T2DM	leukocyte	*MRPL16*	-1.205704337	0.0163751	0.022082136	1
T2DM	leukocyte	*GREM1*	-1.611931881	0.000586359	0.004990048	1

T2DM	leukocyte	*TSC22D4*	-1.264917833	0.001297118	0.004990048	1
T2DM	leukocyte	*MS4A14*	0.179420661	0.020465312	0.02685645	1
T2DM	leukocyte	*DIS3L*	-1.398748069	0.002598091	0.005751212	1
T2DM	leukocyte	*RCCD1*	-2.970256587	2.98E-05	0.003343813	0.117033452
T2DM	leukocyte	*MAPRE2*	-0.884859987	0.001864779	0.005146173	1
T2DM	leukocyte	*NAT16*	-0.609572436	0.002742411	0.005930931	1
T2DM	leukocyte	*GNGT2*	0.362775212	0.015971717	0.02161656	1
T2DM	leukocyte	*SLC27A4*	-0.879325819	0.010001799	0.014296244	1
T2DM	leukocyte	*URM1*	-1.284891463	0.001088325	0.004990048	1
T2DM	leukocyte	*CERCAM*	-0.762047499	0.006842593	0.010372125	1
T2DM	leukocyte	*DOLPP1*	-1.127931173	0.001856891	0.005146173	1
T2DM	leukocyte	*ENDOG*	-1.158664805	0.000840538	0.004990048	1
T2DM	leukocyte	*RNF214*	-1.596415926	0.001456829	0.004990048	1
T2DM	leukocyte	*POP5*	-1.08596641	0.000642636	0.004990048	1
T2DM	leukocyte	*ACAA2*	-1.091732471	0.000422631	0.004990048	1
T2DM	leukocyte	*FN3K*	-1.199474648	0.002745938	0.005931166	1
T2DM	leukocyte	*ZNF23*	-0.847212877	0.003464956	0.006599036	1
T2DM	leukocyte	*ZNF229*	-1.215738431	0.00281669	0.006001505	1
T2DM	leukocyte	*ZNF180*	-0.917791783	0.004186401	0.007242787	1
T2DM	leukocyte	*CA4*	-0.718099658	0.002528022	0.005660135	1
T2DM	leukocyte	*GPX4*	-1.008395133	0.000413363	0.004990048	1
T2DM	leukocyte	*ZNF641*	-1.452886146	0.001990325	0.005227473	1
T2DM	leukocyte	*CACNB3*	-0.861325474	0.040056797	0.049694712	1
T2DM	leukocyte	*TP53I13*	-1.113979874	0.000169555	0.004990048	0.666180856
T2DM	leukocyte	*TUBA1C*	-0.610821573	0.002001483	0.005227473	1
T2DM	leukocyte	*ZNF528*	-3.839917834	0.00018687	0.004990048	0.734212755
T2DM	leukocyte	*RAB4B*	-1.184025714	0.000841405	0.004990048	1
T2DM	leukocyte	*TMC4*	-0.976455251	0.001216668	0.004990048	1
T2DM	leukocyte	*C19orf33*	-0.759575132	0.00405982	0.007184034	1
T2DM	leukocyte	*DNAAF3*	-3.091529722	0.003938597	0.007108734	1
T2DM	leukocyte	*CHAF1A*	-1.493924839	0.00079042	0.004990048	1
T2DM	leukocyte	*UBXN6*	-1.351553565	0.00036378	0.004990048	1
T2DM	leukocyte	*NXN*	-0.703160834	0.001579054	0.005009694	1
T2DM	leukocyte	*KIFC2*	-4.138293495	5.36E-05	0.004896114	0.21066247
T2DM	leukocyte	*SLC43A2*	0.746906879	0.015865051	0.021486999	1
T2DM	leukocyte	*SERPINF2*	0.391256044	0.020142665	0.026486122	1
T2DM	leukocyte	*SRR*	-0.834201601	0.014756364	0.020103243	1
T2DM	leukocyte	*TRPV3*	-4.540437638	0.001995066	0.005227473	1
T2DM	leukocyte	*C19orf48*	-1.583657744	0.002486375	0.005622792	1
T2DM	leukocyte	*ZNF83*	-1.220802688	0.001209996	0.004990048	1
T2DM	leukocyte	*ZNF558*	-0.970904393	0.013813741	0.018990268	1
T2DM	leukocyte	*NDUFV1*	-0.858358187	0.001481832	0.004990048	1
T2DM	leukocyte	*ZNF232*	-0.791550158	0.004703931	0.007720459	1
T2DM	leukocyte	*MIS12*	-1.150831874	0.001613307	0.005033031	1
T2DM	leukocyte	*HID1*	-0.990838329	0.002249929	0.005439981	1
T2DM	leukocyte	*SRP68*	-0.773231395	0.002023381	0.005244224	1
T2DM	leukocyte	*TMEM68*	-1.233526164	0.001859308	0.005146173	1
T2DM	leukocyte	*GSDMA*	-5.891767728	0.005874781	0.009145014	1
T2DM	leukocyte	*MLST8*	-0.721880273	0.00346839	0.006602377	1
T2DM	leukocyte	*ECI1*	-0.737660323	0.001896139	0.005146173	1
T2DM	leukocyte	*ABCA3*	-0.978271876	0.00067897	0.004990048	1
T2DM	leukocyte	*BSCL2*	-0.814322472	0.001161286	0.004990048	1
T2DM	leukocyte	*C2CD3*	-1.703577565	0.001588703	0.005020048	1
T2DM	leukocyte	*TRANK1*	-2.747800707	0.000290167	0.004990048	1
T2DM	leukocyte	*RPSA*	-0.628417573	0.001007409	0.004990048	1
T2DM	leukocyte	*ENTPD3*	-0.567803065	0.004948043	0.008003288	1
T2DM	leukocyte	*ULK4*	-1.314636644	0.000709908	0.004990048	1
T2DM	leukocyte	*LTBP3*	-0.704949293	0.004911531	0.007970068	1
T2DM	leukocyte	*SAC3D1*	-1.091830511	0.001737653	0.005075243	1
T2DM	leukocyte	*CCDC88B*	-2.316313041	0.009738092	0.01395367	1
T2DM	leukocyte	*PBK*	-1.973902597	0.003587879	0.006731985	1
T2DM	leukocyte	*KIAA1586*	-1.312382318	0.000420881	0.004990048	1
T2DM	leukocyte	*FAM83B*	-0.781308482	0.021110353	0.027647525	1
T2DM	leukocyte	*LMBRD1*	-1.17564691	0.000531474	0.004990048	1

T2DM	leukocyte	*TTC39C*	-0.926749587	0.000909652	0.004990048	1
T2DM	leukocyte	*POLR2J3*	-0.934527797	0.003669375	0.006835929	1
T2DM	leukocyte	*COA6*	-1.016471014	0.001615335	0.005033031	1
T2DM	leukocyte	*PXK*	-1.251533061	0.001319454	0.004990048	1
T2DM	leukocyte	*ING5*	-1.721890804	0.000632201	0.004990048	1
T2DM	leukocyte	*BMP1*	-0.795651907	0.023175792	0.030052042	1
T2DM	leukocyte	*HEXIM2*	-1.080094517	0.004184301	0.007242343	1
T2DM	leukocyte	*SERINC2*	-0.634174474	0.002055814	0.00526208	1
T2DM	leukocyte	*TRAPPC11*	-0.738549807	0.003251709	0.006400784	1
T2DM	leukocyte	*ING2*	-0.689482905	0.002208077	0.00539188	1
T2DM	leukocyte	*ADAM9*	-1.447951894	0.008818977	0.012807333	1
T2DM	leukocyte	*NDUFS5*	-0.768257448	0.00051462	0.004990048	1
T2DM	leukocyte	*DNAJC21*	-1.120974297	0.000358083	0.004990048	1
T2DM	leukocyte	*CNNM3*	-0.918680296	0.001733988	0.00507285	1
T2DM	leukocyte	*GSTM4*	-0.978433499	0.005100531	0.008189615	1
T2DM	leukocyte	*SHOX2*	-6.812471475	0.014670683	0.020014276	1
T2DM	leukocyte	*PPIP5K1*	-2.924976234	0.000103505	0.004990048	0.406669438
T2DM	leukocyte	*ADAL*	-2.164157376	0.010034716	0.014326453	1
T2DM	leukocyte	*LCMT2*	-1.250800258	0.006258406	0.009623983	1
T2DM	leukocyte	*ZNF507*	-1.218795246	0.000846804	0.004990048	1
T2DM	leukocyte	*NSG1*	-0.654071497	0.004269282	0.007310294	1
T2DM	leukocyte	*ZBTB49*	-2.478368283	0.002104573	0.005283012	1
T2DM	leukocyte	*GFM1*	-0.782912914	0.001726406	0.005070552	1
T2DM	leukocyte	*FSTL5*	-0.557265061	0.007746658	0.011505329	1
T2DM	leukocyte	*CTRB1*	-0.405400192	0.001935474	0.005184924	1
T2DM	leukocyte	*CTRB2*	-0.373700599	0.00249323	0.005623364	1
T2DM	leukocyte	*TMEM129*	-1.781773233	2.26E-05	0.003059063	0.088712829
T2DM	leukocyte	*PPIC*	-0.443327504	0.009248729	0.013349836	1
T2DM	leukocyte	*STXBP6*	-1.262149703	0.000167716	0.004990048	0.658956787
T2DM	leukocyte	*MFF*	-0.923685092	0.001136151	0.004990048	1
T2DM	leukocyte	*CPLX1*	-0.811547863	0.010307919	0.014679164	1
T2DM	leukocyte	*ROR2*	-2.415549963	0.008139916	0.011987155	1
T2DM	leukocyte	*FAM110B*	-0.92318018	0.003393466	0.006510382	1
T2DM	leukocyte	*ATF5*	-1.147952167	0.001157301	0.004990048	1
T2DM	leukocyte	*XPO6*	-1.773291024	0.000353409	0.004990048	1
T2DM	leukocyte	*RGS14*	-1.280910982	0.003302703	0.006432819	1
T2DM	leukocyte	*TBC1D10B*	-1.226468725	0.000970453	0.004990048	1
T2DM	leukocyte	*PRELID1*	-0.70590814	0.001609136	0.005033031	1
T2DM	leukocyte	*CXCL10*	-151.2856829	0.004227252	0.007272975	1
T2DM	leukocyte	*NMD3*	-0.900167351	0.001596006	0.005024491	1
T2DM	leukocyte	*STK32A*	-1.048871901	0.002925167	0.006103547	1
T2DM	leukocyte	*SNUPN*	-2.817007253	0.000130039	0.004990048	0.510921471
T2DM	leukocyte	*CRADD*	-0.950279779	0.003370986	0.006497619	1
T2DM	leukocyte	*COL22A1*	-8.872209971	0.004409921	0.007423556	1
T2DM	leukocyte	*SDC2*	-2.339104271	0.000486994	0.004990048	1
T2DM	leukocyte	*CLIC4*	-0.514605602	0.004119538	0.007228971	1
T2DM	leukocyte	*SLC38A11*	-7.51265852	0.001894773	0.005146173	1
T2DM	leukocyte	*CCDC8*	-1.11378087	0.03138959	0.039719709	1
T2DM	leukocyte	*HINT1*	-0.659693653	0.000817201	0.004990048	1
T2DM	leukocyte	*DTWD2*	-2.393614844	0.015162527	0.020578089	1
T2DM	leukocyte	*INO80E*	-1.759073169	2.92E-06	0.000882318	0.011470136
T2DM	leukocyte	*C15orf40*	-0.930677341	0.001725302	0.005070552	1
T2DM	leukocyte	*HIC2*	-2.914471466	0.016319557	0.022026637	1
T2DM	leukocyte	*AGPAT2*	-1.006252125	0.000748625	0.004990048	1
T2DM	leukocyte	*FASN*	-1.832256987	0.000154598	0.004990048	0.607417278
T2DM	leukocyte	*MT1E*	-0.5452943	0.002115087	0.005283012	1
T2DM	leukocyte	*LDB2*	-0.931912137	0.006959034	0.010520217	1
T2DM	leukocyte	*RAC3*	-1.345209444	0.001051906	0.004990048	1
T2DM	leukocyte	*LIMS1*	-1.189771932	0.000308825	0.004990048	1
T2DM	leukocyte	*CSGALNACT2*	-2.48839572	0.001066774	0.004990048	1
T2DM	leukocyte	*AVEN*	-2.778287874	0.000420554	0.004990048	1
T2DM	leukocyte	*CTNND2*	-1.055537944	0.00115483	0.004990048	1
T2DM	leukocyte	*WNT10B*	-52.9403424	0.003805399	0.006976862	1
T2DM	leukocyte	*CALML6*	0.860964004	0.019673552	0.025930018	1

T2DM	leukocyte	*TM4SF4*	-0.866361524	0.001422796	0.004990048	1
T2DM	leukocyte	*OTUD3*	-0.696114094	0.001967748	0.005212073	1
T2DM	leukocyte	*MAP3K2*	-1.281315797	0.000325004	0.004990048	1
T2DM	leukocyte	*PUSL1*	-2.130968913	0.000505939	0.004990048	1
T2DM	leukocyte	*IFFO2*	-0.831266966	0.003072943	0.006249984	1
T2DM	leukocyte	*MYRIP*	-1.115106909	0.005923214	0.009205818	1
T2DM	leukocyte	*FAM153A*	-1.612171711	0.016255507	0.021947728	1
T2DM	leukocyte	*ZNF778*	-1.313979084	0.000768884	0.004990048	1
T2DM	leukocyte	*NIPA1*	-1.230205202	0.000600431	0.004990048	1
T2DM	leukocyte	*UBE2E1*	-1.656220988	0.000562565	0.004990048	1
T2DM	leukocyte	*ANKK1*	-22.75887303	0.005285646	0.008421454	1
T2DM	leukocyte	*USP47*	-1.13421842	0.00070269	0.004990048	1
T2DM	leukocyte	*PDCD6IP*	-0.898533279	0.001568013	0.005009694	1
T2DM	leukocyte	*CRTAP*	-1.76764773	0.000472911	0.004990048	1
T2DM	leukocyte	*STX8*	-1.410924662	0.000308712	0.004990048	1
T2DM	leukocyte	*NFRKB*	-1.680485202	0.000925771	0.004990048	1
T2DM	leukocyte	*SETMAR*	-0.875261739	0.002724546	0.005904436	1
T2DM	leukocyte	*CST2*	-2.058848875	0.001149827	0.004990048	1
T2DM	leukocyte	*KRT8*	-0.373345541	0.003708402	0.006872788	1
T2DM	leukocyte	*MGMT*	-1.217776344	0.001217118	0.004990048	1
T2DM	leukocyte	*NFXL1*	-1.669807613	0.000416878	0.004990048	1
T2DM	leukocyte	*KRT4*	-46.1237745	0.009020261	0.01305844	1
T2DM	leukocyte	*SLC23A1*	-6.739266157	0.01111956	0.015704081	1
T2DM	leukocyte	*NPAS2*	-0.707402623	0.004505215	0.007539852	1
T2DM	leukocyte	*NUDT9*	-0.662188635	0.010002641	0.014296244	1
T2DM	leukocyte	*IRX1*	-1.266769452	0.004313727	0.007328649	1
T2DM	leukocyte	*CDH2*	-0.704123506	0.004296604	0.007328649	1
T2DM	leukocyte	*HSPA4*	-1.006044331	0.000877627	0.004990048	1
T2DM	leukocyte	*ARMC10*	-1.182300784	0.003492189	0.006628411	1
T2DM	leukocyte	*ACYP2*	-0.851129931	0.001580744	0.005009694	1
T2DM	leukocyte	*ATF7*	-0.96843867	0.002014617	0.005242007	1
T2DM	leukocyte	*RASA4B*	-1.573140978	0.008659741	0.0126174	1
T2DM	leukocyte	*KCNS3*	-1.170691489	0.004504208	0.007539852	1
T2DM	leukocyte	*FOXN2*	-2.964932265	0.000311274	0.004990048	1
T2DM	leukocyte	*GPR27*	-1.001978022	0.000904688	0.004990048	1
T2DM	leukocyte	*MTSS1*	-1.215749294	0.002835292	0.006015044	1
T2DM	leukocyte	*TMEM43*	-0.912517391	0.002381541	0.005560194	1
T2DM	leukocyte	*RPS9*	-0.601048627	0.000974625	0.004990048	1
T2DM	leukocyte	*NDUFA3*	-1.031950749	0.000782112	0.004990048	1
T2DM	leukocyte	*ZNF160*	-0.95968865	0.001478085	0.004990048	1
T2DM	leukocyte	*ZNF415*	-0.830342566	0.006475664	0.009919253	1
T2DM	leukocyte	*TSNARE1*	0.77092965	0.020539443	0.026935738	1
T2DM	leukocyte	*SOX7*	-1.146413024	0.004310789	0.007328649	1
T2DM	leukocyte	*TRMT61B*	-0.658268055	0.012887553	0.017882851	1
T2DM	leukocyte	*INSR*	-0.652213096	0.002465422	0.005622792	1
T2DM	leukocyte	*MFN1*	-0.872726339	0.006217827	0.009569073	1
T2DM	leukocyte	*KCNMB3*	-1.453535915	0.004545238	0.007568852	1
T2DM	leukocyte	*ATP6V0E2*	-0.806723886	0.001382949	0.004990048	1
T2DM	leukocyte	*JAGN1*	-1.036880469	0.001199988	0.004990048	1
T2DM	leukocyte	*MORN4*	-0.614181095	0.005367637	0.008517547	1
T2DM	leukocyte	*TRIM8*	-1.084412965	0.000951136	0.004990048	1
T2DM	leukocyte	*CDC42BPG*	-0.646699281	0.003674923	0.006839778	1
T2DM	leukocyte	*ZNF440*	-3.856935367	2.98E-05	0.003343813	0.11690834
T2DM	leukocyte	*EXOSC1*	-1.292980566	0.000659169	0.004990048	1
T2DM	leukocyte	*PGAM1*	-0.940360163	0.000297127	0.004990048	1
T2DM	leukocyte	*ESCO2*	-2.46264608	0.038840071	0.048353181	1
T2DM	leukocyte	*MRPL36*	-0.891293974	0.00239871	0.005576645	1
T2DM	leukocyte	*MCC*	-1.155183677	0.00315035	0.006311945	1
T2DM	leukocyte	*ZNF561*	-1.120272161	0.001317578	0.004990048	1
T2DM	leukocyte	*PPID*	-1.166295511	0.008628556	0.012584111	1
T2DM	leukocyte	*RXFP1*	-18.55370939	0.01229086	0.017154809	1
T2DM	leukocyte	*MAP6*	-0.62889606	0.004071987	0.007189	1
T2DM	leukocyte	*NMUR1*	-9.566563372	0.004004087	0.007145551	1
T2DM	leukocyte	*CLSTN1*	-0.848860732	0.000579154	0.004990048	1

T2DM	leukocyte	*PIK3CD*	0.652869713	0.0403489	0.049993954	1
T2DM	leukocyte	*ENC1*	-0.779299653	0.006716891	0.010221016	1
T2DM	leukocyte	*RGS19*	-1.884059364	7.49E-05	0.004990048	0.294167597
T2DM	leukocyte	*ANO5*	-1.070460769	0.004811812	0.007855834	1
T2DM	leukocyte	*GPHN*	-1.673872633	0.000858472	0.004990048	1
T2DM	leukocyte	*VAT1L*	-1.02416132	0.008130167	0.011977287	1
T2DM	leukocyte	*LGALS4*	-0.479517328	0.005243219	0.008370827	1
T2DM	leukocyte	*LRRC34*	-1.089844109	0.003895544	0.007059776	1
T2DM	leukocyte	*SPATA5L1*	-2.072026045	0.000236253	0.004990048	0.928237022
T2DM	leukocyte	*CTPS1*	-0.823882055	0.003998081	0.007143456	1
T2DM	leukocyte	*METTL18*	-1.167431741	0.002939437	0.006123567	1
T2DM	leukocyte	*PWWP2B*	-1.173310587	0.00136191	0.004990048	1
T2DM	leukocyte	*PTEN*	-1.066006507	0.001373851	0.004990048	1
T2DM	leukocyte	*CYP4F11*	-1.929601662	0.016876631	0.022685009	1
T2DM	leukocyte	*TLN2*	-1.723271248	0.004100904	0.00720956	1
T2DM	leukocyte	*TVP23B*	-1.645730519	0.000891893	0.004990048	1
T2DM	leukocyte	*SRGAP2C*	-2.009794345	0.000357939	0.004990048	1
T2DM	leukocyte	*ATPAF2*	-1.029461416	0.006988579	0.010556758	1
T2DM	leukocyte	*ZNF556*	0.702234848	0.020616989	0.027028412	1
T2DM	leukocyte	*ZNF554*	-0.988790865	0.003612906	0.006763117	1
T2DM	leukocyte	*GAP43*	-0.679881553	0.004812673	0.007855834	1
T2DM	leukocyte	*REG1B*	-0.425822061	0.002108197	0.005283012	1
T2DM	leukocyte	*NME6*	-1.593493769	0.002085199	0.005283012	1
T2DM	leukocyte	*TEFM*	-1.202039766	0.001977631	0.005222499	1
T2DM	leukocyte	*MBOAT1*	-1.951930053	0.001858722	0.005146173	1
T2DM	leukocyte	*ZNF131*	-0.959118167	0.0017517	0.005084476	1
T2DM	leukocyte	*BSG*	-0.822186516	0.000972268	0.004990048	1
T2DM	leukocyte	*HINFP*	-2.85469019	0.000686573	0.004990048	1
T2DM	leukocyte	*CERS6*	-0.916773649	0.001240775	0.004990048	1
T2DM	leukocyte	*SPTLC3*	0.961750613	0.01953957	0.025770718	1
T2DM	leukocyte	*COPRS*	-0.655932605	0.00318631	0.006339351	1
T2DM	leukocyte	*SUCLG2*	-1.764818565	0.000468597	0.004990048	1
T2DM	leukocyte	*CSDC2*	-2.582784505	0.003562158	0.006706143	1
T2DM	leukocyte	*ARNT2*	-0.615592927	0.018942412	0.025058833	1
T2DM	leukocyte	*FUT9*	-3.983409621	0.00500911	0.008075829	1
T2DM	leukocyte	*ZNF24*	-1.067520402	0.000502474	0.004990048	1
T2DM	leukocyte	*MANEA*	-1.078276715	0.001701386	0.005060368	1
T2DM	leukocyte	*AFF1*	-1.045899366	0.001259109	0.004990048	1
T2DM	leukocyte	*CTSW*	-1.297525671	4.04E-05	0.004175341	0.158662972
T2DM	leukocyte	*SMPDL3A*	-2.403529781	0.002482924	0.005622792	1
T2DM	leukocyte	*RAD9A*	-2.068342191	0.000130863	0.004990048	0.514158791
T2DM	leukocyte	*EFEMP2*	-0.944317561	0.003347288	0.006472193	1
T2DM	leukocyte	*FUT10*	-2.536994091	0.032872008	0.041488635	1
T2DM	leukocyte	*NAA16*	-0.767507059	0.010647427	0.015091537	1
T2DM	leukocyte	*OVOL1*	-3.039858746	0.000146505	0.004990048	0.575616975
T2DM	leukocyte	*DMXL1*	-1.518704474	0.000971813	0.004990048	1
T2DM	leukocyte	*NADSYN1*	-1.744571349	0.001562753	0.005009694	1
T2DM	leukocyte	*RNASEH2C*	-1.160518068	0.000167405	0.004990048	0.657735293
T2DM	leukocyte	*LCLAT1*	-0.992987978	0.001114044	0.004990048	1
T2DM	leukocyte	*SH3RF3*	-1.379644903	0.003917094	0.007085756	1
T2DM	leukocyte	*DCAKD*	-0.749434959	0.001842654	0.005146173	1
T2DM	leukocyte	*TADA2B*	-1.125822224	0.001067856	0.004990048	1
T2DM	leukocyte	*TRMT112*	-0.822925145	0.001460849	0.004990048	1
T2DM	leukocyte	*NOC3L*	-1.031216665	0.000606939	0.004990048	1
T2DM	leukocyte	*RHOD*	-0.61781187	0.002856967	0.006048134	1
T2DM	leukocyte	*PARP14*	-1.040440931	0.000821415	0.004990048	1
T2DM	leukocyte	*VANGL1*	-0.882735716	0.004136464	0.007236049	1
T2DM	leukocyte	*IQCB1*	-2.59954037	0.000141837	0.004990048	0.557276859
T2DM	leukocyte	*ZNF483*	-0.814692158	0.001276682	0.004990048	1
T2DM	leukocyte	*SLC2A14*	-3.431139289	0.004851079	0.00790211	1
T2DM	leukocyte	*MZT2A*	-0.705022468	0.003069339	0.006249984	1
T2DM	leukocyte	*PPP1R3B*	-3.305220867	1.55E-06	0.00053944	0.006075666
T2DM	leukocyte	*MAP3K11*	-0.912846754	0.001097722	0.004990048	1
T2DM	leukocyte	*NDNF*	0.832790883	0.018545683	0.024608575	1

T2DM	leukocyte	*ARV1*	-0.852774382	0.001995184	0.005227473	1
T2DM	leukocyte	*EHBP1L1*	-0.862104195	0.007841189	0.011622224	1
T2DM	leukocyte	*PPP1R14B*	-0.787595219	0.001156535	0.004990048	1
T2DM	leukocyte	*TNFRSF10D*	-1.021984799	0.006183334	0.009527184	1
T2DM	leukocyte	*GMPPB*	-1.293042144	0.004583111	0.007597909	1
T2DM	leukocyte	*NUDT18*	-0.906566628	0.003518478	0.006649019	1
T2DM	leukocyte	*NUDT4*	-0.808911533	0.003932133	0.007103149	1
T2DM	leukocyte	*PC*	-1.472002731	0.001900041	0.005146173	1
T2DM	leukocyte	*UQCRH*	-0.829941122	0.00074452	0.004990048	1
T2DM	leukocyte	*HEG1*	-0.884991961	0.003536242	0.006673341	1
T2DM	leukocyte	*ENDOV*	-1.97433703	0.000373458	0.004990048	1
T2DM	leukocyte	*DPY19L1*	-1.116013569	0.002044655	0.00526208	1
T2DM	leukocyte	*GPR160*	-1.336498024	0.002877895	0.006055188	1
T2DM	leukocyte	*XXYLT1*	-1.47289918	0.002302967	0.005478715	1
T2DM	leukocyte	*GNG5*	-0.852485077	0.000682846	0.004990048	1
T2DM	leukocyte	*C9orf131*	-1.488704562	0.025405496	0.032641659	1
T2DM	leukocyte	*FAM53A*	-4.638204607	0.001109058	0.004990048	1
T2DM	leukocyte	*SELP*	-5.283850847	0.004043181	0.007168617	1
T2DM	leukocyte	*CTU2*	-0.84446018	0.004343604	0.007358292	1
T2DM	leukocyte	*ADCY6*	-0.955860791	0.004380564	0.007389967	1
T2DM	leukocyte	*PODN*	-0.832177774	0.012684926	0.017627731	1
T2DM	leukocyte	*RALGAPA1*	-0.914975478	0.000946322	0.004990048	1
T2DM	leukocyte	*CNTNAP2*	1.857300612	0.006080337	0.009402422	1
T2DM	leukocyte	*GOLT1A*	-0.91090674	0.001771229	0.005097196	1
T2DM	leukocyte	*NPAS4*	-2.622846402	2.61E-05	0.003310187	0.102412252
T2DM	leukocyte	*ANGEL2*	-1.301788046	0.000977337	0.004990048	1
T2DM	leukocyte	*ZNF266*	-0.815491597	0.000786814	0.004990048	1
T2DM	leukocyte	*BRSK2*	-0.841741338	0.002924105	0.006103547	1
T2DM	leukocyte	*BRMS1*	-0.848761625	0.001784384	0.005100156	1
T2DM	leukocyte	*HRAS*	-0.850294402	0.001547305	0.005009694	1
T2DM	leukocyte	*SRP72*	-0.705140829	0.001808421	0.005122825	1
T2DM	leukocyte	*PCP2*	-3.539778913	0.002874248	0.006055188	1
T2DM	leukocyte	*DENND6A*	-0.838593297	0.003192127	0.006345399	1
T2DM	leukocyte	*YIF1A*	-1.030112933	6.99E-05	0.004990048	0.274636325
T2DM	leukocyte	*NDUFA11*	-0.881275493	0.000776936	0.004990048	1
T2DM	leukocyte	*RSRC1*	-1.966571373	0.000409593	0.004990048	1
T2DM	leukocyte	*DHX36*	-0.971395911	0.000876301	0.004990048	1
T2DM	leukocyte	*PSMD2*	-0.721594297	0.00312136	0.006295597	1
T2DM	leukocyte	*PPM1E*	-0.582246269	0.014177854	0.019436423	1
T2DM	leukocyte	*ZNF408*	-2.84662562	0.001404927	0.004990048	1
T2DM	leukocyte	*ARHGAP1*	-0.839674326	0.003257194	0.006405338	1
T2DM	leukocyte	*MED16*	-0.845220313	0.002182946	0.005356498	1
T2DM	leukocyte	*CHST1*	-1.74250714	0.004302373	0.007328649	1
T2DM	leukocyte	*DOLK*	-1.665701537	0.003229015	0.00638169	1
T2DM	leukocyte	*CCNE2*	-1.076181217	0.0046347	0.007664031	1
T2DM	leukocyte	*ZNF519*	-1.707286586	0.001725485	0.005070552	1
T2DM	leukocyte	*TMEM9B*	-0.939356085	0.00094435	0.004990048	1
T2DM	leukocyte	*EIF3F*	-0.974676361	0.00061698	0.004990048	1
T2DM	leukocyte	*ZNF25*	-0.868305384	0.002246671	0.005438799	1
T2DM	leukocyte	*PPP2R2D*	-0.877844924	0.001422397	0.004990048	1
T2DM	leukocyte	*CLCF1*	-1.075587453	0.011199091	0.015796991	1
T2DM	leukocyte	*MRPL48*	-1.000415258	0.006981015	0.010549388	1
T2DM	leukocyte	*TMEM70*	-0.875408723	0.001671627	0.005048289	1
T2DM	leukocyte	*RPS6KB2*	-1.394615527	0.000838237	0.004990048	1
T2DM	leukocyte	*RMI2*	-2.429832314	0.017428645	0.023291546	1
T2DM	leukocyte	*LINC01106*	-3.978473006	0.013808366	0.018989524	1
T2DM	leukocyte	*SFN*	-1.531676464	0.022885878	0.029705522	1
T2DM	leukocyte	*SWI5*	-1.195927645	0.005097276	0.008187734	1
T2DM	leukocyte	*ZDHHC21*	-1.905826976	0.000284729	0.004990048	1
T2DM	leukocyte	*TUBB6*	-0.433072572	0.007425023	0.011106403	1
T2DM	leukocyte	*CHMP6*	-0.805481127	0.00882436	0.012807872	1
T2DM	leukocyte	*DLEU1*	-1.132110683	0.001713251	0.005068796	1
T2DM	leukocyte	*CCDC57*	-1.467162732	6.72E-05	0.004990048	0.264080744
T2DM	leukocyte	*BNIP3*	-0.688164559	0.001497487	0.004993935	1

T2DM	leukocyte	*SMIM19*	-0.771340486	0.001972587	0.005215543	1
T2DM	leukocyte	*SLC35G1*	-1.270424497	0.00233845	0.005515664	1
T2DM	leukocyte	*COX8A*	-0.711924501	0.000598243	0.004990048	1
T2DM	leukocyte	*CDC26*	-0.976452424	0.001439577	0.004990048	1
T2DM	leukocyte	*RNPEP*	-1.654043387	0.001266403	0.004990048	1
T2DM	leukocyte	*DMRTA1*	-0.774493344	0.005703932	0.008935705	1
T2DM	leukocyte	*CLEC14A*	-0.517972886	0.006727842	0.010230596	1
T2DM	leukocyte	*WDR25*	-2.554437946	0.000207905	0.004990048	0.816858929
T2DM	leukocyte	*DIRAS1*	-1.108060772	0.004267683	0.007310294	1
T2DM	leukocyte	*PHLDB3*	-0.748816191	0.005968456	0.009262768	1
T2DM	leukocyte	*KBTBD11*	-0.769405783	0.003894271	0.007059776	1
T2DM	leukocyte	*RMDN1*	-1.086197274	0.002047854	0.00526208	1
T2DM	leukocyte	*LRRC37A*	-4.909541872	0.002377946	0.005560194	1
T2DM	leukocyte	*ACSF3*	-1.242005563	0.001647806	0.005035955	1
T2DM	leukocyte	*RUFY1*	-2.306702729	0.000600236	0.004990048	1
T2DM	leukocyte	*VSIG10*	-1.506137984	0.000558809	0.004990048	1
T2DM	leukocyte	*WSB2*	-0.795133974	0.002650966	0.005824744	1
T2DM	leukocyte	*ANKLE2*	-0.708738968	0.00428685	0.00732537	1
T2DM	leukocyte	*MUC20*	-0.892979624	0.00414514	0.007238335	1
T2DM	leukocyte	*NFATC2IP*	-1.410383888	0.000844997	0.004990048	1
T2DM	leukocyte	*DPP7*	-0.882538043	0.000944328	0.004990048	1
T2DM	leukocyte	*MTHFR*	-1.030095374	0.001196488	0.004990048	1
T2DM	leukocyte	*DEAF1*	-1.257717512	0.000952659	0.004990048	1
T2DM	leukocyte	*TMEM80*	-0.703456414	0.004921036	0.007973094	1
T2DM	leukocyte	*DSCAML1*	-1.457216242	0.000173295	0.004990048	0.680876638
T2DM	leukocyte	*PUS1*	-1.845568318	0.00270243	0.005880214	1
T2DM	leukocyte	*MAN1B1*	-1.631560654	0.000750498	0.004990048	1
T2DM	leukocyte	*PPFIA3*	-0.719292051	0.003300307	0.006432819	1
T2DM	leukocyte	*PAWR*	-0.825616772	0.003125779	0.006301276	1
T2DM	leukocyte	*GPR4*	-0.71646584	0.002170583	0.005349178	1
T2DM	leukocyte	*ACOT4*	-1.252986105	0.002067628	0.005273744	1
T2DM	leukocyte	*ARIH2*	-0.992831098	0.000324166	0.004990048	1
T2DM	leukocyte	*IRX3*	2.479525057	0.000214266	0.004990048	0.841849721
T2DM	leukocyte	*SAMD12*	-1.257305318	0.002454856	0.005621365	1
T2DM	leukocyte	*CD163*	0.076437943	0.0188306	0.024919309	1
T2DM	leukocyte	*ACAD9*	-0.919927716	0.013717797	0.018871577	1
T2DM	leukocyte	*AGTRAP*	-1.009416714	0.00163287	0.005035955	1
T2DM	leukocyte	*THAP5*	-0.972571787	0.001237134	0.004990048	1
T2DM	leukocyte	*CD151*	-0.75571375	0.001414358	0.004990048	1
T2DM	leukocyte	*CHID1*	-0.841005062	0.00062838	0.004990048	1
T2DM	leukocyte	*ZBTB41*	-1.236783043	0.000922013	0.004990048	1
T2DM	leukocyte	*BET1L*	-1.434471291	0.000681959	0.004990048	1
T2DM	leukocyte	*RIC8A*	-1.213586736	0.001307109	0.004990048	1
T2DM	leukocyte	*IMP3*	-0.885365393	0.005984345	0.009278806	1
T2DM	leukocyte	*GPR150*	-0.691208389	0.036459992	0.045621436	1
T2DM	leukocyte	*ADAMTSL1*	-4.583646515	0.002960796	0.006135605	1
T2DM	leukocyte	*ALS2CL*	-1.244688578	0.003362063	0.006491177	1
T2DM	leukocyte	*STAP2*	-0.638720508	0.005468303	0.008631969	1
T2DM	leukocyte	*DALRD3*	-0.94213439	0.005150368	0.008249407	1
T2DM	leukocyte	*ZNF114*	-2.394914675	0.003613078	0.006763117	1
T2DM	leukocyte	*PLEC*	-1.879929564	0.000200437	0.004990048	0.787515047
T2DM	leukocyte	*WDR6*	-0.795045953	0.010276813	0.014645484	1
T2DM	leukocyte	*AQP11*	-1.108022168	0.004276203	0.007317597	1
T2DM	leukocyte	*ZFAND2A*	-1.234213165	0.000862116	0.004990048	1
T2DM	leukocyte	*DNAJC22*	-0.567435557	0.006162865	0.009503098	1
T2DM	leukocyte	*NT5DC1*	-0.903959342	0.001620099	0.005035955	1
T2DM	leukocyte	*COX14*	-0.880213417	0.003250661	0.006400784	1
T2DM	leukocyte	*P4HTM*	-0.705810092	0.00105344	0.004990048	1
T2DM	leukocyte	*ERBB4*	-8.10389048	0.00099931	0.004990048	1
T2DM	leukocyte	*ERN1*	-1.287562731	0.00071811	0.004990048	1
T2DM	leukocyte	*PARP10*	-1.147207877	0.002692657	0.005864439	1
T2DM	leukocyte	*RPP25*	-1.580734262	0.000661243	0.004990048	1
T2DM	leukocyte	*ZHX2*	-1.020715357	0.002166388	0.005345956	1
T2DM	leukocyte	*HYI*	-1.109360803	0.001193911	0.004990048	1

T2DM	leukocyte	*GAK*	-1.007598032	0.003826465	0.007002413	1
T2DM	leukocyte	*TUFM*	-0.535873705	0.016440662	0.022152044	1
T2DM	leukocyte	*RMI1*	1.075494969	0.035363816	0.044348686	1
T2DM	leukocyte	*C3orf38*	-1.153853862	0.010693997	0.015146617	1
T2DM	leukocyte	*IGSF22*	-0.631232987	0.006661266	0.0101521	1
T2DM	leukocyte	*HTR1F*	-2.804445841	0.000719889	0.004990048	1
T2DM	leukocyte	*TMTC2*	-1.620334079	0.003114168	0.006284318	1
T2DM	leukocyte	*FARSA*	-1.124243726	0.000199112	0.004990048	0.782310684
T2DM	leukocyte	*SPTY2D1*	-0.988548427	0.001307717	0.004990048	1
T2DM	leukocyte	*SAMD4B*	-0.793941729	0.002553381	0.005681968	1
T2DM	leukocyte	*TCAIM*	-1.348474157	0.000956366	0.004990048	1
T2DM	leukocyte	*CALR*	-0.67375109	0.002712146	0.005893818	1
T2DM	leukocyte	*CDH4*	-1.71986147	0.033836145	0.0426223	1
T2DM	leukocyte	*ELMOD2*	-1.713823487	0.000551374	0.004990048	1
T2DM	leukocyte	*LINC00174*	-0.89985544	0.001992013	0.005227473	1
T2DM	leukocyte	*GEMIN4*	-0.880987679	0.004537137	0.007568852	1
T2DM	leukocyte	*LBX2*	0.764693306	0.032436535	0.040978504	1
T2DM	leukocyte	*CDC42EP4*	-0.803013	0.001561267	0.005009694	1
T2DM	leukocyte	*ZBTB42*	-2.035176969	0.003140989	0.006311945	1
T2DM	leukocyte	*SYCN*	-0.486766104	0.003390568	0.006510382	1
T2DM	leukocyte	*CITED4*	-0.598955037	0.003964851	0.007124893	1
T2DM	leukocyte	*B3GNT3*	-2.017155577	0.005261464	0.008396545	1
T2DM	leukocyte	*PUF60*	-0.810545013	0.002829164	0.006011783	1
T2DM	leukocyte	*TSHZ1*	-0.725131963	0.004646495	0.007680302	1
T2DM	leukocyte	*EXOC3*	-1.579898515	0.001694982	0.005060368	1
T2DM	leukocyte	*TH*	-5.582389465	0.000509783	0.004990048	1
T2DM	leukocyte	*ZNF816*	-1.251922347	0.004157281	0.007242343	1
T2DM	leukocyte	*CCDC66*	-2.300722206	0.000741253	0.004990048	1
T2DM	leukocyte	*MCFD2*	-0.721247121	0.001301148	0.004990048	1
T2DM	leukocyte	*SERTM1*	0.580880493	0.03689519	0.046136602	1
T2DM	leukocyte	*GLIPR1L2*	-1.394173687	0.011892024	0.016657313	1
T2DM	leukocyte	*TSPYL5*	-0.732665846	0.001642437	0.005035955	1
T2DM	leukocyte	*SHISA2*	-1.532976385	0.008505642	0.012427917	1
T2DM	leukocyte	*CHST13*	-72.00732348	0.004314371	0.007328649	1
T2DM	leukocyte	*PPA1*	-0.682190165	0.001273201	0.004990048	1
T2DM	leukocyte	*PSMG4*	-0.765220616	0.001727307	0.005070552	1
T2DM	leukocyte	*MAP6D1*	-1.8216795	0.003652414	0.006815321	1
T2DM	leukocyte	*D2HGDH*	-4.171955643	2.20E-05	0.003059063	0.086305646
T2DM	leukocyte	*FAM83H*	-1.340924571	0.003215505	0.006372159	1
T2DM	leukocyte	*LRRC57*	-1.317781212	0.005849596	0.00911303	1
T2DM	leukocyte	*MRPL14*	-0.98545249	0.00075814	0.004990048	1
T2DM	leukocyte	*ZFP82*	-0.844601262	0.003100472	0.00626956	1
T2DM	leukocyte	*FKRP*	-0.823338434	0.003294337	0.006432819	1
T2DM	leukocyte	*RPH3AL*	-0.639583452	0.004476207	0.007504245	1
T2DM	leukocyte	*HIGD1A*	-0.734861199	0.00136029	0.004990048	1
T2DM	leukocyte	*F2R*	-0.558241022	0.024037444	0.031056599	1
T2DM	leukocyte	*ZNF707*	-1.095365507	0.004078463	0.007189	1
T2DM	leukocyte	*OGFOD3*	-0.801123364	0.001459729	0.004990048	1
T2DM	leukocyte	*ZNF467*	-1.165942347	0.00298244	0.006152298	1
T2DM	leukocyte	*ZNF678*	-2.068100332	0.001412811	0.004990048	1
T2DM	leukocyte	*TMEM45A*	-0.701153539	0.004227914	0.007272975	1
T2DM	leukocyte	*SGSH*	-2.407388135	0.006912548	0.010462019	1
T2DM	leukocyte	*GPR135*	-1.016006883	0.003851937	0.007029382	1
T2DM	leukocyte	*ANKRD62*	-1.344221874	0.002386469	0.005560842	1
T2DM	leukocyte	*GPR88*	-17.43621041	0.001281105	0.004990048	1
T2DM	leukocyte	*RFX7*	-2.478893823	0.000173948	0.004990048	0.683442721
T2DM	leukocyte	*ZNF329*	-1.177247887	0.003326377	0.006456709	1
T2DM	leukocyte	*CCDC149*	-1.558394487	0.000612506	0.004990048	1
T2DM	leukocyte	*IDH2*	-0.661457453	0.004522373	0.007557079	1
T2DM	leukocyte	*TNRC18*	-1.147422751	0.001462705	0.004990048	1
T2DM	leukocyte	*TDRKH*	-0.938673711	0.004704401	0.007720459	1
T2DM	leukocyte	*ZNF708*	-0.704976481	0.002653676	0.005824744	1
T2DM	leukocyte	*IST1*	-1.42915478	0.000558061	0.004990048	1
T2DM	leukocyte	*ERCC6L2*	-2.815910316	0.000449268	0.004990048	1

T2DM	leukocyte	*MRPL41*	-0.884678585	0.000948547	0.004990048	1
T2DM	leukocyte	*TP53TG1*	-0.970123899	0.001236657	0.004990048	1
T2DM	leukocyte	*UBA7*	-2.881967263	7.91E-07	0.000310956	0.003109565
T2DM	leukocyte	*ARL6IP4*	-0.602685209	0.006008708	0.009312905	1
T2DM	leukocyte	*FAM153B*	-1.494293524	0.000719489	0.004990048	1
T2DM	leukocyte	*BACE2*	-0.521116682	0.003484507	0.006623429	1
T2DM	leukocyte	*SYNM*	-0.712176105	0.018461231	0.024526286	1
T2DM	leukocyte	*FIGN*	-0.859869469	0.003680487	0.006846891	1
T2DM	leukocyte	*B4GALNT4*	-1.705567302	0.003864524	0.007037843	1
T2DM	leukocyte	*ZNF804B*	-62.99075685	0.00405747	0.007184034	1
T2DM	leukocyte	*YBEY*	-1.04694175	0.002460065	0.005622792	1
T2DM	leukocyte	*NPLOC4*	-0.938806004	0.001626348	0.005035955	1
T2DM	leukocyte	*CAPN12*	-2.027043666	0.000224696	0.004990048	0.882831304
T2DM	leukocyte	*GLRX5*	-0.725478535	0.00560771	0.008817809	1
T2DM	leukocyte	*MXRA7*	-0.819524984	0.000888024	0.004990048	1
T2DM	leukocyte	*ADI1*	-2.013833381	0.000412691	0.004990048	1
T2DM	leukocyte	*SATB1*	-1.446138687	0.000724927	0.004990048	1
T2DM	leukocyte	*EPHB3*	-2.100978047	0.015948504	0.021592582	1
T2DM	leukocyte	*SKA2*	-1.713666485	0.000235612	0.004990048	0.925718204
T2DM	leukocyte	*TTC3*	-0.411872439	0.024427774	0.03151945	1
T2DM	leukocyte	*BRICD5*	-1.614941184	0.021932498	0.028609822	1
T2DM	leukocyte	*TSKU*	-0.942797093	0.00438019	0.007389967	1
T2DM	leukocyte	*ANXA2*	-0.546065023	0.001480241	0.004990048	1
T2DM	leukocyte	*SLC35D3*	-1.662307515	0.00191254	0.00515389	1
T2DM	leukocyte	*GPIHBP1*	-7.728342202	0.00463315	0.007664031	1
T2DM	leukocyte	*VMO1*	0.096244748	0.010293573	0.014664049	1
T2DM	leukocyte	*COL18A1*	-0.450282639	0.002952213	0.006135605	1
T2DM	leukocyte	*C11orf54*	-1.237165079	0.002615306	0.005772773	1
T2DM	leukocyte	*CEP63*	-1.108931885	0.000959996	0.004990048	1
T2DM	leukocyte	*HMGN4*	-1.0593016	0.005915453	0.009197394	1
T2DM	leukocyte	*SPATA13*	-0.863023754	0.001892949	0.005146173	1
T2DM	leukocyte	*ZNF320*	1.07124065	4.40E-05	0.004219101	0.172983145
T2DM	leukocyte	*SLC25A21*	-2.157171538	0.004213299	0.007263735	1
T2DM	leukocyte	*LYSMD4*	-1.028737895	0.003398519	0.006511001	1
T2DM	leukocyte	*BEGAIN*	-1.313845708	0.001888562	0.005146173	1
T2DM	leukocyte	*GPC6*	-1.009829876	0.003334717	0.00646588	1
T2DM	leukocyte	*FANCF*	-3.568264511	0.000137517	0.004990048	0.540304869
T2DM	leukocyte	*SMDT1*	-0.706495554	0.000772156	0.004990048	1
T2DM	leukocyte	*PTTG1IP*	-0.671332467	0.00862844	0.012584111	1
T2DM	leukocyte	*ZNF623*	-0.713065231	0.00843111	0.012341964	1
T2DM	leukocyte	*CCDC125*	-1.526050939	0.027237597	0.034700558	1
T2DM	leukocyte	*BOLA2*	-0.91098447	0.001255185	0.004990048	1
T2DM	leukocyte	*FHL3*	-0.748295361	0.016924399	0.022733662	1
T2DM	leukocyte	*SF3A3*	-0.746161226	0.003795281	0.00696481	1
T2DM	leukocyte	*PCBP3*	-1.592006069	0.00059242	0.004990048	1
T2DM	leukocyte	*SFXN4*	-1.024614424	0.001294288	0.004990048	1
T2DM	leukocyte	*MRPL54*	-0.89286298	0.000875046	0.004990048	1
T2DM	leukocyte	*DGCR6*	-0.942907414	0.002773095	0.005963596	1
T2DM	leukocyte	*NDUFB1*	-0.888964692	0.001130698	0.004990048	1
T2DM	leukocyte	*TRMT12*	-1.052404413	0.001411711	0.004990048	1
T2DM	leukocyte	*FIGLA*	-20.23652911	0.003162954	0.006314657	1
T2DM	leukocyte	*CBX6*	-0.825556585	0.001033765	0.004990048	1
T2DM	leukocyte	*MACC1*	-0.715144884	0.001025227	0.004990048	1
T2DM	leukocyte	*KREMEN1*	-3.107581542	0.00069476	0.004990048	1
T2DM	leukocyte	*NUDT14*	-0.70848157	0.009962727	0.014254754	1
T2DM	leukocyte	*FAM3B*	-0.651355356	0.011690042	0.016403634	1
T2DM	leukocyte	*TOB2*	-0.996203207	0.0021374	0.005317933	1
T2DM	leukocyte	*NPIPA7*	-6.449713566	0.030976073	0.039221718	1
T2DM	leukocyte	*TTC32*	-0.900168687	0.001509128	0.004993935	1
T2DM	leukocyte	*TMEM236*	-0.618587133	0.00819304	0.012051837	1
T2DM	leukocyte	*UQCR10*	-0.752902802	0.001355433	0.004990048	1
T2DM	leukocyte	*NIPSNAP1*	-0.826896458	0.003377169	0.006503419	1
T2DM	leukocyte	*CRELD2*	-0.639196654	0.005713361	0.008943345	1
T2DM	leukocyte	*UBE2F*	-1.400144829	0.001410918	0.004990048	1

T2DM	leukocyte	*GOLGA6L4*	-1.182007319	0.00113352	0.004990048	1
T2DM	leukocyte	*PGP*	-1.554327387	5.48E-05	0.004896114	0.215429008
T2DM	leukocyte	*ACOT1*	-5.458937874	0.003037228	0.006214704	1
T2DM	leukocyte	*OAF*	-1.119392264	0.00161467	0.005033031	1
T2DM	leukocyte	*TM2D3*	-0.883422428	0.001366362	0.004990048	1
T2DM	leukocyte	*TOP1MT*	-2.300814502	0.000578558	0.004990048	1
T2DM	leukocyte	*COPB2*	-1.058401361	0.000638646	0.004990048	1
T2DM	leukocyte	*CCR10*	-1.653124753	0.01183482	0.016598917	1
T2DM	leukocyte	*NCMAP*	-0.830135772	0.00454936	0.007568852	1
T2DM	leukocyte	*TXNRD2*	-0.884847465	0.003147295	0.006311945	1
T2DM	leukocyte	*ZFP1*	-1.177403457	0.001731912	0.005070552	1
T2DM	leukocyte	*XPOT*	-0.856421377	0.00157985	0.005009694	1
T2DM	leukocyte	*C14orf180*	-89.7247995	0.004172038	0.007242343	1
T2DM	leukocyte	*ZBTB40*	-1.115815589	0.018726689	0.024815232	1
T2DM	leukocyte	*UBE2G2*	-0.920983289	0.001880186	0.005146173	1
T2DM	leukocyte	*OSBP2*	-0.988412069	0.004177649	0.007242343	1
T2DM	leukocyte	*TMED9*	-0.780891888	0.001226888	0.004990048	1
T2DM	leukocyte	*BTBD6*	-1.198139929	0.00096713	0.004990048	1
T2DM	leukocyte	*JAG2*	-0.668944852	0.014868916	0.02024254	1
T2DM	leukocyte	*LCN12*	-3.301726179	0.000564782	0.004990048	1
T2DM	leukocyte	*FAM227A*	-0.982753308	0.003088744	0.00625753	1
T2DM	leukocyte	*MUC6*	-0.523793781	0.006269637	0.009633712	1
T2DM	leukocyte	*NOC4L*	-1.524548903	0.000606222	0.004990048	1
T2DM	leukocyte	*NDUFA6*	-0.538787563	0.014945668	0.020325902	1
T2DM	leukocyte	*SIVA1*	-0.878938381	0.001449093	0.004990048	1
T2DM	leukocyte	*BRI3BP*	-1.254840024	0.001443248	0.004990048	1
T2DM	leukocyte	*UBOX5*	-0.861800427	0.003620322	0.00677345	1
T2DM	leukocyte	*MAFF*	-0.528717463	0.006111407	0.009445995	1
T2DM	leukocyte	*SEMA4B*	-0.818876275	0.005397465	0.008554513	1
T2DM	leukocyte	*CIB1*	-0.871243451	0.000913201	0.004990048	1
T2DM	leukocyte	*ANO9*	-1.236372237	0.007979378	0.011786082	1
T2DM	leukocyte	*MFSD6L*	-2.363356557	0.008496678	0.012420662	1
T2DM	leukocyte	*ZNF445*	-3.893548647	0.001031	0.004990048	1
T2DM	leukocyte	*ZNF74*	-1.80463676	0.006892165	0.01043519	1
T2DM	leukocyte	*RBM11*	-0.75665204	0.003025468	0.006200868	1
T2DM	leukocyte	*RGPD2*	-2.578728554	0.004964624	0.008020562	1
T2DM	leukocyte	*GAS2L1*	-2.074669025	0.00538798	0.008542928	1
T2DM	leukocyte	*ATP6V0A2*	-1.454727861	0.001217265	0.004990048	1
T2DM	leukocyte	*RAD51D*	-2.411118815	0.000627942	0.004990048	1
T2DM	leukocyte	*SH3BGR*	-0.552111893	0.004604942	0.007624449	1
T2DM	leukocyte	*MUC1*	-0.543729399	0.006552953	0.010022013	1
T2DM	leukocyte	*IRF7*	-1.449058894	0.003558571	0.006702601	1
T2DM	leukocyte	*FAM131C*	-0.646495023	0.002994181	0.006168923	1
T2DM	leukocyte	*LSAMP*	-0.491055916	0.004335337	0.007353588	1
T2DM	leukocyte	*OLFML2A*	-2.180874915	0.00992366	0.014209205	1
T2DM	leukocyte	*PDIA2*	-0.39300887	0.012304611	0.017167904	1
T2DM	leukocyte	*PCGF3*	-0.894860079	0.003214103	0.006372159	1
T2DM	leukocyte	*P4HB*	-0.604353071	0.001856389	0.005146173	1
T2DM	leukocyte	*PSMD13*	-1.199971464	5.69E-05	0.004970621	0.223677937
T2DM	leukocyte	*BRWD1*	-1.018144936	0.000779239	0.004990048	1
T2DM	leukocyte	*DRG1*	-0.841892081	0.001951757	0.005198138	1
T2DM	leukocyte	*ADAMTSL5*	-5.773035253	0.017460565	0.023326269	1
T2DM	leukocyte	*MORF4L1*	-0.69682556	0.00112431	0.004990048	1
T2DM	leukocyte	*DMWD*	-2.004296925	2.19E-05	0.003059063	0.085914715
T2DM	leukocyte	*PIGP*	-0.764218589	0.000851184	0.004990048	1
T2DM	leukocyte	*NPAP1*	-21.94676388	0.03261288	0.041188044	1
T2DM	leukocyte	*ARL17A*	-0.585626495	0.02244836	0.029205167	1
T2DM	leukocyte	*TRIM69*	-0.845782191	0.006837875	0.010369201	1
T2DM	leukocyte	*IFITM1*	-1.864900665	0.005787439	0.009037699	1
T2DM	leukocyte	*SETD4*	-3.111031148	0.003192887	0.006345399	1
T2DM	leukocyte	*ZNF529*	-1.621808803	0.001340277	0.004990048	1
T2DM	leukocyte	*AIDA*	-0.850391	0.001257899	0.004990048	1
T2DM	leukocyte	*WWOX*	-0.907121591	0.001841007	0.005146173	1
T2DM	leukocyte	*POLR1D*	-0.818261339	0.000942191	0.004990048	1

T2DM	leukocyte	*EDARADD*	-0.934429486	0.002448677	0.005621365	1
T2DM	leukocyte	*ZNF749*	-0.686454263	0.001860876	0.005146173	1
T2DM	leukocyte	*TOR3A*	-0.991779491	0.001063246	0.004990048	1
T2DM	leukocyte	*ZNF555*	-0.71199055	0.003759114	0.00692001	1
T2DM	leukocyte	*NUDT17*	-1.37252592	0.001070023	0.004990048	1
T2DM	leukocyte	*CYP4X1*	1.060021271	0.023240014	0.030105511	1
T2DM	leukocyte	*ZNF197*	-0.835042053	0.002114154	0.005283012	1
T2DM	leukocyte	*RPS23*	-0.644083038	0.000664894	0.004990048	1
T2DM	leukocyte	*BTN3A2*	-0.422781015	0.022572636	0.029328004	1
T2DM	leukocyte	*ZNF396*	-2.394089777	0.002075284	0.005277533	1
T2DM	leukocyte	*CEACAM19*	-0.955918552	0.000129441	0.004990048	0.508573382
T2DM	leukocyte	*ZFP91*	-1.048569755	0.001524078	0.004994246	1
T2DM	leukocyte	*BCR*	-0.861244991	0.001757875	0.005084476	1
T2DM	leukocyte	*HYAL3*	-1.753851487	0.000720427	0.004990048	1
T2DM	leukocyte	*RTN4RL2*	-0.884821303	0.004290022	0.00732537	1
T2DM	leukocyte	*TMEM232*	-1.382058193	0.002478924	0.005622792	1
T2DM	leukocyte	*FAM183A*	-0.719899733	0.004141428	0.007238288	1
T2DM	leukocyte	*EMID1*	-1.118529054	0.003159932	0.006314657	1
T2DM	leukocyte	*ESPN*	-19.10726172	0.000892658	0.004990048	1
T2DM	leukocyte	*TMEM216*	-2.265994197	0.03949684	0.049077509	1
T2DM	leukocyte	*LYPD6*	-3.071636627	0.025578735	0.032828617	1
T2DM	leukocyte	*AKR1C1*	-0.620544223	0.006076084	0.009402422	1
T2DM	leukocyte	*BCAM*	-0.697036513	0.001481963	0.004990048	1
T2DM	leukocyte	*CHP1*	-0.758139691	0.001730815	0.005070552	1
T2DM	leukocyte	*KCNJ11*	-2.918644184	0.001168114	0.004990048	1
T2DM	leukocyte	*ZNF385C*	-3.624860173	0.000198431	0.004990048	0.779636481
T2DM	leukocyte	*EXD3*	-0.929268494	0.00355	0.006692874	1
T2DM	leukocyte	*RGPD1*	-95.8845659	0.004455179	0.007483711	1
T2DM	leukocyte	*DHRS4L2*	-0.947159525	0.001364369	0.004990048	1
T2DM	leukocyte	*PALM3*	-1.083179082	0.00391695	0.007085756	1
T2DM	leukocyte	*KLHL17*	-1.439892246	0.004723182	0.00774192	1
T2DM	leukocyte	*RINL*	-1.177011226	0.001460195	0.004990048	1
T2DM	leukocyte	*ZNF490*	-1.51955664	0.003703315	0.006868344	1
T2DM	leukocyte	*NRN1L*	-2.287168182	0.004336539	0.007353588	1
T2DM	leukocyte	*RAB42*	0.420149417	0.020496255	0.026888076	1
T2DM	leukocyte	*MAPK12*	-0.871021854	0.01766751	0.023570678	1
T2DM	leukocyte	*LINC00265*	-1.814810285	0.004069281	0.007189	1
T2DM	leukocyte	*LAMTOR4*	-0.766086168	0.003627532	0.006781089	1
T2DM	leukocyte	*PRKAR1B*	-1.063012397	0.00338979	0.006510382	1
T2DM	leukocyte	*NUTM2B*	-28.44657379	6.79E-07	0.000296569	0.002669121
T2DM	leukocyte	*AGAP4*	-1.95950769	0.005975289	0.009268422	1
T2DM	leukocyte	*CENPP*	-0.733797515	0.004027728	0.007161461	1
T2DM	leukocyte	*ZNF559*	-0.585295396	0.026643672	0.034043248	1
T2DM	leukocyte	*FOCAD*	-0.96625666	0.002106908	0.005283012	1
T2DM	leukocyte	*ZP3*	-1.201812319	0.004028208	0.007161461	1
T2DM	leukocyte	*SRSF10*	-0.850344854	0.001798106	0.005114303	1
T2DM	leukocyte	*RALGAPA2*	-0.965918359	0.001660899	0.005041098	1
T2DM	leukocyte	*NDOR1*	-2.065289293	0.017080279	0.022921042	1
T2DM	leukocyte	*FBLL1*	-2.074422335	0.000690957	0.004990048	1
T2DM	leukocyte	*ASAH2*	-2.728575462	6.64E-05	0.004990048	0.260896276
T2DM	leukocyte	*S100A16*	-0.468857454	0.003748409	0.006917566	1
T2DM	leukocyte	*PARVB*	-0.699452886	0.000841613	0.004990048	1
T2DM	leukocyte	*UROS*	-1.139646853	0.00092461	0.004990048	1
T2DM	leukocyte	*FAM221A*	-1.531107084	0.000118859	0.004990048	0.466998493
T2DM	leukocyte	*NOXA1*	-1.591794211	0.003740306	0.006909103	1
T2DM	leukocyte	*TMEM198*	-0.704443493	0.005765769	0.009007438	1
T2DM	leukocyte	*ZNF548*	-0.926681448	0.013656631	0.018794011	1
T2DM	leukocyte	*MTF1*	-0.894453101	0.002278362	0.005462378	1
T2DM	leukocyte	*ENTPD8*	-5.486111681	0.004654622	0.007684038	1
T2DM	leukocyte	*RPL14*	-0.591951808	0.001257755	0.004990048	1
T2DM	leukocyte	*MSL1*	-0.908644251	0.003219327	0.006372159	1
T2DM	leukocyte	*NOC2L*	-1.18516461	0.00069042	0.004990048	1
T2DM	leukocyte	*ZNF567*	-0.780499824	0.008497516	0.012420662	1
T2DM	leukocyte	*ARID2*	-1.227510754	0.001053487	0.004990048	1

T2DM	leukocyte	*PLAC9*	-0.931639615	0.002081319	0.005283012	1
T2DM	leukocyte	*CLDN4*	-0.503557171	0.002888329	0.00605886	1
T2DM	leukocyte	*FAM47E*	-1.381187013	0.000396141	0.004990048	1
T2DM	leukocyte	*ZNF527*	-1.039085417	0.01149312	0.01615616	1
T2DM	leukocyte	*ZNF600*	-0.858700849	0.007619975	0.011353388	1
T2DM	leukocyte	*PNRC2*	-0.776485673	0.001873534	0.005146173	1
T2DM	leukocyte	*ZKSCAN3*	-1.749124658	0.004774591	0.007806645	1
T2DM	leukocyte	*RRP7A*	-0.904563479	0.004674001	0.007686961	1
T2DM	leukocyte	*S100A14*	-0.661953772	0.010448065	0.014857201	1
T2DM	leukocyte	*CXCL17*	-0.680855687	0.0071798	0.010795804	1
T2DM	leukocyte	*SPATA41*	-2.911091262	0.027217531	0.034686241	1
T2DM	leukocyte	*BLOC1S2*	-0.959221271	0.001192914	0.004990048	1
T2DM	leukocyte	*ZNF676*	-1.070293852	0.005297774	0.008427107	1
T2DM	leukocyte	*WDSUB1*	-1.090641324	0.004406042	0.007420205	1
T2DM	leukocyte	*ZNF681*	-5.277318764	0.000155258	0.004990048	0.61000834
T2DM	leukocyte	*HRCT1*	0.829605949	0.033694841	0.042472579	1
T2DM	leukocyte	*MPHOSPH8*	-0.888008904	0.001478533	0.004990048	1
T2DM	leukocyte	*SIRPB2*	1.409416118	0.017391605	0.023249955	1
T2DM	leukocyte	*LCOR*	-0.825270634	0.001520862	0.004993935	1
T2DM	leukocyte	*SUPT5H*	-0.801425702	0.001393308	0.004990048	1
T2DM	leukocyte	*XPNPEP3*	-1.493713065	0.000278549	0.004990048	1
T2DM	leukocyte	*PPIA*	-0.725979565	0.001476077	0.004990048	1
T2DM	leukocyte	*ZNF471*	-1.222633458	0.00432002	0.007335073	1
T2DM	leukocyte	*ZNF493*	-1.231534615	0.001180752	0.004990048	1
T2DM	leukocyte	*GTF2IRD2*	-1.30237722	0.000986601	0.004990048	1
T2DM	leukocyte	*SUPT3H*	-0.775326446	0.003040316	0.006215089	1
T2DM	leukocyte	*POM121*	-0.873061829	0.00444214	0.007470068	1
T2DM	leukocyte	*LONP1*	-1.287546276	0.001520485	0.004993935	1
T2DM	leukocyte	*ZNF34*	-1.197499345	0.004127339	0.00723368	1
T2DM	leukocyte	*ZNF781*	-1.114679593	0.013254617	0.018343568	1
T2DM	leukocyte	*EVL*	-1.65478403	0.008639226	0.012594998	1
T2DM	leukocyte	*ZNF124*	-1.513468504	0.00156845	0.005009694	1
T2DM	leukocyte	*XRCC6*	-0.970178489	0.00021594	0.004990048	0.848427746
T2DM	leukocyte	*PIK3R4*	-1.522773826	0.001213932	0.004990048	1
T2DM	leukocyte	*C20orf96*	-1.768175097	0.001758926	0.005084476	1
T2DM	leukocyte	*SULT1A1*	-2.007718901	0.000386199	0.004990048	1
T2DM	leukocyte	*ARL9*	0.917917038	0.02644921	0.033838797	1
T2DM	leukocyte	*TPK1*	-1.8940338	0.012519174	0.017436312	1
T2DM	leukocyte	*AFAP1*	-0.937177955	0.005292834	0.008425434	1
T2DM	leukocyte	*MYO18A*	-0.813898468	0.001889502	0.005146173	1
T2DM	leukocyte	*XRCC2*	-3.299565187	0.002825187	0.006006579	1
T2DM	leukocyte	*MYO6*	-0.659163708	0.002239395	0.005438799	1
T2DM	leukocyte	*ZNF502*	-3.987090878	0.000304896	0.004990048	1
T2DM	leukocyte	*TRAPPC4*	-0.755923277	0.005090883	0.008180809	1
T2DM	leukocyte	*ZNF33B*	-1.754526027	0.000411305	0.004990048	1
T2DM	leukocyte	*AMZ2*	-1.121907863	0.001255722	0.004990048	1
T2DM	leukocyte	*ZNF431*	-0.922187565	0.001756251	0.005084476	1
T2DM	leukocyte	*DAPK1*	-1.567648393	0.001171866	0.004990048	1
T2DM	leukocyte	*HLA-DQA1*	0.060242445	0.039734421	0.049341511	1
T2DM	leukocyte	*CLPSL2*	-0.855839702	0.024938975	0.032105254	1
T2DM	leukocyte	*MVB12B*	-4.234584586	0.006689179	0.010182792	1
T2DM	leukocyte	*ADA*	0.407175881	0.016621141	0.022372204	1
T2DM	leukocyte	*PPTC7*	-1.515158158	0.000313236	0.004990048	1
T2DM	leukocyte	*ZFP28*	-1.293105065	0.001911562	0.00515389	1
T2DM	leukocyte	*ANKRD36B*	-0.901331726	0.010510122	0.014928397	1
T2DM	leukocyte	*SRGAP1*	-1.055408478	0.001857727	0.005146173	1
T2DM	leukocyte	*FAM3C*	-0.746870765	0.001422877	0.004990048	1
T2DM	leukocyte	*AP2A1*	-1.177249767	0.000942115	0.004990048	1
T2DM	leukocyte	*WDR5B*	-2.681032311	0.002031765	0.005251844	1
T2DM	leukocyte	*ZNF138*	-0.589198724	0.017659031	0.023570678	1
T2DM	leukocyte	*ZNF429*	-0.854736644	0.002476874	0.005622792	1
T2DM	leukocyte	*ZNF100*	-1.381870129	0.000348821	0.004990048	1
T2DM	leukocyte	*ZNF763*	-2.183591177	0.00055343	0.004990048	1
T2DM	leukocyte	*ARRDC1*	-0.793658939	0.004306391	0.007328649	1

T2DM	leukocyte	*ZNF682*	-1.718034258	0.001256146	0.004990048	1
T2DM	leukocyte	*ZNF257*	-1.458802554	0.00140436	0.004990048	1
T2DM	leukocyte	*ADAM32*	0.887186851	0.032403057	0.040949376	1
T2DM	leukocyte	*ACSL5*	-1.405937635	0.004252727	0.007299678	1
T2DM	leukocyte	*SND1*	-0.893797831	0.001518523	0.004993935	1
T2DM	leukocyte	*TBC1D9B*	-1.903446289	0.000161616	0.004990048	0.634987616
T2DM	leukocyte	*C6orf141*	-1.853175619	0.001168014	0.004990048	1
T2DM	leukocyte	*GTF2E2*	-0.85683752	0.002175537	0.005349178	1
T2DM	leukocyte	*ZNF165*	-1.007578118	0.001388452	0.004990048	1
T2DM	leukocyte	*SVIL*	-0.817636939	0.002455796	0.005621365	1
T2DM	leukocyte	*MRPL21*	-0.697110381	0.013318567	0.018393198	1
T2DM	leukocyte	*ZNF98*	-1.207918835	0.004375225	0.007387305	1
T2DM	leukocyte	*ZNF517*	-3.627894793	0.001832757	0.005146173	1
T2DM	leukocyte	*SLC22A5*	-1.348298719	0.001907608	0.00515389	1
T2DM	leukocyte	*C5AR1*	0.102665797	0.016913469	0.022726751	1
T2DM	leukocyte	*VEPH1*	-1.520596513	0.012841514	0.017828378	1
T2DM	leukocyte	*SHPK*	-0.881785664	0.034935675	0.043839753	1
T2DM	leukocyte	*PCDHB11*	-2.227241899	6.86E-05	0.004990048	0.269476384
T2DM	leukocyte	*ATG7*	-1.809795524	0.000922697	0.004990048	1
T2DM	leukocyte	*PIGN*	-0.769575861	0.004949857	0.008003288	1
T2DM	leukocyte	*HHLA3*	-0.796841339	0.004443255	0.007470068	1
T2DM	leukocyte	*BCO2*	-0.789316236	0.016359556	0.022073041	1
T2DM	leukocyte	*ENTPD6*	-1.119262678	0.000756197	0.004990048	1
T2DM	leukocyte	*ENPP1*	-0.545517331	0.030802982	0.039016835	1
T2DM	leukocyte	*CCDC154*	3.778833164	0.007219191	0.010842585	1
T2DM	leukocyte	*DPP4*	-0.650355355	0.001314124	0.004990048	1
T2DM	leukocyte	*NMB*	-0.887206256	0.001872235	0.005146173	1
T2DM	leukocyte	*RPS26*	-0.750638956	0.000245688	0.004990048	0.965309009
T2DM	leukocyte	*LHFPL5*	-2.259058825	0.002467448	0.005622792	1
T2DM	leukocyte	*ZNF780A*	-0.789268596	0.00280649	0.006001505	1
T2DM	leukocyte	*ATAD3A*	-1.563314448	0.001251832	0.004990048	1
T2DM	leukocyte	*FAM118B*	-2.069613207	0.00152093	0.004993935	1
T2DM	leukocyte	*SLC9A8*	-3.225103823	0.00043222	0.004990048	1
T2DM	leukocyte	*OCLN*	-0.475885312	0.020376001	0.026748181	1
T2DM	leukocyte	*ZNF181*	-1.218822075	0.002982366	0.006152298	1
T2DM	leukocyte	*TEAD4*	-0.722357006	0.004866125	0.007914645	1
T2DM	leukocyte	*C2orf27A*	-1.552244243	0.000600819	0.004990048	1
T2DM	leukocyte	*PLCG2*	-1.065024474	0.001027835	0.004990048	1
T2DM	leukocyte	*RPL12*	-0.590559727	0.000843175	0.004990048	1
T2DM	leukocyte	*VPS13A*	-0.826906416	0.003052657	0.006227356	1
T2DM	leukocyte	*LEKR1*	-1.544801056	0.00266135	0.00583507	1
T2DM	leukocyte	*ENTPD7*	-1.437156288	0.021761474	0.028424478	1
T2DM	leukocyte	*AGAP9*	-1.813873022	0.002957593	0.006135605	1
T2DM	leukocyte	*ZNF273*	-1.779739181	0.001257158	0.004990048	1
T2DM	leukocyte	*ZNF84*	-1.937009714	0.001136568	0.004990048	1
T2DM	leukocyte	*ZNF667*	-0.643486373	0.003761507	0.00692001	1
T2DM	leukocyte	*CD2AP*	-0.905311151	0.001351135	0.004990048	1
T2DM	leukocyte	*SFI1*	-1.574246888	0.009815346	0.01405924	1
T2DM	leukocyte	*HIBCH*	-0.804056067	0.002749495	0.005935586	1
T2DM	leukocyte	*TMEM229B*	-4.280835378	0.001497088	0.004993935	1
T2DM	leukocyte	*SOWAHC*	-1.616310027	0.000918471	0.004990048	1
T2DM	leukocyte	*SVIP*	-0.811978681	0.001000625	0.004990048	1
T2DM	leukocyte	*DDRGK1*	-1.0055668	0.001022083	0.004990048	1
T2DM	leukocyte	*ZNF607*	-0.924199593	0.012336937	0.017206895	1
T2DM	leukocyte	*HSD17B11*	-1.24953762	0.003146298	0.006311945	1
T2DM	leukocyte	*RPS6KL1*	-1.026642129	0.008441656	0.012352799	1
T2DM	leukocyte	*QRICH1*	-1.454539834	0.001641379	0.005035955	1
T2DM	leukocyte	*DDX42*	-0.673158489	0.007895467	0.011692277	1
T2DM	leukocyte	*HELZ*	-0.986875346	0.001201476	0.004990048	1
T2DM	leukocyte	*ZNF583*	-0.768843161	0.003848981	0.007027251	1
T2DM	leukocyte	*ZNF568*	-0.873235196	0.010391614	0.014787632	1
T2DM	leukocyte	*ZNF587*	-1.040322638	0.0019378	0.00518571	1
T2DM	leukocyte	*TPM2*	-1.006406017	0.001851429	0.005146173	1
T2DM	leukocyte	*ZNF789*	-2.25405706	0.002452266	0.005621365	1

T2DM	leukocyte	*CCDC69*	-0.795040547	0.035363789	0.044348686	1
T2DM	leukocyte	*NCOA6*	-0.825180242	0.001996106	0.005227473	1
T2DM	leukocyte	*STK39*	-0.791497095	0.003291941	0.006432819	1
T2DM	leukocyte	*PAPSS2*	-0.754781937	0.001898446	0.005146173	1
T2DM	leukocyte	*CEP290*	-1.007450401	0.001876156	0.005146173	1
T2DM	leukocyte	*ECI2*	-1.023576252	0.001148562	0.004990048	1
T2DM	leukocyte	*MSRB1*	-1.183750424	0.001780225	0.005098084	1
T2DM	leukocyte	*COLGALT2*	-1.300942491	0.006565838	0.010030007	1
T2DM	leukocyte	*PNP*	-0.478070405	0.017636202	0.023552902	1
T2DM	leukocyte	*DENND4B*	-0.959907351	0.00226566	0.005457772	1
T2DM	leukocyte	*CES1*	-1.619493644	0.014055065	0.019288281	1
T2DM	leukocyte	*TSEN15*	-1.12719002	0.001298444	0.004990048	1
T2DM	leukocyte	*LTN1*	-0.910675614	0.001888636	0.005146173	1
T2DM	leukocyte	*TYW1*	-1.310020794	0.003574082	0.006719453	1
T2DM	leukocyte	*MAP3K3*	-1.069677096	0.000687599	0.004990048	1
T2DM	leukocyte	*C1orf174*	-1.151451946	0.001646885	0.005035955	1
T2DM	leukocyte	*KIAA0753*	-1.995680574	0.000846398	0.004990048	1
T2DM	leukocyte	*ATG9A*	-0.925715554	0.012894405	0.017882851	1
T2DM	leukocyte	*TBKBP1*	-1.230156299	0.000152417	0.004990048	0.598845209
T2DM	leukocyte	*CCDC167*	-0.701730103	0.003311258	0.00644375	1
T2DM	leukocyte	*SOWAHA*	-0.816673774	0.002286778	0.005462378	1
T2DM	leukocyte	*L3MBTL3*	-1.625741958	0.000675703	0.004990048	1
T2DM	leukocyte	*MFAP3L*	-1.428859389	0.003174125	0.006330221	1
T2DM	leukocyte	*PJA2*	-0.793952779	0.001465597	0.004990048	1
T2DM	leukocyte	*INF2*	-1.356983108	0.001522629	0.004993935	1
T2DM	leukocyte	*EFCAB2*	-0.947790945	0.002084734	0.005283012	1
T2DM	leukocyte	*SAMD5*	-1.160822287	0.000927899	0.004990048	1
T2DM	leukocyte	*SPRN*	-4.585796346	0.018048266	0.024021557	1
T2DM	leukocyte	*C6orf163*	-1.526573889	0.00872343	0.012694206	1
T2DM	leukocyte	*PCMTD2*	-0.875272968	0.001880653	0.005146173	1
T2DM	leukocyte	*LIME1*	-1.617216303	0.00091412	0.004990048	1
T2DM	leukocyte	*FOXO6*	-1.777186112	0.002401715	0.005580331	1
T2DM	leukocyte	*SYS1*	-0.82076714	0.005263891	0.008397006	1
T2DM	leukocyte	*INPP5B*	-1.725787139	0.000269811	0.004990048	1
T2DM	leukocyte	*TRAF3IP1*	-2.841451179	0.000183983	0.004990048	0.722870511
T2DM	leukocyte	*CLPSL1*	-0.465004828	0.003938843	0.007108734	1
T2DM	leukocyte	*HSD17B8*	-0.766493887	0.019194823	0.02535007	1
T2DM	leukocyte	*HLA-DOA*	0.135485183	0.014428378	0.019724807	1
T2DM	leukocyte	*HLA-DMA*	0.173186217	0.009137408	0.013208564	1
T2DM	leukocyte	*NOTCH4*	-1.657957596	1.39E-08	1.91E-05	5.45E-05
T2DM	leukocyte	*RNF5*	-1.363808206	5.11E-07	0.000266998	0.002008765
T2DM	leukocyte	*MRPL38*	-0.932708924	0.001567375	0.005009694	1
T2DM	leukocyte	*SMIM5*	-0.687110155	0.005610194	0.008817809	1
T2DM	leukocyte	*EHMT2*	-3.059663047	0.00177626	0.005098084	1
T2DM	leukocyte	*LAYN*	-0.802710072	0.004757486	0.007788402	1
T2DM	leukocyte	*SLC44A4*	-0.653560642	0.003655284	0.006816142	1
T2DM	leukocyte	*MICA*	-0.493033314	0.009969826	0.014259718	1
T2DM	leukocyte	*HLA-C*	-0.672251356	8.75E-05	0.004990048	0.343612634
T2DM	leukocyte	*CCHCR1*	1.104960484	0.017188048	0.023024835	1
T2DM	leukocyte	*PSORS1C1*	-0.693009406	0.004706159	0.007720459	1
T2DM	leukocyte	*DHX16*	-1.060696636	0.00224221	0.005438799	1
T2DM	leukocyte	*ABCF1*	-0.879453573	0.003091338	0.00625753	1
T2DM	leukocyte	*ZNF468*	-2.360136907	0.000538456	0.004990048	1
T2DM	leukocyte	*HLA-G*	-0.640525432	0.033738212	0.04251361	1
T2DM	leukocyte	*ASPDH*	-1.58928782	0.002743203	0.005930931	1
T2DM	leukocyte	*ZNF783*	-4.15926394	0.008921677	0.012934786	1
T2DM	leukocyte	*ZNF425*	-2.82996556	0.0002709	0.004990048	1
T2DM	leukocyte	*PCDHA5*	-1.131894833	0.025564993	0.032825117	1
T2DM	leukocyte	*SPIRE2*	-0.721299971	0.007361738	0.011031377	1
T2DM	leukocyte	*PABPN1L*	-123.7148303	0.002461812	0.005622792	1
T2DM	leukocyte	*PKHD1L1*	-3.453667611	0.005004826	0.008072234	1
T2DM	leukocyte	*SYCE1L*	-1.222358058	0.007536643	0.011246286	1
T2DM	leukocyte	*TMEM231*	-0.882375444	0.00516683	0.008269033	1
T2DM	leukocyte	*LINC00654*	-3.097117135	0.02509905	0.032279597	1

T2DM	leukocyte	*SARNP*	-0.864766954	0.001725071	0.005070552	1
T2DM	leukocyte	*MT1A*	-0.655141081	0.000743304	0.004990048	1
T2DM	leukocyte	*ATP6AP1L*	1.581890566	0.015596067	0.021144564	1
T2DM	leukocyte	*CCDC85C*	-0.924701011	0.003149531	0.006311945	1
T2DM	leukocyte	*RGL3*	-1.68226902	0.001693702	0.005060368	1
T2DM	leukocyte	*CPT1B*	-0.736867713	0.038698483	0.048192183	1
T2DM	leukocyte	*SMN2*	-0.926078785	0.014529949	0.01984985	1
T2DM	leukocyte	*SERF1B*	-8.521975441	0.004457482	0.007484379	1
T2DM	leukocyte	*HMGN1*	-0.747999037	0.000720246	0.004990048	1
T2DM	leukocyte	*LCMT1*	-1.281438732	0.000951265	0.004990048	1
T2DM	leukocyte	*LIN52*	-2.05318686	0.000931236	0.004990048	1
T2DM	leukocyte	*DENND1C*	0.312266086	0.016822248	0.022627392	1
T2DM	leukocyte	*RNPS1*	-0.838467158	0.001898439	0.005146173	1
T2DM	leukocyte	*ZDHHC11B*	-1.507850187	0.003282479	0.006429143	1
T2DM	leukocyte	*HLA-A*	-0.562067715	0.002243945	0.005438799	1
T2DM	leukocyte	*STK38L*	-1.590774832	0.002333954	0.005515664	1
T2DM	leukocyte	*MAP10*	-0.912334038	0.009612523	0.013803948	1
T2DM	leukocyte	*NUP62*	-0.966538628	0.00240985	0.005586019	1
T2DM	leukocyte	*FAM106A*	-1.095370879	0.010435252	0.014844353	1
T2DM	leukocyte	*ZNF254*	-1.209852877	0.000487455	0.004990048	1
T2DM	leukocyte	*LINGO4*	-0.969858194	0.03117188	0.039456932	1
T2DM	leukocyte	*ASIC3*	-0.91570891	0.034732372	0.043598559	1
T2DM	leukocyte	*ARHGEF35*	-0.598720562	0.004579685	0.007595434	1
T2DM	leukocyte	*DNLZ*	0.913955604	0.005465788	0.008631969	1
T2DM	leukocyte	*SUPT4H1*	-1.104563117	0.000988769	0.004990048	1
T2DM	leukocyte	*MXD3*	-0.84964437	0.008304586	0.012183988	1
T2DM	leukocyte	*COG8*	-1.228228176	0.003968716	0.007124893	1
T2DM	leukocyte	*ERV3-1*	-1.160265333	0.017002764	0.022831121	1
T2DM	leukocyte	*RBMXL1*	-0.993996714	0.001561114	0.005009694	1
T2DM	leukocyte	*SRA1*	-0.654207886	0.004314317	0.007328649	1
T2DM	leukocyte	*C8orf82*	-1.762631828	0.000935107	0.004990048	1
T2DM	leukocyte	*LEPROT*	-0.918545098	0.00111231	0.004990048	1
T2DM	leukocyte	*PPP1CB*	-0.739090099	0.00122175	0.004990048	1
T2DM	leukocyte	*NCKIPSD*	-1.329542972	0.001807199	0.005122825	1
T2DM	leukocyte	*ZNF888*	-1.28222639	0.003398845	0.006511001	1
T2DM	leukocyte	*EMP2*	-1.004049098	0.001325264	0.004990048	1
T2DM	leukocyte	*LTB4R*	-1.199453823	0.003511128	0.0066483	1
T2DM	leukocyte	*DNASE1*	-0.723001594	0.010567569	0.014994575	1
T2DM	leukocyte	*NUDT19*	-1.238724633	0.001573864	0.005009694	1
T2DM	leukocyte	*AP1G2*	-3.810255428	0.000310161	0.004990048	1
T2DM	leukocyte	*ZNF90*	-1.078964456	0.003987871	0.007138199	1
T2DM	leukocyte	*REPIN1*	-1.015802629	0.001303688	0.004990048	1
T2DM	leukocyte	*TSPAN4*	-0.895127908	0.009698076	0.013904233	1
T2DM	leukocyte	*CPNE1*	-0.924949951	0.023349512	0.030217467	1
T2DM	leukocyte	*ARL16*	-0.89473237	0.000749374	0.004990048	1
T2DM	leukocyte	*WEE2*	-4.532245946	0.014897743	0.020274761	1
T2DM	leukocyte	*FIS1*	-0.688183804	0.00298298	0.006152298	1
T2DM	leukocyte	*MBLAC1*	-0.957324521	0.035681502	0.04471854	1
T2DM	leukocyte	*MEG3*	-0.573851413	0.001971741	0.005215543	1
T2DM	leukocyte	*EML6*	-1.095932535	0.004011408	0.00714972	1
T2DM	leukocyte	*C12orf71*	-15.07390486	0.002874686	0.006055188	1
T2DM	leukocyte	*IFRD2*	-1.022283315	0.001056525	0.004990048	1
T2DM	leukocyte	*ARHGEF28*	-1.258727395	0.001538969	0.005006447	1
T2DM	leukocyte	*COL28A1*	-4.862041706	0.002070038	0.005273744	1
T2DM	leukocyte	*UBXN2B*	-1.502190387	0.001394967	0.004990048	1
T2DM	leukocyte	*C5orf49*	-0.752137285	0.003096775	0.006265309	1
T2DM	leukocyte	*GOLGA8B*	-0.987444567	0.015993892	0.02163167	1
T2DM	leukocyte	*VPS16*	-1.406776618	0.00484769	0.00790211	1
T2DM	leukocyte	*LINC01139*	-2.813721705	0.001653825	0.005041098	1
T2DM	leukocyte	*ATAD3C*	-0.844477123	0.002342559	0.005519727	1
T2DM	leukocyte	*SYCE3*	-2.008810617	0.000754803	0.004990048	1
T2DM	leukocyte	*PAM16*	-0.843553779	0.001569163	0.005009694	1
T2DM	leukocyte	*LINC00339*	-1.006516357	0.003872597	0.007044182	1
T2DM	leukocyte	*CELA3B*	-0.458415975	0.001786158	0.005100156	1

T2DM	leukocyte	*VAMP2*	-0.686124656	0.0010718	0.004990048	1
T2DM	leukocyte	*PPP3R1*	-1.466895892	0.000600557	0.004990048	1
T2DM	leukocyte	*FANCG*	-1.772913414	0.000955863	0.004990048	1
T2DM	leukocyte	*AP4M1*	-0.669751495	0.008365433	0.012259525	1
T2DM	leukocyte	*CEBPD*	-1.808064488	0.005004431	0.008072234	1
T2DM	leukocyte	*ZNF880*	-0.768076873	0.002479099	0.005622792	1
T2DM	leukocyte	*TRIM16*	-1.215633028	0.003970676	0.007124893	1
T2DM	leukocyte	*TIGD1*	-1.521031765	0.001437651	0.004990048	1
T2DM	leukocyte	*SLC12A8*	-1.604749771	0.000287615	0.004990048	1
T2DM	leukocyte	*UBA52*	-0.703142445	0.000950906	0.004990048	1
T2DM	leukocyte	*EXOSC6*	-1.123275763	0.000387363	0.004990048	1
T2DM	leukocyte	*CKMT1A*	-0.744090962	0.016547143	0.022280235	1
T2DM	leukocyte	*HLA-DPB1*	0.091693911	0.015007798	0.020396277	1
T2DM	leukocyte	*LINC00115*	-2.456392039	0.002338787	0.005515664	1
T2DM	leukocyte	*LINC00937*	-0.910334051	0.010651922	0.015092463	1
T2DM	leukocyte	*TMEM185B*	-0.976572586	0.002565974	0.005695883	1
T2DM	leukocyte	*CYP4F26P*	3.972602344	0.018774468	0.024861774	1
T2DM	leukocyte	*WDR46*	-0.723286695	0.011878383	0.016650077	1
T2DM	leukocyte	*ZNF717*	-0.538742097	0.014407392	0.019702973	1
T2DM	leukocyte	*PROB1*	-1.306233538	0.022983414	0.029822269	1
T2DM	leukocyte	*ARL17B*	-3.097844847	6.88E-05	0.004990048	0.270507287
T2DM	leukocyte	*DHFR*	-1.156010037	0.009700052	0.013904233	1
T2DM	leukocyte	*PSMG3-AS1*	-0.887588404	0.018039728	0.02401833	1
T2DM	leukocyte	*HSBP1*	-0.810407306	0.001218806	0.004990048	1
T2DM	leukocyte	*RPS18*	-0.525052757	0.001979201	0.005222499	1
T2DM	leukocyte	*LINC00865*	-2.083952393	0.005824724	0.009077881	1
T2DM	leukocyte	*HLA-DQB2*	1.629089729	5.86E-06	0.001302453	0.023005665
T2DM	leukocyte	*LINC00665*	-0.65789508	0.004551841	0.007568852	1
T2DM	leukocyte	*LYRM9*	-1.038438861	0.002887639	0.00605886	1
T2DM	leukocyte	*SNHG15*	-0.86421177	0.0021675	0.005345956	1
T2DM	leukocyte	*RPS28*	-0.596345264	0.001012676	0.004990048	1
T2DM	leukocyte	*TRIM26*	-1.281181754	0.000308598	0.004990048	1
T2DM	leukocyte	*ZNF879*	-1.181381197	0.002891221	0.006061691	1
T2DM	leukocyte	*PINLYP*	-2.045858022	0.001127855	0.004990048	1
T2DM	leukocyte	*MCIDAS*	32.60480758	0.012168262	0.01699577	1
T2DM	leukocyte	*ANKRD65*	-0.834144893	0.00212908	0.005307839	1
T2DM	leukocyte	*C12orf75*	-0.516021007	0.00203636	0.005253355	1
T2DM	leukocyte	*B3GALT4*	-0.908980399	0.000223209	0.004990048	0.876987103
T2DM	leukocyte	*ZNF737*	-1.341474264	0.001032343	0.004990048	1
T2DM	leukocyte	*SHISA9*	-6.614470993	0.003502875	0.006639072	1
T2DM	leukocyte	*HLA-DQA2*	0.106096596	0.002630318	0.005799393	1
T2DM	leukocyte	*LRRC37A2*	-0.941597064	0.028851778	0.03665785	1
T2DM	leukocyte	*TXNDC5*	-0.947129092	0.001261997	0.004990048	1
T2DM	leukocyte	*RBM14*	-1.250903121	0.001110784	0.004990048	1
T2DM	leukocyte	*NME1*	-0.667157089	0.002329716	0.005513096	1
T2DM	leukocyte	*CDRT4*	-1.893670782	0.003501191	0.006639072	1
T2DM	leukocyte	*AMY2B*	-0.738178107	0.002651905	0.005824744	1
T2DM	leukocyte	*LY6G5B*	-0.900077797	0.012963811	0.017966425	1
T2DM	leukocyte	*PSMB9*	-0.523952756	0.02585622	0.033134079	1
T2DM	leukocyte	*SMKR1*	-1.125863362	0.003087583	0.00625753	1
T2DM	leukocyte	*LINC00886*	-1.214406425	0.00612136	0.00945394	1
T2DM	leukocyte	*MIF*	-0.563358513	0.001379078	0.004990048	1
T2DM	leukocyte	*NSUN6*	-0.873462901	0.002404422	0.005583319	1
T2DM	leukocyte	*CRCP*	-1.130037308	0.000604271	0.004990048	1
T2DM	leukocyte	*C8orf58*	-1.123880311	0.018480616	0.02453881	1
T2DM	leukocyte	*PI4KA*	-1.52330134	0.001294005	0.004990048	1
T2DM	leukocyte	*AMACR*	-0.672282643	0.008668135	0.012623092	1
T2DM	leukocyte	*BGLAP*	-1.853468106	0.000265895	0.004990048	1
T2DM	leukocyte	*MRPL20*	-0.724728392	0.003159245	0.006314657	1
T2DM	leukocyte	*HLA-DMB*	0.086745605	0.014704812	0.020046914	1
T2DM	leukocyte	*DECR2*	-0.921673581	0.00274432	0.005930931	1
T2DM	leukocyte	*GNG10*	-0.709423716	0.005185523	0.008292193	1
T2DM	leukocyte	*CNTF*	1.762028213	0.013512244	0.018634471	1
T2DM	leukocyte	*EIF4EBP3*	-1.298784753	0.000676996	0.004990048	1

T2DM	leukocyte	*MICAL3*	-0.862148341	0.002488683	0.005622792	1
T2DM	leukocyte	*STON1*	-1.165418763	0.007154201	0.010765551	1
T2DM	leukocyte	*VSIG8*	-2.238591961	0.004014351	0.00714972	1
T2DM	leukocyte	*C4orf48*	-0.681563285	0.00127455	0.004990048	1
T2DM	leukocyte	*IL10RB*	-2.394947145	0.000163305	0.004990048	0.641623898
T2DM	leukocyte	*LEFTY1*	-6.86750909	0.007377549	0.011050853	1
T2DM	leukocyte	*TTC4*	-0.899244728	0.003968606	0.007124893	1
T2DM	leukocyte	*JMJD7*	3.026241729	0.000297498	0.004990048	1
T2DM	leukocyte	*APOBEC3D*	-3.599892946	0.003381032	0.006503419	1
T2DM	leukocyte	*RN7SL832P*	-3.678375319	0.030342778	0.038457024	1
T2DM	leukocyte	*OR2A7*	-3.077083834	0.021557533	0.028195588	1
T2DM	leukocyte	*GSTA1*	-0.404312507	0.021911143	0.028591458	1
T2DM	leukocyte	*GSTA2*	-0.643438344	0.004908057	0.007970068	1
T2DM	leukocyte	*TMEM141*	-1.051579002	0.00294491	0.006128469	1
T2DM	leukocyte	*APOBEC3C*	-1.570228199	0.014590115	0.019911276	1
T2DM	leukocyte	*C4A*	-7.488372484	0.003441016	0.006566174	1
T2DM	leukocyte	*NEAT1*	-0.61506392	0.001794829	0.005114303	1
T2DM	leukocyte	*LINC00920*	-6.521606844	0.014717594	0.02005738	1
T2DM	leukocyte	*UBAP1L*	-0.893570024	0.005459927	0.008629144	1
T2DM	leukocyte	*TWF2*	-1.151994253	0.003071092	0.006249984	1
T2DM	leukocyte	*BCKDHA*	-0.816828188	0.007487522	0.011185732	1
T2DM	leukocyte	*PCDHAC1*	-1.261017222	0.006606627	0.010088394	1
T2DM	leukocyte	*FMN1*	-2.437940294	0.014366283	0.019667292	1
T2DM	leukocyte	*TMEM150C*	-1.147359911	0.001265576	0.004990048	1
T2DM	leukocyte	*LINC01091*	-0.675361649	0.001834364	0.005146173	1
T2DM	leukocyte	*PDCD6*	-0.904142259	0.001135033	0.004990048	1
T2DM	leukocyte	*YJEFN3*	-1.586877537	0.002453723	0.005621365	1
T2DM	leukocyte	*PCDHA10*	-0.824736911	0.003442966	0.006566706	1
T2DM	leukocyte	*ZNF718*	-1.04595972	0.009479433	0.013632757	1
T2DM	leukocyte	*SNHG18*	-0.929659094	0.002219901	0.005407737	1
T2DM	leukocyte	*ZNF550*	-1.073767802	0.001993623	0.005227473	1
T2DM	leukocyte	*LINC01099*	-1.046671354	0.004266932	0.007310294	1
T2DM	leukocyte	*PCDHA12*	-1.422625784	0.004917577	0.007973094	1
T2DM	leukocyte	*ALG11*	-1.708805955	0.001052746	0.004990048	1
T2DM	leukocyte	*ZNF260*	-1.553757152	0.004920056	0.007973094	1
T2DM	leukocyte	*AP5B1*	-1.740230426	0.000908877	0.004990048	1
T2DM	leukocyte	*SIGLEC12*	1.61370917	0.015861815	0.021486999	1
T2DM	leukocyte	*PABPC4L*	-2.448880663	0.004282986	0.007324561	1
T2DM	leukocyte	*NPIPA2*	-9.458640847	0.003532643	0.006669753	1
T2DM	leukocyte	*DPP3*	-0.789908468	0.007651919	0.011388027	1
T2DM	leukocyte	*BRK1*	-0.845402762	0.000155094	0.004990048	0.609365836
T2DM	leukocyte	*EID3*	-2.00382545	0.000789839	0.004990048	1
T2DM	leukocyte	*SNHG9*	-0.842148829	0.001809743	0.005122825	1
T2DM	leukocyte	*PCDHA3*	-1.201097577	0.017768219	0.023686929	1
T2DM	leukocyte	*ZNF432*	-2.070774924	0.000218	0.004990048	0.856521355
T2DM	leukocyte	*LINC01152*	-4.188406983	0.004062847	0.007184034	1
T2DM	leukocyte	*SMLR1*	2.794137908	0.025493777	0.032744377	1
T2DM	leukocyte	*ZNF10*	-1.17285406	0.001520385	0.004993935	1
T2DM	leukocyte	*TAS2R31*	-2.94653174	0.009943694	0.014232705	1
T2DM	leukocyte	*GATC*	-1.289662895	0.000689278	0.004990048	1
T2DM	leukocyte	*TEN1*	-2.465286945	0.001393359	0.004990048	1
T2DM	leukocyte	*CHURC1*	-0.741855799	0.001648699	0.005035955	1
T2DM	leukocyte	*LINC00638*	-6.620899375	0.002359166	0.005540445	1
T2DM	leukocyte	*SLC25A21-AS1*	-0.872787887	0.003958076	0.007124893	1
T2DM	leukocyte	*TMEM179*	-0.632997124	0.004077889	0.007189	1
T2DM	leukocyte	*IQCH-AS1*	-1.115818679	0.036228238	0.045360341	1
T2DM	leukocyte	*PWRN1*	-1.155614189	0.006176481	0.009520358	1
T2DM	leukocyte	*TMEM178B*	-0.827312878	0.003186617	0.006339351	1
T2DM	leukocyte	*LINC01003*	-1.093995761	0.001528844	0.005001523	1
T2DM	leukocyte	*GOLGA8H*	-11.97788044	0.022264913	0.029014542	1
T2DM	leukocyte	*MRPL12*	-1.179285139	0.001138119	0.004990048	1
T2DM	leukocyte	*ZNF234*	-4.970248852	0.00124357	0.004990048	1
T2DM	leukocyte	*LINC00667*	-1.005477284	0.001300671	0.004990048	1
T2DM	leukocyte	*FSBP*	-1.243505938	0.004177894	0.007242343	1

T2DM	leukocyte	*MYO15B*	-1.728859762	0.008847026	0.012831291	1
T2DM	leukocyte	*LINC00663*	-1.296100677	0.007790967	0.011564302	1
T2DM	leukocyte	*ZNF285*	-2.105443343	0.003752385	0.00692001	1
T2DM	leukocyte	*FBXO17*	-0.751493085	0.002485175	0.005622792	1
T2DM	leukocyte	*LINC00528*	0.493105847	0.012891246	0.017882851	1
T2DM	leukocyte	*SNHG8*	-0.688593453	0.001151454	0.004990048	1
T2DM	leukocyte	*MILR1*	0.78206395	0.01001522	0.014303817	1
T2DM	leukocyte	*GTF2H5*	-0.808830908	0.003217602	0.006372159	1
T2DM	mesenchymal_cell	*RBM6*	-0.832993035	2.28E-07	9.23E-05	0.00089423
T2DM	mesenchymal_cell	*CEP68*	0.991739724	3.60E-05	0.005503398	0.141591933
T2DM	mesenchymal_cell	*ERCC1*	-1.153465916	0.000886192	0.04145058	1
T2DM	mesenchymal_cell	*BAK1*	1.043658561	0.000806888	0.039440621	1
T2DM	mesenchymal_cell	*ITIH4*	-1.186516391	0.000202922	0.019302292	0.797278978
T2DM	mesenchymal_cell	*SEC61A2*	-2.103612755	0.000284997	0.024175174	1
T2DM	mesenchymal_cell	*TCF3*	-1.378317002	1.69E-06	0.00044143	0.006621445
T2DM	mesenchymal_cell	*SCARB1*	-1.55221091	0.000484728	0.032693637	1
T2DM	mesenchymal_cell	*ST6GAL1*	0.678213903	0.001099799	0.04910353	1
T2DM	mesenchymal_cell	*TTC38*	-2.28800492	0.000140823	0.017224517	0.553294488
T2DM	mesenchymal_cell	*MARK3*	-1.110702499	0.000254545	0.022224624	1
T2DM	mesenchymal_cell	*CWF19L1*	-0.568030425	6.41E-05	0.008995669	0.251878739
T2DM	mesenchymal_cell	*RBFOX2*	-1.381767956	0.000540163	0.034230682	1
T2DM	mesenchymal_cell	*SAMM50*	-1.93889085	0.000595721	0.03493416	1
T2DM	mesenchymal_cell	*VCPKMT*	-2.151750888	0.00084761	0.040123603	1
T2DM	mesenchymal_cell	*NAGPA*	-2.194073586	0.000187438	0.019302292	0.736443443
T2DM	mesenchymal_cell	*RPAP1*	-2.841836606	0.000726953	0.037577027	1
T2DM	mesenchymal_cell	*ERI1*	-2.227055145	0.000193107	0.019302292	0.758715639
T2DM	mesenchymal_cell	*GCDH*	-2.640186281	9.42E-06	0.001948128	0.03701444
T2DM	mesenchymal_cell	*GTPBP4*	-1.378146135	0.001070366	0.04890078	1
T2DM	mesenchymal_cell	*HSD17B1*	-0.834943316	3.26E-05	0.005331695	0.127960683
T2DM	mesenchymal_cell	*RANGRF*	-1.676075749	0.000939078	0.043407488	1
T2DM	mesenchymal_cell	*NNT*	-0.870618359	0.000564125	0.034810097	1
T2DM	mesenchymal_cell	*FLT3*	0.892234282	0.000206337	0.019302292	0.81069626
T2DM	mesenchymal_cell	*PEPD*	-1.433613259	0.000704367	0.037398103	1
T2DM	mesenchymal_cell	*PRMT1*	-1.095440188	0.000171485	0.018209846	0.67376429
T2DM	mesenchymal_cell	*PTPN12*	-0.544488914	0.000146483	0.017224517	0.575530491
T2DM	mesenchymal_cell	*MAU2*	-1.839981387	2.35E-07	9.23E-05	0.000923051
T2DM	mesenchymal_cell	*NOTCH2*	-0.469993808	1.54E-07	7.57E-05	0.000605691
T2DM	mesenchymal_cell	*RDH5*	-1.817409389	0.000813105	0.039440621	1
T2DM	mesenchymal_cell	*BARD1*	-1.755912631	0.00053993	0.034230682	1
T2DM	mesenchymal_cell	*TLE3*	-1.526438677	0.001098225	0.04910353	1
T2DM	mesenchymal_cell	*MAN2C1*	-0.585036296	3.76E-06	0.000869995	0.014789907
T2DM	mesenchymal_cell	*SCRN2*	-1.168868918	0.000205149	0.019302292	0.806031114
T2DM	mesenchymal_cell	*NPC1*	-2.249177902	4.54E-07	0.000162039	0.001782432
T2DM	mesenchymal_cell	*CNTN4*	-0.559274027	0.000412979	0.030615021	1
T2DM	mesenchymal_cell	*TBC1D7*	-2.80847892	0.000139008	0.017224517	0.546164289
T2DM	mesenchymal_cell	*GIGYF1*	-1.948111793	2.78E-05	0.004743979	0.109111517
T2DM	mesenchymal_cell	*LRP12*	-1.391227627	0.000490945	0.032693637	1
T2DM	mesenchymal_cell	*HSD17B12*	-0.29862748	0.000247506	0.022101199	0.972452734
T2DM	mesenchymal_cell	*APIP*	-0.467343007	0.000436592	0.031766122	1
T2DM	mesenchymal_cell	*DLG5*	-1.500810126	0.00069894	0.037398103	1
T2DM	mesenchymal_cell	*PTPN14*	-0.507679385	4.76E-05	0.006919878	0.1868367
T2DM	mesenchymal_cell	*MED21*	-1.491956268	0.0007642	0.038178998	1
T2DM	mesenchymal_cell	*JAZF1*	-0.728523206	2.26E-05	0.004029478	0.088648525
T2DM	mesenchymal_cell	*NUS1*	-1.574690967	0.000375003	0.029467764	1
T2DM	mesenchymal_cell	*CLEC18A*	-1.745018773	8.05E-08	4.52E-05	0.000316138
T2DM	mesenchymal_cell	*CILP2*	-0.766779226	3.64E-05	0.005503398	0.143088338
T2DM	mesenchymal_cell	*ZNF208*	-0.824008647	0.000649837	0.03596072	1
T2DM	mesenchymal_cell	*COG5*	-2.006553946	0.000637915	0.035805262	1
T2DM	mesenchymal_cell	*RPP25L*	-2.369124644	0.000504119	0.033011362	1
T2DM	mesenchymal_cell	*INPPL1*	-1.681255807	0.000303121	0.024811746	1
T2DM	mesenchymal_cell	*RCCD1*	-1.000728067	0.000575886	0.034810097	1
T2DM	mesenchymal_cell	*POP5*	-1.530652014	0.000455067	0.0322272	1
T2DM	mesenchymal_cell	*TMC8*	-0.603822429	0.00038285	0.029494452	1
T2DM	mesenchymal_cell	*TRANK1*	-1.575310184	0.000833693	0.039946115	1

T2DM	mesenchymal_cell	*DNAJC21*	-1.658127636	0.00010363	0.014040089	0.40716259
T2DM	mesenchymal_cell	*TMEM129*	-0.678232886	0.000588093	0.03493416	1
T2DM	mesenchymal_cell	*SNUPN*	-2.464324064	0.000289191	0.024175174	1
T2DM	mesenchymal_cell	*INO80E*	-1.117707699	2.96E-06	0.000727342	0.011637476
T2DM	mesenchymal_cell	*ZNF440*	-2.00850717	0.000153438	0.017224517	0.602858093
T2DM	mesenchymal_cell	*RGS19*	-0.647135783	0.000700673	0.037398103	1
T2DM	mesenchymal_cell	*RNASEH2C*	-0.781380437	0.000629677	0.035805262	1
T2DM	mesenchymal_cell	*IRX3*	2.559942734	1.64E-18	6.44E-15	6.44E-15
T2DM	mesenchymal_cell	*FARSA*	-1.717183526	7.28E-07	0.000216	0.00286077
T2DM	mesenchymal_cell	*HLA-DQB1*	-0.416255737	3.32E-08	2.61E-05	0.000130308
T2DM	mesenchymal_cell	*D2HGDH*	-1.677046718	0.000459334	0.0322272	1
T2DM	mesenchymal_cell	*UBA7*	-0.493104041	1.12E-05	0.002193575	0.043871498
T2DM	mesenchymal_cell	*ZNF320*	0.294540309	0.000471983	0.032533701	1
T2DM	mesenchymal_cell	*TRMT12*	-1.587585472	0.000767661	0.038178998	1
T2DM	mesenchymal_cell	*PGP*	-1.155218037	0.000150357	0.017224517	0.590753782
T2DM	mesenchymal_cell	*DMWD*	-1.265854161	2.08E-05	0.00389804	0.081858834
T2DM	mesenchymal_cell	*HLA-DRB1*	-0.294321389	4.37E-09	5.73E-06	1.72E-05
T2DM	mesenchymal_cell	*HLA-DQA1*	-0.203574943	0.000410148	0.030615021	1
T2DM	mesenchymal_cell	*PIWIL2*	-0.708460881	0.000143122	0.017224517	0.562325753
T2DM	mesenchymal_cell	*HLA-DRB5*	-0.170683984	0.000164839	0.017990355	0.64765279
T2DM	mesenchymal_cell	*TBKBP1*	-0.564162184	0.000727942	0.037577027	1
T2DM	mesenchymal_cell	*NOTCH4*	-0.401109528	5.41E-07	0.000177081	0.002124967
T2DM	mesenchymal_cell	*RNF5*	-0.675889309	7.70E-07	0.000216	0.003023994
T2DM	mesenchymal_cell	*RNF39*	0.410407318	0.000736429	0.037577027	1
T2DM	mesenchymal_cell	*HCG27*	0.541498994	4.17E-06	0.000911272	0.016402897
T2DM	mesenchymal_cell	*ATF6B*	0.539464648	0.00057024	0.034810097	1
T2DM	mesenchymal_cell	*SLC12A8*	-3.719082151	4.46E-08	2.92E-05	0.000175174
T2DM	mesenchymal_cell	*HLA-DQB2*	1.063346586	1.10E-08	1.08E-05	4.34E-05
T2DM	mesenchymal_cell	*HLA-B*	0.357147446	0.000315463	0.025295016	1
T2DM	mesenchymal_cell	*HLA-DQA2*	0.679592645	3.50E-12	6.88E-09	1.38E-08
T2DM	mesenchymal_cell	*JMJD7*	1.089388615	0.000227778	0.020812572	0.894940598
T2DM	mesenchymal_cell	*ZNF432*	-1.782769077	0.000632666	0.035805262	1
T2DM	PP_cell	*RBM6*	-1.361582632	4.80E-07	0.000269598	0.001887187
T2DM	PP_cell	*CEP68*	1.467898161	0.000183382	0.036540786	0.720508395
T2DM	PP_cell	*TCF3*	-1.625061494	0.000186006	0.036540786	0.730815714
T2DM	PP_cell	*MARK3*	-1.772630664	0.000156907	0.034249235	0.616486224
T2DM	PP_cell	*HSD17B1*	-1.10320676	0.000297373	0.044937675	1
T2DM	PP_cell	*NNT*	-1.540405998	0.000230743	0.040242285	0.906588954
T2DM	PP_cell	*MAU2*	-1.542119812	0.000235575	0.040242285	0.925572548
T2DM	PP_cell	*MAN2C1*	-0.940184891	9.48E-06	0.003385593	0.037241525
T2DM	PP_cell	*HSD17B12*	-0.578912704	7.38E-05	0.020724605	0.290144464
T2DM	PP_cell	*APIP*	-0.92296033	9.62E-05	0.025198778	0.377981672
T2DM	PP_cell	*PTPN14*	-0.947736848	0.000296933	0.044937675	1
T2DM	PP_cell	*JAZF1*	-1.307437947	8.11E-06	0.003186366	0.031863657
T2DM	PP_cell	*CLEC18A*	-3.866515414	3.04E-08	2.39E-05	0.000119263
T2DM	PP_cell	*CILP2*	-1.408555434	0.000146468	0.033851357	0.575473069
T2DM	PP_cell	*INO80E*	-1.549179831	3.62E-05	0.011857222	0.142286666
T2DM	PP_cell	*IRX3*	1.862615791	2.83E-09	5.57E-06	1.11E-05
T2DM	PP_cell	*HLA-DQB1*	-0.793797082	8.16E-08	5.34E-05	0.000320489
T2DM	PP_cell	*ZNF320*	0.590242902	0.000256329	0.041963114	1
T2DM	PP_cell	*HLA-DRB1*	-0.609411662	6.07E-09	7.95E-06	2.38E-05
T2DM	PP_cell	*NOTCH4*	-0.873298737	1.26E-06	0.00055176	0.004965841
T2DM	PP_cell	*RNF5*	-1.138180221	1.07E-06	0.00052359	0.004188719
T2DM	PP_cell	*HCG27*	0.655231386	0.000134552	0.033040892	0.52865427
T2DM	PP_cell	*DNLZ*	0.591883554	0.000234483	0.040242285	0.921284405
T2DM	PP_cell	*PPT2*	1.669573276	6.45E-05	0.019487882	0.253342463
T2DM	PP_cell	*HLA-DQB2*	1.833401553	2.43E-08	2.39E-05	9.55E-05
T2DM	PP_cell	*HLA-DQA2*	1.133319958	5.71E-12	2.24E-08	2.24E-08
T2DM_admission	acinar_cell	*RBM6*	-0.917672389	3.41E-08	1.29E-05	0.000133958
T2DM_admission	acinar_cell	*MLXIPL*	0.581660804	0.000240192	0.019660748	0.943715891
T2DM_admission	acinar_cell	*GIPR*	-0.988885771	0.00023392	0.019660748	0.919070657
T2DM_admission	acinar_cell	*CEP68*	1.383347846	1.72E-08	8.44E-06	6.76E-05
T2DM_admission	acinar_cell	*BAK1*	1.703537331	1.04E-07	3.39E-05	0.000406952
T2DM_admission	acinar_cell	*TCF3*	-1.613032022	4.25E-06	0.000695624	0.016694966

T2DM_admission	acinar_cell	*ST6GAL1*	0.834487685	0.000121353	0.012225528	0.476795584
T2DM_admission	acinar_cell	*MARK3*	-1.837724277	6.00E-06	0.000942178	0.023554457
T2DM_admission	acinar_cell	*CWF19L1*	-0.495311941	0.000559218	0.036275307	1
T2DM_admission	acinar_cell	*CCDC134*	1.316648143	0.000258682	0.020568623	1
T2DM_admission	acinar_cell	*OIP5*	2.34197843	8.25E-05	0.008534212	0.324300047
T2DM_admission	acinar_cell	*GCDH*	-2.378889507	3.41E-05	0.004059448	0.133961794
T2DM_admission	acinar_cell	*HSD17B1*	-0.801494872	6.55E-05	0.006956183	0.257378789
T2DM_admission	acinar_cell	*CNTNAP1*	0.778526985	8.06E-06	0.001218682	0.031685723
T2DM_admission	acinar_cell	*CDK2AP1*	0.757848433	0.000359753	0.02666921	1
T2DM_admission	acinar_cell	*FAM53C*	-1.573862232	0.000234207	0.019660748	0.92019921
T2DM_admission	acinar_cell	*ZNF211*	0.570030816	0.000404415	0.028374015	1
T2DM_admission	acinar_cell	*FLT3*	0.959059462	0.000184549	0.017264156	0.725094549
T2DM_admission	acinar_cell	*OPRL1*	-1.138295147	5.46E-05	0.006131934	0.214617678
T2DM_admission	acinar_cell	*MAU2*	-1.772237155	1.49E-06	0.000307981	0.005851638
T2DM_admission	acinar_cell	*NOTCH2*	-0.818069013	6.03E-12	4.74E-09	2.37E-08
T2DM_admission	acinar_cell	*SPTBN5*	0.888918408	0.000313897	0.024182417	1
T2DM_admission	acinar_cell	*MAN2C1*	-0.554363788	1.01E-05	0.001463258	0.039507953
T2DM_admission	acinar_cell	*NPC1*	-2.337687434	3.76E-06	0.000670899	0.014759771
T2DM_admission	acinar_cell	*GIGYF1*	-1.604713136	0.000794911	0.046615029	1
T2DM_admission	acinar_cell	*HSD17B12*	-0.419032516	7.17E-07	0.000165794	0.002818498
T2DM_admission	acinar_cell	*APIP*	-0.487161411	0.000328599	0.024828216	1
T2DM_admission	acinar_cell	*VIPAS39*	-1.521103227	0.000838423	0.04844358	1
T2DM_admission	acinar_cell	*PTPN14*	-0.581332257	6.08E-05	0.006631241	0.238724675
T2DM_admission	acinar_cell	*POC5*	-0.496734478	2.42E-05	0.003062247	0.094929662
T2DM_admission	acinar_cell	*JAZF1*	-2.169299124	2.11E-16	4.15E-13	8.31E-13
T2DM_admission	acinar_cell	*NUS1*	-1.731001607	0.000489326	0.032790875	1
T2DM_admission	acinar_cell	*CLEC18A*	-3.071796998	9.71E-13	9.54E-10	3.82E-09
T2DM_admission	acinar_cell	*EXTL1*	-1.524619641	0.000631262	0.039368698	1
T2DM_admission	acinar_cell	*CILP2*	-0.896923928	2.59E-05	0.003182263	0.101832401
T2DM_admission	acinar_cell	*SRGAP2*	1.207678053	0.000397462	0.028374015	1
T2DM_admission	acinar_cell	*ICA1L*	-0.530435873	0.000223007	0.019660748	0.876193496
T2DM_admission	acinar_cell	*BDH2*	-1.07991901	2.33E-05	0.003057615	0.091728454
T2DM_admission	acinar_cell	*SGPL1*	-1.259016583	0.000193568	0.017686746	0.760530065
T2DM_admission	acinar_cell	*PCBD1*	-1.314974227	0.00013913	0.01366607	0.546642801
T2DM_admission	acinar_cell	*TMEM129*	-0.736358925	0.000261754	0.020568623	1
T2DM_admission	acinar_cell	*INO80E*	-0.856284687	0.000240072	0.019660748	0.943243401
T2DM_admission	acinar_cell	*SNX32*	0.877004217	0.00067808	0.041627725	1
T2DM_admission	acinar_cell	*PEAK1*	0.766249697	0.000563195	0.036275307	1
T2DM_admission	acinar_cell	*YIF1A*	-0.69040049	0.00074901	0.045274764	1
T2DM_admission	acinar_cell	*IRX3*	3.348519066	1.28E-27	5.03E-24	5.03E-24
T2DM_admission	acinar_cell	*FARSA*	-1.941585931	3.26E-07	8.55E-05	0.00128275
T2DM_admission	acinar_cell	*HLA-DQB1*	-0.465918295	3.61E-08	1.29E-05	0.000141977
T2DM_admission	acinar_cell	*UBA7*	-0.67488792	1.85E-06	0.000353372	0.00727256
T2DM_admission	acinar_cell	*VMO1*	0.654322997	0.000486227	0.032790875	1
T2DM_admission	acinar_cell	*CAMK1D*	1.994993552	0.000403902	0.028374015	1
T2DM_admission	acinar_cell	*HLA-DRB1*	-0.391837494	3.52E-11	2.31E-08	1.38E-07
T2DM_admission	acinar_cell	*HLA-DRB5*	-0.296784376	1.10E-06	0.000240316	0.004325685
T2DM_admission	acinar_cell	*LIME1*	-0.559481216	0.000160273	0.015358852	0.629712948
T2DM_admission	acinar_cell	*NOTCH4*	-0.677536694	2.24E-09	1.26E-06	8.81E-06
T2DM_admission	acinar_cell	*AGER*	1.017394488	3.78E-05	0.004363289	0.14835181
T2DM_admission	acinar_cell	*RNF5*	-0.700274557	2.42E-07	6.79E-05	0.000950124
T2DM_admission	acinar_cell	*HLA-C*	-0.296841946	0.000868684	0.049464647	1
T2DM_admission	acinar_cell	*RNF39*	0.41264696	0.000792894	0.046615029	1
T2DM_admission	acinar_cell	*HCG27*	0.644736633	1.64E-07	4.96E-05	0.000644539
T2DM_admission	acinar_cell	*DNLZ*	0.319975001	0.000492406	0.032790875	1
T2DM_admission	acinar_cell	*ATF6B*	0.678103795	1.28E-05	0.001801329	0.050437218
T2DM_admission	acinar_cell	*SLC12A8*	-2.756983024	6.49E-07	0.000159432	0.002550905
T2DM_admission	acinar_cell	*PPT2*	0.995200738	1.40E-05	0.00189481	0.054949485
T2DM_admission	acinar_cell	*HLA-DQB2*	1.037743804	2.58E-08	1.13E-05	0.000101382
T2DM_admission	acinar_cell	*HLA-B*	0.476511508	1.89E-06	0.000353372	0.007420812
T2DM_admission	acinar_cell	*HLA-DQA2*	0.686929812	1.68E-13	2.20E-10	6.59E-10
T2DM_admission	acinar_cell	*JMJD7*	1.380210135	4.15E-06	0.000695624	0.016313527
T2DM_admission	acinar_cell	*HMBS*	0.472683596	0.000618503	0.039195121	1
T2DM_admission	alpha_cell	*RBM6*	-0.149497121	4.69E-08	1.54E-05	0.000184235

T2DM_admission	alpha_cell	*MLXIPL*	0.099731408	0.000224435	0.016957799	0.881805564
T2DM_admission	alpha_cell	*GIPR*	-0.236088711	2.31E-06	0.000412433	0.009073524
T2DM_admission	alpha_cell	*CEP68*	0.257404438	3.22E-10	1.81E-07	1.27E-06
T2DM_admission	alpha_cell	*BAK1*	0.30734952	8.27E-09	3.61E-06	3.25E-05
T2DM_admission	alpha_cell	*TCF3*	-0.254591248	1.54E-05	0.001832074	0.060458432
T2DM_admission	alpha_cell	*ST6GAL1*	0.132300359	0.000266377	0.018689229	1
T2DM_admission	alpha_cell	*MARK3*	-0.379411094	1.09E-06	0.000213571	0.004271414
T2DM_admission	alpha_cell	*CWF19L1*	-0.080432343	0.000578937	0.032037236	1
T2DM_admission	alpha_cell	*CCDC134*	0.215214016	0.000350063	0.022923293	1
T2DM_admission	alpha_cell	*OIP5*	0.383048926	7.81E-05	0.006977228	0.306998051
T2DM_admission	alpha_cell	*GCDH*	-0.55419366	3.33E-07	8.73E-05	0.001309269
T2DM_admission	alpha_cell	*HSD17B1*	-0.130174058	6.72E-05	0.006291704	0.263910178
T2DM_admission	alpha_cell	*CNTNAP1*	0.126151977	9.26E-06	0.001212892	0.036386767
T2DM_admission	alpha_cell	*CDK2AP1*	0.13903664	0.000117437	0.010030681	0.46141131
T2DM_admission	alpha_cell	*DNAH7*	-0.118400909	0.000562342	0.031563453	1
T2DM_admission	alpha_cell	*FAM53C*	-0.274960102	0.00069799	0.035158991	1
T2DM_admission	alpha_cell	*ZNF211*	0.095452181	0.000381416	0.024566916	1
T2DM_admission	alpha_cell	*POLK*	-0.268490104	0.000296626	0.020446376	1
T2DM_admission	alpha_cell	*FLT3*	0.158052072	0.000127525	0.010660578	0.501047161
T2DM_admission	alpha_cell	*PEPD*	-0.31529553	0.001072009	0.049394903	1
T2DM_admission	alpha_cell	*OPRL1*	-0.186002403	4.62E-05	0.004771893	0.181331924
T2DM_admission	alpha_cell	*MAU2*	-0.300403202	2.57E-06	0.00043896	0.010096077
T2DM_admission	alpha_cell	*PPARG*	-0.426954029	9.38E-05	0.008186863	0.368408843
T2DM_admission	alpha_cell	*NOTCH2*	-0.129957182	3.02E-11	2.38E-08	1.19E-07
T2DM_admission	alpha_cell	*FXYD2*	-0.168490967	0.001053078	0.049256454	1
T2DM_admission	alpha_cell	*SPTBN5*	0.13682184	0.00068474	0.035158991	1
T2DM_admission	alpha_cell	*MAN2C1*	-0.092479579	6.16E-06	0.000896698	0.024210836
T2DM_admission	alpha_cell	*NPC1*	-0.437954417	3.90E-06	0.000613072	0.015326804
T2DM_admission	alpha_cell	*POGZ*	0.17835488	0.00047166	0.029413161	1
T2DM_admission	alpha_cell	*INPP5E*	0.21361291	0.000654484	0.03428626	1
T2DM_admission	alpha_cell	*SEC16A*	0.170509876	0.000980381	0.046408645	1
T2DM_admission	alpha_cell	*HSD17B12*	-0.067706944	9.45E-07	0.00019535	0.003711653
T2DM_admission	alpha_cell	*APIP*	-0.081259321	0.000256079	0.018632083	1
T2DM_admission	alpha_cell	*PTPN14*	-0.089288282	0.000198357	0.015586927	0.779346352
T2DM_admission	alpha_cell	*POC5*	-0.082896037	1.59E-05	0.001842081	0.062630766
T2DM_admission	alpha_cell	*JAZF1*	-0.446904569	6.21E-21	1.22E-17	2.44E-17
T2DM_admission	alpha_cell	*NUS1*	-0.35741459	0.000891992	0.042739447	1
T2DM_admission	alpha_cell	*CLEC18A*	-0.477032154	6.81E-12	6.69E-09	2.68E-08
T2DM_admission	alpha_cell	*AP3S2*	0.447628921	7.46E-06	0.001046474	0.029301273
T2DM_admission	alpha_cell	*EXTL1*	-0.250666195	0.000488563	0.029413161	1
T2DM_admission	alpha_cell	*CILP2*	-0.146930627	1.91E-05	0.002147963	0.075178689
T2DM_admission	alpha_cell	*SRGAP2*	0.215083835	0.000190566	0.015280298	0.748734597
T2DM_admission	alpha_cell	*ICA1L*	-0.084409289	0.00032451	0.021982728	1
T2DM_admission	alpha_cell	*BDH2*	-0.163632648	7.59E-05	0.00693296	0.298117292
T2DM_admission	alpha_cell	*CASP7*	0.085962838	3.49E-05	0.003813143	0.137273134
T2DM_admission	alpha_cell	*CKB*	-0.125681949	0.000334135	0.022251122	1
T2DM_admission	alpha_cell	*SGPL1*	-0.203498448	0.000641113	0.034131535	1
T2DM_admission	alpha_cell	*PCBD1*	-0.246472313	0.000163423	0.013376821	0.642087409
T2DM_admission	alpha_cell	*TMEM129*	-0.121538711	0.000261922	0.018689229	1
T2DM_admission	alpha_cell	*INO80E*	-0.159785034	6.73E-05	0.006291704	0.264251573
T2DM_admission	alpha_cell	*RGS19*	-0.16105548	0.00087681	0.042530682	1
T2DM_admission	alpha_cell	*CTSW*	-0.055854212	0.000521981	0.029722681	1
T2DM_admission	alpha_cell	*SNX32*	0.144599456	0.00050906	0.029413161	1
T2DM_admission	alpha_cell	*RNASEH2C*	-0.148594513	0.000489717	0.029413161	1
T2DM_admission	alpha_cell	*PEAK1*	0.135169659	0.000220457	0.016957799	0.8661754
T2DM_admission	alpha_cell	*YIF1A*	-0.121024016	0.000634303	0.034131535	1
T2DM_admission	alpha_cell	*DSCAML1*	-0.177034314	0.000791004	0.038848205	1
T2DM_admission	alpha_cell	*IRX3*	0.536645624	9.02E-28	3.54E-24	3.54E-24
T2DM_admission	alpha_cell	*FARSA*	-0.332441966	2.65E-07	7.43E-05	0.001039905
T2DM_admission	alpha_cell	*HLA-DQB1*	-0.075349992	4.21E-08	1.54E-05	0.000165524
T2DM_admission	alpha_cell	*TH*	-1.042292572	1.00E-05	0.001268445	0.03932179
T2DM_admission	alpha_cell	*UBA7*	-0.10477671	6.08E-06	0.000896698	0.023879081
T2DM_admission	alpha_cell	*VMO1*	0.104839533	0.000501913	0.029413161	1
T2DM_admission	alpha_cell	*CAMK1D*	0.369614234	4.93E-05	0.004963234	0.193566125

T2DM_admission	alpha_cell	*PGP*	-0.192658065	0.000774329	0.038510624	1
T2DM_admission	alpha_cell	*DMWD*	-0.191704172	0.000418219	0.026502926	1
T2DM_admission	alpha_cell	*NUTM2B*	-1.170460409	5.29E-05	0.005200127	0.208005079
T2DM_admission	alpha_cell	*HLA-DRB1*	-0.064464684	3.79E-11	2.48E-08	1.49E-07
T2DM_admission	alpha_cell	*HLA-DRB5*	-0.049023679	1.49E-06	0.000278314	0.005844588
T2DM_admission	alpha_cell	*LIME1*	-0.080656509	0.000642844	0.034131535	1
T2DM_admission	alpha_cell	*NOTCH4*	-0.109749155	1.97E-09	9.68E-07	7.74E-06
T2DM_admission	alpha_cell	*AGER*	0.173360815	3.62E-05	0.003844321	0.142239888
T2DM_admission	alpha_cell	*RNF5*	-0.111424488	4.36E-07	0.000107128	0.001714049
T2DM_admission	alpha_cell	*RNF39*	0.074349178	0.000236913	0.017562828	0.930829869
T2DM_admission	alpha_cell	*HCG27*	0.106766665	1.20E-07	3.62E-05	0.000470108
T2DM_admission	alpha_cell	*DNLZ*	0.050773195	0.000690128	0.035158991	1
T2DM_admission	alpha_cell	*ATF6B*	0.111944459	1.25E-05	0.001538842	0.049242949
T2DM_admission	alpha_cell	*SLC12A8*	-0.584921018	4.77E-07	0.000110128	0.001872172
T2DM_admission	alpha_cell	*PPT2*	0.174663998	9.12E-06	0.001212892	0.035847154
T2DM_admission	alpha_cell	*EPPK1*	0.184457966	0.001081181	0.049394903	1
T2DM_admission	alpha_cell	*HLA-DQB2*	0.168518054	4.44E-08	1.54E-05	0.000174532
T2DM_admission	alpha_cell	*HLA-B*	0.083202867	7.11E-07	0.000155169	0.002793047
T2DM_admission	alpha_cell	*HLA-DQA2*	0.110993721	3.29E-13	4.30E-10	1.29E-09
T2DM_admission	alpha_cell	*JMJD7*	0.22565654	3.70E-06	0.000606033	0.014544799
T2DM_admission	alpha_cell	*HMBS*	0.078797719	0.000504554	0.029413161	1
T2DM_admission	beta_cell	*RBM6*	-0.089472409	9.63E-08	2.52E-05	0.000378454
T2DM_admission	beta_cell	*MLXIPL*	0.065493865	7.20E-05	0.006022146	0.283040859
T2DM_admission	beta_cell	*GIPR*	-0.140781844	4.78E-06	0.000671393	0.018799009
T2DM_admission	beta_cell	*CEP68*	0.17097216	1.71E-11	1.34E-08	6.72E-08
T2DM_admission	beta_cell	*BAK1*	0.200817984	1.34E-09	5.86E-07	5.27E-06
T2DM_admission	beta_cell	*ANAPC4*	0.5894558	6.83E-05	0.005935502	0.268363874
T2DM_admission	beta_cell	*SPTB*	0.075127425	0.000972432	0.037828564	1
T2DM_admission	beta_cell	*GBA2*	0.242060018	0.001448709	0.049068763	1
T2DM_admission	beta_cell	*TCF3*	-0.152854549	2.70E-05	0.002653239	0.10612956
T2DM_admission	beta_cell	*ST6GAL1*	0.084556304	0.00014122	0.009905263	0.554853856
T2DM_admission	beta_cell	*MARK3*	-0.289370292	2.38E-07	5.49E-05	0.000933981
T2DM_admission	beta_cell	*MGST2*	0.214497263	0.000225562	0.012851644	0.886232907
T2DM_admission	beta_cell	*CWF19L1*	-0.047184134	0.000887258	0.035571797	1
T2DM_admission	beta_cell	*CCDC134*	0.133083445	0.000271066	0.014791934	1
T2DM_admission	beta_cell	*RBL2*	0.147312338	0.000313303	0.015972513	1
T2DM_admission	beta_cell	*OIP5*	0.236580253	5.46E-05	0.00487185	0.214361396
T2DM_admission	beta_cell	*GCDH*	-0.348198932	8.92E-07	0.000166842	0.003503673
T2DM_admission	beta_cell	*TMEM106B*	0.322046765	0.000199984	0.012088287	0.785738668
T2DM_admission	beta_cell	*DHRS11*	0.173553703	0.000313642	0.015972513	1
T2DM_admission	beta_cell	*HSD17B1*	-0.075791847	0.000129509	0.009251644	0.508840394
T2DM_admission	beta_cell	*CNTNAP1*	0.076428915	9.49E-06	0.001165755	0.037304168
T2DM_admission	beta_cell	*PDHX*	0.102276996	0.000281537	0.015152878	1
T2DM_admission	beta_cell	*CDK2AP1*	0.09908492	1.21E-05	0.001403112	0.047705795
T2DM_admission	beta_cell	*SEMA5A*	0.256057274	0.000797535	0.032640766	1
T2DM_admission	beta_cell	*DNAH7*	-0.069282169	0.00095396	0.0374811	1
T2DM_admission	beta_cell	*BCL11A*	0.291355041	0.000920874	0.036546613	1
T2DM_admission	beta_cell	*MTRF1*	0.093708136	0.001354618	0.046686775	1
T2DM_admission	beta_cell	*FAM53C*	-0.18204409	0.001170592	0.043718899	1
T2DM_admission	beta_cell	*IAPP*	0.120854455	0.000156412	0.010172146	0.614542121
T2DM_admission	beta_cell	*ZNF211*	0.059781409	0.000244208	0.013707027	0.9594919
T2DM_admission	beta_cell	*POLK*	-0.187356743	0.000294191	0.015512286	1
T2DM_admission	beta_cell	*FLT3*	0.096194449	0.000118757	0.008803714	0.466596851
T2DM_admission	beta_cell	*BHLHE41*	0.303647006	7.52E-05	0.006152939	0.29534105
T2DM_admission	beta_cell	*OPRL1*	-0.112624089	4.36E-05	0.003982837	0.171262001
T2DM_admission	beta_cell	*BECN1*	0.545672563	0.000183325	0.011433049	0.720282116
T2DM_admission	beta_cell	*ABHD8*	0.144903167	0.000563252	0.025436984	1
T2DM_admission	beta_cell	*MAU2*	-0.188814793	4.23E-06	0.000639524	0.016627624
T2DM_admission	beta_cell	*TRAF3*	0.152214014	0.000317092	0.015972513	1
T2DM_admission	beta_cell	*PPARG*	-0.257976455	0.000157928	0.010172146	0.620500916
T2DM_admission	beta_cell	*NOTCH2*	-0.078596618	3.75E-11	2.03E-08	1.47E-07
T2DM_admission	beta_cell	*OSTF1*	0.303787997	0.000570955	0.025491847	1
T2DM_admission	beta_cell	*SPTBN5*	0.083531269	0.000601802	0.025983316	1
T2DM_admission	beta_cell	*MAN2C1*	-0.054491869	1.15E-05	0.001363485	0.044995007

T2DM_admission	beta_cell	*NPC1*	-0.314073823	2.05E-06	0.000322273	0.008056832
T2DM_admission	beta_cell	*EVI5L*	0.109395572	0.000854907	0.034628121	1
T2DM_admission	beta_cell	*POGZ*	0.129752107	6.95E-05	0.005935502	0.273033113
T2DM_admission	beta_cell	*INPP5E*	0.144423923	0.000196726	0.012077112	0.772935149
T2DM_admission	beta_cell	*SEC16A*	0.127333067	0.000125792	0.009152551	0.494237775
T2DM_admission	beta_cell	*HSD17B12*	-0.040689741	1.27E-06	0.000221668	0.004972986
T2DM_admission	beta_cell	*APIP*	-0.048588944	0.00032732	0.015992755	1
T2DM_admission	beta_cell	*PTPN14*	-0.053574864	0.000259923	0.014383653	1
T2DM_admission	beta_cell	*POC5*	-0.049863539	1.89E-05	0.00199659	0.074189806
T2DM_admission	beta_cell	*JAZF1*	-0.279649462	8.48E-21	1.67E-17	3.33E-17
T2DM_admission	beta_cell	*NUS1*	-0.364154412	0.000153193	0.010172146	0.601896956
T2DM_admission	beta_cell	*CLEC18A*	-0.287486412	9.40E-12	9.24E-09	3.70E-08
T2DM_admission	beta_cell	*AP3S2*	0.529091242	2.89E-11	1.89E-08	1.14E-07
T2DM_admission	beta_cell	*EXTL1*	-0.148429008	0.000626457	0.026466126	1
T2DM_admission	beta_cell	*CILP2*	-0.08918931	1.75E-05	0.001906949	0.068650149
T2DM_admission	beta_cell	*NIPAL1*	0.190027915	0.001274794	0.044324475	1
T2DM_admission	beta_cell	*SRGAP2*	0.147427752	3.68E-05	0.00343967	0.144466153
T2DM_admission	beta_cell	*ICA1L*	-0.050273851	0.000455578	0.021309112	1
T2DM_admission	beta_cell	*BDH2*	-0.098500052	0.000118675	0.008803714	0.466275681
T2DM_admission	beta_cell	*NOXRED1*	0.182993344	0.001237502	0.043758779	1
T2DM_admission	beta_cell	*CASP7*	0.053473924	2.14E-05	0.002160663	0.084265855
T2DM_admission	beta_cell	*CKB*	-0.074524332	0.000723814	0.029935413	1
T2DM_admission	beta_cell	*SGPL1*	-0.127274745	0.001048262	0.039986619	1
T2DM_admission	beta_cell	*PCBD1*	-0.172973032	0.00020885	0.012432913	0.820572242
T2DM_admission	beta_cell	*TMEM129*	-0.071668541	0.000444878	0.021059327	1
T2DM_admission	beta_cell	*INO80E*	-0.097146002	9.53E-05	0.007436229	0.374256362
T2DM_admission	beta_cell	*RGS19*	-0.09684472	0.00099728	0.038414816	1
T2DM_admission	beta_cell	*CTSW*	-0.033312158	0.000614171	0.026229112	1
T2DM_admission	beta_cell	*NAA16*	0.140113658	0.000225697	0.012851644	0.886763456
T2DM_admission	beta_cell	*SNX32*	0.08615544	0.000600844	0.025983316	1
T2DM_admission	beta_cell	*RNASEH2C*	-0.087451046	0.001223398	0.043758779	1
T2DM_admission	beta_cell	*PEAK1*	0.087363557	9.65E-05	0.007436229	0.37924768
T2DM_admission	beta_cell	*YIF1A*	-0.072033708	0.001247387	0.043758779	1
T2DM_admission	beta_cell	*FUT2*	0.018003174	0.001215585	0.043758779	1
T2DM_admission	beta_cell	*DSCAML1*	-0.102738069	0.00138342	0.047264859	1
T2DM_admission	beta_cell	*IRX3*	0.335582039	6.99E-29	2.75E-25	2.75E-25
T2DM_admission	beta_cell	*IMP3*	0.311151925	8.39E-06	0.001063744	0.032976072
T2DM_admission	beta_cell	*COX14*	0.214786618	1.93E-05	0.00199659	0.075870429
T2DM_admission	beta_cell	*FARSA*	-0.205706036	4.33E-07	8.96E-05	0.001703035
T2DM_admission	beta_cell	*HLA-DQB1*	-0.045033617	6.35E-08	1.87E-05	0.000249571
T2DM_admission	beta_cell	*TH*	-0.573273359	8.74E-05	0.007009383	0.343459777
T2DM_admission	beta_cell	*UBA7*	-0.063403913	5.83E-06	0.000763546	0.022906385
T2DM_admission	beta_cell	*ARL6IP4*	0.163437347	1.50E-05	0.001686472	0.059026518
T2DM_admission	beta_cell	*ACTL10*	0.588718588	0.000329706	0.015992755	1
T2DM_admission	beta_cell	*VMO1*	0.064138833	0.000466182	0.021548592	1
T2DM_admission	beta_cell	*CAMK1D*	0.388186819	8.26E-09	2.95E-06	3.25E-05
T2DM_admission	beta_cell	*EIF3C*	0.089266644	0.001244805	0.043758779	1
T2DM_admission	beta_cell	*ZBTB40*	0.194956304	0.001179487	0.043718899	1
T2DM_admission	beta_cell	*DMWD*	-0.117108282	0.000659064	0.027547469	1
T2DM_admission	beta_cell	*NUTM2B*	-0.659427124	0.000148797	0.01007971	0.584623173
T2DM_admission	beta_cell	*ZNF567*	0.217070919	0.00049408	0.02257258	1
T2DM_admission	beta_cell	*HLA-DRB1*	-0.038914123	4.14E-11	2.03E-08	1.63E-07
T2DM_admission	beta_cell	*DPP4*	-0.092386595	0.001217937	0.043758779	1
T2DM_admission	beta_cell	*DDX42*	0.181546603	0.000143701	0.009905263	0.564600006
T2DM_admission	beta_cell	*HLA-DRB5*	-0.029941797	1.30E-06	0.000221668	0.005098372
T2DM_admission	beta_cell	*LIME1*	-0.050855832	0.000436694	0.020924053	1
T2DM_admission	beta_cell	*NOTCH4*	-0.065366129	3.43E-09	1.35E-06	1.35E-05
T2DM_admission	beta_cell	*AGER*	0.106240088	2.95E-05	0.002822459	0.115720809
T2DM_admission	beta_cell	*RNF5*	-0.066392615	7.79E-07	0.000152949	0.003058975
T2DM_admission	beta_cell	*RNF39*	0.045907804	0.000182882	0.011433049	0.718544984
T2DM_admission	beta_cell	*HCG27*	0.065889976	6.67E-08	1.87E-05	0.000262102
T2DM_admission	beta_cell	*DNLZ*	0.031010656	0.000600957	0.025983316	1
T2DM_admission	beta_cell	*ATF6B*	0.071512868	4.70E-06	0.000671393	0.018460966
T2DM_admission	beta_cell	*SLC12A8*	-0.372603521	3.52E-07	7.68E-05	0.00138325

T2DM_admission	beta_cell	*PPT2*	0.108608215	5.80E-06	0.000763546	0.022782134
T2DM_admission	beta_cell	*EPPK1*	0.111320142	0.001068923	0.040382672	1
T2DM_admission	beta_cell	*HLA-DQB2*	0.102661529	3.80E-08	1.24E-05	0.000149188
T2DM_admission	beta_cell	*HLA-B*	0.05496703	1.26E-07	3.10E-05	0.000495203
T2DM_admission	beta_cell	*HLA-DQA2*	0.068739447	1.37E-13	1.79E-10	5.37E-10
T2DM_admission	beta_cell	*JMJD7*	0.143087019	1.37E-06	0.000223998	0.005375952
T2DM_admission	beta_cell	*NEAT1*	0.194897543	0.000327947	0.015992755	1
T2DM_admission	beta_cell	*HMBS*	0.049813462	0.000296111	0.015512286	1
T2DM_admission	beta_cell	*TAS2R31*	1.149306047	0.000222312	0.012851644	0.873462864
T2DM_admission	delta_cell	*RBM6*	-0.697546544	1.45E-07	5.19E-05	0.000571076
T2DM_admission	delta_cell	*GIPR*	-1.016910187	1.62E-06	0.000375439	0.006382465
T2DM_admission	delta_cell	*CEP68*	0.893544316	3.93E-07	0.00012864	0.001543674
T2DM_admission	delta_cell	*BAK1*	1.21828129	1.40E-06	0.00034298	0.005487678
T2DM_admission	delta_cell	*TCF3*	-0.97003699	1.78E-05	0.00291948	0.070067516
T2DM_admission	delta_cell	*CCDC134*	1.089392269	0.000553806	0.049452358	1
T2DM_admission	delta_cell	*HSD17B1*	-0.684494651	2.39E-05	0.003755985	0.093899616
T2DM_admission	delta_cell	*CNTNAP1*	0.587059864	7.40E-05	0.010767235	0.29071535
T2DM_admission	delta_cell	*ZNF211*	0.434321141	0.00045523	0.042585704	1
T2DM_admission	delta_cell	*FLT3*	0.748179855	0.000493521	0.045094024	1
T2DM_admission	delta_cell	*MAU2*	-1.10271183	1.76E-05	0.00291948	0.06924163
T2DM_admission	delta_cell	*NOTCH2*	-0.597067685	1.34E-09	1.05E-06	5.27E-06
T2DM_admission	delta_cell	*SPTBN5*	0.755357012	0.000229525	0.024373023	0.901801853
T2DM_admission	delta_cell	*MAN2C1*	-0.482073537	3.89E-06	0.000764624	0.01529248
T2DM_admission	delta_cell	*HSD17B12*	-0.342785955	1.01E-06	0.000282276	0.003951859
T2DM_admission	delta_cell	*APIP*	-0.433295364	0.000116723	0.014793741	0.458605974
T2DM_admission	delta_cell	*POC5*	-0.458654845	3.65E-06	0.000764624	0.014342599
T2DM_admission	delta_cell	*JAZF1*	-1.729441143	9.16E-17	3.31E-13	3.60E-13
T2DM_admission	delta_cell	*CLEC18A*	-1.825927381	1.81E-08	9.67E-06	7.10E-05
T2DM_admission	delta_cell	*EXTL1*	-1.249493135	0.000189991	0.02073537	0.746473323
T2DM_admission	delta_cell	*CILP2*	-0.686016295	0.00011542	0.014793741	0.453486596
T2DM_admission	delta_cell	*ICA1L*	-0.434319751	0.000149753	0.017169454	0.588378553
T2DM_admission	delta_cell	*CASP7*	0.408223141	0.000145168	0.017169454	0.570364128
T2DM_admission	delta_cell	*TMEM129*	-0.610349057	9.42E-05	0.012755989	0.369923675
T2DM_admission	delta_cell	*INO80E*	-0.772968648	3.08E-05	0.004649185	0.12087882
T2DM_admission	delta_cell	*CTSW*	-0.299383266	0.000445746	0.042585704	1
T2DM_admission	delta_cell	*IRX3*	1.785505209	1.68E-16	3.31E-13	6.62E-13
T2DM_admission	delta_cell	*FARSA*	-1.384289905	7.55E-07	0.000228121	0.002965573
T2DM_admission	delta_cell	*HLA-DQB1*	-0.405567506	1.97E-08	9.67E-06	7.74E-05
T2DM_admission	delta_cell	*UBA7*	-0.530855546	1.49E-05	0.002785186	0.058488899
T2DM_admission	delta_cell	*VMO1*	0.488693456	0.000345278	0.034784577	1
T2DM_admission	delta_cell	*HLA-DRB1*	-0.345343194	1.98E-11	1.94E-08	7.77E-08
T2DM_admission	delta_cell	*HLA-DRB5*	-0.249901815	1.10E-06	0.000289213	0.004338202
T2DM_admission	delta_cell	*LIME1*	-0.443352062	0.000125976	0.015467485	0.494959534
T2DM_admission	delta_cell	*NOTCH4*	-0.54406471	9.05E-09	5.93E-06	3.56E-05
T2DM_admission	delta_cell	*RNF5*	-0.582259518	1.01E-07	3.97E-05	0.00039668
T2DM_admission	delta_cell	*RNF39*	0.364687641	0.000152948	0.017169454	0.600930878
T2DM_admission	delta_cell	*HCG27*	0.364711843	3.88E-06	0.000764624	0.015249476
T2DM_admission	delta_cell	*ATF6B*	0.454474682	0.000377093	0.037039982	1
T2DM_admission	delta_cell	*PPT2*	0.785923934	8.61E-05	0.01208281	0.338318676
T2DM_admission	delta_cell	*HLA-DQB2*	0.861992704	2.58E-08	1.13E-05	0.000101274
T2DM_admission	delta_cell	*HLA-B*	0.313166375	1.78E-05	0.00291948	0.069927771
T2DM_admission	delta_cell	*HLA-DQA2*	0.547934947	4.80E-13	6.28E-10	1.89E-09
T2DM_admission	delta_cell	*JMJD7*	0.873992207	0.000249297	0.025775954	0.979486255
T2DM_admission	duct_epithelial_cell	*RBM6*	-0.455215681	5.52E-06	0.000938953	0.021687736
T2DM_admission	duct_epithelial_cell	*MLXIPL*	0.400919905	8.81E-06	0.00128249	0.034627235
T2DM_admission	duct_epithelial_cell	*QPCTL*	-0.599134472	0.00101381	0.040234944	1
T2DM_admission	duct_epithelial_cell	*CEP68*	0.971470606	1.39E-10	1.10E-07	5.48E-07
T2DM_admission	duct_epithelial_cell	*BAK1*	0.828457847	1.22E-05	0.001593379	0.047801356
T2DM_admission	duct_epithelial_cell	*SPTB*	0.477256632	0.000205537	0.013929731	0.807556191
T2DM_admission	duct_epithelial_cell	*TCF3*	-0.849229647	6.66E-07	0.000153849	0.002615428
T2DM_admission	duct_epithelial_cell	*NLE1*	0.660579568	0.000603365	0.028561705	1
T2DM_admission	duct_epithelial_cell	*POMGNT1*	-1.005103092	0.00022695	0.014861418	0.891685085
T2DM_admission	duct_epithelial_cell	*RBL2*	0.7145644	0.000225706	0.014861418	0.886798004
T2DM_admission	duct_epithelial_cell	*ATP8B4*	6.325634069	0.000443542	0.024895385	1

T2DM_admission	duct_epithelial_cell	*OIP5*	1.51555746	3.51E-06	0.000690332	0.013806646
T2DM_admission	duct_epithelial_cell	*VIPR2*	0.35387087	0.001384687	0.048145436	1
T2DM_admission	duct_epithelial_cell	*HSD17B1*	-0.527428272	2.10E-05	0.00242323	0.082389816
T2DM_admission	duct_epithelial_cell	*CNTNAP1*	0.518293382	5.30E-07	0.00013019	0.002083036
T2DM_admission	duct_epithelial_cell	*PDHX*	0.582207783	0.000145242	0.010877533	0.570655733
T2DM_admission	duct_epithelial_cell	*PRR4*	0.314470561	0.00106636	0.041668898	1
T2DM_admission	duct_epithelial_cell	*CDK2AP1*	0.544495161	3.01E-05	0.003280029	0.118081053
T2DM_admission	duct_epithelial_cell	*ANGPTL1*	-1.292020529	0.000167205	0.012165687	0.656947117
T2DM_admission	duct_epithelial_cell	*SPP1*	-0.136591825	0.001176868	0.044037274	1
T2DM_admission	duct_epithelial_cell	*CCDC77*	-0.951320255	0.001237399	0.045124707	1
T2DM_admission	duct_epithelial_cell	*MTRF1*	0.585008356	0.001071153	0.041668898	1
T2DM_admission	duct_epithelial_cell	*IAPP*	0.185895078	0.001516532	0.049653787	1
T2DM_admission	duct_epithelial_cell	*ZNF211*	0.38312796	0.000173968	0.012427671	0.683521903
T2DM_admission	duct_epithelial_cell	*MOCS1*	-0.478277235	0.000940181	0.038082164	1
T2DM_admission	duct_epithelial_cell	*OPRL1*	-0.590849243	0.000585491	0.028561705	1
T2DM_admission	duct_epithelial_cell	*ABHD8*	0.724559408	0.000559596	0.02855392	1
T2DM_admission	duct_epithelial_cell	*WFIKKN1*	0.542653258	0.00112449	0.042481929	1
T2DM_admission	duct_epithelial_cell	*CGNL1*	-0.333892151	0.000706544	0.031191127	1
T2DM_admission	duct_epithelial_cell	*MAU2*	-0.943573314	3.78E-05	0.003903605	0.148336996
T2DM_admission	duct_epithelial_cell	*SYT5*	0.293694679	0.000335116	0.020256494	1
T2DM_admission	duct_epithelial_cell	*PPARG*	-1.484168775	0.000310885	0.019085423	1
T2DM_admission	duct_epithelial_cell	*NOTCH2*	-0.437455419	2.72E-12	3.49E-09	1.07E-08
T2DM_admission	duct_epithelial_cell	*FXYD2*	-0.545260372	9.92E-05	0.008856282	0.389676387
T2DM_admission	duct_epithelial_cell	*MYPN*	1.66253831	0.0008421	0.034827494	1
T2DM_admission	duct_epithelial_cell	*BARD1*	-0.883475588	0.000498439	0.026826961	1
T2DM_admission	duct_epithelial_cell	*MAN2C1*	-0.314757374	4.99E-05	0.004781497	0.196041364
T2DM_admission	duct_epithelial_cell	*NPC1*	-1.316377076	4.74E-05	0.00466016	0.186406389
T2DM_admission	duct_epithelial_cell	*FN3KRP*	-0.234497612	0.001452347	0.048754417	1
T2DM_admission	duct_epithelial_cell	*CBLC*	-0.351369445	0.000614025	0.02872028	1
T2DM_admission	duct_epithelial_cell	*POGZ*	0.661850791	0.000131211	0.010740163	0.51552781
T2DM_admission	duct_epithelial_cell	*CDCA7*	0.85516248	0.000244167	0.015721334	0.959331978
T2DM_admission	duct_epithelial_cell	*IQGAP2*	-0.230896845	0.000371266	0.021451515	1
T2DM_admission	duct_epithelial_cell	*RAB19*	-1.080547489	0.000863335	0.035333774	1
T2DM_admission	duct_epithelial_cell	*ADHFE1*	-0.978211197	0.000142547	0.010877533	0.560066555
T2DM_admission	duct_epithelial_cell	*SEC16A*	0.66389297	0.000137789	0.010877533	0.541372727
T2DM_admission	duct_epithelial_cell	*HSD17B12*	-0.220686316	1.82E-05	0.002300961	0.071329798
T2DM_admission	duct_epithelial_cell	*APIP*	-0.301149131	0.000365289	0.021421226	1
T2DM_admission	duct_epithelial_cell	*KLHL35*	0.272404384	0.000724813	0.031198389	1
T2DM_admission	duct_epithelial_cell	*VIPAS39*	-0.911487495	0.000117543	0.010039689	0.461825686
T2DM_admission	duct_epithelial_cell	*PTPN14*	-0.37648567	1.02E-05	0.001434694	0.040171424
T2DM_admission	duct_epithelial_cell	*POC5*	-0.303037305	3.45E-05	0.003661289	0.135467677
T2DM_admission	duct_epithelial_cell	*JAZF1*	-1.093299348	3.55E-12	3.49E-09	1.39E-08
T2DM_admission	duct_epithelial_cell	*CLEC18A*	-1.24242675	2.67E-07	7.48E-05	0.001047565
T2DM_admission	duct_epithelial_cell	*AP3S2*	1.344765318	8.72E-06	0.00128249	0.034249948
T2DM_admission	duct_epithelial_cell	*CIB4*	5.079628327	0.000144303	0.010877533	0.56696724
T2DM_admission	duct_epithelial_cell	*EXTL1*	-1.033706446	1.88E-05	0.00230887	0.074016782
T2DM_admission	duct_epithelial_cell	*NPR2*	0.499686046	0.001270341	0.045374278	1
T2DM_admission	duct_epithelial_cell	*CILP2*	-0.606908423	1.02E-06	0.00022217	0.003999058
T2DM_admission	duct_epithelial_cell	*ZNF208*	-0.55243716	0.001095196	0.042186525	1
T2DM_admission	duct_epithelial_cell	*LY6K*	0.56567074	0.001232608	0.045124707	1
T2DM_admission	duct_epithelial_cell	*SRGAP2*	0.67782838	0.001476655	0.048754417	1
T2DM_admission	duct_epithelial_cell	*BDH2*	-0.519519076	4.56E-06	0.000814206	0.017912535
T2DM_admission	duct_epithelial_cell	*NOXRED1*	1.306093369	0.000130405	0.010740163	0.512360815
T2DM_admission	duct_epithelial_cell	*SGPL1*	-0.795168893	8.28E-05	0.007569279	0.325479018
T2DM_admission	duct_epithelial_cell	*PROCA1*	1.9049387	0.000971961	0.03896769	1
T2DM_admission	duct_epithelial_cell	*TMC4*	-0.470946782	0.001109883	0.042337199	1
T2DM_admission	duct_epithelial_cell	*TMC8*	-0.394778629	0.000205631	0.013929731	0.807924393
T2DM_admission	duct_epithelial_cell	*TMEM129*	-0.422408487	0.000768024	0.032101789	1
T2DM_admission	duct_epithelial_cell	*INO80E*	-0.496900947	0.00073053	0.031198389	1
T2DM_admission	duct_epithelial_cell	*SETMAR*	-1.110521327	0.000348731	0.02076009	1
T2DM_admission	duct_epithelial_cell	*RGS19*	-0.677246655	4.43E-05	0.004462198	0.174025709
T2DM_admission	duct_epithelial_cell	*ARNT2*	0.640892573	0.00141205	0.048666184	1
T2DM_admission	duct_epithelial_cell	*CTSW*	-0.215087781	0.000505642	0.026846835	1
T2DM_admission	duct_epithelial_cell	*NAA16*	0.679265879	0.000146732	0.010877533	0.576509248

T2DM_admission	duct_epithelial_cell	*OVOL1*	-0.298241019	0.000697058	0.031191127	1
T2DM_admission	duct_epithelial_cell	*PEAK1*	0.529680392	0.00011557	0.010039689	0.454073705
T2DM_admission	duct_epithelial_cell	*FUT2*	0.119891869	0.00068336	0.031191127	1
T2DM_admission	duct_epithelial_cell	*DSCAML1*	-0.760381345	6.95E-05	0.00649881	0.272950039
T2DM_admission	duct_epithelial_cell	*COX14*	0.56408642	0.000702197	0.031191127	1
T2DM_admission	duct_epithelial_cell	*FARSA*	-1.243778387	1.83E-07	5.99E-05	0.000719087
T2DM_admission	duct_epithelial_cell	*HLA-DQB1*	-0.300419365	1.02E-08	4.99E-06	3.99E-05
T2DM_admission	duct_epithelial_cell	*UBA7*	-0.417785419	2.31E-07	6.99E-05	0.000908189
T2DM_admission	duct_epithelial_cell	*ARL6IP4*	0.528641483	0.000290314	0.018105429	1
T2DM_admission	duct_epithelial_cell	*CAPN12*	-0.336526828	0.001240919	0.045124707	1
T2DM_admission	duct_epithelial_cell	*SKA2*	-0.678570855	0.000526556	0.027221563	1
T2DM_admission	duct_epithelial_cell	*TTC3*	0.210857038	0.001381876	0.048145436	1
T2DM_admission	duct_epithelial_cell	*CAMK1D*	1.732349981	2.46E-06	0.000509197	0.009674735
T2DM_admission	duct_epithelial_cell	*FAM43B*	1.85462575	0.000248084	0.015721334	0.974722705
T2DM_admission	duct_epithelial_cell	*MACC1*	-0.342468461	0.001432362	0.048754417	1
T2DM_admission	duct_epithelial_cell	*EIF3C*	0.575303362	0.000590231	0.028561705	1
T2DM_admission	duct_epithelial_cell	*ZBTB40*	0.885897495	0.000576525	0.028561705	1
T2DM_admission	duct_epithelial_cell	*CYP4X1*	-1.105159374	0.000726651	0.031198389	1
T2DM_admission	duct_epithelial_cell	*HLA-DRB1*	-0.271411315	7.24E-13	1.42E-09	2.84E-09
T2DM_admission	duct_epithelial_cell	*SUPT3H*	-1.282318415	0.001470807	0.048754417	1
T2DM_admission	duct_epithelial_cell	*SULT1A1*	-1.283396342	0.000200722	0.013929731	0.788636253
T2DM_admission	duct_epithelial_cell	*ZNF257*	-0.692927908	0.000515928	0.027027768	1
T2DM_admission	duct_epithelial_cell	*ENPP1*	0.343044762	0.000451595	0.024990389	1
T2DM_admission	duct_epithelial_cell	*DDX42*	0.664541484	0.000592766	0.028561705	1
T2DM_admission	duct_epithelial_cell	*HLA-DRB5*	-0.201741163	4.88E-08	1.92E-05	0.00019176
T2DM_admission	duct_epithelial_cell	*LIME1*	-0.368262517	1.94E-05	0.00230887	0.076192715
T2DM_admission	duct_epithelial_cell	*NOTCH4*	-0.428274275	3.73E-10	2.44E-07	1.46E-06
T2DM_admission	duct_epithelial_cell	*AGER*	0.689961647	5.74E-06	0.000938953	0.022534884
T2DM_admission	duct_epithelial_cell	*RNF5*	-0.423277698	1.48E-07	5.28E-05	0.000580277
T2DM_admission	duct_epithelial_cell	*HCG27*	0.308420398	2.53E-05	0.002836075	0.099262618
T2DM_admission	duct_epithelial_cell	*TAS2R14*	0.300388288	0.001251869	0.045124707	1
T2DM_admission	duct_epithelial_cell	*DNLZ*	0.19656494	0.000602912	0.028561705	1
T2DM_admission	duct_epithelial_cell	*ATF6B*	0.427973984	1.14E-05	0.001548334	0.044901683
T2DM_admission	duct_epithelial_cell	*SLC12A8*	-1.989841527	2.90E-08	1.27E-05	0.000113961
T2DM_admission	duct_epithelial_cell	*PPT2*	0.686041571	4.06E-06	0.000760052	0.015961092
T2DM_admission	duct_epithelial_cell	*HLA-DQB2*	0.67562156	9.58E-09	4.99E-06	3.76E-05
T2DM_admission	duct_epithelial_cell	*HLA-B*	0.313222443	5.08E-07	0.00013019	0.001996931
T2DM_admission	duct_epithelial_cell	*LINC00957*	0.660037204	0.000740011	0.031263462	1
T2DM_admission	duct_epithelial_cell	*HLA-DQA2*	0.456402136	1.67E-14	6.56E-11	6.56E-11
T2DM_admission	duct_epithelial_cell	*JMJD7*	0.829807013	8.05E-06	0.001264843	0.031621076
T2DM_admission	duct_epithelial_cell	*OR2A7*	-0.710918959	0.000397925	0.022658667	1
T2DM_admission	duct_epithelial_cell	*P2RY11*	0.356620863	0.000678696	0.031191127	1
T2DM_admission	duct_epithelial_cell	*HMBS*	0.301635392	0.000478818	0.026128838	1
T2DM_admission	duct_epithelial_cell	*TAS2R31*	5.076739661	0.001322073	0.046796633	1
T2DM_admission	duct_epithelial_cell	*LINC00638*	-1.203527521	0.001471201	0.048754417	1
T2DM_admission	endothelial_cell	*STPG1*	-0.947393964	0.000364913	0.020778905	1
T2DM_admission	endothelial_cell	*RBM6*	-0.811270483	4.68E-09	3.68E-06	1.84E-05
T2DM_admission	endothelial_cell	*ZNF195*	-1.019260226	0.000170881	0.012160846	0.671391076
T2DM_admission	endothelial_cell	*GGNBP2*	-0.96116196	0.000350574	0.020255957	1
T2DM_admission	endothelial_cell	*USH1C*	-0.510800703	0.000856727	0.036109444	1
T2DM_admission	endothelial_cell	*ST7L*	-0.493059249	0.00095333	0.037920116	1
T2DM_admission	endothelial_cell	*BAIAP3*	-0.538617934	0.000863906	0.036109444	1
T2DM_admission	endothelial_cell	*GIPR*	-0.776931599	5.47E-06	0.001022913	0.021481174
T2DM_admission	endothelial_cell	*CEP68*	1.153696526	1.60E-08	8.99E-06	6.30E-05
T2DM_admission	endothelial_cell	*SH2D2A*	-1.152882244	0.000511534	0.027155979	1
T2DM_admission	endothelial_cell	*BAK1*	1.400193703	2.30E-07	6.44E-05	0.000902101
T2DM_admission	endothelial_cell	*LY75*	-2.642110733	0.000764084	0.034068568	1
T2DM_admission	endothelial_cell	*USP36*	-1.415973698	0.001189558	0.043275676	1
T2DM_admission	endothelial_cell	*ITIH4*	-1.01871839	0.000150578	0.011134387	0.591622227
T2DM_admission	endothelial_cell	*HAGH*	-0.990081658	8.15E-05	0.007280483	0.320341263
T2DM_admission	endothelial_cell	*ST6GAL1*	0.488340868	0.00033839	0.019843808	1
T2DM_admission	endothelial_cell	*MARK3*	-1.768967679	5.10E-08	2.22E-05	0.00020023
T2DM_admission	endothelial_cell	*XAB2*	-1.800909843	0.000247902	0.01521887	0.974007695
T2DM_admission	endothelial_cell	*POMGNT1*	-1.239574867	9.23E-05	0.007882797	0.36260864

T2DM_admission	endothelial_cell	*CWF19L1*	-0.443690577	0.000242183	0.015103786	0.95153852
T2DM_admission	endothelial_cell	*AP1B1*	-1.410928123	5.36E-05	0.005694071	0.210680612
T2DM_admission	endothelial_cell	*RPAP1*	-2.566365507	0.000254266	0.015369373	0.999009277
T2DM_admission	endothelial_cell	*HSD17B1*	-0.706761844	6.98E-06	0.001192418	0.027425624
T2DM_admission	endothelial_cell	*CNTNAP1*	0.642616091	1.58E-05	0.00238634	0.062044836
T2DM_admission	endothelial_cell	*CDK2AP1*	0.572409537	0.001317986	0.046505084	1
T2DM_admission	endothelial_cell	*NNT*	-0.701524966	0.000176146	0.012160846	0.692075835
T2DM_admission	endothelial_cell	*CHMP3*	-0.809475651	0.000153031	0.011134387	0.60125689
T2DM_admission	endothelial_cell	*ANGPTL1*	-2.661658956	0.000141483	0.011114854	0.555886464
T2DM_admission	endothelial_cell	*NSUN4*	-1.180872068	0.000233706	0.015052959	0.918230527
T2DM_admission	endothelial_cell	*DNAH7*	-0.56874061	8.85E-05	0.00772634	0.347685307
T2DM_admission	endothelial_cell	*FAM53C*	-1.310390386	3.22E-05	0.003858653	0.126674209
T2DM_admission	endothelial_cell	*ZNF211*	0.464839267	0.000831032	0.035777083	1
T2DM_admission	endothelial_cell	*POLK*	-1.261893061	9.63E-06	0.001576051	0.037825214
T2DM_admission	endothelial_cell	*PEPD*	-1.531871668	3.24E-05	0.003858653	0.127271556
T2DM_admission	endothelial_cell	*PRMT1*	-0.831089675	0.001044954	0.039477155	1
T2DM_admission	endothelial_cell	*EIF2AK4*	-1.046985841	0.000677763	0.03287571	1
T2DM_admission	endothelial_cell	*MAU2*	-1.647081404	5.78E-08	2.27E-05	0.00022721
T2DM_admission	endothelial_cell	*GTPBP3*	-0.525748062	0.001433288	0.049835308	1
T2DM_admission	endothelial_cell	*UCK1*	-1.469528876	0.000965134	0.037920116	1
T2DM_admission	endothelial_cell	*BHMT2*	-0.807319103	0.0001425	0.011114854	0.559882318
T2DM_admission	endothelial_cell	*NOTCH2*	-0.701549428	3.59E-12	3.53E-09	1.41E-08
T2DM_admission	endothelial_cell	*FXYD2*	-0.409240797	0.000725621	0.033740124	1
T2DM_admission	endothelial_cell	*TUBGCP4*	-1.201084633	0.000221563	0.014508679	0.870520759
T2DM_admission	endothelial_cell	*MAN2C1*	-0.482209945	5.05E-06	0.000992512	0.019850231
T2DM_admission	endothelial_cell	*NPC1*	-2.159062843	3.54E-07	9.28E-05	0.001392462
T2DM_admission	endothelial_cell	*FN3KRP*	-0.332813256	0.000764271	0.034068568	1
T2DM_admission	endothelial_cell	*TBC1D7*	-2.125393619	0.000447711	0.024677265	1
T2DM_admission	endothelial_cell	*SCML4*	-1.197437986	0.000944491	0.037920116	1
T2DM_admission	endothelial_cell	*LRP12*	-1.787899368	0.000452218	0.024677265	1
T2DM_admission	endothelial_cell	*HSD17B12*	-0.367295661	9.82E-08	3.22E-05	0.000386007
T2DM_admission	endothelial_cell	*APIP*	-0.452471488	7.83E-05	0.007158157	0.307800768
T2DM_admission	endothelial_cell	*SIDT2*	-0.833967003	0.000786411	0.034331211	1
T2DM_admission	endothelial_cell	*TAGLN*	-0.304003866	0.000837743	0.035777083	1
T2DM_admission	endothelial_cell	*VIPAS39*	-1.305832751	0.000142124	0.011114854	0.558405928
T2DM_admission	endothelial_cell	*POC5*	-0.452558566	1.72E-06	0.00039765	0.006760057
T2DM_admission	endothelial_cell	*JAZF1*	-1.30968796	4.94E-15	9.71E-12	1.94E-11
T2DM_admission	endothelial_cell	*NUS1*	-1.617075535	2.80E-05	0.003858653	0.109841156
T2DM_admission	endothelial_cell	*CC2D1B*	-0.512872069	0.000622255	0.031751159	1
T2DM_admission	endothelial_cell	*EXTL1*	-1.394711225	0.000144619	0.011114854	0.568206742
T2DM_admission	endothelial_cell	*CILP2*	-0.801690303	6.93E-06	0.001192418	0.027230803
T2DM_admission	endothelial_cell	*VPS11*	-0.763088693	0.001003555	0.038478225	1
T2DM_admission	endothelial_cell	*PAQR6*	-0.860425385	0.000518376	0.027155979	1
T2DM_admission	endothelial_cell	*DCAF16*	-1.153958598	0.000643364	0.031997201	1
T2DM_admission	endothelial_cell	*ICA1L*	-0.479835923	2.57E-05	0.003737767	0.100919719
T2DM_admission	endothelial_cell	*PIGX*	-1.261213886	0.000770478	0.034068568	1
T2DM_admission	endothelial_cell	*LMBRD2*	-0.787600357	0.001305288	0.046505084	1
T2DM_admission	endothelial_cell	*CASP7*	0.334440789	6.10E-05	0.006147298	0.239744641
T2DM_admission	endothelial_cell	*TRUB1*	-1.123679328	0.001322266	0.046505084	1
T2DM_admission	endothelial_cell	*CEP57*	-1.095393916	6.60E-05	0.006487616	0.25950463
T2DM_admission	endothelial_cell	*CKB*	-0.440179938	0.000147104	0.011114854	0.577972388
T2DM_admission	endothelial_cell	*SGPL1*	-0.837266929	5.88E-05	0.006079628	0.231025873
T2DM_admission	endothelial_cell	*PCBD1*	-0.501434497	0.000481309	0.025904951	1
T2DM_admission	endothelial_cell	*POP5*	-1.211255282	0.000176424	0.012160846	0.693168247
T2DM_admission	endothelial_cell	*TP53I13*	-0.563042533	0.000241619	0.015103786	0.949321395
T2DM_admission	endothelial_cell	*RAB4B*	-0.903563806	0.001325673	0.046505084	1
T2DM_admission	endothelial_cell	*DNAJC21*	-1.312263765	0.001008719	0.038478225	1
T2DM_admission	endothelial_cell	*TMEM129*	-0.698594592	3.47E-05	0.004008419	0.136286236
T2DM_admission	endothelial_cell	*INO80E*	-0.919107318	2.66E-06	0.000549822	0.010446617
T2DM_admission	endothelial_cell	*ZNF561*	-1.302865432	0.001065408	0.039866566	1
T2DM_admission	endothelial_cell	*CTSW*	-0.2886368	0.000586327	0.03031154	1
T2DM_admission	endothelial_cell	*RNASEH2C*	-0.82963128	3.20E-05	0.003858653	0.125746115
T2DM_admission	endothelial_cell	*PEAK1*	0.422822935	0.001176446	0.043198651	1
T2DM_admission	endothelial_cell	*YIF1A*	-0.619312828	2.90E-05	0.003858653	0.114041902

T2DM_admission	endothelial_cell	*DSCAML1*	-0.508623919	0.001103932	0.040918403	1
T2DM_admission	endothelial_cell	*IRX3*	1.862193191	3.20E-17	1.26E-13	1.26E-13
T2DM_admission	endothelial_cell	*TRIM73*	-0.855138882	0.000677277	0.03287571	1
T2DM_admission	endothelial_cell	*FARSA*	-1.630786483	1.44E-08	8.99E-06	5.67E-05
T2DM_admission	endothelial_cell	*HLA-DQB1*	-0.34795308	9.22E-07	0.000226346	0.003621543
T2DM_admission	endothelial_cell	*HIGD1A*	-0.845340286	0.001007767	0.038478225	1
T2DM_admission	endothelial_cell	*VMO1*	0.537899149	0.000282337	0.01680761	1
T2DM_admission	endothelial_cell	*PGP*	-1.093644219	3.84E-05	0.004311177	0.1508912
T2DM_admission	endothelial_cell	*DMWD*	-1.061027658	6.94E-05	0.006649354	0.272623496
T2DM_admission	endothelial_cell	*NUTM2B*	-4.722214114	3.24E-05	0.003858653	0.127335546
T2DM_admission	endothelial_cell	*HLA-DRB1*	-0.273829592	4.90E-08	2.22E-05	0.000192575
T2DM_admission	endothelial_cell	*SUPT3H*	-1.882920213	0.000686564	0.032896455	1
T2DM_admission	endothelial_cell	*XRCC6*	-0.924098584	0.000104655	0.008748733	0.411190429
T2DM_admission	endothelial_cell	*SULT1A1*	-0.949156235	0.000641894	0.031997201	1
T2DM_admission	endothelial_cell	*DPP4*	-0.432257684	0.000926076	0.037920116	1
T2DM_admission	endothelial_cell	*RPS26*	-0.508230611	0.000771724	0.034068568	1
T2DM_admission	endothelial_cell	*LIME1*	-0.465950502	0.000191494	0.012802692	0.752381656
T2DM_admission	endothelial_cell	*AGER*	0.537477713	0.000192252	0.012802692	0.755358834
T2DM_admission	endothelial_cell	*RNF5*	-0.603988788	9.65E-08	3.22E-05	0.000379185
T2DM_admission	endothelial_cell	*RNF39*	0.335387062	0.000956764	0.037920116	1
T2DM_admission	endothelial_cell	*HCG27*	0.396847607	4.74E-05	0.005170887	0.18615192
T2DM_admission	endothelial_cell	*DNLZ*	0.262085657	0.000729934	0.033740124	1
T2DM_admission	endothelial_cell	*ATF6B*	0.523983386	7.12E-05	0.006659332	0.279691925
T2DM_admission	endothelial_cell	*SLC12A8*	-0.646585786	0.000954247	0.037920116	1
T2DM_admission	endothelial_cell	*PPT2*	0.338282209	0.000719541	0.033740124	1
T2DM_admission	endothelial_cell	*HLA-DQB2*	0.862728881	1.07E-07	3.23E-05	0.000419863
T2DM_admission	endothelial_cell	*HLA-B*	0.36963967	1.87E-06	0.000409116	0.007364094
T2DM_admission	endothelial_cell	*HLA-DQA2*	0.59836796	2.78E-13	3.65E-10	1.09E-09
T2DM_admission	endothelial_cell	*JMJD7*	1.11222595	1.03E-05	0.001620318	0.040507949
T2DM_admission	endothelial_cell	*GATC*	-1.271209676	0.000447138	0.024677265	1
T2DM_admission	epsilon_cell	*CLEC18A*	-13.12636886	1.43E-05	0.00802549	0.05617843
T2DM_admission	epsilon_cell	*CILP2*	-8.928464978	6.22E-05	0.024439789	0.244397894
T2DM_admission	epsilon_cell	*IRX3*	16.63146655	4.96E-14	1.95E-10	1.95E-10
T2DM_admission	epsilon_cell	*HLA-DQB1*	-2.989706767	2.48E-05	0.012157563	0.097260502
T2DM_admission	epsilon_cell	*UBA7*	-6.863896022	6.99E-06	0.005490509	0.027452546
T2DM_admission	epsilon_cell	*HLA-DRB1*	-3.215039152	1.13E-08	1.48E-05	4.45E-05
T2DM_admission	epsilon_cell	*HLA-DRB5*	-3.242192178	3.88E-05	0.016942315	0.152480832
T2DM_admission	epsilon_cell	*NOTCH4*	-4.580842671	3.20E-06	0.00313839	0.012553561
T2DM_admission	epsilon_cell	*HLA-DQB2*	7.958874207	1.10E-05	0.007227829	0.043366972
T2DM_admission	epsilon_cell	*HLA-DQA2*	6.903375205	9.96E-13	1.96E-09	3.91E-09
T2DM_admission	leukocyte	*GCLC*	-1.777226517	0.000230506	0.001064322	0.905658417
T2DM_admission	leukocyte	*NFYA*	-1.295327638	0.000818101	0.001665451	1
T2DM_admission	leukocyte	*STPG1*	-1.194405255	0.000227691	0.001062643	0.894598986
T2DM_admission	leukocyte	*LASP1*	-1.156071019	0.000289798	0.001088708	1
T2DM_admission	leukocyte	*SNX11*	-0.996844458	0.006184704	0.008390781	1
T2DM_admission	leukocyte	*TMEM176A*	-0.704362991	0.00466412	0.006482252	1
T2DM_admission	leukocyte	*M6PR*	-2.662031075	7.86E-05	0.001062643	0.308697687
T2DM_admission	leukocyte	*DBNDD1*	-0.89719355	0.000394896	0.001181681	1
T2DM_admission	leukocyte	*CASP10*	-3.103899826	0.000363905	0.001146348	1
T2DM_admission	leukocyte	*NDUFAF7*	-2.684446827	0.000106089	0.001062643	0.416824957
T2DM_admission	leukocyte	*POLDIP2*	-0.928494899	0.000793606	0.001634654	1
T2DM_admission	leukocyte	*PLXND1*	-2.523457317	0.000276105	0.001088708	1
T2DM_admission	leukocyte	*KDM1A*	-1.238947564	0.000333481	0.00112758	1
T2DM_admission	leukocyte	*RBM6*	-2.183634435	1.97E-09	1.55E-06	7.74E-06
T2DM_admission	leukocyte	*RECQL*	-1.249751091	0.000302522	0.001093914	1
T2DM_admission	leukocyte	*SLC25A13*	-1.17739448	0.000524712	0.001319879	1
T2DM_admission	leukocyte	*MEOX1*	2.739038574	0.003482997	0.004994414	1
T2DM_admission	leukocyte	*LIG3*	-2.520648822	0.010196484	0.013252394	1
T2DM_admission	leukocyte	*PON1*	-1.194037564	0.002974933	0.00434033	1
T2DM_admission	leukocyte	*KMT2E*	-1.308000306	0.000162215	0.001062643	0.63734135
T2DM_admission	leukocyte	*IBTK*	-1.280650379	0.00016302	0.001062643	0.640507146
T2DM_admission	leukocyte	*ZNF195*	-2.149001534	1.16E-05	0.000784902	0.045524327
T2DM_admission	leukocyte	*GGNBP2*	-1.461434866	3.68E-05	0.001037577	0.144455994
T2DM_admission	leukocyte	*TMEM98*	-0.838791773	0.001586475	0.002539896	1

T2DM_admission	leukocyte	*SYNRG*	-1.844374593	0.000123741	0.001062643	0.48617915
T2DM_admission	leukocyte	*SPATA20*	-1.128727211	0.002098282	0.003195406	1
T2DM_admission	leukocyte	*FARP2*	-3.995350583	3.27E-05	0.001037577	0.128659701
T2DM_admission	leukocyte	*USH1C*	-1.391283292	4.39E-05	0.001037577	0.172425123
T2DM_admission	leukocyte	*VPS41*	-1.533464502	0.000640895	0.001440839	1
T2DM_admission	leukocyte	*ELAC2*	-1.223683473	0.000822047	0.001670341	1
T2DM_admission	leukocyte	*CCDC124*	-1.086942979	0.00026249	0.001083674	1
T2DM_admission	leukocyte	*NOS2*	-17.83406838	0.00130735	0.002206435	1
T2DM_admission	leukocyte	*CD79B*	-7.158616004	4.08E-05	0.001037577	0.160273336
T2DM_admission	leukocyte	*ST7L*	-2.316946458	3.65E-06	0.000477601	0.014328042
T2DM_admission	leukocyte	*BAIAP3*	-1.328055872	4.98E-05	0.00105496	0.195765772
T2DM_admission	leukocyte	*FMO3*	-2.910591801	0.03967202	0.047319784	1
T2DM_admission	leukocyte	*CYB561*	-1.110754946	0.000233594	0.001066706	0.917790791
T2DM_admission	leukocyte	*MGST1*	-0.758966088	0.001666895	0.002644017	1
T2DM_admission	leukocyte	*ST3GAL1*	-2.930973286	3.45E-05	0.001037577	0.135391413
T2DM_admission	leukocyte	*IL32*	-0.508145088	0.001242549	0.002152855	1
T2DM_admission	leukocyte	*MED24*	-1.589034104	0.000450283	0.001234587	1
T2DM_admission	leukocyte	*RPS20*	-0.846237704	0.000181242	0.001062643	0.712101733
T2DM_admission	leukocyte	*REV3L*	-0.997794937	0.000551288	0.001352066	1
T2DM_admission	leukocyte	*ETV7*	-2.260666013	0.000304351	0.00109505	1
T2DM_admission	leukocyte	*NCAPD2*	-2.015037791	0.000842499	0.001699271	1
T2DM_admission	leukocyte	*GIPR*	-1.146413114	4.41E-05	0.001037577	0.173223707
T2DM_admission	leukocyte	*FUZ*	-1.781768788	0.000512595	0.001308606	1
T2DM_admission	leukocyte	*LRRC23*	-0.832714164	0.001121265	0.002027359	1
T2DM_admission	leukocyte	*TSPAN9*	-0.940028546	0.001245334	0.002154446	1
T2DM_admission	leukocyte	*BTBD7*	-1.226192838	0.000762922	0.001593931	1
T2DM_admission	leukocyte	*APBA3*	-2.057867319	0.001128782	0.002034397	1
T2DM_admission	leukocyte	*TTC19*	-0.92954171	0.00074854	0.001571055	1
T2DM_admission	leukocyte	*DPF1*	-0.895518721	0.026494113	0.032167914	1
T2DM_admission	leukocyte	*RABGAP1*	-1.148093258	0.000561664	0.001362208	1
T2DM_admission	leukocyte	*QPCTL*	-4.644976554	0.000112659	0.001062643	0.442637755
T2DM_admission	leukocyte	*PPP5C*	-1.151662932	0.000505062	0.001300386	1
T2DM_admission	leukocyte	*ZBTB32*	-18.03485435	0.007378514	0.009853903	1
T2DM_admission	leukocyte	*ERCC1*	-1.533753213	9.89E-05	0.001062643	0.38869646
T2DM_admission	leukocyte	*CD22*	0.551441641	0.003413181	0.004905043	1
T2DM_admission	leukocyte	*LTF*	-3.503531721	6.23E-05	0.001060479	0.24493909
T2DM_admission	leukocyte	*EXTL3*	-1.040078417	0.00056493	0.001362351	1
T2DM_admission	leukocyte	*CALCOCO1*	-3.895077848	0.000285739	0.001088708	1
T2DM_admission	leukocyte	*UBR7*	-1.06259107	0.002307723	0.003475294	1
T2DM_admission	leukocyte	*MAN2B2*	-1.746512749	0.000240878	0.001072513	0.94641157
T2DM_admission	leukocyte	*ANGEL1*	-1.245586312	0.007941201	0.010544434	1
T2DM_admission	leukocyte	*DDX11*	-1.210750865	0.000929601	0.001800431	1
T2DM_admission	leukocyte	*GPRC5A*	-1.191163327	0.006019213	0.008203083	1
T2DM_admission	leukocyte	*UFL1*	-1.187412637	0.000218534	0.001062643	0.85861972
T2DM_admission	leukocyte	*POLA2*	-2.051473124	0.000537295	0.001334229	1
T2DM_admission	leukocyte	*ZC3H3*	-0.905408698	0.005578986	0.007637573	1
T2DM_admission	leukocyte	*MDH1*	-1.181492058	0.000225585	0.001062643	0.886324752
T2DM_admission	leukocyte	*MTMR11*	-4.141769386	0.0006215	0.001422226	1
T2DM_admission	leukocyte	*DPEP1*	-0.678594312	0.000951454	0.001823593	1
T2DM_admission	leukocyte	*NUDCD3*	-1.03233109	0.000566802	0.001364563	1
T2DM_admission	leukocyte	*TTC27*	-2.753760919	0.000160801	0.001062643	0.631785257
T2DM_admission	leukocyte	*SNAI2*	-2.86374324	0.001893925	0.002938875	1
T2DM_admission	leukocyte	*PLEKHB1*	-0.828175685	0.015071818	0.019071554	1
T2DM_admission	leukocyte	*NLRP2*	-1.692402026	0.01566447	0.019789615	1
T2DM_admission	leukocyte	*ZDHHC6*	-1.503465205	0.000325015	0.001118201	1
T2DM_admission	leukocyte	*RNH1*	-1.184590044	0.000162539	0.001062643	0.638614389
T2DM_admission	leukocyte	*NDUFS1*	-1.073984728	0.000285883	0.001088708	1
T2DM_admission	leukocyte	*TYMP*	0.41040322	0.023718231	0.02906704	1
T2DM_admission	leukocyte	*CD44*	-0.606035453	0.000645127	0.001445924	1
T2DM_admission	leukocyte	*KCNG1*	-3.950360151	0.001055528	0.001948857	1
T2DM_admission	leukocyte	*AGPAT4*	-3.126085974	0.0077468	0.010314191	1
T2DM_admission	leukocyte	*SH2D2A*	-7.226293526	1.18E-05	0.000786127	0.046381497
T2DM_admission	leukocyte	*VEZT*	-1.173953634	0.000636145	0.001436444	1
T2DM_admission	leukocyte	*RABEP1*	-1.985737118	0.000382444	0.001165285	1

T2DM_admission	leukocyte	*ARID4A*	-1.708713912	9.21E-05	0.001062643	0.361763381
T2DM_admission	leukocyte	*PNPLA6*	-1.530915609	0.000224527	0.001062643	0.882166515
T2DM_admission	leukocyte	*ABCF2*	-1.63820331	0.000317182	0.001111111	1
T2DM_admission	leukocyte	*UBA6*	-1.91312135	0.000169637	0.001062643	0.666502898
T2DM_admission	leukocyte	*APBA2*	-2.847034854	0.001262481	0.002166065	1
T2DM_admission	leukocyte	*ASTE1*	-1.389096072	0.001717491	0.002713317	1
T2DM_admission	leukocyte	*RNF19A*	-1.042279028	0.000250999	0.001077213	0.986176822
T2DM_admission	leukocyte	*SH3YL1*	-0.701223748	0.001569397	0.002518856	1
T2DM_admission	leukocyte	*FAM136A*	-1.13998647	0.000334021	0.001128435	1
T2DM_admission	leukocyte	*DAPK2*	-1.217216984	0.000526313	0.001319879	1
T2DM_admission	leukocyte	*TIMP2*	-2.534760377	2.68E-05	0.000994138	0.105378619
T2DM_admission	leukocyte	*MYOM2*	1.076891777	0.019873862	0.024725904	1
T2DM_admission	leukocyte	*RPL26L1*	-1.078205333	0.000253593	0.001077213	0.996368042
T2DM_admission	leukocyte	*NSUN2*	-1.824469792	8.56E-05	0.001062643	0.336291815
T2DM_admission	leukocyte	*MRI1*	-1.367050709	0.000524763	0.001319879	1
T2DM_admission	leukocyte	*AGA*	-1.340856698	0.000360412	0.001142338	1
T2DM_admission	leukocyte	*BOD1L1*	-0.994911379	0.00099491	0.001874821	1
T2DM_admission	leukocyte	*DNAH5*	-1.755302426	0.000416455	0.001199564	1
T2DM_admission	leukocyte	*PNKP*	-2.439925673	0.000621017	0.001422226	1
T2DM_admission	leukocyte	*PHLPP2*	-1.005648332	0.00097803	0.001856058	1
T2DM_admission	leukocyte	*CTNS*	-1.369831986	0.001144557	0.002052471	1
T2DM_admission	leukocyte	*INPP4A*	-1.026573218	0.001210971	0.002123116	1
T2DM_admission	leukocyte	*PSMA4*	-1.112121694	0.000221371	0.001062643	0.869764707
T2DM_admission	leukocyte	*LSG1*	-0.674324988	0.009979891	0.013000993	1
T2DM_admission	leukocyte	*SPATA7*	-1.683905222	0.00014378	0.001062643	0.564909784
T2DM_admission	leukocyte	*TRIT1*	-2.437147198	0.000773112	0.001608876	1
T2DM_admission	leukocyte	*CNTLN*	-1.661403181	0.000361687	0.001142338	1
T2DM_admission	leukocyte	*PREX2*	-1.975821467	0.002296037	0.003461677	1
T2DM_admission	leukocyte	*WDR37*	-1.758743163	0.000135001	0.001062643	0.530420329
T2DM_admission	leukocyte	*YTHDC2*	-1.901674973	0.000132564	0.001062643	0.520844053
T2DM_admission	leukocyte	*DTNBP1*	-1.324372344	0.000366352	0.001147001	1
T2DM_admission	leukocyte	*GOPC*	-1.394803609	0.000183343	0.001062643	0.720355442
T2DM_admission	leukocyte	*ZNF800*	-1.373151948	0.000188849	0.001062643	0.741989002
T2DM_admission	leukocyte	*MRPS10*	-1.713380617	4.12E-05	0.001037577	0.161878263
T2DM_admission	leukocyte	*COL9A2*	-1.188866048	0.00087448	0.001741426	1
T2DM_admission	leukocyte	*ERCC8*	-3.151795815	0.009503279	0.012404779	1
T2DM_admission	leukocyte	*H6PD*	-3.3593954	4.33E-05	0.001037577	0.16997902
T2DM_admission	leukocyte	*VAMP3*	-1.414183308	0.000193532	0.001062643	0.760386358
T2DM_admission	leukocyte	*RCN1*	-0.942152666	0.000402648	0.001185781	1
T2DM_admission	leukocyte	*RFC2*	-1.156716251	0.001765172	0.002770339	1
T2DM_admission	leukocyte	*JKAMP*	-1.096092618	0.000394378	0.001181536	1
T2DM_admission	leukocyte	*PTGER3*	-1.02690803	0.001006743	0.001889818	1
T2DM_admission	leukocyte	*BCAR1*	-0.900466871	0.000742009	0.001563191	1
T2DM_admission	leukocyte	*THOC3*	-0.863215588	0.001243275	0.002152855	1
T2DM_admission	leukocyte	*HEBP2*	-1.132769012	9.52E-05	0.001062643	0.374059376
T2DM_admission	leukocyte	*RRP12*	-0.781649838	0.00114124	0.002049538	1
T2DM_admission	leukocyte	*FNIP2*	-2.662655796	0.000160904	0.001062643	0.632192491
T2DM_admission	leukocyte	*MRTO4*	-1.315779889	0.000621016	0.001422226	1
T2DM_admission	leukocyte	*MCF2L2*	-1.89796863	0.000372504	0.001155145	1
T2DM_admission	leukocyte	*LAMA3*	-1.799976149	0.000779795	0.001614233	1
T2DM_admission	leukocyte	*ANAPC4*	-4.202545254	0.003975773	0.005625067	1
T2DM_admission	leukocyte	*LY75*	-5.252901665	2.55E-05	0.000983968	0.100364704
T2DM_admission	leukocyte	*ARID4B*	-0.910239805	0.001386388	0.002298537	1
T2DM_admission	leukocyte	*SDCCAG8*	-1.077581536	0.000866754	0.001732185	1
T2DM_admission	leukocyte	*KIF1B*	-1.139966011	0.000273542	0.001088708	1
T2DM_admission	leukocyte	*TBC1D22A*	-1.190504502	0.000467569	0.001253701	1
T2DM_admission	leukocyte	*SYNE2*	-1.09668232	0.000370576	0.001151894	1
T2DM_admission	leukocyte	*ATP9A*	-1.225504142	0.000288326	0.001088708	1
T2DM_admission	leukocyte	*CHRDL2*	-3.914330824	0.001364379	0.002274982	1
T2DM_admission	leukocyte	*GALC*	-1.709738985	0.00023155	0.001066541	0.909759426
T2DM_admission	leukocyte	*CYFIP2*	-0.93210638	0.000610207	0.001411957	1
T2DM_admission	leukocyte	*USP36*	-1.962635313	5.37E-05	0.001060479	0.211098211
T2DM_admission	leukocyte	*MCOLN3*	-1.046225283	0.001014583	0.001900952	1
T2DM_admission	leukocyte	*ITIH4*	-3.145814509	3.27E-06	0.000443199	0.012852761

T2DM_admission	leukocyte	*PCGF2*	-1.121708506	0.000493558	0.001285934	1
T2DM_admission	leukocyte	*GDI2*	-1.21372164	0.000282909	0.001088708	1
T2DM_admission	leukocyte	*ATP11B*	-2.003055128	0.00016338	0.001062643	0.641918202
T2DM_admission	leukocyte	*LAMC2*	-0.799919165	0.000909588	0.001780653	1
T2DM_admission	leukocyte	*RASGRF1*	-0.806579714	0.001587031	0.002539896	1
T2DM_admission	leukocyte	*CROCC*	-1.496125495	0.003434652	0.004930489	1
T2DM_admission	leukocyte	*FLYWCH1*	-0.813263724	0.000807021	0.0016529	1
T2DM_admission	leukocyte	*OGFR*	-2.586776518	0.003848209	0.005466251	1
T2DM_admission	leukocyte	*GNA15*	1.500361142	0.011398756	0.01468384	1
T2DM_admission	leukocyte	*RIMBP2*	-0.883125043	0.001251587	0.002156134	1
T2DM_admission	leukocyte	*MPC1*	-1.097015848	0.000248398	0.001077213	0.975954259
T2DM_admission	leukocyte	*BCAT1*	0.332376064	0.02149917	0.026521268	1
T2DM_admission	leukocyte	*MRPS35*	-1.195924829	0.00044723	0.001232235	1
T2DM_admission	leukocyte	*ZNF112*	-1.451288524	0.001577422	0.00253068	1
T2DM_admission	leukocyte	*EIF4B*	-1.075082649	0.00026321	0.001083884	1
T2DM_admission	leukocyte	*SLC6A16*	-2.891145417	0.00125034	0.002155971	1
T2DM_admission	leukocyte	*SPHK2*	-1.268767015	0.001722598	0.002719068	1
T2DM_admission	leukocyte	*HAGH*	-1.629486475	1.43E-05	0.000815363	0.056284355
T2DM_admission	leukocyte	*RNF4*	-1.406734698	0.000221557	0.001062643	0.870497955
T2DM_admission	leukocyte	*TM7SF3*	-2.241801656	0.000117326	0.001062643	0.460973836
T2DM_admission	leukocyte	*ST3GAL6*	-1.897151565	0.019828412	0.024684991	1
T2DM_admission	leukocyte	*HIPK2*	-0.946315304	0.000496541	0.001291992	1
T2DM_admission	leukocyte	*TMEM161A*	-1.269288332	0.000757952	0.001588262	1
T2DM_admission	leukocyte	*SNX24*	-2.734411782	0.000133633	0.001062643	0.525042836
T2DM_admission	leukocyte	*ABCA7*	-1.577062857	0.001048638	0.001940644	1
T2DM_admission	leukocyte	*PMS1*	-1.038019183	0.001598048	0.002554405	1
T2DM_admission	leukocyte	*IPO5*	-1.030735239	0.000305692	0.001096936	1
T2DM_admission	leukocyte	*OAT*	-1.065277695	0.000392938	0.001181536	1
T2DM_admission	leukocyte	*WDR18*	-1.320497151	0.000352236	0.001141109	1
T2DM_admission	leukocyte	*MCM10*	1.778552636	0.014309991	0.01821904	1
T2DM_admission	leukocyte	*ERBB3*	-0.928371606	0.001330072	0.002233728	1
T2DM_admission	leukocyte	*ANKRD44*	-4.736159762	0.000661945	0.001464403	1
T2DM_admission	leukocyte	*ADAT1*	-1.091861739	0.001041187	0.001936841	1
T2DM_admission	leukocyte	*MYLK*	-0.72325961	0.016572229	0.02088271	1
T2DM_admission	leukocyte	*GSTO2*	-0.796039939	0.000458674	0.001240899	1
T2DM_admission	leukocyte	*SEC61A2*	-4.215026629	5.74E-06	0.000591121	0.022565957
T2DM_admission	leukocyte	*PRKCQ*	-2.600064422	0.001517649	0.002459919	1
T2DM_admission	leukocyte	*TBC1D1*	-1.154843115	0.000733218	0.001552225	1
T2DM_admission	leukocyte	*CTNNA2*	-1.218907627	0.004181961	0.005880789	1
T2DM_admission	leukocyte	*CD84*	0.157568127	0.002678384	0.003956154	1
T2DM_admission	leukocyte	*ATXN3*	-1.682768003	0.000993841	0.001873994	1
T2DM_admission	leukocyte	*EML1*	-1.834378361	0.000272843	0.001088708	1
T2DM_admission	leukocyte	*STOML1*	-1.016535425	0.00077489	0.001611723	1
T2DM_admission	leukocyte	*DNTTIP2*	-1.025924841	0.000531424	0.001324223	1
T2DM_admission	leukocyte	*RRP15*	-1.171737709	0.000605464	0.00140595	1
T2DM_admission	leukocyte	*REEP1*	-0.738923736	0.017282712	0.02170143	1
T2DM_admission	leukocyte	*POLR1A*	-1.193839998	7.22E-05	0.001062643	0.283508196
T2DM_admission	leukocyte	*LAPTM4A*	-1.044843938	0.000193014	0.001062643	0.758352327
T2DM_admission	leukocyte	*KIF2A*	-1.102144572	0.000383472	0.001166145	1
T2DM_admission	leukocyte	*MAST4*	-1.283138129	0.000255134	0.001077348	1
T2DM_admission	leukocyte	*ADAM7*	-12.58453298	0.031105573	0.037488895	1
T2DM_admission	leukocyte	*DRD4*	-3.037591568	0.005952145	0.008117312	1
T2DM_admission	leukocyte	*PLA2G10*	-4.303997563	0.00051622	0.001313619	1
T2DM_admission	leukocyte	*PIGB*	-3.856460347	0.001835798	0.002861108	1
T2DM_admission	leukocyte	*GNB5*	-1.274452736	0.001705211	0.00269816	1
T2DM_admission	leukocyte	*RAB27A*	-1.059009384	0.00045964	0.001240899	1
T2DM_admission	leukocyte	*PTPN3*	-1.969133898	0.000113466	0.001062643	0.445809334
T2DM_admission	leukocyte	*SPTB*	-0.639069297	0.028695793	0.034733756	1
T2DM_admission	leukocyte	*FGF10*	-2.968624744	0.003523736	0.005041792	1
T2DM_admission	leukocyte	*DGCR2*	-1.593628919	0.000179231	0.001062643	0.704198541
T2DM_admission	leukocyte	*ZXDC*	-3.087626835	5.14E-05	0.00105496	0.201975993
T2DM_admission	leukocyte	*WIPI1*	-3.057868425	4.13E-05	0.001037577	0.162370677
T2DM_admission	leukocyte	*GBA2*	-1.02601368	0.002158819	0.003274904	1
T2DM_admission	leukocyte	*ASNS*	-1.067785054	0.001276678	0.002175489	1

T2DM_admission	leukocyte	*AP3M2*	-1.388100258	0.008717809	0.011474798	1
T2DM_admission	leukocyte	*TESK2*	-4.423708532	0.001296606	0.002195847	1
T2DM_admission	leukocyte	*MAP4K4*	-1.02274178	0.000346999	0.001140861	1
T2DM_admission	leukocyte	*RPL31*	-0.93362439	0.000174808	0.001062643	0.686819314
T2DM_admission	leukocyte	*SEL1L*	-1.053656614	0.000203866	0.001062643	0.80098798
T2DM_admission	leukocyte	*TCF3*	-1.239911544	5.53E-05	0.001060479	0.217127421
T2DM_admission	leukocyte	*CPSF1*	-0.936072376	0.001770513	0.002776984	1
T2DM_admission	leukocyte	*CYBRD1*	0.613252734	0.02542865	0.031027692	1
T2DM_admission	leukocyte	*LNX1*	-0.847562485	0.001058582	0.001952662	1
T2DM_admission	leukocyte	*ALDH3A2*	-0.720851117	0.001442794	0.002362958	1
T2DM_admission	leukocyte	*P4HA2*	-0.835148386	0.001100159	0.001999636	1
T2DM_admission	leukocyte	*TRNT1*	-3.800459015	0.000194998	0.001062643	0.766146907
T2DM_admission	leukocyte	*CRMP1*	-0.863380903	0.001784616	0.002793529	1
T2DM_admission	leukocyte	*DERL2*	-1.217344675	0.000224751	0.001062643	0.883047529
T2DM_admission	leukocyte	*XRCC1*	-1.593235744	0.000584615	0.001382042	1
T2DM_admission	leukocyte	*SCARB1*	-3.542337884	8.41E-06	0.000751223	0.033053797
T2DM_admission	leukocyte	*NLE1*	-0.925370102	0.013434864	0.017177215	1
T2DM_admission	leukocyte	*SMARCE1*	-1.045840222	0.000270036	0.001088175	1
T2DM_admission	leukocyte	*ST6GAL1*	2.946475519	0.000700233	0.001515822	1
T2DM_admission	leukocyte	*MRPS34*	-0.937525273	0.000502584	0.001297407	1
T2DM_admission	leukocyte	*TEAD2*	-1.235331244	0.007985142	0.010595617	1
T2DM_admission	leukocyte	*IPCEF1*	-2.315414333	0.000314561	0.001109126	1
T2DM_admission	leukocyte	*ZZEF1*	-1.75193194	0.00014134	0.001062643	0.555323486
T2DM_admission	leukocyte	*WSCD2*	-1.144965995	0.001524805	0.002467446	1
T2DM_admission	leukocyte	*TIPIN*	-1.044012576	0.020310862	0.025197782	1
T2DM_admission	leukocyte	*SEMA3A*	-3.038998697	0.001479638	0.002406249	1
T2DM_admission	leukocyte	*TTC38*	-2.438334736	6.48E-05	0.001060479	0.254550592
T2DM_admission	leukocyte	*GRAMD4*	-1.712084781	0.000353859	0.001141109	1
T2DM_admission	leukocyte	*CELSR1*	-5.403626274	0.001638506	0.002606352	1
T2DM_admission	leukocyte	*TIMM21*	-1.714703199	0.000376178	0.001159218	1
T2DM_admission	leukocyte	*VPS9D1*	-0.94225174	0.005190968	0.00714622	1
T2DM_admission	leukocyte	*ZNF37A*	-1.214926943	0.000227999	0.001062643	0.895807654
T2DM_admission	leukocyte	*MARK3*	-1.489474946	1.15E-05	0.000784902	0.045123335
T2DM_admission	leukocyte	*CACNG5*	-31.37698597	0.001613412	0.002575821	1
T2DM_admission	leukocyte	*MOCOS*	-2.592837425	0.011010941	0.014230917	1
T2DM_admission	leukocyte	*DLG1*	-1.133027312	0.000353307	0.001141109	1
T2DM_admission	leukocyte	*SEC31B*	1.387066119	0.026266526	0.031921181	1
T2DM_admission	leukocyte	*EXOSC7*	-0.915995257	0.001181262	0.002099858	1
T2DM_admission	leukocyte	*MKRN2*	-1.427905162	0.000394464	0.001181536	1
T2DM_admission	leukocyte	*MLH1*	-1.560055284	0.00050553	0.001300737	1
T2DM_admission	leukocyte	*TPD52*	-0.937155775	0.000312882	0.001108487	1
T2DM_admission	leukocyte	*ACACB*	-1.406391421	0.000394012	0.001181536	1
T2DM_admission	leukocyte	*GPATCH1*	-0.928803593	0.005168784	0.007118175	1
T2DM_admission	leukocyte	*ICAM3*	-1.177273125	0.000554469	0.001356582	1
T2DM_admission	leukocyte	*XAB2*	-1.226894216	0.000230115	0.001064322	0.904121667
T2DM_admission	leukocyte	*ACTL6B*	-0.997349485	0.000828367	0.00167593	1
T2DM_admission	leukocyte	*IL4R*	-0.999073317	0.001326385	0.002228985	1
T2DM_admission	leukocyte	*APBB1IP*	0.419988889	0.005157647	0.007105328	1
T2DM_admission	leukocyte	*GPR137B*	-1.971586673	0.000158983	0.001062643	0.624643461
T2DM_admission	leukocyte	*NAALAD2*	-2.891152386	0.002062866	0.003151674	1
T2DM_admission	leukocyte	*MCCC1*	-1.181702045	0.000675868	0.00148591	1
T2DM_admission	leukocyte	*N4BP2*	-1.773769288	0.000137728	0.001062643	0.541134015
T2DM_admission	leukocyte	*MLLT10*	-1.540646778	0.000300047	0.001093914	1
T2DM_admission	leukocyte	*VDAC3*	-1.362936276	0.000113576	0.001062643	0.446239715
T2DM_admission	leukocyte	*PCM1*	-1.105738399	0.000228716	0.00106363	0.898623815
T2DM_admission	leukocyte	*TP73*	2.695310469	0.010747388	0.013917761	1
T2DM_admission	leukocyte	*TOLLIP*	-1.253002248	0.000237399	0.001070884	0.932739973
T2DM_admission	leukocyte	*CLUL1*	-2.851134351	0.001212494	0.00212484	1
T2DM_admission	leukocyte	*FKBP7*	-1.651692528	0.000186436	0.001062643	0.732506331
T2DM_admission	leukocyte	*LXN*	-0.766907094	0.003504115	0.005019201	1
T2DM_admission	leukocyte	*SAR1A*	-0.933328352	0.000382588	0.001165285	1
T2DM_admission	leukocyte	*CDC14A*	-1.788832671	0.000388286	0.001174424	1
T2DM_admission	leukocyte	*FDFT1*	-1.388024274	8.94E-05	0.001062643	0.351147435
T2DM_admission	leukocyte	*SCGN*	-0.531444846	0.00095912	0.001831992	1

T2DM_admission	leukocyte	*DDX1*	-1.060998994	0.000374125	0.001157429	1
T2DM_admission	leukocyte	*RABL2B*	-1.719171539	0.000530883	0.001324223	1
T2DM_admission	leukocyte	*DCT*	-7.868751649	0.000115704	0.001062643	0.454600304
T2DM_admission	leukocyte	*SCTR*	-1.068976512	0.001656018	0.002629949	1
T2DM_admission	leukocyte	*RIF1*	-2.324270335	4.34E-05	0.001037577	0.170568368
T2DM_admission	leukocyte	*COL5A3*	-5.82642821	0.002066808	0.003154814	1
T2DM_admission	leukocyte	*IGSF9B*	-2.092394167	0.001130718	0.002036018	1
T2DM_admission	leukocyte	*COL4A4*	-2.559079464	0.000702608	0.001519287	1
T2DM_admission	leukocyte	*ZNF506*	-1.113984933	0.000638919	0.001438574	1
T2DM_admission	leukocyte	*CADPS2*	-1.084273781	0.002131476	0.003238426	1
T2DM_admission	leukocyte	*ME2*	-2.811955282	7.74E-05	0.001062643	0.30409743
T2DM_admission	leukocyte	*CCNT2*	-1.167985236	0.004793328	0.006642945	1
T2DM_admission	leukocyte	*SEMA5B*	1.560782599	0.006135351	0.008335337	1
T2DM_admission	leukocyte	*BCKDHB*	-3.001524529	0.00064489	0.001445924	1
T2DM_admission	leukocyte	*DIS3*	-1.173068686	0.000305713	0.001096936	1
T2DM_admission	leukocyte	*PIBF1*	-1.756353994	0.000593915	0.001393962	1
T2DM_admission	leukocyte	*CHMP2B*	-1.051443368	0.000240184	0.001072513	0.943681968
T2DM_admission	leukocyte	*PPIE*	-1.341332719	0.00033199	0.001127387	1
T2DM_admission	leukocyte	*NCOA1*	-1.401500078	0.00019103	0.001062643	0.750557985
T2DM_admission	leukocyte	*AGBL5*	-1.761618295	0.004059809	0.005725409	1
T2DM_admission	leukocyte	*CAD*	-0.987796467	0.013546734	0.017314613	1
T2DM_admission	leukocyte	*CD59*	-0.699551883	0.000861262	0.001725599	1
T2DM_admission	leukocyte	*CD82*	-1.840960193	0.001386493	0.002298537	1
T2DM_admission	leukocyte	*MECOM*	-0.64614616	0.008495847	0.011220229	1
T2DM_admission	leukocyte	*SLC25A24*	-1.127153678	0.000615567	0.001419345	1
T2DM_admission	leukocyte	*PILRA*	0.365009562	0.003611498	0.005159846	1
T2DM_admission	leukocyte	*ZNF213*	-1.258755973	0.000705757	0.001522744	1
T2DM_admission	leukocyte	*CPNE3*	-0.885058063	0.000486203	0.001277166	1
T2DM_admission	leukocyte	*RRN3*	-0.991735372	0.000678903	0.001488511	1
T2DM_admission	leukocyte	*WNT11*	-2.466565449	0.008708526	0.01146642	1
T2DM_admission	leukocyte	*DDHD2*	-1.175866242	0.002627219	0.003884962	1
T2DM_admission	leukocyte	*TTC39A*	-1.028224696	0.0035167	0.005035392	1
T2DM_admission	leukocyte	*EPS15*	-1.506441558	0.000763091	0.001593931	1
T2DM_admission	leukocyte	*ORC1*	-4.401777083	0.000210819	0.001062643	0.828306001
T2DM_admission	leukocyte	*MGST2*	-0.732556231	0.002463545	0.00368033	1
T2DM_admission	leukocyte	*USP40*	-1.963100131	0.000112843	0.001062643	0.443358975
T2DM_admission	leukocyte	*POMGNT1*	-1.726002379	4.13E-05	0.001037577	0.162439754
T2DM_admission	leukocyte	*NFX1*	-1.781413728	0.000582188	0.001382042	1
T2DM_admission	leukocyte	*EIF2AK1*	-1.133408594	0.000805687	0.0016513	1
T2DM_admission	leukocyte	*ITPKC*	-1.386376071	0.000843296	0.001700004	1
T2DM_admission	leukocyte	*TMED2*	-0.891294051	0.000301902	0.001093914	1
T2DM_admission	leukocyte	*ACOX3*	-2.641804118	0.00024002	0.001072513	0.943039422
T2DM_admission	leukocyte	*TRIP6*	-1.072680497	9.62E-05	0.001062643	0.378073479
T2DM_admission	leukocyte	*PGS1*	-1.846652125	0.000284923	0.001088708	1
T2DM_admission	leukocyte	*PSMC5*	-0.981568657	0.000191518	0.001062643	0.752474624
T2DM_admission	leukocyte	*UIMC1*	-1.196448771	0.0009752	0.001854014	1
T2DM_admission	leukocyte	*SF3B2*	-0.969237738	0.000408735	0.001188129	1
T2DM_admission	leukocyte	*KLHL42*	-1.07926389	0.000134038	0.001062643	0.526637187
T2DM_admission	leukocyte	*METTL2A*	-1.852201053	0.000223197	0.001062643	0.876939474
T2DM_admission	leukocyte	*GP6*	1.331487197	0.021300401	0.026300841	1
T2DM_admission	leukocyte	*EDEM2*	-0.821957072	0.035729649	0.042825439	1
T2DM_admission	leukocyte	*SLC15A1*	-1.917761247	0.005599709	0.007663273	1
T2DM_admission	leukocyte	*TGDS*	-1.13436277	0.001073486	0.00196814	1
T2DM_admission	leukocyte	*DOCK3*	-1.250063629	0.001252303	0.002156134	1
T2DM_admission	leukocyte	*ARHGAP28*	-2.823584046	0.001186885	0.00210081	1
T2DM_admission	leukocyte	*PPP1R13B*	-0.801849675	0.001595528	0.002551417	1
T2DM_admission	leukocyte	*ZNF343*	-1.31843291	0.00426707	0.005987614	1
T2DM_admission	leukocyte	*MAVS*	-2.186909273	0.00057666	0.00137565	1
T2DM_admission	leukocyte	*DYNLL1*	-0.958185728	0.000408442	0.001188129	1
T2DM_admission	leukocyte	*RBBP9*	-0.931873921	0.002229739	0.003369478	1
T2DM_admission	leukocyte	*SLC23A2*	-2.029578124	0.00014063	0.001062643	0.552534844
T2DM_admission	leukocyte	*SLC8B1*	0.294226537	0.009896577	0.012901013	1
T2DM_admission	leukocyte	*TMEM230*	-1.081926405	0.000327958	0.001122427	1
T2DM_admission	leukocyte	*KIF16B*	-2.007336844	3.73E-05	0.001037577	0.146451012

T2DM_admission	leukocyte	*ZNF302*	-1.128575858	0.000277506	0.001088708	1
T2DM_admission	leukocyte	*GRAMD1A*	-1.875746387	0.000213545	0.001062643	0.839020176
T2DM_admission	leukocyte	*CDIP1*	-1.210986483	0.000538327	0.001334229	1
T2DM_admission	leukocyte	*MLF2*	-1.284596759	0.000114245	0.001062643	0.448866708
T2DM_admission	leukocyte	*DDX24*	-1.005393766	0.000175326	0.001062643	0.688857688
T2DM_admission	leukocyte	*ANKRD24*	-7.270390555	0.007783273	0.010352227	1
T2DM_admission	leukocyte	*GPATCH2L*	-1.447429749	0.000149942	0.001062643	0.589122588
T2DM_admission	leukocyte	*SLC9A1*	-0.935315796	0.001993444	0.003065456	1
T2DM_admission	leukocyte	*SPTLC1*	-1.078213416	0.000783202	0.001618943	1
T2DM_admission	leukocyte	*YPEL3*	-0.855580465	0.022136152	0.027264245	1
T2DM_admission	leukocyte	*NDUFB2*	-1.025988656	5.06E-05	0.00105496	0.198989844
T2DM_admission	leukocyte	*MAEA*	-0.990684939	0.000927366	0.001800431	1
T2DM_admission	leukocyte	*STRN4*	-1.143026294	0.000337872	0.001134436	1
T2DM_admission	leukocyte	*LYZ*	0.239279882	0.005355628	0.007354862	1
T2DM_admission	leukocyte	*MUL1*	-1.258386175	0.000260314	0.001083674	1
T2DM_admission	leukocyte	*SPG21*	-1.616141746	0.000134942	0.001062643	0.53018864
T2DM_admission	leukocyte	*USP48*	-1.132676532	0.000620252	0.001422226	1
T2DM_admission	leukocyte	*GLG1*	-1.146536041	0.000323498	0.001116892	1
T2DM_admission	leukocyte	*NRCAM*	-0.727189582	0.000940289	0.001810978	1
T2DM_admission	leukocyte	*TF*	-1.42871763	0.009247601	0.012099175	1
T2DM_admission	leukocyte	*MYO15A*	-2.367207131	0.004207349	0.005914143	1
T2DM_admission	leukocyte	*PITPNM3*	-3.071184511	0.001147637	0.002057055	1
T2DM_admission	leukocyte	*SPAG7*	-1.090895446	0.002455679	0.003671371	1
T2DM_admission	leukocyte	*TMEM101*	-1.54525538	0.000151389	0.001062643	0.594806942
T2DM_admission	leukocyte	*PPP2R3C*	-2.041845739	5.81E-05	0.001060479	0.228445543
T2DM_admission	leukocyte	*HAUS4*	-2.260254052	0.010797918	0.013978589	1
T2DM_admission	leukocyte	*OSGEP*	-2.187161816	6.89E-05	0.001062643	0.270567973
T2DM_admission	leukocyte	*SCFD1*	-1.204706257	0.000282764	0.001088708	1
T2DM_admission	leukocyte	*RPGRIP1*	1.073170251	0.0255251	0.031133606	1
T2DM_admission	leukocyte	*EZR*	-0.792401667	0.000475415	0.001261234	1
T2DM_admission	leukocyte	*NUP50*	-2.225846435	5.20E-05	0.00105496	0.20428185
T2DM_admission	leukocyte	*COMT*	-1.042041594	0.00031049	0.00110427	1
T2DM_admission	leukocyte	*ECHDC1*	-0.910342515	0.000869494	0.001735895	1
T2DM_admission	leukocyte	*CBX5*	-1.003780978	0.000218159	0.001062643	0.857145397
T2DM_admission	leukocyte	*SUCO*	-0.821086301	0.000347282	0.001140861	1
T2DM_admission	leukocyte	*MSH2*	-2.489090757	0.000261052	0.001083674	1
T2DM_admission	leukocyte	*TMEM38B*	-1.849744162	4.74E-05	0.00105496	0.186040769
T2DM_admission	leukocyte	*PSMD5*	-1.053221465	0.00072739	0.00154878	1
T2DM_admission	leukocyte	*CRAT*	-2.105776989	6.49E-05	0.001060479	0.25480805
T2DM_admission	leukocyte	*SH2D3C*	-1.437653629	0.002950434	0.004310096	1
T2DM_admission	leukocyte	*CWF19L1*	-1.523875677	2.61E-06	0.000379549	0.01024783
T2DM_admission	leukocyte	*SEMA4G*	-11.44057015	0.000447136	0.001232235	1
T2DM_admission	leukocyte	*IKZF5*	-1.898455397	0.000106091	0.001062643	0.416832586
T2DM_admission	leukocyte	*TLL2*	-4.439664429	0.001272706	0.002175059	1
T2DM_admission	leukocyte	*SRPK1*	-1.448458086	0.000146783	0.001062643	0.576710112
T2DM_admission	leukocyte	*PGC*	-0.90338697	0.003361423	0.004839513	1
T2DM_admission	leukocyte	*EFHC1*	-0.929240653	0.001807462	0.002824788	1
T2DM_admission	leukocyte	*ITPR3*	-1.158384608	0.001154325	0.00206595	1
T2DM_admission	leukocyte	*IFT74*	-1.648618961	0.000211209	0.001062643	0.829839706
T2DM_admission	leukocyte	*ABL1*	-1.204807509	0.000410101	0.001188264	1
T2DM_admission	leukocyte	*SH3GLB1*	-1.23175118	0.000235135	0.001067235	0.923845501
T2DM_admission	leukocyte	*CDC7*	-1.27115333	0.006828211	0.009178255	1
T2DM_admission	leukocyte	*SYDE2*	-1.118549273	0.004236143	0.005948466	1
T2DM_admission	leukocyte	*ABLIM1*	-0.925821119	0.000931159	0.001802228	1
T2DM_admission	leukocyte	*ERMP1*	-1.017938933	0.002018717	0.003094794	1
T2DM_admission	leukocyte	*PALMD*	-1.147407058	7.18E-05	0.001062643	0.28210407
T2DM_admission	leukocyte	*MZF1*	-1.028567735	0.004520324	0.006302467	1
T2DM_admission	leukocyte	*OCEL1*	-1.436969324	0.000999147	0.0018783	1
T2DM_admission	leukocyte	*CATSPERG*	-1.535981071	0.01735816	0.021768341	1
T2DM_admission	leukocyte	*STX1B*	1.478049515	0.00040546	0.001187074	1
T2DM_admission	leukocyte	*HSD3B7*	-3.304388582	0.001359478	0.00226907	1
T2DM_admission	leukocyte	*HNRNPM*	-1.017321587	0.000114604	0.001062643	0.45027913
T2DM_admission	leukocyte	*TIMM13*	-1.193386244	0.000199541	0.001062643	0.783996927
T2DM_admission	leukocyte	*CEP170B*	-1.754533028	0.000286013	0.001088708	1

T2DM_admission	leukocyte	*POLR2E*	-1.632962351	0.000151541	0.001062643	0.595404507
T2DM_admission	leukocyte	*RASSF7*	-1.024178896	0.000492862	0.001284972	1
T2DM_admission	leukocyte	*ARVCF*	-1.9445141	0.001662235	0.002637968	1
T2DM_admission	leukocyte	*TRMT2A*	-1.051457096	0.000877943	0.001743901	1
T2DM_admission	leukocyte	*MED15*	-3.401443989	3.81E-05	0.001037577	0.149721787
T2DM_admission	leukocyte	*SMARCB1*	-1.276554038	0.000228752	0.00106363	0.898767356
T2DM_admission	leukocyte	*DDT*	-1.326872038	9.92E-05	0.001062643	0.389592333
T2DM_admission	leukocyte	*CABIN1*	-1.455249913	0.000779493	0.001614233	1
T2DM_admission	leukocyte	*SPECC1L*	-1.849859844	0.000209944	0.001062643	0.824870689
T2DM_admission	leukocyte	*PES1*	-1.064746602	0.00144433	0.002364489	1
T2DM_admission	leukocyte	*GGT1*	-7.268816778	0.011669093	0.015017316	1
T2DM_admission	leukocyte	*TOP3B*	-1.677372102	0.001713257	0.002707728	1
T2DM_admission	leukocyte	*CARD10*	-0.873099865	0.000511503	0.001308382	1
T2DM_admission	leukocyte	*SLC25A1*	-1.305893498	0.000355988	0.001141109	1
T2DM_admission	leukocyte	*HPS4*	-1.336029015	0.000366375	0.001147001	1
T2DM_admission	leukocyte	*GCAT*	-1.695426657	0.000310601	0.00110427	1
T2DM_admission	leukocyte	*CYP2D6*	2.359779955	0.017096211	0.02150849	1
T2DM_admission	leukocyte	*DDX17*	-1.250597428	0.000133186	0.001062643	0.523286278
T2DM_admission	leukocyte	*HSCB*	-2.13627546	0.00017853	0.001062643	0.701446125
T2DM_admission	leukocyte	*CBY1*	-1.291289348	0.000383869	0.00116645	1
T2DM_admission	leukocyte	*XBP1*	-0.861418859	0.000210797	0.001062643	0.828222477
T2DM_admission	leukocyte	*TIMP3*	-1.305847811	0.000513902	0.001310266	1
T2DM_admission	leukocyte	*CYB5R3*	-1.133816971	0.000294212	0.001089501	1
T2DM_admission	leukocyte	*MIOX*	-2.647424714	0.000986644	0.001867193	1
T2DM_admission	leukocyte	*LMF2*	-1.891402432	0.002294801	0.003461141	1
T2DM_admission	leukocyte	*RHBDD3*	-1.421945198	0.000198781	0.001062643	0.781009793
T2DM_admission	leukocyte	*TOM1*	-1.800848365	0.000557024	0.001357659	1
T2DM_admission	leukocyte	*CHKB*	-3.149890636	0.000167198	0.001062643	0.656920064
T2DM_admission	leukocyte	*TTLL12*	-1.74359837	0.000546772	0.001346876	1
T2DM_admission	leukocyte	*RBFOX2*	-1.166702083	0.00025475	0.001077348	1
T2DM_admission	leukocyte	*SYNGR1*	-3.442958371	5.62E-05	0.001060479	0.220738359
T2DM_admission	leukocyte	*MTMR3*	-1.611788659	0.000145003	0.001062643	0.569718461
T2DM_admission	leukocyte	*SAMM50*	-1.098984847	0.000430106	0.00121662	1
T2DM_admission	leukocyte	*FOXRED2*	-1.205355104	0.002024598	0.003102437	1
T2DM_admission	leukocyte	*SGSM3*	-1.750239801	0.000271187	0.001088708	1
T2DM_admission	leukocyte	*IFT27*	-1.553195103	0.00012971	0.001062643	0.509628963
T2DM_admission	leukocyte	*FAM118A*	-1.387805801	0.002878995	0.004219162	1
T2DM_admission	leukocyte	*ST13*	-1.054994398	0.000177694	0.001062643	0.698158242
T2DM_admission	leukocyte	*POLR3H*	-3.001417063	2.53E-05	0.000982543	0.099236821
T2DM_admission	leukocyte	*PMM1*	-1.562739589	0.000314712	0.001109126	1
T2DM_admission	leukocyte	*DESI1*	-1.490079763	0.000690354	0.001503881	1
T2DM_admission	leukocyte	*KHNYN*	-1.787262113	8.72E-05	0.001062643	0.342554084
T2DM_admission	leukocyte	*RBM23*	-1.183106384	0.000263454	0.001083884	1
T2DM_admission	leukocyte	*PRMT5*	-1.068038832	0.000873473	0.001740302	1
T2DM_admission	leukocyte	*VCPKMT*	-1.735003344	0.000230066	0.001064322	0.903929617
T2DM_admission	leukocyte	*SOS2*	-1.573739835	0.000175807	0.001062643	0.690746995
T2DM_admission	leukocyte	*CDKL1*	-1.941947615	0.00183567	0.002861108	1
T2DM_admission	leukocyte	*NIN*	-2.863731724	7.36E-05	0.001062643	0.289247624
T2DM_admission	leukocyte	*TRIM9*	-0.848571082	0.001335609	0.00223874	1
T2DM_admission	leukocyte	*ATP6V1D*	-1.118097467	0.000186842	0.001062643	0.734101701
T2DM_admission	leukocyte	*PLEK2*	-1.196602527	0.023838935	0.029205854	1
T2DM_admission	leukocyte	*GSTZ1*	-1.735266977	0.00042575	0.001211276	1
T2DM_admission	leukocyte	*TMED8*	-1.438257216	0.000131167	0.001062643	0.515355716
T2DM_admission	leukocyte	*DAAM1*	-1.049220876	0.000398981	0.001183984	1
T2DM_admission	leukocyte	*DHRS7*	-1.330464877	0.00012488	0.001062643	0.490655461
T2DM_admission	leukocyte	*MTHFD1*	-4.80012778	2.21E-05	0.000943039	0.08675955
T2DM_admission	leukocyte	*GSKIP*	-1.146537955	0.000530771	0.001324223	1
T2DM_admission	leukocyte	*TRIP11*	-0.998357873	0.000457192	0.001240899	1
T2DM_admission	leukocyte	*DCAF11*	-1.629243708	0.000143873	0.001062643	0.565278889
T2DM_admission	leukocyte	*EMC9*	-1.054093639	0.003322745	0.004789092	1
T2DM_admission	leukocyte	*NFATC4*	-1.509473569	0.001459549	0.002380476	1
T2DM_admission	leukocyte	*PYGB*	-1.30694931	0.000298034	0.001093914	1
T2DM_admission	leukocyte	*ABHD12*	-2.35788063	6.99E-05	0.001062643	0.274451021
T2DM_admission	leukocyte	*PROCR*	-0.907175885	0.00243376	0.003641372	1

T2DM_admission	leukocyte	*CD40*	-1.289294913	0.000485099	0.001275739	1
T2DM_admission	leukocyte	*IFT52*	-1.14297458	0.000395819	0.001181845	1
T2DM_admission	leukocyte	*MYBL2*	-2.281668364	0.009990382	0.013010346	1
T2DM_admission	leukocyte	*PABPC1L*	-0.869347067	0.033532253	0.040240752	1
T2DM_admission	leukocyte	*SALL4*	1.211765069	0.038588369	0.046055195	1
T2DM_admission	leukocyte	*NELFCD*	-1.09467205	0.000397059	0.001182749	1
T2DM_admission	leukocyte	*MTG2*	-1.337912443	0.000359627	0.001142338	1
T2DM_admission	leukocyte	*DIDO1*	-1.241059267	0.00217387	0.003295191	1
T2DM_admission	leukocyte	*SLC17A9*	0.943544449	0.033294199	0.040005451	1
T2DM_admission	leukocyte	*CDC25B*	-1.951328269	0.002058493	0.003148236	1
T2DM_admission	leukocyte	*ISM1*	1.373536883	0.014620414	0.018590163	1
T2DM_admission	leukocyte	*ARFRP1*	-1.402327115	0.006222167	0.008435756	1
T2DM_admission	leukocyte	*SLC52A3*	-2.618931339	0.002193676	0.00332137	1
T2DM_admission	leukocyte	*RSPO4*	-12.04942398	0.041404899	0.049207456	1
T2DM_admission	leukocyte	*SIRPB1*	0.71231569	0.005535307	0.007588353	1
T2DM_admission	leukocyte	*PLCB4*	-1.026600087	0.000707769	0.001525411	1
T2DM_admission	leukocyte	*POFUT1*	-1.114498699	0.000608624	0.001409123	1
T2DM_admission	leukocyte	*NOP56*	-1.521869022	0.00069665	0.001511098	1
T2DM_admission	leukocyte	*MANBAL*	-1.034279431	0.000709863	0.001527411	1
T2DM_admission	leukocyte	*IDH3B*	-1.438304722	0.000297401	0.001093914	1
T2DM_admission	leukocyte	*MAPRE1*	-0.937752274	0.000603959	0.001404115	1
T2DM_admission	leukocyte	*OXT*	0.837392365	0.014238522	0.018133924	1
T2DM_admission	leukocyte	*TTI1*	-1.808246501	0.000346307	0.001140861	1
T2DM_admission	leukocyte	*CHMP4B*	-0.98951019	0.000285623	0.001088708	1
T2DM_admission	leukocyte	*DHX35*	-1.914773801	0.000730757	0.001550629	1
T2DM_admission	leukocyte	*DNTTIP1*	-2.820829787	4.31E-05	0.001037577	0.169206096
T2DM_admission	leukocyte	*MAP1LC3A*	-0.712624221	0.002131373	0.003238426	1
T2DM_admission	leukocyte	*APMAP*	-1.223424317	0.001061493	0.001956194	1
T2DM_admission	leukocyte	*VAPA*	-0.979221662	0.000215853	0.001062643	0.848085919
T2DM_admission	leukocyte	*MYOM1*	-2.612206171	0.00156202	0.00251112	1
T2DM_admission	leukocyte	*CEP192*	-0.831446828	0.005346475	0.007344861	1
T2DM_admission	leukocyte	*LIPG*	-1.20015367	0.00125479	0.002158524	1
T2DM_admission	leukocyte	*LAMA1*	-5.340278402	0.006090991	0.008286531	1
T2DM_admission	leukocyte	*POLI*	-1.651358981	0.004803903	0.006652991	1
T2DM_admission	leukocyte	*RIOK3*	-1.09416542	0.000195848	0.001062643	0.769486466
T2DM_admission	leukocyte	*NALCN*	-1.03532759	0.001296574	0.002195847	1
T2DM_admission	leukocyte	*FGF14*	-1.197684024	0.000314317	0.001109126	1
T2DM_admission	leukocyte	*FNDC3A*	-0.894998087	0.000439009	0.001222199	1
T2DM_admission	leukocyte	*KLF5*	-0.791693598	0.001215115	0.002128483	1
T2DM_admission	leukocyte	*ACP5*	0.061924463	0.008987999	0.011783065	1
T2DM_admission	leukocyte	*DNAJC3*	-1.046655313	0.000232125	0.001066588	0.912017395
T2DM_admission	leukocyte	*UGGT2*	-1.058260045	0.00065212	0.001450838	1
T2DM_admission	leukocyte	*ARHGEF7*	-1.605608118	0.000176958	0.001062643	0.695267655
T2DM_admission	leukocyte	*PARP4*	-3.188469956	0.000163881	0.001062643	0.643886519
T2DM_admission	leukocyte	*MEDAG*	-1.799120442	0.00092362	0.001797337	1
T2DM_admission	leukocyte	*CLN5*	-1.476995905	0.00019065	0.001062643	0.749063005
T2DM_admission	leukocyte	*OLFM4*	-0.393147603	0.017521939	0.02195271	1
T2DM_admission	leukocyte	*MGRN1*	-1.425700797	0.000577802	0.001376912	1
T2DM_admission	leukocyte	*LONP2*	-1.123009051	0.000426613	0.001212854	1
T2DM_admission	leukocyte	*CBLN1*	-3.024895264	0.006174189	0.008379409	1
T2DM_admission	leukocyte	*DHODH*	-3.469955385	0.000166172	0.001062643	0.652889507
T2DM_admission	leukocyte	*PARD6A*	-0.836941149	0.000779357	0.001614233	1
T2DM_admission	leukocyte	*MMP15*	-2.418418988	0.000634049	0.001434874	1
T2DM_admission	leukocyte	*USB1*	-1.643704448	0.000540153	0.001334756	1
T2DM_admission	leukocyte	*CYB5B*	-2.434150093	3.80E-05	0.001037577	0.149436232
T2DM_admission	leukocyte	*CCDC113*	-1.54115815	0.000686575	0.001497995	1
T2DM_admission	leukocyte	*NME3*	-1.316767506	0.000198574	0.001062643	0.780196044
T2DM_admission	leukocyte	*VAC14*	-1.259032412	0.000437779	0.001222199	1
T2DM_admission	leukocyte	*NPRL3*	-1.968325591	9.49E-05	0.001062643	0.372741685
T2DM_admission	leukocyte	*NAGPA*	-5.896241893	8.73E-05	0.001062643	0.343193199
T2DM_admission	leukocyte	*ZNF500*	-2.358690542	0.000479548	0.001266209	1
T2DM_admission	leukocyte	*NME4*	-1.267850349	0.000305274	0.001096936	1
T2DM_admission	leukocyte	*NOMO3*	-1.995678031	6.31E-05	0.001060479	0.247779015
T2DM_admission	leukocyte	*LMF1*	-1.159613207	0.000724868	0.001544472	1

T2DM_admission	leukocyte	*MTHFSD*	-0.959758877	0.01995769	0.024814483	1
T2DM_admission	leukocyte	*CLCN7*	-2.766540007	0.001723207	0.002719068	1
T2DM_admission	leukocyte	*HAGHL*	-1.97298675	0.000280954	0.001088708	1
T2DM_admission	leukocyte	*METRN*	-1.015175504	0.000127661	0.001062643	0.501579748
T2DM_admission	leukocyte	*PIEZO1*	-1.445911501	0.000391038	0.00117953	1
T2DM_admission	leukocyte	*CLUAP1*	-1.046809396	0.008228709	0.01090408	1
T2DM_admission	leukocyte	*UBFD1*	-1.625907927	0.000428784	0.00121662	1
T2DM_admission	leukocyte	*AQP8*	-1.774550609	0.001364758	0.002274982	1
T2DM_admission	leukocyte	*USP31*	-0.892823253	0.000572979	0.001370771	1
T2DM_admission	leukocyte	*HMOX2*	-1.06032839	0.000526471	0.001319879	1
T2DM_admission	leukocyte	*RBL2*	-1.154488278	0.0063758	0.008617309	1
T2DM_admission	leukocyte	*KAT8*	-1.440987693	0.00024045	0.001072513	0.944726903
T2DM_admission	leukocyte	*NOMO1*	-1.620730822	0.002796275	0.004105592	1
T2DM_admission	leukocyte	*RNF40*	-2.300768506	8.81E-05	0.001062643	0.346213035
T2DM_admission	leukocyte	*KNOP1*	-1.51384267	0.000262374	0.001083674	1
T2DM_admission	leukocyte	*TRIP4*	-1.21687138	0.000851858	0.001707628	1
T2DM_admission	leukocyte	*AP3B2*	-1.222841564	0.000998375	0.001877747	1
T2DM_admission	leukocyte	*IGDCC4*	2.574673866	0.008760215	0.011515184	1
T2DM_admission	leukocyte	*RPAP1*	-1.856272685	0.000291051	0.001088708	1
T2DM_admission	leukocyte	*HOMER2*	-0.900796859	0.000851414	0.001707609	1
T2DM_admission	leukocyte	*ATP8B4*	0.551011462	0.006761475	0.009101006	1
T2DM_admission	leukocyte	*DMXL2*	-2.633031458	0.023693945	0.029046337	1
T2DM_admission	leukocyte	*DNAJC17*	-1.048261199	0.00203992	0.003122261	1
T2DM_admission	leukocyte	*SLC30A4*	-2.770880664	0.004000361	0.005655782	1
T2DM_admission	leukocyte	*ZDHHC2*	-0.771740872	0.000839262	0.001694482	1
T2DM_admission	leukocyte	*TRIM35*	-2.959037003	0.000154468	0.001062643	0.606906527
T2DM_admission	leukocyte	*RIPK2*	-1.573891349	9.29E-05	0.001062643	0.365049188
T2DM_admission	leukocyte	*NBN*	-1.544028597	0.001060548	0.001955371	1
T2DM_admission	leukocyte	*LAPTM4B*	-0.724455534	0.000738798	0.001559773	1
T2DM_admission	leukocyte	*RAB2A*	-0.806009184	0.000682076	0.001492137	1
T2DM_admission	leukocyte	*EIF3E*	-0.973755785	0.000241309	0.001072513	0.948101929
T2DM_admission	leukocyte	*ESRP1*	-0.926016305	0.000893114	0.001764226	1
T2DM_admission	leukocyte	*ARMC1*	-1.314530498	0.000422562	0.001206582	1
T2DM_admission	leukocyte	*SPAG1*	-1.103753325	0.000446215	0.001232235	1
T2DM_admission	leukocyte	*CHRAC1*	-0.947212045	0.000574963	0.001373271	1
T2DM_admission	leukocyte	*SNX16*	-3.366527959	0.000384225	0.001166629	1
T2DM_admission	leukocyte	*GSDMD*	-0.653331091	0.017918344	0.022420756	1
T2DM_admission	leukocyte	*SH2D4A*	-1.28159625	0.00072053	0.001541079	1
T2DM_admission	leukocyte	*INTS10*	-1.626015584	0.000115889	0.001062643	0.455328819
T2DM_admission	leukocyte	*ERI1*	-3.371078226	1.96E-05	0.000917316	0.07705454
T2DM_admission	leukocyte	*LEPROTL1*	-1.417736888	0.000170966	0.001062643	0.67172406
T2DM_admission	leukocyte	*ERICH1*	-1.705504694	0.000191969	0.001062643	0.754246093
T2DM_admission	leukocyte	*ARHGEF10*	-1.171890118	0.001352833	0.002258938	1
T2DM_admission	leukocyte	*KLHDC4*	-1.458498642	0.000612989	0.001415201	1
T2DM_admission	leukocyte	*MCM4*	-1.468672506	0.000449543	0.001234067	1
T2DM_admission	leukocyte	*FGL1*	-1.649848127	0.006904477	0.009271254	1
T2DM_admission	leukocyte	*ECH1*	-1.053460013	0.000494612	0.001287826	1
T2DM_admission	leukocyte	*HNRNPL*	-1.204296839	0.000276899	0.001088708	1
T2DM_admission	leukocyte	*SARS2*	-1.419997357	0.000345346	0.001140861	1
T2DM_admission	leukocyte	*LIN7B*	-1.567434279	0.000282886	0.001088708	1
T2DM_admission	leukocyte	*PPP1R13L*	-1.114549015	0.00367753	0.005246556	1
T2DM_admission	leukocyte	*ERCC2*	-1.09157743	0.003145513	0.004560414	1
T2DM_admission	leukocyte	*KLC3*	-1.125919708	0.000546261	0.001346461	1
T2DM_admission	leukocyte	*AMH*	-1.301485756	0.00065171	0.001450838	1
T2DM_admission	leukocyte	*IL27RA*	-1.357585611	0.004371092	0.006122646	1
T2DM_admission	leukocyte	*PDCD5*	-0.86360704	0.000956865	0.001830343	1
T2DM_admission	leukocyte	*NUMBL*	-0.943425701	0.001070316	0.001964163	1
T2DM_admission	leukocyte	*SLC1A5*	-1.522350653	0.000455861	0.001240899	1
T2DM_admission	leukocyte	*PRKD2*	-1.359297768	0.000364428	0.001146348	1
T2DM_admission	leukocyte	*APLP1*	-0.59415927	0.00041862	0.001200552	1
T2DM_admission	leukocyte	*PLIN3*	-1.290478233	0.000189658	0.001062643	0.745165217
T2DM_admission	leukocyte	*MYH14*	-0.957621696	0.000556004	0.001357223	1
T2DM_admission	leukocyte	*CARD8*	-2.925615213	0.004102412	0.005783415	1
T2DM_admission	leukocyte	*ZNF175*	-1.056760303	0.000901455	0.001772681	1

T2DM_admission	leukocyte	*PLA2G4C*	-1.58282208	0.000351023	0.001141109	1
T2DM_admission	leukocyte	*CAPS*	-1.554546871	0.000500514	0.001295577	1
T2DM_admission	leukocyte	*PPP2R1A*	-0.973184658	0.000653669	0.001453462	1
T2DM_admission	leukocyte	*GCDH*	-1.54475702	8.96E-05	0.001062643	0.351988669
T2DM_admission	leukocyte	*PIK3R2*	-3.820458411	3.26E-05	0.001037577	0.128139788
T2DM_admission	leukocyte	*UPK1A*	-15.72725713	0.001971235	0.003037248	1
T2DM_admission	leukocyte	*COPE*	-1.049670009	0.000185313	0.001062643	0.728093799
T2DM_admission	leukocyte	*ETV2*	-1.248473228	0.005833348	0.007966363	1
T2DM_admission	leukocyte	*PBX4*	-4.463649335	0.001196954	0.002108893	1
T2DM_admission	leukocyte	*SIPA1L3*	-3.286399802	4.44E-05	0.001037577	0.174571559
T2DM_admission	leukocyte	*ETHE1*	-1.679514596	0.000866538	0.001732185	1
T2DM_admission	leukocyte	*SMG9*	-0.971557457	0.002193259	0.00332137	1
T2DM_admission	leukocyte	*RUNDC3B*	-6.699088217	4.84E-05	0.00105496	0.190174834
T2DM_admission	leukocyte	*PMPCB*	-1.411081767	0.00021778	0.001062643	0.855659014
T2DM_admission	leukocyte	*PON2*	-1.056741054	0.000254838	0.001077348	1
T2DM_admission	leukocyte	*ITGB8*	-1.042213449	0.000562603	0.001362351	1
T2DM_admission	leukocyte	*DNAH11*	-3.790504139	0.008803748	0.011560805	1
T2DM_admission	leukocyte	*CAV2*	-0.689281586	0.001113621	0.002017251	1
T2DM_admission	leukocyte	*CAV1*	-0.806446371	0.001231963	0.00214366	1
T2DM_admission	leukocyte	*ANKRD7*	-1.875145231	0.002081125	0.003172969	1
T2DM_admission	leukocyte	*TSPAN12*	-1.827679302	0.000238563	0.00107244	0.937312655
T2DM_admission	leukocyte	*CPVL*	0.131665233	0.008618747	0.011359631	1
T2DM_admission	leukocyte	*ABHD11*	-1.8276312	6.08E-05	0.001060479	0.238842064
T2DM_admission	leukocyte	*CRHR2*	-3.456432059	0.003804893	0.005411731	1
T2DM_admission	leukocyte	*CHCHD2*	-0.81341599	0.00025515	0.001077348	1
T2DM_admission	leukocyte	*EIF3B*	-1.202454527	0.000253204	0.001077213	0.994839733
T2DM_admission	leukocyte	*WASL*	-0.935938887	0.00068666	0.001497995	1
T2DM_admission	leukocyte	*AGFG2*	-1.096744403	0.000812031	0.001657387	1
T2DM_admission	leukocyte	*C1GALT1*	-1.003819095	0.000437305	0.001222199	1
T2DM_admission	leukocyte	*RPA3*	-1.276246841	0.000286456	0.001088708	1
T2DM_admission	leukocyte	*ZNHIT1*	-0.990100847	0.000498166	0.001293956	1
T2DM_admission	leukocyte	*CLDN15*	-0.857101239	0.002969479	0.004333983	1
T2DM_admission	leukocyte	*TMEM106B*	-0.996024385	0.000641223	0.001440839	1
T2DM_admission	leukocyte	*ACTR3C*	-1.853840418	0.000676556	0.00148591	1
T2DM_admission	leukocyte	*TSPAN13*	-0.766543767	0.000564771	0.001362351	1
T2DM_admission	leukocyte	*RARRES2*	-0.527936143	0.001803572	0.00281983	1
T2DM_admission	leukocyte	*AGR2*	-0.712859684	0.004924027	0.00680496	1
T2DM_admission	leukocyte	*TMEM176B*	-0.742186462	0.010932204	0.014143112	1
T2DM_admission	leukocyte	*COA1*	-1.526909865	0.00021953	0.001062643	0.862535208
T2DM_admission	leukocyte	*BLVRA*	-2.108009903	0.000131955	0.001062643	0.518449763
T2DM_admission	leukocyte	*TMEM248*	-1.501290407	0.000101505	0.001062643	0.398814075
T2DM_admission	leukocyte	*RHEB*	-1.03490321	0.000192701	0.001062643	0.757122312
T2DM_admission	leukocyte	*TBL2*	-0.809507233	0.014516761	0.018464342	1
T2DM_admission	leukocyte	*LIMK1*	-3.985246995	0.000584548	0.001382042	1
T2DM_admission	leukocyte	*SPIN1*	-1.825524546	0.000122025	0.001062643	0.479436847
T2DM_admission	leukocyte	*NMRK1*	-1.214806215	0.000608486	0.001409123	1
T2DM_admission	leukocyte	*C5*	-1.201027839	0.010059974	0.013087959	1
T2DM_admission	leukocyte	*OGN*	-2.335332716	0.001920639	0.002974454	1
T2DM_admission	leukocyte	*SUSD1*	-2.123714267	8.62E-05	0.001062643	0.338814241
T2DM_admission	leukocyte	*DNM1*	-0.807056303	0.000934548	0.001804129	1
T2DM_admission	leukocyte	*AK1*	-0.877048263	0.000631927	0.001433512	1
T2DM_admission	leukocyte	*PLGRKT*	-0.977462912	0.000776896	0.001613332	1
T2DM_admission	leukocyte	*DOCK8*	0.426827069	0.016068788	0.020274331	1
T2DM_admission	leukocyte	*MPDZ*	-0.888843813	0.001831433	0.00285657	1
T2DM_admission	leukocyte	*RAPGEF1*	-1.49145861	0.013569589	0.017338184	1
T2DM_admission	leukocyte	*NPDC1*	-0.834890394	0.000324074	0.001116918	1
T2DM_admission	leukocyte	*HPS1*	-1.413786132	0.000244657	0.001077213	0.961255446
T2DM_admission	leukocyte	*CXCL12*	-1.192971016	0.000302005	0.001093914	1
T2DM_admission	leukocyte	*TRDMT1*	-2.922381013	5.25E-05	0.00105496	0.206083247
T2DM_admission	leukocyte	*ATE1*	-2.051988549	7.33E-05	0.001062643	0.288052387
T2DM_admission	leukocyte	*NSMCE4A*	-0.898672379	0.003805705	0.005411731	1
T2DM_admission	leukocyte	*PLEKHA1*	-0.889997402	0.000470822	0.001255055	1
T2DM_admission	leukocyte	*PALD1*	-8.137818544	3.93E-05	0.001037577	0.154462492
T2DM_admission	leukocyte	*SPOCK2*	-1.672349709	0.001290117	0.002190523	1

T2DM_admission	leukocyte	*BMPR1A*	-2.698333303	8.46E-05	0.001062643	0.332292679
T2DM_admission	leukocyte	*LIPA*	0.30116464	0.016928086	0.021303795	1
T2DM_admission	leukocyte	*SFXN3*	-1.819124525	0.000251597	0.001077213	0.98852501
T2DM_admission	leukocyte	*NPM3*	-1.270694166	0.000698541	0.001512992	1
T2DM_admission	leukocyte	*TNKS2*	-1.321669321	0.000199078	0.001062643	0.782177451
T2DM_admission	leukocyte	*ACBD5*	-0.978211275	0.000851226	0.001707609	1
T2DM_admission	leukocyte	*LHPP*	-1.922248683	0.000988441	0.001868906	1
T2DM_admission	leukocyte	*GTPBP4*	-0.984373897	0.00037057	0.001151894	1
T2DM_admission	leukocyte	*NEURL1*	-1.108037639	0.004282771	0.0060075	1
T2DM_admission	leukocyte	*XPNPEP1*	-1.096453993	0.00055339	0.001355918	1
T2DM_admission	leukocyte	*SMC3*	-0.984157457	0.000257346	0.001080629	1
T2DM_admission	leukocyte	*RPL28*	-0.797400352	0.000145806	0.001062643	0.572871619
T2DM_admission	leukocyte	*PBLD*	-0.803391323	0.006058011	0.008250235	1
T2DM_admission	leukocyte	*KRT23*	-0.569910104	0.008375175	0.011075753	1
T2DM_admission	leukocyte	*DHRS11*	-0.853626315	0.004444254	0.006207421	1
T2DM_admission	leukocyte	*CWC25*	-1.415395942	0.000262517	0.001083674	1
T2DM_admission	leukocyte	*RAD51C*	-1.32809728	0.000183525	0.001062643	0.72107126
T2DM_admission	leukocyte	*TRIM37*	-1.108096917	0.000478606	0.001265283	1
T2DM_admission	leukocyte	*RPS6KB1*	-1.19720188	0.001392879	0.002307176	1
T2DM_admission	leukocyte	*TRIM16L*	-1.573724749	0.003035961	0.0044228	1
T2DM_admission	leukocyte	*CDK5RAP3*	-1.795663648	0.000491798	0.001283904	1
T2DM_admission	leukocyte	*CAMTA2*	-1.628769943	0.001555533	0.002503881	1
T2DM_admission	leukocyte	*MED13*	-1.086276565	0.000566479	0.001364563	1
T2DM_admission	leukocyte	*CHRNE*	-1.750186516	0.011324599	0.014593096	1
T2DM_admission	leukocyte	*RAI1*	-0.872431975	0.001431013	0.00235249	1
T2DM_admission	leukocyte	*CCDC47*	-1.107212451	0.000199691	0.001062643	0.784584961
T2DM_admission	leukocyte	*DRG2*	-1.149054863	0.001000561	0.001880059	1
T2DM_admission	leukocyte	*FTSJ3*	-1.161542502	0.000253103	0.001077213	0.994440677
T2DM_admission	leukocyte	*SMARCD2*	-2.836191016	3.24E-05	0.001037577	0.127213381
T2DM_admission	leukocyte	*SYNGR2*	-1.02183286	0.000453642	0.001238727	1
T2DM_admission	leukocyte	*C17orf75*	-1.331117014	0.003736924	0.00532172	1
T2DM_admission	leukocyte	*DHX58*	-2.207822346	0.000803878	0.001649159	1
T2DM_admission	leukocyte	*NAGLU*	-1.120854008	0.001421682	0.002343313	1
T2DM_admission	leukocyte	*HSD17B1*	-1.845849378	1.76E-06	0.000300491	0.00691129
T2DM_admission	leukocyte	*RND2*	-3.009834275	0.000186129	0.001062643	0.731299531
T2DM_admission	leukocyte	*ABCC3*	-0.815159427	0.003041682	0.004429492	1
T2DM_admission	leukocyte	*MPP2*	-0.782034747	0.001433911	0.002354561	1
T2DM_admission	leukocyte	*HLF*	-0.696133411	0.001555344	0.002503881	1
T2DM_admission	leukocyte	*EFNB3*	-1.009223166	0.001185522	0.00210081	1
T2DM_admission	leukocyte	*RANGRF*	-1.533524262	0.000182444	0.001062643	0.716823804
T2DM_admission	leukocyte	*DPH1*	-2.208438844	0.00326706	0.004714021	1
T2DM_admission	leukocyte	*OCIAD1*	-1.008144288	0.000255558	0.001077348	1
T2DM_admission	leukocyte	*UBE2D3*	-0.926988064	0.00057468	0.001373271	1
T2DM_admission	leukocyte	*MAPK10*	-0.939288478	0.000631212	0.001433512	1
T2DM_admission	leukocyte	*ANXA10*	-0.926085907	0.021018377	0.025977101	1
T2DM_admission	leukocyte	*AADAT*	-3.454208563	0.000306971	0.001098442	1
T2DM_admission	leukocyte	*TRIM2*	-1.089591655	0.000461669	0.001244702	1
T2DM_admission	leukocyte	*SH3D19*	-2.353662999	0.000248664	0.001077213	0.977000516
T2DM_admission	leukocyte	*GLRB*	-1.101863651	0.000728689	0.001549254	1
T2DM_admission	leukocyte	*LRP2BP*	-1.209507447	0.001193305	0.002106243	1
T2DM_admission	leukocyte	*UFSP2*	-1.331268495	0.001465516	0.002388226	1
T2DM_admission	leukocyte	*KLHL5*	-1.462580774	0.000262575	0.001083674	1
T2DM_admission	leukocyte	*FAM149A*	-0.851413333	0.008755737	0.011515184	1
T2DM_admission	leukocyte	*DDX25*	-0.873614965	0.011762582	0.01512772	1
T2DM_admission	leukocyte	*HTATIP2*	-1.753897664	4.34E-05	0.001037577	0.1706127
T2DM_admission	leukocyte	*B3GAT1*	-2.759799707	0.000989068	0.001869192	1
T2DM_admission	leukocyte	*P2RX3*	-21.25632707	0.001203284	0.002114239	1
T2DM_admission	leukocyte	*VWA5A*	-1.314392513	0.000618802	0.001422062	1
T2DM_admission	leukocyte	*DTX4*	-1.083602779	0.004524	0.00630499	1
T2DM_admission	leukocyte	*FOXRED1*	-1.841023219	0.001270485	0.002175059	1
T2DM_admission	leukocyte	*ST3GAL4*	-2.236603608	5.48E-05	0.001060479	0.215504129
T2DM_admission	leukocyte	*PRPF19*	-1.039669737	0.000438312	0.001222199	1
T2DM_admission	leukocyte	*CEP164*	-2.143917598	0.000527078	0.001319879	1
T2DM_admission	leukocyte	*PDHX*	-0.957208602	0.020007243	0.02486036	1

T2DM_admission	leukocyte	*SLC1A2*	-1.136139369	0.001565276	0.002514761	1
T2DM_admission	leukocyte	*CD5*	-5.423433126	0.000435505	0.001222199	1
T2DM_admission	leukocyte	*ACCS*	0.556438337	0.004132877	0.005822185	1
T2DM_admission	leukocyte	*MDK*	-1.17991938	0.000200738	0.001062643	0.788699933
T2DM_admission	leukocyte	*MADD*	-1.706643986	8.87E-05	0.001062643	0.348533371
T2DM_admission	leukocyte	*NAA40*	-1.418332584	0.000525284	0.001319879	1
T2DM_admission	leukocyte	*CD81*	-0.72891622	0.001068616	0.001964163	1
T2DM_admission	leukocyte	*SLC35F2*	-1.228594976	0.000579868	0.001379559	1
T2DM_admission	leukocyte	*NUP98*	-1.224864554	0.000350744	0.001141109	1
T2DM_admission	leukocyte	*GTF2H1*	-2.189463155	7.03E-05	0.001062643	0.276313656
T2DM_admission	leukocyte	*PTPN5*	2.033609424	0.006441369	0.008699944	1
T2DM_admission	leukocyte	*PRDM4*	-0.9115608	0.000797995	0.001641531	1
T2DM_admission	leukocyte	*CORO1C*	-1.341059905	0.000484774	0.001275738	1
T2DM_admission	leukocyte	*LIN7A*	-2.479228421	0.001194473	0.002107357	1
T2DM_admission	leukocyte	*KRT18*	-0.388379926	0.00139799	0.002313691	1
T2DM_admission	leukocyte	*METAP2*	-1.122039029	0.000249408	0.001077213	0.979925881
T2DM_admission	leukocyte	*SLC6A12*	-4.579694234	0.026754862	0.032464438	1
T2DM_admission	leukocyte	*WNT5B*	-2.568916303	0.001127338	0.00203366	1
T2DM_admission	leukocyte	*ITFG2*	-5.686198407	6.68E-05	0.001062643	0.262405946
T2DM_admission	leukocyte	*VPS29*	-1.055610373	0.000234505	0.001066706	0.921370438
T2DM_admission	leukocyte	*CREBL2*	-1.4793063	0.000297675	0.001093914	1
T2DM_admission	leukocyte	*GSG1*	-8.727928119	0.000358182	0.001141109	1
T2DM_admission	leukocyte	*LTBR*	-1.364026906	0.000194089	0.001062643	0.762576217
T2DM_admission	leukocyte	*CDK2AP1*	-0.685510709	0.030652637	0.037011128	1
T2DM_admission	leukocyte	*DDX55*	-1.998985225	5.73E-05	0.001060479	0.225084192
T2DM_admission	leukocyte	*STX2*	-1.348644987	0.002495066	0.003720348	1
T2DM_admission	leukocyte	*CAND1*	-1.386031936	0.000149291	0.001062643	0.586563491
T2DM_admission	leukocyte	*NUP107*	-1.523672139	0.000392298	0.001181536	1
T2DM_admission	leukocyte	*KRR1*	-1.234198794	0.000128703	0.001062643	0.505673346
T2DM_admission	leukocyte	*GNB3*	-1.319062775	0.001522192	0.002466266	1
T2DM_admission	leukocyte	*CDCA3*	-2.232253323	0.000845441	0.001700095	1
T2DM_admission	leukocyte	*CHPT1*	-2.225870489	1.14E-05	0.000784902	0.044873671
T2DM_admission	leukocyte	*SPSB2*	-1.135154579	0.00102818	0.001917284	1
T2DM_admission	leukocyte	*ATN1*	-1.264555989	0.000263792	0.00108414	1
T2DM_admission	leukocyte	*NT5DC3*	-1.235341015	0.003059814	0.00444981	1
T2DM_admission	leukocyte	*GOLT1B*	-1.127154927	0.000310304	0.00110427	1
T2DM_admission	leukocyte	*PRKAB1*	-1.030842256	0.000918065	0.001791892	1
T2DM_admission	leukocyte	*C2CD5*	-1.054965623	0.000961376	0.001831992	1
T2DM_admission	leukocyte	*SRSF9*	-1.086180388	9.88E-05	0.001062643	0.388293605
T2DM_admission	leukocyte	*FRK*	-1.184814984	0.000692418	0.001507191	1
T2DM_admission	leukocyte	*RSPH4A*	-0.91971161	0.01302109	0.016659024	1
T2DM_admission	leukocyte	*SMIM8*	-1.368804222	0.000458355	0.001240899	1
T2DM_admission	leukocyte	*NEDD9*	-0.639877215	0.000776458	0.001613276	1
T2DM_admission	leukocyte	*CEP85L*	-1.206627771	0.000624682	0.001426963	1
T2DM_admission	leukocyte	*HDDC2*	-0.930487381	0.000468563	0.001253701	1
T2DM_admission	leukocyte	*TPD52L1*	-0.900408494	0.001905002	0.002952841	1
T2DM_admission	leukocyte	*MTRF1L*	-1.168648121	0.002314908	0.003484779	1
T2DM_admission	leukocyte	*MRPL18*	-1.610416114	0.000303116	0.001093914	1
T2DM_admission	leukocyte	*RNF8*	-1.179939007	0.00040218	0.001185421	1
T2DM_admission	leukocyte	*MDGA1*	1.39677681	0.020717429	0.025637411	1
T2DM_admission	leukocyte	*CD83*	0.173119755	0.020073179	0.024918648	1
T2DM_admission	leukocyte	*GLP1R*	-1.077409694	0.001363266	0.002274426	1
T2DM_admission	leukocyte	*BACH2*	-0.990991706	0.000971405	0.001848256	1
T2DM_admission	leukocyte	*FBXL4*	-1.390857876	0.000444629	0.001230246	1
T2DM_admission	leukocyte	*ASCC3*	-0.925342372	0.000821357	0.001670341	1
T2DM_admission	leukocyte	*WASF1*	-0.975816069	0.001350682	0.002257051	1
T2DM_admission	leukocyte	*ALDH5A1*	-2.619828145	0.006224295	0.008435756	1
T2DM_admission	leukocyte	*C6orf62*	-1.390647439	0.000162831	0.001062643	0.639761964
T2DM_admission	leukocyte	*B3GAT2*	-1.697068733	0.000563629	0.001362351	1
T2DM_admission	leukocyte	*HBS1L*	-1.204495514	0.000300659	0.001093914	1
T2DM_admission	leukocyte	*PEX7*	-1.060999137	0.000730915	0.001550629	1
T2DM_admission	leukocyte	*PHACTR2*	-0.906573833	0.000409147	0.001188129	1
T2DM_admission	leukocyte	*EPM2A*	-1.940422238	0.000318428	0.001113024	1
T2DM_admission	leukocyte	*TFEB*	-1.328619512	0.000982625	0.00186239	1

T2DM_admission	leukocyte	*PRPH2*	-1.56144284	0.000635697	0.001436259	1
T2DM_admission	leukocyte	*DNPH1*	-1.034982184	0.002792209	0.004101156	1
T2DM_admission	leukocyte	*DUSP22*	-1.466392773	0.0004418	0.001226736	1
T2DM_admission	leukocyte	*LAMA4*	-0.880880697	0.001154702	0.00206595	1
T2DM_admission	leukocyte	*FBRSL1*	-1.410995583	0.000359927	0.001142338	1
T2DM_admission	leukocyte	*ENPP5*	-0.954601414	0.0012606	0.002164729	1
T2DM_admission	leukocyte	*NUDT12*	-1.521118844	0.000378301	0.001163156	1
T2DM_admission	leukocyte	*CEP72*	-3.421569193	0.001280852	0.002180445	1
T2DM_admission	leukocyte	*SEMA5A*	-1.314309336	0.001125465	0.002031214	1
T2DM_admission	leukocyte	*GHR*	-2.925649706	0.001202829	0.002114239	1
T2DM_admission	leukocyte	*DAP*	-0.943840258	0.00042309	0.001206582	1
T2DM_admission	leukocyte	*NME5*	-1.179630449	0.023630528	0.028977635	1
T2DM_admission	leukocyte	*BRD8*	-1.363815679	0.000423565	0.001206806	1
T2DM_admission	leukocyte	*NNT*	-2.271988526	2.47E-06	0.000373794	0.009718639
T2DM_admission	leukocyte	*TMCO6*	1.83285184	0.009458668	0.01235065	1
T2DM_admission	leukocyte	*IK*	-1.185951697	0.000206734	0.001062643	0.812258243
T2DM_admission	leukocyte	*PDE8B*	-0.744700075	0.001074713	0.001968161	1
T2DM_admission	leukocyte	*MSH3*	-1.383617976	0.000120158	0.001062643	0.472101847
T2DM_admission	leukocyte	*CCNG1*	-2.302766986	6.37E-05	0.001060479	0.250468893
T2DM_admission	leukocyte	*DROSHA*	-0.838087028	0.004055512	0.005721403	1
T2DM_admission	leukocyte	*GOLPH3*	-1.193698496	0.000234518	0.001066706	0.921422929
T2DM_admission	leukocyte	*NPR3*	-3.142788842	0.001998215	0.003071591	1
T2DM_admission	leukocyte	*RAD50*	-1.522959498	0.000164842	0.001062643	0.647665223
T2DM_admission	leukocyte	*WWC1*	-0.625159124	0.001671364	0.002648967	1
T2DM_admission	leukocyte	*SMAD5*	-1.033339142	0.000530532	0.001324223	1
T2DM_admission	leukocyte	*HMGXB3*	-1.424103961	0.000519466	0.001315067	1
T2DM_admission	leukocyte	*BNIP1*	-0.857197102	0.006748224	0.009086282	1
T2DM_admission	leukocyte	*STC2*	-0.830190958	0.001423975	0.002344844	1
T2DM_admission	leukocyte	*EHHADH*	-1.651866226	0.00029057	0.001088708	1
T2DM_admission	leukocyte	*ACTR8*	-1.097741826	0.000769202	0.001602435	1
T2DM_admission	leukocyte	*TIMMDC1*	-1.281399182	0.000468045	0.001253701	1
T2DM_admission	leukocyte	*CLDN16*	4.106104108	0.023069581	0.028333975	1
T2DM_admission	leukocyte	*NPHP3*	-0.844759627	0.040822402	0.048559255	1
T2DM_admission	leukocyte	*FAM162A*	-1.493198207	5.66E-05	0.001060479	0.222327818
T2DM_admission	leukocyte	*RBP1*	-1.03950343	0.000197142	0.001062643	0.774570943
T2DM_admission	leukocyte	*RNF7*	-1.042730367	0.000547191	0.001347063	1
T2DM_admission	leukocyte	*SERPINI2*	-0.765591945	0.000746897	0.001568868	1
T2DM_admission	leukocyte	*TUSC2*	-1.530932316	0.000197183	0.001062643	0.774732316
T2DM_admission	leukocyte	*CYB561D2*	-1.017823722	0.000737421	0.00155805	1
T2DM_admission	leukocyte	*C3orf14*	-1.012056348	0.000240007	0.001072513	0.942988506
T2DM_admission	leukocyte	*CBLB*	-1.961390988	0.000158653	0.001062643	0.623347203
T2DM_admission	leukocyte	*IFT57*	-1.017415778	0.000556488	0.001357223	1
T2DM_admission	leukocyte	*NCBP2*	-1.085952326	0.000264542	0.001084251	1
T2DM_admission	leukocyte	*C3orf52*	-0.90977287	0.001908005	0.002956054	1
T2DM_admission	leukocyte	*SLC41A3*	-1.168895449	0.000256946	0.001080629	1
T2DM_admission	leukocyte	*ATP6V1A*	-1.386836786	0.000158859	0.001062643	0.624156114
T2DM_admission	leukocyte	*PODXL2*	-0.873330576	0.000638522	0.001438504	1
T2DM_admission	leukocyte	*UPK1B*	-1.183457972	0.024495468	0.029954153	1
T2DM_admission	leukocyte	*NEK11*	-0.970254537	0.000517163	0.00131413	1
T2DM_admission	leukocyte	*PLSCR4*	-1.927935405	0.000273235	0.001088708	1
T2DM_admission	leukocyte	*COMMD2*	-1.115146212	0.000242952	0.001077213	0.954558961
T2DM_admission	leukocyte	*GORASP1*	-2.742669994	0.000181322	0.001062643	0.712415273
T2DM_admission	leukocyte	*AADAC*	-1.09262166	0.000155531	0.001062643	0.611081331
T2DM_admission	leukocyte	*ARHGEF26*	-3.109205832	0.000129445	0.001062643	0.508587559
T2DM_admission	leukocyte	*PLCH1*	-5.389787701	9.43E-05	0.001062643	0.370467839
T2DM_admission	leukocyte	*VIPR1*	-1.543022735	0.005002716	0.006908847	1
T2DM_admission	leukocyte	*NKTR*	-1.088344718	0.000442273	0.001227183	1
T2DM_admission	leukocyte	*NEK4*	-3.426629821	6.61E-05	0.001062643	0.259713669
T2DM_admission	leukocyte	*KANSL3*	-2.5452388	8.90E-05	0.001062643	0.349768662
T2DM_admission	leukocyte	*ACTR1B*	-2.019163103	0.000151884	0.001062643	0.596752628
T2DM_admission	leukocyte	*SLC35F5*	-1.037569495	0.001181139	0.002099858	1
T2DM_admission	leukocyte	*ACTR3*	-1.310398739	0.00015439	0.001062643	0.606599226
T2DM_admission	leukocyte	*TFCP2L1*	-2.435909075	0.031515647	0.037936573	1
T2DM_admission	leukocyte	*POMC*	-1.97770063	0.000129124	0.001062643	0.507329548

T2DM_admission	leukocyte	*TANC1*	-0.76089593	0.009327253	0.012187155	1
T2DM_admission	leukocyte	*REEP6*	-1.361806457	0.001065564	0.001960938	1
T2DM_admission	leukocyte	*INO80B*	-3.809404751	0.000267845	0.001087356	1
T2DM_admission	leukocyte	*MOGS*	-2.048948365	9.70E-05	0.001062643	0.38114229
T2DM_admission	leukocyte	*RTN4*	-0.983263889	0.000151224	0.001062643	0.594159347
T2DM_admission	leukocyte	*HTRA2*	-1.975023645	0.000129862	0.001062643	0.510226926
T2DM_admission	leukocyte	*GALNT3*	-1.266145887	0.00049985	0.001295455	1
T2DM_admission	leukocyte	*MRPL19*	-1.125899227	0.001027874	0.001917284	1
T2DM_admission	leukocyte	*WDR75*	-2.098193289	0.00018441	0.001062643	0.724547516
T2DM_admission	leukocyte	*FANCL*	-2.44703203	0.000300761	0.001093914	1
T2DM_admission	leukocyte	*GGCX*	-1.434332777	0.000453685	0.001238727	1
T2DM_admission	leukocyte	*SF3B1*	-1.336799014	0.00012217	0.001062643	0.480005816
T2DM_admission	leukocyte	*CHMP3*	-1.149163078	4.43E-05	0.001037577	0.173868056
T2DM_admission	leukocyte	*IL1R2*	-1.085212774	0.001694722	0.002682741	1
T2DM_admission	leukocyte	*IL1R1*	-1.084701613	0.000391175	0.00117953	1
T2DM_admission	leukocyte	*IL1RL2*	0.92176812	0.010993524	0.014213082	1
T2DM_admission	leukocyte	*IL18R1*	0.872311251	0.009533837	0.012440533	1
T2DM_admission	leukocyte	*MLPH*	-1.243510844	0.017582558	0.022014617	1
T2DM_admission	leukocyte	*CNPPD1*	-0.958542526	0.000673995	0.001482714	1
T2DM_admission	leukocyte	*HDLBP*	-0.862707992	0.000378779	0.001163156	1
T2DM_admission	leukocyte	*PROC*	1.307339246	0.006163637	0.008367978	1
T2DM_admission	leukocyte	*TAF1B*	-0.969811036	0.002185024	0.003310821	1
T2DM_admission	leukocyte	*STRN*	-1.983060023	0.00048696	0.001278066	1
T2DM_admission	leukocyte	*CEBPZ*	-1.104004592	0.000332277	0.001127387	1
T2DM_admission	leukocyte	*WIPF1*	0.53536103	0.028660333	0.034701525	1
T2DM_admission	leukocyte	*COX7A2L*	-0.961781685	0.000227689	0.001062643	0.89458878
T2DM_admission	leukocyte	*PNO1*	-1.242649185	0.000241185	0.001072513	0.947615674
T2DM_admission	leukocyte	*ORC4*	-1.259132696	0.000216464	0.001062643	0.850487015
T2DM_admission	leukocyte	*SUMO1*	-1.091255855	0.000210525	0.001062643	0.827152733
T2DM_admission	leukocyte	*GRIN3B*	1.655324108	0.018698396	0.023359618	1
T2DM_admission	leukocyte	*ALMS1*	-1.283039516	0.00161951	0.002582409	1
T2DM_admission	leukocyte	*MARK1*	-2.016444989	0.000120367	0.001062643	0.47292011
T2DM_admission	leukocyte	*MORN1*	-1.883199782	0.002562203	0.003798828	1
T2DM_admission	leukocyte	*GPX7*	-1.296257384	2.76E-05	0.000996218	0.108587745
T2DM_admission	leukocyte	*SCP2*	-1.166496651	0.000160307	0.001062643	0.629848064
T2DM_admission	leukocyte	*RALGPS2*	-1.084024377	0.000596612	0.001396953	1
T2DM_admission	leukocyte	*ANGPTL1*	-2.106936959	0.000335995	0.001131077	1
T2DM_admission	leukocyte	*FAM20B*	-1.356834822	0.00026212	0.001083674	1
T2DM_admission	leukocyte	*WRAP73*	-4.665931829	2.49E-05	0.000982543	0.097998175
T2DM_admission	leukocyte	*ICMT*	-1.702680899	5.09E-05	0.00105496	0.199877783
T2DM_admission	leukocyte	*PARK7*	-0.950643089	0.000218102	0.001062643	0.856921744
T2DM_admission	leukocyte	*MECR*	-3.405492484	9.08E-05	0.001062643	0.356741127
T2DM_admission	leukocyte	*EDEM3*	-1.352192302	0.000209662	0.001062643	0.823761382
T2DM_admission	leukocyte	*FBXO6*	-5.74109985	6.03E-05	0.001060479	0.236754076
T2DM_admission	leukocyte	*SMG7*	-1.162603518	0.000352333	0.001141109	1
T2DM_admission	leukocyte	*PDC*	-11.22957046	0.001247028	0.002154446	1
T2DM_admission	leukocyte	*BCAS2*	-1.036150788	0.000348745	0.001141096	1
T2DM_admission	leukocyte	*AGMAT*	-1.658953477	0.001188428	0.00210081	1
T2DM_admission	leukocyte	*TNNI3K*	-2.377200231	0.01012663	0.013165959	1
T2DM_admission	leukocyte	*PLEKHM2*	-2.982399248	9.07E-05	0.001062643	0.356169975
T2DM_admission	leukocyte	*CRYZ*	-0.7737903	0.001046493	0.001939468	1
T2DM_admission	leukocyte	*TMEM9*	-1.155482281	0.000199933	0.001062643	0.785538707
T2DM_admission	leukocyte	*WARS2*	-1.116215	0.001086838	0.001980605	1
T2DM_admission	leukocyte	*C1orf109*	-0.886041885	0.000768979	0.001602435	1
T2DM_admission	leukocyte	*LGALS8*	-1.510303482	0.000159678	0.001062643	0.627376664
T2DM_admission	leukocyte	*HPCAL4*	-2.596703333	0.004028017	0.005688742	1
T2DM_admission	leukocyte	*BMP8B*	-0.978297392	0.001330345	0.002233728	1
T2DM_admission	leukocyte	*MYCL*	0.516033906	0.006093763	0.008287434	1
T2DM_admission	leukocyte	*UAP1*	-0.820570049	0.000683514	0.001493619	1
T2DM_admission	leukocyte	*CTBS*	-1.444627192	0.000137729	0.001062643	0.541138292
T2DM_admission	leukocyte	*ZNHIT6*	-0.891632421	0.000341923	0.001140092	1
T2DM_admission	leukocyte	*CD46*	-0.93195634	0.0002745	0.001088708	1
T2DM_admission	leukocyte	*SLC2A1*	-1.607361961	0.000453487	0.001238727	1
T2DM_admission	leukocyte	*PRDX1*	-0.998646996	0.000233558	0.001066706	0.917647447

T2DM_admission	leukocyte	*CCDC181*	-0.916765635	0.000499453	0.001295455	1
T2DM_admission	leukocyte	*NSUN4*	-4.862028991	1.70E-06	0.000300491	0.006662293
T2DM_admission	leukocyte	*TMED5*	-1.670450046	0.000102911	0.001062643	0.404336322
T2DM_admission	leukocyte	*DR1*	-1.549263585	0.000253371	0.001077213	0.995495454
T2DM_admission	leukocyte	*PTBP2*	-1.179916941	0.000649459	0.001449765	1
T2DM_admission	leukocyte	*NSL1*	-1.863227227	0.000167962	0.001062643	0.659922099
T2DM_admission	leukocyte	*RPA2*	-1.629910305	0.000107356	0.001062643	0.421800419
T2DM_admission	leukocyte	*ESYT2*	-1.29616222	0.00033606	0.001131077	1
T2DM_admission	leukocyte	*DDX59*	-0.934764111	0.001228112	0.002139802	1
T2DM_admission	leukocyte	*TTR*	-0.450858609	0.000404717	0.001185781	1
T2DM_admission	leukocyte	*SPCS2*	-1.092172556	0.00015583	0.001062643	0.612256467
T2DM_admission	leukocyte	*ELOVL4*	-1.622213395	0.000607155	0.001408212	1
T2DM_admission	leukocyte	*FILIP1*	-1.166973644	0.001470828	0.002393904	1
T2DM_admission	leukocyte	*AKAP7*	-2.5829355	7.96E-05	0.001062643	0.312882749
T2DM_admission	leukocyte	*RNF146*	-1.285640034	0.000420414	0.001203063	1
T2DM_admission	leukocyte	*MED28*	-1.884402494	7.28E-05	0.001062643	0.286224319
T2DM_admission	leukocyte	*VAMP8*	-1.312717669	7.92E-05	0.001062643	0.31107182
T2DM_admission	leukocyte	*ARMC2*	-0.938316328	0.001956584	0.003020911	1
T2DM_admission	leukocyte	*PKD2*	-0.921506049	0.000704358	0.001521605	1
T2DM_admission	leukocyte	*SPP1*	-0.56576491	0.000192995	0.001062643	0.758277318
T2DM_admission	leukocyte	*WDR35*	-0.97837335	0.001373537	0.002283803	1
T2DM_admission	leukocyte	*DNAH7*	-1.503355227	9.84E-06	0.000784902	0.038675649
T2DM_admission	leukocyte	*NDUFB3*	-1.080453508	0.000323149	0.001116892	1
T2DM_admission	leukocyte	*GTF3C3*	-1.494006714	0.00209949	0.003196008	1
T2DM_admission	leukocyte	*CPSF3*	-1.790408362	0.000187795	0.001062643	0.737846422
T2DM_admission	leukocyte	*CCDC92*	-1.263133428	0.000235663	0.001067958	0.925919192
T2DM_admission	leukocyte	*CTNNAL1*	-0.859640056	0.00139445	0.002308804	1
T2DM_admission	leukocyte	*NEK6*	-1.050939467	0.007726739	0.010290968	1
T2DM_admission	leukocyte	*BSPRY*	-1.09604911	0.000123847	0.001062643	0.486594049
T2DM_admission	leukocyte	*RBM18*	-1.497903656	0.000313879	0.001109126	1
T2DM_admission	leukocyte	*INVS*	-0.744731292	0.018971373	0.023678058	1
T2DM_admission	leukocyte	*DCAF4*	-1.273805217	0.00238898	0.003585294	1
T2DM_admission	leukocyte	*IFI27L2*	-1.042227883	0.000227461	0.001062643	0.893694823
T2DM_admission	leukocyte	*ACOT2*	-1.61288263	0.002353129	0.003535543	1
T2DM_admission	leukocyte	*ZC2HC1C*	-3.54012098	0.000696898	0.001511098	1
T2DM_admission	leukocyte	*ESRRB*	-3.76291866	0.026716348	0.03242772	1
T2DM_admission	leukocyte	*KLHL29*	-2.484896429	0.033241611	0.039965205	1
T2DM_admission	leukocyte	*AFTPH*	-1.148303596	0.000400036	0.001184732	1
T2DM_admission	leukocyte	*BCL11A*	-1.673284279	0.002618754	0.003874551	1
T2DM_admission	leukocyte	*CRIPT*	-1.39060921	0.000245561	0.001077213	0.964810967
T2DM_admission	leukocyte	*EPCAM*	-0.733050003	0.000342586	0.001140694	1
T2DM_admission	leukocyte	*SLC17A5*	-4.17591212	2.42E-05	0.000982543	0.095239804
T2DM_admission	leukocyte	*CUTC*	-1.240813629	0.001677444	0.002656459	1
T2DM_admission	leukocyte	*PYROXD2*	-1.623882934	0.0021987	0.003327693	1
T2DM_admission	leukocyte	*HELLS*	-1.381811604	0.000459795	0.001240899	1
T2DM_admission	leukocyte	*PRLHR*	-1.497249043	0.001310342	0.002209586	1
T2DM_admission	leukocyte	*WDR11*	-2.058793728	6.20E-05	0.001060479	0.24344166
T2DM_admission	leukocyte	*KANSL1*	-1.324715674	0.000104018	0.001062643	0.4086878
T2DM_admission	leukocyte	*HOXB3*	-1.221591031	0.008526362	0.011249187	1
T2DM_admission	leukocyte	*RCL1*	-2.152997955	0.000110667	0.001062643	0.434809357
T2DM_admission	leukocyte	*CD274*	-3.553891602	0.000146261	0.001062643	0.574658537
T2DM_admission	leukocyte	*PLEKHG1*	-1.115465237	0.000899484	0.001770578	1
T2DM_admission	leukocyte	*PCDHB10*	-1.976149224	0.00095535	0.001829225	1
T2DM_admission	leukocyte	*MRPS14*	-1.96304973	0.000257499	0.001080629	1
T2DM_admission	leukocyte	*SEC16B*	-6.852557318	0.025832603	0.031452215	1
T2DM_admission	leukocyte	*GORAB*	-1.098719337	0.001182379	0.002099858	1
T2DM_admission	leukocyte	*SNX19*	-1.054797096	0.000468145	0.001253701	1
T2DM_admission	leukocyte	*KCNJ5*	0.338707598	0.004470448	0.006239571	1
T2DM_admission	leukocyte	*SLC10A7*	-2.335427234	0.000147859	0.001062643	0.580936275
T2DM_admission	leukocyte	*MASTL*	-1.299700743	8.58E-05	0.001062643	0.337122316
T2DM_admission	leukocyte	*MRC1*	0.274803754	0.015033255	0.019028885	1
T2DM_admission	leukocyte	*IQSEC3*	-1.222404717	0.000655694	0.001454635	1
T2DM_admission	leukocyte	*CCDC77*	-4.447821626	0.006999018	0.009388577	1
T2DM_admission	leukocyte	*DNAJC15*	-1.201637	0.000183474	0.001062643	0.720870934

T2DM_admission	leukocyte	*PROSER1*	-1.57769204	0.000227639	0.001062643	0.894394762
T2DM_admission	leukocyte	*WBP4*	-1.163383692	0.000316279	0.001111111	1
T2DM_admission	leukocyte	*KBTBD7*	-2.555518337	8.28E-05	0.001062643	0.325222243
T2DM_admission	leukocyte	*FAM53C*	-3.010839386	2.08E-06	0.000340493	0.008174348
T2DM_admission	leukocyte	*SIL1*	-1.193157982	0.000211462	0.001062643	0.830832515
T2DM_admission	leukocyte	*PLS1*	-0.903157964	0.001124	0.002030435	1
T2DM_admission	leukocyte	*ZFP30*	-1.472955291	0.000924972	0.001797337	1
T2DM_admission	leukocyte	*UTP20*	-1.774318539	0.008758008	0.011515184	1
T2DM_admission	leukocyte	*TMPO*	-1.714802428	0.000294877	0.001090935	1
T2DM_admission	leukocyte	*APAF1*	-2.720094716	0.014740348	0.018718431	1
T2DM_admission	leukocyte	*PPP3CC*	-1.428409112	0.00034999	0.001141109	1
T2DM_admission	leukocyte	*EPHX2*	-1.378388626	0.000347639	0.00114108	1
T2DM_admission	leukocyte	*CRISPLD1*	-6.32255153	0.000817669	0.001665435	1
T2DM_admission	leukocyte	*PYROXD1*	-0.988440337	0.000272141	0.001088708	1
T2DM_admission	leukocyte	*IAPP*	-0.354839306	0.00253012	0.003761196	1
T2DM_admission	leukocyte	*ZSCAN18*	-0.981449627	0.000515889	0.001313619	1
T2DM_admission	leukocyte	*LHX4*	-2.561106414	0.01000729	0.013028046	1
T2DM_admission	leukocyte	*TRMT1L*	-1.148181845	0.000990556	0.001869863	1
T2DM_admission	leukocyte	*CAT*	-2.82114761	3.02E-05	0.001023384	0.118543044
T2DM_admission	leukocyte	*CSMD2*	1.188666305	0.013797285	0.017611933	1
T2DM_admission	leukocyte	*ACVR2A*	-0.892246742	0.003753427	0.005343193	1
T2DM_admission	leukocyte	*POLK*	-1.990410665	6.06E-06	0.000591121	0.023790281
T2DM_admission	leukocyte	*UBL3*	-1.028154936	0.000264657	0.001084251	1
T2DM_admission	leukocyte	*FMOD*	-1.469660578	0.002706297	0.003991381	1
T2DM_admission	leukocyte	*LDB3*	-1.641819957	0.002299115	0.003463659	1
T2DM_admission	leukocyte	*NAA60*	-1.573025026	0.000152968	0.001062643	0.601010522
T2DM_admission	leukocyte	*TRMT13*	-2.867561606	7.72E-05	0.001062643	0.303455208
T2DM_admission	leukocyte	*RWDD3*	-1.180316695	0.000312871	0.001108487	1
T2DM_admission	leukocyte	*CCDC18*	-1.040243665	0.002723929	0.004011362	1
T2DM_admission	leukocyte	*NBPF14*	-2.905259199	0.001777146	0.002785164	1
T2DM_admission	leukocyte	*PMS2*	-2.279938028	0.000582285	0.001382042	1
T2DM_admission	leukocyte	*EEPD1*	0.509704653	0.018676422	0.023339587	1
T2DM_admission	leukocyte	*KLHL7*	-1.664823773	0.00028603	0.001088708	1
T2DM_admission	leukocyte	*CBX3*	-1.274925145	0.000190986	0.001062643	0.750384386
T2DM_admission	leukocyte	*NPY*	-0.548489929	0.000508545	0.001305383	1
T2DM_admission	leukocyte	*INHBA*	-1.03594135	0.000441793	0.001226736	1
T2DM_admission	leukocyte	*CCZ1*	-1.591348266	0.000733872	0.00155271	1
T2DM_admission	leukocyte	*POLM*	-1.130345551	0.000550809	0.001351735	1
T2DM_admission	leukocyte	*SMU1*	-1.32166197	0.000269967	0.001088175	1
T2DM_admission	leukocyte	*SLC25A51*	-1.074254969	0.008823461	0.01158282	1
T2DM_admission	leukocyte	*ACO1*	-1.059885845	0.000876769	0.001743468	1
T2DM_admission	leukocyte	*PLAU*	-1.352444354	0.005491125	0.007533042	1
T2DM_admission	leukocyte	*RBM19*	-0.968686036	0.001234676	0.002144697	1
T2DM_admission	leukocyte	*BHLHE41*	-1.916678644	0.000625991	0.001429122	1
T2DM_admission	leukocyte	*ITPR2*	-2.883491769	0.000821551	0.001670341	1
T2DM_admission	leukocyte	*EBPL*	-0.962901867	0.000876837	0.001743468	1
T2DM_admission	leukocyte	*ATP7B*	-1.199615242	0.040519269	0.048242487	1
T2DM_admission	leukocyte	*TSFM*	-1.52884364	0.00038064	0.001165285	1
T2DM_admission	leukocyte	*CDK2*	-1.898992123	0.004424872	0.006186948	1
T2DM_admission	leukocyte	*IKZF4*	-1.672939724	0.000651965	0.001450838	1
T2DM_admission	leukocyte	*USP45*	-0.887022722	0.001827387	0.002851392	1
T2DM_admission	leukocyte	*C4BPA*	-1.437022488	0.019826694	0.024684991	1
T2DM_admission	leukocyte	*C4BPB*	-2.601694522	0.004905193	0.006783704	1
T2DM_admission	leukocyte	*RAB38*	-2.639989793	0.009537417	0.012441073	1
T2DM_admission	leukocyte	*CKS2*	-1.036376727	0.000952488	0.001824634	1
T2DM_admission	leukocyte	*SNX21*	-1.197593145	0.000906081	0.001776443	1
T2DM_admission	leukocyte	*ARHGAP40*	-58.94706275	0.000759352	0.001590349	1
T2DM_admission	leukocyte	*NCOA3*	-1.199924368	0.000596353	0.001396953	1
T2DM_admission	leukocyte	*MATN4*	-7.947339205	0.000741472	0.001563191	1
T2DM_admission	leukocyte	*VAPB*	-1.090475316	0.00017374	0.001062643	0.682626023
T2DM_admission	leukocyte	*PARD6B*	-1.493059541	0.000367778	0.001148802	1
T2DM_admission	leukocyte	*ARFGEF2*	-1.480475998	0.000258553	0.001081954	1
T2DM_admission	leukocyte	*ZNFX1*	-4.252620508	4.17E-05	0.001037577	0.163956215
T2DM_admission	leukocyte	*CSE1L*	-1.467302125	0.000151727	0.001062643	0.59613369

T2DM_admission	leukocyte	*BCAS4*	-0.817634049	0.001069497	0.001964163	1
T2DM_admission	leukocyte	*KCNK15*	-0.937531277	0.004857828	0.006722933	1
T2DM_admission	leukocyte	*PEPD*	-2.17441162	9.87E-06	0.000784902	0.038786272
T2DM_admission	leukocyte	*PAIP2B*	-0.682523066	0.009199244	0.012039917	1
T2DM_admission	leukocyte	*SNRNP27*	-1.134451043	0.000408151	0.001188129	1
T2DM_admission	leukocyte	*USP22*	-1.352714598	0.000422422	0.001206582	1
T2DM_admission	leukocyte	*BTN2A2*	-1.704685839	0.00439469	0.006149122	1
T2DM_admission	leukocyte	*MRS2*	-2.013035618	0.000142327	0.001062643	0.559201945
T2DM_admission	leukocyte	*SNRPC*	-1.286046877	0.000163647	0.001062643	0.642970373
T2DM_admission	leukocyte	*SERPINB6*	-1.128785715	0.000162903	0.001062643	0.640047347
T2DM_admission	leukocyte	*ABCC10*	-1.318128962	0.002032632	0.003113533	1
T2DM_admission	leukocyte	*PEX6*	-1.31136354	0.000111873	0.001062643	0.43954816
T2DM_admission	leukocyte	*NQO2*	-3.2245601	1.05E-05	0.000784902	0.041261277
T2DM_admission	leukocyte	*UNC5CL*	-1.068329919	0.000713223	0.001532285	1
T2DM_admission	leukocyte	*ZNF391*	-0.721812392	0.024313259	0.029740596	1
T2DM_admission	leukocyte	*MED20*	-1.757438408	0.000687709	0.00149945	1
T2DM_admission	leukocyte	*TREM1*	0.855141399	0.035208121	0.042213216	1
T2DM_admission	leukocyte	*GLO1*	-1.243288382	0.000293608	0.001088724	1
T2DM_admission	leukocyte	*KCNK17*	-0.75884301	0.004525343	0.00630499	1
T2DM_admission	leukocyte	*ATXN1*	-1.6252523	0.000149801	0.001062643	0.58856807
T2DM_admission	leukocyte	*LRRFIP1*	-0.948471833	0.000361419	0.001142338	1
T2DM_admission	leukocyte	*CXCL6*	-1.464722694	0.010433756	0.013529448	1
T2DM_admission	leukocyte	*SH3TC1*	-1.26846405	0.000498284	0.001293956	1
T2DM_admission	leukocyte	*MT1G*	-0.794720148	0.003556237	0.00508459	1
T2DM_admission	leukocyte	*DOK4*	-0.779005779	0.001727187	0.002722068	1
T2DM_admission	leukocyte	*CLYBL*	-3.130328864	8.79E-05	0.001062643	0.345510206
T2DM_admission	leukocyte	*TM9SF2*	-1.039454013	0.000361028	0.001142338	1
T2DM_admission	leukocyte	*KIF25*	-17.1670307	0.000215541	0.001062643	0.846859759
T2DM_admission	leukocyte	*BMP4*	-2.728282973	0.000172409	0.001062643	0.67739325
T2DM_admission	leukocyte	*GRK4*	-1.036144209	0.020383963	0.02526454	1
T2DM_admission	leukocyte	*HS3ST3B1*	-2.544482696	0.001275324	0.002175489	1
T2DM_admission	leukocyte	*MRPS7*	-0.958969285	0.000526792	0.001319879	1
T2DM_admission	leukocyte	*GGA3*	-2.833563736	0.000139769	0.001062643	0.549152265
T2DM_admission	leukocyte	*DDX31*	-2.057317931	0.000501418	0.001296953	1
T2DM_admission	leukocyte	*PPDPF*	-0.901136667	0.000225682	0.001062643	0.88670341
T2DM_admission	leukocyte	*CHCHD5*	-1.058759837	0.000339538	0.001134896	1
T2DM_admission	leukocyte	*INSIG2*	-1.009712146	0.000401042	0.001184732	1
T2DM_admission	leukocyte	*PSD4*	-1.417687101	0.00224475	0.003390858	1
T2DM_admission	leukocyte	*CLPP*	-1.135056303	0.000488695	0.001280054	1
T2DM_admission	leukocyte	*SH2D3A*	-0.848557393	0.003519791	0.005037981	1
T2DM_admission	leukocyte	*GPR108*	-1.644588611	0.000383055	0.00116578	1
T2DM_admission	leukocyte	*FOSB*	-2.132914543	3.34E-05	0.001037577	0.131154462
T2DM_admission	leukocyte	*SYMPK*	-2.671307296	2.62E-05	0.000984894	0.103031996
T2DM_admission	leukocyte	*NAPB*	-1.061886653	0.00067078	0.001477964	1
T2DM_admission	leukocyte	*PSMF1*	-1.475105915	5.18E-05	0.00105496	0.203711379
T2DM_admission	leukocyte	*RBCK1*	-1.145650674	0.000242868	0.001077213	0.954227133
T2DM_admission	leukocyte	*FLRT3*	-0.939325223	0.001237836	0.002148171	1
T2DM_admission	leukocyte	*MGME1*	-5.36391051	3.37E-05	0.001037577	0.13257979
T2DM_admission	leukocyte	*ITPA*	-1.680628346	0.00011177	0.001062643	0.439142982
T2DM_admission	leukocyte	*MCM8*	-0.850570895	0.040732828	0.04848206	1
T2DM_admission	leukocyte	*BANF2*	-15.07019741	0.00127461	0.002175489	1
T2DM_admission	leukocyte	*MRPS26*	-1.033890296	0.000229196	0.001064322	0.900512547
T2DM_admission	leukocyte	*ZNF436*	-2.986750446	0.000112901	0.001062643	0.443589223
T2DM_admission	leukocyte	*TUBGCP3*	-1.424750916	0.000429587	0.00121662	1
T2DM_admission	leukocyte	*MCF2L*	-0.731536092	0.00420869	0.005914143	1
T2DM_admission	leukocyte	*IGFLR1*	0.323274021	0.014836442	0.018816134	1
T2DM_admission	leukocyte	*NR1D1*	-0.998650702	0.000478869	0.001265283	1
T2DM_admission	leukocyte	*PRMT1*	-1.056206923	9.43E-05	0.001062643	0.370427589
T2DM_admission	leukocyte	*PRR12*	-1.02601996	0.003676795	0.005246556	1
T2DM_admission	leukocyte	*FLRT1*	-2.308406658	0.013585993	0.0173535	1
T2DM_admission	leukocyte	*ASL*	-1.272055412	0.000336673	0.001131556	1
T2DM_admission	leukocyte	*WNK4*	-1.052866515	0.020845151	0.025779225	1
T2DM_admission	leukocyte	*BECN1*	-1.233955639	0.00045962	0.001240899	1
T2DM_admission	leukocyte	*PRKCG*	2.42891225	0.030761113	0.037119291	1

T2DM_admission	leukocyte	*TRAP1*	-0.916579895	0.001291189	0.002191396	1
T2DM_admission	leukocyte	*ZNF384*	-1.476334538	0.000437549	0.001222199	1
T2DM_admission	leukocyte	*RHOJ*	-1.926480232	0.000827975	0.00167593	1
T2DM_admission	leukocyte	*L3HYPDH*	-1.29938682	0.002394188	0.003590368	1
T2DM_admission	leukocyte	*HSPA2*	-1.972957408	0.000399946	0.001184732	1
T2DM_admission	leukocyte	*ZBTB1*	-1.705862985	0.000111699	0.001062643	0.438864665
T2DM_admission	leukocyte	*PLEKHG3*	-1.691839255	0.000373856	0.001157429	1
T2DM_admission	leukocyte	*FGD3*	2.651625135	0.019816273	0.024684991	1
T2DM_admission	leukocyte	*BCL11B*	1.72842016	0.010333068	0.013412166	1
T2DM_admission	leukocyte	*ABHD8*	-1.827179569	0.016188562	0.020412343	1
T2DM_admission	leukocyte	*HELB*	-4.312242619	0.041774801	0.049602053	1
T2DM_admission	leukocyte	*BEST3*	-2.810124524	0.001530493	0.00247563	1
T2DM_admission	leukocyte	*YEATS4*	-1.07947024	0.00056519	0.001362351	1
T2DM_admission	leukocyte	*LRRC61*	-1.742866856	0.000500556	0.001295577	1
T2DM_admission	leukocyte	*IDUA*	-1.138574694	0.000891709	0.001763074	1
T2DM_admission	leukocyte	*FGFRL1*	-1.139263667	0.000570882	0.001370185	1
T2DM_admission	leukocyte	*EMC1*	-1.310123308	0.000693923	0.001507546	1
T2DM_admission	leukocyte	*UBR4*	-1.323099444	0.000219117	0.001062643	0.860909631
T2DM_admission	leukocyte	*SYNGR3*	-1.228042691	0.003190142	0.004611504	1
T2DM_admission	leukocyte	*WFIKKN1*	-0.966529926	0.008369221	0.011071606	1
T2DM_admission	leukocyte	*KDM4B*	-2.973868299	5.31E-05	0.001059005	0.208624049
T2DM_admission	leukocyte	*PNKD*	-1.578678598	0.000118571	0.001062643	0.465863777
T2DM_admission	leukocyte	*PTPN12*	-1.382109607	3.69E-05	0.001037577	0.145068517
T2DM_admission	leukocyte	*POR*	-1.292286402	0.00027649	0.001088708	1
T2DM_admission	leukocyte	*RBM48*	-2.291573668	0.000155257	0.001062643	0.610006318
T2DM_admission	leukocyte	*SRD5A3*	-1.448011213	0.000787513	0.001625926	1
T2DM_admission	leukocyte	*PPAT*	-1.458219192	0.000186948	0.001062643	0.734517318
T2DM_admission	leukocyte	*A4GALT*	-0.887717958	0.004114609	0.005798529	1
T2DM_admission	leukocyte	*NAA38*	-0.984954109	0.000291422	0.001088708	1
T2DM_admission	leukocyte	*PRKRIP1*	-1.111248592	0.000611436	0.001413969	1
T2DM_admission	leukocyte	*VGF*	-0.605521554	0.000459467	0.001240899	1
T2DM_admission	leukocyte	*IRF5*	0.437239383	0.006997001	0.009388577	1
T2DM_admission	leukocyte	*MTX2*	-1.752758537	0.000131716	0.001062643	0.517511447
T2DM_admission	leukocyte	*GAD1*	-4.533065028	0.00018886	0.001062643	0.742030801
T2DM_admission	leukocyte	*ORMDL1*	-1.076037407	0.000724435	0.001544387	1
T2DM_admission	leukocyte	*PSMG2*	-1.331751081	0.000302241	0.001093914	1
T2DM_admission	leukocyte	*TWSG1*	-1.850449473	0.000146902	0.001062643	0.577177733
T2DM_admission	leukocyte	*EIF2AK4*	-1.978004253	2.04E-05	0.000922724	0.080277006
T2DM_admission	leukocyte	*MYO5C*	-0.800318612	0.000940037	0.001810978	1
T2DM_admission	leukocyte	*CGNL1*	-1.457328176	0.000106028	0.001062643	0.416583047
T2DM_admission	leukocyte	*TMOD2*	-1.320208255	0.000422617	0.001206582	1
T2DM_admission	leukocyte	*ELL3*	-3.933657146	8.95E-05	0.001062643	0.351646839
T2DM_admission	leukocyte	*IVD*	-1.547119577	0.001035702	0.001927651	1
T2DM_admission	leukocyte	*CHAC1*	-1.433968895	0.000292406	0.001088708	1
T2DM_admission	leukocyte	*ACKR4*	-1.958764946	0.004903628	0.006783704	1
T2DM_admission	leukocyte	*ANAPC13*	-0.655379513	0.004794959	0.006642945	1
T2DM_admission	leukocyte	*SUMF2*	-1.405398041	0.000178549	0.001062643	0.701519716
T2DM_admission	leukocyte	*TPH1*	-3.326853753	0.005725613	0.007827396	1
T2DM_admission	leukocyte	*DCTD*	-1.494527723	0.000154548	0.001062643	0.607217528
T2DM_admission	leukocyte	*KRI1*	-0.769196429	0.007792518	0.010361017	1
T2DM_admission	leukocyte	*AP1M2*	-1.059014268	0.000444384	0.001230246	1
T2DM_admission	leukocyte	*CDKN2D*	-1.096990179	0.000369118	0.00115009	1
T2DM_admission	leukocyte	*MTUS1*	-0.922010808	0.001010898	0.001894951	1
T2DM_admission	leukocyte	*ADCY4*	-1.113068848	0.003114812	0.004520908	1
T2DM_admission	leukocyte	*DTD2*	-0.973346757	0.001179286	0.002098466	1
T2DM_admission	leukocyte	*HEATR5A*	-2.572281707	8.13E-05	0.001062643	0.319276752
T2DM_admission	leukocyte	*MIS18BP1*	-1.589951983	0.015972755	0.020166116	1
T2DM_admission	leukocyte	*RNASE1*	0.166433314	0.012236205	0.015721403	1
T2DM_admission	leukocyte	*EPB41L4A*	-1.274955862	0.00120976	0.002121941	1
T2DM_admission	leukocyte	*REEP5*	-0.999977326	0.000248872	0.001077213	0.977816683
T2DM_admission	leukocyte	*MAU2*	-3.884143719	2.25E-08	9.82E-06	8.84E-05
T2DM_admission	leukocyte	*SHC2*	-1.346523336	0.000269623	0.001088175	1
T2DM_admission	leukocyte	*SYT5*	-0.581862198	0.012942436	0.016563788	1
T2DM_admission	leukocyte	*CBFA2T3*	-1.086459545	0.00118368	0.002100578	1

T2DM_admission	leukocyte	*ERMARD*	-2.565683585	6.40E-05	0.001060479	0.251410375
T2DM_admission	leukocyte	*PHF10*	-1.208411761	0.000356554	0.001141109	1
T2DM_admission	leukocyte	*SH3BP4*	-0.611022255	0.003889137	0.005520383	1
T2DM_admission	leukocyte	*DOCK6*	-0.866069908	0.001187489	0.00210081	1
T2DM_admission	leukocyte	*ECSIT*	-1.367169678	0.000449329	0.001234067	1
T2DM_admission	leukocyte	*CDC16*	-1.775659291	6.42E-05	0.001060479	0.252200512
T2DM_admission	leukocyte	*DPP6*	-0.6064402	0.001064443	0.001959791	1
T2DM_admission	leukocyte	*GTPBP3*	-1.419289432	5.11E-05	0.00105496	0.2008977
T2DM_admission	leukocyte	*PLVAP*	-0.555871679	0.001532012	0.002477069	1
T2DM_admission	leukocyte	*MRPL34*	-1.135540866	3.20E-05	0.001037577	0.125918259
T2DM_admission	leukocyte	*LSM7*	-0.779253753	0.001373424	0.002283803	1
T2DM_admission	leukocyte	*TULP4*	-1.06282395	0.001840206	0.002865703	1
T2DM_admission	leukocyte	*QRSL1*	-1.253389333	0.000897436	0.001768319	1
T2DM_admission	leukocyte	*RSPH3*	-1.287644316	0.000892082	0.001763074	1
T2DM_admission	leukocyte	*STK33*	-1.006870117	0.000378817	0.001163156	1
T2DM_admission	leukocyte	*NDUFA10*	-1.131054592	0.00044462	0.001230246	1
T2DM_admission	leukocyte	*TRPM4*	-4.51893535	0.001578052	0.00253068	1
T2DM_admission	leukocyte	*UBAC1*	-1.159639506	0.000351977	0.001141109	1
T2DM_admission	leukocyte	*ATXN10*	-1.001103399	0.000324398	0.001117056	1
T2DM_admission	leukocyte	*PNPLA7*	-2.259242687	0.000730598	0.001550629	1
T2DM_admission	leukocyte	*PAK4*	-1.500990042	0.000319281	0.001113024	1
T2DM_admission	leukocyte	*LAMA5*	-0.749036167	0.001630034	0.002594977	1
T2DM_admission	leukocyte	*ADRM1*	-1.147934358	0.000222459	0.001062643	0.874041516
T2DM_admission	leukocyte	*UCK1*	-2.311247511	3.70E-05	0.001037577	0.145346435
T2DM_admission	leukocyte	*MAP3K10*	-2.695001693	0.000283148	0.001088708	1
T2DM_admission	leukocyte	*SMPDL3B*	-0.932337049	0.001650726	0.002622605	1
T2DM_admission	leukocyte	*CLIP1*	-1.335733179	0.00047393	0.001259006	1
T2DM_admission	leukocyte	*ZNF317*	-1.615977533	0.000331197	0.001125668	1
T2DM_admission	leukocyte	*DNMT1*	-1.951672787	0.000209412	0.001062643	0.822779773
T2DM_admission	leukocyte	*ZNF426*	-1.800817883	9.97E-05	0.001062643	0.391890216
T2DM_admission	leukocyte	*LRP3*	-1.753950158	0.000960073	0.001831992	1
T2DM_admission	leukocyte	*HSD17B3*	-15.31944989	0.000292403	0.001088708	1
T2DM_admission	leukocyte	*SYNE1*	-1.048792543	0.000378936	0.001163156	1
T2DM_admission	leukocyte	*EPS8L1*	-3.308142505	0.002009223	0.003086098	1
T2DM_admission	leukocyte	*C1QL1*	-0.475821628	0.002539497	0.003772282	1
T2DM_admission	leukocyte	*F12*	-1.135210202	0.005901273	0.008050729	1
T2DM_admission	leukocyte	*TRAF3*	-0.805014264	0.005126471	0.007069815	1
T2DM_admission	leukocyte	*PDLIM4*	-1.353882779	0.00025312	0.001077213	0.99450767
T2DM_admission	leukocyte	*MGAT1*	-2.757371511	0.001984945	0.003054843	1
T2DM_admission	leukocyte	*DIAPH1*	-0.896100886	0.000592581	0.001392055	1
T2DM_admission	leukocyte	*EXOC4*	-2.19132229	0.000956534	0.001830343	1
T2DM_admission	leukocyte	*C1orf159*	-4.699250011	0.000230526	0.001064322	0.905738398
T2DM_admission	leukocyte	*ANO1*	-2.842668099	0.000588203	0.001384692	1
T2DM_admission	leukocyte	*PPFIA1*	-1.043883272	0.000499707	0.001295455	1
T2DM_admission	leukocyte	*TRAF7*	-1.55748505	0.000366368	0.001147001	1
T2DM_admission	leukocyte	*MAP1B*	-0.706859186	0.001096833	0.001995936	1
T2DM_admission	leukocyte	*CHD1L*	-2.750361171	0.000339268	0.001134896	1
T2DM_admission	leukocyte	*PIAS3*	-0.894911408	0.001078377	0.001970875	1
T2DM_admission	leukocyte	*RBM8A*	-0.907216431	9.37E-05	0.001062643	0.368087993
T2DM_admission	leukocyte	*ZNF304*	-5.557267215	4.48E-05	0.001037577	0.176130471
T2DM_admission	leukocyte	*ZNF132*	1.288392166	0.011060102	0.01427567	1
T2DM_admission	leukocyte	*RHPN2*	-1.073805902	0.001120187	0.002027274	1
T2DM_admission	leukocyte	*GCH1*	-0.7740785	0.001543386	0.002491358	1
T2DM_admission	leukocyte	*CC2D1A*	-1.709428089	0.000199054	0.001062643	0.782083905
T2DM_admission	leukocyte	*CCT6B*	-1.837033481	0.000511294	0.001308382	1
T2DM_admission	leukocyte	*PPARG*	-2.148497042	0.000101856	0.001062643	0.400192484
T2DM_admission	leukocyte	*ENOSF1*	-3.246643708	3.78E-05	0.001037577	0.148399938
T2DM_admission	leukocyte	*IMMT*	-1.116415638	0.001705836	0.00269816	1
T2DM_admission	leukocyte	*MRPL35*	-1.008056157	0.006031177	0.008216538	1
T2DM_admission	leukocyte	*SCLY*	-1.444074488	0.014817448	0.018801362	1
T2DM_admission	leukocyte	*CARD6*	-2.229047351	0.000791306	0.0016329	1
T2DM_admission	leukocyte	*INPP5K*	-1.11360495	0.003225771	0.004659579	1
T2DM_admission	leukocyte	*RPA1*	-1.352652026	0.001142425	0.00205052	1
T2DM_admission	leukocyte	*TMEM128*	-1.868569876	0.000148353	0.001062643	0.582877868

T2DM_admission	leukocyte	*SEC61G*	-0.920162629	0.000209505	0.001062643	0.823145577
T2DM_admission	leukocyte	*FIGNL1*	-3.506099831	0.00118274	0.002099858	1
T2DM_admission	leukocyte	*ITGB4*	-1.69873275	0.024298475	0.029731769	1
T2DM_admission	leukocyte	*TRIM47*	-1.07169433	0.001374948	0.002285182	1
T2DM_admission	leukocyte	*EIF5A*	-1.066540625	0.00012366	0.001062643	0.485859773
T2DM_admission	leukocyte	*PRMT7*	-2.968647785	0.000155389	0.001062643	0.610524729
T2DM_admission	leukocyte	*ANKEF1*	-0.963669932	0.001172498	0.002089227	1
T2DM_admission	leukocyte	*BTBD3*	-0.985525826	0.000404229	0.001185781	1
T2DM_admission	leukocyte	*IGHMBP2*	-1.615933028	0.000108443	0.001062643	0.42607118
T2DM_admission	leukocyte	*LPIN3*	-1.524999753	0.003915938	0.005546402	1
T2DM_admission	leukocyte	*RBM38*	-1.945485769	0.000126439	0.001062643	0.496780193
T2DM_admission	leukocyte	*VSTM2L*	-0.895186335	0.002137943	0.003246996	1
T2DM_admission	leukocyte	*SERINC3*	-1.221958192	0.000169831	0.001062643	0.667266435
T2DM_admission	leukocyte	*BHMT2*	-1.71936342	1.92E-05	0.000917316	0.075627446
T2DM_admission	leukocyte	*CASP9*	-2.320169778	0.00127933	0.002178798	1
T2DM_admission	leukocyte	*WASF3*	-1.151564869	0.001381684	0.002292499	1
T2DM_admission	leukocyte	*SCO1*	-2.117383026	0.000236302	0.001068391	0.928431734
T2DM_admission	leukocyte	*CHI3L1*	-0.859828313	0.009268405	0.012118325	1
T2DM_admission	leukocyte	*SLC41A1*	-1.319978049	0.000619279	0.001422062	1
T2DM_admission	leukocyte	*TRPC4*	-3.436202426	0.000522217	0.001319879	1
T2DM_admission	leukocyte	*POSTN*	0.943266902	0.010286272	0.013355838	1
T2DM_admission	leukocyte	*RFC3*	-0.930907469	0.001231248	0.002143364	1
T2DM_admission	leukocyte	*EPHB2*	-4.670199203	0.000398282	0.001183526	1
T2DM_admission	leukocyte	*BTBD2*	-2.916611625	7.10E-05	0.001062643	0.279061979
T2DM_admission	leukocyte	*CNDP2*	-1.21714235	0.000348745	0.001141096	1
T2DM_admission	leukocyte	*WDR74*	-2.033735576	5.07E-05	0.00105496	0.199149664
T2DM_admission	leukocyte	*GSTT2B*	-1.012965485	0.001737572	0.002734049	1
T2DM_admission	leukocyte	*SLC2A11*	-1.98773599	0.000154486	0.001062643	0.606976195
T2DM_admission	leukocyte	*C1QTNF6*	-1.814308563	0.001297873	0.002196252	1
T2DM_admission	leukocyte	*FAM83F*	-1.297974513	0.000386707	0.001171451	1
T2DM_admission	leukocyte	*GIMAP6*	-2.243865411	0.000437084	0.001222199	1
T2DM_admission	leukocyte	*AGAP3*	-4.225493327	3.19E-05	0.001037577	0.125158115
T2DM_admission	leukocyte	*SFTPD*	-0.817123714	0.0255313	0.031133606	1
T2DM_admission	leukocyte	*IMPA1*	-1.160789172	0.000264923	0.001084251	1
T2DM_admission	leukocyte	*LRRCC1*	-1.807027625	0.000651549	0.001450838	1
T2DM_admission	leukocyte	*RRAS2*	-0.968634583	0.000945687	0.0018187	1
T2DM_admission	leukocyte	*HSD17B4*	-2.278826677	5.48E-05	0.001060479	0.215257166
T2DM_admission	leukocyte	*RNF122*	-1.654311894	0.023195889	0.028480203	1
T2DM_admission	leukocyte	*COX16*	-1.188705985	0.00017167	0.001062643	0.674492986
T2DM_admission	leukocyte	*ELP3*	-2.552828158	0.000140954	0.001062643	0.553807443
T2DM_admission	leukocyte	*PEBP4*	-1.49003182	0.002065923	0.003154688	1
T2DM_admission	leukocyte	*MRO*	-3.146212774	0.000733248	0.001552225	1
T2DM_admission	leukocyte	*MRPS36*	-1.159259245	0.001419797	0.00234189	1
T2DM_admission	leukocyte	*THUMPD3*	-1.43473526	7.68E-05	0.001062643	0.301631291
T2DM_admission	leukocyte	*CHL1*	-1.174186612	0.004160564	0.005855516	1
T2DM_admission	leukocyte	*GSTM1*	1.886652361	0.015026857	0.019026916	1
T2DM_admission	leukocyte	*REG4*	-1.266010709	0.001216263	0.00212877	1
T2DM_admission	leukocyte	*GSTM3*	-0.802086584	0.001446327	0.002366772	1
T2DM_admission	leukocyte	*PTPN22*	-5.380044828	0.006786309	0.009128178	1
T2DM_admission	leukocyte	*SORT1*	-1.549556467	0.000184058	0.001062643	0.723162563
T2DM_admission	leukocyte	*NOTCH2*	-3.595543145	3.59E-15	1.41E-11	1.41E-11
T2DM_admission	leukocyte	*CEPT1*	-2.105618369	0.000251344	0.001077213	0.987529581
T2DM_admission	leukocyte	*NAPG*	-1.218295627	0.000329808	0.001123865	1
T2DM_admission	leukocyte	*SPIRE1*	-1.308213192	0.000155076	0.001062643	0.609292331
T2DM_admission	leukocyte	*FKBP11*	-0.694365568	0.000870979	0.001737977	1
T2DM_admission	leukocyte	*KIDINS220*	-1.317181149	0.000193734	0.001062643	0.761182277
T2DM_admission	leukocyte	*LPIN1*	-0.928835273	0.000244777	0.001077213	0.961727012
T2DM_admission	leukocyte	*IAH1*	-1.546799661	0.000268014	0.001087356	1
T2DM_admission	leukocyte	*ANO3*	1.537128967	0.01227202	0.015762265	1
T2DM_admission	leukocyte	*IL2RA*	0.320234185	0.008893411	0.011670746	1
T2DM_admission	leukocyte	*ECHDC3*	-1.142901338	0.000650162	0.001449765	1
T2DM_admission	leukocyte	*TMEM241*	-1.873997697	0.001765571	0.002770339	1
T2DM_admission	leukocyte	*AGO4*	-2.142453465	0.000296917	0.001093914	1
T2DM_admission	leukocyte	*PRPF38A*	-1.197145723	0.000365397	0.001147001	1

T2DM_admission	leukocyte	*DSC2*	-2.662585988	0.008323846	0.011022321	1
T2DM_admission	leukocyte	*TPGS2*	-1.329248853	0.001042313	0.001936841	1
T2DM_admission	leukocyte	*DAGLA*	-1.280959405	0.00172711	0.002722068	1
T2DM_admission	leukocyte	*TIMM10*	-1.27149185	0.000194546	0.001062643	0.764372527
T2DM_admission	leukocyte	*FADS2*	-1.387887892	0.000379846	0.001164428	1
T2DM_admission	leukocyte	*TMEM165*	-0.98307595	0.000395725	0.001181845	1
T2DM_admission	leukocyte	*CLOCK*	-1.422228349	0.000288402	0.001088708	1
T2DM_admission	leukocyte	*GGACT*	-1.978415701	0.000394526	0.001181536	1
T2DM_admission	leukocyte	*CLDN10*	-0.470291776	0.00403532	0.005694962	1
T2DM_admission	leukocyte	*CARS2*	-1.262071864	0.000116357	0.001062643	0.457164898
T2DM_admission	leukocyte	*TMED7*	-1.095714157	0.000234091	0.001066706	0.919745256
T2DM_admission	leukocyte	*NREP*	-7.059101382	5.48E-05	0.001060479	0.215184128
T2DM_admission	leukocyte	*WDR36*	-1.22505921	0.000664312	0.001467987	1
T2DM_admission	leukocyte	*OSTF1*	-1.591051281	0.000850518	0.001707555	1
T2DM_admission	leukocyte	*GOLM1*	-0.905030727	0.00049712	0.001292643	1
T2DM_admission	leukocyte	*SDS*	0.068840912	0.007928381	0.01053097	1
T2DM_admission	leukocyte	*MSI1*	-2.104712703	0.000302921	0.001093914	1
T2DM_admission	leukocyte	*TBX3*	-1.85547274	0.022651031	0.027872189	1
T2DM_admission	leukocyte	*DTX1*	-2.968236399	0.000527803	0.001320853	1
T2DM_admission	leukocyte	*PNPLA8*	-0.94930456	0.000418982	0.001200714	1
T2DM_admission	leukocyte	*TES*	-0.772854273	0.000762808	0.001593931	1
T2DM_admission	leukocyte	*MDFIC*	-2.935459563	0.000535619	0.001332771	1
T2DM_admission	leukocyte	*MTO1*	-1.0831746	0.00089064	0.001763074	1
T2DM_admission	leukocyte	*LCA5*	-3.39723377	6.37E-05	0.001060479	0.250087316
T2DM_admission	leukocyte	*LMO2*	-1.054358091	0.000518093	0.00131413	1
T2DM_admission	leukocyte	*RDH5*	-1.005020976	0.00092827	0.001800431	1
T2DM_admission	leukocyte	*TFCP2*	-3.872271602	4.06E-05	0.001037577	0.159436432
T2DM_admission	leukocyte	*KRT7*	-0.509179057	0.002374896	0.00356552	1
T2DM_admission	leukocyte	*NHSL1*	-1.249402839	0.007326706	0.00979137	1
T2DM_admission	leukocyte	*AHI1*	-0.694981771	0.003926363	0.005559164	1
T2DM_admission	leukocyte	*HEY2*	-1.668168283	0.000813876	0.001659956	1
T2DM_admission	leukocyte	*PKIB*	-0.827274587	0.000564098	0.001362351	1
T2DM_admission	leukocyte	*SMPD2*	-3.532859914	2.02E-05	0.000922424	0.079328428
T2DM_admission	leukocyte	*SEMA4F*	-1.424014927	0.002939045	0.004295947	1
T2DM_admission	leukocyte	*GNS*	-2.400761455	4.93E-05	0.00105496	0.193716632
T2DM_admission	leukocyte	*MPHOSPH6*	-0.979579146	0.001435869	0.002354561	1
T2DM_admission	leukocyte	*AGT*	-0.717997197	0.00112093	0.002027359	1
T2DM_admission	leukocyte	*NTPCR*	-1.656395741	0.00021747	0.001062643	0.854438404
T2DM_admission	leukocyte	*USP37*	-1.350102105	0.001985756	0.003054843	1
T2DM_admission	leukocyte	*ARMC9*	-5.645997619	0.000382595	0.001165285	1
T2DM_admission	leukocyte	*COX5B*	-0.955574617	0.000248454	0.001077213	0.976174317
T2DM_admission	leukocyte	*REV1*	-1.090222981	0.000573219	0.001370771	1
T2DM_admission	leukocyte	*TSGA10*	-1.097311688	0.003497525	0.005013416	1
T2DM_admission	leukocyte	*TGFBRAP1*	-1.451305028	0.000182926	0.001062643	0.718715771
T2DM_admission	leukocyte	*GCC2*	-1.450160976	5.26E-05	0.00105496	0.2066448
T2DM_admission	leukocyte	*MRPS9*	-1.001699801	0.000803045	0.001648465	1
T2DM_admission	leukocyte	*ANKRD36*	-1.547168286	0.000560272	0.001360512	1
T2DM_admission	leukocyte	*USP44*	-1.506310493	0.001200032	0.002112301	1
T2DM_admission	leukocyte	*DRAM1*	-2.41796005	0.00011213	0.001062643	0.440557988
T2DM_admission	leukocyte	*VILL*	1.254749787	0.019537855	0.024354135	1
T2DM_admission	leukocyte	*FLNB*	-0.849048521	0.00338513	0.004870075	1
T2DM_admission	leukocyte	*NEK3*	-0.643771953	0.012481241	0.016010663	1
T2DM_admission	leukocyte	*VPS36*	-1.912075863	0.000111441	0.001062643	0.437853386
T2DM_admission	leukocyte	*RNASEH2B*	-2.378468283	0.000117485	0.001062643	0.461598651
T2DM_admission	leukocyte	*CKAP2*	-0.842985948	0.015231496	0.019267401	1
T2DM_admission	leukocyte	*TBC1D4*	-1.25880329	0.000410023	0.001188264	1
T2DM_admission	leukocyte	*SUCLA2*	-1.484263248	9.40E-05	0.001062643	0.369412097
T2DM_admission	leukocyte	*RCBTB1*	-1.678425217	0.00162833	0.002593316	1
T2DM_admission	leukocyte	*LMO7*	-0.658064272	0.000830244	0.001678862	1
T2DM_admission	leukocyte	*C7orf25*	-1.054825067	0.00043008	0.00121662	1
T2DM_admission	leukocyte	*RAPGEF5*	-0.789971888	0.001251948	0.002156134	1
T2DM_admission	leukocyte	*KDELR2*	-1.101821505	0.000189568	0.001062643	0.744810935
T2DM_admission	leukocyte	*TBRG4*	-2.837089027	2.82E-05	0.001005591	0.11061503
T2DM_admission	leukocyte	*DBNL*	-3.894429377	2.12E-05	0.000929374	0.083187896

T2DM_admission	leukocyte	*ALPK3*	-0.850068339	0.000641391	0.001440839	1
T2DM_admission	leukocyte	*CIB2*	-1.196078254	0.003410828	0.004903456	1
T2DM_admission	leukocyte	*NMT1*	-0.890646216	0.001886807	0.002928987	1
T2DM_admission	leukocyte	*TEX2*	-0.788257277	0.001486772	0.002415851	1
T2DM_admission	leukocyte	*LIMD2*	0.819875412	0.006514919	0.008790219	1
T2DM_admission	leukocyte	*MRPL47*	-1.149415236	0.000222812	0.001062643	0.875429311
T2DM_admission	leukocyte	*SMPD4*	-3.019632001	4.87E-05	0.00105496	0.191417401
T2DM_admission	leukocyte	*CCDC115*	-1.261181669	0.000585605	0.001382291	1
T2DM_admission	leukocyte	*BIN1*	-2.768970682	0.000248471	0.001077213	0.976241107
T2DM_admission	leukocyte	*HS6ST1*	-1.773041907	0.000199083	0.001062643	0.782198214
T2DM_admission	leukocyte	*GYPC*	-1.082474025	0.001801937	0.002818981	1
T2DM_admission	leukocyte	*NIPSNAP3A*	-1.607497417	0.000638509	0.001438504	1
T2DM_admission	leukocyte	*CDK9*	-1.390788826	0.00040951	0.001188264	1
T2DM_admission	leukocyte	*TXN*	-0.867876425	0.000245505	0.001077213	0.9645884
T2DM_admission	leukocyte	*TOR1B*	-1.427439519	0.007977027	0.010588425	1
T2DM_admission	leukocyte	*TOR1A*	-1.04945802	0.000960081	0.001831992	1
T2DM_admission	leukocyte	*SLC2A8*	-1.245306063	0.000572093	0.001370771	1
T2DM_admission	leukocyte	*ZFP37*	-0.862923655	0.007041011	0.009435242	1
T2DM_admission	leukocyte	*STX17*	-1.126844414	0.00043147	0.001216752	1
T2DM_admission	leukocyte	*PRPF4*	-1.068908761	0.01764321	0.022083521	1
T2DM_admission	leukocyte	*FPGS*	-1.49959651	0.000398485	0.001183526	1
T2DM_admission	leukocyte	*ATP6V1G1*	-0.893341757	0.000356709	0.001141109	1
T2DM_admission	leukocyte	*ARPC5L*	-0.911946888	0.000350549	0.001141109	1
T2DM_admission	leukocyte	*POLR1E*	-0.844902513	0.000559375	0.001360165	1
T2DM_admission	leukocyte	*APTX*	-1.014417326	0.000355911	0.001141109	1
T2DM_admission	leukocyte	*CD72*	-2.135295753	0.00090773	0.001777903	1
T2DM_admission	leukocyte	*TMEM8B*	-1.507948796	0.000541563	0.001337398	1
T2DM_admission	leukocyte	*GRHPR*	-1.207650456	0.00017328	0.001062643	0.680817131
T2DM_admission	leukocyte	*ALDH1B1*	-2.325276179	0.000397514	0.001183205	1
T2DM_admission	leukocyte	*ARHGEF39*	-4.110511506	0.001029444	0.001918342	1
T2DM_admission	leukocyte	*PPIL1*	-2.90707901	9.18E-05	0.001062643	0.360759959
T2DM_admission	leukocyte	*GMPR*	-1.875099205	0.000887283	0.001758011	1
T2DM_admission	leukocyte	*KIAA0319*	-1.183606834	0.001075717	0.001968556	1
T2DM_admission	leukocyte	*LRRC1*	-1.07348821	0.000606881	0.001408212	1
T2DM_admission	leukocyte	*RIPK1*	-1.192116774	0.000517994	0.00131413	1
T2DM_admission	leukocyte	*TUBB2B*	-0.904731196	0.001441915	0.002362504	1
T2DM_admission	leukocyte	*UQCC2*	-1.10353636	0.000559287	0.001360165	1
T2DM_admission	leukocyte	*HMGA1*	-0.830682692	0.000521008	0.00131812	1
T2DM_admission	leukocyte	*TCF19*	0.740616301	0.018904401	0.023601968	1
T2DM_admission	leukocyte	*CLPS*	-0.478683085	0.000478786	0.001265283	1
T2DM_admission	leukocyte	*MTCH1*	-1.060688859	0.000240844	0.001072513	0.946277674
T2DM_admission	leukocyte	*ANKRD42*	-1.821973125	0.000115081	0.001062643	0.452152297
T2DM_admission	leukocyte	*CREBZF*	-1.576281656	0.000208286	0.001062643	0.818355272
T2DM_admission	leukocyte	*PRCP*	-1.502482678	0.000970017	0.001846509	1
T2DM_admission	leukocyte	*NARS2*	-1.342960791	0.00089858	0.001769684	1
T2DM_admission	leukocyte	*TTPA*	-6.827610358	0.012867354	0.016478668	1
T2DM_admission	leukocyte	*GGH*	-1.450602252	0.000612591	0.001415201	1
T2DM_admission	leukocyte	*SDCBP*	-1.913648902	0.000177374	0.001062643	0.696902843
T2DM_admission	leukocyte	*TRPC6*	-2.572769304	0.00629731	0.008525889	1
T2DM_admission	leukocyte	*MMP7*	-0.425749426	0.004019699	0.005679035	1
T2DM_admission	leukocyte	*TRIM29*	-2.588815379	0.036296006	0.043438016	1
T2DM_admission	leukocyte	*FDX1*	-1.234049442	0.000167746	0.001062643	0.659073615
T2DM_admission	leukocyte	*C11orf1*	-1.101146565	0.000722397	0.001544023	1
T2DM_admission	leukocyte	*FXYD2*	-0.719474536	8.28E-05	0.001062643	0.32529608
T2DM_admission	leukocyte	*CASP1*	0.369207546	0.011123331	0.014352567	1
T2DM_admission	leukocyte	*ALKBH8*	-1.538406632	0.00106985	0.001964163	1
T2DM_admission	leukocyte	*NDUFAF1*	-0.910056521	0.024132572	0.029537967	1
T2DM_admission	leukocyte	*TUBGCP4*	-2.176158227	2.24E-05	0.000945312	0.087914044
T2DM_admission	leukocyte	*UACA*	-0.991901576	0.000905502	0.001776194	1
T2DM_admission	leukocyte	*PLCB2*	0.623213361	0.007021127	0.009411808	1
T2DM_admission	leukocyte	*PAK6*	-0.744701687	0.000171244	0.001062643	0.672818401
T2DM_admission	leukocyte	*ADAM10*	-1.490861587	0.000215403	0.001062643	0.84631817
T2DM_admission	leukocyte	*DUOX1*	-1.046311539	0.014886983	0.01886805	1
T2DM_admission	leukocyte	*CYP19A1*	-6.060584827	0.041725615	0.049568731	1

T2DM_admission	leukocyte	*ZNF280D*	-1.479594995	0.00019234	0.001062643	0.755705576
T2DM_admission	leukocyte	*RSL24D1*	-1.158328125	0.000118304	0.001062643	0.464816961
T2DM_admission	leukocyte	*SPTBN5*	2.238051489	0.023412327	0.028736967	1
T2DM_admission	leukocyte	*TTLL7*	-1.100078467	0.000600125	0.001399402	1
T2DM_admission	leukocyte	*SLC44A5*	-1.506439747	0.004682697	0.006501172	1
T2DM_admission	leukocyte	*RTCA*	-2.748943069	0.000353034	0.001141109	1
T2DM_admission	leukocyte	*KHK*	-4.658997144	0.000116076	0.001062643	0.456064063
T2DM_admission	leukocyte	*DYNC2LI1*	-1.323832114	0.000338108	0.001134436	1
T2DM_admission	leukocyte	*ACTR2*	-1.115878296	0.000116172	0.001062643	0.456438157
T2DM_admission	leukocyte	*ATRAID*	-1.111873607	0.000311489	0.001105548	1
T2DM_admission	leukocyte	*LRPPRC*	-1.265795529	0.000446806	0.001232235	1
T2DM_admission	leukocyte	*DUSP5*	-2.261191019	8.35E-05	0.001062643	0.328202187
T2DM_admission	leukocyte	*CALHM2*	0.943767477	0.03038086	0.036705534	1
T2DM_admission	leukocyte	*ARL3*	-1.123928362	0.000120969	0.001062643	0.475289118
T2DM_admission	leukocyte	*PLCE1*	-1.063575097	0.008520223	0.011244863	1
T2DM_admission	leukocyte	*DNAJC13*	-0.940276108	0.002075931	0.003167503	1
T2DM_admission	leukocyte	*FAM149B1*	-1.181630839	0.000934216	0.001804129	1
T2DM_admission	leukocyte	*NCOA4*	-2.518350659	6.49E-05	0.001060479	0.254877796
T2DM_admission	leukocyte	*ASCC1*	-1.330226347	0.000740494	0.001562515	1
T2DM_admission	leukocyte	*RPS24*	-0.833078467	0.000150017	0.001062643	0.589416554
T2DM_admission	leukocyte	*DNA2*	-1.727388378	0.001854982	0.002885283	1
T2DM_admission	leukocyte	*MYPN*	5.630758629	0.000438816	0.001222199	1
T2DM_admission	leukocyte	*ATIC*	-2.027053877	0.000136222	0.001062643	0.535214749
T2DM_admission	leukocyte	*SMARCAL1*	-1.475013669	0.001246252	0.002154446	1
T2DM_admission	leukocyte	*BARD1*	-1.286290619	0.000536477	0.001334061	1
T2DM_admission	leukocyte	*FASTKD1*	-1.495236989	0.000225624	0.001062643	0.886474765
T2DM_admission	leukocyte	*FAM117B*	-1.420298888	0.00052653	0.001319879	1
T2DM_admission	leukocyte	*WDR12*	-1.525126095	0.001019231	0.001906024	1
T2DM_admission	leukocyte	*SLC35A5*	-1.520474211	0.000929729	0.001800431	1
T2DM_admission	leukocyte	*USP8*	-1.491875732	0.000137836	0.001062643	0.541557876
T2DM_admission	leukocyte	*SPPL2A*	-1.301407532	0.000158862	0.001062643	0.624170074
T2DM_admission	leukocyte	*APH1B*	-1.903628823	0.000173017	0.001062643	0.679783598
T2DM_admission	leukocyte	*HCN4*	-1.283716773	0.00515737	0.007105328	1
T2DM_admission	leukocyte	*SEMA7A*	-2.56228247	0.001262475	0.002166065	1
T2DM_admission	leukocyte	*AP1AR*	-0.972301492	0.000623395	0.00142568	1
T2DM_admission	leukocyte	*BBS7*	-1.477146777	0.000232374	0.001066588	0.912999084
T2DM_admission	leukocyte	*RAP1GDS1*	-1.418355453	0.000338507	0.001134808	1
T2DM_admission	leukocyte	*PRDM5*	-1.073607959	0.001435724	0.002354561	1
T2DM_admission	leukocyte	*NUP54*	-0.754425646	0.012867537	0.016478668	1
T2DM_admission	leukocyte	*G3BP2*	-0.982050502	0.000222806	0.001062643	0.875406107
T2DM_admission	leukocyte	*SCARB2*	-1.106321371	0.00024648	0.001077213	0.968419842
T2DM_admission	leukocyte	*CNOT6L*	-1.542352476	0.000102801	0.001062643	0.403905944
T2DM_admission	leukocyte	*USO1*	-0.976630713	0.00027881	0.001088708	1
T2DM_admission	leukocyte	*SHROOM3*	-2.201076836	0.000158099	0.001062643	0.621170776
T2DM_admission	leukocyte	*ANXA3*	-0.825102034	0.000395938	0.001181845	1
T2DM_admission	leukocyte	*PPA2*	-1.153703664	0.000340011	0.001135007	1
T2DM_admission	leukocyte	*MAPK8IP3*	-1.229710916	0.000517255	0.00131413	1
T2DM_admission	leukocyte	*RGS3*	-0.97246758	0.001167216	0.002082648	1
T2DM_admission	leukocyte	*ETV6*	-2.248326134	0.000310416	0.00110427	1
T2DM_admission	leukocyte	*ALG10*	-2.000498707	0.000903472	0.001773984	1
T2DM_admission	leukocyte	*SLCO1C1*	-17.787384	0.000845768	0.001700095	1
T2DM_admission	leukocyte	*ZCRB1*	-1.076330037	0.000199669	0.001062643	0.784499777
T2DM_admission	leukocyte	*C1RL*	-0.954128371	0.026116693	0.031756332	1
T2DM_admission	leukocyte	*CLSTN3*	-1.832690452	0.000371405	0.001152986	1
T2DM_admission	leukocyte	*KLRG1*	-6.887882011	0.001616298	0.002578334	1
T2DM_admission	leukocyte	*TAPBPL*	-0.783652113	0.013705866	0.017500925	1
T2DM_admission	leukocyte	*PEX5*	-1.127408759	0.000671083	0.001477964	1
T2DM_admission	leukocyte	*SLC38A4*	-1.479828505	0.002083931	0.003176014	1
T2DM_admission	leukocyte	*LRIG3*	-1.130988053	0.000811614	0.001657387	1
T2DM_admission	leukocyte	*GLIPR1*	-1.354315633	0.000469168	0.001253988	1
T2DM_admission	leukocyte	*SNRPF*	-0.927579791	0.00041857	0.001200552	1
T2DM_admission	leukocyte	*AMDHD1*	-1.344804387	0.032610662	0.039218638	1
T2DM_admission	leukocyte	*ASCL1*	-1.623816702	0.002263383	0.003416378	1
T2DM_admission	leukocyte	*RITA1*	-1.735045318	0.000290077	0.001088708	1

T2DM_admission	leukocyte	*GLTP*	-0.92624243	0.00052385	0.001319879	1
T2DM_admission	leukocyte	*MTMR6*	-1.484493775	0.000329628	0.001123865	1
T2DM_admission	leukocyte	*PDX1*	-1.610816008	0.000301206	0.001093914	1
T2DM_admission	leukocyte	*SUOX*	-1.026863164	0.010260724	0.013331476	1
T2DM_admission	leukocyte	*CCDC65*	-1.494268627	0.006162619	0.008367978	1
T2DM_admission	leukocyte	*SLC39A5*	-0.589463874	0.008770429	0.011524754	1
T2DM_admission	leukocyte	*KANSL2*	-2.948852334	6.12E-05	0.001060479	0.240409004
T2DM_admission	leukocyte	*CERS5*	-1.719815063	3.98E-05	0.001037577	0.15643039
T2DM_admission	leukocyte	*ZNF740*	-2.361802635	0.000723085	0.001544023	1
T2DM_admission	leukocyte	*WDFY2*	-1.359027915	0.000279569	0.001088708	1
T2DM_admission	leukocyte	*GRTP1*	-2.480013545	0.001005915	0.001889217	1
T2DM_admission	leukocyte	*TSSK4*	-9.407873864	0.003899775	0.005528505	1
T2DM_admission	leukocyte	*TMX1*	-1.742101815	0.000113325	0.001062643	0.445254978
T2DM_admission	leukocyte	*RAB15*	-1.648444242	0.000508805	0.001305383	1
T2DM_admission	leukocyte	*PTGR2*	-0.852743035	0.001735333	0.002732715	1
T2DM_admission	leukocyte	*NIPA2*	-0.951483969	0.000585778	0.001382291	1
T2DM_admission	leukocyte	*SERF2*	-0.960801483	6.31E-05	0.001060479	0.247915902
T2DM_admission	leukocyte	*BNIP2*	-2.015458774	7.51E-05	0.001062643	0.295088867
T2DM_admission	leukocyte	*GTF2A2*	-1.075957273	0.000217668	0.001062643	0.855217186
T2DM_admission	leukocyte	*SRP14*	-0.868669859	0.000247525	0.001077213	0.972525005
T2DM_admission	leukocyte	*BAHD1*	-1.344853681	0.000435788	0.001222199	1
T2DM_admission	leukocyte	*DISP2*	-0.759316893	0.006656438	0.008971919	1
T2DM_admission	leukocyte	*TLE3*	-2.898950157	2.13E-05	0.000929374	0.083643621
T2DM_admission	leukocyte	*SCAPER*	-1.033155065	0.000537953	0.001334229	1
T2DM_admission	leukocyte	*TSPAN3*	-0.924194381	0.000287617	0.001088708	1
T2DM_admission	leukocyte	*MAN2C1*	-1.765243984	5.31E-08	2.09E-05	0.000208769
T2DM_admission	leukocyte	*DNAJA4*	-1.036262369	0.000403325	0.001185781	1
T2DM_admission	leukocyte	*TPM1*	-0.638141619	0.000617273	0.001419945	1
T2DM_admission	leukocyte	*IGF1R*	-0.998020375	0.000290486	0.001088708	1
T2DM_admission	leukocyte	*ADAMTS17*	-2.194978135	0.007398356	0.009873688	1
T2DM_admission	leukocyte	*ULK3*	-1.178723838	0.000471499	0.001255095	1
T2DM_admission	leukocyte	*CELF6*	-1.355372562	0.00068066	0.00149096	1
T2DM_admission	leukocyte	*HAPLN3*	-5.715834522	0.022943881	0.028197219	1
T2DM_admission	leukocyte	*POLG*	-1.856019572	0.000286627	0.001088708	1
T2DM_admission	leukocyte	*WDR93*	-3.643297849	0.006071877	0.008263389	1
T2DM_admission	leukocyte	*UNC45A*	-1.331082497	0.000222244	0.001062643	0.873197708
T2DM_admission	leukocyte	*IQGAP1*	-1.207944568	0.000222882	0.001062643	0.875703158
T2DM_admission	leukocyte	*PMM2*	-1.15046259	0.000457791	0.001240899	1
T2DM_admission	leukocyte	*SLC5A2*	-3.830548362	0.003808338	0.005413516	1
T2DM_admission	leukocyte	*UQCRC2*	-1.28650773	0.000158857	0.001062643	0.624148359
T2DM_admission	leukocyte	*ARHGAP17*	-3.113989739	5.24E-05	0.00105496	0.205701756
T2DM_admission	leukocyte	*TXNL4B*	-2.913567809	7.89E-05	0.001062643	0.309919203
T2DM_admission	leukocyte	*MARVELD3*	-0.872524087	0.002614905	0.003871124	1
T2DM_admission	leukocyte	*KIFC3*	-1.35536879	0.00021976	0.001062643	0.863436028
T2DM_admission	leukocyte	*NUDT7*	-0.975134668	0.00141961	0.00234189	1
T2DM_admission	leukocyte	*GCSH*	-1.12192738	0.000237896	0.001071897	0.934694307
T2DM_admission	leukocyte	*MAP1LC3B*	-0.974285937	0.000225936	0.001062643	0.887703987
T2DM_admission	leukocyte	*OSGIN1*	-0.86520937	0.000996946	0.001875957	1
T2DM_admission	leukocyte	*RPL3L*	-2.009349955	0.010123525	0.013165959	1
T2DM_admission	leukocyte	*RPS2*	-0.767631176	0.000145556	0.001062643	0.571888236
T2DM_admission	leukocyte	*PDPK1*	-1.46206059	0.000376118	0.001159218	1
T2DM_admission	leukocyte	*DEF8*	-1.359892724	0.000161735	0.001062643	0.635458179
T2DM_admission	leukocyte	*GAS8*	-2.230240651	9.13E-05	0.001062643	0.358689341
T2DM_admission	leukocyte	*RANBP10*	-0.931132959	0.005308192	0.007297371	1
T2DM_admission	leukocyte	*PRPSAP2*	-1.346159487	0.000975375	0.001854014	1
T2DM_admission	leukocyte	*TOM1L1*	-1.038024123	0.001152361	0.002063641	1
T2DM_admission	leukocyte	*C17orf80*	-1.658571488	0.002740338	0.004032504	1
T2DM_admission	leukocyte	*VPS53*	-1.387335499	4.72E-05	0.00105496	0.185384912
T2DM_admission	leukocyte	*NPEPPS*	-1.663574231	0.000129884	0.001062643	0.510312546
T2DM_admission	leukocyte	*SCRN2*	-2.38743929	7.51E-05	0.001062643	0.29491828
T2DM_admission	leukocyte	*RHBDL3*	-1.725274541	0.014011357	0.017861979	1
T2DM_admission	leukocyte	*SPACA3*	-1.26002807	6.35E-05	0.001060479	0.249378213
T2DM_admission	leukocyte	*ARSG*	-3.955638264	0.001084188	0.001978529	1
T2DM_admission	leukocyte	*PTRH2*	-1.299380959	0.00041362	0.001193182	1

T2DM_admission	leukocyte	*GNAL*	-1.532355483	0.001922619	0.002976348	1
T2DM_admission	leukocyte	*RPRD1A*	-1.155150813	0.000471165	0.001255055	1
T2DM_admission	leukocyte	*NPC1*	-3.17574767	7.17E-07	0.000156607	0.002818928
T2DM_admission	leukocyte	*SLC14A1*	-1.763733851	0.000986742	0.001867193	1
T2DM_admission	leukocyte	*ZMYND15*	0.217951543	0.01103959	0.014258563	1
T2DM_admission	leukocyte	*SAT2*	-1.478350232	8.31E-05	0.001062643	0.326557984
T2DM_admission	leukocyte	*CCDC40*	-0.954026113	0.00829845	0.010992788	1
T2DM_admission	leukocyte	*ARHGDIA*	-1.044217743	0.000572315	0.001370771	1
T2DM_admission	leukocyte	*TMC6*	-1.762183859	0.001140789	0.002049538	1
T2DM_admission	leukocyte	*TTYH2*	-1.941464794	0.001270828	0.002175059	1
T2DM_admission	leukocyte	*EIF4A3*	-1.267283501	0.000430434	0.001216672	1
T2DM_admission	leukocyte	*TBCD*	-1.731915111	0.001966969	0.003031864	1
T2DM_admission	leukocyte	*FN3KRP*	-1.994306512	5.13E-06	0.00058206	0.020168175
T2DM_admission	leukocyte	*NARF*	-1.684196952	0.00015697	0.001062643	0.616733414
T2DM_admission	leukocyte	*TRIM65*	-1.791914269	0.000434888	0.001222199	1
T2DM_admission	leukocyte	*RNF157*	-1.338657254	0.000539178	0.001334229	1
T2DM_admission	leukocyte	*WDR45B*	-0.993771488	0.000317077	0.001111111	1
T2DM_admission	leukocyte	*TNFRSF11A*	-1.004731991	0.001141357	0.002049538	1
T2DM_admission	leukocyte	*ZCCHC2*	-1.654812454	0.000214564	0.001062643	0.843023495
T2DM_admission	leukocyte	*PMAIP1*	-1.048230369	0.00079382	0.001634654	1
T2DM_admission	leukocyte	*NT5C3B*	-1.187725724	0.000569529	0.001367774	1
T2DM_admission	leukocyte	*PIP4K2B*	-1.056249781	0.001462159	0.002383744	1
T2DM_admission	leukocyte	*TXNL4A*	-1.08207845	0.00015903	0.001062643	0.624828441
T2DM_admission	leukocyte	*PRDM15*	-1.148983772	0.017458504	0.021887193	1
T2DM_admission	leukocyte	*MVB12A*	-1.024512353	0.011796018	0.015165757	1
T2DM_admission	leukocyte	*SH3GL1*	-1.162201159	0.000154263	0.001062643	0.606097776
T2DM_admission	leukocyte	*DUS3L*	-0.571253428	0.031361559	0.037774238	1
T2DM_admission	leukocyte	*SIRT3*	-1.569160481	0.000690507	0.001503881	1
T2DM_admission	leukocyte	*IFITM3*	-0.751621108	0.000449223	0.001234067	1
T2DM_admission	leukocyte	*COL6A1*	-1.715350837	8.00E-05	0.001062643	0.314508198
T2DM_admission	leukocyte	*IFNAR1*	-1.221086614	6.02E-05	0.001060479	0.23669113
T2DM_admission	leukocyte	*TMEM50B*	-0.834687084	0.011684702	0.01503248	1
T2DM_admission	leukocyte	*AKT1*	-1.574514128	0.000871616	0.001738366	1
T2DM_admission	leukocyte	*CBLC*	-1.296777581	0.000144717	0.001062643	0.568592915
T2DM_admission	leukocyte	*EVI5L*	-0.977524978	0.023434634	0.028755365	1
T2DM_admission	leukocyte	*CTU1*	-1.998753488	0.000152551	0.001062643	0.599373177
T2DM_admission	leukocyte	*RERE*	-0.981668501	0.000396455	0.001181845	1
T2DM_admission	leukocyte	*FHAD1*	-1.018344806	0.003907609	0.005536602	1
T2DM_admission	leukocyte	*ARHGEF19*	-2.064105641	0.001736146	0.002732899	1
T2DM_admission	leukocyte	*PGD*	-1.553169377	0.000145289	0.001062643	0.570841031
T2DM_admission	leukocyte	*CNKSR1*	-1.879556239	0.002869946	0.004209039	1
T2DM_admission	leukocyte	*ZNF593*	-1.163080371	0.00017062	0.001062643	0.670367715
T2DM_admission	leukocyte	*FCN3*	-0.554346945	0.004213681	0.005919039	1
T2DM_admission	leukocyte	*CELA3A*	-0.498332796	0.000716722	0.00153628	1
T2DM_admission	leukocyte	*NBPF3*	-1.206762638	0.002931971	0.004287203	1
T2DM_admission	leukocyte	*HSPG2*	-0.695554195	0.00021941	0.001062643	0.862063592
T2DM_admission	leukocyte	*ITGB3BP*	-0.999059486	0.003274579	0.004723135	1
T2DM_admission	leukocyte	*PRKACB*	-1.390004531	0.000193632	0.001062643	0.760781618
T2DM_admission	leukocyte	*PIGK*	-2.058041795	0.00011491	0.001062643	0.451479528
T2DM_admission	leukocyte	*RPS8*	-0.707771215	0.000290926	0.001088708	1
T2DM_admission	leukocyte	*SYPL2*	-3.403521086	0.000746877	0.001568868	1
T2DM_admission	leukocyte	*SLC44A3*	-1.067844931	0.001283311	0.00218274	1
T2DM_admission	leukocyte	*ZNF697*	-1.588666921	0.002716097	0.004001329	1
T2DM_admission	leukocyte	*CELSR2*	-1.318985404	0.001150475	0.002061203	1
T2DM_admission	leukocyte	*ALDH9A1*	-1.023222027	0.000429948	0.00121662	1
T2DM_admission	leukocyte	*ATP1B1*	-0.723241232	0.000271765	0.001088708	1
T2DM_admission	leukocyte	*TIPRL*	-1.034009434	0.000322493	0.001116501	1
T2DM_admission	leukocyte	*NME7*	-2.072911863	1.58E-05	0.000823546	0.062032466
T2DM_admission	leukocyte	*MGST3*	-1.210884339	9.20E-05	0.001062643	0.361458169
T2DM_admission	leukocyte	*LYPLAL1*	-1.40856458	0.000299327	0.001093914	1
T2DM_admission	leukocyte	*SNX27*	-1.827986136	0.000328764	0.001123231	1
T2DM_admission	leukocyte	*CTSK*	-4.20183565	5.03E-05	0.00105496	0.197696363
T2DM_admission	leukocyte	*SELENBP1*	-1.282639974	0.001225924	0.002137886	1
T2DM_admission	leukocyte	*POGZ*	-0.929200113	0.01048804	0.013595351	1

T2DM_admission	leukocyte	*GABPB2*	-1.537660822	0.007071553	0.009472939	1
T2DM_admission	leukocyte	*SLC27A3*	-1.67170688	0.000284853	0.001088708	1
T2DM_admission	leukocyte	*KCNN3*	-1.964402961	0.001071932	0.001966209	1
T2DM_admission	leukocyte	*RIT1*	-2.301885864	0.00022341	0.001062643	0.87777902
T2DM_admission	leukocyte	*SRP9*	-1.156985432	0.000185664	0.001062643	0.729472241
T2DM_admission	leukocyte	*CNIH4*	-1.190461013	0.00023105	0.001065487	0.907794983
T2DM_admission	leukocyte	*PSEN2*	-2.372743932	0.00024964	0.001077213	0.980837293
T2DM_admission	leukocyte	*PYCR2*	-1.28164539	0.000458417	0.001240899	1
T2DM_admission	leukocyte	*LBR*	-1.403051129	0.000431278	0.001216752	1
T2DM_admission	leukocyte	*ARL8A*	-2.059921961	3.40E-05	0.001037577	0.133595061
T2DM_admission	leukocyte	*EML4*	-2.013844524	0.00010264	0.001062643	0.403271339
T2DM_admission	leukocyte	*WDPCP*	-3.012157918	0.000275449	0.001088708	1
T2DM_admission	leukocyte	*REG3G*	-1.396044586	6.17E-05	0.001060479	0.242595904
T2DM_admission	leukocyte	*CIAO1*	-1.721302756	7.90E-05	0.001062643	0.310306635
T2DM_admission	leukocyte	*ZNF514*	-1.39684746	8.59E-05	0.001062643	0.33731471
T2DM_admission	leukocyte	*TPRKB*	-1.052741496	0.000241111	0.001072513	0.947324343
T2DM_admission	leukocyte	*TEX261*	-1.342675458	0.000328386	0.001122913	1
T2DM_admission	leukocyte	*RALB*	-1.191639907	0.000537952	0.001334229	1
T2DM_admission	leukocyte	*RABL2A*	-1.532999515	0.002469624	0.003686608	1
T2DM_admission	leukocyte	*SLC20A1*	-1.803145526	0.000319545	0.001113024	1
T2DM_admission	leukocyte	*LYG1*	-5.086000046	0.007889432	0.010486326	1
T2DM_admission	leukocyte	*PHOSPHO2*	-1.640468732	0.026784241	0.032490053	1
T2DM_admission	leukocyte	*GULP1*	-1.105197846	0.000695868	0.001510534	1
T2DM_admission	leukocyte	*FAM171B*	-1.029190606	0.000517763	0.00131413	1
T2DM_admission	leukocyte	*METTL21A*	-1.184572136	0.000563312	0.001362351	1
T2DM_admission	leukocyte	*SUMF1*	-1.67622753	0.000208064	0.001062643	0.817484838
T2DM_admission	leukocyte	*HES6*	-3.144732111	4.18E-05	0.001037577	0.164102399
T2DM_admission	leukocyte	*ESPNL*	-10.22297641	0.021729763	0.026780502	1
T2DM_admission	leukocyte	*TAMM41*	-1.159958944	0.005552434	0.007609178	1
T2DM_admission	leukocyte	*RAB5A*	-1.11500926	0.000298731	0.001093914	1
T2DM_admission	leukocyte	*CNTN4*	-1.315850986	9.68E-05	0.001062643	0.380516251
T2DM_admission	leukocyte	*POMGNT2*	-2.199956163	9.84E-05	0.001062643	0.38664085
T2DM_admission	leukocyte	*CTDSPL*	-1.073241804	0.000508996	0.001305383	1
T2DM_admission	leukocyte	*IL17RD*	-4.263855649	0.00248512	0.003708332	1
T2DM_admission	leukocyte	*UBA3*	-1.816179111	0.000155729	0.001062643	0.611861149
T2DM_admission	leukocyte	*ARL6IP5*	-1.098313592	0.000224524	0.001062643	0.88215297
T2DM_admission	leukocyte	*LIMD1*	-1.887715891	0.000501955	0.001297006	1
T2DM_admission	leukocyte	*ZNF660*	-1.241162542	0.006375462	0.008617309	1
T2DM_admission	leukocyte	*NXPE3*	-1.065612793	0.000920186	0.001793131	1
T2DM_admission	leukocyte	*PHLDB2*	-0.836731232	0.000767361	0.001600298	1
T2DM_admission	leukocyte	*ABHD10*	-1.156948281	0.002062824	0.003151674	1
T2DM_admission	leukocyte	*TAGLN3*	-0.610153312	0.005280969	0.007267576	1
T2DM_admission	leukocyte	*EIF2A*	-0.979151071	0.000363032	0.001144746	1
T2DM_admission	leukocyte	*ALDH1L1*	-1.531094028	0.001219947	0.002133102	1
T2DM_admission	leukocyte	*TRPC1*	-1.371229193	0.000603216	0.001403431	1
T2DM_admission	leukocyte	*NCEH1*	-0.692310403	0.000990851	0.001869863	1
T2DM_admission	leukocyte	*AMT*	-1.94844889	0.000560055	0.001360512	1
T2DM_admission	leukocyte	*DGKQ*	-1.131984065	0.002505467	0.003733022	1
T2DM_admission	leukocyte	*SCD5*	-0.959552072	0.000642019	0.001441424	1
T2DM_admission	leukocyte	*PYURF*	-0.955406642	0.000307613	0.001098736	1
T2DM_admission	leukocyte	*TBCK*	-1.081704545	0.029448788	0.035623241	1
T2DM_admission	leukocyte	*DDIT4L*	-3.053912935	0.000403871	0.001185781	1
T2DM_admission	leukocyte	*TIFA*	-1.454900506	0.000366135	0.001147001	1
T2DM_admission	leukocyte	*CYP4V2*	-2.729640126	0.000101335	0.001062643	0.398147047
T2DM_admission	leukocyte	*SSBP2*	-0.929715135	0.000693177	0.001507191	1
T2DM_admission	leukocyte	*GTF2H2*	-3.337861488	5.17E-05	0.00105496	0.203200223
T2DM_admission	leukocyte	*BTF3*	-0.906750989	0.000187693	0.001062643	0.73744639
T2DM_admission	leukocyte	*SPATA9*	-5.344456316	0.037824917	0.045185193	1
T2DM_admission	leukocyte	*COMMD10*	-1.340629541	0.000505904	0.00130085	1
T2DM_admission	leukocyte	*ATG12*	-1.137649812	0.000197521	0.001062643	0.776061393
T2DM_admission	leukocyte	*PCYOX1L*	-6.659780723	4.24E-05	0.001037577	0.166709596
T2DM_admission	leukocyte	*TBC1D7*	-4.025830225	0.006121747	0.008319732	1
T2DM_admission	leukocyte	*ZMAT2*	-1.472742309	0.000173545	0.001062643	0.681858303
T2DM_admission	leukocyte	*KLHL3*	-5.651439227	2.47E-05	0.000982543	0.097023021

T2DM_admission	leukocyte	*DCDC2*	-0.835255715	0.001057588	0.001951744	1
T2DM_admission	leukocyte	*TRIM7*	-4.047696277	6.50E-05	0.001060479	0.255575433
T2DM_admission	leukocyte	*ANO7*	-3.261386929	0.000583463	0.001382042	1
T2DM_admission	leukocyte	*RPL7L1*	-1.074749916	0.000280818	0.001088708	1
T2DM_admission	leukocyte	*IRAK1BP1*	-0.935606099	0.001182502	0.002099858	1
T2DM_admission	leukocyte	*MMS22L*	-1.620682348	0.000746052	0.001568868	1
T2DM_admission	leukocyte	*FAXC*	-1.747206274	0.000559437	0.001360165	1
T2DM_admission	leukocyte	*SCML4*	-5.397170559	1.60E-05	0.000823546	0.062695268
T2DM_admission	leukocyte	*TBC1D32*	-3.02095262	0.004670409	0.006486404	1
T2DM_admission	leukocyte	*ABRACL*	-1.102851927	0.001178045	0.002098083	1
T2DM_admission	leukocyte	*DYNLT1*	-1.005914377	0.00015454	0.001062643	0.607187259
T2DM_admission	leukocyte	*WTAP*	-0.993355158	0.000217538	0.001062643	0.854706217
T2DM_admission	leukocyte	*SLC22A3*	-5.579599767	0.017336605	0.021748251	1
T2DM_admission	leukocyte	*VWDE*	-1.077702926	0.001021081	0.001908576	1
T2DM_admission	leukocyte	*GNA12*	-1.848729188	0.00037412	0.001157429	1
T2DM_admission	leukocyte	*SDK1*	-1.317758375	0.001284566	0.002183928	1
T2DM_admission	leukocyte	*CCZ1B*	-1.943959068	0.000197602	0.001062643	0.776377221
T2DM_admission	leukocyte	*RBAK*	-2.282948416	0.001351129	0.002257051	1
T2DM_admission	leukocyte	*POMZP3*	-1.288791903	0.000555435	0.001357223	1
T2DM_admission	leukocyte	*GIGYF1*	-2.907118033	6.32E-06	0.000591121	0.024827101
T2DM_admission	leukocyte	*TRIM4*	-1.077949855	0.00017557	0.001062643	0.689815989
T2DM_admission	leukocyte	*TMEM209*	-1.88031952	0.000117163	0.001062643	0.460334237
T2DM_admission	leukocyte	*NCAPG2*	0.853268087	0.02190513	0.026988164	1
T2DM_admission	leukocyte	*RAB19*	-9.349276857	0.000320616	0.001113794	1
T2DM_admission	leukocyte	*CSGALNACT1*	-1.00732677	0.001069833	0.001964163	1
T2DM_admission	leukocyte	*CCDC25*	-0.9612025	0.000329174	0.001123654	1
T2DM_admission	leukocyte	*BIN3*	-3.410826972	0.000123364	0.001062643	0.484696325
T2DM_admission	leukocyte	*CHMP7*	-1.630734713	0.000814135	0.001659956	1
T2DM_admission	leukocyte	*STAR*	0.686441651	0.011456422	0.014753288	1
T2DM_admission	leukocyte	*LACTB2*	-1.039165054	0.000472339	0.001256287	1
T2DM_admission	leukocyte	*TERF1*	-2.474927629	4.52E-05	0.001037577	0.177425645
T2DM_admission	leukocyte	*LRP12*	-1.193745953	0.000327877	0.001122427	1
T2DM_admission	leukocyte	*ZNF7*	-0.975871233	0.000825049	0.001673837	1
T2DM_admission	leukocyte	*AK3*	-1.054011622	0.00027807	0.001088708	1
T2DM_admission	leukocyte	*CER1*	3.614150525	0.008794658	0.011552729	1
T2DM_admission	leukocyte	*C9orf72*	-1.801768118	0.000105854	0.001062643	0.415901882
T2DM_admission	leukocyte	*FBXO10*	-1.538131447	0.002076734	0.003167503	1
T2DM_admission	leukocyte	*SIGMAR1*	-1.445288982	0.000133405	0.001062643	0.524148991
T2DM_admission	leukocyte	*ZNF462*	-0.997434794	0.000737585	0.00155805	1
T2DM_admission	leukocyte	*UGCG*	-1.243977934	0.000150498	0.001062643	0.591306327
T2DM_admission	leukocyte	*ALAD*	-1.166562446	0.002208475	0.003341201	1
T2DM_admission	leukocyte	*SURF4*	-1.029152294	0.000152154	0.001062643	0.59781394
T2DM_admission	leukocyte	*SURF1*	-1.371770316	0.000107942	0.001062643	0.424104133
T2DM_admission	leukocyte	*SURF6*	-1.741282983	0.000179605	0.001062643	0.705668952
T2DM_admission	leukocyte	*SH3GLB2*	-1.976227268	8.58E-05	0.001062643	0.336929953
T2DM_admission	leukocyte	*LCN2*	-0.466037676	0.001097282	0.001995936	1
T2DM_admission	leukocyte	*GPR107*	-1.560968999	0.000431701	0.001216752	1
T2DM_admission	leukocyte	*SEC16A*	-1.104975242	0.013894192	0.017729874	1
T2DM_admission	leukocyte	*MSRB2*	-1.887815183	8.37E-05	0.001062643	0.328830178
T2DM_admission	leukocyte	*RSU1*	-1.472504436	0.000400481	0.001184732	1
T2DM_admission	leukocyte	*GLUD1*	-1.395666481	0.000214264	0.001062643	0.841843453
T2DM_admission	leukocyte	*FRA10AC1*	-0.863759443	0.000799728	0.001644234	1
T2DM_admission	leukocyte	*DNAJB12*	-1.522643006	0.000182165	0.001062643	0.715724594
T2DM_admission	leukocyte	*LRRC27*	-2.748338635	0.008952611	0.01174451	1
T2DM_admission	leukocyte	*MTG1*	-1.003521254	0.000599212	0.001399402	1
T2DM_admission	leukocyte	*PAOX*	-5.344629191	0.00061305	0.001415201	1
T2DM_admission	leukocyte	*GSTO1*	-1.449110941	0.000398525	0.001183526	1
T2DM_admission	leukocyte	*HSD17B12*	-1.214530076	8.74E-09	4.91E-06	3.44E-05
T2DM_admission	leukocyte	*APIP*	-1.560311378	7.98E-07	0.000164962	0.003134273
T2DM_admission	leukocyte	*PTPRJ*	-1.529329838	0.000518519	0.001314363	1
T2DM_admission	leukocyte	*CCDC82*	-1.330385958	4.19E-05	0.001037577	0.164572304
T2DM_admission	leukocyte	*RPS3*	-0.773802012	0.00018851	0.001062643	0.740654378
T2DM_admission	leukocyte	*AASDHPPT*	-1.236230589	0.000286953	0.001088708	1
T2DM_admission	leukocyte	*GLB1L2*	-1.031453847	0.003500084	0.005015255	1

T2DM_admission	leukocyte	*FADS1*	-0.677632152	0.017202149	0.021620999	1
T2DM_admission	leukocyte	*TMC2*	-67.09675862	0.014683741	0.018658608	1
T2DM_admission	leukocyte	*EML3*	-5.259789936	0.000150114	0.001062643	0.589796159
T2DM_admission	leukocyte	*CCDC15*	-1.443350337	0.000261304	0.001083674	1
T2DM_admission	leukocyte	*CHEK1*	-1.46351377	0.00052494	0.001319879	1
T2DM_admission	leukocyte	*SIDT2*	-1.662964211	5.26E-05	0.00105496	0.206772115
T2DM_admission	leukocyte	*TAGLN*	-1.268064892	5.31E-06	0.00058206	0.020846529
T2DM_admission	leukocyte	*DUSP15*	-1.404043681	0.001042246	0.001936841	1
T2DM_admission	leukocyte	*DSN1*	-1.343994225	0.000762259	0.001593931	1
T2DM_admission	leukocyte	*YTHDF1*	-1.30679638	0.00028185	0.001088708	1
T2DM_admission	leukocyte	*CABLES2*	-1.435668218	0.001407228	0.002326041	1
T2DM_admission	leukocyte	*TRPT1*	-1.094226811	0.001042764	0.001936841	1
T2DM_admission	leukocyte	*MPP7*	-0.905779834	0.001090174	0.001985764	1
T2DM_admission	leukocyte	*DCUN1D2*	-1.28378205	0.002494315	0.003720348	1
T2DM_admission	leukocyte	*TMCO3*	-1.337853675	0.000413253	0.001193	1
T2DM_admission	leukocyte	*TMEM218*	-1.847215946	0.000205728	0.001062643	0.808306477
T2DM_admission	leukocyte	*MIA2*	-1.162884873	0.000202255	0.001062643	0.794660443
T2DM_admission	leukocyte	*LYPD1*	-1.784198903	0.001077244	0.001970434	1
T2DM_admission	leukocyte	*LYPD6B*	-2.912880154	0.001085931	0.00197991	1
T2DM_admission	leukocyte	*WDR17*	-0.888149297	0.002085011	0.003176428	1
T2DM_admission	leukocyte	*CNDP1*	-4.128689994	0.01501327	0.01901584	1
T2DM_admission	leukocyte	*PRSS23*	-0.909005241	0.000414095	0.001193676	1
T2DM_admission	leukocyte	*MTMR12*	-0.967329265	0.000491753	0.001283904	1
T2DM_admission	leukocyte	*DOCK1*	-1.221002989	0.000382471	0.001165285	1
T2DM_admission	leukocyte	*IL18*	0.520542993	0.017974021	0.022483262	1
T2DM_admission	leukocyte	*TMEM86A*	-1.364488505	0.001235289	0.002144697	1
T2DM_admission	leukocyte	*PLBD2*	-2.50201669	0.008057845	0.010681267	1
T2DM_admission	leukocyte	*DLG5*	-1.161179773	0.000279821	0.001088708	1
T2DM_admission	leukocyte	*SLC2A13*	-0.905047208	0.00044978	0.001234067	1
T2DM_admission	leukocyte	*MAGI1*	-1.015300554	0.002509134	0.003735653	1
T2DM_admission	leukocyte	*CSNK1G3*	-1.252557605	0.000933556	0.001804129	1
T2DM_admission	leukocyte	*AKAP6*	-1.398174234	0.000615119	0.001419144	1
T2DM_admission	leukocyte	*MBIP*	-1.315824719	0.000370392	0.001151894	1
T2DM_admission	leukocyte	*MIPOL1*	-1.014348896	0.001455166	0.002374314	1
T2DM_admission	leukocyte	*TMEM18*	-1.256337418	0.000323458	0.001116892	1
T2DM_admission	leukocyte	*NDUFC2*	-0.97095267	0.000165953	0.001062643	0.652029189
T2DM_admission	leukocyte	*VIPAS39*	-2.28086421	2.86E-05	0.001008808	0.112393677
T2DM_admission	leukocyte	*C4orf33*	-2.494759427	0.000379943	0.001164428	1
T2DM_admission	leukocyte	*QDPR*	-0.843393347	0.000735593	0.001555514	1
T2DM_admission	leukocyte	*TEX9*	-2.159312593	0.000187134	0.001062643	0.735249325
T2DM_admission	leukocyte	*ZNF827*	-1.109550351	0.000321168	0.001114222	1
T2DM_admission	leukocyte	*AKR1C2*	-2.427607146	0.001844202	0.002870788	1
T2DM_admission	leukocyte	*KIN*	-2.120544726	0.000151972	0.001062643	0.597097274
T2DM_admission	leukocyte	*TMEM45B*	-1.626351386	0.003048292	0.004437473	1
T2DM_admission	leukocyte	*CENPU*	-1.97678006	0.031421426	0.037834748	1
T2DM_admission	leukocyte	*BICD1*	-0.94853568	0.00126018	0.002164729	1
T2DM_admission	leukocyte	*GUF1*	-3.355660512	1.44E-05	0.000815363	0.056591434
T2DM_admission	leukocyte	*CCL28*	-1.409987039	0.000602635	0.001403431	1
T2DM_admission	leukocyte	*CACUL1*	-1.758610809	9.95E-05	0.001062643	0.390897644
T2DM_admission	leukocyte	*KCNE4*	-1.415072403	0.001051401	0.001942151	1
T2DM_admission	leukocyte	*RABGAP1L*	-1.347470583	0.000267907	0.001087356	1
T2DM_admission	leukocyte	*PTPN14*	-1.426222139	3.93E-06	0.000498523	0.015454216
T2DM_admission	leukocyte	*ARL14EP*	-1.690241152	8.53E-05	0.001062643	0.335163675
T2DM_admission	leukocyte	*EPG5*	-2.031245559	0.000121899	0.001062643	0.478941375
T2DM_admission	leukocyte	*HAUS1*	-1.563480084	0.000409021	0.001188129	1
T2DM_admission	leukocyte	*SPC25*	-1.244057303	0.002537522	0.003770774	1
T2DM_admission	leukocyte	*PDK1*	-1.42432239	0.000550248	0.001351735	1
T2DM_admission	leukocyte	*PDE3B*	-0.748322417	0.000345415	0.001140861	1
T2DM_admission	leukocyte	*TGOLN2*	-1.272010232	8.78E-05	0.001062643	0.344865546
T2DM_admission	leukocyte	*ATG10*	-0.849483474	0.020001725	0.02486036	1
T2DM_admission	leukocyte	*POC5*	-2.508682321	5.75E-09	3.77E-06	2.26E-05
T2DM_admission	leukocyte	*GUCY1A2*	-1.118335662	0.001203608	0.002114239	1
T2DM_admission	leukocyte	*ZNF773*	-0.675518631	0.03790641	0.04526878	1
T2DM_admission	leukocyte	*DCLRE1C*	-3.302011758	0.000665729	0.001470292	1

T2DM_admission	leukocyte	*ZSCAN1*	-1.323024801	0.00012827	0.001062643	0.503973232
T2DM_admission	leukocyte	*USP12*	-1.10581564	0.000349765	0.001141109	1
T2DM_admission	leukocyte	*CCDC50*	-0.867042895	0.001030832	0.001919497	1
T2DM_admission	leukocyte	*PLEKHH2*	-0.93267329	0.009674979	0.012616326	1
T2DM_admission	leukocyte	*GRIA4*	-1.478180535	0.000778117	0.001614162	1
T2DM_admission	leukocyte	*IGSF10*	-2.751854019	0.001024298	0.00191186	1
T2DM_admission	leukocyte	*SPEF2*	-1.04659789	0.010918708	0.014130304	1
T2DM_admission	leukocyte	*GPD1L*	-1.201541717	0.002225674	0.003365925	1
T2DM_admission	leukocyte	*CCNO*	-1.47706486	0.000267907	0.001087356	1
T2DM_admission	leukocyte	*RASGRP3*	-1.217692041	0.002736922	0.004028987	1
T2DM_admission	leukocyte	*CATSPER3*	-6.160649985	0.00118831	0.00210081	1
T2DM_admission	leukocyte	*GPR180*	-1.055977554	0.000608612	0.001409123	1
T2DM_admission	leukocyte	*IFIT5*	-1.194714341	0.001221451	0.002133102	1
T2DM_admission	leukocyte	*ZNF117*	-0.705311889	0.033295451	0.040005451	1
T2DM_admission	leukocyte	*MED21*	-2.026268275	3.85E-05	0.001037577	0.151322836
T2DM_admission	leukocyte	*LGI2*	-1.61202336	0.002904205	0.004252935	1
T2DM_admission	leukocyte	*SRP19*	-1.017467637	0.000218474	0.001062643	0.858385873
T2DM_admission	leukocyte	*DAB2*	-1.611732847	0.000199231	0.001062643	0.782777556
T2DM_admission	leukocyte	*ACMSD*	-1.472496421	0.001939246	0.002997363	1
T2DM_admission	leukocyte	*CAST*	-1.06249581	0.000212789	0.001062643	0.836049284
T2DM_admission	leukocyte	*SYCP2L*	-2.779538905	0.006929449	0.009301608	1
T2DM_admission	leukocyte	*MERTK*	0.51247002	0.041848252	0.049674254	1
T2DM_admission	leukocyte	*TMEM87B*	-1.289881436	0.00056418	0.001362351	1
T2DM_admission	leukocyte	*PLA2R1*	-3.80528952	0.001331979	0.002235518	1
T2DM_admission	leukocyte	*SLC25A27*	-1.727301171	0.000238937	0.001072513	0.93878201
T2DM_admission	leukocyte	*ASAP1*	-1.067378127	0.000598646	0.001399402	1
T2DM_admission	leukocyte	*NMRAL1*	-1.353470804	0.001230549	0.002143098	1
T2DM_admission	leukocyte	*CMTM7*	-1.052119619	0.000407416	0.001188129	1
T2DM_admission	leukocyte	*RPIA*	-1.246043504	0.000330708	0.001124979	1
T2DM_admission	leukocyte	*PTPRD*	-0.763231286	0.035765346	0.042855153	1
T2DM_admission	leukocyte	*GTF2E1*	-3.045558897	0.000102677	0.001062643	0.403417638
T2DM_admission	leukocyte	*CFDP1*	-0.986098887	0.000773102	0.001608876	1
T2DM_admission	leukocyte	*ZDHHC7*	-1.420832697	0.01395508	0.01779601	1
T2DM_admission	leukocyte	*JAZF1*	-2.604488043	8.35E-11	1.09E-07	3.28E-07
T2DM_admission	leukocyte	*CMIP*	-1.24673524	0.000281529	0.001088708	1
T2DM_admission	leukocyte	*PID1*	-2.073288215	0.004668774	0.006486404	1
T2DM_admission	leukocyte	*KCTD15*	-1.576460448	0.001264578	0.002168716	1
T2DM_admission	leukocyte	*LGI4*	1.197666815	0.012930074	0.01655336	1
T2DM_admission	leukocyte	*DDAH1*	-2.723884777	0.000114523	0.001062643	0.449962716
T2DM_admission	leukocyte	*NUS1*	-1.483571542	5.11E-05	0.00105496	0.200706188
T2DM_admission	leukocyte	*PPP2R5E*	-1.276879724	0.0006343	0.001434874	1
T2DM_admission	leukocyte	*ROBO3*	-3.32717089	0.001203755	0.002114239	1
T2DM_admission	leukocyte	*PITPNC1*	-1.360692402	0.000279557	0.001088708	1
T2DM_admission	leukocyte	*CC2D1B*	-2.489019686	0.000236055	0.001068391	0.927458548
T2DM_admission	leukocyte	*CEP112*	-0.956665531	0.000933461	0.001804129	1
T2DM_admission	leukocyte	*ABCA5*	-1.871051334	0.000103345	0.001062643	0.406042832
T2DM_admission	leukocyte	*SRSF12*	-1.961076702	0.002151571	0.003265736	1
T2DM_admission	leukocyte	*PDLIM3*	-0.749158135	0.00167717	0.002656459	1
T2DM_admission	leukocyte	*PDE1C*	-1.242195376	0.007085138	0.009487903	1
T2DM_admission	leukocyte	*RABGEF1*	-1.316895371	0.000207948	0.001062643	0.817028272
T2DM_admission	leukocyte	*MRPL39*	-1.364420709	0.00057789	0.001376912	1
T2DM_admission	leukocyte	*TSEN2*	-2.433564184	0.001556849	0.002504379	1
T2DM_admission	leukocyte	*FLCN*	-1.948246304	0.000170117	0.001062643	0.668387842
T2DM_admission	leukocyte	*MPPE1*	-1.468142051	0.000361342	0.001142338	1
T2DM_admission	leukocyte	*RAB6B*	-1.43973676	0.000616973	0.001419945	1
T2DM_admission	leukocyte	*ACSS1*	-1.373461329	0.012609416	0.016169189	1
T2DM_admission	leukocyte	*ANKRD40*	-1.561285829	0.000605398	0.00140595	1
T2DM_admission	leukocyte	*ATP6V1C1*	-1.108000033	0.000435936	0.001222199	1
T2DM_admission	leukocyte	*MARVELD1*	-4.587680561	4.38E-05	0.001037577	0.171910543
T2DM_admission	leukocyte	*TRMT44*	-1.008193873	0.002111226	0.003212628	1
T2DM_admission	leukocyte	*PPM1J*	-2.754894717	0.00178231	0.00279103	1
T2DM_admission	leukocyte	*DBI*	-1.205349278	0.000114796	0.001062643	0.451034233
T2DM_admission	leukocyte	*HEATR3*	-0.733045361	0.020069623	0.024918648	1
T2DM_admission	leukocyte	*LRGUK*	-3.859319467	0.000648161	0.001447767	1

T2DM_admission	leukocyte	*SETD9*	-1.510326338	4.62E-05	0.001054402	0.181357219
T2DM_admission	leukocyte	*MIER3*	-2.81117627	2.50E-05	0.000982543	0.098247942
T2DM_admission	leukocyte	*TTN*	-3.148263733	9.42E-05	0.001062643	0.370050392
T2DM_admission	leukocyte	*DEPTOR*	-1.061043191	0.007914652	0.010516289	1
T2DM_admission	leukocyte	*RMND1*	-2.066343962	0.000285788	0.001088708	1
T2DM_admission	leukocyte	*TMBIM4*	-1.114161749	0.000162874	0.001062643	0.639931433
T2DM_admission	leukocyte	*PSD3*	-1.012805853	0.000764758	0.001596564	1
T2DM_admission	leukocyte	*FAM161B*	-1.541266533	0.007303299	0.00976341	1
T2DM_admission	leukocyte	*MMP16*	-0.92205008	0.002012898	0.003089327	1
T2DM_admission	leukocyte	*ADK*	-1.308858245	0.000467792	0.001253701	1
T2DM_admission	leukocyte	*BATF*	-16.43774177	0.001139796	0.002049538	1
T2DM_admission	leukocyte	*DRAM2*	-1.85687388	0.000783718	0.001618943	1
T2DM_admission	leukocyte	*CCT8*	-0.849012473	0.001713264	0.002707728	1
T2DM_admission	leukocyte	*CDK20*	-1.035098803	0.001777986	0.002785369	1
T2DM_admission	leukocyte	*TDRD9*	-0.788552894	0.000547822	0.001347773	1
T2DM_admission	leukocyte	*HKDC1*	-2.715928761	0.001845831	0.002872187	1
T2DM_admission	leukocyte	*ZDHHC5*	-1.42157641	0.000317351	0.001111111	1
T2DM_admission	leukocyte	*MED19*	-1.173396653	0.000286792	0.001088708	1
T2DM_admission	leukocyte	*MAPK13*	-1.31679312	0.000891541	0.001763074	1
T2DM_admission	leukocyte	*COX6A2*	-1.297672616	0.003898566	0.005528505	1
T2DM_admission	leukocyte	*MPV17L*	-1.141661049	0.000718915	0.0015393	1
T2DM_admission	leukocyte	*TATDN2*	-2.095556694	0.000270678	0.00108853	1
T2DM_admission	leukocyte	*EXOG*	-1.668753764	6.92E-05	0.001062643	0.272003371
T2DM_admission	leukocyte	*ZFYVE9*	-1.279521208	0.000524586	0.001319879	1
T2DM_admission	leukocyte	*SLC6A1*	-6.528151602	0.001187122	0.00210081	1
T2DM_admission	leukocyte	*NECAP2*	-1.582996266	0.000216346	0.001062643	0.850024067
T2DM_admission	leukocyte	*STEAP2*	-1.610437775	0.001822412	0.00284702	1
T2DM_admission	leukocyte	*GATAD1*	-1.581163918	0.000195265	0.001062643	0.767196767
T2DM_admission	leukocyte	*CLEC18A*	-6.985106048	3.13E-11	6.14E-08	1.23E-07
T2DM_admission	leukocyte	*DHRS4*	-1.43147627	0.000278836	0.001088708	1
T2DM_admission	leukocyte	*DHRS1*	-1.267799642	0.003469	0.004977977	1
T2DM_admission	leukocyte	*AASDH*	-0.877672846	0.002063147	0.003151674	1
T2DM_admission	leukocyte	*SLC38A10*	-1.417150712	0.000227097	0.001062643	0.892263005
T2DM_admission	leukocyte	*SLC37A3*	-0.91401549	0.000807729	0.0016529	1
T2DM_admission	leukocyte	*AP3S2*	-1.213408224	0.00370191	0.005275591	1
T2DM_admission	leukocyte	*SPPL3*	-1.410958972	0.000167506	0.001062643	0.658131783
T2DM_admission	leukocyte	*TNFRSF14*	0.631283373	0.03498844	0.041962631	1
T2DM_admission	leukocyte	*C12orf43*	-1.465304978	0.000508588	0.001305383	1
T2DM_admission	leukocyte	*PEX10*	-2.055621085	0.000128032	0.001062643	0.503035919
T2DM_admission	leukocyte	*LDLRAP1*	-0.987399166	0.000438259	0.001222199	1
T2DM_admission	leukocyte	*KRTCAP3*	-1.167097182	0.000584057	0.001382042	1
T2DM_admission	leukocyte	*PAFAH2*	-3.512677294	0.000110094	0.001062643	0.432559878
T2DM_admission	leukocyte	*EXTL1*	-7.476451428	1.36E-05	0.000815363	0.053486067
T2DM_admission	leukocyte	*TRIM63*	-16.23890482	0.000724199	0.001544387	1
T2DM_admission	leukocyte	*XKR8*	-2.241836613	0.000181387	0.001062643	0.712669125
T2DM_admission	leukocyte	*CNNM4*	-2.101934426	0.000456011	0.001240899	1
T2DM_admission	leukocyte	*ESYT3*	-2.446045641	0.000556459	0.001357223	1
T2DM_admission	leukocyte	*FAIM*	-1.272468676	0.000261847	0.001083674	1
T2DM_admission	leukocyte	*AHCYL2*	-1.407992445	0.000929772	0.001800431	1
T2DM_admission	leukocyte	*COPG2*	-1.375882413	6.14E-05	0.001060479	0.241279383
T2DM_admission	leukocyte	*RNF166*	-1.589406616	0.000550283	0.001351735	1
T2DM_admission	leukocyte	*NIT1*	-1.56019798	0.002430647	0.0036381	1
T2DM_admission	leukocyte	*NPM2*	-1.3748413	0.000684491	0.001494923	1
T2DM_admission	leukocyte	*FGF17*	-1.831402935	0.040298398	0.04799406	1
T2DM_admission	leukocyte	*VWA5B1*	2.762222131	0.010721612	0.013888959	1
T2DM_admission	leukocyte	*CDA*	-13.25277571	0.002350694	0.003534587	1
T2DM_admission	leukocyte	*PINK1*	-1.664523749	3.91E-05	0.001037577	0.153708044
T2DM_admission	leukocyte	*DMTN*	-1.177611249	0.002951985	0.004310096	1
T2DM_admission	leukocyte	*CCAR2*	-1.783394478	0.00020023	0.001062643	0.786705405
T2DM_admission	leukocyte	*EPB41*	-1.16805185	0.000583625	0.001382042	1
T2DM_admission	leukocyte	*MIS18A*	-1.57399542	0.000131834	0.001062643	0.517977079
T2DM_admission	leukocyte	*ALG8*	-1.091983968	0.001045656	0.001938832	1
T2DM_admission	leukocyte	*IFNAR2*	-2.257395958	0.000630885	0.001433512	1
T2DM_admission	leukocyte	*IFNGR2*	-1.305847873	0.000119157	0.001062643	0.468169291

T2DM_admission	leukocyte	*C1QC*	0.061458614	0.007217952	0.009655884	1
T2DM_admission	leukocyte	*GJD2*	-0.771499215	0.0005565	0.001357223	1
T2DM_admission	leukocyte	*CLDN14*	-2.379258711	0.005858792	0.007995552	1
T2DM_admission	leukocyte	*HLCS*	-1.059075656	0.001130716	0.002036018	1
T2DM_admission	leukocyte	*THEM4*	-0.979589181	0.000630594	0.001433512	1
T2DM_admission	leukocyte	*UBR1*	-1.559797479	0.000346551	0.001140861	1
T2DM_admission	leukocyte	*TEPP*	3.505806874	0.021611165	0.026651057	1
T2DM_admission	leukocyte	*ZDHHC1*	-1.264985545	0.000385353	0.001168251	1
T2DM_admission	leukocyte	*ZFYVE28*	-1.537371617	0.001325599	0.002228618	1
T2DM_admission	leukocyte	*LYPD5*	-12.75124368	0.00668818	0.009008522	1
T2DM_admission	leukocyte	*NPR2*	-1.065551107	0.016178837	0.020406629	1
T2DM_admission	leukocyte	*ATAD3B*	-2.962663183	0.000333015	0.00112758	1
T2DM_admission	leukocyte	*ANKLE1*	0.939725533	0.000258993	0.001081954	1
T2DM_admission	leukocyte	*KALRN*	-1.585488642	0.001550761	0.002501207	1
T2DM_admission	leukocyte	*CILP2*	-4.277925754	6.24E-07	0.000144219	0.002451725
T2DM_admission	leukocyte	*TMPRSS3*	-3.222606595	0.002930845	0.004287152	1
T2DM_admission	leukocyte	*RSPH1*	-0.797401527	0.001081912	0.001975294	1
T2DM_admission	leukocyte	*SLC37A1*	-1.192794318	0.001610792	0.002572683	1
T2DM_admission	leukocyte	*NDUFV3*	-0.821386892	0.001859808	0.002891644	1
T2DM_admission	leukocyte	*HSF2BP*	-3.527916446	0.001050136	0.001940726	1
T2DM_admission	leukocyte	*PDXK*	-1.599817091	4.47E-05	0.001037577	0.175578267
T2DM_admission	leukocyte	*CSTB*	-1.373379314	0.000227784	0.001062643	0.894963728
T2DM_admission	leukocyte	*ICOSLG*	0.471226275	0.01717084	0.021595465	1
T2DM_admission	leukocyte	*ZNF66*	-3.31698346	0.000575437	0.001373567	1
T2DM_admission	leukocyte	*FTCD*	-1.812062988	0.000168588	0.001062643	0.66238095
T2DM_admission	leukocyte	*SPATC1L*	-0.956278311	0.000563304	0.001362351	1
T2DM_admission	leukocyte	*LSS*	-1.28591662	0.001265865	0.002169029	1
T2DM_admission	leukocyte	*VAV2*	-1.781940255	0.000825909	0.001674406	1
T2DM_admission	leukocyte	*MCM3AP*	-1.651931859	0.003158215	0.004571187	1
T2DM_admission	leukocyte	*DIP2A*	-0.99178244	0.001191734	0.00210536	1
T2DM_admission	leukocyte	*ZNF208*	-1.162206736	0.000307472	0.001098736	1
T2DM_admission	leukocyte	*ADAMTS13*	-1.391231497	0.02009828	0.024941928	1
T2DM_admission	leukocyte	*SLC2A6*	-2.268560237	0.000165974	0.001062643	0.652110044
T2DM_admission	leukocyte	*RDH13*	-1.389990809	0.000886243	0.001757306	1
T2DM_admission	leukocyte	*MPZL3*	-1.397066856	0.000279933	0.001088708	1
T2DM_admission	leukocyte	*S100A1*	-2.128068139	0.006773537	0.009114118	1
T2DM_admission	leukocyte	*VPS11*	-3.05063363	2.17E-06	0.000340493	0.008512334
T2DM_admission	leukocyte	*PAQR6*	-1.602104869	0.000116949	0.001062643	0.459493111
T2DM_admission	leukocyte	*SLC25A44*	-1.983076932	0.000275902	0.001088708	1
T2DM_admission	leukocyte	*CCR5*	-139.2431028	0.003602379	0.00514869	1
T2DM_admission	leukocyte	*NBEAL2*	-0.844757055	0.001825595	0.002849726	1
T2DM_admission	leukocyte	*LY6E*	-1.394012364	0.000181968	0.001062643	0.714953713
T2DM_admission	leukocyte	*VPS28*	-0.967113013	0.00120611	0.002117283	1
T2DM_admission	leukocyte	*TONSL*	-6.0010195	0.000284979	0.001088708	1
T2DM_admission	leukocyte	*ZNF333*	-4.686519164	7.31E-05	0.001062643	0.287354317
T2DM_admission	leukocyte	*RPL8*	-0.759385973	0.000171089	0.001062643	0.672207467
T2DM_admission	leukocyte	*MAML1*	-2.170607284	0.000539646	0.001334341	1
T2DM_admission	leukocyte	*LRWD1*	-1.641762458	0.001276833	0.002175489	1
T2DM_admission	leukocyte	*CCDC116*	-9.420220801	0.007402757	0.009876208	1
T2DM_admission	leukocyte	*U2AF1L4*	-0.874173734	0.002416872	0.00362099	1
T2DM_admission	leukocyte	*ZNF382*	-1.042570627	0.003991494	0.005645278	1
T2DM_admission	leukocyte	*DUSP14*	-1.550946505	0.000356297	0.001141109	1
T2DM_admission	leukocyte	*PGAP3*	-1.241608917	0.00112341	0.002030303	1
T2DM_admission	leukocyte	*PRPSAP1*	-1.245422629	0.000195627	0.001062643	0.768619863
T2DM_admission	leukocyte	*SIGLEC11*	-15.01396169	0.014729643	0.018710885	1
T2DM_admission	leukocyte	*SPC24*	-3.08138128	0.0024608	0.003677627	1
T2DM_admission	leukocyte	*IP6K3*	3.468228043	0.000253607	0.001077213	0.996421609
T2DM_admission	leukocyte	*LEMD2*	-2.161481418	0.001580523	0.002532576	1
T2DM_admission	leukocyte	*CXCL16*	0.334616801	0.026396299	0.032068973	1
T2DM_admission	leukocyte	*RPL26*	-0.811795838	0.000155426	0.001062643	0.610666952
T2DM_admission	leukocyte	*SNRNP25*	-1.07611176	0.00035762	0.001141109	1
T2DM_admission	leukocyte	*WDR90*	-1.850662527	0.000581812	0.001382042	1
T2DM_admission	leukocyte	*CCDC78*	-3.158055414	0.003474434	0.004983954	1
T2DM_admission	leukocyte	*ZNF75A*	-2.588150178	2.88E-05	0.001008808	0.112986454

T2DM_admission	leukocyte	*ASRGL1*	-0.957333413	0.000779793	0.001614233	1
T2DM_admission	leukocyte	*TAF6L*	-1.496310189	0.001762386	0.002769765	1
T2DM_admission	leukocyte	*ZFPL1*	-1.382162344	0.000336045	0.001131077	1
T2DM_admission	leukocyte	*LRP5*	-2.451492336	8.44E-05	0.001062643	0.331797321
T2DM_admission	leukocyte	*TPCN2*	-1.181149749	0.014084501	0.017943581	1
T2DM_admission	leukocyte	*PDZK1IP1*	-0.845109303	0.000630759	0.001433512	1
T2DM_admission	leukocyte	*CTRC*	-0.60735686	0.000510227	0.001306833	1
T2DM_admission	leukocyte	*SDC3*	-2.262655119	0.000666674	0.001471551	1
T2DM_admission	leukocyte	*OMA1*	-1.648780042	0.001253605	0.002157431	1
T2DM_admission	leukocyte	*TM2D1*	-1.807331822	0.000120171	0.001062643	0.472152962
T2DM_admission	leukocyte	*FUBP1*	-1.071892691	0.000148135	0.001062643	0.582021926
T2DM_admission	leukocyte	*SNX7*	-0.938878629	0.000928852	0.001800431	1
T2DM_admission	leukocyte	*HFM1*	-2.155049284	0.001524677	0.002467446	1
T2DM_admission	leukocyte	*VCAM1*	-0.633323819	0.001321422	0.002223498	1
T2DM_admission	leukocyte	*ZNF496*	-1.177312283	0.000650132	0.001449765	1
T2DM_admission	leukocyte	*TRIM58*	-3.970260769	0.007385401	0.009859749	1
T2DM_admission	leukocyte	*PEA15*	-1.141569885	0.000300193	0.001093914	1
T2DM_admission	leukocyte	*FLVCR1*	-0.942648991	0.000587451	0.001383749	1
T2DM_admission	leukocyte	*TDRD5*	-3.180418924	0.008543937	0.011268589	1
T2DM_admission	leukocyte	*ACP6*	-1.716402652	0.000177955	0.001062643	0.699185715
T2DM_admission	leukocyte	*OXER1*	-3.305103895	0.017532538	0.021958987	1
T2DM_admission	leukocyte	*HAAO*	-1.322966307	0.001116686	0.00202187	1
T2DM_admission	leukocyte	*RFTN2*	-2.329392329	0.003246427	0.004685971	1
T2DM_admission	leukocyte	*KCNJ3*	-0.953468497	0.003153848	0.004569126	1
T2DM_admission	leukocyte	*DUSP19*	-1.985667213	0.000138919	0.001062643	0.54581367
T2DM_admission	leukocyte	*SMC6*	-2.165306676	0.000116154	0.001062643	0.456370942
T2DM_admission	leukocyte	*NOSTRIN*	-1.296122481	0.000731693	0.001551442	1
T2DM_admission	leukocyte	*CTSS*	0.223931536	0.01688082	0.021251118	1
T2DM_admission	leukocyte	*MSX1*	-1.241920695	0.001341362	0.002245911	1
T2DM_admission	leukocyte	*PACRGL*	-1.348041201	0.00063166	0.001433512	1
T2DM_admission	leukocyte	*ERCC3*	-1.556481392	0.000676583	0.00148591	1
T2DM_admission	leukocyte	*TDRD10*	-19.21544151	0.001764119	0.002770339	1
T2DM_admission	leukocyte	*CCNYL1*	-3.134343953	7.11E-05	0.001062643	0.27954506
T2DM_admission	leukocyte	*DCAF16*	-1.722270453	5.66E-05	0.001060479	0.222526642
T2DM_admission	leukocyte	*NPPC*	-13.44379912	3.87E-05	0.001037577	0.152144159
T2DM_admission	leukocyte	*GNPDA2*	-0.832918162	0.006423851	0.008679267	1
T2DM_admission	leukocyte	*NIPAL1*	-0.902699859	0.005040112	0.006955602	1
T2DM_admission	leukocyte	*CGGBP1*	-1.091766849	0.000775383	0.001611893	1
T2DM_admission	leukocyte	*DAPL1*	-4.432776455	1.06E-05	0.000784902	0.041777483
T2DM_admission	leukocyte	*EOGT*	-1.227689923	0.000475731	0.001261234	1
T2DM_admission	leukocyte	*POGLUT1*	-0.979910865	0.000716593	0.00153628	1
T2DM_admission	leukocyte	*IGFN1*	-1.112705409	0.012108257	0.0155621	1
T2DM_admission	leukocyte	*ATP1A1*	-0.809630357	0.000364707	0.001146348	1
T2DM_admission	leukocyte	*ARPC2*	-1.275678741	0.000121496	0.001062643	0.477355909
T2DM_admission	leukocyte	*CCT3*	-0.838654955	0.000896313	0.001768089	1
T2DM_admission	leukocyte	*EOMES*	-4.618098312	0.025055301	0.030610161	1
T2DM_admission	leukocyte	*CWC22*	-1.315476676	0.000407581	0.001188129	1
T2DM_admission	leukocyte	*HDAC11*	-3.2840892	0.000633156	0.001434874	1
T2DM_admission	leukocyte	*SUCLG1*	-1.205756119	0.000205742	0.001062643	0.808361382
T2DM_admission	leukocyte	*EFHB*	-0.592097665	0.021307755	0.026301656	1
T2DM_admission	leukocyte	*ICA1L*	-1.646545885	4.16E-07	0.000116632	0.001632847
T2DM_admission	leukocyte	*RYBP*	-0.869760861	0.000709374	0.001527196	1
T2DM_admission	leukocyte	*GTPBP8*	-2.339371497	2.09E-05	0.000929374	0.082305223
T2DM_admission	leukocyte	*CADPS*	-0.608737719	0.000924204	0.001797337	1
T2DM_admission	leukocyte	*ATXN7*	-1.427653836	0.000157213	0.001062643	0.617688845
T2DM_admission	leukocyte	*PSMD6*	-1.066985963	0.000267272	0.001087356	1
T2DM_admission	leukocyte	*PRICKLE2*	-1.178956293	0.00143203	0.002353177	1
T2DM_admission	leukocyte	*RPL9*	-0.866441362	3.52E-05	0.001037577	0.138320452
T2DM_admission	leukocyte	*IL17RC*	-1.330876146	0.00034681	0.001140861	1
T2DM_admission	leukocyte	*CRELD1*	-0.807019782	0.00298285	0.004348652	1
T2DM_admission	leukocyte	*TTC14*	-2.050700573	9.81E-05	0.001062643	0.385518976
T2DM_admission	leukocyte	*CXCL3*	0.776451318	0.039013724	0.046548716	1
T2DM_admission	leukocyte	*CXCL1*	-0.797304604	0.000701343	0.001517388	1
T2DM_admission	leukocyte	*CCDC158*	-2.947720244	0.001412542	0.002331881	1

T2DM_admission	leukocyte	*TM4SF18*	-1.077441152	0.00305275	0.004442316	1
T2DM_admission	leukocyte	*SNRK*	-0.863411923	0.000467192	0.001253701	1
T2DM_admission	leukocyte	*KIAA1143*	-0.886186343	0.000915655	0.001788965	1
T2DM_admission	leukocyte	*CCR1*	0.823790035	0.020330622	0.025206379	1
T2DM_admission	leukocyte	*NMNAT3*	-0.943126301	0.002707653	0.003991883	1
T2DM_admission	leukocyte	*SMIM12*	-1.172263544	0.000286747	0.001088708	1
T2DM_admission	leukocyte	*TPRA1*	-2.244392185	0.000130987	0.001062643	0.514649526
T2DM_admission	leukocyte	*MEAF6*	-1.057489634	0.000280367	0.001088708	1
T2DM_admission	leukocyte	*DNALI1*	-1.214167976	0.000822629	0.001670341	1
T2DM_admission	leukocyte	*CAMK2N2*	-0.782277209	0.00048351	0.001273265	1
T2DM_admission	leukocyte	*RPN1*	-1.355511899	0.000105778	0.001062643	0.415602997
T2DM_admission	leukocyte	*IFT122*	-1.511462576	0.000900887	0.001772452	1
T2DM_admission	leukocyte	*RFC4*	-1.460514111	0.000637726	0.001438362	1
T2DM_admission	leukocyte	*TKT*	-1.299270013	0.000128597	0.001062643	0.505257585
T2DM_admission	leukocyte	*RFT1*	-2.281783405	8.78E-05	0.001062643	0.344838489
T2DM_admission	leukocyte	*UVSSA*	-1.502267941	0.0083601	0.011063265	1
T2DM_admission	leukocyte	*ARHGEF3*	-1.096239837	0.0013776	0.002288621	1
T2DM_admission	leukocyte	*LRPAP1*	-1.060980509	0.000196065	0.001062643	0.770339424
T2DM_admission	leukocyte	*PIGX*	-1.800960673	5.44E-05	0.001060479	0.213753512
T2DM_admission	leukocyte	*EXO5*	-2.06884535	0.000381652	0.001165285	1
T2DM_admission	leukocyte	*ERMAP*	-4.194032526	3.67E-05	0.001037577	0.144135583
T2DM_admission	leukocyte	*BDH2*	-1.409823235	1.94E-05	0.000917316	0.076244503
T2DM_admission	leukocyte	*CDC25A*	-1.907261646	0.000967203	0.001842046	1
T2DM_admission	leukocyte	*TEX264*	-1.533334026	9.49E-05	0.001062643	0.373053563
T2DM_admission	leukocyte	*PPM1M*	-1.764597161	0.000149746	0.001062643	0.588351348
T2DM_admission	leukocyte	*SAP30*	-1.125091975	0.000777515	0.001613765	1
T2DM_admission	leukocyte	*ANXA5*	-0.784367417	0.000306824	0.001098442	1
T2DM_admission	leukocyte	*CEP44*	-1.359913663	0.000327376	0.001122427	1
T2DM_admission	leukocyte	*HPGD*	-1.469042658	0.018704918	0.023360338	1
T2DM_admission	leukocyte	*NPY1R*	2.41610017	0.019841025	0.024692869	1
T2DM_admission	leukocyte	*ARFIP1*	-1.923211392	5.94E-05	0.001060479	0.233226948
T2DM_admission	leukocyte	*ITGA2*	-0.812241592	0.000978042	0.001856058	1
T2DM_admission	leukocyte	*ELOVL7*	-1.172154786	0.0005639	0.001362351	1
T2DM_admission	leukocyte	*LMBRD2*	-1.489585801	8.85E-05	0.001062643	0.347543575
T2DM_admission	leukocyte	*STARD4*	-1.692451597	0.000123658	0.001062643	0.485853698
T2DM_admission	leukocyte	*CMBL*	-1.267346896	0.000402133	0.001185421	1
T2DM_admission	leukocyte	*PRRC1*	-0.935524685	0.000511831	0.001308382	1
T2DM_admission	leukocyte	*AGGF1*	-1.698005126	8.75E-05	0.001062643	0.343698281
T2DM_admission	leukocyte	*WDR41*	-2.037558416	8.24E-05	0.001062643	0.323651383
T2DM_admission	leukocyte	*SERINC5*	-4.834434479	0.000104959	0.001062643	0.412382787
T2DM_admission	leukocyte	*ERAP1*	-1.307398646	0.030850577	0.037204395	1
T2DM_admission	leukocyte	*RICTOR*	-1.771261863	0.000108502	0.001062643	0.426302798
T2DM_admission	leukocyte	*UTP15*	-1.043763457	0.000826843	0.001675216	1
T2DM_admission	leukocyte	*NSA2*	-0.76070312	0.001043597	0.001936841	1
T2DM_admission	leukocyte	*LEAP2*	-1.753469931	0.001498341	0.002433642	1
T2DM_admission	leukocyte	*DACT2*	-5.017711845	0.000825203	0.001673837	1
T2DM_admission	leukocyte	*PDSS2*	-1.195333633	0.000539261	0.001334229	1
T2DM_admission	leukocyte	*STK17A*	-1.020011603	0.000670091	0.001477434	1
T2DM_admission	leukocyte	*GALNT10*	-2.243729434	0.002018824	0.003094794	1
T2DM_admission	leukocyte	*SAP30L*	-1.660689332	0.000214893	0.001062643	0.844316237
T2DM_admission	leukocyte	*HCN1*	-2.35428548	0.025864792	0.03148165	1
T2DM_admission	leukocyte	*COG5*	-2.197736319	8.64E-05	0.001062643	0.339325598
T2DM_admission	leukocyte	*SLU7*	-1.280033943	0.000194104	0.001062643	0.762635722
T2DM_admission	leukocyte	*CAMLG*	-0.990890728	0.000279692	0.001088708	1
T2DM_admission	leukocyte	*KIF6*	-1.89477151	0.002552798	0.003786313	1
T2DM_admission	leukocyte	*STEAP1*	-1.332056678	0.005670171	0.0077543	1
T2DM_admission	leukocyte	*FNDC1*	-7.272792598	0.002657186	0.003927797	1
T2DM_admission	leukocyte	*CHMP4C*	-1.157179264	0.000416748	0.001199564	1
T2DM_admission	leukocyte	*BRI3*	-1.604736423	2.38E-05	0.000982543	0.093468219
T2DM_admission	leukocyte	*LMTK2*	-1.262452765	0.000254324	0.001077348	0.999237943
T2DM_admission	leukocyte	*DLC1*	-0.860235866	0.026122796	0.031756332	1
T2DM_admission	leukocyte	*PEX2*	-1.034666441	0.000404315	0.001185781	1
T2DM_admission	leukocyte	*MED30*	-1.224918544	0.000318886	0.001113024	1
T2DM_admission	leukocyte	*TNFRSF11B*	-3.517407712	0.001580139	0.002532576	1

T2DM_admission	leukocyte	*CSMD3*	-76.95691984	0.001317259	0.002219344	1
T2DM_admission	leukocyte	*OSGIN2*	-1.727392108	0.000127815	0.001062643	0.502186632
T2DM_admission	leukocyte	*DEFB1*	-0.530694761	0.000907731	0.001777903	1
T2DM_admission	leukocyte	*SUN1*	-0.959750785	0.000371514	0.001152986	1
T2DM_admission	leukocyte	*TMEM184A*	-1.256221637	0.00055061	0.001351735	1
T2DM_admission	leukocyte	*INTS1*	-1.243744671	0.000458562	0.001240899	1
T2DM_admission	leukocyte	*YWHAZ*	-1.088496047	0.000140062	0.001062643	0.550302512
T2DM_admission	leukocyte	*DCAF13*	-1.208517927	0.000514767	0.001311621	1
T2DM_admission	leukocyte	*INTS8*	-1.485536019	0.002697746	0.003980265	1
T2DM_admission	leukocyte	*GEM*	-1.275889073	0.000537094	0.001334229	1
T2DM_admission	leukocyte	*RPP25L*	-2.008009748	0.000135786	0.001062643	0.533503315
T2DM_admission	leukocyte	*FAM219A*	-1.972303547	0.000144097	0.001062643	0.566158803
T2DM_admission	leukocyte	*NUDT2*	-1.410342659	0.000170993	0.001062643	0.671831158
T2DM_admission	leukocyte	*METTL2B*	-0.973882017	0.011016619	0.014233573	1
T2DM_admission	leukocyte	*FXN*	-2.391231695	0.000367288	0.001148802	1
T2DM_admission	leukocyte	*MAMDC2*	-2.657311368	0.000891773	0.001763074	1
T2DM_admission	leukocyte	*PRSS37*	-631.5623973	0.001341602	0.002245911	1
T2DM_admission	leukocyte	*C9orf64*	-1.663526597	0.000216596	0.001062643	0.851004
T2DM_admission	leukocyte	*ANKS6*	-0.910842348	0.008326355	0.011022321	1
T2DM_admission	leukocyte	*CLDN3*	-1.293197969	0.00014358	0.001062643	0.564125942
T2DM_admission	leukocyte	*WNK2*	-2.051011951	0.000110648	0.001062643	0.434734074
T2DM_admission	leukocyte	*NOL6*	-2.381356066	0.000273089	0.001088708	1
T2DM_admission	leukocyte	*PIGO*	-2.496044825	9.05E-05	0.001062643	0.355714904
T2DM_admission	leukocyte	*ARHGAP12*	-1.326465787	0.000289349	0.001088708	1
T2DM_admission	leukocyte	*WRN*	-1.369650154	0.000359514	0.001142338	1
T2DM_admission	leukocyte	*CFL2*	-1.220160275	0.000357463	0.001141109	1
T2DM_admission	leukocyte	*PGM2L1*	-2.426998197	1.99E-05	0.000920455	0.078238646
T2DM_admission	leukocyte	*PHYHIPL*	-1.196173797	0.000647175	0.00144721	1
T2DM_admission	leukocyte	*PKNOX2*	3.754225265	0.019378653	0.024171024	1
T2DM_admission	leukocyte	*RPL36AL*	-0.96773142	8.68E-05	0.001062643	0.340943073
T2DM_admission	leukocyte	*TMEM63C*	-1.064996203	0.000655314	0.001454635	1
T2DM_admission	leukocyte	*OXGR1*	-2.564536738	0.001333371	0.002236898	1
T2DM_admission	leukocyte	*VDAC2*	-1.118475112	0.000110353	0.001062643	0.433577595
T2DM_admission	leukocyte	*SNAPC4*	-1.101283474	0.002281151	0.003441875	1
T2DM_admission	leukocyte	*TSC1*	-1.761143576	0.000212999	0.001062643	0.836873432
T2DM_admission	leukocyte	*ZMYND19*	-1.167159965	0.000682954	0.001493225	1
T2DM_admission	leukocyte	*METTL17*	-1.703167181	0.0003025	0.001093914	1
T2DM_admission	leukocyte	*VWA2*	-3.230878352	0.000385199	0.001168251	1
T2DM_admission	leukocyte	*METTL3*	-1.203914947	0.001434226	0.002354561	1
T2DM_admission	leukocyte	*TRUB1*	-1.862438512	7.48E-05	0.001062643	0.293764887
T2DM_admission	leukocyte	*ARHGAP42*	-1.803726654	0.001018865	0.001906024	1
T2DM_admission	leukocyte	*SLC39A13*	-1.174458042	0.000475695	0.001261234	1
T2DM_admission	leukocyte	*PSMC3*	-1.003192765	0.000237115	0.001070831	0.93162334
T2DM_admission	leukocyte	*CPSF2*	-0.971269949	0.001312982	0.002213088	1
T2DM_admission	leukocyte	*IFI27L1*	-1.328651093	0.000207381	0.001062643	0.814798494
T2DM_admission	leukocyte	*SMCO4*	-1.282165428	0.000451137	0.001236066	1
T2DM_admission	leukocyte	*ABTB2*	-1.400668193	0.007597422	0.010125601	1
T2DM_admission	leukocyte	*LIPC*	-3.756794205	0.02694194	0.032665504	1
T2DM_admission	leukocyte	*CEP57*	-1.170101364	7.75E-05	0.001062643	0.304427756
T2DM_admission	leukocyte	*JAM3*	-0.913333932	0.001522832	0.002466285	1
T2DM_admission	leukocyte	*IKBIP*	-1.36524519	0.000827162	0.001675216	1
T2DM_admission	leukocyte	*ZFYVE19*	-0.969495627	0.005855195	0.007993419	1
T2DM_admission	leukocyte	*CKB*	-1.061306469	1.34E-05	0.000815363	0.052549056
T2DM_admission	leukocyte	*BTRC*	-2.310341855	0.000263243	0.001083884	1
T2DM_admission	leukocyte	*BAG5*	-1.226581178	0.000486292	0.001277166	1
T2DM_admission	leukocyte	*LARP6*	-1.05818875	0.000348365	0.001141096	1
T2DM_admission	leukocyte	*ZNF319*	-3.575353364	0.000139924	0.001062643	0.549761574
T2DM_admission	leukocyte	*SGPL1*	-2.590141115	1.40E-06	0.000261424	0.005489894
T2DM_admission	leukocyte	*FRS2*	-1.408183921	0.000141694	0.001062643	0.556716674
T2DM_admission	leukocyte	*PCBD1*	-1.368516117	1.65E-05	0.000823546	0.064771035
T2DM_admission	leukocyte	*COX11*	-1.45960564	0.000119192	0.001062643	0.468307316
T2DM_admission	leukocyte	*WBP1L*	-2.657657223	0.00018824	0.001062643	0.739594201
T2DM_admission	leukocyte	*CYB5R2*	-1.218896543	0.001106888	0.002007832	1
T2DM_admission	leukocyte	*TUB*	-1.660302698	0.000376872	0.001160448	1

T2DM_admission	leukocyte	*RIC3*	-0.944047993	0.000400509	0.001184732	1
T2DM_admission	leukocyte	*XRRA1*	-1.430292756	9.39E-05	0.001062643	0.368995534
T2DM_admission	leukocyte	*CDYL2*	-1.190854114	0.001250562	0.002155971	1
T2DM_admission	leukocyte	*ZNF143*	-1.180478807	0.000600151	0.001399402	1
T2DM_admission	leukocyte	*CCDC68*	-1.197648311	0.001049007	0.001940644	1
T2DM_admission	leukocyte	*ZSCAN21*	-1.739405836	0.001162961	0.002078832	1
T2DM_admission	leukocyte	*RIMKLB*	-1.159496383	0.000526386	0.001319879	1
T2DM_admission	leukocyte	*GALR1*	1.254327345	0.005644425	0.007721777	1
T2DM_admission	leukocyte	*ATF7IP2*	-1.786307223	6.31E-05	0.001060479	0.247778172
T2DM_admission	leukocyte	*COG1*	-1.245204797	0.000195766	0.001062643	0.769163878
T2DM_admission	leukocyte	*B2M*	-0.804763919	0.00016278	0.001062643	0.639563174
T2DM_admission	leukocyte	*ACSM1*	-2.542457625	0.030746235	0.037112736	1
T2DM_admission	leukocyte	*AP1G1*	-0.890582855	0.000630118	0.001433512	1
T2DM_admission	leukocyte	*SLFN5*	-0.959603359	0.000714246	0.001532645	1
T2DM_admission	leukocyte	*SAAL1*	-1.124726735	0.000896719	0.001768089	1
T2DM_admission	leukocyte	*SCNN1G*	-10.2489531	0.00088648	0.001757306	1
T2DM_admission	leukocyte	*DCTN5*	-1.365347443	0.00020826	0.001062643	0.81825314
T2DM_admission	leukocyte	*STAT6*	-2.066876742	0.000344486	0.001140861	1
T2DM_admission	leukocyte	*PATL1*	-1.148487207	0.000131619	0.001062643	0.517130573
T2DM_admission	leukocyte	*MRPL16*	-1.719504917	0.002902567	0.00425212	1
T2DM_admission	leukocyte	*GREM1*	-1.976147504	0.000246228	0.001077213	0.967430605
T2DM_admission	leukocyte	*TSC22D4*	-1.610269855	0.00036812	0.001148802	1
T2DM_admission	leukocyte	*MS4A14*	0.22571605	0.011203302	0.014446266	1
T2DM_admission	leukocyte	*DIS3L*	-1.928827318	0.0003032	0.001093914	1
T2DM_admission	leukocyte	*RCCD1*	-3.528169267	1.60E-05	0.000823546	0.063014973
T2DM_admission	leukocyte	*MAPRE2*	-1.171618425	0.000339549	0.001134896	1
T2DM_admission	leukocyte	*NAT16*	-0.784982292	0.000793139	0.001634654	1
T2DM_admission	leukocyte	*GNGT2*	0.450363958	0.00926693	0.012118325	1
T2DM_admission	leukocyte	*SLC27A4*	-1.099217771	0.00509664	0.007031144	1
T2DM_admission	leukocyte	*URM1*	-1.760816075	9.86E-05	0.001062643	0.387352738
T2DM_admission	leukocyte	*CERCAM*	-1.012993974	0.001765101	0.002770339	1
T2DM_admission	leukocyte	*DOLPP1*	-1.375568467	0.000961458	0.001831992	1
T2DM_admission	leukocyte	*ENDOG*	-1.51620786	0.000144202	0.001062643	0.566569864
T2DM_admission	leukocyte	*PRR15L*	-1.190907252	0.017243388	0.021665901	1
T2DM_admission	leukocyte	*RNF214*	-2.114135753	0.000245539	0.001077213	0.964724153
T2DM_admission	leukocyte	*POP5*	-1.441295159	8.14E-05	0.001062643	0.319874846
T2DM_admission	leukocyte	*ACAA2*	-1.355621413	0.000140088	0.001062643	0.550406884
T2DM_admission	leukocyte	*FN3K*	-1.425831895	0.001956787	0.003020911	1
T2DM_admission	leukocyte	*ZNF23*	-1.095346741	0.001010876	0.001894951	1
T2DM_admission	leukocyte	*ZNF229*	-1.566849498	0.000811757	0.001657387	1
T2DM_admission	leukocyte	*ZNF180*	-1.189015768	0.001249883	0.002155971	1
T2DM_admission	leukocyte	*CA4*	-0.859384494	0.001667648	0.002644145	1
T2DM_admission	leukocyte	*GPX4*	-1.298511093	7.63E-05	0.001062643	0.299936614
T2DM_admission	leukocyte	*ZNF641*	-1.877737305	0.000509434	0.001305652	1
T2DM_admission	leukocyte	*CACNB3*	-1.107402384	0.021663571	0.026707301	1
T2DM_admission	leukocyte	*TP53I13*	-1.497940381	1.09E-05	0.000784902	0.042799834
T2DM_admission	leukocyte	*TUBA1C*	-0.790744833	0.000502099	0.001297006	1
T2DM_admission	leukocyte	*ZNF528*	-4.966200857	2.63E-05	0.000984894	0.103413857
T2DM_admission	leukocyte	*RAB4B*	-1.583622578	0.000102549	0.001062643	0.402914732
T2DM_admission	leukocyte	*TMC4*	-1.276218712	0.000235232	0.001067235	0.924225727
T2DM_admission	leukocyte	*C19orf33*	-0.984565378	0.001195681	0.002107792	1
T2DM_admission	leukocyte	*DNAAF3*	-4.003524891	0.001163574	0.002078982	1
T2DM_admission	leukocyte	*CHAF1A*	-1.95956971	0.000128496	0.001062643	0.504860383
T2DM_admission	leukocyte	*UBXN6*	-1.745457028	6.20E-05	0.001060479	0.243750447
T2DM_admission	leukocyte	*NXN*	-0.87521019	0.000623762	0.00142569	1
T2DM_admission	leukocyte	*KIFC2*	-5.113235673	1.42E-05	0.000815363	0.055627794
T2DM_admission	leukocyte	*SLC43A2*	0.822763903	0.02082174	0.02575838	1
T2DM_admission	leukocyte	*SERPINF2*	0.483576516	0.012481715	0.016010663	1
T2DM_admission	leukocyte	*SRR*	-1.151448787	0.003415808	0.004907024	1
T2DM_admission	leukocyte	*TRPV3*	-5.830976545	0.00055451	0.001356582	1
T2DM_admission	leukocyte	*C19orf48*	-1.924179819	0.001388033	0.002300119	1
T2DM_admission	leukocyte	*ZNF83*	-1.589945713	0.000245779	0.001077213	0.965665826
T2DM_admission	leukocyte	*ZNF558*	-1.134194383	0.012354388	0.015862873	1
T2DM_admission	leukocyte	*NDUFV1*	-1.124701598	0.000291811	0.001088708	1

T2DM_admission	leukocyte	*ZNF232*	-0.955806768	0.002987516	0.004353839	1
T2DM_admission	leukocyte	*MIS12*	-1.47314116	0.000445821	0.001232235	1
T2DM_admission	leukocyte	*HID1*	-1.284757697	0.000569308	0.001367774	1
T2DM_admission	leukocyte	*SRP68*	-1.005531981	0.000479864	0.001266209	1
T2DM_admission	leukocyte	*TMEM68*	-1.635294084	0.000332563	0.001127387	1
T2DM_admission	leukocyte	*GSDMA*	-7.75289446	0.001615281	0.002577757	1
T2DM_admission	leukocyte	*MLST8*	-0.89779186	0.001566624	0.002515434	1
T2DM_admission	leukocyte	*ECI1*	-0.951646797	0.00049104	0.001283629	1
T2DM_admission	leukocyte	*ABCA3*	-1.306255601	7.92E-05	0.001062643	0.311304753
T2DM_admission	leukocyte	*BSCL2*	-1.062717624	0.000226569	0.001062643	0.890188388
T2DM_admission	leukocyte	*C2CD3*	-2.239934548	0.000303985	0.001094736	1
T2DM_admission	leukocyte	*TRANK1*	-3.193616573	0.000248536	0.001077213	0.976496006
T2DM_admission	leukocyte	*RPSA*	-0.785113676	0.000351877	0.001141109	1
T2DM_admission	leukocyte	*ENTPD3*	-0.793507275	0.00063448	0.001434874	1
T2DM_admission	leukocyte	*ULK4*	-1.60785084	0.000315788	0.001111111	1
T2DM_admission	leukocyte	*LTBP3*	-0.91110141	0.001565565	0.002514761	1
T2DM_admission	leukocyte	*SAC3D1*	-1.409750383	0.000435739	0.001222199	1
T2DM_admission	leukocyte	*CCDC88B*	-3.070143536	0.002878876	0.004219162	1
T2DM_admission	leukocyte	*PBK*	-2.515840027	0.001243035	0.002152855	1
T2DM_admission	leukocyte	*KIAA1586*	-1.670731987	9.41E-05	0.001062643	0.369702719
T2DM_admission	leukocyte	*FAM83B*	-1.240063738	0.001454001	0.002373399	1
T2DM_admission	leukocyte	*LMBRD1*	-1.509322186	0.000109354	0.001062643	0.429651273
T2DM_admission	leukocyte	*TTC39C*	-1.165268292	0.000285599	0.001088708	1
T2DM_admission	leukocyte	*POLR2J3*	-1.259015351	0.000662408	0.001464605	1
T2DM_admission	leukocyte	*COA6*	-1.322649933	0.000358394	0.001141109	1
T2DM_admission	leukocyte	*PXK*	-1.566724148	0.000469914	0.001255055	1
T2DM_admission	leukocyte	*ING5*	-2.249471714	0.000102962	0.001062643	0.404539624
T2DM_admission	leukocyte	*BMP1*	-1.117915694	0.005520114	0.007570167	1
T2DM_admission	leukocyte	*HEXIM2*	-1.398381319	0.0012582	0.002162496	1
T2DM_admission	leukocyte	*SERINC2*	-0.82275479	0.000504823	0.001300386	1
T2DM_admission	leukocyte	*TRAPPC11*	-0.956692108	0.000913101	0.001784988	1
T2DM_admission	leukocyte	*ING2*	-0.872691212	0.00075207	0.001577074	1
T2DM_admission	leukocyte	*ADAM9*	-2.168300488	0.00064595	0.001446812	1
T2DM_admission	leukocyte	*NDUFS5*	-0.982474206	0.000111801	0.001062643	0.439265827
T2DM_admission	leukocyte	*DNAJC21*	-1.445287864	6.26E-05	0.001060479	0.245787879
T2DM_admission	leukocyte	*CNNM3*	-1.178663754	0.000472586	0.001256287	1
T2DM_admission	leukocyte	*GSTM4*	-1.193923714	0.002952015	0.004310096	1
T2DM_admission	leukocyte	*SHOX2*	-9.056925333	0.004769346	0.006614458	1
T2DM_admission	leukocyte	*PPIP5K1*	-3.781724437	1.26E-05	0.000801308	0.049681118
T2DM_admission	leukocyte	*ADAL*	-2.630816323	0.006479032	0.008744801	1
T2DM_admission	leukocyte	*LCMT2*	-1.587445469	0.002542761	0.003774544	1
T2DM_admission	leukocyte	*ZNF507*	-1.582531068	0.000163867	0.001062643	0.643833486
T2DM_admission	leukocyte	*NSG1*	-0.84698109	0.001287304	0.002187637	1
T2DM_admission	leukocyte	*ZBTB49*	-2.931646543	0.001555605	0.002503881	1
T2DM_admission	leukocyte	*GFM1*	-1.052713299	0.000247125	0.001077213	0.970953098
T2DM_admission	leukocyte	*FSTL5*	-0.716121291	0.002913785	0.004263785	1
T2DM_admission	leukocyte	*CTRB1*	-0.526342206	0.000463522	0.001247383	1
T2DM_admission	leukocyte	*CTRB2*	-0.483833702	0.000659523	0.001460691	1
T2DM_admission	leukocyte	*TMEM129*	-2.517988172	1.89E-07	6.20E-05	0.000744365
T2DM_admission	leukocyte	*PPIC*	-0.626773198	0.001370243	0.002282189	1
T2DM_admission	leukocyte	*STXBP6*	-1.535062433	6.85E-05	0.001062643	0.269238058
T2DM_admission	leukocyte	*MFF*	-1.213684997	0.000199444	0.001062643	0.783614881
T2DM_admission	leukocyte	*CPLX1*	-1.055615623	0.003700937	0.005275591	1
T2DM_admission	leukocyte	*ROR2*	-2.863495529	0.00635845	0.008599776	1
T2DM_admission	leukocyte	*FAM110B*	-1.206217531	0.000869376	0.001735895	1
T2DM_admission	leukocyte	*ATF5*	-1.501104798	0.000219146	0.001062643	0.861024893
T2DM_admission	leukocyte	*XPO6*	-2.295435537	5.75E-05	0.001060479	0.225986801
T2DM_admission	leukocyte	*RGS14*	-1.692459799	0.000733207	0.001552225	1
T2DM_admission	leukocyte	*TBC1D10B*	-1.639533994	0.000124994	0.001062643	0.491100254
T2DM_admission	leukocyte	*PRELID1*	-0.925296229	0.000322532	0.001116501	1
T2DM_admission	leukocyte	*CXCL10*	-195.6300815	0.001291768	0.002191432	1
T2DM_admission	leukocyte	*NMD3*	-1.175479094	0.000336243	0.001131077	1
T2DM_admission	leukocyte	*STK32A*	-1.352606961	0.00084446	0.001700095	1
T2DM_admission	leukocyte	*P2RY12*	-54.83960411	0.02995557	0.03620284	1

T2DM_admission	leukocyte	*SNUPN*	-3.689059961	1.31E-05	0.000815363	0.051379311
T2DM_admission	leukocyte	*CRADD*	-1.152306782	0.001985765	0.003054843	1
T2DM_admission	leukocyte	*COL22A1*	-11.31033526	0.00159221	0.002547147	1
T2DM_admission	leukocyte	*SDC2*	-3.106508557	5.59E-05	0.001060479	0.219818442
T2DM_admission	leukocyte	*CLIC4*	-0.663469954	0.001293501	0.002192479	1
T2DM_admission	leukocyte	*SLC38A11*	-9.566700626	0.000580054	0.001379559	1
T2DM_admission	leukocyte	*CCDC8*	-1.26583248	0.033370471	0.040083333	1
T2DM_admission	leukocyte	*HINT1*	-0.867301173	0.000129419	0.001062643	0.508486459
T2DM_admission	leukocyte	*DTWD2*	-3.750684672	0.000932613	0.001804129	1
T2DM_admission	leukocyte	*INO80E*	-2.16849876	5.31E-07	0.000130474	0.002087587
T2DM_admission	leukocyte	*C15orf40*	-1.26772568	0.000204603	0.001062643	0.803886549
T2DM_admission	leukocyte	*HIC2*	-4.397351868	0.001621006	0.002583745	1
T2DM_admission	leukocyte	*AGPAT2*	-1.318005315	0.000122569	0.001062643	0.481571797
T2DM_admission	leukocyte	*FASN*	-2.445704264	1.12E-05	0.000784902	0.043905853
T2DM_admission	leukocyte	*MT1E*	-0.689142771	0.000727798	0.00154878	1
T2DM_admission	leukocyte	*LDB2*	-1.213847036	0.002229597	0.003369478	1
T2DM_admission	leukocyte	*RAC3*	-1.81161701	0.000124032	0.001062643	0.487320468
T2DM_admission	leukocyte	*LIMS1*	-1.541679281	4.77E-05	0.00105496	0.187498153
T2DM_admission	leukocyte	*CSGALNACT2*	-3.120716184	0.00035731	0.001141109	1
T2DM_admission	leukocyte	*AVEN*	-3.695255358	4.50E-05	0.001037577	0.176628351
T2DM_admission	leukocyte	*CTNND2*	-1.332756799	0.000357903	0.001141109	1
T2DM_admission	leukocyte	*WNT10B*	-68.77371524	0.001075	0.001968161	1
T2DM_admission	leukocyte	*CALML6*	1.108703476	0.008980373	0.011776997	1
T2DM_admission	leukocyte	*TM4SF4*	-1.116901753	0.000347154	0.001140861	1
T2DM_admission	leukocyte	*OTUD3*	-0.880994092	0.000656048	0.001454635	1
T2DM_admission	leukocyte	*MAP3K2*	-1.60167061	9.29E-05	0.001062643	0.364919792
T2DM_admission	leukocyte	*PUSL1*	-2.903663989	3.76E-05	0.001037577	0.147621859
T2DM_admission	leukocyte	*IFFO2*	-1.070063746	0.000916949	0.001790602	1
T2DM_admission	leukocyte	*MYRIP*	-1.534760489	0.00098504	0.001866067	1
T2DM_admission	leukocyte	*FAM153A*	-1.885213293	0.014508272	0.018459521	1
T2DM_admission	leukocyte	*ZNF778*	-1.730083421	0.000116865	0.001062643	0.459161279
T2DM_admission	leukocyte	*NIPA1*	-1.548388929	0.000172043	0.001062643	0.675955544
T2DM_admission	leukocyte	*FOXD4*	-11.05726022	0.031142153	0.037521472	1
T2DM_admission	leukocyte	*UBE2E1*	-2.164931502	8.79E-05	0.001062643	0.345430993
T2DM_admission	leukocyte	*ANKK1*	-31.70882499	0.000724308	0.001544387	1
T2DM_admission	leukocyte	*USP47*	-1.481134078	0.000118548	0.001062643	0.465775241
T2DM_admission	leukocyte	*PDCD6IP*	-1.192718348	0.000261419	0.001083674	1
T2DM_admission	leukocyte	*CRTAP*	-2.312949096	6.93E-05	0.001062643	0.272162445
T2DM_admission	leukocyte	*STX8*	-1.719567348	0.000130833	0.001062643	0.51404391
T2DM_admission	leukocyte	*NFRKB*	-2.167056203	0.000202929	0.001062643	0.797306888
T2DM_admission	leukocyte	*SETMAR*	-1.156242966	0.000572873	0.001370771	1
T2DM_admission	leukocyte	*CST5*	-5.389778283	0.021155084	0.026137838	1
T2DM_admission	leukocyte	*CST2*	-2.066268549	0.004537597	0.006319823	1
T2DM_admission	leukocyte	*KRT8*	-0.489235704	0.000940058	0.001810978	1
T2DM_admission	leukocyte	*MGMT*	-1.591985307	0.000234572	0.001066706	0.921634205
T2DM_admission	leukocyte	*NFXL1*	-2.045284314	0.000169783	0.001062643	0.667076808
T2DM_admission	leukocyte	*DNAJC18*	-1.087889488	0.015671919	0.019792661	1
T2DM_admission	leukocyte	*KRT4*	-51.43882419	0.011302308	0.014569149	1
T2DM_admission	leukocyte	*SLC23A1*	-9.163027958	0.002675067	0.003952741	1
T2DM_admission	leukocyte	*NPAS2*	-0.925053721	0.001233597	0.002144697	1
T2DM_admission	leukocyte	*NUDT9*	-0.916193796	0.001935341	0.002992504	1
T2DM_admission	leukocyte	*SMAGP*	-0.650918209	0.016266392	0.020503899	1
T2DM_admission	leukocyte	*IRX1*	-1.638732025	0.001318836	0.002220096	1
T2DM_admission	leukocyte	*CDH2*	-0.911317653	0.001305222	0.002204736	1
T2DM_admission	leukocyte	*HSPA4*	-1.314263107	0.000156349	0.001062643	0.614294538
T2DM_admission	leukocyte	*ARMC10*	-1.622866687	0.00048781	0.001279443	1
T2DM_admission	leukocyte	*ACYP2*	-1.108596645	0.000344073	0.001140861	1
T2DM_admission	leukocyte	*ATF7*	-1.271743531	0.000419518	0.001201375	1
T2DM_admission	leukocyte	*RASA4B*	-1.813044559	0.008493137	0.011220229	1
T2DM_admission	leukocyte	*KCNS3*	-1.500585191	0.001539544	0.002486242	1
T2DM_admission	leukocyte	*FOXN2*	-3.902908978	3.65E-05	0.001037577	0.14330485
T2DM_admission	leukocyte	*GPR27*	-1.257101682	0.000292613	0.001088708	1
T2DM_admission	leukocyte	*MTSS1*	-1.623651867	0.000524762	0.001319879	1
T2DM_admission	leukocyte	*TMEM43*	-1.208573657	0.000465213	0.001251077	1

T2DM_admission	leukocyte	*RPS9*	-0.776518161	0.000210516	0.001062643	0.827118445
T2DM_admission	leukocyte	*NDUFA3*	-1.325547825	0.000174576	0.001062643	0.685909955
T2DM_admission	leukocyte	*ZNF160*	-1.241081497	0.000348805	0.001141096	1
T2DM_admission	leukocyte	*ZNF415*	-1.032373402	0.003233768	0.004669414	1
T2DM_admission	leukocyte	*TSNARE1*	0.893924759	0.019472332	0.024280163	1
T2DM_admission	leukocyte	*SOX7*	-1.487315205	0.001275825	0.002175489	1
T2DM_admission	leukocyte	*TRMT61B*	-0.948627321	0.00182438	0.002848962	1
T2DM_admission	leukocyte	*INSR*	-0.84001851	0.000694108	0.001507546	1
T2DM_admission	leukocyte	*MFN1*	-1.117941195	0.002298111	0.003463474	1
T2DM_admission	leukocyte	*KCNMB3*	-1.867750394	0.001515293	0.002457114	1
T2DM_admission	leukocyte	*ATP6V0E2*	-0.995704263	0.000595437	0.001396699	1
T2DM_admission	leukocyte	*JAGN1*	-1.347013937	0.00025214	0.001077213	0.990658174
T2DM_admission	leukocyte	*MORN4*	-0.766669808	0.002502255	0.003729651	1
T2DM_admission	leukocyte	*TRIM8*	-1.414258645	0.000177611	0.001062643	0.697833999
T2DM_admission	leukocyte	*CDC42BPG*	-0.842605584	0.000993994	0.001873994	1
T2DM_admission	leukocyte	*ZNF440*	-4.720424713	8.79E-06	0.000762289	0.034517466
T2DM_admission	leukocyte	*GAA*	-0.93547868	0.027219078	0.032986971	1
T2DM_admission	leukocyte	*EXOSC1*	-1.556182616	0.000362633	0.001144406	1
T2DM_admission	leukocyte	*PGAM1*	-0.941673888	0.001625039	0.002589124	1
T2DM_admission	leukocyte	*ESCO2*	-3.663441068	0.007511524	0.010017915	1
T2DM_admission	leukocyte	*MRPL36*	-1.168655191	0.0005351	0.001332323	1
T2DM_admission	leukocyte	*MCC*	-1.317196168	0.003404509	0.004896163	1
T2DM_admission	leukocyte	*ZNF561*	-1.478676232	0.000226005	0.001062643	0.887975257
T2DM_admission	leukocyte	*PPID*	-1.463496062	0.004146221	0.005838889	1
T2DM_admission	leukocyte	*RXFP1*	-23.0235108	0.00688134	0.009243345	1
T2DM_admission	leukocyte	*PTGER4*	0.575402101	0.023607382	0.02895829	1
T2DM_admission	leukocyte	*MAP6*	-0.848786004	0.000745236	0.001568308	1
T2DM_admission	leukocyte	*NMUR1*	-12.35503457	0.001224969	0.002137168	1
T2DM_admission	leukocyte	*CLSTN1*	-1.094537194	0.000113522	0.001062643	0.446026254
T2DM_admission	leukocyte	*PIK3CD*	0.827074771	0.023870235	0.029235085	1
T2DM_admission	leukocyte	*ENC1*	-1.000608628	0.002466625	0.00368353	1
T2DM_admission	leukocyte	*RGS19*	-2.41159157	1.04E-05	0.000784902	0.040719109
T2DM_admission	leukocyte	*ANO5*	-1.430881243	0.00104469	0.001937955	1
T2DM_admission	leukocyte	*GPHN*	-2.23473752	0.000108403	0.001062643	0.425916779
T2DM_admission	leukocyte	*VAT1L*	-1.313223986	0.003157493	0.004571187	1
T2DM_admission	leukocyte	*LGALS4*	-0.624733371	0.00155719	0.002504379	1
T2DM_admission	leukocyte	*LRRC34*	-1.309927299	0.002545309	0.003776631	1
T2DM_admission	leukocyte	*SPATA5L1*	-2.298907482	0.000387521	0.001173013	1
T2DM_admission	leukocyte	*CTPS1*	-1.078294201	0.001049578	0.001940644	1
T2DM_admission	leukocyte	*METTL18*	-1.435350759	0.00146934	0.002392473	1
T2DM_admission	leukocyte	*PWWP2B*	-1.514260582	0.000323944	0.001116918	1
T2DM_admission	leukocyte	*FAM90A1*	-2.953888335	0.020536281	0.025437279	1
T2DM_admission	leukocyte	*PTEN*	-1.414251631	0.000221615	0.001062643	0.870725428
T2DM_admission	leukocyte	*CYP4F11*	-2.6416251	0.004435787	0.006197798	1
T2DM_admission	leukocyte	*TLN2*	-2.232812469	0.001216363	0.00212877	1
T2DM_admission	leukocyte	*TVP23B*	-2.123189404	0.000192474	0.001062643	0.756229851
T2DM_admission	leukocyte	*SRGAP2C*	-2.589407057	6.33E-05	0.001060479	0.248648755
T2DM_admission	leukocyte	*ATPAF2*	-1.385986474	0.001583346	0.002536064	1
T2DM_admission	leukocyte	*ZNF556*	0.849534948	0.014848462	0.018825301	1
T2DM_admission	leukocyte	*ZNF554*	-1.321924375	0.000713688	0.001532285	1
T2DM_admission	leukocyte	*GAP43*	-0.885486936	0.001402859	0.002319795	1
T2DM_admission	leukocyte	*REG1B*	-0.543138087	0.000646628	0.001446812	1
T2DM_admission	leukocyte	*NME6*	-2.130193105	0.000344891	0.001140861	1
T2DM_admission	leukocyte	*SLFN12*	0.949366891	0.017468491	0.021892728	1
T2DM_admission	leukocyte	*TEFM*	-1.595408364	0.000354786	0.001141109	1
T2DM_admission	leukocyte	*MBOAT1*	-2.548611782	0.000408649	0.001188129	1
T2DM_admission	leukocyte	*ZNF131*	-1.241616981	0.000425206	0.001210604	1
T2DM_admission	leukocyte	*BSG*	-1.07155044	0.000184465	0.001062643	0.72476164
T2DM_admission	leukocyte	*HINFP*	-3.740021595	0.00010931	0.001062643	0.429478663
T2DM_admission	leukocyte	*CERS6*	-1.180061426	0.000299393	0.001093914	1
T2DM_admission	leukocyte	*SPTLC3*	1.242562244	0.008683657	0.011437509	1
T2DM_admission	leukocyte	*COPRS*	-0.892515946	0.000481825	0.001270529	1
T2DM_admission	leukocyte	*SUCLG2*	-2.311277193	6.75E-05	0.001062643	0.265060623
T2DM_admission	leukocyte	*CSDC2*	-3.477749986	0.000640759	0.001440839	1

T2DM_admission	leukocyte	*ARNT2*	-0.79340176	0.00851637	0.011243554	1
T2DM_admission	leukocyte	*FUT9*	-3.846298714	0.018413641	0.023025842	1
T2DM_admission	leukocyte	*ZNF24*	-1.395163294	7.64E-05	0.001062643	0.299988486
T2DM_admission	leukocyte	*MANEA*	-1.447657527	0.00024768	0.001077213	0.973134565
T2DM_admission	leukocyte	*AFF1*	-1.380616818	0.000212852	0.001062643	0.836294701
T2DM_admission	leukocyte	*CTSW*	-1.613310663	8.99E-06	0.000762289	0.035322435
T2DM_admission	leukocyte	*SMPDL3A*	-2.499379607	0.006206966	0.008418077	1
T2DM_admission	leukocyte	*RAD9A*	-2.225706262	0.000343324	0.001140861	1
T2DM_admission	leukocyte	*EFEMP2*	-1.228250785	0.00090195	0.001772767	1
T2DM_admission	leukocyte	*ZNF738*	-1.097930822	0.015350105	0.019411188	1
T2DM_admission	leukocyte	*FUT10*	-3.360521063	0.013954098	0.01779601	1
T2DM_admission	leukocyte	*NAA16*	-0.944571987	0.006252618	0.008471192	1
T2DM_admission	leukocyte	*SNX32*	2.303848545	0.025358696	0.030951947	1
T2DM_admission	leukocyte	*OVOL1*	-3.590332715	9.58E-05	0.001062643	0.376552401
T2DM_admission	leukocyte	*DMXL1*	-1.980416165	0.000182839	0.001062643	0.71837607
T2DM_admission	leukocyte	*NADSYN1*	-2.589837533	4.42E-05	0.001037577	0.17379775
T2DM_admission	leukocyte	*RNASEH2C*	-1.627705843	4.39E-06	0.000522919	0.017256316
T2DM_admission	leukocyte	*LCLAT1*	-1.266058666	0.000299642	0.001093914	1
T2DM_admission	leukocyte	*SH3RF3*	-1.689417445	0.002120783	0.003225922	1
T2DM_admission	leukocyte	*DCAKD*	-0.953269196	0.000568881	0.001367774	1
T2DM_admission	leukocyte	*TADA2B*	-1.467284721	0.000207701	0.001062643	0.816057272
T2DM_admission	leukocyte	*TRMT112*	-1.066580118	0.000333433	0.00112758	1
T2DM_admission	leukocyte	*NOC3L*	-1.277508906	0.000220224	0.001062643	0.865258392
T2DM_admission	leukocyte	*RHOD*	-0.769268084	0.001235231	0.002144697	1
T2DM_admission	leukocyte	*PARP14*	-1.333169013	0.000192341	0.001062643	0.755708282
T2DM_admission	leukocyte	*VANGL1*	-1.142805827	0.001241012	0.002152731	1
T2DM_admission	leukocyte	*IQCB1*	-3.30401219	2.59E-05	0.000984894	0.101796696
T2DM_admission	leukocyte	*ZNF483*	-1.064338787	0.000251631	0.001077213	0.988658231
T2DM_admission	leukocyte	*SLC2A14*	-4.498007751	0.001317889	0.002219454	1
T2DM_admission	leukocyte	*MZT2A*	-0.927192919	0.000706638	0.001523809	1
T2DM_admission	leukocyte	*PPP1R3B*	-4.008002979	4.01E-07	0.000116632	0.001574769
T2DM_admission	leukocyte	*MAP3K11*	-1.163031432	0.000297332	0.001093914	1
T2DM_admission	leukocyte	*NDNF*	1.070652664	0.008460583	0.011181174	1
T2DM_admission	leukocyte	*ARV1*	-1.091432391	0.00057921	0.001379223	1
T2DM_admission	leukocyte	*EHBP1L1*	-1.143752579	0.002151944	0.003265736	1
T2DM_admission	leukocyte	*PPP1R14B*	-1.025034279	0.000234472	0.001066706	0.921241124
T2DM_admission	leukocyte	*TNFRSF10D*	-1.328345741	0.001964607	0.003029411	1
T2DM_admission	leukocyte	*GMPPB*	-1.782857158	0.000673398	0.001482231	1
T2DM_admission	leukocyte	*NUDT18*	-1.162929375	0.001128049	0.002034008	1
T2DM_admission	leukocyte	*NUDT4*	-1.055630721	0.001062857	0.00195779	1
T2DM_admission	leukocyte	*PC*	-1.856408852	0.000657462	0.001456947	1
T2DM_admission	leukocyte	*UQCRH*	-1.077384505	0.000139859	0.001062643	0.54950494
T2DM_admission	leukocyte	*HEG1*	-1.148768423	0.000989593	0.001869283	1
T2DM_admission	leukocyte	*ENDOV*	-2.618524279	4.04E-05	0.001037577	0.158857052
T2DM_admission	leukocyte	*DPY19L1*	-1.459187921	0.000452844	0.001238727	1
T2DM_admission	leukocyte	*GPR160*	-1.814609797	0.000431398	0.001216752	1
T2DM_admission	leukocyte	*XXYLT1*	-1.887699995	0.000678484	0.001488422	1
T2DM_admission	leukocyte	*GNG5*	-1.068940443	0.000211779	0.001062643	0.832077937
T2DM_admission	leukocyte	*C9orf131*	-2.189280042	0.004246013	0.005960195	1
T2DM_admission	leukocyte	*FAM53A*	-6.673183901	4.47E-05	0.001037577	0.175769511
T2DM_admission	leukocyte	*SELP*	-6.861387868	0.001165241	0.002080069	1
T2DM_admission	leukocyte	*CTU2*	-1.129625065	0.000904419	0.001774955	1
T2DM_admission	leukocyte	*ADCY6*	-1.224370076	0.001499318	0.002434224	1
T2DM_admission	leukocyte	*PODN*	-0.903703593	0.018548692	0.02318734	1
T2DM_admission	leukocyte	*RALGAPA1*	-1.160102459	0.000266121	0.001086558	1
T2DM_admission	leukocyte	*CNTNAP2*	2.140173902	0.005962189	0.008128189	1
T2DM_admission	leukocyte	*GOLT1A*	-1.188390728	0.000388646	0.001174609	1
T2DM_admission	leukocyte	*NPAS4*	-3.090799315	1.63E-05	0.000823546	0.063924955
T2DM_admission	leukocyte	*ANGEL2*	-1.695024037	0.000188283	0.001062643	0.739764298
T2DM_admission	leukocyte	*ZNF266*	-1.201834523	1.68E-05	0.000823546	0.065883669
T2DM_admission	leukocyte	*BRSK2*	-1.090582435	0.000797779	0.001641531	1
T2DM_admission	leukocyte	*BRMS1*	-1.10434234	0.000406713	0.001188129	1
T2DM_admission	leukocyte	*HRAS*	-1.108637755	0.000330239	0.001124357	1
T2DM_admission	leukocyte	*SRP72*	-0.934912463	0.000320244	0.001113485	1

T2DM_admission	leukocyte	*PCP2*	-4.538483928	0.000885684	0.001757306	1
T2DM_admission	leukocyte	*BTC*	-1.223298913	0.023936123	0.029306647	1
T2DM_admission	leukocyte	*DENND6A*	-1.135802748	0.000512918	0.001308606	1
T2DM_admission	leukocyte	*YIF1A*	-1.354090271	5.44E-06	0.00058206	0.021375196
T2DM_admission	leukocyte	*NDUFA11*	-1.138245836	0.000159382	0.001062643	0.626210511
T2DM_admission	leukocyte	*RSRC1*	-2.705583436	2.35E-05	0.000980682	0.092184077
T2DM_admission	leukocyte	*DHX36*	-1.267564478	0.000158751	0.001062643	0.623733252
T2DM_admission	leukocyte	*PSMD2*	-1.00292699	0.000352584	0.001141109	1
T2DM_admission	leukocyte	*PPM1E*	-0.787725332	0.003895789	0.005527828	1
T2DM_admission	leukocyte	*ZNF408*	-4.657685898	5.48E-06	0.00058206	0.021536203
T2DM_admission	leukocyte	*ARHGAP1*	-1.20927914	0.000227743	0.001062643	0.894802673
T2DM_admission	leukocyte	*MED16*	-1.030061692	0.00116105	0.002076362	1
T2DM_admission	leukocyte	*CHST1*	-2.261831542	0.001265353	0.002169029	1
T2DM_admission	leukocyte	*DOLK*	-2.396932581	0.000227442	0.001062643	0.893618444
T2DM_admission	leukocyte	*CCNE2*	-1.390021365	0.00146684	0.002389393	1
T2DM_admission	leukocyte	*ZNF519*	-2.312502586	0.000222204	0.001062643	0.873041316
T2DM_admission	leukocyte	*TMEM9B*	-1.244048487	0.000139383	0.001062643	0.547636976
T2DM_admission	leukocyte	*EIF3F*	-1.272882801	0.000100361	0.001062643	0.394317004
T2DM_admission	leukocyte	*ZNF25*	-1.114233535	0.000648013	0.001447767	1
T2DM_admission	leukocyte	*PPP2R2D*	-1.143314644	0.000301401	0.001093914	1
T2DM_admission	leukocyte	*CLCF1*	-1.473462864	0.00250693	0.003733786	1
T2DM_admission	leukocyte	*UCP2*	0.698159211	0.029719973	0.035929161	1
T2DM_admission	leukocyte	*MRPL48*	-1.325122177	0.001881418	0.002921775	1
T2DM_admission	leukocyte	*TMEM70*	-1.109230973	0.000531509	0.001324223	1
T2DM_admission	leukocyte	*RPS6KB2*	-1.593263703	0.000903314	0.001773984	1
T2DM_admission	leukocyte	*RMI2*	-3.365733507	0.00417377	0.005871371	1
T2DM_admission	leukocyte	*LINC01106*	-5.549078687	0.002814391	0.004130646	1
T2DM_admission	leukocyte	*SFN*	-1.61567429	0.03681858	0.04402319	1
T2DM_admission	leukocyte	*SWI5*	-1.73374715	0.000412462	0.001192427	1
T2DM_admission	leukocyte	*ZDHHC21*	-2.522307322	2.95E-05	0.001015174	0.115729869
T2DM_admission	leukocyte	*LRRN1*	-1.099549218	0.040105684	0.04779352	1
T2DM_admission	leukocyte	*TUBB6*	-0.545794096	0.003336584	0.004805512	1
T2DM_admission	leukocyte	*CHMP6*	-1.07711738	0.002317512	0.003487363	1
T2DM_admission	leukocyte	*DLEU1*	-1.542335655	0.000202204	0.001062643	0.794459724
T2DM_admission	leukocyte	*CCDC57*	-1.707282229	5.47E-05	0.001060479	0.214827262
T2DM_admission	leukocyte	*BNIP3*	-0.911839746	0.000252516	0.001077213	0.9921368
T2DM_admission	leukocyte	*SMIM19*	-0.996845833	0.000504112	0.001299643	1
T2DM_admission	leukocyte	*SLC35G1*	-1.646849026	0.000599461	0.001399402	1
T2DM_admission	leukocyte	*COX8A*	-0.904942918	0.000147508	0.001062643	0.579559786
T2DM_admission	leukocyte	*CDC26*	-1.318514808	0.000181881	0.001062643	0.714611021
T2DM_admission	leukocyte	*CRLF3*	-3.500118841	0.030584613	0.036940346	1
T2DM_admission	leukocyte	*RNPEP*	-2.123212796	0.000319015	0.001113024	1
T2DM_admission	leukocyte	*DMRTA1*	-0.94529979	0.003336554	0.004805512	1
T2DM_admission	leukocyte	*CLEC14A*	-0.677241564	0.002054759	0.00314375	1
T2DM_admission	leukocyte	*WDR25*	-3.360620283	2.19E-05	0.000943039	0.085960713
T2DM_admission	leukocyte	*DIRAS1*	-1.521285809	0.000642999	0.001442799	1
T2DM_admission	leukocyte	*PHLDB3*	-1.01567001	0.001178536	0.002098083	1
T2DM_admission	leukocyte	*PRR15*	1.280819452	0.02284815	0.028105943	1
T2DM_admission	leukocyte	*KBTBD11*	-0.990007471	0.001234086	0.002144697	1
T2DM_admission	leukocyte	*RMDN1*	-1.342980343	0.000913165	0.001784988	1
T2DM_admission	leukocyte	*LRRC37A*	-6.377237393	0.000596559	0.001396953	1
T2DM_admission	leukocyte	*ACSF3*	-1.623656315	0.000344914	0.001140861	1
T2DM_admission	leukocyte	*RUFY1*	-3.029055823	8.87E-05	0.001062643	0.348349672
T2DM_admission	leukocyte	*VSIG10*	-2.010631667	6.14E-05	0.001060479	0.241357176
T2DM_admission	leukocyte	*WSB2*	-1.031735022	0.000692894	0.001507191	1
T2DM_admission	leukocyte	*ANKLE2*	-0.927855119	0.001143244	0.002051052	1
T2DM_admission	leukocyte	*FUT2*	0.484416051	0.034574005	0.041478249	1
T2DM_admission	leukocyte	*MUC20*	-1.143141799	0.001410775	0.002330561	1
T2DM_admission	leukocyte	*NFATC2IP*	-1.81225401	0.000191082	0.001062643	0.75075993
T2DM_admission	leukocyte	*DPP7*	-1.150269409	0.000177633	0.001062643	0.697918634
T2DM_admission	leukocyte	*MTHFR*	-1.307946975	0.000345899	0.001140861	1
T2DM_admission	leukocyte	*DEAF1*	-1.640497966	0.000177614	0.001062643	0.697845796
T2DM_admission	leukocyte	*TMEM80*	-0.928533494	0.001242775	0.002152855	1
T2DM_admission	leukocyte	*DSCAML1*	-1.856832088	3.15E-05	0.001037577	0.123866891

T2DM_admission	leukocyte	*PUS1*	-2.506442467	0.000394547	0.001181536	1
T2DM_admission	leukocyte	*MAN1B1*	-2.13637584	0.000123564	0.001062643	0.485483188
T2DM_admission	leukocyte	*PPFIA3*	-0.929960626	0.00095148	0.001823593	1
T2DM_admission	leukocyte	*PAWR*	-1.080606126	0.000767029	0.001600298	1
T2DM_admission	leukocyte	*GPR4*	-1.012002184	0.000165267	0.001062643	0.64933435
T2DM_admission	leukocyte	*ACOT4*	-1.516211897	0.001185365	0.00210081	1
T2DM_admission	leukocyte	*ARIH2*	-1.248026773	8.45E-05	0.001062643	0.332145982
T2DM_admission	leukocyte	*IRX3*	3.044270526	7.71E-05	0.001062643	0.302933631
T2DM_admission	leukocyte	*SAMD12*	-1.663458018	0.000490696	0.001283585	1
T2DM_admission	leukocyte	*CD163*	0.099614432	0.00775154	0.010317005	1
T2DM_admission	leukocyte	*ACAD9*	-0.990432506	0.020991121	0.025951578	1
T2DM_admission	leukocyte	*IL17RA*	-2.084381882	0.001959754	0.003024302	1
T2DM_admission	leukocyte	*AGTRAP*	-1.321461545	0.000334392	0.001128716	1
T2DM_admission	leukocyte	*THAP5*	-1.266902697	0.000252063	0.001077213	0.990354673
T2DM_admission	leukocyte	*CD151*	-0.989975925	0.000275848	0.001088708	1
T2DM_admission	leukocyte	*CHID1*	-1.078075497	0.000137574	0.001062643	0.540527769
T2DM_admission	leukocyte	*ZBTB41*	-1.626005306	0.000151167	0.001062643	0.593936392
T2DM_admission	leukocyte	*BET1L*	-1.901150597	9.00E-05	0.001062643	0.353796905
T2DM_admission	leukocyte	*RIC8A*	-1.580200746	0.00027181	0.001088708	1
T2DM_admission	leukocyte	*IMP3*	-1.177390285	0.001474995	0.002399691	1
T2DM_admission	leukocyte	*DPY19L2*	-0.885028003	0.01601504	0.020213007	1
T2DM_admission	leukocyte	*GPR150*	-1.0685029	0.004912076	0.006790833	1
T2DM_admission	leukocyte	*ADAMTSL1*	-6.307461396	0.000374789	0.001157716	1
T2DM_admission	leukocyte	*ALS2CL*	-1.616311542	0.000924582	0.001797337	1
T2DM_admission	leukocyte	*STAP2*	-0.919502379	0.000503553	0.001299056	1
T2DM_admission	leukocyte	*DALRD3*	-1.211807432	0.001748321	0.002749861	1
T2DM_admission	leukocyte	*ZNF114*	-3.126763048	0.000949933	0.001823593	1
T2DM_admission	leukocyte	*PLEC*	-2.424678783	3.02E-05	0.001023384	0.118712585
T2DM_admission	leukocyte	*WDR6*	-1.135720559	0.001427796	0.002348599	1
T2DM_admission	leukocyte	*AQP11*	-1.497553873	0.000782449	0.001618875	1
T2DM_admission	leukocyte	*ZFAND2A*	-1.638569799	0.000119079	0.001062643	0.4678601
T2DM_admission	leukocyte	*DNAJC22*	-0.581443321	0.014632145	0.018599061	1
T2DM_admission	leukocyte	*NT5DC1*	-1.181190541	0.000339689	0.001134896	1
T2DM_admission	leukocyte	*COX14*	-1.09471094	0.001453383	0.002373399	1
T2DM_admission	leukocyte	*P4HTM*	-0.89187589	0.00031731	0.001111111	1
T2DM_admission	leukocyte	*ERBB4*	-10.56991689	0.000188808	0.001062643	0.741828503
T2DM_admission	leukocyte	*ERN1*	-1.727323843	7.90E-05	0.001062643	0.310493651
T2DM_admission	leukocyte	*PARP10*	-1.25303375	0.004357112	0.006107418	1
T2DM_admission	leukocyte	*RPP25*	-2.087161602	9.19E-05	0.001062643	0.361145293
T2DM_admission	leukocyte	*ZHX2*	-1.395218816	0.000266316	0.001086558	1
T2DM_admission	leukocyte	*HYI*	-1.529071181	0.000102311	0.001062643	0.401978716
T2DM_admission	leukocyte	*GAK*	-1.268478825	0.001539584	0.002486242	1
T2DM_admission	leukocyte	*TUFM*	-0.700918415	0.006342999	0.008584789	1
T2DM_admission	leukocyte	*RMI1*	1.505395216	0.010408068	0.013500594	1
T2DM_admission	leukocyte	*C3orf38*	-1.535880637	0.00312163	0.00452913	1
T2DM_admission	leukocyte	*IGSF22*	-0.830322713	0.00190518	0.002952841	1
T2DM_admission	leukocyte	*HTR1F*	-3.664832958	0.000120893	0.001062643	0.474987846
T2DM_admission	leukocyte	*TMTC2*	-2.041172685	0.001197776	0.002109396	1
T2DM_admission	leukocyte	*FARSA*	-1.504283262	1.49E-05	0.000823546	0.058604833
T2DM_admission	leukocyte	*SPTY2D1*	-1.272998576	0.000317582	0.001111111	1
T2DM_admission	leukocyte	*SAMD4B*	-1.035389452	0.000620658	0.001422226	1
T2DM_admission	leukocyte	*TCAIM*	-1.730274234	0.000227165	0.001062643	0.892529947
T2DM_admission	leukocyte	*FUCA1*	0.464747681	0.039690366	0.047327299	1
T2DM_admission	leukocyte	*CALR*	-0.822370659	0.001453798	0.002373399	1
T2DM_admission	leukocyte	*CDH4*	-2.17499285	0.019577765	0.024396142	1
T2DM_admission	leukocyte	*NSUN7*	-1.552177597	0.019932018	0.024790408	1
T2DM_admission	leukocyte	*ELMOD2*	-2.181335944	0.000130968	0.001062643	0.514575223
T2DM_admission	leukocyte	*LINC00174*	-1.132501102	0.000713471	0.001532285	1
T2DM_admission	leukocyte	*GEMIN4*	-1.15171133	0.0012488	0.00215577	1
T2DM_admission	leukocyte	*LBX2*	0.993621923	0.015621049	0.019747459	1
T2DM_admission	leukocyte	*CDC42EP4*	-1.029779077	0.000418069	0.001200552	1
T2DM_admission	leukocyte	*ZBTB42*	-2.631223179	0.000894783	0.001766634	1
T2DM_admission	leukocyte	*SYCN*	-0.630617031	0.000960647	0.001831992	1
T2DM_admission	leukocyte	*CITED4*	-0.768543637	0.001301771	0.002200323	1

T2DM_admission	leukocyte	*ZNF154*	-0.806209087	0.031078945	0.037468296	1
T2DM_admission	leukocyte	*B3GNT3*	-2.790898515	0.000783569	0.001618943	1
T2DM_admission	leukocyte	*PUF60*	-1.031618455	0.000947958	0.001821284	1
T2DM_admission	leukocyte	*TSHZ1*	-0.939521048	0.001421853	0.002343313	1
T2DM_admission	leukocyte	*ANKRD18A*	-0.618663299	0.026945507	0.032665504	1
T2DM_admission	leukocyte	*EXOC3*	-2.227441501	0.000118263	0.001062643	0.464653435
T2DM_admission	leukocyte	*TH*	-8.011435656	1.43E-05	0.000815363	0.056292998
T2DM_admission	leukocyte	*FAHD1*	-0.91652975	0.007364565	0.009838618	1
T2DM_admission	leukocyte	*ZNF816*	-1.62330718	0.001226853	0.002138556	1
T2DM_admission	leukocyte	*CCDC66*	-3.071433027	8.93E-05	0.001062643	0.350943157
T2DM_admission	leukocyte	*MCFD2*	-0.942864691	0.000255428	0.001077348	1
T2DM_admission	leukocyte	*SERTM1*	0.727721588	0.022951747	0.028198065	1
T2DM_admission	leukocyte	*GLIPR1L2*	-1.871809288	0.003307723	0.00476919	1
T2DM_admission	leukocyte	*TSPYL5*	-0.935605896	0.000470326	0.001255055	1
T2DM_admission	leukocyte	*SHISA2*	-1.666029119	0.012863052	0.016478668	1
T2DM_admission	leukocyte	*CHST13*	-93.1325014	0.001321995	0.00222351	1
T2DM_admission	leukocyte	*PPA1*	-0.890773507	0.000252618	0.001077213	0.992537004
T2DM_admission	leukocyte	*PSMG4*	-1.002826599	0.000354155	0.001141109	1
T2DM_admission	leukocyte	*MAP6D1*	-2.189977696	0.002368633	0.003557476	1
T2DM_admission	leukocyte	*ZNF792*	-1.98986186	0.010966083	0.014182271	1
T2DM_admission	leukocyte	*D2HGDH*	-5.01441204	9.12E-06	0.000762289	0.035827577
T2DM_admission	leukocyte	*FAM83H*	-1.767356225	0.000730242	0.001550629	1
T2DM_admission	leukocyte	*LRRC57*	-1.919883605	0.000477927	0.001265283	1
T2DM_admission	leukocyte	*MRPL14*	-1.268946232	0.00016187	0.001062643	0.635985676
T2DM_admission	leukocyte	*ZFP82*	-1.095473102	0.000847251	0.001701865	1
T2DM_admission	leukocyte	*FKRP*	-0.980119533	0.002340855	0.00352114	1
T2DM_admission	leukocyte	*RPH3AL*	-0.841891029	0.001135162	0.002043083	1
T2DM_admission	leukocyte	*HIGD1A*	-0.987642116	0.000180742	0.001062643	0.710134249
T2DM_admission	leukocyte	*F2R*	-0.58110218	0.041027208	0.048788106	1
T2DM_admission	leukocyte	*ZNF707*	-1.419400939	0.001206565	0.002117283	1
T2DM_admission	leukocyte	*OGFOD3*	-1.002901836	0.00052889	0.001321888	1
T2DM_admission	leukocyte	*ZNF467*	-1.525093844	0.000728073	0.00154878	1
T2DM_admission	leukocyte	*ZNF678*	-2.561065504	0.000584998	0.001382115	1
T2DM_admission	leukocyte	*TMEM45A*	-0.907675975	0.001276087	0.002175489	1
T2DM_admission	leukocyte	*SGSH*	-2.87520171	0.005014598	0.006922824	1
T2DM_admission	leukocyte	*GPR135*	-1.327847581	0.001017872	0.001906024	1
T2DM_admission	leukocyte	*ANKRD62*	-1.47520447	0.003736988	0.00532172	1
T2DM_admission	leukocyte	*RFX7*	-3.172938219	2.92E-05	0.001014102	0.11459356
T2DM_admission	leukocyte	*ZNF329*	-1.525579621	0.000934896	0.001804129	1
T2DM_admission	leukocyte	*CCDC149*	-2.018029329	0.000113785	0.001062643	0.447061732
T2DM_admission	leukocyte	*IDH2*	-0.881575509	0.000996157	0.001875957	1
T2DM_admission	leukocyte	*TNRC18*	-1.534425515	0.000214371	0.001062643	0.842265349
T2DM_admission	leukocyte	*TDRKH*	-1.183993505	0.001926379	0.002980994	1
T2DM_admission	leukocyte	*ZNF708*	-0.916147045	0.00068078	0.00149096	1
T2DM_admission	leukocyte	*IST1*	-1.86791749	8.72E-05	0.001062643	0.342519126
T2DM_admission	leukocyte	*ERCC6L2*	-3.655271071	7.42E-05	0.001062643	0.291353538
T2DM_admission	leukocyte	*MRPL41*	-1.153367941	0.000177926	0.001062643	0.699069693
T2DM_admission	leukocyte	*TP53TG1*	-1.29782835	0.000170531	0.001062643	0.670017084
T2DM_admission	leukocyte	*UBA7*	-3.381418837	4.67E-07	0.000122423	0.001836338
T2DM_admission	leukocyte	*ARL6IP4*	-0.765809608	0.002392577	0.003589322	1
T2DM_admission	leukocyte	*FAM153B*	-1.881380748	0.000212224	0.001062643	0.833829314
T2DM_admission	leukocyte	*BACE2*	-0.675997514	0.000978951	0.001856322	1
T2DM_admission	leukocyte	*SYNM*	-0.989862969	0.00438883	0.006143112	1
T2DM_admission	leukocyte	*FIGN*	-1.110064244	0.001108409	0.002009662	1
T2DM_admission	leukocyte	*B4GALNT4*	-2.245733975	0.000936459	0.001806258	1
T2DM_admission	leukocyte	*ZNF804B*	-80.25308503	0.001448937	0.002370055	1
T2DM_admission	leukocyte	*YBEY*	-1.352088513	0.000669219	0.001476341	1
T2DM_admission	leukocyte	*NPLOC4*	-1.233311448	0.000317067	0.001111111	1
T2DM_admission	leukocyte	*CAPN12*	-2.39031727	0.000154005	0.001062643	0.605087025
T2DM_admission	leukocyte	*GLRX5*	-0.890411556	0.003104643	0.004507814	1
T2DM_admission	leukocyte	*MXRA7*	-1.026724992	0.000292064	0.001088708	1
T2DM_admission	leukocyte	*ADI1*	-2.63798955	5.71E-05	0.001060479	0.224426415
T2DM_admission	leukocyte	*SATB1*	-1.885497607	0.000126399	0.001062643	0.496620196
T2DM_admission	leukocyte	*EPHB3*	-2.061069158	0.039722297	0.047351003	1

T2DM_admission	leukocyte	*SKA2*	-2.306397677	1.67E-05	0.000823546	0.065523347
T2DM_admission	leukocyte	*TTC3*	-0.501304712	0.017194497	0.021618297	1
T2DM_admission	leukocyte	*BRICD5*	-2.223139072	0.006069129	0.008262511	1
T2DM_admission	leukocyte	*TSKU*	-1.234533663	0.001172207	0.002089227	1
T2DM_admission	leukocyte	*ANXA2*	-0.707046304	0.000343614	0.001140861	1
T2DM_admission	leukocyte	*SLC35D3*	-1.901711156	0.002012349	0.003089327	1
T2DM_admission	leukocyte	*GPIHBP1*	-10.12804589	0.001247484	0.002154446	1
T2DM_admission	leukocyte	*VMO1*	0.122276902	0.004574177	0.006361749	1
T2DM_admission	leukocyte	*COL18A1*	-0.601360556	0.000553549	0.001355918	1
T2DM_admission	leukocyte	*C11orf54*	-1.487364036	0.001646977	0.002617707	1
T2DM_admission	leukocyte	*CEP63*	-1.420802931	0.000233164	0.001066706	0.916100579
T2DM_admission	leukocyte	*HMGN4*	-1.48192168	0.000809715	0.00165524	1
T2DM_admission	leukocyte	*SPATA13*	-1.095879136	0.000600546	0.001399493	1
T2DM_admission	leukocyte	*ZNF320*	1.167157426	0.000108035	0.001062643	0.424471463
T2DM_admission	leukocyte	*SLC25A21*	-2.79546508	0.0012558	0.002159317	1
T2DM_admission	leukocyte	*CAMK1D*	-1.825586404	0.036757661	0.043963729	1
T2DM_admission	leukocyte	*LYSMD4*	-1.367630679	0.000705666	0.001522744	1
T2DM_admission	leukocyte	*BEGAIN*	-1.733829581	0.000361291	0.001142338	1
T2DM_admission	leukocyte	*GPC6*	-1.324814039	0.000809649	0.00165524	1
T2DM_admission	leukocyte	*FANCF*	-4.61198721	1.82E-05	0.000880427	0.071314623
T2DM_admission	leukocyte	*CALN1*	-3.786782836	0.032113142	0.038632129	1
T2DM_admission	leukocyte	*SMDT1*	-0.770500682	0.001422832	0.002343944	1
T2DM_admission	leukocyte	*PTTG1IP*	-0.870128362	0.003064707	0.004453119	1
T2DM_admission	leukocyte	*PLGLB1*	1.841577787	0.030832648	0.037194189	1
T2DM_admission	leukocyte	*ZNF623*	-0.964654728	0.001935322	0.002992504	1
T2DM_admission	leukocyte	*CCDC125*	-2.155502785	0.006667828	0.008984189	1
T2DM_admission	leukocyte	*BOLA2*	-1.170928334	0.000309822	0.00110427	1
T2DM_admission	leukocyte	*FHL3*	-1.064259746	0.003129045	0.004538213	1
T2DM_admission	leukocyte	*SF3A3*	-0.965222655	0.001125039	0.002031214	1
T2DM_admission	leukocyte	*MX2*	1.105026017	0.033403027	0.040110176	1
T2DM_admission	leukocyte	*PCBP3*	-1.662858192	0.001802311	0.002818981	1
T2DM_admission	leukocyte	*SFXN4*	-1.332038995	0.000274503	0.001088708	1
T2DM_admission	leukocyte	*MRPL54*	-1.17701341	0.000135586	0.001062643	0.532718844
T2DM_admission	leukocyte	*DGCR6*	-1.220915145	0.000752211	0.001577074	1
T2DM_admission	leukocyte	*NDUFB1*	-1.115170084	0.000381201	0.001165285	1
T2DM_admission	leukocyte	*TRMT12*	-1.350935277	0.000364539	0.001146348	1
T2DM_admission	leukocyte	*FIGLA*	-28.5588364	0.000290999	0.001088708	1
T2DM_admission	leukocyte	*CBX6*	-1.04055655	0.000321307	0.001114222	1
T2DM_admission	leukocyte	*MACC1*	-0.907341776	0.000290283	0.001088708	1
T2DM_admission	leukocyte	*KREMEN1*	-3.343142276	0.001504086	0.002439948	1
T2DM_admission	leukocyte	*NUDT14*	-1.007024463	0.001440644	0.002361407	1
T2DM_admission	leukocyte	*FAM3B*	-0.917787238	0.001999031	0.003071644	1
T2DM_admission	leukocyte	*TOB2*	-1.306122069	0.000462164	0.001244702	1
T2DM_admission	leukocyte	*NPIPA7*	-9.694237372	0.004792429	0.006642945	1
T2DM_admission	leukocyte	*TTC32*	-1.164209436	0.000357543	0.001141109	1
T2DM_admission	leukocyte	*TMEM236*	-0.804685422	0.002772944	0.004074381	1
T2DM_admission	leukocyte	*UQCR10*	-1.004138264	0.000201581	0.001062643	0.792013383
T2DM_admission	leukocyte	*EIF3C*	-0.723450041	0.02571067	0.03131346	1
T2DM_admission	leukocyte	*NIPSNAP1*	-1.073844898	0.000929134	0.001800431	1
T2DM_admission	leukocyte	*CRELD2*	-0.833132777	0.001727051	0.002722068	1
T2DM_admission	leukocyte	*UBE2F*	-1.885567312	0.000184225	0.001062643	0.723819126
T2DM_admission	leukocyte	*GOLGA6L4*	-1.362189066	0.001101942	0.002000708	1
T2DM_admission	leukocyte	*PGP*	-2.066880204	3.07E-06	0.000430544	0.012055222
T2DM_admission	leukocyte	*ACOT1*	-5.789016905	0.006254754	0.008471192	1
T2DM_admission	leukocyte	*OAF*	-1.515294394	0.000204824	0.001062643	0.80475315
T2DM_admission	leukocyte	*TM2D3*	-1.149715454	0.000289511	0.001088708	1
T2DM_admission	leukocyte	*TOP1MT*	-2.86817216	0.000190111	0.001062643	0.746946813
T2DM_admission	leukocyte	*COPB2*	-1.381792921	0.000105387	0.001062643	0.414066686
T2DM_admission	leukocyte	*CCR10*	-2.043018205	0.006812645	0.009160466	1
T2DM_admission	leukocyte	*NCMAP*	-1.075943012	0.001379546	0.002289918	1
T2DM_admission	leukocyte	*WDR27*	-1.589284789	0.037333996	0.044625881	1
T2DM_admission	leukocyte	*TXNRD2*	-1.147732526	0.000862763	0.001726874	1
T2DM_admission	leukocyte	*ZFP1*	-1.525972782	0.000412752	0.001192427	1
T2DM_admission	leukocyte	*XPOT*	-1.102671867	0.000402118	0.001185421	1

T2DM_admission	leukocyte	*C14orf180*	-116.0093991	0.001272704	0.002175059	1
T2DM_admission	leukocyte	*ZBTB40*	-1.549569882	0.004512184	0.006293468	1
T2DM_admission	leukocyte	*UBE2G2*	-1.254686065	0.000229825	0.001064322	0.902981351
T2DM_admission	leukocyte	*OSBP2*	-1.280679381	0.001244716	0.0021544	1
T2DM_admission	leukocyte	*TMED9*	-1.01397052	0.000261051	0.001083674	1
T2DM_admission	leukocyte	*BTBD6*	-1.555971152	0.000193184	0.001062643	0.759020771
T2DM_admission	leukocyte	*JAG2*	-0.932371127	0.003148082	0.004562455	1
T2DM_admission	leukocyte	*LCN12*	-4.202507353	0.000134785	0.001062643	0.529569668
T2DM_admission	leukocyte	*FAM227A*	-1.234797872	0.001221552	0.002133102	1
T2DM_admission	leukocyte	*MUC6*	-0.676844748	0.002123689	0.003229092	1
T2DM_admission	leukocyte	*NOC4L*	-1.94718446	0.000139281	0.001062643	0.547236084
T2DM_admission	leukocyte	*NDUFA6*	-0.653164418	0.010276483	0.013347538	1
T2DM_admission	leukocyte	*SIVA1*	-1.146659831	0.000301445	0.001093914	1
T2DM_admission	leukocyte	*BRI3BP*	-1.644437812	0.000281585	0.001088708	1
T2DM_admission	leukocyte	*UBOX5*	-1.132940048	0.000877526	0.001743901	1
T2DM_admission	leukocyte	*MAFF*	-0.689395502	0.001872574	0.00290919	1
T2DM_admission	leukocyte	*SEMA4B*	-1.077331806	0.001452038	0.002373152	1
T2DM_admission	leukocyte	*CIB1*	-1.135254977	0.000170913	0.001062643	0.671518025
T2DM_admission	leukocyte	*ANO9*	-1.493856836	0.005298665	0.007289375	1
T2DM_admission	leukocyte	*INPP5J*	-5.600277778	0.019336302	0.024125859	1
T2DM_admission	leukocyte	*MFSD6L*	-3.140281072	0.002352167	0.003535449	1
T2DM_admission	leukocyte	*ZNF445*	-5.105975591	0.000181251	0.001062643	0.7121358
T2DM_admission	leukocyte	*ZNF74*	-2.474007279	0.001272196	0.002175059	1
T2DM_admission	leukocyte	*RBM11*	-0.977317559	0.000863888	0.001728216	1
T2DM_admission	leukocyte	*RGPD2*	-3.089009509	0.003419291	0.004910231	1
T2DM_admission	leukocyte	*CDK10*	-0.950564993	0.024768421	0.030278508	1
T2DM_admission	leukocyte	*GAS2L1*	-2.580003941	0.002609518	0.003864604	1
T2DM_admission	leukocyte	*ATP6V0A2*	-1.851408664	0.000342115	0.001140092	1
T2DM_admission	leukocyte	*RAD51D*	-3.149469752	0.000102292	0.001062643	0.401903466
T2DM_admission	leukocyte	*SH3BGR*	-0.589055473	0.008547841	0.011269955	1
T2DM_admission	leukocyte	*MUC1*	-0.711816531	0.001961288	0.003025481	1
T2DM_admission	leukocyte	*IRF7*	-1.90093109	0.000880389	0.001747877	1
T2DM_admission	leukocyte	*FAM131C*	-0.833114341	0.000876637	0.001743468	1
T2DM_admission	leukocyte	*LSAMP*	-0.619259146	0.001752349	0.002755094	1
T2DM_admission	leukocyte	*OLFML2A*	-2.950485112	0.002409884	0.003612528	1
T2DM_admission	leukocyte	*PDIA2*	-0.508488334	0.004842221	0.006703695	1
T2DM_admission	leukocyte	*PCGF3*	-1.171375668	0.000793564	0.001634654	1
T2DM_admission	leukocyte	*P4HB*	-0.791240466	0.000393419	0.001181536	1
T2DM_admission	leukocyte	*PSMD13*	-1.503095269	1.16E-05	0.000784902	0.045394405
T2DM_admission	leukocyte	*BRWD1*	-1.29100791	0.000210466	0.001062643	0.826920112
T2DM_admission	leukocyte	*DRG1*	-1.095136602	0.000456838	0.001240899	1
T2DM_admission	leukocyte	*ADAMTSL5*	-6.249663676	0.025205185	0.030783703	1
T2DM_admission	leukocyte	*MORF4L1*	-0.907836789	0.000222893	0.001062643	0.875745133
T2DM_admission	leukocyte	*DMWD*	-2.635494384	1.20E-06	0.000236063	0.004721267
T2DM_admission	leukocyte	*PIGP*	-0.935612292	0.000381967	0.001165285	1
T2DM_admission	leukocyte	*NPAP1*	-34.04242575	0.003937051	0.005572289	1
T2DM_admission	leukocyte	*ARL17A*	-0.718674892	0.01482001	0.018801362	1
T2DM_admission	leukocyte	*TRIM69*	-1.084091021	0.002565069	0.003800209	1
T2DM_admission	leukocyte	*IFITM1*	-2.626470986	0.000722889	0.001544023	1
T2DM_admission	leukocyte	*SETD4*	-3.155018498	0.009293099	0.012146569	1
T2DM_admission	leukocyte	*ZNF529*	-2.070773622	0.000367831	0.001148802	1
T2DM_admission	leukocyte	*AIDA*	-1.065948817	0.00043669	0.001222199	1
T2DM_admission	leukocyte	*GSAP*	1.01359245	0.036262779	0.043411474	1
T2DM_admission	leukocyte	*WWOX*	-1.184503018	0.000403325	0.001185781	1
T2DM_admission	leukocyte	*POLR1D*	-1.061198534	0.000190741	0.001062643	0.749420339
T2DM_admission	leukocyte	*EDARADD*	-1.196223484	0.000741698	0.001563191	1
T2DM_admission	leukocyte	*ZNF749*	-1.028917956	4.97E-05	0.00105496	0.195316419
T2DM_admission	leukocyte	*TOR3A*	-1.297218834	0.000195978	0.001062643	0.769996454
T2DM_admission	leukocyte	*ZNF555*	-0.931334722	0.000976779	0.001855786	1
T2DM_admission	leukocyte	*NUDT17*	-1.723077356	0.000353569	0.001141109	1
T2DM_admission	leukocyte	*CYP4X1*	1.178114197	0.028231559	0.034192909	1
T2DM_admission	leukocyte	*ZNF197*	-1.083940707	0.000519192	0.001315067	1
T2DM_admission	leukocyte	*RPS23*	-0.836884324	0.000119577	0.001062643	0.469816405
T2DM_admission	leukocyte	*BTN3A2*	-0.540984918	0.011134532	0.014362303	1

T2DM_admission	leukocyte	*ZNF396*	-2.9556285	0.000943774	0.001816677	1
T2DM_admission	leukocyte	*SMYD4*	-0.83999768	0.038426465	0.045875899	1
T2DM_admission	leukocyte	*CEACAM19*	-1.104079968	0.000120328	0.001062643	0.472768327
T2DM_admission	leukocyte	*ZFP91*	-1.423100443	0.000182226	0.001062643	0.715967413
T2DM_admission	leukocyte	*BCR*	-1.083782441	0.00061619	0.001419945	1
T2DM_admission	leukocyte	*FSCN2*	-1.411749822	0.029671202	0.035881242	1
T2DM_admission	leukocyte	*HYAL3*	-1.690959384	0.00456662	0.006353488	1
T2DM_admission	leukocyte	*RTN4RL2*	-1.146229274	0.001289515	0.002190448	1
T2DM_admission	leukocyte	*TMEM232*	-1.753744715	0.000837789	0.001692377	1
T2DM_admission	leukocyte	*FAM183A*	-0.929439745	0.0012819	0.002181285	1
T2DM_admission	leukocyte	*EMID1*	-1.316523349	0.002510225	0.003735861	1
T2DM_admission	leukocyte	*ESPN*	-22.85668979	0.000546053	0.001346461	1
T2DM_admission	leukocyte	*TMEM216*	-2.57392235	0.041910459	0.04973307	1
T2DM_admission	leukocyte	*LYPD6*	-4.38488134	0.005566157	0.007625325	1
T2DM_admission	leukocyte	*AKR1C1*	-0.81493114	0.001723189	0.002719068	1
T2DM_admission	leukocyte	*BCAM*	-0.907318985	0.000319908	0.001113304	1
T2DM_admission	leukocyte	*CHP1*	-0.986171297	0.000392777	0.001181536	1
T2DM_admission	leukocyte	*KCNJ11*	-3.777764439	0.000256841	0.001080629	1
T2DM_admission	leukocyte	*ZNF385C*	-3.769828188	0.000762094	0.001593931	1
T2DM_admission	leukocyte	*EXD3*	-1.203719659	0.001019037	0.001906024	1
T2DM_admission	leukocyte	*C17orf97*	-0.710542864	0.02003966	0.024892768	1
T2DM_admission	leukocyte	*RGPD1*	-124.0718327	0.001368853	0.002280841	1
T2DM_admission	leukocyte	*DHRS4L2*	-1.234396017	0.000283338	0.001088708	1
T2DM_admission	leukocyte	*TRPV2*	-6.501321282	0.027388071	0.033181538	1
T2DM_admission	leukocyte	*MCRS1*	-1.232144052	0.026432292	0.032102775	1
T2DM_admission	leukocyte	*PALM3*	-1.404343039	0.001140371	0.002049538	1
T2DM_admission	leukocyte	*KLHL17*	-1.917047903	0.001067599	0.001963762	1
T2DM_admission	leukocyte	*RINL*	-1.534775514	0.00030645	0.001098442	1
T2DM_admission	leukocyte	*ZNF490*	-2.067432217	0.00059265	0.001392055	1
T2DM_admission	leukocyte	*NRN1L*	-2.962984086	0.001306466	0.002205889	1
T2DM_admission	leukocyte	*RAB42*	0.536621321	0.010042956	0.013070147	1
T2DM_admission	leukocyte	*MAPK12*	-1.174624278	0.005384099	0.007391378	1
T2DM_admission	leukocyte	*LINC00265*	-2.352949688	0.001195792	0.002107792	1
T2DM_admission	leukocyte	*LAMTOR4*	-1.035411936	0.000626514	0.001429485	1
T2DM_admission	leukocyte	*PRKAR1B*	-1.462169714	0.000455385	0.001240899	1
T2DM_admission	leukocyte	*NUTM2B*	-35.21701602	8.84E-08	3.16E-05	0.000347214
T2DM_admission	leukocyte	*AGAP4*	-2.650571357	0.001217516	0.00212984	1
T2DM_admission	leukocyte	*CENPP*	-0.868473856	0.003067831	0.00445601	1
T2DM_admission	leukocyte	*FOCAD*	-1.252064968	0.000528817	0.001321888	1
T2DM_admission	leukocyte	*ZP3*	-1.441700859	0.002690702	0.003971363	1
T2DM_admission	leukocyte	*SRSF10*	-1.095551642	0.000467907	0.001253701	1
T2DM_admission	leukocyte	*RALGAPA2*	-1.2446626	0.000423179	0.001206582	1
T2DM_admission	leukocyte	*NDOR1*	-2.764801708	0.005481748	0.007522804	1
T2DM_admission	leukocyte	*FBLL1*	-2.710995229	0.000114639	0.001062643	0.450415556
T2DM_admission	leukocyte	*ASAH2*	-3.109735936	7.67E-05	0.001062643	0.301435004
T2DM_admission	leukocyte	*S100A16*	-0.607917714	0.001078489	0.001970875	1
T2DM_admission	leukocyte	*PARVB*	-0.780470935	0.001192619	0.002105978	1
T2DM_admission	leukocyte	*UROS*	-1.485971944	0.000171911	0.001062643	0.675439891
T2DM_admission	leukocyte	*FAM221A*	-1.741622808	0.000140144	0.001062643	0.550627049
T2DM_admission	leukocyte	*NOXA1*	-2.063245968	0.001079586	0.001971964	1
T2DM_admission	leukocyte	*TMEM198*	-0.904827813	0.002037814	0.003120254	1
T2DM_admission	leukocyte	*ZNF548*	-1.203859086	0.005321688	0.007313365	1
T2DM_admission	leukocyte	*MTF1*	-1.124656163	0.00084582	0.001700095	1
T2DM_admission	leukocyte	*ZDHHC11*	-1.905047542	0.042045519	0.049878274	1
T2DM_admission	leukocyte	*ENTPD8*	-7.052387011	0.001553263	0.002503187	1
T2DM_admission	leukocyte	*RPL14*	-0.776668227	0.000232128	0.001066588	0.912030225
T2DM_admission	leukocyte	*FBF1*	0.752503743	0.031949539	0.038447087	1
T2DM_admission	leukocyte	*MSL1*	-1.317669806	0.000202272	0.001062643	0.794728337
T2DM_admission	leukocyte	*NOC2L*	-1.462976828	0.000268775	0.001088175	1
T2DM_admission	leukocyte	*ZNF567*	-1.020931455	0.002750419	0.00404431	1
T2DM_admission	leukocyte	*ARID2*	-1.605700275	0.000193418	0.001062643	0.759938016
T2DM_admission	leukocyte	*PLAC9*	-1.297209781	0.000192612	0.001062643	0.756772468
T2DM_admission	leukocyte	*CLDN4*	-0.649104939	0.00083554	0.001688702	1
T2DM_admission	leukocyte	*FAM47E*	-1.81338515	5.21E-05	0.00105496	0.204778436

T2DM_admission	leukocyte	*ZNF527*	-1.368541751	0.003785739	0.005387239	1
T2DM_admission	leukocyte	*ZNF600*	-1.122207986	0.002417373	0.00362099	1
T2DM_admission	leukocyte	*PNRC2*	-1.015135217	0.000405841	0.001187305	1
T2DM_admission	leukocyte	*ZKSCAN3*	-2.578943427	0.000295553	0.001092406	1
T2DM_admission	leukocyte	*RRP7A*	-1.205292515	0.001043215	0.001936841	1
T2DM_admission	leukocyte	*S100A14*	-0.882552712	0.002980929	0.004347464	1
T2DM_admission	leukocyte	*CXCL17*	-0.846815711	0.003631067	0.005184034	1
T2DM_admission	leukocyte	*SPATA41*	-4.52602981	0.002820299	0.004137772	1
T2DM_admission	leukocyte	*BLOC1S2*	-1.345228416	7.71E-05	0.001062643	0.302956869
T2DM_admission	leukocyte	*ZNF676*	-1.516548278	0.000588672	0.001384966	1
T2DM_admission	leukocyte	*WDSUB1*	-1.379454948	0.001730967	0.002726932	1
T2DM_admission	leukocyte	*ZNF681*	-6.90602675	1.66E-05	0.000823546	0.06539431
T2DM_admission	leukocyte	*HRCT1*	1.085653395	0.01562798	0.019749866	1
T2DM_admission	leukocyte	*MPHOSPH8*	-1.175454428	0.000251959	0.001077213	0.989945552
T2DM_admission	leukocyte	*SIRPB2*	1.818155955	0.007612583	0.010142367	1
T2DM_admission	leukocyte	*LCOR*	-1.119789123	0.000182316	0.001062643	0.716318926
T2DM_admission	leukocyte	*SUPT5H*	-1.103224624	0.000129689	0.001062643	0.509549855
T2DM_admission	leukocyte	*XPNPEP3*	-1.902099791	5.68E-05	0.001060479	0.223035205
T2DM_admission	leukocyte	*ZNF107*	-0.928099907	0.013206596	0.016890858	1
T2DM_admission	leukocyte	*PPIA*	-0.923210038	0.000436548	0.001222199	1
T2DM_admission	leukocyte	*ZNF471*	-1.579828263	0.00133821	0.002242144	1
T2DM_admission	leukocyte	*ZNF493*	-1.603981878	0.000238169	0.001071897	0.93576579
T2DM_admission	leukocyte	*GTF2IRD2*	-1.652034799	0.000278024	0.001088708	1
T2DM_admission	leukocyte	*SUPT3H*	-1.008315906	0.00080043	0.001644817	1
T2DM_admission	leukocyte	*POM121*	-1.10280023	0.001772647	0.002779222	1
T2DM_admission	leukocyte	*LONP1*	-1.669277955	0.0003491	0.001141108	1
T2DM_admission	leukocyte	*ZNF34*	-1.601774951	0.000845937	0.001700095	1
T2DM_admission	leukocyte	*ZNF781*	-1.666776259	0.001274512	0.002175489	1
T2DM_admission	leukocyte	*EVL*	-2.467119996	0.00066012	0.00146119	1
T2DM_admission	leukocyte	*ZNF124*	-1.969449232	0.000345092	0.001140861	1
T2DM_admission	leukocyte	*XRCC6*	-1.247954317	3.48E-05	0.001037577	0.136722944
T2DM_admission	leukocyte	*PIK3R4*	-2.081090121	0.000119841	0.001062643	0.470854157
T2DM_admission	leukocyte	*ZNF605*	-1.743702551	0.037753716	0.045113853	1
T2DM_admission	leukocyte	*C20orf96*	-2.529453	9.92E-05	0.001062643	0.389584622
T2DM_admission	leukocyte	*SULT1A1*	-2.840127228	1.26E-05	0.000801308	0.049374414
T2DM_admission	leukocyte	*ARL9*	1.100118893	0.020670921	0.025595981	1
T2DM_admission	leukocyte	*TPK1*	-2.075558233	0.017295219	0.021704452	1
T2DM_admission	leukocyte	*AFAP1*	-1.241730576	0.001308836	0.002207994	1
T2DM_admission	leukocyte	*MYO18A*	-1.029724708	0.000627528	0.001430967	1
T2DM_admission	leukocyte	*XRCC2*	-4.283951736	0.000744929	0.001568308	1
T2DM_admission	leukocyte	*MYO6*	-0.857384073	0.000543678	0.001341777	1
T2DM_admission	leukocyte	*ZNF502*	-5.324954432	2.73E-05	0.000996218	0.107121511
T2DM_admission	leukocyte	*TRAPPC4*	-1.025475433	0.000947811	0.001821284	1
T2DM_admission	leukocyte	*ZNF33B*	-2.285785475	6.24E-05	0.001060479	0.245247423
T2DM_admission	leukocyte	*AMZ2*	-1.450151504	0.000286617	0.001088708	1
T2DM_admission	leukocyte	*ZNF431*	-1.21723087	0.000327913	0.001122427	1
T2DM_admission	leukocyte	*DAPK1*	-2.023508309	0.000268172	0.001087356	1
T2DM_admission	leukocyte	*HLA-DQA1*	0.081961747	0.014940438	0.018929694	1
T2DM_admission	leukocyte	*CLPSL2*	-0.98177963	0.025249596	0.03082836	1
T2DM_admission	leukocyte	*MVB12B*	-6.449666797	0.000326887	0.001122427	1
T2DM_admission	leukocyte	*ADA*	0.509160861	0.009184413	0.012024512	1
T2DM_admission	leukocyte	*PPTC7*	-1.905105834	8.08E-05	0.001062643	0.317569142
T2DM_admission	leukocyte	*ZFP28*	-1.682730698	0.000442736	0.001227599	1
T2DM_admission	leukocyte	*ANKRD36B*	-1.169845766	0.003869081	0.005493899	1
T2DM_admission	leukocyte	*SRGAP1*	-1.373399841	0.000427132	0.00121345	1
T2DM_admission	leukocyte	*FAM3C*	-0.972258093	0.000303587	0.001094307	1
T2DM_admission	leukocyte	*AP2A1*	-1.534755218	0.000176471	0.001062643	0.693352625
T2DM_admission	leukocyte	*WDR5B*	-3.616868823	0.000293596	0.001088724	1
T2DM_admission	leukocyte	*ZNF138*	-0.823911323	0.003900488	0.005528505	1
T2DM_admission	leukocyte	*ZNF429*	-1.109885897	0.000630536	0.001433512	1
T2DM_admission	leukocyte	*ZNF100*	-1.617957212	0.000270398	0.00108852	1
T2DM_admission	leukocyte	*ZNF763*	-2.558273687	0.000432348	0.001217703	1
T2DM_admission	leukocyte	*ARRDC1*	-1.020278816	0.001411149	0.002330561	1
T2DM_admission	leukocyte	*ZNF682*	-2.152312638	0.000439232	0.001222199	1

T2DM_admission	leukocyte	*ZNF257*	-1.967252043	0.000179376	0.001062643	0.704766546
T2DM_admission	leukocyte	*ADAM32*	1.221966143	0.010370269	0.013456007	1
T2DM_admission	leukocyte	*ACSL5*	-2.002812321	0.00039639	0.001181845	1
T2DM_admission	leukocyte	*SND1*	-1.180397834	0.00026953	0.001088175	1
T2DM_admission	leukocyte	*TBC1D9B*	-2.514910275	1.45E-05	0.000815363	0.057075439
T2DM_admission	leukocyte	*C6orf141*	-2.358437312	0.000325568	0.001119124	1
T2DM_admission	leukocyte	*GTF2E2*	-1.120576001	0.000488538	0.001280054	1
T2DM_admission	leukocyte	*ZNF165*	-1.214220671	0.000804222	0.001649159	1
T2DM_admission	leukocyte	*SVIL*	-1.064528834	0.000603308	0.001403431	1
T2DM_admission	leukocyte	*MRPL21*	-0.892696563	0.005831659	0.007966363	1
T2DM_admission	leukocyte	*ZNF98*	-1.56686078	0.001302049	0.002200323	1
T2DM_admission	leukocyte	*ZNF517*	-4.664270886	0.00049235	0.001284491	1
T2DM_admission	leukocyte	*SLC22A5*	-1.758282666	0.000429332	0.00121662	1
T2DM_admission	leukocyte	*C5AR1*	0.131988615	0.007558634	0.010077324	1
T2DM_admission	leukocyte	*VEPH1*	-2.044367472	0.003616055	0.005164478	1
T2DM_admission	leukocyte	*SHPK*	-1.073244284	0.025550428	0.031147264	1
T2DM_admission	leukocyte	*PCDHB11*	-2.24222229	0.000489698	0.001281828	1
T2DM_admission	leukocyte	*MIB2*	-0.779218003	0.020325447	0.025206379	1
T2DM_admission	leukocyte	*ATG7*	-2.343631582	0.000189942	0.001062643	0.746283975
T2DM_admission	leukocyte	*PIGN*	-1.007581061	0.001371489	0.002283297	1
T2DM_admission	leukocyte	*HHLA3*	-1.042894178	0.001200501	0.002112301	1
T2DM_admission	leukocyte	*BCO2*	-1.131350507	0.00275964	0.004056351	1
T2DM_admission	leukocyte	*ENTPD6*	-1.364908047	0.000352841	0.001141109	1
T2DM_admission	leukocyte	*ENPP1*	-0.691231285	0.017296167	0.021704452	1
T2DM_admission	leukocyte	*CCDC154*	3.971803916	0.014038508	0.017890788	1
T2DM_admission	leukocyte	*DPP4*	-0.872352031	0.000177716	0.001062643	0.698245185
T2DM_admission	leukocyte	*NMB*	-1.147265542	0.000468742	0.001253701	1
T2DM_admission	leukocyte	*RPS26*	-0.991744722	2.51E-05	0.000982543	0.098560903
T2DM_admission	leukocyte	*LHFPL5*	-3.010280316	0.000449743	0.001234067	1
T2DM_admission	leukocyte	*ZNF780A*	-1.015748561	0.000822309	0.001670341	1
T2DM_admission	leukocyte	*ATAD3A*	-2.119534608	0.000141487	0.001062643	0.555903068
T2DM_admission	leukocyte	*FAM118B*	-2.865160733	0.000134416	0.001062643	0.528121022
T2DM_admission	leukocyte	*SLC9A8*	-4.188347725	7.01E-05	0.001062643	0.27540394
T2DM_admission	leukocyte	*OCLN*	-0.633173702	0.007267206	0.009718466	1
T2DM_admission	leukocyte	*ZNF181*	-1.62289152	0.000582859	0.001382042	1
T2DM_admission	leukocyte	*TEAD4*	-0.940080207	0.0014347	0.002354561	1
T2DM_admission	leukocyte	*C2orf27A*	-1.923183519	0.000217329	0.001062643	0.853885941
T2DM_admission	leukocyte	*PLCG2*	-1.401947332	0.000170623	0.001062643	0.670376538
T2DM_admission	leukocyte	*RPL12*	-0.751822206	0.000218396	0.001062643	0.85807976
T2DM_admission	leukocyte	*VPS13A*	-1.053831934	0.001023321	0.001910945	1
T2DM_admission	leukocyte	*LEKR1*	-2.022804315	0.000621523	0.001422226	1
T2DM_admission	leukocyte	*ENTPD7*	-1.800835343	0.012385022	0.015897011	1
T2DM_admission	leukocyte	*AGAP9*	-2.634657559	0.000173195	0.001062643	0.680483761
T2DM_admission	leukocyte	*ZNF273*	-2.391428943	0.000163135	0.001062643	0.640956892
T2DM_admission	leukocyte	*ZNF84*	-2.509648706	0.00024473	0.001077213	0.961542927
T2DM_admission	leukocyte	*ZNF667*	-0.833130892	0.001101475	0.002000708	1
T2DM_admission	leukocyte	*CD2AP*	-1.177623262	0.000287423	0.001088708	1
T2DM_admission	leukocyte	*SFI1*	-1.981772842	0.004688365	0.006506741	1
T2DM_admission	leukocyte	*HIBCH*	-1.055006927	0.000631489	0.001433512	1
T2DM_admission	leukocyte	*TMEM229B*	-5.71691622	0.000225484	0.001062643	0.885928159
T2DM_admission	leukocyte	*SOWAHC*	-2.1167336	0.000159572	0.001062643	0.626956877
T2DM_admission	leukocyte	*SVIP*	-1.036473675	0.000258705	0.001081954	1
T2DM_admission	leukocyte	*DDRGK1*	-1.314182978	0.000188623	0.001062643	0.741099511
T2DM_admission	leukocyte	*ZNF607*	-1.209103424	0.004403277	0.006158945	1
T2DM_admission	leukocyte	*HSD17B11*	-1.721694556	0.000401005	0.001184732	1
T2DM_admission	leukocyte	*RPS6KL1*	-1.352441081	0.002542942	0.003774544	1
T2DM_admission	leukocyte	*QRICH1*	-1.958486922	0.00022638	0.001062643	0.889445749
T2DM_admission	leukocyte	*DDX42*	-0.874424789	0.002684754	0.003964074	1
T2DM_admission	leukocyte	*HELZ*	-1.292914962	0.00022365	0.001062643	0.878722365
T2DM_admission	leukocyte	*ZNF583*	-0.999147513	0.001085952	0.00197991	1
T2DM_admission	leukocyte	*ZNF568*	-1.183655086	0.002515851	0.003742817	1
T2DM_admission	leukocyte	*ZNF587*	-1.351652314	0.000459849	0.001240899	1
T2DM_admission	leukocyte	*TPM2*	-1.311335302	0.000418014	0.001200552	1
T2DM_admission	leukocyte	*NBR2*	-1.49377426	0.021282535	0.026287042	1

T2DM_admission	leukocyte	*ZNF789*	-3.119015383	0.000266255	0.001086558	1
T2DM_admission	leukocyte	*NCOA6*	-1.066256224	0.000512657	0.001308606	1
T2DM_admission	leukocyte	*STK39*	-1.037825814	0.00080156	0.001646277	1
T2DM_admission	leukocyte	*PAPSS2*	-0.98188416	0.000440813	0.00122573	1
T2DM_admission	leukocyte	*CEP290*	-1.324863431	0.000375592	0.001159218	1
T2DM_admission	leukocyte	*ECI2*	-1.357403343	0.000176387	0.001062643	0.693026403
T2DM_admission	leukocyte	*MSRB1*	-1.56956626	0.000313494	0.001109126	1
T2DM_admission	leukocyte	*COLGALT2*	-1.582299618	0.004031055	0.005690987	1
T2DM_admission	leukocyte	*PNP*	-0.658495858	0.004463203	0.006231672	1
T2DM_admission	leukocyte	*DENND4B*	-1.277647225	0.000408145	0.001188129	1
T2DM_admission	leukocyte	*CES1*	-1.815604284	0.016622971	0.020933222	1
T2DM_admission	leukocyte	*TSEN15*	-1.458770959	0.000293725	0.001088724	1
T2DM_admission	leukocyte	*LTN1*	-1.165987238	0.000538967	0.001334229	1
T2DM_admission	leukocyte	*TYW1*	-1.82585336	0.000412012	0.001192043	1
T2DM_admission	leukocyte	*ITPRIPL1*	-1.387111191	0.009356954	0.012221899	1
T2DM_admission	leukocyte	*MAP3K3*	-1.392465817	0.000121164	0.001062643	0.476051442
T2DM_admission	leukocyte	*C1orf174*	-1.491613659	0.000390095	0.00117808	1
T2DM_admission	leukocyte	*KIAA0753*	-2.367907654	0.000572531	0.001370771	1
T2DM_admission	leukocyte	*ATG9A*	-1.323837955	0.001979182	0.003048297	1
T2DM_admission	leukocyte	*TBKBP1*	-1.483471158	7.11E-05	0.001062643	0.279303269
T2DM_admission	leukocyte	*CCDC167*	-0.928947126	0.000718761	0.0015393	1
T2DM_admission	leukocyte	*SOWAHA*	-1.058627014	0.000583116	0.001382042	1
T2DM_admission	leukocyte	*L3MBTL3*	-2.083789924	0.000150658	0.001062643	0.591934601
T2DM_admission	leukocyte	*MFAP3L*	-1.820766059	0.001074274	0.001968161	1
T2DM_admission	leukocyte	*PJA2*	-1.001390807	0.000482229	0.001270743	1
T2DM_admission	leukocyte	*INF2*	-1.706243919	0.000525219	0.001319879	1
T2DM_admission	leukocyte	*EFCAB2*	-1.169843818	0.00095111	0.001823593	1
T2DM_admission	leukocyte	*SAMD5*	-1.501130769	0.000195278	0.001062643	0.767245636
T2DM_admission	leukocyte	*DDO*	-1.819629079	0.035980531	0.043086713	1
T2DM_admission	leukocyte	*C6orf163*	-2.163496703	0.001223888	0.002136231	1
T2DM_admission	leukocyte	*PCMTD2*	-1.136515178	0.000446457	0.001232235	1
T2DM_admission	leukocyte	*LIME1*	-2.538334174	5.98E-06	0.000591121	0.023507009
T2DM_admission	leukocyte	*FOXO6*	-2.225596449	0.000944173	0.001816677	1
T2DM_admission	leukocyte	*SYS1*	-1.185789997	0.000453543	0.001238727	1
T2DM_admission	leukocyte	*INPP5B*	-2.448837417	6.92E-06	0.000632075	0.027179205
T2DM_admission	leukocyte	*TRAF3IP1*	-3.816928115	1.24E-05	0.000801308	0.048767472
T2DM_admission	leukocyte	*C2orf72*	-2.944760636	0.016581508	0.020887703	1
T2DM_admission	leukocyte	*CLPSL1*	-0.589231697	0.001483943	0.002412251	1
T2DM_admission	leukocyte	*HSD17B8*	-1.134802918	0.002563207	0.003798883	1
T2DM_admission	leukocyte	*HLA-DOA*	0.181542729	0.004351744	0.006102071	1
T2DM_admission	leukocyte	*HLA-DMA*	0.225381317	0.003167051	0.004581496	1
T2DM_admission	leukocyte	*NOTCH4*	-2.053037299	9.78E-10	9.61E-07	3.84E-06
T2DM_admission	leukocyte	*RNF5*	-1.754115473	1.92E-08	9.45E-06	7.56E-05
T2DM_admission	leukocyte	*MRPL38*	-1.253838138	0.000217818	0.001062643	0.855805103
T2DM_admission	leukocyte	*SMIM5*	-0.914404772	0.001344621	0.002250006	1
T2DM_admission	leukocyte	*EHMT2*	-4.119046858	0.00025234	0.001077213	0.991445749
T2DM_admission	leukocyte	*LAYN*	-1.047804555	0.001348633	0.0022548	1
T2DM_admission	leukocyte	*SLC44A4*	-0.902994332	0.00047736	0.001264698	1
T2DM_admission	leukocyte	*MICA*	-0.694176129	0.001599441	0.002555593	1
T2DM_admission	leukocyte	*HLA-C*	-0.889884631	6.27E-06	0.000591121	0.024617451
T2DM_admission	leukocyte	*POU5F1*	1.162174592	0.025624695	0.03122811	1
T2DM_admission	leukocyte	*CCHCR1*	1.587022421	0.002912718	0.004263785	1
T2DM_admission	leukocyte	*PSORS1C1*	-0.853757464	0.002453971	0.003670214	1
T2DM_admission	leukocyte	*DHX16*	-1.359712847	0.000654818	0.00145437	1
T2DM_admission	leukocyte	*ABCF1*	-1.179434511	0.000557856	0.001358844	1
T2DM_admission	leukocyte	*LILRB3*	1.0774421	0.006356045	0.008599484	1
T2DM_admission	leukocyte	*ZNF468*	-3.06663026	9.17E-05	0.001062643	0.360166742
T2DM_admission	leukocyte	*HLA-G*	-0.797913551	0.021408992	0.02641832	1
T2DM_admission	leukocyte	*HLA-F*	-0.387928407	0.041330829	0.049134289	1
T2DM_admission	leukocyte	*ASPDH*	-2.063059035	0.000719524	0.001539766	1
T2DM_admission	leukocyte	*ZNF783*	-5.645921916	0.002016482	0.003093619	1
T2DM_admission	leukocyte	*ZNF425*	-3.058128404	0.000619055	0.001422062	1
T2DM_admission	leukocyte	*PCDHA5*	-1.184475726	0.042111777	0.049941797	1
T2DM_admission	leukocyte	*SPIRE2*	-0.877877402	0.004549493	0.006334146	1

T2DM_admission	leukocyte	*PABPN1L*	-160.4110726	0.000637294	0.001438212	1
T2DM_admission	leukocyte	*PKHD1L1*	-4.534359442	0.001348607	0.0022548	1
T2DM_admission	leukocyte	*SYCE1L*	-1.717512783	0.001090835	0.001986047	1
T2DM_admission	leukocyte	*TMEM231*	-1.128337792	0.001867122	0.002901868	1
T2DM_admission	leukocyte	*LINC00654*	-4.319638988	0.006578289	0.008872673	1
T2DM_admission	leukocyte	*SAP25*	-0.897331859	0.005578238	0.007637573	1
T2DM_admission	leukocyte	*SARNP*	-1.111563708	0.000458243	0.001240899	1
T2DM_admission	leukocyte	*MT1A*	-0.58819192	0.008431635	0.011146666	1
T2DM_admission	leukocyte	*ATP6AP1L*	1.911019546	0.011047445	0.01426402	1
T2DM_admission	leukocyte	*CCDC85C*	-1.193006965	0.000920526	0.001793131	1
T2DM_admission	leukocyte	*RGL3*	-2.093787655	0.000676991	0.001485976	1
T2DM_admission	leukocyte	*CPT1B*	-1.108240685	0.006836991	0.009186915	1
T2DM_admission	leukocyte	*SMN2*	-1.142501184	0.00872478	0.011480128	1
T2DM_admission	leukocyte	*SERF1B*	-9.641342252	0.00513136	0.007074074	1
T2DM_admission	leukocyte	*HMGN1*	-0.956223505	0.000169537	0.001062643	0.66611242
T2DM_admission	leukocyte	*LCMT1*	-1.664731779	0.000188225	0.001062643	0.739535909
T2DM_admission	leukocyte	*LIN52*	-2.67334193	0.000177187	0.001062643	0.696167054
T2DM_admission	leukocyte	*DENND1C*	0.397977841	0.008043251	0.010665519	1
T2DM_admission	leukocyte	*DEFB134*	-126.8839692	0.009088221	0.01190254	1
T2DM_admission	leukocyte	*RNPS1*	-1.105369483	0.000368785	0.001149965	1
T2DM_admission	leukocyte	*ZDHHC11B*	-1.968397781	0.000842389	0.001699271	1
T2DM_admission	leukocyte	*HLA-A*	-0.675455802	0.001400948	0.00231761	1
T2DM_admission	leukocyte	*STK38L*	-1.906838653	0.001503349	0.002439759	1
T2DM_admission	leukocyte	*MAP10*	-1.273724397	0.00166241	0.002637968	1
T2DM_admission	leukocyte	*NUP62*	-1.283461251	0.000456535	0.001240899	1
T2DM_admission	leukocyte	*FAM106A*	-1.557416912	0.001537599	0.002485079	1
T2DM_admission	leukocyte	*ZNF254*	-1.544648986	0.000107415	0.001062643	0.422034245
T2DM_admission	leukocyte	*ASIC3*	-1.461978257	0.003363832	0.004841208	1
T2DM_admission	leukocyte	*ARHGEF35*	-0.795517664	0.001049598	0.001940644	1
T2DM_admission	leukocyte	*DNLZ*	0.843614386	0.025702941	0.03131346	1
T2DM_admission	leukocyte	*SUPT4H1*	-1.433306589	0.000200976	0.001062643	0.78963277
T2DM_admission	leukocyte	*MXD3*	-1.091943738	0.003170602	0.004584945	1
T2DM_admission	leukocyte	*COG8*	-1.68218127	0.00059938	0.001399402	1
T2DM_admission	leukocyte	*ERV3-1*	-1.518520597	0.006586003	0.008880029	1
T2DM_admission	leukocyte	*CCL14*	-1.463499289	0.009058573	0.011871625	1
T2DM_admission	leukocyte	*RBMXL1*	-1.177897618	0.001112188	0.002015584	1
T2DM_admission	leukocyte	*SRA1*	-0.829796622	0.001637436	0.002605704	1
T2DM_admission	leukocyte	*C8orf82*	-2.39953838	8.90E-05	0.001062643	0.349535996
T2DM_admission	leukocyte	*LEPROT*	-1.201028774	0.000208594	0.001062643	0.819565958
T2DM_admission	leukocyte	*PPP1CB*	-0.961524207	0.000252713	0.001077213	0.99290743
T2DM_admission	leukocyte	*NCKIPSD*	-1.651633988	0.000747099	0.001568868	1
T2DM_admission	leukocyte	*ZNF888*	-1.663123084	0.000950229	0.001823593	1
T2DM_admission	leukocyte	*EMP2*	-1.296503206	0.000310848	0.00110427	1
T2DM_admission	leukocyte	*LTB4R*	-1.519322012	0.001298522	0.002196252	1
T2DM_admission	leukocyte	*DNASE1*	-0.997022802	0.002163195	0.003280275	1
T2DM_admission	leukocyte	*NUDT19*	-1.577700341	0.000462209	0.001244702	1
T2DM_admission	leukocyte	*AP1G2*	-5.017113124	3.61E-05	0.001037577	0.141900553
T2DM_admission	leukocyte	*ZNF90*	-1.396545233	0.001188623	0.00210081	1
T2DM_admission	leukocyte	*REPIN1*	-1.321317564	0.000274851	0.001088708	1
T2DM_admission	leukocyte	*TSPAN4*	-1.059281027	0.007758345	0.010322566	1
T2DM_admission	leukocyte	*CPNE1*	-1.071078259	0.022360094	0.027531435	1
T2DM_admission	leukocyte	*ARL16*	-0.983284688	0.001271335	0.002175059	1
T2DM_admission	leukocyte	*WEE2*	-6.008691389	0.004985646	0.006887694	1
T2DM_admission	leukocyte	*FIS1*	-0.910187983	0.000634719	0.001434874	1
T2DM_admission	leukocyte	*MBLAC1*	-1.166208608	0.02602328	0.031654943	1
T2DM_admission	leukocyte	*MEG3*	-0.745451959	0.000471085	0.001255055	1
T2DM_admission	leukocyte	*EML6*	-1.401294014	0.00137335	0.002283803	1
T2DM_admission	leukocyte	*C12orf71*	-18.59740713	0.001379027	0.002289918	1
T2DM_admission	leukocyte	*IFRD2*	-1.310878662	0.000259129	0.001081954	1
T2DM_admission	leukocyte	*ARHGEF28*	-1.65078205	0.000302464	0.001093914	1
T2DM_admission	leukocyte	*COL28A1*	-5.356884138	0.003158761	0.004571187	1
T2DM_admission	leukocyte	*UBXN2B*	-2.001500338	0.000212397	0.001062643	0.834509526
T2DM_admission	leukocyte	*C5orf49*	-0.97562299	0.00084517	0.001700095	1
T2DM_admission	leukocyte	*GOLGA8B*	-1.564899211	0.00089687	0.001768089	1

T2DM_admission	leukocyte	*VPS16*	-1.719286995	0.002746813	0.004040519	1
T2DM_admission	leukocyte	*LINC01139*	-3.740431151	0.00027457	0.001088708	1
T2DM_admission	leukocyte	*ATAD3C*	-1.096658308	0.000587286	0.001383749	1
T2DM_admission	leukocyte	*SYCE3*	-2.447454202	0.000356519	0.001141109	1
T2DM_admission	leukocyte	*PAM16*	-1.094025213	0.000361466	0.001142338	1
T2DM_admission	leukocyte	*LINC00339*	-1.342114293	0.000807525	0.0016529	1
T2DM_admission	leukocyte	*CELA3B*	-0.595339461	0.000417534	0.001200552	1
T2DM_admission	leukocyte	*FAM228B*	-0.915860187	0.014781995	0.018765254	1
T2DM_admission	leukocyte	*VAMP2*	-0.874870838	0.000285613	0.001088708	1
T2DM_admission	leukocyte	*PPP3R1*	-1.917971454	9.51E-05	0.001062643	0.373805191
T2DM_admission	leukocyte	*FANCG*	-1.85675868	0.002619192	0.003874551	1
T2DM_admission	leukocyte	*AP4M1*	-0.827680698	0.004587289	0.006377728	1
T2DM_admission	leukocyte	*CEBPD*	-2.395002754	0.001220472	0.002133102	1
T2DM_admission	leukocyte	*ZNF880*	-1.041696169	0.000357299	0.001141109	1
T2DM_admission	leukocyte	*TRIM16*	-1.57288474	0.001187005	0.00210081	1
T2DM_admission	leukocyte	*TIGD1*	-1.818262039	0.000919803	0.001793131	1
T2DM_admission	leukocyte	*SLC12A8*	-2.0365524	6.25E-05	0.001060479	0.245672064
T2DM_admission	leukocyte	*UBA52*	-0.924793909	0.000156343	0.001062643	0.61427195
T2DM_admission	leukocyte	*EXOSC6*	-1.572063801	1.56E-05	0.000823546	0.061296166
T2DM_admission	leukocyte	*CKMT1A*	-0.900440546	0.011643214	0.014988921	1
T2DM_admission	leukocyte	*HLA-DPB1*	0.12353616	0.004366718	0.0061187	1
T2DM_admission	leukocyte	*C4B*	9.838317641	0.028849496	0.034909045	1
T2DM_admission	leukocyte	*LINC00115*	-3.281844941	0.000404653	0.001185781	1
T2DM_admission	leukocyte	*LINC00937*	-1.174281962	0.004161008	0.005855516	1
T2DM_admission	leukocyte	*TMEM185B*	-1.271993517	0.000634268	0.001434874	1
T2DM_admission	leukocyte	*CYP4F26P*	5.101007142	0.008670024	0.011423382	1
T2DM_admission	leukocyte	*WDR46*	-0.950941513	0.004013074	0.005671715	1
T2DM_admission	leukocyte	*ZNF717*	-0.57583955	0.022901668	0.028154147	1
T2DM_admission	leukocyte	*PROB1*	-1.751433683	0.00800372	0.010616683	1
T2DM_admission	leukocyte	*ARL17B*	-3.492727982	9.48E-05	0.001062643	0.372380393
T2DM_admission	leukocyte	*DHFR*	-1.451362075	0.004734509	0.006568462	1
T2DM_admission	leukocyte	*PSMG3-AS1*	-1.112366454	0.009937544	0.012950119	1
T2DM_admission	leukocyte	*HSBP1*	-1.055349601	0.000248401	0.001077213	0.975968932
T2DM_admission	leukocyte	*RPS18*	-0.689322909	0.000411446	0.001191282	1
T2DM_admission	leukocyte	*DIRC3*	-3.162012347	0.02478574	0.030290256	1
T2DM_admission	leukocyte	*LINC00865*	-2.697418603	0.001904447	0.002952841	1
T2DM_admission	leukocyte	*HLA-DQB2*	1.901553831	4.20E-06	0.000516024	0.016512773
T2DM_admission	leukocyte	*GOLGA8N*	-0.896326801	0.035917485	0.043024329	1
T2DM_admission	leukocyte	*LINC00665*	-0.857710838	0.001293158	0.002192479	1
T2DM_admission	leukocyte	*LYRM9*	-1.318820729	0.00099658	0.001875957	1
T2DM_admission	leukocyte	*SNHG15*	-1.067351948	0.00098706	0.001867193	1
T2DM_admission	leukocyte	*RPS28*	-0.776832842	0.000195632	0.001062643	0.76864009
T2DM_admission	leukocyte	*TRIM26*	-1.667353336	4.42E-05	0.001037577	0.173600309
T2DM_admission	leukocyte	*ZNF879*	-1.518938127	0.000862778	0.001726874	1
T2DM_admission	leukocyte	*PINLYP*	-2.675762631	0.000211464	0.001062643	0.830842707
T2DM_admission	leukocyte	*MCIDAS*	40.22496741	0.007131095	0.009546192	1
T2DM_admission	leukocyte	*ANKRD65*	-1.075855395	0.000568707	0.001367774	1
T2DM_admission	leukocyte	*C12orf75*	-0.666953245	0.000523752	0.001319879	1
T2DM_admission	leukocyte	*B3GALT4*	-1.025066448	0.0002936	0.001088724	1
T2DM_admission	leukocyte	*ARHGEF38*	-1.325691121	0.025954985	0.031581646	1
T2DM_admission	leukocyte	*ZNF737*	-1.708762809	0.000276838	0.001088708	1
T2DM_admission	leukocyte	*SHISA9*	-8.62926828	0.000921557	0.00179425	1
T2DM_admission	leukocyte	*HLA-DQA2*	0.133093678	0.001029723	0.001918342	1
T2DM_admission	leukocyte	*LRRC37A2*	-1.013119767	0.040806102	0.048554566	1
T2DM_admission	leukocyte	*TXNDC5*	-1.224087515	0.000288896	0.001088708	1
T2DM_admission	leukocyte	*RBM14*	-1.60331009	0.000277333	0.001088708	1
T2DM_admission	leukocyte	*NME1*	-0.864483136	0.000599739	0.001399402	1
T2DM_admission	leukocyte	*CDRT4*	-2.461683886	0.000960674	0.001831992	1
T2DM_admission	leukocyte	*AMY2B*	-0.956584882	0.000704454	0.001521605	1
T2DM_admission	leukocyte	*LY6G5B*	-1.374908536	0.000961195	0.001831992	1
T2DM_admission	leukocyte	*PSMB9*	-0.690788369	0.01060095	0.013737182	1
T2DM_admission	leukocyte	*PCDHGC3*	-1.032220271	0.014377331	0.018298844	1
T2DM_admission	leukocyte	*SMKR1*	-1.490724565	0.000654576	0.00145437	1
T2DM_admission	leukocyte	*LINC00886*	-1.419907469	0.00530385	0.007293954	1

T2DM_admission	leukocyte	*MIF*	-0.73746817	0.000269793	0.001088175	1
T2DM_admission	leukocyte	*NSUN6*	-1.137411409	0.000586128	0.001382291	1
T2DM_admission	leukocyte	*HLA-DOB*	1.373041079	0.04012867	0.047806411	1
T2DM_admission	leukocyte	*CRCP*	-1.445470219	0.000135563	0.001062643	0.532627546
T2DM_admission	leukocyte	*C8orf58*	-1.493703042	0.006457602	0.008718872	1
T2DM_admission	leukocyte	*PI4KA*	-2.015883929	0.000212564	0.001062643	0.835164878
T2DM_admission	leukocyte	*AMACR*	-0.867708839	0.003208627	0.004636519	1
T2DM_admission	leukocyte	*ARHGDIG*	-0.956400518	0.036743413	0.04396007	1
T2DM_admission	leukocyte	*BGLAP*	-2.197752433	0.000169172	0.001062643	0.664677722
T2DM_admission	leukocyte	*MRPL20*	-0.928187212	0.001007197	0.001889818	1
T2DM_admission	leukocyte	*LINC00960*	-0.676222612	0.022452353	0.02763637	1
T2DM_admission	leukocyte	*HLA-DMB*	0.112090143	0.006104672	0.008299397	1
T2DM_admission	leukocyte	*DECR2*	-1.199396062	0.000698208	0.001512992	1
T2DM_admission	leukocyte	*GNG10*	-0.923383754	0.001551644	0.002501604	1
T2DM_admission	leukocyte	*CNTF*	2.428749645	0.003061297	0.00444981	1
T2DM_admission	leukocyte	*EIF4EBP3*	-1.743678953	7.21E-05	0.001062643	0.283393924
T2DM_admission	leukocyte	*MICAL3*	-1.117833746	0.000646334	0.001446812	1
T2DM_admission	leukocyte	*STON1*	-1.598139995	0.001335433	0.00223874	1
T2DM_admission	leukocyte	*VSIG8*	-2.931631651	0.001047648	0.001940644	1
T2DM_admission	leukocyte	*C4orf48*	-0.887153825	0.000264569	0.001084251	1
T2DM_admission	leukocyte	*IL10RB*	-2.60830257	0.000355018	0.001141109	1
T2DM_admission	leukocyte	*LEFTY1*	-8.836893835	0.002708851	0.003992152	1
T2DM_admission	leukocyte	*TTC4*	-1.183078835	0.00097834	0.001856058	1
T2DM_admission	leukocyte	*JMJD7*	3.915993246	4.66E-05	0.00105496	0.183231631
T2DM_admission	leukocyte	*APOBEC3D*	-4.683153184	0.000911411	0.001783334	1
T2DM_admission	leukocyte	*RN7SL832P*	-3.97595982	0.041734121	0.049568731	1
T2DM_admission	leukocyte	*OR2A7*	-4.367177741	0.004552301	0.006335809	1
T2DM_admission	leukocyte	*GSTA1*	-0.575940482	0.004512267	0.006293468	1
T2DM_admission	leukocyte	*GSTA2*	-0.803233339	0.00225348	0.003402738	1
T2DM_admission	leukocyte	*TMEM141*	-1.376349329	0.000711028	0.00152908	1
T2DM_admission	leukocyte	*APOBEC3C*	-2.145408463	0.003698613	0.005274718	1
T2DM_admission	leukocyte	*C4A*	-9.795909819	0.000872342	0.001738931	1
T2DM_admission	leukocyte	*NEAT1*	-0.805588396	0.000374806	0.001157716	1
T2DM_admission	leukocyte	*LINC00920*	-7.121723127	0.020500159	0.025400544	1
T2DM_admission	leukocyte	*UBAP1L*	-1.145295136	0.001949274	0.003011678	1
T2DM_admission	leukocyte	*TWF2*	-1.713223903	0.000128204	0.001062643	0.503712707
T2DM_admission	leukocyte	*BCKDHA*	-1.093906146	0.001836756	0.002861464	1
T2DM_admission	leukocyte	*PCDHAC1*	-1.519056362	0.004426629	0.006187203	1
T2DM_admission	leukocyte	*FMN1*	-3.458587435	0.0025204	0.003748166	1
T2DM_admission	leukocyte	*TMEM150C*	-1.411466785	0.000561343	0.001362208	1
T2DM_admission	leukocyte	*LINC01091*	-0.843744187	0.000708981	0.001527186	1
T2DM_admission	leukocyte	*PDCD6*	-1.186853246	0.000202059	0.001062643	0.793889895
T2DM_admission	leukocyte	*YJEFN3*	-2.062197862	0.000617021	0.001419945	1
T2DM_admission	leukocyte	*PCDHA10*	-1.074194988	0.000919367	0.001793131	1
T2DM_admission	leukocyte	*ZNF718*	-1.245308625	0.00721516	0.009655437	1
T2DM_admission	leukocyte	*SNHG18*	-1.139573115	0.001106825	0.002007832	1
T2DM_admission	leukocyte	*ZNF550*	-1.404622501	0.000435752	0.001222199	1
T2DM_admission	leukocyte	*LINC01099*	-1.35428029	0.001298112	0.002196252	1
T2DM_admission	leukocyte	*PCDHA12*	-1.345477128	0.020683168	0.025603077	1
T2DM_admission	leukocyte	*ALG11*	-2.250412161	0.000174784	0.001062643	0.686725808
T2DM_admission	leukocyte	*ZNF260*	-1.925898437	0.00242817	0.003635778	1
T2DM_admission	leukocyte	*AP5B1*	-2.281540775	0.000154824	0.001062643	0.608301941
T2DM_admission	leukocyte	*SIGLEC12*	2.073687066	0.007012422	0.009403346	1
T2DM_admission	leukocyte	*PABPC4L*	-3.119492878	0.001549555	0.002500288	1
T2DM_admission	leukocyte	*NPIPA2*	-12.16490505	0.001100334	0.001999636	1
T2DM_admission	leukocyte	*BRK1*	-0.985399504	0.000125367	0.001062643	0.49256504
T2DM_admission	leukocyte	*EID3*	-2.612210552	0.000141253	0.001062643	0.554983004
T2DM_admission	leukocyte	*SNHG9*	-1.089269932	0.000447867	0.001233124	1
T2DM_admission	leukocyte	*TAS2R43*	2.047477514	0.022858588	0.028109982	1
T2DM_admission	leukocyte	*TAS2R20*	6.620813507	0.01727824	0.02170143	1
T2DM_admission	leukocyte	*ZNF432*	-2.648361038	3.90E-05	0.001037577	0.153391628
T2DM_admission	leukocyte	*LINC01152*	-5.439552091	0.001170724	0.002087959	1
T2DM_admission	leukocyte	*SMLR1*	3.581537349	0.012748577	0.016342303	1
T2DM_admission	leukocyte	*ZNF10*	-1.532053215	0.000314756	0.001109126	1

T2DM_admission	leukocyte	*TAS2R31*	-3.798072186	0.003848218	0.005466251	1
T2DM_admission	leukocyte	*GATC*	-1.759726508	5.62E-05	0.001060479	0.220855921
T2DM_admission	leukocyte	*TEN1*	-3.718081662	2.75E-05	0.000996218	0.108211879
T2DM_admission	leukocyte	*CHURC1*	-0.993826069	0.000245015	0.001077213	0.962662807
T2DM_admission	leukocyte	*LINC00638*	-9.264564295	0.000214501	0.001062643	0.842772921
T2DM_admission	leukocyte	*SLC25A21-AS1*	-1.143609908	0.001022526	0.001910369	1
T2DM_admission	leukocyte	*TMEM179*	-0.822773481	0.001164965	0.002080069	1
T2DM_admission	leukocyte	*IQCH-AS1*	-1.688925576	0.005824122	0.007959296	1
T2DM_admission	leukocyte	*SH3RF3-AS1*	2.056180122	0.033446595	0.040150221	1
T2DM_admission	leukocyte	*PWRN1*	-1.414462243	0.003551836	0.005080148	1
T2DM_admission	leukocyte	*TMEM178B*	-1.028412384	0.001428049	0.002348599	1
T2DM_admission	leukocyte	*LINC01003*	-1.435367977	0.000298098	0.001093914	1
T2DM_admission	leukocyte	*GOLGA8H*	-17.16109903	0.004388782	0.006143112	1
T2DM_admission	leukocyte	*MRPL12*	-1.53592783	0.000227357	0.001062643	0.893287558
T2DM_admission	leukocyte	*ZNF234*	-6.078769183	0.000592748	0.001392055	1
T2DM_admission	leukocyte	*LINC00667*	-1.313566531	0.000257712	0.001080629	1
T2DM_admission	leukocyte	*FSBP*	-1.610944854	0.001247059	0.002154446	1
T2DM_admission	leukocyte	*MYO15B*	-2.421083333	0.001427598	0.002348599	1
T2DM_admission	leukocyte	*LINC00663*	-1.762832754	0.001642552	0.00261173	1
T2DM_admission	leukocyte	*ZNF285*	-2.659241853	0.00145145	0.002373152	1
T2DM_admission	leukocyte	*FBXO17*	-0.97004186	0.000681935	0.001492137	1
T2DM_admission	leukocyte	*LINC00528*	0.675104565	0.003061147	0.00444981	1
T2DM_admission	leukocyte	*SNHG8*	-0.907683019	0.000193558	0.001062643	0.760491319
T2DM_admission	leukocyte	*MILR1*	0.911067779	0.009062625	0.011872975	1
T2DM_admission	leukocyte	*GTF2H5*	-1.056586612	0.000814703	0.001660254	1
T2DM_admission	mesenchymal_cell	*RBM6*	-1.008795298	4.98E-08	1.78E-05	0.000195838
T2DM_admission	mesenchymal_cell	*ST7L*	-0.689117811	0.001139084	0.042716695	1
T2DM_admission	mesenchymal_cell	*QPCTL*	-0.844205023	0.001333765	0.047893638	1
T2DM_admission	mesenchymal_cell	*CEP68*	1.411373039	3.15E-07	7.72E-05	0.001235698
T2DM_admission	mesenchymal_cell	*ERCC1*	-1.311098627	0.00101286	0.039795261	1
T2DM_admission	mesenchymal_cell	*BAK1*	1.460407929	4.54E-05	0.005098244	0.178330183
T2DM_admission	mesenchymal_cell	*ITIH4*	-1.214657596	0.000937995	0.037605957	1
T2DM_admission	mesenchymal_cell	*SEC61A2*	-2.505298192	0.000170549	0.013173055	0.670085247
T2DM_admission	mesenchymal_cell	*TCF3*	-1.590072643	1.55E-06	0.000320518	0.006089847
T2DM_admission	mesenchymal_cell	*SCARB1*	-1.930855512	0.000159777	0.013173055	0.627762549
T2DM_admission	mesenchymal_cell	*ST6GAL1*	0.863967727	0.000298616	0.020584892	1
T2DM_admission	mesenchymal_cell	*TTC38*	-2.388892258	0.00054556	0.028966309	1
T2DM_admission	mesenchymal_cell	*MARK3*	-1.312050078	0.000170992	0.013173055	0.671825824
T2DM_admission	mesenchymal_cell	*CWF19L1*	-0.577171169	0.00041099	0.025230903	1
T2DM_admission	mesenchymal_cell	*SAMM50*	-2.285792513	0.000429759	0.025274167	1
T2DM_admission	mesenchymal_cell	*VCPKMT*	-2.373582974	0.001365931	0.048349037	1
T2DM_admission	mesenchymal_cell	*NAGPA*	-2.317770218	0.000598168	0.030923723	1
T2DM_admission	mesenchymal_cell	*RPAP1*	-3.637474632	0.000168278	0.013173055	0.66116543
T2DM_admission	mesenchymal_cell	*ERI1*	-2.483738988	0.000298635	0.020584892	1
T2DM_admission	mesenchymal_cell	*GCDH*	-3.972603966	6.70E-09	3.76E-06	2.63E-05
T2DM_admission	mesenchymal_cell	*GTPBP4*	-1.553203469	0.001340876	0.047893638	1
T2DM_admission	mesenchymal_cell	*HSD17B1*	-1.066687834	3.89E-06	0.000694796	0.015285513
T2DM_admission	mesenchymal_cell	*RANGRF*	-1.987004976	0.000646088	0.032132659	1
T2DM_admission	mesenchymal_cell	*CHPT1*	-0.854228114	0.000902206	0.03663557	1
T2DM_admission	mesenchymal_cell	*PNO1*	-1.769137744	0.000851535	0.03663557	1
T2DM_admission	mesenchymal_cell	*NSUN4*	-1.55310097	0.000465454	0.026125274	1
T2DM_admission	mesenchymal_cell	*FAM53C*	-1.379782599	0.000904467	0.03663557	1
T2DM_admission	mesenchymal_cell	*FLT3*	1.095587496	7.37E-05	0.007622994	0.289673777
T2DM_admission	mesenchymal_cell	*ARHGAP40*	-174.5105007	0.000706683	0.032874904	1
T2DM_admission	mesenchymal_cell	*PEPD*	-1.923956908	7.65E-05	0.00762496	0.300752721
T2DM_admission	mesenchymal_cell	*NQO2*	-1.409808506	0.000866784	0.03663557	1
T2DM_admission	mesenchymal_cell	*MOCS1*	-0.715443941	0.001183951	0.043474254	1
T2DM_admission	mesenchymal_cell	*OPRL1*	-1.128018206	0.00033848	0.021681357	1
T2DM_admission	mesenchymal_cell	*FOSB*	-0.507505117	0.001200012	0.043655975	1
T2DM_admission	mesenchymal_cell	*PRMT1*	-1.115317911	0.000874754	0.03663557	1
T2DM_admission	mesenchymal_cell	*MAU2*	-2.191713434	8.50E-08	2.52E-05	0.000333945
T2DM_admission	mesenchymal_cell	*UCK1*	-2.135998941	0.000493592	0.026790626	1
T2DM_admission	mesenchymal_cell	*ZNF426*	-1.820043046	0.001143608	0.042716695	1
T2DM_admission	mesenchymal_cell	*BHMT2*	-0.649636482	0.000833477	0.03663557	1

T2DM_admission	mesenchymal_cell	*NOTCH2*	-0.608528816	3.42E-09	2.32E-06	1.34E-05
T2DM_admission	mesenchymal_cell	*BARD1*	-2.082293504	0.000357953	0.022323789	1
T2DM_admission	mesenchymal_cell	*TLE3*	-1.719034397	0.001386054	0.048623257	1
T2DM_admission	mesenchymal_cell	*MAN2C1*	-0.690285984	2.08E-06	0.000388467	0.008157813
T2DM_admission	mesenchymal_cell	*NPC1*	-2.82101115	3.71E-08	1.62E-05	0.00014576
T2DM_admission	mesenchymal_cell	*FN3KRP*	-0.457124282	0.000896703	0.03663557	1
T2DM_admission	mesenchymal_cell	*SH3GL1*	-1.131238232	0.000655464	0.032191457	1
T2DM_admission	mesenchymal_cell	*IQGAP2*	-0.675302946	0.00084731	0.03663557	1
T2DM_admission	mesenchymal_cell	*TBC1D7*	-3.197388834	0.000161269	0.013173055	0.63362742
T2DM_admission	mesenchymal_cell	*GIGYF1*	-2.077441306	0.000101229	0.009469765	0.397730118
T2DM_admission	mesenchymal_cell	*LRP12*	-1.618228156	0.000420676	0.025274167	1
T2DM_admission	mesenchymal_cell	*HSD17B12*	-0.445768363	1.95E-06	0.000382824	0.007656471
T2DM_admission	mesenchymal_cell	*APIP*	-0.568257186	0.000199362	0.01477912	0.783293371
T2DM_admission	mesenchymal_cell	*DLG5*	-1.743000967	0.000615492	0.031406096	1
T2DM_admission	mesenchymal_cell	*PTPN14*	-0.584013607	4.70E-05	0.005130932	0.184713554
T2DM_admission	mesenchymal_cell	*POC5*	-0.54142268	4.07E-05	0.004993422	0.159789489
T2DM_admission	mesenchymal_cell	*MED21*	-1.743194581	0.000625175	0.031491163	1
T2DM_admission	mesenchymal_cell	*JAZF1*	-1.003695675	3.80E-07	8.29E-05	0.001492316
T2DM_admission	mesenchymal_cell	*NUS1*	-2.052080203	5.53E-05	0.005867264	0.217088774
T2DM_admission	mesenchymal_cell	*CLEC18A*	-2.317742641	5.65E-10	7.41E-07	2.22E-06
T2DM_admission	mesenchymal_cell	*FAIM*	-2.057489394	0.000872893	0.03663557	1
T2DM_admission	mesenchymal_cell	*PINK1*	-1.385046949	0.000711247	0.032874904	1
T2DM_admission	mesenchymal_cell	*CILP2*	-0.908959207	2.06E-05	0.00270434	0.081130189
T2DM_admission	mesenchymal_cell	*VPS11*	-1.123211476	0.00032667	0.021391465	1
T2DM_admission	mesenchymal_cell	*PIGX*	-1.886835969	0.000497764	0.026790626	1
T2DM_admission	mesenchymal_cell	*BDH2*	-0.541377308	0.000594138	0.030923723	1
T2DM_admission	mesenchymal_cell	*WDR41*	-1.866888069	0.000485432	0.026790626	1
T2DM_admission	mesenchymal_cell	*COG5*	-2.371053993	0.000447834	0.025875581	1
T2DM_admission	mesenchymal_cell	*RPP25L*	-2.803855323	0.000342134	0.021681357	1
T2DM_admission	mesenchymal_cell	*INPPL1*	-1.738992166	0.001152448	0.042716695	1
T2DM_admission	mesenchymal_cell	*NOXRED1*	1.974160866	0.001050685	0.0408727	1
T2DM_admission	mesenchymal_cell	*PATL1*	-1.424883953	0.000193107	0.014590691	0.758715931
T2DM_admission	mesenchymal_cell	*POP5*	-2.050475597	4.40E-05	0.005098244	0.172915693
T2DM_admission	mesenchymal_cell	*TMC8*	-0.635350995	0.001151033	0.042716695	1
T2DM_admission	mesenchymal_cell	*DNAJC21*	-1.940408167	7.76E-05	0.00762496	0.304998417
T2DM_admission	mesenchymal_cell	*TMEM129*	-1.006184883	9.19E-06	0.001388494	0.036100839
T2DM_admission	mesenchymal_cell	*SNUPN*	-3.016471702	0.000113599	0.010143837	0.446328813
T2DM_admission	mesenchymal_cell	*INO80E*	-1.260106438	4.58E-06	0.000778604	0.017993579
T2DM_admission	mesenchymal_cell	*SETMAR*	-1.545171202	0.000899826	0.03663557	1
T2DM_admission	mesenchymal_cell	*ZNF440*	-2.063461802	0.000717243	0.032874904	1
T2DM_admission	mesenchymal_cell	*RGS19*	-0.74680199	0.000668206	0.032412114	1
T2DM_admission	mesenchymal_cell	*CTSW*	-0.392843582	0.000719583	0.032874904	1
T2DM_admission	mesenchymal_cell	*RNASEH2C*	-1.199992217	4.95E-06	0.000778604	0.019465095
T2DM_admission	mesenchymal_cell	*YIF1A*	-0.811797212	0.000248022	0.017717767	0.974477174
T2DM_admission	mesenchymal_cell	*ZNF408*	-1.175637079	0.000116269	0.010151571	0.456820701
T2DM_admission	mesenchymal_cell	*IRX3*	3.241867635	3.98E-22	1.56E-18	1.56E-18
T2DM_admission	mesenchymal_cell	*FARSA*	-2.352928212	3.54E-09	2.32E-06	1.39E-05
T2DM_admission	mesenchymal_cell	*HLA-DQB1*	-0.463173241	8.96E-08	2.52E-05	0.000352182
T2DM_admission	mesenchymal_cell	*UBA7*	-0.554616559	1.72E-05	0.002380813	0.067636404
T2DM_admission	mesenchymal_cell	*SKA2*	-1.485169919	0.000313577	0.02124212	1
T2DM_admission	mesenchymal_cell	*PGP*	-1.504349716	1.76E-05	0.002380813	0.069043565
T2DM_admission	mesenchymal_cell	*DMWD*	-1.564270928	4.77E-06	0.000778604	0.018743432
T2DM_admission	mesenchymal_cell	*HLA-DRB1*	-0.350155193	1.25E-09	1.22E-06	4.90E-06
T2DM_admission	mesenchymal_cell	*SULT1A1*	-2.439646225	8.14E-05	0.007796154	0.319642304
T2DM_admission	mesenchymal_cell	*DPP4*	-1.013606485	0.000692469	0.032874904	1
T2DM_admission	mesenchymal_cell	*HLA-DRB5*	-0.218868179	2.64E-05	0.003346348	0.103736796
T2DM_admission	mesenchymal_cell	*MAP3K3*	-1.008570719	0.001152186	0.042716695	1
T2DM_admission	mesenchymal_cell	*LIME1*	-0.64063141	0.000212419	0.015455469	0.834595348
T2DM_admission	mesenchymal_cell	*INPP5B*	-0.623404616	0.000995374	0.039503293	1
T2DM_admission	mesenchymal_cell	*NOTCH4*	-0.472180443	2.88E-07	7.53E-05	0.001129655
T2DM_admission	mesenchymal_cell	*AGER*	1.058241946	0.000430992	0.025274167	1
T2DM_admission	mesenchymal_cell	*RNF5*	-0.847548481	6.98E-08	2.29E-05	0.000274267
T2DM_admission	mesenchymal_cell	*HLA-C*	-0.360993814	0.000462515	0.026125274	1
T2DM_admission	mesenchymal_cell	*RNF39*	0.529068459	0.000153498	0.013110726	0.60309338

T2DM_admission	mesenchymal_cell	*HCG27*	0.688441419	3.58E-07	8.28E-05	0.001407206
T2DM_admission	mesenchymal_cell	*ATF6B*	0.695832921	0.000110757	0.010120089	0.435163814
T2DM_admission	mesenchymal_cell	*SLC12A8*	-4.340615803	2.78E-08	1.36E-05	0.000109063
T2DM_admission	mesenchymal_cell	*EXOSC6*	-0.99676793	0.00031978	0.0212952	1
T2DM_admission	mesenchymal_cell	*HLA-DQB2*	1.169254538	4.62E-08	1.78E-05	0.000181522
T2DM_admission	mesenchymal_cell	*HLA-B*	0.464807414	4.54E-05	0.005098244	0.178438523
T2DM_admission	mesenchymal_cell	*HLA-DQA2*	0.795977063	1.37E-12	2.70E-09	5.39E-09
T2DM_admission	mesenchymal_cell	*JMJD7*	1.462676188	1.67E-05	0.002380813	0.065599331
T2DM_admission	mesenchymal_cell	*ZNF432*	-1.996733166	0.000870782	0.03663557	1
T2DM_admission	PP_cell	*RBM6*	-1.81883063	4.93E-09	3.88E-06	1.94E-05
T2DM_admission	PP_cell	*CEP68*	1.893350509	2.70E-05	0.004616313	0.106175194
T2DM_admission	PP_cell	*HAGH*	-1.472834914	0.000365496	0.033396163	1
T2DM_admission	PP_cell	*TCF3*	-2.179196473	1.30E-05	0.00273703	0.051218292
T2DM_admission	PP_cell	*MARK3*	-2.451700438	5.43E-06	0.001523051	0.021322721
T2DM_admission	PP_cell	*XAB2*	-2.39607294	0.000661987	0.046561223	1
T2DM_admission	PP_cell	*POMGNT1*	-2.53178483	0.000132269	0.015284802	0.51968327
T2DM_admission	PP_cell	*AP1B1*	-1.969309206	0.000589187	0.045359675	1
T2DM_admission	PP_cell	*HSD17B1*	-1.523242402	1.39E-05	0.00273703	0.054740591
T2DM_admission	PP_cell	*NNT*	-1.870085171	0.00010062	0.013412311	0.395337315
T2DM_admission	PP_cell	*CHMP3*	-1.55574781	0.000546905	0.042975832	1
T2DM_admission	PP_cell	*NSUN4*	-2.049785755	0.000503321	0.040358146	1
T2DM_admission	PP_cell	*FAM53C*	-2.048141818	0.00018952	0.019595389	0.744624796
T2DM_admission	PP_cell	*POLK*	-2.224356017	7.18E-05	0.010840837	0.281913855
T2DM_admission	PP_cell	*FLT3*	1.458972123	0.000674584	0.046561223	1
T2DM_admission	PP_cell	*PEPD*	-2.133657388	0.000463072	0.039552409	1
T2DM_admission	PP_cell	*OPRL1*	-2.418518409	4.18E-05	0.006850405	0.16440972
T2DM_admission	PP_cell	*MAU2*	-2.197272522	5.17E-06	0.001523051	0.020299609
T2DM_admission	PP_cell	*NOTCH2*	-0.684526113	5.24E-05	0.008241355	0.206033879
T2DM_admission	PP_cell	*FXYD2*	-1.318802502	0.000196573	0.019803442	0.772334251
T2DM_admission	PP_cell	*SPTBN5*	1.782461617	0.000647098	0.046561223	1
T2DM_admission	PP_cell	*MAN2C1*	-1.171138855	1.60E-06	0.000522792	0.006273501
T2DM_admission	PP_cell	*NPC1*	-2.57407646	0.000131096	0.015284802	0.51507776
T2DM_admission	PP_cell	*GIGYF1*	-2.274522151	0.000435619	0.038034418	1
T2DM_admission	PP_cell	*HSD17B12*	-0.869213497	2.26E-07	9.86E-05	0.000887526
T2DM_admission	PP_cell	*APIP*	-1.184587531	1.34E-05	0.00273703	0.052499345
T2DM_admission	PP_cell	*TAGLN*	-0.689191748	0.000675487	0.046561223	1
T2DM_admission	PP_cell	*VIPAS39*	-2.336440006	0.000282381	0.027736872	1
T2DM_admission	PP_cell	*PTPN14*	-1.156551955	0.000122763	0.015072995	0.482335854
T2DM_admission	PP_cell	*JAZF1*	-1.771361636	1.45E-07	7.12E-05	0.000569428
T2DM_admission	PP_cell	*CLEC18A*	-5.518590188	6.10E-12	1.20E-08	2.39E-08
T2DM_admission	PP_cell	*CILP2*	-1.656738941	0.00010241	0.013412311	0.402369342
T2DM_admission	PP_cell	*PAQR6*	-2.228599221	0.00032287	0.030940393	1
T2DM_admission	PP_cell	*ICA1L*	-1.107101253	7.73E-05	0.010840837	0.303543428
T2DM_admission	PP_cell	*BDH2*	-1.970561528	1.09E-05	0.002516086	0.042773464
T2DM_admission	PP_cell	*PATL1*	-1.827613105	0.00043279	0.038034418	1
T2DM_admission	PP_cell	*TMEM129*	-1.48152618	2.41E-05	0.004517046	0.09485797
T2DM_admission	PP_cell	*INO80E*	-1.952126939	5.97E-06	0.001564084	0.02346126
T2DM_admission	PP_cell	*SNX32*	1.726198841	0.000746807	0.047731588	1
T2DM_admission	PP_cell	*RNASEH2C*	-1.285030227	0.00015765	0.016740709	0.619406237
T2DM_admission	PP_cell	*FUT2*	0.383651065	0.000753209	0.047731588	1
T2DM_admission	PP_cell	*IRX3*	2.404052491	2.56E-11	3.35E-08	1.00E-07
T2DM_admission	PP_cell	*HLA-DQB1*	-0.87279556	2.90E-07	0.000113882	0.001138825
T2DM_admission	PP_cell	*UBA7*	-0.840128832	7.62E-05	0.010840837	0.299343966
T2DM_admission	PP_cell	*ZNF320*	0.636280663	0.000607757	0.045359675	1
T2DM_admission	PP_cell	*PGP*	-1.610840837	0.000141805	0.01564521	0.557150979
T2DM_admission	PP_cell	*DMWD*	-2.203318608	2.70E-05	0.004616313	0.105940259
T2DM_admission	PP_cell	*HLA-DRB1*	-0.725289247	1.74E-09	1.71E-06	6.86E-06
T2DM_admission	PP_cell	*XRCC6*	-1.465951481	0.000500782	0.040358146	1
T2DM_admission	PP_cell	*RPS26*	-1.012129253	0.000495987	0.040358146	1
T2DM_admission	PP_cell	*HLA-DRB5*	-0.598208043	0.000113004	0.014322283	0.44399076
T2DM_admission	PP_cell	*MAP3K3*	-2.078330593	0.000729982	0.047731588	1
T2DM_admission	PP_cell	*NOTCH4*	-1.028697056	6.88E-07	0.000245654	0.002702198
T2DM_admission	PP_cell	*AGER*	1.467823985	0.000611876	0.045359675	1
T2DM_admission	PP_cell	*RNF5*	-1.49014384	2.75E-08	1.80E-05	0.000108129

T2DM_admission	PP_cell	*HLA-C*	-0.602623735	0.000143351	0.01564521	0.563227568
T2DM_admission	PP_cell	*RNF39*	0.779320437	0.00034997	0.032738873	1
T2DM_admission	PP_cell	*HCG27*	0.870171256	1.03E-05	0.002516086	0.040507031
T2DM_admission	PP_cell	*PPT2*	1.621381669	0.000736949	0.047731588	1
T2DM_admission	PP_cell	*HLA-DQB2*	2.024079354	8.46E-08	4.75E-05	0.000332538
T2DM_admission	PP_cell	*HLA-B*	0.655651613	0.000701038	0.047489257	1
T2DM_admission	PP_cell	*HLA-DQA2*	1.329526658	2.10E-12	8.25E-09	8.25E-09
T2DM_insulin	acinar_cell	*CEP68*	0.381914223	8.86E-05	0.03165813	0.348239435
T2DM_insulin	acinar_cell	*CXCL11*	0.160901485	2.24E-05	0.008797388	0.087973877
T2DM_insulin	acinar_cell	*IRX3*	0.755101666	6.24E-10	8.18E-07	2.45E-06
T2DM_insulin	acinar_cell	*HLA-DQB1*	-0.171964483	3.05E-07	0.000239955	0.001199775
T2DM_insulin	acinar_cell	*HLA-DRB1*	-0.10058692	1.86E-05	0.008797388	0.073019871
T2DM_insulin	acinar_cell	*HLA-DRB5*	-0.117977391	1.07E-06	0.000702563	0.004215378
T2DM_insulin	acinar_cell	*HLA-DMA*	0.095423592	2.02E-05	0.008797388	0.079331571
T2DM_insulin	acinar_cell	*NOTCH4*	-0.254798396	1.49E-08	1.47E-05	5.86E-05
T2DM_insulin	acinar_cell	*HLA-DQB2*	0.542625012	2.28E-13	8.94E-10	8.94E-10
T2DM_insulin	acinar_cell	*HLA-DQA2*	0.255276726	5.23E-12	1.03E-08	2.06E-08
T2DM_insulin	acinar_cell	*HLA-DMB*	0.471322054	1.43E-06	0.00080286	0.005620018
T2DM_insulin	alpha_cell	*SEC61A2*	-0.187098915	5.36E-05	0.01913977	0.210537472
T2DM_insulin	alpha_cell	*BTNL8*	-0.598152286	0.000141156	0.046216718	0.554600621
T2DM_insulin	alpha_cell	*CXCL11*	0.025145981	3.88E-05	0.015262061	0.152620611
T2DM_insulin	alpha_cell	*IRX3*	0.111838978	9.93E-09	9.76E-06	3.90E-05
T2DM_insulin	alpha_cell	*HLA-DQB1*	-0.029615844	5.77E-08	4.54E-05	0.000226881
T2DM_insulin	alpha_cell	*HLA-DRB1*	-0.017340854	7.50E-06	0.003684049	0.029472395
T2DM_insulin	alpha_cell	*HLA-DRB5*	-0.020268158	5.42E-07	0.000354838	0.002129028
T2DM_insulin	alpha_cell	*HLA-DMA*	0.015113966	2.93E-05	0.012790155	0.115111395
T2DM_insulin	alpha_cell	*NOTCH4*	-0.042507972	4.85E-09	6.35E-06	1.90E-05
T2DM_insulin	alpha_cell	*HLA-DQB2*	0.09129261	8.30E-14	3.26E-10	3.26E-10
T2DM_insulin	alpha_cell	*HLA-DQA2*	0.041771879	5.15E-12	1.01E-08	2.02E-08
T2DM_insulin	alpha_cell	*HLA-DMB*	0.078286206	1.54E-06	0.000865916	0.006061409
T2DM_insulin	beta_cell	*CEP68*	0.039071458	0.000107856	0.038524224	0.423766459
T2DM_insulin	beta_cell	*SEC61A2*	-0.120962664	0.000158967	0.044612922	0.624580914
T2DM_insulin	beta_cell	*BTNL8*	-0.354562033	0.000184842	0.048416252	0.726243778
T2DM_insulin	beta_cell	*CXCL11*	0.015726761	2.07E-05	0.00812182	0.081218203
T2DM_insulin	beta_cell	*IRX3*	0.073100721	9.52E-10	1.25E-06	3.74E-06
T2DM_insulin	beta_cell	*HLA-DQB1*	-0.017843727	6.79E-08	5.33E-05	0.000266663
T2DM_insulin	beta_cell	*ZBTB40*	0.090516727	0.000149051	0.044612922	0.585620484
T2DM_insulin	beta_cell	*HLA-DRB1*	-0.010548052	6.66E-06	0.003270615	0.026164918
T2DM_insulin	beta_cell	*HLA-DRB5*	-0.01276389	2.04E-07	0.000133431	0.000800588
T2DM_insulin	beta_cell	*HLA-DMA*	0.00938426	1.86E-05	0.008111444	0.073002998
T2DM_insulin	beta_cell	*NOTCH4*	-0.025365906	7.70E-09	7.56E-06	3.03E-05
T2DM_insulin	beta_cell	*PPT2*	0.036166097	0.000143706	0.044612922	0.564619317
T2DM_insulin	beta_cell	*HLA-DQB2*	0.056215027	3.34E-14	1.31E-10	1.31E-10
T2DM_insulin	beta_cell	*HLA-DQA2*	0.026276884	1.06E-12	2.07E-09	4.15E-09
T2DM_insulin	beta_cell	*HLA-DMB*	0.049667965	5.22E-07	0.00029293	0.00205051
T2DM_insulin	delta_cell	*CXCL11*	0.141559167	1.07E-05	0.005998557	0.041989899
T2DM_insulin	delta_cell	*IRX3*	0.459690304	9.10E-08	8.93E-05	0.000357363
T2DM_insulin	delta_cell	*HLA-DQB1*	-0.149022494	2.04E-07	0.000160672	0.000803362
T2DM_insulin	delta_cell	*HLA-DRB1*	-0.088555554	1.48E-05	0.00727725	0.058218
T2DM_insulin	delta_cell	*HLA-DRB5*	-0.095340319	2.86E-06	0.001871247	0.011227481
T2DM_insulin	delta_cell	*HLA-DMA*	0.080545045	1.73E-05	0.007530916	0.067778242
T2DM_insulin	delta_cell	*NOTCH4*	-0.20812461	3.08E-08	4.03E-05	0.000121029
T2DM_insulin	delta_cell	*PPT2*	0.315168022	7.33E-05	0.026169415	0.287863566
T2DM_insulin	delta_cell	*HLA-DQB2*	0.434498027	1.57E-12	6.19E-09	6.19E-09
T2DM_insulin	delta_cell	*HLA-DQA2*	0.19381382	1.19E-10	2.33E-07	4.67E-07
T2DM_insulin	delta_cell	*HLA-DMB*	0.290826069	5.22E-05	0.020521897	0.205218972
T2DM_insulin	duct_epithelial_cell	*CEP68*	0.234199001	9.81E-05	0.038535138	0.385351383
T2DM_insulin	duct_epithelial_cell	*CXCL11*	0.098927834	2.18E-05	0.009516526	0.085648735
T2DM_insulin	duct_epithelial_cell	*HLA-DQB1*	-0.110042694	1.27E-07	0.000124289	0.000497156
T2DM_insulin	duct_epithelial_cell	*HLA-DRB1*	-0.068210132	5.61E-06	0.003146666	0.022026661
T2DM_insulin	duct_epithelial_cell	*HLA-DRB5*	-0.07149655	1.12E-06	0.000734954	0.004409722
T2DM_insulin	duct_epithelial_cell	*HLA-DMA*	0.060305274	1.29E-05	0.006346573	0.050772587
T2DM_insulin	duct_epithelial_cell	*NOTCH4*	-0.152321809	2.01E-08	2.63E-05	7.89E-05
T2DM_insulin	duct_epithelial_cell	*PPT2*	0.228012512	0.000114864	0.041027155	0.451298709

T2DM_insulin	duct_epithelial_cell	*HLA-DQB2*	0.351949515	5.18E-14	2.03E-10	2.03E-10
T2DM_insulin	duct_epithelial_cell	*HLA-DQA2*	0.168431933	9.93E-13	1.95E-09	3.90E-09
T2DM_insulin	duct_epithelial_cell	*HLA-DMB*	0.310217195	6.49E-07	0.000510102	0.00255051
T2DM_insulin	endothelial_cell	*CEP68*	0.340240382	2.71E-05	0.017776184	0.106657104
T2DM_insulin	endothelial_cell	*ALOX5*	-0.275167177	0.000131013	0.039596298	0.514751877
T2DM_insulin	endothelial_cell	*EXTL1*	-0.602864096	3.53E-05	0.018233293	0.138565562
T2DM_insulin	endothelial_cell	*CXCL11*	0.131191384	3.71E-05	0.018233293	0.145866343
T2DM_insulin	endothelial_cell	*IRX3*	0.346737335	7.59E-05	0.027117923	0.298297152
T2DM_insulin	endothelial_cell	*HLA-DQB1*	-0.11929613	2.26E-05	0.017776184	0.088941071
T2DM_insulin	endothelial_cell	*ZBTB40*	0.3601792	7.53E-05	0.027117923	0.295886741
T2DM_insulin	endothelial_cell	*AGAP4*	0.669036674	0.000144909	0.04019126	0.569347519
T2DM_insulin	endothelial_cell	*HLA-DMA*	0.089261146	1.78E-06	0.001750242	0.007000969
T2DM_insulin	endothelial_cell	*AGER*	0.224427589	8.82E-05	0.028865377	0.34638453
T2DM_insulin	endothelial_cell	*PPT2*	0.158199687	6.81E-05	0.027117923	0.267472909
T2DM_insulin	endothelial_cell	*HLA-DQB2*	0.471006405	2.74E-13	1.07E-09	1.07E-09
T2DM_insulin	endothelial_cell	*B3GALT4*	-0.182651218	0.000153441	0.04019126	0.602868905
T2DM_insulin	endothelial_cell	*HLA-DQA2*	0.232079786	9.73E-13	1.91E-09	3.82E-09
T2DM_insulin	endothelial_cell	*HLA-DMB*	0.394982205	1.38E-08	1.81E-05	5.43E-05
T2DM_insulin	epsilon_cell	*HLA-DQB1*	-1.201763728	1.97E-05	0.019314483	0.077257934
T2DM_insulin	epsilon_cell	*HLA-DRB1*	-0.908622035	4.84E-05	0.037992038	0.19007735
T2DM_insulin	epsilon_cell	*HLA-DRB5*	-1.258157023	5.80E-05	0.037992038	0.227952228
T2DM_insulin	epsilon_cell	*NOTCH4*	-1.852077016	2.11E-06	0.002766217	0.008298652
T2DM_insulin	epsilon_cell	*HLA-DQB2*	4.468038356	5.15E-10	1.01E-06	2.02E-06
T2DM_insulin	epsilon_cell	*HLA-DQA2*	2.557526941	2.88E-11	1.13E-07	1.13E-07
T2DM_insulin	leukocyte	*GCLC*	-0.565541505	0.003164023	0.011330438	1
T2DM_insulin	leukocyte	*NFYA*	-0.434345602	0.004714976	0.011330438	1
T2DM_insulin	leukocyte	*STPG1*	-0.376850996	0.003403169	0.011330438	1
T2DM_insulin	leukocyte	*LASP1*	-0.361359054	0.004333866	0.011330438	1
T2DM_insulin	leukocyte	*SNX11*	-0.396423699	0.006110524	0.011570498	1
T2DM_insulin	leukocyte	*TMEM176A*	-0.286176937	0.003793655	0.011330438	1
T2DM_insulin	leukocyte	*M6PR*	-0.792072099	0.003089237	0.011330438	1
T2DM_insulin	leukocyte	*CASP10*	-0.914302735	0.008182764	0.013396068	1
T2DM_insulin	leukocyte	*NDUFAF7*	-0.825323439	0.002690484	0.011330438	1
T2DM_insulin	leukocyte	*POLDIP2*	-0.322509693	0.003339436	0.011330438	1
T2DM_insulin	leukocyte	*PLXND1*	-0.745386836	0.006827051	0.012014984	1
T2DM_insulin	leukocyte	*KDM1A*	-0.37889072	0.005726878	0.011570498	1
T2DM_insulin	leukocyte	*RBM6*	-0.526035123	0.000272626	0.011330438	1
T2DM_insulin	leukocyte	*RECQL*	-0.414611963	0.00253992	0.011330438	1
T2DM_insulin	leukocyte	*SLC25A13*	-0.385068578	0.004289336	0.011330438	1
T2DM_insulin	leukocyte	*MEOX1*	0.857972498	0.021192548	0.028613581	1
T2DM_insulin	leukocyte	*PON1*	-0.461247917	0.003859138	0.011330438	1
T2DM_insulin	leukocyte	*KMT2E*	-0.409758839	0.002926449	0.011330438	1
T2DM_insulin	leukocyte	*IBTK*	-0.397196615	0.003231935	0.011330438	1
T2DM_insulin	leukocyte	*ZNF195*	-0.584019673	0.002690302	0.011330438	1
T2DM_insulin	leukocyte	*GGNBP2*	-0.396044914	0.004857462	0.011330438	1
T2DM_insulin	leukocyte	*TMEM98*	-0.250010414	0.017757714	0.024532369	1
T2DM_insulin	leukocyte	*SYNRG*	-0.550514809	0.003910735	0.011330438	1
T2DM_insulin	leukocyte	*SPATA20*	-0.314380853	0.030969845	0.039764876	1
T2DM_insulin	leukocyte	*FARP2*	-1.11190739	0.003604456	0.011330438	1
T2DM_insulin	leukocyte	*USH1C*	-0.400769519	0.003037007	0.011330438	1
T2DM_insulin	leukocyte	*VPS41*	-0.485304902	0.006516872	0.011788577	1
T2DM_insulin	leukocyte	*ELAC2*	-0.425635384	0.003387624	0.011330438	1
T2DM_insulin	leukocyte	*CCDC124*	-0.345255932	0.003507059	0.011330438	1
T2DM_insulin	leukocyte	*NOS2*	-6.065858118	0.005902199	0.011570498	1
T2DM_insulin	leukocyte	*CD79B*	-1.74382916	0.011839486	0.0176246	1
T2DM_insulin	leukocyte	*BAIAP3*	-0.371764103	0.004243011	0.011330438	1
T2DM_insulin	leukocyte	*CYB561*	-0.3441002	0.004097735	0.011330438	1
T2DM_insulin	leukocyte	*MGST1*	-0.223368061	0.019802598	0.026987307	1
T2DM_insulin	leukocyte	*ST3GAL1*	-0.907296126	0.001243759	0.011330438	1
T2DM_insulin	leukocyte	*IL32*	-0.171577065	0.006041374	0.011570498	1
T2DM_insulin	leukocyte	*MED24*	-0.542514635	0.002556039	0.011330438	1
T2DM_insulin	leukocyte	*RPS20*	-0.266174948	0.003022765	0.011330438	1
T2DM_insulin	leukocyte	*REV3L*	-0.310480558	0.006791066	0.012013551	1
T2DM_insulin	leukocyte	*MLXIPL*	-0.331994018	0.011089547	0.016732269	1

T2DM_insulin	leukocyte	*ETV7*	-0.709318058	0.004322448	0.011330438	1
T2DM_insulin	leukocyte	*NCAPD2*	-0.60054977	0.012225749	0.017949483	1
T2DM_insulin	leukocyte	*GIPR*	-0.319635333	0.004129497	0.011330438	1
T2DM_insulin	leukocyte	*FUZ*	-0.573565242	0.004859126	0.011330438	1
T2DM_insulin	leukocyte	*LRRC23*	-0.274271085	0.006883294	0.012078812	1
T2DM_insulin	leukocyte	*TSPAN9*	-0.31756978	0.006025039	0.011570498	1
T2DM_insulin	leukocyte	*BTBD7*	-0.372211147	0.010084117	0.015574095	1
T2DM_insulin	leukocyte	*APBA3*	-0.74055094	0.003168337	0.011330438	1
T2DM_insulin	leukocyte	*TTC19*	-0.318106906	0.003669982	0.011330438	1
T2DM_insulin	leukocyte	*DPF1*	-0.365723636	0.022490797	0.030128313	1
T2DM_insulin	leukocyte	*RABGAP1*	-0.374494497	0.004604647	0.011330438	1
T2DM_insulin	leukocyte	*PPP5C*	-0.376317404	0.004210505	0.011330438	1
T2DM_insulin	leukocyte	*ERCC1*	-0.465525391	0.002923165	0.011330438	1
T2DM_insulin	leukocyte	*CD22*	0.175413819	0.019009025	0.026005146	1
T2DM_insulin	leukocyte	*LTF*	-1.070221822	0.002070942	0.011330438	1
T2DM_insulin	leukocyte	*EXTL3*	-0.312862549	0.00900676	0.014309095	1
T2DM_insulin	leukocyte	*CALCOCO1*	-1.020532469	0.016681697	0.023299818	1
T2DM_insulin	leukocyte	*UBR7*	-0.371351745	0.007318782	0.012496955	1
T2DM_insulin	leukocyte	*MAN2B2*	-0.55529429	0.003283936	0.011330438	1
T2DM_insulin	leukocyte	*ANGEL1*	-0.465720013	0.012439556	0.018194523	1
T2DM_insulin	leukocyte	*DDX11*	-0.389733309	0.007276555	0.012446489	1
T2DM_insulin	leukocyte	*GPRC5A*	-0.406012227	0.018390774	0.025300193	1
T2DM_insulin	leukocyte	*UFL1*	-0.370080091	0.003716544	0.011330438	1
T2DM_insulin	leukocyte	*POLA2*	-0.634877884	0.00698426	0.01217983	1
T2DM_insulin	leukocyte	*ZC3H3*	-0.31981356	0.013689534	0.019673073	1
T2DM_insulin	leukocyte	*MDH1*	-0.382241053	0.002655411	0.011330438	1
T2DM_insulin	leukocyte	*MTMR11*	-1.406200409	0.003435771	0.011330438	1
T2DM_insulin	leukocyte	*DPEP1*	-0.215938684	0.00809639	0.013304764	1
T2DM_insulin	leukocyte	*NUDCD3*	-0.318850073	0.007338873	0.012522047	1
T2DM_insulin	leukocyte	*TTC27*	-0.765637141	0.00823678	0.013448692	1
T2DM_insulin	leukocyte	*PLEKHB1*	-0.295347788	0.029044721	0.037637437	1
T2DM_insulin	leukocyte	*OSBPL5*	-1.16049184	0.000119308	0.011330438	0.468759837
T2DM_insulin	leukocyte	*NLRP2*	-0.9059345	0.001123434	0.011330438	1
T2DM_insulin	leukocyte	*ZDHHC6*	-0.58473328	0.000430801	0.011330438	1
T2DM_insulin	leukocyte	*RNH1*	-0.35649908	0.004264118	0.011330438	1
T2DM_insulin	leukocyte	*NDUFS1*	-0.323850858	0.005873766	0.011570498	1
T2DM_insulin	leukocyte	*TYMP*	0.153270882	0.033417786	0.042546495	1
T2DM_insulin	leukocyte	*CD44*	-0.198636909	0.004860536	0.011330438	1
T2DM_insulin	leukocyte	*KCNG1*	-1.10782075	0.020721912	0.02807079	1
T2DM_insulin	leukocyte	*AGPAT4*	-1.265036237	0.006654477	0.01190541	1
T2DM_insulin	leukocyte	*VEZT*	-0.389037632	0.004365315	0.011330438	1
T2DM_insulin	leukocyte	*RABEP1*	-0.638305864	0.004038003	0.011330438	1
T2DM_insulin	leukocyte	*ARID4A*	-0.527802367	0.002351271	0.011330438	1
T2DM_insulin	leukocyte	*PNPLA6*	-0.49108763	0.002877526	0.011330438	1
T2DM_insulin	leukocyte	*ABCF2*	-0.527002217	0.003533519	0.011330438	1
T2DM_insulin	leukocyte	*UBA6*	-0.603807036	0.002801447	0.011330438	1
T2DM_insulin	leukocyte	*APBA2*	-0.964134218	0.005964901	0.011570498	1
T2DM_insulin	leukocyte	*ASTE1*	-0.455323953	0.009656486	0.015078655	1
T2DM_insulin	leukocyte	*RNF19A*	-0.329936647	0.003516425	0.011330438	1
T2DM_insulin	leukocyte	*SH3YL1*	-0.244608489	0.005482751	0.011427973	1
T2DM_insulin	leukocyte	*FAM136A*	-0.369799965	0.003384319	0.011330438	1
T2DM_insulin	leukocyte	*DAPK2*	-0.388250913	0.00535617	0.011421159	1
T2DM_insulin	leukocyte	*TIMP2*	-0.736351647	0.002128416	0.011330438	1
T2DM_insulin	leukocyte	*MYOM2*	0.413552602	0.024320921	0.032293645	1
T2DM_insulin	leukocyte	*RPL26L1*	-0.339650369	0.003704413	0.011330438	1
T2DM_insulin	leukocyte	*NSUN2*	-0.569782392	0.002006361	0.011330438	1
T2DM_insulin	leukocyte	*MRI1*	-0.365599738	0.019520455	0.026658279	1
T2DM_insulin	leukocyte	*AGA*	-0.446437114	0.00277938	0.011330438	1
T2DM_insulin	leukocyte	*BOD1L1*	-0.294072151	0.014273622	0.020385699	1
T2DM_insulin	leukocyte	*DNAH5*	-0.576730161	0.003497438	0.011330438	1
T2DM_insulin	leukocyte	*PHLPP2*	-0.336745235	0.005436408	0.011421159	1
T2DM_insulin	leukocyte	*CTNS*	-0.461268403	0.005817315	0.011570498	1
T2DM_insulin	leukocyte	*INPP4A*	-0.348730257	0.005631766	0.01154792	1
T2DM_insulin	leukocyte	*PSMA4*	-0.36211056	0.002459396	0.011330438	1

T2DM_insulin	leukocyte	*SPATA7*	-0.440550864	0.012259358	0.017972767	1
T2DM_insulin	leukocyte	*TRIT1*	-0.783851926	0.006465558	0.011733569	1
T2DM_insulin	leukocyte	*CNTLN*	-0.519219029	0.005002628	0.011330438	1
T2DM_insulin	leukocyte	*PREX2*	-0.685375596	0.007735809	0.012941946	1
T2DM_insulin	leukocyte	*WDR37*	-0.545828979	0.002852106	0.011330438	1
T2DM_insulin	leukocyte	*YTHDC2*	-0.610991555	0.00198788	0.011330438	1
T2DM_insulin	leukocyte	*DTNBP1*	-0.427846932	0.003746212	0.011330438	1
T2DM_insulin	leukocyte	*ANO2*	-4.867917879	0.017964674	0.024774729	1
T2DM_insulin	leukocyte	*GOPC*	-0.445451195	0.002624715	0.011330438	1
T2DM_insulin	leukocyte	*ZNF800*	-0.432109543	0.003090495	0.011330438	1
T2DM_insulin	leukocyte	*MRPS10*	-0.536632052	0.001219757	0.011330438	1
T2DM_insulin	leukocyte	*COL9A2*	-0.417395122	0.003256655	0.011330438	1
T2DM_insulin	leukocyte	*ERCC8*	-1.223354224	0.011248158	0.016874384	1
T2DM_insulin	leukocyte	*H6PD*	-0.985549449	0.002518651	0.011330438	1
T2DM_insulin	leukocyte	*VAMP3*	-0.445625208	0.003099357	0.011330438	1
T2DM_insulin	leukocyte	*EPN3*	-0.7491647	0.027776318	0.036220762	1
T2DM_insulin	leukocyte	*RCN1*	-0.301213831	0.004389626	0.011330438	1
T2DM_insulin	leukocyte	*RFC2*	-0.426890974	0.003657863	0.011330438	1
T2DM_insulin	leukocyte	*JKAMP*	-0.372789394	0.002403979	0.011330438	1
T2DM_insulin	leukocyte	*PTGER3*	-0.265156172	0.032487459	0.041509993	1
T2DM_insulin	leukocyte	*BCAR1*	-0.307673952	0.003699131	0.011330438	1
T2DM_insulin	leukocyte	*THOC3*	-0.286583298	0.006946968	0.012141743	1
T2DM_insulin	leukocyte	*HEBP2*	-0.340630919	0.003125104	0.011330438	1
T2DM_insulin	leukocyte	*RRP12*	-0.263228275	0.005801061	0.011570498	1
T2DM_insulin	leukocyte	*FNIP2*	-0.683252357	0.014749587	0.020951239	1
T2DM_insulin	leukocyte	*MRTO4*	-0.420668392	0.005864173	0.011570498	1
T2DM_insulin	leukocyte	*MCF2L2*	-0.612887534	0.003803962	0.011330438	1
T2DM_insulin	leukocyte	*LAMA3*	-0.564312791	0.007987558	0.013208382	1
T2DM_insulin	leukocyte	*ANAPC4*	-1.244912853	0.031677574	0.040593995	1
T2DM_insulin	leukocyte	*LY75*	-1.591816297	0.001315154	0.011330438	1
T2DM_insulin	leukocyte	*ARID4B*	-0.343326814	0.002388908	0.011330438	1
T2DM_insulin	leukocyte	*SDCCAG8*	-0.361321372	0.004919529	0.011330438	1
T2DM_insulin	leukocyte	*KIF1B*	-0.362057415	0.003607504	0.011330438	1
T2DM_insulin	leukocyte	*TBC1D22A*	-0.386655301	0.004216516	0.011330438	1
T2DM_insulin	leukocyte	*SYNE2*	-0.354859357	0.003719953	0.011330438	1
T2DM_insulin	leukocyte	*ATP9A*	-0.381496919	0.004480967	0.011330438	1
T2DM_insulin	leukocyte	*CHRDL2*	-1.297402577	0.007524998	0.012715195	1
T2DM_insulin	leukocyte	*GALC*	-0.584664734	0.001521355	0.011330438	1
T2DM_insulin	leukocyte	*CYFIP2*	-0.308726378	0.004257862	0.011330438	1
T2DM_insulin	leukocyte	*USP36*	-0.602465533	0.001796019	0.011330438	1
T2DM_insulin	leukocyte	*MCOLN3*	-0.37024749	0.003402705	0.011330438	1
T2DM_insulin	leukocyte	*ITIH4*	-0.987894074	0.000233559	0.011330438	0.917654856
T2DM_insulin	leukocyte	*PCGF2*	-0.357606764	0.005154314	0.011330438	1
T2DM_insulin	leukocyte	*GDI2*	-0.397760676	0.002734295	0.011330438	1
T2DM_insulin	leukocyte	*ATP11B*	-0.601480369	0.004363187	0.011330438	1
T2DM_insulin	leukocyte	*LAMC2*	-0.273444115	0.004297445	0.011330438	1
T2DM_insulin	leukocyte	*RASGRF1*	-0.265999609	0.008718738	0.013987718	1
T2DM_insulin	leukocyte	*CROCC*	-0.521477218	0.010223956	0.0157344	1
T2DM_insulin	leukocyte	*FLYWCH1*	-0.285770156	0.003030177	0.011330438	1
T2DM_insulin	leukocyte	*OGFR*	-0.745576973	0.035916598	0.045360435	1
T2DM_insulin	leukocyte	*GNA15*	0.55956578	0.017484518	0.024206016	1
T2DM_insulin	leukocyte	*RIMBP2*	-0.298711084	0.005985854	0.011570498	1
T2DM_insulin	leukocyte	*MPC1*	-0.349302197	0.003305072	0.011330438	1
T2DM_insulin	leukocyte	*MRPS35*	-0.388167476	0.004113265	0.011330438	1
T2DM_insulin	leukocyte	*ZNF112*	-0.413535096	0.023359502	0.03112224	1
T2DM_insulin	leukocyte	*EIF4B*	-0.310153268	0.008025396	0.01322829	1
T2DM_insulin	leukocyte	*SLC6A16*	-0.987475839	0.005508542	0.011434531	1
T2DM_insulin	leukocyte	*SPHK2*	-0.444225815	0.005718494	0.011563542	1
T2DM_insulin	leukocyte	*HAGH*	-0.454978063	0.002282787	0.011330438	1
T2DM_insulin	leukocyte	*RNF4*	-0.448922798	0.002998824	0.011330438	1
T2DM_insulin	leukocyte	*TM7SF3*	-0.639263637	0.005677957	0.011549238	1
T2DM_insulin	leukocyte	*HIPK2*	-0.305393369	0.00465116	0.011330438	1
T2DM_insulin	leukocyte	*TMEM161A*	-0.424596059	0.00455519	0.011330438	1
T2DM_insulin	leukocyte	*SNX24*	-0.78706839	0.005628821	0.01154792	1

T2DM_insulin	leukocyte	*PMS1*	-0.359229998	0.005947601	0.011570498	1
T2DM_insulin	leukocyte	*IPO5*	-0.328233724	0.003788234	0.011330438	1
T2DM_insulin	leukocyte	*OAT*	-0.340654793	0.004309874	0.011330438	1
T2DM_insulin	leukocyte	*WDR18*	-0.447150973	0.002309531	0.011330438	1
T2DM_insulin	leukocyte	*MCM10*	0.656194117	0.022862666	0.030553543	1
T2DM_insulin	leukocyte	*ERBB3*	-0.297732898	0.00954347	0.014974558	1
T2DM_insulin	leukocyte	*ANKRD44*	-1.164609586	0.034997664	0.04437103	1
T2DM_insulin	leukocyte	*ADAT1*	-0.384101038	0.003673355	0.011330438	1
T2DM_insulin	leukocyte	*MYLK*	-0.330584942	0.005816777	0.011570498	1
T2DM_insulin	leukocyte	*GSTO2*	-0.253738239	0.004916493	0.011330438	1
T2DM_insulin	leukocyte	*SEC61A2*	-1.446070557	8.91E-05	0.011330438	0.350063187
T2DM_insulin	leukocyte	*PRKCQ*	-0.771572298	0.017794584	0.024566029	1
T2DM_insulin	leukocyte	*TBC1D1*	-0.368220527	0.00670018	0.011939628	1
T2DM_insulin	leukocyte	*CTNNA2*	-0.450894315	0.007630073	0.012827794	1
T2DM_insulin	leukocyte	*CD84*	0.056434339	0.006769689	0.011991254	1
T2DM_insulin	leukocyte	*ATXN3*	-0.541817688	0.007597907	0.012795617	1
T2DM_insulin	leukocyte	*EML1*	-0.588503213	0.00327594	0.011330438	1
T2DM_insulin	leukocyte	*STOML1*	-0.337175812	0.004987021	0.011330438	1
T2DM_insulin	leukocyte	*DNTTIP2*	-0.37131139	0.001591168	0.011330438	1
T2DM_insulin	leukocyte	*RRP15*	-0.385567512	0.004489714	0.011330438	1
T2DM_insulin	leukocyte	*REEP1*	-0.328495017	0.007695741	0.012910575	1
T2DM_insulin	leukocyte	*LAPTM4A*	-0.32935271	0.00308402	0.011330438	1
T2DM_insulin	leukocyte	*KIF2A*	-0.356034361	0.003866565	0.011330438	1
T2DM_insulin	leukocyte	*MAST4*	-0.418760869	0.002651296	0.011330438	1
T2DM_insulin	leukocyte	*ADAM7*	-5.222734489	0.024262919	0.032238421	1
T2DM_insulin	leukocyte	*DRD4*	-1.177370247	0.007262027	0.012437884	1
T2DM_insulin	leukocyte	*PLA2G10*	-1.387181789	0.004830417	0.011330438	1
T2DM_insulin	leukocyte	*GNB5*	-0.401632515	0.012783538	0.018608509	1
T2DM_insulin	leukocyte	*RAB27A*	-0.36226585	0.002546028	0.011330438	1
T2DM_insulin	leukocyte	*PTPN3*	-0.611848429	0.002526301	0.011330438	1
T2DM_insulin	leukocyte	*FGF10*	-1.015595399	0.01194299	0.017706833	1
T2DM_insulin	leukocyte	*DGCR2*	-0.509439924	0.002560756	0.011330438	1
T2DM_insulin	leukocyte	*ZXDC*	-0.913397356	0.00255775	0.011330438	1
T2DM_insulin	leukocyte	*WIPI1*	-0.878030415	0.003030999	0.011330438	1
T2DM_insulin	leukocyte	*GBA2*	-0.352243108	0.008002877	0.013211473	1
T2DM_insulin	leukocyte	*ASNS*	-0.360546811	0.006163178	0.011570498	1
T2DM_insulin	leukocyte	*MAP4K4*	-0.33198617	0.003452479	0.011330438	1
T2DM_insulin	leukocyte	*RPL31*	-0.29341493	0.002977024	0.011330438	1
T2DM_insulin	leukocyte	*SEL1L*	-0.321025056	0.004376178	0.011330438	1
T2DM_insulin	leukocyte	*TCF3*	-0.377166134	0.002010309	0.011330438	1
T2DM_insulin	leukocyte	*CPSF1*	-0.30351695	0.010690394	0.016273754	1
T2DM_insulin	leukocyte	*CYBRD1*	0.233802265	0.03190454	0.04084488	1
T2DM_insulin	leukocyte	*LNX1*	-0.288410352	0.005017169	0.011330438	1
T2DM_insulin	leukocyte	*ALDH3A2*	-0.257153708	0.004208665	0.011330438	1
T2DM_insulin	leukocyte	*P4HA2*	-0.264332667	0.009289389	0.014663725	1
T2DM_insulin	leukocyte	*TRNT1*	-1.201064552	0.003028507	0.011330438	1
T2DM_insulin	leukocyte	*CRMP1*	-0.310088491	0.004722294	0.011330438	1
T2DM_insulin	leukocyte	*DERL2*	-0.389817998	0.002928785	0.011330438	1
T2DM_insulin	leukocyte	*XRCC1*	-0.449712663	0.014516946	0.020652657	1
T2DM_insulin	leukocyte	*SCARB1*	-0.990169088	0.001715778	0.011330438	1
T2DM_insulin	leukocyte	*NLE1*	-0.326187909	0.028211626	0.036678848	1
T2DM_insulin	leukocyte	*SMARCE1*	-0.335665933	0.003240326	0.011330438	1
T2DM_insulin	leukocyte	*MRPS34*	-0.311762202	0.003572298	0.011330438	1
T2DM_insulin	leukocyte	*TEAD2*	-0.394719738	0.03280473	0.041847333	1
T2DM_insulin	leukocyte	*IPCEF1*	-0.541249248	0.033927041	0.043138946	1
T2DM_insulin	leukocyte	*ZZEF1*	-0.545623029	0.002837459	0.011330438	1
T2DM_insulin	leukocyte	*WSCD2*	-0.342053509	0.01709009	0.023777254	1
T2DM_insulin	leukocyte	*SEMA3A*	-1.02216679	0.007095311	0.01228624	1
T2DM_insulin	leukocyte	*TTC38*	-0.688943818	0.0044794	0.011330438	1
T2DM_insulin	leukocyte	*GRAMD4*	-0.492503438	0.009659704	0.015078655	1
T2DM_insulin	leukocyte	*CELSR1*	-1.564189888	0.021707528	0.029248586	1
T2DM_insulin	leukocyte	*TIMM21*	-0.553625554	0.003834688	0.011330438	1
T2DM_insulin	leukocyte	*VPS9D1*	-0.35556062	0.00790921	0.013134103	1
T2DM_insulin	leukocyte	*ZNF37A*	-0.338179163	0.009778132	0.01522119	1

T2DM_insulin	leukocyte	*MARK3*	-0.445912746	0.000941489	0.011330438	1
T2DM_insulin	leukocyte	*CACNG5*	-11.55243731	0.003457887	0.011330438	1
T2DM_insulin	leukocyte	*MOCOS*	-0.888595857	0.028226674	0.036686272	1
T2DM_insulin	leukocyte	*DLG1*	-0.367609914	0.003511113	0.011330438	1
T2DM_insulin	leukocyte	*EXOSC7*	-0.309798886	0.00573872	0.011570498	1
T2DM_insulin	leukocyte	*MKRN2*	-0.449149195	0.004999663	0.011330438	1
T2DM_insulin	leukocyte	*MLH1*	-0.504468673	0.004625569	0.011330438	1
T2DM_insulin	leukocyte	*TPD52*	-0.298078989	0.003889298	0.011330438	1
T2DM_insulin	leukocyte	*ACACB*	-0.505727361	0.001330679	0.011330438	1
T2DM_insulin	leukocyte	*GPATCH1*	-0.339826419	0.009982929	0.015458818	1
T2DM_insulin	leukocyte	*ICAM3*	-0.449843956	0.000891959	0.011330438	1
T2DM_insulin	leukocyte	*XAB2*	-0.380742281	0.003995237	0.011330438	1
T2DM_insulin	leukocyte	*ACTL6B*	-0.296918875	0.012198801	0.017937534	1
T2DM_insulin	leukocyte	*IL4R*	-0.328313565	0.007894112	0.013125674	1
T2DM_insulin	leukocyte	*APBB1IP*	0.154934569	0.009366553	0.01477366	1
T2DM_insulin	leukocyte	*MCCC1*	-0.409341958	0.003025195	0.011330438	1
T2DM_insulin	leukocyte	*N4BP2*	-0.545566856	0.003149813	0.011330438	1
T2DM_insulin	leukocyte	*MLLT10*	-0.494446326	0.003480112	0.011330438	1
T2DM_insulin	leukocyte	*VDAC3*	-0.40218662	0.004129941	0.011330438	1
T2DM_insulin	leukocyte	*PCM1*	-0.342027895	0.004099719	0.011330438	1
T2DM_insulin	leukocyte	*TP73*	0.999854502	0.017177872	0.023872903	1
T2DM_insulin	leukocyte	*TOLLIP*	-0.407644421	0.002602169	0.011330438	1
T2DM_insulin	leukocyte	*CLUL1*	-0.967545463	0.005686417	0.011549238	1
T2DM_insulin	leukocyte	*FKBP7*	-0.494335249	0.004858104	0.011330438	1
T2DM_insulin	leukocyte	*LXN*	-0.272973082	0.008870324	0.014148275	1
T2DM_insulin	leukocyte	*SAR1A*	-0.301339492	0.003879464	0.011330438	1
T2DM_insulin	leukocyte	*CDC14A*	-0.538141729	0.007192568	0.012373634	1
T2DM_insulin	leukocyte	*FDFT1*	-0.4207961	0.002781168	0.011330438	1
T2DM_insulin	leukocyte	*SCGN*	-0.173222476	0.006717872	0.011954039	1
T2DM_insulin	leukocyte	*DDX1*	-0.339142095	0.004186956	0.011330438	1
T2DM_insulin	leukocyte	*RABL2B*	-0.561983756	0.004343391	0.011330438	1
T2DM_insulin	leukocyte	*DCT*	-2.378300036	0.003344327	0.011330438	1
T2DM_insulin	leukocyte	*SCTR*	-0.378140095	0.005073487	0.011330438	1
T2DM_insulin	leukocyte	*RIF1*	-0.73358011	0.001155898	0.011330438	1
T2DM_insulin	leukocyte	*IGSF9B*	-0.654035219	0.01038376	0.015911777	1
T2DM_insulin	leukocyte	*COL4A4*	-0.998158544	0.000873662	0.011330438	1
T2DM_insulin	leukocyte	*ZNF506*	-0.360731669	0.005362683	0.011421159	1
T2DM_insulin	leukocyte	*CADPS2*	-0.3790714	0.006852324	0.012045987	1
T2DM_insulin	leukocyte	*ME2*	-0.82576655	0.003469261	0.011330438	1
T2DM_insulin	leukocyte	*CCNT2*	-0.408420868	0.013000509	0.018823927	1
T2DM_insulin	leukocyte	*SEMA5B*	0.541401911	0.016672626	0.02329543	1
T2DM_insulin	leukocyte	*BCKDHB*	-0.827396218	0.017859878	0.024647509	1
T2DM_insulin	leukocyte	*DIS3*	-0.381406063	0.003115757	0.011330438	1
T2DM_insulin	leukocyte	*PIBF1*	-0.564037872	0.005479412	0.011427973	1
T2DM_insulin	leukocyte	*CHMP2B*	-0.336276712	0.003098431	0.011330438	1
T2DM_insulin	leukocyte	*PPIE*	-0.472130794	0.001466655	0.011330438	1
T2DM_insulin	leukocyte	*NCOA1*	-0.441963593	0.00305097	0.011330438	1
T2DM_insulin	leukocyte	*AGBL5*	-0.591816644	0.015058598	0.021336182	1
T2DM_insulin	leukocyte	*CD59*	-0.237188925	0.004438014	0.011330438	1
T2DM_insulin	leukocyte	*CD82*	-0.659741138	0.003907293	0.011330438	1
T2DM_insulin	leukocyte	*MECOM*	-0.252955093	0.009474171	0.014889607	1
T2DM_insulin	leukocyte	*SLC25A24*	-0.36863357	0.004795	0.011330438	1
T2DM_insulin	leukocyte	*PILRA*	0.114968145	0.020980744	0.028366601	1
T2DM_insulin	leukocyte	*ZNF213*	-0.415288491	0.004889193	0.011330438	1
T2DM_insulin	leukocyte	*CPNE3*	-0.289279783	0.004090418	0.011330438	1
T2DM_insulin	leukocyte	*RRN3*	-0.329593582	0.004458098	0.011330438	1
T2DM_insulin	leukocyte	*WNT11*	-0.87941585	0.018508352	0.025435228	1
T2DM_insulin	leukocyte	*DDHD2*	-0.449413889	0.003787071	0.011330438	1
T2DM_insulin	leukocyte	*TTC39A*	-0.322128739	0.021305848	0.028756673	1
T2DM_insulin	leukocyte	*EPS15*	-0.417044901	0.018950806	0.025970602	1
T2DM_insulin	leukocyte	*ORC1*	-1.371513276	0.003642034	0.011330438	1
T2DM_insulin	leukocyte	*MGST2*	-0.258429149	0.007149298	0.012352504	1
T2DM_insulin	leukocyte	*USP40*	-0.594310005	0.003243732	0.011330438	1
T2DM_insulin	leukocyte	*POMGNT1*	-0.516240759	0.002015314	0.011330438	1

T2DM_insulin	leukocyte	*NFX1*	-0.517932376	0.011787111	0.017562214	1
T2DM_insulin	leukocyte	*EIF2AK1*	-0.343580668	0.010529395	0.016078505	1
T2DM_insulin	leukocyte	*ITPKC*	-0.474166455	0.004038809	0.011330438	1
T2DM_insulin	leukocyte	*TMED2*	-0.2870407	0.003382424	0.011330438	1
T2DM_insulin	leukocyte	*ACOX3*	-0.741635364	0.009421248	0.014830162	1
T2DM_insulin	leukocyte	*TRIP6*	-0.314886729	0.003939952	0.011330438	1
T2DM_insulin	leukocyte	*PGS1*	-0.59621858	0.003175137	0.011330438	1
T2DM_insulin	leukocyte	*PSMC5*	-0.29178857	0.005235188	0.011389288	1
T2DM_insulin	leukocyte	*UIMC1*	-0.42939284	0.002880127	0.011330438	1
T2DM_insulin	leukocyte	*SH3BP2*	0.428448654	0.030111886	0.038853727	1
T2DM_insulin	leukocyte	*SF3B2*	-0.290759688	0.007584987	0.01278254	1
T2DM_insulin	leukocyte	*KLHL42*	-0.315062145	0.004994363	0.011330438	1
T2DM_insulin	leukocyte	*METTL2A*	-0.587381844	0.00320061	0.011330438	1
T2DM_insulin	leukocyte	*GP6*	0.489585412	0.032997854	0.04205273	1
T2DM_insulin	leukocyte	*SLC15A1*	-0.688437246	0.012266726	0.017976861	1
T2DM_insulin	leukocyte	*TGDS*	-0.304669748	0.026965004	0.035279887	1
T2DM_insulin	leukocyte	*DOCK3*	-0.421348298	0.006166137	0.011570498	1
T2DM_insulin	leukocyte	*ARHGAP28*	-0.950990743	0.005964885	0.011570498	1
T2DM_insulin	leukocyte	*PPP1R13B*	-0.270033395	0.007427306	0.012600123	1
T2DM_insulin	leukocyte	*ZNF343*	-0.48684168	0.007876379	0.013101733	1
T2DM_insulin	leukocyte	*MAVS*	-0.6661146	0.00828078	0.013483293	1
T2DM_insulin	leukocyte	*DYNLL1*	-0.302080968	0.005014278	0.011330438	1
T2DM_insulin	leukocyte	*RBBP9*	-0.331925785	0.006092507	0.011570498	1
T2DM_insulin	leukocyte	*SLC23A2*	-0.65276168	0.002046311	0.011330438	1
T2DM_insulin	leukocyte	*SLC8B1*	0.101438846	0.025126781	0.033251304	1
T2DM_insulin	leukocyte	*TMEM230*	-0.340816075	0.004378451	0.011330438	1
T2DM_insulin	leukocyte	*DZANK1*	0.829987872	0.039205179	0.049166022	1
T2DM_insulin	leukocyte	*KIF16B*	-0.53514013	0.005632591	0.01154792	1
T2DM_insulin	leukocyte	*ZNF302*	-0.349740634	0.004553185	0.011330438	1
T2DM_insulin	leukocyte	*GRAMD1A*	-0.591564405	0.003277635	0.011330438	1
T2DM_insulin	leukocyte	*CDIP1*	-0.412438375	0.00299454	0.011330438	1
T2DM_insulin	leukocyte	*MLF2*	-0.403170759	0.002293895	0.011330438	1
T2DM_insulin	leukocyte	*DDX24*	-0.31722076	0.002870602	0.011330438	1
T2DM_insulin	leukocyte	*ANKRD24*	-3.392234056	0.001766176	0.011330438	1
T2DM_insulin	leukocyte	*GPATCH2L*	-0.451999682	0.002870087	0.011330438	1
T2DM_insulin	leukocyte	*SLC9A1*	-0.338323529	0.00486595	0.011330438	1
T2DM_insulin	leukocyte	*SPTLC1*	-0.371578499	0.003559204	0.011330438	1
T2DM_insulin	leukocyte	*NDUFB2*	-0.299818573	0.00286119	0.011330438	1
T2DM_insulin	leukocyte	*MAEA*	-0.349604132	0.003250809	0.011330438	1
T2DM_insulin	leukocyte	*STRN4*	-0.358898127	0.004594557	0.011330438	1
T2DM_insulin	leukocyte	*LYZ*	0.082626442	0.015451226	0.021810191	1
T2DM_insulin	leukocyte	*MUL1*	-0.378461136	0.005678355	0.011549238	1
T2DM_insulin	leukocyte	*SPG21*	-0.492792464	0.003377584	0.011330438	1
T2DM_insulin	leukocyte	*USP48*	-0.377280095	0.004093751	0.011330438	1
T2DM_insulin	leukocyte	*GLG1*	-0.372831423	0.003235166	0.011330438	1
T2DM_insulin	leukocyte	*NRCAM*	-0.242327124	0.005506349	0.011434531	1
T2DM_insulin	leukocyte	*TF*	-0.786117147	0.000310481	0.011330438	1
T2DM_insulin	leukocyte	*MYO15A*	-0.8659475	0.008373473	0.013599474	1
T2DM_insulin	leukocyte	*PITPNM3*	-1.032927834	0.005888205	0.011570498	1
T2DM_insulin	leukocyte	*SPAG7*	-0.363306054	0.011082454	0.016732269	1
T2DM_insulin	leukocyte	*TMEM101*	-0.498662468	0.002077387	0.011330438	1
T2DM_insulin	leukocyte	*PPP2R3C*	-0.5652262	0.00507099	0.011330438	1
T2DM_insulin	leukocyte	*OSGEP*	-0.658175636	0.002559763	0.011330438	1
T2DM_insulin	leukocyte	*SCFD1*	-0.38168289	0.003771763	0.011330438	1
T2DM_insulin	leukocyte	*RPGRIP1*	0.414057357	0.03001153	0.038749688	1
T2DM_insulin	leukocyte	*EZR*	-0.258259637	0.00413246	0.011330438	1
T2DM_insulin	leukocyte	*NUP50*	-0.63472853	0.003663209	0.011330438	1
T2DM_insulin	leukocyte	*COMT*	-0.332778309	0.003728466	0.011330438	1
T2DM_insulin	leukocyte	*ECHDC1*	-0.294282162	0.006717382	0.011954039	1
T2DM_insulin	leukocyte	*CBX5*	-0.283432254	0.008569027	0.01382216	1
T2DM_insulin	leukocyte	*SUCO*	-0.263283483	0.003870652	0.011330438	1
T2DM_insulin	leukocyte	*MSH2*	-0.79317213	0.003390638	0.011330438	1
T2DM_insulin	leukocyte	*TMEM38B*	-0.562776848	0.001827051	0.011330438	1
T2DM_insulin	leukocyte	*PSMD5*	-0.345546367	0.005242456	0.011392572	1

T2DM_insulin	leukocyte	*CRAT*	-0.622212737	0.00295694	0.011330438	1
T2DM_insulin	leukocyte	*SH2D3C*	-0.527450231	0.006021447	0.011570498	1
T2DM_insulin	leukocyte	*CWF19L1*	-0.384482141	0.00282779	0.011330438	1
T2DM_insulin	leukocyte	*IKZF5*	-0.595127583	0.002213786	0.011330438	1
T2DM_insulin	leukocyte	*TLL2*	-1.478850211	0.006877082	0.012075281	1
T2DM_insulin	leukocyte	*SRPK1*	-0.451534134	0.002879817	0.011330438	1
T2DM_insulin	leukocyte	*PGC*	-0.268709195	0.028047069	0.036525334	1
T2DM_insulin	leukocyte	*EFHC1*	-0.311230367	0.008497156	0.013744474	1
T2DM_insulin	leukocyte	*ITPR3*	-0.384107357	0.006652623	0.01190541	1
T2DM_insulin	leukocyte	*IFT74*	-0.499145795	0.004728288	0.011330438	1
T2DM_insulin	leukocyte	*ABL1*	-0.38229773	0.004750584	0.011330438	1
T2DM_insulin	leukocyte	*SH3GLB1*	-0.384878197	0.003803641	0.011330438	1
T2DM_insulin	leukocyte	*CDC7*	-0.431776515	0.020674028	0.028029074	1
T2DM_insulin	leukocyte	*SYDE2*	-0.423013481	0.006454876	0.01173015	1
T2DM_insulin	leukocyte	*ABLIM1*	-0.281746146	0.011179792	0.016816769	1
T2DM_insulin	leukocyte	*ERMP1*	-0.340816605	0.009237524	0.01459945	1
T2DM_insulin	leukocyte	*PALMD*	-0.238850462	0.037413253	0.047138726	1
T2DM_insulin	leukocyte	*MZF1*	-0.42624986	0.003045367	0.011330438	1
T2DM_insulin	leukocyte	*OCEL1*	-0.609521027	0.000439623	0.011330438	1
T2DM_insulin	leukocyte	*CATSPERG*	-0.530818271	0.03841391	0.048297041	1
T2DM_insulin	leukocyte	*HSD3B7*	-1.272088491	0.001901227	0.011330438	1
T2DM_insulin	leukocyte	*HNRNPM*	-0.313229745	0.002782093	0.011330438	1
T2DM_insulin	leukocyte	*TIMM13*	-0.376770909	0.003103998	0.011330438	1
T2DM_insulin	leukocyte	*CEP170B*	-0.573154414	0.002843013	0.011330438	1
T2DM_insulin	leukocyte	*POLR2E*	-0.510272444	0.002871156	0.011330438	1
T2DM_insulin	leukocyte	*RASSF7*	-0.325480266	0.005292398	0.011421159	1
T2DM_insulin	leukocyte	*ARVCF*	-0.720225117	0.003354943	0.011330438	1
T2DM_insulin	leukocyte	*TRMT2A*	-0.329449652	0.008662858	0.013926501	1
T2DM_insulin	leukocyte	*MED15*	-0.977374415	0.002880363	0.011330438	1
T2DM_insulin	leukocyte	*LZTR1*	-0.733528692	0.001065262	0.011330438	1
T2DM_insulin	leukocyte	*SMARCB1*	-0.39291042	0.004288162	0.011330438	1
T2DM_insulin	leukocyte	*DDT*	-0.418049637	0.002012132	0.011330438	1
T2DM_insulin	leukocyte	*CABIN1*	-0.500184783	0.00363511	0.011330438	1
T2DM_insulin	leukocyte	*SPECC1L*	-0.570797083	0.003973589	0.011330438	1
T2DM_insulin	leukocyte	*PES1*	-0.447349049	0.000750326	0.011330438	1
T2DM_insulin	leukocyte	*GGT1*	-2.561983811	0.02518975	0.03330179	1
T2DM_insulin	leukocyte	*TOP3B*	-0.508564895	0.016652973	0.023284531	1
T2DM_insulin	leukocyte	*CARD10*	-0.243231909	0.014784782	0.020989512	1
T2DM_insulin	leukocyte	*SLC25A1*	-0.430481928	0.003035231	0.011330438	1
T2DM_insulin	leukocyte	*HPS4*	-0.463245125	0.001863203	0.011330438	1
T2DM_insulin	leukocyte	*GCAT*	-0.516185106	0.005693638	0.011549238	1
T2DM_insulin	leukocyte	*CYP2D6*	0.898544489	0.022220528	0.029827281	1
T2DM_insulin	leukocyte	*DDX17*	-0.382981169	0.003216187	0.011330438	1
T2DM_insulin	leukocyte	*HSCB*	-0.701899572	0.001930294	0.011330438	1
T2DM_insulin	leukocyte	*CBY1*	-0.41567525	0.00399552	0.011330438	1
T2DM_insulin	leukocyte	*XBP1*	-0.271104019	0.003315147	0.011330438	1
T2DM_insulin	leukocyte	*TIMP3*	-0.423085548	0.004598189	0.011330438	1
T2DM_insulin	leukocyte	*CYB5R3*	-0.342822883	0.005840791	0.011570498	1
T2DM_insulin	leukocyte	*MIOX*	-0.90614853	0.004518991	0.011330438	1
T2DM_insulin	leukocyte	*LMF2*	-0.757618488	0.002100102	0.011330438	1
T2DM_insulin	leukocyte	*RHBDD3*	-0.459675233	0.002455187	0.011330438	1
T2DM_insulin	leukocyte	*TOM1*	-0.576127543	0.005422199	0.011421159	1
T2DM_insulin	leukocyte	*CHKB*	-0.845653562	0.010935682	0.016550349	1
T2DM_insulin	leukocyte	*BIK*	-0.752054001	0.003885368	0.011330438	1
T2DM_insulin	leukocyte	*ARSA*	-0.327582379	0.023970402	0.031871306	1
T2DM_insulin	leukocyte	*TTLL12*	-0.575288334	0.00407748	0.011330438	1
T2DM_insulin	leukocyte	*RBFOX2*	-0.374271999	0.003130684	0.011330438	1
T2DM_insulin	leukocyte	*SYNGR1*	-1.03660307	0.002256621	0.011330438	1
T2DM_insulin	leukocyte	*MTMR3*	-0.451198602	0.007397133	0.012565211	1
T2DM_insulin	leukocyte	*APOL4*	-1.068913195	0.023322073	0.031082912	1
T2DM_insulin	leukocyte	*SAMM50*	-0.358505864	0.003823604	0.011330438	1
T2DM_insulin	leukocyte	*FOXRED2*	-0.404555847	0.009086208	0.014418301	1
T2DM_insulin	leukocyte	*SGSM3*	-0.574446908	0.00261566	0.011330438	1
T2DM_insulin	leukocyte	*IFT27*	-0.470225843	0.003526654	0.011330438	1

T2DM_insulin	leukocyte	*ST13*	-0.331411305	0.003021577	0.011330438	1
T2DM_insulin	leukocyte	*POLR3H*	-0.788138561	0.005343911	0.011421159	1
T2DM_insulin	leukocyte	*PMM1*	-0.507283918	0.003227823	0.011330438	1
T2DM_insulin	leukocyte	*DESI1*	-0.500817462	0.004077637	0.011330438	1
T2DM_insulin	leukocyte	*KHNYN*	-0.551791062	0.002284054	0.011330438	1
T2DM_insulin	leukocyte	*RBM23*	-0.377308835	0.003385743	0.011330438	1
T2DM_insulin	leukocyte	*PRMT5*	-0.346045316	0.006614218	0.011871102	1
T2DM_insulin	leukocyte	*VCPKMT*	-0.573839473	0.002155512	0.011330438	1
T2DM_insulin	leukocyte	*SOS2*	-0.492174983	0.00313188	0.011330438	1
T2DM_insulin	leukocyte	*CDKL1*	-0.633232808	0.01051625	0.016070925	1
T2DM_insulin	leukocyte	*NIN*	-0.858506491	0.002764773	0.011330438	1
T2DM_insulin	leukocyte	*TRIM9*	-0.284861483	0.006685931	0.011938173	1
T2DM_insulin	leukocyte	*ATP6V1D*	-0.339290662	0.004302941	0.011330438	1
T2DM_insulin	leukocyte	*PLEK2*	-0.518123156	0.013741184	0.019732863	1
T2DM_insulin	leukocyte	*GSTZ1*	-0.560262593	0.004171742	0.011330438	1
T2DM_insulin	leukocyte	*TMED8*	-0.435244929	0.003564484	0.011330438	1
T2DM_insulin	leukocyte	*DAAM1*	-0.347049478	0.003185137	0.011330438	1
T2DM_insulin	leukocyte	*DHRS7*	-0.406598489	0.00315317	0.011330438	1
T2DM_insulin	leukocyte	*MTHFD1*	-1.444546752	0.001303173	0.011330438	1
T2DM_insulin	leukocyte	*TELO2*	-0.375320606	0.033551041	0.04269447	1
T2DM_insulin	leukocyte	*GSKIP*	-0.375629486	0.004256702	0.011330438	1
T2DM_insulin	leukocyte	*TRIP11*	-0.325117274	0.004052375	0.011330438	1
T2DM_insulin	leukocyte	*DCAF11*	-0.457072415	0.007240305	0.012418942	1
T2DM_insulin	leukocyte	*EMC9*	-0.326020025	0.022202503	0.029813272	1
T2DM_insulin	leukocyte	*NFATC4*	-0.503400733	0.007521986	0.012715195	1
T2DM_insulin	leukocyte	*PYGB*	-0.300262626	0.036385021	0.045922502	1
T2DM_insulin	leukocyte	*ABHD12*	-0.742740717	0.001607238	0.011330438	1
T2DM_insulin	leukocyte	*PROCR*	-0.29768486	0.01224417	0.017963907	1
T2DM_insulin	leukocyte	*CD40*	-0.420728879	0.004143784	0.011330438	1
T2DM_insulin	leukocyte	*IFT52*	-0.366728647	0.004202105	0.011330438	1
T2DM_insulin	leukocyte	*MYBL2*	-1.288397491	0.000249019	0.011330438	0.978396877
T2DM_insulin	leukocyte	*NELFCD*	-0.356850861	0.003640897	0.011330438	1
T2DM_insulin	leukocyte	*MTG2*	-0.418441209	0.004951303	0.011330438	1
T2DM_insulin	leukocyte	*DIDO1*	-0.389300593	0.015459124	0.021810191	1
T2DM_insulin	leukocyte	*CDC25B*	-0.809665977	0.001281867	0.011330438	1
T2DM_insulin	leukocyte	*ISM1*	0.523936992	0.019006359	0.026005146	1
T2DM_insulin	leukocyte	*ARFRP1*	-0.558916202	0.006034574	0.011570498	1
T2DM_insulin	leukocyte	*SLC52A3*	-0.85171196	0.012134148	0.017862521	1
T2DM_insulin	leukocyte	*SIRPB1*	0.249406983	0.014445255	0.020587417	1
T2DM_insulin	leukocyte	*PLCB4*	-0.273349509	0.023161966	0.030911469	1
T2DM_insulin	leukocyte	*POFUT1*	-0.366580245	0.004522933	0.011330438	1
T2DM_insulin	leukocyte	*NOP56*	-0.529716821	0.002956834	0.011330438	1
T2DM_insulin	leukocyte	*MANBAL*	-0.342919879	0.004700695	0.011330438	1
T2DM_insulin	leukocyte	*IDH3B*	-0.483813559	0.002181469	0.011330438	1
T2DM_insulin	leukocyte	*MAPRE1*	-0.299940076	0.005734687	0.011570498	1
T2DM_insulin	leukocyte	*OXT*	0.321099867	0.017936624	0.024744731	1
T2DM_insulin	leukocyte	*TTI1*	-0.583982611	0.00361596	0.011330438	1
T2DM_insulin	leukocyte	*CHMP4B*	-0.317947401	0.003328954	0.011330438	1
T2DM_insulin	leukocyte	*DHX35*	-0.529512267	0.018660082	0.025625817	1
T2DM_insulin	leukocyte	*DNTTIP1*	-0.913622962	0.000849382	0.011330438	1
T2DM_insulin	leukocyte	*APMAP*	-0.32469175	0.028676636	0.037197261	1
T2DM_insulin	leukocyte	*VAPA*	-0.310180972	0.003165587	0.011330438	1
T2DM_insulin	leukocyte	*MYOM1*	-0.821429252	0.012257705	0.017972767	1
T2DM_insulin	leukocyte	*CEP192*	-0.301747031	0.010909147	0.016529903	1
T2DM_insulin	leukocyte	*LIPG*	-0.406186971	0.005967207	0.011570498	1
T2DM_insulin	leukocyte	*RIOK3*	-0.338792663	0.003684611	0.011330438	1
T2DM_insulin	leukocyte	*NALCN*	-0.357138074	0.005201553	0.011378137	1
T2DM_insulin	leukocyte	*FGF14*	-0.358277186	0.006641187	0.011903843	1
T2DM_insulin	leukocyte	*FNDC3A*	-0.283009861	0.005120693	0.011330438	1
T2DM_insulin	leukocyte	*KLF5*	-0.262271981	0.00695439	0.01214931	1
T2DM_insulin	leukocyte	*ACP5*	0.023219064	0.013632035	0.019604782	1
T2DM_insulin	leukocyte	*DNAJC3*	-0.33487729	0.00301717	0.011330438	1
T2DM_insulin	leukocyte	*UGGT2*	-0.36284928	0.003246161	0.011330438	1
T2DM_insulin	leukocyte	*ARHGEF7*	-0.504899513	0.002983619	0.011330438	1

T2DM_insulin	leukocyte	*PARP4*	-0.980167824	0.003526026	0.011330438	1
T2DM_insulin	leukocyte	*MEDAG*	-0.56216776	0.009140759	0.014469799	1
T2DM_insulin	leukocyte	*CLN5*	-0.468882852	0.002856726	0.011330438	1
T2DM_insulin	leukocyte	*OLFM4*	-0.185166659	0.004839151	0.011330438	1
T2DM_insulin	leukocyte	*MGRN1*	-0.490485568	0.002865333	0.011330438	1
T2DM_insulin	leukocyte	*LONP2*	-0.36878124	0.003575189	0.011330438	1
T2DM_insulin	leukocyte	*CBLN1*	-1.220835331	0.00538328	0.011421159	1
T2DM_insulin	leukocyte	*DHODH*	-1.034804621	0.004685842	0.011330438	1
T2DM_insulin	leukocyte	*PARD6A*	-0.250953614	0.011177743	0.016816769	1
T2DM_insulin	leukocyte	*MMP15*	-0.793961846	0.004733698	0.011330438	1
T2DM_insulin	leukocyte	*USB1*	-0.501089317	0.007902171	0.013129703	1
T2DM_insulin	leukocyte	*CYB5B*	-0.736887476	0.001688551	0.011330438	1
T2DM_insulin	leukocyte	*CCDC113*	-0.471972155	0.008841559	0.014115597	1
T2DM_insulin	leukocyte	*NME3*	-0.389554504	0.005571288	0.011495826	1
T2DM_insulin	leukocyte	*VAC14*	-0.396000952	0.005352502	0.011421159	1
T2DM_insulin	leukocyte	*NPRL3*	-0.549499806	0.006067331	0.011570498	1
T2DM_insulin	leukocyte	*ZNF500*	-0.697658333	0.00929655	0.014669134	1
T2DM_insulin	leukocyte	*NME4*	-0.410377325	0.003248917	0.011330438	1
T2DM_insulin	leukocyte	*NOMO3*	-0.627940359	0.001522976	0.011330438	1
T2DM_insulin	leukocyte	*LMF1*	-0.386525084	0.004552365	0.011330438	1
T2DM_insulin	leukocyte	*CLCN7*	-0.845400496	0.015872253	0.022312015	1
T2DM_insulin	leukocyte	*HAGHL*	-0.632027648	0.003388431	0.011330438	1
T2DM_insulin	leukocyte	*METRN*	-0.316322207	0.002647164	0.011330438	1
T2DM_insulin	leukocyte	*PIEZO1*	-0.435367963	0.007171258	0.012363261	1
T2DM_insulin	leukocyte	*UBFD1*	-0.486972976	0.007903219	0.013129703	1
T2DM_insulin	leukocyte	*AQP8*	-0.62979078	0.004213624	0.011330438	1
T2DM_insulin	leukocyte	*USP31*	-0.273661454	0.007851538	0.013077022	1
T2DM_insulin	leukocyte	*HMOX2*	-0.34097445	0.004992742	0.011330438	1
T2DM_insulin	leukocyte	*RBL2*	-0.45129475	0.007248203	0.01242504	1
T2DM_insulin	leukocyte	*KAT8*	-0.456171068	0.003417362	0.011330438	1
T2DM_insulin	leukocyte	*NOMO1*	-0.557703655	0.009588166	0.015025219	1
T2DM_insulin	leukocyte	*RNF40*	-0.703600578	0.002530191	0.011330438	1
T2DM_insulin	leukocyte	*KNOP1*	-0.483700633	0.003316591	0.011330438	1
T2DM_insulin	leukocyte	*TRIP4*	-0.436313259	0.002598953	0.011330438	1
T2DM_insulin	leukocyte	*AP3B2*	-0.409386402	0.005528654	0.011449692	1
T2DM_insulin	leukocyte	*IGDCC4*	0.93605991	0.016400367	0.0229804	1
T2DM_insulin	leukocyte	*RPAP1*	-0.599477226	0.003213587	0.011330438	1
T2DM_insulin	leukocyte	*HOMER2*	-0.29017935	0.006813683	0.012014984	1
T2DM_insulin	leukocyte	*ATP8B4*	0.198765334	0.013882969	0.019921908	1
T2DM_insulin	leukocyte	*DNAJC17*	-0.346756394	0.010190289	0.015699813	1
T2DM_insulin	leukocyte	*ZDHHC2*	-0.256848114	0.005128109	0.011330438	1
T2DM_insulin	leukocyte	*TRIM35*	-0.928329508	0.002796007	0.011330438	1
T2DM_insulin	leukocyte	*RIPK2*	-0.459233943	0.004081553	0.011330438	1
T2DM_insulin	leukocyte	*NBN*	-0.606578761	0.001199823	0.011330438	1
T2DM_insulin	leukocyte	*LAPTM4B*	-0.239436987	0.004972911	0.011330438	1
T2DM_insulin	leukocyte	*RAB2A*	-0.283026057	0.002668337	0.011330438	1
T2DM_insulin	leukocyte	*EIF3E*	-0.308615297	0.003388411	0.011330438	1
T2DM_insulin	leukocyte	*ESRP1*	-0.307056926	0.005535537	0.011449692	1
T2DM_insulin	leukocyte	*ARMC1*	-0.393935347	0.007800134	0.01301113	1
T2DM_insulin	leukocyte	*SPAG1*	-0.360758585	0.003853345	0.011330438	1
T2DM_insulin	leukocyte	*CHRAC1*	-0.312315186	0.004251168	0.011330438	1
T2DM_insulin	leukocyte	*SNX16*	-1.184185446	0.001659828	0.011330438	1
T2DM_insulin	leukocyte	*GSDMD*	-0.285535427	0.009176301	0.014520212	1
T2DM_insulin	leukocyte	*SH2D4A*	-0.455675886	0.002463314	0.011330438	1
T2DM_insulin	leukocyte	*INTS10*	-0.5023353	0.002710264	0.011330438	1
T2DM_insulin	leukocyte	*ERI1*	-0.991783708	0.001561649	0.011330438	1
T2DM_insulin	leukocyte	*LEPROTL1*	-0.460183212	0.002125354	0.011330438	1
T2DM_insulin	leukocyte	*ERICH1*	-0.565694123	0.00183944	0.011330438	1
T2DM_insulin	leukocyte	*ARHGEF10*	-0.429840945	0.003077097	0.011330438	1
T2DM_insulin	leukocyte	*KLHDC4*	-0.475587949	0.004906897	0.011330438	1
T2DM_insulin	leukocyte	*MCM4*	-0.477988089	0.004027445	0.011330438	1
T2DM_insulin	leukocyte	*FGL1*	-0.714927812	0.003199838	0.011330438	1
T2DM_insulin	leukocyte	*ECH1*	-0.347181222	0.003838459	0.011330438	1
T2DM_insulin	leukocyte	*HNRNPL*	-0.390867833	0.002960807	0.011330438	1

T2DM_insulin	leukocyte	*SARS2*	-0.49384191	0.001723605	0.011330438	1
T2DM_insulin	leukocyte	*LIN7B*	-0.444087352	0.009591054	0.015025219	1
T2DM_insulin	leukocyte	*PPP1R13L*	-0.322650398	0.034222526	0.043466792	1
T2DM_insulin	leukocyte	*ERCC2*	-0.424878993	0.003796119	0.011330438	1
T2DM_insulin	leukocyte	*KLC3*	-0.366065987	0.00464928	0.011330438	1
T2DM_insulin	leukocyte	*AMH*	-0.390141939	0.01006797	0.015561391	1
T2DM_insulin	leukocyte	*IL27RA*	-0.45018901	0.017312459	0.024010113	1
T2DM_insulin	leukocyte	*PDCD5*	-0.283019015	0.006414816	0.01170553	1
T2DM_insulin	leukocyte	*NUMBL*	-0.317193004	0.005609994	0.011546185	1
T2DM_insulin	leukocyte	*SLC1A5*	-0.433432785	0.011960788	0.017706833	1
T2DM_insulin	leukocyte	*PRKD2*	-0.530286293	0.000461984	0.011330438	1
T2DM_insulin	leukocyte	*APLP1*	-0.194028323	0.003716102	0.011330438	1
T2DM_insulin	leukocyte	*PLIN3*	-0.479409329	0.000479898	0.011330438	1
T2DM_insulin	leukocyte	*MYH14*	-0.297566452	0.006905548	0.012100405	1
T2DM_insulin	leukocyte	*ZNF175*	-0.354567416	0.005034542	0.011330438	1
T2DM_insulin	leukocyte	*PLA2G4C*	-0.581254921	0.000948139	0.011330438	1
T2DM_insulin	leukocyte	*CAPS*	-0.502252539	0.004629566	0.011330438	1
T2DM_insulin	leukocyte	*PPP2R1A*	-0.322360535	0.004468708	0.011330438	1
T2DM_insulin	leukocyte	*GCDH*	-0.484263738	0.001985648	0.011330438	1
T2DM_insulin	leukocyte	*PIK3R2*	-1.070636558	0.003370375	0.011330438	1
T2DM_insulin	leukocyte	*UPK1A*	-5.077792385	0.011869113	0.017638109	1
T2DM_insulin	leukocyte	*COPE*	-0.330554937	0.003031622	0.011330438	1
T2DM_insulin	leukocyte	*ETV2*	-0.447870476	0.012752161	0.018577398	1
T2DM_insulin	leukocyte	*PBX4*	-1.696481594	0.001931212	0.011330438	1
T2DM_insulin	leukocyte	*SIPA1L3*	-0.957434276	0.002739451	0.011330438	1
T2DM_insulin	leukocyte	*SMG9*	-0.338748903	0.007163494	0.012363261	1
T2DM_insulin	leukocyte	*RUNDC3B*	-1.97289297	0.002583682	0.011330438	1
T2DM_insulin	leukocyte	*PMPCB*	-0.447244004	0.00316606	0.011330438	1
T2DM_insulin	leukocyte	*PON2*	-0.314408099	0.006139663	0.011570498	1
T2DM_insulin	leukocyte	*ITGB8*	-0.336755519	0.005009738	0.011330438	1
T2DM_insulin	leukocyte	*CAV2*	-0.232806436	0.005556517	0.011472179	1
T2DM_insulin	leukocyte	*CAV1*	-0.272803579	0.005910735	0.011570498	1
T2DM_insulin	leukocyte	*ANKRD7*	-0.630736506	0.009118969	0.014446947	1
T2DM_insulin	leukocyte	*TSPAN12*	-0.587897238	0.002918331	0.011330438	1
T2DM_insulin	leukocyte	*CPVL*	0.048595012	0.014628652	0.020794491	1
T2DM_insulin	leukocyte	*ABHD11*	-0.487736287	0.007045837	0.012237751	1
T2DM_insulin	leukocyte	*CHCHD2*	-0.259837127	0.003263117	0.011330438	1
T2DM_insulin	leukocyte	*EIF3B*	-0.38219711	0.003403732	0.011330438	1
T2DM_insulin	leukocyte	*WASL*	-0.308149004	0.004882524	0.011330438	1
T2DM_insulin	leukocyte	*AGFG2*	-0.354304589	0.006445847	0.01173015	1
T2DM_insulin	leukocyte	*C1GALT1*	-0.325114214	0.004130289	0.011330438	1
T2DM_insulin	leukocyte	*RPA3*	-0.408457736	0.003462529	0.011330438	1
T2DM_insulin	leukocyte	*ZNHIT1*	-0.356434983	0.001597245	0.011330438	1
T2DM_insulin	leukocyte	*TMEM106B*	-0.316807949	0.006253483	0.011570498	1
T2DM_insulin	leukocyte	*ACTR3C*	-0.627027151	0.003791544	0.011330438	1
T2DM_insulin	leukocyte	*TSPAN13*	-0.249573148	0.004698924	0.011330438	1
T2DM_insulin	leukocyte	*RARRES2*	-0.194625381	0.003764899	0.011330438	1
T2DM_insulin	leukocyte	*AGR2*	-0.240073693	0.017089931	0.023777254	1
T2DM_insulin	leukocyte	*TMEM176B*	-0.289234245	0.012509024	0.018258541	1
T2DM_insulin	leukocyte	*COA1*	-0.465953056	0.004514265	0.011330438	1
T2DM_insulin	leukocyte	*BLVRA*	-0.639198668	0.00351128	0.011330438	1
T2DM_insulin	leukocyte	*TMEM248*	-0.482302931	0.001663315	0.011330438	1
T2DM_insulin	leukocyte	*RHEB*	-0.329571192	0.00279003	0.011330438	1
T2DM_insulin	leukocyte	*TBL2*	-0.286628111	0.029303174	0.037934817	1
T2DM_insulin	leukocyte	*LIMK1*	-1.265413112	0.00596733	0.011570498	1
T2DM_insulin	leukocyte	*SPIN1*	-0.564485199	0.002774174	0.011330438	1
T2DM_insulin	leukocyte	*NMRK1*	-0.401221556	0.004358955	0.011330438	1
T2DM_insulin	leukocyte	*OGN*	-0.768908703	0.010106815	0.015598378	1
T2DM_insulin	leukocyte	*SUSD1*	-0.658698991	0.002164003	0.011330438	1
T2DM_insulin	leukocyte	*DNM1*	-0.269619197	0.005365164	0.011421159	1
T2DM_insulin	leukocyte	*AK1*	-0.308629693	0.00245802	0.011330438	1
T2DM_insulin	leukocyte	*PLGRKT*	-0.302487693	0.008815257	0.014103329	1
T2DM_insulin	leukocyte	*DOCK8*	0.160544787	0.022593718	0.03024556	1
T2DM_insulin	leukocyte	*MPDZ*	-0.306389427	0.006828572	0.012014984	1

T2DM_insulin	leukocyte	*NPDC1*	-0.261407754	0.004586218	0.011330438	1
T2DM_insulin	leukocyte	*HPS1*	-0.436317631	0.004365264	0.011330438	1
T2DM_insulin	leukocyte	*CXCL12*	-0.387336409	0.003131284	0.011330438	1
T2DM_insulin	leukocyte	*TRDMT1*	-0.867370971	0.002503889	0.011330438	1
T2DM_insulin	leukocyte	*ATE1*	-0.61887747	0.002598768	0.011330438	1
T2DM_insulin	leukocyte	*NSMCE4A*	-0.334474662	0.006682587	0.011938173	1
T2DM_insulin	leukocyte	*PLEKHA1*	-0.286443021	0.004595086	0.011330438	1
T2DM_insulin	leukocyte	*PALD1*	-2.379624257	0.002465348	0.011330438	1
T2DM_insulin	leukocyte	*SPOCK2*	-0.564108074	0.006264017	0.011570498	1
T2DM_insulin	leukocyte	*BMPR1A*	-0.822659823	0.002543935	0.011330438	1
T2DM_insulin	leukocyte	*LIPA*	0.114779164	0.021898479	0.029455366	1
T2DM_insulin	leukocyte	*SFXN3*	-0.636895386	0.001249065	0.011330438	1
T2DM_insulin	leukocyte	*NPM3*	-0.410257536	0.005845515	0.011570498	1
T2DM_insulin	leukocyte	*TNKS2*	-0.413529924	0.003376674	0.011330438	1
T2DM_insulin	leukocyte	*ACBD5*	-0.324035284	0.005395131	0.011421159	1
T2DM_insulin	leukocyte	*LHPP*	-0.52614122	0.023188934	0.030936952	1
T2DM_insulin	leukocyte	*GTPBP4*	-0.319456891	0.003619769	0.011330438	1
T2DM_insulin	leukocyte	*XPNPEP1*	-0.365559884	0.003737708	0.011330438	1
T2DM_insulin	leukocyte	*SMC3*	-0.312506623	0.003472074	0.011330438	1
T2DM_insulin	leukocyte	*RPL28*	-0.236783966	0.004509535	0.011330438	1
T2DM_insulin	leukocyte	*PBLD*	-0.321454403	0.005683776	0.011549238	1
T2DM_insulin	leukocyte	*DHRS11*	-0.324537331	0.006457703	0.01173015	1
T2DM_insulin	leukocyte	*CWC25*	-0.467802507	0.002384136	0.011330438	1
T2DM_insulin	leukocyte	*RAD51C*	-0.388196339	0.005898268	0.011570498	1
T2DM_insulin	leukocyte	*TRIM37*	-0.361336054	0.00413243	0.011330438	1
T2DM_insulin	leukocyte	*RPS6KB1*	-0.438688086	0.003185712	0.011330438	1
T2DM_insulin	leukocyte	*TRIM16L*	-0.525121925	0.012750249	0.018577398	1
T2DM_insulin	leukocyte	*CDK5RAP3*	-0.452883292	0.026862354	0.03518073	1
T2DM_insulin	leukocyte	*CAMTA2*	-0.612408751	0.002736154	0.011330438	1
T2DM_insulin	leukocyte	*MED13*	-0.396282717	0.001541122	0.011330438	1
T2DM_insulin	leukocyte	*RAI1*	-0.29553625	0.00653212	0.011799861	1
T2DM_insulin	leukocyte	*CCDC47*	-0.347831244	0.003256434	0.011330438	1
T2DM_insulin	leukocyte	*DRG2*	-0.406327892	0.003388971	0.011330438	1
T2DM_insulin	leukocyte	*FTSJ3*	-0.26603219	0.034822643	0.044188081	1
T2DM_insulin	leukocyte	*SMARCD2*	-0.808923579	0.002835511	0.011330438	1
T2DM_insulin	leukocyte	*SYNGR2*	-0.334823473	0.003808855	0.011330438	1
T2DM_insulin	leukocyte	*C17orf75*	-0.542103853	0.002942518	0.011330438	1
T2DM_insulin	leukocyte	*DHX58*	-0.808854439	0.001988301	0.011330438	1
T2DM_insulin	leukocyte	*NAGLU*	-0.371193154	0.007802042	0.01301113	1
T2DM_insulin	leukocyte	*HSD17B1*	-0.453709079	0.003093626	0.011330438	1
T2DM_insulin	leukocyte	*RND2*	-0.947926447	0.00303749	0.011330438	1
T2DM_insulin	leukocyte	*MPP2*	-0.263149157	0.006909306	0.012100405	1
T2DM_insulin	leukocyte	*HLF*	-0.234924297	0.007166891	0.012363261	1
T2DM_insulin	leukocyte	*EFNB3*	-0.342354577	0.00560962	0.011546185	1
T2DM_insulin	leukocyte	*RANGRF*	-0.445016244	0.006244546	0.011570498	1
T2DM_insulin	leukocyte	*DPH1*	-0.761590748	0.010636892	0.016217442	1
T2DM_insulin	leukocyte	*OCIAD1*	-0.327259345	0.002797704	0.011330438	1
T2DM_insulin	leukocyte	*UBE2D3*	-0.286974204	0.007267265	0.012441431	1
T2DM_insulin	leukocyte	*MAPK10*	-0.356826267	0.001076692	0.011330438	1
T2DM_insulin	leukocyte	*AADAT*	-1.065122042	0.005067889	0.011330438	1
T2DM_insulin	leukocyte	*TRIM2*	-0.357814794	0.003778268	0.011330438	1
T2DM_insulin	leukocyte	*SH3D19*	-0.73705854	0.003863272	0.011330438	1
T2DM_insulin	leukocyte	*GLRB*	-0.355396705	0.006066341	0.011570498	1
T2DM_insulin	leukocyte	*LRP2BP*	-0.427511793	0.003922531	0.011330438	1
T2DM_insulin	leukocyte	*UFSP2*	-0.445006467	0.007405254	0.012573571	1
T2DM_insulin	leukocyte	*KLHL5*	-0.469207423	0.00319368	0.011330438	1
T2DM_insulin	leukocyte	*FAM149A*	-0.402580343	0.001799895	0.011330438	1
T2DM_insulin	leukocyte	*DDX25*	-0.337768108	0.014176376	0.020276295	1
T2DM_insulin	leukocyte	*HTATIP2*	-0.417896825	0.01416225	0.020263467	1
T2DM_insulin	leukocyte	*B3GAT1*	-0.797999212	0.016473865	0.023066934	1
T2DM_insulin	leukocyte	*P2RX3*	-7.271840225	0.005277282	0.011419801	1
T2DM_insulin	leukocyte	*VWA5A*	-0.42196077	0.005649707	0.011549238	1
T2DM_insulin	leukocyte	*DTX4*	-0.401812235	0.008022295	0.01322829	1
T2DM_insulin	leukocyte	*FOXRED1*	-0.509517765	0.024708238	0.032752586	1

T2DM_insulin	leukocyte	*ST3GAL4*	-0.644150279	0.003437384	0.011330438	1
T2DM_insulin	leukocyte	*PRPF19*	-0.334305545	0.004414305	0.011330438	1
T2DM_insulin	leukocyte	*CEP164*	-0.730362777	0.002940136	0.011330438	1
T2DM_insulin	leukocyte	*PDHX*	-0.358692429	0.02815391	0.03662805	1
T2DM_insulin	leukocyte	*SLC1A2*	-0.429091756	0.002633316	0.011330438	1
T2DM_insulin	leukocyte	*ACCS*	0.212196981	0.00588614	0.011570498	1
T2DM_insulin	leukocyte	*MDK*	-0.369404693	0.003375135	0.011330438	1
T2DM_insulin	leukocyte	*MADD*	-0.516587095	0.002811053	0.011330438	1
T2DM_insulin	leukocyte	*NAA40*	-0.453139851	0.005274513	0.011419801	1
T2DM_insulin	leukocyte	*CD81*	-0.2285803	0.009774226	0.015221139	1
T2DM_insulin	leukocyte	*SLC35F2*	-0.468819725	0.000944901	0.011330438	1
T2DM_insulin	leukocyte	*NUP98*	-0.395108384	0.003687721	0.011330438	1
T2DM_insulin	leukocyte	*GTF2H1*	-0.630959097	0.003916689	0.011330438	1
T2DM_insulin	leukocyte	*PTPN5*	0.737218575	0.012877588	0.018704637	1
T2DM_insulin	leukocyte	*PRDM4*	-0.311732589	0.003877646	0.011330438	1
T2DM_insulin	leukocyte	*CORO1C*	-0.442472565	0.003744308	0.011330438	1
T2DM_insulin	leukocyte	*LIN7A*	-0.854896938	0.004898804	0.011330438	1
T2DM_insulin	leukocyte	*KRT18*	-0.131258702	0.006543282	0.011804448	1
T2DM_insulin	leukocyte	*METAP2*	-0.353623225	0.0036488	0.011330438	1
T2DM_insulin	leukocyte	*SLC6A12*	-2.453276506	0.002807123	0.011330438	1
T2DM_insulin	leukocyte	*WNT5B*	-0.866298807	0.005682284	0.011549238	1
T2DM_insulin	leukocyte	*ITFG2*	-1.294567137	0.022249391	0.029855825	1
T2DM_insulin	leukocyte	*VPS29*	-0.361497835	0.001512074	0.011330438	1
T2DM_insulin	leukocyte	*CREBL2*	-0.474929991	0.003449979	0.011330438	1
T2DM_insulin	leukocyte	*GSG1*	-3.15523743	0.001157251	0.011330438	1
T2DM_insulin	leukocyte	*LTBR*	-0.466103773	0.001342379	0.011330438	1
T2DM_insulin	leukocyte	*CDK2AP1*	-0.269803627	0.032163152	0.041122365	1
T2DM_insulin	leukocyte	*DDX55*	-0.596788642	0.002485907	0.011330438	1
T2DM_insulin	leukocyte	*STX2*	-0.524384571	0.003067554	0.011330438	1
T2DM_insulin	leukocyte	*CAND1*	-0.432188188	0.00290352	0.011330438	1
T2DM_insulin	leukocyte	*NUP107*	-0.506960328	0.002973769	0.011330438	1
T2DM_insulin	leukocyte	*KRR1*	-0.355910443	0.005427012	0.011421159	1
T2DM_insulin	leukocyte	*GNB3*	-0.451610818	0.006267428	0.011570498	1
T2DM_insulin	leukocyte	*CDCA3*	-0.725989768	0.006268755	0.011570498	1
T2DM_insulin	leukocyte	*CHPT1*	-0.704217992	0.000471827	0.011330438	1
T2DM_insulin	leukocyte	*SPSB2*	-0.379569121	0.005706991	0.011549238	1
T2DM_insulin	leukocyte	*ATN1*	-0.416590893	0.002471801	0.011330438	1
T2DM_insulin	leukocyte	*NT5DC3*	-0.416316662	0.011955986	0.017706833	1
T2DM_insulin	leukocyte	*GOLT1B*	-0.36078003	0.003649102	0.011330438	1
T2DM_insulin	leukocyte	*PRKAB1*	-0.339648784	0.005957321	0.011570498	1
T2DM_insulin	leukocyte	*C2CD5*	-0.354503738	0.005208598	0.011381858	1
T2DM_insulin	leukocyte	*SRSF9*	-0.342617078	0.001983476	0.011330438	1
T2DM_insulin	leukocyte	*FRK*	-0.394079035	0.004489367	0.011330438	1
T2DM_insulin	leukocyte	*SMIM8*	-0.446642972	0.003986446	0.011330438	1
T2DM_insulin	leukocyte	*NEDD9*	-0.21981801	0.003641889	0.011330438	1
T2DM_insulin	leukocyte	*CEP85L*	-0.376495621	0.007193021	0.012373634	1
T2DM_insulin	leukocyte	*HDDC2*	-0.301902462	0.004261145	0.011330438	1
T2DM_insulin	leukocyte	*TPD52L1*	-0.316349862	0.006016755	0.011570498	1
T2DM_insulin	leukocyte	*MTRF1L*	-0.413666405	0.006615107	0.011871102	1
T2DM_insulin	leukocyte	*MRPL18*	-0.506577208	0.004213634	0.011330438	1
T2DM_insulin	leukocyte	*RNF8*	-0.391856096	0.003082626	0.011330438	1
T2DM_insulin	leukocyte	*MDGA1*	0.579194949	0.015718251	0.022135129	1
T2DM_insulin	leukocyte	*CD83*	0.071919961	0.015003052	0.021272822	1
T2DM_insulin	leukocyte	*GLP1R*	-0.37223282	0.005334916	0.011421159	1
T2DM_insulin	leukocyte	*BACH2*	-0.364842607	0.002226395	0.011330438	1
T2DM_insulin	leukocyte	*FBXL4*	-0.452496412	0.004010429	0.011330438	1
T2DM_insulin	leukocyte	*ASCC3*	-0.310404647	0.004712389	0.011330438	1
T2DM_insulin	leukocyte	*WASF1*	-0.331064814	0.006177398	0.011570498	1
T2DM_insulin	leukocyte	*ALDH5A1*	-0.909773973	0.016739724	0.023364254	1
T2DM_insulin	leukocyte	*C6orf62*	-0.432779083	0.003126565	0.011330438	1
T2DM_insulin	leukocyte	*B3GAT2*	-0.567219551	0.003701155	0.011330438	1
T2DM_insulin	leukocyte	*HBS1L*	-0.386478167	0.003492135	0.011330438	1
T2DM_insulin	leukocyte	*PEX7*	-0.351798568	0.004797674	0.011330438	1
T2DM_insulin	leukocyte	*PHACTR2*	-0.294508675	0.003838333	0.011330438	1

T2DM_insulin	leukocyte	*EPM2A*	-0.637894905	0.002881586	0.011330438	1
T2DM_insulin	leukocyte	*TFEB*	-0.472977938	0.003133999	0.011330438	1
T2DM_insulin	leukocyte	*DNPH1*	-0.30459741	0.02670841	0.03503751	1
T2DM_insulin	leukocyte	*DUSP22*	-0.491119459	0.003041582	0.011330438	1
T2DM_insulin	leukocyte	*LAMA4*	-0.316810748	0.003246894	0.011330438	1
T2DM_insulin	leukocyte	*FBRSL1*	-0.464714328	0.003085215	0.011330438	1
T2DM_insulin	leukocyte	*ENPP5*	-0.364172667	0.001948167	0.011330438	1
T2DM_insulin	leukocyte	*NUDT12*	-0.448864248	0.008252684	0.013448692	1
T2DM_insulin	leukocyte	*CEP72*	-0.976936616	0.020591616	0.027936622	1
T2DM_insulin	leukocyte	*SEMA5A*	-0.407005972	0.011085331	0.016732269	1
T2DM_insulin	leukocyte	*GHR*	-0.99167914	0.005708481	0.011549238	1
T2DM_insulin	leukocyte	*DAP*	-0.299282131	0.004878829	0.011330438	1
T2DM_insulin	leukocyte	*NME5*	-0.708080336	0.000624238	0.011330438	1
T2DM_insulin	leukocyte	*BRD8*	-0.442842708	0.003947587	0.011330438	1
T2DM_insulin	leukocyte	*NNT*	-0.581736209	0.002388512	0.011330438	1
T2DM_insulin	leukocyte	*IK*	-0.373893065	0.003219683	0.011330438	1
T2DM_insulin	leukocyte	*PDE8B*	-0.253333743	0.005087455	0.011330438	1
T2DM_insulin	leukocyte	*MSH3*	-0.462111429	0.001218372	0.011330438	1
T2DM_insulin	leukocyte	*CCNG1*	-0.666101872	0.003584313	0.011330438	1
T2DM_insulin	leukocyte	*DROSHA*	-0.294361287	0.011027492	0.016664237	1
T2DM_insulin	leukocyte	*GOLPH3*	-0.373693304	0.003731589	0.011330438	1
T2DM_insulin	leukocyte	*NPR3*	-0.973674473	0.015902696	0.022346814	1
T2DM_insulin	leukocyte	*RAD50*	-0.477329334	0.002943009	0.011330438	1
T2DM_insulin	leukocyte	*WWC1*	-0.218848027	0.005590903	0.011524093	1
T2DM_insulin	leukocyte	*SMAD5*	-0.360071455	0.002362911	0.011330438	1
T2DM_insulin	leukocyte	*HMGXB3*	-0.522731144	0.00133689	0.011330438	1
T2DM_insulin	leukocyte	*BNIP1*	-0.275838046	0.028146526	0.03662805	1
T2DM_insulin	leukocyte	*STC2*	-0.287448584	0.005415614	0.011421159	1
T2DM_insulin	leukocyte	*EHHADH*	-0.453666562	0.012208192	0.017937915	1
T2DM_insulin	leukocyte	*ACTR8*	-0.364174978	0.004953199	0.011330438	1
T2DM_insulin	leukocyte	*TIMMDC1*	-0.413455259	0.004475242	0.011330438	1
T2DM_insulin	leukocyte	*NPHP3*	-0.436829585	0.007735136	0.012941946	1
T2DM_insulin	leukocyte	*FAM162A*	-0.489389995	0.000889515	0.011330438	1
T2DM_insulin	leukocyte	*RBP1*	-0.328248189	0.003074189	0.011330438	1
T2DM_insulin	leukocyte	*RNF7*	-0.353596472	0.003160369	0.011330438	1
T2DM_insulin	leukocyte	*SERPINI2*	-0.24271276	0.007106147	0.012294167	1
T2DM_insulin	leukocyte	*TUSC2*	-0.472909484	0.003782343	0.011330438	1
T2DM_insulin	leukocyte	*CYB561D2*	-0.276497559	0.020943875	0.0283265	1
T2DM_insulin	leukocyte	*C3orf14*	-0.368733969	0.000752545	0.011330438	1
T2DM_insulin	leukocyte	*CBLB*	-0.585053553	0.004551365	0.011330438	1
T2DM_insulin	leukocyte	*IFT57*	-0.325360834	0.005437404	0.011421159	1
T2DM_insulin	leukocyte	*NCBP2*	-0.354333661	0.002724287	0.011330438	1
T2DM_insulin	leukocyte	*C3orf52*	-0.308831057	0.007964284	0.013183733	1
T2DM_insulin	leukocyte	*SLC41A3*	-0.323851088	0.010765074	0.016355753	1
T2DM_insulin	leukocyte	*ATP6V1A*	-0.428986077	0.003261433	0.011330438	1
T2DM_insulin	leukocyte	*PODXL2*	-0.287796529	0.004601127	0.011330438	1
T2DM_insulin	leukocyte	*UPK1B*	-0.619182613	0.003041659	0.011330438	1
T2DM_insulin	leukocyte	*NEK11*	-0.328628147	0.00306511	0.011330438	1
T2DM_insulin	leukocyte	*PLSCR4*	-0.618419193	0.003284071	0.011330438	1
T2DM_insulin	leukocyte	*COMMD2*	-0.346998251	0.004034438	0.011330438	1
T2DM_insulin	leukocyte	*GORASP1*	-0.821998088	0.004720749	0.011330438	1
T2DM_insulin	leukocyte	*ARHGEF26*	-0.967369173	0.002710012	0.011330438	1
T2DM_insulin	leukocyte	*PLCH1*	-1.786826065	0.001114391	0.011330438	1
T2DM_insulin	leukocyte	*VIPR1*	-0.575932919	0.008333442	0.013546584	1
T2DM_insulin	leukocyte	*NKTR*	-0.369245445	0.002683856	0.011330438	1
T2DM_insulin	leukocyte	*NEK4*	-1.064432526	0.001801762	0.011330438	1
T2DM_insulin	leukocyte	*KANSL3*	-0.792571631	0.002119876	0.011330438	1
T2DM_insulin	leukocyte	*ACTR1B*	-0.638565528	0.002555215	0.011330438	1
T2DM_insulin	leukocyte	*SLC35F5*	-0.374960501	0.003160842	0.011330438	1
T2DM_insulin	leukocyte	*ACTR3*	-0.398157991	0.003788516	0.011330438	1
T2DM_insulin	leukocyte	*POMC*	-0.611284473	0.002885869	0.011330438	1
T2DM_insulin	leukocyte	*TANC1*	-0.337107878	0.003724807	0.011330438	1
T2DM_insulin	leukocyte	*REEP6*	-0.42416665	0.01026069	0.015772399	1
T2DM_insulin	leukocyte	*INO80B*	-1.409980431	0.000681249	0.011330438	1

T2DM_insulin	leukocyte	*MOGS*	-0.628386502	0.002608811	0.011330438	1
T2DM_insulin	leukocyte	*RTN4*	-0.308250654	0.002777948	0.011330438	1
T2DM_insulin	leukocyte	*HTRA2*	-0.600246278	0.00340352	0.011330438	1
T2DM_insulin	leukocyte	*GALNT3*	-0.413342633	0.004215366	0.011330438	1
T2DM_insulin	leukocyte	*MRPL19*	-0.381244702	0.005122072	0.011330438	1
T2DM_insulin	leukocyte	*WDR75*	-0.665273422	0.002828431	0.011330438	1
T2DM_insulin	leukocyte	*FANCL*	-0.797927754	0.002996041	0.011330438	1
T2DM_insulin	leukocyte	*GGCX*	-0.460266606	0.004601164	0.011330438	1
T2DM_insulin	leukocyte	*SF3B1*	-0.413934797	0.002738803	0.011330438	1
T2DM_insulin	leukocyte	*CHMP3*	-0.330328726	0.003113216	0.011330438	1
T2DM_insulin	leukocyte	*IL1R2*	-0.376024743	0.006161515	0.011570498	1
T2DM_insulin	leukocyte	*IL1R1*	-0.346260022	0.004364949	0.011330438	1
T2DM_insulin	leukocyte	*IL1RL2*	0.333946697	0.020342674	0.027646616	1
T2DM_insulin	leukocyte	*IL18R1*	0.363944297	0.006457352	0.01173015	1
T2DM_insulin	leukocyte	*MLPH*	-0.569617079	0.006165863	0.011570498	1
T2DM_insulin	leukocyte	*CNPPD1*	-0.308762114	0.005817226	0.011570498	1
T2DM_insulin	leukocyte	*HDLBP*	-0.280890731	0.003564365	0.011330438	1
T2DM_insulin	leukocyte	*TAF1B*	-0.323078309	0.010160807	0.015674052	1
T2DM_insulin	leukocyte	*STRN*	-0.674863734	0.002798592	0.011330438	1
T2DM_insulin	leukocyte	*CEBPZ*	-0.368593594	0.002550589	0.011330438	1
T2DM_insulin	leukocyte	*WIPF1*	0.210456607	0.030301132	0.039046621	1
T2DM_insulin	leukocyte	*COX7A2L*	-0.305243057	0.003218929	0.011330438	1
T2DM_insulin	leukocyte	*PNO1*	-0.394656171	0.003321377	0.011330438	1
T2DM_insulin	leukocyte	*ORC4*	-0.432950865	0.001360445	0.011330438	1
T2DM_insulin	leukocyte	*SUMO1*	-0.300465759	0.010180232	0.015697854	1
T2DM_insulin	leukocyte	*GRIN3B*	0.674744095	0.015788467	0.022226043	1
T2DM_insulin	leukocyte	*ALMS1*	-0.468524174	0.003745378	0.011330438	1
T2DM_insulin	leukocyte	*MARK1*	-0.640166464	0.002110239	0.011330438	1
T2DM_insulin	leukocyte	*MORN1*	-0.609514944	0.013967271	0.020020945	1
T2DM_insulin	leukocyte	*GPX7*	-0.416839348	0.000686968	0.011330438	1
T2DM_insulin	leukocyte	*SCP2*	-0.374551955	0.002273078	0.011330438	1
T2DM_insulin	leukocyte	*RALGPS2*	-0.356299743	0.004488596	0.011330438	1
T2DM_insulin	leukocyte	*ANGPTL1*	-0.676616255	0.003733272	0.011330438	1
T2DM_insulin	leukocyte	*FAM20B*	-0.425569882	0.003938948	0.011330438	1
T2DM_insulin	leukocyte	*WRAP73*	-1.272405824	0.003794504	0.011330438	1
T2DM_insulin	leukocyte	*ICMT*	-0.416779232	0.012510051	0.018258541	1
T2DM_insulin	leukocyte	*PARK7*	-0.302986543	0.003004443	0.011330438	1
T2DM_insulin	leukocyte	*MECR*	-1.028877964	0.002903308	0.011330438	1
T2DM_insulin	leukocyte	*EDEM3*	-0.425100086	0.003337509	0.011330438	1
T2DM_insulin	leukocyte	*FBXO6*	-1.502975024	0.008174317	0.0133932	1
T2DM_insulin	leukocyte	*SMG7*	-0.37348163	0.003842577	0.011330438	1
T2DM_insulin	leukocyte	*PDC*	-4.893622002	0.000396668	0.011330438	1
T2DM_insulin	leukocyte	*RGS2*	0.190214401	0.030438997	0.039211416	1
T2DM_insulin	leukocyte	*BCAS2*	-0.334785438	0.003617622	0.011330438	1
T2DM_insulin	leukocyte	*AGMAT*	-0.636225176	0.001744044	0.011330438	1
T2DM_insulin	leukocyte	*TNNI3K*	-1.018633166	0.005523632	0.011446386	1
T2DM_insulin	leukocyte	*PLEKHM2*	-0.81062677	0.00737938	0.012556771	1
T2DM_insulin	leukocyte	*CRYZ*	-0.273694341	0.003505796	0.011330438	1
T2DM_insulin	leukocyte	*TMEM9*	-0.274678155	0.025990816	0.0341989	1
T2DM_insulin	leukocyte	*WARS2*	-0.382951302	0.004764099	0.011330438	1
T2DM_insulin	leukocyte	*C1orf109*	-0.286698773	0.006127751	0.011570498	1
T2DM_insulin	leukocyte	*LGALS8*	-0.468286233	0.003199008	0.011330438	1
T2DM_insulin	leukocyte	*HPCAL4*	-0.891084817	0.012944934	0.018774693	1
T2DM_insulin	leukocyte	*BMP8B*	-0.355555546	0.003310712	0.011330438	1
T2DM_insulin	leukocyte	*MYCL*	0.196936006	0.008392722	0.013614784	1
T2DM_insulin	leukocyte	*MFAP2*	-0.306443585	0.007872068	0.013100109	1
T2DM_insulin	leukocyte	*UAP1*	-0.276427872	0.003964145	0.011330438	1
T2DM_insulin	leukocyte	*CTBS*	-0.465126739	0.001995372	0.011330438	1
T2DM_insulin	leukocyte	*ZNHIT6*	-0.280215789	0.004593194	0.011330438	1
T2DM_insulin	leukocyte	*CD46*	-0.295845116	0.003632651	0.011330438	1
T2DM_insulin	leukocyte	*SLC2A1*	-0.545557713	0.002723417	0.011330438	1
T2DM_insulin	leukocyte	*PRDX1*	-0.31246056	0.003740648	0.011330438	1
T2DM_insulin	leukocyte	*CCDC181*	-0.298396	0.004327397	0.011330438	1
T2DM_insulin	leukocyte	*NSUN4*	-1.34982018	0.000818493	0.011330438	1

T2DM_insulin	leukocyte	*TMED5*	-0.51384095	0.00262675	0.011330438	1
T2DM_insulin	leukocyte	*DR1*	-0.459917965	0.006233864	0.011570498	1
T2DM_insulin	leukocyte	*PTBP2*	-0.353998358	0.009984304	0.015458818	1
T2DM_insulin	leukocyte	*NSL1*	-0.582159913	0.003069494	0.011330438	1
T2DM_insulin	leukocyte	*RPA2*	-0.476728418	0.004332145	0.011330438	1
T2DM_insulin	leukocyte	*ESYT2*	-0.448543779	0.001779092	0.011330438	1
T2DM_insulin	leukocyte	*DDX59*	-0.316448223	0.005859842	0.011570498	1
T2DM_insulin	leukocyte	*TTR*	-0.146073919	0.003904589	0.011330438	1
T2DM_insulin	leukocyte	*SPCS2*	-0.347721534	0.002430468	0.011330438	1
T2DM_insulin	leukocyte	*ELOVL4*	-0.537066511	0.004259247	0.011330438	1
T2DM_insulin	leukocyte	*FILIP1*	-0.370775056	0.01093949	0.016550349	1
T2DM_insulin	leukocyte	*AKAP7*	-0.759583661	0.003479523	0.011330438	1
T2DM_insulin	leukocyte	*RNF146*	-0.416049851	0.004050446	0.011330438	1
T2DM_insulin	leukocyte	*MED28*	-0.547950497	0.003676076	0.011330438	1
T2DM_insulin	leukocyte	*VAMP8*	-0.370499777	0.005028291	0.011330438	1
T2DM_insulin	leukocyte	*ARMC2*	-0.354049166	0.003255776	0.011330438	1
T2DM_insulin	leukocyte	*PKD2*	-0.304938675	0.004755074	0.011330438	1
T2DM_insulin	leukocyte	*SPP1*	-0.151233882	0.012090587	0.017818423	1
T2DM_insulin	leukocyte	*WDR35*	-0.326122744	0.00722632	0.012409183	1
T2DM_insulin	leukocyte	*DNAH7*	-0.448547763	0.000895678	0.011330438	1
T2DM_insulin	leukocyte	*NDUFB3*	-0.339571797	0.004426711	0.011330438	1
T2DM_insulin	leukocyte	*GTF3C3*	-0.484719457	0.011971485	0.017715828	1
T2DM_insulin	leukocyte	*CPSF3*	-0.399770364	0.035723856	0.045204196	1
T2DM_insulin	leukocyte	*CCDC92*	-0.404907794	0.002992497	0.011330438	1
T2DM_insulin	leukocyte	*CTNNAL1*	-0.275000662	0.010038661	0.015522195	1
T2DM_insulin	leukocyte	*NEK6*	-0.415227333	0.008040798	0.013235147	1
T2DM_insulin	leukocyte	*BSPRY*	-0.289011688	0.010810761	0.016411836	1
T2DM_insulin	leukocyte	*RBM18*	-0.481315466	0.003546527	0.011330438	1
T2DM_insulin	leukocyte	*DCAF4*	-0.455531418	0.006237141	0.011570498	1
T2DM_insulin	leukocyte	*IFI27L2*	-0.313334673	0.005256432	0.011402654	1
T2DM_insulin	leukocyte	*ACOT2*	-0.502229054	0.01707643	0.023775087	1
T2DM_insulin	leukocyte	*ZC2HC1C*	-0.967066616	0.019670587	0.026832745	1
T2DM_insulin	leukocyte	*KLHL29*	-1.038358011	0.025059381	0.033184465	1
T2DM_insulin	leukocyte	*AFTPH*	-0.39729848	0.002039831	0.011330438	1
T2DM_insulin	leukocyte	*BCL11A*	-0.58684687	0.00786667	0.013096672	1
T2DM_insulin	leukocyte	*CRIPT*	-0.418638416	0.005438361	0.011421159	1
T2DM_insulin	leukocyte	*EPCAM*	-0.236089609	0.003682837	0.011330438	1
T2DM_insulin	leukocyte	*SLC17A5*	-0.985605259	0.012099738	0.017825223	1
T2DM_insulin	leukocyte	*CUTC*	-0.435538605	0.005479916	0.011427973	1
T2DM_insulin	leukocyte	*PYROXD2*	-0.526089794	0.012487102	0.018238596	1
T2DM_insulin	leukocyte	*HELLS*	-0.457949725	0.003459714	0.011330438	1
T2DM_insulin	leukocyte	*PRLHR*	-0.505662091	0.006272609	0.011570498	1
T2DM_insulin	leukocyte	*WDR11*	-0.715739353	0.000454178	0.011330438	1
T2DM_insulin	leukocyte	*KANSL1*	-0.436891348	0.001267503	0.011330438	1
T2DM_insulin	leukocyte	*HOXB3*	-0.383792754	0.037420638	0.047138726	1
T2DM_insulin	leukocyte	*RCL1*	-0.661210874	0.002791391	0.011330438	1
T2DM_insulin	leukocyte	*CD274*	-1.022404786	0.005941154	0.011570498	1
T2DM_insulin	leukocyte	*PLEKHG1*	-0.4114543	0.002041721	0.011330438	1
T2DM_insulin	leukocyte	*PCDHB10*	-0.708116194	0.002874748	0.011330438	1
T2DM_insulin	leukocyte	*MRPS14*	-0.691822695	0.001180855	0.011330438	1
T2DM_insulin	leukocyte	*GORAB*	-0.370431705	0.005897083	0.011570498	1
T2DM_insulin	leukocyte	*SNX19*	-0.369647094	0.002019897	0.011330438	1
T2DM_insulin	leukocyte	*KCNJ5*	0.120072287	0.011151669	0.016813088	1
T2DM_insulin	leukocyte	*SLC10A7*	-0.813411496	0.000874074	0.011330438	1
T2DM_insulin	leukocyte	*MASTL*	-0.369759752	0.004895644	0.011330438	1
T2DM_insulin	leukocyte	*MRC1*	0.09701072	0.030646738	0.039440233	1
T2DM_insulin	leukocyte	*IQSEC3*	-0.402649884	0.004706873	0.011330438	1
T2DM_insulin	leukocyte	*MTRF1*	-0.735887312	0.017410567	0.024120634	1
T2DM_insulin	leukocyte	*DNAJC15*	-0.3642826	0.004293672	0.011330438	1
T2DM_insulin	leukocyte	*PROSER1*	-0.52205623	0.002128983	0.011330438	1
T2DM_insulin	leukocyte	*WBP4*	-0.373813547	0.00356584	0.011330438	1
T2DM_insulin	leukocyte	*KBTBD7*	-0.770246243	0.002811609	0.011330438	1
T2DM_insulin	leukocyte	*FAM53C*	-0.704090287	0.005197331	0.011378137	1
T2DM_insulin	leukocyte	*SIL1*	-0.39902703	0.001807469	0.011330438	1

T2DM_insulin	leukocyte	*PLS1*	-0.276717171	0.011959303	0.017706833	1
T2DM_insulin	leukocyte	*ZFP30*	-0.573125406	0.001171879	0.011330438	1
T2DM_insulin	leukocyte	*UTP20*	-0.824222564	0.002166145	0.011330438	1
T2DM_insulin	leukocyte	*TMPO*	-0.594264428	0.001583451	0.011330438	1
T2DM_insulin	leukocyte	*PPP3CC*	-0.448741557	0.004678522	0.011330438	1
T2DM_insulin	leukocyte	*EPHX2*	-0.445617606	0.003590721	0.011330438	1
T2DM_insulin	leukocyte	*CRISPLD1*	-2.091618184	0.005301842	0.011421159	1
T2DM_insulin	leukocyte	*TRIM6*	0.427546555	0.029382847	0.038012909	1
T2DM_insulin	leukocyte	*PYROXD1*	-0.305352843	0.004624747	0.011330438	1
T2DM_insulin	leukocyte	*IAPP*	-0.115970631	0.012944363	0.018774693	1
T2DM_insulin	leukocyte	*ZSCAN18*	-0.35919134	0.001373235	0.011330438	1
T2DM_insulin	leukocyte	*LHX4*	-0.954824137	0.015603605	0.021989442	1
T2DM_insulin	leukocyte	*TRMT1L*	-0.367028156	0.008026532	0.01322829	1
T2DM_insulin	leukocyte	*CAT*	-0.793249258	0.003134776	0.011330438	1
T2DM_insulin	leukocyte	*CSMD2*	0.461216787	0.016122916	0.022599692	1
T2DM_insulin	leukocyte	*ACVR2A*	-0.315282214	0.009911464	0.01538607	1
T2DM_insulin	leukocyte	*POLK*	-0.581888522	0.000865742	0.011330438	1
T2DM_insulin	leukocyte	*UBL3*	-0.329674497	0.003226065	0.011330438	1
T2DM_insulin	leukocyte	*LDB3*	-0.649938516	0.002372946	0.011330438	1
T2DM_insulin	leukocyte	*NAA60*	-0.507191551	0.002110512	0.011330438	1
T2DM_insulin	leukocyte	*TRMT13*	-0.793510862	0.005878264	0.011570498	1
T2DM_insulin	leukocyte	*RWDD3*	-0.401128383	0.002037526	0.011330438	1
T2DM_insulin	leukocyte	*CCDC18*	-0.441663989	0.001352213	0.011330438	1
T2DM_insulin	leukocyte	*NBPF14*	-1.004265412	0.006520592	0.011788627	1
T2DM_insulin	leukocyte	*PMS2*	-0.751375096	0.004308299	0.011330438	1
T2DM_insulin	leukocyte	*EEPD1*	0.182293674	0.034155581	0.04341549	1
T2DM_insulin	leukocyte	*KLHL7*	-0.527466814	0.003799871	0.011330438	1
T2DM_insulin	leukocyte	*CBX3*	-0.402113322	0.003045755	0.011330438	1
T2DM_insulin	leukocyte	*NPY*	-0.180579403	0.00395095	0.011330438	1
T2DM_insulin	leukocyte	*INHBA*	-0.337296389	0.003963964	0.011330438	1
T2DM_insulin	leukocyte	*CCZ1*	-0.512228371	0.006200341	0.011570498	1
T2DM_insulin	leukocyte	*POLM*	-0.321747635	0.013272584	0.01915093	1
T2DM_insulin	leukocyte	*SMU1*	-0.449281751	0.001819871	0.011330438	1
T2DM_insulin	leukocyte	*SLC25A51*	-0.453995179	0.005318884	0.011421159	1
T2DM_insulin	leukocyte	*ACO1*	-0.356217634	0.004860835	0.011330438	1
T2DM_insulin	leukocyte	*PLAU*	-0.430974253	0.025865484	0.034056798	1
T2DM_insulin	leukocyte	*RBM19*	-0.331326889	0.00539023	0.011421159	1
T2DM_insulin	leukocyte	*BHLHE41*	-0.602731999	0.006761369	0.011982597	1
T2DM_insulin	leukocyte	*ITPR2*	-0.923368484	0.0069807	0.012179027	1
T2DM_insulin	leukocyte	*EBPL*	-0.327078143	0.004424872	0.011330438	1
T2DM_insulin	leukocyte	*TSFM*	-0.514709226	0.002592139	0.011330438	1
T2DM_insulin	leukocyte	*CDK2*	-0.574338713	0.030180711	0.038904203	1
T2DM_insulin	leukocyte	*IKZF4*	-0.518242423	0.007828833	0.013044735	1
T2DM_insulin	leukocyte	*USP45*	-0.307679014	0.006476588	0.011737322	1
T2DM_insulin	leukocyte	*C4BPA*	-0.563868367	0.021338523	0.028790885	1
T2DM_insulin	leukocyte	*C4BPB*	-0.947651275	0.009868388	0.015333268	1
T2DM_insulin	leukocyte	*RAB38*	-1.074038824	0.007914713	0.013137687	1
T2DM_insulin	leukocyte	*CKS2*	-0.322504863	0.009617503	0.015048653	1
T2DM_insulin	leukocyte	*SNX21*	-0.369467769	0.009940992	0.015425812	1
T2DM_insulin	leukocyte	*ARHGAP40*	-19.40751693	0.005242797	0.011392572	1
T2DM_insulin	leukocyte	*NCOA3*	-0.352120444	0.011176993	0.016816769	1
T2DM_insulin	leukocyte	*MATN4*	-2.459016	0.00856981	0.01382216	1
T2DM_insulin	leukocyte	*VAPB*	-0.33937835	0.00325642	0.011330438	1
T2DM_insulin	leukocyte	*PARD6B*	-0.479375217	0.003975234	0.011330438	1
T2DM_insulin	leukocyte	*ARFGEF2*	-0.474831549	0.003168186	0.011330438	1
T2DM_insulin	leukocyte	*ZNFX1*	-1.165719555	0.004675276	0.011330438	1
T2DM_insulin	leukocyte	*CSE1L*	-0.48787321	0.001514475	0.011330438	1
T2DM_insulin	leukocyte	*BCAS4*	-0.281909711	0.004503436	0.011330438	1
T2DM_insulin	leukocyte	*KCNK15*	-0.333954554	0.011528292	0.017228854	1
T2DM_insulin	leukocyte	*PEPD*	-0.671067556	0.000592362	0.011330438	1
T2DM_insulin	leukocyte	*SNRNP27*	-0.353514202	0.005540481	0.011451105	1
T2DM_insulin	leukocyte	*USP22*	-0.447938179	0.003282037	0.011330438	1
T2DM_insulin	leukocyte	*BTN2A2*	-0.81287246	0.000625447	0.011330438	1
T2DM_insulin	leukocyte	*MRS2*	-0.622564826	0.00305012	0.011330438	1

T2DM_insulin	leukocyte	*SNRPC*	-0.405074226	0.002791477	0.011330438	1
T2DM_insulin	leukocyte	*SERPINB6*	-0.369966884	0.001857828	0.011330438	1
T2DM_insulin	leukocyte	*ABCC10*	-0.46405767	0.006229991	0.011570498	1
T2DM_insulin	leukocyte	*PEX6*	-0.353041439	0.008815927	0.014103329	1
T2DM_insulin	leukocyte	*NQO2*	-0.853726501	0.003303956	0.011330438	1
T2DM_insulin	leukocyte	*UNC5CL*	-0.357747384	0.004317224	0.011330438	1
T2DM_insulin	leukocyte	*MED20*	-0.551037578	0.007358277	0.01254259	1
T2DM_insulin	leukocyte	*GLO1*	-0.375863301	0.005841356	0.011570498	1
T2DM_insulin	leukocyte	*KCNK17*	-0.258492875	0.014877736	0.021110374	1
T2DM_insulin	leukocyte	*ATXN1*	-0.498466282	0.003408645	0.011330438	1
T2DM_insulin	leukocyte	*LRRFIP1*	-0.327422863	0.00193088	0.011330438	1
T2DM_insulin	leukocyte	*CXCL6*	-0.587494964	0.009685331	0.015106656	1
T2DM_insulin	leukocyte	*SH3TC1*	-0.399546304	0.005750525	0.011570498	1
T2DM_insulin	leukocyte	*DOK4*	-0.277734417	0.004903336	0.011330438	1
T2DM_insulin	leukocyte	*CLYBL*	-1.197767802	0.00015761	0.011330438	0.619248013
T2DM_insulin	leukocyte	*TM9SF2*	-0.338824071	0.003411338	0.011330438	1
T2DM_insulin	leukocyte	*KIF25*	-5.272867074	0.004210899	0.011330438	1
T2DM_insulin	leukocyte	*BMP4*	-0.885837979	0.002131073	0.011330438	1
T2DM_insulin	leukocyte	*GRK4*	-0.392857378	0.026803376	0.035115194	1
T2DM_insulin	leukocyte	*HS3ST3B1*	-0.858406489	0.006203871	0.011570498	1
T2DM_insulin	leukocyte	*MRPS7*	-0.318714237	0.003714411	0.011330438	1
T2DM_insulin	leukocyte	*GGA3*	-0.784447034	0.007927444	0.013147711	1
T2DM_insulin	leukocyte	*DDX31*	-0.697660692	0.00296073	0.011330438	1
T2DM_insulin	leukocyte	*PPDPF*	-0.286013842	0.00319833	0.011330438	1
T2DM_insulin	leukocyte	*CHCHD5*	-0.345398756	0.003244247	0.011330438	1
T2DM_insulin	leukocyte	*INSIG2*	-0.334334302	0.003164748	0.011330438	1
T2DM_insulin	leukocyte	*PSD4*	-0.538642515	0.003458532	0.011330438	1
T2DM_insulin	leukocyte	*CLPP*	-0.381408472	0.003171922	0.011330438	1
T2DM_insulin	leukocyte	*GPR108*	-0.547626658	0.002900775	0.011330438	1
T2DM_insulin	leukocyte	*SYMPK*	-0.627860011	0.01283694	0.018659392	1
T2DM_insulin	leukocyte	*NAPB*	-0.363476647	0.00337014	0.011330438	1
T2DM_insulin	leukocyte	*PSMF1*	-0.416483371	0.004008266	0.011330438	1
T2DM_insulin	leukocyte	*RBCK1*	-0.327938251	0.008163838	0.013387195	1
T2DM_insulin	leukocyte	*FLRT3*	-0.314730066	0.006423294	0.01170553	1
T2DM_insulin	leukocyte	*MGME1*	-1.38033372	0.007206758	0.012381002	1
T2DM_insulin	leukocyte	*ITPA*	-0.519091641	0.002655507	0.011330438	1
T2DM_insulin	leukocyte	*BANF2*	-5.098721679	0.006054316	0.011570498	1
T2DM_insulin	leukocyte	*MRPS26*	-0.319160447	0.004180701	0.011330438	1
T2DM_insulin	leukocyte	*ZNF436*	-0.982559711	0.001380938	0.011330438	1
T2DM_insulin	leukocyte	*TUBGCP3*	-0.474933467	0.003118181	0.011330438	1
T2DM_insulin	leukocyte	*MCF2L*	-0.288365353	0.004495895	0.011330438	1
T2DM_insulin	leukocyte	*IGFLR1*	0.119541563	0.023284509	0.031043378	1
T2DM_insulin	leukocyte	*NR1D1*	-0.35160186	0.001959391	0.011330438	1
T2DM_insulin	leukocyte	*PRMT1*	-0.328937777	0.002195526	0.011330438	1
T2DM_insulin	leukocyte	*PRR12*	-0.359349352	0.010412468	0.01593712	1
T2DM_insulin	leukocyte	*FLRT1*	-0.85678716	0.021066873	0.028463461	1
T2DM_insulin	leukocyte	*ASL*	-0.397342354	0.004799229	0.011330438	1
T2DM_insulin	leukocyte	*WNK4*	-0.417818348	0.020930352	0.028317959	1
T2DM_insulin	leukocyte	*BECN1*	-0.398998228	0.00433658	0.011330438	1
T2DM_insulin	leukocyte	*PRKCG*	0.950701884	0.033239223	0.042332871	1
T2DM_insulin	leukocyte	*TRAP1*	-0.319134384	0.004780363	0.011330438	1
T2DM_insulin	leukocyte	*ZNF384*	-0.479884117	0.003998137	0.011330438	1
T2DM_insulin	leukocyte	*RHOJ*	-0.630068225	0.005895611	0.011570498	1
T2DM_insulin	leukocyte	*L3HYPDH*	-0.366339994	0.031102258	0.039908809	1
T2DM_insulin	leukocyte	*HSPA2*	-0.606311968	0.006150867	0.011570498	1
T2DM_insulin	leukocyte	*ZBTB1*	-0.550525579	0.001689545	0.011330438	1
T2DM_insulin	leukocyte	*PLEKHG3*	-0.530594679	0.004955695	0.011330438	1
T2DM_insulin	leukocyte	*FGD3*	1.021883191	0.023757581	0.031609731	1
T2DM_insulin	leukocyte	*BCL11B*	0.631671749	0.018268081	0.025166652	1
T2DM_insulin	leukocyte	*ABHD8*	-0.925703557	0.002155881	0.011330438	1
T2DM_insulin	leukocyte	*BEST3*	-0.760517777	0.030801373	0.039574426	1
T2DM_insulin	leukocyte	*YEATS4*	-0.349337792	0.004957622	0.011330438	1
T2DM_insulin	leukocyte	*LRRC61*	-0.568218002	0.004270228	0.011330438	1
T2DM_insulin	leukocyte	*IDUA*	-0.373064325	0.006114467	0.011570498	1

T2DM_insulin	leukocyte	*FGFRL1*	-0.342963138	0.009010104	0.014309095	1
T2DM_insulin	leukocyte	*EMC1*	-0.440724866	0.004060208	0.011330438	1
T2DM_insulin	leukocyte	*UBR4*	-0.405708391	0.004318864	0.011330438	1
T2DM_insulin	leukocyte	*SYNGR3*	-0.449693221	0.006543672	0.011804448	1
T2DM_insulin	leukocyte	*WFIKKN1*	-0.369304345	0.011176563	0.016816769	1
T2DM_insulin	leukocyte	*KDM4B*	-0.850898529	0.00359064	0.011330438	1
T2DM_insulin	leukocyte	*PNKD*	-0.486590895	0.002811901	0.011330438	1
T2DM_insulin	leukocyte	*PTPN12*	-0.484782356	0.000268045	0.011330438	1
T2DM_insulin	leukocyte	*POR*	-0.399903185	0.004600543	0.011330438	1
T2DM_insulin	leukocyte	*RBM48*	-0.720133033	0.002759561	0.011330438	1
T2DM_insulin	leukocyte	*SRD5A3*	-0.456030836	0.007756461	0.012968143	1
T2DM_insulin	leukocyte	*PPAT*	-0.438748381	0.004644202	0.011330438	1
T2DM_insulin	leukocyte	*A4GALT*	-0.33821976	0.005907386	0.011570498	1
T2DM_insulin	leukocyte	*NAA38*	-0.315144601	0.003511521	0.011330438	1
T2DM_insulin	leukocyte	*PRKRIP1*	-0.366345019	0.00444645	0.011330438	1
T2DM_insulin	leukocyte	*VGF*	-0.203311552	0.003054782	0.011330438	1
T2DM_insulin	leukocyte	*IRF5*	0.143334133	0.025986728	0.0341989	1
T2DM_insulin	leukocyte	*MTX2*	-0.544656551	0.002773786	0.011330438	1
T2DM_insulin	leukocyte	*GAD1*	-1.356648176	0.004896973	0.011330438	1
T2DM_insulin	leukocyte	*ORMDL1*	-0.335273803	0.007997089	0.013211473	1
T2DM_insulin	leukocyte	*PSMG2*	-0.443020841	0.002470116	0.011330438	1
T2DM_insulin	leukocyte	*TWSG1*	-0.587870167	0.002388933	0.011330438	1
T2DM_insulin	leukocyte	*EIF2AK4*	-0.540638185	0.003365563	0.011330438	1
T2DM_insulin	leukocyte	*MYO5C*	-0.264037461	0.00599231	0.011570498	1
T2DM_insulin	leukocyte	*CGNL1*	-0.483822533	0.001192046	0.011330438	1
T2DM_insulin	leukocyte	*TMOD2*	-0.428651094	0.003944196	0.011330438	1
T2DM_insulin	leukocyte	*IVD*	-0.548399866	0.003407245	0.011330438	1
T2DM_insulin	leukocyte	*CHAC1*	-0.467771354	0.002927055	0.011330438	1
T2DM_insulin	leukocyte	*ANAPC13*	-0.248066463	0.007179136	0.012365991	1
T2DM_insulin	leukocyte	*SUMF2*	-0.446521145	0.002714053	0.011330438	1
T2DM_insulin	leukocyte	*TPH1*	-1.444369607	0.002520195	0.011330438	1
T2DM_insulin	leukocyte	*DCTD*	-0.457009947	0.003573072	0.011330438	1
T2DM_insulin	leukocyte	*KRI1*	-0.261573814	0.022685113	0.030347228	1
T2DM_insulin	leukocyte	*AP1M2*	-0.340618937	0.004444643	0.011330438	1
T2DM_insulin	leukocyte	*CDKN2D*	-0.367765362	0.002642567	0.011330438	1
T2DM_insulin	leukocyte	*MTUS1*	-0.317171277	0.004401639	0.011330438	1
T2DM_insulin	leukocyte	*ADCY4*	-0.400538554	0.007387544	0.012565211	1
T2DM_insulin	leukocyte	*DTD2*	-0.32106972	0.007048225	0.012237751	1
T2DM_insulin	leukocyte	*HEATR5A*	-0.771170033	0.002929405	0.011330438	1
T2DM_insulin	leukocyte	*RNASE1*	0.062154081	0.018472137	0.025394341	1
T2DM_insulin	leukocyte	*EPB41L4A*	-0.429992576	0.005981001	0.011570498	1
T2DM_insulin	leukocyte	*REEP5*	-0.315659916	0.003589701	0.011330438	1
T2DM_insulin	leukocyte	*MAU2*	-1.077235628	9.41E-05	0.011330438	0.369891368
T2DM_insulin	leukocyte	*SHC2*	-0.428345819	0.003520295	0.011330438	1
T2DM_insulin	leukocyte	*SYT5*	-0.223847386	0.016050115	0.02251371	1
T2DM_insulin	leukocyte	*CBFA2T3*	-0.366589701	0.005862513	0.011570498	1
T2DM_insulin	leukocyte	*ERMARD*	-0.54999317	0.030929426	0.039725961	1
T2DM_insulin	leukocyte	*PHF10*	-0.398723843	0.003011108	0.011330438	1
T2DM_insulin	leukocyte	*SH3BP4*	-0.221804119	0.008312321	0.01352344	1
T2DM_insulin	leukocyte	*DOCK6*	-0.282881454	0.007664417	0.012869015	1
T2DM_insulin	leukocyte	*ECSIT*	-0.443679075	0.004132441	0.011330438	1
T2DM_insulin	leukocyte	*CDC16*	-0.567254805	0.001302723	0.011330438	1
T2DM_insulin	leukocyte	*DPP6*	-0.203667962	0.005640229	0.01154792	1
T2DM_insulin	leukocyte	*GTPBP3*	-0.460070193	0.000945288	0.011330438	1
T2DM_insulin	leukocyte	*PLVAP*	-0.202163067	0.003708947	0.011330438	1
T2DM_insulin	leukocyte	*MRPL34*	-0.227611962	0.035817005	0.045274858	1
T2DM_insulin	leukocyte	*LSM7*	-0.283732008	0.003343354	0.011330438	1
T2DM_insulin	leukocyte	*TULP4*	-0.407568552	0.002629794	0.011330438	1
T2DM_insulin	leukocyte	*QRSL1*	-0.423071105	0.004761364	0.011330438	1
T2DM_insulin	leukocyte	*RSPH3*	-0.416396678	0.006816704	0.012014984	1
T2DM_insulin	leukocyte	*STK33*	-0.352455982	0.001728792	0.011330438	1
T2DM_insulin	leukocyte	*NDUFA10*	-0.404452917	0.001563668	0.011330438	1
T2DM_insulin	leukocyte	*TRPM4*	-1.519167353	0.007470252	0.012656584	1
T2DM_insulin	leukocyte	*UBAC1*	-0.363622482	0.004774087	0.011330438	1

T2DM_insulin	leukocyte	*ATXN10*	-0.327098546	0.003096744	0.011330438	1
T2DM_insulin	leukocyte	*PNPLA7*	-0.748160347	0.004849545	0.011330438	1
T2DM_insulin	leukocyte	*PAK4*	-0.479206241	0.003808582	0.011330438	1
T2DM_insulin	leukocyte	*CEP85*	0.545672407	0.017469477	0.024193717	1
T2DM_insulin	leukocyte	*LAMA5*	-0.267459201	0.004613154	0.011330438	1
T2DM_insulin	leukocyte	*ADRM1*	-0.364085043	0.003189914	0.011330438	1
T2DM_insulin	leukocyte	*UCK1*	-0.691716263	0.001875758	0.011330438	1
T2DM_insulin	leukocyte	*MAP3K10*	-0.807444544	0.006163438	0.011570498	1
T2DM_insulin	leukocyte	*SMPDL3B*	-0.313097247	0.007787316	0.013008659	1
T2DM_insulin	leukocyte	*CLIP1*	-0.409995895	0.006901894	0.012100405	1
T2DM_insulin	leukocyte	*ZNF317*	-0.511434518	0.004225063	0.011330438	1
T2DM_insulin	leukocyte	*DNMT1*	-0.600002564	0.004102409	0.011330438	1
T2DM_insulin	leukocyte	*ZNF426*	-0.588792613	0.001355992	0.011330438	1
T2DM_insulin	leukocyte	*LRP3*	-0.597516	0.00461557	0.011330438	1
T2DM_insulin	leukocyte	*HSD17B3*	-4.534844037	0.006935014	0.012128132	1
T2DM_insulin	leukocyte	*SYNE1*	-0.34044098	0.003667824	0.011330438	1
T2DM_insulin	leukocyte	*EPS8L1*	-1.269865257	0.002827863	0.011330438	1
T2DM_insulin	leukocyte	*C1QL1*	-0.169651198	0.006722703	0.011957221	1
T2DM_insulin	leukocyte	*F12*	-0.355426797	0.029949931	0.038682866	1
T2DM_insulin	leukocyte	*TRAF3*	-0.266252171	0.01974129	0.026913091	1
T2DM_insulin	leukocyte	*PDLIM4*	-0.428832153	0.003516124	0.011330438	1
T2DM_insulin	leukocyte	*DIAPH1*	-0.292952095	0.00468578	0.011330438	1
T2DM_insulin	leukocyte	*EXOC4*	-0.809857728	0.00211187	0.011330438	1
T2DM_insulin	leukocyte	*C1orf159*	-1.086820815	0.031954617	0.040882347	1
T2DM_insulin	leukocyte	*ANO1*	-0.933759052	0.004468508	0.011330438	1
T2DM_insulin	leukocyte	*PPFIA1*	-0.339652999	0.004342465	0.011330438	1
T2DM_insulin	leukocyte	*TRAF7*	-0.516572582	0.002919711	0.011330438	1
T2DM_insulin	leukocyte	*MAP1B*	-0.263598087	0.002172447	0.011330438	1
T2DM_insulin	leukocyte	*CHD1L*	-0.869362457	0.004340794	0.011330438	1
T2DM_insulin	leukocyte	*PIAS3*	-0.269650296	0.013114871	0.018958178	1
T2DM_insulin	leukocyte	*RBM8A*	-0.256438367	0.005425419	0.011421159	1
T2DM_insulin	leukocyte	*ZNF304*	-1.650069347	0.002277186	0.011330438	1
T2DM_insulin	leukocyte	*RHPN2*	-0.403728365	0.002034698	0.011330438	1
T2DM_insulin	leukocyte	*GCH1*	-0.26819332	0.005733562	0.011570498	1
T2DM_insulin	leukocyte	*CC2D1A*	-0.511001003	0.005102516	0.011330438	1
T2DM_insulin	leukocyte	*CCT6B*	-0.573575026	0.006293454	0.011592584	1
T2DM_insulin	leukocyte	*PPARG*	-0.658276665	0.002714241	0.011330438	1
T2DM_insulin	leukocyte	*ENOSF1*	-0.962308309	0.002099959	0.011330438	1
T2DM_insulin	leukocyte	*IMMT*	-0.440949359	0.001806589	0.011330438	1
T2DM_insulin	leukocyte	*MRPL35*	-0.435602041	0.002805897	0.011330438	1
T2DM_insulin	leukocyte	*SCLY*	-0.641221303	0.00643377	0.011719185	1
T2DM_insulin	leukocyte	*CARD6*	-0.746216405	0.004668734	0.011330438	1
T2DM_insulin	leukocyte	*INPP5K*	-0.372965895	0.012987208	0.01881517	1
T2DM_insulin	leukocyte	*RPA1*	-0.434355233	0.008528351	0.013783584	1
T2DM_insulin	leukocyte	*TMEM128*	-0.582476438	0.002900371	0.011330438	1
T2DM_insulin	leukocyte	*SEC61G*	-0.300120457	0.002327158	0.011330438	1
T2DM_insulin	leukocyte	*FIGNL1*	-0.960158249	0.025321335	0.033452429	1
T2DM_insulin	leukocyte	*ITGB4*	-0.754416607	0.01176968	0.017542896	1
T2DM_insulin	leukocyte	*TRIM47*	-0.407693342	0.002173653	0.011330438	1
T2DM_insulin	leukocyte	*EIF5A*	-0.332492101	0.00258145	0.011330438	1
T2DM_insulin	leukocyte	*PRMT7*	-0.744146098	0.016961867	0.023640715	1
T2DM_insulin	leukocyte	*ANKEF1*	-0.324582005	0.005909121	0.011570498	1
T2DM_insulin	leukocyte	*BTBD3*	-0.315124106	0.004395696	0.011330438	1
T2DM_insulin	leukocyte	*LPIN3*	-0.469895722	0.025190359	0.03330179	1
T2DM_insulin	leukocyte	*RBM38*	-0.674710704	0.000814609	0.011330438	1
T2DM_insulin	leukocyte	*VSTM2L*	-0.332854922	0.004041079	0.011330438	1
T2DM_insulin	leukocyte	*SERINC3*	-0.338895006	0.008637826	0.013903325	1
T2DM_insulin	leukocyte	*BHMT2*	-0.525155982	0.001012806	0.011330438	1
T2DM_insulin	leukocyte	*WASF3*	-0.385122884	0.007066757	0.012258405	1
T2DM_insulin	leukocyte	*SCO1*	-0.625331706	0.006249616	0.011570498	1
T2DM_insulin	leukocyte	*CHI3L1*	-0.281779882	0.03176648	0.040694652	1
T2DM_insulin	leukocyte	*SLC41A1*	-0.440309069	0.004035272	0.011330438	1
T2DM_insulin	leukocyte	*TRPC4*	-1.111157428	0.00472902	0.011330438	1
T2DM_insulin	leukocyte	*POSTN*	0.347994626	0.017127185	0.023820429	1

T2DM_insulin	leukocyte	*RFC3*	-0.313325346	0.00616235	0.011570498	1
T2DM_insulin	leukocyte	*EPHB2*	-1.327843221	0.011229491	0.016859255	1
T2DM_insulin	leukocyte	*BTBD2*	-0.879816418	0.002545173	0.011330438	1
T2DM_insulin	leukocyte	*CNDP2*	-0.396045892	0.003386465	0.011330438	1
T2DM_insulin	leukocyte	*WDR74*	-0.606977654	0.002321278	0.011330438	1
T2DM_insulin	leukocyte	*GSTT2B*	-0.349491762	0.006508568	0.011778978	1
T2DM_insulin	leukocyte	*SLC2A11*	-0.599059618	0.004083623	0.011330438	1
T2DM_insulin	leukocyte	*C1QTNF6*	-0.543559644	0.015238676	0.021555956	1
T2DM_insulin	leukocyte	*FAM83F*	-0.419867442	0.003840291	0.011330438	1
T2DM_insulin	leukocyte	*GIMAP6*	-0.767142854	0.002464942	0.011330438	1
T2DM_insulin	leukocyte	*AGAP3*	-1.196193841	0.003022483	0.011330438	1
T2DM_insulin	leukocyte	*SFTPD*	-0.353802367	0.014889157	0.021118953	1
T2DM_insulin	leukocyte	*IMPA1*	-0.365586117	0.003818653	0.011330438	1
T2DM_insulin	leukocyte	*LRRCC1*	-0.569551189	0.006811495	0.012014984	1
T2DM_insulin	leukocyte	*RRAS2*	-0.336719728	0.003800791	0.011330438	1
T2DM_insulin	leukocyte	*HSD17B4*	-0.706709728	0.001629765	0.011330438	1
T2DM_insulin	leukocyte	*COX16*	-0.374288031	0.002888887	0.011330438	1
T2DM_insulin	leukocyte	*ELP3*	-0.794547381	0.002850236	0.011330438	1
T2DM_insulin	leukocyte	*PEBP4*	-0.563381385	0.00335523	0.011330438	1
T2DM_insulin	leukocyte	*MRO*	-1.079605091	0.003523045	0.011330438	1
T2DM_insulin	leukocyte	*MRPS36*	-0.373887019	0.009559814	0.014994215	1
T2DM_insulin	leukocyte	*THUMPD3*	-0.444920676	0.002015358	0.011330438	1
T2DM_insulin	leukocyte	*CHL1*	-0.37423793	0.0214443	0.028923671	1
T2DM_insulin	leukocyte	*REG4*	-0.433082732	0.005322814	0.011421159	1
T2DM_insulin	leukocyte	*GSTM3*	-0.285062341	0.004361806	0.011330438	1
T2DM_insulin	leukocyte	*SORT1*	-0.487596438	0.003041003	0.011330438	1
T2DM_insulin	leukocyte	*NOTCH2*	-0.766084709	2.42E-05	0.011330438	0.095227714
T2DM_insulin	leukocyte	*CEPT1*	-0.690500975	0.002501193	0.011330438	1
T2DM_insulin	leukocyte	*NAPG*	-0.387882753	0.00399114	0.011330438	1
T2DM_insulin	leukocyte	*SPIRE1*	-0.406532479	0.003074123	0.011330438	1
T2DM_insulin	leukocyte	*FKBP11*	-0.219772988	0.007965941	0.013183733	1
T2DM_insulin	leukocyte	*KIDINS220*	-0.415401421	0.003076985	0.011330438	1
T2DM_insulin	leukocyte	*LPIN1*	-0.291712591	0.003723416	0.011330438	1
T2DM_insulin	leukocyte	*IAH1*	-0.505318873	0.002716242	0.011330438	1
T2DM_insulin	leukocyte	*ANO3*	0.592814456	0.015012335	0.021278306	1
T2DM_insulin	leukocyte	*IL2RA*	0.118918448	0.014427105	0.020582461	1
T2DM_insulin	leukocyte	*ECHDC3*	-0.393037512	0.003148162	0.011330438	1
T2DM_insulin	leukocyte	*TMEM241*	-0.694565364	0.003515808	0.011330438	1
T2DM_insulin	leukocyte	*AGO4*	-0.766439522	0.001116769	0.011330438	1
T2DM_insulin	leukocyte	*PRPF38A*	-0.383107991	0.004078004	0.011330438	1
T2DM_insulin	leukocyte	*DSC2*	-0.878225244	0.028401949	0.036901871	1
T2DM_insulin	leukocyte	*TPGS2*	-0.43656694	0.006691385	0.011938173	1
T2DM_insulin	leukocyte	*DAGLA*	-0.408026623	0.011952944	0.017706833	1
T2DM_insulin	leukocyte	*TIMM10*	-0.387563225	0.004236374	0.011330438	1
T2DM_insulin	leukocyte	*FADS2*	-0.450499275	0.003674852	0.011330438	1
T2DM_insulin	leukocyte	*TMEM165*	-0.323311856	0.003343971	0.011330438	1
T2DM_insulin	leukocyte	*CLOCK*	-0.425902358	0.006256363	0.011570498	1
T2DM_insulin	leukocyte	*GGACT*	-0.621475297	0.005059132	0.011330438	1
T2DM_insulin	leukocyte	*CLDN10*	-0.144558423	0.02603377	0.034243952	1
T2DM_insulin	leukocyte	*CARS2*	-0.3683072	0.004625477	0.011330438	1
T2DM_insulin	leukocyte	*TMED7*	-0.34812527	0.003244743	0.011330438	1
T2DM_insulin	leukocyte	*NREP*	-1.897873876	0.006307309	0.011612661	1
T2DM_insulin	leukocyte	*WDR36*	-0.406576318	0.004443411	0.011330438	1
T2DM_insulin	leukocyte	*OSTF1*	-0.535750744	0.004674836	0.011330438	1
T2DM_insulin	leukocyte	*GOLM1*	-0.326857238	0.001539842	0.011330438	1
T2DM_insulin	leukocyte	*SDS*	0.025387089	0.013673725	0.019657544	1
T2DM_insulin	leukocyte	*MSI1*	-0.674591565	0.003545608	0.011330438	1
T2DM_insulin	leukocyte	*DTX1*	-0.974732692	0.004151366	0.011330438	1
T2DM_insulin	leukocyte	*PNPLA8*	-0.319043537	0.002829205	0.011330438	1
T2DM_insulin	leukocyte	*TES*	-0.263718194	0.003824123	0.011330438	1
T2DM_insulin	leukocyte	*MDFIC*	-0.71540406	0.03359747	0.04273372	1
T2DM_insulin	leukocyte	*MTO1*	-0.362603838	0.005090053	0.011330438	1
T2DM_insulin	leukocyte	*LCA5*	-1.037010293	0.002112914	0.011330438	1
T2DM_insulin	leukocyte	*LMO2*	-0.340416279	0.004768332	0.011330438	1

T2DM_insulin	leukocyte	*RDH5*	-0.32751783	0.00657785	0.011838925	1
T2DM_insulin	leukocyte	*TFCP2*	-1.142994346	0.002283225	0.011330438	1
T2DM_insulin	leukocyte	*KRT7*	-0.175195634	0.008462539	0.013705406	1
T2DM_insulin	leukocyte	*NHSL1*	-0.470368547	0.011012152	0.016647459	1
T2DM_insulin	leukocyte	*AHI1*	-0.297325335	0.001889416	0.011330438	1
T2DM_insulin	leukocyte	*HEY2*	-0.646977484	0.001075833	0.011330438	1
T2DM_insulin	leukocyte	*PKIB*	-0.272094654	0.004288856	0.011330438	1
T2DM_insulin	leukocyte	*SEMA4F*	-0.542124082	0.004355462	0.011330438	1
T2DM_insulin	leukocyte	*GNS*	-0.707179048	0.002605503	0.011330438	1
T2DM_insulin	leukocyte	*MPHOSPH6*	-0.329121292	0.007001948	0.012199846	1
T2DM_insulin	leukocyte	*AGT*	-0.257102949	0.003302228	0.011330438	1
T2DM_insulin	leukocyte	*NTPCR*	-0.523162607	0.003270314	0.011330438	1
T2DM_insulin	leukocyte	*USP37*	-0.479721434	0.005657624	0.011549238	1
T2DM_insulin	leukocyte	*ARMC9*	-1.365597458	0.030514547	0.039295856	1
T2DM_insulin	leukocyte	*COX5B*	-0.292675514	0.004715011	0.011330438	1
T2DM_insulin	leukocyte	*REV1*	-0.32598141	0.009509178	0.014932678	1
T2DM_insulin	leukocyte	*TSGA10*	-0.328360302	0.027766156	0.036219531	1
T2DM_insulin	leukocyte	*TGFBRAP1*	-0.459983515	0.002824319	0.011330438	1
T2DM_insulin	leukocyte	*GCC2*	-0.432383327	0.0023955	0.011330438	1
T2DM_insulin	leukocyte	*MRPS9*	-0.344589972	0.003689397	0.011330438	1
T2DM_insulin	leukocyte	*ANKRD36*	-0.507349542	0.004384686	0.011330438	1
T2DM_insulin	leukocyte	*USP44*	-0.483297672	0.00887283	0.014148275	1
T2DM_insulin	leukocyte	*PLXNC1*	0.635493624	0.032715616	0.041747209	1
T2DM_insulin	leukocyte	*DRAM1*	-0.750225035	0.002543788	0.011330438	1
T2DM_insulin	leukocyte	*VILL*	0.493857891	0.020642988	0.027996651	1
T2DM_insulin	leukocyte	*FLNB*	-0.301386456	0.008806255	0.014103329	1
T2DM_insulin	leukocyte	*VPS36*	-0.598350726	0.002325649	0.011330438	1
T2DM_insulin	leukocyte	*RNASEH2B*	-0.733557393	0.002779114	0.011330438	1
T2DM_insulin	leukocyte	*CKAP2*	-0.362436995	0.008600709	0.013849257	1
T2DM_insulin	leukocyte	*TBC1D4*	-0.413277697	0.003484653	0.011330438	1
T2DM_insulin	leukocyte	*SUCLA2*	-0.449080959	0.002923286	0.011330438	1
T2DM_insulin	leukocyte	*RCBTB1*	-0.49847322	0.018451713	0.025375143	1
T2DM_insulin	leukocyte	*LMO7*	-0.218385796	0.005216612	0.011389288	1
T2DM_insulin	leukocyte	*C7orf25*	-0.377391508	0.001512846	0.011330438	1
T2DM_insulin	leukocyte	*RAPGEF5*	-0.266683198	0.006085441	0.011570498	1
T2DM_insulin	leukocyte	*KDELR2*	-0.347685328	0.003016845	0.011330438	1
T2DM_insulin	leukocyte	*TBRG4*	-0.802345626	0.002859048	0.011330438	1
T2DM_insulin	leukocyte	*DBNL*	-1.095741113	0.00258868	0.011330438	1
T2DM_insulin	leukocyte	*ALPK3*	-0.280066325	0.004625155	0.011330438	1
T2DM_insulin	leukocyte	*NMT1*	-0.30917423	0.006598289	0.011859412	1
T2DM_insulin	leukocyte	*MYCBPAP*	-0.575550068	0.039628049	0.049648789	1
T2DM_insulin	leukocyte	*TEX2*	-0.268655234	0.006392535	0.011698309	1
T2DM_insulin	leukocyte	*LIMD2*	0.265091449	0.026743834	0.035059586	1
T2DM_insulin	leukocyte	*MRPL47*	-0.370592648	0.002723549	0.011330438	1
T2DM_insulin	leukocyte	*SMPD4*	-0.889909515	0.002575331	0.011330438	1
T2DM_insulin	leukocyte	*CCDC115*	-0.394118162	0.006816675	0.012014984	1
T2DM_insulin	leukocyte	*BIN1*	-0.785506398	0.008861539	0.014141749	1
T2DM_insulin	leukocyte	*HS6ST1*	-0.559616083	0.003107936	0.011330438	1
T2DM_insulin	leukocyte	*NIPSNAP3A*	-0.555406729	0.002967832	0.011330438	1
T2DM_insulin	leukocyte	*CDK9*	-0.455354587	0.003572433	0.011330438	1
T2DM_insulin	leukocyte	*TXN*	-0.275863325	0.003333812	0.011330438	1
T2DM_insulin	leukocyte	*TOR1B*	-0.602072565	0.004833289	0.011330438	1
T2DM_insulin	leukocyte	*TOR1A*	-0.357055208	0.004668762	0.011330438	1
T2DM_insulin	leukocyte	*SLC2A8*	-0.408741844	0.00441254	0.011330438	1
T2DM_insulin	leukocyte	*ZFP37*	-0.333804867	0.00866032	0.013926501	1
T2DM_insulin	leukocyte	*STX17*	-0.3567617	0.00500898	0.011330438	1
T2DM_insulin	leukocyte	*PRPF4*	-0.48139405	0.007117869	0.012309025	1
T2DM_insulin	leukocyte	*FPGS*	-0.485935046	0.003857486	0.011330438	1
T2DM_insulin	leukocyte	*ATP6V1G1*	-0.285256648	0.004094072	0.011330438	1
T2DM_insulin	leukocyte	*ARPC5L*	-0.292173419	0.003925076	0.011330438	1
T2DM_insulin	leukocyte	*POLR1E*	-0.240808026	0.013264829	0.019146772	1
T2DM_insulin	leukocyte	*APTX*	-0.333030693	0.003156488	0.011330438	1
T2DM_insulin	leukocyte	*CD72*	-0.619999849	0.015273151	0.021585687	1
T2DM_insulin	leukocyte	*TMEM8B*	-0.510157466	0.003207138	0.011330438	1

T2DM_insulin	leukocyte	*GRHPR*	-0.445081857	0.000491958	0.011330438	1
T2DM_insulin	leukocyte	*ALDH1B1*	-0.735668343	0.004776262	0.011330438	1
T2DM_insulin	leukocyte	*ARHGEF39*	-1.139369099	0.021956068	0.029522721	1
T2DM_insulin	leukocyte	*PPIL1*	-0.891844959	0.002514068	0.011330438	1
T2DM_insulin	leukocyte	*GMPR*	-0.624206987	0.005327502	0.011421159	1
T2DM_insulin	leukocyte	*KIAA0319*	-0.395210578	0.005968343	0.011570498	1
T2DM_insulin	leukocyte	*LRRC1*	-0.356427604	0.004148258	0.011330438	1
T2DM_insulin	leukocyte	*RIPK1*	-0.385587187	0.004692661	0.011330438	1
T2DM_insulin	leukocyte	*TUBB2B*	-0.298414965	0.008134719	0.013356168	1
T2DM_insulin	leukocyte	*UQCC2*	-0.315162148	0.013077567	0.018918174	1
T2DM_insulin	leukocyte	*HMGA1*	-0.275820064	0.003717152	0.011330438	1
T2DM_insulin	leukocyte	*TCF19*	0.317902931	0.011168282	0.016816769	1
T2DM_insulin	leukocyte	*CLPS*	-0.121904259	0.025112447	0.033243532	1
T2DM_insulin	leukocyte	*MTCH1*	-0.344050931	0.002706634	0.011330438	1
T2DM_insulin	leukocyte	*FCHSD2*	0.570944103	0.022523923	0.030162404	1
T2DM_insulin	leukocyte	*ANKRD42*	-0.547880032	0.003497671	0.011330438	1
T2DM_insulin	leukocyte	*CREBZF*	-0.498348257	0.003149901	0.011330438	1
T2DM_insulin	leukocyte	*PRCP*	-0.405829832	0.02483244	0.032906123	1
T2DM_insulin	leukocyte	*NARS2*	-0.446197612	0.005466208	0.011427973	1
T2DM_insulin	leukocyte	*GGH*	-0.592158012	0.000428553	0.011330438	1
T2DM_insulin	leukocyte	*SDCBP*	-0.515815086	0.010931157	0.016550349	1
T2DM_insulin	leukocyte	*NEK1*	-0.594917839	0.022333153	0.02993755	1
T2DM_insulin	leukocyte	*TRPC6*	-0.961907664	0.01010778	0.015598378	1
T2DM_insulin	leukocyte	*MMP7*	-0.150094737	0.010655114	0.016227482	1
T2DM_insulin	leukocyte	*FDX1*	-0.388497415	0.002850786	0.011330438	1
T2DM_insulin	leukocyte	*C11orf1*	-0.360914658	0.005261647	0.011402654	1
T2DM_insulin	leukocyte	*FXYD2*	-0.219543804	0.00248926	0.011330438	1
T2DM_insulin	leukocyte	*CASP1*	0.137783213	0.017037806	0.023738134	1
T2DM_insulin	leukocyte	*ALKBH8*	-0.515806042	0.00574126	0.011570498	1
T2DM_insulin	leukocyte	*TUBGCP4*	-0.625409348	0.002152186	0.011330438	1
T2DM_insulin	leukocyte	*UACA*	-0.301423575	0.011105119	0.016749333	1
T2DM_insulin	leukocyte	*PLCB2*	0.212691315	0.020510355	0.027845607	1
T2DM_insulin	leukocyte	*PAK6*	-0.194204064	0.013589664	0.019553021	1
T2DM_insulin	leukocyte	*ADAM10*	-0.503980141	0.001633107	0.011330438	1
T2DM_insulin	leukocyte	*DUOX1*	-0.47545423	0.005327499	0.011421159	1
T2DM_insulin	leukocyte	*CYP19A1*	-3.133711061	0.008016375	0.013228197	1
T2DM_insulin	leukocyte	*ZNF280D*	-0.46652118	0.003068955	0.011330438	1
T2DM_insulin	leukocyte	*RSL24D1*	-0.369623444	0.001978568	0.011330438	1
T2DM_insulin	leukocyte	*TTLL7*	-0.366665638	0.003973316	0.011330438	1
T2DM_insulin	leukocyte	*SLC44A5*	-0.57571944	0.00649371	0.011762927	1
T2DM_insulin	leukocyte	*RTCA*	-0.832722531	0.006419692	0.01170553	1
T2DM_insulin	leukocyte	*DYNC2LI1*	-0.485639499	0.000929361	0.011330438	1
T2DM_insulin	leukocyte	*ACTR2*	-0.34982288	0.002345381	0.011330438	1
T2DM_insulin	leukocyte	*ATRAID*	-0.343201821	0.005069227	0.011330438	1
T2DM_insulin	leukocyte	*LRPPRC*	-0.395904902	0.00568891	0.011549238	1
T2DM_insulin	leukocyte	*MYOF*	-0.228404235	0.011207015	0.01684018	1
T2DM_insulin	leukocyte	*DUSP5*	-0.709090151	0.001891986	0.011330438	1
T2DM_insulin	leukocyte	*CALHM2*	0.434426892	0.012082884	0.017813752	1
T2DM_insulin	leukocyte	*ARL3*	-0.335797003	0.003825091	0.011330438	1
T2DM_insulin	leukocyte	*PLCE1*	-0.472214236	0.003268066	0.011330438	1
T2DM_insulin	leukocyte	*DNAJC13*	-0.332670185	0.006081121	0.011570498	1
T2DM_insulin	leukocyte	*FAM149B1*	-0.470016116	0.000915869	0.011330438	1
T2DM_insulin	leukocyte	*NCOA4*	-0.757830343	0.002470802	0.011330438	1
T2DM_insulin	leukocyte	*ASCC1*	-0.42095989	0.007169074	0.012363261	1
T2DM_insulin	leukocyte	*RPS24*	-0.260213765	0.00286433	0.011330438	1
T2DM_insulin	leukocyte	*DNA2*	-0.517990088	0.018754009	0.025727828	1
T2DM_insulin	leukocyte	*MYPN*	1.90015633	0.002812337	0.011330438	1
T2DM_insulin	leukocyte	*ATIC*	-0.687857878	0.001114182	0.011330438	1
T2DM_insulin	leukocyte	*SMARCAL1*	-0.497938489	0.006065427	0.011570498	1
T2DM_insulin	leukocyte	*BARD1*	-0.405238591	0.006023979	0.011570498	1
T2DM_insulin	leukocyte	*FASTKD1*	-0.408266976	0.011208214	0.01684018	1
T2DM_insulin	leukocyte	*FAM117B*	-0.494107566	0.002387677	0.011330438	1
T2DM_insulin	leukocyte	*WDR12*	-0.532980984	0.0038393	0.011330438	1
T2DM_insulin	leukocyte	*SLC35A5*	-0.502641844	0.005845463	0.011570498	1

T2DM_insulin	leukocyte	*USP8*	-0.462484062	0.002921556	0.011330438	1
T2DM_insulin	leukocyte	*SPPL2A*	-0.410223961	0.00271793	0.011330438	1
T2DM_insulin	leukocyte	*APH1B*	-0.580333548	0.003938231	0.011330438	1
T2DM_insulin	leukocyte	*HCN4*	-0.545577741	0.002757786	0.011330438	1
T2DM_insulin	leukocyte	*SEMA7A*	-0.863777609	0.006195285	0.011570498	1
T2DM_insulin	leukocyte	*AP1AR*	-0.335824823	0.002923186	0.011330438	1
T2DM_insulin	leukocyte	*BBS7*	-0.4370972	0.006090107	0.011570498	1
T2DM_insulin	leukocyte	*RAP1GDS1*	-0.46117313	0.003341169	0.011330438	1
T2DM_insulin	leukocyte	*PRDM5*	-0.362192551	0.006772358	0.011991254	1
T2DM_insulin	leukocyte	*NUP54*	-0.288349889	0.016659805	0.023285796	1
T2DM_insulin	leukocyte	*G3BP2*	-0.316355166	0.002746937	0.011330438	1
T2DM_insulin	leukocyte	*SCARB2*	-0.394042997	0.001008861	0.011330438	1
T2DM_insulin	leukocyte	*CNOT6L*	-0.462806155	0.003337758	0.011330438	1
T2DM_insulin	leukocyte	*USO1*	-0.316732581	0.002997002	0.011330438	1
T2DM_insulin	leukocyte	*SHROOM3*	-0.684709558	0.003080778	0.011330438	1
T2DM_insulin	leukocyte	*ANXA3*	-0.262529855	0.004530624	0.011330438	1
T2DM_insulin	leukocyte	*PPA2*	-0.368485003	0.003956416	0.011330438	1
T2DM_insulin	leukocyte	*EGF*	-1.042037141	0.003933695	0.011330438	1
T2DM_insulin	leukocyte	*MAPK8IP3*	-0.407702204	0.003749578	0.011330438	1
T2DM_insulin	leukocyte	*RGS3*	-0.372289274	0.001747667	0.011330438	1
T2DM_insulin	leukocyte	*ETV6*	-0.677828977	0.00618112	0.011570498	1
T2DM_insulin	leukocyte	*ALG10*	-0.66759482	0.005284336	0.011419801	1
T2DM_insulin	leukocyte	*SLCO1C1*	-6.163891783	0.003587287	0.011330438	1
T2DM_insulin	leukocyte	*ZCRB1*	-0.338301545	0.003240498	0.011330438	1
T2DM_insulin	leukocyte	*C1RL*	-0.423956346	0.01280758	0.018623605	1
T2DM_insulin	leukocyte	*CLSTN3*	-0.463085595	0.023512287	0.031315178	1
T2DM_insulin	leukocyte	*KLRG1*	-1.825404623	0.035361567	0.044774605	1
T2DM_insulin	leukocyte	*VAMP1*	0.304506649	0.010536303	0.016082803	1
T2DM_insulin	leukocyte	*TAPBPL*	-0.327935797	0.009390089	0.014787038	1
T2DM_insulin	leukocyte	*PEX5*	-0.361983643	0.005960283	0.011570498	1
T2DM_insulin	leukocyte	*SLC38A4*	-0.534017567	0.005156229	0.011330438	1
T2DM_insulin	leukocyte	*LRIG3*	-0.394543558	0.00326244	0.011330438	1
T2DM_insulin	leukocyte	*GLIPR1*	-0.441702411	0.004069273	0.011330438	1
T2DM_insulin	leukocyte	*SNRPF*	-0.285596386	0.006228594	0.011570498	1
T2DM_insulin	leukocyte	*ASCL1*	-0.559301226	0.008088711	0.013302865	1
T2DM_insulin	leukocyte	*GLTP*	-0.303174333	0.004253295	0.011330438	1
T2DM_insulin	leukocyte	*MTMR6*	-0.476415731	0.00370785	0.011330438	1
T2DM_insulin	leukocyte	*PDX1*	-0.516516772	0.003517674	0.011330438	1
T2DM_insulin	leukocyte	*SUOX*	-0.489569672	0.002057217	0.011330438	1
T2DM_insulin	leukocyte	*CCDC65*	-0.529888108	0.014447953	0.020587417	1
T2DM_insulin	leukocyte	*SLC39A5*	-0.200706255	0.024625779	0.032654299	1
T2DM_insulin	leukocyte	*KANSL2*	-0.901176322	0.002038252	0.011330438	1
T2DM_insulin	leukocyte	*CERS5*	-0.43465262	0.008927125	0.014211781	1
T2DM_insulin	leukocyte	*ZNF740*	-0.740944222	0.007567178	0.012760276	1
T2DM_insulin	leukocyte	*WDFY2*	-0.443429491	0.002830929	0.011330438	1
T2DM_insulin	leukocyte	*GRTP1*	-0.768752285	0.01024981	0.015761841	1
T2DM_insulin	leukocyte	*TSSK4*	-2.717042255	0.03581348	0.045274858	1
T2DM_insulin	leukocyte	*TMX1*	-0.53781707	0.002691404	0.011330438	1
T2DM_insulin	leukocyte	*RAB15*	-0.611212646	0.001171768	0.011330438	1
T2DM_insulin	leukocyte	*PTGR2*	-0.270510459	0.012346115	0.01808646	1
T2DM_insulin	leukocyte	*NIPA2*	-0.312660179	0.004440694	0.011330438	1
T2DM_insulin	leukocyte	*SERF2*	-0.279682917	0.00335894	0.011330438	1
T2DM_insulin	leukocyte	*BNIP2*	-0.610952881	0.002506766	0.011330438	1
T2DM_insulin	leukocyte	*GTF2A2*	-0.3406286	0.003200401	0.011330438	1
T2DM_insulin	leukocyte	*SRP14*	-0.272306528	0.003815703	0.011330438	1
T2DM_insulin	leukocyte	*BAHD1*	-0.462175165	0.002334275	0.011330438	1
T2DM_insulin	leukocyte	*DISP2*	-0.320730551	0.003897015	0.011330438	1
T2DM_insulin	leukocyte	*TLE3*	-0.834580049	0.002057482	0.011330438	1
T2DM_insulin	leukocyte	*SCAPER*	-0.345422488	0.003567792	0.011330438	1
T2DM_insulin	leukocyte	*TSPAN3*	-0.287701694	0.0044737	0.011330438	1
T2DM_insulin	leukocyte	*MAN2C1*	-0.398117299	0.002002643	0.011330438	1
T2DM_insulin	leukocyte	*DNAJA4*	-0.326831803	0.004955237	0.011330438	1
T2DM_insulin	leukocyte	*TPM1*	-0.208321646	0.004881979	0.011330438	1
T2DM_insulin	leukocyte	*IGF1R*	-0.311763621	0.00436457	0.011330438	1

T2DM_insulin	leukocyte	*ULK3*	-0.373606802	0.005258175	0.011402654	1
T2DM_insulin	leukocyte	*CELF6*	-0.448228055	0.004666427	0.011330438	1
T2DM_insulin	leukocyte	*POLG*	-0.586166019	0.003918539	0.011330438	1
T2DM_insulin	leukocyte	*WDR93*	-1.215286476	0.021174523	0.028599073	1
T2DM_insulin	leukocyte	*UNC45A*	-0.448583188	0.001727362	0.011330438	1
T2DM_insulin	leukocyte	*IQGAP1*	-0.36381092	0.005112719	0.011330438	1
T2DM_insulin	leukocyte	*PMM2*	-0.376990708	0.003831385	0.011330438	1
T2DM_insulin	leukocyte	*SLC5A2*	-1.445472142	0.005964912	0.011570498	1
T2DM_insulin	leukocyte	*UQCRC2*	-0.369055828	0.006365395	0.011665654	1
T2DM_insulin	leukocyte	*ARHGAP17*	-0.923233851	0.002528572	0.011330438	1
T2DM_insulin	leukocyte	*TXNL4B*	-0.872233072	0.002918902	0.011330438	1
T2DM_insulin	leukocyte	*MARVELD3*	-0.287071945	0.012643828	0.018433247	1
T2DM_insulin	leukocyte	*KIFC3*	-0.420732352	0.003870591	0.011330438	1
T2DM_insulin	leukocyte	*NUDT7*	-0.332567495	0.006139498	0.011570498	1
T2DM_insulin	leukocyte	*GCSH*	-0.355981073	0.003320912	0.011330438	1
T2DM_insulin	leukocyte	*MAP1LC3B*	-0.309315037	0.003192407	0.011330438	1
T2DM_insulin	leukocyte	*ADAD2*	-4.616328537	0.031338017	0.040198194	1
T2DM_insulin	leukocyte	*OSGIN1*	-0.290659234	0.005362043	0.011421159	1
T2DM_insulin	leukocyte	*RPS2*	-0.23765556	0.003061809	0.011330438	1
T2DM_insulin	leukocyte	*PDPK1*	-0.444504392	0.006474553	0.011737322	1
T2DM_insulin	leukocyte	*DEF8*	-0.433960844	0.002433845	0.011330438	1
T2DM_insulin	leukocyte	*GAS8*	-0.612470201	0.006813743	0.012014984	1
T2DM_insulin	leukocyte	*RANBP10*	-0.346575591	0.008976579	0.014278939	1
T2DM_insulin	leukocyte	*PRPSAP2*	-0.428789785	0.008168022	0.013388469	1
T2DM_insulin	leukocyte	*TOM1L1*	-0.356535541	0.004931885	0.011330438	1
T2DM_insulin	leukocyte	*C17orf80*	-0.4989399	0.023256166	0.031016115	1
T2DM_insulin	leukocyte	*VPS53*	-0.370746313	0.006174219	0.011570498	1
T2DM_insulin	leukocyte	*NPEPPS*	-0.505448626	0.00341291	0.011330438	1
T2DM_insulin	leukocyte	*ARSG*	-1.159688955	0.015844156	0.02228049	1
T2DM_insulin	leukocyte	*PTRH2*	-0.421401265	0.003927702	0.011330438	1
T2DM_insulin	leukocyte	*GNAL*	-0.54814979	0.00520173	0.011378137	1
T2DM_insulin	leukocyte	*RPRD1A*	-0.375843122	0.004171026	0.011330438	1
T2DM_insulin	leukocyte	*NPC1*	-0.788049583	0.001952964	0.011330438	1
T2DM_insulin	leukocyte	*SLC14A1*	-0.538056878	0.011381545	0.017041955	1
T2DM_insulin	leukocyte	*ZMYND15*	0.082696382	0.015169563	0.021477915	1
T2DM_insulin	leukocyte	*SAT2*	-0.455902113	0.002242751	0.011330438	1
T2DM_insulin	leukocyte	*CCDC40*	-0.316774201	0.027301851	0.035684954	1
T2DM_insulin	leukocyte	*ARHGDIA*	-0.33637781	0.005203999	0.011378137	1
T2DM_insulin	leukocyte	*TMC6*	-0.591292312	0.005978406	0.011570498	1
T2DM_insulin	leukocyte	*TTYH2*	-0.65521257	0.006168821	0.011570498	1
T2DM_insulin	leukocyte	*EIF4A3*	-0.454589225	0.001471211	0.011330438	1
T2DM_insulin	leukocyte	*TBCD*	-0.634746742	0.004281538	0.011330438	1
T2DM_insulin	leukocyte	*FN3KRP*	-0.53622622	0.002021448	0.011330438	1
T2DM_insulin	leukocyte	*NARF*	-0.536615551	0.002423634	0.011330438	1
T2DM_insulin	leukocyte	*TRIM65*	-0.470339972	0.020055713	0.027303845	1
T2DM_insulin	leukocyte	*RNF157*	-0.451132164	0.003316589	0.011330438	1
T2DM_insulin	leukocyte	*WDR45B*	-0.309174788	0.004785012	0.011330438	1
T2DM_insulin	leukocyte	*TNFRSF11A*	-0.343461747	0.005103196	0.011330438	1
T2DM_insulin	leukocyte	*ZCCHC2*	-0.505356875	0.004422233	0.011330438	1
T2DM_insulin	leukocyte	*PMAIP1*	-0.34812995	0.005018329	0.011330438	1
T2DM_insulin	leukocyte	*NT5C3B*	-0.417742388	0.00227405	0.011330438	1
T2DM_insulin	leukocyte	*PIP4K2B*	-0.355181615	0.007047586	0.012237751	1
T2DM_insulin	leukocyte	*TXNL4A*	-0.332992193	0.003426907	0.011330438	1
T2DM_insulin	leukocyte	*MVB12A*	-0.340656726	0.034983381	0.044367238	1
T2DM_insulin	leukocyte	*SH3GL1*	-0.36623676	0.00267431	0.011330438	1
T2DM_insulin	leukocyte	*DUS3L*	-0.253978736	0.015956828	0.022398848	1
T2DM_insulin	leukocyte	*SIRT3*	-0.523113936	0.004389567	0.011330438	1
T2DM_insulin	leukocyte	*IFITM3*	-0.233107337	0.006128947	0.011570498	1
T2DM_insulin	leukocyte	*COL6A1*	-0.549626211	0.001459855	0.011330438	1
T2DM_insulin	leukocyte	*IFNAR1*	-0.388070067	0.001323952	0.011330438	1
T2DM_insulin	leukocyte	*TMEM50B*	-0.371653116	0.004693995	0.011330438	1
T2DM_insulin	leukocyte	*AKT1*	-0.521030953	0.005535138	0.011449692	1
T2DM_insulin	leukocyte	*CBLC*	-0.385350527	0.004461019	0.011330438	1
T2DM_insulin	leukocyte	*CTU1*	-0.661017034	0.001611746	0.011330438	1

T2DM_insulin	leukocyte	*RERE*	-0.31814621	0.003839294	0.011330438	1
T2DM_insulin	leukocyte	*FHAD1*	-0.352160966	0.011975882	0.017715828	1
T2DM_insulin	leukocyte	*PGD*	-0.523085152	0.001273277	0.011330438	1
T2DM_insulin	leukocyte	*CNKSR1*	-0.684314544	0.006267912	0.011570498	1
T2DM_insulin	leukocyte	*ZNF593*	-0.364038543	0.003048654	0.011330438	1
T2DM_insulin	leukocyte	*FCN3*	-0.170502251	0.026656782	0.034981461	1
T2DM_insulin	leukocyte	*CELA3A*	-0.173635269	0.002993084	0.011330438	1
T2DM_insulin	leukocyte	*HSPG2*	-0.267597514	0.000343122	0.011330438	1
T2DM_insulin	leukocyte	*ITGB3BP*	-0.281636931	0.036834317	0.046444812	1
T2DM_insulin	leukocyte	*PRKACB*	-0.432084845	0.003527139	0.011330438	1
T2DM_insulin	leukocyte	*PIGK*	-0.635408405	0.002712496	0.011330438	1
T2DM_insulin	leukocyte	*RPS8*	-0.222505796	0.004125345	0.011330438	1
T2DM_insulin	leukocyte	*SYPL2*	-1.108549531	0.00568288	0.011549238	1
T2DM_insulin	leukocyte	*SLC44A3*	-0.360243567	0.00623325	0.011570498	1
T2DM_insulin	leukocyte	*ZNF697*	-0.499907053	0.017511487	0.024234812	1
T2DM_insulin	leukocyte	*CELSR2*	-0.437962375	0.006562276	0.011821724	1
T2DM_insulin	leukocyte	*ALDH9A1*	-0.333912613	0.003809817	0.011330438	1
T2DM_insulin	leukocyte	*ATP1B1*	-0.231337691	0.00336095	0.011330438	1
T2DM_insulin	leukocyte	*TIPRL*	-0.33960932	0.002933346	0.011330438	1
T2DM_insulin	leukocyte	*NME7*	-0.559428802	0.003344815	0.011330438	1
T2DM_insulin	leukocyte	*MGST3*	-0.34343253	0.005219437	0.011389288	1
T2DM_insulin	leukocyte	*LYPLAL1*	-0.446475212	0.003898932	0.011330438	1
T2DM_insulin	leukocyte	*SNX27*	-0.622974872	0.002054282	0.011330438	1
T2DM_insulin	leukocyte	*CTSK*	-1.104437086	0.007286352	0.012457822	1
T2DM_insulin	leukocyte	*SELENBP1*	-0.43315574	0.005973539	0.011570498	1
T2DM_insulin	leukocyte	*POGZ*	-0.33125534	0.021585119	0.029103614	1
T2DM_insulin	leukocyte	*GABPB2*	-0.583304597	0.010081907	0.015574095	1
T2DM_insulin	leukocyte	*SLC27A3*	-0.455552045	0.012769292	0.018595459	1
T2DM_insulin	leukocyte	*KCNN3*	-0.690379164	0.003793503	0.011330438	1
T2DM_insulin	leukocyte	*RIT1*	-0.560142433	0.023711609	0.031559739	1
T2DM_insulin	leukocyte	*SRP9*	-0.365633915	0.002933885	0.011330438	1
T2DM_insulin	leukocyte	*CNIH4*	-0.369856204	0.00396411	0.011330438	1
T2DM_insulin	leukocyte	*PSEN2*	-0.705628689	0.006089523	0.011570498	1
T2DM_insulin	leukocyte	*PYCR2*	-0.416226896	0.00416222	0.011330438	1
T2DM_insulin	leukocyte	*LBR*	-0.457533777	0.003843419	0.011330438	1
T2DM_insulin	leukocyte	*ARL8A*	-0.598263413	0.002434064	0.011330438	1
T2DM_insulin	leukocyte	*EML4*	-0.593501924	0.003942776	0.011330438	1
T2DM_insulin	leukocyte	*WDPCP*	-0.979957395	0.002882823	0.011330438	1
T2DM_insulin	leukocyte	*REG3G*	-0.433433218	0.001735703	0.011330438	1
T2DM_insulin	leukocyte	*CIAO1*	-0.551225466	0.001455717	0.011330438	1
T2DM_insulin	leukocyte	*ZNF514*	-0.436291074	0.0020076	0.011330438	1
T2DM_insulin	leukocyte	*TPRKB*	-0.329892926	0.00376396	0.011330438	1
T2DM_insulin	leukocyte	*TEX261*	-0.432082811	0.003605607	0.011330438	1
T2DM_insulin	leukocyte	*RALB*	-0.412937117	0.002525238	0.011330438	1
T2DM_insulin	leukocyte	*RABL2A*	-0.518822308	0.009884997	0.015351048	1
T2DM_insulin	leukocyte	*SLC20A1*	-0.569600995	0.004197736	0.011330438	1
T2DM_insulin	leukocyte	*LYG1*	-1.623504592	0.032709476	0.041747209	1
T2DM_insulin	leukocyte	*GULP1*	-0.369492473	0.004302897	0.011330438	1
T2DM_insulin	leukocyte	*FAM171B*	-0.327405235	0.005420429	0.011421159	1
T2DM_insulin	leukocyte	*METTL21A*	-0.382401445	0.00505439	0.011330438	1
T2DM_insulin	leukocyte	*SUMF1*	-0.535704949	0.002835609	0.011330438	1
T2DM_insulin	leukocyte	*HES6*	-0.913639519	0.002719343	0.011330438	1
T2DM_insulin	leukocyte	*TAMM41*	-0.385898975	0.020166528	0.027426199	1
T2DM_insulin	leukocyte	*RAB5A*	-0.35367034	0.003869248	0.011330438	1
T2DM_insulin	leukocyte	*CNTN4*	-0.469134922	0.000465254	0.011330438	1
T2DM_insulin	leukocyte	*POMGNT2*	-0.694388094	0.001964678	0.011330438	1
T2DM_insulin	leukocyte	*CTDSPL*	-0.34928384	0.004389224	0.011330438	1
T2DM_insulin	leukocyte	*IL17RD*	-1.408312198	0.01186402	0.017638109	1
T2DM_insulin	leukocyte	*UBA3*	-0.52890423	0.005548074	0.011460768	1
T2DM_insulin	leukocyte	*ARL6IP5*	-0.348489114	0.003196885	0.011330438	1
T2DM_insulin	leukocyte	*LIMD1*	-0.654986272	0.002362431	0.011330438	1
T2DM_insulin	leukocyte	*NXPE3*	-0.29849482	0.019409079	0.026525186	1
T2DM_insulin	leukocyte	*PHLDB2*	-0.277562443	0.004948203	0.011330438	1
T2DM_insulin	leukocyte	*ABHD10*	-0.468103702	0.001694	0.011330438	1

T2DM_insulin	leukocyte	*TAGLN3*	-0.217708819	0.012198769	0.017937534	1
T2DM_insulin	leukocyte	*EIF2A*	-0.317651446	0.003580659	0.011330438	1
T2DM_insulin	leukocyte	*ALDH1L1*	-0.517906636	0.005870655	0.011570498	1
T2DM_insulin	leukocyte	*TRPC1*	-0.449940132	0.004591737	0.011330438	1
T2DM_insulin	leukocyte	*NCEH1*	-0.238341174	0.004304179	0.011330438	1
T2DM_insulin	leukocyte	*AMT*	-0.641950349	0.004201916	0.011330438	1
T2DM_insulin	leukocyte	*DGKQ*	-0.359269173	0.015699092	0.022116074	1
T2DM_insulin	leukocyte	*SCD5*	-0.316125598	0.004629857	0.011330438	1
T2DM_insulin	leukocyte	*PYURF*	-0.304515852	0.003772962	0.011330438	1
T2DM_insulin	leukocyte	*DDIT4L*	-0.873066718	0.010872922	0.016483075	1
T2DM_insulin	leukocyte	*TIFA*	-0.478391552	0.003171893	0.011330438	1
T2DM_insulin	leukocyte	*CYP4V2*	-0.736003638	0.0083023	0.013512733	1
T2DM_insulin	leukocyte	*SSBP2*	-0.30739021	0.004737	0.011330438	1
T2DM_insulin	leukocyte	*IQGAP2*	0.220452038	0.016901014	0.023572625	1
T2DM_insulin	leukocyte	*GTF2H2*	-0.986241063	0.002597369	0.011330438	1
T2DM_insulin	leukocyte	*BTF3*	-0.286686252	0.0029416	0.011330438	1
T2DM_insulin	leukocyte	*SPATA9*	-2.522025812	0.013591067	0.019553021	1
T2DM_insulin	leukocyte	*COMMD10*	-0.438012982	0.004219835	0.011330438	1
T2DM_insulin	leukocyte	*ATG12*	-0.357295311	0.003241993	0.011330438	1
T2DM_insulin	leukocyte	*PCYOX1L*	-1.833838425	0.004529217	0.011330438	1
T2DM_insulin	leukocyte	*ZMAT2*	-0.458653177	0.003233544	0.011330438	1
T2DM_insulin	leukocyte	*KLHL3*	-1.271159493	0.016898931	0.023572625	1
T2DM_insulin	leukocyte	*DCDC2*	-0.283904266	0.005062746	0.011330438	1
T2DM_insulin	leukocyte	*TRIM7*	-1.448836108	0.000318412	0.011330438	1
T2DM_insulin	leukocyte	*ANO7*	-0.957640616	0.010989601	0.016619762	1
T2DM_insulin	leukocyte	*RPL7L1*	-0.345641996	0.003263617	0.011330438	1
T2DM_insulin	leukocyte	*IRAK1BP1*	-0.322390522	0.004891247	0.011330438	1
T2DM_insulin	leukocyte	*MMS22L*	-0.572632237	0.00269678	0.011330438	1
T2DM_insulin	leukocyte	*FAXC*	-0.582879048	0.003745892	0.011330438	1
T2DM_insulin	leukocyte	*TBC1D32*	-1.357199145	0.001371865	0.011330438	1
T2DM_insulin	leukocyte	*ABRACL*	-0.416697815	0.002023635	0.011330438	1
T2DM_insulin	leukocyte	*DYNLT1*	-0.323836723	0.002159197	0.011330438	1
T2DM_insulin	leukocyte	*WTAP*	-0.333713068	0.001758601	0.011330438	1
T2DM_insulin	leukocyte	*SLC22A3*	-2.232886056	0.016486172	0.023075942	1
T2DM_insulin	leukocyte	*VWDE*	-0.357589252	0.006059453	0.011570498	1
T2DM_insulin	leukocyte	*GNA12*	-0.583029289	0.004722009	0.011330438	1
T2DM_insulin	leukocyte	*SDK1*	-0.407964298	0.01207629	0.017813386	1
T2DM_insulin	leukocyte	*CCZ1B*	-0.543042573	0.008835973	0.014115597	1
T2DM_insulin	leukocyte	*RBAK*	-0.819941238	0.003747698	0.011330438	1
T2DM_insulin	leukocyte	*POMZP3*	-0.373672749	0.011709209	0.017459386	1
T2DM_insulin	leukocyte	*GIGYF1*	-0.881796562	0.000562569	0.011330438	1
T2DM_insulin	leukocyte	*TRIM4*	-0.345228058	0.002479636	0.011330438	1
T2DM_insulin	leukocyte	*TMEM209*	-0.589445664	0.002359268	0.011330438	1
T2DM_insulin	leukocyte	*RAB19*	-3.69904795	0.000337227	0.011330438	1
T2DM_insulin	leukocyte	*CSGALNACT1*	-0.331047338	0.006780192	0.011999719	1
T2DM_insulin	leukocyte	*CCDC25*	-0.314933371	0.003046172	0.011330438	1
T2DM_insulin	leukocyte	*BIN3*	-0.963958472	0.006286735	0.011585639	1
T2DM_insulin	leukocyte	*CHMP7*	-0.600044697	0.00192066	0.011330438	1
T2DM_insulin	leukocyte	*STAR*	0.247238967	0.021829315	0.029382453	1
T2DM_insulin	leukocyte	*LACTB2*	-0.340244954	0.003945221	0.011330438	1
T2DM_insulin	leukocyte	*TERF1*	-0.733938049	0.00231611	0.011330438	1
T2DM_insulin	leukocyte	*LRP12*	-0.38168704	0.003823501	0.011330438	1
T2DM_insulin	leukocyte	*ZNF7*	-0.324681958	0.005079482	0.011330438	1
T2DM_insulin	leukocyte	*AK3*	-0.337032032	0.003423005	0.011330438	1
T2DM_insulin	leukocyte	*CER1*	1.300058342	0.017632378	0.024384939	1
T2DM_insulin	leukocyte	*C9orf72*	-0.569451182	0.002032258	0.011330438	1
T2DM_insulin	leukocyte	*FBXO10*	-0.498194894	0.012025316	0.017759823	1
T2DM_insulin	leukocyte	*SIGMAR1*	-0.429120106	0.004287119	0.011330438	1
T2DM_insulin	leukocyte	*ZNF462*	-0.337227652	0.004057433	0.011330438	1
T2DM_insulin	leukocyte	*UGCG*	-0.364597823	0.005150618	0.011330438	1
T2DM_insulin	leukocyte	*ALAD*	-0.39201923	0.009595762	0.015026604	1
T2DM_insulin	leukocyte	*SURF4*	-0.327381663	0.002412776	0.011330438	1
T2DM_insulin	leukocyte	*SURF1*	-0.354701825	0.011694451	0.017450626	1
T2DM_insulin	leukocyte	*SURF6*	-0.546704591	0.00305831	0.011330438	1

T2DM_insulin	leukocyte	*SH3GLB2*	-0.612594695	0.002169436	0.011330438	1
T2DM_insulin	leukocyte	*LCN2*	-0.156437907	0.005791384	0.011570498	1
T2DM_insulin	leukocyte	*GPR107*	-0.47995759	0.00642179	0.01170553	1
T2DM_insulin	leukocyte	*INPP5E*	-0.52932459	0.037506605	0.047231876	1
T2DM_insulin	leukocyte	*SEC16A*	-0.420633031	0.018363951	0.025280997	1
T2DM_insulin	leukocyte	*MSRB2*	-0.616272887	0.001222098	0.011330438	1
T2DM_insulin	leukocyte	*RSU1*	-0.489544364	0.003041568	0.011330438	1
T2DM_insulin	leukocyte	*GLUD1*	-0.441813198	0.003169566	0.011330438	1
T2DM_insulin	leukocyte	*FRA10AC1*	-0.280016678	0.006196536	0.011570498	1
T2DM_insulin	leukocyte	*DNAJB12*	-0.466595113	0.003876396	0.011330438	1
T2DM_insulin	leukocyte	*MTG1*	-0.300394955	0.009680131	0.015104542	1
T2DM_insulin	leukocyte	*GSTO1*	-0.466922055	0.004062897	0.011330438	1
T2DM_insulin	leukocyte	*HSD17B12*	-0.286040659	0.000644306	0.011330438	1
T2DM_insulin	leukocyte	*APIP*	-0.418677371	0.000852188	0.011330438	1
T2DM_insulin	leukocyte	*PTPRJ*	-0.511493919	0.003462188	0.011330438	1
T2DM_insulin	leukocyte	*CCDC82*	-0.408122795	0.001551558	0.011330438	1
T2DM_insulin	leukocyte	*RPS3*	-0.246337929	0.00275894	0.011330438	1
T2DM_insulin	leukocyte	*AASDHPPT*	-0.395782652	0.003455703	0.011330438	1
T2DM_insulin	leukocyte	*GLB1L2*	-0.306422782	0.028925257	0.037494997	1
T2DM_insulin	leukocyte	*FADS1*	-0.254121412	0.024459418	0.032455608	1
T2DM_insulin	leukocyte	*TMC2*	-23.12039674	0.034229131	0.043466792	1
T2DM_insulin	leukocyte	*EML3*	-1.334214472	0.015454808	0.021810191	1
T2DM_insulin	leukocyte	*CHEK1*	-0.471389747	0.00491309	0.011330438	1
T2DM_insulin	leukocyte	*SIDT2*	-0.539552116	0.000953716	0.011330438	1
T2DM_insulin	leukocyte	*TAGLN*	-0.424764986	0.000123018	0.011330438	0.483337044
T2DM_insulin	leukocyte	*DUSP15*	-0.46355847	0.006408551	0.01170553	1
T2DM_insulin	leukocyte	*DSN1*	-0.458712772	0.003813873	0.011330438	1
T2DM_insulin	leukocyte	*YTHDF1*	-0.41805711	0.00343894	0.011330438	1
T2DM_insulin	leukocyte	*CABLES2*	-0.619611946	0.000519617	0.011330438	1
T2DM_insulin	leukocyte	*TRPT1*	-0.360660068	0.006500686	0.011770137	1
T2DM_insulin	leukocyte	*MPP7*	-0.307171541	0.005282616	0.011419801	1
T2DM_insulin	leukocyte	*DCUN1D2*	-0.413378119	0.014202539	0.020306323	1
T2DM_insulin	leukocyte	*TMCO3*	-0.417880628	0.005473492	0.011427973	1
T2DM_insulin	leukocyte	*TMEM218*	-0.587860868	0.002932581	0.011330438	1
T2DM_insulin	leukocyte	*MIA2*	-0.357275903	0.004038456	0.011330438	1
T2DM_insulin	leukocyte	*LYPD1*	-0.598119703	0.005778164	0.011570498	1
T2DM_insulin	leukocyte	*LYPD6B*	-0.977676872	0.005752819	0.011570498	1
T2DM_insulin	leukocyte	*WDR17*	-0.313424072	0.006234177	0.011570498	1
T2DM_insulin	leukocyte	*CNDP1*	-1.469002732	0.029322954	0.037947921	1
T2DM_insulin	leukocyte	*PRSS23*	-0.310645559	0.002376219	0.011330438	1
T2DM_insulin	leukocyte	*MTMR12*	-0.320251473	0.00366507	0.011330438	1
T2DM_insulin	leukocyte	*DOCK1*	-0.397461318	0.003595691	0.011330438	1
T2DM_insulin	leukocyte	*IL18*	0.200304253	0.021857519	0.02941034	1
T2DM_insulin	leukocyte	*TMEM86A*	-0.461290576	0.005953587	0.011570498	1
T2DM_insulin	leukocyte	*PLBD2*	-0.89744972	0.016696268	0.023311883	1
T2DM_insulin	leukocyte	*DLG5*	-0.376949624	0.002976407	0.011330438	1
T2DM_insulin	leukocyte	*SLC2A13*	-0.296838403	0.003753065	0.011330438	1
T2DM_insulin	leukocyte	*MAGI1*	-0.354947947	0.007798014	0.01301113	1
T2DM_insulin	leukocyte	*CSNK1G3*	-0.42829251	0.004372078	0.011330438	1
T2DM_insulin	leukocyte	*AKAP6*	-0.476973754	0.003258932	0.011330438	1
T2DM_insulin	leukocyte	*MBIP*	-0.412186496	0.004976622	0.011330438	1
T2DM_insulin	leukocyte	*MIPOL1*	-0.348049131	0.005945903	0.011570498	1
T2DM_insulin	leukocyte	*TMEM18*	-0.403443878	0.003640021	0.011330438	1
T2DM_insulin	leukocyte	*NDUFC2*	-0.309766108	0.00248209	0.011330438	1
T2DM_insulin	leukocyte	*VIPAS39*	-0.655276379	0.0024683	0.011330438	1
T2DM_insulin	leukocyte	*C4orf33*	-0.653947524	0.018988712	0.026005146	1
T2DM_insulin	leukocyte	*QDPR*	-0.260311131	0.008691742	0.013955805	1
T2DM_insulin	leukocyte	*TEX9*	-0.691307838	0.002596648	0.011330438	1
T2DM_insulin	leukocyte	*ZNF827*	-0.292523741	0.0169182	0.023588221	1
T2DM_insulin	leukocyte	*AKR1C2*	-1.169492231	0.000158058	0.011330438	0.621008552
T2DM_insulin	leukocyte	*DPYSL4*	-0.401648757	0.030762229	0.039537062	1
T2DM_insulin	leukocyte	*KIN*	-0.657689699	0.00309228	0.011330438	1
T2DM_insulin	leukocyte	*TMEM45B*	-0.547928033	0.011947651	0.017706833	1
T2DM_insulin	leukocyte	*CENPU*	-0.753909802	0.038780115	0.048695133	1

T2DM_insulin	leukocyte	*BICD1*	-0.32507456	0.00538292	0.011421159	1
T2DM_insulin	leukocyte	*GUF1*	-0.892851859	0.003656805	0.011330438	1
T2DM_insulin	leukocyte	*CCL28*	-0.474868457	0.003621348	0.011330438	1
T2DM_insulin	leukocyte	*CACUL1*	-0.539451301	0.00264452	0.011330438	1
T2DM_insulin	leukocyte	*RABGAP1L*	-0.42896996	0.00348082	0.011330438	1
T2DM_insulin	leukocyte	*PTPN14*	-0.323029887	0.008484965	0.013736063	1
T2DM_insulin	leukocyte	*ARL14EP*	-0.521824839	0.002253755	0.011330438	1
T2DM_insulin	leukocyte	*EPG5*	-0.595461907	0.004561825	0.011330438	1
T2DM_insulin	leukocyte	*HAUS1*	-0.556765385	0.001527957	0.011330438	1
T2DM_insulin	leukocyte	*SPC25*	-0.538705101	0.000995138	0.011330438	1
T2DM_insulin	leukocyte	*PDK1*	-0.475077126	0.003707497	0.011330438	1
T2DM_insulin	leukocyte	*PDE3B*	-0.194865767	0.018940634	0.02596572	1
T2DM_insulin	leukocyte	*TGOLN2*	-0.397790974	0.002010137	0.011330438	1
T2DM_insulin	leukocyte	*POC5*	-0.535496108	0.001745404	0.011330438	1
T2DM_insulin	leukocyte	*GUCY1A2*	-0.377481922	0.005917362	0.011570498	1
T2DM_insulin	leukocyte	*ZNF773*	-0.285118923	0.027355924	0.03574374	1
T2DM_insulin	leukocyte	*DCLRE1C*	-0.841717283	0.028913349	0.03749193	1
T2DM_insulin	leukocyte	*ZSCAN1*	-0.411974269	0.002672526	0.011330438	1
T2DM_insulin	leukocyte	*USP12*	-0.328244009	0.007527499	0.012715195	1
T2DM_insulin	leukocyte	*CCDC50*	-0.30024461	0.004210119	0.011330438	1
T2DM_insulin	leukocyte	*PLEKHH2*	-0.351754203	0.014002178	0.02006366	1
T2DM_insulin	leukocyte	*GRIA4*	-0.481693426	0.005821967	0.011570498	1
T2DM_insulin	leukocyte	*IGSF10*	-0.843614584	0.011243144	0.016873305	1
T2DM_insulin	leukocyte	*GPD1L*	-0.426092977	0.00631117	0.011614327	1
T2DM_insulin	leukocyte	*CCNO*	-0.345882436	0.031624355	0.040539018	1
T2DM_insulin	leukocyte	*RASGRP3*	-0.431422186	0.007520333	0.012715195	1
T2DM_insulin	leukocyte	*CATSPER3*	-2.066498621	0.006176679	0.011570498	1
T2DM_insulin	leukocyte	*GPR180*	-0.360056155	0.003248359	0.011330438	1
T2DM_insulin	leukocyte	*IFIT5*	-0.390864721	0.007716102	0.012933688	1
T2DM_insulin	leukocyte	*PRDM8*	0.438129831	0.014465998	0.020593083	1
T2DM_insulin	leukocyte	*ZNF117*	-0.279669529	0.033555746	0.04269447	1
T2DM_insulin	leukocyte	*MED21*	-0.602997993	0.002037626	0.011330438	1
T2DM_insulin	leukocyte	*LGI2*	-0.521665902	0.015241142	0.021555956	1
T2DM_insulin	leukocyte	*SRP19*	-0.32124025	0.003291874	0.011330438	1
T2DM_insulin	leukocyte	*DAB2*	-0.50944838	0.003066024	0.011330438	1
T2DM_insulin	leukocyte	*ACMSD*	-0.50446431	0.007497189	0.012691278	1
T2DM_insulin	leukocyte	*CAST*	-0.336202359	0.003168035	0.011330438	1
T2DM_insulin	leukocyte	*SYCP2L*	-0.883160875	0.030731225	0.039510139	1
T2DM_insulin	leukocyte	*TMEM87B*	-0.413843861	0.005335969	0.011421159	1
T2DM_insulin	leukocyte	*PLA2R1*	-1.301776435	0.005701281	0.011549238	1
T2DM_insulin	leukocyte	*SLC25A27*	-0.638184157	0.000630436	0.011330438	1
T2DM_insulin	leukocyte	*ASAP1*	-0.353048599	0.004252105	0.011330438	1
T2DM_insulin	leukocyte	*NMRAL1*	-0.414007003	0.012797735	0.01861618	1
T2DM_insulin	leukocyte	*CMTM7*	-0.302633083	0.010445139	0.015974679	1
T2DM_insulin	leukocyte	*RPIA*	-0.392333359	0.004420467	0.011330438	1
T2DM_insulin	leukocyte	*PTPRD*	-0.322373477	0.025533853	0.033699197	1
T2DM_insulin	leukocyte	*GTF2E1*	-0.936728512	0.002625953	0.011330438	1
T2DM_insulin	leukocyte	*CFDP1*	-0.333549718	0.004183903	0.011330438	1
T2DM_insulin	leukocyte	*JAZF1*	-0.578341542	0.00028188	0.011330438	1
T2DM_insulin	leukocyte	*CMIP*	-0.398616024	0.003454751	0.011330438	1
T2DM_insulin	leukocyte	*KCTD15*	-0.517351376	0.007715348	0.012933688	1
T2DM_insulin	leukocyte	*LGI4*	0.528884913	0.005706326	0.011549238	1
T2DM_insulin	leukocyte	*DDAH1*	-0.863863558	0.002064397	0.011330438	1
T2DM_insulin	leukocyte	*NUS1*	-0.452896705	0.001846235	0.011330438	1
T2DM_insulin	leukocyte	*PPP2R5E*	-0.457439281	0.002054719	0.011330438	1
T2DM_insulin	leukocyte	*THY1*	0.395461013	0.020726231	0.02807079	1
T2DM_insulin	leukocyte	*ROBO3*	-1.122611444	0.005937349	0.011570498	1
T2DM_insulin	leukocyte	*PITPNC1*	-0.442862354	0.002900724	0.011330438	1
T2DM_insulin	leukocyte	*CEP112*	-0.308581172	0.007176963	0.012365991	1
T2DM_insulin	leukocyte	*ABCA5*	-0.565260679	0.003138887	0.011330438	1
T2DM_insulin	leukocyte	*SRSF12*	-0.604743531	0.017179799	0.023872903	1
T2DM_insulin	leukocyte	*PDLIM3*	-0.268208941	0.004613694	0.011330438	1
T2DM_insulin	leukocyte	*PDE1C*	-0.484107117	0.008223787	0.013443435	1
T2DM_insulin	leukocyte	*RABGEF1*	-0.427074606	0.002452276	0.011330438	1

T2DM_insulin	leukocyte	*MRPL39*	-0.487051396	0.001975445	0.011330438	1
T2DM_insulin	leukocyte	*TSEN2*	-0.946003995	0.001953755	0.011330438	1
T2DM_insulin	leukocyte	*FLCN*	-0.618839923	0.002635413	0.011330438	1
T2DM_insulin	leukocyte	*MPPE1*	-0.590154734	0.000305529	0.011330438	1
T2DM_insulin	leukocyte	*RAB6B*	-0.473583892	0.004564305	0.011330438	1
T2DM_insulin	leukocyte	*ANKRD40*	-0.46199588	0.010609645	0.016188464	1
T2DM_insulin	leukocyte	*ATP6V1C1*	-0.360216091	0.003981966	0.011330438	1
T2DM_insulin	leukocyte	*MARVELD1*	-1.287145066	0.003884788	0.011330438	1
T2DM_insulin	leukocyte	*TRMT44*	-0.342324104	0.008575847	0.013826222	1
T2DM_insulin	leukocyte	*PPM1J*	-0.93487919	0.007586888	0.01278254	1
T2DM_insulin	leukocyte	*DBI*	-0.351151529	0.004660716	0.011330438	1
T2DM_insulin	leukocyte	*HEATR3*	-0.357000045	0.004352045	0.011330438	1
T2DM_insulin	leukocyte	*LRGUK*	-1.204118229	0.007368727	0.012552105	1
T2DM_insulin	leukocyte	*SETD9*	-0.545652125	0.000209953	0.011330438	0.824906532
T2DM_insulin	leukocyte	*MIER3*	-0.866245229	0.001073432	0.011330438	1
T2DM_insulin	leukocyte	*TTN*	-0.950813499	0.002978291	0.011330438	1
T2DM_insulin	leukocyte	*DEPTOR*	-0.399308955	0.011842439	0.0176246	1
T2DM_insulin	leukocyte	*RMND1*	-0.645026773	0.004347143	0.011330438	1
T2DM_insulin	leukocyte	*TMBIM4*	-0.358751835	0.002232859	0.011330438	1
T2DM_insulin	leukocyte	*PSD3*	-0.337481157	0.004745627	0.011330438	1
T2DM_insulin	leukocyte	*FAM161B*	-0.480233189	0.035296194	0.044706237	1
T2DM_insulin	leukocyte	*MMP16*	-0.302775395	0.010655868	0.016227482	1
T2DM_insulin	leukocyte	*ADK*	-0.419000433	0.004796867	0.011330438	1
T2DM_insulin	leukocyte	*BATF*	-5.549494021	0.005673737	0.011549238	1
T2DM_insulin	leukocyte	*DRAM2*	-0.595294648	0.006700656	0.011939628	1
T2DM_insulin	leukocyte	*ART3*	-2.418196674	0.000291117	0.011330438	1
T2DM_insulin	leukocyte	*CCT8*	-0.31408483	0.00348458	0.011330438	1
T2DM_insulin	leukocyte	*CDK20*	-0.347598136	0.008225507	0.013443435	1
T2DM_insulin	leukocyte	*SFXN2*	-0.278923491	0.038732577	0.048654903	1
T2DM_insulin	leukocyte	*TDRD9*	-0.246008645	0.006621867	0.01187463	1
T2DM_insulin	leukocyte	*HKDC1*	-0.901977145	0.009206611	0.014556448	1
T2DM_insulin	leukocyte	*ZDHHC5*	-0.478196771	0.002287329	0.011330438	1
T2DM_insulin	leukocyte	*MED19*	-0.376039948	0.003422256	0.011330438	1
T2DM_insulin	leukocyte	*MAPK13*	-0.419135739	0.007737498	0.012941946	1
T2DM_insulin	leukocyte	*COX6A2*	-0.483134425	0.006809146	0.012014984	1
T2DM_insulin	leukocyte	*MPV17L*	-0.389489044	0.003663513	0.011330438	1
T2DM_insulin	leukocyte	*TATDN2*	-0.660820173	0.003827663	0.011330438	1
T2DM_insulin	leukocyte	*EXOG*	-0.487109301	0.00344694	0.011330438	1
T2DM_insulin	leukocyte	*ZFYVE9*	-0.427789594	0.003503384	0.011330438	1
T2DM_insulin	leukocyte	*SLC6A1*	-2.210740022	0.005697247	0.011549238	1
T2DM_insulin	leukocyte	*NECAP2*	-0.502784574	0.003089724	0.011330438	1
T2DM_insulin	leukocyte	*STEAP2*	-0.598315918	0.003535162	0.011330438	1
T2DM_insulin	leukocyte	*GATAD1*	-0.508267616	0.002566415	0.011330438	1
T2DM_insulin	leukocyte	*CLEC18A*	-1.83091237	1.17E-05	0.011330438	0.045954047
T2DM_insulin	leukocyte	*DHRS4*	-0.453733596	0.003721783	0.011330438	1
T2DM_insulin	leukocyte	*DHRS1*	-0.443799638	0.009980412	0.015458818	1
T2DM_insulin	leukocyte	*AASDH*	-0.279398233	0.013515641	0.019473031	1
T2DM_insulin	leukocyte	*SLC38A10*	-0.433937311	0.004471104	0.011330438	1
T2DM_insulin	leukocyte	*SLC37A3*	-0.310910537	0.004108342	0.011330438	1
T2DM_insulin	leukocyte	*AP3S2*	-0.449802996	0.006739042	0.011963834	1
T2DM_insulin	leukocyte	*SPPL3*	-0.437509172	0.003295018	0.011330438	1
T2DM_insulin	leukocyte	*TNFRSF14*	0.253286936	0.033140316	0.042220591	1
T2DM_insulin	leukocyte	*C12orf43*	-0.462926245	0.005682523	0.011549238	1
T2DM_insulin	leukocyte	*PEX10*	-0.620606785	0.003591204	0.011330438	1
T2DM_insulin	leukocyte	*LDLRAP1*	-0.312498749	0.005076665	0.011330438	1
T2DM_insulin	leukocyte	*KRTCAP3*	-0.382860303	0.004499189	0.011330438	1
T2DM_insulin	leukocyte	*PAFAH2*	-1.091355922	0.002480948	0.011330438	1
T2DM_insulin	leukocyte	*EXTL1*	-3.064453867	7.12E-06	0.009328675	0.027986024
T2DM_insulin	leukocyte	*TRIM63*	-5.836546447	0.002217401	0.011330438	1
T2DM_insulin	leukocyte	*XKR8*	-0.69643567	0.003404615	0.011330438	1
T2DM_insulin	leukocyte	*ESYT3*	-0.663718368	0.018335926	0.025251264	1
T2DM_insulin	leukocyte	*FAIM*	-0.408186856	0.003190046	0.011330438	1
T2DM_insulin	leukocyte	*AHCYL2*	-0.522484342	0.001974658	0.011330438	1
T2DM_insulin	leukocyte	*COPG2*	-0.36009087	0.008264452	0.013462285	1

T2DM_insulin	leukocyte	*RNF166*	-0.516491164	0.00469421	0.011330438	1
T2DM_insulin	leukocyte	*NIT1*	-0.51795305	0.011256834	0.016880954	1
T2DM_insulin	leukocyte	*NPM2*	-0.442558467	0.005912175	0.011570498	1
T2DM_insulin	leukocyte	*VWA5B1*	1.022524956	0.017375888	0.024081081	1
T2DM_insulin	leukocyte	*CDA*	-4.722123183	0.006343997	0.011637991	1
T2DM_insulin	leukocyte	*PINK1*	-0.478022874	0.002937502	0.011330438	1
T2DM_insulin	leukocyte	*DMTN*	-0.432214832	0.006004406	0.011570498	1
T2DM_insulin	leukocyte	*CCAR2*	-0.563250643	0.003100146	0.011330438	1
T2DM_insulin	leukocyte	*EPB41*	-0.39310644	0.003560592	0.011330438	1
T2DM_insulin	leukocyte	*MIS18A*	-0.465996889	0.004368789	0.011330438	1
T2DM_insulin	leukocyte	*ALG8*	-0.367802119	0.005430462	0.011421159	1
T2DM_insulin	leukocyte	*IFNAR2*	-0.540949744	0.039151526	0.049114415	1
T2DM_insulin	leukocyte	*IFNGR2*	-0.399197707	0.003054851	0.011330438	1
T2DM_insulin	leukocyte	*C1QC*	0.022194789	0.014555101	0.020697427	1
T2DM_insulin	leukocyte	*GJD2*	-0.237101409	0.007550408	0.012742935	1
T2DM_insulin	leukocyte	*CLDN14*	-0.868600063	0.011299228	0.016931605	1
T2DM_insulin	leukocyte	*HLCS*	-0.346528756	0.007303826	0.012482268	1
T2DM_insulin	leukocyte	*THEM4*	-0.329634073	0.003773879	0.011330438	1
T2DM_insulin	leukocyte	*UBR1*	-0.501982654	0.003737258	0.011330438	1
T2DM_insulin	leukocyte	*TEPP*	1.351496883	0.025745806	0.033956116	1
T2DM_insulin	leukocyte	*ZDHHC1*	-0.425670929	0.002628268	0.011330438	1
T2DM_insulin	leukocyte	*ZFYVE28*	-0.552547041	0.00366495	0.011330438	1
T2DM_insulin	leukocyte	*NPR2*	-0.365674088	0.037683103	0.047423738	1
T2DM_insulin	leukocyte	*ATAD3B*	-0.907636997	0.005636686	0.01154792	1
T2DM_insulin	leukocyte	*ANKLE1*	0.225351963	0.027375915	0.035757969	1
T2DM_insulin	leukocyte	*KALRN*	-0.50059701	0.011852826	0.017633379	1
T2DM_insulin	leukocyte	*CILP2*	-0.903447851	0.008040065	0.013235147	1
T2DM_insulin	leukocyte	*TMPRSS3*	-1.091059708	0.011201419	0.01684018	1
T2DM_insulin	leukocyte	*RSPH1*	-0.289986568	0.002761647	0.011330438	1
T2DM_insulin	leukocyte	*SLC37A1*	-0.46048703	0.002167886	0.011330438	1
T2DM_insulin	leukocyte	*NDUFV3*	-0.303846239	0.003746816	0.011330438	1
T2DM_insulin	leukocyte	*HSF2BP*	-1.075279632	0.011902769	0.01766754	1
T2DM_insulin	leukocyte	*PDXK*	-0.496676605	0.001416963	0.011330438	1
T2DM_insulin	leukocyte	*CSTB*	-0.423341935	0.004219872	0.011330438	1
T2DM_insulin	leukocyte	*ICOSLG*	0.168408026	0.031979884	0.040901356	1
T2DM_insulin	leukocyte	*ZNF66*	-1.048665273	0.006124202	0.011570498	1
T2DM_insulin	leukocyte	*FTCD*	-0.528304637	0.005745235	0.011570498	1
T2DM_insulin	leukocyte	*LSS*	-0.480062775	0.002440963	0.011330438	1
T2DM_insulin	leukocyte	*VAV2*	-0.59288871	0.005081835	0.011330438	1
T2DM_insulin	leukocyte	*DIP2A*	-0.289282703	0.017291128	0.023988997	1
T2DM_insulin	leukocyte	*ZNF208*	-0.37459832	0.003397884	0.011330438	1
T2DM_insulin	leukocyte	*SLC2A6*	-0.674328009	0.004818753	0.011330438	1
T2DM_insulin	leukocyte	*RDH13*	-0.43742184	0.008427202	0.013659437	1
T2DM_insulin	leukocyte	*MPZL3*	-0.418060716	0.006184642	0.011570498	1
T2DM_insulin	leukocyte	*S100A1*	-0.72507826	0.020162147	0.027426199	1
T2DM_insulin	leukocyte	*VPS11*	-0.750161213	0.003351872	0.011330438	1
T2DM_insulin	leukocyte	*PAQR6*	-0.444621746	0.007095266	0.01228624	1
T2DM_insulin	leukocyte	*SLC25A44*	-0.629188354	0.003662667	0.011330438	1
T2DM_insulin	leukocyte	*NBEAL2*	-0.243122638	0.023869945	0.031748481	1
T2DM_insulin	leukocyte	*LY6K*	-0.710907274	0.006794557	0.012014318	1
T2DM_insulin	leukocyte	*LY6E*	-0.387662726	0.008765064	0.014050565	1
T2DM_insulin	leukocyte	*VPS28*	-0.310615397	0.008833383	0.014115597	1
T2DM_insulin	leukocyte	*TONSL*	-1.658740058	0.011546086	0.017248887	1
T2DM_insulin	leukocyte	*ZNF333*	-1.213350747	0.009720181	0.015148984	1
T2DM_insulin	leukocyte	*RPL8*	-0.239674356	0.002816812	0.011330438	1
T2DM_insulin	leukocyte	*MAML1*	-0.708992459	0.004424457	0.011330438	1
T2DM_insulin	leukocyte	*LRWD1*	-0.566493522	0.005127468	0.011330438	1
T2DM_insulin	leukocyte	*U2AF1L4*	-0.299748319	0.008807585	0.014103329	1
T2DM_insulin	leukocyte	*ZNF382*	-0.403856081	0.004981329	0.011330438	1
T2DM_insulin	leukocyte	*DUSP14*	-0.501357385	0.003654565	0.011330438	1
T2DM_insulin	leukocyte	*PGAP3*	-0.411329223	0.006573409	0.011836354	1
T2DM_insulin	leukocyte	*PRPSAP1*	-0.430422272	0.001188807	0.011330438	1
T2DM_insulin	leukocyte	*SIGLEC11*	-6.970993462	0.004348883	0.011330438	1
T2DM_insulin	leukocyte	*DBF4B*	-0.61446535	0.002733766	0.011330438	1

T2DM_insulin	leukocyte	*LEMD2*	-0.796922865	0.003352358	0.011330438	1
T2DM_insulin	leukocyte	*CXCL16*	0.142592095	0.017188056	0.023872903	1
T2DM_insulin	leukocyte	*RPL26*	-0.253888615	0.00289386	0.011330438	1
T2DM_insulin	leukocyte	*SNRNP25*	-0.354184418	0.003090902	0.011330438	1
T2DM_insulin	leukocyte	*WDR90*	-0.554014916	0.009507715	0.014932678	1
T2DM_insulin	leukocyte	*CCDC78*	-1.329084303	0.0019542	0.011330438	1
T2DM_insulin	leukocyte	*AMDHD2*	0.338864378	0.035786064	0.045268334	1
T2DM_insulin	leukocyte	*ZNF75A*	-0.74881333	0.002305786	0.011330438	1
T2DM_insulin	leukocyte	*ASRGL1*	-0.310471276	0.006070473	0.011570498	1
T2DM_insulin	leukocyte	*TAF6L*	-0.457165713	0.016112744	0.022593495	1
T2DM_insulin	leukocyte	*ZFPL1*	-0.428230351	0.005146911	0.011330438	1
T2DM_insulin	leukocyte	*LRP5*	-0.701507544	0.004611466	0.011330438	1
T2DM_insulin	leukocyte	*PDZK1IP1*	-0.282710085	0.00398448	0.011330438	1
T2DM_insulin	leukocyte	*CTRC*	-0.193199799	0.0053709	0.011421159	1
T2DM_insulin	leukocyte	*SDC3*	-0.757293704	0.004129414	0.011330438	1
T2DM_insulin	leukocyte	*OMA1*	-0.518475585	0.010620982	0.016199471	1
T2DM_insulin	leukocyte	*TM2D1*	-0.559613538	0.002711556	0.011330438	1
T2DM_insulin	leukocyte	*FUBP1*	-0.319654367	0.004381225	0.011330438	1
T2DM_insulin	leukocyte	*SNX7*	-0.310647614	0.005798689	0.011570498	1
T2DM_insulin	leukocyte	*HFM1*	-0.691729429	0.010398763	0.015922346	1
T2DM_insulin	leukocyte	*VCAM1*	-0.215194843	0.006000145	0.011570498	1
T2DM_insulin	leukocyte	*ZNF496*	-0.385387615	0.004941923	0.011330438	1
T2DM_insulin	leukocyte	*TRIM58*	-1.497755759	0.010928751	0.016550349	1
T2DM_insulin	leukocyte	*PEA15*	-0.354985986	0.004641362	0.011330438	1
T2DM_insulin	leukocyte	*FLVCR1*	-0.312544641	0.004104826	0.011330438	1
T2DM_insulin	leukocyte	*TDRD5*	-1.233444	0.010218876	0.015732745	1
T2DM_insulin	leukocyte	*C1orf115*	-0.270924882	0.01192899	0.017699775	1
T2DM_insulin	leukocyte	*ACP6*	-0.474025609	0.009136466	0.014468834	1
T2DM_insulin	leukocyte	*HAAO*	-0.438209432	0.006554132	0.011815361	1
T2DM_insulin	leukocyte	*RFTN2*	-0.926778276	0.003187884	0.011330438	1
T2DM_insulin	leukocyte	*KCNJ3*	-0.339825065	0.008054367	0.01325193	1
T2DM_insulin	leukocyte	*DUSP19*	-0.557673345	0.00704619	0.012237751	1
T2DM_insulin	leukocyte	*SMC6*	-0.669443651	0.002694174	0.011330438	1
T2DM_insulin	leukocyte	*NOSTRIN*	-0.331881293	0.029421391	0.038050246	1
T2DM_insulin	leukocyte	*CTSS*	0.094867565	0.010803454	0.016407719	1
T2DM_insulin	leukocyte	*MSX1*	-0.417264062	0.006660242	0.01190541	1
T2DM_insulin	leukocyte	*PACRGL*	-0.460582736	0.00327642	0.011330438	1
T2DM_insulin	leukocyte	*ERCC3*	-0.510958298	0.004957256	0.011330438	1
T2DM_insulin	leukocyte	*TDRD10*	-5.703590787	0.019409496	0.026525186	1
T2DM_insulin	leukocyte	*CCNYL1*	-0.947236697	0.002501513	0.011330438	1
T2DM_insulin	leukocyte	*DCAF16*	-0.5362442	0.00159406	0.011330438	1
T2DM_insulin	leukocyte	*NPPC*	-3.842139012	0.003061418	0.011330438	1
T2DM_insulin	leukocyte	*GNPDA2*	-0.262238147	0.030713528	0.039500311	1
T2DM_insulin	leukocyte	*NIPAL1*	-0.293428675	0.021696364	0.029243572	1
T2DM_insulin	leukocyte	*CGGBP1*	-0.3870249	0.002693154	0.011330438	1
T2DM_insulin	leukocyte	*DAPL1*	-1.28795251	0.00127205	0.011330438	1
T2DM_insulin	leukocyte	*EOGT*	-0.396517854	0.00448535	0.011330438	1
T2DM_insulin	leukocyte	*POGLUT1*	-0.302528801	0.008532223	0.013784171	1
T2DM_insulin	leukocyte	*ATP1A1*	-0.265936112	0.003195317	0.011330438	1
T2DM_insulin	leukocyte	*ARPC2*	-0.395002785	0.002729737	0.011330438	1
T2DM_insulin	leukocyte	*CCT3*	-0.267563307	0.007623729	0.01282334	1
T2DM_insulin	leukocyte	*EOMES*	-2.258760509	0.005785824	0.011570498	1
T2DM_insulin	leukocyte	*CWC22*	-0.41597801	0.00487682	0.011330438	1
T2DM_insulin	leukocyte	*HDAC11*	-0.995966707	0.009065429	0.014391139	1
T2DM_insulin	leukocyte	*SUCLG1*	-0.373625447	0.00377429	0.011330438	1
T2DM_insulin	leukocyte	*ICA1L*	-0.338972896	0.008688239	0.013955805	1
T2DM_insulin	leukocyte	*RYBP*	-0.304724342	0.002814385	0.011330438	1
T2DM_insulin	leukocyte	*GTPBP8*	-0.629638667	0.003931259	0.011330438	1
T2DM_insulin	leukocyte	*CADPS*	-0.197830273	0.006707722	0.0119468	1
T2DM_insulin	leukocyte	*ATXN7*	-0.444116834	0.003069617	0.011330438	1
T2DM_insulin	leukocyte	*PSMD6*	-0.344743313	0.003018834	0.011330438	1
T2DM_insulin	leukocyte	*PRICKLE2*	-0.425563567	0.00375562	0.011330438	1
T2DM_insulin	leukocyte	*RPL9*	-0.243371301	0.003431722	0.011330438	1
T2DM_insulin	leukocyte	*IL17RC*	-0.443210259	0.002697713	0.011330438	1

T2DM_insulin	leukocyte	*CRELD1*	-0.239446139	0.026505757	0.034818161	1
T2DM_insulin	leukocyte	*TTC14*	-0.629319784	0.002610898	0.011330438	1
T2DM_insulin	leukocyte	*CXCL3*	0.319215589	0.032605824	0.041634151	1
T2DM_insulin	leukocyte	*CXCL1*	-0.252259119	0.006929696	0.01212774	1
T2DM_insulin	leukocyte	*CCDC158*	-1.018807153	0.005464875	0.011427973	1
T2DM_insulin	leukocyte	*TM4SF18*	-0.402330797	0.005342098	0.011421159	1
T2DM_insulin	leukocyte	*SNRK*	-0.274337769	0.005116814	0.011330438	1
T2DM_insulin	leukocyte	*KIAA1143*	-0.295609055	0.005355124	0.011421159	1
T2DM_insulin	leukocyte	*CCR1*	0.328862419	0.019675525	0.026832745	1
T2DM_insulin	leukocyte	*NMNAT3*	-0.354256752	0.004555984	0.011330438	1
T2DM_insulin	leukocyte	*SMIM12*	-0.370648803	0.003878506	0.011330438	1
T2DM_insulin	leukocyte	*TPRA1*	-0.698360303	0.00272807	0.011330438	1
T2DM_insulin	leukocyte	*MEAF6*	-0.332337145	0.004040166	0.011330438	1
T2DM_insulin	leukocyte	*DNALI1*	-0.354487984	0.013918344	0.01996538	1
T2DM_insulin	leukocyte	*CAMK2N2*	-0.258548778	0.003678575	0.011330438	1
T2DM_insulin	leukocyte	*RPN1*	-0.393824711	0.004561017	0.011330438	1
T2DM_insulin	leukocyte	*IFT122*	-0.508234257	0.004937721	0.011330438	1
T2DM_insulin	leukocyte	*RFC4*	-0.489057982	0.003980633	0.011330438	1
T2DM_insulin	leukocyte	*TKT*	-0.403443006	0.002751823	0.011330438	1
T2DM_insulin	leukocyte	*RFT1*	-0.733270709	0.001503502	0.011330438	1
T2DM_insulin	leukocyte	*UVSSA*	-0.56692256	0.012204651	0.017937915	1
T2DM_insulin	leukocyte	*ARHGEF3*	-0.35223382	0.009634749	0.01506364	1
T2DM_insulin	leukocyte	*LRPAP1*	-0.286666579	0.01128224	0.016912599	1
T2DM_insulin	leukocyte	*PIGX*	-0.537204736	0.002432949	0.011330438	1
T2DM_insulin	leukocyte	*ERMAP*	-1.260737254	0.001781679	0.011330438	1
T2DM_insulin	leukocyte	*BDH2*	-0.393608978	0.002671363	0.011330438	1
T2DM_insulin	leukocyte	*CDC25A*	-0.673132863	0.00335888	0.011330438	1
T2DM_insulin	leukocyte	*TEX264*	-0.464220527	0.002922636	0.011330438	1
T2DM_insulin	leukocyte	*PPM1M*	-0.530856109	0.004074402	0.011330438	1
T2DM_insulin	leukocyte	*SAP30*	-0.362376402	0.006414542	0.01170553	1
T2DM_insulin	leukocyte	*ANXA5*	-0.242297808	0.004987871	0.011330438	1
T2DM_insulin	leukocyte	*CEP44*	-0.393237866	0.008894759	0.014171739	1
T2DM_insulin	leukocyte	*HPGD*	-0.58804244	0.017777174	0.024550621	1
T2DM_insulin	leukocyte	*NPY1R*	0.87241736	0.03416983	0.043419555	1
T2DM_insulin	leukocyte	*ARFIP1*	-0.562639086	0.003094933	0.011330438	1
T2DM_insulin	leukocyte	*ITGA2*	-0.277964342	0.004495674	0.011330438	1
T2DM_insulin	leukocyte	*ELOVL7*	-0.422487185	0.001748107	0.011330438	1
T2DM_insulin	leukocyte	*LMBRD2*	-0.437360998	0.003748705	0.011330438	1
T2DM_insulin	leukocyte	*STARD4*	-0.510806887	0.003527325	0.011330438	1
T2DM_insulin	leukocyte	*CMBL*	-0.404329414	0.00446913	0.011330438	1
T2DM_insulin	leukocyte	*PRRC1*	-0.310630957	0.003670198	0.011330438	1
T2DM_insulin	leukocyte	*AGGF1*	-0.520550038	0.002457601	0.011330438	1
T2DM_insulin	leukocyte	*WDR41*	-0.600335236	0.003484972	0.011330438	1
T2DM_insulin	leukocyte	*SERINC5*	-1.237008545	0.012443035	0.018194523	1
T2DM_insulin	leukocyte	*RICTOR*	-0.545709167	0.002672519	0.011330438	1
T2DM_insulin	leukocyte	*UTP15*	-0.347258761	0.005088912	0.011330438	1
T2DM_insulin	leukocyte	*KLKB1*	-0.895488398	0.000706106	0.011330438	1
T2DM_insulin	leukocyte	*NSA2*	-0.246795441	0.007395085	0.012565211	1
T2DM_insulin	leukocyte	*LEAP2*	-0.610668572	0.005360994	0.011421159	1
T2DM_insulin	leukocyte	*DACT2*	-1.630995857	0.006193081	0.011570498	1
T2DM_insulin	leukocyte	*PDSS2*	-0.391328118	0.004332732	0.011330438	1
T2DM_insulin	leukocyte	*STK17A*	-0.333093726	0.005153927	0.011330438	1
T2DM_insulin	leukocyte	*GALNT10*	-0.63457573	0.027884054	0.036349186	1
T2DM_insulin	leukocyte	*SAP30L*	-0.508331899	0.004335175	0.011330438	1
T2DM_insulin	leukocyte	*COG5*	-0.67443415	0.002413593	0.011330438	1
T2DM_insulin	leukocyte	*SLU7*	-0.400688257	0.003307859	0.011330438	1
T2DM_insulin	leukocyte	*CAMLG*	-0.301263053	0.005405322	0.011421159	1
T2DM_insulin	leukocyte	*KIF6*	-0.723656254	0.00371173	0.011330438	1
T2DM_insulin	leukocyte	*STEAP1*	-0.547924804	0.004164478	0.011330438	1
T2DM_insulin	leukocyte	*FNDC1*	-2.148555803	0.025388589	0.033530006	1
T2DM_insulin	leukocyte	*CHMP4C*	-0.37526769	0.003949616	0.011330438	1
T2DM_insulin	leukocyte	*BRI3*	-0.456590766	0.002462321	0.011330438	1
T2DM_insulin	leukocyte	*LMTK2*	-0.404587685	0.003156922	0.011330438	1
T2DM_insulin	leukocyte	*DLC1*	-0.391812489	0.010731237	0.016316962	1

T2DM_insulin	leukocyte	*PEX2*	-0.33548661	0.003873795	0.011330438	1
T2DM_insulin	leukocyte	*MED30*	-0.373194281	0.005752009	0.011570498	1
T2DM_insulin	leukocyte	*TNFRSF11B*	-1.049593972	0.017586545	0.024330118	1
T2DM_insulin	leukocyte	*CSMD3*	-26.01378869	0.006250441	0.011570498	1
T2DM_insulin	leukocyte	*OSGIN2*	-0.536088182	0.002756014	0.011330438	1
T2DM_insulin	leukocyte	*DEFB1*	-0.174803713	0.005924885	0.011570498	1
T2DM_insulin	leukocyte	*SUN1*	-0.307110376	0.004126842	0.011330438	1
T2DM_insulin	leukocyte	*TMEM184A*	-0.419100316	0.003701682	0.011330438	1
T2DM_insulin	leukocyte	*INTS1*	-0.405038982	0.004059765	0.011330438	1
T2DM_insulin	leukocyte	*YWHAZ*	-0.336182368	0.003059057	0.011330438	1
T2DM_insulin	leukocyte	*DCAF13*	-0.402538154	0.003579135	0.011330438	1
T2DM_insulin	leukocyte	*INTS8*	-0.43068713	0.028493211	0.036983755	1
T2DM_insulin	leukocyte	*GEM*	-0.402297495	0.005986455	0.011570498	1
T2DM_insulin	leukocyte	*RPP25L*	-0.614017754	0.003300845	0.011330438	1
T2DM_insulin	leukocyte	*FAM219A*	-0.613524941	0.002905907	0.011330438	1
T2DM_insulin	leukocyte	*NUDT2*	-0.456156917	0.002204617	0.011330438	1
T2DM_insulin	leukocyte	*NIPSNAP3B*	0.689142634	0.031780008	0.040698713	1
T2DM_insulin	leukocyte	*FXN*	-0.774801812	0.003650786	0.011330438	1
T2DM_insulin	leukocyte	*MAMDC2*	-0.88630404	0.005259091	0.011402654	1
T2DM_insulin	leukocyte	*PRSS37*	-214.900987	0.005997088	0.011570498	1
T2DM_insulin	leukocyte	*C9orf64*	-0.541199589	0.002443	0.011330438	1
T2DM_insulin	leukocyte	*ANKS6*	-0.38620189	0.004843056	0.011330438	1
T2DM_insulin	leukocyte	*CLDN3*	-0.394568295	0.003487324	0.011330438	1
T2DM_insulin	leukocyte	*WNK2*	-0.635616513	0.002552572	0.011330438	1
T2DM_insulin	leukocyte	*NOL6*	-0.762769838	0.003327706	0.011330438	1
T2DM_insulin	leukocyte	*PIGO*	-0.857800213	0.000704467	0.011330438	1
T2DM_insulin	leukocyte	*ARHGAP12*	-0.419360165	0.003905286	0.011330438	1
T2DM_insulin	leukocyte	*WRN*	-0.442209108	0.003719059	0.011330438	1
T2DM_insulin	leukocyte	*CFL2*	-0.394395991	0.003665459	0.011330438	1
T2DM_insulin	leukocyte	*PGM2L1*	-0.69319338	0.002153388	0.011330438	1
T2DM_insulin	leukocyte	*PHYHIPL*	-0.396197484	0.004439621	0.011330438	1
T2DM_insulin	leukocyte	*RPL36AL*	-0.279814087	0.004265608	0.011330438	1
T2DM_insulin	leukocyte	*TMEM63C*	-0.34952437	0.00485826	0.011330438	1
T2DM_insulin	leukocyte	*AKR1E2*	-1.560388847	0.015442927	0.021810191	1
T2DM_insulin	leukocyte	*OXGR1*	-0.865874525	0.006368752	0.011665654	1
T2DM_insulin	leukocyte	*VDAC2*	-0.350479671	0.002279672	0.011330438	1
T2DM_insulin	leukocyte	*SNAPC4*	-0.396869855	0.0056275	0.01154792	1
T2DM_insulin	leukocyte	*TSC1*	-0.560317122	0.003008556	0.011330438	1
T2DM_insulin	leukocyte	*ZMYND19*	-0.38568912	0.004709429	0.011330438	1
T2DM_insulin	leukocyte	*METTL17*	-0.536309177	0.00416882	0.011330438	1
T2DM_insulin	leukocyte	*VWA2*	-0.952425783	0.008405285	0.013629535	1
T2DM_insulin	leukocyte	*METTL3*	-0.421676421	0.004929603	0.011330438	1
T2DM_insulin	leukocyte	*TRUB1*	-0.613676225	0.001015015	0.011330438	1
T2DM_insulin	leukocyte	*ARHGAP42*	-0.564883821	0.009571913	0.015007201	1
T2DM_insulin	leukocyte	*SLC39A13*	-0.383186654	0.004094464	0.011330438	1
T2DM_insulin	leukocyte	*PSMC3*	-0.296744414	0.00618016	0.011570498	1
T2DM_insulin	leukocyte	*CPSF2*	-0.33509177	0.005245396	0.011392572	1
T2DM_insulin	leukocyte	*IFI27L1*	-0.409061699	0.004024051	0.011330438	1
T2DM_insulin	leukocyte	*SMCO4*	-0.419313505	0.003862396	0.011330438	1
T2DM_insulin	leukocyte	*ABTB2*	-0.522955469	0.012078101	0.017813386	1
T2DM_insulin	leukocyte	*LIPC*	-1.772988074	0.008555995	0.013816894	1
T2DM_insulin	leukocyte	*CEP57*	-0.348170839	0.003063118	0.011330438	1
T2DM_insulin	leukocyte	*JAM3*	-0.319099017	0.005282573	0.011419801	1
T2DM_insulin	leukocyte	*IKBIP*	-0.442128347	0.006396781	0.01170063	1
T2DM_insulin	leukocyte	*ZFYVE19*	-0.382225582	0.006219203	0.011570498	1
T2DM_insulin	leukocyte	*CKB*	-0.318330342	0.001006933	0.011330438	1
T2DM_insulin	leukocyte	*BTRC*	-0.696241864	0.005619308	0.011547208	1
T2DM_insulin	leukocyte	*BAG5*	-0.406639603	0.0035898	0.011330438	1
T2DM_insulin	leukocyte	*LARP6*	-0.350596709	0.002845899	0.011330438	1
T2DM_insulin	leukocyte	*ZNF319*	-1.119392955	0.002677278	0.011330438	1
T2DM_insulin	leukocyte	*SGPL1*	-0.610628846	0.004173998	0.011330438	1
T2DM_insulin	leukocyte	*FRS2*	-0.438992464	0.002815126	0.011330438	1
T2DM_insulin	leukocyte	*PCBD1*	-0.349375363	0.005614792	0.011547208	1
T2DM_insulin	leukocyte	*COX11*	-0.448860689	0.002884726	0.011330438	1

T2DM_insulin	leukocyte	*WBP1L*	-0.847273005	0.002717757	0.011330438	1
T2DM_insulin	leukocyte	*CYB5R2*	-0.432891322	0.003531479	0.011330438	1
T2DM_insulin	leukocyte	*TUB*	-0.473127314	0.010721684	0.016308748	1
T2DM_insulin	leukocyte	*RIC3*	-0.305750179	0.003889852	0.011330438	1
T2DM_insulin	leukocyte	*XRRA1*	-0.44047128	0.00245376	0.011330438	1
T2DM_insulin	leukocyte	*TRIM66*	-0.409733892	0.03483087	0.044188081	1
T2DM_insulin	leukocyte	*CDYL2*	-0.425430196	0.003693717	0.011330438	1
T2DM_insulin	leukocyte	*ZNF143*	-0.387815047	0.00452676	0.011330438	1
T2DM_insulin	leukocyte	*CCDC68*	-0.372170251	0.010334708	0.015871143	1
T2DM_insulin	leukocyte	*ZSCAN21*	-0.649878938	0.002244941	0.011330438	1
T2DM_insulin	leukocyte	*RIMKLB*	-0.389237133	0.003380696	0.011330438	1
T2DM_insulin	leukocyte	*GALR1*	0.45634572	0.011220651	0.016852423	1
T2DM_insulin	leukocyte	*ATF7IP2*	-0.578481588	0.00110284	0.011330438	1
T2DM_insulin	leukocyte	*COG1*	-0.405902819	0.002233554	0.011330438	1
T2DM_insulin	leukocyte	*B2M*	-0.258643731	0.00227492	0.011330438	1
T2DM_insulin	leukocyte	*AP1G1*	-0.288273365	0.005330394	0.011421159	1
T2DM_insulin	leukocyte	*SLFN5*	-0.313948803	0.005302727	0.011421159	1
T2DM_insulin	leukocyte	*SAAL1*	-0.373847908	0.005438316	0.011421159	1
T2DM_insulin	leukocyte	*SCNN1G*	-3.400900227	0.005472142	0.011427973	1
T2DM_insulin	leukocyte	*DCTN5*	-0.489581162	0.000811187	0.011330438	1
T2DM_insulin	leukocyte	*STAT6*	-0.64832844	0.004693768	0.011330438	1
T2DM_insulin	leukocyte	*PATL1*	-0.341070056	0.004245456	0.011330438	1
T2DM_insulin	leukocyte	*MRPL16*	-0.566883292	0.013426599	0.019358939	1
T2DM_insulin	leukocyte	*GREM1*	-0.603120831	0.004837303	0.011330438	1
T2DM_insulin	leukocyte	*TSC22D4*	-0.468250517	0.009098639	0.014422203	1
T2DM_insulin	leukocyte	*MS4A14*	0.082140011	0.020110635	0.027369132	1
T2DM_insulin	leukocyte	*DIS3L*	-0.623434539	0.00327785	0.011330438	1
T2DM_insulin	leukocyte	*MS4A8*	-1.226604317	0.003568434	0.011330438	1
T2DM_insulin	leukocyte	*RCCD1*	-1.05024455	0.001221718	0.011330438	1
T2DM_insulin	leukocyte	*MAPRE2*	-0.322612004	0.012971593	0.018806416	1
T2DM_insulin	leukocyte	*NAT16*	-0.278836722	0.00268877	0.011330438	1
T2DM_insulin	leukocyte	*GNGT2*	0.169820767	0.013480916	0.019430124	1
T2DM_insulin	leukocyte	*SLC27A4*	-0.373441554	0.016565185	0.02317828	1
T2DM_insulin	leukocyte	*URM1*	-0.490283327	0.0063255	0.011629804	1
T2DM_insulin	leukocyte	*CERCAM*	-0.313556586	0.014787215	0.020989512	1
T2DM_insulin	leukocyte	*DOLPP1*	-0.444169493	0.007261272	0.012437884	1
T2DM_insulin	leukocyte	*ENDOG*	-0.50146401	0.001547599	0.011330438	1
T2DM_insulin	leukocyte	*PRR15L*	-0.508970593	0.010375595	0.01590871	1
T2DM_insulin	leukocyte	*RNF214*	-0.636925935	0.005403632	0.011421159	1
T2DM_insulin	leukocyte	*POP5*	-0.464917386	0.001371243	0.011330438	1
T2DM_insulin	leukocyte	*ACAA2*	-0.423888821	0.002712932	0.011330438	1
T2DM_insulin	leukocyte	*FN3K*	-0.492483117	0.007070213	0.012258988	1
T2DM_insulin	leukocyte	*ZNF23*	-0.364270149	0.005902344	0.011570498	1
T2DM_insulin	leukocyte	*ZNF229*	-0.523681898	0.004824736	0.011330438	1
T2DM_insulin	leukocyte	*ZNF180*	-0.401684968	0.006041373	0.011570498	1
T2DM_insulin	leukocyte	*CA4*	-0.23208988	0.032511834	0.041527632	1
T2DM_insulin	leukocyte	*GPX4*	-0.39931714	0.002189546	0.011330438	1
T2DM_insulin	leukocyte	*ZNF641*	-0.596251344	0.005447329	0.011427973	1
T2DM_insulin	leukocyte	*CACNB3*	-0.518868135	0.006742027	0.011963834	1
T2DM_insulin	leukocyte	*TP53I13*	-0.417545482	0.002017771	0.011330438	1
T2DM_insulin	leukocyte	*TUBA1C*	-0.245764355	0.006461362	0.011731372	1
T2DM_insulin	leukocyte	*ZNF528*	-1.273386879	0.006647988	0.011905364	1
T2DM_insulin	leukocyte	*RAB4B*	-0.496656693	0.002156118	0.011330438	1
T2DM_insulin	leukocyte	*TMC4*	-0.384316004	0.005286999	0.011419801	1
T2DM_insulin	leukocyte	*C19orf33*	-0.331774012	0.005971782	0.011570498	1
T2DM_insulin	leukocyte	*DNAAF3*	-1.338566582	0.006249165	0.011570498	1
T2DM_insulin	leukocyte	*CHAF1A*	-0.491689807	0.015536755	0.021910951	1
T2DM_insulin	leukocyte	*UBXN6*	-0.548414889	0.001530924	0.011330438	1
T2DM_insulin	leukocyte	*NXN*	-0.253824953	0.012472159	0.018223545	1
T2DM_insulin	leukocyte	*KIFC2*	-1.603203147	0.000608472	0.011330438	1
T2DM_insulin	leukocyte	*SERPINF2*	0.18079335	0.018670684	0.025631418	1
T2DM_insulin	leukocyte	*TRPV3*	-2.013613347	0.002675485	0.011330438	1
T2DM_insulin	leukocyte	*ZNF83*	-0.506003361	0.003297576	0.011330438	1
T2DM_insulin	leukocyte	*NDUFV1*	-0.353815111	0.004108447	0.011330438	1

T2DM_insulin	leukocyte	*ZNF232*	-0.357879901	0.005118586	0.011330438	1
T2DM_insulin	leukocyte	*MIS12*	-0.423294481	0.011060241	0.0167073	1
T2DM_insulin	leukocyte	*HID1*	-0.429881154	0.003690569	0.011330438	1
T2DM_insulin	leukocyte	*SRP68*	-0.330762526	0.003823025	0.011330438	1
T2DM_insulin	leukocyte	*TMEM68*	-0.521648066	0.003943423	0.011330438	1
T2DM_insulin	leukocyte	*MLST8*	-0.291467122	0.009734893	0.015165898	1
T2DM_insulin	leukocyte	*ECI1*	-0.312224759	0.003979104	0.011330438	1
T2DM_insulin	leukocyte	*ABCA3*	-0.377699104	0.0040564	0.011330438	1
T2DM_insulin	leukocyte	*BSCL2*	-0.293444163	0.010346792	0.015879901	1
T2DM_insulin	leukocyte	*C2CD3*	-0.721369485	0.003398192	0.011330438	1
T2DM_insulin	leukocyte	*TRANK1*	-0.983329303	0.004500204	0.011330438	1
T2DM_insulin	leukocyte	*RPSA*	-0.261376767	0.002734542	0.011330438	1
T2DM_insulin	leukocyte	*ENTPD3*	-0.276270812	0.002740996	0.011330438	1
T2DM_insulin	leukocyte	*LTBP3*	-0.342448835	0.002761603	0.011330438	1
T2DM_insulin	leukocyte	*SAC3D1*	-0.461838238	0.003710106	0.011330438	1
T2DM_insulin	leukocyte	*PBK*	-0.881527516	0.004385711	0.011330438	1
T2DM_insulin	leukocyte	*KIAA1586*	-0.567663735	0.000833351	0.011330438	1
T2DM_insulin	leukocyte	*FAM83B*	-0.408805507	0.008216769	0.013442029	1
T2DM_insulin	leukocyte	*LMBRD1*	-0.452287749	0.003505909	0.011330438	1
T2DM_insulin	leukocyte	*TTC39C*	-0.354904841	0.005392458	0.011421159	1
T2DM_insulin	leukocyte	*POLR2J3*	-0.412002293	0.005020768	0.011330438	1
T2DM_insulin	leukocyte	*COA6*	-0.427280956	0.003691379	0.011330438	1
T2DM_insulin	leukocyte	*PXK*	-0.525778115	0.003120724	0.011330438	1
T2DM_insulin	leukocyte	*ING5*	-0.691084276	0.002660361	0.011330438	1
T2DM_insulin	leukocyte	*BMP1*	-0.383058499	0.016642856	0.023278669	1
T2DM_insulin	leukocyte	*HEXIM2*	-0.472463728	0.006065926	0.011570498	1
T2DM_insulin	leukocyte	*SERINC2*	-0.260755972	0.005504041	0.011434531	1
T2DM_insulin	leukocyte	*TRAPPC11*	-0.30865433	0.00705804	0.012248692	1
T2DM_insulin	leukocyte	*ING2*	-0.279099773	0.006648096	0.011905364	1
T2DM_insulin	leukocyte	*ADAM9*	-0.559074485	0.026752008	0.035059586	1
T2DM_insulin	leukocyte	*NDUFS5*	-0.318959517	0.001585707	0.011330438	1
T2DM_insulin	leukocyte	*DNAJC21*	-0.44089136	0.002104658	0.011330438	1
T2DM_insulin	leukocyte	*CNNM3*	-0.38539215	0.003996315	0.011330438	1
T2DM_insulin	leukocyte	*GSTM4*	-0.420494444	0.008376362	0.013599474	1
T2DM_insulin	leukocyte	*PPIP5K1*	-1.118636481	0.001144655	0.011330438	1
T2DM_insulin	leukocyte	*ADAL*	-1.086508599	0.004633936	0.011330438	1
T2DM_insulin	leukocyte	*LCMT2*	-0.642776998	0.002087009	0.011330438	1
T2DM_insulin	leukocyte	*ZNF507*	-0.492142039	0.003160737	0.011330438	1
T2DM_insulin	leukocyte	*NSG1*	-0.286136645	0.006174833	0.011570498	1
T2DM_insulin	leukocyte	*ZBTB49*	-1.130873226	0.002114816	0.011330438	1
T2DM_insulin	leukocyte	*GFM1*	-0.336292592	0.003193083	0.011330438	1
T2DM_insulin	leukocyte	*FSTL5*	-0.231018323	0.015598211	0.021989442	1
T2DM_insulin	leukocyte	*CTRB1*	-0.171545473	0.004060609	0.011330438	1
T2DM_insulin	leukocyte	*CTRB2*	-0.15630148	0.005593394	0.011524093	1
T2DM_insulin	leukocyte	*TMEM129*	-0.521509432	0.006587852	0.011847079	1
T2DM_insulin	leukocyte	*PPIC*	-0.191958234	0.013562968	0.019526897	1
T2DM_insulin	leukocyte	*STXBP6*	-0.44370613	0.003755839	0.011330438	1
T2DM_insulin	leukocyte	*MFF*	-0.39023505	0.002597048	0.011330438	1
T2DM_insulin	leukocyte	*CPLX1*	-0.372069366	0.009985869	0.015458818	1
T2DM_insulin	leukocyte	*ROR2*	-1.046575002	0.012023369	0.017759823	1
T2DM_insulin	leukocyte	*FAM110B*	-0.410256516	0.004346117	0.011330438	1
T2DM_insulin	leukocyte	*ATF5*	-0.469307138	0.003617104	0.011330438	1
T2DM_insulin	leukocyte	*XPO6*	-0.699777946	0.00201325	0.011330438	1
T2DM_insulin	leukocyte	*RGS14*	-0.545991175	0.006082335	0.011570498	1
T2DM_insulin	leukocyte	*TBC1D10B*	-0.503594271	0.003005201	0.011330438	1
T2DM_insulin	leukocyte	*PRELID1*	-0.303287545	0.002991778	0.011330438	1
T2DM_insulin	leukocyte	*CXCL10*	-66.23312583	0.006079991	0.011570498	1
T2DM_insulin	leukocyte	*CXCL11*	0.505924112	0.000837989	0.011330438	1
T2DM_insulin	leukocyte	*NMD3*	-0.383279139	0.003238183	0.011330438	1
T2DM_insulin	leukocyte	*STK32A*	-0.478872735	0.002922229	0.011330438	1
T2DM_insulin	leukocyte	*P2RY12*	-22.24136849	0.026625431	0.034951994	1
T2DM_insulin	leukocyte	*SNUPN*	-1.085430348	0.001239623	0.011330438	1
T2DM_insulin	leukocyte	*CRADD*	-0.397386506	0.007241485	0.012418942	1
T2DM_insulin	leukocyte	*COL22A1*	-3.77316749	0.007991111	0.013208698	1

T2DM_insulin	leukocyte	*SDC2*	-0.774095796	0.011458711	0.017150962	1
T2DM_insulin	leukocyte	*CLIC4*	-0.22190576	0.006730691	0.011961858	1
T2DM_insulin	leukocyte	*SLC38A11*	-2.801342717	0.011174177	0.016816769	1
T2DM_insulin	leukocyte	*HINT1*	-0.278175699	0.001991596	0.011330438	1
T2DM_insulin	leukocyte	*INO80E*	-0.652204731	0.000145849	0.011330438	0.573042456
T2DM_insulin	leukocyte	*C15orf40*	-0.403335229	0.002929553	0.011330438	1
T2DM_insulin	leukocyte	*HIC2*	-1.429474088	0.009869645	0.015333268	1
T2DM_insulin	leukocyte	*AGPAT2*	-0.405140343	0.002947395	0.011330438	1
T2DM_insulin	leukocyte	*FASN*	-0.704599481	0.001436789	0.011330438	1
T2DM_insulin	leukocyte	*MT1E*	-0.221750356	0.006182477	0.011570498	1
T2DM_insulin	leukocyte	*LDB2*	-0.380063402	0.015908535	0.022347027	1
T2DM_insulin	leukocyte	*RAC3*	-0.565997707	0.002531027	0.011330438	1
T2DM_insulin	leukocyte	*LIMS1*	-0.440586005	0.003427615	0.011330438	1
T2DM_insulin	leukocyte	*CSGALNACT2*	-0.8683514	0.012371949	0.0181108	1
T2DM_insulin	leukocyte	*AVEN*	-1.07139535	0.002889704	0.011330438	1
T2DM_insulin	leukocyte	*CTNND2*	-0.390847185	0.008390186	0.013614784	1
T2DM_insulin	leukocyte	*WNT10B*	-22.43087438	0.007234287	0.012417437	1
T2DM_insulin	leukocyte	*CALML6*	0.392235096	0.019926208	0.02713694	1
T2DM_insulin	leukocyte	*TM4SF4*	-0.367713913	0.003018642	0.011330438	1
T2DM_insulin	leukocyte	*OTUD3*	-0.302630805	0.003203919	0.011330438	1
T2DM_insulin	leukocyte	*MAP3K2*	-0.472579099	0.003683687	0.011330438	1
T2DM_insulin	leukocyte	*PUSL1*	-0.803890414	0.004056652	0.011330438	1
T2DM_insulin	leukocyte	*IFFO2*	-0.339212288	0.008140504	0.013360083	1
T2DM_insulin	leukocyte	*MYRIP*	-0.505578356	0.00627263	0.011570498	1
T2DM_insulin	leukocyte	*FAM153A*	-0.806008112	0.008493435	0.013744113	1
T2DM_insulin	leukocyte	*ZNF778*	-0.550872262	0.002007522	0.011330438	1
T2DM_insulin	leukocyte	*NIPA1*	-0.465922901	0.00441612	0.011330438	1
T2DM_insulin	leukocyte	*FOXD4*	-4.719343257	0.020532051	0.027865433	1
T2DM_insulin	leukocyte	*UBE2E1*	-0.660899391	0.002571218	0.011330438	1
T2DM_insulin	leukocyte	*ANKK1*	-10.26234136	0.005871055	0.011570498	1
T2DM_insulin	leukocyte	*USP47*	-0.458009188	0.002723515	0.011330438	1
T2DM_insulin	leukocyte	*PDCD6IP*	-0.375534371	0.003795834	0.011330438	1
T2DM_insulin	leukocyte	*CRTAP*	-0.698585042	0.002476464	0.011330438	1
T2DM_insulin	leukocyte	*STX8*	-0.553624943	0.001928292	0.011330438	1
T2DM_insulin	leukocyte	*NFRKB*	-0.687542077	0.002993486	0.011330438	1
T2DM_insulin	leukocyte	*SETMAR*	-0.381800987	0.004183998	0.011330438	1
T2DM_insulin	leukocyte	*CST2*	-0.914131437	0.001567204	0.011330438	1
T2DM_insulin	leukocyte	*KRT8*	-0.163504727	0.00537056	0.011421159	1
T2DM_insulin	leukocyte	*MGMT*	-0.508473964	0.003089417	0.011330438	1
T2DM_insulin	leukocyte	*NFXL1*	-0.728889653	0.000739404	0.011330438	1
T2DM_insulin	leukocyte	*DNAJC18*	-0.370962045	0.037984892	0.047788229	1
T2DM_insulin	leukocyte	*KRT4*	-23.45540345	0.003627706	0.011330438	1
T2DM_insulin	leukocyte	*SLC23A1*	-3.136630693	0.009639694	0.015065376	1
T2DM_insulin	leukocyte	*NPAS2*	-0.30171064	0.007960885	0.013183733	1
T2DM_insulin	leukocyte	*NUDT9*	-0.31296437	0.007661664	0.012869015	1
T2DM_insulin	leukocyte	*SMAGP*	-0.246005569	0.022201564	0.029813272	1
T2DM_insulin	leukocyte	*IRX1*	-0.553811322	0.006268213	0.011570498	1
T2DM_insulin	leukocyte	*CDH2*	-0.30822971	0.006178444	0.011570498	1
T2DM_insulin	leukocyte	*HSPA4*	-0.417030227	0.002518881	0.011330438	1
T2DM_insulin	leukocyte	*ARMC10*	-0.495009009	0.007391048	0.012565211	1
T2DM_insulin	leukocyte	*ACYP2*	-0.353763255	0.004020356	0.011330438	1
T2DM_insulin	leukocyte	*ATF7*	-0.412607876	0.003951144	0.011330438	1
T2DM_insulin	leukocyte	*KCNS3*	-0.509024524	0.006821251	0.012014984	1
T2DM_insulin	leukocyte	*FOXN2*	-1.065141233	0.004547183	0.011330438	1
T2DM_insulin	leukocyte	*GPR27*	-0.409873375	0.002942552	0.011330438	1
T2DM_insulin	leukocyte	*MTSS1*	-0.570029877	0.002170411	0.011330438	1
T2DM_insulin	leukocyte	*TMEM43*	-0.383677432	0.005140766	0.011330438	1
T2DM_insulin	leukocyte	*RPS9*	-0.250260597	0.002631542	0.011330438	1
T2DM_insulin	leukocyte	*NDUFA3*	-0.40892478	0.00354856	0.011330438	1
T2DM_insulin	leukocyte	*ZNF160*	-0.400507314	0.003659608	0.011330438	1
T2DM_insulin	leukocyte	*ZNF415*	-0.372622503	0.007441356	0.01261851	1
T2DM_insulin	leukocyte	*TSNARE1*	0.377729142	0.012915607	0.018752927	1
T2DM_insulin	leukocyte	*SOX7*	-0.502103131	0.00617052	0.011570498	1
T2DM_insulin	leukocyte	*TRMT61B*	-0.339662557	0.004940432	0.011330438	1

T2DM_insulin	leukocyte	*INSR*	-0.272971934	0.005508869	0.011434531	1
T2DM_insulin	leukocyte	*MFN1*	-0.421618572	0.003786161	0.011330438	1
T2DM_insulin	leukocyte	*KCNMB3*	-0.73855048	0.00158685	0.011330438	1
T2DM_insulin	leukocyte	*ATP6V0E2*	-0.256138196	0.026126213	0.034342552	1
T2DM_insulin	leukocyte	*JAGN1*	-0.431695334	0.003138198	0.011330438	1
T2DM_insulin	leukocyte	*MORN4*	-0.313150598	0.001874092	0.011330438	1
T2DM_insulin	leukocyte	*TRIM8*	-0.445410962	0.002946648	0.011330438	1
T2DM_insulin	leukocyte	*CDC42BPG*	-0.27395463	0.007028353	0.012235001	1
T2DM_insulin	leukocyte	*ZNF440*	-1.490590022	0.000408232	0.011330438	1
T2DM_insulin	leukocyte	*GAA*	-0.535221889	0.001463817	0.011330438	1
T2DM_insulin	leukocyte	*EXOSC1*	-0.463775202	0.007447489	0.012623462	1
T2DM_insulin	leukocyte	*PGAM1*	-0.258285634	0.029504274	0.038132332	1
T2DM_insulin	leukocyte	*ESCO2*	-1.377568917	0.01136028	0.017016599	1
T2DM_insulin	leukocyte	*MRPL36*	-0.401429604	0.002743396	0.011330438	1
T2DM_insulin	leukocyte	*MCC*	-0.501421712	0.004993281	0.011330438	1
T2DM_insulin	leukocyte	*ZNF561*	-0.466272268	0.003405283	0.011330438	1
T2DM_insulin	leukocyte	*PPID*	-0.497850687	0.014054796	0.020131715	1
T2DM_insulin	leukocyte	*PTGER4*	0.223306443	0.026961799	0.035279887	1
T2DM_insulin	leukocyte	*MAP6*	-0.255274079	0.010645991	0.016225019	1
T2DM_insulin	leukocyte	*ZNF584*	-0.555314022	0.012638746	0.018432677	1
T2DM_insulin	leukocyte	*NMUR1*	-4.222725203	0.005392539	0.011421159	1
T2DM_insulin	leukocyte	*CLSTN1*	-0.363582826	0.001243694	0.011330438	1
T2DM_insulin	leukocyte	*PIK3CD*	0.31521382	0.030138006	0.038874664	1
T2DM_insulin	leukocyte	*RGS19*	-0.681144014	0.001711128	0.011330438	1
T2DM_insulin	leukocyte	*ANO5*	-0.457957741	0.008238711	0.013448692	1
T2DM_insulin	leukocyte	*GPHN*	-0.693300925	0.002492935	0.011330438	1
T2DM_insulin	leukocyte	*VAT1L*	-0.430802212	0.014742683	0.020949006	1
T2DM_insulin	leukocyte	*LGALS4*	-0.20498604	0.008944173	0.014233153	1
T2DM_insulin	leukocyte	*LRRC34*	-0.422976865	0.014128623	0.020230087	1
T2DM_insulin	leukocyte	*SPATA5L1*	-0.84738071	0.000988606	0.011330438	1
T2DM_insulin	leukocyte	*CTPS1*	-0.342954864	0.008676029	0.013941971	1
T2DM_insulin	leukocyte	*METTL18*	-0.50497683	0.004833625	0.011330438	1
T2DM_insulin	leukocyte	*PWWP2B*	-0.486895935	0.003600333	0.011330438	1
T2DM_insulin	leukocyte	*PTEN*	-0.511495145	0.000769611	0.011330438	1
T2DM_insulin	leukocyte	*CYP4F11*	-0.957103912	0.009428273	0.014830386	1
T2DM_insulin	leukocyte	*TLN2*	-0.756178618	0.005798688	0.011570498	1
T2DM_insulin	leukocyte	*TVP23B*	-0.68590019	0.00241844	0.011330438	1
T2DM_insulin	leukocyte	*SRGAP2C*	-0.802817726	0.001789179	0.011330438	1
T2DM_insulin	leukocyte	*ATPAF2*	-0.519686802	0.002856337	0.011330438	1
T2DM_insulin	leukocyte	*ZNF556*	0.364769474	0.008437781	0.013670944	1
T2DM_insulin	leukocyte	*ZNF554*	-0.428970623	0.00568274	0.011549238	1
T2DM_insulin	leukocyte	*GAP43*	-0.303513313	0.005833652	0.011570498	1
T2DM_insulin	leukocyte	*REG1B*	-0.181080686	0.004183306	0.011330438	1
T2DM_insulin	leukocyte	*NME6*	-0.678219411	0.004110865	0.011330438	1
T2DM_insulin	leukocyte	*TEFM*	-0.52495125	0.003082134	0.011330438	1
T2DM_insulin	leukocyte	*MBOAT1*	-0.786628361	0.006011699	0.011570498	1
T2DM_insulin	leukocyte	*ZNF131*	-0.408751402	0.003484413	0.011330438	1
T2DM_insulin	leukocyte	*BSG*	-0.337292762	0.003035827	0.011330438	1
T2DM_insulin	leukocyte	*HINFP*	-1.155906451	0.00260241	0.011330438	1
T2DM_insulin	leukocyte	*CERS6*	-0.396469312	0.002219286	0.011330438	1
T2DM_insulin	leukocyte	*SPTLC3*	0.4702501	0.012384151	0.018121911	1
T2DM_insulin	leukocyte	*COPRS*	-0.286131917	0.004831139	0.011330438	1
T2DM_insulin	leukocyte	*SUCLG2*	-0.698376633	0.00242723	0.011330438	1
T2DM_insulin	leukocyte	*CSDC2*	-1.298379813	0.001329879	0.011330438	1
T2DM_insulin	leukocyte	*ARNT2*	-0.302434473	0.011555593	0.017256528	1
T2DM_insulin	leukocyte	*ZNF24*	-0.435679175	0.001866995	0.011330438	1
T2DM_insulin	leukocyte	*MANEA*	-0.483003283	0.002076853	0.011330438	1
T2DM_insulin	leukocyte	*AFF1*	-0.455296512	0.002102277	0.011330438	1
T2DM_insulin	leukocyte	*CTSW*	-0.304718103	0.034696574	0.044046152	1
T2DM_insulin	leukocyte	*PDE3A*	-0.607523448	0.003214231	0.011330438	1
T2DM_insulin	leukocyte	*RAD9A*	-0.722276389	0.00343613	0.011330438	1
T2DM_insulin	leukocyte	*EFEMP2*	-0.415968375	0.004640716	0.011330438	1
T2DM_insulin	leukocyte	*ZNF738*	-0.39410355	0.028443045	0.03694305	1
T2DM_insulin	leukocyte	*FUT10*	-1.292894237	0.017226304	0.023915953	1

T2DM_insulin	leukocyte	*NAA16*	-0.369647542	0.007048621	0.012237751	1
T2DM_insulin	leukocyte	*OVOL1*	-0.937244567	0.010337046	0.015871143	1
T2DM_insulin	leukocyte	*DMXL1*	-0.574884316	0.006235508	0.011570498	1
T2DM_insulin	leukocyte	*NADSYN1*	-0.6940019	0.005850482	0.011570498	1
T2DM_insulin	leukocyte	*RNASEH2C*	-0.393416741	0.005191815	0.011376821	1
T2DM_insulin	leukocyte	*LCLAT1*	-0.417805985	0.002658624	0.011330438	1
T2DM_insulin	leukocyte	*SH3RF3*	-0.602511968	0.005786159	0.011570498	1
T2DM_insulin	leukocyte	*DCAKD*	-0.296716584	0.006910998	0.012100405	1
T2DM_insulin	leukocyte	*TADA2B*	-0.463168259	0.00319114	0.011330438	1
T2DM_insulin	leukocyte	*TRMT112*	-0.350535158	0.002983979	0.011330438	1
T2DM_insulin	leukocyte	*NOC3L*	-0.364312512	0.007972699	0.013189362	1
T2DM_insulin	leukocyte	*RHOD*	-0.260170521	0.005933194	0.011570498	1
T2DM_insulin	leukocyte	*PARP14*	-0.386695036	0.006455969	0.01173015	1
T2DM_insulin	leukocyte	*VANGL1*	-0.401426768	0.004283251	0.011330438	1
T2DM_insulin	leukocyte	*IQCB1*	-0.959133235	0.002103446	0.011330438	1
T2DM_insulin	leukocyte	*ZNF483*	-0.328765047	0.004407417	0.011330438	1
T2DM_insulin	leukocyte	*SLC2A14*	-1.514148344	0.006471729	0.011737322	1
T2DM_insulin	leukocyte	*MZT2A*	-0.2967081	0.006344764	0.011637991	1
T2DM_insulin	leukocyte	*PPP1R3B*	-0.872110158	0.005481386	0.011427973	1
T2DM_insulin	leukocyte	*MAP3K11*	-0.366653342	0.004079055	0.011330438	1
T2DM_insulin	leukocyte	*NDNF*	0.392141326	0.01515631	0.021466886	1
T2DM_insulin	leukocyte	*ARV1*	-0.367731128	0.003503923	0.011330438	1
T2DM_insulin	leukocyte	*EHBP1L1*	-0.339073548	0.021979593	0.029544243	1
T2DM_insulin	leukocyte	*PPP1R14B*	-0.335139516	0.002455239	0.011330438	1
T2DM_insulin	leukocyte	*PEAK1*	-0.248051825	0.039106414	0.049073491	1
T2DM_insulin	leukocyte	*TNFRSF10D*	-0.481059842	0.004756847	0.011330438	1
T2DM_insulin	leukocyte	*GMPPB*	-0.533979375	0.010331867	0.015871143	1
T2DM_insulin	leukocyte	*NUDT18*	-0.395982839	0.005233009	0.011389288	1
T2DM_insulin	leukocyte	*NUDT4*	-0.321103467	0.012163186	0.01789856	1
T2DM_insulin	leukocyte	*PC*	-0.630829351	0.003554348	0.011330438	1
T2DM_insulin	leukocyte	*UQCRH*	-0.338955189	0.002550785	0.011330438	1
T2DM_insulin	leukocyte	*HEG1*	-0.384790682	0.005468475	0.011427973	1
T2DM_insulin	leukocyte	*HAP1*	-0.464531946	0.019512291	0.026656394	1
T2DM_insulin	leukocyte	*ENDOV*	-0.682060198	0.007089531	0.01228624	1
T2DM_insulin	leukocyte	*DPY19L1*	-0.483068036	0.003457426	0.011330438	1
T2DM_insulin	leukocyte	*TUBB8*	-11.11014481	0.028589115	0.037095981	1
T2DM_insulin	leukocyte	*GPR160*	-0.558806391	0.006337688	0.011637991	1
T2DM_insulin	leukocyte	*XXYLT1*	-0.539871716	0.01439186	0.020539636	1
T2DM_insulin	leukocyte	*GNG5*	-0.346042594	0.002527846	0.011330438	1
T2DM_insulin	leukocyte	*FAM53A*	-1.771314737	0.00636738	0.011665654	1
T2DM_insulin	leukocyte	*SELP*	-2.305806808	0.005995265	0.011570498	1
T2DM_insulin	leukocyte	*CTU2*	-0.385855411	0.004308474	0.011330438	1
T2DM_insulin	leukocyte	*SNX31*	-2.918590283	0.017713548	0.024479962	1
T2DM_insulin	leukocyte	*ADCY6*	-0.445995667	0.003588054	0.011330438	1
T2DM_insulin	leukocyte	*PODN*	-0.362873686	0.017275635	0.023975969	1
T2DM_insulin	leukocyte	*RALGAPA1*	-0.391215724	0.001958767	0.011330438	1
T2DM_insulin	leukocyte	*CNTNAP2*	0.71645744	0.020439616	0.027759161	1
T2DM_insulin	leukocyte	*GOLT1A*	-0.387705408	0.003561207	0.011330438	1
T2DM_insulin	leukocyte	*NPAS4*	-0.858475851	0.002568772	0.011330438	1
T2DM_insulin	leukocyte	*ANGEL2*	-0.538749476	0.002800016	0.011330438	1
T2DM_insulin	leukocyte	*ZNF266*	-0.241186494	0.029621794	0.038271631	1
T2DM_insulin	leukocyte	*BRSK2*	-0.362389844	0.005012834	0.011330438	1
T2DM_insulin	leukocyte	*BRMS1*	-0.359020169	0.003796728	0.011330438	1
T2DM_insulin	leukocyte	*HRAS*	-0.358991279	0.003414828	0.011330438	1
T2DM_insulin	leukocyte	*SRP72*	-0.30311158	0.003304915	0.011330438	1
T2DM_insulin	leukocyte	*BTC*	-0.631057733	0.003351562	0.011330438	1
T2DM_insulin	leukocyte	*DENND6A*	-0.350270935	0.0069791	0.012179027	1
T2DM_insulin	leukocyte	*YIF1A*	-0.333605519	0.00478687	0.011330438	1
T2DM_insulin	leukocyte	*NDUFA11*	-0.35342381	0.003152509	0.011330438	1
T2DM_insulin	leukocyte	*RSRC1*	-0.771494222	0.002391131	0.011330438	1
T2DM_insulin	leukocyte	*DHX36*	-0.395904426	0.002971239	0.011330438	1
T2DM_insulin	leukocyte	*PSMD2*	-0.36447357	0.001075252	0.011330438	1
T2DM_insulin	leukocyte	*PPM1E*	-0.317724339	0.00336958	0.011330438	1
T2DM_insulin	leukocyte	*ARHGAP1*	-0.379725776	0.003559644	0.011330438	1

T2DM_insulin	leukocyte	*MED16*	-0.370404053	0.00326676	0.011330438	1
T2DM_insulin	leukocyte	*CHST1*	-0.763438426	0.006141929	0.011570498	1
T2DM_insulin	leukocyte	*DOLK*	-0.721072963	0.005227256	0.011389288	1
T2DM_insulin	leukocyte	*CCNE2*	-0.45432017	0.008836427	0.014115597	1
T2DM_insulin	leukocyte	*ZNF519*	-0.798390606	0.001326497	0.011330438	1
T2DM_insulin	leukocyte	*TMEM9B*	-0.387195517	0.002830402	0.011330438	1
T2DM_insulin	leukocyte	*SCUBE2*	-0.225423603	0.031916595	0.040847004	1
T2DM_insulin	leukocyte	*EIF3F*	-0.360699877	0.005508516	0.011434531	1
T2DM_insulin	leukocyte	*ZNF25*	-0.377088176	0.003652389	0.011330438	1
T2DM_insulin	leukocyte	*PPP2R2D*	-0.367873165	0.003407357	0.011330438	1
T2DM_insulin	leukocyte	*CLCF1*	-0.564173706	0.003564675	0.011330438	1
T2DM_insulin	leukocyte	*UCP2*	0.276587426	0.030107355	0.038853727	1
T2DM_insulin	leukocyte	*MRPL48*	-0.447268011	0.008241297	0.013448692	1
T2DM_insulin	leukocyte	*TMEM70*	-0.372967846	0.003353014	0.011330438	1
T2DM_insulin	leukocyte	*RPS6KB2*	-0.533559327	0.005126234	0.011330438	1
T2DM_insulin	leukocyte	*RMI2*	-1.061371942	0.022910774	0.03059702	1
T2DM_insulin	leukocyte	*LINC01106*	-1.533901077	0.037574015	0.047301604	1
T2DM_insulin	leukocyte	*SWI5*	-0.520289161	0.007604428	0.012801112	1
T2DM_insulin	leukocyte	*ZDHHC21*	-0.700064362	0.003501649	0.011330438	1
T2DM_insulin	leukocyte	*TUBB6*	-0.210464178	0.004372489	0.011330438	1
T2DM_insulin	leukocyte	*CHMP6*	-0.39465118	0.00494429	0.011330438	1
T2DM_insulin	leukocyte	*DLEU1*	-0.483501072	0.003349281	0.011330438	1
T2DM_insulin	leukocyte	*CCDC57*	-0.586568505	0.00048168	0.011330438	1
T2DM_insulin	leukocyte	*BNIP3*	-0.296019692	0.002772765	0.011330438	1
T2DM_insulin	leukocyte	*SMIM19*	-0.331568341	0.003572065	0.011330438	1
T2DM_insulin	leukocyte	*SLC35G1*	-0.542348792	0.004425853	0.011330438	1
T2DM_insulin	leukocyte	*COX8A*	-0.28311788	0.002789273	0.011330438	1
T2DM_insulin	leukocyte	*CDC26*	-0.396205643	0.004621055	0.011330438	1
T2DM_insulin	leukocyte	*RNPEP*	-0.694361419	0.003034441	0.011330438	1
T2DM_insulin	leukocyte	*DMRTA1*	-0.330373416	0.009794458	0.015234531	1
T2DM_insulin	leukocyte	*CLEC14A*	-0.206989596	0.017679851	0.024441989	1
T2DM_insulin	leukocyte	*ZNF575*	0.43815385	0.025245581	0.033363569	1
T2DM_insulin	leukocyte	*WDR25*	-0.975288309	0.001921069	0.011330438	1
T2DM_insulin	leukocyte	*DIRAS1*	-0.477282755	0.007011102	0.012210382	1
T2DM_insulin	leukocyte	*PHLDB3*	-0.320404547	0.009964255	0.015455806	1
T2DM_insulin	leukocyte	*PRR15*	0.49133965	0.027902954	0.03636176	1
T2DM_insulin	leukocyte	*KBTBD11*	-0.338134464	0.005454858	0.011427973	1
T2DM_insulin	leukocyte	*RMDN1*	-0.427933897	0.007794866	0.01301113	1
T2DM_insulin	leukocyte	*LRRC37A*	-2.097470603	0.004461958	0.011330438	1
T2DM_insulin	leukocyte	*ACSF3*	-0.518387763	0.00400821	0.011330438	1
T2DM_insulin	leukocyte	*RUFY1*	-0.922656931	0.002641592	0.011330438	1
T2DM_insulin	leukocyte	*VSIG10*	-0.618869846	0.001896051	0.011330438	1
T2DM_insulin	leukocyte	*WSB2*	-0.362251095	0.002704689	0.011330438	1
T2DM_insulin	leukocyte	*ANKLE2*	-0.274293368	0.015459756	0.021810191	1
T2DM_insulin	leukocyte	*MUC20*	-0.393781462	0.005617309	0.011547208	1
T2DM_insulin	leukocyte	*NFATC2IP*	-0.590645697	0.002200286	0.011330438	1
T2DM_insulin	leukocyte	*DPP7*	-0.361409759	0.003015336	0.011330438	1
T2DM_insulin	leukocyte	*MTHFR*	-0.423551167	0.003523072	0.011330438	1
T2DM_insulin	leukocyte	*DEAF1*	-0.515736083	0.002998328	0.011330438	1
T2DM_insulin	leukocyte	*TMEM80*	-0.318280248	0.005317479	0.011421159	1
T2DM_insulin	leukocyte	*DSCAML1*	-0.524301961	0.003081186	0.011330438	1
T2DM_insulin	leukocyte	*PUS1*	-0.822145466	0.00342091	0.011330438	1
T2DM_insulin	leukocyte	*MAN1B1*	-0.666964284	0.002543742	0.011330438	1
T2DM_insulin	leukocyte	*PPFIA3*	-0.307885694	0.005868755	0.011570498	1
T2DM_insulin	leukocyte	*UBE2U*	-1.751350449	0.01222977	0.017949483	1
T2DM_insulin	leukocyte	*PAWR*	-0.359789424	0.004788909	0.011330438	1
T2DM_insulin	leukocyte	*ACOT4*	-0.482082669	0.00942894	0.014830386	1
T2DM_insulin	leukocyte	*ARIH2*	-0.413616448	0.001034846	0.011330438	1
T2DM_insulin	leukocyte	*SAMD12*	-0.397560118	0.035913357	0.045360435	1
T2DM_insulin	leukocyte	*CD163*	0.036451809	0.014140686	0.020239984	1
T2DM_insulin	leukocyte	*ACAD9*	-0.39315852	0.021038653	0.028435111	1
T2DM_insulin	leukocyte	*AGTRAP*	-0.422970929	0.003836492	0.011330438	1
T2DM_insulin	leukocyte	*THAP5*	-0.403388339	0.003337886	0.011330438	1
T2DM_insulin	leukocyte	*CD151*	-0.319008166	0.003164337	0.011330438	1

T2DM_insulin	leukocyte	*CHID1*	-0.370234368	0.000976893	0.011330438	1
T2DM_insulin	leukocyte	*ZBTB41*	-0.509277439	0.002802521	0.011330438	1
T2DM_insulin	leukocyte	*BET1L*	-0.555036615	0.003989728	0.011330438	1
T2DM_insulin	leukocyte	*RIC8A*	-0.518190793	0.002642347	0.011330438	1
T2DM_insulin	leukocyte	*IMP3*	-0.412856224	0.004990619	0.011330438	1
T2DM_insulin	leukocyte	*ADAMTSL1*	-1.477882219	0.035825791	0.045274858	1
T2DM_insulin	leukocyte	*ALS2CL*	-0.547692716	0.004704276	0.011330438	1
T2DM_insulin	leukocyte	*STAP2*	-0.306548789	0.00349304	0.011330438	1
T2DM_insulin	leukocyte	*DALRD3*	-0.408100358	0.007944789	0.013170918	1
T2DM_insulin	leukocyte	*ZNF114*	-1.027425282	0.006242973	0.011570498	1
T2DM_insulin	leukocyte	*PLEC*	-0.829289548	0.000326703	0.011330438	1
T2DM_insulin	leukocyte	*WDR6*	-0.305091384	0.030984656	0.039770897	1
T2DM_insulin	leukocyte	*AQP11*	-0.397042146	0.024923174	0.033015223	1
T2DM_insulin	leukocyte	*ZFAND2A*	-0.485247792	0.004109137	0.011330438	1
T2DM_insulin	leukocyte	*ZNF223*	-0.474287827	0.015955534	0.022398848	1
T2DM_insulin	leukocyte	*NT5DC1*	-0.381218586	0.003590969	0.011330438	1
T2DM_insulin	leukocyte	*COX14*	-0.36690426	0.00720544	0.012381002	1
T2DM_insulin	leukocyte	*P4HTM*	-0.304523666	0.00196134	0.011330438	1
T2DM_insulin	leukocyte	*ERBB4*	-3.46078935	0.00208142	0.011330438	1
T2DM_insulin	leukocyte	*ERN1*	-0.492196467	0.004619167	0.011330438	1
T2DM_insulin	leukocyte	*PARP10*	-0.447371973	0.010366132	0.01590337	1
T2DM_insulin	leukocyte	*RPP25*	-0.634611127	0.002748375	0.011330438	1
T2DM_insulin	leukocyte	*ZHX2*	-0.419039971	0.005822957	0.011570498	1
T2DM_insulin	leukocyte	*HYI*	-0.476677802	0.002289019	0.011330438	1
T2DM_insulin	leukocyte	*GAK*	-0.408620922	0.01019349	0.015699813	1
T2DM_insulin	leukocyte	*TUFM*	-0.224527964	0.027677836	0.036128312	1
T2DM_insulin	leukocyte	*RMI1*	0.71299633	0.002245518	0.011330438	1
T2DM_insulin	leukocyte	*C3orf38*	-0.519460992	0.01182827	0.017616858	1
T2DM_insulin	leukocyte	*IGSF22*	-0.284547396	0.007378721	0.012556771	1
T2DM_insulin	leukocyte	*HTR1F*	-1.129733171	0.002842751	0.011330438	1
T2DM_insulin	leukocyte	*TMTC2*	-0.711054974	0.004486731	0.011330438	1
T2DM_insulin	leukocyte	*FARSA*	-0.46115118	0.00082987	0.011330438	1
T2DM_insulin	leukocyte	*SPTY2D1*	-0.400354737	0.004350963	0.011330438	1
T2DM_insulin	leukocyte	*SAMD4B*	-0.294462936	0.01424323	0.020357093	1
T2DM_insulin	leukocyte	*TCAIM*	-0.556890582	0.002807481	0.011330438	1
T2DM_insulin	leukocyte	*CALR*	-0.269074635	0.008709477	0.013978569	1
T2DM_insulin	leukocyte	*NSUN7*	-0.648808423	0.014281454	0.020389474	1
T2DM_insulin	leukocyte	*ELMOD2*	-0.644409725	0.004437332	0.011330438	1
T2DM_insulin	leukocyte	*LINC00174*	-0.387058856	0.003582136	0.011330438	1
T2DM_insulin	leukocyte	*GEMIN4*	-0.382915771	0.006887812	0.012081345	1
T2DM_insulin	leukocyte	*LBX2*	0.418005848	0.010429722	0.015957312	1
T2DM_insulin	leukocyte	*CDC42EP4*	-0.326502651	0.004844525	0.011330438	1
T2DM_insulin	leukocyte	*ZBTB42*	-0.977153236	0.001893751	0.011330438	1
T2DM_insulin	leukocyte	*SYCN*	-0.205474833	0.006744691	0.011963834	1
T2DM_insulin	leukocyte	*CITED4*	-0.239249405	0.011708825	0.017459386	1
T2DM_insulin	leukocyte	*ABCA13*	-7.613769403	0.013184544	0.019044879	1
T2DM_insulin	leukocyte	*B3GNT3*	-0.785172184	0.017341243	0.024041546	1
T2DM_insulin	leukocyte	*PUF60*	-0.315719125	0.010848938	0.016457713	1
T2DM_insulin	leukocyte	*TSHZ1*	-0.328813695	0.004929506	0.011330438	1
T2DM_insulin	leukocyte	*EXOC3*	-0.698833266	0.002353939	0.011330438	1
T2DM_insulin	leukocyte	*TH*	-1.963095063	0.007422432	0.012597294	1
T2DM_insulin	leukocyte	*FAHD1*	-0.284332831	0.036295596	0.045824356	1
T2DM_insulin	leukocyte	*ZNF816*	-0.570297651	0.004238369	0.011330438	1
T2DM_insulin	leukocyte	*CCDC66*	-0.92812874	0.002869591	0.011330438	1
T2DM_insulin	leukocyte	*MCFD2*	-0.304645428	0.002926637	0.011330438	1
T2DM_insulin	leukocyte	*SERTM1*	0.290340036	0.022316639	0.029925623	1
T2DM_insulin	leukocyte	*TSPYL5*	-0.297256826	0.005142414	0.011330438	1
T2DM_insulin	leukocyte	*SHISA2*	-0.739322181	0.005438786	0.011421159	1
T2DM_insulin	leukocyte	*CHST13*	-31.45259824	0.006315263	0.011616417	1
T2DM_insulin	leukocyte	*PPA1*	-0.284970465	0.003196051	0.011330438	1
T2DM_insulin	leukocyte	*PSMG4*	-0.319837221	0.004118625	0.011330438	1
T2DM_insulin	leukocyte	*MAP6D1*	-0.719789885	0.011874789	0.017638109	1
T2DM_insulin	leukocyte	*ZNF792*	-0.687456752	0.026891909	0.035207701	1
T2DM_insulin	leukocyte	*D2HGDH*	-1.444056986	0.001291882	0.011330438	1

T2DM_insulin	leukocyte	*FAM83H*	-0.561524362	0.006878208	0.012075281	1
T2DM_insulin	leukocyte	*LRRC57*	-0.64904548	0.00294387	0.011330438	1
T2DM_insulin	leukocyte	*MRPL14*	-0.409754968	0.00215976	0.011330438	1
T2DM_insulin	leukocyte	*ZFP82*	-0.359561186	0.005814166	0.011570498	1
T2DM_insulin	leukocyte	*LSMEM1*	-0.497795825	0.031605006	0.040527438	1
T2DM_insulin	leukocyte	*FKRP*	-0.437325559	0.000627561	0.011330438	1
T2DM_insulin	leukocyte	*RPH3AL*	-0.286822196	0.005233452	0.011389288	1
T2DM_insulin	leukocyte	*HIGD1A*	-0.303588304	0.003749279	0.011330438	1
T2DM_insulin	leukocyte	*F2R*	-0.302897021	0.007317884	0.012496955	1
T2DM_insulin	leukocyte	*ZNF707*	-0.479150408	0.005923221	0.011570498	1
T2DM_insulin	leukocyte	*SLC25A41*	-2.16713561	0.006540054	0.011804448	1
T2DM_insulin	leukocyte	*OGFOD3*	-0.350240407	0.002304918	0.011330438	1
T2DM_insulin	leukocyte	*ZNF467*	-0.470016172	0.008735443	0.014008798	1
T2DM_insulin	leukocyte	*TMEM45A*	-0.307217674	0.006039377	0.011570498	1
T2DM_insulin	leukocyte	*GPR135*	-0.434901378	0.0067314	0.011961858	1
T2DM_insulin	leukocyte	*ANKRD62*	-0.610868344	0.002497963	0.011330438	1
T2DM_insulin	leukocyte	*RFX7*	-0.899583253	0.002842547	0.011330438	1
T2DM_insulin	leukocyte	*ZNF329*	-0.53601877	0.003409886	0.011330438	1
T2DM_insulin	leukocyte	*CCDC149*	-0.641853332	0.00199505	0.011330438	1
T2DM_insulin	leukocyte	*IDH2*	-0.289895072	0.006415634	0.01170553	1
T2DM_insulin	leukocyte	*TNRC18*	-0.471001506	0.004220817	0.011330438	1
T2DM_insulin	leukocyte	*TDRKH*	-0.455084011	0.002683387	0.011330438	1
T2DM_insulin	leukocyte	*ZNF708*	-0.308047833	0.004020608	0.011330438	1
T2DM_insulin	leukocyte	*IST1*	-0.570102611	0.002563584	0.011330438	1
T2DM_insulin	leukocyte	*ERCC6L2*	-1.08629148	0.003022039	0.011330438	1
T2DM_insulin	leukocyte	*MRPL41*	-0.36271875	0.002991709	0.011330438	1
T2DM_insulin	leukocyte	*TP53TG1*	-0.429016554	0.001752747	0.011330438	1
T2DM_insulin	leukocyte	*UBA7*	-0.607278084	0.02266661	0.030332803	1
T2DM_insulin	leukocyte	*ARL6IP4*	-0.269591471	0.007103179	0.012294167	1
T2DM_insulin	leukocyte	*FAM153B*	-0.516123162	0.010502269	0.016055804	1
T2DM_insulin	leukocyte	*BACE2*	-0.229937733	0.004747622	0.011330438	1
T2DM_insulin	leukocyte	*SYNM*	-0.379445821	0.00595998	0.011570498	1
T2DM_insulin	leukocyte	*FIGN*	-0.355177417	0.008592016	0.013846609	1
T2DM_insulin	leukocyte	*ZNF804B*	-27.79333892	0.005478722	0.011427973	1
T2DM_insulin	leukocyte	*YBEY*	-0.43471954	0.005880819	0.011570498	1
T2DM_insulin	leukocyte	*NPLOC4*	-0.392656067	0.003888992	0.011330438	1
T2DM_insulin	leukocyte	*CAPN12*	-0.69597781	0.005522049	0.011446386	1
T2DM_insulin	leukocyte	*GLRX5*	-0.276382395	0.020805392	0.028168292	1
T2DM_insulin	leukocyte	*MXRA7*	-0.330230828	0.003347997	0.011330438	1
T2DM_insulin	leukocyte	*ADI1*	-0.788570428	0.002450006	0.011330438	1
T2DM_insulin	leukocyte	*SATB1*	-0.602064639	0.002053171	0.011330438	1
T2DM_insulin	leukocyte	*SKA2*	-0.688336584	0.001213597	0.011330438	1
T2DM_insulin	leukocyte	*TSKU*	-0.419546931	0.005476941	0.011427973	1
T2DM_insulin	leukocyte	*ANXA2*	-0.217769334	0.005491767	0.011434531	1
T2DM_insulin	leukocyte	*SLC35D3*	-0.742181816	0.002403266	0.011330438	1
T2DM_insulin	leukocyte	*GPIHBP1*	-3.397099897	0.006403567	0.01170553	1
T2DM_insulin	leukocyte	*VMO1*	0.042759594	0.012522983	0.018270627	1
T2DM_insulin	leukocyte	*ALG12*	-0.548850736	0.024288174	0.032261067	1
T2DM_insulin	leukocyte	*COL18A1*	-0.154319945	0.025636747	0.033823634	1
T2DM_insulin	leukocyte	*C11orf54*	-0.485637878	0.009655755	0.015078655	1
T2DM_insulin	leukocyte	*CEP63*	-0.466784585	0.002329638	0.011330438	1
T2DM_insulin	leukocyte	*HMGN4*	-0.511872599	0.003574689	0.011330438	1
T2DM_insulin	leukocyte	*SPATA13*	-0.364559812	0.004046104	0.011330438	1
T2DM_insulin	leukocyte	*ZNF320*	0.400707154	0.000815771	0.011330438	1
T2DM_insulin	leukocyte	*SLC25A21*	-0.948918873	0.005823913	0.011570498	1
T2DM_insulin	leukocyte	*LYSMD4*	-0.433740873	0.006823756	0.012014984	1
T2DM_insulin	leukocyte	*BEGAIN*	-0.542844185	0.004920048	0.011330438	1
T2DM_insulin	leukocyte	*GPC6*	-0.422445612	0.007153708	0.01235469	1
T2DM_insulin	leukocyte	*FANCF*	-1.310354623	0.00216298	0.011330438	1
T2DM_insulin	leukocyte	*SMDT1*	-0.214706359	0.025189167	0.03330179	1
T2DM_insulin	leukocyte	*PTTG1IP*	-0.318264799	0.006381585	0.011683713	1
T2DM_insulin	leukocyte	*ZNF623*	-0.324695272	0.008598972	0.013849257	1
T2DM_insulin	leukocyte	*CCDC125*	-0.692507142	0.028168705	0.036635168	1
T2DM_insulin	leukocyte	*BOLA2*	-0.383023783	0.002966467	0.011330438	1

T2DM_insulin	leukocyte	*FHL3*	-0.318886126	0.025780827	0.033972625	1
T2DM_insulin	leukocyte	*SF3A3*	-0.337940023	0.004081178	0.011330438	1
T2DM_insulin	leukocyte	*MX2*	0.468675642	0.023087276	0.030822259	1
T2DM_insulin	leukocyte	*PCBP3*	-0.451303076	0.032919538	0.041980157	1
T2DM_insulin	leukocyte	*SFXN4*	-0.427613779	0.003269612	0.011330438	1
T2DM_insulin	leukocyte	*MRPL54*	-0.343665356	0.005018123	0.011330438	1
T2DM_insulin	leukocyte	*DGCR6*	-0.44044187	0.002203877	0.011330438	1
T2DM_insulin	leukocyte	*NDUFB1*	-0.344997386	0.005643368	0.01154833	1
T2DM_insulin	leukocyte	*TRMT12*	-0.454489673	0.002529034	0.011330438	1
T2DM_insulin	leukocyte	*FIGLA*	-9.367362161	0.002765244	0.011330438	1
T2DM_insulin	leukocyte	*CBX6*	-0.307162521	0.007490357	0.012685177	1
T2DM_insulin	leukocyte	*MACC1*	-0.276492076	0.005422085	0.011421159	1
T2DM_insulin	leukocyte	*KREMEN1*	-1.31871176	0.00161763	0.011330438	1
T2DM_insulin	leukocyte	*NUDT14*	-0.330926458	0.008368044	0.013597207	1
T2DM_insulin	leukocyte	*FAM3B*	-0.296166641	0.012028223	0.017759823	1
T2DM_insulin	leukocyte	*TOB2*	-0.421864699	0.004396446	0.011330438	1
T2DM_insulin	leukocyte	*TTC32*	-0.394406812	0.002324645	0.011330438	1
T2DM_insulin	leukocyte	*LRRC55*	-4.226014083	0.010874047	0.016483075	1
T2DM_insulin	leukocyte	*TMEM236*	-0.24972324	0.019380678	0.026504241	1
T2DM_insulin	leukocyte	*UQCR10*	-0.331942648	0.00197286	0.011330438	1
T2DM_insulin	leukocyte	*NIPSNAP1*	-0.369775832	0.004088462	0.011330438	1
T2DM_insulin	leukocyte	*CRELD2*	-0.288653576	0.006257378	0.011570498	1
T2DM_insulin	leukocyte	*UBE2F*	-0.620194176	0.001951181	0.011330438	1
T2DM_insulin	leukocyte	*GOLGA6L4*	-0.54392796	0.001033654	0.011330438	1
T2DM_insulin	leukocyte	*PGP*	-0.594555501	0.000724498	0.011330438	1
T2DM_insulin	leukocyte	*ACOT1*	-1.913000408	0.022892694	0.03058327	1
T2DM_insulin	leukocyte	*OAF*	-0.476851811	0.003256507	0.011330438	1
T2DM_insulin	leukocyte	*TM2D3*	-0.368106125	0.003474376	0.011330438	1
T2DM_insulin	leukocyte	*MROH7*	-0.693702738	0.005069518	0.011330438	1
T2DM_insulin	leukocyte	*TOP1MT*	-0.810472433	0.007918974	0.013139209	1
T2DM_insulin	leukocyte	*COPB2*	-0.421207876	0.002913454	0.011330438	1
T2DM_insulin	leukocyte	*LRRC19*	-1.420270692	0.000463591	0.011330438	1
T2DM_insulin	leukocyte	*CCR10*	-0.883051748	0.003227301	0.011330438	1
T2DM_insulin	leukocyte	*NCMAP*	-0.360358238	0.006975839	0.012179027	1
T2DM_insulin	leukocyte	*WDR27*	-0.885195094	0.003496396	0.011330438	1
T2DM_insulin	leukocyte	*TXNRD2*	-0.382417649	0.005180237	0.011364127	1
T2DM_insulin	leukocyte	*ZFP1*	-0.515834805	0.002643123	0.011330438	1
T2DM_insulin	leukocyte	*XPOT*	-0.346590197	0.005094979	0.011330438	1
T2DM_insulin	leukocyte	*C14orf180*	-39.27993671	0.006008628	0.011570498	1
T2DM_insulin	leukocyte	*UBE2G2*	-0.378528235	0.005131638	0.011330438	1
T2DM_insulin	leukocyte	*OSBP2*	-0.432097596	0.006087732	0.011570498	1
T2DM_insulin	leukocyte	*TMED9*	-0.325012148	0.003207231	0.011330438	1
T2DM_insulin	leukocyte	*BTBD6*	-0.490562833	0.003080107	0.011330438	1
T2DM_insulin	leukocyte	*JAG2*	-0.335985246	0.007370248	0.012552105	1
T2DM_insulin	leukocyte	*LCN12*	-1.236458084	0.004677223	0.011330438	1
T2DM_insulin	leukocyte	*FAM227A*	-0.403392231	0.007805711	0.013011726	1
T2DM_insulin	leukocyte	*MUC6*	-0.221094108	0.011494306	0.017184601	1
T2DM_insulin	leukocyte	*NOC4L*	-0.568304968	0.00511306	0.011330438	1
T2DM_insulin	leukocyte	*SIVA1*	-0.364011069	0.003862839	0.011330438	1
T2DM_insulin	leukocyte	*BRI3BP*	-0.524736726	0.003519705	0.011330438	1
T2DM_insulin	leukocyte	*UBOX5*	-0.37599118	0.005426416	0.011421159	1
T2DM_insulin	leukocyte	*MAFF*	-0.232256954	0.008333047	0.013546584	1
T2DM_insulin	leukocyte	*SEMA4B*	-0.367704088	0.006205334	0.011570498	1
T2DM_insulin	leukocyte	*CIB1*	-0.356884175	0.002925933	0.011330438	1
T2DM_insulin	leukocyte	*MFSD6L*	-0.994750163	0.015249311	0.021559749	1
T2DM_insulin	leukocyte	*AQP12B*	-2.361272454	0.009789489	0.015232833	1
T2DM_insulin	leukocyte	*ZNF445*	-1.425170523	0.008501895	0.01374648	1
T2DM_insulin	leukocyte	*RBM11*	-0.347778571	0.002832307	0.011330438	1
T2DM_insulin	leukocyte	*RGPD2*	-1.210370701	0.003871736	0.011330438	1
T2DM_insulin	leukocyte	*ATP6V0A2*	-0.573295052	0.005230469	0.011389288	1
T2DM_insulin	leukocyte	*RAD51D*	-0.965820896	0.002697702	0.011330438	1
T2DM_insulin	leukocyte	*SH3BGR*	-0.288339665	0.00118922	0.011330438	1
T2DM_insulin	leukocyte	*MUC1*	-0.247973847	0.006606399	0.011868561	1
T2DM_insulin	leukocyte	*IRF7*	-0.562450881	0.013199319	0.019059215	1

T2DM_insulin	leukocyte	*FAM131C*	-0.276600058	0.005403581	0.011421159	1
T2DM_insulin	leukocyte	*LSAMP*	-0.194952791	0.013107648	0.018954711	1
T2DM_insulin	leukocyte	*OLFML2A*	-0.968621206	0.012120574	0.017849227	1
T2DM_insulin	leukocyte	*PDIA2*	-0.184519304	0.010037751	0.015522195	1
T2DM_insulin	leukocyte	*PCGF3*	-0.410187578	0.003090921	0.011330438	1
T2DM_insulin	leukocyte	*P4HB*	-0.247139038	0.005304979	0.011421159	1
T2DM_insulin	leukocyte	*PSMD13*	-0.380722041	0.005150126	0.011330438	1
T2DM_insulin	leukocyte	*BRWD1*	-0.424554276	0.002146679	0.011330438	1
T2DM_insulin	leukocyte	*DRG1*	-0.359721299	0.003742224	0.011330438	1
T2DM_insulin	leukocyte	*ZNF696*	-1.072648625	0.005121459	0.011330438	1
T2DM_insulin	leukocyte	*MORF4L1*	-0.288169278	0.003169234	0.011330438	1
T2DM_insulin	leukocyte	*DMWD*	-0.767392145	0.000370609	0.011330438	1
T2DM_insulin	leukocyte	*PIGP*	-0.341266042	0.001103028	0.011330438	1
T2DM_insulin	leukocyte	*NPAP1*	-12.21267383	0.009195844	0.014545279	1
T2DM_insulin	leukocyte	*ARL17A*	-0.246858467	0.035051283	0.044424674	1
T2DM_insulin	leukocyte	*TRIM69*	-0.406685307	0.004388924	0.011330438	1
T2DM_insulin	leukocyte	*IFITM1*	-0.998013997	0.001216467	0.011330438	1
T2DM_insulin	leukocyte	*SETD4*	-1.122246569	0.019811763	0.026990436	1
T2DM_insulin	leukocyte	*ZNF529*	-0.704284576	0.002281809	0.011330438	1
T2DM_insulin	leukocyte	*AIDA*	-0.35683787	0.003029502	0.011330438	1
T2DM_insulin	leukocyte	*WWOX*	-0.382603689	0.004005154	0.011330438	1
T2DM_insulin	leukocyte	*POLR1D*	-0.339376214	0.002658352	0.011330438	1
T2DM_insulin	leukocyte	*EDARADD*	-0.391297904	0.005451952	0.011427973	1
T2DM_insulin	leukocyte	*ZNF749*	-0.291659399	0.003779995	0.011330438	1
T2DM_insulin	leukocyte	*TOR3A*	-0.393687081	0.004424954	0.011330438	1
T2DM_insulin	leukocyte	*ZNF555*	-0.299630041	0.007557367	0.012749204	1
T2DM_insulin	leukocyte	*NUDT17*	-0.514850262	0.007185974	0.012372346	1
T2DM_insulin	leukocyte	*CYP4X1*	0.529742388	0.012977034	0.018807365	1
T2DM_insulin	leukocyte	*ZNF197*	-0.355301397	0.004172373	0.011330438	1
T2DM_insulin	leukocyte	*RPS23*	-0.262373407	0.002388004	0.011330438	1
T2DM_insulin	leukocyte	*ZNF396*	-0.887822912	0.01237172	0.0181108	1
T2DM_insulin	leukocyte	*CEACAM19*	-0.234761186	0.03948901	0.049506165	1
T2DM_insulin	leukocyte	*ZFP91*	-0.469967432	0.001856194	0.011330438	1
T2DM_insulin	leukocyte	*BCR*	-0.371199902	0.003141419	0.011330438	1
T2DM_insulin	leukocyte	*HYAL3*	-0.900307662	0.000143192	0.011330438	0.562602546
T2DM_insulin	leukocyte	*RTN4RL2*	-0.387187443	0.00618863	0.011570498	1
T2DM_insulin	leukocyte	*TMEM232*	-0.610694351	0.003401999	0.011330438	1
T2DM_insulin	leukocyte	*FAM183A*	-0.310822351	0.006693763	0.011938173	1
T2DM_insulin	leukocyte	*EMID1*	-0.515967953	0.002857774	0.011330438	1
T2DM_insulin	leukocyte	*ESPN*	-9.513394721	0.000290637	0.011330438	1
T2DM_insulin	leukocyte	*AKR1C1*	-0.289446671	0.00505807	0.011330438	1
T2DM_insulin	leukocyte	*BCAM*	-0.264855573	0.008161045	0.013387195	1
T2DM_insulin	leukocyte	*CHP1*	-0.321719033	0.003588813	0.011330438	1
T2DM_insulin	leukocyte	*KCNJ11*	-1.222408147	0.002896176	0.011330438	1
T2DM_insulin	leukocyte	*ZNF385C*	-1.257369253	0.004693662	0.011330438	1
T2DM_insulin	leukocyte	*EXD3*	-0.405463035	0.005325236	0.011421159	1
T2DM_insulin	leukocyte	*RGPD1*	-41.84265848	0.006555736	0.011815361	1
T2DM_insulin	leukocyte	*DHRS4L2*	-0.397527708	0.003240977	0.011330438	1
T2DM_insulin	leukocyte	*TRPV2*	-2.426590169	0.038134128	0.047960624	1
T2DM_insulin	leukocyte	*FANCA*	-0.690416315	0.001990282	0.011330438	1
T2DM_insulin	leukocyte	*PALM3*	-0.472112284	0.005882613	0.011570498	1
T2DM_insulin	leukocyte	*KLHL17*	-0.645988848	0.00549326	0.011434531	1
T2DM_insulin	leukocyte	*RINL*	-0.486848357	0.003932348	0.011330438	1
T2DM_insulin	leukocyte	*ZNF490*	-0.701677969	0.0033271	0.011330438	1
T2DM_insulin	leukocyte	*NRN1L*	-1.000419772	0.006272425	0.011570498	1
T2DM_insulin	leukocyte	*NWD1*	-0.960640745	0.030710953	0.039500311	1
T2DM_insulin	leukocyte	*RAB42*	0.170336011	0.039610076	0.049642102	1
T2DM_insulin	leukocyte	*MAPK12*	-0.491658757	0.003351247	0.011330438	1
T2DM_insulin	leukocyte	*LINC00265*	-0.793635308	0.005924983	0.011570498	1
T2DM_insulin	leukocyte	*LAMTOR4*	-0.322317971	0.007343038	0.012522047	1
T2DM_insulin	leukocyte	*PRKAR1B*	-0.433133859	0.008918916	0.014204468	1
T2DM_insulin	leukocyte	*NUTM2B*	-5.82873248	0.025784263	0.033972625	1
T2DM_insulin	leukocyte	*CENPP*	-0.274924919	0.018259219	0.025163265	1
T2DM_insulin	leukocyte	*ZNF559*	-0.315357697	0.008889588	0.014169246	1

T2DM_insulin	leukocyte	*FOCAD*	-0.406811966	0.004572967	0.011330438	1
T2DM_insulin	leukocyte	*ZP3*	-0.473171118	0.013126205	0.018967583	1
T2DM_insulin	leukocyte	*SERPINA5*	-0.235624607	0.026595122	0.034923875	1
T2DM_insulin	leukocyte	*SRSF10*	-0.340208282	0.00622249	0.011570498	1
T2DM_insulin	leukocyte	*RALGAPA2*	-0.409434277	0.003498212	0.011330438	1
T2DM_insulin	leukocyte	*NDOR1*	-1.100472486	0.005373477	0.011421159	1
T2DM_insulin	leukocyte	*FBLL1*	-0.846044951	0.002434118	0.011330438	1
T2DM_insulin	leukocyte	*ASAH2*	-1.182125383	0.000153423	0.011330438	0.602797695
T2DM_insulin	leukocyte	*S100A16*	-0.20429716	0.005663364	0.011549238	1
T2DM_insulin	leukocyte	*PARVB*	-0.213647032	0.025488856	0.033651114	1
T2DM_insulin	leukocyte	*UROS*	-0.466559593	0.002972357	0.011330438	1
T2DM_insulin	leukocyte	*FAM221A*	-0.606436802	0.000840856	0.011330438	1
T2DM_insulin	leukocyte	*NOXA1*	-0.704037211	0.004970256	0.011330438	1
T2DM_insulin	leukocyte	*TMEM198*	-0.302575506	0.009387871	0.014787038	1
T2DM_insulin	leukocyte	*ZNF548*	-0.419268727	0.01451836	0.020652657	1
T2DM_insulin	leukocyte	*MTF1*	-0.388008713	0.003737907	0.011330438	1
T2DM_insulin	leukocyte	*RPL14*	-0.253517041	0.00247875	0.011330438	1
T2DM_insulin	leukocyte	*FBF1*	0.395945093	0.004479916	0.011330438	1
T2DM_insulin	leukocyte	*MSL1*	-0.365519834	0.009433391	0.01483145	1
T2DM_insulin	leukocyte	*NOC2L*	-0.473403126	0.00298649	0.011330438	1
T2DM_insulin	leukocyte	*ZNF567*	-0.378746554	0.005152142	0.011330438	1
T2DM_insulin	leukocyte	*ARID2*	-0.501870033	0.003348049	0.011330438	1
T2DM_insulin	leukocyte	*PLAC9*	-0.347582608	0.011875805	0.017638109	1
T2DM_insulin	leukocyte	*CLDN4*	-0.220213153	0.004316051	0.011330438	1
T2DM_insulin	leukocyte	*FAM47E*	-0.742157502	3.05E-05	0.011330438	0.119762241
T2DM_insulin	leukocyte	*ZNF527*	-0.547897269	0.003509691	0.011330438	1
T2DM_insulin	leukocyte	*ZNF600*	-0.335519257	0.022375512	0.029984102	1
T2DM_insulin	leukocyte	*PNRC2*	-0.312086343	0.006186569	0.011570498	1
T2DM_insulin	leukocyte	*ZKSCAN3*	-1.123973507	7.12E-05	0.011330438	0.279911498
T2DM_insulin	leukocyte	*RRP7A*	-0.362819878	0.012943323	0.018774693	1
T2DM_insulin	leukocyte	*S100A14*	-0.268816124	0.022736312	0.030405368	1
T2DM_insulin	leukocyte	*CXCL17*	-0.2903567	0.012025357	0.017759823	1
T2DM_insulin	leukocyte	*BLOC1S2*	-0.377897686	0.005165054	0.011343487	1
T2DM_insulin	leukocyte	*ZNF676*	-0.593674909	0.000704098	0.011330438	1
T2DM_insulin	leukocyte	*WDSUB1*	-0.489857659	0.005085306	0.011330438	1
T2DM_insulin	leukocyte	*ZNF681*	-1.70261127	0.0075137	0.01271375	1
T2DM_insulin	leukocyte	*HRCT1*	0.416385363	0.019549495	0.026687509	1
T2DM_insulin	leukocyte	*MPHOSPH8*	-0.377530551	0.003072169	0.011330438	1
T2DM_insulin	leukocyte	*SIRPB2*	0.668146961	0.013521193	0.019473889	1
T2DM_insulin	leukocyte	*LCOR*	-0.350236971	0.003202826	0.011330438	1
T2DM_insulin	leukocyte	*SUPT5H*	-0.328551297	0.004103324	0.011330438	1
T2DM_insulin	leukocyte	*XPNPEP3*	-0.577477189	0.002084592	0.011330438	1
T2DM_insulin	leukocyte	*ZNF107*	-0.433659939	0.003546155	0.011330438	1
T2DM_insulin	leukocyte	*SFTA2*	-0.517330401	0.001226094	0.011330438	1
T2DM_insulin	leukocyte	*PPIA*	-0.319795301	0.002156123	0.011330438	1
T2DM_insulin	leukocyte	*ZNF471*	-0.539652948	0.00579371	0.011570498	1
T2DM_insulin	leukocyte	*ZNF493*	-0.509784833	0.003271213	0.011330438	1
T2DM_insulin	leukocyte	*GTF2IRD2*	-0.54168307	0.002687578	0.011330438	1
T2DM_insulin	leukocyte	*SUPT3H*	-0.330426749	0.005662698	0.011549238	1
T2DM_insulin	leukocyte	*POM121*	-0.36093775	0.009984817	0.015458818	1
T2DM_insulin	leukocyte	*LONP1*	-0.546569657	0.003193912	0.011330438	1
T2DM_insulin	leukocyte	*ZNF34*	-0.52326592	0.006042847	0.011570498	1
T2DM_insulin	leukocyte	*ZNF781*	-0.688335414	0.000806899	0.011330438	1
T2DM_insulin	leukocyte	*EVL*	-0.716196247	0.012787726	0.018608509	1
T2DM_insulin	leukocyte	*ZNF124*	-0.634711137	0.003678361	0.011330438	1
T2DM_insulin	leukocyte	*XRCC6*	-0.351288938	0.003342337	0.011330438	1
T2DM_insulin	leukocyte	*PIK3R4*	-0.625638221	0.003591506	0.011330438	1
T2DM_insulin	leukocyte	*C20orf96*	-0.85959903	0.000864504	0.011330438	1
T2DM_insulin	leukocyte	*SULT1A1*	-0.836079749	0.001204895	0.011330438	1
T2DM_insulin	leukocyte	*ARL9*	0.413636237	0.028457146	0.036949149	1
T2DM_insulin	leukocyte	*TPK1*	-0.961016617	0.00551209	0.011434531	1
T2DM_insulin	leukocyte	*AFAP1*	-0.465150963	0.00243028	0.011330438	1
T2DM_insulin	leukocyte	*MYO18A*	-0.338477348	0.00464805	0.011330438	1
T2DM_insulin	leukocyte	*XRCC2*	-1.417950988	0.004938616	0.011330438	1

T2DM_insulin	leukocyte	*MYO6*	-0.280577351	0.004373228	0.011330438	1
T2DM_insulin	leukocyte	*ZNF502*	-1.425937896	0.004669587	0.011330438	1
T2DM_insulin	leukocyte	*TRAPPC4*	-0.293521732	0.01718922	0.023872903	1
T2DM_insulin	leukocyte	*ZNF33B*	-0.739572597	0.001106486	0.011330438	1
T2DM_insulin	leukocyte	*AMZ2*	-0.463066436	0.003538038	0.011330438	1
T2DM_insulin	leukocyte	*ZNF431*	-0.394935029	0.00333554	0.011330438	1
T2DM_insulin	leukocyte	*DAPK1*	-0.598306887	0.006658589	0.01190541	1
T2DM_insulin	leukocyte	*HLA-DQA1*	0.031017801	0.020370785	0.027675247	1
T2DM_insulin	leukocyte	*CLPSL2*	-0.386777618	0.026430573	0.034731011	1
T2DM_insulin	leukocyte	*ADA*	0.191154451	0.013780659	0.019782319	1
T2DM_insulin	leukocyte	*PPTC7*	-0.647836868	0.000736538	0.011330438	1
T2DM_insulin	leukocyte	*KRT39*	-2.617445017	0.029087598	0.037680572	1
T2DM_insulin	leukocyte	*ZFP28*	-0.542855245	0.004315959	0.011330438	1
T2DM_insulin	leukocyte	*ANKRD36B*	-0.363736024	0.023711975	0.031559739	1
T2DM_insulin	leukocyte	*SRGAP1*	-0.448181814	0.00379294	0.011330438	1
T2DM_insulin	leukocyte	*FAM3C*	-0.312104055	0.003499966	0.011330438	1
T2DM_insulin	leukocyte	*AP2A1*	-0.482552963	0.002982254	0.011330438	1
T2DM_insulin	leukocyte	*WDR5B*	-1.050371471	0.008096638	0.013304764	1
T2DM_insulin	leukocyte	*ZNF138*	-0.294902358	0.009284727	0.014662255	1
T2DM_insulin	leukocyte	*ZNF429*	-0.362618257	0.0049195	0.011330438	1
T2DM_insulin	leukocyte	*ZNF100*	-0.559173761	0.001525504	0.011330438	1
T2DM_insulin	leukocyte	*ZNF763*	-0.688561892	0.017057582	0.023757263	1
T2DM_insulin	leukocyte	*ARRDC1*	-0.335259241	0.008251165	0.013448692	1
T2DM_insulin	leukocyte	*ZNF682*	-0.801537949	0.000978287	0.011330438	1
T2DM_insulin	leukocyte	*ZNF257*	-0.643211648	0.002037917	0.011330438	1
T2DM_insulin	leukocyte	*ACSL5*	-0.657638585	0.003398323	0.011330438	1
T2DM_insulin	leukocyte	*SND1*	-0.370457775	0.003987927	0.011330438	1
T2DM_insulin	leukocyte	*SULT1A2*	-1.307756356	0.008645183	0.013909469	1
T2DM_insulin	leukocyte	*TBC1D9B*	-0.681115216	0.003106155	0.011330438	1
T2DM_insulin	leukocyte	*C6orf141*	-0.682511434	0.008814089	0.014103329	1
T2DM_insulin	leukocyte	*GTF2E2*	-0.35181142	0.005836297	0.011570498	1
T2DM_insulin	leukocyte	*ZNF165*	-0.316057875	0.028037736	0.036525287	1
T2DM_insulin	leukocyte	*SVIL*	-0.357950851	0.003678401	0.011330438	1
T2DM_insulin	leukocyte	*MRPL21*	-0.392805947	0.002249957	0.011330438	1
T2DM_insulin	leukocyte	*ZNF98*	-0.521883528	0.006995385	0.01219382	1
T2DM_insulin	leukocyte	*ZNF517*	-1.598885584	0.002627339	0.011330438	1
T2DM_insulin	leukocyte	*SLC22A5*	-0.579268391	0.003484115	0.011330438	1
T2DM_insulin	leukocyte	*C5AR1*	0.047349833	0.01581625	0.022249211	1
T2DM_insulin	leukocyte	*VEPH1*	-0.629379949	0.024076894	0.03200207	1
T2DM_insulin	leukocyte	*OGDHL*	-0.33717463	0.037250685	0.046954745	1
T2DM_insulin	leukocyte	*PCDHB11*	-0.820283717	0.001319015	0.011330438	1
T2DM_insulin	leukocyte	*ATG7*	-0.750961332	0.002600439	0.011330438	1
T2DM_insulin	leukocyte	*PIGN*	-0.295717131	0.018006185	0.024823263	1
T2DM_insulin	leukocyte	*HHLA3*	-0.281737744	0.027571723	0.036001761	1
T2DM_insulin	leukocyte	*BCO2*	-0.386549577	0.010010391	0.015490676	1
T2DM_insulin	leukocyte	*ENTPD6*	-0.382728034	0.011635004	0.017368514	1
T2DM_insulin	leukocyte	*ENPP1*	-0.286217648	0.013062888	0.0189039	1
T2DM_insulin	leukocyte	*DPP4*	-0.258299512	0.005186672	0.011371894	1
T2DM_insulin	leukocyte	*NMB*	-0.365008887	0.005069755	0.011330438	1
T2DM_insulin	leukocyte	*RPS26*	-0.244103088	0.009002729	0.014308949	1
T2DM_insulin	leukocyte	*LHFPL5*	-0.955705633	0.005024911	0.011330438	1
T2DM_insulin	leukocyte	*ZNF780A*	-0.301344512	0.012449393	0.018197048	1
T2DM_insulin	leukocyte	*ATAD3A*	-0.679585279	0.002121491	0.011330438	1
T2DM_insulin	leukocyte	*FAM118B*	-0.815604387	0.006198804	0.011570498	1
T2DM_insulin	leukocyte	*CCDC180*	-1.030556352	0.001698503	0.011330438	1
T2DM_insulin	leukocyte	*SLC9A8*	-1.175820672	0.004941312	0.011330438	1
T2DM_insulin	leukocyte	*ZNF181*	-0.541957702	0.003822741	0.011330438	1
T2DM_insulin	leukocyte	*TEAD4*	-0.320773695	0.006162821	0.011570498	1
T2DM_insulin	leukocyte	*C2orf27A*	-0.770557068	0.000190598	0.011330438	0.748859662
T2DM_insulin	leukocyte	*PLCG2*	-0.426202	0.004005785	0.011330438	1
T2DM_insulin	leukocyte	*RPL12*	-0.258510761	0.001369832	0.011330438	1
T2DM_insulin	leukocyte	*VPS13A*	-0.340015444	0.007624159	0.01282334	1
T2DM_insulin	leukocyte	*LEKR1*	-0.68518361	0.003511536	0.011330438	1
T2DM_insulin	leukocyte	*ENTPD7*	-0.63606405	0.026114544	0.034338702	1

T2DM_insulin	leukocyte	*AGAP9*	-0.699352468	0.012067713	0.017811436	1
T2DM_insulin	leukocyte	*ZNF273*	-0.836214773	0.000900687	0.011330438	1
T2DM_insulin	leukocyte	*ZNF84*	-0.718545831	0.008182887	0.013396068	1
T2DM_insulin	leukocyte	*ZNF667*	-0.279648292	0.005810083	0.011570498	1
T2DM_insulin	leukocyte	*CD2AP*	-0.37434493	0.003697072	0.011330438	1
T2DM_insulin	leukocyte	*SFI1*	-0.821886126	0.003145331	0.011330438	1
T2DM_insulin	leukocyte	*HIBCH*	-0.330956595	0.006936094	0.012128132	1
T2DM_insulin	leukocyte	*TMEM229B*	-1.782062334	0.003785929	0.011330438	1
T2DM_insulin	leukocyte	*SOWAHC*	-0.676239656	0.002385013	0.011330438	1
T2DM_insulin	leukocyte	*SVIP*	-0.312510677	0.005536883	0.011449692	1
T2DM_insulin	leukocyte	*DDRGK1*	-0.406929526	0.003597657	0.011330438	1
T2DM_insulin	leukocyte	*ZNF607*	-0.459293548	0.006447892	0.01173015	1
T2DM_insulin	leukocyte	*HSD17B11*	-0.522308401	0.006852076	0.012045987	1
T2DM_insulin	leukocyte	*RPS6KL1*	-0.470065201	0.008249404	0.013448692	1
T2DM_insulin	leukocyte	*QRICH1*	-0.637283544	0.002513601	0.011330438	1
T2DM_insulin	leukocyte	*DDX42*	-0.310139081	0.007340148	0.012522047	1
T2DM_insulin	leukocyte	*HELZ*	-0.404942066	0.003604046	0.011330438	1
T2DM_insulin	leukocyte	*ZNF583*	-0.335431529	0.005741534	0.011570498	1
T2DM_insulin	leukocyte	*ZNF568*	-0.357035432	0.021729677	0.029268393	1
T2DM_insulin	leukocyte	*ZNF587*	-0.44011988	0.004072719	0.011330438	1
T2DM_insulin	leukocyte	*TPM2*	-0.444341056	0.002605638	0.011330438	1
T2DM_insulin	leukocyte	*CNGA1*	-0.538646993	0.019555443	0.026687509	1
T2DM_insulin	leukocyte	*ZNF789*	-0.843708224	0.013002835	0.018823927	1
T2DM_insulin	leukocyte	*CCDC69*	-0.384554944	0.025784235	0.033972625	1
T2DM_insulin	leukocyte	*NCOA6*	-0.350429717	0.004037417	0.011330438	1
T2DM_insulin	leukocyte	*STK39*	-0.336633739	0.006177557	0.011570498	1
T2DM_insulin	leukocyte	*PAPSS2*	-0.321193519	0.003791594	0.011330438	1
T2DM_insulin	leukocyte	*CEP290*	-0.43400103	0.003346379	0.011330438	1
T2DM_insulin	leukocyte	*ECI2*	-0.410749791	0.004262661	0.011330438	1
T2DM_insulin	leukocyte	*MSRB1*	-0.501094713	0.003762835	0.011330438	1
T2DM_insulin	leukocyte	*OXCT2*	-0.408378873	0.011878452	0.017638109	1
T2DM_insulin	leukocyte	*PNP*	-0.206830295	0.024509644	0.032511274	1
T2DM_insulin	leukocyte	*PAX9*	9.2144022	0.018773371	0.025745402	1
T2DM_insulin	leukocyte	*DENND4B*	-0.375795149	0.008840655	0.014115597	1
T2DM_insulin	leukocyte	*CES1*	-0.760843896	0.01149256	0.017184601	1
T2DM_insulin	leukocyte	*TSEN15*	-0.466093409	0.00357652	0.011330438	1
T2DM_insulin	leukocyte	*LTN1*	-0.369029439	0.005813337	0.011570498	1
T2DM_insulin	leukocyte	*TYW1*	-0.560918052	0.006282885	0.011583977	1
T2DM_insulin	leukocyte	*MAP3K3*	-0.430490888	0.002767233	0.011330438	1
T2DM_insulin	leukocyte	*C1orf174*	-0.467815108	0.005091994	0.011330438	1
T2DM_insulin	leukocyte	*ATG9A*	-0.440086494	0.009609781	0.015042561	1
T2DM_insulin	leukocyte	*TBKBP1*	-0.486520764	0.001034731	0.011330438	1
T2DM_insulin	leukocyte	*CCDC167*	-0.310808936	0.004374792	0.011330438	1
T2DM_insulin	leukocyte	*SOWAHA*	-0.347485439	0.004470154	0.011330438	1
T2DM_insulin	leukocyte	*L3MBTL3*	-0.783338136	0.000333475	0.011330438	1
T2DM_insulin	leukocyte	*MFAP3L*	-0.639141094	0.003841795	0.011330438	1
T2DM_insulin	leukocyte	*PJA2*	-0.314237749	0.005811565	0.011570498	1
T2DM_insulin	leukocyte	*INF2*	-0.552113725	0.004719644	0.011330438	1
T2DM_insulin	leukocyte	*EFCAB2*	-0.36350752	0.009714423	0.015146019	1
T2DM_insulin	leukocyte	*SAMD5*	-0.472818107	0.00313046	0.011330438	1
T2DM_insulin	leukocyte	*PCMTD2*	-0.369843421	0.0040125	0.011330438	1
T2DM_insulin	leukocyte	*LIME1*	-0.508926311	0.022275027	0.029880021	1
T2DM_insulin	leukocyte	*FOXO6*	-0.729703044	0.006330824	0.011634148	1
T2DM_insulin	leukocyte	*SYS1*	-0.428397614	0.001420755	0.011330438	1
T2DM_insulin	leukocyte	*TRAF3IP1*	-1.120430582	0.001235771	0.011330438	1
T2DM_insulin	leukocyte	*HLA-DOA*	0.060652508	0.016438781	0.023026015	1
T2DM_insulin	leukocyte	*HLA-DMA*	0.111033216	0.000251167	0.011330438	0.986833637
T2DM_insulin	leukocyte	*NOTCH4*	-0.767082432	8.84E-09	3.47E-05	3.47E-05
T2DM_insulin	leukocyte	*RNF5*	-0.53270214	1.73E-05	0.011330438	0.068046959
T2DM_insulin	leukocyte	*MRPL38*	-0.408727452	0.002403922	0.011330438	1
T2DM_insulin	leukocyte	*SMIM5*	-0.355905597	0.001674324	0.011330438	1
T2DM_insulin	leukocyte	*EHMT2*	-1.093109944	0.014451539	0.020587417	1
T2DM_insulin	leukocyte	*LAYN*	-0.357895834	0.005831052	0.011570498	1
T2DM_insulin	leukocyte	*SLC44A4*	-0.323303968	0.001635604	0.011330438	1

T2DM_insulin	leukocyte	*HLA-C*	-0.205662859	0.008564152	0.01382216	1
T2DM_insulin	leukocyte	*POU5F1*	0.427890717	0.038517994	0.048412411	1
T2DM_insulin	leukocyte	*CCHCR1*	0.565686713	0.007539713	0.012730353	1
T2DM_insulin	leukocyte	*PSORS1C1*	-0.461964562	3.67E-05	0.011330438	0.144213616
T2DM_insulin	leukocyte	*DHX16*	-0.43858722	0.005638366	0.01154792	1
T2DM_insulin	leukocyte	*ABCF1*	-0.358664669	0.008217805	0.013442029	1
T2DM_insulin	leukocyte	*LILRB3*	0.474608558	0.002469842	0.011330438	1
T2DM_insulin	leukocyte	*ZNF468*	-0.829221755	0.007731708	0.012941946	1
T2DM_insulin	leukocyte	*HLA-F*	-0.196955787	0.009094763	0.014422203	1
T2DM_insulin	leukocyte	*ASPDH*	-0.682054049	0.004867715	0.011330438	1
T2DM_insulin	leukocyte	*ZNF425*	-1.015976152	0.004185605	0.011330438	1
T2DM_insulin	leukocyte	*PCDHA5*	-0.625486943	0.00687742	0.012075281	1
T2DM_insulin	leukocyte	*SPIRE2*	-0.421763052	0.000597543	0.011330438	1
T2DM_insulin	leukocyte	*PABPN1L*	-52.46063309	0.004913665	0.011330438	1
T2DM_insulin	leukocyte	*PKHD1L1*	-1.420167652	0.011466008	0.017155348	1
T2DM_insulin	leukocyte	*SYCE1L*	-0.565621415	0.006757905	0.011981863	1
T2DM_insulin	leukocyte	*TMEM231*	-0.31811524	0.027214216	0.035594093	1
T2DM_insulin	leukocyte	*SARNP*	-0.365427409	0.003720954	0.011330438	1
T2DM_insulin	leukocyte	*ATP6AP1L*	0.763897868	0.010526892	0.016078505	1
T2DM_insulin	leukocyte	*CCDC85C*	-0.414382581	0.003749417	0.011330438	1
T2DM_insulin	leukocyte	*RGL3*	-0.760587101	0.001876829	0.011330438	1
T2DM_insulin	leukocyte	*CPT1B*	-0.391780954	0.016048708	0.02251371	1
T2DM_insulin	leukocyte	*SMN2*	-0.401623294	0.020251028	0.027531588	1
T2DM_insulin	leukocyte	*HMGN1*	-0.307894511	0.00229431	0.011330438	1
T2DM_insulin	leukocyte	*LCMT1*	-0.530714455	0.002718093	0.011330438	1
T2DM_insulin	leukocyte	*LIN52*	-0.841231856	0.002966512	0.011330438	1
T2DM_insulin	leukocyte	*DENND1C*	0.143919653	0.015800908	0.02223559	1
T2DM_insulin	leukocyte	*RNPS1*	-0.347866056	0.004765537	0.011330438	1
T2DM_insulin	leukocyte	*ZDHHC11B*	-0.630974367	0.007040946	0.012237751	1
T2DM_insulin	leukocyte	*GOLGA8O*	-0.926363234	0.029454057	0.038079957	1
T2DM_insulin	leukocyte	*HLA-A*	-0.18540097	0.027241386	0.035617773	1
T2DM_insulin	leukocyte	*STK38L*	-0.646945311	0.006691425	0.011938173	1
T2DM_insulin	leukocyte	*MAP10*	-0.400078897	0.012867366	0.018696702	1
T2DM_insulin	leukocyte	*NUP62*	-0.409691194	0.004840043	0.011330438	1
T2DM_insulin	leukocyte	*FAM106A*	-0.477512039	0.01445998	0.020591976	1
T2DM_insulin	leukocyte	*ZNF254*	-0.491622209	0.001907146	0.011330438	1
T2DM_insulin	leukocyte	*ASIC3*	-0.558929182	0.004755687	0.011330438	1
T2DM_insulin	leukocyte	*ARHGEF35*	-0.26352774	0.006264819	0.011570498	1
T2DM_insulin	leukocyte	*SUPT4H1*	-0.449291087	0.003338352	0.011330438	1
T2DM_insulin	leukocyte	*MXD3*	-0.325520495	0.026751582	0.035059586	1
T2DM_insulin	leukocyte	*COG8*	-0.57794153	0.002985665	0.011330438	1
T2DM_insulin	leukocyte	*CCL14*	-0.490252866	0.027694148	0.036137598	1
T2DM_insulin	leukocyte	*RBMXL1*	-0.417357315	0.003623072	0.011330438	1
T2DM_insulin	leukocyte	*SRA1*	-0.288660253	0.005803089	0.011570498	1
T2DM_insulin	leukocyte	*C8orf82*	-0.78630796	0.001221274	0.011330438	1
T2DM_insulin	leukocyte	*LEPROT*	-0.377109027	0.003365785	0.011330438	1
T2DM_insulin	leukocyte	*PPP1CB*	-0.303362437	0.003643427	0.011330438	1
T2DM_insulin	leukocyte	*NCKIPSD*	-0.606992416	0.001805956	0.011330438	1
T2DM_insulin	leukocyte	*ZNF888*	-0.555699487	0.005423041	0.011421159	1
T2DM_insulin	leukocyte	*EMP2*	-0.457019798	0.00136924	0.011330438	1
T2DM_insulin	leukocyte	*LTB4R*	-0.497469548	0.008002195	0.013211473	1
T2DM_insulin	leukocyte	*DNASE1*	-0.270123314	0.036402017	0.045929199	1
T2DM_insulin	leukocyte	*NUDT19*	-0.48022597	0.007272107	0.012444298	1
T2DM_insulin	leukocyte	*AP1G2*	-1.394297832	0.003839769	0.011330438	1
T2DM_insulin	leukocyte	*ZNF90*	-0.471548767	0.005845952	0.011570498	1
T2DM_insulin	leukocyte	*REPIN1*	-0.424433699	0.00325328	0.011330438	1
T2DM_insulin	leukocyte	*TSPAN4*	-0.443326949	0.005016395	0.011330438	1
T2DM_insulin	leukocyte	*CPNE1*	-0.475029873	0.01073797	0.016320884	1
T2DM_insulin	leukocyte	*ARL16*	-0.339273563	0.005111851	0.011330438	1
T2DM_insulin	leukocyte	*FIS1*	-0.324136951	0.002185838	0.011330438	1
T2DM_insulin	leukocyte	*VSTM5*	-1.309638953	0.008993536	0.014300123	1
T2DM_insulin	leukocyte	*AS3MT*	0.445814483	0.035526359	0.04496877	1
T2DM_insulin	leukocyte	*MEG3*	-0.243124575	0.004080852	0.011330438	1
T2DM_insulin	leukocyte	*EML6*	-0.463692017	0.007660241	0.012869015	1

T2DM_insulin	leukocyte	*IFRD2*	-0.420834933	0.003144344	0.011330438	1
T2DM_insulin	leukocyte	*ARHGEF28*	-0.493533492	0.0065229	0.011788627	1
T2DM_insulin	leukocyte	*COL28A1*	-1.582428358	0.028103024	0.036586077	1
T2DM_insulin	leukocyte	*UBXN2B*	-0.610198218	0.00446171	0.011330438	1
T2DM_insulin	leukocyte	*C5orf49*	-0.324582957	0.005170691	0.011349522	1
T2DM_insulin	leukocyte	*GOLGA8B*	-0.470145032	0.011986499	0.01772486	1
T2DM_insulin	leukocyte	*VPS16*	-0.687365875	0.002569351	0.011330438	1
T2DM_insulin	leukocyte	*LINC01139*	-0.873774834	0.032337636	0.041332002	1
T2DM_insulin	leukocyte	*ATAD3C*	-0.362797424	0.004187811	0.011330438	1
T2DM_insulin	leukocyte	*SYCE3*	-0.708425691	0.009255718	0.014622322	1
T2DM_insulin	leukocyte	*PAM16*	-0.351189111	0.003936204	0.011330438	1
T2DM_insulin	leukocyte	*LINC00339*	-0.43759451	0.005946134	0.011570498	1
T2DM_insulin	leukocyte	*CELA3B*	-0.193289085	0.003913085	0.011330438	1
T2DM_insulin	leukocyte	*FAM228B*	-0.415700725	0.005331701	0.011421159	1
T2DM_insulin	leukocyte	*VAMP2*	-0.274599157	0.004135924	0.011330438	1
T2DM_insulin	leukocyte	*PPP3R1*	-0.58653996	0.002651754	0.011330438	1
T2DM_insulin	leukocyte	*FANCG*	-0.723395841	0.00315344	0.011330438	1
T2DM_insulin	leukocyte	*AP4M1*	-0.345600552	0.002876377	0.011330438	1
T2DM_insulin	leukocyte	*ZNF880*	-0.299189459	0.009825673	0.015277036	1
T2DM_insulin	leukocyte	*TRIM16*	-0.539388521	0.005115002	0.011330438	1
T2DM_insulin	leukocyte	*TIGD1*	-0.512098217	0.018753447	0.025727828	1
T2DM_insulin	leukocyte	*SLC12A8*	-0.572653467	0.004589103	0.011330438	1
T2DM_insulin	leukocyte	*UBA52*	-0.292513227	0.002599994	0.011330438	1
T2DM_insulin	leukocyte	*EXOSC6*	-0.453304905	0.001707418	0.011330438	1
T2DM_insulin	leukocyte	*CKMT1A*	-0.380900872	0.007201043	0.012381002	1
T2DM_insulin	leukocyte	*HLA-DPB1*	0.037553481	0.029109656	0.037696717	1
T2DM_insulin	leukocyte	*C4B*	4.1925103	0.019009107	0.026005146	1
T2DM_insulin	leukocyte	*LINC00115*	-1.118074238	0.002409351	0.011330438	1
T2DM_insulin	leukocyte	*LINC00937*	-0.422733054	0.009380101	0.014783159	1
T2DM_insulin	leukocyte	*TMEM185B*	-0.397774597	0.007134677	0.012332664	1
T2DM_insulin	leukocyte	*CYP4F26P*	1.872775862	0.01523544	0.021555956	1
T2DM_insulin	leukocyte	*WDR46*	-0.328880113	0.012218816	0.017946814	1
T2DM_insulin	leukocyte	*PROB1*	-0.541810364	0.038838184	0.048752468	1
T2DM_insulin	leukocyte	*ARL17B*	-1.005463166	0.004657294	0.011330438	1
T2DM_insulin	leukocyte	*PSMG3-AS1*	-0.488562338	0.004353362	0.011330438	1
T2DM_insulin	leukocyte	*HSBP1*	-0.340063522	0.002948509	0.011330438	1
T2DM_insulin	leukocyte	*RPS18*	-0.230868585	0.002887662	0.011330438	1
T2DM_insulin	leukocyte	*DIRC3*	-1.282970241	0.021814954	0.029373186	1
T2DM_insulin	leukocyte	*LINC00865*	-0.956306035	0.005573823	0.011495826	1
T2DM_insulin	leukocyte	*HLA-DQB2*	0.923627024	1.82E-08	3.58E-05	7.17E-05
T2DM_insulin	leukocyte	*GOLGA8N*	-0.43928232	0.009622369	0.015050274	1
T2DM_insulin	leukocyte	*LINC00665*	-0.295119643	0.00530576	0.011421159	1
T2DM_insulin	leukocyte	*LYRM9*	-0.475123583	0.002825278	0.011330438	1
T2DM_insulin	leukocyte	*SNHG15*	-0.375445591	0.003522952	0.011330438	1
T2DM_insulin	leukocyte	*RPS28*	-0.247473055	0.002808497	0.011330438	1
T2DM_insulin	leukocyte	*TRIM26*	-0.540227584	0.00086055	0.011330438	1
T2DM_insulin	leukocyte	*ZNF879*	-0.489946374	0.006802398	0.012014984	1
T2DM_insulin	leukocyte	*PINLYP*	-0.934757375	0.001116856	0.011330438	1
T2DM_insulin	leukocyte	*MCIDAS*	13.9724884	0.018592545	0.025541996	1
T2DM_insulin	leukocyte	*ANKRD65*	-0.349417476	0.004825047	0.011330438	1
T2DM_insulin	leukocyte	*C12orf75*	-0.219070074	0.004120292	0.011330438	1
T2DM_insulin	leukocyte	*B3GALT4*	-0.430354274	0.000129127	0.011330438	0.507338592
T2DM_insulin	leukocyte	*ZNF737*	-0.562125629	0.002593935	0.011330438	1
T2DM_insulin	leukocyte	*SHISA9*	-2.858655668	0.005706998	0.011549238	1
T2DM_insulin	leukocyte	*HLA-DQA2*	0.049393661	0.002158542	0.011330438	1
T2DM_insulin	leukocyte	*TXNDC5*	-0.410464912	0.002205425	0.011330438	1
T2DM_insulin	leukocyte	*RBM14*	-0.494399809	0.004755591	0.011330438	1
T2DM_insulin	leukocyte	*NME1*	-0.271645803	0.006616878	0.011871102	1
T2DM_insulin	leukocyte	*CDRT4*	-0.818750653	0.005684462	0.011549238	1
T2DM_insulin	leukocyte	*AMY2B*	-0.316518865	0.004759224	0.011330438	1
T2DM_insulin	leukocyte	*LY6G5B*	-0.46830038	0.004627321	0.011330438	1
T2DM_insulin	leukocyte	*PCDHGC3*	-0.412663642	0.013731586	0.019726289	1
T2DM_insulin	leukocyte	*SMKR1*	-0.483159177	0.005410826	0.011421159	1
T2DM_insulin	leukocyte	*C1QTNF9*	-6.569878705	0.005509345	0.011434531	1

T2DM_insulin	leukocyte	*LINC00886*	-0.538344923	0.007770921	0.012986793	1
T2DM_insulin	leukocyte	*MIF*	-0.229114712	0.004373402	0.011330438	1
T2DM_insulin	leukocyte	*NSUN6*	-0.372880993	0.004536381	0.011330438	1
T2DM_insulin	leukocyte	*HLA-DOB*	1.051727575	7.53E-05	0.011330438	0.2957142
T2DM_insulin	leukocyte	*CRCP*	-0.478426315	0.001469144	0.011330438	1
T2DM_insulin	leukocyte	*ARHGAP8*	-0.378175263	0.03295012	0.042005522	1
T2DM_insulin	leukocyte	*C8orf58*	-0.450156287	0.038735693	0.048654903	1
T2DM_insulin	leukocyte	*PI4KA*	-0.555344402	0.010190313	0.015699813	1
T2DM_insulin	leukocyte	*AMACR*	-0.322296846	0.005842147	0.011570498	1
T2DM_insulin	leukocyte	*BGLAP*	-0.536761777	0.020712155	0.02807079	1
T2DM_insulin	leukocyte	*MRPL20*	-0.291203132	0.00937426	0.014779882	1
T2DM_insulin	leukocyte	*LINC00960*	-0.26934178	0.022048848	0.029627197	1
T2DM_insulin	leukocyte	*HLA-DMB*	0.042961937	0.00812928	0.013352818	1
T2DM_insulin	leukocyte	*DECR2*	-0.403386704	0.004086854	0.011330438	1
T2DM_insulin	leukocyte	*GNG10*	-0.35734606	0.002040353	0.011330438	1
T2DM_insulin	leukocyte	*EIF4EBP3*	-0.54263243	0.001867623	0.011330438	1
T2DM_insulin	leukocyte	*PSG4*	1.171562885	0.024404605	0.032393815	1
T2DM_insulin	leukocyte	*MICAL3*	-0.3697521	0.004489043	0.011330438	1
T2DM_insulin	leukocyte	*STON1*	-0.413768793	0.03646214	0.045990288	1
T2DM_insulin	leukocyte	*VSIG8*	-0.98042627	0.005777578	0.011570498	1
T2DM_insulin	leukocyte	*C4orf48*	-0.283041961	0.003382105	0.011330438	1
T2DM_insulin	leukocyte	*IL10RB*	-1.051503705	0.000288167	0.011330438	1
T2DM_insulin	leukocyte	*PLA2G4B*	-0.547849311	0.010814519	0.016411836	1
T2DM_insulin	leukocyte	*LEFTY1*	-3.312597981	0.004640363	0.011330438	1
T2DM_insulin	leukocyte	*TTC4*	-0.392479993	0.005885718	0.011570498	1
T2DM_insulin	leukocyte	*APOBEC3D*	-1.436796555	0.010396906	0.015922346	1
T2DM_insulin	leukocyte	*OR2A7*	-1.513413683	0.013290739	0.019170086	1
T2DM_insulin	leukocyte	*GSTA1*	-0.214533079	0.007723566	0.012940678	1
T2DM_insulin	leukocyte	*GSTA2*	-0.225763337	0.030615025	0.039412331	1
T2DM_insulin	leukocyte	*TMEM141*	-0.441952788	0.006192631	0.011570498	1
T2DM_insulin	leukocyte	*APOBEC3C*	-0.795157797	0.006742885	0.011963834	1
T2DM_insulin	leukocyte	*C4A*	-3.545617072	0.002412801	0.011330438	1
T2DM_insulin	leukocyte	*NEAT1*	-0.264683523	0.003248406	0.011330438	1
T2DM_insulin	leukocyte	*LINC00920*	-2.877695001	0.018386941	0.025300193	1
T2DM_insulin	leukocyte	*UBAP1L*	-0.389151902	0.008032804	0.013233077	1
T2DM_insulin	leukocyte	*TWF2*	-0.469240763	0.008252299	0.013448692	1
T2DM_insulin	leukocyte	*BCKDHA*	-0.37166958	0.007685644	0.012899144	1
T2DM_insulin	leukocyte	*PCDHAC1*	-0.594819068	0.005009925	0.011330438	1
T2DM_insulin	leukocyte	*FMN1*	-1.160384626	0.010701243	0.016283959	1
T2DM_insulin	leukocyte	*TMEM150C*	-0.467430959	0.004016792	0.011330438	1
T2DM_insulin	leukocyte	*LINC01091*	-0.256544372	0.009523845	0.014949735	1
T2DM_insulin	leukocyte	*PDCD6*	-0.372920405	0.003275469	0.011330438	1
T2DM_insulin	leukocyte	*YJEFN3*	-0.693340196	0.003743563	0.011330438	1
T2DM_insulin	leukocyte	*PCDHA10*	-0.359477746	0.005224238	0.011389288	1
T2DM_insulin	leukocyte	*ZNF718*	-0.514863321	0.005153241	0.011330438	1
T2DM_insulin	leukocyte	*SNHG18*	-0.42575361	0.002148721	0.011330438	1
T2DM_insulin	leukocyte	*ZNF550*	-0.443592861	0.005152505	0.011330438	1
T2DM_insulin	leukocyte	*LINC01099*	-0.458828804	0.006066812	0.011570498	1
T2DM_insulin	leukocyte	*PCDHA12*	-0.533551846	0.020860099	0.028232631	1
T2DM_insulin	leukocyte	*ALG11*	-0.715540503	0.002656244	0.011330438	1
T2DM_insulin	leukocyte	*ZNF260*	-0.657872546	0.00909968	0.014422203	1
T2DM_insulin	leukocyte	*AP5B1*	-0.676295516	0.004743013	0.011330438	1
T2DM_insulin	leukocyte	*SIGLEC12*	0.750997584	0.013933833	0.019980303	1
T2DM_insulin	leukocyte	*PABPC4L*	-1.038637158	0.007957079	0.013183733	1
T2DM_insulin	leukocyte	*NPIPA2*	-4.197011854	0.004576772	0.011330438	1
T2DM_insulin	leukocyte	*DPP3*	-0.437181807	0.001223074	0.011330438	1
T2DM_insulin	leukocyte	*BRK1*	-0.345043681	0.000719742	0.011330438	1
T2DM_insulin	leukocyte	*EID3*	-0.817568395	0.002702579	0.011330438	1
T2DM_insulin	leukocyte	*SNHG9*	-0.359543451	0.003526569	0.011330438	1
T2DM_insulin	leukocyte	*PCDHA3*	-0.566801301	0.014272664	0.020385699	1
T2DM_insulin	leukocyte	*ZNF432*	-0.81558778	0.001423981	0.011330438	1
T2DM_insulin	leukocyte	*LINC01152*	-1.816432384	0.006342273	0.011637991	1
T2DM_insulin	leukocyte	*SMLR1*	1.339436849	0.018991134	0.026005146	1
T2DM_insulin	leukocyte	*ZNF10*	-0.498285801	0.003169122	0.011330438	1

T2DM_insulin	leukocyte	*TAS2R31*	-1.337406762	0.010377711	0.01590871	1
T2DM_insulin	leukocyte	*GATC*	-0.565792069	0.001108311	0.011330438	1
T2DM_insulin	leukocyte	*TEN1*	-1.267859522	0.000317619	0.011330438	1
T2DM_insulin	leukocyte	*CHURC1*	-0.314503749	0.003471107	0.011330438	1
T2DM_insulin	leukocyte	*LINC00638*	-2.552447193	0.010231243	0.015739449	1
T2DM_insulin	leukocyte	*SLC25A21-AS1*	-0.388256683	0.004987372	0.011330438	1
T2DM_insulin	leukocyte	*TMEM179*	-0.275057153	0.006261158	0.011570498	1
T2DM_insulin	leukocyte	*IQCH-AS1*	-0.726085978	0.002832662	0.011330438	1
T2DM_insulin	leukocyte	*PWRN1*	-0.523448731	0.006588411	0.011847079	1
T2DM_insulin	leukocyte	*TMEM178B*	-0.359689963	0.004974657	0.011330438	1
T2DM_insulin	leukocyte	*LINC01003*	-0.468000233	0.002980379	0.011330438	1
T2DM_insulin	leukocyte	*GOLGA8H*	-5.851381256	0.014442598	0.020587417	1
T2DM_insulin	leukocyte	*MRPL12*	-0.488581669	0.003146133	0.011330438	1
T2DM_insulin	leukocyte	*ZNF234*	-1.480198237	0.035180684	0.044574302	1
T2DM_insulin	leukocyte	*LINC00667*	-0.416509144	0.003522296	0.011330438	1
T2DM_insulin	leukocyte	*FSBP*	-0.543430247	0.006105397	0.011570498	1
T2DM_insulin	leukocyte	*MYO15B*	-0.671975565	0.025818969	0.034006949	1
T2DM_insulin	leukocyte	*LINC00663*	-0.50643063	0.022751971	0.030415956	1
T2DM_insulin	leukocyte	*ZNF285*	-0.923740002	0.005345829	0.011421159	1
T2DM_insulin	leukocyte	*FBXO17*	-0.310002953	0.006264802	0.011570498	1
T2DM_insulin	leukocyte	*LINC00528*	0.25440588	0.00494796	0.011330438	1
T2DM_insulin	leukocyte	*SNHG8*	-0.286956147	0.003003806	0.011330438	1
T2DM_insulin	leukocyte	*MILR1*	0.300556946	0.030158494	0.038888324	1
T2DM_insulin	leukocyte	*GTF2H5*	-0.360603902	0.004011862	0.011330438	1
T2DM_insulin	mesenchymal_cell	*CEP68*	0.436056151	6.91E-05	0.027162253	0.271622528
T2DM_insulin	mesenchymal_cell	*ART3*	-0.534017296	0.00012061	0.036452112	0.473877459
T2DM_insulin	mesenchymal_cell	*CLEC18A*	-0.574388243	0.000109248	0.036452112	0.429234216
T2DM_insulin	mesenchymal_cell	*CXCL11*	0.192913695	9.37E-06	0.005258949	0.036812643
T2DM_insulin	mesenchymal_cell	*IRX3*	0.680752573	3.15E-07	0.00031844	0.001238045
T2DM_insulin	mesenchymal_cell	*HLA-DQB1*	-0.173538259	4.55E-07	0.000357208	0.001786042
T2DM_insulin	mesenchymal_cell	*HLA-DRB1*	-0.097067272	2.23E-05	0.010964775	0.0877182
T2DM_insulin	mesenchymal_cell	*HLA-DRB5*	-0.095556022	3.83E-06	0.00250842	0.015050521
T2DM_insulin	mesenchymal_cell	*HLA-DMA*	0.107214345	3.68E-05	0.016059429	0.144534863
T2DM_insulin	mesenchymal_cell	*NOTCH4*	-0.18667715	3.24E-07	0.00031844	0.001273758
T2DM_insulin	mesenchymal_cell	*PSORS1C1*	-0.123193155	0.000111982	0.036452112	0.439977742
T2DM_insulin	mesenchymal_cell	*HLA-DQB2*	0.635383115	7.49E-14	2.94E-10	2.94E-10
T2DM_insulin	mesenchymal_cell	*HLA-DQA2*	0.295092303	3.69E-11	7.24E-08	1.45E-07
T2DM_insulin	PP_cell	*CEP68*	0.726964944	4.94E-05	0.021584462	0.194260157
T2DM_insulin	PP_cell	*CXCL11*	0.351345787	2.99E-06	0.00348512	0.011756182
T2DM_insulin	PP_cell	*IRX3*	0.663567975	3.55E-06	0.00348512	0.013940481
T2DM_insulin	PP_cell	*HLA-DQB1*	-0.299322458	9.41E-06	0.006162119	0.036972712
T2DM_insulin	PP_cell	*HLA-DRB5*	-0.240279488	9.42E-05	0.036993087	0.369930867
T2DM_insulin	PP_cell	*HLA-DMA*	0.207347426	6.52E-06	0.005126934	0.02563467
T2DM_insulin	PP_cell	*NOTCH4*	-0.346599657	2.53E-05	0.014177966	0.099245764
T2DM_insulin	PP_cell	*HLA-DQB2*	1.132702893	4.37E-14	1.72E-10	1.72E-10
T2DM_insulin	PP_cell	*HLA-DQA2*	0.53818504	7.84E-13	1.54E-09	3.08E-09
T2DM_insulin	PP_cell	*PSMB9*	0.445144878	0.000121512	0.043402021	0.47742223
T2DM_insulin	PP_cell	*HLA-DMB*	0.523790308	4.32E-05	0.021236049	0.169888393

c. Identified trait-associated genes from CTS TWAS analysis in subcutaneous adipose tissue

disorder	celltype	gene	Estimates	Pvalues	Pvalues_BH	Pvalue_Bonferroni
CAD	adipocyte	*SEMA3F*	-0.221590916	0.000214112	0.040791534	1
CAD	adipocyte	*UHRF1BP1*	0.298009851	5.89E-06	0.004627148	0.037017185
CAD	adipocyte	*ATP2A3*	0.776529784	6.04E-05	0.019972965	0.379486343
CAD	adipocyte	*TIPIN*	0.183881257	0.000172384	0.037604821	1
CAD	adipocyte	*KCNH4*	0.381232879	0.000323666	0.048449646	1
CAD	adipocyte	*ABHD12*	0.67395607	7.58E-05	0.022684977	0.476384519
CAD	adipocyte	*MAP1LC3A*	0.467660301	4.09E-05	0.016877267	0.257029754
CAD	adipocyte	*PIGU*	0.894812704	0.000242112	0.043490239	1
CAD	adipocyte	*ICAM2*	0.669562833	9.07E-05	0.025854379	0.570353888
CAD	adipocyte	*BCHE*	-0.393518576	0.000298047	0.047841246	1
CAD	adipocyte	*UMPS*	0.946003793	0.000110083	0.026618961	0.692092993
CAD	adipocyte	*PLXNA1*	0.480526332	9.65E-05	0.025854379	0.6067602
CAD	adipocyte	*GGCX*	0.496745831	8.00E-06	0.005589586	0.050306278
CAD	adipocyte	*EIF2B2*	0.411332606	0.000263931	0.046092571	1
CAD	adipocyte	*HIF3A*	0.433052568	0.000176049	0.037604821	1
CAD	adipocyte	*SNRPC*	-0.474334587	1.41E-05	0.008893002	0.088930016
CAD	adipocyte	*GRK4*	0.141948429	0.000283195	0.04685383	1
CAD	adipocyte	*CARF*	-0.646418916	1.87E-07	0.000426176	0.00117815
CAD	adipocyte	*TMEM91*	0.63717179	4.01E-05	0.016877267	0.252224167
CAD	adipocyte	*NBEAL1*	-0.444578326	2.71E-07	0.000426176	0.001704705
CAD	adipocyte	*AMT*	-0.204062848	0.000132447	0.030840525	0.832694187
CAD	adipocyte	*PSD2*	2.17767934	2.96E-05	0.014308366	0.18600876
CAD	adipocyte	*DISP1*	0.450123067	4.30E-05	0.016877267	0.270036268
CAD	adipocyte	*LMOD1*	-0.377136493	0.000235932	0.043490239	1
CAD	adipocyte	*ICA1L*	0.951207371	2.86E-08	0.000180026	0.000180026
CAD	adipocyte	*APEH*	-0.555952414	4.69E-06	0.004215517	0.029508622
CAD	adipocyte	*RNF123*	0.313329824	0.000179441	0.037604821	1
CAD	adipocyte	*IRS1*	-0.19917963	2.62E-07	0.000426176	0.001649937
CAD	adipocyte	*PCSK9*	2.689171795	1.68E-05	0.009473447	0.105762348
CAD	adipocyte	*LPL*	-0.268188618	5.10E-07	0.000640972	0.003204859
CAD	adipocyte	*CRLF3*	0.943074885	5.72E-05	0.019972965	0.359459753
CAD	adipocyte	*TMEM136*	0.507744052	0.000100568	0.025854379	0.632271404
CAD	adipocyte	*HLA-DRB1*	-0.307984642	0.000278516	0.04685383	1
CAD	adipocyte	*C6orf106*	0.419851132	2.07E-06	0.00217275	0.013036501
CAD	adipocyte	*WDR5B*	0.650672199	0.000310899	0.047841246	1
CAD	adipocyte	*TMEM116*	-0.240956522	0.000311992	0.047841246	1
CAD	adipocyte	*CENPW*	0.955158191	1.81E-05	0.009473447	0.113681364
CAD	adipocyte	*SLC2A1-AS1*	-0.762757322	0.000197736	0.039838503	1
CAD	adipocyte	*HLA-B*	0.338224767	0.000102809	0.025854379	0.646359484
CAD	adipocyte	*LY6G5B*	-0.132040605	0.000202773	0.039838503	1
CAD	adipocyte	*BCKDHA*	-1.129618427	5.77E-05	0.019972965	0.362990495
CAD	adipocyte	*HMBS*	0.315926285	7.04E-05	0.022136592	0.442731837
CAD	ASPC	*SEMA3F*	-0.103732254	4.86E-05	0.015457113	0.305567695
CAD	ASPC	*UHRF1BP1*	0.124191885	9.58E-06	0.004631262	0.060206403
CAD	ASPC	*TIE1*	-0.177831755	5.34E-05	0.015534196	0.335549187
CAD	ASPC	*TIPIN*	0.078025641	0.000170755	0.029820393	1
CAD	ASPC	*TXNDC16*	-0.33173762	0.000197394	0.033230556	1
CAD	ASPC	*ABHD12*	0.28063681	0.000316538	0.041459946	1
CAD	ASPC	*ACTR5*	-1.033868473	5.51E-05	0.015534196	0.346311173
CAD	ASPC	*MAP1LC3A*	0.226139623	7.43E-06	0.00389067	0.046688039
CAD	ASPC	*TRPV4*	-0.343788772	0.000345196	0.044290788	1
CAD	ASPC	*FAM162A*	-0.375655998	0.000384067	0.04829254	1
CAD	ASPC	*BCHE*	-0.184491979	7.31E-05	0.016420961	0.459786899
CAD	ASPC	*UMPS*	0.427901908	0.000149645	0.027671181	0.940820153
CAD	ASPC	*PLXNA1*	0.195331479	0.000207577	0.033230556	1
CAD	ASPC	*GGCX*	0.207094791	1.50E-05	0.006300934	0.094514008
CAD	ASPC	*SNRPC*	-0.213002031	5.08E-06	0.002903781	0.031941586
CAD	ASPC	*GRK4*	0.061333311	0.000211837	0.033230556	1

CAD	ASPC	*KLHDC10*	-0.823757593	6.63E-07	0.000595882	0.004171176
CAD	ASPC	*CARF*	-0.306024165	1.99E-08	6.25E-05	0.000124963
CAD	ASPC	*TMEM91*	0.255929869	0.000147902	0.027671181	0.929860174
CAD	ASPC	*NME7*	-0.725379606	1.14E-05	0.005125916	0.071762817
CAD	ASPC	*NBEAL1*	-0.209114158	3.02E-08	6.33E-05	0.000189878
CAD	ASPC	*AMT*	-0.089613284	7.17E-05	0.016420961	0.450533169
CAD	ASPC	*PSD2*	0.87523765	6.78E-05	0.01639443	0.426255178
CAD	ASPC	*DNAJB12*	-0.78380191	0.000259089	0.035410716	1
CAD	ASPC	*HSD17B12*	-0.096571969	0.000397366	0.048985109	1
CAD	ASPC	*MIA3*	-1.065284976	1.25E-06	0.000953986	0.00782848
CAD	ASPC	*DISP1*	0.180375159	0.000146245	0.027671181	0.919441536
CAD	ASPC	*LMOD1*	-0.176370233	6.50E-05	0.01639443	0.408586436
CAD	ASPC	*ICA1L*	0.417197428	8.62E-08	0.000135474	0.000541897
CAD	ASPC	*APEH*	-0.249414271	1.37E-06	0.000953986	0.008585874
CAD	ASPC	*RNF123*	0.130583737	0.00024005	0.034297727	1
CAD	ASPC	*OXGR1*	-0.702937453	0.000207575	0.033230556	1
CAD	ASPC	*SP2*	-0.736479984	0.000132346	0.027028612	0.832060354
CAD	ASPC	*IRS1*	-0.083623143	4.92E-07	0.000595882	0.003091012
CAD	ASPC	*PCSK9*	1.130722607	1.79E-05	0.006638179	0.112849039
CAD	ASPC	*C3AR1*	-0.313060551	5.68E-05	0.015534196	0.357286508
CAD	ASPC	*DHX36*	-0.954814542	0.000168117	0.029820393	1
CAD	ASPC	*LPL*	-0.114242134	6.03E-07	0.000595882	0.003793195
CAD	ASPC	*CRLF3*	0.480703962	6.77E-05	0.01639443	0.425724706
CAD	ASPC	*TMEM136*	0.20562618	0.000229205	0.033511954	1
CAD	ASPC	*FES*	-2.641727231	1.69E-10	1.06E-06	1.06E-06
CAD	ASPC	*MSL1*	-0.146200964	0.000221995	0.033230556	1
CAD	ASPC	*HLA-DRB1*	-0.144947497	3.89E-05	0.013589761	0.244615706
CAD	ASPC	*MYO6*	-0.350440827	0.000286279	0.038294408	1
CAD	ASPC	*C6orf106*	0.182447629	1.99E-06	0.001248183	0.012481826
CAD	ASPC	*TMEM116*	-0.105032181	0.000218257	0.033230556	1
CAD	ASPC	*CENPW*	0.407364533	1.61E-05	0.006307341	0.100917452
CAD	ASPC	*SLC2A1-AS1*	-0.403038027	0.000133273	0.027028612	0.837886985
CAD	ASPC	*LY6G5B*	-0.054745207	0.00024549	0.034297727	1
CAD	ASPC	*BCKDHA*	-0.497624099	0.000102667	0.022257458	0.645466277
CAD	ASPC	*HMBS*	0.136985474	4.92E-05	0.015457113	0.309142265
CAD	dendritic_cell	*SEMA3F*	-1.391341494	2.79E-05	0.023097142	0.175119318
CAD	dendritic_cell	*SNRPC*	-3.689527095	7.72E-06	0.008089947	0.048539681
CAD	dendritic_cell	*CARF*	-3.367792099	1.64E-06	0.003646194	0.010284978
CAD	dendritic_cell	*NBEAL1*	-1.862827742	5.96E-06	0.007500221	0.037501107
CAD	dendritic_cell	*AMT*	-1.25360808	2.94E-05	0.023097142	0.18477714
CAD	dendritic_cell	*APEH*	-3.590223364	1.74E-06	0.003646194	0.010938581
CAD	dendritic_cell	*IRS1*	-1.257473217	5.34E-07	0.003355722	0.003355722
CAD	dendritic_cell	*LPL*	-1.282476941	5.05E-06	0.007500221	0.031772337
CAD	dendritic_cell	*TMEM136*	3.648367324	5.58E-05	0.035109913	0.351099134
CAD	dendritic_cell	*TMEM116*	-1.926988759	3.72E-05	0.02599999	0.233999913
CAD	endothelial	*IRS1*	-0.274259811	5.15E-06	0.032401592	0.032401592
CAD	macrophage	*SEMA3F*	-0.263144251	0.000144074	0.032349767	0.905793488
CAD	macrophage	*MDH1*	0.78203347	0.000121008	0.029252727	0.760779337
CAD	macrophage	*UHRF1BP1*	0.354523512	2.64E-06	0.003080364	0.016591875
CAD	macrophage	*ATP2A3*	0.749544586	0.000220828	0.041674027	1
CAD	macrophage	*TIPIN*	0.223714337	7.67E-05	0.026786429	0.482155727
CAD	macrophage	*MAP1LC3A*	0.599096317	2.80E-06	0.003080364	0.017595837
CAD	macrophage	*PIGU*	0.979480105	0.000179371	0.036377522	1
CAD	macrophage	*ICAM2*	0.786207857	0.000119309	0.029252727	0.750093104
CAD	macrophage	*UMPS*	1.113638595	2.85E-05	0.012820952	0.17949333
CAD	macrophage	*PLXNA1*	0.540850653	0.000106247	0.029252727	0.667972403
CAD	macrophage	*GGCX*	0.59278735	2.94E-06	0.003080364	0.018482183
CAD	macrophage	*EIF2B2*	0.490354702	0.000125628	0.029252727	0.789823635
CAD	macrophage	*SNRPC*	-0.513523004	4.17E-05	0.015436555	0.262421439
CAD	macrophage	*GRK4*	0.17360833	0.000121463	0.029252727	0.763640727
CAD	macrophage	*POLR1E*	0.396736085	0.000279306	0.04856531	1

CAD	macrophage	*CARF*	-0.635769084	4.94E-06	0.004435187	0.031046311
CAD	macrophage	*TMEM91*	0.665303042	0.000123444	0.029252727	0.776090143
CAD	macrophage	*NBEAL1*	-0.437086757	5.82E-06	0.004570773	0.036566187
CAD	macrophage	*AMT*	-0.229171098	0.000190142	0.037356887	1
CAD	macrophage	*PSD2*	2.40181989	3.36E-05	0.014097326	0.211459892
CAD	macrophage	*DISP1*	0.436238582	0.000273893	0.04856531	1
CAD	macrophage	*ICA1L*	0.99225737	5.63E-08	0.000354242	0.000354242
CAD	macrophage	*APEH*	-0.621867313	8.56E-06	0.005380989	0.053809885
CAD	macrophage	*RNF123*	0.37806941	8.69E-05	0.028754713	0.546339543
CAD	macrophage	*OXGR1*	-1.882530782	0.000285815	0.04856531	1
CAD	macrophage	*IRS1*	-0.220309976	1.17E-06	0.002455802	0.007367405
CAD	macrophage	*PCSK9*	3.087734753	1.33E-05	0.007623321	0.083856532
CAD	macrophage	*LPL*	-0.27562718	6.82E-06	0.004767267	0.042905401
CAD	macrophage	*CRLF3*	0.998024257	3.97E-05	0.015436555	0.249595005
CAD	macrophage	*TMEM136*	0.560939249	0.000108357	0.029252727	0.681241564
CAD	macrophage	*C6orf106*	0.508731156	4.56E-07	0.001434038	0.002868076
CAD	macrophage	*WDR5B*	0.78221233	0.000151789	0.032906705	0.954294459
CAD	macrophage	*CENPW*	1.017399875	1.76E-05	0.009236475	0.110837703
CAD	macrophage	*FAM216A*	1.298266167	0.000172868	0.036227388	1
CAD	macrophage	*HLA-B*	0.404780832	0.000101934	0.029252727	0.640856696
CAD	macrophage	*LY6G5B*	-0.152006742	0.000225373	0.041674027	1
CAD	macrophage	*HMBS*	0.388676293	2.38E-05	0.011497335	0.14946536
CAD	mast_cell	*UHRF1BP1*	2.04877728	8.46E-06	0.013302876	0.053211506
CAD	mast_cell	*NBEAL1*	-2.379430572	2.61E-05	0.027390538	0.164343229
CAD	mast_cell	*ICA1L*	4.616567135	4.31E-06	0.009781714	0.027106953
CAD	mast_cell	*IRS1*	-1.411102537	1.43E-06	0.009003131	0.009003131
CAD	mast_cell	*C6orf106*	2.758628743	4.67E-06	0.009781714	0.029345141
CAD	mast_cell	*CENPW*	6.793961742	2.51E-05	0.027390538	0.157900886
CAD	monocyte	*UHRF1BP1*	2.46799926	4.18E-06	0.024892658	0.026282733
CAD	monocyte	*GGCX*	3.97377307	7.92E-06	0.024892658	0.049785316
CAD	monocyte	*PSD2*	19.22397939	2.29E-05	0.036005012	0.14402005
CAD	monocyte	*C6orf106*	2.635714297	1.88E-05	0.036005012	0.11816803
CAD	neutrophil	*APEH*	-7.136494515	4.90E-06	0.030794764	0.030794764
CAD	nk_cell	*UHRF1BP1*	1.180005646	3.12E-06	0.011458714	0.019630595
CAD	nk_cell	*ICA1L*	2.115676742	1.44E-05	0.030089982	0.090269947
CAD	nk_cell	*C6orf106*	1.493949951	3.65E-06	0.011458714	0.022917428
CAD	nk_cell	*HMBS*	1.420051413	2.15E-05	0.033769955	0.135079821
CAD	pericyte	*SCYL3*	2.404764574	0.000691333	0.034565119	1
CAD	pericyte	*C1orf112*	4.195217761	0.002666086	0.04058519	1
CAD	pericyte	*SEMA3F*	-0.840001029	0.003161089	0.042947791	1
CAD	pericyte	*MAD1L1*	3.691983979	0.004510872	0.048175643	1
CAD	pericyte	*GCFC2*	3.796914094	0.001195597	0.034565119	1
CAD	pericyte	*SYNRG*	3.674134832	0.001671349	0.036359072	1
CAD	pericyte	*FARP2*	2.68234475	0.002302145	0.0387427	1
CAD	pericyte	*TRAPPC6A*	4.701123488	0.002812169	0.041045659	1
CAD	pericyte	*SEC62*	3.600776409	0.002706132	0.040799638	1
CAD	pericyte	*CEP68*	1.800167874	0.00240101	0.039224575	1
CAD	pericyte	*ZBTB32*	8.249767128	0.001967337	0.037807709	1
CAD	pericyte	*MDH1*	3.795762311	6.44E-06	0.006066092	0.040481254
CAD	pericyte	*WWTR1*	2.953378199	0.002317549	0.0387427	1
CAD	pericyte	*OSBPL5*	4.540502771	0.001841116	0.036727251	1
CAD	pericyte	*UBR2*	2.89255571	0.003654215	0.045313356	1
CAD	pericyte	*TYMP*	2.480346308	0.004371098	0.047876468	1
CAD	pericyte	*VRK2*	3.008917209	0.002408253	0.039224575	1
CAD	pericyte	*ARID4A*	3.697075733	0.001706429	0.036710139	1
CAD	pericyte	*FUT8*	3.077198524	0.003729778	0.045499723	1
CAD	pericyte	*CUL3*	3.059680566	0.004504653	0.048175643	1
CAD	pericyte	*CNTLN*	3.010213113	0.00453137	0.048175643	1
CAD	pericyte	*POLR2B*	4.037871857	0.001508311	0.035381167	1
CAD	pericyte	*CP*	6.049222457	0.001033014	0.034565119	1
CAD	pericyte	*ANO2*	1.401616476	0.001542189	0.035381167	1

CAD	pericyte	*NFE2L3*	5.837800876	0.001442871	0.035044707	1
CAD	pericyte	*FOXN3*	2.699326958	0.002594985	0.040259323	1
CAD	pericyte	*ANAPC4*	4.071455837	0.00076225	0.034565119	1
CAD	pericyte	*GINM1*	3.993290461	0.001225319	0.034565119	1
CAD	pericyte	*DCBLD2*	3.746807166	0.001326446	0.034565119	1
CAD	pericyte	*GDI2*	3.206624442	0.000840795	0.034565119	1
CAD	pericyte	*ATP11B*	1.543743004	0.0039128	0.046327258	1
CAD	pericyte	*CDK14*	2.749751761	0.002233093	0.0387427	1
CAD	pericyte	*QSER1*	3.65541932	0.00400081	0.046493697	1
CAD	pericyte	*VMP1*	3.250988474	0.00225513	0.0387427	1
CAD	pericyte	*EPN1*	5.133347195	0.000758704	0.034565119	1
CAD	pericyte	*UHRF1BP1*	1.430436014	5.95E-06	0.006066092	0.037409797
CAD	pericyte	*IPO5*	4.204587674	0.003064059	0.042566384	1
CAD	pericyte	*TIE1*	-1.548642603	0.003619156	0.045269918	1
CAD	pericyte	*GOLGA5*	4.308104802	0.002587681	0.040259323	1
CAD	pericyte	*ZFAT*	3.559244673	0.001860157	0.036727251	1
CAD	pericyte	*SP100*	3.484208914	0.001148585	0.034565119	1
CAD	pericyte	*RASSF1*	3.430331184	0.000554357	0.03183793	1
CAD	pericyte	*IFI35*	1.726455197	0.004081215	0.046721986	1
CAD	pericyte	*CYFIP1*	3.515955182	0.002885572	0.041307015	1
CAD	pericyte	*DNAJA2*	5.164818912	0.00136537	0.034565119	1
CAD	pericyte	*PDE8A*	2.566185777	0.000544939	0.031722516	1
CAD	pericyte	*LMAN1*	4.393925693	0.001068614	0.034565119	1
CAD	pericyte	*TIPIN*	1.019859543	5.59E-05	0.01463766	0.351303829
CAD	pericyte	*SRI*	4.934207568	0.000930888	0.034565119	1
CAD	pericyte	*EIF4G3*	2.832928012	0.002618989	0.04035683	1
CAD	pericyte	*VPS9D1*	5.036455231	0.003661394	0.045313356	1
CAD	pericyte	*SLC25A3*	3.894230231	0.000636717	0.033638992	1
CAD	pericyte	*PLD1*	3.803857057	0.000801745	0.034565119	1
CAD	pericyte	*RBMS2*	3.840440959	0.000974969	0.034565119	1
CAD	pericyte	*GPATCH1*	3.385502205	0.001348387	0.034565119	1
CAD	pericyte	*DYNC1I2*	3.57259653	0.001191409	0.034565119	1
CAD	pericyte	*SIRT6*	8.445021878	0.001066758	0.034565119	1
CAD	pericyte	*PIK3C3*	3.68475275	0.001391933	0.034565119	1
CAD	pericyte	*SYNJ2*	3.259704286	0.0008359	0.034565119	1
CAD	pericyte	*PCM1*	2.929488247	0.001881052	0.036727251	1
CAD	pericyte	*OSBPL6*	5.266993336	0.001217616	0.034565119	1
CAD	pericyte	*XRCC5*	3.364299597	0.002148529	0.038369728	1
CAD	pericyte	*REXO1*	5.702562963	0.001270341	0.034565119	1
CAD	pericyte	*DDX1*	4.111486557	0.002040657	0.037841263	1
CAD	pericyte	*DNM2*	3.278581816	0.003731554	0.045499723	1
CAD	pericyte	*PSEN1*	2.850044375	0.001751286	0.036710139	1
CAD	pericyte	*ZNF506*	3.853885075	0.001800908	0.036710139	1
CAD	pericyte	*ERC1*	2.781947333	0.002989156	0.042197406	1
CAD	pericyte	*PDS5B*	3.626441193	0.000874403	0.034565119	1
CAD	pericyte	*GSTP1*	4.689993827	0.001824293	0.036727251	1
CAD	pericyte	*WBP11*	4.976813369	0.002263436	0.0387427	1
CAD	pericyte	*NCOA1*	2.828170316	0.002720757	0.040835321	1
CAD	pericyte	*TAF9*	7.345843502	0.001150591	0.034565119	1
CAD	pericyte	*USP40*	2.933215626	0.001092664	0.034565119	1
CAD	pericyte	*GMCL1*	4.552259288	0.000242308	0.024799812	1
CAD	pericyte	*ERGIC2*	3.863485844	0.0028637	0.041199267	1
CAD	pericyte	*AAMDC*	1.020439608	0.003647829	0.045313356	1
CAD	pericyte	*ANAPC5*	3.497721369	0.001871093	0.036727251	1
CAD	pericyte	*SLC23A2*	4.373012875	0.000611756	0.033530993	1
CAD	pericyte	*OAS1*	5.190702367	0.004615517	0.048380004	1
CAD	pericyte	*FUS*	3.548043758	0.001195498	0.034565119	1
CAD	pericyte	*ZNF302*	1.417183543	0.001610433	0.035525597	1
CAD	pericyte	*KCNH4*	2.033516584	0.000276123	0.024799812	1
CAD	pericyte	*PAPOLA*	3.492393349	0.001802942	0.036710139	1
CAD	pericyte	*USP48*	2.310012955	0.002015796	0.03783077	1

CAD	pericyte	*TXNL1*	4.217870654	0.000937342	0.034565119	1
CAD	pericyte	*PPP2R3C*	3.344782097	0.003306185	0.043629875	1
CAD	pericyte	*WAC*	2.558871747	0.004711952	0.048688525	1
CAD	pericyte	*PCSK5*	3.175746228	0.001122483	0.034565119	1
CAD	pericyte	*NRP1*	3.040647372	0.000943238	0.034565119	1
CAD	pericyte	*CDC34*	4.670760258	0.002817569	0.041045659	1
CAD	pericyte	*MTAP*	5.930281529	0.000728695	0.034565119	1
CAD	pericyte	*TIMP3*	2.739839051	0.003097889	0.042805339	1
CAD	pericyte	*TTLL1*	5.901324077	0.001796617	0.036710139	1
CAD	pericyte	*AP1B1*	3.565266524	0.00132675	0.034565119	1
CAD	pericyte	*RBFOX2*	3.360857466	0.001383213	0.034565119	1
CAD	pericyte	*RBM23*	4.355239287	0.000312898	0.026229157	1
CAD	pericyte	*SOS2*	3.075931611	0.003315514	0.043629875	1
CAD	pericyte	*CNIH1*	4.864110871	0.000102384	0.019164322	0.643690792
CAD	pericyte	*ABHD12*	2.54308014	0.000168857	0.023591244	1
CAD	pericyte	*NDRG3*	5.043301573	0.000275639	0.024799812	1
CAD	pericyte	*SLC17A9*	4.532982307	0.002701184	0.040799638	1
CAD	pericyte	*RNF24*	2.984916104	0.001415442	0.034895087	1
CAD	pericyte	*MYL9*	3.133467718	0.000220697	0.024799812	1
CAD	pericyte	*MAP1LC3A*	2.357713297	7.72E-06	0.006066092	0.04852874
CAD	pericyte	*SMCHD1*	2.713185508	0.004467859	0.048175643	1
CAD	pericyte	*STK24*	3.428107551	0.00295084	0.041877951	1
CAD	pericyte	*ARHGEF7*	3.11697757	0.001810621	0.036720571	1
CAD	pericyte	*VWA8*	2.540636448	0.002031749	0.037841263	1
CAD	pericyte	*TSC22D1*	3.963397298	0.000180824	0.024188147	1
CAD	pericyte	*MGRN1*	2.144440914	0.001002788	0.034565119	1
CAD	pericyte	*NFAT5*	2.714121643	0.002206691	0.0387427	1
CAD	pericyte	*SLC7A5*	16.93761836	0.001301763	0.034565119	1
CAD	pericyte	*KNOP1*	3.233316422	0.000347469	0.02668961	1
CAD	pericyte	*CSK*	5.686466947	0.002830764	0.041045659	1
CAD	pericyte	*HERC1*	3.316922177	0.000820416	0.034565119	1
CAD	pericyte	*DNAJC17*	4.427413679	0.001468671	0.035377516	1
CAD	pericyte	*RIPK2*	3.21990022	0.004666904	0.048577522	1
CAD	pericyte	*IKBKB*	3.705364279	0.002458476	0.039429683	1
CAD	pericyte	*GDAP1*	3.71487096	0.004138385	0.046816261	1
CAD	pericyte	*ERICH1*	3.573615736	0.001579865	0.035381167	1
CAD	pericyte	*FCGRT*	1.965607948	0.003616842	0.045269918	1
CAD	pericyte	*AES*	4.351227853	0.002721489	0.040835321	1
CAD	pericyte	*RPS16*	3.609493574	0.000469948	0.029253131	1
CAD	pericyte	*PRKD2*	2.482379388	0.00137544	0.034565119	1
CAD	pericyte	*FZR1*	5.541650955	0.00496253	0.049919086	1
CAD	pericyte	*CYTH2*	5.40018269	0.000119173	0.020812171	0.749238147
CAD	pericyte	*TFPT*	3.767571397	0.003030023	0.04236944	1
CAD	pericyte	*ZNF14*	4.95682183	0.002254938	0.0387427	1
CAD	pericyte	*EPHB6*	2.54849866	0.004243112	0.047403738	1
CAD	pericyte	*USP42*	3.963756349	0.003331675	0.043637999	1
CAD	pericyte	*AP1S1*	10.0467973	0.001280214	0.034565119	1
CAD	pericyte	*PHF14*	2.755171989	0.004930161	0.04975268	1
CAD	pericyte	*LIMK1*	4.789382296	0.003310079	0.043629875	1
CAD	pericyte	*PDLIM1*	1.775273258	0.003504641	0.04451248	1
CAD	pericyte	*CCNJ*	3.548703756	0.003446096	0.044125465	1
CAD	pericyte	*DNMBP*	3.525342879	0.004307933	0.04750524	1
CAD	pericyte	*DDX50*	4.39362338	0.000929892	0.034565119	1
CAD	pericyte	*MAPK8*	2.934278606	0.003155595	0.042947791	1
CAD	pericyte	*CCSER2*	3.217179942	0.002915369	0.041562188	1
CAD	pericyte	*LIPA*	2.183278497	0.001852848	0.036727251	1
CAD	pericyte	*MLLT6*	4.648598126	0.000725689	0.034565119	1
CAD	pericyte	*RPS6KB1*	4.425609713	0.000938312	0.034565119	1
CAD	pericyte	*NUP88*	3.691075763	0.000618155	0.033530993	1
CAD	pericyte	*ICAM2*	3.773813364	1.91E-05	0.008013521	0.120202812
CAD	pericyte	*UTP6*	4.061802187	0.002237323	0.0387427	1

CAD	pericyte	*CYTH1*	2.153337772	0.003629082	0.045269918	1
CAD	pericyte	*RAB5C*	2.531031766	0.004476245	0.048175643	1
CAD	pericyte	*WFS1*	5.134169596	0.003931524	0.046378327	1
CAD	pericyte	*SH3D19*	1.652067099	0.004956657	0.049919086	1
CAD	pericyte	*RAPGEF2*	3.129770025	0.0003532	0.02668961	1
CAD	pericyte	*UFSP2*	3.946964647	0.004087364	0.046721986	1
CAD	pericyte	*KLF3*	3.396408771	0.004398726	0.047928574	1
CAD	pericyte	*KLHL5*	2.434609927	0.000258268	0.024799812	1
CAD	pericyte	*NDUFS8*	5.679724576	0.001977392	0.037807709	1
CAD	pericyte	*CAPRIN2*	4.171559637	0.000369817	0.02668961	1
CAD	pericyte	*LTA4H*	4.59916246	0.001450604	0.035076726	1
CAD	pericyte	*VPS29*	3.856802249	0.002439553	0.039413454	1
CAD	pericyte	*CNOT2*	2.997514074	0.004252426	0.047403738	1
CAD	pericyte	*GNPTAB*	3.322095745	0.003401378	0.04373101	1
CAD	pericyte	*PHC1*	6.972203899	0.001741883	0.036710139	1
CAD	pericyte	*COL12A1*	3.147418103	0.001804252	0.036710139	1
CAD	pericyte	*SMIM8*	3.414275129	0.000251703	0.024799812	1
CAD	pericyte	*MDN1*	3.713876383	2.82E-05	0.009733533	0.177208387
CAD	pericyte	*ASCC3*	2.930084401	0.004140264	0.046816261	1
CAD	pericyte	*PACRG*	5.459163417	0.002089184	0.037961558	1
CAD	pericyte	*TFEB*	4.383481269	0.003836891	0.045686612	1
CAD	pericyte	*SENP6*	3.370569789	0.00097229	0.034565119	1
CAD	pericyte	*DAP*	3.865739654	0.003032646	0.04236944	1
CAD	pericyte	*SMAD5*	3.236212799	0.004697781	0.048657243	1
CAD	pericyte	*BNIP1*	1.159325533	0.000539065	0.031722516	1
CAD	pericyte	*BCHE*	-1.755679842	0.000952712	0.034565119	1
CAD	pericyte	*PRKAR2A*	1.96163462	0.000696822	0.034565119	1
CAD	pericyte	*UMPS*	2.428337985	0.00204645	0.037841263	1
CAD	pericyte	*PLXNA1*	2.733729442	1.32E-05	0.007247662	0.082706997
CAD	pericyte	*ABTB1*	4.039819271	0.003768322	0.045499723	1
CAD	pericyte	*COMMD2*	3.072830064	0.003392661	0.04373101	1
CAD	pericyte	*ABCC5*	2.962910953	0.004906039	0.049710001	1
CAD	pericyte	*MOB1A*	4.824648085	0.001506997	0.035381167	1
CAD	pericyte	*STAM2*	4.037041714	0.001345612	0.034565119	1
CAD	pericyte	*PPM1G*	3.57118261	0.00282777	0.041045659	1
CAD	pericyte	*GGCX*	2.886440983	3.52E-07	0.000630892	0.002216029
CAD	pericyte	*SF3B1*	2.99683344	0.002817236	0.041045659	1
CAD	pericyte	*STRN*	3.69161126	0.002301201	0.0387427	1
CAD	pericyte	*CEBPZ*	2.832741396	0.00084954	0.034565119	1
CAD	pericyte	*PRKD3*	3.953454577	0.000223257	0.024799812	1
CAD	pericyte	*RMDN2*	4.281700567	0.003348499	0.043652553	1
CAD	pericyte	*BCL9*	3.620276981	0.001540266	0.035381167	1
CAD	pericyte	*TMEM59*	3.195770435	0.002451195	0.039413454	1
CAD	pericyte	*DOCK7*	3.611866859	0.001355326	0.034565119	1
CAD	pericyte	*TMED5*	3.711086359	0.003129063	0.042947791	1
CAD	pericyte	*PRRC2C*	3.768798157	0.00010364	0.019164322	0.651586934
CAD	pericyte	*RCOR3*	3.728377314	0.001310196	0.034565119	1
CAD	pericyte	*MTFR1L*	4.16001967	0.003165282	0.042947791	1
CAD	pericyte	*KMT2A*	3.256681657	0.003159688	0.042947791	1
CAD	pericyte	*SENP5*	3.460121535	0.002144069	0.038369728	1
CAD	pericyte	*PTBP3*	3.438015323	0.003468474	0.044321742	1
CAD	pericyte	*CNTRL*	3.585048376	0.002647132	0.040572748	1
CAD	pericyte	*EIF2B2*	2.448029269	1.23E-05	0.007247662	0.077214998
CAD	pericyte	*CNRIP1*	3.231902415	0.001595456	0.035443925	1
CAD	pericyte	*CAAP1*	3.280540886	0.002504235	0.039558105	1
CAD	pericyte	*DNAJC15*	4.483060294	0.001067563	0.034565119	1
CAD	pericyte	*SMAD9*	5.137230669	0.001581376	0.035381167	1
CAD	pericyte	*SIL1*	2.667869854	0.004042147	0.046642937	1
CAD	pericyte	*LIAS*	3.907281532	0.001880837	0.036727251	1
CAD	pericyte	*ACVR2A*	3.224030048	0.00353772	0.044751802	1
CAD	pericyte	*POLK*	2.96620409	0.004483075	0.048175643	1

CAD	pericyte	*SPATA1*	3.81622916	0.002365266	0.039029998	1
CAD	pericyte	*PMS2*	4.450256	0.001822924	0.036727251	1
CAD	pericyte	*KLHL7*	4.273744335	0.001248573	0.034565119	1
CAD	pericyte	*FAM126A*	3.045214971	0.001311659	0.034565119	1
CAD	pericyte	*FKBP9*	2.395829026	0.004310887	0.04750524	1
CAD	pericyte	*PKN1*	3.713590471	0.00483306	0.049407237	1
CAD	pericyte	*SPATS2*	4.356807576	0.000186222	0.02439126	1
CAD	pericyte	*TTC21B*	3.345600731	0.003260157	0.043609808	1
CAD	pericyte	*PLA2G12A*	5.035488941	0.000385709	0.027075465	1
CAD	pericyte	*AGO2*	4.003009683	0.002087928	0.037961558	1
CAD	pericyte	*RNF114*	4.216647936	9.57E-05	0.019164322	0.601424773
CAD	pericyte	*HIF3A*	2.67661951	1.48E-05	0.007247662	0.092772625
CAD	pericyte	*SNRPC*	-1.95404542	0.000390443	0.027075465	1
CAD	pericyte	*NUP153*	3.719042932	0.001272805	0.034565119	1
CAD	pericyte	*CNOT1*	3.000800584	0.003346505	0.043652553	1
CAD	pericyte	*GRK4*	0.718504444	0.000264292	0.024799812	1
CAD	pericyte	*MIF4GD*	7.111473896	0.002007319	0.03783077	1
CAD	pericyte	*SYMPK*	4.781206362	0.000100082	0.019164322	0.629212563
CAD	pericyte	*PANK2*	4.542451716	0.00081655	0.034565119	1
CAD	pericyte	*PSMF1*	2.753160324	0.004385239	0.047915501	1
CAD	pericyte	*DSTN*	3.376728596	0.003710298	0.045499723	1
CAD	pericyte	*MMP24*	3.139574244	0.000511458	0.030624163	1
CAD	pericyte	*KLC1*	3.658202346	0.00122408	0.034565119	1
CAD	pericyte	*SBDS*	4.501009353	0.000801157	0.034565119	1
CAD	pericyte	*BECN1*	4.321553903	0.000704235	0.034565119	1
CAD	pericyte	*RAP1B*	2.905937027	0.003277247	0.043629875	1
CAD	pericyte	*HIP1*	3.143105329	0.001940643	0.037540992	1
CAD	pericyte	*PTPN12*	3.048039231	0.00316968	0.042947791	1
CAD	pericyte	*SUMF2*	3.610861493	0.002705766	0.040799638	1
CAD	pericyte	*SPCS3*	4.53156904	0.001199236	0.034565119	1
CAD	pericyte	*SERGEF*	4.55584959	0.00036759	0.02668961	1
CAD	pericyte	*SLC44A2*	4.145688195	0.002993485	0.042197406	1
CAD	pericyte	*RPL36*	4.872481976	7.59E-05	0.017044725	0.4771149
CAD	pericyte	*BST2*	5.26484452	0.001848781	0.036727251	1
CAD	pericyte	*TUBGCP2*	3.380731399	0.001272522	0.034565119	1
CAD	pericyte	*PAK4*	5.379457115	0.002765539	0.040951088	1
CAD	pericyte	*POMT1*	3.197509904	0.004430709	0.048110312	1
CAD	pericyte	*TBC1D5*	3.004262577	0.000968262	0.034565119	1
CAD	pericyte	*RFTN1*	2.683854827	0.002658815	0.040572748	1
CAD	pericyte	*EXOC4*	2.240351284	0.003080816	0.042663195	1
CAD	pericyte	*ACAP3*	3.162912595	0.001105115	0.034565119	1
CAD	pericyte	*NINJ1*	6.054795201	0.000348836	0.02668961	1
CAD	pericyte	*NPHP4*	5.846101908	0.001026575	0.034565119	1
CAD	pericyte	*MAP1B*	0.829582041	0.004880433	0.049649327	1
CAD	pericyte	*LGALS3*	3.165126372	0.003867295	0.045886261	1
CAD	pericyte	*TERF2*	3.70798481	0.003931867	0.046378327	1
CAD	pericyte	*PTPRA*	3.424787121	0.001192258	0.034565119	1
CAD	pericyte	*MUTYH*	5.031306702	0.000808747	0.034565119	1
CAD	pericyte	*POMP*	4.142657365	0.003363162	0.043686357	1
CAD	pericyte	*FAM83F*	14.60253017	0.002133601	0.038369728	1
CAD	pericyte	*SWAP70*	4.006896955	0.001167534	0.034565119	1
CAD	pericyte	*SBF2*	2.607812576	0.003106103	0.042824708	1
CAD	pericyte	*AP4B1*	4.295968362	0.000734679	0.034565119	1
CAD	pericyte	*NARS*	5.11439336	0.000623246	0.033530993	1
CAD	pericyte	*FBXO18*	4.556672659	0.000624006	0.033530993	1
CAD	pericyte	*ZCCHC11*	2.856926287	0.002760293	0.040951088	1
CAD	pericyte	*B4GALNT1*	5.524660666	0.000391899	0.027075465	1
CAD	pericyte	*SP110*	3.386616997	0.002319299	0.0387427	1
CAD	pericyte	*USP37*	3.32305998	0.002559324	0.04022617	1
CAD	pericyte	*GCC2*	3.12738949	0.000222414	0.024799812	1
CAD	pericyte	*SLC41A2*	3.696464144	0.002258815	0.0387427	1

CAD	pericyte	*LRCH1*	3.636385442	0.001260111	0.034565119	1
CAD	pericyte	*NUPL2*	3.442438126	0.002496347	0.039558105	1
CAD	pericyte	*UGGT1*	4.32934789	0.001303159	0.034565119	1
CAD	pericyte	*STX17*	4.328440272	0.00114673	0.034565119	1
CAD	pericyte	*GARNL3*	4.447571015	0.000849967	0.034565119	1
CAD	pericyte	*POLR1E*	1.520237938	0.0003326	0.02668961	1
CAD	pericyte	*KIF13A*	3.153543798	0.001552697	0.035381167	1
CAD	pericyte	*CCDC90B*	3.308421828	0.00317788	0.042966298	1
CAD	pericyte	*NARS2*	4.214122074	0.001373231	0.034565119	1
CAD	pericyte	*TGS1*	2.310075679	0.0014407	0.035044707	1
CAD	pericyte	*DDX60*	4.879464436	0.001572799	0.035381167	1
CAD	pericyte	*FDX1*	3.364202183	0.004260198	0.047403738	1
CAD	pericyte	*RMDN3*	4.137442004	0.000153828	0.023345686	0.967113914
CAD	pericyte	*UACA*	2.814770892	0.004486999	0.048175643	1
CAD	pericyte	*RTCA*	2.128005169	0.003242378	0.043464458	1
CAD	pericyte	*IFT172*	4.192255227	0.000287491	0.02510354	1
CAD	pericyte	*DYNC2LI1*	1.678222111	0.004494161	0.048175643	1
CAD	pericyte	*MYOF*	1.74698411	0.000921189	0.034565119	1
CAD	pericyte	*ARL3*	4.165583606	6.96E-05	0.017044725	0.43763544
CAD	pericyte	*RBP4*	3.530692465	0.002498219	0.039558105	1
CAD	pericyte	*DNAJC13*	3.719460461	0.002075726	0.037961558	1
CAD	pericyte	*RPS24*	3.636815578	0.00394327	0.046425731	1
CAD	pericyte	*CARF*	-2.236187403	0.000273785	0.024799812	1
CAD	pericyte	*NAB1*	4.476168076	0.001112811	0.034565119	1
CAD	pericyte	*COX17*	4.350826067	0.003769344	0.045499723	1
CAD	pericyte	*USP8*	2.672618544	0.004278378	0.04743944	1
CAD	pericyte	*ARHGAP24*	2.873769406	0.001300198	0.034565119	1
CAD	pericyte	*FAM13A*	2.189972261	0.002329369	0.0387427	1
CAD	pericyte	*HERC3*	3.586617781	0.002293256	0.0387427	1
CAD	pericyte	*BBS7*	4.89830935	0.001804268	0.036710139	1
CAD	pericyte	*KIAA1109*	3.079702443	0.002322515	0.0387427	1
CAD	pericyte	*NUP54*	3.531837738	4.16E-05	0.011764439	0.261701757
CAD	pericyte	*SCARB2*	3.331922826	0.00364002	0.045313356	1
CAD	pericyte	*GAS2L3*	5.595436093	0.001514734	0.035381167	1
CAD	pericyte	*LPAR6*	3.259539316	0.002472896	0.039525186	1
CAD	pericyte	*TMX1*	4.560906624	0.00229943	0.0387427	1
CAD	pericyte	*C14orf79*	6.531394012	0.0015749	0.035381167	1
CAD	pericyte	*TCF12*	2.832696453	0.002002524	0.03783077	1
CAD	pericyte	*SRP14*	3.810800023	0.000234057	0.024799812	1
CAD	pericyte	*IGF1R*	3.26219708	0.002547339	0.040138142	1
CAD	pericyte	*CRTC3*	3.712593612	0.001327138	0.034565119	1
CAD	pericyte	*ZFHX3*	2.749070033	0.004832366	0.049407237	1
CAD	pericyte	*CMTM3*	4.948967545	0.003484451	0.044401111	1
CAD	pericyte	*MYO19*	4.31673453	9.03E-05	0.018940359	0.567683036
CAD	pericyte	*ASXL3*	3.951623917	0.003126806	0.042947791	1
CAD	pericyte	*NARF*	2.868612566	0.003952732	0.046447144	1
CAD	pericyte	*SIRT3*	2.196636073	0.004314537	0.04750524	1
CAD	pericyte	*IFITM3*	4.765690638	0.001091014	0.034565119	1
CAD	pericyte	*RCN3*	4.371571947	0.004071619	0.046721986	1
CAD	pericyte	*GPN2*	6.090005453	0.000269721	0.024799812	1
CAD	pericyte	*MOB3C*	4.421824269	0.002862034	0.041199267	1
CAD	pericyte	*GPR161*	3.556351995	0.00380667	0.045499723	1
CAD	pericyte	*TIPRL*	4.690862192	0.001276686	0.034565119	1
CAD	pericyte	*TMCO1*	3.688361678	0.004532253	0.048175643	1
CAD	pericyte	*MGST3*	3.469895402	0.002267714	0.0387427	1
CAD	pericyte	*SEMA6C*	4.409712812	0.001258013	0.034565119	1
CAD	pericyte	*TP53BP2*	4.570292395	0.001783878	0.036710139	1
CAD	pericyte	*NVL*	3.738117475	0.003191505	0.04305792	1
CAD	pericyte	*GUK1*	4.578691831	0.001969665	0.037807709	1
CAD	pericyte	*WDPCP*	0.908228056	0.001519591	0.035381167	1
CAD	pericyte	*METTL21A*	4.49767137	0.001379445	0.034565119	1

CAD	pericyte	*NBEAL1*	-1.432755448	0.000561128	0.03183793	1
CAD	pericyte	*FANCD2*	8.225913785	3.68E-05	0.011577693	0.231553869
CAD	pericyte	*TAMM41*	4.947286774	2.94E-05	0.009733533	0.18493712
CAD	pericyte	*CTDSPL*	2.997417223	0.004083241	0.046721986	1
CAD	pericyte	*LIMD1*	2.769471922	0.002244623	0.0387427	1
CAD	pericyte	*AMT*	-1.038125222	0.00022673	0.024799812	1
CAD	pericyte	*NDUFS6*	4.530473502	0.003353616	0.043652553	1
CAD	pericyte	*LHFPL2*	3.834336123	0.003289019	0.043629875	1
CAD	pericyte	*SSBP2*	3.052396807	0.001092001	0.034565119	1
CAD	pericyte	*PPIP5K2*	2.629181347	0.003770401	0.045499723	1
CAD	pericyte	*PSD2*	6.366845172	0.002567888	0.040259323	1
CAD	pericyte	*FAM193B*	3.796985675	0.002791336	0.041045659	1
CAD	pericyte	*IRAK1BP1*	4.023954539	0.001283455	0.034565119	1
CAD	pericyte	*PHIP*	2.629051789	0.003818257	0.045551014	1
CAD	pericyte	*TACC1*	2.917098954	0.001733612	0.036710139	1
CAD	pericyte	*AK3*	3.266846624	0.001895285	0.036871353	1
CAD	pericyte	*ZNF462*	3.95217111	0.002160471	0.038369728	1
CAD	pericyte	*ASTN2*	3.164435489	0.003578692	0.045088657	1
CAD	pericyte	*WDR31*	6.113602844	9.04E-05	0.018940359	0.568210755
CAD	pericyte	*ADIRF*	4.202985337	0.000306383	0.026030132	1
CAD	pericyte	*PDCD11*	3.954712176	0.000167542	0.023591244	1
CAD	pericyte	*TTC12*	1.05104256	0.001111543	0.034565119	1
CAD	pericyte	*COMMD7*	5.132706378	0.001866658	0.036727251	1
CAD	pericyte	*ARID5B*	2.231262545	0.003397302	0.04373101	1
CAD	pericyte	*SAP18*	3.66828496	0.002154865	0.038369728	1
CAD	pericyte	*DOCK1*	2.671084915	0.004341295	0.047633025	1
CAD	pericyte	*PIP4K2A*	2.62288063	0.004919361	0.049723513	1
CAD	pericyte	*BTBD11*	5.492264959	0.001594264	0.035443925	1
CAD	pericyte	*UBE3B*	1.607811848	0.004614695	0.048380004	1
CAD	pericyte	*DLG5*	1.722073118	0.003960805	0.046447144	1
CAD	pericyte	*FAM177A1*	4.570446205	0.000358804	0.02668961	1
CAD	pericyte	*MIPOL1*	2.514529209	0.000958083	0.034565119	1
CAD	pericyte	*C4orf33*	3.783398771	0.001347713	0.034565119	1
CAD	pericyte	*VPS26B*	4.538053034	0.001309506	0.034565119	1
CAD	pericyte	*ASAP2*	3.357674781	0.000693334	0.034565119	1
CAD	pericyte	*BICD1*	3.444159652	0.002830325	0.041045659	1
CAD	pericyte	*RNF219*	4.622785372	0.002039455	0.037841263	1
CAD	pericyte	*TGOLN2*	4.183189095	0.000881975	0.034565119	1
CAD	pericyte	*CWF19L2*	3.657490214	0.000361841	0.02668961	1
CAD	pericyte	*MBNL1*	2.361716282	0.002748895	0.040951088	1
CAD	pericyte	*ZNF117*	0.532316627	0.00362587	0.045269918	1
CAD	pericyte	*CAST*	1.682488583	0.004910118	0.049710001	1
CAD	pericyte	*TUBGCP5*	3.427655571	0.003317175	0.043629875	1
CAD	pericyte	*CFDP1*	3.121525519	0.004389904	0.047915501	1
CAD	pericyte	*ROBO4*	2.574286029	0.004508324	0.048175643	1
CAD	pericyte	*CEP112*	1.909034034	0.004627327	0.048406	1
CAD	pericyte	*DISP1*	2.516570645	7.01E-06	0.006066092	0.044063478
CAD	pericyte	*TSEN2*	2.183563441	0.001619291	0.035596085	1
CAD	pericyte	*TTC39B*	3.044763772	0.001659459	0.036225767	1
CAD	pericyte	*DEPTOR*	4.390581024	0.001396459	0.034565119	1
CAD	pericyte	*DCK*	3.456647247	0.00234551	0.038878835	1
CAD	pericyte	*DRAM2*	2.524377985	0.004648746	0.048468767	1
CAD	pericyte	*MED19*	1.468612102	0.001283055	0.034565119	1
CAD	pericyte	*VPS8*	2.371974915	0.002115206	0.03832363	1
CAD	pericyte	*RPUSD3*	6.45472002	0.001012058	0.034565119	1
CAD	pericyte	*FCHO2*	3.191090996	0.002603228	0.040259323	1
CAD	pericyte	*DYRK1A*	3.22168275	0.001281253	0.034565119	1
CAD	pericyte	*AUTS2*	2.597851626	0.002398997	0.039224575	1
CAD	pericyte	*MITD1*	2.629006749	0.004191708	0.047143595	1
CAD	pericyte	*DYNC1I1*	4.852560715	0.001248224	0.034565119	1
CAD	pericyte	*ELK4*	5.166087504	0.000223333	0.024799812	1

CAD	pericyte	*NIT1*	1.963739034	0.000689459	0.034565119	1
CAD	pericyte	*RCAN1*	3.23629042	0.001392516	0.034565119	1
CAD	pericyte	*PSMD4*	4.939954011	0.000869191	0.034565119	1
CAD	pericyte	*CCDC107*	2.865391125	0.000965899	0.034565119	1
CAD	pericyte	*ORAI2*	3.542122937	0.00348881	0.044401111	1
CAD	pericyte	*DVL3*	4.719185762	0.00026382	0.024799812	1
CAD	pericyte	*PGAP3*	7.307081413	0.001420891	0.034895087	1
CAD	pericyte	*ALDH16A1*	6.17315118	7.18E-05	0.017044725	0.451351658
CAD	pericyte	*LARP4*	1.909409901	0.002483291	0.039525186	1
CAD	pericyte	*JMJD8*	2.040324181	0.000876619	0.034565119	1
CAD	pericyte	*RBP7*	5.37624543	0.00265384	0.040572748	1
CAD	pericyte	*NFIA*	2.447366609	0.003233348	0.043436019	1
CAD	pericyte	*ZNF496*	5.202218944	0.001864239	0.036727251	1
CAD	pericyte	*HAAO*	4.331462091	0.000847494	0.034565119	1
CAD	pericyte	*SMC6*	3.011246322	0.004892074	0.049687353	1
CAD	pericyte	*PDLIM5*	3.314496056	0.001162704	0.034565119	1
CAD	pericyte	*DCAF16*	4.663366424	0.001482205	0.035381167	1
CAD	pericyte	*PBXIP1*	3.582026732	0.003020791	0.04236944	1
CAD	pericyte	*LRRC58*	4.260607164	0.004687923	0.048657243	1
CAD	pericyte	*ICA1L*	5.205607431	1.43E-10	9.02E-07	9.02E-07
CAD	pericyte	*GMPS*	3.921825358	0.004267618	0.047403738	1
CAD	pericyte	*FAM208A*	2.488056698	0.004414376	0.048015883	1
CAD	pericyte	*ABLIM2*	4.389299737	0.004105537	0.046759984	1
CAD	pericyte	*RNF123*	1.89376635	1.50E-05	0.007247662	0.094219612
CAD	pericyte	*ANXA5*	2.583722139	0.003801288	0.045499723	1
CAD	pericyte	*CEP44*	3.913936931	0.004644921	0.048468767	1
CAD	pericyte	*PRRC1*	4.759746456	0.000584995	0.03283807	1
CAD	pericyte	*UBLCP1*	2.136551619	0.002349922	0.038878835	1
CAD	pericyte	*SAP30L*	4.25204543	0.002213158	0.0387427	1
CAD	pericyte	*RPS14*	3.309296935	0.002860511	0.041199267	1
CAD	pericyte	*BRI3*	2.579891237	0.004259717	0.047403738	1
CAD	pericyte	*SLC4A2*	6.357169276	0.001774032	0.036710139	1
CAD	pericyte	*FAM219A*	4.462591411	0.001092026	0.034565119	1
CAD	pericyte	*HGSNAT*	3.205750646	0.000893034	0.034565119	1
CAD	pericyte	*HNRNPK*	4.260110329	0.000897632	0.034565119	1
CAD	pericyte	*TRMT10B*	4.098016419	0.002087684	0.037961558	1
CAD	pericyte	*VCP*	2.920182606	0.000702762	0.034565119	1
CAD	pericyte	*PIGO*	5.35602936	0.000886781	0.034565119	1
CAD	pericyte	*CFL2*	4.53061333	0.000250297	0.024799812	1
CAD	pericyte	*ZCCHC24*	4.74493911	0.000255589	0.024799812	1
CAD	pericyte	*PGM2L1*	3.733817168	0.000750694	0.034565119	1
CAD	pericyte	*OXGR1*	-8.580191245	0.000274715	0.024799812	1
CAD	pericyte	*QSOX2*	7.880868466	0.001219607	0.034565119	1
CAD	pericyte	*TSC1*	3.689631196	0.000846561	0.034565119	1
CAD	pericyte	*JAM3*	2.543445403	0.004137472	0.046816261	1
CAD	pericyte	*COX11*	2.173081871	0.004772654	0.048973259	1
CAD	pericyte	*STXBP4*	2.512519949	0.004517567	0.048175643	1
CAD	pericyte	*ILK*	3.703393302	0.002171598	0.038458701	1
CAD	pericyte	*TMEM41B*	4.47306786	0.001204535	0.034565119	1
CAD	pericyte	*C16orf45*	3.076320001	0.003385983	0.04373101	1
CAD	pericyte	*RPL13*	3.165097033	0.004520319	0.048175643	1
CAD	pericyte	*ZNF641*	1.547706478	0.00074729	0.034565119	1
CAD	pericyte	*TMC4*	6.400121385	0.000505431	0.030554299	1
CAD	pericyte	*LENG8*	3.47849893	0.000303537	0.026030132	1
CAD	pericyte	*KIFC2*	1.407585209	0.001087866	0.034565119	1
CAD	pericyte	*CYB5D2*	4.829639065	0.000882558	0.034565119	1
CAD	pericyte	*CD320*	6.367250175	0.002078383	0.037961558	1
CAD	pericyte	*SPRYD3*	6.818258775	0.001181923	0.034565119	1
CAD	pericyte	*NDUFV1*	4.294883634	0.000403435	0.027401174	1
CAD	pericyte	*DDB1*	4.029068447	0.004542106	0.048175643	1
CAD	pericyte	*UBTD2*	2.80747068	0.002444124	0.039413454	1

CAD	pericyte	*MLKL*	1.170000313	0.001038053	0.034565119	1
CAD	pericyte	*STX18*	3.985171954	0.001776575	0.036710139	1
CAD	pericyte	*IRS1*	-0.769770763	2.54E-05	0.009404623	0.159878585
CAD	pericyte	*SLC38A11*	4.817348977	0.003734253	0.045499723	1
CAD	pericyte	*MT1E*	4.8579075	0.004567754	0.048296703	1
CAD	pericyte	*LIMS1*	1.941256035	0.00177014	0.036710139	1
CAD	pericyte	*MAP3K2*	3.270818469	0.002066709	0.037961558	1
CAD	pericyte	*NFXL1*	5.314073113	0.000159107	0.023345686	1
CAD	pericyte	*TSNARE1*	1.795758602	0.001057294	0.034565119	1
CAD	pericyte	*MFN1*	3.162051545	0.001020591	0.034565119	1
CAD	pericyte	*KCNMB3*	2.016339482	0.001527235	0.035381167	1
CAD	pericyte	*ATP6V0E2*	7.018598501	0.001323853	0.034565119	1
CAD	pericyte	*LRRC8C*	5.311808206	0.003697865	0.045496047	1
CAD	pericyte	*TBCA*	3.201602502	0.000481918	0.029498045	1
CAD	pericyte	*BCL2L1*	3.769659866	0.000418796	0.027715494	1
CAD	pericyte	*GPHN*	1.741425049	0.004043328	0.046642937	1
CAD	pericyte	*MTBP*	4.396134538	0.00367724	0.045338952	1
CAD	pericyte	*SPTLC3*	4.357486631	0.002327238	0.0387427	1
CAD	pericyte	*ZMAT3*	4.858261622	0.00380272	0.045499723	1
CAD	pericyte	*ZNF738*	1.535812418	0.002478189	0.039525186	1
CAD	pericyte	*LCLAT1*	3.185480555	0.004716289	0.048688525	1
CAD	pericyte	*ABLIM3*	3.14774859	0.003776256	0.045499723	1
CAD	pericyte	*GLRX*	3.670569502	0.001542134	0.035381167	1
CAD	pericyte	*MAP3K11*	5.19680289	0.002310491	0.0387427	1
CAD	pericyte	*OLR1*	11.394545	0.002989048	0.042197406	1
CAD	pericyte	*RNF213*	2.933704632	0.004221856	0.047313387	1
CAD	pericyte	*GOLIM4*	3.67694795	0.000107773	0.019359081	0.677567833
CAD	pericyte	*USMG5*	3.966200661	0.001579948	0.035381167	1
CAD	pericyte	*MGA*	2.266363506	0.00460391	0.048380004	1
CAD	pericyte	*ANKRD36C*	3.013946714	0.004000776	0.046493697	1
CAD	pericyte	*LARP7*	3.717767429	0.004190701	0.047143595	1
CAD	pericyte	*FGFBP3*	6.605565741	0.002746413	0.040951088	1
CAD	pericyte	*RSRC1*	1.912780807	0.003806721	0.045499723	1
CAD	pericyte	*TP53I11*	4.20375773	0.001226967	0.034565119	1
CAD	pericyte	*LPL*	-0.970330091	0.000132461	0.021915375	0.832784233
CAD	pericyte	*IP6K1*	4.042201555	0.000197649	0.024799812	1
CAD	pericyte	*TCP11L1*	2.425898278	0.002593555	0.040259323	1
CAD	pericyte	*CRLF3*	4.881265311	4.01E-07	0.000630892	0.002523569
CAD	pericyte	*ZNF843*	8.853645608	0.003971798	0.046447144	1
CAD	pericyte	*ANKLE2*	4.583680175	0.000838087	0.034565119	1
CAD	pericyte	*DPP7*	4.370121227	0.0028909	0.041307015	1
CAD	pericyte	*TBL1XR1*	2.494373098	0.003518516	0.044598608	1
CAD	pericyte	*RPLP2*	3.325944979	0.003868255	0.045886261	1
CAD	pericyte	*ZNF354C*	4.359602467	0.002158885	0.038369728	1
CAD	pericyte	*IMP3*	5.197800643	0.002201547	0.0387427	1
CAD	pericyte	*MLF1*	4.8230641	0.000251198	0.024799812	1
CAD	pericyte	*LCORL*	2.945021425	0.004875315	0.049649327	1
CAD	pericyte	*GAK*	3.012425185	0.004854059	0.049541343	1
CAD	pericyte	*HLA-DQB1*	-0.694463762	0.004739529	0.048768277	1
CAD	pericyte	*TIGD5*	3.504630002	0.000159673	0.023345686	1
CAD	pericyte	*PDXDC1*	3.024274546	0.002145919	0.038369728	1
CAD	pericyte	*ZNF609*	2.909497172	0.000734346	0.034565119	1
CAD	pericyte	*ZNF594*	4.49837042	0.001550229	0.035381167	1
CAD	pericyte	*D2HGDH*	2.801083356	0.001649909	0.036142776	1
CAD	pericyte	*MRPL14*	4.125239637	4.30E-05	0.011764439	0.2705821
CAD	pericyte	*ZFP82*	4.895864489	0.000441179	0.028579527	1
CAD	pericyte	*ZBTB20*	2.289460932	0.002737571	0.040951088	1
CAD	pericyte	*TNRC18*	3.490482467	0.002023802	0.037841263	1
CAD	pericyte	*IST1*	2.952757778	0.004544004	0.048175643	1
CAD	pericyte	*TRAK1*	3.550469286	0.000149662	0.023345686	0.94092692
CAD	pericyte	*ACBD3*	3.644057134	0.002833437	0.041045659	1

CAD	pericyte	*PLCXD3*	2.169902144	7.59E-05	0.017044725	0.477252294
CAD	pericyte	*ZNF721*	2.08278047	0.00398938	0.046493697	1
CAD	pericyte	*WBP2NL*	5.448575769	0.001130628	0.034565119	1
CAD	pericyte	*BMP8A*	3.738499192	0.001281931	0.034565119	1
CAD	pericyte	*CBX6*	5.48465981	0.002606258	0.040259323	1
CAD	pericyte	*PP2D1*	2.271926299	0.0046975	0.048657243	1
CAD	pericyte	*CSF1*	2.056088561	0.00040533	0.027401174	1
CAD	pericyte	*WDR27*	3.228987893	0.004159408	0.046948296	1
CAD	pericyte	*ZNF93*	7.47474192	0.00099913	0.034565119	1
CAD	pericyte	*ZFP90*	4.605398575	0.001443706	0.035044707	1
CAD	pericyte	*KIAA0825*	3.602732079	0.001371893	0.034565119	1
CAD	pericyte	*SP1*	2.282234066	0.001784315	0.036710139	1
CAD	pericyte	*C16orf52*	4.047427873	0.001091844	0.034565119	1
CAD	pericyte	*ZNF529*	2.60066073	0.004570787	0.048296703	1
CAD	pericyte	*TOR3A*	6.962793684	0.000738184	0.034565119	1
CAD	pericyte	*KANK3*	4.77801562	0.004130867	0.046816261	1
CAD	pericyte	*DNAH17*	6.612273004	0.000562114	0.03183793	1
CAD	pericyte	*TMEM220*	4.050754365	0.001073547	0.034565119	1
CAD	pericyte	*SRSF10*	3.296896947	0.003742515	0.045499723	1
CAD	pericyte	*DPYD*	2.67671768	0.002768286	0.040951088	1
CAD	pericyte	*FAM196A*	3.551557026	0.002635485	0.040511729	1
CAD	pericyte	*NELFB*	5.077245121	0.001774939	0.036710139	1
CAD	pericyte	*ADAT2*	3.230468773	0.000483267	0.029498045	1
CAD	pericyte	*ZNF527*	4.335257906	0.001732782	0.036710139	1
CAD	pericyte	*ZKSCAN3*	3.612903674	0.004617147	0.048380004	1
CAD	pericyte	*HLA-DRB1*	-1.289824696	0.002602991	0.040259323	1
CAD	pericyte	*MPHOSPH8*	3.462505422	0.00093578	0.034565119	1
CAD	pericyte	*SUPT5H*	4.722758103	0.000427361	0.027987662	1
CAD	pericyte	*SUPT3H*	3.470581501	0.001900162	0.036871353	1
CAD	pericyte	*ZNF565*	2.307038224	0.003045297	0.042451851	1
CAD	pericyte	*ASB13*	4.908318374	0.002888159	0.041307015	1
CAD	pericyte	*HDAC2*	4.068177824	0.00320024	0.043083312	1
CAD	pericyte	*HRH1*	3.270751559	0.004775029	0.048973259	1
CAD	pericyte	*TOMM7*	4.581025293	0.001702013	0.036710139	1
CAD	pericyte	*ZNF431*	3.576047794	0.000737881	0.034565119	1
CAD	pericyte	*STRN3*	3.220815693	0.001517584	0.035381167	1
CAD	pericyte	*C6orf106*	2.152385103	3.59E-07	0.000630892	0.00225739
CAD	pericyte	*NHLRC2*	3.202288745	0.003325636	0.043637999	1
CAD	pericyte	*KPNA5*	3.457953298	0.002689469	0.040799638	1
CAD	pericyte	*ANKRD36B*	2.266576107	0.004037464	0.046642937	1
CAD	pericyte	*SRGAP1*	3.854319287	0.000209295	0.024799812	1
CAD	pericyte	*PCDHB16*	0.998989102	0.003797193	0.045499723	1
CAD	pericyte	*WDR5B*	3.825592757	1.72E-05	0.007744296	0.10842014
CAD	pericyte	*ZMYM1*	4.400470382	0.002004403	0.03783077	1
CAD	pericyte	*IGF2R*	2.864486511	0.003755802	0.045499723	1
CAD	pericyte	*ZNF682*	4.56621341	0.001978485	0.037807709	1
CAD	pericyte	*KANK2*	3.806203596	0.004200169	0.047154399	1
CAD	pericyte	*GTF2E2*	3.999741379	0.000373578	0.02668961	1
CAD	pericyte	*RAMP2-AS1*	3.22909061	0.000671252	0.034565119	1
CAD	pericyte	*ZNF517*	8.691643751	0.002407879	0.039224575	1
CAD	pericyte	*ZNF665*	1.708177087	0.002015005	0.03783077	1
CAD	pericyte	*ZNF624*	6.821127258	0.000455112	0.028612907	1
CAD	pericyte	*ZNF71*	2.437020037	0.0004116	0.027529057	1
CAD	pericyte	*NOL8*	4.138965355	0.002178041	0.038464447	1
CAD	pericyte	*MRPL42*	2.450027811	0.000286026	0.02510354	1
CAD	pericyte	*HIBCH*	2.701356111	0.000240298	0.024799812	1
CAD	pericyte	*MIER1*	3.827007101	0.000612679	0.033530993	1
CAD	pericyte	*DDRGK1*	5.648809134	0.001540181	0.035381167	1
CAD	pericyte	*QRICH1*	1.801601747	0.000445615	0.028579527	1
CAD	pericyte	*TMEM116*	-0.931292619	0.004028805	0.046642937	1
CAD	pericyte	*GFPT1*	3.268438067	0.000362535	0.02668961	1

CAD	pericyte	*MGEA5*	2.151236093	0.004733765	0.048768277	1
CAD	pericyte	*ZNF808*	2.907952974	0.000914681	0.034565119	1
CAD	pericyte	*LRBA*	3.20191687	0.00109497	0.034565119	1
CAD	pericyte	*MDM4*	3.340283357	0.002448805	0.039413454	1
CAD	pericyte	*PAPSS2*	2.708535894	0.00175745	0.036710139	1
CAD	pericyte	*FAN1*	4.938337323	0.001056788	0.034565119	1
CAD	pericyte	*ZNF652*	3.65770237	0.002407831	0.039224575	1
CAD	pericyte	*SFT2D1*	4.907739119	0.000543662	0.031722516	1
CAD	pericyte	*APRT*	5.346361213	0.002431577	0.039413454	1
CAD	pericyte	*GIGYF2*	3.134016675	0.003067055	0.042566384	1
CAD	pericyte	*PRRT1*	1.48079951	0.00397464	0.046447144	1
CAD	pericyte	*GPANK1*	1.963770721	0.003301698	0.043629875	1
CAD	pericyte	*BAG6*	2.722636176	4.21E-05	0.011764439	0.264445996
CAD	pericyte	*HLA-F*	1.674338485	0.002274939	0.0387427	1
CAD	pericyte	*TRIM13*	3.042914182	0.003586566	0.045097485	1
CAD	pericyte	*C1QTNF9B*	103.5436118	0.001245677	0.034565119	1
CAD	pericyte	*NYNRIN*	7.058282963	0.000122576	0.020828029	0.770637071
CAD	pericyte	*ZDHHC11B*	2.766246091	0.001606312	0.035525597	1
CAD	pericyte	*HCG27*	0.896686186	0.003009462	0.042327712	1
CAD	pericyte	*TAS2R14*	1.23168468	0.004094769	0.046721986	1
CAD	pericyte	*SIPA1*	3.355260063	0.000138213	0.022280634	0.868944715
CAD	pericyte	*ERV3-1*	0.546794345	0.00329795	0.043629875	1
CAD	pericyte	*HEXA*	4.815038311	0.001342803	0.034565119	1
CAD	pericyte	*ATF6B*	1.395555111	0.004341198	0.047633025	1
CAD	pericyte	*CLIC1*	4.338273689	0.000321382	0.026240618	1
CAD	pericyte	*LYRM4*	3.919191255	0.001094517	0.034565119	1
CAD	pericyte	*NPEPL1*	4.718929779	0.001482203	0.035381167	1
CAD	pericyte	*VPS52*	4.726416383	0.000339524	0.02668961	1
CAD	pericyte	*ERCC6*	4.832539015	0.000450035	0.028579527	1
CAD	pericyte	*NOL7*	4.288877401	0.00315438	0.042947791	1
CAD	pericyte	*TAS2R46*	-2.123746346	0.004052953	0.046668347	1
CAD	pericyte	*RPL41*	7.289181563	0.000633183	0.033638992	1
CAD	pericyte	*PATL2*	4.412844325	0.00357491	0.045088657	1
CAD	pericyte	*RPS18*	1.874068814	0.004592177	0.048380004	1
CAD	pericyte	*GOLGA8N*	1.18408029	0.003677885	0.045338952	1
CAD	pericyte	*HLA-B*	1.990746341	1.04E-05	0.007247662	0.065381515
CAD	pericyte	*HLA-DQA2*	0.790246572	0.002922552	0.041570331	1
CAD	pericyte	*LY6G5B*	-0.487358664	0.00344317	0.044125465	1
CAD	pericyte	*LINC00886*	3.646827178	0.000770118	0.034565119	1
CAD	pericyte	*ATP5J2*	3.875994622	0.001226934	0.034565119	1
CAD	pericyte	*ARFGAP3*	3.727519562	0.002009334	0.03783077	1
CAD	pericyte	*AP5Z1*	4.243302583	0.001569204	0.035381167	1
CAD	pericyte	*FGF14-IT1*	20.30203132	0.001749884	0.036710139	1
CAD	pericyte	*APOBEC3C*	9.836927512	0.000851356	0.034565119	1
CAD	pericyte	*UBE2V1*	5.880509351	0.003384064	0.04373101	1
CAD	pericyte	*SHANK3*	1.618754259	0.000320212	0.026240618	1
CAD	pericyte	*ZNF260*	5.236772316	0.000180541	0.024188147	1
CAD	pericyte	*FPGT*	5.227068267	0.002803991	0.041045659	1
CAD	pericyte	*HMBS*	1.731297513	2.52E-05	0.009404623	0.158595615
CAD	pericyte	*ZNF253*	3.880334046	0.004288976	0.047473231	1
CAD	pericyte	*IQCH-AS1*	1.690887274	0.001874232	0.036727251	1
CAD	pericyte	*HCG17*	2.962373842	0.001726688	0.036710139	1
CAD	SMC	*SEMA3F*	-0.874892695	6.25E-05	0.026695616	0.392878817
CAD	SMC	*UHRF1BP1*	0.968415398	3.02E-05	0.023734972	0.189879774
CAD	SMC	*TIE1*	-1.355144352	0.000141175	0.049309179	0.887565229
CAD	SMC	*MAP1LC3A*	1.545607634	8.52E-05	0.031506883	0.535617018
CAD	SMC	*BCHE*	-1.588307505	4.94E-05	0.026695616	0.310339763
CAD	SMC	*GGCX*	1.581107544	4.98E-05	0.026695616	0.313217273
CAD	SMC	*SNRPC*	-1.66935696	1.20E-05	0.015148454	0.075742272
CAD	SMC	*CARF*	-2.12352287	3.45E-07	0.001646819	0.00216688
CAD	SMC	*NBEAL1*	-1.324082782	2.35E-06	0.003698719	0.014794877

CAD	SMC	*AMT*	-0.768482982	5.96E-05	0.026695616	0.374903169
CAD	SMC	*PSD2*	7.329073662	5.39E-05	0.026695616	0.338785099
CAD	SMC	*ICA1L*	2.128041663	4.99E-05	0.026695616	0.31400899
CAD	SMC	*APEH*	-1.624248794	1.60E-05	0.016742249	0.100453491
CAD	SMC	*IRS1*	-0.708415481	5.24E-07	0.001646819	0.003293639
CAD	SMC	*LPL*	-0.873115565	1.67E-06	0.003502718	0.010508153
CAD	SMC	*HLA-DRB1*	-1.165749013	6.44E-05	0.026695616	0.405120765
CAD	SMC	*C6orf106*	1.26103989	2.92E-05	0.023734972	0.183853564
CAD	SMC	*HMBS*	1.141207005	6.79E-05	0.026695616	0.427129864
CAD	t_cell	*UHRF1BP1*	1.977884802	4.34E-06	0.013386897	0.027297398
CAD	t_cell	*TIPIN*	1.354168686	5.68E-05	0.035679599	0.356795986
CAD	t_cell	*MAP1LC3A*	1.803879275	0.000101043	0.048866166	0.635260163
CAD	t_cell	*PLXNA1*	2.998193277	8.63E-05	0.046372005	0.542740491
CAD	t_cell	*GGCX*	3.229618247	6.84E-06	0.013386897	0.043027462
CAD	t_cell	*SNRPC*	-3.036346637	5.52E-05	0.035679599	0.347224131
CAD	t_cell	*GRK4*	1.105180925	4.69E-05	0.035679599	0.294765933
CAD	t_cell	*DISP1*	2.571158793	5.19E-05	0.035679599	0.326350902
CAD	t_cell	*ICA1L*	3.527256797	1.06E-05	0.013386897	0.066934487
CAD	t_cell	*APEH*	-3.662138562	3.23E-06	0.013386897	0.02030928
CAD	t_cell	*TMEM136*	3.18648354	8.85E-05	0.046372005	0.556464058
CAD	t_cell	*C6orf106*	2.582831971	8.94E-06	0.013386897	0.056212832
CAD	t_cell	*HMBS*	2.361056464	2.28E-05	0.023891527	0.143349162
T2DM	adipocyte	*CFH*	0.764379222	9.89E-07	0.000141342	0.006219028
T2DM	adipocyte	*SEMA3F*	-0.190854127	3.12E-05	0.001056656	0.196089012
T2DM	adipocyte	*TMEM176A*	0.219908802	0.001354056	0.012355519	1
T2DM	adipocyte	*M6PR*	0.419785141	0.010413941	0.04844569	1
T2DM	adipocyte	*RBM6*	-0.17523276	2.68E-07	6.01E-05	0.001683482
T2DM	adipocyte	*RECQL*	1.175220336	0.000281424	0.00440128	1
T2DM	adipocyte	*WDR54*	0.70627487	0.00270842	0.020040713	1
T2DM	adipocyte	*ABCB4*	1.001079099	0.001327592	0.012202593	1
T2DM	adipocyte	*GGNBP2*	-0.424286223	0.000120741	0.002547302	0.759095875
T2DM	adipocyte	*TMEM132A*	1.60654627	8.38E-05	0.001941003	0.527046922
T2DM	adipocyte	*RALA*	0.820748405	0.005177898	0.030234016	1
T2DM	adipocyte	*NOS2*	3.880728858	0.005261284	0.030505142	1
T2DM	adipocyte	*TSR3*	1.541420111	0.00019984	0.003539133	1
T2DM	adipocyte	*STARD3NL*	0.839936845	0.001254863	0.01176218	1
T2DM	adipocyte	*CD4*	0.980551654	0.000119054	0.002528693	0.748493043
T2DM	adipocyte	*SYT7*	0.55652033	0.003183525	0.022463322	1
T2DM	adipocyte	*LARS2*	0.551611562	0.004555499	0.028028472	1
T2DM	adipocyte	*PPP5C*	0.518226947	0.008359992	0.041846552	1
T2DM	adipocyte	*CEP68*	0.616962994	5.39E-11	3.39E-08	3.39E-07
T2DM	adipocyte	*ZBTB32*	-0.942418472	0.003261523	0.022707856	1
T2DM	adipocyte	*ALOX5*	0.646167739	0.001585896	0.013733509	1
T2DM	adipocyte	*PSMC4*	0.33698729	0.010647862	0.049259093	1
T2DM	adipocyte	*ANGEL1*	1.447897452	1.39E-05	0.00073252	0.0873849
T2DM	adipocyte	*NPC1L1*	-0.471021975	0.010113087	0.047519414	1
T2DM	adipocyte	*IL20RA*	4.104859967	6.79E-05	0.001674964	0.427115719
T2DM	adipocyte	*WWTR1*	0.633493032	0.003699172	0.024415616	1
T2DM	adipocyte	*OSBPL5*	1.080298787	0.000637107	0.007514999	1
T2DM	adipocyte	*AQR*	0.66982432	0.003788725	0.024853488	1
T2DM	adipocyte	*AKAP11*	0.818175845	0.007214815	0.038407743	1
T2DM	adipocyte	*NR1H3*	0.526236778	0.002056225	0.01648866	1
T2DM	adipocyte	*KPNA6*	0.550717486	0.005367302	0.030981545	1
T2DM	adipocyte	*VIM*	0.614120093	0.001878237	0.01547857	1
T2DM	adipocyte	*CD44*	0.526024245	0.000654659	0.007678812	1
T2DM	adipocyte	*ANK1*	1.423116226	1.79E-05	0.000809781	0.112497183
T2DM	adipocyte	*BAK1*	0.190650893	0.003936908	0.02530812	1
T2DM	adipocyte	*GRN*	0.905312668	0.000175866	0.003310394	1
T2DM	adipocyte	*ARID4A*	0.819788873	0.002392262	0.018520378	1
T2DM	adipocyte	*FUT8*	0.743157927	0.003354501	0.02302506	1
T2DM	adipocyte	*PIAS1*	0.689994919	0.002530596	0.019099469	1

T2DM	adipocyte	*SLC4A7*	0.718593705	0.000776328	0.008653858	1
T2DM	adipocyte	*ADSS*	1.052472473	0.000228959	0.003808107	1
T2DM	adipocyte	*CUL3*	0.779974163	0.000922927	0.009622628	1
T2DM	adipocyte	*TUBG2*	-0.26452018	3.39E-09	1.71E-06	2.13E-05
T2DM	adipocyte	*MAT2B*	1.010371431	0.000890194	0.009413933	1
T2DM	adipocyte	*C6*	-0.546502407	0.000159428	0.003103179	1
T2DM	adipocyte	*RAB27B*	0.278389779	0.000254202	0.0040489	1
T2DM	adipocyte	*THAP3*	-0.890113302	0.00579028	0.032892943	1
T2DM	adipocyte	*MED17*	0.739541107	0.000574488	0.00702686	1
T2DM	adipocyte	*LCP2*	0.484788301	0.002197241	0.017354337	1
T2DM	adipocyte	*CNTLN*	0.811551415	7.33E-05	0.001763187	0.461054154
T2DM	adipocyte	*EPHA3*	0.867238019	1.76E-05	0.000808689	0.110790407
T2DM	adipocyte	*DSG2*	0.755850368	0.002727662	0.020127714	1
T2DM	adipocyte	*YTHDC2*	0.788873807	0.002213593	0.017461556	1
T2DM	adipocyte	*POLR2B*	0.837200965	0.00423846	0.026727379	1
T2DM	adipocyte	*CP*	1.344824929	2.74E-05	0.000990203	0.172295278
T2DM	adipocyte	*DTNBP1*	0.975384042	0.000358553	0.005239142	1
T2DM	adipocyte	*TSPAN17*	0.298367294	0.002280457	0.017854583	1
T2DM	adipocyte	*SNX29*	0.66116833	0.000779621	0.008675178	1
T2DM	adipocyte	*RFC2*	0.733499593	0.006349311	0.034985203	1
T2DM	adipocyte	*DKK3*	0.790824293	4.89E-05	0.001371325	0.30717688
T2DM	adipocyte	*NFE2L3*	1.54164017	5.52E-06	0.000480968	0.034733837
T2DM	adipocyte	*MAPK9*	0.539318011	0.003840951	0.024997991	1
T2DM	adipocyte	*CYBA*	0.89991572	4.93E-05	0.001378479	0.31015769
T2DM	adipocyte	*ALX4*	2.577028198	0.001115823	0.010825898	1
T2DM	adipocyte	*ANAPC4*	0.860883176	0.000246189	0.003980202	1
T2DM	adipocyte	*OPN3*	0.190354052	0.006797818	0.036779587	1
T2DM	adipocyte	*TBC1D22A*	0.724654501	0.000788248	0.008709522	1
T2DM	adipocyte	*ITIH4*	0.152838918	4.95E-06	0.000450593	0.031090909
T2DM	adipocyte	*IL17RB*	-1.353714564	0.006273714	0.03462936	1
T2DM	adipocyte	*GDI2*	0.441787055	0.008914577	0.043649492	1
T2DM	adipocyte	*CDK14*	0.674537563	0.006246445	0.034509139	1
T2DM	adipocyte	*YBX3*	0.842289866	0.000316771	0.004833828	1
T2DM	adipocyte	*PIGV*	-0.417823727	0.001365861	0.012445176	1
T2DM	adipocyte	*QSER1*	0.940243491	0.001473338	0.013046308	1
T2DM	adipocyte	*VMP1*	0.701413668	0.00334571	0.02302506	1
T2DM	adipocyte	*EPN1*	1.136931632	0.00092171	0.009622628	1
T2DM	adipocyte	*CASP8*	0.545284989	0.006331166	0.034915828	1
T2DM	adipocyte	*LPAR2*	0.576507786	0.000663823	0.007725842	1
T2DM	adipocyte	*SUGP2*	0.40964013	0.004031716	0.025812014	1
T2DM	adipocyte	*BTBD1*	1.139223691	2.74E-05	0.000990203	0.172148999
T2DM	adipocyte	*ANKRD44*	0.574626748	0.001349826	0.012351141	1
T2DM	adipocyte	*NDUFB4*	0.98445736	0.002421655	0.018680915	1
T2DM	adipocyte	*MAP2K4*	0.705229424	0.008589326	0.042824024	1
T2DM	adipocyte	*TBC1D1*	0.934350217	5.66E-05	0.001496027	0.356054526
T2DM	adipocyte	*NGEF*	3.376260606	8.60E-05	0.001965411	0.540488082
T2DM	adipocyte	*CD84*	0.312654823	0.002907422	0.021024181	1
T2DM	adipocyte	*SP100*	0.822949264	0.000184469	0.003395587	1
T2DM	adipocyte	*TP53BP1*	0.514800611	0.006438641	0.035384382	1
T2DM	adipocyte	*HDAC4*	0.756187092	0.005314677	0.030739072	1
T2DM	adipocyte	*RASSF1*	0.456071698	0.00585076	0.03304917	1
T2DM	adipocyte	*IFI35*	0.281330371	0.007811263	0.040586289	1
T2DM	adipocyte	*CYFIP1*	0.825653207	0.001756928	0.014708134	1
T2DM	adipocyte	*PITX1*	-1.692220578	0.000809597	0.008852065	1
T2DM	adipocyte	*NUCKS1*	0.666278828	0.005082777	0.029948844	1
T2DM	adipocyte	*GAL*	2.16886325	6.18E-05	0.001599534	0.388686728
T2DM	adipocyte	*NEDD4*	0.436746677	0.000518157	0.006450799	1
T2DM	adipocyte	*GNB5*	0.312437273	0.0076886	0.040114714	1
T2DM	adipocyte	*ASNS*	0.790365161	0.000767918	0.008576883	1
T2DM	adipocyte	*TESK2*	0.675530933	0.007722798	0.040259728	1
T2DM	adipocyte	*CDC42*	0.896228628	0.002625271	0.019596375	1

T2DM	adipocyte	*OSBPL3*	0.481152252	0.004578058	0.028052873	1
T2DM	adipocyte	*SNX13*	0.774845634	0.003217307	0.022589029	1
T2DM	adipocyte	*VASH1*	0.732362097	0.004566608	0.028037365	1
T2DM	adipocyte	*TRIP13*	-2.005608658	0.008027182	0.041298601	1
T2DM	adipocyte	*CYBRD1*	0.372224028	0.002712684	0.020040713	1
T2DM	adipocyte	*EPN2*	0.594862383	0.009162562	0.044546498	1
T2DM	adipocyte	*LIMS2*	0.385313162	0.004394274	0.027428067	1
T2DM	adipocyte	*TRHDE*	-0.332164558	0.005901356	0.033072047	1
T2DM	adipocyte	*NFATC3*	0.435217387	0.004242869	0.026728373	1
T2DM	adipocyte	*ALPK1*	0.771948225	0.001571757	0.013648668	1
T2DM	adipocyte	*SDHA*	0.424472736	0.000599499	0.007210362	1
T2DM	adipocyte	*NTN4*	0.694217484	0.000187367	0.003398243	1
T2DM	adipocyte	*SRI*	1.030843077	0.003622072	0.024071846	1
T2DM	adipocyte	*EIF4G3*	0.524750291	0.008161683	0.041544539	1
T2DM	adipocyte	*CELSR1*	1.431501887	9.02E-05	0.002025613	0.567171542
T2DM	adipocyte	*ADD2*	1.380294361	5.62E-05	0.001489992	0.353128009
T2DM	adipocyte	*SLC25A3*	0.759484579	0.000392846	0.005550157	1
T2DM	adipocyte	*PLD1*	0.830097306	0.00052476	0.006520091	1
T2DM	adipocyte	*MCM6*	0.542705765	0.01026032	0.047996007	1
T2DM	adipocyte	*NT5C2*	0.746235806	0.000215349	0.00364933	1
T2DM	adipocyte	*XAB2*	-0.48547129	0.009518505	0.045716457	1
T2DM	adipocyte	*STXBP2*	0.530917414	2.81E-05	0.00099459	0.176736318
T2DM	adipocyte	*CTTNBP2*	-0.434086241	0.004311195	0.027050381	1
T2DM	adipocyte	*DNAJC10*	1.018560533	0.000791894	0.008730615	1
T2DM	adipocyte	*DYNC1I2*	0.647079526	0.00375519	0.024695481	1
T2DM	adipocyte	*LRCH4*	-0.29646909	0.002442471	0.018795368	1
T2DM	adipocyte	*FBLN1*	0.254986415	0.00258478	0.019415187	1
T2DM	adipocyte	*SYNJ2*	0.466604419	0.003536994	0.023782977	1
T2DM	adipocyte	*FGF20*	6.040854257	1.79E-05	0.000809781	0.112559625
T2DM	adipocyte	*XRCC5*	0.809099199	0.001504447	0.013241122	1
T2DM	adipocyte	*DNM2*	0.805017403	0.003875434	0.025066724	1
T2DM	adipocyte	*DCT*	-0.608937308	0.000495701	0.006341444	1
T2DM	adipocyte	*MAGI3*	0.745646169	0.004178907	0.026484666	1
T2DM	adipocyte	*STK17B*	0.935480056	0.000187879	0.003398243	1
T2DM	adipocyte	*HSPB11*	0.815089593	0.004437814	0.027624292	1
T2DM	adipocyte	*COBLL1*	-0.392292339	1.00E-07	2.74E-05	0.000631291
T2DM	adipocyte	*GSK3B*	0.687534539	0.002503212	0.0190529	1
T2DM	adipocyte	*ERC1*	0.624853406	0.005228882	0.030410711	1
T2DM	adipocyte	*PDS5B*	0.720265589	0.001957609	0.015997616	1
T2DM	adipocyte	*RPS5*	0.914464519	0.001650366	0.014136036	1
T2DM	adipocyte	*GSTP1*	1.086662925	0.000267929	0.004221729	1
T2DM	adipocyte	*APOB*	-0.472356908	0.000417312	0.005670594	1
T2DM	adipocyte	*NCOA1*	0.677165604	0.001705566	0.014412493	1
T2DM	adipocyte	*CD59*	0.667750992	0.000169408	0.003217726	1
T2DM	adipocyte	*CD82*	0.37047083	0.002573876	0.019356409	1
T2DM	adipocyte	*SCAMP1*	0.758073932	0.000321544	0.0048855	1
T2DM	adipocyte	*CPNE3*	0.361956193	0.001768754	0.014787441	1
T2DM	adipocyte	*EPS15*	0.27662111	0.009279367	0.044980246	1
T2DM	adipocyte	*ATG16L1*	1.071698881	0.000758308	0.00851336	1
T2DM	adipocyte	*B4GALT1*	0.869701109	0.000666042	0.007725842	1
T2DM	adipocyte	*SNX10*	-0.066533515	0.00356262	0.023853237	1
T2DM	adipocyte	*LPCAT2*	0.370471582	0.009498041	0.045678294	1
T2DM	adipocyte	*KLHL42*	0.506807019	0.008728394	0.043068751	1
T2DM	adipocyte	*C3orf18*	0.7174714	1.06E-06	0.000145821	0.006675688
T2DM	adipocyte	*FKBP1A*	1.060387943	0.000416952	0.005670594	1
T2DM	adipocyte	*XRN2*	0.813792406	0.002731427	0.020131868	1
T2DM	adipocyte	*TESC*	1.517006739	1.08E-05	0.000656895	0.067660206
T2DM	adipocyte	*SLC23A2*	0.857380962	0.002238588	0.017614524	1
T2DM	adipocyte	*OAS1*	1.235067132	0.000162424	0.00313738	1
T2DM	adipocyte	*FUS*	0.793764919	0.001272858	0.011908421	1
T2DM	adipocyte	*FXYD5*	0.960672319	2.85E-05	0.001002409	0.179431168

T2DM	adipocyte	*GMIP*	1.396835781	4.25E-05	0.001220134	0.26720942
T2DM	adipocyte	*PAPOLA*	0.713195312	0.003349764	0.02302506	1
T2DM	adipocyte	*RGS1*	1.637143415	9.06E-06	0.000586394	0.056934528
T2DM	adipocyte	*YPEL3*	0.170050086	0.003865621	0.025034168	1
T2DM	adipocyte	*LYZ*	0.47339008	0.000599812	0.007210362	1
T2DM	adipocyte	*PABPC4*	-0.544410526	7.15E-07	0.000115199	0.004492766
T2DM	adipocyte	*PDPR*	0.841234029	0.000662755	0.007725842	1
T2DM	adipocyte	*EXOC1*	0.741068517	0.005400241	0.031090947	1
T2DM	adipocyte	*TXNL1*	0.811639033	0.006643138	0.036170411	1
T2DM	adipocyte	*IL5RA*	3.446024549	0.001165317	0.011138941	1
T2DM	adipocyte	*SEL1L3*	1.052914472	3.03E-06	0.000307456	0.019062268
T2DM	adipocyte	*MYO15A*	0.603381346	0.006688796	0.036377559	1
T2DM	adipocyte	*NLRP1*	0.629521203	0.003510858	0.023694794	1
T2DM	adipocyte	*RGS17*	-0.358517217	0.008959638	0.043835988	1
T2DM	adipocyte	*PPP2R3C*	0.727715421	0.001789167	0.014898665	1
T2DM	adipocyte	*OSGEP*	0.965173375	0.003973687	0.025492416	1
T2DM	adipocyte	*DPYSL2*	0.684478201	0.000186024	0.003395587	1
T2DM	adipocyte	*COMT*	0.896829204	0.005780324	0.032892943	1
T2DM	adipocyte	*VNN3*	1.842480246	0.000485084	0.006236657	1
T2DM	adipocyte	*SUCO*	0.776361091	0.008621256	0.042949155	1
T2DM	adipocyte	*MSH2*	1.197510096	0.000579698	0.007060862	1
T2DM	adipocyte	*TMEM38B*	1.385424986	0.001592145	0.013763932	1
T2DM	adipocyte	*CWF19L1*	-0.219023162	0.000689935	0.00792984	1
T2DM	adipocyte	*CREM*	0.933986768	0.001678244	0.014340975	1
T2DM	adipocyte	*TPSD1*	4.618711421	8.90E-07	0.000133232	0.005595729
T2DM	adipocyte	*ITPR3*	0.963868155	1.40E-05	0.00073252	0.088044726
T2DM	adipocyte	*NRP1*	0.67754133	0.000818938	0.008923161	1
T2DM	adipocyte	*STX1B*	-0.378202731	0.003922893	0.02524384	1
T2DM	adipocyte	*MARCH2*	0.655916617	0.002009011	0.016276616	1
T2DM	adipocyte	*P2RX6*	-0.532809909	0.000212255	0.00360662	1
T2DM	adipocyte	*GGT5*	1.144281104	2.80E-06	0.000294676	0.017619489
T2DM	adipocyte	*PPM1F*	0.739679172	0.003230084	0.022589029	1
T2DM	adipocyte	*CYTH4*	1.048193259	1.89E-05	0.000811251	0.118583918
T2DM	adipocyte	*CARD10*	0.43846241	0.004814827	0.028912435	1
T2DM	adipocyte	*SH3BP1*	1.316999528	0.000176999	0.003311883	1
T2DM	adipocyte	*TIMP3*	0.675128371	0.002007054	0.016276616	1
T2DM	adipocyte	*RBFOX2*	0.647579076	0.008656931	0.042990619	1
T2DM	adipocyte	*KIAA0930*	0.391161992	0.007400835	0.039028017	1
T2DM	adipocyte	*KCTD17*	2.115888198	2.22E-05	0.000837403	0.139846262
T2DM	adipocyte	*DESI1*	0.218738712	0.00368312	0.024400185	1
T2DM	adipocyte	*ATP6V1D*	-0.237885876	0.008226822	0.041544539	1
T2DM	adipocyte	*PLTP*	0.229999721	0.000410487	0.005646146	1
T2DM	adipocyte	*CD40*	0.656988348	0.007351583	0.038905221	1
T2DM	adipocyte	*NDRG3*	0.879694589	0.001530309	0.013362569	1
T2DM	adipocyte	*BMP7*	1.40148894	1.47E-05	0.000753745	0.092713048
T2DM	adipocyte	*DIDO1*	0.560285318	0.000921055	0.009622628	1
T2DM	adipocyte	*SLC17A9*	0.827625597	0.000211149	0.00360662	1
T2DM	adipocyte	*CCM2L*	1.252679923	5.57E-06	0.000480968	0.035039513
T2DM	adipocyte	*METTL4*	1.173531699	0.001301862	0.012083315	1
T2DM	adipocyte	*LPIN2*	0.915705159	0.002058788	0.01648866	1
T2DM	adipocyte	*SMCHD1*	0.724734368	0.001106765	0.010771256	1
T2DM	adipocyte	*MYL12A*	0.86622856	0.002793565	0.020448966	1
T2DM	adipocyte	*HTR2A*	2.666267786	1.57E-05	0.000762794	0.098400458
T2DM	adipocyte	*UGGT2*	0.497491732	0.002523791	0.019099469	1
T2DM	adipocyte	*SGCG*	-0.308387529	0.010739493	0.049500875	1
T2DM	adipocyte	*PARP4*	0.858361675	0.000922772	0.009622628	1
T2DM	adipocyte	*MEDAG*	1.126795294	1.38E-05	0.00073252	0.087022641
T2DM	adipocyte	*TSC22D1*	0.817516052	1.98E-05	0.00081257	0.124611157
T2DM	adipocyte	*CTCF*	0.612030164	0.00510579	0.029999308	1
T2DM	adipocyte	*COG4*	-0.349994898	0.009627281	0.045962686	1
T2DM	adipocyte	*HCFC1R1*	1.658250251	9.11E-05	0.002031326	0.572833803

T2DM	adipocyte	*LMF1*	0.786001341	0.004133895	0.026252322	1
T2DM	adipocyte	*SLC7A5*	3.794377007	0.000151891	0.003021954	0.954937616
T2DM	adipocyte	*NUBP1*	1.020484999	0.010157678	0.047657704	1
T2DM	adipocyte	*UBE2I*	0.642222544	0.005991369	0.033423016	1
T2DM	adipocyte	*RBL2*	0.434718079	9.51E-06	0.000597863	0.059786299
T2DM	adipocyte	*KAT8*	0.672799301	0.007269238	0.038599407	1
T2DM	adipocyte	*SYT17*	-0.298796919	0.009887741	0.046765514	1
T2DM	adipocyte	*TMC5*	-0.687403667	0.000367401	0.005285702	1
T2DM	adipocyte	*CSK*	1.382143911	5.20E-05	0.001416342	0.327174962
T2DM	adipocyte	*HERC1*	0.773147105	0.000649606	0.007633777	1
T2DM	adipocyte	*RASL12*	1.357354471	1.12E-05	0.000673225	0.070688618
T2DM	adipocyte	*FAH*	-0.485420726	0.002511024	0.019089246	1
T2DM	adipocyte	*EHD4*	0.681245114	0.002174737	0.01721986	1
T2DM	adipocyte	*DTWD1*	0.513574469	0.00198019	0.01612624	1
T2DM	adipocyte	*PDGFRL*	0.590157678	6.35E-05	0.001613043	0.398922286
T2DM	adipocyte	*EYA1*	0.188187795	0.001020307	0.010362093	1
T2DM	adipocyte	*CPQ*	0.27527137	0.007398758	0.039028017	1
T2DM	adipocyte	*NIPAL2*	0.634344275	3.27E-05	0.00106579	0.205718058
T2DM	adipocyte	*IKBKB*	1.100782326	2.02E-05	0.000815077	0.127121943
T2DM	adipocyte	*PLAT*	1.099421187	1.99E-06	0.000232105	0.012533667
T2DM	adipocyte	*GDAP1*	0.561125515	0.003390039	0.023141342	1
T2DM	adipocyte	*ZC2HC1A*	0.559802735	0.003919029	0.02524384	1
T2DM	adipocyte	*CHRAC1*	0.813296474	0.00480571	0.028912435	1
T2DM	adipocyte	*MTMR9*	0.963292594	0.003479172	0.02351995	1
T2DM	adipocyte	*LEPROTL1*	1.202418843	0.000184977	0.003395587	1
T2DM	adipocyte	*ERICH1*	0.820863426	0.001710054	0.014420117	1
T2DM	adipocyte	*TUSC3*	0.913826001	7.67E-06	0.000560411	0.048195372
T2DM	adipocyte	*DMPK*	-0.633729011	0.001008486	0.0102761	1
T2DM	adipocyte	*AES*	1.127924545	0.000209299	0.003599172	1
T2DM	adipocyte	*CCDC61*	-0.393706857	0.00654905	0.035865745	1
T2DM	adipocyte	*IL27RA*	0.623349306	0.000734851	0.008338307	1
T2DM	adipocyte	*CCNE1*	-1.031300076	0.002627604	0.019596375	1
T2DM	adipocyte	*CCDC9*	0.87557837	0.003297773	0.022858988	1
T2DM	adipocyte	*FZR1*	1.363492573	0.003211271	0.022583065	1
T2DM	adipocyte	*TGFB1*	0.685904041	0.001948181	0.015948197	1
T2DM	adipocyte	*CEACAM4*	3.867447631	4.38E-05	0.001246899	0.27556471
T2DM	adipocyte	*BABAM1*	1.386788022	0.009738066	0.046311059	1
T2DM	adipocyte	*TYK2*	0.628629227	0.00328245	0.022786903	1
T2DM	adipocyte	*GRIN2D*	1.843591306	0.000345553	0.005139885	1
T2DM	adipocyte	*PPP2R1A*	0.956618108	0.009258536	0.044913899	1
T2DM	adipocyte	*GCDH*	-0.814793763	1.60E-06	0.00020495	0.010042538
T2DM	adipocyte	*LILRB5*	1.043640293	3.37E-05	0.001075499	0.212118229
T2DM	adipocyte	*ELL*	0.660309572	0.000930504	0.009685559	1
T2DM	adipocyte	*COPE*	0.85076177	0.008755044	0.043103336	1
T2DM	adipocyte	*ATP13A1*	0.626073601	2.11E-08	7.36E-06	0.000132473
T2DM	adipocyte	*SIPA1L3*	0.659517115	0.004077407	0.026030006	1
T2DM	adipocyte	*ZNF85*	0.550982683	0.007447249	0.039180632	1
T2DM	adipocyte	*PON3*	1.434003395	0.000598767	0.007210362	1
T2DM	adipocyte	*OGDH*	0.853498091	0.00150587	0.013241122	1
T2DM	adipocyte	*ADAP1*	2.935422501	2.16E-05	0.000829507	0.135652422
T2DM	adipocyte	*MET*	0.596700903	0.002220618	0.017495019	1
T2DM	adipocyte	*CPVL*	0.884052789	3.54E-09	1.71E-06	2.22E-05
T2DM	adipocyte	*NOD1*	0.87843836	0.000869102	0.009308421	1
T2DM	adipocyte	*SNX8*	0.919718244	0.00954735	0.045746239	1
T2DM	adipocyte	*USP42*	0.967372248	0.001649967	0.014136036	1
T2DM	adipocyte	*C1GALT1*	0.673472347	0.010716402	0.049466976	1
T2DM	adipocyte	*PHF14*	0.711288099	0.00256435	0.019307868	1
T2DM	adipocyte	*MEST*	-0.155737546	0.004895119	0.029226604	1
T2DM	adipocyte	*POU6F2*	-0.499549368	0.001392089	0.012592899	1
T2DM	adipocyte	*TSPAN13*	1.261935624	0.007048994	0.037630362	1
T2DM	adipocyte	*TMEM176B*	0.169417322	0.001142359	0.010981664	1

T2DM	adipocyte	*POLD2*	-0.89755853	1.38E-05	0.00073252	0.086688834
T2DM	adipocyte	*GCK*	-8.371810539	0.00106838	0.010577802	1
T2DM	adipocyte	*CLIP2*	1.193949724	0.000126474	0.002615595	0.795140953
T2DM	adipocyte	*LIMK1*	1.520733399	9.07E-05	0.002030351	0.570528668
T2DM	adipocyte	*SLC1A1*	0.131986568	0.00788161	0.040841088	1
T2DM	adipocyte	*TNFSF8*	0.802986079	0.001593788	0.013763932	1
T2DM	adipocyte	*KDM4C*	0.66596964	0.007819808	0.040597139	1
T2DM	adipocyte	*PTGDS*	2.246511405	0.000124015	0.002586077	0.779682675
T2DM	adipocyte	*DNMBP*	0.937996929	0.000794477	0.008732301	1
T2DM	adipocyte	*MAPK8*	0.662292363	0.005430961	0.031125299	1
T2DM	adipocyte	*PLEKHA1*	1.028647866	0.000135134	0.002758392	0.849584688
T2DM	adipocyte	*MICU1*	0.855149992	0.000417605	0.005670594	1
T2DM	adipocyte	*CCSER2*	0.77416386	0.001786488	0.014898665	1
T2DM	adipocyte	*SFXN3*	0.787579871	0.001402236	0.012666465	1
T2DM	adipocyte	*ACBD5*	0.596322728	0.0096915	0.046159439	1
T2DM	adipocyte	*SHOC2*	0.701470644	0.004461108	0.027714414	1
T2DM	adipocyte	*DHRS11*	-0.220256912	0.000104531	0.002281892	0.657184821
T2DM	adipocyte	*MLLT6*	1.050115026	0.000250208	0.004012908	1
T2DM	adipocyte	*PSMB3*	0.843422682	0.000296604	0.004604319	1
T2DM	adipocyte	*RGS9*	2.076251829	0.000206857	0.003597496	1
T2DM	adipocyte	*WNT3*	-0.657698936	0.001711056	0.014420117	1
T2DM	adipocyte	*TRIM37*	0.852432454	0.0019914	0.016196549	1
T2DM	adipocyte	*GALK1*	2.097169553	0.0001894	0.00340696	1
T2DM	adipocyte	*RNF167*	0.731998878	0.002930904	0.021107208	1
T2DM	adipocyte	*C1QBP*	1.148463733	0.002160888	0.017153409	1
T2DM	adipocyte	*ICAM2*	0.56103263	1.84E-05	0.000811251	0.115397001
T2DM	adipocyte	*CYTH1*	0.685765043	9.65E-07	0.000141146	0.006069257
T2DM	adipocyte	*HSD17B1*	-0.184124619	9.38E-08	2.68E-05	0.000589879
T2DM	adipocyte	*DLX4*	-3.960192685	0.000153333	0.003025687	0.964007162
T2DM	adipocyte	*VAT1*	0.765386333	1.69E-06	0.000210988	0.010630286
T2DM	adipocyte	*MPP2*	1.746431803	6.55E-06	0.000515083	0.041206604
T2DM	adipocyte	*EFTUD2*	0.579679096	0.008632635	0.042959913	1
T2DM	adipocyte	*FAM20A*	0.82677242	2.10E-05	0.000819911	0.132005746
T2DM	adipocyte	*UBE2D3*	0.805770098	0.00102648	0.010375372	1
T2DM	adipocyte	*NDUFC1*	1.009751646	0.009417381	0.045543902	1
T2DM	adipocyte	*CPE*	0.769568268	0.000444926	0.005901368	1
T2DM	adipocyte	*WFS1*	1.01169357	0.000395929	0.005568695	1
T2DM	adipocyte	*CPZ*	15.63312756	3.33E-05	0.00107474	0.209574232
T2DM	adipocyte	*SLC2A9*	0.92330994	3.19E-07	6.46E-05	0.00200306
T2DM	adipocyte	*STIM2*	0.69519742	0.001378728	0.012508027	1
T2DM	adipocyte	*RAPGEF2*	0.716120982	0.000253796	0.0040489	1
T2DM	adipocyte	*KLF3*	0.915196911	0.000692889	0.007949253	1
T2DM	adipocyte	*UGDH*	0.879599022	0.001960665	0.015997616	1
T2DM	adipocyte	*NRXN2*	2.33109861	3.20E-05	0.00106579	0.201275181
T2DM	adipocyte	*EIF4G2*	0.869008347	0.003377874	0.023102217	1
T2DM	adipocyte	*MADD*	0.887418004	0.000220303	0.003723247	1
T2DM	adipocyte	*CARS*	0.49879453	0.003981077	0.025513793	1
T2DM	adipocyte	*RPS13*	0.476250767	0.003021886	0.021564811	1
T2DM	adipocyte	*TCIRG1*	0.851892952	2.16E-05	0.000829507	0.136039172
T2DM	adipocyte	*PPFIBP1*	0.497262648	0.000663963	0.007725842	1
T2DM	adipocyte	*COQ5*	0.07234904	0.005893757	0.033072047	1
T2DM	adipocyte	*GLI1*	1.921890507	1.40E-05	0.00073252	0.08813191
T2DM	adipocyte	*LTA4H*	1.026242544	0.000850992	0.009161281	1
T2DM	adipocyte	*WNT5B*	0.945398661	0.000453831	0.005967377	1
T2DM	adipocyte	*PRR4*	0.275086819	0.001570089	0.013648668	1
T2DM	adipocyte	*PARP11*	0.958170076	0.001505179	0.013241122	1
T2DM	adipocyte	*CREBL2*	0.780273017	7.64E-05	0.001825352	0.480067492
T2DM	adipocyte	*CDK2AP1*	0.174198826	0.000121527	0.002555327	0.764042895
T2DM	adipocyte	*MGP*	0.325767265	0.000185657	0.003395587	1
T2DM	adipocyte	*ENDOU*	-0.313010383	0.001204705	0.011392753	1
T2DM	adipocyte	*GNB3*	-0.073961703	0.008669948	0.043009506	1

T2DM	adipocyte	*GNPTAB*	0.859509296	0.000480017	0.006196857	1
T2DM	adipocyte	*ST8SIA1*	0.54236406	0.001090066	0.010645331	1
T2DM	adipocyte	*C2CD5*	0.863780173	0.000246196	0.003980202	1
T2DM	adipocyte	*PHC1*	1.85674003	3.13E-05	0.001056656	0.196538082
T2DM	adipocyte	*RNGTT*	0.777139745	0.003219344	0.022589029	1
T2DM	adipocyte	*UST*	0.403078725	0.001076875	0.010621557	1
T2DM	adipocyte	*PHACTR1*	0.753646532	1.73E-05	0.000808689	0.108674985
T2DM	adipocyte	*CAP2*	-0.275429925	0.004615713	0.028228589	1
T2DM	adipocyte	*ASCC3*	0.660605435	0.004558154	0.028028472	1
T2DM	adipocyte	*BVES*	-0.176448178	0.004183426	0.026486608	1
T2DM	adipocyte	*VNN2*	0.479537313	0.002043513	0.016459063	1
T2DM	adipocyte	*PHACTR2*	0.723003338	0.002016815	0.016318811	1
T2DM	adipocyte	*TFEB*	1.068375586	0.000496261	0.006341444	1
T2DM	adipocyte	*FAM120B*	0.38156168	0.002530404	0.019099469	1
T2DM	adipocyte	*TMEM30A*	0.616413414	0.005956173	0.033285742	1
T2DM	adipocyte	*SENP6*	0.697663815	0.00310228	0.022013581	1
T2DM	adipocyte	*FBRSL1*	0.662871483	0.008260009	0.041544539	1
T2DM	adipocyte	*LY86*	1.028509732	0.002517407	0.019099469	1
T2DM	adipocyte	*DAP*	0.634438771	0.005837915	0.033035977	1
T2DM	adipocyte	*LOX*	1.113430365	0.002296339	0.017956569	1
T2DM	adipocyte	*RNF130*	0.656120365	0.008681667	0.043011538	1
T2DM	adipocyte	*TXNDC15*	0.7144802	0.010737058	0.049500875	1
T2DM	adipocyte	*SMAD5*	0.770651254	0.002631266	0.019600438	1
T2DM	adipocyte	*HGD*	-1.320979504	0.007573462	0.039657458	1
T2DM	adipocyte	*NPHP3*	0.107808574	0.005000643	0.02954797	1
T2DM	adipocyte	*IFT57*	1.030840522	0.008172419	0.041544539	1
T2DM	adipocyte	*NKTR*	0.756883814	4.96E-05	0.001380058	0.311893204
T2DM	adipocyte	*MOB1A*	1.126577681	0.000265271	0.004190353	1
T2DM	adipocyte	*KCNIP3*	0.848582684	0.001279126	0.011931548	1
T2DM	adipocyte	*SLC35F5*	0.484347175	0.003438628	0.023295965	1
T2DM	adipocyte	*ACTR3*	0.89744095	0.000168363	0.003217315	1
T2DM	adipocyte	*DNAJC27*	-0.502851118	0.004800039	0.028912435	1
T2DM	adipocyte	*STAM2*	0.774731675	0.008357332	0.041846552	1
T2DM	adipocyte	*NRBP1*	-0.578067683	0.000232277	0.003853101	1
T2DM	adipocyte	*ITGA4*	0.579071796	0.000291766	0.004540422	1
T2DM	adipocyte	*PPM1G*	-0.593110357	0.007948496	0.040960814	1
T2DM	adipocyte	*POLE4*	1.488679429	0.000348008	0.005151093	1
T2DM	adipocyte	*GLS*	0.685697292	0.002793966	0.020448966	1
T2DM	adipocyte	*GNLY*	0.690811035	0.001819419	0.015070735	1
T2DM	adipocyte	*SF3B1*	0.610823683	0.008250096	0.041544539	1
T2DM	adipocyte	*ST3GAL5*	0.56364018	0.003425143	0.023240953	1
T2DM	adipocyte	*PLCD4*	-0.775197862	0.010527706	0.048849149	1
T2DM	adipocyte	*CHMP3*	-0.380248432	0.002363577	0.018345446	1
T2DM	adipocyte	*MLPH*	1.117931276	8.05E-06	0.000574639	0.050592825
T2DM	adipocyte	*STRN*	0.832764151	0.002937113	0.021127722	1
T2DM	adipocyte	*SDC1*	0.632385185	0.002868686	0.020898528	1
T2DM	adipocyte	*WIPF1*	0.542276035	0.003373119	0.023101091	1
T2DM	adipocyte	*THADA*	0.66518505	0.008891201	0.043578441	1
T2DM	adipocyte	*CD207*	10.86255209	2.33E-06	0.000261239	0.014629362
T2DM	adipocyte	*GPX7*	-0.160542639	0.000750496	0.008440726	1
T2DM	adipocyte	*RALGPS2*	0.607115106	0.000502354	0.006370945	1
T2DM	adipocyte	*TMEM59*	0.775426282	0.002349983	0.018271006	1
T2DM	adipocyte	*MRPL37*	0.519530518	0.003856688	0.025028633	1
T2DM	adipocyte	*QSOX1*	0.778094028	0.001145861	0.010998516	1
T2DM	adipocyte	*RAP1A*	0.771611773	0.000392096	0.005550157	1
T2DM	adipocyte	*LEPR*	0.444714963	0.009651803	0.046040127	1
T2DM	adipocyte	*SMG7*	0.742583318	0.004078186	0.026030006	1
T2DM	adipocyte	*NCF2*	1.133497521	4.22E-05	0.001215782	0.265040475
T2DM	adipocyte	*RGS2*	1.20677052	0.001351612	0.012351141	1
T2DM	adipocyte	*OLFML3*	0.626549858	0.004602447	0.028174864	1
T2DM	adipocyte	*PLA2G2D*	-1.901428539	0.000736084	0.008338307	1

T2DM	adipocyte	*DR1*	0.943373092	0.004788141	0.028912435	1
T2DM	adipocyte	*PRRC2C*	0.63517815	0.0021692	0.017197683	1
T2DM	adipocyte	*TNFSF4*	-0.523472503	0.000262631	0.004159091	1
T2DM	adipocyte	*CD3EAP*	-0.478245255	6.39E-05	0.001613043	0.401647662
T2DM	adipocyte	*KMT2A*	0.817625442	0.001038438	0.010445535	1
T2DM	adipocyte	*ATF6*	0.695232144	0.003144761	0.022214732	1
T2DM	adipocyte	*ELOVL4*	0.206042391	0.000404379	0.00563134	1
T2DM	adipocyte	*SGIP1*	0.54975099	0.001300303	0.012083315	1
T2DM	adipocyte	*FOXO3*	0.661244695	0.005195516	0.030297489	1
T2DM	adipocyte	*ABCG2*	1.236278879	0.000118017	0.002518058	0.741973759
T2DM	adipocyte	*SPP1*	0.583013732	0.007468857	0.039228656	1
T2DM	adipocyte	*MFSD1*	0.852838238	0.000864594	0.009291798	1
T2DM	adipocyte	*DNAH7*	-0.203154649	1.23E-05	0.000707371	0.077313341
T2DM	adipocyte	*SENP5*	0.722725923	0.005000249	0.02954797	1
T2DM	adipocyte	*PTBP3*	0.913911923	3.64E-05	0.001121192	0.228723166
T2DM	adipocyte	*FKBP15*	0.79537558	0.000339965	0.005088951	1
T2DM	adipocyte	*CNTRL*	0.753415218	0.003595876	0.023999231	1
T2DM	adipocyte	*BSPRY*	6.071620623	0.00205408	0.01648866	1
T2DM	adipocyte	*HDHD3*	-0.066646452	0.007886286	0.040841088	1
T2DM	adipocyte	*TTLL5*	0.837862338	0.002755386	0.020260946	1
T2DM	adipocyte	*ATAD2B*	0.722177739	0.004470427	0.027717528	1
T2DM	adipocyte	*HOXB3*	-0.341458288	0.001419412	0.012784877	1
T2DM	adipocyte	*GORAB*	0.727427844	0.008230563	0.041544539	1
T2DM	adipocyte	*MRC1*	0.41501117	0.001905733	0.015641442	1
T2DM	adipocyte	*IQSEC3*	-0.793795321	0.009406835	0.045527921	1
T2DM	adipocyte	*DNAJC15*	0.841462783	0.008087307	0.041430225	1
T2DM	adipocyte	*SMAD9*	1.161151873	1.89E-05	0.000811251	0.118855822
T2DM	adipocyte	*APAF1*	0.444069844	0.009099593	0.044377023	1
T2DM	adipocyte	*ADRA1A*	0.058121312	0.001136188	0.010939068	1
T2DM	adipocyte	*CRISPLD1*	1.352290637	7.15E-06	0.000534934	0.04493448
T2DM	adipocyte	*TEX14*	0.600179752	8.71E-05	0.001970146	0.547700578
T2DM	adipocyte	*ECHDC2*	-0.143086218	0.004342014	0.02716243	1
T2DM	adipocyte	*A1BG*	1.109928622	0.000234735	0.003883633	1
T2DM	adipocyte	*SEC22A*	0.420200402	0.000242343	0.003967742	1
T2DM	adipocyte	*POPDC2*	0.921445903	0.008643914	0.042959913	1
T2DM	adipocyte	*RNF115*	0.840287616	0.000615093	0.00733793	1
T2DM	adipocyte	*TNFSF10*	0.64785499	0.00392214	0.02524384	1
T2DM	adipocyte	*GPSM2*	0.54971554	0.001502767	0.013241122	1
T2DM	adipocyte	*ACVR2A*	0.876413839	0.000458125	0.006000481	1
T2DM	adipocyte	*FLT3*	2.183967441	3.75E-07	7.37E-05	0.002358153
T2DM	adipocyte	*ODF2L*	0.355329756	0.008258315	0.041544539	1
T2DM	adipocyte	*PMS2*	0.707442328	0.003608708	0.024033845	1
T2DM	adipocyte	*SMU1*	0.810590182	0.008768643	0.043128627	1
T2DM	adipocyte	*SSPN*	0.266867213	0.003546426	0.023800752	1
T2DM	adipocyte	*PKN1*	0.613973643	0.009503294	0.045678294	1
T2DM	adipocyte	*EBPL*	0.211910655	0.00825341	0.041544539	1
T2DM	adipocyte	*NLN*	0.256203225	0.006509391	0.03571077	1
T2DM	adipocyte	*ATF1*	0.632181746	0.004768237	0.0289083	1
T2DM	adipocyte	*ARHGAP9*	0.618237316	0.008112178	0.041444425	1
T2DM	adipocyte	*SPATS2*	0.597203904	0.009483103	0.045678294	1
T2DM	adipocyte	*IKZF4*	-0.603113614	0.009133045	0.044442304	1
T2DM	adipocyte	*SARDH*	3.628396033	3.28E-05	0.00106579	0.206327659
T2DM	adipocyte	*NMI*	0.654669894	0.008114854	0.041444425	1
T2DM	adipocyte	*RAB38*	1.183407398	0.007918186	0.040871623	1
T2DM	adipocyte	*AGO2*	0.849838776	0.004500805	0.027823562	1
T2DM	adipocyte	*MOGAT1*	-0.278551903	0.008131253	0.041460817	1
T2DM	adipocyte	*PEPD*	0.376602151	0.010184763	0.047713563	1
T2DM	adipocyte	*ATP8A1*	0.297328833	0.001493067	0.013202408	1
T2DM	adipocyte	*HIF3A*	0.262376207	0.002991146	0.021369695	1
T2DM	adipocyte	*SERPINB6*	0.882890409	0.005371383	0.030981545	1
T2DM	adipocyte	*GLO1*	0.971871514	0.000112857	0.002446652	0.709529196

T2DM	adipocyte	*EREG*	4.13997679	0.000987361	0.010109996	1
T2DM	adipocyte	*CNOT1*	0.604734678	0.003912098	0.02524384	1
T2DM	adipocyte	*GOT2*	0.934959598	0.000367185	0.005285702	1
T2DM	adipocyte	*KIF25*	0.704615511	0.010535939	0.048849149	1
T2DM	adipocyte	*GRK4*	0.098315549	0.001022503	0.010362093	1
T2DM	adipocyte	*GGA3*	0.98251968	0.00554258	0.031707189	1
T2DM	adipocyte	*OPRL1*	-0.418742811	6.00E-06	0.000483445	0.037717875
T2DM	adipocyte	*PPDPF*	1.225300117	0.00032492	0.004910514	1
T2DM	adipocyte	*INSIG2*	1.124793193	0.000905164	0.009519534	1
T2DM	adipocyte	*GPCPD1*	0.777295187	0.002899595	0.021024181	1
T2DM	adipocyte	*PANK2*	0.90090037	0.004560701	0.028028472	1
T2DM	adipocyte	*DSTN*	0.860448261	0.000581809	0.007061457	1
T2DM	adipocyte	*ITPA*	1.018302034	0.008256892	0.041544539	1
T2DM	adipocyte	*FAM110A*	1.564570044	0.00494167	0.029476547	1
T2DM	adipocyte	*ZNF436*	0.407753671	0.000549389	0.006785877	1
T2DM	adipocyte	*KLC1*	0.631933376	0.005423931	0.031125299	1
T2DM	adipocyte	*MCF2L*	0.360426531	0.000225547	0.003776376	1
T2DM	adipocyte	*F10*	1.097500609	2.79E-05	0.00099459	0.175114341
T2DM	adipocyte	*CAPNS1*	0.539251922	0.006124787	0.034016372	1
T2DM	adipocyte	*COX6B1*	1.030038626	0.010341288	0.048231217	1
T2DM	adipocyte	*PRR12*	0.397632483	0.000189914	0.00340696	1
T2DM	adipocyte	*BECN1*	1.085849189	0.001298695	0.012083315	1
T2DM	adipocyte	*RHOJ*	0.839152956	3.79E-05	0.001141194	0.238473742
T2DM	adipocyte	*PZP*	0.780000146	0.001756605	0.014708134	1
T2DM	adipocyte	*AIF1L*	1.067660929	7.59E-06	0.000560411	0.047690493
T2DM	adipocyte	*CANX*	0.844961384	0.001176336	0.011188536	1
T2DM	adipocyte	*FGD3*	1.133544362	2.04E-05	0.000815077	0.128505435
T2DM	adipocyte	*RAP1B*	0.684772549	0.002389112	0.018520378	1
T2DM	adipocyte	*PTPN12*	0.695260378	0.001897194	0.015591714	1
T2DM	adipocyte	*FGL2*	0.394612055	0.000784899	0.008687779	1
T2DM	adipocyte	*STEAP4*	0.456891704	0.004532133	0.027934825	1
T2DM	adipocyte	*MGAT3*	-0.650617663	0.000905469	0.009519534	1
T2DM	adipocyte	*BAIAP2L2*	0.509808503	0.003856235	0.025028633	1
T2DM	adipocyte	*RIBC2*	0.233952283	0.001532644	0.013364399	1
T2DM	adipocyte	*EMC4*	0.603082085	0.000768059	0.008576883	1
T2DM	adipocyte	*OPN1SW*	-0.160429842	0.007334765	0.038848919	1
T2DM	adipocyte	*PDE11A*	-0.161246721	0.005179264	0.030234016	1
T2DM	adipocyte	*VPS13C*	0.510340372	0.005872664	0.033072047	1
T2DM	adipocyte	*SERGEF*	1.038827253	0.000412918	0.005655801	1
T2DM	adipocyte	*PLD2*	0.558363022	0.000435679	0.005815531	1
T2DM	adipocyte	*SLC44A2*	0.952090445	0.000726986	0.008280007	1
T2DM	adipocyte	*EPB41L4A*	0.765835795	0.0029121	0.021024181	1
T2DM	adipocyte	*CDO1*	-0.40359913	0.005110428	0.029999308	1
T2DM	adipocyte	*SHC2*	0.297148466	0.003252883	0.022672808	1
T2DM	adipocyte	*RPL36*	0.781329372	0.000420526	0.005685695	1
T2DM	adipocyte	*GTPBP3*	-0.462592469	0.002624401	0.019596375	1
T2DM	adipocyte	*BST2*	1.048125462	3.79E-05	0.001141194	0.238509597
T2DM	adipocyte	*PAK4*	1.043171752	0.010861781	0.049988844	1
T2DM	adipocyte	*TMEM160*	1.937026736	0.000211878	0.00360662	1
T2DM	adipocyte	*CLIP1*	0.574440429	0.010128656	0.047557029	1
T2DM	adipocyte	*C12orf65*	0.678791037	0.00024627	0.003980202	1
T2DM	adipocyte	*PKDREJ*	-0.249177658	0.004478121	0.027737878	1
T2DM	adipocyte	*LILRB2*	1.408989614	3.43E-05	0.001075499	0.215877464
T2DM	adipocyte	*COX4I2*	1.376794788	0.004792403	0.028912435	1
T2DM	adipocyte	*PPT1*	0.610538964	0.010547135	0.048865025	1
T2DM	adipocyte	*TBC1D5*	0.553180826	0.007432807	0.0391374	1
T2DM	adipocyte	*AOC2*	-0.763259584	0.001455074	0.012939249	1
T2DM	adipocyte	*EXOC4*	0.658793218	0.000123694	0.002586077	0.777663355
T2DM	adipocyte	*NPHP4*	1.197406636	0.004641039	0.028355893	1
T2DM	adipocyte	*GCH1*	0.521856907	0.000634652	0.007500112	1
T2DM	adipocyte	*MATN3*	-1.591784376	0.000716352	0.008203473	1

T2DM	adipocyte	*EMILIN2*	0.249336372	0.008408904	0.042024469	1
T2DM	adipocyte	*TRIM5*	0.675029965	0.003857602	0.025028633	1
T2DM	adipocyte	*TRIM22*	0.592382583	0.002898197	0.021024181	1
T2DM	adipocyte	*CARD6*	0.705657401	0.008082889	0.041430225	1
T2DM	adipocyte	*XAF1*	0.388507107	0.000341717	0.005103031	1
T2DM	adipocyte	*TERF2*	0.729236749	0.010059221	0.04740804	1
T2DM	adipocyte	*PTPRA*	0.673529154	0.003584843	0.02395102	1
T2DM	adipocyte	*CTNNBL1*	0.734522821	0.009480767	0.045678294	1
T2DM	adipocyte	*DMGDH*	-0.67194466	0.001737461	0.014603498	1
T2DM	adipocyte	*TRPC4*	-0.759377869	0.001063107	0.010575556	1
T2DM	adipocyte	*CNDP2*	1.358591353	0.000349155	0.005151093	1
T2DM	adipocyte	*FAM83F*	-1.283697185	0.000398325	0.005589884	1
T2DM	adipocyte	*TMTC1*	0.649846888	0.005128847	0.030051316	1
T2DM	adipocyte	*IPO8*	0.893384724	0.003547209	0.023800752	1
T2DM	adipocyte	*SWAP70*	0.860759671	0.000168882	0.003217451	1
T2DM	adipocyte	*SBF2*	0.594211795	0.005220051	0.030387463	1
T2DM	adipocyte	*MICAL2*	0.268351631	0.009073723	0.044290759	1
T2DM	adipocyte	*EIF2S1*	0.801483423	0.009613637	0.045962686	1
T2DM	adipocyte	*EDEM1*	0.946486543	0.005495095	0.031464171	1
T2DM	adipocyte	*SYT6*	1.752143904	0.000972241	0.010020462	1
T2DM	adipocyte	*VAV3*	0.557335153	0.000465393	0.006057822	1
T2DM	adipocyte	*AP4B1*	0.638956533	0.003969031	0.025488558	1
T2DM	adipocyte	*PPHLN1*	0.442721579	0.006878716	0.037121449	1
T2DM	adipocyte	*YWHAQ*	0.849034948	0.000379469	0.005434446	1
T2DM	adipocyte	*KIAA1468*	0.817873095	0.000560221	0.006892587	1
T2DM	adipocyte	*ECHDC3*	-0.260312995	0.006976399	0.037359985	1
T2DM	adipocyte	*IL15RA*	1.206360335	0.000176681	0.003311883	1
T2DM	adipocyte	*KLRD1*	0.765582541	0.00830355	0.04166354	1
T2DM	adipocyte	*PRH2*	-0.562464026	0.001578295	0.013686543	1
T2DM	adipocyte	*DDB2*	0.804660762	6.37E-05	0.001613043	0.400562279
T2DM	adipocyte	*ACP2*	0.370871459	0.008245188	0.041544539	1
T2DM	adipocyte	*APLNR*	0.785415136	0.000359857	0.005239142	1
T2DM	adipocyte	*TMEM165*	0.410898204	0.009492052	0.045678294	1
T2DM	adipocyte	*WDR36*	0.88618615	0.004274647	0.026874704	1
T2DM	adipocyte	*OSTF1*	1.089476395	8.65E-05	0.001970098	0.543746972
T2DM	adipocyte	*P2RX4*	0.453859961	1.17E-05	0.000693307	0.07349055
T2DM	adipocyte	*SRPK2*	0.680627156	0.003077591	0.021863065	1
T2DM	adipocyte	*ANKRD6*	0.718622608	0.004758197	0.028875276	1
T2DM	adipocyte	*NT5E*	0.499932231	2.11E-05	0.000820178	0.132868758
T2DM	adipocyte	*ITGA7*	-0.104717847	0.009628258	0.045962686	1
T2DM	adipocyte	*FAIM2*	1.446440877	0.00562669	0.032129882	1
T2DM	adipocyte	*MICAL1*	0.870820028	4.86E-06	0.000449002	0.030532108
T2DM	adipocyte	*SMYD5*	-0.508064275	0.009529926	0.045736368	1
T2DM	adipocyte	*KCNMB4*	0.441537428	0.004854535	0.029094817	1
T2DM	adipocyte	*USP15*	0.609289161	0.003341737	0.02302506	1
T2DM	adipocyte	*MPHOSPH6*	1.001510418	0.000452964	0.005967377	1
T2DM	adipocyte	*SP110*	0.599646638	0.001347475	0.012351141	1
T2DM	adipocyte	*USP37*	0.583732651	0.006696731	0.036389239	1
T2DM	adipocyte	*TMBIM1*	-0.329310004	0.003799593	0.024853488	1
T2DM	adipocyte	*ARMC9*	1.008604993	0.000726094	0.008280007	1
T2DM	adipocyte	*ANKRD36*	0.720039266	0.001678854	0.014340975	1
T2DM	adipocyte	*APPL2*	0.671058719	0.009553811	0.045746239	1
T2DM	adipocyte	*SLC41A2*	0.759587865	0.006393332	0.035166124	1
T2DM	adipocyte	*RNASEH2B*	0.437165499	0.008217411	0.041544539	1
T2DM	adipocyte	*LRCH1*	0.862564406	1.37E-05	0.00073252	0.085982155
T2DM	adipocyte	*RCBTB2*	0.734564137	0.007132519	0.03800182	1
T2DM	adipocyte	*KDELR2*	1.165659101	0.001684773	0.014365585	1
T2DM	adipocyte	*NACAD*	-1.136271143	0.001255356	0.01176218	1
T2DM	adipocyte	*ALPK3*	-0.407247126	0.002480623	0.018972842	1
T2DM	adipocyte	*TM6SF1*	0.737156122	2.51E-06	0.000272372	0.015797588
T2DM	adipocyte	*EPRS*	0.883153268	0.008640647	0.042959913	1

T2DM	adipocyte	*IL1RN*	1.333343051	3.23E-05	0.00106579	0.20310753
T2DM	adipocyte	*PRPF4*	0.303764808	0.001349073	0.012351141	1
T2DM	adipocyte	*GARNL3*	0.98159536	0.000186333	0.003395587	1
T2DM	adipocyte	*LMX1B*	2.072385723	0.003031484	0.021584304	1
T2DM	adipocyte	*NOV*	0.442510907	0.005298346	0.030672835	1
T2DM	adipocyte	*TLN1*	0.619275051	0.002653101	0.01973607	1
T2DM	adipocyte	*DENND4C*	0.73176609	0.000892181	0.009413933	1
T2DM	adipocyte	*PPIL1*	2.636399447	0.001878503	0.01547857	1
T2DM	adipocyte	*TCF19*	0.307686947	0.008069553	0.041430225	1
T2DM	adipocyte	*FLOT1*	0.648525548	9.90E-05	0.002191322	0.622335484
T2DM	adipocyte	*MTCH1*	1.035741836	0.001126925	0.010905597	1
T2DM	adipocyte	*FHDC1*	1.78308491	4.09E-05	0.001200631	0.256934995
T2DM	adipocyte	*ARRB1*	0.804232577	0.0004395	0.005854102	1
T2DM	adipocyte	*PRCP*	0.731365655	6.67E-05	0.001671925	0.419653295
T2DM	adipocyte	*SULF1*	0.400842079	0.001051044	0.010522157	1
T2DM	adipocyte	*NEK1*	0.546322052	0.010404338	0.04844569	1
T2DM	adipocyte	*DDX60*	0.991386099	1.81E-05	0.000811251	0.113729208
T2DM	adipocyte	*CASP1*	1.14623416	0.000671285	0.007766803	1
T2DM	adipocyte	*MAP2K5*	0.681537338	0.003866419	0.025034168	1
T2DM	adipocyte	*NDUFAF1*	-0.291752875	0.009861135	0.046754867	1
T2DM	adipocyte	*UACA*	0.654316253	0.006943269	0.037214266	1
T2DM	adipocyte	*SPTBN5*	0.194785005	6.86E-05	0.001683725	0.431033646
T2DM	adipocyte	*DNASE2B*	2.657413221	0.000749371	0.008440726	1
T2DM	adipocyte	*IFT172*	0.706498571	0.002136226	0.017000572	1
T2DM	adipocyte	*ADCY3*	0.736702629	0.006896604	0.037133048	1
T2DM	adipocyte	*LHCGR*	-0.428163129	0.000468471	0.006072732	1
T2DM	adipocyte	*CENPO*	1.847249263	0.001248107	0.011729218	1
T2DM	adipocyte	*MYOF*	0.310240051	0.000140222	0.002831013	0.881575368
T2DM	adipocyte	*BTBD16*	-0.416541805	0.001516538	0.013316309	1
T2DM	adipocyte	*DNAJC13*	0.866155967	0.001306337	0.012095643	1
T2DM	adipocyte	*RPS24*	0.810214443	0.001452027	0.012939249	1
T2DM	adipocyte	*BARD1*	0.834899484	0.005264527	0.030505142	1
T2DM	adipocyte	*CARF*	0.332538407	0.000462058	0.006039415	1
T2DM	adipocyte	*FAM117B*	1.058595135	6.89E-05	0.001685133	0.433079084
T2DM	adipocyte	*SENP7*	0.781045944	0.000304682	0.004674872	1
T2DM	adipocyte	*COX17*	1.165384951	0.000587069	0.007111569	1
T2DM	adipocyte	*HERC3*	0.779862665	0.004705123	0.028691668	1
T2DM	adipocyte	*PRKG2*	0.550382208	0.00423088	0.026706366	1
T2DM	adipocyte	*BBS7*	1.054639996	0.003380633	0.023102217	1
T2DM	adipocyte	*KIAA1109*	0.684294447	0.00204261	0.016459063	1
T2DM	adipocyte	*PRDM5*	0.768402701	0.000149915	0.002992119	0.942517403
T2DM	adipocyte	*NAAA*	0.433146056	0.002602092	0.019521902	1
T2DM	adipocyte	*NUP54*	0.398713172	0.008191483	0.041544539	1
T2DM	adipocyte	*ANXA3*	1.216698876	6.81E-06	0.000524448	0.042826518
T2DM	adipocyte	*PPA2*	0.565121159	0.000982695	0.010086671	1
T2DM	adipocyte	*ETV6*	0.814435169	0.000195635	0.003484289	1
T2DM	adipocyte	*PRICKLE1*	0.700574794	6.79E-05	0.001674964	0.426635906
T2DM	adipocyte	*C1RL*	0.356901821	0.003835483	0.024988274	1
T2DM	adipocyte	*CLSTN3*	0.38545978	0.006621746	0.036169344	1
T2DM	adipocyte	*POC1B*	0.9308265	0.006389436	0.035166124	1
T2DM	adipocyte	*GAS2L3*	1.257514668	6.76E-05	0.001674964	0.425309236
T2DM	adipocyte	*ACVRL1*	0.31651146	0.008512664	0.042475493	1
T2DM	adipocyte	*LPAR6*	0.7772991	0.001077806	0.010621557	1
T2DM	adipocyte	*RHOF*	-0.379467331	0.005881161	0.033072047	1
T2DM	adipocyte	*TMX1*	0.958625931	0.006136646	0.034031853	1
T2DM	adipocyte	*STON2*	0.661449096	0.000224782	0.003776376	1
T2DM	adipocyte	*PTGR2*	-0.107727273	0.009117781	0.044402393	1
T2DM	adipocyte	*AK7*	2.940897851	0.000202395	0.003564307	1
T2DM	adipocyte	*FBLN5*	0.899606498	0.000304363	0.004674872	1
T2DM	adipocyte	*TCF12*	0.649315222	0.001618013	0.013915793	1
T2DM	adipocyte	*SRP14*	0.679047423	8.38E-05	0.001941003	0.527019318

T2DM	adipocyte	*UBE2Q2*	0.912089745	0.000361578	0.005239142	1
T2DM	adipocyte	*HMG20A*	1.020282125	2.84E-06	0.000294676	0.017850812
T2DM	adipocyte	*MAN2C1*	-0.113730888	5.27E-05	0.00142143	0.331193085
T2DM	adipocyte	*TPM1*	0.791972636	2.82E-05	0.00099459	0.177037022
T2DM	adipocyte	*IGF1R*	0.76177836	1.85E-05	0.000811251	0.116030552
T2DM	adipocyte	*ZFHX3*	0.67836978	0.004339959	0.02716243	1
T2DM	adipocyte	*NUDT7*	-0.268510534	0.006785617	0.036776874	1
T2DM	adipocyte	*CMTM3*	1.148711714	0.002020306	0.016326047	1
T2DM	adipocyte	*MYO19*	1.1178276	1.37E-06	0.000179833	0.00863198
T2DM	adipocyte	*RASL10B*	-0.395535951	0.002416832	0.018666613	1
T2DM	adipocyte	*LRRC46*	-0.54402862	6.62E-07	0.00011251	0.004162887
T2DM	adipocyte	*ASXL3*	0.989336668	0.002394953	0.018520378	1
T2DM	adipocyte	*C18orf8*	-0.419876368	0.007761436	0.040394163	1
T2DM	adipocyte	*TMC6*	0.197214851	0.005199776	0.030297489	1
T2DM	adipocyte	*TBCD*	0.512749427	0.005907414	0.033072047	1
T2DM	adipocyte	*FN3KRP*	-0.129665377	0.008273855	0.041575074	1
T2DM	adipocyte	*SECTM1*	0.653730029	0.006493449	0.035654421	1
T2DM	adipocyte	*WDR45B*	0.566734529	0.002132114	0.017000572	1
T2DM	adipocyte	*RNF165*	0.607092928	0.005791972	0.032892943	1
T2DM	adipocyte	*DUS3L*	0.246383802	0.003693318	0.024415616	1
T2DM	adipocyte	*CCDC97*	1.124771898	0.00146974	0.013032798	1
T2DM	adipocyte	*SIRT3*	0.440611585	0.000939595	0.009764021	1
T2DM	adipocyte	*IFITM3*	1.141323712	0.000188101	0.003398243	1
T2DM	adipocyte	*GEMIN7*	-0.630234768	0.008483049	0.042361343	1
T2DM	adipocyte	*MYO1F*	0.609039875	9.89E-05	0.002191322	0.621551
T2DM	adipocyte	*CACNG8*	3.41823286	4.43E-06	0.000428187	0.027832156
T2DM	adipocyte	*RCN3*	1.14882009	6.29E-05	0.001608673	0.395604388
T2DM	adipocyte	*SH3BGRL3*	0.879748078	0.0005888	0.007118824	1
T2DM	adipocyte	*SERBP1*	0.808330824	0.007237723	0.038464552	1
T2DM	adipocyte	*GPR161*	0.711687758	0.002913011	0.021024181	1
T2DM	adipocyte	*ADCY10*	-0.732998793	0.00411214	0.026167029	1
T2DM	adipocyte	*RGL1*	0.73171285	0.000507114	0.006389226	1
T2DM	adipocyte	*TARS2*	0.489176846	0.007955799	0.040964871	1
T2DM	adipocyte	*SNX27*	0.829107696	0.004795995	0.028912435	1
T2DM	adipocyte	*IKBKE*	0.877549185	0.005159797	0.030176414	1
T2DM	adipocyte	*TP53BP2*	1.094271449	0.000386186	0.005518078	1
T2DM	adipocyte	*KCNN3*	0.819117951	0.000165391	0.003170158	1
T2DM	adipocyte	*RIT1*	0.915153976	0.001961852	0.015997616	1
T2DM	adipocyte	*CEP170*	0.456935913	0.009168629	0.044546498	1
T2DM	adipocyte	*GUK1*	1.025093359	0.001134355	0.010938173	1
T2DM	adipocyte	*CDC42BPA*	0.508447581	0.003251363	0.022672808	1
T2DM	adipocyte	*CNIH3*	0.519923548	0.008796636	0.043206604	1
T2DM	adipocyte	*WNT9A*	0.609776518	1.73E-06	0.000210988	0.010851002
T2DM	adipocyte	*PLEKHA6*	0.607310075	0.000242158	0.003967742	1
T2DM	adipocyte	*EML4*	0.657256367	0.010079482	0.047432414	1
T2DM	adipocyte	*EXOC6B*	0.556256467	0.010530439	0.048849149	1
T2DM	adipocyte	*TEX261*	1.506282879	0.008875518	0.043560015	1
T2DM	adipocyte	*SLC20A1*	0.965085602	0.000129511	0.002660892	0.814232849
T2DM	adipocyte	*ZNF385B*	0.437622444	0.000471018	0.006093191	1
T2DM	adipocyte	*GULP1*	0.540285036	0.000245287	0.003980202	1
T2DM	adipocyte	*METTL21A*	1.000656225	0.001788103	0.014898665	1
T2DM	adipocyte	*NBEAL1*	0.246547929	0.000195241	0.003484289	1
T2DM	adipocyte	*FANCD2*	1.337625442	0.000256402	0.00407071	1
T2DM	adipocyte	*TAMM41*	0.743986374	0.001817708	0.015070735	1
T2DM	adipocyte	*OSBPL10*	0.611020099	9.02E-06	0.000586394	0.056719715
T2DM	adipocyte	*STAC*	1.337692689	0.000510905	0.006411295	1
T2DM	adipocyte	*ARL6IP5*	0.815485138	0.005324015	0.030764784	1
T2DM	adipocyte	*LIMD1*	0.721025791	0.000981723	0.010086671	1
T2DM	adipocyte	*PLA1A*	-0.19324565	0.008078923	0.041430225	1
T2DM	adipocyte	*LYAR*	1.402324803	0.003318577	0.02297786	1
T2DM	adipocyte	*ATP10D*	0.403886085	0.000510858	0.006411295	1

T2DM	adipocyte	*CISD2*	0.655709912	0.002704142	0.020040713	1
T2DM	adipocyte	*USP53*	0.729158593	0.001703746	0.014412493	1
T2DM	adipocyte	*SSBP2*	0.665651213	0.001607709	0.013865111	1
T2DM	adipocyte	*BHMT*	-0.149650564	3.67E-05	0.001125274	0.23068112
T2DM	adipocyte	*YIPF5*	1.041556095	0.006644954	0.036170411	1
T2DM	adipocyte	*PSD2*	1.035864073	0.009457434	0.045667347	1
T2DM	adipocyte	*FGD2*	0.583922411	0.002858739	0.020850222	1
T2DM	adipocyte	*TPBG*	1.177504268	2.06E-05	0.000815077	0.129750605
T2DM	adipocyte	*PHIP*	0.805904672	0.000410222	0.005646146	1
T2DM	adipocyte	*CCDC25*	0.895451347	2.19E-05	0.000830044	0.13771129
T2DM	adipocyte	*DOCK5*	0.910104674	0.000128092	0.002640372	0.80531354
T2DM	adipocyte	*ADHFE1*	-0.100549272	0.002346416	0.018271006	1
T2DM	adipocyte	*MTDH*	0.702854709	0.00705083	0.037630362	1
T2DM	adipocyte	*MAL2*	-0.258812623	0.002625128	0.019596375	1
T2DM	adipocyte	*FAM135B*	1.53732109	0.002855575	0.020850222	1
T2DM	adipocyte	*ZNF7*	0.669299841	0.000740302	0.008371002	1
T2DM	adipocyte	*SLC39A4*	-0.514129359	0.003358355	0.02302506	1
T2DM	adipocyte	*ZNF462*	0.829990131	0.006585376	0.036001965	1
T2DM	adipocyte	*SEC16A*	0.372538297	0.005150028	0.030147322	1
T2DM	adipocyte	*RSU1*	0.735810951	0.001625295	0.01395933	1
T2DM	adipocyte	*GLUD1*	0.874357775	0.000124224	0.002586077	0.780995261
T2DM	adipocyte	*HTR7*	1.677230592	5.89E-06	0.000480968	0.037034185
T2DM	adipocyte	*HSD17B12*	-0.255688562	1.92E-07	4.65E-05	0.001208565
T2DM	adipocyte	*APIP*	-0.091999262	0.000185045	0.003395587	1
T2DM	adipocyte	*PTPRJ*	0.663039157	7.20E-05	0.001748913	0.452968523
T2DM	adipocyte	*C11orf49*	0.574555771	0.003802935	0.024853488	1
T2DM	adipocyte	*ENDOD1*	1.232872896	1.65E-05	0.000780926	0.103714757
T2DM	adipocyte	*CAPN5*	0.67824038	0.000227879	0.003800196	1
T2DM	adipocyte	*ZC3H12C*	0.97131676	0.000389873	0.00554555	1
T2DM	adipocyte	*C11orf52*	-2.552265357	0.000207141	0.003597496	1
T2DM	adipocyte	*TMC2*	-1.157335125	0.004666382	0.028483051	1
T2DM	adipocyte	*MS4A3*	-19.30758114	0.004819506	0.028912435	1
T2DM	adipocyte	*PLCH2*	6.316497239	8.41E-05	0.001941003	0.528909605
T2DM	adipocyte	*FEZ1*	1.43974977	5.89E-06	0.000480968	0.037034511
T2DM	adipocyte	*DUSP15*	-0.347916772	0.010869214	0.049988844	1
T2DM	adipocyte	*DOC2A*	-0.504302092	0.000605694	0.007267173	1
T2DM	adipocyte	*TMCO3*	1.071840931	0.000395081	0.005568695	1
T2DM	adipocyte	*SAP18*	1.016675254	0.002787268	0.020447552	1
T2DM	adipocyte	*LYPD6B*	-0.857145976	0.000402499	0.00563134	1
T2DM	adipocyte	*PDCD4*	0.889178639	0.00034582	0.005139885	1
T2DM	adipocyte	*CCDC102B*	0.86061998	0.000876744	0.009344489	1
T2DM	adipocyte	*RGS18*	1.223820944	5.45E-05	0.001458864	0.342833054
T2DM	adipocyte	*C11orf53*	-2.414625293	0.009784821	0.046465231	1
T2DM	adipocyte	*DOCK1*	0.487441472	0.009540318	0.045746239	1
T2DM	adipocyte	*TEX12*	-5.3479217	0.004045928	0.025876652	1
T2DM	adipocyte	*PIP4K2A*	0.534444983	0.002485481	0.0189869	1
T2DM	adipocyte	*PRSS53*	-0.347911058	0.006796022	0.036779587	1
T2DM	adipocyte	*IPMK*	1.025272771	0.000156726	0.003069586	0.985337166
T2DM	adipocyte	*GXYLT1*	0.848371123	0.004574178	0.028052873	1
T2DM	adipocyte	*FAM177A1*	1.08906613	7.98E-05	0.00187981	0.501909399
T2DM	adipocyte	*ANKRD50*	0.954152208	0.004088439	0.026055765	1
T2DM	adipocyte	*PTPRO*	0.642552893	0.001799942	0.014968563	1
T2DM	adipocyte	*VTI1A*	0.600162142	0.000947462	0.009829531	1
T2DM	adipocyte	*AKR1C2*	-0.494163244	0.001088052	0.010645331	1
T2DM	adipocyte	*INPP1*	0.811100112	7.26E-05	0.001755852	0.456521498
T2DM	adipocyte	*MFSD6*	0.762173772	0.007857667	0.040760025	1
T2DM	adipocyte	*ASAP2*	0.756268701	0.000847216	0.009161281	1
T2DM	adipocyte	*BICD1*	0.756158255	0.001133186	0.010938173	1
T2DM	adipocyte	*RABGAP1L*	0.62035515	8.57E-05	0.001965411	0.539062506
T2DM	adipocyte	*ARL14EP*	-0.644559217	0.000153522	0.003025687	0.965194031
T2DM	adipocyte	*G6PC2*	29.7675433	1.52E-05	0.000758566	0.095579353

T2DM	adipocyte	*POC5*	-0.315334081	0.005424765	0.031125299	1
T2DM	adipocyte	*CWF19L2*	0.636158133	0.004790059	0.028912435	1
T2DM	adipocyte	*ZFP36L2*	1.096258459	6.95E-07	0.000115022	0.004370837
T2DM	adipocyte	*TMEM123*	0.805062834	0.002497385	0.0190529	1
T2DM	adipocyte	*CLEC4F*	2.120937655	1.98E-05	0.00081257	0.124355278
T2DM	adipocyte	*SAR1B*	0.625702834	0.000205822	0.003597496	1
T2DM	adipocyte	*TMEM87B*	0.439035202	0.006003735	0.033462306	1
T2DM	adipocyte	*PTPRR*	0.80050968	0.002091497	0.016708059	1
T2DM	adipocyte	*PLA2R1*	0.595974087	0.000531234	0.006574546	1
T2DM	adipocyte	*FRMD1*	-0.537788999	0.000419737	0.005685695	1
T2DM	adipocyte	*ASAP1*	0.476136703	0.010176803	0.047711827	1
T2DM	adipocyte	*LPCAT1*	1.102209875	0.000101976	0.002241686	0.641122203
T2DM	adipocyte	*ZDHHC7*	0.824175101	0.000662476	0.007725842	1
T2DM	adipocyte	*FAM92B*	-7.971437932	0.000627426	0.007442698	1
T2DM	adipocyte	*JAZF1*	-0.687593177	8.43E-05	0.001941003	0.529893923
T2DM	adipocyte	*MCOLN2*	0.298601313	0.006014725	0.033476156	1
T2DM	adipocyte	*PPP2R5E*	0.448186375	0.001615986	0.013915793	1
T2DM	adipocyte	*ANKRD29*	0.635800463	1.63E-05	0.000780895	0.102297184
T2DM	adipocyte	*THY1*	0.874920889	2.37E-05	0.000879907	0.148704348
T2DM	adipocyte	*UBASH3B*	0.645231737	0.008210358	0.041544539	1
T2DM	adipocyte	*ROBO4*	0.398039711	0.002623483	0.019596375	1
T2DM	adipocyte	*PRKCA*	0.707253467	0.000571195	0.007000196	1
T2DM	adipocyte	*LRRK1*	0.921454054	3.82E-05	0.001144351	0.240313649
T2DM	adipocyte	*UCHL1*	-0.620795896	0.002432522	0.018741746	1
T2DM	adipocyte	*GBP5*	0.466328341	0.006542274	0.035859878	1
T2DM	adipocyte	*PDLIM3*	0.319294432	0.004967843	0.029508585	1
T2DM	adipocyte	*L3MBTL4*	0.901687691	0.000254385	0.0040489	1
T2DM	adipocyte	*PDE1C*	0.645970095	0.003420819	0.023240953	1
T2DM	adipocyte	*TSEN2*	0.471576751	1.49E-05	0.000753745	0.093464409
T2DM	adipocyte	*PPP4R1*	0.884541067	0.001827511	0.015117845	1
T2DM	adipocyte	*CDK19*	0.679582199	0.004962888	0.029508585	1
T2DM	adipocyte	*TTC39B*	0.495653445	0.001090439	0.010645331	1
T2DM	adipocyte	*PDIA4*	1.318608462	5.43E-05	0.001458864	0.34139159
T2DM	adipocyte	*DEPTOR*	0.830002631	7.35E-05	0.001763187	0.461954897
T2DM	adipocyte	*UGT2B4*	-0.565452863	0.000236192	0.003897484	1
T2DM	adipocyte	*BATF*	1.677742192	3.25E-05	0.00106579	0.204382481
T2DM	adipocyte	*NDUFAF6*	-0.491914766	0.001053909	0.010534064	1
T2DM	adipocyte	*ANKRD9*	-0.696056893	0.001084953	0.010641344	1
T2DM	adipocyte	*GDF6*	1.374229753	3.96E-05	0.00117329	0.248737547
T2DM	adipocyte	*NODAL*	-0.977897297	0.00471417	0.028718982	1
T2DM	adipocyte	*VPS8*	0.586561007	4.20E-05	0.001215782	0.264128323
T2DM	adipocyte	*NTAN1*	0.550792948	0.009900572	0.046765514	1
T2DM	adipocyte	*FCHO2*	0.818108346	0.000184991	0.003395587	1
T2DM	adipocyte	*TIMP4*	0.47206275	0.006091647	0.03386223	1
T2DM	adipocyte	*TMED6*	-2.295466403	0.000372668	0.005349235	1
T2DM	adipocyte	*CLEC18A*	-0.583201732	1.48E-08	5.47E-06	9.31E-05
T2DM	adipocyte	*IL34*	1.354643133	0.001081247	0.010621557	1
T2DM	adipocyte	*DYRK1A*	0.595312142	0.005101858	0.029999308	1
T2DM	adipocyte	*KCNJ15*	-0.271561055	0.004971767	0.029508585	1
T2DM	adipocyte	*CREB3L1*	0.312686005	0.009894447	0.046765514	1
T2DM	adipocyte	*SLC37A3*	0.851852563	0.005121844	0.030038276	1
T2DM	adipocyte	*AP3S2*	0.342180425	2.25E-09	1.29E-06	1.41E-05
T2DM	adipocyte	*C12orf43*	0.378483751	0.001815122	0.015070735	1
T2DM	adipocyte	*TRIM63*	0.273161379	0.000275625	0.00432134	1
T2DM	adipocyte	*GALNT14*	2.070954725	4.29E-05	0.001226752	0.269885417
T2DM	adipocyte	*LRRC43*	-1.49108461	0.000672044	0.007766803	1
T2DM	adipocyte	*MRAS*	-0.172376841	0.006580497	0.036001965	1
T2DM	adipocyte	*CLSTN2*	-0.308781836	0.008129879	0.041460817	1
T2DM	adipocyte	*COLEC12*	0.73384098	3.72E-05	0.001129902	0.233889771
T2DM	adipocyte	*AUTS2*	0.599232275	0.001127507	0.010905597	1
T2DM	adipocyte	*CD1E*	0.846022462	2.31E-05	0.000864716	0.145272239

T2DM	adipocyte	*NCF1*	1.711637034	1.99E-05	0.00081257	0.12513576
T2DM	adipocyte	*ELK4*	0.943977741	0.001016771	0.010343757	1
T2DM	adipocyte	*RNF166*	0.697724627	0.002454044	0.01883831	1
T2DM	adipocyte	*CDC42SE2*	0.81515593	0.000392345	0.005550157	1
T2DM	adipocyte	*IFNAR2*	0.225237393	0.005413524	0.031125299	1
T2DM	adipocyte	*ARHGAP27*	1.252384942	0.000387482	0.005524036	1
T2DM	adipocyte	*STARD9*	0.509456394	0.000878415	0.009344489	1
T2DM	adipocyte	*ACE*	0.758565397	0.000349852	0.005151093	1
T2DM	adipocyte	*EFCAB14*	0.992421369	0.00174404	0.014639223	1
T2DM	adipocyte	*CALM3*	0.702303402	0.00232867	0.018186764	1
T2DM	adipocyte	*SLC37A1*	0.96796504	8.94E-05	0.002015343	0.562280806
T2DM	adipocyte	*C21orf33*	-0.464048343	0.009833842	0.046660651	1
T2DM	adipocyte	*ZNF66*	0.520648933	0.005394243	0.031084883	1
T2DM	adipocyte	*VAV2*	0.614502689	0.010831468	0.049888235	1
T2DM	adipocyte	*MPZL3*	1.456612847	8.13E-06	0.000574639	0.051142869
T2DM	adipocyte	*FDPS*	0.647206018	7.92E-05	0.001875094	0.498196445
T2DM	adipocyte	*VPS28*	0.389379533	0.0002733	0.004295599	1
T2DM	adipocyte	*RPL8*	0.839247034	0.000949191	0.009831245	1
T2DM	adipocyte	*MFSD12*	1.072374407	0.001367976	0.012446407	1
T2DM	adipocyte	*YDJC*	3.345103903	9.12E-06	0.000586394	0.057333373
T2DM	adipocyte	*AP2M1*	0.798398536	0.000848521	0.009161281	1
T2DM	adipocyte	*IKZF3*	0.633615561	0.001527028	0.013352465	1
T2DM	adipocyte	*ASGR2*	3.454988775	5.72E-06	0.000480968	0.035950989
T2DM	adipocyte	*JMJD8*	0.258676182	0.008092304	0.041430225	1
T2DM	adipocyte	*ZG16B*	21.34553997	0.000207732	0.003597825	1
T2DM	adipocyte	*RBP7*	1.333645457	1.97E-05	0.00081257	0.123934031
T2DM	adipocyte	*LAPTM5*	0.622265265	5.82E-05	0.001523337	0.365600973
T2DM	adipocyte	*PEF1*	1.516198987	0.002274619	0.017853345	1
T2DM	adipocyte	*CAMK2N1*	1.52649623	8.81E-06	0.000586394	0.055393238
T2DM	adipocyte	*NFIA*	0.559664339	0.004750963	0.02885923	1
T2DM	adipocyte	*KCNT2*	0.725083155	6.84E-06	0.000524448	0.043004767
T2DM	adipocyte	*VCAM1*	0.422801952	0.005662398	0.032304441	1
T2DM	adipocyte	*EXTL2*	-0.233622473	0.009992411	0.047128496	1
T2DM	adipocyte	*ZNF496*	0.991969947	0.000115616	0.002489307	0.726877698
T2DM	adipocyte	*LMX1A*	0.207099458	0.001434404	0.012846296	1
T2DM	adipocyte	*DISC1*	0.518529586	0.004090511	0.026055765	1
T2DM	adipocyte	*FAM84A*	-1.009402764	0.000407858	0.005639191	1
T2DM	adipocyte	*CCDC74A*	4.552711132	6.29E-05	0.001608673	0.39573355
T2DM	adipocyte	*PDLIM5*	0.749725326	0.000570374	0.007000196	1
T2DM	adipocyte	*RNF149*	0.823338958	0.000850809	0.009161281	1
T2DM	adipocyte	*TDRD10*	0.947723329	1.94E-05	0.00081257	0.12226285
T2DM	adipocyte	*PBXIP1*	1.083702139	0.006933592	0.037194107	1
T2DM	adipocyte	*LMOD3*	-0.946359077	0.010375097	0.048353027	1
T2DM	adipocyte	*SLC22A15*	0.595184364	0.000610637	0.007298626	1
T2DM	adipocyte	*LMOD1*	-0.202531392	0.009898886	0.046765514	1
T2DM	adipocyte	*ARPC2*	0.74152321	0.002814256	0.020573521	1
T2DM	adipocyte	*SPTA1*	-0.101018681	0.000551795	0.006802222	1
T2DM	adipocyte	*AIM2*	1.178674646	4.62E-06	0.000440302	0.029059958
T2DM	adipocyte	*CADPS*	1.612863307	6.29E-05	0.001608673	0.395671678
T2DM	adipocyte	*GMPS*	0.985579515	0.003227171	0.022589029	1
T2DM	adipocyte	*TIPARP*	0.553265267	0.0061384	0.034031853	1
T2DM	adipocyte	*DNASE1L3*	0.794755251	3.42E-05	0.001075499	0.215287667
T2DM	adipocyte	*GYG1*	0.679400875	0.006927667	0.037194059	1
T2DM	adipocyte	*HPS3*	0.531042605	0.009682545	0.04615175	1
T2DM	adipocyte	*PLB1*	1.460982445	1.90E-05	0.000811251	0.119253961
T2DM	adipocyte	*CDCP1*	1.362816944	1.34E-05	0.00073252	0.084515187
T2DM	adipocyte	*DTX3L*	0.612824696	0.000311964	0.004772055	1
T2DM	adipocyte	*YEATS2*	1.00133127	0.000289944	0.004523265	1
T2DM	adipocyte	*MEAF6*	1.153183021	0.001000838	0.010214718	1
T2DM	adipocyte	*RPN1*	0.972862306	0.001693371	0.014406257	1
T2DM	adipocyte	*RFT1*	-0.63346933	2.07E-05	0.000815077	0.130412398

T2DM	adipocyte	*DNAJB14*	0.598200369	0.001169586	0.011158103	1
T2DM	adipocyte	*BDH2*	-0.203240409	0.002916031	0.021024181	1
T2DM	adipocyte	*ATRIP*	-2.833899613	0.000190209	0.00340696	1
T2DM	adipocyte	*SHISA5*	0.790833282	0.008335753	0.041791768	1
T2DM	adipocyte	*RNF123*	0.32241205	4.77E-07	8.82E-05	0.002998102
T2DM	adipocyte	*GRM2*	-6.724349312	0.004979896	0.029508585	1
T2DM	adipocyte	*NPY1R*	-0.398811617	0.009785266	0.046465231	1
T2DM	adipocyte	*IL15*	0.72767915	0.00068043	0.00783958	1
T2DM	adipocyte	*ITGA2*	1.00884688	0.000463999	0.006052207	1
T2DM	adipocyte	*PRRC1*	0.900958549	0.001713572	0.014421988	1
T2DM	adipocyte	*RICTOR*	0.720287621	0.004201324	0.026573166	1
T2DM	adipocyte	*KLKB1*	0.159077342	0.004315691	0.027051592	1
T2DM	adipocyte	*MB21D1*	1.232617948	4.13E-05	0.001202961	0.259839665
T2DM	adipocyte	*DCBLD1*	0.57425	0.001413879	0.012753312	1
T2DM	adipocyte	*TMEM200A*	0.9118899	5.09E-05	0.001392051	0.320171735
T2DM	adipocyte	*PDSS2*	0.688102657	0.007932894	0.040913952	1
T2DM	adipocyte	*STXBP5*	0.690996857	0.003739099	0.024615411	1
T2DM	adipocyte	*PI16*	0.865858014	1.82E-05	0.000811251	0.114402364
T2DM	adipocyte	*RPS14*	0.592491347	0.003341938	0.02302506	1
T2DM	adipocyte	*CAMLG*	0.462689541	0.000767242	0.008576883	1
T2DM	adipocyte	*SUN1*	0.513272448	0.00051439	0.006418731	1
T2DM	adipocyte	*OXR1*	0.518713693	0.004883135	0.02918324	1
T2DM	adipocyte	*COX6C*	0.981210616	0.0048604	0.029102223	1
T2DM	adipocyte	*DCAF13*	1.25073111	0.0008804	0.009349786	1
T2DM	adipocyte	*INTS8*	0.528337831	0.001696653	0.014410427	1
T2DM	adipocyte	*TMEM65*	0.679624591	0.003353831	0.02302506	1
T2DM	adipocyte	*SYK*	0.602976206	0.001062483	0.010575556	1
T2DM	adipocyte	*HNRNPK*	0.724852434	0.009734395	0.046311059	1
T2DM	adipocyte	*MARCH8*	0.980402223	0.001453024	0.012939249	1
T2DM	adipocyte	*PGM2L1*	0.742851123	4.11E-05	0.001202864	0.258615685
T2DM	adipocyte	*TAF3*	0.875659114	0.000142107	0.002845314	0.893428674
T2DM	adipocyte	*SLC18A2*	0.666578298	0.003700983	0.024415616	1
T2DM	adipocyte	*PDZD8*	0.373810982	0.002134958	0.017000572	1
T2DM	adipocyte	*QSOX2*	1.707068339	0.000348577	0.005151093	1
T2DM	adipocyte	*SLC39A13*	1.728447244	8.70E-05	0.001970146	0.547017198
T2DM	adipocyte	*LIPC*	0.382530717	0.007405825	0.039028017	1
T2DM	adipocyte	*JAM3*	0.659704788	0.000205779	0.003597496	1
T2DM	adipocyte	*TRMT61A*	-0.595228678	2.12E-07	4.95E-05	0.001335348
T2DM	adipocyte	*ASPG*	-0.988695728	0.009969636	0.047056383	1
T2DM	adipocyte	*PCBD1*	-0.197592139	7.93E-05	0.001875094	0.498774903
T2DM	adipocyte	*ZNF202*	1.139821371	0.002447059	0.018807652	1
T2DM	adipocyte	*C2*	1.356936051	1.70E-14	2.13E-11	1.07E-10
T2DM	adipocyte	*ILK*	0.877976456	0.001102246	0.010743911	1
T2DM	adipocyte	*DCHS1*	0.926094849	5.95E-05	0.001546933	0.374357706
T2DM	adipocyte	*NETO1*	0.472284336	0.00328375	0.022786903	1
T2DM	adipocyte	*LMO1*	0.686484898	0.007049181	0.037630362	1
T2DM	adipocyte	*RPL27A*	0.715202499	0.00204462	0.016459063	1
T2DM	adipocyte	*AKIP1*	1.385954465	0.005816759	0.032945911	1
T2DM	adipocyte	*C16orf46*	-4.325466012	0.000965127	0.009963466	1
T2DM	adipocyte	*CLEC4E*	0.193806214	0.009881113	0.046765514	1
T2DM	adipocyte	*SEC11C*	1.656934143	2.87E-05	0.00100347	0.180624615
T2DM	adipocyte	*SAAL1*	-1.143359087	0.001424751	0.012814606	1
T2DM	adipocyte	*TSC22D4*	1.218491553	0.000608824	0.007290808	1
T2DM	adipocyte	*AKTIP*	1.228173057	0.002951245	0.021180912	1
T2DM	adipocyte	*EVA1C*	0.282044057	0.001222572	0.01150645	1
T2DM	adipocyte	*SNRPD1*	1.061352999	0.01009615	0.047475315	1
T2DM	adipocyte	*GATAD2A*	1.153292684	1.79E-06	0.00021176	0.011223265
T2DM	adipocyte	*RPL13*	0.62893005	0.005670783	0.032322949	1
T2DM	adipocyte	*ZNF641*	0.241751299	0.000919236	0.009622628	1
T2DM	adipocyte	*CD320*	1.493991849	1.43E-05	0.000736344	0.089833997
T2DM	adipocyte	*SPRYD3*	1.441309212	0.003707865	0.024435377	1

T2DM	adipocyte	*PRDX2*	1.007686971	0.00043405	0.005815531	1
T2DM	adipocyte	*TMC8*	0.643675423	7.06E-06	0.00053468	0.044378444
T2DM	adipocyte	*KCTD5*	1.376818755	0.000805607	0.008823777	1
T2DM	adipocyte	*BSCL2*	-1.140561591	0.000426581	0.005742857	1
T2DM	adipocyte	*TTC21A*	0.930610372	0.004818414	0.028912435	1
T2DM	adipocyte	*LTBP3*	0.576039944	1.39E-07	3.49E-05	0.00087268
T2DM	adipocyte	*UBTD2*	0.602864235	0.001159099	0.011108623	1
T2DM	adipocyte	*PXK*	0.605674101	2.76E-05	0.000991922	0.173586403
T2DM	adipocyte	*RFWD3*	0.338899414	0.005904888	0.033072047	1
T2DM	adipocyte	*RHOH*	0.495173603	0.001203427	0.011392753	1
T2DM	adipocyte	*FNTA*	0.678725396	0.008370575	0.041866194	1
T2DM	adipocyte	*WFDC13*	1.720045629	0.004283067	0.026900743	1
T2DM	adipocyte	*NPNT*	0.603992159	0.007522385	0.039476824	1
T2DM	adipocyte	*INPP5D*	0.868359934	1.76E-05	0.000808689	0.110582397
T2DM	adipocyte	*STXBP6*	0.648330724	0.000153032	0.003025687	0.962112518
T2DM	adipocyte	*FEM1B*	0.658575253	0.000170238	0.003223745	1
T2DM	adipocyte	*IRS1*	-0.328047295	1.62E-28	1.02E-24	1.02E-24
T2DM	adipocyte	*FAM110B*	0.893178887	0.003558713	0.02385248	1
T2DM	adipocyte	*XPO6*	1.085224831	0.000162683	0.00313738	1
T2DM	adipocyte	*PGM2*	1.133916743	0.00063916	0.007525092	1
T2DM	adipocyte	*PTK2*	0.5386819	0.005796943	0.032892943	1
T2DM	adipocyte	*RNASE6*	2.103075752	8.80E-06	0.000586394	0.055335973
T2DM	adipocyte	*INO80E*	-0.393220926	0.002473584	0.018950029	1
T2DM	adipocyte	*CSGALNACT2*	0.924553483	0.000330701	0.004973968	1
T2DM	adipocyte	*GUSB*	0.955661344	0.004224866	0.026695207	1
T2DM	adipocyte	*SEMA3E*	0.973314737	4.98E-07	8.95E-05	0.003132297
T2DM	adipocyte	*METTL7B*	-1.061263747	0.000249662	0.004012908	1
T2DM	adipocyte	*DLGAP1*	0.883849423	3.29E-05	0.00106579	0.206763313
T2DM	adipocyte	*HSPA4*	0.947131599	0.0076448	0.039952498	1
T2DM	adipocyte	*GTSF1*	2.628623377	0.000983479	0.010086671	1
T2DM	adipocyte	*TMEM133*	-1.588163696	0.004159336	0.026387231	1
T2DM	adipocyte	*TTLL6*	-4.003436965	0.005239568	0.030444698	1
T2DM	adipocyte	*KCNS3*	0.14911544	0.003222904	0.022589029	1
T2DM	adipocyte	*AKAP13*	0.631998552	3.21E-05	0.00106579	0.201748085
T2DM	adipocyte	*MTSS1*	0.616549926	0.000443447	0.005894191	1
T2DM	adipocyte	*SOX7*	0.773530485	0.005924079	0.033135839	1
T2DM	adipocyte	*ALK*	-0.315866881	0.008721476	0.043068751	1
T2DM	adipocyte	*MFN1*	0.527389817	0.002474626	0.018950029	1
T2DM	adipocyte	*MCC*	0.637054516	0.002766633	0.020319888	1
T2DM	adipocyte	*HOPX*	0.937878308	0.006892188	0.037133048	1
T2DM	adipocyte	*LRRC8C*	1.423193812	0.000118153	0.002518058	0.742827234
T2DM	adipocyte	*RSL1D1*	0.822047205	0.009210927	0.044717451	1
T2DM	adipocyte	*BCL2L1*	0.577301706	0.002526009	0.019099469	1
T2DM	adipocyte	*NMUR1*	-0.591328069	0.010072257	0.047432414	1
T2DM	adipocyte	*P2RY6*	1.058400233	0.000493994	0.006338247	1
T2DM	adipocyte	*RGS19*	-0.300975515	1.26E-05	0.000715288	0.079396947
T2DM	adipocyte	*TCEA2*	1.542145457	0.002087381	0.01669639	1
T2DM	adipocyte	*CAMTA1*	0.728845234	0.003635871	0.024138034	1
T2DM	adipocyte	*FAM90A1*	4.473581946	0.009332141	0.045201211	1
T2DM	adipocyte	*AQP4*	-0.541617074	0.000360308	0.005239142	1
T2DM	adipocyte	*SRGAP2C*	0.621303189	0.001686305	0.014365585	1
T2DM	adipocyte	*ANKRD20A4*	-1.758564063	0.006145269	0.034039917	1
T2DM	adipocyte	*FRMD3*	0.799311503	0.000126426	0.002615595	0.794839576
T2DM	adipocyte	*MALT1*	0.738384346	0.003399212	0.023153678	1
T2DM	adipocyte	*MBOAT1*	0.737172638	0.004750949	0.02885923	1
T2DM	adipocyte	*C1QTNF4*	-0.087690876	0.000206375	0.003597496	1
T2DM	adipocyte	*SPTLC3*	0.945913832	0.003796126	0.024853488	1
T2DM	adipocyte	*SUCLG2*	0.530149811	0.002709799	0.020040713	1
T2DM	adipocyte	*RCAN2*	0.82308841	0.000119568	0.002531055	0.751723382
T2DM	adipocyte	*PRSS27*	1.407412261	0.001040068	0.010445535	1
T2DM	adipocyte	*FGGY*	0.479155345	0.010293991	0.04811771	1

T2DM	adipocyte	*FIBP*	1.829169315	1.07E-07	2.81E-05	0.000674133
T2DM	adipocyte	*CTSW*	-0.059942727	0.001521365	0.013340055	1
T2DM	adipocyte	*PDE3A*	0.526138074	0.000454648	0.005967377	1
T2DM	adipocyte	*AGAP5*	-22.08900287	0.001303084	0.012083315	1
T2DM	adipocyte	*CFL1*	0.950387865	0.001205055	0.011392753	1
T2DM	adipocyte	*RAB37*	0.838153084	0.001211859	0.011439876	1
T2DM	adipocyte	*DCP2*	0.789755246	0.003354941	0.02302506	1
T2DM	adipocyte	*SNX32*	0.388836355	6.74E-05	0.001674964	0.424025182
T2DM	adipocyte	*RNASEH2C*	-0.34933528	0.002889538	0.021024181	1
T2DM	adipocyte	*MYEOV*	-0.096652008	0.000875886	0.009344489	1
T2DM	adipocyte	*ANKRD13D*	0.570013439	0.007669757	0.040049637	1
T2DM	adipocyte	*LCLAT1*	0.613614643	0.006804206	0.036782496	1
T2DM	adipocyte	*HPSE2*	0.25317182	0.004397565	0.027428067	1
T2DM	adipocyte	*ABLIM3*	0.784356173	3.45E-05	0.001075499	0.216774434
T2DM	adipocyte	*MAP3K11*	1.169583767	0.000622001	0.007406292	1
T2DM	adipocyte	*C1QA*	1.085445581	5.50E-05	0.001465574	0.345875434
T2DM	adipocyte	*OLR1*	1.960011507	7.20E-05	0.001748913	0.452411491
T2DM	adipocyte	*SMARCC1*	0.809208443	0.002153219	0.017114141	1
T2DM	adipocyte	*VEGFB*	-0.343145057	6.07E-06	0.000483445	0.038192146
T2DM	adipocyte	*PEAK1*	0.627878506	0.004410112	0.027479064	1
T2DM	adipocyte	*C1orf100*	2.038951947	0.005846527	0.03304917	1
T2DM	adipocyte	*RNF213*	0.707358722	0.001834272	0.015153835	1
T2DM	adipocyte	*PLK3*	1.098552802	3.43E-05	0.001075499	0.215856446
T2DM	adipocyte	*GPR160*	0.974242617	0.000432573	0.005811082	1
T2DM	adipocyte	*USMG5*	0.638846501	0.010696184	0.049409926	1
T2DM	adipocyte	*HOXB2*	-0.41194558	0.000211654	0.00360662	1
T2DM	adipocyte	*SLC25A30*	1.040767131	0.003130974	0.022160689	1
T2DM	adipocyte	*LEMD3*	0.737978147	0.006779517	0.036775516	1
T2DM	adipocyte	*FAM53A*	-0.779784815	0.000792935	0.008730615	1
T2DM	adipocyte	*LINGO2*	1.019061709	0.000113427	0.002450574	0.713116961
T2DM	adipocyte	*ANKRD36C*	0.659525382	0.000504393	0.006370945	1
T2DM	adipocyte	*IL20RB*	0.256516478	0.003801043	0.024853488	1
T2DM	adipocyte	*KLF14*	-4.144368103	1.22E-11	9.61E-09	7.68E-08
T2DM	adipocyte	*SH3PXD2B*	0.26581017	0.000184859	0.003395587	1
T2DM	adipocyte	*C4orf32*	0.652723597	0.003027507	0.021580426	1
T2DM	adipocyte	*NLRP6*	-1.18558318	0.006775718	0.036775516	1
T2DM	adipocyte	*CHST2*	2.479023329	2.19E-06	0.000250757	0.013791616
T2DM	adipocyte	*TP53I11*	0.836347139	2.47E-05	0.000914004	0.155380665
T2DM	adipocyte	*TMEM9B*	1.255912575	1.19E-05	0.00069764	0.074647431
T2DM	adipocyte	*LPL*	-0.108594705	0.007895865	0.040844634	1
T2DM	adipocyte	*A2M*	0.78179535	3.48E-05	0.00107846	0.218927389
T2DM	adipocyte	*B3GALT6*	0.84336391	0.00543029	0.031125299	1
T2DM	adipocyte	*TCP11L1*	0.409984402	0.001461597	0.012978897	1
T2DM	adipocyte	*COX8A*	1.56364293	0.000633701	0.007500112	1
T2DM	adipocyte	*B3GNT4*	-4.277927175	0.000528743	0.006556624	1
T2DM	adipocyte	*CDC26*	-0.211909475	0.008893135	0.043578441	1
T2DM	adipocyte	*SLCO3A1*	0.793720353	1.74E-05	0.000808689	0.109337141
T2DM	adipocyte	*RNF152*	0.730239805	0.007575729	0.039657458	1
T2DM	adipocyte	*ZNF843*	-1.359888779	0.008042997	0.041346132	1
T2DM	adipocyte	*ANKLE2*	1.052052596	0.000886688	0.009400688	1
T2DM	adipocyte	*GCNT4*	3.722898889	8.35E-06	0.00058364	0.052527596
T2DM	adipocyte	*DSCAML1*	0.890115753	7.65E-07	0.000120195	0.004807798
T2DM	adipocyte	*B3GNT8*	3.518552654	1.06E-05	0.000654035	0.066711538
T2DM	adipocyte	*MAN1B1*	1.103276009	0.008279305	0.041575074	1
T2DM	adipocyte	*C8orf31*	2.095438606	0.000802543	0.008805568	1
T2DM	adipocyte	*TIMM22*	1.051019158	0.003512573	0.023694794	1
T2DM	adipocyte	*PAWR*	0.90695264	0.007899964	0.040844634	1
T2DM	adipocyte	*RBM44*	0.666748865	0.003533736	0.023782977	1
T2DM	adipocyte	*ATOX1*	1.044967196	0.000103152	0.002259646	0.648518533
T2DM	adipocyte	*RPLP2*	0.726758339	0.010426293	0.048447972	1
T2DM	adipocyte	*C12orf54*	-1.220315466	0.000177723	0.003315555	1

T2DM	adipocyte	*FAM87B*	-0.66042153	0.001926052	0.015787597	1
T2DM	adipocyte	*RIC8A*	-0.640988779	0.007276517	0.038605453	1
T2DM	adipocyte	*IMP3*	1.439465427	5.01E-05	0.001382515	0.315251067
T2DM	adipocyte	*C2orf73*	-0.810372662	0.001174204	0.011185187	1
T2DM	adipocyte	*GRAMD1C*	1.273161531	3.03E-05	0.001045532	0.1902868
T2DM	adipocyte	*LCORL*	0.789784818	0.001064891	0.010576279	1
T2DM	adipocyte	*SH2B1*	-0.168994256	0.004807134	0.028912435	1
T2DM	adipocyte	*ZC3H12D*	1.834672502	4.60E-05	0.00130246	0.289146215
T2DM	adipocyte	*PLEC*	0.703530256	0.001378611	0.012508027	1
T2DM	adipocyte	*SH2D4B*	3.934799115	0.000164304	0.003158959	1
T2DM	adipocyte	*PRSS36*	1.86333488	1.24E-05	0.000707371	0.077681096
T2DM	adipocyte	*CSRNP3*	0.634026458	0.003412822	0.023221226	1
T2DM	adipocyte	*STX19*	0.793239662	3.20E-06	0.000319674	0.020139452
T2DM	adipocyte	*GDPD4*	1.95710504	0.004469026	0.027717528	1
T2DM	adipocyte	*TRIM73*	-0.413378367	0.005897379	0.033072047	1
T2DM	adipocyte	*SNX18*	0.626891154	0.005617811	0.032108344	1
T2DM	adipocyte	*FARSA*	-0.768161049	3.10E-05	0.001056656	0.195035001
T2DM	adipocyte	*CALR*	1.095036139	8.00E-09	3.35E-06	5.03E-05
T2DM	adipocyte	*HLA-DQB1*	-0.278126995	2.31E-14	2.42E-11	1.45E-10
T2DM	adipocyte	*EGR3*	1.544769408	1.24E-05	0.000707371	0.077810771
T2DM	adipocyte	*CIITA*	0.512456488	0.000449166	0.005932583	1
T2DM	adipocyte	*SERTAD2*	0.942401585	0.000407803	0.005639191	1
T2DM	adipocyte	*B3GNT3*	0.836203141	0.000361772	0.005239142	1
T2DM	adipocyte	*HCLS1*	1.105307163	1.98E-05	0.00081257	0.124402675
T2DM	adipocyte	*RNF182*	-0.264910014	0.001324646	0.012193337	1
T2DM	adipocyte	*SSTR2*	-0.873074047	0.000504649	0.006370945	1
T2DM	adipocyte	*SHISA2*	16.18860592	4.75E-06	0.000445914	0.029876251
T2DM	adipocyte	*SRSF8*	1.428092523	0.002948299	0.021180912	1
T2DM	adipocyte	*ZDHHC20*	0.839646425	0.001538672	0.013398383	1
T2DM	adipocyte	*ANKRD34A*	-0.507232716	0.004525394	0.027920657	1
T2DM	adipocyte	*SLC26A11*	0.968335242	0.000748523	0.008440726	1
T2DM	adipocyte	*SLC25A41*	-2.802729414	0.000826912	0.008978922	1
T2DM	adipocyte	*DDX60L*	0.635981382	0.000680835	0.00783958	1
T2DM	adipocyte	*SGSH*	1.28785795	0.005977016	0.033372555	1
T2DM	adipocyte	*SETD2*	0.499550205	0.006898584	0.037133048	1
T2DM	adipocyte	*ATG9B*	1.792505125	4.80E-05	0.001353931	0.30192658
T2DM	adipocyte	*ZBTB20*	0.517679724	0.00412989	0.026252322	1
T2DM	adipocyte	*RFX7*	0.729246173	0.003187754	0.022467949	1
T2DM	adipocyte	*CHST15*	0.561373795	0.001311	0.012116168	1
T2DM	adipocyte	*TNRC18*	0.787209783	0.00225429	0.017715898	1
T2DM	adipocyte	*SYNM*	-0.638896134	0.010418097	0.04844569	1
T2DM	adipocyte	*CAPN12*	1.048067136	4.07E-05	0.001200631	0.255952316
T2DM	adipocyte	*FES*	0.973279841	0.003395732	0.023153678	1
T2DM	adipocyte	*CLEC4G*	1.20980179	0.004977429	0.029508585	1
T2DM	adipocyte	*TTC3*	0.110542788	0.002004762	0.016276616	1
T2DM	adipocyte	*ZNF320*	0.10905931	0.001165806	0.011138941	1
T2DM	adipocyte	*BEGAIN*	-1.92598974	0.00119412	0.011340529	1
T2DM	adipocyte	*GPR19*	1.801685374	9.44E-06	0.000597863	0.059366531
T2DM	adipocyte	*URAD*	-1.100499544	0.000208751	0.003599172	1
T2DM	adipocyte	*GPR132*	0.62261515	0.009439018	0.045613457	1
T2DM	adipocyte	*RP1L1*	0.417553178	0.000817087	0.008918444	1
T2DM	adipocyte	*BMP8A*	1.117345023	6.01E-07	0.000105007	0.003780258
T2DM	adipocyte	*CBX6*	1.37028163	8.35E-05	0.001941003	0.524727468
T2DM	adipocyte	*KCTD16*	0.468541623	0.002961827	0.021232615	1
T2DM	adipocyte	*ZNF703*	-0.438673504	0.000408117	0.005639191	1
T2DM	adipocyte	*TBX1*	1.279941101	3.04E-05	0.001045558	0.191337077
T2DM	adipocyte	*EIF3C*	0.207605752	0.001079593	0.010621557	1
T2DM	adipocyte	*CNTN2*	-0.81596607	0.00445839	0.027714414	1
T2DM	adipocyte	*PCDH9*	-0.163765126	0.008734832	0.043068751	1
T2DM	adipocyte	*MROH7*	0.564526514	0.004720045	0.028726937	1
T2DM	adipocyte	*CSF1*	0.238451586	0.006016869	0.033476156	1

T2DM	adipocyte	*WDR27*	0.612319876	0.007913199	0.040871623	1
T2DM	adipocyte	*PROS1*	0.481060884	0.000877935	0.009344489	1
T2DM	adipocyte	*TMEM173*	0.612356294	0.000414158	0.005660454	1
T2DM	adipocyte	*ZNF93*	1.541737566	0.002185708	0.017284962	1
T2DM	adipocyte	*SEPT5*	2.59471611	1.65E-05	0.000780926	0.103863217
T2DM	adipocyte	*OSBP2*	0.743552346	0.003567863	0.023862929	1
T2DM	adipocyte	*TMED9*	0.838771318	0.000304867	0.004674872	1
T2DM	adipocyte	*ZFP90*	1.088623147	0.000411315	0.005646146	1
T2DM	adipocyte	*NDUFA6*	0.091868924	0.008741169	0.043068751	1
T2DM	adipocyte	*TMEM106A*	0.914911505	9.02E-06	0.000586394	0.056692166
T2DM	adipocyte	*TNFAIP8L1*	0.916799617	0.000100433	0.00221551	0.631420448
T2DM	adipocyte	*SV2B*	0.454948862	0.009614555	0.045962686	1
T2DM	adipocyte	*LSAMP*	0.349959433	0.005809995	0.032937275	1
T2DM	adipocyte	*AHNAK2*	0.808922939	0.000221672	0.003736334	1
T2DM	adipocyte	*C16orf52*	0.833010369	0.00298186	0.021351882	1
T2DM	adipocyte	*ADARB2*	0.747931715	0.003527746	0.02377164	1
T2DM	adipocyte	*PIGP*	-0.073395017	0.00524789	0.030464898	1
T2DM	adipocyte	*POMK*	0.846539624	0.008773901	0.043128627	1
T2DM	adipocyte	*LINC00839*	0.47795353	8.23E-05	0.001930952	0.517495226
T2DM	adipocyte	*RTN4RL1*	-0.566014263	0.00588638	0.033072047	1
T2DM	adipocyte	*RASA3*	-0.420031756	0.008207096	0.041544539	1
T2DM	adipocyte	*GSAP*	0.561280611	0.000513388	0.006418731	1
T2DM	adipocyte	*BPIFB4*	11.57705254	0.002552861	0.019244405	1
T2DM	adipocyte	*TOR3A*	1.322290961	0.005891982	0.033072047	1
T2DM	adipocyte	*CYP4X1*	0.428234372	0.007542743	0.039550645	1
T2DM	adipocyte	*KPNA4*	0.769440287	0.008189514	0.041544539	1
T2DM	adipocyte	*RGS7BP*	-0.476156107	0.000729797	0.008296988	1
T2DM	adipocyte	*ZNF396*	-0.303331117	0.007370663	0.038973391	1
T2DM	adipocyte	*TNFRSF18*	2.615952489	5.88E-06	0.000480968	0.036936708
T2DM	adipocyte	*C3orf70*	0.238294431	0.00361928	0.024071846	1
T2DM	adipocyte	*ANGPTL5*	1.459609997	0.002503149	0.0190529	1
T2DM	adipocyte	*EPOR*	0.554063078	0.005054329	0.029809163	1
T2DM	adipocyte	*GJA4*	0.535892448	0.001524602	0.013349825	1
T2DM	adipocyte	*IFT140*	1.13799096	0.00022585	0.003776376	1
T2DM	adipocyte	*TET3*	0.989974879	5.92E-05	0.001545232	0.372400866
T2DM	adipocyte	*TRPV2*	1.219979816	1.56E-05	0.000762794	0.097900691
T2DM	adipocyte	*ADH1A*	-0.294064835	0.000483992	0.006235371	1
T2DM	adipocyte	*SEMA4D*	0.894404031	0.000580637	0.007060862	1
T2DM	adipocyte	*MCRS1*	0.582417773	0.000446794	0.005913671	1
T2DM	adipocyte	*GFRAL*	0.286125553	0.00810307	0.041444425	1
T2DM	adipocyte	*FAM166A*	-1.427467166	0.00015628	0.003069586	0.982531971
T2DM	adipocyte	*NUTM2B*	-4.29072942	0.001058984	0.010567984	1
T2DM	adipocyte	*NCR3LG1*	2.626047421	5.76E-06	0.000480968	0.036214359
T2DM	adipocyte	*RUFY4*	-2.101814439	0.004266982	0.026853368	1
T2DM	adipocyte	*PLSCR1*	0.94445442	9.14E-06	0.000586394	0.05746665
T2DM	adipocyte	*CERKL*	0.709652594	0.00619427	0.034250988	1
T2DM	adipocyte	*SRSF10*	0.697683675	0.003269384	0.022737411	1
T2DM	adipocyte	*FAM72B*	3.367369414	9.74E-06	0.00060642	0.061248469
T2DM	adipocyte	*SUMO2*	1.272837086	0.000162662	0.00313738	1
T2DM	adipocyte	*DPYD*	0.657709361	0.000302179	0.004667817	1
T2DM	adipocyte	*TMEM201*	0.741925288	0.001066544	0.010576279	1
T2DM	adipocyte	*BEND4*	1.797202197	0.000339005	0.005086696	1
T2DM	adipocyte	*LRRK2*	0.475004763	0.000134049	0.002745173	0.842768051
T2DM	adipocyte	*C9orf152*	-1.098978411	0.001048747	0.010515902	1
T2DM	adipocyte	*ALKBH2*	0.422730281	0.001889217	0.015546476	1
T2DM	adipocyte	*SP6*	-3.610627343	0.007790635	0.040512589	1
T2DM	adipocyte	*S100A13*	0.893473229	0.000245391	0.003980202	1
T2DM	adipocyte	*LINC00994*	-0.343449302	0.003110988	0.022050487	1
T2DM	adipocyte	*HLA-DRB1*	-0.502807016	9.14E-15	1.91E-11	5.74E-11
T2DM	adipocyte	*WDSUB1*	0.38191761	0.005905496	0.033072047	1
T2DM	adipocyte	*SIRPB2*	1.025772399	0.00010676	0.002322483	0.67119759

T2DM	adipocyte	*GTF2IRD2*	0.254824154	0.004515556	0.027887328	1
T2DM	adipocyte	*SUPT3H*	0.730695756	0.003775605	0.024803791	1
T2DM	adipocyte	*GIMAP5*	0.388644393	0.002908494	0.021024181	1
T2DM	adipocyte	*NTNG2*	1.372858608	0.000324482	0.004910514	1
T2DM	adipocyte	*ASB13*	1.220925097	0.009470525	0.045678294	1
T2DM	adipocyte	*ZNF124*	0.883399408	0.010691133	0.049409926	1
T2DM	adipocyte	*RFX8*	0.391359864	0.002350219	0.018271006	1
T2DM	adipocyte	*SULT1A1*	-0.182331866	0.003351075	0.02302506	1
T2DM	adipocyte	*TPK1*	0.491638427	0.000201679	0.00356168	1
T2DM	adipocyte	*SULF2*	0.351290111	0.003828468	0.024968444	1
T2DM	adipocyte	*HRH1*	0.770856311	1.88E-05	0.000811251	0.118394548
T2DM	adipocyte	*FAM180B*	-0.229117488	0.00774328	0.040333057	1
T2DM	adipocyte	*TOMM7*	1.148598791	0.000404862	0.00563134	1
T2DM	adipocyte	*NF1*	0.465058056	0.007234515	0.038464552	1
T2DM	adipocyte	*HLA-DQA1*	-0.271327555	2.86E-06	0.000294676	0.017975214
T2DM	adipocyte	*MVB12B*	0.853404216	0.002047451	0.016460777	1
T2DM	adipocyte	*SRGAP1*	0.764812672	0.000504542	0.006370945	1
T2DM	adipocyte	*FUT11*	0.894453394	7.82E-06	0.00056523	0.049175051
T2DM	adipocyte	*ANXA4*	0.81371009	0.000503954	0.006370945	1
T2DM	adipocyte	*GAL3ST4*	1.049560038	1.51E-05	0.00075802	0.094752491
T2DM	adipocyte	*SLC25A29*	0.466301618	0.003052549	0.021709701	1
T2DM	adipocyte	*FAM110D*	0.888543349	0.001039245	0.010445535	1
T2DM	adipocyte	*C5AR1*	0.875908347	3.91E-05	0.001165288	0.245875773
T2DM	adipocyte	*SLC2A10*	0.386753973	0.008674416	0.043009506	1
T2DM	adipocyte	*TTC30A*	-0.347302231	0.002519432	0.019099469	1
T2DM	adipocyte	*HOXA4*	-0.70493413	0.008739149	0.043068751	1
T2DM	adipocyte	*CCDC154*	0.633386346	0.008969509	0.043850159	1
T2DM	adipocyte	*MFAP5*	0.771258881	0.000404732	0.00563134	1
T2DM	adipocyte	*PSAP*	0.722661284	0.000868865	0.009308421	1
T2DM	adipocyte	*AC122129.1*	-1.520997823	0.000823497	0.008957307	1
T2DM	adipocyte	*SGTB*	0.779630279	0.000892429	0.009413933	1
T2DM	adipocyte	*PRIM1*	-0.473658544	0.009073436	0.044290759	1
T2DM	adipocyte	*SFI1*	1.18351339	0.00035854	0.005239142	1
T2DM	adipocyte	*MIER1*	0.950159608	0.000117748	0.002518058	0.740279664
T2DM	adipocyte	*ZNF251*	0.667401615	0.00325026	0.022672808	1
T2DM	adipocyte	*HSD17B11*	1.089528713	1.93E-05	0.00081257	0.121080489
T2DM	adipocyte	*ANTXRL*	49.05056692	0.000577508	0.007050089	1
T2DM	adipocyte	*TMEM116*	-0.151914443	0.002988643	0.021369695	1
T2DM	adipocyte	*ZNF568*	0.809304801	2.59E-05	0.000951173	0.162824078
T2DM	adipocyte	*HLA-DRB5*	-0.146844218	2.91E-12	2.61E-09	1.83E-08
T2DM	adipocyte	*MAFK*	1.417604824	0.000362498	0.005239142	1
T2DM	adipocyte	*ARC*	3.961974817	0.002655752	0.01973607	1
T2DM	adipocyte	*LRBA*	0.626660561	0.005785799	0.032892943	1
T2DM	adipocyte	*ZNF534*	-0.419219839	0.006919307	0.037180926	1
T2DM	adipocyte	*APCDD1L*	1.450452342	0.007290411	0.038646554	1
T2DM	adipocyte	*CAPZA2*	0.894335203	0.000781054	0.008675772	1
T2DM	adipocyte	*RASGEF1A*	4.631596808	3.45E-05	0.001075499	0.216674704
T2DM	adipocyte	*APRT*	1.51376987	0.001114095	0.010825837	1
T2DM	adipocyte	*AP001007.1*	-3.38839742	0.008740581	0.043068751	1
T2DM	adipocyte	*CHML*	0.267620331	0.001278961	0.011931548	1
T2DM	adipocyte	*CENPW*	-0.804076636	2.42E-06	0.000267384	0.01524086
T2DM	adipocyte	*FAM229B*	-0.188462982	0.001319407	0.012162916	1
T2DM	adipocyte	*HIST2H2BF*	-2.608753817	0.006179019	0.034196737	1
T2DM	adipocyte	*PCMTD2*	0.87476091	0.000783987	0.008687779	1
T2DM	adipocyte	*SYS1*	1.818272839	0.00013989	0.002831013	0.879490359
T2DM	adipocyte	*SYT15*	0.487306406	0.004812494	0.028912435	1
T2DM	adipocyte	*OXLD1*	0.351114616	0.00819164	0.041544539	1
T2DM	adipocyte	*HLA-DRA*	0.54641325	1.44E-14	2.13E-11	9.08E-11
T2DM	adipocyte	*NOTCH4*	-0.048997122	7.28E-08	2.18E-05	0.000457512
T2DM	adipocyte	*AGER*	0.363928118	0.000173491	0.003275486	1
T2DM	adipocyte	*PRRT1*	0.293665451	0.000209527	0.003599172	1

T2DM	adipocyte	*DXO*	-0.232100148	0.002914245	0.021024181	1
T2DM	adipocyte	*NELFE*	0.367855265	5.25E-05	0.00142143	0.330366371
T2DM	adipocyte	*BAG6*	0.368017155	0.000247283	0.003986336	1
T2DM	adipocyte	*NFKBIL1*	0.347997864	0.004362491	0.027263402	1
T2DM	adipocyte	*PSORS1C1*	0.036261917	0.007386071	0.039022039	1
T2DM	adipocyte	*LINC01123*	-4.321343682	0.006992317	0.037413357	1
T2DM	adipocyte	*HLA-E*	0.239039498	0.003608419	0.024033845	1
T2DM	adipocyte	*TBC1D8*	0.643347496	0.001442026	0.012896188	1
T2DM	adipocyte	*GABBR1*	0.912357782	0.000498387	0.006355699	1
T2DM	adipocyte	*FAM71F2*	-0.610359779	0.001079258	0.010621557	1
T2DM	adipocyte	*AC016586.1*	1.87905792	0.008152069	0.041533273	1
T2DM	adipocyte	*MT1M*	1.968696954	2.19E-05	0.000830044	0.137787289
T2DM	adipocyte	*SAMD9*	0.680965183	0.001430805	0.012832337	1
T2DM	adipocyte	*SERF1B*	-0.425481635	0.004998023	0.02954797	1
T2DM	adipocyte	*DENND6B*	1.847001735	0.000135873	0.002764505	0.854232184
T2DM	adipocyte	*EIF3CL*	0.928507677	0.004974449	0.029508585	1
T2DM	adipocyte	*LIN52*	0.436809431	0.000158918	0.003102843	0.999115573
T2DM	adipocyte	*NYNRIN*	1.04348915	8.62E-06	0.000586394	0.054219238
T2DM	adipocyte	*HCG27*	0.298011338	4.12E-11	2.88E-08	2.59E-07
T2DM	adipocyte	*COLQ*	-0.752882762	0.0066446	0.036170411	1
T2DM	adipocyte	*RNF208*	-1.958249612	0.001389922	0.012591415	1
T2DM	adipocyte	*FGFR1OP*	0.584031652	0.001703486	0.014412493	1
T2DM	adipocyte	*CCDC183*	-1.273508416	0.001698445	0.014410427	1
T2DM	adipocyte	*DNLZ*	0.170490289	0.000140492	0.002831013	0.883275902
T2DM	adipocyte	*SUPT4H1*	0.546793635	0.005102332	0.029999308	1
T2DM	adipocyte	*ARHGAP19*	0.893379115	0.001446042	0.012913729	1
T2DM	adipocyte	*SIPA1*	0.585200173	5.87E-06	0.000480968	0.03688265
T2DM	adipocyte	*HEXA*	1.002333791	0.001312408	0.012116168	1
T2DM	adipocyte	*LBH*	1.255990692	3.46E-05	0.001075499	0.217250747
T2DM	adipocyte	*ATF6B*	0.383303086	1.31E-05	0.00073252	0.082364985
T2DM	adipocyte	*SLC35F6*	1.123356018	0.001023518	0.010362093	1
T2DM	adipocyte	*PPM1N*	1.13417759	1.41E-05	0.00073252	0.088634939
T2DM	adipocyte	*TAX1BP3*	0.835779078	0.003469046	0.023476742	1
T2DM	adipocyte	*AP1G2*	1.44283898	1.32E-05	0.00073252	0.083260242
T2DM	adipocyte	*TM6SF2*	7.793426366	5.04E-05	0.001382515	0.316595862
T2DM	adipocyte	*NEURL1B*	1.062067614	1.09E-05	0.000658243	0.068457236
T2DM	adipocyte	*STARD10*	0.205254919	4.61E-07	8.78E-05	0.002897928
T2DM	adipocyte	*UBE2QL1*	-0.72027187	0.000321711	0.0048855	1
T2DM	adipocyte	*KCNU1*	-0.575383117	6.67E-09	2.99E-06	4.19E-05
T2DM	adipocyte	*MYL5*	-1.145183156	0.010305725	0.048136772	1
T2DM	adipocyte	*AC016773.1*	-0.126425555	0.004484752	0.027751613	1
T2DM	adipocyte	*RNU6ATAC35P*	-1.63956826	0.000198178	0.00351961	1
T2DM	adipocyte	*OR2A25*	-4.879314571	0.006916954	0.037180926	1
T2DM	adipocyte	*SLC12A8*	-0.296450436	0.006628662	0.036170411	1
T2DM	adipocyte	*UBA52*	0.544507094	0.000435267	0.005815531	1
T2DM	adipocyte	*AC062017.1*	-0.43499878	4.98E-05	0.00138011	0.313284889
T2DM	adipocyte	*ADRA2B*	3.683808588	1.55E-05	0.000762794	0.097408865
T2DM	adipocyte	*LINC00205*	-0.315635917	0.009105513	0.044377023	1
T2DM	adipocyte	*LINC01133*	1.068094997	2.06E-05	0.000815077	0.129606343
T2DM	adipocyte	*LINC01117*	0.593624599	0.000141662	0.002845314	0.890631344
T2DM	adipocyte	*LINC00184*	-0.731623967	2.04E-05	0.000815077	0.128165778
T2DM	adipocyte	*LINC00237*	3.868591965	2.60E-05	0.000951173	0.163601693
T2DM	adipocyte	*NOL7*	1.096669996	0.001038228	0.010445535	1
T2DM	adipocyte	*ELFN1*	2.469362438	3.65E-06	0.000358488	0.022943226
T2DM	adipocyte	*ZNF503-AS1*	-0.727986228	0.006826789	0.036872873	1
T2DM	adipocyte	*C14orf132*	0.531057602	0.001429689	0.012832337	1
T2DM	adipocyte	*AC108463.2*	0.533912224	0.010622448	0.049177709	1
T2DM	adipocyte	*LINC01013*	1.978791086	1.07E-06	0.000145821	0.006707778
T2DM	adipocyte	*LINC00954*	2.380491555	2.95E-05	0.001026284	0.185757444
T2DM	adipocyte	*PATL2*	0.995209793	0.000665206	0.007725842	1
T2DM	adipocyte	*U91324.1*	-1.454020211	0.002351081	0.018271006	1

T2DM	adipocyte	*CISD3*	1.95834008	0.000359065	0.005239142	1
T2DM	adipocyte	*KLHL7-AS1*	-1.254075414	0.00051456	0.006418731	1
T2DM	adipocyte	*TAPBP*	0.458038068	0.000297797	0.004611451	1
T2DM	adipocyte	*HLA-DQB2*	0.121208435	1.18E-08	4.63E-06	7.41E-05
T2DM	adipocyte	*TNF*	0.384831809	0.000624122	0.007417495	1
T2DM	adipocyte	*HLA-B*	0.341252922	3.08E-07	6.46E-05	0.001935017
T2DM	adipocyte	*HLA-DQA2*	0.348931095	6.50E-20	2.04E-16	4.08E-16
T2DM	adipocyte	*TXNDC5*	1.407660784	0.008704479	0.043068751	1
T2DM	adipocyte	*ASB14*	0.399320632	0.005008862	0.02956875	1
T2DM	adipocyte	*LY6G5B*	-0.087987012	0.001220469	0.01150388	1
T2DM	adipocyte	*AQP1*	0.60624312	0.003133586	0.022160689	1
T2DM	adipocyte	*ARHGEF25*	0.428670073	0.007637621	0.03994819	1
T2DM	adipocyte	*ARFGAP3*	0.84166245	0.010220351	0.047844634	1
T2DM	adipocyte	*C22orf39*	1.284009584	0.003880551	0.025074024	1
T2DM	adipocyte	*MRPL20*	0.753455254	0.004883214	0.02918324	1
T2DM	adipocyte	*DECR2*	-0.758914556	0.007465371	0.039228656	1
T2DM	adipocyte	*FGF14-IT1*	2.976190043	1.61E-05	0.000777737	0.101105782
T2DM	adipocyte	*NAT6*	0.570802532	0.0007228	0.008262261	1
T2DM	adipocyte	*IL10RB*	0.491910824	0.002279214	0.017854583	1
T2DM	adipocyte	*PLA2G4B*	0.361756813	4.76E-08	1.57E-05	0.000299179
T2DM	adipocyte	*JMJD7*	0.389662243	5.66E-08	1.78E-05	0.000355575
T2DM	adipocyte	*APOBEC3C*	1.691517613	6.57E-05	0.001651245	0.41281126
T2DM	adipocyte	*SRP14-AS1*	-0.090384607	0.002755356	0.020260946	1
T2DM	adipocyte	*FMN1*	0.212776739	0.003699321	0.024415616	1
T2DM	adipocyte	*DNAH10OS*	-3.545076407	1.75E-06	0.000210988	0.010971375
T2DM	adipocyte	*LINC00989*	1.262272662	0.000845497	0.009161281	1
T2DM	adipocyte	*LINC01021*	-0.85663683	0.000237303	0.003905567	1
T2DM	adipocyte	*FAM218A*	-0.956086447	0.000326677	0.004925224	1
T2DM	adipocyte	*SNHG18*	-0.387157483	0.00096151	0.009942461	1
T2DM	adipocyte	*TRNP1*	4.394937353	3.21E-05	0.00106579	0.202001671
T2DM	adipocyte	*PRKDC*	0.721544836	0.0070668	0.037683607	1
T2DM	adipocyte	*TAS2R20*	0.171306383	0.00495688	0.029508585	1
T2DM	adipocyte	*CTSO*	1.208350575	7.72E-05	0.001839518	0.485632843
T2DM	adipocyte	*HMBS*	0.251004888	3.71E-05	0.001129902	0.232985147
T2DM	adipocyte	*KIAA1147*	0.501300782	0.010317158	0.048154397	1
T2DM	adipocyte	*ZBED6*	-1.545672783	0.00099243	0.010145383	1
T2DM	adipocyte	*FNTB*	-0.97784501	0.003822201	0.024953455	1
T2DM	adipocyte	*LINC00638*	-0.975924438	0.003196497	0.022504342	1
T2DM	adipocyte	*LINC00639*	0.388641127	1.27E-06	0.000170304	0.00800429
T2DM	adipocyte	*LINC00924*	0.593163606	0.003426811	0.023240953	1
T2DM	adipocyte	*UBE2Q2L*	-0.869992386	0.002679385	0.019888182	1
T2DM	adipocyte	*AC083843.1*	-5.068093842	3.10E-07	6.46E-05	0.001947622
T2DM	adipocyte	*AC009120.5*	-0.766769313	0.000467578	0.006072732	1
T2DM	adipocyte	*AC139100.2*	-3.246647442	0.003675859	0.02437777	1
T2DM	adipocyte	*LINC00906*	-4.487878605	0.000423263	0.005710415	1
T2DM	adipocyte	*AC009060.1*	-1.344609235	8.40E-07	0.000128759	0.005279123
T2DM	adipocyte	*LINC01050*	3.679485564	5.74E-05	0.001511181	0.361172254
T2DM	ASPC	*SEMA3F*	-0.096741292	7.48E-07	0.000120508	0.004699814
T2DM	ASPC	*RBM6*	-0.079597947	3.97E-08	1.08E-05	0.000249439
T2DM	ASPC	*ZNF195*	-0.153620091	0.001442074	0.047717469	1
T2DM	ASPC	*GGNBP2*	-0.25000736	5.91E-07	0.000103162	0.003713832
T2DM	ASPC	*CEP68*	0.245456724	2.88E-09	1.21E-06	1.81E-05
T2DM	ASPC	*TUBG2*	-0.110336912	6.95E-09	2.57E-06	4.37E-05
T2DM	ASPC	*ITIH4*	0.062848257	8.50E-06	0.000937955	0.053463458
T2DM	ASPC	*MARK3*	-0.749038275	1.34E-08	4.20E-06	8.41E-05
T2DM	ASPC	*CTTNBP2*	-0.317636302	7.86E-06	0.000898377	0.049410749
T2DM	ASPC	*LRCH4*	-0.14215588	0.000604895	0.02535315	1
T2DM	ASPC	*KCNN2*	-1.047576559	0.000195433	0.011591383	1
T2DM	ASPC	*COBLL1*	-0.185926792	3.28E-08	9.37E-06	0.000206172
T2DM	ASPC	*C3orf18*	0.312414855	7.18E-07	0.000118807	0.004514649
T2DM	ASPC	*PABPC4*	-0.282447305	3.69E-09	1.45E-06	2.32E-05

T2DM	ASPC	*SLC26A4*	-2.626610255	1.30E-05	0.001338862	0.081670593
T2DM	ASPC	*ALKBH5*	-0.172363312	0.001388297	0.046181083	1
T2DM	ASPC	*MAP3K1*	-0.457906806	0.000186854	0.011188076	1
T2DM	ASPC	*CWF19L1*	-0.109131886	7.91E-05	0.006092493	0.49758588
T2DM	ASPC	*PPP6R2*	-0.582986119	1.72E-05	0.001693128	0.108360169
T2DM	ASPC	*RBL2*	0.16452262	0.000172124	0.01071429	1
T2DM	ASPC	*EYA1*	0.097524976	8.27E-05	0.006186681	0.519681226
T2DM	ASPC	*FCER2*	-0.844183701	0.001344165	0.045191249	1
T2DM	ASPC	*DMPK*	-0.350039494	0.000131028	0.008684047	0.823772703
T2DM	ASPC	*GCDH*	-0.406548977	3.68E-07	7.47E-05	0.002315465
T2DM	ASPC	*ATP13A1*	0.241990386	5.01E-07	9.27E-05	0.003151374
T2DM	ASPC	*CPVL*	0.336552916	0.000205491	0.011847527	1
T2DM	ASPC	*POLD2*	-0.456998034	7.54E-06	0.000877953	0.047409467
T2DM	ASPC	*DHRS11*	-0.080503842	0.001141685	0.040099288	1
T2DM	ASPC	*HSD17B1*	-0.073961377	4.37E-07	8.33E-05	0.002749998
T2DM	ASPC	*VAT1*	0.246105557	0.000981559	0.036088085	1
T2DM	ASPC	*OCIAD1*	-0.824101951	9.11E-05	0.00665014	0.57297608
T2DM	ASPC	*WFS1*	1.686905901	9.50E-16	1.19E-12	5.97E-12
T2DM	ASPC	*CDK2AP1*	0.073023402	0.000133714	0.008756845	0.840657101
T2DM	ASPC	*ENDOU*	-0.134817063	0.000964786	0.035891167	1
T2DM	ASPC	*MAPK14*	-0.460714536	0.000474577	0.021691757	1
T2DM	ASPC	*BVES*	-0.086748399	0.000890731	0.033533095	1
T2DM	ASPC	*DNAJC27*	-0.30780762	0.000207289	0.011847527	1
T2DM	ASPC	*NRBP1*	-0.361965393	5.54E-07	9.96E-05	0.003484782
T2DM	ASPC	*PPM1G*	-0.584331956	2.60E-07	5.44E-05	0.001631963
T2DM	ASPC	*CHMP3*	-0.252924967	5.22E-06	0.000643271	0.032792114
T2DM	ASPC	*GPX7*	-0.070032292	0.000498356	0.022334612	1
T2DM	ASPC	*CD3EAP*	-0.218131156	2.66E-05	0.002535376	0.167334825
T2DM	ASPC	*ELOVL4*	0.079166003	0.001260062	0.043054409	1
T2DM	ASPC	*DNAH7*	-0.085607986	1.42E-05	0.001443717	0.089510469
T2DM	ASPC	*CCDC92*	-0.173238351	0.000778477	0.030976472	1
T2DM	ASPC	*HOXB3*	-0.196695	3.46E-05	0.003192162	0.217726579
T2DM	ASPC	*ADRA1A*	0.028195977	0.00020533	0.011847527	1
T2DM	ASPC	*TEX14*	0.237116055	0.000363997	0.018019309	1
T2DM	ASPC	*RASL11A*	-0.480398725	0.000810268	0.031838487	1
T2DM	ASPC	*PMS2*	0.380866758	0.001458645	0.047893484	1
T2DM	ASPC	*CBX3*	-0.35222834	0.001382543	0.046181083	1
T2DM	ASPC	*GRK4*	0.043976745	0.000521425	0.022765292	1
T2DM	ASPC	*OPRL1*	-0.177965771	5.22E-06	0.000643271	0.032806816
T2DM	ASPC	*ZNF436*	0.191511649	0.000180319	0.010900652	1
T2DM	ASPC	*PRR12*	0.154633602	0.000864612	0.03274588	1
T2DM	ASPC	*DPP6*	-0.517957771	7.50E-06	0.000877953	0.047138105
T2DM	ASPC	*GTPBP3*	-0.23137619	0.000580262	0.02473877	1
T2DM	ASPC	*C12orf65*	0.295382239	0.000516883	0.022724794	1
T2DM	ASPC	*DMGDH*	-0.631110652	6.34E-05	0.005313786	0.398533929
T2DM	ASPC	*DSTYK*	-0.373550831	0.000336908	0.017055971	1
T2DM	ASPC	*TMCC2*	-0.152706805	0.001272106	0.043230992	1
T2DM	ASPC	*TMBIM1*	-0.169384473	0.000771763	0.030904937	1
T2DM	ASPC	*AOAH*	-0.184587414	8.50E-05	0.006286584	0.534359649
T2DM	ASPC	*SYTL2*	-0.44852495	0.000111133	0.00784068	0.698695832
T2DM	ASPC	*DCUN1D5*	-0.171995794	0.000995465	0.036386559	1
T2DM	ASPC	*SPTBN5*	0.083403987	5.45E-05	0.004692591	0.34255913
T2DM	ASPC	*CARF*	0.137213106	0.001011208	0.036537162	1
T2DM	ASPC	*HMG20A*	0.530863258	8.33E-06	0.000935353	0.052379763
T2DM	ASPC	*MAN2C1*	-0.050077148	2.55E-05	0.002465384	0.160249978
T2DM	ASPC	*MYO19*	0.558207061	1.13E-05	0.001184543	0.071072584
T2DM	ASPC	*VPS53*	-0.232949511	0.000815381	0.03184039	1
T2DM	ASPC	*LRRC46*	-0.196642538	6.76E-05	0.005518794	0.424947108
T2DM	ASPC	*C18orf8*	-0.286005601	7.37E-05	0.00594361	0.463601572
T2DM	ASPC	*NPC1*	-0.468582225	2.92E-06	0.000374823	0.01836632
T2DM	ASPC	*SIRT3*	0.191940441	0.001249311	0.042920317	1

T2DM	ASPC	*WNT9A*	0.190400532	0.001118194	0.039494863	1
T2DM	ASPC	*NBEAL1*	0.097861943	0.000707487	0.028882929	1
T2DM	ASPC	*PDGFC*	-0.547543016	0.000106867	0.007634933	0.671874086
T2DM	ASPC	*BHMT*	-0.058974412	0.000130695	0.008684047	0.821679856
T2DM	ASPC	*PPIP5K2*	-0.373742703	7.77E-05	0.006092493	0.488374861
T2DM	ASPC	*CCDC25*	0.337373839	0.000976051	0.036088085	1
T2DM	ASPC	*HSD17B12*	-0.118720526	1.29E-08	4.20E-06	8.10E-05
T2DM	ASPC	*APIP*	-0.039787202	0.000131221	0.008684047	0.824984453
T2DM	ASPC	*EIF3M*	-0.317052308	0.000382375	0.018645994	1
T2DM	ASPC	*MS4A3*	-23.83923076	0.000752483	0.030326033	1
T2DM	ASPC	*DOC2A*	-0.227282965	0.000254849	0.013464191	1
T2DM	ASPC	*ARL14EP*	-0.419477803	1.40E-07	3.03E-05	0.000879158
T2DM	ASPC	*POC5*	-0.18697486	0.0001768	0.010875031	1
T2DM	ASPC	*ZFP36L2*	0.699977937	0.000638253	0.026399305	1
T2DM	ASPC	*SAR1B*	0.280721637	0.000500892	0.022334612	1
T2DM	ASPC	*JAZF1*	-0.94577446	1.03E-19	3.25E-16	6.50E-16
T2DM	ASPC	*LONRF1*	-0.391282685	4.59E-05	0.004007116	0.288512359
T2DM	ASPC	*TSEN2*	0.231567756	2.59E-06	0.000347135	0.016287986
T2DM	ASPC	*NDUFAF6*	-0.35428124	8.25E-07	0.000129675	0.005186994
T2DM	ASPC	*TIMP4*	0.350323437	3.50E-05	0.003192162	0.220259182
T2DM	ASPC	*CLEC18A*	-0.233539788	8.32E-08	1.94E-05	0.000523226
T2DM	ASPC	*KCNJ15*	-0.147562737	0.000359647	0.017945254	1
T2DM	ASPC	*AP3S2*	0.155672466	2.10E-10	1.20E-07	1.32E-06
T2DM	ASPC	*CD1E*	0.290247583	0.000738419	0.029951242	1
T2DM	ASPC	*ZNF276*	-0.403333172	0.000476135	0.021691757	1
T2DM	ASPC	*DIP2A*	-0.213117459	0.000633287	0.026367398	1
T2DM	ASPC	*SPTA1*	-0.03980573	0.001324195	0.044759217	1
T2DM	ASPC	*ICA1L*	-0.205210514	0.000582366	0.02473877	1
T2DM	ASPC	*RFT1*	-0.311220657	2.36E-06	0.000329077	0.014808448
T2DM	ASPC	*BDH2*	-0.104622491	0.000339112	0.017055971	1
T2DM	ASPC	*ATRIP*	-2.073539478	0.000404757	0.019574666	1
T2DM	ASPC	*RNF123*	0.132246554	1.18E-06	0.00017043	0.007440407
T2DM	ASPC	*CCDC171*	-0.669134995	0.001002207	0.036421235	1
T2DM	ASPC	*REEP3*	-0.270809409	0.000675714	0.027766099	1
T2DM	ASPC	*TRMT61A*	-0.242105443	1.19E-06	0.00017043	0.007498926
T2DM	ASPC	*PCBD1*	-0.082948836	9.20E-05	0.00665014	0.578562153
T2DM	ASPC	*C2*	0.569757758	9.57E-12	7.52E-09	6.02E-08
T2DM	ASPC	*SAAL1*	-1.388346099	4.86E-11	3.06E-08	3.06E-07
T2DM	ASPC	*VKORC1*	-0.139161516	0.001216508	0.042022988	1
T2DM	ASPC	*GATAD2A*	0.523224161	0.00108684	0.038604294	1
T2DM	ASPC	*PRDX2*	0.540195284	0.000298362	0.015250408	1
T2DM	ASPC	*LTBP3*	0.212134933	1.10E-05	0.001167508	0.068882965
T2DM	ASPC	*KIF5C*	-0.447183754	0.000832691	0.032117356	1
T2DM	ASPC	*PXK*	0.245461256	0.000244671	0.013035983	1
T2DM	ASPC	*TAP1*	-0.41895812	6.11E-07	0.000103819	0.003841287
T2DM	ASPC	*IRS1*	-0.136490968	7.89E-27	4.96E-23	4.96E-23
T2DM	ASPC	*HINT1*	-0.468028148	0.000858678	0.032718236	1
T2DM	ASPC	*INO80E*	-0.201037743	0.000449833	0.021263909	1
T2DM	ASPC	*EMB*	-0.102186082	0.000153486	0.009747156	0.96496846
T2DM	ASPC	*TTLL6*	-3.192859463	1.08E-05	0.001167508	0.068169643
T2DM	ASPC	*RGS19*	-0.156173857	1.21E-07	2.71E-05	0.000760006
T2DM	ASPC	*GPHN*	-0.157305904	0.00102897	0.036756448	1
T2DM	ASPC	*LAMB2*	-0.224872252	3.00E-05	0.002815123	0.188613252
T2DM	ASPC	*C1QTNF4*	-0.036808658	0.000219036	0.012406138	1
T2DM	ASPC	*FIBP*	0.56002451	0.000577571	0.02473877	1
T2DM	ASPC	*CTSW*	-0.030025635	0.000234254	0.012806576	1
T2DM	ASPC	*SNX32*	0.134265567	0.000948163	0.035482749	1
T2DM	ASPC	*RNASEH2C*	-0.180398813	0.000382588	0.018645994	1
T2DM	ASPC	*VEGFB*	-0.147195656	7.13E-06	0.000862185	0.044833627
T2DM	ASPC	*HOXB2*	-0.197602128	3.78E-05	0.003347352	0.237662018
T2DM	ASPC	*TMEM81*	0.899130936	0.001505259	0.048781267	1

T2DM	ASPC	*KLF14*	-2.083697308	1.17E-09	5.24E-07	7.33E-06
T2DM	ASPC	*NLRP6*	-0.706375427	0.000178166	0.010875031	1
T2DM	ASPC	*DHX36*	-0.675761309	0.000504352	0.022334612	1
T2DM	ASPC	*PACS1*	-0.741476592	0.000206936	0.011847527	1
T2DM	ASPC	*C12orf54*	-0.451445786	0.001474048	0.048017301	1
T2DM	ASPC	*RIC8A*	-0.378162587	0.000530866	0.023017606	1
T2DM	ASPC	*IMP3*	0.775609035	6.46E-05	0.005345211	0.406236063
T2DM	ASPC	*STX19*	0.305841237	5.73E-05	0.004868127	0.360241431
T2DM	ASPC	*FARSA*	-0.405925595	8.79E-07	0.000131546	0.005524923
T2DM	ASPC	*CALR*	0.440759087	8.12E-05	0.006147697	0.510258832
T2DM	ASPC	*HLA-DQB1*	-0.118991923	1.63E-15	1.70E-12	1.02E-11
T2DM	ASPC	*B3GNT3*	0.3303422	0.000831996	0.032117356	1
T2DM	ASPC	*ZNF320*	0.047357455	0.000852887	0.032695734	1
T2DM	ASPC	*RP1L1*	0.193375194	0.000260628	0.013654734	1
T2DM	ASPC	*BMP8A*	0.673547317	3.87E-11	2.70E-08	2.43E-07
T2DM	ASPC	*ZNF703*	-0.208699789	7.83E-05	0.006092493	0.492174465
T2DM	ASPC	*EIF3C*	0.093357971	0.000504456	0.022334612	1
T2DM	ASPC	*LINC00839*	0.172278781	0.000801426	0.031689102	1
T2DM	ASPC	*RASA3*	-0.32193868	3.60E-05	0.003235473	0.226483132
T2DM	ASPC	*RGS7BP*	-0.222756493	0.000227461	0.012655278	1
T2DM	ASPC	*HLA-DRB1*	-0.238742615	8.66E-19	1.82E-15	5.45E-15
T2DM	ASPC	*MYO6*	-0.291850842	7.95E-05	0.006092493	0.499584436
T2DM	ASPC	*FAM180B*	-0.12815577	0.000465897	0.021691757	1
T2DM	ASPC	*ZNF33B*	-0.148576799	0.000227356	0.012655278	1
T2DM	ASPC	*HLA-DQA1*	-0.138715743	1.16E-08	4.06E-06	7.32E-05
T2DM	ASPC	*SLC39A10*	-0.793589939	0.000410171	0.019685073	1
T2DM	ASPC	*PRIM1*	-0.312002109	0.000128334	0.008684047	0.806837869
T2DM	ASPC	*ZNF568*	0.301340994	0.001212077	0.042022988	1
T2DM	ASPC	*HLA-DRB5*	-0.062002939	1.85E-12	1.66E-09	1.16E-08
T2DM	ASPC	*MAP3K3*	-0.869466303	0.000244486	0.013035983	1
T2DM	ASPC	*CENPW*	-0.339110382	2.72E-06	0.00035681	0.017126884
T2DM	ASPC	*HLA-DMA*	-0.332674388	0.001213993	0.042022988	1
T2DM	ASPC	*HLA-DRA*	0.19011728	6.14E-10	2.97E-07	3.86E-06
T2DM	ASPC	*NOTCH4*	-0.020647335	7.74E-08	1.94E-05	0.000486327
T2DM	ASPC	*PRRT1*	0.117580679	0.000417274	0.019874264	1
T2DM	ASPC	*NELFE*	0.143158185	0.000234067	0.012806576	1
T2DM	ASPC	*LINC01123*	-2.328555628	0.000604284	0.02535315	1
T2DM	ASPC	*LIN52*	0.190494094	0.00026619	0.013830896	1
T2DM	ASPC	*HCG27*	0.120987579	2.64E-10	1.38E-07	1.66E-06
T2DM	ASPC	*COLQ*	-0.862679598	0.000112241	0.00784068	0.705661221
T2DM	ASPC	*FGFR1OP*	0.298580269	0.00045562	0.02137676	1
T2DM	ASPC	*DNLZ*	0.07159585	0.000149358	0.009581745	0.939010991
T2DM	ASPC	*ATF6B*	0.145022118	0.000158677	0.009976045	0.997604454
T2DM	ASPC	*CLIC1*	-0.393610867	0.000469645	0.021691757	1
T2DM	ASPC	*STARD10*	0.084608787	8.49E-07	0.000130182	0.005337448
T2DM	ASPC	*KCNU1*	-0.27096725	2.60E-06	0.000347135	0.016315343
T2DM	ASPC	*HLA-DQB2*	0.050391484	4.47E-08	1.17E-05	0.000281054
T2DM	ASPC	*HLA-B*	0.117846814	0.00013626	0.008831647	0.856669738
T2DM	ASPC	*HLA-DQA2*	0.140298349	4.61E-18	7.24E-15	2.90E-14
T2DM	ASPC	*LY6G5B*	-0.0363689	0.001462629	0.047893484	1
T2DM	ASPC	*NAT6*	0.238077074	0.001028377	0.036756448	1
T2DM	ASPC	*PLA2G4B*	0.149892606	8.10E-08	1.94E-05	0.000509264
T2DM	ASPC	*JMJD7*	0.170357159	1.93E-08	5.77E-06	0.000121084
T2DM	ASPC	*ZNF286B*	-0.471647557	0.000289228	0.014904718	1
T2DM	ASPC	*DNAH10OS*	-1.424994459	0.000237501	0.012872126	1
T2DM	ASPC	*HMBS*	0.111089802	1.71E-05	0.001693128	0.107272389
T2DM	ASPC	*AC083843.1*	-1.926762604	0.000128697	0.008684047	0.80912068
T2DM	ASPC	*AC009060.1*	-0.639968192	3.97E-07	7.80E-05	0.002495959
T2DM	b_cell	*SCYL3*	-3.375263656	0.010003819	0.01514059	1
T2DM	b_cell	*CFH*	-1.1806019	0.00104436	0.00415349	1
T2DM	b_cell	*NFYA*	-4.039083573	0.018639561	0.025420156	1

T2DM	b_cell	*NIPAL3*	-3.266449655	0.002880379	0.0061721	1
T2DM	b_cell	*SEMA3F*	-3.973851516	2.40E-08	1.88E-05	0.000150607
T2DM	b_cell	*KRIT1*	-2.710701776	0.019041882	0.025867829	1
T2DM	b_cell	*RAD52*	-2.238958496	0.001223614	0.004253251	1
T2DM	b_cell	*BAD*	-3.677071297	0.001210941	0.00424133	1
T2DM	b_cell	*HS3ST1*	2.578205087	0.001104201	0.004159248	1
T2DM	b_cell	*LASP1*	-2.824958158	0.005398709	0.009426623	1
T2DM	b_cell	*CYP26B1*	-3.045661746	0.001389574	0.004333459	1
T2DM	b_cell	*ICA1*	-1.487035948	0.000851116	0.00415349	1
T2DM	b_cell	*DBNDD1*	2.181357913	0.00444587	0.008235471	1
T2DM	b_cell	*ALS2*	-2.274568891	0.001710345	0.004728644	1
T2DM	b_cell	*POLDIP2*	-2.349978097	0.00108122	0.00415349	1
T2DM	b_cell	*CD38*	3.248700956	0.01941137	0.026290238	1
T2DM	b_cell	*RBM6*	-3.42204252	1.48E-08	1.55E-05	9.31E-05
T2DM	b_cell	*ARHGAP33*	-4.455217316	0.000208002	0.00415349	1
T2DM	b_cell	*PDK4*	-2.081572066	0.002146238	0.005293604	1
T2DM	b_cell	*SLC22A16*	-4.386290566	0.007662136	0.012198493	1
T2DM	b_cell	*CDC27*	-2.144328387	0.015250718	0.02144515	1
T2DM	b_cell	*MEOX1*	-2.187270768	0.001922643	0.005009388	1
T2DM	b_cell	*LIG3*	-2.514069238	0.008021819	0.012633561	1
T2DM	b_cell	*CIAPIN1*	-3.635111282	0.003100894	0.006438348	1
T2DM	b_cell	*COPZ2*	-1.588428947	0.006325575	0.010529227	1
T2DM	b_cell	*GCFC2*	-2.218846763	0.002142824	0.005287258	1
T2DM	b_cell	*WDR54*	-3.781275549	0.001065054	0.00415349	1
T2DM	b_cell	*ABCB4*	1.922821413	0.000742859	0.00415349	1
T2DM	b_cell	*ZNF195*	-3.270109455	0.000119501	0.00415349	0.751305386
T2DM	b_cell	*PDK2*	-2.103741776	0.000925732	0.00415349	1
T2DM	b_cell	*GGNBP2*	-3.189891986	4.27E-05	0.003541347	0.268475418
T2DM	b_cell	*TMEM98*	-2.24372607	0.002343299	0.005521859	1
T2DM	b_cell	*ABCC8*	-6.048137671	0.000964859	0.00415349	1
T2DM	b_cell	*CCL18*	3.972838018	0.001834341	0.004898539	1
T2DM	b_cell	*SYNRG*	-2.468006121	0.003747306	0.007300686	1
T2DM	b_cell	*TMEM132A*	-6.495409801	0.001395315	0.00434199	1
T2DM	b_cell	*SPATA20*	-2.525180943	0.000878632	0.00415349	1
T2DM	b_cell	*TNFRSF12A*	4.822493343	0.009839563	0.014920726	1
T2DM	b_cell	*MAP3K9*	1.082665317	0.000360191	0.00415349	1
T2DM	b_cell	*RALA*	-2.843955707	0.007117334	0.011508915	1
T2DM	b_cell	*BAIAP2L1*	-2.047313338	0.000998419	0.00415349	1
T2DM	b_cell	*FARP2*	-2.556586218	0.008364551	0.013052353	1
T2DM	b_cell	*IFRD1*	-1.645502067	0.000897586	0.00415349	1
T2DM	b_cell	*GTF2IRD1*	-1.891849131	0.001013956	0.00415349	1
T2DM	b_cell	*VPS41*	-2.686919567	0.020970588	0.028207549	1
T2DM	b_cell	*ELAC2*	-2.391471404	0.002111624	0.00524942	1
T2DM	b_cell	*CCDC124*	-3.85119685	0.015004414	0.021165077	1
T2DM	b_cell	*NOS2*	-15.98288291	0.001775796	0.004814329	1
T2DM	b_cell	*TRAPPC6A*	-4.554402357	0.02130711	0.028592914	1
T2DM	b_cell	*CD79B*	0.232925934	0.000701233	0.00415349	1
T2DM	b_cell	*ST7L*	-2.677675129	0.000488072	0.00415349	1
T2DM	b_cell	*PAX6*	-3.913913266	0.01796005	0.024653894	1
T2DM	b_cell	*RPUSD1*	-5.044526256	0.000779644	0.00415349	1
T2DM	b_cell	*CDK11A*	-3.868048133	0.001297076	0.004268027	1
T2DM	b_cell	*DLEC1*	-2.315228832	0.000927561	0.00415349	1
T2DM	b_cell	*SS18L2*	-2.677206982	0.000507615	0.00415349	1
T2DM	b_cell	*MPND*	2.94859826	0.00042565	0.00415349	1
T2DM	b_cell	*MGST1*	-1.042667076	0.000946275	0.00415349	1
T2DM	b_cell	*NFIX*	-1.53218653	0.001330317	0.004274973	1
T2DM	b_cell	*MMP25*	-2.899163257	0.002628143	0.005859268	1
T2DM	b_cell	*IL32*	-2.945921372	0.001341134	0.004286583	1
T2DM	b_cell	*PKD1*	-2.088304133	0.00371661	0.00726565	1
T2DM	b_cell	*MAPK8IP2*	1.596017048	0.000853241	0.00415349	1
T2DM	b_cell	*MED24*	-2.939715125	0.00084004	0.00415349	1

T2DM	b_cell	*RHOBTB2*	3.911629621	0.011022478	0.016348443	1
T2DM	b_cell	*RPS20*	-3.081203518	0.032823921	0.041825979	1
T2DM	b_cell	*POMT2*	-4.078848147	0.002230419	0.005401538	1
T2DM	b_cell	*MLXIPL*	-0.718446051	0.037450066	0.04702388	1
T2DM	b_cell	*METTL13*	-2.345193347	0.001079567	0.00415349	1
T2DM	b_cell	*DYRK4*	-2.446932595	0.013366462	0.019212266	1
T2DM	b_cell	*STARD3NL*	-2.342626144	0.001416019	0.004364365	1
T2DM	b_cell	*ZNF200*	-3.462914396	0.014451067	0.02052267	1
T2DM	b_cell	*CD4*	-1.901323826	0.000745872	0.00415349	1
T2DM	b_cell	*LRRC23*	3.516713244	0.015691275	0.021981071	1
T2DM	b_cell	*HFE*	-3.375158171	0.000956007	0.00415349	1
T2DM	b_cell	*SCMH1*	-2.236646391	0.008962375	0.013820567	1
T2DM	b_cell	*MRC2*	-1.49232407	0.00098002	0.00415349	1
T2DM	b_cell	*TSPAN9*	-1.764373503	0.000230135	0.00415349	1
T2DM	b_cell	*MKS1*	-4.737729966	0.006855629	0.011183534	1
T2DM	b_cell	*UTP18*	-2.432326072	0.001069542	0.00415349	1
T2DM	b_cell	*TTC19*	-1.933632222	0.004075104	0.007751945	1
T2DM	b_cell	*DPF1*	-10.20033597	0.006872962	0.011205995	1
T2DM	b_cell	*SYT7*	-4.648227963	0.0103724	0.015574702	1
T2DM	b_cell	*LARS2*	-2.591390435	0.016038971	0.022393296	1
T2DM	b_cell	*PPP5C*	-2.786891216	0.008152092	0.012781098	1
T2DM	b_cell	*ZBTB32*	-3.130699379	0.000688848	0.00415349	1
T2DM	b_cell	*TMEM159*	-2.177566502	0.000619447	0.00415349	1
T2DM	b_cell	*ERCC1*	-1.838305293	0.000945041	0.00415349	1
T2DM	b_cell	*LTF*	-4.638232474	0.013132177	0.018936238	1
T2DM	b_cell	*ALOX5*	1.740307136	0.000891398	0.00415349	1
T2DM	b_cell	*MAP4K5*	-2.381295893	0.002123183	0.005267208	1
T2DM	b_cell	*PSMC4*	-1.797899757	0.003777338	0.007340997	1
T2DM	b_cell	*MAN2B2*	-4.020697113	0.007545642	0.012052706	1
T2DM	b_cell	*CLDN11*	-1.590566017	0.000824354	0.00415349	1
T2DM	b_cell	*ANGEL1*	-3.08866868	0.005363945	0.009387268	1
T2DM	b_cell	*RNF14*	-2.210952155	0.001342844	0.004286796	1
T2DM	b_cell	*DDX11*	-3.053783316	0.001209975	0.00424031	1
T2DM	b_cell	*HEBP1*	-4.801202375	0.002814648	0.006087269	1
T2DM	b_cell	*TACC3*	-2.404970915	0.000501719	0.00415349	1
T2DM	b_cell	*MDH1*	-1.685775095	0.000763601	0.00415349	1
T2DM	b_cell	*COX15*	-3.459713139	0.022995191	0.030609944	1
T2DM	b_cell	*CCDC88C*	-2.989973396	0.02563645	0.033543467	1
T2DM	b_cell	*NPC1L1*	4.387833726	0.003086627	0.006415083	1
T2DM	b_cell	*NUDCD3*	-2.632038839	0.004161301	0.007865091	1
T2DM	b_cell	*CHDH*	-6.433667218	0.001603796	0.004609717	1
T2DM	b_cell	*IL20RA*	-6.943303926	0.000751221	0.00415349	1
T2DM	b_cell	*GLT8D1*	-1.887087914	0.000779985	0.00415349	1
T2DM	b_cell	*RUFY3*	-2.339029469	0.00353152	0.007021717	1
T2DM	b_cell	*WWTR1*	-1.845877254	0.001613681	0.004616359	1
T2DM	b_cell	*TTC27*	-2.812811002	0.007265561	0.01168549	1
T2DM	b_cell	*VSIG2*	-6.330866366	0.002904939	0.006192483	1
T2DM	b_cell	*PHLDB1*	-1.298161017	0.000258962	0.00415349	1
T2DM	b_cell	*MARCO*	-1.388063793	0.010332213	0.015536623	1
T2DM	b_cell	*MNAT1*	-1.887251552	0.001570278	0.004559973	1
T2DM	b_cell	*OSBPL5*	-2.433953906	0.001424752	0.004380342	1
T2DM	b_cell	*AQR*	-2.125181851	0.002176438	0.005332528	1
T2DM	b_cell	*CPS1*	-2.17602579	0.000490567	0.00415349	1
T2DM	b_cell	*NLRP2*	-3.700348126	0.003230197	0.006613998	1
T2DM	b_cell	*ZDHHC6*	-2.593363832	0.001141689	0.00418468	1
T2DM	b_cell	*RNH1*	-2.634630075	0.00537885	0.009404013	1
T2DM	b_cell	*NDUFS1*	-2.709285765	0.0049986	0.008943142	1
T2DM	b_cell	*BIRC3*	1.051560382	0.000778761	0.00415349	1
T2DM	b_cell	*GLRX2*	-3.740968543	0.011534011	0.016962609	1
T2DM	b_cell	*SNAPC1*	-2.726717109	0.001078022	0.00415349	1
T2DM	b_cell	*GCLM*	-1.764947048	0.001286502	0.004268027	1

T2DM	b_cell	*UBR2*	-2.384996486	0.010049134	0.015187237	1
T2DM	b_cell	*NR1H3*	-1.588607937	0.00280211	0.006072688	1
T2DM	b_cell	*TYMP*	-2.577251298	0.005022125	0.008977566	1
T2DM	b_cell	*TOMM34*	3.545594833	0.001410649	0.004364365	1
T2DM	b_cell	*KPNA6*	-2.582369575	0.011669075	0.017109019	1
T2DM	b_cell	*VIM*	-2.110101171	0.008071794	0.012693189	1
T2DM	b_cell	*FAS*	-1.947716263	0.003020431	0.006329816	1
T2DM	b_cell	*CD44*	-1.908609151	0.001318865	0.004274973	1
T2DM	b_cell	*BTN3A1*	-2.880136388	0.00427266	0.008004235	1
T2DM	b_cell	*MIPEP*	-2.471340039	0.001529085	0.004502743	1
T2DM	b_cell	*INSRR*	-6.676747217	0.01015616	0.015315849	1
T2DM	b_cell	*VRK2*	-2.576439501	0.013350987	0.019194524	1
T2DM	b_cell	*VEZT*	-2.526134791	0.003632061	0.007178486	1
T2DM	b_cell	*SNX1*	-2.741909463	0.005981568	0.010125504	1
T2DM	b_cell	*ARNTL2*	-3.967726799	0.007871521	0.012459278	1
T2DM	b_cell	*SLC39A9*	-3.082592711	0.003850067	0.007434083	1
T2DM	b_cell	*ANK1*	-2.892260708	0.001228252	0.004253251	1
T2DM	b_cell	*RABEP1*	-2.220522349	0.020281496	0.02737436	1
T2DM	b_cell	*CENPQ*	-3.205128247	0.00018568	0.00415349	1
T2DM	b_cell	*IFT88*	-2.562380418	0.002816217	0.006088569	1
T2DM	b_cell	*ZCCHC8*	-3.050103272	0.008789046	0.013606681	1
T2DM	b_cell	*FUT8*	-2.435062639	0.032860224	0.04186266	1
T2DM	b_cell	*UBA6*	-2.954010317	0.000777081	0.00415349	1
T2DM	b_cell	*PIAS1*	-2.326265063	0.010213889	0.015384456	1
T2DM	b_cell	*SLC4A7*	-2.641597464	0.012276602	0.017841654	1
T2DM	b_cell	*ASTE1*	-2.607703962	0.003900729	0.007513983	1
T2DM	b_cell	*MYOC*	-1.463884977	0.006961459	0.011309223	1
T2DM	b_cell	*SH3YL1*	-2.738724483	0.000187687	0.00415349	1
T2DM	b_cell	*VCL*	-2.68387919	0.001130924	0.004182741	1
T2DM	b_cell	*ADSS*	-3.197886346	0.026373101	0.0343928	1
T2DM	b_cell	*STAP1*	0.25188342	0.000605455	0.00415349	1
T2DM	b_cell	*TBC1D23*	-2.491596873	0.002745658	0.006006247	1
T2DM	b_cell	*CUL3*	-2.356236859	0.037467096	0.047035869	1
T2DM	b_cell	*MYOM2*	-3.756971728	0.015610917	0.021878251	1
T2DM	b_cell	*TUBG2*	-3.024972643	2.14E-08	1.88E-05	0.000134363
T2DM	b_cell	*NSUN2*	-3.758345923	0.0044698	0.008272485	1
T2DM	b_cell	*MRI1*	-2.473534778	0.00060246	0.00415349	1
T2DM	b_cell	*AGA*	-2.97126949	0.001015977	0.00415349	1
T2DM	b_cell	*MAT2B*	-3.733670385	0.01035767	0.015560018	1
T2DM	b_cell	*CLEC16A*	-2.704596156	0.037539342	0.047107754	1
T2DM	b_cell	*SKIV2L2*	-2.420676626	0.005740307	0.009822893	1
T2DM	b_cell	*C6*	-1.714968331	0.000460429	0.00415349	1
T2DM	b_cell	*RAI14*	-1.999815837	0.001407669	0.004357467	1
T2DM	b_cell	*PNKP*	-2.274916129	0.000249704	0.00415349	1
T2DM	b_cell	*SPDL1*	-2.962968416	0.003159562	0.006514977	1
T2DM	b_cell	*PQLC2*	-2.99513993	0.001085135	0.00415349	1
T2DM	b_cell	*PSMA4*	-2.891097762	0.001576205	0.004562749	1
T2DM	b_cell	*LSG1*	-2.402239773	0.014507729	0.020589185	1
T2DM	b_cell	*THAP3*	-3.717361098	0.000702111	0.00415349	1
T2DM	b_cell	*SPATA7*	-2.695217624	0.00213692	0.005285137	1
T2DM	b_cell	*MED17*	-3.035306507	0.012020882	0.017522672	1
T2DM	b_cell	*RETSAT*	-1.363716572	0.000671938	0.00415349	1
T2DM	b_cell	*CAPG*	2.784638865	0.026154122	0.034135554	1
T2DM	b_cell	*LCP2*	-1.660103299	0.00084096	0.00415349	1
T2DM	b_cell	*CUL7*	-3.138082833	0.007272638	0.011687903	1
T2DM	b_cell	*CTNNA1*	-2.256383438	0.00498091	0.00892419	1
T2DM	b_cell	*CNTLN*	-2.309619979	0.003258064	0.006651595	1
T2DM	b_cell	*EPHA3*	-1.549378681	0.000792268	0.00415349	1
T2DM	b_cell	*DSG2*	-2.42168621	0.001070355	0.00415349	1
T2DM	b_cell	*WDR37*	-2.594907393	0.00337911	0.006811307	1
T2DM	b_cell	*YTHDC2*	-2.521710381	0.021064776	0.028316068	1

T2DM	b_cell	*ATP6V1H*	-2.451364308	0.00222806	0.005400083	1
T2DM	b_cell	*POLR2B*	-2.731568646	0.004161181	0.007865091	1
T2DM	b_cell	*CP*	-4.200895852	0.006782402	0.011092862	1
T2DM	b_cell	*ANO2*	-1.344218187	0.001073884	0.00415349	1
T2DM	b_cell	*GOPC*	-2.945710036	0.00392822	0.007554824	1
T2DM	b_cell	*TSPAN17*	-2.012135803	0.025945804	0.033919998	1
T2DM	b_cell	*CC2D2A*	-2.039254526	0.001556921	0.004533747	1
T2DM	b_cell	*RRM2B*	-3.607131523	0.006089447	0.010252906	1
T2DM	b_cell	*SNX29*	-2.236204533	0.021951678	0.029320204	1
T2DM	b_cell	*LMO3*	-1.271904307	0.000951189	0.00415349	1
T2DM	b_cell	*MRPS10*	-2.186893457	0.000676945	0.00415349	1
T2DM	b_cell	*CELF2*	-1.831900146	0.003668273	0.007211517	1
T2DM	b_cell	*ERCC8*	-2.296253939	0.004912292	0.008839032	1
T2DM	b_cell	*ADAMTS6*	-2.743270112	0.01670006	0.023192684	1
T2DM	b_cell	*H6PD*	-2.717205323	0.001054771	0.00415349	1
T2DM	b_cell	*UTS2*	-3.603877751	0.001037905	0.00415349	1
T2DM	b_cell	*RFC2*	4.865347958	0.003052258	0.006376763	1
T2DM	b_cell	*JKAMP*	-2.182103419	0.001929866	0.005023674	1
T2DM	b_cell	*DKK3*	-2.919592969	0.002028635	0.005163442	1
T2DM	b_cell	*ARHGEF5*	3.525003147	0.000796448	0.00415349	1
T2DM	b_cell	*MCUR1*	-3.595902536	0.002934235	0.006222119	1
T2DM	b_cell	*LIMA1*	-0.966068128	0.00064196	0.00415349	1
T2DM	b_cell	*PTGER3*	-1.148257139	0.000786975	0.00415349	1
T2DM	b_cell	*PIK3CB*	-2.208883387	0.001120383	0.004182741	1
T2DM	b_cell	*THOC3*	-6.389288991	0.010654997	0.015915412	1
T2DM	b_cell	*RRP12*	-2.742986272	0.004870605	0.0087917	1
T2DM	b_cell	*ALX4*	4.273299062	0.00086429	0.00415349	1
T2DM	b_cell	*FOXN3*	-2.150765954	0.003655534	0.007196818	1
T2DM	b_cell	*AKR7A2*	-3.276509929	0.001848652	0.004914366	1
T2DM	b_cell	*MRTO4*	-4.897283562	0.003675271	0.007220759	1
T2DM	b_cell	*MCF2L2*	-3.22533897	0.001315804	0.004274973	1
T2DM	b_cell	*ANAPC4*	-2.628172964	0.007622746	0.012151167	1
T2DM	b_cell	*SDCCAG8*	-1.846394094	0.005782021	0.009883514	1
T2DM	b_cell	*TBC1D22A*	-2.225614381	0.029561211	0.038060892	1
T2DM	b_cell	*PLEKHH1*	-2.730045123	0.000539268	0.00415349	1
T2DM	b_cell	*ATP9A*	-1.381158965	0.000654912	0.00415349	1
T2DM	b_cell	*GALC*	-3.603693337	0.019018909	0.025842205	1
T2DM	b_cell	*FAM114A2*	-2.969820068	0.002021705	0.005157371	1
T2DM	b_cell	*MRPL43*	-3.685158132	0.004177205	0.00787705	1
T2DM	b_cell	*ITIH4*	2.396217227	0.003433041	0.006885994	1
T2DM	b_cell	*TRAF1*	-4.49079585	0.007487931	0.011975737	1
T2DM	b_cell	*PCGF2*	-1.691742803	0.011818237	0.017267315	1
T2DM	b_cell	*IL17RB*	-5.456494484	0.000363204	0.00415349	1
T2DM	b_cell	*TRAF3IP2*	-2.363155854	0.001500766	0.004478079	1
T2DM	b_cell	*DCBLD2*	-1.862267133	0.001035146	0.00415349	1
T2DM	b_cell	*TMCC3*	-3.830165129	0.006075381	0.010240193	1
T2DM	b_cell	*PITHD1*	-2.694386737	0.001508251	0.004480341	1
T2DM	b_cell	*ATP11B*	-1.844838414	0.036902263	0.046438056	1
T2DM	b_cell	*LAMC2*	-1.540460168	0.029552054	0.038056895	1
T2DM	b_cell	*ZC3H11A*	-5.272275561	0.001306404	0.004274973	1
T2DM	b_cell	*RIOK2*	-3.782501173	0.013231037	0.019051081	1
T2DM	b_cell	*FLYWCH1*	-2.549381675	0.001162783	0.004195495	1
T2DM	b_cell	*DNAJC25*	-2.629482888	0.00107537	0.00415349	1
T2DM	b_cell	*YBX3*	-2.680536903	0.004678166	0.008562338	1
T2DM	b_cell	*OGFR*	-2.846783235	0.004665251	0.008548654	1
T2DM	b_cell	*PIGV*	2.374084755	0.025607131	0.033519057	1
T2DM	b_cell	*QSER1*	-2.567191581	0.00233441	0.005509725	1
T2DM	b_cell	*NCKAP1*	-1.632910346	0.001399408	0.004344868	1
T2DM	b_cell	*SFSWAP*	-2.508139186	0.003696584	0.00724956	1
T2DM	b_cell	*TNK2*	-2.809461644	0.031779416	0.040592684	1
T2DM	b_cell	*VMP1*	-2.274865245	0.003549335	0.007050448	1

T2DM	b_cell	*EIF4B*	-2.956108414	0.022027188	0.029408565	1
T2DM	b_cell	*SLC6A16*	-2.788624195	0.000676226	0.00415349	1
T2DM	b_cell	*ISOC2*	-6.098943102	0.005814914	0.009915476	1
T2DM	b_cell	*EPN1*	-3.87823696	0.026274523	0.034271354	1
T2DM	b_cell	*ADCK1*	-2.205988491	0.000994795	0.00415349	1
T2DM	b_cell	*HAGH*	-2.372669898	0.00083513	0.00415349	1
T2DM	b_cell	*CASP8*	-2.836585927	0.023702895	0.031369643	1
T2DM	b_cell	*DMRT3*	-3.78824253	0.001265548	0.004257394	1
T2DM	b_cell	*ST3GAL6*	-1.698973648	0.000288232	0.00415349	1
T2DM	b_cell	*ATP2C2*	-4.167179453	0.002170872	0.005323038	1
T2DM	b_cell	*CDON*	-1.29631533	0.000760911	0.00415349	1
T2DM	b_cell	*TAF2*	-2.442655287	0.028472723	0.036832924	1
T2DM	b_cell	*TMEM161A*	-2.873231705	0.001362996	0.004298348	1
T2DM	b_cell	*LPAR2*	-2.635730514	0.003028629	0.006342768	1
T2DM	b_cell	*ABCA7*	3.501944263	0.005451016	0.009490594	1
T2DM	b_cell	*DDX20*	-3.604981632	0.001207912	0.004235439	1
T2DM	b_cell	*BTBD1*	-2.649210406	0.006154166	0.010342486	1
T2DM	b_cell	*CHI3L2*	3.758746356	0.000569804	0.00415349	1
T2DM	b_cell	*PMS1*	-2.795981728	0.005580449	0.009641728	1
T2DM	b_cell	*TAF11*	-3.281789951	0.001462538	0.004424917	1
T2DM	b_cell	*ANKS1A*	-2.376742567	0.003230728	0.006613998	1
T2DM	b_cell	*UHRF1BP1*	-1.596795655	0.005365064	0.009387268	1
T2DM	b_cell	*IPO5*	-3.234921069	0.017888009	0.024587212	1
T2DM	b_cell	*WDR18*	-3.195481403	0.000151732	0.00415349	0.953941209
T2DM	b_cell	*MCM10*	1.340029832	0.000536589	0.00415349	1
T2DM	b_cell	*ZC3H15*	-3.212298057	0.006373444	0.010586483	1
T2DM	b_cell	*TMEM206*	-2.953101848	0.001303941	0.004274973	1
T2DM	b_cell	*SEC61A2*	-2.150404601	0.000889126	0.00415349	1
T2DM	b_cell	*ASB1*	-3.59392663	0.00052815	0.00415349	1
T2DM	b_cell	*PPP2R5A*	-2.099609452	0.002093683	0.005240042	1
T2DM	b_cell	*TIE1*	-1.885157245	0.001455721	0.004416626	1
T2DM	b_cell	*NFYC*	-2.961784898	0.013779049	0.019692858	1
T2DM	b_cell	*ZMYND12*	-2.212060983	0.031157302	0.039911564	1
T2DM	b_cell	*SLC9A3*	-3.27860902	0.023615237	0.031282975	1
T2DM	b_cell	*NGEF*	-10.39759385	0.017292079	0.023882974	1
T2DM	b_cell	*CD84*	-1.787192959	0.0029587	0.006264583	1
T2DM	b_cell	*ZNRD1*	-3.855274972	0.002875551	0.006165959	1
T2DM	b_cell	*ATXN3*	-1.910225376	0.014278862	0.020305634	1
T2DM	b_cell	*GOLGA5*	-2.200037842	0.00088787	0.00415349	1
T2DM	b_cell	*EML1*	-1.685196725	0.000516996	0.00415349	1
T2DM	b_cell	*ZFAT*	2.165447189	0.001689828	0.004704627	1
T2DM	b_cell	*MTFR1*	-3.121017984	0.002912824	0.006193075	1
T2DM	b_cell	*STAG3*	-4.369091274	0.023806783	0.031477023	1
T2DM	b_cell	*MYO9A*	-2.134637339	0.00455116	0.00839095	1
T2DM	b_cell	*STOML1*	3.382351063	0.001010011	0.00415349	1
T2DM	b_cell	*METTL22*	-4.146247478	0.016753931	0.023252089	1
T2DM	b_cell	*TP53BP1*	-2.487992581	0.01132362	0.016699882	1
T2DM	b_cell	*RRP15*	-1.826119588	0.006583053	0.01084014	1
T2DM	b_cell	*DHX8*	-2.546280448	0.001762446	0.004791201	1
T2DM	b_cell	*SYT1*	-2.452742656	0.000360627	0.00415349	1
T2DM	b_cell	*ROGDI*	3.778977459	0.00092501	0.00415349	1
T2DM	b_cell	*HDAC4*	-2.399610102	0.002983927	0.006295285	1
T2DM	b_cell	*RASSF1*	-3.079005349	0.023706292	0.031369643	1
T2DM	b_cell	*IFI35*	-2.296577802	0.010980516	0.016304795	1
T2DM	b_cell	*HEATR6*	-3.535050067	0.003267316	0.006662866	1
T2DM	b_cell	*POLR1A*	-2.660826519	0.000124011	0.00415349	0.779659076
T2DM	b_cell	*STON1-GTF2A1L*	-1.142081436	0.001485967	0.004462053	1
T2DM	b_cell	*SRBD1*	-2.703650054	0.016198246	0.022590589	1
T2DM	b_cell	*CYFIP1*	-2.360147262	0.002265043	0.005437315	1
T2DM	b_cell	*PSME4*	-2.595561417	0.015706535	0.02198775	1
T2DM	b_cell	*PITX1*	-7.165558835	0.000741905	0.00415349	1

T2DM	b_cell	*SDK2*	1.621458273	0.000623138	0.00415349	1
T2DM	b_cell	*NUCKS1*	-2.496921854	0.007673105	0.012209772	1
T2DM	b_cell	*DNAJA2*	-2.649121908	0.001094327	0.00415349	1
T2DM	b_cell	*KCNAB2*	-2.492443684	0.000523804	0.00415349	1
T2DM	b_cell	*GAL*	2.167610245	0.000669577	0.00415349	1
T2DM	b_cell	*NEDD4*	-1.86553069	0.003007773	0.006311705	1
T2DM	b_cell	*PIGB*	-3.228820499	0.036660254	0.04619824	1
T2DM	b_cell	*PFN2*	-3.425283654	0.000573776	0.00415349	1
T2DM	b_cell	*DAPP1*	3.074260299	0.00751724	0.012013444	1
T2DM	b_cell	*FGF10*	-1.441495766	0.003652474	0.007196562	1
T2DM	b_cell	*TMEM260*	-2.875449618	0.0073503	0.011794624	1
T2DM	b_cell	*CLTCL1*	-1.880027446	0.001836733	0.004898539	1
T2DM	b_cell	*DGCR2*	-2.966135098	0.002748956	0.006007705	1
T2DM	b_cell	*ZXDC*	-2.617388622	0.003483209	0.006956459	1
T2DM	b_cell	*WIPI1*	-2.141766371	0.001095351	0.00415349	1
T2DM	b_cell	*NDST1*	-2.795811508	0.002520702	0.005742541	1
T2DM	b_cell	*ASNS*	-3.975327475	0.00897753	0.013835751	1
T2DM	b_cell	*AP3M2*	-3.728964721	0.016367825	0.022801798	1
T2DM	b_cell	*ST6GALNAC2*	-2.68584872	0.00129586	0.004268027	1
T2DM	b_cell	*EIF2B3*	-1.747048527	0.001148981	0.004187618	1
T2DM	b_cell	*CAMK2A*	4.557039322	0.000829285	0.00415349	1
T2DM	b_cell	*CDC42*	-2.900678973	0.030190841	0.038800044	1
T2DM	b_cell	*OSBPL3*	-2.470308005	0.00124716	0.004253251	1
T2DM	b_cell	*RAD18*	-2.982058541	0.002574439	0.005806222	1
T2DM	b_cell	*SNX13*	-2.550780046	0.006050885	0.010210172	1
T2DM	b_cell	*VASH1*	-3.504445154	0.001785746	0.004835048	1
T2DM	b_cell	*TRIP13*	-12.67843471	0.003218644	0.006598061	1
T2DM	b_cell	*MBD3*	-4.539487558	0.000821791	0.00415349	1
T2DM	b_cell	*HLTF*	-3.109251255	0.000796574	0.00415349	1
T2DM	b_cell	*CPSF1*	-2.322519286	0.000897467	0.00415349	1
T2DM	b_cell	*CYBRD1*	-1.815934763	0.006211962	0.010403464	1
T2DM	b_cell	*EPN2*	-2.39969645	0.002311145	0.005492478	1
T2DM	b_cell	*LIMS2*	-1.614655213	0.000973881	0.00415349	1
T2DM	b_cell	*AFF4*	-2.478440846	0.006215872	0.010405595	1
T2DM	b_cell	*TRHDE*	-1.146611411	0.000770688	0.00415349	1
T2DM	b_cell	*TRNT1*	-2.440879166	0.001630391	0.004644435	1
T2DM	b_cell	*ACADVL*	-3.110623584	0.006891717	0.011227838	1
T2DM	b_cell	*CRMP1*	-1.966584968	0.000489627	0.00415349	1
T2DM	b_cell	*AP1M1*	-2.83570507	0.023412211	0.031033643	1
T2DM	b_cell	*PVR*	-2.950051717	0.002909259	0.006192483	1
T2DM	b_cell	*XRCC1*	-4.591490419	0.026998987	0.035121587	1
T2DM	b_cell	*MOV10L1*	-2.899217545	0.000720181	0.00415349	1
T2DM	b_cell	*TP63*	-1.782083486	0.001034028	0.00415349	1
T2DM	b_cell	*ALPK1*	-2.550521985	0.007153937	0.01153843	1
T2DM	b_cell	*LLGL2*	-2.700146086	0.009522326	0.014506146	1
T2DM	b_cell	*PDE8A*	-1.868320312	0.004486785	0.008299035	1
T2DM	b_cell	*NLE1*	2.961892927	0.000398595	0.00415349	1
T2DM	b_cell	*SMARCE1*	-2.325413101	0.001903949	0.004987601	1
T2DM	b_cell	*GSDMB*	-1.599790725	0.000749424	0.00415349	1
T2DM	b_cell	*PICALM*	-2.02164284	0.004604411	0.008469259	1
T2DM	b_cell	*GLI2*	-1.815633987	0.001125342	0.004182741	1
T2DM	b_cell	*CLNS1A*	-1.771723279	0.034247022	0.043400731	1
T2DM	b_cell	*ATP2A3*	3.499140209	0.006575487	0.010833356	1
T2DM	b_cell	*CA12*	-2.254236633	0.000921372	0.00415349	1
T2DM	b_cell	*NTN4*	-1.467246206	0.000779032	0.00415349	1
T2DM	b_cell	*LMAN1*	-3.090945676	0.010574688	0.015806719	1
T2DM	b_cell	*ENO1*	-3.357731407	0.003375013	0.006805229	1
T2DM	b_cell	*SRI*	-2.669245887	0.001536115	0.004508014	1
T2DM	b_cell	*EIF4G3*	-2.295832861	0.00982756	0.014909718	1
T2DM	b_cell	*TTC38*	-1.685140649	0.000727429	0.00415349	1
T2DM	b_cell	*GRAMD4*	-3.568624215	0.028143574	0.036437119	1

T2DM	b_cell	*CELSR1*	-5.307758293	0.011806534	0.017254226	1
T2DM	b_cell	*ADD2*	6.417120915	0.004164608	0.007865091	1
T2DM	b_cell	*VPS9D1*	-4.182321698	0.00778116	0.012350455	1
T2DM	b_cell	*MARK3*	-2.643960891	0.000819829	0.00415349	1
T2DM	b_cell	*SLC25A3*	-2.814945611	0.024941912	0.032709596	1
T2DM	b_cell	*FSCN1*	-3.039245636	0.000947764	0.00415349	1
T2DM	b_cell	*MOCOS*	2.385659013	0.000956093	0.00415349	1
T2DM	b_cell	*PLD1*	-2.029662855	0.001709943	0.004728644	1
T2DM	b_cell	*BCAP29*	-2.636940291	0.004994722	0.008941292	1
T2DM	b_cell	*TUBA3D*	-8.888597431	0.001195449	0.004217141	1
T2DM	b_cell	*MKRN2*	-2.319769855	0.000545435	0.00415349	1
T2DM	b_cell	*MCM6*	4.041597342	0.001011184	0.00415349	1
T2DM	b_cell	*RBMS2*	-1.719347166	0.001036544	0.00415349	1
T2DM	b_cell	*MLH1*	-2.693600859	0.001913564	0.004996087	1
T2DM	b_cell	*FMO4*	-2.564372869	0.001288659	0.004268027	1
T2DM	b_cell	*RGS11*	-1.976755087	0.000682361	0.00415349	1
T2DM	b_cell	*TPD52*	1.020310663	0.000547649	0.00415349	1
T2DM	b_cell	*GPATCH1*	-2.310300961	0.00222303	0.005396212	1
T2DM	b_cell	*ICAM3*	3.816748555	0.00056781	0.00415349	1
T2DM	b_cell	*NT5C2*	-2.222842859	0.028873959	0.037275249	1
T2DM	b_cell	*CAMSAP3*	-17.56000254	0.000705411	0.00415349	1
T2DM	b_cell	*XAB2*	-3.491634615	0.000119179	0.00415349	0.749280959
T2DM	b_cell	*STXBP2*	-2.50908899	0.024524711	0.032263414	1
T2DM	b_cell	*MAP2K7*	-2.524789306	0.001087787	0.00415349	1
T2DM	b_cell	*CTTNBP2*	-2.560505123	0.000147947	0.00415349	0.930144415
T2DM	b_cell	*TM9SF3*	-2.643319646	0.006557527	0.010809431	1
T2DM	b_cell	*DYNC1I2*	-2.652262492	0.019643439	0.026593089	1
T2DM	b_cell	*APBB1IP*	-1.826103006	0.002265004	0.005437315	1
T2DM	b_cell	*LRCH4*	-8.729537457	2.15E-05	0.002553516	0.135336327
T2DM	b_cell	*CAPZB*	-2.500969339	0.004038786	0.007696831	1
T2DM	b_cell	*GPR137B*	-2.244697737	0.001627065	0.004639164	1
T2DM	b_cell	*NAALAD2*	-1.903749743	0.006836064	0.011163204	1
T2DM	b_cell	*FGFR1*	-1.892910833	0.000988058	0.00415349	1
T2DM	b_cell	*FBLN1*	-1.13247148	0.001505108	0.004478191	1
T2DM	b_cell	*PIAS2*	-2.308841913	0.001721244	0.004747873	1
T2DM	b_cell	*AMPH*	-1.525412752	0.003007023	0.006311705	1
T2DM	b_cell	*MCCC1*	-1.949228478	0.003700068	0.00724956	1
T2DM	b_cell	*FAP*	-1.580855575	0.001322819	0.004274973	1
T2DM	b_cell	*ACER3*	-1.68682931	0.000470368	0.00415349	1
T2DM	b_cell	*PIK3C3*	-2.821647838	0.015568516	0.02182856	1
T2DM	b_cell	*N4BP2*	-3.157735712	0.010950873	0.016268464	1
T2DM	b_cell	*TULP3*	-2.314024444	0.001808392	0.004858702	1
T2DM	b_cell	*SYNJ2*	-1.865725934	0.000875011	0.00415349	1
T2DM	b_cell	*ZCWPW1*	-2.484752723	0.004752984	0.00865764	1
T2DM	b_cell	*FGF20*	-23.86789365	0.00073649	0.00415349	1
T2DM	b_cell	*PCM1*	-2.36615375	0.009431719	0.014389036	1
T2DM	b_cell	*MYH7B*	-3.792308087	0.014193958	0.020216904	1
T2DM	b_cell	*FKBP7*	-1.943734748	0.004825135	0.008742254	1
T2DM	b_cell	*OSBPL6*	-2.885306677	0.001837875	0.004898539	1
T2DM	b_cell	*XRCC5*	-2.592685065	0.014784276	0.02090615	1
T2DM	b_cell	*LXN*	-2.47524569	0.001498463	0.004475552	1
T2DM	b_cell	*REXO1*	-4.004429831	0.004419444	0.008201016	1
T2DM	b_cell	*SAR1A*	-2.803594211	0.003848469	0.00743328	1
T2DM	b_cell	*RAPGEF3*	-1.101073567	0.031232294	0.039999477	1
T2DM	b_cell	*FDFT1*	-2.733899273	0.002850746	0.00613369	1
T2DM	b_cell	*SCGN*	-12.04419182	0.004044766	0.007705893	1
T2DM	b_cell	*DDX1*	-2.720068722	0.002797384	0.006066627	1
T2DM	b_cell	*DNM2*	-2.860401328	0.01205136	0.017563027	1
T2DM	b_cell	*RABL2B*	-2.023346441	0.000838038	0.00415349	1
T2DM	b_cell	*DDX43*	-3.323762909	0.000164913	0.00415349	1
T2DM	b_cell	*DCT*	-3.985777737	0.000853124	0.00415349	1

T2DM	b_cell	*SLC35C2*	-2.724242452	0.001561909	0.004541962	1
T2DM	b_cell	*SCTR*	-1.926483723	0.000836334	0.00415349	1
T2DM	b_cell	*RIF1*	-2.575204541	0.003180732	0.006549103	1
T2DM	b_cell	*KCNN2*	-5.77417447	0.000274179	0.00415349	1
T2DM	b_cell	*PSEN1*	-2.656777566	0.008386388	0.013079936	1
T2DM	b_cell	*CPOX*	-2.817023804	0.000781846	0.00415349	1
T2DM	b_cell	*MOK*	-2.367866918	0.001144846	0.00418468	1
T2DM	b_cell	*RSBN1*	-3.241801636	0.003762111	0.007320455	1
T2DM	b_cell	*MAGI3*	-2.144299985	0.002141696	0.005287258	1
T2DM	b_cell	*COL4A4*	0.822508531	0.000811902	0.00415349	1
T2DM	b_cell	*PKP1*	-3.784700613	0.011215438	0.016575331	1
T2DM	b_cell	*ZNF506*	-3.088900647	0.000836359	0.00415349	1
T2DM	b_cell	*KIAA0141*	-2.525152266	0.000999544	0.00415349	1
T2DM	b_cell	*PCDHA6*	-40.96342839	0.000320069	0.00415349	1
T2DM	b_cell	*PCDHGA2*	4.285410689	0.008246815	0.012910291	1
T2DM	b_cell	*HSPB11*	-2.355051387	0.001908151	0.004988168	1
T2DM	b_cell	*WDR70*	-2.192059278	0.003801907	0.007368246	1
T2DM	b_cell	*PGR*	-1.849642434	0.00037183	0.00415349	1
T2DM	b_cell	*COBLL1*	-1.446402746	3.16E-05	0.003200692	0.198442887
T2DM	b_cell	*GEMIN5*	-3.222810791	0.010695232	0.015967923	1
T2DM	b_cell	*GSK3B*	-2.224421813	0.006230697	0.010416354	1
T2DM	b_cell	*ERC1*	-2.193010429	0.016853774	0.023364741	1
T2DM	b_cell	*LYRM2*	-2.096328934	0.003011497	0.006317412	1
T2DM	b_cell	*ULK2*	-2.084443082	0.001377874	0.004323793	1
T2DM	b_cell	*PLOD1*	-3.083633601	0.001505664	0.004478191	1
T2DM	b_cell	*PIBF1*	-2.39503615	0.005789816	0.00989146	1
T2DM	b_cell	*TDRD3*	-2.472593635	0.018938116	0.025749121	1
T2DM	b_cell	*PDS5B*	-2.566416795	0.021044678	0.0282951	1
T2DM	b_cell	*OXCT1*	-2.132761399	0.002428501	0.005628918	1
T2DM	b_cell	*ZNF324*	-2.787206076	0.000879212	0.00415349	1
T2DM	b_cell	*ZNF586*	-2.372895594	0.014544902	0.020623319	1
T2DM	b_cell	*ZNF446*	5.52041346	0.039855283	0.049765673	1
T2DM	b_cell	*CHMP2B*	-2.880881751	0.001231482	0.004253251	1
T2DM	b_cell	*PPIE*	-3.359071099	0.002749192	0.006007705	1
T2DM	b_cell	*ZMPSTE24*	-2.395432027	0.001123239	0.004182741	1
T2DM	b_cell	*NOA1*	2.117757224	0.001921176	0.005007644	1
T2DM	b_cell	*GSTP1*	-2.350578203	0.001455717	0.004416626	1
T2DM	b_cell	*WBP11*	-3.197491258	0.002358334	0.005540753	1
T2DM	b_cell	*COL16A1*	-1.863269003	0.00026702	0.00415349	1
T2DM	b_cell	*APOB*	-3.186748825	0.000513309	0.00415349	1
T2DM	b_cell	*NCOA1*	-2.199654566	0.010783176	0.016064888	1
T2DM	b_cell	*CD59*	-2.776110975	0.014465345	0.020538307	1
T2DM	b_cell	*TAF9*	-5.232103916	0.017847394	0.024536804	1
T2DM	b_cell	*FCN1*	-2.36281841	0.000439142	0.00415349	1
T2DM	b_cell	*SCAMP1*	-2.763894925	0.018350729	0.025102488	1
T2DM	b_cell	*OVGP1*	3.060131608	0.021918027	0.02929131	1
T2DM	b_cell	*ABCB1*	-1.778598993	0.001726815	0.004752113	1
T2DM	b_cell	*ZNF213*	-1.914111284	0.002274933	0.005445221	1
T2DM	b_cell	*CPNE3*	-1.821880473	0.014090839	0.020083678	1
T2DM	b_cell	*RRN3*	-3.064266946	0.002814288	0.006087269	1
T2DM	b_cell	*CTTN*	-1.64355228	0.00091826	0.00415349	1
T2DM	b_cell	*EPS15*	-1.792388197	0.016612443	0.0230812	1
T2DM	b_cell	*USP40*	-2.253994111	0.002276225	0.005445221	1
T2DM	b_cell	*MAST2*	-2.732574525	0.002560361	0.005798627	1
T2DM	b_cell	*B4GALT1*	-2.808983655	0.017619707	0.024271494	1
T2DM	b_cell	*EIF2AK1*	-2.543430506	0.006191893	0.010383684	1
T2DM	b_cell	*SNX10*	-1.947534493	0.000487552	0.00415349	1
T2DM	b_cell	*SEPHS1*	-2.378754269	0.001684719	0.004704627	1
T2DM	b_cell	*ITPKC*	-1.764065779	0.000907704	0.00415349	1
T2DM	b_cell	*CEACAM6*	19.19164661	0.004132034	0.007836531	1
T2DM	b_cell	*TMED2*	-3.750220723	0.010987715	0.016307783	1

T2DM	b_cell	*ACOX3*	-2.953573252	0.019864859	0.026852369	1
T2DM	b_cell	*HSD17B14*	-3.40985085	0.000211722	0.00415349	1
T2DM	b_cell	*TRIP6*	-3.625112552	0.001177491	0.004202203	1
T2DM	b_cell	*ACHE*	-3.326927427	0.007381339	0.01183235	1
T2DM	b_cell	*SRRT*	-3.186275394	0.00226736	0.005440798	1
T2DM	b_cell	*PIGS*	-2.091528376	0.005422023	0.009463702	1
T2DM	b_cell	*ATXN7L3*	2.823194272	0.000721295	0.00415349	1
T2DM	b_cell	*PGS1*	-3.494663639	0.00391995	0.007541225	1
T2DM	b_cell	*PSMC5*	-2.304313328	0.00044889	0.00415349	1
T2DM	b_cell	*LPCAT2*	-2.20451751	0.000841343	0.00415349	1
T2DM	b_cell	*OGFOD1*	-3.87595762	0.00128836	0.004268027	1
T2DM	b_cell	*TXNDC16*	-2.930519989	0.030572225	0.039234043	1
T2DM	b_cell	*GMCL1*	-2.979930331	0.008835664	0.013662031	1
T2DM	b_cell	*PTHLH*	-3.026742399	0.001237728	0.004253251	1
T2DM	b_cell	*PHACTR3*	-1.776973108	0.00324785	0.006636086	1
T2DM	b_cell	*ERGIC2*	-2.911358797	0.002664602	0.005916635	1
T2DM	b_cell	*AAMDC*	-1.979453837	0.000203484	0.00415349	1
T2DM	b_cell	*METTL2A*	-4.339864195	0.001347304	0.004288861	1
T2DM	b_cell	*DDX18*	-2.246363881	0.002894895	0.006192483	1
T2DM	b_cell	*ASAP3*	-2.092504327	0.000837651	0.00415349	1
T2DM	b_cell	*EDEM2*	-4.470580018	0.001476018	0.004446444	1
T2DM	b_cell	*DOCK3*	-1.637675367	0.000630441	0.00415349	1
T2DM	b_cell	*C3orf18*	2.945655293	2.52E-05	0.002754251	0.158145663
T2DM	b_cell	*FKBP1A*	-4.311739379	0.009638394	0.014665194	1
T2DM	b_cell	*NSFL1C*	-3.53001098	0.016388517	0.022820511	1
T2DM	b_cell	*ZNF343*	-2.656815786	0.002445342	0.005640408	1
T2DM	b_cell	*CPXM1*	-2.673899587	0.000736746	0.00415349	1
T2DM	b_cell	*MAVS*	-2.6313504	0.004690865	0.008580584	1
T2DM	b_cell	*LZTS3*	-8.82034779	0.001671925	0.004700981	1
T2DM	b_cell	*XRN2*	-2.685864174	0.004653459	0.008534509	1
T2DM	b_cell	*TESC*	-3.018078231	0.000794543	0.00415349	1
T2DM	b_cell	*ANAPC5*	-2.809220353	0.022651298	0.030184127	1
T2DM	b_cell	*SLC23A2*	-2.92207721	0.006323609	0.010529227	1
T2DM	b_cell	*TMEM230*	-2.620971639	0.035473089	0.044818993	1
T2DM	b_cell	*DZANK1*	-3.008270784	0.00087049	0.00415349	1
T2DM	b_cell	*TASP1*	-2.418990235	0.002276047	0.005445221	1
T2DM	b_cell	*KIF16B*	-2.307985937	0.001743921	0.004783999	1
T2DM	b_cell	*FUS*	-2.451511404	0.007850695	0.012432574	1
T2DM	b_cell	*FXYD5*	-3.829498498	0.006054049	0.010212451	1
T2DM	b_cell	*ZNF302*	-2.555866246	0.001605059	0.00460987	1
T2DM	b_cell	*FXYD3*	-10.48123361	0.003125631	0.006466219	1
T2DM	b_cell	*GANAB*	-2.994980449	0.003206122	0.006581388	1
T2DM	b_cell	*GMIP*	-4.329441426	0.001693335	0.004705788	1
T2DM	b_cell	*ANKRD24*	-2.89810452	0.0023322	0.005509725	1
T2DM	b_cell	*GPATCH2L*	-2.403383734	0.005439243	0.009478105	1
T2DM	b_cell	*SLC9A1*	-2.005013072	0.000961402	0.00415349	1
T2DM	b_cell	*SPTLC1*	-2.934642416	0.003552879	0.007055259	1
T2DM	b_cell	*PAPOLA*	-2.527949654	0.037362434	0.046951354	1
T2DM	b_cell	*RGS1*	-3.279919591	0.000785618	0.00415349	1
T2DM	b_cell	*MUL1*	-4.141756851	0.000572285	0.00415349	1
T2DM	b_cell	*SPG21*	-3.571581874	0.032706048	0.041691591	1
T2DM	b_cell	*GNPTG*	-3.196561887	0.003023275	0.006333666	1
T2DM	b_cell	*ZNF268*	-2.95951786	0.00048593	0.00415349	1
T2DM	b_cell	*PABPC4*	-5.954913462	0.000886336	0.00415349	1
T2DM	b_cell	*CERS4*	-3.656888064	0.01483073	0.020957698	1
T2DM	b_cell	*USP48*	-2.657333502	0.004158858	0.007865091	1
T2DM	b_cell	*EXOC1*	-2.445399259	0.004054729	0.007722533	1
T2DM	b_cell	*NLRC4*	-4.485171803	0.007199755	0.011606375	1
T2DM	b_cell	*LAMB1*	-1.658092564	0.000379509	0.00415349	1
T2DM	b_cell	*SLC26A4*	-4.463649678	0.000496259	0.00415349	1
T2DM	b_cell	*DLD*	-2.652119617	0.0028379	0.006114421	1

T2DM	b_cell	*TXNL1*	-2.770182685	0.005432211	0.009475664	1
T2DM	b_cell	*IL5RA*	-5.836255743	0.00064688	0.00415349	1
T2DM	b_cell	*ITGA6*	-1.537133664	0.00065836	0.00415349	1
T2DM	b_cell	*RAPGEF4*	-1.36463148	0.00085731	0.00415349	1
T2DM	b_cell	*SEL1L3*	0.741786954	0.000587248	0.00415349	1
T2DM	b_cell	*TF*	-2.527424472	0.000792931	0.00415349	1
T2DM	b_cell	*MYO15A*	-2.975227988	0.00341914	0.006872165	1
T2DM	b_cell	*ALKBH5*	-3.580572913	0.000115285	0.00415349	0.724797561
T2DM	b_cell	*NLRP1*	-2.55300229	0.004175448	0.00787705	1
T2DM	b_cell	*SPAG7*	-2.718123081	0.008251624	0.012911389	1
T2DM	b_cell	*ZFHX4*	-1.290898879	0.000712636	0.00415349	1
T2DM	b_cell	*RGS17*	-3.165775833	0.000507757	0.00415349	1
T2DM	b_cell	*TMEM101*	-3.083233416	0.00059441	0.00415349	1
T2DM	b_cell	*CMA1*	-7.734924999	0.003344625	0.006768457	1
T2DM	b_cell	*PPP2R3C*	-2.557419076	0.0083468	0.013034961	1
T2DM	b_cell	*OSGEP*	-3.575151378	0.023629668	0.031295496	1
T2DM	b_cell	*SLC22A17*	-4.187575298	0.000211767	0.00415349	1
T2DM	b_cell	*SCFD1*	-2.082583587	0.014865234	0.020997018	1
T2DM	b_cell	*RPGRIP1*	-3.000056861	0.006358275	0.010566872	1
T2DM	b_cell	*TOX4*	-3.105534597	0.005737082	0.009822722	1
T2DM	b_cell	*RFFL*	-3.493663368	0.001264471	0.004257394	1
T2DM	b_cell	*MFSD11*	-2.461600023	0.007993276	0.012601236	1
T2DM	b_cell	*DPYSL2*	-1.891802993	0.001345973	0.004286796	1
T2DM	b_cell	*COMT*	-2.859224403	0.002820106	0.00609273	1
T2DM	b_cell	*VNN3*	2.155433581	0.000456095	0.00415349	1
T2DM	b_cell	*SEC22C*	-3.129650106	0.026834312	0.034943521	1
T2DM	b_cell	*FMO2*	-1.338245281	0.002059848	0.005205091	1
T2DM	b_cell	*PDE6C*	4.245244076	0.004253757	0.007980713	1
T2DM	b_cell	*CWF19L1*	-4.403292261	0.000320068	0.00415349	1
T2DM	b_cell	*IKZF5*	-3.181055364	0.003066428	0.006387091	1
T2DM	b_cell	*BLNK*	2.822264498	0.004956587	0.008893283	1
T2DM	b_cell	*WAC*	-2.464293489	0.005717965	0.009800666	1
T2DM	b_cell	*CREM*	-2.3849626	0.002260803	0.005435438	1
T2DM	b_cell	*TPSD1*	-17.29627764	0.002663718	0.005916635	1
T2DM	b_cell	*ITPR3*	-1.79387546	0.000807996	0.00415349	1
T2DM	b_cell	*IFT74*	-2.258174909	0.003990601	0.007635091	1
T2DM	b_cell	*IL12RB1*	4.34811806	0.000628177	0.00415349	1
T2DM	b_cell	*ACOT7*	-3.049031406	0.000533232	0.00415349	1
T2DM	b_cell	*CDC7*	-3.254095299	0.008882677	0.013721226	1
T2DM	b_cell	*PCSK5*	-1.299541134	0.000935792	0.00415349	1
T2DM	b_cell	*RAB18*	-2.079692789	0.00772619	0.01228181	1
T2DM	b_cell	*NRP1*	-1.890492816	0.002845038	0.006127699	1
T2DM	b_cell	*PRTFDC1*	-2.020372622	0.00073579	0.00415349	1
T2DM	b_cell	*PALMD*	-1.08316833	0.000998089	0.00415349	1
T2DM	b_cell	*TSPAN15*	-1.528080626	0.000732688	0.00415349	1
T2DM	b_cell	*CATSPERG*	-3.319787403	0.035851209	0.045242182	1
T2DM	b_cell	*STX1B*	-4.890370015	0.000115939	0.00415349	0.728905463
T2DM	b_cell	*HSD3B7*	-2.443497043	0.00160046	0.004605077	1
T2DM	b_cell	*MARCH2*	-2.410422548	0.005093773	0.009038822	1
T2DM	b_cell	*CDC34*	-2.46380286	0.000665842	0.00415349	1
T2DM	b_cell	*MTAP*	-3.291157584	0.001884376	0.004965245	1
T2DM	b_cell	*POLR2E*	-1.965749834	0.000615794	0.00415349	1
T2DM	b_cell	*MADCAM1*	-4.569969387	0.006722513	0.011026465	1
T2DM	b_cell	*TRMT2A*	-3.084138788	0.000829007	0.00415349	1
T2DM	b_cell	*ZDHHC8*	-2.198621692	0.001461094	0.0044241	1
T2DM	b_cell	*MED15*	-2.468291204	0.002900221	0.006192483	1
T2DM	b_cell	*LZTR1*	-3.40738675	0.002117312	0.005257323	1
T2DM	b_cell	*CECR2*	-1.780722316	0.000644988	0.00415349	1
T2DM	b_cell	*SMARCB1*	-2.792791533	0.012776153	0.018479173	1
T2DM	b_cell	*P2RX6*	-3.352308958	0.000545863	0.00415349	1
T2DM	b_cell	*SLC7A4*	-6.931451597	0.018173526	0.024892584	1

T2DM	b_cell	*DDT*	-5.208945248	0.020756624	0.027955633	1
T2DM	b_cell	*SUSD2*	-1.971267861	0.039014613	0.048783785	1
T2DM	b_cell	*GGT5*	-2.287361563	0.0011173	0.004182741	1
T2DM	b_cell	*SPECC1L*	-3.452793186	0.019131241	0.025972385	1
T2DM	b_cell	*YPEL1*	-3.841970089	0.004946484	0.008877689	1
T2DM	b_cell	*PRODH*	-23.01017197	0.006324216	0.010529227	1
T2DM	b_cell	*TOP3B*	-2.483422138	0.000943123	0.00415349	1
T2DM	b_cell	*CRYBB3*	-6.530549943	0.002514388	0.005735834	1
T2DM	b_cell	*CYTH4*	-2.897381551	0.00159912	0.004603327	1
T2DM	b_cell	*CARD10*	-2.972883806	0.00190003	0.004985596	1
T2DM	b_cell	*LRP5L*	-4.805292787	0.002187508	0.005349464	1
T2DM	b_cell	*SLC25A1*	-2.452801195	0.001163087	0.004195495	1
T2DM	b_cell	*LGALS2*	3.547738772	0.001773594	0.004810434	1
T2DM	b_cell	*GGA1*	-2.394884114	0.000374741	0.00415349	1
T2DM	b_cell	*SH3BP1*	-3.047354232	0.000711855	0.00415349	1
T2DM	b_cell	*HPS4*	-2.801639615	0.002549342	0.00579036	1
T2DM	b_cell	*PIK3IP1*	-3.548578573	0.017209644	0.023784794	1
T2DM	b_cell	*TRIOBP*	-2.271247697	0.007008268	0.011379386	1
T2DM	b_cell	*GCAT*	-3.75076969	0.001787418	0.004835411	1
T2DM	b_cell	*EIF3L*	-3.898586865	0.038613878	0.048304545	1
T2DM	b_cell	*CCDC134*	5.268238947	0.006168537	0.010360188	1
T2DM	b_cell	*PICK1*	-2.954430652	0.002687727	0.005933194	1
T2DM	b_cell	*SLC16A8*	-5.679312412	0.00063548	0.00415349	1
T2DM	b_cell	*KDELR3*	-2.354010398	0.002102627	0.005241681	1
T2DM	b_cell	*CYP2D6*	2.788404475	0.021277433	0.028565283	1
T2DM	b_cell	*DDX17*	-2.023460723	0.008271416	0.012939138	1
T2DM	b_cell	*FBXO7*	-2.984027694	0.021301925	0.028592059	1
T2DM	b_cell	*POLDIP3*	-2.362470292	0.000956474	0.00415349	1
T2DM	b_cell	*TIMP3*	-1.509317601	0.001358767	0.004298348	1
T2DM	b_cell	*PPP6R2*	-2.941186835	0.002373455	0.00555544	1
T2DM	b_cell	*CYB5R3*	-2.627492333	0.001909899	0.004990664	1
T2DM	b_cell	*RHBDD3*	-2.175941225	0.000988139	0.00415349	1
T2DM	b_cell	*TTLL1*	-4.789640176	0.002988448	0.006296833	1
T2DM	b_cell	*RASL10A*	-3.587222137	0.002568986	0.005799671	1
T2DM	b_cell	*HMGXB4*	-3.167232583	0.005558292	0.009618899	1
T2DM	b_cell	*TOM1*	-1.622338116	0.003003042	0.006308941	1
T2DM	b_cell	*NEFH*	4.285176179	0.001218004	0.004251853	1
T2DM	b_cell	*BIK*	-11.52453267	0.001285851	0.004268027	1
T2DM	b_cell	*MCAT*	-3.63204921	0.000585571	0.00415349	1
T2DM	b_cell	*ARSA*	-1.246022982	0.032131844	0.040992878	1
T2DM	b_cell	*TSPO*	-3.252967519	0.011050494	0.016370042	1
T2DM	b_cell	*TTLL12*	-3.156250838	0.000682159	0.00415349	1
T2DM	b_cell	*RBFOX2*	-1.77063323	0.001475248	0.004446254	1
T2DM	b_cell	*MTMR3*	-2.20014695	0.002666042	0.005916635	1
T2DM	b_cell	*APOL4*	-3.000467888	0.00691238	0.011255668	1
T2DM	b_cell	*SAMM50*	-1.893101802	0.0008909	0.00415349	1
T2DM	b_cell	*IFT27*	-2.620044121	0.000980687	0.00415349	1
T2DM	b_cell	*KIAA0930*	-2.695832794	0.006232913	0.010416354	1
T2DM	b_cell	*NCF4*	-2.145222266	0.002219868	0.005393569	1
T2DM	b_cell	*KCTD17*	-7.586352817	0.001163154	0.004195495	1
T2DM	b_cell	*EP300*	-2.552092304	0.01860552	0.025386012	1
T2DM	b_cell	*ACO2*	-2.416515475	0.004135671	0.007838698	1
T2DM	b_cell	*POLR3H*	-2.713253322	0.000944161	0.00415349	1
T2DM	b_cell	*ZBED4*	-3.320553868	0.003001088	0.006308941	1
T2DM	b_cell	*HDAC10*	-4.144000442	0.001532328	0.004505963	1
T2DM	b_cell	*SDR39U1*	-3.504155294	0.001811104	0.004861831	1
T2DM	b_cell	*RBM23*	-2.842852928	0.002607602	0.005849897	1
T2DM	b_cell	*VCPKMT*	-2.935033291	0.005522565	0.009579216	1
T2DM	b_cell	*SOS2*	-2.46830877	0.015508528	0.021774108	1
T2DM	b_cell	*PYGL*	-1.083262673	0.00284829	0.006132603	1
T2DM	b_cell	*CNIH1*	-3.477658762	0.00262015	0.005854674	1

T2DM	b_cell	*ATP6V1D*	-2.086422347	0.000110228	0.00415349	0.693002723
T2DM	b_cell	*GSTZ1*	-2.519983972	0.001094962	0.00415349	1
T2DM	b_cell	*TMED8*	-4.316405287	0.009728285	0.014780504	1
T2DM	b_cell	*DAAM1*	-1.617342317	0.001281207	0.004266508	1
T2DM	b_cell	*ISM2*	8.070929796	3.06E-05	0.003163865	0.192425653
T2DM	b_cell	*PPM1A*	-2.316559774	0.016550838	0.023010862	1
T2DM	b_cell	*SLC10A1*	-2.104883543	0.000718933	0.00415349	1
T2DM	b_cell	*DICER1*	-2.319166011	0.004676331	0.008561472	1
T2DM	b_cell	*ZFYVE21*	-3.439576718	0.014961165	0.021123029	1
T2DM	b_cell	*MTHFD1*	-1.574350559	0.000756741	0.00415349	1
T2DM	b_cell	*VRK1*	-3.507565145	0.00712769	0.011510862	1
T2DM	b_cell	*CCNB1IP1*	-2.988382345	0.001959249	0.005073227	1
T2DM	b_cell	*TRIP11*	-2.343986434	0.002408778	0.00560266	1
T2DM	b_cell	*PCK2*	-4.260609334	0.002203446	0.005373572	1
T2DM	b_cell	*KIAA0391*	7.363580634	0.014979237	0.02113871	1
T2DM	b_cell	*PLTP*	-1.326846786	0.018610924	0.025386609	1
T2DM	b_cell	*GSS*	4.205003358	0.027524716	0.035724172	1
T2DM	b_cell	*PYGB*	-2.065057043	0.000938136	0.00415349	1
T2DM	b_cell	*ABHD12*	-2.316014417	0.005692338	0.009767394	1
T2DM	b_cell	*PROCR*	-1.953334453	0.000284532	0.00415349	1
T2DM	b_cell	*NINL*	-1.867965228	0.002293725	0.005477692	1
T2DM	b_cell	*CD40*	4.197494407	0.016516584	0.022973399	1
T2DM	b_cell	*UQCC1*	-3.186081643	0.001151038	0.004188961	1
T2DM	b_cell	*IFT52*	-2.666803059	0.010509003	0.015721688	1
T2DM	b_cell	*DOK5*	-2.27781799	0.000984231	0.00415349	1
T2DM	b_cell	*CSTF1*	-4.141089299	0.002834337	0.006110932	1
T2DM	b_cell	*BMP7*	-2.89032556	0.00080467	0.00415349	1
T2DM	b_cell	*TPD52L2*	-2.24883263	0.001095539	0.00415349	1
T2DM	b_cell	*NELFCD*	-3.246371479	0.003314657	0.006731024	1
T2DM	b_cell	*CTSZ*	-3.333973768	0.00824312	0.01290772	1
T2DM	b_cell	*PRPF6*	-2.703742468	0.001394068	0.00434199	1
T2DM	b_cell	*MTG2*	-2.755487199	0.003067063	0.006387091	1
T2DM	b_cell	*TCFL5*	-3.756750324	0.036791979	0.046336372	1
T2DM	b_cell	*DIDO1*	-2.280779227	0.018005695	0.024711156	1
T2DM	b_cell	*SLC17A9*	7.365269293	0.009561755	0.014558413	1
T2DM	b_cell	*BIRC7*	2.048612739	0.006051074	0.010210172	1
T2DM	b_cell	*NKAIN4*	-7.735347706	0.000252422	0.00415349	1
T2DM	b_cell	*PTK6*	1.435285451	0.000607601	0.00415349	1
T2DM	b_cell	*GMEB2*	-4.603109931	0.009440742	0.014399307	1
T2DM	b_cell	*RNF24*	-2.889239231	0.002255765	0.005429181	1
T2DM	b_cell	*NDUFAF5*	-1.169470137	0.031718193	0.040539191	1
T2DM	b_cell	*TRIB3*	-4.149476652	0.000429021	0.00415349	1
T2DM	b_cell	*SLC52A3*	-6.168425173	0.020531043	0.027681464	1
T2DM	b_cell	*ANGPT4*	-3.439620738	0.0005927	0.00415349	1
T2DM	b_cell	*RSPO4*	3.483520492	0.001684971	0.004704627	1
T2DM	b_cell	*SIRPB1*	1.173668143	0.000556243	0.00415349	1
T2DM	b_cell	*SEC23B*	-2.293469655	0.002312869	0.005492478	1
T2DM	b_cell	*CCM2L*	-2.710879946	0.000869925	0.00415349	1
T2DM	b_cell	*MYL9*	-2.535144337	0.006004335	0.010153108	1
T2DM	b_cell	*MROH8*	-4.068050022	0.002366332	0.005545309	1
T2DM	b_cell	*TTI1*	-3.820767227	0.016252711	0.022661521	1
T2DM	b_cell	*RPRD1B*	-2.458179996	0.001196749	0.004217466	1
T2DM	b_cell	*PXMP4*	-4.793144551	0.000618686	0.00415349	1
T2DM	b_cell	*ACTR5*	-2.862744847	0.000705848	0.00415349	1
T2DM	b_cell	*WFDC2*	6.238655467	0.001244622	0.004253251	1
T2DM	b_cell	*DHX35*	-3.498299306	0.00370593	0.007256052	1
T2DM	b_cell	*DNTTIP1*	-3.290409598	0.00401921	0.007671152	1
T2DM	b_cell	*PIGU*	-2.972429425	0.023276823	0.030891682	1
T2DM	b_cell	*APMAP*	-1.822801302	0.001042821	0.00415349	1
T2DM	b_cell	*CELF4*	-2.586804958	0.000646694	0.00415349	1
T2DM	b_cell	*METTL4*	-3.703767385	0.014260737	0.020293629	1

T2DM	b_cell	*LPIN2*	-2.779565007	0.00479125	0.008705951	1
T2DM	b_cell	*MYL12A*	-3.348225409	0.006682032	0.010976978	1
T2DM	b_cell	*LIPG*	-8.529539768	0.001385718	0.004327874	1
T2DM	b_cell	*NALCN*	-2.406503193	0.000970361	0.00415349	1
T2DM	b_cell	*HTR2A*	-6.204314653	0.000795384	0.00415349	1
T2DM	b_cell	*KLF5*	-3.845685986	0.001247395	0.004253251	1
T2DM	b_cell	*STK24*	-2.938601033	0.004866208	0.008790778	1
T2DM	b_cell	*DNAJC3*	-3.024550565	0.012837654	0.018558366	1
T2DM	b_cell	*UGGT2*	-1.856889092	0.004164012	0.007865091	1
T2DM	b_cell	*ARHGEF7*	-2.197970524	0.002579408	0.005814536	1
T2DM	b_cell	*SGCG*	-1.553494436	0.000771683	0.00415349	1
T2DM	b_cell	*PARP4*	-2.509583259	0.003842848	0.007424704	1
T2DM	b_cell	*RGCC*	-1.039358779	0.007651937	0.012191439	1
T2DM	b_cell	*VWA8*	-1.958009921	0.005369454	0.009390197	1
T2DM	b_cell	*KATNAL1*	-2.452576625	0.001241319	0.004253251	1
T2DM	b_cell	*DHRS12*	-1.832201842	0.000932102	0.00415349	1
T2DM	b_cell	*MEDAG*	-1.707948507	0.000914992	0.00415349	1
T2DM	b_cell	*TSC22D1*	-2.222883379	0.004306192	0.008050261	1
T2DM	b_cell	*MGRN1*	-2.049715481	0.002985615	0.006296733	1
T2DM	b_cell	*CORO1A*	2.96120032	0.019278384	0.026143917	1
T2DM	b_cell	*GDPD3*	-2.607928959	0.001472922	0.004442975	1
T2DM	b_cell	*NFAT5*	-1.967914595	0.002672434	0.005916798	1
T2DM	b_cell	*LONP2*	-2.625788565	0.004795869	0.008709882	1
T2DM	b_cell	*PLLP*	-4.187960293	0.001760496	0.004789372	1
T2DM	b_cell	*ACD*	-3.978716542	0.000791621	0.00415349	1
T2DM	b_cell	*USB1*	-5.37574589	0.004929092	0.008859119	1
T2DM	b_cell	*SETD6*	-2.33284321	0.001099317	0.00415349	1
T2DM	b_cell	*COG4*	-2.976526751	0.000195994	0.00415349	1
T2DM	b_cell	*SLC7A6OS*	-1.264345027	0.000702014	0.00415349	1
T2DM	b_cell	*HCFC1R1*	-2.51556755	0.000784844	0.00415349	1
T2DM	b_cell	*NPRL3*	-1.962989744	0.007402421	0.011857075	1
T2DM	b_cell	*NAGPA*	-3.52024079	0.002550471	0.00579083	1
T2DM	b_cell	*CRISPLD2*	-1.707310485	0.002766562	0.006028396	1
T2DM	b_cell	*NME4*	-2.684794517	0.000664975	0.00415349	1
T2DM	b_cell	*NOMO3*	-3.69788154	0.00029631	0.00415349	1
T2DM	b_cell	*LMF1*	-2.743244286	0.008276307	0.012943568	1
T2DM	b_cell	*NARFL*	-4.126152844	0.026510764	0.034543663	1
T2DM	b_cell	*MTHFSD*	-2.224550025	0.002639316	0.005882091	1
T2DM	b_cell	*CLCN7*	-4.23762877	0.004107975	0.007801017	1
T2DM	b_cell	*HAGHL*	5.700646563	0.018536551	0.025307705	1
T2DM	b_cell	*SLC7A5*	-10.19872452	0.00173731	0.004775894	1
T2DM	b_cell	*FBXO31*	-2.396686373	0.000778953	0.00415349	1
T2DM	b_cell	*STUB1*	-4.416544107	0.001160957	0.004195495	1
T2DM	b_cell	*RHBDL1*	-5.387922955	0.009401935	0.014357534	1
T2DM	b_cell	*UBE2I*	-2.42509653	0.002247668	0.005418361	1
T2DM	b_cell	*EARS2*	-2.230011578	0.000589078	0.00415349	1
T2DM	b_cell	*BFAR*	-2.878428049	0.001591733	0.004593791	1
T2DM	b_cell	*RRN3P2*	-3.96462591	0.001683289	0.004704627	1
T2DM	b_cell	*STX4*	3.328473522	0.03703728	0.046598634	1
T2DM	b_cell	*KAT8*	-3.159954015	0.023323823	0.030930259	1
T2DM	b_cell	*NOMO1*	-2.711693195	0.000533092	0.00415349	1
T2DM	b_cell	*SYT17*	-2.334816745	0.000228955	0.00415349	1
T2DM	b_cell	*TMC5*	-3.395833851	0.00034721	0.00415349	1
T2DM	b_cell	*KNOP1*	-3.411339837	0.018541004	0.025307705	1
T2DM	b_cell	*IQCH*	-2.355517384	0.001174234	0.004202203	1
T2DM	b_cell	*LACTB*	-2.866284126	0.001327678	0.004274973	1
T2DM	b_cell	*HERC1*	-2.197963872	0.009016851	0.01388074	1
T2DM	b_cell	*TRIP4*	-3.100789119	0.017844894	0.024536804	1
T2DM	b_cell	*RASL12*	-3.423587248	0.000883762	0.00415349	1
T2DM	b_cell	*AP3B2*	1.304166441	0.018135572	0.024859113	1
T2DM	b_cell	*TTC23*	-2.523075612	0.002817691	0.00608966	1

T2DM	b_cell	*FAH*	-1.806215268	0.000766762	0.00415349	1
T2DM	b_cell	*RPAP1*	-2.243183526	0.000538327	0.00415349	1
T2DM	b_cell	*EHD4*	-2.597879328	0.002671479	0.005916798	1
T2DM	b_cell	*ATP8B4*	-1.365073955	0.000768642	0.00415349	1
T2DM	b_cell	*DTWD1*	-1.760066603	0.004294511	0.008039295	1
T2DM	b_cell	*DNAJC17*	-2.849131826	0.002007811	0.005131344	1
T2DM	b_cell	*PDGFRL*	-1.63888045	0.001755248	0.004789372	1
T2DM	b_cell	*ZFAND1*	-2.10586449	0.001507133	0.00448007	1
T2DM	b_cell	*RIPK2*	-3.27673178	0.003478289	0.006953391	1
T2DM	b_cell	*NBN*	-2.782971996	0.002884646	0.006179137	1
T2DM	b_cell	*CPQ*	-1.691481565	0.004647675	0.008526388	1
T2DM	b_cell	*NIPAL2*	-1.832086341	0.00349882	0.006971457	1
T2DM	b_cell	*IKBKB*	-2.751697017	0.018123865	0.024857055	1
T2DM	b_cell	*PLAT*	-1.817724361	0.000961922	0.00415349	1
T2DM	b_cell	*RAB2A*	-1.621843057	0.01699049	0.023523279	1
T2DM	b_cell	*ZC2HC1A*	-2.781424971	0.008309921	0.012989675	1
T2DM	b_cell	*ARMC1*	-2.168534299	0.000309199	0.00415349	1
T2DM	b_cell	*SPAG1*	-2.491054008	0.005063209	0.009007468	1
T2DM	b_cell	*CHRAC1*	-2.905684984	0.001378964	0.004323793	1
T2DM	b_cell	*SNX16*	-2.714099115	0.001804607	0.00485268	1
T2DM	b_cell	*TSTA3*	-5.4952586	0.001297544	0.004268027	1
T2DM	b_cell	*MTMR9*	-2.352536437	0.001464867	0.004429831	1
T2DM	b_cell	*LEPROTL1*	-3.198438692	0.003952919	0.007588398	1
T2DM	b_cell	*TNFRSF10A*	-1.657658321	0.009701808	0.014750972	1
T2DM	b_cell	*ERICH1*	-2.458305651	0.003950256	0.007585602	1
T2DM	b_cell	*TUSC3*	-1.69512199	0.000853972	0.00415349	1
T2DM	b_cell	*ARHGEF10*	-1.682147501	0.002073484	0.005226942	1
T2DM	b_cell	*MCM4*	-3.854965445	0.0034366	0.006887461	1
T2DM	b_cell	*MAN2B1*	-2.169556229	0.004168907	0.007870846	1
T2DM	b_cell	*TULP2*	4.309709561	0.000326074	0.00415349	1
T2DM	b_cell	*NUCB1*	-2.773573932	0.003309746	0.00672757	1
T2DM	b_cell	*SARS2*	-4.91459233	0.004250461	0.007980713	1
T2DM	b_cell	*SNRNP70*	-2.982160211	0.005740113	0.009822893	1
T2DM	b_cell	*FCGRT*	-2.968809251	0.002905755	0.006192483	1
T2DM	b_cell	*PEX11G*	-4.884140415	0.006729338	0.011034781	1
T2DM	b_cell	*ERCC2*	-4.82881336	0.001489448	0.004462053	1
T2DM	b_cell	*PLEKHJ1*	-3.535265735	0.001328987	0.004274973	1
T2DM	b_cell	*CD37*	0.69673569	0.000618435	0.00415349	1
T2DM	b_cell	*STX10*	4.770372633	0.013410625	0.019249452	1
T2DM	b_cell	*FCER2*	0.757720419	0.000664872	0.00415349	1
T2DM	b_cell	*DMPK*	-5.838222598	0.002031573	0.005166868	1
T2DM	b_cell	*IL4I1*	-2.761835883	0.005433398	0.009475664	1
T2DM	b_cell	*AES*	-3.06649751	0.004431496	0.008216105	1
T2DM	b_cell	*C19orf53*	-4.832549877	0.021424755	0.028715467	1
T2DM	b_cell	*TIMM44*	-2.991244994	0.007075589	0.011462054	1
T2DM	b_cell	*OLFM2*	-1.719532364	0.001043909	0.00415349	1
T2DM	b_cell	*RASAL3*	-2.829973031	0.00108989	0.00415349	1
T2DM	b_cell	*SYDE1*	-3.305183375	0.000650346	0.00415349	1
T2DM	b_cell	*AURKC*	1.637588099	0.000333013	0.00415349	1
T2DM	b_cell	*CCNE1*	-5.441570269	0.000695626	0.00415349	1
T2DM	b_cell	*PDCD5*	-3.271050408	0.025975298	0.033951496	1
T2DM	b_cell	*ANKRD27*	-2.038727502	0.000834919	0.00415349	1
T2DM	b_cell	*PRX*	2.90036599	0.001355589	0.004296951	1
T2DM	b_cell	*CCDC94*	-4.526817486	0.002799774	0.006069716	1
T2DM	b_cell	*POLR2I*	-3.726606425	0.01042743	0.015642389	1
T2DM	b_cell	*CLIP3*	-2.601563677	0.000424863	0.00415349	1
T2DM	b_cell	*SLC1A5*	-2.033765251	0.001254338	0.004255815	1
T2DM	b_cell	*PRKD2*	-3.499168365	0.034610474	0.043808346	1
T2DM	b_cell	*CACTIN*	-3.176510662	0.000779752	0.00415349	1
T2DM	b_cell	*CCDC9*	3.594703856	0.000638093	0.00415349	1
T2DM	b_cell	*FZR1*	4.993671446	0.026613789	0.03467072	1

T2DM	b_cell	*BBC3*	-7.294402098	0.016430556	0.022868919	1
T2DM	b_cell	*TGFB1*	-2.888309365	0.005111926	0.009060806	1
T2DM	b_cell	*PLIN3*	-2.314088019	0.00096329	0.00415349	1
T2DM	b_cell	*MRPL4*	-3.289454256	0.003182432	0.006549248	1
T2DM	b_cell	*ICAM5*	-24.14618897	1.95E-05	0.002553516	0.122475857
T2DM	b_cell	*CD33*	-2.937779931	0.001672855	0.004701493	1
T2DM	b_cell	*TYK2*	-2.960378516	0.015168863	0.021353928	1
T2DM	b_cell	*RABAC1*	-2.49197783	0.000678823	0.00415349	1
T2DM	b_cell	*PTPRS*	-1.346113863	0.00184301	0.004901439	1
T2DM	b_cell	*GRIN2D*	-3.081597883	0.000760627	0.00415349	1
T2DM	b_cell	*CARD8*	-2.739330649	0.002188332	0.005349464	1
T2DM	b_cell	*LIG1*	1.942751446	0.000861856	0.00415349	1
T2DM	b_cell	*ZNF175*	-4.866636538	0.000960193	0.00415349	1
T2DM	b_cell	*PLA2G4C*	-2.592148089	0.003198426	0.006575704	1
T2DM	b_cell	*CAPS*	-2.951817396	0.017309084	0.023901211	1
T2DM	b_cell	*MIER2*	-4.952147018	0.003804082	0.007370189	1
T2DM	b_cell	*PPP2R1A*	-3.47461645	0.009430759	0.014389036	1
T2DM	b_cell	*TNPO2*	-2.945076913	0.029304378	0.037768886	1
T2DM	b_cell	*GCDH*	-2.364779193	8.73E-05	0.00415349	0.548926274
T2DM	b_cell	*LILRB5*	-1.702928171	0.000781406	0.00415349	1
T2DM	b_cell	*MAST1*	-6.4073774	0.000778133	0.00415349	1
T2DM	b_cell	*PRPF31*	-3.629411881	0.001433699	0.004395857	1
T2DM	b_cell	*TFPT*	4.912634511	0.010900137	0.016208411	1
T2DM	b_cell	*ELL*	-2.086528205	0.002849775	0.00613369	1
T2DM	b_cell	*UPK1A*	-9.563144271	0.005934915	0.010062787	1
T2DM	b_cell	*COPE*	3.941713664	0.007094116	0.01148319	1
T2DM	b_cell	*LSR*	-2.388676212	0.000800522	0.00415349	1
T2DM	b_cell	*KXD1*	-2.648898794	0.001057707	0.00415349	1
T2DM	b_cell	*HPN*	-3.508040605	0.000196382	0.00415349	1
T2DM	b_cell	*SIPA1L3*	2.626765924	0.018966458	0.02578208	1
T2DM	b_cell	*SMG9*	-2.64593443	0.001377547	0.004323793	1
T2DM	b_cell	*GTPBP10*	-2.446876664	0.005316507	0.009320937	1
T2DM	b_cell	*PON3*	-3.00471603	0.000819334	0.00415349	1
T2DM	b_cell	*PON2*	-2.683382198	0.002448905	0.005641723	1
T2DM	b_cell	*HBP1*	-2.642424537	0.002909907	0.006192483	1
T2DM	b_cell	*WDR91*	-3.238573003	0.001919632	0.005005693	1
T2DM	b_cell	*DLX5*	29.21440982	0.001639404	0.004657671	1
T2DM	b_cell	*STEAP1B*	5.097095729	0.003298889	0.00670977	1
T2DM	b_cell	*PTN*	-2.844380329	0.001109303	0.004166182	1
T2DM	b_cell	*MPP6*	-2.018884942	0.001063104	0.00415349	1
T2DM	b_cell	*DFNA5*	-2.135892911	0.00148589	0.004462053	1
T2DM	b_cell	*OGDH*	-2.746199691	0.008112475	0.012728507	1
T2DM	b_cell	*ADAP1*	-8.647902349	0.001611467	0.004615623	1
T2DM	b_cell	*H2AFV*	-3.137119249	0.008840873	0.013666725	1
T2DM	b_cell	*CAV1*	-1.284595869	0.000932087	0.00415349	1
T2DM	b_cell	*MET*	-1.722042478	0.000738492	0.00415349	1
T2DM	b_cell	*RNF32*	-2.279818101	0.000481139	0.00415349	1
T2DM	b_cell	*LMBR1*	-2.842393325	0.007906077	0.01250453	1
T2DM	b_cell	*HOXA2*	-7.580133757	0.001505789	0.004478191	1
T2DM	b_cell	*HOXA5*	-2.013278282	0.000572819	0.00415349	1
T2DM	b_cell	*HOXA6*	-2.189534707	0.0005229	0.00415349	1
T2DM	b_cell	*CPED1*	-2.168980228	0.003490508	0.00696378	1
T2DM	b_cell	*HIBADH*	-2.203302346	0.002317293	0.005497668	1
T2DM	b_cell	*CPVL*	-1.604608769	0.003960298	0.007597923	1
T2DM	b_cell	*ABHD11*	-3.491238852	0.020440112	0.027576605	1
T2DM	b_cell	*PLEKHA8*	-2.129604778	0.00081538	0.00415349	1
T2DM	b_cell	*NOD1*	-2.718118699	0.003728331	0.007274989	1
T2DM	b_cell	*CHCHD2*	-3.120342242	0.027287386	0.03543809	1
T2DM	b_cell	*CCL24*	-4.81138075	0.039499999	0.049361259	1
T2DM	b_cell	*NPTX2*	-90.55829759	0.002402724	0.005596247	1
T2DM	b_cell	*CYP3A5*	-2.866649402	0.001541217	0.004509332	1

T2DM	b_cell	*ZKSCAN1*	-2.482179933	0.003491305	0.00696378	1
T2DM	b_cell	*EIF3B*	-2.847447929	0.001546525	0.004516025	1
T2DM	b_cell	*SNX8*	-3.100911743	0.003124687	0.006466219	1
T2DM	b_cell	*TAF6*	-2.771579686	0.002170823	0.005323038	1
T2DM	b_cell	*AIMP2*	-2.468536458	0.00100756	0.00415349	1
T2DM	b_cell	*RBM28*	-1.525661398	0.000438288	0.00415349	1
T2DM	b_cell	*USP42*	-2.508949075	0.002585532	0.005820315	1
T2DM	b_cell	*IMPDH1*	-3.752832947	0.00141682	0.004364365	1
T2DM	b_cell	*LSM5*	-3.209085436	0.001560594	0.004540237	1
T2DM	b_cell	*AP1S1*	-6.087466593	0.002003822	0.005129117	1
T2DM	b_cell	*PLOD3*	-3.761047029	0.002697639	0.005946726	1
T2DM	b_cell	*RPA3*	-3.028108041	0.005027018	0.008978654	1
T2DM	b_cell	*ZNHIT1*	-3.479807236	0.005869287	0.009978422	1
T2DM	b_cell	*PHF14*	-2.351131093	0.008592043	0.013360914	1
T2DM	b_cell	*ZNF862*	-2.859539737	0.009709316	0.014758818	1
T2DM	b_cell	*MEST*	-1.534375668	0.000161587	0.00415349	1
T2DM	b_cell	*ACTR3C*	-3.411256815	0.019239627	0.026096986	1
T2DM	b_cell	*POU6F2*	-2.022807241	0.000764724	0.00415349	1
T2DM	b_cell	*TSPAN13*	2.304097606	0.000900895	0.00415349	1
T2DM	b_cell	*RARRES2*	-1.701407995	0.00116156	0.004195495	1
T2DM	b_cell	*COA1*	-2.093026593	0.015088496	0.021274137	1
T2DM	b_cell	*BLVRA*	-2.866028803	0.001798291	0.004852128	1
T2DM	b_cell	*URGCP*	-2.361467774	0.001323393	0.004274973	1
T2DM	b_cell	*POLD2*	-5.716579706	0.000142915	0.00415349	0.898507226
T2DM	b_cell	*GCK*	-29.96085443	0.000674614	0.00415349	1
T2DM	b_cell	*YKT6*	-2.59011713	0.00106616	0.00415349	1
T2DM	b_cell	*TBL2*	-1.991376743	0.023904486	0.031588461	1
T2DM	b_cell	*LIMK1*	-4.576190723	0.006839766	0.011166349	1
T2DM	b_cell	*SPATA6L*	-2.066019077	0.000678202	0.00415349	1
T2DM	b_cell	*SLC1A1*	-1.143651516	0.018152304	0.024868933	1
T2DM	b_cell	*FKTN*	-3.069438371	0.003701461	0.00724956	1
T2DM	b_cell	*NMRK1*	-1.892603823	0.003338976	0.006765112	1
T2DM	b_cell	*TMEM245*	-2.544996555	0.003815655	0.007388059	1
T2DM	b_cell	*MEGF9*	-2.882447962	0.008853493	0.013682868	1
T2DM	b_cell	*TRIM14*	-1.874639333	0.000389554	0.00415349	1
T2DM	b_cell	*CORO2A*	-2.613608532	0.00048421	0.00415349	1
T2DM	b_cell	*C5*	-2.007046626	0.001189752	0.004210557	1
T2DM	b_cell	*OGN*	-2.632929699	0.00171359	0.004735535	1
T2DM	b_cell	*ECM2*	-1.361193253	0.000908176	0.00415349	1
T2DM	b_cell	*PTGR1*	-1.607448747	0.001907736	0.004988168	1
T2DM	b_cell	*SUSD1*	-2.321967828	0.013466792	0.019316843	1
T2DM	b_cell	*TNFSF8*	-2.405900887	0.00061375	0.00415349	1
T2DM	b_cell	*PLGRKT*	-2.410278224	0.000869055	0.00415349	1
T2DM	b_cell	*DOCK8*	-1.741688439	0.036428241	0.045915066	1
T2DM	b_cell	*ELAVL2*	-5.596532804	0.000109473	0.00415349	0.688258843
T2DM	b_cell	*FUBP3*	-2.681245469	0.00246212	0.005658298	1
T2DM	b_cell	*MPDZ*	-1.526295165	0.00094022	0.00415349	1
T2DM	b_cell	*PTGDS*	-5.222324725	0.001838493	0.004898539	1
T2DM	b_cell	*SHB*	-11.6498139	0.024882974	0.032652736	1
T2DM	b_cell	*DVL1*	-3.006506891	0.001054232	0.00415349	1
T2DM	b_cell	*PDLIM1*	-2.215239951	0.001257685	0.004257394	1
T2DM	b_cell	*CCNJ*	-3.613433861	0.019894166	0.026886204	1
T2DM	b_cell	*ATRNL1*	-2.054827458	0.00133606	0.004282824	1
T2DM	b_cell	*HPS1*	-3.697362726	0.010144019	0.015301212	1
T2DM	b_cell	*PHYH*	-2.11687967	0.000893365	0.00415349	1
T2DM	b_cell	*DNMBP*	-2.043279198	0.001245509	0.004253251	1
T2DM	b_cell	*TRDMT1*	-3.644730036	0.007021689	0.011392351	1
T2DM	b_cell	*DDX50*	-2.898105134	0.004659412	0.008540445	1
T2DM	b_cell	*MAPK8*	-2.323061949	0.005680492	0.009761317	1
T2DM	b_cell	*ATE1*	-2.203241625	0.007126567	0.011510862	1
T2DM	b_cell	*NSMCE4A*	-2.42383207	0.001076014	0.00415349	1

T2DM	b_cell	*PLEKHA1*	-2.614833873	0.005682587	0.009761317	1
T2DM	b_cell	*SPOCK2*	-2.688452508	0.000945186	0.00415349	1
T2DM	b_cell	*MICU1*	-2.282086915	0.007794424	0.012363109	1
T2DM	b_cell	*CCSER2*	-2.365043401	0.004753646	0.00865764	1
T2DM	b_cell	*BMPR1A*	-1.315362064	0.001789192	0.00483813	1
T2DM	b_cell	*SFXN3*	-3.429580439	0.002033283	0.00516834	1
T2DM	b_cell	*KAZALD1*	-6.882134042	0.000243208	0.00415349	1
T2DM	b_cell	*ANKRD26*	-2.147777862	0.012768807	0.018475832	1
T2DM	b_cell	*ACBD5*	-2.861078028	0.009031848	0.013898681	1
T2DM	b_cell	*MTPAP*	-3.634049171	0.008780641	0.013603719	1
T2DM	b_cell	*PITRM1*	-3.657981665	0.005209774	0.00917989	1
T2DM	b_cell	*MAP3K8*	-2.732974852	0.001721714	0.004747873	1
T2DM	b_cell	*SHOC2*	-2.437495259	0.011576287	0.017008049	1
T2DM	b_cell	*TFAM*	-3.262544584	0.000628561	0.00415349	1
T2DM	b_cell	*PBLD*	-1.501398197	0.010610052	0.015852042	1
T2DM	b_cell	*DHRS11*	-3.350133776	8.63E-06	0.001459962	0.054250397
T2DM	b_cell	*MLLT6*	-3.08758265	0.004639224	0.008518341	1
T2DM	b_cell	*CASC3*	-2.899099431	0.002641695	0.005883223	1
T2DM	b_cell	*RAPGEFL1*	-2.767893098	0.000776033	0.00415349	1
T2DM	b_cell	*RGS9*	-2.939818973	0.00079474	0.00415349	1
T2DM	b_cell	*WNT3*	-5.396627944	0.000244867	0.00415349	1
T2DM	b_cell	*ASPA*	-1.506586956	0.000776133	0.00415349	1
T2DM	b_cell	*RPS6KB1*	-2.763276629	0.00295925	0.006264583	1
T2DM	b_cell	*TRIM16L*	-2.626938769	0.000389977	0.00415349	1
T2DM	b_cell	*CDK5RAP3*	-2.89310652	0.001084707	0.00415349	1
T2DM	b_cell	*CBX1*	-2.41420642	0.002684359	0.005929925	1
T2DM	b_cell	*RECQL5*	-3.366530874	0.003063878	0.006386805	1
T2DM	b_cell	*PIGL*	-2.440495671	0.002640386	0.005882391	1
T2DM	b_cell	*GALK1*	-5.168404161	0.001835795	0.004898539	1
T2DM	b_cell	*MED13*	-2.466588374	0.011305125	0.016680431	1
T2DM	b_cell	*HOXB6*	-2.51405328	0.000217998	0.00415349	1
T2DM	b_cell	*C1QBP*	-2.684710253	0.001268118	0.004257394	1
T2DM	b_cell	*GOSR1*	-2.379551001	0.001983393	0.005095868	1
T2DM	b_cell	*DRG2*	-2.254919243	0.000772983	0.00415349	1
T2DM	b_cell	*FTSJ3*	-2.425156622	0.007024562	0.011394071	1
T2DM	b_cell	*AKAP10*	-2.617471194	0.012872605	0.018600337	1
T2DM	b_cell	*ALDH3A1*	4.464634225	0.004701809	0.008595602	1
T2DM	b_cell	*SMARCD2*	-2.443893666	0.000715132	0.00415349	1
T2DM	b_cell	*ICAM2*	-1.803274466	0.002959932	0.006264583	1
T2DM	b_cell	*SYNGR2*	-3.671353672	0.000135658	0.00415349	0.85288485
T2DM	b_cell	*DDX5*	-1.645715607	0.006463983	0.010691676	1
T2DM	b_cell	*PEX12*	-5.324333995	0.000355719	0.00415349	1
T2DM	b_cell	*DHX58*	-4.04489077	0.002257846	0.005430406	1
T2DM	b_cell	*RAB5C*	-2.723993245	0.001950471	0.005052579	1
T2DM	b_cell	*NAGLU*	-2.155232565	0.000702495	0.00415349	1
T2DM	b_cell	*HSD17B1*	-3.602720923	3.55E-05	0.003315867	0.223109368
T2DM	b_cell	*EZH1*	-2.220399884	0.008604784	0.013373053	1
T2DM	b_cell	*DLX4*	-27.89909689	0.000690794	0.00415349	1
T2DM	b_cell	*VAT1*	-1.894632968	0.00400394	0.007651297	1
T2DM	b_cell	*LRRC59*	-5.064682428	0.004671802	0.008555671	1
T2DM	b_cell	*ALOX12*	-5.127907124	0.001699813	0.004717848	1
T2DM	b_cell	*MPP2*	-4.173974865	0.000858961	0.00415349	1
T2DM	b_cell	*EFNB3*	12.35120387	0.000488194	0.00415349	1
T2DM	b_cell	*FAM20A*	-2.092329562	0.001415836	0.004364365	1
T2DM	b_cell	*YWHAE*	-2.509999492	0.00382751	0.007404171	1
T2DM	b_cell	*DPH1*	-3.779522437	0.001184613	0.004202203	1
T2DM	b_cell	*DHRS7B*	-2.371109537	0.001814027	0.004867153	1
T2DM	b_cell	*RCVRN*	-9.777939495	0.009179487	0.014086267	1
T2DM	b_cell	*SLC9A3R1*	-3.902248674	0.016785285	0.023285324	1
T2DM	b_cell	*MYH3*	-2.905352773	0.002123807	0.005267208	1
T2DM	b_cell	*TMEM104*	-3.370726996	0.003911052	0.007531021	1

T2DM	b_cell	*TMEM97*	3.043279804	0.000234131	0.00415349	1
T2DM	b_cell	*RAB34*	-2.709902872	0.000726469	0.00415349	1
T2DM	b_cell	*SLAIN2*	-2.277619701	0.005131484	0.009087785	1
T2DM	b_cell	*OCIAD1*	-3.138169606	0.010348213	0.015549526	1
T2DM	b_cell	*CWH43*	-21.13677122	0.006070631	0.010234931	1
T2DM	b_cell	*KIAA1211*	2.717587058	0.005803692	0.009904519	1
T2DM	b_cell	*AREG*	2.208403215	0.000619817	0.00415349	1
T2DM	b_cell	*UBE2D3*	-2.282703061	0.035038402	0.044305397	1
T2DM	b_cell	*TBC1D9*	-2.584514578	0.004523262	0.008354216	1
T2DM	b_cell	*CPE*	-2.01955216	0.001802813	0.004852128	1
T2DM	b_cell	*WFS1*	-1.92361648	0.003516031	0.006997559	1
T2DM	b_cell	*GRPEL1*	-3.413557172	0.001245704	0.004253251	1
T2DM	b_cell	*AADAT*	-2.792458707	0.000971607	0.00415349	1
T2DM	b_cell	*CPZ*	-55.57194883	0.000703053	0.00415349	1
T2DM	b_cell	*TRIM2*	-1.811754752	0.000972976	0.00415349	1
T2DM	b_cell	*SLC2A9*	-2.056772441	0.003092175	0.006423584	1
T2DM	b_cell	*SH3D19*	-1.026633746	0.001161322	0.004195495	1
T2DM	b_cell	*RAPGEF2*	-2.032788096	0.006586255	0.010842572	1
T2DM	b_cell	*LRP2BP*	-3.013104597	0.023718954	0.031374093	1
T2DM	b_cell	*UFSP2*	-3.455745833	0.008995004	0.013857288	1
T2DM	b_cell	*KLF3*	-2.861572757	0.01248997	0.018108734	1
T2DM	b_cell	*KLHL5*	-2.095167336	0.001759483	0.004789372	1
T2DM	b_cell	*FAM149A*	-1.143002599	0.030363716	0.039006269	1
T2DM	b_cell	*UGDH*	-2.287998492	0.001159728	0.004195495	1
T2DM	b_cell	*HTATIP2*	-2.598342017	0.013048202	0.018819465	1
T2DM	b_cell	*CTSC*	-1.840443715	0.000824453	0.00415349	1
T2DM	b_cell	*ELP4*	-2.316185379	0.003031363	0.006344326	1
T2DM	b_cell	*C11orf63*	-2.045943483	0.000974923	0.00415349	1
T2DM	b_cell	*SNX15*	-3.523803048	0.005506712	0.009566611	1
T2DM	b_cell	*DTX4*	-4.046114362	0.030825557	0.039518817	1
T2DM	b_cell	*PUS3*	-2.796003819	0.002522531	0.005743989	1
T2DM	b_cell	*DCPS*	4.433319336	0.026973426	0.035095598	1
T2DM	b_cell	*FOXRED1*	-2.086241222	0.006923075	0.011270163	1
T2DM	b_cell	*NRXN2*	-8.49252357	0.027405232	0.035576439	1
T2DM	b_cell	*MS4A4A*	-1.55382395	0.002149293	0.00529906	1
T2DM	b_cell	*CCDC86*	-6.70433606	0.000741921	0.00415349	1
T2DM	b_cell	*PRPF19*	-2.880853331	0.001822631	0.004880273	1
T2DM	b_cell	*HPX*	-3.466101275	0.025858735	0.033813201	1
T2DM	b_cell	*CHORDC1*	-3.152425191	0.005156494	0.00911667	1
T2DM	b_cell	*PANX1*	-2.798325112	0.000986956	0.00415349	1
T2DM	b_cell	*EIF4G2*	-2.962966413	0.014519345	0.020591726	1
T2DM	b_cell	*GALNT18*	-1.602827274	0.000769066	0.00415349	1
T2DM	b_cell	*CBL*	-1.971912384	0.004442243	0.008231177	1
T2DM	b_cell	*KIAA1549L*	-3.734572772	0.002565695	0.005799421	1
T2DM	b_cell	*MADD*	-3.202978835	0.00620625	0.010399439	1
T2DM	b_cell	*NAA40*	-2.348392405	0.000874605	0.00415349	1
T2DM	b_cell	*CARS*	-2.185898461	0.012273332	0.017841026	1
T2DM	b_cell	*SLC22A18*	-1.753410911	0.013313217	0.019162133	1
T2DM	b_cell	*C11orf21*	-6.161266677	0.011676624	0.017116095	1
T2DM	b_cell	*ELMOD1*	-7.231971099	0.000287042	0.00415349	1
T2DM	b_cell	*NDUFS8*	-4.211002766	0.017429338	0.024044445	1
T2DM	b_cell	*TCIRG1*	-3.005085774	0.005188977	0.009153507	1
T2DM	b_cell	*GTF2H1*	-3.043441145	0.007237109	0.011651653	1
T2DM	b_cell	*PPFIBP1*	-2.048928203	0.003238886	0.006622074	1
T2DM	b_cell	*PRDM4*	-3.210894458	0.003057955	0.006378687	1
T2DM	b_cell	*ASIC1*	-4.898730399	0.000147743	0.00415349	0.928859746
T2DM	b_cell	*CAPRIN2*	-3.560490511	0.006642384	0.010920677	1
T2DM	b_cell	*KCTD10*	-2.393176117	0.001035337	0.00415349	1
T2DM	b_cell	*CYP27B1*	2.862520396	0.000560853	0.00415349	1
T2DM	b_cell	*ACSS3*	-1.005052951	0.003640341	0.007185816	1
T2DM	b_cell	*GLI1*	-4.838202763	0.000783139	0.00415349	1

T2DM	b_cell	*METAP2*	-3.086712002	0.022515569	0.030009621	1
T2DM	b_cell	*LTA4H*	-3.225879053	0.004545328	0.008382656	1
T2DM	b_cell	*TRPV4*	-6.206534964	0.010283803	0.015478638	1
T2DM	b_cell	*ITFG2*	-2.891737008	0.005059219	0.009005468	1
T2DM	b_cell	*GPN3*	-4.491610592	0.023593294	0.031260492	1
T2DM	b_cell	*VPS29*	-2.099444595	0.001296006	0.004268027	1
T2DM	b_cell	*KCNA1*	-6.993348112	0.000778274	0.00415349	1
T2DM	b_cell	*CREBL2*	-2.060398848	0.003442575	0.006894407	1
T2DM	b_cell	*LTBR*	-2.742645442	0.002662256	0.005916438	1
T2DM	b_cell	*MGP*	-1.433561142	0.006700724	0.011002208	1
T2DM	b_cell	*RASAL1*	2.69705815	0.000908371	0.00415349	1
T2DM	b_cell	*EIF2B1*	-3.353156203	0.003610812	0.007151285	1
T2DM	b_cell	*DDX55*	-2.803023278	0.00026941	0.00415349	1
T2DM	b_cell	*ENDOU*	-7.154481256	3.06E-06	0.000686434	0.019220161
T2DM	b_cell	*STX2*	-2.31787418	0.01048307	0.015699228	1
T2DM	b_cell	*CAND1*	-2.384048047	0.000982954	0.00415349	1
T2DM	b_cell	*NUP107*	-2.125114341	0.002078068	0.005230315	1
T2DM	b_cell	*CNOT2*	-2.588079492	0.006236149	0.010418993	1
T2DM	b_cell	*KRR1*	-3.543745311	0.003047142	0.006373048	1
T2DM	b_cell	*COPS7A*	-3.54504864	0.00079857	0.00415349	1
T2DM	b_cell	*GNB3*	-2.381377925	0.000958674	0.00415349	1
T2DM	b_cell	*CDCA3*	-4.963642342	0.00362033	0.007162057	1
T2DM	b_cell	*CHPT1*	-1.833403334	0.001178963	0.004202203	1
T2DM	b_cell	*GNPTAB*	-2.415026956	0.004355776	0.008109199	1
T2DM	b_cell	*SPSB2*	-3.100219863	0.00107955	0.00415349	1
T2DM	b_cell	*ATN1*	-2.220882847	0.001322162	0.004274973	1
T2DM	b_cell	*NT5DC3*	-1.968622212	0.001201064	0.004223203	1
T2DM	b_cell	*PRKAB1*	2.964847831	0.004631391	0.008508929	1
T2DM	b_cell	*ST8SIA1*	-1.697904618	0.000756756	0.00415349	1
T2DM	b_cell	*C2CD5*	-2.58715319	0.030030475	0.038601839	1
T2DM	b_cell	*COL12A1*	-1.22020005	0.000748097	0.00415349	1
T2DM	b_cell	*TDP2*	-3.217048583	0.002350156	0.005533109	1
T2DM	b_cell	*RWDD1*	-2.654090652	0.001290514	0.004268027	1
T2DM	b_cell	*RSPH4A*	-2.321364091	0.039614619	0.049494656	1
T2DM	b_cell	*SMIM8*	-1.842099141	0.001224327	0.004253251	1
T2DM	b_cell	*MCM9*	-2.637791045	0.004898435	0.00882673	1
T2DM	b_cell	*FAM184A*	-1.509049883	0.001603314	0.004609717	1
T2DM	b_cell	*GABRR2*	-3.629228799	0.001242707	0.004253251	1
T2DM	b_cell	*HDDC2*	-2.703349671	0.001876777	0.004957688	1
T2DM	b_cell	*TPD52L1*	-1.77785774	0.001662633	0.004679659	1
T2DM	b_cell	*UST*	-1.222682284	0.000939926	0.00415349	1
T2DM	b_cell	*TULP1*	-21.87127735	0.001261901	0.004257394	1
T2DM	b_cell	*SLC26A8*	-1.937289356	0.01738441	0.023989417	1
T2DM	b_cell	*MAPK14*	-2.596733012	0.001171943	0.004202203	1
T2DM	b_cell	*RNF8*	-1.880550106	0.000714409	0.00415349	1
T2DM	b_cell	*PHACTR1*	-2.547220933	0.006128846	0.010302689	1
T2DM	b_cell	*MDGA1*	3.096544505	0.002300903	0.005481934	1
T2DM	b_cell	*CD83*	-3.901040989	0.000382373	0.00415349	1
T2DM	b_cell	*MDN1*	-2.714699752	0.003666133	0.007210886	1
T2DM	b_cell	*CAP2*	-1.527290858	0.000390426	0.00415349	1
T2DM	b_cell	*GPR63*	-3.436918537	0.005791993	0.009892492	1
T2DM	b_cell	*ASCC3*	-2.528369688	0.011539357	0.016962609	1
T2DM	b_cell	*BVES*	-5.948613494	4.12E-05	0.003541347	0.25906912
T2DM	b_cell	*WASF1*	-1.604138271	0.001077182	0.00415349	1
T2DM	b_cell	*GPLD1*	-1.315344358	0.008366653	0.013052394	1
T2DM	b_cell	*ALDH5A1*	-2.408619717	0.015105423	0.021293228	1
T2DM	b_cell	*VNN1*	-2.220079209	0.00393953	0.007571944	1
T2DM	b_cell	*VNN2*	3.661233567	0.000214484	0.00415349	1
T2DM	b_cell	*ACOT13*	-2.60692866	0.00155919	0.004538253	1
T2DM	b_cell	*C6orf62*	-2.261433452	0.005366286	0.009387268	1
T2DM	b_cell	*B3GAT2*	-5.453929399	0.014163876	0.020178629	1

T2DM	b_cell	*HBS1L*	-2.817188521	0.009280917	0.014213206	1
T2DM	b_cell	*PEX7*	-1.97417118	0.034206553	0.043366928	1
T2DM	b_cell	*SLC16A10*	-1.874115853	0.003141661	0.006486859	1
T2DM	b_cell	*PHACTR2*	-2.373787145	0.004427801	0.008213295	1
T2DM	b_cell	*PACRG*	-2.773279738	0.000654985	0.00415349	1
T2DM	b_cell	*TFEB*	-3.462868674	0.011394238	0.016792212	1
T2DM	b_cell	*TBP*	-3.510049822	0.026222679	0.034210829	1
T2DM	b_cell	*PRPH2*	-3.870121085	0.000191884	0.00415349	1
T2DM	b_cell	*DUSP22*	-2.711891369	0.001883499	0.004965013	1
T2DM	b_cell	*EXOC2*	-2.82357638	0.023032224	0.030639779	1
T2DM	b_cell	*TMEM30A*	-2.714321183	0.003390038	0.006828956	1
T2DM	b_cell	*SENP6*	-2.282999821	0.007144169	0.01153155	1
T2DM	b_cell	*TTK*	1.46099654	0.001148942	0.004187618	1
T2DM	b_cell	*LAMA4*	-0.96293489	0.00067751	0.00415349	1
T2DM	b_cell	*CLIC5*	-1.905795369	0.0011901	0.004210557	1
T2DM	b_cell	*LY86*	1.90066135	0.000866449	0.00415349	1
T2DM	b_cell	*TBX18*	-1.729728922	0.001035116	0.00415349	1
T2DM	b_cell	*PCDHB2*	-2.502324023	0.031398272	0.040179308	1
T2DM	b_cell	*NUDT12*	-3.720427996	0.001644639	0.004657671	1
T2DM	b_cell	*C7*	-1.969279601	0.00062602	0.00415349	1
T2DM	b_cell	*PAPD7*	-2.866243266	0.005278677	0.009275306	1
T2DM	b_cell	*GHR*	-0.803556098	0.003839122	0.007422066	1
T2DM	b_cell	*NME5*	-1.982475754	0.0031119	0.006452677	1
T2DM	b_cell	*NNT*	-2.571135035	0.001606246	0.004610639	1
T2DM	b_cell	*PFDN1*	-2.121919927	0.00407306	0.007750402	1
T2DM	b_cell	*LOX*	-1.7849671	0.000781312	0.00415349	1
T2DM	b_cell	*APBB3*	3.189533837	0.023168542	0.030775539	1
T2DM	b_cell	*TMCO6*	1.950994122	0.001177611	0.004202203	1
T2DM	b_cell	*SPARC*	-1.539381537	0.001429392	0.004387981	1
T2DM	b_cell	*IK*	-3.623429262	0.002974151	0.006280982	1
T2DM	b_cell	*PDE8B*	-1.441795765	0.001155748	0.004195495	1
T2DM	b_cell	*CLK4*	-2.609722504	0.002725402	0.005972326	1
T2DM	b_cell	*PCDHB15*	-2.89788564	0.002134725	0.005283865	1
T2DM	b_cell	*HAVCR1*	2.949947956	0.001683798	0.004704627	1
T2DM	b_cell	*RNF130*	-1.868412888	0.001613927	0.004616359	1
T2DM	b_cell	*ARSB*	-2.684380919	0.014600267	0.020683163	1
T2DM	b_cell	*CLINT1*	-2.186752029	0.004023303	0.007676633	1
T2DM	b_cell	*BTNL8*	-2.443795442	0.002510936	0.005734251	1
T2DM	b_cell	*TTC1*	-2.938834044	0.010186761	0.015354632	1
T2DM	b_cell	*MSH3*	-2.446581497	0.000568925	0.00415349	1
T2DM	b_cell	*RASGRF2*	-1.276676749	0.000753486	0.00415349	1
T2DM	b_cell	*POLR3G*	-2.088298667	0.000590728	0.00415349	1
T2DM	b_cell	*DROSHA*	-2.391064766	0.011539538	0.016962609	1
T2DM	b_cell	*CDH6*	-1.926609846	0.005034218	0.008986408	1
T2DM	b_cell	*SLC27A6*	-4.755554572	0.000648915	0.00415349	1
T2DM	b_cell	*TARS*	-2.493141004	0.001384417	0.00432604	1
T2DM	b_cell	*LNPEP*	-2.052136709	0.014101915	0.02009491	1
T2DM	b_cell	*SLC12A7*	-3.679402465	0.000943402	0.00415349	1
T2DM	b_cell	*IL4*	3.87452784	0.002036174	0.005172292	1
T2DM	b_cell	*GNPDA1*	-3.550874431	0.003650906	0.007196562	1
T2DM	b_cell	*PPWD1*	-2.686572394	0.008691162	0.013481702	1
T2DM	b_cell	*TRIM23*	-3.331312882	0.004816322	0.008733839	1
T2DM	b_cell	*TRAPPC13*	-2.732189517	0.017316022	0.02390554	1
T2DM	b_cell	*TXNDC15*	-2.713388853	0.001094596	0.00415349	1
T2DM	b_cell	*SMAD5*	-2.598037786	0.006088228	0.010252906	1
T2DM	b_cell	*HMGXB3*	-3.094312583	0.009295132	0.014228998	1
T2DM	b_cell	*ATP6V0E1*	-2.93240931	0.002899974	0.006192483	1
T2DM	b_cell	*CNTN3*	-1.593735306	0.000780145	0.00415349	1
T2DM	b_cell	*ACTR8*	4.399238711	0.033638268	0.042749907	1
T2DM	b_cell	*HGD*	7.209160087	0.001889022	0.004970573	1
T2DM	b_cell	*NIT2*	-2.494621441	0.001040441	0.00415349	1

T2DM	b_cell	*FAM162A*	-2.539437565	0.00034879	0.00415349	1
T2DM	b_cell	*PCCB*	-2.605642331	0.001388576	0.004333459	1
T2DM	b_cell	*RBP1*	-5.636014522	0.000748587	0.00415349	1
T2DM	b_cell	*RNF7*	-3.427208338	0.009717833	0.014768193	1
T2DM	b_cell	*BCHE*	-2.972750837	0.001007798	0.00415349	1
T2DM	b_cell	*PDCD10*	-3.080633987	0.005057426	0.009004825	1
T2DM	b_cell	*WNT5A*	-5.911079934	0.000852234	0.00415349	1
T2DM	b_cell	*PRKAR2A*	-2.623673301	0.000951228	0.00415349	1
T2DM	b_cell	*HYAL1*	-2.023332119	0.005399279	0.009426623	1
T2DM	b_cell	*CYB561D2*	-3.928483387	0.000562308	0.00415349	1
T2DM	b_cell	*IFT57*	3.535205348	0.009060753	0.013931268	1
T2DM	b_cell	*IQCG*	-3.048727731	0.003433687	0.006885994	1
T2DM	b_cell	*GBE1*	-1.228046818	0.000917108	0.00415349	1
T2DM	b_cell	*UMPS*	-3.202083183	0.000325404	0.00415349	1
T2DM	b_cell	*NCBP2*	-3.440638288	0.007957149	0.012564143	1
T2DM	b_cell	*SLC41A3*	-2.282530036	0.000669043	0.00415349	1
T2DM	b_cell	*PLXNA1*	-2.532979189	0.002433225	0.005628918	1
T2DM	b_cell	*ABTB1*	-3.862407843	0.004752872	0.00865764	1
T2DM	b_cell	*EFCC1*	-5.032147658	5.68E-05	0.00415349	0.357051481
T2DM	b_cell	*NEK11*	-2.572658258	0.002221942	0.005395656	1
T2DM	b_cell	*PLSCR4*	-1.879456996	0.00217617	0.005332528	1
T2DM	b_cell	*WDR48*	-2.659330361	0.034065016	0.043213631	1
T2DM	b_cell	*COMMD2*	-2.442775146	0.001105544	0.004159248	1
T2DM	b_cell	*ABCC5*	-2.228431652	0.000691313	0.00415349	1
T2DM	b_cell	*ARHGEF26*	-1.819687381	0.001236965	0.004253251	1
T2DM	b_cell	*KLHL24*	-2.035440256	0.003545344	0.007044747	1
T2DM	b_cell	*PLCH1*	6.653995392	0.000652977	0.00415349	1
T2DM	b_cell	*NKTR*	-2.042638633	0.023097997	0.030704248	1
T2DM	b_cell	*CLCN2*	-6.719941987	0.007394315	0.01185013	1
T2DM	b_cell	*MOB1A*	-3.322893037	0.004860715	0.008786462	1
T2DM	b_cell	*LMAN2L*	-2.551108514	0.020207098	0.027285658	1
T2DM	b_cell	*PIKFYVE*	-2.671064325	0.033390998	0.042461408	1
T2DM	b_cell	*KCNIP3*	-3.725016799	0.001604273	0.004609717	1
T2DM	b_cell	*FAHD2A*	4.73688396	0.004227632	0.007948302	1
T2DM	b_cell	*NCL*	-3.29879143	0.018229102	0.024952398	1
T2DM	b_cell	*ACTR1B*	-3.657413795	0.004917936	0.008844125	1
T2DM	b_cell	*SLC35F5*	-2.363664006	0.023698062	0.031369643	1
T2DM	b_cell	*ACTR3*	-2.677746377	0.0100317	0.015168181	1
T2DM	b_cell	*STEAP3*	-1.926140699	0.014515218	0.020590518	1
T2DM	b_cell	*TFCP2L1*	-3.36954788	0.00334381	0.006768457	1
T2DM	b_cell	*TP53I3*	-2.110467369	0.001077159	0.00415349	1
T2DM	b_cell	*DNAJC27*	-5.184777975	0.002163017	0.005312843	1
T2DM	b_cell	*STAM2*	-2.815437648	0.005732359	0.009817309	1
T2DM	b_cell	*GPD2*	-2.590055779	0.003634705	0.007180327	1
T2DM	b_cell	*CENPA*	-2.831929393	0.000828	0.00415349	1
T2DM	b_cell	*TANC1*	-1.398969168	0.000441953	0.00415349	1
T2DM	b_cell	*NRBP1*	-5.502505186	0.000378761	0.00415349	1
T2DM	b_cell	*ITGB6*	-5.385867188	0.0001827	0.00415349	1
T2DM	b_cell	*PPM1G*	-5.118786276	0.000933119	0.00415349	1
T2DM	b_cell	*PCSK4*	-4.091980257	0.002767518	0.006028396	1
T2DM	b_cell	*INO80B*	-2.847154606	0.001683346	0.004704627	1
T2DM	b_cell	*MOGS*	-1.737234696	0.000231945	0.00415349	1
T2DM	b_cell	*GRB14*	-2.141265465	0.000699267	0.00415349	1
T2DM	b_cell	*CLIP4*	-1.769346191	0.001068231	0.00415349	1
T2DM	b_cell	*SPTBN1*	-1.210782594	0.000730498	0.00415349	1
T2DM	b_cell	*RTN4*	-2.153328179	0.000466719	0.00415349	1
T2DM	b_cell	*GALNT3*	1.443192712	0.000901951	0.00415349	1
T2DM	b_cell	*POLE4*	-3.602033313	0.001931883	0.005024672	1
T2DM	b_cell	*MRPL19*	-2.202160869	0.000824712	0.00415349	1
T2DM	b_cell	*LANCL1*	-3.352652647	0.013226546	0.019050469	1
T2DM	b_cell	*WDR75*	-3.269201325	0.005608608	0.009663282	1

T2DM	b_cell	*EFEMP1*	-1.206470215	0.00067803	0.00415349	1
T2DM	b_cell	*GLS*	-2.277038844	0.009341686	0.014282874	1
T2DM	b_cell	*GGCX*	-2.996063169	0.018429567	0.025188411	1
T2DM	b_cell	*GNLY*	-1.749568378	0.000735448	0.00415349	1
T2DM	b_cell	*SF3B1*	-2.432125421	0.013390461	0.019233683	1
T2DM	b_cell	*ST3GAL5*	-1.541678373	0.000668345	0.00415349	1
T2DM	b_cell	*PLCD4*	-2.670367668	0.000697013	0.00415349	1
T2DM	b_cell	*CHMP3*	-6.584488908	7.15E-05	0.00415349	0.449400994
T2DM	b_cell	*ZNF142*	-3.497639875	0.011273838	0.016638174	1
T2DM	b_cell	*MLPH*	-2.467910791	0.000814993	0.00415349	1
T2DM	b_cell	*HDLBP*	-2.52381883	0.009093564	0.01397488	1
T2DM	b_cell	*STK25*	-2.228199597	0.000795542	0.00415349	1
T2DM	b_cell	*TPO*	-1.675407776	0.000906472	0.00415349	1
T2DM	b_cell	*TAF1B*	-3.066120009	0.005016541	0.008972687	1
T2DM	b_cell	*STRN*	-2.694500985	0.00522868	0.009202887	1
T2DM	b_cell	*CEBPZ*	-2.646479755	0.001608003	0.004610639	1
T2DM	b_cell	*PRKD3*	-2.454388649	0.003626529	0.007172063	1
T2DM	b_cell	*SLC25A12*	-2.340354218	0.001016686	0.00415349	1
T2DM	b_cell	*SDC1*	11.46841039	0.036675737	0.046208488	1
T2DM	b_cell	*SOS1*	-1.914760294	0.00241656	0.005613599	1
T2DM	b_cell	*WIPF1*	-2.223360459	0.001704171	0.004725848	1
T2DM	b_cell	*PNO1*	-2.247733863	0.000784446	0.00415349	1
T2DM	b_cell	*ORC4*	-2.731905545	0.034484616	0.043663005	1
T2DM	b_cell	*THADA*	-2.44668957	0.009694475	0.014743387	1
T2DM	b_cell	*ARID3A*	-2.981175177	0.002442479	0.0056392	1
T2DM	b_cell	*CD207*	-48.84425168	0.003236832	0.006620026	1
T2DM	b_cell	*VAX2*	-4.767930397	0.000998332	0.00415349	1
T2DM	b_cell	*MSH6*	-3.57836944	0.005575752	0.009640408	1
T2DM	b_cell	*ALMS1*	-2.259045853	0.001643978	0.004657671	1
T2DM	b_cell	*DNAJC16*	-3.109280047	0.033135057	0.042187142	1
T2DM	b_cell	*MORN1*	-2.171483354	0.001524702	0.004498266	1
T2DM	b_cell	*SCP2*	-2.101717737	0.002567407	0.005799671	1
T2DM	b_cell	*RALGPS2*	0.415190076	0.000573988	0.00415349	1
T2DM	b_cell	*CEP104*	-1.995839705	0.001615118	0.004617667	1
T2DM	b_cell	*TMEM59*	-2.551371718	0.003797036	0.007363346	1
T2DM	b_cell	*WRAP73*	-3.799945771	0.001264154	0.004257394	1
T2DM	b_cell	*MRPL37*	-2.065831042	0.002472179	0.005676621	1
T2DM	b_cell	*ICMT*	-4.297744841	0.00078716	0.00415349	1
T2DM	b_cell	*QSOX1*	-3.816961402	0.011065312	0.016384271	1
T2DM	b_cell	*KCNC4*	-3.252316044	0.000378118	0.00415349	1
T2DM	b_cell	*EDEM3*	-2.893317613	0.006230718	0.010416354	1
T2DM	b_cell	*RAP1A*	-2.434043513	0.026056892	0.034022779	1
T2DM	b_cell	*CAPZA1*	-2.967559198	0.006750627	0.011063918	1
T2DM	b_cell	*DOCK7*	-2.274362495	0.002621051	0.005854674	1
T2DM	b_cell	*FBXO6*	-4.054411172	0.002585675	0.005820315	1
T2DM	b_cell	*C1orf21*	-1.463010109	0.000359853	0.00415349	1
T2DM	b_cell	*LEPR*	-2.633953673	0.017529602	0.024168554	1
T2DM	b_cell	*MFN2*	-2.645219471	0.000994056	0.00415349	1
T2DM	b_cell	*MIIP*	-4.580106132	0.000823754	0.00415349	1
T2DM	b_cell	*SMG7*	-2.75221234	0.005203451	0.009176464	1
T2DM	b_cell	*NCF2*	-1.94525508	0.000767442	0.00415349	1
T2DM	b_cell	*RGS2*	-4.921110911	0.003056585	0.006378687	1
T2DM	b_cell	*OLFML3*	-2.043752997	0.000764747	0.00415349	1
T2DM	b_cell	*TNNI3K*	-3.914975175	0.032999866	0.042032042	1
T2DM	b_cell	*CFHR3*	-3.230928039	0.031540822	0.0403453	1
T2DM	b_cell	*CRYZ*	-3.47599271	0.008014588	0.012625336	1
T2DM	b_cell	*TTF2*	-3.147193233	0.002671872	0.005916798	1
T2DM	b_cell	*TMEM9*	-2.345404416	0.000498629	0.00415349	1
T2DM	b_cell	*WARS2*	-2.303513026	0.000968232	0.00415349	1
T2DM	b_cell	*OSCP1*	-2.044975071	0.001296277	0.004268027	1
T2DM	b_cell	*GNPAT*	-3.630675381	0.011111417	0.016433188	1

T2DM	b_cell	*C1orf109*	-2.942400937	0.000497264	0.00415349	1
T2DM	b_cell	*TBCE*	-3.615931156	0.001537852	0.004509332	1
T2DM	b_cell	*LGALS8*	-2.424060959	0.000798222	0.00415349	1
T2DM	b_cell	*MTR*	-2.44979179	0.004247702	0.007978877	1
T2DM	b_cell	*KCNQ4*	-3.99357694	0.00013129	0.00415349	0.825419198
T2DM	b_cell	*ACADM*	-2.558956326	0.006949396	0.011300363	1
T2DM	b_cell	*PADI2*	3.508580771	0.001045716	0.00415349	1
T2DM	b_cell	*MFAP2*	2.999633556	0.027032554	0.035150707	1
T2DM	b_cell	*CTBS*	-3.297591432	0.033222159	0.042272356	1
T2DM	b_cell	*ZNHIT6*	-2.996725897	0.00415296	0.007865091	1
T2DM	b_cell	*PLA2G2D*	3.82041253	0.001322831	0.004274973	1
T2DM	b_cell	*RBBP5*	-2.285318593	0.020426782	0.027564538	1
T2DM	b_cell	*KIF17*	-5.153590182	0.001487521	0.004462053	1
T2DM	b_cell	*CD46*	-1.939511899	0.002620805	0.005854674	1
T2DM	b_cell	*PRDX1*	-3.363446423	0.025192933	0.033025014	1
T2DM	b_cell	*NSUN4*	-4.318894314	0.018804093	0.025607386	1
T2DM	b_cell	*TMED5*	-2.45361015	0.00209753	0.005241324	1
T2DM	b_cell	*DR1*	-2.962966441	0.002623289	0.005854674	1
T2DM	b_cell	*CNN3*	-2.054260931	0.001094953	0.00415349	1
T2DM	b_cell	*PRRC2C*	-2.282303923	0.021829063	0.029187435	1
T2DM	b_cell	*ABCD3*	-2.991622959	0.012328776	0.017909199	1
T2DM	b_cell	*VAMP4*	-2.549062727	0.007234228	0.011651653	1
T2DM	b_cell	*PTBP2*	-1.766444058	0.034050847	0.043213631	1
T2DM	b_cell	*TNFSF4*	-4.36670182	4.30E-05	0.003541347	0.270371555
T2DM	b_cell	*DARS2*	-3.820347871	0.000982082	0.00415349	1
T2DM	b_cell	*DIEXF*	-4.34590255	0.000911291	0.00415349	1
T2DM	b_cell	*RCAN3*	-2.850049064	0.001340003	0.004286583	1
T2DM	b_cell	*RCOR3*	-2.533163464	0.004968445	0.008909473	1
T2DM	b_cell	*NSL1*	-2.83353828	0.004628075	0.008505322	1
T2DM	b_cell	*RPA2*	-3.727763829	0.009545661	0.014538172	1
T2DM	b_cell	*SLC5A9*	5.405636794	0.002495688	0.005713909	1
T2DM	b_cell	*OSBPL9*	-2.519162588	0.010821131	0.016109981	1
T2DM	b_cell	*TXNDC12*	-3.37144205	0.016411049	0.022846826	1
T2DM	b_cell	*ESYT2*	-2.23155513	0.002425087	0.005623947	1
T2DM	b_cell	*CD3EAP*	-2.861670901	3.64E-05	0.003315867	0.228794843
T2DM	b_cell	*A4GNT*	-4.022141334	0.003065769	0.006387091	1
T2DM	b_cell	*KMT2A*	-2.5665764	0.008012746	0.012625336	1
T2DM	b_cell	*IFT46*	-3.856828079	0.001646611	0.004658975	1
T2DM	b_cell	*CAMSAP2*	-2.544024064	0.010949265	0.016268464	1
T2DM	b_cell	*ATF6*	-2.505122985	0.006512338	0.01075468	1
T2DM	b_cell	*LRMP*	1.39338076	0.00070634	0.00415349	1
T2DM	b_cell	*ATP10B*	-4.75328095	0.000844185	0.00415349	1
T2DM	b_cell	*ELOVL4*	4.422870266	0.0031193	0.006465696	1
T2DM	b_cell	*FILIP1*	-1.65012217	0.000695115	0.00415349	1
T2DM	b_cell	*SGIP1*	-1.907047984	0.00100945	0.00415349	1
T2DM	b_cell	*AKAP7*	-2.368703812	0.000669839	0.00415349	1
T2DM	b_cell	*ALDH8A1*	-1.623825059	0.032153808	0.041001711	1
T2DM	b_cell	*MED28*	-3.44363845	0.003263489	0.006657222	1
T2DM	b_cell	*SLC16A7*	-2.166080994	0.003243325	0.006628995	1
T2DM	b_cell	*VAMP8*	-2.4089548	0.00105595	0.00415349	1
T2DM	b_cell	*FOXO3*	-2.243276564	0.005251658	0.009235574	1
T2DM	b_cell	*ABCG2*	-2.652849222	0.000716803	0.00415349	1
T2DM	b_cell	*SPP1*	2.553759481	0.035282633	0.044596283	1
T2DM	b_cell	*FAM47E-STBD1*	-2.39758851	0.001704823	0.004725848	1
T2DM	b_cell	*MFSD1*	-2.040308238	0.001345252	0.004286796	1
T2DM	b_cell	*RAB3GAP2*	-2.612939245	0.016717734	0.023212101	1
T2DM	b_cell	*UBN1*	-2.581609828	0.003499589	0.006971457	1
T2DM	b_cell	*UCHL3*	-3.15357996	0.000878716	0.00415349	1
T2DM	b_cell	*DNAH7*	-2.700036802	8.75E-07	0.000261873	0.005499342
T2DM	b_cell	*NDUFB3*	-4.290008958	0.013140263	0.018943552	1
T2DM	b_cell	*TRPM6*	-2.603076903	0.001714463	0.004735864	1

T2DM	b_cell	*GDA*	5.666563429	0.006239175	0.010421279	1
T2DM	b_cell	*C2orf40*	-2.7868853	0.001389278	0.004333459	1
T2DM	b_cell	*CPSF3*	-2.536497488	0.001626118	0.004638568	1
T2DM	b_cell	*PIGZ*	-1.644348664	0.00238953	0.00557776	1
T2DM	b_cell	*SENP5*	-2.416724844	0.006896909	0.011233385	1
T2DM	b_cell	*CCDC92*	-2.848558285	0.000121522	0.00415349	0.764005755
T2DM	b_cell	*FKBP15*	-2.661245405	0.008666318	0.013453122	1
T2DM	b_cell	*CTNNAL1*	-1.136547365	0.013578872	0.019450984	1
T2DM	b_cell	*WDR34*	2.599624689	0.017462559	0.024081401	1
T2DM	b_cell	*TRIM32*	-4.424807795	0.003055497	0.006378687	1
T2DM	b_cell	*PHF19*	-2.775164855	0.000664202	0.00415349	1
T2DM	b_cell	*BSPRY*	3.592264276	0.000683124	0.00415349	1
T2DM	b_cell	*PPP6C*	-2.300421817	0.001228208	0.004253251	1
T2DM	b_cell	*HDHD3*	-1.72814791	5.59E-05	0.00415349	0.351655286
T2DM	b_cell	*HSDL2*	-1.332128811	0.001400914	0.004345113	1
T2DM	b_cell	*INVS*	-2.310146509	0.006764132	0.011080276	1
T2DM	b_cell	*KDSR*	-2.167692865	0.001194547	0.004217141	1
T2DM	b_cell	*DNAL1*	-2.424376653	0.001975556	0.005092436	1
T2DM	b_cell	*ACOT2*	-1.062733775	0.023272917	0.030891682	1
T2DM	b_cell	*TTLL5*	-2.776507417	0.021676978	0.029008762	1
T2DM	b_cell	*FLVCR2*	-2.068800456	0.000750772	0.00415349	1
T2DM	b_cell	*PPP4R4*	2.368834269	0.002969165	0.00627678	1
T2DM	b_cell	*ZC2HC1C*	-2.566068123	0.000875494	0.00415349	1
T2DM	b_cell	*ALDH6A1*	-2.500858228	0.000958497	0.00415349	1
T2DM	b_cell	*ESRRB*	-2.451917832	0.000766291	0.00415349	1
T2DM	b_cell	*COQ6*	3.847653299	0.003742631	0.0072961	1
T2DM	b_cell	*GPR75*	-12.38847224	0.003393339	0.006833415	1
T2DM	b_cell	*KLHL29*	-2.017396067	0.001756635	0.004789372	1
T2DM	b_cell	*ATAD2B*	-2.420797277	0.005539872	0.009597457	1
T2DM	b_cell	*ATL2*	-2.649807	0.005095891	0.00904003	1
T2DM	b_cell	*CNRIP1*	-3.483058799	0.000821625	0.00415349	1
T2DM	b_cell	*CRIPT*	-4.311108572	0.003901008	0.007513983	1
T2DM	b_cell	*EPCAM*	-6.565237157	8.54E-05	0.00415349	0.536721219
T2DM	b_cell	*SLC17A5*	-2.863008059	0.010802958	0.016090546	1
T2DM	b_cell	*OGFRL1*	-2.450354606	0.006775447	0.011087255	1
T2DM	b_cell	*CUTC*	-2.672071691	0.004858731	0.008785402	1
T2DM	b_cell	*PYROXD2*	-1.130666119	0.023837326	0.031510779	1
T2DM	b_cell	*MXI1*	-3.292205124	0.005239665	0.00921964	1
T2DM	b_cell	*C10orf88*	-2.55166707	0.001071504	0.00415349	1
T2DM	b_cell	*HELLS*	2.588883146	0.000961049	0.00415349	1
T2DM	b_cell	*AVPI1*	-4.065183163	0.00650947	0.010752769	1
T2DM	b_cell	*WDR11*	-2.71596649	0.012540485	0.018170553	1
T2DM	b_cell	*C10orf76*	-2.304585184	0.00336317	0.006790062	1
T2DM	b_cell	*KCNIP2*	-1.286301139	0.005861979	0.009968694	1
T2DM	b_cell	*KANSL1*	-1.93228895	0.007948015	0.012555067	1
T2DM	b_cell	*HOXB5*	-3.25377919	0.000179608	0.00415349	1
T2DM	b_cell	*HOXB3*	-3.166965642	4.33E-05	0.003541347	0.272190538
T2DM	b_cell	*CD274*	-2.821369509	0.0008521	0.00415349	1
T2DM	b_cell	*NUP43*	-2.825330587	0.005977397	0.010123894	1
T2DM	b_cell	*LRP11*	-1.997253235	0.000363345	0.00415349	1
T2DM	b_cell	*CCDC170*	-1.438787796	0.001219062	0.004252746	1
T2DM	b_cell	*CYSTM1*	-2.36123335	0.003487135	0.006959878	1
T2DM	b_cell	*PCDHB10*	1.515253367	0.000604554	0.00415349	1
T2DM	b_cell	*PCDHB14*	-6.364385849	0.003780152	0.007342551	1
T2DM	b_cell	*SEC16B*	-5.298623517	0.002771125	0.006028396	1
T2DM	b_cell	*ACAT2*	-4.036727159	0.025530177	0.033425286	1
T2DM	b_cell	*SNX19*	-2.74139202	0.012560328	0.018195112	1
T2DM	b_cell	*MASTL*	-3.243424834	5.76E-05	0.00415349	0.36207734
T2DM	b_cell	*MRC1*	-1.26514285	0.000880874	0.00415349	1
T2DM	b_cell	*IQSEC3*	-5.767730268	0.002441065	0.005638124	1
T2DM	b_cell	*CCDC77*	-5.080854859	0.002233815	0.005401538	1

T2DM	b_cell	*ENOX1*	-2.135477985	0.001008078	0.00415349	1
T2DM	b_cell	*WBP4*	-2.371218511	0.002507555	0.005732727	1
T2DM	b_cell	*SMAD9*	-1.975198499	0.000793945	0.00415349	1
T2DM	b_cell	*SIL1*	-2.506858716	0.012137753	0.017668454	1
T2DM	b_cell	*ARL1*	-2.427046424	0.001948064	0.005048426	1
T2DM	b_cell	*APAF1*	-2.691490937	0.003754141	0.007309385	1
T2DM	b_cell	*EPHX2*	-1.967407745	0.000266952	0.00415349	1
T2DM	b_cell	*ZNF706*	-2.881410727	0.01615135	0.022535183	1
T2DM	b_cell	*CRISPLD1*	-2.965840186	0.00085648	0.00415349	1
T2DM	b_cell	*RDH10*	-1.597314745	0.006218199	0.010405595	1
T2DM	b_cell	*SCPEP1*	-1.674072635	0.028066073	0.036344263	1
T2DM	b_cell	*TRIM6*	-3.575233124	0.000451844	0.00415349	1
T2DM	b_cell	*CEP89*	-2.170475488	0.018425214	0.025187937	1
T2DM	b_cell	*ECHDC2*	-1.60575332	7.78E-05	0.00415349	0.4893596
T2DM	b_cell	*PRB2*	-2.656539515	0.001992251	0.005104028	1
T2DM	b_cell	*PYROXD1*	-3.257545324	0.037217684	0.046797516	1
T2DM	b_cell	*ZSCAN18*	-3.319406899	0.0083	0.012977394	1
T2DM	b_cell	*PDZRN3*	-1.039086857	0.000780357	0.00415349	1
T2DM	b_cell	*CSTA*	-2.48134839	0.001132587	0.00418468	1
T2DM	b_cell	*POPDC2*	3.836382643	0.000949717	0.00415349	1
T2DM	b_cell	*B4GALT4*	3.888274705	0.00748252	0.011970128	1
T2DM	b_cell	*CD80*	2.619353772	0.000770999	0.00415349	1
T2DM	b_cell	*CAT*	-1.502003248	0.000938839	0.00415349	1
T2DM	b_cell	*GJB6*	-101.253067	0.000816978	0.00415349	1
T2DM	b_cell	*TMEM39B*	-2.785994651	0.000818015	0.00415349	1
T2DM	b_cell	*RNF115*	-2.657903744	0.011107511	0.016431276	1
T2DM	b_cell	*POLR3GL*	4.947444528	0.004710112	0.008602378	1
T2DM	b_cell	*TNFSF10*	-2.559126298	0.002310778	0.005492478	1
T2DM	b_cell	*LIAS*	-1.793660858	0.000719097	0.00415349	1
T2DM	b_cell	*CLCC1*	-3.959679108	0.004456779	0.008250815	1
T2DM	b_cell	*GPSM2*	-2.083061422	0.003981449	0.007624542	1
T2DM	b_cell	*CXCR4*	1.063092046	0.000834358	0.00415349	1
T2DM	b_cell	*ACVR2A*	-2.535963971	0.005221939	0.009196171	1
T2DM	b_cell	*POLK*	-2.552854201	0.008123868	0.012743202	1
T2DM	b_cell	*FLT3*	2.67866812	0.00071352	0.00415349	1
T2DM	b_cell	*MTIF3*	-2.209487949	0.005813514	0.009915476	1
T2DM	b_cell	*RASL11A*	-5.043479395	0.000151944	0.00415349	0.955274923
T2DM	b_cell	*FMOD*	-2.109617825	0.000619541	0.00415349	1
T2DM	b_cell	*SERAC1*	-2.153544636	0.000589099	0.00415349	1
T2DM	b_cell	*LDB3*	-1.895197882	0.005595265	0.009653521	1
T2DM	b_cell	*FAM35A*	-2.451256569	0.028709332	0.037100837	1
T2DM	b_cell	*FAM213A*	-1.410146971	0.000558586	0.00415349	1
T2DM	b_cell	*NAA60*	-2.666982509	0.000827061	0.00415349	1
T2DM	b_cell	*ODF2L*	-2.066427787	0.037977559	0.047591172	1
T2DM	b_cell	*SPATA1*	-2.879313571	0.006315427	0.010520692	1
T2DM	b_cell	*LRRC39*	-1.960301086	0.005219643	0.009194703	1
T2DM	b_cell	*RWDD3*	6.506375737	0.03170764	0.040533943	1
T2DM	b_cell	*CCDC18*	-2.452127482	0.0025595	0.005798627	1
T2DM	b_cell	*RPAP2*	-2.381223141	0.003060212	0.006381278	1
T2DM	b_cell	*BBS9*	-2.549037339	0.002605577	0.005849897	1
T2DM	b_cell	*KIAA0087*	-8.870259009	0.000163374	0.00415349	1
T2DM	b_cell	*KLHL7*	-3.102300989	0.020768058	0.027959054	1
T2DM	b_cell	*HERPUD2*	-2.881119296	0.00293732	0.006226208	1
T2DM	b_cell	*CBX3*	-3.677818523	0.000988382	0.00415349	1
T2DM	b_cell	*FAM126A*	-2.486520256	0.014275331	0.020305206	1
T2DM	b_cell	*FKBP9*	-1.837345622	0.001175376	0.004202203	1
T2DM	b_cell	*CCZ1*	-2.846606319	0.027031062	0.035150707	1
T2DM	b_cell	*POLM*	-2.204929701	0.000417201	0.00415349	1
T2DM	b_cell	*SMU1*	-3.182965397	0.025356029	0.033224959	1
T2DM	b_cell	*GLIPR2*	-3.472456416	0.004622091	0.008496808	1
T2DM	b_cell	*DCAF10*	-2.370734917	0.003563875	0.007074861	1

T2DM	b_cell	*BICC1*	-1.217594364	0.001037626	0.00415349	1
T2DM	b_cell	*CISD1*	-2.419488829	0.000499812	0.00415349	1
T2DM	b_cell	*SLC25A16*	-1.34170596	0.003430559	0.006885994	1
T2DM	b_cell	*RBM19*	-3.358706096	0.030747033	0.039442276	1
T2DM	b_cell	*ACADS*	-1.985297409	0.000679137	0.00415349	1
T2DM	b_cell	*SSPN*	-1.680183655	0.018277293	0.025012918	1
T2DM	b_cell	*CCDC91*	-2.204998685	0.022098175	0.029490814	1
T2DM	b_cell	*PKN1*	-3.124263975	0.002623015	0.005854674	1
T2DM	b_cell	*ATP7B*	-1.415991943	0.004581843	0.008437623	1
T2DM	b_cell	*NLN*	-1.430676179	0.0036449	0.007192558	1
T2DM	b_cell	*CENPK*	1.619807751	0.003283106	0.006688769	1
T2DM	b_cell	*OPTN*	-2.33066638	0.00051231	0.00415349	1
T2DM	b_cell	*ATF1*	-2.767526746	0.037728053	0.047325672	1
T2DM	b_cell	*TSFM*	-2.764006455	0.002931424	0.006221257	1
T2DM	b_cell	*ARHGAP9*	-4.518982965	0.003138873	0.006486603	1
T2DM	b_cell	*SPATS2*	-2.776103925	0.004037952	0.007696831	1
T2DM	b_cell	*PDE1B*	-4.486950437	0.001431985	0.004393796	1
T2DM	b_cell	*SMUG1*	-1.347017462	0.033153057	0.042201512	1
T2DM	b_cell	*SARDH*	-6.228489623	0.000774276	0.00415349	1
T2DM	b_cell	*DBH*	-15.40306296	0.01029352	0.015489555	1
T2DM	b_cell	*STIL*	-4.159773394	0.001768781	0.004801523	1
T2DM	b_cell	*NDUFAF4*	-1.983769469	0.009957394	0.015073955	1
T2DM	b_cell	*TTC21B*	-2.714961785	0.012115873	0.017652303	1
T2DM	b_cell	*NMI*	-3.533042986	0.010410473	0.015624407	1
T2DM	b_cell	*ACVR1C*	-1.252371807	0.001276473	0.004257394	1
T2DM	b_cell	*SLC36A1*	-1.932743408	0.017919382	0.024603441	1
T2DM	b_cell	*PLA2G12A*	-3.396359831	0.02804748	0.03632767	1
T2DM	b_cell	*C4BPB*	-3.858930555	0.001141692	0.00418468	1
T2DM	b_cell	*RAB38*	-3.40334134	0.000562443	0.00415349	1
T2DM	b_cell	*AGO2*	-2.864661605	0.005154735	0.00911667	1
T2DM	b_cell	*CHPF*	-2.910721345	0.001250393	0.004253251	1
T2DM	b_cell	*DNPEP*	-2.864491263	0.001622931	0.004634377	1
T2DM	b_cell	*MOGAT1*	-2.004087611	0.000952656	0.00415349	1
T2DM	b_cell	*FAM124B*	-3.086517267	0.021234156	0.028522663	1
T2DM	b_cell	*FAM210B*	-2.436345926	0.004933496	0.008861968	1
T2DM	b_cell	*SNX21*	-1.545771519	0.000782193	0.00415349	1
T2DM	b_cell	*WFDC3*	-1.926507062	0.001456285	0.004416626	1
T2DM	b_cell	*SLC12A5*	-6.746398934	0.001106325	0.004159248	1
T2DM	b_cell	*SDC4*	-2.850803115	0.000366199	0.00415349	1
T2DM	b_cell	*VAPB*	-3.170707507	0.00090174	0.00415349	1
T2DM	b_cell	*TOX2*	-1.641191595	0.002582499	0.005819416	1
T2DM	b_cell	*ARFGEF2*	-2.560095275	0.006197482	0.010387515	1
T2DM	b_cell	*CDH26*	-2.578689958	0.000800821	0.00415349	1
T2DM	b_cell	*STX16*	-2.940383109	0.004008878	0.007653756	1
T2DM	b_cell	*RNF114*	-3.1886052	0.002659178	0.005911687	1
T2DM	b_cell	*KCNK15*	2.383181579	0.000661471	0.00415349	1
T2DM	b_cell	*PEPD*	-1.566173374	0.002902158	0.006192483	1
T2DM	b_cell	*CHST8*	-7.884668299	0.004782039	0.00869424	1
T2DM	b_cell	*STAMBP*	-3.232362199	0.015572246	0.021828921	1
T2DM	b_cell	*USP22*	-2.884028419	0.019327932	0.026193118	1
T2DM	b_cell	*HIF3A*	-1.486091871	0.002366481	0.005545309	1
T2DM	b_cell	*BTN2A2*	1.82054532	0.000623458	0.00415349	1
T2DM	b_cell	*MRS2*	-2.783655464	0.007552109	0.012059971	1
T2DM	b_cell	*SERPINB6*	-2.583959779	0.002113683	0.005250385	1
T2DM	b_cell	*HIST1H1D*	-4.232709092	0.016306413	0.022721281	1
T2DM	b_cell	*PEX6*	-1.87389138	0.000887522	0.00415349	1
T2DM	b_cell	*NQO2*	-2.437523453	0.001576315	0.004562749	1
T2DM	b_cell	*RPS10*	3.161495308	0.000637496	0.00415349	1
T2DM	b_cell	*HIST1H2BJ*	3.543831124	0.000100393	0.00415349	0.631172505
T2DM	b_cell	*MED20*	-2.962186573	0.000401221	0.00415349	1
T2DM	b_cell	*KLHDC3*	-2.313007071	0.000637283	0.00415349	1

T2DM	b_cell	*MEA1*	-3.581344926	0.001126725	0.004182741	1
T2DM	b_cell	*KLHL31*	-1.455800908	0.002304557	0.005484718	1
T2DM	b_cell	*COL21A1*	-1.850715916	0.001035005	0.00415349	1
T2DM	b_cell	*RPP40*	-2.958935497	0.021349314	0.028637324	1
T2DM	b_cell	*EEF1E1*	-3.556983974	0.000789724	0.00415349	1
T2DM	b_cell	*EREG*	3.035003836	0.000673398	0.00415349	1
T2DM	b_cell	*MYRF*	-4.362561785	0.022424094	0.029894037	1
T2DM	b_cell	*CNOT1*	-2.469560536	0.031299117	0.040068734	1
T2DM	b_cell	*MT1G*	-19.40280996	0.000358648	0.00415349	1
T2DM	b_cell	*CLYBL*	-2.994208531	0.0058962	0.010013347	1
T2DM	b_cell	*TMTC4*	-2.096506157	0.000859434	0.00415349	1
T2DM	b_cell	*KIF25*	-5.341675287	0.005396642	0.009426623	1
T2DM	b_cell	*TEKT3*	-2.48995554	0.009309822	0.014244549	1
T2DM	b_cell	*MRPS7*	-4.351957357	0.011035422	0.016358965	1
T2DM	b_cell	*GGA3*	-2.873015915	0.001890351	0.004970573	1
T2DM	b_cell	*NUP85*	-2.796055149	0.002701366	0.005946726	1
T2DM	b_cell	*NT5C*	-3.887356951	0.029425287	0.037909175	1
T2DM	b_cell	*PPP1R12C*	-3.967989403	0.015269654	0.021466975	1
T2DM	b_cell	*OPRL1*	-8.372908773	7.03E-08	4.02E-05	0.000441671
T2DM	b_cell	*PLGLB2*	-30.95093466	0.032158333	0.041001711	1
T2DM	b_cell	*CHCHD5*	-4.927615682	0.038278883	0.047911474	1
T2DM	b_cell	*PAX8*	-2.68616972	0.008978823	0.013835751	1
T2DM	b_cell	*POLR1B*	-2.435227776	0.000816746	0.00415349	1
T2DM	b_cell	*SLC25A23*	-2.414781466	0.024524283	0.032263414	1
T2DM	b_cell	*GTF2F1*	-3.7039432	0.007633206	0.012164757	1
T2DM	b_cell	*CLPP*	-2.26238209	0.00232628	0.005506523	1
T2DM	b_cell	*SH2D3A*	-3.32352947	0.000630116	0.00415349	1
T2DM	b_cell	*TRIP10*	-1.808493267	0.001269521	0.004257394	1
T2DM	b_cell	*GPR108*	-3.214954972	0.003470519	0.006942141	1
T2DM	b_cell	*RTN2*	-3.162776637	0.00058851	0.00415349	1
T2DM	b_cell	*SDCBP2*	-6.127888269	0.018384659	0.02514343	1
T2DM	b_cell	*PANK2*	-2.772263107	0.00328321	0.006688769	1
T2DM	b_cell	*PSMF1*	-2.858612602	0.000835718	0.00415349	1
T2DM	b_cell	*DTD1*	-3.228925789	0.024190118	0.031896659	1
T2DM	b_cell	*RRBP1*	-2.596499525	0.003323342	0.006745057	1
T2DM	b_cell	*BMP2*	-3.655852044	0.000790375	0.00415349	1
T2DM	b_cell	*DSTN*	-2.444477585	0.002566246	0.005799421	1
T2DM	b_cell	*MGME1*	-1.887290772	0.0007855	0.00415349	1
T2DM	b_cell	*MCM8*	-1.903672306	0.02267889	0.030208089	1
T2DM	b_cell	*TMEM74B*	-5.090612994	0.03037273	0.039009878	1
T2DM	b_cell	*FAM110A*	-3.045311459	0.000797902	0.00415349	1
T2DM	b_cell	*MRPS26*	-1.94187464	0.009096471	0.013975932	1
T2DM	b_cell	*NCLN*	-4.14595086	0.001252847	0.004255347	1
T2DM	b_cell	*ZNF436*	-1.72640595	0.013498047	0.019352844	1
T2DM	b_cell	*MMP24*	-2.391819705	0.005062227	0.009007468	1
T2DM	b_cell	*UROD*	-3.560568293	0.021887659	0.02925956	1
T2DM	b_cell	*ST3GAL3*	-2.410511341	0.005104202	0.009050883	1
T2DM	b_cell	*KLC1*	-2.739462238	0.015129707	0.021313123	1
T2DM	b_cell	*XRCC3*	-2.40323865	0.001860375	0.004935095	1
T2DM	b_cell	*MCF2L*	-1.659919261	0.003895962	0.007508864	1
T2DM	b_cell	*F10*	-1.855754058	0.00079427	0.00415349	1
T2DM	b_cell	*PCID2*	-2.67873717	0.001269493	0.004257394	1
T2DM	b_cell	*PROZ*	3.810884155	0.000605906	0.00415349	1
T2DM	b_cell	*IGFLR1*	8.578908151	0.001142231	0.00418468	1
T2DM	b_cell	*TSKS*	-3.015111054	0.001029	0.00415349	1
T2DM	b_cell	*FLRT1*	-2.772802508	0.001651558	0.004668493	1
T2DM	b_cell	*DNAJC8*	-3.248342471	0.000917015	0.00415349	1
T2DM	b_cell	*ZNF384*	-3.930583337	0.019661189	0.026611388	1
T2DM	b_cell	*EMG1*	-4.134218746	0.011782406	0.017226973	1
T2DM	b_cell	*ATG14*	-2.223992224	0.001905549	0.004988168	1
T2DM	b_cell	*RHOJ*	-1.605225842	0.00073256	0.00415349	1

T2DM	b_cell	*L3HYPDH*	-1.491929434	0.002436329	0.005631324	1
T2DM	b_cell	*ZBTB1*	-2.374218115	0.003004449	0.006308941	1
T2DM	b_cell	*PZP*	-1.712672971	0.001036108	0.00415349	1
T2DM	b_cell	*RHOT1*	-2.159792586	0.004901238	0.00882925	1
T2DM	b_cell	*WDR60*	-2.098903282	0.00205292	0.005193845	1
T2DM	b_cell	*AIF1L*	-2.249131933	0.00085753	0.00415349	1
T2DM	b_cell	*MAP2K2*	-3.086765482	0.001087177	0.00415349	1
T2DM	b_cell	*CANX*	-2.948302999	0.015528618	0.021796924	1
T2DM	b_cell	*IPPK*	-2.974966793	0.000593601	0.00415349	1
T2DM	b_cell	*FGD3*	-2.608842482	0.001094285	0.00415349	1
T2DM	b_cell	*ABHD8*	-3.377307249	0.005803761	0.009904519	1
T2DM	b_cell	*MASP1*	-3.013168892	0.000453339	0.00415349	1
T2DM	b_cell	*ATP13A4*	-3.080259019	0.003718232	0.007266559	1
T2DM	b_cell	*HRASLS*	-4.473414163	0.002554697	0.005795069	1
T2DM	b_cell	*HELB*	-3.537856665	0.00121511	0.004245409	1
T2DM	b_cell	*RAP1B*	-2.312464787	0.013369439	0.019212266	1
T2DM	b_cell	*TAS2R4*	-3.976267295	0.038232033	0.047871897	1
T2DM	b_cell	*LRRC61*	-1.936529152	0.016018585	0.022374772	1
T2DM	b_cell	*IDUA*	3.787385772	0.002249901	0.005421667	1
T2DM	b_cell	*EMC1*	-3.515483632	0.00692513	0.011270591	1
T2DM	b_cell	*PLA2G5*	-2.310522164	0.010044381	0.015183703	1
T2DM	b_cell	*UBR4*	-2.738490477	0.000694207	0.00415349	1
T2DM	b_cell	*SIN3B*	-3.24301504	0.011921475	0.017406018	1
T2DM	b_cell	*WFIKKN1*	-29.40014337	0.000194111	0.00415349	1
T2DM	b_cell	*WDR24*	-6.080284202	0.000410548	0.00415349	1
T2DM	b_cell	*CHTF18*	6.026345571	0.021920728	0.02929131	1
T2DM	b_cell	*SMARCA4*	-3.146817712	0.001594843	0.004599438	1
T2DM	b_cell	*TICAM1*	-4.309910007	0.003384924	0.006820839	1
T2DM	b_cell	*AAMP*	-2.914157088	0.000525137	0.00415349	1
T2DM	b_cell	*PNKD*	-3.368277586	0.001882109	0.004963761	1
T2DM	b_cell	*ZNF835*	-4.026663422	0.000937959	0.00415349	1
T2DM	b_cell	*HIP1*	-2.057022748	0.002509311	0.005734251	1
T2DM	b_cell	*PTPN12*	-2.28571625	0.003423405	0.006878539	1
T2DM	b_cell	*POR*	-1.883253847	0.001032833	0.00415349	1
T2DM	b_cell	*FGL2*	-2.245346086	0.008105744	0.012724298	1
T2DM	b_cell	*STEAP4*	-2.979562668	0.003399225	0.006843076	1
T2DM	b_cell	*GNAI1*	-1.337127279	0.000579118	0.00415349	1
T2DM	b_cell	*SGCE*	-1.867293565	0.00114452	0.00418468	1
T2DM	b_cell	*RBM48*	-1.951325499	0.000691607	0.00415349	1
T2DM	b_cell	*SRD5A3*	-2.872705083	0.001709558	0.004728644	1
T2DM	b_cell	*DGCR6L*	3.479844857	0.001638092	0.004657671	1
T2DM	b_cell	*VPREB3*	-29.44487946	0.007986523	0.012595482	1
T2DM	b_cell	*MGAT3*	-2.636145346	0.000730522	0.00415349	1
T2DM	b_cell	*ATF4*	-3.695960984	0.018518341	0.025293246	1
T2DM	b_cell	*A4GALT*	-1.962683015	0.002903354	0.006192483	1
T2DM	b_cell	*CDC42EP1*	-2.510980837	0.00099712	0.00415349	1
T2DM	b_cell	*TPST2*	-1.818110307	0.000969628	0.00415349	1
T2DM	b_cell	*TST*	-2.169334634	0.002142048	0.005287258	1
T2DM	b_cell	*APOL5*	-13.90452253	0.000836223	0.00415349	1
T2DM	b_cell	*RIBC2*	3.22195524	2.07E-05	0.002553516	0.130421625
T2DM	b_cell	*EMC4*	-2.643210233	0.010073606	0.015216906	1
T2DM	b_cell	*SPECC1*	-1.743740692	0.001084987	0.00415349	1
T2DM	b_cell	*POT1*	-2.966672376	0.027761633	0.036009364	1
T2DM	b_cell	*CDHR3*	-1.54971589	0.001758265	0.004789372	1
T2DM	b_cell	*PRKRIP1*	-2.833704059	0.001466257	0.004431902	1
T2DM	b_cell	*FOXP2*	-1.221182755	0.000327926	0.00415349	1
T2DM	b_cell	*STRIP2*	4.713785465	0.024578965	0.032314503	1
T2DM	b_cell	*FLNC*	-2.67041952	0.001978817	0.005092436	1
T2DM	b_cell	*IRF5*	-1.75803375	0.020951533	0.028187949	1
T2DM	b_cell	*KLHDC10*	-2.14857325	0.002574797	0.005806222	1
T2DM	b_cell	*OPN1SW*	-4.204634985	0.003447123	0.006899733	1

T2DM	b_cell	*HOXD3*	-1.895698873	0.001038585	0.00415349	1
T2DM	b_cell	*PDE11A*	-3.801876678	0.000425489	0.00415349	1
T2DM	b_cell	*CHN1*	-1.542553839	0.00086598	0.00415349	1
T2DM	b_cell	*GAD1*	3.054293693	0.000704688	0.00415349	1
T2DM	b_cell	*ORMDL1*	-1.647643116	0.009833823	0.01491562	1
T2DM	b_cell	*HOXD9*	-4.458143566	0.014711191	0.020812164	1
T2DM	b_cell	*PSMG2*	-2.406060699	0.002487495	0.005702562	1
T2DM	b_cell	*TWSG1*	-3.512080066	0.012485609	0.018108734	1
T2DM	b_cell	*WDFY4*	1.341069212	0.001311366	0.004274973	1
T2DM	b_cell	*EIF2AK4*	-3.450911061	0.000661949	0.00415349	1
T2DM	b_cell	*TMOD2*	-2.782432024	0.001126097	0.004182741	1
T2DM	b_cell	*IVD*	-3.293868161	0.011516512	0.016952543	1
T2DM	b_cell	*ARPP19*	-3.346416007	0.004509893	0.008336871	1
T2DM	b_cell	*VPS13C*	-2.338133727	0.012458407	0.018076622	1
T2DM	b_cell	*CALML4*	-9.504612481	0.002445642	0.005640408	1
T2DM	b_cell	*ACKR4*	-2.966082345	0.004319419	0.008068011	1
T2DM	b_cell	*ANAPC13*	-2.058311069	0.001365795	0.004299827	1
T2DM	b_cell	*SUMF2*	-2.88653654	0.003575379	0.007093217	1
T2DM	b_cell	*SPCS3*	-3.578107609	0.017602514	0.024253124	1
T2DM	b_cell	*RPAIN*	-2.88923673	0.000534918	0.00415349	1
T2DM	b_cell	*PLD2*	3.696803858	0.000469896	0.00415349	1
T2DM	b_cell	*ATP1B2*	-6.253034289	0.007511082	0.012006654	1
T2DM	b_cell	*CCL4*	-2.137866203	0.001273186	0.004257394	1
T2DM	b_cell	*PHF20L1*	-2.570596153	0.004003449	0.007651297	1
T2DM	b_cell	*PUS7L*	-2.969193202	0.034244147	0.043400731	1
T2DM	b_cell	*CDKN2D*	1.995579961	0.000950346	0.00415349	1
T2DM	b_cell	*MTUS1*	-2.398164576	0.001247954	0.004253251	1
T2DM	b_cell	*NGDN*	-2.717083927	0.001437947	0.004399209	1
T2DM	b_cell	*RIPK3*	-3.325593266	0.002089379	0.005231352	1
T2DM	b_cell	*ADCY4*	-2.354657069	0.00168529	0.004704627	1
T2DM	b_cell	*RAB2B*	-2.499870903	0.001340626	0.004286583	1
T2DM	b_cell	*MIS18BP1*	-2.026215697	0.021145398	0.028418366	1
T2DM	b_cell	*RNASE1*	-3.072875881	0.007980896	0.012594351	1
T2DM	b_cell	*TEP1*	-4.034180991	0.013182544	0.018998056	1
T2DM	b_cell	*EPB41L4A*	-1.33225142	0.000861657	0.00415349	1
T2DM	b_cell	*CDO1*	-1.90391342	0.000808237	0.00415349	1
T2DM	b_cell	*REEP5*	-2.704689738	0.005651127	0.009724561	1
T2DM	b_cell	*QRICH2*	-2.463901407	0.001686877	0.004704627	1
T2DM	b_cell	*SEC14L1*	-2.152059531	0.001505744	0.004478191	1
T2DM	b_cell	*RHBDF2*	2.627498781	0.00043941	0.00415349	1
T2DM	b_cell	*AANAT*	4.384476811	0.000314425	0.00415349	1
T2DM	b_cell	*TMEM8A*	-3.73725296	0.001684068	0.004704627	1
T2DM	b_cell	*SHC2*	-1.88908054	0.005522419	0.009579216	1
T2DM	b_cell	*ERMARD*	-3.407672087	0.001592154	0.004593791	1
T2DM	b_cell	*PHF10*	-3.534296567	0.009405741	0.014359857	1
T2DM	b_cell	*GALNT8*	-3.407383162	0.000136575	0.00415349	0.858647105
T2DM	b_cell	*DOCK6*	-1.573253217	0.001361654	0.004298348	1
T2DM	b_cell	*ECSIT*	-3.942967643	0.011514921	0.016952543	1
T2DM	b_cell	*ELOF1*	-3.763534975	0.000804031	0.00415349	1
T2DM	b_cell	*CDC16*	-2.53424995	0.005065762	0.009009461	1
T2DM	b_cell	*THEM6*	-3.035722353	0.000387436	0.00415349	1
T2DM	b_cell	*TOMM40*	-4.192427636	0.00383874	0.007422066	1
T2DM	b_cell	*DPP6*	-3.324911122	0.000197695	0.00415349	1
T2DM	b_cell	*FAM98C*	3.567761705	0.011627471	0.017055842	1
T2DM	b_cell	*ATP8B3*	-3.037532837	0.002646808	0.005892522	1
T2DM	b_cell	*GTPBP3*	-6.137383896	0.011707868	0.017144273	1
T2DM	b_cell	*PLVAP*	-1.356953446	0.003646866	0.007194179	1
T2DM	b_cell	*BST2*	-2.766301464	0.00123678	0.004253251	1
T2DM	b_cell	*DDA1*	-2.510620557	0.000754581	0.00415349	1
T2DM	b_cell	*TULP4*	-2.542778425	0.003512031	0.00699181	1
T2DM	b_cell	*RTN4IP1*	-3.082341449	0.004705661	0.008599361	1

T2DM	b_cell	*RSPH3*	-2.340248424	0.012015824	0.017521682	1
T2DM	b_cell	*ACTN4*	-2.755884666	0.003788212	0.007350767	1
T2DM	b_cell	*NDUFA10*	-1.788772357	0.018320089	0.025066028	1
T2DM	b_cell	*PXDN*	-1.29423725	0.00077909	0.00415349	1
T2DM	b_cell	*PGPEP1*	4.15305438	0.005685226	0.009763184	1
T2DM	b_cell	*LSM4*	-2.908996009	0.013766317	0.019683611	1
T2DM	b_cell	*CAMSAP1*	-2.888923841	0.001862829	0.0049373	1
T2DM	b_cell	*UBAC1*	-3.489999613	0.010139607	0.015298227	1
T2DM	b_cell	*TNNT3*	-3.32915027	0.000274594	0.00415349	1
T2DM	b_cell	*ATXN10*	-2.511421412	0.031488236	0.040286231	1
T2DM	b_cell	*TUBGCP2*	-2.311476556	0.002220225	0.005393569	1
T2DM	b_cell	*CYP2E1*	5.529261419	0.007091902	0.011482562	1
T2DM	b_cell	*PAK4*	-2.662463562	0.000994102	0.00415349	1
T2DM	b_cell	*ZNF337*	-2.425769025	0.001802637	0.004852128	1
T2DM	b_cell	*POMT1*	-3.214339861	0.00305163	0.006376763	1
T2DM	b_cell	*UCK1*	2.633472572	0.011120228	0.016442351	1
T2DM	b_cell	*MAP3K10*	-2.663197806	0.00076077	0.00415349	1
T2DM	b_cell	*SMPDL3B*	4.917875167	0.000595175	0.00415349	1
T2DM	b_cell	*ATPIF1*	-3.905038526	0.001654133	0.00466975	1
T2DM	b_cell	*THEMIS2*	-1.836611384	0.002868234	0.006154645	1
T2DM	b_cell	*CLIP1*	-2.457251999	0.010503711	0.015719312	1
T2DM	b_cell	*ZNF317*	-2.164724447	0.000905939	0.00415349	1
T2DM	b_cell	*PPAN*	5.92268815	0.002086364	0.005230315	1
T2DM	b_cell	*C12orf65*	-2.596395302	0.037936419	0.047549096	1
T2DM	b_cell	*PKDREJ*	-7.684208752	0.000780036	0.00415349	1
T2DM	b_cell	*HSD17B3*	-2.772834844	0.00072583	0.00415349	1
T2DM	b_cell	*HABP4*	-1.959321108	0.000785261	0.00415349	1
T2DM	b_cell	*EPS8L1*	4.071358507	0.000742531	0.00415349	1
T2DM	b_cell	*LILRB2*	-2.799481389	0.000741477	0.00415349	1
T2DM	b_cell	*COX4I2*	2.384390916	0.000714448	0.00415349	1
T2DM	b_cell	*ATP6V1E1*	-2.713206084	0.000511428	0.00415349	1
T2DM	b_cell	*PPT1*	-2.161875852	0.000885905	0.00415349	1
T2DM	b_cell	*HAUS8*	-3.222609324	0.000366753	0.00415349	1
T2DM	b_cell	*TBC1D5*	-2.108522306	0.009249905	0.014177023	1
T2DM	b_cell	*RFTN1*	-1.63776523	0.001961497	0.005076957	1
T2DM	b_cell	*KCNC3*	-3.864980448	0.003577153	0.007094499	1
T2DM	b_cell	*KIF3A*	-2.827062523	0.003172583	0.006535397	1
T2DM	b_cell	*AOC3*	-1.734644881	0.000617526	0.00415349	1
T2DM	b_cell	*ACLY*	-3.313928253	0.006941204	0.011290906	1
T2DM	b_cell	*AOC2*	-4.83385892	0.000850949	0.00415349	1
T2DM	b_cell	*DIAPH1*	-3.121680134	0.004198444	0.00790288	1
T2DM	b_cell	*EXOC4*	-1.788409393	0.014247453	0.020283907	1
T2DM	b_cell	*ACAP3*	-2.185715678	0.00171893	0.004746118	1
T2DM	b_cell	*C1orf159*	-3.113320056	0.002275116	0.005445221	1
T2DM	b_cell	*PPFIA1*	-2.423525594	0.002276665	0.005445221	1
T2DM	b_cell	*NINJ1*	-2.766508356	0.000715088	0.00415349	1
T2DM	b_cell	*NPHP4*	-2.399297035	0.000945983	0.00415349	1
T2DM	b_cell	*MAP1B*	-1.03692284	0.00645822	0.010687768	1
T2DM	b_cell	*CKMT2*	-2.004082513	0.000743414	0.00415349	1
T2DM	b_cell	*ZCCHC9*	-3.421644218	0.010905006	0.016211816	1
T2DM	b_cell	*KHDRBS3*	-1.73253915	0.000937738	0.00415349	1
T2DM	b_cell	*CHD1L*	-3.283747255	0.007614829	0.012141625	1
T2DM	b_cell	*RBM8A*	-2.074897008	0.000350903	0.00415349	1
T2DM	b_cell	*ZSCAN5A*	-3.355842738	0.007066929	0.011450974	1
T2DM	b_cell	*ZNF132*	-2.581472917	0.024270593	0.031982648	1
T2DM	b_cell	*RHPN2*	-4.073722896	0.000110755	0.00415349	0.696318957
T2DM	b_cell	*C19orf12*	-1.370201995	0.001110091	0.004166654	1
T2DM	b_cell	*ACTR10*	-2.981476041	0.018753048	0.025547219	1
T2DM	b_cell	*GCH1*	-2.224897625	0.002418277	0.005614368	1
T2DM	b_cell	*LGALS3*	-2.567890011	0.003691969	0.007246772	1
T2DM	b_cell	*DNAJB1*	-2.370389612	0.001010966	0.00415349	1

T2DM	b_cell	*FBXW9*	-2.985542803	0.006125873	0.010300445	1
T2DM	b_cell	*C19orf57*	-5.341525869	0.001296772	0.004268027	1
T2DM	b_cell	*MATN3*	-11.14742095	0.000907669	0.00415349	1
T2DM	b_cell	*CCT6B*	-3.596108583	0.001878185	0.004959322	1
T2DM	b_cell	*RAF1*	-2.807388936	0.015242445	0.021438312	1
T2DM	b_cell	*ENOSF1*	-1.799803582	0.001364586	0.004299827	1
T2DM	b_cell	*EMILIN2*	-1.847365297	0.002553221	0.005794981	1
T2DM	b_cell	*TRIM5*	-3.093104555	0.009806571	0.014881466	1
T2DM	b_cell	*CNGA4*	-4.729602058	0.000716237	0.00415349	1
T2DM	b_cell	*RRP8*	-4.153044097	0.034054371	0.043213631	1
T2DM	b_cell	*IMMT*	-2.174991848	0.005682395	0.009761317	1
T2DM	b_cell	*CARD6*	-4.024203102	0.007269634	0.011686062	1
T2DM	b_cell	*INPP5K*	-2.260583293	0.000652643	0.00415349	1
T2DM	b_cell	*MYBBP1A*	-2.459056214	0.001029053	0.00415349	1
T2DM	b_cell	*RPA1*	-2.54805234	0.001329487	0.004274973	1
T2DM	b_cell	*SERPINF1*	-1.912763701	0.001294809	0.004268027	1
T2DM	b_cell	*TMEM128*	-4.855758037	0.011230038	0.016585212	1
T2DM	b_cell	*SEC61G*	-2.476650701	0.002328266	0.005509149	1
T2DM	b_cell	*FIGNL1*	-4.102347372	0.003653024	0.007196562	1
T2DM	b_cell	*WBP2*	-3.068400892	0.001689652	0.004704627	1
T2DM	b_cell	*EIF5A*	-2.857071031	0.00047959	0.00415349	1
T2DM	b_cell	*KDM6B*	-4.400902933	0.008487929	0.013218631	1
T2DM	b_cell	*XAF1*	-1.562780602	0.001937987	0.005028529	1
T2DM	b_cell	*REEP2*	-3.439798404	0.005916272	0.01004201	1
T2DM	b_cell	*PRMT7*	-3.173221177	0.008080505	0.012700534	1
T2DM	b_cell	*BTBD3*	-1.922446865	0.000789813	0.00415349	1
T2DM	b_cell	*PCNA*	5.496606086	0.017631052	0.024281801	1
T2DM	b_cell	*PTPRA*	-2.465540774	0.0105103	0.015721688	1
T2DM	b_cell	*IGHMBP2*	-3.692685143	0.001363198	0.004298348	1
T2DM	b_cell	*MMACHC*	-3.471652512	0.00045131	0.00415349	1
T2DM	b_cell	*CTNNBL1*	-3.111649527	0.009503153	0.014483958	1
T2DM	b_cell	*LPIN3*	-2.390085583	0.000964397	0.00415349	1
T2DM	b_cell	*SERINC3*	-2.916135516	0.000716398	0.00415349	1
T2DM	b_cell	*DMGDH*	-1.70519456	0.00043228	0.00415349	1
T2DM	b_cell	*BHMT2*	-1.147448228	0.001284114	0.004268027	1
T2DM	b_cell	*KANK4*	-1.572325736	0.00166006	0.004679659	1
T2DM	b_cell	*ATP8A2*	-3.001406284	0.000772093	0.00415349	1
T2DM	b_cell	*USPL1*	-3.053015809	0.018866496	0.025664149	1
T2DM	b_cell	*POMP*	-3.543449435	0.019690944	0.026645924	1
T2DM	b_cell	*WASF3*	-1.441393918	0.00075239	0.00415349	1
T2DM	b_cell	*RNF17*	4.284521797	0.006229212	0.010416354	1
T2DM	b_cell	*PEMT*	-2.072228413	0.000852121	0.00415349	1
T2DM	b_cell	*SCO1*	-3.674657085	0.001817238	0.004869982	1
T2DM	b_cell	*TMCC2*	-6.252197041	8.37E-06	0.001459962	0.052614512
T2DM	b_cell	*COG6*	-2.504729257	0.027818287	0.036075406	1
T2DM	b_cell	*TRPC4*	-3.525716307	0.000809574	0.00415349	1
T2DM	b_cell	*RFC3*	-2.446251893	0.00202522	0.00516315	1
T2DM	b_cell	*PRAM1*	-3.04536007	0.000166793	0.00415349	1
T2DM	b_cell	*ZNF414*	-2.68063959	0.000781971	0.00415349	1
T2DM	b_cell	*CNDP2*	-2.941908743	0.001590603	0.004593531	1
T2DM	b_cell	*RTN3*	-1.87358227	0.001723631	0.004750754	1
T2DM	b_cell	*C1QTNF6*	-2.492625542	0.000893266	0.00415349	1
T2DM	b_cell	*FAM83F*	-5.616160089	0.000655027	0.00415349	1
T2DM	b_cell	*GIMAP4*	-2.244766951	0.000353173	0.00415349	1
T2DM	b_cell	*ADCK2*	-3.381276858	0.001379039	0.004323793	1
T2DM	b_cell	*AGAP3*	-2.601123755	0.001328542	0.004274973	1
T2DM	b_cell	*ACTR3B*	-3.924902211	0.004556419	0.00839637	1
T2DM	b_cell	*DYDC2*	-13.08560353	0.000916549	0.00415349	1
T2DM	b_cell	*TMTC1*	-1.267328734	0.000912737	0.00415349	1
T2DM	b_cell	*IPO8*	-3.202775443	0.010407571	0.015623782	1
T2DM	b_cell	*LARS*	-2.123851736	0.008329819	0.013014308	1

T2DM	b_cell	*SPINK5*	-4.74757619	0.007013997	0.011382808	1
T2DM	b_cell	*IMPA1*	-3.12737456	0.018826476	0.025625038	1
T2DM	b_cell	*LRRCC1*	-2.542845107	0.006346334	0.010555397	1
T2DM	b_cell	*AMPD3*	-2.227072367	0.000616958	0.00415349	1
T2DM	b_cell	*SBF2*	-1.976721577	0.004368072	0.008127276	1
T2DM	b_cell	*MICAL2*	-2.527641928	0.033736035	0.042865492	1
T2DM	b_cell	*HSD17B4*	-2.504786522	0.002157047	0.00530985	1
T2DM	b_cell	*DPF2*	-2.635807091	0.001457078	0.004416899	1
T2DM	b_cell	*GSC*	-6.999022296	0.00076662	0.00415349	1
T2DM	b_cell	*CATSPERB*	-1.834320842	0.000337355	0.00415349	1
T2DM	b_cell	*COX16*	-3.714940853	0.012576464	0.018214289	1
T2DM	b_cell	*MED6*	-1.663379313	0.001828836	0.004890949	1
T2DM	b_cell	*EIF2S1*	-3.316588513	0.028207857	0.036512826	1
T2DM	b_cell	*ELP3*	-2.695037802	0.002548413	0.005790341	1
T2DM	b_cell	*ADAMDEC1*	-9.664414298	0.007774655	0.012350455	1
T2DM	b_cell	*MRO*	-3.963871893	0.001316183	0.004274973	1
T2DM	b_cell	*THUMPD3*	-3.226699168	0.000863548	0.00415349	1
T2DM	b_cell	*CNTN6*	-2.211966795	0.000660622	0.00415349	1
T2DM	b_cell	*KATNBL1*	-2.251053081	0.003372135	0.00680379	1
T2DM	b_cell	*GSTM1*	2.987440095	0.027095726	0.035225564	1
T2DM	b_cell	*GSTM5*	-2.311545534	0.006385406	0.010597953	1
T2DM	b_cell	*SYT6*	-7.778472568	0.000953655	0.00415349	1
T2DM	b_cell	*VAV3*	2.763880967	0.008438676	0.013145182	1
T2DM	b_cell	*PSRC1*	2.870600959	0.00208456	0.005230315	1
T2DM	b_cell	*WNT2B*	-2.453083048	0.001274684	0.004257394	1
T2DM	b_cell	*CEPT1*	-2.196084415	0.002179389	0.005337677	1
T2DM	b_cell	*AP4B1*	-3.05893396	0.005425554	0.009467238	1
T2DM	b_cell	*NAPG*	-2.894049176	0.00348177	0.006955795	1
T2DM	b_cell	*SPIRE1*	-2.374596012	0.000610173	0.00415349	1
T2DM	b_cell	*PPHLN1*	-2.206246309	0.039253876	0.049073199	1
T2DM	b_cell	*FKBP11*	-3.958132334	0.03535704	0.044681349	1
T2DM	b_cell	*YWHAQ*	-2.770193326	0.03208475	0.040941104	1
T2DM	b_cell	*LPIN1*	-2.231975011	0.000582745	0.00415349	1
T2DM	b_cell	*LDHA*	-2.566113711	0.001753046	0.004789372	1
T2DM	b_cell	*ANO3*	-1.38230312	0.000897697	0.00415349	1
T2DM	b_cell	*FST*	-2.891566663	0.000527142	0.00415349	1
T2DM	b_cell	*NARS*	-2.742996928	0.001826503	0.004888559	1
T2DM	b_cell	*FBXO18*	-3.041984091	0.020227332	0.027307115	1
T2DM	b_cell	*ECHDC3*	-1.935002472	0.000731779	0.00415349	1
T2DM	b_cell	*IL15RA*	-2.107454435	0.000953886	0.00415349	1
T2DM	b_cell	*TMEM241*	-1.93502678	0.00135478	0.004296951	1
T2DM	b_cell	*EMP1*	-1.963816046	0.001795459	0.004852128	1
T2DM	b_cell	*KLRD1*	-1.841008791	0.001171049	0.004202203	1
T2DM	b_cell	*PRH2*	-8.380945723	3.36E-05	0.003301266	0.211281053
T2DM	b_cell	*DDB2*	-2.561321735	0.031628406	0.040449102	1
T2DM	b_cell	*ACP2*	4.737673813	0.031119902	0.03987178	1
T2DM	b_cell	*HOOK1*	2.503928497	0.000645627	0.00415349	1
T2DM	b_cell	*ZCCHC11*	-2.462513584	0.017901659	0.024589847	1
T2DM	b_cell	*PRPF38A*	-4.073080497	0.008224216	0.012881327	1
T2DM	b_cell	*FHOD3*	-1.542642938	0.000437475	0.00415349	1
T2DM	b_cell	*TPGS2*	-2.289092739	0.00286411	0.006149816	1
T2DM	b_cell	*SLC43A3*	-4.60108621	0.019288356	0.0261518	1
T2DM	b_cell	*TIMM10*	-3.829803224	0.00016575	0.00415349	1
T2DM	b_cell	*DHX34*	-3.379330551	0.000517389	0.00415349	1
T2DM	b_cell	*APLNR*	-3.121007176	0.001452123	0.004416445	1
T2DM	b_cell	*FADS2*	-2.30987193	0.001248419	0.004253251	1
T2DM	b_cell	*TMEM165*	-1.931199343	0.005728033	0.009813953	1
T2DM	b_cell	*CLOCK*	-2.686838125	0.015214834	0.021404266	1
T2DM	b_cell	*CLDN10*	1.9830185	0.000724359	0.00415349	1
T2DM	b_cell	*DZIP1*	-2.10533887	0.001008346	0.00415349	1
T2DM	b_cell	*BIVM*	-4.545071601	0.007268487	0.011686062	1

T2DM	b_cell	*ERCC5*	-6.556128562	0.003344934	0.006768457	1
T2DM	b_cell	*CARS2*	-2.386764896	0.0007558	0.00415349	1
T2DM	b_cell	*STT3A*	-2.618467432	0.001876471	0.004957688	1
T2DM	b_cell	*KLB*	-1.262694001	0.002588902	0.005823408	1
T2DM	b_cell	*WDR36*	-2.816778133	0.003200629	0.006578083	1
T2DM	b_cell	*NAA35*	-2.848352617	0.018106213	0.024838264	1
T2DM	b_cell	*TMEM2*	-1.894505638	0.001281242	0.004266508	1
T2DM	b_cell	*AGTPBP1*	-2.373704371	0.00234573	0.005525518	1
T2DM	b_cell	*GOLM1*	-3.230157597	0.001165756	0.004200062	1
T2DM	b_cell	*ADAM19*	0.924390378	0.000206512	0.00415349	1
T2DM	b_cell	*USP30*	-2.372185397	0.01706689	0.023618652	1
T2DM	b_cell	*HRK*	2.759343825	0.000735654	0.00415349	1
T2DM	b_cell	*P2RX4*	-1.777786245	0.018134625	0.024859113	1
T2DM	b_cell	*DTX1*	-1.934525976	0.000246648	0.00415349	1
T2DM	b_cell	*DMTF1*	-2.187689876	0.029220958	0.037676818	1
T2DM	b_cell	*CCDC146*	-2.094402741	0.003337746	0.006765112	1
T2DM	b_cell	*TMEM60*	4.377980428	0.027133422	0.035252702	1
T2DM	b_cell	*POM121C*	-3.147763023	0.000287394	0.00415349	1
T2DM	b_cell	*SRPK2*	-2.277522369	0.013970697	0.01994409	1
T2DM	b_cell	*TES*	-3.189200895	0.004917059	0.008844125	1
T2DM	b_cell	*MDFIC*	-1.748463935	0.001380285	0.004323793	1
T2DM	b_cell	*ANKRD6*	-3.096153186	0.03175449	0.040577334	1
T2DM	b_cell	*KHDC1*	-2.081270638	0.004103332	0.007798563	1
T2DM	b_cell	*SNX14*	-2.760116931	0.010673996	0.015940004	1
T2DM	b_cell	*NT5E*	-1.633560358	0.003230243	0.006613998	1
T2DM	b_cell	*PRR5L*	-1.93848078	0.001061693	0.00415349	1
T2DM	b_cell	*NAT10*	-2.868736472	0.002608283	0.005849897	1
T2DM	b_cell	*ITGA7*	-1.368140334	0.000210575	0.00415349	1
T2DM	b_cell	*RDH5*	-1.353064974	0.001074471	0.00415349	1
T2DM	b_cell	*BLOC1S1*	-3.807857337	0.002417053	0.005613599	1
T2DM	b_cell	*PPP1R1A*	-2.836681567	0.001575433	0.004562749	1
T2DM	b_cell	*B4GALNT1*	3.831126234	0.001764332	0.004791515	1
T2DM	b_cell	*FAIM2*	-5.56623375	0.000564108	0.00415349	1
T2DM	b_cell	*KRT7*	2.19688025	0.000262479	0.00415349	1
T2DM	b_cell	*SLC26A10*	-8.936858455	0.000349064	0.00415349	1
T2DM	b_cell	*MAP7*	-1.632714629	0.001151348	0.004188961	1
T2DM	b_cell	*AHI1*	-1.408974818	0.008109743	0.012727398	1
T2DM	b_cell	*PKIB*	-2.715581654	0.000748564	0.00415349	1
T2DM	b_cell	*SMPD2*	3.133588362	0.008033256	0.012648405	1
T2DM	b_cell	*MICAL1*	-3.237533605	0.009422731	0.014382304	1
T2DM	b_cell	*PRADC1*	4.10490284	0.00305754	0.006378687	1
T2DM	b_cell	*SMYD5*	-3.303244171	0.000188732	0.00415349	1
T2DM	b_cell	*DYSF*	-2.207133254	0.00055407	0.00415349	1
T2DM	b_cell	*KCNMB4*	2.4613805	0.001092844	0.00415349	1
T2DM	b_cell	*USP15*	-2.350425637	0.017246861	0.023825757	1
T2DM	b_cell	*KLHL36*	-2.448333409	0.002322241	0.005505253	1
T2DM	b_cell	*MPHOSPH6*	4.486635415	0.013562755	0.019432325	1
T2DM	b_cell	*FHOD1*	-2.112857425	0.000622461	0.00415349	1
T2DM	b_cell	*CCDC102A*	-3.802915527	0.000594152	0.00415349	1
T2DM	b_cell	*ZNF670*	2.516337312	0.006956809	0.01130459	1
T2DM	b_cell	*KCNK1*	-1.130231855	0.038262543	0.047900559	1
T2DM	b_cell	*NTPCR*	-2.716122392	0.002774341	0.006030781	1
T2DM	b_cell	*RNASEL*	-2.240374819	0.004700923	0.008595602	1
T2DM	b_cell	*PIGC*	-2.600836197	0.004113105	0.007807696	1
T2DM	b_cell	*SP110*	2.88814323	0.017847432	0.024536804	1
T2DM	b_cell	*SLC19A3*	-1.107010541	0.001756088	0.004789372	1
T2DM	b_cell	*TMBIM1*	-2.503185179	0.000104931	0.00415349	0.65970417
T2DM	b_cell	*ARMC9*	-3.147993759	0.002808976	0.006081279	1
T2DM	b_cell	*TMEM127*	-3.177932619	0.001802481	0.004852128	1
T2DM	b_cell	*GCC2*	-2.731676442	0.011696619	0.017137415	1
T2DM	b_cell	*ANKRD36*	-2.585549552	0.004587289	0.008445179	1

T2DM	b_cell	*USP44*	-3.397143359	0.000661223	0.00415349	1
T2DM	b_cell	*APPL2*	-2.449756921	0.00307692	0.006405496	1
T2DM	b_cell	*DRAM1*	-2.522628975	0.00222733	0.005400083	1
T2DM	b_cell	*SLC41A2*	-2.563345685	0.002325085	0.005505767	1
T2DM	b_cell	*NEK3*	-2.2937332	0.019061182	0.025888454	1
T2DM	b_cell	*VPS36*	-2.607611173	0.007986727	0.012595482	1
T2DM	b_cell	*TBC1D4*	-2.148144906	0.002563862	0.005799421	1
T2DM	b_cell	*SUCLA2*	-2.276241752	0.001355424	0.004296951	1
T2DM	b_cell	*RCBTB1*	-3.502115663	0.000226669	0.00415349	1
T2DM	b_cell	*MED4*	-3.366587883	0.006335539	0.010543022	1
T2DM	b_cell	*RCBTB2*	-2.208510555	0.001371779	0.004316504	1
T2DM	b_cell	*SETDB2*	-2.87645904	0.006891709	0.011227838	1
T2DM	b_cell	*SPDYE1*	-2.399280266	0.00168432	0.004704627	1
T2DM	b_cell	*CHST12*	-3.051082076	0.002167881	0.005319854	1
T2DM	b_cell	*GPNMB*	-1.666070264	0.000935809	0.00415349	1
T2DM	b_cell	*KDELR2*	-3.751306412	0.008917913	0.01376551	1
T2DM	b_cell	*NUPL2*	-2.814263322	0.002142035	0.005287258	1
T2DM	b_cell	*ZDHHC4*	-3.337246616	0.002006188	0.005130808	1
T2DM	b_cell	*AOAH*	-1.727337772	0.000114157	0.00415349	0.717706652
T2DM	b_cell	*TBRG4*	3.476110907	0.003404901	0.006850117	1
T2DM	b_cell	*DDX56*	-4.415362281	0.008102003	0.012721602	1
T2DM	b_cell	*HUS1*	-2.13395844	0.001839672	0.004898539	1
T2DM	b_cell	*NACAD*	-13.6801777	1.64E-05	0.002349192	0.103364449
T2DM	b_cell	*TTC5*	-3.417642804	0.000883865	0.00415349	1
T2DM	b_cell	*MTHFS*	-1.854764604	0.020600026	0.027762564	1
T2DM	b_cell	*ALPK3*	-1.966516526	0.000461898	0.00415349	1
T2DM	b_cell	*TM6SF1*	-1.879753825	0.002129049	0.005276048	1
T2DM	b_cell	*RSAD1*	-3.28481083	0.001378319	0.004323793	1
T2DM	b_cell	*NMT1*	-1.606660951	0.023100222	0.030704248	1
T2DM	b_cell	*MYCBPAP*	-2.170423671	0.023324377	0.030930259	1
T2DM	b_cell	*BRIP1*	2.514392382	0.000795272	0.00415349	1
T2DM	b_cell	*RTP4*	-6.374417257	0.02473917	0.03248437	1
T2DM	b_cell	*MRPL47*	-4.663031074	0.006505706	0.010749375	1
T2DM	b_cell	*BLK*	0.107248443	0.000596316	0.00415349	1
T2DM	b_cell	*EPRS*	-3.169293269	0.007298665	0.011723738	1
T2DM	b_cell	*RPS6KC1*	-2.359056903	0.003721022	0.007268006	1
T2DM	b_cell	*CBWD2*	-3.883037518	0.007653827	0.012191439	1
T2DM	b_cell	*IL1RN*	2.646739442	0.001200433	0.004223203	1
T2DM	b_cell	*SAP130*	-3.655084448	0.005938266	0.010065752	1
T2DM	b_cell	*GYPC*	-2.199204032	0.00179918	0.004852128	1
T2DM	b_cell	*LRRC8A*	-2.420730657	0.001140016	0.00418468	1
T2DM	b_cell	*TXN*	-2.791636707	0.001293664	0.004268027	1
T2DM	b_cell	*ODF2*	-3.816136155	0.016881393	0.023392841	1
T2DM	b_cell	*TOR1B*	-2.513623081	0.001176227	0.004202203	1
T2DM	b_cell	*TOR1A*	-3.092045967	0.000215792	0.00415349	1
T2DM	b_cell	*SLC2A8*	-3.415277204	0.001780387	0.004822616	1
T2DM	b_cell	*ZFP37*	-2.428509586	0.002103554	0.005241681	1
T2DM	b_cell	*ZNF189*	-3.37399592	0.003656217	0.007196818	1
T2DM	b_cell	*ALDOB*	-4.392585582	0.002724304	0.005972002	1
T2DM	b_cell	*STX17*	-3.161990659	0.010768187	0.01604636	1
T2DM	b_cell	*PRPF4*	5.027421728	0.001514732	0.004480664	1
T2DM	b_cell	*FPGS*	-2.618800577	0.000795122	0.00415349	1
T2DM	b_cell	*TEX10*	-2.379783617	0.001318945	0.004274973	1
T2DM	b_cell	*GARNL3*	-2.788528372	0.002904305	0.006192483	1
T2DM	b_cell	*GABBR2*	-5.207256931	0.000819611	0.00415349	1
T2DM	b_cell	*PSMB7*	-2.91660185	0.00598859	0.010134661	1
T2DM	b_cell	*RABEPK*	-2.267827488	0.00100898	0.00415349	1
T2DM	b_cell	*GOLGA1*	-1.920079397	0.001035108	0.00415349	1
T2DM	b_cell	*LMX1B*	-6.635856855	0.000670082	0.00415349	1
T2DM	b_cell	*DSCC1*	-1.485439268	0.002649663	0.005895393	1
T2DM	b_cell	*DERL1*	-3.739518899	0.029907271	0.038459196	1

T2DM	b_cell	*NOV*	-3.613207582	0.003137882	0.006486603	1
T2DM	b_cell	*POLR1E*	-2.335637617	0.012851254	0.018573755	1
T2DM	b_cell	*IL11RA*	-2.743349131	0.001982519	0.005095706	1
T2DM	b_cell	*APTX*	-2.64909815	0.003430253	0.006885994	1
T2DM	b_cell	*TLN1*	-1.882961872	0.002151504	0.005302433	1
T2DM	b_cell	*SPAG8*	6.357293505	0.001893287	0.00497413	1
T2DM	b_cell	*CD72*	-3.813823212	0.009349055	0.014290666	1
T2DM	b_cell	*TMEM8B*	4.282589129	0.034460154	0.043644438	1
T2DM	b_cell	*GRHPR*	-3.466474626	0.000670422	0.00415349	1
T2DM	b_cell	*ARHGEF39*	2.563600051	0.012285288	0.017850151	1
T2DM	b_cell	*DENND4C*	-2.163870253	0.006439817	0.010668545	1
T2DM	b_cell	*PPIL1*	4.582634952	0.001051978	0.00415349	1
T2DM	b_cell	*KIF13A*	-1.840940777	0.001906654	0.004988168	1
T2DM	b_cell	*TMEM63B*	-2.090936602	0.002618282	0.005854674	1
T2DM	b_cell	*FRS3*	5.354708101	0.010337889	0.015541442	1
T2DM	b_cell	*SLC22A23*	-1.820740842	0.001161632	0.004195495	1
T2DM	b_cell	*LRRC1*	-1.876788135	0.000566673	0.00415349	1
T2DM	b_cell	*FOXF2*	-6.26417658	0.038000038	0.047593002	1
T2DM	b_cell	*BPHL*	-1.356840855	0.002434322	0.005628918	1
T2DM	b_cell	*RIPK1*	-2.396862324	0.001986853	0.005098507	1
T2DM	b_cell	*TCF19*	-3.409479342	0.018876564	0.025670984	1
T2DM	b_cell	*FLOT1*	-2.190187406	0.00724707	0.011664702	1
T2DM	b_cell	*TPMT*	-4.889284757	0.013964477	0.019939737	1
T2DM	b_cell	*MTCH1*	-3.298062981	0.026848619	0.034954912	1
T2DM	b_cell	*FHDC1*	-2.819327169	0.000795021	0.00415349	1
T2DM	b_cell	*MYO7A*	-2.335180332	0.001459605	0.004422426	1
T2DM	b_cell	*ARRB1*	-2.283086717	0.00361094	0.007151285	1
T2DM	b_cell	*CCDC90B*	-2.57005949	0.001353106	0.004296454	1
T2DM	b_cell	*SYTL2*	-1.853473531	0.000336674	0.00415349	1
T2DM	b_cell	*PI15*	3.716220006	0.000860475	0.00415349	1
T2DM	b_cell	*TTPA*	-1.750889185	0.000109299	0.00415349	0.68716342
T2DM	b_cell	*GGH*	-2.338607496	0.001072658	0.00415349	1
T2DM	b_cell	*SULF1*	-1.198573309	0.001036981	0.00415349	1
T2DM	b_cell	*TGS1*	-2.666462548	0.023592587	0.031260492	1
T2DM	b_cell	*NEK1*	-2.281002966	0.004799347	0.008710593	1
T2DM	b_cell	*DDX60*	-3.391275512	0.005688802	0.009764542	1
T2DM	b_cell	*DCUN1D5*	-1.847916374	0.00019955	0.00415349	1
T2DM	b_cell	*TRIM29*	-3.922371448	0.000990863	0.00415349	1
T2DM	b_cell	*POU2F3*	2.444668768	0.001321923	0.004274973	1
T2DM	b_cell	*PPP2R1B*	-1.176261094	0.000766213	0.00415349	1
T2DM	b_cell	*FDX1*	-2.798743809	0.002186115	0.005349464	1
T2DM	b_cell	*FXYD6*	-5.580562733	0.028879869	0.037275249	1
T2DM	b_cell	*TMPRSS13*	-7.710194496	0.013519835	0.019379663	1
T2DM	b_cell	*MAP2K5*	-2.311481032	0.019990017	0.027004134	1
T2DM	b_cell	*NDUFAF1*	-4.473947778	9.80E-05	0.00415349	0.616373777
T2DM	b_cell	*ITGA11*	-1.545300234	0.000528881	0.00415349	1
T2DM	b_cell	*PAQR5*	-3.521350134	0.000971397	0.00415349	1
T2DM	b_cell	*ITPKA*	4.195093288	0.000893254	0.00415349	1
T2DM	b_cell	*UACA*	-1.926294902	0.001882234	0.004963761	1
T2DM	b_cell	*PLCB2*	-1.477520484	0.011536694	0.016962609	1
T2DM	b_cell	*RSL24D1*	-3.686918355	0.014555025	0.02063302	1
T2DM	b_cell	*RABGGTB*	-3.947783382	0.008036127	0.012649758	1
T2DM	b_cell	*DNASE2B*	-18.03079914	0.001807127	0.004857377	1
T2DM	b_cell	*DBT*	-3.126063636	0.007979942	0.012594351	1
T2DM	b_cell	*RTCA*	-2.202906312	0.032824779	0.041825979	1
T2DM	b_cell	*IFT172*	-2.495197067	0.007578502	0.012092904	1
T2DM	b_cell	*KHK*	-2.224380738	0.00232204	0.005505253	1
T2DM	b_cell	*ADCY3*	-2.858168758	0.001145663	0.004185233	1
T2DM	b_cell	*DYNC2LI1*	-2.64718591	0.001050017	0.00415349	1
T2DM	b_cell	*LHCGR*	-2.641038131	0.000567292	0.00415349	1
T2DM	b_cell	*THUMPD2*	-2.74785646	0.002741899	0.006000111	1

T2DM	b_cell	*PREB*	-2.364452856	0.000563291	0.00415349	1
T2DM	b_cell	*ABCG5*	-3.030497015	0.005583425	0.009641728	1
T2DM	b_cell	*ATRAID*	-3.607673058	0.013218788	0.019043657	1
T2DM	b_cell	*LRPPRC*	-2.656844484	0.00505611	0.009004825	1
T2DM	b_cell	*DTNB*	-2.172633604	0.002384625	0.005571214	1
T2DM	b_cell	*CYP2C9*	2.260552838	0.00876558	0.013583732	1
T2DM	b_cell	*CH25H*	-2.311041337	0.012348374	0.017925243	1
T2DM	b_cell	*ATAD1*	-3.029511264	0.005964094	0.010106808	1
T2DM	b_cell	*BTBD16*	-6.444061394	0.000141387	0.00415349	0.888899315
T2DM	b_cell	*TACC2*	-1.339820994	0.0013179	0.004274973	1
T2DM	b_cell	*CALHM2*	-2.237662085	0.00076256	0.00415349	1
T2DM	b_cell	*ARL3*	-2.605636762	0.002154675	0.005308166	1
T2DM	b_cell	*PLCE1*	-1.756853322	0.000565038	0.00415349	1
T2DM	b_cell	*RBP4*	-2.016601971	0.00134232	0.004286796	1
T2DM	b_cell	*DNAJC13*	-2.73835387	0.006998162	0.011365912	1
T2DM	b_cell	*FAM149B1*	-2.885046795	0.002301507	0.005481934	1
T2DM	b_cell	*ATIC*	-3.778256543	0.000723584	0.00415349	1
T2DM	b_cell	*SMARCAL1*	-3.265717732	0.029999401	0.038569781	1
T2DM	b_cell	*BARD1*	-3.238608682	0.001528728	0.004502743	1
T2DM	b_cell	*STAT4*	-1.810752708	0.000388302	0.00415349	1
T2DM	b_cell	*ASNSD1*	-2.292189206	0.000363602	0.00415349	1
T2DM	b_cell	*METTL5*	-3.778297523	0.007060679	0.011446748	1
T2DM	b_cell	*NAB1*	-3.16078966	0.008493436	0.013223931	1
T2DM	b_cell	*MDH1B*	2.272270516	0.000762522	0.00415349	1
T2DM	b_cell	*ITGAV*	-2.271433271	0.005587851	0.009643377	1
T2DM	b_cell	*COX17*	3.969861845	0.016763149	0.023259748	1
T2DM	b_cell	*USP8*	-2.137655281	0.002350713	0.005533109	1
T2DM	b_cell	*SPPL2A*	-2.891672165	0.012877482	0.018601696	1
T2DM	b_cell	*UBL7*	-3.373253106	0.001581782	0.004576467	1
T2DM	b_cell	*ARHGAP24*	-1.985327886	0.005918314	0.010042765	1
T2DM	b_cell	*FAM13A*	-1.504500201	0.003353536	0.006777924	1
T2DM	b_cell	*HERC3*	-2.703340258	0.008540945	0.013291317	1
T2DM	b_cell	*AP1AR*	-3.621613436	0.005836636	0.009939039	1
T2DM	b_cell	*PRKG2*	-2.01954635	0.000626333	0.00415349	1
T2DM	b_cell	*BBS7*	-2.480485291	0.001250768	0.004253251	1
T2DM	b_cell	*KIAA1109*	-2.343054143	0.02222628	0.029649188	1
T2DM	b_cell	*MMRN1*	-2.305801004	0.001440924	0.004405146	1
T2DM	b_cell	*PRDM5*	-1.672981631	0.001376596	0.004323793	1
T2DM	b_cell	*TRPC3*	-4.365160313	0.001320001	0.004274973	1
T2DM	b_cell	*NAAA*	-2.465915029	0.006676823	0.010971559	1
T2DM	b_cell	*NUP54*	-2.147617057	0.007278497	0.01169433	1
T2DM	b_cell	*BMP2K*	-2.361404561	0.001348678	0.004290463	1
T2DM	b_cell	*G3BP2*	-3.443670032	0.001362329	0.004298348	1
T2DM	b_cell	*SCARB2*	-2.71386275	0.005994924	0.010139921	1
T2DM	b_cell	*CNOT6L*	-3.021885946	0.010825186	0.016112202	1
T2DM	b_cell	*ANXA3*	-2.280233488	0.000868304	0.00415349	1
T2DM	b_cell	*ENPEP*	-2.251986429	0.000641087	0.00415349	1
T2DM	b_cell	*PAPSS1*	-2.100411973	0.007307467	0.011730073	1
T2DM	b_cell	*SLC39A8*	-3.07504758	0.005316292	0.009320937	1
T2DM	b_cell	*RNF185*	-3.150081778	0.002135878	0.005284637	1
T2DM	b_cell	*B4GALNT3*	-6.576249432	0.000565277	0.00415349	1
T2DM	b_cell	*ERP27*	2.943133306	0.001725948	0.004752113	1
T2DM	b_cell	*ETV6*	-2.300517022	0.005606427	0.009662174	1
T2DM	b_cell	*KIF21A*	-1.311255092	0.002606411	0.005849897	1
T2DM	b_cell	*CPNE8*	-2.287009018	0.000992652	0.00415349	1
T2DM	b_cell	*ALG10*	-3.956681079	0.00109163	0.00415349	1
T2DM	b_cell	*SLCO1C1*	-2.853605043	0.002488011	0.005702562	1
T2DM	b_cell	*PRICKLE1*	-1.26092767	0.001175111	0.004202203	1
T2DM	b_cell	*C1RL*	-1.778153981	0.006432023	0.010661251	1
T2DM	b_cell	*CLSTN3*	-3.447888921	0.024259641	0.031974919	1
T2DM	b_cell	*KLRG1*	-2.587821003	0.000393747	0.00415349	1

T2DM	b_cell	*VAMP1*	-2.346807944	0.019331329	0.026193118	1
T2DM	b_cell	*TAPBPL*	-3.069828007	0.020850232	0.028063671	1
T2DM	b_cell	*CD27*	-3.526645496	0.00088007	0.00415349	1
T2DM	b_cell	*PEX5*	3.027463231	0.005104888	0.009050883	1
T2DM	b_cell	*PPFIA2*	-1.36634773	0.000960383	0.00415349	1
T2DM	b_cell	*LGR5*	-3.475395036	0.000704018	0.00415349	1
T2DM	b_cell	*POC1B*	-3.363144688	0.007195271	0.01160212	1
T2DM	b_cell	*SNRPF*	-4.192410528	0.016121649	0.022498736	1
T2DM	b_cell	*AMDHD1*	-2.983058912	0.007698436	0.012243883	1
T2DM	b_cell	*GAS2L3*	-1.930505522	0.000775402	0.00415349	1
T2DM	b_cell	*TMEM132B*	-4.130220751	0.002358367	0.005540753	1
T2DM	b_cell	*TDG*	-2.723564243	0.002393328	0.005579107	1
T2DM	b_cell	*MMAB*	-2.686051869	0.000421042	0.00415349	1
T2DM	b_cell	*GLTP*	-2.521555279	0.001234374	0.004253251	1
T2DM	b_cell	*MTMR6*	-2.202519936	0.000923709	0.00415349	1
T2DM	b_cell	*SLC46A3*	-1.877174802	0.006530975	0.010776966	1
T2DM	b_cell	*CCDC65*	-2.660751231	0.000539556	0.00415349	1
T2DM	b_cell	*ACVRL1*	-2.827240645	0.002706049	0.005952834	1
T2DM	b_cell	*GPR84*	-41.20241497	2.55E-05	0.002754251	0.160621577
T2DM	b_cell	*KANSL2*	-1.704017622	0.00109177	0.00415349	1
T2DM	b_cell	*CERS5*	-3.115028057	0.002433424	0.005628918	1
T2DM	b_cell	*LMBR1L*	-3.956673399	0.010862003	0.01615935	1
T2DM	b_cell	*ZNF740*	-2.495050102	0.001746365	0.004788224	1
T2DM	b_cell	*WDFY2*	-2.574349748	0.026440308	0.034466144	1
T2DM	b_cell	*HNRNPA1L2*	6.648396955	0.002555103	0.005795069	1
T2DM	b_cell	*LPAR6*	-2.390800323	0.001988807	0.005101414	1
T2DM	b_cell	*VPS33A*	-3.720472404	0.001178565	0.004202203	1
T2DM	b_cell	*METTL21C*	9.94631631	0.007125797	0.011510862	1
T2DM	b_cell	*GRTP1*	-1.908624837	0.002098741	0.005241681	1
T2DM	b_cell	*CBLN3*	1.205482453	0.011912525	0.01739699	1
T2DM	b_cell	*TSSK4*	-11.35702262	0.003013504	0.006319514	1
T2DM	b_cell	*TMX1*	-3.11668851	0.00201096	0.005137305	1
T2DM	b_cell	*RTN1*	-1.336057926	0.000902054	0.00415349	1
T2DM	b_cell	*C14orf37*	-2.501482927	0.002617453	0.005854674	1
T2DM	b_cell	*RAB15*	-2.488824544	0.000416802	0.00415349	1
T2DM	b_cell	*STON2*	-1.799108744	0.000904439	0.00415349	1
T2DM	b_cell	*PTGR2*	-2.665839965	0.000151425	0.00415349	0.952007968
T2DM	b_cell	*AK7*	-4.283486956	0.000835628	0.00415349	1
T2DM	b_cell	*FBLN5*	-1.449058493	0.000880857	0.00415349	1
T2DM	b_cell	*C14orf79*	-2.73008636	0.000932714	0.00415349	1
T2DM	b_cell	*DUOXA1*	-3.460516375	0.01443742	0.020507921	1
T2DM	b_cell	*MFAP1*	-3.400222439	0.001437194	0.004399046	1
T2DM	b_cell	*TCF12*	-2.035340546	0.033327062	0.042388679	1
T2DM	b_cell	*SORD*	5.776604338	0.010816368	0.016106705	1
T2DM	b_cell	*ZSCAN29*	-3.51335868	0.001272674	0.004257394	1
T2DM	b_cell	*LYSMD2*	-4.82673012	0.004261992	0.007989011	1
T2DM	b_cell	*BAHD1*	-3.071293677	0.001056931	0.00415349	1
T2DM	b_cell	*DISP2*	1.78077475	0.029832564	0.038378827	1
T2DM	b_cell	*ANP32A*	-2.365880222	0.001289724	0.004268027	1
T2DM	b_cell	*COMMD4*	-5.122395834	0.004153775	0.007865091	1
T2DM	b_cell	*UBE2Q2*	-2.70182006	0.003312357	0.006729773	1
T2DM	b_cell	*PSTPIP1*	-2.840963105	0.002456884	0.005651273	1
T2DM	b_cell	*HMG20A*	-2.051450543	0.010474782	0.01569776	1
T2DM	b_cell	*SCAPER*	-2.314156349	0.007117727	0.011508915	1
T2DM	b_cell	*NEIL1*	-2.026478583	0.000294205	0.00415349	1
T2DM	b_cell	*MAN2C1*	-2.587038128	1.57E-07	7.05E-05	0.000986842
T2DM	b_cell	*DNAJA4*	-2.617719514	0.000887185	0.00415349	1
T2DM	b_cell	*TPM1*	-2.310600647	0.002859051	0.006143148	1
T2DM	b_cell	*IGF1R*	-1.833641258	0.001598278	0.004603327	1
T2DM	b_cell	*CYP11A1*	-2.260624284	0.015163154	0.021350672	1
T2DM	b_cell	*BBS4*	-2.807590933	0.005556287	0.009618899	1

T2DM	b_cell	*ADAMTS17*	-1.728507144	0.000628444	0.00415349	1
T2DM	b_cell	*ULK3*	-4.682732494	0.009204933	0.014111538	1
T2DM	b_cell	*SCAMP2*	-3.720519709	0.000280617	0.00415349	1
T2DM	b_cell	*HAPLN3*	-2.961430995	0.000724905	0.00415349	1
T2DM	b_cell	*POLG*	-3.408926714	0.002423837	0.005623123	1
T2DM	b_cell	*WDR93*	-1.836482707	0.000325348	0.00415349	1
T2DM	b_cell	*TICRR*	-4.961413346	0.000624757	0.00415349	1
T2DM	b_cell	*MFGE8*	-1.782833621	0.000765952	0.00415349	1
T2DM	b_cell	*UNC45A*	-2.741573863	0.009496071	0.014476673	1
T2DM	b_cell	*ST8SIA2*	-17.87136709	0.000548192	0.00415349	1
T2DM	b_cell	*IQGAP1*	-2.198408854	0.008736599	0.013545498	1
T2DM	b_cell	*CRTC3*	-2.471879605	0.004068491	0.007746397	1
T2DM	b_cell	*SH3GL3*	2.077203579	0.007905883	0.01250453	1
T2DM	b_cell	*SEC11A*	-2.50658806	0.002232002	0.005401538	1
T2DM	b_cell	*TGFB1I1*	-2.826878844	0.001237399	0.004253251	1
T2DM	b_cell	*PARN*	-2.365928716	0.003983802	0.007626725	1
T2DM	b_cell	*FTO*	-2.217019747	0.003864006	0.007456417	1
T2DM	b_cell	*TXNL4B*	-3.742004944	0.001700436	0.004717848	1
T2DM	b_cell	*MARVELD3*	2.07518945	0.000495653	0.00415349	1
T2DM	b_cell	*ZFHX3*	-1.937913044	0.002131689	0.005278429	1
T2DM	b_cell	*KIFC3*	-3.28303727	0.002656011	0.005906736	1
T2DM	b_cell	*ADAMTS18*	-1.867898698	0.001019609	0.00415349	1
T2DM	b_cell	*NUDT7*	-4.820310411	0.000441058	0.00415349	1
T2DM	b_cell	*GCSH*	-2.339180918	0.007865096	0.012452243	1
T2DM	b_cell	*CMTM3*	-2.070741498	0.00083707	0.00415349	1
T2DM	b_cell	*MAP1LC3B*	-2.911812621	0.004710989	0.008602378	1
T2DM	b_cell	*TLDC1*	-4.061118115	0.021332441	0.028620797	1
T2DM	b_cell	*RPS2*	-3.461653108	0.003971847	0.007613111	1
T2DM	b_cell	*NDUFB10*	-4.506489514	0.005165412	0.009127303	1
T2DM	b_cell	*TIGD7*	-1.891737211	0.009520024	0.014506146	1
T2DM	b_cell	*DEF8*	-2.267121347	0.00032291	0.00415349	1
T2DM	b_cell	*GALNS*	-3.516678656	0.000978233	0.00415349	1
T2DM	b_cell	*GAS8*	-1.783179932	0.026063287	0.034024063	1
T2DM	b_cell	*COPS3*	-2.490025358	0.001053331	0.00415349	1
T2DM	b_cell	*PRPSAP2*	3.086942773	0.03799251	0.047593002	1
T2DM	b_cell	*DDX52*	-3.123320992	0.008077564	0.012699085	1
T2DM	b_cell	*RASL10B*	-8.784215917	0.00031851	0.00415349	1
T2DM	b_cell	*UNC45B*	-12.17135217	0.002971566	0.006279742	1
T2DM	b_cell	*TOM1L1*	-1.973683396	0.001451698	0.004416445	1
T2DM	b_cell	*C17orf80*	-2.81717983	0.002227295	0.005400083	1
T2DM	b_cell	*VPS53*	-2.882526982	0.000452213	0.00415349	1
T2DM	b_cell	*SPATA22*	-1.292703322	0.008927694	0.013777224	1
T2DM	b_cell	*NPEPPS*	-2.449750389	0.003052975	0.006376763	1
T2DM	b_cell	*LRRC46*	-10.18784906	3.03E-07	0.000118892	0.001902273
T2DM	b_cell	*SCRN2*	-1.239499644	0.005651908	0.009724561	1
T2DM	b_cell	*RHBDL3*	-5.668837294	0.00370007	0.00724956	1
T2DM	b_cell	*ARSG*	-3.31593192	0.000815446	0.00415349	1
T2DM	b_cell	*ABCA8*	-1.138001377	0.001036839	0.00415349	1
T2DM	b_cell	*G6PC3*	-6.02585033	0.004716058	0.008609133	1
T2DM	b_cell	*IMPA2*	-3.124276744	0.002080289	0.005230315	1
T2DM	b_cell	*GNAL*	-2.498804433	0.001496568	0.004474047	1
T2DM	b_cell	*RPRD1A*	-3.05947999	0.003438521	0.006889095	1
T2DM	b_cell	*C18orf21*	-3.194096297	0.003611654	0.007151285	1
T2DM	b_cell	*GALNT1*	-1.98099541	0.001038329	0.00415349	1
T2DM	b_cell	*ASXL3*	-2.626681826	0.00112697	0.004182741	1
T2DM	b_cell	*ADCYAP1*	11.36745673	0.003666805	0.007210886	1
T2DM	b_cell	*ESCO1*	-2.928938683	0.031039798	0.039785364	1
T2DM	b_cell	*GREB1L*	-1.337078728	0.000400435	0.00415349	1
T2DM	b_cell	*C18orf8*	-4.92130791	0.000616005	0.00415349	1
T2DM	b_cell	*NPC1*	-3.544592192	0.000679492	0.00415349	1
T2DM	b_cell	*SLC14A1*	4.796848855	0.003411959	0.006859926	1

T2DM	b_cell	*SLC13A5*	-19.44969786	0.001517397	0.004483023	1
T2DM	b_cell	*CCDC40*	-1.778349165	0.002366466	0.005545309	1
T2DM	b_cell	*CARD14*	-2.491382442	0.00075891	0.00415349	1
T2DM	b_cell	*EIF4A3*	4.145292751	0.032020632	0.040883501	1
T2DM	b_cell	*TBCD*	-2.444145738	0.003730752	0.007277456	1
T2DM	b_cell	*FN3KRP*	-4.065331941	0.00015539	0.00415349	0.976933957
T2DM	b_cell	*NARF*	-3.522077335	0.005800608	0.009904515	1
T2DM	b_cell	*CBX8*	1.132781629	0.000750317	0.00415349	1
T2DM	b_cell	*SECTM1*	-4.51277725	0.001721831	0.004747873	1
T2DM	b_cell	*RNF157*	-1.631420503	0.001585955	0.004584321	1
T2DM	b_cell	*RNF165*	-2.21042233	0.000830271	0.00415349	1
T2DM	b_cell	*SMAD4*	-3.260058709	0.00936217	0.014303758	1
T2DM	b_cell	*PIP4K2B*	-2.612377629	0.000985558	0.00415349	1
T2DM	b_cell	*GRB7*	2.730495212	0.001083148	0.00415349	1
T2DM	b_cell	*FKBP10*	-2.38415863	0.000613128	0.00415349	1
T2DM	b_cell	*TXNL4A*	-2.863177367	0.00071686	0.00415349	1
T2DM	b_cell	*NFIC*	-1.653221368	0.000816968	0.00415349	1
T2DM	b_cell	*TPGS1*	-2.515166072	0.001412145	0.004364365	1
T2DM	b_cell	*PRDM15*	-1.979522739	0.028698727	0.037094757	1
T2DM	b_cell	*PFKL*	-4.07333039	0.003132886	0.006479096	1
T2DM	b_cell	*MVB12A*	-3.082181693	0.003798804	0.007364502	1
T2DM	b_cell	*DUS3L*	5.687405448	0.000126796	0.00415349	0.797167409
T2DM	b_cell	*TMEM91*	-6.004163001	0.002403351	0.005596247	1
T2DM	b_cell	*SIRT3*	-1.930072334	0.015787996	0.02209195	1
T2DM	b_cell	*IFITM3*	-2.268675774	0.001150961	0.004188961	1
T2DM	b_cell	*COL6A1*	-1.344195171	0.000395938	0.00415349	1
T2DM	b_cell	*IFNAR1*	-3.227447548	0.000772329	0.00415349	1
T2DM	b_cell	*AKT1*	-2.831158996	0.00100038	0.00415349	1
T2DM	b_cell	*SAE1*	-2.365684006	0.004353835	0.008107986	1
T2DM	b_cell	*GEMIN7*	3.110322609	0.006056625	0.010214055	1
T2DM	b_cell	*WTIP*	-1.825267932	0.000725611	0.00415349	1
T2DM	b_cell	*CAPN10*	-2.235834351	0.002453643	0.005646432	1
T2DM	b_cell	*MYO1F*	-1.494893994	0.001309387	0.004274973	1
T2DM	b_cell	*CACNG8*	-6.40561757	0.000912255	0.00415349	1
T2DM	b_cell	*FBN3*	-3.855335468	0.004886855	0.008810915	1
T2DM	b_cell	*EVI5L*	4.430735338	0.019968104	0.026980328	1
T2DM	b_cell	*CTU1*	-3.381292986	0.000736728	0.00415349	1
T2DM	b_cell	*RCN3*	-1.955497746	0.000826321	0.00415349	1
T2DM	b_cell	*RERE*	-2.008763839	0.016005683	0.022361718	1
T2DM	b_cell	*ARHGEF19*	-1.801570219	0.00343661	0.006887461	1
T2DM	b_cell	*PEX14*	-1.854137324	0.000891724	0.00415349	1
T2DM	b_cell	*IL22RA1*	-5.387029887	0.005167799	0.009128955	1
T2DM	b_cell	*ZNF593*	-5.821334541	0.000845848	0.00415349	1
T2DM	b_cell	*C1orf216*	-2.870105775	0.001292744	0.004268027	1
T2DM	b_cell	*GPN2*	-2.15988537	0.00057269	0.00415349	1
T2DM	b_cell	*NBPF3*	-2.693771591	0.000273027	0.00415349	1
T2DM	b_cell	*HSPG2*	-1.716031433	0.00100613	0.00415349	1
T2DM	b_cell	*ITGB3BP*	-2.201167053	0.011630093	0.017055842	1
T2DM	b_cell	*SERBP1*	-3.186363008	0.018856781	0.025660733	1
T2DM	b_cell	*PIGK*	-3.386402194	0.008630409	0.013403998	1
T2DM	b_cell	*RPS8*	-2.869491001	0.026221075	0.034210829	1
T2DM	b_cell	*MOB3C*	2.698925504	0.000792641	0.00415349	1
T2DM	b_cell	*CYP4B1*	-1.405802617	0.010023468	0.01515938	1
T2DM	b_cell	*SYPL2*	-1.961678998	0.015556521	0.021821474	1
T2DM	b_cell	*ZNF697*	-2.531448768	0.000904288	0.00415349	1
T2DM	b_cell	*CELSR2*	-6.950922277	0.003139611	0.006486603	1
T2DM	b_cell	*ATP1B1*	-2.025734998	0.000557388	0.00415349	1
T2DM	b_cell	*TIPRL*	-3.420512058	0.003348031	0.006770366	1
T2DM	b_cell	*NME7*	-3.298993746	0.003722941	0.007268984	1
T2DM	b_cell	*CREG1*	-3.20958665	0.002649973	0.005895393	1
T2DM	b_cell	*GPA33*	-7.262696879	0.00074398	0.00415349	1

T2DM	b_cell	*RXRG*	14.64213081	0.005052723	0.00900155	1
T2DM	b_cell	*TMCO1*	-3.210705391	0.01732051	0.023906487	1
T2DM	b_cell	*MGST3*	-2.040376413	0.001357934	0.004298348	1
T2DM	b_cell	*ADCY10*	-2.824822889	0.000517667	0.00415349	1
T2DM	b_cell	*RFWD2*	-2.391546339	0.009000794	0.013862811	1
T2DM	b_cell	*PPOX*	-2.687786847	0.006080733	0.010246468	1
T2DM	b_cell	*RRNAD1*	-4.074514489	0.000435723	0.00415349	1
T2DM	b_cell	*PIGM*	-2.641159646	0.001319598	0.004274973	1
T2DM	b_cell	*RGL1*	-1.442532953	0.00100044	0.00415349	1
T2DM	b_cell	*TUFT1*	-2.793182392	0.000273207	0.00415349	1
T2DM	b_cell	*TARS2*	-2.265220149	0.004904304	0.008832242	1
T2DM	b_cell	*SNX27*	-3.218610412	0.014626853	0.020706828	1
T2DM	b_cell	*SELENBP1*	-1.704683155	0.000967567	0.00415349	1
T2DM	b_cell	*SEMA6C*	-5.257311573	0.001978289	0.005092436	1
T2DM	b_cell	*ARNT*	-2.777261899	0.005044357	0.008996844	1
T2DM	b_cell	*POGZ*	-2.34782919	0.005833774	0.009937291	1
T2DM	b_cell	*GABPB2*	-1.8777424	0.013681111	0.019570681	1
T2DM	b_cell	*DYRK3*	-3.413142752	0.001744063	0.004783999	1
T2DM	b_cell	*EIF2D*	-3.40007494	0.001753165	0.004789372	1
T2DM	b_cell	*HHIPL2*	-6.978922182	0.009132025	0.014023704	1
T2DM	b_cell	*TP53BP2*	-3.105503313	0.002337297	0.005511847	1
T2DM	b_cell	*RAB13*	-2.583297307	0.000496272	0.00415349	1
T2DM	b_cell	*S100A8*	2.259582486	0.001488793	0.004462053	1
T2DM	b_cell	*HAX1*	-3.647040097	0.011705136	0.017144273	1
T2DM	b_cell	*CREB3L4*	-3.611698394	0.000660644	0.00415349	1
T2DM	b_cell	*KCNN3*	-2.211395318	0.001096934	0.00415349	1
T2DM	b_cell	*GATAD2B*	-2.432223055	0.029879856	0.038431804	1
T2DM	b_cell	*RIT1*	-3.075080469	0.0018955	0.004975787	1
T2DM	b_cell	*FLG*	-2.408043536	0.00329466	0.006703407	1
T2DM	b_cell	*GALNT2*	-2.49240753	0.002708436	0.00595502	1
T2DM	b_cell	*SCCPDH*	-3.01253715	0.002515454	0.005736184	1
T2DM	b_cell	*CEP170*	-2.52805778	0.008673002	0.013456852	1
T2DM	b_cell	*NVL*	-2.70366571	0.003251334	0.006641045	1
T2DM	b_cell	*SDE2*	-5.538589286	0.009686022	0.014734096	1
T2DM	b_cell	*FBXO28*	-2.961330117	0.020766914	0.027959054	1
T2DM	b_cell	*GUK1*	-2.160995831	0.001025868	0.00415349	1
T2DM	b_cell	*CDC42BPA*	-1.663495259	0.002210204	0.005381337	1
T2DM	b_cell	*CNIH3*	-2.409463514	0.003707645	0.007257149	1
T2DM	b_cell	*PYCR2*	4.767466441	0.004158358	0.007865091	1
T2DM	b_cell	*WNT9A*	-1.988683165	0.00726139	0.011681771	1
T2DM	b_cell	*PLEKHA6*	-1.82902939	0.002095493	0.005240399	1
T2DM	b_cell	*ABCG8*	-6.796602836	0.039983083	0.049905428	1
T2DM	b_cell	*EML4*	-2.502532543	0.011789736	0.017233683	1
T2DM	b_cell	*CHAC2*	-4.986766257	0.005775971	0.009875857	1
T2DM	b_cell	*WDPCP*	-0.901591253	0.000763196	0.00415349	1
T2DM	b_cell	*ASXL2*	-2.693290752	0.011179032	0.016525411	1
T2DM	b_cell	*CIAO1*	-3.409299236	0.00148831	0.004462053	1
T2DM	b_cell	*ZNF514*	-2.147510012	0.000464575	0.00415349	1
T2DM	b_cell	*EXOC6B*	-2.104689091	0.010331633	0.015536623	1
T2DM	b_cell	*TEX261*	-5.509796319	0.02142584	0.028715467	1
T2DM	b_cell	*THNSL2*	-1.11289579	0.008419604	0.01312523	1
T2DM	b_cell	*TMEM177*	-3.674428653	0.000796266	0.00415349	1
T2DM	b_cell	*RABL2A*	-4.058828411	0.002314602	0.005493356	1
T2DM	b_cell	*FBLN7*	-1.879292251	0.007131531	0.011514107	1
T2DM	b_cell	*ZC3H8*	-2.899508847	0.007779525	0.012350455	1
T2DM	b_cell	*FAHD2B*	-3.918946068	0.000941691	0.00415349	1
T2DM	b_cell	*LYG1*	-2.45087312	0.000401739	0.00415349	1
T2DM	b_cell	*AFF3*	1.62829122	0.018137228	0.024859113	1
T2DM	b_cell	*UBXN4*	-2.61649168	0.005364084	0.009387268	1
T2DM	b_cell	*THSD7B*	-1.203656626	0.004133992	0.007837879	1
T2DM	b_cell	*POLR2D*	-3.713888959	0.00175654	0.004789372	1

T2DM	b_cell	*ZNF385B*	-1.4992655	0.001455583	0.004416626	1
T2DM	b_cell	*TMEFF2*	-1.785501539	0.001446854	0.004415713	1
T2DM	b_cell	*GULP1*	-1.404988394	0.001234293	0.004253251	1
T2DM	b_cell	*FAM171B*	-2.458435283	0.001243932	0.004253251	1
T2DM	b_cell	*HSPD1*	-2.867588756	0.006312534	0.010520692	1
T2DM	b_cell	*PTH2R*	-1.735137781	0.002224693	0.005398166	1
T2DM	b_cell	*SUMF1*	-2.145744804	0.006846208	0.011171064	1
T2DM	b_cell	*ESPNL*	-20.97842319	0.015076308	0.02126172	1
T2DM	b_cell	*FANCD2*	1.117384309	0.000558719	0.00415349	1
T2DM	b_cell	*OSBPL10*	2.304481624	0.000968677	0.00415349	1
T2DM	b_cell	*SLC25A38*	2.132088492	0.00123874	0.004253251	1
T2DM	b_cell	*CTDSPL*	-3.297356445	0.005682262	0.009761317	1
T2DM	b_cell	*STAC*	-2.042078781	0.000769299	0.00415349	1
T2DM	b_cell	*ARL6IP5*	-2.60151402	0.003628642	0.007173984	1
T2DM	b_cell	*TMF1*	-2.803702534	0.002574221	0.005806222	1
T2DM	b_cell	*COL8A1*	-1.225673879	0.00070237	0.00415349	1
T2DM	b_cell	*NXPE3*	-4.264973467	0.002924491	0.006211579	1
T2DM	b_cell	*PLA1A*	-6.925889166	8.63E-06	0.001459962	0.054273343
T2DM	b_cell	*RABL3*	-3.381481189	0.004937932	0.008867403	1
T2DM	b_cell	*ADPRH*	-2.702684282	0.00402715	0.007681642	1
T2DM	b_cell	*IGSF11*	-3.946187399	0.014318148	0.020352295	1
T2DM	b_cell	*BOC*	-1.566419779	0.000720254	0.00415349	1
T2DM	b_cell	*AGTR1*	-1.661864667	0.000713945	0.00415349	1
T2DM	b_cell	*EIF2A*	-2.167813759	0.004187429	0.007886869	1
T2DM	b_cell	*TCTA*	-2.530430474	0.00318132	0.006549103	1
T2DM	b_cell	*EAF2*	1.458866437	0.000945068	0.00415349	1
T2DM	b_cell	*ILDR1*	2.36066766	0.005585563	0.009642074	1
T2DM	b_cell	*MUC4*	-2.316388224	0.002542522	0.005783227	1
T2DM	b_cell	*DGKQ*	-2.767417118	0.000978115	0.00415349	1
T2DM	b_cell	*LYAR*	-2.381659939	0.000811432	0.00415349	1
T2DM	b_cell	*CENPC*	-2.474369839	0.018675437	0.025463559	1
T2DM	b_cell	*ATP10D*	-2.291112911	0.014270958	0.020303579	1
T2DM	b_cell	*PYURF*	-5.387013404	0.005038084	0.008990003	1
T2DM	b_cell	*CISD2*	-3.279997339	0.015281374	0.021478649	1
T2DM	b_cell	*CCNA2*	3.640025524	0.005141118	0.009099722	1
T2DM	b_cell	*USP53*	-1.75830297	0.001972283	0.005091111	1
T2DM	b_cell	*MARCH1*	2.091028916	0.027903328	0.036170767	1
T2DM	b_cell	*PDGFC*	-1.648689334	0.000615618	0.00415349	1
T2DM	b_cell	*CBR4*	-2.392770619	0.007778721	0.012350455	1
T2DM	b_cell	*NDUFS6*	-4.420749156	0.008350519	0.013036929	1
T2DM	b_cell	*FAM105A*	-2.585821597	0.000359752	0.00415349	1
T2DM	b_cell	*LHFPL2*	-3.120713431	0.037420592	0.046996257	1
T2DM	b_cell	*SSBP2*	-2.153828671	0.01511588	0.021299033	1
T2DM	b_cell	*BHMT*	-3.244199097	3.58E-07	0.000124872	0.00224769
T2DM	b_cell	*CRHBP*	-3.028139574	0.000285458	0.00415349	1
T2DM	b_cell	*RASA1*	-1.955340578	0.006500479	0.010743562	1
T2DM	b_cell	*PPIP5K2*	-3.158879866	0.000871639	0.00415349	1
T2DM	b_cell	*SPATA9*	-1.519350227	0.001855731	0.004929017	1
T2DM	b_cell	*TSLP*	-3.502919249	0.002016004	0.005148098	1
T2DM	b_cell	*FEM1C*	-3.619156624	0.00780636	0.012371713	1
T2DM	b_cell	*COMMD10*	-2.374320787	0.008400039	0.013097978	1
T2DM	b_cell	*YIPF5*	-2.896467848	0.001515012	0.004480664	1
T2DM	b_cell	*SLC25A48*	-3.381295087	0.018438169	0.02519469	1
T2DM	b_cell	*GABRB2*	-2.267598993	0.00033327	0.00415349	1
T2DM	b_cell	*MYLK4*	-4.761565992	0.001262379	0.004257394	1
T2DM	b_cell	*TBC1D7*	-3.937026208	0.00816355	0.012795871	1
T2DM	b_cell	*GFOD1*	-1.233282957	0.001879436	0.004960544	1
T2DM	b_cell	*PSD2*	5.156820308	0.000205275	0.00415349	1
T2DM	b_cell	*ZMAT2*	-2.129073588	0.000919631	0.00415349	1
T2DM	b_cell	*KLHL3*	-1.654761972	0.001133617	0.00418468	1
T2DM	b_cell	*TRIM7*	3.030571314	0.001547917	0.004517818	1

T2DM	b_cell	*HIGD2A*	-4.14486112	0.000871178	0.00415349	1
T2DM	b_cell	*FAM193B*	-2.716285304	0.003204946	0.006581388	1
T2DM	b_cell	*MUT*	-3.052915364	0.001863562	0.0049373	1
T2DM	b_cell	*DOK3*	-2.495436312	0.005115298	0.009064228	1
T2DM	b_cell	*ABT1*	-2.643444084	0.002298224	0.005481387	1
T2DM	b_cell	*MLIP*	-3.732476373	0.008060071	0.012684272	1
T2DM	b_cell	*TPBG*	-2.143689315	0.000795897	0.00415349	1
T2DM	b_cell	*IRAK1BP1*	-2.518147945	0.002680888	0.005924338	1
T2DM	b_cell	*PHIP*	-2.29193495	0.009279647	0.014213206	1
T2DM	b_cell	*PM20D2*	-4.494557721	0.022955957	0.03056419	1
T2DM	b_cell	*RNF217*	-2.104155556	0.00169061	0.004704627	1
T2DM	b_cell	*SLC18B1*	-1.551317845	0.028374667	0.036713631	1
T2DM	b_cell	*AIG1*	-1.623753231	0.001222093	0.004252746	1
T2DM	b_cell	*DYNLT1*	-2.151267623	0.000784088	0.00415349	1
T2DM	b_cell	*PNLDC1*	-4.77830098	0.028879	0.037275249	1
T2DM	b_cell	*SLC22A3*	-1.619845175	0.000769197	0.00415349	1
T2DM	b_cell	*GNA12*	-2.874813863	0.011356641	0.016740727	1
T2DM	b_cell	*C7orf50*	-2.231994455	0.002229006	0.005400293	1
T2DM	b_cell	*SDK1*	-1.249932521	0.000668461	0.00415349	1
T2DM	b_cell	*RBAK*	-5.4563899	0.006232095	0.010416354	1
T2DM	b_cell	*EGFR*	-1.21965986	0.000349598	0.00415349	1
T2DM	b_cell	*POMZP3*	-3.60499258	0.002591326	0.00582516	1
T2DM	b_cell	*CCT6A*	-3.33442415	0.007797517	0.012363932	1
T2DM	b_cell	*PSPH*	-1.873139309	0.011578567	0.017008049	1
T2DM	b_cell	*GIGYF1*	-4.358264084	0.010095436	0.015238897	1
T2DM	b_cell	*TRIM4*	-2.483405825	0.002324611	0.005505767	1
T2DM	b_cell	*TMEM209*	-1.898231944	0.001613546	0.004616359	1
T2DM	b_cell	*AGBL3*	-1.87940193	0.001014846	0.00415349	1
T2DM	b_cell	*EPHA1*	-4.674970144	0.005174041	0.009136916	1
T2DM	b_cell	*NCAPG2*	-2.74273134	0.006380225	0.010594949	1
T2DM	b_cell	*FBXO25*	-2.564531142	0.001489717	0.004462053	1
T2DM	b_cell	*CSGALNACT1*	-2.905369945	0.007679867	0.01221744	1
T2DM	b_cell	*SLC25A37*	-2.441285193	0.001929225	0.005023674	1
T2DM	b_cell	*DOCK5*	-2.982756789	0.006040826	0.010198355	1
T2DM	b_cell	*ST18*	-2.269690115	0.000568711	0.00415349	1
T2DM	b_cell	*RGS20*	-2.187930747	0.000948587	0.00415349	1
T2DM	b_cell	*TACC1*	-2.019281079	0.001985685	0.005097591	1
T2DM	b_cell	*ADHFE1*	-1.589857377	2.58E-05	0.002754251	0.162500803
T2DM	b_cell	*SLC26A7*	-1.872528206	0.00143453	0.004395857	1
T2DM	b_cell	*ATP6V0D2*	-3.400546318	0.000769838	0.00415349	1
T2DM	b_cell	*MTDH*	-2.549540018	0.015550867	0.021818412	1
T2DM	b_cell	*MAL2*	-2.450910572	0.00037746	0.00415349	1
T2DM	b_cell	*FAM135B*	3.242738254	0.000855412	0.00415349	1
T2DM	b_cell	*ZNF7*	-2.452974166	0.01703017	0.023573024	1
T2DM	b_cell	*SLC39A4*	-11.73746627	0.005118262	0.009066925	1
T2DM	b_cell	*AK3*	-2.490373239	0.001748266	0.004789372	1
T2DM	b_cell	*CER1*	-5.998825586	0.013544082	0.019409993	1
T2DM	b_cell	*PLIN2*	-2.833351376	0.00369184	0.007246772	1
T2DM	b_cell	*HAUS6*	-3.520201706	0.003949805	0.007585602	1
T2DM	b_cell	*C9orf72*	-2.582897537	0.011096984	0.016423432	1
T2DM	b_cell	*SIGMAR1*	-3.323990196	0.00074131	0.00415349	1
T2DM	b_cell	*CBWD5*	-2.329169517	0.000876585	0.00415349	1
T2DM	b_cell	*ZNF462*	-2.117754124	0.001365474	0.004299827	1
T2DM	b_cell	*ALAD*	-1.868100315	0.013596095	0.019466784	1
T2DM	b_cell	*ASTN2*	-3.003601408	0.009032927	0.013898681	1
T2DM	b_cell	*WDR31*	-3.481382976	0.004144957	0.007853931	1
T2DM	b_cell	*POLE3*	4.145351651	0.007587237	0.01210377	1
T2DM	b_cell	*SURF1*	-3.884389072	0.003970977	0.007613111	1
T2DM	b_cell	*SURF6*	3.521826567	0.003231791	0.006614021	1
T2DM	b_cell	*ASB6*	-3.188967242	0.001011951	0.00415349	1
T2DM	b_cell	*NTMT1*	-3.681625263	0.008329642	0.013014308	1

T2DM	b_cell	*INPP5E*	-4.6056781	0.001641937	0.004657671	1
T2DM	b_cell	*DPH7*	-3.461722531	0.001679468	0.004704627	1
T2DM	b_cell	*SLC39A12*	-5.616842503	0.010209098	0.015380925	1
T2DM	b_cell	*RSU1*	-2.509964667	0.00520573	0.00917791	1
T2DM	b_cell	*PARD3*	-1.218863192	0.000816818	0.00415349	1
T2DM	b_cell	*NRBF2*	-2.517525303	0.000227303	0.00415349	1
T2DM	b_cell	*ADIRF*	-2.236249719	0.002104321	0.005241681	1
T2DM	b_cell	*HTR7*	-2.97456167	0.000827465	0.00415349	1
T2DM	b_cell	*RPP30*	-3.232018047	0.005601239	0.009655878	1
T2DM	b_cell	*DNAJB12*	-3.20119436	0.003034254	0.006348205	1
T2DM	b_cell	*PLEKHS1*	4.821546397	0.004338833	0.008089632	1
T2DM	b_cell	*MTG1*	-2.264314316	0.002728484	0.005976996	1
T2DM	b_cell	*PAOX*	2.334122424	0.000874361	0.00415349	1
T2DM	b_cell	*GSTO1*	-2.539762154	0.000503462	0.00415349	1
T2DM	b_cell	*PDCD11*	-2.372098015	0.001311551	0.004274973	1
T2DM	b_cell	*RGS10*	-2.198268754	0.000509283	0.00415349	1
T2DM	b_cell	*GAS2*	-3.743717632	0.002332301	0.005509725	1
T2DM	b_cell	*LIN7C*	-4.142201704	0.010066058	0.015209158	1
T2DM	b_cell	*ZNF215*	1.81735271	0.001549668	0.004517818	1
T2DM	b_cell	*HSD17B12*	-5.87741537	3.17E-08	1.99E-05	0.000199456
T2DM	b_cell	*APIP*	-2.559157027	1.46E-06	0.000397014	0.009156278
T2DM	b_cell	*GLYAT*	-1.275453442	0.004335344	0.008085527	1
T2DM	b_cell	*PTPRJ*	-1.965577604	0.005293862	0.009291769	1
T2DM	b_cell	*CCDC82*	-2.59097601	0.000498397	0.00415349	1
T2DM	b_cell	*SERPINH1*	-1.963641488	0.001885273	0.004965526	1
T2DM	b_cell	*CAPN5*	-2.594080762	0.001977815	0.005092436	1
T2DM	b_cell	*ZC3H12C*	-2.796442805	0.004822464	0.008739934	1
T2DM	b_cell	*DRD2*	-11.73897648	0.001501744	0.004478191	1
T2DM	b_cell	*C11orf52*	-20.42499785	0.000763737	0.00415349	1
T2DM	b_cell	*NPAT*	-3.025364288	0.010491645	0.015704993	1
T2DM	b_cell	*GRIK4*	-2.214150878	0.010266943	0.015456961	1
T2DM	b_cell	*ADAM33*	-1.860634083	0.000598253	0.00415349	1
T2DM	b_cell	*MTA2*	-4.68239045	0.029214805	0.037676611	1
T2DM	b_cell	*TMC2*	-8.443923635	0.021486722	0.02878479	1
T2DM	b_cell	*EML3*	-2.785592844	0.00184114	0.004898539	1
T2DM	b_cell	*INCENP*	-3.082576481	0.000841612	0.00415349	1
T2DM	b_cell	*MS4A3*	-53.31500836	0.000626499	0.00415349	1
T2DM	b_cell	*PLCH2*	-9.13954135	0.000815644	0.00415349	1
T2DM	b_cell	*CPSF7*	-3.14222021	0.009106701	0.013988231	1
T2DM	b_cell	*FEZ1*	-2.678519181	0.001144629	0.00418468	1
T2DM	b_cell	*COMMD7*	-2.873345292	0.001568931	0.004558164	1
T2DM	b_cell	*KIAA1755*	-2.255162304	0.003735151	0.007283776	1
T2DM	b_cell	*CABLES2*	2.986994337	0.001382608	0.00432604	1
T2DM	b_cell	*TRPT1*	-2.867948406	0.000937662	0.00415349	1
T2DM	b_cell	*DOC2A*	-9.23606195	2.17E-06	0.000505081	0.013637198
T2DM	b_cell	*TAOK2*	-3.982011911	0.00175277	0.004789372	1
T2DM	b_cell	*MPP7*	-2.84180318	0.00016581	0.00415349	1
T2DM	b_cell	*CWC15*	-3.347371494	0.003824547	0.007400716	1
T2DM	b_cell	*TMCO3*	-3.301188435	0.013403518	0.019243644	1
T2DM	b_cell	*SAP18*	-3.645806523	0.004689892	0.008580584	1
T2DM	b_cell	*LYPD6B*	-4.147080759	0.000516268	0.00415349	1
T2DM	b_cell	*WDR17*	-4.423525895	0.021978495	0.029349787	1
T2DM	b_cell	*CCDC102B*	-1.732039907	0.000944498	0.00415349	1
T2DM	b_cell	*RGS18*	-2.829005341	0.00079404	0.00415349	1
T2DM	b_cell	*PRSS23*	-1.242425199	0.000810419	0.00415349	1
T2DM	b_cell	*MTMR12*	-2.380443827	0.002477906	0.005683544	1
T2DM	b_cell	*PPP1R1C*	7.864047083	0.000438378	0.00415349	1
T2DM	b_cell	*C11orf53*	20.40444414	0.002490504	0.005706196	1
T2DM	b_cell	*CCT5*	-2.447425869	0.001684817	0.004704627	1
T2DM	b_cell	*FAM173B*	-1.878927082	0.000552759	0.00415349	1
T2DM	b_cell	*DOCK1*	-1.987038493	0.001962395	0.005077193	1

T2DM	b_cell	*TEX12*	-18.12237358	0.000743588	0.00415349	1
T2DM	b_cell	*PIP4K2A*	-2.588165714	0.008754342	0.013569661	1
T2DM	b_cell	*CRIM1*	-1.14843401	0.000622211	0.00415349	1
T2DM	b_cell	*ABCB9*	-6.133236019	0.000160648	0.00415349	1
T2DM	b_cell	*RILPL2*	-2.781038438	0.002037207	0.005172827	1
T2DM	b_cell	*PRSS53*	-11.4155732	0.000107481	0.00415349	0.675733104
T2DM	b_cell	*GPR158*	-2.708769501	0.002334644	0.005509725	1
T2DM	b_cell	*THRB*	-1.63039062	0.001530756	0.004503445	1
T2DM	b_cell	*TMEM86A*	-3.691267162	0.002247495	0.005418361	1
T2DM	b_cell	*BTBD11*	-2.229780329	0.000764222	0.00415349	1
T2DM	b_cell	*UBE3B*	-3.045591738	0.012134756	0.017668183	1
T2DM	b_cell	*ANK3*	-1.852087806	0.00608655	0.010252906	1
T2DM	b_cell	*IPMK*	-2.635400615	0.002085581	0.005230315	1
T2DM	b_cell	*DLG5*	-1.960915363	0.000461923	0.00415349	1
T2DM	b_cell	*GXYLT1*	-3.296973024	0.033809265	0.042949858	1
T2DM	b_cell	*MIPOL1*	-1.557179659	0.001453416	0.004416445	1
T2DM	b_cell	*EXT2*	-2.56551698	0.014966212	0.02112541	1
T2DM	b_cell	*TMEM18*	-4.040101192	0.002928077	0.006217095	1
T2DM	b_cell	*KCTD14*	8.478584624	0.004177198	0.00787705	1
T2DM	b_cell	*THRSP*	-2.012249602	0.000576632	0.00415349	1
T2DM	b_cell	*NDUFC2*	-4.675778456	0.012228277	0.017783757	1
T2DM	b_cell	*ME3*	-2.108772214	0.003772326	0.007333523	1
T2DM	b_cell	*ADAMTS12*	-1.15725487	0.000895027	0.00415349	1
T2DM	b_cell	*PTPRO*	-3.422819538	0.001815409	0.004867153	1
T2DM	b_cell	*VPS26B*	-3.859413059	0.006578006	0.010834667	1
T2DM	b_cell	*NCAPD3*	-2.932010581	0.000837949	0.00415349	1
T2DM	b_cell	*QDPR*	-2.001557462	0.00035814	0.00415349	1
T2DM	b_cell	*FAM160B1*	-2.13276358	0.001297143	0.004268027	1
T2DM	b_cell	*ANO4*	-1.990766462	0.000981513	0.00415349	1
T2DM	b_cell	*AKR1C2*	-1.539833162	0.000571431	0.00415349	1
T2DM	b_cell	*DPYSL4*	2.327079079	0.015699297	0.021987409	1
T2DM	b_cell	*ADAM8*	-3.070650309	0.000860837	0.00415349	1
T2DM	b_cell	*ITIH2*	1.309823599	0.000647013	0.00415349	1
T2DM	b_cell	*PIGF*	-3.068112667	0.003204129	0.006581388	1
T2DM	b_cell	*INPP1*	-2.334322547	0.001571217	0.004560592	1
T2DM	b_cell	*MFSD6*	-3.082238526	0.006023983	0.010177114	1
T2DM	b_cell	*RNF144A*	-1.376001839	0.000862097	0.00415349	1
T2DM	b_cell	*ASAP2*	-1.990119267	0.00210601	0.005241681	1
T2DM	b_cell	*ADAM17*	-2.68596385	0.012798051	0.018505369	1
T2DM	b_cell	*TMEM45B*	-7.62661839	0.000948411	0.00415349	1
T2DM	b_cell	*ACSL1*	-1.065998161	0.001930523	0.005023674	1
T2DM	b_cell	*CCDC122*	-1.957950488	0.004234049	0.007955609	1
T2DM	b_cell	*GABRA2*	-3.234050605	0.000741699	0.00415349	1
T2DM	b_cell	*CENPJ*	-1.782405341	0.001075537	0.00415349	1
T2DM	b_cell	*PARP8*	-1.716447411	0.003290134	0.006696366	1
T2DM	b_cell	*CACUL1*	-2.768570054	0.006878606	0.011212288	1
T2DM	b_cell	*BEND6*	-2.205228987	0.001122957	0.004182741	1
T2DM	b_cell	*PTPN14*	-1.357371058	0.000711401	0.00415349	1
T2DM	b_cell	*ARL14EP*	-4.116970227	7.99E-05	0.00415349	0.502061196
T2DM	b_cell	*EPG5*	-3.258842288	0.007439445	0.011907279	1
T2DM	b_cell	*SPC25*	-1.487842098	0.008902611	0.013745264	1
T2DM	b_cell	*G6PC2*	-129.8404747	0.000735525	0.00415349	1
T2DM	b_cell	*TGOLN2*	-3.19252448	0.02430088	0.032009156	1
T2DM	b_cell	*POC5*	-3.857219895	6.82E-05	0.00415349	0.428817201
T2DM	b_cell	*TADA1*	-4.026917831	0.003462809	0.006928923	1
T2DM	b_cell	*GUCY1A2*	-1.592181535	0.000770926	0.00415349	1
T2DM	b_cell	*CWF19L2*	-2.254304056	0.005479982	0.009527833	1
T2DM	b_cell	*ZNF547*	2.526872744	0.00081339	0.00415349	1
T2DM	b_cell	*ZNF773*	-5.325607811	0.000279864	0.00415349	1
T2DM	b_cell	*NMT2*	-2.070080115	0.000582844	0.00415349	1
T2DM	b_cell	*ZNF837*	2.386076655	0.000864859	0.00415349	1

T2DM	b_cell	*ZFP36L2*	-2.253058155	0.010446588	0.015663654	1
T2DM	b_cell	*PLEKHH2*	-0.837033418	0.022397663	0.029865134	1
T2DM	b_cell	*TMEM123*	-3.186290274	0.004930984	0.008859987	1
T2DM	b_cell	*IGSF10*	-2.053164938	0.00084623	0.00415349	1
T2DM	b_cell	*SPEF2*	-2.271790112	0.001416839	0.004364365	1
T2DM	b_cell	*SPARCL1*	-1.182505352	0.00082201	0.00415349	1
T2DM	b_cell	*CCNO*	16.95256394	0.004771306	0.008682258	1
T2DM	b_cell	*CLEC4F*	-7.452449587	0.000745704	0.00415349	1
T2DM	b_cell	*PELO*	-2.811066805	0.000803136	0.00415349	1
T2DM	b_cell	*GPR180*	-2.04965028	0.001136568	0.00418468	1
T2DM	b_cell	*WDR78*	-2.594799926	0.000964805	0.00415349	1
T2DM	b_cell	*IFIT5*	-3.501969844	0.007119162	0.011508915	1
T2DM	b_cell	*PRDM8*	1.898051487	0.002357417	0.005540753	1
T2DM	b_cell	*HHEX*	-2.90261709	0.000765529	0.00415349	1
T2DM	b_cell	*ZNF117*	-0.83984852	0.01219759	0.017747339	1
T2DM	b_cell	*RAB3C*	-1.999682021	0.000963757	0.00415349	1
T2DM	b_cell	*RAD17*	-2.92734376	0.001617102	0.004621237	1
T2DM	b_cell	*MED21*	-3.035019048	0.002714917	0.005958936	1
T2DM	b_cell	*SRP19*	-3.347377049	0.00193617	0.005026068	1
T2DM	b_cell	*CENPH*	-2.192407461	0.015549607	0.021818412	1
T2DM	b_cell	*CARHSP1*	-2.494992795	0.001183774	0.004202203	1
T2DM	b_cell	*TEKT5*	-6.035760901	0.00231	0.005492478	1
T2DM	b_cell	*BANK1*	0.285783067	0.000589609	0.00415349	1
T2DM	b_cell	*CAST*	-2.101544469	0.010955748	0.016271862	1
T2DM	b_cell	*SCOC*	-2.250199731	0.001483499	0.004462053	1
T2DM	b_cell	*CLGN*	-17.53940553	0.00164431	0.004657671	1
T2DM	b_cell	*SYCP2L*	-2.327478841	0.000896494	0.00415349	1
T2DM	b_cell	*AHCTF1*	-2.437345282	0.003158545	0.006514977	1
T2DM	b_cell	*MERTK*	-0.958294745	0.007458182	0.011934231	1
T2DM	b_cell	*TMEM87B*	-2.708128996	0.004867447	0.008790778	1
T2DM	b_cell	*PTPRR*	-3.277151045	0.001068254	0.00415349	1
T2DM	b_cell	*PLA2R1*	-1.375308006	0.001973224	0.005091111	1
T2DM	b_cell	*SLC25A27*	-1.606586365	0.000532785	0.00415349	1
T2DM	b_cell	*FRMD1*	-3.02441433	0.000777164	0.00415349	1
T2DM	b_cell	*ASAP1*	-1.917515819	0.00193623	0.005026068	1
T2DM	b_cell	*INO80C*	-3.1413363	0.000793032	0.00415349	1
T2DM	b_cell	*LPCAT1*	-3.809689703	0.003169544	0.006531275	1
T2DM	b_cell	*C16orf89*	-2.46149781	0.002923217	0.006210972	1
T2DM	b_cell	*TEX29*	-3.203695998	0.00149586	0.004474047	1
T2DM	b_cell	*ADPRHL1*	-3.945390928	0.000971556	0.00415349	1
T2DM	b_cell	*TUBGCP5*	-3.329322312	0.01404933	0.020029056	1
T2DM	b_cell	*LURAP1L*	-1.22022194	0.002855974	0.006140735	1
T2DM	b_cell	*CFDP1*	-2.475214877	0.004108158	0.007801017	1
T2DM	b_cell	*ZDHHC7*	-2.297820755	0.002232532	0.005401538	1
T2DM	b_cell	*FAM92B*	9.391818755	0.000626578	0.00415349	1
T2DM	b_cell	*JAZF1*	-3.805325815	0.001360872	0.004298348	1
T2DM	b_cell	*MCOLN2*	1.748532598	0.000379874	0.00415349	1
T2DM	b_cell	*CACNA2D1*	-1.134951523	0.000716507	0.00415349	1
T2DM	b_cell	*ZUFSP*	-2.928067184	0.002021135	0.005157371	1
T2DM	b_cell	*HS3ST3A1*	-3.314567428	0.004518996	0.008348788	1
T2DM	b_cell	*PPP2R5E*	-2.02307191	0.036187027	0.045638291	1
T2DM	b_cell	*ANKRD29*	-1.697866691	0.001680489	0.004704627	1
T2DM	b_cell	*THY1*	-1.784549663	0.000904696	0.00415349	1
T2DM	b_cell	*TBCEL*	-2.035101357	0.001054618	0.00415349	1
T2DM	b_cell	*UBASH3B*	-2.37268643	0.000946112	0.00415349	1
T2DM	b_cell	*ROBO4*	-1.887325374	0.001227516	0.004253251	1
T2DM	b_cell	*ROBO3*	-2.14414072	0.000557519	0.00415349	1
T2DM	b_cell	*CC2D1B*	-3.134422657	0.024231171	0.031944091	1
T2DM	b_cell	*PRKCA*	-2.667390966	0.006122324	0.010297514	1
T2DM	b_cell	*LRRK1*	-2.903930022	0.005689123	0.009764542	1
T2DM	b_cell	*CEP112*	-1.439163047	0.001327077	0.004274973	1

T2DM	b_cell	*UCHL1*	-2.654160258	0.000715993	0.00415349	1
T2DM	b_cell	*MIA3*	-3.078110647	0.005523247	0.009579216	1
T2DM	b_cell	*DISP1*	3.769616498	0.01685075	0.023364741	1
T2DM	b_cell	*FAM167A*	2.84204038	0.000544755	0.00415349	1
T2DM	b_cell	*NEIL2*	4.324465369	0.002033795	0.00516834	1
T2DM	b_cell	*LONRF1*	-2.311394063	0.000258717	0.00415349	1
T2DM	b_cell	*CCSAP*	-4.187002425	0.002748024	0.006007705	1
T2DM	b_cell	*GBP5*	-2.750068452	0.001106795	0.004159248	1
T2DM	b_cell	*FAM69A*	-2.118998731	0.000980861	0.00415349	1
T2DM	b_cell	*PDLIM3*	-1.994465816	0.002105641	0.005241681	1
T2DM	b_cell	*NCAM2*	-1.624533211	0.000965656	0.00415349	1
T2DM	b_cell	*L3MBTL4*	-2.262926168	0.004191363	0.007891914	1
T2DM	b_cell	*PDE1C*	-1.728289969	0.000824662	0.00415349	1
T2DM	b_cell	*RABGEF1*	-2.443375837	0.001840589	0.004898539	1
T2DM	b_cell	*FLCN*	-2.637372436	0.00251187	0.005734251	1
T2DM	b_cell	*CXXC1*	-3.51045755	0.001646209	0.004658975	1
T2DM	b_cell	*PPP4R1*	-2.564783508	0.0026758	0.00592142	1
T2DM	b_cell	*APCDD1*	-1.342260442	0.001828952	0.004890949	1
T2DM	b_cell	*EPHB1*	-3.715147338	0.004130663	0.007836293	1
T2DM	b_cell	*ANKRD40*	-2.547134212	0.00060489	0.00415349	1
T2DM	b_cell	*CYP2U1*	-3.525907118	0.002110599	0.005248946	1
T2DM	b_cell	*FBXL18*	-2.938859451	0.001867202	0.004940699	1
T2DM	b_cell	*PROM2*	-11.79959471	0.000925185	0.00415349	1
T2DM	b_cell	*AK9*	-2.223660564	0.001652202	0.004668493	1
T2DM	b_cell	*CDK19*	-2.637026842	0.017109165	0.023661531	1
T2DM	b_cell	*GTF3C6*	3.410248043	0.003653798	0.007196562	1
T2DM	b_cell	*TTC39B*	-2.758568641	0.029124693	0.037568106	1
T2DM	b_cell	*AGPAT5*	3.280696503	0.028839738	0.037246392	1
T2DM	b_cell	*MMS19*	-2.873495796	0.001515633	0.004480664	1
T2DM	b_cell	*GOLGA7B*	2.389452872	0.002212437	0.005382969	1
T2DM	b_cell	*TRMT44*	-2.315676585	0.001105067	0.004159248	1
T2DM	b_cell	*C16orf87*	3.734318402	0.002236593	0.005404098	1
T2DM	b_cell	*HEATR3*	-2.116058653	0.014206592	0.020230316	1
T2DM	b_cell	*CNOT8*	-3.145519674	0.015335374	0.021544916	1
T2DM	b_cell	*LRGUK*	-2.494204199	0.019092842	0.025925853	1
T2DM	b_cell	*SETD9*	-2.144904482	7.51E-05	0.00415349	0.47184207
T2DM	b_cell	*FAM126B*	-2.390434924	0.002671029	0.005916798	1
T2DM	b_cell	*ALS2CR12*	-2.235879557	0.000380854	0.00415349	1
T2DM	b_cell	*SPAG17*	-1.340177536	0.000858964	0.00415349	1
T2DM	b_cell	*DEPTOR*	-2.524325356	0.001640634	0.004657671	1
T2DM	b_cell	*RRAGA*	-5.469018808	0.004126781	0.007831293	1
T2DM	b_cell	*TMBIM4*	-2.552165061	0.011954906	0.017446118	1
T2DM	b_cell	*FAM161B*	-2.984210764	0.002711873	0.005955133	1
T2DM	b_cell	*WIF1*	6.523682805	0.000318477	0.00415349	1
T2DM	b_cell	*UGT2B4*	-3.944489934	0.000773565	0.00415349	1
T2DM	b_cell	*MMP16*	-1.529461466	0.000328938	0.00415349	1
T2DM	b_cell	*KCNMA1*	-1.339255559	0.000727168	0.00415349	1
T2DM	b_cell	*BATF*	6.172341521	0.021758041	0.02909866	1
T2DM	b_cell	*DCK*	2.932168199	0.002104619	0.005241681	1
T2DM	b_cell	*DPY19L4*	-2.750761678	0.001643211	0.004657671	1
T2DM	b_cell	*NDUFAF6*	-3.320631627	0.000157637	0.00415349	0.991063433
T2DM	b_cell	*DRAM2*	-2.400920213	0.006208963	0.010401212	1
T2DM	b_cell	*ADAMTSL3*	-1.254349516	0.001521384	0.004492692	1
T2DM	b_cell	*WHAMM*	-3.654174686	0.000112083	0.00415349	0.704664708
T2DM	b_cell	*CXCL13*	-4.436461081	0.000840811	0.00415349	1
T2DM	b_cell	*N6AMT1*	-2.3437714	0.001263134	0.004257394	1
T2DM	b_cell	*RWDD2B*	-2.057619724	0.002889227	0.006184116	1
T2DM	b_cell	*ANKRD9*	-3.309285109	0.000308926	0.00415349	1
T2DM	b_cell	*SFXN2*	-2.171558063	0.001708365	0.004728644	1
T2DM	b_cell	*C14orf2*	-4.028909452	0.002448369	0.005641723	1
T2DM	b_cell	*SH3RF2*	-1.502840627	0.003750646	0.007304929	1

T2DM	b_cell	*GDF6*	-5.769948101	0.004270659	0.008002871	1
T2DM	b_cell	*NODAL*	-3.452651263	0.000784616	0.00415349	1
T2DM	b_cell	*MED19*	-2.252699766	0.002081902	0.005230315	1
T2DM	b_cell	*WDYHV1*	-3.764811231	0.001095467	0.00415349	1
T2DM	b_cell	*ATAD2*	-3.613636466	0.013480678	0.01933235	1
T2DM	b_cell	*SASS6*	-3.138357432	0.000703287	0.00415349	1
T2DM	b_cell	*ITGAD*	-2.631710301	0.004925083	0.008854445	1
T2DM	b_cell	*VPS8*	-1.977809702	0.023073359	0.030688007	1
T2DM	b_cell	*MPV17L*	-2.287518524	0.029436779	0.037916212	1
T2DM	b_cell	*RPUSD3*	-3.561373499	0.001980891	0.005095689	1
T2DM	b_cell	*GHRL*	-2.854946838	0.001032023	0.00415349	1
T2DM	b_cell	*EXOG*	-3.493546807	0.004184832	0.007886524	1
T2DM	b_cell	*SLC6A1*	-5.869322975	0.00039679	0.00415349	1
T2DM	b_cell	*FCHO2*	-2.242541766	0.003338223	0.006765112	1
T2DM	b_cell	*TIMP4*	-1.846186095	0.005534098	0.009595387	1
T2DM	b_cell	*CPT2*	3.772475992	0.001759719	0.004789372	1
T2DM	b_cell	*GATAD1*	-1.86738726	0.005485073	0.009534048	1
T2DM	b_cell	*TMED6*	-11.49925373	0.00072031	0.00415349	1
T2DM	b_cell	*CLEC18A*	-15.58335124	1.07E-07	5.60E-05	0.000672084
T2DM	b_cell	*DHRS1*	-2.507799642	0.005344666	0.009365082	1
T2DM	b_cell	*AASDH*	-2.97500771	0.009941124	0.015058607	1
T2DM	b_cell	*RNF111*	-1.86864765	0.001933304	0.005024672	1
T2DM	b_cell	*DYRK1A*	-2.316331538	0.029771716	0.038308387	1
T2DM	b_cell	*KCNJ15*	-6.497013324	0.004161339	0.007865091	1
T2DM	b_cell	*LCA5L*	-2.068275324	0.031766161	0.040583997	1
T2DM	b_cell	*CREB3L1*	-2.606824369	0.002910575	0.006192483	1
T2DM	b_cell	*C2CD2*	-1.428898395	0.002871394	0.006159146	1
T2DM	b_cell	*SLC38A10*	-3.244661502	0.010161151	0.015319701	1
T2DM	b_cell	*C9orf43*	-4.55724165	0.000409933	0.00415349	1
T2DM	b_cell	*WDR19*	-2.674655737	0.002379383	0.005562581	1
T2DM	b_cell	*SLC37A3*	-2.590844679	0.002047708	0.005191106	1
T2DM	b_cell	*SPPL3*	-2.618600894	0.005834029	0.009937291	1
T2DM	b_cell	*RADIL*	-2.055738441	0.000576956	0.00415349	1
T2DM	b_cell	*LDLRAP1*	-4.806603431	0.014597038	0.020683163	1
T2DM	b_cell	*PAFAH2*	-4.42380213	0.001176983	0.004202203	1
T2DM	b_cell	*TRIM63*	4.246996513	8.82E-06	0.001459962	0.055478563
T2DM	b_cell	*WDR66*	-2.501608273	0.000474103	0.00415349	1
T2DM	b_cell	*UBXN11*	-3.151942782	0.001892558	0.00497413	1
T2DM	b_cell	*GALNT14*	6.970275852	0.004349813	0.008105298	1
T2DM	b_cell	*RHPN1*	-3.673614771	0.003780473	0.007342551	1
T2DM	b_cell	*TPRG1L*	-4.487768162	0.000722209	0.00415349	1
T2DM	b_cell	*LRRC43*	-6.980479379	0.00074285	0.00415349	1
T2DM	b_cell	*XKR8*	-4.078630244	0.000619466	0.00415349	1
T2DM	b_cell	*EYA3*	-2.757844698	0.01107784	0.01639896	1
T2DM	b_cell	*MRAS*	-1.552633386	0.000199448	0.00415349	1
T2DM	b_cell	*ABHD3*	-1.843093803	0.006009749	0.01015953	1
T2DM	b_cell	*CLSTN2*	-1.238795119	0.000698732	0.00415349	1
T2DM	b_cell	*COLEC12*	-1.321193817	0.000812435	0.00415349	1
T2DM	b_cell	*RNF207*	-5.415984905	0.003084176	0.006414229	1
T2DM	b_cell	*AUTS2*	-1.683377148	0.002125615	0.005269615	1
T2DM	b_cell	*MITD1*	-3.257701951	0.017789178	0.024478127	1
T2DM	b_cell	*TSPAN33*	-2.819882607	0.03205197	0.04090758	1
T2DM	b_cell	*AHCYL2*	-2.447917722	0.011768191	0.017218203	1
T2DM	b_cell	*B4GALT5*	-1.953563114	0.00112418	0.004182741	1
T2DM	b_cell	*CD1D*	-9.289950222	0.00029189	0.00415349	1
T2DM	b_cell	*FAM86C1*	9.551293411	0.014256012	0.020291498	1
T2DM	b_cell	*NCF1*	2.014491469	0.000692732	0.00415349	1
T2DM	b_cell	*PPP1R9A*	-1.267017367	0.000669214	0.00415349	1
T2DM	b_cell	*ZFAND2B*	-3.984580373	0.004261072	0.007989011	1
T2DM	b_cell	*GDPD5*	-1.655837088	0.000689846	0.00415349	1
T2DM	b_cell	*DYNC1I1*	-2.945899309	0.002240106	0.005409545	1

T2DM	b_cell	*TMED4*	-2.910569616	0.001798069	0.004852128	1
T2DM	b_cell	*COPG2*	-1.558521802	0.00039266	0.00415349	1
T2DM	b_cell	*ELK4*	-3.432802741	0.009282555	0.014213206	1
T2DM	b_cell	*DUSP23*	-3.273892044	0.000925075	0.00415349	1
T2DM	b_cell	*RNF166*	-4.121640867	0.030224595	0.038835486	1
T2DM	b_cell	*F11R*	-2.166676504	0.001545731	0.004515804	1
T2DM	b_cell	*NIT1*	-1.781191273	0.004779321	0.00869424	1
T2DM	b_cell	*ZNF276*	-3.225301132	0.000354974	0.00415349	1
T2DM	b_cell	*CDA*	-3.796824411	0.012776995	0.018479173	1
T2DM	b_cell	*B4GALT3*	-2.938516593	0.004315283	0.008064858	1
T2DM	b_cell	*DMTN*	-1.69226696	0.012217586	0.017772319	1
T2DM	b_cell	*FAM160B2*	-2.692492519	0.001183936	0.004202203	1
T2DM	b_cell	*NDUFS2*	-3.019549028	0.003755259	0.007309385	1
T2DM	b_cell	*SLC5A11*	-1.739049985	0.004980206	0.00892419	1
T2DM	b_cell	*CCAR2*	-3.44067186	0.003355002	0.006777924	1
T2DM	b_cell	*EPB41*	-1.671809456	0.005384615	0.009411475	1
T2DM	b_cell	*ALG8*	-3.544895335	0.007560371	0.0120701	1
T2DM	b_cell	*MRPL10*	-2.831862432	0.000842941	0.00415349	1
T2DM	b_cell	*SV2A*	-10.8591521	0.002672709	0.005916798	1
T2DM	b_cell	*RCAN1*	-2.385316506	0.002467903	0.005668874	1
T2DM	b_cell	*UBE2Z*	-3.858575849	0.003403763	0.006850019	1
T2DM	b_cell	*CLIC6*	-2.760652857	0.024540175	0.032270253	1
T2DM	b_cell	*CBR3*	-3.054836335	0.023259707	0.030890109	1
T2DM	b_cell	*SIM2*	4.982189217	0.001662691	0.004679659	1
T2DM	b_cell	*HLCS*	-1.991788742	0.005770454	0.009869109	1
T2DM	b_cell	*ARHGAP27*	-2.509670005	0.00108273	0.00415349	1
T2DM	b_cell	*PADI4*	-6.877818543	0.001286341	0.004268027	1
T2DM	b_cell	*PSMD4*	-3.600924575	0.010715021	0.015989873	1
T2DM	b_cell	*STARD9*	-1.457914096	0.001079965	0.00415349	1
T2DM	b_cell	*THEM4*	-2.1694898	0.002276998	0.005445221	1
T2DM	b_cell	*TCHH*	2.587653784	0.002357633	0.005540753	1
T2DM	b_cell	*MED8*	-2.305832031	0.004215941	0.007929499	1
T2DM	b_cell	*RSPRY1*	-3.449079541	0.009305301	0.014241097	1
T2DM	b_cell	*CCDC17*	-5.72040005	0.000678947	0.00415349	1
T2DM	b_cell	*ACE*	-2.147735979	0.000725459	0.00415349	1
T2DM	b_cell	*TEPP*	15.88903369	0.027957529	0.036233557	1
T2DM	b_cell	*EFCAB14*	-3.19965421	0.008785519	0.013604571	1
T2DM	b_cell	*LYPD5*	-5.455213819	0.008091267	0.012711094	1
T2DM	b_cell	*CCDC117*	-4.332365846	0.03475101	0.043959678	1
T2DM	b_cell	*CCDC107*	-2.359455588	0.000394149	0.00415349	1
T2DM	b_cell	*NPR2*	-2.497019263	0.001349172	0.004290463	1
T2DM	b_cell	*ZNF235*	-2.744364976	0.001406359	0.004357467	1
T2DM	b_cell	*DFFA*	-2.608035501	0.007099156	0.01148839	1
T2DM	b_cell	*ATAD3B*	-4.129361376	0.017908735	0.024594193	1
T2DM	b_cell	*SSU72*	-2.411177446	0.001157779	0.004195495	1
T2DM	b_cell	*NR2F6*	-2.738155638	0.003700626	0.00724956	1
T2DM	b_cell	*ANKLE1*	-5.666936747	0.033234514	0.04227952	1
T2DM	b_cell	*CILP2*	-21.59349666	0.000906488	0.00415349	1
T2DM	b_cell	*RSPH1*	-6.534232369	0.002462399	0.005658298	1
T2DM	b_cell	*U2AF1*	-5.851754798	0.010523244	0.015737306	1
T2DM	b_cell	*HSF2BP*	-2.857098137	0.016598812	0.023072458	1
T2DM	b_cell	*CSTB*	-2.287768564	0.000418267	0.00415349	1
T2DM	b_cell	*C21orf33*	-10.20757509	0.000239707	0.00415349	1
T2DM	b_cell	*ICOSLG*	4.932735736	0.004738392	0.008639869	1
T2DM	b_cell	*LRRC3*	-4.551785391	0.003877076	0.007477048	1
T2DM	b_cell	*ITGB2*	-2.577421252	0.005034108	0.008986408	1
T2DM	b_cell	*LSS*	-2.050031196	0.000743532	0.00415349	1
T2DM	b_cell	*VAV2*	3.236683813	0.033604216	0.042715266	1
T2DM	b_cell	*MCM3AP*	-2.071165947	0.025465787	0.033354876	1
T2DM	b_cell	*PCNT*	-2.079307044	0.012678063	0.018348752	1
T2DM	b_cell	*DIP2A*	-3.807927279	0.001180447	0.004202203	1

T2DM	b_cell	*PRMT2*	-2.735410477	0.000643462	0.00415349	1
T2DM	b_cell	*ZNF208*	-1.753387739	0.000963083	0.00415349	1
T2DM	b_cell	*ADAMTS13*	-2.624230036	0.001215288	0.004245409	1
T2DM	b_cell	*ZNF714*	-3.113598841	0.014829102	0.020957698	1
T2DM	b_cell	*GPSM1*	-3.794711156	0.000105494	0.00415349	0.663238559
T2DM	b_cell	*C19orf47*	-3.253453225	0.000994078	0.00415349	1
T2DM	b_cell	*HIPK4*	5.103047495	0.011519379	0.016952793	1
T2DM	b_cell	*RDH13*	-4.949105909	0.005090281	0.009037019	1
T2DM	b_cell	*MPZL3*	-3.127227353	0.000829362	0.00415349	1
T2DM	b_cell	*PCSK7*	-3.556861275	0.009369391	0.014311312	1
T2DM	b_cell	*S100A1*	2.185683552	0.000354984	0.00415349	1
T2DM	b_cell	*ZBTB7B*	-4.229066703	0.010013665	0.015150372	1
T2DM	b_cell	*VPS11*	-3.635829208	0.000768804	0.00415349	1
T2DM	b_cell	*FDPS*	-2.053832744	0.009628739	0.014654051	1
T2DM	b_cell	*PAQR6*	1.457417143	0.001508992	0.004480341	1
T2DM	b_cell	*SLC25A44*	-3.235336653	0.000719719	0.00415349	1
T2DM	b_cell	*NBEAL2*	-4.018207818	0.017124521	0.02367756	1
T2DM	b_cell	*FCRL3*	0.247268661	0.000688334	0.00415349	1
T2DM	b_cell	*FGFR4*	6.366103897	0.000568109	0.00415349	1
T2DM	b_cell	*NACC1*	2.974182518	0.000832857	0.00415349	1
T2DM	b_cell	*LY6K*	9.491267365	0.001762693	0.004791201	1
T2DM	b_cell	*TONSL*	-4.139060411	0.000946635	0.00415349	1
T2DM	b_cell	*MUM1*	-1.878727017	0.002378849	0.005562581	1
T2DM	b_cell	*ZNF333*	-3.532671858	0.01880536	0.025607386	1
T2DM	b_cell	*PPP1R16A*	-1.945292152	0.002507162	0.005732727	1
T2DM	b_cell	*ORAI2*	1.914868517	0.000902701	0.00415349	1
T2DM	b_cell	*LRWD1*	3.367872937	0.00299911	0.006308941	1
T2DM	b_cell	*FBXL13*	-2.591323437	0.001098816	0.00415349	1
T2DM	b_cell	*MFSD12*	-4.03522387	0.005571013	0.009636761	1
T2DM	b_cell	*YDJC*	11.64466786	0.027230072	0.035370963	1
T2DM	b_cell	*CCDC116*	3.622106885	0.003913439	0.007533311	1
T2DM	b_cell	*DVL3*	-3.195798458	0.018091046	0.024822873	1
T2DM	b_cell	*FBXO27*	-1.819731655	0.002770426	0.006028396	1
T2DM	b_cell	*DMKN*	3.592561015	0.006194319	0.010384982	1
T2DM	b_cell	*U2AF1L4*	1.554351401	0.009337062	0.014279278	1
T2DM	b_cell	*BDH1*	-2.864548354	0.000791049	0.00415349	1
T2DM	b_cell	*COX7A1*	-2.995063401	0.000423889	0.00415349	1
T2DM	b_cell	*DUSP14*	-1.965578691	0.001582713	0.004577055	1
T2DM	b_cell	*LRRC56*	2.479629843	0.007499225	0.01199075	1
T2DM	b_cell	*PGAP3*	-4.534820317	0.001809396	0.004859322	1
T2DM	b_cell	*IKZF3*	0.524756296	0.000593644	0.00415349	1
T2DM	b_cell	*ACOX1*	-2.134420737	0.000752231	0.00415349	1
T2DM	b_cell	*PRPSAP1*	-2.898378417	0.001091198	0.00415349	1
T2DM	b_cell	*CYGB*	-3.123412089	0.000454953	0.00415349	1
T2DM	b_cell	*ZNF577*	-2.341454375	0.007786587	0.01235595	1
T2DM	b_cell	*ALDH16A1*	-3.907483114	0.003498938	0.006971457	1
T2DM	b_cell	*SIGLEC11*	-5.658964094	0.000537031	0.00415349	1
T2DM	b_cell	*MPP3*	-2.287855697	0.000636546	0.00415349	1
T2DM	b_cell	*ASB16*	3.84180257	0.003974329	0.007615547	1
T2DM	b_cell	*PLCD3*	-2.143806383	0.002537462	0.005775896	1
T2DM	b_cell	*LARP4*	-2.40860372	0.011042421	0.016361937	1
T2DM	b_cell	*SPC24*	-11.1343531	0.024896891	0.03266418	1
T2DM	b_cell	*IP6K3*	-14.48957876	0.000430164	0.00415349	1
T2DM	b_cell	*SCIMP*	2.452500333	0.010879048	0.016180879	1
T2DM	b_cell	*ASGR2*	-13.70802094	0.002983567	0.006295285	1
T2DM	b_cell	*TNFSF13*	6.527957867	0.000432997	0.00415349	1
T2DM	b_cell	*SENP3*	2.754040988	0.001395172	0.00434199	1
T2DM	b_cell	*SNRNP25*	-5.091749174	0.000870354	0.00415349	1
T2DM	b_cell	*WDR90*	-2.912619828	0.001312368	0.004274973	1
T2DM	b_cell	*JMJD8*	5.472444295	0.000309581	0.00415349	1
T2DM	b_cell	*MEIOB*	-4.914093327	0.00214029	0.005287258	1

T2DM	b_cell	*FLYWCH2*	-4.490837644	0.023018455	0.030627942	1
T2DM	b_cell	*ZG16B*	-39.67287119	0.000685917	0.00415349	1
T2DM	b_cell	*NXF1*	-2.480144382	0.002475412	0.005679896	1
T2DM	b_cell	*TPCN2*	-1.773141906	0.001142941	0.00418468	1
T2DM	b_cell	*CYP4A22*	13.85686537	0.000164089	0.00415349	1
T2DM	b_cell	*PDZK1IP1*	50.0294544	0.005261659	0.009250573	1
T2DM	b_cell	*COA7*	3.319526397	0.000711564	0.00415349	1
T2DM	b_cell	*C1orf123*	-3.903861328	0.000160237	0.00415349	1
T2DM	b_cell	*ACOT11*	-2.96955266	0.00028419	0.00415349	1
T2DM	b_cell	*USP24*	-2.262944625	0.005539703	0.009597457	1
T2DM	b_cell	*ZSWIM5*	-2.771276853	0.024042397	0.031746467	1
T2DM	b_cell	*RBP7*	-2.28418746	0.000952701	0.00415349	1
T2DM	b_cell	*FBLIM1*	-2.441557966	0.001425385	0.004380342	1
T2DM	b_cell	*SLC25A34*	6.510478124	0.03678094	0.046331751	1
T2DM	b_cell	*PDPN*	-2.512638168	0.000987781	0.00415349	1
T2DM	b_cell	*LRRC38*	-13.85302421	0.003186431	0.006555332	1
T2DM	b_cell	*LAPTM5*	3.661378134	0.016989302	0.023523279	1
T2DM	b_cell	*PEF1*	-2.406564506	0.001015127	0.00415349	1
T2DM	b_cell	*SYNC*	-2.546584315	0.002507278	0.005732727	1
T2DM	b_cell	*RBBP4*	-3.048800822	0.033869444	0.043008926	1
T2DM	b_cell	*TSSK3*	-2.710240312	0.011625167	0.017055842	1
T2DM	b_cell	*CAMK2N1*	-3.388937261	0.000886169	0.00415349	1
T2DM	b_cell	*TTLL10*	-6.865654613	0.001821167	0.00487843	1
T2DM	b_cell	*NFIA*	-1.81320088	0.004394389	0.008166575	1
T2DM	b_cell	*FUBP1*	-2.591940564	0.029694131	0.038216377	1
T2DM	b_cell	*NEXN*	-1.903188242	0.001031711	0.00415349	1
T2DM	b_cell	*B3GALT2*	-3.591853436	0.000641107	0.00415349	1
T2DM	b_cell	*NTNG1*	-1.700839003	0.008515063	0.013254321	1
T2DM	b_cell	*HFM1*	-1.89749287	0.000762199	0.00415349	1
T2DM	b_cell	*KCNT2*	-1.397063097	0.001003615	0.00415349	1
T2DM	b_cell	*VCAM1*	-2.378101645	0.000917022	0.00415349	1
T2DM	b_cell	*EXTL2*	-3.426499345	0.000135037	0.00415349	0.848980689
T2DM	b_cell	*ZNF496*	-4.172011702	0.011005533	0.016330373	1
T2DM	b_cell	*TRIM58*	1.565429874	0.000750414	0.00415349	1
T2DM	b_cell	*IGSF8*	-2.442678606	0.000777949	0.00415349	1
T2DM	b_cell	*VANGL2*	-5.449040586	0.004321138	0.008068011	1
T2DM	b_cell	*FCRLB*	-6.731204671	0.006109893	0.010281826	1
T2DM	b_cell	*KLHDC9*	2.637182803	0.007089299	0.011481305	1
T2DM	b_cell	*FLVCR1*	-2.246098376	0.000776495	0.00415349	1
T2DM	b_cell	*TDRD5*	-3.758746429	0.004335106	0.008085527	1
T2DM	b_cell	*SNED1*	-1.962151161	0.0022631	0.005437295	1
T2DM	b_cell	*C1orf115*	-2.238220439	0.009163635	0.014068809	1
T2DM	b_cell	*CNST*	-2.785169888	0.005084681	0.009030336	1
T2DM	b_cell	*PM20D1*	-4.019811256	0.000653793	0.00415349	1
T2DM	b_cell	*HAAO*	-3.495073974	0.039778051	0.049679103	1
T2DM	b_cell	*B3GALNT2*	-2.990450332	0.024980091	0.032752832	1
T2DM	b_cell	*PUS10*	-2.376357645	0.001512564	0.004480664	1
T2DM	b_cell	*RFTN2*	-2.136115489	0.001167257	0.004202203	1
T2DM	b_cell	*DISC1*	-2.892603288	0.002405944	0.005600211	1
T2DM	b_cell	*FAM84A*	-5.354689141	0.000735964	0.00415349	1
T2DM	b_cell	*NUP35*	-3.783130161	0.002437745	0.005632525	1
T2DM	b_cell	*FBXO41*	5.844560531	0.025493334	0.033384002	1
T2DM	b_cell	*CCDC74A*	-11.77841635	0.000732703	0.00415349	1
T2DM	b_cell	*NOSTRIN*	-1.73258055	0.000679255	0.00415349	1
T2DM	b_cell	*SGPP2*	7.111813532	0.008362011	0.01305163	1
T2DM	b_cell	*BBS5*	-2.893298526	0.001982236	0.005095706	1
T2DM	b_cell	*HPGDS*	-2.841876969	0.003652468	0.007196562	1
T2DM	b_cell	*PDLIM5*	-2.251269794	0.005712164	0.009793394	1
T2DM	b_cell	*RPRD2*	-2.693060544	0.010449475	0.015664246	1
T2DM	b_cell	*PACRGL*	-2.628995276	0.019724944	0.026686189	1
T2DM	b_cell	*C1QTNF7*	-1.316385576	0.000840174	0.00415349	1

T2DM	b_cell	*LYSMD1*	-3.135994665	0.000602588	0.00415349	1
T2DM	b_cell	*RNF149*	-2.576748517	0.007415932	0.011872667	1
T2DM	b_cell	*DHX57*	-2.494207306	0.001650417	0.004667644	1
T2DM	b_cell	*S100A9*	3.569102349	0.002122004	0.005266891	1
T2DM	b_cell	*S100A12*	-23.71319269	0.001530052	0.004503445	1
T2DM	b_cell	*TDRD10*	-2.57564385	0.001220227	0.004252746	1
T2DM	b_cell	*DCAF16*	-2.593353548	0.001181207	0.004202203	1
T2DM	b_cell	*NIPAL1*	-2.753908439	0.000783484	0.00415349	1
T2DM	b_cell	*ANTXR2*	-1.915861798	0.003484657	0.006957141	1
T2DM	b_cell	*CGGBP1*	-1.2451399	0.020071308	0.027108123	1
T2DM	b_cell	*DCST2*	-4.059286689	0.001014237	0.00415349	1
T2DM	b_cell	*LMOD3*	-3.194602312	0.0007689	0.00415349	1
T2DM	b_cell	*NBPF10*	2.191623925	0.001399452	0.004344868	1
T2DM	b_cell	*SLC22A15*	-1.781161277	0.001313826	0.004274973	1
T2DM	b_cell	*SLC15A2*	1.897214677	0.000754768	0.00415349	1
T2DM	b_cell	*LRRC58*	-3.234927995	0.002934401	0.006222119	1
T2DM	b_cell	*FSTL1*	-1.375422863	0.001206541	0.004235305	1
T2DM	b_cell	*LMOD1*	-1.906568657	7.45E-05	0.00415349	0.468584183
T2DM	b_cell	*TMEM183A*	-3.399905733	0.0012072	0.004235305	1
T2DM	b_cell	*IGFBP7*	-1.883961302	0.001277684	0.004259172	1
T2DM	b_cell	*ARPC2*	-2.770623491	0.011022912	0.016348443	1
T2DM	b_cell	*GLB1L*	3.460743273	0.005075396	0.009021491	1
T2DM	b_cell	*NUAK2*	1.007148654	0.000537553	0.00415349	1
T2DM	b_cell	*SPTA1*	-2.72375554	9.67E-06	0.001520543	0.060821708
T2DM	b_cell	*PYHIN1*	-2.11309112	0.00020172	0.00415349	1
T2DM	b_cell	*AIM2*	2.259900685	0.000719332	0.00415349	1
T2DM	b_cell	*EFHB*	-1.025024174	0.016826764	0.023337715	1
T2DM	b_cell	*ICA1L*	-2.920860187	0.000141636	0.00415349	0.890465267
T2DM	b_cell	*CD200R1*	-2.008518584	0.000563676	0.00415349	1
T2DM	b_cell	*GTPBP8*	-2.510758402	0.001144349	0.00418468	1
T2DM	b_cell	*SPICE1*	-2.664072524	0.004353743	0.008107986	1
T2DM	b_cell	*CADPS*	3.705738002	0.001756031	0.004789372	1
T2DM	b_cell	*GMPS*	-3.217200516	0.007987623	0.012595482	1
T2DM	b_cell	*DCLK3*	4.244141066	0.000323872	0.00415349	1
T2DM	b_cell	*DNASE1L3*	-2.950979415	0.002520982	0.005742541	1
T2DM	b_cell	*CRELD1*	-2.22506811	0.004173246	0.007876672	1
T2DM	b_cell	*PCOLCE2*	-1.199785169	0.000847194	0.00415349	1
T2DM	b_cell	*TTC14*	-2.305309768	0.008347185	0.013034961	1
T2DM	b_cell	*RCHY1*	-3.456846119	0.030822047	0.039518817	1
T2DM	b_cell	*CCDC158*	-2.556563937	0.002233582	0.005401538	1
T2DM	b_cell	*GYG1*	-3.346548651	0.003959972	0.007597923	1
T2DM	b_cell	*RYK*	-2.5000412	0.00490877	0.008837754	1
T2DM	b_cell	*PLB1*	-2.549462822	0.00077854	0.00415349	1
T2DM	b_cell	*CDCP1*	-2.601469265	0.000773297	0.00415349	1
T2DM	b_cell	*LZTFL1*	-2.088392653	0.000777402	0.00415349	1
T2DM	b_cell	*FYCO1*	-3.488070695	0.005177374	0.009138167	1
T2DM	b_cell	*LRRC2*	-1.77326771	0.001760086	0.004789372	1
T2DM	b_cell	*DTX3L*	-2.945853979	0.016028412	0.022383524	1
T2DM	b_cell	*NMNAT3*	-2.597184999	0.011352317	0.016738277	1
T2DM	b_cell	*SMIM12*	-3.021864568	0.002070217	0.005222896	1
T2DM	b_cell	*YEATS2*	-2.999820333	0.010368051	0.015571891	1
T2DM	b_cell	*MEAF6*	-3.237976499	0.002932016	0.006221257	1
T2DM	b_cell	*DNALI1*	-3.689811156	0.003004152	0.006308941	1
T2DM	b_cell	*RPN1*	-3.591876745	0.016857399	0.023364741	1
T2DM	b_cell	*RFC4*	2.006990758	0.000555692	0.00415349	1
T2DM	b_cell	*RFT1*	-4.921638407	2.12E-05	0.002553516	0.133380651
T2DM	b_cell	*GNL3*	-2.870121453	0.001657898	0.004678277	1
T2DM	b_cell	*FAM208A*	-2.797250515	0.022672093	0.030205435	1
T2DM	b_cell	*LRPAP1*	-3.656716989	0.011313075	0.016688246	1
T2DM	b_cell	*SLC51A*	-2.146178503	0.000819258	0.00415349	1
T2DM	b_cell	*PIGX*	-3.170227873	0.001227113	0.004253251	1

T2DM	b_cell	*ABLIM2*	-2.146998425	0.000824886	0.00415349	1
T2DM	b_cell	*EXO5*	2.755354151	0.030570739	0.039234043	1
T2DM	b_cell	*ERMAP*	-2.575746355	0.001222164	0.004252746	1
T2DM	b_cell	*METAP1*	-3.71957509	0.005599561	0.009655634	1
T2DM	b_cell	*SLC9B2*	-2.707869426	0.000678038	0.00415349	1
T2DM	b_cell	*BDH2*	-2.139842978	2.13E-05	0.002553516	0.134178794
T2DM	b_cell	*ZNF589*	-3.520468317	0.001234435	0.004253251	1
T2DM	b_cell	*ATRIP*	-7.433397301	0.00040168	0.00415349	1
T2DM	b_cell	*SHISA5*	-3.013563685	0.001453341	0.004416445	1
T2DM	b_cell	*RNF123*	2.866237907	0.026702554	0.03477915	1
T2DM	b_cell	*RAD54L2*	-2.912685577	0.005828553	0.009935761	1
T2DM	b_cell	*GRM2*	4.121973072	0.000576181	0.00415349	1
T2DM	b_cell	*POC1A*	4.83035528	0.001021429	0.00415349	1
T2DM	b_cell	*PPM1M*	-3.389440043	0.000965907	0.00415349	1
T2DM	b_cell	*C4orf3*	-3.346885604	0.002773716	0.006030781	1
T2DM	b_cell	*ANXA5*	-2.192132412	0.003479743	0.006953955	1
T2DM	b_cell	*MAP9*	-2.480783977	0.000766969	0.00415349	1
T2DM	b_cell	*CEP44*	-2.837120021	0.002380045	0.005562581	1
T2DM	b_cell	*C4orf45*	-1.995600795	0.000499439	0.00415349	1
T2DM	b_cell	*TMEM144*	-1.799267204	0.000901271	0.00415349	1
T2DM	b_cell	*NPY1R*	-1.350808901	0.00076854	0.00415349	1
T2DM	b_cell	*IL15*	-2.446743384	0.001344837	0.004286796	1
T2DM	b_cell	*ARFIP1*	-2.536413612	0.007780077	0.012350455	1
T2DM	b_cell	*OTUD4*	-3.189547513	0.010347527	0.015549526	1
T2DM	b_cell	*ITGA2*	-2.540607821	0.000893056	0.00415349	1
T2DM	b_cell	*NDUFAF2*	-2.392651921	0.000786062	0.00415349	1
T2DM	b_cell	*LMBRD2*	-3.252194422	0.003122704	0.006466219	1
T2DM	b_cell	*PGGT1B*	-3.169955677	0.035086008	0.044356673	1
T2DM	b_cell	*CMBL*	-2.345820088	0.000783093	0.00415349	1
T2DM	b_cell	*C5orf63*	-2.347816582	0.000653531	0.00415349	1
T2DM	b_cell	*PRRC1*	-2.443111921	0.002511424	0.005734251	1
T2DM	b_cell	*F2RL1*	-5.779156681	0.03414589	0.043298752	1
T2DM	b_cell	*WDR41*	-2.272679068	0.000512185	0.00415349	1
T2DM	b_cell	*NDUFS4*	-2.579677453	0.003635276	0.007180327	1
T2DM	b_cell	*RHOBTB3*	-1.054529397	0.002244464	0.005416869	1
T2DM	b_cell	*TIGD6*	-3.795057536	0.000823477	0.00415349	1
T2DM	b_cell	*SERINC5*	-2.424228717	0.004158923	0.007865091	1
T2DM	b_cell	*ENPP6*	-7.705723322	0.002006785	0.005130808	1
T2DM	b_cell	*PRIMPOL*	-3.067774984	0.001048055	0.00415349	1
T2DM	b_cell	*ERAP1*	-1.846325352	0.002290315	0.005472904	1
T2DM	b_cell	*RICTOR*	-2.510606059	0.012122359	0.01765422	1
T2DM	b_cell	*ANKRA2*	-2.448032537	0.001231509	0.004253251	1
T2DM	b_cell	*UBLCP1*	-2.576056874	0.008249352	0.012911047	1
T2DM	b_cell	*UTP15*	-3.271224769	0.000799577	0.00415349	1
T2DM	b_cell	*TLR3*	-2.431320791	0.000912937	0.00415349	1
T2DM	b_cell	*CCDC127*	-3.26174108	0.00112476	0.004182741	1
T2DM	b_cell	*GRIK2*	-1.953816674	0.000419027	0.00415349	1
T2DM	b_cell	*MB21D1*	3.778320011	0.011267455	0.016632658	1
T2DM	b_cell	*TXLNB*	-6.095416346	0.004407258	0.008188071	1
T2DM	b_cell	*DCBLD1*	-1.838173009	0.001707286	0.004728644	1
T2DM	b_cell	*TMEM200A*	-2.999512917	0.001259763	0.004257394	1
T2DM	b_cell	*PDSS2*	-2.480820298	0.026045045	0.034020664	1
T2DM	b_cell	*STXBP5*	-2.466501249	0.020611253	0.027771742	1
T2DM	b_cell	*PI16*	-1.400657356	0.000866259	0.00415349	1
T2DM	b_cell	*STK17A*	-3.047714246	0.003121954	0.006466219	1
T2DM	b_cell	*GALNT10*	-3.017711851	0.023081083	0.030691788	1
T2DM	b_cell	*SAP30L*	-3.791789199	0.002779951	0.006035067	1
T2DM	b_cell	*HCN1*	-3.207414741	0.001833909	0.004898539	1
T2DM	b_cell	*RP9*	-4.504492301	0.003354028	0.006777924	1
T2DM	b_cell	*KCNK5*	-4.214872308	0.000786096	0.00415349	1
T2DM	b_cell	*KIAA1324L*	-1.844101855	0.000449069	0.00415349	1

T2DM	b_cell	*USP49*	-2.548095777	0.002048621	0.00519116	1
T2DM	b_cell	*ZNF704*	-1.542340084	0.001323809	0.004274973	1
T2DM	b_cell	*FNDC1*	-1.842462357	0.001607452	0.004610639	1
T2DM	b_cell	*BRI3*	-2.713560286	0.019796821	0.02677767	1
T2DM	b_cell	*LMTK2*	-2.79585164	0.005049711	0.009000686	1
T2DM	b_cell	*CTSB*	-2.051827706	0.002889921	0.006184116	1
T2DM	b_cell	*PEX2*	-2.652755053	0.001237387	0.004253251	1
T2DM	b_cell	*TNFRSF11B*	-2.75024945	0.001031673	0.00415349	1
T2DM	b_cell	*DEFB1*	-4.217548622	0.000543858	0.00415349	1
T2DM	b_cell	*SUN1*	-1.905892029	0.008797654	0.013615691	1
T2DM	b_cell	*OXR1*	-2.178993531	0.010601265	0.015842679	1
T2DM	b_cell	*TMEM184A*	1.698552115	0.001121097	0.004182741	1
T2DM	b_cell	*SLC4A2*	-4.574966016	0.007308207	0.011730073	1
T2DM	b_cell	*FASTK*	-2.257749839	0.000841612	0.00415349	1
T2DM	b_cell	*ALDH7A1*	-2.296368578	0.008000943	0.012610159	1
T2DM	b_cell	*COX6C*	-2.82686743	0.00208212	0.005230315	1
T2DM	b_cell	*YWHAZ*	-2.245854936	0.02175749	0.02909866	1
T2DM	b_cell	*SLC25A32*	-2.968790525	0.001215053	0.004245409	1
T2DM	b_cell	*DCAF13*	-3.892963743	0.008633724	0.013405834	1
T2DM	b_cell	*INTS8*	-2.295534143	0.006535255	0.010781199	1
T2DM	b_cell	*FREM1*	-1.272757416	0.000591027	0.00415349	1
T2DM	b_cell	*RPP25L*	5.569348884	0.035607032	0.044961118	1
T2DM	b_cell	*FAM219A*	-3.144339013	0.014625529	0.020706828	1
T2DM	b_cell	*TMEM65*	-3.044931398	0.015135458	0.021316448	1
T2DM	b_cell	*CCDC171*	-2.529262579	0.00113101	0.004182741	1
T2DM	b_cell	*NIPSNAP3B*	-1.831774639	0.000424439	0.00415349	1
T2DM	b_cell	*TMC1*	-2.629606823	0.00130479	0.004274973	1
T2DM	b_cell	*HGSNAT*	-2.600803	0.028590828	0.036978098	1
T2DM	b_cell	*C9orf64*	-2.227099126	0.002693561	0.005942501	1
T2DM	b_cell	*SVEP1*	-1.125578516	0.000874529	0.00415349	1
T2DM	b_cell	*ZNF367*	4.431814718	0.000497619	0.00415349	1
T2DM	b_cell	*NOL6*	-3.686759344	0.001572548	0.004562116	1
T2DM	b_cell	*AQP3*	-4.867149376	0.004108346	0.007801017	1
T2DM	b_cell	*TRMT10B*	-2.940868735	0.005048917	0.009000686	1
T2DM	b_cell	*VCP*	-2.777145047	0.010725263	0.015993768	1
T2DM	b_cell	*PIGO*	-4.246827092	0.000884044	0.00415349	1
T2DM	b_cell	*LRFN5*	-1.453359344	0.004081215	0.007761223	1
T2DM	b_cell	*WRN*	-2.405709336	0.003815588	0.007388059	1
T2DM	b_cell	*CFL2*	3.935455943	0.018741294	0.025536739	1
T2DM	b_cell	*ZCCHC24*	-2.010147724	0.001255966	0.004257394	1
T2DM	b_cell	*PGM2L1*	3.865984847	0.001323971	0.004274973	1
T2DM	b_cell	*GJB2*	5.572377096	0.006565229	0.010819291	1
T2DM	b_cell	*CRYL1*	-3.192847759	0.004593753	0.008454603	1
T2DM	b_cell	*REEP3*	-2.601216446	0.000408795	0.00415349	1
T2DM	b_cell	*LRR1*	-2.745401115	0.001222318	0.004252746	1
T2DM	b_cell	*RPL36AL*	-2.776170023	0.028924735	0.037325494	1
T2DM	b_cell	*KLHDC2*	-2.438334042	0.00421434	0.007929499	1
T2DM	b_cell	*AKR1E2*	-2.310217063	0.000192899	0.00415349	1
T2DM	b_cell	*ATP5C1*	-3.332728149	0.007659466	0.012197332	1
T2DM	b_cell	*TAF3*	-2.490771772	0.014840372	0.02096661	1
T2DM	b_cell	*VDAC2*	-2.23516037	0.00142502	0.004380342	1
T2DM	b_cell	*SLC18A2*	-2.591810591	0.001861942	0.004937169	1
T2DM	b_cell	*PDZD8*	-2.036308838	0.017737795	0.024412767	1
T2DM	b_cell	*QSOX2*	2.204167545	0.000837813	0.00415349	1
T2DM	b_cell	*CLEC1B*	1.345831876	0.016000533	0.022359491	1
T2DM	b_cell	*SNAPC4*	-2.626873309	0.015312201	0.021517168	1
T2DM	b_cell	*SDCCAG3*	-3.508633574	0.003873656	0.007472745	1
T2DM	b_cell	*AK8*	-3.999094696	0.001136398	0.00418468	1
T2DM	b_cell	*TSC1*	-2.936996873	0.011899085	0.017381401	1
T2DM	b_cell	*ZMYND19*	-6.522962419	0.001864638	0.004938071	1
T2DM	b_cell	*STOX1*	-1.392801944	0.002050552	0.005192035	1

T2DM	b_cell	*RET*	1.979515268	0.000768399	0.00415349	1
T2DM	b_cell	*STK32C*	-2.632518892	0.00091926	0.00415349	1
T2DM	b_cell	*NSMF*	-2.77107269	0.000836352	0.00415349	1
T2DM	b_cell	*METTL3*	-3.120446443	0.002784247	0.006042306	1
T2DM	b_cell	*TRUB1*	-3.430381187	0.00234045	0.005517214	1
T2DM	b_cell	*HSPA12A*	-1.286653014	0.003152001	0.006503654	1
T2DM	b_cell	*UBTD1*	-2.700221997	0.001434754	0.004395857	1
T2DM	b_cell	*ARHGAP42*	-1.646316269	0.001001437	0.00415349	1
T2DM	b_cell	*OTOGL*	-8.497172763	0.0155089	0.021774108	1
T2DM	b_cell	*TTC7B*	-2.208529533	0.001078247	0.00415349	1
T2DM	b_cell	*SLC39A13*	-3.427430024	0.00156479	0.004548235	1
T2DM	b_cell	*PSMC3*	-2.505263935	0.002825023	0.006097224	1
T2DM	b_cell	*TC2N*	-1.465891003	0.000765772	0.00415349	1
T2DM	b_cell	*CLMN*	-1.50701031	0.000836408	0.00415349	1
T2DM	b_cell	*PTER*	-2.68246924	0.001315702	0.004274973	1
T2DM	b_cell	*ABTB2*	-2.454845941	0.001087602	0.00415349	1
T2DM	b_cell	*LIPC*	-2.404468431	0.001544764	0.004515077	1
T2DM	b_cell	*CEP57*	-3.523061214	0.00118213	0.004202203	1
T2DM	b_cell	*TCP11L2*	-2.988055253	0.003787627	0.007350767	1
T2DM	b_cell	*JAM3*	-2.777092669	0.004815829	0.008733839	1
T2DM	b_cell	*GPT2*	-1.703801376	0.001118523	0.004182741	1
T2DM	b_cell	*IKBIP*	-3.294910048	0.006820265	0.01114609	1
T2DM	b_cell	*HIF1AN*	-2.790707523	0.001203847	0.004229472	1
T2DM	b_cell	*NDUFB8*	-4.198128827	0.001886516	0.004966719	1
T2DM	b_cell	*AVPR1A*	-2.386347968	0.009064151	0.013933085	1
T2DM	b_cell	*TRMT61A*	-8.550116694	3.55E-07	0.000124872	0.002231469
T2DM	b_cell	*BTRC*	-2.156920319	0.002356569	0.005540753	1
T2DM	b_cell	*LARP6*	-1.996875869	0.001213924	0.004245409	1
T2DM	b_cell	*ASPG*	-9.034077269	0.001536611	0.004508014	1
T2DM	b_cell	*GABRB3*	4.326163425	0.004097785	0.007790375	1
T2DM	b_cell	*TBATA*	-13.56933477	0.038986494	0.048758323	1
T2DM	b_cell	*PCBD1*	-3.788448162	6.05E-07	0.000190271	0.003805422
T2DM	b_cell	*COX11*	-2.09902807	0.001096807	0.00415349	1
T2DM	b_cell	*ZNF202*	-4.288029109	0.021928606	0.029295611	1
T2DM	b_cell	*FAM227B*	-1.774502554	0.002943343	0.006236871	1
T2DM	b_cell	*ILK*	-2.325682492	0.002301225	0.005481934	1
T2DM	b_cell	*TAF10*	-2.847645603	0.000749219	0.00415349	1
T2DM	b_cell	*DCHS1*	-2.612448438	0.000965165	0.00415349	1
T2DM	b_cell	*NETO1*	0.641539089	0.000512436	0.00415349	1
T2DM	b_cell	*USP54*	-2.165997368	0.002766871	0.006028396	1
T2DM	b_cell	*C11orf74*	-1.977205577	0.000558391	0.00415349	1
T2DM	b_cell	*CYB5R2*	2.56911625	0.000958303	0.00415349	1
T2DM	b_cell	*TUB*	-2.752316769	0.000232335	0.00415349	1
T2DM	b_cell	*RIC3*	-3.352876182	0.001296872	0.004268027	1
T2DM	b_cell	*LMO1*	-5.656328835	0.001693844	0.004705788	1
T2DM	b_cell	*XRRA1*	-2.281747068	0.000540991	0.00415349	1
T2DM	b_cell	*PRTG*	-3.323481797	0.001441289	0.004405146	1
T2DM	b_cell	*AKIP1*	-4.847108576	0.00660203	0.010862853	1
T2DM	b_cell	*C16orf46*	-35.38745058	0.000463193	0.00415349	1
T2DM	b_cell	*TMEM41B*	-2.828289722	0.002963502	0.006266914	1
T2DM	b_cell	*TMX3*	-2.930247931	0.004229105	0.007948694	1
T2DM	b_cell	*CCDC68*	-2.569607989	0.002088819	0.005231352	1
T2DM	b_cell	*ZNF3*	-3.048817594	0.002056553	0.005198853	1
T2DM	b_cell	*CLEC4D*	-7.960683487	0.011037813	0.016358965	1
T2DM	b_cell	*ZSCAN21*	-1.803699899	0.024498653	0.032242628	1
T2DM	b_cell	*RIMKLB*	-2.140170682	0.001022636	0.00415349	1
T2DM	b_cell	*BEAN1*	-2.767302277	0.001687926	0.004704627	1
T2DM	b_cell	*TK2*	-2.2856759	0.000216071	0.00415349	1
T2DM	b_cell	*SEC11C*	3.383482165	0.001435945	0.004397363	1
T2DM	b_cell	*GALR1*	-3.843978373	0.000600384	0.00415349	1
T2DM	b_cell	*CENPV*	-2.285077786	0.000731652	0.00415349	1

T2DM	b_cell	*SPDYE6*	-4.616367671	0.005846771	0.009953602	1
T2DM	b_cell	*ATF7IP2*	-2.865688839	0.005266788	0.009257002	1
T2DM	b_cell	*ZNF592*	-2.418921866	0.001118424	0.004182741	1
T2DM	b_cell	*ACSM1*	1.378690801	0.000494287	0.00415349	1
T2DM	b_cell	*AP1G1*	-2.780561479	0.004297016	0.008039295	1
T2DM	b_cell	*SLFN5*	-2.115691352	0.001055094	0.00415349	1
T2DM	b_cell	*CATSPER2*	-2.540643836	0.002029404	0.005163442	1
T2DM	b_cell	*C16orf45*	-2.687632747	0.00473145	0.008629715	1
T2DM	b_cell	*SAAL1*	-5.924701085	0.000642557	0.00415349	1
T2DM	b_cell	*YPEL4*	-6.995007687	0.006383132	0.010596976	1
T2DM	b_cell	*FAM111A*	-2.698746042	0.00735995	0.011804082	1
T2DM	b_cell	*SCNN1G*	-14.66349402	0.00178714	0.004835411	1
T2DM	b_cell	*ANKDD1A*	-2.913043676	0.002081777	0.005230315	1
T2DM	b_cell	*DCTN5*	-2.147333656	0.009060135	0.013931268	1
T2DM	b_cell	*STX3*	-2.514440675	0.000973322	0.00415349	1
T2DM	b_cell	*NYAP1*	-3.972106022	0.003085724	0.006415083	1
T2DM	b_cell	*TSC22D4*	-2.045595228	0.001040041	0.00415349	1
T2DM	b_cell	*MS4A6E*	-2.938392456	0.001333679	0.004280164	1
T2DM	b_cell	*MS4A7*	-1.705713436	0.000306033	0.00415349	1
T2DM	b_cell	*MS4A14*	-2.429595711	0.00033497	0.00415349	1
T2DM	b_cell	*DIS3L*	-1.810511495	0.00103111	0.00415349	1
T2DM	b_cell	*CCNDBP1*	-2.818539488	0.00160854	0.004610639	1
T2DM	b_cell	*SMAD3*	-2.015814308	0.0026281	0.005859268	1
T2DM	b_cell	*RCCD1*	-2.648750603	0.001021223	0.00415349	1
T2DM	b_cell	*AKTIP*	-3.396049106	0.003544883	0.007044747	1
T2DM	b_cell	*EVA1C*	-1.488273268	0.005855005	0.009962224	1
T2DM	b_cell	*CNPY4*	-1.901063628	0.036823804	0.046367165	1
T2DM	b_cell	*NKX3-1*	-33.34760944	0.002878545	0.006170274	1
T2DM	b_cell	*DUSP18*	-4.022616064	0.003216926	0.006598061	1
T2DM	b_cell	*TEF*	-1.338191808	0.012651449	0.018318668	1
T2DM	b_cell	*MEI1*	3.170972575	0.001249068	0.004253251	1
T2DM	b_cell	*PBX3*	-2.465518621	0.008820939	0.013645975	1
T2DM	b_cell	*SNRPD1*	-2.387878533	0.000966304	0.00415349	1
T2DM	b_cell	*URM1*	-3.936852231	0.001550822	0.004518081	1
T2DM	b_cell	*CERCAM*	-1.837445966	0.004162739	0.007865091	1
T2DM	b_cell	*ENDOG*	2.12418002	0.006603811	0.010862941	1
T2DM	b_cell	*ISLR2*	-3.680409448	0.002078338	0.005230315	1
T2DM	b_cell	*SP2*	-3.775719899	0.001043838	0.00415349	1
T2DM	b_cell	*GPRC5B*	-2.795059356	0.001200221	0.004223203	1
T2DM	b_cell	*KATNAL2*	-1.354151022	0.003517476	0.006998218	1
T2DM	b_cell	*RNF214*	-2.421693962	0.001574878	0.004562749	1
T2DM	b_cell	*ENGASE*	-2.047105791	0.002821049	0.00609273	1
T2DM	b_cell	*RBFOX3*	-3.834984253	0.001127387	0.004182741	1
T2DM	b_cell	*MYO5B*	-1.524331543	0.011774766	0.017219808	1
T2DM	b_cell	*RRM1*	-3.172374527	0.002410381	0.005604315	1
T2DM	b_cell	*TRIM68*	-4.163539403	0.00055	0.00415349	1
T2DM	b_cell	*FN3K*	-2.168015407	0.001067435	0.00415349	1
T2DM	b_cell	*ZNF229*	-6.945447857	0.034097168	0.043245692	1
T2DM	b_cell	*ZNF646*	-4.008907535	0.025630227	0.033542306	1
T2DM	b_cell	*VKORC1*	-2.789114454	4.34E-05	0.003541347	0.27268372
T2DM	b_cell	*SMG8*	-7.287317017	0.000873216	0.00415349	1
T2DM	b_cell	*RAB8A*	-4.267612415	0.011485341	0.016917561	1
T2DM	b_cell	*PROCA1*	-4.161139905	0.000732582	0.00415349	1
T2DM	b_cell	*CACNB3*	1.749311892	0.000942134	0.00415349	1
T2DM	b_cell	*TP53I13*	-1.97422078	0.003428716	0.006885994	1
T2DM	b_cell	*ZNF528*	-2.34022893	0.001027653	0.00415349	1
T2DM	b_cell	*ZNF701*	-3.942312301	0.00670394	0.011004614	1
T2DM	b_cell	*SERTAD3*	-5.849055589	0.002711665	0.005955133	1
T2DM	b_cell	*TMC4*	-3.67925255	0.000942856	0.00415349	1
T2DM	b_cell	*TTYH1*	-2.740147547	0.001473454	0.004442975	1
T2DM	b_cell	*LENG8*	-2.320993244	0.002805346	0.006076587	1

T2DM	b_cell	*CDC42EP5*	-2.924332291	0.000593952	0.00415349	1
T2DM	b_cell	*ZNF283*	3.501323872	0.014492226	0.020571828	1
T2DM	b_cell	*PPP1R14A*	-5.359387124	0.011926991	0.017410028	1
T2DM	b_cell	*SPINT2*	-2.351373407	0.000909341	0.00415349	1
T2DM	b_cell	*UBXN6*	-3.144246838	0.000459418	0.00415349	1
T2DM	b_cell	*ZNF444*	-2.625055473	0.000899207	0.00415349	1
T2DM	b_cell	*GLOD4*	-3.718901827	0.011584862	0.017013322	1
T2DM	b_cell	*KIFC2*	-2.789110931	9.20E-05	0.00415349	0.57822136
T2DM	b_cell	*SERPINF2*	5.100325797	0.008951194	0.0138101	1
T2DM	b_cell	*CYB5D2*	-2.894339082	0.005082354	0.009028754	1
T2DM	b_cell	*ZNF83*	-2.198919629	0.001643935	0.004657671	1
T2DM	b_cell	*CD320*	-4.521274675	0.005910152	0.010034331	1
T2DM	b_cell	*SPRYD3*	-2.780672648	0.000904163	0.00415349	1
T2DM	b_cell	*SOAT2*	-3.306504673	0.010723205	0.015993768	1
T2DM	b_cell	*ZNF558*	-2.487943984	0.014919139	0.021068425	1
T2DM	b_cell	*NDUFV1*	-2.793005036	0.003285421	0.006688938	1
T2DM	b_cell	*CDK2AP2*	-3.812246461	0.001696409	0.004710831	1
T2DM	b_cell	*NUDT8*	-3.322741153	0.007118573	0.011508915	1
T2DM	b_cell	*MIS12*	-3.155435258	0.001328859	0.004274973	1
T2DM	b_cell	*HID1*	-3.310637187	0.002429357	0.005628918	1
T2DM	b_cell	*ATP5H*	-4.148624276	0.006754972	0.011068154	1
T2DM	b_cell	*MGAT5B*	-7.944749639	0.013044908	0.018819031	1
T2DM	b_cell	*TMC8*	-2.058301992	0.004783746	0.008694829	1
T2DM	b_cell	*TMEM68*	-3.8829746	0.005161028	0.009122121	1
T2DM	b_cell	*GSDMA*	-5.545198703	0.001324909	0.004274973	1
T2DM	b_cell	*KRT24*	-7.355358998	0.000358361	0.00415349	1
T2DM	b_cell	*ZNF598*	-2.995764444	0.000630196	0.00415349	1
[truncated: 13,933,499 more chars]
