# Supplementary material for: CausalCellInfer: Prioritizing Cell‐Type‐Specific Disease Associations From Genetically Predicted Transcriptomes in Biobank Cohorts
Source: Adv Sci (Weinh). 2026 Oct 14:e78197. Online ahead of print. doi: 10.1002/advs.78197 (PMC13669230; doi:10.1002/advs.78197)
Supplement: Supplementary file 2 [file ADVS-9999-e78197-s002.zip › advs78197-sup-00013-S12.pdf]

Table S12 Comparison of genes identified from causal inference and CTS-TWAS analysis

a. Comparison of genes identified from causal inference and CTS-TWAS analysis with adjustment for multiple testing correction(FDR<=0.05)

Tissue	Disorder	Cell type	No. of causal genes	No. of TWAS genes	No. of novel genes from causal inference	No. of novel genes from TWAS	No. of common genes
Frontal Cortex	Anxiety	Astros	12	0	12	0	0
Frontal Cortex	Anxiety	Ex	60	0	60	0	0
Frontal Cortex	Anxiety	Inhib	53	0	53	0	0
Frontal Cortex	Anxiety	Micro_Macro	4	0	4	0	0
Frontal Cortex	Anxiety	Oligos	19	0	19	0	0
Frontal Cortex	Anxiety	OPCs	13	0	13	0	0
Frontal Cortex	Bipolar	Astros	7	0	7	0	0
Frontal Cortex	Bipolar	Ex	63	185	44	166	19
Frontal Cortex	Bipolar	Inhib	42	1	41	0	1
Frontal Cortex	Bipolar	Micro_Macro	5	0	5	0	0
Frontal Cortex	Bipolar	Oligos	13	0	13	0	0
Frontal Cortex	Bipolar	OPCs	10	0	10	0	0
Frontal Cortex	Depression	Astros	13	2	12	1	1
Frontal Cortex	Depression	Ex	78	2	77	1	1
Frontal Cortex	Depression	Inhib	72	2	71	1	1
Frontal Cortex	Depression	Micro_Macro	9	2	7	0	2
Frontal Cortex	Depression	Oligos	21	1	20	0	1
Frontal Cortex	Depression	OPCs	16	1	15	0	1
Frontal Cortex	Psychosis	Astros	6	0	6	0	0
Frontal Cortex	Psychosis	Ex	53	0	53	0	0
Frontal Cortex	Psychosis	Inhib	63	0	63	0	0
Frontal Cortex	Psychosis	Micro_Macro	6	0	6	0	0
Frontal Cortex	Psychosis	Oligos	21	0	21	0	0
Frontal Cortex	Psychosis	OPCs	9	0	9	0	0
Pancreas	Prediabetes	acinar_cell	16	0	16	0	0
Pancreas	Prediabetes	alpha_cell	27	0	27	0	0
Pancreas	Prediabetes	beta_cell	63	0	63	0	0
Pancreas	Prediabetes	delta_cell	10	0	10	0	0
Pancreas	Prediabetes	duct_epithelial_cell	12	1275	2	1265	10
Pancreas	Prediabetes	endothelial_cell	13	0	13	0	0
Pancreas	Prediabetes	epsilon_cell	4	0	4	0	0
Pancreas	Prediabetes	leukocyte	5	0	5	0	0
Pancreas	Prediabetes	mesenchymal_cell	10	0	10	0	0
Pancreas	Prediabetes	PP_cell	8	1636	0	1628	8
Pancreas	T1DM	acinar_cell	24	34	18	28	6
Pancreas	T1DM	alpha_cell	50	35	45	30	5
Pancreas	T1DM	beta_cell	114	36	107	29	7
Pancreas	T1DM	delta_cell	12	29	8	25	4
Pancreas	T1DM	duct_epithelial_cell	15	32	12	29	3
Pancreas	T1DM	endothelial_cell	10	70	7	67	3
Pancreas	T1DM	epsilon_cell	6	16	3	13	3
Pancreas	T1DM	leukocyte	5	3632	2	3629	3
Pancreas	T1DM	mesenchymal_cell	11	37	7	33	4
Pancreas	T1DM	PP_cell	7	29	3	25	4
Pancreas	T2DM	acinar_cell	44	54	17	27	27
Pancreas	T2DM	alpha_cell	81	72	39	30	42
Pancreas	T2DM	beta_cell	163	91	102	30	61
Pancreas	T2DM	delta_cell	19	33	9	23	10
Pancreas	T2DM	duct_epithelial_cell	31	48	14	31	17
Pancreas	T2DM	endothelial_cell	27	167	6	146	21
Pancreas	T2DM	epsilon_cell	6	5	4	3	2
Pancreas	T2DM	leukocyte	12	3171	0	3159	12
Pancreas	T2DM	mesenchymal_cell	23	88	8	73	15
Pancreas	T2DM	PP_cell	17	26	6	15	11
Pancreas	T2DM_admission	acinar_cell	51	69	15	33	36
Pancreas	T2DM_admission	alpha_cell	82	86	34	38	48
Pancreas	T2DM_admission	beta_cell	185	116	105	36	80
Pancreas	T2DM_admission	delta_cell	22	44	8	30	14
Pancreas	T2DM_admission	duct_epithelial_cell	34	120	8	94	26
Pancreas	T2DM_admission	endothelial_cell	29	113	9	93	20
Pancreas	T2DM_admission	epsilon_cell	7	10	3	6	4
Pancreas	T2DM_admission	leukocyte	11	3313	0	3302	11
Pancreas	T2DM_admission	mesenchymal_cell	24	112	6	94	18
Pancreas	T2DM_admission	PP_cell	18	62	5	49	13
Pancreas	T2DM_insulin	acinar_cell	37	11	33	7	4
Pancreas	T2DM_insulin	alpha_cell	57	12	52	7	5
Pancreas	T2DM_insulin	beta_cell	102	15	95	8	7
Pancreas	T2DM_insulin	delta_cell	13	11	10	8	3
Pancreas	T2DM_insulin	duct_epithelial_cell	23	11	20	8	3
Pancreas	T2DM_insulin	endothelial_cell	22	15	17	10	5
Pancreas	T2DM_insulin	epsilon_cell	9	6	8	5	1
Pancreas	T2DM_insulin	leukocyte	7	3136	0	3129	7
Pancreas	T2DM_insulin	mesenchymal_cell	17	13	13	9	4
Pancreas	T2DM_insulin	PP_cell	12	11	9	8	3
SAT	Obesity	adipocyte	123	2398	5	2280	118
SAT	Obesity	ASPC	331	422	117	208	214
SAT	Obesity	b_cell	14	645	0	631	14
SAT	Obesity	dendritic_cell	22	113	2	93	20
SAT	Obesity	endothelial	25	4730	0	4705	25
SAT	Obesity	LEC	16	5102	0	5086	16
SAT	Obesity	macrophage	88	2189	4	2105	84
SAT	Obesity	mast_cell	28	5036	0	5008	28
SAT	Obesity	monocyte	16	251	5	240	11
SAT	Obesity	neutrophil	11	5195	0	5184	11
SAT	Obesity	nk_cell	26	2495	0	2469	26
SAT	Obesity	pericyte	41	4814	2	4775	39
SAT	Obesity	SMC	51	252	6	207	45
SAT	Obesity	t_cell	33	3948	0	3915	33
SAT	CAD	adipocyte	97	42	70	15	27
SAT	CAD	ASPC	193	51	155	13	38
SAT	CAD	b_cell	8	0	8	0	0
SAT	CAD	dendritic_cell	11	10	9	8	2
SAT	CAD	endothelial	14	1	13	0	1
SAT	CAD	LEC	13	0	13	0	0
SAT	CAD	macrophage	72	37	49	14	23
SAT	CAD	mast_cell	16	6	12	2	4
SAT	CAD	monocyte	11	4	8	1	3
SAT	CAD	neutrophil	7	1	6	0	1
SAT	CAD	nk_cell	16	4	14	2	2
SAT	CAD	pericyte	29	625	7	603	22
SAT	CAD	SMC	35	18	25	8	10
SAT	CAD	t_cell	18	13	13	8	5
SAT	T2DM	adipocyte	104	1367	11	1274	93

SAT	T2DM	ASPC	226	194	115	83	111
SAT	T2DM	b_cell	10	5037	1	5028	9
SAT	T2DM	dendritic_cell	16	4976	0	4960	16
SAT	T2DM	endothelial	21	3956	1	3936	20
SAT	T2DM	LEC	15	4698	2	4685	13
SAT	T2DM	macrophage	68	1686	7	1625	61
SAT	T2DM	mast_cell	28	4943	1	4916	27
SAT	T2DM	monocyte	13	4953	1	4941	12
SAT	T2DM	neutrophil	8	11	4	7	4
SAT	T2DM	nk_cell	20	50	4	34	16
SAT	T2DM	pericyte	29	3829	2	3802	27
SAT	T2DM	SMC	44	83	20	59	24
SAT	T2DM	t_cell	27	394	6	373	21
SAT	HTN	adipocyte	134	381	20	267	114
SAT	HTN	ASPC	329	446	92	209	237
SAT	HTN	b_cell	10	5114	0	5104	10
SAT	HTN	dendritic_cell	27	115	0	88	27
SAT	HTN	endothelial	26	242	8	224	18
SAT	HTN	LEC	9	168	0	159	9
SAT	HTN	macrophage	87	271	28	212	59
SAT	HTN	mast_cell	26	148	8	130	18
SAT	HTN	monocyte	16	49	4	37	12
SAT	HTN	neutrophil	8	14	1	7	7
SAT	HTN	nk_cell	43	3014	0	2971	43
SAT	HTN	pericyte	60	299	6	245	54
SAT	HTN	SMC	75	330	4	259	71
SAT	HTN	t_cell	38	2001	6	1969	32
SAT	Stroke	adipocyte	44	0	44	0	0
SAT	Stroke	ASPC	127	2	125	0	2
SAT	Stroke	b_cell	4	0	4	0	0
SAT	Stroke	dendritic_cell	7	0	7	0	0
SAT	Stroke	endothelial	10	0	10	0	0
SAT	Stroke	LEC	8	0	8	0	0
SAT	Stroke	macrophage	31	0	31	0	0
SAT	Stroke	mast_cell	6	0	6	0	0
SAT	Stroke	monocyte	6	0	6	0	0
SAT	Stroke	neutrophil	3	0	3	0	0
SAT	Stroke	nk_cell	10	0	10	0	0
SAT	Stroke	pericyte	10	0	10	0	0
SAT	Stroke	SMC	15	4	14	3	1
SAT	Stroke	t_cell	15	0	15	0	0
SAT	Heart_Failure	adipocyte	76	36	59	19	17
SAT	Heart_Failure	ASPC	154	36	131	13	23
SAT	Heart_Failure	b_cell	6	3834	1	3829	5
SAT	Heart_Failure	dendritic_cell	24	5680	0	5656	24
SAT	Heart_Failure	endothelial	18	14	13	9	5
SAT	Heart_Failure	LEC	12	9	11	8	1
SAT	Heart_Failure	macrophage	56	22	47	13	9
SAT	Heart_Failure	mast_cell	17	25	12	20	5
SAT	Heart_Failure	monocyte	9	1139	1	1131	8
SAT	Heart_Failure	neutrophil	4	1	4	1	0
SAT	Heart_Failure	nk_cell	10	23	4	17	6
SAT	Heart_Failure	pericyte	31	1159	5	1133	26
SAT	Heart_Failure	SMC	40	20	32	12	8
SAT	Heart_Failure	t_cell	18	23	12	17	6
VAT	Obesity	adipocyte	121	1146	4	1029	117
VAT	Obesity	ASPC	172	305	42	175	130
VAT	Obesity	b_cell	12	28	4	20	8
VAT	Obesity	dendritic_cell	20	161	3	144	17
VAT	Obesity	endometrium	19	4232	0	4213	19
VAT	Obesity	endothelial	22	3424	0	3402	22
VAT	Obesity	LEC	52	175	9	132	43
VAT	Obesity	macrophage	69	167	17	115	52
VAT	Obesity	mast_cell	24	3983	0	3959	24
VAT	Obesity	mesothelium	173	288	48	163	125
VAT	Obesity	monocyte	15	1865	0	1850	15
VAT	Obesity	nk_cell	14	4178	0	4164	14
VAT	Obesity	pericyte	42	149	8	115	34
VAT	Obesity	SMC	36	131	9	104	27
VAT	Obesity	t_cell	39	2024	1	1986	38
VAT	CAD	adipocyte	66	1220	15	1169	51
VAT	CAD	ASPC	114	56	86	28	28
VAT	CAD	b_cell	6	0	6	0	0
VAT	CAD	dendritic_cell	16	0	16	0	0
VAT	CAD	endometrium	7	0	7	0	0
VAT	CAD	endothelial	21	0	21	0	0
VAT	CAD	LEC	23	3	21	1	2
VAT	CAD	macrophage	34	1169	6	1141	28
VAT	CAD	mast_cell	15	0	15	0	0
VAT	CAD	mesothelium	106	143	61	98	45
VAT	CAD	monocyte	10	0	10	0	0
VAT	CAD	nk_cell	9	0	9	0	0
VAT	CAD	pericyte	33	0	33	0	0
VAT	CAD	SMC	19	0	19	0	0
VAT	CAD	t_cell	20	78	9	67	11
VAT	T2DM	adipocyte	74	183	34	143	40
VAT	T2DM	ASPC	102	178	48	124	54
VAT	T2DM	b_cell	8	4211	2	4205	6
VAT	T2DM	dendritic_cell	21	99	6	84	15
VAT	T2DM	endometrium	13	4284	2	4273	11
VAT	T2DM	endothelial	22	3323	1	3302	21
VAT	T2DM	LEC	34	59	16	41	18
VAT	T2DM	macrophage	52	64	24	36	28
VAT	T2DM	mast_cell	21	4140	3	4122	18
VAT	T2DM	mesothelium	119	115	63	59	56
VAT	T2DM	monocyte	13	19	9	15	4
VAT	T2DM	nk_cell	11	3964	1	3954	10
VAT	T2DM	pericyte	39	55	16	32	23
VAT	T2DM	SMC	26	68	11	53	15
VAT	T2DM	t_cell	34	1353	2	1321	32
VAT	HTN	adipocyte	134	214	27	107	107
VAT	HTN	ASPC	199	1139	18	958	181
VAT	HTN	b_cell	13	18	7	12	6
VAT	HTN	dendritic_cell	30	58	9	37	21
VAT	HTN	endometrium	17	3714	0	3697	17
VAT	HTN	endothelial	42	65	10	33	32
VAT	HTN	LEC	56	946	4	894	52

VAT	HTN	macrophage	89	187	19	117	70
VAT	HTN	mast_cell	27	34	7	14	20
VAT	HTN	mesothelium	193	361	45	213	148
VAT	HTN	monocyte	24	2828	1	2805	23
VAT	HTN	nk_cell	14	4001	0	3987	14
VAT	HTN	pericyte	60	1265	2	1207	58
VAT	HTN	SMC	42	2859	2	2819	40
VAT	HTN	t_cell	67	911	1	845	66
VAT	Stroke	adipocyte	51	0	51	0	0
VAT	Stroke	ASPC	70	806	29	765	41

b. Comparison of genes identified from causal inference and CTS-TWAS analysis with adjustment for multiple testing correction(Bonferroni<=0.05)

Tissue	Disorder	Cell type	No. of causal genes	No. of TWAS genes	No. of novel genes from causal inference	No. of novel genes from TWAS	No. of common genes
Frontal Cortex	Anxiety	Astros	12	0	12	0	0
Frontal Cortex	Anxiety	Ex	60	0	60	0	0
Frontal Cortex	Anxiety	Inhib	53	0	53	0	0
Frontal Cortex	Anxiety	Micro_Macro	4	0	4	0	0
Frontal Cortex	Anxiety	Oligos	19	0	19	0	0
Frontal Cortex	Anxiety	OPCs	13	0	13	0	0
Frontal Cortex	Bipolar	Astros	7	0	7	0	0
Frontal Cortex	Bipolar	Ex	63	0	63	0	0
Frontal Cortex	Bipolar	Inhib	42	1	41	0	1
Frontal Cortex	Bipolar	Micro_Macro	5	0	5	0	0
Frontal Cortex	Bipolar	Oligos	13	0	13	0	0
Frontal Cortex	Bipolar	OPCs	10	0	10	0	0
Frontal Cortex	Depression	Astros	13	2	12	1	1
Frontal Cortex	Depression	Ex	78	2	77	1	1
Frontal Cortex	Depression	Inhib	72	1	71	0	1
Frontal Cortex	Depression	Micro_Macro	9	1	8	0	1
Frontal Cortex	Depression	Oligos	21	1	20	0	1
Frontal Cortex	Depression	OPCs	16	1	15	0	1
Frontal Cortex	Psychosis	Astros	6	0	6	0	0
Frontal Cortex	Psychosis	Ex	53	0	53	0	0
Frontal Cortex	Psychosis	Inhib	63	0	63	0	0
Frontal Cortex	Psychosis	Micro_Macro	6	0	6	0	0
Frontal Cortex	Psychosis	Oligos	21	0	21	0	0
Frontal Cortex	Psychosis	OPCs	9	0	9	0	0
Pancreas	Prediabetes	acinar_cell	16	0	16	0	0
Pancreas	Prediabetes	alpha_cell	27	0	27	0	0
Pancreas	Prediabetes	beta_cell	63	0	63	0	0
Pancreas	Prediabetes	delta_cell	10	0	10	0	0
Pancreas	Prediabetes	duct_epithelial_cell	12	3	11	2	1
Pancreas	Prediabetes	endothelial_cell	13	0	13	0	0
Pancreas	Prediabetes	epsilon_cell	4	0	4	0	0
Pancreas	Prediabetes	leukocyte	5	0	5	0	0
Pancreas	Prediabetes	mesenchymal_cell	10	0	10	0	0
Pancreas	Prediabetes	PP_cell	8	2	7	1	1
Pancreas	T1DM	acinar_cell	24	27	19	22	5
Pancreas	T1DM	alpha_cell	50	27	45	22	5
Pancreas	T1DM	beta_cell	114	27	108	21	6
Pancreas	T1DM	delta_cell	12	26	8	22	4
Pancreas	T1DM	duct_epithelial_cell	15	27	12	24	3
Pancreas	T1DM	endothelial_cell	10	23	8	21	2
Pancreas	T1DM	epsilon_cell	6	15	3	12	3
Pancreas	T1DM	leukocyte	5	3168	2	3165	3
Pancreas	T1DM	mesenchymal_cell	11	28	8	25	3
Pancreas	T1DM	PP_cell	7	21	3	17	4
Pancreas	T2DM	acinar_cell	44	20	33	9	11
Pancreas	T2DM	alpha_cell	81	22	67	8	14
Pancreas	T2DM	beta_cell	163	24	148	9	15
Pancreas	T2DM	delta_cell	19	12	12	5	7
Pancreas	T2DM	duct_epithelial_cell	31	17	24	10	7
Pancreas	T2DM	endothelial_cell	27	20	16	9	11
Pancreas	T2DM	epsilon_cell	6	5	4	3	2
Pancreas	T2DM	leukocyte	12	21	7	16	5
Pancreas	T2DM	mesenchymal_cell	23	20	16	13	7
Pancreas	T2DM	PP_cell	17	11	13	7	4
Pancreas	T2DM_admission	acinar_cell	51	27	35	11	16
Pancreas	T2DM_admission	alpha_cell	82	32	65	15	17
Pancreas	T2DM_admission	beta_cell	185	34	164	13	21
Pancreas	T2DM_admission	delta_cell	22	20	12	10	10
Pancreas	T2DM_admission	duct_epithelial_cell	34	30	23	19	11
Pancreas	T2DM_admission	endothelial_cell	29	25	17	13	12
Pancreas	T2DM_admission	epsilon_cell	7	6	4	3	3
Pancreas	T2DM_admission	leukocyte	11	62	5	56	6
Pancreas	T2DM_admission	mesenchymal_cell	24	26	12	14	12
Pancreas	T2DM_admission	PP_cell	18	17	12	11	6
Pancreas	T2DM_insulin	acinar_cell	37	7	35	5	2
Pancreas	T2DM_insulin	alpha_cell	57	8	55	6	2
Pancreas	T2DM_insulin	beta_cell	102	8	100	6	2
Pancreas	T2DM_insulin	delta_cell	13	7	10	4	3
Pancreas	T2DM_insulin	duct_epithelial_cell	23	7	22	6	1
Pancreas	T2DM_insulin	endothelial_cell	22	4	22	4	0
Pancreas	T2DM_insulin	epsilon_cell	9	3	8	2	1
Pancreas	T2DM_insulin	leukocyte	7	4	5	2	2
Pancreas	T2DM_insulin	mesenchymal_cell	17	7	15	5	2
Pancreas	T2DM_insulin	PP_cell	12	6	11	5	1
SAT	Obesity	adipocyte	123	365	85	327	38
SAT	Obesity	ASPC	331	112	271	52	60
SAT	Obesity	b_cell	14	32	4	22	10
SAT	Obesity	dendritic_cell	22	45	10	33	12
SAT	Obesity	endothelial	25	1634	7	1616	18
SAT	Obesity	LEC	16	464	5	453	11
SAT	Obesity	macrophage	88	186	49	147	39
SAT	Obesity	mast_cell	28	2777	10	2759	18
SAT	Obesity	monocyte	16	47	6	37	10
SAT	Obesity	neutrophil	11	25	6	20	5
SAT	Obesity	nk_cell	26	106	10	90	16
SAT	Obesity	pericyte	41	2417	17	2393	24
SAT	Obesity	SMC	51	69	29	47	22
SAT	Obesity	t_cell	33	378	8	353	25
SAT	CAD	adipocyte	97	8	92	3	5
SAT	CAD	ASPC	193	12	185	4	8
SAT	CAD	b_cell	8	0	8	0	0
SAT	CAD	dendritic_cell	11	6	9	4	2
SAT	CAD	endothelial	14	1	13	0	1
SAT	CAD	LEC	13	0	13	0	0
SAT	CAD	macrophage	72	9	65	2	7
SAT	CAD	mast_cell	16	3	14	1	2
SAT	CAD	monocyte	11	2	9	0	2
SAT	CAD	neutrophil	7	1	6	0	1
SAT	CAD	nk_cell	16	2	15	1	1
SAT	CAD	pericyte	29	8	24	3	5
SAT	CAD	SMC	35	4	32	1	3
SAT	CAD	t_cell	18	3	16	1	2
SAT	T2DM	adipocyte	104	87	82	65	22
SAT	T2DM	ASPC	226	55	193	22	33

SAT	T2DM	b_cell	10	33	6	29	4
SAT	T2DM	dendritic_cell	16	361	8	353	8
SAT	T2DM	endothelial	21	1004	10	993	11
SAT	T2DM	LEC	15	75	7	67	8
SAT	T2DM	macrophage	68	277	52	261	16
SAT	T2DM	mast_cell	28	2628	14	2614	14
SAT	T2DM	monocyte	13	138	6	131	7
SAT	T2DM	neutrophil	8	8	5	5	3
SAT	T2DM	nk_cell	20	23	11	14	9
SAT	T2DM	pericyte	29	135	15	121	14
SAT	T2DM	SMC	44	29	32	17	12
SAT	T2DM	t_cell	27	32	18	23	9
SAT	HTN	adipocyte	134	88	82	36	52
SAT	HTN	ASPC	329	112	257	40	72
SAT	HTN	b_cell	10	92	4	86	6
SAT	HTN	dendritic_cell	27	30	12	15	15
SAT	HTN	endothelial	26	37	18	29	8
SAT	HTN	LEC	9	18	3	12	6
SAT	HTN	macrophage	87	73	59	45	28
SAT	HTN	mast_cell	26	36	16	26	10
SAT	HTN	monocyte	16	18	11	13	5
SAT	HTN	neutrophil	8	5	5	2	3
SAT	HTN	nk_cell	43	85	21	63	22
SAT	HTN	pericyte	60	55	41	36	19
SAT	HTN	SMC	75	70	41	36	34
SAT	HTN	t_cell	38	71	26	59	12
SAT	Stroke	adipocyte	44	0	44	0	0
SAT	Stroke	ASPC	127	2	125	0	2
SAT	Stroke	b_cell	4	0	4	0	0
SAT	Stroke	dendritic_cell	7	0	7	0	0
SAT	Stroke	endothelial	10	0	10	0	0
SAT	Stroke	LEC	8	0	8	0	0
SAT	Stroke	macrophage	31	0	31	0	0
SAT	Stroke	mast_cell	6	0	6	0	0
SAT	Stroke	monocyte	6	0	6	0	0
SAT	Stroke	neutrophil	3	0	3	0	0
SAT	Stroke	nk_cell	10	0	10	0	0
SAT	Stroke	pericyte	10	0	10	0	0
SAT	Stroke	SMC	15	3	14	2	1
SAT	Stroke	t_cell	15	0	15	0	0
SAT	Heart_Failure	adipocyte	76	15	73	12	3
SAT	Heart_Failure	ASPC	154	17	147	10	7
SAT	Heart_Failure	b_cell	6	23	3	20	3
SAT	Heart_Failure	dendritic_cell	24	4757	9	4742	15
SAT	Heart_Failure	endothelial	18	6	16	4	2
SAT	Heart_Failure	LEC	12	7	12	7	0
SAT	Heart_Failure	macrophage	56	14	53	11	3
SAT	Heart_Failure	mast_cell	17	9	15	7	2
SAT	Heart_Failure	monocyte	9	10	7	8	2
SAT	Heart_Failure	neutrophil	4	1	4	1	0
SAT	Heart_Failure	nk_cell	10	9	7	6	3
SAT	Heart_Failure	pericyte	31	31	25	25	6
SAT	Heart_Failure	SMC	40	11	38	9	2
SAT	Heart_Failure	t_cell	18	13	16	11	2
VAT	Obesity	adipocyte	121	112	80	71	41
VAT	Obesity	ASPC	172	91	123	42	49
VAT	Obesity	b_cell	12	18	4	10	8
VAT	Obesity	dendritic_cell	20	42	5	27	15
VAT	Obesity	endometrium	19	2868	8	2857	11
VAT	Obesity	endothelial	22	739	6	723	16
VAT	Obesity	LEC	52	55	30	33	22
VAT	Obesity	macrophage	69	62	44	37	25
VAT	Obesity	mast_cell	24	136	11	123	13
VAT	Obesity	mesothelium	173	87	127	41	46
VAT	Obesity	monocyte	15	37	4	26	11
VAT	Obesity	nk_cell	14	2798	4	2788	10
VAT	Obesity	pericyte	42	51	23	32	19
VAT	Obesity	SMC	36	48	19	31	17
VAT	Obesity	t_cell	39	409	17	387	22
VAT	CAD	adipocyte	66	17	58	9	8
VAT	CAD	ASPC	114	2	112	0	2
VAT	CAD	b_cell	6	0	6	0	0
VAT	CAD	dendritic_cell	16	0	16	0	0
VAT	CAD	endometrium	7	0	7	0	0
VAT	CAD	endothelial	21	0	21	0	0
VAT	CAD	LEC	23	1	22	0	1
VAT	CAD	macrophage	34	12	29	7	5
VAT	CAD	mast_cell	15	0	15	0	0
VAT	CAD	mesothelium	106	12	97	3	9
VAT	CAD	monocyte	10	0	10	0	0
VAT	CAD	nk_cell	9	0	9	0	0
VAT	CAD	pericyte	33	0	33	0	0
VAT	CAD	SMC	19	0	19	0	0
VAT	CAD	t_cell	20	6	17	3	3
VAT	T2DM	adipocyte	74	33	58	17	16
VAT	T2DM	ASPC	102	34	83	15	19
VAT	T2DM	b_cell	8	21	6	19	2
VAT	T2DM	dendritic_cell	21	17	14	10	7
VAT	T2DM	endometrium	13	3007	7	3001	6
VAT	T2DM	endothelial	22	758	10	746	12
VAT	T2DM	LEC	34	22	25	13	9
VAT	T2DM	macrophage	52	29	34	11	18
VAT	T2DM	mast_cell	21	257	14	250	7
VAT	T2DM	mesothelium	119	41	99	21	20
VAT	T2DM	monocyte	13	12	10	9	3
VAT	T2DM	nk_cell	11	296	4	289	7
VAT	T2DM	pericyte	39	24	26	11	13
VAT	T2DM	SMC	26	23	17	14	9
VAT	T2DM	t_cell	34	52	24	42	10
VAT	HTN	adipocyte	134	60	83	9	51
VAT	HTN	ASPC	199	216	126	143	73
VAT	HTN	b_cell	13	6	10	3	3
VAT	HTN	dendritic_cell	30	13	21	4	9
VAT	HTN	endometrium	17	56	11	50	6
VAT	HTN	endothelial	42	27	23	8	19
VAT	HTN	LEC	56	60	30	34	26
VAT	HTN	macrophage	89	49	53	13	36

VAT	HTN	mast_cell	27	13	16	2	11
VAT	HTN	mesothelium	193	81	133	21	60
VAT	HTN	monocyte	24	38	11	25	13
VAT	HTN	nk_cell	14	1681	5	1672	9
VAT	HTN	pericyte	60	72	30	42	30
VAT	HTN	SMC	42	150	19	127	23
VAT	HTN	t_cell	67	59	36	28	31
VAT	Stroke	adipocyte	51	0	51	0	0
VAT	Stroke	ASPC	70	3	68	1	2
