# Supplementary material for: CausalCellInfer: Prioritizing Cell‐Type‐Specific Disease Associations From Genetically Predicted Transcriptomes in Biobank Cohorts
Source: Adv Sci (Weinh). 2026 Oct 14:e78197. Online ahead of print. doi: 10.1002/advs.78197 (PMC13669230; doi:10.1002/advs.78197)
Supplement: Supplementary file 2 [file ADVS-9999-e78197-s002.zip › advs78197-sup-00014-S13.pdf]

Table S13 Sensitivity and robustness of the analyses

a. Comparision of CTS-TWAS results identified from basic and covariate adjustment analyses

Tissue	Trait	Celltype	N_genes	Pearson_r	Abs_Pearson_r	P_value
SAT	CAD	ASPC	6287	0.984	0.984	2.22E-16
SAT	CAD	LEC	6287	0.9695	0.9695	2.22E-16
SAT	CAD	SMC	6287	0.9823	0.9823	2.22E-16
SAT	CAD	adipocyte	6287	0.9835	0.9835	2.22E-16
SAT	CAD	b_cell	6287	0.9938	0.9938	2.22E-16
SAT	CAD	dendritic_cell	6287	0.9945	0.9945	2.22E-16
SAT	CAD	endothelial	6287	0.9399	0.9399	2.22E-16
SAT	CAD	macrophage	6287	0.9745	0.9745	2.22E-16
SAT	CAD	mast_cell	6287	0.9522	0.9522	2.22E-16
SAT	CAD	monocyte	6287	0.9781	0.9781	2.22E-16
SAT	CAD	neutrophil	6287	0.9833	0.9833	2.22E-16
SAT	CAD	nk_cell	6287	0.9389	0.9389	2.22E-16
SAT	CAD	pericyte	6287	0.9615	0.9615	2.22E-16
SAT	CAD	t_cell	6287	0.9853	0.9853	2.22E-16
SAT	HTN	ASPC	6287	0.9503	0.9503	2.22E-16
SAT	HTN	LEC	6287	0.959	0.959	2.22E-16
SAT	HTN	SMC	6287	0.9567	0.9567	2.22E-16
SAT	HTN	adipocyte	6287	0.9397	0.9397	2.22E-16
SAT	HTN	b_cell	6287	0.9936	0.9936	2.22E-16
SAT	HTN	dendritic_cell	6287	0.9615	0.9615	2.22E-16
SAT	HTN	endothelial	6287	0.9296	0.9296	2.22E-16
SAT	HTN	macrophage	6287	0.928	0.928	2.22E-16
SAT	HTN	mast_cell	6287	0.8955	0.8955	2.22E-16
SAT	HTN	monocyte	6287	0.8884	0.8884	2.22E-16
SAT	HTN	neutrophil	6287	0.6296	0.6296	2.22E-16
SAT	HTN	nk_cell	6287	0.9279	0.9279	2.22E-16
SAT	HTN	pericyte	6287	0.8914	0.8914	2.22E-16
SAT	HTN	t_cell	6287	0.9765	0.9765	2.22E-16
SAT	Heart_Failure	ASPC	6287	0.9875	0.9875	2.22E-16
SAT	Heart_Failure	LEC	6287	0.9868	0.9868	2.22E-16
SAT	Heart_Failure	SMC	6287	0.9892	0.9892	2.22E-16
SAT	Heart_Failure	adipocyte	6287	0.9878	0.9878	2.22E-16
SAT	Heart_Failure	b_cell	6287	0.9992	0.9992	2.22E-16
SAT	Heart_Failure	dendritic_cell	6287	0.9998	0.9998	2.22E-16
SAT	Heart_Failure	endothelial	6287	0.9721	0.9721	2.22E-16
SAT	Heart_Failure	macrophage	6287	0.9858	0.9858	2.22E-16
SAT	Heart_Failure	mast_cell	6287	0.9638	0.9638	2.22E-16
SAT	Heart_Failure	monocyte	6287	0.9949	0.9949	2.22E-16
SAT	Heart_Failure	neutrophil	6287	0.9929	0.9929	2.22E-16
SAT	Heart_Failure	nk_cell	6287	0.94	0.94	2.22E-16
SAT	Heart_Failure	pericyte	6287	0.9923	0.9923	2.22E-16
SAT	Heart_Failure	t_cell	6287	0.9833	0.9833	2.22E-16
SAT	Obesity	ASPC	6287	0.4247	0.4247	2.22E-16
SAT	Obesity	LEC	6287	0.8905	0.8905	2.22E-16
SAT	Obesity	SMC	6287	0.3996	0.3996	2.22E-16
SAT	Obesity	adipocyte	6287	0.6546	0.6546	2.22E-16
SAT	Obesity	b_cell	6287	0.5991	0.5991	2.22E-16
SAT	Obesity	dendritic_cell	6287	0.7434	0.7434	2.22E-16
SAT	Obesity	endothelial	6287	0.8368	0.8368	2.22E-16
SAT	Obesity	macrophage	6287	0.5374	0.5374	2.22E-16
SAT	Obesity	mast_cell	6287	0.4892	0.4892	2.22E-16
SAT	Obesity	monocyte	6287	-0.0854	0.0854	1.18E-11
SAT	Obesity	neutrophil	6287	-0.5932	0.5932	2.22E-16
SAT	Obesity	nk_cell	6287	-0.4392	0.4392	2.22E-16
SAT	Obesity	pericyte	6287	0.4996	0.4996	2.22E-16

SAT	Obesity	t_cell	6287	0.2227	0.2227	2.22E-16
SAT	Stroke	ASPC	6287	0.9934	0.9934	2.22E-16
SAT	Stroke	LEC	6287	0.9882	0.9882	2.22E-16
SAT	Stroke	SMC	6287	0.9957	0.9957	2.22E-16
SAT	Stroke	adipocyte	6287	0.9898	0.9898	2.22E-16
SAT	Stroke	b_cell	6287	0.9752	0.9752	2.22E-16
SAT	Stroke	dendritic_cell	6287	0.9976	0.9976	2.22E-16
SAT	Stroke	endothelial	6287	0.9815	0.9815	2.22E-16
SAT	Stroke	macrophage	6287	0.9872	0.9872	2.22E-16
SAT	Stroke	mast_cell	6287	0.9891	0.9891	2.22E-16
SAT	Stroke	monocyte	6287	0.996	0.996	2.22E-16
SAT	Stroke	neutrophil	6287	0.9889	0.9889	2.22E-16
SAT	Stroke	nk_cell	6287	0.9857	0.9857	2.22E-16
SAT	Stroke	pericyte	6287	0.9799	0.9799	2.22E-16
SAT	Stroke	t_cell	6287	0.9928	0.9928	2.22E-16
SAT	T2DM	ASPC	6287	0.9348	0.9348	2.22E-16
SAT	T2DM	LEC	6287	0.9922	0.9922	2.22E-16
SAT	T2DM	SMC	6287	0.9399	0.9399	2.22E-16
SAT	T2DM	adipocyte	6287	0.9588	0.9588	2.22E-16
SAT	T2DM	b_cell	6287	0.9916	0.9916	2.22E-16
SAT	T2DM	dendritic_cell	6287	0.993	0.993	2.22E-16
SAT	T2DM	endothelial	6287	0.9895	0.9895	2.22E-16
SAT	T2DM	macrophage	6287	0.9709	0.9709	2.22E-16
SAT	T2DM	mast_cell	6287	0.9892	0.9892	2.22E-16
SAT	T2DM	monocyte	6287	0.9857	0.9857	2.22E-16
SAT	T2DM	neutrophil	6287	0.9698	0.9698	2.22E-16
SAT	T2DM	nk_cell	6287	0.6972	0.6972	2.22E-16
SAT	T2DM	pericyte	6287	0.9231	0.9231	2.22E-16
SAT	T2DM	t_cell	6287	0.9448	0.9448	2.22E-16
VAT	CAD	ASPC	4852	0.9868	0.9868	2.22E-16
VAT	CAD	LEC	4852	0.9871	0.9871	2.22E-16
VAT	CAD	SMC	4852	0.9757	0.9757	2.22E-16
VAT	CAD	adipocyte	4852	0.9878	0.9878	2.22E-16
VAT	CAD	b_cell	4852	0.9962	0.9962	2.22E-16
VAT	CAD	dendritic_cell	4852	0.9838	0.9838	2.22E-16
VAT	CAD	endometrium	4852	0.9257	0.9257	2.22E-16
VAT	CAD	endothelial	4852	0.9733	0.9733	2.22E-16
VAT	CAD	macrophage	4852	0.9919	0.9919	2.22E-16
VAT	CAD	mast_cell	4852	0.926	0.926	2.22E-16
VAT	CAD	mesothelium	4852	0.9883	0.9883	2.22E-16
VAT	CAD	monocyte	4852	0.9957	0.9957	2.22E-16
VAT	CAD	nk_cell	4852	0.8251	0.8251	2.22E-16
VAT	CAD	pericyte	4852	0.9815	0.9815	2.22E-16
VAT	CAD	t_cell	4852	0.963	0.963	2.22E-16
VAT	HTN	ASPC	4852	0.9685	0.9685	2.22E-16
VAT	HTN	LEC	4852	0.9741	0.9741	2.22E-16
VAT	HTN	SMC	4852	0.9659	0.9659	2.22E-16
VAT	HTN	adipocyte	4852	0.9219	0.9219	2.22E-16
VAT	HTN	b_cell	4852	0.9798	0.9798	2.22E-16
VAT	HTN	dendritic_cell	4852	0.858	0.858	2.22E-16
VAT	HTN	endometrium	4852	0.9828	0.9828	2.22E-16
VAT	HTN	endothelial	4852	0.8554	0.8554	2.22E-16
VAT	HTN	macrophage	4852	0.9607	0.9607	2.22E-16
VAT	HTN	mast_cell	4852	0.7085	0.7085	2.22E-16
VAT	HTN	mesothelium	4852	0.9505	0.9505	2.22E-16
VAT	HTN	monocyte	4852	0.9908	0.9908	2.22E-16
VAT	HTN	nk_cell	4852	0.9851	0.9851	2.22E-16
VAT	HTN	pericyte	4852	0.9741	0.9741	2.22E-16

VAT	HTN	t_cell	4852	0.9437	0.9437	2.22E-16
VAT	Heart_Failure	ASPC	4852	0.9926	0.9926	2.22E-16
VAT	Heart_Failure	LEC	4852	0.99	0.99	2.22E-16
VAT	Heart_Failure	SMC	4852	0.9911	0.9911	2.22E-16
VAT	Heart_Failure	adipocyte	4852	0.9933	0.9933	2.22E-16
VAT	Heart_Failure	b_cell	4852	0.9984	0.9984	2.22E-16
VAT	Heart_Failure	dendritic_cell	4852	0.9993	0.9993	2.22E-16
VAT	Heart_Failure	endometrium	4852	0.994	0.994	2.22E-16
VAT	Heart_Failure	endothelial	4852	0.984	0.984	2.22E-16
VAT	Heart_Failure	macrophage	4852	0.9935	0.9935	2.22E-16
VAT	Heart_Failure	mast_cell	4852	0.9783	0.9783	2.22E-16
VAT	Heart_Failure	mesothelium	4852	0.9935	0.9935	2.22E-16
VAT	Heart_Failure	monocyte	4852	0.994	0.994	2.22E-16
VAT	Heart_Failure	nk_cell	4852	0.7658	0.7658	2.22E-16
VAT	Heart_Failure	pericyte	4852	0.9903	0.9903	2.22E-16
VAT	Heart_Failure	t_cell	4852	0.9836	0.9836	2.22E-16
VAT	Obesity	ASPC	4852	0.401	0.401	2.22E-16
VAT	Obesity	LEC	4852	0.4607	0.4607	2.22E-16
VAT	Obesity	SMC	4852	0.0282	0.0282	0.049507817
VAT	Obesity	adipocyte	4852	0.5561	0.5561	2.22E-16
VAT	Obesity	b_cell	4852	0.8981	0.8981	2.22E-16
VAT	Obesity	dendritic_cell	4852	-0.2048	0.2048	2.22E-16
VAT	Obesity	endometrium	4852	0.746	0.746	2.22E-16
VAT	Obesity	endothelial	4852	0.7846	0.7846	2.22E-16
VAT	Obesity	macrophage	4852	0.4192	0.4192	2.22E-16
VAT	Obesity	mast_cell	4852	0.0224	0.0224	0.118737478
VAT	Obesity	mesothelium	4852	0.4403	0.4403	2.22E-16
VAT	Obesity	monocyte	4852	0.8864	0.8864	2.22E-16
VAT	Obesity	nk_cell	4852	-0.6421	0.6421	2.22E-16
VAT	Obesity	pericyte	4852	0.4717	0.4717	2.22E-16
VAT	Obesity	t_cell	4852	0.3863	0.3863	2.22E-16
VAT	Stroke	ASPC	4852	0.9965	0.9965	2.22E-16
VAT	Stroke	LEC	4852	0.994	0.994	2.22E-16
VAT	Stroke	SMC	4852	0.9958	0.9958	2.22E-16
VAT	Stroke	adipocyte	4852	0.9901	0.9901	2.22E-16
VAT	Stroke	b_cell	4852	0.987	0.987	2.22E-16
VAT	Stroke	dendritic_cell	4852	0.9969	0.9969	2.22E-16
VAT	Stroke	endometrium	4852	0.9967	0.9967	2.22E-16
VAT	Stroke	endothelial	4852	0.9833	0.9833	2.22E-16
VAT	Stroke	macrophage	4852	0.9938	0.9938	2.22E-16
VAT	Stroke	mast_cell	4852	0.9923	0.9923	2.22E-16
VAT	Stroke	mesothelium	4852	0.9955	0.9955	2.22E-16
VAT	Stroke	monocyte	4852	0.9939	0.9939	2.22E-16
VAT	Stroke	nk_cell	4852	0.9311	0.9311	2.22E-16
VAT	Stroke	pericyte	4852	0.985	0.985	2.22E-16
VAT	Stroke	t_cell	4852	0.9873	0.9873	2.22E-16
VAT	T2DM	ASPC	4852	0.9413	0.9413	2.22E-16
VAT	T2DM	LEC	4852	0.9448	0.9448	2.22E-16
VAT	T2DM	SMC	4852	0.9288	0.9288	2.22E-16
VAT	T2DM	adipocyte	4852	0.9099	0.9099	2.22E-16
VAT	T2DM	b_cell	4852	0.9953	0.9953	2.22E-16
VAT	T2DM	dendritic_cell	4852	0.974	0.974	2.22E-16
VAT	T2DM	endometrium	4852	0.9952	0.9952	2.22E-16
VAT	T2DM	endothelial	4852	0.9889	0.9889	2.22E-16
VAT	T2DM	macrophage	4852	0.9429	0.9429	2.22E-16
VAT	T2DM	mast_cell	4852	0.987	0.987	2.22E-16
VAT	T2DM	mesothelium	4852	0.9402	0.9402	2.22E-16
VAT	T2DM	monocyte	4852	0.8909	0.8909	2.22E-16

VAT	T2DM	nk_cell	4852	0.9628	0.9628	2.22E-16
VAT	T2DM	pericyte	4852	0.9283	0.9283	2.22E-16
VAT	T2DM	t_cell	4852	0.9676	0.9676	2.22E-16
Frontal Cortex	Anxiety	Astros	2857	0.9991	0.9991	2.22E-16
Frontal Cortex	Anxiety	Ex	2857	0.9992	0.9992	2.22E-16
Frontal Cortex	Anxiety	Inhib	2857	0.9982	0.9982	2.22E-16
Frontal Cortex	Anxiety	Micro_Macro	2857	0.9996	0.9996	2.22E-16
Frontal Cortex	Anxiety	OPCs	2857	0.9996	0.9996	2.22E-16
Frontal Cortex	Anxiety	Oligos	2857	0.9956	0.9956	2.22E-16
Frontal Cortex	Bipolar	Astros	2857	0.9986	0.9986	2.22E-16
Frontal Cortex	Bipolar	Ex	2857	0.9994	0.9994	2.22E-16
Frontal Cortex	Bipolar	Inhib	2857	0.9984	0.9984	2.22E-16
Frontal Cortex	Bipolar	Micro_Macro	2857	0.9956	0.9956	2.22E-16
Frontal Cortex	Bipolar	OPCs	2857	0.9984	0.9984	2.22E-16
Frontal Cortex	Bipolar	Oligos	2857	0.9968	0.9968	2.22E-16
Frontal Cortex	Depression	Astros	2857	0.9978	0.9978	2.22E-16
Frontal Cortex	Depression	Ex	2857	0.997	0.997	2.22E-16
Frontal Cortex	Depression	Inhib	2857	0.9921	0.9921	2.22E-16
Frontal Cortex	Depression	Micro_Macro	2857	0.9917	0.9917	2.22E-16
Frontal Cortex	Depression	OPCs	2857	0.9969	0.9969	2.22E-16
Frontal Cortex	Depression	Oligos	2857	0.9854	0.9854	2.22E-16
Frontal Cortex	Psychosis	Astros	2857	0.9985	0.9985	2.22E-16
Frontal Cortex	Psychosis	Ex	2857	0.9975	0.9975	2.22E-16
Frontal Cortex	Psychosis	Inhib	2857	0.9969	0.9969	2.22E-16
Frontal Cortex	Psychosis	Micro_Macro	2857	0.9929	0.9929	2.22E-16
Frontal Cortex	Psychosis	OPCs	2857	0.9979	0.9979	2.22E-16
Frontal Cortex	Psychosis	Oligos	2857	0.995	0.995	2.22E-16
Pancreas	Prediabetes	PP_cell	3929	0.9978	0.9978	2.22E-16
Pancreas	Prediabetes	acinar_cell	3929	0.9923	0.9923	2.22E-16
Pancreas	Prediabetes	alpha_cell	3929	0.9875	0.9875	2.22E-16
Pancreas	Prediabetes	beta_cell	3929	0.9877	0.9877	2.22E-16
Pancreas	Prediabetes	delta_cell	3929	0.9893	0.9893	2.22E-16
Pancreas	Prediabetes	duct_epithelial_cell	3929	0.9972	0.9972	2.22E-16
Pancreas	Prediabetes	endothelial_cell	3929	0.9883	0.9883	2.22E-16
Pancreas	Prediabetes	epsilon_cell	3929	0.9691	0.9691	2.22E-16
Pancreas	Prediabetes	leukocyte	3929	0.9974	0.9974	2.22E-16
Pancreas	Prediabetes	mesenchymal_cell	3929	0.9928	0.9928	2.22E-16
Pancreas	T1DM	PP_cell	3929	0.9965	0.9965	2.22E-16
Pancreas	T1DM	acinar_cell	3929	0.9917	0.9917	2.22E-16
Pancreas	T1DM	alpha_cell	3929	0.9919	0.9919	2.22E-16
Pancreas	T1DM	beta_cell	3929	0.9911	0.9911	2.22E-16
Pancreas	T1DM	delta_cell	3929	0.9947	0.9947	2.22E-16
Pancreas	T1DM	duct_epithelial_cell	3929	0.9897	0.9897	2.22E-16
Pancreas	T1DM	endothelial_cell	3929	0.9975	0.9975	2.22E-16
Pancreas	T1DM	epsilon_cell	3929	0.9961	0.9961	2.22E-16
Pancreas	T1DM	leukocyte	3929	0.9997	0.9997	2.22E-16
Pancreas	T1DM	mesenchymal_cell	3929	0.995	0.995	2.22E-16
Pancreas	T2DM	PP_cell	3929	0.9555	0.9555	2.22E-16
Pancreas	T2DM	acinar_cell	3929	0.9632	0.9632	2.22E-16
Pancreas	T2DM	alpha_cell	3929	0.9398	0.9398	2.22E-16
Pancreas	T2DM	beta_cell	3929	0.9384	0.9384	2.22E-16
Pancreas	T2DM	delta_cell	3929	0.9532	0.9532	2.22E-16
Pancreas	T2DM	duct_epithelial_cell	3929	0.9593	0.9593	2.22E-16
Pancreas	T2DM	endothelial_cell	3929	0.9742	0.9742	2.22E-16
Pancreas	T2DM	epsilon_cell	3929	0.9675	0.9675	2.22E-16
Pancreas	T2DM	leukocyte	3929	0.9889	0.9889	2.22E-16
Pancreas	T2DM	mesenchymal_cell	3929	0.955	0.955	2.22E-16
Pancreas	T2DM_admission	PP_cell	3929	0.9739	0.9739	2.22E-16

Pancreas	T2DM_admission	acinar_cell	3929	0.9518	0.9518	2.22E-16
Pancreas	T2DM_admission	alpha_cell	3929	0.9279	0.9279	2.22E-16
Pancreas	T2DM_admission	beta_cell	3929	0.9273	0.9273	2.22E-16
Pancreas	T2DM_admission	delta_cell	3929	0.9554	0.9554	2.22E-16
Pancreas	T2DM_admission	duct_epithelial_cell	3929	0.9719	0.9719	2.22E-16
Pancreas	T2DM_admission	endothelial_cell	3929	0.9654	0.9654	2.22E-16
Pancreas	T2DM_admission	epsilon_cell	3929	0.9375	0.9375	2.22E-16
Pancreas	T2DM_admission	leukocyte	3929	0.9892	0.9892	2.22E-16
Pancreas	T2DM_admission	mesenchymal_cell	3929	0.9524	0.9524	2.22E-16
Pancreas	T2DM_insulin	PP_cell	3929	0.9862	0.9862	2.22E-16
Pancreas	T2DM_insulin	acinar_cell	3929	0.9791	0.9791	2.22E-16
Pancreas	T2DM_insulin	alpha_cell	3929	0.9795	0.9795	2.22E-16
Pancreas	T2DM_insulin	beta_cell	3929	0.9785	0.9785	2.22E-16
Pancreas	T2DM_insulin	delta_cell	3929	0.9761	0.9761	2.22E-16
Pancreas	T2DM_insulin	duct_epithelial_cell	3929	0.9748	0.9748	2.22E-16
Pancreas	T2DM_insulin	endothelial_cell	3929	0.992	0.992	2.22E-16
Pancreas	T2DM_insulin	epsilon_cell	3929	0.975	0.975	2.22E-16
Pancreas	T2DM_insulin	leukocyte	3929	0.9967	0.9967	2.22E-16
Pancreas	T2DM_insulin	mesenchymal_cell	3929	0.991	0.991	2.22E-16

Note: 2.22E-16 indicates the p value <2.22E-16□

b. Comparision of CTS-TWAS results identified from full and European-only analyses

Tissue	Trait	Celltype	N_genes	Pearson_r	Abs_Pearson_r	P_vaule
SAT	CAD	ASPC	6339	0.9796	0.9796	2.22E-16
SAT	CAD	LEC	6339	0.9859	0.9859	2.22E-16
SAT	CAD	SMC	6339	0.9737	0.9737	2.22E-16
SAT	CAD	adipocyte	6339	0.9822	0.9822	2.22E-16
SAT	CAD	b_cell	6339	0.9956	0.9956	2.22E-16
SAT	CAD	dendritic_cell	6339	0.9868	0.9868	2.22E-16
SAT	CAD	endothelial	6339	0.9797	0.9797	2.22E-16
SAT	CAD	macrophage	6339	0.9807	0.9807	2.22E-16
SAT	CAD	mast_cell	6339	0.9875	0.9875	2.22E-16
SAT	CAD	monocyte	6339	0.9831	0.9831	2.22E-16
SAT	CAD	neutrophil	6339	0.9923	0.9923	2.22E-16
SAT	CAD	nk_cell	6339	0.96	0.96	2.22E-16
SAT	CAD	pericyte	6339	0.9871	0.9871	2.22E-16
SAT	CAD	t_cell	6339	0.9839	0.9839	2.22E-16
SAT	HTN	ASPC	6339	0.9895	0.9895	2.22E-16
SAT	HTN	LEC	6339	0.9974	0.9974	2.22E-16
SAT	HTN	SMC	6339	0.991	0.991	2.22E-16
SAT	HTN	adipocyte	6339	0.9905	0.9905	2.22E-16
SAT	HTN	b_cell	6339	0.9991	0.9991	2.22E-16
SAT	HTN	dendritic_cell	6339	0.9932	0.9932	2.22E-16
SAT	HTN	endothelial	6339	0.9958	0.9958	2.22E-16
SAT	HTN	macrophage	6339	0.9914	0.9914	2.22E-16
SAT	HTN	mast_cell	6339	0.9955	0.9955	2.22E-16
SAT	HTN	monocyte	6339	0.9918	0.9918	2.22E-16
SAT	HTN	neutrophil	6339	0.9776	0.9776	2.22E-16
SAT	HTN	nk_cell	6339	0.9972	0.9972	2.22E-16
SAT	HTN	pericyte	6339	0.9947	0.9947	2.22E-16
SAT	HTN	t_cell	6339	0.9978	0.9978	2.22E-16
SAT	Heart_Failure	ASPC	6339	0.978	0.978	2.22E-16
SAT	Heart_Failure	LEC	6339	0.9828	0.9828	2.22E-16
SAT	Heart_Failure	SMC	6339	0.9731	0.9731	2.22E-16
SAT	Heart_Failure	adipocyte	6339	0.9818	0.9818	2.22E-16
SAT	Heart_Failure	b_cell	6339	0.9989	0.9989	2.22E-16
SAT	Heart_Failure	dendritic_cell	6339	0.9996	0.9996	2.22E-16
SAT	Heart_Failure	endothelial	6339	0.9795	0.9795	2.22E-16
SAT	Heart_Failure	macrophage	6339	0.9809	0.9809	2.22E-16
SAT	Heart_Failure	mast_cell	6339	0.9614	0.9614	2.22E-16
SAT	Heart_Failure	monocyte	6339	0.9724	0.9724	2.22E-16
SAT	Heart_Failure	neutrophil	6339	0.9944	0.9944	2.22E-16
SAT	Heart_Failure	nk_cell	6339	0.9634	0.9634	2.22E-16
SAT	Heart_Failure	pericyte	6339	0.9946	0.9946	2.22E-16
SAT	Heart_Failure	t_cell	6339	0.9879	0.9879	2.22E-16
SAT	Obesity	ASPC	6339	0.9876	0.9876	2.22E-16
SAT	Obesity	LEC	6339	0.9987	0.9987	2.22E-16
SAT	Obesity	SMC	6339	0.9894	0.9894	2.22E-16
SAT	Obesity	adipocyte	6339	0.9943	0.9943	2.22E-16
SAT	Obesity	b_cell	6339	0.9983	0.9983	2.22E-16
SAT	Obesity	dendritic_cell	6339	0.9747	0.9747	2.22E-16
SAT	Obesity	endothelial	6339	0.9987	0.9987	2.22E-16
SAT	Obesity	macrophage	6339	0.9944	0.9944	2.22E-16
SAT	Obesity	mast_cell	6339	0.998	0.998	2.22E-16
SAT	Obesity	monocyte	6339	0.9843	0.9843	2.22E-16
SAT	Obesity	neutrophil	6339	0.9988	0.9988	2.22E-16

SAT	Obesity	nk_cell	6339	0.998	0.998	2.22E-16
SAT	Obesity	pericyte	6339	0.998	0.998	2.22E-16
SAT	Obesity	t_cell	6339	0.9973	0.9973	2.22E-16
SAT	Stroke	ASPC	6339	0.9753	0.9753	2.22E-16
SAT	Stroke	LEC	6339	0.9904	0.9904	2.22E-16
SAT	Stroke	SMC	6339	0.9829	0.9829	2.22E-16
SAT	Stroke	adipocyte	6339	0.9781	0.9781	2.22E-16
SAT	Stroke	b_cell	6339	0.9905	0.9905	2.22E-16
SAT	Stroke	dendritic_cell	6339	0.9857	0.9857	2.22E-16
SAT	Stroke	endothelial	6339	0.9832	0.9832	2.22E-16
SAT	Stroke	macrophage	6339	0.9766	0.9766	2.22E-16
SAT	Stroke	mast_cell	6339	0.9876	0.9876	2.22E-16
SAT	Stroke	monocyte	6339	0.9891	0.9891	2.22E-16
SAT	Stroke	neutrophil	6339	0.9713	0.9713	2.22E-16
SAT	Stroke	nk_cell	6339	0.981	0.981	2.22E-16
SAT	Stroke	pericyte	6339	0.9794	0.9794	2.22E-16
SAT	Stroke	t_cell	6339	0.9868	0.9868	2.22E-16
SAT	T2DM	ASPC	6339	0.9738	0.9738	2.22E-16
SAT	T2DM	LEC	6339	0.998	0.998	2.22E-16
SAT	T2DM	SMC	6339	0.982	0.982	2.22E-16
SAT	T2DM	adipocyte	6339	0.986	0.986	2.22E-16
SAT	T2DM	b_cell	6339	0.9979	0.9979	2.22E-16
SAT	T2DM	dendritic_cell	6339	0.9974	0.9974	2.22E-16
SAT	T2DM	endothelial	6339	0.9972	0.9972	2.22E-16
SAT	T2DM	macrophage	6339	0.9906	0.9906	2.22E-16
SAT	T2DM	mast_cell	6339	0.997	0.997	2.22E-16
SAT	T2DM	monocyte	6339	0.996	0.996	2.22E-16
SAT	T2DM	neutrophil	6339	0.9963	0.9963	2.22E-16
SAT	T2DM	nk_cell	6339	0.9863	0.9863	2.22E-16
SAT	T2DM	pericyte	6339	0.9928	0.9928	2.22E-16
SAT	T2DM	t_cell	6339	0.9884	0.9884	2.22E-16
VAT	CAD	ASPC	4928	0.9806	0.9806	2.22E-16
VAT	CAD	LEC	4928	0.9813	0.9813	2.22E-16
VAT	CAD	SMC	4928	0.9766	0.9766	2.22E-16
VAT	CAD	adipocyte	4928	0.986	0.986	2.22E-16
VAT	CAD	b_cell	4928	0.9941	0.9941	2.22E-16
VAT	CAD	dendritic_cell	4928	0.9904	0.9904	2.22E-16
VAT	CAD	endometrium	4928	0.9873	0.9873	2.22E-16
VAT	CAD	endothelial	4928	0.9834	0.9834	2.22E-16
VAT	CAD	macrophage	4928	0.9863	0.9863	2.22E-16
VAT	CAD	mast_cell	4928	0.9706	0.9706	2.22E-16
VAT	CAD	mesothelium	4928	0.9843	0.9843	2.22E-16
VAT	CAD	monocyte	4928	0.9974	0.9974	2.22E-16
VAT	CAD	nk_cell	4928	0.9654	0.9654	2.22E-16
VAT	CAD	pericyte	4928	0.981	0.981	2.22E-16
VAT	CAD	t_cell	4928	0.9891	0.9891	2.22E-16
VAT	HTN	ASPC	4928	0.9933	0.9933	2.22E-16
VAT	HTN	LEC	4928	0.9938	0.9938	2.22E-16
VAT	HTN	SMC	4928	0.9943	0.9943	2.22E-16
VAT	HTN	adipocyte	4928	0.9893	0.9893	2.22E-16
VAT	HTN	b_cell	4928	0.996	0.996	2.22E-16
VAT	HTN	dendritic_cell	4928	0.986	0.986	2.22E-16
VAT	HTN	endometrium	4928	0.9983	0.9983	2.22E-16
VAT	HTN	endothelial	4928	0.9864	0.9864	2.22E-16
VAT	HTN	macrophage	4928	0.9913	0.9913	2.22E-16
VAT	HTN	mast_cell	4928	0.9894	0.9894	2.22E-16

VAT	HTN	mesothelium	4928	0.9901	0.9901	2.22E-16
VAT	HTN	monocyte	4928	0.9978	0.9978	2.22E-16
VAT	HTN	nk_cell	4928	0.9991	0.9991	2.22E-16
VAT	HTN	pericyte	4928	0.9947	0.9947	2.22E-16
VAT	HTN	t_cell	4928	0.9954	0.9954	2.22E-16
VAT	Heart_Failure	ASPC	4928	0.9879	0.9879	2.22E-16
VAT	Heart_Failure	LEC	4928	0.9597	0.9597	2.22E-16
VAT	Heart_Failure	SMC	4928	0.9837	0.9837	2.22E-16
VAT	Heart_Failure	adipocyte	4928	0.9894	0.9894	2.22E-16
VAT	Heart_Failure	b_cell	4928	0.9963	0.9963	2.22E-16
VAT	Heart_Failure	dendritic_cell	4928	0.9989	0.9989	2.22E-16
VAT	Heart_Failure	endometrium	4928	0.9968	0.9968	2.22E-16
VAT	Heart_Failure	endothelial	4928	0.9612	0.9612	2.22E-16
VAT	Heart_Failure	macrophage	4928	0.9889	0.9889	2.22E-16
VAT	Heart_Failure	mast_cell	4928	0.9831	0.9831	2.22E-16
VAT	Heart_Failure	mesothelium	4928	0.9888	0.9888	2.22E-16
VAT	Heart_Failure	monocyte	4928	0.99	0.99	2.22E-16
VAT	Heart_Failure	nk_cell	4928	0.9413	0.9413	2.22E-16
VAT	Heart_Failure	pericyte	4928	0.9788	0.9788	2.22E-16
VAT	Heart_Failure	t_cell	4928	0.9914	0.9914	2.22E-16
VAT	Obesity	ASPC	4928	0.9891	0.9891	2.22E-16
VAT	Obesity	LEC	4928	0.9621	0.9621	2.22E-16
VAT	Obesity	SMC	4928	0.9844	0.9844	2.22E-16
VAT	Obesity	adipocyte	4928	0.9937	0.9937	2.22E-16
VAT	Obesity	b_cell	4928	0.9979	0.9979	2.22E-16
VAT	Obesity	dendritic_cell	4928	0.9912	0.9912	2.22E-16
VAT	Obesity	endometrium	4928	0.9989	0.9989	2.22E-16
VAT	Obesity	endothelial	4928	0.998	0.998	2.22E-16
VAT	Obesity	macrophage	4928	0.9685	0.9685	2.22E-16
VAT	Obesity	mast_cell	4928	0.9978	0.9978	2.22E-16
VAT	Obesity	mesothelium	4928	0.9869	0.9869	2.22E-16
VAT	Obesity	monocyte	4928	0.9979	0.9979	2.22E-16
VAT	Obesity	nk_cell	4928	0.9995	0.9995	2.22E-16
VAT	Obesity	pericyte	4928	0.9885	0.9885	2.22E-16
VAT	Obesity	t_cell	4928	0.9977	0.9977	2.22E-16
VAT	Stroke	ASPC	4928	0.9875	0.9875	2.22E-16
VAT	Stroke	LEC	4928	0.9646	0.9646	2.22E-16
VAT	Stroke	SMC	4928	0.9841	0.9841	2.22E-16
VAT	Stroke	adipocyte	4928	0.9816	0.9816	2.22E-16
VAT	Stroke	b_cell	4928	0.9897	0.9897	2.22E-16
VAT	Stroke	dendritic_cell	4928	0.986	0.986	2.22E-16
VAT	Stroke	endometrium	4928	0.9955	0.9955	2.22E-16
VAT	Stroke	endothelial	4928	0.9807	0.9807	2.22E-16
VAT	Stroke	macrophage	4928	0.9825	0.9825	2.22E-16
VAT	Stroke	mast_cell	4928	0.9852	0.9852	2.22E-16
VAT	Stroke	mesothelium	4928	0.985	0.985	2.22E-16
VAT	Stroke	monocyte	4928	0.9757	0.9757	2.22E-16
VAT	Stroke	nk_cell	4928	0.9701	0.9701	2.22E-16
VAT	Stroke	pericyte	4928	0.9527	0.9527	2.22E-16
VAT	Stroke	t_cell	4928	0.9613	0.9613	2.22E-16
VAT	T2DM	ASPC	4928	0.9767	0.9767	2.22E-16
VAT	T2DM	LEC	4928	0.932	0.932	2.22E-16
VAT	T2DM	SMC	4928	0.981	0.981	2.22E-16
VAT	T2DM	adipocyte	4928	0.977	0.977	2.22E-16
VAT	T2DM	b_cell	4928	0.9984	0.9984	2.22E-16
VAT	T2DM	dendritic_cell	4928	0.991	0.991	2.22E-16

VAT	T2DM	endometrium	4928	0.9988	0.9988	2.22E-16
VAT	T2DM	endothelial	4928	0.9967	0.9967	2.22E-16
VAT	T2DM	macrophage	4928	0.9594	0.9594	2.22E-16
VAT	T2DM	mast_cell	4928	0.9974	0.9974	2.22E-16
VAT	T2DM	mesothelium	4928	0.975	0.975	2.22E-16
VAT	T2DM	monocyte	4928	0.9817	0.9817	2.22E-16
VAT	T2DM	nk_cell	4928	0.998	0.998	2.22E-16
VAT	T2DM	pericyte	4928	0.9745	0.9745	2.22E-16
VAT	T2DM	t_cell	4928	0.9937	0.9937	2.22E-16
Frontal Cortex	Anxiety	Astros	2875	0.9802	0.9802	2.22E-16
Frontal Cortex	Anxiety	Ex	2875	0.9817	0.9817	2.22E-16
Frontal Cortex	Anxiety	Inhib	2875	0.9835	0.9835	2.22E-16
Frontal Cortex	Anxiety	Micro_Macro	2875	0.9964	0.9964	2.22E-16
Frontal Cortex	Anxiety	OPCs	2875	0.9886	0.9886	2.22E-16
Frontal Cortex	Anxiety	Oligos	2875	0.9741	0.9741	2.22E-16
Frontal Cortex	Bipolar	Astros	2875	0.9682	0.9682	2.22E-16
Frontal Cortex	Bipolar	Ex	2875	0.9782	0.9782	2.22E-16
Frontal Cortex	Bipolar	Inhib	2875	0.9693	0.9693	2.22E-16
Frontal Cortex	Bipolar	Micro_Macro	2875	0.9652	0.9652	2.22E-16
Frontal Cortex	Bipolar	OPCs	2875	0.9597	0.9597	2.22E-16
Frontal Cortex	Bipolar	Oligos	2875	0.9703	0.9703	2.22E-16
Frontal Cortex	Depression	Astros	2875	0.982	0.982	2.22E-16
Frontal Cortex	Depression	Ex	2875	0.9818	0.9818	2.22E-16
Frontal Cortex	Depression	Inhib	2875	0.9791	0.9791	2.22E-16
Frontal Cortex	Depression	Micro_Macro	2875	0.9764	0.9764	2.22E-16
Frontal Cortex	Depression	OPCs	2875	0.9893	0.9893	2.22E-16
Frontal Cortex	Depression	Oligos	2875	0.9736	0.9736	2.22E-16
Frontal Cortex	Psychosis	Astros	2875	0.8146	0.8146	2.22E-16
Frontal Cortex	Psychosis	Ex	2875	0.9274	0.9274	2.22E-16
Frontal Cortex	Psychosis	Inhib	2875	0.9456	0.9456	2.22E-16
Frontal Cortex	Psychosis	Micro_Macro	2875	0.93	0.93	2.22E-16
Frontal Cortex	Psychosis	OPCs	2875	0.87	0.87	2.22E-16
Frontal Cortex	Psychosis	Oligos	2875	0.9576	0.9576	2.22E-16
Pancreas	Prediabetes	PP_cell	3958	0.9841	0.9841	2.22E-16
Pancreas	Prediabetes	acinar_cell	3958	0.9519	0.9519	2.22E-16
Pancreas	Prediabetes	alpha_cell	3958	0.9307	0.9307	2.22E-16
Pancreas	Prediabetes	beta_cell	3958	0.938	0.938	2.22E-16
Pancreas	Prediabetes	delta_cell	3958	0.9491	0.9491	2.22E-16
Pancreas	Prediabetes	duct_epithelial_cell	3958	0.9807	0.9807	2.22E-16
Pancreas	Prediabetes	endothelial_cell	3958	0.9479	0.9479	2.22E-16
Pancreas	Prediabetes	epsilon_cell	3958	0.959	0.959	2.22E-16
Pancreas	Prediabetes	leukocyte	3958	0.9872	0.9872	2.22E-16
Pancreas	Prediabetes	mesenchymal_cell	3958	0.9672	0.9672	2.22E-16
Pancreas	T1DM	PP_cell	3958	0.9692	0.9692	2.22E-16
Pancreas	T1DM	acinar_cell	3958	0.9703	0.9703	2.22E-16
Pancreas	T1DM	alpha_cell	3958	0.9685	0.9685	2.22E-16
Pancreas	T1DM	beta_cell	3958	0.964	0.964	2.22E-16
Pancreas	T1DM	delta_cell	3958	0.9747	0.9747	2.22E-16
Pancreas	T1DM	duct_epithelial_cell	3958	0.9311	0.9311	2.22E-16
Pancreas	T1DM	endothelial_cell	3958	0.9917	0.9917	2.22E-16
Pancreas	T1DM	epsilon_cell	3958	0.9852	0.9852	2.22E-16
Pancreas	T1DM	leukocyte	3958	0.9989	0.9989	2.22E-16
Pancreas	T1DM	mesenchymal_cell	3958	0.9846	0.9846	2.22E-16
Pancreas	T2DM	PP_cell	3958	0.9803	0.9803	2.22E-16
Pancreas	T2DM	acinar_cell	3958	0.9707	0.9707	2.22E-16
Pancreas	T2DM	alpha_cell	3958	0.9699	0.9699	2.22E-16

Pancreas	T2DM	beta_cell	3958	0.964	0.964	2.22E-16
Pancreas	T2DM	delta_cell	3958	0.9725	0.9725	2.22E-16
Pancreas	T2DM	duct_epithelial_cell	3958	0.9607	0.9607	2.22E-16
Pancreas	T2DM	endothelial_cell	3958	0.9832	0.9832	2.22E-16
Pancreas	T2DM	epsilon_cell	3958	0.9888	0.9888	2.22E-16
Pancreas	T2DM	leukocyte	3958	0.9927	0.9927	2.22E-16
Pancreas	T2DM	mesenchymal_cell	3958	0.9794	0.9794	2.22E-16
Pancreas	T2DM_admission	PP_cell	3958	0.9898	0.9898	2.22E-16
Pancreas	T2DM_admission	acinar_cell	3958	0.9719	0.9719	2.22E-16
Pancreas	T2DM_admission	alpha_cell	3958	0.9722	0.9722	2.22E-16
Pancreas	T2DM_admission	beta_cell	3958	0.9682	0.9682	2.22E-16
Pancreas	T2DM_admission	delta_cell	3958	0.9824	0.9824	2.22E-16
Pancreas	T2DM_admission	duct_epithelial_cell	3958	0.9788	0.9788	2.22E-16
Pancreas	T2DM_admission	endothelial_cell	3958	0.9863	0.9863	2.22E-16
Pancreas	T2DM_admission	epsilon_cell	3958	0.986	0.986	2.22E-16
Pancreas	T2DM_admission	leukocyte	3958	0.9943	0.9943	2.22E-16
Pancreas	T2DM_admission	mesenchymal_cell	3958	0.9844	0.9844	2.22E-16
Pancreas	T2DM_insulin	PP_cell	3958	0.9799	0.9799	2.22E-16
Pancreas	T2DM_insulin	acinar_cell	3958	0.9531	0.9531	2.22E-16
Pancreas	T2DM_insulin	alpha_cell	3958	0.9558	0.9558	2.22E-16
Pancreas	T2DM_insulin	beta_cell	3958	0.9466	0.9466	2.22E-16
Pancreas	T2DM_insulin	delta_cell	3958	0.9627	0.9627	2.22E-16
Pancreas	T2DM_insulin	duct_epithelial_cell	3958	0.8953	0.8953	2.22E-16
Pancreas	T2DM_insulin	endothelial_cell	3958	0.985	0.985	2.22E-16
Pancreas	T2DM_insulin	epsilon_cell	3958	0.8957	0.8957	2.22E-16
Pancreas	T2DM_insulin	leukocyte	3958	0.9934	0.9934	2.22E-16
Pancreas	T2DM_insulin	mesenchymal_cell	3958	0.9761	0.9761	2.22E-16

Note: 2.22E-16 indicates the p value <2.22E-16□

c. Comparision of causal inference results identified from basic and covariate adjustment analyses

Tissue	Trait	Celltype	N_genes	Pearson_r	Abs_Pearson_r	P_vaule
SAT	CAD	ASPC	163	0.941	0.941	2.22E-16
SAT	CAD	LEC	11	0.5268	0.5268	9.59E-02
SAT	CAD	SMC	30	0.8959	0.8959	2.25E-11
SAT	CAD	adipocyte	77	0.9385	0.9385	2.22E-16
SAT	CAD	b_cell	7	0.9256	0.9256	2.79E-03
SAT	CAD	dendritic_cell	10	0.8198	0.8198	3.69E-03
SAT	CAD	endothelial	8	0.8238	0.8238	1.19E-02
SAT	CAD	macrophage	62	0.9288	0.9288	2.22E-16
SAT	CAD	mast_cell	13	0.9499	0.9499	6.83E-07
SAT	CAD	monocyte	8	0.749	0.749	3.25E-02
SAT	CAD	neutrophil	6	0.9504	0.9504	3.63E-03
SAT	CAD	nk_cell	13	0.8088	0.8088	8.15E-04
SAT	CAD	pericyte	24	0.8183	0.8183	1.03E-06
SAT	CAD	t_cell	17	0.8694	0.8694	5.85E-06
SAT	HTN	ASPC	283	0.9479	0.9479	2.22E-16
SAT	HTN	LEC	18	0.9353	0.9353	1.26E-08
SAT	HTN	SMC	64	0.916	0.916	2.22E-16
SAT	HTN	adipocyte	114	0.9276	0.9276	2.22E-16
SAT	HTN	b_cell	9	0.5912	0.5912	9.36E-02
SAT	HTN	dendritic_cell	26	0.9214	0.9214	2.45E-11
SAT	HTN	endothelial	28	0.903	0.903	4.92E-11
SAT	HTN	macrophage	95	0.8993	0.8993	2.22E-16
SAT	HTN	mast_cell	33	0.8944	0.8944	2.32E-12
SAT	HTN	monocyte	15	0.9264	0.9264	7.09E-07
SAT	HTN	neutrophil	9	0.8709	0.8709	2.24E-03
SAT	HTN	nk_cell	38	0.8694	0.8694	1.43E-12
SAT	HTN	pericyte	52	0.846	0.846	2.91E-15
SAT	HTN	t_cell	39	0.8448	0.8448	1.37E-11
SAT	Heart_Failure	ASPC	142	0.9774	0.9774	2.22E-16
SAT	Heart_Failure	LEC	12	0.9844	0.9844	7.09E-09
SAT	Heart_Failure	SMC	40	0.9939	0.9939	2.22E-16
SAT	Heart_Failure	adipocyte	67	0.9716	0.9716	2.22E-16
SAT	Heart_Failure	b_cell	6	0.9281	0.9281	7.57E-03
SAT	Heart_Failure	dendritic_cell	22	0.9901	0.9901	2.22E-16
SAT	Heart_Failure	endothelial	17	0.9795	0.9795	7.53E-12
SAT	Heart_Failure	macrophage	54	0.9927	0.9927	2.22E-16
SAT	Heart_Failure	mast_cell	16	0.9796	0.9796	3.74E-11
SAT	Heart_Failure	monocyte	8	0.9971	0.9971	6.08E-08
SAT	Heart_Failure	neutrophil	3	0.9997	0.9997	1.56E-02
SAT	Heart_Failure	nk_cell	10	0.989	0.989	6.32E-08
SAT	Heart_Failure	pericyte	27	0.9835	0.9835	2.22E-16
SAT	Heart_Failure	t_cell	16	0.9397	0.9397	6.62E-08
SAT	Obesity	ASPC	326	0.9988	0.9988	2.22E-16
SAT	Obesity	LEC	16	0.9994	0.9994	2.22E-16
SAT	Obesity	SMC	51	0.9992	0.9992	2.22E-16
SAT	Obesity	adipocyte	121	0.9989	0.9989	2.22E-16
SAT	Obesity	b_cell	14	0.9996	0.9996	2.22E-16
SAT	Obesity	dendritic_cell	22	0.9995	0.9995	2.22E-16
SAT	Obesity	endothelial	25	0.9995	0.9995	2.22E-16
SAT	Obesity	macrophage	85	0.999	0.999	2.22E-16
SAT	Obesity	mast_cell	26	0.9993	0.9993	2.22E-16
SAT	Obesity	monocyte	18	0.9991	0.9991	2.22E-16
SAT	Obesity	neutrophil	10	0.9998	0.9998	7.00E-15

SAT	Obesity	nk_cell	25	0.9993	0.9993	2.22E-16
SAT	Obesity	pericyte	41	0.9994	0.9994	2.22E-16
SAT	Obesity	t_cell	33	0.999	0.999	2.22E-16
SAT	Stroke	ASPC	105	0.9425	0.9425	2.22E-16
SAT	Stroke	LEC	6	0.9872	0.9872	2.45E-04
SAT	Stroke	SMC	10	0.8134	0.8134	4.21E-03
SAT	Stroke	adipocyte	32	0.9105	0.9105	4.92E-13
SAT	Stroke	b_cell	4	0.9804	0.9804	1.96E-02
SAT	Stroke	dendritic_cell	6	0.993	0.993	7.33E-05
SAT	Stroke	endothelial	6	0.9669	0.9669	1.63E-03
SAT	Stroke	macrophage	22	0.8939	0.8939	2.09E-08
SAT	Stroke	mast_cell	4	0.9959	0.9959	4.10E-03
SAT	Stroke	monocyte	5	0.9814	0.9814	3.04E-03
SAT	Stroke	neutrophil	2			
SAT	Stroke	nk_cell	7	0.9659	0.9659	4.05E-04
SAT	Stroke	pericyte	8	0.8866	0.8866	3.34E-03
SAT	Stroke	t_cell	14	0.8817	0.8817	3.05E-05
SAT	T2DM	ASPC	205	0.9861	0.9861	2.22E-16
SAT	T2DM	LEC	15	0.9908	0.9908	1.12E-12
SAT	T2DM	SMC	37	0.9895	0.9895	2.22E-16
SAT	T2DM	adipocyte	96	0.9894	0.9894	2.22E-16
SAT	T2DM	b_cell	9	0.9938	0.9938	6.14E-08
SAT	T2DM	dendritic_cell	15	0.9919	0.9919	4.90E-13
SAT	T2DM	endothelial	20	0.9952	0.9952	2.22E-16
SAT	T2DM	macrophage	59	0.9888	0.9888	2.22E-16
SAT	T2DM	mast_cell	28	0.9944	0.9944	2.22E-16
SAT	T2DM	monocyte	13	0.952	0.952	5.42E-07
SAT	T2DM	neutrophil	8	0.976	0.976	3.39E-05
SAT	T2DM	nk_cell	16	0.994	0.994	7.39E-15
SAT	T2DM	pericyte	27	0.9885	0.9885	2.22E-16
SAT	T2DM	t_cell	23	0.9929	0.9929	2.22E-16
VAT	CAD	ASPC	87	0.8262	0.8262	2.22E-16
VAT	CAD	LEC	19	0.466	0.466	4.43E-02
VAT	CAD	SMC	16	0.5521	0.5521	2.66E-02
VAT	CAD	adipocyte	48	0.7621	0.7621	3.13E-10
VAT	CAD	b_cell	7	0.8302	0.8302	2.08E-02
VAT	CAD	dendritic_cell	11	0.6169	0.6169	4.32E-02
VAT	CAD	endometrium	8	0.5074	0.5074	1.99E-01
VAT	CAD	endothelial	14	0.6685	0.6685	8.96E-03
VAT	CAD	macrophage	33	0.6615	0.6615	2.77E-05
VAT	CAD	mast_cell	13	0.781	0.781	1.62E-03
VAT	CAD	mesothelium	95	0.7183	0.7183	2.53E-16
VAT	CAD	monocyte	9	0.9026	0.9026	8.62E-04
VAT	CAD	nk_cell	9	0.7611	0.7611	1.72E-02
VAT	CAD	pericyte	25	0.5745	0.5745	2.67E-03
VAT	CAD	t_cell	17	0.8372	0.8372	2.77E-05
VAT	HTN	ASPC	162	0.7735	0.7735	2.22E-16
VAT	HTN	LEC	39	0.6629	0.6629	4.26E-06
VAT	HTN	SMC	33	0.7317	0.7317	1.31E-06
VAT	HTN	adipocyte	112	0.7897	0.7897	2.22E-16
VAT	HTN	b_cell	6	0.6481	0.6481	1.64E-01
VAT	HTN	dendritic_cell	21	0.6078	0.6078	3.47E-03
VAT	HTN	endometrium	10	0.8509	0.8509	1.80E-03
VAT	HTN	endothelial	22	0.718	0.718	1.68E-04
VAT	HTN	macrophage	63	0.7628	0.7628	3.72E-13
VAT	HTN	mast_cell	20	0.337	0.337	1.46E-01

VAT	HTN	mesothelium	162	0.807	0.807	2.22E-16
VAT	HTN	monocyte	12	0.7519	0.7519	4.80E-03
VAT	HTN	nk_cell	8	0.8134	0.8134	1.41E-02
VAT	HTN	pericyte	49	0.8119	0.8119	1.46E-12
VAT	HTN	t_cell	50	0.7195	0.7195	3.93E-09
VAT	Heart_Failure	ASPC	80	0.9075	0.9075	2.22E-16
VAT	Heart_Failure	LEC	16	0.9123	0.9123	8.46E-07
VAT	Heart_Failure	SMC	14	0.783	0.783	9.27E-04
VAT	Heart_Failure	adipocyte	56	0.881	0.881	2.22E-16
VAT	Heart_Failure	b_cell	2			
VAT	Heart_Failure	dendritic_cell	11	0.9198	0.9198	6.10E-05
VAT	Heart_Failure	endometrium	6	0.9595	0.9595	2.43E-03
VAT	Heart_Failure	endothelial	8	0.9156	0.9156	1.41E-03
VAT	Heart_Failure	macrophage	27	0.9531	0.9531	1.74E-14
VAT	Heart_Failure	mast_cell	5	0.7437	0.7437	1.50E-01
VAT	Heart_Failure	mesothelium	83	0.8744	0.8744	2.22E-16
VAT	Heart_Failure	monocyte	1			
VAT	Heart_Failure	nk_cell	6	0.3407	0.3407	5.09E-01
VAT	Heart_Failure	pericyte	13	0.9593	0.9593	2.22E-07
VAT	Heart_Failure	t_cell	17	0.7505	0.7505	5.19E-04
VAT	Obesity	ASPC	10	-0.3362	0.3362	3.42E-01
VAT	Obesity	LEC	3	-0.068	0.068	9.57E-01
VAT	Obesity	SMC	2			
VAT	Obesity	adipocyte	5	-0.7689	0.7689	1.29E-01
VAT	Obesity	b_cell	0			
VAT	Obesity	dendritic_cell	2			
VAT	Obesity	endometrium	1			
VAT	Obesity	endothelial	1			
VAT	Obesity	macrophage	3	-0.4896	0.4896	6.74E-01
VAT	Obesity	mast_cell	2			
VAT	Obesity	mesothelium	7	-0.5555	0.5555	1.95E-01
VAT	Obesity	monocyte	3	0.1508	0.1508	9.04E-01
VAT	Obesity	nk_cell	0			
VAT	Obesity	pericyte	2			
VAT	Obesity	t_cell	0			
VAT	Stroke	ASPC	64	0.9106	0.9106	2.22E-16
VAT	Stroke	LEC	15	0.8846	0.8846	1.19E-05
VAT	Stroke	SMC	9	0.9173	0.9173	4.94E-04
VAT	Stroke	adipocyte	40	0.9379	0.9379	2.22E-16
VAT	Stroke	b_cell	1			
VAT	Stroke	dendritic_cell	6	0.982	0.982	4.83E-04
VAT	Stroke	endometrium	3	0.9987	0.9987	3.25E-02
VAT	Stroke	endothelial	7	0.9018	0.9018	5.50E-03
VAT	Stroke	macrophage	17	0.9286	0.9286	7.53E-08
VAT	Stroke	mast_cell	6	0.9796	0.9796	6.20E-04
VAT	Stroke	mesothelium	67	0.9288	0.9288	2.22E-16
VAT	Stroke	monocyte	4	0.988	0.988	1.20E-02
VAT	Stroke	nk_cell	8	0.9593	0.9593	1.63E-04
VAT	Stroke	pericyte	13	0.9512	0.9512	5.92E-07
VAT	Stroke	t_cell	12	0.9369	0.9369	7.08E-06
VAT	T2DM	ASPC	65	0.7368	0.7368	2.62E-12
VAT	T2DM	LEC	14	0.3734	0.3734	1.88E-01
VAT	T2DM	SMC	15	0.1961	0.1961	4.84E-01
VAT	T2DM	adipocyte	49	0.6278	0.6278	1.38E-06
VAT	T2DM	b_cell	3	0.9592	0.9592	1.82E-01
VAT	T2DM	dendritic_cell	12	0.4579	0.4579	1.34E-01

VAT	T2DM	endometrium	6	0.8097	0.8097	5.09E-02
VAT	T2DM	endothelial	14	0.5743	0.5743	3.17E-02
VAT	T2DM	macrophage	31	0.6546	0.6546	6.47E-05
VAT	T2DM	mast_cell	11	0.438	0.438	1.78E-01
VAT	T2DM	mesothelium	74	0.7436	0.7436	3.23E-14
VAT	T2DM	monocyte	6	0.5793	0.5793	2.28E-01
VAT	T2DM	nk_cell	6	0.554	0.554	2.54E-01
VAT	T2DM	pericyte	19	0.4221	0.4221	7.18E-02
VAT	T2DM	t_cell	17	0.7365	0.7365	7.47E-04
Frontal Cortex	Anxiety	Astros	11	0.9929	0.9929	1.24E-09
Frontal Cortex	Anxiety	Ex	51	0.9967	0.9967	2.22E-16
Frontal Cortex	Anxiety	Inhib	44	0.9969	0.9969	2.22E-16
Frontal Cortex	Anxiety	Micro_Macro	3	0.9903	0.9903	8.87E-02
Frontal Cortex	Anxiety	OPCs	8	0.9734	0.9734	4.61E-05
Frontal Cortex	Anxiety	Oligos	17	0.9959	0.9959	2.22E-16
Frontal Cortex	Bipolar	Astros	5	0.9991	0.9991	3.24E-05
Frontal Cortex	Bipolar	Ex	58	0.9995	0.9995	2.22E-16
Frontal Cortex	Bipolar	Inhib	40	0.9996	0.9996	2.22E-16
Frontal Cortex	Bipolar	Micro_Macro	4	1	1	5.00E-16
Frontal Cortex	Bipolar	OPCs	8	0.9992	0.9992	1.28E-09
Frontal Cortex	Bipolar	Oligos	12	0.989	0.989	1.25E-09
Frontal Cortex	Depression	Astros	9	0.9811	0.9811	3.00E-06
Frontal Cortex	Depression	Ex	62	0.9742	0.9742	2.22E-16
Frontal Cortex	Depression	Inhib	54	0.98	0.98	2.22E-16
Frontal Cortex	Depression	Micro_Macro	6	0.9965	0.9965	1.84E-05
Frontal Cortex	Depression	OPCs	12	0.9696	0.9696	1.94E-07
Frontal Cortex	Depression	Oligos	16	0.9864	0.9864	2.23E-12
Frontal Cortex	Psychosis	Astros	3	0.9956	0.9956	5.97E-02
Frontal Cortex	Psychosis	Ex	46	0.9986	0.9986	2.22E-16
Frontal Cortex	Psychosis	Inhib	53	0.9984	0.9984	2.22E-16
Frontal Cortex	Psychosis	Micro_Macro	3	0.9999	0.9999	9.00E-03
Frontal Cortex	Psychosis	OPCs	5	0.9958	0.9958	3.27E-04
Frontal Cortex	Psychosis	Oligos	17	0.9986	0.9986	2.22E-16
Pancreas	Prediabetes	PP_cell	8	0.9982	0.9982	1.46E-08
Pancreas	Prediabetes	acinar_cell	14	0.9975	0.9975	3.51E-15
Pancreas	Prediabetes	alpha_cell	26	0.9994	0.9994	2.22E-16
Pancreas	Prediabetes	beta_cell	62	0.9986	0.9986	2.22E-16
Pancreas	Prediabetes	delta_cell	10	0.998	0.998	6.98E-11
Pancreas	Prediabetes	duct_epithelial_cell	12	0.999	0.999	7.86E-15
Pancreas	Prediabetes	endothelial_cell	13	0.9991	0.9991	2.22E-16
Pancreas	Prediabetes	epsilon_cell	4	0.9999	0.9999	1.00E-04
Pancreas	Prediabetes	leukocyte	5	1	1	2.22E-16
Pancreas	Prediabetes	mesenchymal_cell	9	0.9945	0.9945	4.04E-08
Pancreas	T1DM	PP_cell	4	0.9997	0.9997	3.00E-04
Pancreas	T1DM	acinar_cell	20	0.9996	0.9996	2.22E-16
Pancreas	T1DM	alpha_cell	44	0.9993	0.9993	2.22E-16
Pancreas	T1DM	beta_cell	103	0.9989	0.9989	2.22E-16
Pancreas	T1DM	delta_cell	9	0.9999	0.9999	3.29E-14
Pancreas	T1DM	duct_epithelial_cell	10	0.9998	0.9998	7.00E-15
Pancreas	T1DM	endothelial_cell	7	0.9998	0.9998	1.09E-09
Pancreas	T1DM	epsilon_cell	3	0.9999	0.9999	9.00E-03
Pancreas	T1DM	leukocyte	3	0.9999	0.9999	9.00E-03
Pancreas	T1DM	mesenchymal_cell	7	0.9999	0.9999	1.92E-10
Pancreas	T2DM	PP_cell	15	0.8176	0.8176	1.97E-04
Pancreas	T2DM	acinar_cell	38	0.991	0.991	2.22E-16
Pancreas	T2DM	alpha_cell	75	0.9905	0.9905	2.22E-16

Pancreas	T2DM	beta_cell	150	0.9879	0.9879	2.22E-16
Pancreas	T2DM	delta_cell	17	0.9901	0.9901	3.31E-14
Pancreas	T2DM	duct_epithelial_cell	28	0.9637	0.9637	2.22E-16
Pancreas	T2DM	endothelial_cell	23	0.9832	0.9832	2.22E-16
Pancreas	T2DM	epsilon_cell	5	0.998	0.998	1.07E-04
Pancreas	T2DM	leukocyte	10	0.9951	0.9951	2.51E-09
Pancreas	T2DM	mesenchymal_cell	21	0.9922	0.9922	2.22E-16
Pancreas	T2DM_admission	PP_cell	15	0.9722	0.9722	1.41E-09
Pancreas	T2DM_admission	acinar_cell	46	0.993	0.993	2.22E-16
Pancreas	T2DM_admission	alpha_cell	78	0.9916	0.9916	2.22E-16
Pancreas	T2DM_admission	beta_cell	166	0.9895	0.9895	2.22E-16
Pancreas	T2DM_admission	delta_cell	21	0.989	0.989	2.22E-16
Pancreas	T2DM_admission	duct_epithelial_cell	33	0.9745	0.9745	2.22E-16
Pancreas	T2DM_admission	endothelial_cell	25	0.9878	0.9878	2.22E-16
Pancreas	T2DM_admission	epsilon_cell	6	0.9947	0.9947	4.21E-05
Pancreas	T2DM_admission	leukocyte	11	0.9918	0.9918	2.37E-09
Pancreas	T2DM_admission	mesenchymal_cell	24	0.9881	0.9881	2.22E-16
Pancreas	T2DM_insulin	PP_cell	11	0.9946	0.9946	3.63E-10
Pancreas	T2DM_insulin	acinar_cell	32	0.9977	0.9977	2.22E-16
Pancreas	T2DM_insulin	alpha_cell	51	0.9949	0.9949	2.22E-16
Pancreas	T2DM_insulin	beta_cell	100	0.9958	0.9958	2.22E-16
Pancreas	T2DM_insulin	delta_cell	11	0.9958	0.9958	1.17E-10
Pancreas	T2DM_insulin	duct_epithelial_cell	22	0.9718	0.9718	5.11E-14
Pancreas	T2DM_insulin	endothelial_cell	20	0.9884	0.9884	3.46E-16
Pancreas	T2DM_insulin	epsilon_cell	5	0.9973	0.9973	1.68E-04
Pancreas	T2DM_insulin	leukocyte	7	0.9903	0.9903	1.77E-05
Pancreas	T2DM_insulin	mesenchymal_cell	15	0.9954	0.9954	1.25E-14

Note: 2.22E-16 indicates the p value <2.22E-16, null indicates there are no identified common genes for the correlation analysis□

d. Comparision of causal inference results identified from full and European-only analyses

Tissue	Trait	Celltype	N_genes	Pearson_r	Abs_Pearson_r	P_vaule
SAT	CAD	ASPC	163	0.9118	0.9118	2.2E-16
SAT	CAD	LEC	9	0.7811	0.7811	1.3E-02
SAT	CAD	SMC	32	0.8824	0.8824	2.4E-11
SAT	CAD	adipocyte	76	0.925	0.925	2.2E-16
SAT	CAD	b_cell	5	0.8554	0.8554	6.5E-02
SAT	CAD	dendritic_cell	9	0.7635	0.7635	1.7E-02
SAT	CAD	endothelial	8	0.8702	0.8702	4.9E-03
SAT	CAD	macrophage	55	0.907	0.907	2.2E-16
SAT	CAD	mast_cell	13	0.9261	0.9261	5.5E-06
SAT	CAD	monocyte	9	0.6181	0.6181	7.6E-02
SAT	CAD	neutrophil	6	0.6221	0.6221	1.9E-01
SAT	CAD	nk_cell	12	0.767	0.767	3.6E-03
SAT	CAD	pericyte	27	0.7746	0.7746	2.1E-06
SAT	CAD	t_cell	20	0.681	0.681	9.5E-04
SAT	HTN	ASPC	306	0.9701	0.9701	2.2E-16
SAT	HTN	LEC	20	0.9806	0.9806	3.4E-14
SAT	HTN	SMC	67	0.9658	0.9658	2.2E-16
SAT	HTN	adipocyte	127	0.9717	0.9717	2.2E-16
SAT	HTN	b_cell	11	0.7811	0.7811	4.5E-03
SAT	HTN	dendritic_cell	28	0.9684	0.9684	2.2E-16
SAT	HTN	endothelial	31	0.952	0.952	2.2E-16
SAT	HTN	macrophage	109	0.9532	0.9532	2.2E-16
SAT	HTN	mast_cell	35	0.9634	0.9634	2.2E-16
SAT	HTN	monocyte	18	0.9781	0.9781	2.5E-12
SAT	HTN	neutrophil	9	0.8596	0.8596	3.0E-03
SAT	HTN	nk_cell	42	0.932	0.932	2.2E-16
SAT	HTN	pericyte	57	0.9157	0.9157	2.2E-16
SAT	HTN	t_cell	46	0.9435	0.9435	2.2E-16
SAT	Heart_Failure	ASPC	129	0.8935	0.8935	2.2E-16
SAT	Heart_Failure	LEC	9	0.8256	0.8256	6.1E-03
SAT	Heart_Failure	SMC	30	0.9317	0.9317	7.7E-14
SAT	Heart_Failure	adipocyte	61	0.8932	0.8932	2.2E-16
SAT	Heart_Failure	b_cell	4	0.9279	0.9279	7.2E-02
SAT	Heart_Failure	dendritic_cell	18	0.9599	0.9599	3.0E-10
SAT	Heart_Failure	endothelial	14	0.9118	0.9118	5.6E-06
SAT	Heart_Failure	macrophage	45	0.932	0.932	2.2E-16
SAT	Heart_Failure	mast_cell	16	0.9395	0.9395	6.8E-08
SAT	Heart_Failure	monocyte	8	0.7283	0.7283	4.0E-02
SAT	Heart_Failure	neutrophil	3	0.9963	0.9963	5.5E-02
SAT	Heart_Failure	nk_cell	9	0.9542	0.9542	6.5E-05
SAT	Heart_Failure	pericyte	22	0.9263	0.9263	6.3E-10
SAT	Heart_Failure	t_cell	15	0.8316	0.8316	1.2E-04
SAT	Obesity	ASPC	279	0.9663	0.9663	2.2E-16
SAT	Obesity	LEC	15	0.9839	0.9839	4.2E-11
SAT	Obesity	SMC	45	0.9798	0.9798	2.2E-16
SAT	Obesity	adipocyte	108	0.9503	0.9503	2.2E-16
SAT	Obesity	b_cell	14	0.9842	0.9842	2.2E-10
SAT	Obesity	dendritic_cell	20	0.9803	0.9803	4.0E-14
SAT	Obesity	endothelial	20	0.9703	0.9703	1.5E-12
SAT	Obesity	macrophage	77	0.9588	0.9588	2.2E-16
SAT	Obesity	mast_cell	26	0.9807	0.9807	2.2E-16
SAT	Obesity	monocyte	14	0.9734	0.9734	4.8E-09
SAT	Obesity	neutrophil	10	0.9963	0.9963	8.2E-10

SAT	Obesity	nk_cell	25	0.9879	0.9879	2.2E-16
SAT	Obesity	pericyte	37	0.9794	0.9794	2.2E-16
SAT	Obesity	t_cell	31	0.968	0.968	2.2E-16
SAT	Stroke	ASPC	94	0.8895	0.8895	2.2E-16
SAT	Stroke	LEC	5	0.9912	0.9912	9.9E-04
SAT	Stroke	SMC	8	0.1773	0.1773	6.7E-01
SAT	Stroke	adipocyte	25	0.7109	0.7109	6.8E-05
SAT	Stroke	b_cell	4	0.8773	0.8773	1.2E-01
SAT	Stroke	dendritic_cell	6	0.5177	0.5177	2.9E-01
SAT	Stroke	endothelial	4	0.882	0.882	1.2E-01
SAT	Stroke	macrophage	19	0.5321	0.5321	1.9E-02
SAT	Stroke	mast_cell	2			
SAT	Stroke	monocyte	4	0.9192	0.9192	8.1E-02
SAT	Stroke	neutrophil	0			
SAT	Stroke	nk_cell	7	0.434	0.434	3.3E-01
SAT	Stroke	pericyte	6	0.5978	0.5978	2.1E-01
SAT	Stroke	t_cell	9	0.8977	0.8977	1.0E-03
SAT	T2DM	ASPC	186	0.9524	0.9524	2.2E-16
SAT	T2DM	LEC	12	0.9675	0.9675	2.7E-07
SAT	T2DM	SMC	32	0.9642	0.9642	2.2E-16
SAT	T2DM	adipocyte	85	0.9541	0.9541	2.2E-16
SAT	T2DM	b_cell	11	0.893	0.893	2.1E-04
SAT	T2DM	dendritic_cell	18	0.9252	0.9252	3.9E-08
SAT	T2DM	endothelial	17	0.9806	0.9806	5.0E-12
SAT	T2DM	macrophage	50	0.9618	0.9618	2.2E-16
SAT	T2DM	mast_cell	24	0.9741	0.9741	1.1E-15
SAT	T2DM	monocyte	12	0.6346	0.6346	2.7E-02
SAT	T2DM	neutrophil	8	0.7214	0.7214	4.3E-02
SAT	T2DM	nk_cell	16	0.9536	0.9536	1.1E-08
SAT	T2DM	pericyte	24	0.9435	0.9435	5.0E-12
SAT	T2DM	t_cell	21	0.9718	0.9718	2.2E-13
VAT	CAD	ASPC	94	0.863	0.863	2.2E-16
VAT	CAD	LEC	20	0.8558	0.8558	1.5E-06
VAT	CAD	SMC	18	0.8434	0.8434	1.1E-05
VAT	CAD	adipocyte	53	0.8732	0.8732	2.2E-16
VAT	CAD	b_cell	7	0.6615	0.6615	1.1E-01
VAT	CAD	dendritic_cell	15	0.856	0.856	4.7E-05
VAT	CAD	endometrium	9	0.7151	0.7151	3.0E-02
VAT	CAD	endothelial	18	0.7498	0.7498	3.4E-04
VAT	CAD	macrophage	37	0.8473	0.8473	3.8E-11
VAT	CAD	mast_cell	10	0.4884	0.4884	1.5E-01
VAT	CAD	mesothelium	97	0.849	0.849	2.2E-16
VAT	CAD	monocyte	7	0.5752	0.5752	1.8E-01
VAT	CAD	nk_cell	10	0.6519	0.6519	4.1E-02
VAT	CAD	pericyte	25	0.8981	0.8981	1.1E-09
VAT	CAD	t_cell	18	0.7088	0.7088	9.9E-04
VAT	HTN	ASPC	190	0.9711	0.9711	2.2E-16
VAT	HTN	LEC	53	0.9703	0.9703	2.2E-16
VAT	HTN	SMC	46	0.9706	0.9706	2.2E-16
VAT	HTN	adipocyte	136	0.9824	0.9824	2.2E-16
VAT	HTN	b_cell	13	0.9657	0.9657	8.8E-08
VAT	HTN	dendritic_cell	27	0.9554	0.9554	9.4E-15
VAT	HTN	endometrium	16	0.9505	0.9505	1.7E-08
VAT	HTN	endothelial	37	0.9666	0.9666	2.2E-16
VAT	HTN	macrophage	80	0.9717	0.9717	2.2E-16

VAT	HTN	mast_cell	29	0.9677	0.9677	2.2E-16
VAT	HTN	mesothelium	191	0.9795	0.9795	2.2E-16
VAT	HTN	monocyte	21	0.9256	0.9256	1.9E-09
VAT	HTN	nk_cell	15	0.9401	0.9401	1.9E-07
VAT	HTN	pericyte	64	0.971	0.971	2.2E-16
VAT	HTN	t_cell	66	0.9688	0.9688	2.2E-16
VAT	Heart_Failure	ASPC	80	0.8735	0.8735	2.2E-16
VAT	Heart_Failure	LEC	14	0.8606	0.8606	7.8E-05
VAT	Heart_Failure	SMC	14	0.9003	0.9003	1.1E-05
VAT	Heart_Failure	adipocyte	58	0.8842	0.8842	2.2E-16
VAT	Heart_Failure	b_cell	3	0.9952	0.9952	6.2E-02
VAT	Heart_Failure	dendritic_cell	10	0.9511	0.9511	2.4E-05
VAT	Heart_Failure	endometrium	7	0.8906	0.8906	7.2E-03
VAT	Heart_Failure	endothelial	9	0.9584	0.9584	4.6E-05
VAT	Heart_Failure	macrophage	30	0.9238	0.9238	3.4E-13
VAT	Heart_Failure	mast_cell	6	0.9484	0.9484	3.9E-03
VAT	Heart_Failure	mesothelium	84	0.8565	0.8565	2.2E-16
VAT	Heart_Failure	monocyte	2			
VAT	Heart_Failure	nk_cell	8	0.9625	0.9625	1.3E-04
VAT	Heart_Failure	pericyte	15	0.9225	0.9225	9.8E-07
VAT	Heart_Failure	t_cell	16	0.9314	0.9314	1.6E-07
VAT	Obesity	ASPC	161	0.9705	0.9705	2.2E-16
VAT	Obesity	LEC	44	0.9612	0.9612	2.2E-16
VAT	Obesity	SMC	32	0.95	0.95	2.2E-16
VAT	Obesity	adipocyte	109	0.9763	0.9763	2.2E-16
VAT	Obesity	b_cell	10	0.9932	0.9932	9.3E-09
VAT	Obesity	dendritic_cell	20	0.9874	0.9874	7.3E-16
VAT	Obesity	endometrium	16	0.9584	0.9584	5.2E-09
VAT	Obesity	endothelial	24	0.9176	0.9176	2.8E-10
VAT	Obesity	macrophage	62	0.9791	0.9791	2.2E-16
VAT	Obesity	mast_cell	24	0.9899	0.9899	2.2E-16
VAT	Obesity	mesothelium	163	0.9689	0.9689	2.2E-16
VAT	Obesity	monocyte	15	0.9736	0.9736	1.0E-09
VAT	Obesity	nk_cell	12	0.9943	0.9943	4.7E-11
VAT	Obesity	pericyte	36	0.9413	0.9413	2.2E-16
VAT	Obesity	t_cell	32	0.944	0.944	5.5E-16
VAT	Stroke	ASPC	56	0.7524	0.7524	2.3E-11
VAT	Stroke	LEC	13	0.5785	0.5785	3.8E-02
VAT	Stroke	SMC	10	0.3315	0.3315	3.5E-01
VAT	Stroke	adipocyte	38	0.7819	0.7819	6.8E-09
VAT	Stroke	b_cell	1			
VAT	Stroke	dendritic_cell	5	0.5409	0.5409	3.5E-01
VAT	Stroke	endometrium	2			
VAT	Stroke	endothelial	5	0.3882	0.3882	5.2E-01
VAT	Stroke	macrophage	12	0.8608	0.8608	3.2E-04
VAT	Stroke	mast_cell	6	0.8924	0.8924	1.7E-02
VAT	Stroke	mesothelium	60	0.826	0.826	4.5E-16
VAT	Stroke	monocyte	4	0.9369	0.9369	6.3E-02
VAT	Stroke	nk_cell	6	0.7905	0.7905	6.1E-02
VAT	Stroke	pericyte	15	0.9014	0.9014	4.5E-06
VAT	Stroke	t_cell	8	0.8376	0.8376	9.4E-03
VAT	T2DM	ASPC	85	0.9328	0.9328	2.2E-16
VAT	T2DM	LEC	28	0.8803	0.8803	6.6E-10
VAT	T2DM	SMC	25	0.8237	0.8237	4.2E-07
VAT	T2DM	adipocyte	66	0.9135	0.9135	2.2E-16

VAT	T2DM	b_cell	7	0.9742	0.9742	2.0E-04
VAT	T2DM	dendritic_cell	18	0.848	0.848	8.8E-06
VAT	T2DM	endometrium	8	0.9266	0.9266	9.3E-04
VAT	T2DM	endothelial	17	0.9362	0.9362	3.3E-08
VAT	T2DM	macrophage	44	0.9222	0.9222	2.2E-16
VAT	T2DM	mast_cell	17	0.919	0.919	1.9E-07
VAT	T2DM	mesothelium	98	0.9292	0.9292	2.2E-16
VAT	T2DM	monocyte	10	0.9307	0.9307	9.3E-05
VAT	T2DM	nk_cell	11	0.9227	0.9227	5.2E-05
VAT	T2DM	pericyte	33	0.8975	0.8975	1.5E-12
VAT	T2DM	t_cell	29	0.9507	0.9507	3.0E-15
Frontal Cortex	Anxiety	Astros	12	0.1507	0.1507	6.4E-01
Frontal Cortex	Anxiety	Ex	47	0.8462	0.8462	6.9E-14
Frontal Cortex	Anxiety	Inhib	36	0.8152	0.8152	1.4E-09
Frontal Cortex	Anxiety	Micro_Macro	3	0.9886	0.9886	9.6E-02
Frontal Cortex	Anxiety	OPCs	10	0.9035	0.9035	3.4E-04
Frontal Cortex	Anxiety	Oligos	15	0.0492	0.0492	8.6E-01
Frontal Cortex	Bipolar	Astros	5	0.8451	0.8451	7.1E-02
Frontal Cortex	Bipolar	Ex	43	0.81	0.81	4.7E-11
Frontal Cortex	Bipolar	Inhib	34	0.69	0.69	6.3E-06
Frontal Cortex	Bipolar	Micro_Macro	3	0.8509	0.8509	3.5E-01
Frontal Cortex	Bipolar	OPCs	7	0.8749	0.8749	9.9E-03
Frontal Cortex	Bipolar	Oligos	9	0.7794	0.7794	1.3E-02
Frontal Cortex	Depression	Astros	10	0.8233	0.8233	3.4E-03
Frontal Cortex	Depression	Ex	62	0.8272	0.8272	2.2E-16
Frontal Cortex	Depression	Inhib	49	0.8444	0.8444	2.5E-14
Frontal Cortex	Depression	Micro_Macro	6	0.9672	0.9672	1.6E-03
Frontal Cortex	Depression	OPCs	11	0.9013	0.9013	1.5E-04
Frontal Cortex	Depression	Oligos	17	0.9092	0.9092	4.3E-07
Frontal Cortex	Psychosis	Astros	2			
Frontal Cortex	Psychosis	Ex	33	0.6848	0.6848	1.1E-05
Frontal Cortex	Psychosis	Inhib	31	0.6729	0.6729	3.4E-05
Frontal Cortex	Psychosis	Micro_Macro	2			
Frontal Cortex	Psychosis	OPCs	3	0.8566	0.8566	3.5E-01
Frontal Cortex	Psychosis	Oligos	10	0.842	0.842	2.2E-03
Pancreas	Prediabetes	PP_cell	5	0.7791	0.7791	1.2E-01
Pancreas	Prediabetes	acinar_cell	12	0.24	0.24	4.5E-01
Pancreas	Prediabetes	alpha_cell	20	0.7162	0.7162	3.8E-04
Pancreas	Prediabetes	beta_cell	37	0.6265	0.6265	3.4E-05
Pancreas	Prediabetes	delta_cell	6	0.5959	0.5959	2.1E-01
Pancreas	Prediabetes	duct_epithelial_cell	7	0.8741	0.8741	1.0E-02
Pancreas	Prediabetes	endothelial_cell	9	0.8444	0.8444	4.2E-03
Pancreas	Prediabetes	epsilon_cell	4	0.9863	0.9863	1.4E-02
Pancreas	Prediabetes	leukocyte	4	0.9221	0.9221	7.8E-02
Pancreas	Prediabetes	mesenchymal_cell	9	0.4285	0.4285	2.5E-01
Pancreas	T1DM	PP_cell	6	0.9795	0.9795	6.3E-04
Pancreas	T1DM	acinar_cell	16	0.9915	0.9915	8.4E-14
Pancreas	T1DM	alpha_cell	35	0.9894	0.9894	2.2E-16
Pancreas	T1DM	beta_cell	72	0.9785	0.9785	2.2E-16
Pancreas	T1DM	delta_cell	10	0.9845	0.9845	2.5E-07
Pancreas	T1DM	duct_epithelial_cell	9	0.9961	0.9961	1.2E-08
Pancreas	T1DM	endothelial_cell	8	0.9946	0.9946	3.9E-07
Pancreas	T1DM	epsilon_cell	5	0.8734	0.8734	5.3E-02
Pancreas	T1DM	leukocyte	5	0.9902	0.9902	1.2E-03
Pancreas	T1DM	mesenchymal_cell	8	0.7096	0.7096	4.9E-02

Pancreas	T2DM	PP_cell	13	0.552	0.552	5.0E-02
Pancreas	T2DM	acinar_cell	40	0.9536	0.9536	2.2E-16
Pancreas	T2DM	alpha_cell	67	0.9535	0.9535	2.2E-16
Pancreas	T2DM	beta_cell	139	0.946	0.946	2.2E-16
Pancreas	T2DM	delta_cell	13	0.9501	0.9501	6.7E-07
Pancreas	T2DM	duct_epithelial_cell	25	0.9038	0.9038	6.0E-10
Pancreas	T2DM	endothelial_cell	19	0.9408	0.9408	2.1E-09
Pancreas	T2DM	epsilon_cell	5	0.9866	0.9866	1.9E-03
Pancreas	T2DM	leukocyte	8	0.9471	0.9471	3.6E-04
Pancreas	T2DM	mesenchymal_cell	21	0.9513	0.9513	3.7E-11
Pancreas	T2DM_admission	PP_cell	11	0.9162	0.9162	7.4E-05
Pancreas	T2DM_admission	acinar_cell	43	0.9817	0.9817	2.2E-16
Pancreas	T2DM_admission	alpha_cell	76	0.9776	0.9776	2.2E-16
Pancreas	T2DM_admission	beta_cell	158	0.9653	0.9653	2.2E-16
Pancreas	T2DM_admission	delta_cell	18	0.9778	0.9778	2.8E-12
Pancreas	T2DM_admission	duct_epithelial_cell	28	0.9505	0.9505	1.0E-14
Pancreas	T2DM_admission	endothelial_cell	23	0.969	0.969	3.1E-14
Pancreas	T2DM_admission	epsilon_cell	6	0.9931	0.9931	7.1E-05
Pancreas	T2DM_admission	leukocyte	9	0.9795	0.9795	4.0E-06
Pancreas	T2DM_admission	mesenchymal_cell	23	0.975	0.975	3.4E-15
Pancreas	T2DM_insulin	PP_cell	8	0.7965	0.7965	1.8E-02
Pancreas	T2DM_insulin	acinar_cell	22	0.8608	0.8608	2.7E-07
Pancreas	T2DM_insulin	alpha_cell	36	0.8787	0.8787	1.8E-12
Pancreas	T2DM_insulin	beta_cell	71	0.8633	0.8633	2.2E-16
Pancreas	T2DM_insulin	delta_cell	9	0.9712	0.9712	1.3E-05
Pancreas	T2DM_insulin	duct_epithelial_cell	17	0.472	0.472	5.6E-02
Pancreas	T2DM_insulin	endothelial_cell	16	0.8218	0.8218	9.4E-05
Pancreas	T2DM_insulin	epsilon_cell	5	0.5682	0.5682	3.2E-01
Pancreas	T2DM_insulin	leukocyte	5	0.7139	0.7139	1.8E-01
Pancreas	T2DM_insulin	mesenchymal_cell	11	0.9224	0.9224	5.3E-05

Note: 2.2E-16 indicates the p value <2.2E-16, null indicates there are no identified common genes for the correlation analysis
