# Supplementary material for: CausalCellInfer: Prioritizing Cell‐Type‐Specific Disease Associations From Genetically Predicted Transcriptomes in Biobank Cohorts
Source: Adv Sci (Weinh). 2026 Oct 14:e78197. Online ahead of print. doi: 10.1002/advs.78197 (PMC13669230; doi:10.1002/advs.78197)
Supplement: Supplementary file 2 [file ADVS-9999-e78197-s002.zip › advs78197-sup-0002-S1.pdf]

Table S1 Performance benchmark analysis across full and UKBB specific gene panels

Gene Panel	Methods	Cortex			Adipose SAT			Adipose VAT			Pancreas		
		CCC	RMSE	MAE	CCC	RMSE	MAE	CCC	RMSE	MAE	CCC	RMSE	MAE
	Scaden	0.8890	0.0932	0.0704	0.8127	0.0606	0.0353	0.7829	0.0556	0.0331	0.9346	0.0426	0.0279
	CausalCellInfer (scI-GCM)	0.9834	0.0309	**0.0236**	**0.8921**	**0.0397**	**0.0234**	**0.8982**	**0.0378**	**0.0218**	0.9530	**0.0382**	**0.0256**
	CausalCellInfer (PC-simple)	**0.9847**	**0.0302**	0.0237	0.8874	0.0414	0.0245	0.8764	0.0411	0.0227	**0.9624**	0.0385	0.0264
Full	MuSiC	0.9651	0.0460	0.0342	0.8695	0.0490	0.0278	0.8722	0.0428	0.0247	0.9567	0.0385	0.0271
	NNLS	0.6784	0.1749	0.1417	0.8168	0.0596	0.0376	0.7833	0.0591	0.0367	0.8204	0.0870	0.0578
	DWLS	0.9603	0.0482	0.0368	0.8777	0.0479	0.0284	0.8576	0.0433	0.0252	0.9396	0.0463	0.0298
	DISSECT	0.6708	0.1512	0.1110	0.7474	0.0721	0.0415	0.7333	0.0618	0.0340	0.8256	0.0801	0.0514
	Scaden	0.8736	0.0964	0.0743	0.8228	0.0578	0.0347	0.7650	0.0555	0.0331	0.9268	0.0448	0.0296
	CausalCellInfer (scI-GCM)	0.9738	0.0403	0.0305	0.8875	**0.0434**	**0.0261**	**0.8463**	**0.0437**	**0.0257**	**0.9578**	0.0429	**0.0291**
	CausalCellInfer (PC-simple)	**0.9767**	**0.0381**	**0.0289**	**0.8934**	0.0437	0.0261	0.8468	0.0450	0.0259	0.9547	**0.0422**	0.0291
UKBB	MuSiC	0.9586	0.0508	0.0371	0.8639	0.0506	0.0300	0.8418	0.0485	0.0279	0.8925	0.0664	0.0476
	NNLS	0.9560	0.0536	0.0415	0.7857	0.0665	0.0429	0.7937	0.0587	0.0376	0.7410	0.1105	0.0781
	DWLS	0.9350	0.0633	0.0499	0.8563	0.0541	0.0337	0.7983	0.0520	0.0306	0.8832	0.0633	0.0423
	DISSECT	0.3333	0.1775	0.1287	0.7398	0.0706	0.0389	0.6904	0.0656	0.0357	0.6529	0.1128	0.0736
