# Supplementary material for: CausalCellInfer: Prioritizing Cell‐Type‐Specific Disease Associations From Genetically Predicted Transcriptomes in Biobank Cohorts
Source: Adv Sci (Weinh). 2026 Oct 14:e78197. Online ahead of print. doi: 10.1002/advs.78197 (PMC13669230; doi:10.1002/advs.78197)
Supplement: Supplementary file 2 [file ADVS-9999-e78197-s002.zip › advs78197-sup-0003-S2.pdf]

Table S2 Comparison of differentially expressed genes identified

Tissue	Cell Type	PPV	scRef Control Count	scRef Condition Count	Overall count
Pancreas	Acinar Cell	0.96	25437	6193	31630
	Alpha Cell	0.831	27877	13541	41418
	Beta Cell	0.941	21866	6234	28100
	Delta Cell	0.624	2387	706	3093
	Duct Epithelial Cell	0.701	6148	3530	9678
	Endothelial Cell	0.473	2064	893	2957
	Epsilon Cell	0.036	68	19	87
	Leukocyte	0.081	493	258	751
	Mesenchymal Cell	0.627	3881	1887	5768
	PP cell	0.163	814	287	1101
Frontal cortex	Astrocytes	0.103	172	459	631
	Excitatory neurons	0.701	10336	16368	26704
	Inhibitory neurons	0.47	3582	5893	9475
	Microglia/Macrophage	0.055	248	428	676
	Oligodendrocytes	0.183	1887	1574	3461
	Oligodendrocyte progenitor cells	0.191	819	942	1761
SAT	Adipocyte	0.906	6242	6194	12436
	Adipose stem and progenitor cells (ASPC)	0.797	5314	9417	14731
	B cell	0.074	55	227	282
	Dendritic Cell	0.229	156	287	443
	Endothelial Cell	0.707	2457	4740	7197
	Lymphatic endothelial cells (LEC)	0.198	105	430	535
	Macrophage	0.88	2857	4409	7266
	Mast Cell	0.129	183	572	755
	Monocyte	0.13	168	629	797
	Neutrophil	0.052	52	26	78
	NK cell	0.279	351	855	1206
	T cell	0.214	606	1766	2372
	Smooth muscle cells (SMC)	0.371	398	850	1248
	Pericyte	0.204	232	300	532
VAT	Adipocyte	0.705	4425	4943	9368
	Adipose stem and progenitor cells (ASPC)	0.621	4553	6914	11467
	B cell	0.024	25	42	67
	Dendritic Cell	0.102	116	300	416
	Endometrium	0.093	43	262	305
	Endothelial Cell	0.509	2081	2983	5064
	Lymphatic endothelial cells (LEC)	0.248	577	1077	1654
	Macrophage	0.478	1597	5731	7328
	Mast Cell	0.053	81	127	208
	Mesothelium	0.723	5030	19658	24688
	Monocyte	0.071	90	187	277
	NK cell	0.097	219	276	495
	Pericyte	0.138	326	281	607
	T cell	0.264	686	1835	2521
	Smooth muscle cells (SMC)	0.121	388	692	1080

Note: SAT is the abbreviation for subcutaneous adipose tissue, VAT is the abbreviation for visceral adipose tissue
