# Supplementary material for: CausalCellInfer: Prioritizing Cell‐Type‐Specific Disease Associations From Genetically Predicted Transcriptomes in Biobank Cohorts
Source: Adv Sci (Weinh). 2026 Oct 14:e78197. Online ahead of print. doi: 10.1002/advs.78197 (PMC13669230; doi:10.1002/advs.78197)
Supplement: Supplementary file 2 [file ADVS-9999-e78197-s002.zip › advs78197-sup-0004-S3.pdf]

Table S3 Estimated associations between estimated cell proportions and disease onset

Disease	celltype	beta	pvalue	qvalue	mean proportion	median proportion	SD of proportions
Depression	Astrocyte	0.349344479	0.157297493	0.188756991	0.01296	0.01285	0.00136
Depression	Excitory neuron	0.035768245	0.018036671	0.027055006	0.42306	0.42396	0.02215
Depression	Inhibitory neuron	0.077088977	0.000498927	0.001496782	0.31986	0.31962	0.01514
Depression	Microglia/Macrophage	-0.510795951	0.003812823	0.007625646	0.0095	0.00918	0.00189
Depression	Oligodendrocyte precursor	0.056779997	0.27037295	0.27037295	0.05012	0.04972	0.00651
Depression	Oligodendrocyte	-0.085276671	1.45E-07	8.69E-07	0.18451	0.18284	0.02063
T2DM	Acinar cell	0.355020726	0.001615201	0.003553442	0.04317	0.04298	0.00301
T2DM	Duct epithelial cell	0.373331282	3.81E-06	1.40E-05	0.06984	0.06963	0.00419
T2DM	Endothelial cell	0.095142994	0.336647671	0.431430617	0.05138	0.05134	0.00342
T2DM	Leukocyte	-0.06040816	0.837904613	0.913066206	0.01026	0.0102	0.00115
T2DM	Mesenchymal cell	0.656294611	4.58E-08	5.04E-07	0.03702	0.03694	0.00282
T2DM	Alpha cell	0.002653652	0.913066206	0.913066206	0.26851	0.26842	0.01394
T2DM	Delta cell	0.188143407	0.000636075	0.001749206	0.05051	0.04981	0.00615
T2DM	PP cell	0.215849341	0.07091862	0.111443546	0.02054	0.02008	0.00284
T2DM	Epsilon cell	-0.881452643	0.352988686	0.431430617	0.00398	0.00395	0.00036
T2DM	Beta cell	-0.110780929	7.76E-07	4.27E-06	0.44479	0.44561	0.01511
T2DM	Alpha/beta ratio	0.017953392	0.014327101	0.026266352	*	*	*
SAT BMI	Adipose stem and precursor cell	1.260269017	0.076820149	0.097771099	0.37681	0.37758	0.00965
SAT BMI	Lymphatic endothelial cell	1.07717506	0.44254914	0.476591381	0.03356	0.03263	0.0049
SAT BMI	Smooth muscle cell	9.57666729	0.000186335	0.000372671	0.04363	0.04335	0.00268
SAT BMI	Adipocyte	0.599559889	0.622823291	0.622823291	0.15886	0.15869	0.00564
SAT BMI	B cell	-29.08081915	2.46E-02	3.45E-02	0.00599	0.00599	0.00053
SAT BMI	Dendritic cell	-7.40076279	0.107269022	0.125147193	0.01866	0.01857	0.0015
SAT BMI	Endothelial	-1.510014365	4.12E-04	7.20E-04	0.09079	0.09173	0.01608
SAT BMI	Macrophage	4.013504669	4.05526E-05	9.46227E-05	0.13716	0.13693	0.00703
SAT BMI	Mast cell	16.66351679	1.73983E-05	4.87153E-05	0.02086	0.02059	0.00177
SAT BMI	Monocyte	12.1057829	1.76E-03	2.73E-03	0.01757	0.01739	0.00178
SAT BMI	Neutrophil	56.30990704	1.94992E-15	1.36494E-14	0.00743	0.00735	0.00097
SAT BMI	NK cell	-20.94766299	3.28034E-32	4.59247E-31	0.03707	0.03789	0.00388
SAT BMI	Pericyte	18.7857525	1.94857E-06	6.82001E-06	0.02986	0.02971	0.00174
SAT BMI	T cell	25.81402129	1.41858E-13	6.62004E-13	0.02175	0.02163	0.00197
VAT BMI	Adipose stem and precursor cell	-1.062876878	1.42E-01	1.93E-01	0.2685	0.26851	0.0095
VAT BMI	Lymphatic endothelial cell	-2.255397112	0.462572815	0.533737863	0.03539	0.03513	0.00224
VAT BMI	Smooth muscle cell	45.16195	4.57948E-10	2.28974E-09	0.01668	0.01654	0.00095
VAT BMI	Adipocyte	4.153365712	0.003342695	0.007162919	0.1293	0.12912	0.00486
VAT BMI	B cell	-0.925319782	0.931587331	0.931587331	0.00612	0.00609	0.00064
VAT BMI	Dendritic cell	46.03050829	1.06245E-09	3.98418E-09	0.01488	0.0148	0.00091
VAT BMI	Endometrium	9.167961312	4.16E-01	5.20E-01	0.00831	0.00825	0.00061
VAT BMI	Endothelial	-1.605687331	0.041210599	0.068684332	0.06182	0.06296	0.00874
VAT BMI	Macrophage	0.33648321	8.40E-01	9.00E-01	0.07979	0.07955	0.00412
VAT BMI	Mast cell	37.32441676	7.21365E-05	0.000180341	0.01267	0.01258	0.00073
VAT BMI	Mesothelium	4.289093879	5.17983E-09	1.55395E-08	0.29028	0.29011	0.00936
VAT BMI	Monocyte	-8.539173391	1.25E-01	1.88E-01	0.01415	0.01409	0.00123
VAT BMI	NK cell	-27.67223717	4.56164E-36	6.84246E-35	0.02567	0.02677	0.00311
VAT BMI	Pericyte	28.95595643	0.011895107	0.022303326	0.01521	0.01518	6.00E-04
VAT BMI	T cell	-50.07611457	2.72392E-20	2.04294E-19	0.02122	0.02115	0.00127

Note:qvalue indicates the qvaule after multiple testing correction by Benjamini-Hochberg method;Astros is the abbreviation for Astrocytes, Ex is the abbreviation for Excitatory neuron, Inhib is the abbreviation for Inhibitory neuron, MicroMacro is the abbreviation for Microglia/Macrophage, OPCs is the abbreviation for Oligodendrocyte precursor; ASPC is the abbreviation for Adipose stem and precursor cell, LEC is the abbreviation for Lymphatic endothelial cell, SMC is the abbreviation for Smooth muscle cell.* indicates cells that are not applicable for current estimation
