# Supplementary material for: CausalCellInfer: Prioritizing Cell‐Type‐Specific Disease Associations From Genetically Predicted Transcriptomes in Biobank Cohorts
Source: Adv Sci (Weinh). 2026 Oct 14:e78197. Online ahead of print. doi: 10.1002/advs.78197 (PMC13669230; doi:10.1002/advs.78197)
Supplement: Supplementary file 2 [file ADVS-9999-e78197-s002.zip › advs78197-sup-0005-S4.pdf]

Table S4 Cell type interaction effects on disorder status

a. Cell type interaction effects on depression in the frontal cortex

Tissue	Trait	Cell type interaction	Pvalue
Frontal cortex	Depression	Ex:Inhib	0.9755645
Frontal cortex	Depression	Ex:Micro_Macro	0.70178907
Frontal cortex	Depression	Ex:OPCs	0.68071621
Frontal cortex	Depression	Ex:Oligos	**0.01111706**
Frontal cortex	Depression	Inhib:Micro_Macro	0.58126025
Frontal cortex	Depression	Inhib:OPCs	0.35885833
Frontal cortex	Depression	Inhib:Oligos	***0.0805781***
Frontal cortex	Depression	Micro_Macro:OPCs	0.67716352
Frontal cortex	Depression	Micro_Macro:Oligos	0.81555145
Frontal cortex	Depression	OPCs:Oligos	0.71161884

Note: Astros is the abbreviation for Astrocytes; MicroMacro is the abbreviation for Microglia/Macrophage; OPCs is the abbreviation for Oligodendrocyte precursor

b. Cell type interaction effects on T2DM in the pancreas□

Tissue	Trait	Cell type interaction	Pvalue
Pancreas	T2DM	duct_epithelial_cell:endothelial_cell	***0.05030018***
Pancreas	T2DM	duct_epithelial_cell:leukocyte	***0.05670365***
Pancreas	T2DM	duct_epithelial_cell:mesenchymal_cell	**0.00322339**
Pancreas	T2DM	duct_epithelial_cell:alpha_cell	0.9123302
Pancreas	T2DM	duct_epithelial_cell:delta_cell	3.32E-01
Pancreas	T2DM	duct_epithelial_cell:PP_cell	0.7080235
Pancreas	T2DM	duct_epithelial_cell:epsilon_cell	0.34485085
Pancreas	T2DM	duct_epithelial_cell:beta_cell	0.87473075
Pancreas	T2DM	endothelial_cell:leukocyte	0.57353405
Pancreas	T2DM	endothelial_cell:mesenchymal_cell	0.16214993
Pancreas	T2DM	endothelial_cell:alpha_cell	0.35015599
Pancreas	T2DM	endothelial_cell:delta_cell	0.73737204
Pancreas	T2DM	endothelial_cell:PP_cell	1.62E-01
Pancreas	T2DM	endothelial_cell:epsilon_cell	2.33E-01
Pancreas	T2DM	endothelial_cell:beta_cell	0.21542553
Pancreas	T2DM	leukocyte:mesenchymal_cell	0.22532
Pancreas	T2DM	leukocyte:alpha_cell	***0.0785611***
Pancreas	T2DM	leukocyte:delta_cell	0.67711279
Pancreas	T2DM	leukocyte:PP_cell	0.27246319
Pancreas	T2DM	leukocyte:epsilon_cell	0.19485244
Pancreas	T2DM	leukocyte:beta_cell	**0.00318546**
Pancreas	T2DM	mesenchymal_cell:alpha_cell	**3.06E-02**
Pancreas	T2DM	mesenchymal_cell:delta_cell	0.82653135
Pancreas	T2DM	mesenchymal_cell:PP_cell	**0.00565235**
Pancreas	T2DM	mesenchymal_cell:epsilon_cell	0.11614666
Pancreas	T2DM	mesenchymal_cell:beta_cell	***0.08513141***
Pancreas	T2DM	alpha_cell:delta_cell	0.51248043
Pancreas	T2DM	alpha_cell:PP_cell	**0.00327598**
Pancreas	T2DM	alpha_cell:epsilon_cell	0.49339086
Pancreas	T2DM	alpha_cell:beta_cell	0.43256464
Pancreas	T2DM	delta_cell:PP_cell	0.80508856
Pancreas	T2DM	delta_cell:epsilon_cell	7.83E-01
Pancreas	T2DM	delta_cell:beta_cell	***0.05781763***
Pancreas	T2DM	PP_cell:epsilon_cell	0.59060423
Pancreas	T2DM	PP_cell:beta_cell	0.10990806
Pancreas	T2DM	epsilon_cell:beta_cell	9.57E-01

c. Cell type interaction effects on obesity in the subcutaneous adipose tissue

Tissue	Trait	Cell type interaction	Pvalue
SAT	Obesity	ASPC:LEC	**4.73E-03**
SAT	Obesity	ASPC:SMC	0.24506652
SAT	Obesity	ASPC:adipocyte	0.57539881
SAT	Obesity	ASPC:b_cell	0.13532347
SAT	Obesity	ASPC:dendritic_cell	0.23602351
SAT	Obesity	ASPC:macrophage	0.85546771
SAT	Obesity	ASPC:mast_cell	**1.78E-03**
SAT	Obesity	ASPC:monocyte	0.6860553
SAT	Obesity	ASPC:neutrophil	9.40E-01
SAT	Obesity	ASPC:nk_cell	**0.00031683**
SAT	Obesity	ASPC:pericyte	**1.43E-03**
SAT	Obesity	ASPC:t_cell	**4.13E-07**
SAT	Obesity	LEC:SMC	**0.01814628**
SAT	Obesity	LEC:adipocyte	**1.88E-02**
SAT	Obesity	LEC:b_cell	**0.00025249**
SAT	Obesity	LEC:dendritic_cell	**0.00176223**
SAT	Obesity	LEC:macrophage	3.52E-01
SAT	Obesity	LEC:mast_cell	**0.00974052**
SAT	Obesity	LEC:monocyte	0.24483908
SAT	Obesity	LEC:neutrophil	4.79E-01
SAT	Obesity	LEC:nk_cell	**9.25E-06**
SAT	Obesity	LEC:pericyte	**4.09E-02**
SAT	Obesity	LEC:t_cell	**4.59E-03**
SAT	Obesity	SMC:adipocyte	**4.13E-03**
SAT	Obesity	SMC:b_cell	0.24747253
SAT	Obesity	SMC:dendritic_cell	2.67E-01
SAT	Obesity	SMC:macrophage	**0.00921008**
SAT	Obesity	SMC:mast_cell	**2.89E-02**
SAT	Obesity	SMC:monocyte	0.55301892
SAT	Obesity	SMC:neutrophil	**2.40E-02**
SAT	Obesity	SMC:nk_cell	**4.25E-05**
SAT	Obesity	SMC:pericyte	**1.31E-06**
SAT	Obesity	SMC:t_cell	**0.00346203**
SAT	Obesity	adipocyte:b_cell	0.10736334
SAT	Obesity	adipocyte:dendritic_cell	0.80851702
SAT	Obesity	adipocyte:macrophage	1.71E-01
SAT	Obesity	adipocyte:mast_cell	**0.00424343**

SAT	Obesity	adipocyte:monocyte	4.25E-01
SAT	Obesity	adipocyte:neutrophil	**6.03E-02**
SAT	Obesity	adipocyte:nk_cell	0.31759755
SAT	Obesity	adipocyte:pericyte	9.39E-01
SAT	Obesity	adipocyte:t_cell	**0.00689157**
SAT	Obesity	b_cell:dendritic_cell	0.95177954
SAT	Obesity	b_cell:macrophage	4.51E-01
SAT	Obesity	b_cell:mast_cell	**4.21E-03**
SAT	Obesity	b_cell:monocyte	0.23358375
SAT	Obesity	b_cell:neutrophil	**0.03839472**
SAT	Obesity	b_cell:nk_cell	**0.01046318**
SAT	Obesity	b_cell:pericyte	**7.65E-03**
SAT	Obesity	b_cell:t_cell	**1.97E-04**
SAT	Obesity	dendritic_cell:macrophage	***5.17E-02***
SAT	Obesity	dendritic_cell:mast_cell	3.09E-01
SAT	Obesity	dendritic_cell:monocyte	0.59036409
SAT	Obesity	dendritic_cell:neutrophil	**0.00238595**
SAT	Obesity	dendritic_cell:nk_cell	0.57412387
SAT	Obesity	dendritic_cell:pericyte	***0.06021844***
SAT	Obesity	dendritic_cell:t_cell	***8.19E-02***
SAT	Obesity	macrophage:mast_cell	0.13139713
SAT	Obesity	macrophage:monocyte	0.30064477
SAT	Obesity	macrophage:neutrophil	***0.08357006***
SAT	Obesity	macrophage:nk_cell	**0.00424697**
SAT	Obesity	macrophage:pericyte	**1.24E-02**
SAT	Obesity	macrophage:t_cell	**2.48E-04**
SAT	Obesity	mast_cell:monocyte	0.4251265
SAT	Obesity	mast_cell:neutrophil	4.81E-01
SAT	Obesity	mast_cell:nk_cell	**0.00131752**
SAT	Obesity	mast_cell:pericyte	**0.02824009**
SAT	Obesity	mast_cell:t_cell	***9.94E-02***

SAT	Obesity	monocyte:neutrophil	0.44130355
SAT	Obesity	monocyte:nk_cell	0.46298214
SAT	Obesity	monocyte:pericyte	1.19E-01
SAT	Obesity	monocyte:t_cell	0.46989198
SAT	Obesity	neutrophil:nk_cell	**7.85E-07**
SAT	Obesity	neutrophil:pericyte	**1.37E-03**
SAT	Obesity	neutrophil:t_cell	0.36684085
SAT	Obesity	nk_cell:pericyte	**0.02456507**
SAT	Obesity	nk_cell:t_cell	***0.07740871***
SAT	Obesity	pericyte:t_cell	**0.02233631**

d. Cell type interaction effects on obesity in the visceral adipose tissue

Tissue	Trait	Cell type interaction	Pvalue
VAT	Obesity	ASPC:LEC	7.64E-01
VAT	Obesity	ASPC:SMC	***6.44E-02***
VAT	Obesity	ASPC:adipocyte	**7.95E-03**
VAT	Obesity	ASPC:b_cell	**1.36E-02**
VAT	Obesity	ASPC:dendritic_cell	6.01E-01
VAT	Obesity	ASPC:endometrium	**5.41E-03**
VAT	Obesity	ASPC:macrophage	2.57E-01
VAT	Obesity	ASPC:mast_cell	2.88E-01
VAT	Obesity	ASPC:mesothelium	**3.07E-02**
VAT	Obesity	ASPC:monocyte	8.68E-01
VAT	Obesity	ASPC:nk_cell	8.36E-01
VAT	Obesity	ASPC:pericyte	4.18E-01
VAT	Obesity	ASPC:t_cell	5.92E-01
VAT	Obesity	LEC:SMC	2.99E-01
VAT	Obesity	LEC:adipocyte	**9.66E-03**
VAT	Obesity	LEC:b_cell	***7.48E-02***
VAT	Obesity	LEC:dendritic_cell	***8.02E-02***
VAT	Obesity	LEC:endometrium	**5.57E-08**
VAT	Obesity	LEC:macrophage	**4.76E-02**
VAT	Obesity	LEC:mast_cell	9.68E-01
VAT	Obesity	LEC:mesothelium	**5.68E-03**
VAT	Obesity	LEC:monocyte	9.46E-01
VAT	Obesity	LEC:nk_cell	**3.75E-03**
VAT	Obesity	LEC:pericyte	**1.67E-04**
VAT	Obesity	LEC:t_cell	3.47E-01
VAT	Obesity	SMC:adipocyte	2.42E-01
VAT	Obesity	SMC:b_cell	1.13E-01
VAT	Obesity	SMC:dendritic_cell	6.17E-01
VAT	Obesity	SMC:endometrium	**1.03E-04**
VAT	Obesity	SMC:macrophage	5.82E-01
VAT	Obesity	SMC:mast_cell	1.49E-01
VAT	Obesity	SMC:mesothelium	5.21E-01
VAT	Obesity	SMC:monocyte	***7.26E-02***
VAT	Obesity	SMC:nk_cell	**1.28E-04**
VAT	Obesity	SMC:pericyte	**3.45E-05**
VAT	Obesity	SMC:t_cell	3.92E-01
VAT	Obesity	adipocyte:b_cell	1.24E-01
VAT	Obesity	adipocyte:dendritic_cell	2.50E-01
VAT	Obesity	adipocyte:endometrium	**2.78E-09**

VAT	Obesity	adipocyte:macrophage	2.09E-01
VAT	Obesity	adipocyte:mast_cell	2.81E-01
VAT	Obesity	adipocyte:mesothelium	**2.23E-02**
VAT	Obesity	adipocyte:monocyte	6.39E-01
VAT	Obesity	adipocyte:nk_cell	**3.19E-05**
VAT	Obesity	adipocyte:pericyte	**2.73E-05**
VAT	Obesity	adipocyte:t_cell	***6.71E-02***
VAT	Obesity	b_cell:dendritic_cell	1.21E-01
VAT	Obesity	b_cell:endometrium	**2.91E-08**
VAT	Obesity	b_cell:macrophage	2.86E-01
VAT	Obesity	b_cell:mast_cell	2.14E-01
VAT	Obesity	b_cell:mesothelium	**1.70E-04**
VAT	Obesity	b_cell:monocyte	***7.49E-02***
VAT	Obesity	b_cell:nk_cell	**1.46E-06**
VAT	Obesity	b_cell:pericyte	**1.22E-07**
VAT	Obesity	b_cell:t_cell	1.37E-01
VAT	Obesity	dendritic_cell:endometrium	**1.53E-02**
VAT	Obesity	dendritic_cell:macrophage	**4.10E-02**
VAT	Obesity	dendritic_cell:mast_cell	4.94E-01
VAT	Obesity	dendritic_cell:mesothelium	**7.23E-03**
VAT	Obesity	dendritic_cell:monocyte	6.98E-01
VAT	Obesity	dendritic_cell:nk_cell	***8.91E-02***
VAT	Obesity	dendritic_cell:pericyte	**1.41E-03**
VAT	Obesity	dendritic_cell:t_cell	3.51E-01
VAT	Obesity	endometrium:macrophage	**5.64E-04**
VAT	Obesity	endometrium:mast_cell	4.12E-01
VAT	Obesity	endometrium:mesothelium	2.78E-01
VAT	Obesity	endometrium:monocyte	4.62E-01
VAT	Obesity	endometrium:nk_cell	**2.82E-07**
VAT	Obesity	endometrium:pericyte	***7.23E-02***
VAT	Obesity	endometrium:t_cell	***5.21E-02***
VAT	Obesity	macrophage:mast_cell	1.78E-01
VAT	Obesity	macrophage:mesothelium	8.85E-01
VAT	Obesity	macrophage:monocyte	1.63E-01
VAT	Obesity	macrophage:nk_cell	9.19E-01
VAT	Obesity	macrophage:pericyte	**1.90E-02**
VAT	Obesity	macrophage:t_cell	2.10E-01
VAT	Obesity	mast_cell:mesothelium	1.03E-01
VAT	Obesity	mast_cell:monocyte	**2.42E-02**
VAT	Obesity	mast_cell:nk_cell	5.62E-01
VAT	Obesity	mast_cell:pericyte	3.16E-01

VAT	Obesity	mast_cell:t_cell	1.29E-01
VAT	Obesity	mesothelium:monocyte	4.62E-01
VAT	Obesity	mesothelium:nk_cell	**1.27E-04**
VAT	Obesity	mesothelium:pericyte	5.85E-01
VAT	Obesity	mesothelium:t_cell	6.89E-01
VAT	Obesity	monocyte:nk_cell	3.69E-01
VAT	Obesity	monocyte:pericyte	3.07E-01
VAT	Obesity	monocyte:t_cell	5.20E-01
VAT	Obesity	nk_cell:pericyte	**9.47E-04**
VAT	Obesity	nk_cell:t_cell	8.20E-01
VAT	Obesity	pericyte:t_cell	2.24E-01

e. Cross tissue cell type interactions□

Trait	First tissue	Second tissue	Variable	Pvalue
Depression	Frontal cortex	Pancreas	Ex	0.77972662
Depression	Frontal cortex	Pancreas	Inhib	0.82393658
Depression	Frontal cortex	Pancreas	MicroMacro	0.91598358
Depression	Frontal cortex	Pancreas	OPCs	0.73195791
Depression	Frontal cortex	Pancreas	Oligos	0.74543721
Depression	Frontal cortex	Pancreas	duct_epithelial_cell	0.78472255
Depression	Frontal cortex	Pancreas	endothelial_cell	0.63059751
Depression	Frontal cortex	Pancreas	leukocyte	0.68635775
Depression	Frontal cortex	Pancreas	mesenchymal_cell	0.48448636
Depression	Frontal cortex	Pancreas	alpha_cell	0.94239018
Depression	Frontal cortex	Pancreas	delta_cell	0.90215208
Depression	Frontal cortex	Pancreas	PP_cell	0.26641881
Depression	Frontal cortex	Pancreas	epsilon_cell	0.49130186
Depression	Frontal cortex	Pancreas	beta_cell	0.68523411
Depression	Frontal cortex	Pancreas	Ex:Inhib	0.99103426
Depression	Frontal cortex	Pancreas	Ex:MicroMacro	0.71797195
Depression	Frontal cortex	Pancreas	Ex:OPCs	0.62986962
Depression	Frontal cortex	Pancreas	Ex:Oligos	**0.02913141**
Depression	Frontal cortex	Pancreas	Ex:duct_epithelial_cell	0.70081449
Depression	Frontal cortex	Pancreas	Ex:endothelial_cell	0.71122987
Depression	Frontal cortex	Pancreas	Ex:leukocyte	0.68047182
Depression	Frontal cortex	Pancreas	Ex:mesenchymal_cell	0.4396112
Depression	Frontal cortex	Pancreas	Ex:alpha_cell	0.91598342
Depression	Frontal cortex	Pancreas	Ex:delta_cell	0.81748885
Depression	Frontal cortex	Pancreas	Ex:PP_cell	0.29209388
Depression	Frontal cortex	Pancreas	Ex:epsilon_cell	0.67799809
Depression	Frontal cortex	Pancreas	Ex:beta_cell	0.66573396
Depression	Frontal cortex	Pancreas	Inhib:MicroMacro	0.58517127
Depression	Frontal cortex	Pancreas	Inhib:OPCs	0.31725008
Depression	Frontal cortex	Pancreas	Inhib:Oligos	0.18159341
Depression	Frontal cortex	Pancreas	Inhib:duct_epithelial_cell	0.72814371
Depression	Frontal cortex	Pancreas	Inhib:endothelial_cell	0.63183261
Depression	Frontal cortex	Pancreas	Inhib:leukocyte	0.67643079
Depression	Frontal cortex	Pancreas	Inhib:mesenchymal_cell	0.48385566
Depression	Frontal cortex	Pancreas	Inhib:alpha_cell	0.97475649
Depression	Frontal cortex	Pancreas	Inhib:delta_cell	0.87002002
Depression	Frontal cortex	Pancreas	Inhib:PP_cell	0.27700243
Depression	Frontal cortex	Pancreas	Inhib:epsilon_cell	0.54713128
Depression	Frontal cortex	Pancreas	Inhib:beta_cell	0.70346486
Depression	Frontal cortex	Pancreas	MicroMacro:OPCs	0.69879793
Depression	Frontal cortex	Pancreas	MicroMacro:Oligos	0.76581727
Depression	Frontal cortex	Pancreas	MicroMacro:duct_epithelial_cell	0.52647261
Depression	Frontal cortex	Pancreas	MicroMacro:endothelial_cell	0.80108911

Depression	Frontal cortex	Pancreas	MicroMacro:leukocyte	0.82036589
Depression	Frontal cortex	Pancreas	MicroMacro:mesenchymal_cell	0.21652239
Depression	Frontal cortex	Pancreas	MicroMacro:alpha_cell	0.92825213
Depression	Frontal cortex	Pancreas	MicroMacro:delta_cell	0.61997013
Depression	Frontal cortex	Pancreas	MicroMacro:PP_cell	0.28528554
Depression	Frontal cortex	Pancreas	MicroMacro:epsilon_cell	0.72819515
Depression	Frontal cortex	Pancreas	MicroMacro:beta_cell	0.95994792
Depression	Frontal cortex	Pancreas	OPCs:Oligos	0.59002932
Depression	Frontal cortex	Pancreas	OPCs:duct_epithelial_cell	0.59200821
Depression	Frontal cortex	Pancreas	OPCs:endothelial_cell	0.75927277
Depression	Frontal cortex	Pancreas	OPCs:leukocyte	0.72876039
Depression	Frontal cortex	Pancreas	OPCs:mesenchymal_cell	0.4803858
Depression	Frontal cortex	Pancreas	OPCs:alpha_cell	0.89033379
Depression	Frontal cortex	Pancreas	OPCs:delta_cell	0.78524398
Depression	Frontal cortex	Pancreas	OPCs:PP_cell	0.25818782
Depression	Frontal cortex	Pancreas	OPCs:epsilon_cell	0.73411885
Depression	Frontal cortex	Pancreas	OPCs:beta_cell	0.65957138
Depression	Frontal cortex	Pancreas	Oligos:duct_epithelial_cell	0.65570306
Depression	Frontal cortex	Pancreas	Oligos:endothelial_cell	0.70541023
Depression	Frontal cortex	Pancreas	Oligos:leukocyte	0.63301816
Depression	Frontal cortex	Pancreas	Oligos:mesenchymal_cell	0.42129957
Depression	Frontal cortex	Pancreas	Oligos:alpha_cell	0.88629613
Depression	Frontal cortex	Pancreas	Oligos:delta_cell	0.7679387
Depression	Frontal cortex	Pancreas	Oligos:PP_cell	0.28486914
Depression	Frontal cortex	Pancreas	Oligos:epsilon_cell	0.63662798
Depression	Frontal cortex	Pancreas	Oligos:beta_cell	0.62069494
Depression	Frontal cortex	Pancreas	duct_epithelial_cell:endothelial_c	0.36678455
Depression	Frontal cortex	Pancreas	duct_epithelial_cell:leukocyte	0.42735462
Depression	Frontal cortex	Pancreas	duct_epithelial_cell:mesenchymal	0.50826743
Depression	Frontal cortex	Pancreas	duct_epithelial_cell:alpha_cell	0.48260383
Depression	Frontal cortex	Pancreas	duct_epithelial_cell:delta_cell	0.38773058
Depression	Frontal cortex	Pancreas	duct_epithelial_cell:PP_cell	0.94296843
Depression	Frontal cortex	Pancreas	duct_epithelial_cell:epsilon_cell	0.50261319
Depression	Frontal cortex	Pancreas	duct_epithelial_cell:beta_cell	0.38625726
Depression	Frontal cortex	Pancreas	endothelial_cell:leukocyte	0.9634569
Depression	Frontal cortex	Pancreas	endothelial_cell:mesenchymal_ce	0.54449106
Depression	Frontal cortex	Pancreas	endothelial_cell:alpha_cell	0.80651466
Depression	Frontal cortex	Pancreas	endothelial_cell:delta_cell	0.74597747
Depression	Frontal cortex	Pancreas	endothelial_cell:PP_cell	0.520598
Depression	Frontal cortex	Pancreas	endothelial_cell:epsilon_cell	0.76118736
Depression	Frontal cortex	Pancreas	endothelial_cell:beta_cell	0.62273765
Depression	Frontal cortex	Pancreas	leukocyte:mesenchymal_cell	0.82565192
Depression	Frontal cortex	Pancreas	leukocyte:alpha_cell	0.79572581
Depression	Frontal cortex	Pancreas	leukocyte:delta_cell	0.51225656
Depression	Frontal cortex	Pancreas	leukocyte:PP_cell	0.43518179
Depression	Frontal cortex	Pancreas	leukocyte:epsilon_cell	***0.05438721***

Depression	Frontal cortex	Pancreas	leukocyte:beta_cell	0.88285545
Depression	Frontal cortex	Pancreas	mesenchymal_cell:alpha_cell	0.90412403
Depression	Frontal cortex	Pancreas	mesenchymal_cell:delta_cell	0.45813856
Depression	Frontal cortex	Pancreas	mesenchymal_cell:PP_cell	0.9602634
Depression	Frontal cortex	Pancreas	mesenchymal_cell:epsilon_cell	0.27522734
Depression	Frontal cortex	Pancreas	mesenchymal_cell:beta_cell	0.59006751
Depression	Frontal cortex	Pancreas	alpha_cell:delta_cell	0.23329265
Depression	Frontal cortex	Pancreas	alpha_cell:PP_cell	0.7843447
Depression	Frontal cortex	Pancreas	alpha_cell:epsilon_cell	0.65385421
Depression	Frontal cortex	Pancreas	alpha_cell:beta_cell	0.48237566
Depression	Frontal cortex	Pancreas	delta_cell:PP_cell	0.33832434
Depression	Frontal cortex	Pancreas	delta_cell:epsilon_cell	0.82189991
Depression	Frontal cortex	Pancreas	delta_cell:beta_cell	0.17709732
Depression	Frontal cortex	Pancreas	PP_cell:epsilon_cell	0.31407615
Depression	Frontal cortex	Pancreas	PP_cell:beta_cell	0.71154434
Depression	Frontal cortex	Pancreas	epsilon_cell:beta_cell	0.49666533
Depression	Frontal cortex	SAT	Ex	0.71514166
Depression	Frontal cortex	SAT	Inhib	0.75717705
Depression	Frontal cortex	SAT	MicroMacro	0.91749027
Depression	Frontal cortex	SAT	OPCs	0.61880318
Depression	Frontal cortex	SAT	Oligos	0.80515319
Depression	Frontal cortex	SAT	ASPC	0.5390469
Depression	Frontal cortex	SAT	LEC	0.79653786
Depression	Frontal cortex	SAT	SMC	***0.0507791***
Depression	Frontal cortex	SAT	adipocyte	0.8053499
Depression	Frontal cortex	SAT	b_cell	0.94401259
Depression	Frontal cortex	SAT	dendritic_cell	0.81799245
Depression	Frontal cortex	SAT	macrophage	0.77838479
Depression	Frontal cortex	SAT	mast_cell	0.94801254
Depression	Frontal cortex	SAT	monocyte	0.51914066
Depression	Frontal cortex	SAT	neutrophil	0.89712814
Depression	Frontal cortex	SAT	nk_cell	0.79409881
Depression	Frontal cortex	SAT	pericyte	0.75915093
Depression	Frontal cortex	SAT	t_cell	0.3570095
Depression	Frontal cortex	SAT	Ex:Inhib	0.86751388
Depression	Frontal cortex	SAT	Ex:MicroMacro	0.96455162
Depression	Frontal cortex	SAT	Ex:OPCs	0.67442311
Depression	Frontal cortex	SAT	Ex:Oligos	0.47123777
Depression	Frontal cortex	SAT	Ex:ASPC	0.52898904
Depression	Frontal cortex	SAT	Ex:LEC	0.81946707
Depression	Frontal cortex	SAT	Ex:SMC	0.0488227
Depression	Frontal cortex	SAT	Ex:adipocyte	0.80417435
Depression	Frontal cortex	SAT	Ex:b_cell	0.85079094
Depression	Frontal cortex	SAT	Ex:dendritic_cell	0.9524783
Depression	Frontal cortex	SAT	Ex:macrophage	0.8040401
Depression	Frontal cortex	SAT	Ex:mast_cell	0.9675978

Depression	Frontal cortex	SAT	Ex:monocyte	0.56823908
Depression	Frontal cortex	SAT	Ex:neutrophil	0.73325973
Depression	Frontal cortex	SAT	Ex:nk_cell	0.62354932
Depression	Frontal cortex	SAT	Ex:pericyte	0.58921938
Depression	Frontal cortex	SAT	Ex:t_cell	0.27017588
Depression	Frontal cortex	SAT	Inhib:MicroMacro	0.89685474
Depression	Frontal cortex	SAT	Inhib:OPCs	0.34001228
Depression	Frontal cortex	SAT	Inhib:Oligos	0.37677095
Depression	Frontal cortex	SAT	Inhib:ASPC	0.58296609
Depression	Frontal cortex	SAT	Inhib:LEC	0.79647992
Depression	Frontal cortex	SAT	Inhib:SMC	0.05046435
Depression	Frontal cortex	SAT	Inhib:adipocyte	0.67412999
Depression	Frontal cortex	SAT	Inhib:b_cell	0.85700621
Depression	Frontal cortex	SAT	Inhib:dendritic_cell	0.94825064
Depression	Frontal cortex	SAT	Inhib:macrophage	0.74060609
Depression	Frontal cortex	SAT	Inhib:mast_cell	0.94265592
Depression	Frontal cortex	SAT	Inhib:monocyte	0.49095357
Depression	Frontal cortex	SAT	Inhib:neutrophil	0.7110502
Depression	Frontal cortex	SAT	Inhib:nk_cell	0.73966163
Depression	Frontal cortex	SAT	Inhib:pericyte	0.52410218
Depression	Frontal cortex	SAT	Inhib:t_cell	0.28994241
Depression	Frontal cortex	SAT	MicroMacro:OPCs	0.56951091
Depression	Frontal cortex	SAT	MicroMacro:Oligos	0.9264086
Depression	Frontal cortex	SAT	MicroMacro:ASPC	0.76231768
Depression	Frontal cortex	SAT	MicroMacro:LEC	0.68016308
Depression	Frontal cortex	SAT	MicroMacro:SMC	0.33462081
Depression	Frontal cortex	SAT	MicroMacro:adipocyte	0.56271663
Depression	Frontal cortex	SAT	MicroMacro:b_cell	0.5087087
Depression	Frontal cortex	SAT	MicroMacro:dendritic_cell	0.80362074
Depression	Frontal cortex	SAT	MicroMacro:macrophage	0.69061333
Depression	Frontal cortex	SAT	MicroMacro:mast_cell	0.66844033
Depression	Frontal cortex	SAT	MicroMacro:monocyte	0.85617912
Depression	Frontal cortex	SAT	MicroMacro:neutrophil	0.84167034
Depression	Frontal cortex	SAT	MicroMacro:nk_cell	0.8551138
Depression	Frontal cortex	SAT	MicroMacro:pericyte	0.23862515
Depression	Frontal cortex	SAT	MicroMacro:t_cell	0.0928086
Depression	Frontal cortex	SAT	OPCs:Oligos	0.53173169
Depression	Frontal cortex	SAT	OPCs:ASPC	0.54779196
Depression	Frontal cortex	SAT	OPCs:LEC	0.80011699
Depression	Frontal cortex	SAT	OPCs:SMC	0.05427109
Depression	Frontal cortex	SAT	OPCs:adipocyte	0.97568576
Depression	Frontal cortex	SAT	OPCs:b_cell	0.91651241
Depression	Frontal cortex	SAT	OPCs:dendritic_cell	0.9768748
Depression	Frontal cortex	SAT	OPCs:macrophage	0.75339458
Depression	Frontal cortex	SAT	OPCs:mast_cell	0.8467622
Depression	Frontal cortex	SAT	OPCs:monocyte	0.43576125

Depression	Frontal cortex	SAT	OPCs:neutrophil	0.52524644
Depression	Frontal cortex	SAT	OPCs:nk_cell	0.44801769
Depression	Frontal cortex	SAT	OPCs:pericyte	0.48226167
Depression	Frontal cortex	SAT	OPCs:t_cell	0.2829173
Depression	Frontal cortex	SAT	Oligos:ASPC	0.61630409
Depression	Frontal cortex	SAT	Oligos:LEC	0.7971185
Depression	Frontal cortex	SAT	Oligos:SMC	0.04564157
Depression	Frontal cortex	SAT	Oligos:adipocyte	0.71927306
Depression	Frontal cortex	SAT	Oligos:b_cell	0.77126064
Depression	Frontal cortex	SAT	Oligos:dendritic_cell	0.89986759
Depression	Frontal cortex	SAT	Oligos:macrophage	0.82822948
Depression	Frontal cortex	SAT	Oligos:mast_cell	0.99237962
Depression	Frontal cortex	SAT	Oligos:monocyte	0.51643498
Depression	Frontal cortex	SAT	Oligos:neutrophil	0.64952236
Depression	Frontal cortex	SAT	Oligos:nk_cell	0.63105383
Depression	Frontal cortex	SAT	Oligos:pericyte	0.56936286
Depression	Frontal cortex	SAT	Oligos:t_cell	0.3077139
Depression	Frontal cortex	SAT	ASPC:LEC	0.96394394
Depression	Frontal cortex	SAT	ASPC:SMC	0.64504332
Depression	Frontal cortex	SAT	ASPC:adipocyte	0.78566173
Depression	Frontal cortex	SAT	ASPC:b_cell	0.48587412
Depression	Frontal cortex	SAT	ASPC:dendritic_cell	0.46362859
Depression	Frontal cortex	SAT	ASPC:macrophage	0.88127704
Depression	Frontal cortex	SAT	ASPC:mast_cell	0.41758334
Depression	Frontal cortex	SAT	ASPC:monocyte	0.79638099
Depression	Frontal cortex	SAT	ASPC:neutrophil	0.34772146
Depression	Frontal cortex	SAT	ASPC:nk_cell	0.24863903
Depression	Frontal cortex	SAT	ASPC:pericyte	0.23512227
Depression	Frontal cortex	SAT	ASPC:t_cell	0.40397702
Depression	Frontal cortex	SAT	LEC:SMC	0.74558144
Depression	Frontal cortex	SAT	LEC:adipocyte	0.62891682
Depression	Frontal cortex	SAT	LEC:b_cell	0.05889586
Depression	Frontal cortex	SAT	LEC:dendritic_cell	0.98197928
Depression	Frontal cortex	SAT	LEC:macrophage	0.53729488
Depression	Frontal cortex	SAT	LEC:mast_cell	0.64686872
Depression	Frontal cortex	SAT	LEC:monocyte	0.76666571
Depression	Frontal cortex	SAT	LEC:neutrophil	0.25815038
Depression	Frontal cortex	SAT	LEC:nk_cell	0.49198868
Depression	Frontal cortex	SAT	LEC:pericyte	0.78628903
Depression	Frontal cortex	SAT	LEC:t_cell	0.75114937
Depression	Frontal cortex	SAT	SMC:adipocyte	0.82897551
Depression	Frontal cortex	SAT	SMC:b_cell	0.54965878
Depression	Frontal cortex	SAT	SMC:dendritic_cell	0.43040182
Depression	Frontal cortex	SAT	SMC:macrophage	0.46725396
Depression	Frontal cortex	SAT	SMC:mast_cell	0.8190997
Depression	Frontal cortex	SAT	SMC:monocyte	0.17848367

Depression	Frontal cortex	SAT	SMC:neutrophil	0.68309292
Depression	Frontal cortex	SAT	SMC:nk_cell	0.68229912
Depression	Frontal cortex	SAT	SMC:pericyte	0.1573641
Depression	Frontal cortex	SAT	SMC:t_cell	0.98621158
Depression	Frontal cortex	SAT	adipocyte:b_cell	0.56040819
Depression	Frontal cortex	SAT	adipocyte:dendritic_cell	0.38824077
Depression	Frontal cortex	SAT	adipocyte:macrophage	0.68366593
Depression	Frontal cortex	SAT	adipocyte:mast_cell	0.81508571
Depression	Frontal cortex	SAT	adipocyte:monocyte	0.38478645
Depression	Frontal cortex	SAT	adipocyte:neutrophil	0.10685972
Depression	Frontal cortex	SAT	adipocyte:nk_cell	0.33507572
Depression	Frontal cortex	SAT	adipocyte:pericyte	***0.06975813***
Depression	Frontal cortex	SAT	adipocyte:t_cell	0.29899958
Depression	Frontal cortex	SAT	b_cell:dendritic_cell	0.34051921
Depression	Frontal cortex	SAT	b_cell:macrophage	0.32649462
Depression	Frontal cortex	SAT	b_cell:mast_cell	0.75051681
Depression	Frontal cortex	SAT	b_cell:monocyte	0.99152915
Depression	Frontal cortex	SAT	b_cell:neutrophil	0.21680802
Depression	Frontal cortex	SAT	b_cell:nk_cell	0.04662689
Depression	Frontal cortex	SAT	b_cell:pericyte	***0.05719241***
Depression	Frontal cortex	SAT	b_cell:t_cell	0.20859647
Depression	Frontal cortex	SAT	dendritic_cell:macrophage	0.47189577
Depression	Frontal cortex	SAT	dendritic_cell:mast_cell	0.72594595
Depression	Frontal cortex	SAT	dendritic_cell:monocyte	0.30024762
Depression	Frontal cortex	SAT	dendritic_cell:neutrophil	0.68882511
Depression	Frontal cortex	SAT	dendritic_cell:nk_cell	0.65686821
Depression	Frontal cortex	SAT	dendritic_cell:pericyte	0.59682601
Depression	Frontal cortex	SAT	dendritic_cell:t_cell	0.89023556
Depression	Frontal cortex	SAT	macrophage:mast_cell	0.46890482
Depression	Frontal cortex	SAT	macrophage:monocyte	0.89139344
Depression	Frontal cortex	SAT	macrophage:neutrophil	***0.08712037***
Depression	Frontal cortex	SAT	macrophage:nk_cell	0.53692264
Depression	Frontal cortex	SAT	macrophage:pericyte	0.28249348
Depression	Frontal cortex	SAT	macrophage:t_cell	0.80323022
Depression	Frontal cortex	SAT	mast_cell:monocyte	0.71402465
Depression	Frontal cortex	SAT	mast_cell:neutrophil	0.35820097
Depression	Frontal cortex	SAT	mast_cell:nk_cell	0.20320996
Depression	Frontal cortex	SAT	mast_cell:pericyte	0.16265483
Depression	Frontal cortex	SAT	mast_cell:t_cell	0.82298204
Depression	Frontal cortex	SAT	monocyte:neutrophil	0.47728495
Depression	Frontal cortex	SAT	monocyte:nk_cell	0.43648405
Depression	Frontal cortex	SAT	monocyte:pericyte	0.13072367
Depression	Frontal cortex	SAT	monocyte:t_cell	0.28034269
Depression	Frontal cortex	SAT	neutrophil:nk_cell	**0.00397429**
Depression	Frontal cortex	SAT	neutrophil:pericyte	0.22825061
Depression	Frontal cortex	SAT	neutrophil:t_cell	0.73237156

Depression	Frontal cortex	SAT	nk_cell:pericyte	0.71568582
Depression	Frontal cortex	SAT	nk_cell:t_cell	0.93813562
Depression	Frontal cortex	SAT	pericyte:t_cell	0.65974165
Depression	Frontal cortex	VAT	Ex	0.71514166
Depression	Frontal cortex	VAT	Inhib	0.75717705
Depression	Frontal cortex	VAT	MicroMacro	0.91749027
Depression	Frontal cortex	VAT	OPCs	0.61880318
Depression	Frontal cortex	VAT	Oligos	0.80515319
Depression	Frontal cortex	VAT	ASPC	0.5390469
Depression	Frontal cortex	VAT	LEC	0.79653786
Depression	Frontal cortex	VAT	SMC	0.0507791
Depression	Frontal cortex	VAT	adipocyte	0.8053499
Depression	Frontal cortex	VAT	b_cell	0.94401259
Depression	Frontal cortex	VAT	dendritic_cell	0.81799245
Depression	Frontal cortex	VAT	macrophage	0.77838479
Depression	Frontal cortex	VAT	mast_cell	0.94801254
Depression	Frontal cortex	VAT	monocyte	0.51914066
Depression	Frontal cortex	VAT	neutrophil	0.89712814
Depression	Frontal cortex	VAT	nk_cell	0.79409881
Depression	Frontal cortex	VAT	pericyte	0.75915093
Depression	Frontal cortex	VAT	t_cell	0.3570095
Depression	Frontal cortex	VAT	Ex:Inhib	0.86751388
Depression	Frontal cortex	VAT	Ex:MicroMacro	0.96455162
Depression	Frontal cortex	VAT	Ex:OPCs	0.67442311
Depression	Frontal cortex	VAT	Ex:Oligos	0.47123777
Depression	Frontal cortex	VAT	Ex:ASPC	0.52898904
Depression	Frontal cortex	VAT	Ex:LEC	0.81946707
Depression	Frontal cortex	VAT	Ex:SMC	**0.0488227**
Depression	Frontal cortex	VAT	Ex:adipocyte	0.80417435
Depression	Frontal cortex	VAT	Ex:b_cell	0.85079094
Depression	Frontal cortex	VAT	Ex:dendritic_cell	0.9524783
Depression	Frontal cortex	VAT	Ex:macrophage	0.8040401
Depression	Frontal cortex	VAT	Ex:mast_cell	0.9675978
Depression	Frontal cortex	VAT	Ex:monocyte	0.56823908
Depression	Frontal cortex	VAT	Ex:neutrophil	0.73325973
Depression	Frontal cortex	VAT	Ex:nk_cell	0.62354932
Depression	Frontal cortex	VAT	Ex:pericyte	0.58921938
Depression	Frontal cortex	VAT	Ex:t_cell	0.27017588
Depression	Frontal cortex	VAT	Inhib:MicroMacro	0.89685474
Depression	Frontal cortex	VAT	Inhib:OPCs	0.34001228
Depression	Frontal cortex	VAT	Inhib:Oligos	0.37677095
Depression	Frontal cortex	VAT	Inhib:ASPC	0.58296609
Depression	Frontal cortex	VAT	Inhib:LEC	0.79647992
Depression	Frontal cortex	VAT	Inhib:SMC	0.05046435
Depression	Frontal cortex	VAT	Inhib:adipocyte	0.67412999
Depression	Frontal cortex	VAT	Inhib:b_cell	0.85700621

Depression	Frontal cortex	VAT	Inhib:dendritic_cell	0.94825064
Depression	Frontal cortex	VAT	Inhib:macrophage	0.74060609
Depression	Frontal cortex	VAT	Inhib:mast_cell	0.94265592
Depression	Frontal cortex	VAT	Inhib:monocyte	0.49095357
Depression	Frontal cortex	VAT	Inhib:neutrophil	0.7110502
Depression	Frontal cortex	VAT	Inhib:nk_cell	0.73966163
Depression	Frontal cortex	VAT	Inhib:pericyte	0.52410218
Depression	Frontal cortex	VAT	Inhib:t_cell	0.28994241
Depression	Frontal cortex	VAT	MicroMacro:OPCs	0.56951091
Depression	Frontal cortex	VAT	MicroMacro:Oligos	0.9264086
Depression	Frontal cortex	VAT	MicroMacro:ASPC	0.76231768
Depression	Frontal cortex	VAT	MicroMacro:LEC	0.68016308
Depression	Frontal cortex	VAT	MicroMacro:SMC	0.33462081
Depression	Frontal cortex	VAT	MicroMacro:adipocyte	0.56271663
Depression	Frontal cortex	VAT	MicroMacro:b_cell	0.5087087
Depression	Frontal cortex	VAT	MicroMacro:dendritic_cell	0.80362074
Depression	Frontal cortex	VAT	MicroMacro:macrophage	0.69061333
Depression	Frontal cortex	VAT	MicroMacro:mast_cell	0.66844033
Depression	Frontal cortex	VAT	MicroMacro:monocyte	0.85617912
Depression	Frontal cortex	VAT	MicroMacro:neutrophil	0.84167034
Depression	Frontal cortex	VAT	MicroMacro:nk_cell	0.8551138
Depression	Frontal cortex	VAT	MicroMacro:pericyte	0.23862515
Depression	Frontal cortex	VAT	MicroMacro:t_cell	***0.0928086***
Depression	Frontal cortex	VAT	OPCs:Oligos	0.53173169
Depression	Frontal cortex	VAT	OPCs:ASPC	0.54779196
Depression	Frontal cortex	VAT	OPCs:LEC	0.80011699
Depression	Frontal cortex	VAT	OPCs:SMC	***0.05427109***
Depression	Frontal cortex	VAT	OPCs:adipocyte	0.97568576
Depression	Frontal cortex	VAT	OPCs:b_cell	0.91651241
Depression	Frontal cortex	VAT	OPCs:dendritic_cell	0.9768748
Depression	Frontal cortex	VAT	OPCs:macrophage	0.75339458
Depression	Frontal cortex	VAT	OPCs:mast_cell	0.8467622
Depression	Frontal cortex	VAT	OPCs:monocyte	0.43576125
Depression	Frontal cortex	VAT	OPCs:neutrophil	0.52524644
Depression	Frontal cortex	VAT	OPCs:nk_cell	0.44801769
Depression	Frontal cortex	VAT	OPCs:pericyte	0.48226167
Depression	Frontal cortex	VAT	OPCs:t_cell	0.2829173
Depression	Frontal cortex	VAT	Oligos:ASPC	0.61630409
Depression	Frontal cortex	VAT	Oligos:LEC	0.7971185
Depression	Frontal cortex	VAT	Oligos:SMC	**0.04564157**
Depression	Frontal cortex	VAT	Oligos:adipocyte	0.71927306
Depression	Frontal cortex	VAT	Oligos:b_cell	0.77126064
Depression	Frontal cortex	VAT	Oligos:dendritic_cell	0.89986759
Depression	Frontal cortex	VAT	Oligos:macrophage	0.82822948
Depression	Frontal cortex	VAT	Oligos:mast_cell	0.99237962
Depression	Frontal cortex	VAT	Oligos:monocyte	0.51643498

Depression	Frontal cortex	VAT	Oligos:neutrophil	0.64952236
Depression	Frontal cortex	VAT	Oligos:nk_cell	0.63105383
Depression	Frontal cortex	VAT	Oligos:pericyte	0.56936286
Depression	Frontal cortex	VAT	Oligos:t_cell	0.3077139
Depression	Frontal cortex	VAT	ASPC:LEC	0.96394394
Depression	Frontal cortex	VAT	ASPC:SMC	0.64504332
Depression	Frontal cortex	VAT	ASPC:adipocyte	0.78566173
Depression	Frontal cortex	VAT	ASPC:b_cell	0.48587412
Depression	Frontal cortex	VAT	ASPC:dendritic_cell	0.46362859
Depression	Frontal cortex	VAT	ASPC:macrophage	0.88127704
Depression	Frontal cortex	VAT	ASPC:mast_cell	0.41758334
Depression	Frontal cortex	VAT	ASPC:monocyte	0.79638099
Depression	Frontal cortex	VAT	ASPC:neutrophil	0.34772146
Depression	Frontal cortex	VAT	ASPC:nk_cell	0.24863903
Depression	Frontal cortex	VAT	ASPC:pericyte	0.23512227
Depression	Frontal cortex	VAT	ASPC:t_cell	0.40397702
Depression	Frontal cortex	VAT	LEC:SMC	0.74558144
Depression	Frontal cortex	VAT	LEC:adipocyte	0.62891682
Depression	Frontal cortex	VAT	LEC:b_cell	0.05889586
Depression	Frontal cortex	VAT	LEC:dendritic_cell	0.98197928
Depression	Frontal cortex	VAT	LEC:macrophage	0.53729488
Depression	Frontal cortex	VAT	LEC:mast_cell	0.64686872
Depression	Frontal cortex	VAT	LEC:monocyte	0.76666571
Depression	Frontal cortex	VAT	LEC:neutrophil	0.25815038
Depression	Frontal cortex	VAT	LEC:nk_cell	0.49198868
Depression	Frontal cortex	VAT	LEC:pericyte	0.78628903
Depression	Frontal cortex	VAT	LEC:t_cell	0.75114937
Depression	Frontal cortex	VAT	SMC:adipocyte	0.82897551
Depression	Frontal cortex	VAT	SMC:b_cell	0.54965878
Depression	Frontal cortex	VAT	SMC:dendritic_cell	0.43040182
Depression	Frontal cortex	VAT	SMC:macrophage	0.46725396
Depression	Frontal cortex	VAT	SMC:mast_cell	0.8190997
Depression	Frontal cortex	VAT	SMC:monocyte	0.17848367
Depression	Frontal cortex	VAT	SMC:neutrophil	0.68309292
Depression	Frontal cortex	VAT	SMC:nk_cell	0.68229912
Depression	Frontal cortex	VAT	SMC:pericyte	0.1573641
Depression	Frontal cortex	VAT	SMC:t_cell	0.98621158
Depression	Frontal cortex	VAT	adipocyte:b_cell	0.56040819
Depression	Frontal cortex	VAT	adipocyte:dendritic_cell	0.38824077
Depression	Frontal cortex	VAT	adipocyte:macrophage	0.68366593
Depression	Frontal cortex	VAT	adipocyte:mast_cell	0.81508571
Depression	Frontal cortex	VAT	adipocyte:monocyte	0.38478645
Depression	Frontal cortex	VAT	adipocyte:neutrophil	0.10685972
Depression	Frontal cortex	VAT	adipocyte:nk_cell	0.33507572
Depression	Frontal cortex	VAT	adipocyte:pericyte	0.06975813
Depression	Frontal cortex	VAT	adipocyte:t_cell	0.29899958

Depression	Frontal cortex	VAT	b_cell:dendritic_cell	0.34051921
Depression	Frontal cortex	VAT	b_cell:macrophage	0.32649462
Depression	Frontal cortex	VAT	b_cell:mast_cell	0.75051681
Depression	Frontal cortex	VAT	b_cell:monocyte	0.99152915
Depression	Frontal cortex	VAT	b_cell:neutrophil	0.21680802
Depression	Frontal cortex	VAT	b_cell:nk_cell	0.04662689
Depression	Frontal cortex	VAT	b_cell:pericyte	0.05719241
Depression	Frontal cortex	VAT	b_cell:t_cell	0.20859647
Depression	Frontal cortex	VAT	dendritic_cell:macrophage	0.47189577
Depression	Frontal cortex	VAT	dendritic_cell:mast_cell	0.72594595
Depression	Frontal cortex	VAT	dendritic_cell:monocyte	0.30024762
Depression	Frontal cortex	VAT	dendritic_cell:neutrophil	0.68882511
Depression	Frontal cortex	VAT	dendritic_cell:nk_cell	0.65686821
Depression	Frontal cortex	VAT	dendritic_cell:pericyte	0.59682601
Depression	Frontal cortex	VAT	dendritic_cell:t_cell	0.89023556
Depression	Frontal cortex	VAT	macrophage:mast_cell	0.46890482
Depression	Frontal cortex	VAT	macrophage:monocyte	0.89139344
Depression	Frontal cortex	VAT	macrophage:neutrophil	0.08712037
Depression	Frontal cortex	VAT	macrophage:nk_cell	0.53692264
Depression	Frontal cortex	VAT	macrophage:pericyte	0.28249348
Depression	Frontal cortex	VAT	macrophage:t_cell	0.80323022
Depression	Frontal cortex	VAT	mast_cell:monocyte	0.71402465
Depression	Frontal cortex	VAT	mast_cell:neutrophil	0.35820097
Depression	Frontal cortex	VAT	mast_cell:nk_cell	0.20320996
Depression	Frontal cortex	VAT	mast_cell:pericyte	0.16265483
Depression	Frontal cortex	VAT	mast_cell:t_cell	0.82298204
Depression	Frontal cortex	VAT	monocyte:neutrophil	0.47728495
Depression	Frontal cortex	VAT	monocyte:nk_cell	0.43648405
Depression	Frontal cortex	VAT	monocyte:pericyte	0.13072367
Depression	Frontal cortex	VAT	monocyte:t_cell	0.28034269
Depression	Frontal cortex	VAT	neutrophil:nk_cell	0.00397429
Depression	Frontal cortex	VAT	neutrophil:pericyte	0.22825061
Depression	Frontal cortex	VAT	neutrophil:t_cell	0.73237156
Depression	Frontal cortex	VAT	nk_cell:pericyte	0.71568582
Depression	Frontal cortex	VAT	nk_cell:t_cell	0.93813562
Depression	Frontal cortex	VAT	pericyte:t_cell	0.65974165
T2DM	Pancreas	SAT	duct_epithelial_cell	0.96348802
T2DM	Pancreas	SAT	endothelial_cell	0.000121
T2DM	Pancreas	SAT	leukocyte	0.68724051
T2DM	Pancreas	SAT	mesenchymal_cell	0.15992551
T2DM	Pancreas	SAT	alpha_cell	0.58297637
T2DM	Pancreas	SAT	delta_cell	0.53356158
T2DM	Pancreas	SAT	PP_cell	0.27282945
T2DM	Pancreas	SAT	epsilon_cell	0.12414599
T2DM	Pancreas	SAT	beta_cell	0.42718069
T2DM	Pancreas	SAT	ASPC	0.76036518

T2DM	Pancreas	SAT	LEC	0.76118893
T2DM	Pancreas	SAT	SMC	0.33267956
T2DM	Pancreas	SAT	adipocyte	0.00679661
T2DM	Pancreas	SAT	b_cell	0.17941452
T2DM	Pancreas	SAT	dendritic_cell	0.36903864
T2DM	Pancreas	SAT	macrophage	0.99211718
T2DM	Pancreas	SAT	mast_cell	0.34731047
T2DM	Pancreas	SAT	monocyte	0.94736611
T2DM	Pancreas	SAT	neutrophil	0.79777987
T2DM	Pancreas	SAT	nk_cell	0.20549195
T2DM	Pancreas	SAT	pericyte	0.47318617
T2DM	Pancreas	SAT	t_cell	0.48153555
T2DM	Pancreas	SAT	duct_epithelial_cell:endothelial_c	0.32997503
T2DM	Pancreas	SAT	duct_epithelial_cell:leukocyte	0.61614605
T2DM	Pancreas	SAT	duct_epithelial_cell:mesenchymal	0.18611637
T2DM	Pancreas	SAT	duct_epithelial_cell:alpha_cell	0.95796137
T2DM	Pancreas	SAT	duct_epithelial_cell:delta_cell	0.40195852
T2DM	Pancreas	SAT	duct_epithelial_cell:PP_cell	0.90363127
T2DM	Pancreas	SAT	duct_epithelial_cell:epsilon_cell	0.40215133
T2DM	Pancreas	SAT	duct_epithelial_cell:beta_cell	0.43418157
T2DM	Pancreas	SAT	duct_epithelial_cell:ASPC	0.83514629
T2DM	Pancreas	SAT	duct_epithelial_cell:LEC	0.13890262
T2DM	Pancreas	SAT	duct_epithelial_cell:SMC	0.28105392
T2DM	Pancreas	SAT	duct_epithelial_cell:adipocyte	**0.02318777**
T2DM	Pancreas	SAT	duct_epithelial_cell:b_cell	0.89934261
T2DM	Pancreas	SAT	duct_epithelial_cell:dendritic_cel	0.85276286
T2DM	Pancreas	SAT	duct_epithelial_cell:macrophage	0.93470887
T2DM	Pancreas	SAT	duct_epithelial_cell:mast_cell	0.81536183
T2DM	Pancreas	SAT	duct_epithelial_cell:monocyte	0.46963148
T2DM	Pancreas	SAT	duct_epithelial_cell:neutrophil	0.72438364
T2DM	Pancreas	SAT	duct_epithelial_cell:nk_cell	0.35514209
T2DM	Pancreas	SAT	duct_epithelial_cell:pericyte	0.31399246
T2DM	Pancreas	SAT	duct_epithelial_cell:t_cell	0.74338113
T2DM	Pancreas	SAT	endothelial_cell:leukocyte	0.03573003
T2DM	Pancreas	SAT	endothelial_cell:mesenchymal_ce	0.11992245
T2DM	Pancreas	SAT	endothelial_cell:alpha_cell	**0.00344697**
T2DM	Pancreas	SAT	endothelial_cell:delta_cell	**0.00201649**
T2DM	Pancreas	SAT	endothelial_cell:PP_cell	0.12120876
T2DM	Pancreas	SAT	endothelial_cell:epsilon_cell	0.88931359
T2DM	Pancreas	SAT	endothelial_cell:beta_cell	**0.00026039**
T2DM	Pancreas	SAT	endothelial_cell:ASPC	**0.0490978**
T2DM	Pancreas	SAT	endothelial_cell:LEC	0.89542088
T2DM	Pancreas	SAT	endothelial_cell:SMC	0.27077759
T2DM	Pancreas	SAT	endothelial_cell:adipocyte	0.79067967
T2DM	Pancreas	SAT	endothelial_cell:b_cell	0.29341569
T2DM	Pancreas	SAT	endothelial_cell:dendritic_cell	0.32297288

T2DM	Pancreas	SAT	endothelial_cell:macrophage	0.22577514
T2DM	Pancreas	SAT	endothelial_cell:mast_cell	0.74824625
T2DM	Pancreas	SAT	endothelial_cell:monocyte	0.37677253
T2DM	Pancreas	SAT	endothelial_cell:neutrophil	**0.0389968**
T2DM	Pancreas	SAT	endothelial_cell:nk_cell	**0.0060122**
T2DM	Pancreas	SAT	endothelial_cell:pericyte	**0.02256553**
T2DM	Pancreas	SAT	endothelial_cell:t_cell	***0.08179028***
T2DM	Pancreas	SAT	leukocyte:mesenchymal_cell	0.83145597
T2DM	Pancreas	SAT	leukocyte:alpha_cell	0.88451886
T2DM	Pancreas	SAT	leukocyte:delta_cell	0.68503079
T2DM	Pancreas	SAT	leukocyte:PP_cell	0.46590921
T2DM	Pancreas	SAT	leukocyte:epsilon_cell	0.28943154
T2DM	Pancreas	SAT	leukocyte:beta_cell	0.86672317
T2DM	Pancreas	SAT	leukocyte:ASPC	0.461492
T2DM	Pancreas	SAT	leukocyte:LEC	0.98401611
T2DM	Pancreas	SAT	leukocyte:SMC	0.88432805
T2DM	Pancreas	SAT	leukocyte:adipocyte	0.2961349
T2DM	Pancreas	SAT	leukocyte:b_cell	0.43021527
T2DM	Pancreas	SAT	leukocyte:dendritic_cell	0.15361512
T2DM	Pancreas	SAT	leukocyte:macrophage	0.61660868
T2DM	Pancreas	SAT	leukocyte:mast_cell	0.17559163
T2DM	Pancreas	SAT	leukocyte:monocyte	0.29776933
T2DM	Pancreas	SAT	leukocyte:neutrophil	0.51463911
T2DM	Pancreas	SAT	leukocyte:nk_cell	0.61567035
T2DM	Pancreas	SAT	leukocyte:pericyte	0.50406664
T2DM	Pancreas	SAT	leukocyte:t_cell	0.76962909
T2DM	Pancreas	SAT	mesenchymal_cell:alpha_cell	0.43266364
T2DM	Pancreas	SAT	mesenchymal_cell:delta_cell	0.77483438
T2DM	Pancreas	SAT	mesenchymal_cell:PP_cell	0.26462523
T2DM	Pancreas	SAT	mesenchymal_cell:epsilon_cell	0.52767765
T2DM	Pancreas	SAT	mesenchymal_cell:beta_cell	0.37973062
T2DM	Pancreas	SAT	mesenchymal_cell:ASPC	0.44796197
T2DM	Pancreas	SAT	mesenchymal_cell:LEC	0.12190628
T2DM	Pancreas	SAT	mesenchymal_cell:SMC	0.58850023
T2DM	Pancreas	SAT	mesenchymal_cell:adipocyte	0.90278043
T2DM	Pancreas	SAT	mesenchymal_cell:b_cell	0.42072091
T2DM	Pancreas	SAT	mesenchymal_cell:dendritic_cell	0.88197174
T2DM	Pancreas	SAT	mesenchymal_cell:macrophage	0.72586491
T2DM	Pancreas	SAT	mesenchymal_cell:mast_cell	0.78484502
T2DM	Pancreas	SAT	mesenchymal_cell:monocyte	0.17974576
T2DM	Pancreas	SAT	mesenchymal_cell:neutrophil	0.12236134
T2DM	Pancreas	SAT	mesenchymal_cell:nk_cell	**0.03670891**
T2DM	Pancreas	SAT	mesenchymal_cell:pericyte	0.21107796
T2DM	Pancreas	SAT	mesenchymal_cell:t_cell	0.39198268
T2DM	Pancreas	SAT	alpha_cell:delta_cell	***0.09451958***
T2DM	Pancreas	SAT	alpha_cell:PP_cell	0.60379232

T2DM	Pancreas	SAT	alpha_cell:epsilon_cell	0.16224869
T2DM	Pancreas	SAT	alpha_cell:beta_cell	0.16015178
T2DM	Pancreas	SAT	alpha_cell:ASPC	0.80561448
T2DM	Pancreas	SAT	alpha_cell:LEC	0.62474367
T2DM	Pancreas	SAT	alpha_cell:SMC	0.3593575
T2DM	Pancreas	SAT	alpha_cell:adipocyte	**0.01329023**
T2DM	Pancreas	SAT	alpha_cell:b_cell	0.55563113
T2DM	Pancreas	SAT	alpha_cell:dendritic_cell	0.70830232
T2DM	Pancreas	SAT	alpha_cell:macrophage	0.65439971
T2DM	Pancreas	SAT	alpha_cell:mast_cell	0.1485094
T2DM	Pancreas	SAT	alpha_cell:monocyte	0.52806743
T2DM	Pancreas	SAT	alpha_cell:neutrophil	0.23760405
T2DM	Pancreas	SAT	alpha_cell:nk_cell	0.46947705
T2DM	Pancreas	SAT	alpha_cell:pericyte	0.27026983
T2DM	Pancreas	SAT	alpha_cell:t_cell	0.85311208
T2DM	Pancreas	SAT	delta_cell:PP_cell	0.86696666
T2DM	Pancreas	SAT	delta_cell:epsilon_cell	0.18472022
T2DM	Pancreas	SAT	delta_cell:beta_cell	0.06963777
T2DM	Pancreas	SAT	delta_cell:ASPC	0.30627782
T2DM	Pancreas	SAT	delta_cell:LEC	0.61606291
T2DM	Pancreas	SAT	delta_cell:SMC	0.16775962
T2DM	Pancreas	SAT	delta_cell:adipocyte	**0.0122907**
T2DM	Pancreas	SAT	delta_cell:b_cell	0.29261235
T2DM	Pancreas	SAT	delta_cell:dendritic_cell	0.46455625
T2DM	Pancreas	SAT	delta_cell:macrophage	0.83272949
T2DM	Pancreas	SAT	delta_cell:mast_cell	**0.03810678**
T2DM	Pancreas	SAT	delta_cell:monocyte	0.90698398
T2DM	Pancreas	SAT	delta_cell:neutrophil	0.36292943
T2DM	Pancreas	SAT	delta_cell:nk_cell	0.83339262
T2DM	Pancreas	SAT	delta_cell:pericyte	**0.02716698**
T2DM	Pancreas	SAT	delta_cell:t_cell	0.33280378
T2DM	Pancreas	SAT	PP_cell:epsilon_cell	***0.09081388***
T2DM	Pancreas	SAT	PP_cell:beta_cell	0.83677567
T2DM	Pancreas	SAT	PP_cell:ASPC	0.35688684
T2DM	Pancreas	SAT	PP_cell:LEC	0.99158559
T2DM	Pancreas	SAT	PP_cell:SMC	0.1993489
T2DM	Pancreas	SAT	PP_cell:adipocyte	**0.00764883**
T2DM	Pancreas	SAT	PP_cell:b_cell	0.28743724
T2DM	Pancreas	SAT	PP_cell:dendritic_cell	0.74882022
T2DM	Pancreas	SAT	PP_cell:macrophage	0.77744561
T2DM	Pancreas	SAT	PP_cell:mast_cell	***0.0786356***
T2DM	Pancreas	SAT	PP_cell:monocyte	0.80934329
T2DM	Pancreas	SAT	PP_cell:neutrophil	0.20753636
T2DM	Pancreas	SAT	PP_cell:nk_cell	0.8125891
T2DM	Pancreas	SAT	PP_cell:pericyte	0.05333262
T2DM	Pancreas	SAT	PP_cell:t_cell	0.88536955

T2DM	Pancreas	SAT	epsilon_cell:beta_cell	0.15208521
T2DM	Pancreas	SAT	epsilon_cell:ASPC	0.41633605
T2DM	Pancreas	SAT	epsilon_cell:LEC	***0.05309682***
T2DM	Pancreas	SAT	epsilon_cell:SMC	0.76341049
T2DM	Pancreas	SAT	epsilon_cell:adipocyte	0.25447149
T2DM	Pancreas	SAT	epsilon_cell:b_cell	0.19870482
T2DM	Pancreas	SAT	epsilon_cell:dendritic_cell	**0.04753288**
T2DM	Pancreas	SAT	epsilon_cell:macrophage	0.77884542
T2DM	Pancreas	SAT	epsilon_cell:mast_cell	***0.097459***
T2DM	Pancreas	SAT	epsilon_cell:monocyte	0.16348134
T2DM	Pancreas	SAT	epsilon_cell:neutrophil	0.14791614
T2DM	Pancreas	SAT	epsilon_cell:nk_cell	0.4502471
T2DM	Pancreas	SAT	epsilon_cell:pericyte	0.15688054
T2DM	Pancreas	SAT	epsilon_cell:t_cell	0.69701501
T2DM	Pancreas	SAT	beta_cell:ASPC	0.60216297
T2DM	Pancreas	SAT	beta_cell:LEC	0.66680427
T2DM	Pancreas	SAT	beta_cell:SMC	0.32362217
T2DM	Pancreas	SAT	beta_cell:adipocyte	0.00887285
T2DM	Pancreas	SAT	beta_cell:b_cell	0.21318757
T2DM	Pancreas	SAT	beta_cell:dendritic_cell	0.51691788
T2DM	Pancreas	SAT	beta_cell:macrophage	0.95856785
T2DM	Pancreas	SAT	beta_cell:mast_cell	0.14583174
T2DM	Pancreas	SAT	beta_cell:monocyte	0.6378686
T2DM	Pancreas	SAT	beta_cell:neutrophil	0.22384218
T2DM	Pancreas	SAT	beta_cell:nk_cell	0.34073849
T2DM	Pancreas	SAT	beta_cell:pericyte	0.22378215
T2DM	Pancreas	SAT	beta_cell:t_cell	0.70066687
T2DM	Pancreas	SAT	ASPC:LEC	**0.01828592**
T2DM	Pancreas	SAT	ASPC:SMC	0.25762784
T2DM	Pancreas	SAT	ASPC:adipocyte	0.69317723
T2DM	Pancreas	SAT	ASPC:b_cell	***0.07544378***
T2DM	Pancreas	SAT	ASPC:dendritic_cell	0.23257235
T2DM	Pancreas	SAT	ASPC:macrophage	0.91222797
T2DM	Pancreas	SAT	ASPC:mast_cell	0.80415197
T2DM	Pancreas	SAT	ASPC:monocyte	0.29884771
T2DM	Pancreas	SAT	ASPC:neutrophil	0.19439491
T2DM	Pancreas	SAT	ASPC:nk_cell	0.30017419
T2DM	Pancreas	SAT	ASPC:pericyte	0.2261871
T2DM	Pancreas	SAT	ASPC:t_cell	0.27499603
T2DM	Pancreas	SAT	LEC:SMC	0.63284265
T2DM	Pancreas	SAT	LEC:adipocyte	0.47695014
T2DM	Pancreas	SAT	LEC:b_cell	**0.00794548**
T2DM	Pancreas	SAT	LEC:dendritic_cell	0.54004226
T2DM	Pancreas	SAT	LEC:macrophage	0.21195816
T2DM	Pancreas	SAT	LEC:mast_cell	0.94392561
T2DM	Pancreas	SAT	LEC:monocyte	0.4759678

T2DM	Pancreas	SAT	LEC:neutrophil	0.33226424
T2DM	Pancreas	SAT	LEC:nk_cell	**0.00583383**
T2DM	Pancreas	SAT	LEC:pericyte	0.26603199
T2DM	Pancreas	SAT	LEC:t_cell	0.3719512
T2DM	Pancreas	SAT	SMC:adipocyte	0.11448421
T2DM	Pancreas	SAT	SMC:b_cell	0.34808955
T2DM	Pancreas	SAT	SMC:dendritic_cell	0.25045711
T2DM	Pancreas	SAT	SMC:macrophage	**0.01652624**
T2DM	Pancreas	SAT	SMC:mast_cell	0.41672
T2DM	Pancreas	SAT	SMC:monocyte	0.36827814
T2DM	Pancreas	SAT	SMC:neutrophil	***0.05250651***
T2DM	Pancreas	SAT	SMC:nk_cell	0.13900647
T2DM	Pancreas	SAT	SMC:pericyte	**0.00198425**
T2DM	Pancreas	SAT	SMC:t_cell	**0.0303994**
T2DM	Pancreas	SAT	adipocyte:b_cell	0.19834354
T2DM	Pancreas	SAT	adipocyte:dendritic_cell	0.96614959
T2DM	Pancreas	SAT	adipocyte:macrophage	**0.02831957**
T2DM	Pancreas	SAT	adipocyte:mast_cell	0.10579177
T2DM	Pancreas	SAT	adipocyte:monocyte	0.15107268
T2DM	Pancreas	SAT	adipocyte:neutrophil	**0.00419385**
T2DM	Pancreas	SAT	adipocyte:nk_cell	0.58328826
T2DM	Pancreas	SAT	adipocyte:pericyte	**0.03746546**
T2DM	Pancreas	SAT	adipocyte:t_cell	0.73179449
T2DM	Pancreas	SAT	b_cell:dendritic_cell	**0.00984223**
T2DM	Pancreas	SAT	b_cell:macrophage	0.1745086
T2DM	Pancreas	SAT	b_cell:mast_cell	***0.08808755***
T2DM	Pancreas	SAT	b_cell:monocyte	0.69580254
T2DM	Pancreas	SAT	b_cell:neutrophil	**7.91E-06**
T2DM	Pancreas	SAT	b_cell:nk_cell	0.34283673
T2DM	Pancreas	SAT	b_cell:pericyte	**0.02558668**
T2DM	Pancreas	SAT	b_cell:t_cell	0.50619276
T2DM	Pancreas	SAT	dendritic_cell:macrophage	0.12179187
T2DM	Pancreas	SAT	dendritic_cell:mast_cell	0.16603902
T2DM	Pancreas	SAT	dendritic_cell:monocyte	0.32733716
T2DM	Pancreas	SAT	dendritic_cell:neutrophil	0.1586055
T2DM	Pancreas	SAT	dendritic_cell:nk_cell	0.93197384
T2DM	Pancreas	SAT	dendritic_cell:pericyte	0.51965566
T2DM	Pancreas	SAT	dendritic_cell:t_cell	0.69921381
T2DM	Pancreas	SAT	macrophage:mast_cell	0.39443955
T2DM	Pancreas	SAT	macrophage:monocyte	0.99869765
T2DM	Pancreas	SAT	macrophage:neutrophil	0.24111
T2DM	Pancreas	SAT	macrophage:nk_cell	0.28302848
T2DM	Pancreas	SAT	macrophage:pericyte	0.91033296
T2DM	Pancreas	SAT	macrophage:t_cell	***0.07417619***
T2DM	Pancreas	SAT	mast_cell:monocyte	0.56029284
T2DM	Pancreas	SAT	mast_cell:neutrophil	0.65921315

T2DM	Pancreas	SAT	mast_cell:nk_cell	0.9863878
T2DM	Pancreas	SAT	mast_cell:pericyte	0.77392751
T2DM	Pancreas	SAT	mast_cell:t_cell	0.27041492
T2DM	Pancreas	SAT	monocyte:neutrophil	0.39988367
T2DM	Pancreas	SAT	monocyte:nk_cell	0.20427782
T2DM	Pancreas	SAT	monocyte:pericyte	0.29685812
T2DM	Pancreas	SAT	monocyte:t_cell	***0.0654075***
T2DM	Pancreas	SAT	neutrophil:nk_cell	**0.01071561**
T2DM	Pancreas	SAT	neutrophil:pericyte	**0.03955965**
T2DM	Pancreas	SAT	neutrophil:t_cell	**0.03977717**
T2DM	Pancreas	SAT	nk_cell:pericyte	0.3729832
T2DM	Pancreas	SAT	nk_cell:t_cell	0.28358561
T2DM	Pancreas	SAT	pericyte:t_cell	0.93082578
T2DM	Pancreas	VAT	duct_epithelial_cell	***0.07809328***
T2DM	Pancreas	VAT	endothelial_cell	**0.04682661**
T2DM	Pancreas	VAT	leukocyte	0.65813829
T2DM	Pancreas	VAT	mesenchymal_cell	0.652908
T2DM	Pancreas	VAT	alpha_cell	***0.09755256***
T2DM	Pancreas	VAT	delta_cell	0.23204584
T2DM	Pancreas	VAT	PP_cell	0.17239847
T2DM	Pancreas	VAT	epsilon_cell	0.90540356
T2DM	Pancreas	VAT	beta_cell	0.13418268
T2DM	Pancreas	VAT	ASPC	0.13264185
T2DM	Pancreas	VAT	LEC	0.25880323
T2DM	Pancreas	VAT	SMC	0.20815095
T2DM	Pancreas	VAT	adipocyte	***0.07969284***
T2DM	Pancreas	VAT	b_cell	**0.00305949**
T2DM	Pancreas	VAT	dendritic_cell	0.79775727
T2DM	Pancreas	VAT	endometrium	0.71990995
T2DM	Pancreas	VAT	macrophage	0.98212345
T2DM	Pancreas	VAT	mast_cell	0.21342428
T2DM	Pancreas	VAT	mesothelium	0.11876995
T2DM	Pancreas	VAT	monocyte	0.7554106
T2DM	Pancreas	VAT	nk_cell	**0.00055607**
T2DM	Pancreas	VAT	pericyte	0.8519925
T2DM	Pancreas	VAT	t_cell	**0.01281705**
T2DM	Pancreas	VAT	duct_epithelial_cell:endothelial_c	0.28218308
T2DM	Pancreas	VAT	duct_epithelial_cell:leukocyte	0.94173824
T2DM	Pancreas	VAT	duct_epithelial_cell:mesenchymal	0.26677191
T2DM	Pancreas	VAT	duct_epithelial_cell:alpha_cell	0.99602893
T2DM	Pancreas	VAT	duct_epithelial_cell:delta_cell	0.35984935
T2DM	Pancreas	VAT	duct_epithelial_cell:PP_cell	0.95996981
T2DM	Pancreas	VAT	duct_epithelial_cell:epsilon_cell	0.27706535
T2DM	Pancreas	VAT	duct_epithelial_cell:beta_cell	0.384384
T2DM	Pancreas	VAT	duct_epithelial_cell:ASPC	**0.01697742**
T2DM	Pancreas	VAT	duct_epithelial_cell:LEC	0.9909473

T2DM	Pancreas	VAT	duct_epithelial_cell:SMC	0.44163217
T2DM	Pancreas	VAT	duct_epithelial_cell:adipocyte	**0.04657927**
T2DM	Pancreas	VAT	duct_epithelial_cell:b_cell	**0.00034608**
T2DM	Pancreas	VAT	duct_epithelial_cell:dendritic_cel	0.70877395
T2DM	Pancreas	VAT	duct_epithelial_cell:endometrium	0.41377739
T2DM	Pancreas	VAT	duct_epithelial_cell:macrophage	0.90745635
T2DM	Pancreas	VAT	duct_epithelial_cell:mast_cell	0.74723944
T2DM	Pancreas	VAT	duct_epithelial_cell:mesothelium	**0.0103271**
T2DM	Pancreas	VAT	duct_epithelial_cell:monocyte	0.922322
T2DM	Pancreas	VAT	duct_epithelial_cell:nk_cell	2.03E-05
T2DM	Pancreas	VAT	duct_epithelial_cell:pericyte	0.60051389
T2DM	Pancreas	VAT	duct_epithelial_cell:t_cell	**0.0094299**
T2DM	Pancreas	VAT	endothelial_cell:leukocyte	**0.04659236**
T2DM	Pancreas	VAT	endothelial_cell:mesenchymal_ce	***0.05802082***
T2DM	Pancreas	VAT	endothelial_cell:alpha_cell	**0.00136219**
T2DM	Pancreas	VAT	endothelial_cell:delta_cell	**0.00052322**
T2DM	Pancreas	VAT	endothelial_cell:PP_cell	0.11548804
T2DM	Pancreas	VAT	endothelial_cell:epsilon_cell	0.78214809
T2DM	Pancreas	VAT	endothelial_cell:beta_cell	**6.76E-05**
T2DM	Pancreas	VAT	endothelial_cell:ASPC	0.67080626
T2DM	Pancreas	VAT	endothelial_cell:LEC	**0.0251075**
T2DM	Pancreas	VAT	endothelial_cell:SMC	0.54381246
T2DM	Pancreas	VAT	endothelial_cell:adipocyte	0.99361037
T2DM	Pancreas	VAT	endothelial_cell:b_cell	0.32971092
T2DM	Pancreas	VAT	endothelial_cell:dendritic_cell	0.71760299
T2DM	Pancreas	VAT	endothelial_cell:endometrium	0.51025662
T2DM	Pancreas	VAT	endothelial_cell:macrophage	0.34483827
T2DM	Pancreas	VAT	endothelial_cell:mast_cell	0.48774691
T2DM	Pancreas	VAT	endothelial_cell:mesothelium	0.93340323
T2DM	Pancreas	VAT	endothelial_cell:monocyte	0.30853692
T2DM	Pancreas	VAT	endothelial_cell:nk_cell	0.41193483
T2DM	Pancreas	VAT	endothelial_cell:pericyte	0.81283548
T2DM	Pancreas	VAT	endothelial_cell:t_cell	0.26677339
T2DM	Pancreas	VAT	leukocyte:mesenchymal_cell	0.32481768
T2DM	Pancreas	VAT	leukocyte:alpha_cell	0.59977003
T2DM	Pancreas	VAT	leukocyte:delta_cell	0.09580941
T2DM	Pancreas	VAT	leukocyte:PP_cell	0.6093176
T2DM	Pancreas	VAT	leukocyte:epsilon_cell	0.3524143
T2DM	Pancreas	VAT	leukocyte:beta_cell	0.34140521
T2DM	Pancreas	VAT	leukocyte:ASPC	0.42942941
T2DM	Pancreas	VAT	leukocyte:LEC	0.20285164
T2DM	Pancreas	VAT	leukocyte:SMC	0.26344646
T2DM	Pancreas	VAT	leukocyte:adipocyte	0.53003148
T2DM	Pancreas	VAT	leukocyte:b_cell	0.35294782
T2DM	Pancreas	VAT	leukocyte:dendritic_cell	0.59166957
T2DM	Pancreas	VAT	leukocyte:endometrium	0.64281266

T2DM	Pancreas	VAT	leukocyte:macrophage	0.46818609
T2DM	Pancreas	VAT	leukocyte:mast_cell	0.1913251
T2DM	Pancreas	VAT	leukocyte:mesothelium	0.62796363
T2DM	Pancreas	VAT	leukocyte:monocyte	0.54573875
T2DM	Pancreas	VAT	leukocyte:nk_cell	0.22390733
T2DM	Pancreas	VAT	leukocyte:pericyte	0.97081939
T2DM	Pancreas	VAT	leukocyte:t_cell	0.04566505
T2DM	Pancreas	VAT	mesenchymal_cell:alpha_cell	0.57453048
T2DM	Pancreas	VAT	mesenchymal_cell:delta_cell	0.88150498
T2DM	Pancreas	VAT	mesenchymal_cell:PP_cell	0.36522754
T2DM	Pancreas	VAT	mesenchymal_cell:epsilon_cell	0.75115525
T2DM	Pancreas	VAT	mesenchymal_cell:beta_cell	0.38366435
T2DM	Pancreas	VAT	mesenchymal_cell:ASPC	0.30341468
T2DM	Pancreas	VAT	mesenchymal_cell:LEC	0.17166597
T2DM	Pancreas	VAT	mesenchymal_cell:SMC	0.31947178
T2DM	Pancreas	VAT	mesenchymal_cell:adipocyte	0.97324771
T2DM	Pancreas	VAT	mesenchymal_cell:b_cell	0.12227294
T2DM	Pancreas	VAT	mesenchymal_cell:dendritic_cell	0.45650506
T2DM	Pancreas	VAT	mesenchymal_cell:endometrium	0.14842188
T2DM	Pancreas	VAT	mesenchymal_cell:macrophage	0.66589376
T2DM	Pancreas	VAT	mesenchymal_cell:mast_cell	0.71744886
T2DM	Pancreas	VAT	mesenchymal_cell:mesothelium	0.08148733
T2DM	Pancreas	VAT	mesenchymal_cell:monocyte	0.71977789
T2DM	Pancreas	VAT	mesenchymal_cell:nk_cell	0.05127272
T2DM	Pancreas	VAT	mesenchymal_cell:pericyte	0.32538963
T2DM	Pancreas	VAT	mesenchymal_cell:t_cell	***0.05655125***
T2DM	Pancreas	VAT	alpha_cell:delta_cell	0.10075931
T2DM	Pancreas	VAT	alpha_cell:PP_cell	0.76628939
T2DM	Pancreas	VAT	alpha_cell:epsilon_cell	0.10167912
T2DM	Pancreas	VAT	alpha_cell:beta_cell	0.18101797
T2DM	Pancreas	VAT	alpha_cell:ASPC	***0.06421838***
T2DM	Pancreas	VAT	alpha_cell:LEC	0.66803107
T2DM	Pancreas	VAT	alpha_cell:SMC	0.59032758
T2DM	Pancreas	VAT	alpha_cell:adipocyte	0.11894493
T2DM	Pancreas	VAT	alpha_cell:b_cell	**0.00275569**
T2DM	Pancreas	VAT	alpha_cell:dendritic_cell	0.6517685
T2DM	Pancreas	VAT	alpha_cell:endometrium	0.95406352
T2DM	Pancreas	VAT	alpha_cell:macrophage	0.71546863
T2DM	Pancreas	VAT	alpha_cell:mast_cell	0.52975426
T2DM	Pancreas	VAT	alpha_cell:mesothelium	0.05607374
T2DM	Pancreas	VAT	alpha_cell:monocyte	0.85595202
T2DM	Pancreas	VAT	alpha_cell:nk_cell	**0.00118179**
T2DM	Pancreas	VAT	alpha_cell:pericyte	0.46282141
T2DM	Pancreas	VAT	alpha_cell:t_cell	**0.02231581**
T2DM	Pancreas	VAT	delta_cell:PP_cell	0.63141188
T2DM	Pancreas	VAT	delta_cell:epsilon_cell	***0.07099051***

T2DM	Pancreas	VAT	delta_cell:beta_cell	***0.07344051***
T2DM	Pancreas	VAT	delta_cell:ASPC	***0.0724172***
T2DM	Pancreas	VAT	delta_cell:LEC	0.62687413
T2DM	Pancreas	VAT	delta_cell:SMC	0.84933548
T2DM	Pancreas	VAT	delta_cell:adipocyte	0.24403061
T2DM	Pancreas	VAT	delta_cell:b_cell	**0.02705046**
T2DM	Pancreas	VAT	delta_cell:dendritic_cell	0.52523745
T2DM	Pancreas	VAT	delta_cell:endometrium	0.63937646
T2DM	Pancreas	VAT	delta_cell:macrophage	0.92682532
T2DM	Pancreas	VAT	delta_cell:mast_cell	0.20819836
T2DM	Pancreas	VAT	delta_cell:mesothelium	0.0990026
T2DM	Pancreas	VAT	delta_cell:monocyte	0.77730783
T2DM	Pancreas	VAT	delta_cell:nk_cell	0.00364014
T2DM	Pancreas	VAT	delta_cell:pericyte	0.24548818
T2DM	Pancreas	VAT	delta_cell:t_cell	**0.04060968**
T2DM	Pancreas	VAT	PP_cell:epsilon_cell	***0.07073076***
T2DM	Pancreas	VAT	PP_cell:beta_cell	0.61889084
T2DM	Pancreas	VAT	PP_cell:ASPC	0.13410537
T2DM	Pancreas	VAT	PP_cell:LEC	0.61567533
T2DM	Pancreas	VAT	PP_cell:SMC	0.53709744
T2DM	Pancreas	VAT	PP_cell:adipocyte	0.52950587
T2DM	Pancreas	VAT	PP_cell:b_cell	***0.07695005***
T2DM	Pancreas	VAT	PP_cell:dendritic_cell	0.80516253
T2DM	Pancreas	VAT	PP_cell:endometrium	0.10408507
T2DM	Pancreas	VAT	PP_cell:macrophage	0.7018505
T2DM	Pancreas	VAT	PP_cell:mast_cell	0.94114526
T2DM	Pancreas	VAT	PP_cell:mesothelium	0.23444261
T2DM	Pancreas	VAT	PP_cell:monocyte	0.96573889
T2DM	Pancreas	VAT	PP_cell:nk_cell	**0.01133093**
T2DM	Pancreas	VAT	PP_cell:pericyte	0.71173355
T2DM	Pancreas	VAT	PP_cell:t_cell	0.38199992
T2DM	Pancreas	VAT	epsilon_cell:beta_cell	***0.0848577***
T2DM	Pancreas	VAT	epsilon_cell:ASPC	**0.03696924**
T2DM	Pancreas	VAT	epsilon_cell:LEC	0.27078956
T2DM	Pancreas	VAT	epsilon_cell:SMC	**0.00499444**
T2DM	Pancreas	VAT	epsilon_cell:adipocyte	0.22210833
T2DM	Pancreas	VAT	epsilon_cell:b_cell	0.28715126
T2DM	Pancreas	VAT	epsilon_cell:dendritic_cell	0.682497
T2DM	Pancreas	VAT	epsilon_cell:endometrium	0.52105331
T2DM	Pancreas	VAT	epsilon_cell:macrophage	0.51037673
T2DM	Pancreas	VAT	epsilon_cell:mast_cell	0.93178586
T2DM	Pancreas	VAT	epsilon_cell:mesothelium	***0.08227178***
T2DM	Pancreas	VAT	epsilon_cell:monocyte	***0.07749121***
T2DM	Pancreas	VAT	epsilon_cell:nk_cell	0.2523439
T2DM	Pancreas	VAT	epsilon_cell:pericyte	0.91224114
T2DM	Pancreas	VAT	epsilon_cell:t_cell	0.53556408

T2DM	Pancreas	VAT	beta_cell:ASPC	0.10338746
T2DM	Pancreas	VAT	beta_cell:LEC	0.4365188
T2DM	Pancreas	VAT	beta_cell:SMC	0.92410549
T2DM	Pancreas	VAT	beta_cell:adipocyte	0.19475614
T2DM	Pancreas	VAT	beta_cell:b_cell	**0.00302432**
T2DM	Pancreas	VAT	beta_cell:dendritic_cell	0.7136117
T2DM	Pancreas	VAT	beta_cell:endometrium	0.87585576
T2DM	Pancreas	VAT	beta_cell:macrophage	0.96829026
T2DM	Pancreas	VAT	beta_cell:mast_cell	0.22917949
T2DM	Pancreas	VAT	beta_cell:mesothelium	***0.07422136***
T2DM	Pancreas	VAT	beta_cell:monocyte	0.91218422
T2DM	Pancreas	VAT	beta_cell:nk_cell	**0.00346773**
T2DM	Pancreas	VAT	beta_cell:pericyte	0.39138067
T2DM	Pancreas	VAT	beta_cell:t_cell	**0.02274358**
T2DM	Pancreas	VAT	ASPC:LEC	0.47401717
T2DM	Pancreas	VAT	ASPC:SMC	**0.00790156**
T2DM	Pancreas	VAT	ASPC:adipocyte	**0.02285139**
T2DM	Pancreas	VAT	ASPC:b_cell	0.65021814
T2DM	Pancreas	VAT	ASPC:dendritic_cell	0.35084117
T2DM	Pancreas	VAT	ASPC:endometrium	0.43185478
T2DM	Pancreas	VAT	ASPC:macrophage	0.96858651
T2DM	Pancreas	VAT	ASPC:mast_cell	0.70876975
T2DM	Pancreas	VAT	ASPC:mesothelium	0.64276565
T2DM	Pancreas	VAT	ASPC:monocyte	0.48324922
T2DM	Pancreas	VAT	ASPC:nk_cell	0.12320917
T2DM	Pancreas	VAT	ASPC:pericyte	0.25369284
T2DM	Pancreas	VAT	ASPC:t_cell	0.38261655
T2DM	Pancreas	VAT	LEC:SMC	***0.05291415***
T2DM	Pancreas	VAT	LEC:adipocyte	0.55187537
T2DM	Pancreas	VAT	LEC:b_cell	0.64771721
T2DM	Pancreas	VAT	LEC:dendritic_cell	***0.0503179***
T2DM	Pancreas	VAT	LEC:endometrium	0.16829462
T2DM	Pancreas	VAT	LEC:macrophage	0.23342018
T2DM	Pancreas	VAT	LEC:mast_cell	0.36273323
T2DM	Pancreas	VAT	LEC:mesothelium	0.11591034
T2DM	Pancreas	VAT	LEC:monocyte	0.31721287
T2DM	Pancreas	VAT	LEC:nk_cell	0.24949828
T2DM	Pancreas	VAT	LEC:pericyte	**0.03467235**
T2DM	Pancreas	VAT	LEC:t_cell	0.89829889
T2DM	Pancreas	VAT	SMC:adipocyte	***0.06749887***
T2DM	Pancreas	VAT	SMC:b_cell	**0.00343835**
T2DM	Pancreas	VAT	SMC:dendritic_cell	0.45376708
T2DM	Pancreas	VAT	SMC:endometrium	0.16097557
T2DM	Pancreas	VAT	SMC:macrophage	**0.00686134**
T2DM	Pancreas	VAT	SMC:mast_cell	0.21399553
T2DM	Pancreas	VAT	SMC:mesothelium	**0.01328534**

T2DM	Pancreas	VAT	SMC:monocyte	**0.04992094**
T2DM	Pancreas	VAT	SMC:nk_cell	0.27662675
T2DM	Pancreas	VAT	SMC:pericyte	0.96216014
T2DM	Pancreas	VAT	SMC:t_cell	0.61588723
T2DM	Pancreas	VAT	adipocyte:b_cell	0.15108652
T2DM	Pancreas	VAT	adipocyte:dendritic_cell	0.34425329
T2DM	Pancreas	VAT	adipocyte:endometrium	0.17031061
T2DM	Pancreas	VAT	adipocyte:macrophage	**0.0093081**
T2DM	Pancreas	VAT	adipocyte:mast_cell	***0.09927229***
T2DM	Pancreas	VAT	adipocyte:mesothelium	0.85568307
T2DM	Pancreas	VAT	adipocyte:monocyte	**0.02556316**
T2DM	Pancreas	VAT	adipocyte:nk_cell	**0.00282693**
T2DM	Pancreas	VAT	adipocyte:pericyte	***0.08490673***
T2DM	Pancreas	VAT	adipocyte:t_cell	0.36594408
T2DM	Pancreas	VAT	b_cell:dendritic_cell	0.24945421
T2DM	Pancreas	VAT	b_cell:endometrium	0.16188815
T2DM	Pancreas	VAT	b_cell:macrophage	**0.00764174**
T2DM	Pancreas	VAT	b_cell:mast_cell	0.10089324
T2DM	Pancreas	VAT	b_cell:mesothelium	0.53308568
T2DM	Pancreas	VAT	b_cell:monocyte	**0.00190559**
T2DM	Pancreas	VAT	b_cell:nk_cell	0.25922822
T2DM	Pancreas	VAT	b_cell:pericyte	***0.06839117***
T2DM	Pancreas	VAT	b_cell:t_cell	0.69971966
T2DM	Pancreas	VAT	dendritic_cell:endometrium	0.3030885
T2DM	Pancreas	VAT	dendritic_cell:macrophage	0.3834226
T2DM	Pancreas	VAT	dendritic_cell:mast_cell	0.2368336
T2DM	Pancreas	VAT	dendritic_cell:mesothelium	0.16460869
T2DM	Pancreas	VAT	dendritic_cell:monocyte	0.04524262
T2DM	Pancreas	VAT	dendritic_cell:nk_cell	0.12784787
T2DM	Pancreas	VAT	dendritic_cell:pericyte	0.75218023
T2DM	Pancreas	VAT	dendritic_cell:t_cell	0.80543254
T2DM	Pancreas	VAT	endometrium:macrophage	0.22618651
T2DM	Pancreas	VAT	endometrium:mast_cell	0.32156948
T2DM	Pancreas	VAT	endometrium:mesothelium	0.21031176
T2DM	Pancreas	VAT	endometrium:monocyte	0.53933523
T2DM	Pancreas	VAT	endometrium:nk_cell	0.58024444
T2DM	Pancreas	VAT	endometrium:pericyte	0.6273574
T2DM	Pancreas	VAT	endometrium:t_cell	0.78099385
T2DM	Pancreas	VAT	macrophage:mast_cell	0.41179425
T2DM	Pancreas	VAT	macrophage:mesothelium	0.14838199
T2DM	Pancreas	VAT	macrophage:monocyte	0.5109686
T2DM	Pancreas	VAT	macrophage:nk_cell	0.14050227
T2DM	Pancreas	VAT	macrophage:pericyte	0.61260784
T2DM	Pancreas	VAT	macrophage:t_cell	0.14660742
T2DM	Pancreas	VAT	mast_cell:mesothelium	0.23407552
T2DM	Pancreas	VAT	mast_cell:monocyte	0.46204858

T2DM	Pancreas	VAT	mast_cell:nk_cell	0.95480475
T2DM	Pancreas	VAT	mast_cell:pericyte	0.2127622
T2DM	Pancreas	VAT	mast_cell:t_cell	***0.05878174***
T2DM	Pancreas	VAT	mesothelium:monocyte	**0.04467139**
T2DM	Pancreas	VAT	mesothelium:nk_cell	0.81910548
T2DM	Pancreas	VAT	mesothelium:pericyte	0.10063517
T2DM	Pancreas	VAT	mesothelium:t_cell	0.45671048
T2DM	Pancreas	VAT	monocyte:nk_cell	***0.09301825***
T2DM	Pancreas	VAT	monocyte:pericyte	0.65982395
T2DM	Pancreas	VAT	monocyte:t_cell	**0.03169538**
T2DM	Pancreas	VAT	nk_cell:pericyte	0.1732244
T2DM	Pancreas	VAT	nk_cell:t_cell	0.9134381
T2DM	Pancreas	VAT	pericyte:t_cell	0.49692325
Obesity	SAT	VAT	ASPC_SAT:LEC_SAT	0.139886882
Obesity	SAT	VAT	ASPC_SAT:SMC_SAT	0.25099973
Obesity	SAT	VAT	ASPC_SAT:adipocyte_SAT	***0.074404683***
Obesity	SAT	VAT	ASPC_SAT:b_cell_SAT	0.74005422
Obesity	SAT	VAT	ASPC_SAT:dendritic_cell_SAT	0.71718851
Obesity	SAT	VAT	ASPC_SAT:macrophage_SAT	0.523372342
Obesity	SAT	VAT	ASPC_SAT:mast_cell_SAT	**0.007274007**
Obesity	SAT	VAT	ASPC_SAT:monocyte_SAT	0.084956974
Obesity	SAT	VAT	ASPC_SAT:nk_cell_SAT	**0.015089054**
Obesity	SAT	VAT	ASPC_SAT:pericyte_SAT	0.822458654
Obesity	SAT	VAT	ASPC_SAT:t_cell_SAT	**0.000649308**
Obesity	SAT	VAT	ASPC_SAT:ASPC_VAT	**0.011339492**
Obesity	SAT	VAT	ASPC_SAT:LEC_VAT	**0.024187599**
Obesity	SAT	VAT	ASPC_SAT:SMC_VAT	0.785940583
Obesity	SAT	VAT	ASPC_SAT:adipocyte_VAT	***0.0833612***
Obesity	SAT	VAT	ASPC_SAT:b_cell_VAT	0.474037861
Obesity	SAT	VAT	ASPC_SAT:dendritic_cell_VAT	0.464150051
Obesity	SAT	VAT	ASPC_SAT:macrophage_VAT	0.641273776
Obesity	SAT	VAT	ASPC_SAT:mast_cell_VAT	0.515731796
Obesity	SAT	VAT	ASPC_SAT:monocyte_VAT	0.39663188
Obesity	SAT	VAT	ASPC_SAT:nk_cell_VAT	**0.001656984**
Obesity	SAT	VAT	ASPC_SAT:pericyte_VAT	**0.016745403**
Obesity	SAT	VAT	ASPC_SAT:t_cell_VAT	0.687139626
Obesity	SAT	VAT	LEC_SAT:SMC_SAT	0.188717305
Obesity	SAT	VAT	LEC_SAT:adipocyte_SAT	0.159578622
Obesity	SAT	VAT	LEC_SAT:b_cell_SAT	0.456924067
Obesity	SAT	VAT	LEC_SAT:dendritic_cell_SAT	0.97158446
Obesity	SAT	VAT	LEC_SAT:macrophage_SAT	0.557310431
Obesity	SAT	VAT	LEC_SAT:mast_cell_SAT	**0.041021157**
Obesity	SAT	VAT	LEC_SAT:monocyte_SAT	0.30102665
Obesity	SAT	VAT	LEC_SAT:nk_cell_SAT	**0.047380066**
Obesity	SAT	VAT	LEC_SAT:pericyte_SAT	0.111762136
Obesity	SAT	VAT	LEC_SAT:t_cell_SAT	***0.062695689***

Obesity	SAT	VAT	LEC_SAT:ASPC_VAT	0.144062083
Obesity	SAT	VAT	LEC_SAT:LEC_VAT	**0.041976758**
Obesity	SAT	VAT	LEC_SAT:SMC_VAT	0.213718846
Obesity	SAT	VAT	LEC_SAT:adipocyte_VAT	**0.010299179**
Obesity	SAT	VAT	LEC_SAT:b_cell_VAT	**0.00691476**
Obesity	SAT	VAT	LEC_SAT:dendritic_cell_VAT	**0.006699365**
Obesity	SAT	VAT	LEC_SAT:macrophage_VAT	0.736130895
Obesity	SAT	VAT	LEC_SAT:mast_cell_VAT	0.856837866
Obesity	SAT	VAT	LEC_SAT:monocyte_VAT	0.780200627
Obesity	SAT	VAT	LEC_SAT:nk_cell_VAT	0.712713757
Obesity	SAT	VAT	LEC_SAT:pericyte_VAT	0.838848373
Obesity	SAT	VAT	LEC_SAT:t_cell_VAT	0.368323341
Obesity	SAT	VAT	SMC_SAT:adipocyte_SAT	***0.085512836***
Obesity	SAT	VAT	SMC_SAT:b_cell_SAT	0.611367546
Obesity	SAT	VAT	SMC_SAT:dendritic_cell_SAT	0.95782989
Obesity	SAT	VAT	SMC_SAT:macrophage_SAT	***0.063166895***
Obesity	SAT	VAT	SMC_SAT:mast_cell_SAT	0.168103691
Obesity	SAT	VAT	SMC_SAT:monocyte_SAT	0.326959236
Obesity	SAT	VAT	SMC_SAT:nk_cell_SAT	0.166240582
Obesity	SAT	VAT	SMC_SAT:pericyte_SAT	***0.076261492***
Obesity	SAT	VAT	SMC_SAT:t_cell_SAT	***0.050007071***
Obesity	SAT	VAT	SMC_SAT:ASPC_VAT	0.696128311
Obesity	SAT	VAT	SMC_SAT:LEC_VAT	0.215970577
Obesity	SAT	VAT	SMC_SAT:SMC_VAT	0.854794108
Obesity	SAT	VAT	SMC_SAT:adipocyte_VAT	***0.099048187***
Obesity	SAT	VAT	SMC_SAT:b_cell_VAT	0.162602859
Obesity	SAT	VAT	SMC_SAT:dendritic_cell_VAT	0.718715205
Obesity	SAT	VAT	SMC_SAT:macrophage_VAT	0.828063409
Obesity	SAT	VAT	SMC_SAT:mast_cell_VAT	0.332014562
Obesity	SAT	VAT	SMC_SAT:monocyte_VAT	0.880672727
Obesity	SAT	VAT	SMC_SAT:nk_cell_VAT	0.896403404
Obesity	SAT	VAT	SMC_SAT:pericyte_VAT	0.429567076
Obesity	SAT	VAT	SMC_SAT:t_cell_VAT	0.684622679
Obesity	SAT	VAT	adipocyte_SAT:b_cell_SAT	0.494297399
Obesity	SAT	VAT	adipocyte_SAT:dendritic_cell_SA	0.398654351
Obesity	SAT	VAT	adipocyte_SAT:macrophage_SA	0.327451836
Obesity	SAT	VAT	adipocyte_SAT:mast_cell_SAT	0.106293401
Obesity	SAT	VAT	adipocyte_SAT:monocyte_SAT	***0.051837511***
Obesity	SAT	VAT	adipocyte_SAT:nk_cell_SAT	0.108170654
Obesity	SAT	VAT	adipocyte_SAT:pericyte_SAT	0.103632268
Obesity	SAT	VAT	adipocyte_SAT:t_cell_SAT	0.610404428
Obesity	SAT	VAT	adipocyte_SAT:ASPC_VAT	0.73026057
Obesity	SAT	VAT	adipocyte_SAT:LEC_VAT	0.31193788
Obesity	SAT	VAT	adipocyte_SAT:SMC_VAT	**0.026485201**
Obesity	SAT	VAT	adipocyte_SAT:adipocyte_VAT	0.208417533
Obesity	SAT	VAT	adipocyte_SAT:b_cell_VAT	0.191119915

Obesity	SAT	VAT	adipocyte_SAT:dendritic_cell_V/	0.830946148
Obesity	SAT	VAT	adipocyte_SAT:macrophage_VA'	0.268924114
Obesity	SAT	VAT	adipocyte_SAT:mast_cell_VAT	0.112012949
Obesity	SAT	VAT	adipocyte_SAT:monocyte_VAT	0.712311917
Obesity	SAT	VAT	adipocyte_SAT:nk_cell_VAT	**0.001283656**
Obesity	SAT	VAT	adipocyte_SAT:pericyte_VAT	**0.004184319**
Obesity	SAT	VAT	adipocyte_SAT:t_cell_VAT	0.446602771
Obesity	SAT	VAT	b_cell_SAT:dendritic_cell_SAT	0.375752314
Obesity	SAT	VAT	b_cell_SAT:macrophage_SAT	0.841839128
Obesity	SAT	VAT	b_cell_SAT:mast_cell_SAT	0.268884537
Obesity	SAT	VAT	b_cell_SAT:monocyte_SAT	**0.013754236**
Obesity	SAT	VAT	b_cell_SAT:nk_cell_SAT	0.127014476
Obesity	SAT	VAT	b_cell_SAT:pericyte_SAT	0.427608166
Obesity	SAT	VAT	b_cell_SAT:t_cell_SAT	0.628019826
Obesity	SAT	VAT	b_cell_SAT:ASPC_VAT	0.106146675
Obesity	SAT	VAT	b_cell_SAT:LEC_VAT	0.312375871
Obesity	SAT	VAT	b_cell_SAT:SMC_VAT	0.121025397
Obesity	SAT	VAT	b_cell_SAT:adipocyte_VAT	0.647478307
Obesity	SAT	VAT	b_cell_SAT:b_cell_VAT	0.051830401
Obesity	SAT	VAT	b_cell_SAT:dendritic_cell_VAT	0.059983975
Obesity	SAT	VAT	b_cell_SAT:macrophage_VAT	0.89196523
Obesity	SAT	VAT	b_cell_SAT:mast_cell_VAT	0.354341204
Obesity	SAT	VAT	b_cell_SAT:monocyte_VAT	0.256007969
Obesity	SAT	VAT	b_cell_SAT:nk_cell_VAT	**0.000172856**
Obesity	SAT	VAT	b_cell_SAT:pericyte_VAT	**0.005405607**
Obesity	SAT	VAT	b_cell_SAT:t_cell_VAT	0.13427837
Obesity	SAT	VAT	dendritic_cell_SAT:macrophage_	0.9415823
Obesity	SAT	VAT	dendritic_cell_SAT:mast_cell_S/	0.548379602
Obesity	SAT	VAT	dendritic_cell_SAT:monocyte_S/	0.11227383
Obesity	SAT	VAT	dendritic_cell_SAT:nk_cell_SAT	0.527962611
Obesity	SAT	VAT	dendritic_cell_SAT:pericyte_SAT	0.14241599
Obesity	SAT	VAT	dendritic_cell_SAT:t_cell_SAT	0.887292027
Obesity	SAT	VAT	dendritic_cell_SAT:ASPC_VAT	0.718006678
Obesity	SAT	VAT	dendritic_cell_SAT:LEC_VAT	0.481540126
Obesity	SAT	VAT	dendritic_cell_SAT:SMC_VAT	0.778402798
Obesity	SAT	VAT	dendritic_cell_SAT:adipocyte_V/	0.017304898
Obesity	SAT	VAT	dendritic_cell_SAT:b_cell_VAT	0.121432717
Obesity	SAT	VAT	dendritic_cell_SAT:dendritic_cel	***0.066649316***
Obesity	SAT	VAT	dendritic_cell_SAT:macrophage_	0.113397125
Obesity	SAT	VAT	dendritic_cell_SAT:mast_cell_V/	0.281961623
Obesity	SAT	VAT	dendritic_cell_SAT:monocyte_V/	0.96449162
Obesity	SAT	VAT	dendritic_cell_SAT:nk_cell_VAT	0.552875762
Obesity	SAT	VAT	dendritic_cell_SAT:pericyte_VA'	0.645446192
Obesity	SAT	VAT	dendritic_cell_SAT:t_cell_VAT	0.11308887
Obesity	SAT	VAT	macrophage_SAT:mast_cell_SAT	0.127998641
Obesity	SAT	VAT	macrophage_SAT:monocyte_SA'	0.161953908

Obesity	SAT	VAT	macrophage_SAT:nk_cell_SAT	**0.000401693**
Obesity	SAT	VAT	macrophage_SAT:pericyte_SAT	0.933379155
Obesity	SAT	VAT	macrophage_SAT:t_cell_SAT	0.114298162
Obesity	SAT	VAT	macrophage_SAT:ASPC_VAT	0.275412406
Obesity	SAT	VAT	macrophage_SAT:LEC_VAT	0.577611367
Obesity	SAT	VAT	macrophage_SAT:SMC_VAT	0.233827614
Obesity	SAT	VAT	macrophage_SAT:adipocyte_VA	0.119273735
Obesity	SAT	VAT	macrophage_SAT:b_cell_VAT	0.294025613
Obesity	SAT	VAT	macrophage_SAT:dendritic_cell_	0.148864891
Obesity	SAT	VAT	macrophage_SAT:macrophage_V	0.304880887
Obesity	SAT	VAT	macrophage_SAT:mast_cell_VA	0.38410412
Obesity	SAT	VAT	macrophage_SAT:monocyte_VA	0.145783605
Obesity	SAT	VAT	macrophage_SAT:nk_cell_VAT	**7.03E-05**
Obesity	SAT	VAT	macrophage_SAT:pericyte_VAT	**0.006387076**
Obesity	SAT	VAT	macrophage_SAT:t_cell_VAT	0.372696992
Obesity	SAT	VAT	mast_cell_SAT:monocyte_SAT	0.356001563
Obesity	SAT	VAT	mast_cell_SAT:nk_cell_SAT	0.293662247
Obesity	SAT	VAT	mast_cell_SAT:pericyte_SAT	0.274887221
Obesity	SAT	VAT	mast_cell_SAT:t_cell_SAT	0.338656501
Obesity	SAT	VAT	mast_cell_SAT:ASPC_VAT	0.243959966
Obesity	SAT	VAT	mast_cell_SAT:LEC_VAT	**0.014554844**
Obesity	SAT	VAT	mast_cell_SAT:SMC_VAT	0.75218811
Obesity	SAT	VAT	mast_cell_SAT:adipocyte_VAT	**0.001269535**
Obesity	SAT	VAT	mast_cell_SAT:b_cell_VAT	0.371387579
Obesity	SAT	VAT	mast_cell_SAT:dendritic_cell_V	0.895288729
Obesity	SAT	VAT	mast_cell_SAT:macrophage_VA	0.392496545
Obesity	SAT	VAT	mast_cell_SAT:mast_cell_VAT	**0.014604581**
Obesity	SAT	VAT	mast_cell_SAT:monocyte_VAT	***0.07704629***
Obesity	SAT	VAT	mast_cell_SAT:nk_cell_VAT	0.499588032
Obesity	SAT	VAT	mast_cell_SAT:pericyte_VAT	**0.005582189**
Obesity	SAT	VAT	mast_cell_SAT:t_cell_VAT	**0.017382586**
Obesity	SAT	VAT	monocyte_SAT:nk_cell_SAT	0.196552771
Obesity	SAT	VAT	monocyte_SAT:pericyte_SAT	0.101710601
Obesity	SAT	VAT	monocyte_SAT:t_cell_SAT	0.156666624
Obesity	SAT	VAT	monocyte_SAT:ASPC_VAT	0.401955977
Obesity	SAT	VAT	monocyte_SAT:LEC_VAT	0.385931313
Obesity	SAT	VAT	monocyte_SAT:SMC_VAT	0.618987479
Obesity	SAT	VAT	monocyte_SAT:adipocyte_VAT	**0.00149331**
Obesity	SAT	VAT	monocyte_SAT:b_cell_VAT	0.122284369
Obesity	SAT	VAT	monocyte_SAT:dendritic_cell_V	0.038212353
Obesity	SAT	VAT	monocyte_SAT:macrophage_VA	0.656918845
Obesity	SAT	VAT	monocyte_SAT:mast_cell_VAT	0.595456052
Obesity	SAT	VAT	monocyte_SAT:monocyte_VAT	***0.093954309***
Obesity	SAT	VAT	monocyte_SAT:nk_cell_VAT	**0.028440375**
Obesity	SAT	VAT	monocyte_SAT:pericyte_VAT	***0.099924536***
Obesity	SAT	VAT	monocyte_SAT:t_cell_VAT	0.200040201

Obesity	SAT	VAT	nk_cell_SAT:pericyte_SAT	0.165414234
Obesity	SAT	VAT	nk_cell_SAT:t_cell_SAT	0.315329781
Obesity	SAT	VAT	nk_cell_SAT:ASPC_VAT	**0.00890831**
Obesity	SAT	VAT	nk_cell_SAT:LEC_VAT	0.999667441
Obesity	SAT	VAT	nk_cell_SAT:SMC_VAT	0.273690258
Obesity	SAT	VAT	nk_cell_SAT:adipocyte_VAT	0.300103344
Obesity	SAT	VAT	nk_cell_SAT:b_cell_VAT	0.554110131
Obesity	SAT	VAT	nk_cell_SAT:dendritic_cell_VAT	0.863313336
Obesity	SAT	VAT	nk_cell_SAT:macrophage_VAT	0.951511822
Obesity	SAT	VAT	nk_cell_SAT:mast_cell_VAT	0.114634409
Obesity	SAT	VAT	nk_cell_SAT:monocyte_VAT	0.141800371
Obesity	SAT	VAT	nk_cell_SAT:nk_cell_VAT	**0.000769738**
Obesity	SAT	VAT	nk_cell_SAT:pericyte_VAT	**0.002933408**
Obesity	SAT	VAT	nk_cell_SAT:t_cell_VAT	0.146690097
Obesity	SAT	VAT	pericyte_SAT:t_cell_SAT	0.844868205
Obesity	SAT	VAT	pericyte_SAT:ASPC_VAT	0.833322666
Obesity	SAT	VAT	pericyte_SAT:LEC_VAT	0.523766211
Obesity	SAT	VAT	pericyte_SAT:SMC_VAT	0.644500362
Obesity	SAT	VAT	pericyte_SAT:adipocyte_VAT	0.351519905
Obesity	SAT	VAT	pericyte_SAT:b_cell_VAT	0.342620982
Obesity	SAT	VAT	pericyte_SAT:dendritic_cell_VAT	0.279917256
Obesity	SAT	VAT	pericyte_SAT:macrophage_VAT	0.576528651
Obesity	SAT	VAT	pericyte_SAT:mast_cell_VAT	**0.01078661**
Obesity	SAT	VAT	pericyte_SAT:monocyte_VAT	***0.051482444***
Obesity	SAT	VAT	pericyte_SAT:nk_cell_VAT	0.114021343
Obesity	SAT	VAT	pericyte_SAT:pericyte_VAT	0.28873594
Obesity	SAT	VAT	pericyte_SAT:t_cell_VAT	0.328213881
Obesity	SAT	VAT	t_cell_SAT:ASPC_VAT	0.258946974
Obesity	SAT	VAT	t_cell_SAT:LEC_VAT	0.308157919
Obesity	SAT	VAT	t_cell_SAT:SMC_VAT	0.352711188
Obesity	SAT	VAT	t_cell_SAT:adipocyte_VAT	**9.78E-06**
Obesity	SAT	VAT	t_cell_SAT:b_cell_VAT	0.362500559
Obesity	SAT	VAT	t_cell_SAT:dendritic_cell_VAT	0.663371045
Obesity	SAT	VAT	t_cell_SAT:macrophage_VAT	0.031095081
Obesity	SAT	VAT	t_cell_SAT:mast_cell_VAT	0.682840301
Obesity	SAT	VAT	t_cell_SAT:monocyte_VAT	0.667225994
Obesity	SAT	VAT	t_cell_SAT:nk_cell_VAT	**0.028879768**
Obesity	SAT	VAT	t_cell_SAT:pericyte_VAT	**0.001900505**
Obesity	SAT	VAT	t_cell_SAT:t_cell_VAT	0.201970288
Obesity	SAT	VAT	ASPC_VAT:LEC_VAT	**0.009320911**
Obesity	SAT	VAT	ASPC_VAT:SMC_VAT	**0.030477553**
Obesity	SAT	VAT	ASPC_VAT:adipocyte_VAT	**0.000338604**
Obesity	SAT	VAT	ASPC_VAT:b_cell_VAT	**1.97E-09**
Obesity	SAT	VAT	ASPC_VAT:dendritic_cell_VAT	**0.001131651**
Obesity	SAT	VAT	ASPC_VAT:macrophage_VAT	0.686925993
Obesity	SAT	VAT	ASPC_VAT:mast_cell_VAT	0.146792576

Obesity	SAT	VAT	ASPC_VAT:monocyte_VAT	0.600775065
Obesity	SAT	VAT	ASPC_VAT:nk_cell_VAT	0.286842808
Obesity	SAT	VAT	ASPC_VAT:pericyte_VAT	0.166510366
Obesity	SAT	VAT	ASPC_VAT:t_cell_VAT	**0.022665254**
Obesity	SAT	VAT	LEC_VAT:SMC_VAT	0.648068081
Obesity	SAT	VAT	LEC_VAT:adipocyte_VAT	0.296929529
Obesity	SAT	VAT	LEC_VAT:b_cell_VAT	0.312555809
Obesity	SAT	VAT	LEC_VAT:dendritic_cell_VAT	0.213991908
Obesity	SAT	VAT	LEC_VAT:macrophage_VAT	0.801660969
Obesity	SAT	VAT	LEC_VAT:mast_cell_VAT	0.24934883
Obesity	SAT	VAT	LEC_VAT:monocyte_VAT	**0.027720115**
Obesity	SAT	VAT	LEC_VAT:nk_cell_VAT	**0.007178859**
Obesity	SAT	VAT	LEC_VAT:pericyte_VAT	**0.077948698**
Obesity	SAT	VAT	LEC_VAT:t_cell_VAT	0.253330356
Obesity	SAT	VAT	SMC_VAT:adipocyte_VAT	**0.007790049**
Obesity	SAT	VAT	SMC_VAT:b_cell_VAT	**0.043087747**
Obesity	SAT	VAT	SMC_VAT:dendritic_cell_VAT	0.814846243
Obesity	SAT	VAT	SMC_VAT:macrophage_VAT	0.770444202
Obesity	SAT	VAT	SMC_VAT:mast_cell_VAT	0.121023271
Obesity	SAT	VAT	SMC_VAT:monocyte_VAT	0.11756166
Obesity	SAT	VAT	SMC_VAT:nk_cell_VAT	0.433360755
Obesity	SAT	VAT	SMC_VAT:pericyte_VAT	**0.003509705**
Obesity	SAT	VAT	SMC_VAT:t_cell_VAT	0.367739613
Obesity	SAT	VAT	adipocyte_VAT:b_cell_VAT	0.844825107
Obesity	SAT	VAT	adipocyte_VAT:dendritic_cell_V	0.526634155
Obesity	SAT	VAT	adipocyte_VAT:macrophage_VA	**0.030842076**
Obesity	SAT	VAT	adipocyte_VAT:mast_cell_VAT	0.301812603
Obesity	SAT	VAT	adipocyte_VAT:monocyte_VAT	0.241468826
Obesity	SAT	VAT	adipocyte_VAT:nk_cell_VAT	0.052966861
Obesity	SAT	VAT	adipocyte_VAT:pericyte_VAT	0.008628862
Obesity	SAT	VAT	adipocyte_VAT:t_cell_VAT	0.883291987
Obesity	SAT	VAT	b_cell_VAT:dendritic_cell_VAT	0.166482247
Obesity	SAT	VAT	b_cell_VAT:macrophage_VAT	0.130268429
Obesity	SAT	VAT	b_cell_VAT:mast_cell_VAT	0.889517962
Obesity	SAT	VAT	b_cell_VAT:monocyte_VAT	**0.007997572**
Obesity	SAT	VAT	b_cell_VAT:nk_cell_VAT	**0.030289561**
Obesity	SAT	VAT	b_cell_VAT:pericyte_VAT	**9.38E-05**
Obesity	SAT	VAT	b_cell_VAT:t_cell_VAT	***0.050626336***
Obesity	SAT	VAT	dendritic_cell_VAT:macrophage_	0.154146182
Obesity	SAT	VAT	dendritic_cell_VAT:mast_cell_V	0.982544755
Obesity	SAT	VAT	dendritic_cell_VAT:monocyte_V	0.542564088
Obesity	SAT	VAT	dendritic_cell_VAT:nk_cell_VA	0.617219184
Obesity	SAT	VAT	dendritic_cell_VAT:pericyte_VA	0.677454009
Obesity	SAT	VAT	dendritic_cell_VAT:t_cell_VAT	0.562413046
Obesity	SAT	VAT	macrophage_VAT:mast_cell_VA	0.590375674
Obesity	SAT	VAT	macrophage_VAT:monocyte_VA	0.792171094

Obesity	SAT	VAT	macrophage_VAT:nk_cell_VAT	0.570160431
Obesity	SAT	VAT	macrophage_VAT:pericyte_VAT	0.126548169
Obesity	SAT	VAT	macrophage_VAT:t_cell_VAT	0.505378935
Obesity	SAT	VAT	mast_cell_VAT:monocyte_VAT	***0.053237842***
Obesity	SAT	VAT	mast_cell_VAT:nk_cell_VAT	**0.041613926**
Obesity	SAT	VAT	mast_cell_VAT:pericyte_VAT	0.650789506
Obesity	SAT	VAT	mast_cell_VAT:t_cell_VAT	0.723983297
Obesity	SAT	VAT	monocyte_VAT:nk_cell_VAT	0.583831841
Obesity	SAT	VAT	monocyte_VAT:pericyte_VAT	0.110081913
Obesity	SAT	VAT	monocyte_VAT:t_cell_VAT	***0.053467944***
Obesity	SAT	VAT	nk_cell_VAT:pericyte_VAT	0.713059534
Obesity	SAT	VAT	nk_cell_VAT:t_cell_VAT	**0.041916389**
Obesity	SAT	VAT	pericyte_VAT:t_cell_VAT	0.306876684
