# Supplementary material for: CausalCellInfer: Prioritizing Cell‐Type‐Specific Disease Associations From Genetically Predicted Transcriptomes in Biobank Cohorts
Source: Adv Sci (Weinh). 2026 Oct 14:e78197. Online ahead of print. doi: 10.1002/advs.78197 (PMC13669230; doi:10.1002/advs.78197)
Supplement: Supplementary file 2 [file ADVS-9999-e78197-s002.zip › advs78197-sup-0006-S5.pdf]

Table S5 Identified causal genes from inferred CTS expression profiles

a. Identified cell type specific causal genes for depression and related disorders from frontal cortex

disorder	celltype	gene	assoc	zMin
Anxiety	Astros	*ITIH4*	TRUE	2.214292376
Anxiety	Astros	*KCNN4*	TRUE	2.249295278
Anxiety	Astros	*CHRNE*	TRUE	2.477637898
Anxiety	Astros	*SGCA*	TRUE	2.411862304
Anxiety	Astros	*S1PR4*	TRUE	2.14671706
Anxiety	Astros	*ZSCAN9*	TRUE	2.442976446
Anxiety	Astros	*ZNF503*	TRUE	2.153121139
Anxiety	Astros	*KLK5*	TRUE	1.998220687
Anxiety	Astros	*FAHD1*	TRUE	2.573587046
Anxiety	Astros	*CLCNKA*	TRUE	2.417504283
Anxiety	Astros	*ZKSCAN3*	TRUE	2.774118275
Anxiety	Astros	*RP11.63A1.2*	TRUE	2.760243442
Anxiety	Ex	*ITIH4*	TRUE	2.202417902
Anxiety	Ex	*F7*	TRUE	2.123384478
Anxiety	Ex	*APBB1IP*	TRUE	2.114672621
Anxiety	Ex	*SLC5A4*	TRUE	2.252436482
Anxiety	Ex	*ZC3H14*	TRUE	2.058084983
Anxiety	Ex	*CD40*	TRUE	2.192334067
Anxiety	Ex	*KAT8*	TRUE	2.013787833
Anxiety	Ex	*KCNN4*	TRUE	2.261680954
Anxiety	Ex	*HAS1*	TRUE	3.023398156
Anxiety	Ex	*RPL18A*	TRUE	2.146794906
Anxiety	Ex	*SUSD1*	TRUE	2.908752771
Anxiety	Ex	*CHRNE*	TRUE	2.740870039
Anxiety	Ex	*SGCA*	TRUE	2.794979197
Anxiety	Ex	*RCVRN*	TRUE	2.378387677
Anxiety	Ex	*GNB3*	TRUE	2.165877691
Anxiety	Ex	*NME5*	TRUE	2.904763868
Anxiety	Ex	*CNN3*	TRUE	2.459891366
Anxiety	Ex	*DIEXF*	TRUE	1.969520023
Anxiety	Ex	*SOHLH2*	TRUE	2.00086708
Anxiety	Ex	*POLM*	TRUE	2.310027669
Anxiety	Ex	*WFDC3*	TRUE	2.298650999
Anxiety	Ex	*PAK4*	TRUE	2.138155231
Anxiety	Ex	*ZSCAN9*	TRUE	2.550143144
Anxiety	Ex	*ETV6*	TRUE	2.349465916
Anxiety	Ex	*C18orf8*	TRUE	2.133512974
Anxiety	Ex	*TCTE1*	TRUE	2.070871703
Anxiety	Ex	*CBR3*	TRUE	2.294728708
Anxiety	Ex	*ADCK3*	TRUE	2.839657212
Anxiety	Ex	*TMEM183A*	TRUE	2.035957136
Anxiety	Ex	*CCDC127*	TRUE	2.448333275
Anxiety	Ex	*ZNF503*	TRUE	2.721059895
Anxiety	Ex	*FAM96B*	TRUE	2.273376372
Anxiety	Ex	*KLK5*	TRUE	2.222649804
Anxiety	Ex	*NADSYN1*	TRUE	2.029578349
Anxiety	Ex	*MAMSTR*	TRUE	2.169196911
Anxiety	Ex	*C9orf50*	TRUE	2.363321545
Anxiety	Ex	*C2orf82*	TRUE	2.137249191
Anxiety	Ex	*NPIPA5*	TRUE	2.012724922
Anxiety	Ex	*NUDT17*	TRUE	2.353783772
Anxiety	Ex	*CLCNKA*	TRUE	3.068949799
Anxiety	Ex	*HYAL3*	TRUE	2.174818917
Anxiety	Ex	*DHRS4L2*	TRUE	2.160944944
Anxiety	Ex	*ZFP69*	TRUE	2.863923067
Anxiety	Ex	*NWD1*	TRUE	1.991927904

Anxiety	Ex	*JAKMIP3*	TRUE	2.026530355
Anxiety	Ex	*NBR1*	TRUE	2.303043882
Anxiety	Ex	*ZKSCAN3*	TRUE	2.655464339
Anxiety	Ex	*TRAPPC4*	TRUE	1.96045222
Anxiety	Ex	*TPM2*	TRUE	2.201206539
Anxiety	Ex	*HLA.DRB5*	TRUE	2.177416827
Anxiety	Ex	*SLC2A1.AS1*	TRUE	2.118159959
Anxiety	Ex	*AC093642.3*	TRUE	2.167348805
Anxiety	Ex	*RP11.108M9.4*	TRUE	2.455361025
Anxiety	Ex	*RP11.584P21.2*	TRUE	2.114699488
Anxiety	Ex	*RP13.977J11.2*	TRUE	3.103136033
Anxiety	Ex	*RP11.63A1.2*	TRUE	3.101558302
Anxiety	Ex	*RP11.250B2.6*	TRUE	2.864290769
Anxiety	Ex	*CASC15*	TRUE	2.094504548
Anxiety	Ex	*AP000350.5*	TRUE	1.975106351
Anxiety	Ex	*RP11.686O6.2*	TRUE	2.226459048
Anxiety	Inhib	*ITIH4*	TRUE	2.205369407
Anxiety	Inhib	*F7*	TRUE	1.985963481
Anxiety	Inhib	*APBB1IP*	TRUE	2.275552881
Anxiety	Inhib	*SLC5A4*	TRUE	2.297307057
Anxiety	Inhib	*ZC3H14*	TRUE	2.058766978
Anxiety	Inhib	*CD40*	TRUE	2.085764742
Anxiety	Inhib	*KCNN4*	TRUE	2.289672591
Anxiety	Inhib	*HAS1*	TRUE	2.987728376
Anxiety	Inhib	*RPL18A*	TRUE	2.122287219
Anxiety	Inhib	*SUSD1*	TRUE	2.773019084
Anxiety	Inhib	*DHRS11*	TRUE	2.061535132
Anxiety	Inhib	*CHRNE*	TRUE	2.69787944
Anxiety	Inhib	*SGCA*	TRUE	2.827457121
Anxiety	Inhib	*RCVRN*	TRUE	2.521011604
Anxiety	Inhib	*GNB3*	TRUE	2.042569715
Anxiety	Inhib	*NME5*	TRUE	2.739897443
Anxiety	Inhib	*CNN3*	TRUE	2.204814371
Anxiety	Inhib	*SOHLH2*	TRUE	2.147777906
Anxiety	Inhib	*POLM*	TRUE	2.234292027
Anxiety	Inhib	*WFDC3*	TRUE	2.29975841
Anxiety	Inhib	*PAK4*	TRUE	2.048037267
Anxiety	Inhib	*ZSCAN9*	TRUE	2.557136734
Anxiety	Inhib	*ETV6*	TRUE	2.411365185
Anxiety	Inhib	*C18orf8*	TRUE	2.036752186
Anxiety	Inhib	*TCTE1*	TRUE	2.124518805
Anxiety	Inhib	*CBR3*	TRUE	2.005885414
Anxiety	Inhib	*CCDC64B*	TRUE	2.091561984
Anxiety	Inhib	*ADCK3*	TRUE	2.687321106
Anxiety	Inhib	*CCDC127*	TRUE	2.13249636
Anxiety	Inhib	*ZNF503*	TRUE	2.513293592
Anxiety	Inhib	*KLK5*	TRUE	2.156021646
Anxiety	Inhib	*MAMSTR*	TRUE	2.175189593
Anxiety	Inhib	*C9orf50*	TRUE	2.202027076
Anxiety	Inhib	*C2orf82*	TRUE	2.093847539
Anxiety	Inhib	*NPIPA5*	TRUE	1.961464244
Anxiety	Inhib	*NUDT17*	TRUE	2.165821821
Anxiety	Inhib	*CLCNKA*	TRUE	2.748149413
Anxiety	Inhib	*HYAL3*	TRUE	2.093268208

Anxiety	Inhib	*DHRS4L2*	TRUE	2.019314041
Anxiety	Inhib	*ZFP69*	TRUE	2.784097492
Anxiety	Inhib	*NWD1*	TRUE	1.972463218
Anxiety	Inhib	*NBR1*	TRUE	1.991412109
Anxiety	Inhib	*ZKSCAN3*	TRUE	2.662382971
Anxiety	Inhib	*TPM2*	TRUE	2.140683857
Anxiety	Inhib	*HLA.DRB5*	TRUE	1.97375404
Anxiety	Inhib	*SLC2A1.AS1*	TRUE	2.053499737
Anxiety	Inhib	*AC093642.3*	TRUE	2.086487135
Anxiety	Inhib	*RP11.108M9.4*	TRUE	2.352713379
Anxiety	Inhib	*GS1.259H13.2*	TRUE	1.974644829
Anxiety	Inhib	*RP11.584P21.2*	TRUE	2.070745071
Anxiety	Inhib	*RP13.977J11.2*	TRUE	2.951074635
Anxiety	Inhib	*RP11.250B2.6*	TRUE	2.430786209
Anxiety	Inhib	*CASC15*	TRUE	1.994218117
Anxiety	Micro_Macro	*SGCA*	TRUE	2.24228395
Anxiety	Micro_Macro	*ZSCAN9*	TRUE	2.658650527
Anxiety	Micro_Macro	*ZKSCAN3*	TRUE	2.300699798
Anxiety	Micro_Macro	*RP11.63A1.2*	TRUE	2.836933203
Anxiety	Oligos	*KCNN4*	TRUE	2.148672972
Anxiety	Oligos	*HAS1*	TRUE	1.961602234
Anxiety	Oligos	*SUSD1*	TRUE	2.023288867
Anxiety	Oligos	*SGCA*	TRUE	2.839026115
Anxiety	Oligos	*NME5*	TRUE	2.633878255
Anxiety	Oligos	*S1PR4*	TRUE	1.972747858
Anxiety	Oligos	*PAK4*	TRUE	1.98247722
Anxiety	Oligos	*ZSCAN9*	TRUE	2.594384304
Anxiety	Oligos	*ADCK3*	TRUE	2.433805788
Anxiety	Oligos	*ZNF503*	TRUE	2.505925754
Anxiety	Oligos	*FAHD1*	TRUE	2.204420477
Anxiety	Oligos	*C2orf82*	TRUE	2.004907253
Anxiety	Oligos	*CLCNKA*	TRUE	2.775728151
Anxiety	Oligos	*HYAL3*	TRUE	2.176181195
Anxiety	Oligos	*ZFP69*	TRUE	2.08929014
Anxiety	Oligos	*ZKSCAN3*	TRUE	2.487597856
Anxiety	Oligos	*AC093642.3*	TRUE	2.114189766
Anxiety	Oligos	*RP13.977J11.2*	TRUE	2.273672568
Anxiety	Oligos	*RP11.63A1.2*	TRUE	2.815649417
Anxiety	OPCs	*CHRNE*	TRUE	2.024351039
Anxiety	OPCs	*GNB3*	TRUE	1.999178755
Anxiety	OPCs	*NME5*	TRUE	2.393184107
Anxiety	OPCs	*ZSCAN9*	TRUE	2.586230891
Anxiety	OPCs	*ADCK3*	TRUE	2.02099209
Anxiety	OPCs	*ZNF503*	TRUE	2.375489311
Anxiety	OPCs	*HYAL3*	TRUE	2.181230302
Anxiety	OPCs	*ZFP69*	TRUE	2.137494463
Anxiety	OPCs	*ZKSCAN3*	TRUE	2.770429641
Anxiety	OPCs	*TPM2*	TRUE	2.221973159
Anxiety	OPCs	*RP11.584P21.2*	TRUE	2.004428804
Anxiety	OPCs	*RP13.977J11.2*	TRUE	2.862100691
Anxiety	OPCs	*RP11.63A1.2*	TRUE	3.066347264

Bipolar	Astros	*PLA2G4C*	TRUE	2.074189322
Bipolar	Astros	*ZNF623*	TRUE	2.275630145
Bipolar	Astros	*NUTM2B*	TRUE	2.424265837
Bipolar	Astros	*AC093159.1*	TRUE	2.094660726
Bipolar	Astros	*LILRA4*	TRUE	2.573358251
Bipolar	Astros	*HLA.DOB*	TRUE	2.442964642
Bipolar	Astros	*CTD.3074O7.2*	TRUE	2.126353658
Bipolar	Ex	*SCYL3*	TRUE	2.092474046
Bipolar	Ex	*LIG3*	TRUE	1.990917801
Bipolar	Ex	*LRRC23*	TRUE	2.158737466
Bipolar	Ex	*DRD4*	TRUE	2.311704076
Bipolar	Ex	*IFT74*	TRUE	2.962816383
Bipolar	Ex	*PCK2*	TRUE	2.076675051
Bipolar	Ex	*POLI*	TRUE	2.276661012
Bipolar	Ex	*PLA2G4C*	TRUE	2.731974226
Bipolar	Ex	*HAS1*	TRUE	2.136882594
Bipolar	Ex	*TSPAN11*	TRUE	2.430850887
Bipolar	Ex	*AKAP3*	TRUE	2.154087921
Bipolar	Ex	*DDX55*	TRUE	2.053275977
Bipolar	Ex	*TFG*	TRUE	2.328855245
Bipolar	Ex	*ABCC5*	TRUE	3.004309798
Bipolar	Ex	*SMYD1*	TRUE	2.045775065
Bipolar	Ex	*DNAJC16*	TRUE	1.999946415
Bipolar	Ex	*MORN1*	TRUE	2.066804557
Bipolar	Ex	*DNAL1*	TRUE	3.12423148
Bipolar	Ex	*ZNF549*	TRUE	2.172592997
Bipolar	Ex	*SARDH*	TRUE	2.265055871
Bipolar	Ex	*PEX6*	TRUE	2.5144419
Bipolar	Ex	*POT1*	TRUE	2.081731923
Bipolar	Ex	*NINJ1*	TRUE	2.495498481
Bipolar	Ex	*ZNF304*	TRUE	1.984130533
Bipolar	Ex	*RAF1*	TRUE	2.261890501
Bipolar	Ex	*CCDC64*	TRUE	2.872320568
Bipolar	Ex	*DSCC1*	TRUE	2.173942761
Bipolar	Ex	*TMEM8B*	TRUE	2.136791671
Bipolar	Ex	*BPHL*	TRUE	1.976151874
Bipolar	Ex	*POLG*	TRUE	2.53023666
Bipolar	Ex	*DUS3L*	TRUE	2.237614276
Bipolar	Ex	*C7orf50*	TRUE	2.603757595
Bipolar	Ex	*PSPH*	TRUE	2.11677712
Bipolar	Ex	*REXO4*	TRUE	1.961563952
Bipolar	Ex	*TTC12*	TRUE	1.989797782
Bipolar	Ex	*FADS1*	TRUE	2.198882289
Bipolar	Ex	*ZNF208*	TRUE	2.614542492
Bipolar	Ex	*ZNF75A*	TRUE	1.975926066
Bipolar	Ex	*PPP1R21*	TRUE	2.532621447
Bipolar	Ex	*EOGT*	TRUE	2.057084903
Bipolar	Ex	*ADORA1*	TRUE	1.990546757
Bipolar	Ex	*YWHAZ*	TRUE	3.190620074
Bipolar	Ex	*CCDC171*	TRUE	2.248805216
Bipolar	Ex	*TRPV6*	TRUE	2.149790289

Bipolar	Ex	*HIF1AN*	TRUE	2.205743589
Bipolar	Ex	*THAP5*	TRUE	2.00695914
Bipolar	Ex	*ZNF623*	TRUE	3.518583817
Bipolar	Ex	*COA5*	TRUE	2.102002585
Bipolar	Ex	*ACSM5*	TRUE	2.378089663
Bipolar	Ex	*CIB1*	TRUE	2.400579383
Bipolar	Ex	*CDK10*	TRUE	2.031885555
Bipolar	Ex	*ZKSCAN4*	TRUE	2.23645745
Bipolar	Ex	*NWD1*	TRUE	2.63255267
Bipolar	Ex	*NUTM2B*	TRUE	2.428808051
Bipolar	Ex	*TCEA3*	TRUE	2.090238757
Bipolar	Ex	*AC093159.1*	TRUE	2.222305901
Bipolar	Ex	*LRRC37A2*	TRUE	2.121248063
Bipolar	Ex	*LILRA4*	TRUE	2.259933064
Bipolar	Ex	*HLA.DOB*	TRUE	2.543215338
Bipolar	Ex	*RP11.332J15.2*	TRUE	2.258129293
Bipolar	Ex	*HPR*	TRUE	2.209439259
Bipolar	Ex	*CTD.2619J13.19*	TRUE	2.023725571
Bipolar	Ex	*RP11.438L19.1*	TRUE	2.104467107
Bipolar	Inhib	*TSSC1*	TRUE	2.941220771
Bipolar	Inhib	*PTGER3*	TRUE	2.279605657
Bipolar	Inhib	*DRD4*	TRUE	2.084332915
Bipolar	Inhib	*IFT74*	TRUE	2.920586315
Bipolar	Inhib	*AKAP3*	TRUE	2.290416234
Bipolar	Inhib	*DNAJC16*	TRUE	2.02124449
Bipolar	Inhib	*MORN1*	TRUE	2.414579038
Bipolar	Inhib	*DNAL1*	TRUE	2.711042388
Bipolar	Inhib	*ZNF549*	TRUE	2.427565184
Bipolar	Inhib	*SARDH*	TRUE	2.461551855
Bipolar	Inhib	*PEX6*	TRUE	1.97628592
Bipolar	Inhib	*NINJ1*	TRUE	3.074285764
Bipolar	Inhib	*ZNF304*	TRUE	2.321009722
Bipolar	Inhib	*RAF1*	TRUE	2.641051712
Bipolar	Inhib	*CCDC64*	TRUE	2.335587481
Bipolar	Inhib	*DSCC1*	TRUE	2.211405678
Bipolar	Inhib	*TMEM8B*	TRUE	2.385511842
Bipolar	Inhib	*POLG*	TRUE	2.71921971
Bipolar	Inhib	*TRIM65*	TRUE	2.265764297
Bipolar	Inhib	*DUS3L*	TRUE	2.025266749
Bipolar	Inhib	*PSPH*	TRUE	2.047234053
Bipolar	Inhib	*FADS1*	TRUE	2.783974636
Bipolar	Inhib	*MRPL39*	TRUE	2.711065139
Bipolar	Inhib	*TRMT44*	TRUE	2.022563786
Bipolar	Inhib	*JMJD8*	TRUE	2.071809501
Bipolar	Inhib	*TRPV6*	TRUE	2.277924719
Bipolar	Inhib	*AFAP1L2*	TRUE	2.050671673
Bipolar	Inhib	*METTL18*	TRUE	2.110021399
Bipolar	Inhib	*DPY19L1*	TRUE	2.159401313
Bipolar	Inhib	*THAP5*	TRUE	2.201051894
Bipolar	Inhib	*STAP2*	TRUE	2.060319707
Bipolar	Inhib	*ZNF623*	TRUE	3.077543457

Bipolar	Inhib	*CIB1*	TRUE	2.359015318
Bipolar	Inhib	*NWD1*	TRUE	2.330950103
Bipolar	Inhib	*NUTM2B*	TRUE	2.48614767
Bipolar	Inhib	*ASAH2*	TRUE	1.991612002
Bipolar	Inhib	*AC093159.1*	TRUE	2.223648027
Bipolar	Inhib	*LILRA4*	TRUE	2.710552242
Bipolar	Inhib	*HLA.DOB*	TRUE	2.464249135
Bipolar	Inhib	*ZNF585B*	TRUE	2.265875107
Bipolar	Inhib	*HPR*	TRUE	2.787415371
Bipolar	Inhib	*RP11.438L19.1*	TRUE	2.280396265
Bipolar	Micro_Macro	*IFT74*	TRUE	2.277108701
Bipolar	Micro_Macro	*DSCC1*	TRUE	2.006200992
Bipolar	Micro_Macro	*NUTM2B*	TRUE	2.348890926
Bipolar	Micro_Macro	*HLA.DOB*	TRUE	2.143565398
Bipolar	Micro_Macro	*HPR*	TRUE	1.96468158
Bipolar	Oligos	*IFT74*	TRUE	2.779262803
Bipolar	Oligos	*CDCA3*	TRUE	2.14437385
Bipolar	Oligos	*NINJ1*	TRUE	2.253277335
Bipolar	Oligos	*RAF1*	TRUE	2.081297355
Bipolar	Oligos	*DSCC1*	TRUE	2.191501797
Bipolar	Oligos	*POLG*	TRUE	2.274467895
Bipolar	Oligos	*ZNF623*	TRUE	2.446017346
Bipolar	Oligos	*CIB1*	TRUE	2.008476793
Bipolar	Oligos	*NWD1*	TRUE	1.975034086
Bipolar	Oligos	*NUTM2B*	TRUE	2.398737599
Bipolar	Oligos	*AC093159.1*	TRUE	2.007460151
Bipolar	Oligos	*HLA.DOB*	TRUE	2.593403872
Bipolar	Oligos	*HPR*	TRUE	2.583861769
Bipolar	OPCs	*IFT74*	TRUE	2.665099153
Bipolar	OPCs	*PLA2G4C*	TRUE	2.335260797
Bipolar	OPCs	*NINJ1*	TRUE	2.094319254
Bipolar	OPCs	*POLG*	TRUE	2.092644501
Bipolar	OPCs	*ZNF623*	TRUE	2.244416968
Bipolar	OPCs	*NUTM2B*	TRUE	2.445958882
Bipolar	OPCs	*AC093159.1*	TRUE	1.999833034
Bipolar	OPCs	*HLA.DOB*	TRUE	2.523254839
Bipolar	OPCs	*CTD.3074O7.2*	TRUE	2.133668144
Bipolar	OPCs	*HPR*	TRUE	2.193457768
Depression	Astros	*PPP2R3C*	TRUE	1.972889809
Depression	Astros	*CLEC4M*	TRUE	2.453037479
Depression	Astros	*ENPP5*	TRUE	2.111052117
Depression	Astros	*ZSCAN9*	TRUE	3.139634959
Depression	Astros	*NBEAL2*	TRUE	1.971706998
Depression	Astros	*RP9*	TRUE	1.980994358
Depression	Astros	*ZSCAN21*	TRUE	2.30180792
Depression	Astros	*EHBP1L1*	TRUE	2.21087923
Depression	Astros	*FAHD1*	TRUE	2.042151719
Depression	Astros	*YBEY*	TRUE	1.993333074
Depression	Astros	*RP3.470B24.5*	TRUE	2.153885738
Depression	Astros	*CTD.3074O7.2*	TRUE	2.884027491
Depression	Astros	*CTD.3126B10.4*	TRUE	2.814599909

Depression	Ex	*ITGA2B*	TRUE	2.121082813
Depression	Ex	*MED24*	TRUE	3.129876769
Depression	Ex	*DNAH5*	TRUE	2.237105889
Depression	Ex	*CDH3*	TRUE	2.096221084
Depression	Ex	*NFYC*	TRUE	2.108751089
Depression	Ex	*NUCKS1*	TRUE	2.836011253
Depression	Ex	*SLC4A11*	TRUE	2.312291446
Depression	Ex	*MYO15A*	TRUE	2.463660253
Depression	Ex	*PPP2R3C*	TRUE	3.003122933
Depression	Ex	*POLI*	TRUE	2.563500983
Depression	Ex	*KLC3*	TRUE	2.117875385
Depression	Ex	*CLEC4M*	TRUE	2.4339663
Depression	Ex	*MTPAP*	TRUE	2.132898601
Depression	Ex	*ENPP5*	TRUE	2.769576503
Depression	Ex	*C1orf109*	TRUE	2.118036974
Depression	Ex	*AVPI1*	TRUE	2.7312129
Depression	Ex	*PRB2*	TRUE	2.105208127
Depression	Ex	*MAP1B*	TRUE	2.301770167
Depression	Ex	*NIP7*	TRUE	2.890800453
Depression	Ex	*GSTT2B*	TRUE	2.07877267
Depression	Ex	*SMYD5*	TRUE	2.68880157
Depression	Ex	*ZSCAN9*	TRUE	3.365628192
Depression	Ex	*PPA2*	TRUE	2.421193787
Depression	Ex	*COL6A2*	TRUE	2.062932022
Depression	Ex	*GIGYF1*	TRUE	1.970765663
Depression	Ex	*MTG1*	TRUE	2.022974219
Depression	Ex	*TRPT1*	TRUE	2.380215459
Depression	Ex	*GUF1*	TRUE	2.014131365
Depression	Ex	*HEATR3*	TRUE	2.633793217
Depression	Ex	*SCUBE1*	TRUE	2.025980673
Depression	Ex	*SPATC1L*	TRUE	1.993138303
Depression	Ex	*CCDC64B*	TRUE	2.717961478
Depression	Ex	*PPP1R21*	TRUE	2.078350648
Depression	Ex	*ANXA5*	TRUE	2.407850547
Depression	Ex	*RP9*	TRUE	3.366126397
Depression	Ex	*SLC18A2*	TRUE	2.063801972
Depression	Ex	*ZSCAN21*	TRUE	2.494756757
Depression	Ex	*SAMD14*	TRUE	2.570833349
Depression	Ex	*FAM21C*	TRUE	1.967014753
Depression	Ex	*ZNF738*	TRUE	2.310031767
Depression	Ex	*SLC2A14*	TRUE	2.233628098
Depression	Ex	*EHBP1L1*	TRUE	2.53245312
Depression	Ex	*RNF26*	TRUE	2.140391866
Depression	Ex	*PAAF1*	TRUE	2.591980204
Depression	Ex	*ZNF619*	TRUE	2.171166103
Depression	Ex	*PFAS*	TRUE	2.079263469
Depression	Ex	*PCED1B*	TRUE	2.427204548
Depression	Ex	*FAHD1*	TRUE	2.274934164
Depression	Ex	*GLIPR1L2*	TRUE	2.451457784
Depression	Ex	*FAM89A*	TRUE	2.60432917
Depression	Ex	*ALG12*	TRUE	2.446452991

Depression	Ex	*IRS2*	TRUE	2.517275433
Depression	Ex	*ZNF749*	TRUE	2.350372563
Depression	Ex	*NUDT17*	TRUE	1.992718764
Depression	Ex	*GPR179*	TRUE	2.821801606
Depression	Ex	*FAM180B*	TRUE	2.667303864
Depression	Ex	*RPL12*	TRUE	2.304615627
Depression	Ex	*HLA.C*	TRUE	2.246727887
Depression	Ex	*MALRD1*	TRUE	2.158272437
Depression	Ex	*LINC00487*	TRUE	2.168261062
Depression	Ex	*C19orf73*	TRUE	2.261547652
Depression	Ex	*RP11.197K6.1*	TRUE	2.466033816
Depression	Ex	*CTA.150C2.13*	TRUE	2.115531547
Depression	Ex	*KIAA0040*	TRUE	2.704880449
Depression	Ex	*RP3.470B24.5*	TRUE	2.508093999
Depression	Ex	*RP1.156L9.1*	TRUE	2.124834762
Depression	Ex	*LILRA4*	TRUE	2.440473888
Depression	Ex	*PPIL3*	TRUE	2.401306509
Depression	Ex	*CTD.3074O7.2*	TRUE	2.044744105
Depression	Ex	*CTC.498J12.1*	TRUE	2.296115071
Depression	Ex	*AC133633.2*	TRUE	2.033341737
Depression	Ex	*RP11.665C16.6*	TRUE	2.249779506
Depression	Ex	*RP5.1021I20.2*	TRUE	2.129262194
Depression	Ex	*SMIM6*	TRUE	2.009539945
Depression	Ex	*CTD.3126B10.4*	TRUE	2.700701481
Depression	Ex	*CTC.548K16.1*	TRUE	1.99384064
Depression	Ex	*RP11.678G14.3*	TRUE	2.531851272
Depression	Ex	*RP11.54O7.17*	TRUE	3.4616737
Depression	Inhib	*MED24*	TRUE	2.518394768
Depression	Inhib	*DNAH5*	TRUE	2.16576143
Depression	Inhib	*CDH3*	TRUE	2.257666273
Depression	Inhib	*NFYC*	TRUE	2.302283615
Depression	Inhib	*NUCKS1*	TRUE	2.99580921
Depression	Inhib	*NAALAD2*	TRUE	1.97358534
Depression	Inhib	*SLC4A11*	TRUE	2.161798269
Depression	Inhib	*MYO15A*	TRUE	2.341398062
Depression	Inhib	*PPP2R3C*	TRUE	2.626876841
Depression	Inhib	*OSGEP*	TRUE	1.985911107
Depression	Inhib	*POLI*	TRUE	2.635507079
Depression	Inhib	*TAF1C*	TRUE	2.005967979
Depression	Inhib	*CLEC4M*	TRUE	2.393077354
Depression	Inhib	*SYT10*	TRUE	1.994279489
Depression	Inhib	*ENPP5*	TRUE	2.883216352
Depression	Inhib	*ZNHIT6*	TRUE	2.025594177
Depression	Inhib	*HEATR1*	TRUE	2.203339771
Depression	Inhib	*AVPI1*	TRUE	2.200443405
Depression	Inhib	*AKAP1*	TRUE	2.063966218
Depression	Inhib	*PRB2*	TRUE	2.167263006
Depression	Inhib	*RIBC2*	TRUE	1.99492613
Depression	Inhib	*MAP1B*	TRUE	2.175927783
Depression	Inhib	*DLG4*	TRUE	1.993902497
Depression	Inhib	*NIP7*	TRUE	3.059982239

Depression	Inhib	*SMYD5*	TRUE	2.869568925
Depression	Inhib	*ZSCAN9*	TRUE	3.781321087
Depression	Inhib	*PPA2*	TRUE	2.439878451
Depression	Inhib	*NARF*	TRUE	2.039984879
Depression	Inhib	*COL6A2*	TRUE	2.093757845
Depression	Inhib	*MTG1*	TRUE	2.026431731
Depression	Inhib	*HEATR3*	TRUE	2.487550656
Depression	Inhib	*SPATC1L*	TRUE	2.014116609
Depression	Inhib	*NBEAL2*	TRUE	2.074353284
Depression	Inhib	*CCDC64B*	TRUE	2.900846865
Depression	Inhib	*PPP1R21*	TRUE	2.044066663
Depression	Inhib	*ANXA5*	TRUE	2.133370675
Depression	Inhib	*RP9*	TRUE	3.585573274
Depression	Inhib	*WBSCR27*	TRUE	2.138725419
Depression	Inhib	*ZSCAN21*	TRUE	2.200543927
Depression	Inhib	*SAMD14*	TRUE	2.339285377
Depression	Inhib	*ZNF738*	TRUE	2.181269645
Depression	Inhib	*EHBP1L1*	TRUE	2.437220723
Depression	Inhib	*PAAF1*	TRUE	2.353194446
Depression	Inhib	*ZNF619*	TRUE	2.166362249
Depression	Inhib	*PCED1B*	TRUE	2.457975543
Depression	Inhib	*FAHD1*	TRUE	2.323599205
Depression	Inhib	*GLIPR1L2*	TRUE	2.513983099
Depression	Inhib	*ALG12*	TRUE	2.209730665
Depression	Inhib	*IRS2*	TRUE	2.532709244
Depression	Inhib	*ZNF749*	TRUE	2.387616579
Depression	Inhib	*NUDT17*	TRUE	2.054086168
Depression	Inhib	*GPR179*	TRUE	3.027295224
Depression	Inhib	*RPL12*	TRUE	2.089251465
Depression	Inhib	*HLA.C*	TRUE	2.499773416
Depression	Inhib	*LINC00487*	TRUE	2.073132489
Depression	Inhib	*C19orf73*	TRUE	2.289961705
Depression	Inhib	*RP11.197K6.1*	TRUE	2.434267788
Depression	Inhib	*CTA.150C2.13*	TRUE	2.068048402
Depression	Inhib	*KIAA0040*	TRUE	2.833257423
Depression	Inhib	*RP3.470B24.5*	TRUE	2.25748251
Depression	Inhib	*RP1.156L9.1*	TRUE	2.213425945
Depression	Inhib	*LILRA4*	TRUE	2.098940235
Depression	Inhib	*PPIL3*	TRUE	2.391453402
Depression	Inhib	*RP11.826N14.2*	TRUE	1.984165486
Depression	Inhib	*CTC.498J12.1*	TRUE	2.275481407
Depression	Inhib	*AC133633.2*	TRUE	1.974789464
Depression	Inhib	*RP5.1021I20.2*	TRUE	1.993938642
Depression	Inhib	*SMIM6*	TRUE	2.000619583
Depression	Inhib	*CTD.3126B10.4*	TRUE	2.832868389
Depression	Inhib	*RP11.107F6.3*	TRUE	1.96422538
Depression	Inhib	*RP11.678G14.3*	TRUE	2.578409635
Depression	Inhib	*RP11.54O7.17*	TRUE	3.174358534
Depression	Micro_Macro	*GSTT2B*	TRUE	1.975810774
Depression	Micro_Macro	*ZSCAN9*	TRUE	3.496382076
Depression	Micro_Macro	*NBEAL2*	TRUE	1.992032249

Depression	Micro_Macro	*CCDC64B*	TRUE	2.804148048
Depression	Micro_Macro	*ZSCAN21*	TRUE	2.302935546
Depression	Micro_Macro	*YBEY*	TRUE	1.975802536
Depression	Micro_Macro	*BTN3A2*	TRUE	2.280824811
Depression	Micro_Macro	*CTD.3074O7.2*	TRUE	2.724176897
Depression	Micro_Macro	*CTD.3126B10.4*	TRUE	2.453527115
Depression	Oligos	*CLEC4M*	TRUE	2.21730073
Depression	Oligos	*ENPP5*	TRUE	2.592662501
Depression	Oligos	*NIP7*	TRUE	2.723463775
Depression	Oligos	*ZSCAN9*	TRUE	3.660236702
Depression	Oligos	*HEATR3*	TRUE	2.277434742
Depression	Oligos	*CCDC64B*	TRUE	2.575787066
Depression	Oligos	*RP9*	TRUE	3.018742068
Depression	Oligos	*ZSCAN21*	TRUE	2.782536971
Depression	Oligos	*ZNF738*	TRUE	2.033151445
Depression	Oligos	*EHBP1L1*	TRUE	2.435126781
Depression	Oligos	*PCED1B*	TRUE	1.990756859
Depression	Oligos	*FAHD1*	TRUE	2.241884518
Depression	Oligos	*ZNF749*	TRUE	2.156167841
Depression	Oligos	*HLA.C*	TRUE	2.358467499
Depression	Oligos	*C19orf73*	TRUE	1.971232342
Depression	Oligos	*CTA.150C2.13*	TRUE	1.986288775
Depression	Oligos	*KIAA0040*	TRUE	2.458168324
Depression	Oligos	*RP1.156L9.1*	TRUE	2.025964453
Depression	Oligos	*PPIL3*	TRUE	2.048416462
Depression	Oligos	*CTD.3126B10.4*	TRUE	2.688639672
Depression	Oligos	*RP11.678G14.3*	TRUE	2.179811068
Depression	OPCs	*ENPP5*	TRUE	2.424660161
Depression	OPCs	*GSTT2B*	TRUE	2.735171095
Depression	OPCs	*ZSCAN9*	TRUE	3.746482394
Depression	OPCs	*COL6A2*	TRUE	1.979425345
Depression	OPCs	*SPATC1L*	TRUE	2.412703051
Depression	OPCs	*CCDC64B*	TRUE	1.973296649
Depression	OPCs	*RP9*	TRUE	2.869410294
Depression	OPCs	*ZSCAN21*	TRUE	2.175805195
Depression	OPCs	*ZNF738*	TRUE	2.246745443
Depression	OPCs	*EHBP1L1*	TRUE	1.972020626
Depression	OPCs	*PAAF1*	TRUE	2.153388342
Depression	OPCs	*FAHD1*	TRUE	2.29378294
Depression	OPCs	*ZNF749*	TRUE	2.088208668
Depression	OPCs	*FAM180B*	TRUE	2.421544208
Depression	OPCs	*CTD.3074O7.2*	TRUE	2.285637397
Depression	OPCs	*CTD.3126B10.4*	TRUE	2.859233629
Psychosis	Astros	*GP6*	TRUE	2.223118573
Psychosis	Astros	*SSH3*	TRUE	2.408339967
Psychosis	Astros	*CCDC163P*	TRUE	2.042823605
Psychosis	Astros	*RP11.430H10.1*	TRUE	3.014324467
Psychosis	Astros	*CTD.3126B10.4*	TRUE	2.110603529
Psychosis	Astros	*RP13.539J13.1*	TRUE	1.987879669
Psychosis	Ex	*LIG3*	TRUE	2.182419421
Psychosis	Ex	*RPS20*	TRUE	2.385336225

Psychosis	Ex	*ALOX5*	TRUE	2.756109838
Psychosis	Ex	*RNASET2*	TRUE	2.078898276
Psychosis	Ex	*ERCC8*	TRUE	2.472180377
Psychosis	Ex	*GP6*	TRUE	2.898575377
Psychosis	Ex	*ACR*	TRUE	2.411333571
Psychosis	Ex	*CLUAP1*	TRUE	2.364753561
Psychosis	Ex	*TMEM176B*	TRUE	2.003921377
Psychosis	Ex	*SYT10*	TRUE	2.585684327
Psychosis	Ex	*BRD8*	TRUE	1.99225968
Psychosis	Ex	*NID1*	TRUE	2.362772513
Psychosis	Ex	*DIEXF*	TRUE	2.254247387
Psychosis	Ex	*PCDHB10*	TRUE	3.4526547
Psychosis	Ex	*SLC2A8*	TRUE	2.532524931
Psychosis	Ex	*FRAS1*	TRUE	1.967518195
Psychosis	Ex	*DUS3L*	TRUE	2.517329954
Psychosis	Ex	*RSPH10B*	TRUE	2.070715619
Psychosis	Ex	*SETD9*	TRUE	2.123908336
Psychosis	Ex	*DRAM2*	TRUE	2.375114694
Psychosis	Ex	*DRC1*	TRUE	2.219675108
Psychosis	Ex	*HIST1H4H*	TRUE	2.268725046
Psychosis	Ex	*C1orf123*	TRUE	2.38413498
Psychosis	Ex	*RNF149*	TRUE	2.433279422
Psychosis	Ex	*DUS2*	TRUE	2.924717314
Psychosis	Ex	*KLK5*	TRUE	2.07294361
Psychosis	Ex	*ANO5*	TRUE	2.713812361
Psychosis	Ex	*SSH3*	TRUE	2.784028773
Psychosis	Ex	*SLCO4C1*	TRUE	2.040206336
Psychosis	Ex	*ZNF619*	TRUE	2.251489579
Psychosis	Ex	*CCDC125*	TRUE	2.035680543
Psychosis	Ex	*ACSM5*	TRUE	1.966663401
Psychosis	Ex	*GOLGA6L4*	TRUE	1.987856262
Psychosis	Ex	*ACOT1*	TRUE	2.262876504
Psychosis	Ex	*KIF24*	TRUE	2.293780733
Psychosis	Ex	*RDM1*	TRUE	2.648970171
Psychosis	Ex	*KBTBD12*	TRUE	2.81007284
Psychosis	Ex	*S100A6*	TRUE	2.333317509
Psychosis	Ex	*SH3BGRL2*	TRUE	1.979650071
Psychosis	Ex	*SH3D21*	TRUE	2.243880448
Psychosis	Ex	*RP1.60O19.2*	TRUE	2.342959932
Psychosis	Ex	*LINC00443*	TRUE	2.14131147
Psychosis	Ex	*CCDC163P*	TRUE	2.473170475
Psychosis	Ex	*AC079922.3*	TRUE	2.165520799
Psychosis	Ex	*RP11.430H10.1*	TRUE	3.540475542
Psychosis	Ex	*RP11.377D9.3*	TRUE	2.397437194
Psychosis	Ex	*CHURC1*	TRUE	3.04607523
Psychosis	Ex	*RP11.410E4.1*	TRUE	2.663023994
Psychosis	Ex	*CTD.3126B10.4*	TRUE	2.233774257
Psychosis	Ex	*GOLGA8H*	TRUE	2.194008599
Psychosis	Ex	*CTD.2623N2.11*	TRUE	2.206742012
Psychosis	Ex	*RP13.539J13.1*	TRUE	2.144646102
Psychosis	Ex	*AP000350.5*	TRUE	2.033804716

Psychosis	Inhib	*RPS20*	TRUE	2.8141125
Psychosis	Inhib	*LARS2*	TRUE	2.245562637
Psychosis	Inhib	*ALOX5*	TRUE	2.32677981
Psychosis	Inhib	*RNASET2*	TRUE	1.976659446
Psychosis	Inhib	*ERCC8*	TRUE	2.684710229
Psychosis	Inhib	*GP6*	TRUE	3.068156829
Psychosis	Inhib	*FMO2*	TRUE	1.965456991
Psychosis	Inhib	*ACR*	TRUE	2.372223786
Psychosis	Inhib	*CLUAP1*	TRUE	2.502770386
Psychosis	Inhib	*RIPK2*	TRUE	2.34239322
Psychosis	Inhib	*TMEM176B*	TRUE	1.996988758
Psychosis	Inhib	*SYT10*	TRUE	2.443002942
Psychosis	Inhib	*BRD8*	TRUE	2.14037127
Psychosis	Inhib	*PCDHB10*	TRUE	3.181965756
Psychosis	Inhib	*SLC2A8*	TRUE	2.751757186
Psychosis	Inhib	*CAPN11*	TRUE	2.194768184
Psychosis	Inhib	*DIAPH3*	TRUE	2.139495776
Psychosis	Inhib	*GAS8*	TRUE	2.19980757
Psychosis	Inhib	*DUS3L*	TRUE	2.451288134
Psychosis	Inhib	*SETD9*	TRUE	2.249618434
Psychosis	Inhib	*MIER3*	TRUE	2.258922943
Psychosis	Inhib	*RMND1*	TRUE	2.0535126
Psychosis	Inhib	*DRC1*	TRUE	2.141558804
Psychosis	Inhib	*RNF149*	TRUE	2.747266135
Psychosis	Inhib	*ELP6*	TRUE	2.034375669
Psychosis	Inhib	*SCN3B*	TRUE	2.306581122
Psychosis	Inhib	*AKIP1*	TRUE	2.257995742
Psychosis	Inhib	*SLC33A1*	TRUE	2.096305929
Psychosis	Inhib	*FRMPD2*	TRUE	2.012271688
Psychosis	Inhib	*ANO5*	TRUE	2.403864302
Psychosis	Inhib	*FIBP*	TRUE	2.186252779
Psychosis	Inhib	*TMEM134*	TRUE	2.293507985
Psychosis	Inhib	*SSH3*	TRUE	2.936458332
Psychosis	Inhib	*SLCO4C1*	TRUE	2.346077618
Psychosis	Inhib	*TOM1L2*	TRUE	2.105699622
Psychosis	Inhib	*ZNF619*	TRUE	2.321181244
Psychosis	Inhib	*PAK2*	TRUE	2.231172114
Psychosis	Inhib	*FAM87A*	TRUE	2.355812883
Psychosis	Inhib	*TTC3*	TRUE	2.239857459
Psychosis	Inhib	*ACOT1*	TRUE	2.188719054
Psychosis	Inhib	*TOR3A*	TRUE	2.305876468
Psychosis	Inhib	*RDM1*	TRUE	2.550814089
Psychosis	Inhib	*KBTBD12*	TRUE	2.954629593
Psychosis	Inhib	*P2RX2*	TRUE	2.028218913
Psychosis	Inhib	*S100A6*	TRUE	2.79115632
Psychosis	Inhib	*C6orf47*	TRUE	2.258290322
Psychosis	Inhib	*SH3D21*	TRUE	2.336989
Psychosis	Inhib	*C19orf73*	TRUE	1.99253968
Psychosis	Inhib	*RP1.60O19.2*	TRUE	2.477611995
Psychosis	Inhib	*ANKRD65*	TRUE	2.203088275
Psychosis	Inhib	*CCDC163P*	TRUE	2.074156907

Psychosis	Inhib	*AC079922.3*	TRUE	2.284076801
Psychosis	Inhib	*APOBEC3G*	TRUE	2.103310088
Psychosis	Inhib	*RP11.430H10.1*	TRUE	3.320403003
Psychosis	Inhib	*RP11.377D9.3*	TRUE	2.658971013
Psychosis	Inhib	*CHURC1*	TRUE	2.870699474
Psychosis	Inhib	*RP11.410E4.1*	TRUE	2.551245343
Psychosis	Inhib	*CTD.3126B10.4*	TRUE	2.23181891
Psychosis	Inhib	*GOLGA8H*	TRUE	2.136492641
Psychosis	Inhib	*CTD.2623N2.11*	TRUE	2.037797311
Psychosis	Inhib	*XXbac.BPGBPG55C20.3*	TRUE	2.198201773
Psychosis	Inhib	*RP13.539J13.1*	TRUE	1.962545887
Psychosis	Inhib	*AP000350.5*	TRUE	2.116199506
Psychosis	Micro_Macro	*RDM1*	TRUE	2.337963705
Psychosis	Micro_Macro	*CCDC163P*	TRUE	2.751237169
Psychosis	Micro_Macro	*AC079922.3*	TRUE	2.016716411
Psychosis	Micro_Macro	*CTD.3074O7.2*	TRUE	2.146294343
Psychosis	Micro_Macro	*RP11.410E4.1*	TRUE	2.394342928
Psychosis	Micro_Macro	*RP13.539J13.1*	TRUE	2.329079819
Psychosis	Oligos	*ALOX5*	TRUE	2.051135007
Psychosis	Oligos	*ERCC8*	TRUE	2.193703149
Psychosis	Oligos	*ACR*	TRUE	2.09469818
Psychosis	Oligos	*PCDHB10*	TRUE	2.819810567
Psychosis	Oligos	*SLC2A8*	TRUE	2.129319824
Psychosis	Oligos	*DUS3L*	TRUE	2.305893857
Psychosis	Oligos	*ANO5*	TRUE	2.172426136
Psychosis	Oligos	*SSH3*	TRUE	2.614607168
Psychosis	Oligos	*ACOT1*	TRUE	2.065262327
Psychosis	Oligos	*RDM1*	TRUE	2.117119849
Psychosis	Oligos	*KBTBD12*	TRUE	2.012339301
Psychosis	Oligos	*C6orf47*	TRUE	2.062275154
Psychosis	Oligos	*RFPL3S*	TRUE	2.286518101
Psychosis	Oligos	*ARL2*	TRUE	2.021997123
Psychosis	Oligos	*CCDC163P*	TRUE	2.292788821
Psychosis	Oligos	*AC079922.3*	TRUE	1.979485824
Psychosis	Oligos	*RP11.430H10.1*	TRUE	3.13052125
Psychosis	Oligos	*CHURC1*	TRUE	2.812558452
Psychosis	Oligos	*RP11.410E4.1*	TRUE	2.515768088
Psychosis	Oligos	*CTD.3126B10.4*	TRUE	2.440320157
Psychosis	Oligos	*RP13.539J13.1*	TRUE	2.301337952
Psychosis	OPCs	*ERCC8*	TRUE	2.133233881
Psychosis	OPCs	*SLC2A8*	TRUE	2.3328283
Psychosis	OPCs	*DUS3L*	TRUE	2.358269793
Psychosis	OPCs	*ANO5*	TRUE	2.204222184
Psychosis	OPCs	*CCDC163P*	TRUE	2.269603691
Psychosis	OPCs	*RP11.430H10.1*	TRUE	2.66218067
Psychosis	OPCs	*RP11.410E4.1*	TRUE	2.592622509
Psychosis	OPCs	*CTD.3126B10.4*	TRUE	2.094242033
Psychosis	OPCs	*RP13.539J13.1*	TRUE	2.066785655
Depression_admission_freq	Astros	*OBP2A*	TRUE	2.048776242
Depression_admission_freq	Astros	*AHI1*	TRUE	2.226903742
Depression_admission_freq	Astros	*EFHB*	TRUE	2.415744431

Depression_admission_freq	Astros	*RP11.826N14.2*	TRUE	2.133455818
Depression_admission_freq	Astros	*CTD.3126B10.4*	TRUE	2.184480486
Depression_admission_freq	Astros	*CTA.246H3.12*	TRUE	2.206822357
Depression_admission_freq	Ex	*STAG3*	TRUE	2.174797955
Depression_admission_freq	Ex	*NUCKS1*	TRUE	2.299737767
Depression_admission_freq	Ex	*FBXW11*	TRUE	2.320496663
Depression_admission_freq	Ex	*PVR*	TRUE	2.27100136
Depression_admission_freq	Ex	*ICAM3*	TRUE	2.076031046
Depression_admission_freq	Ex	*HBQ1*	TRUE	1.980686971
Depression_admission_freq	Ex	*SPAG7*	TRUE	2.091440554
Depression_admission_freq	Ex	*ARSB*	TRUE	2.098487042
Depression_admission_freq	Ex	*ACOT2*	TRUE	2.132028041
Depression_admission_freq	Ex	*PAX8*	TRUE	2.389779957
Depression_admission_freq	Ex	*TPH1*	TRUE	2.108528853
Depression_admission_freq	Ex	*KLK10*	TRUE	1.962825338
Depression_admission_freq	Ex	*QRSL1*	TRUE	2.139609896
Depression_admission_freq	Ex	*PSRC1*	TRUE	3.115073088
Depression_admission_freq	Ex	*DHX34*	TRUE	2.400152581
Depression_admission_freq	Ex	*AHI1*	TRUE	2.649882913
Depression_admission_freq	Ex	*CIB2*	TRUE	2.136337491
Depression_admission_freq	Ex	*TUBGCP4*	TRUE	1.984683164
Depression_admission_freq	Ex	*CNKSR1*	TRUE	2.908231754
Depression_admission_freq	Ex	*FBXL17*	TRUE	2.043538098
Depression_admission_freq	Ex	*ASAP1*	TRUE	2.109129748
Depression_admission_freq	Ex	*NRG1*	TRUE	2.133075717
Depression_admission_freq	Ex	*EYA3*	TRUE	2.074351702
Depression_admission_freq	Ex	*POGLUT1*	TRUE	2.224800125
Depression_admission_freq	Ex	*EFHB*	TRUE	2.442621059
Depression_admission_freq	Ex	*FAM178B*	TRUE	2.018877574
Depression_admission_freq	Ex	*RNASE2*	TRUE	2.165712034
Depression_admission_freq	Ex	*PLEKHG5*	TRUE	1.985388862
Depression_admission_freq	Ex	*TMEM134*	TRUE	2.512525676
Depression_admission_freq	Ex	*RNF26*	TRUE	2.076019294
Depression_admission_freq	Ex	*GEN1*	TRUE	2.406259928
Depression_admission_freq	Ex	*RESP18*	TRUE	2.121591714
Depression_admission_freq	Ex	*ARHGEF37*	TRUE	2.175244885
Depression_admission_freq	Ex	*ZNF681*	TRUE	2.093558734
Depression_admission_freq	Ex	*TNFRSF25*	TRUE	2.040536019
Depression_admission_freq	Ex	*ZNF737*	TRUE	2.341087619
Depression_admission_freq	Ex	*HLA.DQA2*	TRUE	2.051477932
Depression_admission_freq	Ex	*RP11.826N14.2*	TRUE	2.54530363
Depression_admission_freq	Ex	*RP11.377D9.3*	TRUE	2.159313924
Depression_admission_freq	Ex	*CTD.3126B10.4*	TRUE	2.098706008
Depression_admission_freq	Ex	*RP13.539J13.1*	TRUE	2.045256755
Depression_admission_freq	Ex	*CTA.246H3.12*	TRUE	2.358782181
Depression_admission_freq	Inhib	*THOC3*	TRUE	2.203874009
Depression_admission_freq	Inhib	*PVR*	TRUE	2.002037071
Depression_admission_freq	Inhib	*ICAM3*	TRUE	2.075543205
Depression_admission_freq	Inhib	*SDR39U1*	TRUE	1.975398036
Depression_admission_freq	Inhib	*CCDC170*	TRUE	2.135445961
Depression_admission_freq	Inhib	*ATP5S*	TRUE	1.965318486

Depression_admission_freq	Inhib	*PAX8*	TRUE	2.578379052
Depression_admission_freq	Inhib	*AP5S1*	TRUE	2.321901989
Depression_admission_freq	Inhib	*TPH1*	TRUE	2.243696451
Depression_admission_freq	Inhib	*CHIT1*	TRUE	2.238717475
Depression_admission_freq	Inhib	*PSRC1*	TRUE	2.991714226
Depression_admission_freq	Inhib	*DHX34*	TRUE	2.096053968
Depression_admission_freq	Inhib	*AHI1*	TRUE	2.966019404
Depression_admission_freq	Inhib	*CIB2*	TRUE	1.974831735
Depression_admission_freq	Inhib	*CNKSR1*	TRUE	3.009047637
Depression_admission_freq	Inhib	*FBXL17*	TRUE	2.553637144
Depression_admission_freq	Inhib	*TTC12*	TRUE	2.063161669
Depression_admission_freq	Inhib	*FBXL2*	TRUE	2.286192127
Depression_admission_freq	Inhib	*VPS11*	TRUE	2.078659527
Depression_admission_freq	Inhib	*EFHB*	TRUE	2.564524727
Depression_admission_freq	Inhib	*SLC38A8*	TRUE	2.902027846
Depression_admission_freq	Inhib	*RNASE2*	TRUE	2.100582151
Depression_admission_freq	Inhib	*FAM21C*	TRUE	2.209009274
Depression_admission_freq	Inhib	*TLR1*	TRUE	2.173774589
Depression_admission_freq	Inhib	*PPP1R42*	TRUE	2.136052066
Depression_admission_freq	Inhib	*GEN1*	TRUE	2.037897507
Depression_admission_freq	Inhib	*ATOH7*	TRUE	2.29057225
Depression_admission_freq	Inhib	*RESP18*	TRUE	2.130406989
Depression_admission_freq	Inhib	*ARHGEF37*	TRUE	2.45256013
Depression_admission_freq	Inhib	*COA5*	TRUE	2.231708131
Depression_admission_freq	Inhib	*RDM1*	TRUE	2.064198889
Depression_admission_freq	Inhib	*NWD1*	TRUE	1.97239086
Depression_admission_freq	Inhib	*ZNF737*	TRUE	1.982487033
Depression_admission_freq	Inhib	*GS1.259H13.2*	TRUE	2.049745873
Depression_admission_freq	Inhib	*RP11.826N14.2*	TRUE	2.355618867
Depression_admission_freq	Inhib	*RP11.377D9.3*	TRUE	1.982557333
Depression_admission_freq	Inhib	*CTD.3126B10.4*	TRUE	2.134301556
Depression_admission_freq	Inhib	*XXbac.BPG181B23.7*	TRUE	1.986659795
Depression_admission_freq	Inhib	*RP13.539J13.1*	TRUE	2.076930138
Depression_admission_freq	Inhib	*CTA.246H3.12*	TRUE	2.470126949
Depression_admission_freq	Micro_Macro	*TIPIN*	TRUE	2.014102675
Depression_admission_freq	Micro_Macro	*AHI1*	TRUE	1.982940487
Depression_admission_freq	Micro_Macro	*EFHB*	TRUE	2.225109033
Depression_admission_freq	Oligos	*AP5S1*	TRUE	2.02531113
Depression_admission_freq	Oligos	*TPH1*	TRUE	2.00400125
Depression_admission_freq	Oligos	*AHI1*	TRUE	2.393921578
Depression_admission_freq	Oligos	*CNKSR1*	TRUE	2.757219636
Depression_admission_freq	Oligos	*VPS11*	TRUE	2.08283695
Depression_admission_freq	Oligos	*EFHB*	TRUE	2.398870175
Depression_admission_freq	Oligos	*SLC38A8*	TRUE	2.051462907
Depression_admission_freq	Oligos	*RNASE2*	TRUE	1.996661321
Depression_admission_freq	Oligos	*POLE*	TRUE	2.016746352
Depression_admission_freq	Oligos	*RP11.826N14.2*	TRUE	2.177054766
Depression_admission_freq	Oligos	*CTD.3126B10.4*	TRUE	2.153833448
Depression_admission_freq	Oligos	*CTA.246H3.12*	TRUE	2.058065861
Depression_admission_freq	Oligos	*RBAKDN*	TRUE	1.974889788
Depression_admission_freq	OPCs	*ICAM3*	TRUE	2.09623747

Depression_admission_freq	OPCs	*PAX8*	TRUE	2.440730624
Depression_admission_freq	OPCs	*PSRC1*	TRUE	2.326777402
Depression_admission_freq	OPCs	*AHI1*	TRUE	2.788946833
Depression_admission_freq	OPCs	*CNKSR1*	TRUE	2.862668697
Depression_admission_freq	OPCs	*FBXL17*	TRUE	2.12682389
Depression_admission_freq	OPCs	*TTC12*	TRUE	2.011869859
Depression_admission_freq	OPCs	*EFHB*	TRUE	2.125157358
Depression_admission_freq	OPCs	*RNASE2*	TRUE	2.018038033
Depression_admission_freq	OPCs	*POLE*	TRUE	2.067859779
Depression_admission_freq	OPCs	*HLA.DQA2*	TRUE	2.234249592
Depression_admission_freq	OPCs	*RP11.826N14.2*	TRUE	2.212079688
Depression_admission_freq	OPCs	*CTD.3126B10.4*	TRUE	2.022196628
Depression_admission_freq	OPCs	*XXbac.BPG181B23.7*	TRUE	1.990507432
TRD	Astros	*TNFRSF10C*	TRUE	1.961284831
TRD	Astros	*ZNF664*	TRUE	1.974287076
TRD	Astros	*FAHD1*	TRUE	1.96552785
TRD	Astros	*AC159540.1*	TRUE	2.043864819
TRD	Astros	*LILRA4*	TRUE	2.190605943
TRD	Astros	*SMIM22*	TRUE	2.331348197
TRD	Ex	*SLC7A9*	TRUE	2.090892779
TRD	Ex	*VCL*	TRUE	2.023023341
TRD	Ex	*NUCKS1*	TRUE	2.418340358
TRD	Ex	*CPSF1*	TRUE	2.500437462
TRD	Ex	*MRVI1*	TRUE	2.302924112
TRD	Ex	*SEC31B*	TRUE	2.201682273
TRD	Ex	*ABCB1*	TRUE	2.176050035
TRD	Ex	*FCGBP*	TRUE	2.163079771
TRD	Ex	*CLUAP1*	TRUE	2.104188136
TRD	Ex	*PYCRL*	TRUE	2.036507102
TRD	Ex	*CHCHD2*	TRUE	2.124352769
TRD	Ex	*PNPO*	TRUE	2.133743081
TRD	Ex	*LRRC42*	TRUE	2.315225746
TRD	Ex	*DNAL1*	TRUE	2.244562677
TRD	Ex	*CCDC170*	TRUE	1.966011328
TRD	Ex	*PCDHB10*	TRUE	2.616024208
TRD	Ex	*MRS2*	TRUE	1.96266658
TRD	Ex	*ZNF391*	TRUE	2.079786172
TRD	Ex	*GPR108*	TRUE	2.403346321
TRD	Ex	*MASP1*	TRUE	3.051550944
TRD	Ex	*CCT6B*	TRUE	2.008083188
TRD	Ex	*SCLY*	TRUE	1.973266227
TRD	Ex	*TSGA10*	TRUE	2.691392204
TRD	Ex	*NEDD1*	TRUE	2.069007849
TRD	Ex	*THNSL2*	TRUE	1.962174963
TRD	Ex	*EXOG*	TRUE	2.075684237
TRD	Ex	*TCHH*	TRUE	1.970731719
TRD	Ex	*PRKCD*	TRUE	2.115869258
TRD	Ex	*RFT1*	TRUE	2.023612496
TRD	Ex	*ANXA5*	TRUE	2.206540471
TRD	Ex	*OR51E2*	TRUE	2.061918044
TRD	Ex	*CCDC88B*	TRUE	2.043401828

TRD	Ex	*FGGY*	TRUE	2.299010553
TRD	Ex	*PARP14*	TRUE	2.460984316
TRD	Ex	*AHSA2*	TRUE	2.212207727
TRD	Ex	*TNFRSF10C*	TRUE	2.326697522
TRD	Ex	*ZNF613*	TRUE	2.052952123
TRD	Ex	*ZNF664*	TRUE	2.583641627
TRD	Ex	*C11orf54*	TRUE	2.033855368
TRD	Ex	*CCDC125*	TRUE	2.613400648
TRD	Ex	*PCGF3*	TRUE	2.042184458
TRD	Ex	*KIF24*	TRUE	2.337819837
TRD	Ex	*FAM26F*	TRUE	2.245372491
TRD	Ex	*APRT*	TRUE	2.794235012
TRD	Ex	*ZNF891*	TRUE	2.224804595
TRD	Ex	*AC004540.5*	TRUE	2.123516743
TRD	Ex	*LINC00152*	TRUE	1.969929397
TRD	Ex	*LINC00937*	TRUE	1.972535294
TRD	Ex	*RP11.15I11.3*	TRUE	2.131749911
TRD	Ex	*AC097713.3*	TRUE	2.43995092
TRD	Ex	*LILRA4*	TRUE	2.031394782
TRD	Ex	*AF146191.4*	TRUE	2.159059301
TRD	Ex	*LINC00926*	TRUE	1.963730898
TRD	Ex	*CTD.2589M5.5*	TRUE	2.450375092
TRD	Ex	*CTD.2349P21.9*	TRUE	2.864837982
TRD	Ex	*CTD.2623N2.11*	TRUE	2.576775484
TRD	Ex	*RP11.147L13.8*	TRUE	2.167018653
TRD	Inhib	*SLC7A9*	TRUE	2.066329419
TRD	Inhib	*VCL*	TRUE	2.126358549
TRD	Inhib	*CASP8*	TRUE	2.082896977
TRD	Inhib	*NUCKS1*	TRUE	2.087543289
TRD	Inhib	*CPSF1*	TRUE	2.114021955
TRD	Inhib	*MRVI1*	TRUE	2.164092861
TRD	Inhib	*SEC31B*	TRUE	2.033760244
TRD	Inhib	*FCGBP*	TRUE	2.16694986
TRD	Inhib	*CDC7*	TRUE	1.996637742
TRD	Inhib	*CLUAP1*	TRUE	2.043045294
TRD	Inhib	*PYCRL*	TRUE	2.225122257
TRD	Inhib	*CHCHD2*	TRUE	2.083555438
TRD	Inhib	*PNPO*	TRUE	1.993101446
TRD	Inhib	*LRRC42*	TRUE	2.424096966
TRD	Inhib	*DNAL1*	TRUE	2.198032006
TRD	Inhib	*CCDC170*	TRUE	2.053817875
TRD	Inhib	*PCDHB10*	TRUE	2.321102506
TRD	Inhib	*ZNF391*	TRUE	2.030014139
TRD	Inhib	*GPR108*	TRUE	2.458375514
TRD	Inhib	*MASP1*	TRUE	3.070999119
TRD	Inhib	*SCLY*	TRUE	2.156662492
TRD	Inhib	*TSGA10*	TRUE	2.477714127
TRD	Inhib	*THNSL2*	TRUE	2.163529496
TRD	Inhib	*RMND1*	TRUE	1.980831491
TRD	Inhib	*TCHH*	TRUE	2.015329243
TRD	Inhib	*PRKCD*	TRUE	2.2069345

TRD	Inhib	*ANXA5*	TRUE	2.031044439
TRD	Inhib	*OR51E2*	TRUE	2.362556832
TRD	Inhib	*CCDC88B*	TRUE	1.970230242
TRD	Inhib	*FGGY*	TRUE	2.313804297
TRD	Inhib	*PARP14*	TRUE	2.464459399
TRD	Inhib	*TNFRSF10C*	TRUE	2.284828796
TRD	Inhib	*ZNF613*	TRUE	2.222340345
TRD	Inhib	*MUC20*	TRUE	1.967925032
TRD	Inhib	*ZNF664*	TRUE	2.646092608
TRD	Inhib	*CCDC125*	TRUE	2.30330677
TRD	Inhib	*KIF24*	TRUE	2.32578127
TRD	Inhib	*FAM26F*	TRUE	2.526880375
TRD	Inhib	*APRT*	TRUE	2.767036837
TRD	Inhib	*ZNF891*	TRUE	2.23583854
TRD	Inhib	*RP1.137D17.1*	TRUE	2.070956203
TRD	Inhib	*RP11.15I11.3*	TRUE	2.13086465
TRD	Inhib	*AC097713.3*	TRUE	2.446733928
TRD	Inhib	*LILRA4*	TRUE	2.229780456
TRD	Inhib	*CTD.2589M5.5*	TRUE	2.188907837
TRD	Inhib	*RP11.107F6.3*	TRUE	2.319068847
TRD	Inhib	*CTD.2349P21.9*	TRUE	2.497158082
TRD	Inhib	*CTD.2623N2.11*	TRUE	2.526896337
TRD	Inhib	*RP11.147L13.8*	TRUE	2.167280916
TRD	Micro_Macro	*MASP1*	TRUE	2.533299743
TRD	Micro_Macro	*KIF24*	TRUE	2.044612772
TRD	Oligos	*MRVI1*	TRUE	2.214566822
TRD	Oligos	*PYCRL*	TRUE	1.968327893
TRD	Oligos	*CHCHD2*	TRUE	1.979683587
TRD	Oligos	*GPR108*	TRUE	2.026774986
TRD	Oligos	*MASP1*	TRUE	2.497831437
TRD	Oligos	*FGGY*	TRUE	2.108542107
TRD	Oligos	*PARP14*	TRUE	2.417444666
TRD	Oligos	*ZNF664*	TRUE	2.261107021
TRD	Oligos	*APRT*	TRUE	2.30780318
TRD	Oligos	*ZNF891*	TRUE	2.003515685
TRD	OPCs	*PYCRL*	TRUE	2.305825469
TRD	OPCs	*LRRC42*	TRUE	2.316820291
TRD	OPCs	*SCLY*	TRUE	2.101094152
TRD	OPCs	*ZNF664*	TRUE	2.456162801
TRD	OPCs	*FAHD1*	TRUE	2.006768152
TRD	OPCs	*APRT*	TRUE	2.301357597
TRD	OPCs	*AC097713.3*	TRUE	2.187766808
TRD	OPCs	*RP11.384P7.7*	TRUE	2.047554975
TRD	OPCs	*CTD.2349P21.9*	TRUE	1.975612165

b. Identified cell type specific causal genes for T2DM and related disorders from pancreas

disorder	celltype	gene	assoc	zMin
Prediabetes	acinar_cell	*STPG1*	TRUE	1.968466597
Prediabetes	acinar_cell	*OTX1*	TRUE	2.679296292
Prediabetes	acinar_cell	*KIAA0087*	TRUE	2.320711183
Prediabetes	acinar_cell	*SH2D3A*	TRUE	2.676365519
Prediabetes	acinar_cell	*A4GALT*	TRUE	2.388182485
Prediabetes	acinar_cell	*NEK3*	TRUE	1.967639749
Prediabetes	acinar_cell	*SPTBN5*	TRUE	1.974824951
Prediabetes	acinar_cell	*RGS3*	TRUE	2.515002227
Prediabetes	acinar_cell	*CAPN5*	TRUE	2.071145345
Prediabetes	acinar_cell	*CNNM4*	TRUE	2.389037647
Prediabetes	acinar_cell	*RPRML*	TRUE	1.999195573
Prediabetes	acinar_cell	*GPR88*	TRUE	2.444648854
Prediabetes	acinar_cell	*FAM166A*	TRUE	2.091930808
Prediabetes	acinar_cell	*ZNF559*	TRUE	1.985066148
Prediabetes	acinar_cell	*PIWIL2*	TRUE	2.225069491
Prediabetes	acinar_cell	*NOTCH4*	TRUE	2.12222411
Prediabetes	alpha_cell	*STPG1*	TRUE	2.65342763
Prediabetes	alpha_cell	*ST3GAL6*	TRUE	1.962215815
Prediabetes	alpha_cell	*TESK2*	TRUE	2.210712172
Prediabetes	alpha_cell	*OTX1*	TRUE	3.575564914
Prediabetes	alpha_cell	*KIAA0087*	TRUE	2.514262081
Prediabetes	alpha_cell	*SH2D3A*	TRUE	3.103958974
Prediabetes	alpha_cell	*A4GALT*	TRUE	2.56571506
Prediabetes	alpha_cell	*NEK3*	TRUE	2.140550366
Prediabetes	alpha_cell	*CIB2*	TRUE	1.99502752
Prediabetes	alpha_cell	*SPTBN5*	TRUE	2.502567281
Prediabetes	alpha_cell	*RGS3*	TRUE	2.304939402
Prediabetes	alpha_cell	*ZMYND15*	TRUE	2.019468352
Prediabetes	alpha_cell	*CAPN5*	TRUE	2.30737618
Prediabetes	alpha_cell	*PDE3B*	TRUE	1.973540161
Prediabetes	alpha_cell	*CNNM4*	TRUE	2.334619736
Prediabetes	alpha_cell	*NBEAL2*	TRUE	2.028503001
Prediabetes	alpha_cell	*CPSF2*	TRUE	2.021651792
Prediabetes	alpha_cell	*RAD9A*	TRUE	2.138245866
Prediabetes	alpha_cell	*HAP1*	TRUE	2.652267508
Prediabetes	alpha_cell	*RPRML*	TRUE	2.127133663
Prediabetes	alpha_cell	*GPR88*	TRUE	2.461672629
Prediabetes	alpha_cell	*SETD4*	TRUE	2.081376
Prediabetes	alpha_cell	*KBTBD12*	TRUE	2.590319436
Prediabetes	alpha_cell	*FAM166A*	TRUE	3.081240949
Prediabetes	alpha_cell	*PIWIL2*	TRUE	2.730164018
Prediabetes	alpha_cell	*NOTCH4*	TRUE	2.222706115
Prediabetes	alpha_cell	*PINLYP*	TRUE	2.129869085

Prediabetes	beta_cell	*STPG1*	TRUE	3.018156693
Prediabetes	beta_cell	*APBA3*	TRUE	2.069528056
Prediabetes	beta_cell	*ST3GAL6*	TRUE	2.324661521
Prediabetes	beta_cell	*FGF10*	TRUE	2.042036362
Prediabetes	beta_cell	*TESK2*	TRUE	2.040689514
Prediabetes	beta_cell	*NDE1*	TRUE	2.238593079
Prediabetes	beta_cell	*CACNG5*	TRUE	2.000730554
Prediabetes	beta_cell	*APBB1IP*	TRUE	2.013320169
Prediabetes	beta_cell	*ZNF500*	TRUE	2.048778999
Prediabetes	beta_cell	*NOMO3*	TRUE	2.050889145
Prediabetes	beta_cell	*NAGLU*	TRUE	2.662969516
Prediabetes	beta_cell	*UFSP2*	TRUE	2.131499378
Prediabetes	beta_cell	*OTX1*	TRUE	3.607728808
Prediabetes	beta_cell	*WDR77*	TRUE	2.247634983
Prediabetes	beta_cell	*FILIP1*	TRUE	2.10616405
Prediabetes	beta_cell	*ZC2HC1C*	TRUE	2.228508854
Prediabetes	beta_cell	*SLC17A5*	TRUE	2.6978888
Prediabetes	beta_cell	*KIAA0087*	TRUE	2.608001358
Prediabetes	beta_cell	*INSIG2*	TRUE	1.982283858
Prediabetes	beta_cell	*SH2D3A*	TRUE	3.11109603
Prediabetes	beta_cell	*A4GALT*	TRUE	2.573562365
Prediabetes	beta_cell	*MAP3K10*	TRUE	2.102170261
Prediabetes	beta_cell	*FLNB*	TRUE	2.099500373
Prediabetes	beta_cell	*NEK3*	TRUE	2.298526848
Prediabetes	beta_cell	*CIB2*	TRUE	2.190703649
Prediabetes	beta_cell	*BLK*	TRUE	1.966236314
Prediabetes	beta_cell	*SPTBN5*	TRUE	2.481874391
Prediabetes	beta_cell	*RGS3*	TRUE	2.759294036
Prediabetes	beta_cell	*ZMYND15*	TRUE	2.181473688
Prediabetes	beta_cell	*IQGAP2*	TRUE	2.105743178
Prediabetes	beta_cell	*TRIM7*	TRUE	2.655369286
Prediabetes	beta_cell	*MTG1*	TRUE	1.996069326
Prediabetes	beta_cell	*CAPN5*	TRUE	2.358826958
Prediabetes	beta_cell	*DCUN1D2*	TRUE	1.96152049
Prediabetes	beta_cell	*PDE3B*	TRUE	2.257430702
Prediabetes	beta_cell	*DDAH1*	TRUE	2.892198065
Prediabetes	beta_cell	*LRGUK*	TRUE	2.095378722
Prediabetes	beta_cell	*CNNM4*	TRUE	2.488239044
Prediabetes	beta_cell	*NBEAL2*	TRUE	2.178097191
Prediabetes	beta_cell	*SPC24*	TRUE	1.994893066
Prediabetes	beta_cell	*ERCC3*	TRUE	1.969730102
Prediabetes	beta_cell	*RYBP*	TRUE	2.000409044
Prediabetes	beta_cell	*RPL9*	TRUE	2.003227356
Prediabetes	beta_cell	*CPSF2*	TRUE	2.052783398
Prediabetes	beta_cell	*KIFC2*	TRUE	2.167171415

Prediabetes	beta_cell	*HAP1*	TRUE	2.54687089
Prediabetes	beta_cell	*PFN4*	TRUE	2.064549681
Prediabetes	beta_cell	*TMEM80*	TRUE	2.259600496
Prediabetes	beta_cell	*RPRML*	TRUE	2.109693144
Prediabetes	beta_cell	*GPR88*	TRUE	2.599469743
Prediabetes	beta_cell	*CALN1*	TRUE	2.240710456
Prediabetes	beta_cell	*SH3BGR*	TRUE	1.987525007
Prediabetes	beta_cell	*SETD4*	TRUE	2.627092815
Prediabetes	beta_cell	*KBTBD12*	TRUE	2.822638228
Prediabetes	beta_cell	*FAM166A*	TRUE	2.336513445
Prediabetes	beta_cell	*ZNF763*	TRUE	2.225679808
Prediabetes	beta_cell	*PIWIL2*	TRUE	2.925023622
Prediabetes	beta_cell	*OGDHL*	TRUE	1.977139651
Prediabetes	beta_cell	*ENPP1*	TRUE	2.145023107
Prediabetes	beta_cell	*NOTCH4*	TRUE	2.3162522
Prediabetes	beta_cell	*LINC00865*	TRUE	2.193767651
Prediabetes	beta_cell	*PINLYP*	TRUE	2.348198758
Prediabetes	beta_cell	*LRRC37A2*	TRUE	2.068454661
Prediabetes	delta_cell	*OTX1*	TRUE	3.150436428
Prediabetes	delta_cell	*SH2D3A*	TRUE	3.047455399
Prediabetes	delta_cell	*RGS3*	TRUE	2.543411379
Prediabetes	delta_cell	*PDE3B*	TRUE	2.00392137
Prediabetes	delta_cell	*CNNM4*	TRUE	2.142621429
Prediabetes	delta_cell	*NBEAL2*	TRUE	2.074286704
Prediabetes	delta_cell	*CALN1*	TRUE	2.134376263
Prediabetes	delta_cell	*FAM166A*	TRUE	2.061360089
Prediabetes	delta_cell	*PIWIL2*	TRUE	1.994118079
Prediabetes	delta_cell	*PINLYP*	TRUE	2.445515608
Prediabetes	duct_epithelial_cell	*STPG1*	TRUE	1.986533264
Prediabetes	duct_epithelial_cell	*ST3GAL6*	TRUE	1.983396217
Prediabetes	duct_epithelial_cell	*NAGLU*	TRUE	2.055169201
Prediabetes	duct_epithelial_cell	*OTX1*	TRUE	3.170004028
Prediabetes	duct_epithelial_cell	*KIAA0087*	TRUE	2.309600546
Prediabetes	duct_epithelial_cell	*MAP3K10*	TRUE	2.115670188
Prediabetes	duct_epithelial_cell	*CIB2*	TRUE	2.014884646
Prediabetes	duct_epithelial_cell	*RPRML*	TRUE	2.33776216
Prediabetes	duct_epithelial_cell	*KBTBD12*	TRUE	2.415083287
Prediabetes	duct_epithelial_cell	*ZNF559*	TRUE	2.143719424
Prediabetes	duct_epithelial_cell	*PIWIL2*	TRUE	2.578528929
Prediabetes	duct_epithelial_cell	*BGLAP*	TRUE	1.974513813
Prediabetes	endothelial_cell	*ST3GAL6*	TRUE	2.017885735
Prediabetes	endothelial_cell	*OTX1*	TRUE	3.425651481
Prediabetes	endothelial_cell	*SLC17A5*	TRUE	1.993181825
Prediabetes	endothelial_cell	*KIAA0087*	TRUE	2.491446777
Prediabetes	endothelial_cell	*SH2D3A*	TRUE	3.08875326

Prediabetes	endothelial_cell	*TRIM7*	TRUE	2.361309123
Prediabetes	endothelial_cell	*CAPN5*	TRUE	2.19366652
Prediabetes	endothelial_cell	*HAP1*	TRUE	2.189330856
Prediabetes	endothelial_cell	*RPRML*	TRUE	2.303360667
Prediabetes	endothelial_cell	*GPR88*	TRUE	2.393190655
Prediabetes	endothelial_cell	*KBTBD12*	TRUE	2.175450756
Prediabetes	endothelial_cell	*FAM166A*	TRUE	2.333497457
Prediabetes	endothelial_cell	*PIWIL2*	TRUE	2.741115252
Prediabetes	epsilon_cell	*OTX1*	TRUE	3.231007925
Prediabetes	epsilon_cell	*SPTBN5*	TRUE	2.359788808
Prediabetes	epsilon_cell	*RPRML*	TRUE	2.020362268
Prediabetes	epsilon_cell	*GPR88*	TRUE	2.171435491
Prediabetes	leukocyte	*OTX1*	TRUE	3.310923318
Prediabetes	leukocyte	*KIAA0087*	TRUE	2.12990719
Prediabetes	leukocyte	*SH2D3A*	TRUE	2.091193932
Prediabetes	leukocyte	*GPR88*	TRUE	2.293530854
Prediabetes	leukocyte	*FAM166A*	TRUE	1.972793728
Prediabetes	mesenchymal_cell	*OTX1*	TRUE	2.467097891
Prediabetes	mesenchymal_cell	*KIAA0087*	TRUE	2.252648072
Prediabetes	mesenchymal_cell	*SH2D3A*	TRUE	2.662575857
Prediabetes	mesenchymal_cell	*CIB2*	TRUE	2.035322709
Prediabetes	mesenchymal_cell	*ZMYND15*	TRUE	2.035935239
Prediabetes	mesenchymal_cell	*RPRML*	TRUE	2.282905061
Prediabetes	mesenchymal_cell	*FAM166A*	TRUE	2.537194537
Prediabetes	mesenchymal_cell	*ZNF559*	TRUE	2.114099354
Prediabetes	mesenchymal_cell	*PIWIL2*	TRUE	2.392253974
Prediabetes	mesenchymal_cell	*HLA.DQA2*	TRUE	2.35416309
Prediabetes	PP_cell	*NAGLU*	TRUE	1.989406272
Prediabetes	PP_cell	*OTX1*	TRUE	3.216783482
Prediabetes	PP_cell	*SH2D3A*	TRUE	2.464207433
Prediabetes	PP_cell	*SPTBN5*	TRUE	2.292526115
Prediabetes	PP_cell	*RGS3*	TRUE	2.49350808
Prediabetes	PP_cell	*GPR88*	TRUE	2.212160255
Prediabetes	PP_cell	*ZNF559*	TRUE	2.13706378
Prediabetes	PP_cell	*NOTCH4*	TRUE	2.026833656
T1DM	acinar_cell	*CEP68*	TRUE	2.039816542
T1DM	acinar_cell	*ARHGAP22*	TRUE	2.338562117
T1DM	acinar_cell	*ARHGEF19*	TRUE	2.056157083
T1DM	acinar_cell	*CLEC18A*	TRUE	2.208844175
T1DM	acinar_cell	*EXTL1*	TRUE	2.486988106
T1DM	acinar_cell	*RFTN2*	TRUE	2.280101024
T1DM	acinar_cell	*ZNF589*	TRUE	2.003447813
T1DM	acinar_cell	*ENGASE*	TRUE	3.022904695
T1DM	acinar_cell	*MORN4*	TRUE	1.977746871
T1DM	acinar_cell	*PCP2*	TRUE	2.130160503

T1DM	acinar_cell	*IRX3*	TRUE	2.256876722
T1DM	acinar_cell	*RPRML*	TRUE	2.59619996
T1DM	acinar_cell	*CDK10*	TRUE	2.09906341
T1DM	acinar_cell	*FAM228A*	TRUE	2.59118772
T1DM	acinar_cell	*SFTA2*	TRUE	2.681572037
T1DM	acinar_cell	*CCDC154*	TRUE	2.219157151
T1DM	acinar_cell	*HSD17B8*	TRUE	8.330615626
T1DM	acinar_cell	*NOTCH4*	TRUE	3.472857145
T1DM	acinar_cell	*PSORS1C1*	TRUE	2.057809738
T1DM	acinar_cell	*HLA.DQB2*	TRUE	8.115633058
T1DM	acinar_cell	*PSMB9*	TRUE	2.136022887
T1DM	acinar_cell	*HLA.DOB*	TRUE	6.198798961
T1DM	acinar_cell	*HLA.DMB*	TRUE	5.269886796
T1DM	acinar_cell	*GOLGA8K*	TRUE	2.281748653
T1DM	alpha_cell	*CEP68*	TRUE	2.414224708
T1DM	alpha_cell	*ZNF213*	TRUE	2.478459952
T1DM	alpha_cell	*STX1B*	TRUE	2.024618568
T1DM	alpha_cell	*KHNYN*	TRUE	1.976084697
T1DM	alpha_cell	*CTSH*	TRUE	2.822831157
T1DM	alpha_cell	*FTSJ3*	TRUE	2.362316392
T1DM	alpha_cell	*GPRC5D*	TRUE	1.97285872
T1DM	alpha_cell	*VIPR1*	TRUE	2.357327369
T1DM	alpha_cell	*ARHGAP22*	TRUE	2.481264638
T1DM	alpha_cell	*EPO*	TRUE	2.993773722
T1DM	alpha_cell	*PTPN22*	TRUE	2.466363149
T1DM	alpha_cell	*ULK3*	TRUE	2.239548473
T1DM	alpha_cell	*ARHGEF19*	TRUE	1.962610812
T1DM	alpha_cell	*NBPF3*	TRUE	1.974147033
T1DM	alpha_cell	*CABLES2*	TRUE	2.838390836
T1DM	alpha_cell	*SLC25A27*	TRUE	2.086856361
T1DM	alpha_cell	*CDK20*	TRUE	2.513258955
T1DM	alpha_cell	*SFXN2*	TRUE	1.971594739
T1DM	alpha_cell	*CLEC18A*	TRUE	2.580312432
T1DM	alpha_cell	*EXTL1*	TRUE	2.477253402
T1DM	alpha_cell	*SLC5A11*	TRUE	2.147199784
T1DM	alpha_cell	*RFTN2*	TRUE	2.402269383
T1DM	alpha_cell	*GNPDA2*	TRUE	2.000176054
T1DM	alpha_cell	*SRGAP2*	TRUE	2.035861656
T1DM	alpha_cell	*LMBRD2*	TRUE	1.993756954
T1DM	alpha_cell	*ENGASE*	TRUE	3.097226652
T1DM	alpha_cell	*EVPL*	TRUE	2.050724425
T1DM	alpha_cell	*MORN4*	TRUE	2.258744306
T1DM	alpha_cell	*SNTB1*	TRUE	2.249962881
T1DM	alpha_cell	*PCP2*	TRUE	2.160643134
T1DM	alpha_cell	*UBE2U*	TRUE	2.353229262

T1DM	alpha_cell	*IRX3*	TRUE	2.429888303
T1DM	alpha_cell	*RPRML*	TRUE	2.804592848
T1DM	alpha_cell	*F2R*	TRUE	2.085562033
T1DM	alpha_cell	*ANO9*	TRUE	1.998095872
T1DM	alpha_cell	*CDK10*	TRUE	2.308407765
T1DM	alpha_cell	*FAM228A*	TRUE	2.54874806
T1DM	alpha_cell	*ZKSCAN3*	TRUE	2.000283978
T1DM	alpha_cell	*SFTA2*	TRUE	2.461808135
T1DM	alpha_cell	*ZNF517*	TRUE	2.119405728
T1DM	alpha_cell	*CCDC154*	TRUE	2.322190197
T1DM	alpha_cell	*HSPB9*	TRUE	2.147530455
T1DM	alpha_cell	*HLA.DRB5*	TRUE	2.533092923
T1DM	alpha_cell	*HSD17B8*	TRUE	7.523365461
T1DM	alpha_cell	*NOTCH4*	TRUE	2.593408811
T1DM	alpha_cell	*HLA.DQB2*	TRUE	8.938393619
T1DM	alpha_cell	*PSMB9*	TRUE	2.291112894
T1DM	alpha_cell	*HLA.DOB*	TRUE	6.269361722
T1DM	alpha_cell	*HLA.DMB*	TRUE	5.836751043
T1DM	alpha_cell	*GOLGA8K*	TRUE	2.782592619
T1DM	beta_cell	*USH1C*	TRUE	2.059272889
T1DM	beta_cell	*BAIAP3*	TRUE	2.058337341
T1DM	beta_cell	*CEP68*	TRUE	3.032492485
T1DM	beta_cell	*ZDHHC6*	TRUE	2.052419674
T1DM	beta_cell	*RNASET2*	TRUE	2.371258778
T1DM	beta_cell	*RNF19A*	TRUE	1.973906797
T1DM	beta_cell	*SPTB*	TRUE	2.056260109
T1DM	beta_cell	*GPATCH1*	TRUE	1.96856575
T1DM	beta_cell	*ZNF213*	TRUE	2.856989636
T1DM	beta_cell	*PPP1R13B*	TRUE	2.145889441
T1DM	beta_cell	*KIF16B*	TRUE	2.182137334
T1DM	beta_cell	*STX1B*	TRUE	2.028384796
T1DM	beta_cell	*KHNYN*	TRUE	2.375870158
T1DM	beta_cell	*POLI*	TRUE	2.067977134
T1DM	beta_cell	*CCDC113*	TRUE	2.18814712
T1DM	beta_cell	*CTSH*	TRUE	2.837237992
T1DM	beta_cell	*ECH1*	TRUE	2.204543819
T1DM	beta_cell	*KLC3*	TRUE	2.116563357
T1DM	beta_cell	*OGN*	TRUE	1.98516172
T1DM	beta_cell	*BACH2*	TRUE	2.252146711
T1DM	beta_cell	*DNPH1*	TRUE	1.968354809
T1DM	beta_cell	*VIPR1*	TRUE	2.825924131
T1DM	beta_cell	*FANCL*	TRUE	1.965996158
T1DM	beta_cell	*PYROXD2*	TRUE	1.963253347
T1DM	beta_cell	*HELLS*	TRUE	2.050575522
T1DM	beta_cell	*YEATS4*	TRUE	2.043820396

T1DM	beta_cell	*ARHGAP22*	TRUE	2.56171327
T1DM	beta_cell	*TPH1*	TRUE	1.961424769
T1DM	beta_cell	*MAU2*	TRUE	2.122299491
T1DM	beta_cell	*SYT5*	TRUE	2.45141832
T1DM	beta_cell	*EPO*	TRUE	2.774312911
T1DM	beta_cell	*PIAS3*	TRUE	2.529801733
T1DM	beta_cell	*RHPN2*	TRUE	2.565120382
T1DM	beta_cell	*EIF5A*	TRUE	2.21895126
T1DM	beta_cell	*PTPN22*	TRUE	2.454072041
T1DM	beta_cell	*NOTCH2*	TRUE	2.020438851
T1DM	beta_cell	*AVIL*	TRUE	2.306220225
T1DM	beta_cell	*TBRG4*	TRUE	2.179573793
T1DM	beta_cell	*PLCB2*	TRUE	1.966678987
T1DM	beta_cell	*SPTBN5*	TRUE	2.028268733
T1DM	beta_cell	*CALHM2*	TRUE	2.193108523
T1DM	beta_cell	*ULK3*	TRUE	2.50514336
T1DM	beta_cell	*ARHGEF19*	TRUE	2.231805854
T1DM	beta_cell	*NBPF3*	TRUE	2.021023454
T1DM	beta_cell	*GIGYF1*	TRUE	2.145361135
T1DM	beta_cell	*EML3*	TRUE	2.417431081
T1DM	beta_cell	*CABLES2*	TRUE	3.057641678
T1DM	beta_cell	*AKR1C2*	TRUE	2.068118499
T1DM	beta_cell	*SPC25*	TRUE	2.271936221
T1DM	beta_cell	*SLC25A27*	TRUE	2.260578095
T1DM	beta_cell	*PTPRD*	TRUE	2.605638962
T1DM	beta_cell	*MRPL39*	TRUE	2.030552915
T1DM	beta_cell	*CCT8*	TRUE	2.058733547
T1DM	beta_cell	*CDK20*	TRUE	2.699009513
T1DM	beta_cell	*CLEC18A*	TRUE	2.602509591
T1DM	beta_cell	*EXTL1*	TRUE	2.513024275
T1DM	beta_cell	*AHCYL2*	TRUE	2.074207776
T1DM	beta_cell	*SLC5A11*	TRUE	2.169704279
T1DM	beta_cell	*OMA1*	TRUE	2.083602976
T1DM	beta_cell	*RFTN2*	TRUE	3.067113012
T1DM	beta_cell	*GNPDA2*	TRUE	2.429226593
T1DM	beta_cell	*NIPAL1*	TRUE	2.271920482
T1DM	beta_cell	*SRGAP2*	TRUE	2.665570781
T1DM	beta_cell	*ZNF589*	TRUE	2.037575323
T1DM	beta_cell	*LMBRD2*	TRUE	2.46346972
T1DM	beta_cell	*ENGASE*	TRUE	3.241125211
T1DM	beta_cell	*CACNB3*	TRUE	2.014524062
T1DM	beta_cell	*EVPL*	TRUE	2.079122765
T1DM	beta_cell	*SLC38A11*	TRUE	2.264769971
T1DM	beta_cell	*KCNMB3*	TRUE	1.996778123
T1DM	beta_cell	*MORN4*	TRUE	2.399524914

T1DM	beta_cell	*ZNF440*	TRUE	2.260219385
T1DM	beta_cell	*PPID*	TRUE	2.011925583
T1DM	beta_cell	*CLSTN1*	TRUE	2.589899802
T1DM	beta_cell	*PTEN*	TRUE	2.180546234
T1DM	beta_cell	*SNTB1*	TRUE	2.466644196
T1DM	beta_cell	*B3GALT1*	TRUE	1.970166439
T1DM	beta_cell	*SMPDL3A*	TRUE	2.152379726
T1DM	beta_cell	*PCP2*	TRUE	2.155665704
T1DM	beta_cell	*CHRNA7*	TRUE	2.199927865
T1DM	beta_cell	*UBE2U*	TRUE	2.361705473
T1DM	beta_cell	*IRX3*	TRUE	2.630705317
T1DM	beta_cell	*SAMD12*	TRUE	1.984957998
T1DM	beta_cell	*IL17RA*	TRUE	2.586700226
T1DM	beta_cell	*DALRD3*	TRUE	2.371862453
T1DM	beta_cell	*TMTC2*	TRUE	2.021975247
T1DM	beta_cell	*RPRML*	TRUE	2.798134769
T1DM	beta_cell	*ZNF154*	TRUE	2.247548433
T1DM	beta_cell	*TH*	TRUE	1.977401528
T1DM	beta_cell	*F2R*	TRUE	2.461712183
T1DM	beta_cell	*CAMK1D*	TRUE	2.298335054
T1DM	beta_cell	*ANO9*	TRUE	2.097187314
T1DM	beta_cell	*RBM11*	TRUE	2.280463346
T1DM	beta_cell	*CDK10*	TRUE	2.551725632
T1DM	beta_cell	*FAM228A*	TRUE	2.610270717
T1DM	beta_cell	*LYPD6*	TRUE	2.099192019
T1DM	beta_cell	*BCAM*	TRUE	1.978429637
T1DM	beta_cell	*ZKSCAN3*	TRUE	1.985122616
T1DM	beta_cell	*SFTA2*	TRUE	2.701207225
T1DM	beta_cell	*MVB12B*	TRUE	2.447950535
T1DM	beta_cell	*ZNF517*	TRUE	2.356242474
T1DM	beta_cell	*CCDC154*	TRUE	2.259594379
T1DM	beta_cell	*HSPB9*	TRUE	1.96651729
T1DM	beta_cell	*RPS26*	TRUE	3.089749274
T1DM	beta_cell	*C2orf27A*	TRUE	2.070515242
T1DM	beta_cell	*HLA.DRB5*	TRUE	2.524555138
T1DM	beta_cell	*HSD17B8*	TRUE	7.656909119
T1DM	beta_cell	*NOTCH4*	TRUE	2.584206907
T1DM	beta_cell	*PSORS1C1*	TRUE	1.982120884
T1DM	beta_cell	*HLA.DQB2*	TRUE	9.028860491
T1DM	beta_cell	*PSMB9*	TRUE	2.388460186
T1DM	beta_cell	*HLA.DOB*	TRUE	6.262548882
T1DM	beta_cell	*HLA.DMB*	TRUE	5.880130148
T1DM	beta_cell	*GOLGA8K*	TRUE	3.014647256
T1DM	delta_cell	*ARHGAP22*	TRUE	2.303790005
T1DM	delta_cell	*ZNF589*	TRUE	2.842909926

T1DM	delta_cell	*ENGASE*	TRUE	2.304319419
T1DM	delta_cell	*FAM228A*	TRUE	2.178024995
T1DM	delta_cell	*SFTA2*	TRUE	2.977025014
T1DM	delta_cell	*HLA.DRB5*	TRUE	2.158470401
T1DM	delta_cell	*HSD17B8*	TRUE	6.917263192
T1DM	delta_cell	*NOTCH4*	TRUE	4.173463039
T1DM	delta_cell	*HLA.DQB2*	TRUE	7.910569819
T1DM	delta_cell	*PSMB9*	TRUE	2.228337488
T1DM	delta_cell	*HLA.DOB*	TRUE	6.359277496
T1DM	delta_cell	*TAS2R43*	TRUE	2.055118216
T1DM	duct_epithelial_cell	*ARHGAP22*	TRUE	2.304773146
T1DM	duct_epithelial_cell	*CDK20*	TRUE	2.191666092
T1DM	duct_epithelial_cell	*ZNF589*	TRUE	2.200697662
T1DM	duct_epithelial_cell	*ENGASE*	TRUE	2.487327545
T1DM	duct_epithelial_cell	*MORN4*	TRUE	1.97230932
T1DM	duct_epithelial_cell	*RPRML*	TRUE	1.961464783
T1DM	duct_epithelial_cell	*FAM228A*	TRUE	1.985759106
T1DM	duct_epithelial_cell	*SFTA2*	TRUE	2.755481765
T1DM	duct_epithelial_cell	*HLA.DRB5*	TRUE	2.317664633
T1DM	duct_epithelial_cell	*HSD17B8*	TRUE	7.044755751
T1DM	duct_epithelial_cell	*NOTCH4*	TRUE	2.273477623
T1DM	duct_epithelial_cell	*HLA.DQB2*	TRUE	9.023931516
T1DM	duct_epithelial_cell	*HLA.DOB*	TRUE	5.521327075
T1DM	duct_epithelial_cell	*HLA.DMB*	TRUE	5.665865059
T1DM	duct_epithelial_cell	*GOLGA8K*	TRUE	2.092175859
T1DM	endothelial_cell	*ARHGAP22*	TRUE	2.017191669
T1DM	endothelial_cell	*VPS11*	TRUE	2.261124675
T1DM	endothelial_cell	*ENGASE*	TRUE	2.877059185
T1DM	endothelial_cell	*SNTB1*	TRUE	2.008521637
T1DM	endothelial_cell	*HSD17B8*	TRUE	5.798181612
T1DM	endothelial_cell	*ATF6B*	TRUE	2.80913099
T1DM	endothelial_cell	*HLA.DQB2*	TRUE	9.604418917
T1DM	endothelial_cell	*HLA.DOB*	TRUE	7.117175505
T1DM	endothelial_cell	*HLA.DMB*	TRUE	4.256928155
T1DM	endothelial_cell	*TAS2R43*	TRUE	2.069265431
T1DM	epsilon_cell	*SFTA2*	TRUE	3.265879749
T1DM	epsilon_cell	*HLA.DMA*	TRUE	3.232776767
T1DM	epsilon_cell	*NOTCH4*	TRUE	2.743097643
T1DM	epsilon_cell	*PSORS1C1*	TRUE	2.23303672
T1DM	epsilon_cell	*HLA.DQB2*	TRUE	2.997978075
T1DM	epsilon_cell	*HLA.DOB*	TRUE	6.830137518
T1DM	leukocyte	*SFTA2*	TRUE	2.530126897
T1DM	leukocyte	*PSORS1C1*	TRUE	2.518054906
T1DM	leukocyte	*HLA.DQB2*	TRUE	2.128458218
T1DM	leukocyte	*HLA.DOB*	TRUE	4.128528944

T1DM	leukocyte	*C4A*	TRUE	4.644546888
T1DM	mesenchymal_cell	*CTSH*	TRUE	2.19684091
T1DM	mesenchymal_cell	*ENGASE*	TRUE	3.087012529
T1DM	mesenchymal_cell	*RPRML*	TRUE	2.133280828
T1DM	mesenchymal_cell	*CDK10*	TRUE	2.054201903
T1DM	mesenchymal_cell	*LYPD6*	TRUE	1.997658674
T1DM	mesenchymal_cell	*SFTA2*	TRUE	3.646186202
T1DM	mesenchymal_cell	*HSD17B8*	TRUE	4.741012407
T1DM	mesenchymal_cell	*HLA.DMA*	TRUE	4.958232859
T1DM	mesenchymal_cell	*PSORS1C1*	TRUE	1.991733606
T1DM	mesenchymal_cell	*HLA.DQB2*	TRUE	4.687684094
T1DM	mesenchymal_cell	*HLA.DOB*	TRUE	6.282607679
T1DM	PP_cell	*SFTA2*	TRUE	2.572871659
T1DM	PP_cell	*HLA.DMA*	TRUE	3.500176417
T1DM	PP_cell	*NOTCH4*	TRUE	3.736808286
T1DM	PP_cell	*PSORS1C1*	TRUE	2.408580169
T1DM	PP_cell	*ATF6B*	TRUE	2.456807306
T1DM	PP_cell	*HLA.DQB2*	TRUE	7.451785622
T1DM	PP_cell	*HLA.DOB*	TRUE	6.777560107
T2DM	acinar_cell	*RBM6*	TRUE	2.576545433
T2DM	acinar_cell	*MLXIPL*	TRUE	2.457919973
T2DM	acinar_cell	*QPCTL*	TRUE	2.083857893
T2DM	acinar_cell	*CEP68*	TRUE	3.294729016
T2DM	acinar_cell	*BAK1*	TRUE	2.65831765
T2DM	acinar_cell	*TCF3*	TRUE	2.699186947
T2DM	acinar_cell	*ST6GAL1*	TRUE	2.133222151
T2DM	acinar_cell	*CWF19L1*	TRUE	2.970279289
T2DM	acinar_cell	*CCDC134*	TRUE	2.494564823
T2DM	acinar_cell	*SLC1A1*	TRUE	2.182805078
T2DM	acinar_cell	*CNTNAP1*	TRUE	2.089252718
T2DM	acinar_cell	*BTNL8*	TRUE	2.13
T2DM	acinar_cell	*DNAH7*	TRUE	2.228588852
T2DM	acinar_cell	*ZNF211*	TRUE	2.054908908
T2DM	acinar_cell	*FLT3*	TRUE	3.260980927
T2DM	acinar_cell	*OPRL1*	TRUE	3.085659707
T2DM	acinar_cell	*RIBC2*	TRUE	2.483235675
T2DM	acinar_cell	*NOTCH2*	TRUE	4.902154906
T2DM	acinar_cell	*LRP4*	TRUE	2.689001752
T2DM	acinar_cell	*MAN2C1*	TRUE	3.295470717
T2DM	acinar_cell	*HSD17B12*	TRUE	2.790330299
T2DM	acinar_cell	*APIP*	TRUE	2.274434877
T2DM	acinar_cell	*PRSS53*	TRUE	2.130611577
T2DM	acinar_cell	*PTPN14*	TRUE	2.760929446
T2DM	acinar_cell	*POC5*	TRUE	2.016237569
T2DM	acinar_cell	*JAZF1*	TRUE	5.412746096

T2DM	acinar_cell	*CLEC18A*	TRUE	5.32058161
T2DM	acinar_cell	*EXTL1*	TRUE	2.100875034
T2DM	acinar_cell	*ANKLE1*	TRUE	2.454977248
T2DM	acinar_cell	*ICA1L*	TRUE	2.036983589
T2DM	acinar_cell	*PPM1M*	TRUE	2.142453458
T2DM	acinar_cell	*TMC8*	TRUE	2.045990126
T2DM	acinar_cell	*IRX3*	TRUE	9.566213635
T2DM	acinar_cell	*GPR88*	TRUE	1.962244756
T2DM	acinar_cell	*VMO1*	TRUE	3.18168685
T2DM	acinar_cell	*ZNF320*	TRUE	2.56206333
T2DM	acinar_cell	*FAM43B*	TRUE	3.117137547
T2DM	acinar_cell	*PIWIL2*	TRUE	2.416840807
T2DM	acinar_cell	*NOTCH4*	TRUE	3.220598918
T2DM	acinar_cell	*HCG27*	TRUE	2.659216208
T2DM	acinar_cell	*DNLZ*	TRUE	3.274865271
T2DM	acinar_cell	*SLC12A8*	TRUE	2.757676871
T2DM	acinar_cell	*HLA.DQA2*	TRUE	3.749651891
T2DM	acinar_cell	*HMBS*	TRUE	2.184343307
T2DM	alpha_cell	*RBM6*	TRUE	2.976751944
T2DM	alpha_cell	*MLXIPL*	TRUE	3.058946996
T2DM	alpha_cell	*GIPR*	TRUE	2.307092364
T2DM	alpha_cell	*CEP68*	TRUE	4.203392815
T2DM	alpha_cell	*LTF*	TRUE	2.237029979
T2DM	alpha_cell	*BAK1*	TRUE	3.434895929
T2DM	alpha_cell	*ITIH4*	TRUE	2.734774845
T2DM	alpha_cell	*TCF3*	TRUE	3.133363765
T2DM	alpha_cell	*ST6GAL1*	TRUE	2.440809565
T2DM	alpha_cell	*MARK3*	TRUE	2.689954655
T2DM	alpha_cell	*CWF19L1*	TRUE	3.436667661
T2DM	alpha_cell	*CCDC134*	TRUE	2.888777255
T2DM	alpha_cell	*SLC1A1*	TRUE	2.199326536
T2DM	alpha_cell	*CDK2AP1*	TRUE	2.210137074
T2DM	alpha_cell	*NNT*	TRUE	2.196550625
T2DM	alpha_cell	*BTNL8*	TRUE	2.746462089
T2DM	alpha_cell	*DNAH7*	TRUE	3.311372097
T2DM	alpha_cell	*ZNF211*	TRUE	2.338087774
T2DM	alpha_cell	*FLT3*	TRUE	3.148931401
T2DM	alpha_cell	*PTPN12*	TRUE	2.166191181
T2DM	alpha_cell	*RIBC2*	TRUE	2.651603696
T2DM	alpha_cell	*MAU2*	TRUE	2.765915027
T2DM	alpha_cell	*SYT5*	TRUE	2.17717667
T2DM	alpha_cell	*PPARG*	TRUE	2.445737115
T2DM	alpha_cell	*NOTCH2*	TRUE	5.189354821
T2DM	alpha_cell	*LRP4*	TRUE	2.442637036
T2DM	alpha_cell	*MYPN*	TRUE	2.585576929

T2DM	alpha_cell	*MAN2C1*	TRUE	3.88376582
T2DM	alpha_cell	*ZNF514*	TRUE	2.167530562
T2DM	alpha_cell	*CDCA7*	TRUE	2.497253106
T2DM	alpha_cell	*CNTN4*	TRUE	2.771421753
T2DM	alpha_cell	*RAB19*	TRUE	2.602875183
T2DM	alpha_cell	*ADHFE1*	TRUE	2.075832787
T2DM	alpha_cell	*HSD17B12*	TRUE	3.299178686
T2DM	alpha_cell	*APIP*	TRUE	2.801287099
T2DM	alpha_cell	*KLHL35*	TRUE	2.13791723
T2DM	alpha_cell	*CCDC15*	TRUE	2.102551542
T2DM	alpha_cell	*PRSS53*	TRUE	2.253966079
T2DM	alpha_cell	*PTPN14*	TRUE	3.137631812
T2DM	alpha_cell	*POC5*	TRUE	2.446938423
T2DM	alpha_cell	*JAZF1*	TRUE	7.106285034
T2DM	alpha_cell	*CC2D1B*	TRUE	2.567656439
T2DM	alpha_cell	*CLEC18A*	TRUE	5.307842964
T2DM	alpha_cell	*CIB4*	TRUE	2.552236291
T2DM	alpha_cell	*EXTL1*	TRUE	2.613552467
T2DM	alpha_cell	*ANKLE1*	TRUE	2.396666484
T2DM	alpha_cell	*ICA1L*	TRUE	2.505810632
T2DM	alpha_cell	*BDH2*	TRUE	2.150220062
T2DM	alpha_cell	*PPM1M*	TRUE	2.119514454
T2DM	alpha_cell	*CASP7*	TRUE	3.295959952
T2DM	alpha_cell	*CYB5R2*	TRUE	2.145537665
T2DM	alpha_cell	*TMC8*	TRUE	2.803663337
T2DM	alpha_cell	*INO80E*	TRUE	3.067875584
T2DM	alpha_cell	*CTSW*	TRUE	1.97071294
T2DM	alpha_cell	*ZNF738*	TRUE	2.253214722
T2DM	alpha_cell	*TUBB8*	TRUE	2.162365059
T2DM	alpha_cell	*PHYHD1*	TRUE	2.905977225
T2DM	alpha_cell	*DSCAML1*	TRUE	2.472192286
T2DM	alpha_cell	*IRX3*	TRUE	9.385655156
T2DM	alpha_cell	*FARSA*	TRUE	2.159906949
T2DM	alpha_cell	*GPR88*	TRUE	2.601678955
T2DM	alpha_cell	*CAPN12*	TRUE	2.117372409
T2DM	alpha_cell	*VMO1*	TRUE	3.155844566
T2DM	alpha_cell	*ZNF320*	TRUE	3.308018729
T2DM	alpha_cell	*CAMK1D*	TRUE	2.202904901
T2DM	alpha_cell	*FAM43B*	TRUE	2.697097222
T2DM	alpha_cell	*CEACAM19*	TRUE	2.066055129
T2DM	alpha_cell	*ZNF385C*	TRUE	1.967267386
T2DM	alpha_cell	*NUTM2B*	TRUE	3.306569017
T2DM	alpha_cell	*ZDHHC11*	TRUE	2.022305292
T2DM	alpha_cell	*PIWIL2*	TRUE	3.076017766
T2DM	alpha_cell	*PCDHB11*	TRUE	2.146399703

T2DM	alpha_cell	*TBKBP1*	TRUE	2.111870126
T2DM	alpha_cell	*NOTCH4*	TRUE	3.416745696
T2DM	alpha_cell	*HCG27*	TRUE	2.827369438
T2DM	alpha_cell	*DNLZ*	TRUE	3.416659019
T2DM	alpha_cell	*SLC12A8*	TRUE	3.712861387
T2DM	alpha_cell	*LINC00957*	TRUE	2.126418598
T2DM	alpha_cell	*HLA.DQA2*	TRUE	3.674970447
T2DM	alpha_cell	*JMJD7*	TRUE	2.18597019
T2DM	alpha_cell	*OR2A7*	TRUE	2.006470987
T2DM	beta_cell	*RBM6*	TRUE	2.441592207
T2DM	beta_cell	*BAIAP3*	TRUE	2.129697803
T2DM	beta_cell	*MLXIPL*	TRUE	3.77544523
T2DM	beta_cell	*GIPR*	TRUE	2.530736973
T2DM	beta_cell	*CEP68*	TRUE	5.24414491
T2DM	beta_cell	*LTF*	TRUE	2.646812768
T2DM	beta_cell	*ANGEL1*	TRUE	2.012180226
T2DM	beta_cell	*BAK1*	TRUE	3.771321536
T2DM	beta_cell	*ANAPC4*	TRUE	2.072619006
T2DM	beta_cell	*ITIH4*	TRUE	3.164791684
T2DM	beta_cell	*MYLK*	TRUE	1.990882616
T2DM	beta_cell	*SPTB*	TRUE	2.596246142
T2DM	beta_cell	*TCF3*	TRUE	3.674320977
T2DM	beta_cell	*NLE1*	TRUE	2.516969538
T2DM	beta_cell	*ST6GAL1*	TRUE	3.223649501
T2DM	beta_cell	*MARK3*	TRUE	3.962145402
T2DM	beta_cell	*SEC31B*	TRUE	1.986481092
T2DM	beta_cell	*MECOM*	TRUE	2.024841031
T2DM	beta_cell	*MGST2*	TRUE	2.40069319
T2DM	beta_cell	*SH3BP2*	TRUE	2.072719838
T2DM	beta_cell	*CWF19L1*	TRUE	3.695147595
T2DM	beta_cell	*CCDC134*	TRUE	2.905795672
T2DM	beta_cell	*AP1B1*	TRUE	2.067602322
T2DM	beta_cell	*TOM1*	TRUE	2.309383724
T2DM	beta_cell	*BIK*	TRUE	2.344073754
T2DM	beta_cell	*DIDO1*	TRUE	2.029953786
T2DM	beta_cell	*RBL2*	TRUE	2.732297786
T2DM	beta_cell	*NOMO1*	TRUE	2.81315716
T2DM	beta_cell	*CTSH*	TRUE	2.045562937
T2DM	beta_cell	*VIPR2*	TRUE	2.014607138
T2DM	beta_cell	*SLC1A1*	TRUE	2.918811709
T2DM	beta_cell	*DHRS11*	TRUE	2.840751719
T2DM	beta_cell	*SLC2A9*	TRUE	2.193192156
T2DM	beta_cell	*COQ5*	TRUE	2.201440098
T2DM	beta_cell	*CDK2AP1*	TRUE	2.431949565
T2DM	beta_cell	*SEMA5A*	TRUE	2.23036194

T2DM	beta_cell	*NNT*	TRUE	2.937351507
T2DM	beta_cell	*TMCO6*	TRUE	2.459942326
T2DM	beta_cell	*BTNL8*	TRUE	2.772132391
T2DM	beta_cell	*ANGPTL1*	TRUE	2.271778762
T2DM	beta_cell	*WDR77*	TRUE	2.219304979
T2DM	beta_cell	*DNAH7*	TRUE	3.635122577
T2DM	beta_cell	*BCL11A*	TRUE	2.360782465
T2DM	beta_cell	*IAPP*	TRUE	3.203147668
T2DM	beta_cell	*ZNF211*	TRUE	2.458947401
T2DM	beta_cell	*FLT3*	TRUE	3.493880844
T2DM	beta_cell	*GRK4*	TRUE	1.980130144
T2DM	beta_cell	*OPRL1*	TRUE	2.046735465
T2DM	beta_cell	*WFIKKN1*	TRUE	2.17495517
T2DM	beta_cell	*PTPN12*	TRUE	2.722958139
T2DM	beta_cell	*RIBC2*	TRUE	2.687890027
T2DM	beta_cell	*MAU2*	TRUE	2.082740369
T2DM	beta_cell	*SYT5*	TRUE	3.062069215
T2DM	beta_cell	*GTPBP3*	TRUE	2.113430679
T2DM	beta_cell	*TRAF3*	TRUE	2.452724921
T2DM	beta_cell	*PPARG*	TRUE	3.002579107
T2DM	beta_cell	*INPP5K*	TRUE	2.092543641
T2DM	beta_cell	*ITGB4*	TRUE	2.486212013
T2DM	beta_cell	*BHMT2*	TRUE	1.972308535
T2DM	beta_cell	*NOTCH2*	TRUE	5.435588748
T2DM	beta_cell	*LRP4*	TRUE	2.912522301
T2DM	beta_cell	*NHSL1*	TRUE	2.174443554
T2DM	beta_cell	*MYPN*	TRUE	2.63823858
T2DM	beta_cell	*NUP54*	TRUE	2.259209687
T2DM	beta_cell	*C1RL*	TRUE	2.256722116
T2DM	beta_cell	*VPS53*	TRUE	2.088386379
T2DM	beta_cell	*SPACA3*	TRUE	2.13102598
T2DM	beta_cell	*NPC1*	TRUE	3.016381366
T2DM	beta_cell	*DUS3L*	TRUE	2.332948287
T2DM	beta_cell	*EVI5L*	TRUE	2.222016381
T2DM	beta_cell	*POGZ*	TRUE	3.047356489
T2DM	beta_cell	*ZNF514*	TRUE	2.606459952
T2DM	beta_cell	*CDCA7*	TRUE	2.744222896
T2DM	beta_cell	*TAMM41*	TRUE	2.042140218
T2DM	beta_cell	*CNTN4*	TRUE	3.230010464
T2DM	beta_cell	*TAGLN3*	TRUE	2.185912202
T2DM	beta_cell	*GIGYF1*	TRUE	2.131332064
T2DM	beta_cell	*RAB19*	TRUE	2.609048847
T2DM	beta_cell	*ADHFE1*	TRUE	2.358774295
T2DM	beta_cell	*SEC16A*	TRUE	2.363857685
T2DM	beta_cell	*HSD17B12*	TRUE	3.603719245

T2DM	beta_cell	*APIP*	TRUE	2.019585872
T2DM	beta_cell	*KLHL35*	TRUE	2.519515016
T2DM	beta_cell	*CCDC15*	TRUE	2.058777425
T2DM	beta_cell	*MAGI1*	TRUE	2.173883982
T2DM	beta_cell	*PTPN14*	TRUE	3.381098644
T2DM	beta_cell	*POC5*	TRUE	2.878412644
T2DM	beta_cell	*NMRAL1*	TRUE	2.013367593
T2DM	beta_cell	*JAZF1*	TRUE	7.630344523
T2DM	beta_cell	*CC2D1B*	TRUE	2.866417137
T2DM	beta_cell	*CLEC18A*	TRUE	5.524846709
T2DM	beta_cell	*AP3S2*	TRUE	4.776205894
T2DM	beta_cell	*CIB4*	TRUE	2.927378729
T2DM	beta_cell	*EXTL1*	TRUE	2.596282703
T2DM	beta_cell	*ANKLE1*	TRUE	2.483222887
T2DM	beta_cell	*ZNF208*	TRUE	2.179766127
T2DM	beta_cell	*NIPAL1*	TRUE	2.184418214
T2DM	beta_cell	*SRGAP2*	TRUE	2.658663811
T2DM	beta_cell	*ICA1L*	TRUE	2.849605838
T2DM	beta_cell	*BDH2*	TRUE	2.409353023
T2DM	beta_cell	*PPM1M*	TRUE	2.136586721
T2DM	beta_cell	*ERAP2*	TRUE	2.022466624
T2DM	beta_cell	*HCN1*	TRUE	2.136158142
T2DM	beta_cell	*RELL2*	TRUE	2.153895392
T2DM	beta_cell	*DLC1*	TRUE	1.991810871
T2DM	beta_cell	*NOXRED1*	TRUE	1.999319533
T2DM	beta_cell	*CASP7*	TRUE	3.62965135
T2DM	beta_cell	*CYB5R2*	TRUE	2.346922522
T2DM	beta_cell	*RCCD1*	TRUE	2.055667093
T2DM	beta_cell	*PRR15L*	TRUE	2.126814769
T2DM	beta_cell	*SLC43A2*	TRUE	2.130235476
T2DM	beta_cell	*TMC8*	TRUE	3.026210362
T2DM	beta_cell	*TRANK1*	TRUE	2.217446916
T2DM	beta_cell	*INO80E*	TRUE	2.802534281
T2DM	beta_cell	*SMAGP*	TRUE	2.813176286
T2DM	beta_cell	*ARNT2*	TRUE	2.186418939
T2DM	beta_cell	*ZNF738*	TRUE	2.709228418
T2DM	beta_cell	*PPP1R3B*	TRUE	2.802608535
T2DM	beta_cell	*PEAK1*	TRUE	2.514474874
T2DM	beta_cell	*TUBB8*	TRUE	2.312027609
T2DM	beta_cell	*BTC*	TRUE	2.203884076
T2DM	beta_cell	*PHYHD1*	TRUE	3.268872941
T2DM	beta_cell	*TCP11L1*	TRUE	2.081418306
T2DM	beta_cell	*DSCAML1*	TRUE	2.428576244
T2DM	beta_cell	*IRX3*	TRUE	10.06812724
T2DM	beta_cell	*IL17RA*	TRUE	2.061974221

T2DM	beta_cell	*IMP3*	TRUE	2.227981749
T2DM	beta_cell	*FARSA*	TRUE	2.178523232
T2DM	beta_cell	*GPR88*	TRUE	2.617856792
T2DM	beta_cell	*CAPN12*	TRUE	2.26962379
T2DM	beta_cell	*VMO1*	TRUE	3.44641396
T2DM	beta_cell	*ZNF320*	TRUE	3.349355416
T2DM	beta_cell	*CAMK1D*	TRUE	5.494862163
T2DM	beta_cell	*FAM43B*	TRUE	3.328959003
T2DM	beta_cell	*ZBTB40*	TRUE	3.012108404
T2DM	beta_cell	*DMWD*	TRUE	2.173099771
T2DM	beta_cell	*CEACAM19*	TRUE	2.388643044
T2DM	beta_cell	*NUTM2B*	TRUE	3.221623874
T2DM	beta_cell	*NDOR1*	TRUE	2.32124926
T2DM	beta_cell	*ZDHHC11*	TRUE	2.284286497
T2DM	beta_cell	*ZNF567*	TRUE	2.428848
T2DM	beta_cell	*ZNF605*	TRUE	2.356593992
T2DM	beta_cell	*PIWIL2*	TRUE	3.142947475
T2DM	beta_cell	*PCDHB11*	TRUE	2.290515957
T2DM	beta_cell	*ENPP1*	TRUE	2.054216016
T2DM	beta_cell	*DDX42*	TRUE	2.320247464
T2DM	beta_cell	*KIAA0753*	TRUE	2.186058552
T2DM	beta_cell	*TBKBP1*	TRUE	2.350272733
T2DM	beta_cell	*NOTCH4*	TRUE	2.894665577
T2DM	beta_cell	*ZNF425*	TRUE	2.121965272
T2DM	beta_cell	*HCG27*	TRUE	2.893580463
T2DM	beta_cell	*DNLZ*	TRUE	3.31262243
T2DM	beta_cell	*SLC12A8*	TRUE	4.409199149
T2DM	beta_cell	*EPPK1*	TRUE	1.994168766
T2DM	beta_cell	*LINC00957*	TRUE	2.596825954
T2DM	beta_cell	*HLA.DQA2*	TRUE	3.921472999
T2DM	beta_cell	*JMJD7*	TRUE	2.465950996
T2DM	beta_cell	*OR2A7*	TRUE	2.062279896
T2DM	beta_cell	*P2RY11*	TRUE	2.391698933
T2DM	beta_cell	*BCKDHA*	TRUE	2.235185854
T2DM	beta_cell	*FPGT*	TRUE	2.394562033
T2DM	beta_cell	*HMBS*	TRUE	2.901305206
T2DM	beta_cell	*TAS2R31*	TRUE	2.026106102
T2DM	delta_cell	*RBM6*	TRUE	2.713325941
T2DM	delta_cell	*SH2D2A*	TRUE	2.262813531
T2DM	delta_cell	*CCDC134*	TRUE	2.438162093
T2DM	delta_cell	*FLT3*	TRUE	2.267514043
T2DM	delta_cell	*NOTCH2*	TRUE	3.140123462
T2DM	delta_cell	*MAN2C1*	TRUE	2.88374485
T2DM	delta_cell	*HSD17B12*	TRUE	2.085401854
T2DM	delta_cell	*POC5*	TRUE	1.970862954

T2DM	delta_cell	*JAZF1*	TRUE	5.318832384
T2DM	delta_cell	*CLEC18A*	TRUE	2.002918578
T2DM	delta_cell	*CILP2*	TRUE	2.251151281
T2DM	delta_cell	*CASP7*	TRUE	2.160497509
T2DM	delta_cell	*TMC8*	TRUE	2.481810904
T2DM	delta_cell	*CTSW*	TRUE	2.497908153
T2DM	delta_cell	*IRX3*	TRUE	6.060713716
T2DM	delta_cell	*FAM43B*	TRUE	2.724330389
T2DM	delta_cell	*NOTCH4*	TRUE	2.832601259
T2DM	delta_cell	*HLA.DQA2*	TRUE	3.444935089
T2DM	delta_cell	*TAS2R20*	TRUE	2.238574174
T2DM	duct_epithelial_cell	*CEP68*	TRUE	3.829672282
T2DM	duct_epithelial_cell	*BAK1*	TRUE	2.203461808
T2DM	duct_epithelial_cell	*CWF19L1*	TRUE	2.812842676
T2DM	duct_epithelial_cell	*CNTNAP1*	TRUE	2.080349419
T2DM	duct_epithelial_cell	*BTNL8*	TRUE	2.280811284
T2DM	duct_epithelial_cell	*ZNF211*	TRUE	2.275895098
T2DM	duct_epithelial_cell	*RIBC2*	TRUE	2.535798516
T2DM	duct_epithelial_cell	*NOTCH2*	TRUE	3.337420104
T2DM	duct_epithelial_cell	*LRP4*	TRUE	2.030118736
T2DM	duct_epithelial_cell	*MAN2C1*	TRUE	3.2034066
T2DM	duct_epithelial_cell	*CDCA7*	TRUE	2.064024502
T2DM	duct_epithelial_cell	*CNTN4*	TRUE	2.67933297
T2DM	duct_epithelial_cell	*HSD17B12*	TRUE	2.160866115
T2DM	duct_epithelial_cell	*APIP*	TRUE	2.62840324
T2DM	duct_epithelial_cell	*PTPN14*	TRUE	1.968476853
T2DM	duct_epithelial_cell	*POC5*	TRUE	2.190607144
T2DM	duct_epithelial_cell	*JAZF1*	TRUE	4.585462214
T2DM	duct_epithelial_cell	*CC2D1B*	TRUE	2.261532524
T2DM	duct_epithelial_cell	*CLEC18A*	TRUE	2.503503956
T2DM	duct_epithelial_cell	*ANKLE1*	TRUE	2.402139977
T2DM	duct_epithelial_cell	*CTSW*	TRUE	2.292876031
T2DM	duct_epithelial_cell	*FARSA*	TRUE	2.007238479
T2DM	duct_epithelial_cell	*VMO1*	TRUE	2.219127719
T2DM	duct_epithelial_cell	*ZNF320*	TRUE	3.351384334
T2DM	duct_epithelial_cell	*CAMK1D*	TRUE	3.161653471
T2DM	duct_epithelial_cell	*PIWIL2*	TRUE	2.980899584
T2DM	duct_epithelial_cell	*NOTCH4*	TRUE	3.340958694
T2DM	duct_epithelial_cell	*DNLZ*	TRUE	3.33287889
T2DM	duct_epithelial_cell	*SLC12A8*	TRUE	2.070471381
T2DM	duct_epithelial_cell	*HLA.DQA2*	TRUE	3.741807637
T2DM	duct_epithelial_cell	*HMBS*	TRUE	2.212612901
T2DM	endothelial_cell	*RBM6*	TRUE	3.89599485
T2DM	endothelial_cell	*CEP68*	TRUE	3.760714201
T2DM	endothelial_cell	*BAK1*	TRUE	2.713624189

T2DM	endothelial_cell	*ITIH4*	TRUE	2.269227592
T2DM	endothelial_cell	*CWF19L1*	TRUE	3.425850317
T2DM	endothelial_cell	*ZNF211*	TRUE	2.318015403
T2DM	endothelial_cell	*MAU2*	TRUE	2.366976448
T2DM	endothelial_cell	*NOTCH2*	TRUE	4.796408821
T2DM	endothelial_cell	*MAN2C1*	TRUE	3.582469158
T2DM	endothelial_cell	*HSD17B12*	TRUE	2.443312044
T2DM	endothelial_cell	*APIP*	TRUE	2.830194306
T2DM	endothelial_cell	*JAZF1*	TRUE	4.20460242
T2DM	endothelial_cell	*CC2D1B*	TRUE	2.023897167
T2DM	endothelial_cell	*EXTL1*	TRUE	2.4344008
T2DM	endothelial_cell	*ANKLE1*	TRUE	2.106224384
T2DM	endothelial_cell	*INO80E*	TRUE	2.200510148
T2DM	endothelial_cell	*IRX3*	TRUE	6.580233749
T2DM	endothelial_cell	*GPR88*	TRUE	2.132412371
T2DM	endothelial_cell	*CAPN12*	TRUE	2.139109919
T2DM	endothelial_cell	*ZNF320*	TRUE	3.247204716
T2DM	endothelial_cell	*FAM43B*	TRUE	3.199212766
T2DM	endothelial_cell	*PIWIL2*	TRUE	2.946186508
T2DM	endothelial_cell	*PCDHB11*	TRUE	2.020121776
T2DM	endothelial_cell	*RNF5*	TRUE	2.865388819
T2DM	endothelial_cell	*DNLZ*	TRUE	3.156286918
T2DM	endothelial_cell	*HLA.DQA2*	TRUE	4.668022347
T2DM	endothelial_cell	*JMJD7*	TRUE	3.200757257
T2DM	epsilon_cell	*BTNL8*	TRUE	2.166451722
T2DM	epsilon_cell	*CILP2*	TRUE	2.890407974
T2DM	epsilon_cell	*IRX3*	TRUE	5.917803869
T2DM	epsilon_cell	*UBA7*	TRUE	4.270001918
T2DM	epsilon_cell	*NOTCH4*	TRUE	2.033136859
T2DM	epsilon_cell	*HLA.DQA2*	TRUE	5.47048731
T2DM	leukocyte	*QPCTL*	TRUE	2.089579782
T2DM	leukocyte	*NOTCH2*	TRUE	4.757076166
T2DM	leukocyte	*CC2D1B*	TRUE	1.983036503
T2DM	leukocyte	*ART3*	TRUE	2.05294946
T2DM	leukocyte	*EXTL1*	TRUE	2.079576681
T2DM	leukocyte	*CILP2*	TRUE	2.629291141
T2DM	leukocyte	*IRX3*	TRUE	3.328168402
T2DM	leukocyte	*GPR88*	TRUE	1.960048263
T2DM	leukocyte	*UBA7*	TRUE	2.63499007
T2DM	leukocyte	*NUTM2B*	TRUE	2.343700505
T2DM	leukocyte	*NOTCH4*	TRUE	2.278348873
T2DM	leukocyte	*DNLZ*	TRUE	2.256858481
T2DM	mesenchymal_cell	*CEP68*	TRUE	2.846267998
T2DM	mesenchymal_cell	*ITIH4*	TRUE	2.114961144
T2DM	mesenchymal_cell	*CWF19L1*	TRUE	2.625458722

T2DM	mesenchymal_cell	*SLC1A1*	TRUE	1.995996574
T2DM	mesenchymal_cell	*HSD17B1*	TRUE	2.917811432
T2DM	mesenchymal_cell	*BTNL8*	TRUE	2.076689893
T2DM	mesenchymal_cell	*ZNF211*	TRUE	2.132072481
T2DM	mesenchymal_cell	*RIBC2*	TRUE	2.169389544
T2DM	mesenchymal_cell	*NOTCH2*	TRUE	1.962938942
T2DM	mesenchymal_cell	*MAN2C1*	TRUE	3.537466593
T2DM	mesenchymal_cell	*CDCA7*	TRUE	2.398782989
T2DM	mesenchymal_cell	*CNTN4*	TRUE	2.364365324
T2DM	mesenchymal_cell	*CLEC18A*	TRUE	2.273054733
T2DM	mesenchymal_cell	*PPM1M*	TRUE	2.287880911
T2DM	mesenchymal_cell	*INO80E*	TRUE	2.693714751
T2DM	mesenchymal_cell	*CTSW*	TRUE	2.36046353
T2DM	mesenchymal_cell	*IRX3*	TRUE	7.59264719
T2DM	mesenchymal_cell	*ZNF320*	TRUE	3.214740665
T2DM	mesenchymal_cell	*PIWIL2*	TRUE	2.281711582
T2DM	mesenchymal_cell	*LIME1*	TRUE	2.106711172
T2DM	mesenchymal_cell	*HCG27*	TRUE	2.06824214
T2DM	mesenchymal_cell	*SLC12A8*	TRUE	3.154212449
T2DM	mesenchymal_cell	*JMJD7*	TRUE	2.464442083
T2DM	PP_cell	*RBM6*	TRUE	2.409900913
T2DM	PP_cell	*CEP68*	TRUE	2.486734822
T2DM	PP_cell	*CCDC134*	TRUE	2.389161863
T2DM	PP_cell	*OPRL1*	TRUE	3.125195299
T2DM	PP_cell	*RIBC2*	TRUE	2.593177169
T2DM	PP_cell	*SPTBN5*	TRUE	2.253293458
T2DM	PP_cell	*MAN2C1*	TRUE	2.309690263
T2DM	PP_cell	*HSD17B12*	TRUE	2.377174632
T2DM	PP_cell	*APIP*	TRUE	2.25847276
T2DM	PP_cell	*PTPN14*	TRUE	2.319081518
T2DM	PP_cell	*CLEC18A*	TRUE	2.979866604
T2DM	PP_cell	*CILP2*	TRUE	2.564722325
T2DM	PP_cell	*TMC8*	TRUE	2.640148361
T2DM	PP_cell	*IRX3*	TRUE	2.915446669
T2DM	PP_cell	*VMO1*	TRUE	2.069487155
T2DM	PP_cell	*ZNF320*	TRUE	2.715362701
T2DM	PP_cell	*DNLZ*	TRUE	2.006827921
T2DM_admission	acinar_cell	*RBM6*	TRUE	3.050975925
T2DM_admission	acinar_cell	*MLXIPL*	TRUE	2.22303948
T2DM_admission	acinar_cell	*QPCTL*	TRUE	2.166003561
T2DM_admission	acinar_cell	*CEP68*	TRUE	4.46563948
T2DM_admission	acinar_cell	*BAK1*	TRUE	3.332516336
T2DM_admission	acinar_cell	*TCF3*	TRUE	2.530763563
T2DM_admission	acinar_cell	*ST6GAL1*	TRUE	2.416874089
T2DM_admission	acinar_cell	*CWF19L1*	TRUE	2.321376805

T2DM_admission	acinar_cell	*CCDC134*	TRUE	2.41736115
T2DM_admission	acinar_cell	*CRHR2*	TRUE	2.079899981
T2DM_admission	acinar_cell	*SLC1A1*	TRUE	2.051158548
T2DM_admission	acinar_cell	*CNTNAP1*	TRUE	2.158522779
T2DM_admission	acinar_cell	*ZNF211*	TRUE	2.382631787
T2DM_admission	acinar_cell	*FLT3*	TRUE	3.516854694
T2DM_admission	acinar_cell	*MOCS1*	TRUE	2.167923285
T2DM_admission	acinar_cell	*OPRL1*	TRUE	3.420476055
T2DM_admission	acinar_cell	*RIBC2*	TRUE	2.178797841
T2DM_admission	acinar_cell	*NOTCH2*	TRUE	5.8768114
T2DM_admission	acinar_cell	*MAN2C1*	TRUE	3.296907327
T2DM_admission	acinar_cell	*HSD17B12*	TRUE	3.723518302
T2DM_admission	acinar_cell	*APIP*	TRUE	2.252838726
T2DM_admission	acinar_cell	*CCDC15*	TRUE	2.389136349
T2DM_admission	acinar_cell	*PTPN14*	TRUE	2.733776043
T2DM_admission	acinar_cell	*POC5*	TRUE	3.096895716
T2DM_admission	acinar_cell	*JAZF1*	TRUE	6.529028673
T2DM_admission	acinar_cell	*CLEC18A*	TRUE	6.517472336
T2DM_admission	acinar_cell	*EXTL1*	TRUE	2.751624592
T2DM_admission	acinar_cell	*ANKLE1*	TRUE	2.688459536
T2DM_admission	acinar_cell	*SRGAP2*	TRUE	2.060759663
T2DM_admission	acinar_cell	*ICA1L*	TRUE	2.317550956
T2DM_admission	acinar_cell	*BDH2*	TRUE	2.765511805
T2DM_admission	acinar_cell	*TMEM129*	TRUE	1.984566089
T2DM_admission	acinar_cell	*PEAK1*	TRUE	2.304625651
T2DM_admission	acinar_cell	*GPR4*	TRUE	2.106721233
T2DM_admission	acinar_cell	*IRX3*	TRUE	10.55301929
T2DM_admission	acinar_cell	*FARSA*	TRUE	2.597134172
T2DM_admission	acinar_cell	*VMO1*	TRUE	3.047950918
T2DM_admission	acinar_cell	*ZNF320*	TRUE	2.344073943
T2DM_admission	acinar_cell	*FAM43B*	TRUE	2.255205803
T2DM_admission	acinar_cell	*PLGLB1*	TRUE	1.969755154
T2DM_admission	acinar_cell	*LIME1*	TRUE	3.11265782
T2DM_admission	acinar_cell	*NOTCH4*	TRUE	3.309785579
T2DM_admission	acinar_cell	*HCG27*	TRUE	3.000021117
T2DM_admission	acinar_cell	*DNLZ*	TRUE	2.668558564
T2DM_admission	acinar_cell	*SLC12A8*	TRUE	2.35007725
T2DM_admission	acinar_cell	*HLA.DQA2*	TRUE	3.528563443
T2DM_admission	acinar_cell	*CNTF*	TRUE	2.110730023
T2DM_admission	acinar_cell	*JMJD7*	TRUE	2.901593377
T2DM_admission	acinar_cell	*OR2A7*	TRUE	2.164755598
T2DM_admission	acinar_cell	*P2RY11*	TRUE	1.964266442
T2DM_admission	acinar_cell	*HMBS*	TRUE	2.40491079
T2DM_admission	alpha_cell	*RBM6*	TRUE	3.157918094
T2DM_admission	alpha_cell	*MLXIPL*	TRUE	2.926750745

T2DM_admission	alpha_cell	*GIPR*	TRUE	3.476131629
T2DM_admission	alpha_cell	*CEP68*	TRUE	5.192093176
T2DM_admission	alpha_cell	*BAK1*	TRUE	3.908055401
T2DM_admission	alpha_cell	*ITIH4*	TRUE	2.110622284
T2DM_admission	alpha_cell	*TCF3*	TRUE	3.08980857
T2DM_admission	alpha_cell	*ST6GAL1*	TRUE	2.896973148
T2DM_admission	alpha_cell	*MYH7B*	TRUE	2.195841922
T2DM_admission	alpha_cell	*DCT*	TRUE	1.996379008
T2DM_admission	alpha_cell	*CWF19L1*	TRUE	2.785163155
T2DM_admission	alpha_cell	*PALMD*	TRUE	2.149128283
T2DM_admission	alpha_cell	*CCDC134*	TRUE	3.114514894
T2DM_admission	alpha_cell	*CRHR2*	TRUE	2.279581276
T2DM_admission	alpha_cell	*SLC1A1*	TRUE	2.068350606
T2DM_admission	alpha_cell	*CNTNAP1*	TRUE	2.055681687
T2DM_admission	alpha_cell	*CDK2AP1*	TRUE	2.497477342
T2DM_admission	alpha_cell	*CD83*	TRUE	2.138814329
T2DM_admission	alpha_cell	*BTNL8*	TRUE	2.215709072
T2DM_admission	alpha_cell	*DNAH7*	TRUE	2.593801556
T2DM_admission	alpha_cell	*ZNF211*	TRUE	2.650981161
T2DM_admission	alpha_cell	*FLT3*	TRUE	3.438520401
T2DM_admission	alpha_cell	*MOCS1*	TRUE	2.428218971
T2DM_admission	alpha_cell	*OPRL1*	TRUE	2.483461667
T2DM_admission	alpha_cell	*RIBC2*	TRUE	2.339222061
T2DM_admission	alpha_cell	*PPARG*	TRUE	2.452525463
T2DM_admission	alpha_cell	*NOTCH2*	TRUE	6.060722301
T2DM_admission	alpha_cell	*MYPN*	TRUE	2.685694276
T2DM_admission	alpha_cell	*MAN2C1*	TRUE	3.607010228
T2DM_admission	alpha_cell	*SPACA3*	TRUE	2.278468514
T2DM_admission	alpha_cell	*FN3KRP*	TRUE	2.644073236
T2DM_admission	alpha_cell	*POGZ*	TRUE	1.987544223
T2DM_admission	alpha_cell	*CDCA7*	TRUE	2.848268829
T2DM_admission	alpha_cell	*ADHFE1*	TRUE	2.113374368
T2DM_admission	alpha_cell	*INPP5E*	TRUE	1.962994936
T2DM_admission	alpha_cell	*HSD17B12*	TRUE	4.265878884
T2DM_admission	alpha_cell	*APIP*	TRUE	2.405102563
T2DM_admission	alpha_cell	*KLHL35*	TRUE	2.106643205
T2DM_admission	alpha_cell	*CCDC15*	TRUE	2.778526454
T2DM_admission	alpha_cell	*PTPN14*	TRUE	3.039690922
T2DM_admission	alpha_cell	*POC5*	TRUE	3.053674361
T2DM_admission	alpha_cell	*JAZF1*	TRUE	8.155551661
T2DM_admission	alpha_cell	*CC2D1B*	TRUE	2.153688483
T2DM_admission	alpha_cell	*CLEC18A*	TRUE	6.430380032
T2DM_admission	alpha_cell	*AP3S2*	TRUE	2.18180227
T2DM_admission	alpha_cell	*CIB4*	TRUE	2.466792899
T2DM_admission	alpha_cell	*EXTL1*	TRUE	3.364259831

T2DM_admission	alpha_cell	*ANKLE1*	TRUE	2.512900566
T2DM_admission	alpha_cell	*SRGAP2*	TRUE	2.608495365
T2DM_admission	alpha_cell	*ICA1L*	TRUE	2.807559815
T2DM_admission	alpha_cell	*BDH2*	TRUE	2.874833389
T2DM_admission	alpha_cell	*CASP7*	TRUE	3.715004099
T2DM_admission	alpha_cell	*EVPL*	TRUE	2.129867839
T2DM_admission	alpha_cell	*TMC8*	TRUE	2.620978906
T2DM_admission	alpha_cell	*TMEM129*	TRUE	2.621308456
T2DM_admission	alpha_cell	*INO80E*	TRUE	2.743875515
T2DM_admission	alpha_cell	*PEAK1*	TRUE	2.838792101
T2DM_admission	alpha_cell	*ZNF408*	TRUE	2.260929568
T2DM_admission	alpha_cell	*FUT2*	TRUE	2.338818447
T2DM_admission	alpha_cell	*DSCAML1*	TRUE	2.59151451
T2DM_admission	alpha_cell	*GPR4*	TRUE	2.165556485
T2DM_admission	alpha_cell	*IRX3*	TRUE	10.34683147
T2DM_admission	alpha_cell	*TH*	TRUE	3.190226721
T2DM_admission	alpha_cell	*VMO1*	TRUE	2.969598698
T2DM_admission	alpha_cell	*ZNF320*	TRUE	2.961615518
T2DM_admission	alpha_cell	*PLGLB1*	TRUE	1.979378768
T2DM_admission	alpha_cell	*EIF3C*	TRUE	2.000405415
T2DM_admission	alpha_cell	*NUTM2B*	TRUE	3.559829011
T2DM_admission	alpha_cell	*PIWIL2*	TRUE	2.379400011
T2DM_admission	alpha_cell	*LIME1*	TRUE	2.715563458
T2DM_admission	alpha_cell	*INPP5B*	TRUE	1.980471019
T2DM_admission	alpha_cell	*NOTCH4*	TRUE	3.445004954
T2DM_admission	alpha_cell	*HCG27*	TRUE	3.258688654
T2DM_admission	alpha_cell	*DNLZ*	TRUE	2.951411338
T2DM_admission	alpha_cell	*SLC12A8*	TRUE	3.628170823
T2DM_admission	alpha_cell	*EPPK1*	TRUE	2.90130265
T2DM_admission	alpha_cell	*HLA.DQA2*	TRUE	3.426463187
T2DM_admission	alpha_cell	*CNTF*	TRUE	2.239555208
T2DM_admission	alpha_cell	*JMJD7*	TRUE	2.932501712
T2DM_admission	alpha_cell	*OR2A7*	TRUE	2.462014081
T2DM_admission	alpha_cell	*P2RY11*	TRUE	2.190673107
T2DM_admission	alpha_cell	*HMBS*	TRUE	2.320933808
T2DM_admission	beta_cell	*STPG1*	TRUE	2.270062378
T2DM_admission	beta_cell	*RBM6*	TRUE	2.938137549
T2DM_admission	beta_cell	*ZNF195*	TRUE	2.117381393
T2DM_admission	beta_cell	*USH1C*	TRUE	2.196782181
T2DM_admission	beta_cell	*ST7L*	TRUE	2.354559669
T2DM_admission	beta_cell	*BAIAP3*	TRUE	2.24630044
T2DM_admission	beta_cell	*MLXIPL*	TRUE	3.655157808
T2DM_admission	beta_cell	*GIPR*	TRUE	3.819763309
T2DM_admission	beta_cell	*CEP68*	TRUE	6.314835567
T2DM_admission	beta_cell	*LTF*	TRUE	2.377333816

T2DM_admission	beta_cell	*BAK1*	TRUE	4.63977353
T2DM_admission	beta_cell	*ERCC8*	TRUE	2.268743876
T2DM_admission	beta_cell	*ANAPC4*	TRUE	2.256920361
T2DM_admission	beta_cell	*ITIH4*	TRUE	2.73374573
T2DM_admission	beta_cell	*MYLK*	TRUE	2.623408425
T2DM_admission	beta_cell	*SPTB*	TRUE	2.948937666
T2DM_admission	beta_cell	*GBA2*	TRUE	2.067759884
T2DM_admission	beta_cell	*TCF3*	TRUE	3.815196928
T2DM_admission	beta_cell	*SCARB1*	TRUE	1.975561607
T2DM_admission	beta_cell	*NLE1*	TRUE	2.396994092
T2DM_admission	beta_cell	*ST6GAL1*	TRUE	3.652726999
T2DM_admission	beta_cell	*MARK3*	TRUE	3.928684289
T2DM_admission	beta_cell	*MYH7B*	TRUE	2.166055767
T2DM_admission	beta_cell	*DCT*	TRUE	2.160757078
T2DM_admission	beta_cell	*MGST2*	TRUE	2.494252473
T2DM_admission	beta_cell	*LYZ*	TRUE	2.180699259
T2DM_admission	beta_cell	*RPGRIP1*	TRUE	2.096359059
T2DM_admission	beta_cell	*CWF19L1*	TRUE	3.133611115
T2DM_admission	beta_cell	*PALMD*	TRUE	2.450057575
T2DM_admission	beta_cell	*CCDC134*	TRUE	2.354883237
T2DM_admission	beta_cell	*ARSA*	TRUE	2.256984212
T2DM_admission	beta_cell	*DIDO1*	TRUE	2.061891228
T2DM_admission	beta_cell	*OLFM4*	TRUE	2.200266817
T2DM_admission	beta_cell	*RBL2*	TRUE	2.875156406
T2DM_admission	beta_cell	*ATP8B4*	TRUE	1.996998064
T2DM_admission	beta_cell	*VIPR2*	TRUE	2.518140093
T2DM_admission	beta_cell	*CRHR2*	TRUE	2.331138857
T2DM_admission	beta_cell	*SLC1A1*	TRUE	2.728405414
T2DM_admission	beta_cell	*DHRS11*	TRUE	2.752876723
T2DM_admission	beta_cell	*CNTNAP1*	TRUE	2.247315579
T2DM_admission	beta_cell	*FAM149A*	TRUE	2.243598058
T2DM_admission	beta_cell	*CDK2AP1*	TRUE	2.163474784
T2DM_admission	beta_cell	*CD83*	TRUE	2.348361036
T2DM_admission	beta_cell	*SEMA5A*	TRUE	1.980734004
T2DM_admission	beta_cell	*NNT*	TRUE	2.473913647
T2DM_admission	beta_cell	*TMCO6*	TRUE	2.008003889
T2DM_admission	beta_cell	*BTNL8*	TRUE	2.305316974
T2DM_admission	beta_cell	*ANGPTL1*	TRUE	2.226351765
T2DM_admission	beta_cell	*WDR77*	TRUE	2.06348739
T2DM_admission	beta_cell	*ATP10B*	TRUE	2.168192502
T2DM_admission	beta_cell	*DNAH7*	TRUE	3.040766901
T2DM_admission	beta_cell	*BCL11A*	TRUE	2.240311529
T2DM_admission	beta_cell	*CCDC77*	TRUE	2.524247099
T2DM_admission	beta_cell	*FAM53C*	TRUE	2.30319617
T2DM_admission	beta_cell	*IAPP*	TRUE	2.503418815

T2DM_admission	beta_cell	*ZNF211*	TRUE	2.731840145
T2DM_admission	beta_cell	*FLT3*	TRUE	3.758027051
T2DM_admission	beta_cell	*BHLHE41*	TRUE	2.249515023
T2DM_admission	beta_cell	*MOCS1*	TRUE	2.651564829
T2DM_admission	beta_cell	*GRK4*	TRUE	2.12448699
T2DM_admission	beta_cell	*OPRL1*	TRUE	2.612717375
T2DM_admission	beta_cell	*ABHD8*	TRUE	2.701952919
T2DM_admission	beta_cell	*RIBC2*	TRUE	2.370111037
T2DM_admission	beta_cell	*CGNL1*	TRUE	2.070766385
T2DM_admission	beta_cell	*MAU2*	TRUE	2.37782754
T2DM_admission	beta_cell	*SYT5*	TRUE	2.509282491
T2DM_admission	beta_cell	*GTPBP3*	TRUE	2.196317747
T2DM_admission	beta_cell	*TULP4*	TRUE	2.110453981
T2DM_admission	beta_cell	*TRAF3*	TRUE	2.88661416
T2DM_admission	beta_cell	*PPARG*	TRUE	3.328506507
T2DM_admission	beta_cell	*BHMT2*	TRUE	2.595216804
T2DM_admission	beta_cell	*NOTCH2*	TRUE	6.461922382
T2DM_admission	beta_cell	*NHSL1*	TRUE	2.321550539
T2DM_admission	beta_cell	*NEK1*	TRUE	2.123457495
T2DM_admission	beta_cell	*BTBD16*	TRUE	2.059974836
T2DM_admission	beta_cell	*MYPN*	TRUE	2.715314056
T2DM_admission	beta_cell	*NUP54*	TRUE	2.482727877
T2DM_admission	beta_cell	*C1RL*	TRUE	2.163189185
T2DM_admission	beta_cell	*SPACA3*	TRUE	2.539108818
T2DM_admission	beta_cell	*NPC1*	TRUE	3.828866605
T2DM_admission	beta_cell	*FN3KRP*	TRUE	2.383489236
T2DM_admission	beta_cell	*DUS3L*	TRUE	2.343535178
T2DM_admission	beta_cell	*EVI5L*	TRUE	2.95743037
T2DM_admission	beta_cell	*POGZ*	TRUE	3.375319263
T2DM_admission	beta_cell	*ZNF514*	TRUE	2.093545233
T2DM_admission	beta_cell	*CDCA7*	TRUE	3.085745007
T2DM_admission	beta_cell	*IQGAP2*	TRUE	2.358228707
T2DM_admission	beta_cell	*SCML4*	TRUE	2.737726652
T2DM_admission	beta_cell	*SLC22A3*	TRUE	1.961277257
T2DM_admission	beta_cell	*ADHFE1*	TRUE	2.562151861
T2DM_admission	beta_cell	*INPP5E*	TRUE	2.019971137
T2DM_admission	beta_cell	*HSD17B12*	TRUE	4.691160776
T2DM_admission	beta_cell	*KLHL35*	TRUE	2.50735322
T2DM_admission	beta_cell	*CCDC15*	TRUE	2.743325562
T2DM_admission	beta_cell	*PTPN14*	TRUE	3.464755464
T2DM_admission	beta_cell	*POC5*	TRUE	3.147154136
T2DM_admission	beta_cell	*PTPRD*	TRUE	2.079974969
T2DM_admission	beta_cell	*ZDHHC7*	TRUE	2.431381279
T2DM_admission	beta_cell	*JAZF1*	TRUE	8.931030687
T2DM_admission	beta_cell	*NUS1*	TRUE	2.122682242

T2DM_admission	beta_cell	*CC2D1B*	TRUE	2.472508563
T2DM_admission	beta_cell	*CLEC18A*	TRUE	6.663642948
T2DM_admission	beta_cell	*AP3S2*	TRUE	5.501512794
T2DM_admission	beta_cell	*CIB4*	TRUE	2.856934943
T2DM_admission	beta_cell	*EXTL1*	TRUE	3.307543136
T2DM_admission	beta_cell	*ANKLE1*	TRUE	2.715422104
T2DM_admission	beta_cell	*LY6K*	TRUE	2.182349266
T2DM_admission	beta_cell	*CLHC1*	TRUE	2.191285079
T2DM_admission	beta_cell	*DUSP19*	TRUE	2.007571306
T2DM_admission	beta_cell	*NIPAL1*	TRUE	2.539769498
T2DM_admission	beta_cell	*SRGAP2*	TRUE	3.684447564
T2DM_admission	beta_cell	*ICA1L*	TRUE	3.289760335
T2DM_admission	beta_cell	*BDH2*	TRUE	3.116144577
T2DM_admission	beta_cell	*ERAP2*	TRUE	1.972993946
T2DM_admission	beta_cell	*RELL2*	TRUE	1.988449784
T2DM_admission	beta_cell	*NOXRED1*	TRUE	2.328893486
T2DM_admission	beta_cell	*CASP7*	TRUE	4.077540696
T2DM_admission	beta_cell	*CEP57*	TRUE	2.145443643
T2DM_admission	beta_cell	*PROCA1*	TRUE	2.066227982
T2DM_admission	beta_cell	*TP53I13*	TRUE	2.368004647
T2DM_admission	beta_cell	*SLC43A2*	TRUE	2.007085692
T2DM_admission	beta_cell	*EVPL*	TRUE	2.286218197
T2DM_admission	beta_cell	*TMC8*	TRUE	2.806402658
T2DM_admission	beta_cell	*TMEM129*	TRUE	2.407497371
T2DM_admission	beta_cell	*INO80E*	TRUE	2.809920619
T2DM_admission	beta_cell	*KCNAB3*	TRUE	2.142322152
T2DM_admission	beta_cell	*SMAGP*	TRUE	2.301254529
T2DM_admission	beta_cell	*ARNT2*	TRUE	2.58409781
T2DM_admission	beta_cell	*RNASEH2C*	TRUE	2.22746217
T2DM_admission	beta_cell	*PPP1R3B*	TRUE	2.550916569
T2DM_admission	beta_cell	*PEAK1*	TRUE	3.562995579
T2DM_admission	beta_cell	*ZNF266*	TRUE	2.274862994
T2DM_admission	beta_cell	*YIF1A*	TRUE	1.98878481
T2DM_admission	beta_cell	*FUT1*	TRUE	1.96453563
T2DM_admission	beta_cell	*ZNF408*	TRUE	2.789657219
T2DM_admission	beta_cell	*PHYHD1*	TRUE	2.147383424
T2DM_admission	beta_cell	*TCP11L1*	TRUE	2.197292972
T2DM_admission	beta_cell	*FUT2*	TRUE	2.308608072
T2DM_admission	beta_cell	*DSCAML1*	TRUE	2.550250618
T2DM_admission	beta_cell	*GPR4*	TRUE	2.159236756
T2DM_admission	beta_cell	*IRX3*	TRUE	10.99429859
T2DM_admission	beta_cell	*COX14*	TRUE	2.935952566
T2DM_admission	beta_cell	*TRIM73*	TRUE	2.227040946
T2DM_admission	beta_cell	*RMI1*	TRUE	2.104690822
T2DM_admission	beta_cell	*FARSA*	TRUE	2.25443105

T2DM_admission	beta_cell	*TH*	TRUE	3.248704845
T2DM_admission	beta_cell	*ACTL10*	TRUE	2.56542579
T2DM_admission	beta_cell	*TTC3*	TRUE	2.237209565
T2DM_admission	beta_cell	*VMO1*	TRUE	3.351475086
T2DM_admission	beta_cell	*ZNF320*	TRUE	3.010949229
T2DM_admission	beta_cell	*CAMK1D*	TRUE	4.977343257
T2DM_admission	beta_cell	*FAM43B*	TRUE	2.653848071
T2DM_admission	beta_cell	*EIF3C*	TRUE	2.079356829
T2DM_admission	beta_cell	*PGP*	TRUE	2.296974787
T2DM_admission	beta_cell	*ZBTB40*	TRUE	2.500907914
T2DM_admission	beta_cell	*NDUFA6*	TRUE	2.055715085
T2DM_admission	beta_cell	*DMWD*	TRUE	2.495873315
T2DM_admission	beta_cell	*CYP4X1*	TRUE	2.113784877
T2DM_admission	beta_cell	*NUTM2B*	TRUE	3.431703162
T2DM_admission	beta_cell	*NDOR1*	TRUE	2.034856784
T2DM_admission	beta_cell	*ZNF567*	TRUE	2.533778107
T2DM_admission	beta_cell	*ZNF605*	TRUE	2.100927209
T2DM_admission	beta_cell	*PIWIL2*	TRUE	2.524745754
T2DM_admission	beta_cell	*ENPP1*	TRUE	2.383637419
T2DM_admission	beta_cell	*DPP4*	TRUE	2.728950956
T2DM_admission	beta_cell	*DDX42*	TRUE	2.319995914
T2DM_admission	beta_cell	*KIAA0753*	TRUE	2.05675933
T2DM_admission	beta_cell	*LIME1*	TRUE	2.55641902
T2DM_admission	beta_cell	*INPP5B*	TRUE	2.328401053
T2DM_admission	beta_cell	*AGAP6*	TRUE	1.966079918
T2DM_admission	beta_cell	*NOTCH4*	TRUE	3.427135561
T2DM_admission	beta_cell	*NYNRIN*	TRUE	2.078600015
T2DM_admission	beta_cell	*HCG27*	TRUE	3.326923897
T2DM_admission	beta_cell	*DNLZ*	TRUE	2.890356864
T2DM_admission	beta_cell	*SLC12A8*	TRUE	4.561866859
T2DM_admission	beta_cell	*EPPK1*	TRUE	3.001394741
T2DM_admission	beta_cell	*PSMG3.AS1*	TRUE	2.037884028
T2DM_admission	beta_cell	*LINC00957*	TRUE	2.432470031
T2DM_admission	beta_cell	*HLA.DQA2*	TRUE	3.632292361
T2DM_admission	beta_cell	*CNTF*	TRUE	2.099050699
T2DM_admission	beta_cell	*JMJD7*	TRUE	3.122839795
T2DM_admission	beta_cell	*OR2A7*	TRUE	2.536860944
T2DM_admission	beta_cell	*P2RY11*	TRUE	2.642159191
T2DM_admission	beta_cell	*HMBS*	TRUE	2.589847297
T2DM_admission	beta_cell	*TAS2R31*	TRUE	2.401313237
T2DM_admission	delta_cell	*RBM6*	TRUE	2.581248072
T2DM_admission	delta_cell	*CEP68*	TRUE	2.711119889
T2DM_admission	delta_cell	*SH2D2A*	TRUE	2.264729431
T2DM_admission	delta_cell	*BAK1*	TRUE	2.272588017
T2DM_admission	delta_cell	*CCDC134*	TRUE	2.487485482

T2DM_admission	delta_cell	*FLT3*	TRUE	2.212432964
T2DM_admission	delta_cell	*NOTCH2*	TRUE	3.671907689
T2DM_admission	delta_cell	*MAN2C1*	TRUE	2.803871775
T2DM_admission	delta_cell	*HSD17B12*	TRUE	2.803749514
T2DM_admission	delta_cell	*POC5*	TRUE	3.04382622
T2DM_admission	delta_cell	*JAZF1*	TRUE	6.121770142
T2DM_admission	delta_cell	*CLEC18A*	TRUE	2.572153153
T2DM_admission	delta_cell	*CILP2*	TRUE	2.200147185
T2DM_admission	delta_cell	*CASP7*	TRUE	2.452095648
T2DM_admission	delta_cell	*TMC8*	TRUE	2.088212961
T2DM_admission	delta_cell	*FUT2*	TRUE	2.409324895
T2DM_admission	delta_cell	*IRX3*	TRUE	6.437678318
T2DM_admission	delta_cell	*FAM43B*	TRUE	2.007260658
T2DM_admission	delta_cell	*EPPK1*	TRUE	2.096118881
T2DM_admission	delta_cell	*HLA.DQA2*	TRUE	2.175152578
T2DM_admission	delta_cell	*TAS2R43*	TRUE	2.317160735
T2DM_admission	delta_cell	*TAS2R20*	TRUE	2.487124538
T2DM_admission	duct_epithelial_cell	*QPCTL*	TRUE	2.143002278
T2DM_admission	duct_epithelial_cell	*CEP68*	TRUE	4.96889593
T2DM_admission	duct_epithelial_cell	*BAK1*	TRUE	2.601577923
T2DM_admission	duct_epithelial_cell	*CWF19L1*	TRUE	2.012769764
T2DM_admission	duct_epithelial_cell	*PALMD*	TRUE	2.000732393
T2DM_admission	duct_epithelial_cell	*CNTNAP1*	TRUE	2.697028203
T2DM_admission	duct_epithelial_cell	*CDK2AP1*	TRUE	2.023911184
T2DM_admission	duct_epithelial_cell	*ZNF211*	TRUE	2.810278826
T2DM_admission	duct_epithelial_cell	*RIBC2*	TRUE	2.194382164
T2DM_admission	duct_epithelial_cell	*NOTCH2*	TRUE	4.500853905
T2DM_admission	duct_epithelial_cell	*MAN2C1*	TRUE	3.022399638
T2DM_admission	duct_epithelial_cell	*FN3KRP*	TRUE	2.460691336
T2DM_admission	duct_epithelial_cell	*CDCA7*	TRUE	2.452502437
T2DM_admission	duct_epithelial_cell	*HSD17B12*	TRUE	2.82059998
T2DM_admission	duct_epithelial_cell	*CCDC15*	TRUE	2.471828083
T2DM_admission	duct_epithelial_cell	*PTPN14*	TRUE	2.045035383
T2DM_admission	duct_epithelial_cell	*POC5*	TRUE	3.21835657
T2DM_admission	duct_epithelial_cell	*JAZF1*	TRUE	4.993858523
T2DM_admission	duct_epithelial_cell	*CLEC18A*	TRUE	3.045318241
T2DM_admission	duct_epithelial_cell	*ANKLE1*	TRUE	2.575019094
T2DM_admission	duct_epithelial_cell	*TMEM129*	TRUE	1.969731622
T2DM_admission	duct_epithelial_cell	*CTSW*	TRUE	2.08454582
T2DM_admission	duct_epithelial_cell	*PEAK1*	TRUE	2.571794989
T2DM_admission	duct_epithelial_cell	*FUT2*	TRUE	2.268402666
T2DM_admission	duct_epithelial_cell	*FARSA*	TRUE	2.912451942
T2DM_admission	duct_epithelial_cell	*ZNF320*	TRUE	3.017879079
T2DM_admission	duct_epithelial_cell	*CAMK1D*	TRUE	2.590951858
T2DM_admission	duct_epithelial_cell	*PIWIL2*	TRUE	2.309896038

T2DM_admission	duct_epithelial_cell	*LIME1*	TRUE	2.237626297
T2DM_admission	duct_epithelial_cell	*NOTCH4*	TRUE	3.57527807
T2DM_admission	duct_epithelial_cell	*DNLZ*	TRUE	2.862182718
T2DM_admission	duct_epithelial_cell	*SLC12A8*	TRUE	2.132343481
T2DM_admission	duct_epithelial_cell	*HLA.DQA2*	TRUE	2.865186915
T2DM_admission	duct_epithelial_cell	*HMBS*	TRUE	2.671370075
T2DM_admission	endothelial_cell	*RBM6*	TRUE	3.938336456
T2DM_admission	endothelial_cell	*CEP68*	TRUE	4.549017279
T2DM_admission	endothelial_cell	*BAK1*	TRUE	3.295943692
T2DM_admission	endothelial_cell	*CWF19L1*	TRUE	2.774716331
T2DM_admission	endothelial_cell	*CRHR2*	TRUE	2.006427447
T2DM_admission	endothelial_cell	*ZNF211*	TRUE	2.712361695
T2DM_admission	endothelial_cell	*NOTCH2*	TRUE	5.657559486
T2DM_admission	endothelial_cell	*MAN2C1*	TRUE	3.689987623
T2DM_admission	endothelial_cell	*NPC1*	TRUE	2.044114061
T2DM_admission	endothelial_cell	*FN3KRP*	TRUE	1.976488435
T2DM_admission	endothelial_cell	*HSD17B12*	TRUE	3.163671452
T2DM_admission	endothelial_cell	*APIP*	TRUE	2.935210798
T2DM_admission	endothelial_cell	*CCDC15*	TRUE	2.389564971
T2DM_admission	endothelial_cell	*POC5*	TRUE	2.465142653
T2DM_admission	endothelial_cell	*JAZF1*	TRUE	4.89160374
T2DM_admission	endothelial_cell	*EXTL1*	TRUE	2.41460634
T2DM_admission	endothelial_cell	*ANKLE1*	TRUE	2.248397417
T2DM_admission	endothelial_cell	*TMEM129*	TRUE	2.136718595
T2DM_admission	endothelial_cell	*FUT2*	TRUE	2.446079716
T2DM_admission	endothelial_cell	*IRX3*	TRUE	7.49326076
T2DM_admission	endothelial_cell	*ZNF320*	TRUE	2.974051218
T2DM_admission	endothelial_cell	*FAM43B*	TRUE	2.41608965
T2DM_admission	endothelial_cell	*PIWIL2*	TRUE	2.234692993
T2DM_admission	endothelial_cell	*LIME1*	TRUE	3.135335432
T2DM_admission	endothelial_cell	*RNF5*	TRUE	3.250279004
T2DM_admission	endothelial_cell	*DNLZ*	TRUE	2.63349165
T2DM_admission	endothelial_cell	*HLA.DQA2*	TRUE	5.013940934
T2DM_admission	endothelial_cell	*JMJD7*	TRUE	3.716840544
T2DM_admission	endothelial_cell	*TAS2R43*	TRUE	2.461439019
T2DM_admission	epsilon_cell	*OPRL1*	TRUE	2.13782944
T2DM_admission	epsilon_cell	*CILP2*	TRUE	3.012035436
T2DM_admission	epsilon_cell	*IP6K3*	TRUE	2.177416845
T2DM_admission	epsilon_cell	*IRX3*	TRUE	5.895579204
T2DM_admission	epsilon_cell	*UBA7*	TRUE	4.251084578
T2DM_admission	epsilon_cell	*NOTCH4*	TRUE	2.227472467
T2DM_admission	epsilon_cell	*TAS2R43*	TRUE	2.383287683
T2DM_admission	leukocyte	*QPCTL*	TRUE	2.384226893
T2DM_admission	leukocyte	*NOTCH2*	TRUE	5.540252879
T2DM_admission	leukocyte	*POC5*	TRUE	2.580255736

T2DM_admission	leukocyte	*CLEC18A*	TRUE	2.059824543
T2DM_admission	leukocyte	*CILP2*	TRUE	2.807600299
T2DM_admission	leukocyte	*IRX3*	TRUE	3.466935934
T2DM_admission	leukocyte	*UBA7*	TRUE	2.469102687
T2DM_admission	leukocyte	*PLGLB1*	TRUE	2.073396928
T2DM_admission	leukocyte	*NUTM2B*	TRUE	2.163305278
T2DM_admission	leukocyte	*OR2A7*	TRUE	2.326391462
T2DM_admission	leukocyte	*TAS2R20*	TRUE	2.133428638
T2DM_admission	mesenchymal_cell	*RBM6*	TRUE	3.307895251
T2DM_admission	mesenchymal_cell	*CEP68*	TRUE	3.695609535
T2DM_admission	mesenchymal_cell	*BAK1*	TRUE	2.158783948
T2DM_admission	mesenchymal_cell	*CWF19L1*	TRUE	1.991038981
T2DM_admission	mesenchymal_cell	*HSD17B1*	TRUE	3.310378806
T2DM_admission	mesenchymal_cell	*ZNF211*	TRUE	2.421576816
T2DM_admission	mesenchymal_cell	*NOTCH2*	TRUE	2.448952326
T2DM_admission	mesenchymal_cell	*MAN2C1*	TRUE	3.499004503
T2DM_admission	mesenchymal_cell	*FN3KRP*	TRUE	2.30472128
T2DM_admission	mesenchymal_cell	*CDCA7*	TRUE	2.725011987
T2DM_admission	mesenchymal_cell	*HSD17B12*	TRUE	2.771742704
T2DM_admission	mesenchymal_cell	*CCDC15*	TRUE	2.568755337
T2DM_admission	mesenchymal_cell	*POC5*	TRUE	2.285083072
T2DM_admission	mesenchymal_cell	*CLEC18A*	TRUE	2.964138922
T2DM_admission	mesenchymal_cell	*TMEM129*	TRUE	2.255389963
T2DM_admission	mesenchymal_cell	*INO80E*	TRUE	2.302083746
T2DM_admission	mesenchymal_cell	*FUT2*	TRUE	2.576370037
T2DM_admission	mesenchymal_cell	*IRX3*	TRUE	8.348023457
T2DM_admission	mesenchymal_cell	*ZNF320*	TRUE	2.760535735
T2DM_admission	mesenchymal_cell	*LIME1*	TRUE	3.238258732
T2DM_admission	mesenchymal_cell	*HCG27*	TRUE	2.397921671
T2DM_admission	mesenchymal_cell	*SLC12A8*	TRUE	2.8741022
T2DM_admission	mesenchymal_cell	*JMJD7*	TRUE	2.871151081
T2DM_admission	mesenchymal_cell	*TAS2R43*	TRUE	2.437445876
T2DM_admission	PP_cell	*RBM6*	TRUE	2.125509595
T2DM_admission	PP_cell	*CEP68*	TRUE	2.661860631
T2DM_admission	PP_cell	*CCDC134*	TRUE	2.270347953
T2DM_admission	PP_cell	*OPRL1*	TRUE	3.504839914
T2DM_admission	PP_cell	*RIBC2*	TRUE	2.258778266
T2DM_admission	PP_cell	*SPTBN5*	TRUE	2.870454911
T2DM_admission	PP_cell	*MAN2C1*	TRUE	2.019125429
T2DM_admission	PP_cell	*HSD17B12*	TRUE	3.152729812
T2DM_admission	PP_cell	*APIP*	TRUE	2.022742486
T2DM_admission	PP_cell	*CCDC15*	TRUE	2.049961059
T2DM_admission	PP_cell	*PTPN14*	TRUE	2.15611758
T2DM_admission	PP_cell	*JAZF1*	TRUE	2.513048958
T2DM_admission	PP_cell	*CLEC18A*	TRUE	4.047278555

T2DM_admission	PP_cell	*CILP2*	TRUE	2.417643417
T2DM_admission	PP_cell	*IP6K3*	TRUE	2.118816149
T2DM_admission	PP_cell	*TMC8*	TRUE	2.40700774
T2DM_admission	PP_cell	*IRX3*	TRUE	3.222702474
T2DM_admission	PP_cell	*ZNF320*	TRUE	2.349159114
T2DM_insulin	acinar_cell	*CEP68*	TRUE	2.923357142
T2DM_insulin	acinar_cell	*ALOX5*	TRUE	2.641552327
T2DM_insulin	acinar_cell	*SPTB*	TRUE	1.965425146
T2DM_insulin	acinar_cell	*TF*	TRUE	2.227346976
T2DM_insulin	acinar_cell	*OIP5*	TRUE	2.022327701
T2DM_insulin	acinar_cell	*BTNL8*	TRUE	3.492986939
T2DM_insulin	acinar_cell	*ZNF211*	TRUE	2.217596256
T2DM_insulin	acinar_cell	*PKDREJ*	TRUE	2.274117011
T2DM_insulin	acinar_cell	*NOTCH2*	TRUE	2.321346937
T2DM_insulin	acinar_cell	*LRP4*	TRUE	1.968865811
T2DM_insulin	acinar_cell	*HSPG2*	TRUE	2.020773685
T2DM_insulin	acinar_cell	*RNF175*	TRUE	2.570075572
T2DM_insulin	acinar_cell	*TAGLN*	TRUE	1.967232553
T2DM_insulin	acinar_cell	*CLEC18A*	TRUE	3.447296132
T2DM_insulin	acinar_cell	*EXTL1*	TRUE	3.579706928
T2DM_insulin	acinar_cell	*NIPAL1*	TRUE	2.166237779
T2DM_insulin	acinar_cell	*ZNF589*	TRUE	2.111142252
T2DM_insulin	acinar_cell	*CXCL11*	TRUE	2.424840719
T2DM_insulin	acinar_cell	*IRX3*	TRUE	5.858406783
T2DM_insulin	acinar_cell	*ZNF223*	TRUE	2.706213999
T2DM_insulin	acinar_cell	*DNAJC22*	TRUE	2.115995261
T2DM_insulin	acinar_cell	*RMI1*	TRUE	2.298870016
T2DM_insulin	acinar_cell	*RPRML*	TRUE	2.146972128
T2DM_insulin	acinar_cell	*ZNF320*	TRUE	2.125595062
T2DM_insulin	acinar_cell	*EIF3C*	TRUE	2.508569026
T2DM_insulin	acinar_cell	*LRRC19*	TRUE	2.499023323
T2DM_insulin	acinar_cell	*HYAL3*	TRUE	2.53914026
T2DM_insulin	acinar_cell	*FAM47E*	TRUE	1.968177808
T2DM_insulin	acinar_cell	*PIWIL2*	TRUE	2.221433182
T2DM_insulin	acinar_cell	*PAX9*	TRUE	2.528663609
T2DM_insulin	acinar_cell	*NOTCH4*	TRUE	2.5724545
T2DM_insulin	acinar_cell	*PSORS1C1*	TRUE	2.228650836
T2DM_insulin	acinar_cell	*LILRB3*	TRUE	1.999880119
T2DM_insulin	acinar_cell	*HLA.DQB2*	TRUE	3.141611163
T2DM_insulin	acinar_cell	*HLA.DOB*	TRUE	2.186789301
T2DM_insulin	acinar_cell	*CDK3*	TRUE	2.874500562
T2DM_insulin	acinar_cell	*TAS2R43*	TRUE	2.284242451
T2DM_insulin	alpha_cell	*CEP68*	TRUE	2.808518176
T2DM_insulin	alpha_cell	*ALOX5*	TRUE	2.967492067
T2DM_insulin	alpha_cell	*OSBPL5*	TRUE	2.30280234

T2DM_insulin	alpha_cell	*ZDHHC6*	TRUE	2.12770309
T2DM_insulin	alpha_cell	*EPHA3*	TRUE	2.205257736
T2DM_insulin	alpha_cell	*EPN3*	TRUE	1.986557149
T2DM_insulin	alpha_cell	*SEC61A2*	TRUE	2.628137066
T2DM_insulin	alpha_cell	*SPTB*	TRUE	1.994253775
T2DM_insulin	alpha_cell	*ANO8*	TRUE	2.548224378
T2DM_insulin	alpha_cell	*TF*	TRUE	2.315106095
T2DM_insulin	alpha_cell	*MYBL2*	TRUE	2.277161321
T2DM_insulin	alpha_cell	*SLC17A9*	TRUE	1.961409409
T2DM_insulin	alpha_cell	*OIP5*	TRUE	2.20590932
T2DM_insulin	alpha_cell	*BTNL8*	TRUE	3.348280479
T2DM_insulin	alpha_cell	*PDC*	TRUE	1.990954685
T2DM_insulin	alpha_cell	*ZNF211*	TRUE	2.206325293
T2DM_insulin	alpha_cell	*MAU2*	TRUE	2.028733095
T2DM_insulin	alpha_cell	*PKDREJ*	TRUE	2.865637717
T2DM_insulin	alpha_cell	*RHPN2*	TRUE	1.967283745
T2DM_insulin	alpha_cell	*NOTCH2*	TRUE	2.787415683
T2DM_insulin	alpha_cell	*EVI5L*	TRUE	1.988247817
T2DM_insulin	alpha_cell	*HSPG2*	TRUE	2.130134458
T2DM_insulin	alpha_cell	*RNF175*	TRUE	2.252096891
T2DM_insulin	alpha_cell	*RAB19*	TRUE	2.329418213
T2DM_insulin	alpha_cell	*CABLES2*	TRUE	2.139512304
T2DM_insulin	alpha_cell	*SETD9*	TRUE	2.143109219
T2DM_insulin	alpha_cell	*CLEC18A*	TRUE	3.421138588
T2DM_insulin	alpha_cell	*EXTL1*	TRUE	3.605321192
T2DM_insulin	alpha_cell	*NIPAL1*	TRUE	1.973347302
T2DM_insulin	alpha_cell	*ZNF589*	TRUE	2.06433603
T2DM_insulin	alpha_cell	*CXCL11*	TRUE	2.291951351
T2DM_insulin	alpha_cell	*FUT10*	TRUE	2.268679568
T2DM_insulin	alpha_cell	*IRX3*	TRUE	5.429803187
T2DM_insulin	alpha_cell	*ZNF223*	TRUE	2.728335556
T2DM_insulin	alpha_cell	*DNAJC22*	TRUE	2.32509577
T2DM_insulin	alpha_cell	*RMI1*	TRUE	2.294825135
T2DM_insulin	alpha_cell	*RPRML*	TRUE	2.160083782
T2DM_insulin	alpha_cell	*ZNF320*	TRUE	2.281124886
T2DM_insulin	alpha_cell	*EIF3C*	TRUE	2.313723561
T2DM_insulin	alpha_cell	*PLA2G6*	TRUE	2.172005509
T2DM_insulin	alpha_cell	*LRRC19*	TRUE	2.815960061
T2DM_insulin	alpha_cell	*ZBTB40*	TRUE	2.212955946
T2DM_insulin	alpha_cell	*HYAL3*	TRUE	2.403067252
T2DM_insulin	alpha_cell	*FAM47E*	TRUE	2.167148616
T2DM_insulin	alpha_cell	*ZNF676*	TRUE	1.98566799
T2DM_insulin	alpha_cell	*PIWIL2*	TRUE	2.972154473
T2DM_insulin	alpha_cell	*HSPB9*	TRUE	2.148282375
T2DM_insulin	alpha_cell	*PAX9*	TRUE	2.966914067

T2DM_insulin	alpha_cell	*L3MBTL3*	TRUE	2.034432946
T2DM_insulin	alpha_cell	*NOTCH4*	TRUE	2.542758172
T2DM_insulin	alpha_cell	*PSORS1C1*	TRUE	2.265996009
T2DM_insulin	alpha_cell	*LILRB3*	TRUE	1.982953349
T2DM_insulin	alpha_cell	*GMNC*	TRUE	2.022459986
T2DM_insulin	alpha_cell	*HLA.DQB2*	TRUE	3.39185583
T2DM_insulin	alpha_cell	*HLA.DOB*	TRUE	2.15216655
T2DM_insulin	alpha_cell	*OR2A7*	TRUE	2.137523128
T2DM_insulin	alpha_cell	*TAS2R43*	TRUE	2.30722153
T2DM_insulin	beta_cell	*CEP68*	TRUE	3.61954482
T2DM_insulin	beta_cell	*ALOX5*	TRUE	2.615268808
T2DM_insulin	beta_cell	*OSBPL5*	TRUE	2.350707524
T2DM_insulin	beta_cell	*ZDHHC6*	TRUE	2.609783731
T2DM_insulin	beta_cell	*BAK1*	TRUE	2.027724088
T2DM_insulin	beta_cell	*EPHA3*	TRUE	2.178943412
T2DM_insulin	beta_cell	*PTGER3*	TRUE	2.119054754
T2DM_insulin	beta_cell	*SEC61A2*	TRUE	2.492805019
T2DM_insulin	beta_cell	*SPTB*	TRUE	2.118820431
T2DM_insulin	beta_cell	*ANO8*	TRUE	2.754284299
T2DM_insulin	beta_cell	*ACACB*	TRUE	2.107232283
T2DM_insulin	beta_cell	*CAD*	TRUE	1.97975518
T2DM_insulin	beta_cell	*DZANK1*	TRUE	1.984839527
T2DM_insulin	beta_cell	*TF*	TRUE	2.321345778
T2DM_insulin	beta_cell	*OCEL1*	TRUE	2.116429464
T2DM_insulin	beta_cell	*MYBL2*	TRUE	2.192597437
T2DM_insulin	beta_cell	*MAP1LC3A*	TRUE	2.174156986
T2DM_insulin	beta_cell	*OIP5*	TRUE	2.367267723
T2DM_insulin	beta_cell	*ETHE1*	TRUE	2.118968155
T2DM_insulin	beta_cell	*SLC35F2*	TRUE	2.002031308
T2DM_insulin	beta_cell	*BACH2*	TRUE	2.232569706
T2DM_insulin	beta_cell	*SEMA5A*	TRUE	2.200989008
T2DM_insulin	beta_cell	*BTNL8*	TRUE	3.694425977
T2DM_insulin	beta_cell	*PDC*	TRUE	2.003577519
T2DM_insulin	beta_cell	*BMP8B*	TRUE	2.149884965
T2DM_insulin	beta_cell	*IAPP*	TRUE	2.486910432
T2DM_insulin	beta_cell	*ZNF211*	TRUE	2.259005695
T2DM_insulin	beta_cell	*PEPD*	TRUE	2.133709426
T2DM_insulin	beta_cell	*CLYBL*	TRUE	2.199168238
T2DM_insulin	beta_cell	*PTPN12*	TRUE	2.149694053
T2DM_insulin	beta_cell	*MAU2*	TRUE	2.061447476
T2DM_insulin	beta_cell	*PKDREJ*	TRUE	2.81475999
T2DM_insulin	beta_cell	*F12*	TRUE	2.363832664
T2DM_insulin	beta_cell	*TRAF3*	TRUE	2.547919213
T2DM_insulin	beta_cell	*RHPN2*	TRUE	2.053310557
T2DM_insulin	beta_cell	*NOTCH2*	TRUE	2.875405319

T2DM_insulin	beta_cell	*LRP4*	TRUE	2.137069129
T2DM_insulin	beta_cell	*GRHPR*	TRUE	2.119013659
T2DM_insulin	beta_cell	*EVI5L*	TRUE	2.612178545
T2DM_insulin	beta_cell	*HSPG2*	TRUE	2.156179786
T2DM_insulin	beta_cell	*CNTN4*	TRUE	2.025968218
T2DM_insulin	beta_cell	*NXPE3*	TRUE	2.197163824
T2DM_insulin	beta_cell	*RNF175*	TRUE	2.544833807
T2DM_insulin	beta_cell	*RAB19*	TRUE	2.354027017
T2DM_insulin	beta_cell	*TAGLN*	TRUE	2.294533938
T2DM_insulin	beta_cell	*CABLES2*	TRUE	2.295079009
T2DM_insulin	beta_cell	*ZNF827*	TRUE	1.972030719
T2DM_insulin	beta_cell	*AKR1C2*	TRUE	2.070165099
T2DM_insulin	beta_cell	*KCNE4*	TRUE	2.145601814
T2DM_insulin	beta_cell	*JAZF1*	TRUE	2.274886558
T2DM_insulin	beta_cell	*SETD9*	TRUE	2.34592684
T2DM_insulin	beta_cell	*TMED6*	TRUE	2.086477511
T2DM_insulin	beta_cell	*CLEC18A*	TRUE	3.563505599
T2DM_insulin	beta_cell	*EXTL1*	TRUE	3.57431253
T2DM_insulin	beta_cell	*TONSL*	TRUE	2.13771782
T2DM_insulin	beta_cell	*NIPAL1*	TRUE	3.148011784
T2DM_insulin	beta_cell	*SRGAP2*	TRUE	2.34458633
T2DM_insulin	beta_cell	*CRELD1*	TRUE	2.61269523
T2DM_insulin	beta_cell	*ZNF589*	TRUE	2.068102852
T2DM_insulin	beta_cell	*KLKB1*	TRUE	2.452060537
T2DM_insulin	beta_cell	*LIPC*	TRUE	2.234870547
T2DM_insulin	beta_cell	*KIAA1586*	TRUE	2.664952916
T2DM_insulin	beta_cell	*SHOX2*	TRUE	2.211276872
T2DM_insulin	beta_cell	*CXCL11*	TRUE	2.419444813
T2DM_insulin	beta_cell	*FUT10*	TRUE	2.525512256
T2DM_insulin	beta_cell	*ANKLE2*	TRUE	2.186545816
T2DM_insulin	beta_cell	*IRX3*	TRUE	6.031667436
T2DM_insulin	beta_cell	*SAMD12*	TRUE	2.415745929
T2DM_insulin	beta_cell	*ZNF223*	TRUE	2.692923732
T2DM_insulin	beta_cell	*DNAJC22*	TRUE	2.684920317
T2DM_insulin	beta_cell	*RMI1*	TRUE	2.584424094
T2DM_insulin	beta_cell	*RPRML*	TRUE	2.12933326
T2DM_insulin	beta_cell	*TTC3*	TRUE	2.083405819
T2DM_insulin	beta_cell	*ZNF320*	TRUE	2.300132792
T2DM_insulin	beta_cell	*KREMEN1*	TRUE	2.120424534
T2DM_insulin	beta_cell	*TMEM236*	TRUE	2.192737172
T2DM_insulin	beta_cell	*EIF3C*	TRUE	2.974327286
T2DM_insulin	beta_cell	*PLA2G6*	TRUE	2.441058406
T2DM_insulin	beta_cell	*LRRC19*	TRUE	2.867440492
T2DM_insulin	beta_cell	*ZBTB40*	TRUE	3.352137578
T2DM_insulin	beta_cell	*NDUFA6*	TRUE	2.167404307

T2DM_insulin	beta_cell	*ZNF74*	TRUE	2.256036796
T2DM_insulin	beta_cell	*HYAL3*	TRUE	2.586081613
T2DM_insulin	beta_cell	*C17orf97*	TRUE	2.033838116
T2DM_insulin	beta_cell	*AGAP4*	TRUE	2.000151779
T2DM_insulin	beta_cell	*FAM47E*	TRUE	2.370235214
T2DM_insulin	beta_cell	*S100A14*	TRUE	2.346913695
T2DM_insulin	beta_cell	*ZNF676*	TRUE	2.049031613
T2DM_insulin	beta_cell	*PIWIL2*	TRUE	2.931273379
T2DM_insulin	beta_cell	*HSPB9*	TRUE	2.296087163
T2DM_insulin	beta_cell	*OCLN*	TRUE	2.46475167
T2DM_insulin	beta_cell	*C2orf27A*	TRUE	2.034675468
T2DM_insulin	beta_cell	*PAX9*	TRUE	3.088941254
T2DM_insulin	beta_cell	*L3MBTL3*	TRUE	2.137251524
T2DM_insulin	beta_cell	*NOTCH4*	TRUE	2.288387159
T2DM_insulin	beta_cell	*PSORS1C1*	TRUE	2.266947455
T2DM_insulin	beta_cell	*LILRB3*	TRUE	2.149463254
T2DM_insulin	beta_cell	*GMNC*	TRUE	2.1802163
T2DM_insulin	beta_cell	*C1QTNF9*	TRUE	1.996616496
T2DM_insulin	beta_cell	*HLA.DMB*	TRUE	2.288611696
T2DM_insulin	beta_cell	*OR2A7*	TRUE	2.14609967
T2DM_insulin	beta_cell	*LIMS3*	TRUE	2.167372994
T2DM_insulin	delta_cell	*ALOX5*	TRUE	2.701911976
T2DM_insulin	delta_cell	*OSBPL5*	TRUE	2.033626
T2DM_insulin	delta_cell	*BTNL8*	TRUE	3.050105957
T2DM_insulin	delta_cell	*PKDREJ*	TRUE	2.442704138
T2DM_insulin	delta_cell	*CXCL11*	TRUE	2.556342416
T2DM_insulin	delta_cell	*IRX3*	TRUE	4.128126599
T2DM_insulin	delta_cell	*DNAJC22*	TRUE	2.163775298
T2DM_insulin	delta_cell	*PAX9*	TRUE	2.461609601
T2DM_insulin	delta_cell	*NOTCH4*	TRUE	2.351335313
T2DM_insulin	delta_cell	*PSORS1C1*	TRUE	2.144091687
T2DM_insulin	delta_cell	*HLA.DQB2*	TRUE	3.22824947
T2DM_insulin	delta_cell	*HLA.DOB*	TRUE	2.212442772
T2DM_insulin	delta_cell	*TAS2R43*	TRUE	2.407662862
T2DM_insulin	duct_epithelial_cell	*CEP68*	TRUE	2.937238345
T2DM_insulin	duct_epithelial_cell	*ALOX5*	TRUE	2.737343295
T2DM_insulin	duct_epithelial_cell	*MYBL2*	TRUE	2.044566184
T2DM_insulin	duct_epithelial_cell	*BTNL8*	TRUE	3.540506248
T2DM_insulin	duct_epithelial_cell	*ZNF211*	TRUE	2.201249803
T2DM_insulin	duct_epithelial_cell	*PKDREJ*	TRUE	2.630054275
T2DM_insulin	duct_epithelial_cell	*EVI5L*	TRUE	1.967652593
T2DM_insulin	duct_epithelial_cell	*CLEC18A*	TRUE	2.520366052
T2DM_insulin	duct_epithelial_cell	*EXTL1*	TRUE	2.22749084
T2DM_insulin	duct_epithelial_cell	*CXCL11*	TRUE	3.298171863
T2DM_insulin	duct_epithelial_cell	*ZNF223*	TRUE	2.476746358

T2DM_insulin	duct_epithelial_cell	*RMI1*	TRUE	2.068529256
T2DM_insulin	duct_epithelial_cell	*FARSA*	TRUE	2.034168167
T2DM_insulin	duct_epithelial_cell	*ZNF320*	TRUE	2.363002805
T2DM_insulin	duct_epithelial_cell	*EIF3C*	TRUE	2.128590763
T2DM_insulin	duct_epithelial_cell	*LRRC19*	TRUE	2.394593519
T2DM_insulin	duct_epithelial_cell	*PIWIL2*	TRUE	2.848488487
T2DM_insulin	duct_epithelial_cell	*PAX9*	TRUE	2.856088011
T2DM_insulin	duct_epithelial_cell	*NOTCH4*	TRUE	2.102690855
T2DM_insulin	duct_epithelial_cell	*LILRB3*	TRUE	2.023325126
T2DM_insulin	duct_epithelial_cell	*HLA.DMB*	TRUE	2.400203306
T2DM_insulin	duct_epithelial_cell	*CDK3*	TRUE	2.891470531
T2DM_insulin	duct_epithelial_cell	*TAS2R43*	TRUE	2.008837879
T2DM_insulin	endothelial_cell	*CEP68*	TRUE	3.05224816
T2DM_insulin	endothelial_cell	*ALOX5*	TRUE	2.098203906
T2DM_insulin	endothelial_cell	*ANO8*	TRUE	2.393460336
T2DM_insulin	endothelial_cell	*ZNF211*	TRUE	2.745045574
T2DM_insulin	endothelial_cell	*PKDREJ*	TRUE	2.55012924
T2DM_insulin	endothelial_cell	*NOTCH2*	TRUE	2.45656319
T2DM_insulin	endothelial_cell	*EVI5L*	TRUE	2.11004747
T2DM_insulin	endothelial_cell	*EXTL1*	TRUE	3.156029862
T2DM_insulin	endothelial_cell	*CXCL11*	TRUE	2.153051988
T2DM_insulin	endothelial_cell	*IRX3*	TRUE	3.413372235
T2DM_insulin	endothelial_cell	*ZNF223*	TRUE	2.687847408
T2DM_insulin	endothelial_cell	*DNAJC22*	TRUE	2.018739629
T2DM_insulin	endothelial_cell	*RMI1*	TRUE	2.39998013
T2DM_insulin	endothelial_cell	*ZNF320*	TRUE	2.263913173
T2DM_insulin	endothelial_cell	*EIF3C*	TRUE	2.431898352
T2DM_insulin	endothelial_cell	*LRRC19*	TRUE	2.713412235
T2DM_insulin	endothelial_cell	*PIWIL2*	TRUE	2.7899545
T2DM_insulin	endothelial_cell	*PAX9*	TRUE	2.516727995
T2DM_insulin	endothelial_cell	*AS3MT*	TRUE	2.109962146
T2DM_insulin	endothelial_cell	*HLA.DQA2*	TRUE	2.046481131
T2DM_insulin	endothelial_cell	*HLA.DMB*	TRUE	3.153317823
T2DM_insulin	endothelial_cell	*TAS2R43*	TRUE	2.343103561
T2DM_insulin	epsilon_cell	*BTNL8*	TRUE	3.144691964
T2DM_insulin	epsilon_cell	*EXTL1*	TRUE	2.703609234
T2DM_insulin	epsilon_cell	*IRX3*	TRUE	1.974812986
T2DM_insulin	epsilon_cell	*PAX9*	TRUE	2.228789101
T2DM_insulin	epsilon_cell	*NOTCH4*	TRUE	2.349703456
T2DM_insulin	epsilon_cell	*PSORS1C1*	TRUE	1.963065715
T2DM_insulin	epsilon_cell	*HLA.DQA2*	TRUE	2.326644284
T2DM_insulin	epsilon_cell	*HLA.DOB*	TRUE	2.265349732
T2DM_insulin	epsilon_cell	*TAS2R43*	TRUE	2.072568412
T2DM_insulin	leukocyte	*NOTCH2*	TRUE	2.49567305
T2DM_insulin	leukocyte	*EXTL1*	TRUE	2.651736108

T2DM_insulin	leukocyte	*LRRC19*	TRUE	2.745431409
T2DM_insulin	leukocyte	*PAX9*	TRUE	2.122054854
T2DM_insulin	leukocyte	*NOTCH4*	TRUE	2.030662236
T2DM_insulin	leukocyte	*PSORS1C1*	TRUE	2.280476807
T2DM_insulin	leukocyte	*OR2A7*	TRUE	2.170735233
T2DM_insulin	mesenchymal_cell	*CEP68*	TRUE	2.84436493
T2DM_insulin	mesenchymal_cell	*BTNL8*	TRUE	3.194459796
T2DM_insulin	mesenchymal_cell	*ZNF211*	TRUE	2.617466005
T2DM_insulin	mesenchymal_cell	*PKDREJ*	TRUE	2.256831337
T2DM_insulin	mesenchymal_cell	*EXTL1*	TRUE	2.566662535
T2DM_insulin	mesenchymal_cell	*CXCL11*	TRUE	2.335972926
T2DM_insulin	mesenchymal_cell	*IRX3*	TRUE	4.656986298
T2DM_insulin	mesenchymal_cell	*ZNF223*	TRUE	2.365210155
T2DM_insulin	mesenchymal_cell	*RPRML*	TRUE	2.054840684
T2DM_insulin	mesenchymal_cell	*ZNF320*	TRUE	2.164964419
T2DM_insulin	mesenchymal_cell	*LRRC19*	TRUE	2.54655849
T2DM_insulin	mesenchymal_cell	*PIWIL2*	TRUE	2.415876105
T2DM_insulin	mesenchymal_cell	*PAX9*	TRUE	2.839985556
T2DM_insulin	mesenchymal_cell	*PSORS1C1*	TRUE	2.292280476
T2DM_insulin	mesenchymal_cell	*AS3MT*	TRUE	1.960978167
T2DM_insulin	mesenchymal_cell	*HLA.DQB2*	TRUE	2.209343188
T2DM_insulin	mesenchymal_cell	*CDK3*	TRUE	2.050967333
T2DM_insulin	PP_cell	*CEP68*	TRUE	2.847814471
T2DM_insulin	PP_cell	*TF*	TRUE	1.974419471
T2DM_insulin	PP_cell	*BTNL8*	TRUE	2.959087583
T2DM_insulin	PP_cell	*CLEC18A*	TRUE	2.024758692
T2DM_insulin	PP_cell	*CXCL11*	TRUE	3.151811679
T2DM_insulin	PP_cell	*ZNF223*	TRUE	2.014153393
T2DM_insulin	PP_cell	*EIF3C*	TRUE	2.029697028
T2DM_insulin	PP_cell	*PAX9*	TRUE	2.199184259
T2DM_insulin	PP_cell	*NOTCH4*	TRUE	2.414044795
T2DM_insulin	PP_cell	*PSORS1C1*	TRUE	2.081042135
T2DM_insulin	PP_cell	*GMNC*	TRUE	2.0314107
T2DM_insulin	PP_cell	*CDK3*	TRUE	2.904075662
T2DM_admission_freq	acinar_cell	*SLC7A9*	TRUE	2.45726525
T2DM_admission_freq	acinar_cell	*SLC4A11*	TRUE	2.003881646
T2DM_admission_freq	acinar_cell	*BTNL8*	TRUE	2.073589224
T2DM_admission_freq	acinar_cell	*CYB561D2*	TRUE	2.098335825
T2DM_admission_freq	acinar_cell	*VIPR1*	TRUE	2.206324096
T2DM_admission_freq	acinar_cell	*CXCL6*	TRUE	2.881969025
T2DM_admission_freq	acinar_cell	*IRF5*	TRUE	2.119655901
T2DM_admission_freq	acinar_cell	*FAM149B1*	TRUE	2.040451771
T2DM_admission_freq	acinar_cell	*PIK3C2G*	TRUE	2.67471584
T2DM_admission_freq	acinar_cell	*PLEKHH2*	TRUE	2.009837307
T2DM_admission_freq	acinar_cell	*C17orf50*	TRUE	1.97985277

T2DM_admission_freq	acinar_cell	*STOX1*	TRUE	2.380706194
T2DM_admission_freq	acinar_cell	*KCNAB3*	TRUE	2.094918522
T2DM_admission_freq	acinar_cell	*PDE3A*	TRUE	2.658578325
T2DM_admission_freq	acinar_cell	*TUBB8*	TRUE	2.509135392
T2DM_admission_freq	acinar_cell	*ZNF404*	TRUE	1.964319876
T2DM_admission_freq	acinar_cell	*GEN1*	TRUE	1.970924705
T2DM_admission_freq	acinar_cell	*NPIPA5*	TRUE	2.018301656
T2DM_admission_freq	acinar_cell	*RHCE*	TRUE	1.978207019
T2DM_admission_freq	acinar_cell	*ZNF79*	TRUE	2.251788654
T2DM_admission_freq	acinar_cell	*PAX9*	TRUE	4.082714005
T2DM_admission_freq	alpha_cell	*SLC7A9*	TRUE	2.777775469
T2DM_admission_freq	alpha_cell	*MRPS10*	TRUE	2.029049384
T2DM_admission_freq	alpha_cell	*SLC4A11*	TRUE	2.289261853
T2DM_admission_freq	alpha_cell	*VIPR2*	TRUE	2.31420984
T2DM_admission_freq	alpha_cell	*APOA5*	TRUE	2.02122652
T2DM_admission_freq	alpha_cell	*CHPT1*	TRUE	2.201601274
T2DM_admission_freq	alpha_cell	*BTNL8*	TRUE	2.01803416
T2DM_admission_freq	alpha_cell	*CYB561D2*	TRUE	2.064985137
T2DM_admission_freq	alpha_cell	*VIPR1*	TRUE	2.594607523
T2DM_admission_freq	alpha_cell	*CXCL6*	TRUE	2.705824028
T2DM_admission_freq	alpha_cell	*GRK4*	TRUE	1.98890765
T2DM_admission_freq	alpha_cell	*IRF5*	TRUE	2.331236014
T2DM_admission_freq	alpha_cell	*STK33*	TRUE	2.703510346
T2DM_admission_freq	alpha_cell	*CEP85*	TRUE	2.050474133
T2DM_admission_freq	alpha_cell	*FAM149B1*	TRUE	2.152282176
T2DM_admission_freq	alpha_cell	*PIK3C2G*	TRUE	2.481701436
T2DM_admission_freq	alpha_cell	*SPC25*	TRUE	2.284883959
T2DM_admission_freq	alpha_cell	*PLEKHH2*	TRUE	2.037971234
T2DM_admission_freq	alpha_cell	*C17orf50*	TRUE	1.975492811
T2DM_admission_freq	alpha_cell	*CDK20*	TRUE	2.308229072
T2DM_admission_freq	alpha_cell	*ICOSLG*	TRUE	1.99773095
T2DM_admission_freq	alpha_cell	*SIGLEC11*	TRUE	2.063715709
T2DM_admission_freq	alpha_cell	*STOX1*	TRUE	2.292258387
T2DM_admission_freq	alpha_cell	*SERPINF2*	TRUE	2.072294723
T2DM_admission_freq	alpha_cell	*MIS12*	TRUE	2.01257062
T2DM_admission_freq	alpha_cell	*KCNAB3*	TRUE	2.177296149
T2DM_admission_freq	alpha_cell	*PAQR8*	TRUE	1.964562581
T2DM_admission_freq	alpha_cell	*PDE3A*	TRUE	3.032708503
T2DM_admission_freq	alpha_cell	*TUBB8*	TRUE	2.545154548
T2DM_admission_freq	alpha_cell	*ZNF404*	TRUE	2.004337647
T2DM_admission_freq	alpha_cell	*GEN1*	TRUE	1.991287929
T2DM_admission_freq	alpha_cell	*AQP11*	TRUE	2.096533452
T2DM_admission_freq	alpha_cell	*NPIPA5*	TRUE	2.073449058
T2DM_admission_freq	alpha_cell	*NWD1*	TRUE	1.970485775
T2DM_admission_freq	alpha_cell	*RHCE*	TRUE	2.067044397

T2DM_admission_freq	alpha_cell	*ZNF79*	TRUE	2.241173342
T2DM_admission_freq	alpha_cell	*ZNF311*	TRUE	2.228100101
T2DM_admission_freq	alpha_cell	*ENTPD7*	TRUE	2.032696835
T2DM_admission_freq	alpha_cell	*PAX9*	TRUE	4.459744591
T2DM_admission_freq	alpha_cell	*PCDHGC3*	TRUE	2.082851942
T2DM_admission_freq	alpha_cell	*CFHR1*	TRUE	2.244854883
T2DM_admission_freq	beta_cell	*SLC7A9*	TRUE	2.8294167
T2DM_admission_freq	beta_cell	*ADRB1*	TRUE	2.134680057
T2DM_admission_freq	beta_cell	*LMO3*	TRUE	2.037886971
T2DM_admission_freq	beta_cell	*MRPS10*	TRUE	2.058359822
T2DM_admission_freq	beta_cell	*ZNF37A*	TRUE	2.23352282
T2DM_admission_freq	beta_cell	*TTC39A*	TRUE	2.176975804
T2DM_admission_freq	beta_cell	*SLC4A11*	TRUE	2.226561381
T2DM_admission_freq	beta_cell	*VIPR2*	TRUE	2.444006644
T2DM_admission_freq	beta_cell	*AK1*	TRUE	2.18949765
T2DM_admission_freq	beta_cell	*APOA5*	TRUE	1.964173572
T2DM_admission_freq	beta_cell	*CEP164*	TRUE	1.965117903
T2DM_admission_freq	beta_cell	*CHPT1*	TRUE	2.66199674
T2DM_admission_freq	beta_cell	*BTNL8*	TRUE	2.049435445
T2DM_admission_freq	beta_cell	*CYB561D2*	TRUE	2.145281643
T2DM_admission_freq	beta_cell	*VIPR1*	TRUE	2.743950906
T2DM_admission_freq	beta_cell	*CXCL6*	TRUE	2.845349055
T2DM_admission_freq	beta_cell	*GRK4*	TRUE	2.190303556
T2DM_admission_freq	beta_cell	*IRF5*	TRUE	2.325556741
T2DM_admission_freq	beta_cell	*STK33*	TRUE	2.989964863
T2DM_admission_freq	beta_cell	*CEP85*	TRUE	2.239686926
T2DM_admission_freq	beta_cell	*TSGA10*	TRUE	2.004670174
T2DM_admission_freq	beta_cell	*MRPS9*	TRUE	2.21419663
T2DM_admission_freq	beta_cell	*TMEM8B*	TRUE	2.000671569
T2DM_admission_freq	beta_cell	*DUSP5*	TRUE	2.038377603
T2DM_admission_freq	beta_cell	*FAM149B1*	TRUE	2.211650785
T2DM_admission_freq	beta_cell	*PIK3C2G*	TRUE	2.718851369
T2DM_admission_freq	beta_cell	*RSU1*	TRUE	2.459007291
T2DM_admission_freq	beta_cell	*CABLES2*	TRUE	1.999032709
T2DM_admission_freq	beta_cell	*SPC25*	TRUE	2.318984354
T2DM_admission_freq	beta_cell	*PLEKHH2*	TRUE	2.329319862
T2DM_admission_freq	beta_cell	*C17orf50*	TRUE	2.019019976
T2DM_admission_freq	beta_cell	*CDK20*	TRUE	2.544178126
T2DM_admission_freq	beta_cell	*CLEC18A*	TRUE	1.980040305
T2DM_admission_freq	beta_cell	*ICOSLG*	TRUE	2.081092519
T2DM_admission_freq	beta_cell	*SIGLEC11*	TRUE	2.088461113
T2DM_admission_freq	beta_cell	*OMA1*	TRUE	2.036355298
T2DM_admission_freq	beta_cell	*NMNAT3*	TRUE	2.198718912
T2DM_admission_freq	beta_cell	*C9orf64*	TRUE	2.051531726
T2DM_admission_freq	beta_cell	*STOX1*	TRUE	2.347574627

T2DM_admission_freq	beta_cell	*SERPINF2*	TRUE	2.056384732
T2DM_admission_freq	beta_cell	*MIS12*	TRUE	2.541556454
T2DM_admission_freq	beta_cell	*PXK*	TRUE	2.214533541
T2DM_admission_freq	beta_cell	*KCNAB3*	TRUE	2.1755355
T2DM_admission_freq	beta_cell	*PAQR8*	TRUE	2.018378332
T2DM_admission_freq	beta_cell	*ATP6V0E2*	TRUE	2.143477789
T2DM_admission_freq	beta_cell	*SPTLC3*	TRUE	2.144541807
T2DM_admission_freq	beta_cell	*PDE3A*	TRUE	3.114569758
T2DM_admission_freq	beta_cell	*TUBB8*	TRUE	2.552866156
T2DM_admission_freq	beta_cell	*GEN1*	TRUE	2.019369793
T2DM_admission_freq	beta_cell	*AQP11*	TRUE	2.08443091
T2DM_admission_freq	beta_cell	*FKRP*	TRUE	1.973721148
T2DM_admission_freq	beta_cell	*NPIPA5*	TRUE	2.022654924
T2DM_admission_freq	beta_cell	*SETD4*	TRUE	2.211854752
T2DM_admission_freq	beta_cell	*AKR1C1*	TRUE	2.219624366
T2DM_admission_freq	beta_cell	*RHCE*	TRUE	2.065736649
T2DM_admission_freq	beta_cell	*ZNF311*	TRUE	2.107444524
T2DM_admission_freq	beta_cell	*ENTPD7*	TRUE	2.42309866
T2DM_admission_freq	beta_cell	*COLGALT2*	TRUE	1.979960269
T2DM_admission_freq	beta_cell	*PAX9*	TRUE	4.455086344
T2DM_admission_freq	beta_cell	*PCDHGC3*	TRUE	2.161988918
T2DM_admission_freq	beta_cell	*CFHR1*	TRUE	2.249470706
T2DM_admission_freq	beta_cell	*FMN1*	TRUE	2.117809481
T2DM_admission_freq	beta_cell	*GTF2H5*	TRUE	2.051400478
T2DM_admission_freq	delta_cell	*SLC7A9*	TRUE	2.56807932
T2DM_admission_freq	delta_cell	*CYB561D2*	TRUE	2.346107328
T2DM_admission_freq	delta_cell	*CXCL6*	TRUE	2.634444563
T2DM_admission_freq	delta_cell	*FAM149B1*	TRUE	2.045004105
T2DM_admission_freq	delta_cell	*PIK3C2G*	TRUE	2.502908273
T2DM_admission_freq	delta_cell	*STOX1*	TRUE	2.150889806
T2DM_admission_freq	delta_cell	*PDE3A*	TRUE	2.152709398
T2DM_admission_freq	delta_cell	*AQP11*	TRUE	2.087845067
T2DM_admission_freq	delta_cell	*ZNF311*	TRUE	2.116664335
T2DM_admission_freq	delta_cell	*PAX9*	TRUE	4.469011244
T2DM_admission_freq	delta_cell	*PCDHGC3*	TRUE	1.991531351
T2DM_admission_freq	duct_epithelial_cell	*SLC7A9*	TRUE	2.755849187
T2DM_admission_freq	duct_epithelial_cell	*COQ5*	TRUE	1.962755566
T2DM_admission_freq	duct_epithelial_cell	*BTNL8*	TRUE	1.984791336
T2DM_admission_freq	duct_epithelial_cell	*CYB561D2*	TRUE	2.393233252
T2DM_admission_freq	duct_epithelial_cell	*IRF5*	TRUE	2.029297856
T2DM_admission_freq	duct_epithelial_cell	*FAM149B1*	TRUE	2.061606936
T2DM_admission_freq	duct_epithelial_cell	*PIK3C2G*	TRUE	2.340034213
T2DM_admission_freq	duct_epithelial_cell	*PLEKHH2*	TRUE	2.227090786
T2DM_admission_freq	duct_epithelial_cell	*CDK20*	TRUE	2.41229218
T2DM_admission_freq	duct_epithelial_cell	*STOX1*	TRUE	2.245372169

T2DM_admission_freq	duct_epithelial_cell	*SERPINF2*	TRUE	2.047469493
T2DM_admission_freq	duct_epithelial_cell	*PDE3A*	TRUE	2.392028265
T2DM_admission_freq	duct_epithelial_cell	*TUBB8*	TRUE	2.1377933
T2DM_admission_freq	duct_epithelial_cell	*GEN1*	TRUE	2.006984073
T2DM_admission_freq	duct_epithelial_cell	*AQP11*	TRUE	2.074416232
T2DM_admission_freq	duct_epithelial_cell	*NPIPA5*	TRUE	1.9704183
T2DM_admission_freq	duct_epithelial_cell	*ZNF79*	TRUE	2.003524169
T2DM_admission_freq	duct_epithelial_cell	*ENTPD7*	TRUE	2.183037991
T2DM_admission_freq	duct_epithelial_cell	*PAX9*	TRUE	4.419885742
T2DM_admission_freq	duct_epithelial_cell	*CFHR1*	TRUE	2.049653266
T2DM_admission_freq	endothelial_cell	*SLC7A9*	TRUE	2.819155397
T2DM_admission_freq	endothelial_cell	*CYB561D2*	TRUE	2.20697701
T2DM_admission_freq	endothelial_cell	*IRF5*	TRUE	1.97477706
T2DM_admission_freq	endothelial_cell	*PIK3C2G*	TRUE	2.73382645
T2DM_admission_freq	endothelial_cell	*STOX1*	TRUE	2.222618567
T2DM_admission_freq	endothelial_cell	*SERPINF2*	TRUE	2.062221026
T2DM_admission_freq	endothelial_cell	*PDE3A*	TRUE	2.975940356
T2DM_admission_freq	endothelial_cell	*TUBB8*	TRUE	2.308122446
T2DM_admission_freq	endothelial_cell	*ZNF404*	TRUE	1.983211423
T2DM_admission_freq	endothelial_cell	*AQP11*	TRUE	2.097203735
T2DM_admission_freq	endothelial_cell	*ZNF79*	TRUE	2.253023796
T2DM_admission_freq	endothelial_cell	*ZNF311*	TRUE	2.185482966
T2DM_admission_freq	endothelial_cell	*PAX9*	TRUE	4.560888079
T2DM_admission_freq	endothelial_cell	*CFHR1*	TRUE	2.064700095
T2DM_admission_freq	epsilon_cell	*BTNL8*	TRUE	1.977112405
T2DM_admission_freq	epsilon_cell	*SERPINF2*	TRUE	1.992861787
T2DM_admission_freq	epsilon_cell	*ZNF311*	TRUE	2.03616426
T2DM_admission_freq	epsilon_cell	*PAX9*	TRUE	4.3611974
T2DM_admission_freq	epsilon_cell	*PSORS1C1*	TRUE	2.385539001
T2DM_admission_freq	leukocyte	*SLC7A9*	TRUE	2.716188623
T2DM_admission_freq	leukocyte	*SLC4A11*	TRUE	2.137269122
T2DM_admission_freq	leukocyte	*STOX1*	TRUE	2.248319976
T2DM_admission_freq	leukocyte	*PDE3A*	TRUE	2.039114966
T2DM_admission_freq	leukocyte	*ZNF79*	TRUE	1.998291858
T2DM_admission_freq	leukocyte	*PAX9*	TRUE	4.361544712
T2DM_admission_freq	mesenchymal_cell	*SLC7A9*	TRUE	2.872078227
T2DM_admission_freq	mesenchymal_cell	*BTNL8*	TRUE	2.038045726
T2DM_admission_freq	mesenchymal_cell	*CYB561D2*	TRUE	2.521897408
T2DM_admission_freq	mesenchymal_cell	*CXCL6*	TRUE	2.803631042
T2DM_admission_freq	mesenchymal_cell	*FAM149B1*	TRUE	2.249834218
T2DM_admission_freq	mesenchymal_cell	*C17orf50*	TRUE	2.018521126
T2DM_admission_freq	mesenchymal_cell	*STOX1*	TRUE	2.21377874
T2DM_admission_freq	mesenchymal_cell	*MIS12*	TRUE	2.318743514
T2DM_admission_freq	mesenchymal_cell	*ZNF79*	TRUE	1.995231025
T2DM_admission_freq	mesenchymal_cell	*PAX9*	TRUE	4.064631936

T2DM_admission_freq	mesenchymal_cell	*PSORS1C1*	TRUE	2.250775727
T2DM_admission_freq	PP_cell	*SLC7A9*	TRUE	2.152253392
T2DM_admission_freq	PP_cell	*CYB561D2*	TRUE	2.38815041
T2DM_admission_freq	PP_cell	*PLEKHH2*	TRUE	2.255877568
T2DM_admission_freq	PP_cell	*SERPINF2*	TRUE	2.223770627
T2DM_admission_freq	PP_cell	*TUBB8*	TRUE	2.099450363
T2DM_admission_freq	PP_cell	*ZNF404*	TRUE	2.004698334
T2DM_admission_freq	PP_cell	*GEN1*	TRUE	2.055868907
T2DM_admission_freq	PP_cell	*ZNF311*	TRUE	2.145312351
T2DM_admission_freq	PP_cell	*PAX9*	TRUE	3.97832369
T2DM_admission_freq	PP_cell	*HLA.DQB2*	TRUE	1.972976461
T2DM_mortality	acinar_cell	*KCNG1*	TRUE	2.336291458
T2DM_mortality	acinar_cell	*BAK1*	TRUE	1.997587658
T2DM_mortality	acinar_cell	*TCF3*	TRUE	2.024741563
T2DM_mortality	acinar_cell	*CDHR5*	TRUE	2.028563438
T2DM_mortality	acinar_cell	*MROH8*	TRUE	2.492701063
T2DM_mortality	acinar_cell	*MLPH*	TRUE	2.653231294
T2DM_mortality	acinar_cell	*RIBC2*	TRUE	2.007532631
T2DM_mortality	acinar_cell	*PLVAP*	TRUE	2.159202038
T2DM_mortality	acinar_cell	*LPIN3*	TRUE	1.987898488
T2DM_mortality	acinar_cell	*NOTCH2*	TRUE	1.981506894
T2DM_mortality	acinar_cell	*MYOF*	TRUE	1.971366688
T2DM_mortality	acinar_cell	*CYP4B1*	TRUE	2.812600608
T2DM_mortality	acinar_cell	*TRIM58*	TRUE	2.260465779
T2DM_mortality	acinar_cell	*HCN1*	TRUE	2.227737917
T2DM_mortality	acinar_cell	*FUT9*	TRUE	2.789249632
T2DM_mortality	acinar_cell	*C9orf131*	TRUE	2.064150522
T2DM_mortality	acinar_cell	*IRX3*	TRUE	3.332544952
T2DM_mortality	acinar_cell	*GPR88*	TRUE	2.362701171
T2DM_mortality	acinar_cell	*DDO*	TRUE	2.150080293
T2DM_mortality	acinar_cell	*C6orf163*	TRUE	2.288392693
T2DM_mortality	acinar_cell	*SMTNL1*	TRUE	2.594586696
T2DM_mortality	alpha_cell	*KCNG1*	TRUE	2.066742539
T2DM_mortality	alpha_cell	*BAK1*	TRUE	2.172526633
T2DM_mortality	alpha_cell	*ITIH4*	TRUE	1.980951798
T2DM_mortality	alpha_cell	*MSH4*	TRUE	1.997251844
T2DM_mortality	alpha_cell	*TCF3*	TRUE	2.369359859
T2DM_mortality	alpha_cell	*CDHR5*	TRUE	2.026825261
T2DM_mortality	alpha_cell	*MROH8*	TRUE	2.620616056
T2DM_mortality	alpha_cell	*CHPT1*	TRUE	1.996999649
T2DM_mortality	alpha_cell	*MLPH*	TRUE	2.747906914
T2DM_mortality	alpha_cell	*FLRT1*	TRUE	1.974355853
T2DM_mortality	alpha_cell	*RIBC2*	TRUE	2.10550909
T2DM_mortality	alpha_cell	*PLVAP*	TRUE	2.248880543
T2DM_mortality	alpha_cell	*LPIN3*	TRUE	2.209954167

T2DM_mortality	alpha_cell	*CASP9*	TRUE	2.111886693
T2DM_mortality	alpha_cell	*SFTPD*	TRUE	2.274666839
T2DM_mortality	alpha_cell	*NOTCH2*	TRUE	2.213543334
T2DM_mortality	alpha_cell	*NDUFAF1*	TRUE	2.22277711
T2DM_mortality	alpha_cell	*MYOF*	TRUE	2.001609096
T2DM_mortality	alpha_cell	*CCDC65*	TRUE	2.006979488
T2DM_mortality	alpha_cell	*NPC1*	TRUE	2.573748671
T2DM_mortality	alpha_cell	*TRIM65*	TRUE	2.189349719
T2DM_mortality	alpha_cell	*CYP4B1*	TRUE	2.646554701
T2DM_mortality	alpha_cell	*SPATA9*	TRUE	2.206348255
T2DM_mortality	alpha_cell	*TRIM63*	TRUE	2.113098934
T2DM_mortality	alpha_cell	*FGF17*	TRUE	2.069347053
T2DM_mortality	alpha_cell	*TRIM58*	TRUE	2.162316548
T2DM_mortality	alpha_cell	*TUB*	TRUE	2.143877173
T2DM_mortality	alpha_cell	*FAM153A*	TRUE	2.159312206
T2DM_mortality	alpha_cell	*FUT9*	TRUE	2.836150252
T2DM_mortality	alpha_cell	*MRPL48*	TRUE	2.114229423
T2DM_mortality	alpha_cell	*IRX3*	TRUE	3.138244594
T2DM_mortality	alpha_cell	*GPR88*	TRUE	2.479487998
T2DM_mortality	alpha_cell	*SLC35D3*	TRUE	2.108271315
T2DM_mortality	alpha_cell	*NUTM2B*	TRUE	1.965278427
T2DM_mortality	alpha_cell	*AGAP4*	TRUE	2.381829327
T2DM_mortality	alpha_cell	*EVL*	TRUE	2.209752311
T2DM_mortality	alpha_cell	*ACSL5*	TRUE	2.163152345
T2DM_mortality	alpha_cell	*DDO*	TRUE	2.515933592
T2DM_mortality	alpha_cell	*RBM20*	TRUE	1.995356372
T2DM_mortality	alpha_cell	*C6orf163*	TRUE	2.36315963
T2DM_mortality	alpha_cell	*TREX1*	TRUE	2.080636306
T2DM_mortality	alpha_cell	*SMTNL1*	TRUE	2.569508868
T2DM_mortality	alpha_cell	*WDR46*	TRUE	1.960857262
T2DM_mortality	alpha_cell	*OR2A7*	TRUE	2.013204932
T2DM_mortality	beta_cell	*SYNRG*	TRUE	2.588671542
T2DM_mortality	beta_cell	*KCNG1*	TRUE	2.468370806
T2DM_mortality	beta_cell	*BAK1*	TRUE	2.885198314
T2DM_mortality	beta_cell	*PNPLA6*	TRUE	2.148847478
T2DM_mortality	beta_cell	*GALC*	TRUE	2.235984458
T2DM_mortality	beta_cell	*ITIH4*	TRUE	2.321016213
T2DM_mortality	beta_cell	*MSH4*	TRUE	2.030234084
T2DM_mortality	beta_cell	*TCF3*	TRUE	2.442665069
T2DM_mortality	beta_cell	*EXOSC7*	TRUE	2.074915013
T2DM_mortality	beta_cell	*GPR137B*	TRUE	2.712059575
T2DM_mortality	beta_cell	*ZNF343*	TRUE	1.978331328
T2DM_mortality	beta_cell	*CDHR5*	TRUE	2.327791086
T2DM_mortality	beta_cell	*ARFRP1*	TRUE	1.964128508
T2DM_mortality	beta_cell	*MROH8*	TRUE	2.666101709

T2DM_mortality	beta_cell	*DHX35*	TRUE	2.023158122
T2DM_mortality	beta_cell	*NOMO1*	TRUE	2.221889609
T2DM_mortality	beta_cell	*LIMK1*	TRUE	1.970037008
T2DM_mortality	beta_cell	*GLRB*	TRUE	2.332165859
T2DM_mortality	beta_cell	*CHPT1*	TRUE	2.151836755
T2DM_mortality	beta_cell	*LAMA4*	TRUE	2.103512275
T2DM_mortality	beta_cell	*DNAH6*	TRUE	2.00271778
T2DM_mortality	beta_cell	*MLPH*	TRUE	2.513895269
T2DM_mortality	beta_cell	*CEP104*	TRUE	1.969037573
T2DM_mortality	beta_cell	*CDK2*	TRUE	2.333274089
T2DM_mortality	beta_cell	*MT1G*	TRUE	2.228056042
T2DM_mortality	beta_cell	*RIBC2*	TRUE	2.135000279
T2DM_mortality	beta_cell	*PLVAP*	TRUE	2.299124606
T2DM_mortality	beta_cell	*LAMA5*	TRUE	2.068275175
T2DM_mortality	beta_cell	*LPIN3*	TRUE	2.221740546
T2DM_mortality	beta_cell	*CASP9*	TRUE	2.434329886
T2DM_mortality	beta_cell	*SFTPD*	TRUE	2.810405042
T2DM_mortality	beta_cell	*NOTCH2*	TRUE	2.27013323
T2DM_mortality	beta_cell	*SPIRE1*	TRUE	2.102954274
T2DM_mortality	beta_cell	*FLNB*	TRUE	2.133163559
T2DM_mortality	beta_cell	*NDUFAF1*	TRUE	2.617711897
T2DM_mortality	beta_cell	*PRDM5*	TRUE	2.232238898
T2DM_mortality	beta_cell	*NUP54*	TRUE	2.593842178
T2DM_mortality	beta_cell	*CCDC65*	TRUE	2.008920321
T2DM_mortality	beta_cell	*NPC1*	TRUE	2.751478569
T2DM_mortality	beta_cell	*TRIM65*	TRUE	2.215377032
T2DM_mortality	beta_cell	*CELA3A*	TRUE	2.283238287
T2DM_mortality	beta_cell	*CYP4B1*	TRUE	2.857589707
T2DM_mortality	beta_cell	*RABL2A*	TRUE	2.340370424
T2DM_mortality	beta_cell	*SPATA9*	TRUE	2.42873501
T2DM_mortality	beta_cell	*UGCG*	TRUE	1.962389682
T2DM_mortality	beta_cell	*POC5*	TRUE	1.975158814
T2DM_mortality	beta_cell	*CC2D1B*	TRUE	1.995345237
T2DM_mortality	beta_cell	*CDK20*	TRUE	1.975757845
T2DM_mortality	beta_cell	*TRIM63*	TRUE	2.240519795
T2DM_mortality	beta_cell	*FGF17*	TRUE	2.097408417
T2DM_mortality	beta_cell	*VAV2*	TRUE	2.063807023
T2DM_mortality	beta_cell	*TRIM58*	TRUE	2.267772271
T2DM_mortality	beta_cell	*CCDC141*	TRUE	2.216256295
T2DM_mortality	beta_cell	*CADPS*	TRUE	2.261775722
T2DM_mortality	beta_cell	*PPM1M*	TRUE	1.963403918
T2DM_mortality	beta_cell	*HCN1*	TRUE	2.383120245
T2DM_mortality	beta_cell	*TUB*	TRUE	2.222325328
T2DM_mortality	beta_cell	*C19orf48*	TRUE	1.966430129
T2DM_mortality	beta_cell	*FAM153A*	TRUE	2.702792242

T2DM_mortality	beta_cell	*FOXD4*	TRUE	2.056880664
T2DM_mortality	beta_cell	*PIK3CD*	TRUE	2.305194279
T2DM_mortality	beta_cell	*FUT9*	TRUE	2.9318779
T2DM_mortality	beta_cell	*RAB43*	TRUE	2.042983438
T2DM_mortality	beta_cell	*C9orf131*	TRUE	2.314391226
T2DM_mortality	beta_cell	*BTC*	TRUE	1.977962517
T2DM_mortality	beta_cell	*DENND6A*	TRUE	2.606806343
T2DM_mortality	beta_cell	*CLCF1*	TRUE	2.226807571
T2DM_mortality	beta_cell	*MRPL48*	TRUE	2.188214328
T2DM_mortality	beta_cell	*PHLDB3*	TRUE	2.014453137
T2DM_mortality	beta_cell	*IRX3*	TRUE	3.522611759
T2DM_mortality	beta_cell	*SMCO3*	TRUE	2.058984288
T2DM_mortality	beta_cell	*GPR88*	TRUE	2.524244959
T2DM_mortality	beta_cell	*SLC35D3*	TRUE	2.026600982
T2DM_mortality	beta_cell	*CAMK1D*	TRUE	2.903759129
T2DM_mortality	beta_cell	*LYPD6*	TRUE	1.964023491
T2DM_mortality	beta_cell	*AGAP4*	TRUE	2.215030019
T2DM_mortality	beta_cell	*ADAT2*	TRUE	2.132601615
T2DM_mortality	beta_cell	*EVL*	TRUE	2.300475441
T2DM_mortality	beta_cell	*ACSL5*	TRUE	2.235187583
T2DM_mortality	beta_cell	*DDO*	TRUE	2.627896265
T2DM_mortality	beta_cell	*RBM20*	TRUE	2.006518278
T2DM_mortality	beta_cell	*C6orf163*	TRUE	2.324881059
T2DM_mortality	beta_cell	*TMEM231*	TRUE	2.73843178
T2DM_mortality	beta_cell	*TREX1*	TRUE	2.114609014
T2DM_mortality	beta_cell	*SMTNL1*	TRUE	2.4673685
T2DM_mortality	beta_cell	*LINC00205*	TRUE	2.209904026
T2DM_mortality	beta_cell	*WDR46*	TRUE	2.774699526
T2DM_mortality	beta_cell	*OR2A7*	TRUE	2.031831762
T2DM_mortality	beta_cell	*LIMS3*	TRUE	2.082918482
T2DM_mortality	delta_cell	*MROH8*	TRUE	2.065353102
T2DM_mortality	delta_cell	*LPIN3*	TRUE	2.005739547
T2DM_mortality	delta_cell	*CYP4B1*	TRUE	2.815051621
T2DM_mortality	delta_cell	*FGF17*	TRUE	1.973240208
T2DM_mortality	delta_cell	*IRX3*	TRUE	2.0843938
T2DM_mortality	delta_cell	*DDO*	TRUE	2.040323006
T2DM_mortality	delta_cell	*SMTNL1*	TRUE	2.31344799
T2DM_mortality	duct_epithelial_cell	*CDHR5*	TRUE	2.026949759
T2DM_mortality	duct_epithelial_cell	*MROH8*	TRUE	2.047737506
T2DM_mortality	duct_epithelial_cell	*RIBC2*	TRUE	2.079362306
T2DM_mortality	duct_epithelial_cell	*LPIN3*	TRUE	1.985514364
T2DM_mortality	duct_epithelial_cell	*CASP9*	TRUE	2.077501675
T2DM_mortality	duct_epithelial_cell	*NDUFAF1*	TRUE	2.149219213
T2DM_mortality	duct_epithelial_cell	*TRIM58*	TRUE	2.145309542
T2DM_mortality	duct_epithelial_cell	*FUT9*	TRUE	2.385110207

T2DM_mortality	duct_epithelial_cell	*DDO*	TRUE	2.16900015
T2DM_mortality	duct_epithelial_cell	*C6orf163*	TRUE	2.23446136
T2DM_mortality	duct_epithelial_cell	*TMEM231*	TRUE	2.075737712
T2DM_mortality	duct_epithelial_cell	*TREX1*	TRUE	2.012047175
T2DM_mortality	duct_epithelial_cell	*HLA.DQA2*	TRUE	2.127182565
T2DM_mortality	endothelial_cell	*BAK1*	TRUE	2.17595251
T2DM_mortality	endothelial_cell	*ITIH4*	TRUE	2.033995717
T2DM_mortality	endothelial_cell	*MROH8*	TRUE	2.311272502
T2DM_mortality	endothelial_cell	*MLPH*	TRUE	2.521244444
T2DM_mortality	endothelial_cell	*CASP9*	TRUE	2.03531936
T2DM_mortality	endothelial_cell	*NOTCH2*	TRUE	2.120601659
T2DM_mortality	endothelial_cell	*MYOF*	TRUE	1.98624805
T2DM_mortality	endothelial_cell	*NPC1*	TRUE	2.378417506
T2DM_mortality	endothelial_cell	*TRIM65*	TRUE	2.012838021
T2DM_mortality	endothelial_cell	*FUT9*	TRUE	2.837503689
T2DM_mortality	endothelial_cell	*GPR88*	TRUE	2.396895737
T2DM_mortality	endothelial_cell	*SMTNL1*	TRUE	2.488469871
T2DM_mortality	epsilon_cell	*CYP4B1*	TRUE	2.908042065
T2DM_mortality	epsilon_cell	*FUT9*	TRUE	2.358538248
T2DM_mortality	epsilon_cell	*GPR88*	TRUE	2.228369147
T2DM_mortality	epsilon_cell	*SMTNL1*	TRUE	2.324589228
T2DM_mortality	leukocyte	MROH8	TRUE	2.121033155
T2DM_mortality	leukocyte	FUT9	TRUE	2.184595168
T2DM_mortality	leukocyte	GPR88	TRUE	2.324811726
T2DM_mortality	leukocyte	SMTNL1	TRUE	2.148790799
T2DM_mortality	mesenchymal_cell	MROH8	TRUE	2.409097015
T2DM_mortality	mesenchymal_cell	NDUFAF1	TRUE	2.101914614
T2DM_mortality	mesenchymal_cell	TRIM63	TRUE	2.182656755
T2DM_mortality	mesenchymal_cell	FUT9	TRUE	1.996096189
T2DM_mortality	mesenchymal_cell	IRX3	TRUE	2.623270538
T2DM_mortality	mesenchymal_cell	LYPD6	TRUE	1.97114281
T2DM_mortality	mesenchymal_cell	DDO	TRUE	2.290532938
T2DM_mortality	mesenchymal_cell	C6orf163	TRUE	2.240441843
T2DM_mortality	mesenchymal_cell	TREX1	TRUE	1.975736469
T2DM_mortality	mesenchymal_cell	SMTNL1	TRUE	2.181080219
T2DM_mortality	PP_cell	MLPH	TRUE	2.1446663
T2DM_mortality	PP_cell	RIBC2	TRUE	2.303896452
T2DM_mortality	PP_cell	CYP4B1	TRUE	2.559864217
T2DM_mortality	PP_cell	GPR88	TRUE	2.214974098
T2DM_mortality	PP_cell	SMTNL1	TRUE	2.093450854

c. Identified cell type specific causal genes for obesity and related disorders from SAT

disorder	celltype	gene	assoc	zMin
CAD	adipocyte	*DLX6*	TRUE	3.05768278
CAD	adipocyte	*LAMC2*	TRUE	2.079349762
CAD	adipocyte	*ATP2A3*	TRUE	2.152364946
CAD	adipocyte	*TIPIN*	TRUE	3.389545984
CAD	adipocyte	*TXNDC16*	TRUE	2.035752693
CAD	adipocyte	*RPGRIP1*	TRUE	2.250177492
CAD	adipocyte	*SUSD2*	TRUE	2.225892949
CAD	adipocyte	*FOXRED2*	TRUE	2.214976703
CAD	adipocyte	*ABHD12*	TRUE	2.269059626
CAD	adipocyte	*MAP1LC3A*	TRUE	2.938530229
CAD	adipocyte	*CTSH*	TRUE	2.006163569
CAD	adipocyte	*AMH*	TRUE	2.045671176
CAD	adipocyte	*MAG*	TRUE	2.156847402
CAD	adipocyte	*STEAP1B*	TRUE	2.552130938
CAD	adipocyte	*ICAM2*	TRUE	2.040423616
CAD	adipocyte	*ENDOU*	TRUE	2.595457735
CAD	adipocyte	*BVES*	TRUE	2.002075557
CAD	adipocyte	*APBB3*	TRUE	2.204270282
CAD	adipocyte	*BNIP1*	TRUE	2.669579675
CAD	adipocyte	*BCHE*	TRUE	3.229938448
CAD	adipocyte	*UMPS*	TRUE	2.197242896
CAD	adipocyte	*PLXNA1*	TRUE	3.09421374
CAD	adipocyte	*GGCX*	TRUE	3.628574866
CAD	adipocyte	*TNFSF4*	TRUE	2.644293724
CAD	adipocyte	*EIF2B2*	TRUE	2.914922339
CAD	adipocyte	*CKS2*	TRUE	1.989006238
CAD	adipocyte	*HIF3A*	TRUE	2.196026373
CAD	adipocyte	*GRK4*	TRUE	3.316240964
CAD	adipocyte	*REEP2*	TRUE	1.986100995
CAD	adipocyte	*B4GALNT1*	TRUE	2.538602116
CAD	adipocyte	*TMBIM1*	TRUE	2.05694508
CAD	adipocyte	*POLR1E*	TRUE	2.775350236
CAD	adipocyte	*PIANP*	TRUE	2.025595455
CAD	adipocyte	*HORMAD1*	TRUE	2.29651234
CAD	adipocyte	*NKD2*	TRUE	2.030286464
CAD	adipocyte	*PSD2*	TRUE	3.576874189
CAD	adipocyte	*SLC18B1*	TRUE	2.659822255
CAD	adipocyte	*HSD17B12*	TRUE	2.996461567
CAD	adipocyte	*TTC12*	TRUE	2.312263904
CAD	adipocyte	*DUSP15*	TRUE	2.602588602
CAD	adipocyte	*DISP1*	TRUE	3.221600725
CAD	adipocyte	*MED19*	TRUE	2.032124753
CAD	adipocyte	*CLEC18A*	TRUE	2.696215425
CAD	adipocyte	*MX1*	TRUE	1.982178471
CAD	adipocyte	*U2AF1*	TRUE	2.066401218
CAD	adipocyte	*NAGS*	TRUE	2.236997308

CAD	adipocyte	*JMJD8*	TRUE	2.095011659
CAD	adipocyte	*LMOD1*	TRUE	3.135623885
CAD	adipocyte	*ICA1L*	TRUE	2.457228877
CAD	adipocyte	*APEH*	TRUE	2.862504721
CAD	adipocyte	*GPER1*	TRUE	2.509650895
CAD	adipocyte	*OXGR1*	TRUE	3.439545116
CAD	adipocyte	*KIFC2*	TRUE	2.202290995
CAD	adipocyte	*ULK4*	TRUE	2.729962589
CAD	adipocyte	*MLKL*	TRUE	2.165113775
CAD	adipocyte	*IRS1*	TRUE	4.667595416
CAD	adipocyte	*PCSK9*	TRUE	4.051216077
CAD	adipocyte	*CALML6*	TRUE	1.997065246
CAD	adipocyte	*C3AR1*	TRUE	2.131530386
CAD	adipocyte	*ZNF738*	TRUE	1.995108244
CAD	adipocyte	*TLR6*	TRUE	2.115002253
CAD	adipocyte	*LPL*	TRUE	4.532917016
CAD	adipocyte	*PAAF1*	TRUE	2.595644471
CAD	adipocyte	*RIMKLA*	TRUE	2.419740863
CAD	adipocyte	*PPFIA3*	TRUE	1.997343838
CAD	adipocyte	*C12orf54*	TRUE	2.21756941
CAD	adipocyte	*MPI*	TRUE	2.02998061
CAD	adipocyte	*TIGD5*	TRUE	2.221339656
CAD	adipocyte	*LYNX1*	TRUE	2.288578216
CAD	adipocyte	*ZNF443*	TRUE	2.302078699
CAD	adipocyte	*MRPL14*	TRUE	2.101945164
CAD	adipocyte	*ADIPOQ*	TRUE	2.345544328
CAD	adipocyte	*TMEM136*	TRUE	3.098515473
CAD	adipocyte	*PLCXD3*	TRUE	2.591928019
CAD	adipocyte	*ACOT1*	TRUE	2.446667294
CAD	adipocyte	*GDF3*	TRUE	1.969648528
CAD	adipocyte	*A3GALT2*	TRUE	2.31935041
CAD	adipocyte	*MSL1*	TRUE	2.937947718
CAD	adipocyte	*C6orf106*	TRUE	3.238989676
CAD	adipocyte	*KIAA1211L*	TRUE	1.965755825
CAD	adipocyte	*WDR5B*	TRUE	2.063410386
CAD	adipocyte	*RPS26*	TRUE	2.178447697
CAD	adipocyte	*ZNF71*	TRUE	2.059794643
CAD	adipocyte	*ZNF334*	TRUE	2.435719305
CAD	adipocyte	*TMEM116*	TRUE	3.238650643
CAD	adipocyte	*TBKBP1*	TRUE	2.341659237
CAD	adipocyte	*CENPW*	TRUE	3.701389562
CAD	adipocyte	*PRCD*	TRUE	2.03389422
CAD	adipocyte	*PROB1*	TRUE	2.188790768
CAD	adipocyte	*AC112229.4*	TRUE	2.212225006
CAD	adipocyte	*LINC00957*	TRUE	2.195781915
CAD	adipocyte	*LY6G5B*	TRUE	2.268335802
CAD	adipocyte	*SHANK3*	TRUE	2.023691892
CAD	adipocyte	*ARMS2*	TRUE	2.538403523

CAD	adipocyte	*ZNF10*	TRUE	2.506854166
CAD	adipocyte	*HMBS*	TRUE	3.607661975
CAD	adipocyte	*GALNT4*	TRUE	2.471979892
CAD	ASPC	*TNFRSF12A*	TRUE	1.972073829
CAD	ASPC	*DLX6*	TRUE	3.014625977
CAD	ASPC	*MAPK8IP2*	TRUE	2.147446193
CAD	ASPC	*MTMR11*	TRUE	2.345198182
CAD	ASPC	*NPC1L1*	TRUE	2.967140149
CAD	ASPC	*LAMC2*	TRUE	2.128126088
CAD	ASPC	*SDK2*	TRUE	2.001922829
CAD	ASPC	*ASNS*	TRUE	2.381549242
CAD	ASPC	*ATP2A3*	TRUE	2.535126438
CAD	ASPC	*TIPIN*	TRUE	3.683140554
CAD	ASPC	*FBLN1*	TRUE	2.327983516
CAD	ASPC	*SCGN*	TRUE	2.096824193
CAD	ASPC	*COL16A1*	TRUE	2.227620815
CAD	ASPC	*TXNDC16*	TRUE	2.646310593
CAD	ASPC	*RPGRIP1*	TRUE	2.513912429
CAD	ASPC	*MARCH2*	TRUE	2.271872075
CAD	ASPC	*SUSD2*	TRUE	2.245453618
CAD	ASPC	*SLC25A1*	TRUE	2.111749214
CAD	ASPC	*FOXRED2*	TRUE	2.507883921
CAD	ASPC	*TRIM9*	TRUE	1.985537663
CAD	ASPC	*ABHD12*	TRUE	3.021598966
CAD	ASPC	*ACTR5*	TRUE	2.58858499
CAD	ASPC	*MAP1LC3A*	TRUE	3.62442116
CAD	ASPC	*PIGU*	TRUE	1.962832113
CAD	ASPC	*DHODH*	TRUE	2.130101538
CAD	ASPC	*NAGPA*	TRUE	2.624476957
CAD	ASPC	*CTSH*	TRUE	2.5298685
CAD	ASPC	*AMH*	TRUE	2.177995007
CAD	ASPC	*DMPK*	TRUE	2.566073937
CAD	ASPC	*MAG*	TRUE	2.215008883
CAD	ASPC	*STEAP1B*	TRUE	2.947188931
CAD	ASPC	*COA1*	TRUE	2.483443759
CAD	ASPC	*AK1*	TRUE	2.220231719
CAD	ASPC	*ICAM2*	TRUE	3.121948999
CAD	ASPC	*VAT1*	TRUE	2.077427895
CAD	ASPC	*KLHL5*	TRUE	2.096934792
CAD	ASPC	*TRPV4*	TRUE	2.578201767
CAD	ASPC	*MDN1*	TRUE	2.276191214
CAD	ASPC	*BVES*	TRUE	2.296545735
CAD	ASPC	*APBB3*	TRUE	2.363618403
CAD	ASPC	*BNIP1*	TRUE	2.942649648
CAD	ASPC	*NIT2*	TRUE	2.104036153
CAD	ASPC	*BCHE*	TRUE	3.741208038
CAD	ASPC	*UMPS*	TRUE	3.42475634
CAD	ASPC	*PLXNA1*	TRUE	3.527471265

CAD	ASPC	*GGCX*	TRUE	4.091885175
CAD	ASPC	*ZNF142*	TRUE	2.287200102
CAD	ASPC	*CFHR3*	TRUE	2.00038848
CAD	ASPC	*TNFSF4*	TRUE	2.939128095
CAD	ASPC	*DIEXF*	TRUE	2.024488832
CAD	ASPC	*EIF2B2*	TRUE	2.089881485
CAD	ASPC	*CKS2*	TRUE	2.568516441
CAD	ASPC	*HIF3A*	TRUE	3.080203608
CAD	ASPC	*GRK4*	TRUE	3.65185118
CAD	ASPC	*YEATS4*	TRUE	2.271775546
CAD	ASPC	*SMARCA4*	TRUE	2.064672999
CAD	ASPC	*EMC4*	TRUE	2.143774999
CAD	ASPC	*KLHDC10*	TRUE	3.262597965
CAD	ASPC	*PLVAP*	TRUE	2.032648114
CAD	ASPC	*REEP2*	TRUE	2.015461509
CAD	ASPC	*SPIRE1*	TRUE	2.011600327
CAD	ASPC	*HRK*	TRUE	2.052233094
CAD	ASPC	*TMBIM1*	TRUE	1.966820123
CAD	ASPC	*POLR1E*	TRUE	3.396231439
CAD	ASPC	*RMDN3*	TRUE	2.116718814
CAD	ASPC	*BTBD16*	TRUE	1.99433635
CAD	ASPC	*PLCE1*	TRUE	2.131536219
CAD	ASPC	*NUP54*	TRUE	2.379116049
CAD	ASPC	*GPR84*	TRUE	2.047630685
CAD	ASPC	*TICRR*	TRUE	2.517480026
CAD	ASPC	*TGFB1I1*	TRUE	2.08075758
CAD	ASPC	*UNC45B*	TRUE	2.021223791
CAD	ASPC	*TMEM91*	TRUE	2.099884847
CAD	ASPC	*NME7*	TRUE	2.88821873
CAD	ASPC	*WNT9A*	TRUE	2.251808282
CAD	ASPC	*AFF3*	TRUE	2.123665712
CAD	ASPC	*NKD2*	TRUE	2.043138525
CAD	ASPC	*PSD2*	TRUE	3.548732891
CAD	ASPC	*SLC18B1*	TRUE	3.027091511
CAD	ASPC	*DNAJB12*	TRUE	2.021157195
CAD	ASPC	*PDCD11*	TRUE	2.304896774
CAD	ASPC	*HSD17B12*	TRUE	3.354442977
CAD	ASPC	*TTC12*	TRUE	2.665661248
CAD	ASPC	*DUSP15*	TRUE	2.64460057
CAD	ASPC	*TMEM86A*	TRUE	1.988210006
CAD	ASPC	*ARL14EP*	TRUE	2.196838525
CAD	ASPC	*PTPRR*	TRUE	2.154645336
CAD	ASPC	*ADPRHL1*	TRUE	2.018740912
CAD	ASPC	*MIA3*	TRUE	2.928588189
CAD	ASPC	*DISP1*	TRUE	3.688850449
CAD	ASPC	*MED19*	TRUE	2.361384603
CAD	ASPC	*CLEC18A*	TRUE	1.997963167
CAD	ASPC	*KCNJ15*	TRUE	2.122762826

CAD	ASPC	*MX1*	TRUE	2.313953501
CAD	ASPC	*EPB41*	TRUE	2.581701397
CAD	ASPC	*U2AF1*	TRUE	2.37992496
CAD	ASPC	*S100A1*	TRUE	2.01779496
CAD	ASPC	*FBXO27*	TRUE	2.337603361
CAD	ASPC	*ALDH16A1*	TRUE	2.061203875
CAD	ASPC	*MPP3*	TRUE	2.341293732
CAD	ASPC	*LARP4*	TRUE	2.048996596
CAD	ASPC	*JMJD8*	TRUE	2.52324085
CAD	ASPC	*CGGBP1*	TRUE	1.965483167
CAD	ASPC	*LMOD1*	TRUE	3.618345545
CAD	ASPC	*C1orf50*	TRUE	2.068300736
CAD	ASPC	*APEH*	TRUE	2.883932128
CAD	ASPC	*GPER1*	TRUE	2.906907724
CAD	ASPC	*PIGO*	TRUE	2.155284322
CAD	ASPC	*OXGR1*	TRUE	3.635140514
CAD	ASPC	*STK32C*	TRUE	2.190109904
CAD	ASPC	*RIMKLB*	TRUE	2.030493917
CAD	ASPC	*ANKDD1A*	TRUE	2.141276754
CAD	ASPC	*SP2*	TRUE	2.001108553
CAD	ASPC	*KIFC2*	TRUE	2.146697878
CAD	ASPC	*ULK4*	TRUE	3.010441786
CAD	ASPC	*MLKL*	TRUE	2.588371847
CAD	ASPC	*LGALS9*	TRUE	2.191520583
CAD	ASPC	*IRS1*	TRUE	4.942158062
CAD	ASPC	*PCSK9*	TRUE	4.127945484
CAD	ASPC	*CALML6*	TRUE	2.432296603
CAD	ASPC	*TSNARE1*	TRUE	2.289729254
CAD	ASPC	*KCNMB3*	TRUE	2.062404604
CAD	ASPC	*RSL1D1*	TRUE	2.111140182
CAD	ASPC	*C3AR1*	TRUE	3.453714936
CAD	ASPC	*TLN2*	TRUE	2.123077148
CAD	ASPC	*SULT1B1*	TRUE	2.040567443
CAD	ASPC	*GOLIM4*	TRUE	2.060382993
CAD	ASPC	*TLR6*	TRUE	2.282893324
CAD	ASPC	*LPL*	TRUE	4.734183314
CAD	ASPC	*PAAF1*	TRUE	2.296333552
CAD	ASPC	*TEX26*	TRUE	2.059885184
CAD	ASPC	*CHMP6*	TRUE	2.1057001
CAD	ASPC	*CRLF3*	TRUE	3.425020419
CAD	ASPC	*RIMKLA*	TRUE	2.160730746
CAD	ASPC	*FBXO39*	TRUE	2.010098178
CAD	ASPC	*AC024940.1*	TRUE	2.064669214
CAD	ASPC	*PPFIA3*	TRUE	2.195862908
CAD	ASPC	*C12orf54*	TRUE	2.515493397
CAD	ASPC	*TIGD5*	TRUE	2.471029452
CAD	ASPC	*TMEM86B*	TRUE	2.019172576
CAD	ASPC	*LYNX1*	TRUE	2.503222444

CAD	ASPC	*ZNF443*	TRUE	2.800832458
CAD	ASPC	*MRPL14*	TRUE	2.760179848
CAD	ASPC	*ADIPOQ*	TRUE	2.404821778
CAD	ASPC	*TMEM136*	TRUE	3.285341473
CAD	ASPC	*FES*	TRUE	4.516768607
CAD	ASPC	*PLCXD3*	TRUE	3.041031504
CAD	ASPC	*ZNF320*	TRUE	2.083137147
CAD	ASPC	*PP2D1*	TRUE	2.407688378
CAD	ASPC	*ACOT1*	TRUE	2.66668853
CAD	ASPC	*GDF3*	TRUE	2.209181033
CAD	ASPC	*CSF1*	TRUE	2.439543268
CAD	ASPC	*A3GALT2*	TRUE	2.366366117
CAD	ASPC	*TCN2*	TRUE	2.244835734
CAD	ASPC	*SP1*	TRUE	2.051931919
CAD	ASPC	*ADH1A*	TRUE	2.078592736
CAD	ASPC	*C2orf66*	TRUE	2.328907594
CAD	ASPC	*MSL1*	TRUE	3.37696159
CAD	ASPC	*SULT1A1*	TRUE	2.054086576
CAD	ASPC	*MYO6*	TRUE	2.592502931
CAD	ASPC	*C6orf106*	TRUE	3.272080359
CAD	ASPC	*ZFP28*	TRUE	2.16552546
CAD	ASPC	*KIAA1211L*	TRUE	2.1708891
CAD	ASPC	*ZNF100*	TRUE	2.485095766
CAD	ASPC	*ZNF665*	TRUE	2.261237783
CAD	ASPC	*MRPL42*	TRUE	2.195922075
CAD	ASPC	*HIBCH*	TRUE	2.047621128
CAD	ASPC	*ZNF334*	TRUE	2.746243527
CAD	ASPC	*TMEM116*	TRUE	3.484630193
CAD	ASPC	*TBKBP1*	TRUE	2.027455882
CAD	ASPC	*CENPW*	TRUE	4.121036616
CAD	ASPC	*FAM216A*	TRUE	2.058713543
CAD	ASPC	*ZNF425*	TRUE	2.139864547
CAD	ASPC	*ARHGEF35*	TRUE	2.105094545
CAD	ASPC	*SIPA1*	TRUE	2.478695442
CAD	ASPC	*PRCD*	TRUE	2.710010679
CAD	ASPC	*LINC00863*	TRUE	2.037602416
CAD	ASPC	*PROB1*	TRUE	2.079404
CAD	ASPC	*AC112229.4*	TRUE	2.256054836
CAD	ASPC	*LINC00865*	TRUE	1.993745885
CAD	ASPC	*GOLGA8N*	TRUE	2.326215895
CAD	ASPC	*HLA.B*	TRUE	2.170809172
CAD	ASPC	*LINC00957*	TRUE	2.375288923
CAD	ASPC	*B3GALT4*	TRUE	2.038983754
CAD	ASPC	*LY6G5B*	TRUE	2.157875353
CAD	ASPC	*TDGF1*	TRUE	2.008638762
CAD	ASPC	*C22orf39*	TRUE	2.457907485
CAD	ASPC	*BCKDHA*	TRUE	2.046975367
CAD	ASPC	*ZNF10*	TRUE	2.767849077

CAD	ASPC	*HMBS*	TRUE	3.968547686
CAD	ASPC	*GALNT4*	TRUE	2.491186628
CAD	ASPC	*IQCH.AS1*	TRUE	2.241557153
CAD	ASPC	*AC144652.1*	TRUE	2.149410631
CAD	b_cell	*DLX6*	TRUE	2.023318715
CAD	b_cell	*MAG*	TRUE	1.975578797
CAD	b_cell	*BNIP1*	TRUE	2.127887229
CAD	b_cell	*AMT*	TRUE	1.975563106
CAD	b_cell	*DISP1*	TRUE	2.648513381
CAD	b_cell	*CLEC18A*	TRUE	2.656290534
CAD	b_cell	*OXGR1*	TRUE	2.823102342
CAD	b_cell	*LY6G5B*	TRUE	3.182475375
CAD	dendritic_cell	*SUSD2*	TRUE	2.23171107
CAD	dendritic_cell	*STEAP1B*	TRUE	2.125954466
CAD	dendritic_cell	*GGCX*	TRUE	2.29362084
CAD	dendritic_cell	*SNRPC*	TRUE	2.405908264
CAD	dendritic_cell	*HORMAD1*	TRUE	2.231064927
CAD	dendritic_cell	*SLC18B1*	TRUE	2.282181041
CAD	dendritic_cell	*HSD17B12*	TRUE	2.301192854
CAD	dendritic_cell	*LMOD1*	TRUE	2.044784769
CAD	dendritic_cell	*IRS1*	TRUE	2.197510581
CAD	dendritic_cell	*RPS26*	TRUE	2.126907427
CAD	dendritic_cell	*LY6G5B*	TRUE	2.562710878
CAD	endothelial	*MAPK8IP2*	TRUE	2.055647747
CAD	endothelial	*MTMR11*	TRUE	2.00881158
CAD	endothelial	*TIPIN*	TRUE	2.090121365
CAD	endothelial	*BCHE*	TRUE	2.767159137
CAD	endothelial	*SNRPC*	TRUE	2.031101176
CAD	endothelial	*GRK4*	TRUE	2.785583095
CAD	endothelial	*REEP2*	TRUE	2.040257069
CAD	endothelial	*POLR1E*	TRUE	2.271453174
CAD	endothelial	*AMT*	TRUE	2.155172436
CAD	endothelial	*IRS1*	TRUE	3.084076827
CAD	endothelial	*ZNF443*	TRUE	2.162994259
CAD	endothelial	*PROB1*	TRUE	2.183659754
CAD	endothelial	*LY6G5B*	TRUE	2.690644156
CAD	endothelial	*TDGF1*	TRUE	2.306845259
CAD	LEC	*SEMA3F*	TRUE	2.350681446
CAD	LEC	*TIPIN*	TRUE	2.674204855
CAD	LEC	*KCNH4*	TRUE	2.717109524
CAD	LEC	*SUSD2*	TRUE	2.245179744
CAD	LEC	*BNIP1*	TRUE	2.149195351
CAD	LEC	*SNRPC*	TRUE	2.858267946
CAD	LEC	*GRK4*	TRUE	2.856727826
CAD	LEC	*POLR1E*	TRUE	2.410053175
CAD	LEC	*CLEC18A*	TRUE	2.486157797
CAD	LEC	*IRS1*	TRUE	2.802293763
CAD	LEC	*ACOT1*	TRUE	2.219057042

CAD	LEC	*TAS2R14*	TRUE	2.092158661
CAD	LEC	*LY6G5B*	TRUE	2.510296679
CAD	macrophage	*DLX6*	TRUE	2.930276455
CAD	macrophage	*UHRF1BP1*	TRUE	2.308445902
CAD	macrophage	*TIE1*	TRUE	2.310768448
CAD	macrophage	*TIPIN*	TRUE	3.386678977
CAD	macrophage	*SUSD2*	TRUE	2.248024667
CAD	macrophage	*MAP1LC3A*	TRUE	3.014619225
CAD	macrophage	*CTSH*	TRUE	2.09898779
CAD	macrophage	*MAG*	TRUE	2.175770046
CAD	macrophage	*STEAP1B*	TRUE	1.972830136
CAD	macrophage	*ENDOU*	TRUE	2.464887648
CAD	macrophage	*BVES*	TRUE	2.122105146
CAD	macrophage	*BNIP1*	TRUE	2.720277316
CAD	macrophage	*BCHE*	TRUE	2.962551905
CAD	macrophage	*PLXNA1*	TRUE	2.942534072
CAD	macrophage	*GGCX*	TRUE	3.474000563
CAD	macrophage	*EIF2B2*	TRUE	2.615341747
CAD	macrophage	*GRK4*	TRUE	3.290424662
CAD	macrophage	*REEP2*	TRUE	2.014952345
CAD	macrophage	*POLR1E*	TRUE	3.153632038
CAD	macrophage	*TMEM91*	TRUE	2.615429246
CAD	macrophage	*HORMAD1*	TRUE	2.087007064
CAD	macrophage	*NKD2*	TRUE	1.98893723
CAD	macrophage	*PSD2*	TRUE	2.95189354
CAD	macrophage	*SLC18B1*	TRUE	1.993059478
CAD	macrophage	*HSD17B12*	TRUE	2.785605496
CAD	macrophage	*TTC12*	TRUE	2.289087677
CAD	macrophage	*DUSP15*	TRUE	2.594143569
CAD	macrophage	*DISP1*	TRUE	2.591068098
CAD	macrophage	*CLEC18A*	TRUE	2.689571402
CAD	macrophage	*MX1*	TRUE	2.030848638
CAD	macrophage	*LMOD1*	TRUE	2.241234559
CAD	macrophage	*ICA1L*	TRUE	3.436455347
CAD	macrophage	*APEH*	TRUE	2.604573955
CAD	macrophage	*GPER1*	TRUE	2.483142643
CAD	macrophage	*OXGR1*	TRUE	3.389402635
CAD	macrophage	*MEI1*	TRUE	2.005891976
CAD	macrophage	*ZNF641*	TRUE	2.390036252
CAD	macrophage	*KIFC2*	TRUE	2.368234425
CAD	macrophage	*ULK4*	TRUE	2.457117488
CAD	macrophage	*MLKL*	TRUE	2.009027475
CAD	macrophage	*IRS1*	TRUE	4.538399626
CAD	macrophage	*PCSK9*	TRUE	3.477465989
CAD	macrophage	*LPL*	TRUE	3.969263325
CAD	macrophage	*PAAF1*	TRUE	2.405030655
CAD	macrophage	*RIMKLA*	TRUE	2.143152329
CAD	macrophage	*TIGD5*	TRUE	1.965063697

CAD	macrophage	*ZNF443*	TRUE	2.613962978
CAD	macrophage	*TMEM136*	TRUE	2.361721821
CAD	macrophage	*PLCXD3*	TRUE	2.018455444
CAD	macrophage	*ZNF320*	TRUE	1.996363798
CAD	macrophage	*BMP8A*	TRUE	2.026544514
CAD	macrophage	*ACOT1*	TRUE	2.264665172
CAD	macrophage	*GDF3*	TRUE	2.01753766
CAD	macrophage	*A3GALT2*	TRUE	2.129018775
CAD	macrophage	*MSL1*	TRUE	2.497255846
CAD	macrophage	*C6orf106*	TRUE	2.680975981
CAD	macrophage	*KIAA1211L*	TRUE	2.018634659
CAD	macrophage	*WDR5B*	TRUE	2.760916881
CAD	macrophage	*ZNF334*	TRUE	2.081625606
CAD	macrophage	*TMEM116*	TRUE	2.806013242
CAD	macrophage	*TBKBP1*	TRUE	2.361449035
CAD	macrophage	*CENPW*	TRUE	2.615036568
CAD	macrophage	*PROB1*	TRUE	2.088870011
CAD	macrophage	*AC112229.4*	TRUE	2.08572987
CAD	macrophage	*GOLGA8N*	TRUE	1.988686136
CAD	macrophage	*LINC00957*	TRUE	1.984304024
CAD	macrophage	*LY6G5B*	TRUE	2.784369296
CAD	macrophage	*ARMS2*	TRUE	2.140304427
CAD	macrophage	*ZNF10*	TRUE	2.000533004
CAD	macrophage	*HMBS*	TRUE	3.629335938
CAD	macrophage	*GALNT4*	TRUE	2.342183723
CAD	macrophage	*AC144652.1*	TRUE	1.97647797
CAD	mast_cell	*DLX6*	TRUE	2.017693815
CAD	mast_cell	*UHRF1BP1*	TRUE	1.981500504
CAD	mast_cell	*TIPIN*	TRUE	2.57328573
CAD	mast_cell	*BCHE*	TRUE	2.549755995
CAD	mast_cell	*GRK4*	TRUE	3.015009824
CAD	mast_cell	*POLR1E*	TRUE	2.730811721
CAD	mast_cell	*APEH*	TRUE	2.058192528
CAD	mast_cell	*OXGR1*	TRUE	2.70052901
CAD	mast_cell	*IRS1*	TRUE	4.141678725
CAD	mast_cell	*LPL*	TRUE	2.694073594
CAD	mast_cell	*ZNF443*	TRUE	2.156038737
CAD	mast_cell	*C6orf106*	TRUE	1.988811002
CAD	mast_cell	*RPS26*	TRUE	1.976798981
CAD	mast_cell	*TMEM116*	TRUE	1.999004096
CAD	mast_cell	*CENPW*	TRUE	3.062914363
CAD	mast_cell	*LY6G5B*	TRUE	2.968903839
CAD	monocyte	*UHRF1BP1*	TRUE	2.461823054
CAD	monocyte	*MAG*	TRUE	2.102730688
CAD	monocyte	*GGCX*	TRUE	2.809831934
CAD	monocyte	*WFDC3*	TRUE	2.176435791
CAD	monocyte	*PSD2*	TRUE	3.47306611
CAD	monocyte	*DUSP15*	TRUE	2.390614082

CAD	monocyte	*DISP1*	TRUE	2.22382412
CAD	monocyte	*OXGR1*	TRUE	2.803042257
CAD	monocyte	*RPS26*	TRUE	2.186482648
CAD	monocyte	*LY6G5B*	TRUE	2.632236354
CAD	monocyte	*HMBS*	TRUE	2.83041155
CAD	neutrophil	*TIPIN*	TRUE	2.161490597
CAD	neutrophil	*CTSH*	TRUE	1.964151594
CAD	neutrophil	*CLEC18A*	TRUE	2.230786239
CAD	neutrophil	*APEH*	TRUE	2.227861318
CAD	neutrophil	*OXGR1*	TRUE	2.465778307
CAD	neutrophil	*ACOT1*	TRUE	2.182341051
CAD	neutrophil	*LY6G5B*	TRUE	2.731785267
CAD	nk_cell	*UHRF1BP1*	TRUE	2.213446403
CAD	nk_cell	*SUSD2*	TRUE	2.196145852
CAD	nk_cell	*BNIP1*	TRUE	2.215638145
CAD	nk_cell	*GRK4*	TRUE	3.132143275
CAD	nk_cell	*POLR1E*	TRUE	2.481015759
CAD	nk_cell	*CLEC18A*	TRUE	2.69241437
CAD	nk_cell	*OXGR1*	TRUE	3.195215735
CAD	nk_cell	*KIFC2*	TRUE	2.027537601
CAD	nk_cell	*IRS1*	TRUE	2.980911345
CAD	nk_cell	*C8orf46*	TRUE	1.995719019
CAD	nk_cell	*PPFIA3*	TRUE	2.169129644
CAD	nk_cell	*BMP8A*	TRUE	1.985923514
CAD	nk_cell	*TMEM116*	TRUE	2.408320388
CAD	nk_cell	*LY6G5B*	TRUE	2.687885556
CAD	nk_cell	*ARMS2*	TRUE	2.376374411
CAD	nk_cell	*HMBS*	TRUE	3.437293498
CAD	pericyte	*TIPIN*	TRUE	2.891701056
CAD	pericyte	*KCNH4*	TRUE	2.275439972
CAD	pericyte	*MAP1LC3A*	TRUE	2.305001676
CAD	pericyte	*AMH*	TRUE	2.178911667
CAD	pericyte	*BNIP1*	TRUE	2.714138397
CAD	pericyte	*PLXNA1*	TRUE	2.570512706
CAD	pericyte	*GGCX*	TRUE	3.284086892
CAD	pericyte	*EIF2B2*	TRUE	2.535011136
CAD	pericyte	*HIF3A*	TRUE	2.240865204
CAD	pericyte	*GRK4*	TRUE	2.323336927
CAD	pericyte	*B4GALNT1*	TRUE	2.426697937
CAD	pericyte	*HSD17B12*	TRUE	2.459785247
CAD	pericyte	*TTC12*	TRUE	2.198577907
CAD	pericyte	*DISP1*	TRUE	2.839889488
CAD	pericyte	*ICA1L*	TRUE	4.301414328
CAD	pericyte	*RNF123*	TRUE	3.062461261
CAD	pericyte	*OXGR1*	TRUE	3.054938133
CAD	pericyte	*KIFC2*	TRUE	2.307829131
CAD	pericyte	*IRS1*	TRUE	2.737092815
CAD	pericyte	*LPL*	TRUE	2.448487243

CAD	pericyte	*PAAF1*	TRUE	2.049830411
CAD	pericyte	*PLCXD3*	TRUE	2.28958668
CAD	pericyte	*A3GALT2*	TRUE	2.226877844
CAD	pericyte	*C6orf106*	TRUE	2.376115362
CAD	pericyte	*WDR5B*	TRUE	2.366441057
CAD	pericyte	*RPS26*	TRUE	2.102459967
CAD	pericyte	*ZNF717*	TRUE	2.063464115
CAD	pericyte	*HMBS*	TRUE	3.094397254
CAD	pericyte	*GALNT4*	TRUE	1.961010224
CAD	SMC	*DLX6*	TRUE	2.341780013
CAD	SMC	*TIE1*	TRUE	2.323356877
CAD	SMC	*TIPIN*	TRUE	2.677980488
CAD	SMC	*MAP1LC3A*	TRUE	2.538028985
CAD	SMC	*CTSH*	TRUE	1.980312181
CAD	SMC	*MAG*	TRUE	2.064346172
CAD	SMC	*ENDOU*	TRUE	2.364815093
CAD	SMC	*BNIP1*	TRUE	2.084100945
CAD	SMC	*BCHE*	TRUE	3.046769744
CAD	SMC	*GGCX*	TRUE	3.007453187
CAD	SMC	*EIF2B2*	TRUE	2.01165929
CAD	SMC	*GRK4*	TRUE	3.248969846
CAD	SMC	*POLR1E*	TRUE	2.926446278
CAD	SMC	*CARF*	TRUE	1.984887838
CAD	SMC	*PSD2*	TRUE	3.227535884
CAD	SMC	*SLC18B1*	TRUE	2.329688168
CAD	SMC	*HSD17B12*	TRUE	2.668953365
CAD	SMC	*DISP1*	TRUE	2.827228609
CAD	SMC	*CLEC18A*	TRUE	2.800506576
CAD	SMC	*GPER1*	TRUE	2.193387273
CAD	SMC	*OXGR1*	TRUE	3.19744978
CAD	SMC	*ZNF641*	TRUE	1.991044827
CAD	SMC	*KIFC2*	TRUE	2.153501086
CAD	SMC	*ULK4*	TRUE	2.153978901
CAD	SMC	*IRS1*	TRUE	4.293967954
CAD	SMC	*LPL*	TRUE	3.440138753
CAD	SMC	*PAAF1*	TRUE	2.189294592
CAD	SMC	*PLCXD3*	TRUE	2.100344035
CAD	SMC	*A3GALT2*	TRUE	2.164096169
CAD	SMC	*C6orf106*	TRUE	2.614582032
CAD	SMC	*RPS26*	TRUE	2.103965675
CAD	SMC	*TMEM116*	TRUE	2.816241195
CAD	SMC	*LY6G5B*	TRUE	2.137342533
CAD	SMC	*HMBS*	TRUE	3.457274449
CAD	SMC	*GALNT4*	TRUE	2.08190783
CAD	t_cell	*DLX6*	TRUE	2.288933742
CAD	t_cell	*TIPIN*	TRUE	2.686978623
CAD	t_cell	*MAG*	TRUE	2.099578452
CAD	t_cell	*BNIP1*	TRUE	2.197307988

CAD	t_cell	*GGCX*	TRUE	2.666313952
CAD	t_cell	*GRK4*	TRUE	2.821626948
CAD	t_cell	*HORMAD1*	TRUE	1.996406938
CAD	t_cell	*SLC18B1*	TRUE	2.301510607
CAD	t_cell	*CLEC18A*	TRUE	2.142923865
CAD	t_cell	*APEH*	TRUE	3.459601377
CAD	t_cell	*OXGR1*	TRUE	2.598095029
CAD	t_cell	*ULK4*	TRUE	2.08382799
CAD	t_cell	*IRS1*	TRUE	2.676964775
CAD	t_cell	*BANF1*	TRUE	2.149693563
CAD	t_cell	*RIMKLA*	TRUE	2.056546569
CAD	t_cell	*GDF3*	TRUE	2.092093497
CAD	t_cell	*LY6G5B*	TRUE	2.484267661
CAD	t_cell	*HMBS*	TRUE	3.094333325
T2DM	adipocyte	*RBM6*	TRUE	2.123004519
T2DM	adipocyte	*CEP68*	TRUE	5.137788013
T2DM	adipocyte	*BAK1*	TRUE	2.069604233
T2DM	adipocyte	*TUBG2*	TRUE	2.56955796
T2DM	adipocyte	*RAB27B*	TRUE	2.363349114
T2DM	adipocyte	*TSPAN17*	TRUE	2.065109323
T2DM	adipocyte	*ITIH4*	TRUE	2.626923602
T2DM	adipocyte	*ASNS*	TRUE	1.986379847
T2DM	adipocyte	*STXBP2*	TRUE	2.33279497
T2DM	adipocyte	*LRCH4*	TRUE	2.631248262
T2DM	adipocyte	*COBLL1*	TRUE	2.908374924
T2DM	adipocyte	*SNX10*	TRUE	2.582655729
T2DM	adipocyte	*C3orf18*	TRUE	1.961481327
T2DM	adipocyte	*CWF19L1*	TRUE	2.627690609
T2DM	adipocyte	*ISM2*	TRUE	2.263778199
T2DM	adipocyte	*RBL2*	TRUE	2.836879663
T2DM	adipocyte	*IGDCC4*	TRUE	2.057743866
T2DM	adipocyte	*EYA1*	TRUE	2.549025243
T2DM	adipocyte	*TLE6*	TRUE	2.02033537
T2DM	adipocyte	*ATP13A1*	TRUE	4.092632628
T2DM	adipocyte	*CDK2AP1*	TRUE	2.199772431
T2DM	adipocyte	*ENDOU*	TRUE	2.732664831
T2DM	adipocyte	*BVES*	TRUE	2.49930332
T2DM	adipocyte	*NPHP3*	TRUE	2.361135099
T2DM	adipocyte	*SDC1*	TRUE	2.266824152
T2DM	adipocyte	*GPX7*	TRUE	2.947060504
T2DM	adipocyte	*PLA2G2D*	TRUE	2.19195994
T2DM	adipocyte	*CD3EAP*	TRUE	2.452388762
T2DM	adipocyte	*ELOVL4*	TRUE	2.900031604
T2DM	adipocyte	*DNAH7*	TRUE	3.611225987
T2DM	adipocyte	*ADRA1A*	TRUE	2.895577027
T2DM	adipocyte	*TEX14*	TRUE	2.718853519
T2DM	adipocyte	*GJB6*	TRUE	2.177984587
T2DM	adipocyte	*GRK4*	TRUE	2.949232707

T2DM	adipocyte	*ZNF436*	TRUE	2.45691385
T2DM	adipocyte	*PRR12*	TRUE	2.533385322
T2DM	adipocyte	*RIBC2*	TRUE	2.755455083
T2DM	adipocyte	*OPN1SW*	TRUE	2.205358165
T2DM	adipocyte	*PKDREJ*	TRUE	2.479969347
T2DM	adipocyte	*P2RX4*	TRUE	2.314869115
T2DM	adipocyte	*NACAD*	TRUE	2.578596147
T2DM	adipocyte	*MYOF*	TRUE	2.010753219
T2DM	adipocyte	*LRRC46*	TRUE	2.674047196
T2DM	adipocyte	*FN3KRP*	TRUE	2.128025078
T2DM	adipocyte	*DUS3L*	TRUE	2.290355502
T2DM	adipocyte	*ARHGEF19*	TRUE	2.232602028
T2DM	adipocyte	*PLA1A*	TRUE	2.338996578
T2DM	adipocyte	*BHMT*	TRUE	2.803779577
T2DM	adipocyte	*PSD2*	TRUE	2.174117218
T2DM	adipocyte	*HSD17B12*	TRUE	4.716345533
T2DM	adipocyte	*APIP*	TRUE	3.31384862
T2DM	adipocyte	*DUSP15*	TRUE	2.279950585
T2DM	adipocyte	*PPP1R1C*	TRUE	2.042785557
T2DM	adipocyte	*PRSS53*	TRUE	1.994926147
T2DM	adipocyte	*TSEN2*	TRUE	2.939756845
T2DM	adipocyte	*CLEC18A*	TRUE	2.570465636
T2DM	adipocyte	*KCNJ15*	TRUE	2.181156136
T2DM	adipocyte	*AP3S2*	TRUE	5.348464272
T2DM	adipocyte	*C12orf43*	TRUE	2.02060351
T2DM	adipocyte	*CD1E*	TRUE	2.773513974
T2DM	adipocyte	*VPS28*	TRUE	2.158849177
T2DM	adipocyte	*LMX1A*	TRUE	2.561715303
T2DM	adipocyte	*SPTA1*	TRUE	2.648783257
T2DM	adipocyte	*BDH2*	TRUE	2.264412595
T2DM	adipocyte	*TRMT61A*	TRUE	3.606485223
T2DM	adipocyte	*PCBD1*	TRUE	3.311256647
T2DM	adipocyte	*LTBP3*	TRUE	3.145677764
T2DM	adipocyte	*PXK*	TRUE	2.206381394
T2DM	adipocyte	*IRS1*	TRUE	9.972414838
T2DM	adipocyte	*C1QTNF4*	TRUE	2.760470913
T2DM	adipocyte	*FIBP*	TRUE	2.064705239
T2DM	adipocyte	*VEGFB*	TRUE	3.087011103
T2DM	adipocyte	*IL20RB*	TRUE	2.022100075
T2DM	adipocyte	*KLF14*	TRUE	3.018199544
T2DM	adipocyte	*STX19*	TRUE	3.056439546
T2DM	adipocyte	*B3GNT3*	TRUE	3.074123683
T2DM	adipocyte	*ZNF320*	TRUE	2.96271273
T2DM	adipocyte	*DGCR6*	TRUE	2.060394564
T2DM	adipocyte	*RP1L1*	TRUE	2.954753953
T2DM	adipocyte	*MROH7*	TRUE	2.211693686
T2DM	adipocyte	*NDUFA6*	TRUE	2.323298882
T2DM	adipocyte	*LINC00839*	TRUE	3.260223073

T2DM	adipocyte	*RGS7BP*	TRUE	2.250172338
T2DM	adipocyte	*MCRS1*	TRUE	2.079953887
T2DM	adipocyte	*NUTM2B*	TRUE	2.627287164
T2DM	adipocyte	*TMEM201*	TRUE	2.047988178
T2DM	adipocyte	*ALKBH2*	TRUE	2.149698453
T2DM	adipocyte	*TMEM116*	TRUE	2.12283746
T2DM	adipocyte	*HLA.DRB5*	TRUE	2.23923545
T2DM	adipocyte	*CENPW*	TRUE	3.81589737
T2DM	adipocyte	*FAM229B*	TRUE	2.237720207
T2DM	adipocyte	*NELFE*	TRUE	2.239326743
T2DM	adipocyte	*LINC01123*	TRUE	2.261323516
T2DM	adipocyte	*AC016586.1*	TRUE	1.990290456
T2DM	adipocyte	*SERF1B*	TRUE	2.392719752
T2DM	adipocyte	*LIN52*	TRUE	2.147077541
T2DM	adipocyte	*DNLZ*	TRUE	3.627030801
T2DM	adipocyte	*SIPA1*	TRUE	2.039938586
T2DM	adipocyte	*STARD10*	TRUE	4.79453349
T2DM	adipocyte	*AC073133.1*	TRUE	2.096561035
T2DM	adipocyte	*GS1.124K5.4*	TRUE	2.216529451
T2DM	adipocyte	*HLA.DQA2*	TRUE	4.734210266
T2DM	adipocyte	*HMBS*	TRUE	3.694390722
T2DM	adipocyte	*LINC00639*	TRUE	2.249279336
T2DM	ASPC	*RBM6*	TRUE	2.663426375
T2DM	ASPC	*ZNF195*	TRUE	2.657226648
T2DM	ASPC	*GGNBP2*	TRUE	2.442958539
T2DM	ASPC	*CEP68*	TRUE	5.536380814
T2DM	ASPC	*BAK1*	TRUE	2.032409976
T2DM	ASPC	*TUBG2*	TRUE	2.83320425
T2DM	ASPC	*RAB27B*	TRUE	2.526378425
T2DM	ASPC	*MED17*	TRUE	2.015385772
T2DM	ASPC	*TSPAN17*	TRUE	2.348158857
T2DM	ASPC	*EPN3*	TRUE	2.176081041
T2DM	ASPC	*ITIH4*	TRUE	2.466739413
T2DM	ASPC	*ASNS*	TRUE	2.145536354
T2DM	ASPC	*SDHA*	TRUE	2.471446738
T2DM	ASPC	*MARK3*	TRUE	2.94832361
T2DM	ASPC	*XAB2*	TRUE	2.630670586
T2DM	ASPC	*STXBP2*	TRUE	2.415610893
T2DM	ASPC	*CTTNBP2*	TRUE	3.526144533
T2DM	ASPC	*LRCH4*	TRUE	3.121305496
T2DM	ASPC	*KCNN2*	TRUE	2.015510347
T2DM	ASPC	*COBLL1*	TRUE	4.902917954
T2DM	ASPC	*OVGP1*	TRUE	2.248177245
T2DM	ASPC	*CPNE3*	TRUE	2.005357523
T2DM	ASPC	*EPDR1*	TRUE	2.393225228
T2DM	ASPC	*CWF19L1*	TRUE	3.359276589
T2DM	ASPC	*PPP6R2*	TRUE	2.144886535
T2DM	ASPC	*ATP6V1D*	TRUE	2.089395066

T2DM	ASPC	*ISM2*	TRUE	2.580599705
T2DM	ASPC	*PSMB5*	TRUE	2.029037659
T2DM	ASPC	*DHODH*	TRUE	1.974950378
T2DM	ASPC	*RBL2*	TRUE	2.530265162
T2DM	ASPC	*IGDCC4*	TRUE	2.046926698
T2DM	ASPC	*EYA1*	TRUE	3.805854641
T2DM	ASPC	*DMPK*	TRUE	2.921438368
T2DM	ASPC	*TLE6*	TRUE	2.487911002
T2DM	ASPC	*CCDC61*	TRUE	2.560609701
T2DM	ASPC	*ICAM5*	TRUE	2.583365958
T2DM	ASPC	*GCDH*	TRUE	2.347344294
T2DM	ASPC	*ATP13A1*	TRUE	4.140529164
T2DM	ASPC	*CRHR2*	TRUE	1.978554951
T2DM	ASPC	*POLD2*	TRUE	3.613585573
T2DM	ASPC	*SYNGR2*	TRUE	1.975134284
T2DM	ASPC	*WFS1*	TRUE	7.14971946
T2DM	ASPC	*C11orf21*	TRUE	2.51461039
T2DM	ASPC	*CDK2AP1*	TRUE	2.211904207
T2DM	ASPC	*ENDOU*	TRUE	3.107038141
T2DM	ASPC	*BVES*	TRUE	3.198866622
T2DM	ASPC	*NPHP3*	TRUE	2.597643549
T2DM	ASPC	*UMPS*	TRUE	2.005694943
T2DM	ASPC	*EFCC1*	TRUE	2.264244776
T2DM	ASPC	*DNAJC27*	TRUE	2.837074822
T2DM	ASPC	*ITGB6*	TRUE	2.010610616
T2DM	ASPC	*RTN4*	TRUE	2.022913203
T2DM	ASPC	*CHMP3*	TRUE	4.006200453
T2DM	ASPC	*SDC1*	TRUE	2.357808281
T2DM	ASPC	*GPX7*	TRUE	3.121032696
T2DM	ASPC	*PLA2G2D*	TRUE	2.620685534
T2DM	ASPC	*TNFSF4*	TRUE	2.524679669
T2DM	ASPC	*CD3EAP*	TRUE	3.577499201
T2DM	ASPC	*ELOVL4*	TRUE	2.778092431
T2DM	ASPC	*DNAH7*	TRUE	2.991953888
T2DM	ASPC	*HDHD3*	TRUE	2.321641835
T2DM	ASPC	*EPCAM*	TRUE	1.966401788
T2DM	ASPC	*MASTL*	TRUE	2.312245396
T2DM	ASPC	*ADRA1A*	TRUE	3.678564059
T2DM	ASPC	*TEX14*	TRUE	2.898684408
T2DM	ASPC	*ECHDC2*	TRUE	2.122374175
T2DM	ASPC	*SEC22A*	TRUE	2.151451203
T2DM	ASPC	*GJB6*	TRUE	2.27572471
T2DM	ASPC	*RASL11A*	TRUE	2.714050952
T2DM	ASPC	*PMS2*	TRUE	2.349750432
T2DM	ASPC	*SSPN*	TRUE	1.998560193
T2DM	ASPC	*EBPL*	TRUE	2.011894473
T2DM	ASPC	*GLO1*	TRUE	2.05931237
T2DM	ASPC	*GRK4*	TRUE	3.385712553

T2DM	ASPC	*ZNF436*	TRUE	3.315588578
T2DM	ASPC	*PRR12*	TRUE	2.95062673
T2DM	ASPC	*WFIKKN1*	TRUE	1.983213474
T2DM	ASPC	*BAIAP2L2*	TRUE	2.047230424
T2DM	ASPC	*RIBC2*	TRUE	2.689305391
T2DM	ASPC	*OPN1SW*	TRUE	2.654722159
T2DM	ASPC	*DPP6*	TRUE	3.40494568
T2DM	ASPC	*GTPBP3*	TRUE	2.721038307
T2DM	ASPC	*HBZ*	TRUE	2.051224474
T2DM	ASPC	*PKDREJ*	TRUE	2.617817447
T2DM	ASPC	*P2RX4*	TRUE	2.173129482
T2DM	ASPC	*SMYD5*	TRUE	2.136792826
T2DM	ASPC	*TMBIM1*	TRUE	1.971681108
T2DM	ASPC	*AOAH*	TRUE	3.153689369
T2DM	ASPC	*NACAD*	TRUE	2.282655989
T2DM	ASPC	*SYTL2*	TRUE	2.529137029
T2DM	ASPC	*DCUN1D5*	TRUE	2.566297413
T2DM	ASPC	*MYOF*	TRUE	2.498686393
T2DM	ASPC	*BTBD16*	TRUE	2.090996514
T2DM	ASPC	*C1RL*	TRUE	2.114353595
T2DM	ASPC	*GPR84*	TRUE	2.618601791
T2DM	ASPC	*HMG20A*	TRUE	3.27001049
T2DM	ASPC	*SCAMP2*	TRUE	2.341661367
T2DM	ASPC	*VPS53*	TRUE	2.316384137
T2DM	ASPC	*SPATA22*	TRUE	2.012623739
T2DM	ASPC	*LRRC46*	TRUE	2.182981589
T2DM	ASPC	*NPC1*	TRUE	2.481166281
T2DM	ASPC	*FN3KRP*	TRUE	2.891783543
T2DM	ASPC	*DUS3L*	TRUE	2.561126727
T2DM	ASPC	*GEMIN7*	TRUE	2.32554949
T2DM	ASPC	*ARHGEF19*	TRUE	2.21211627
T2DM	ASPC	*TARS2*	TRUE	2.307456709
T2DM	ASPC	*WNT9A*	TRUE	2.051341078
T2DM	ASPC	*THSD7B*	TRUE	1.982553031
T2DM	ASPC	*PLA1A*	TRUE	2.997447039
T2DM	ASPC	*PPIP5K2*	TRUE	2.469512357
T2DM	ASPC	*CCDC25*	TRUE	2.615269033
T2DM	ASPC	*ZNF7*	TRUE	2.09656394
T2DM	ASPC	*HSD17B12*	TRUE	5.459795253
T2DM	ASPC	*APIP*	TRUE	3.739425888
T2DM	ASPC	*EIF3M*	TRUE	2.47729353
T2DM	ASPC	*DUSP15*	TRUE	2.688093884
T2DM	ASPC	*PPP1R1C*	TRUE	2.065735741
T2DM	ASPC	*ABCB9*	TRUE	2.615457
T2DM	ASPC	*ARL14EP*	TRUE	4.330037758
T2DM	ASPC	*POC5*	TRUE	3.170648278
T2DM	ASPC	*SAR1B*	TRUE	2.132328025
T2DM	ASPC	*PLA2R1*	TRUE	2.109145237

T2DM	ASPC	*JAZF1*	TRUE	7.709060541
T2DM	ASPC	*LONRF1*	TRUE	2.818210522
T2DM	ASPC	*TSEN2*	TRUE	3.628872863
T2DM	ASPC	*NDUFAF6*	TRUE	3.895361262
T2DM	ASPC	*WHAMM*	TRUE	2.444200085
T2DM	ASPC	*TIMP4*	TRUE	2.678692835
T2DM	ASPC	*CLEC18A*	TRUE	2.529193428
T2DM	ASPC	*KCNJ15*	TRUE	3.350286121
T2DM	ASPC	*AP3S2*	TRUE	6.199059306
T2DM	ASPC	*C12orf43*	TRUE	2.410123166
T2DM	ASPC	*CD1E*	TRUE	2.487014411
T2DM	ASPC	*ZNF276*	TRUE	2.286960593
T2DM	ASPC	*DIP2A*	TRUE	2.542576915
T2DM	ASPC	*FDPS*	TRUE	2.093872772
T2DM	ASPC	*EXTL2*	TRUE	2.139424753
T2DM	ASPC	*LMX1A*	TRUE	2.360235506
T2DM	ASPC	*LMOD1*	TRUE	2.656928622
T2DM	ASPC	*SPTA1*	TRUE	2.563237143
T2DM	ASPC	*RFT1*	TRUE	2.681163826
T2DM	ASPC	*BDH2*	TRUE	3.219077895
T2DM	ASPC	*REEP3*	TRUE	2.245860274
T2DM	ASPC	*CEP57*	TRUE	2.030073774
T2DM	ASPC	*PCBD1*	TRUE	3.762238211
T2DM	ASPC	*SAAL1*	TRUE	4.878734999
T2DM	ASPC	*VKORC1*	TRUE	2.019245899
T2DM	ASPC	*PSCA*	TRUE	2.496223137
T2DM	ASPC	*LTBP3*	TRUE	3.154510034
T2DM	ASPC	*KIF5C*	TRUE	2.559351142
T2DM	ASPC	*PXK*	TRUE	2.773738183
T2DM	ASPC	*IRS1*	TRUE	10.6087442
T2DM	ASPC	*LRRC45*	TRUE	2.283266069
T2DM	ASPC	*EMB*	TRUE	3.415411977
T2DM	ASPC	*KCNS3*	TRUE	1.970117057
T2DM	ASPC	*ZNF561*	TRUE	2.107739844
T2DM	ASPC	*RGS19*	TRUE	2.693442939
T2DM	ASPC	*LAMB2*	TRUE	2.329410065
T2DM	ASPC	*C1QTNF4*	TRUE	2.721007792
T2DM	ASPC	*MYEOV*	TRUE	2.366480602
T2DM	ASPC	*HPSE2*	TRUE	2.116301482
T2DM	ASPC	*VEGFB*	TRUE	4.011853487
T2DM	ASPC	*IL20RB*	TRUE	2.324865748
T2DM	ASPC	*KLF14*	TRUE	5.06575773
T2DM	ASPC	*LPL*	TRUE	2.075509463
T2DM	ASPC	*HORMAD2*	TRUE	2.242496232
T2DM	ASPC	*C12orf54*	TRUE	2.089778284
T2DM	ASPC	*GOLGA8Q*	TRUE	1.960515554
T2DM	ASPC	*STX19*	TRUE	3.548276829
T2DM	ASPC	*B3GNT3*	TRUE	2.850446481

T2DM	ASPC	*GRID1*	TRUE	2.084839716
T2DM	ASPC	*ZNF320*	TRUE	3.247320772
T2DM	ASPC	*DGCR6*	TRUE	2.053940504
T2DM	ASPC	*RP1L1*	TRUE	2.948365911
T2DM	ASPC	*BMP8A*	TRUE	3.447068112
T2DM	ASPC	*EIF3C*	TRUE	2.341012062
T2DM	ASPC	*NDUFA6*	TRUE	2.420088915
T2DM	ASPC	*SH3BGR*	TRUE	2.019486642
T2DM	ASPC	*RASA3*	TRUE	2.958923857
T2DM	ASPC	*RGS7BP*	TRUE	3.228725591
T2DM	ASPC	*CEACAM19*	TRUE	2.083475121
T2DM	ASPC	*ADH1A*	TRUE	2.088671441
T2DM	ASPC	*MCRS1*	TRUE	2.597668916
T2DM	ASPC	*NUTM2B*	TRUE	2.416830026
T2DM	ASPC	*TMEM201*	TRUE	2.589753832
T2DM	ASPC	*ALKBH2*	TRUE	2.560986976
T2DM	ASPC	*GTF2IRD2*	TRUE	2.611126767
T2DM	ASPC	*ZNF775*	TRUE	1.976030759
T2DM	ASPC	*MYO6*	TRUE	2.818077315
T2DM	ASPC	*FAM180B*	TRUE	2.389941946
T2DM	ASPC	*TRPV1*	TRUE	2.19110306
T2DM	ASPC	*ZNF33B*	TRUE	2.272240747
T2DM	ASPC	*C6orf106*	TRUE	1.985985744
T2DM	ASPC	*TBC1D9B*	TRUE	2.408509376
T2DM	ASPC	*TTC30A*	TRUE	2.119830778
T2DM	ASPC	*PRIM1*	TRUE	3.139404227
T2DM	ASPC	*TMEM116*	TRUE	2.844059813
T2DM	ASPC	*ZNF568*	TRUE	2.484986223
T2DM	ASPC	*CENPW*	TRUE	4.258509016
T2DM	ASPC	*FAM229B*	TRUE	2.84779676
T2DM	ASPC	*ASAH2B*	TRUE	2.044204013
T2DM	ASPC	*AGAP6*	TRUE	2.116494743
T2DM	ASPC	*OXLD1*	TRUE	2.698211851
T2DM	ASPC	*NELFE*	TRUE	2.186369767
T2DM	ASPC	*LINC01123*	TRUE	3.096652264
T2DM	ASPC	*SERF1B*	TRUE	2.703285016
T2DM	ASPC	*LIN52*	TRUE	2.893965293
T2DM	ASPC	*HCG27*	TRUE	2.603986188
T2DM	ASPC	*FGFR1OP*	TRUE	2.908418156
T2DM	ASPC	*DNLZ*	TRUE	3.479743981
T2DM	ASPC	*STARD10*	TRUE	4.874119353
T2DM	ASPC	*KCNU1*	TRUE	2.597346709
T2DM	ASPC	*AC062017.1*	TRUE	2.624284821
T2DM	ASPC	*AC073133.1*	TRUE	2.241501026
T2DM	ASPC	*GS1.124K5.4*	TRUE	2.720421854
T2DM	ASPC	*HLA.DQA2*	TRUE	1.9640459
T2DM	ASPC	*NME1.NME2*	TRUE	2.481532077
T2DM	ASPC	*JMJD7*	TRUE	2.064874966

T2DM	ASPC	*CCDC13*	TRUE	2.183105272
T2DM	ASPC	*ZNF286B*	TRUE	2.042455404
T2DM	ASPC	*DNAH10OS*	TRUE	2.207458985
T2DM	ASPC	*HMBS*	TRUE	4.127560543
T2DM	ASPC	*LINC00639*	TRUE	2.693230294
T2DM	ASPC	*AC083843.1*	TRUE	3.224863475
T2DM	ASPC	*SH3RF3.AS1*	TRUE	2.043556027
T2DM	ASPC	*LINC01140*	TRUE	2.102249238
T2DM	b_cell	*HSD17B1*	TRUE	2.781436496
T2DM	b_cell	*ELOVL4*	TRUE	2.274684984
T2DM	b_cell	*CLEC18A*	TRUE	4.731854925
T2DM	b_cell	*IRS1*	TRUE	4.947658968
T2DM	b_cell	*C1QTNF4*	TRUE	2.027687111
T2DM	b_cell	*HLA.DRB5*	TRUE	3.573328328
T2DM	b_cell	*HCG27*	TRUE	2.04413938
T2DM	b_cell	*DNLZ*	TRUE	3.277140028
T2DM	b_cell	*STARD10*	TRUE	3.135235559
T2DM	b_cell	*PLA2G4B*	TRUE	3.033452217
T2DM	dendritic_cell	*TUBG2*	TRUE	2.154334835
T2DM	dendritic_cell	*ITIH4*	TRUE	2.243828394
T2DM	dendritic_cell	*BVES*	TRUE	2.616038881
T2DM	dendritic_cell	*GPX7*	TRUE	2.96729314
T2DM	dendritic_cell	*ADRA1A*	TRUE	2.072001007
T2DM	dendritic_cell	*SLC12A5*	TRUE	2.189298263
T2DM	dendritic_cell	*MAN2C1*	TRUE	2.710871506
T2DM	dendritic_cell	*HSD17B12*	TRUE	4.188215128
T2DM	dendritic_cell	*KCNJ15*	TRUE	2.598510303
T2DM	dendritic_cell	*TRMT61A*	TRUE	3.191888447
T2DM	dendritic_cell	*IRS1*	TRUE	7.014624126
T2DM	dendritic_cell	*ZNF320*	TRUE	2.978021522
T2DM	dendritic_cell	*NOTCH4*	TRUE	5.01507201
T2DM	dendritic_cell	*DNLZ*	TRUE	3.319620962
T2DM	dendritic_cell	*STARD10*	TRUE	4.456834413
T2DM	dendritic_cell	*PLA2G4B*	TRUE	2.911348345
T2DM	endothelial	*RBM6*	TRUE	2.513309128
T2DM	endothelial	*TUBG2*	TRUE	3.88361392
T2DM	endothelial	*IGDCC4*	TRUE	2.226054887
T2DM	endothelial	*CDK2AP1*	TRUE	3.236833785
T2DM	endothelial	*GNB3*	TRUE	2.238358743
T2DM	endothelial	*GPX7*	TRUE	2.448051897
T2DM	endothelial	*DNAH7*	TRUE	2.049098036
T2DM	endothelial	*MAN2C1*	TRUE	2.407121864
T2DM	endothelial	*LRRC46*	TRUE	2.533174871
T2DM	endothelial	*BHMT*	TRUE	2.647080953
T2DM	endothelial	*APIP*	TRUE	2.369083356
T2DM	endothelial	*CLEC18A*	TRUE	2.577332424
T2DM	endothelial	*AP3S2*	TRUE	3.623424637
T2DM	endothelial	*IRS1*	TRUE	8.936685996

T2DM	endothelial	*RGS19*	TRUE	2.459362287
T2DM	endothelial	*C1QTNF4*	TRUE	2.884938911
T2DM	endothelial	*CTSW*	TRUE	2.486470295
T2DM	endothelial	*ZNF320*	TRUE	2.105499684
T2DM	endothelial	*DNLZ*	TRUE	3.027718399
T2DM	endothelial	*HLA.DQA2*	TRUE	3.496705018
T2DM	endothelial	*JMJD7*	TRUE	2.421783512
T2DM	LEC	*ITIH4*	TRUE	2.838004095
T2DM	LEC	*CDK2AP1*	TRUE	2.761094619
T2DM	LEC	*ADRA1A*	TRUE	2.367124293
T2DM	LEC	*GRK4*	TRUE	2.346105552
T2DM	LEC	*BHMT*	TRUE	2.882790717
T2DM	LEC	*HSD17B12*	TRUE	2.601725479
T2DM	LEC	*CLEC18A*	TRUE	4.462760124
T2DM	LEC	*AP3S2*	TRUE	4.48815426
T2DM	LEC	*IRS1*	TRUE	5.593120424
T2DM	LEC	*ZNF320*	TRUE	2.369142412
T2DM	LEC	*HLA.DRB5*	TRUE	2.481371155
T2DM	LEC	*DNLZ*	TRUE	2.360591976
T2DM	LEC	*STARD10*	TRUE	2.157439832
T2DM	LEC	*HLA.DQA2*	TRUE	5.471976001
T2DM	LEC	*JMJD7*	TRUE	2.713109768
T2DM	macrophage	*CEP68*	TRUE	3.937092829
T2DM	macrophage	*BAK1*	TRUE	1.987434141
T2DM	macrophage	*RAB27B*	TRUE	2.312886769
T2DM	macrophage	*ITIH4*	TRUE	2.177687168
T2DM	macrophage	*LRCH4*	TRUE	2.225328836
T2DM	macrophage	*SNX10*	TRUE	2.614854829
T2DM	macrophage	*CWF19L1*	TRUE	2.359613004
T2DM	macrophage	*ISM2*	TRUE	2.390754878
T2DM	macrophage	*EYA1*	TRUE	2.787650698
T2DM	macrophage	*TLE6*	TRUE	2.156751213
T2DM	macrophage	*ICAM5*	TRUE	2.070108255
T2DM	macrophage	*ATP13A1*	TRUE	3.450155194
T2DM	macrophage	*WFS1*	TRUE	3.504447116
T2DM	macrophage	*C11orf21*	TRUE	2.162142614
T2DM	macrophage	*COQ5*	TRUE	2.210126034
T2DM	macrophage	*ENDOU*	TRUE	2.698423245
T2DM	macrophage	*BVES*	TRUE	2.894382242
T2DM	macrophage	*NPHP3*	TRUE	2.034424878
T2DM	macrophage	*CHMP3*	TRUE	2.361120107
T2DM	macrophage	*GPX7*	TRUE	2.285750663
T2DM	macrophage	*SERPINC1*	TRUE	2.15398942
T2DM	macrophage	*ELOVL4*	TRUE	2.624432831
T2DM	macrophage	*DNAH7*	TRUE	2.8739288
T2DM	macrophage	*ADRA1A*	TRUE	3.309982996
T2DM	macrophage	*GRK4*	TRUE	2.99158606
T2DM	macrophage	*HBZ*	TRUE	2.039164141

T2DM	macrophage	*PKDREJ*	TRUE	2.546209489
T2DM	macrophage	*FN3KRP*	TRUE	2.264648592
T2DM	macrophage	*ARHGEF19*	TRUE	2.1968391
T2DM	macrophage	*PLA1A*	TRUE	2.669005774
T2DM	macrophage	*BHMT*	TRUE	2.518089515
T2DM	macrophage	*HSD17B12*	TRUE	4.525547696
T2DM	macrophage	*APIP*	TRUE	3.134255406
T2DM	macrophage	*DUSP15*	TRUE	2.373699975
T2DM	macrophage	*TSEN2*	TRUE	2.399427798
T2DM	macrophage	*CLEC18A*	TRUE	4.796961889
T2DM	macrophage	*KCNJ15*	TRUE	2.014266129
T2DM	macrophage	*AP3S2*	TRUE	5.450561353
T2DM	macrophage	*TRIM63*	TRUE	2.815454981
T2DM	macrophage	*CD1E*	TRUE	2.452894248
T2DM	macrophage	*TRMT61A*	TRUE	2.414300942
T2DM	macrophage	*PCBD1*	TRUE	2.878256692
T2DM	macrophage	*IRS1*	TRUE	9.813354449
T2DM	macrophage	*C8orf46*	TRUE	2.505019139
T2DM	macrophage	*RGS19*	TRUE	3.71956081
T2DM	macrophage	*C1QTNF4*	TRUE	3.14626282
T2DM	macrophage	*SNX32*	TRUE	2.168754252
T2DM	macrophage	*VEGFB*	TRUE	2.846563653
T2DM	macrophage	*HORMAD2*	TRUE	2.050197163
T2DM	macrophage	*TRIM73*	TRUE	2.727806471
T2DM	macrophage	*B3GNT3*	TRUE	2.422038405
T2DM	macrophage	*ZNF320*	TRUE	2.943651788
T2DM	macrophage	*DGCR6*	TRUE	1.994846286
T2DM	macrophage	*RP1L1*	TRUE	2.729501668
T2DM	macrophage	*BMP8A*	TRUE	5.018950449
T2DM	macrophage	*ZNF703*	TRUE	2.202140358
T2DM	macrophage	*NDUFA6*	TRUE	2.090987303
T2DM	macrophage	*LINC00839*	TRUE	2.720423862
T2DM	macrophage	*NUTM2B*	TRUE	2.071047795
T2DM	macrophage	*HLA.DRB1*	TRUE	2.247708682
T2DM	macrophage	*ZNF775*	TRUE	1.994009761
T2DM	macrophage	*FAM180B*	TRUE	2.239753395
T2DM	macrophage	*HCG27*	TRUE	2.98397739
T2DM	macrophage	*DNLZ*	TRUE	3.601924991
T2DM	macrophage	*STARD10*	TRUE	4.647814089
T2DM	macrophage	*GS1.124K5.4*	TRUE	2.435598898
T2DM	macrophage	*HLA.DQA2*	TRUE	3.035355499
T2DM	macrophage	*HMBS*	TRUE	3.835300507
T2DM	mast_cell	*CEP68*	TRUE	2.698612296
T2DM	mast_cell	*IGDCC4*	TRUE	2.027708126
T2DM	mast_cell	*ATP13A1*	TRUE	2.249330403
T2DM	mast_cell	*HSD17B1*	TRUE	3.560554199
T2DM	mast_cell	*CDK2AP1*	TRUE	2.139288648
T2DM	mast_cell	*NPHP3*	TRUE	1.963716205

T2DM	mast_cell	*GPX7*	TRUE	2.364795805
T2DM	mast_cell	*ELOVL4*	TRUE	2.257076703
T2DM	mast_cell	*DNAH7*	TRUE	2.551278599
T2DM	mast_cell	*ADRA1A*	TRUE	2.74737956
T2DM	mast_cell	*GRK4*	TRUE	2.935141647
T2DM	mast_cell	*OPRL1*	TRUE	2.376273539
T2DM	mast_cell	*PKDREJ*	TRUE	2.480193447
T2DM	mast_cell	*MAN2C1*	TRUE	2.265964908
T2DM	mast_cell	*HSD17B12*	TRUE	2.462446757
T2DM	mast_cell	*CLEC18A*	TRUE	2.079484857
T2DM	mast_cell	*AP3S2*	TRUE	4.834457856
T2DM	mast_cell	*RNF123*	TRUE	3.538327002
T2DM	mast_cell	*PCBD1*	TRUE	2.582360683
T2DM	mast_cell	*IRS1*	TRUE	9.448609989
T2DM	mast_cell	*C8orf46*	TRUE	2.405020577
T2DM	mast_cell	*ZNF320*	TRUE	2.566642403
T2DM	mast_cell	*CENPW*	TRUE	2.559829737
T2DM	mast_cell	*HCG27*	TRUE	2.213629564
T2DM	mast_cell	*DNLZ*	TRUE	3.59552318
T2DM	mast_cell	*HLA.DQA2*	TRUE	3.371848849
T2DM	mast_cell	*JMJD7*	TRUE	3.302683265
T2DM	mast_cell	*HMBS*	TRUE	2.126405114
T2DM	monocyte	*ITIH4*	TRUE	3.554075564
T2DM	monocyte	*C3orf18*	TRUE	2.336481097
T2DM	monocyte	*ATP13A1*	TRUE	3.57573678
T2DM	monocyte	*CLEC18A*	TRUE	3.004384099
T2DM	monocyte	*AP3S2*	TRUE	4.015930577
T2DM	monocyte	*IRS1*	TRUE	2.435763228
T2DM	monocyte	*C1QTNF4*	TRUE	2.957373351
T2DM	monocyte	*ZNF320*	TRUE	2.77183161
T2DM	monocyte	*HCG27*	TRUE	3.073206014
T2DM	monocyte	*DNLZ*	TRUE	3.468993802
T2DM	monocyte	*HLA.DQB2*	TRUE	2.27156721
T2DM	monocyte	*JMJD7*	TRUE	2.466033244
T2DM	monocyte	*HMBS*	TRUE	3.011010432
T2DM	neutrophil	*CDK2AP1*	TRUE	2.520547201
T2DM	neutrophil	*CLEC18A*	TRUE	4.596507401
T2DM	neutrophil	*C1QTNF4*	TRUE	3.465896503
T2DM	neutrophil	*HLA.DQB1*	TRUE	3.323520229
T2DM	neutrophil	*NOTCH4*	TRUE	3.634106395
T2DM	neutrophil	*DNLZ*	TRUE	3.669687852
T2DM	neutrophil	*STARD10*	TRUE	4.102438953
T2DM	neutrophil	*HLA.DQA2*	TRUE	3.987521252
T2DM	nk_cell	*TUBG2*	TRUE	2.812356349
T2DM	nk_cell	*ITIH4*	TRUE	3.469890839
T2DM	nk_cell	*SDC1*	TRUE	2.139015064
T2DM	nk_cell	*GRK4*	TRUE	2.447314707
T2DM	nk_cell	*MAN2C1*	TRUE	2.070244065

T2DM	nk_cell	*HSD17B12*	TRUE	2.846566039
T2DM	nk_cell	*APIP*	TRUE	2.076227027
T2DM	nk_cell	*CLEC18A*	TRUE	4.713698458
T2DM	nk_cell	*AP3S2*	TRUE	3.362098118
T2DM	nk_cell	*RNF123*	TRUE	1.997017389
T2DM	nk_cell	*IRS1*	TRUE	7.961525761
T2DM	nk_cell	*RGS19*	TRUE	2.057835025
T2DM	nk_cell	*C1QTNF4*	TRUE	3.020503137
T2DM	nk_cell	*ZNF320*	TRUE	2.319009137
T2DM	nk_cell	*BMP8A*	TRUE	2.907438252
T2DM	nk_cell	*EIF3C*	TRUE	2.160861682
T2DM	nk_cell	*DNLZ*	TRUE	3.499354622
T2DM	nk_cell	*STARD10*	TRUE	4.455253775
T2DM	nk_cell	*PLA2G4B*	TRUE	2.676418365
T2DM	nk_cell	*HMBS*	TRUE	3.238033788
T2DM	pericyte	*CEP68*	TRUE	3.507213883
T2DM	pericyte	*ITIH4*	TRUE	4.190404549
T2DM	pericyte	*ATP13A1*	TRUE	3.282342938
T2DM	pericyte	*WFS1*	TRUE	2.529199
T2DM	pericyte	*CDK2AP1*	TRUE	2.414709139
T2DM	pericyte	*NPHP3*	TRUE	2.105422064
T2DM	pericyte	*CD3EAP*	TRUE	2.034968743
T2DM	pericyte	*ELOVL4*	TRUE	2.03694906
T2DM	pericyte	*ADRA1A*	TRUE	3.127992843
T2DM	pericyte	*GRK4*	TRUE	2.233906855
T2DM	pericyte	*MAN2C1*	TRUE	2.080365396
T2DM	pericyte	*ARHGEF19*	TRUE	2.198817812
T2DM	pericyte	*PLA1A*	TRUE	2.541453933
T2DM	pericyte	*HSD17B12*	TRUE	4.237729372
T2DM	pericyte	*TSEN2*	TRUE	2.163078625
T2DM	pericyte	*CLEC18A*	TRUE	4.344083473
T2DM	pericyte	*AP3S2*	TRUE	3.892042002
T2DM	pericyte	*RNF123*	TRUE	2.999903613
T2DM	pericyte	*PCBD1*	TRUE	3.101046772
T2DM	pericyte	*IRS1*	TRUE	7.021617613
T2DM	pericyte	*C1QTNF4*	TRUE	1.970626271
T2DM	pericyte	*ZNF320*	TRUE	2.676670124
T2DM	pericyte	*HLA.DRB5*	TRUE	2.188527319
T2DM	pericyte	*HCG27*	TRUE	2.75764131
T2DM	pericyte	*DNLZ*	TRUE	3.338512508
T2DM	pericyte	*STARD10*	TRUE	4.57679239
T2DM	pericyte	*HLA.DQA2*	TRUE	4.985181301
T2DM	pericyte	*JMJD7*	TRUE	2.683016334
T2DM	pericyte	*HMBS*	TRUE	3.319200284
T2DM	SMC	*CEP68*	TRUE	3.698301756
T2DM	SMC	*TUBG2*	TRUE	2.033609582
T2DM	SMC	*LRCH4*	TRUE	2.476100524
T2DM	SMC	*COBLL1*	TRUE	2.205954558

T2DM	SMC	*IGDCC4*	TRUE	2.0022171
T2DM	SMC	*ATP13A1*	TRUE	2.156349473
T2DM	SMC	*CDK2AP1*	TRUE	2.504036229
T2DM	SMC	*ENDOU*	TRUE	2.69598799
T2DM	SMC	*GPX7*	TRUE	2.850440429
T2DM	SMC	*SERPINC1*	TRUE	2.313291482
T2DM	SMC	*DNAH7*	TRUE	2.101134048
T2DM	SMC	*ADRA1A*	TRUE	2.193465531
T2DM	SMC	*GRK4*	TRUE	2.862898861
T2DM	SMC	*RIBC2*	TRUE	2.478354563
T2DM	SMC	*OPN1SW*	TRUE	2.156838946
T2DM	SMC	*PKDREJ*	TRUE	2.113593795
T2DM	SMC	*MAN2C1*	TRUE	3.022859229
T2DM	SMC	*FN3KRP*	TRUE	2.127449338
T2DM	SMC	*PLA1A*	TRUE	2.799520647
T2DM	SMC	*HSD17B12*	TRUE	4.59826092
T2DM	SMC	*APIP*	TRUE	2.434387262
T2DM	SMC	*CLEC18A*	TRUE	3.985012014
T2DM	SMC	*AP3S2*	TRUE	5.247681874
T2DM	SMC	*CD1E*	TRUE	2.110296247
T2DM	SMC	*BDH2*	TRUE	2.113905121
T2DM	SMC	*RNF123*	TRUE	1.994011378
T2DM	SMC	*TRMT61A*	TRUE	3.536623995
T2DM	SMC	*PCBD1*	TRUE	2.015305998
T2DM	SMC	*IRS1*	TRUE	9.659558283
T2DM	SMC	*C8orf46*	TRUE	2.310066014
T2DM	SMC	*RGS19*	TRUE	2.421213584
T2DM	SMC	*C1QTNF4*	TRUE	2.848511046
T2DM	SMC	*B3GNT3*	TRUE	1.960446181
T2DM	SMC	*ZNF320*	TRUE	2.952353176
T2DM	SMC	*ZNF703*	TRUE	2.808347007
T2DM	SMC	*NDUFA6*	TRUE	2.030867869
T2DM	SMC	*TMEM116*	TRUE	1.995513354
T2DM	SMC	*HCG27*	TRUE	2.619058766
T2DM	SMC	*DNLZ*	TRUE	3.597636933
T2DM	SMC	*STARD10*	TRUE	4.453426907
T2DM	SMC	*GS1.124K5.4*	TRUE	2.550748167
T2DM	SMC	*NME1.NME2*	TRUE	2.05323325
T2DM	SMC	*JMJD7*	TRUE	2.25041293
T2DM	SMC	*HMBS*	TRUE	3.417269306
T2DM	t_cell	*CEP68*	TRUE	3.502300635
T2DM	t_cell	*EPN3*	TRUE	2.051840615
T2DM	t_cell	*ITIH4*	TRUE	3.447098804
T2DM	t_cell	*PABPC4*	TRUE	2.254602911
T2DM	t_cell	*CDK2AP1*	TRUE	2.052274354
T2DM	t_cell	*GNB3*	TRUE	2.263373793
T2DM	t_cell	*ADRA1A*	TRUE	2.627815325
T2DM	t_cell	*GRK4*	TRUE	2.087751121

T2DM	t_cell	*RIBC2*	TRUE	2.207018188
T2DM	t_cell	*MAN2C1*	TRUE	1.983108991
T2DM	t_cell	*CLEC18A*	TRUE	4.391325352
T2DM	t_cell	*KCNJ15*	TRUE	2.214816428
T2DM	t_cell	*AP3S2*	TRUE	5.074153104
T2DM	t_cell	*IRS1*	TRUE	7.472346283
T2DM	t_cell	*C8orf46*	TRUE	2.520911718
T2DM	t_cell	*C1QTNF4*	TRUE	2.013621181
T2DM	t_cell	*KLF14*	TRUE	3.854777999
T2DM	t_cell	*BANF1*	TRUE	1.969327413
T2DM	t_cell	*ZNF320*	TRUE	2.745709155
T2DM	t_cell	*DGCR6*	TRUE	2.022817088
T2DM	t_cell	*EIF3C*	TRUE	2.317633868
T2DM	t_cell	*HCG27*	TRUE	3.277533942
T2DM	t_cell	*DNLZ*	TRUE	3.517519232
T2DM	t_cell	*STARD10*	TRUE	4.260740725
T2DM	t_cell	*GS1.124K5.4*	TRUE	2.190471633
T2DM	t_cell	*HLA.DQA2*	TRUE	2.598593743
T2DM	t_cell	*HMBS*	TRUE	3.038366206
HTN	adipocyte	*WNT16*	TRUE	2.265284722
HTN	adipocyte	*COPZ2*	TRUE	2.365426091
HTN	adipocyte	*HFE*	TRUE	3.344896765
HTN	adipocyte	*CEP68*	TRUE	3.373523315
HTN	adipocyte	*SDCCAG8*	TRUE	2.015726735
HTN	adipocyte	*PIGV*	TRUE	3.31109912
HTN	adipocyte	*POLR1A*	TRUE	2.896716806
HTN	adipocyte	*SCGN*	TRUE	2.596328683
HTN	adipocyte	*SNX10*	TRUE	3.883118588
HTN	adipocyte	*PABPC4*	TRUE	2.721580599
HTN	adipocyte	*SLC7A4*	TRUE	2.403749691
HTN	adipocyte	*DNTTIP1*	TRUE	2.407954387
HTN	adipocyte	*POLI*	TRUE	3.28664977
HTN	adipocyte	*MTHFSD*	TRUE	2.430511193
HTN	adipocyte	*IGDCC4*	TRUE	2.187388001
HTN	adipocyte	*EYA1*	TRUE	2.592630617
HTN	adipocyte	*ICAM5*	TRUE	2.36266001
HTN	adipocyte	*ATP13A1*	TRUE	2.944721264
HTN	adipocyte	*HOXA2*	TRUE	2.492898805
HTN	adipocyte	*MEST*	TRUE	2.121031865
HTN	adipocyte	*KCTD10*	TRUE	2.466810314
HTN	adipocyte	*ENDOU*	TRUE	2.533007733
HTN	adipocyte	*PCDHB6*	TRUE	2.442652558
HTN	adipocyte	*NPHP3*	TRUE	2.187273491
HTN	adipocyte	*DNAJC27*	TRUE	2.261867099
HTN	adipocyte	*IRF6*	TRUE	2.430150209
HTN	adipocyte	*TCF21*	TRUE	3.002125576
HTN	adipocyte	*HDHD3*	TRUE	2.376835078
HTN	adipocyte	*VSX2*	TRUE	2.231733603

HTN	adipocyte	*EPHX2*	TRUE	1.966473974
HTN	adipocyte	*TEX14*	TRUE	4.170060218
HTN	adipocyte	*GJB6*	TRUE	2.708246832
HTN	adipocyte	*FAM124B*	TRUE	3.303191098
HTN	adipocyte	*TOX2*	TRUE	2.505080683
HTN	adipocyte	*ZNF391*	TRUE	2.774231165
HTN	adipocyte	*GRK4*	TRUE	2.874312409
HTN	adipocyte	*TRAP1*	TRUE	4.252065582
HTN	adipocyte	*APOL5*	TRUE	2.750041092
HTN	adipocyte	*ATP8B3*	TRUE	2.894428538
HTN	adipocyte	*HAUS8*	TRUE	2.952924272
HTN	adipocyte	*REEP2*	TRUE	2.250202721
HTN	adipocyte	*RSG1*	TRUE	2.025371417
HTN	adipocyte	*ACP2*	TRUE	6.831272431
HTN	adipocyte	*AGT*	TRUE	3.679371814
HTN	adipocyte	*NMT1*	TRUE	4.603658276
HTN	adipocyte	*FBN2*	TRUE	3.312575342
HTN	adipocyte	*GPR84*	TRUE	2.024839957
HTN	adipocyte	*SCRN2*	TRUE	2.55778411
HTN	adipocyte	*CREB3L4*	TRUE	2.588145711
HTN	adipocyte	*SLC10A4*	TRUE	3.53597715
HTN	adipocyte	*SLC18B1*	TRUE	2.923084441
HTN	adipocyte	*NRBF2*	TRUE	2.796610467
HTN	adipocyte	*PDCD11*	TRUE	6.262518634
HTN	adipocyte	*HSD17B12*	TRUE	4.998584698
HTN	adipocyte	*TTC12*	TRUE	2.060440994
HTN	adipocyte	*DUSP15*	TRUE	3.761428869
HTN	adipocyte	*NEIL2*	TRUE	3.089789194
HTN	adipocyte	*CLEC18A*	TRUE	2.469651553
HTN	adipocyte	*SLC5A11*	TRUE	4.46034057
HTN	adipocyte	*S100B*	TRUE	3.461702476
HTN	adipocyte	*ABCF3*	TRUE	3.736887129
HTN	adipocyte	*TNFSF13*	TRUE	4.242560668
HTN	adipocyte	*LMOD1*	TRUE	4.677496554
HTN	adipocyte	*EXO5*	TRUE	3.199544965
HTN	adipocyte	*C1orf50*	TRUE	2.649509237
HTN	adipocyte	*BDH2*	TRUE	2.080925217
HTN	adipocyte	*RNF123*	TRUE	2.876272827
HTN	adipocyte	*ERAP1*	TRUE	3.251981347
HTN	adipocyte	*TRMT61A*	TRUE	3.484207075
HTN	adipocyte	*XRRA1*	TRUE	1.974699109
HTN	adipocyte	*TRIM66*	TRUE	2.865516899
HTN	adipocyte	*RIMKLB*	TRUE	3.840002888
HTN	adipocyte	*KIR3DL1*	TRUE	3.829604544
HTN	adipocyte	*SRR*	TRUE	3.163297762
HTN	adipocyte	*CYP7A1*	TRUE	3.105603443
HTN	adipocyte	*HRASLS5*	TRUE	2.024915327
HTN	adipocyte	*IRS1*	TRUE	5.046458719

HTN	adipocyte	*INO80E*	TRUE	2.380230785
HTN	adipocyte	*CALML6*	TRUE	2.851232063
HTN	adipocyte	*RGS19*	TRUE	4.443010076
HTN	adipocyte	*HOXC5*	TRUE	2.026225545
HTN	adipocyte	*VEGFB*	TRUE	4.5793333
HTN	adipocyte	*NRROS*	TRUE	1.999833118
HTN	adipocyte	*TLR6*	TRUE	2.532777381
HTN	adipocyte	*SNX31*	TRUE	3.533145167
HTN	adipocyte	*GLIS1*	TRUE	2.028405695
HTN	adipocyte	*RSRC1*	TRUE	1.974578475
HTN	adipocyte	*NRIP3*	TRUE	2.357789428
HTN	adipocyte	*PPFIA3*	TRUE	2.419687781
HTN	adipocyte	*COX14*	TRUE	2.553212281
HTN	adipocyte	*CPNE7*	TRUE	1.970459913
HTN	adipocyte	*MPI*	TRUE	4.786839121
HTN	adipocyte	*HYI*	TRUE	2.783769731
HTN	adipocyte	*CTC1*	TRUE	2.141183034
HTN	adipocyte	*DEFB124*	TRUE	2.749713851
HTN	adipocyte	*ZNF443*	TRUE	2.341831593
HTN	adipocyte	*CLN8*	TRUE	2.373046106
HTN	adipocyte	*ZNF320*	TRUE	3.139293635
HTN	adipocyte	*RP1L1*	TRUE	3.888295454
HTN	adipocyte	*KREMEN1*	TRUE	3.080464327
HTN	adipocyte	*EIF3C*	TRUE	2.634839299
HTN	adipocyte	*A3GALT2*	TRUE	4.123063891
HTN	adipocyte	*LPAR5*	TRUE	2.239965449
HTN	adipocyte	*NUTM2A*	TRUE	2.104189549
HTN	adipocyte	*DDX51*	TRUE	2.538609038
HTN	adipocyte	*CDK10*	TRUE	2.074652758
HTN	adipocyte	*TCN2*	TRUE	1.986531752
HTN	adipocyte	*ARL17A*	TRUE	2.184865836
HTN	adipocyte	*MCRS1*	TRUE	3.130674817
HTN	adipocyte	*ZNF559*	TRUE	2.500650055
HTN	adipocyte	*FBF1*	TRUE	4.133579171
HTN	adipocyte	*HLA.DRB1*	TRUE	2.632261933
HTN	adipocyte	*C6orf106*	TRUE	2.810414712
HTN	adipocyte	*HIST1H3E*	TRUE	2.038193719
HTN	adipocyte	*ZNF100*	TRUE	2.361459946
HTN	adipocyte	*RPS26*	TRUE	2.143893944
HTN	adipocyte	*TMEM116*	TRUE	5.898335079
HTN	adipocyte	*PNP*	TRUE	2.622352359
HTN	adipocyte	*AL590822.1*	TRUE	2.526779598
HTN	adipocyte	*HSD17B8*	TRUE	3.294593518
HTN	adipocyte	*STK19*	TRUE	2.991144873
HTN	adipocyte	*MXD3*	TRUE	2.322728811
HTN	adipocyte	*SIPA1*	TRUE	3.887884793
HTN	adipocyte	*ATF6B*	TRUE	3.485932695
HTN	adipocyte	*AC016773.1*	TRUE	2.187561934

HTN	adipocyte	*LINC00092*	TRUE	2.021849892
HTN	adipocyte	*AC017074.2*	TRUE	2.264387615
HTN	adipocyte	*PROB1*	TRUE	2.860985231
HTN	adipocyte	*AC067945.3*	TRUE	2.201906932
HTN	adipocyte	*AC112229.4*	TRUE	2.124678698
HTN	adipocyte	*PLA2G4B*	TRUE	1.98346037
HTN	adipocyte	*ZNF10*	TRUE	3.337434455
HTN	adipocyte	*HMBS*	TRUE	3.541612296
HTN	adipocyte	*AC144652.1*	TRUE	2.916058619
HTN	ASPC	*WNT16*	TRUE	2.55568668
HTN	ASPC	*M6PR*	TRUE	2.418109157
HTN	ASPC	*ARHGAP33*	TRUE	2.077504322
HTN	ASPC	*COPZ2*	TRUE	3.231588878
HTN	ASPC	*HFE*	TRUE	3.723046191
HTN	ASPC	*CEP68*	TRUE	5.001044329
HTN	ASPC	*NR1H3*	TRUE	2.514515712
HTN	ASPC	*SNX1*	TRUE	2.324111487
HTN	ASPC	*EPN3*	TRUE	2.371462793
HTN	ASPC	*LIMA1*	TRUE	2.325317499
HTN	ASPC	*ANAPC4*	TRUE	2.235832829
HTN	ASPC	*ITIH4*	TRUE	2.189340332
HTN	ASPC	*PITHD1*	TRUE	2.145894556
HTN	ASPC	*PIGV*	TRUE	3.362275374
HTN	ASPC	*HAGH*	TRUE	2.886853375
HTN	ASPC	*RRP15*	TRUE	2.129287137
HTN	ASPC	*POLR1A*	TRUE	2.982924087
HTN	ASPC	*ASNS*	TRUE	2.534650763
HTN	ASPC	*ATP2A3*	TRUE	3.642385719
HTN	ASPC	*TIPIN*	TRUE	2.414704877
HTN	ASPC	*SCGN*	TRUE	2.949622875
HTN	ASPC	*DDX43*	TRUE	2.449668865
HTN	ASPC	*CCNT2*	TRUE	2.777207076
HTN	ASPC	*COL16A1*	TRUE	2.351237453
HTN	ASPC	*FMO2*	TRUE	2.202507325
HTN	ASPC	*MARCH2*	TRUE	4.189501186
HTN	ASPC	*SLC7A4*	TRUE	3.08792333
HTN	ASPC	*CARD10*	TRUE	1.967551552
HTN	ASPC	*TXN2*	TRUE	2.10603708
HTN	ASPC	*FAM118A*	TRUE	2.144850138
HTN	ASPC	*MTG2*	TRUE	2.079800615
HTN	ASPC	*MANBAL*	TRUE	2.089770493
HTN	ASPC	*DNTTIP1*	TRUE	2.856951311
HTN	ASPC	*POLI*	TRUE	3.76006783
HTN	ASPC	*VWA8*	TRUE	2.55001851
HTN	ASPC	*CRISPLD2*	TRUE	2.571701771
HTN	ASPC	*MTHFSD*	TRUE	3.184481358
HTN	ASPC	*KAT8*	TRUE	3.25727258
HTN	ASPC	*ZFAND1*	TRUE	2.014695372

HTN	ASPC	*EYA1*	TRUE	3.876907708
HTN	ASPC	*PEX11G*	TRUE	2.340128949
HTN	ASPC	*CCDC94*	TRUE	2.547179541
HTN	ASPC	*ICAM5*	TRUE	2.838468003
HTN	ASPC	*ATP13A1*	TRUE	3.705262517
HTN	ASPC	*HOXA2*	TRUE	4.389903009
HTN	ASPC	*CRHR2*	TRUE	2.408194558
HTN	ASPC	*EPHB6*	TRUE	2.758873961
HTN	ASPC	*MEST*	TRUE	3.197733799
HTN	ASPC	*ACTR3C*	TRUE	3.186157936
HTN	ASPC	*SLC1A1*	TRUE	2.110285891
HTN	ASPC	*WNT3*	TRUE	2.813542441
HTN	ASPC	*ICAM2*	TRUE	2.780909258
HTN	ASPC	*DDX5*	TRUE	3.386157021
HTN	ASPC	*CYTH1*	TRUE	2.385620889
HTN	ASPC	*SLC2A9*	TRUE	2.158324069
HTN	ASPC	*CAND1*	TRUE	3.855195994
HTN	ASPC	*BVES*	TRUE	2.050421153
HTN	ASPC	*GUCA1B*	TRUE	2.261538559
HTN	ASPC	*CEP72*	TRUE	2.542475949
HTN	ASPC	*NME5*	TRUE	1.967147053
HTN	ASPC	*PCDHB6*	TRUE	2.370074589
HTN	ASPC	*BNIP1*	TRUE	2.421096851
HTN	ASPC	*NPHP3*	TRUE	2.765452087
HTN	ASPC	*PCCB*	TRUE	4.420690315
HTN	ASPC	*TP53I3*	TRUE	3.00878378
HTN	ASPC	*DNAJC27*	TRUE	4.189173501
HTN	ASPC	*RTN4*	TRUE	2.119942191
HTN	ASPC	*ZNF142*	TRUE	2.571388388
HTN	ASPC	*CD207*	TRUE	2.441246151
HTN	ASPC	*C1orf21*	TRUE	2.970209482
HTN	ASPC	*GBP3*	TRUE	2.053699142
HTN	ASPC	*NSUN4*	TRUE	2.292551588
HTN	ASPC	*IRF6*	TRUE	2.840339466
HTN	ASPC	*DDX59*	TRUE	2.00045948
HTN	ASPC	*FILIP1*	TRUE	2.212290043
HTN	ASPC	*TCF21*	TRUE	3.101680656
HTN	ASPC	*TRPM6*	TRUE	3.059474598
HTN	ASPC	*HDHD3*	TRUE	2.73348774
HTN	ASPC	*HSDL2*	TRUE	2.019823522
HTN	ASPC	*VSX2*	TRUE	2.114376857
HTN	ASPC	*HELLS*	TRUE	2.086667102
HTN	ASPC	*EPHX2*	TRUE	3.255297759
HTN	ASPC	*TEX14*	TRUE	5.194404465
HTN	ASPC	*TRIM6*	TRUE	2.944483039
HTN	ASPC	*GJB6*	TRUE	1.990293349
HTN	ASPC	*SLC25A51*	TRUE	2.148512392
HTN	ASPC	*NDUFAF4*	TRUE	2.739241623

HTN	ASPC	*ACVR1C*	TRUE	3.747528467
HTN	ASPC	*FAM124B*	TRUE	3.4684445
HTN	ASPC	*TOX2*	TRUE	2.71550888
HTN	ASPC	*PEPD*	TRUE	2.050534794
HTN	ASPC	*ZNF391*	TRUE	2.537109473
HTN	ASPC	*GRK4*	TRUE	3.439640141
HTN	ASPC	*GPR108*	TRUE	2.198094911
HTN	ASPC	*TRAP1*	TRUE	4.688737157
HTN	ASPC	*RHOT1*	TRUE	2.337540671
HTN	ASPC	*YEATS4*	TRUE	2.283759082
HTN	ASPC	*STYXL1*	TRUE	2.136738047
HTN	ASPC	*APOL5*	TRUE	3.262101764
HTN	ASPC	*KLHDC10*	TRUE	3.335054907
HTN	ASPC	*RIPK3*	TRUE	2.03468886
HTN	ASPC	*GALNT8*	TRUE	2.165051661
HTN	ASPC	*CDC16*	TRUE	3.530079752
HTN	ASPC	*ATP8B3*	TRUE	3.241153184
HTN	ASPC	*DDA1*	TRUE	2.321206649
HTN	ASPC	*PGPEP1*	TRUE	2.896499576
HTN	ASPC	*CHMP1A*	TRUE	2.615773776
HTN	ASPC	*HAUS8*	TRUE	4.659946303
HTN	ASPC	*EXOC4*	TRUE	2.477264034
HTN	ASPC	*ANO1*	TRUE	2.650593132
HTN	ASPC	*KDM6B*	TRUE	2.73145058
HTN	ASPC	*REEP2*	TRUE	2.157149176
HTN	ASPC	*RSG1*	TRUE	2.394436334
HTN	ASPC	*WNT2B*	TRUE	4.374728681
HTN	ASPC	*ACP2*	TRUE	7.611350447
HTN	ASPC	*APLNR*	TRUE	2.087696821
HTN	ASPC	*TMEM165*	TRUE	4.079992979
HTN	ASPC	*TMEM60*	TRUE	2.166341001
HTN	ASPC	*POM121C*	TRUE	2.061645901
HTN	ASPC	*BLOC1S1*	TRUE	2.062901727
HTN	ASPC	*AGT*	TRUE	3.484660746
HTN	ASPC	*NACAD*	TRUE	2.29430418
HTN	ASPC	*CIB2*	TRUE	2.605804217
HTN	ASPC	*NMT1*	TRUE	5.158630488
HTN	ASPC	*RTP4*	TRUE	2.468568701
HTN	ASPC	*SLC2A8*	TRUE	2.449137881
HTN	ASPC	*PAQR5*	TRUE	2.065213494
HTN	ASPC	*RMDN3*	TRUE	4.702780601
HTN	ASPC	*IFT172*	TRUE	3.202488103
HTN	ASPC	*DYNC2LI1*	TRUE	3.025257334
HTN	ASPC	*TRPC3*	TRUE	1.986472152
HTN	ASPC	*G3BP2*	TRUE	2.447169348
HTN	ASPC	*FBN2*	TRUE	3.591401302
HTN	ASPC	*GLTP*	TRUE	2.596524683
HTN	ASPC	*GPR84*	TRUE	2.736024551

HTN	ASPC	*TICRR*	TRUE	2.877173919
HTN	ASPC	*SCRN2*	TRUE	2.683005279
HTN	ASPC	*GREB1L*	TRUE	2.983802166
HTN	ASPC	*RERE*	TRUE	2.457608596
HTN	ASPC	*SYPL2*	TRUE	2.423867163
HTN	ASPC	*RGS5*	TRUE	3.124144393
HTN	ASPC	*ARNT*	TRUE	2.145496698
HTN	ASPC	*WNT9A*	TRUE	2.815500557
HTN	ASPC	*RABL2A*	TRUE	2.449166864
HTN	ASPC	*GPR17*	TRUE	2.071800574
HTN	ASPC	*PHOSPHO2*	TRUE	2.043389085
HTN	ASPC	*SLC10A4*	TRUE	3.362404823
HTN	ASPC	*TSLP*	TRUE	2.637817729
HTN	ASPC	*GFOD1*	TRUE	2.287668736
HTN	ASPC	*PSD2*	TRUE	2.218369614
HTN	ASPC	*SLC18B1*	TRUE	3.493754954
HTN	ASPC	*CCDC25*	TRUE	2.455198364
HTN	ASPC	*SURF1*	TRUE	2.184845269
HTN	ASPC	*NRBF2*	TRUE	2.237740372
HTN	ASPC	*PDCD11*	TRUE	8.595116013
HTN	ASPC	*HSD17B12*	TRUE	5.602301646
HTN	ASPC	*TTC12*	TRUE	2.678083867
HTN	ASPC	*DUSP15*	TRUE	3.895683986
HTN	ASPC	*ARID5B*	TRUE	2.796809667
HTN	ASPC	*ARL14EP*	TRUE	2.592540351
HTN	ASPC	*CWF19L2*	TRUE	4.838018259
HTN	ASPC	*PRDM8*	TRUE	3.090749735
HTN	ASPC	*HS3ST3A1*	TRUE	2.156621262
HTN	ASPC	*DISP1*	TRUE	2.495531277
HTN	ASPC	*NEIL2*	TRUE	4.301411783
HTN	ASPC	*TSEN2*	TRUE	2.098779242
HTN	ASPC	*PROM2*	TRUE	2.592577399
HTN	ASPC	*AK9*	TRUE	2.437044663
HTN	ASPC	*TTC39B*	TRUE	2.062859031
HTN	ASPC	*DCK*	TRUE	2.150429921
HTN	ASPC	*NDUFAF6*	TRUE	4.23201128
HTN	ASPC	*C8orf37*	TRUE	2.073968886
HTN	ASPC	*ANKRD9*	TRUE	2.461846081
HTN	ASPC	*MED19*	TRUE	2.034161732
HTN	ASPC	*VPS8*	TRUE	2.732773593
HTN	ASPC	*STEAP2*	TRUE	1.98174008
HTN	ASPC	*CLEC18A*	TRUE	2.063080097
HTN	ASPC	*KCNJ15*	TRUE	2.65050185
HTN	ASPC	*ZNF276*	TRUE	2.289191353
HTN	ASPC	*SLC5A11*	TRUE	4.65708226
HTN	ASPC	*S100B*	TRUE	3.616090345
HTN	ASPC	*FDPS*	TRUE	2.322412686
HTN	ASPC	*PMF1*	TRUE	1.973884039

HTN	ASPC	*ABCF3*	TRUE	4.539128123
HTN	ASPC	*TNFSF13*	TRUE	4.160443745
HTN	ASPC	*LMOD1*	TRUE	5.639208269
HTN	ASPC	*FAM208A*	TRUE	3.641791725
HTN	ASPC	*EXO5*	TRUE	3.831478401
HTN	ASPC	*C1orf50*	TRUE	3.55784781
HTN	ASPC	*BDH2*	TRUE	3.016502594
HTN	ASPC	*RNF123*	TRUE	2.988048113
HTN	ASPC	*ERAP1*	TRUE	4.169878036
HTN	ASPC	*TMEM184A*	TRUE	2.40279785
HTN	ASPC	*C9orf64*	TRUE	2.148635446
HTN	ASPC	*ZNF367*	TRUE	2.784627156
HTN	ASPC	*REEP3*	TRUE	2.387185089
HTN	ASPC	*HSPA12A*	TRUE	1.996602389
HTN	ASPC	*XRRA1*	TRUE	2.849508187
HTN	ASPC	*TRIM66*	TRUE	3.033042258
HTN	ASPC	*RIMKLB*	TRUE	4.676888259
HTN	ASPC	*ZNF641*	TRUE	2.705955167
HTN	ASPC	*KIR3DL1*	TRUE	3.785066416
HTN	ASPC	*SRR*	TRUE	3.688221285
HTN	ASPC	*CYP7A1*	TRUE	2.948998085
HTN	ASPC	*HRASLS5*	TRUE	3.087956706
HTN	ASPC	*PXK*	TRUE	2.080460344
HTN	ASPC	*TNIP2*	TRUE	2.39683721
HTN	ASPC	*IRS1*	TRUE	5.79324694
HTN	ASPC	*CXCL10*	TRUE	2.018222694
HTN	ASPC	*STK32A*	TRUE	1.991175384
HTN	ASPC	*INO80E*	TRUE	2.564797705
HTN	ASPC	*FAM103A1*	TRUE	2.042622365
HTN	ASPC	*LIMS1*	TRUE	2.451008053
HTN	ASPC	*CALML6*	TRUE	3.810023465
HTN	ASPC	*HOXD4*	TRUE	2.346505555
HTN	ASPC	*RNF34*	TRUE	2.094208079
HTN	ASPC	*PAQR8*	TRUE	4.500278546
HTN	ASPC	*SOX7*	TRUE	2.240024486
HTN	ASPC	*GIMAP8*	TRUE	2.220831774
HTN	ASPC	*DLK2*	TRUE	2.791637225
HTN	ASPC	*RGS19*	TRUE	5.58276287
HTN	ASPC	*CTSW*	TRUE	3.050546543
HTN	ASPC	*ZNF738*	TRUE	2.252273172
HTN	ASPC	*HOXC5*	TRUE	3.005593503
HTN	ASPC	*MYEOV*	TRUE	2.839930594
HTN	ASPC	*AHSA2*	TRUE	2.206162534
HTN	ASPC	*DMRT2*	TRUE	3.196565893
HTN	ASPC	*VEGFB*	TRUE	5.360586395
HTN	ASPC	*CCS*	TRUE	3.035283464
HTN	ASPC	*NRROS*	TRUE	2.899739466
HTN	ASPC	*TLR6*	TRUE	4.157076536

HTN	ASPC	*CTU2*	TRUE	2.238514236
HTN	ASPC	*SNX31*	TRUE	3.903210748
HTN	ASPC	*GLIS1*	TRUE	3.344894702
HTN	ASPC	*KLF14*	TRUE	4.340130657
HTN	ASPC	*RSRC1*	TRUE	3.989020396
HTN	ASPC	*NRIP3*	TRUE	2.748841548
HTN	ASPC	*PPP2R2D*	TRUE	3.089890062
HTN	ASPC	*CRLF3*	TRUE	2.554957668
HTN	ASPC	*MTHFR*	TRUE	5.204580584
HTN	ASPC	*TOP3A*	TRUE	2.488063526
HTN	ASPC	*PPFIA3*	TRUE	2.782165405
HTN	ASPC	*GEN1*	TRUE	1.97257341
HTN	ASPC	*COX14*	TRUE	5.173036654
HTN	ASPC	*MPI*	TRUE	3.304614647
HTN	ASPC	*HYI*	TRUE	2.190368034
HTN	ASPC	*CTC1*	TRUE	3.313122777
HTN	ASPC	*DEFB124*	TRUE	3.530822163
HTN	ASPC	*ZNF443*	TRUE	3.186915268
HTN	ASPC	*TMEM136*	TRUE	2.433959257
HTN	ASPC	*CLDN7*	TRUE	3.092270216
HTN	ASPC	*ADO*	TRUE	2.55061892
HTN	ASPC	*FAM89A*	TRUE	2.485224655
HTN	ASPC	*CLN8*	TRUE	4.763915278
HTN	ASPC	*FES*	TRUE	7.519877567
HTN	ASPC	*MXRA7*	TRUE	2.167156608
HTN	ASPC	*COL18A1*	TRUE	2.884896145
HTN	ASPC	*ZNF320*	TRUE	3.425138589
HTN	ASPC	*FAM43B*	TRUE	2.424470928
HTN	ASPC	*KLRC4*	TRUE	2.128494531
HTN	ASPC	*RP1L1*	TRUE	4.121847666
HTN	ASPC	*BMP8A*	TRUE	2.040389392
HTN	ASPC	*KREMEN1*	TRUE	3.529013888
HTN	ASPC	*EIF3C*	TRUE	2.597690583
HTN	ASPC	*A3GALT2*	TRUE	4.208984409
HTN	ASPC	*CCR10*	TRUE	3.087351309
HTN	ASPC	*LPAR5*	TRUE	2.562318273
HTN	ASPC	*NUTM2A*	TRUE	2.103004132
HTN	ASPC	*DDX51*	TRUE	3.213097107
HTN	ASPC	*TCN2*	TRUE	2.721352961
HTN	ASPC	*LSAMP*	TRUE	2.248588646
HTN	ASPC	*ARL17A*	TRUE	3.201383224
HTN	ASPC	*ZNF396*	TRUE	2.072304673
HTN	ASPC	*FOXI2*	TRUE	2.063560547
HTN	ASPC	*RHD*	TRUE	2.014366997
HTN	ASPC	*MCRS1*	TRUE	4.610890481
HTN	ASPC	*ZNF559*	TRUE	2.902592215
HTN	ASPC	*ZDHHC11*	TRUE	2.71882805
HTN	ASPC	*FBF1*	TRUE	3.614114805

HTN	ASPC	*FAM47E*	TRUE	2.448349751
HTN	ASPC	*HLA.DRB1*	TRUE	3.120463955
HTN	ASPC	*ZNF34*	TRUE	2.07161108
HTN	ASPC	*C6orf106*	TRUE	3.135159324
HTN	ASPC	*ZFP28*	TRUE	2.137648279
HTN	ASPC	*KIAA1211L*	TRUE	2.070744797
HTN	ASPC	*HCAR1*	TRUE	2.021406747
HTN	ASPC	*HIST1H3E*	TRUE	2.555262382
HTN	ASPC	*FUT11*	TRUE	2.411190982
HTN	ASPC	*ZNF100*	TRUE	3.298676605
HTN	ASPC	*C6orf141*	TRUE	2.020254735
HTN	ASPC	*SLC22A5*	TRUE	2.352275608
HTN	ASPC	*RPS26*	TRUE	2.195900234
HTN	ASPC	*RPS6KL1*	TRUE	2.801477461
HTN	ASPC	*TMEM116*	TRUE	4.717998428
HTN	ASPC	*GFPT1*	TRUE	3.066504382
HTN	ASPC	*PNP*	TRUE	2.836270088
HTN	ASPC	*PRMT6*	TRUE	2.100323302
HTN	ASPC	*TGM2*	TRUE	2.257067001
HTN	ASPC	*AL590822.1*	TRUE	3.236953111
HTN	ASPC	*CENPW*	TRUE	2.673104654
HTN	ASPC	*INPP5B*	TRUE	2.182807511
HTN	ASPC	*HSD17B8*	TRUE	3.227207561
HTN	ASPC	*STK19*	TRUE	3.722350679
HTN	ASPC	*NOTCH2NL*	TRUE	2.025181653
HTN	ASPC	*MXD3*	TRUE	2.914093863
HTN	ASPC	*SIPA1*	TRUE	5.144951364
HTN	ASPC	*ATF6B*	TRUE	4.010474589
HTN	ASPC	*PPM1N*	TRUE	3.002853996
HTN	ASPC	*GALT*	TRUE	2.376941929
HTN	ASPC	*REPIN1*	TRUE	1.961378665
HTN	ASPC	*WEE2*	TRUE	2.387519739
HTN	ASPC	*PPME1*	TRUE	2.149227945
HTN	ASPC	*STARD10*	TRUE	2.007654624
HTN	ASPC	*SLC12A8*	TRUE	2.495433014
HTN	ASPC	*LINC00092*	TRUE	2.155465946
HTN	ASPC	*SLC26A6*	TRUE	2.179210284
HTN	ASPC	*AC017074.2*	TRUE	2.225586847
HTN	ASPC	*AC108463.2*	TRUE	2.35603797
HTN	ASPC	*PROB1*	TRUE	3.292174645
HTN	ASPC	*AC067945.3*	TRUE	2.086821214
HTN	ASPC	*AC112229.4*	TRUE	2.204196332
HTN	ASPC	*GOLGA8N*	TRUE	2.049143547
HTN	ASPC	*PSG4*	TRUE	2.980393371
HTN	ASPC	*ZNF10*	TRUE	2.967919738
HTN	ASPC	*HMBS*	TRUE	4.22319156
HTN	ASPC	*SH3RF3.AS1*	TRUE	2.267056629
HTN	ASPC	*LINC00668*	TRUE	2.217563487

HTN	ASPC	*SRXN1*	TRUE	2.201647414
HTN	ASPC	*AC144652.1*	TRUE	3.53088287
HTN	b_cell	*SNX10*	TRUE	3.149642544
HTN	b_cell	*ACP2*	TRUE	3.520003386
HTN	b_cell	*HSD17B12*	TRUE	2.684642805
HTN	b_cell	*CLEC18A*	TRUE	3.66680268
HTN	b_cell	*ZNF589*	TRUE	2.366421763
HTN	b_cell	*KIR3DL1*	TRUE	3.0404597
HTN	b_cell	*RPP25*	TRUE	3.883862359
HTN	b_cell	*A3GALT2*	TRUE	2.103218258
HTN	b_cell	*RPS26*	TRUE	2.266187686
HTN	b_cell	*NOTCH2NL*	TRUE	2.435476115
HTN	dendritic_cell	*ITIH4*	TRUE	2.706980525
HTN	dendritic_cell	*SNX10*	TRUE	4.16335725
HTN	dendritic_cell	*SNRPC*	TRUE	2.351101936
HTN	dendritic_cell	*ACP2*	TRUE	6.019122053
HTN	dendritic_cell	*AGT*	TRUE	2.386527081
HTN	dendritic_cell	*SLC10A4*	TRUE	1.991917614
HTN	dendritic_cell	*SLC18B1*	TRUE	2.548771744
HTN	dendritic_cell	*HSD17B12*	TRUE	4.117686029
HTN	dendritic_cell	*TNFSF13*	TRUE	2.467389434
HTN	dendritic_cell	*LMOD1*	TRUE	3.63310135
HTN	dendritic_cell	*RNF123*	TRUE	2.60059514
HTN	dendritic_cell	*TRMT61A*	TRUE	2.878261745
HTN	dendritic_cell	*TRIM66*	TRUE	2.376966737
HTN	dendritic_cell	*KIR3DL1*	TRUE	2.789886591
HTN	dendritic_cell	*SNX31*	TRUE	2.222995727
HTN	dendritic_cell	*RPP25*	TRUE	2.327653381
HTN	dendritic_cell	*HYI*	TRUE	3.644615209
HTN	dendritic_cell	*CLDN7*	TRUE	3.901980528
HTN	dendritic_cell	*ZNF320*	TRUE	2.866782701
HTN	dendritic_cell	*EIF3C*	TRUE	2.917819398
HTN	dendritic_cell	*A3GALT2*	TRUE	2.283244954
HTN	dendritic_cell	*FBF1*	TRUE	2.220692979
HTN	dendritic_cell	*HIST1H3E*	TRUE	3.10994969
HTN	dendritic_cell	*RPS26*	TRUE	3.780348306
HTN	dendritic_cell	*TMEM116*	TRUE	3.599475562
HTN	dendritic_cell	*HSD17B8*	TRUE	2.659727563
HTN	dendritic_cell	*LTB4R*	TRUE	2.483783194
HTN	endothelial	*PCDHA6*	TRUE	2.034750289
HTN	endothelial	*IGDCC4*	TRUE	2.105680469
HTN	endothelial	*NDUFAF4*	TRUE	2.104698292
HTN	endothelial	*ATP8B3*	TRUE	2.208106237
HTN	endothelial	*PKDREJ*	TRUE	3.105727211
HTN	endothelial	*RSG1*	TRUE	2.033473903
HTN	endothelial	*CIB2*	TRUE	2.172463141
HTN	endothelial	*FBN2*	TRUE	1.974366342
HTN	endothelial	*TTC12*	TRUE	1.97213686

HTN	endothelial	*DOC2A*	TRUE	2.953640799
HTN	endothelial	*NEIL2*	TRUE	1.989651627
HTN	endothelial	*SLC5A11*	TRUE	2.593477878
HTN	endothelial	*TNFSF13*	TRUE	2.906996839
HTN	endothelial	*RGS19*	TRUE	2.799183536
HTN	endothelial	*RPP25*	TRUE	3.237285574
HTN	endothelial	*CPNE7*	TRUE	2.419992243
HTN	endothelial	*TRIM73*	TRUE	2.318728717
HTN	endothelial	*HYI*	TRUE	2.073684763
HTN	endothelial	*NPIPA1*	TRUE	2.013876367
HTN	endothelial	*EIF3C*	TRUE	2.291125579
HTN	endothelial	*HIST1H3E*	TRUE	3.360754349
HTN	endothelial	*RPS26*	TRUE	2.718626643
HTN	endothelial	*HSD17B8*	TRUE	2.180555017
HTN	endothelial	*AC017074.2*	TRUE	2.202429675
HTN	endothelial	*PROB1*	TRUE	2.214900645
HTN	endothelial	*PSG4*	TRUE	2.320758883
HTN	LEC	*HFE*	TRUE	2.409055182
HTN	LEC	*HSD17B12*	TRUE	2.605661059
HTN	LEC	*DUSP15*	TRUE	2.076730017
HTN	LEC	*CLEC18A*	TRUE	2.418108346
HTN	LEC	*SLC5A11*	TRUE	3.081738621
HTN	LEC	*TNFSF13*	TRUE	2.489860735
HTN	LEC	*HIST1H3E*	TRUE	2.535525402
HTN	LEC	*RPS26*	TRUE	2.860556585
HTN	LEC	*PLA2G4B*	TRUE	2.053947055
HTN	macrophage	*COPZ2*	TRUE	2.732645099
HTN	macrophage	*HFE*	TRUE	3.249489247
HTN	macrophage	*ITIH4*	TRUE	2.285676677
HTN	macrophage	*SCGN*	TRUE	2.467615524
HTN	macrophage	*PCDHA6*	TRUE	2.29635273
HTN	macrophage	*SNX10*	TRUE	2.935789289
HTN	macrophage	*POLI*	TRUE	1.999099352
HTN	macrophage	*MTHFSD*	TRUE	2.967222774
HTN	macrophage	*EYA1*	TRUE	2.369689628
HTN	macrophage	*ICAM5*	TRUE	2.022567544
HTN	macrophage	*ENDOU*	TRUE	2.463217988
HTN	macrophage	*IRF6*	TRUE	2.184504468
HTN	macrophage	*ALDH8A1*	TRUE	2.021076415
HTN	macrophage	*TCF21*	TRUE	2.868906749
HTN	macrophage	*HDHD3*	TRUE	2.131246833
HTN	macrophage	*VSX2*	TRUE	2.124607534
HTN	macrophage	*TEX14*	TRUE	3.102155228
HTN	macrophage	*TRIM6*	TRUE	2.016147712
HTN	macrophage	*NDUFAF4*	TRUE	2.68985873
HTN	macrophage	*FAM124B*	TRUE	2.197620636
HTN	macrophage	*TOX2*	TRUE	3.583861044
HTN	macrophage	*TCP11*	TRUE	2.624661847

HTN	macrophage	*TRAP1*	TRUE	2.563017351
HTN	macrophage	*APOL5*	TRUE	2.87059002
HTN	macrophage	*AANAT*	TRUE	2.232539204
HTN	macrophage	*GALNT8*	TRUE	2.318222455
HTN	macrophage	*ATP8B3*	TRUE	3.418700427
HTN	macrophage	*PKDREJ*	TRUE	3.350492389
HTN	macrophage	*RSG1*	TRUE	2.275416888
HTN	macrophage	*ACP2*	TRUE	2.531303394
HTN	macrophage	*AGT*	TRUE	2.406039196
HTN	macrophage	*CIB2*	TRUE	2.875954697
HTN	macrophage	*NMT1*	TRUE	3.46432182
HTN	macrophage	*RTP4*	TRUE	3.479368269
HTN	macrophage	*SLC10A4*	TRUE	2.098801473
HTN	macrophage	*SLC18B1*	TRUE	2.011444068
HTN	macrophage	*NRBF2*	TRUE	2.666943673
HTN	macrophage	*PDCD11*	TRUE	2.821884557
HTN	macrophage	*HSD17B12*	TRUE	3.561315562
HTN	macrophage	*TTC12*	TRUE	3.007221664
HTN	macrophage	*DUSP15*	TRUE	3.576450165
HTN	macrophage	*DOC2A*	TRUE	2.661209686
HTN	macrophage	*NEIL2*	TRUE	2.542344942
HTN	macrophage	*SLC5A11*	TRUE	3.368233889
HTN	macrophage	*S100B*	TRUE	2.632303144
HTN	macrophage	*FCN2*	TRUE	2.736329306
HTN	macrophage	*PPP1R16A*	TRUE	2.251263538
HTN	macrophage	*ABCF3*	TRUE	3.406473178
HTN	macrophage	*TNFSF13*	TRUE	3.077874986
HTN	macrophage	*LMOD1*	TRUE	2.186319272
HTN	macrophage	*EXO5*	TRUE	2.016039341
HTN	macrophage	*RNF123*	TRUE	2.582904336
HTN	macrophage	*ERAP1*	TRUE	2.967932215
HTN	macrophage	*KIR3DL1*	TRUE	2.624622714
HTN	macrophage	*C19orf33*	TRUE	2.241093741
HTN	macrophage	*CYP7A1*	TRUE	2.453758721
HTN	macrophage	*HRASLS5*	TRUE	2.618520063
HTN	macrophage	*RGS19*	TRUE	3.888510174
HTN	macrophage	*NIPAL4*	TRUE	2.151168535
HTN	macrophage	*ZNF738*	TRUE	2.032543373
HTN	macrophage	*VEGFB*	TRUE	2.212837481
HTN	macrophage	*PPFIA3*	TRUE	2.729190169
HTN	macrophage	*ZNF223*	TRUE	2.297810992
HTN	macrophage	*CPNE7*	TRUE	2.644535441
HTN	macrophage	*MPI*	TRUE	2.064981462
HTN	macrophage	*TRIM73*	TRUE	2.753463062
HTN	macrophage	*CTC1*	TRUE	2.437378895
HTN	macrophage	*NPIPA1*	TRUE	2.130048653
HTN	macrophage	*RP1L1*	TRUE	2.723271186
HTN	macrophage	*A3GALT2*	TRUE	2.488770959

HTN	macrophage	*DDX51*	TRUE	2.75286854
HTN	macrophage	*MCRS1*	TRUE	2.513499855
HTN	macrophage	*C6orf106*	TRUE	2.008947711
HTN	macrophage	*HIST1H3E*	TRUE	2.033995518
HTN	macrophage	*RPS26*	TRUE	3.232054613
HTN	macrophage	*TMEM116*	TRUE	3.4849253
HTN	macrophage	*TGM2*	TRUE	2.512703418
HTN	macrophage	*HSD17B8*	TRUE	2.384426411
HTN	macrophage	*STK19*	TRUE	3.208894585
HTN	macrophage	*LTB4R*	TRUE	2.182565612
HTN	macrophage	*SLC26A6*	TRUE	3.142691922
HTN	macrophage	*PROB1*	TRUE	2.383938049
HTN	macrophage	*PSG4*	TRUE	2.348055096
HTN	macrophage	*PLA2G4B*	TRUE	2.629501201
HTN	macrophage	*ZNF10*	TRUE	2.035694359
HTN	macrophage	*RTEL1*	TRUE	3.01189
HTN	macrophage	*LINC00668*	TRUE	2.08764697
HTN	mast_cell	*HFE*	TRUE	2.168077585
HTN	mast_cell	*MTHFSD*	TRUE	2.23670788
HTN	mast_cell	*IGDCC4*	TRUE	2.150324861
HTN	mast_cell	*HDHD3*	TRUE	2.192251955
HTN	mast_cell	*TEX14*	TRUE	2.663033142
HTN	mast_cell	*NDUFAF4*	TRUE	2.179524173
HTN	mast_cell	*FAM124B*	TRUE	2.065033932
HTN	mast_cell	*PKDREJ*	TRUE	3.361616505
HTN	mast_cell	*ACP2*	TRUE	3.081987356
HTN	mast_cell	*AGT*	TRUE	1.997218531
HTN	mast_cell	*NMT1*	TRUE	2.092719681
HTN	mast_cell	*NRBF2*	TRUE	2.101159012
HTN	mast_cell	*HSD17B12*	TRUE	2.316693431
HTN	mast_cell	*S100B*	TRUE	2.476887016
HTN	mast_cell	*ABCF3*	TRUE	2.642754582
HTN	mast_cell	*RNF123*	TRUE	2.328539992
HTN	mast_cell	*ERAP1*	TRUE	2.420444951
HTN	mast_cell	*RGS19*	TRUE	2.414179743
HTN	mast_cell	*HYI*	TRUE	2.008415625
HTN	mast_cell	*NPIPA1*	TRUE	2.341257772
HTN	mast_cell	*DDX51*	TRUE	2.245873538
HTN	mast_cell	*RPS26*	TRUE	2.775466064
HTN	mast_cell	*TMEM116*	TRUE	2.669229506
HTN	mast_cell	*TGM2*	TRUE	2.645897761
HTN	mast_cell	*PLA2G4B*	TRUE	2.019821233
HTN	mast_cell	*RTEL1*	TRUE	2.79828856
HTN	monocyte	*ITIH4*	TRUE	2.071379152
HTN	monocyte	*YPEL3*	TRUE	2.120914535
HTN	monocyte	*HOXA2*	TRUE	2.514193278
HTN	monocyte	*SNX15*	TRUE	2.022709612
HTN	monocyte	*TEX14*	TRUE	3.026650403

HTN	monocyte	*PKDREJ*	TRUE	2.476539369
HTN	monocyte	*ACP2*	TRUE	4.495321234
HTN	monocyte	*AGT*	TRUE	2.667112824
HTN	monocyte	*CREB3L4*	TRUE	2.26090023
HTN	monocyte	*DUSP15*	TRUE	3.043552274
HTN	monocyte	*ABCF3*	TRUE	2.588991749
HTN	monocyte	*NME6*	TRUE	3.924315107
HTN	monocyte	*HYI*	TRUE	2.053196
HTN	monocyte	*RPS26*	TRUE	3.248102003
HTN	monocyte	*PLA2G4B*	TRUE	2.23332595
HTN	monocyte	*RTEL1*	TRUE	2.493177384
HTN	neutrophil	*ZNF391*	TRUE	2.610806572
HTN	neutrophil	*ACP2*	TRUE	4.056735712
HTN	neutrophil	*CIB2*	TRUE	2.834389962
HTN	neutrophil	*CLEC18A*	TRUE	2.438975258
HTN	neutrophil	*S100B*	TRUE	2.198913137
HTN	neutrophil	*CDC25A*	TRUE	2.46705797
HTN	neutrophil	*CTSW*	TRUE	2.469343259
HTN	neutrophil	*HSD17B8*	TRUE	2.521472897
HTN	nk_cell	*ITIH4*	TRUE	2.376419586
HTN	nk_cell	*SNX10*	TRUE	3.733070241
HTN	nk_cell	*POLI*	TRUE	2.33619102
HTN	nk_cell	*HDHD3*	TRUE	2.009483531
HTN	nk_cell	*GRK4*	TRUE	2.130849417
HTN	nk_cell	*ATP8B3*	TRUE	1.970442618
HTN	nk_cell	*ACP2*	TRUE	4.775808462
HTN	nk_cell	*AGT*	TRUE	1.974294023
HTN	nk_cell	*KHK*	TRUE	2.096494473
HTN	nk_cell	*SCRN2*	TRUE	2.225118221
HTN	nk_cell	*CREB3L4*	TRUE	2.634861217
HTN	nk_cell	*SLC10A4*	TRUE	2.65455238
HTN	nk_cell	*SLC18B1*	TRUE	2.158453243
HTN	nk_cell	*HSD17B12*	TRUE	3.529785241
HTN	nk_cell	*NEIL2*	TRUE	2.688018608
HTN	nk_cell	*CLEC18A*	TRUE	3.793958103
HTN	nk_cell	*SLC5A11*	TRUE	3.250437292
HTN	nk_cell	*S100B*	TRUE	3.28537304
HTN	nk_cell	*TNFSF13*	TRUE	4.38780516
HTN	nk_cell	*ZNF589*	TRUE	3.396916449
HTN	nk_cell	*RNF123*	TRUE	3.321363589
HTN	nk_cell	*CHMP4C*	TRUE	1.968290381
HTN	nk_cell	*TRIM66*	TRUE	2.685802982
HTN	nk_cell	*SRR*	TRUE	2.320901279
HTN	nk_cell	*IRS1*	TRUE	3.461647426
HTN	nk_cell	*RGS19*	TRUE	2.197372501
HTN	nk_cell	*VEGFB*	TRUE	2.864365219
HTN	nk_cell	*PPFIA3*	TRUE	1.96144941
HTN	nk_cell	*HYI*	TRUE	2.593434699

HTN	nk_cell	*ZNF320*	TRUE	2.443652913
HTN	nk_cell	*KREMEN1*	TRUE	1.980340974
HTN	nk_cell	*EIF3C*	TRUE	2.544772736
HTN	nk_cell	*ZNF793*	TRUE	2.004486353
HTN	nk_cell	*FBF1*	TRUE	2.735828462
HTN	nk_cell	*SLC22A5*	TRUE	2.786248871
HTN	nk_cell	*TMEM116*	TRUE	4.474721737
HTN	nk_cell	*PNP*	TRUE	2.392678796
HTN	nk_cell	*HSD17B8*	TRUE	2.362600677
HTN	nk_cell	*LTB4R2*	TRUE	3.186127051
HTN	nk_cell	*AC017074.2*	TRUE	2.367184171
HTN	nk_cell	*ZNF709*	TRUE	2.189142487
HTN	nk_cell	*PLA2G4B*	TRUE	2.707129065
HTN	nk_cell	*HMBS*	TRUE	2.791731744
HTN	pericyte	*CEP68*	TRUE	2.278780309
HTN	pericyte	*ITIH4*	TRUE	3.270761432
HTN	pericyte	*UHRF1BP1*	TRUE	2.092454688
HTN	pericyte	*TIE1*	TRUE	2.001150902
HTN	pericyte	*SNX10*	TRUE	2.903178382
HTN	pericyte	*SLC7A4*	TRUE	2.09385111
HTN	pericyte	*POLI*	TRUE	2.858864287
HTN	pericyte	*IGDCC4*	TRUE	2.067690225
HTN	pericyte	*ATP13A1*	TRUE	2.165146099
HTN	pericyte	*MEST*	TRUE	2.015188255
HTN	pericyte	*KCTD10*	TRUE	2.041622779
HTN	pericyte	*PCDHB6*	TRUE	2.306190103
HTN	pericyte	*NPHP3*	TRUE	1.996482248
HTN	pericyte	*TCF21*	TRUE	2.073051957
HTN	pericyte	*TRAP1*	TRUE	3.341127316
HTN	pericyte	*APOL5*	TRUE	2.578746017
HTN	pericyte	*ATP8B3*	TRUE	2.648091539
HTN	pericyte	*HAUS8*	TRUE	2.068780909
HTN	pericyte	*AGT*	TRUE	3.540472092
HTN	pericyte	*CIB2*	TRUE	2.287340947
HTN	pericyte	*NMT1*	TRUE	4.045661036
HTN	pericyte	*FBN2*	TRUE	3.27629336
HTN	pericyte	*SCRN2*	TRUE	2.832781337
HTN	pericyte	*SLC10A4*	TRUE	3.653282611
HTN	pericyte	*NRBF2*	TRUE	2.58402898
HTN	pericyte	*PDCD11*	TRUE	4.20399074
HTN	pericyte	*HSD17B12*	TRUE	4.725927926
HTN	pericyte	*PRDM8*	TRUE	2.096454411
HTN	pericyte	*NEIL2*	TRUE	2.876836895
HTN	pericyte	*CLEC18A*	TRUE	3.10662856
HTN	pericyte	*SLC5A11*	TRUE	3.179823219
HTN	pericyte	*S100B*	TRUE	3.24144946
HTN	pericyte	*ABCF3*	TRUE	2.642407692
HTN	pericyte	*TNFSF13*	TRUE	3.947533042

HTN	pericyte	*EXO5*	TRUE	2.758083024
HTN	pericyte	*RNF123*	TRUE	3.962377687
HTN	pericyte	*ERAP1*	TRUE	2.148327187
HTN	pericyte	*TRIM66*	TRUE	2.48019585
HTN	pericyte	*RIMKLB*	TRUE	2.047373133
HTN	pericyte	*CYP7A1*	TRUE	2.061004387
HTN	pericyte	*HRASLS5*	TRUE	2.086174658
HTN	pericyte	*IRS1*	TRUE	2.235244034
HTN	pericyte	*C8orf46*	TRUE	2.447865813
HTN	pericyte	*NME6*	TRUE	2.360222278
HTN	pericyte	*VEGFB*	TRUE	2.240806792
HTN	pericyte	*ZNF320*	TRUE	2.593474793
HTN	pericyte	*A3GALT2*	TRUE	4.052998375
HTN	pericyte	*FBF1*	TRUE	3.352245017
HTN	pericyte	*HLA.DRB1*	TRUE	2.976443164
HTN	pericyte	*SULT1A2*	TRUE	2.289678566
HTN	pericyte	*SLC22A5*	TRUE	3.569445419
HTN	pericyte	*RPS26*	TRUE	3.825664461
HTN	pericyte	*TMEM116*	TRUE	3.674694949
HTN	pericyte	*PNP*	TRUE	1.999255051
HTN	pericyte	*HSD17B8*	TRUE	2.884964674
HTN	pericyte	*STK19*	TRUE	2.618295431
HTN	pericyte	*NOTCH2NL*	TRUE	2.371080313
HTN	pericyte	*SIPA1*	TRUE	3.354240298
HTN	pericyte	*ZNF10*	TRUE	2.977063314
HTN	pericyte	*HMBS*	TRUE	2.912500833
HTN	SMC	*HFE*	TRUE	3.151677291
HTN	SMC	*POLR1A*	TRUE	2.094201847
HTN	SMC	*SNX10*	TRUE	2.752741485
HTN	SMC	*AAMDC*	TRUE	2.486152338
HTN	SMC	*PABPC4*	TRUE	2.431622664
HTN	SMC	*SLC7A4*	TRUE	2.141870222
HTN	SMC	*POLI*	TRUE	3.137645346
HTN	SMC	*IGDCC4*	TRUE	2.120860482
HTN	SMC	*ENDOU*	TRUE	2.236826347
HTN	SMC	*IRF6*	TRUE	2.758401247
HTN	SMC	*HDHD3*	TRUE	2.333086306
HTN	SMC	*ACOT2*	TRUE	1.968129569
HTN	SMC	*TOX2*	TRUE	2.43841944
HTN	SMC	*SNRPC*	TRUE	3.31738425
HTN	SMC	*GRK4*	TRUE	2.614233419
HTN	SMC	*TRAP1*	TRUE	3.041544586
HTN	SMC	*ATP8B3*	TRUE	2.656473958
HTN	SMC	*ACP2*	TRUE	5.229379928
HTN	SMC	*AGT*	TRUE	3.31372937
HTN	SMC	*NMT1*	TRUE	2.97239367
HTN	SMC	*FBN2*	TRUE	3.055070514
HTN	SMC	*SCRN2*	TRUE	2.975756565

HTN	SMC	*SLC10A4*	TRUE	2.414946477
HTN	SMC	*SLC18B1*	TRUE	2.739093815
HTN	SMC	*PDCD11*	TRUE	2.26677731
HTN	SMC	*HSD17B12*	TRUE	4.866335757
HTN	SMC	*DUSP15*	TRUE	2.312254545
HTN	SMC	*NEIL2*	TRUE	2.436047959
HTN	SMC	*CLEC18A*	TRUE	2.5014285
HTN	SMC	*SLC5A11*	TRUE	4.453838785
HTN	SMC	*S100B*	TRUE	2.77577618
HTN	SMC	*ABCF3*	TRUE	2.784231136
HTN	SMC	*TNFSF13*	TRUE	3.069670304
HTN	SMC	*C1orf50*	TRUE	2.683845294
HTN	SMC	*ZNF589*	TRUE	3.051153509
HTN	SMC	*RNF123*	TRUE	2.000740745
HTN	SMC	*ERAP1*	TRUE	2.988662177
HTN	SMC	*TRMT61A*	TRUE	3.317219958
HTN	SMC	*TRIM66*	TRUE	2.677468149
HTN	SMC	*RIMKLB*	TRUE	3.622458652
HTN	SMC	*KIR3DL1*	TRUE	2.415537429
HTN	SMC	*SRR*	TRUE	2.566971771
HTN	SMC	*CYP7A1*	TRUE	2.026543288
HTN	SMC	*IRS1*	TRUE	4.925208671
HTN	SMC	*INO80E*	TRUE	3.568516704
HTN	SMC	*CALML6*	TRUE	2.050048392
HTN	SMC	*PAQR8*	TRUE	2.034497899
HTN	SMC	*RGS19*	TRUE	4.909768972
HTN	SMC	*CTSW*	TRUE	3.000243531
HTN	SMC	*VEGFB*	TRUE	3.377497093
HTN	SMC	*NRIP3*	TRUE	2.187574834
HTN	SMC	*MTHFR*	TRUE	2.168910976
HTN	SMC	*CPNE7*	TRUE	1.984258796
HTN	SMC	*HYI*	TRUE	2.673861499
HTN	SMC	*DEFB124*	TRUE	2.837765946
HTN	SMC	*ZNF320*	TRUE	2.937202464
HTN	SMC	*KREMEN1*	TRUE	2.763502909
HTN	SMC	*EIF3C*	TRUE	2.340788486
HTN	SMC	*A3GALT2*	TRUE	3.956647603
HTN	SMC	*CDK10*	TRUE	2.516476506
HTN	SMC	*ZNF559*	TRUE	2.33467433
HTN	SMC	*FBF1*	TRUE	3.251524908
HTN	SMC	*HLA.DRB1*	TRUE	3.395629523
HTN	SMC	*HIST1H3E*	TRUE	2.382473294
HTN	SMC	*SLC22A5*	TRUE	2.489625629
HTN	SMC	*RPS26*	TRUE	2.013652681
HTN	SMC	*TMEM116*	TRUE	5.750878345
HTN	SMC	*PNP*	TRUE	2.486208821
HTN	SMC	*AL590822.1*	TRUE	2.388436702
HTN	SMC	*HSD17B8*	TRUE	3.396552752

HTN	SMC	*MXD3*	TRUE	2.188777713
HTN	SMC	*LTB4R*	TRUE	2.064703239
HTN	SMC	*AC016773.1*	TRUE	2.321679561
HTN	SMC	*ZNF10*	TRUE	2.711843981
HTN	SMC	*HMBS*	TRUE	3.083476874
HTN	t_cell	*EPN3*	TRUE	2.189409954
HTN	t_cell	*PCDHA6*	TRUE	2.499326966
HTN	t_cell	*SNX10*	TRUE	2.392132214
HTN	t_cell	*YPEL3*	TRUE	3.069158761
HTN	t_cell	*MTHFSD*	TRUE	2.63442834
HTN	t_cell	*TEX14*	TRUE	3.284059199
HTN	t_cell	*NDUFAF4*	TRUE	2.386558673
HTN	t_cell	*TOX2*	TRUE	2.834139953
HTN	t_cell	*TCP11*	TRUE	2.475926521
HTN	t_cell	*APOL5*	TRUE	2.245699896
HTN	t_cell	*AANAT*	TRUE	2.256400542
HTN	t_cell	*GALNT8*	TRUE	1.984266498
HTN	t_cell	*ATP8B3*	TRUE	2.776061104
HTN	t_cell	*PKDREJ*	TRUE	2.635820221
HTN	t_cell	*ACP2*	TRUE	3.118593573
HTN	t_cell	*AGT*	TRUE	2.448813379
HTN	t_cell	*NMT1*	TRUE	2.482210364
HTN	t_cell	*SLC18B1*	TRUE	2.582970126
HTN	t_cell	*PLEKHS1*	TRUE	2.574344936
HTN	t_cell	*SLC5A11*	TRUE	2.236513909
HTN	t_cell	*TNFSF13*	TRUE	4.192692858
HTN	t_cell	*LMOD1*	TRUE	2.224273396
HTN	t_cell	*ERAP1*	TRUE	2.492496628
HTN	t_cell	*KIR3DL1*	TRUE	2.115310759
HTN	t_cell	*C19orf33*	TRUE	2.121625747
HTN	t_cell	*NME6*	TRUE	2.453243186
HTN	t_cell	*NRIP3*	TRUE	2.192787457
HTN	t_cell	*TRIM73*	TRUE	2.08544761
HTN	t_cell	*NPIPA1*	TRUE	2.162389084
HTN	t_cell	*RP1L1*	TRUE	2.067665643
HTN	t_cell	*NUTM2A*	TRUE	2.137130892
HTN	t_cell	*HLA.DRB1*	TRUE	1.999633621
HTN	t_cell	*HIST1H3E*	TRUE	4.131201165
HTN	t_cell	*RPS26*	TRUE	3.182888426
HTN	t_cell	*TGM2*	TRUE	2.341690626
HTN	t_cell	*STK19*	TRUE	2.176424899
HTN	t_cell	*PLA2G4B*	TRUE	2.802422209
HTN	t_cell	*ZNF10*	TRUE	2.052590307
Obesity	adipocyte	*RBM6*	TRUE	3.17408595
Obesity	adipocyte	*MAPK8IP2*	TRUE	2.0539476
Obesity	adipocyte	*LSG1*	TRUE	2.239377791
Obesity	adipocyte	*ITIH4*	TRUE	4.444239586
Obesity	adipocyte	*UHRF1BP1*	TRUE	3.802116996

Obesity	adipocyte	*TIPIN*	TRUE	3.576317138
Obesity	adipocyte	*PCDHGA2*	TRUE	2.271461094
Obesity	adipocyte	*EPYC*	TRUE	2.188074689
Obesity	adipocyte	*COL16A1*	TRUE	2.02633097
Obesity	adipocyte	*PABPC4*	TRUE	2.966969899
Obesity	adipocyte	*STX1B*	TRUE	2.28080814
Obesity	adipocyte	*SMARCB1*	TRUE	2.05929429
Obesity	adipocyte	*RASD2*	TRUE	2.033685219
Obesity	adipocyte	*DESI1*	TRUE	2.954839623
Obesity	adipocyte	*ABHD12*	TRUE	2.405681172
Obesity	adipocyte	*SLC52A3*	TRUE	2.070004161
Obesity	adipocyte	*ELMO3*	TRUE	2.169561439
Obesity	adipocyte	*CLUAP1*	TRUE	2.430465136
Obesity	adipocyte	*KAT8*	TRUE	3.489762782
Obesity	adipocyte	*MCM4*	TRUE	2.1098627
Obesity	adipocyte	*PPP1R37*	TRUE	2.076924502
Obesity	adipocyte	*ICAM5*	TRUE	1.974244072
Obesity	adipocyte	*ATP13A1*	TRUE	2.33584097
Obesity	adipocyte	*SLC26A8*	TRUE	2.113557817
Obesity	adipocyte	*BNIP1*	TRUE	2.597631098
Obesity	adipocyte	*MFAP2*	TRUE	2.03574362
Obesity	adipocyte	*SERPINC1*	TRUE	4.265469368
Obesity	adipocyte	*COLEC11*	TRUE	2.194717624
Obesity	adipocyte	*CCDC92*	TRUE	4.897412465
Obesity	adipocyte	*DCAF4*	TRUE	2.625282398
Obesity	adipocyte	*HOXB3*	TRUE	2.889940035
Obesity	adipocyte	*ADRA1A*	TRUE	3.061410095
Obesity	adipocyte	*GJB6*	TRUE	3.388384178
Obesity	adipocyte	*SMUG1*	TRUE	2.069420848
Obesity	adipocyte	*GRK4*	TRUE	3.469494025
Obesity	adipocyte	*PCSK2*	TRUE	2.463475303
Obesity	adipocyte	*RIBC2*	TRUE	3.444154759
Obesity	adipocyte	*ATP8B3*	TRUE	4.967241449
Obesity	adipocyte	*PKDREJ*	TRUE	3.088333295
Obesity	adipocyte	*EMILIN2*	TRUE	3.53723634
Obesity	adipocyte	*LARS*	TRUE	1.970011063
Obesity	adipocyte	*GSTM5*	TRUE	2.418126751
Obesity	adipocyte	*P2RX4*	TRUE	1.980998161
Obesity	adipocyte	*TMEM60*	TRUE	2.021779112
Obesity	adipocyte	*POM121C*	TRUE	2.03155076
Obesity	adipocyte	*EPHA7*	TRUE	2.596926902
Obesity	adipocyte	*DSCC1*	TRUE	2.264005249
Obesity	adipocyte	*CARF*	TRUE	2.150496251
Obesity	adipocyte	*UBL7*	TRUE	1.972696746
Obesity	adipocyte	*KLRG1*	TRUE	2.186340471
Obesity	adipocyte	*RITA1*	TRUE	2.429310323
Obesity	adipocyte	*PTGR2*	TRUE	1.988807172
Obesity	adipocyte	*WDR93*	TRUE	2.049982802

Obesity	adipocyte	*C18orf8*	TRUE	3.267288583
Obesity	adipocyte	*TRIM65*	TRUE	3.164099467
Obesity	adipocyte	*TUFT1*	TRUE	2.265422209
Obesity	adipocyte	*SLC25A48*	TRUE	2.938627939
Obesity	adipocyte	*ATP6V0D2*	TRUE	2.61479925
Obesity	adipocyte	*NRBF2*	TRUE	2.263013426
Obesity	adipocyte	*HSD17B12*	TRUE	5.656222854
Obesity	adipocyte	*TTC12*	TRUE	3.401575632
Obesity	adipocyte	*CCDC15*	TRUE	3.289691551
Obesity	adipocyte	*DUSP15*	TRUE	2.190844169
Obesity	adipocyte	*DOC2A*	TRUE	3.80317559
Obesity	adipocyte	*DPYSL4*	TRUE	3.032690995
Obesity	adipocyte	*BEND6*	TRUE	2.120255892
Obesity	adipocyte	*POC5*	TRUE	2.613164557
Obesity	adipocyte	*TCTEX1D1*	TRUE	2.309398402
Obesity	adipocyte	*FAM167A*	TRUE	1.997766422
Obesity	adipocyte	*STEAP2*	TRUE	2.93634563
Obesity	adipocyte	*CLEC18A*	TRUE	3.629458568
Obesity	adipocyte	*CD1E*	TRUE	2.421751958
Obesity	adipocyte	*SLC5A11*	TRUE	4.892872874
Obesity	adipocyte	*NAGS*	TRUE	2.025045507
Obesity	adipocyte	*CLHC1*	TRUE	2.527582701
Obesity	adipocyte	*LMOD1*	TRUE	6.336067239
Obesity	adipocyte	*DNALI1*	TRUE	2.97804976
Obesity	adipocyte	*TEX264*	TRUE	2.038819876
Obesity	adipocyte	*LRFN5*	TRUE	2.089352204
Obesity	adipocyte	*TRMT61A*	TRUE	2.362938763
Obesity	adipocyte	*SRR*	TRUE	3.8985683
Obesity	adipocyte	*BTNL3*	TRUE	2.04451703
Obesity	adipocyte	*RAB24*	TRUE	3.0313158
Obesity	adipocyte	*TRMT61B*	TRUE	3.481564557
Obesity	adipocyte	*C1QTNF4*	TRUE	5.256647561
Obesity	adipocyte	*FIBP*	TRUE	3.276496477
Obesity	adipocyte	*FUT10*	TRUE	2.529809384
Obesity	adipocyte	*HPSE2*	TRUE	4.300359329
Obesity	adipocyte	*CADM2*	TRUE	2.513577382
Obesity	adipocyte	*RUFY1*	TRUE	2.179427673
Obesity	adipocyte	*PRSS42*	TRUE	1.993861418
Obesity	adipocyte	*STX19*	TRUE	3.495972643
Obesity	adipocyte	*B3GNT3*	TRUE	3.278047913
Obesity	adipocyte	*AMIGO1*	TRUE	2.124083155
Obesity	adipocyte	*CAV3*	TRUE	2.52739837
Obesity	adipocyte	*BRICD5*	TRUE	2.51039822
Obesity	adipocyte	*LYSMD4*	TRUE	2.169249217
Obesity	adipocyte	*CCBE1*	TRUE	2.21100448
Obesity	adipocyte	*PSMG1*	TRUE	3.581270856
Obesity	adipocyte	*RP1L1*	TRUE	4.480474674
Obesity	adipocyte	*PLA2G6*	TRUE	2.385416483

Obesity	adipocyte	*KLHL33*	TRUE	2.226979615
Obesity	adipocyte	*CENPP*	TRUE	2.345395308
Obesity	adipocyte	*RPS26*	TRUE	3.023417962
Obesity	adipocyte	*ZNF780A*	TRUE	2.452367803
Obesity	adipocyte	*ADH4*	TRUE	3.255308344
Obesity	adipocyte	*ANKRD13B*	TRUE	2.48764608
Obesity	adipocyte	*CENPW*	TRUE	2.417274442
Obesity	adipocyte	*CCHCR1*	TRUE	1.983609688
Obesity	adipocyte	*HLA.E*	TRUE	3.795603531
Obesity	adipocyte	*EIF3CL*	TRUE	3.341459278
Obesity	adipocyte	*ASIC3*	TRUE	3.35060965
Obesity	adipocyte	*NOTCH2NL*	TRUE	2.066675658
Obesity	adipocyte	*SLC12A8*	TRUE	2.514072755
Obesity	adipocyte	*PLA2G4B*	TRUE	2.20488535
Obesity	adipocyte	*PCDHAC1*	TRUE	2.817779061
Obesity	adipocyte	*MPV17L2*	TRUE	3.86267166
Obesity	adipocyte	*HMBS*	TRUE	5.876746714
Obesity	adipocyte	*HP*	TRUE	3.10039738
Obesity	adipocyte	*DUXA*	TRUE	2.424032088
Obesity	adipocyte	*SH3RF3.AS1*	TRUE	3.179465097
Obesity	adipocyte	*LINC00562*	TRUE	2.855000689
Obesity	adipocyte	*GTF2H5*	TRUE	2.045034791
Obesity	ASPC	*WNT16*	TRUE	2.138115464
Obesity	ASPC	*RBM6*	TRUE	2.888663473
Obesity	ASPC	*SLC22A16*	TRUE	2.438850062
Obesity	ASPC	*COPZ2*	TRUE	2.419984198
Obesity	ASPC	*MAPK8IP2*	TRUE	2.268841382
Obesity	ASPC	*HFE*	TRUE	2.320205815
Obesity	ASPC	*CENPQ*	TRUE	2.57071184
Obesity	ASPC	*ZCCHC8*	TRUE	2.23805265
Obesity	ASPC	*SH3YL1*	TRUE	3.813700602
Obesity	ASPC	*VCL*	TRUE	2.167084358
Obesity	ASPC	*TUBG2*	TRUE	2.11630152
Obesity	ASPC	*LSG1*	TRUE	2.661735622
Obesity	ASPC	*TSPAN17*	TRUE	2.094728086
Obesity	ASPC	*UTS2*	TRUE	2.072791509
Obesity	ASPC	*RCN1*	TRUE	2.254413909
Obesity	ASPC	*JKAMP*	TRUE	2.132954807
Obesity	ASPC	*ITIH4*	TRUE	2.548863226
Obesity	ASPC	*ATP11B*	TRUE	2.278554601
Obesity	ASPC	*MBD3*	TRUE	2.145765723
Obesity	ASPC	*TIPIN*	TRUE	3.258485278
Obesity	ASPC	*MARK3*	TRUE	2.025780717
Obesity	ASPC	*XAB2*	TRUE	2.424517213
Obesity	ASPC	*DYNC1I2*	TRUE	2.235898558
Obesity	ASPC	*MYH7B*	TRUE	2.229352802
Obesity	ASPC	*PCDHGA2*	TRUE	2.441697305
Obesity	ASPC	*CCNT2*	TRUE	3.459132712

Obesity	ASPC	*COBLL1*	TRUE	2.977722648
Obesity	ASPC	*DOPEY1*	TRUE	2.373052067
Obesity	ASPC	*EPYC*	TRUE	2.502000125
Obesity	ASPC	*COL16A1*	TRUE	3.89553944
Obesity	ASPC	*SCAMP1*	TRUE	2.085485921
Obesity	ASPC	*OGFOD1*	TRUE	4.34744839
Obesity	ASPC	*PABPC4*	TRUE	3.156307747
Obesity	ASPC	*RGS17*	TRUE	2.721975404
Obesity	ASPC	*PDE6C*	TRUE	2.579862316
Obesity	ASPC	*OCEL1*	TRUE	2.000148638
Obesity	ASPC	*SMARCB1*	TRUE	2.658411374
Obesity	ASPC	*TFIP11*	TRUE	2.267158349
Obesity	ASPC	*FBXO7*	TRUE	1.986890247
Obesity	ASPC	*RASD2*	TRUE	2.09923308
Obesity	ASPC	*DESI1*	TRUE	3.352041415
Obesity	ASPC	*DICER1*	TRUE	2.028688257
Obesity	ASPC	*PSMB5*	TRUE	2.041435432
Obesity	ASPC	*ABHD12*	TRUE	3.350052699
Obesity	ASPC	*ANGPT4*	TRUE	2.216487901
Obesity	ASPC	*CEP192*	TRUE	2.104700871
Obesity	ASPC	*CORO1A*	TRUE	3.422172857
Obesity	ASPC	*NAGPA*	TRUE	2.314638664
Obesity	ASPC	*CLUAP1*	TRUE	2.855171267
Obesity	ASPC	*KAT8*	TRUE	4.148402851
Obesity	ASPC	*KNOP1*	TRUE	3.463667103
Obesity	ASPC	*ZDHHC2*	TRUE	3.726189874
Obesity	ASPC	*RAB2A*	TRUE	2.074880336
Obesity	ASPC	*MCM4*	TRUE	2.509759773
Obesity	ASPC	*SNRNP70*	TRUE	2.338621407
Obesity	ASPC	*PPP1R37*	TRUE	2.688662457
Obesity	ASPC	*ICAM5*	TRUE	3.15272668
Obesity	ASPC	*ZNF175*	TRUE	2.300292388
Obesity	ASPC	*ELL*	TRUE	2.072428034
Obesity	ASPC	*ATP13A1*	TRUE	3.84990908
Obesity	ASPC	*DLX5*	TRUE	1.99092707
Obesity	ASPC	*HOXA2*	TRUE	2.40495951
Obesity	ASPC	*MEST*	TRUE	2.538032244
Obesity	ASPC	*PLGRKT*	TRUE	2.304770656
Obesity	ASPC	*ACBD5*	TRUE	1.974287557
Obesity	ASPC	*RAD51C*	TRUE	2.578429505
Obesity	ASPC	*FOXRED1*	TRUE	2.177107939
Obesity	ASPC	*TRPV4*	TRUE	3.079464187
Obesity	ASPC	*GABRR2*	TRUE	2.271131931
Obesity	ASPC	*SLC26A8*	TRUE	2.463141375
Obesity	ASPC	*CAP2*	TRUE	2.998391302
Obesity	ASPC	*NME5*	TRUE	3.323869488
Obesity	ASPC	*BNIP1*	TRUE	3.004209189
Obesity	ASPC	*C3orf14*	TRUE	2.397804288

Obesity	ASPC	*KLHL24*	TRUE	2.356218185
Obesity	ASPC	*PLCH1*	TRUE	2.759479858
Obesity	ASPC	*FAHD2A*	TRUE	2.025261396
Obesity	ASPC	*ACTR1B*	TRUE	2.840225605
Obesity	ASPC	*TFCP2L1*	TRUE	2.006911773
Obesity	ASPC	*ITGB6*	TRUE	2.107824141
Obesity	ASPC	*CHMP3*	TRUE	4.660358566
Obesity	ASPC	*CD207*	TRUE	2.668143604
Obesity	ASPC	*DNAJC16*	TRUE	2.492995156
Obesity	ASPC	*MFAP2*	TRUE	2.647078714
Obesity	ASPC	*SERPINC1*	TRUE	3.216111489
Obesity	ASPC	*CD3EAP*	TRUE	2.072122994
Obesity	ASPC	*COLEC11*	TRUE	2.735661588
Obesity	ASPC	*DNAH7*	TRUE	2.010366013
Obesity	ASPC	*CCDC92*	TRUE	4.743219261
Obesity	ASPC	*HDHD3*	TRUE	2.256531134
Obesity	ASPC	*INVS*	TRUE	1.999482295
Obesity	ASPC	*DCAF4*	TRUE	3.480410926
Obesity	ASPC	*CNRIP1*	TRUE	2.14458506
Obesity	ASPC	*CRIPT*	TRUE	2.525575575
Obesity	ASPC	*HOXB3*	TRUE	1.962841352
Obesity	ASPC	*SNX19*	TRUE	3.416831199
Obesity	ASPC	*ADRA1A*	TRUE	3.883932314
Obesity	ASPC	*SEC22A*	TRUE	2.091019523
Obesity	ASPC	*GJB6*	TRUE	3.455318963
Obesity	ASPC	*SLC25A51*	TRUE	2.302783703
Obesity	ASPC	*ATP7B*	TRUE	2.184352019
Obesity	ASPC	*ARHGAP9*	TRUE	2.780088735
Obesity	ASPC	*SMUG1*	TRUE	2.238251999
Obesity	ASPC	*CHST8*	TRUE	1.963461399
Obesity	ASPC	*SNRPC*	TRUE	2.656325963
Obesity	ASPC	*EEF1E1*	TRUE	2.624254882
Obesity	ASPC	*GRK4*	TRUE	4.018539352
Obesity	ASPC	*PCSK2*	TRUE	4.361413845
Obesity	ASPC	*YEATS4*	TRUE	2.821350608
Obesity	ASPC	*RIBC2*	TRUE	3.431454916
Obesity	ASPC	*RPAIN*	TRUE	2.680544597
Obesity	ASPC	*ATP8B3*	TRUE	5.482420812
Obesity	ASPC	*PGPEP1*	TRUE	2.358764089
Obesity	ASPC	*CYP2E1*	TRUE	2.078794283
Obesity	ASPC	*PKDREJ*	TRUE	3.17872432
Obesity	ASPC	*EMILIN2*	TRUE	4.426287586
Obesity	ASPC	*IGHMBP2*	TRUE	2.827486719
Obesity	ASPC	*PEMT*	TRUE	2.128372905
Obesity	ASPC	*BTBD2*	TRUE	2.055498307
Obesity	ASPC	*LARS*	TRUE	3.396644856
Obesity	ASPC	*GSTM5*	TRUE	2.385698552
Obesity	ASPC	*P2RX4*	TRUE	2.561976819

Obesity	ASPC	*TMEM60*	TRUE	3.89512269
Obesity	ASPC	*POM121C*	TRUE	5.729142504
Obesity	ASPC	*EPHA7*	TRUE	2.666001444
Obesity	ASPC	*MRPS9*	TRUE	2.367403445
Obesity	ASPC	*NMT1*	TRUE	2.311055661
Obesity	ASPC	*DSCC1*	TRUE	2.843318083
Obesity	ASPC	*TMEM63B*	TRUE	2.307217227
Obesity	ASPC	*TTPA*	TRUE	2.152007737
Obesity	ASPC	*MAP2K5*	TRUE	2.654707652
Obesity	ASPC	*SLC44A5*	TRUE	2.081537226
Obesity	ASPC	*IFT172*	TRUE	3.32540492
Obesity	ASPC	*CARF*	TRUE	2.544262563
Obesity	ASPC	*UBL7*	TRUE	2.298851338
Obesity	ASPC	*MMRN1*	TRUE	2.406494797
Obesity	ASPC	*KLRG1*	TRUE	2.447491378
Obesity	ASPC	*RITA1*	TRUE	2.8397358
Obesity	ASPC	*GPR84*	TRUE	2.07142371
Obesity	ASPC	*PTGR2*	TRUE	2.350790459
Obesity	ASPC	*CYP11A1*	TRUE	2.63388696
Obesity	ASPC	*WDR93*	TRUE	3.1025107
Obesity	ASPC	*SH3GL3*	TRUE	1.976084397
Obesity	ASPC	*MYO19*	TRUE	2.423664413
Obesity	ASPC	*C18orf8*	TRUE	2.319823005
Obesity	ASPC	*TRIM65*	TRUE	2.671518699
Obesity	ASPC	*CBX8*	TRUE	2.154136664
Obesity	ASPC	*TUFT1*	TRUE	2.739799523
Obesity	ASPC	*SEMA6C*	TRUE	1.96679243
Obesity	ASPC	*S100A8*	TRUE	2.832202554
Obesity	ASPC	*HAX1*	TRUE	2.511174431
Obesity	ASPC	*FBLN7*	TRUE	2.255248186
Obesity	ASPC	*AFF3*	TRUE	2.799176008
Obesity	ASPC	*THSD7B*	TRUE	2.30167677
Obesity	ASPC	*PHOSPHO2*	TRUE	2.028613699
Obesity	ASPC	*EIF2A*	TRUE	2.162365451
Obesity	ASPC	*MARCH1*	TRUE	2.731984755
Obesity	ASPC	*SLC25A48*	TRUE	2.978449337
Obesity	ASPC	*PCYOX1L*	TRUE	3.445175414
Obesity	ASPC	*RPL7L1*	TRUE	1.974506404
Obesity	ASPC	*SDK1*	TRUE	2.278245228
Obesity	ASPC	*ATP6V0D2*	TRUE	4.110941965
Obesity	ASPC	*NRBF2*	TRUE	2.248873628
Obesity	ASPC	*HSD17B12*	TRUE	6.715533498
Obesity	ASPC	*TTC12*	TRUE	3.818286609
Obesity	ASPC	*CCDC15*	TRUE	4.152984727
Obesity	ASPC	*DUSP15*	TRUE	2.543828314
Obesity	ASPC	*TRPT1*	TRUE	3.332931294
Obesity	ASPC	*DOC2A*	TRUE	2.584374109
Obesity	ASPC	*DPYSL4*	TRUE	3.312771499

Obesity	ASPC	*BEND6*	TRUE	2.516317249
Obesity	ASPC	*ARL14EP*	TRUE	3.474093646
Obesity	ASPC	*POC5*	TRUE	4.962664876
Obesity	ASPC	*TCTEX1D1*	TRUE	2.498164187
Obesity	ASPC	*JAZF1*	TRUE	2.153925429
Obesity	ASPC	*CEP112*	TRUE	2.031800845
Obesity	ASPC	*ZKSCAN2*	TRUE	2.256164554
Obesity	ASPC	*TIMP4*	TRUE	2.15521834
Obesity	ASPC	*STEAP2*	TRUE	3.555743505
Obesity	ASPC	*CLEC18A*	TRUE	3.506476152
Obesity	ASPC	*HPD*	TRUE	2.258062834
Obesity	ASPC	*CD1E*	TRUE	2.264264654
Obesity	ASPC	*COPG2*	TRUE	2.268373616
Obesity	ASPC	*SLC5A11*	TRUE	5.12977861
Obesity	ASPC	*SIM2*	TRUE	2.123500962
Obesity	ASPC	*DFFA*	TRUE	1.991424177
Obesity	ASPC	*C21orf33*	TRUE	2.28581157
Obesity	ASPC	*C19orf47*	TRUE	2.606099086
Obesity	ASPC	*ZBTB7B*	TRUE	3.981428244
Obesity	ASPC	*FBXO27*	TRUE	2.01878252
Obesity	ASPC	*FUBP1*	TRUE	5.926101093
Obesity	ASPC	*ACP6*	TRUE	3.033256628
Obesity	ASPC	*ANTXR2*	TRUE	2.957751198
Obesity	ASPC	*SLC15A2*	TRUE	2.614118118
Obesity	ASPC	*LMOD1*	TRUE	7.880652908
Obesity	ASPC	*GYG1*	TRUE	2.624931604
Obesity	ASPC	*DNALI1*	TRUE	3.697727676
Obesity	ASPC	*RFT1*	TRUE	2.342808107
Obesity	ASPC	*SLC51A*	TRUE	3.289061458
Obesity	ASPC	*C1orf50*	TRUE	2.242355453
Obesity	ASPC	*BDH2*	TRUE	2.057645225
Obesity	ASPC	*TEX264*	TRUE	3.02479331
Obesity	ASPC	*ERAP1*	TRUE	3.489907281
Obesity	ASPC	*TMEM184A*	TRUE	2.624103816
Obesity	ASPC	*YWHAZ*	TRUE	3.374695544
Obesity	ASPC	*LRFN5*	TRUE	2.17206155
Obesity	ASPC	*RIMKLB*	TRUE	2.584049177
Obesity	ASPC	*SAAL1*	TRUE	2.112692929
Obesity	ASPC	*MS4A6E*	TRUE	2.147862434
Obesity	ASPC	*URM1*	TRUE	2.926511361
Obesity	ASPC	*SP2*	TRUE	1.980572199
Obesity	ASPC	*VKORC1*	TRUE	3.553232827
Obesity	ASPC	*PSCA*	TRUE	3.348691474
Obesity	ASPC	*UBXN6*	TRUE	3.392692281
Obesity	ASPC	*SRR*	TRUE	4.256588212
Obesity	ASPC	*MLST8*	TRUE	2.165124707
Obesity	ASPC	*VWCE*	TRUE	2.264108711
Obesity	ASPC	*REEP4*	TRUE	1.962076019

Obesity	ASPC	*COL4A3*	TRUE	2.022931493
Obesity	ASPC	*RAB24*	TRUE	3.646839757
Obesity	ASPC	*CTNND2*	TRUE	2.014062973
Obesity	ASPC	*HOXD4*	TRUE	2.110981968
Obesity	ASPC	*ADORA2B*	TRUE	2.988001669
Obesity	ASPC	*SOX7*	TRUE	2.471642046
Obesity	ASPC	*CYP4F22*	TRUE	2.226218688
Obesity	ASPC	*ZNF554*	TRUE	2.037291606
Obesity	ASPC	*CXCR6*	TRUE	2.143717238
Obesity	ASPC	*C1QTNF4*	TRUE	6.20158116
Obesity	ASPC	*CTSW*	TRUE	2.140405815
Obesity	ASPC	*SNTG2*	TRUE	3.845385168
Obesity	ASPC	*CCL19*	TRUE	2.297299872
Obesity	ASPC	*FUT10*	TRUE	2.077832412
Obesity	ASPC	*HPSE2*	TRUE	5.369950168
Obesity	ASPC	*KLF14*	TRUE	2.60297733
Obesity	ASPC	*HRAS*	TRUE	2.49982106
Obesity	ASPC	*SRP72*	TRUE	2.291447468
Obesity	ASPC	*RSRC1*	TRUE	2.344102588
Obesity	ASPC	*CADM2*	TRUE	3.120429916
Obesity	ASPC	*PSMD2*	TRUE	2.313677486
Obesity	ASPC	*TMEM9B*	TRUE	3.16080157
Obesity	ASPC	*CCDC57*	TRUE	3.014946706
Obesity	ASPC	*SMIM19*	TRUE	2.093848828
Obesity	ASPC	*RUFY1*	TRUE	3.210263141
Obesity	ASPC	*TALDO1*	TRUE	2.494677789
Obesity	ASPC	*AP006621.1*	TRUE	2.719888842
Obesity	ASPC	*PPFIA3*	TRUE	2.492404151
Obesity	ASPC	*FAM87B*	TRUE	2.633256794
Obesity	ASPC	*RPP25*	TRUE	2.227568579
Obesity	ASPC	*STX19*	TRUE	4.505674349
Obesity	ASPC	*CTC1*	TRUE	2.17599314
Obesity	ASPC	*FARSA*	TRUE	1.992434233
Obesity	ASPC	*GIPC3*	TRUE	2.049179588
Obesity	ASPC	*B3GNT3*	TRUE	3.194237247
Obesity	ASPC	*ZNF322*	TRUE	2.664848741
Obesity	ASPC	*AMIGO1*	TRUE	2.192811832
Obesity	ASPC	*IZUMO1*	TRUE	2.087671527
Obesity	ASPC	*FBXL6*	TRUE	2.473281804
Obesity	ASPC	*CAV3*	TRUE	3.02893621
Obesity	ASPC	*BRICD5*	TRUE	2.796553601
Obesity	ASPC	*GRID1*	TRUE	3.401966582
Obesity	ASPC	*LYSMD4*	TRUE	2.680207931
Obesity	ASPC	*CCBE1*	TRUE	2.217888448
Obesity	ASPC	*LRIT3*	TRUE	2.177594331
Obesity	ASPC	*PSMG1*	TRUE	3.689081254
Obesity	ASPC	*RP1L1*	TRUE	3.182829825
Obesity	ASPC	*MAATS1*	TRUE	2.09273425

Obesity	ASPC	*PLA2G6*	TRUE	3.889255146
Obesity	ASPC	*SNN*	TRUE	2.285715337
Obesity	ASPC	*NOC4L*	TRUE	2.45699001
Obesity	ASPC	*MAFF*	TRUE	2.569349987
Obesity	ASPC	*GP1BA*	TRUE	2.371845324
Obesity	ASPC	*KLHL33*	TRUE	2.249823677
Obesity	ASPC	*SRL*	TRUE	2.035531008
Obesity	ASPC	*TRABD2A*	TRUE	2.415871605
Obesity	ASPC	*ADH1A*	TRUE	2.199512951
Obesity	ASPC	*HYKK*	TRUE	2.416302063
Obesity	ASPC	*CENPP*	TRUE	2.763200558
Obesity	ASPC	*JAKMIP3*	TRUE	2.023487376
Obesity	ASPC	*FAM47E*	TRUE	3.200529899
Obesity	ASPC	*HCAR1*	TRUE	2.490158631
Obesity	ASPC	*SLC25A29*	TRUE	2.051862508
Obesity	ASPC	*SULT1A2*	TRUE	2.3401438
Obesity	ASPC	*PCDHB11*	TRUE	2.540594848
Obesity	ASPC	*C14orf178*	TRUE	1.994137818
Obesity	ASPC	*ZNF780A*	TRUE	3.132926575
Obesity	ASPC	*LEKR1*	TRUE	2.130638947
Obesity	ASPC	*MRPL42*	TRUE	1.9873508
Obesity	ASPC	*ZNF649*	TRUE	2.42353615
Obesity	ASPC	*ADH4*	TRUE	3.383313222
Obesity	ASPC	*ZKSCAN8*	TRUE	2.068860265
Obesity	ASPC	*ANKRD13B*	TRUE	2.410880022
Obesity	ASPC	*TYW1*	TRUE	2.169715293
Obesity	ASPC	*PRMT6*	TRUE	3.096065722
Obesity	ASPC	*CENPW*	TRUE	2.764297281
Obesity	ASPC	*SYT15*	TRUE	2.029616309
Obesity	ASPC	*NOTCH4*	TRUE	2.046417823
Obesity	ASPC	*SKIV2L*	TRUE	2.624415977
Obesity	ASPC	*HLA.E*	TRUE	4.08217347
Obesity	ASPC	*VGLL3*	TRUE	1.987498615
Obesity	ASPC	*COLQ*	TRUE	1.997175851
Obesity	ASPC	*ASIC3*	TRUE	3.784679251
Obesity	ASPC	*NOTCH2NL*	TRUE	2.63323079
Obesity	ASPC	*CPNE1*	TRUE	2.338352048
Obesity	ASPC	*C17orf67*	TRUE	1.968700268
Obesity	ASPC	*ATAD3C*	TRUE	2.531089409
Obesity	ASPC	*SLC12A8*	TRUE	3.463751061
Obesity	ASPC	*EXOSC6*	TRUE	2.827645598
Obesity	ASPC	*AC069213.1*	TRUE	2.011471384
Obesity	ASPC	*LINC00240*	TRUE	2.181921467
Obesity	ASPC	*RPL41*	TRUE	2.117195571
Obesity	ASPC	*RPS18*	TRUE	2.036754072
Obesity	ASPC	*LINC00484*	TRUE	2.176324954
Obesity	ASPC	*LINC00545*	TRUE	2.283571967
Obesity	ASPC	*TXNDC5*	TRUE	1.98448542

Obesity	ASPC	*CCDC169*	TRUE	2.170804705
Obesity	ASPC	*PLA2G4B*	TRUE	2.266760926
Obesity	ASPC	*SRP14.AS1*	TRUE	2.180315224
Obesity	ASPC	*SNHG18*	TRUE	2.657449426
Obesity	ASPC	*PCDHGA6*	TRUE	2.005830032
Obesity	ASPC	*MPV17L2*	TRUE	4.185548729
Obesity	ASPC	*HMBS*	TRUE	6.258539697
Obesity	ASPC	*AC093668.2*	TRUE	2.511956641
Obesity	ASPC	*PGA5*	TRUE	2.031211731
Obesity	ASPC	*HP*	TRUE	2.687319925
Obesity	ASPC	*DUXA*	TRUE	2.504506423
Obesity	ASPC	*UBE2Q2L*	TRUE	2.217134023
Obesity	ASPC	*AC083843.1*	TRUE	2.700745198
Obesity	ASPC	*SH3RF3.AS1*	TRUE	3.674419062
Obesity	ASPC	*LINC00562*	TRUE	3.184523157
Obesity	ASPC	*LINC01140*	TRUE	2.620250287
Obesity	ASPC	*GTF2H5*	TRUE	2.748847804
Obesity	b_cell	*ITIH4*	TRUE	2.862143514
Obesity	b_cell	*CHRNE*	TRUE	1.969396231
Obesity	b_cell	*SNRPC*	TRUE	4.389287651
Obesity	b_cell	*PKDREJ*	TRUE	2.409191935
Obesity	b_cell	*PRSS53*	TRUE	2.192760586
Obesity	b_cell	*DPYSL4*	TRUE	2.339348316
Obesity	b_cell	*CLEC18A*	TRUE	7.527964202
Obesity	b_cell	*RNF123*	TRUE	3.703288117
Obesity	b_cell	*C1QTNF4*	TRUE	6.353463238
Obesity	b_cell	*CTSW*	TRUE	2.2651154
Obesity	b_cell	*TRIM73*	TRUE	4.951161845
Obesity	b_cell	*SULT1A2*	TRUE	4.104755643
Obesity	b_cell	*POU5F1*	TRUE	2.816018375
Obesity	b_cell	*NOTCH2NL*	TRUE	3.080096999
Obesity	dendritic_cell	*ITIH4*	TRUE	5.923736035
Obesity	dendritic_cell	*CHRNE*	TRUE	2.364528414
Obesity	dendritic_cell	*SERPINC1*	TRUE	5.367454686
Obesity	dendritic_cell	*ADRA1A*	TRUE	2.35937321
Obesity	dendritic_cell	*PKDREJ*	TRUE	2.174270524
Obesity	dendritic_cell	*IGHMBP2*	TRUE	2.536241535
Obesity	dendritic_cell	*DSCC1*	TRUE	1.968621701
Obesity	dendritic_cell	*HSD17B12*	TRUE	5.077962654
Obesity	dendritic_cell	*TTC12*	TRUE	2.39691368
Obesity	dendritic_cell	*DPYSL4*	TRUE	3.092604396
Obesity	dendritic_cell	*LMOD1*	TRUE	4.582431836
Obesity	dendritic_cell	*RNF123*	TRUE	2.080452253
Obesity	dendritic_cell	*TRMT61A*	TRUE	2.142219066
Obesity	dendritic_cell	*C8orf46*	TRUE	2.424513171
Obesity	dendritic_cell	*C1QTNF4*	TRUE	5.379203865
Obesity	dendritic_cell	*TRIM73*	TRUE	4.023350055
Obesity	dendritic_cell	*EIF3C*	TRUE	4.096527092

Obesity	dendritic_cell	*BTN3A2*	TRUE	2.821839546
Obesity	dendritic_cell	*RPS26*	TRUE	3.133241542
Obesity	dendritic_cell	*ZNF780A*	TRUE	3.26235296
Obesity	dendritic_cell	*NOTCH4*	TRUE	2.438208846
Obesity	dendritic_cell	*SKIV2L*	TRUE	2.997162837
Obesity	endothelial	*RBM6*	TRUE	3.115452345
Obesity	endothelial	*ITIH4*	TRUE	3.447589415
Obesity	endothelial	*CHRNE*	TRUE	2.336186325
Obesity	endothelial	*MFAP2*	TRUE	2.612754373
Obesity	endothelial	*SERPINC1*	TRUE	2.552047395
Obesity	endothelial	*HIST1H2BJ*	TRUE	2.324827522
Obesity	endothelial	*ATP8B3*	TRUE	3.899754769
Obesity	endothelial	*PKDREJ*	TRUE	2.456682375
Obesity	endothelial	*DOC2A*	TRUE	3.52563015
Obesity	endothelial	*DPYSL4*	TRUE	2.348900733
Obesity	endothelial	*SLC5A11*	TRUE	3.878947677
Obesity	endothelial	*LMOD1*	TRUE	1.969703798
Obesity	endothelial	*ADORA2B*	TRUE	3.448810034
Obesity	endothelial	*C1QTNF4*	TRUE	7.150885439
Obesity	endothelial	*CTSW*	TRUE	4.253129836
Obesity	endothelial	*TRIM73*	TRUE	4.815546663
Obesity	endothelial	*PSMG1*	TRUE	2.081799308
Obesity	endothelial	*CCL4L2*	TRUE	1.976491793
Obesity	endothelial	*ZNF780A*	TRUE	2.91879801
Obesity	endothelial	*ADH4*	TRUE	2.14332473
Obesity	endothelial	*MRPL38*	TRUE	1.999684249
Obesity	endothelial	*SKIV2L*	TRUE	2.211457739
Obesity	endothelial	*ZFP57*	TRUE	2.098240821
Obesity	endothelial	*HP*	TRUE	2.330981273
Obesity	endothelial	*DUXA*	TRUE	2.045677855
Obesity	LEC	*ITIH4*	TRUE	4.253499999
Obesity	LEC	*TIPIN*	TRUE	2.227676722
Obesity	LEC	*SERPINC1*	TRUE	2.498888174
Obesity	LEC	*ADRA1A*	TRUE	2.304673154
Obesity	LEC	*GRK4*	TRUE	2.905162028
Obesity	LEC	*HSD17B12*	TRUE	2.724131222
Obesity	LEC	*DPYSL4*	TRUE	3.257270845
Obesity	LEC	*CLEC18A*	TRUE	7.543023477
Obesity	LEC	*SLC5A11*	TRUE	3.487644911
Obesity	LEC	*C1QTNF4*	TRUE	3.854333081
Obesity	LEC	*BTN3A2*	TRUE	2.848294317
Obesity	LEC	*RPS26*	TRUE	2.382566948
Obesity	LEC	*ZNF780A*	TRUE	2.076364321
Obesity	LEC	*SKIV2L*	TRUE	2.328456539
Obesity	LEC	*C17orf107*	TRUE	2.015390582
Obesity	LEC	*NOTCH2NL*	TRUE	2.785030839
Obesity	macrophage	*WNT16*	TRUE	2.346449499
Obesity	macrophage	*RBM6*	TRUE	3.201714157

Obesity	macrophage	*ITIH4*	TRUE	4.75746538
Obesity	macrophage	*TIPIN*	TRUE	3.580041723
Obesity	macrophage	*DESI1*	TRUE	3.18314756
Obesity	macrophage	*CORO1A*	TRUE	2.148748791
Obesity	macrophage	*ELMO3*	TRUE	1.962452553
Obesity	macrophage	*CLUAP1*	TRUE	2.332283872
Obesity	macrophage	*ICAM5*	TRUE	2.393108438
Obesity	macrophage	*ATP13A1*	TRUE	2.391810374
Obesity	macrophage	*DLX5*	TRUE	2.022761766
Obesity	macrophage	*BNIP1*	TRUE	2.259663879
Obesity	macrophage	*CHMP3*	TRUE	2.435342454
Obesity	macrophage	*SERPINC1*	TRUE	5.679609483
Obesity	macrophage	*CCDC92*	TRUE	3.164863258
Obesity	macrophage	*DCAF4*	TRUE	2.403107566
Obesity	macrophage	*ADRA1A*	TRUE	3.529216681
Obesity	macrophage	*GJB6*	TRUE	2.788493285
Obesity	macrophage	*SMUG1*	TRUE	2.114380287
Obesity	macrophage	*GRK4*	TRUE	3.575481917
Obesity	macrophage	*OPRL1*	TRUE	2.406675487
Obesity	macrophage	*YEATS4*	TRUE	2.110982386
Obesity	macrophage	*ATP8B3*	TRUE	5.122799078
Obesity	macrophage	*PKDREJ*	TRUE	3.11662732
Obesity	macrophage	*TMEM60*	TRUE	2.04852031
Obesity	macrophage	*EPHA7*	TRUE	2.470458901
Obesity	macrophage	*DSCC1*	TRUE	2.5512707
Obesity	macrophage	*CARF*	TRUE	2.62276932
Obesity	macrophage	*C18orf8*	TRUE	2.910776983
Obesity	macrophage	*TRIM65*	TRUE	2.629369835
Obesity	macrophage	*SLC25A48*	TRUE	2.650843508
Obesity	macrophage	*PCYOX1L*	TRUE	2.354358962
Obesity	macrophage	*MTG1*	TRUE	2.565510125
Obesity	macrophage	*HSD17B12*	TRUE	5.479955641
Obesity	macrophage	*TTC12*	TRUE	3.303428689
Obesity	macrophage	*CCDC15*	TRUE	2.986732967
Obesity	macrophage	*DUSP15*	TRUE	2.239154346
Obesity	macrophage	*DOC2A*	TRUE	2.52209808
Obesity	macrophage	*DPYSL4*	TRUE	3.73156787
Obesity	macrophage	*POC5*	TRUE	2.390230822
Obesity	macrophage	*TCTEX1D1*	TRUE	2.290992068
Obesity	macrophage	*NEIL2*	TRUE	2.257305482
Obesity	macrophage	*CLEC18A*	TRUE	5.741623713
Obesity	macrophage	*HPD*	TRUE	3.424298527
Obesity	macrophage	*CD1E*	TRUE	2.122406707
Obesity	macrophage	*SLC5A11*	TRUE	4.655745782
Obesity	macrophage	*CLHC1*	TRUE	2.422393962
Obesity	macrophage	*LMOD1*	TRUE	4.838537359
Obesity	macrophage	*ZNF646*	TRUE	2.940108208
Obesity	macrophage	*VKORC1*	TRUE	2.754559905

Obesity	macrophage	*PSCA*	TRUE	2.619081208
Obesity	macrophage	*SRR*	TRUE	3.961862717
Obesity	macrophage	*C8orf46*	TRUE	3.306456141
Obesity	macrophage	*RAB24*	TRUE	2.661831851
Obesity	macrophage	*FAM103A1*	TRUE	2.589662013
Obesity	macrophage	*TRMT61B*	TRUE	3.264702748
Obesity	macrophage	*C1QTNF4*	TRUE	6.842600008
Obesity	macrophage	*EFEMP2*	TRUE	3.163270442
Obesity	macrophage	*FUT10*	TRUE	1.999976557
Obesity	macrophage	*HPSE2*	TRUE	3.452402368
Obesity	macrophage	*STX19*	TRUE	2.641910635
Obesity	macrophage	*TRIM73*	TRUE	4.56067287
Obesity	macrophage	*B3GNT3*	TRUE	2.632710121
Obesity	macrophage	*ZNF322*	TRUE	2.273195407
Obesity	macrophage	*ZFP41*	TRUE	1.963685884
Obesity	macrophage	*BRICD5*	TRUE	2.299049251
Obesity	macrophage	*PSMG1*	TRUE	3.664990547
Obesity	macrophage	*RP1L1*	TRUE	4.198414012
Obesity	macrophage	*PLA2G6*	TRUE	3.675619925
Obesity	macrophage	*CENPP*	TRUE	1.963648533
Obesity	macrophage	*FAM47E*	TRUE	2.657990403
Obesity	macrophage	*HCAR1*	TRUE	2.297421714
Obesity	macrophage	*CCL4L2*	TRUE	2.236482575
Obesity	macrophage	*RPS26*	TRUE	3.446396085
Obesity	macrophage	*ZNF780A*	TRUE	2.569967686
Obesity	macrophage	*ADH4*	TRUE	2.854052539
Obesity	macrophage	*CCHCR1*	TRUE	2.100785767
Obesity	macrophage	*HLA.E*	TRUE	3.620654394
Obesity	macrophage	*EIF3CL*	TRUE	3.292862465
Obesity	macrophage	*ASIC3*	TRUE	3.691357908
Obesity	macrophage	*NOTCH2NL*	TRUE	2.114733021
Obesity	macrophage	*LINC00545*	TRUE	2.044931392
Obesity	macrophage	*PLA2G4B*	TRUE	2.142953941
Obesity	macrophage	*PCDHAC1*	TRUE	2.490681411
Obesity	macrophage	*MPV17L2*	TRUE	3.742098851
Obesity	macrophage	*HMBS*	TRUE	6.04572154
Obesity	macrophage	*DUXA*	TRUE	2.503250547
Obesity	macrophage	*SH3RF3.AS1*	TRUE	2.716810578
Obesity	mast_cell	*ITIH4*	TRUE	2.979242143
Obesity	mast_cell	*UHRF1BP1*	TRUE	7.824117756
Obesity	mast_cell	*TIPIN*	TRUE	2.458324998
Obesity	mast_cell	*DESI1*	TRUE	2.111638523
Obesity	mast_cell	*CORO1A*	TRUE	3.095193997
Obesity	mast_cell	*DLX5*	TRUE	1.973047628
Obesity	mast_cell	*SERPINC1*	TRUE	3.730264663
Obesity	mast_cell	*ADRA1A*	TRUE	1.960144202
Obesity	mast_cell	*GRK4*	TRUE	3.271221288
Obesity	mast_cell	*PKDREJ*	TRUE	3.137193706

Obesity	mast_cell	*IGHMBP2*	TRUE	2.250257829
Obesity	mast_cell	*DSCC1*	TRUE	2.283725649
Obesity	mast_cell	*HSD17B12*	TRUE	2.811009012
Obesity	mast_cell	*DPYSL4*	TRUE	3.340432809
Obesity	mast_cell	*CLEC18A*	TRUE	2.241636101
Obesity	mast_cell	*HPD*	TRUE	3.278803862
Obesity	mast_cell	*ZNF646*	TRUE	2.45526027
Obesity	mast_cell	*PSCA*	TRUE	2.729501709
Obesity	mast_cell	*SRR*	TRUE	3.082450719
Obesity	mast_cell	*C8orf46*	TRUE	2.927291977
Obesity	mast_cell	*TRMT61B*	TRUE	2.122835821
Obesity	mast_cell	*C1QTNF4*	TRUE	5.687877558
Obesity	mast_cell	*TRIM73*	TRUE	2.960252459
Obesity	mast_cell	*PSMG1*	TRUE	3.368247659
Obesity	mast_cell	*BTN3A2*	TRUE	2.194768637
Obesity	mast_cell	*SULT1A2*	TRUE	3.153972542
Obesity	mast_cell	*RPS26*	TRUE	2.117454817
Obesity	mast_cell	*HMBS*	TRUE	2.27667498
Obesity	monocyte	*RBM6*	TRUE	2.90977825
Obesity	monocyte	*CENPQ*	TRUE	2.337001379
Obesity	monocyte	*ITIH4*	TRUE	3.007340976
Obesity	monocyte	*UHRF1BP1*	TRUE	3.806003657
Obesity	monocyte	*DESI1*	TRUE	1.978035625
Obesity	monocyte	*DLX5*	TRUE	2.486748751
Obesity	monocyte	*PRSS16*	TRUE	3.340050671
Obesity	monocyte	*GSTM1*	TRUE	2.005036147
Obesity	monocyte	*SLC25A48*	TRUE	2.171924161
Obesity	monocyte	*CLEC18A*	TRUE	2.501562948
Obesity	monocyte	*C1QTNF4*	TRUE	4.803922712
Obesity	monocyte	*TRIM73*	TRUE	2.872122002
Obesity	monocyte	*EIF3C*	TRUE	3.028591506
Obesity	monocyte	*TRABD2A*	TRUE	2.852850954
Obesity	monocyte	*SP6*	TRUE	2.841357392
Obesity	monocyte	*POU5F1*	TRUE	2.216417625
Obesity	neutrophil	*TIPIN*	TRUE	2.771740374
Obesity	neutrophil	*SERPINC1*	TRUE	1.967708414
Obesity	neutrophil	*CLEC18A*	TRUE	7.61942465
Obesity	neutrophil	*C1QTNF4*	TRUE	7.198257012
Obesity	neutrophil	*CTSW*	TRUE	3.031529387
Obesity	neutrophil	*TRIM73*	TRUE	2.723150093
Obesity	neutrophil	*EIF3C*	TRUE	4.918312713
Obesity	neutrophil	*HYAL3*	TRUE	2.909261002
Obesity	neutrophil	*NOTCH4*	TRUE	2.354542619
Obesity	neutrophil	*SKIV2L*	TRUE	2.592395946
Obesity	neutrophil	*AC084018.1*	TRUE	2.282049134
Obesity	nk_cell	*ITIH4*	TRUE	5.508391392
Obesity	nk_cell	*UHRF1BP1*	TRUE	2.940670732
Obesity	nk_cell	*PRSS16*	TRUE	2.361485905

Obesity	nk_cell	*MFAP2*	TRUE	2.221757499
Obesity	nk_cell	*GRK4*	TRUE	2.775357337
Obesity	nk_cell	*ATP8B3*	TRUE	4.113992947
Obesity	nk_cell	*PKDREJ*	TRUE	2.218126151
Obesity	nk_cell	*HSD17B12*	TRUE	3.136835257
Obesity	nk_cell	*DPYSL4*	TRUE	2.908459632
Obesity	nk_cell	*CLEC18A*	TRUE	7.772949403
Obesity	nk_cell	*HPD*	TRUE	2.824402375
Obesity	nk_cell	*SLC5A11*	TRUE	2.799347276
Obesity	nk_cell	*SRR*	TRUE	3.325849765
Obesity	nk_cell	*FAM103A1*	TRUE	1.968588751
Obesity	nk_cell	*ADORA2B*	TRUE	3.948645586
Obesity	nk_cell	*C1QTNF4*	TRUE	5.21358165
Obesity	nk_cell	*TRIM73*	TRUE	4.365831149
Obesity	nk_cell	*PSMG1*	TRUE	2.601459789
Obesity	nk_cell	*BMP8A*	TRUE	3.247260783
Obesity	nk_cell	*HCAR1*	TRUE	2.2168667
Obesity	nk_cell	*ZNF780A*	TRUE	2.641471911
Obesity	nk_cell	*NOTCH4*	TRUE	2.67107901
Obesity	nk_cell	*SKIV2L*	TRUE	3.158113287
Obesity	nk_cell	*POU5F1*	TRUE	2.433227678
Obesity	nk_cell	*C17orf107*	TRUE	2.164286114
Obesity	nk_cell	*HMBS*	TRUE	5.190198766
Obesity	pericyte	*ITIH4*	TRUE	5.961262843
Obesity	pericyte	*UHRF1BP1*	TRUE	6.908488353
Obesity	pericyte	*TIPIN*	TRUE	2.942201601
Obesity	pericyte	*YPEL3*	TRUE	4.024346239
Obesity	pericyte	*STX1B*	TRUE	2.128645178
Obesity	pericyte	*KAT8*	TRUE	1.973066402
Obesity	pericyte	*ATP13A1*	TRUE	2.086091589
Obesity	pericyte	*BNIP1*	TRUE	2.155086309
Obesity	pericyte	*SERPINC1*	TRUE	4.40357426
Obesity	pericyte	*CCDC92*	TRUE	2.465484247
Obesity	pericyte	*ADRA1A*	TRUE	2.998754292
Obesity	pericyte	*SMUG1*	TRUE	2.003780283
Obesity	pericyte	*GRK4*	TRUE	2.330673676
Obesity	pericyte	*RIBC2*	TRUE	2.14628658
Obesity	pericyte	*ATP8B3*	TRUE	4.878092177
Obesity	pericyte	*PKDREJ*	TRUE	2.234804785
Obesity	pericyte	*IGHMBP2*	TRUE	2.8703246
Obesity	pericyte	*EPHA7*	TRUE	2.192368651
Obesity	pericyte	*HSD17B12*	TRUE	5.64989039
Obesity	pericyte	*TTC12*	TRUE	3.175097653
Obesity	pericyte	*DPYSL4*	TRUE	3.461168664
Obesity	pericyte	*TCTEX1D1*	TRUE	2.259658753
Obesity	pericyte	*CLEC18A*	TRUE	6.702127013
Obesity	pericyte	*SLC5A11*	TRUE	2.863298179
Obesity	pericyte	*SRR*	TRUE	2.430056557

Obesity	pericyte	*C8orf46*	TRUE	2.158418261
Obesity	pericyte	*TRMT61B*	TRUE	2.670261311
Obesity	pericyte	*C1QTNF4*	TRUE	5.103130038
Obesity	pericyte	*TRIM73*	TRUE	4.364505389
Obesity	pericyte	*CAV3*	TRUE	2.188595302
Obesity	pericyte	*CENPP*	TRUE	2.498955639
Obesity	pericyte	*SULT1A2*	TRUE	2.846313131
Obesity	pericyte	*RPS26*	TRUE	2.873072597
Obesity	pericyte	*HLA.E*	TRUE	2.990419439
Obesity	pericyte	*ASIC3*	TRUE	2.516258668
Obesity	pericyte	*NOTCH2NL*	TRUE	2.555377338
Obesity	pericyte	*LINC00240*	TRUE	2.164128663
Obesity	pericyte	*DPP3*	TRUE	2.26014111
Obesity	pericyte	*HMBS*	TRUE	5.342325351
Obesity	pericyte	*SH3RF3.AS1*	TRUE	2.343096775
Obesity	pericyte	*LINC00562*	TRUE	2.048248241
Obesity	SMC	*WNT16*	TRUE	2.30892939
Obesity	SMC	*ITIH4*	TRUE	2.397866031
Obesity	SMC	*TIPIN*	TRUE	2.702208822
Obesity	SMC	*COL16A1*	TRUE	2.198319152
Obesity	SMC	*PABPC4*	TRUE	3.303666026
Obesity	SMC	*DESI1*	TRUE	2.708401852
Obesity	SMC	*ELMO3*	TRUE	2.416173534
Obesity	SMC	*MFAP2*	TRUE	2.289294238
Obesity	SMC	*SERPINC1*	TRUE	5.448194497
Obesity	SMC	*CCDC92*	TRUE	3.442064773
Obesity	SMC	*GJB6*	TRUE	2.34465217
Obesity	SMC	*GRK4*	TRUE	3.484720993
Obesity	SMC	*RIBC2*	TRUE	3.166005972
Obesity	SMC	*ATP8B3*	TRUE	4.708860496
Obesity	SMC	*PKDREJ*	TRUE	2.570510285
Obesity	SMC	*IGHMBP2*	TRUE	2.553674518
Obesity	SMC	*EPHA7*	TRUE	2.064455335
Obesity	SMC	*DSCC1*	TRUE	2.499177495
Obesity	SMC	*HSD17B12*	TRUE	5.578113859
Obesity	SMC	*TTC12*	TRUE	2.752180395
Obesity	SMC	*DPYSL4*	TRUE	3.448581369
Obesity	SMC	*CLEC18A*	TRUE	6.382257294
Obesity	SMC	*HPD*	TRUE	2.796478687
Obesity	SMC	*SLC5A11*	TRUE	4.807086797
Obesity	SMC	*CLHC1*	TRUE	1.968045087
Obesity	SMC	*RNF123*	TRUE	2.998270867
Obesity	SMC	*TRMT61A*	TRUE	2.654926412
Obesity	SMC	*SRR*	TRUE	3.643160751
Obesity	SMC	*C8orf46*	TRUE	3.125770396
Obesity	SMC	*TRMT61B*	TRUE	2.193269518
Obesity	SMC	*C1QTNF4*	TRUE	6.515763481
Obesity	SMC	*CAV3*	TRUE	2.056619971

Obesity	SMC	*PSMG1*	TRUE	3.562162464
Obesity	SMC	*RP1L1*	TRUE	2.442202622
Obesity	SMC	*PLA2G6*	TRUE	2.28914915
Obesity	SMC	*GP1BA*	TRUE	1.964190316
Obesity	SMC	*BTN3A2*	TRUE	2.182163729
Obesity	SMC	*CENPP*	TRUE	2.874190329
Obesity	SMC	*CCL4L2*	TRUE	2.830560877
Obesity	SMC	*PCDHB11*	TRUE	1.983107056
Obesity	SMC	*RPS26*	TRUE	3.095146866
Obesity	SMC	*ZNF780A*	TRUE	3.014186564
Obesity	SMC	*ADH4*	TRUE	3.326919178
Obesity	SMC	*SKIV2L*	TRUE	2.491631773
Obesity	SMC	*HLA.E*	TRUE	2.670864895
Obesity	SMC	*ASIC3*	TRUE	2.338992941
Obesity	SMC	*NOTCH2NL*	TRUE	2.557855287
Obesity	SMC	*PCDHAC1*	TRUE	2.633528207
Obesity	SMC	*MPV17L2*	TRUE	3.008788922
Obesity	SMC	*HMBS*	TRUE	5.647514339
Obesity	SMC	*DUXA*	TRUE	2.361024527
Obesity	t_cell	*RBM6*	TRUE	3.060033121
Obesity	t_cell	*ITIH4*	TRUE	3.074054585
Obesity	t_cell	*TIPIN*	TRUE	2.808990312
Obesity	t_cell	*ELMO3*	TRUE	2.024358978
Obesity	t_cell	*SERPINC1*	TRUE	4.23059354
Obesity	t_cell	*CCDC92*	TRUE	2.745316682
Obesity	t_cell	*ADRA1A*	TRUE	3.018932337
Obesity	t_cell	*GRK4*	TRUE	2.907611074
Obesity	t_cell	*PCSK2*	TRUE	2.297374774
Obesity	t_cell	*RIBC2*	TRUE	3.059302428
Obesity	t_cell	*ATP8B3*	TRUE	3.735872742
Obesity	t_cell	*SH3GL3*	TRUE	2.015447949
Obesity	t_cell	*HSD17B12*	TRUE	2.12324404
Obesity	t_cell	*DPYSL4*	TRUE	3.691188801
Obesity	t_cell	*CLEC18A*	TRUE	6.258795416
Obesity	t_cell	*HPD*	TRUE	2.041686793
Obesity	t_cell	*SLC5A11*	TRUE	2.489103147
Obesity	t_cell	*LMOD1*	TRUE	3.823614165
Obesity	t_cell	*PSCA*	TRUE	2.115312729
Obesity	t_cell	*SRR*	TRUE	3.567663736
Obesity	t_cell	*C8orf46*	TRUE	3.468893243
Obesity	t_cell	*ADORA2B*	TRUE	3.916407456
Obesity	t_cell	*TRMT61B*	TRUE	2.524077436
Obesity	t_cell	*C1QTNF4*	TRUE	5.115189612
Obesity	t_cell	*TRIM73*	TRUE	4.659679169
Obesity	t_cell	*PSMG1*	TRUE	3.464047622
Obesity	t_cell	*RP1L1*	TRUE	3.926154931
Obesity	t_cell	*PLA2G6*	TRUE	2.264651661
Obesity	t_cell	*CENPP*	TRUE	2.578296415

Obesity	t_cell	*SULT1A2*	TRUE	3.950010917
Obesity	t_cell	*RPS26*	TRUE	3.206728536
Obesity	t_cell	*ASIC3*	TRUE	2.711412812
Obesity	t_cell	*HMBS*	TRUE	5.444626684
Stroke	adipocyte	*CENPQ*	TRUE	3.090126877
Stroke	adipocyte	*CYP2D6*	TRUE	2.820884266
Stroke	adipocyte	*MGRN1*	TRUE	2.049953032
Stroke	adipocyte	*IGDCC4*	TRUE	2.091191773
Stroke	adipocyte	*C17orf75*	TRUE	2.730624137
Stroke	adipocyte	*BVES*	TRUE	2.319668743
Stroke	adipocyte	*PCDHB6*	TRUE	2.414111173
Stroke	adipocyte	*MFAP2*	TRUE	2.14261014
Stroke	adipocyte	*EPCAM*	TRUE	2.553048338
Stroke	adipocyte	*STIL*	TRUE	2.309127539
Stroke	adipocyte	*BTN2A2*	TRUE	2.212874994
Stroke	adipocyte	*TRAP1*	TRUE	2.365871642
Stroke	adipocyte	*LARS*	TRUE	1.984747441
Stroke	adipocyte	*ITGA7*	TRUE	2.015906974
Stroke	adipocyte	*LYG1*	TRUE	2.455783419
Stroke	adipocyte	*NBEAL1*	TRUE	1.984102884
Stroke	adipocyte	*ILDR1*	TRUE	2.082802691
Stroke	adipocyte	*C1orf50*	TRUE	2.478533073
Stroke	adipocyte	*TMEM184A*	TRUE	2.145897345
Stroke	adipocyte	*KIR3DL1*	TRUE	2.017483632
Stroke	adipocyte	*CHAF1A*	TRUE	2.085252021
Stroke	adipocyte	*FAM57A*	TRUE	2.485336003
Stroke	adipocyte	*ULK4*	TRUE	2.359389018
Stroke	adipocyte	*KCNAB3*	TRUE	2.427912562
Stroke	adipocyte	*GEN1*	TRUE	2.00433866
Stroke	adipocyte	*FUCA1*	TRUE	2.059965239
Stroke	adipocyte	*FJX1*	TRUE	2.041168552
Stroke	adipocyte	*TMEM86B*	TRUE	2.103404809
Stroke	adipocyte	*CLDN7*	TRUE	2.864433202
Stroke	adipocyte	*RP1L1*	TRUE	2.324008629
Stroke	adipocyte	*PDE6G*	TRUE	2.104661623
Stroke	adipocyte	*FFAR4*	TRUE	1.982886066
Stroke	adipocyte	*ZNF749*	TRUE	2.896579015
Stroke	adipocyte	*GPAT2*	TRUE	2.15015192
Stroke	adipocyte	*TMEM116*	TRUE	2.556173173
Stroke	adipocyte	*ANKRD13B*	TRUE	2.227647787
Stroke	adipocyte	*COL15A1*	TRUE	2.633510247
Stroke	adipocyte	*DNLZ*	TRUE	2.027312298
Stroke	adipocyte	*NUTM2D*	TRUE	2.040389815
Stroke	adipocyte	*LILRA2*	TRUE	2.22551278
Stroke	adipocyte	*TDGF1*	TRUE	3.247932047
Stroke	adipocyte	*LINC00942*	TRUE	2.085812264
Stroke	adipocyte	*SHANK3*	TRUE	2.024870114
Stroke	adipocyte	*ARMS2*	TRUE	2.059704776

Stroke	ASPC	*ZDHHC6*	TRUE	1.964118155
Stroke	ASPC	*NR1H3*	TRUE	2.079218967
Stroke	ASPC	*CENPQ*	TRUE	3.471412158
Stroke	ASPC	*FSCN1*	TRUE	2.102640051
Stroke	ASPC	*GPR137B*	TRUE	2.240283502
Stroke	ASPC	*EPDR1*	TRUE	2.078730168
Stroke	ASPC	*NECAP1*	TRUE	2.359791266
Stroke	ASPC	*SPTLC1*	TRUE	2.305127014
Stroke	ASPC	*TPSD1*	TRUE	2.306317577
Stroke	ASPC	*MARCH2*	TRUE	2.973924125
Stroke	ASPC	*CYP2D6*	TRUE	3.019762085
Stroke	ASPC	*CNIH1*	TRUE	2.410647749
Stroke	ASPC	*MGRN1*	TRUE	2.13044261
Stroke	ASPC	*IGDCC4*	TRUE	2.073073238
Stroke	ASPC	*RAB2A*	TRUE	2.324263853
Stroke	ASPC	*CAV1*	TRUE	2.270158566
Stroke	ASPC	*TBL2*	TRUE	2.015409524
Stroke	ASPC	*C17orf75*	TRUE	3.86761454
Stroke	ASPC	*TMEM104*	TRUE	1.96203411
Stroke	ASPC	*BVES*	TRUE	2.648322587
Stroke	ASPC	*CEP72*	TRUE	2.015808052
Stroke	ASPC	*PCDHB6*	TRUE	2.444180242
Stroke	ASPC	*MRPL37*	TRUE	2.273854047
Stroke	ASPC	*MFAP2*	TRUE	2.191013329
Stroke	ASPC	*GPR75*	TRUE	2.108782443
Stroke	ASPC	*EPCAM*	TRUE	2.850179364
Stroke	ASPC	*MRC1*	TRUE	2.403313149
Stroke	ASPC	*FMOD*	TRUE	1.989634939
Stroke	ASPC	*FAM213A*	TRUE	2.288538645
Stroke	ASPC	*CCZ1*	TRUE	2.101766869
Stroke	ASPC	*SLC25A16*	TRUE	1.970823653
Stroke	ASPC	*STIL*	TRUE	3.068072574
Stroke	ASPC	*BTN2A2*	TRUE	2.014193654
Stroke	ASPC	*TRAP1*	TRUE	2.561104502
Stroke	ASPC	*RHOT1*	TRUE	2.010213442
Stroke	ASPC	*SHC2*	TRUE	2.134615326
Stroke	ASPC	*QRSL1*	TRUE	2.246955575
Stroke	ASPC	*EMILIN2*	TRUE	2.45918648
Stroke	ASPC	*LARS*	TRUE	2.527710193
Stroke	ASPC	*CNTN6*	TRUE	2.109478792
Stroke	ASPC	*WNT2B*	TRUE	1.967672646
Stroke	ASPC	*PRR5L*	TRUE	2.094593523
Stroke	ASPC	*ITGA7*	TRUE	2.289007097
Stroke	ASPC	*KLHL36*	TRUE	2.662175242
Stroke	ASPC	*RNASEL*	TRUE	2.355326526
Stroke	ASPC	*SPAG8*	TRUE	2.176653139
Stroke	ASPC	*RMDN3*	TRUE	2.457196543
Stroke	ASPC	*SLC35A5*	TRUE	2.115934323

Stroke	ASPC	*C14orf79*	TRUE	2.212099043
Stroke	ASPC	*RPRD1A*	TRUE	2.245481899
Stroke	ASPC	*FKBP10*	TRUE	2.818494335
Stroke	ASPC	*CELSR2*	TRUE	2.068357297
Stroke	ASPC	*ARNT*	TRUE	2.189517627
Stroke	ASPC	*LYG1*	TRUE	2.743053505
Stroke	ASPC	*PLA1A*	TRUE	2.126466939
Stroke	ASPC	*ILDR1*	TRUE	2.107817093
Stroke	ASPC	*KLHL3*	TRUE	2.659979465
Stroke	ASPC	*ATP6V0D2*	TRUE	2.324042049
Stroke	ASPC	*NTMT1*	TRUE	2.145330525
Stroke	ASPC	*GSTO1*	TRUE	4.696306051
Stroke	ASPC	*GLYAT*	TRUE	1.997406691
Stroke	ASPC	*NMT2*	TRUE	2.162979501
Stroke	ASPC	*WDYHV1*	TRUE	2.269350316
Stroke	ASPC	*SLC6A1*	TRUE	2.142091263
Stroke	ASPC	*CLEC18A*	TRUE	1.982921349
Stroke	ASPC	*B4GALT3*	TRUE	2.42322188
Stroke	ASPC	*CBR3*	TRUE	2.551681497
Stroke	ASPC	*DIP2A*	TRUE	2.066718029
Stroke	ASPC	*ZBTB7B*	TRUE	2.116619128
Stroke	ASPC	*FDPS*	TRUE	2.005066769
Stroke	ASPC	*RFC4*	TRUE	2.548941949
Stroke	ASPC	*C1orf50*	TRUE	2.693059833
Stroke	ASPC	*TMEM184A*	TRUE	2.838755876
Stroke	ASPC	*YWHAZ*	TRUE	2.540420321
Stroke	ASPC	*HSPA12A*	TRUE	2.524716461
Stroke	ASPC	*ZFYVE19*	TRUE	1.987690166
Stroke	ASPC	*KIR3DL1*	TRUE	1.982073075
Stroke	ASPC	*CHAF1A*	TRUE	2.393479352
Stroke	ASPC	*FAM57A*	TRUE	2.615454914
Stroke	ASPC	*SRR*	TRUE	2.012287765
Stroke	ASPC	*ULK4*	TRUE	2.635779605
Stroke	ASPC	*RFWD3*	TRUE	2.008267081
Stroke	ASPC	*IRS1*	TRUE	2.008167111
Stroke	ASPC	*UPF3A*	TRUE	2.564131811
Stroke	ASPC	*SH3TC2*	TRUE	2.024337884
Stroke	ASPC	*GAA*	TRUE	2.125785314
Stroke	ASPC	*MRPL52*	TRUE	1.983275636
Stroke	ASPC	*FAM192A*	TRUE	1.984350821
Stroke	ASPC	*GMPPB*	TRUE	1.964890361
Stroke	ASPC	*DDX23*	TRUE	2.254921073
Stroke	ASPC	*CTDNEP1*	TRUE	1.977606876
Stroke	ASPC	*CRLF3*	TRUE	3.211616774
Stroke	ASPC	*ACSF3*	TRUE	1.985692214
Stroke	ASPC	*IRX3*	TRUE	2.158747962
Stroke	ASPC	*GEN1*	TRUE	2.372130221
Stroke	ASPC	*FUCA1*	TRUE	2.596042974

Stroke	ASPC	*FJX1*	TRUE	2.104821591
Stroke	ASPC	*TMEM86B*	TRUE	2.232626785
Stroke	ASPC	*SPNS3*	TRUE	2.246238666
Stroke	ASPC	*GRID1*	TRUE	2.082495277
Stroke	ASPC	*PLGLB1*	TRUE	1.976384999
Stroke	ASPC	*RP1L1*	TRUE	2.246625364
Stroke	ASPC	*PDE6G*	TRUE	2.197205898
Stroke	ASPC	*FFAR4*	TRUE	2.965187587
Stroke	ASPC	*ZNF749*	TRUE	3.009114693
Stroke	ASPC	*ADH1A*	TRUE	2.281154399
Stroke	ASPC	*BEND4*	TRUE	2.004667876
Stroke	ASPC	*ZFP28*	TRUE	2.179479998
Stroke	ASPC	*PIWIL2*	TRUE	2.107190299
Stroke	ASPC	*TMEM116*	TRUE	2.303809973
Stroke	ASPC	*SH3BGRL2*	TRUE	2.277304477
Stroke	ASPC	*COL15A1*	TRUE	3.529724364
Stroke	ASPC	*LRRC10B*	TRUE	2.471656095
Stroke	ASPC	*DNLZ*	TRUE	1.993223497
Stroke	ASPC	*NUTM2D*	TRUE	2.155379533
Stroke	ASPC	*PPP3R1*	TRUE	2.120146823
Stroke	ASPC	*CDKN2AIPNL*	TRUE	1.987232819
Stroke	ASPC	*IRGM*	TRUE	1.998495858
Stroke	ASPC	*LILRA2*	TRUE	2.195145889
Stroke	ASPC	*TDGF1*	TRUE	2.898546993
Stroke	ASPC	*MRPL20*	TRUE	2.228287531
Stroke	ASPC	*NME1.NME2*	TRUE	1.96297386
Stroke	ASPC	*LINC00942*	TRUE	2.259336658
Stroke	ASPC	*SHANK3*	TRUE	2.424833018
Stroke	ASPC	*TMEM200B*	TRUE	2.417649185
Stroke	ASPC	*ARMS2*	TRUE	2.325858471
Stroke	ASPC	*GOLGA8H*	TRUE	2.016858338
Stroke	b_cell	*IGDCC4*	TRUE	2.005338368
Stroke	b_cell	*FAM57A*	TRUE	2.182553552
Stroke	b_cell	*LILRA2*	TRUE	2.119379776
Stroke	b_cell	*TDGF1*	TRUE	2.933708497
Stroke	dendritic_cell	*BVES*	TRUE	2.587831683
Stroke	dendritic_cell	*HORMAD1*	TRUE	1.965764724
Stroke	dendritic_cell	*FJX1*	TRUE	1.960904124
Stroke	dendritic_cell	*CLDN7*	TRUE	2.777175877
Stroke	dendritic_cell	*PIGP*	TRUE	2.002291571
Stroke	dendritic_cell	*ZNF749*	TRUE	2.643628506
Stroke	dendritic_cell	*TDGF1*	TRUE	2.975802058
Stroke	endothelial	*CYP2D6*	TRUE	2.04591217
Stroke	endothelial	*IGDCC4*	TRUE	2.062800179
Stroke	endothelial	*PCDHB6*	TRUE	2.097757101
Stroke	endothelial	*EPCAM*	TRUE	1.972900962
Stroke	endothelial	*BTN2A2*	TRUE	2.004354151
Stroke	endothelial	*HBZ*	TRUE	1.995744808

Stroke	endothelial	*FKBP10*	TRUE	2.223981072
Stroke	endothelial	*GPAT2*	TRUE	2.049170273
Stroke	endothelial	*HLA.DQB2*	TRUE	2.05288553
Stroke	endothelial	*TDGF1*	TRUE	3.218915041
Stroke	LEC	*CENPQ*	TRUE	2.643385581
Stroke	LEC	*MFAP2*	TRUE	2.109118075
Stroke	LEC	*FKBP10*	TRUE	3.335414018
Stroke	LEC	*ILDR1*	TRUE	2.060704258
Stroke	LEC	*CLEC18A*	TRUE	2.033996229
Stroke	LEC	*FAM57A*	TRUE	1.96623733
Stroke	LEC	*NUTM2D*	TRUE	2.149951579
Stroke	LEC	*TDGF1*	TRUE	2.024559132
Stroke	macrophage	*CENPQ*	TRUE	2.989593953
Stroke	macrophage	*CYP2D6*	TRUE	2.956790449
Stroke	macrophage	*IGDCC4*	TRUE	2.047070092
Stroke	macrophage	*DLX5*	TRUE	2.005950829
Stroke	macrophage	*BVES*	TRUE	2.438633809
Stroke	macrophage	*PCDHB6*	TRUE	2.126024832
Stroke	macrophage	*MFAP2*	TRUE	2.109137501
Stroke	macrophage	*EPCAM*	TRUE	2.62534582
Stroke	macrophage	*TRAP1*	TRUE	2.235034721
Stroke	macrophage	*FKBP10*	TRUE	2.548818316
Stroke	macrophage	*LYG1*	TRUE	2.176287847
Stroke	macrophage	*NBEAL1*	TRUE	2.288888738
Stroke	macrophage	*PLA1A*	TRUE	2.04588254
Stroke	macrophage	*GSTO1*	TRUE	2.157192047
Stroke	macrophage	*C1orf50*	TRUE	2.297858957
Stroke	macrophage	*TMEM184A*	TRUE	2.110759212
Stroke	macrophage	*CHAF1A*	TRUE	2.195040733
Stroke	macrophage	*FAM57A*	TRUE	2.366994262
Stroke	macrophage	*ULK4*	TRUE	2.209281787
Stroke	macrophage	*KCNAB3*	TRUE	2.306413658
Stroke	macrophage	*RP1L1*	TRUE	2.023696005
Stroke	macrophage	*FFAR4*	TRUE	2.700951755
Stroke	macrophage	*ZNF749*	TRUE	2.762856386
Stroke	macrophage	*GPAT2*	TRUE	2.287747925
Stroke	macrophage	*TMEM116*	TRUE	2.341927844
Stroke	macrophage	*AC084018.1*	TRUE	2.106044374
Stroke	macrophage	*DNLZ*	TRUE	2.006160776
Stroke	macrophage	*NUTM2D*	TRUE	2.086276145
Stroke	macrophage	*TDGF1*	TRUE	3.277241326
Stroke	macrophage	*LINC00942*	TRUE	2.087975532
Stroke	macrophage	*ARMS2*	TRUE	2.100336122
Stroke	mast_cell	*IGDCC4*	TRUE	2.176126754
Stroke	mast_cell	*DLX5*	TRUE	1.99216303
Stroke	mast_cell	*FKBP10*	TRUE	2.725217782
Stroke	mast_cell	*ULK4*	TRUE	2.150862146
Stroke	mast_cell	*TMEM116*	TRUE	1.995528707

Stroke	mast_cell	*TDGF1*	TRUE	2.29433021
Stroke	monocyte	*CENPQ*	TRUE	3.169078912
Stroke	monocyte	*IGDCC4*	TRUE	1.99096254
Stroke	monocyte	*ILDR1*	TRUE	2.105999427
Stroke	monocyte	*CLDN7*	TRUE	2.187004435
Stroke	monocyte	*HLA.DQB2*	TRUE	2.57931495
Stroke	monocyte	*TDGF1*	TRUE	2.354665124
Stroke	neutrophil	*ILDR1*	TRUE	1.984321431
Stroke	neutrophil	*HLA.DQB1*	TRUE	2.048778893
Stroke	neutrophil	*TDGF1*	TRUE	2.421380753
Stroke	nk_cell	*CENPQ*	TRUE	2.456694505
Stroke	nk_cell	*DLX5*	TRUE	2.009003425
Stroke	nk_cell	*BVES*	TRUE	2.086089847
Stroke	nk_cell	*BTN2A2*	TRUE	2.128629958
Stroke	nk_cell	*FKBP10*	TRUE	2.116152718
Stroke	nk_cell	*ILDR1*	TRUE	2.079434397
Stroke	nk_cell	*CHAF1A*	TRUE	1.965442752
Stroke	nk_cell	*FAM57A*	TRUE	2.302483351
Stroke	nk_cell	*TMEM116*	TRUE	2.121293412
Stroke	nk_cell	*TDGF1*	TRUE	2.681727465
Stroke	pericyte	*CENPQ*	TRUE	1.997981936
Stroke	pericyte	*CYP2D6*	TRUE	2.960426939
Stroke	pericyte	*IGDCC4*	TRUE	1.998189945
Stroke	pericyte	*PCDHB6*	TRUE	2.115777935
Stroke	pericyte	*FKBP10*	TRUE	2.444341501
Stroke	pericyte	*FFAR4*	TRUE	2.593083978
Stroke	pericyte	*COL15A1*	TRUE	2.469609871
Stroke	pericyte	*NUTM2D*	TRUE	2.084051255
Stroke	pericyte	*TDGF1*	TRUE	3.077453391
Stroke	pericyte	*TMEM200B*	TRUE	2.086188066
Stroke	SMC	*CENPQ*	TRUE	2.208000864
Stroke	SMC	*CYP2D6*	TRUE	2.476125279
Stroke	SMC	*FKBP10*	TRUE	2.463491233
Stroke	SMC	*LYG1*	TRUE	2.226549435
Stroke	SMC	*PLA1A*	TRUE	2.111719285
Stroke	SMC	*C1orf50*	TRUE	2.264934601
Stroke	SMC	*ULK4*	TRUE	2.176226171
Stroke	SMC	*HLA.DQB1*	TRUE	2.163332804
Stroke	SMC	*CLDN7*	TRUE	2.813012916
Stroke	SMC	*YBEY*	TRUE	2.094117005
Stroke	SMC	*PIGP*	TRUE	2.01049075
Stroke	SMC	*ZNF749*	TRUE	2.812407475
Stroke	SMC	*TMEM116*	TRUE	2.36322714
Stroke	SMC	*NUTM2D*	TRUE	1.967703905
Stroke	SMC	*TDGF1*	TRUE	2.770674506
Stroke	t_cell	*CD79B*	TRUE	2.109913838
Stroke	t_cell	*CENPQ*	TRUE	2.578620214
Stroke	t_cell	*MFAP2*	TRUE	1.977363704

Stroke	t_cell	*EPCAM*	TRUE	2.163846527
Stroke	t_cell	*TRAP1*	TRUE	2.180167487
Stroke	t_cell	*FKBP10*	TRUE	2.93074714
Stroke	t_cell	*CHAF1A*	TRUE	2.190116637
Stroke	t_cell	*FAM57A*	TRUE	2.029555247
Stroke	t_cell	*ULK4*	TRUE	2.076064436
Stroke	t_cell	*RP1L1*	TRUE	2.106079489
Stroke	t_cell	*GPAT2*	TRUE	3.029271396
Stroke	t_cell	*LRRC10B*	TRUE	2.401628848
Stroke	t_cell	*DNLZ*	TRUE	2.126795894
Stroke	t_cell	*LILRA2*	TRUE	2.153649675
Stroke	t_cell	*TDGF1*	TRUE	2.16824111
Heart_Failure	adipocyte	*MAP3K9*	TRUE	1.981314771
Heart_Failure	adipocyte	*SLC7A9*	TRUE	2.550724617
Heart_Failure	adipocyte	*LAMC2*	TRUE	2.701560396
Heart_Failure	adipocyte	*TIPIN*	TRUE	2.031170154
Heart_Failure	adipocyte	*OXCT1*	TRUE	2.034362405
Heart_Failure	adipocyte	*COL16A1*	TRUE	3.282580561
Heart_Failure	adipocyte	*BIRC7*	TRUE	2.759953212
Heart_Failure	adipocyte	*NDUFAF5*	TRUE	2.126263197
Heart_Failure	adipocyte	*CORO1A*	TRUE	1.968980698
Heart_Failure	adipocyte	*CTSH*	TRUE	2.203848311
Heart_Failure	adipocyte	*TIMM44*	TRUE	2.261165791
Heart_Failure	adipocyte	*CDK2AP1*	TRUE	3.247369391
Heart_Failure	adipocyte	*PCDHB6*	TRUE	2.900218304
Heart_Failure	adipocyte	*BNIP1*	TRUE	2.294912428
Heart_Failure	adipocyte	*HEMK1*	TRUE	2.735344285
Heart_Failure	adipocyte	*GRIN3B*	TRUE	2.145949348
Heart_Failure	adipocyte	*CFHR3*	TRUE	2.944966137
Heart_Failure	adipocyte	*ALDH8A1*	TRUE	1.959996484
Heart_Failure	adipocyte	*CTNNAL1*	TRUE	2.363846508
Heart_Failure	adipocyte	*LDB3*	TRUE	2.007132051
Heart_Failure	adipocyte	*TBCC*	TRUE	2.000847912
Heart_Failure	adipocyte	*GRK4*	TRUE	1.991281074
Heart_Failure	adipocyte	*UCK1*	TRUE	2.344368753
Heart_Failure	adipocyte	*CHMP1A*	TRUE	2.568479226
Heart_Failure	adipocyte	*MRPL35*	TRUE	2.263687088
Heart_Failure	adipocyte	*REEP2*	TRUE	3.583390573
Heart_Failure	adipocyte	*ADAM19*	TRUE	2.036492714
Heart_Failure	adipocyte	*POM121C*	TRUE	2.531975602
Heart_Failure	adipocyte	*LGR5*	TRUE	2.462444605
Heart_Failure	adipocyte	*FLG*	TRUE	2.495768755
Heart_Failure	adipocyte	*CISD2*	TRUE	2.005609206
Heart_Failure	adipocyte	*BHMT*	TRUE	2.898113139
Heart_Failure	adipocyte	*TSLP*	TRUE	3.477019667
Heart_Failure	adipocyte	*SLC18B1*	TRUE	1.967903094
Heart_Failure	adipocyte	*DUSP15*	TRUE	3.22275141
Heart_Failure	adipocyte	*DPYSL4*	TRUE	2.187716721

Heart_Failure	adipocyte	*GAL3ST2*	TRUE	2.903948753
Heart_Failure	adipocyte	*SLFN13*	TRUE	1.967599034
Heart_Failure	adipocyte	*WHAMM*	TRUE	1.993811394
Heart_Failure	adipocyte	*FCRL3*	TRUE	2.481975674
Heart_Failure	adipocyte	*EFHB*	TRUE	2.779848421
Heart_Failure	adipocyte	*DCLK3*	TRUE	2.743499044
Heart_Failure	adipocyte	*HDHD2*	TRUE	2.608844042
Heart_Failure	adipocyte	*GSTM4*	TRUE	1.960227786
Heart_Failure	adipocyte	*RGS14*	TRUE	2.69456646
Heart_Failure	adipocyte	*ORMDL3*	TRUE	5.562462294
Heart_Failure	adipocyte	*DCAKD*	TRUE	2.000262566
Heart_Failure	adipocyte	*NRROS*	TRUE	2.603283175
Heart_Failure	adipocyte	*CHST1*	TRUE	2.479480804
Heart_Failure	adipocyte	*PAAF1*	TRUE	2.235091347
Heart_Failure	adipocyte	*FBXO39*	TRUE	2.3575031
Heart_Failure	adipocyte	*NIM1K*	TRUE	2.33286384
Heart_Failure	adipocyte	*ZNF454*	TRUE	2.85841248
Heart_Failure	adipocyte	*KLHDC7A*	TRUE	2.498555058
Heart_Failure	adipocyte	*HLA.DQB1*	TRUE	3.866291603
Heart_Failure	adipocyte	*FKRP*	TRUE	2.379261108
Heart_Failure	adipocyte	*TDRKH*	TRUE	2.450670864
Heart_Failure	adipocyte	*ACOT1*	TRUE	2.048876453
Heart_Failure	adipocyte	*A3GALT2*	TRUE	2.225591158
Heart_Failure	adipocyte	*MUC1*	TRUE	3.68142814
Heart_Failure	adipocyte	*MYBL1*	TRUE	2.04076106
Heart_Failure	adipocyte	*MCRS1*	TRUE	3.163280416
Heart_Failure	adipocyte	*SLC22A5*	TRUE	2.251906415
Heart_Failure	adipocyte	*KLHL14*	TRUE	2.262738338
Heart_Failure	adipocyte	*RPS26*	TRUE	2.748888673
Heart_Failure	adipocyte	*MICA*	TRUE	2.962858574
Heart_Failure	adipocyte	*FAM153C*	TRUE	2.35126502
Heart_Failure	adipocyte	*ATF6B*	TRUE	2.065005048
Heart_Failure	adipocyte	*CPNE1*	TRUE	2.733121011
Heart_Failure	adipocyte	*TBC1D26*	TRUE	2.031188866
Heart_Failure	adipocyte	*PROB1*	TRUE	2.022620854
Heart_Failure	adipocyte	*ZBTB22*	TRUE	2.187794809
Heart_Failure	adipocyte	*HLA.DQA2*	TRUE	3.064180109
Heart_Failure	adipocyte	*UPK3B*	TRUE	2.243119541
Heart_Failure	adipocyte	*TTC4*	TRUE	2.772428659
Heart_Failure	adipocyte	*MPV17L2*	TRUE	2.111458007
Heart_Failure	ASPC	*RBM6*	TRUE	2.236295116
Heart_Failure	ASPC	*MAPK8IP2*	TRUE	2.005099058
Heart_Failure	ASPC	*MED24*	TRUE	2.44739495
Heart_Failure	ASPC	*SLC7A9*	TRUE	2.597639749
Heart_Failure	ASPC	*PHLPP2*	TRUE	2.567756367
Heart_Failure	ASPC	*RRP12*	TRUE	2.180787945
Heart_Failure	ASPC	*LAMC2*	TRUE	2.939809786
Heart_Failure	ASPC	*TAF2*	TRUE	2.762505488

Heart_Failure	ASPC	*LIMS2*	TRUE	2.70024634
Heart_Failure	ASPC	*OXCT1*	TRUE	3.6192932
Heart_Failure	ASPC	*COL16A1*	TRUE	4.246267782
Heart_Failure	ASPC	*KCNH4*	TRUE	2.416414229
Heart_Failure	ASPC	*PABPC4*	TRUE	2.193371589
Heart_Failure	ASPC	*ISM2*	TRUE	2.075369451
Heart_Failure	ASPC	*PRPF6*	TRUE	2.279403354
Heart_Failure	ASPC	*SLC17A9*	TRUE	2.900459339
Heart_Failure	ASPC	*BIRC7*	TRUE	2.854060636
Heart_Failure	ASPC	*NDUFAF5*	TRUE	2.472924962
Heart_Failure	ASPC	*CORO1A*	TRUE	3.044136506
Heart_Failure	ASPC	*DHODH*	TRUE	2.118669936
Heart_Failure	ASPC	*CTSH*	TRUE	2.787063467
Heart_Failure	ASPC	*TIMM44*	TRUE	2.948978676
Heart_Failure	ASPC	*GRWD1*	TRUE	2.211330408
Heart_Failure	ASPC	*HOXA6*	TRUE	2.509410544
Heart_Failure	ASPC	*RGP1*	TRUE	2.083271618
Heart_Failure	ASPC	*PEX12*	TRUE	2.108721218
Heart_Failure	ASPC	*CTSC*	TRUE	1.974269317
Heart_Failure	ASPC	*CDK2AP1*	TRUE	3.108943611
Heart_Failure	ASPC	*CHPT1*	TRUE	2.397129766
Heart_Failure	ASPC	*PCDHB6*	TRUE	2.489617634
Heart_Failure	ASPC	*BNIP1*	TRUE	2.421823793
Heart_Failure	ASPC	*HEMK1*	TRUE	2.458040591
Heart_Failure	ASPC	*INO80B*	TRUE	2.427047345
Heart_Failure	ASPC	*CD207*	TRUE	2.020511147
Heart_Failure	ASPC	*CFHR3*	TRUE	3.117077055
Heart_Failure	ASPC	*OSCP1*	TRUE	2.293226501
Heart_Failure	ASPC	*CTNNAL1*	TRUE	2.650576646
Heart_Failure	ASPC	*ARHGAP9*	TRUE	3.004130138
Heart_Failure	ASPC	*IKZF4*	TRUE	1.971272297
Heart_Failure	ASPC	*TBCC*	TRUE	2.589108559
Heart_Failure	ASPC	*GRK4*	TRUE	2.156450541
Heart_Failure	ASPC	*KRI1*	TRUE	2.110030821
Heart_Failure	ASPC	*TOMM40*	TRUE	2.828831188
Heart_Failure	ASPC	*UCK1*	TRUE	2.526646373
Heart_Failure	ASPC	*CHMP1A*	TRUE	2.502670826
Heart_Failure	ASPC	*MRPL35*	TRUE	1.984178344
Heart_Failure	ASPC	*REEP2*	TRUE	4.001663926
Heart_Failure	ASPC	*PCNA*	TRUE	2.444868352
Heart_Failure	ASPC	*BTBD2*	TRUE	2.451310071
Heart_Failure	ASPC	*SPIRE1*	TRUE	2.275591802
Heart_Failure	ASPC	*GOLM1*	TRUE	2.593089275
Heart_Failure	ASPC	*ADAM19*	TRUE	2.374179868
Heart_Failure	ASPC	*POM121C*	TRUE	4.003968692
Heart_Failure	ASPC	*AHI1*	TRUE	2.17840331
Heart_Failure	ASPC	*ZDHHC4*	TRUE	2.003657214
Heart_Failure	ASPC	*RMDN3*	TRUE	2.785107795

Heart_Failure	ASPC	*SLC35A5*	TRUE	2.022985643
Heart_Failure	ASPC	*GSTCD*	TRUE	2.483338837
Heart_Failure	ASPC	*LGR5*	TRUE	3.180237872
Heart_Failure	ASPC	*MFGE8*	TRUE	2.037215762
Heart_Failure	ASPC	*FN3KRP*	TRUE	1.97560936
Heart_Failure	ASPC	*DUS3L*	TRUE	2.050658705
Heart_Failure	ASPC	*NME7*	TRUE	2.238489592
Heart_Failure	ASPC	*GPA33*	TRUE	2.251056442
Heart_Failure	ASPC	*ADCY10*	TRUE	2.310493911
Heart_Failure	ASPC	*FLG*	TRUE	2.541971739
Heart_Failure	ASPC	*CISD2*	TRUE	2.667779141
Heart_Failure	ASPC	*TSLP*	TRUE	4.279650486
Heart_Failure	ASPC	*SLC18B1*	TRUE	2.393096764
Heart_Failure	ASPC	*EPHA1*	TRUE	2.139525613
Heart_Failure	ASPC	*HSD17B12*	TRUE	2.358662182
Heart_Failure	ASPC	*DUSP15*	TRUE	3.364525767
Heart_Failure	ASPC	*DPYSL4*	TRUE	2.287873944
Heart_Failure	ASPC	*POC5*	TRUE	2.139757288
Heart_Failure	ASPC	*GAL3ST2*	TRUE	2.879916039
Heart_Failure	ASPC	*TTC39B*	TRUE	2.530128276
Heart_Failure	ASPC	*NDUFAF6*	TRUE	2.992517786
Heart_Failure	ASPC	*WHAMM*	TRUE	2.739030641
Heart_Failure	ASPC	*EPB41*	TRUE	2.202549362
Heart_Failure	ASPC	*TEPP*	TRUE	2.158623123
Heart_Failure	ASPC	*C21orf33*	TRUE	2.810993459
Heart_Failure	ASPC	*FCRL3*	TRUE	2.752433519
Heart_Failure	ASPC	*FUBP1*	TRUE	2.765536398
Heart_Failure	ASPC	*EFHB*	TRUE	2.793993102
Heart_Failure	ASPC	*DCLK3*	TRUE	2.765882076
Heart_Failure	ASPC	*DNALI1*	TRUE	2.791857839
Heart_Failure	ASPC	*SAP30L*	TRUE	2.557739333
Heart_Failure	ASPC	*AKR1E2*	TRUE	2.233510002
Heart_Failure	ASPC	*HSPA12A*	TRUE	2.118155664
Heart_Failure	ASPC	*CHRFAM7A*	TRUE	2.055339449
Heart_Failure	ASPC	*HDHD2*	TRUE	2.727433696
Heart_Failure	ASPC	*UBXN6*	TRUE	1.962487923
Heart_Failure	ASPC	*HRASLS5*	TRUE	2.179285585
Heart_Failure	ASPC	*GSTM4*	TRUE	2.226213757
Heart_Failure	ASPC	*RGS14*	TRUE	2.886121879
Heart_Failure	ASPC	*CALML6*	TRUE	2.340088775
Heart_Failure	ASPC	*RPS9*	TRUE	2.181740895
Heart_Failure	ASPC	*LRRC34*	TRUE	2.462765181
Heart_Failure	ASPC	*ORMDL3*	TRUE	5.932102175
Heart_Failure	ASPC	*DCAKD*	TRUE	2.390525026
Heart_Failure	ASPC	*PARP15*	TRUE	1.986090306
Heart_Failure	ASPC	*GOLIM4*	TRUE	2.580683144
Heart_Failure	ASPC	*NRROS*	TRUE	3.069431899
Heart_Failure	ASPC	*CHST1*	TRUE	2.851653912

Heart_Failure	ASPC	*PAAF1*	TRUE	2.207764166
Heart_Failure	ASPC	*ACSF3*	TRUE	2.1977151
Heart_Failure	ASPC	*FBXO39*	TRUE	2.442455086
Heart_Failure	ASPC	*NIM1K*	TRUE	2.67975606
Heart_Failure	ASPC	*ZNF454*	TRUE	2.912444461
Heart_Failure	ASPC	*KLHDC7A*	TRUE	2.6149323
Heart_Failure	ASPC	*HLA.DQB1*	TRUE	3.202219159
Heart_Failure	ASPC	*HIGD1A*	TRUE	2.446551315
Heart_Failure	ASPC	*TDRKH*	TRUE	2.785805396
Heart_Failure	ASPC	*NPLOC4*	TRUE	2.090469891
Heart_Failure	ASPC	*GLRX5*	TRUE	2.102524671
Heart_Failure	ASPC	*GRID1*	TRUE	1.97387918
Heart_Failure	ASPC	*ZNF623*	TRUE	2.444321008
Heart_Failure	ASPC	*ACOT1*	TRUE	2.663506708
Heart_Failure	ASPC	*CSF1*	TRUE	2.221223797
Heart_Failure	ASPC	*A3GALT2*	TRUE	2.308732071
Heart_Failure	ASPC	*FAM227A*	TRUE	2.043365551
Heart_Failure	ASPC	*MUC1*	TRUE	3.317743361
Heart_Failure	ASPC	*MYBL1*	TRUE	2.529173247
Heart_Failure	ASPC	*MRPS21*	TRUE	2.549116446
Heart_Failure	ASPC	*ADH1A*	TRUE	2.471911283
Heart_Failure	ASPC	*MCRS1*	TRUE	3.497494
Heart_Failure	ASPC	*ZNF626*	TRUE	1.972898588
Heart_Failure	ASPC	*MSL1*	TRUE	2.197089479
Heart_Failure	ASPC	*UTS2B*	TRUE	2.407307059
Heart_Failure	ASPC	*FAM47E*	TRUE	1.977089896
Heart_Failure	ASPC	*FAM180B*	TRUE	2.156053748
Heart_Failure	ASPC	*SLC22A5*	TRUE	2.979126149
Heart_Failure	ASPC	*KLHL14*	TRUE	2.710865024
Heart_Failure	ASPC	*RPS26*	TRUE	3.108544454
Heart_Failure	ASPC	*MICB*	TRUE	2.072523596
Heart_Failure	ASPC	*MICA*	TRUE	2.168219489
Heart_Failure	ASPC	*FAM153C*	TRUE	2.979843797
Heart_Failure	ASPC	*CLLU1OS*	TRUE	2.158081673
Heart_Failure	ASPC	*FAM205A*	TRUE	2.360260514
Heart_Failure	ASPC	*SERF1B*	TRUE	2.157372685
Heart_Failure	ASPC	*ATF6B*	TRUE	2.153131437
Heart_Failure	ASPC	*CPNE1*	TRUE	3.502565491
Heart_Failure	ASPC	*TBC1D26*	TRUE	2.138724279
Heart_Failure	ASPC	*CYB5RL*	TRUE	2.21999671
Heart_Failure	ASPC	*PROB1*	TRUE	2.086292763
Heart_Failure	ASPC	*ZNF736*	TRUE	2.198990538
Heart_Failure	ASPC	*ZBTB22*	TRUE	2.191884393
Heart_Failure	ASPC	*HLA.DQA2*	TRUE	2.882010287
Heart_Failure	ASPC	*EIF6*	TRUE	2.109781547
Heart_Failure	ASPC	*UPK3B*	TRUE	2.245671092
Heart_Failure	ASPC	*TTC4*	TRUE	2.955862477
Heart_Failure	ASPC	*ARMS2*	TRUE	2.053528263

Heart_Failure	ASPC	*MPV17L2*	TRUE	2.176418079
Heart_Failure	ASPC	*GTF2H5*	TRUE	2.06019633
Heart_Failure	b_cell	*PDLIM4*	TRUE	2.986175029
Heart_Failure	b_cell	*REEP2*	TRUE	2.275937724
Heart_Failure	b_cell	*HLA.DQB1*	TRUE	2.060475793
Heart_Failure	b_cell	*RPS26*	TRUE	2.34099437
Heart_Failure	b_cell	*FAM205A*	TRUE	2.549119659
Heart_Failure	b_cell	*UPK3B*	TRUE	2.193125993
Heart_Failure	dendritic_cell	*SEMA3F*	TRUE	3.010085151
Heart_Failure	dendritic_cell	*DBNDD1*	TRUE	2.175274426
Heart_Failure	dendritic_cell	*BIRC7*	TRUE	2.636806843
Heart_Failure	dendritic_cell	*CDK2AP1*	TRUE	2.350337051
Heart_Failure	dendritic_cell	*IL4*	TRUE	2.286034605
Heart_Failure	dendritic_cell	*CFHR3*	TRUE	2.138173493
Heart_Failure	dendritic_cell	*REEP2*	TRUE	3.301403548
Heart_Failure	dendritic_cell	*CYP2C9*	TRUE	2.215453219
Heart_Failure	dendritic_cell	*FLG*	TRUE	2.447901685
Heart_Failure	dendritic_cell	*DUSP15*	TRUE	2.007189457
Heart_Failure	dendritic_cell	*KLHDC9*	TRUE	1.974839467
Heart_Failure	dendritic_cell	*DCLK3*	TRUE	2.333656854
Heart_Failure	dendritic_cell	*ORMDL3*	TRUE	5.587455059
Heart_Failure	dendritic_cell	*DCAKD*	TRUE	2.582325493
Heart_Failure	dendritic_cell	*CPNE7*	TRUE	1.973130685
Heart_Failure	dendritic_cell	*KLHDC7A*	TRUE	2.508857343
Heart_Failure	dendritic_cell	*SH3BGR*	TRUE	2.047083943
Heart_Failure	dendritic_cell	*RPS26*	TRUE	3.367973886
Heart_Failure	dendritic_cell	*MICB*	TRUE	3.268231994
Heart_Failure	dendritic_cell	*FAM153C*	TRUE	2.313058472
Heart_Failure	dendritic_cell	*FAM205A*	TRUE	2.886753831
Heart_Failure	dendritic_cell	*CPNE1*	TRUE	2.303635231
Heart_Failure	dendritic_cell	*ZBTB22*	TRUE	2.621439862
Heart_Failure	dendritic_cell	*UPK3B*	TRUE	2.448546682
Heart_Failure	endothelial	*SLC7A9*	TRUE	2.021259839
Heart_Failure	endothelial	*CDK2AP1*	TRUE	2.765000599
Heart_Failure	endothelial	*PCDHB6*	TRUE	2.538659714
Heart_Failure	endothelial	*CFHR3*	TRUE	2.244421932
Heart_Failure	endothelial	*REEP2*	TRUE	3.459851261
Heart_Failure	endothelial	*BHMT*	TRUE	2.237699873
Heart_Failure	endothelial	*TSLP*	TRUE	3.124376247
Heart_Failure	endothelial	*GAL3ST2*	TRUE	2.686389526
Heart_Failure	endothelial	*ORMDL3*	TRUE	2.627435758
Heart_Failure	endothelial	*CPNE7*	TRUE	1.961821011
Heart_Failure	endothelial	*TRIM73*	TRUE	2.192504606
Heart_Failure	endothelial	*HLA.DQB1*	TRUE	2.918494983
Heart_Failure	endothelial	*SH3BGR*	TRUE	2.068769137
Heart_Failure	endothelial	*MUC1*	TRUE	2.152675959
Heart_Failure	endothelial	*SLC22A5*	TRUE	2.120727035
Heart_Failure	endothelial	*RPS26*	TRUE	3.202063403

Heart_Failure	endothelial	*FAM205A*	TRUE	2.28380071
Heart_Failure	endothelial	*HLA.DQA2*	TRUE	2.860994578
Heart_Failure	LEC	*KCNH4*	TRUE	2.156905613
Heart_Failure	LEC	*CDK2AP1*	TRUE	1.970493012
Heart_Failure	LEC	*FLG*	TRUE	2.479572537
Heart_Failure	LEC	*BHMT*	TRUE	2.344148772
Heart_Failure	LEC	*ROM1*	TRUE	2.606208765
Heart_Failure	LEC	*DUSP15*	TRUE	2.051829
Heart_Failure	LEC	*HLA.DQB1*	TRUE	3.888114689
Heart_Failure	LEC	*RPS26*	TRUE	3.365954627
Heart_Failure	LEC	*MICA*	TRUE	2.603708998
Heart_Failure	LEC	*FAM205A*	TRUE	2.38875791
Heart_Failure	LEC	*CPNE1*	TRUE	2.046257391
Heart_Failure	LEC	*HLA.DQA2*	TRUE	3.381435866
Heart_Failure	macrophage	*SLC7A9*	TRUE	2.494481847
Heart_Failure	macrophage	*LAMC2*	TRUE	2.454837365
Heart_Failure	macrophage	*COL16A1*	TRUE	2.31080587
Heart_Failure	macrophage	*BIRC7*	TRUE	2.7752976
Heart_Failure	macrophage	*NDUFAF5*	TRUE	2.006716643
Heart_Failure	macrophage	*CORO1A*	TRUE	2.018781463
Heart_Failure	macrophage	*CTSH*	TRUE	1.998098922
Heart_Failure	macrophage	*CDK2AP1*	TRUE	3.189940243
Heart_Failure	macrophage	*PCDHB6*	TRUE	2.339548336
Heart_Failure	macrophage	*BNIP1*	TRUE	2.243671778
Heart_Failure	macrophage	*HEMK1*	TRUE	2.635601526
Heart_Failure	macrophage	*GRIN3B*	TRUE	2.052824232
Heart_Failure	macrophage	*CFHR3*	TRUE	2.785850513
Heart_Failure	macrophage	*ALDH8A1*	TRUE	2.050890699
Heart_Failure	macrophage	*UCK1*	TRUE	2.216088503
Heart_Failure	macrophage	*CHMP1A*	TRUE	2.005961632
Heart_Failure	macrophage	*MRPL35*	TRUE	2.325496128
Heart_Failure	macrophage	*REEP2*	TRUE	3.756347658
Heart_Failure	macrophage	*FLG*	TRUE	2.819276828
Heart_Failure	macrophage	*BHMT*	TRUE	2.758642784
Heart_Failure	macrophage	*TSLP*	TRUE	4.29321349
Heart_Failure	macrophage	*DUSP15*	TRUE	3.064357801
Heart_Failure	macrophage	*DPYSL4*	TRUE	2.335503783
Heart_Failure	macrophage	*GAL3ST2*	TRUE	2.956099579
Heart_Failure	macrophage	*C21orf33*	TRUE	2.334087619
Heart_Failure	macrophage	*FCRL3*	TRUE	2.344691136
Heart_Failure	macrophage	*EFHB*	TRUE	2.669601215
Heart_Failure	macrophage	*DCLK3*	TRUE	2.046773515
Heart_Failure	macrophage	*CHRFAM7A*	TRUE	1.992419909
Heart_Failure	macrophage	*HDHD2*	TRUE	2.522176763
Heart_Failure	macrophage	*HRASLS5*	TRUE	2.04316415
Heart_Failure	macrophage	*RGS14*	TRUE	2.411414162
Heart_Failure	macrophage	*ORMDL3*	TRUE	5.752058705
Heart_Failure	macrophage	*DCAKD*	TRUE	2.186849931

Heart_Failure	macrophage	*CHST1*	TRUE	2.201910609
Heart_Failure	macrophage	*PAAF1*	TRUE	2.277544257
Heart_Failure	macrophage	*FBXO39*	TRUE	2.259175909
Heart_Failure	macrophage	*NIM1K*	TRUE	2.010776843
Heart_Failure	macrophage	*KLHDC7A*	TRUE	2.492558585
Heart_Failure	macrophage	*HLA.DQB1*	TRUE	3.691619788
Heart_Failure	macrophage	*FKRP*	TRUE	2.366514329
Heart_Failure	macrophage	*ACOT1*	TRUE	2.273378709
Heart_Failure	macrophage	*A3GALT2*	TRUE	2.142675487
Heart_Failure	macrophage	*MUC1*	TRUE	3.295025883
Heart_Failure	macrophage	*MCRS1*	TRUE	2.760447928
Heart_Failure	macrophage	*SLC22A5*	TRUE	2.710539987
Heart_Failure	macrophage	*RPS26*	TRUE	2.835549651
Heart_Failure	macrophage	*MICB*	TRUE	2.183537102
Heart_Failure	macrophage	*MICA*	TRUE	2.308540824
Heart_Failure	macrophage	*FAM205A*	TRUE	2.716728954
Heart_Failure	macrophage	*CPNE1*	TRUE	2.890533834
Heart_Failure	macrophage	*ZBTB22*	TRUE	2.105449712
Heart_Failure	macrophage	*HLA.DQA2*	TRUE	2.747223844
Heart_Failure	macrophage	*UPK3B*	TRUE	2.218091169
Heart_Failure	macrophage	*TTC4*	TRUE	2.665898152
Heart_Failure	macrophage	*ARMS2*	TRUE	2.074308474
Heart_Failure	mast_cell	*SLC7A9*	TRUE	2.219555544
Heart_Failure	mast_cell	*LAMC2*	TRUE	2.376630421
Heart_Failure	mast_cell	*REEP2*	TRUE	2.420062785
Heart_Failure	mast_cell	*FLG*	TRUE	2.683797252
Heart_Failure	mast_cell	*GAL3ST2*	TRUE	1.989659747
Heart_Failure	mast_cell	*ORMDL3*	TRUE	5.589273831
Heart_Failure	mast_cell	*KLHDC7A*	TRUE	2.116679677
Heart_Failure	mast_cell	*HLA.DQB1*	TRUE	2.177428596
Heart_Failure	mast_cell	*FKRP*	TRUE	2.255880438
Heart_Failure	mast_cell	*MUC1*	TRUE	3.02839433
Heart_Failure	mast_cell	*SLC22A5*	TRUE	3.012986483
Heart_Failure	mast_cell	*RPS26*	TRUE	3.188573592
Heart_Failure	mast_cell	*MICB*	TRUE	2.324885624
Heart_Failure	mast_cell	*FAM205A*	TRUE	2.735828357
Heart_Failure	mast_cell	*ZBTB22*	TRUE	2.334915743
Heart_Failure	mast_cell	*HLA.DQA2*	TRUE	3.133462918
Heart_Failure	mast_cell	*TNFSF12*	TRUE	1.99938631
Heart_Failure	monocyte	*BIRC7*	TRUE	2.248910996
Heart_Failure	monocyte	*DHODH*	TRUE	2.076510716
Heart_Failure	monocyte	*UCK1*	TRUE	1.989988026
Heart_Failure	monocyte	*DUSP15*	TRUE	2.572324922
Heart_Failure	monocyte	*RPS26*	TRUE	3.510164098
Heart_Failure	monocyte	*MICA*	TRUE	2.803020529
Heart_Failure	monocyte	*FAM205A*	TRUE	2.293260845
Heart_Failure	monocyte	*ATF6B*	TRUE	2.982123602
Heart_Failure	monocyte	*HLA.DQA2*	TRUE	1.999220437

Heart_Failure	neutrophil	*NRROS*	TRUE	2.528332176
Heart_Failure	neutrophil	*HLA.DQB1*	TRUE	8.350750182
Heart_Failure	neutrophil	*ACOT1*	TRUE	1.968139932
Heart_Failure	neutrophil	*FAM205A*	TRUE	2.607496799
Heart_Failure	nk_cell	*MRPL35*	TRUE	2.177644571
Heart_Failure	nk_cell	*REEP2*	TRUE	2.098362051
Heart_Failure	nk_cell	*RNF123*	TRUE	2.554327282
Heart_Failure	nk_cell	*HDHD2*	TRUE	2.482721519
Heart_Failure	nk_cell	*ORMDL3*	TRUE	3.812469253
Heart_Failure	nk_cell	*HLA.DQB1*	TRUE	3.217759652
Heart_Failure	nk_cell	*BMP8A*	TRUE	2.117855194
Heart_Failure	nk_cell	*SLC22A5*	TRUE	3.625326882
Heart_Failure	nk_cell	*RPS26*	TRUE	2.623848851
Heart_Failure	nk_cell	*HLA.DQA2*	TRUE	2.840685623
Heart_Failure	pericyte	*DBNDD1*	TRUE	1.979540965
Heart_Failure	pericyte	*KCNH4*	TRUE	2.102500056
Heart_Failure	pericyte	*CDK2AP1*	TRUE	2.611727674
Heart_Failure	pericyte	*PCDHB6*	TRUE	2.502092151
Heart_Failure	pericyte	*BNIP1*	TRUE	2.329373963
Heart_Failure	pericyte	*HEMK1*	TRUE	2.681153047
Heart_Failure	pericyte	*UCK1*	TRUE	2.108857379
Heart_Failure	pericyte	*REEP2*	TRUE	2.965010338
Heart_Failure	pericyte	*ADAM19*	TRUE	2.04656636
Heart_Failure	pericyte	*NMT1*	TRUE	2.556369797
Heart_Failure	pericyte	*ROM1*	TRUE	2.595303328
Heart_Failure	pericyte	*DPYSL4*	TRUE	2.25445109
Heart_Failure	pericyte	*TTC39B*	TRUE	1.965987579
Heart_Failure	pericyte	*NIT1*	TRUE	2.034776849
Heart_Failure	pericyte	*EFHB*	TRUE	2.3247683
Heart_Failure	pericyte	*RNF123*	TRUE	3.227493097
Heart_Failure	pericyte	*SAP30L*	TRUE	2.09084574
Heart_Failure	pericyte	*HDHD2*	TRUE	2.106080113
Heart_Failure	pericyte	*RGS14*	TRUE	2.122915998
Heart_Failure	pericyte	*ORMDL3*	TRUE	2.967658992
Heart_Failure	pericyte	*KLHDC7A*	TRUE	2.30156134
Heart_Failure	pericyte	*HLA.DQB1*	TRUE	2.698171415
Heart_Failure	pericyte	*FKRP*	TRUE	2.296610733
Heart_Failure	pericyte	*A3GALT2*	TRUE	2.124258044
Heart_Failure	pericyte	*SLC22A5*	TRUE	5.097878071
Heart_Failure	pericyte	*RPS26*	TRUE	3.487318421
Heart_Failure	pericyte	*MICB*	TRUE	2.460621653
Heart_Failure	pericyte	*FAM205A*	TRUE	2.84339239
Heart_Failure	pericyte	*CPNE1*	TRUE	1.999971671
Heart_Failure	pericyte	*HLA.DQA2*	TRUE	3.273883476
Heart_Failure	pericyte	*TMEM200B*	TRUE	2.380135909
Heart_Failure	SMC	*COL16A1*	TRUE	2.840955693
Heart_Failure	SMC	*BIRC7*	TRUE	2.889716705
Heart_Failure	SMC	*CTSH*	TRUE	2.057044922

Heart_Failure	SMC	*CDK2AP1*	TRUE	2.615560462
Heart_Failure	SMC	*HEMK1*	TRUE	2.135927822
Heart_Failure	SMC	*GRIN3B*	TRUE	2.03292185
Heart_Failure	SMC	*CFHR3*	TRUE	2.748028331
Heart_Failure	SMC	*ALDH8A1*	TRUE	2.108529053
Heart_Failure	SMC	*CTNNAL1*	TRUE	2.233562298
Heart_Failure	SMC	*UCK1*	TRUE	2.183917206
Heart_Failure	SMC	*MRPL35*	TRUE	2.118973
Heart_Failure	SMC	*REEP2*	TRUE	3.274675517
Heart_Failure	SMC	*ADAM19*	TRUE	2.070352649
Heart_Failure	SMC	*ROM1*	TRUE	2.05615311
Heart_Failure	SMC	*DUSP15*	TRUE	2.214233617
Heart_Failure	SMC	*DPYSL4*	TRUE	2.120337661
Heart_Failure	SMC	*GAL3ST2*	TRUE	2.313300602
Heart_Failure	SMC	*EFHB*	TRUE	2.543868763
Heart_Failure	SMC	*DCLK3*	TRUE	2.593922823
Heart_Failure	SMC	*CHRFAM7A*	TRUE	1.966790979
Heart_Failure	SMC	*ORMDL3*	TRUE	5.624567621
Heart_Failure	SMC	*DCAKD*	TRUE	2.399910861
Heart_Failure	SMC	*CHST1*	TRUE	2.082750794
Heart_Failure	SMC	*PAAF1*	TRUE	2.108200413
Heart_Failure	SMC	*FBXO39*	TRUE	2.253431609
Heart_Failure	SMC	*ZNF454*	TRUE	2.510937706
Heart_Failure	SMC	*KLHDC7A*	TRUE	2.324244361
Heart_Failure	SMC	*HLA.DQB1*	TRUE	3.83211278
Heart_Failure	SMC	*FKRP*	TRUE	2.123975736
Heart_Failure	SMC	*TDRKH*	TRUE	2.303973209
Heart_Failure	SMC	*A3GALT2*	TRUE	2.108127763
Heart_Failure	SMC	*MUC1*	TRUE	3.183947436
Heart_Failure	SMC	*MCRS1*	TRUE	2.013181621
Heart_Failure	SMC	*SLC22A5*	TRUE	3.551192329
Heart_Failure	SMC	*RPS26*	TRUE	2.868309124
Heart_Failure	SMC	*MICA*	TRUE	2.489056297
Heart_Failure	SMC	*FAM205A*	TRUE	2.923976629
Heart_Failure	SMC	*CPNE1*	TRUE	2.660625619
Heart_Failure	SMC	*HLA.DQA2*	TRUE	2.441840001
Heart_Failure	SMC	*TTC4*	TRUE	2.438696157
Heart_Failure	t_cell	*SLC7A9*	TRUE	2.21887474
Heart_Failure	t_cell	*UCK1*	TRUE	1.976584542
Heart_Failure	t_cell	*MRPL35*	TRUE	2.099063555
Heart_Failure	t_cell	*REEP2*	TRUE	3.029638434
Heart_Failure	t_cell	*DPYSL4*	TRUE	2.342065051
Heart_Failure	t_cell	*ORMDL3*	TRUE	3.768566614
Heart_Failure	t_cell	*CHST1*	TRUE	2.712045958
Heart_Failure	t_cell	*KLHDC7A*	TRUE	1.96543216
Heart_Failure	t_cell	*HLA.DQB1*	TRUE	3.951443346
Heart_Failure	t_cell	*FKRP*	TRUE	2.004536115
Heart_Failure	t_cell	*MUC1*	TRUE	2.551222509

Heart_Failure	t_cell	*SLC22A5*	TRUE	2.927958151
Heart_Failure	t_cell	*RPS26*	TRUE	3.219138437
Heart_Failure	t_cell	*MICB*	TRUE	2.223862981
Heart_Failure	t_cell	*FAM205A*	TRUE	2.735326238
Heart_Failure	t_cell	*ATF6B*	TRUE	2.310607017
Heart_Failure	t_cell	*CPNE1*	TRUE	2.164168444
Heart_Failure	t_cell	*ZBTB22*	TRUE	2.355309645
CAD_admission_freq	adipocyte	*F7*	TRUE	2.90847062
CAD_admission_freq	adipocyte	*LAMC2*	TRUE	2.01741734
CAD_admission_freq	adipocyte	*CLTCL1*	TRUE	2.42525931
CAD_admission_freq	adipocyte	*RPGRIP1*	TRUE	2.28268388
CAD_admission_freq	adipocyte	*IGDCC4*	TRUE	2.0306909
CAD_admission_freq	adipocyte	*CCDC61*	TRUE	2.22724831
CAD_admission_freq	adipocyte	*CAPS*	TRUE	2.19675446
CAD_admission_freq	adipocyte	*CYP27B1*	TRUE	2.03384363
CAD_admission_freq	adipocyte	*TOX2*	TRUE	2.55184934
CAD_admission_freq	adipocyte	*NRM*	TRUE	2.36444441
CAD_admission_freq	adipocyte	*KHK*	TRUE	2.38746746
CAD_admission_freq	adipocyte	*DMTN*	TRUE	2.26400921
CAD_admission_freq	adipocyte	*GSTM4*	TRUE	1.97650472
CAD_admission_freq	adipocyte	*RAB24*	TRUE	1.99666243
CAD_admission_freq	adipocyte	*HOXD4*	TRUE	2.13716925
CAD_admission_freq	adipocyte	*CSDC2*	TRUE	1.98479415
CAD_admission_freq	adipocyte	*SLC22A1*	TRUE	3.12157785
CAD_admission_freq	adipocyte	*PAAF1*	TRUE	3.12881035
CAD_admission_freq	adipocyte	*GOLGA8Q*	TRUE	2.67057785
CAD_admission_freq	adipocyte	*ACTL10*	TRUE	2.28384808
CAD_admission_freq	adipocyte	*PLA2G6*	TRUE	2.29691344
CAD_admission_freq	adipocyte	*SHISA4*	TRUE	2.01908593
CAD_admission_freq	adipocyte	*PSORS1C1*	TRUE	2.0652492
CAD_admission_freq	adipocyte	*AC084018.1*	TRUE	2.05943039
CAD_admission_freq	adipocyte	*GS1.124K5.4*	TRUE	2.26677472
CAD_admission_freq	adipocyte	*ZNF286B*	TRUE	2.20606858
CAD_admission_freq	adipocyte	*HMBS*	TRUE	2.11348006
CAD_admission_freq	adipocyte	*SRXN1*	TRUE	2.46994373
CAD_admission_freq	ASPC	*TYMP*	TRUE	2.1130286
CAD_admission_freq	ASPC	*MED17*	TRUE	2.10998282
CAD_admission_freq	ASPC	*F7*	TRUE	2.40314068
CAD_admission_freq	ASPC	*LAMC2*	TRUE	2.04879382
CAD_admission_freq	ASPC	*CLTCL1*	TRUE	2.56584873
CAD_admission_freq	ASPC	*TPD52*	TRUE	2.27580725
CAD_admission_freq	ASPC	*STX7*	TRUE	2.05525908
CAD_admission_freq	ASPC	*GEMIN5*	TRUE	2.10560566
CAD_admission_freq	ASPC	*TXNDC16*	TRUE	2.29400936
CAD_admission_freq	ASPC	*RPGRIP1*	TRUE	2.44847445
CAD_admission_freq	ASPC	*TMED8*	TRUE	2.02513224
CAD_admission_freq	ASPC	*MON1B*	TRUE	2.592485
CAD_admission_freq	ASPC	*TRIP4*	TRUE	2.20697284

CAD_admission_freq	ASPC	*CCDC61*	TRUE	2.6146719
CAD_admission_freq	ASPC	*CAPS*	TRUE	2.54239619
CAD_admission_freq	ASPC	*ELAVL2*	TRUE	2.00238693
CAD_admission_freq	ASPC	*AKAP10*	TRUE	2.10382961
CAD_admission_freq	ASPC	*CYP27B1*	TRUE	2.23112368
CAD_admission_freq	ASPC	*CHPT1*	TRUE	2.03963585
CAD_admission_freq	ASPC	*EFCC1*	TRUE	2.33570152
CAD_admission_freq	ASPC	*SDC1*	TRUE	1.98856316
CAD_admission_freq	ASPC	*DNAJC6*	TRUE	2.05772062
CAD_admission_freq	ASPC	*MED28*	TRUE	2.24058252
CAD_admission_freq	ASPC	*C4BPB*	TRUE	2.09242299
CAD_admission_freq	ASPC	*TOX2*	TRUE	2.60676534
CAD_admission_freq	ASPC	*CLPP*	TRUE	1.960804
CAD_admission_freq	ASPC	*CCL4*	TRUE	2.21214409
CAD_admission_freq	ASPC	*MRPL34*	TRUE	2.01157261
CAD_admission_freq	ASPC	*DHX34*	TRUE	1.99326027
CAD_admission_freq	ASPC	*TOR1B*	TRUE	1.99691793
CAD_admission_freq	ASPC	*NRM*	TRUE	2.36575854
CAD_admission_freq	ASPC	*KHK*	TRUE	2.3672599
CAD_admission_freq	ASPC	*LHCGR*	TRUE	2.1527545
CAD_admission_freq	ASPC	*ABCG5*	TRUE	2.08189511
CAD_admission_freq	ASPC	*ARL3*	TRUE	2.03562157
CAD_admission_freq	ASPC	*PAPSS1*	TRUE	2.33912158
CAD_admission_freq	ASPC	*PPFIA2*	TRUE	2.1782352
CAD_admission_freq	ASPC	*LYSMD2*	TRUE	2.29094351
CAD_admission_freq	ASPC	*KIFC3*	TRUE	2.3017993
CAD_admission_freq	ASPC	*MAP3K6*	TRUE	2.36217672
CAD_admission_freq	ASPC	*SCCPDH*	TRUE	2.69632409
CAD_admission_freq	ASPC	*SNAP47*	TRUE	2.20899469
CAD_admission_freq	ASPC	*ESPNL*	TRUE	2.60472891
CAD_admission_freq	ASPC	*TSLP*	TRUE	2.02324337
CAD_admission_freq	ASPC	*ATP6V0D2*	TRUE	2.10439258
CAD_admission_freq	ASPC	*VIPAS39*	TRUE	2.08735755
CAD_admission_freq	ASPC	*FAM213B*	TRUE	1.98624333
CAD_admission_freq	ASPC	*DMTN*	TRUE	3.0004541
CAD_admission_freq	ASPC	*CCDC127*	TRUE	1.96594995
CAD_admission_freq	ASPC	*FAM227B*	TRUE	2.02818923
CAD_admission_freq	ASPC	*GPRC5B*	TRUE	2.08967627
CAD_admission_freq	ASPC	*CX3CR1*	TRUE	1.96288317
CAD_admission_freq	ASPC	*GSTM4*	TRUE	1.97091518
CAD_admission_freq	ASPC	*RAB24*	TRUE	2.36072078
CAD_admission_freq	ASPC	*HOXD4*	TRUE	2.29847007
CAD_admission_freq	ASPC	*GRIK1*	TRUE	1.97561259
CAD_admission_freq	ASPC	*CSDC2*	TRUE	1.99787743
CAD_admission_freq	ASPC	*DMRT2*	TRUE	2.31166917
CAD_admission_freq	ASPC	*SLC22A1*	TRUE	3.83873534
CAD_admission_freq	ASPC	*ZNF25*	TRUE	2.02887206
CAD_admission_freq	ASPC	*PAAF1*	TRUE	2.92883611

CAD_admission_freq	ASPC	*TMEM80*	TRUE	2.07636027
CAD_admission_freq	ASPC	*CD163L1*	TRUE	1.98557466
CAD_admission_freq	ASPC	*GOLGA8Q*	TRUE	2.71655971
CAD_admission_freq	ASPC	*TMPRSS9*	TRUE	2.53995487
CAD_admission_freq	ASPC	*HYI*	TRUE	2.08947594
CAD_admission_freq	ASPC	*CLEC4G*	TRUE	2.41245845
CAD_admission_freq	ASPC	*ACTL10*	TRUE	2.33725374
CAD_admission_freq	ASPC	*PLA2G6*	TRUE	3.00825682
CAD_admission_freq	ASPC	*RASA3*	TRUE	2.18733612
CAD_admission_freq	ASPC	*GSAP*	TRUE	2.27536363
CAD_admission_freq	ASPC	*ISG15*	TRUE	1.97305194
CAD_admission_freq	ASPC	*SEMA4A*	TRUE	2.27515916
CAD_admission_freq	ASPC	*GM2A*	TRUE	1.97158516
CAD_admission_freq	ASPC	*SHISA4*	TRUE	2.05894671
CAD_admission_freq	ASPC	*PSORS1C1*	TRUE	2.08082945
CAD_admission_freq	ASPC	*PCDHA5*	TRUE	2.38634751
CAD_admission_freq	ASPC	*GIMAP1*	TRUE	2.09420083
CAD_admission_freq	ASPC	*GS1.124K5.4*	TRUE	1.96211214
CAD_admission_freq	ASPC	*ZNF286B*	TRUE	2.39016173
CAD_admission_freq	ASPC	*HMBS*	TRUE	2.2639158
CAD_admission_freq	ASPC	*ZNF578*	TRUE	1.98149039
CAD_admission_freq	ASPC	*HOXB7*	TRUE	1.97725432
CAD_admission_freq	ASPC	*SRXN1*	TRUE	2.73738404
CAD_admission_freq	b_cell	*F7*	TRUE	2.63818105
CAD_admission_freq	b_cell	*MICB*	TRUE	2.66677283
CAD_admission_freq	b_cell	*SRXN1*	TRUE	2.22217696
CAD_admission_freq	dendritic_cell	*SNX15*	TRUE	1.99434912
CAD_admission_freq	dendritic_cell	*KHK*	TRUE	2.15827213
CAD_admission_freq	dendritic_cell	*NUDT13*	TRUE	2.10907034
CAD_admission_freq	dendritic_cell	*PAAF1*	TRUE	2.42736327
CAD_admission_freq	dendritic_cell	*GOLGA8Q*	TRUE	2.32177767
CAD_admission_freq	dendritic_cell	*SMDT1*	TRUE	2.15880403
CAD_admission_freq	dendritic_cell	*FAM228A*	TRUE	1.997153
CAD_admission_freq	dendritic_cell	*MICB*	TRUE	2.34241892
CAD_admission_freq	dendritic_cell	*PCDHA5*	TRUE	2.03585185
CAD_admission_freq	dendritic_cell	*GS1.124K5.4*	TRUE	2.13329679
CAD_admission_freq	dendritic_cell	*SRXN1*	TRUE	2.7141939
CAD_admission_freq	endothelial	*F7*	TRUE	2.33139437
CAD_admission_freq	endothelial	*LAMC2*	TRUE	2.04412644
CAD_admission_freq	endothelial	*KHK*	TRUE	2.27095979
CAD_admission_freq	endothelial	*PAAF1*	TRUE	2.91410124
CAD_admission_freq	endothelial	*GOLGA8Q*	TRUE	2.21989649
CAD_admission_freq	endothelial	*MICB*	TRUE	2.70358901
CAD_admission_freq	endothelial	*PSORS1C1*	TRUE	2.15150417
CAD_admission_freq	endothelial	*AC084018.1*	TRUE	1.98884019
CAD_admission_freq	endothelial	*GS1.124K5.4*	TRUE	1.96854138
CAD_admission_freq	endothelial	*SRXN1*	TRUE	2.06615117
CAD_admission_freq	LEC	*KHK*	TRUE	2.28797042

CAD_admission_freq	LEC	*PAAF1*	TRUE	2.48924406
CAD_admission_freq	LEC	*GOLGA8Q*	TRUE	2.26711375
CAD_admission_freq	LEC	*SMDT1*	TRUE	2.14717705
CAD_admission_freq	LEC	*HYAL3*	TRUE	1.98907162
CAD_admission_freq	LEC	*GS1.124K5.4*	TRUE	2.03817197
CAD_admission_freq	LEC	*SRXN1*	TRUE	2.45124589
CAD_admission_freq	macrophage	*F7*	TRUE	2.16543393
CAD_admission_freq	macrophage	*CLTCL1*	TRUE	2.57509824
CAD_admission_freq	macrophage	*IGDCC4*	TRUE	1.96978336
CAD_admission_freq	macrophage	*CCDC61*	TRUE	2.05529177
CAD_admission_freq	macrophage	*CAPS*	TRUE	1.99170551
CAD_admission_freq	macrophage	*TOX2*	TRUE	2.47426058
CAD_admission_freq	macrophage	*NRM*	TRUE	2.29425265
CAD_admission_freq	macrophage	*KHK*	TRUE	2.36818135
CAD_admission_freq	macrophage	*DMTN*	TRUE	2.72590747
CAD_admission_freq	macrophage	*RAB24*	TRUE	2.04389008
CAD_admission_freq	macrophage	*HOXD4*	TRUE	2.21631976
CAD_admission_freq	macrophage	*SLC22A1*	TRUE	3.34171705
CAD_admission_freq	macrophage	*PAAF1*	TRUE	3.12178205
CAD_admission_freq	macrophage	*GOLGA8Q*	TRUE	2.89346058
CAD_admission_freq	macrophage	*HYI*	TRUE	1.9622752
CAD_admission_freq	macrophage	*ACTL10*	TRUE	1.9687866
CAD_admission_freq	macrophage	*SMDT1*	TRUE	1.96503618
CAD_admission_freq	macrophage	*PLA2G6*	TRUE	2.41249947
CAD_admission_freq	macrophage	*FAM228A*	TRUE	1.99066354
CAD_admission_freq	macrophage	*PSORS1C1*	TRUE	2.19042399
CAD_admission_freq	macrophage	*AC084018.1*	TRUE	2.05425055
CAD_admission_freq	macrophage	*GS1.124K5.4*	TRUE	2.30708577
CAD_admission_freq	macrophage	*ZNF286B*	TRUE	2.1245186
CAD_admission_freq	macrophage	*HMBS*	TRUE	2.19530084
CAD_admission_freq	macrophage	*SRXN1*	TRUE	2.68107173
CAD_admission_freq	mast_cell	*F7*	TRUE	2.86525807
CAD_admission_freq	mast_cell	*IGDCC4*	TRUE	2.08581928
CAD_admission_freq	mast_cell	*NRM*	TRUE	2.33143309
CAD_admission_freq	mast_cell	*SLC22A1*	TRUE	2.55842953
CAD_admission_freq	mast_cell	*PAAF1*	TRUE	2.51584241
CAD_admission_freq	mast_cell	*GOLGA8Q*	TRUE	2.3976914
CAD_admission_freq	mast_cell	*HYI*	TRUE	1.97328163
CAD_admission_freq	mast_cell	*SMN2*	TRUE	2.04786338
CAD_admission_freq	mast_cell	*SRXN1*	TRUE	2.50524045
CAD_admission_freq	monocyte	*SLC22A1*	TRUE	2.13439621
CAD_admission_freq	monocyte	*GOLGA8Q*	TRUE	2.38951796
CAD_admission_freq	monocyte	*SMDT1*	TRUE	1.96776148
CAD_admission_freq	monocyte	*MICB*	TRUE	2.1024977
CAD_admission_freq	monocyte	*SRXN1*	TRUE	2.06227881
CAD_admission_freq	neutrophil	*SRXN1*	TRUE	2.15799376
CAD_admission_freq	nk_cell	*F7*	TRUE	2.42492538
CAD_admission_freq	nk_cell	*SDC1*	TRUE	1.99475271

CAD_admission_freq	nk_cell	*TOX2*	TRUE	2.1970605
CAD_admission_freq	nk_cell	*NRM*	TRUE	3.04958673
CAD_admission_freq	nk_cell	*KHK*	TRUE	2.25441241
CAD_admission_freq	nk_cell	*SLC22A1*	TRUE	2.630457
CAD_admission_freq	nk_cell	*PAAF1*	TRUE	2.75622112
CAD_admission_freq	nk_cell	*GOLGA8Q*	TRUE	2.37439554
CAD_admission_freq	pericyte	*F7*	TRUE	2.67747432
CAD_admission_freq	pericyte	*IGDCC4*	TRUE	2.04297819
CAD_admission_freq	pericyte	*TOX2*	TRUE	1.9945387
CAD_admission_freq	pericyte	*KHK*	TRUE	2.13887286
CAD_admission_freq	pericyte	*PAAF1*	TRUE	3.13542164
CAD_admission_freq	pericyte	*GOLGA8Q*	TRUE	2.6145165
CAD_admission_freq	pericyte	*SMDT1*	TRUE	2.11679727
CAD_admission_freq	pericyte	*HYAL3*	TRUE	2.02613444
CAD_admission_freq	pericyte	*MICB*	TRUE	2.20562597
CAD_admission_freq	pericyte	*PSORS1C1*	TRUE	2.09847078
CAD_admission_freq	pericyte	*GS1.124K5.4*	TRUE	2.1914403
CAD_admission_freq	pericyte	*SRXN1*	TRUE	2.57002766
CAD_admission_freq	SMC	*F7*	TRUE	2.4348322
CAD_admission_freq	SMC	*IGDCC4*	TRUE	2.1162553
CAD_admission_freq	SMC	*TOX2*	TRUE	2.48295478
CAD_admission_freq	SMC	*NRM*	TRUE	2.79868634
CAD_admission_freq	SMC	*KHK*	TRUE	2.07061469
CAD_admission_freq	SMC	*DMTN*	TRUE	2.72350333
CAD_admission_freq	SMC	*HOXD4*	TRUE	2.33837521
CAD_admission_freq	SMC	*PAAF1*	TRUE	2.91363234
CAD_admission_freq	SMC	*GOLGA8Q*	TRUE	2.73790959
CAD_admission_freq	SMC	*GPANK1*	TRUE	2.24949577
CAD_admission_freq	SMC	*GSTM2*	TRUE	2.08838341
CAD_admission_freq	SMC	*GS1.124K5.4*	TRUE	2.34965988
CAD_admission_freq	SMC	*HMBS*	TRUE	2.09296632
CAD_admission_freq	SMC	*SRXN1*	TRUE	2.51100181
CAD_admission_freq	t_cell	*F7*	TRUE	2.59222378
CAD_admission_freq	t_cell	*TOX2*	TRUE	1.99843811
CAD_admission_freq	t_cell	*KHK*	TRUE	2.19318604
CAD_admission_freq	t_cell	*DMTN*	TRUE	2.26794839
CAD_admission_freq	t_cell	*SLC22A1*	TRUE	2.76500155
CAD_admission_freq	t_cell	*PAAF1*	TRUE	2.81136504
CAD_admission_freq	t_cell	*GOLGA8Q*	TRUE	2.61634109
CAD_admission_freq	t_cell	*MICB*	TRUE	2.10788285
CAD_admission_freq	t_cell	*GS1.124K5.4*	TRUE	2.03997287
CAD_admission_freq	t_cell	*HMBS*	TRUE	1.99987137
CAD_admission_freq	t_cell	*SRXN1*	TRUE	2.70145589
CAD_mortality	adipocyte	*ICAM3*	TRUE	2.38686283
CAD_mortality	adipocyte	*LYRM2*	TRUE	1.98825774
CAD_mortality	adipocyte	*MANBAL*	TRUE	2.15814287
CAD_mortality	adipocyte	*TNFRSF10A*	TRUE	2.41101905
CAD_mortality	adipocyte	*IRF6*	TRUE	2.50156458

CAD_mortality	adipocyte	*EPCAM*	TRUE	2.09732392
CAD_mortality	adipocyte	*TOX2*	TRUE	1.96521034
CAD_mortality	adipocyte	*TOR1B*	TRUE	2.65914709
CAD_mortality	adipocyte	*KANSL2*	TRUE	2.48081336
CAD_mortality	adipocyte	*POMZP3*	TRUE	2.48272946
CAD_mortality	adipocyte	*MPP7*	TRUE	2.09367765
CAD_mortality	adipocyte	*RCAN1*	TRUE	2.83542178
CAD_mortality	adipocyte	*PLCD3*	TRUE	2.25448698
CAD_mortality	adipocyte	*PDZK1IP1*	TRUE	2.59125367
CAD_mortality	adipocyte	*TRIM66*	TRUE	2.45257163
CAD_mortality	adipocyte	*PCSK9*	TRUE	2.20768142
CAD_mortality	adipocyte	*STK32A*	TRUE	2.20764376
CAD_mortality	adipocyte	*TSNARE1*	TRUE	2.89198711
CAD_mortality	adipocyte	*COL24A1*	TRUE	2.2546817
CAD_mortality	adipocyte	*HAP1*	TRUE	2.15912654
CAD_mortality	adipocyte	*TMEM81*	TRUE	2.20140719
CAD_mortality	adipocyte	*BANF1*	TRUE	1.96005109
CAD_mortality	adipocyte	*TACSTD2*	TRUE	2.03221492
CAD_mortality	adipocyte	*GDF3*	TRUE	2.60805853
CAD_mortality	adipocyte	*TOP1MT*	TRUE	1.98368198
CAD_mortality	adipocyte	*PDE6G*	TRUE	2.15519134
CAD_mortality	adipocyte	*FCAR*	TRUE	3.47437567
CAD_mortality	adipocyte	*JAKMIP3*	TRUE	2.92290728
CAD_mortality	adipocyte	*XPNPEP3*	TRUE	2.22754278
CAD_mortality	adipocyte	*KIAA1211L*	TRUE	2.97394114
CAD_mortality	adipocyte	*ZNF100*	TRUE	2.09456147
CAD_mortality	adipocyte	*TBKBP1*	TRUE	2.00671856
CAD_mortality	adipocyte	*NELFE*	TRUE	2.08129317
CAD_mortality	adipocyte	*AC112229.4*	TRUE	2.35558507
CAD_mortality	adipocyte	*PCDHGA11*	TRUE	2.0991968
CAD_mortality	ASPC	*RHOBTB2*	TRUE	2.06181802
CAD_mortality	ASPC	*ATP2A3*	TRUE	1.97293076
CAD_mortality	ASPC	*ICAM3*	TRUE	2.36420512
CAD_mortality	ASPC	*NMRK2*	TRUE	1.97157952
CAD_mortality	ASPC	*LYRM2*	TRUE	2.13010642
CAD_mortality	ASPC	*MRPS33*	TRUE	2.64975844
CAD_mortality	ASPC	*TPD52L2*	TRUE	2.50810737
CAD_mortality	ASPC	*MANBAL*	TRUE	2.6438733
CAD_mortality	ASPC	*TNFRSF10A*	TRUE	2.44798754
CAD_mortality	ASPC	*ALDH3A1*	TRUE	1.96804877
CAD_mortality	ASPC	*CAND1*	TRUE	2.33355444
CAD_mortality	ASPC	*CHMP3*	TRUE	2.3890642
CAD_mortality	ASPC	*CD207*	TRUE	1.97049017
CAD_mortality	ASPC	*IRF6*	TRUE	2.61378524
CAD_mortality	ASPC	*EPCAM*	TRUE	2.47117283
CAD_mortality	ASPC	*RWDD3*	TRUE	2.09232987
CAD_mortality	ASPC	*EBPL*	TRUE	2.44079904
CAD_mortality	ASPC	*TOX2*	TRUE	2.11196893

CAD_mortality	ASPC	*CHCHD5*	TRUE	2.07691164
CAD_mortality	ASPC	*LRRC8A*	TRUE	2.19014469
CAD_mortality	ASPC	*TOR1B*	TRUE	2.75663058
CAD_mortality	ASPC	*PRPF4*	TRUE	2.10155733
CAD_mortality	ASPC	*ERP27*	TRUE	1.99200003
CAD_mortality	ASPC	*KANSL2*	TRUE	2.45123919
CAD_mortality	ASPC	*TRIM65*	TRUE	2.0336088
CAD_mortality	ASPC	*PYCR2*	TRUE	2.18696301
CAD_mortality	ASPC	*LYG1*	TRUE	1.98070851
CAD_mortality	ASPC	*ZNF660*	TRUE	1.99256649
CAD_mortality	ASPC	*POMZP3*	TRUE	2.62384003
CAD_mortality	ASPC	*GRIK4*	TRUE	2.24469793
CAD_mortality	ASPC	*MPP7*	TRUE	2.09537727
CAD_mortality	ASPC	*ATG10*	TRUE	2.12834298
CAD_mortality	ASPC	*RAD17*	TRUE	2.00488195
CAD_mortality	ASPC	*WDR66*	TRUE	2.21398629
CAD_mortality	ASPC	*RCAN1*	TRUE	2.86659765
CAD_mortality	ASPC	*TEPP*	TRUE	2.41533783
CAD_mortality	ASPC	*MUM1*	TRUE	2.96192264
CAD_mortality	ASPC	*PLCD3*	TRUE	2.86216756
CAD_mortality	ASPC	*PDZK1IP1*	TRUE	2.79887205
CAD_mortality	ASPC	*COA7*	TRUE	2.20347605
CAD_mortality	ASPC	*TSSK3*	TRUE	2.30405347
CAD_mortality	ASPC	*ARPC5*	TRUE	2.03107708
CAD_mortality	ASPC	*LRRC2*	TRUE	1.99976539
CAD_mortality	ASPC	*AKR1E2*	TRUE	2.05432235
CAD_mortality	ASPC	*TRUB1*	TRUE	2.09304711
CAD_mortality	ASPC	*TRIM66*	TRUE	2.35183095
CAD_mortality	ASPC	*VKORC1*	TRUE	2.00887414
CAD_mortality	ASPC	*LRP1B*	TRUE	2.03067093
CAD_mortality	ASPC	*LGALS9*	TRUE	2.13141231
CAD_mortality	ASPC	*PCSK9*	TRUE	2.18200078
CAD_mortality	ASPC	*STK32A*	TRUE	2.31070272
CAD_mortality	ASPC	*TSNARE1*	TRUE	2.92156149
CAD_mortality	ASPC	*HOPX*	TRUE	2.80672679
CAD_mortality	ASPC	*COL24A1*	TRUE	2.6426626
CAD_mortality	ASPC	*RAB43*	TRUE	2.86563135
CAD_mortality	ASPC	*RHOD*	TRUE	1.99272751
CAD_mortality	ASPC	*HAP1*	TRUE	2.25802397
CAD_mortality	ASPC	*LPL*	TRUE	2.36695186
CAD_mortality	ASPC	*ETV4*	TRUE	2.32499626
CAD_mortality	ASPC	*TMEM39A*	TRUE	3.51055536
CAD_mortality	ASPC	*CTC1*	TRUE	2.26366277
CAD_mortality	ASPC	*SF3A3*	TRUE	2.4331797
CAD_mortality	ASPC	*B3GALT5*	TRUE	2.21290885
CAD_mortality	ASPC	*TACSTD2*	TRUE	2.06680745
CAD_mortality	ASPC	*GDF3*	TRUE	2.62033415
CAD_mortality	ASPC	*TMED9*	TRUE	2.10421253

CAD_mortality	ASPC	*PDE6G*	TRUE	2.10940809
CAD_mortality	ASPC	*FCAR*	TRUE	3.61277606
CAD_mortality	ASPC	*HYKK*	TRUE	2.02922502
CAD_mortality	ASPC	*JAKMIP3*	TRUE	3.63983718
CAD_mortality	ASPC	*SEMA4A*	TRUE	1.96683636
CAD_mortality	ASPC	*XPNPEP3*	TRUE	2.57501492
CAD_mortality	ASPC	*MYO18A*	TRUE	2.05093368
CAD_mortality	ASPC	*KIAA1211L*	TRUE	2.63569726
CAD_mortality	ASPC	*ZNF429*	TRUE	2.14610789
CAD_mortality	ASPC	*ADH4*	TRUE	2.07746507
CAD_mortality	ASPC	*RPS6KL1*	TRUE	2.14190211
CAD_mortality	ASPC	*ITPRIPL1*	TRUE	2.47594656
CAD_mortality	ASPC	*TBKBP1*	TRUE	2.29986047
CAD_mortality	ASPC	*ZNF525*	TRUE	2.05189707
CAD_mortality	ASPC	*ASAH2B*	TRUE	2.11440185
CAD_mortality	ASPC	*NELFE*	TRUE	2.09380032
CAD_mortality	ASPC	*AC112229.4*	TRUE	2.34635985
CAD_mortality	ASPC	*LINC00484*	TRUE	2.20569626
CAD_mortality	b_cell	*IRF6*	TRUE	2.05574148
CAD_mortality	b_cell	*RAB43*	TRUE	3.13063779
CAD_mortality	b_cell	*HAP1*	TRUE	2.36661602
CAD_mortality	b_cell	*GDF3*	TRUE	2.12819624
CAD_mortality	dendritic_cell	*ICAM3*	TRUE	2.00934735
CAD_mortality	dendritic_cell	*IRF6*	TRUE	2.01101247
CAD_mortality	dendritic_cell	*TRIM66*	TRUE	2.11715466
CAD_mortality	dendritic_cell	*TSNARE1*	TRUE	2.22492902
CAD_mortality	dendritic_cell	*FCAR*	TRUE	2.90318768
CAD_mortality	endothelial	*EPCAM*	TRUE	2.11010774
CAD_mortality	endothelial	*PLCD3*	TRUE	2.03154125
CAD_mortality	endothelial	*PDZK1IP1*	TRUE	2.1623807
CAD_mortality	endothelial	*TRIM66*	TRUE	2.20216535
CAD_mortality	endothelial	*COL24A1*	TRUE	1.96873613
CAD_mortality	endothelial	*HAP1*	TRUE	2.02172463
CAD_mortality	LEC	*OPN3*	TRUE	1.99645181
CAD_mortality	LEC	*RAB43*	TRUE	2.83476278
CAD_mortality	LEC	*KIAA1211L*	TRUE	2.46615361
CAD_mortality	LEC	*AC112229.4*	TRUE	2.27115807
CAD_mortality	LEC	*HLA.DQA2*	TRUE	2.0327369
CAD_mortality	macrophage	*F7*	TRUE	2.02992231
CAD_mortality	macrophage	*ICAM3*	TRUE	2.1047245
CAD_mortality	macrophage	*MANBAL*	TRUE	2.26553938
CAD_mortality	macrophage	*TNFRSF10A*	TRUE	2.22305739
CAD_mortality	macrophage	*ALDH3A1*	TRUE	1.98563688
CAD_mortality	macrophage	*IRF6*	TRUE	2.43236659
CAD_mortality	macrophage	*EPCAM*	TRUE	2.16746153
CAD_mortality	macrophage	*TOX2*	TRUE	1.96399852
CAD_mortality	macrophage	*TOR1B*	TRUE	2.37891929
CAD_mortality	macrophage	*KANSL2*	TRUE	2.1490865

CAD_mortality	macrophage	*ZNF660*	TRUE	1.98222945
CAD_mortality	macrophage	*POMZP3*	TRUE	2.27973218
CAD_mortality	macrophage	*ANKLE1*	TRUE	1.98193553
CAD_mortality	macrophage	*PDZK1IP1*	TRUE	2.23494239
CAD_mortality	macrophage	*TRIM66*	TRUE	2.34688936
CAD_mortality	macrophage	*PCSK9*	TRUE	2.04049594
CAD_mortality	macrophage	*STK32A*	TRUE	2.01727665
CAD_mortality	macrophage	*TSNARE1*	TRUE	2.54943386
CAD_mortality	macrophage	*COL24A1*	TRUE	2.31876469
CAD_mortality	macrophage	*HAP1*	TRUE	2.22975207
CAD_mortality	macrophage	*GOLGA8Q*	TRUE	2.08025615
CAD_mortality	macrophage	*B3GALT5*	TRUE	2.07347327
CAD_mortality	macrophage	*TACSTD2*	TRUE	1.98983978
CAD_mortality	macrophage	*GDF3*	TRUE	2.61144305
CAD_mortality	macrophage	*FCAR*	TRUE	3.32929212
CAD_mortality	macrophage	*JAKMIP3*	TRUE	3.42581578
CAD_mortality	macrophage	*XPNPEP3*	TRUE	2.11113921
CAD_mortality	macrophage	*KIAA1211L*	TRUE	2.83984492
CAD_mortality	macrophage	*RPS6KL1*	TRUE	2.05112964
CAD_mortality	macrophage	*TBKBP1*	TRUE	2.2423836
CAD_mortality	macrophage	*AC112229.4*	TRUE	2.3562552
CAD_mortality	macrophage	*LINC00484*	TRUE	2.14021043
CAD_mortality	macrophage	*PCDHGA11*	TRUE	1.99926997
CAD_mortality	macrophage	*LINC00668*	TRUE	1.98096096
CAD_mortality	mast_cell	*F7*	TRUE	2.00280275
CAD_mortality	mast_cell	*TNFRSF10A*	TRUE	2.18515677
CAD_mortality	mast_cell	*TOR1B*	TRUE	2.19964186
CAD_mortality	mast_cell	*POMZP3*	TRUE	1.9898792
CAD_mortality	mast_cell	*RCAN1*	TRUE	2.17419711
CAD_mortality	mast_cell	*HAP1*	TRUE	2.30016012
CAD_mortality	mast_cell	*KIAA1211L*	TRUE	2.42915273
CAD_mortality	mast_cell	*HLA.DQA2*	TRUE	2.18632774
CAD_mortality	monocyte	*TNFRSF10A*	TRUE	2.19120755
CAD_mortality	monocyte	*RAB43*	TRUE	2.92017959
CAD_mortality	monocyte	*GDF3*	TRUE	2.04864477
CAD_mortality	monocyte	*HLA.DQB2*	TRUE	2.07679835
CAD_mortality	neutrophil	*JAKMIP3*	TRUE	3.05102547
CAD_mortality	nk_cell	*TSNARE1*	TRUE	2.1074391
CAD_mortality	nk_cell	*HAP1*	TRUE	2.1325662
CAD_mortality	nk_cell	*FCAR*	TRUE	2.45141521
CAD_mortality	nk_cell	*HLA.DQA2*	TRUE	2.13593032
CAD_mortality	pericyte	*IRF6*	TRUE	2.11371466
CAD_mortality	pericyte	*RCAN1*	TRUE	2.72010697
CAD_mortality	pericyte	*TRIM66*	TRUE	2.08829377
CAD_mortality	pericyte	*TSNARE1*	TRUE	2.33544115
CAD_mortality	pericyte	*RAB43*	TRUE	2.94302636
CAD_mortality	pericyte	*HAP1*	TRUE	2.2616866
CAD_mortality	pericyte	*TMEM80*	TRUE	2.05195802

CAD_mortality	pericyte	*GDF3*	TRUE	2.11894492
CAD_mortality	pericyte	*FCAR*	TRUE	3.14988467
CAD_mortality	pericyte	*JAKMIP3*	TRUE	2.08004644
CAD_mortality	pericyte	*KIAA1211L*	TRUE	2.4851677
CAD_mortality	pericyte	*CHML*	TRUE	2.12625192
CAD_mortality	pericyte	*AC112229.4*	TRUE	2.13136821
CAD_mortality	pericyte	*LINC00484*	TRUE	2.25289355
CAD_mortality	pericyte	*HLA.DQA2*	TRUE	2.1133294
CAD_mortality	SMC	*IRF6*	TRUE	2.48492436
CAD_mortality	SMC	*TOR1B*	TRUE	2.03480841
CAD_mortality	SMC	*KANSL2*	TRUE	2.03907808
CAD_mortality	SMC	*POMZP3*	TRUE	2.13191864
CAD_mortality	SMC	*PDZK1IP1*	TRUE	1.97693434
CAD_mortality	SMC	*TRIM66*	TRUE	2.16496946
CAD_mortality	SMC	*TSNARE1*	TRUE	2.51231531
CAD_mortality	SMC	*COL24A1*	TRUE	2.04632399
CAD_mortality	SMC	*RAB43*	TRUE	2.74601319
CAD_mortality	SMC	*B3GALT5*	TRUE	2.12744742
CAD_mortality	SMC	*GDF3*	TRUE	2.00301893
CAD_mortality	SMC	*JAKMIP3*	TRUE	3.23377229
CAD_mortality	SMC	*NELFE*	TRUE	2.02911988
CAD_mortality	SMC	*LINC00484*	TRUE	2.10667612
CAD_mortality	SMC	*HLA.DQA2*	TRUE	2.14389011
CAD_mortality	t_cell	*ALDH3A1*	TRUE	2.0372145
CAD_mortality	t_cell	*TOR1B*	TRUE	1.98272868
CAD_mortality	t_cell	*TRIM66*	TRUE	2.0636015
CAD_mortality	t_cell	*TSNARE1*	TRUE	2.2341198
CAD_mortality	t_cell	*RAB43*	TRUE	2.66885942
CAD_mortality	t_cell	*HAP1*	TRUE	1.96541843
CAD_mortality	t_cell	*BANF1*	TRUE	2.07805983
CAD_mortality	t_cell	*GDF3*	TRUE	2.65008727
CAD_mortality	t_cell	*FCAR*	TRUE	3.15387315
CAD_mortality	t_cell	*KIAA1211L*	TRUE	2.45217158
CAD_mortality	t_cell	*CHML*	TRUE	2.00939497

d. Identified cell type specific causal genes for obesity and related disorders from VAT

disorder	celltype	gene	assoc	zMin
CAD	adipocyte	*UHRF1BP1*	TRUE	3.393733818
CAD	adipocyte	*ZMYND12*	TRUE	1.996840264
CAD	adipocyte	*TIPIN*	TRUE	3.070426959
CAD	adipocyte	*CACNG4*	TRUE	2.055818263
CAD	adipocyte	*FMO4*	TRUE	3.579666418
CAD	adipocyte	*ITPR3*	TRUE	2.682334956
CAD	adipocyte	*MTAP*	TRUE	2.20116992
CAD	adipocyte	*SUSD2*	TRUE	2.658618336
CAD	adipocyte	*LIPA*	TRUE	2.338472599
CAD	adipocyte	*RAB5C*	TRUE	1.994469361
CAD	adipocyte	*BVES*	TRUE	2.159419095
CAD	adipocyte	*BNIP1*	TRUE	2.995144556
CAD	adipocyte	*PCCB*	TRUE	2.277385404
CAD	adipocyte	*GGCX*	TRUE	2.742268414
CAD	adipocyte	*CFHR3*	TRUE	2.217021664
CAD	adipocyte	*EIF2B2*	TRUE	1.999905089
CAD	adipocyte	*PTGIS*	TRUE	2.378844758
CAD	adipocyte	*TCP11*	TRUE	2.859153123
CAD	adipocyte	*GRK4*	TRUE	3.536755833
CAD	adipocyte	*FAM98C*	TRUE	1.962006333
CAD	adipocyte	*DDC*	TRUE	2.491223789
CAD	adipocyte	*B4GALNT1*	TRUE	2.562923995
CAD	adipocyte	*POLR1E*	TRUE	2.567974698
CAD	adipocyte	*TMEM91*	TRUE	3.157018071
CAD	adipocyte	*EVA1B*	TRUE	2.3390181
CAD	adipocyte	*WDPCP*	TRUE	2.416576767
CAD	adipocyte	*FBXO10*	TRUE	3.265674117
CAD	adipocyte	*HSD17B12*	TRUE	2.981459042
CAD	adipocyte	*TTC12*	TRUE	2.22049331
CAD	adipocyte	*MMP16*	TRUE	2.083691884
CAD	adipocyte	*CLEC18A*	TRUE	2.144680577
CAD	adipocyte	*S100A1*	TRUE	2.015175436
CAD	adipocyte	*CPSF4*	TRUE	2.124093999
CAD	adipocyte	*ALDH16A1*	TRUE	1.995363272
CAD	adipocyte	*DAPL1*	TRUE	2.061587535
CAD	adipocyte	*RNF123*	TRUE	2.533791488
CAD	adipocyte	*PIGO*	TRUE	2.040986574
CAD	adipocyte	*OXGR1*	TRUE	1.992245247
CAD	adipocyte	*C16orf46*	TRUE	2.8653684
CAD	adipocyte	*ENGASE*	TRUE	2.200127193
CAD	adipocyte	*LGALS9*	TRUE	2.210893193
CAD	adipocyte	*NPIPB3*	TRUE	2.141111863
CAD	adipocyte	*MYO7B*	TRUE	2.15234102
CAD	adipocyte	*TTLL6*	TRUE	2.099445201

CAD	adipocyte	*RGS19*	TRUE	2.213111459
CAD	adipocyte	*NXNL1*	TRUE	2.227692043
CAD	adipocyte	*C3AR1*	TRUE	2.264236812
CAD	adipocyte	*NME6*	TRUE	2.071016274
CAD	adipocyte	*ZNF738*	TRUE	2.051063992
CAD	adipocyte	*CCNE2*	TRUE	2.419117342
CAD	adipocyte	*PAAF1*	TRUE	2.231659426
CAD	adipocyte	*SLC25A22*	TRUE	2.380243806
CAD	adipocyte	*LYNX1*	TRUE	2.501051025
CAD	adipocyte	*ADIPOQ*	TRUE	2.536176622
CAD	adipocyte	*TMEM136*	TRUE	2.021535376
CAD	adipocyte	*FES*	TRUE	2.61765554
CAD	adipocyte	*VSIG10L*	TRUE	2.298535218
CAD	adipocyte	*COLCA1*	TRUE	2.087290561
CAD	adipocyte	*RPS26*	TRUE	2.003267326
CAD	adipocyte	*MRPL42*	TRUE	2.092995621
CAD	adipocyte	*TMEM116*	TRUE	3.505752212
CAD	adipocyte	*TBKBP1*	TRUE	2.298571707
CAD	adipocyte	*AP001468.1*	TRUE	2.124479614
CAD	adipocyte	*LINC00707*	TRUE	1.990800831
CAD	adipocyte	*LY6G5B*	TRUE	1.973817124
CAD	adipocyte	*GOLGA8K*	TRUE	2.048830393
CAD	ASPC	*UHRF1BP1*	TRUE	2.410792779
CAD	ASPC	*TIE1*	TRUE	2.140619418
CAD	ASPC	*ZMYND12*	TRUE	2.011425463
CAD	ASPC	*TIPIN*	TRUE	3.304212238
CAD	ASPC	*ADD2*	TRUE	2.140456604
CAD	ASPC	*CACNG4*	TRUE	2.057018036
CAD	ASPC	*FMO4*	TRUE	3.507972285
CAD	ASPC	*RPH3A*	TRUE	2.392641836
CAD	ASPC	*PABPC4*	TRUE	1.997777681
CAD	ASPC	*ITPR3*	TRUE	2.860495615
CAD	ASPC	*SCD*	TRUE	2.361646608
CAD	ASPC	*MTAP*	TRUE	2.423961832
CAD	ASPC	*SUSD2*	TRUE	2.710782133
CAD	ASPC	*THOC5*	TRUE	2.034597367
CAD	ASPC	*RABAC1*	TRUE	1.99545928
CAD	ASPC	*LIPA*	TRUE	2.667275143
CAD	ASPC	*RAB5C*	TRUE	2.583051865
CAD	ASPC	*BVES*	TRUE	2.337598439
CAD	ASPC	*BNIP1*	TRUE	3.054808929
CAD	ASPC	*PCCB*	TRUE	2.76894673
CAD	ASPC	*C3orf14*	TRUE	2.223439407
CAD	ASPC	*STEAP3*	TRUE	2.024068334
CAD	ASPC	*GGCX*	TRUE	3.015213233
CAD	ASPC	*CFHR3*	TRUE	2.725334726

CAD	ASPC	*C2orf40*	TRUE	2.006476151
CAD	ASPC	*POPDC2*	TRUE	2.312062161
CAD	ASPC	*PTGIS*	TRUE	2.8054053
CAD	ASPC	*TCP11*	TRUE	2.741814583
CAD	ASPC	*GRK4*	TRUE	3.702971649
CAD	ASPC	*TRAP1*	TRUE	2.067358767
CAD	ASPC	*ABHD8*	TRUE	2.091624942
CAD	ASPC	*OPN1SW*	TRUE	2.060516429
CAD	ASPC	*SERGEF*	TRUE	2.193348255
CAD	ASPC	*FAM98C*	TRUE	2.065514957
CAD	ASPC	*PLVAP*	TRUE	2.130578178
CAD	ASPC	*CCT6B*	TRUE	2.157350845
CAD	ASPC	*TIMM10B*	TRUE	2.484736857
CAD	ASPC	*DDC*	TRUE	2.526290221
CAD	ASPC	*B4GALNT1*	TRUE	2.739199777
CAD	ASPC	*POLR1E*	TRUE	2.642651422
CAD	ASPC	*TCF19*	TRUE	2.691819765
CAD	ASPC	*DIAPH3*	TRUE	2.165372943
CAD	ASPC	*TMEM91*	TRUE	3.258414387
CAD	ASPC	*SIRT3*	TRUE	1.990221875
CAD	ASPC	*EVA1B*	TRUE	2.521251891
CAD	ASPC	*ARNT*	TRUE	2.490805465
CAD	ASPC	*TBC1D7*	TRUE	2.184010355
CAD	ASPC	*IRAK1BP1*	TRUE	2.23702871
CAD	ASPC	*CSGALNACT1*	TRUE	2.207945461
CAD	ASPC	*FBXO10*	TRUE	3.328130187
CAD	ASPC	*LRRC27*	TRUE	2.049315044
CAD	ASPC	*HSD17B12*	TRUE	3.121418942
CAD	ASPC	*TTC12*	TRUE	2.348583155
CAD	ASPC	*ROBO3*	TRUE	2.523151368
CAD	ASPC	*MMP16*	TRUE	2.472879986
CAD	ASPC	*CLEC18A*	TRUE	2.394790801
CAD	ASPC	*S100A1*	TRUE	2.328768608
CAD	ASPC	*CPSF4*	TRUE	2.697098824
CAD	ASPC	*ABCF3*	TRUE	2.101593164
CAD	ASPC	*ALDH16A1*	TRUE	2.043037328
CAD	ASPC	*DAPL1*	TRUE	2.581503886
CAD	ASPC	*RNF123*	TRUE	2.333062222
CAD	ASPC	*DAGLB*	TRUE	2.009004335
CAD	ASPC	*PIGO*	TRUE	2.366805363
CAD	ASPC	*OXGR1*	TRUE	2.105484964
CAD	ASPC	*C16orf46*	TRUE	2.880321801
CAD	ASPC	*CCDC68*	TRUE	2.111514638
CAD	ASPC	*ENGASE*	TRUE	2.451353743
CAD	ASPC	*ATOH8*	TRUE	2.359642143
CAD	ASPC	*LGALS9*	TRUE	2.524091298

CAD	ASPC	*IRS1*	TRUE	2.043354338
CAD	ASPC	*NPIPB3*	TRUE	2.244854193
CAD	ASPC	*MYO7B*	TRUE	2.227566
CAD	ASPC	*DNAJC18*	TRUE	2.36717926
CAD	ASPC	*TTLL6*	TRUE	1.99641588
CAD	ASPC	*RGS19*	TRUE	2.506306464
CAD	ASPC	*NXNL1*	TRUE	2.535974271
CAD	ASPC	*C3AR1*	TRUE	2.744694311
CAD	ASPC	*NME6*	TRUE	2.129988226
CAD	ASPC	*ZNF738*	TRUE	2.149164898
CAD	ASPC	*MAP3K11*	TRUE	1.978040483
CAD	ASPC	*CCNE2*	TRUE	2.545255959
CAD	ASPC	*PAAF1*	TRUE	2.288921408
CAD	ASPC	*SPRYD4*	TRUE	2.112902306
CAD	ASPC	*SLC25A22*	TRUE	2.462152342
CAD	ASPC	*LYNX1*	TRUE	2.576182568
CAD	ASPC	*DEFB119*	TRUE	2.900666365
CAD	ASPC	*MRPL14*	TRUE	2.179490916
CAD	ASPC	*ADIPOQ*	TRUE	2.494678497
CAD	ASPC	*TMEM136*	TRUE	2.886163578
CAD	ASPC	*CLN8*	TRUE	1.966162526
CAD	ASPC	*FES*	TRUE	3.144389387
CAD	ASPC	*A3GALT2*	TRUE	2.057044082
CAD	ASPC	*SEPT5*	TRUE	2.036087275
CAD	ASPC	*NBPF12*	TRUE	2.113185461
CAD	ASPC	*VSIG10L*	TRUE	2.426965315
CAD	ASPC	*COLCA1*	TRUE	2.099422729
CAD	ASPC	*SEMA4A*	TRUE	1.968724698
CAD	ASPC	*ZNF605*	TRUE	2.173625021
CAD	ASPC	*WDR5B*	TRUE	1.978151695
CAD	ASPC	*RPS26*	TRUE	2.106354066
CAD	ASPC	*MRPL42*	TRUE	2.281399655
CAD	ASPC	*TMEM116*	TRUE	3.459136594
CAD	ASPC	*TBKBP1*	TRUE	2.395636399
CAD	ASPC	*STK19*	TRUE	1.98809866
CAD	ASPC	*BAG6*	TRUE	1.985300054
CAD	ASPC	*LILRB3*	TRUE	2.722265493
CAD	ASPC	*LINC00484*	TRUE	2.019441813
CAD	ASPC	*AP001468.1*	TRUE	2.205786069
CAD	ASPC	*LINC00707*	TRUE	2.455809833
CAD	ASPC	*SRP14.AS1*	TRUE	2.417361575
CAD	ASPC	*GOLGA8K*	TRUE	2.580828882
CAD	ASPC	*HMBS*	TRUE	2.293670369
CAD	ASPC	*GRIN2B*	TRUE	2.610355257
CAD	b_cell	*TCP11*	TRUE	3.25351524
CAD	b_cell	*CLEC18A*	TRUE	2.344965584

CAD	b_cell	*NLRP6*	TRUE	2.212029786
CAD	b_cell	*HYAL3*	TRUE	1.99281422
CAD	b_cell	*LY6G5B*	TRUE	2.14962493
CAD	b_cell	*LINC00960*	TRUE	2.495317062
CAD	dendritic_cell	*SUSD2*	TRUE	2.30585158
CAD	dendritic_cell	*CENPM*	TRUE	2.053607737
CAD	dendritic_cell	*TCP11*	TRUE	2.755376598
CAD	dendritic_cell	*GRK4*	TRUE	3.045944862
CAD	dendritic_cell	*TMEM91*	TRUE	3.021310057
CAD	dendritic_cell	*FBXO10*	TRUE	3.266754364
CAD	dendritic_cell	*CLEC18A*	TRUE	2.311915623
CAD	dendritic_cell	*ENGASE*	TRUE	2.055253466
CAD	dendritic_cell	*LGALS9*	TRUE	2.117923127
CAD	dendritic_cell	*NXNL1*	TRUE	2.600241435
CAD	dendritic_cell	*CCNE2*	TRUE	2.446750699
CAD	dendritic_cell	*TMEM116*	TRUE	2.133996328
CAD	dendritic_cell	*TBKBP1*	TRUE	2.034757962
CAD	dendritic_cell	*LINC00960*	TRUE	2.691754616
CAD	dendritic_cell	*GOLGA8K*	TRUE	1.987483877
CAD	dendritic_cell	*TAS2R20*	TRUE	2.646892121
CAD	endometrium	*TIPIN*	TRUE	2.323194231
CAD	endometrium	*SUSD2*	TRUE	2.303242901
CAD	endometrium	*TCP11*	TRUE	3.245432244
CAD	endometrium	*TCF19*	TRUE	1.990697005
CAD	endometrium	*TMEM91*	TRUE	2.127358395
CAD	endometrium	*TMEM116*	TRUE	2.65330628
CAD	endometrium	*LINC00960*	TRUE	1.991233887
CAD	endothelial	*SUSD2*	TRUE	2.124490566
CAD	endothelial	*CENPM*	TRUE	2.04587885
CAD	endothelial	*WFDC3*	TRUE	2.055221206
CAD	endothelial	*TCP11*	TRUE	2.639379715
CAD	endothelial	*GRK4*	TRUE	2.974882003
CAD	endothelial	*B4GALNT1*	TRUE	1.998678296
CAD	endothelial	*TCF19*	TRUE	1.961969151
CAD	endothelial	*SLC37A4*	TRUE	2.179367156
CAD	endothelial	*TMEM91*	TRUE	2.420130087
CAD	endothelial	*AMT*	TRUE	2.150444458
CAD	endothelial	*FBXO10*	TRUE	2.69000207
CAD	endothelial	*HSD17B12*	TRUE	2.138457968
CAD	endothelial	*CLEC18A*	TRUE	2.082602339
CAD	endothelial	*RGS19*	TRUE	1.966047494
CAD	endothelial	*NXNL1*	TRUE	2.54363595
CAD	endothelial	*KCNK7*	TRUE	2.432331824
CAD	endothelial	*LYNX1*	TRUE	2.05394471
CAD	endothelial	*TMEM116*	TRUE	2.361174248
CAD	endothelial	*LY6G5B*	TRUE	2.054102624

CAD	endothelial	*LINC00960*	TRUE	2.599558125
CAD	endothelial	*TAS2R20*	TRUE	2.637160725
CAD	LEC	*TIPIN*	TRUE	2.730015303
CAD	LEC	*ITPR3*	TRUE	3.24790752
CAD	LEC	*SUSD2*	TRUE	2.488634172
CAD	LEC	*BNIP1*	TRUE	2.155806515
CAD	LEC	*C3orf14*	TRUE	2.158881348
CAD	LEC	*EIF2B2*	TRUE	2.477770758
CAD	LEC	*TCP11*	TRUE	2.663643531
CAD	LEC	*GRK4*	TRUE	3.297236057
CAD	LEC	*POLR1E*	TRUE	1.989397532
CAD	LEC	*TCF19*	TRUE	2.762799171
CAD	LEC	*SLC37A4*	TRUE	2.643763016
CAD	LEC	*TMEM91*	TRUE	2.70763168
CAD	LEC	*HSD17B12*	TRUE	2.65988103
CAD	LEC	*RNF123*	TRUE	2.20300982
CAD	LEC	*RGS19*	TRUE	2.204871271
CAD	LEC	*NXNL1*	TRUE	2.526556519
CAD	LEC	*ZNF738*	TRUE	2.017432809
CAD	LEC	*NLRP6*	TRUE	2.429436074
CAD	LEC	*RPS26*	TRUE	2.005193842
CAD	LEC	*TMEM116*	TRUE	2.775350324
CAD	LEC	*TBKBP1*	TRUE	2.155998817
CAD	LEC	*LY6G5B*	TRUE	2.100550252
CAD	LEC	*GOLGA8K*	TRUE	2.230285986
CAD	macrophage	*UHRF1BP1*	TRUE	3.023247317
CAD	macrophage	*ZMYND12*	TRUE	2.041704316
CAD	macrophage	*TIPIN*	TRUE	2.728041884
CAD	macrophage	*FMO4*	TRUE	2.926906502
CAD	macrophage	*ITPR3*	TRUE	2.298787511
CAD	macrophage	*SUSD2*	TRUE	2.670269092
CAD	macrophage	*CENPM*	TRUE	2.009909618
CAD	macrophage	*BVES*	TRUE	2.290861759
CAD	macrophage	*BNIP1*	TRUE	2.815033442
CAD	macrophage	*PCCB*	TRUE	2.195062788
CAD	macrophage	*GGCX*	TRUE	2.052896966
CAD	macrophage	*CFHR3*	TRUE	2.248454586
CAD	macrophage	*EIF2B2*	TRUE	2.192688561
CAD	macrophage	*POPDC2*	TRUE	2.12821945
CAD	macrophage	*TCP11*	TRUE	2.665605177
CAD	macrophage	*GRK4*	TRUE	3.442537195
CAD	macrophage	*DDC*	TRUE	2.491653301
CAD	macrophage	*POLR1E*	TRUE	2.483546397
CAD	macrophage	*TCF19*	TRUE	2.318537017
CAD	macrophage	*TMEM91*	TRUE	3.162314946
CAD	macrophage	*FBXO10*	TRUE	2.691010456

CAD	macrophage	*HSD17B12*	TRUE	2.920455926
CAD	macrophage	*CLEC18A*	TRUE	2.36001278
CAD	macrophage	*RNF123*	TRUE	2.475510207
CAD	macrophage	*C16orf46*	TRUE	2.700225524
CAD	macrophage	*ATOH8*	TRUE	2.090515913
CAD	macrophage	*NXNL1*	TRUE	2.558705796
CAD	macrophage	*KCNK7*	TRUE	2.080696539
CAD	macrophage	*PAAF1*	TRUE	1.993847308
CAD	macrophage	*SPRYD4*	TRUE	1.968771963
CAD	macrophage	*TMEM116*	TRUE	2.95414163
CAD	macrophage	*TBKBP1*	TRUE	2.228664881
CAD	macrophage	*GOLGA8K*	TRUE	2.330492968
CAD	macrophage	*HMBS*	TRUE	3.120412193
CAD	mast_cell	*ITPR3*	TRUE	2.153893386
CAD	mast_cell	*SUSD2*	TRUE	2.536932538
CAD	mast_cell	*BVES*	TRUE	2.169784022
CAD	mast_cell	*BNIP1*	TRUE	2.4983257
CAD	mast_cell	*GRK4*	TRUE	2.07475856
CAD	mast_cell	*EVA1B*	TRUE	2.310089199
CAD	mast_cell	*AMT*	TRUE	2.676966336
CAD	mast_cell	*SLC39A4*	TRUE	2.082652092
CAD	mast_cell	*CLEC18A*	TRUE	2.281561045
CAD	mast_cell	*RGS19*	TRUE	2.095937497
CAD	mast_cell	*NXNL1*	TRUE	2.561265907
CAD	mast_cell	*SEPT5*	TRUE	1.986664009
CAD	mast_cell	*AP001468.1*	TRUE	2.024996493
CAD	mast_cell	*LINC00960*	TRUE	2.1006242
CAD	mast_cell	*TAS2R20*	TRUE	2.148723453
CAD	mesothelium	*PAX6*	TRUE	2.235399868
CAD	mesothelium	*CYB561*	TRUE	2.060803492
CAD	mesothelium	*TUBG2*	TRUE	1.965385074
CAD	mesothelium	*LAMC2*	TRUE	1.99101651
CAD	mesothelium	*TIE1*	TRUE	3.001128639
CAD	mesothelium	*ZMYND12*	TRUE	2.081282256
CAD	mesothelium	*TIPIN*	TRUE	2.993314081
CAD	mesothelium	*FMO4*	TRUE	2.844061939
CAD	mesothelium	*TXNDC16*	TRUE	2.365445571
CAD	mesothelium	*PABPC4*	TRUE	2.606394978
CAD	mesothelium	*ITPR3*	TRUE	2.861735714
CAD	mesothelium	*SCD*	TRUE	2.902522971
CAD	mesothelium	*SUSD2*	TRUE	2.730404356
CAD	mesothelium	*THOC5*	TRUE	2.40747584
CAD	mesothelium	*ZNF500*	TRUE	2.056367053
CAD	mesothelium	*CTSH*	TRUE	2.053290568
CAD	mesothelium	*NFKBIB*	TRUE	2.308108567
CAD	mesothelium	*LIPA*	TRUE	2.033111408

CAD	mesothelium	*ST3GAL4*	TRUE	2.234961622
CAD	mesothelium	*KCTD10*	TRUE	2.254820784
CAD	mesothelium	*BVES*	TRUE	2.46016649
CAD	mesothelium	*BNIP1*	TRUE	2.825575902
CAD	mesothelium	*PCCB*	TRUE	2.208113397
CAD	mesothelium	*C3orf14*	TRUE	2.050335429
CAD	mesothelium	*GGCX*	TRUE	2.276033951
CAD	mesothelium	*PASK*	TRUE	2.618028183
CAD	mesothelium	*CFHR3*	TRUE	2.818654711
CAD	mesothelium	*HSDL2*	TRUE	2.100786175
CAD	mesothelium	*EIF2B2*	TRUE	2.270538932
CAD	mesothelium	*POPDC2*	TRUE	2.479157392
CAD	mesothelium	*PTGIS*	TRUE	2.755746562
CAD	mesothelium	*SNRPC*	TRUE	3.370312878
CAD	mesothelium	*TCP11*	TRUE	2.434374015
CAD	mesothelium	*GRK4*	TRUE	3.473175295
CAD	mesothelium	*ABHD8*	TRUE	2.54251339
CAD	mesothelium	*OPN1SW*	TRUE	2.197459305
CAD	mesothelium	*CCT6B*	TRUE	2.422250825
CAD	mesothelium	*B4GALNT1*	TRUE	2.135251525
CAD	mesothelium	*RCBTB1*	TRUE	1.996430622
CAD	mesothelium	*POLR1E*	TRUE	2.447144751
CAD	mesothelium	*TCF19*	TRUE	2.235374159
CAD	mesothelium	*POU2F3*	TRUE	1.97746396
CAD	mesothelium	*ASCC1*	TRUE	2.528912262
CAD	mesothelium	*GFOD2*	TRUE	2.179133656
CAD	mesothelium	*TMEM91*	TRUE	3.102626517
CAD	mesothelium	*EVA1B*	TRUE	2.532910297
CAD	mesothelium	*NME7*	TRUE	2.78774796
CAD	mesothelium	*ARNT*	TRUE	3.471845434
CAD	mesothelium	*NBEAL1*	TRUE	2.853948892
CAD	mesothelium	*AMT*	TRUE	2.337855021
CAD	mesothelium	*FBXO10*	TRUE	3.177792784
CAD	mesothelium	*HSD17B12*	TRUE	3.347100753
CAD	mesothelium	*MIA3*	TRUE	2.467846696
CAD	mesothelium	*MMP16*	TRUE	2.710735031
CAD	mesothelium	*WDYHV1*	TRUE	2.061916062
CAD	mesothelium	*MX1*	TRUE	2.104836323
CAD	mesothelium	*S100A1*	TRUE	2.40233796
CAD	mesothelium	*DAPL1*	TRUE	2.195846541
CAD	mesothelium	*DAGLB*	TRUE	2.228773717
CAD	mesothelium	*OXGR1*	TRUE	2.232732384
CAD	mesothelium	*C16orf46*	TRUE	2.858191864
CAD	mesothelium	*CCDC68*	TRUE	2.551061083
CAD	mesothelium	*MS4A7*	TRUE	1.974938267
CAD	mesothelium	*ENGASE*	TRUE	1.993604224

CAD	mesothelium	*ULK4*	TRUE	2.354150678
CAD	mesothelium	*ATOH8*	TRUE	2.121147968
CAD	mesothelium	*LGALS9*	TRUE	2.717313012
CAD	mesothelium	*IRS1*	TRUE	2.237081032
CAD	mesothelium	*NPIPB3*	TRUE	2.418179875
CAD	mesothelium	*DNAJC18*	TRUE	2.768179674
CAD	mesothelium	*TTLL6*	TRUE	2.250260672
CAD	mesothelium	*RGS19*	TRUE	2.676880555
CAD	mesothelium	*NXNL1*	TRUE	2.465367893
CAD	mesothelium	*C3AR1*	TRUE	3.076809468
CAD	mesothelium	*C11orf80*	TRUE	2.981187712
CAD	mesothelium	*PAAF1*	TRUE	2.351510987
CAD	mesothelium	*MYPOP*	TRUE	2.469939641
CAD	mesothelium	*SPRYD4*	TRUE	2.331341817
CAD	mesothelium	*SLC25A22*	TRUE	2.560266708
CAD	mesothelium	*MPI*	TRUE	2.018149106
CAD	mesothelium	*LINC00174*	TRUE	2.166365476
CAD	mesothelium	*LYNX1*	TRUE	2.857126963
CAD	mesothelium	*DEFB119*	TRUE	2.254126763
CAD	mesothelium	*ADIPOQ*	TRUE	2.706969115
CAD	mesothelium	*TMEM136*	TRUE	2.390590056
CAD	mesothelium	*FES*	TRUE	3.638613238
CAD	mesothelium	*A3GALT2*	TRUE	2.11332493
CAD	mesothelium	*SEPT5*	TRUE	2.599726982
CAD	mesothelium	*PSMD13*	TRUE	2.254614426
CAD	mesothelium	*VSIG10L*	TRUE	2.588095488
CAD	mesothelium	*MSL1*	TRUE	2.323497871
CAD	mesothelium	*COLCA1*	TRUE	2.751737307
CAD	mesothelium	*MYO6*	TRUE	2.107919764
CAD	mesothelium	*ZNF100*	TRUE	2.223420652
CAD	mesothelium	*ZNF334*	TRUE	2.161692574
CAD	mesothelium	*TMEM116*	TRUE	3.451695607
CAD	mesothelium	*SHISA4*	TRUE	2.252456409
CAD	mesothelium	*TBKBP1*	TRUE	2.52186229
CAD	mesothelium	*C6orf163*	TRUE	2.513399126
CAD	mesothelium	*STK19*	TRUE	2.339463809
CAD	mesothelium	*LINC00484*	TRUE	2.088556655
CAD	mesothelium	*LINC00707*	TRUE	2.356137965
CAD	mesothelium	*LINC00960*	TRUE	2.285998106
CAD	mesothelium	*GOLGA8K*	TRUE	2.578261987
CAD	mesothelium	*HMBS*	TRUE	2.004027779
CAD	mesothelium	*GRIN2B*	TRUE	2.143718842
CAD	monocyte	*SUSD2*	TRUE	2.631443608
CAD	monocyte	*BNIP1*	TRUE	2.38120635
CAD	monocyte	*DDC*	TRUE	2.133255471
CAD	monocyte	*TMEM91*	TRUE	2.732560903

CAD	monocyte	*CLEC18A*	TRUE	2.083805981
CAD	monocyte	*NXNL1*	TRUE	2.378024921
CAD	monocyte	*HYAL3*	TRUE	1.970637507
CAD	monocyte	*TMEM116*	TRUE	2.301442065
CAD	monocyte	*LY6G5B*	TRUE	2.214645298
CAD	monocyte	*TAS2R20*	TRUE	2.318215274
CAD	nk_cell	*TIPIN*	TRUE	2.273623791
CAD	nk_cell	*EIF2B2*	TRUE	2.038682079
CAD	nk_cell	*TCP11*	TRUE	3.033180395
CAD	nk_cell	*GRK4*	TRUE	1.980298227
CAD	nk_cell	*POLR1E*	TRUE	2.577604288
CAD	nk_cell	*CLEC18A*	TRUE	2.112658436
CAD	nk_cell	*NXNL1*	TRUE	2.625422548
CAD	nk_cell	*LINC00960*	TRUE	2.193111563
CAD	nk_cell	*TAS2R20*	TRUE	2.317742548
CAD	pericyte	*PAX6*	TRUE	2.055373349
CAD	pericyte	*ZMYND12*	TRUE	2.174454846
CAD	pericyte	*TIPIN*	TRUE	2.095938378
CAD	pericyte	*CENPM*	TRUE	2.361538856
CAD	pericyte	*GGCX*	TRUE	2.176373711
CAD	pericyte	*CFHR3*	TRUE	2.363659098
CAD	pericyte	*EIF2B2*	TRUE	2.188532903
CAD	pericyte	*TCP11*	TRUE	2.542357892
CAD	pericyte	*GRK4*	TRUE	3.286907144
CAD	pericyte	*DDC*	TRUE	2.154642674
CAD	pericyte	*POLR1E*	TRUE	2.459111539
CAD	pericyte	*TMEM91*	TRUE	3.115810328
CAD	pericyte	*SLC39A4*	TRUE	2.223688509
CAD	pericyte	*HSD17B12*	TRUE	2.909797032
CAD	pericyte	*MMP16*	TRUE	2.200684798
CAD	pericyte	*CLEC18A*	TRUE	2.086827312
CAD	pericyte	*C16orf46*	TRUE	2.305531422
CAD	pericyte	*ENGASE*	TRUE	1.969352602
CAD	pericyte	*LGALS9*	TRUE	2.288208061
CAD	pericyte	*RGS19*	TRUE	2.08734614
CAD	pericyte	*NXNL1*	TRUE	2.185583381
CAD	pericyte	*C3AR1*	TRUE	2.093826062
CAD	pericyte	*CCNE2*	TRUE	2.060631518
CAD	pericyte	*LYNX1*	TRUE	2.097122881
CAD	pericyte	*ADIPOQ*	TRUE	2.164177328
CAD	pericyte	*FES*	TRUE	2.035567804
CAD	pericyte	*TMEM116*	TRUE	3.125909749
CAD	pericyte	*TBKBP1*	TRUE	2.174421731
CAD	pericyte	*C6orf163*	TRUE	2.23363571
CAD	pericyte	*LINC00960*	TRUE	2.041086901
CAD	pericyte	*SRP14.AS1*	TRUE	2.014954483

CAD	pericyte	*GOLGA8K*	TRUE	2.193024449
CAD	pericyte	*TAS2R20*	TRUE	2.173256843
CAD	SMC	*CD79B*	TRUE	2.000524187
CAD	SMC	*ZMYND12*	TRUE	2.103738427
CAD	SMC	*TIPIN*	TRUE	2.51173508
CAD	SMC	*BNIP1*	TRUE	2.437485508
CAD	SMC	*SNRPC*	TRUE	2.65851186
CAD	SMC	*TCP11*	TRUE	2.514877675
CAD	SMC	*GRK4*	TRUE	3.423735611
CAD	SMC	*POLR1E*	TRUE	2.716693115
CAD	SMC	*TMEM91*	TRUE	2.718340013
CAD	SMC	*HSD17B12*	TRUE	3.006230149
CAD	SMC	*MMP16*	TRUE	2.369209601
CAD	SMC	*NXNL1*	TRUE	2.435898276
CAD	SMC	*FES*	TRUE	2.138328139
CAD	SMC	*HYAL3*	TRUE	2.04362283
CAD	SMC	*TMEM116*	TRUE	3.302983084
CAD	SMC	*TBKBP1*	TRUE	2.050075008
CAD	SMC	*LY6G5B*	TRUE	2.167210613
CAD	SMC	*LINC00960*	TRUE	2.216913402
CAD	SMC	*GOLGA8K*	TRUE	2.26720405
CAD	t_cell	*UHRF1BP1*	TRUE	3.169648187
CAD	t_cell	*ZMYND12*	TRUE	2.075230686
CAD	t_cell	*TIPIN*	TRUE	2.749645177
CAD	t_cell	*ITPR3*	TRUE	3.113609876
CAD	t_cell	*SUSD2*	TRUE	2.151334229
CAD	t_cell	*EIF2B2*	TRUE	2.318163426
CAD	t_cell	*TCP11*	TRUE	2.741738324
CAD	t_cell	*GRK4*	TRUE	3.393135747
CAD	t_cell	*DDC*	TRUE	2.371912757
CAD	t_cell	*POLR1E*	TRUE	2.595880163
CAD	t_cell	*TMEM91*	TRUE	2.037674131
CAD	t_cell	*EVA1B*	TRUE	2.494499641
CAD	t_cell	*HSD17B12*	TRUE	2.541350102
CAD	t_cell	*RNF123*	TRUE	2.861079256
CAD	t_cell	*MS4A14*	TRUE	2.059113024
CAD	t_cell	*LGALS9*	TRUE	2.211502162
CAD	t_cell	*NXNL1*	TRUE	2.463490152
CAD	t_cell	*C3AR1*	TRUE	2.046330377
CAD	t_cell	*LYNX1*	TRUE	2.218861296
CAD	t_cell	*HMBS*	TRUE	2.058633731
T2DM	adipocyte	*SNX11*	TRUE	2.078921042
T2DM	adipocyte	*CEP68*	TRUE	4.194511934
T2DM	adipocyte	*CD5L*	TRUE	2.023003415
T2DM	adipocyte	*TACR2*	TRUE	2.653055369
T2DM	adipocyte	*OVGP1*	TRUE	2.232586597

T2DM	adipocyte	*PABPC4*	TRUE	4.398579411
T2DM	adipocyte	*CWF19L1*	TRUE	2.709550751
T2DM	adipocyte	*NOMO3*	TRUE	2.547306824
T2DM	adipocyte	*TLE6*	TRUE	2.588178512
T2DM	adipocyte	*CHN2*	TRUE	3.407812546
T2DM	adipocyte	*CNTNAP1*	TRUE	3.124377412
T2DM	adipocyte	*GNRHR*	TRUE	1.982818949
T2DM	adipocyte	*C11orf21*	TRUE	2.40816879
T2DM	adipocyte	*CDK2AP1*	TRUE	2.180630591
T2DM	adipocyte	*BVES*	TRUE	2.943122791
T2DM	adipocyte	*NPHP3*	TRUE	2.027705234
T2DM	adipocyte	*GPX7*	TRUE	2.882133565
T2DM	adipocyte	*APOA1*	TRUE	2.277946862
T2DM	adipocyte	*ELOVL4*	TRUE	2.555063777
T2DM	adipocyte	*ADRA1A*	TRUE	3.707836732
T2DM	adipocyte	*CD80*	TRUE	2.047474244
T2DM	adipocyte	*GRK4*	TRUE	2.952493239
T2DM	adipocyte	*RIBC2*	TRUE	2.119836241
T2DM	adipocyte	*OPN1SW*	TRUE	2.401356537
T2DM	adipocyte	*PKDREJ*	TRUE	2.200399385
T2DM	adipocyte	*IRF4*	TRUE	3.06457889
T2DM	adipocyte	*TCF19*	TRUE	1.976117337
T2DM	adipocyte	*SLC37A4*	TRUE	2.904047725
T2DM	adipocyte	*MAN2C1*	TRUE	3.190679243
T2DM	adipocyte	*RPL3L*	TRUE	2.045784852
T2DM	adipocyte	*FN3KRP*	TRUE	2.01155088
T2DM	adipocyte	*DUS3L*	TRUE	2.9067452
T2DM	adipocyte	*ARHGEF19*	TRUE	2.446417123
T2DM	adipocyte	*HSD17B12*	TRUE	4.726625119
T2DM	adipocyte	*APIP*	TRUE	3.001354123
T2DM	adipocyte	*JAZF1*	TRUE	4.602493767
T2DM	adipocyte	*PCDH1*	TRUE	1.970651107
T2DM	adipocyte	*CLEC18A*	TRUE	5.170874308
T2DM	adipocyte	*KRTCAP3*	TRUE	3.971695008
T2DM	adipocyte	*FTCD*	TRUE	1.96261682
T2DM	adipocyte	*SPTA1*	TRUE	2.974753598
T2DM	adipocyte	*RFT1*	TRUE	2.776138782
T2DM	adipocyte	*ANPEP*	TRUE	2.384444048
T2DM	adipocyte	*ZSWIM1*	TRUE	2.576274528
T2DM	adipocyte	*IRS1*	TRUE	3.468094074
T2DM	adipocyte	*TTLL6*	TRUE	4.658522723
T2DM	adipocyte	*RGS19*	TRUE	3.287370809
T2DM	adipocyte	*RNASEH2C*	TRUE	2.06962675
T2DM	adipocyte	*CCNE2*	TRUE	3.573494825
T2DM	adipocyte	*SLC25A22*	TRUE	2.166301624
T2DM	adipocyte	*ZNF223*	TRUE	2.025086187

T2DM	adipocyte	*STX19*	TRUE	2.005718484
T2DM	adipocyte	*TRIM73*	TRUE	2.49373911
T2DM	adipocyte	*RPRML*	TRUE	2.726597408
T2DM	adipocyte	*TTC3*	TRUE	2.211870169
T2DM	adipocyte	*ZNF320*	TRUE	2.671600392
T2DM	adipocyte	*NDUFA6*	TRUE	2.460519615
T2DM	adipocyte	*GP1BA*	TRUE	2.295664705
T2DM	adipocyte	*ZKSCAN7*	TRUE	2.428907523
T2DM	adipocyte	*CCDC154*	TRUE	2.05351616
T2DM	adipocyte	*ZNF780A*	TRUE	1.987123247
T2DM	adipocyte	*TMEM116*	TRUE	2.035475547
T2DM	adipocyte	*SYCE1L*	TRUE	2.031050147
T2DM	adipocyte	*LIN52*	TRUE	2.445861194
T2DM	adipocyte	*HCG27*	TRUE	2.583277709
T2DM	adipocyte	*ASIC3*	TRUE	1.979490588
T2DM	adipocyte	*DNLZ*	TRUE	3.49712892
T2DM	adipocyte	*STARD10*	TRUE	5.280456696
T2DM	adipocyte	*AC016773.1*	TRUE	2.01793066
T2DM	adipocyte	*GS1.124K5.4*	TRUE	2.27495518
T2DM	adipocyte	*HLA.DQA2*	TRUE	3.647546555
T2DM	adipocyte	*NME1.NME2*	TRUE	2.477292423
T2DM	adipocyte	*SRP14.AS1*	TRUE	2.064689588
T2DM	adipocyte	*AP001172.2*	TRUE	2.649471837
T2DM	ASPC	*RBM6*	TRUE	2.023745927
T2DM	ASPC	*TRAF3IP3*	TRUE	2.002399204
T2DM	ASPC	*CEP68*	TRUE	4.298846306
T2DM	ASPC	*SEC61A2*	TRUE	2.24104952
T2DM	ASPC	*CD5L*	TRUE	2.072630807
T2DM	ASPC	*TACR2*	TRUE	2.624954626
T2DM	ASPC	*OVGP1*	TRUE	2.327461213
T2DM	ASPC	*PABPC4*	TRUE	5.156154884
T2DM	ASPC	*CWF19L1*	TRUE	3.121510562
T2DM	ASPC	*NOMO3*	TRUE	2.81597165
T2DM	ASPC	*TLE6*	TRUE	2.674707473
T2DM	ASPC	*ICAM5*	TRUE	2.489018655
T2DM	ASPC	*GCDH*	TRUE	2.365403311
T2DM	ASPC	*CHN2*	TRUE	2.306132354
T2DM	ASPC	*GNRHR*	TRUE	1.97139657
T2DM	ASPC	*C11orf21*	TRUE	2.705304218
T2DM	ASPC	*CDK2AP1*	TRUE	2.389480218
T2DM	ASPC	*BVES*	TRUE	3.320155808
T2DM	ASPC	*NPHP3*	TRUE	1.963953778
T2DM	ASPC	*GPX7*	TRUE	3.149166965
T2DM	ASPC	*APOA1*	TRUE	2.289862563
T2DM	ASPC	*ELOVL4*	TRUE	2.162749124
T2DM	ASPC	*TECTB*	TRUE	2.0871882

T2DM	ASPC	*ADRA1A*	TRUE	3.764035988
T2DM	ASPC	*PDZRN3*	TRUE	2.211243588
T2DM	ASPC	*CD80*	TRUE	2.257209975
T2DM	ASPC	*KCNK17*	TRUE	3.029197996
T2DM	ASPC	*GRK4*	TRUE	2.975580668
T2DM	ASPC	*ABHD8*	TRUE	2.221150296
T2DM	ASPC	*RIBC2*	TRUE	2.096576658
T2DM	ASPC	*OPN1SW*	TRUE	2.798359824
T2DM	ASPC	*STK33*	TRUE	2.273044581
T2DM	ASPC	*PKDREJ*	TRUE	2.67145531
T2DM	ASPC	*MICAL1*	TRUE	2.078890721
T2DM	ASPC	*IRF4*	TRUE	3.187630086
T2DM	ASPC	*TCF19*	TRUE	3.065099628
T2DM	ASPC	*SLC37A4*	TRUE	3.014873765
T2DM	ASPC	*TRPC3*	TRUE	2.377680568
T2DM	ASPC	*MAN2C1*	TRUE	2.665081199
T2DM	ASPC	*RPL3L*	TRUE	2.264931607
T2DM	ASPC	*C18orf8*	TRUE	2.585376891
T2DM	ASPC	*FN3KRP*	TRUE	1.966391232
T2DM	ASPC	*DUS3L*	TRUE	2.920879996
T2DM	ASPC	*ARHGEF19*	TRUE	2.497836553
T2DM	ASPC	*BHMT*	TRUE	2.155087632
T2DM	ASPC	*HSD17B12*	TRUE	5.01780832
T2DM	ASPC	*APIP*	TRUE	3.167036009
T2DM	ASPC	*POC5*	TRUE	2.570536013
T2DM	ASPC	*JAZF1*	TRUE	4.705151748
T2DM	ASPC	*PCDH1*	TRUE	1.973617318
T2DM	ASPC	*CLEC18A*	TRUE	5.653811432
T2DM	ASPC	*KRTCAP3*	TRUE	4.04861657
T2DM	ASPC	*FTCD*	TRUE	2.074710272
T2DM	ASPC	*SPTA1*	TRUE	3.107351642
T2DM	ASPC	*RFT1*	TRUE	3.168236119
T2DM	ASPC	*ZSWIM1*	TRUE	2.709565602
T2DM	ASPC	*IRS1*	TRUE	4.668822625
T2DM	ASPC	*C8orf46*	TRUE	2.556957422
T2DM	ASPC	*INO80E*	TRUE	1.98049075
T2DM	ASPC	*SEMA3E*	TRUE	2.194471312
T2DM	ASPC	*TTLL6*	TRUE	4.40148998
T2DM	ASPC	*RGS19*	TRUE	3.600653971
T2DM	ASPC	*RNASEH2C*	TRUE	2.525464805
T2DM	ASPC	*CCNE2*	TRUE	3.970532781
T2DM	ASPC	*SPRYD4*	TRUE	2.221966993
T2DM	ASPC	*SLC25A22*	TRUE	2.236311221
T2DM	ASPC	*ZNF223*	TRUE	2.148602439
T2DM	ASPC	*STX19*	TRUE	2.383148323
T2DM	ASPC	*TRIM73*	TRUE	2.893599541

T2DM	ASPC	*RPRML*	TRUE	2.856062284
T2DM	ASPC	*ZNF320*	TRUE	2.616044865
T2DM	ASPC	*EMILIN3*	TRUE	2.019741628
T2DM	ASPC	*ACOT1*	TRUE	2.04798351
T2DM	ASPC	*NDUFA6*	TRUE	2.485482393
T2DM	ASPC	*GP1BA*	TRUE	2.78024748
T2DM	ASPC	*EVI2B*	TRUE	2.106235006
T2DM	ASPC	*FFAR4*	TRUE	5.420390861
T2DM	ASPC	*ZKSCAN7*	TRUE	2.286722341
T2DM	ASPC	*FAM110D*	TRUE	2.136119373
T2DM	ASPC	*CCDC154*	TRUE	2.005132683
T2DM	ASPC	*ZNF780A*	TRUE	2.02365776
T2DM	ASPC	*TMEM116*	TRUE	2.318494297
T2DM	ASPC	*NOTCH4*	TRUE	2.352627232
T2DM	ASPC	*FAM153C*	TRUE	2.215380332
T2DM	ASPC	*LIN52*	TRUE	2.242021795
T2DM	ASPC	*HCG27*	TRUE	2.520013746
T2DM	ASPC	*ASIC3*	TRUE	1.96049014
T2DM	ASPC	*DNLZ*	TRUE	3.487046752
T2DM	ASPC	*ARHGAP19*	TRUE	3.043443865
T2DM	ASPC	*STARD10*	TRUE	5.346118964
T2DM	ASPC	*EVPLL*	TRUE	2.097412255
T2DM	ASPC	*AC016773.1*	TRUE	2.347828977
T2DM	ASPC	*POLR2J2*	TRUE	2.20339205
T2DM	ASPC	*LINC00941*	TRUE	2.759029716
T2DM	ASPC	*GS1.124K5.4*	TRUE	2.233202863
T2DM	ASPC	*HLA.DQA2*	TRUE	3.904232775
T2DM	ASPC	*NME1.NME2*	TRUE	2.409127103
T2DM	ASPC	*PLA2G4B*	TRUE	2.084380109
T2DM	ASPC	*SRP14.AS1*	TRUE	2.191556879
T2DM	ASPC	*MC1R*	TRUE	2.28554455
T2DM	ASPC	*AP001172.2*	TRUE	2.500504563
T2DM	ASPC	*YTHDF3.AS1*	TRUE	2.666587873
T2DM	b_cell	*ITIH4*	TRUE	2.478392217
T2DM	b_cell	*PKDREJ*	TRUE	2.092639669
T2DM	b_cell	*CLEC18A*	TRUE	5.484473331
T2DM	b_cell	*CTSW*	TRUE	3.48575657
T2DM	b_cell	*TRIM73*	TRUE	2.313255375
T2DM	b_cell	*HLA.DRB5*	TRUE	2.807325517
T2DM	b_cell	*POLR2J2*	TRUE	2.167102953
T2DM	b_cell	*HLA.DQA2*	TRUE	5.565624227
T2DM	dendritic_cell	*RBM6*	TRUE	2.369591725
T2DM	dendritic_cell	*PABPC4*	TRUE	2.548553545
T2DM	dendritic_cell	*CWF19L1*	TRUE	2.134464871
T2DM	dendritic_cell	*OPN1SW*	TRUE	2.02871465
T2DM	dendritic_cell	*PKDREJ*	TRUE	2.064807699

T2DM	dendritic_cell	*SPTBN5*	TRUE	2.864409607
T2DM	dendritic_cell	*APIP*	TRUE	2.176878568
T2DM	dendritic_cell	*CLEC18A*	TRUE	5.222258316
T2DM	dendritic_cell	*SPTA1*	TRUE	3.020374555
T2DM	dendritic_cell	*C1QTNF4*	TRUE	2.44399065
T2DM	dendritic_cell	*CTSW*	TRUE	1.967524954
T2DM	dendritic_cell	*CCNE2*	TRUE	2.8514478
T2DM	dendritic_cell	*TRIM73*	TRUE	1.960697998
T2DM	dendritic_cell	*NOTCH4*	TRUE	2.785077148
T2DM	dendritic_cell	*DNLZ*	TRUE	3.12314523
T2DM	dendritic_cell	*TMEM110*	TRUE	2.187720316
T2DM	dendritic_cell	*STARD10*	TRUE	3.344349492
T2DM	dendritic_cell	*POLR2J2*	TRUE	2.211143648
T2DM	dendritic_cell	*LINC00941*	TRUE	2.343783792
T2DM	dendritic_cell	*NME1.NME2*	TRUE	2.623632573
T2DM	dendritic_cell	*TAS2R20*	TRUE	2.312874616
T2DM	endometrium	*ADRA1A*	TRUE	2.015704569
T2DM	endometrium	*TCF19*	TRUE	2.682302651
T2DM	endometrium	*MAN2C1*	TRUE	2.318173086
T2DM	endometrium	*KRTCAP3*	TRUE	3.576869811
T2DM	endometrium	*ZSWIM1*	TRUE	2.26158804
T2DM	endometrium	*GP1BA*	TRUE	2.057841979
T2DM	endometrium	*HLA.DRB5*	TRUE	3.074164763
T2DM	endometrium	*DNLZ*	TRUE	3.316193336
T2DM	endometrium	*STARD10*	TRUE	2.435837607
T2DM	endometrium	*POLR2J2*	TRUE	2.266633218
T2DM	endometrium	*HLA.DQA2*	TRUE	5.74557069
T2DM	endometrium	*PLA2G4B*	TRUE	3.486212388
T2DM	endometrium	*AP001172.2*	TRUE	2.285222336
T2DM	endothelial	*PABPC4*	TRUE	2.240831542
T2DM	endothelial	*BVES*	TRUE	2.51882362
T2DM	endothelial	*GPX7*	TRUE	2.500919175
T2DM	endothelial	*PKDREJ*	TRUE	2.330883737
T2DM	endothelial	*TCF19*	TRUE	2.649444744
T2DM	endothelial	*MAN2C1*	TRUE	2.575921981
T2DM	endothelial	*HSD17B12*	TRUE	2.684787212
T2DM	endothelial	*APIP*	TRUE	2.163764338
T2DM	endothelial	*CLEC18A*	TRUE	5.354913073
T2DM	endothelial	*KRTCAP3*	TRUE	2.620734309
T2DM	endothelial	*SPTA1*	TRUE	3.08866963
T2DM	endothelial	*IRS1*	TRUE	2.845254122
T2DM	endothelial	*TTLL6*	TRUE	3.944206291
T2DM	endothelial	*RGS19*	TRUE	2.766168895
T2DM	endothelial	*CTSW*	TRUE	3.927879441
T2DM	endothelial	*CCNE2*	TRUE	2.145642845
T2DM	endothelial	*DNLZ*	TRUE	2.377159339

T2DM	endothelial	*STARD10*	TRUE	3.029250062
T2DM	endothelial	*HLA.DQA2*	TRUE	3.677523866
T2DM	endothelial	*TDGF1*	TRUE	2.015755802
T2DM	endothelial	*PLA2G4B*	TRUE	4.617331245
T2DM	endothelial	*TAS2R20*	TRUE	2.800157315
T2DM	LEC	*PABPC4*	TRUE	2.929777279
T2DM	LEC	*TLE6*	TRUE	2.451383669
T2DM	LEC	*GPX7*	TRUE	2.244997743
T2DM	LEC	*ADRA1A*	TRUE	2.570164223
T2DM	LEC	*GRK4*	TRUE	2.548484734
T2DM	LEC	*OPN1SW*	TRUE	1.995026364
T2DM	LEC	*PKDREJ*	TRUE	1.993111892
T2DM	LEC	*TCF19*	TRUE	3.368132877
T2DM	LEC	*SLC37A4*	TRUE	2.400841238
T2DM	LEC	*MAN2C1*	TRUE	3.163551052
T2DM	LEC	*ARHGEF19*	TRUE	2.420069891
T2DM	LEC	*BHMT*	TRUE	2.010447498
T2DM	LEC	*HSD17B12*	TRUE	4.243875272
T2DM	LEC	*APIP*	TRUE	2.306640986
T2DM	LEC	*CLEC18A*	TRUE	4.765170054
T2DM	LEC	*KRTCAP3*	TRUE	3.486629855
T2DM	LEC	*RFT1*	TRUE	2.262430402
T2DM	LEC	*MST1R*	TRUE	2.266864243
T2DM	LEC	*ZSWIM1*	TRUE	2.557022615
T2DM	LEC	*IRS1*	TRUE	2.638379647
T2DM	LEC	*C8orf46*	TRUE	2.306919484
T2DM	LEC	*INO80E*	TRUE	1.979747812
T2DM	LEC	*TTLL6*	TRUE	3.461426221
T2DM	LEC	*RGS19*	TRUE	3.131613544
T2DM	LEC	*TRIM73*	TRUE	2.211597103
T2DM	LEC	*RPRML*	TRUE	2.130527406
T2DM	LEC	*ZNF320*	TRUE	2.286762657
T2DM	LEC	*HLA.DRB5*	TRUE	2.348013112
T2DM	LEC	*NOTCH4*	TRUE	2.078772017
T2DM	LEC	*DNLZ*	TRUE	3.333233956
T2DM	LEC	*STARD10*	TRUE	5.089798388
T2DM	LEC	*POLR2J2*	TRUE	2.224678833
T2DM	LEC	*GS1.124K5.4*	TRUE	2.355815185
T2DM	LEC	*HLA.DQA2*	TRUE	2.714659563
T2DM	macrophage	*RBM6*	TRUE	2.138304909
T2DM	macrophage	*CEP68*	TRUE	3.250942003
T2DM	macrophage	*OVGP1*	TRUE	2.039950802
T2DM	macrophage	*PABPC4*	TRUE	2.606257897
T2DM	macrophage	*CWF19L1*	TRUE	2.423013966
T2DM	macrophage	*NOMO3*	TRUE	2.322638464
T2DM	macrophage	*TLE6*	TRUE	2.625897081

T2DM	macrophage	*CDK2AP1*	TRUE	3.044051429
T2DM	macrophage	*BVES*	TRUE	3.058791922
T2DM	macrophage	*GPX7*	TRUE	2.184317018
T2DM	macrophage	*ADRA1A*	TRUE	2.818472965
T2DM	macrophage	*GRK4*	TRUE	2.658805108
T2DM	macrophage	*PKDREJ*	TRUE	2.392327324
T2DM	macrophage	*TCF19*	TRUE	2.391820863
T2DM	macrophage	*MAN2C1*	TRUE	3.104566308
T2DM	macrophage	*RPL3L*	TRUE	2.130697124
T2DM	macrophage	*FN3KRP*	TRUE	2.139552186
T2DM	macrophage	*DUS3L*	TRUE	2.739603107
T2DM	macrophage	*ARHGEF19*	TRUE	2.045580238
T2DM	macrophage	*HSD17B12*	TRUE	4.629983575
T2DM	macrophage	*APIP*	TRUE	2.629440908
T2DM	macrophage	*JAZF1*	TRUE	4.423416328
T2DM	macrophage	*CLEC18A*	TRUE	5.58401526
T2DM	macrophage	*KRTCAP3*	TRUE	3.856090564
T2DM	macrophage	*PAQR6*	TRUE	2.085666349
T2DM	macrophage	*SPTA1*	TRUE	3.49079786
T2DM	macrophage	*RFT1*	TRUE	2.78637183
T2DM	macrophage	*RAPSN*	TRUE	2.216516085
T2DM	macrophage	*ANPEP*	TRUE	2.399509306
T2DM	macrophage	*ZSWIM1*	TRUE	2.973887301
T2DM	macrophage	*IRS1*	TRUE	2.99157252
T2DM	macrophage	*C8orf46*	TRUE	1.98731983
T2DM	macrophage	*TTLL6*	TRUE	3.648546527
T2DM	macrophage	*CCNE2*	TRUE	2.82438912
T2DM	macrophage	*TRIM73*	TRUE	2.56857574
T2DM	macrophage	*RPRML*	TRUE	2.22874237
T2DM	macrophage	*ZNF320*	TRUE	2.533788303
T2DM	macrophage	*NDUFA6*	TRUE	2.280057715
T2DM	macrophage	*GP1BA*	TRUE	2.140223909
T2DM	macrophage	*NOTCH4*	TRUE	2.290863347
T2DM	macrophage	*SYCE1L*	TRUE	2.00598743
T2DM	macrophage	*HCG27*	TRUE	2.153407309
T2DM	macrophage	*DNLZ*	TRUE	3.559817109
T2DM	macrophage	*STARD10*	TRUE	5.064403829
T2DM	macrophage	*AC016773.1*	TRUE	1.985209804
T2DM	macrophage	*POLR2J2*	TRUE	2.19516844
T2DM	macrophage	*GS1.124K5.4*	TRUE	2.126753514
T2DM	macrophage	*HLA.DQA2*	TRUE	4.419101108
T2DM	macrophage	*NME1.NME2*	TRUE	2.686825973
T2DM	macrophage	*PLA2G4B*	TRUE	2.085607672
T2DM	macrophage	*AP001172.2*	TRUE	2.105449616
T2DM	macrophage	*YTHDF3.AS1*	TRUE	2.178346911
T2DM	mast_cell	*GNB3*	TRUE	2.354845523

T2DM	mast_cell	*BVES*	TRUE	2.482596398
T2DM	mast_cell	*GPX7*	TRUE	2.623828349
T2DM	mast_cell	*ADRA1A*	TRUE	3.391373627
T2DM	mast_cell	*PKDREJ*	TRUE	2.170016172
T2DM	mast_cell	*IRF4*	TRUE	2.112658322
T2DM	mast_cell	*CLEC18A*	TRUE	5.304514374
T2DM	mast_cell	*SPTA1*	TRUE	3.011750288
T2DM	mast_cell	*RAPSN*	TRUE	2.931151028
T2DM	mast_cell	*ANPEP*	TRUE	2.662455801
T2DM	mast_cell	*ZSWIM1*	TRUE	2.452089611
T2DM	mast_cell	*RGS19*	TRUE	3.469603905
T2DM	mast_cell	*RPRML*	TRUE	2.383208263
T2DM	mast_cell	*ACOT1*	TRUE	2.189195496
T2DM	mast_cell	*HLA.DRB5*	TRUE	2.305585046
T2DM	mast_cell	*DNLZ*	TRUE	3.418652813
T2DM	mast_cell	*TMEM110*	TRUE	2.029520239
T2DM	mast_cell	*STARD10*	TRUE	3.935899718
T2DM	mast_cell	*HLA.DQA2*	TRUE	6.409162694
T2DM	mast_cell	*NME1.NME2*	TRUE	2.092377388
T2DM	mast_cell	*PLA2G4B*	TRUE	2.801461669
T2DM	mesothelium	*RBM6*	TRUE	4.141768141
T2DM	mesothelium	*CEP68*	TRUE	3.978079428
T2DM	mesothelium	*TUBG2*	TRUE	2.97710571
T2DM	mesothelium	*PIGV*	TRUE	2.176447961
T2DM	mesothelium	*SEC61A2*	TRUE	3.402223655
T2DM	mesothelium	*CD5L*	TRUE	1.999271437
T2DM	mesothelium	*TACR2*	TRUE	2.571210332
T2DM	mesothelium	*DCT*	TRUE	2.185020761
T2DM	mesothelium	*OVGP1*	TRUE	2.533139996
T2DM	mesothelium	*SNX10*	TRUE	2.258958901
T2DM	mesothelium	*PABPC4*	TRUE	6.069441518
T2DM	mesothelium	*CWF19L1*	TRUE	3.555958842
T2DM	mesothelium	*SCD*	TRUE	2.017603581
T2DM	mesothelium	*NOMO3*	TRUE	3.121441946
T2DM	mesothelium	*TLE6*	TRUE	2.546865843
T2DM	mesothelium	*ICAM5*	TRUE	2.477401328
T2DM	mesothelium	*GCDH*	TRUE	2.350297837
T2DM	mesothelium	*RNF32*	TRUE	2.308086383
T2DM	mesothelium	*C11orf21*	TRUE	3.056023308
T2DM	mesothelium	*CDK2AP1*	TRUE	3.565920676
T2DM	mesothelium	*BVES*	TRUE	3.288002723
T2DM	mesothelium	*CHMP3*	TRUE	2.092661869
T2DM	mesothelium	*GPX7*	TRUE	3.03059546
T2DM	mesothelium	*APOA1*	TRUE	2.169314158
T2DM	mesothelium	*ELOVL4*	TRUE	3.026444685
T2DM	mesothelium	*TECTB*	TRUE	2.135349981

T2DM	mesothelium	*ADRA1A*	TRUE	3.728027566
T2DM	mesothelium	*TEX14*	TRUE	2.071248598
T2DM	mesothelium	*PDZRN3*	TRUE	2.53163324
T2DM	mesothelium	*CD80*	TRUE	2.478932965
T2DM	mesothelium	*RPS10*	TRUE	2.062304121
T2DM	mesothelium	*KCNK17*	TRUE	3.074819818
T2DM	mesothelium	*GRK4*	TRUE	2.969377267
T2DM	mesothelium	*XRCC3*	TRUE	2.099084039
T2DM	mesothelium	*ABHD8*	TRUE	2.842729624
T2DM	mesothelium	*RIBC2*	TRUE	1.973966094
T2DM	mesothelium	*OPN1SW*	TRUE	2.742268944
T2DM	mesothelium	*PKDREJ*	TRUE	2.67703097
T2DM	mesothelium	*MICAL1*	TRUE	2.012715808
T2DM	mesothelium	*RCBTB1*	TRUE	2.070213613
T2DM	mesothelium	*IRF4*	TRUE	3.104074284
T2DM	mesothelium	*TCF19*	TRUE	2.708464659
T2DM	mesothelium	*SLC37A4*	TRUE	2.915989903
T2DM	mesothelium	*TRPC3*	TRUE	2.37381824
T2DM	mesothelium	*GCSH*	TRUE	2.065246239
T2DM	mesothelium	*RPL3L*	TRUE	2.041590634
T2DM	mesothelium	*VPS53*	TRUE	2.208007397
T2DM	mesothelium	*C18orf8*	TRUE	3.629226267
T2DM	mesothelium	*FN3KRP*	TRUE	2.756685508
T2DM	mesothelium	*DUS3L*	TRUE	2.85686726
T2DM	mesothelium	*SAE1*	TRUE	2.412894438
T2DM	mesothelium	*GEMIN7*	TRUE	2.0097632
T2DM	mesothelium	*ARHGEF19*	TRUE	2.535522224
T2DM	mesothelium	*PLEKHA6*	TRUE	2.308247597
T2DM	mesothelium	*BHMT*	TRUE	2.130356098
T2DM	mesothelium	*HSD17B12*	TRUE	5.350269618
T2DM	mesothelium	*APIP*	TRUE	3.424179838
T2DM	mesothelium	*CCDC15*	TRUE	1.965518656
T2DM	mesothelium	*POC5*	TRUE	2.939734911
T2DM	mesothelium	*JAZF1*	TRUE	5.350626432
T2DM	mesothelium	*PCDH1*	TRUE	2.140259572
T2DM	mesothelium	*CLEC18A*	TRUE	5.196138473
T2DM	mesothelium	*KRTCAP3*	TRUE	3.72489215
T2DM	mesothelium	*SPATA2L*	TRUE	2.174413722
T2DM	mesothelium	*FTCD*	TRUE	2.212845761
T2DM	mesothelium	*PAQR6*	TRUE	2.237631825
T2DM	mesothelium	*SPTA1*	TRUE	3.129158939
T2DM	mesothelium	*RFT1*	TRUE	3.731624745
T2DM	mesothelium	*TMEM63C*	TRUE	2.003925419
T2DM	mesothelium	*RAPSN*	TRUE	2.522754398
T2DM	mesothelium	*ANPEP*	TRUE	2.810389461
T2DM	mesothelium	*SERINC2*	TRUE	2.043263744

T2DM	mesothelium	*ZSWIM1*	TRUE	2.623254512
T2DM	mesothelium	*IRS1*	TRUE	4.710583862
T2DM	mesothelium	*INO80E*	TRUE	3.118572994
T2DM	mesothelium	*LRRC45*	TRUE	2.133015532
T2DM	mesothelium	*SEMA3E*	TRUE	1.984065241
T2DM	mesothelium	*EMB*	TRUE	3.085509476
T2DM	mesothelium	*TTLL6*	TRUE	5.075730352
T2DM	mesothelium	*RGS19*	TRUE	3.829221971
T2DM	mesothelium	*CTSW*	TRUE	2.386787842
T2DM	mesothelium	*RNASEH2C*	TRUE	2.612160665
T2DM	mesothelium	*HOXB2*	TRUE	2.290383734
T2DM	mesothelium	*SPRYD4*	TRUE	2.26698801
T2DM	mesothelium	*SLC25A22*	TRUE	2.756393594
T2DM	mesothelium	*STX19*	TRUE	2.213569362
T2DM	mesothelium	*ZNF852*	TRUE	2.344545448
T2DM	mesothelium	*RPRML*	TRUE	2.769557182
T2DM	mesothelium	*ZNF320*	TRUE	2.566996872
T2DM	mesothelium	*ACOT1*	TRUE	2.063347136
T2DM	mesothelium	*NDUFA6*	TRUE	2.149922776
T2DM	mesothelium	*GP1BA*	TRUE	2.531137178
T2DM	mesothelium	*DMWD*	TRUE	2.588503412
T2DM	mesothelium	*EVI2B*	TRUE	2.053880575
T2DM	mesothelium	*RASA3*	TRUE	2.510081953
T2DM	mesothelium	*FFAR4*	TRUE	4.13460683
T2DM	mesothelium	*COLCA1*	TRUE	2.022914213
T2DM	mesothelium	*ZNF780A*	TRUE	2.153574154
T2DM	mesothelium	*TMEM116*	TRUE	2.521258446
T2DM	mesothelium	*HLA.DRB5*	TRUE	2.653547224
T2DM	mesothelium	*SHISA4*	TRUE	2.486425884
T2DM	mesothelium	*LIN52*	TRUE	1.997623218
T2DM	mesothelium	*HCG27*	TRUE	2.378137758
T2DM	mesothelium	*DNLZ*	TRUE	3.624306497
T2DM	mesothelium	*ARHGAP19*	TRUE	3.366030913
T2DM	mesothelium	*STARD10*	TRUE	5.2731063
T2DM	mesothelium	*AC016773.1*	TRUE	2.426065758
T2DM	mesothelium	*AC108463.2*	TRUE	2.797641394
T2DM	mesothelium	*POLR2J2*	TRUE	2.190514536
T2DM	mesothelium	*LINC00332*	TRUE	1.997187955
T2DM	mesothelium	*LINC00957*	TRUE	2.294355418
T2DM	mesothelium	*LINC00941*	TRUE	2.561988407
T2DM	mesothelium	*GS1.124K5.4*	TRUE	2.49558881
T2DM	mesothelium	*HLA.DQA2*	TRUE	3.530644534
T2DM	mesothelium	*NME1.NME2*	TRUE	2.713494861
T2DM	mesothelium	*SMIM6*	TRUE	1.974591594
T2DM	mesothelium	*LINC00675*	TRUE	2.269833366
T2DM	mesothelium	*AP001172.2*	TRUE	2.593210422

T2DM	mesothelium	*YTHDF3.AS1*	TRUE	2.567172367
T2DM	monocyte	*ITIH4*	TRUE	3.273797569
T2DM	monocyte	*NOMO3*	TRUE	2.244670157
T2DM	monocyte	*TLE6*	TRUE	2.301988594
T2DM	monocyte	*IRF4*	TRUE	2.396271329
T2DM	monocyte	*CLEC18A*	TRUE	5.054702533
T2DM	monocyte	*SPTA1*	TRUE	3.42986924
T2DM	monocyte	*C1QTNF4*	TRUE	2.057875725
T2DM	monocyte	*HLA.DRB5*	TRUE	2.075650421
T2DM	monocyte	*NOTCH4*	TRUE	2.467129398
T2DM	monocyte	*DNLZ*	TRUE	2.991949016
T2DM	monocyte	*STARD10*	TRUE	4.62310333
T2DM	monocyte	*HLA.DQA2*	TRUE	3.477285722
T2DM	monocyte	*TAS2R20*	TRUE	2.154656928
T2DM	nk_cell	*RIBC2*	TRUE	1.963101166
T2DM	nk_cell	*IRF4*	TRUE	2.881432962
T2DM	nk_cell	*SPTBN5*	TRUE	2.502915333
T2DM	nk_cell	*ARHGEF19*	TRUE	2.075541683
T2DM	nk_cell	*HSD17B12*	TRUE	2.759275946
T2DM	nk_cell	*APIP*	TRUE	2.012390725
T2DM	nk_cell	*CLEC18A*	TRUE	4.635218185
T2DM	nk_cell	*SPTA1*	TRUE	2.444894547
T2DM	nk_cell	*IRS1*	TRUE	2.656902717
T2DM	nk_cell	*HLA.DQA1*	TRUE	2.998391541
T2DM	nk_cell	*NOTCH4*	TRUE	2.256742135
T2DM	pericyte	*RBM6*	TRUE	3.333646094
T2DM	pericyte	*OVGP1*	TRUE	2.097630949
T2DM	pericyte	*PABPC4*	TRUE	3.178466948
T2DM	pericyte	*CHN2*	TRUE	2.110381863
T2DM	pericyte	*CDK2AP1*	TRUE	2.073647373
T2DM	pericyte	*BVES*	TRUE	2.563512935
T2DM	pericyte	*GPX7*	TRUE	2.70180145
T2DM	pericyte	*ADRA1A*	TRUE	2.290473199
T2DM	pericyte	*GRK4*	TRUE	2.430863734
T2DM	pericyte	*PKDREJ*	TRUE	2.308956589
T2DM	pericyte	*IRF4*	TRUE	2.667861955
T2DM	pericyte	*TCF19*	TRUE	2.188710889
T2DM	pericyte	*MAN2C1*	TRUE	2.608083955
T2DM	pericyte	*DUS3L*	TRUE	2.521267993
T2DM	pericyte	*ARHGEF19*	TRUE	1.992386061
T2DM	pericyte	*HSD17B12*	TRUE	4.392532143
T2DM	pericyte	*APIP*	TRUE	2.602917265
T2DM	pericyte	*JAZF1*	TRUE	3.019632301
T2DM	pericyte	*CLEC18A*	TRUE	5.235651894
T2DM	pericyte	*KRTCAP3*	TRUE	3.01532231
T2DM	pericyte	*SPTA1*	TRUE	2.928377562

T2DM	pericyte	*RFT1*	TRUE	2.458000224
T2DM	pericyte	*RAPSN*	TRUE	2.180885858
T2DM	pericyte	*ZSWIM1*	TRUE	2.422130305
T2DM	pericyte	*IRS1*	TRUE	2.233034833
T2DM	pericyte	*RGS19*	TRUE	2.524118573
T2DM	pericyte	*CTSW*	TRUE	2.236886419
T2DM	pericyte	*CCNE2*	TRUE	2.319348749
T2DM	pericyte	*TRIM73*	TRUE	2.344287717
T2DM	pericyte	*RPRML*	TRUE	2.356103964
T2DM	pericyte	*ZNF320*	TRUE	2.320847958
T2DM	pericyte	*NOTCH4*	TRUE	2.260839262
T2DM	pericyte	*DNLZ*	TRUE	3.402514008
T2DM	pericyte	*STARD10*	TRUE	4.957298202
T2DM	pericyte	*POLR2J2*	TRUE	2.2062748
T2DM	pericyte	*GS1.124K5.4*	TRUE	2.092955554
T2DM	pericyte	*HLA.DQA2*	TRUE	3.082852897
T2DM	pericyte	*NME1.NME2*	TRUE	2.008966787
T2DM	pericyte	*JMJD7*	TRUE	1.983864382
T2DM	SMC	*RBM6*	TRUE	3.324282676
T2DM	SMC	*PABPC4*	TRUE	3.414110872
T2DM	SMC	*TLE6*	TRUE	2.167987154
T2DM	SMC	*GPX7*	TRUE	2.507171468
T2DM	SMC	*ADRA1A*	TRUE	3.014844642
T2DM	SMC	*GRK4*	TRUE	2.738470141
T2DM	SMC	*TCF19*	TRUE	2.094974707
T2DM	SMC	*MAN2C1*	TRUE	3.183459746
T2DM	SMC	*ARHGEF19*	TRUE	2.316599896
T2DM	SMC	*HSD17B12*	TRUE	4.594166729
T2DM	SMC	*APIP*	TRUE	2.02381446
T2DM	SMC	*JAZF1*	TRUE	3.061958504
T2DM	SMC	*SPTA1*	TRUE	3.26666004
T2DM	SMC	*ANPEP*	TRUE	2.420988439
T2DM	SMC	*ZSWIM1*	TRUE	2.348507993
T2DM	SMC	*IRS1*	TRUE	2.481853146
T2DM	SMC	*TTLL6*	TRUE	4.139676013
T2DM	SMC	*RPRML*	TRUE	2.034523689
T2DM	SMC	*PIGP*	TRUE	2.233470248
T2DM	SMC	*HLA.DRB5*	TRUE	2.578939892
T2DM	SMC	*FAM153C*	TRUE	2.005794598
T2DM	SMC	*DNLZ*	TRUE	3.427427628
T2DM	SMC	*STARD10*	TRUE	3.850756309
T2DM	SMC	*AC108463.2*	TRUE	2.125506491
T2DM	SMC	*LINC00941*	TRUE	2.097066558
T2DM	SMC	*HLA.DQA2*	TRUE	2.824716737
T2DM	t_cell	*PABPC4*	TRUE	3.725922852
T2DM	t_cell	*CWF19L1*	TRUE	2.282378626

T2DM	t_cell	*NOMO3*	TRUE	2.374105364
T2DM	t_cell	*TLE6*	TRUE	2.340801189
T2DM	t_cell	*CHN2*	TRUE	3.734175578
T2DM	t_cell	*GNB3*	TRUE	2.442193779
T2DM	t_cell	*BVES*	TRUE	2.483380206
T2DM	t_cell	*ADRA1A*	TRUE	2.075030165
T2DM	t_cell	*GRK4*	TRUE	2.135511374
T2DM	t_cell	*OPN1SW*	TRUE	2.148293968
T2DM	t_cell	*PKDREJ*	TRUE	2.532716516
T2DM	t_cell	*IRF4*	TRUE	2.232430929
T2DM	t_cell	*FN3KRP*	TRUE	2.086380454
T2DM	t_cell	*ARHGEF19*	TRUE	2.402035481
T2DM	t_cell	*HSD17B12*	TRUE	4.52819912
T2DM	t_cell	*APIP*	TRUE	2.619054451
T2DM	t_cell	*PCDH1*	TRUE	2.031951365
T2DM	t_cell	*CLEC18A*	TRUE	4.166157791
T2DM	t_cell	*KRTCAP3*	TRUE	3.403905298
T2DM	t_cell	*PAQR6*	TRUE	1.988300666
T2DM	t_cell	*SPTA1*	TRUE	3.214796173
T2DM	t_cell	*ANPEP*	TRUE	2.141011457
T2DM	t_cell	*ZSWIM1*	TRUE	2.928800443
T2DM	t_cell	*IRS1*	TRUE	3.88418071
T2DM	t_cell	*TTLL6*	TRUE	3.800914212
T2DM	t_cell	*CCNE2*	TRUE	3.051492164
T2DM	t_cell	*TRIM73*	TRUE	2.297609891
T2DM	t_cell	*ACOT1*	TRUE	2.179657613
T2DM	t_cell	*NDUFA6*	TRUE	2.102368412
T2DM	t_cell	*NOTCH4*	TRUE	2.470051652
T2DM	t_cell	*DNLZ*	TRUE	3.391589639
T2DM	t_cell	*STARD10*	TRUE	4.886955038
T2DM	t_cell	*GS1.124K5.4*	TRUE	2.238174483
T2DM	t_cell	*HLA.DQA2*	TRUE	2.380565196
HTN	adipocyte	*CD79B*	TRUE	2.435092866
HTN	adipocyte	*CEP68*	TRUE	2.76916073
HTN	adipocyte	*SDCCAG8*	TRUE	3.38780082
HTN	adipocyte	*PIGV*	TRUE	3.795722243
HTN	adipocyte	*UHRF1BP1*	TRUE	3.197984687
HTN	adipocyte	*POLR1A*	TRUE	2.117674676
HTN	adipocyte	*DUSP13*	TRUE	2.078074017
HTN	adipocyte	*SNX10*	TRUE	3.687943819
HTN	adipocyte	*AAMDC*	TRUE	2.069794283
HTN	adipocyte	*MUL1*	TRUE	2.257011178
HTN	adipocyte	*PABPC4*	TRUE	2.668444721
HTN	adipocyte	*SCD*	TRUE	2.369149381
HTN	adipocyte	*MARCH2*	TRUE	3.477110574
HTN	adipocyte	*BIRC7*	TRUE	2.872976852

HTN	adipocyte	*POLI*	TRUE	3.053940802
HTN	adipocyte	*MTHFSD*	TRUE	3.048813068
HTN	adipocyte	*ICAM5*	TRUE	2.276591
HTN	adipocyte	*SGCA*	TRUE	2.650832229
HTN	adipocyte	*CCND1*	TRUE	2.357709158
HTN	adipocyte	*BNIP1*	TRUE	1.970892959
HTN	adipocyte	*NPHP3*	TRUE	1.966213487
HTN	adipocyte	*PCCB*	TRUE	3.865370109
HTN	adipocyte	*IRF6*	TRUE	2.630044212
HTN	adipocyte	*ACOT2*	TRUE	2.465798248
HTN	adipocyte	*ZFP30*	TRUE	2.091720716
HTN	adipocyte	*ADRA1A*	TRUE	1.997146288
HTN	adipocyte	*TBX2*	TRUE	5.040321368
HTN	adipocyte	*TEX14*	TRUE	2.474015244
HTN	adipocyte	*BTN2A2*	TRUE	3.213263832
HTN	adipocyte	*ZNF391*	TRUE	2.339470288
HTN	adipocyte	*TCP11*	TRUE	1.987801258
HTN	adipocyte	*GRK4*	TRUE	2.710977402
HTN	adipocyte	*TSKS*	TRUE	2.347658368
HTN	adipocyte	*TRAP1*	TRUE	4.072804472
HTN	adipocyte	*ABHD8*	TRUE	2.538008495
HTN	adipocyte	*ADCY4*	TRUE	2.880117164
HTN	adipocyte	*ATP8B3*	TRUE	2.955910493
HTN	adipocyte	*PGPEP1*	TRUE	2.360267283
HTN	adipocyte	*HAUS8*	TRUE	4.374981136
HTN	adipocyte	*RSG1*	TRUE	2.35208348
HTN	adipocyte	*ACP2*	TRUE	4.284909786
HTN	adipocyte	*AGT*	TRUE	3.007240763
HTN	adipocyte	*NMT1*	TRUE	3.896399033
HTN	adipocyte	*SLC37A4*	TRUE	2.789949245
HTN	adipocyte	*NUSAP1*	TRUE	3.022577511
HTN	adipocyte	*FBN2*	TRUE	3.811396437
HTN	adipocyte	*FITM1*	TRUE	2.537215287
HTN	adipocyte	*RPL3L*	TRUE	2.157454702
HTN	adipocyte	*DEF8*	TRUE	2.440502614
HTN	adipocyte	*SCRN2*	TRUE	2.275412295
HTN	adipocyte	*C18orf8*	TRUE	2.169186584
HTN	adipocyte	*MMEL1*	TRUE	2.210119178
HTN	adipocyte	*CREB3L4*	TRUE	3.4330296
HTN	adipocyte	*PLEKHA6*	TRUE	2.053864052
HTN	adipocyte	*IRAK1BP1*	TRUE	2.157212283
HTN	adipocyte	*ZNF215*	TRUE	2.238505387
HTN	adipocyte	*HSD17B12*	TRUE	5.355486765
HTN	adipocyte	*TNKS1BP1*	TRUE	4.352803049
HTN	adipocyte	*NEIL2*	TRUE	3.543290603
HTN	adipocyte	*ART3*	TRUE	2.615897567

HTN	adipocyte	*SFXN2*	TRUE	4.918352001
HTN	adipocyte	*CLEC18A*	TRUE	2.794213121
HTN	adipocyte	*SLC5A11*	TRUE	4.60305368
HTN	adipocyte	*S100B*	TRUE	3.50860745
HTN	adipocyte	*ABCF3*	TRUE	4.746788753
HTN	adipocyte	*MRPL55*	TRUE	5.6538278
HTN	adipocyte	*LYSMD1*	TRUE	3.387814337
HTN	adipocyte	*RFT1*	TRUE	2.383548685
HTN	adipocyte	*RNF123*	TRUE	4.649806432
HTN	adipocyte	*ERAP1*	TRUE	1.963188832
HTN	adipocyte	*CYB5R2*	TRUE	2.664788959
HTN	adipocyte	*TRIM66*	TRUE	3.565939864
HTN	adipocyte	*RIMKLB*	TRUE	3.486477515
HTN	adipocyte	*SRR*	TRUE	3.412489298
HTN	adipocyte	*SOAT2*	TRUE	2.551907865
HTN	adipocyte	*SERINC2*	TRUE	2.453963585
HTN	adipocyte	*CPT1C*	TRUE	3.479782686
HTN	adipocyte	*INO80E*	TRUE	4.383188723
HTN	adipocyte	*RGPD8*	TRUE	2.923416626
HTN	adipocyte	*MUC3A*	TRUE	2.010132333
HTN	adipocyte	*MYO7B*	TRUE	3.523402652
HTN	adipocyte	*TTLL6*	TRUE	5.448183147
HTN	adipocyte	*PAQR8*	TRUE	2.028002237
HTN	adipocyte	*MRPL36*	TRUE	2.720585085
HTN	adipocyte	*RGS19*	TRUE	4.848111934
HTN	adipocyte	*NME6*	TRUE	2.49253504
HTN	adipocyte	*CTSW*	TRUE	2.738687899
HTN	adipocyte	*MST1*	TRUE	3.143805034
HTN	adipocyte	*C11orf80*	TRUE	3.046223075
HTN	adipocyte	*CTU2*	TRUE	2.209061382
HTN	adipocyte	*SNX31*	TRUE	4.299490352
HTN	adipocyte	*CCNE2*	TRUE	4.403567401
HTN	adipocyte	*ORAI3*	TRUE	3.97471976
HTN	adipocyte	*ODF3B*	TRUE	2.246363037
HTN	adipocyte	*GEN1*	TRUE	2.475295645
HTN	adipocyte	*MPI*	TRUE	6.17800662
HTN	adipocyte	*TRIM73*	TRUE	2.388117597
HTN	adipocyte	*HYI*	TRUE	3.581730225
HTN	adipocyte	*CLDN7*	TRUE	2.264310892
HTN	adipocyte	*ADO*	TRUE	5.473750758
HTN	adipocyte	*CLN8*	TRUE	3.519391604
HTN	adipocyte	*FES*	TRUE	6.868552594
HTN	adipocyte	*ZNF320*	TRUE	3.46879707
HTN	adipocyte	*A3GALT2*	TRUE	3.75863654
HTN	adipocyte	*NUTM2A*	TRUE	3.581737117
HTN	adipocyte	*FOXI2*	TRUE	2.20791163

HTN	adipocyte	*KLHL17*	TRUE	2.173838145
HTN	adipocyte	*ZNF559*	TRUE	2.530353649
HTN	adipocyte	*FBF1*	TRUE	3.081039701
HTN	adipocyte	*COLCA1*	TRUE	2.541918762
HTN	adipocyte	*HRCT1*	TRUE	2.496053821
HTN	adipocyte	*HIST1H3E*	TRUE	4.990549733
HTN	adipocyte	*WDR5B*	TRUE	2.542163695
HTN	adipocyte	*ZNF100*	TRUE	2.163465088
HTN	adipocyte	*SULT1A2*	TRUE	3.320129162
HTN	adipocyte	*C6orf141*	TRUE	1.967762752
HTN	adipocyte	*RPS26*	TRUE	3.875630571
HTN	adipocyte	*ZNF311*	TRUE	2.316166789
HTN	adipocyte	*AGAP9*	TRUE	2.082262357
HTN	adipocyte	*TMEM116*	TRUE	6.680691784
HTN	adipocyte	*PNP*	TRUE	2.761130468
HTN	adipocyte	*SHISA4*	TRUE	4.072941023
HTN	adipocyte	*HSD17B8*	TRUE	3.030170478
HTN	adipocyte	*HLA.C*	TRUE	2.294475863
HTN	adipocyte	*ATF6B*	TRUE	3.362110165
HTN	adipocyte	*PPM1N*	TRUE	2.425504633
HTN	adipocyte	*AC016773.1*	TRUE	2.419413981
HTN	adipocyte	*SLC12A8*	TRUE	2.625957542
HTN	adipocyte	*TIAF1*	TRUE	2.827603431
HTN	adipocyte	*OSER1.AS1*	TRUE	2.042850127
HTN	adipocyte	*HLA.DQA2*	TRUE	2.404768676
HTN	adipocyte	*TNFSF12*	TRUE	3.065930791
HTN	adipocyte	*ZNF709*	TRUE	2.278011027
HTN	adipocyte	*LINC00638*	TRUE	2.876215969
HTN	ASPC	*ARHGAP33*	TRUE	2.003063637
HTN	ASPC	*CD79B*	TRUE	2.242524
HTN	ASPC	*CEP68*	TRUE	3.88742929
HTN	ASPC	*CHDH*	TRUE	2.924319778
HTN	ASPC	*RNASET2*	TRUE	2.062312632
HTN	ASPC	*ANAPC4*	TRUE	2.185407242
HTN	ASPC	*SDCCAG8*	TRUE	3.971006044
HTN	ASPC	*PLEKHH1*	TRUE	2.138705105
HTN	ASPC	*PIGV*	TRUE	3.867925667
HTN	ASPC	*POLR1A*	TRUE	2.313341261
HTN	ASPC	*DUSP13*	TRUE	2.158903529
HTN	ASPC	*SNX10*	TRUE	3.23253013
HTN	ASPC	*AAMDC*	TRUE	2.540652533
HTN	ASPC	*TBX5*	TRUE	1.971722261
HTN	ASPC	*MUL1*	TRUE	1.98366033
HTN	ASPC	*PABPC4*	TRUE	2.883441265
HTN	ASPC	*RGS17*	TRUE	2.715097992
HTN	ASPC	*SCD*	TRUE	3.006486127

HTN	ASPC	*SETD1A*	TRUE	2.297780012
HTN	ASPC	*MARCH2*	TRUE	4.229499534
HTN	ASPC	*BIRC7*	TRUE	2.802382501
HTN	ASPC	*POLI*	TRUE	3.346142375
HTN	ASPC	*MTHFSD*	TRUE	3.28822488
HTN	ASPC	*SNX16*	TRUE	2.826944373
HTN	ASPC	*ICAM5*	TRUE	2.837813145
HTN	ASPC	*MEST*	TRUE	1.984623823
HTN	ASPC	*SLC1A1*	TRUE	2.261975913
HTN	ASPC	*NAGLU*	TRUE	2.022590844
HTN	ASPC	*SGCA*	TRUE	2.637253485
HTN	ASPC	*CCND1*	TRUE	3.501579281
HTN	ASPC	*BVES*	TRUE	2.077614259
HTN	ASPC	*NME5*	TRUE	2.688649369
HTN	ASPC	*BNIP1*	TRUE	2.17927391
HTN	ASPC	*CLDN16*	TRUE	2.00801914
HTN	ASPC	*NPHP3*	TRUE	2.06952678
HTN	ASPC	*PCCB*	TRUE	4.64802231
HTN	ASPC	*KIAA1324*	TRUE	1.990372228
HTN	ASPC	*IRF6*	TRUE	2.566142212
HTN	ASPC	*ACOT2*	TRUE	2.979134039
HTN	ASPC	*ZFP30*	TRUE	2.774895381
HTN	ASPC	*ADRA1A*	TRUE	2.173577963
HTN	ASPC	*TBX2*	TRUE	5.386652229
HTN	ASPC	*TEX14*	TRUE	2.430603883
HTN	ASPC	*TOX2*	TRUE	1.970543283
HTN	ASPC	*BTN2A2*	TRUE	3.475253825
HTN	ASPC	*ZNF391*	TRUE	2.088656247
HTN	ASPC	*GRK4*	TRUE	2.772909101
HTN	ASPC	*TSKS*	TRUE	2.89924096
HTN	ASPC	*TRAP1*	TRUE	4.551716359
HTN	ASPC	*ABHD8*	TRUE	3.325656133
HTN	ASPC	*GFER*	TRUE	2.062963176
HTN	ASPC	*GAD1*	TRUE	2.272947768
HTN	ASPC	*ADCY4*	TRUE	4.33054174
HTN	ASPC	*CDC16*	TRUE	2.134970213
HTN	ASPC	*ATP8B3*	TRUE	3.073507351
HTN	ASPC	*PGPEP1*	TRUE	2.58991653
HTN	ASPC	*HAUS8*	TRUE	4.576343145
HTN	ASPC	*GCH1*	TRUE	2.4717296
HTN	ASPC	*RSG1*	TRUE	2.081442288
HTN	ASPC	*SLC2A11*	TRUE	2.241338702
HTN	ASPC	*ACP2*	TRUE	5.374876581
HTN	ASPC	*CLDN10*	TRUE	2.361448106
HTN	ASPC	*AGT*	TRUE	3.388903173
HTN	ASPC	*NMT1*	TRUE	4.344448427

HTN	ASPC	*SLC37A4*	TRUE	2.955342656
HTN	ASPC	*PPP2R1B*	TRUE	2.306759065
HTN	ASPC	*NUSAP1*	TRUE	2.743251032
HTN	ASPC	*G3BP2*	TRUE	2.063538941
HTN	ASPC	*ENPEP*	TRUE	2.219765032
HTN	ASPC	*FBN2*	TRUE	4.00025587
HTN	ASPC	*FITM1*	TRUE	2.739186453
HTN	ASPC	*RTN1*	TRUE	2.984496353
HTN	ASPC	*RPL3L*	TRUE	2.40113064
HTN	ASPC	*DEF8*	TRUE	3.927666187
HTN	ASPC	*LRRC46*	TRUE	1.963220952
HTN	ASPC	*SCRN2*	TRUE	2.4596661
HTN	ASPC	*C18orf8*	TRUE	2.383746039
HTN	ASPC	*MMEL1*	TRUE	2.199667277
HTN	ASPC	*CREB3L4*	TRUE	3.21851299
HTN	ASPC	*CISD2*	TRUE	2.005465318
HTN	ASPC	*TSLP*	TRUE	2.154473051
HTN	ASPC	*IRAK1BP1*	TRUE	3.651353415
HTN	ASPC	*ZNF215*	TRUE	2.301190514
HTN	ASPC	*HSD17B12*	TRUE	5.398453172
HTN	ASPC	*TNKS1BP1*	TRUE	4.553516485
HTN	ASPC	*TTC12*	TRUE	2.265241577
HTN	ASPC	*PRDM8*	TRUE	2.929587621
HTN	ASPC	*MED21*	TRUE	1.965777734
HTN	ASPC	*NEIL2*	TRUE	2.062730005
HTN	ASPC	*MMP16*	TRUE	2.380364357
HTN	ASPC	*ART3*	TRUE	2.615377469
HTN	ASPC	*SFXN2*	TRUE	5.096879542
HTN	ASPC	*CLEC18A*	TRUE	2.835321149
HTN	ASPC	*ETS2*	TRUE	2.023237169
HTN	ASPC	*SLC5A11*	TRUE	4.50148411
HTN	ASPC	*LRRC36*	TRUE	2.180937448
HTN	ASPC	*ATAD3B*	TRUE	1.998769551
HTN	ASPC	*S100B*	TRUE	3.717466598
HTN	ASPC	*CCR5*	TRUE	2.030703412
HTN	ASPC	*ABCF3*	TRUE	5.05303829
HTN	ASPC	*AQP5*	TRUE	1.984045397
HTN	ASPC	*MRPL55*	TRUE	6.38707822
HTN	ASPC	*LYSMD1*	TRUE	3.558209958
HTN	ASPC	*DNALI1*	TRUE	2.103639922
HTN	ASPC	*RFT1*	TRUE	2.245868828
HTN	ASPC	*RNF123*	TRUE	4.807521271
HTN	ASPC	*ERAP1*	TRUE	2.852447473
HTN	ASPC	*SOX17*	TRUE	2.232612747
HTN	ASPC	*DCSTAMP*	TRUE	3.013615469
HTN	ASPC	*AQP7*	TRUE	2.175888833

HTN	ASPC	*STOX1*	TRUE	2.107868095
HTN	ASPC	*CYB5R2*	TRUE	2.894303686
HTN	ASPC	*TRIM66*	TRUE	3.523247457
HTN	ASPC	*GALR1*	TRUE	2.110262053
HTN	ASPC	*CHAF1A*	TRUE	2.003913013
HTN	ASPC	*SRR*	TRUE	3.546288759
HTN	ASPC	*C8orf46*	TRUE	2.441338261
HTN	ASPC	*CPT1C*	TRUE	4.56197495
HTN	ASPC	*INO80E*	TRUE	3.292211657
HTN	ASPC	*RGPD8*	TRUE	3.135707744
HTN	ASPC	*MYO7B*	TRUE	3.830710136
HTN	ASPC	*DNAJC18*	TRUE	2.800649513
HTN	ASPC	*TTLL6*	TRUE	4.739370465
HTN	ASPC	*PAQR8*	TRUE	2.477022264
HTN	ASPC	*GIMAP8*	TRUE	1.968855277
HTN	ASPC	*MRPL36*	TRUE	2.872347073
HTN	ASPC	*RGS19*	TRUE	4.822157804
HTN	ASPC	*NME6*	TRUE	2.599712961
HTN	ASPC	*ZNF738*	TRUE	1.989495463
HTN	ASPC	*PPP1R14B*	TRUE	2.211246029
HTN	ASPC	*MST1*	TRUE	2.585974174
HTN	ASPC	*C11orf80*	TRUE	2.987250528
HTN	ASPC	*GPR160*	TRUE	2.48729336
HTN	ASPC	*CTU2*	TRUE	2.445883562
HTN	ASPC	*SNX31*	TRUE	4.195645949
HTN	ASPC	*CCNE2*	TRUE	4.863893347
HTN	ASPC	*TMEM9B*	TRUE	2.088599656
HTN	ASPC	*CTDNEP1*	TRUE	2.059974193
HTN	ASPC	*ORAI3*	TRUE	2.890542405
HTN	ASPC	*ODF3B*	TRUE	2.671182563
HTN	ASPC	*GEN1*	TRUE	2.291595294
HTN	ASPC	*MPI*	TRUE	2.407426235
HTN	ASPC	*TRIM73*	TRUE	2.71195608
HTN	ASPC	*HYI*	TRUE	3.91420354
HTN	ASPC	*CTC1*	TRUE	2.679816678
HTN	ASPC	*ZFP41*	TRUE	1.978204395
HTN	ASPC	*ADO*	TRUE	5.725346825
HTN	ASPC	*CLN8*	TRUE	4.157622145
HTN	ASPC	*FES*	TRUE	7.326148597
HTN	ASPC	*ZNF320*	TRUE	3.488457809
HTN	ASPC	*KREMEN1*	TRUE	2.131720054
HTN	ASPC	*ALDH1A3*	TRUE	2.761609486
HTN	ASPC	*A3GALT2*	TRUE	3.889054933
HTN	ASPC	*NUTM2A*	TRUE	2.76596477
HTN	ASPC	*FFAR4*	TRUE	2.489218242
HTN	ASPC	*FOXI2*	TRUE	2.382723749

HTN	ASPC	*KLHL17*	TRUE	3.054816921
HTN	ASPC	*ZNF559*	TRUE	2.662794272
HTN	ASPC	*FZD9*	TRUE	2.581995064
HTN	ASPC	*FAM26F*	TRUE	1.968495273
HTN	ASPC	*FBF1*	TRUE	3.338645358
HTN	ASPC	*FAM47E*	TRUE	3.01099499
HTN	ASPC	*COLCA1*	TRUE	3.234005836
HTN	ASPC	*HRCT1*	TRUE	2.091075404
HTN	ASPC	*ZNF605*	TRUE	2.016307062
HTN	ASPC	*HIST1H3E*	TRUE	5.149133872
HTN	ASPC	*WDR5B*	TRUE	3.317775563
HTN	ASPC	*ZNF100*	TRUE	2.343476888
HTN	ASPC	*SULT1A2*	TRUE	3.282477932
HTN	ASPC	*CCL4L2*	TRUE	2.18670714
HTN	ASPC	*MFAP5*	TRUE	2.90608183
HTN	ASPC	*RPS26*	TRUE	3.831808852
HTN	ASPC	*ZNF311*	TRUE	2.625379818
HTN	ASPC	*AGAP9*	TRUE	2.2598391
HTN	ASPC	*RPS6KL1*	TRUE	2.780186058
HTN	ASPC	*TMEM116*	TRUE	6.722541838
HTN	ASPC	*ZNF442*	TRUE	2.022896238
HTN	ASPC	*PNP*	TRUE	2.642982416
HTN	ASPC	*SHISA4*	TRUE	4.066847069
HTN	ASPC	*MFAP3L*	TRUE	2.045073341
HTN	ASPC	*TGM2*	TRUE	2.35113833
HTN	ASPC	*HSD17B8*	TRUE	2.786625858
HTN	ASPC	*HLA.C*	TRUE	2.446631325
HTN	ASPC	*TMEM170B*	TRUE	2.528542498
HTN	ASPC	*MXD3*	TRUE	2.005155024
HTN	ASPC	*ATF6B*	TRUE	3.562731444
HTN	ASPC	*PPM1N*	TRUE	2.981636131
HTN	ASPC	*GALT*	TRUE	2.062038692
HTN	ASPC	*UBXN2B*	TRUE	2.402985785
HTN	ASPC	*SLC12A8*	TRUE	3.120167895
HTN	ASPC	*TIAF1*	TRUE	4.015108613
HTN	ASPC	*OSER1.AS1*	TRUE	2.224902996
HTN	ASPC	*AC108463.2*	TRUE	2.334457184
HTN	ASPC	*HLA.DQA2*	TRUE	2.625141519
HTN	ASPC	*TNFSF12*	TRUE	3.639806675
HTN	ASPC	*PNMA2*	TRUE	2.061772174
HTN	ASPC	*PLA2G4B*	TRUE	2.136763069
HTN	ASPC	*LINC00638*	TRUE	3.066042024
HTN	ASPC	*ZNF285*	TRUE	1.97377213
HTN	b_cell	*ITIH4*	TRUE	2.337731564
HTN	b_cell	*BIRC7*	TRUE	2.721211191
HTN	b_cell	*CLEC18A*	TRUE	3.979641447

HTN	b_cell	*CTSW*	TRUE	2.961350462
HTN	b_cell	*SNX31*	TRUE	3.01334377
HTN	b_cell	*RPP25*	TRUE	2.334647301
HTN	b_cell	*TRIM73*	TRUE	2.350750298
HTN	b_cell	*ADO*	TRUE	3.073902526
HTN	b_cell	*HIST1H3E*	TRUE	3.788638347
HTN	b_cell	*HLA.DRB5*	TRUE	2.181184461
HTN	b_cell	*POU5F1*	TRUE	2.042488774
HTN	b_cell	*HLA.DQA2*	TRUE	2.378828057
HTN	b_cell	*WFDC6*	TRUE	2.727738113
HTN	dendritic_cell	*CD79B*	TRUE	2.038968464
HTN	dendritic_cell	*MARCH2*	TRUE	2.272451943
HTN	dendritic_cell	*BIRC7*	TRUE	2.647868331
HTN	dendritic_cell	*IRF6*	TRUE	2.588257825
HTN	dendritic_cell	*ACOT2*	TRUE	2.008847351
HTN	dendritic_cell	*TBX2*	TRUE	2.087508422
HTN	dendritic_cell	*TCP11*	TRUE	2.111491818
HTN	dendritic_cell	*RIPK3*	TRUE	2.073288999
HTN	dendritic_cell	*ATP8B3*	TRUE	2.004154768
HTN	dendritic_cell	*HAUS8*	TRUE	3.097272008
HTN	dendritic_cell	*ART3*	TRUE	2.258863779
HTN	dendritic_cell	*CLEC18A*	TRUE	4.272709235
HTN	dendritic_cell	*S100B*	TRUE	2.774752896
HTN	dendritic_cell	*SRR*	TRUE	2.343183585
HTN	dendritic_cell	*RGPD8*	TRUE	2.852558715
HTN	dendritic_cell	*RGS19*	TRUE	3.271962106
HTN	dendritic_cell	*NME6*	TRUE	4.303552563
HTN	dendritic_cell	*CTSW*	TRUE	2.807312867
HTN	dendritic_cell	*SNX31*	TRUE	2.283252563
HTN	dendritic_cell	*CCNE2*	TRUE	3.468665761
HTN	dendritic_cell	*TRIM73*	TRUE	2.724952587
HTN	dendritic_cell	*CLDN7*	TRUE	2.929402979
HTN	dendritic_cell	*ADO*	TRUE	4.821504762
HTN	dendritic_cell	*ZNF320*	TRUE	2.53629936
HTN	dendritic_cell	*A3GALT2*	TRUE	2.097573369
HTN	dendritic_cell	*NUTM2A*	TRUE	2.338858648
HTN	dendritic_cell	*FOXI2*	TRUE	2.072242822
HTN	dendritic_cell	*RPS26*	TRUE	3.378467226
HTN	dendritic_cell	*TMEM116*	TRUE	3.04478332
HTN	dendritic_cell	*TMEM110*	TRUE	2.082355853
HTN	endometrium	*BIRC7*	TRUE	2.911644373
HTN	endometrium	*SGCA*	TRUE	2.215087499
HTN	endometrium	*TCP11*	TRUE	2.313901133
HTN	endometrium	*TNKS1BP1*	TRUE	2.605026664
HTN	endometrium	*SLC5A11*	TRUE	2.848249799
HTN	endometrium	*LYSMD1*	TRUE	2.585950251

HTN	endometrium	*RNF123*	TRUE	2.12927781
HTN	endometrium	*TRIM66*	TRUE	2.203638872
HTN	endometrium	*MST1*	TRUE	1.986628308
HTN	endometrium	*RPP25*	TRUE	2.783645052
HTN	endometrium	*TRIM73*	TRUE	2.485229901
HTN	endometrium	*NUTM2A*	TRUE	2.970117728
HTN	endometrium	*HIST1H3E*	TRUE	3.982078319
HTN	endometrium	*TMEM116*	TRUE	5.535716198
HTN	endometrium	*PSORS1C2*	TRUE	2.549470777
HTN	endometrium	*WFDC6*	TRUE	2.830936326
HTN	endometrium	*PLA2G4B*	TRUE	2.203686355
HTN	endothelial	*BIRC7*	TRUE	2.420947371
HTN	endothelial	*SGCA*	TRUE	2.480957392
HTN	endothelial	*ACOT2*	TRUE	2.096649876
HTN	endothelial	*TBX2*	TRUE	3.457755059
HTN	endothelial	*BTN2A2*	TRUE	1.971083338
HTN	endothelial	*TCP11*	TRUE	2.337068479
HTN	endothelial	*AGT*	TRUE	2.10319135
HTN	endothelial	*FBN2*	TRUE	3.09952747
HTN	endothelial	*HSD17B12*	TRUE	3.230942993
HTN	endothelial	*NEIL2*	TRUE	2.957606198
HTN	endothelial	*ART3*	TRUE	2.067022194
HTN	endothelial	*SFXN2*	TRUE	2.292553073
HTN	endothelial	*CLEC18A*	TRUE	3.873919891
HTN	endothelial	*SLC5A11*	TRUE	2.606118974
HTN	endothelial	*S100B*	TRUE	2.478893813
HTN	endothelial	*LYSMD1*	TRUE	2.210840508
HTN	endothelial	*RNF123*	TRUE	2.340620671
HTN	endothelial	*NYAP1*	TRUE	2.030209941
HTN	endothelial	*SRR*	TRUE	2.511976184
HTN	endothelial	*CPT1C*	TRUE	2.054424293
HTN	endothelial	*INO80E*	TRUE	2.18583022
HTN	endothelial	*RGPD8*	TRUE	2.603448053
HTN	endothelial	*TTLL6*	TRUE	4.031628652
HTN	endothelial	*RGS19*	TRUE	3.826551118
HTN	endothelial	*NME6*	TRUE	2.682821071
HTN	endothelial	*CTSW*	TRUE	3.637866512
HTN	endothelial	*MST1*	TRUE	2.576882222
HTN	endothelial	*SNX31*	TRUE	3.560378166
HTN	endothelial	*TRIM73*	TRUE	2.001007844
HTN	endothelial	*HYI*	TRUE	2.067287729
HTN	endothelial	*ADO*	TRUE	3.955479914
HTN	endothelial	*CLN8*	TRUE	2.333484378
HTN	endothelial	*ZNF320*	TRUE	2.478275386
HTN	endothelial	*A3GALT2*	TRUE	2.106961196
HTN	endothelial	*NUTM2A*	TRUE	3.532025975

HTN	endothelial	*HIST1H3E*	TRUE	4.701108485
HTN	endothelial	*RPS26*	TRUE	2.964321273
HTN	endothelial	*TMEM116*	TRUE	4.1157047
HTN	endothelial	*PRRT1*	TRUE	3.262299271
HTN	endothelial	*AC016773.1*	TRUE	1.986506711
HTN	endothelial	*HLA.DQA2*	TRUE	2.279608058
HTN	endothelial	*PLA2G4B*	TRUE	2.305004428
HTN	LEC	*CD79B*	TRUE	2.080200043
HTN	LEC	*PIGV*	TRUE	1.974051569
HTN	LEC	*MARCH2*	TRUE	2.688593991
HTN	LEC	*BIRC7*	TRUE	2.438204851
HTN	LEC	*IRF6*	TRUE	2.341086258
HTN	LEC	*ZNF391*	TRUE	2.866849744
HTN	LEC	*RSG1*	TRUE	2.09256159
HTN	LEC	*AGT*	TRUE	3.129571125
HTN	LEC	*SLC37A4*	TRUE	2.608320586
HTN	LEC	*FBN2*	TRUE	2.781802631
HTN	LEC	*SCRN2*	TRUE	2.032052422
HTN	LEC	*HSD17B12*	TRUE	4.477639126
HTN	LEC	*NEIL2*	TRUE	3.342979615
HTN	LEC	*PROM2*	TRUE	2.257522235
HTN	LEC	*SFXN2*	TRUE	4.505455698
HTN	LEC	*CLEC18A*	TRUE	2.142800493
HTN	LEC	*SLC5A11*	TRUE	3.200786124
HTN	LEC	*S100B*	TRUE	2.767443346
HTN	LEC	*ABCF3*	TRUE	2.634212031
HTN	LEC	*LYSMD1*	TRUE	3.362868657
HTN	LEC	*TRIM66*	TRUE	2.116265539
HTN	LEC	*SRR*	TRUE	2.826534542
HTN	LEC	*CPT1C*	TRUE	4.261980169
HTN	LEC	*INO80E*	TRUE	4.154307605
HTN	LEC	*RGPD8*	TRUE	2.931196736
HTN	LEC	*MYO7B*	TRUE	2.829383353
HTN	LEC	*ADORA2B*	TRUE	1.981358505
HTN	LEC	*TTLL6*	TRUE	3.312274512
HTN	LEC	*MRPL36*	TRUE	2.300258918
HTN	LEC	*RGS19*	TRUE	4.51696302
HTN	LEC	*NME6*	TRUE	2.142975104
HTN	LEC	*CTSW*	TRUE	2.621487881
HTN	LEC	*MST1*	TRUE	2.636756456
HTN	LEC	*CTU2*	TRUE	2.366371241
HTN	LEC	*SNX31*	TRUE	2.858046028
HTN	LEC	*CCNE2*	TRUE	2.728835333
HTN	LEC	*ORAI3*	TRUE	3.428765229
HTN	LEC	*MPI*	TRUE	3.692143859
HTN	LEC	*TRIM73*	TRUE	2.305710866

HTN	LEC	*HYI*	TRUE	2.21242126
HTN	LEC	*ADO*	TRUE	2.00406746
HTN	LEC	*CLN8*	TRUE	3.386705831
HTN	LEC	*FES*	TRUE	4.576868508
HTN	LEC	*ZNF320*	TRUE	2.649463575
HTN	LEC	*NPIPA1*	TRUE	1.988556935
HTN	LEC	*A3GALT2*	TRUE	2.663506074
HTN	LEC	*NUTM2A*	TRUE	3.240745568
HTN	LEC	*HRCT1*	TRUE	2.737732512
HTN	LEC	*HIST1H3E*	TRUE	3.546277477
HTN	LEC	*RPS26*	TRUE	3.643225299
HTN	LEC	*TMEM116*	TRUE	5.232096279
HTN	LEC	*HLA.DRB5*	TRUE	2.695689624
HTN	LEC	*PNP*	TRUE	2.428348078
HTN	LEC	*HLA.C*	TRUE	2.174518335
HTN	LEC	*TIAF1*	TRUE	3.278877787
HTN	LEC	*TNFSF12*	TRUE	2.946806843
HTN	macrophage	*SDCCAG8*	TRUE	2.125033662
HTN	macrophage	*PIGV*	TRUE	3.690352898
HTN	macrophage	*UHRF1BP1*	TRUE	3.17700991
HTN	macrophage	*MUL1*	TRUE	2.02341487
HTN	macrophage	*SCD*	TRUE	2.409761406
HTN	macrophage	*MARCH2*	TRUE	3.089468026
HTN	macrophage	*BIRC7*	TRUE	3.00803116
HTN	macrophage	*POLI*	TRUE	3.025046681
HTN	macrophage	*MTHFSD*	TRUE	2.527488935
HTN	macrophage	*ICAM5*	TRUE	2.273076076
HTN	macrophage	*SGCA*	TRUE	2.738929947
HTN	macrophage	*PCCB*	TRUE	3.270492755
HTN	macrophage	*IRF6*	TRUE	2.231491999
HTN	macrophage	*ACOT2*	TRUE	2.478366166
HTN	macrophage	*TBX2*	TRUE	4.731890892
HTN	macrophage	*BTN2A2*	TRUE	3.008632919
HTN	macrophage	*ZNF391*	TRUE	1.982117966
HTN	macrophage	*GRK4*	TRUE	2.185842901
HTN	macrophage	*TSKS*	TRUE	2.023923825
HTN	macrophage	*TRAP1*	TRUE	2.658803742
HTN	macrophage	*ABHD8*	TRUE	2.352271473
HTN	macrophage	*ATP8B3*	TRUE	2.856808181
HTN	macrophage	*PGPEP1*	TRUE	2.087707422
HTN	macrophage	*HAUS8*	TRUE	3.692602002
HTN	macrophage	*NMT1*	TRUE	2.675295333
HTN	macrophage	*SLC37A4*	TRUE	2.452969994
HTN	macrophage	*FBN2*	TRUE	2.607295654
HTN	macrophage	*RPL3L*	TRUE	1.9831937
HTN	macrophage	*SCRN2*	TRUE	2.350563491

HTN	macrophage	*MMEL1*	TRUE	2.023657695
HTN	macrophage	*CREB3L4*	TRUE	3.252057361
HTN	macrophage	*TSLP*	TRUE	2.191329544
HTN	macrophage	*ZNF215*	TRUE	2.042782127
HTN	macrophage	*HSD17B12*	TRUE	5.252932834
HTN	macrophage	*NEIL2*	TRUE	4.973662718
HTN	macrophage	*ART3*	TRUE	2.747915843
HTN	macrophage	*SFXN2*	TRUE	3.96744111
HTN	macrophage	*CLEC18A*	TRUE	3.03443237
HTN	macrophage	*SLC5A11*	TRUE	4.467514943
HTN	macrophage	*S100B*	TRUE	3.547471445
HTN	macrophage	*ABCF3*	TRUE	4.60867629
HTN	macrophage	*MRPL55*	TRUE	4.565656013
HTN	macrophage	*LYSMD1*	TRUE	2.903942403
HTN	macrophage	*RFT1*	TRUE	2.247536501
HTN	macrophage	*RNF123*	TRUE	4.468003947
HTN	macrophage	*RPP25L*	TRUE	2.017080706
HTN	macrophage	*CYB5R2*	TRUE	2.0516688
HTN	macrophage	*TRIM66*	TRUE	3.313059354
HTN	macrophage	*RIMKLB*	TRUE	2.914676305
HTN	macrophage	*SRR*	TRUE	3.347950416
HTN	macrophage	*INO80E*	TRUE	4.73221386
HTN	macrophage	*RGPD8*	TRUE	3.073000684
HTN	macrophage	*MUC3A*	TRUE	2.170610946
HTN	macrophage	*TTLL6*	TRUE	4.269999224
HTN	macrophage	*MRPL36*	TRUE	2.482288698
HTN	macrophage	*CTSW*	TRUE	2.660826873
HTN	macrophage	*MST1*	TRUE	2.994033367
HTN	macrophage	*CTU2*	TRUE	2.378634967
HTN	macrophage	*SNX31*	TRUE	4.298224057
HTN	macrophage	*CCNE2*	TRUE	4.267682815
HTN	macrophage	*ORAI3*	TRUE	3.748747995
HTN	macrophage	*MPI*	TRUE	6.14290418
HTN	macrophage	*TRIM73*	TRUE	2.438889537
HTN	macrophage	*HYI*	TRUE	3.535858986
HTN	macrophage	*ADO*	TRUE	5.240328578
HTN	macrophage	*ZNF320*	TRUE	3.296769715
HTN	macrophage	*A3GALT2*	TRUE	3.440268603
HTN	macrophage	*NUTM2A*	TRUE	3.326293316
HTN	macrophage	*FOXI2*	TRUE	2.041594452
HTN	macrophage	*KLHL17*	TRUE	2.492852279
HTN	macrophage	*ZNF559*	TRUE	2.440255937
HTN	macrophage	*HIST1H3E*	TRUE	4.955654048
HTN	macrophage	*WDR5B*	TRUE	2.101679217
HTN	macrophage	*SULT1A2*	TRUE	2.665587352
HTN	macrophage	*C6orf141*	TRUE	2.226302529

HTN	macrophage	*RPS26*	TRUE	3.528943225
HTN	macrophage	*ZNF311*	TRUE	2.01545452
HTN	macrophage	*TMEM116*	TRUE	6.257741832
HTN	macrophage	*SHISA4*	TRUE	4.23325889
HTN	macrophage	*HSD17B8*	TRUE	2.874842862
HTN	macrophage	*HLA.C*	TRUE	2.871395337
HTN	macrophage	*MXD3*	TRUE	2.052007359
HTN	macrophage	*ATF6B*	TRUE	2.787946033
HTN	macrophage	*AC016773.1*	TRUE	2.395263267
HTN	macrophage	*TIAF1*	TRUE	3.238551484
HTN	macrophage	*LINC00092*	TRUE	1.981753568
HTN	macrophage	*HLA.DQA2*	TRUE	2.537300245
HTN	macrophage	*TNFSF12*	TRUE	3.648512839
HTN	macrophage	*LINC00638*	TRUE	2.089196995
HTN	mast_cell	*SGCA*	TRUE	2.137589653
HTN	mast_cell	*TEX14*	TRUE	2.999314022
HTN	mast_cell	*BTN2A2*	TRUE	3.466737603
HTN	mast_cell	*RIPK3*	TRUE	1.974092161
HTN	mast_cell	*HAUS8*	TRUE	3.329138521
HTN	mast_cell	*FBN2*	TRUE	3.151726252
HTN	mast_cell	*NEIL2*	TRUE	2.266136228
HTN	mast_cell	*CLEC18A*	TRUE	4.33944633
HTN	mast_cell	*SLC5A11*	TRUE	4.397678502
HTN	mast_cell	*S100B*	TRUE	2.902600954
HTN	mast_cell	*SRR*	TRUE	2.572133529
HTN	mast_cell	*RGS19*	TRUE	4.510679984
HTN	mast_cell	*CTSW*	TRUE	3.014250972
HTN	mast_cell	*SNX31*	TRUE	2.362241674
HTN	mast_cell	*SEZ6L2*	TRUE	1.999520193
HTN	mast_cell	*ORAI3*	TRUE	2.302779354
HTN	mast_cell	*RPP25*	TRUE	2.829928162
HTN	mast_cell	*ZNF320*	TRUE	1.987669414
HTN	mast_cell	*ACOT1*	TRUE	2.11354592
HTN	mast_cell	*A3GALT2*	TRUE	2.146520614
HTN	mast_cell	*NUTM2A*	TRUE	3.274808122
HTN	mast_cell	*HIST1H3E*	TRUE	3.99715597
HTN	mast_cell	*PCDHB11*	TRUE	1.966532706
HTN	mast_cell	*TMEM116*	TRUE	3.324055673
HTN	mast_cell	*PPM1N*	TRUE	2.129165037
HTN	mast_cell	*HLA.DQA2*	TRUE	2.197077505
HTN	mast_cell	*TNFSF12*	TRUE	3.165886478
HTN	mesothelium	*M6PR*	TRUE	2.265014751
HTN	mesothelium	*ARHGAP33*	TRUE	2.144287037
HTN	mesothelium	*CD79B*	TRUE	2.372758825
HTN	mesothelium	*CEP68*	TRUE	3.300829473
HTN	mesothelium	*RNASET2*	TRUE	2.819208402

HTN	mesothelium	*CENPQ*	TRUE	2.112719443
HTN	mesothelium	*SDCCAG8*	TRUE	3.891994141
HTN	mesothelium	*PIGV*	TRUE	4.226725255
HTN	mesothelium	*POLR1A*	TRUE	3.067920596
HTN	mesothelium	*SNX10*	TRUE	4.346132808
HTN	mesothelium	*AAMDC*	TRUE	3.257907752
HTN	mesothelium	*MUL1*	TRUE	2.762118826
HTN	mesothelium	*PABPC4*	TRUE	3.956733537
HTN	mesothelium	*RGS17*	TRUE	2.111850996
HTN	mesothelium	*ITPR3*	TRUE	2.126780502
HTN	mesothelium	*SCD*	TRUE	3.980970734
HTN	mesothelium	*MARCH2*	TRUE	3.554366062
HTN	mesothelium	*THOC5*	TRUE	2.345655541
HTN	mesothelium	*FAM118A*	TRUE	1.961280969
HTN	mesothelium	*BIRC7*	TRUE	2.920585028
HTN	mesothelium	*SIRPB1*	TRUE	2.244215523
HTN	mesothelium	*POLI*	TRUE	3.59386901
HTN	mesothelium	*MTHFSD*	TRUE	3.187962983
HTN	mesothelium	*LIN7B*	TRUE	2.61281731
HTN	mesothelium	*PEX11G*	TRUE	2.023086148
HTN	mesothelium	*ICAM5*	TRUE	2.869019542
HTN	mesothelium	*MEST*	TRUE	3.019700606
HTN	mesothelium	*SLC1A1*	TRUE	1.993114069
HTN	mesothelium	*SGCA*	TRUE	2.66764968
HTN	mesothelium	*CCND1*	TRUE	4.257719269
HTN	mesothelium	*C11orf21*	TRUE	2.052950368
HTN	mesothelium	*BVES*	TRUE	2.037778699
HTN	mesothelium	*NME5*	TRUE	2.389406963
HTN	mesothelium	*BNIP1*	TRUE	2.073267871
HTN	mesothelium	*NPHP3*	TRUE	2.169113153
HTN	mesothelium	*PCCB*	TRUE	4.284053651
HTN	mesothelium	*IRF6*	TRUE	2.752773345
HTN	mesothelium	*ELOVL4*	TRUE	2.046448684
HTN	mesothelium	*ACOT2*	TRUE	2.909619278
HTN	mesothelium	*ZFP30*	TRUE	2.15909632
HTN	mesothelium	*ADRA1A*	TRUE	2.227150551
HTN	mesothelium	*TBX2*	TRUE	5.273790542
HTN	mesothelium	*TEX14*	TRUE	3.711703078
HTN	mesothelium	*TOX2*	TRUE	2.284230523
HTN	mesothelium	*BTN2A2*	TRUE	3.247350346
HTN	mesothelium	*ZNF391*	TRUE	2.402738471
HTN	mesothelium	*GRK4*	TRUE	2.872207393
HTN	mesothelium	*UROD*	TRUE	2.113083779
HTN	mesothelium	*TSKS*	TRUE	2.590018496
HTN	mesothelium	*TRAP1*	TRUE	4.257279353
HTN	mesothelium	*ABHD8*	TRUE	3.587489268

HTN	mesothelium	*GFER*	TRUE	2.009637668
HTN	mesothelium	*STYXL1*	TRUE	2.067873585
HTN	mesothelium	*GAD1*	TRUE	2.324817519
HTN	mesothelium	*EIF2AK4*	TRUE	2.147480875
HTN	mesothelium	*ATP8B3*	TRUE	3.225429908
HTN	mesothelium	*PGPEP1*	TRUE	2.539348241
HTN	mesothelium	*HAUS8*	TRUE	5.150969613
HTN	mesothelium	*RSG1*	TRUE	2.138982719
HTN	mesothelium	*ACP2*	TRUE	4.209128271
HTN	mesothelium	*AGT*	TRUE	3.208536548
HTN	mesothelium	*SLC37A4*	TRUE	2.582526565
HTN	mesothelium	*PPP2R1B*	TRUE	2.937058548
HTN	mesothelium	*NUSAP1*	TRUE	2.850573539
HTN	mesothelium	*ENPEP*	TRUE	2.224083796
HTN	mesothelium	*FBN2*	TRUE	3.872248945
HTN	mesothelium	*FITM1*	TRUE	2.320644678
HTN	mesothelium	*RTN1*	TRUE	2.175010545
HTN	mesothelium	*HAPLN3*	TRUE	2.415673463
HTN	mesothelium	*RPL3L*	TRUE	2.265278574
HTN	mesothelium	*DEF8*	TRUE	2.475615461
HTN	mesothelium	*VPS53*	TRUE	2.050403341
HTN	mesothelium	*LRRC46*	TRUE	2.360758277
HTN	mesothelium	*SCRN2*	TRUE	2.657303623
HTN	mesothelium	*C18orf8*	TRUE	3.173706524
HTN	mesothelium	*CREB3L4*	TRUE	2.591243039
HTN	mesothelium	*PLEKHA6*	TRUE	2.833566862
HTN	mesothelium	*TSLP*	TRUE	2.450540339
HTN	mesothelium	*IRAK1BP1*	TRUE	3.251435664
HTN	mesothelium	*SLC18B1*	TRUE	2.596004277
HTN	mesothelium	*ZNF215*	TRUE	2.239531843
HTN	mesothelium	*HSD17B12*	TRUE	5.831481044
HTN	mesothelium	*TNKS1BP1*	TRUE	4.592866128
HTN	mesothelium	*TTC12*	TRUE	2.201297683
HTN	mesothelium	*DUSP15*	TRUE	2.022543175
HTN	mesothelium	*TRPT1*	TRUE	2.01140731
HTN	mesothelium	*PRDM8*	TRUE	2.708981459
HTN	mesothelium	*NEIL2*	TRUE	3.67279926
HTN	mesothelium	*PROM2*	TRUE	2.310777291
HTN	mesothelium	*MMP16*	TRUE	2.395653427
HTN	mesothelium	*ART3*	TRUE	2.681255732
HTN	mesothelium	*SFXN2*	TRUE	5.04246131
HTN	mesothelium	*SLC5A11*	TRUE	4.753307468
HTN	mesothelium	*HSF2BP*	TRUE	1.99111381
HTN	mesothelium	*S100B*	TRUE	3.746622007
HTN	mesothelium	*ZNF208*	TRUE	2.160863206
HTN	mesothelium	*ABCF3*	TRUE	4.89676831

HTN	mesothelium	*MRPL55*	TRUE	5.970439209
HTN	mesothelium	*LYSMD1*	TRUE	3.036686752
HTN	mesothelium	*DCAF16*	TRUE	3.463302357
HTN	mesothelium	*RFT1*	TRUE	2.91326618
HTN	mesothelium	*ZNF589*	TRUE	2.355012266
HTN	mesothelium	*RNF123*	TRUE	3.821670113
HTN	mesothelium	*ERAP1*	TRUE	4.181951695
HTN	mesothelium	*RPP25L*	TRUE	2.766904829
HTN	mesothelium	*OXGR1*	TRUE	2.159258012
HTN	mesothelium	*CASP7*	TRUE	2.081899399
HTN	mesothelium	*ARL5B*	TRUE	3.096952794
HTN	mesothelium	*CYB5R2*	TRUE	2.685304652
HTN	mesothelium	*TRIM66*	TRUE	3.977733185
HTN	mesothelium	*RIMKLB*	TRUE	4.772893138
HTN	mesothelium	*GALR1*	TRUE	2.071509847
HTN	mesothelium	*ZNF23*	TRUE	2.150566708
HTN	mesothelium	*CHAF1A*	TRUE	2.221654445
HTN	mesothelium	*SRR*	TRUE	3.400853518
HTN	mesothelium	*SOAT2*	TRUE	2.771917559
HTN	mesothelium	*ZNF598*	TRUE	2.519151086
HTN	mesothelium	*SERINC2*	TRUE	2.623954594
HTN	mesothelium	*TCTN2*	TRUE	1.998122515
HTN	mesothelium	*CPT1C*	TRUE	3.361721952
HTN	mesothelium	*RAB24*	TRUE	2.85770103
HTN	mesothelium	*INO80E*	TRUE	4.904382954
HTN	mesothelium	*RGPD8*	TRUE	3.305098168
HTN	mesothelium	*MUC3A*	TRUE	2.043563003
HTN	mesothelium	*MYO7B*	TRUE	3.304810865
HTN	mesothelium	*DNAJC18*	TRUE	3.299218843
HTN	mesothelium	*SMAGP*	TRUE	1.970706015
HTN	mesothelium	*TTLL6*	TRUE	5.144097006
HTN	mesothelium	*PAQR8*	TRUE	2.71065598
HTN	mesothelium	*MRPL36*	TRUE	2.7907448
HTN	mesothelium	*RGS19*	TRUE	5.317990145
HTN	mesothelium	*CTSW*	TRUE	3.056707339
HTN	mesothelium	*MST1*	TRUE	2.733658889
HTN	mesothelium	*C11orf80*	TRUE	4.170399444
HTN	mesothelium	*CTU2*	TRUE	2.279777808
HTN	mesothelium	*SNX31*	TRUE	4.453537491
HTN	mesothelium	*ORAI3*	TRUE	4.240134955
HTN	mesothelium	*MTHFR*	TRUE	3.57555457
HTN	mesothelium	*ODF3B*	TRUE	2.457935974
HTN	mesothelium	*GEN1*	TRUE	2.866119181
HTN	mesothelium	*MPI*	TRUE	7.049378809
HTN	mesothelium	*HYI*	TRUE	3.438494912
HTN	mesothelium	*CTC1*	TRUE	2.190241116

HTN	mesothelium	*CLDN7*	TRUE	2.871481279
HTN	mesothelium	*ADO*	TRUE	5.636459714
HTN	mesothelium	*CLN8*	TRUE	3.820611085
HTN	mesothelium	*FES*	TRUE	8.126442456
HTN	mesothelium	*ZNF320*	TRUE	3.513871467
HTN	mesothelium	*NPIPA1*	TRUE	2.10732923
HTN	mesothelium	*ACOT1*	TRUE	2.429985604
HTN	mesothelium	*ALDH1A3*	TRUE	2.048312091
HTN	mesothelium	*A3GALT2*	TRUE	3.917055524
HTN	mesothelium	*NUTM2A*	TRUE	3.069988445
HTN	mesothelium	*FOXI2*	TRUE	2.371788932
HTN	mesothelium	*KLHL17*	TRUE	1.986023316
HTN	mesothelium	*ZNF559*	TRUE	2.772896236
HTN	mesothelium	*FAM26F*	TRUE	2.551136536
HTN	mesothelium	*FBF1*	TRUE	3.90600605
HTN	mesothelium	*MSL1*	TRUE	1.980770889
HTN	mesothelium	*COLCA1*	TRUE	3.231820363
HTN	mesothelium	*HRCT1*	TRUE	3.010000524
HTN	mesothelium	*HIST1H3E*	TRUE	5.006394582
HTN	mesothelium	*WDR5B*	TRUE	2.863339783
HTN	mesothelium	*ZNF100*	TRUE	3.143261928
HTN	mesothelium	*SULT1A2*	TRUE	3.35693663
HTN	mesothelium	*C6orf141*	TRUE	2.418830318
HTN	mesothelium	*PCDHB11*	TRUE	1.985192912
HTN	mesothelium	*RPS26*	TRUE	3.875741755
HTN	mesothelium	*ZNF311*	TRUE	2.34911183
HTN	mesothelium	*AGAP9*	TRUE	2.472549298
HTN	mesothelium	*TMEM116*	TRUE	6.599013798
HTN	mesothelium	*PNP*	TRUE	2.910954223
HTN	mesothelium	*SHISA4*	TRUE	5.249397533
HTN	mesothelium	*AL590822.1*	TRUE	2.48249547
HTN	mesothelium	*HSD17B8*	TRUE	3.015218973
HTN	mesothelium	*HLA.C*	TRUE	3.180796091
HTN	mesothelium	*TMEM170B*	TRUE	2.02296282
HTN	mesothelium	*ATF6B*	TRUE	3.317193777
HTN	mesothelium	*PPM1N*	TRUE	2.617416632
HTN	mesothelium	*LTB4R*	TRUE	2.572503815
HTN	mesothelium	*AC016773.1*	TRUE	1.982013011
HTN	mesothelium	*SLC12A8*	TRUE	2.532098871
HTN	mesothelium	*TIAF1*	TRUE	3.651650085
HTN	mesothelium	*OSER1.AS1*	TRUE	2.160095723
HTN	mesothelium	*AL049840.1*	TRUE	2.711612665
HTN	mesothelium	*PLEKHM1*	TRUE	2.1002566
HTN	mesothelium	*LINC00092*	TRUE	2.015537973
HTN	mesothelium	*AC108463.2*	TRUE	2.317732837
HTN	mesothelium	*HLA.DQA2*	TRUE	2.149108734

HTN	mesothelium	*LINC00211*	TRUE	2.178782343
HTN	mesothelium	*TNFSF12*	TRUE	3.222864054
HTN	mesothelium	*ZNF709*	TRUE	3.238936469
HTN	mesothelium	*LINC00638*	TRUE	2.818521138
HTN	monocyte	*ITIH4*	TRUE	2.076290437
HTN	monocyte	*PIGV*	TRUE	2.870135536
HTN	monocyte	*BIRC7*	TRUE	2.211461404
HTN	monocyte	*MTHFSD*	TRUE	1.981564632
HTN	monocyte	*SGCA*	TRUE	2.396120266
HTN	monocyte	*WFDC3*	TRUE	2.093977117
HTN	monocyte	*BTN2A2*	TRUE	2.367654335
HTN	monocyte	*ZNF391*	TRUE	2.205224463
HTN	monocyte	*AGT*	TRUE	2.947221424
HTN	monocyte	*SFXN2*	TRUE	4.03803994
HTN	monocyte	*CLEC18A*	TRUE	3.382491918
HTN	monocyte	*CDC25A*	TRUE	3.760130428
HTN	monocyte	*TRIM66*	TRUE	1.996957122
HTN	monocyte	*TTLL6*	TRUE	2.027189089
HTN	monocyte	*MST1*	TRUE	2.611964611
HTN	monocyte	*ORAI3*	TRUE	2.380487509
HTN	monocyte	*MPI*	TRUE	2.099034953
HTN	monocyte	*HYI*	TRUE	2.86603087
HTN	monocyte	*CLN8*	TRUE	2.910777192
HTN	monocyte	*ZNF320*	TRUE	2.209155187
HTN	monocyte	*NUTM2A*	TRUE	3.172552487
HTN	monocyte	*HIST1H3E*	TRUE	3.124326084
HTN	monocyte	*TMEM116*	TRUE	4.094161259
HTN	monocyte	*HLA.DQA2*	TRUE	2.171903058
HTN	nk_cell	*BTN2A2*	TRUE	1.970700341
HTN	nk_cell	*HSD17B12*	TRUE	2.064445544
HTN	nk_cell	*PROM2*	TRUE	2.245570601
HTN	nk_cell	*CLEC18A*	TRUE	3.227513839
HTN	nk_cell	*ABCF3*	TRUE	3.442639232
HTN	nk_cell	*INO80E*	TRUE	2.444106294
HTN	nk_cell	*RGPD8*	TRUE	2.678321476
HTN	nk_cell	*SNX31*	TRUE	3.771776745
HTN	nk_cell	*MPI*	TRUE	3.54179157
HTN	nk_cell	*TRIM73*	TRUE	2.04783346
HTN	nk_cell	*ADO*	TRUE	4.117028702
HTN	nk_cell	*TMEM116*	TRUE	3.933150263
HTN	nk_cell	*PRRT1*	TRUE	2.115499025
HTN	nk_cell	*TIAF1*	TRUE	2.550622039
HTN	pericyte	*PIGV*	TRUE	2.62694283
HTN	pericyte	*BIRC7*	TRUE	2.863172675
HTN	pericyte	*POLI*	TRUE	2.916434869
HTN	pericyte	*IRF6*	TRUE	2.289746756

HTN	pericyte	*BTN2A2*	TRUE	2.769105244
HTN	pericyte	*TRAP1*	TRUE	2.741459131
HTN	pericyte	*ATP8B3*	TRUE	2.442505336
HTN	pericyte	*HAUS8*	TRUE	2.941553722
HTN	pericyte	*ACP2*	TRUE	2.687864004
HTN	pericyte	*AGT*	TRUE	3.079003464
HTN	pericyte	*FBN2*	TRUE	3.286760654
HTN	pericyte	*SCRN2*	TRUE	2.253706413
HTN	pericyte	*CREB3L4*	TRUE	2.750866756
HTN	pericyte	*TSLP*	TRUE	2.188873181
HTN	pericyte	*HSD17B12*	TRUE	4.990231152
HTN	pericyte	*TNKS1BP1*	TRUE	2.406338707
HTN	pericyte	*ART3*	TRUE	2.527941033
HTN	pericyte	*SFXN2*	TRUE	4.160144999
HTN	pericyte	*SLC5A11*	TRUE	3.52126529
HTN	pericyte	*S100B*	TRUE	3.43717788
HTN	pericyte	*ABCF3*	TRUE	4.154620577
HTN	pericyte	*MRPL55*	TRUE	2.589384938
HTN	pericyte	*LYSMD1*	TRUE	2.760634358
HTN	pericyte	*TRIM66*	TRUE	2.943141171
HTN	pericyte	*RIMKLB*	TRUE	2.48088179
HTN	pericyte	*SRR*	TRUE	2.540858189
HTN	pericyte	*CPT1C*	TRUE	3.420395051
HTN	pericyte	*INO80E*	TRUE	3.399426563
HTN	pericyte	*RGPD8*	TRUE	2.857518001
HTN	pericyte	*TTLL6*	TRUE	2.796788506
HTN	pericyte	*PAQR8*	TRUE	2.206501093
HTN	pericyte	*MRPL36*	TRUE	1.96296958
HTN	pericyte	*RGS19*	TRUE	4.383390943
HTN	pericyte	*CTSW*	TRUE	3.035770685
HTN	pericyte	*MST1*	TRUE	3.76470951
HTN	pericyte	*CCNE2*	TRUE	3.875491386
HTN	pericyte	*ORAI3*	TRUE	3.275103142
HTN	pericyte	*GEN1*	TRUE	2.081561107
HTN	pericyte	*MPI*	TRUE	5.965724376
HTN	pericyte	*ADO*	TRUE	3.939246675
HTN	pericyte	*CLN8*	TRUE	2.55265505
HTN	pericyte	*FES*	TRUE	4.786941114
HTN	pericyte	*ZNF320*	TRUE	3.125634288
HTN	pericyte	*A3GALT2*	TRUE	3.101456706
HTN	pericyte	*NUTM2A*	TRUE	3.60377747
HTN	pericyte	*KLHL17*	TRUE	2.179213432
HTN	pericyte	*ZNF559*	TRUE	2.110899784
HTN	pericyte	*FAM26F*	TRUE	2.165826888
HTN	pericyte	*HRCT1*	TRUE	2.552921827
HTN	pericyte	*HIST1H3E*	TRUE	5.024049823

HTN	pericyte	*C6orf141*	TRUE	2.207564642
HTN	pericyte	*RPS26*	TRUE	3.703787658
HTN	pericyte	*TMEM116*	TRUE	5.887087301
HTN	pericyte	*PNP*	TRUE	2.48071754
HTN	pericyte	*HSD17B8*	TRUE	2.671991996
HTN	pericyte	*HLA.C*	TRUE	2.509483937
HTN	pericyte	*ATF6B*	TRUE	2.036166664
HTN	pericyte	*AC016773.1*	TRUE	2.767947295
HTN	pericyte	*TIAF1*	TRUE	2.932268141
HTN	pericyte	*LINC00960*	TRUE	2.170227653
HTN	SMC	*CD79B*	TRUE	2.393903716
HTN	SMC	*POLI*	TRUE	2.864734473
HTN	SMC	*ACOT2*	TRUE	2.288525816
HTN	SMC	*ZNF391*	TRUE	2.850517536
HTN	SMC	*GRK4*	TRUE	2.31512947
HTN	SMC	*ATP8B3*	TRUE	2.707846648
HTN	SMC	*HAUS8*	TRUE	2.340055123
HTN	SMC	*AGT*	TRUE	3.047170908
HTN	SMC	*LRRC46*	TRUE	2.168406963
HTN	SMC	*MMEL1*	TRUE	2.179011124
HTN	SMC	*CREB3L4*	TRUE	2.44613212
HTN	SMC	*HSD17B12*	TRUE	5.069684963
HTN	SMC	*TNKS1BP1*	TRUE	2.075589383
HTN	SMC	*NEIL2*	TRUE	3.904919661
HTN	SMC	*SFXN2*	TRUE	3.058294937
HTN	SMC	*SLC5A11*	TRUE	3.746520686
HTN	SMC	*S100B*	TRUE	3.06807955
HTN	SMC	*ABCF3*	TRUE	3.619118777
HTN	SMC	*ZNF589*	TRUE	3.125423388
HTN	SMC	*TRIM66*	TRUE	2.786000358
HTN	SMC	*SRR*	TRUE	2.653228625
HTN	SMC	*INO80E*	TRUE	2.67864931
HTN	SMC	*RGPD8*	TRUE	2.938679843
HTN	SMC	*TTLL6*	TRUE	4.651732067
HTN	SMC	*RGS19*	TRUE	3.271131807
HTN	SMC	*CTSW*	TRUE	2.312283187
HTN	SMC	*MST1*	TRUE	2.411468799
HTN	SMC	*SNX31*	TRUE	2.704548375
HTN	SMC	*CCNE2*	TRUE	2.447543306
HTN	SMC	*MPI*	TRUE	3.691834227
HTN	SMC	*HYI*	TRUE	3.521057719
HTN	SMC	*FES*	TRUE	5.490366263
HTN	SMC	*A3GALT2*	TRUE	2.452549273
HTN	SMC	*NUTM2A*	TRUE	3.330076026
HTN	SMC	*CDK10*	TRUE	2.700499509
HTN	SMC	*GLDN*	TRUE	2.218114887

HTN	SMC	*HIST1H3E*	TRUE	3.302897741
HTN	SMC	*TMEM116*	TRUE	6.310479446
HTN	SMC	*HLA.DRB5*	TRUE	2.271412979
HTN	SMC	*HLA.C*	TRUE	3.089690102
HTN	SMC	*TNFSF12*	TRUE	2.989565026
HTN	SMC	*LINC00960*	TRUE	2.541200641
HTN	t_cell	*CD79B*	TRUE	2.291898988
HTN	t_cell	*SNX10*	TRUE	2.089697113
HTN	t_cell	*BIRC7*	TRUE	2.048853456
HTN	t_cell	*POLI*	TRUE	2.430620125
HTN	t_cell	*MTHFSD*	TRUE	2.182403438
HTN	t_cell	*SGCA*	TRUE	2.633968686
HTN	t_cell	*IRF6*	TRUE	2.110620785
HTN	t_cell	*ACOT2*	TRUE	2.719533499
HTN	t_cell	*TBX2*	TRUE	3.163538512
HTN	t_cell	*TEX14*	TRUE	2.477880551
HTN	t_cell	*WFDC3*	TRUE	2.145726159
HTN	t_cell	*BTN2A2*	TRUE	3.10004195
HTN	t_cell	*GRK4*	TRUE	2.079890052
HTN	t_cell	*TRAP1*	TRUE	2.453196698
HTN	t_cell	*ATP8B3*	TRUE	2.601334389
HTN	t_cell	*HAUS8*	TRUE	2.045820372
HTN	t_cell	*ACP2*	TRUE	3.523655288
HTN	t_cell	*AGT*	TRUE	2.698265642
HTN	t_cell	*NMT1*	TRUE	2.945546775
HTN	t_cell	*FBN2*	TRUE	2.502722349
HTN	t_cell	*FITM1*	TRUE	2.360046399
HTN	t_cell	*SCRN2*	TRUE	2.153708281
HTN	t_cell	*HSD17B12*	TRUE	4.141415511
HTN	t_cell	*NEIL2*	TRUE	2.361677516
HTN	t_cell	*ART3*	TRUE	2.346479933
HTN	t_cell	*SFXN2*	TRUE	4.298197733
HTN	t_cell	*CLEC18A*	TRUE	3.202464767
HTN	t_cell	*SLC5A11*	TRUE	4.057225693
HTN	t_cell	*S100B*	TRUE	3.153383096
HTN	t_cell	*ABCF3*	TRUE	3.645802883
HTN	t_cell	*MRPL55*	TRUE	3.607566949
HTN	t_cell	*LYSMD1*	TRUE	2.025804774
HTN	t_cell	*RNF123*	TRUE	4.462405975
HTN	t_cell	*TRIM66*	TRUE	2.778203088
HTN	t_cell	*SRR*	TRUE	2.977505453
HTN	t_cell	*RGPD8*	TRUE	2.483917984
HTN	t_cell	*TTLL6*	TRUE	3.332673876
HTN	t_cell	*MRPL36*	TRUE	2.508288957
HTN	t_cell	*RGS19*	TRUE	2.963722989
HTN	t_cell	*CTSW*	TRUE	2.159636197

HTN	t_cell	*ZNF738*	TRUE	2.03283858
HTN	t_cell	*MST1*	TRUE	3.059341676
HTN	t_cell	*SNX31*	TRUE	3.046882003
HTN	t_cell	*CCNE2*	TRUE	2.799331528
HTN	t_cell	*ORAI3*	TRUE	2.920243449
HTN	t_cell	*MPI*	TRUE	5.254785307
HTN	t_cell	*TRIM73*	TRUE	2.797769472
HTN	t_cell	*HYI*	TRUE	3.186738339
HTN	t_cell	*ADO*	TRUE	4.775944117
HTN	t_cell	*CLN8*	TRUE	2.581138619
HTN	t_cell	*FES*	TRUE	3.575089992
HTN	t_cell	*ZNF320*	TRUE	2.912225181
HTN	t_cell	*ACOT1*	TRUE	2.433393711
HTN	t_cell	*A3GALT2*	TRUE	2.514633134
HTN	t_cell	*NUTM2A*	TRUE	3.115762736
HTN	t_cell	*CDK10*	TRUE	1.965285607
HTN	t_cell	*HIST1H3E*	TRUE	4.92856209
HTN	t_cell	*C6orf141*	TRUE	2.145391374
HTN	t_cell	*RPS26*	TRUE	3.327948987
HTN	t_cell	*TMEM116*	TRUE	3.850873264
HTN	t_cell	*HLA.DRB5*	TRUE	2.173674639
HTN	t_cell	*HSD17B8*	TRUE	2.767825328
HTN	t_cell	*TMEM110*	TRUE	2.273769763
HTN	t_cell	*ATF6B*	TRUE	2.315774399
HTN	t_cell	*AC016773.1*	TRUE	2.711983075
HTN	t_cell	*TIAF1*	TRUE	3.301714122
HTN	t_cell	*TNFSF12*	TRUE	3.317957145
Obesity	adipocyte	*CENPQ*	TRUE	2.176942112
Obesity	adipocyte	*LSG1*	TRUE	2.267938048
Obesity	adipocyte	*CD5L*	TRUE	2.637644991
Obesity	adipocyte	*TIPIN*	TRUE	2.261014229
Obesity	adipocyte	*PABPC4*	TRUE	3.658270538
Obesity	adipocyte	*RGS17*	TRUE	2.109532667
Obesity	adipocyte	*TBX15*	TRUE	3.258994568
Obesity	adipocyte	*ITPR3*	TRUE	2.367324261
Obesity	adipocyte	*ANKRD54*	TRUE	2.495216458
Obesity	adipocyte	*CENPM*	TRUE	3.630221322
Obesity	adipocyte	*ELMO3*	TRUE	2.3318332
Obesity	adipocyte	*CLUAP1*	TRUE	2.400924933
Obesity	adipocyte	*PYCARD*	TRUE	2.197951225
Obesity	adipocyte	*SLC22A18*	TRUE	2.491545181
Obesity	adipocyte	*BTN3A3*	TRUE	2.005599252
Obesity	adipocyte	*GABRR2*	TRUE	2.17909303
Obesity	adipocyte	*GLP1R*	TRUE	2.035829984
Obesity	adipocyte	*NME5*	TRUE	1.988185328
Obesity	adipocyte	*BNIP1*	TRUE	2.099090311

Obesity	adipocyte	*PCCB*	TRUE	2.667449365
Obesity	adipocyte	*CHMP3*	TRUE	2.074033113
Obesity	adipocyte	*SERPINC1*	TRUE	4.698451302
Obesity	adipocyte	*INVS*	TRUE	1.988106944
Obesity	adipocyte	*SNX19*	TRUE	2.187374258
Obesity	adipocyte	*ADRA1A*	TRUE	2.295289843
Obesity	adipocyte	*OPN4*	TRUE	3.264768856
Obesity	adipocyte	*SLC25A51*	TRUE	2.112110436
Obesity	adipocyte	*BTN2A2*	TRUE	2.612576549
Obesity	adipocyte	*GRK4*	TRUE	3.065989573
Obesity	adipocyte	*RIBC2*	TRUE	3.233830514
Obesity	adipocyte	*AP1M2*	TRUE	2.772965389
Obesity	adipocyte	*ATP8B3*	TRUE	5.838034815
Obesity	adipocyte	*PKDREJ*	TRUE	2.653526926
Obesity	adipocyte	*NDUFA2*	TRUE	2.135948064
Obesity	adipocyte	*MYBBP1A*	TRUE	2.193726252
Obesity	adipocyte	*GSTM5*	TRUE	2.206286835
Obesity	adipocyte	*MICAL1*	TRUE	2.018070326
Obesity	adipocyte	*DSCC1*	TRUE	2.213073359
Obesity	adipocyte	*SLC37A4*	TRUE	4.065268679
Obesity	adipocyte	*NDUFAF1*	TRUE	2.04399428
Obesity	adipocyte	*CENPO*	TRUE	6.452184182
Obesity	adipocyte	*CYP2C8*	TRUE	2.221996099
Obesity	adipocyte	*VPS33A*	TRUE	2.903236525
Obesity	adipocyte	*DIAPH3*	TRUE	2.680071175
Obesity	adipocyte	*FITM1*	TRUE	2.349872066
Obesity	adipocyte	*CYP11A1*	TRUE	2.442033457
Obesity	adipocyte	*MYO19*	TRUE	2.565784479
Obesity	adipocyte	*C18orf8*	TRUE	4.655108456
Obesity	adipocyte	*CBX8*	TRUE	2.537168251
Obesity	adipocyte	*COL6A1*	TRUE	2.221701375
Obesity	adipocyte	*MMEL1*	TRUE	2.131955705
Obesity	adipocyte	*FAHD2B*	TRUE	2.058922708
Obesity	adipocyte	*ADHFE1*	TRUE	2.203343188
Obesity	adipocyte	*RGR*	TRUE	2.355362247
Obesity	adipocyte	*HSD17B12*	TRUE	5.695887748
Obesity	adipocyte	*TNKS1BP1*	TRUE	3.028530796
Obesity	adipocyte	*TTC12*	TRUE	2.704924932
Obesity	adipocyte	*RGS18*	TRUE	2.729461732
Obesity	adipocyte	*DPYSL4*	TRUE	2.696966043
Obesity	adipocyte	*POC5*	TRUE	2.759325171
Obesity	adipocyte	*MMP16*	TRUE	1.982026923
Obesity	adipocyte	*CLEC18A*	TRUE	5.861585408
Obesity	adipocyte	*ETS2*	TRUE	2.395624755
Obesity	adipocyte	*SLC5A11*	TRUE	4.773016064
Obesity	adipocyte	*CILP2*	TRUE	2.28810914

Obesity	adipocyte	*ABCF3*	TRUE	2.461063483
Obesity	adipocyte	*HS3ST6*	TRUE	2.114773592
Obesity	adipocyte	*CAMK2N1*	TRUE	2.554219517
Obesity	adipocyte	*CLHC1*	TRUE	2.340462223
Obesity	adipocyte	*DNALI1*	TRUE	3.460386383
Obesity	adipocyte	*RFT1*	TRUE	2.51808696
Obesity	adipocyte	*MST1R*	TRUE	2.822537433
Obesity	adipocyte	*RAPSN*	TRUE	2.669484807
Obesity	adipocyte	*SMG8*	TRUE	2.246586575
Obesity	adipocyte	*SRR*	TRUE	3.781611545
Obesity	adipocyte	*FNTA*	TRUE	2.117762734
Obesity	adipocyte	*NPNT*	TRUE	1.983152146
Obesity	adipocyte	*GPRIN1*	TRUE	3.005486488
Obesity	adipocyte	*INO80E*	TRUE	3.629925055
Obesity	adipocyte	*RGPD8*	TRUE	2.261941735
Obesity	adipocyte	*MYO7B*	TRUE	2.248049827
Obesity	adipocyte	*ADORA2B*	TRUE	4.785461654
Obesity	adipocyte	*TTLL6*	TRUE	6.66805566
Obesity	adipocyte	*TSNARE1*	TRUE	2.58163944
Obesity	adipocyte	*RGS19*	TRUE	2.43216149
Obesity	adipocyte	*FUT10*	TRUE	2.528392662
Obesity	adipocyte	*MRGPRF*	TRUE	2.055719064
Obesity	adipocyte	*CADM2*	TRUE	4.233592355
Obesity	adipocyte	*ORAI3*	TRUE	5.057489954
Obesity	adipocyte	*SPRYD4*	TRUE	2.114852431
Obesity	adipocyte	*STX19*	TRUE	3.462054952
Obesity	adipocyte	*MPI*	TRUE	1.98062859
Obesity	adipocyte	*ZNF852*	TRUE	2.526178986
Obesity	adipocyte	*C3orf38*	TRUE	3.93457845
Obesity	adipocyte	*RPRML*	TRUE	3.974131918
Obesity	adipocyte	*PPP1R27*	TRUE	3.423830151
Obesity	adipocyte	*PSMG1*	TRUE	2.44632929
Obesity	adipocyte	*NPIPA5*	TRUE	2.312484069
Obesity	adipocyte	*EMILIN3*	TRUE	3.221647589
Obesity	adipocyte	*ACOT1*	TRUE	2.131821737
Obesity	adipocyte	*NUTM2A*	TRUE	3.067894416
Obesity	adipocyte	*GP1BA*	TRUE	2.71070471
Obesity	adipocyte	*CENPP*	TRUE	2.941225374
Obesity	adipocyte	*SKOR1*	TRUE	2.650370713
Obesity	adipocyte	*ZNF605*	TRUE	2.072387875
Obesity	adipocyte	*SULT1A2*	TRUE	9.393845978
Obesity	adipocyte	*DNAH10*	TRUE	4.067429145
Obesity	adipocyte	*RPS26*	TRUE	2.948047025
Obesity	adipocyte	*ZNF780A*	TRUE	3.735740575
Obesity	adipocyte	*SHISA4*	TRUE	4.667379691
Obesity	adipocyte	*C6orf163*	TRUE	2.085232855

Obesity	adipocyte	*GPANK1*	TRUE	3.387634764
Obesity	adipocyte	*C15orf59*	TRUE	2.287450891
Obesity	adipocyte	*SMN2*	TRUE	2.213046378
Obesity	adipocyte	*AC084018.1*	TRUE	3.655705184
Obesity	adipocyte	*ASIC3*	TRUE	2.662468263
Obesity	adipocyte	*SLC23A3*	TRUE	2.349933524
Obesity	adipocyte	*LINC00960*	TRUE	2.313040568
Obesity	adipocyte	*PLA2G4B*	TRUE	2.084926142
Obesity	adipocyte	*PDF*	TRUE	2.132444627
Obesity	adipocyte	*AP001172.2*	TRUE	3.084424453
Obesity	ASPC	*RBM6*	TRUE	3.189373737
Obesity	ASPC	*CENPQ*	TRUE	2.35307605
Obesity	ASPC	*FLT4*	TRUE	2.353188643
Obesity	ASPC	*LSG1*	TRUE	2.502374353
Obesity	ASPC	*CD5L*	TRUE	2.64391232
Obesity	ASPC	*TIPIN*	TRUE	2.777862254
Obesity	ASPC	*FDFT1*	TRUE	3.837924004
Obesity	ASPC	*GSTP1*	TRUE	2.382957394
Obesity	ASPC	*CD82*	TRUE	2.06666315
Obesity	ASPC	*PABPC4*	TRUE	4.543790978
Obesity	ASPC	*RGS17*	TRUE	4.1413682
Obesity	ASPC	*TBX15*	TRUE	3.724511575
Obesity	ASPC	*ITPR3*	TRUE	2.368990631
Obesity	ASPC	*IL12RB1*	TRUE	2.01029235
Obesity	ASPC	*CENPM*	TRUE	3.24586488
Obesity	ASPC	*ABHD12*	TRUE	2.563467539
Obesity	ASPC	*ELMO3*	TRUE	2.478749786
Obesity	ASPC	*CLCN7*	TRUE	2.098585859
Obesity	ASPC	*CLUAP1*	TRUE	2.605472152
Obesity	ASPC	*PYCARD*	TRUE	3.015305188
Obesity	ASPC	*MEST*	TRUE	2.198512354
Obesity	ASPC	*SLC22A18*	TRUE	2.547732166
Obesity	ASPC	*BTN3A3*	TRUE	2.694183573
Obesity	ASPC	*GABRR2*	TRUE	2.823235326
Obesity	ASPC	*GLP1R*	TRUE	2.109875108
Obesity	ASPC	*NME5*	TRUE	3.294257749
Obesity	ASPC	*BNIP1*	TRUE	2.284417871
Obesity	ASPC	*PCCB*	TRUE	3.754221242
Obesity	ASPC	*CHMP3*	TRUE	2.933860205
Obesity	ASPC	*SERPINC1*	TRUE	6.15171884
Obesity	ASPC	*INVS*	TRUE	2.53489522
Obesity	ASPC	*SNX19*	TRUE	2.707156404
Obesity	ASPC	*ADRA1A*	TRUE	2.805645841
Obesity	ASPC	*TEX14*	TRUE	2.637380594
Obesity	ASPC	*OPN4*	TRUE	3.184267399
Obesity	ASPC	*SLC25A51*	TRUE	2.778751868

Obesity	ASPC	*NEURL2*	TRUE	2.262991439
Obesity	ASPC	*BTN2A2*	TRUE	2.420524225
Obesity	ASPC	*MED20*	TRUE	2.085561075
Obesity	ASPC	*TREM1*	TRUE	2.330402217
Obesity	ASPC	*KCNK17*	TRUE	2.313483386
Obesity	ASPC	*GRK4*	TRUE	3.449731917
Obesity	ASPC	*RIBC2*	TRUE	3.313711055
Obesity	ASPC	*AP1M2*	TRUE	2.906842713
Obesity	ASPC	*ATP8B3*	TRUE	6.239212258
Obesity	ASPC	*STK33*	TRUE	3.147716502
Obesity	ASPC	*PGPEP1*	TRUE	2.334200116
Obesity	ASPC	*PKDREJ*	TRUE	2.974302576
Obesity	ASPC	*MYBBP1A*	TRUE	2.414340219
Obesity	ASPC	*LARS*	TRUE	2.136772526
Obesity	ASPC	*MICAL1*	TRUE	2.385151731
Obesity	ASPC	*SCN2A*	TRUE	3.016797482
Obesity	ASPC	*DSCC1*	TRUE	2.254536059
Obesity	ASPC	*IRF4*	TRUE	2.004124262
Obesity	ASPC	*SLC37A4*	TRUE	3.462570399
Obesity	ASPC	*NDUFAF1*	TRUE	3.102610683
Obesity	ASPC	*CENPO*	TRUE	6.724166973
Obesity	ASPC	*CYP2C8*	TRUE	2.320830506
Obesity	ASPC	*KLRG1*	TRUE	2.683939018
Obesity	ASPC	*VPS33A*	TRUE	3.33244857
Obesity	ASPC	*DIAPH3*	TRUE	3.009884773
Obesity	ASPC	*FITM1*	TRUE	2.501984161
Obesity	ASPC	*DUOXA1*	TRUE	1.973724576
Obesity	ASPC	*CYP11A1*	TRUE	2.268695355
Obesity	ASPC	*MYO19*	TRUE	3.585807729
Obesity	ASPC	*C18orf8*	TRUE	5.595124917
Obesity	ASPC	*CBX8*	TRUE	2.937125635
Obesity	ASPC	*COL6A1*	TRUE	2.162868867
Obesity	ASPC	*SAE1*	TRUE	3.629055667
Obesity	ASPC	*MMEL1*	TRUE	2.539515536
Obesity	ASPC	*SEMA6C*	TRUE	2.58615713
Obesity	ASPC	*FAHD2B*	TRUE	2.499039782
Obesity	ASPC	*RGR*	TRUE	2.942774653
Obesity	ASPC	*MTG1*	TRUE	2.075946549
Obesity	ASPC	*HSD17B12*	TRUE	5.987696349
Obesity	ASPC	*TNKS1BP1*	TRUE	4.026746913
Obesity	ASPC	*TTC12*	TRUE	3.027160677
Obesity	ASPC	*RGS18*	TRUE	3.183914507
Obesity	ASPC	*DPYSL4*	TRUE	2.70288147
Obesity	ASPC	*POC5*	TRUE	3.488323826
Obesity	ASPC	*JAZF1*	TRUE	2.294818502
Obesity	ASPC	*MMP16*	TRUE	2.827705124

Obesity	ASPC	*RWDD2B*	TRUE	2.261588864
Obesity	ASPC	*STEAP2*	TRUE	2.296693324
Obesity	ASPC	*TMED6*	TRUE	2.821300965
Obesity	ASPC	*CLEC18A*	TRUE	4.333813028
Obesity	ASPC	*ETS2*	TRUE	2.783843908
Obesity	ASPC	*SLC5A11*	TRUE	4.783947221
Obesity	ASPC	*CILP2*	TRUE	3.308370538
Obesity	ASPC	*ABCF3*	TRUE	2.715011877
Obesity	ASPC	*HS3ST6*	TRUE	2.270568291
Obesity	ASPC	*CLHC1*	TRUE	2.52194847
Obesity	ASPC	*DCST2*	TRUE	2.269925153
Obesity	ASPC	*DCLK3*	TRUE	2.045278967
Obesity	ASPC	*DNALI1*	TRUE	3.963446778
Obesity	ASPC	*RFT1*	TRUE	2.655427934
Obesity	ASPC	*RASEF*	TRUE	2.087579804
Obesity	ASPC	*CCDC68*	TRUE	2.665061875
Obesity	ASPC	*SPDYE6*	TRUE	2.332937214
Obesity	ASPC	*PIP4K2C*	TRUE	2.20552413
Obesity	ASPC	*PIP5KL1*	TRUE	2.058209204
Obesity	ASPC	*SRR*	TRUE	3.964121743
Obesity	ASPC	*FNTA*	TRUE	2.592675984
Obesity	ASPC	*NPNT*	TRUE	1.980368514
Obesity	ASPC	*CPLX1*	TRUE	2.302186992
Obesity	ASPC	*IRS1*	TRUE	2.292531351
Obesity	ASPC	*GPRIN1*	TRUE	2.513332248
Obesity	ASPC	*INO80E*	TRUE	3.23872436
Obesity	ASPC	*RGPD8*	TRUE	2.550875836
Obesity	ASPC	*MYO7B*	TRUE	2.501209941
Obesity	ASPC	*CNGB3*	TRUE	2.011029306
Obesity	ASPC	*ADORA2B*	TRUE	4.86155004
Obesity	ASPC	*TTLL6*	TRUE	5.804848569
Obesity	ASPC	*TSNARE1*	TRUE	3.041767576
Obesity	ASPC	*TRMT61B*	TRUE	2.564089484
Obesity	ASPC	*RGS19*	TRUE	2.777850281
Obesity	ASPC	*CTSW*	TRUE	2.724528288
Obesity	ASPC	*FUT10*	TRUE	2.81470052
Obesity	ASPC	*HOXB2*	TRUE	1.964715078
Obesity	ASPC	*CADM2*	TRUE	4.965731005
Obesity	ASPC	*ORAI3*	TRUE	5.010343317
Obesity	ASPC	*SPRYD4*	TRUE	2.240535029
Obesity	ASPC	*RUFY1*	TRUE	2.459213166
Obesity	ASPC	*AP006621.1*	TRUE	3.408866513
Obesity	ASPC	*STX19*	TRUE	3.897228319
Obesity	ASPC	*MPI*	TRUE	2.031599434
Obesity	ASPC	*ZNF852*	TRUE	3.014013143
Obesity	ASPC	*C3orf38*	TRUE	4.411246395

Obesity	ASPC	*RPRML*	TRUE	3.949357778
Obesity	ASPC	*FKRP*	TRUE	2.0437254
Obesity	ASPC	*ZFP41*	TRUE	2.532570551
Obesity	ASPC	*CLN8*	TRUE	2.082683605
Obesity	ASPC	*PPP1R27*	TRUE	3.086581733
Obesity	ASPC	*PSMG1*	TRUE	2.884338087
Obesity	ASPC	*UPP1*	TRUE	2.119465566
Obesity	ASPC	*NPIPA5*	TRUE	2.648706539
Obesity	ASPC	*EMILIN3*	TRUE	3.299931701
Obesity	ASPC	*ACOT1*	TRUE	3.366049141
Obesity	ASPC	*NUTM2A*	TRUE	2.011178678
Obesity	ASPC	*MAFF*	TRUE	2.118074749
Obesity	ASPC	*GP1BA*	TRUE	3.507731043
Obesity	ASPC	*TRABD2A*	TRUE	2.149206283
Obesity	ASPC	*CENPP*	TRUE	3.353263269
Obesity	ASPC	*OSTN*	TRUE	2.462015046
Obesity	ASPC	*SKOR1*	TRUE	2.2245205
Obesity	ASPC	*FBF1*	TRUE	2.362354849
Obesity	ASPC	*CPNE4*	TRUE	3.328900232
Obesity	ASPC	*ZNF605*	TRUE	2.750242199
Obesity	ASPC	*SULT1A2*	TRUE	8.892268688
Obesity	ASPC	*MFAP5*	TRUE	2.197489969
Obesity	ASPC	*DNAH10*	TRUE	4.463094555
Obesity	ASPC	*RPS26*	TRUE	3.027004761
Obesity	ASPC	*ZNF780A*	TRUE	3.764948765
Obesity	ASPC	*ZNF560*	TRUE	1.984894313
Obesity	ASPC	*ADH4*	TRUE	2.071837519
Obesity	ASPC	*SHISA4*	TRUE	5.056061272
Obesity	ASPC	*C6orf163*	TRUE	2.341244714
Obesity	ASPC	*GPANK1*	TRUE	3.405344706
Obesity	ASPC	*C15orf59*	TRUE	2.322847078
Obesity	ASPC	*SMN2*	TRUE	2.459004913
Obesity	ASPC	*AC084018.1*	TRUE	3.444338373
Obesity	ASPC	*ASIC3*	TRUE	2.653668928
Obesity	ASPC	*SLC23A3*	TRUE	2.149307351
Obesity	ASPC	*GALT*	TRUE	2.240901119
Obesity	ASPC	*RNU6ATAC35P*	TRUE	2.057784142
Obesity	ASPC	*GS1.124K5.4*	TRUE	2.094832122
Obesity	ASPC	*ISY1*	TRUE	2.549545934
Obesity	ASPC	*LINC00960*	TRUE	2.352385616
Obesity	ASPC	*PLA2G4B*	TRUE	1.99750839
Obesity	ASPC	*BRK1*	TRUE	2.226169167
Obesity	ASPC	*AP001172.2*	TRUE	3.009606817
Obesity	ASPC	*GTF2H5*	TRUE	2.128285333
Obesity	b_cell	*ITIH4*	TRUE	5.279223765
Obesity	b_cell	*MFAP2*	TRUE	2.004646682

Obesity	b_cell	*ATP8B3*	TRUE	3.320941987
Obesity	b_cell	*DPYSL4*	TRUE	2.410297212
Obesity	b_cell	*CLEC18A*	TRUE	7.864561823
Obesity	b_cell	*RNF123*	TRUE	3.296715155
Obesity	b_cell	*RAPSN*	TRUE	2.050231336
Obesity	b_cell	*CTSW*	TRUE	6.306802679
Obesity	b_cell	*TRIM73*	TRUE	5.98076843
Obesity	b_cell	*RPRML*	TRUE	2.414309775
Obesity	b_cell	*ZFP57*	TRUE	2.646839299
Obesity	b_cell	*LINC00664*	TRUE	2.278340764
Obesity	dendritic_cell	*RBM6*	TRUE	3.652415122
Obesity	dendritic_cell	*UHRF1BP1*	TRUE	2.823350055
Obesity	dendritic_cell	*PABPC4*	TRUE	2.863415527
Obesity	dendritic_cell	*RIBC2*	TRUE	3.059004571
Obesity	dendritic_cell	*ATP8B3*	TRUE	4.82741285
Obesity	dendritic_cell	*TBX6*	TRUE	4.467539959
Obesity	dendritic_cell	*DPYSL4*	TRUE	2.919583327
Obesity	dendritic_cell	*CLEC18A*	TRUE	7.928725366
Obesity	dendritic_cell	*SRR*	TRUE	2.035721958
Obesity	dendritic_cell	*RGPD8*	TRUE	2.059055077
Obesity	dendritic_cell	*CTSW*	TRUE	3.771209606
Obesity	dendritic_cell	*TRIM73*	TRUE	6.236058019
Obesity	dendritic_cell	*RPRML*	TRUE	2.655886934
Obesity	dendritic_cell	*NUTM2A*	TRUE	2.272509657
Obesity	dendritic_cell	*GP1BA*	TRUE	2.750525266
Obesity	dendritic_cell	*SULT1A2*	TRUE	6.832152515
Obesity	dendritic_cell	*RPS26*	TRUE	2.764802587
Obesity	dendritic_cell	*NOTCH4*	TRUE	2.079328741
Obesity	dendritic_cell	*TMEM110*	TRUE	2.713817671
Obesity	dendritic_cell	*LINC00664*	TRUE	2.363376144
Obesity	endometrium	*CENPM*	TRUE	2.310906006
Obesity	endometrium	*SNRPC*	TRUE	1.990312292
Obesity	endometrium	*TBX6*	TRUE	3.62807079
Obesity	endometrium	*DPYSL4*	TRUE	2.788635387
Obesity	endometrium	*RNF123*	TRUE	3.667925188
Obesity	endometrium	*ADORA2B*	TRUE	3.076478125
Obesity	endometrium	*C1QTNF4*	TRUE	3.642877735
Obesity	endometrium	*CTSW*	TRUE	2.110772
Obesity	endometrium	*ORAI3*	TRUE	3.18249664
Obesity	endometrium	*TRIM73*	TRUE	5.363718462
Obesity	endometrium	*RPRML*	TRUE	2.794633059
Obesity	endometrium	*EMILIN3*	TRUE	2.654691051
Obesity	endometrium	*NUTM2A*	TRUE	2.762594193
Obesity	endometrium	*GP1BA*	TRUE	2.867860952
Obesity	endometrium	*SULT1A2*	TRUE	3.425971339
Obesity	endometrium	*DNAH10*	TRUE	3.053257615

Obesity	endometrium	*ZFP57*	TRUE	2.756972498
Obesity	endometrium	*LINC00664*	TRUE	2.336520203
Obesity	endometrium	*AP001172.2*	TRUE	2.547276039
Obesity	endothelial	*SNRPC*	TRUE	4.587986162
Obesity	endothelial	*AP1M2*	TRUE	2.156876408
Obesity	endothelial	*ATP8B3*	TRUE	4.748823602
Obesity	endothelial	*PKDREJ*	TRUE	2.475092685
Obesity	endothelial	*CBX8*	TRUE	2.141068725
Obesity	endothelial	*HSD17B12*	TRUE	3.062129726
Obesity	endothelial	*DPYSL4*	TRUE	2.392804969
Obesity	endothelial	*CLEC18A*	TRUE	4.812351196
Obesity	endothelial	*SLC5A11*	TRUE	3.199567434
Obesity	endothelial	*CILP2*	TRUE	3.285862073
Obesity	endothelial	*RAPSN*	TRUE	2.593842596
Obesity	endothelial	*SRR*	TRUE	2.224644777
Obesity	endothelial	*ADORA2B*	TRUE	3.789445286
Obesity	endothelial	*TTLL6*	TRUE	5.263222447
Obesity	endothelial	*CTSW*	TRUE	5.965572309
Obesity	endothelial	*RPP25*	TRUE	2.564452033
Obesity	endothelial	*TRIM73*	TRUE	4.473860447
Obesity	endothelial	*RPRML*	TRUE	2.297071716
Obesity	endothelial	*NUTM2A*	TRUE	3.351475578
Obesity	endothelial	*BTN3A2*	TRUE	2.211654693
Obesity	endothelial	*ZNF780A*	TRUE	3.250551014
Obesity	endothelial	*AC084018.1*	TRUE	2.884729101
Obesity	LEC	*ITIH4*	TRUE	3.286197227
Obesity	LEC	*ITPR3*	TRUE	2.263583378
Obesity	LEC	*CENPM*	TRUE	2.042137275
Obesity	LEC	*BTN3A3*	TRUE	2.008089938
Obesity	LEC	*SERPINC1*	TRUE	2.211407672
Obesity	LEC	*OPN4*	TRUE	2.607939696
Obesity	LEC	*SLC25A51*	TRUE	2.448066608
Obesity	LEC	*GRK4*	TRUE	2.49022637
Obesity	LEC	*AP1M2*	TRUE	2.359877755
Obesity	LEC	*ATP8B3*	TRUE	3.26818699
Obesity	LEC	*PKDREJ*	TRUE	2.361979823
Obesity	LEC	*IGHMBP2*	TRUE	2.37269295
Obesity	LEC	*SLC37A4*	TRUE	3.80188218
Obesity	LEC	*CYP2C8*	TRUE	2.007380156
Obesity	LEC	*CBX8*	TRUE	2.719209182
Obesity	LEC	*MTG1*	TRUE	2.228726978
Obesity	LEC	*HSD17B12*	TRUE	4.918814109
Obesity	LEC	*DPYSL4*	TRUE	2.235895011
Obesity	LEC	*CLEC18A*	TRUE	6.354389826
Obesity	LEC	*ETS2*	TRUE	2.397690607
Obesity	LEC	*SLC5A11*	TRUE	3.191916858

Obesity	LEC	*CILP2*	TRUE	2.158823739
Obesity	LEC	*DNALI1*	TRUE	2.325214884
Obesity	LEC	*MST1R*	TRUE	3.758459283
Obesity	LEC	*SRR*	TRUE	3.338419902
Obesity	LEC	*C8orf46*	TRUE	2.080789669
Obesity	LEC	*GPRIN1*	TRUE	2.714306246
Obesity	LEC	*INO80E*	TRUE	2.614508884
Obesity	LEC	*RGPD8*	TRUE	2.140296632
Obesity	LEC	*ADORA2B*	TRUE	4.676641059
Obesity	LEC	*TTLL6*	TRUE	4.005793107
Obesity	LEC	*C1QTNF4*	TRUE	2.965596748
Obesity	LEC	*CTSW*	TRUE	3.440903713
Obesity	LEC	*ORAI3*	TRUE	4.952153865
Obesity	LEC	*STX19*	TRUE	1.988177241
Obesity	LEC	*TRIM73*	TRUE	5.061358114
Obesity	LEC	*RPRML*	TRUE	2.794574982
Obesity	LEC	*PPP1R27*	TRUE	3.325008303
Obesity	LEC	*ACOT1*	TRUE	2.043192657
Obesity	LEC	*NUTM2A*	TRUE	3.015279083
Obesity	LEC	*SULT1A2*	TRUE	5.680361108
Obesity	LEC	*DNAH10*	TRUE	4.985772722
Obesity	LEC	*RPS26*	TRUE	2.788658106
Obesity	LEC	*ZNF780A*	TRUE	3.496466868
Obesity	LEC	*GPANK1*	TRUE	2.294777189
Obesity	LEC	*C15orf59*	TRUE	1.976057102
Obesity	LEC	*SMN2*	TRUE	2.047407162
Obesity	LEC	*AC084018.1*	TRUE	2.76370559
Obesity	LEC	*ASIC3*	TRUE	2.58187143
Obesity	LEC	*GS1.124K5.4*	TRUE	2.197820867
Obesity	LEC	*DPP3*	TRUE	2.372758189
Obesity	LEC	*LINC00664*	TRUE	2.360878503
Obesity	macrophage	*RBM6*	TRUE	3.116525178
Obesity	macrophage	*CENPQ*	TRUE	2.010492387
Obesity	macrophage	*TBX15*	TRUE	3.164117177
Obesity	macrophage	*CENPM*	TRUE	2.976265003
Obesity	macrophage	*ELMO3*	TRUE	2.485664186
Obesity	macrophage	*BTN3A3*	TRUE	2.448459362
Obesity	macrophage	*PCCB*	TRUE	2.361819802
Obesity	macrophage	*MFAP2*	TRUE	2.147511358
Obesity	macrophage	*SERPINC1*	TRUE	5.145205232
Obesity	macrophage	*SLC25A51*	TRUE	2.447260423
Obesity	macrophage	*GRK4*	TRUE	2.764854231
Obesity	macrophage	*RIBC2*	TRUE	2.232812273
Obesity	macrophage	*AP1M2*	TRUE	2.332809671
Obesity	macrophage	*ATP8B3*	TRUE	5.889859484
Obesity	macrophage	*PKDREJ*	TRUE	2.797387663

Obesity	macrophage	*IGHMBP2*	TRUE	2.044268285
Obesity	macrophage	*MICAL1*	TRUE	2.069370344
Obesity	macrophage	*CENPO*	TRUE	2.16150987
Obesity	macrophage	*VPS33A*	TRUE	2.433386104
Obesity	macrophage	*DIAPH3*	TRUE	2.249538705
Obesity	macrophage	*C18orf8*	TRUE	3.590905756
Obesity	macrophage	*CBX8*	TRUE	2.783635706
Obesity	macrophage	*MMEL1*	TRUE	2.000545327
Obesity	macrophage	*HSD17B12*	TRUE	5.468788781
Obesity	macrophage	*DPYSL4*	TRUE	2.736493708
Obesity	macrophage	*POC5*	TRUE	2.489933778
Obesity	macrophage	*NEIL2*	TRUE	2.43585996
Obesity	macrophage	*CLEC18A*	TRUE	5.75746055
Obesity	macrophage	*SLC5A11*	TRUE	4.45413658
Obesity	macrophage	*CILP2*	TRUE	3.156787502
Obesity	macrophage	*ABCF3*	TRUE	2.266300327
Obesity	macrophage	*DNALI1*	TRUE	2.239754553
Obesity	macrophage	*RFT1*	TRUE	2.685815894
Obesity	macrophage	*SRR*	TRUE	3.664891293
Obesity	macrophage	*GPRIN1*	TRUE	2.58630778
Obesity	macrophage	*INO80E*	TRUE	3.203424422
Obesity	macrophage	*RGPD8*	TRUE	2.389764283
Obesity	macrophage	*ADORA2B*	TRUE	3.668018165
Obesity	macrophage	*TTLL6*	TRUE	4.913203571
Obesity	macrophage	*C1QTNF4*	TRUE	2.983417274
Obesity	macrophage	*CTSW*	TRUE	3.511992446
Obesity	macrophage	*CADM2*	TRUE	2.039008588
Obesity	macrophage	*ORAI3*	TRUE	5.119400024
Obesity	macrophage	*STX19*	TRUE	1.969418914
Obesity	macrophage	*C3orf38*	TRUE	3.462061318
Obesity	macrophage	*RPRML*	TRUE	3.473468562
Obesity	macrophage	*ZFP41*	TRUE	2.490320327
Obesity	macrophage	*NPIPA5*	TRUE	2.599689085
Obesity	macrophage	*ACOT1*	TRUE	2.879853135
Obesity	macrophage	*NUTM2A*	TRUE	2.995458833
Obesity	macrophage	*GP1BA*	TRUE	2.982026938
Obesity	macrophage	*CENPP*	TRUE	2.397611347
Obesity	macrophage	*SKOR1*	TRUE	2.407599962
Obesity	macrophage	*CPNE4*	TRUE	2.529430609
Obesity	macrophage	*SULT1A2*	TRUE	8.776585569
Obesity	macrophage	*DNAH10*	TRUE	3.382555137
Obesity	macrophage	*RPS26*	TRUE	2.825369595
Obesity	macrophage	*ZNF780A*	TRUE	3.640109712
Obesity	macrophage	*SHISA4*	TRUE	5.190388275
Obesity	macrophage	*C6orf163*	TRUE	2.026274672
Obesity	macrophage	*GPANK1*	TRUE	2.725299928

Obesity	macrophage	*C15orf59*	TRUE	2.134822302
Obesity	macrophage	*SMN2*	TRUE	2.104714797
Obesity	macrophage	*AC084018.1*	TRUE	3.30510914
Obesity	macrophage	*ASIC3*	TRUE	2.34547195
Obesity	macrophage	*GS1.124K5.4*	TRUE	2.077824566
Obesity	macrophage	*ISY1*	TRUE	2.130697781
Obesity	macrophage	*LINC00664*	TRUE	2.276661311
Obesity	macrophage	*AP001172.2*	TRUE	2.518040369
Obesity	mast_cell	*RBM6*	TRUE	1.979941287
Obesity	mast_cell	*CENPM*	TRUE	2.400026998
Obesity	mast_cell	*SERPINC1*	TRUE	3.098673746
Obesity	mast_cell	*TEX14*	TRUE	2.171176379
Obesity	mast_cell	*BTN2A2*	TRUE	3.187502863
Obesity	mast_cell	*RIBC2*	TRUE	2.146528353
Obesity	mast_cell	*PKDREJ*	TRUE	2.504732875
Obesity	mast_cell	*TBX6*	TRUE	4.301816865
Obesity	mast_cell	*DPYSL4*	TRUE	2.686247318
Obesity	mast_cell	*CLEC18A*	TRUE	8.175526768
Obesity	mast_cell	*SLC5A11*	TRUE	4.41747633
Obesity	mast_cell	*SPDYE6*	TRUE	2.123616678
Obesity	mast_cell	*SRR*	TRUE	2.984523643
Obesity	mast_cell	*ADORA2B*	TRUE	2.550161545
Obesity	mast_cell	*CTSW*	TRUE	2.887517867
Obesity	mast_cell	*ORAI3*	TRUE	3.788170914
Obesity	mast_cell	*TRIM73*	TRUE	4.791383115
Obesity	mast_cell	*RPRML*	TRUE	2.914753548
Obesity	mast_cell	*ACOT1*	TRUE	3.423248556
Obesity	mast_cell	*NUTM2A*	TRUE	3.251352239
Obesity	mast_cell	*BTN3A2*	TRUE	3.533217888
Obesity	mast_cell	*SULT1A2*	TRUE	6.145792127
Obesity	mast_cell	*C17orf107*	TRUE	2.373775121
Obesity	mast_cell	*LINC00664*	TRUE	2.338418835
Obesity	mesothelium	*RBM6*	TRUE	3.470363239
Obesity	mesothelium	*FMO3*	TRUE	2.067526957
Obesity	mesothelium	*CENPQ*	TRUE	2.473119334
Obesity	mesothelium	*FLT4*	TRUE	2.341943698
Obesity	mesothelium	*LSG1*	TRUE	2.539776222
Obesity	mesothelium	*CD5L*	TRUE	2.630677601
Obesity	mesothelium	*TIPIN*	TRUE	2.746314282
Obesity	mesothelium	*NT5C2*	TRUE	2.144084574
Obesity	mesothelium	*FDFT1*	TRUE	3.834854008
Obesity	mesothelium	*GSTP1*	TRUE	2.544487634
Obesity	mesothelium	*CD82*	TRUE	1.971277889
Obesity	mesothelium	*OGFOD1*	TRUE	2.433582933
Obesity	mesothelium	*PABPC4*	TRUE	5.048926233
Obesity	mesothelium	*RGS17*	TRUE	4.464359758

Obesity	mesothelium	*TBX15*	TRUE	3.417853561
Obesity	mesothelium	*ITPR3*	TRUE	2.911399666
Obesity	mesothelium	*IL12RB1*	TRUE	2.213892687
Obesity	mesothelium	*CENPM*	TRUE	3.289251072
Obesity	mesothelium	*ABHD12*	TRUE	2.544643989
Obesity	mesothelium	*ELMO3*	TRUE	2.561792788
Obesity	mesothelium	*CLCN7*	TRUE	2.082140474
Obesity	mesothelium	*CLUAP1*	TRUE	2.56857971
Obesity	mesothelium	*PYCARD*	TRUE	3.111933449
Obesity	mesothelium	*MEST*	TRUE	2.578940812
Obesity	mesothelium	*SLC22A18*	TRUE	2.361909133
Obesity	mesothelium	*BTN3A3*	TRUE	2.435185332
Obesity	mesothelium	*GABRR2*	TRUE	2.790850916
Obesity	mesothelium	*GLP1R*	TRUE	2.100707297
Obesity	mesothelium	*PRPH2*	TRUE	1.978926736
Obesity	mesothelium	*NME5*	TRUE	3.029942188
Obesity	mesothelium	*BNIP1*	TRUE	2.437499441
Obesity	mesothelium	*PCCB*	TRUE	3.858242908
Obesity	mesothelium	*CHMP3*	TRUE	3.469916163
Obesity	mesothelium	*SERPINC1*	TRUE	6.187865208
Obesity	mesothelium	*INVS*	TRUE	2.867727726
Obesity	mesothelium	*SNX19*	TRUE	3.15052845
Obesity	mesothelium	*ADRA1A*	TRUE	2.832305264
Obesity	mesothelium	*TEX14*	TRUE	2.525343599
Obesity	mesothelium	*OPN4*	TRUE	3.220910828
Obesity	mesothelium	*SLC25A51*	TRUE	2.765335236
Obesity	mesothelium	*NEURL2*	TRUE	2.285851988
Obesity	mesothelium	*BTN2A2*	TRUE	2.460845832
Obesity	mesothelium	*MED20*	TRUE	2.033916017
Obesity	mesothelium	*TREM1*	TRUE	2.150979309
Obesity	mesothelium	*KCNK17*	TRUE	2.224451012
Obesity	mesothelium	*GRK4*	TRUE	3.543376948
Obesity	mesothelium	*RIBC2*	TRUE	3.175080841
Obesity	mesothelium	*AP1M2*	TRUE	2.733985008
Obesity	mesothelium	*ATP8B3*	TRUE	6.380437238
Obesity	mesothelium	*PGPEP1*	TRUE	2.503814021
Obesity	mesothelium	*PKDREJ*	TRUE	2.942102106
Obesity	mesothelium	*MYBBP1A*	TRUE	2.286028052
Obesity	mesothelium	*LARS*	TRUE	2.471696848
Obesity	mesothelium	*GSTM5*	TRUE	2.564082349
Obesity	mesothelium	*POM121C*	TRUE	2.581661629
Obesity	mesothelium	*MICAL1*	TRUE	2.550458108
Obesity	mesothelium	*SCN2A*	TRUE	2.494431685
Obesity	mesothelium	*DSCC1*	TRUE	2.357539107
Obesity	mesothelium	*SLC37A4*	TRUE	3.465661438
Obesity	mesothelium	*NDUFAF1*	TRUE	2.842981927

Obesity	mesothelium	*CENPO*	TRUE	7.388959186
Obesity	mesothelium	*CYP2C8*	TRUE	2.167493999
Obesity	mesothelium	*KLRG1*	TRUE	2.64337465
Obesity	mesothelium	*VPS33A*	TRUE	3.347491675
Obesity	mesothelium	*NOVA1*	TRUE	2.195408751
Obesity	mesothelium	*FITM1*	TRUE	2.543514038
Obesity	mesothelium	*CYP11A1*	TRUE	2.28561875
Obesity	mesothelium	*MYO19*	TRUE	3.878455311
Obesity	mesothelium	*C18orf8*	TRUE	5.935076043
Obesity	mesothelium	*CBX8*	TRUE	2.734493577
Obesity	mesothelium	*COL6A1*	TRUE	2.531106162
Obesity	mesothelium	*SAE1*	TRUE	3.684745221
Obesity	mesothelium	*MMEL1*	TRUE	2.506346606
Obesity	mesothelium	*SEMA6C*	TRUE	2.871792496
Obesity	mesothelium	*FAHD2B*	TRUE	2.645491352
Obesity	mesothelium	*RGR*	TRUE	3.106567205
Obesity	mesothelium	*HSD17B12*	TRUE	6.155975119
Obesity	mesothelium	*TNKS1BP1*	TRUE	3.669533738
Obesity	mesothelium	*TTC12*	TRUE	2.880551678
Obesity	mesothelium	*RGS18*	TRUE	3.045013346
Obesity	mesothelium	*DPYSL4*	TRUE	2.672582903
Obesity	mesothelium	*POC5*	TRUE	3.584634758
Obesity	mesothelium	*PLA2R1*	TRUE	2.218854061
Obesity	mesothelium	*JAZF1*	TRUE	2.472015139
Obesity	mesothelium	*MMP16*	TRUE	2.868959193
Obesity	mesothelium	*RWDD2B*	TRUE	2.280239157
Obesity	mesothelium	*STEAP2*	TRUE	2.604104137
Obesity	mesothelium	*TMED6*	TRUE	2.673632011
Obesity	mesothelium	*CLEC18A*	TRUE	4.471797046
Obesity	mesothelium	*ETS2*	TRUE	2.680621612
Obesity	mesothelium	*SLC5A11*	TRUE	4.920302856
Obesity	mesothelium	*CILP2*	TRUE	3.234984993
Obesity	mesothelium	*GPSM1*	TRUE	2.362898019
Obesity	mesothelium	*ABCF3*	TRUE	2.679541272
Obesity	mesothelium	*CAMK2N1*	TRUE	3.116515432
Obesity	mesothelium	*CLHC1*	TRUE	2.494812145
Obesity	mesothelium	*ANTXR2*	TRUE	2.140066939
Obesity	mesothelium	*DCST2*	TRUE	2.334242791
Obesity	mesothelium	*DCLK3*	TRUE	1.965770567
Obesity	mesothelium	*DNALI1*	TRUE	3.72275255
Obesity	mesothelium	*RFT1*	TRUE	2.910841964
Obesity	mesothelium	*TEX264*	TRUE	2.378722331
Obesity	mesothelium	*CCDC68*	TRUE	2.444741311
Obesity	mesothelium	*CHRFAM7A*	TRUE	2.009835265
Obesity	mesothelium	*SPDYE6*	TRUE	2.2995722
Obesity	mesothelium	*SRR*	TRUE	3.927296002

Obesity	mesothelium	*FNTA*	TRUE	2.734296001
Obesity	mesothelium	*IRS1*	TRUE	2.436370134
Obesity	mesothelium	*GPRIN1*	TRUE	2.522672248
Obesity	mesothelium	*INO80E*	TRUE	4.251230597
Obesity	mesothelium	*RGPD8*	TRUE	2.584465307
Obesity	mesothelium	*CALML6*	TRUE	2.049878888
Obesity	mesothelium	*MYO7B*	TRUE	2.303135222
Obesity	mesothelium	*ADORA2B*	TRUE	4.912083298
Obesity	mesothelium	*TTLL6*	TRUE	5.808223398
Obesity	mesothelium	*TSNARE1*	TRUE	3.130918547
Obesity	mesothelium	*RGS19*	TRUE	2.751961088
Obesity	mesothelium	*CTSW*	TRUE	3.631631535
Obesity	mesothelium	*FUT10*	TRUE	2.920092632
Obesity	mesothelium	*HOXB2*	TRUE	2.170983653
Obesity	mesothelium	*CADM2*	TRUE	4.739622895
Obesity	mesothelium	*ORAI3*	TRUE	5.089886703
Obesity	mesothelium	*SPRYD4*	TRUE	2.492311138
Obesity	mesothelium	*RUFY1*	TRUE	2.443742928
Obesity	mesothelium	*AP006621.1*	TRUE	3.34518941
Obesity	mesothelium	*STX19*	TRUE	3.728932908
Obesity	mesothelium	*ZNF852*	TRUE	3.1482199
Obesity	mesothelium	*C3orf38*	TRUE	4.489466093
Obesity	mesothelium	*RPRML*	TRUE	4.074701633
Obesity	mesothelium	*FKRP*	TRUE	2.035558824
Obesity	mesothelium	*ZFP41*	TRUE	2.212473442
Obesity	mesothelium	*CLDN7*	TRUE	2.420807213
Obesity	mesothelium	*CLN8*	TRUE	2.063347143
Obesity	mesothelium	*PPP1R27*	TRUE	2.968407832
Obesity	mesothelium	*PSMG1*	TRUE	2.738638021
Obesity	mesothelium	*NPIPA5*	TRUE	2.636604697
Obesity	mesothelium	*EMILIN3*	TRUE	2.993578301
Obesity	mesothelium	*ACOT1*	TRUE	3.726208185
Obesity	mesothelium	*OAF*	TRUE	2.114249316
Obesity	mesothelium	*NUTM2A*	TRUE	2.408177577
Obesity	mesothelium	*GP1BA*	TRUE	3.619518691
Obesity	mesothelium	*TRABD2A*	TRUE	2.094601282
Obesity	mesothelium	*CENPP*	TRUE	2.901280745
Obesity	mesothelium	*JAKMIP3*	TRUE	1.986678228
Obesity	mesothelium	*OSTN*	TRUE	2.334119579
Obesity	mesothelium	*SKOR1*	TRUE	2.296491458
Obesity	mesothelium	*FBF1*	TRUE	2.070215725
Obesity	mesothelium	*CPNE4*	TRUE	3.317823007
Obesity	mesothelium	*ZNF605*	TRUE	2.727959579
Obesity	mesothelium	*SULT1A2*	TRUE	8.476164186
Obesity	mesothelium	*MFAP5*	TRUE	2.446849655
Obesity	mesothelium	*DNAH10*	TRUE	4.482720193

Obesity	mesothelium	*RPS26*	TRUE	3.157749808
Obesity	mesothelium	*ZNF780A*	TRUE	3.859022904
Obesity	mesothelium	*ADH4*	TRUE	2.264974466
Obesity	mesothelium	*SH3BGRL2*	TRUE	1.968569149
Obesity	mesothelium	*SHISA4*	TRUE	5.504562917
Obesity	mesothelium	*C6orf163*	TRUE	2.339239171
Obesity	mesothelium	*GPANK1*	TRUE	3.72705833
Obesity	mesothelium	*C15orf59*	TRUE	2.062019686
Obesity	mesothelium	*SMN2*	TRUE	2.471806811
Obesity	mesothelium	*AC084018.1*	TRUE	3.625620799
Obesity	mesothelium	*ASIC3*	TRUE	2.46196668
Obesity	mesothelium	*SLC23A3*	TRUE	2.087762164
Obesity	mesothelium	*CPNE1*	TRUE	1.963107686
Obesity	mesothelium	*AL049840.1*	TRUE	1.986953956
Obesity	mesothelium	*GS1.124K5.4*	TRUE	2.086207169
Obesity	mesothelium	*ISY1*	TRUE	2.655217912
Obesity	mesothelium	*LINC00960*	TRUE	2.328554384
Obesity	mesothelium	*PLA2G4B*	TRUE	2.117493309
Obesity	mesothelium	*BRK1*	TRUE	2.580774185
Obesity	mesothelium	*AP001172.2*	TRUE	3.177271403
Obesity	mesothelium	*GTF2H5*	TRUE	2.037820937
Obesity	monocyte	*RBM6*	TRUE	3.47065819
Obesity	monocyte	*ITIH4*	TRUE	4.469042551
Obesity	monocyte	*MFAP2*	TRUE	2.099209111
Obesity	monocyte	*ATP8B3*	TRUE	2.950605883
Obesity	monocyte	*DPYSL4*	TRUE	2.070777806
Obesity	monocyte	*CLEC18A*	TRUE	7.372504141
Obesity	monocyte	*RNF123*	TRUE	2.177177512
Obesity	monocyte	*TTLL6*	TRUE	2.214241091
Obesity	monocyte	*C1QTNF4*	TRUE	4.90451996
Obesity	monocyte	*ORAI3*	TRUE	3.665439882
Obesity	monocyte	*NUTM2A*	TRUE	3.440048479
Obesity	monocyte	*BTN3A2*	TRUE	3.147276531
Obesity	monocyte	*NOTCH4*	TRUE	2.181698155
Obesity	monocyte	*AC084018.1*	TRUE	3.087258658
Obesity	monocyte	*LINC00664*	TRUE	2.495978227
Obesity	nk_cell	*RBM6*	TRUE	5.713580655
Obesity	nk_cell	*ITIH4*	TRUE	3.330537017
Obesity	nk_cell	*BTN3A3*	TRUE	2.000511744
Obesity	nk_cell	*MFAP2*	TRUE	2.109178044
Obesity	nk_cell	*RIBC2*	TRUE	3.092226424
Obesity	nk_cell	*HSD17B12*	TRUE	2.061994654
Obesity	nk_cell	*CLEC18A*	TRUE	6.772615042
Obesity	nk_cell	*GPRIN1*	TRUE	2.318803905
Obesity	nk_cell	*INO80E*	TRUE	3.261369134
Obesity	nk_cell	*ADORA2B*	TRUE	2.265095034

Obesity	nk_cell	*TRIM73*	TRUE	4.841060096
Obesity	nk_cell	*SULT1A2*	TRUE	6.946175093
Obesity	nk_cell	*ZNF780A*	TRUE	2.342826832
Obesity	nk_cell	*ZFP57*	TRUE	2.204394541
Obesity	pericyte	*RBM6*	TRUE	3.198840644
Obesity	pericyte	*ITIH4*	TRUE	2.968237967
Obesity	pericyte	*PABPC4*	TRUE	2.033359221
Obesity	pericyte	*CENPM*	TRUE	2.91577482
Obesity	pericyte	*SERPINC1*	TRUE	3.286651943
Obesity	pericyte	*GRK4*	TRUE	2.468690187
Obesity	pericyte	*RIBC2*	TRUE	2.422237556
Obesity	pericyte	*ATP8B3*	TRUE	5.233210711
Obesity	pericyte	*PKDREJ*	TRUE	2.650460567
Obesity	pericyte	*CBX8*	TRUE	2.302611618
Obesity	pericyte	*HSD17B12*	TRUE	5.213581751
Obesity	pericyte	*DPYSL4*	TRUE	2.708135747
Obesity	pericyte	*POC5*	TRUE	2.012108956
Obesity	pericyte	*CLEC18A*	TRUE	3.840103701
Obesity	pericyte	*ETS2*	TRUE	2.052983809
Obesity	pericyte	*SLC5A11*	TRUE	3.25386019
Obesity	pericyte	*CILP2*	TRUE	2.79651672
Obesity	pericyte	*HS3ST6*	TRUE	2.036136852
Obesity	pericyte	*RAPSN*	TRUE	4.000848786
Obesity	pericyte	*SRR*	TRUE	2.937407721
Obesity	pericyte	*GPRIN1*	TRUE	2.125503257
Obesity	pericyte	*ADORA2B*	TRUE	4.02271791
Obesity	pericyte	*TTLL6*	TRUE	2.755989899
Obesity	pericyte	*CTSW*	TRUE	4.553706212
Obesity	pericyte	*CADM2*	TRUE	2.042228361
Obesity	pericyte	*ORAI3*	TRUE	5.019397922
Obesity	pericyte	*RPP25*	TRUE	2.174432005
Obesity	pericyte	*TRIM73*	TRUE	2.134256096
Obesity	pericyte	*C3orf38*	TRUE	2.387609363
Obesity	pericyte	*RPRML*	TRUE	3.199979558
Obesity	pericyte	*ZFP41*	TRUE	2.065070682
Obesity	pericyte	*NUTM2A*	TRUE	3.957462897
Obesity	pericyte	*SULT1A2*	TRUE	6.858564297
Obesity	pericyte	*DNAH10*	TRUE	4.323641795
Obesity	pericyte	*RPS26*	TRUE	2.778904717
Obesity	pericyte	*ZNF780A*	TRUE	3.150381417
Obesity	pericyte	*GPANK1*	TRUE	2.274047347
Obesity	pericyte	*C17orf107*	TRUE	2.047175849
Obesity	pericyte	*SLC23A3*	TRUE	2.119190305
Obesity	pericyte	*GS1.124K5.4*	TRUE	2.014149574
Obesity	pericyte	*LINC00960*	TRUE	2.02668045
Obesity	pericyte	*LINC00664*	TRUE	2.32685937

Obesity	SMC	*CD79B*	TRUE	2.062686203
Obesity	SMC	*CENPM*	TRUE	2.130556552
Obesity	SMC	*SLC22A18*	TRUE	2.220370982
Obesity	SMC	*SERPINC1*	TRUE	2.170842969
Obesity	SMC	*GRK4*	TRUE	2.977234901
Obesity	SMC	*RIBC2*	TRUE	2.878985629
Obesity	SMC	*ATP8B3*	TRUE	5.73994882
Obesity	SMC	*PKDREJ*	TRUE	2.416837524
Obesity	SMC	*SLC37A4*	TRUE	2.561681696
Obesity	SMC	*C18orf8*	TRUE	2.231556983
Obesity	SMC	*MMEL1*	TRUE	2.319958472
Obesity	SMC	*HSD17B12*	TRUE	5.31995192
Obesity	SMC	*DPYSL4*	TRUE	2.96260229
Obesity	SMC	*MMP16*	TRUE	2.152371438
Obesity	SMC	*SLC5A11*	TRUE	3.91373291
Obesity	SMC	*SRR*	TRUE	3.018483985
Obesity	SMC	*INO80E*	TRUE	2.600772818
Obesity	SMC	*RGPD8*	TRUE	2.25886015
Obesity	SMC	*ADORA2B*	TRUE	3.841590414
Obesity	SMC	*TTLL6*	TRUE	5.812309868
Obesity	SMC	*CTSW*	TRUE	3.837113795
Obesity	SMC	*RPRML*	TRUE	2.813834845
Obesity	SMC	*PPP1R27*	TRUE	2.730555767
Obesity	SMC	*EMILIN3*	TRUE	2.829115671
Obesity	SMC	*ACOT1*	TRUE	2.645551626
Obesity	SMC	*NUTM2A*	TRUE	3.792541638
Obesity	SMC	*GP1BA*	TRUE	2.336892224
Obesity	SMC	*BTN3A2*	TRUE	2.977553063
Obesity	SMC	*SULT1A2*	TRUE	5.701033895
Obesity	SMC	*DNAH10*	TRUE	4.034161381
Obesity	SMC	*ZNF780A*	TRUE	3.145522079
Obesity	SMC	*GPANK1*	TRUE	2.388530318
Obesity	SMC	*C15orf59*	TRUE	2.077389606
Obesity	SMC	*AC084018.1*	TRUE	1.965495112
Obesity	SMC	*TMEM110*	TRUE	2.354456935
Obesity	SMC	*LINC00960*	TRUE	2.271910601
Obesity	t_cell	*RBM6*	TRUE	2.78566623
Obesity	t_cell	*PABPC4*	TRUE	2.309091304
Obesity	t_cell	*BTN3A3*	TRUE	3.134817571
Obesity	t_cell	*SERPINC1*	TRUE	1.976790546
Obesity	t_cell	*GRK4*	TRUE	2.22783984
Obesity	t_cell	*AP1M2*	TRUE	2.573651146
Obesity	t_cell	*ATP8B3*	TRUE	5.961838724
Obesity	t_cell	*CYP2E1*	TRUE	2.053949744
Obesity	t_cell	*PKDREJ*	TRUE	2.733683636
Obesity	t_cell	*FITM1*	TRUE	2.310243011

Obesity	t_cell	*HSD17B12*	TRUE	5.227429744
Obesity	t_cell	*DPYSL4*	TRUE	2.610996406
Obesity	t_cell	*CLEC18A*	TRUE	6.294185899
Obesity	t_cell	*SLC5A11*	TRUE	4.130911147
Obesity	t_cell	*CILP2*	TRUE	3.229613369
Obesity	t_cell	*SRR*	TRUE	3.102639602
Obesity	t_cell	*INO80E*	TRUE	2.510046781
Obesity	t_cell	*RGPD8*	TRUE	2.06926909
Obesity	t_cell	*ADORA2B*	TRUE	4.332597046
Obesity	t_cell	*TTLL6*	TRUE	5.066265337
Obesity	t_cell	*C1QTNF4*	TRUE	3.805577175
Obesity	t_cell	*ORAI3*	TRUE	3.401162834
Obesity	t_cell	*RPP25*	TRUE	2.475366053
Obesity	t_cell	*C3orf38*	TRUE	2.469920005
Obesity	t_cell	*RPRML*	TRUE	2.173089864
Obesity	t_cell	*PPP1R27*	TRUE	2.151296092
Obesity	t_cell	*ACOT1*	TRUE	3.499853422
Obesity	t_cell	*NUTM2A*	TRUE	2.658004672
Obesity	t_cell	*GP1BA*	TRUE	3.604849723
Obesity	t_cell	*SULT1A2*	TRUE	5.221392215
Obesity	t_cell	*MRPL21*	TRUE	2.482669448
Obesity	t_cell	*RPS26*	TRUE	2.552709744
Obesity	t_cell	*ZNF780A*	TRUE	2.562994878
Obesity	t_cell	*GPANK1*	TRUE	2.766575661
Obesity	t_cell	*AC084018.1*	TRUE	1.982904992
Obesity	t_cell	*TMEM110*	TRUE	3.907825656
Obesity	t_cell	*GS1.124K5.4*	TRUE	2.216484613
Obesity	t_cell	*DNAH10OS*	TRUE	2.929107254
Obesity	t_cell	*PDF*	TRUE	2.320133176
Stroke	adipocyte	*SNX11*	TRUE	2.012468032
Stroke	adipocyte	*CD79B*	TRUE	1.999049511
Stroke	adipocyte	*CENPQ*	TRUE	3.017709695
Stroke	adipocyte	*TBX5*	TRUE	2.778310385
Stroke	adipocyte	*CHRD*	TRUE	2.326567185
Stroke	adipocyte	*MARCH2*	TRUE	2.102573247
Stroke	adipocyte	*OSM*	TRUE	2.092672975
Stroke	adipocyte	*MLC1*	TRUE	2.399614835
Stroke	adipocyte	*C17orf75*	TRUE	2.184675179
Stroke	adipocyte	*BVES*	TRUE	2.509157244
Stroke	adipocyte	*DR1*	TRUE	2.480430579
Stroke	adipocyte	*EPCAM*	TRUE	2.271837387
Stroke	adipocyte	*BTN2A2*	TRUE	2.003715348
Stroke	adipocyte	*TRAP1*	TRUE	2.190876566
Stroke	adipocyte	*SLC2A11*	TRUE	2.162209434
Stroke	adipocyte	*LARS*	TRUE	2.157534722
Stroke	adipocyte	*TPMT*	TRUE	2.179238556

Stroke	adipocyte	*NOVA1*	TRUE	2.000243365
Stroke	adipocyte	*DOPEY2*	TRUE	2.473945209
Stroke	adipocyte	*LYG1*	TRUE	2.139365905
Stroke	adipocyte	*CLIC6*	TRUE	2.083482936
Stroke	adipocyte	*ICOSLG*	TRUE	2.470569425
Stroke	adipocyte	*ALDH16A1*	TRUE	2.517941215
Stroke	adipocyte	*DNASE1L3*	TRUE	2.232315187
Stroke	adipocyte	*CHRFAM7A*	TRUE	3.025986026
Stroke	adipocyte	*FAM57A*	TRUE	2.562223636
Stroke	adipocyte	*SCARA5*	TRUE	2.066956606
Stroke	adipocyte	*GPRIN1*	TRUE	2.186420536
Stroke	adipocyte	*GPR156*	TRUE	2.333041222
Stroke	adipocyte	*MYPOP*	TRUE	2.337999359
Stroke	adipocyte	*ZNF404*	TRUE	2.060679726
Stroke	adipocyte	*GEN1*	TRUE	2.450795607
Stroke	adipocyte	*YBEY*	TRUE	2.119328751
Stroke	adipocyte	*CLN8*	TRUE	2.114405798
Stroke	adipocyte	*EMILIN3*	TRUE	2.045474253
Stroke	adipocyte	*ZNF749*	TRUE	2.95382722
Stroke	adipocyte	*FAM86B1*	TRUE	1.98138865
Stroke	adipocyte	*QRFPR*	TRUE	2.051439423
Stroke	adipocyte	*JAKMIP3*	TRUE	2.277674462
Stroke	adipocyte	*WDR5B*	TRUE	2.386801174
Stroke	adipocyte	*CCDC154*	TRUE	2.275273611
Stroke	adipocyte	*TMEM116*	TRUE	2.828800698
Stroke	adipocyte	*AC084018.1*	TRUE	2.079999382
Stroke	adipocyte	*DNLZ*	TRUE	2.237046908
Stroke	adipocyte	*LINC01166*	TRUE	2.251292122
Stroke	adipocyte	*LILRA2*	TRUE	2.092771545
Stroke	adipocyte	*PNMA2*	TRUE	2.243035499
Stroke	adipocyte	*SPRR2F*	TRUE	2.06475897
Stroke	adipocyte	*ARMS2*	TRUE	1.977043317
Stroke	adipocyte	*LIN37*	TRUE	2.017534439
Stroke	adipocyte	*AC026740.1*	TRUE	2.15058548
Stroke	ASPC	*AOC1*	TRUE	2.259683176
Stroke	ASPC	*CD79B*	TRUE	2.005201507
Stroke	ASPC	*CENPQ*	TRUE	3.32602858
Stroke	ASPC	*TIPIN*	TRUE	2.148041987
Stroke	ASPC	*TBX5*	TRUE	2.544368641
Stroke	ASPC	*MARCH2*	TRUE	2.641228205
Stroke	ASPC	*OSM*	TRUE	2.111934527
Stroke	ASPC	*MLC1*	TRUE	2.615924258
Stroke	ASPC	*PYCARD*	TRUE	2.195463919
Stroke	ASPC	*TBL2*	TRUE	2.093470893
Stroke	ASPC	*ENO3*	TRUE	2.328516652
Stroke	ASPC	*BVES*	TRUE	2.441454143

Stroke	ASPC	*RASGRF2*	TRUE	2.16808069
Stroke	ASPC	*DR1*	TRUE	2.118289798
Stroke	ASPC	*EPCAM*	TRUE	2.599682933
Stroke	ASPC	*TRAP1*	TRUE	2.60110013
Stroke	ASPC	*QRSL1*	TRUE	2.193388546
Stroke	ASPC	*RGS22*	TRUE	2.00888748
Stroke	ASPC	*SLC2A11*	TRUE	2.797347204
Stroke	ASPC	*KLHL36*	TRUE	2.674719246
Stroke	ASPC	*CAPN9*	TRUE	2.475079471
Stroke	ASPC	*TPMT*	TRUE	2.100401922
Stroke	ASPC	*NOVA1*	TRUE	2.337631164
Stroke	ASPC	*DOPEY2*	TRUE	3.118876778
Stroke	ASPC	*LYG1*	TRUE	1.964144704
Stroke	ASPC	*NBEAL1*	TRUE	2.59974047
Stroke	ASPC	*PLA2R1*	TRUE	2.223551744
Stroke	ASPC	*NEIL2*	TRUE	2.05291467
Stroke	ASPC	*CLIC6*	TRUE	2.256599136
Stroke	ASPC	*ICOSLG*	TRUE	2.54371399
Stroke	ASPC	*CCR5*	TRUE	2.008453055
Stroke	ASPC	*ZNF577*	TRUE	2.287043439
Stroke	ASPC	*ALDH16A1*	TRUE	2.361746046
Stroke	ASPC	*DNASE1L3*	TRUE	2.584181133
Stroke	ASPC	*HSPA12A*	TRUE	2.221778398
Stroke	ASPC	*CHRFAM7A*	TRUE	2.433777933
Stroke	ASPC	*CNPY4*	TRUE	2.410375198
Stroke	ASPC	*PROCA1*	TRUE	2.016533825
Stroke	ASPC	*CHAF1A*	TRUE	2.07099775
Stroke	ASPC	*FAM57A*	TRUE	2.827203792
Stroke	ASPC	*GPRIN1*	TRUE	2.149772632
Stroke	ASPC	*GPR156*	TRUE	2.078787175
Stroke	ASPC	*CTDNEP1*	TRUE	2.603344447
Stroke	ASPC	*ZNF404*	TRUE	1.991497447
Stroke	ASPC	*GEN1*	TRUE	2.316788405
Stroke	ASPC	*MRPL14*	TRUE	2.040242322
Stroke	ASPC	*CLN8*	TRUE	2.445025212
Stroke	ASPC	*EMILIN3*	TRUE	1.971713249
Stroke	ASPC	*TMEM106A*	TRUE	2.131545626
Stroke	ASPC	*ZNF749*	TRUE	2.755363625
Stroke	ASPC	*FAM86B1*	TRUE	2.121486032
Stroke	ASPC	*QRFPR*	TRUE	2.200673525
Stroke	ASPC	*JAKMIP3*	TRUE	2.19634468
Stroke	ASPC	*STK31*	TRUE	2.150292776
Stroke	ASPC	*WDR5B*	TRUE	3.040565067
Stroke	ASPC	*CCDC154*	TRUE	2.147982166
Stroke	ASPC	*ZNF181*	TRUE	2.271354663
Stroke	ASPC	*TMEM116*	TRUE	3.158273127

Stroke	ASPC	*C20orf203*	TRUE	2.169186504
Stroke	ASPC	*COL15A1*	TRUE	2.005173686
Stroke	ASPC	*AC084018.1*	TRUE	2.04337204
Stroke	ASPC	*TCTEX1D2*	TRUE	1.986279229
Stroke	ASPC	*DNLZ*	TRUE	2.215252903
Stroke	ASPC	*AC009495.3*	TRUE	2.194857787
Stroke	ASPC	*LINC01166*	TRUE	2.573571577
Stroke	ASPC	*PSMB9*	TRUE	2.131351418
Stroke	ASPC	*PNMA2*	TRUE	2.593107159
Stroke	ASPC	*SPRR2F*	TRUE	2.192194464
Stroke	ASPC	*ARMS2*	TRUE	2.101184919
Stroke	ASPC	*LIN37*	TRUE	2.346222825
Stroke	b_cell	*CHRFAM7A*	TRUE	2.784975996
Stroke	dendritic_cell	*CENPQ*	TRUE	2.711239831
Stroke	dendritic_cell	*MLC1*	TRUE	2.057967631
Stroke	dendritic_cell	*DOPEY2*	TRUE	2.257406619
Stroke	dendritic_cell	*CHRFAM7A*	TRUE	2.800368867
Stroke	dendritic_cell	*ZNF404*	TRUE	1.973760712
Stroke	dendritic_cell	*YBEY*	TRUE	2.423911712
Stroke	dendritic_cell	*DNLZ*	TRUE	2.013551633
Stroke	endometrium	*MLC1*	TRUE	2.004405514
Stroke	endometrium	*ICOSLG*	TRUE	2.332899967
Stroke	endometrium	*CHRFAM7A*	TRUE	3.072563104
Stroke	endometrium	*CCDC154*	TRUE	1.99293583
Stroke	endometrium	*TMEM116*	TRUE	2.698889565
Stroke	endometrium	*DNLZ*	TRUE	2.117780284
Stroke	endothelial	*CENPQ*	TRUE	2.585174257
Stroke	endothelial	*OSM*	TRUE	2.062636396
Stroke	endothelial	*BVES*	TRUE	2.120169524
Stroke	endothelial	*ICOSLG*	TRUE	2.254266888
Stroke	endothelial	*CHRFAM7A*	TRUE	2.543229275
Stroke	endothelial	*YBEY*	TRUE	1.960418426
Stroke	endothelial	*CLN8*	TRUE	2.216821536
Stroke	endothelial	*ZNF749*	TRUE	2.359671666
Stroke	endothelial	*TMEM116*	TRUE	2.35209193
Stroke	endothelial	*HLA.DQB2*	TRUE	2.075279634
Stroke	endothelial	*LINC01166*	TRUE	2.031174736
Stroke	endothelial	*LIN37*	TRUE	2.167406849
Stroke	LEC	*CENPQ*	TRUE	2.521038418
Stroke	LEC	*CHRD*	TRUE	1.980823252
Stroke	LEC	*OSM*	TRUE	2.285327918
Stroke	LEC	*BVES*	TRUE	2.150087601
Stroke	LEC	*TPMT*	TRUE	2.280658424
Stroke	LEC	*RASL10B*	TRUE	2.155218929
Stroke	LEC	*DOPEY2*	TRUE	1.985644176
Stroke	LEC	*ICOSLG*	TRUE	2.335058276

Stroke	LEC	*CHRFAM7A*	TRUE	2.90675144
Stroke	LEC	*FAM57A*	TRUE	2.182511416
Stroke	LEC	*GPRIN1*	TRUE	2.244151257
Stroke	LEC	*GPR156*	TRUE	2.27539799
Stroke	LEC	*GEN1*	TRUE	2.384216766
Stroke	LEC	*YBEY*	TRUE	2.085051774
Stroke	LEC	*ZNF749*	TRUE	2.704636829
Stroke	LEC	*CCDC154*	TRUE	2.103641797
Stroke	LEC	*TMEM116*	TRUE	2.486336194
Stroke	LEC	*DNLZ*	TRUE	2.091820814
Stroke	LEC	*LINC01166*	TRUE	2.385913849
Stroke	macrophage	*CENPQ*	TRUE	3.377216357
Stroke	macrophage	*CHRD*	TRUE	2.1632069
Stroke	macrophage	*MARCH2*	TRUE	2.018044299
Stroke	macrophage	*MLC1*	TRUE	2.411228005
Stroke	macrophage	*BVES*	TRUE	2.429495733
Stroke	macrophage	*SLC2A11*	TRUE	2.092302736
Stroke	macrophage	*CLIC6*	TRUE	1.98297975
Stroke	macrophage	*ICOSLG*	TRUE	2.213730266
Stroke	macrophage	*DNASE1L3*	TRUE	2.261137959
Stroke	macrophage	*CHRFAM7A*	TRUE	2.9563358
Stroke	macrophage	*CHAF1A*	TRUE	1.988259842
Stroke	macrophage	*FAM57A*	TRUE	2.158172956
Stroke	macrophage	*ZNF404*	TRUE	2.038202607
Stroke	macrophage	*GEN1*	TRUE	2.085108792
Stroke	macrophage	*ZNF749*	TRUE	2.932017904
Stroke	macrophage	*QRFPR*	TRUE	2.226052668
Stroke	macrophage	*WDR5B*	TRUE	2.582463838
Stroke	macrophage	*TMEM116*	TRUE	2.977607066
Stroke	macrophage	*AC084018.1*	TRUE	2.027060802
Stroke	macrophage	*DNLZ*	TRUE	2.160286302
Stroke	macrophage	*HLA.DQB2*	TRUE	2.192756444
Stroke	macrophage	*SPRR2F*	TRUE	2.141169069
Stroke	macrophage	*AC026740.1*	TRUE	1.981173343
Stroke	mast_cell	*CENPQ*	TRUE	3.273189823
Stroke	mast_cell	*MLC1*	TRUE	1.988890932
Stroke	mast_cell	*BVES*	TRUE	2.291070513
Stroke	mast_cell	*BTN2A2*	TRUE	2.01134538
Stroke	mast_cell	*ICOSLG*	TRUE	2.392710827
Stroke	mast_cell	*ALDH16A1*	TRUE	2.512854714
Stroke	mast_cell	*CHRFAM7A*	TRUE	2.995220559
Stroke	mast_cell	*CHAF1A*	TRUE	1.987196366
Stroke	mast_cell	*YBEY*	TRUE	2.555691884
Stroke	mast_cell	*DNLZ*	TRUE	2.1399111
Stroke	mast_cell	*AC026740.1*	TRUE	1.961185954
Stroke	mesothelium	*AOC1*	TRUE	2.142053683

Stroke	mesothelium	*SNX11*	TRUE	2.039317235
Stroke	mesothelium	*CENPQ*	TRUE	2.878815569
Stroke	mesothelium	*ALDH18A1*	TRUE	2.010159414
Stroke	mesothelium	*TIPIN*	TRUE	1.961059866
Stroke	mesothelium	*TBX5*	TRUE	2.735083835
Stroke	mesothelium	*SPTLC1*	TRUE	2.06974791
Stroke	mesothelium	*CHRD*	TRUE	2.360072604
Stroke	mesothelium	*MARCH2*	TRUE	2.359462408
Stroke	mesothelium	*OSM*	TRUE	2.135487746
Stroke	mesothelium	*SLC35E4*	TRUE	2.009641693
Stroke	mesothelium	*MLC1*	TRUE	2.391362987
Stroke	mesothelium	*ZNF500*	TRUE	2.047877128
Stroke	mesothelium	*ZDHHC2*	TRUE	2.044636441
Stroke	mesothelium	*ENO3*	TRUE	2.178912394
Stroke	mesothelium	*C17orf75*	TRUE	2.28498408
Stroke	mesothelium	*BVES*	TRUE	2.539647687
Stroke	mesothelium	*SLC41A3*	TRUE	2.101896449
Stroke	mesothelium	*DR1*	TRUE	2.724036761
Stroke	mesothelium	*EPCAM*	TRUE	2.440237435
Stroke	mesothelium	*BTN2A2*	TRUE	2.087722743
Stroke	mesothelium	*TRAP1*	TRUE	2.350443866
Stroke	mesothelium	*SLC2A11*	TRUE	2.52552104
Stroke	mesothelium	*LARS*	TRUE	2.467921259
Stroke	mesothelium	*CAPN9*	TRUE	2.35774878
Stroke	mesothelium	*TPMT*	TRUE	2.416626753
Stroke	mesothelium	*ASCC1*	TRUE	2.012722472
Stroke	mesothelium	*NOVA1*	TRUE	2.246524597
Stroke	mesothelium	*RASL10B*	TRUE	2.400060173
Stroke	mesothelium	*SCRN2*	TRUE	2.13012386
Stroke	mesothelium	*MIEN1*	TRUE	2.199612399
Stroke	mesothelium	*DOPEY2*	TRUE	2.837448599
Stroke	mesothelium	*ARNT*	TRUE	2.320968838
Stroke	mesothelium	*LYG1*	TRUE	2.093493211
Stroke	mesothelium	*RGR*	TRUE	2.06761107
Stroke	mesothelium	*NMT2*	TRUE	2.442846514
Stroke	mesothelium	*ATP5G3*	TRUE	1.993340903
Stroke	mesothelium	*CLIC6*	TRUE	2.168311173
Stroke	mesothelium	*ICOSLG*	TRUE	2.460131185
Stroke	mesothelium	*CCR5*	TRUE	2.104510343
Stroke	mesothelium	*ALDH16A1*	TRUE	2.560495859
Stroke	mesothelium	*DNASE1L3*	TRUE	2.438144699
Stroke	mesothelium	*CHRFAM7A*	TRUE	3.08070772
Stroke	mesothelium	*CNPY4*	TRUE	2.160012211
Stroke	mesothelium	*CHAF1A*	TRUE	2.075066753
Stroke	mesothelium	*FAM57A*	TRUE	2.690725854
Stroke	mesothelium	*ULK4*	TRUE	2.314035844

Stroke	mesothelium	*SCARA5*	TRUE	2.423983391
Stroke	mesothelium	*GPRIN1*	TRUE	2.229287881
Stroke	mesothelium	*USP38*	TRUE	2.192043121
Stroke	mesothelium	*GAA*	TRUE	2.064359627
Stroke	mesothelium	*RGS19*	TRUE	2.001797278
Stroke	mesothelium	*GPR156*	TRUE	2.571512575
Stroke	mesothelium	*MYPOP*	TRUE	2.914287565
Stroke	mesothelium	*GEN1*	TRUE	2.576841615
Stroke	mesothelium	*ZFPM1*	TRUE	2.076068694
Stroke	mesothelium	*CHST13*	TRUE	2.020374007
Stroke	mesothelium	*YBEY*	TRUE	2.122809641
Stroke	mesothelium	*CLN8*	TRUE	2.305221825
Stroke	mesothelium	*EMILIN3*	TRUE	2.061643826
Stroke	mesothelium	*PIGP*	TRUE	2.004826998
Stroke	mesothelium	*ZNF749*	TRUE	2.995868542
Stroke	mesothelium	*CLCNKA*	TRUE	2.00381894
Stroke	mesothelium	*JAKMIP3*	TRUE	2.316148759
Stroke	mesothelium	*FAM26F*	TRUE	2.113190963
Stroke	mesothelium	*WDR5B*	TRUE	2.753928134
Stroke	mesothelium	*PIWIL2*	TRUE	2.127236229
Stroke	mesothelium	*CCDC154*	TRUE	2.162345097
Stroke	mesothelium	*ZNF181*	TRUE	1.998793061
Stroke	mesothelium	*TMEM116*	TRUE	2.619317763
Stroke	mesothelium	*COL15A1*	TRUE	2.070614276
Stroke	mesothelium	*AC084018.1*	TRUE	2.104571973
Stroke	mesothelium	*DNLZ*	TRUE	2.007352723
Stroke	mesothelium	*LTB4R*	TRUE	1.990394763
Stroke	mesothelium	*NBPF1*	TRUE	2.256499192
Stroke	mesothelium	*AC009495.3*	TRUE	2.252402334
Stroke	mesothelium	*LINC01166*	TRUE	2.462895803
Stroke	mesothelium	*LINC00342*	TRUE	1.962976103
Stroke	mesothelium	*CDKN2AIPNL*	TRUE	2.077594688
Stroke	mesothelium	*LILRA2*	TRUE	2.151204672
Stroke	mesothelium	*PNMA2*	TRUE	2.133470616
Stroke	mesothelium	*SPRR2F*	TRUE	2.22307037
Stroke	mesothelium	*ARMS2*	TRUE	2.050572199
Stroke	mesothelium	*LIN37*	TRUE	2.266804835
Stroke	mesothelium	*AC026740.1*	TRUE	2.047656151
Stroke	monocyte	*ICOSLG*	TRUE	2.442432255
Stroke	monocyte	*HLA.DQB1*	TRUE	1.978442934
Stroke	monocyte	*ZNF749*	TRUE	2.931938618
Stroke	monocyte	*TMEM116*	TRUE	2.28389238
Stroke	monocyte	*DNLZ*	TRUE	2.130602669
Stroke	nk_cell	*CENPQ*	TRUE	3.136545856
Stroke	nk_cell	*ICOSLG*	TRUE	2.029325904
Stroke	nk_cell	*CHRFAM7A*	TRUE	2.836707382

Stroke	nk_cell	*CHAF1A*	TRUE	2.012822691
Stroke	nk_cell	*GPRIN1*	TRUE	2.065784252
Stroke	nk_cell	*YBEY*	TRUE	2.043144141
Stroke	nk_cell	*ZNF749*	TRUE	2.184687172
Stroke	nk_cell	*LINC01166*	TRUE	2.194841329
Stroke	pericyte	*CENPQ*	TRUE	3.057503769
Stroke	pericyte	*OSM*	TRUE	1.976294466
Stroke	pericyte	*MLC1*	TRUE	2.071735415
Stroke	pericyte	*BVES*	TRUE	2.334980432
Stroke	pericyte	*EPCAM*	TRUE	2.070681988
Stroke	pericyte	*BTN2A2*	TRUE	2.005538793
Stroke	pericyte	*TRAP1*	TRUE	2.096083059
Stroke	pericyte	*TPMT*	TRUE	1.986029058
Stroke	pericyte	*NOVA1*	TRUE	1.996088636
Stroke	pericyte	*DOPEY2*	TRUE	2.469757963
Stroke	pericyte	*CLIC6*	TRUE	2.036503865
Stroke	pericyte	*ICOSLG*	TRUE	2.228773092
Stroke	pericyte	*ALDH16A1*	TRUE	2.292841168
Stroke	pericyte	*CHRFAM7A*	TRUE	2.998998369
Stroke	pericyte	*GEN1*	TRUE	2.072686241
Stroke	pericyte	*ZNF749*	TRUE	2.84019226
Stroke	pericyte	*TMEM116*	TRUE	2.647540303
Stroke	pericyte	*DNLZ*	TRUE	2.168058165
Stroke	pericyte	*LINC01166*	TRUE	2.415858981
Stroke	pericyte	*PNMA2*	TRUE	2.245530092
Stroke	pericyte	*SPRR2F*	TRUE	2.197149465
Stroke	SMC	*CENPQ*	TRUE	2.98468532
Stroke	SMC	*C17orf75*	TRUE	2.042551716
Stroke	SMC	*DR1*	TRUE	2.372960992
Stroke	SMC	*TPMT*	TRUE	2.033528384
Stroke	SMC	*LYG1*	TRUE	2.022892242
Stroke	SMC	*ALDH16A1*	TRUE	2.42314239
Stroke	SMC	*CHRFAM7A*	TRUE	2.625824307
Stroke	SMC	*ZNF404*	TRUE	1.974108196
Stroke	SMC	*GEN1*	TRUE	2.255810729
Stroke	SMC	*YBEY*	TRUE	2.607224659
Stroke	SMC	*ZNF749*	TRUE	2.962934123
Stroke	SMC	*TMEM116*	TRUE	3.105257644
Stroke	SMC	*CR1*	TRUE	2.079201394
Stroke	SMC	*DNLZ*	TRUE	2.109806782
Stroke	t_cell	*CENPQ*	TRUE	2.786970162
Stroke	t_cell	*MLC1*	TRUE	2.103903081
Stroke	t_cell	*BTN2A2*	TRUE	1.999667841
Stroke	t_cell	*SLC2A11*	TRUE	2.001696587
Stroke	t_cell	*SCRN2*	TRUE	1.963655516
Stroke	t_cell	*DOPEY2*	TRUE	2.54692767

Stroke	t_cell	*ICOSLG*	TRUE	2.152815318
Stroke	t_cell	*CHRFAM7A*	TRUE	2.828556413
Stroke	t_cell	*GEN1*	TRUE	2.13135521
Stroke	t_cell	*ZNF749*	TRUE	2.561807651
Stroke	t_cell	*CCDC154*	TRUE	2.054078681
Stroke	t_cell	*TMEM116*	TRUE	2.404924816
Stroke	t_cell	*DNLZ*	TRUE	2.223558505
Heart_Failure	adipocyte	*SLC7A9*	TRUE	2.476123053
Heart_Failure	adipocyte	*LAMC2*	TRUE	2.515965089
Heart_Failure	adipocyte	*CROCC*	TRUE	2.024056745
Heart_Failure	adipocyte	*MAST2*	TRUE	2.608176231
Heart_Failure	adipocyte	*SETD1A*	TRUE	2.78116359
Heart_Failure	adipocyte	*SMARCB1*	TRUE	2.59315539
Heart_Failure	adipocyte	*TOX3*	TRUE	2.600949122
Heart_Failure	adipocyte	*CTSH*	TRUE	2.224099378
Heart_Failure	adipocyte	*TIMM44*	TRUE	2.256766455
Heart_Failure	adipocyte	*CD33*	TRUE	1.996417998
Heart_Failure	adipocyte	*RAB5C*	TRUE	2.009944357
Heart_Failure	adipocyte	*KIF20A*	TRUE	3.697263857
Heart_Failure	adipocyte	*BNIP1*	TRUE	2.491604059
Heart_Failure	adipocyte	*IL18R1*	TRUE	3.847624129
Heart_Failure	adipocyte	*CTNNAL1*	TRUE	2.84153713
Heart_Failure	adipocyte	*GRK4*	TRUE	2.082833785
Heart_Failure	adipocyte	*ANGPTL6*	TRUE	2.437436854
Heart_Failure	adipocyte	*NMT1*	TRUE	2.954100037
Heart_Failure	adipocyte	*ALG10*	TRUE	1.96533639
Heart_Failure	adipocyte	*PNMT*	TRUE	2.90810301
Heart_Failure	adipocyte	*EVA1B*	TRUE	2.199510271
Heart_Failure	adipocyte	*FLG*	TRUE	2.821792245
Heart_Failure	adipocyte	*TSLP*	TRUE	4.27586407
Heart_Failure	adipocyte	*ROM1*	TRUE	3.103881609
Heart_Failure	adipocyte	*DPYSL4*	TRUE	2.130959346
Heart_Failure	adipocyte	*FZD7*	TRUE	2.13354253
Heart_Failure	adipocyte	*FCRL3*	TRUE	2.597895847
Heart_Failure	adipocyte	*FGFR4*	TRUE	2.669693778
Heart_Failure	adipocyte	*KLHDC9*	TRUE	2.687052015
Heart_Failure	adipocyte	*EFHB*	TRUE	2.7583911
Heart_Failure	adipocyte	*MST1R*	TRUE	2.463217477
Heart_Failure	adipocyte	*GPER1*	TRUE	2.515929689
Heart_Failure	adipocyte	*HSPA12A*	TRUE	2.203905443
Heart_Failure	adipocyte	*HDHD2*	TRUE	2.714087395
Heart_Failure	adipocyte	*ZNF23*	TRUE	2.95421401
Heart_Failure	adipocyte	*TRPV3*	TRUE	2.286519824
Heart_Failure	adipocyte	*GSDMA*	TRUE	2.143603203
Heart_Failure	adipocyte	*CHRNB1*	TRUE	2.527115784
Heart_Failure	adipocyte	*TMEM133*	TRUE	2.306843484

Heart_Failure	adipocyte	*PAAF1*	TRUE	2.025768319
Heart_Failure	adipocyte	*RPS6KB2*	TRUE	2.712724808
Heart_Failure	adipocyte	*OR4N2*	TRUE	2.003350542
Heart_Failure	adipocyte	*DPY19L2*	TRUE	2.207415407
Heart_Failure	adipocyte	*ZNF454*	TRUE	2.708512492
Heart_Failure	adipocyte	*LINC00314*	TRUE	2.32635356
Heart_Failure	adipocyte	*TRIM73*	TRUE	2.113618122
Heart_Failure	adipocyte	*HLA.DQB1*	TRUE	4.161773076
Heart_Failure	adipocyte	*D2HGDH*	TRUE	2.710643939
Heart_Failure	adipocyte	*FKRP*	TRUE	2.051652489
Heart_Failure	adipocyte	*A3GALT2*	TRUE	2.277809488
Heart_Failure	adipocyte	*QRFPR*	TRUE	2.517079897
Heart_Failure	adipocyte	*TMEM232*	TRUE	2.489641262
Heart_Failure	adipocyte	*WDR5B*	TRUE	2.182178509
Heart_Failure	adipocyte	*PDCD1LG2*	TRUE	2.826753591
Heart_Failure	adipocyte	*RPS26*	TRUE	2.990053597
Heart_Failure	adipocyte	*MICA*	TRUE	3.566591027
Heart_Failure	adipocyte	*ATF6B*	TRUE	2.728278958
Heart_Failure	adipocyte	*CPNE1*	TRUE	2.310527217
Heart_Failure	adipocyte	*OR2A1*	TRUE	2.88559975
Heart_Failure	adipocyte	*ZNF736*	TRUE	2.495496521
Heart_Failure	adipocyte	*ZBTB22*	TRUE	2.721540426
Heart_Failure	adipocyte	*TTC4*	TRUE	2.702398086
Heart_Failure	adipocyte	*LILRA6*	TRUE	2.366427753
Heart_Failure	adipocyte	*MC1R*	TRUE	2.101035743
Heart_Failure	adipocyte	*GTF2H5*	TRUE	2.382966672
Heart_Failure	ASPC	*CLCA4*	TRUE	2.054357424
Heart_Failure	ASPC	*SLC7A9*	TRUE	2.536497227
Heart_Failure	ASPC	*LAMC2*	TRUE	2.633414899
Heart_Failure	ASPC	*CROCC*	TRUE	2.128700626
Heart_Failure	ASPC	*BPIFB2*	TRUE	2.019187336
Heart_Failure	ASPC	*MAST2*	TRUE	2.820241975
Heart_Failure	ASPC	*FXYD5*	TRUE	2.006474279
Heart_Failure	ASPC	*PABPC4*	TRUE	2.52088209
Heart_Failure	ASPC	*SETD1A*	TRUE	2.812154019
Heart_Failure	ASPC	*SMARCB1*	TRUE	2.62897569
Heart_Failure	ASPC	*TOX3*	TRUE	2.566661704
Heart_Failure	ASPC	*CTSH*	TRUE	2.399828084
Heart_Failure	ASPC	*TIMM44*	TRUE	2.167476311
Heart_Failure	ASPC	*CD33*	TRUE	2.139314139
Heart_Failure	ASPC	*RAB5C*	TRUE	2.234126084
Heart_Failure	ASPC	*PRDM4*	TRUE	2.272160443
Heart_Failure	ASPC	*VNN2*	TRUE	2.074966297
Heart_Failure	ASPC	*KIF20A*	TRUE	3.701543596
Heart_Failure	ASPC	*BNIP1*	TRUE	2.517869745
Heart_Failure	ASPC	*IL18R1*	TRUE	4.085580936

Heart_Failure	ASPC	*ALDH8A1*	TRUE	2.204299082
Heart_Failure	ASPC	*CTNNAL1*	TRUE	2.864217402
Heart_Failure	ASPC	*ITIH5*	TRUE	2.525075905
Heart_Failure	ASPC	*GRK4*	TRUE	2.173976
Heart_Failure	ASPC	*PROZ*	TRUE	2.035669691
Heart_Failure	ASPC	*ANGPTL6*	TRUE	2.486540067
Heart_Failure	ASPC	*ADAM19*	TRUE	2.313603307
Heart_Failure	ASPC	*NMT1*	TRUE	2.889192497
Heart_Failure	ASPC	*ALG10*	TRUE	2.508591273
Heart_Failure	ASPC	*PNMT*	TRUE	3.348022671
Heart_Failure	ASPC	*EVA1B*	TRUE	2.832306462
Heart_Failure	ASPC	*FLG*	TRUE	3.106300913
Heart_Failure	ASPC	*GPR17*	TRUE	2.156620592
Heart_Failure	ASPC	*BHMT*	TRUE	2.039880947
Heart_Failure	ASPC	*TSLP*	TRUE	4.667439173
Heart_Failure	ASPC	*ROM1*	TRUE	3.042609004
Heart_Failure	ASPC	*DPYSL4*	TRUE	2.132430361
Heart_Failure	ASPC	*FZD7*	TRUE	2.720827023
Heart_Failure	ASPC	*SIM2*	TRUE	2.165211706
Heart_Failure	ASPC	*FCRL3*	TRUE	2.698886332
Heart_Failure	ASPC	*FGFR4*	TRUE	2.803452938
Heart_Failure	ASPC	*KLHDC9*	TRUE	2.960151358
Heart_Failure	ASPC	*NOSTRIN*	TRUE	2.170529993
Heart_Failure	ASPC	*EFHB*	TRUE	2.759092833
Heart_Failure	ASPC	*DNALI1*	TRUE	2.179172155
Heart_Failure	ASPC	*MST1R*	TRUE	2.824465019
Heart_Failure	ASPC	*SAP30L*	TRUE	2.011812374
Heart_Failure	ASPC	*GPER1*	TRUE	2.559817273
Heart_Failure	ASPC	*HSPA12A*	TRUE	2.084940293
Heart_Failure	ASPC	*PIP4K2C*	TRUE	2.366313779
Heart_Failure	ASPC	*HDHD2*	TRUE	2.126866229
Heart_Failure	ASPC	*ZNF23*	TRUE	2.925406088
Heart_Failure	ASPC	*TRPV3*	TRUE	2.12775905
Heart_Failure	ASPC	*GSDMA*	TRUE	2.22104226
Heart_Failure	ASPC	*ZSWIM1*	TRUE	2.041632888
Heart_Failure	ASPC	*CHRNB1*	TRUE	2.607453057
Heart_Failure	ASPC	*TMEM133*	TRUE	2.29278841
Heart_Failure	ASPC	*LGALS4*	TRUE	1.985356257
Heart_Failure	ASPC	*PARP15*	TRUE	2.096117526
Heart_Failure	ASPC	*PAAF1*	TRUE	2.600860957
Heart_Failure	ASPC	*RPS6KB2*	TRUE	2.581868252
Heart_Failure	ASPC	*OR4N2*	TRUE	2.225691556
Heart_Failure	ASPC	*DPY19L2*	TRUE	2.779047125
Heart_Failure	ASPC	*ZNF454*	TRUE	2.802565899
Heart_Failure	ASPC	*LINC00314*	TRUE	2.282086249
Heart_Failure	ASPC	*HLA.DQB1*	TRUE	4.110523959

Heart_Failure	ASPC	*LBX2*	TRUE	2.120252474
Heart_Failure	ASPC	*D2HGDH*	TRUE	2.733138195
Heart_Failure	ASPC	*FKRP*	TRUE	2.236523966
Heart_Failure	ASPC	*PSMG1*	TRUE	2.006433151
Heart_Failure	ASPC	*A3GALT2*	TRUE	2.391020385
Heart_Failure	ASPC	*SIGIRR*	TRUE	2.292293316
Heart_Failure	ASPC	*C14orf80*	TRUE	1.97619241
Heart_Failure	ASPC	*GOLGA8R*	TRUE	2.064828075
Heart_Failure	ASPC	*QRFPR*	TRUE	2.881672444
Heart_Failure	ASPC	*TMEM232*	TRUE	2.948374329
Heart_Failure	ASPC	*MRPS21*	TRUE	2.485022553
Heart_Failure	ASPC	*SLC15A5*	TRUE	2.014609183
Heart_Failure	ASPC	*WDR5B*	TRUE	2.232682818
Heart_Failure	ASPC	*PDCD1LG2*	TRUE	2.873469204
Heart_Failure	ASPC	*RPS26*	TRUE	2.902225703
Heart_Failure	ASPC	*MICA*	TRUE	3.332231963
Heart_Failure	ASPC	*ATF6B*	TRUE	2.390904079
Heart_Failure	ASPC	*ZNF891*	TRUE	2.10667481
Heart_Failure	ASPC	*CPNE1*	TRUE	2.540193077
Heart_Failure	ASPC	*OR2A1*	TRUE	2.850038581
Heart_Failure	ASPC	*ZNF736*	TRUE	2.712221046
Heart_Failure	ASPC	*ZBTB22*	TRUE	2.394869611
Heart_Failure	ASPC	*TTC4*	TRUE	3.082561614
Heart_Failure	ASPC	*LILRA6*	TRUE	2.36844137
Heart_Failure	ASPC	*MEX3A*	TRUE	2.013334352
Heart_Failure	ASPC	*MC1R*	TRUE	2.146435084
Heart_Failure	ASPC	*PWRN1*	TRUE	2.260170694
Heart_Failure	ASPC	*GTF2H5*	TRUE	2.674366584
Heart_Failure	b_cell	*TSLP*	TRUE	3.898578905
Heart_Failure	b_cell	*GSDMA*	TRUE	2.270986701
Heart_Failure	b_cell	*HLA.DQA2*	TRUE	6.850664572
Heart_Failure	dendritic_cell	*RBM6*	TRUE	2.862298577
Heart_Failure	dendritic_cell	*SLC7A9*	TRUE	2.000039966
Heart_Failure	dendritic_cell	*IL18R1*	TRUE	2.046401711
Heart_Failure	dendritic_cell	*FLG*	TRUE	2.825255203
Heart_Failure	dendritic_cell	*TSLP*	TRUE	2.852377133
Heart_Failure	dendritic_cell	*ROM1*	TRUE	2.188731939
Heart_Failure	dendritic_cell	*KLHDC9*	TRUE	2.21724931
Heart_Failure	dendritic_cell	*GSDMA*	TRUE	5.308672637
Heart_Failure	dendritic_cell	*ZNF454*	TRUE	2.30794781
Heart_Failure	dendritic_cell	*ZNF385C*	TRUE	1.984048852
Heart_Failure	dendritic_cell	*RPS26*	TRUE	2.76495863
Heart_Failure	dendritic_cell	*PSMB9*	TRUE	2.906506457
Heart_Failure	endometrium	*TSLP*	TRUE	4.159514581
Heart_Failure	endometrium	*ROM1*	TRUE	2.654664394
Heart_Failure	endometrium	*HDHD2*	TRUE	2.516169456

Heart_Failure	endometrium	*GSDMA*	TRUE	2.653378162
Heart_Failure	endometrium	*LINC00314*	TRUE	2.130818436
Heart_Failure	endometrium	*HLA.DQB1*	TRUE	3.587299127
Heart_Failure	endometrium	*HLA.DQB2*	TRUE	3.41265777
Heart_Failure	endometrium	*ZNF736*	TRUE	2.668998611
Heart_Failure	endothelial	*SLC7A9*	TRUE	2.260449439
Heart_Failure	endothelial	*CDK2AP1*	TRUE	2.395548332
Heart_Failure	endothelial	*TSLP*	TRUE	3.148163269
Heart_Failure	endothelial	*FCRL3*	TRUE	2.389640702
Heart_Failure	endothelial	*MST1R*	TRUE	3.469881499
Heart_Failure	endothelial	*GSDMA*	TRUE	4.810977516
Heart_Failure	endothelial	*CHRNB1*	TRUE	2.52291724
Heart_Failure	endothelial	*HLA.DQB1*	TRUE	3.350352835
Heart_Failure	endothelial	*RPS26*	TRUE	2.611286754
Heart_Failure	endothelial	*LILRA6*	TRUE	2.00724219
Heart_Failure	LEC	*SLC7A9*	TRUE	2.409006223
Heart_Failure	LEC	*LAMC2*	TRUE	2.321946986
Heart_Failure	LEC	*PNMT*	TRUE	2.19839101
Heart_Failure	LEC	*FLG*	TRUE	2.430723407
Heart_Failure	LEC	*TSLP*	TRUE	3.992547053
Heart_Failure	LEC	*FGFR4*	TRUE	2.46739806
Heart_Failure	LEC	*KLHDC9*	TRUE	2.290575804
Heart_Failure	LEC	*MST1R*	TRUE	3.004428547
Heart_Failure	LEC	*GSDMA*	TRUE	2.777852508
Heart_Failure	LEC	*CHRNB1*	TRUE	2.453757665
Heart_Failure	LEC	*DPY19L2*	TRUE	2.163916079
Heart_Failure	LEC	*ZNF454*	TRUE	2.729620981
Heart_Failure	LEC	*LINC00314*	TRUE	2.139572666
Heart_Failure	LEC	*HLA.DQB1*	TRUE	4.403319376
Heart_Failure	LEC	*RPS26*	TRUE	3.162160176
Heart_Failure	LEC	*MICA*	TRUE	2.675637223
Heart_Failure	macrophage	*SLC7A9*	TRUE	2.532419314
Heart_Failure	macrophage	*MAST2*	TRUE	2.048207897
Heart_Failure	macrophage	*SETD1A*	TRUE	2.21104569
Heart_Failure	macrophage	*SMARCB1*	TRUE	2.340669692
Heart_Failure	macrophage	*KIF20A*	TRUE	2.493122154
Heart_Failure	macrophage	*BNIP1*	TRUE	2.33921991
Heart_Failure	macrophage	*ALDH8A1*	TRUE	2.077396016
Heart_Failure	macrophage	*ANGPTL6*	TRUE	2.15543009
Heart_Failure	macrophage	*NMT1*	TRUE	2.326999813
Heart_Failure	macrophage	*PNMT*	TRUE	5.31643103
Heart_Failure	macrophage	*EVA1B*	TRUE	2.933684511
Heart_Failure	macrophage	*FLG*	TRUE	2.683023821
Heart_Failure	macrophage	*TSLP*	TRUE	4.518360384
Heart_Failure	macrophage	*ROM1*	TRUE	2.256484906
Heart_Failure	macrophage	*DPYSL4*	TRUE	2.124075162

Heart_Failure	macrophage	*SIM2*	TRUE	1.960583222
Heart_Failure	macrophage	*FCRL3*	TRUE	2.203986793
Heart_Failure	macrophage	*FGFR4*	TRUE	1.985776101
Heart_Failure	macrophage	*KLHDC9*	TRUE	2.535304969
Heart_Failure	macrophage	*EFHB*	TRUE	2.679817183
Heart_Failure	macrophage	*HDHD2*	TRUE	2.591653403
Heart_Failure	macrophage	*CHRNB1*	TRUE	2.509517184
Heart_Failure	macrophage	*PAAF1*	TRUE	2.182367415
Heart_Failure	macrophage	*RPS6KB2*	TRUE	2.192475484
Heart_Failure	macrophage	*LINC00314*	TRUE	1.984079969
Heart_Failure	macrophage	*TRIM73*	TRUE	2.119701697
Heart_Failure	macrophage	*FKRP*	TRUE	2.084606155
Heart_Failure	macrophage	*GOLGA8R*	TRUE	2.012784394
Heart_Failure	macrophage	*QRFPR*	TRUE	2.808453054
Heart_Failure	macrophage	*WDR5B*	TRUE	1.984677592
Heart_Failure	macrophage	*PDCD1LG2*	TRUE	2.587381506
Heart_Failure	macrophage	*RPS26*	TRUE	2.942637938
Heart_Failure	macrophage	*HSD17B8*	TRUE	1.965651283
Heart_Failure	macrophage	*MICA*	TRUE	2.940135048
Heart_Failure	macrophage	*ATF6B*	TRUE	1.989648224
Heart_Failure	macrophage	*CPNE1*	TRUE	2.477075264
Heart_Failure	macrophage	*ZNF736*	TRUE	2.361354104
Heart_Failure	macrophage	*ZBTB22*	TRUE	2.442405017
Heart_Failure	macrophage	*HLA.DQA2*	TRUE	3.624910918
Heart_Failure	macrophage	*TTC4*	TRUE	2.481236699
Heart_Failure	mast_cell	*PNMT*	TRUE	2.576738464
Heart_Failure	mast_cell	*EVA1B*	TRUE	3.395010061
Heart_Failure	mast_cell	*TSLP*	TRUE	3.090753323
Heart_Failure	mast_cell	*EFHB*	TRUE	2.527361842
Heart_Failure	mast_cell	*GSDMA*	TRUE	2.731641155
Heart_Failure	mast_cell	*HLA.DQB1*	TRUE	5.516632707
Heart_Failure	mesothelium	*RBM6*	TRUE	3.221842511
Heart_Failure	mesothelium	*CYB561*	TRUE	2.106496628
Heart_Failure	mesothelium	*CLCA4*	TRUE	2.216150054
Heart_Failure	mesothelium	*SLC7A9*	TRUE	2.490195568
Heart_Failure	mesothelium	*LAMC2*	TRUE	2.950672218
Heart_Failure	mesothelium	*AHRR*	TRUE	2.099067051
Heart_Failure	mesothelium	*ADD2*	TRUE	2.109115959
Heart_Failure	mesothelium	*BPIFB2*	TRUE	2.144605656
Heart_Failure	mesothelium	*OXCT1*	TRUE	2.346141509
Heart_Failure	mesothelium	*MLF2*	TRUE	2.049961417
Heart_Failure	mesothelium	*PABPC4*	TRUE	3.510457096
Heart_Failure	mesothelium	*SMARCB1*	TRUE	2.177333623
Heart_Failure	mesothelium	*NDUFAF5*	TRUE	2.499124859
Heart_Failure	mesothelium	*TOX3*	TRUE	2.545307172
Heart_Failure	mesothelium	*CTSH*	TRUE	2.643896179

Heart_Failure	mesothelium	*CD33*	TRUE	2.236931996
Heart_Failure	mesothelium	*RGP1*	TRUE	1.972895879
Heart_Failure	mesothelium	*KCTD10*	TRUE	2.755500694
Heart_Failure	mesothelium	*CDK2AP1*	TRUE	2.532644691
Heart_Failure	mesothelium	*KIF20A*	TRUE	3.756807541
Heart_Failure	mesothelium	*BNIP1*	TRUE	2.215963631
Heart_Failure	mesothelium	*IL18R1*	TRUE	3.444695559
Heart_Failure	mesothelium	*CTNNAL1*	TRUE	2.229612447
Heart_Failure	mesothelium	*ITIH5*	TRUE	3.06343408
Heart_Failure	mesothelium	*PROZ*	TRUE	2.031682824
Heart_Failure	mesothelium	*ZNF384*	TRUE	2.037814329
Heart_Failure	mesothelium	*ANGPTL6*	TRUE	2.445740404
Heart_Failure	mesothelium	*LRRC41*	TRUE	2.00770317
Heart_Failure	mesothelium	*FIGNL1*	TRUE	2.441570811
Heart_Failure	mesothelium	*ADAM19*	TRUE	2.047810148
Heart_Failure	mesothelium	*POM121C*	TRUE	2.994134495
Heart_Failure	mesothelium	*TSGA10*	TRUE	2.010372865
Heart_Failure	mesothelium	*ALG10*	TRUE	3.020772782
Heart_Failure	mesothelium	*FN3KRP*	TRUE	2.46574147
Heart_Failure	mesothelium	*PNMT*	TRUE	3.020118672
Heart_Failure	mesothelium	*EVA1B*	TRUE	2.789405229
Heart_Failure	mesothelium	*NME7*	TRUE	2.084331452
Heart_Failure	mesothelium	*FLG*	TRUE	3.060191
Heart_Failure	mesothelium	*GPR17*	TRUE	2.193000608
Heart_Failure	mesothelium	*TSLP*	TRUE	4.159175006
Heart_Failure	mesothelium	*SLC18B1*	TRUE	2.369303517
Heart_Failure	mesothelium	*HSD17B12*	TRUE	2.250341947
Heart_Failure	mesothelium	*ROM1*	TRUE	2.803250529
Heart_Failure	mesothelium	*DPYSL4*	TRUE	2.133437549
Heart_Failure	mesothelium	*POC5*	TRUE	2.083309081
Heart_Failure	mesothelium	*FZD7*	TRUE	2.031364247
Heart_Failure	mesothelium	*WHAMM*	TRUE	2.288061432
Heart_Failure	mesothelium	*DHRS1*	TRUE	2.033249626
Heart_Failure	mesothelium	*RNF166*	TRUE	2.231852237
Heart_Failure	mesothelium	*CDC42SE2*	TRUE	2.003590286
Heart_Failure	mesothelium	*SIM2*	TRUE	1.984631292
Heart_Failure	mesothelium	*C21orf33*	TRUE	2.102958283
Heart_Failure	mesothelium	*FCRL3*	TRUE	2.754065281
Heart_Failure	mesothelium	*FGFR4*	TRUE	2.981270374
Heart_Failure	mesothelium	*KLHDC9*	TRUE	2.551125782
Heart_Failure	mesothelium	*EFHB*	TRUE	2.603771575
Heart_Failure	mesothelium	*RCHY1*	TRUE	2.133801665
Heart_Failure	mesothelium	*RAD54L2*	TRUE	2.03948695
Heart_Failure	mesothelium	*ZFYVE19*	TRUE	2.045027004
Heart_Failure	mesothelium	*C16orf46*	TRUE	1.968214417
Heart_Failure	mesothelium	*TMEM170A*	TRUE	2.869026914

Heart_Failure	mesothelium	*KATNAL2*	TRUE	2.467046357
Heart_Failure	mesothelium	*ZNF23*	TRUE	2.536311997
Heart_Failure	mesothelium	*TRPV3*	TRUE	2.082517567
Heart_Failure	mesothelium	*NUDT8*	TRUE	1.978206962
Heart_Failure	mesothelium	*GSDMA*	TRUE	2.429416425
Heart_Failure	mesothelium	*DTWD2*	TRUE	2.053963664
Heart_Failure	mesothelium	*CHRNB1*	TRUE	2.74223888
Heart_Failure	mesothelium	*DNAJC18*	TRUE	2.217775573
Heart_Failure	mesothelium	*TMEM133*	TRUE	2.381391753
Heart_Failure	mesothelium	*C11orf80*	TRUE	3.385734662
Heart_Failure	mesothelium	*GPR160*	TRUE	2.175036302
Heart_Failure	mesothelium	*PAAF1*	TRUE	2.710554976
Heart_Failure	mesothelium	*SPRYD4*	TRUE	2.501400636
Heart_Failure	mesothelium	*DPY19L2*	TRUE	2.388176277
Heart_Failure	mesothelium	*ZNF454*	TRUE	2.578855826
Heart_Failure	mesothelium	*LINC00314*	TRUE	2.223323257
Heart_Failure	mesothelium	*HLA.DQB1*	TRUE	3.580698544
Heart_Failure	mesothelium	*FKRP*	TRUE	1.994339864
Heart_Failure	mesothelium	*PSMG1*	TRUE	3.114963353
Heart_Failure	mesothelium	*FAM19A1*	TRUE	2.048417493
Heart_Failure	mesothelium	*ZNF703*	TRUE	2.309193604
Heart_Failure	mesothelium	*A3GALT2*	TRUE	2.41783828
Heart_Failure	mesothelium	*CDK10*	TRUE	2.30163432
Heart_Failure	mesothelium	*GOLGA8R*	TRUE	2.073843291
Heart_Failure	mesothelium	*TMEM232*	TRUE	2.209330414
Heart_Failure	mesothelium	*MRPS21*	TRUE	2.175337788
Heart_Failure	mesothelium	*ZNF385C*	TRUE	2.200412843
Heart_Failure	mesothelium	*ZP3*	TRUE	2.028287048
Heart_Failure	mesothelium	*PDCD1LG2*	TRUE	2.609657488
Heart_Failure	mesothelium	*RPS26*	TRUE	3.158214627
Heart_Failure	mesothelium	*TATDN3*	TRUE	2.158084832
Heart_Failure	mesothelium	*MICA*	TRUE	2.887538962
Heart_Failure	mesothelium	*ZNF155*	TRUE	2.141033315
Heart_Failure	mesothelium	*SLFN12L*	TRUE	2.192551366
Heart_Failure	mesothelium	*CPNE1*	TRUE	2.99412135
Heart_Failure	mesothelium	*OR2A1*	TRUE	2.537173299
Heart_Failure	mesothelium	*ZNF736*	TRUE	2.137211159
Heart_Failure	mesothelium	*ZBTB22*	TRUE	2.122415117
Heart_Failure	mesothelium	*TTC4*	TRUE	3.511751741
Heart_Failure	mesothelium	*LILRA6*	TRUE	2.689710888
Heart_Failure	mesothelium	*MEX3A*	TRUE	2.162229542
Heart_Failure	mesothelium	*GTF2H5*	TRUE	2.177880622
Heart_Failure	monocyte	*GSDMA*	TRUE	2.016396854
Heart_Failure	monocyte	*HLA.DQB1*	TRUE	4.962505119
Heart_Failure	monocyte	*HLA.DQA2*	TRUE	2.454520512
Heart_Failure	nk_cell	*EVA1B*	TRUE	2.876060026

Heart_Failure	nk_cell	*TSLP*	TRUE	2.260108876
Heart_Failure	nk_cell	*KLHDC9*	TRUE	2.103994415
Heart_Failure	nk_cell	*GSDMA*	TRUE	2.53309544
Heart_Failure	nk_cell	*GPRIN1*	TRUE	2.114271457
Heart_Failure	nk_cell	*CHRNB1*	TRUE	2.803839469
Heart_Failure	nk_cell	*ZNF454*	TRUE	2.582543778
Heart_Failure	nk_cell	*HLA.DQB1*	TRUE	5.01288932
Heart_Failure	nk_cell	*MICB*	TRUE	2.137545957
Heart_Failure	pericyte	*RBM6*	TRUE	2.452426973
Heart_Failure	pericyte	*PNMT*	TRUE	2.770873292
Heart_Failure	pericyte	*FLG*	TRUE	2.821562717
Heart_Failure	pericyte	*TSLP*	TRUE	4.334520143
Heart_Failure	pericyte	*DPYSL4*	TRUE	2.040610402
Heart_Failure	pericyte	*SIM2*	TRUE	2.061909918
Heart_Failure	pericyte	*FCRL3*	TRUE	2.085886021
Heart_Failure	pericyte	*FGFR4*	TRUE	1.979706133
Heart_Failure	pericyte	*EFHB*	TRUE	2.395052992
Heart_Failure	pericyte	*HDHD2*	TRUE	2.095980804
Heart_Failure	pericyte	*GSDMA*	TRUE	3.15602552
Heart_Failure	pericyte	*CHRNB1*	TRUE	2.765917606
Heart_Failure	pericyte	*ZNF454*	TRUE	2.205288204
Heart_Failure	pericyte	*HLA.DQB1*	TRUE	4.261080535
Heart_Failure	pericyte	*LBX2*	TRUE	2.186225188
Heart_Failure	pericyte	*PDCD1LG2*	TRUE	1.967806679
Heart_Failure	pericyte	*RPS26*	TRUE	3.03730821
Heart_Failure	pericyte	*CPNE1*	TRUE	2.442457543
Heart_Failure	pericyte	*ZBTB22*	TRUE	2.483699118
Heart_Failure	pericyte	*LILRA6*	TRUE	2.064809456
Heart_Failure	SMC	*RBM6*	TRUE	3.5425593
Heart_Failure	SMC	*ADAM11*	TRUE	2.230573437
Heart_Failure	SMC	*INO80B*	TRUE	1.96463558
Heart_Failure	SMC	*ALG10*	TRUE	1.965156291
Heart_Failure	SMC	*FLG*	TRUE	1.999751448
Heart_Failure	SMC	*TSLP*	TRUE	2.296157806
Heart_Failure	SMC	*ROM1*	TRUE	2.264983105
Heart_Failure	SMC	*DPYSL4*	TRUE	2.082322165
Heart_Failure	SMC	*FCRL3*	TRUE	2.307129699
Heart_Failure	SMC	*KLHDC9*	TRUE	2.792228903
Heart_Failure	SMC	*GSDMA*	TRUE	3.515322819
Heart_Failure	SMC	*PAAF1*	TRUE	2.083185541
Heart_Failure	SMC	*HLA.DQB1*	TRUE	4.560790967
Heart_Failure	SMC	*PDCD1LG2*	TRUE	2.065741041
Heart_Failure	SMC	*MICA*	TRUE	2.811040122
Heart_Failure	SMC	*CPNE1*	TRUE	2.631082159
Heart_Failure	SMC	*ZBTB22*	TRUE	2.563814188
Heart_Failure	t_cell	*SLC7A9*	TRUE	2.415374168

Heart_Failure	t_cell	*KIF20A*	TRUE	2.855633402
Heart_Failure	t_cell	*ALDH8A1*	TRUE	1.995055764
Heart_Failure	t_cell	*NMT1*	TRUE	2.353751294
Heart_Failure	t_cell	*EVA1B*	TRUE	3.355637471
Heart_Failure	t_cell	*FLG*	TRUE	2.385178999
Heart_Failure	t_cell	*TSLP*	TRUE	2.454209
Heart_Failure	t_cell	*DPYSL4*	TRUE	1.971371077
Heart_Failure	t_cell	*SIM2*	TRUE	2.025480735
Heart_Failure	t_cell	*EFHB*	TRUE	2.245699667
Heart_Failure	t_cell	*RNF123*	TRUE	1.993468197
Heart_Failure	t_cell	*HDHD2*	TRUE	2.567370427
Heart_Failure	t_cell	*ZNF23*	TRUE	2.416410173
Heart_Failure	t_cell	*GSDMA*	TRUE	2.875359446
Heart_Failure	t_cell	*CHRNB1*	TRUE	2.416645608
Heart_Failure	t_cell	*PAAF1*	TRUE	1.996805213
Heart_Failure	t_cell	*HLA.DQB1*	TRUE	4.435107578
Heart_Failure	t_cell	*LBX2*	TRUE	2.076232588
Heart_Failure	t_cell	*RPS26*	TRUE	3.247469366
Heart_Failure	t_cell	*MICA*	TRUE	2.236690086
Heart_Failure	t_cell	*CPNE1*	TRUE	2.122053855
CAD_admission_freq	adipocyte	*ZNF200*	TRUE	3.0341879
CAD_admission_freq	adipocyte	*LAMC2*	TRUE	2.046419116
CAD_admission_freq	adipocyte	*GNA15*	TRUE	4.123063719
CAD_admission_freq	adipocyte	*NAALAD2*	TRUE	2.055434266
CAD_admission_freq	adipocyte	*SLC7A5*	TRUE	2.061024632
CAD_admission_freq	adipocyte	*EPHX3*	TRUE	2.231185907
CAD_admission_freq	adipocyte	*CCL24*	TRUE	2.185277474
CAD_admission_freq	adipocyte	*DNAJC12*	TRUE	2.029139222
CAD_admission_freq	adipocyte	*PEX12*	TRUE	2.111799899
CAD_admission_freq	adipocyte	*TAS2R10*	TRUE	2.898793682
CAD_admission_freq	adipocyte	*TOX2*	TRUE	2.144678676
CAD_admission_freq	adipocyte	*WDFY4*	TRUE	2.40725209
CAD_admission_freq	adipocyte	*ENPEP*	TRUE	2.596832402
CAD_admission_freq	adipocyte	*ADPRH*	TRUE	2.487361989
CAD_admission_freq	adipocyte	*TMEM236*	TRUE	2.629599972
CAD_admission_freq	adipocyte	*PROM2*	TRUE	2.081436564
CAD_admission_freq	adipocyte	*POLR2J3*	TRUE	2.857090954
CAD_admission_freq	adipocyte	*TTLL6*	TRUE	2.182592713
CAD_admission_freq	adipocyte	*SMPDL3A*	TRUE	2.204385488
CAD_admission_freq	adipocyte	*PAAF1*	TRUE	2.948931768
CAD_admission_freq	adipocyte	*B3GNT3*	TRUE	2.094658537
CAD_admission_freq	adipocyte	*PPP1R27*	TRUE	3.082048292
CAD_admission_freq	adipocyte	*AGAP9*	TRUE	2.486530794
CAD_admission_freq	adipocyte	*MICB*	TRUE	2.39887364
CAD_admission_freq	adipocyte	*AC084018.1*	TRUE	2.013355713
CAD_admission_freq	adipocyte	*ARHGAP19*	TRUE	2.003418702

CAD_admission_freq	adipocyte	*ACSM4*	TRUE	2.114812267
CAD_admission_freq	adipocyte	*ZNF883*	TRUE	2.566464576
CAD_admission_freq	adipocyte	*JMJD7*	TRUE	2.040271433
CAD_admission_freq	adipocyte	*RNASE4*	TRUE	2.129617423
CAD_admission_freq	adipocyte	*AC004466.1*	TRUE	2.905662374
CAD_admission_freq	ASPC	*ZNF200*	TRUE	3.051659672
CAD_admission_freq	ASPC	*RNASET2*	TRUE	1.963994531
CAD_admission_freq	ASPC	*LAMC2*	TRUE	2.15339074
CAD_admission_freq	ASPC	*GNA15*	TRUE	3.417502461
CAD_admission_freq	ASPC	*NAALAD2*	TRUE	2.123690359
CAD_admission_freq	ASPC	*PPP1R37*	TRUE	1.961337949
CAD_admission_freq	ASPC	*EPHX3*	TRUE	2.286274869
CAD_admission_freq	ASPC	*CCL24*	TRUE	2.106481513
CAD_admission_freq	ASPC	*DNAJC12*	TRUE	2.214099809
CAD_admission_freq	ASPC	*PEX12*	TRUE	2.27958199
CAD_admission_freq	ASPC	*TAS2R10*	TRUE	2.675164099
CAD_admission_freq	ASPC	*TOX2*	TRUE	2.341583818
CAD_admission_freq	ASPC	*WDFY4*	TRUE	2.809519033
CAD_admission_freq	ASPC	*ENPEP*	TRUE	2.701059206
CAD_admission_freq	ASPC	*ADPRH*	TRUE	2.43174877
CAD_admission_freq	ASPC	*TMEM236*	TRUE	2.658569278
CAD_admission_freq	ASPC	*PROM2*	TRUE	1.979142324
CAD_admission_freq	ASPC	*CBR1*	TRUE	1.97530937
CAD_admission_freq	ASPC	*GPER1*	TRUE	2.015114744
CAD_admission_freq	ASPC	*NUDT13*	TRUE	2.134823933
CAD_admission_freq	ASPC	*DCTN5*	TRUE	1.972682819
CAD_admission_freq	ASPC	*POLR2J3*	TRUE	2.809109235
CAD_admission_freq	ASPC	*PRNP*	TRUE	1.984491343
CAD_admission_freq	ASPC	*SMPDL3A*	TRUE	2.332347845
CAD_admission_freq	ASPC	*SEZ6L2*	TRUE	2.233505743
CAD_admission_freq	ASPC	*PAAF1*	TRUE	3.059907834
CAD_admission_freq	ASPC	*B3GNT3*	TRUE	2.139446142
CAD_admission_freq	ASPC	*CLEC4G*	TRUE	2.141742203
CAD_admission_freq	ASPC	*PPP1R27*	TRUE	3.185415804
CAD_admission_freq	ASPC	*ZFP62*	TRUE	2.263735202
CAD_admission_freq	ASPC	*AGAP9*	TRUE	2.645260556
CAD_admission_freq	ASPC	*INPP5B*	TRUE	2.016935321
CAD_admission_freq	ASPC	*MICB*	TRUE	2.383427775
CAD_admission_freq	ASPC	*SERF1B*	TRUE	2.092635909
CAD_admission_freq	ASPC	*AC084018.1*	TRUE	1.994400221
CAD_admission_freq	ASPC	*ARHGAP19*	TRUE	2.32540185
CAD_admission_freq	ASPC	*ACSM4*	TRUE	2.024979282
CAD_admission_freq	ASPC	*TMEM242*	TRUE	1.972823948
CAD_admission_freq	ASPC	*ZNF883*	TRUE	2.554363266
CAD_admission_freq	ASPC	*JMJD7*	TRUE	2.15434909
CAD_admission_freq	ASPC	*RNASE4*	TRUE	1.991580471

CAD_admission_freq	ASPC	*AC004466.1*	TRUE	2.813313115
CAD_admission_freq	ASPC	*DOC2B*	TRUE	2.101616256
CAD_admission_freq	dendritic_cell	*ZNF200*	TRUE	2.476083176
CAD_admission_freq	dendritic_cell	*EPHX3*	TRUE	1.992856387
CAD_admission_freq	dendritic_cell	*TAS2R10*	TRUE	2.740876545
CAD_admission_freq	dendritic_cell	*SPTBN5*	TRUE	2.069112773
CAD_admission_freq	dendritic_cell	*DPYSL4*	TRUE	1.984005563
CAD_admission_freq	dendritic_cell	*POLR2J3*	TRUE	2.665742903
CAD_admission_freq	dendritic_cell	*PAAF1*	TRUE	2.321236442
CAD_admission_freq	dendritic_cell	*ZNF575*	TRUE	2.011328502
CAD_admission_freq	dendritic_cell	*AC004466.1*	TRUE	2.459597271
CAD_admission_freq	endometrium	*TAS2R10*	TRUE	2.870032441
CAD_admission_freq	endometrium	*PPP1R27*	TRUE	1.983305317
CAD_admission_freq	endothelial	*GNA15*	TRUE	3.329026416
CAD_admission_freq	endothelial	*EPHX3*	TRUE	2.027179645
CAD_admission_freq	endothelial	*TAS2R10*	TRUE	2.394104387
CAD_admission_freq	endothelial	*PROM2*	TRUE	2.13203202
CAD_admission_freq	endothelial	*PAAF1*	TRUE	2.105956561
CAD_admission_freq	endothelial	*MICB*	TRUE	2.420769143
CAD_admission_freq	LEC	*ZNF200*	TRUE	2.950100381
CAD_admission_freq	LEC	*GNA15*	TRUE	3.185129233
CAD_admission_freq	LEC	*TAS2R10*	TRUE	3.033096482
CAD_admission_freq	LEC	*WDFY4*	TRUE	2.288244172
CAD_admission_freq	LEC	*ADPRH*	TRUE	2.06897996
CAD_admission_freq	LEC	*PROM2*	TRUE	1.977278325
CAD_admission_freq	LEC	*POLR2J3*	TRUE	2.717901908
CAD_admission_freq	LEC	*PAAF1*	TRUE	2.565307037
CAD_admission_freq	LEC	*B3GNT3*	TRUE	2.049838014
CAD_admission_freq	LEC	*PPP1R27*	TRUE	3.125898965
CAD_admission_freq	LEC	*MICB*	TRUE	2.307368738
CAD_admission_freq	LEC	*ZNF883*	TRUE	1.985075271
CAD_admission_freq	LEC	*AC004466.1*	TRUE	2.344926724
CAD_admission_freq	macrophage	*ZNF200*	TRUE	2.932291875
CAD_admission_freq	macrophage	*EPHX3*	TRUE	2.139949192
CAD_admission_freq	macrophage	*TAS2R10*	TRUE	2.713767401
CAD_admission_freq	macrophage	*TOX2*	TRUE	2.042203808
CAD_admission_freq	macrophage	*ENPEP*	TRUE	2.019859816
CAD_admission_freq	macrophage	*POLR2J3*	TRUE	2.706983106
CAD_admission_freq	macrophage	*PAAF1*	TRUE	2.99756031
CAD_admission_freq	macrophage	*PPP1R27*	TRUE	2.317819328
CAD_admission_freq	macrophage	*AGAP9*	TRUE	2.347993258
CAD_admission_freq	macrophage	*MICB*	TRUE	2.469960826
CAD_admission_freq	macrophage	*SERF1B*	TRUE	2.046385161
CAD_admission_freq	macrophage	*ARHGAP19*	TRUE	2.103454294
CAD_admission_freq	macrophage	*JMJD7*	TRUE	2.02521718
CAD_admission_freq	macrophage	*AC004466.1*	TRUE	2.806581167

CAD_admission_freq	macrophage	*DOC2B*	TRUE	1.990986747
CAD_admission_freq	mast_cell	*ZNF200*	TRUE	2.465132261
CAD_admission_freq	mast_cell	*GNA15*	TRUE	3.725660203
CAD_admission_freq	mast_cell	*EPHX3*	TRUE	1.974788486
CAD_admission_freq	mast_cell	*TAS2R10*	TRUE	2.770589085
CAD_admission_freq	mast_cell	*TMEM236*	TRUE	2.386512017
CAD_admission_freq	mast_cell	*POLR2J3*	TRUE	2.665457576
CAD_admission_freq	mast_cell	*ZNF575*	TRUE	2.158847643
CAD_admission_freq	mast_cell	*GPAT2*	TRUE	2.06016334
CAD_admission_freq	mast_cell	*MICB*	TRUE	2.320382089
CAD_admission_freq	mast_cell	*JMJD7*	TRUE	2.081331181
CAD_admission_freq	mast_cell	*AC004466.1*	TRUE	2.364962609
CAD_admission_freq	mesothelium	*SPATA20*	TRUE	2.04357797
CAD_admission_freq	mesothelium	*ZNF200*	TRUE	3.076645065
CAD_admission_freq	mesothelium	*RNASET2*	TRUE	2.03419978
CAD_admission_freq	mesothelium	*CHPF2*	TRUE	1.9621745
CAD_admission_freq	mesothelium	*PNKP*	TRUE	2.07407616
CAD_admission_freq	mesothelium	*LAMC2*	TRUE	2.06011015
CAD_admission_freq	mesothelium	*GNA15*	TRUE	3.47205811
CAD_admission_freq	mesothelium	*NAALAD2*	TRUE	2.360221345
CAD_admission_freq	mesothelium	*SLC7A5*	TRUE	2.030413663
CAD_admission_freq	mesothelium	*PPP1R37*	TRUE	2.044422151
CAD_admission_freq	mesothelium	*EPHX3*	TRUE	2.242727824
CAD_admission_freq	mesothelium	*CCL24*	TRUE	2.222763144
CAD_admission_freq	mesothelium	*LIPA*	TRUE	1.980804271
CAD_admission_freq	mesothelium	*DNAJC12*	TRUE	1.979663044
CAD_admission_freq	mesothelium	*PEX12*	TRUE	2.351946497
CAD_admission_freq	mesothelium	*TAS2R10*	TRUE	2.64678314
CAD_admission_freq	mesothelium	*TOX2*	TRUE	2.300669233
CAD_admission_freq	mesothelium	*L3HYPDH*	TRUE	1.989991171
CAD_admission_freq	mesothelium	*WDFY4*	TRUE	2.898415915
CAD_admission_freq	mesothelium	*ENPEP*	TRUE	2.617601792
CAD_admission_freq	mesothelium	*ADPRH*	TRUE	2.51712726
CAD_admission_freq	mesothelium	*TMEM236*	TRUE	2.676406134
CAD_admission_freq	mesothelium	*PROM2*	TRUE	2.003801397
CAD_admission_freq	mesothelium	*GPER1*	TRUE	2.111148922
CAD_admission_freq	mesothelium	*NUDT13*	TRUE	2.165109418
CAD_admission_freq	mesothelium	*DCTN5*	TRUE	2.0998912
CAD_admission_freq	mesothelium	*POLR2J3*	TRUE	2.84341621
CAD_admission_freq	mesothelium	*TTLL6*	TRUE	2.131662046
CAD_admission_freq	mesothelium	*SMPDL3A*	TRUE	2.266349303
CAD_admission_freq	mesothelium	*PAAF1*	TRUE	3.07063268
CAD_admission_freq	mesothelium	*B3GNT3*	TRUE	2.121005811
CAD_admission_freq	mesothelium	*CLEC4G*	TRUE	2.274765078
CAD_admission_freq	mesothelium	*PPP1R27*	TRUE	3.052773039
CAD_admission_freq	mesothelium	*RNPC3*	TRUE	2.196597121

CAD_admission_freq	mesothelium	*ZFP62*	TRUE	2.609967784
CAD_admission_freq	mesothelium	*AGAP9*	TRUE	2.551358774
CAD_admission_freq	mesothelium	*INPP5B*	TRUE	1.977096103
CAD_admission_freq	mesothelium	*MICB*	TRUE	2.391650268
CAD_admission_freq	mesothelium	*SERF1B*	TRUE	2.033445181
CAD_admission_freq	mesothelium	*AC084018.1*	TRUE	2.090845338
CAD_admission_freq	mesothelium	*ARHGAP19*	TRUE	2.242926842
CAD_admission_freq	mesothelium	*ACSM4*	TRUE	2.200594232
CAD_admission_freq	mesothelium	*TMEM242*	TRUE	1.985129414
CAD_admission_freq	mesothelium	*ZNF883*	TRUE	2.42382764
CAD_admission_freq	mesothelium	*JMJD7*	TRUE	2.001092734
CAD_admission_freq	mesothelium	*RNASE4*	TRUE	2.317689413
CAD_admission_freq	mesothelium	*AC004466.1*	TRUE	2.848915962
CAD_admission_freq	mesothelium	*DOC2B*	TRUE	2.143250368
CAD_admission_freq	monocyte	*TAS2R10*	TRUE	2.414446834
CAD_admission_freq	monocyte	*ENPEP*	TRUE	2.420856402
CAD_admission_freq	monocyte	*PPP1R27*	TRUE	2.605043582
CAD_admission_freq	monocyte	*MICB*	TRUE	2.323064512
CAD_admission_freq	nk_cell	*ZNF200*	TRUE	2.694214175
CAD_admission_freq	nk_cell	*TAS2R10*	TRUE	2.657182242
CAD_admission_freq	nk_cell	*WDFY4*	TRUE	2.070364369
CAD_admission_freq	nk_cell	*PROM2*	TRUE	2.604276067
CAD_admission_freq	nk_cell	*POLR2J3*	TRUE	2.2727187
CAD_admission_freq	nk_cell	*PAAF1*	TRUE	2.670278013
CAD_admission_freq	nk_cell	*MICB*	TRUE	2.658667525
CAD_admission_freq	nk_cell	*JMJD7*	TRUE	1.98914701
CAD_admission_freq	nk_cell	*AC004466.1*	TRUE	2.783936474
CAD_admission_freq	pericyte	*ZNF200*	TRUE	2.772551076
CAD_admission_freq	pericyte	*GNA15*	TRUE	3.571452235
CAD_admission_freq	pericyte	*EPHX3*	TRUE	2.18183592
CAD_admission_freq	pericyte	*TAS2R10*	TRUE	2.577283194
CAD_admission_freq	pericyte	*TOX2*	TRUE	1.993780897
CAD_admission_freq	pericyte	*WDFY4*	TRUE	2.493543795
CAD_admission_freq	pericyte	*ADPRH*	TRUE	2.155678371
CAD_admission_freq	pericyte	*POLR2J3*	TRUE	2.285700264
CAD_admission_freq	pericyte	*SMPDL3A*	TRUE	1.991440669
CAD_admission_freq	pericyte	*PAAF1*	TRUE	2.707982503
CAD_admission_freq	pericyte	*PPP1R27*	TRUE	2.315324943
CAD_admission_freq	pericyte	*AGAP9*	TRUE	2.184833724
CAD_admission_freq	pericyte	*MICB*	TRUE	2.281238136
CAD_admission_freq	pericyte	*SERF1B*	TRUE	2.001103237
CAD_admission_freq	pericyte	*JMJD7*	TRUE	2.067190152
CAD_admission_freq	pericyte	*AC004466.1*	TRUE	2.593534481
CAD_admission_freq	SMC	*ZNF200*	TRUE	2.846113867
CAD_admission_freq	SMC	*GNA15*	TRUE	3.56779177
CAD_admission_freq	SMC	*TAS2R10*	TRUE	2.701998442

CAD_admission_freq	SMC	*WDFY4*	TRUE	1.960613948
CAD_admission_freq	SMC	*ADPRH*	TRUE	2.103553378
CAD_admission_freq	SMC	*TMEM236*	TRUE	2.462447073
CAD_admission_freq	SMC	*POLR2J3*	TRUE	2.40404501
CAD_admission_freq	SMC	*PAAF1*	TRUE	2.88267979
CAD_admission_freq	SMC	*ZNF575*	TRUE	2.030394057
CAD_admission_freq	SMC	*PPP1R27*	TRUE	2.515041341
CAD_admission_freq	SMC	*MICB*	TRUE	2.45771392
CAD_admission_freq	SMC	*JMJD7*	TRUE	2.070535676
CAD_admission_freq	SMC	*DOC2B*	TRUE	2.09920863
CAD_admission_freq	t_cell	*ZNF200*	TRUE	2.519115673
CAD_admission_freq	t_cell	*GNA15*	TRUE	3.528552556
CAD_admission_freq	t_cell	*EPHX3*	TRUE	2.095666384
CAD_admission_freq	t_cell	*CCL24*	TRUE	2.044613792
CAD_admission_freq	t_cell	*TAS2R10*	TRUE	2.107355427
CAD_admission_freq	t_cell	*WDFY4*	TRUE	2.33647352
CAD_admission_freq	t_cell	*ENPEP*	TRUE	1.989464246
CAD_admission_freq	t_cell	*TMEM236*	TRUE	2.091928419
CAD_admission_freq	t_cell	*POLR2J3*	TRUE	2.439315603
CAD_admission_freq	t_cell	*SEZ6L2*	TRUE	1.969105818
CAD_admission_freq	t_cell	*PAAF1*	TRUE	2.823779947
CAD_admission_freq	t_cell	*ZNF575*	TRUE	2.1769168
CAD_admission_freq	t_cell	*B3GNT3*	TRUE	2.123251177
CAD_admission_freq	t_cell	*CLEC4G*	TRUE	2.120181965
CAD_admission_freq	t_cell	*MICB*	TRUE	2.553762985
CAD_admission_freq	t_cell	*ACSM4*	TRUE	2.074910122
CAD_admission_freq	t_cell	*JMJD7*	TRUE	2.03011916
CAD_admission_freq	t_cell	*AC004466.1*	TRUE	2.513903623
CAD_mortality	adipocyte	*BAK1*	TRUE	1.983031575
CAD_mortality	adipocyte	*ARHGEF5*	TRUE	2.062229679
CAD_mortality	adipocyte	*OPN3*	TRUE	2.203990173
CAD_mortality	adipocyte	*CDC6*	TRUE	2.103946287
CAD_mortality	adipocyte	*CYB5R3*	TRUE	2.015080003
CAD_mortality	adipocyte	*PPP1R37*	TRUE	1.990922829
CAD_mortality	adipocyte	*MTRF1L*	TRUE	2.446584132
CAD_mortality	adipocyte	*IRF6*	TRUE	2.283838458
CAD_mortality	adipocyte	*OPN4*	TRUE	2.164757524
CAD_mortality	adipocyte	*LYG1*	TRUE	2.318197259
CAD_mortality	adipocyte	*ZNF660*	TRUE	1.985063464
CAD_mortality	adipocyte	*POMZP3*	TRUE	2.531018167
CAD_mortality	adipocyte	*POLE3*	TRUE	2.004067178
CAD_mortality	adipocyte	*ROM1*	TRUE	2.414586322
CAD_mortality	adipocyte	*UVSSA*	TRUE	2.049204498
CAD_mortality	adipocyte	*TRIM66*	TRUE	2.328409132
CAD_mortality	adipocyte	*ENDOG*	TRUE	2.037018499
CAD_mortality	adipocyte	*RAB43*	TRUE	2.521850047

CAD_mortality	adipocyte	*GOLGA6L4*	TRUE	2.716687086
CAD_mortality	adipocyte	*C11orf87*	TRUE	2.324297454
CAD_mortality	adipocyte	*TBKBP1*	TRUE	1.98216485
CAD_mortality	adipocyte	*GOLGA8B*	TRUE	2.000876628
CAD_mortality	adipocyte	*LINC00937*	TRUE	2.046479827
CAD_mortality	adipocyte	*TDGF1*	TRUE	2.314582255
CAD_mortality	adipocyte	*LEFTY1*	TRUE	2.247858496
CAD_mortality	ASPC	*BAK1*	TRUE	2.030363319
CAD_mortality	ASPC	*ARHGEF5*	TRUE	2.157653046
CAD_mortality	ASPC	*OPN3*	TRUE	2.310398336
CAD_mortality	ASPC	*SYNJ2*	TRUE	2.144529111
CAD_mortality	ASPC	*CDC6*	TRUE	2.200653082
CAD_mortality	ASPC	*CYB5R3*	TRUE	2.094959475
CAD_mortality	ASPC	*TNFRSF10A*	TRUE	2.131618004
CAD_mortality	ASPC	*PPP1R37*	TRUE	2.098471052
CAD_mortality	ASPC	*MTRF1L*	TRUE	2.439147159
CAD_mortality	ASPC	*IRF6*	TRUE	2.299290499
CAD_mortality	ASPC	*OPN4*	TRUE	2.090224715
CAD_mortality	ASPC	*GTF2F1*	TRUE	2.14952767
CAD_mortality	ASPC	*NAAA*	TRUE	2.11509565
CAD_mortality	ASPC	*CIAO1*	TRUE	2.023354142
CAD_mortality	ASPC	*LYG1*	TRUE	2.339137072
CAD_mortality	ASPC	*POMZP3*	TRUE	2.842935111
CAD_mortality	ASPC	*POLE3*	TRUE	2.183692843
CAD_mortality	ASPC	*ROM1*	TRUE	2.503408112
CAD_mortality	ASPC	*GBP5*	TRUE	1.971820936
CAD_mortality	ASPC	*STEAP2*	TRUE	1.989075738
CAD_mortality	ASPC	*LRRC56*	TRUE	1.960666127
CAD_mortality	ASPC	*DCD*	TRUE	2.029376029
CAD_mortality	ASPC	*UVSSA*	TRUE	2.33275295
CAD_mortality	ASPC	*TRIM66*	TRUE	2.240976548
CAD_mortality	ASPC	*ACSM1*	TRUE	2.027633279
CAD_mortality	ASPC	*ENDOG*	TRUE	2.165811513
CAD_mortality	ASPC	*IRS1*	TRUE	2.025871251
CAD_mortality	ASPC	*ZNF703*	TRUE	2.072612044
CAD_mortality	ASPC	*GOLGA6L4*	TRUE	2.777578164
CAD_mortality	ASPC	*C11orf87*	TRUE	2.319336636
CAD_mortality	ASPC	*ZNF100*	TRUE	2.118761892
CAD_mortality	ASPC	*RPS6KL1*	TRUE	2.051461541
CAD_mortality	ASPC	*GOLGA8B*	TRUE	2.152170999
CAD_mortality	ASPC	*LINC00937*	TRUE	1.995372664
CAD_mortality	ASPC	*TDGF1*	TRUE	2.362459731
CAD_mortality	ASPC	*LEFTY1*	TRUE	2.514879715
CAD_mortality	ASPC	*TMEM158*	TRUE	2.027394727
CAD_mortality	b_cell	*IRF6*	TRUE	1.990434098
CAD_mortality	b_cell	*RAB43*	TRUE	3.23392267

CAD_mortality	dendritic_cell	*TNFRSF10A*	TRUE	2.004646502
CAD_mortality	dendritic_cell	*IRF6*	TRUE	2.126269477
CAD_mortality	dendritic_cell	*ROM1*	TRUE	2.336407524
CAD_mortality	dendritic_cell	*PROM2*	TRUE	2.271652051
CAD_mortality	dendritic_cell	*CYP4A22*	TRUE	1.979793383
CAD_mortality	dendritic_cell	*ENDOG*	TRUE	2.063326084
CAD_mortality	dendritic_cell	*RAB43*	TRUE	2.839976187
CAD_mortality	dendritic_cell	*GOLGA6L4*	TRUE	2.044138174
CAD_mortality	dendritic_cell	*TDGF1*	TRUE	2.608573668
CAD_mortality	dendritic_cell	*ITGB3*	TRUE	2.040612615
CAD_mortality	endometrium	*OPN3*	TRUE	2.093892514
CAD_mortality	endometrium	*ROM1*	TRUE	2.458566295
CAD_mortality	endometrium	*C11orf87*	TRUE	2.091915478
CAD_mortality	endometrium	*TDGF1*	TRUE	2.194413658
CAD_mortality	endothelial	*MTRF1L*	TRUE	2.309143219
CAD_mortality	endothelial	*PROM2*	TRUE	2.339488806
CAD_mortality	endothelial	*TRIM66*	TRUE	2.045514404
CAD_mortality	endothelial	*RAB43*	TRUE	2.888742201
CAD_mortality	endothelial	*TOP1MT*	TRUE	1.981348651
CAD_mortality	endothelial	*TDGF1*	TRUE	2.580937133
CAD_mortality	LEC	*OPN3*	TRUE	2.022355789
CAD_mortality	LEC	*IRF6*	TRUE	2.152262517
CAD_mortality	LEC	*ZNF660*	TRUE	2.075547595
CAD_mortality	LEC	*POMZP3*	TRUE	2.373706582
CAD_mortality	LEC	*POLE3*	TRUE	2.02833565
CAD_mortality	LEC	*UVSSA*	TRUE	2.280225611
CAD_mortality	LEC	*GOLGA6L4*	TRUE	2.505662087
CAD_mortality	LEC	*TBKBP1*	TRUE	2.052518169
CAD_mortality	LEC	*TDGF1*	TRUE	2.452358372
CAD_mortality	macrophage	*ARHGEF5*	TRUE	1.962751101
CAD_mortality	macrophage	*TNFRSF10A*	TRUE	2.062722377
CAD_mortality	macrophage	*PPP1R37*	TRUE	2.095541095
CAD_mortality	macrophage	*MTRF1L*	TRUE	2.063838585
CAD_mortality	macrophage	*IRF6*	TRUE	2.182605886
CAD_mortality	macrophage	*POMZP3*	TRUE	2.233122934
CAD_mortality	macrophage	*POLE3*	TRUE	2.038765277
CAD_mortality	macrophage	*ROM1*	TRUE	2.201174152
CAD_mortality	macrophage	*CYP4A22*	TRUE	2.242527222
CAD_mortality	macrophage	*UVSSA*	TRUE	2.03539153
CAD_mortality	macrophage	*TRIM66*	TRUE	2.178060005
CAD_mortality	macrophage	*ENDOG*	TRUE	1.992420832
CAD_mortality	macrophage	*GOLGA6L4*	TRUE	2.325755801
CAD_mortality	macrophage	*C11orf87*	TRUE	2.221740958
CAD_mortality	macrophage	*HLA.DQA2*	TRUE	1.981703029
CAD_mortality	macrophage	*LEFTY1*	TRUE	2.472790388
CAD_mortality	mast_cell	*POMZP3*	TRUE	2.37025147

CAD_mortality	mast_cell	*CYP4A22*	TRUE	2.123607136
CAD_mortality	mast_cell	*HLA.DQB1*	TRUE	2.005568104
CAD_mortality	mast_cell	*GOLGA6L4*	TRUE	2.11212547
CAD_mortality	mast_cell	*C11orf87*	TRUE	2.194528577
CAD_mortality	mast_cell	*HCG20*	TRUE	2.1100468
CAD_mortality	mast_cell	*TDGF1*	TRUE	2.412649935
CAD_mortality	mesothelium	*OPN3*	TRUE	2.272437896
CAD_mortality	mesothelium	*SYNJ2*	TRUE	2.412913439
CAD_mortality	mesothelium	*MRPS33*	TRUE	1.9632844
CAD_mortality	mesothelium	*CDC6*	TRUE	2.12653001
CAD_mortality	mesothelium	*TOP3B*	TRUE	2.136669063
CAD_mortality	mesothelium	*CYB5R3*	TRUE	2.465297827
CAD_mortality	mesothelium	*MANBAL*	TRUE	2.185613675
CAD_mortality	mesothelium	*ACP5*	TRUE	2.020617855
CAD_mortality	mesothelium	*TNFRSF10A*	TRUE	2.117393346
CAD_mortality	mesothelium	*PPP1R37*	TRUE	1.981361662
CAD_mortality	mesothelium	*UST*	TRUE	2.079981395
CAD_mortality	mesothelium	*MTRF1L*	TRUE	2.73448391
CAD_mortality	mesothelium	*IRF6*	TRUE	2.320792431
CAD_mortality	mesothelium	*EPCAM*	TRUE	2.043569782
CAD_mortality	mesothelium	*OPN4*	TRUE	2.019557717
CAD_mortality	mesothelium	*EBPL*	TRUE	2.103115222
CAD_mortality	mesothelium	*PRPF4*	TRUE	2.039103286
CAD_mortality	mesothelium	*CENPO*	TRUE	2.329793392
CAD_mortality	mesothelium	*NAAA*	TRUE	1.999792501
CAD_mortality	mesothelium	*CCDC40*	TRUE	2.024610646
CAD_mortality	mesothelium	*LYG1*	TRUE	2.520788347
CAD_mortality	mesothelium	*ZNF660*	TRUE	2.287945405
CAD_mortality	mesothelium	*POMZP3*	TRUE	2.642432478
CAD_mortality	mesothelium	*POLE3*	TRUE	2.138484628
CAD_mortality	mesothelium	*TMEM236*	TRUE	2.038484041
CAD_mortality	mesothelium	*GBP5*	TRUE	1.977826892
CAD_mortality	mesothelium	*STEAP2*	TRUE	2.362713223
CAD_mortality	mesothelium	*DIP2A*	TRUE	2.127767326
CAD_mortality	mesothelium	*LRRC56*	TRUE	2.110142931
CAD_mortality	mesothelium	*DCD*	TRUE	1.964549357
CAD_mortality	mesothelium	*CYP4A22*	TRUE	2.313674122
CAD_mortality	mesothelium	*UVSSA*	TRUE	2.238372791
CAD_mortality	mesothelium	*TRIM66*	TRUE	2.008737735
CAD_mortality	mesothelium	*ENDOG*	TRUE	2.096953889
CAD_mortality	mesothelium	*IRS1*	TRUE	2.048034203
CAD_mortality	mesothelium	*TTLL6*	TRUE	1.977211414
CAD_mortality	mesothelium	*SCAND1*	TRUE	1.96715089
CAD_mortality	mesothelium	*RAB43*	TRUE	2.556132216
CAD_mortality	mesothelium	*CHRNA7*	TRUE	2.085413285
CAD_mortality	mesothelium	*RBM44*	TRUE	2.095556818

CAD_mortality	mesothelium	*ODF3B*	TRUE	2.128324538
CAD_mortality	mesothelium	*CDH4*	TRUE	2.213689009
CAD_mortality	mesothelium	*GOLGA6L4*	TRUE	2.824743691
CAD_mortality	mesothelium	*C11orf87*	TRUE	2.323610597
CAD_mortality	mesothelium	*XPNPEP3*	TRUE	2.090564677
CAD_mortality	mesothelium	*ZNF100*	TRUE	1.980555372
CAD_mortality	mesothelium	*RPS6KL1*	TRUE	1.965685388
CAD_mortality	mesothelium	*TBKBP1*	TRUE	2.021865366
CAD_mortality	mesothelium	*LINC00937*	TRUE	2.216300988
CAD_mortality	mesothelium	*TDGF1*	TRUE	2.168210337
CAD_mortality	mesothelium	*LEFTY1*	TRUE	2.348746991
CAD_mortality	monocyte	*CYP4A22*	TRUE	2.016130002
CAD_mortality	monocyte	*TRIM66*	TRUE	2.04930788
CAD_mortality	monocyte	*HLA.DQB1*	TRUE	2.195058476
CAD_mortality	monocyte	*C11orf87*	TRUE	2.29342431
CAD_mortality	monocyte	*TBKBP1*	TRUE	2.255019217
CAD_mortality	nk_cell	*LYG1*	TRUE	2.11337816
CAD_mortality	nk_cell	*PROM2*	TRUE	2.26477583
CAD_mortality	nk_cell	*CYP4A22*	TRUE	2.062160124
CAD_mortality	nk_cell	*RAB43*	TRUE	3.381391989
CAD_mortality	nk_cell	*HLA.DQB1*	TRUE	2.042916423
CAD_mortality	nk_cell	*C11orf87*	TRUE	2.023011807
CAD_mortality	pericyte	*MTRF1L*	TRUE	2.020710477
CAD_mortality	pericyte	*IRF6*	TRUE	2.229029494
CAD_mortality	pericyte	*ROM1*	TRUE	2.331266958
CAD_mortality	pericyte	*CYP4A22*	TRUE	2.188833332
CAD_mortality	pericyte	*GOLGA6L4*	TRUE	2.565498853
CAD_mortality	pericyte	*C11orf87*	TRUE	2.120299468
CAD_mortality	pericyte	*TDGF1*	TRUE	2.359245024
CAD_mortality	pericyte	*LEFTY1*	TRUE	1.960510453
CAD_mortality	SMC	*ARHGEF5*	TRUE	1.971590998
CAD_mortality	SMC	*IRF6*	TRUE	2.031926644
CAD_mortality	SMC	*LYG1*	TRUE	2.396458894
CAD_mortality	SMC	*ZNF660*	TRUE	2.038445265
CAD_mortality	SMC	*ROM1*	TRUE	2.265194053
CAD_mortality	SMC	*CYP4A22*	TRUE	2.069950649
CAD_mortality	SMC	*TRIM66*	TRUE	2.236989221
CAD_mortality	SMC	*RAB43*	TRUE	2.192207838
CAD_mortality	SMC	*C11orf87*	TRUE	2.084557937
CAD_mortality	SMC	*TBKBP1*	TRUE	2.09242149
CAD_mortality	SMC	*TDGF1*	TRUE	2.381968003
CAD_mortality	t_cell	*CDC6*	TRUE	1.998990462
CAD_mortality	t_cell	*MTRF1L*	TRUE	2.138817861
CAD_mortality	t_cell	*IRF6*	TRUE	2.237830807
CAD_mortality	t_cell	*LYG1*	TRUE	2.043063417
CAD_mortality	t_cell	*DCD*	TRUE	2.033533308

CAD_mortality	t_cell	*CYP4A22*	TRUE	2.135861497
CAD_mortality	t_cell	*TRIM66*	TRUE	2.209307281
CAD_mortality	t_cell	*RAB43*	TRUE	2.871455049
CAD_mortality	t_cell	*TOP1MT*	TRUE	2.112937424
CAD_mortality	t_cell	*TBKBP1*	TRUE	2.175042622
CAD_mortality	t_cell	*TDGF1*	TRUE	2.314855544
