# Supplementary material for: CausalCellInfer: Prioritizing Cell‐Type‐Specific Disease Associations From Genetically Predicted Transcriptomes in Biobank Cohorts
Source: Adv Sci (Weinh). 2026 Oct 14:e78197. Online ahead of print. doi: 10.1002/advs.78197 (PMC13669230; doi:10.1002/advs.78197)
Supplement: Supplementary file 2 [file ADVS-9999-e78197-s002.zip › advs78197-sup-0007-S6.pdf]

Table S6 Enriched pathways based on identified putative causal genes for studied traits from UK Biobank

Anxiety

pathway name	set size	candidatescontained	p-value	q-value	pathway source
Viral myocarditis - Homo sapiens (human)	60	3 (5.1%)	0.000331	0.17	KEGG
Asthma - Homo sapiens (human)	31	2 (6.7%)	0.00215	0.222	KEGG
Allograft rejection - Homo sapiens (human)	38	2 (5.4%)	0.00326	0.222	KEGG
Intestinal immune network for IgA production - Homo sapiens (human)	49	2 (4.3%)	0.00522	0.222	KEGG
Autoimmune thyroid disease - Homo sapiens (human)	53	2 (3.8%)	0.00636	0.222	KEGG
Signaling by membrane-tethered fusions of PDGFRA or PDGFRB	5	1 (20.0%)	0.0115	0.222	Reactome
Extrinsic Pathway of Fibrin Clot Formation	5	1 (20.0%)	0.0115	0.222	Reactome
NAD biosynthesis from 2-amino-3-carboxymuconate semialdehyde	5	1 (20.0%)	0.0115	0.222	HumanCyc
Highly sodium permeable postsynaptic acetylcholine nicotinic receptors	7	1 (14.3%)	0.016	0.222	Reactome
CS/DS degradation	7	1 (14.3%)	0.016	0.222	Reactome
Hypertrophic cardiomyopathy - Homo sapiens (human)	90	2 (2.2%)	0.0182	0.222	KEGG
NAD Biosynthesis II (from tryptophan)	8	1 (12.5%)	0.0183	0.222	Wikipathways
Allograft Rejection	93	2 (2.2%)	0.0194	0.222	Wikipathways
Dilated cardiomyopathy - Homo sapiens (human)	96	2 (2.1%)	0.0201	0.222	KEGG
cd40l signaling pathway	9	1 (11.1%)	0.0206	0.222	BioCarta
Ca2+ activated K+ channels	9	1 (11.1%)	0.0206	0.222	Reactome
Platelet activation, signaling and aggregation	263	3 (1.1%)	0.0223	0.222	Reactome
NLR Proteins	10	1 (10.0%)	0.0228	0.222	Wikipathways
Gamma-carboxylation of protein precursors	10	1 (10.0%)	0.0228	0.222	Reactome
Removal of aminoterminal propeptides from gamma-carboxylated proteins	10	1 (10.0%)	0.0228	0.222	Reactome
Pexophagy	10	1 (10.0%)	0.0228	0.222	Reactome
Non-homologous end joining	10	1 (10.0%)	0.0228	0.222	Wikipathways
Potassium Channels	103	2 (1.9%)	0.0234	0.222	Reactome
Gamma-carboxylation, transport, and amino-terminal cleavage of proteins	11	1 (9.1%)	0.0251	0.222	Reactome
CMP phosphorylation	11	1 (9.1%)	0.0251	0.222	HumanCyc
Presynaptic nicotinic acetylcholine receptors	12	1 (8.3%)	0.0274	0.222	Reactome
pyrimidine deoxyribonucleotide phosphorylation	12	1 (8.3%)	0.0274	0.222	HumanCyc
guanosine deoxyribonucleotides de novo biosynthesis	12	1 (8.3%)	0.0274	0.222	HumanCyc
adenosine deoxyribonucleotides de novo biosynthesis	12	1 (8.3%)	0.0274	0.222	HumanCyc
NAD de novo biosynthesis	12	1 (8.3%)	0.0274	0.222	HumanCyc
Toxoplasmosis - Homo sapiens (human)	112	2 (1.8%)	0.0274	0.222	KEGG
extrinsic prothrombin activation pathway	13	1 (7.7%)	0.0296	0.222	BioCarta
BMAL1:CLOCK,NPAS2 activates circadian gene expression	13	1 (7.7%)	0.0296	0.222	Reactome
visual signal transduction	13	1 (7.7%)	0.0296	0.222	BioCarta
purine deoxyribonucleosides salvage	13	1 (7.7%)	0.0296	0.222	HumanCyc
guanosine ribonucleotides de novo biosynthesis	13	1 (7.7%)	0.0296	0.222	HumanCyc
UTP and CTP de novo biosynthesis	13	1 (7.7%)	0.0296	0.222	HumanCyc
Activation of RAC1	13	1 (7.7%)	0.0296	0.222	Reactome
Non-homologous end-joining - Homo sapiens (human)	13	1 (7.7%)	0.0296	0.222	KEGG
DNA-PK pathway in nonhomologous end joining	13	1 (7.7%)	0.0296	0.222	PID
Postsynaptic nicotinic acetylcholine receptors	14	1 (7.1%)	0.0318	0.222	Reactome
Acetylcholine binding and downstream events	14	1 (7.1%)	0.0318	0.222	Reactome
S1P4 pathway	14	1 (7.1%)	0.0318	0.222	PID
Lysosphingolipid and LPA receptors	14	1 (7.1%)	0.0318	0.222	Reactome
Nicotinate and Nicotinamide Metabolism	14	1 (7.1%)	0.0318	0.222	SMPDB
superpathway of pyrimidine ribonucleotides de novo biosynthesis	15	1 (6.7%)	0.0341	0.222	HumanCyc
p130Cas linkage to MAPK signaling for integrins	15	1 (6.7%)	0.0341	0.222	Reactome
GRB2:SOS provides linkage to MAPK signaling for Integrins	15	1 (6.7%)	0.0341	0.222	Reactome
pyrimidine deoxyribonucleotides biosynthesis from CTP	16	1 (6.2%)	0.0363	0.222	HumanCyc
guanosine nucleotides de novo biosynthesis	16	1 (6.2%)	0.0363	0.222	HumanCyc
pyrimidine deoxyribonucleotides de novo biosynthesis	16	1 (6.2%)	0.0363	0.222	HumanCyc
Systemic lupus erythematosus - Homo sapiens (human)	136	2 (1.5%)	0.038	0.222	KEGG
Doxorubicin Metabolism Pathway	17	1 (5.9%)	0.0385	0.222	SMPDB
TNF receptor superfamily (TNFSF) members mediating non-canonical NF-kB pathway	17	1 (5.9%)	0.0385	0.222	Reactome
superpathway of pyrimidine deoxyribonucleoside salvage	17	1 (5.9%)	0.0385	0.222	HumanCyc
Signaling by FLT3 fusion proteins	18	1 (5.6%)	0.0408	0.222	Reactome
G beta:gamma signalling through BTK	18	1 (5.6%)	0.0408	0.222	Reactome

NRF2 pathway	**143**	**2 (1.4%)**	0.0428	0.222	**Wikipathways**
Hematopoietic Stem Cell Gene Regulation by GABP alpha-beta Complex	**19**	**1 (5.3%)**	0.043	0.222	**Wikipathways**
Prostacyclin signalling through prostacyclin receptor	**19**	**1 (5.3%)**	0.043	0.222	**Reactome**
Glycosaminoglycan degradation - Homo sapiens (human)	**19**	**1 (5.3%)**	0.043	0.222	**KEGG**
Small Ligand GPCRs	**19**	**1 (5.3%)**	0.043	0.222	**Wikipathways**
Signaling by PDGFR in disease	**20**	**1 (5.0%)**	0.0452	0.222	**Reactome**
Bivalirudin Action Pathway	**20**	**1 (5.0%)**	0.0452	0.222	**SMPDB**
Argatroban Action Pathway	**20**	**1 (5.0%)**	0.0452	0.222	**SMPDB**
Ximelagatran Action Pathway	**20**	**1 (5.0%)**	0.0452	0.222	**SMPDB**
Lepirudin Action Pathway	**20**	**1 (5.0%)**	0.0452	0.222	**SMPDB**
Coagulation	**20**	**1 (5.0%)**	0.0452	0.222	**SMPDB**
Aminocaproic Acid Action Pathway	**20**	**1 (5.0%)**	0.0452	0.222	**SMPDB**
Tranexamic Acid Action Pathway	**20**	**1 (5.0%)**	0.0452	0.222	**SMPDB**
Urokinase Action Pathway	**20**	**1 (5.0%)**	0.0452	0.222	**SMPDB**
Reteplase Action Pathway	**20**	**1 (5.0%)**	0.0452	0.222	**SMPDB**
Streptokinase Action Pathway	**20**	**1 (5.0%)**	0.0452	0.222	**SMPDB**
Tenecteplase Action Pathway	**20**	**1 (5.0%)**	0.0452	0.222	**SMPDB**
Alteplase Action Pathway	**20**	**1 (5.0%)**	0.0452	0.222	**SMPDB**
Anistreplase Action Pathway	**20**	**1 (5.0%)**	0.0452	0.222	**SMPDB**
Aprotinin Action Pathway	**20**	**1 (5.0%)**	0.0452	0.222	**SMPDB**
Phenindione Action Pathway	**20**	**1 (5.0%)**	0.0452	0.222	**SMPDB**
Dicoumarol Action Pathway	**20**	**1 (5.0%)**	0.0452	0.222	**SMPDB**
Warfarin Action Pathway	**20**	**1 (5.0%)**	0.0452	0.222	**SMPDB**
Acenocoumarol Action Pathway	**20**	**1 (5.0%)**	0.0452	0.222	**SMPDB**
Phenprocoumon Action Pathway	**20**	**1 (5.0%)**	0.0452	0.222	**SMPDB**
Dicumarol Action Pathway	**20**	**1 (5.0%)**	0.0452	0.222	**SMPDB**
G beta:gamma signalling through PLC beta	**20**	**1 (5.0%)**	0.0452	0.222	**Reactome**
G beta:gamma signalling through CDC42	**20**	**1 (5.0%)**	0.0452	0.222	**Reactome**
Cellular hexose transport	**20**	**1 (5.0%)**	0.0452	0.222	**Reactome**
Syndecan interactions	**20**	**1 (5.0%)**	0.0452	0.222	**Reactome**
Cell adhesion molecules - Homo sapiens (human)	**149**	**2 (1.4%)**	0.0455	0.222	**KEGG**
FLT3 signaling in disease	**21**	**1 (4.8%)**	0.0474	0.222	**Reactome**
Ardeparin Action Pathway	**21**	**1 (4.8%)**	0.0474	0.222	**SMPDB**
Heparin Action Pathway	**21**	**1 (4.8%)**	0.0474	0.222	**SMPDB**
Fondaparinux Action Pathway	**21**	**1 (4.8%)**	0.0474	0.222	**SMPDB**
Enoxaparin Action Pathway	**21**	**1 (4.8%)**	0.0474	0.222	**SMPDB**
Presynaptic function of Kainate receptors	**21**	**1 (4.8%)**	0.0474	0.222	**Reactome**
Sphingosine 1-phosphate (S1P) pathway	**21**	**1 (4.8%)**	0.0474	0.222	**PID**
ADP signalling through P2Y purinoceptor 12	**22**	**1 (4.5%)**	0.0496	0.222	**Reactome**
superpathway of pyrimidine deoxyribonucleotides de novo biosynthesis	**22**	**1 (4.5%)**	0.0496	0.222	**HumanCyc**
NAD+ biosynthetic pathways	**22**	**1 (4.5%)**	0.0496	0.222	**Wikipathways**
Citric acid cycle (TCA cycle)	**22**	**1 (4.5%)**	0.0496	0.222	**Reactome**

Bipolar

pathway name	set size	candidatescontained	p-value	q-value	pathway source
GP1b-IX-V activation signalling	10	2 (20.0%)	0.000464	0.162	Reactome
eicosapentaenoate biosynthesis	13	2 (15.4%)	0.000799	0.162	HumanCyc
Rap1 signalling	16	2 (12.5%)	0.00122	0.162	Reactome
p38 signaling mediated by MAPKAP kinases	21	2 (9.5%)	0.00212	0.162	PID
3-Phosphoglycerate dehydrogenase deficiency	27	2 (7.4%)	0.00349	0.162	SMPDB
Non Ketotic Hyperglycinemia	27	2 (7.4%)	0.00349	0.162	SMPDB
Glycine and Serine Metabolism	27	2 (7.4%)	0.00349	0.162	SMPDB
Dimethylglycine Dehydrogenase Deficiency	27	2 (7.4%)	0.00349	0.162	SMPDB
Hyperglycinemia, non-ketotic	27	2 (7.4%)	0.00349	0.162	SMPDB
Dimethylglycine Dehydrogenase Deficiency	27	2 (7.4%)	0.00349	0.162	SMPDB
Sarcosinemia	27	2 (7.4%)	0.00349	0.162	SMPDB
Dihydropyrimidine Dehydrogenase Deficiency	27	2 (7.4%)	0.00349	0.162	SMPDB
Polymerase switching on the C-strand of the	27	2 (7.4%)	0.00349	0.162	Reactome
EGF	28	2 (7.1%)	0.00375	0.162	INOH
IGF1 pathway	30	2 (6.7%)	0.0043	0.173	PID
Herpes simplex virus 1 infection - Homo sapiens	498	6 (1.2%)	0.00537	0.195	KEGG
Class I PI3K signaling events mediated by Akt	35	2 (5.7%)	0.00582	0.195	PID
Telomere C-strand (Lagging Strand) Synthesis	35	2 (5.7%)	0.00582	0.195	Reactome
GMCSF-mediated signaling events	37	2 (5.4%)	0.00649	0.197	PID
Hyaluronan biosynthesis and export	2	1 (50.0%)	0.00654	0.197	Reactome
Sphingolipid signaling pathway - Homo sapiens	119	3 (2.5%)	0.00689	0.198	KEGG
Glycine, serine and threonine metabolism - Homo sapiens (human)	40	2 (5.0%)	0.00756	0.207	KEGG
Common Pathways Underlying Drug Addiction	41	2 (4.9%)	0.00793	0.208	Wikipathways
Hydrolysis of LPE	3	1 (33.3%)	0.00979	0.246	Reactome
IL5	48	2 (4.2%)	0.0107	0.254	NetPath
IL3	51	2 (3.9%)	0.0121	0.254	NetPath
Extension of Telomeres	51	2 (3.9%)	0.0121	0.254	Reactome
Resolution of AP sites via the single-nucleotide replacement pathway	4	1 (25.0%)	0.013	0.254	Reactome
Linoleic acid metabolism known to be affected by coronavirus infection	4	1 (25.0%)	0.013	0.254	Wikipathways
serine biosynthesis (phosphorylated route)	4	1 (25.0%)	0.013	0.254	HumanCyc
PTK6 Activates STAT3	4	1 (25.0%)	0.013	0.254	Reactome
Regulation of lipolysis in adipocytes - Homo sapiens (human)	57	2 (3.6%)	0.0139	0.263	KEGG
VEGF signaling pathway - Homo sapiens (human)	59	2 (3.4%)	0.0159	0.268	KEGG
Adenosine P1 receptors	5	1 (20.0%)	0.0163	0.268	Reactome
glycine betaine degradation	5	1 (20.0%)	0.0163	0.268	HumanCyc
Dopamine receptors	5	1 (20.0%)	0.0163	0.268	Reactome
Long-term depression - Homo sapiens (human)	60	2 (3.3%)	0.0165	0.268	KEGG
mTOR signaling pathway	64	2 (3.1%)	0.0186	0.279	PID
Choline catabolism	6	1 (16.7%)	0.0195	0.279	Reactome
Alpha Linolenic Acid and Linoleic Acid Metabolism	6	1 (16.7%)	0.0195	0.279	SMPDB
Pilocytic astrocytoma	6	1 (16.7%)	0.0195	0.279	Wikipathways
Fc epsilon RI signaling pathway - Homo sapiens	68	2 (3.0%)	0.0203	0.279	KEGG
FGF	67	2 (3.0%)	0.0203	0.279	INOH
Prolactin	70	2 (2.9%)	0.022	0.279	NetPath
superpathway of choline degradation to L-serine	7	1 (14.3%)	0.0227	0.279	HumanCyc
APEX1-Independent Resolution of AP Sites via the Single Nucleotide Replacement Pathway	7	1 (14.3%)	0.0227	0.279	Reactome
serine and glycine biosynthesis	7	1 (14.3%)	0.0227	0.279	HumanCyc
Negative feedback regulation of MAPK pathway	7	1 (14.3%)	0.0227	0.279	Reactome
MicroRNA for Targeting Cancer Growth and Vascularization in Glioblastoma	7	1 (14.3%)	0.0227	0.279	Wikipathways
Linoleate metabolism	72	2 (2.8%)	0.0232	0.279	EHMN
Prolactin Signaling Pathway	76	2 (2.6%)	0.0256	0.279	Wikipathways
oleate biosynthesis	8	1 (12.5%)	0.0259	0.279	HumanCyc
Linoleic acid (LA) metabolism	8	1 (12.5%)	0.0259	0.279	Reactome
MAPK and NFkB signaling pathways inhibited by Yersinia YopJ	8	1 (12.5%)	0.0259	0.279	Wikipathways
Conjugation of salicylate with glycine	8	1 (12.5%)	0.0259	0.279	Reactome
B cell receptor signaling pathway - Homo sapiens (human)	82	2 (2.5%)	0.0282	0.279	KEGG
Serine biosynthesis	9	1 (11.1%)	0.0291	0.279	Reactome
Prostanoid ligand receptors	9	1 (11.1%)	0.0291	0.279	Reactome

Signaling by MRAS-complex mutants	9	1 (11.1%)	0.0291	0.279	Reactome
MAP kinase cascade	9	1 (11.1%)	0.0291	0.279	HumanCyc
Conjugation of carboxylic acids	9	1 (11.1%)	0.0291	0.279	Reactome
Amino Acid conjugation	9	1 (11.1%)	0.0291	0.279	Reactome
sphingosine and sphingosine-1-phosphate	9	1 (11.1%)	0.0291	0.279	HumanCyc
HDR through MMEJ (alt-NHEJ)	10	1 (10.0%)	0.0323	0.293	Reactome
regulation of splicing through sam68	10	1 (10.0%)	0.0323	0.293	BioCarta
cAMP signaling pathway - Homo sapiens (human)	216	3 (1.4%)	0.0335	0.293	KEGG
Nucleotide GPCRs	11	1 (9.1%)	0.0355	0.293	Wikipathways
Hydrolysis of LPC	11	1 (9.1%)	0.0355	0.293	Reactome
Purine metabolism	222	3 (1.4%)	0.0359	0.293	EHMN
GnRH signaling pathway - Homo sapiens (human)	93	2 (2.2%)	0.0372	0.293	KEGG
Base Excision Repair	94	2 (2.2%)	0.0372	0.293	Reactome
Stimuli-sensing channels	94	2 (2.1%)	0.0379	0.293	Reactome
Scavenging of heme from plasma	12	1 (8.3%)	0.0386	0.293	Reactome
Omega-9 FA synthesis	12	1 (8.3%)	0.0386	0.293	Wikipathways
Negative regulation of NOTCH4 signaling	12	1 (8.3%)	0.0386	0.293	Reactome
Regulation of localization of FOXO transcription	12	1 (8.3%)	0.0386	0.293	Reactome
Hyaluronan metabolism	12	1 (8.3%)	0.0386	0.293	Reactome
Fc gamma R-mediated phagocytosis - Homo sapiens (human)	97	2 (2.1%)	0.0394	0.293	KEGG
Choline metabolism in cancer - Homo sapiens	98	2 (2.0%)	0.0409	0.293	KEGG
sarcosine oncometabolite pathway	13	1 (7.7%)	0.0418	0.293	SMPDB
alpha-linolenic acid (ALA) metabolism	13	1 (7.7%)	0.0418	0.293	Reactome
alpha-linolenic (omega3) and linoleic (omega6) acid metabolism	13	1 (7.7%)	0.0418	0.293	Reactome
melanocyte development and pigmentation pathway	13	1 (7.7%)	0.0418	0.293	BioCarta
multiple antiapoptotic pathways from igf-1r signaling lead to bad phosphorylation	13	1 (7.7%)	0.0418	0.293	BioCarta
Chk1/Chk2(Cds1) mediated inactivation of Cyclin B:Cdk1 complex	13	1 (7.7%)	0.0418	0.293	Reactome
Telomere C-strand synthesis initiation	13	1 (7.7%)	0.0418	0.293	Reactome
Omega-3-Omega-6 FA synthesis	14	1 (7.1%)	0.0449	0.295	Wikipathways
Ras signaling in the CD4+ TCR pathway	14	1 (7.1%)	0.0449	0.295	PID
MAPK pathway in congenital thyroid cancer	14	1 (7.1%)	0.0449	0.295	Wikipathways
phosphorylation of mek1 by cdk5/p35 down regulates the map kinase pathway	14	1 (7.1%)	0.0449	0.295	BioCarta
γ-linolenate biosynthesis	14	1 (7.1%)	0.0449	0.295	HumanCyc
Telomere Maintenance	105	2 (1.9%)	0.0455	0.295	Reactome
ErbB1 downstream signaling	106	2 (1.9%)	0.0471	0.295	PID
Eicosanoid ligand-binding receptors	15	1 (6.7%)	0.048	0.295	Reactome
ERK Pathway in Huntington,s Disease	15	1 (6.7%)	0.048	0.295	Wikipathways
Activation of BAD and translocation to mitochondria	15	1 (6.7%)	0.048	0.295	Reactome
Mercaptopurine Metabolism Pathway	15	1 (6.7%)	0.048	0.295	SMPDB

Depression

pathway name	set size	candidatescontained	p-value	q-value	pathway source
Eptifibatide Action Pathway	2	1 (50.0%)	0.00624	0.307	SMPDB
Tirofiban Action Pathway	2	1 (50.0%)	0.00624	0.307	SMPDB
Abciximab Action Pathway	2	1 (50.0%)	0.00624	0.307	SMPDB
Pyrophosphate hydrolysis	2	1 (50.0%)	0.00624	0.307	Reactome
5-aminoimidazole ribonucleotide biosynthesis	3	1 (33.3%)	0.00935	0.307	HumanCyc
Cocaine addiction - Homo sapiens (human)	49	2 (4.1%)	0.0102	0.307	KEGG
IRS activation	4	1 (25.0%)	0.0124	0.307	Reactome
Neurexins and neuroligins	57	2 (3.5%)	0.0137	0.307	Reactome
Focal Adhesion-PI3K-Akt-mTOR-signaling pathway	302	4 (1.3%)	0.0143	0.307	Wikipathways
tRNA splicing	5	1 (20.0%)	0.0155	0.307	HumanCyc
Human papillomavirus infection - Homo sapiens (human)	331	4 (1.2%)	0.0194	0.307	KEGG
NrCAM interactions	7	1 (14.3%)	0.0217	0.307	Reactome
overview of telomerase rna component gene hterc transcriptional regulation	7	1 (14.3%)	0.0217	0.307	BioCarta
SOS-mediated signalling	7	1 (14.3%)	0.0217	0.307	Reactome
Erythropoietin activates Phospholipase C gamma (PLCG)	7	1 (14.3%)	0.0217	0.307	Reactome
Erythropoietin activates STAT5	7	1 (14.3%)	0.0217	0.307	Reactome
Axon guidance	356	4 (1.1%)	0.0246	0.307	Reactome
Antigen processing and presentation - Homo sapiens (human)	78	2 (2.6%)	0.0247	0.307	KEGG
PI3K/AKT activation	9	1 (11.1%)	0.0278	0.307	Reactome
Nervous system development	379	4 (1.1%)	0.03	0.307	Reactome
Protein-protein interactions at synapses	88	2 (2.3%)	0.0308	0.307	Reactome
ECM-receptor interaction - Homo sapiens (human)	88	2 (2.3%)	0.0308	0.307	KEGG
IL-13 signaling	10	1 (10.0%)	0.0308	0.307	INOH
Signal attenuation	10	1 (10.0%)	0.0308	0.307	Reactome
Signaling by Leptin	11	1 (9.1%)	0.0339	0.307	Reactome
insulin signaling pathway	11	1 (9.1%)	0.0339	0.307	BioCarta
RHO GTPases activate KTN1	11	1 (9.1%)	0.0339	0.307	Reactome
Complement system	93	2 (2.2%)	0.0341	0.307	Wikipathways
RAF/MAP kinase cascade	235	3 (1.3%)	0.0368	0.307	Reactome
Activation of Ca-permeable Kainate Receptor	12	1 (8.3%)	0.0369	0.307	Reactome
Ionotropic activity of kainate receptors	12	1 (8.3%)	0.0369	0.307	Reactome
Purine ribonucleoside monophosphate biosynthesis	12	1 (8.3%)	0.0369	0.307	Reactome
Erythropoietin activates Phosphoinositide-3-kinase (PI3K)	12	1 (8.3%)	0.0369	0.307	Reactome
Aspartate and asparagine metabolism	12	1 (8.3%)	0.0369	0.307	Reactome
Butyrophilin (BTN) family interactions	12	1 (8.3%)	0.0369	0.307	Reactome
MAPK1/MAPK3 signaling	236	3 (1.3%)	0.0372	0.307	Reactome
Endosomal/Vacuolar pathway	13	1 (7.7%)	0.0399	0.307	Reactome
L1CAM interactions	102	2 (2.0%)	0.0404	0.307	Reactome
LGI-ADAM interactions	14	1 (7.1%)	0.0429	0.307	Reactome
Erythropoietin activates RAS	14	1 (7.1%)	0.0429	0.307	Reactome
il-7 signal transduction	15	1 (6.7%)	0.0459	0.307	BioCarta
p130Cas linkage to MAPK signaling for integrins	15	1 (6.7%)	0.0459	0.307	Reactome
GRB2:SOS provides linkage to MAPK signaling for Integrins	15	1 (6.7%)	0.0459	0.307	Reactome
FOXO-mediated transcription of cell death genes	15	1 (6.7%)	0.0459	0.307	Reactome
Nucleobase biosynthesis	15	1 (6.7%)	0.0459	0.307	Reactome
Leucine Stimulation on Insulin Signaling	15	1 (6.7%)	0.0459	0.307	SMPDB
RHO GTPases activate CIT	16	1 (6.2%)	0.0489	0.307	Reactome
FOXA2 pathway	16	1 (6.2%)	0.0489	0.307	Wikipathways

Psychosis

pathway name	set size	candidatescontained	p-value	q-value	pathway source
Transport of vitamins, nucleosides, and related molecules	44	3 (6.8%)	0.00032	0.13	Reactome
eicosapentaenoate biosynthesis	13	2 (15.4%)	0.000694	0.141	HumanCyc
fas signaling pathway (cd95)	20	2 (10.0%)	0.00167	0.181	BioCarta
Biosynthesis of DPAn-3-derived 13-series resolvins	1	1 (100.0%)	0.00305	0.181	Reactome
Biosynthesis of DPAn-3-derived maresins	2	1 (50.0%)	0.00609	0.181	Reactome
Biosynthesis of electrophilic ?-3 PUFA oxo-derivatives	2	1 (50.0%)	0.00609	0.181	Reactome
aspirin triggered resolvin E biosynthesis	2	1 (50.0%)	0.00609	0.181	HumanCyc
aspirin triggered resolvin D biosynthesis	2	1 (50.0%)	0.00609	0.181	HumanCyc
aspirin-triggered lipoxin biosynthesis	2	1 (50.0%)	0.00609	0.181	HumanCyc
Biosynthesis of DPAn-3-derived protectins and resolvins	2	1 (50.0%)	0.00609	0.181	Reactome
resolvin D biosynthesis	2	1 (50.0%)	0.00609	0.181	HumanCyc
Stimulation of the cell death response by PAK-2p34	2	1 (50.0%)	0.00609	0.181	Reactome
Regulation of PAK-2p34 activity by PS-GAP/RHG10	2	1 (50.0%)	0.00609	0.181	Reactome
SLC-mediated transmembrane transport	243	4 (1.6%)	0.00626	0.181	Reactome
Fas ligand pathway and stress induction of heat shock proteins	43	2 (4.7%)	0.00758	0.183	Wikipathways
Biosynthesis of aspirin-triggered D-series resolvins	3	1 (33.3%)	0.00913	0.183	Reactome
lipoxin biosynthesis	3	1 (33.3%)	0.00913	0.183	HumanCyc
FMO oxidises nucleophiles	3	1 (33.3%)	0.00913	0.183	Reactome
Ovarian steroidogenesis - Homo sapiens (human)	51	2 (3.9%)	0.0105	0.183	KEGG
Regulation of Actin Cytoskeleton	151	3 (2.0%)	0.0109	0.183	Wikipathways
Apoptosis-related network due to altered Notch3 in ovarian cancer	53	2 (3.8%)	0.0114	0.183	Wikipathways
Biosynthesis of E-series 18(R)-resolvins	4	1 (25.0%)	0.0122	0.183	Reactome
Biosynthesis of DPAn-3 SPMs	4	1 (25.0%)	0.0122	0.183	Reactome
Biosynthesis of DPA-derived SPMs	4	1 (25.0%)	0.0122	0.183	Reactome
Biosynthesis of D-series resolvins	4	1 (25.0%)	0.0122	0.183	Reactome
Resolution of AP sites via the single-nucleotide replacement pathway	4	1 (25.0%)	0.0122	0.183	Reactome
Regulation of Apoptosis	4	1 (25.0%)	0.0122	0.183	Reactome
RHOJ GTPase cycle	56	2 (3.6%)	0.0126	0.183	Reactome
RHOQ GTPase cycle	60	2 (3.3%)	0.0144	0.192	Reactome
Biosynthesis of E-series 18(S)-resolvins	5	1 (20.0%)	0.0152	0.192	Reactome
Catalytic cycle of mammalian Flavin-containing MonoOxygenases (FMOs)	5	1 (20.0%)	0.0152	0.192	Wikipathways
hiv-1 defeats host-mediated resistance by cem15	5	1 (20.0%)	0.0152	0.192	BioCarta
Gap-filling DNA repair synthesis and ligation in TC-NER	66	2 (3.0%)	0.0172	0.192	Reactome
Ciliopathies	183	3 (1.6%)	0.0181	0.192	Wikipathways
Acrosome Reaction and Sperm:Oocyte Membrane Binding	6	1 (16.7%)	0.0182	0.192	Reactome
Synthesis of Lipoxins (LX)	6	1 (16.7%)	0.0182	0.192	Reactome
Biosynthesis of EPA-derived SPMs	6	1 (16.7%)	0.0182	0.192	Reactome
leukotriene biosynthesis	6	1 (16.7%)	0.0182	0.192	HumanCyc
CDC42 signaling events	71	2 (2.8%)	0.0198	0.192	PID
TCR signaling	73	2 (2.7%)	0.0208	0.192	Reactome
APEX1-Independent Resolution of AP Sites via the Single Nucleotide Replacement Pathway	7	1 (14.3%)	0.0212	0.192	Reactome
APOBEC3G mediated resistance to HIV-1 infection	7	1 (14.3%)	0.0212	0.192	Reactome
RHOG GTPase cycle	74	2 (2.7%)	0.0214	0.192	Reactome
Fibroblast growth factor-1	74	2 (2.7%)	0.0214	0.192	NetPath
Transcription-Coupled Nucleotide Excision Repair (TC-NER)	79	2 (2.5%)	0.0241	0.192	Reactome
Biosynthesis of maresins	8	1 (12.5%)	0.0242	0.192	Reactome
Nef and signal transduction	8	1 (12.5%)	0.0242	0.192	Reactome
Conjugation of salicylate with glycine	8	1 (12.5%)	0.0242	0.192	Reactome
Dual hijack model of Vif in HIV infection	8	1 (12.5%)	0.0242	0.192	Wikipathways
oleate biosynthesis	8	1 (12.5%)	0.0242	0.192	HumanCyc
palmitate biosynthesis	9	1 (12.5%)	0.0242	0.192	HumanCyc
Synthesis of 5-eicosatetraenoic acids	9	1 (11.1%)	0.0271	0.2	Reactome
p75NTR recruits signalling complexes	9	1 (11.1%)	0.0271	0.2	Reactome
Conjugation of carboxylic acids	9	1 (11.1%)	0.0271	0.2	Reactome

Amino Acid conjugation	9	1 (11.1%)	0.0271	0.2	Reactome
RAC2 GTPase cycle	85	2 (2.4%)	0.0277	0.2	Reactome
Regulation of actin cytoskeleton - Homo sapiens (human)	218	3 (1.4%)	0.0286	0.2	KEGG
ECM-receptor interaction - Homo sapiens (human)	88	2 (2.3%)	0.0295	0.2	KEGG
Host Interactions of HIV factors	89	2 (2.2%)	0.0301	0.2	Reactome
HDR through MMEJ (alt-NHEJ)	10	1 (10.0%)	0.0301	0.2	Reactome
Vif-mediated degradation of APOBEC3G	10	1 (10.0%)	0.0301	0.2	Reactome
3-oxo-10R-octadecatrienoate beta-oxidation	11	1 (9.1%)	0.0331	0.215	EHMN
RAC3 GTPase cycle	94	2 (2.1%)	0.0333	0.215	Reactome
Nucleotide Excision Repair	97	2 (2.1%)	0.0353	0.216	Reactome
Transport of nucleosides and free purine and pyrimidine bases across the plasma membrane	12	1 (8.3%)	0.036	0.216	Reactome
CD28 dependent Vav1 pathway	12	1 (8.3%)	0.036	0.216	Reactome
Transport of organic anions	12	1 (8.3%)	0.036	0.216	Reactome
Integrin-mediated Cell Adhesion	101	2 (2.0%)	0.038	0.216	Wikipathways
Leukotriene metabolism	102	2 (2.0%)	0.0386	0.216	EHMN
Activation of RAC1	13	1 (7.7%)	0.039	0.216	Reactome
Ganglio Sphingolipid Metabolism	13	1 (7.7%)	0.039	0.216	Wikipathways
Platelet Adhesion to exposed collagen	13	1 (7.7%)	0.039	0.216	Reactome
stearate biosynthesis	13	1 (7.7%)	0.039	0.216	HumanCyc
Degradation of the extracellular matrix	103	2 (1.9%)	0.0393	0.216	Reactome
γ-linolenate biosynthesis	14	1 (7.1%)	0.0419	0.227	HumanCyc
Transport of small molecules	641	5 (0.8%)	0.0442	0.231	Reactome
Glycosphingolipid biosynthesis - ganglio series - Homo sapiens (human)	15	1 (6.7%)	0.0448	0.231	KEGG
Sema3A PAK dependent Axon repulsion	16	1 (6.2%)	0.0478	0.231	Reactome
p75NTR signals via NF-kB	16	1 (6.2%)	0.0478	0.231	Reactome
Elevation of cytosolic Ca2+ levels	16	1 (6.2%)	0.0478	0.231	Reactome

Prediabetes

pathway name	set size	candidatescontained	p-value	q-value	pathway source
Pre-NOTCH Processing in Golgi	12	2 (16.7%)	0.000773	0.355	Reactome
Ephrin B reverse signaling	24	2 (8.3%)	0.00315	0.355	PID
Glycosphingolipid biosynthesis - lacto and neolacto series - Homo sapiens (human)	27	2 (7.4%)	0.00398	0.355	KEGG
Sialic acid metabolism	33	2 (6.1%)	0.0059	0.355	Reactome
PDE3B signalling	2	1 (50.0%)	0.00699	0.355	Reactome
PKB-mediated events	2	1 (50.0%)	0.00699	0.355	Reactome
PI3K Cascade	44	2 (4.5%)	0.0103	0.355	Reactome
DNA IR-double strand breaks and cellular response via ATM	3	1 (33.3%)	0.0105	0.355	Wikipathways
IRS-mediated signalling	48	2 (4.2%)	0.0122	0.355	Reactome
IRS-related events triggered by IGF1R	52	2 (3.8%)	0.0142	0.355	Reactome
IGF1R signaling cascade	53	2 (3.8%)	0.0147	0.355	Reactome
Signaling by Type 1 Insulin-like Growth Factor 1 Receptor (IGF1R)	53	2 (3.8%)	0.0147	0.355	Reactome
Insulin receptor signalling cascade	54	2 (3.7%)	0.0153	0.355	Reactome
Class IB PI3K non-lipid kinase events	5	1 (20.0%)	0.0174	0.355	PID
Vitamin B2 (riboflavin) metabolism	5	1 (20.0%)	0.0174	0.355	EHMN
Riboflavin Metabolism	5	1 (20.0%)	0.0174	0.355	SMPDB
Synthesis of substrates in N-glycan biosythesis	63	2 (3.2%)	0.0204	0.355	Reactome
RUNX1 regulates transcription of genes involved in BCR signaling	6	1 (16.7%)	0.0208	0.355	Reactome
FGFR1b ligand binding and activation	6	1 (16.7%)	0.0208	0.355	Reactome
Pre-NOTCH Processing in the Endoplasmic Reticulum	6	1 (16.7%)	0.0208	0.355	Reactome
Pantothenate and CoA Biosynthesis	6	1 (16.7%)	0.0208	0.355	SMPDB
Vitamin B2 (riboflavin) metabolism	7	1 (14.3%)	0.0242	0.355	Reactome
Gene expression (Transcription)	1439	10 (0.7%)	0.0248	0.355	Reactome
NOTCH4 Intracellular Domain Regulates Transcription	8	1 (12.5%)	0.0277	0.355	Reactome
Riboflavin metabolism - Homo sapiens (human)	8	1 (12.5%)	0.0277	0.355	KEGG
Biosynthesis of the N-glycan precursor (dolichol lipid-linked oligosaccharide, LLO) and transfer to a nascent protein	77	2 (2.6%)	0.0297	0.355	Reactome
Signaling by Insulin receptor	78	2 (2.6%)	0.0304	0.355	Reactome
NOTCH4 Activation and Transmission of Signal to the Nucleus	9	1 (11.1%)	0.0311	0.355	Reactome
ATR Signaling	9	1 (11.1%)	0.0311	0.355	Wikipathways
polyadenylation of mrna	9	1 (11.1%)	0.0311	0.355	BioCarta
Focal Adhesion	198	3 (1.5%)	0.0318	0.355	Wikipathways
Gamma-carboxylation of protein precursors	10	1 (10.0%)	0.0345	0.355	Reactome
Removal of aminoterminal propeptides from gamma-carboxylated proteins	10	1 (10.0%)	0.0345	0.355	Reactome
FGFR2b ligand binding and activation	10	1 (10.0%)	0.0345	0.355	Reactome
Organic anion transporters	10	1 (10.0%)	0.0345	0.355	Reactome
RNA Polymerase II Transcription	1307	9 (0.7%)	0.035	0.355	Reactome
Rap1 signaling pathway - Homo sapiens (human)	210	3 (1.4%)	0.0369	0.355	KEGG
Gamma-carboxylation, transport, and amino-terminal cleavage of proteins	11	1 (9.1%)	0.0378	0.355	Reactome
PTF1A related regulatory pathway	11	1 (9.1%)	0.0378	0.355	Wikipathways
regulators of bone mineralization	11	1 (9.1%)	0.0378	0.355	BioCarta
Regulation of actin cytoskeleton - Homo sapiens (human)	218	3 (1.4%)	0.0405	0.355	KEGG
Negative regulation of NOTCH4 signaling	12	1 (8.3%)	0.0412	0.355	Reactome
Transport of Mature mRNA Derived from an Intronless Transcript	12	1 (8.3%)	0.0412	0.355	Reactome
Amplification of signal from unattached kinetochores via a MAD2 inhibitory signal	96	2 (2.1%)	0.0444	0.355	Reactome
Amplification of signal from the kinetochores	96	2 (2.1%)	0.0444	0.355	Reactome
FGFRL1 modulation of FGFR1 signaling	13	1 (7.7%)	0.0446	0.355	Reactome
RHO GTPases activate IQGAPs	13	1 (7.7%)	0.0446	0.355	Reactome
eNOS activation	13	1 (7.7%)	0.0446	0.355	Reactome
NO metabolism in cystic fibrosis	13	1 (7.7%)	0.0446	0.355	Wikipathways

Transport of Mature mRNAs Derived from Intronless Transcripts	**13**	**1 (7.7%)**	0.0446	0.355	**Reactome**
EML4 and NUDC in mitotic spindle formation	**100**	**2 (2.0%)**	0.0478	0.355	**Reactome**
Osteoblast Signaling	**14**	**1 (7.1%)**	0.0479	0.355	**Wikipathways**
Nicotinate and Nicotinamide Metabolism	**14**	**1 (7.1%)**	0.0479	0.355	**SMPDB**
PIWI-interacting RNA (piRNA) biogenesis	**14**	**1 (7.1%)**	0.0479	0.355	**Reactome**
RAF/MAP kinase cascade	**235**	**3 (1.3%)**	0.0488	0.355	**Reactome**
MAPK1/MAPK3 signaling	**236**	**3 (1.3%)**	0.0493	0.355	**Reactome**
Generic Transcription Pathway	**1178**	**8 (0.7%)**	0.0497	0.355	

T1DM

pathway name	set size	candidatescontained	p-value	q-value	pathway source
MHC class II antigen presentation	60	6 (10.0%)	0.00000191	0.00148	Reactome
Asthma - Homo sapiens (human)	31	4 (13.3%)	0.0000344	0.0134	KEGG
Allograft rejection - Homo sapiens (human)	38	4 (10.8%)	0.0000803	0.0208	KEGG
Graft-versus-host disease - Homo sapiens (human)	42	4 (9.5%)	0.000133	0.0208	KEGG
Type I diabetes mellitus - Homo sapiens (human)	43	4 (9.3%)	0.000146	0.0208	KEGG
Ebola Virus Pathway on Host	130	6 (4.6%)	0.00016	0.0208	Wikipathways
Intestinal immune network for IgA production - Homo sapiens (human)	49	4 (8.5%)	0.000207	0.0217	KEGG
Th1 and Th2 cell differentiation - Homo sapiens (human)	92	5 (5.4%)	0.000272	0.0217	KEGG
Allograft Rejection	93	5 (5.4%)	0.000286	0.0217	Wikipathways
Staphylococcus aureus infection - Homo sapiens (human)	96	5 (5.3%)	0.000301	0.0217	KEGG
Autoimmune thyroid disease - Homo sapiens (human)	53	4 (7.7%)	0.000306	0.0217	KEGG
Hematopoietic cell lineage - Homo sapiens (human)	99	5 (5.1%)	0.000365	0.0237	KEGG
Viral myocarditis - Homo sapiens (human)	60	4 (6.8%)	0.000498	0.0298	KEGG
Pre-NOTCH Processing in the Endoplasmic Reticulum	6	2 (33.3%)	0.00057	0.0317	Reactome
Translocation of ZAP-70 to Immunological synapse	28	3 (10.7%)	0.00069	0.035	Reactome
Inflammatory bowel disease - Homo sapiens (human)	65	4 (6.2%)	0.000721	0.035	KEGG
Phosphorylation of CD3 and TCR zeta chains	31	3 (9.7%)	0.000934	0.0428	Reactome
TCR signaling	73	4 (5.5%)	0.00112	0.0482	Reactome
Leishmaniasis - Homo sapiens (human)	77	4 (5.3%)	0.0013	0.0531	KEGG
Antigen processing and presentation - Homo sapiens (human)	78	4 (5.1%)	0.00143	0.0555	KEGG
Systemic lupus erythematosus - Homo sapiens (human)	136	5 (3.7%)	0.00151	0.0558	KEGG
Pre-NOTCH Processing in Golgi	12	2 (16.7%)	0.00245	0.0815	Reactome
Human T-cell leukemia virus 1 infection - Homo sapiens (human)	219	6 (2.7%)	0.00248	0.0815	KEGG
Renin-angiotensin-aldosterone system (RAAS)	44	3 (6.8%)	0.0026	0.0815	Wikipathways
Rheumatoid arthritis - Homo sapiens (human)	93	4 (4.3%)	0.00262	0.0815	KEGG
Sudden Infant Death Syndrome (SIDS) Susceptibility Pathways	159	5 (3.1%)	0.00318	0.095	Wikipathways
Biogenic Amine Synthesis	15	2 (13.3%)	0.00385	0.111	Wikipathways
Downstream TCR signaling	52	3 (5.8%)	0.00418	0.116	Reactome
Metabolism of amine-derived hormones	16	2 (12.5%)	0.00438	0.117	Reactome
Th17 cell differentiation - Homo sapiens (human)	107	4 (3.7%)	0.0045	0.117	KEGG
Toxoplasmosis - Homo sapiens (human)	112	4 (3.6%)	0.0053	0.133	KEGG
NCAM signaling for neurite out-growth	59	3 (5.1%)	0.00596	0.145	Reactome
Folate biosynthesis - Homo sapiens (human)	26	2 (7.7%)	0.0114	0.26	KEGG
Canonical and non-canonical Notch signaling	27	2 (7.4%)	0.0123	0.26	Wikipathways
Herpes simplex virus 1 infection - Homo sapiens (human)	498	8 (1.6%)	0.0125	0.26	KEGG
Notch	78	3 (3.8%)	0.0128	0.26	INOH
Regulatory circuits of the STAT3 signaling pathway	78	3 (3.8%)	0.0128	0.26	Wikipathways
Notch-HLH transcription pathway	28	2 (7.1%)	0.0131	0.26	Reactome
Cell adhesion molecules - Homo sapiens (human)	149	4 (2.7%)	0.0138	0.26	KEGG
Adaptive Immune System	719	10 (1.4%)	0.014	0.26	Reactome
Synthesis of IP3 and IP4 in the cytosol	29	2 (6.9%)	0.0141	0.26	Reactome
Interaction between L1 and Ankyrins	29	2 (6.9%)	0.0141	0.26	Reactome
Phagosome - Homo sapiens (human)	152	4 (2.6%)	0.0148	0.267	KEGG
C-MYB transcription factor network	85	3 (3.5%)	0.0161	0.281	PID
Insulin secretion - Homo sapiens (human)	86	3 (3.5%)	0.0166	0.281	KEGG
PD-1 signaling	32	2 (6.2%)	0.017	0.281	Reactome
Alpha-synuclein signaling	32	2 (6.2%)	0.017	0.281	PID
Role of Osx and miRNAs in tooth development	33	2 (6.1%)	0.018	0.284	Wikipathways
Toxicity of botulinum toxin type C (botC)	3	1 (33.3%)	0.0186	0.284	Reactome
Inositol transporters	3	1 (33.3%)	0.0186	0.284	Reactome
N-acetylglucosamine degradation I	3	1 (33.3%)	0.0186	0.284	HumanCyc
Influenza A - Homo sapiens (human)	172	4 (2.3%)	0.0227	0.306	KEGG
Aldosterone synthesis and secretion - Homo sapiens (human)	98	3 (3.1%)	0.0234	0.306	KEGG
eIF5A regulation in response to inhibition of the nuclear export system	4	1 (25.0%)	0.0248	0.306	Wikipathways
Hypusine synthesis from eIF5A-lysine	4	1 (25.0%)	0.0248	0.306	Reactome
Fosphenytoin (Antiarrhythmic) Metabolism Pathway	4	1 (25.0%)	0.0248	0.306	SMPDB

Catecholamine biosynthesis	4	1 (25.0%)	0.0248	0.306	Reactome
catecholamine biosynthesis	4	1 (25.0%)	0.0248	0.306	HumanCyc
Tyrosine hydroxylase deficiency	4	1 (25.0%)	0.0248	0.306	SMPDB
Catecholamine Biosynthesis	4	1 (25.0%)	0.0248	0.306	SMPDB
Aromatic L-Aminoacid Decarboxylase Deficiency	4	1 (25.0%)	0.0248	0.306	SMPDB
Regulation of Apoptosis	4	1 (25.0%)	0.0248	0.306	Reactome
N-acetylglucosamine degradation II	4	1 (25.0%)	0.0248	0.306	HumanCyc
Tuberculosis - Homo sapiens (human)	180	4 (2.2%)	0.0258	0.313	KEGG
Inositol phosphate metabolism	41	2 (4.9%)	0.0271	0.324	INOH
Generation of second messenger molecules	42	2 (4.8%)	0.0283	0.334	Reactome
PAR1-mediated thrombin signaling events	43	2 (4.7%)	0.0296	0.334	PID
Kinesins	43	2 (4.7%)	0.0296	0.334	Reactome
Serotonin and melatonin biosynthesis	5	1 (20.0%)	0.0309	0.334	Reactome
serotonin and melatonin biosynthesis	5	1 (20.0%)	0.0309	0.334	HumanCyc
The fatty acid cycling model	5	1 (20.0%)	0.0309	0.334	Reactome
The proton buffering model	5	1 (20.0%)	0.0309	0.334	Reactome
Notch Signaling	45	2 (4.4%)	0.0322	0.343	Wikipathways
Activation of C3 and C5	6	1 (16.7%)	0.0369	0.366	Reactome
allopregnanolone biosynthesis	6	1 (16.7%)	0.0369	0.366	HumanCyc
Somatroph axis (GH) and its relationship to dietary restriction and aging	6	1 (16.7%)	0.0369	0.366	Wikipathways
Mitochondrial Uncoupling	6	1 (16.7%)	0.0369	0.366	Reactome
methionine degradation	6	1 (16.7%)	0.0369	0.366	HumanCyc
Epstein-Barr virus infection - Homo sapiens (human)	202	4 (2.0%)	0.0372	0.366	KEGG
Cocaine addiction - Homo sapiens (human)	49	2 (4.1%)	0.0376	0.366	KEGG
Thyroid hormone signaling pathway - Homo sapiens (human)	121	3 (2.5%)	0.0401	0.384	KEGG
Inositol phosphate metabolism	51	2 (3.9%)	0.0404	0.384	Reactome
Signaling events mediated by Stem cell factor receptor (c-Kit)	52	2 (3.8%)	0.0419	0.388	PID
Erythropoietin activates Phospholipase C gamma (PLCG)	7	1 (14.3%)	0.043	0.388	Reactome
Erythropoietin activates STAT5	7	1 (14.3%)	0.043	0.388	Reactome
Apoptosis-related network due to altered Notch3 in ovarian cancer	53	2 (3.8%)	0.0433	0.388	Wikipathways
Notch signaling pathway	53	2 (3.8%)	0.0433	0.388	PID
Fanconi anemia pathway - Homo sapiens (human)	54	2 (3.7%)	0.0448	0.396	KEGG
The alternative pathway of fetal androgen synthesis	8	1 (12.5%)	0.049	0.401	Wikipathways
NOTCH4 Intracellular Domain Regulates Transcription	8	1 (12.5%)	0.049	0.401	Reactome
Fatty Acids bound to GPR40 (FFAR1) regulate insulin secretion	8	1 (12.5%)	0.049	0.401	Reactome
Regulation of PTEN localization	8	1 (12.5%)	0.049	0.401	Reactome
Inactivation of CDC42 and RAC1	8	1 (12.5%)	0.049	0.401	Reactome
cysteine biosynthesis	8	1 (12.5%)	0.049	0.401	HumanCyc
FTO Obesity Variant Mechanism	8	1 (12.5%)	0.049	0.401	Wikipathways
Dopaminergic synapse - Homo sapiens (human)	132	3 (2.3%)	0.0497	0.403	KEGG

T2DM

pathway name	set size	candidates contained	p-value	q-value	pathway source
Vitamin B3 (nicotinate and nicotinamide) metabolism	47	4 (8.5%)	0.000803	0.544	EHMN
Pre-NOTCH Processing in the Endoplasmic Reticulum	6	2 (33.3%)	0.00116	0.544	Reactome
Cytochrome c-mediated apoptotic response	12	2 (16.7%)	0.00493	0.544	Reactome
Pre-NOTCH Processing in Golgi	12	2 (16.7%)	0.00493	0.544	Reactome
Lysosome - Homo sapiens (human)	128	5 (3.9%)	0.00578	0.544	KEGG
role of mitochondria in apoptotic signaling	13	2 (15.4%)	0.00579	0.544	BioCarta
Nicotinate and Nicotinamide Metabolism	14	2 (14.3%)	0.00671	0.544	SMPDB
Transcriptional misregulation in cancer - Homo sapiens (human)	192	6 (3.1%)	0.00732	0.544	KEGG
Notch Signaling	45	3 (6.7%)	0.00751	0.544	Wikipathways
Apoptotic factor-mediated response	16	2 (12.5%)	0.00875	0.544	Reactome
FLT3 mutants bind TKIs	1	1 (100.0%)	0.00893	0.544	Reactome
Mtb iron assimilation by chelation	1	1 (100.0%)	0.00893	0.544	Reactome
Latent infection - Other responses of Mtb to phagocytosis	1	1 (100.0%)	0.00893	0.544	Reactome
Apoptosis Modulation and Signaling	92	4 (4.3%)	0.00925	0.544	Wikipathways
Intrinsic Pathway for Apoptosis	52	3 (5.8%)	0.0112	0.544	Reactome
Nanomaterial induced apoptosis	20	2 (10.0%)	0.0135	0.544	Wikipathways
Herpes simplex virus 1 infection - Homo sapiens (human)	498	10 (2.0%)	0.0137	0.544	KEGG
Regulation of apoptosis by parathyroid hormone-related protein	21	2 (9.5%)	0.0149	0.544	Wikipathways
Notch	58	3 (5.2%)	0.015	0.544	NetPath
SHC1 events in ERBB2 signaling	22	2 (9.1%)	0.0163	0.544	Reactome
Wnt-beta-catenin signaling pathway in leukemia	22	2 (9.1%)	0.0163	0.544	Wikipathways
MHC class II antigen presentation	60	3 (5.0%)	0.0164	0.544	Reactome
Steroid hormone biosynthesis - Homo sapiens (human)	61	3 (4.9%)	0.0172	0.544	KEGG
Negative regulators of DDX58/IFIH1 signaling	23	2 (8.7%)	0.0177	0.544	Reactome
GPCRs, Class B Secretin-like	23	2 (8.7%)	0.0177	0.544	Wikipathways
Activation and oligomerization of BAK protein	2	1 (50.0%)	0.0178	0.544	Reactome
GDP-L-fucose biosynthesis II (from L-fucose)	2	1 (50.0%)	0.0178	0.544	HumanCyc
NAD phosphorylation and dephosphorylation	2	1 (50.0%)	0.0178	0.544	HumanCyc
Cytosolic iron-sulfur cluster assembly	2	1 (50.0%)	0.0178	0.544	Reactome
ChREBP activates metabolic gene expression	2	1 (50.0%)	0.0178	0.544	Reactome
Pathways in cancer - Homo sapiens (human)	531	10 (1.9%)	0.0205	0.544	KEGG
ID	26	2 (7.7%)	0.0224	0.544	NetPath
Canonical and non-canonical Notch signaling	27	2 (7.4%)	0.024	0.544	Wikipathways
Sulfur amino acid metabolism	27	2 (7.4%)	0.024	0.544	Reactome
Notch-HLH transcription pathway	28	2 (7.1%)	0.0257	0.544	Reactome
methionine salvage	3	1 (33.3%)	0.0266	0.544	HumanCyc
Interconversion of 2-oxoglutarate and 2-hydroxyglutarate	3	1 (33.3%)	0.0266	0.544	Reactome
Downregulation of ERBB2 signaling	29	2 (6.9%)	0.0275	0.544	Reactome
Interaction between L1 and Ankyrins	29	2 (6.9%)	0.0275	0.544	Reactome
Human papillomavirus infection - Homo sapiens (human)	331	7 (2.1%)	0.0289	0.544	KEGG
EGFR downregulation	30	2 (6.7%)	0.0292	0.544	Reactome
ADORA2B mediated anti-inflammatory cytokines production	132	4 (3.0%)	0.0306	0.544	Reactome
Vitamin B5 - CoA biosynthesis from pantothenate	31	2 (6.5%)	0.0311	0.544	EHMN
Nuclear Envelope (NE) Reassembly	78	3 (3.8%)	0.0326	0.544	Reactome
Apoptosis - multiple species - Homo sapiens (human)	32	2 (6.2%)	0.033	0.544	KEGG
White fat cell differentiation	32	2 (6.2%)	0.033	0.544	Wikipathways
IL17 signaling pathway	32	2 (6.2%)	0.033	0.544	Wikipathways
Apoptosis - Homo sapiens (human)	136	4 (2.9%)	0.0336	0.544	KEGG
Glucagon-type ligand receptors	33	2 (6.1%)	0.0349	0.544	Reactome
Role of Osx and miRNAs in tooth development	33	2 (6.1%)	0.0349	0.544	Wikipathways
Sealing of the nuclear envelope (NE) by ESCRT-III	33	2 (6.1%)	0.0349	0.544	Reactome
2-oxoisovalerate decarboxylation to isobutanoyl-CoA	4	1 (25.0%)	0.0353	0.544	HumanCyc
Lysosomal oligosaccharide catabolism	4	1 (25.0%)	0.0353	0.544	Reactome
phospholipase c delta in phospholipid associated cell signaling	4	1 (25.0%)	0.0353	0.544	BioCarta

S-methyl-5-thio-α-D-ribose 1-phosphate degradation	**4**	**1 (25.0%)**	0.0353	0.544	**HumanCyc**
HCN channels	**4**	**1 (25.0%)**	0.0353	0.544	**Reactome**
RHOU GTPase cycle	**34**	**2 (5.9%)**	0.0369	0.544	**Reactome**
Nicotinate and nicotinamide metabolism - Homo sapiens (human)	**35**	**2 (5.7%)**	0.0389	0.544	**KEGG**
G alpha (s) signalling events	**143**	**4 (2.8%)**	0.0392	0.544	**Reactome**
Asparagine N-linked glycosylation	**283**	**6 (2.1%)**	0.0414	0.544	**Reactome**
Apoptosis	**87**	**3 (3.4%)**	0.0429	0.544	**Wikipathways**
Thyroid cancer - Homo sapiens (human)	**37**	**2 (5.4%)**	0.043	0.544	**KEGG**
Activation of caspases through apoptosome-mediated cleavage	**5**	**1 (20.0%)**	0.0439	0.544	**Reactome**
FLT3 signaling through SRC family kinases	**5**	**1 (20.0%)**	0.0439	0.544	**Reactome**
tetrapyrrole biosynthesis	**5**	**1 (20.0%)**	0.0439	0.544	**HumanCyc**
Vitamin B2 (riboflavin) metabolism	**5**	**1 (20.0%)**	0.0439	0.544	**EHMN**
Riboflavin Metabolism	**5**	**1 (20.0%)**	0.0439	0.544	**SMPDB**
MECP2 regulates transcription factors	**5**	**1 (20.0%)**	0.0439	0.544	**Reactome**
basic mechanism of action of ppara pparb(d) and pparg and effects on gene expression	**5**	**1 (20.0%)**	0.0439	0.544	**BioCarta**
role of ppar-gamma coactivators in obesity and thermogenesis	**5**	**1 (20.0%)**	0.0439	0.544	**BioCarta**
PKA-mediated phosphorylation of key metabolic factors	**5**	**1 (20.0%)**	0.0439	0.544	**Reactome**
ketolysis	**5**	**1 (20.0%)**	0.0439	0.544	**HumanCyc**
Ubiquinone Biosynthesis	**5**	**1 (20.0%)**	0.0439	0.544	**SMPDB**
Class B/2 (Secretin family receptors)	**91**	**3 (3.3%)**	0.0479	0.544	**Reactome**
Oxidative Damage	**40**	**2 (5.0%)**	0.0495	0.544	**Wikipathways**
Class C/3 (Metabotropic glutamate/pheromone receptors)	**40**	**2 (5.0%)**	0.0495	0.544	**Reactome**

T2DM_related_admission

pathway name	set size	candidatescontained	p-value	q-value	pathway source
ABO blood group biosynthesis	3	2 (66.7%)	0.000299	0.327	Reactome
Globo Sphingolipid Metabolism	19	3 (15.8%)	0.000855	0.415	Wikipathways
Pre-NOTCH Processing in the Endoplasmic Reticulum	6	2 (33.3%)	0.00146	0.415	Reactome
GPCRs, Class B Secretin-like	23	3 (13.0%)	0.00152	0.415	Wikipathways
Glycosphingolipid biosynthesis - lactoseries	7	2 (28.6%)	0.00204	0.446	EHMN
Macrophage markers	9	2 (22.2%)	0.00345	0.594	Wikipathways
Cytochrome c-mediated apoptotic response	12	2 (16.7%)	0.00619	0.594	Reactome
Pre-NOTCH Processing in Golgi	12	2 (16.7%)	0.00619	0.594	Reactome
Class C/3 (Metabotropic glutamate/pheromone receptors)	40	3 (7.5%)	0.00747	0.594	Reactome
Nicotinate and Nicotinamide Metabolism	14	2 (14.3%)	0.00843	0.594	SMPDB
Glycosphingolipid biosynthesis - globo and isoglobo series - Homo sapiens (human)	15	2 (13.3%)	0.00966	0.594	KEGG
FLT3 mutants bind TKIs	1	1 (100.0%)	0.01	0.594	Reactome
Mtb iron assimilation by chelation	1	1 (100.0%)	0.01	0.594	Reactome
Latent infection - Other responses of Mtb to phagocytosis	1	1 (100.0%)	0.01	0.594	Reactome
Molybdenum cofactor (Moco) biosynthesis	1	1 (100.0%)	0.01	0.594	Wikipathways
ADORA2B mediated anti-inflammatory cytokines production	132	5 (3.8%)	0.0107	0.594	Reactome
Apoptotic factor-mediated response	16	2 (12.5%)	0.011	0.594	Reactome
Glycosphingolipid biosynthesis - neolactoseries	16	2 (12.5%)	0.011	0.594	EHMN
Vitamin B3 (nicotinate and nicotinamide) metabolism	47	3 (6.4%)	0.0117	0.594	EHMN
Transcriptional misregulation in cancer - Homo sapiens (human)	192	6 (3.1%)	0.0127	0.594	KEGG
Class B/2 (Secretin family receptors)	91	4 (4.4%)	0.0133	0.594	Reactome
Glycosphingolipid biosynthesis - globoseries	18	2 (11.1%)	0.0138	0.594	EHMN
Other glycan degradation - Homo sapiens (human)	18	2 (11.1%)	0.0138	0.594	KEGG
G alpha (s) signalling events	143	5 (3.5%)	0.0147	0.594	Reactome
Intrinsic Pathway for Apoptosis	52	3 (5.8%)	0.0153	0.594	Reactome
Nanomaterial induced apoptosis	20	2 (10.0%)	0.0169	0.594	Wikipathways
Blood group systems biosynthesis	20	2 (10.0%)	0.0169	0.594	Reactome
3-phosphoinositide degradation	20	2 (10.0%)	0.0169	0.594	HumanCyc
p38 signaling mediated by MAPKAP kinases	21	2 (9.5%)	0.0186	0.594	PID
Leukotriene metabolism	102	4 (3.9%)	0.0194	0.594	EHMN
C21-steroid hormone biosynthesis and metabolism	57	3 (5.3%)	0.0196	0.594	EHMN
Activation and oligomerization of BAK protein	2	1 (50.0%)	0.02	0.594	Reactome
NAD phosphorylation and dephosphorylation	2	1 (50.0%)	0.02	0.594	HumanCyc
Cytosolic iron-sulfur cluster assembly	2	1 (50.0%)	0.02	0.594	Reactome
ChREBP activates metabolic gene expression	2	1 (50.0%)	0.02	0.594	Reactome
Wnt-beta-catenin signaling pathway in leukemia	22	2 (9.1%)	0.0203	0.594	Wikipathways
Notch	58	3 (5.2%)	0.0205	0.594	NetPath
GPCR ligand binding	476	10 (2.1%)	0.0218	0.594	Reactome
Negative regulators of DDX58/IFIH1 signaling	23	2 (8.7%)	0.0221	0.594	Reactome
Steroid hormone biosynthesis - Homo sapiens (human)	61	3 (4.9%)	0.0234	0.594	KEGG
ID	26	2 (7.7%)	0.0278	0.594	NetPath
Folate biosynthesis - Homo sapiens (human)	26	2 (7.7%)	0.0278	0.594	KEGG
SARS-CoV-2 and COVID-19 Pathway	3	1 (33.3%)	0.0298	0.594	Wikipathways
Melanin biosynthesis	3	1 (33.3%)	0.0298	0.594	Reactome
methionine salvage	3	1 (33.3%)	0.0298	0.594	HumanCyc
ARL13B-mediated ciliary trafficking of INPP5E	3	1 (33.3%)	0.0298	0.594	Reactome
Interconversion of 2-oxoglutarate and 2-hydroxyglutarate	3	1 (33.3%)	0.0298	0.594	Reactome
Glycosphingolipid biosynthesis - lacto and neolacto series - Homo sapiens (human)	27	2 (7.4%)	0.0299	0.594	KEGG
Canonical and non-canonical Notch signaling	27	2 (7.4%)	0.0299	0.594	Wikipathways
Sulfur amino acid metabolism	27	2 (7.4%)	0.0299	0.594	Reactome
Notch-HLH transcription pathway	28	2 (7.1%)	0.032	0.594	Reactome
NOTCH-Ncore	28	2 (7.1%)	0.032	0.594	Signalink
Plasma lipoprotein clearance	29	2 (6.9%)	0.0341	0.594	Reactome
Interaction between L1 and Ankyrins	29	2 (6.9%)	0.0341	0.594	Reactome
Parkinson,s disease pathway	71	3 (4.2%)	0.0346	0.594	Wikipathways
Vitamin B5 - CoA biosynthesis from pantothenate	31	2 (6.5%)	0.0385	0.594	EHMN

eumelanin biosynthesis	4	1 (25.0%)	0.0396	0.594	HumanCyc
Lysosomal oligosaccharide catabolism	4	1 (25.0%)	0.0396	0.594	Reactome
Catecholamine biosynthesis	4	1 (25.0%)	0.0396	0.594	Reactome
catecholamine biosynthesis	4	1 (25.0%)	0.0396	0.594	HumanCyc
Tyrosine hydroxylase deficiency	4	1 (25.0%)	0.0396	0.594	SMPDB
Catecholamine Biosynthesis	4	1 (25.0%)	0.0396	0.594	SMPDB
Aromatic L-Aminoacid Decarboxylase Deficiency	4	1 (25.0%)	0.0396	0.594	SMPDB
S-methyl-5-thio-α-D-ribose 1-phosphate degradation	4	1 (25.0%)	0.0396	0.594	HumanCyc
Lysosome - Homo sapiens (human)	128	4 (3.1%)	0.0401	0.594	KEGG
Apoptosis - multiple species - Homo sapiens (human)	32	2 (6.2%)	0.0409	0.594	KEGG
Monoamine Transport	32	2 (6.2%)	0.0409	0.594	Wikipathways
Glucagon-type ligand receptors	33	2 (6.1%)	0.0432	0.594	Reactome
Role of Osx and miRNAs in tooth development	33	2 (6.1%)	0.0432	0.594	Wikipathways
Tyrosine metabolism	33	2 (6.1%)	0.0432	0.594	INOH
RHOU GTPase cycle	34	2 (5.9%)	0.0456	0.594	Reactome
Nicotinate and nicotinamide metabolism - Homo sapiens (human)	35	2 (5.7%)	0.0481	0.594	KEGG
Histidine metabolism	35	2 (5.7%)	0.0481	0.594	EHMN
Degradation pathway of sphingolipids, including diseases	5	1 (20.0%)	0.0493	0.594	Wikipathways
Activation of caspases through apoptosome-mediated cleavage	5	1 (20.0%)	0.0493	0.594	Reactome
HDL clearance	5	1 (20.0%)	0.0493	0.594	Reactome
FLT3 signaling through SRC family kinases	5	1 (20.0%)	0.0493	0.594	Reactome
tetrapyrrole biosynthesis	5	1 (20.0%)	0.0493	0.594	HumanCyc
Vitamin B2 (riboflavin) metabolism	5	1 (20.0%)	0.0493	0.594	EHMN
Riboflavin Metabolism	5	1 (20.0%)	0.0493	0.594	SMPDB
MECP2 regulates transcription factors	5	1 (20.0%)	0.0493	0.594	Reactome
basic mechanism of action of ppara pparb(d) and pparg and effects on gene expression	5	1 (20.0%)	0.0493	0.594	BioCarta
role of ppar-gamma coactivators in obesity and thermogenesis	5	1 (20.0%)	0.0493	0.594	BioCarta
Abacavir transmembrane transport	5	1 (20.0%)	0.0493	0.594	Reactome
PKA-mediated phosphorylation of key metabolic factors	5	1 (20.0%)	0.0493	0.594	Reactome
ketolysis	5	1 (20.0%)	0.0493	0.594	HumanCyc
bupropion degradation	5	1 (20.0%)	0.0493	0.594	HumanCyc
Metabolism	1954	27 (1.4%)	0.0499	0.594	Reactome

T2DM_requires_insulin

pathway name	set size	candidatescontained	p-value	q-value	pathway source
Defective factor XII causes hereditary angioedema	2	2 (100.0%)	0.0000299	0.0214	Reactome
Herpes simplex virus 1 infection - Homo sapiens (human)	498	11 (2.2%)	0.0000821	0.0294	KEGG
Defects of contact activation system (CAS) and kallikrein/kinin system (KKS)	5	2 (40.0%)	0.000296	0.0469	Reactome
Diseases of hemostasis	5	2 (40.0%)	0.000296	0.0469	Reactome
MHC class II antigen presentation	60	4 (6.7%)	0.000328	0.0469	Reactome
Pre-NOTCH Processing in the Endoplasmic Reticulum	6	2 (33.3%)	0.000443	0.0528	Reactome
Asthma - Homo sapiens (human)	31	3 (10.0%)	0.000585	0.0599	KEGG
Allograft rejection - Homo sapiens (human)	38	3 (8.1%)	0.00109	0.0895	KEGG
Graft-versus-host disease - Homo sapiens (human)	42	3 (7.1%)	0.00158	0.0895	KEGG
Th1 and Th2 cell differentiation - Homo sapiens (human)	92	4 (4.3%)	0.00164	0.0895	KEGG
Type I diabetes mellitus - Homo sapiens (human)	43	3 (7.0%)	0.00169	0.0895	KEGG
Allograft Rejection	93	4 (4.3%)	0.00171	0.0895	Wikipathways
Pre-NOTCH Processing in Golgi	12	2 (16.7%)	0.00191	0.0895	Reactome
Intestinal immune network for IgA production - Homo sapiens (human)	49	3 (6.4%)	0.00219	0.0895	KEGG
Influenza A - Homo sapiens (human)	172	5 (2.9%)	0.00256	0.0895	KEGG
Autoimmune thyroid disease - Homo sapiens (human)	53	3 (5.8%)	0.00292	0.0895	KEGG
Toxoplasmosis - Homo sapiens (human)	112	4 (3.6%)	0.00336	0.0895	KEGG
Intrinsic Pathway of Fibrin Clot Formation	17	2 (11.8%)	0.00386	0.0895	Reactome
Viral myocarditis - Homo sapiens (human)	60	3 (5.1%)	0.00418	0.0895	KEGG
Epstein-Barr virus infection - Homo sapiens (human)	202	5 (2.5%)	0.00499	0.0895	KEGG
Bivalirudin Action Pathway	20	2 (10.0%)	0.00533	0.0895	SMPDB
Argatroban Action Pathway	20	2 (10.0%)	0.00533	0.0895	SMPDB
Ximelagatran Action Pathway	20	2 (10.0%)	0.00533	0.0895	SMPDB
Lepirudin Action Pathway	20	2 (10.0%)	0.00533	0.0895	SMPDB
Coagulation	20	2 (10.0%)	0.00533	0.0895	SMPDB
Aminocaproic Acid Action Pathway	20	2 (10.0%)	0.00533	0.0895	SMPDB
Tranexamic Acid Action Pathway	20	2 (10.0%)	0.00533	0.0895	SMPDB
Urokinase Action Pathway	20	2 (10.0%)	0.00533	0.0895	SMPDB
Reteplase Action Pathway	20	2 (10.0%)	0.00533	0.0895	SMPDB
Streptokinase Action Pathway	20	2 (10.0%)	0.00533	0.0895	SMPDB
Tenecteplase Action Pathway	20	2 (10.0%)	0.00533	0.0895	SMPDB
Alteplase Action Pathway	20	2 (10.0%)	0.00533	0.0895	SMPDB
Anistreplase Action Pathway	20	2 (10.0%)	0.00533	0.0895	SMPDB
Aprotinin Action Pathway	20	2 (10.0%)	0.00533	0.0895	SMPDB
Phenindione Action Pathway	20	2 (10.0%)	0.00533	0.0895	SMPDB
Dicoumarol Action Pathway	20	2 (10.0%)	0.00533	0.0895	SMPDB
Warfarin Action Pathway	20	2 (10.0%)	0.00533	0.0895	SMPDB
Acenocoumarol Action Pathway	20	2 (10.0%)	0.00533	0.0895	SMPDB
Phenprocoumon Action Pathway	20	2 (10.0%)	0.00533	0.0895	SMPDB
Dicumarol Action Pathway	20	2 (10.0%)	0.00533	0.0895	SMPDB
Inflammatory bowel disease - Homo sapiens (human)	65	3 (4.6%)	0.00549	0.0895	KEGG
Biosynthesis of DPAn-3-derived 13-series resolvins	1	1 (100.0%)	0.00551	0.0895	Reactome
Ebola Virus Pathway on Host	130	4 (3.1%)	0.00571	0.0895	Wikipathways
Ardeparin Action Pathway	21	2 (9.5%)	0.00587	0.0895	SMPDB
Heparin Action Pathway	21	2 (9.5%)	0.00587	0.0895	SMPDB
Fondaparinux Action Pathway	21	2 (9.5%)	0.00587	0.0895	SMPDB
Enoxaparin Action Pathway	21	2 (9.5%)	0.00587	0.0895	SMPDB
eicosanoid metabolism	23	2 (8.7%)	0.00703	0.0987	BioCarta
intrinsic prothrombin activation pathway	23	2 (8.7%)	0.00703	0.0987	BioCarta
Blood Clotting Cascade	23	2 (8.7%)	0.00703	0.0987	Wikipathways
Eicosanoid Synthesis	23	2 (8.7%)	0.00703	0.0987	Wikipathways
Leishmaniasis - Homo sapiens (human)	77	3 (3.9%)	0.00846	0.116	KEGG
Cell adhesion molecules - Homo sapiens (human)	149	4 (2.7%)	0.00897	0.116	KEGG
Antigen processing and presentation - Homo sapiens (human)	78	3 (3.8%)	0.00908	0.116	KEGG
Phagosome - Homo sapiens (human)	152	4 (2.6%)	0.0096	0.116	KEGG
Canonical and non-canonical Notch signaling	27	2 (7.4%)	0.00961	0.116	Wikipathways
Translocation of ZAP-70 to Immunological synapse	28	2 (7.1%)	0.0103	0.116	Reactome
RAS and bradykinin pathways in COVID-19	28	2 (7.1%)	0.0103	0.116	Wikipathways
Notch-HLH transcription pathway	28	2 (7.1%)	0.0103	0.116	Reactome
Biosynthesis of DPAn-3-derived maresins	2	1 (50.0%)	0.011	0.116	Reactome

Biosynthesis of electrophilic ?-3 PUFA oxo-derivatives	2 1 (50.0%)	0.011	0.116	Reactome
aspirin triggered resolvin E biosynthesis	2 1 (50.0%)	0.011	0.116	HumanCyc
aspirin triggered resolvin D biosynthesis	2 1 (50.0%)	0.011	0.116	HumanCyc
aspirin-triggered lipoxin biosynthesis	2 1 (50.0%)	0.011	0.116	HumanCyc
Biosynthesis of DPAn-3-derived protectins and resolvins	2 1 (50.0%)	0.011	0.116	Reactome
resolvin D biosynthesis	2 1 (50.0%)	0.011	0.116	HumanCyc
Activation and oligomerization of BAK protein	2 1 (50.0%)	0.011	0.116	Reactome
UMP biosynthesis	2 1 (50.0%)	0.011	0.116	HumanCyc
Phosphorylation of CD3 and TCR zeta chains	31 2 (6.5%)	0.0126	0.13	Reactome
PD-1 signaling	32 2 (6.2%)	0.0133	0.136	Reactome
Role of Osx and miRNAs in tooth development	33 2 (6.1%)	0.0142	0.141	Wikipathways
Rheumatoid arthritis - Homo sapiens (human)	93 3 (3.3%)	0.0142	0.141	KEGG
Staphylococcus aureus infection - Homo sapiens (human)	96 3 (3.2%)	0.015	0.147	KEGG
biotin-carboxyl carrier protein assembly	3 1 (33.3%)	0.0164	0.153	HumanCyc
Biosynthesis of aspirin-triggered D-series resolvins	3 1 (33.3%)	0.0164	0.153	Reactome
lipoxin biosynthesis	3 1 (33.3%)	0.0164	0.153	HumanCyc
Pyrimidine biosynthesis	3 1 (33.3%)	0.0164	0.153	Reactome
Hematopoietic cell lineage - Homo sapiens (human)	99 3 (3.1%)	0.0168	0.154	KEGG
Formation of Fibrin Clot (Clotting Cascade)	37 2 (5.4%)	0.0176	0.16	Reactome
Oxidative Damage	40 2 (5.0%)	0.0204	0.176	Wikipathways
Ferroptosis	40 2 (5.0%)	0.0204	0.176	Wikipathways
Th17 cell differentiation - Homo sapiens (human)	107 3 (2.8%)	0.0212	0.176	KEGG
Ferroptosis - Homo sapiens (human)	41 2 (4.9%)	0.0214	0.176	KEGG
fatty acid biosynthesis initiation	4 1 (25.0%)	0.0219	0.176	HumanCyc
Biosynthesis of E-series 18(R)-resolvins	4 1 (25.0%)	0.0219	0.176	Reactome
Biosynthesis of DPAn-3 SPMs	4 1 (25.0%)	0.0219	0.176	Reactome
Biosynthesis of DPA-derived SPMs	4 1 (25.0%)	0.0219	0.176	Reactome
Biosynthesis of D-series resolvins	4 1 (25.0%)	0.0219	0.176	Reactome
arsenate detoxification I (glutaredoxin)	4 1 (25.0%)	0.0219	0.176	HumanCyc
Generation of second messenger molecules	42 2 (4.8%)	0.0224	0.178	Reactome
Prostaglandin Synthesis and Regulation	44 2 (4.5%)	0.0244	0.188	Wikipathways
CD4 T cell receptor signaling-ERK cascade	44 2 (4.5%)	0.0244	0.188	INOH
Neurodegeneration with brain iron accumulation (NBIA) subtypes pathway	44 2 (4.5%)	0.0244	0.188	Wikipathways
Notch Signaling	45 2 (4.4%)	0.0254	0.194	Wikipathways
Generic Transcription Pathway	1178 12 (1.0%)	0.0265	0.199	Reactome
Biosynthesis of E-series 18(S)-resolvins	5 1 (20.0%)	0.0272	0.199	Reactome
Sulfide oxidation to sulfate	5 1 (20.0%)	0.0272	0.199	Reactome
RUNX1 regulates expression of components of tight junctions	5 1 (20.0%)	0.0272	0.199	Reactome
Pyruvate metabolism - Homo sapiens (human)	47 2 (4.3%)	0.0276	0.199	KEGG
Chondroitin sulfate/dermatan sulfate metabolism	50 2 (4.0%)	0.0309	0.219	Reactome
Cholesterol metabolism - Homo sapiens (human)	50 2 (4.0%)	0.0309	0.219	KEGG
Synthesis of Lipoxins (LX)	6 1 (16.7%)	0.0326	0.22	Reactome
Biosynthesis of EPA-derived SPMs	6 1 (16.7%)	0.0326	0.22	Reactome
leukotriene biosynthesis	6 1 (16.7%)	0.0326	0.22	HumanCyc
allopregnanolone biosynthesis	6 1 (16.7%)	0.0326	0.22	HumanCyc
Acyl chain remodeling of CL	6 1 (16.7%)	0.0326	0.22	Reactome
Downstream TCR signaling	52 2 (3.8%)	0.0332	0.222	Reactome
Notch signaling pathway	53 2 (3.8%)	0.0344	0.228	PID
Systemic lupus erythematosus - Homo sapiens (human)	136 3 (2.2%)	0.0377	0.241	KEGG
TFAP2A acts as a transcriptional repressor during retinoic acid induced cell differentiation	7 1 (14.3%)	0.0379	0.241	Reactome
Activation of NLRP3 Inflammasome by SARS-CoV-2	7 1 (14.3%)	0.0379	0.241	Wikipathways
CS/DS degradation	7 1 (14.3%)	0.0379	0.241	Reactome
ECM proteoglycans	56 2 (3.6%)	0.0381	0.241	Reactome
CD4 T cell receptor signaling-JNK cascade	57 2 (3.5%)	0.0393	0.246	INOH
Notch	58 2 (3.4%)	0.0406	0.246	NetPath
Complement and Coagulation Cascades	59 2 (3.4%)	0.0419	0.246	Wikipathways
Notch signaling pathway - Homo sapiens (human)	59 2 (3.4%)	0.0419	0.246	KEGG
SARS-CoV-2 innate immunity evasion and cell-specific immune response	60 2 (3.4%)	0.0419	0.246	Wikipathways
Biosynthesis of maresins	8 1 (12.5%)	0.0432	0.246	Reactome
The alternative pathway of fetal androgen synthesis	8 1 (12.5%)	0.0432	0.246	Wikipathways
NOTCH4 Intracellular Domain Regulates Transcription	8 1 (12.5%)	0.0432	0.246	Reactome

TICAM1-dependent activation of IRF3/IRF7	8	1 (12.5%)	0.0432	0.246	Reactome
yaci and bcma stimulation of b cell immune responses	8	1 (12.5%)	0.0432	0.246	BioCarta
Inactivation of CDC42 and RAC1	8	1 (12.5%)	0.0432	0.246	Reactome
Phase 3 - rapid repolarisation	8	1 (12.5%)	0.0432	0.246	Reactome
FTO Obesity Variant Mechanism	8	1 (12.5%)	0.0432	0.246	Wikipathways
Notch Signaling Pathway Netpath	61	2 (3.3%)	0.0445	0.249	Wikipathways
Arachidonic acid metabolism - Homo sapiens (human)	61	2 (3.3%)	0.0445	0.249	KEGG
Breast cancer - Homo sapiens (human)	147	3 (2.0%)	0.0474	0.257	KEGG
Synthesis of 5-eicosatetraenoic acids	9	1 (11.1%)	0.0485	0.257	Reactome
Benzo(a)pyrene metabolism	9	1 (11.1%)	0.0485	0.257	Wikipathways
NOTCH4 Activation and Transmission of Signal to the Nucleus	9	1 (11.1%)	0.0485	0.257	Reactome
Prostanoid ligand receptors	9	1 (11.1%)	0.0485	0.257	Reactome
cd40l signaling pathway	9	1 (11.1%)	0.0485	0.257	BioCarta
RHO GTPases Activate Rhotekin and Rhophilins	9	1 (11.1%)	0.0485	0.257	Reactome
Gene expression (Transcription)	1439	13 (0.9%)	0.0492	0.259	Reactome

CAD_SAT

pathway name	set size	candidatescontained	p-value	q-value	pathway source
Ganglio Sphingolipid Metabolism	13	3 (23.1%)	0.000359	0.21	Wikipathways
Pyrimidine biosynthesis	3	2 (66.7%)	0.000369	0.21	Reactome
Alanine, aspartate and glutamate metabolism - Homo sapiens (human)	37	4 (10.8%)	0.00074	0.281	KEGG
Vitamin A and carotenoid metabolism	43	4 (9.3%)	0.00131	0.373	Wikipathways
Metabolic pathway of LDL, HDL and TG, including diseases	6	2 (33.3%)	0.0018	0.41	Wikipathways
visceral fat deposits and the metabolic syndrome	8	2 (25.0%)	0.00332	0.629	BioCarta
spliceosomal assembly	9	2 (22.2%)	0.00423	0.632	BioCarta
Factors and pathways affecting insulin-like growth factor (IGF1)-Akt signaling	36	3 (8.3%)	0.00744	0.632	Wikipathways
Ammonia Recycling	12	2 (16.7%)	0.0076	0.632	SMPDB
valine degradation	13	2 (15.4%)	0.00891	0.632	HumanCyc
Herpes simplex virus 1 infection - Homo sapiens (human)	498	12 (2.4%)	0.00996	0.632	KEGG
SEMA3A-Plexin repulsion signaling by inhibiting Integrin adhesion	14	2 (14.3%)	0.0103	0.632	Reactome
serotonin degradation	14	2 (14.3%)	0.0103	0.632	HumanCyc
D-mannose degradation	1	1 (100.0%)	0.0112	0.632	HumanCyc
Intestinal lipid absorption	1	1 (100.0%)	0.0112	0.632	Reactome
Nucleobase biosynthesis	15	2 (13.3%)	0.0118	0.632	Reactome
superpathway of pyrimidine ribonucleotides de novo biosynthesis	15	2 (13.3%)	0.0118	0.632	HumanCyc
Glycosphingolipid biosynthesis - ganglio series - Homo sapiens (human)	15	2 (13.3%)	0.0118	0.632	KEGG
Glutamate and glutamine metabolism	15	2 (13.3%)	0.0118	0.632	Reactome
Sema3A PAK dependent Axon repulsion	16	2 (12.5%)	0.0134	0.632	Reactome
CRMPs in Sema3A signaling	16	2 (12.5%)	0.0134	0.632	Reactome
Regulation of TLR by endogenous ligand	16	2 (12.5%)	0.0134	0.632	Reactome
Pyrimidine metabolism	84	4 (4.8%)	0.0145	0.632	Wikipathways
Sulfation Biotransformation Reaction	17	2 (11.8%)	0.0151	0.632	Wikipathways
Rett syndrome causing genes	48	3 (6.2%)	0.0164	0.632	Wikipathways
Pyrimidine nucleotides nucleosides metabolism	51	3 (5.9%)	0.0192	0.632	INOH
UMP biosynthesis	2	1 (50.0%)	0.0222	0.632	HumanCyc
SDK interactions	2	1 (50.0%)	0.0222	0.632	Reactome
Proprotein convertase subtilisin-kexin type 9 (PCSK9) mediated LDL receptor degradation	2	1 (50.0%)	0.0222	0.632	Wikipathways
Evolocumab Mechanism	2	1 (50.0%)	0.0222	0.632	Wikipathways
Glycosphingolipid biosynthesis - ganglioseries	21	2 (9.5%)	0.0226	0.632	EHMN
Branched-chain amino acid catabolism	21	2 (9.5%)	0.0226	0.632	Reactome
Transcription factor regulation in adipogenesis	22	2 (9.1%)	0.0247	0.632	Wikipathways
Type II diabetes mellitus	22	2 (9.1%)	0.0247	0.632	Wikipathways
superpathway of pyrimidine deoxyribonucleotides de novo biosynthesis	22	2 (9.1%)	0.0247	0.632	HumanCyc
Pyrimidine metabolism - Homo sapiens (human)	57	3 (5.3%)	0.0257	0.632	KEGG
Estrogen signaling pathway	23	2 (8.7%)	0.0269	0.632	Wikipathways
Cytosolic sulfonation of small molecules	25	2 (8.0%)	0.0314	0.632	Reactome
glycine cleavage	3	1 (33.3%)	0.0331	0.632	HumanCyc
glutamine degradation/glutamate biosynthesis	3	1 (33.3%)	0.0331	0.632	HumanCyc
asparagine biosynthesis	3	1 (33.3%)	0.0331	0.632	HumanCyc
Heroin metabolism	3	1 (33.3%)	0.0331	0.632	Wikipathways
Integration of viral DNA into host genomic DNA	3	1 (33.3%)	0.0331	0.632	Reactome
Autointegration results in viral DNA circles	3	1 (33.3%)	0.0331	0.632	Reactome
EGR2 and SOX10-mediated initiation of Schwann cell myelination	26	2 (7.7%)	0.0338	0.632	Reactome
Glycosylphosphatidylinositol (GPI)-anchor biosynthesis - Homo sapiens (human)	26	2 (7.7%)	0.0338	0.632	KEGG
IL4-mediated signaling events	65	3 (4.6%)	0.036	0.632	PID
Fatty acid elongation - Homo sapiens (human)	27	2 (7.4%)	0.0362	0.632	KEGG
Biosynthesis of unsaturated fatty acids - Homo sapiens (human)	27	2 (7.4%)	0.0362	0.632	KEGG
Metabolism of amino acids and derivatives	339	8 (2.4%)	0.0364	0.632	Reactome

One-carbon metabolism	28	2 (7.1%)	0.0387	0.632	Wikipathways
Felbamate Metabolism Pathway	4	1 (25.0%)	0.0439	0.632	SMPDB
Glycine degradation	4	1 (25.0%)	0.0439	0.632	Reactome
Heroin Metabolism Pathway	4	1 (25.0%)	0.0439	0.632	SMPDB
2-oxoisovalerate decarboxylation to isobutanoyl-CoA	4	1 (25.0%)	0.0439	0.632	HumanCyc
Vitamin K Metabolism	4	1 (25.0%)	0.0439	0.632	SMPDB
IRS activation	4	1 (25.0%)	0.0439	0.632	Reactome
Composition of Lipid Particles	4	1 (25.0%)	0.0439	0.632	Wikipathways
Intestinal absorption	4	1 (25.0%)	0.0439	0.632	Reactome
Regulation of gene expression by Hypoxia-inducible Factor	4	1 (25.0%)	0.0439	0.632	Reactome
Microbial modulation of RIPK1-mediated regulated necrosis	4	1 (25.0%)	0.0439	0.632	Reactome
3-Methylglutaconic Aciduria Type I	30	2 (6.7%)	0.0439	0.632	SMPDB
Valine, Leucine and Isoleucine Degradation	30	2 (6.7%)	0.0439	0.632	SMPDB
2-Methyl-3-Hydroxybutryl CoA Dehydrogenase Deficiency	30	2 (6.7%)	0.0439	0.632	SMPDB
Isovaleric Aciduria	30	2 (6.7%)	0.0439	0.632	SMPDB
3-Methylcrotonyl Coa Carboxylase Deficiency Type I	30	2 (6.7%)	0.0439	0.632	SMPDB
Propionic Acidemia	30	2 (6.7%)	0.0439	0.632	SMPDB
Maple Syrup Urine Disease	30	2 (6.7%)	0.0439	0.632	SMPDB
3-Hydroxy-3-Methylglutaryl-CoA Lyase Deficiency	30	2 (6.7%)	0.0439	0.632	SMPDB
Isobutyryl-coa dehydrogenase deficiency	30	2 (6.7%)	0.0439	0.632	SMPDB
3-hydroxyisobutyric aciduria	30	2 (6.7%)	0.0439	0.632	SMPDB
3-hydroxyisobutyric acid dehydrogenase deficiency	30	2 (6.7%)	0.0439	0.632	SMPDB
Isovaleric acidemia	30	2 (6.7%)	0.0439	0.632	SMPDB
Methylmalonate Semialdehyde Dehydrogenase Deficiency	30	2 (6.7%)	0.0439	0.632	SMPDB
Methylmalonic Aciduria	30	2 (6.7%)	0.0439	0.632	SMPDB
3-Methylglutaconic Aciduria Type IV	30	2 (6.7%)	0.0439	0.632	SMPDB
3-Methylglutaconic Aciduria Type III	30	2 (6.7%)	0.0439	0.632	SMPDB
Beta-Ketothiolase Deficiency	30	2 (6.7%)	0.0439	0.632	SMPDB
Folate metabolism	31	2 (6.5%)	0.0466	0.632	INOH
MECP2 and Associated Rett Syndrome	73	3 (4.1%)	0.0482	0.632	Wikipathways
Glyoxylate metabolism and glycine degradation	32	2 (6.2%)	0.0494	0.632	Reactome
Constitutive Androstane Receptor Pathway	32	2 (6.2%)	0.0494	0.632	Wikipathways

T2DM_SAT

pathway name	set size	candidatescontained	p-value	q-value	pathway source
Translocation of ZAP-70 to Immunological synapse	28	5 (17.9%)	0.0000218	0.0191	Reactome
Phosphorylation of CD3 and TCR zeta chains	31	5 (16.1%)	0.0000366	0.0191	Reactome
PD-1 signaling	32	5 (15.6%)	0.0000429	0.0191	Reactome
MHC class II antigen presentation	60	6 (10.0%)	0.0000969	0.0323	Reactome
Generation of second messenger molecules	42	5 (11.9%)	0.000164	0.0437	Reactome
Downstream TCR signaling	52	5 (9.6%)	0.000453	0.0817	Reactome
Pyrimidine biosynthesis	3	2 (66.7%)	0.000457	0.0817	Reactome
Asthma - Homo sapiens (human)	31	4 (13.3%)	0.000491	0.0817	KEGG
Allograft rejection - Homo sapiens (human)	38	4 (10.8%)	0.0011	0.164	KEGG
Graft-versus-host disease - Homo sapiens (human)	42	4 (9.5%)	0.00178	0.212	KEGG
Type I diabetes mellitus - Homo sapiens (human)	43	4 (9.3%)	0.00195	0.212	KEGG
CD4 T cell receptor signaling-ERK cascade	44	4 (9.1%)	0.00212	0.212	INOH
TCR signaling	73	5 (6.8%)	0.00213	0.212	Reactome
Alpha Linolenic Acid and Linoleic Acid Metabolism	6	2 (33.3%)	0.00223	0.212	SMPDB
Acyl chain remodelling of PS	22	3 (13.6%)	0.00244	0.213	Reactome
Costimulation by the CD28 family	77	5 (6.5%)	0.00269	0.213	Reactome
Intestinal immune network for IgA production - Homo sapiens (human)	49	4 (8.5%)	0.00271	0.213	KEGG
Pyrimidine metabolism	84	5 (6.0%)	0.00392	0.275	Wikipathways
Autoimmune thyroid disease - Homo sapiens (human)	53	4 (7.7%)	0.00392	0.275	KEGG
CD4 T cell receptor signaling-JNK cascade	57	4 (7.0%)	0.00545	0.349	INOH
Ebola Virus Pathway on Host	130	6 (4.6%)	0.00567	0.349	Wikipathways
Rheumatoid arthritis - Homo sapiens (human)	93	5 (5.4%)	0.00576	0.349	KEGG
Viral myocarditis - Homo sapiens (human)	60	4 (6.8%)	0.00616	0.357	KEGG
Interferon gamma signaling	96	5 (5.2%)	0.00689	0.382	Reactome
Hematopoietic cell lineage - Homo sapiens (human)	99	5 (5.1%)	0.0075	0.387	KEGG
CD4 T cell receptor signaling-NFkB cascade	99	5 (5.1%)	0.00782	0.387	INOH
Ubiquinone and other terpenoid-quinone biosynthesis - Homo sapiens (human)	11	2 (18.2%)	0.00785	0.387	KEGG
Inflammatory bowel disease - Homo sapiens (human)	65	4 (6.2%)	0.00866	0.412	KEGG
Visual phototransduction	103	5 (4.9%)	0.00921	0.423	Reactome
antigen processing and presentation	13	2 (15.4%)	0.011	0.448	BioCarta
Phagosome - Homo sapiens (human)	152	6 (4.0%)	0.0114	0.448	KEGG
ALKBH2 mediated reversal of alkylation damage	1	1 (100.0%)	0.0124	0.448	Reactome
Mismatch repair (MMR) directed by MSH2:MSH3 (MutSbeta)	14	2 (14.3%)	0.0127	0.448	Reactome
Mismatch repair (MMR) directed by MSH2:MSH6 (MutSalpha)	14	2 (14.3%)	0.0127	0.448	Reactome
Removal of the Flap Intermediate	14	2 (14.3%)	0.0127	0.448	Reactome
Polymerase switching	14	2 (14.3%)	0.0127	0.448	Reactome
Leading Strand Synthesis	14	2 (14.3%)	0.0127	0.448	Reactome
Toxoplasmosis - Homo sapiens (human)	112	5 (4.5%)	0.0129	0.448	KEGG
Endocytosis - Homo sapiens (human)	252	8 (3.2%)	0.0135	0.448	KEGG
Mismatch Repair	15	2 (13.3%)	0.0145	0.448	Reactome
Processive synthesis on the lagging strand	15	2 (13.3%)	0.0145	0.448	Reactome
Nucleobase biosynthesis	15	2 (13.3%)	0.0145	0.448	Reactome
superpathway of pyrimidine ribonucleotides de novo biosynthesis	15	2 (13.3%)	0.0145	0.448	HumanCyc
Leishmaniasis - Homo sapiens (human)	77	4 (5.3%)	0.0148	0.448	KEGG
Antigen processing and presentation - Homo sapiens (human)	78	4 (5.1%)	0.0161	0.466	KEGG
Deregulation of Rab and Rab Effector Genes in Bladder Cancer	16	2 (12.5%)	0.0164	0.466	Wikipathways
Transcription of E2F targets under negative control by DREAM complex	16	2 (12.5%)	0.0164	0.466	Reactome
Beta5 beta6 beta7 and beta8 integrin cell surface interactions	17	2 (11.8%)	0.0185	0.503	PID
Serotonin and anxiety	17	2 (11.8%)	0.0185	0.503	Wikipathways
Influenza A - Homo sapiens (human)	172	6 (3.5%)	0.0205	0.539	KEGG
Acyl chain remodelling of PG	18	2 (11.1%)	0.0206	0.539	Reactome
Herpes simplex virus 1 infection - Homo sapiens (human)	498	12 (2.4%)	0.0217	0.542	KEGG

Metabolism of fat-soluble vitamins	49	3 (6.1%)	0.0229	0.542	Reactome
Glycerophospholipid biosynthesis	130	5 (3.8%)	0.0231	0.542	Reactome
Platelet degranulation	131	5 (3.8%)	0.0238	0.542	Reactome
CD4 T cell receptor signaling	132	5 (3.8%)	0.0245	0.542	INOH
Activation and oligomerization of BAK protein	2	1 (50.0%)	0.0247	0.542	Reactome
UMP biosynthesis	2	1 (50.0%)	0.0247	0.542	HumanCyc
Lagging Strand Synthesis	20	2 (10.0%)	0.0252	0.542	Reactome
Pyrimidine nucleotides nucleosides metabolism	51	3 (5.9%)	0.0254	0.542	INOH
Response to elevated platelet cytosolic Ca2+	136	5 (3.7%)	0.0274	0.542	Reactome
Ardeparin Action Pathway	21	2 (9.5%)	0.0276	0.542	SMPDB
Heparin Action Pathway	21	2 (9.5%)	0.0276	0.542	SMPDB
Fondaparinux Action Pathway	21	2 (9.5%)	0.0276	0.542	SMPDB
Enoxaparin Action Pathway	21	2 (9.5%)	0.0276	0.542	SMPDB
HS-GAG degradation	21	2 (9.5%)	0.0276	0.542	Reactome
Methionine De Novo and Salvage Pathway	21	2 (9.5%)	0.0276	0.542	Wikipathways
Th1 and Th2 cell differentiation - Homo sapiens (human)	92	4 (4.3%)	0.0277	0.542	KEGG
Allograft Rejection	93	4 (4.3%)	0.0286	0.553	Wikipathways
Staphylococcus aureus infection - Homo sapiens (human)	96	4 (4.3%)	0.0296	0.564	KEGG
superpathway of pyrimidine deoxyribonucleotides de novo biosynthesis	22	2 (9.1%)	0.0302	0.564	HumanCyc
Mismatch repair - Homo sapiens (human)	23	2 (8.7%)	0.0328	0.564	KEGG
DNA Mismatch Repair	23	2 (8.7%)	0.0328	0.564	Wikipathways
Butanoate metabolism	23	2 (8.7%)	0.0328	0.564	EHMN
Pyrimidine metabolism - Homo sapiens (human)	57	3 (5.3%)	0.0339	0.564	KEGG
Synthesis of very long-chain fatty acyl-CoAs	24	2 (8.3%)	0.0354	0.564	Reactome
glutamine degradation/glutamate biosynthesis	3	1 (33.3%)	0.0368	0.564	HumanCyc
asparagine biosynthesis	3	1 (33.3%)	0.0368	0.564	HumanCyc
methionine salvage	3	1 (33.3%)	0.0368	0.564	HumanCyc
methylglyoxal degradation I	3	1 (33.3%)	0.0368	0.564	HumanCyc
Pyruvaldehyde Degradation	3	1 (33.3%)	0.0368	0.564	SMPDB
Integration of viral DNA into host genomic DNA	3	1 (33.3%)	0.0368	0.564	Reactome
Autointegration results in viral DNA circles	3	1 (33.3%)	0.0368	0.564	Reactome
Metabolism of vitamin K	3	1 (33.3%)	0.0368	0.564	Reactome
Direct Reversal Repair	3	1 (33.3%)	0.0368	0.564	Wikipathways
Cell adhesion molecules - Homo sapiens (human)	149	5 (3.4%)	0.0374	0.564	KEGG
G0 and Early G1	25	2 (8.0%)	0.0382	0.564	Reactome
alpha-Linolenic acid metabolism - Homo sapiens (human)	25	2 (8.0%)	0.0382	0.564	KEGG
Potassium Channels	103	4 (3.9%)	0.0395	0.564	Reactome
Arachidonic acid metabolism - Homo sapiens (human)	61	3 (4.9%)	0.0402	0.564	KEGG
Calcium Regulation in the Cardiac Cell	151	5 (3.3%)	0.0403	0.564	Wikipathways
Fertilization	26	2 (7.7%)	0.0411	0.564	Reactome
Polymerase switching on the C-strand of the telomere	27	2 (7.4%)	0.044	0.564	Reactome
Fatty acid elongation - Homo sapiens (human)	27	2 (7.4%)	0.044	0.564	KEGG
Biosynthesis of unsaturated fatty acids - Homo sapiens (human)	27	2 (7.4%)	0.044	0.564	KEGG
Acyl chain remodelling of PC	27	2 (7.4%)	0.044	0.564	Reactome
Sulfur amino acid metabolism	27	2 (7.4%)	0.044	0.564	Reactome
Th17 cell differentiation - Homo sapiens (human)	107	4 (3.7%)	0.0444	0.564	KEGG
Phospholipid Biosynthesis	28	2 (7.1%)	0.047	0.564	SMPDB
Felbamate Metabolism Pathway	4	1 (25.0%)	0.0488	0.564	SMPDB
trans, trans-farnesyl diphosphate biosynthesis	4	1 (25.0%)	0.0488	0.564	HumanCyc
glutaryl-CoA degradation	4	1 (25.0%)	0.0488	0.564	HumanCyc
IRS activation	4	1 (25.0%)	0.0488	0.564	Reactome
Composition of Lipid Particles	4	1 (25.0%)	0.0488	0.564	Wikipathways
Lysosomal oligosaccharide catabolism	4	1 (25.0%)	0.0488	0.564	Reactome
phenylalanine degradation/tyrosine biosynthesis	4	1 (25.0%)	0.0488	0.564	HumanCyc
Defective pyroptosis	4	1 (25.0%)	0.0488	0.564	Reactome
Proteoglycan syndecan-mediated signaling events	4	1 (25.0%)	0.0488	0.564	PID
S-methyl-5-thio-α-D-ribose 1-phosphate degradation	4	1 (25.0%)	0.0488	0.564	HumanCyc
Vitamin K Metabolism	4	1 (25.0%)	0.0488	0.564	SMPDB

HTN_SAT

pathway name	set size	candidatescontained	p-value	q-value	pathway source
Felbamate Metabolism Pathway	4	2 (50.0%)	0.00162	0.588	SMPDB
Fatty Acid Omega Oxidation	15	3 (20.0%)	0.00179	0.588	Wikipathways
Chemical carcinogenesis - Homo sapiens (human)	83	6 (7.2%)	0.00261	0.588	KEGG
Benzene metabolism	6	2 (33.3%)	0.00397	0.588	Wikipathways
Drug metabolism - cytochrome P450 - Homo sapiens (human)	72	5 (7.1%)	0.0062	0.588	KEGG
tRNA processing	103	6 (5.8%)	0.00751	0.588	Reactome
Ion transport by P-type ATPases	47	4 (8.5%)	0.00766	0.588	Reactome
Disulfiram Action Pathway	25	3 (12.0%)	0.00803	0.588	SMPDB
Metabolism of xenobiotics by cytochrome P450 - Homo sapiens (human)	78	5 (6.6%)	0.00873	0.588	KEGG
Surfactant metabolism	26	3 (11.5%)	0.00896	0.588	Reactome
spliceosomal assembly	9	2 (22.2%)	0.00922	0.588	BioCarta
Ethanol metabolism resulting in production of ROS by CYP2E1	9	2 (22.2%)	0.00922	0.588	Wikipathways
acetone degradation I (to methylglyoxal)	10	2 (20.0%)	0.0114	0.588	HumanCyc
GP1b-IX-V activation signalling	10	2 (20.0%)	0.0114	0.588	Reactome
Linoleic acid metabolism - Homo sapiens (human)	29	3 (10.3%)	0.0121	0.588	KEGG
Molecules associated with elastic fibres	30	3 (10.0%)	0.0133	0.588	Reactome
Eicosanoid metabolism via cyclooxygenases (COX)	30	3 (10.0%)	0.0133	0.588	Wikipathways
C-MYB transcription factor network	85	5 (5.9%)	0.0138	0.588	PID
Phenylalanine and Tyrosine Metabolism	11	2 (18.2%)	0.0138	0.588	SMPDB
Phenylketonuria	11	2 (18.2%)	0.0138	0.588	SMPDB
Tyrosinemia Type 3 (TYRO3)	11	2 (18.2%)	0.0138	0.588	SMPDB
Tyrosinemia Type 2 (or Richner-Hanhart syndrome)	11	2 (18.2%)	0.0138	0.588	SMPDB
Ubiquinone and other terpenoid-quinone biosynthesis - Homo sapiens (human)	11	2 (18.2%)	0.0138	0.588	KEGG
Translation	307	11 (3.6%)	0.0141	0.588	Reactome
Transferrin endocytosis and recycling	31	3 (9.7%)	0.0146	0.588	Reactome
C21-steroid hormone biosynthesis and metabolism	57	4 (7.0%)	0.0149	0.588	EHMN
Ethanol oxidation	12	2 (16.7%)	0.0164	0.588	Reactome
Negative regulation of NOTCH4 signaling	12	2 (16.7%)	0.0164	0.588	Reactome
Metabolism of Dichloroethylene by CYP450	1	1 (100.0%)	0.0167	0.588	Wikipathways
Acrylamide Biotransformation and Exposure Biomarkers	1	1 (100.0%)	0.0167	0.588	Wikipathways
pregnenolone biosynthesis	1	1 (100.0%)	0.0167	0.588	HumanCyc
Vitamin E	1	1 (100.0%)	0.0167	0.588	Reactome
dicer pathway	1	1 (100.0%)	0.0167	0.588	BioCarta
Formation of the ternary complex, and subsequently, the 43S complex	59	4 (6.8%)	0.0168	0.588	Reactome
Tyrosine metabolism	33	3 (9.1%)	0.0173	0.588	INOH
SNARE interactions in vesicular transport - Homo sapiens (human)	33	3 (9.1%)	0.0173	0.588	KEGG
noradrenaline and adrenaline degradation	13	2 (15.4%)	0.0191	0.588	HumanCyc
Elastic fibre formation	35	3 (8.6%)	0.0202	0.588	Reactome
Ion channel transport	165	7 (4.2%)	0.0207	0.588	Reactome
Glycerophospholipid biosynthesis	130	6 (4.6%)	0.0217	0.588	Reactome
Celecoxib Action Pathway	36	3 (8.3%)	0.0218	0.588	SMPDB
Tyrosine metabolism - Homo sapiens (human)	36	3 (8.3%)	0.0218	0.588	KEGG
serotonin degradation	14	2 (14.3%)	0.0221	0.588	HumanCyc
Omega-3-Omega-6 FA synthesis	14	2 (14.3%)	0.0221	0.588	Wikipathways
Platelet degranulation	131	6 (4.6%)	0.0225	0.588	Reactome
Integrin cell surface interactions	66	4 (6.1%)	0.0243	0.588	Reactome
Ribosomal scanning and start codon recognition	66	4 (6.1%)	0.0243	0.588	Reactome
Translation initiation complex formation	66	4 (6.1%)	0.0243	0.588	Reactome
MAP2K and MAPK activation	38	3 (7.9%)	0.0251	0.588	Reactome
Activation of the mRNA upon binding of the cap-binding complex and eIFs, and subsequent binding to 43S	67	4 (6.0%)	0.0255	0.588	Reactome
Response to elevated platelet cytosolic Ca2+	136	6 (4.4%)	0.0264	0.588	Reactome
Integrin-mediated Cell Adhesion	101	5 (5.0%)	0.0269	0.588	Wikipathways
Phase I - Functionalization of compounds	101	5 (5.0%)	0.0269	0.588	Reactome
Smooth Muscle Contraction	39	3 (7.7%)	0.0269	0.588	Reactome

mRNA decay by 3, to 5, exoribonuclease	16	2 (12.5%)	0.0285	0.588	Reactome
Biological oxidations	219	8 (3.7%)	0.0306	0.588	Reactome
tRNA modification in the nucleus and cytosol	41	3 (7.3%)	0.0306	0.588	Reactome
phospholipases	41	3 (7.3%)	0.0306	0.588	HumanCyc
Late Phase of HIV Life Cycle	141	6 (4.3%)	0.0308	0.588	Reactome
Platelet activation, signaling and aggregation	263	9 (3.4%)	0.0324	0.588	Reactome
Signaling downstream of RAS mutants	42	3 (7.1%)	0.0326	0.588	Reactome
Signaling by RAS mutants	42	3 (7.1%)	0.0326	0.588	Reactome
Paradoxical activation of RAF signaling by kinase inactive BRAF	42	3 (7.1%)	0.0326	0.588	Reactome
Signaling by moderate kinase activity BRAF mutants	42	3 (7.1%)	0.0326	0.588	Reactome
Signaling by RAF1 mutants	42	3 (7.1%)	0.0326	0.588	Reactome
Felbamate Metabolism	2	1 (50.0%)	0.0331	0.588	Wikipathways
Signalling to ERK5	2	1 (50.0%)	0.0331	0.588	Reactome
Insulin effects increased synthesis of Xylulose-5-Phosphate	2	1 (50.0%)	0.0331	0.588	Reactome
Hypoxia-mediated EMT and Stemness	2	1 (50.0%)	0.0331	0.588	Wikipathways
7-(3-amino-3-carboxypropyl)-wyosine biosynthesis	2	1 (50.0%)	0.0331	0.588	HumanCyc
SDK interactions	2	1 (50.0%)	0.0331	0.588	Reactome
Neutrophil degranulation	487	14 (2.9%)	0.0333	0.588	Reactome
Thermogenesis	108	5 (4.6%)	0.0345	0.588	Wikipathways
Vitamin A and carotenoid metabolism	43	3 (7.0%)	0.0346	0.588	Wikipathways
Deposition of new CENPA-containing nucleosomes at the centromere	43	3 (7.0%)	0.0346	0.588	Reactome
Nucleosome assembly	43	3 (7.0%)	0.0346	0.588	Reactome
Metabolism of RNA	584	16 (2.7%)	0.035	0.588	Reactome
Tyrosinemia, transient, of the newborn	18	2 (11.1%)	0.0355	0.588	SMPDB
Dopamine beta-hydroxylase deficiency	18	2 (11.1%)	0.0355	0.588	SMPDB
Tyrosine Metabolism	18	2 (11.1%)	0.0355	0.588	SMPDB
Alkaptonuria	18	2 (11.1%)	0.0355	0.588	SMPDB
Monoamine oxidase-a deficiency (MAO-A)	18	2 (11.1%)	0.0355	0.588	SMPDB
Hawkinsinuria	18	2 (11.1%)	0.0355	0.588	SMPDB
Tyrosinemia Type I	18	2 (11.1%)	0.0355	0.588	SMPDB
Tie2 Signaling	18	2 (11.1%)	0.0355	0.588	Reactome
Metapathway biotransformation Phase I and II	186	7 (3.8%)	0.0364	0.588	Wikipathways
Glycosaminoglycan degradation - Homo sapiens (human)	19	2 (10.5%)	0.0393	0.588	KEGG
Formation of a pool of free 40S subunits	113	5 (4.4%)	0.0407	0.588	Reactome
HIV Life Cycle	151	6 (4.0%)	0.0409	0.588	Reactome
Golgi-to-ER retrograde transport	116	5 (4.3%)	0.0447	0.588	Reactome
Ardeparin Action Pathway	21	2 (9.5%)	0.0472	0.588	SMPDB
Heparin Action Pathway	21	2 (9.5%)	0.0472	0.588	SMPDB
Fondaparinux Action Pathway	21	2 (9.5%)	0.0472	0.588	SMPDB
Enoxaparin Action Pathway	21	2 (9.5%)	0.0472	0.588	SMPDB
Formation of HIV elongation complex in the absence of HIV Tat	49	3 (6.1%)	0.0481	0.588	Reactome
methylglyoxal degradation III	3	1 (33.3%)	0.0492	0.588	HumanCyc
Hydroxycarboxylic acid-binding receptors	3	1 (33.3%)	0.0492	0.588	Reactome
wybutosine biosynthesis	3	1 (33.3%)	0.0492	0.588	HumanCyc
Metabolism of vitamin K	3	1 (33.3%)	0.0492	0.588	Reactome
Toxicity of botulinum toxin type C (botC)	3	1 (33.3%)	0.0492	0.588	Reactome
Inositol transporters	3	1 (33.3%)	0.0492	0.588	Reactome

Obesity_SAT

pathway name	set size	candidatescontained	p-value	q-value	pathway source
Felbamate Metabolism Pathway	4	2 (50.0%)	0.00162	0.588	SMPDB
Fatty Acid Omega Oxidation	15	3 (20.0%)	0.00179	0.588	Wikipathways
Chemical carcinogenesis - Homo sapiens (human)	83	6 (7.2%)	0.00261	0.588	KEGG
Benzene metabolism	6	2 (33.3%)	0.00397	0.588	Wikipathways
Drug metabolism - cytochrome P450 - Homo sapiens (human)	72	5 (7.1%)	0.0062	0.588	KEGG
tRNA processing	103	6 (5.8%)	0.00751	0.588	Reactome
Ion transport by P-type ATPases	47	4 (8.5%)	0.00766	0.588	Reactome
Disulfiram Action Pathway	25	3 (12.0%)	0.00803	0.588	SMPDB
Metabolism of xenobiotics by cytochrome P450 - Homo sapiens (human)	78	5 (6.6%)	0.00873	0.588	KEGG
Surfactant metabolism	26	3 (11.5%)	0.00896	0.588	Reactome
spliceosomal assembly	9	2 (22.2%)	0.00922	0.588	BioCarta
Ethanol metabolism resulting in production of ROS by CYP2E1	9	2 (22.2%)	0.00922	0.588	Wikipathways
acetone degradation I (to methylglyoxal)	10	2 (20.0%)	0.0114	0.588	HumanCyc
GP1b-IX-V activation signalling	10	2 (20.0%)	0.0114	0.588	Reactome
Linoleic acid metabolism - Homo sapiens (human)	29	3 (10.3%)	0.0121	0.588	KEGG
Molecules associated with elastic fibres	30	3 (10.0%)	0.0133	0.588	Reactome
Eicosanoid metabolism via cyclooxygenases (COX)	30	3 (10.0%)	0.0133	0.588	Wikipathways
C-MYB transcription factor network	85	5 (5.9%)	0.0138	0.588	PID
Phenylalanine and Tyrosine Metabolism	11	2 (18.2%)	0.0138	0.588	SMPDB
Phenylketonuria	11	2 (18.2%)	0.0138	0.588	SMPDB
Tyrosinemia Type 3 (TYRO3)	11	2 (18.2%)	0.0138	0.588	SMPDB
Tyrosinemia Type 2 (or Richner-Hanhart syndrome)	11	2 (18.2%)	0.0138	0.588	SMPDB
Ubiquinone and other terpenoid-quinone biosynthesis - Homo sapiens (human)	11	2 (18.2%)	0.0138	0.588	KEGG
Translation	307	11 (3.6%)	0.0141	0.588	Reactome
Transferrin endocytosis and recycling	31	3 (9.7%)	0.0146	0.588	Reactome
C21-steroid hormone biosynthesis and metabolism	57	4 (7.0%)	0.0149	0.588	EHMN
Ethanol oxidation	12	2 (16.7%)	0.0164	0.588	Reactome
Negative regulation of NOTCH4 signaling	12	2 (16.7%)	0.0164	0.588	Reactome
Metabolism of Dichloroethylene by CYP450	1	1 (100.0%)	0.0167	0.588	Wikipathways
Acrylamide Biotransformation and Exposure Biomarkers	1	1 (100.0%)	0.0167	0.588	Wikipathways
pregnenolone biosynthesis	1	1 (100.0%)	0.0167	0.588	HumanCyc
Vitamin E	1	1 (100.0%)	0.0167	0.588	Reactome
dicer pathway	1	1 (100.0%)	0.0167	0.588	BioCarta
Formation of the ternary complex, and subsequently, the 43S complex	59	4 (6.8%)	0.0168	0.588	Reactome
Tyrosine metabolism	33	3 (9.1%)	0.0173	0.588	INOH
SNARE interactions in vesicular transport - Homo sapiens (human)	33	3 (9.1%)	0.0173	0.588	KEGG
noradrenaline and adrenaline degradation	13	2 (15.4%)	0.0191	0.588	HumanCyc
Elastic fibre formation	35	3 (8.6%)	0.0202	0.588	Reactome
Ion channel transport	165	7 (4.2%)	0.0207	0.588	Reactome
Glycerophospholipid biosynthesis	130	6 (4.6%)	0.0217	0.588	Reactome
Celecoxib Action Pathway	36	3 (8.3%)	0.0218	0.588	SMPDB
Tyrosine metabolism - Homo sapiens (human)	36	3 (8.3%)	0.0218	0.588	KEGG
serotonin degradation	14	2 (14.3%)	0.0221	0.588	HumanCyc
Omega-3-Omega-6 FA synthesis	14	2 (14.3%)	0.0221	0.588	Wikipathways
Platelet degranulation	131	6 (4.6%)	0.0225	0.588	Reactome
Integrin cell surface interactions	66	4 (6.1%)	0.0243	0.588	Reactome
Ribosomal scanning and start codon recognition	66	4 (6.1%)	0.0243	0.588	Reactome
Translation initiation complex formation	66	4 (6.1%)	0.0243	0.588	Reactome
MAP2K and MAPK activation	38	3 (7.9%)	0.0251	0.588	Reactome
Activation of the mRNA upon binding of the cap-binding complex and eIFs, and subsequent binding to 43S	67	4 (6.0%)	0.0255	0.588	Reactome
Response to elevated platelet cytosolic Ca2+	136	6 (4.4%)	0.0264	0.588	Reactome
Integrin-mediated Cell Adhesion	101	5 (5.0%)	0.0269	0.588	Wikipathways
Phase I - Functionalization of compounds	101	5 (5.0%)	0.0269	0.588	Reactome
Smooth Muscle Contraction	39	3 (7.7%)	0.0269	0.588	Reactome

mRNA decay by 3, to 5, exoribonuclease	16	2 (12.5%)	0.0285	0.588	Reactome
Biological oxidations	219	8 (3.7%)	0.0306	0.588	Reactome
tRNA modification in the nucleus and cytosol	41	3 (7.3%)	0.0306	0.588	Reactome
phospholipases	41	3 (7.3%)	0.0306	0.588	HumanCyc
Late Phase of HIV Life Cycle	141	6 (4.3%)	0.0308	0.588	Reactome
Platelet activation, signaling and aggregation	263	9 (3.4%)	0.0324	0.588	Reactome
Signaling downstream of RAS mutants	42	3 (7.1%)	0.0326	0.588	Reactome
Signaling by RAS mutants	42	3 (7.1%)	0.0326	0.588	Reactome
Paradoxical activation of RAF signaling by kinase inactive BRAF	42	3 (7.1%)	0.0326	0.588	Reactome
Signaling by moderate kinase activity BRAF mutants	42	3 (7.1%)	0.0326	0.588	Reactome
Signaling by RAF1 mutants	42	3 (7.1%)	0.0326	0.588	Reactome
Felbamate Metabolism	2	1 (50.0%)	0.0331	0.588	Wikipathways
Signalling to ERK5	2	1 (50.0%)	0.0331	0.588	Reactome
Insulin effects increased synthesis of Xylulose-5-Phosphate	2	1 (50.0%)	0.0331	0.588	Reactome
Hypoxia-mediated EMT and Stemness	2	1 (50.0%)	0.0331	0.588	Wikipathways
7-(3-amino-3-carboxypropyl)-wyosine biosynthesis	2	1 (50.0%)	0.0331	0.588	HumanCyc
SDK interactions	2	1 (50.0%)	0.0331	0.588	Reactome
Neutrophil degranulation	487	14 (2.9%)	0.0333	0.588	Reactome
Thermogenesis	108	5 (4.6%)	0.0345	0.588	Wikipathways
Vitamin A and carotenoid metabolism	43	3 (7.0%)	0.0346	0.588	Wikipathways
Deposition of new CENPA-containing nucleosomes at the centromere	43	3 (7.0%)	0.0346	0.588	Reactome
Nucleosome assembly	43	3 (7.0%)	0.0346	0.588	Reactome
Metabolism of RNA	584	16 (2.7%)	0.035	0.588	Reactome
Tyrosinemia, transient, of the newborn	18	2 (11.1%)	0.0355	0.588	SMPDB
Dopamine beta-hydroxylase deficiency	18	2 (11.1%)	0.0355	0.588	SMPDB
Tyrosine Metabolism	18	2 (11.1%)	0.0355	0.588	SMPDB
Alkaptonuria	18	2 (11.1%)	0.0355	0.588	SMPDB
Monoamine oxidase-a deficiency (MAO-A)	18	2 (11.1%)	0.0355	0.588	SMPDB
Hawkinsinuria	18	2 (11.1%)	0.0355	0.588	SMPDB
Tyrosinemia Type I	18	2 (11.1%)	0.0355	0.588	SMPDB
Tie2 Signaling	18	2 (11.1%)	0.0355	0.588	Reactome
Metapathway biotransformation Phase I and II	186	7 (3.8%)	0.0364	0.588	Wikipathways
Glycosaminoglycan degradation - Homo sapiens (human)	19	2 (10.5%)	0.0393	0.588	KEGG
Formation of a pool of free 40S subunits	113	5 (4.4%)	0.0407	0.588	Reactome
HIV Life Cycle	151	6 (4.0%)	0.0409	0.588	Reactome
Golgi-to-ER retrograde transport	116	5 (4.3%)	0.0447	0.588	Reactome
Ardeparin Action Pathway	21	2 (9.5%)	0.0472	0.588	SMPDB
Heparin Action Pathway	21	2 (9.5%)	0.0472	0.588	SMPDB
Fondaparinux Action Pathway	21	2 (9.5%)	0.0472	0.588	SMPDB
Enoxaparin Action Pathway	21	2 (9.5%)	0.0472	0.588	SMPDB
Formation of HIV elongation complex in the absence of HIV Tat	49	3 (6.1%)	0.0481	0.588	Reactome
methylglyoxal degradation III	3	1 (33.3%)	0.0492	0.588	HumanCyc
Hydroxycarboxylic acid-binding receptors	3	1 (33.3%)	0.0492	0.588	Reactome
wybutosine biosynthesis	3	1 (33.3%)	0.0492	0.588	HumanCyc
Metabolism of vitamin K	3	1 (33.3%)	0.0492	0.588	Reactome
Toxicity of botulinum toxin type C (botC)	3	1 (33.3%)	0.0492	0.588	Reactome
Inositol transporters	3	1 (33.3%)	0.0492	0.588	Reactome

Stroke_SAT

pathway name	set size	candidatescontained	p-value	q-value	pathway source
Celecoxib Metabolism Pathway	8	2 (25.0%)	0.00132	0.441	SMPDB
Metabolism of xenobiotics by cytochrome P450 - Homo sapiens (human)	78	4 (5.3%)	0.00196	0.441	KEGG
Biological oxidations	219	6 (2.7%)	0.00434	0.441	Reactome
Fatty Acid Omega Oxidation	15	2 (13.3%)	0.00479	0.441	Wikipathways
Activation of BAD and translocation to mitochondria	15	2 (13.3%)	0.00479	0.441	Reactome
Phase I - Functionalization of compounds	101	4 (4.0%)	0.00547	0.441	Reactome
CD4 T cell receptor signaling-JNK cascade	57	3 (5.3%)	0.00739	0.441	INOH
Integrin	124	4 (3.2%)	0.0111	0.441	INOH
Adaptive Immune System	719	11 (1.5%)	0.0115	0.441	Reactome
Drug metabolism - cytochrome P450 - Homo sapiens (human)	72	3 (4.3%)	0.013	0.441	KEGG
Aripiprazole Metabolic Pathway	2	1 (50.0%)	0.0139	0.441	Wikipathways
ascorbate recycling (cytosolic)	2	1 (50.0%)	0.0139	0.441	HumanCyc
L-glutamine tRNA biosynthesis	2	1 (50.0%)	0.0139	0.441	HumanCyc
N-glycan antennae elongation in the medial/trans-Golgi	26	2 (7.7%)	0.0141	0.441	Reactome
Canonical and non-canonical Notch signaling	27	2 (7.4%)	0.0152	0.441	Wikipathways
Translocation of ZAP-70 to Immunological synapse	28	2 (7.1%)	0.0163	0.441	Reactome
EGF	28	2 (7.1%)	0.0163	0.441	INOH
Keratan sulfate biosynthesis	28	2 (7.1%)	0.0163	0.441	Reactome
insulin	78	3 (3.8%)	0.0173	0.441	INOH
VEGFR1 specific signals	29	2 (6.9%)	0.0174	0.441	PID
B cell receptor signaling pathway - Homo sapiens (human)	82	3 (3.8%)	0.0185	0.441	KEGG
vegf hypoxia and angiogenesis	30	2 (6.7%)	0.0186	0.441	BioCarta
Activation of BH3-only proteins	30	2 (6.7%)	0.0186	0.441	Reactome
IGF1 pathway	30	2 (6.7%)	0.0186	0.441	PID
insulin Mam	82	3 (3.7%)	0.0197	0.441	INOH
Phosphorylation of CD3 and TCR zeta chains	31	2 (6.5%)	0.0197	0.441	Reactome
Chemical carcinogenesis - Homo sapiens (human)	83	3 (3.6%)	0.0204	0.441	KEGG
Desipramine Metabolism Pathway	3	1 (33.3%)	0.0208	0.441	SMPDB
Phenylacetate Metabolism	3	1 (33.3%)	0.0208	0.441	SMPDB
PD-1 signaling	32	2 (6.2%)	0.021	0.441	Reactome
15q13.3 copy number variation syndrome	32	2 (6.2%)	0.021	0.441	Wikipathways
Keratan sulfate/keratin metabolism	33	2 (6.1%)	0.0222	0.441	Reactome
Mitochondrial translation termination	88	3 (3.4%)	0.0237	0.441	Reactome
Mitochondrial translation elongation	88	3 (3.4%)	0.0237	0.441	Reactome
Mitochondrial translation initiation	88	3 (3.4%)	0.0237	0.441	Reactome
Hepatitis C - Homo sapiens (human)	157	4 (2.5%)	0.0243	0.441	KEGG
Celecoxib Action Pathway	36	2 (5.6%)	0.0262	0.441	SMPDB
IGF signaling	36	2 (5.6%)	0.0262	0.441	INOH
Inactivation, recovery and regulation of the phototransduction cascade	36	2 (5.6%)	0.0262	0.441	Reactome
ATR signaling pathway	37	2 (5.4%)	0.0275	0.441	PID
The phototransduction cascade	37	2 (5.4%)	0.0275	0.441	Reactome
Felbamate Metabolism Pathway	4	1 (25.0%)	0.0277	0.441	SMPDB
ahr signal transduction pathway	4	1 (25.0%)	0.0277	0.441	BioCarta
Regulation of gene expression by Hypoxia-inducible Factor	4	1 (25.0%)	0.0277	0.441	Reactome
Clomipramine Metabolism Pathway	4	1 (25.0%)	0.0277	0.441	SMPDB
Codeine Metabolism Pathway	4	1 (25.0%)	0.0277	0.441	SMPDB
trans, trans-farnesyl diphosphate biosynthesis	4	1 (25.0%)	0.0277	0.441	HumanCyc
IRS activation	4	1 (25.0%)	0.0277	0.441	Reactome
Reuptake of GABA	4	1 (25.0%)	0.0277	0.441	Reactome
arsenate detoxification I (glutaredoxin)	4	1 (25.0%)	0.0277	0.441	HumanCyc
Amino acid conjugation of benzoic acid	4	1 (25.0%)	0.0277	0.441	Wikipathways
Mitochondrial translation	94	3 (3.2%)	0.0281	0.441	Reactome
Signaling events regulated by Ret tyrosine kinase	39	2 (5.1%)	0.0304	0.441	PID
Influenza A - Homo sapiens (human)	172	4 (2.3%)	0.0325	0.441	KEGG
Abacavir metabolism	5	1 (20.0%)	0.0345	0.441	Reactome
Doxepin Metabolism Pathway	5	1 (20.0%)	0.0345	0.441	SMPDB

Cross-presentation of soluble exogenous antigens (endosomes)	5	1 (20.0%)	0.0345	0.441	Reactome
NR1H2 & NR1H3 regulate gene expression linked to triglyceride lipolysis in adipose	5	1 (20.0%)	0.0345	0.441	Reactome
NR1H2 & NR1H3 regulate gene expression linked to gluconeogenesis	5	1 (20.0%)	0.0345	0.441	Reactome
NR1H2 & NR1H3 regulate gene expression to limit cholesterol uptake	5	1 (20.0%)	0.0345	0.441	Reactome
Synthesis of GDP-mannose	5	1 (20.0%)	0.0345	0.441	Reactome
RHOT1 GTPase cycle	5	1 (20.0%)	0.0345	0.441	Reactome
Free fatty acid receptors	5	1 (20.0%)	0.0345	0.441	Reactome
Adefovir Dipivoxil Metabolism Pathway	5	1 (20.0%)	0.0345	0.441	SMPDB
Tenofovir Metabolism Pathway	5	1 (20.0%)	0.0345	0.441	SMPDB
Graft-versus-host disease - Homo sapiens (human)	42	2 (4.8%)	0.0348	0.441	KEGG
Generation of second messenger molecules	42	2 (4.8%)	0.0348	0.441	Reactome
Tuberculosis - Homo sapiens (human)	180	4 (2.2%)	0.0368	0.441	KEGG
Galactose metabolism	44	2 (4.5%)	0.0379	0.441	EHMN
CD4 T cell receptor signaling-ERK cascade	44	2 (4.5%)	0.0379	0.441	INOH
Insulin Pathway	46	2 (4.3%)	0.0411	0.441	PID
Cholesterol metabolism (includes both Bloch and Kandutsch-Russell pathways)	46	2 (4.3%)	0.0411	0.441	Wikipathways
NOSTRIN mediated eNOS trafficking	6	1 (16.7%)	0.0413	0.441	Reactome
Biosynthesis of maresin-like SPMs	6	1 (16.7%)	0.0413	0.441	Reactome
Venlafaxine Metabolism Pathway	6	1 (16.7%)	0.0413	0.441	SMPDB
Imipramine Metabolism Pathway	6	1 (16.7%)	0.0413	0.441	SMPDB
Fluoxetine Metabolism Pathway	6	1 (16.7%)	0.0413	0.441	SMPDB
Activated NTRK3 signals through PI3K	6	1 (16.7%)	0.0413	0.441	Reactome
Conjugation of benzoate with glycine	6	1 (16.7%)	0.0413	0.441	Reactome
GDP-mannose biosynthesis	6	1 (16.7%)	0.0413	0.441	HumanCyc
Metapathway biotransformation Phase I and II	186	4 (2.2%)	0.0415	0.441	Wikipathways
Notch-mediated HES/HEY network	48	2 (4.2%)	0.0444	0.441	PID
Aryl hydrocarbon receptor signalling	7	1 (14.3%)	0.048	0.441	Reactome
Tramadol Metabolism Pathway	7	1 (14.3%)	0.048	0.441	SMPDB
SOS-mediated signalling	7	1 (14.3%)	0.048	0.441	Reactome
Modulation by Mtb of host immune system	7	1 (14.3%)	0.048	0.441	Reactome
ceramide de novo biosynthesis	7	1 (14.3%)	0.048	0.441	HumanCyc
serine and glycine biosynthesis	7	1 (14.3%)	0.048	0.441	

Heart_failure_SAT

pathway name	set size	candidatescontained	p-value	q-value	pathway source
MHC class II antigen presentation	60	4 (6.7%)	0.00172	0.497	Reactome
Celecoxib Metabolism Pathway	8	2 (25.0%)	0.00197	0.497	SMPDB
Asthma - Homo sapiens (human)	31	3 (10.0%)	0.0021	0.497	KEGG
Allograft rejection - Homo sapiens (human)	38	3 (8.1%)	0.00384	0.497	KEGG
Intestinal immune network for IgA production - Homo sapiens (human)	49	3 (6.4%)	0.00754	0.497	KEGG
Allograft Rejection	93	4 (4.3%)	0.00829	0.497	Wikipathways
Metabolism of Tetrahydrocannabinol (THC)	1	1 (100.0%)	0.00856	0.497	Wikipathways
Biosynthesis of DHA-derived SPMs	17	2 (11.8%)	0.00908	0.497	Reactome
Pyrimidine nucleotides nucleosides metabolism	51	3 (5.9%)	0.00944	0.497	INOH
Hematopoietic cell lineage - Homo sapiens (human)	99	4 (4.1%)	0.00993	0.497	KEGG
Autoimmune thyroid disease - Homo sapiens (human)	53	3 (5.8%)	0.00996	0.497	KEGG
Biosynthesis of specialized proresolving mediators (SPMs)	19	2 (10.5%)	0.0113	0.497	Reactome
Translesion Synthesis by POLH	19	2 (10.5%)	0.0113	0.497	Reactome
Pyrimidine metabolism - Homo sapiens (human)	57	3 (5.3%)	0.0128	0.497	KEGG
RA biosynthesis pathway	22	2 (9.1%)	0.015	0.497	Reactome
Toxoplasmosis - Homo sapiens (human)	112	4 (3.6%)	0.0156	0.497	KEGG
Biosynthesis of maresin conjugates in tissue regeneration (MCTR)	2	1 (50.0%)	0.017	0.497	Reactome
Biosynthesis of DHA-derived sulfido conjugates	2	1 (50.0%)	0.017	0.497	Reactome
Fatty acid metabolism	177	5 (2.8%)	0.0176	0.497	Reactome
Synthesis of very long-chain fatty acyl-CoAs	24	2 (8.3%)	0.0177	0.497	Reactome
Inflammatory bowel disease - Homo sapiens (human)	65	3 (4.6%)	0.0182	0.497	KEGG
Amphetamine addiction - Homo sapiens (human)	69	3 (4.3%)	0.0213	0.497	KEGG
Metapathway biotransformation Phase I and II	186	5 (2.7%)	0.0218	0.497	Wikipathways
Drug metabolism - cytochrome P450 - Homo sapiens (human)	72	3 (4.3%)	0.0221	0.497	KEGG
Fatty acid elongation - Homo sapiens (human)	27	2 (7.4%)	0.0222	0.497	KEGG
Biosynthesis of unsaturated fatty acids - Homo sapiens (human)	27	2 (7.4%)	0.0222	0.497	KEGG
Cytokines and Inflammatory Response	29	2 (7.1%)	0.0238	0.497	Wikipathways
Translocation of ZAP-70 to Immunological synapse	28	2 (7.1%)	0.0238	0.497	Reactome
Ebola Virus Pathway on Host	130	4 (3.1%)	0.0254	0.497	Wikipathways
methionine salvage	3	1 (33.3%)	0.0255	0.497	HumanCyc
Rosiglitazone Metabolism Pathway	3	1 (33.3%)	0.0255	0.497	SMPDB
Pyrimidine biosynthesis	3	1 (33.3%)	0.0255	0.497	Reactome
Metabolism of xenobiotics by cytochrome P450 - Homo sapiens (human)	78	3 (3.9%)	0.0273	0.497	KEGG
Leishmaniasis - Homo sapiens (human)	77	3 (3.9%)	0.0273	0.497	KEGG
Phosphorylation of CD3 and TCR zeta chains	31	2 (6.5%)	0.0287	0.497	Reactome
PD-1 signaling	32	2 (6.2%)	0.0305	0.497	Reactome
Development and heterogeneity of the ILC family	32	2 (6.2%)	0.0305	0.497	Wikipathways
Drug metabolism - other enzymes - Homo sapiens (human)	80	3 (3.8%)	0.0312	0.497	KEGG
Inflammatory Response Pathway	33	2 (6.1%)	0.0323	0.497	Wikipathways
Late Phase of HIV Life Cycle	141	4 (2.8%)	0.0329	0.497	Reactome
Felbamate Metabolism Pathway	4	1 (25.0%)	0.0338	0.497	SMPDB
Activation, myristolyation of BID and translocation to mitochondria	4	1 (25.0%)	0.0338	0.497	Reactome
Utilization of Ketone Bodies	4	1 (25.0%)	0.0338	0.497	Reactome
HCMV Late Events	4	1 (25.0%)	0.0338	0.497	Reactome
Invadopodia formation	4	1 (25.0%)	0.0338	0.497	Reactome
pyrimidine ribonucleosides salvage I	4	1 (25.0%)	0.0338	0.497	HumanCyc
Chemical carcinogenesis - Homo sapiens (human)	83	3 (3.6%)	0.0343	0.497	KEGG
Pyrimidine metabolism	84	3 (3.6%)	0.0353	0.497	Wikipathways
Celecoxib Action Pathway	36	2 (5.6%)	0.0379	0.497	SMPDB
Inactivation, recovery and regulation of the phototransduction cascade	36	2 (5.6%)	0.0379	0.497	Reactome
The phototransduction cascade	37	2 (5.4%)	0.0398	0.497	Reactome
Fatty acyl-CoA biosynthesis	37	2 (5.4%)	0.0398	0.497	Reactome
HIV Life Cycle	151	4 (2.6%)	0.0407	0.497	Reactome
Translesion synthesis by Y family DNA polymerases bypasses lesions on DNA template	38	2 (5.3%)	0.0418	0.497	Reactome
Abacavir metabolism	5	1 (20.0%)	0.0421	0.497	Reactome
glycine betaine degradation	5	1 (20.0%)	0.0421	0.497	HumanCyc
Doxepin Metabolism Pathway	5	1 (20.0%)	0.0421	0.497	SMPDB
Succinyl CoA: 3-ketoacid CoA transferase deficiency	5	1 (20.0%)	0.0421	0.497	SMPDB
Ketone Body Metabolism	5	1 (20.0%)	0.0421	0.497	SMPDB
ketolysis	5	1 (20.0%)	0.0421	0.497	HumanCyc
Synthesis and Degradation of Ketone Bodies	5	1 (20.0%)	0.0421	0.497	Wikipathways
Cross-presentation of soluble exogenous antigens (endosomes)	5	1 (20.0%)	0.0421	0.497	Reactome
SUMOylation of DNA replication proteins	39	2 (5.1%)	0.0438	0.497	Reactome
Rheumatoid arthritis - Homo sapiens (human)	93	3 (3.3%)	0.0443	0.497	KEGG
Th1 and Th2 cell differentiation - Homo sapiens (human)	92	3 (3.3%)	0.0443	0.497	KEGG
Glycine Serine metabolism	40	2 (5.0%)	0.0459	0.497	INOH
Ribosome - Homo sapiens (human)	158	4 (2.5%)	0.0467	0.497	KEGG
Dual Incision in GG-NER	41	2 (4.9%)	0.048	0.497	Reactome
Metabolism of lipids	646	10 (1.6%)	0.0497	0.497	Reactome

CAD_VAT

pathway name	set size	candidatescontained	p-value	q-value	pathway source
Folate metabolism	31	3 (9.7%)	0.00168	0.426	INOH
CMP phosphorylation	11	2 (18.2%)	0.00306	0.426	HumanCyc
pyrimidine deoxyribonucleotide phosphorylation	12	2 (16.7%)	0.00365	0.426	HumanCyc
guanosine deoxyribonucleotides de novo biosynthesis	12	2 (16.7%)	0.00365	0.426	HumanCyc
adenosine deoxyribonucleotides de novo biosynthesis	12	2 (16.7%)	0.00365	0.426	HumanCyc
Ganglio Sphingolipid Metabolism	13	2 (15.4%)	0.0043	0.426	Wikipathways
UTP and CTP de novo biosynthesis	13	2 (15.4%)	0.0043	0.426	HumanCyc
purine deoxyribonucleosides salvage	13	2 (15.4%)	0.0043	0.426	HumanCyc
guanosine ribonucleotides de novo biosynthesis	13	2 (15.4%)	0.0043	0.426	HumanCyc
superpathway of pyrimidine ribonucleotides de novo biosynthesis	15	2 (13.3%)	0.00573	0.426	HumanCyc
pyrimidine deoxyribonucleotides biosynthesis from CTP	16	2 (12.5%)	0.00651	0.426	HumanCyc
pyrimidine deoxyribonucleotides de novo biosynthesis	16	2 (12.5%)	0.00651	0.426	HumanCyc
guanosine nucleotides de novo biosynthesis	16	2 (12.5%)	0.00651	0.426	HumanCyc
superpathway of pyrimidine deoxyribonucleoside salvage	17	2 (11.8%)	0.00735	0.426	HumanCyc
D-mannose degradation	1	1 (100.0%)	0.00767	0.426	HumanCyc
S-methyl-5,-thioadenosine degradation	1	1 (100.0%)	0.00767	0.426	HumanCyc
TNFalpha	234	6 (2.6%)	0.00921	0.426	NetPath
Glycosphingolipid biosynthesis - ganglioseries	21	2 (9.5%)	0.0111	0.426	EHMN
Transcription factor regulation in adipogenesis	22	2 (9.1%)	0.0122	0.426	Wikipathways
Type II diabetes mellitus	22	2 (9.1%)	0.0122	0.426	Wikipathways
superpathway of pyrimidine deoxyribonucleotides de novo biosynthesis	22	2 (9.1%)	0.0122	0.426	HumanCyc
Sterols are 12-hydroxylated by CYP8B1	2	1 (50.0%)	0.0153	0.426	Reactome
Inhibition of Host mRNA Processing and RNA Silencing	2	1 (50.0%)	0.0153	0.426	Reactome
Adipocytokine signaling pathway - Homo sapiens (human)	69	3 (4.3%)	0.0159	0.426	KEGG
Vitamin B9 (folate) metabolism	26	2 (7.7%)	0.0168	0.426	EHMN
CDC42 signaling events	71	3 (4.2%)	0.0172	0.426	PID
Biosynthesis of unsaturated fatty acids - Homo sapiens (human)	27	2 (7.4%)	0.018	0.426	KEGG
Effects of PIP2 hydrolysis	27	2 (7.4%)	0.018	0.426	Reactome
Adipogenesis	131	4 (3.1%)	0.0182	0.426	Wikipathways
Reelin signaling pathway	28	2 (7.1%)	0.0193	0.426	PID
B cell receptor signaling	134	4 (3.0%)	0.0196	0.426	INOH
Glyoxylate and dicarboxylate metabolism - Homo sapiens (human)	30	2 (6.7%)	0.022	0.426	KEGG
Trafficking of AMPA receptors	30	2 (6.7%)	0.022	0.426	Reactome
Glutamate binding, activation of AMPA receptors and synaptic plasticity	30	2 (6.7%)	0.022	0.426	Reactome
glycine cleavage	3	1 (33.3%)	0.0228	0.426	HumanCyc
Tandem of pore domain in a weak inwardly rectifying K+ channels (TWIK)	3	1 (33.3%)	0.0228	0.426	Reactome
B cell receptor signaling pathway - Homo sapiens (human)	82	3 (3.8%)	0.0235	0.426	KEGG
Disruption of postsynaptic signaling by CNV	33	2 (6.1%)	0.0263	0.426	Wikipathways
Alpha 6 Beta 4 signaling pathway	33	2 (6.1%)	0.0263	0.426	Wikipathways
Pyrimidine metabolism	84	3 (3.6%)	0.0267	0.426	Wikipathways
Vitamin D-sensitive calcium signaling in depression	35	2 (5.7%)	0.0294	0.426	Wikipathways
Glycine degradation	4	1 (25.0%)	0.0303	0.426	Reactome
ahr signal transduction pathway	4	1 (25.0%)	0.0303	0.426	BioCarta
Regulation of gene expression by Hypoxia-inducible Factor	4	1 (25.0%)	0.0303	0.426	Reactome
tryptophan degradation X (mammalian, via tryptamine)	4	1 (25.0%)	0.0303	0.426	HumanCyc
Catecholamine biosynthesis	4	1 (25.0%)	0.0303	0.426	Reactome
catecholamine biosynthesis	4	1 (25.0%)	0.0303	0.426	HumanCyc
Tyrosine hydroxylase deficiency	4	1 (25.0%)	0.0303	0.426	SMPDB
Catecholamine Biosynthesis	4	1 (25.0%)	0.0303	0.426	SMPDB
Aromatic L-Aminoacid Decarboxylase Deficiency	4	1 (25.0%)	0.0303	0.426	SMPDB

Vitamin K Metabolism	**4 1 (25.0%)**	0.0303	0.426	**SMPDB**
IRS activation	**4 1 (25.0%)**	0.0303	0.426	**Reactome**
propionyl-CoA degradation	**4 1 (25.0%)**	0.0303	0.426	**HumanCyc**
Phosphorylation of proteins involved in G1/S transition by active Cyclin E:Cdk2 complexes	**4 1 (25.0%)**	0.0303	0.426	**Reactome**
ALKBH3 mediated reversal of alkylation damage	**4 1 (25.0%)**	0.0303	0.426	**Reactome**
Factors and pathways affecting insulin-like growth factor (IGF1)-Akt signaling	**36 2 (5.6%)**	0.031	0.426	**Wikipathways**
Cushing syndrome - Homo sapiens (human)	**155 4 (2.6%)**	0.0313	0.426	**KEGG**
Fatty acyl-CoA biosynthesis	**37 2 (5.4%)**	0.0326	0.426	**Reactome**
TBC/RABGAPs	**38 2 (5.3%)**	0.0342	0.426	**Reactome**
G alpha (i) signalling events	**316 6 (1.9%)**	0.0346	0.426	**Reactome**
Association of TriC/CCT with target proteins during biosynthesis	**39 2 (5.1%)**	0.0359	0.426	**Reactome**
Small cell lung cancer	**96 3 (3.1%)**	0.0375	0.426	**Wikipathways**
Serotonin and melatonin biosynthesis	**5 1 (20.0%)**	0.0378	0.426	**Reactome**
serotonin and melatonin biosynthesis	**5 1 (20.0%)**	0.0378	0.426	**HumanCyc**
Catalytic cycle of mammalian Flavin-containing MonoOxygenases (FMOs)	**5 1 (20.0%)**	0.0378	0.426	**Wikipathways**
tetrapyrrole biosynthesis	**5 1 (20.0%)**	0.0378	0.426	**HumanCyc**
Synthesis of GDP-mannose	**5 1 (20.0%)**	0.0378	0.426	**Reactome**
Propionyl-CoA catabolism	**5 1 (20.0%)**	0.0378	0.426	**Reactome**
Ultraconserved region 339 modulation of tumor suppressor microRNAs in cancer	**5 1 (20.0%)**	0.0378	0.426	**Wikipathways**
Arachidonate production from DAG	**5 1 (20.0%)**	0.0378	0.426	**Reactome**
CD4 T cell receptor signaling-NFkB cascade	**99 3 (3.0%)**	0.0405	0.426	**INOH**
RHOF GTPase cycle	**42 2 (4.8%)**	0.0411	0.426	**Reactome**
Activated NTRK3 signals through PI3K	**6 1 (16.7%)**	0.0451	0.426	**Reactome**
GDP-mannose biosynthesis	**6 1 (16.7%)**	0.0451	0.426	**HumanCyc**
Methionine salvage pathway	**6 1 (16.7%)**	0.0451	0.426	**Reactome**
Mitochondrial fatty acid synthesis pathway	**6 1 (16.7%)**	0.0451	0.426	**Wikipathways**
chondroitin and dermatan biosynthesis	**6 1 (16.7%)**	0.0451	0.426	**HumanCyc**
Fatty acid metabolism	**177 4 (2.3%)**	0.0464	0.426	**Reactome**
Cardiac conduction	**105 3 (2.9%)**	0.0468	0.426	**Reactome**
Type II diabetes mellitus - Homo sapiens (human)	**46 2 (4.3%)**	0.0484	0.426	**KEGG**

T2DM_VAT

pathway name	set size	candidatescontained	p-value	q-value	pathway source
Biosynthesis of unsaturated fatty acids - Homo sapiens (human)	27	4 (14.8%)	0.0000294	0.0285	KEGG
Allograft rejection - Homo sapiens (human)	38	4 (10.8%)	0.000105	0.0462	KEGG
Graft-versus-host disease - Homo sapiens (human)	42	4 (9.5%)	0.000174	0.0462	KEGG
Type I diabetes mellitus - Homo sapiens (human)	43	4 (9.3%)	0.00019	0.0462	KEGG
Intestinal immune network for IgA production - Homo sapiens (human)	49	4 (8.5%)	0.00027	0.0524	KEGG
Autoimmune thyroid disease - Homo sapiens (human)	53	4 (7.7%)	0.000399	0.0606	KEGG
Metabolism of amino acids and derivatives	339	9 (2.7%)	0.000437	0.0606	Reactome
Hematopoietic cell lineage - Homo sapiens (human)	99	5 (5.1%)	0.000502	0.0609	KEGG
Viral myocarditis - Homo sapiens (human)	60	4 (6.8%)	0.000647	0.0613	KEGG
Alpha Linolenic Acid and Linoleic Acid Metabolism	6	2 (33.3%)	0.000654	0.0613	SMPDB
G alpha (q) signalling events	221	7 (3.2%)	0.000697	0.0613	Reactome
Fatty acid elongation - Homo sapiens (human)	27	3 (11.1%)	0.000757	0.0613	KEGG
Translocation of ZAP-70 to Immunological synapse	28	3 (10.7%)	0.000844	0.063	Reactome
Asthma - Homo sapiens (human)	31	3 (10.0%)	0.00104	0.0716	KEGG
Phosphorylation of CD3 and TCR zeta chains	31	3 (9.7%)	0.00114	0.0716	Reactome
oleate biosynthesis	8	2 (25.0%)	0.00121	0.0716	HumanCyc
PD-1 signaling	32	3 (9.4%)	0.00125	0.0716	Reactome
Costimulation by the CD28 family	77	4 (5.2%)	0.00176	0.0947	Reactome
Fatty acyl-CoA biosynthesis	37	3 (8.1%)	0.00192	0.0979	Reactome
Generation of second messenger molecules	42	3 (7.1%)	0.00276	0.134	Reactome
Cell adhesion molecules - Homo sapiens (human)	149	5 (3.4%)	0.00315	0.141	KEGG
GPCR downstream signalling	633	11 (1.7%)	0.00329	0.141	Reactome
Rheumatoid arthritis - Homo sapiens (human)	93	4 (4.3%)	0.00336	0.141	KEGG
Allograft Rejection	93	4 (4.3%)	0.00349	0.141	Wikipathways
Interferon gamma signaling	96	4 (4.2%)	0.00391	0.152	Reactome
Downstream TCR signaling	52	3 (5.8%)	0.00507	0.189	Reactome
Th17 cell differentiation - Homo sapiens (human)	107	4 (3.7%)	0.00575	0.207	KEGG
er associated degradation (erad) pathway	18	2 (11.1%)	0.00633	0.212	BioCarta
Synthesis of glycosylphosphatidylinositol (GPI)	18	2 (11.1%)	0.00633	0.212	Reactome
CD4 T cell receptor signaling-JNK cascade	57	3 (5.3%)	0.00655	0.212	INOH
Signaling by GPCR	706	11 (1.6%)	0.00739	0.229	Reactome
MHC class II antigen presentation	60	3 (5.0%)	0.00756	0.229	Reactome
Platelet activation, signaling and aggregation	263	6 (2.3%)	0.00841	0.238	Reactome
Methionine De Novo and Salvage Pathway	21	2 (9.5%)	0.00858	0.238	Wikipathways
Metabolism	1954	22 (1.1%)	0.00858	0.238	Reactome
Inflammatory bowel disease - Homo sapiens (human)	65	3 (4.6%)	0.00942	0.254	KEGG
Synthesis of very long-chain fatty acyl-CoAs	24	2 (8.3%)	0.0111	0.292	Reactome
G0 and Early G1	25	2 (8.0%)	0.012	0.297	Reactome
Systemic lupus erythematosus - Homo sapiens (human)	136	4 (3.0%)	0.0125	0.297	KEGG
TCR signaling	73	3 (4.1%)	0.0129	0.297	Reactome
Glycosylphosphatidylinositol (GPI)-anchor biosynthesis - Homo sapiens (human)	26	2 (7.7%)	0.013	0.297	KEGG
Vitamin B9 (folate) metabolism	26	2 (7.7%)	0.013	0.297	EHMN
TWIK-related alkaline pH activated K+ channel (TALK)	2	1 (50.0%)	0.0134	0.297	Reactome
Peptide GPCRs	75	3 (4.0%)	0.0139	0.297	Wikipathways
Sulfur amino acid metabolism	27	2 (7.4%)	0.014	0.297	Reactome
GPCR ligand binding	476	8 (1.7%)	0.0144	0.297	Reactome
Leishmaniasis - Homo sapiens (human)	77	3 (3.9%)	0.0144	0.297	KEGG
One-carbon metabolism	28	2 (7.1%)	0.015	0.303	Wikipathways
Antigen processing and presentation - Homo sapiens (human)	78	3 (3.8%)	0.0154	0.305	KEGG
Interaction between L1 and Ankyrins	29	2 (6.9%)	0.016	0.311	Reactome
G alpha (12/13) signalling events	80	3 (3.8%)	0.0165	0.314	Reactome
Folate metabolism	31	2 (6.5%)	0.0182	0.334	INOH
Phagosome - Homo sapiens (human)	152	4 (2.6%)	0.0186	0.334	KEGG
Calcium Regulation in the Cardiac Cell	151	4 (2.6%)	0.0186	0.334	Wikipathways
methionine salvage	3	1 (33.3%)	0.02	0.34	HumanCyc
Melanin biosynthesis	3	1 (33.3%)	0.02	0.34	Reactome
Signaling by MST1	3	1 (33.3%)	0.02	0.34	Reactome
Interferon Signaling	162	4 (2.5%)	0.0234	0.357	Reactome
Th1 and Th2 cell differentiation - Homo sapiens (human)	92	3 (3.3%)	0.0238	0.357	KEGG

Staphylococcus aureus infection - Homo sapiens (human)	96	3 (3.2%)	0.0252	0.357	KEGG
Post-translational modification: synthesis of GPI-anchored proteins	94	3 (3.2%)	0.0252	0.357	Reactome
eumelanin biosynthesis	4	1 (25.0%)	0.0265	0.357	HumanCyc
glutaryl-CoA degradation	4	1 (25.0%)	0.0265	0.357	HumanCyc
Glycine degradation	4	1 (25.0%)	0.0265	0.357	Reactome
IRS activation	4	1 (25.0%)	0.0265	0.357	Reactome
Lysosomal oligosaccharide catabolism	4	1 (25.0%)	0.0265	0.357	Reactome
Phosphorylation of proteins involved in G1/S transition by active Cyclin E:Cdk2 complexes	4	1 (25.0%)	0.0265	0.357	Reactome
Amino Acid Metabolism Pathway Excerpt (Histidine catabolism extension)	4	1 (25.0%)	0.0265	0.357	Wikipathways
S-methyl-5-thio-α-D-ribose 1-phosphate degradation	4	1 (25.0%)	0.0265	0.357	HumanCyc
Vitamin B6-dependent and responsive disorders	4	1 (25.0%)	0.0265	0.357	Wikipathways
Class A/1 (Rhodopsin-like receptors)	346	6 (1.7%)	0.0286	0.357	Reactome
CD4 T cell receptor signaling-NFkB cascade	99	3 (3.0%)	0.0288	0.357	INOH
Glycine Serine metabolism	40	2 (5.0%)	0.0294	0.357	INOH
Glycine, serine and threonine metabolism - Homo sapiens (human)	40	2 (5.0%)	0.0294	0.357	KEGG
Fatty acid metabolism	177	4 (2.3%)	0.0305	0.357	Reactome
L1CAM interactions	102	3 (2.9%)	0.031	0.357	Reactome
Visual phototransduction	103	3 (2.9%)	0.0318	0.357	Reactome
HDL clearance	5	1 (20.0%)	0.0331	0.357	Reactome
glycine betaine degradation	5	1 (20.0%)	0.0331	0.357	HumanCyc
Tachykinin receptors bind tachykinins	5	1 (20.0%)	0.0331	0.357	Reactome
Ultraconserved region 339 modulation of tumor suppressor microRNAs in cancer	5	1 (20.0%)	0.0331	0.357	Wikipathways
SUMO is conjugated to E1 (UBA2:SAE1)	5	1 (20.0%)	0.0331	0.357	Reactome
Trafficking of myristoylated proteins to the cilium	5	1 (20.0%)	0.0331	0.357	Reactome
Free fatty acid receptors	5	1 (20.0%)	0.0331	0.357	Reactome
Adefovir Dipivoxil Metabolism Pathway	5	1 (20.0%)	0.0331	0.357	SMPDB
Tenofovir Metabolism Pathway	5	1 (20.0%)	0.0331	0.357	SMPDB
De novo fatty acid biosynthesis	43	2 (4.7%)	0.0336	0.357	EHMN
CD4 T cell receptor signaling-ERK cascade	44	2 (4.5%)	0.035	0.357	INOH
Cholesterol metabolism (includes both Bloch and Kandutsch-Russell pathways)	46	2 (4.3%)	0.038	0.357	Wikipathways
Toxoplasmosis - Homo sapiens (human)	112	3 (2.7%)	0.0393	0.357	KEGG
Choline catabolism	6	1 (16.7%)	0.0395	0.357	Reactome
Activated NTRK3 signals through PI3K	6	1 (16.7%)	0.0395	0.357	Reactome
Pre-NOTCH Processing in the Endoplasmic Reticulum	6	1 (16.7%)	0.0395	0.357	Reactome
SUMO is transferred from E1 to E2 (UBE2I, UBC9)	6	1 (16.7%)	0.0395	0.357	Reactome
basic mechanisms of sumoylation	6	1 (16.7%)	0.0395	0.357	BioCarta
histidine degradation	6	1 (16.7%)	0.0395	0.357	HumanCyc
Methionine salvage pathway	6	1 (16.7%)	0.0395	0.357	Reactome
Mitochondrial fatty acid synthesis pathway	6	1 (16.7%)	0.0395	0.357	Wikipathways
Proton-coupled monocarboxylate transport	6	1 (16.7%)	0.0395	0.357	Reactome
Cysteine and methionine metabolism - Homo sapiens (human)	50	2 (4.0%)	0.0442	0.357	KEGG
Ovarian steroidogenesis - Homo sapiens (human)	51	2 (3.9%)	0.0458	0.357	KEGG
Scavenging by Class B Receptors	7	1 (14.3%)	0.046	0.357	Reactome
HDL assembly	7	1 (14.3%)	0.046	0.357	Reactome
The retinoid cycle in cones (daylight vision)	7	1 (14.3%)	0.046	0.357	Reactome
superpathway of choline degradation to L-serine	7	1 (14.3%)	0.046	0.357	HumanCyc
SOS-mediated signalling	7	1 (14.3%)	0.046	0.357	Reactome
Neurofascin interactions	7	1 (14.3%)	0.046	0.357	Reactome
Epstein-Barr virus infection - Homo sapiens (human)	202	4 (2.0%)	0.046	0.357	KEGG
AMPK signaling pathway - Homo sapiens (human)	120	3 (2.5%)	0.0466	0.357	KEGG
One-carbon metabolism and related pathways	52	2 (3.8%)	0.0474	0.357	Wikipathways
SRP-dependent cotranslational protein targeting to membrane	123	3 (2.4%)	0.0495	0.357	Reactome

HTN_VAT

pathway name	set size	candidatescontained	p-value	q-value	pathway source
Biosynthesis of unsaturated fatty acids - Homo sapiens (human)	27	5 (18.5%)	0.0000227	0.0333	KEGG
oleate biosynthesis	8	3 (37.5%)	0.000116	0.068	HumanCyc
Human T-cell leukemia virus 1 infection - Homo sapiens (human)	219	11 (5.0%)	0.000139	0.068	KEGG
Cushing syndrome - Homo sapiens (human)	155	9 (5.8%)	0.000194	0.0711	KEGG
Fatty acid elongation - Homo sapiens (human)	27	4 (14.8%)	0.000386	0.113	KEGG
Folate metabolism	31	4 (12.9%)	0.000665	0.163	INOH
Herpes simplex virus 1 infection - Homo sapiens (human)	498	16 (3.2%)	0.000802	0.168	KEGG
Viral myocarditis - Homo sapiens (human)	60	5 (8.5%)	0.001	0.184	KEGG
Fatty acyl-CoA biosynthesis	37	4 (10.8%)	0.00131	0.201	Reactome
G1 to S cell cycle control	64	5 (7.8%)	0.00145	0.201	Wikipathways
Cortisol synthesis and secretion - Homo sapiens (human)	65	5 (7.7%)	0.00156	0.201	KEGG
Taurine and Hypotaurine Metabolism	5	2 (40.0%)	0.00164	0.201	SMPDB
Type I diabetes mellitus - Homo sapiens (human)	43	4 (9.3%)	0.00231	0.246	KEGG
Alpha Linolenic Acid and Linoleic Acid Metabolism	6	2 (33.3%)	0.00245	0.246	SMPDB
Renin-angiotensin-aldosterone system (RAAS)	44	4 (9.1%)	0.00252	0.246	Wikipathways
Cholesterol metabolism (includes both Bloch and Kandutsch-Russell pathways)	46	4 (8.7%)	0.00296	0.272	Wikipathways
Myometrial relaxation and contraction pathways	155	7 (4.5%)	0.00418	0.324	Wikipathways
Ovarian steroidogenesis - Homo sapiens (human)	51	4 (7.8%)	0.00432	0.324	KEGG
palmitate biosynthesis	9	2 (25.0%)	0.00449	0.324	HumanCyc
AMPK signaling pathway - Homo sapiens (human)	120	6 (5.0%)	0.00482	0.324	KEGG
btg family proteins and cell cycle regulation	9	2 (22.2%)	0.00572	0.324	BioCarta
Serine biosynthesis	9	2 (22.2%)	0.00572	0.324	Reactome
Cyclin E associated events during G1/S transition	29	3 (10.3%)	0.00619	0.324	Reactome
Cyclin A:Cdk2-associated events at S phase entry	29	3 (10.3%)	0.00619	0.324	Reactome
CDK-mediated phosphorylation and removal of Cdc6	29	3 (10.3%)	0.00619	0.324	Reactome
-arrestins in gpcr desensitization	30	3 (10.0%)	0.00681	0.324	BioCarta
Oocyte meiosis - Homo sapiens (human)	129	6 (4.7%)	0.00682	0.324	KEGG
Tight junction interactions	31	3 (9.7%)	0.00747	0.324	Reactome
activation of camp-dependent protein kinase pka	31	3 (9.7%)	0.00747	0.324	BioCarta
Vascular smooth muscle contraction - Homo sapiens (human)	133	6 (4.5%)	0.00788	0.324	KEGG
role of -arrestins in the activation and targeting of map kinases	32	3 (9.4%)	0.00817	0.324	BioCarta
Fatty acid metabolism	177	7 (4.0%)	0.00825	0.324	Reactome
G alpha (i) signalling events	316	10 (3.2%)	0.00847	0.324	Reactome
Nucleotide GPCRs	11	2 (18.2%)	0.00859	0.324	Wikipathways
Taurine and hypotaurine metabolism - Homo sapiens (human)	11	2 (18.2%)	0.00859	0.324	KEGG
CMP phosphorylation	11	2 (18.2%)	0.00859	0.324	HumanCyc
pertussis toxin-insensitive ccr5 signaling in macrophage	11	2 (18.2%)	0.00859	0.324	BioCarta
Autosomal recessive Osteopetrosis pathways	11	2 (18.2%)	0.00859	0.324	Wikipathways
Aldosterone synthesis and secretion - Homo sapiens (human)	98	5 (5.1%)	0.00908	0.324	KEGG
Human cytomegalovirus infection - Homo sapiens (human)	225	8 (3.6%)	0.00932	0.324	KEGG
pyrimidine deoxyribonucleotide phosphorylation	12	2 (16.7%)	0.0102	0.324	HumanCyc
guanosine deoxyribonucleotides de novo biosynthesis	12	2 (16.7%)	0.0102	0.324	HumanCyc
adenosine deoxyribonucleotides de novo biosynthesis	12	2 (16.7%)	0.0102	0.324	HumanCyc
g-protein signaling through tubby proteins	12	2 (16.7%)	0.0102	0.324	BioCarta
roles of arrestin dependent recruitment of src kinases in gpcr signaling	36	3 (8.3%)	0.0113	0.324	BioCarta
Mitochondrial Fatty Acid Beta-Oxidation	37	3 (8.3%)	0.0113	0.324	Reactome
S Phase	104	5 (4.8%)	0.0116	0.324	Reactome
Passive transport by Aquaporins	13	2 (15.4%)	0.012	0.324	Reactome
MFAP5-mediated ovarian cancer cell motility and invasiveness	13	2 (15.4%)	0.012	0.324	Wikipathways
purine deoxyribonucleosides salvage	13	2 (15.4%)	0.012	0.324	HumanCyc
guanosine ribonucleotides de novo biosynthesis	13	2 (15.4%)	0.012	0.324	HumanCyc
UTP and CTP de novo biosynthesis	13	2 (15.4%)	0.012	0.324	HumanCyc

activation of pkc through g-protein coupled receptors	13 2 (15.4%)	0.012	0.324	BioCarta
stearate biosynthesis	13 2 (15.4%)	0.012	0.324	HumanCyc
Allograft rejection - Homo sapiens (human)	38 3 (8.1%)	0.0122	0.324	KEGG
Parathyroid hormone synthesis, secretion and action - Homo sapiens (human)	106 5 (4.7%)	0.0125	0.324	KEGG
Cell adhesion molecules - Homo sapiens (human)	149 6 (4.1%)	0.0129	0.324	KEGG
Purine nucleotides nucleosides metabolism	107 5 (4.7%)	0.013	0.324	INOH
D-mannose degradation	1 1 (100.0%)	0.013	0.324	HumanCyc
Adrenergic signaling in cardiomyocytes - Homo sapiens (human)	150 6 (4.0%)	0.0137	0.333	KEGG
Omega-3-Omega-6 FA synthesis	14 2 (14.3%)	0.0139	0.333	Wikipathways
Calcium Regulation in the Cardiac Cell	151 6 (4.0%)	0.0142	0.335	Wikipathways
superpathway of pyrimidine ribonucleotides de novo biosynthesis	15 2 (13.3%)	0.0158	0.369	HumanCyc
Thyroid hormone synthesis - Homo sapiens (human)	75 4 (5.3%)	0.0165	0.371	KEGG
Hepatitis C - Homo sapiens (human)	157 6 (3.8%)	0.0169	0.371	KEGG
Graft-versus-host disease - Homo sapiens (human)	42 3 (7.1%)	0.0172	0.371	KEGG
chrebp regulation by carbohydrates and camp	42 3 (7.1%)	0.0172	0.371	BioCarta
G1/S Transition	76 4 (5.3%)	0.0173	0.371	Reactome
pyrimidine deoxyribonucleotides biosynthesis from CTP	16 2 (12.5%)	0.018	0.371	HumanCyc
guanosine nucleotides de novo biosynthesis	16 2 (12.5%)	0.018	0.371	HumanCyc
pyrimidine deoxyribonucleotides de novo biosynthesis	16 2 (12.5%)	0.018	0.371	HumanCyc
De novo fatty acid biosynthesis	43 3 (7.0%)	0.0183	0.373	EHMN
Acute viral myocarditis	78 4 (5.1%)	0.0188	0.378	Wikipathways
superpathway of pyrimidine deoxyribonucleoside salvage	17 2 (11.8%)	0.0202	0.398	HumanCyc
Cell cycle	120 5 (4.2%)	0.0203	0.398	Wikipathways
activation of csk by camp-dependent protein kinase inhibits signaling through the t cell receptor	46 3 (6.5%)	0.0219	0.403	BioCarta
Metabolism of Angiotensinogen to Angiotensins	18 2 (11.1%)	0.0225	0.403	Reactome
SCF(Skp2)-mediated degradation of p27/p21	18 2 (11.1%)	0.0225	0.403	Reactome
Cell cycle - Homo sapiens (human)	124 5 (4.0%)	0.023	0.403	KEGG
Switching of origins to a post-replicative state	47 3 (6.4%)	0.0232	0.403	Reactome
ion channels and their functional role in vascular endothelium	47 3 (6.4%)	0.0232	0.403	BioCarta
Tight junction - Homo sapiens (human)	169 6 (3.6%)	0.0233	0.403	KEGG
Amino sugar and nucleotide sugar metabolism - Homo sapiens (human)	48 3 (6.2%)	0.0245	0.403	KEGG
G alpha (q) signalling events	221 7 (3.2%)	0.0257	0.403	Reactome
Aquaporin-mediated transport	49 3 (6.1%)	0.0258	0.403	Reactome
Insulin secretion - Homo sapiens (human)	86 4 (4.7%)	0.0259	0.403	KEGG
Transport of glycerol from adipocytes to the liver by Aquaporins	2 1 (50.0%)	0.0259	0.403	Reactome
GABA synthesis	2 1 (50.0%)	0.0259	0.403	Reactome
purine deoxyribonucleosides degradation	2 1 (50.0%)	0.0259	0.403	HumanCyc
guanine and guanosine salvage	2 1 (50.0%)	0.0259	0.403	HumanCyc
choline degradation	2 1 (50.0%)	0.0259	0.403	HumanCyc
Neurotransmitter release cycle	50 3 (6.0%)	0.0272	0.403	Reactome
S-Adenosylhomocysteine (SAH) Hydrolase Deficiency	20 2 (10.0%)	0.0275	0.403	SMPDB
Methionine Metabolism	20 2 (10.0%)	0.0275	0.403	SMPDB
Methionine Adenosyltransferase Deficiency	20 2 (10.0%)	0.0275	0.403	SMPDB
Glycine N-methyltransferase Deficiency	20 2 (10.0%)	0.0275	0.403	SMPDB
Hypermethioninemia	20 2 (10.0%)	0.0275	0.403	SMPDB
Methylenetetrahydrofolate Reductase Deficiency (MTHFRD)	20 2 (10.0%)	0.0275	0.403	SMPDB
Homocystinuria-megaloblastic anemia due to defect in cobalamin metabolism, cblG complementation type	20 2 (10.0%)	0.0275	0.403	SMPDB
Cystathionine Beta-Synthase Deficiency	20 2 (10.0%)	0.0275	0.403	SMPDB
ncRNAs involved in Wnt signaling in hepatocellular carcinoma	90 4 (4.4%)	0.0299	0.405	Wikipathways
Phosphorylation of the APC/C	21 2 (9.5%)	0.0301	0.405	Reactome
G1/S DNA Damage Checkpoints	21 2 (9.5%)	0.0301	0.405	Reactome
Conversion from APC/C:Cdc20 to APC/C:Cdh1 in late anaphase	21 2 (9.5%)	0.0301	0.405	Reactome
Autoimmune thyroid disease - Homo sapiens (human)	53 3 (5.8%)	0.0301	0.405	KEGG
One-carbon metabolism and related pathways	52 3 (5.8%)	0.0301	0.405	Wikipathways

Netrin-UNC5B signaling pathway	52	3 (5.8%)	0.0301	0.405	Wikipathways
Cardiac Progenitor Differentiation	53	3 (5.7%)	0.0317	0.405	Wikipathways
Bile acid biosynthesis	53	3 (5.7%)	0.0317	0.405	EHMN
superpathway of pyrimidine deoxyribonucleotides de novo biosynthesis	22	2 (9.1%)	0.0329	0.405	HumanCyc
cystic fibrosis transmembrane conductance regulator (cftr) and beta 2 adrenergic receptor (b2ar) pathway	22	2 (9.1%)	0.0329	0.405	BioCarta
Inactivation of APC/C via direct inhibition of the APC/C complex	22	2 (9.1%)	0.0329	0.405	Reactome
Inhibition of the proteolytic activity of APC/C required for the onset of anaphase by mitotic spindle checkpoint components	22	2 (9.1%)	0.0329	0.405	Reactome
Allograft Rejection	93	4 (4.3%)	0.0332	0.405	Wikipathways
Salivary secretion - Homo sapiens (human)	93	4 (4.3%)	0.0332	0.405	KEGG
Renin-angiotensin system - Homo sapiens (human)	23	2 (8.7%)	0.0357	0.405	KEGG
cyclins and cell cycle regulation	23	2 (8.7%)	0.0357	0.405	BioCarta
induction of apoptosis through dr3 and dr4/5 death receptors	23	2 (8.7%)	0.0357	0.405	BioCarta
Autodegradation of Cdh1 by Cdh1:APC/C	23	2 (8.7%)	0.0357	0.405	Reactome
Interferon gamma signaling	96	4 (4.2%)	0.0367	0.405	Reactome
GPCR downstream signalling	633	14 (2.2%)	0.0371	0.405	Reactome
Class A/1 (Rhodopsin-like receptors)	346	9 (2.6%)	0.0375	0.405	Reactome
LncRNA involvement in canonical Wnt signaling and colorectal cancer	97	4 (4.1%)	0.0379	0.405	Wikipathways
Anchoring of the basal body to the plasma membrane	97	4 (4.1%)	0.0379	0.405	Reactome
glutamate dependent acid resistance	3	1 (33.3%)	0.0386	0.405	HumanCyc
MECP2 regulates transcription of genes involved in GABA signaling	3	1 (33.3%)	0.0386	0.405	Reactome
tetrahydrobiopterin de novo biosynthesis	3	1 (33.3%)	0.0386	0.405	HumanCyc
Signaling by MST1	3	1 (33.3%)	0.0386	0.405	Reactome
Glycine Metabolism	3	1 (33.3%)	0.0386	0.405	Wikipathways
adenine and adenosine salvage III	3	1 (33.3%)	0.0386	0.405	HumanCyc
purine ribonucleosides degradation to ribose-1-phosphate	3	1 (33.3%)	0.0386	0.405	HumanCyc
taurine biosynthesis	3	1 (33.3%)	0.0386	0.405	HumanCyc
Inositol transporters	3	1 (33.3%)	0.0386	0.405	Reactome
Cyclin A/B1/B2 associated events during G2/M transition	24	2 (8.3%)	0.0386	0.405	Reactome
tumor suppressor arf inhibits ribosomal biogenesis	24	2 (8.3%)	0.0386	0.405	BioCarta
APC/C:Cdc20 mediated degradation of Securin	24	2 (8.3%)	0.0386	0.405	Reactome
Synthesis of very long-chain fatty acyl-CoAs	24	2 (8.3%)	0.0386	0.405	Reactome
superpathway of purine nucleotide salvage	59	3 (5.1%)	0.0415	0.405	HumanCyc
cell cycle: g1/s check point	25	2 (8.0%)	0.0416	0.405	BioCarta
G0 and Early G1	25	2 (8.0%)	0.0416	0.405	Reactome
APC/C:Cdc20 mediated degradation of Cyclin B	25	2 (8.0%)	0.0416	0.405	Reactome
Progesterone-mediated oocyte maturation - Homo sapiens (human)	100	4 (4.0%)	0.0416	0.405	KEGG
Signaling by GPCR	706	15 (2.1%)	0.0425	0.405	Reactome
Wnt signaling pathway and pluripotency	101	4 (4.0%)	0.0429	0.405	Wikipathways
Long-term depression - Homo sapiens (human)	60	3 (5.0%)	0.0433	0.405	KEGG
Mitotic G1 phase and G1/S transition	104	4 (3.8%)	0.0469	0.405	Reactome
corticosteroids and cardioprotection	27	2 (7.4%)	0.0479	0.405	BioCarta
APC-Cdc20 mediated degradation of Nek2A	27	2 (7.4%)	0.0479	0.405	Reactome
Viral carcinogenesis - Homo sapiens (human)	204	6 (3.0%)	0.0498	0.405	KEGG

Heart_failure_VAT

pathway name	set size	candidatescontained	p-value	q-value	pathway source
PD-1 signaling	32	4 (12.5%)	0.0000696	0.0533	Reactome
Interleukin-37 signaling	5	2 (40.0%)	0.000478	0.183	Reactome
MHC class II antigen presentation	60	4 (6.7%)	0.000812	0.183	Reactome
Translocation of ZAP-70 to Immunological synapse	28	3 (10.7%)	0.000957	0.183	Reactome
Phosphorylation of CD3 and TCR zeta chains	31	3 (9.7%)	0.00129	0.198	Reactome
Costimulation by the CD28 family	77	4 (5.2%)	0.00206	0.263	Reactome
Generation of second messenger molecules	42	3 (7.1%)	0.00313	0.342	Reactome
CD4 T cell receptor signaling-ERK cascade	44	3 (6.8%)	0.00357	0.342	INOH
Adaptive Immune System	719	12 (1.7%)	0.00427	0.363	Reactome
CD4 T cell receptor signaling-NFkB cascade	99	4 (4.0%)	0.0051	0.391	INOH
Downstream TCR signaling	52	3 (5.8%)	0.00572	0.399	Reactome
FGFR4 mutant receptor activation	1	1 (100.0%)	0.007	0.435	Reactome
CD4 T cell receptor signaling-JNK cascade	57	3 (5.3%)	0.00739	0.435	INOH
Inflammatory bowel disease - Homo sapiens (human)	65	3 (4.6%)	0.0106	0.507	KEGG
CD4 T cell receptor signaling	132	4 (3.0%)	0.0138	0.507	INOH
TCR signaling	73	3 (4.1%)	0.0145	0.507	Reactome
Asthma - Homo sapiens (human)	31	2 (6.7%)	0.0186	0.507	KEGG
methionine salvage	3	1 (33.3%)	0.0208	0.507	HumanCyc
betaKlotho-mediated ligand binding	3	1 (33.3%)	0.0208	0.507	Reactome
Signaling by MST1	3	1 (33.3%)	0.0208	0.507	Reactome
Interconversion of 2-oxoglutarate and 2-hydroxyglutarate	3	1 (33.3%)	0.0208	0.507	Reactome
Inactivation, recovery and regulation of the phototransduction cascade	36	2 (5.6%)	0.0262	0.507	Reactome
Allograft Rejection	93	3 (3.2%)	0.0274	0.507	Wikipathways
The phototransduction cascade	37	2 (5.4%)	0.0275	0.507	Reactome
Allograft rejection - Homo sapiens (human)	38	2 (5.4%)	0.0275	0.507	KEGG
Fatty acyl-CoA biosynthesis	37	2 (5.4%)	0.0275	0.507	Reactome
Activation, myristolyation of BID and translocation to mitochondria	4	1 (25.0%)	0.0277	0.507	Reactome
Utilization of Ketone Bodies	4	1 (25.0%)	0.0277	0.507	Reactome
Catecholamine biosynthesis	4	1 (25.0%)	0.0277	0.507	Reactome
catecholamine biosynthesis	4	1 (25.0%)	0.0277	0.507	HumanCyc
Tyrosine hydroxylase deficiency	4	1 (25.0%)	0.0277	0.507	SMPDB
Catecholamine Biosynthesis	4	1 (25.0%)	0.0277	0.507	SMPDB
Aromatic L-Aminoacid Decarboxylase Deficiency	4	1 (25.0%)	0.0277	0.507	SMPDB
Invadopodia formation	4	1 (25.0%)	0.0277	0.507	Reactome
Interleukin-18 signaling	4	1 (25.0%)	0.0277	0.507	Reactome
Synthesis of PIPs in the nucleus	4	1 (25.0%)	0.0277	0.507	Reactome
Interferon gamma signaling	96	3 (3.1%)	0.0297	0.507	Reactome
Hematopoietic cell lineage - Homo sapiens (human)	99	3 (3.1%)	0.0313	0.507	KEGG
Glycine Serine metabolism	40	2 (5.0%)	0.0318	0.507	INOH
Gamma carboxylation, hypusine formation and arylsulfatase activation	41	2 (4.9%)	0.0333	0.507	Reactome
glycine betaine degradation	5	1 (20.0%)	0.0345	0.507	HumanCyc
Leukotriene receptors	5	1 (20.0%)	0.0345	0.507	Reactome
Succinyl CoA: 3-ketoacid CoA transferase deficiency	5	1 (20.0%)	0.0345	0.507	SMPDB
Ketone Body Metabolism	5	1 (20.0%)	0.0345	0.507	SMPDB
ketolysis	5	1 (20.0%)	0.0345	0.507	HumanCyc
Synthesis and Degradation of Ketone Bodies	5	1 (20.0%)	0.0345	0.507	Wikipathways
Graft-versus-host disease - Homo sapiens (human)	42	2 (4.8%)	0.0348	0.507	KEGG
Type I diabetes mellitus - Homo sapiens (human)	43	2 (4.7%)	0.0363	0.507	KEGG
a6b1 and a6b4 Integrin signaling	45	2 (4.4%)	0.0395	0.507	PID
Choline catabolism	6	1 (16.7%)	0.0413	0.507	Reactome
p75NTR negatively regulates cell cycle via SC1	6	1 (16.7%)	0.0413	0.507	Reactome
Mitochondrial fatty acid synthesis pathway	6	1 (16.7%)	0.0413	0.507	Wikipathways
NOSTRIN mediated eNOS trafficking	6	1 (16.7%)	0.0413	0.507	Reactome
Intestinal immune network for IgA production - Homo sapiens (human)	49	2 (4.3%)	0.0427	0.507	KEGG
Toxoplasmosis - Homo sapiens (human)	112	3 (2.7%)	0.0438	0.507	KEGG
superpathway of choline degradation to L-serine	7	1 (14.3%)	0.048	0.507	HumanCyc
Anchoring fibril formation	7	1 (14.3%)	0.048	0.507	Reactome
Aryl hydrocarbon receptor signalling	7	1 (14.3%)	0.048	0.507	Reactome
RHOD GTPase cycle	51	2 (3.9%)	0.0495	0.507	Reactome

Obesity_VAT

pathway name	set size	candidatescontained	p-value	q-value	pathway source
Drug metabolism - cytochrome P450 - Homo sapiens (human)	72	6 (8.6%)	0.0000944	0.0879	KEGG
Butyrophilin (BTN) family interactions	12	3 (25.0%)	0.000237	0.0879	Reactome
Chemical carcinogenesis - Homo sapiens (human)	83	6 (7.2%)	0.000243	0.0879	KEGG
Metapathway biotransformation Phase I and II	186	8 (4.3%)	0.000804	0.0879	Wikipathways
Deposition of new CENPA-containing nucleosomes at the centromere	43	4 (9.3%)	0.00107	0.0879	Reactome
Nucleosome assembly	43	4 (9.3%)	0.00107	0.0879	Reactome
Myometrial relaxation and contraction pathways	155	7 (4.5%)	0.00129	0.0879	Wikipathways
Tamoxifen metabolism	21	3 (14.3%)	0.00134	0.0879	Wikipathways
Biosynthesis of maresin-like SPMs	6	2 (33.3%)	0.00162	0.0879	Reactome
Arachidonate Epoxygenase - Epoxide Hydrolase	7	2 (28.6%)	0.00225	0.0879	Wikipathways
Caffeine Metabolism	7	2 (28.6%)	0.00225	0.0879	SMPDB
Biological oxidations	219	8 (3.7%)	0.00227	0.0879	Reactome
Biosynthesis of maresins	8	2 (25.0%)	0.00298	0.0879	Reactome
NOTCH4 Intracellular Domain Regulates Transcription	8	2 (25.0%)	0.00298	0.0879	Reactome
C21-steroid hormone biosynthesis and metabolism	57	4 (7.0%)	0.00306	0.0879	EHMN
Linoleic acid metabolism - Homo sapiens (human)	29	3 (10.3%)	0.00345	0.0879	KEGG
Ethanol metabolism resulting in production of ROS by CYP2E1	9	2 (22.2%)	0.0038	0.0879	Wikipathways
Ketoprofen Action Pathway	30	3 (10.0%)	0.00381	0.0879	SMPDB
Acetylsalicylic Acid Action Pathway	30	3 (10.0%)	0.00381	0.0879	SMPDB
Diflunisal Action Pathway	30	3 (10.0%)	0.00381	0.0879	SMPDB
Acetaminophen Action Pathway	30	3 (10.0%)	0.00381	0.0879	SMPDB
Sulindac Action Pathway	30	3 (10.0%)	0.00381	0.0879	SMPDB
Ketorolac Action Pathway	30	3 (10.0%)	0.00381	0.0879	SMPDB
Naproxen Action Pathway	30	3 (10.0%)	0.00381	0.0879	SMPDB
Flurbiprofen Action Pathway	30	3 (10.0%)	0.00381	0.0879	SMPDB
Antrafenine Action Pathway	30	3 (10.0%)	0.00381	0.0879	SMPDB
Trisalicylate-choline Action Pathway	30	3 (10.0%)	0.00381	0.0879	SMPDB
Nepafenac Action Pathway	30	3 (10.0%)	0.00381	0.0879	SMPDB
Phenylbutazone Action Pathway	30	3 (10.0%)	0.00381	0.0879	SMPDB
Lornoxicam Action Pathway	30	3 (10.0%)	0.00381	0.0879	SMPDB
Salsalate Action Pathway	30	3 (10.0%)	0.00381	0.0879	SMPDB
Salicylic Acid Action Pathway	30	3 (10.0%)	0.00381	0.0879	SMPDB
Salicylate-sodium Action Pathway	30	3 (10.0%)	0.00381	0.0879	SMPDB
Oxaprozin Action Pathway	30	3 (10.0%)	0.00381	0.0879	SMPDB
Nabumetone Action Pathway	30	3 (10.0%)	0.00381	0.0879	SMPDB
Bromfenac Action Pathway	30	3 (10.0%)	0.00381	0.0879	SMPDB
Mefenamic Acid Action Pathway	30	3 (10.0%)	0.00381	0.0879	SMPDB
Piroxicam Action Pathway	30	3 (10.0%)	0.00381	0.0879	SMPDB
Carprofen Action Pathway	30	3 (10.0%)	0.00381	0.0879	SMPDB
Fenoprofen Action Pathway	30	3 (10.0%)	0.00381	0.0879	SMPDB
Antipyrine Action Pathway	30	3 (10.0%)	0.00381	0.0879	SMPDB
Magnesium salicylate Action Pathway	30	3 (10.0%)	0.00381	0.0879	SMPDB
Tenoxicam Action Pathway	30	3 (10.0%)	0.00381	0.0879	SMPDB
Tiaprofenic Acid Action Pathway	30	3 (10.0%)	0.00381	0.0879	SMPDB
Tolmetin Action Pathway	30	3 (10.0%)	0.00381	0.0879	SMPDB
Suprofen Action Pathway	30	3 (10.0%)	0.00381	0.0879	SMPDB
Etodolac Action Pathway	30	3 (10.0%)	0.00381	0.0879	SMPDB
Rofecoxib Action Pathway	30	3 (10.0%)	0.00381	0.0879	SMPDB
Diclofenac Action Pathway	30	3 (10.0%)	0.00381	0.0879	SMPDB
Etoricoxib Action Pathway	30	3 (10.0%)	0.00381	0.0879	SMPDB
Lumiracoxib Action Pathway	30	3 (10.0%)	0.00381	0.0879	SMPDB
Valdecoxib Action Pathway	30	3 (10.0%)	0.00381	0.0879	SMPDB
Meloxicam Action Pathway	30	3 (10.0%)	0.00381	0.0879	SMPDB
Leukotriene C4 Synthesis Deficiency	30	3 (10.0%)	0.00381	0.0879	SMPDB
Arachidonic Acid Metabolism	30	3 (10.0%)	0.00381	0.0879	SMPDB
Indomethacin Action Pathway	31	3 (9.7%)	0.00419	0.0948	SMPDB
Phase I - Functionalization of compounds	101	5 (5.0%)	0.00433	0.0963	Reactome
Opsins	10	2 (20.0%)	0.00472	0.103	Reactome
Calcium Regulation in the Cardiac Cell	151	6 (4.0%)	0.00534	0.115	Wikipathways
CYP2E1 reactions	11	2 (18.2%)	0.00573	0.121	Reactome
Celecoxib Action Pathway	36	3 (8.3%)	0.0064	0.133	SMPDB

MFAP5-mediated ovarian cancer cell motility and invasiveness	13	2 (15.4%)	0.00802	0.164	Wikipathways
Metabolism of xenobiotics by cytochrome P450 - Homo sapiens (human)	78	4 (5.3%)	0.00853	0.172	KEGG
G alpha (q) signalling events	221	7 (3.2%)	0.00907	0.18	Reactome
RHO GTPases Activate Formins	123	5 (4.1%)	0.00982	0.19	Reactome
Drug metabolism - other enzymes - Homo sapiens (human)	80	4 (5.0%)	0.0102	0.19	KEGG
Metabolism of Dichloroethylene by CYP450	1	1 (100.0%)	0.0106	0.19	Wikipathways
Acrylamide Biotransformation and Exposure Biomarkers	1	1 (100.0%)	0.0106	0.19	Wikipathways
pregnenolone biosynthesis	1	1 (100.0%)	0.0106	0.19	HumanCyc
D-mannose degradation	1	1 (100.0%)	0.0106	0.19	HumanCyc
Fatty Acid Omega Oxidation	15	2 (13.3%)	0.0106	0.19	Wikipathways
Renin-angiotensin-aldosterone system (RAAS)	44	3 (6.8%)	0.0112	0.197	Wikipathways
Metabolism	1954	31 (1.6%)	0.0119	0.208	Reactome
Chromosome Maintenance	134	5 (3.8%)	0.0134	0.224	Reactome
Vascular smooth muscle contraction - Homo sapiens (human)	133	5 (3.8%)	0.0134	0.224	KEGG
Calcium signaling pathway - Homo sapiens (human)	240	7 (2.9%)	0.0135	0.224	KEGG
Biosynthesis of DHA-derived SPMs	17	2 (11.8%)	0.0136	0.224	Reactome
Ibuprofen Action Pathway	50	3 (6.0%)	0.0158	0.257	SMPDB
Ovarian steroidogenesis - Homo sapiens (human)	51	3 (5.9%)	0.0166	0.262	KEGG
Pyrimidine nucleotides nucleosides metabolism	51	3 (5.9%)	0.0166	0.262	INOH
Biosynthesis of specialized proresolving mediators (SPMs)	19	2 (10.5%)	0.0169	0.262	Reactome
Phosphatidylinositol phosphate metabolism	93	4 (4.3%)	0.0169	0.262	EHMN
Mitotic Anaphase	195	6 (3.1%)	0.0174	0.267	Reactome
Mitotic Metaphase and Anaphase	196	6 (3.1%)	0.0178	0.269	Reactome
Notch signaling pathway	53	3 (5.7%)	0.0184	0.274	PID
Amplification of signal from unattached kinetochores via a MAD2 inhibitory signal	96	4 (4.2%)	0.0188	0.274	Reactome
Amplification of signal from the kinetochores	96	4 (4.2%)	0.0188	0.274	Reactome
Inflammatory mediator regulation of TRP channels - Homo sapiens (human)	98	4 (4.1%)	0.0201	0.29	KEGG
Xenobiotics	21	2 (9.5%)	0.0204	0.29	Reactome
Felbamate Metabolism	2	1 (50.0%)	0.021	0.29	Wikipathways
epoxysqualene biosynthesis	2	1 (50.0%)	0.021	0.29	HumanCyc
TWIK-related alkaline pH activated K+ channel (TALK)	2	1 (50.0%)	0.021	0.29	Reactome
EML4 and NUDC in mitotic spindle formation	100	4 (4.0%)	0.0215	0.293	Reactome
glutathione-mediated detoxification	22	2 (9.1%)	0.0223	0.298	HumanCyc
Cytochrome P450 - arranged by substrate type	57	3 (5.3%)	0.0223	0.298	Reactome
Leukotriene metabolism	102	4 (3.9%)	0.0229	0.303	EHMN
Arachidonic acid metabolism - Homo sapiens (human)	61	3 (4.9%)	0.0267	0.345	KEGG
Steroid hormone biosynthesis - Homo sapiens (human)	61	3 (4.9%)	0.0267	0.345	KEGG
Oxidation by Cytochrome P450	62	3 (4.8%)	0.0278	0.356	Wikipathways
Resolution of Sister Chromatid Cohesion	109	4 (3.7%)	0.0284	0.36	Reactome
Rosiglitazone Metabolism Pathway	3	1 (33.3%)	0.0314	0.362	SMPDB
methylglyoxal degradation III	3	1 (33.3%)	0.0314	0.362	HumanCyc
FMO oxidises nucleophiles	3	1 (33.3%)	0.0314	0.362	Reactome
Signaling by MST1	3	1 (33.3%)	0.0314	0.362	Reactome
pyrimidine ribonucleosides degradation	3	1 (33.3%)	0.0314	0.362	HumanCyc
The AIM2 inflammasome	3	1 (33.3%)	0.0314	0.362	Reactome
Inositol transporters	3	1 (33.3%)	0.0314	0.362	Reactome
Interconversion of 2-oxoglutarate and 2-hydroxyglutarate	3	1 (33.3%)	0.0314	0.362	Reactome
Mitotic Spindle Checkpoint	114	4 (3.5%)	0.0327	0.362	Reactome
Signaling by NOTCH4	27	2 (7.4%)	0.0328	0.362	Reactome
Canonical and non-canonical Notch signaling	27	2 (7.4%)	0.0328	0.362	Wikipathways
Effects of PIP2 hydrolysis	27	2 (7.4%)	0.0328	0.362	Reactome
Fatty acid elongation - Homo sapiens (human)	27	2 (7.4%)	0.0328	0.362	KEGG
Biosynthesis of unsaturated fatty acids - Homo sapiens (human)	27	2 (7.4%)	0.0328	0.362	KEGG
D-myo-inositol (1,4,5)-trisphosphate biosynthesis	27	2 (7.4%)	0.0328	0.362	HumanCyc
Acetaminophen Metabolism Pathway	30	2 (6.7%)	0.0398	0.416	SMPDB
Molecules associated with elastic fibres	30	2 (6.7%)	0.0398	0.416	Reactome
Metabolism of lipids	646	12 (1.9%)	0.0406	0.416	Reactome
Linoleate metabolism	72	3 (4.2%)	0.0406	0.416	EHMN

Felbamate Metabolism Pathway	4	1 (25.0%)	0.0416	0.416	SMPDB
IRS activation	4	1 (25.0%)	0.0416	0.416	Reactome
propionyl-CoA degradation	4	1 (25.0%)	0.0416	0.416	HumanCyc
pyrimidine deoxyribonucleosides degradation	4	1 (25.0%)	0.0416	0.416	HumanCyc
guanosine nucleotides degradation	4	1 (25.0%)	0.0416	0.416	HumanCyc
Nanomaterial-induced inflammasome activation	4	1 (25.0%)	0.0416	0.416	Wikipathways
CLEC7A/inflammasome pathway	4	1 (25.0%)	0.0416	0.416	Reactome
Synthesis of PIPs in the nucleus	4	1 (25.0%)	0.0416	0.416	Reactome
Metabolism of steroid hormones	31	2 (6.5%)	0.0422	0.419	Reactome
Tyrosine metabolism	33	2 (6.1%)	0.0473	0.462	INOH
SNARE interactions in vesicular transport - Homo sapiens (human)	33	2 (6.1%)	0.0473	0.462	KEGG
Separation of Sister Chromatids	130	4 (3.1%)	0.049	0.464	Reactome

Stroke_VAT

pathway name	set size	candidatescontained	p-value	q-value	pathway source
Costimulation by the CD28 family	77	3 (3.9%)	0.00639	0.392	Reactome
Translocation of ZAP-70 to Immunological synapse	28	2 (7.1%)	0.00828	0.392	Reactome
histamine degradation	2	1 (50.0%)	0.0098	0.392	HumanCyc
L-glutamine tRNA biosynthesis	2	1 (50.0%)	0.0098	0.392	HumanCyc
Phosphorylation of CD3 and TCR zeta chains	31	2 (6.5%)	0.0101	0.392	Reactome
PD-1 signaling	32	2 (6.2%)	0.0107	0.392	Reactome
CD4 T cell receptor signaling-NFkB cascade	99	3 (3.0%)	0.0127	0.392	INOH
The AIM2 inflammasome	3	1 (33.3%)	0.0147	0.392	Reactome
Generation of second messenger molecules	42	2 (4.8%)	0.018	0.392	Reactome
ahr signal transduction pathway	4	1 (25.0%)	0.0195	0.392	BioCarta
Regulation of gene expression by Hypoxia-inducible Factor	4	1 (25.0%)	0.0195	0.392	Reactome
proline biosynthesis	4	1 (25.0%)	0.0195	0.392	HumanCyc
Nanomaterial-induced inflammasome activation	4	1 (25.0%)	0.0195	0.392	Wikipathways
CLEC7A/inflammasome pathway	4	1 (25.0%)	0.0195	0.392	Reactome
ALKBH3 mediated reversal of alkylation damage	4	1 (25.0%)	0.0195	0.392	Reactome
CD4 T cell receptor signaling-ERK cascade	44	2 (4.5%)	0.0197	0.392	INOH
Galactose metabolism	44	2 (4.5%)	0.0197	0.392	EHMN
Intestinal immune network for IgA production - Homo sapiens (human)	49	2 (4.3%)	0.0223	0.392	KEGG
Notch-mediated HES/HEY network	48	2 (4.2%)	0.0232	0.392	PID
Binding and entry of HIV virion	5	1 (20.0%)	0.0243	0.392	Reactome
Leukotriene receptors	5	1 (20.0%)	0.0243	0.392	Reactome
ornithine de novo biosynthesis	5	1 (20.0%)	0.0243	0.392	HumanCyc
Arginine and proline metabolism - Homo sapiens (human)	51	2 (4.0%)	0.025	0.392	KEGG
Downstream TCR signaling	52	2 (3.8%)	0.0269	0.392	Reactome
CD4 T cell receptor signaling	132	3 (2.3%)	0.0271	0.392	INOH
CD4 T cell receptor signaling-JNK cascade	57	2 (3.5%)	0.0319	0.392	INOH
Legionellosis - Homo sapiens (human)	57	2 (3.5%)	0.0319	0.392	KEGG
Aryl hydrocarbon receptor signalling	7	1 (14.3%)	0.0339	0.392	Reactome
ceramide de novo biosynthesis	7	1 (14.3%)	0.0339	0.392	HumanCyc
Activation of NLRP3 Inflammasome by SARS-CoV-2	7	1 (14.3%)	0.0339	0.392	Wikipathways
Reversal of alkylation damage by DNA dioxygenases	7	1 (14.3%)	0.0339	0.392	Reactome
APEX1-Independent Resolution of AP Sites via the Single Nucleotide Replacement Pathway	7	1 (14.3%)	0.0339	0.392	Reactome
MHC class II antigen presentation	60	2 (3.3%)	0.0351	0.392	Reactome
TYROBP causal network in microglia	60	2 (3.3%)	0.0351	0.392	Wikipathways
DNA Damage Reversal	8	1 (12.5%)	0.0386	0.392	Reactome
Orexin and neuropeptides FF and QRFP bind to their respective receptors	8	1 (12.5%)	0.0386	0.392	Reactome
YAP1- and WWTR1 (TAZ)-stimulated gene expression	9	1 (11.1%)	0.0434	0.392	Reactome
Methylation Pathways	9	1 (11.1%)	0.0434	0.392	Wikipathways
btg family proteins and cell cycle regulation	9	1 (11.1%)	0.0434	0.392	BioCarta
RUNX1 and FOXP3 control the development of regulatory T lymphocytes (Tregs)	10	1 (10.0%)	0.0481	0.392	Reactome
Opsins	10	1 (10.0%)	0.0481	0.392	Reactome
The NLRP3 inflammasome	10	1 (10.0%)	0.0481	0.392	Reactome
TCR signaling	73	2 (2.7%)	0.05	0.392	Reactome

Depression-related admission

pathway name	set size	candidatescontained	p-value	q-value	pathway source
DNA Double-Strand Break Repair	169	4 (2.4%)	0.000817	0.248	Reactome
Ebola Virus Pathway on Host	130	3 (2.3%)	0.00426	0.248	Wikipathways
HDR through Homologous Recombination (HRR) or Single Strand Annealing (SSA)	134	3 (2.3%)	0.00454	0.248	Reactome
L-glutamine tRNA biosynthesis	2	1 (50.0%)	0.00506	0.248	HumanCyc
Homology Directed Repair	140	3 (2.2%)	0.00513	0.248	Reactome
Cell adhesion molecules - Homo sapiens (human)	149	3 (2.0%)	0.00611	0.248	KEGG
TNFR2 non-canonical NF-kB pathway	50	2 (4.0%)	0.00706	0.248	Reactome
DNA Repair	323	4 (1.2%)	0.00852	0.248	Reactome
NRAGE signals death through JNK	59	2 (3.4%)	0.00972	0.248	Reactome
Linoleic acid metabolism known to be affected by coronavirus infection	4	1 (25.0%)	0.0101	0.248	Wikipathways
GRB7 events in ERBB2 signaling	5	1 (20.0%)	0.0126	0.248	Reactome
Serotonin and melatonin biosynthesis	5	1 (20.0%)	0.0126	0.248	Reactome
serotonin and melatonin biosynthesis	5	1 (20.0%)	0.0126	0.248	HumanCyc
HDR through Homologous Recombination (HRR)	68	2 (2.9%)	0.0128	0.248	Reactome
neuroregulin receptor degredation protein-1 controls erbb3 receptor recycling	6	1 (16.7%)	0.0151	0.248	BioCarta
Pre-NOTCH Processing in the Endoplasmic Reticulum	6	1 (16.7%)	0.0151	0.248	Reactome
Cell death signalling via NRAGE, NRIF and NADE	76	2 (2.6%)	0.0158	0.248	Reactome
G alpha (12/13) signalling events	80	2 (2.5%)	0.0174	0.248	Reactome
chondroitin sulfate degradation (metazoa)	7	1 (14.3%)	0.0176	0.248	HumanCyc
CS/DS degradation	7	1 (14.3%)	0.0176	0.248	Reactome
Nectin/Necl trans heterodimerization	7	1 (14.3%)	0.0176	0.248	Reactome
C-type lectin receptors (CLRs)	84	2 (2.4%)	0.019	0.248	Reactome
DNA replication initiation	8	1 (12.5%)	0.0201	0.248	Reactome
oleate biosynthesis	8	1 (12.5%)	0.0201	0.248	HumanCyc
palmitate biosynthesis	9	1 (12.5%)	0.0201	0.248	HumanCyc
Dectin-1 mediated noncanonical NF-kB signaling	8	1 (12.5%)	0.0201	0.248	Reactome
NIK-->noncanonical NF-kB signaling	8	1 (12.5%)	0.0201	0.248	Reactome
MyD88:MAL(TIRAP) cascade initiated on plasma membrane	91	2 (2.2%)	0.0221	0.248	Reactome
Toll Like Receptor TLR1:TLR2 Cascade	91	2 (2.2%)	0.0221	0.248	Reactome
Toll Like Receptor TLR6:TLR2 Cascade	91	2 (2.2%)	0.0221	0.248	Reactome
Toll Like Receptor 2 (TLR2) Cascade	91	2 (2.2%)	0.0221	0.248	Reactome
p75 NTR receptor-mediated signalling	96	2 (2.1%)	0.0244	0.248	Reactome
Digestion of dietary carbohydrate	10	1 (10.0%)	0.025	0.248	Reactome
PI3K events in ERBB4 signaling	10	1 (10.0%)	0.025	0.248	Reactome
15q11.2 copy number variation syndrome	10	1 (10.0%)	0.025	0.248	Wikipathways
Nucleotide Excision Repair	11	1 (9.1%)	0.0275	0.248	SMPDB
Regulation of BACH1 activity	11	1 (9.1%)	0.0275	0.248	Reactome
Omega-9 FA synthesis	12	1 (8.3%)	0.03	0.248	Wikipathways
The activation of arylsulfatases	13	1 (7.7%)	0.0324	0.248	Reactome
ERBB2 Activates PTK6 Signaling	13	1 (7.7%)	0.0324	0.248	Reactome
Downregulation of ERBB2:ERBB3 signaling	13	1 (7.7%)	0.0324	0.248	Reactome
stearate biosynthesis	13	1 (7.7%)	0.0324	0.248	HumanCyc
Toll Like Receptor 4 (TLR4) Cascade	112	2 (1.8%)	0.0325	0.248	Reactome
Immune System	1828	9 (0.5%)	0.034	0.248	Reactome
SHC1 events in ERBB4 signaling	14	1 (7.1%)	0.0349	0.248	Reactome
Omega-3-Omega-6 FA synthesis	14	1 (7.1%)	0.0349	0.248	Wikipathways
ERBB2 Regulates Cell Motility	15	1 (6.7%)	0.0373	0.248	Reactome
ErbB receptor signaling network	15	1 (6.7%)	0.0373	0.248	PID
Biogenic Amine Synthesis	15	1 (6.7%)	0.0373	0.248	Wikipathways
PI3K events in ERBB2 signaling	16	1 (6.2%)	0.0398	0.248	Reactome
GRB2 events in ERBB2 signaling	16	1 (6.2%)	0.0398	0.248	Reactome
Regulation of TLR by endogenous ligand	16	1 (6.2%)	0.0398	0.248	Reactome
Metabolism of amine-derived hormones	16	1 (6.2%)	0.0398	0.248	Reactome
ID signaling pathway	16	1 (6.2%)	0.0398	0.248	Wikipathways
MAP3K8 (TPL2)-dependent MAPK1/3 activation	16	1 (6.2%)	0.0398	0.248	Reactome
Oocyte meiosis - Homo sapiens (human)	129	2 (1.6%)	0.042	0.248	KEGG
Glycosaminoglycan degradation	17	1 (5.9%)	0.0422	0.248	Wikipathways
Sialuria or French Type Sialuria	17	1 (5.9%)	0.0422	0.248	SMPDB

Sialuria or French Type Sialuria	17	1 (5.9%)	0.0422	0.248	**SMPDB**
Amino Sugar Metabolism	17	1 (5.9%)	0.0422	0.248	**SMPDB**
G(M2)-Gangliosidosis: Variant B, Tay-sachs disease	17	1 (5.9%)	0.0422	0.248	**SMPDB**
Tay-Sachs Disease	17	1 (5.9%)	0.0422	0.248	**SMPDB**
Salla Disease/Infantile Sialic Acid Storage Disease	17	1 (5.9%)	0.0422	0.248	**SMPDB**
Simplified Depiction of MYD88 Distinct Input-Output Pathway	17	1 (5.9%)	0.0422	0.248	**Wikipathways**
Regulation of HMOX1 expression and activity	17	1 (5.9%)	0.0422	0.248	**Reactome**
Toll-like Receptor Cascades	130	2 (1.5%)	0.0426	0.248	**Reactome**
Activation of NF-kappaB in B cells	18	1 (5.6%)	0.0446	0.248	**Reactome**
Death Receptor Signalling	134	2 (1.5%)	0.045	0.248	**Reactome**
Glycosaminoglycan degradation - Homo sapiens (human)	19	1 (5.3%)	0.047	0.248	**KEGG**
CD209 (DC-SIGN) signaling	19	1 (5.3%)	0.047	0.248	**Reactome**
Tryptophan Metabolism	19	1 (5.3%)	0.047	0.248	**SMPDB**
G2/M Transition	138	2 (1.4%)	0.0475	0.248	**Reactome**
Innate Immune System	1065	6 (0.6%)	0.0484	0.248	**Reactome**
Mitotic G2-G2/M phases	140	2 (1.4%)	0.0487	0.248	**Reactome**
Arf1 pathway	20	1 (5.0%)	0.0495	0.248	**PID**

TRD

pathway name	set size	candidatescontained	p-value	q-value	pathway source
C-type lectin receptors (CLRs)	84	3 (3.6%)	0.00103	0.207	Reactome
TRAIL signaling pathway	25	2 (8.0%)	0.00159	0.207	PID
adenine and adenosine salvage I	1	1 (100.0%)	0.00238	0.207	HumanCyc
Apoptotic cleavage of cellular proteins	38	2 (5.3%)	0.00366	0.207	Reactome
CLEC7A (Dectin-1) signaling	41	2 (4.9%)	0.00425	0.207	Reactome
RAGE	45	2 (4.4%)	0.00511	0.207	NetPath
Ceramide signaling pathway	48	2 (4.2%)	0.00579	0.207	PID
Apoptotic execution phase	52	2 (3.8%)	0.00677	0.207	Reactome
hiv-1 nef: negative effector of fas and tnf	53	2 (3.8%)	0.00702	0.207	BioCarta
Erlotinib Action Pathway	3	1 (33.3%)	0.00713	0.207	SMPDB
Vitamins B6 activation to pyridoxal phosphate	3	1 (33.3%)	0.00713	0.207	Reactome
Proximal tubule transport	57	2 (3.5%)	0.00809	0.207	Wikipathways
Cilium Assembly	178	3 (1.7%)	0.00856	0.207	Reactome
Activation, myristolyation of BID and translocation to mitochondria	4	1 (25.0%)	0.0095	0.207	Reactome
CLEC7A/inflammasome pathway	4	1 (25.0%)	0.0095	0.207	Reactome
Microbial modulation of RIPK1-mediated regulated necrosis	4	1 (25.0%)	0.0095	0.207	Reactome
Vitamin B6 metabolism	4	1 (25.0%)	0.0095	0.207	INOH
Vitamin B6-dependent and responsive disorders	4	1 (25.0%)	0.0095	0.207	Wikipathways
Integrins in angiogenesis	63	2 (3.2%)	0.00981	0.207	PID
AGE-RAGE pathway	66	2 (3.0%)	0.0107	0.207	Wikipathways
Signaling events mediated by VEGFR1 and VEGFR2	68	2 (2.9%)	0.0114	0.207	PID
FasL/ CD95L signaling	5	1 (20.0%)	0.0119	0.207	Reactome
Abacavir transmembrane transport	5	1 (20.0%)	0.0119	0.207	Reactome
Ficolins bind to repetitive carbohydrate structures on the target cell surface	5	1 (20.0%)	0.0119	0.207	Reactome
Hypophosphatasia	5	1 (20.0%)	0.0119	0.207	SMPDB
Vitamin B6 Metabolism	5	1 (20.0%)	0.0119	0.207	SMPDB
pyridoxal 5,-phosphate salvage	5	1 (20.0%)	0.0119	0.207	HumanCyc
TLR3-mediated TICAM1-dependent programmed cell death	6	1 (16.7%)	0.0142	0.207	Reactome
multi-drug resistance factors	6	1 (16.7%)	0.0142	0.207	BioCarta
Venlafaxine Metabolism Pathway	6	1 (16.7%)	0.0142	0.207	SMPDB
Vitamin B6 metabolism - Homo sapiens (human)	6	1 (16.7%)	0.0142	0.207	KEGG
16p11.2 proximal deletion syndrome	79	2 (2.5%)	0.0151	0.207	Wikipathways
TNFR1-induced proapoptotic signaling	7	1 (14.3%)	0.0166	0.207	Reactome
TRAIL signaling	7	1 (14.3%)	0.0166	0.207	Reactome
Paclitaxel Action Pathway	7	1 (14.3%)	0.0166	0.207	SMPDB
Docetaxel Action Pathway	7	1 (14.3%)	0.0166	0.207	SMPDB
Organelle biogenesis and maintenance	231	3 (1.3%)	0.0172	0.207	Reactome
HuR (ELAVL1) binds and stabilizes mRNA	8	1 (12.5%)	0.0189	0.207	Reactome
Lectin pathway of complement activation	8	1 (12.5%)	0.0189	0.207	Reactome
Apoptosis Modulation and Signaling	92	2 (2.2%)	0.0201	0.207	Wikipathways
Allograft Rejection	93	2 (2.2%)	0.0206	0.207	Wikipathways
TRIF-mediated programmed cell death	9	1 (11.1%)	0.0212	0.207	Reactome
Clopidogrel Metabolism Pathway	9	1 (11.1%)	0.0212	0.207	SMPDB
Clopidogrel Action Pathway	9	1 (11.1%)	0.0212	0.207	SMPDB
ROS sensing by NFE2L2	9	1 (11.1%)	0.0212	0.207	Reactome
polyadenylation of mrna	9	1 (11.1%)	0.0212	0.207	BioCarta
Metabolism of ingested SeMet, Sec, MeSec into H2Se	9	1 (11.1%)	0.0212	0.207	Reactome
Anchoring of the basal body to the plasma membrane	97	2 (2.1%)	0.0222	0.207	Reactome
Dimerization of procaspase-8	10	1 (10.0%)	0.0236	0.207	Reactome
Nanoparticle triggered regulated necrosis	10	1 (10.0%)	0.0236	0.207	Wikipathways
Abacavir transport and metabolism	10	1 (10.0%)	0.0236	0.207	Reactome
Vindesine Action Pathway	10	1 (10.0%)	0.0236	0.207	SMPDB
Vincristine Action Pathway	10	1 (10.0%)	0.0236	0.207	SMPDB
Folding of actin by CCT/TriC	10	1 (10.0%)	0.0236	0.207	Reactome
Maturation of nucleoprotein	10	1 (10.0%)	0.0236	0.207	Reactome
Maturation of nucleoprotein	10	1 (10.0%)	0.0236	0.207	Reactome
Platelet activation, signaling and aggregation	263	3 (1.1%)	0.0242	0.207	Reactome

C-type lectin receptor signaling pathway - Homo sapiens (human)	104 2 (1.9%)	0.0253	0.207	KEGG
Regulation by c-FLIP	11 1 (9.1%)	0.0259	0.207	Reactome
CASP8 activity is inhibited	11 1 (9.1%)	0.0259	0.207	Reactome
Vinblastine Action Pathway	11 1 (9.1%)	0.0259	0.207	SMPDB
Vinorelbine Action Pathway	11 1 (9.1%)	0.0259	0.207	SMPDB
MET activates RAS signaling	11 1 (9.1%)	0.0259	0.207	Reactome
Caspase-mediated cleavage of cytoskeletal proteins	12 1 (8.3%)	0.0282	0.207	Reactome
NF-kB activation through FADD/RIP-1 pathway mediated by caspase-8 and -10	12 1 (8.3%)	0.0282	0.207	Reactome
LncRNA-mediated mechanisms of therapeutic resistance	12 1 (8.3%)	0.0282	0.207	Wikipathways
lectin induced complement pathway	12 1 (8.3%)	0.0282	0.207	BioCarta
TP53 Regulates Transcription of Death Receptors and Ligands	12 1 (8.3%)	0.0282	0.207	Reactome
Transport of Mature mRNA Derived from an Intronless Transcript	12 1 (8.3%)	0.0282	0.207	Reactome
Selenoamino Acid Metabolism	12 1 (8.3%)	0.0282	0.207	SMPDB
Purine salvage	13 1 (7.7%)	0.0305	0.207	Reactome
Transport of Mature mRNAs Derived from Intronless Transcripts	13 1 (7.7%)	0.0305	0.207	Reactome
Apoptosis	127 2 (1.6%)	0.0366	0.207	Reactome
Innate Immune System	1065 6 (0.6%)	0.0372	0.207	Reactome
Caspase activation via Death Receptors in the presence of ligand	16 1 (6.2%)	0.0375	0.207	Reactome
Mycophenolic Acid Metabolism Pathway	16 1 (6.2%)	0.0375	0.207	SMPDB
Codeine and Morphine Metabolism	16 1 (6.2%)	0.0375	0.207	Wikipathways
Platelet degranulation	131 2 (1.5%)	0.0387	0.207	Reactome
tnfr1 signaling pathway	17 1 (5.9%)	0.0398	0.207	BioCarta
Drug Induction of Bile Acid Pathway	17 1 (5.9%)	0.0398	0.207	Wikipathways
Doxorubicin Metabolism Pathway	17 1 (5.9%)	0.0398	0.207	SMPDB
Regulation of HMOX1 expression and activity	17 1 (5.9%)	0.0398	0.207	Reactome
Selenocompound metabolism - Homo sapiens (human)	17 1 (5.9%)	0.0398	0.207	KEGG
Response to elevated platelet cytosolic Ca2+	136 2 (1.5%)	0.0414	0.207	Reactome
Simplified Interaction Map Between LOXL4 and Oxidative Stress Pathway	18 1 (5.6%)	0.042	0.207	Wikipathways
internal ribosome entry pathway	18 1 (5.6%)	0.042	0.207	BioCarta
Lamivudine Metabolism Pathway	18 1 (5.6%)	0.042	0.207	SMPDB
MFAP5 effect on permeability and motility of endothelial cells via cytoskeleton rearrangement	18 1 (5.6%)	0.042	0.207	Wikipathways
Apoptosis Modulation by HSP70	19 1 (5.3%)	0.0443	0.207	Wikipathways
Etoposide Action Pathway	19 1 (5.3%)	0.0443	0.207	SMPDB
Etoposide Metabolism Pathway	19 1 (5.3%)	0.0443	0.207	SMPDB
Scavenging by Class A Receptors	19 1 (5.3%)	0.0443	0.207	Reactome
Processing of Intronless Pre-mRNAs	19 1 (5.3%)	0.0443	0.207	Reactome
Programmed Cell Death	142 2 (1.4%)	0.0448	0.207	Reactome
Regucalcin in proximal tubule epithelial kidney cells	20 1 (5.0%)	0.0466	0.207	Wikipathways
Nanomaterial induced apoptosis	20 1 (5.0%)	0.0466	0.207	Wikipathways
fas signaling pathway (cd95)	20 1 (5.0%)	0.0466	0.207	BioCarta
VEGFR2 mediated cell proliferation	20 1 (5.0%)	0.0466	0.207	Reactome
Host-pathogen interaction of human coronaviruses - apoptosis	21 1 (4.8%)	0.0489	0.207	Wikipathways
Imatinib and Chronic Myeloid Leukemia	21 1 (4.8%)	0.0489	0.207	Wikipathways
NGF	21 1 (4.8%)	0.0489	0.207	INOH
Galanin receptor pathway	21 1 (4.8%)	0.0489	0.207	Wikipathways

T2DM_admission_freq

pathway name	set size	candidatescontained	p-value	q-value	pathway source
G alpha (s) signalling events	143	4 (2.8%)	0.00167	0.313	Reactome
GPCRs, Class B Secretin-like	23	2 (8.7%)	0.00301	0.313	Wikipathways
Glucagon-type ligand receptors	33	2 (6.1%)	0.00615	0.313	Reactome
ADORA2B mediated anti-inflammatory cytokines production	132	3 (2.3%)	0.0116	0.313	Reactome
Regulation of Apoptosis	4	1 (25.0%)	0.0142	0.313	Reactome
NAD salvage	4	1 (25.0%)	0.0142	0.313	HumanCyc
Adefovir Dipivoxil Metabolism Pathway	5	1 (20.0%)	0.0177	0.313	SMPDB
Tenofovir Metabolism Pathway	5	1 (20.0%)	0.0177	0.313	SMPDB
Ubiquinone Biosynthesis	5	1 (20.0%)	0.0177	0.313	SMPDB
NAD biosynthesis from 2-amino-3-carboxymuconate semialdehyde	5	1 (20.0%)	0.0177	0.313	HumanCyc
allopregnanolone biosynthesis	6	1 (16.7%)	0.0213	0.313	HumanCyc
Phosphonate and phosphinate metabolism - Homo sapiens (human)	6	1 (16.7%)	0.0213	0.313	KEGG
ceramide de novo biosynthesis	7	1 (14.3%)	0.0248	0.313	HumanCyc
phosphatidylcholine biosynthesis	7	1 (14.3%)	0.0248	0.313	HumanCyc
ubiquinol-10 biosynthesis	7	1 (14.3%)	0.0248	0.313	HumanCyc
Renin secretion - Homo sapiens (human)	69	2 (2.9%)	0.0252	0.313	KEGG
Metabolism of vitamins and cofactors	180	3 (1.7%)	0.0263	0.313	Reactome
Endoderm differentiation	71	2 (2.8%)	0.0266	0.313	Wikipathways
Regulation of cytoskeletal remodeling and cell spreading by IPP complex components	8	1 (12.5%)	0.0282	0.313	Reactome
Phosphate bond hydrolysis by NTPDase proteins	8	1 (12.5%)	0.0282	0.313	Reactome
Ubiquinol biosynthesis	8	1 (12.5%)	0.0282	0.313	Reactome
Costimulation by the CD28 family	77	2 (2.6%)	0.0309	0.313	Reactome
Mitotic Prometaphase	192	3 (1.6%)	0.0311	0.313	Reactome
Adrenoceptors	9	1 (11.1%)	0.0317	0.313	Reactome
Benzo(a)pyrene metabolism	9	1 (11.1%)	0.0317	0.313	Wikipathways
Mitotic Anaphase	195	3 (1.5%)	0.0323	0.313	Reactome
Mitotic Metaphase and Anaphase	196	3 (1.5%)	0.0327	0.313	Reactome
Complement and coagulation cascades - Homo sapiens (human)	85	2 (2.4%)	0.037	0.313	KEGG
Synthesis of bile acids and bile salts via 27-hydroxycholesterol	11	1 (9.1%)	0.0386	0.313	Reactome
cxcr4 signaling pathway	11	1 (9.1%)	0.0386	0.313	BioCarta
Dissolution of Fibrin Clot	11	1 (9.1%)	0.0386	0.313	Reactome
Ubiquinone and other terpenoid-quinone biosynthesis - Homo sapiens (human)	11	1 (9.1%)	0.0386	0.313	KEGG
Mitochondrial translation termination	88	2 (2.3%)	0.0394	0.313	Reactome
Mitochondrial translation elongation	88	2 (2.3%)	0.0394	0.313	Reactome
Mitochondrial translation initiation	88	2 (2.3%)	0.0394	0.313	Reactome
Gap junction - Homo sapiens (human)	88	2 (2.3%)	0.0394	0.313	KEGG
cAMP signaling pathway - Homo sapiens (human)	216	3 (1.4%)	0.0418	0.313	KEGG
Morphine addiction - Homo sapiens (human)	91	2 (2.2%)	0.0419	0.313	KEGG
Class B/2 (Secretin family receptors)	91	2 (2.2%)	0.0419	0.313	Reactome
Butyrophilin (BTN) family interactions	12	1 (8.3%)	0.0421	0.313	Reactome
NAD de novo biosynthesis	12	1 (8.3%)	0.0421	0.313	HumanCyc
Rheumatoid arthritis - Homo sapiens (human)	93	2 (2.2%)	0.0427	0.313	KEGG
Mitochondrial translation	94	2 (2.1%)	0.0444	0.313	Reactome
attenuation of gpcr signaling	13	1 (7.7%)	0.0455	0.313	BioCarta
Passive transport by Aquaporins	13	1 (7.7%)	0.0455	0.313	Reactome
Interferon gamma signaling	96	2 (2.1%)	0.0461	0.313	Reactome
Amplification of signal from unattached kinetochores via a MAD2 inhibitory signal	96	2 (2.1%)	0.0461	0.313	Reactome
Amplification of signal from the kinetochores	96	2 (2.1%)	0.0461	0.313	Reactome
M Phase	383	4 (1.0%)	0.047	0.313	Reactome
Synthesis of bile acids and bile salts via 24-hydroxycholesterol	14	1 (7.1%)	0.0489	0.313	Reactome
Kennedy pathway from sphingolipids	14	1 (7.1%)	0.0489	0.313	Wikipathways
EML4 and NUDC in mitotic spindle formation	100	2 (2.0%)	0.0496	0.313	Reactome

CAD_admission_freq_SAT

pathway name	set size	candidatescontained	p-value	q-value	pathway source
Lysosome - Homo sapiens (human)	128	4 (3.1%)	0.00254	0.258	KEGG
Norepinephrine Neurotransmitter Release Cycle	18	2 (11.1%)	0.00287	0.258	Reactome
Lamivudine Metabolism Pathway	18	2 (11.1%)	0.00287	0.258	SMPDB
glutathione-mediated detoxification	22	2 (9.1%)	0.00428	0.258	HumanCyc
snRNP Assembly	24	2 (8.3%)	0.00508	0.258	Reactome
Metabolism of non-coding RNA	24	2 (8.3%)	0.00508	0.258	Reactome
Drug metabolism - other enzymes - Homo sapiens (human)	80	3 (3.8%)	0.00545	0.258	KEGG
Activation of Na-permeable kainate receptors	2	1 (50.0%)	0.00891	0.258	Reactome
Biosynthesis of maresin conjugates in tissue regeneration (MCTR)	2	1 (50.0%)	0.00891	0.258	Reactome
Biosynthesis of DHA-derived sulfido conjugates	2	1 (50.0%)	0.00891	0.258	Reactome
sulfate activation for sulfonation	2	1 (50.0%)	0.00891	0.258	HumanCyc
Nuclear Receptors in Lipid Metabolism and Toxicity	35	2 (5.7%)	0.0106	0.258	Wikipathways
Glutathione conjugation	37	2 (5.4%)	0.0118	0.258	Reactome
1,25-dihydroxyvitamin D3 biosynthesis	3	1 (33.3%)	0.0133	0.258	HumanCyc
Phase II - Conjugation of compounds	111	3 (2.7%)	0.0134	0.258	Reactome
Non-integrin membrane-ECM interactions	42	2 (4.8%)	0.0151	0.258	Reactome
Glycosaminoglycan metabolism	119	3 (2.5%)	0.0161	0.258	Reactome
Biological oxidations	219	4 (1.8%)	0.0165	0.258	Reactome
pyrimidine deoxyribonucleosides degradation	4	1 (25.0%)	0.0177	0.258	HumanCyc
Polyol Pathway	4	1 (25.0%)	0.0177	0.258	Wikipathways
Proteoglycan syndecan-mediated signaling events	4	1 (25.0%)	0.0177	0.258	PID
Metabolism of ingested H2SeO4 and H2SeO3 into H2Se	4	1 (25.0%)	0.0177	0.258	Reactome
Transcriptional regulation of white adipocyte differentiation	47	2 (4.3%)	0.0186	0.258	Reactome
Ether lipid metabolism - Homo sapiens (human)	49	2 (4.1%)	0.0202	0.258	KEGG
Chondroitin sulfate/dermatan sulfate metabolism	50	2 (4.0%)	0.0209	0.258	Reactome
Neurotransmitter release cycle	50	2 (4.0%)	0.0209	0.258	Reactome
Trafficking of myristoylated proteins to the cilium	5	1 (20.0%)	0.0221	0.258	Reactome
Capecitabine Action Pathway	5	1 (20.0%)	0.0221	0.258	SMPDB
Capecitabine Metabolism Pathway	5	1 (20.0%)	0.0221	0.258	SMPDB
Extrinsic Pathway of Fibrin Clot Formation	5	1 (20.0%)	0.0221	0.258	Reactome
Degradation pathway of sphingolipids, including diseases	5	1 (20.0%)	0.0221	0.258	Wikipathways
tetrapyrrole biosynthesis	5	1 (20.0%)	0.0221	0.258	HumanCyc
Fructose catabolism	5	1 (20.0%)	0.0221	0.258	Reactome
Abacavir transmembrane transport	5	1 (20.0%)	0.0221	0.258	Reactome
Fluid shear stress and atherosclerosis - Homo sapiens (human)	139	3 (2.2%)	0.0242	0.258	KEGG
Clathrin-mediated endocytosis	142	3 (2.1%)	0.0255	0.258	Reactome
Golgi Associated Vesicle Biogenesis	56	2 (3.6%)	0.0259	0.258	Reactome
NRF2 pathway	143	3 (2.1%)	0.026	0.258	Wikipathways
Vitamins	6	1 (16.7%)	0.0265	0.258	Reactome
Acyl chain remodeling of CL	6	1 (16.7%)	0.0265	0.258	Reactome
Transport and synthesis of PAPS	6	1 (16.7%)	0.0265	0.258	Reactome
Phosphonate and phosphinate metabolism - Homo sapiens (human)	6	1 (16.7%)	0.0265	0.258	KEGG
Glutathione metabolism - Homo sapiens (human)	57	2 (3.5%)	0.0268	0.258	KEGG
Porphyrin metabolism	7	1 (14.3%)	0.0309	0.258	INOH
Fructose metabolism	7	1 (14.3%)	0.0309	0.258	Reactome
Anchoring fibril formation	7	1 (14.3%)	0.0309	0.258	Reactome
Tramadol Metabolism Pathway	7	1 (14.3%)	0.0309	0.258	SMPDB
CS/DS degradation	7	1 (14.3%)	0.0309	0.258	Reactome
phosphatidylcholine biosynthesis	7	1 (14.3%)	0.0309	0.258	HumanCyc
Chemokine receptors bind chemokines	62	2 (3.2%)	0.0313	0.258	Reactome
sucrose degradation	8	1 (12.5%)	0.0352	0.258	HumanCyc
Organic cation transport	8	1 (12.5%)	0.0352	0.258	Reactome
Non-genomic actions of 1,25 dihydroxyvitamin D3	68	2 (2.9%)	0.037	0.258	Wikipathways
Metabolism of carbohydrates	285	4 (1.4%)	0.0379	0.258	Reactome
Drug metabolism - cytochrome P450 - Homo sapiens (human)	72	2 (2.9%)	0.039	0.258	KEGG

Vitamin D (calciferol) metabolism	9	1 (11.1%)	0.0395	0.258	Reactome
heme biosynthesis	9	1 (11.1%)	0.0395	0.258	HumanCyc
Heme Biosynthesis	9	1 (11.1%)	0.0395	0.258	Wikipathways
Type I hemidesmosome assembly	9	1 (11.1%)	0.0395	0.258	Reactome
Formation of annular gap junctions	9	1 (11.1%)	0.0395	0.258	Reactome
Bile acids synthesis and enterohepatic circulation	9	1 (11.1%)	0.0395	0.258	Wikipathways
trans-Golgi Network Vesicle Budding	72	2 (2.8%)	0.0411	0.258	Reactome
Cytosolic DNA-sensing pathway	73	2 (2.7%)	0.0421	0.258	Wikipathways
Membrane Trafficking	578	6 (1.0%)	0.0435	0.258	Reactome
Vitamin D Metabolism	10	1 (10.0%)	0.0438	0.258	Wikipathways
Gamma-carboxylation of protein precursors	10	1 (10.0%)	0.0438	0.258	Reactome
Removal of aminoterminal propeptides from gamma-carboxylated proteins	10	1 (10.0%)	0.0438	0.258	Reactome
Intracellular Signalling Through LHCGR Receptor and Luteinizing Hormone/Choriogonadotropin	10	1 (10.0%)	0.0438	0.258	SMPDB
Neurotransmitter clearance	10	1 (10.0%)	0.0438	0.258	Reactome
Abacavir transport and metabolism	10	1 (10.0%)	0.0438	0.258	Reactome
Gap junction degradation	10	1 (10.0%)	0.0438	0.258	Reactome
Sulfur metabolism - Homo sapiens (human)	10	1 (10.0%)	0.0438	0.258	KEGG
Liver X receptor pathway	10	1 (10.0%)	0.0438	0.258	Wikipathways
Peptide GPCRs	75	2 (2.7%)	0.0442	0.258	Wikipathways
Metabolism of xenobiotics by cytochrome P450 - Homo sapiens (human)	78	2 (2.6%)	0.0453	0.258	KEGG
Metabolism	1954	14 (0.7%)	0.0458	0.258	Reactome
Synaptic vesicle cycle - Homo sapiens (human)	78	2 (2.6%)	0.0474	0.258	KEGG
Pyrimidine salvage	11	1 (9.1%)	0.0481	0.258	Reactome
Gamma-carboxylation, transport, and amino-terminal cleavage of proteins	11	1 (9.1%)	0.0481	0.258	Reactome
Alpha6 beta4 integrin-ligand interactions	11	1 (9.1%)	0.0481	0.258	PID
pertussis toxin-insensitive ccr5 signaling in macrophage	11	1 (9.1%)	0.0481	0.258	BioCarta

CAD_mortality_SAT

pathway name	set size	candidatescontained	p-value	q-value	pathway source
Metabolic pathway of LDL, HDL and TG, including diseases	6	2 (33.3%)	0.000311	0.078	Wikipathways
Familial lipoprotein lipase deficiency	13	2 (15.4%)	0.00158	0.078	SMPDB
Glycerolipid Metabolism	13	2 (15.4%)	0.00158	0.078	SMPDB
Glycerol Kinase Deficiency	13	2 (15.4%)	0.00158	0.078	SMPDB
D-glyceric acidura	13	2 (15.4%)	0.00158	0.078	SMPDB
Bivalirudin Action Pathway	20	2 (10.0%)	0.00377	0.078	SMPDB
Argatroban Action Pathway	20	2 (10.0%)	0.00377	0.078	SMPDB
Ximelagatran Action Pathway	20	2 (10.0%)	0.00377	0.078	SMPDB
Lepirudin Action Pathway	20	2 (10.0%)	0.00377	0.078	SMPDB
Coagulation	20	2 (10.0%)	0.00377	0.078	SMPDB
Aminocaproic Acid Action Pathway	20	2 (10.0%)	0.00377	0.078	SMPDB
Tranexamic Acid Action Pathway	20	2 (10.0%)	0.00377	0.078	SMPDB
Urokinase Action Pathway	20	2 (10.0%)	0.00377	0.078	SMPDB
Reteplase Action Pathway	20	2 (10.0%)	0.00377	0.078	SMPDB
Streptokinase Action Pathway	20	2 (10.0%)	0.00377	0.078	SMPDB
Tenecteplase Action Pathway	20	2 (10.0%)	0.00377	0.078	SMPDB
Alteplase Action Pathway	20	2 (10.0%)	0.00377	0.078	SMPDB
Anistreplase Action Pathway	20	2 (10.0%)	0.00377	0.078	SMPDB
Aprotinin Action Pathway	20	2 (10.0%)	0.00377	0.078	SMPDB
Phenindione Action Pathway	20	2 (10.0%)	0.00377	0.078	SMPDB
Dicoumarol Action Pathway	20	2 (10.0%)	0.00377	0.078	SMPDB
Warfarin Action Pathway	20	2 (10.0%)	0.00377	0.078	SMPDB
Acenocoumarol Action Pathway	20	2 (10.0%)	0.00377	0.078	SMPDB
Phenprocoumon Action Pathway	20	2 (10.0%)	0.00377	0.078	SMPDB
Dicumarol Action Pathway	20	2 (10.0%)	0.00377	0.078	SMPDB
Ardeparin Action Pathway	21	2 (9.5%)	0.00416	0.078	SMPDB
Heparin Action Pathway	21	2 (9.5%)	0.00416	0.078	SMPDB
Fondaparinux Action Pathway	21	2 (9.5%)	0.00416	0.078	SMPDB
Enoxaparin Action Pathway	21	2 (9.5%)	0.00416	0.078	SMPDB
Translocation of ZAP-70 to Immunological synapse	28	2 (7.1%)	0.00733	0.133	Reactome
Phosphorylation of CD3 and TCR zeta chains	31	2 (6.5%)	0.00894	0.152	Reactome
Proprotein convertase subtilisin-kexin type 9 (PCSK9) mediated LDL receptor degradation	2	1 (50.0%)	0.00921	0.152	Wikipathways
Evolocumab Mechanism	2	1 (50.0%)	0.00921	0.152	Wikipathways
PD-1 signaling	32	2 (6.2%)	0.00951	0.152	Reactome
Interferon gamma signaling	96	3 (3.1%)	0.00986	0.153	Reactome
Tyrosine metabolism	33	2 (6.1%)	0.0101	0.153	INOH
Tyrosine metabolism - Homo sapiens (human)	36	2 (5.6%)	0.0119	0.175	KEGG
Integration of viral DNA into host genomic DNA	3	1 (33.3%)	0.0138	0.187	Reactome
Autointegration results in viral DNA circles	3	1 (33.3%)	0.0138	0.187	Reactome
Metabolism of vitamin K	3	1 (33.3%)	0.0138	0.187	Reactome
Generation of second messenger molecules	42	2 (4.8%)	0.016	0.204	Reactome
Vitamin A and carotenoid metabolism	43	2 (4.7%)	0.0168	0.204	Wikipathways
CD4 T cell receptor signaling-ERK cascade	44	2 (4.5%)	0.0175	0.204	INOH
Thyroid hormone signaling pathway - Homo sapiens (human)	121	3 (2.5%)	0.0183	0.204	KEGG
Felbamate Metabolism Pathway	4	1 (25.0%)	0.0183	0.204	SMPDB
Composition of Lipid Particles	4	1 (25.0%)	0.0183	0.204	Wikipathways
proline biosynthesis	4	1 (25.0%)	0.0183	0.204	HumanCyc
Vitamin K Metabolism	4	1 (25.0%)	0.0183	0.204	SMPDB
hop pathway in cardiac development	4	1 (25.0%)	0.0183	0.204	BioCarta
Rett syndrome causing genes	48	2 (4.2%)	0.0206	0.222	Wikipathways
Metabolism of fat-soluble vitamins	49	2 (4.1%)	0.0214	0.222	Reactome
Ebola Virus Pathway on Host	130	3 (2.3%)	0.0221	0.222	Wikipathways
Cholesterol metabolism - Homo sapiens (human)	50	2 (4.0%)	0.0223	0.222	KEGG
Extrinsic Pathway of Fibrin Clot Formation	5	1 (20.0%)	0.0229	0.222	Reactome
role of ppar-gamma coactivators in obesity and thermogenesis	5	1 (20.0%)	0.0229	0.222	BioCarta
Cross-presentation of soluble exogenous antigens (endosomes)	5	1 (20.0%)	0.0229	0.222	Reactome
Downstream TCR signaling	52	2 (3.8%)	0.024	0.229	Reactome

2,-deoxy-α-D-ribose 1-phosphate degradation	6	1 (16.7%)	0.0274	0.257	HumanCyc
CD4 T cell receptor signaling-JNK cascade	57	2 (3.5%)	0.0284	0.262	INOH
MHC class II antigen presentation	60	2 (3.3%)	0.0313	0.271	Reactome
Plasma lipoprotein assembly, remodeling, and clearance	60	2 (3.3%)	0.0313	0.271	Reactome
TRAIL signaling	7	1 (14.3%)	0.0319	0.271	Reactome
2-LTR circle formation	7	1 (14.3%)	0.0319	0.271	Reactome
APOBEC3G mediated resistance to HIV-1 infection	7	1 (14.3%)	0.0319	0.271	Reactome
HIV Life Cycle	151	3 (2.0%)	0.0325	0.272	Reactome
RAB geranylgeranylation	65	2 (3.1%)	0.0362	0.282	Reactome
Ifosfamide Action Pathway	8	1 (12.5%)	0.0363	0.282	SMPDB
Ifosfamide Metabolism Pathway	8	1 (12.5%)	0.0363	0.282	SMPDB
Hypothetical Craniofacial Development Pathway	8	1 (12.5%)	0.0363	0.282	Wikipathways
visceral fat deposits and the metabolic syndrome	8	1 (12.5%)	0.0363	0.282	BioCarta
Glycolysis / Gluconeogenesis - Homo sapiens (human)	67	2 (3.0%)	0.0382	0.283	KEGG
Interferon Signaling	162	3 (1.9%)	0.0388	0.283	Reactome
Cyclophosphamide Action Pathway	9	1 (11.1%)	0.0408	0.283	SMPDB
Cyclophosphamide Metabolism Pathway	9	1 (11.1%)	0.0408	0.283	SMPDB
Chylomicron remodeling	9	1 (11.1%)	0.0408	0.283	Reactome
Integration of provirus	9	1 (11.1%)	0.0408	0.283	Reactome
Drug metabolism - cytochrome P450 - Homo sapiens (human)	72	2 (2.9%)	0.0414	0.283	KEGG
Endoderm differentiation	71	2 (2.8%)	0.0425	0.283	Wikipathways
TCR signaling	73	2 (2.7%)	0.0447	0.283	Reactome
putrescine degradation III	10	1 (10.0%)	0.0452	0.283	HumanCyc
Reduction of cytosolic Ca++ levels	10	1 (10.0%)	0.0452	0.283	Reactome
Gamma-carboxylation of protein precursors	10	1 (10.0%)	0.0452	0.283	Reactome
Removal of aminoterminal propeptides from gamma-carboxylated proteins	10	1 (10.0%)	0.0452	0.283	Reactome
Activation of the phototransduction cascade	10	1 (10.0%)	0.0452	0.283	Reactome
Dimerization of procaspase-8	10	1 (10.0%)	0.0452	0.283	Reactome
Opsins	10	1 (10.0%)	0.0452	0.283	Reactome
VLDLR internalisation and degradation	10	1 (10.0%)	0.0452	0.283	Reactome
Metabolism of xenobiotics by cytochrome P450 - Homo sapiens (human)	78	2 (2.6%)	0.048	0.285	KEGG
Costimulation by the CD28 family	77	2 (2.6%)	0.0492	0.285	Reactome
Gamma-carboxylation, transport, and amino-terminal cleavage of proteins	11	1 (9.1%)	0.0496	0.285	Reactome
Regulation by c-FLIP	11	1 (9.1%)	0.0496	0.285	Reactome
CASP8 activity is inhibited	11	1 (9.1%)	0.0496	0.285	Reactome
Ubiquinone and other terpenoid-quinone biosynthesis - Homo sapiens (human)	11	1 (9.1%)	0.0496	0.285	KEGG
Membrane Trafficking	578	6 (1.0%)	0.0498	0.285	Reactome

T2DM_mortality

pathway name	set size	candidatescontained	p-value	q-value	pathway source
Small cell lung cancer - Homo sapiens (human)	92	6 (6.5%)	0.00000477	0.00272	KEGG
Small cell lung cancer	96	6 (6.2%)	0.00000611	0.00272	Wikipathways
Ebola Virus Pathway on Host	130	5 (3.8%)	0.000379	0.112	Wikipathways
Genes related to primary cilium development (based on CRISPR)	103	4 (3.9%)	0.00146	0.133	Wikipathways
PI3K/AKT Signaling in Cancer	106	4 (3.8%)	0.00162	0.133	Reactome
role of mitochondria in apoptotic signaling	13	2 (15.4%)	0.00168	0.133	BioCarta
Ciliopathies	183	5 (2.7%)	0.00177	0.133	Wikipathways
Toxoplasmosis - Homo sapiens (human)	112	4 (3.6%)	0.00198	0.133	KEGG
Hepatitis C and Hepatocellular Carcinoma	56	3 (5.4%)	0.00239	0.133	Wikipathways
Focal Adhesion	198	5 (2.5%)	0.00249	0.133	Wikipathways
Endometrial cancer - Homo sapiens (human)	58	3 (5.2%)	0.00264	0.133	KEGG
Epstein-Barr virus infection - Homo sapiens (human)	202	5 (2.5%)	0.00266	0.133	KEGG
Focal adhesion - Homo sapiens (human)	201	5 (2.5%)	0.00266	0.133	KEGG
regulation of cell cycle progression by plk3	18	2 (11.1%)	0.00325	0.133	BioCarta
Endometrial cancer	63	3 (4.8%)	0.00334	0.133	Wikipathways
Pathways in cancer - Homo sapiens (human)	531	8 (1.5%)	0.00357	0.133	KEGG
prion pathway	19	2 (10.5%)	0.00363	0.133	BioCarta
Regucalcin in proximal tubule epithelial kidney cells	20	2 (10.0%)	0.00402	0.133	Wikipathways
Nanomaterial induced apoptosis	20	2 (10.0%)	0.00402	0.133	Wikipathways
Measles - Homo sapiens (human)	139	4 (2.9%)	0.00431	0.133	KEGG
Glycosphingolipid metabolism	69	3 (4.3%)	0.00432	0.133	EHMN
Regulation by TREX1	1	1 (100.0%)	0.00476	0.133	Reactome
Human papillomavirus infection - Homo sapiens (human)	331	6 (1.8%)	0.00483	0.133	KEGG
Sphingolipid Metabolism	22	2 (9.1%)	0.00485	0.133	SMPDB
Gaucher Disease	22	2 (9.1%)	0.00485	0.133	SMPDB
Globoid Cell Leukodystrophy	22	2 (9.1%)	0.00485	0.133	SMPDB
Metachromatic Leukodystrophy (MLD)	22	2 (9.1%)	0.00485	0.133	SMPDB
Fabry disease	22	2 (9.1%)	0.00485	0.133	SMPDB
Krabbe disease	22	2 (9.1%)	0.00485	0.133	SMPDB
Non-small cell lung cancer	72	3 (4.2%)	0.00487	0.133	Wikipathways
Non-small cell lung cancer - Homo sapiens (human)	72	3 (4.2%)	0.00487	0.133	KEGG
Melanoma - Homo sapiens (human)	72	3 (4.2%)	0.00487	0.133	KEGG
Breast cancer - Homo sapiens (human)	147	4 (2.7%)	0.00526	0.133	KEGG
Laminin interactions	23	2 (8.7%)	0.0053	0.133	Reactome
PI3K-Akt signaling pathway	340	6 (1.8%)	0.00541	0.133	Wikipathways
Glioma - Homo sapiens (human)	75	3 (4.0%)	0.00546	0.133	KEGG
Gastric cancer - Homo sapiens (human)	149	4 (2.7%)	0.00552	0.133	KEGG
Pancreatic cancer - Homo sapiens (human)	76	3 (3.9%)	0.00566	0.133	KEGG
Breast cancer pathway	154	4 (2.6%)	0.00619	0.14	Wikipathways
Constitutive Signaling by Aberrant PI3K in Cancer	79	3 (3.8%)	0.0063	0.14	Reactome
PI3K-Akt signaling pathway - Homo sapiens (human)	354	6 (1.7%)	0.00657	0.14	KEGG
Hepatitis C - Homo sapiens (human)	157	4 (2.5%)	0.00662	0.14	KEGG
ID	26	2 (7.7%)	0.00675	0.14	NetPath
Colorectal cancer - Homo sapiens (human)	86	3 (3.5%)	0.00797	0.161	KEGG
Synthesis of PC	29	2 (6.9%)	0.00835	0.165	Reactome
Pancreatic adenocarcinoma pathway	89	3 (3.4%)	0.00875	0.169	Wikipathways
Influenza A - Homo sapiens (human)	172	4 (2.3%)	0.00908	0.172	KEGG
Downstream signaling of activated FGFR1	31	2 (6.5%)	0.00951	0.173	Reactome
Activation and oligomerization of BAK protein	2	1 (50.0%)	0.00951	0.173	Reactome
Apoptosis - multiple species - Homo sapiens (human)	32	2 (6.2%)	0.0101	0.18	KEGG
Fc gamma R-mediated phagocytosis - Homo sapiens (human)	97	3 (3.1%)	0.0108	0.187	KEGG
Prostate cancer - Homo sapiens (human)	97	3 (3.1%)	0.0111	0.187	KEGG
Metabolism of lipids	646	8 (1.2%)	0.0112	0.187	Reactome
Glycerophospholipid metabolism - Homo sapiens (human)	98	3 (3.1%)	0.0114	0.187	KEGG
PI5P, PP2A and IER3 Regulate PI3K/AKT Signaling	100	3 (3.0%)	0.012	0.194	Reactome
Amoebiasis - Homo sapiens (human)	102	3 (3.0%)	0.0123	0.196	KEGG
Leukotriene metabolism	102	3 (2.9%)	0.0127	0.198	EHMN
G13 Signaling Pathway	38	2 (5.3%)	0.0141	0.214	Wikipathways
Focal Adhesion-PI3K-Akt-mTOR-signaling pathway	302	5 (1.7%)	0.0144	0.214	Wikipathways
Negative regulation of the PI3K/AKT network	107	3 (2.8%)	0.0144	0.214	Reactome
Oxidative Damage	40	2 (5.0%)	0.0155	0.223	Wikipathways
Microglia Pathogen Phagocytosis Pathway	40	2 (5.0%)	0.0155	0.223	Wikipathways

Generation of second messenger molecules	42	2 (4.8%)	0.017	0.233	Reactome
FOXM1 transcription factor network	42	2 (4.8%)	0.017	0.233	PID
Non-integrin membrane-ECM interactions	42	2 (4.8%)	0.017	0.233	Reactome
Rap1 signaling pathway - Homo sapiens (human)	210	4 (1.9%)	0.0178	0.239	KEGG
Human immunodeficiency virus 1 infection - Homo sapiens (human)	212	4 (1.9%)	0.0183	0.239	KEGG
Phospholipid metabolism	213	4 (1.9%)	0.0186	0.239	Reactome
Phosphorylation of proteins involved in G1/S transition by active Cyclin E:Cdk2 complexes	4	1 (25.0%)	0.0189	0.239	Reactome
HCN channels	4	1 (25.0%)	0.0189	0.239	Reactome
a6b1 and a6b4 Integrin signaling	45	2 (4.4%)	0.0194	0.239	PID
Thyroid hormone signaling pathway - Homo sapiens (human)	121	3 (2.5%)	0.0199	0.239	KEGG
PIP3 activates AKT signaling	218	4 (1.8%)	0.0201	0.239	Reactome
Regulation of actin cytoskeleton - Homo sapiens (human)	218	4 (1.8%)	0.0201	0.239	KEGG
Glycosphingolipid metabolism	46	2 (4.3%)	0.0202	0.239	Reactome
Human T-cell leukemia virus 1 infection - Homo sapiens (human)	219	4 (1.8%)	0.0204	0.239	KEGG
agrin in postsynaptic differentiation	47	2 (4.3%)	0.021	0.243	BioCarta
Sphingolipid metabolism - Homo sapiens (human)	49	2 (4.1%)	0.0228	0.256	KEGG
Retrograde transport at the Trans-Golgi-Network	49	2 (4.1%)	0.0228	0.256	Reactome
BCR	129	3 (2.3%)	0.0236	0.256	NetPath
Activation of caspases through apoptosome-mediated cleavage	5	1 (20.0%)	0.0236	0.256	Reactome
G2 Phase	5	1 (20.0%)	0.0236	0.256	Reactome
Signaling by FGFR1	51	2 (3.9%)	0.0245	0.263	Reactome
Intrinsic Pathway for Apoptosis	52	2 (3.8%)	0.0254	0.269	Reactome
Nuclear Envelope Breakdown	53	2 (3.8%)	0.0263	0.27	Reactome
Apoptosis - Homo sapiens (human)	136	3 (2.2%)	0.027	0.27	KEGG
Yersinia infection - Homo sapiens (human)	137	3 (2.2%)	0.027	0.27	KEGG
Glycerophospholipid metabolism	136	3 (2.2%)	0.027	0.27	EHMN
Signaling by PTK6	54	2 (3.7%)	0.0273	0.27	Reactome
Signaling by Non-Receptor Tyrosine Kinases	54	2 (3.7%)	0.0273	0.27	Reactome
PTK6 Regulates Cell Cycle	6	1 (16.7%)	0.0283	0.27	Reactome
Pre-NOTCH Processing in the Endoplasmic Reticulum	6	1 (16.7%)	0.0283	0.27	Reactome
Phosphonate and phosphinate metabolism - Homo sapiens (human)	6	1 (16.7%)	0.0283	0.27	KEGG
TCR	244	4 (1.6%)	0.0285	0.27	NetPath
ECM proteoglycans	56	2 (3.6%)	0.0292	0.273	Reactome
Caspase Cascade in Apoptosis	57	2 (3.5%)	0.0301	0.277	PID
C21-steroid hormone biosynthesis and metabolism	57	2 (3.5%)	0.0301	0.277	EHMN
Notch	58	2 (3.4%)	0.0311	0.279	NetPath
VEGF signaling pathway - Homo sapiens (human)	59	2 (3.4%)	0.0321	0.279	KEGG
Viral myocarditis - Homo sapiens (human)	60	2 (3.4%)	0.0321	0.279	KEGG
SMAC (DIABLO) binds to IAPs	7	1 (14.3%)	0.0329	0.279	Reactome
SMAC(DIABLO)-mediated dissociation of IAP:caspase complexes	7	1 (14.3%)	0.0329	0.279	Reactome
FGFR3b ligand binding and activation	7	1 (14.3%)	0.0329	0.279	Reactome
Glycerophospholipid catabolism	7	1 (14.3%)	0.0329	0.279	Reactome
phosphatidylcholine biosynthesis	7	1 (14.3%)	0.0329	0.279	HumanCyc
Intracellular signaling by second messengers	259	4 (1.5%)	0.0348	0.288	Reactome
Regulation of Actin Cytoskeleton	151	3 (2.0%)	0.0352	0.288	Wikipathways
Regulation of retinoblastoma protein	64	2 (3.1%)	0.0373	0.288	PID
GnRH secretion - Homo sapiens (human)	64	2 (3.1%)	0.0373	0.288	KEGG
Inhibition of Signaling by Overexpressed EGFR	8	1 (12.5%)	0.0375	0.288	Reactome
Signaling by Overexpressed Wild-Type EGFR in Cancer	8	1 (12.5%)	0.0375	0.288	Reactome
west nile virus	8	1 (12.5%)	0.0375	0.288	BioCarta
SMAC, XIAP-regulated apoptotic response	8	1 (12.5%)	0.0375	0.288	Reactome
EPH-ephrin mediated repulsion of cells	8	1 (12.5%)	0.0375	0.288	Reactome
fatty acid activation	8	1 (12.5%)	0.0375	0.288	HumanCyc
FTO Obesity Variant Mechanism	8	1 (12.5%)	0.0375	0.288	Wikipathways
Beta1 integrin cell surface interactions	66	2 (3.0%)	0.0394	0.289	PID
Fc epsilon RI signaling pathway - Homo sapiens (human)	68	2 (3.0%)	0.0405	0.289	KEGG
FGF	67	2 (3.0%)	0.0405	0.289	INOH

Diseases of signal transduction by growth factor receptors and second messengers	274	4 (1.5%)	0.0415	0.289	Reactome
DNA damage response	68	2 (2.9%)	0.0416	0.289	Wikipathways
Melanoma	68	2 (2.9%)	0.0416	0.289	Wikipathways
Hepatitis B - Homo sapiens (human)	162	3 (1.9%)	0.042	0.289	KEGG
EGFR interacts with phospholipase C-gamma	9	1 (11.1%)	0.0421	0.289	Reactome
cyclin e destruction pathway	9	1 (11.1%)	0.0421	0.289	BioCarta
Alpha6Beta4Integrin	71	2 (2.8%)	0.045	0.289	NetPath
CDC42 signaling events	71	2 (2.8%)	0.045	0.289	PID
Joubert Syndrome	72	2 (2.8%)	0.0461	0.289	Wikipathways
PI3K events in ERBB4 signaling	10	1 (10.0%)	0.0466	0.289	Reactome
MTHFR deficiency	10	1 (10.0%)	0.0466	0.289	Wikipathways
Regulation of the apoptosome activity	10	1 (10.0%)	0.0466	0.289	Reactome
Formation of apoptosome	10	1 (10.0%)	0.0466	0.289	Reactome
Caspase activation via Dependence Receptors in the absence of ligand	10	1 (10.0%)	0.0466	0.289	Reactome
e2f1 destruction pathway	10	1 (10.0%)	0.0466	0.289	BioCarta
Chromosomal and microsatellite instability in colorectal cancer	73	2 (2.7%)	0.0473	0.289	Wikipathways
p53 signaling pathway - Homo sapiens (human)	73	2 (2.7%)	0.0473	0.289	KEGG
TCR signaling	73	2 (2.7%)	0.0473	0.289	Reactome
EPH-Ephrin signaling	74	2 (2.7%)	0.0485	0.289	Reactome

CAD_admission_freq_VAT

pathway name	set size	candidatescontained	p-value	q-value	pathway source
prion pathway	19	2 (10.5%)	0.00103	0.148	BioCarta
NCAM signaling for neurite out-growth	59	2 (3.4%)	0.00972	0.148	Reactome
chondroitin and dermatan biosynthesis	6	1 (16.7%)	0.0151	0.148	HumanCyc
Anchoring fibril formation	7	1 (14.3%)	0.0176	0.148	Reactome
APEX1-Independent Resolution of AP Sites via the Single Nucleotide Replacement Pathway	7	1 (14.3%)	0.0176	0.148	Reactome
COPI-mediated anterograde transport	83	2 (2.4%)	0.0186	0.148	Reactome
Metabolism	1954	10 (0.5%)	0.0194	0.148	Reactome
Fatty Acids bound to GPR40 (FFAR1) regulate insulin secretion	8	1 (12.5%)	0.0201	0.148	Reactome
Phase I biotransformations, non P450	8	1 (12.5%)	0.0201	0.148	Wikipathways
Conjugation of salicylate with glycine	8	1 (12.5%)	0.0201	0.148	Reactome
Sepiapterin reductase deficiency	9	1 (11.1%)	0.0226	0.148	SMPDB
Segawa syndrome	9	1 (11.1%)	0.0226	0.148	SMPDB
Pterine Biosynthesis	9	1 (11.1%)	0.0226	0.148	SMPDB
Dopa-responsive dystonia	9	1 (11.1%)	0.0226	0.148	SMPDB
Hyperphenylalaniemia due to guanosine triphosphate cyclohydrolase deficiency	9	1 (11.1%)	0.0226	0.148	SMPDB
Hyperphenylalaninemia due to 6-pyruvoyltetrahydropterin synthase deficiency (ptps)	9	1 (11.1%)	0.0226	0.148	SMPDB
Hyperphenylalaninemia due to dhpr-deficiency	9	1 (11.1%)	0.0226	0.148	SMPDB
Type I hemidesmosome assembly	9	1 (11.1%)	0.0226	0.148	Reactome
Conjugation of carboxylic acids	9	1 (11.1%)	0.0226	0.148	Reactome
Amino Acid conjugation	9	1 (11.1%)	0.0226	0.148	Reactome
Complement system	93	2 (2.2%)	0.023	0.148	Wikipathways
Acetylcholine regulates insulin secretion	10	1 (10.0%)	0.025	0.148	Reactome
Amoebiasis - Homo sapiens (human)	102	2 (2.0%)	0.0269	0.148	KEGG
Free fatty acids regulate insulin secretion	11	1 (9.1%)	0.0275	0.148	Reactome
Alpha6 beta4 integrin-ligand interactions	11	1 (9.1%)	0.0275	0.148	PID
Aspartate and asparagine metabolism	12	1 (8.3%)	0.03	0.148	Reactome
D-myo-inositol (1,4,5)-trisphosphate degradation	13	1 (7.7%)	0.0324	0.148	HumanCyc
DNA-PK pathway in nonhomologous end joining	13	1 (7.7%)	0.0324	0.148	PID
eicosapentaenoate biosynthesis	13	1 (7.7%)	0.0324	0.148	HumanCyc
Synthesis of IP2, IP, and Ins in the cytosol	14	1 (7.1%)	0.0349	0.148	Reactome
Tryptophan catabolism	14	1 (7.1%)	0.0349	0.148	Reactome
Triglyceride biosynthesis	14	1 (7.1%)	0.0349	0.148	Reactome
γ-linolenate biosynthesis	14	1 (7.1%)	0.0349	0.148	HumanCyc
Glycosaminoglycan metabolism	119	2 (1.7%)	0.0363	0.148	Reactome
PAR4-mediated thrombin signaling events	15	1 (6.7%)	0.0373	0.148	PID
1D-myo-inositol hexakisphosphate biosynthesis II (mammalian)	15	1 (6.7%)	0.0373	0.148	HumanCyc
D-myo-inositol (1,3,4)-trisphosphate biosynthesis	15	1 (6.7%)	0.0373	0.148	HumanCyc
Leucine Stimulation on Insulin Signaling	15	1 (6.7%)	0.0373	0.148	SMPDB
Synthesis of Prostaglandins (PG) and Thromboxanes (TX)	16	1 (6.2%)	0.0398	0.148	Reactome
CRMPs in Sema3A signaling	16	1 (6.2%)	0.0398	0.148	Reactome
Doxorubicin Metabolism Pathway	17	1 (5.9%)	0.0422	0.148	SMPDB
Bile Acid Biosynthesis	17	1 (5.9%)	0.0422	0.148	SMPDB
27-Hydroxylase Deficiency	17	1 (5.9%)	0.0422	0.148	SMPDB
Congenital Bile Acid Synthesis Defect Type II	17	1 (5.9%)	0.0422	0.148	SMPDB
Cerebrotendinous Xanthomatosis (CTX)	17	1 (5.9%)	0.0422	0.148	SMPDB
Zellweger Syndrome	17	1 (5.9%)	0.0422	0.148	SMPDB
Familial Hypercholanemia (FHCA)	17	1 (5.9%)	0.0422	0.148	SMPDB
Congenital Bile Acid Synthesis Defect Type III	17	1 (5.9%)	0.0422	0.148	SMPDB
dermatan sulfate biosynthesis	17	1 (5.9%)	0.0422	0.148	HumanCyc
Metabolism of Angiotensinogen to Angiotensins	18	1 (5.6%)	0.0446	0.148	Reactome
LDL clearance	18	1 (5.6%)	0.0446	0.148	Reactome
G alpha (i) signalling events	316	3 (0.9%)	0.0451	0.148	Reactome
ER to Golgi Anterograde Transport	136	2 (1.5%)	0.0462	0.148	Reactome
superpathway of D-myo-inositol (1,4,5)-trisphosphate metabolism	20	1 (5.0%)	0.0495	0.148	HumanCyc
3-phosphoinositide degradation	20	1 (5.0%)	0.0495	0.148	HumanCyc

Steroid biosynthesis - Homo sapiens (human)	**20**	**1 (5.0%)**	0.0495	0.148	**KEGG**
Class I peroxisomal membrane protein import	**20**	**1 (5.0%)**	0.0495	0.148	**Reactome**
Glycosaminoglycan biosynthesis - chondroitin sulfate / dermatan sulfate - Homo sapiens (human)	**20**	**1 (5.0%)**	0.0495	0.148	**KEGG**
Chondroitin sulfate biosynthesis	**20**	**1 (5.0%)**	0.0495	0.148	**Reactome**

CAD_mortality_VAT

pathway name	set size	candidatescontained	p-value	q-value	pathway source
Opsins	10	2 (20.0%)	0.000443	0.189	Reactome
Regulation of signaling by NODAL	12	2 (16.7%)	0.000647	0.189	Reactome
Osteoclast Signaling	16	2 (12.5%)	0.00117	0.214	Wikipathways
Nanomaterial induced apoptosis	20	2 (10.0%)	0.00183	0.214	Wikipathways
Signaling by NODAL	20	2 (10.0%)	0.00183	0.214	Reactome
Apoptosis Modulation and Signaling	92	3 (3.3%)	0.00315	0.249	Wikipathways
Interferon gamma signaling	96	3 (3.1%)	0.00355	0.249	Reactome
the information processing pathway at the ifn beta enhancer	29	2 (6.9%)	0.00385	0.249	BioCarta
Activation of the pre-replicative complex	33	2 (6.1%)	0.00496	0.249	Reactome
DNA Replication Pre-Initiation	37	2 (5.4%)	0.00621	0.249	Reactome
Activation and oligomerization of BAK protein	2	1 (50.0%)	0.00639	0.249	Reactome
Eptifibatide Action Pathway	2	1 (50.0%)	0.00639	0.249	SMPDB
Tirofiban Action Pathway	2	1 (50.0%)	0.00639	0.249	SMPDB
Abciximab Action Pathway	2	1 (50.0%)	0.00639	0.249	SMPDB
Conjugation of phenylacetate with glutamine	2	1 (50.0%)	0.00639	0.249	Reactome
Apoptosis - Homo sapiens (human)	136	3 (2.2%)	0.00932	0.278	KEGG
Phenylacetate Metabolism	3	1 (33.3%)	0.00957	0.278	SMPDB
Signaling events mediated by PTP1B	52	2 (3.8%)	0.012	0.278	PID
IRS activation	4	1 (25.0%)	0.0127	0.278	Reactome
dermatan sulfate biosynthesis (late stages)	4	1 (25.0%)	0.0127	0.278	HumanCyc
Lipoic acid metabolism - Homo sapiens (human)	4	1 (25.0%)	0.0127	0.278	KEGG
Interferon Signaling	162	3 (1.9%)	0.0149	0.278	Reactome
Oxidation by Cytochrome P450	62	2 (3.2%)	0.0168	0.278	Wikipathways
Integrins in angiogenesis	63	2 (3.2%)	0.0173	0.278	PID
Influenza A - Homo sapiens (human)	172	3 (1.7%)	0.0175	0.278	KEGG
IL4-mediated signaling events	65	2 (3.1%)	0.0183	0.278	PID
Gap-filling DNA repair synthesis and ligation in TC-NER	66	2 (3.0%)	0.0189	0.278	Reactome
Activated NTRK3 signals through PI3K	6	1 (16.7%)	0.0191	0.278	Reactome
Conjugation of benzoate with glycine	6	1 (16.7%)	0.0191	0.278	Reactome
Dual incision in TC-NER	67	2 (3.0%)	0.0194	0.278	Reactome
Vitamin B2 (riboflavin) metabolism	7	1 (14.3%)	0.0222	0.278	Reactome
SOS-mediated signalling	7	1 (14.3%)	0.0222	0.278	Reactome
TRAIL signaling	7	1 (14.3%)	0.0222	0.278	Reactome
Primary focal segmental glomerulosclerosis (FSGS)	73	2 (2.7%)	0.0228	0.278	Wikipathways
Synthesis of DNA	76	2 (2.6%)	0.0246	0.278	Reactome
G1/S Transition	76	2 (2.6%)	0.0246	0.278	Reactome
Riboflavin metabolism - Homo sapiens (human)	8	1 (12.5%)	0.0253	0.278	KEGG
CDC6 association with the ORC:origin complex	8	1 (12.5%)	0.0253	0.278	Reactome
Vitamin C (ascorbate) metabolism	8	1 (12.5%)	0.0253	0.278	Reactome
Hypothetical Craniofacial Development Pathway	8	1 (12.5%)	0.0253	0.278	Wikipathways
DNA replication initiation	8	1 (12.5%)	0.0253	0.278	Reactome
Mitochondrial Beta-Oxidation of Medium Chain Saturated Fatty Acids	8	1 (12.5%)	0.0253	0.278	SMPDB
Butyrate Metabolism	8	1 (12.5%)	0.0253	0.278	SMPDB
Eicosanoid metabolism via cytochrome P450 monooxygenases (CYP) pathway	8	1 (12.5%)	0.0253	0.278	Wikipathways
Transcription-Coupled Nucleotide Excision Repair (TC-NER)	79	2 (2.5%)	0.0264	0.278	Reactome
DNA Replication	81	2 (2.5%)	0.0276	0.278	Reactome
Signaling by NTRK3 (TRKC)	9	1 (11.1%)	0.0284	0.278	Reactome
PI3K/AKT activation	9	1 (11.1%)	0.0284	0.278	Reactome
Conjugation of carboxylic acids	9	1 (11.1%)	0.0284	0.278	Reactome
Amino Acid conjugation	9	1 (11.1%)	0.0284	0.278	Reactome
Cell surface interactions at the vascular wall	215	3 (1.4%)	0.0312	0.278	Reactome
Apoptosis	87	2 (2.3%)	0.0315	0.278	Wikipathways
IL-13 signaling	10	1 (10.0%)	0.0316	0.278	INOH
Signal attenuation	10	1 (10.0%)	0.0316	0.278	Reactome
Dimerization of procaspase-8	10	1 (10.0%)	0.0316	0.278	Reactome
Mitochondrial translation termination	88	2 (2.3%)	0.0322	0.278	Reactome
Biological oxidations	219	3 (1.4%)	0.0327	0.278	Reactome

Highly calcium permeable postsynaptic nicotinic acetylcholine receptors	11	1 (9.1%)	0.0347	0.278	Reactome
apoptotic dna-fragmentation and tissue homeostasis	11	1 (9.1%)	0.0347	0.278	BioCarta
Signaling by Leptin	11	1 (9.1%)	0.0347	0.278	Reactome
insulin signaling pathway	11	1 (9.1%)	0.0347	0.278	BioCarta
Serotonin Transporter Activity	11	1 (9.1%)	0.0347	0.278	Wikipathways
Regulation by c-FLIP	11	1 (9.1%)	0.0347	0.278	Reactome
CASP8 activity is inhibited	11	1 (9.1%)	0.0347	0.278	Reactome
Dermatan sulfate biosynthesis	11	1 (9.1%)	0.0347	0.278	Reactome
Valproic Acid Metabolism Pathway	11	1 (9.1%)	0.0347	0.278	SMPDB
Rheumatoid arthritis - Homo sapiens (human)	93	2 (2.2%)	0.0349	0.278	KEGG
Mitochondrial translation	94	2 (2.1%)	0.0363	0.278	Reactome
carm1 and regulation of the estrogen receptor	12	1 (8.3%)	0.0378	0.278	BioCarta
PECAM1 interactions	12	1 (8.3%)	0.0378	0.278	Reactome
POU5F1 (OCT4), SOX2, NANOG activate genes related to proliferation	12	1 (8.3%)	0.0378	0.278	Reactome
TP53 Regulates Transcription of Death Receptors and Ligands	12	1 (8.3%)	0.0378	0.278	Reactome
Eicosanoids	12	1 (8.3%)	0.0378	0.278	Reactome
Miscellaneous substrates	12	1 (8.3%)	0.0378	0.278	Reactome
Nucleotide Excision Repair	97	2 (2.1%)	0.0385	0.278	Reactome
Hematopoietic cell lineage - Homo sapiens (human)	99	2 (2.0%)	0.0392	0.278	KEGG
role of mitochondria in apoptotic signaling	13	1 (7.7%)	0.0408	0.278	BioCarta
multiple antiapoptotic pathways from igf-1r signaling lead to bad phosphorylation	13	1 (7.7%)	0.0408	0.278	BioCarta
Osteopontin Signaling	13	1 (7.7%)	0.0408	0.278	Wikipathways
MFAP5-mediated ovarian cancer cell motility and invasiveness	13	1 (7.7%)	0.0408	0.278	Wikipathways
D-myo-inositol (1,4,5)-trisphosphate degradation	13	1 (7.7%)	0.0408	0.278	HumanCyc
chondroitin sulfate biosynthesis (late stages)	13	1 (7.7%)	0.0408	0.278	HumanCyc
Valproic acid pathway	13	1 (7.7%)	0.0408	0.278	Wikipathways
eicosapentaenoate biosynthesis	13	1 (7.7%)	0.0408	0.278	HumanCyc
Phase I - Functionalization of compounds	101	2 (2.0%)	0.0414	0.278	Reactome
S Phase	104	2 (1.9%)	0.0436	0.278	Reactome
Mitotic G1 phase and G1/S transition	104	2 (1.9%)	0.0436	0.278	Reactome
CDT1 association with the CDC6:ORC:origin complex	14	1 (7.1%)	0.0439	0.278	Reactome
Postsynaptic nicotinic acetylcholine receptors	14	1 (7.1%)	0.0439	0.278	Reactome
Acetylcholine binding and downstream events	14	1 (7.1%)	0.0439	0.278	Reactome
Osteoblast Signaling	14	1 (7.1%)	0.0439	0.278	Wikipathways
γ-linolenate biosynthesis	14	1 (7.1%)	0.0439	0.278	HumanCyc
Leucine Stimulation on Insulin Signaling	15	1 (6.7%)	0.047	0.278	SMPDB
p130Cas linkage to MAPK signaling for integrins	15	1 (6.7%)	0.047	0.278	Reactome
GRB2:SOS provides linkage to MAPK signaling for Integrins	15	1 (6.7%)	0.047	0.278	Reactome
1D-myo-inositol hexakisphosphate biosynthesis II (mammalian)	15	1 (6.7%)	0.047	0.278	HumanCyc
D-myo-inositol (1,3,4)-trisphosphate biosynthesis	15	1 (6.7%)	0.047	0.278	HumanCyc
Fatty acids	15	1 (6.7%)	0.047	0.278	Reactome
DNA damage response (only ATM dependent)	110	2 (1.8%)	0.0483	0.278	Wikipathways
Metabolism of water-soluble vitamins and cofactors	111	2 (1.8%)	0.0491	0.278	Reactome
