# Supplementary material for: CausalCellInfer: Prioritizing Cell‐Type‐Specific Disease Associations From Genetically Predicted Transcriptomes in Biobank Cohorts
Source: Adv Sci (Weinh). 2026 Oct 14:e78197. Online ahead of print. doi: 10.1002/advs.78197 (PMC13669230; doi:10.1002/advs.78197)
Supplement: Supplementary file 2 [file ADVS-9999-e78197-s002.zip › advs78197-sup-0008-S7.pdf]

Table S7 Genes identified from TWAS analysis with adjustment for estimated cell proportions

tissue	disorder	Genes	Estimates_cellprop	Pvalues_cellprop	Pvalue_Bonferroni_cellprop	Pvalue_BH_cellprop	gene_name
Frontal Cortex	Depression	ENSG00000137185.7	-0.007446263	1.27E-07	0.000255456	0.000255456	ZSCAN9
Frontal Cortex	Depression	ENSG00000186470.9	0.006024303	6.26E-06	0.012601627	0.006300813	BTN3A2
Frontal Cortex	Depression	ENSG00000244731.3	0.007779012	6.25E-05	0.125838035	0.041946012	C4A
Pancreas	T1DM	ENSG00000011523.9	0.001320601	0.000993831	1	0.04640285	CEP68
Pancreas	T1DM	ENSG00000076650.2	0.003483687	0.001078733	1	0.049467622	GPATCH1
Pancreas	T1DM	ENSG00000099785.6	0.001726473	0.000873976	1	0.042346594	MARCH2
Pancreas	T1DM	ENSG00000124508.12	-0.002359585	0.000255627	0.656451124	0.014587803	BTN2A2
Pancreas	T1DM	ENSG00000137288.5	0.002643162	0.000293663	0.754127073	0.016394067	UQCC2
Pancreas	T1DM	ENSG00000167280.12	0.001354907	0.000775642	1	0.03830479	ENGASE
Pancreas	T1DM	ENSG00000179344.12	-0.005476016	2.96E-21	7.61E-18	6.34E-19	HLA-DQB1
Pancreas	T1DM	ENSG00000182477.5	0.001240491	5.71E-05	0.146623614	0.003491038	OR2B8P
Pancreas	T1DM	ENSG00000186470.9	-0.000958064	1.28E-05	0.032914354	0.000843958	BTN3A2
Pancreas	T1DM	ENSG00000189298.9	-0.004961783	1.41E-09	3.62E-06	1.58E-07	ZKSCAN3
Pancreas	T1DM	ENSG00000196260.3	-0.003202614	3.94E-11	1.01E-07	5.06E-09	SFTA2
Pancreas	T1DM	ENSG00000197062.7	-0.002154522	2.03E-08	5.22E-05	2.01E-06	RP5-874C20.3
Pancreas	T1DM	ENSG00000197728.5	0.00118086	0.000466793	1	0.024973406	RPS26
Pancreas	T1DM	ENSG00000198502.5	-0.003063224	1.81E-39	4.64E-36	1.16E-36	HLA-DRB5
Pancreas	T1DM	ENSG00000204228.3	0.0047664	3.40E-28	8.73E-25	1.25E-25	HSD17B8
Pancreas	T1DM	ENSG00000204257.10	0.0031622	1.01E-29	2.58E-26	5.17E-27	HLA-DMA
Pancreas	T1DM	ENSG00000204301.5	-0.006629097	9.03E-26	2.32E-22	2.32E-23	NOTCH4
Pancreas	T1DM	ENSG00000204305.9	0.003713229	0.000235442	0.604614121	0.01374123	AGER
Pancreas	T1DM	ENSG00000204308.6	-0.002142507	1.90E-14	4.88E-11	2.87E-12	RNF5
Pancreas	T1DM	ENSG00000204338.4	-0.003504122	4.50E-29	1.16E-25	1.93E-26	CYP21A1P
Pancreas	T1DM	ENSG00000204351.7	-0.002830754	2.10E-23	5.40E-20	4.91E-21	SKIV2L
Pancreas	T1DM	ENSG00000204371.7	0.004453456	2.63E-05	0.067635975	0.001690899	EHMT2
Pancreas	T1DM	ENSG00000204385.6	-0.001364878	0.000328449	0.843457414	0.017945902	SLC44A4
Pancreas	T1DM	ENSG00000204520.8	0.001432358	3.86E-07	0.000990548	3.20E-05	MICA
Pancreas	T1DM	ENSG00000204525.10	0.000970859	3.03E-05	0.077936275	0.001900885	HLA-C
Pancreas	T1DM	ENSG00000204536.9	0.003534407	5.25E-09	1.35E-05	5.40E-07	CCHCR1
Pancreas	T1DM	ENSG00000204540.6	-0.002118881	1.52E-11	3.91E-08	2.06E-09	PSORS1C1
Pancreas	T1DM	ENSG00000204616.6	-0.002149228	1.71E-07	0.000438408	1.51E-05	TRIM31
Pancreas	T1DM	ENSG00000204618.4	0.004892466	6.37E-16	1.64E-12	1.02E-13	RNF39
Pancreas	T1DM	ENSG00000204644.5	0.001914015	1.84E-07	0.000472293	1.57E-05	ZFP57
Pancreas	T1DM	ENSG00000206344.6	0.002092894	3.72E-06	0.009546279	0.000260615	HCG27
Pancreas	T1DM	ENSG00000213676.6	0.002074921	4.48E-09	1.15E-05	4.80E-07	ATF6B
Pancreas	T1DM	ENSG00000213760.6	-0.002161277	0.000760322	1	0.038284453	ATP6V1G2
Pancreas	T1DM	ENSG00000219392.1	-0.002945179	3.84E-06	0.009864654	0.000260615	RP1-265C24.5
Pancreas	T1DM	ENSG00000221988.8	0.00623609	8.79E-11	2.26E-07	1.03E-08	PPT2
Pancreas	T1DM	ENSG00000224389.4	0.005486048	3.86E-06	0.009903386	0.000260615	C4B
Pancreas	T1DM	ENSG00000224796.1	0.005930985	1.33E-17	3.42E-14	2.28E-15	RPL32P1
Pancreas	T1DM	ENSG00000229391.3	0.005878019	1.51E-53	3.88E-50	3.88E-50	HLA-DRB6
Pancreas	T1DM	ENSG00000229759.1	-0.000990963	0.00096253	1	0.045773668	MRPS18AP1
Pancreas	T1DM	ENSG00000229836.1	0.002601908	2.55E-14	6.56E-11	3.64E-12	XXbac-BPG248L24.10
Pancreas	T1DM	ENSG00000231402.1	0.001939407	6.79E-11	1.74E-07	8.30E-09	WASF5P
Pancreas	T1DM	ENSG00000232629.4	0.004904399	1.57E-47	4.02E-44	2.01E-44	HLA-DQB2
Pancreas	T1DM	ENSG00000234127.4	-0.001865492	0.000676758	1	0.034758299	TRIM26
Pancreas	T1DM	ENSG00000235109.3	0.001098206	1.96E-06	0.005031361	0.000143753	ZSCAN31
Pancreas	T1DM	ENSG00000235863.2	-0.001878393	0.000528441	1	0.027694627	B3GALT4
Pancreas	T1DM	ENSG00000236529.1	0.005179134	9.10E-05	0.233721676	0.005435388	RP13-254B10.1
Pancreas	T1DM	ENSG00000237541.3	0.005887442	6.44E-27	1.65E-23	1.84E-24	HLA-DQA2
Pancreas	T1DM	ENSG00000237669.1	0.002010631	7.94E-07	0.002039598	6.37E-05	HCG4P3
Pancreas	T1DM	ENSG00000240065.3	0.008117937	6.51E-20	1.67E-16	1.29E-17	PSMB9
Pancreas	T1DM	ENSG00000241106.2	0.002981786	1.15E-19	2.94E-16	2.10E-17	HLA-DOB
Pancreas	T1DM	ENSG00000244731.3	-0.005878779	4.52E-46	1.16E-42	3.87E-43	C4A
Pancreas	T1DM	ENSG00000256166.1	0.004916826	6.19E-27	1.59E-23	1.84E-24	XXbac-BPG248L24.13
Pancreas	T1DM	ENSG00000260302.1	0.003374929	1.17E-06	0.00301007	9.12E-05	RP11-973H7.1
Pancreas	T1DM	ENSG00000271821.1	0.003872823	1.56E-06	0.004004901	0.000117791	XXbac-BPG299F13.14
Pancreas	T1DM	ENSG00000272221.1	0.001179828	9.10E-08	0.000233599	8.34E-06	XXbac-BPG181B23.7
Pancreas	T1DM	ENSG00000273340.1	-0.001647747	2.43E-08	6.24E-05	2.31E-06	MICE
Pancreas	T2DM	ENSG00000004534.10	-0.003393908	2.04E-07	0.000523424	5.23E-05	RBM6
Pancreas	T2DM	ENSG00000009950.11	0.005485409	7.27E-05	0.186710079	0.006438279	MLXIPL
Pancreas	T2DM	ENSG00000010310.4	-0.00764268	0.00119303	1	0.040849362	GIPR
Pancreas	T2DM	ENSG00000011523.9	0.005797831	2.76E-08	7.08E-05	8.85E-06	CEP68
Pancreas	T2DM	ENSG00000030110.8	0.011017341	2.02E-06	0.00519481	0.000431985	BAK1
Pancreas	T2DM	ENSG00000066294.10	0.005912261	0.000436359	1	0.020225759	CD84
Pancreas	T2DM	ENSG00000071564.10	-0.010878643	3.16E-05	0.081240829	0.003692765	TCF3
Pancreas	T2DM	ENSG00000073849.10	0.008251104	0.000914691	1	0.035306133	ST6GAL1
Pancreas	T2DM	ENSG00000075413.13	-0.015487526	1.45E-08	3.73E-05	6.22E-06	MARK3
Pancreas	T2DM	ENSG00000095485.12	-0.002744305	9.36E-05	0.240323682	0.007510115	CWF19L1
Pancreas	T2DM	ENSG00000103479.10	0.005922199	0.00093469	1	0.035306133	RBL2
Pancreas	T2DM	ENSG00000103512.10	0.008459255	0.001261266	1	0.041428975	NOMO1
Pancreas	T2DM	ENSG00000108272.9	0.008331831	0.000663435	1	0.028395025	DHRS11
Pancreas	T2DM	ENSG00000108786.6	-0.004851247	0.00044106	1	0.020225759	HSD17B1
Pancreas	T2DM	ENSG00000108797.7	0.007219314	7.79E-05	0.199995047	0.006666502	CNTNAP1
Pancreas	T2DM	ENSG00000111215.7	0.005412917	0.001254073	1	0.041428975	PRR4
Pancreas	T2DM	ENSG00000111328.2	0.005531994	0.000100877	0.259052616	0.007669642	CDK2AP1
Pancreas	T2DM	ENSG00000112992.12	-0.005734172	0.000396943	1	0.01997046	NNT
Pancreas	T2DM	ENSG00000118997.9	-0.005724881	7.19E-05	0.184518162	0.006438279	DNAH7
Pancreas	T2DM	ENSG00000122025.10	0.010222286	0.000313939	0.806195284	0.017915451	FLT3
Pancreas	T2DM	ENSG00000125510.11	-0.006830479	0.000348317	0.894477061	0.018634939	OPRL1
Pancreas	T2DM	ENSG00000127947.11	-0.006256751	0.001469256	1	0.045626317	PTPN12
Pancreas	T2DM	ENSG00000127957.12	-0.009593219	0.000181782	0.466817424	0.011385791	PMS2P3
Pancreas	T2DM	ENSG00000129933.16	-0.008815974	7.45E-06	0.019127487	0.001195468	MAU2
Pancreas	T2DM	ENSG00000129990.10	0.006880814	0.000934898	1	0.035306133	SYT5
Pancreas	T2DM	ENSG00000134250.13	-0.007991324	2.28E-08	5.85E-05	8.36E-06	NOTCH2
Pancreas	T2DM	ENSG00000137731.9	-0.009595427	0.000810902	1	0.03305389	FXYD2
Pancreas	T2DM	ENSG00000140400.10	-0.002869621	1.43E-05	0.036608117	0.002096587	MAN2C1
Pancreas	T2DM	ENSG00000141699.6	0.007479622	0.001499514	1	0.045626317	FAM134C
Pancreas	T2DM	ENSG00000143442.17	0.006702144	0.000420847	1	0.020225759	POGZ
Pancreas	T2DM	ENSG00000144619.10	-0.005594357	0.000623919	1	0.027624536	CNTN4

Pancreas	T2DM	ENSG00000146830.9	-0.010270288	0.000309471	0.794721893	0.017915451	GIGYF1
Pancreas	T2DM	ENSG00000148396.14	0.005482624	0.000902131	1	0.035306133	SEC16A
Pancreas	T2DM	ENSG00000149084.7	-0.002294707	0.000124611	0.320000702	0.009142877	HSD17B12
Pancreas	T2DM	ENSG00000149089.8	-0.002348758	0.000404386	1	0.01997046	APIP
Pancreas	T2DM	ENSG00000152104.7	-0.0067859	0.000325618	0.836187396	0.018177987	PTPN14
Pancreas	T2DM	ENSG00000153814.7	-0.014963784	1.35E-16	3.46E-13	1.73E-13	JAZF1
Pancreas	T2DM	ENSG00000154222.10	-0.003491239	0.001510217	1	0.045626317	CC2D1B
Pancreas	T2DM	ENSG00000158008.5	-0.007988942	0.001004224	1	0.036840659	EXTL1
Pancreas	T2DM	ENSG00000160161.5	-0.00635158	9.02E-05	0.231587465	0.007470563	CILP2
Pancreas	T2DM	ENSG00000165806.15	0.004285223	0.00016621	0.426826694	0.010998318	CASP7
Pancreas	T2DM	ENSG00000166165.8	-0.006736253	0.000782146	1	0.032927049	CKB
Pancreas	T2DM	ENSG00000166228.4	-0.005793195	0.000914678	1	0.035306133	PCBD1
Pancreas	T2DM	ENSG00000169592.10	-0.006715716	3.58E-05	0.091878487	0.003994717	INO80E
Pancreas	T2DM	ENSG00000171282.9	0.009477228	0.001166528	1	0.040481675	RP11-1055B8.7
Pancreas	T2DM	ENSG00000172543.3	-0.002823507	0.001482795	1	0.045626317	CTSW
Pancreas	T2DM	ENSG00000175287.14	0.005441456	0.000639173	1	0.027820271	PHYHD1
Pancreas	T2DM	ENSG00000177508.11	0.034536061	1.01E-24	2.59E-21	2.59E-21	IRX3
Pancreas	T2DM	ENSG00000179115.6	-0.009705202	6.68E-05	0.171549729	0.006353694	FARSA
Pancreas	T2DM	ENSG00000179344.12	-0.005587074	0.000205921	0.528805957	0.012297813	HLA-DQB1
Pancreas	T2DM	ENSG00000182179.6	-0.006125673	3.55E-06	0.00911166	0.000607444	UBA7
Pancreas	T2DM	ENSG00000182196.9	0.006299	0.001046325	1	0.037844535	ARL6IP4
Pancreas	T2DM	ENSG00000182853.7	0.009116853	0.000428728	1	0.020225759	VMO1
Pancreas	T2DM	ENSG00000182986.8	0.002505139	0.00049976	1	0.022515516	ZNF320
Pancreas	T2DM	ENSG00000196126.6	-0.007545411	2.19E-06	0.005615803	0.000431985	HLA-DRB1
Pancreas	T2DM	ENSG00000197181.7	-0.006174536	0.000981695	1	0.036536121	PIWIL2
Pancreas	T2DM	ENSG00000198231.8	0.005185353	0.001290622	1	0.041428975	DDX42
Pancreas	T2DM	ENSG00000204301.5	-0.008497019	2.33E-07	0.00059716	5.43E-05	NOTCH4
Pancreas	T2DM	ENSG00000204308.6	-0.003071697	2.44E-05	0.06277343	0.003239188	RNF5
Pancreas	T2DM	ENSG00000204618.4	0.00587238	0.000192334	0.493914241	0.011759863	RNF39
Pancreas	T2DM	ENSG00000206344.6	0.004772884	5.00E-05	0.128287656	0.005131506	HCG27
Pancreas	T2DM	ENSG00000212127.5	0.004598987	0.001135688	1	0.040083532	TAS2R14
Pancreas	T2DM	ENSG00000213221.4	0.003469077	0.000156025	0.400672926	0.010998318	DNLZ
Pancreas	T2DM	ENSG00000213676.6	0.003302169	0.000333084	0.855358434	0.018199116	ATF6B
Pancreas	T2DM	ENSG00000221988.8	0.010536438	2.52E-05	0.064783756	0.003239188	PPT2
Pancreas	T2DM	ENSG00000226232.4	-0.005851814	0.000172863	0.443911521	0.011097788	RP11-419C5.2
Pancreas	T2DM	ENSG00000229358.3	0.008032456	0.001283993	1	0.041428975	DPY19L1P1
Pancreas	T2DM	ENSG00000229391.3	0.005808094	4.89E-09	1.26E-05	3.14E-06	HLA-DRB6
Pancreas	T2DM	ENSG00000232629.4	0.003697468	2.71E-05	0.069568765	0.003312798	HLA-DQB2
Pancreas	T2DM	ENSG00000232709.1	0.01625128	1.64E-07	0.000420512	4.67E-05	MARK2P9
Pancreas	T2DM	ENSG00000233609.3	0.006472533	0.001447058	1	0.045626317	RP11-62H7.2
Pancreas	T2DM	ENSG00000234745.5	0.003641763	0.00016404	0.421253663	0.010998318	HLA-B
Pancreas	T2DM	ENSG00000237541.3	0.008534871	2.15E-09	5.52E-06	1.84E-06	HLA-DQA2
Pancreas	T2DM	ENSG00000242142.1	0.005630956	1.47E-05	0.037738565	0.002096587	SERBP1P3
Pancreas	T2DM	ENSG00000243753.1	0.003968251	0.000167031	0.428934408	0.010998318	HLA-L
Pancreas	T2DM	ENSG00000243789.6	0.010325273	3.85E-05	0.098896375	0.004120682	JMJD7
Pancreas	T2DM	ENSG00000244731.3	-0.003821207	0.000370817	0.952258785	0.019433853	C4A
Pancreas	T2DM	ENSG00000247157.2	0.002652802	0.001267528	1	0.041428975	RP11-434C1.1
Pancreas	T2DM	ENSG00000248278.1	0.010742648	7.85E-09	2.02E-05	4.03E-06	SUMO2P17
Pancreas	T2DM	ENSG00000251417.1	-0.011279226	0.000101545	0.260767811	0.007669642	RP11-1348G14.4
Pancreas	T2DM	ENSG00000255837.1	0.005339102	0.000390917	1	0.01997046	TAS2R20
Pancreas	T2DM	ENSG00000260274.1	-0.00863994	3.34E-06	0.008587928	0.000607444	RP11-817O13.8
Pancreas	T2DM	ENSG00000260814.2	-0.009833664	6.40E-05	0.164258423	0.006317632	RP11-107F6.3
Pancreas	T2DM	ENSG00000260911.1	0.003020388	0.001139446	1	0.040083532	RP11-196G11.2
Pancreas	T2DM	ENSG00000268225.1	0.005456772	0.00080574	1	0.03305389	CTD-2331H12.5
Pancreas	T2DM_admission	ENSG00000004534.10	-0.004083911	5.37E-08	0.000137948	1.25E-05	RBM6
Pancreas	T2DM_admission	ENSG00000009950.11	0.006173441	0.000102825	0.26405371	0.005063095	MLXIPL
Pancreas	T2DM_admission	ENSG00000010310.4	-0.012730988	2.66E-06	0.00682726	0.00031033	GIPR
Pancreas	T2DM_admission	ENSG00000011523.9	0.00800987	2.44E-11	6.25E-08	1.56E-08	CEP68
Pancreas	T2DM_admission	ENSG00000030110.8	0.015031688	1.71E-08	4.40E-05	5.50E-06	BAK1
Pancreas	T2DM_admission	ENSG00000066294.10	0.007160392	0.000211199	0.542359703	0.00904011	CD84
Pancreas	T2DM_admission	ENSG00000070182.13	0.007574605	0.001475735	1	0.033836506	SPTB
Pancreas	T2DM_admission	ENSG00000071564.10	-0.012807298	2.03E-05	0.052127595	0.001468422	TCF3
Pancreas	T2DM_admission	ENSG00000073849.10	0.010792528	0.000161688	0.415215244	0.007414558	ST6GAL1
Pancreas	T2DM_admission	ENSG00000075413.13	-0.01756302	2.27E-08	5.83E-05	6.47E-06	MARK3
Pancreas	T2DM_admission	ENSG00000095485.12	-0.002692489	0.000855789	1	0.022656349	CWF19L1
Pancreas	T2DM_admission	ENSG00000103479.10	0.007222671	0.000446474	1	0.014331807	RBL2
Pancreas	T2DM_admission	ENSG00000106460.14	0.014501798	0.000110616	0.284062315	0.005260413	TMEM106B
Pancreas	T2DM_admission	ENSG00000108272.9	0.009844182	0.000467426	1	0.014819145	DHRS11
Pancreas	T2DM_admission	ENSG00000108786.6	-0.006265681	7.88E-05	0.20223339	0.004302838	HSD17B1
Pancreas	T2DM_admission	ENSG00000108797.7	0.009343987	8.66E-06	0.022243711	0.000794418	CNTNAP1
Pancreas	T2DM_admission	ENSG00000109332.15	0.008572443	0.001747972	1	0.038040607	UBE2D3
Pancreas	T2DM_admission	ENSG00000110435.7	0.005635664	0.000406141	1	0.013545063	PDHX
Pancreas	T2DM_admission	ENSG00000111328.2	0.00700922	1.82E-05	0.046783846	0.001443011	CDK2AP1
Pancreas	T2DM_admission	ENSG00000112992.12	-0.005681812	0.00226552	1	0.046778499	NNT
Pancreas	T2DM_admission	ENSG00000118997.9	-0.005559873	0.000796877	1	0.021449685	DNAH7
Pancreas	T2DM_admission	ENSG00000120662.11	0.007685578	0.001543586	1	0.034468939	MTRF1
Pancreas	T2DM_admission	ENSG00000120709.6	-0.009370029	0.000391155	1	0.013393155	FAM53C
Pancreas	T2DM_admission	ENSG00000121417.9	0.004836372	0.000284686	0.731073748	0.010911548	ZNF211
Pancreas	T2DM_admission	ENSG00000122008.11	-0.01264184	7.06E-05	0.181426286	0.004031695	POLK
Pancreas	T2DM_admission	ENSG00000122025.10	0.012704947	9.78E-05	0.251273667	0.004964677	FLT3
Pancreas	T2DM_admission	ENSG00000125510.11	-0.008957477	4.51E-05	0.1157578	0.002756138	OPRL1
Pancreas	T2DM_admission	ENSG00000127220.5	0.011263469	0.000969452	1	0.02417042	ABHD8
Pancreas	T2DM_admission	ENSG00000127957.12	-0.011578497	8.50E-05	0.218336501	0.004548677	PMS2P3
Pancreas	T2DM_admission	ENSG00000129933.16	-0.01085965	1.58E-06	0.004056744	0.000213513	MAU2
Pancreas	T2DM_admission	ENSG00000130338.8	0.006364416	0.002335635	1	0.047333554	TULP4
Pancreas	T2DM_admission	ENSG00000131323.10	0.010107775	0.000377244	0.968763585	0.013188885	TRAF3
Pancreas	T2DM_admission	ENSG00000134250.13	-0.01084365	4.18E-11	1.07E-07	2.15E-08	NOTCH2
Pancreas	T2DM_admission	ENSG00000137731.9	-0.011652882	0.000403358	1	0.013545063	FXYD2
Pancreas	T2DM_admission	ENSG00000137877.8	0.006688412	0.000603246	1	0.017806153	SPTBN5
Pancreas	T2DM_admission	ENSG00000140105.13	-0.00801426	0.002276991	1	0.046778499	WARS
Pancreas	T2DM_admission	ENSG00000140400.10	-0.003381778	8.63E-06	0.022165888	0.000794418	MAN2C1
Pancreas	T2DM_admission	ENSG00000141560.10	-0.002701629	0.002461443	1	0.048622958	FN3KRP
Pancreas	T2DM_admission	ENSG00000141699.6	0.009972005	0.000231525	0.59455591	0.009437395	FAM134C

Pancreas	T2DM_admission	ENSG00000142459.4	0.011132138	0.000980269	1	0.024205102	EVI5L
Pancreas	T2DM_admission	ENSG00000143442.17	0.008559146	8.94E-05	0.229564541	0.004684991	POGZ
Pancreas	T2DM_admission	ENSG00000144354.9	0.008407045	0.00152402	1	0.03433055	CDCA7
Pancreas	T2DM_admission	ENSG00000146830.9	-0.009931678	0.00241051	1	0.048360847	GIGYF1
Pancreas	T2DM_admission	ENSG00000148384.11	0.011727967	0.000228947	0.587935065	0.009437395	INPP5E
Pancreas	T2DM_admission	ENSG00000148396.14	0.007144747	0.000168028	0.43149599	0.007570105	SEC16A
Pancreas	T2DM_admission	ENSG00000149084.7	-0.003366714	9.74E-07	0.002501751	0.000156359	HSD17B12
Pancreas	T2DM_admission	ENSG00000149089.8	-0.002765679	0.000291134	0.74763117	0.010994576	APIP
Pancreas	T2DM_admission	ENSG00000152104.7	-0.007931525	0.000258075	0.662737678	0.01004148	PTPN14
Pancreas	T2DM_admission	ENSG00000152359.10	-0.004003392	1.58E-05	0.040614291	0.001310138	POC5
Pancreas	T2DM_admission	ENSG00000153814.7	-0.019917769	1.02E-21	2.62E-18	1.31E-18	JAZF1
Pancreas	T2DM_admission	ENSG00000158008.5	-0.009607447	0.000579648	1	0.017308569	EXTL1
Pancreas	T2DM_admission	ENSG00000160161.5	-0.007941133	2.06E-05	0.05286319	0.001468422	CILP2
Pancreas	T2DM_admission	ENSG00000163486.8	0.011089539	5.68E-05	0.145772969	0.003390069	SRGAP2
Pancreas	T2DM_admission	ENSG00000163596.12	-0.003561478	0.000375795	0.965040757	0.013188885	ICA1L
Pancreas	T2DM_admission	ENSG00000164039.10	-0.012681047	0.000116967	0.300371847	0.005461306	BDH2
Pancreas	T2DM_admission	ENSG00000165806.15	0.005547722	2.23E-05	0.057269899	0.001547835	CASP7
Pancreas	T2DM_admission	ENSG00000166037.6	-0.006004083	0.001510123	1	0.034318558	CEP57
Pancreas	T2DM_admission	ENSG00000166165.8	-0.00819247	0.000380054	0.975977497	0.013188885	CKB
Pancreas	T2DM_admission	ENSG00000166224.12	-0.006799718	0.000638376	1	0.018419672	SGPL1
Pancreas	T2DM_admission	ENSG00000166228.4	-0.008258884	3.93E-05	0.100905808	0.002522645	PCBD1
Pancreas	T2DM_admission	ENSG00000167543.11	-0.007448719	0.002050509	1	0.043161544	TP53I13
Pancreas	T2DM_admission	ENSG00000168936.6	-0.004770644	0.000318695	0.818407761	0.011691539	TMEM129
Pancreas	T2DM_admission	ENSG00000169085.7	0.002628433	0.001820281	1	0.039281359	C8orf46
Pancreas	T2DM_admission	ENSG00000169592.10	-0.007456002	6.56E-05	0.168518426	0.003829964	INO80E
Pancreas	T2DM_admission	ENSG00000171282.9	0.011583739	0.000556064	1	0.016799675	RP11-1055B8.7
Pancreas	T2DM_admission	ENSG00000171700.9	-0.012516904	0.00096127	1	0.02417042	RGS19
Pancreas	T2DM_admission	ENSG00000171962.13	0.006632691	0.002493368	1	0.048877622	LRRC48
Pancreas	T2DM_admission	ENSG00000172379.14	0.010953569	0.002178824	1	0.045489587	ARNT2
Pancreas	T2DM_admission	ENSG00000172543.3	-0.0034789	0.000658865	1	0.018799603	CTSW
Pancreas	T2DM_admission	ENSG00000172766.14	0.007166531	0.00023918	0.614214725	0.009597105	NAA16
Pancreas	T2DM_admission	ENSG00000172803.13	0.006369343	0.00053312	1	0.016386194	SNX32
Pancreas	T2DM_admission	ENSG00000172922.4	-0.007626802	0.000535997	1	0.016386194	RNASEH2C
Pancreas	T2DM_admission	ENSG00000173517.6	0.009606885	9.86E-05	0.253198519	0.004964677	PEAK1
Pancreas	T2DM_admission	ENSG00000174851.10	-0.004649186	0.000710318	1	0.019613942	YIF1A
Pancreas	T2DM_admission	ENSG00000175213.2	-0.008626582	0.001712257	1	0.037581849	ZNF408
Pancreas	T2DM_admission	ENSG00000176920.10	0.002119068	0.001226974	1	0.028907053	FUT2
Pancreas	T2DM_admission	ENSG00000177103.9	-0.01109344	0.001188154	1	0.028251653	DSCAML1
Pancreas	T2DM_admission	ENSG00000177508.11	0.043414047	3.06E-29	7.87E-26	7.87E-26	IRX3
Pancreas	T2DM_admission	ENSG00000178449.4	0.013288302	3.78E-05	0.097093342	0.002489573	COX14
Pancreas	T2DM_admission	ENSG00000179115.6	-0.014549899	1.99E-07	0.000511908	3.59E-05	FARSA
Pancreas	T2DM_admission	ENSG00000179218.9	0.006814805	0.002437495	1	0.048523163	CALR
Pancreas	T2DM_admission	ENSG00000179344.12	-0.006158764	0.000372283	0.956022431	0.013188885	HLA-DQB1
Pancreas	T2DM_admission	ENSG00000182179.6	-0.006853497	6.42E-06	0.016486244	0.000634086	UBA7
Pancreas	T2DM_admission	ENSG00000182196.9	0.009413479	2.04E-05	0.052265626	0.001468422	ARL6IP4
Pancreas	T2DM_admission	ENSG00000182853.7	0.010182242	0.000622931	1	0.018178262	VMO1
Pancreas	T2DM_admission	ENSG00000182986.8	0.002605872	0.001634655	1	0.036187889	ZNF320
Pancreas	T2DM_admission	ENSG00000184110.10	0.006701508	0.001251815	1	0.029224201	EIF3C
Pancreas	T2DM_admission	ENSG00000184207.8	-0.00888625	0.000748824	1	0.020457225	PGP
Pancreas	T2DM_admission	ENSG00000185800.7	-0.008368071	0.000419679	1	0.013642227	DMWD
Pancreas	T2DM_admission	ENSG00000189398.5	-0.009125156	0.001107595	1	0.02658228	OR7E12P
Pancreas	T2DM_admission	ENSG00000196126.6	-0.009983638	5.03E-08	0.000129215	1.25E-05	HLA-DRB1
Pancreas	T2DM_admission	ENSG00000197062.7	-0.003698379	0.001283949	1	0.029704334	RP5-874C20.3
Pancreas	T2DM_admission	ENSG00000198231.8	0.006915491	0.000188997	0.48534387	0.008367998	DDX42
Pancreas	T2DM_admission	ENSG00000198502.5	-0.003050984	1.20E-05	0.030809414	0.00102698	HLA-DRB5
Pancreas	T2DM_admission	ENSG00000203896.5	-0.005821256	0.000417944	1	0.013642227	LIME1
Pancreas	T2DM_admission	ENSG00000204301.5	-0.010163301	7.43E-08	0.000190837	1.59E-05	NOTCH4
Pancreas	T2DM_admission	ENSG00000204305.9	0.010503577	0.000504287	1	0.015792794	AGER
Pancreas	T2DM_admission	ENSG00000204308.6	-0.003832033	4.66E-06	0.011978467	0.000479139	RNF5
Pancreas	T2DM_admission	ENSG00000204536.9	0.005460227	0.0025594	1	0.049791969	CCHCR1
Pancreas	T2DM_admission	ENSG00000204618.4	0.007550476	3.04E-05	0.078039375	0.002053668	RNF39
Pancreas	T2DM_admission	ENSG00000206344.6	0.006293701	3.28E-06	0.008433577	0.000351399	HCG27
Pancreas	T2DM_admission	ENSG00000213221.4	0.003583132	0.000680445	1	0.019202006	DNLZ
Pancreas	T2DM_admission	ENSG00000213676.6	0.004333867	4.20E-05	0.107791619	0.002629064	ATF6B
Pancreas	T2DM_admission	ENSG00000221988.8	0.011155718	0.000104495	0.268344037	0.005063095	PPT2
Pancreas	T2DM_admission	ENSG00000224997.1	-0.010115069	0.002340873	1	0.047333554	AL049840.1
Pancreas	T2DM_admission	ENSG00000226232.4	-0.00933826	1.85E-07	0.000476149	3.59E-05	RP11-419C5.2
Pancreas	T2DM_admission	ENSG00000228536.1	-0.011339387	0.000801857	1	0.021449685	RP11-392O17.1
Pancreas	T2DM_admission	ENSG00000228782.3	0.010024914	0.00202592	1	0.042996391	MRPL45P2
Pancreas	T2DM_admission	ENSG00000229358.3	0.010599914	0.000219259	0.563056426	0.009230433	DPY19L1P1
Pancreas	T2DM_admission	ENSG00000229391.3	0.008072664	1.51E-12	3.87E-09	1.29E-09	HLA-DRB6
Pancreas	T2DM_admission	ENSG00000232629.4	0.004006725	7.63E-05	0.195854535	0.004257707	HLA-DQB2
Pancreas	T2DM_admission	ENSG00000232709.1	0.018519455	2.10E-07	0.000538064	3.59E-05	MARK2P9
Pancreas	T2DM_admission	ENSG00000233609.3	0.007748022	0.00091124	1	0.023400641	RP11-62H7.2
Pancreas	T2DM_admission	ENSG00000234745.5	0.004756387	1.85E-05	0.047619377	0.001443011	HLA-B
Pancreas	T2DM_admission	ENSG00000235728.1	0.01061569	0.000900044	1	0.023400641	AC007349.5
Pancreas	T2DM_admission	ENSG00000237541.3	0.010257914	3.89E-10	1.00E-06	1.67E-07	HLA-DQA2
Pancreas	T2DM_admission	ENSG00000242142.1	0.006560262	1.13E-05	0.028925455	0.000997429	SERBP1P3
Pancreas	T2DM_admission	ENSG00000243753.1	0.005638929	3.27E-06	0.008400931	0.000351399	HLA-L
Pancreas	T2DM_admission	ENSG00000243789.6	0.013769293	1.80E-06	0.004617304	0.000230865	JMJD7
Pancreas	T2DM_admission	ENSG00000244731.3	-0.004520624	0.000248734	0.638747882	0.00982689	C4A
Pancreas	T2DM_admission	ENSG00000248278.1	0.012578111	4.14E-09	1.06E-05	1.52E-06	SUMO2P17
Pancreas	T2DM_admission	ENSG00000251417.1	-0.016108067	1.38E-06	0.003531518	0.000196195	RP11-1348G14.4
Pancreas	T2DM_admission	ENSG00000255837.1	0.005865702	0.000701718	1	0.019587077	TAS2R20
Pancreas	T2DM_admission	ENSG00000256166.1	0.005070308	0.000211218	0.542406597	0.00904011	XXbac-BPG248L24.13
Pancreas	T2DM_admission	ENSG00000256269.2	0.003973338	0.000305163	0.783659739	0.011357388	HMBS
Pancreas	T2DM_admission	ENSG00000259015.1	0.010991632	0.00090317	1	0.023400641	RP11-109N23.6
Pancreas	T2DM_admission	ENSG00000259982.1	0.00376819	0.001090039	1	0.02640773	CDC37P1
Pancreas	T2DM_admission	ENSG00000260274.1	-0.010065856	2.47E-06	0.006334161	0.000301627	RP11-817O13.8
Pancreas	T2DM_admission	ENSG00000260814.2	-0.013790315	1.08E-06	0.002770896	0.000162994	RP11-107F6.3
Pancreas	T2DM_admission	ENSG00000260911.1	0.00351099	0.001002146	1	0.024509633	RP11-196G11.2
Pancreas	T2DM_admission	ENSG00000268225.1	0.00579192	0.001976261	1	0.042291985	CTD-2331H12.5
Pancreas	T2DM_admission	ENSG00000271751.1	-0.006650697	0.000955898	1	0.02417042	RP11-110I1.14

Pancreas	T2DM_insulin	ENSG00000011523.9	0.001833919	0.000118228	0.303610495	0.0202407	CEP68
Pancreas	T2DM_insulin	ENSG00000156219.12	-0.001326335	3.08E-05	0.07912119	0.006593433	ART3
Pancreas	T2DM_insulin	ENSG00000158008.5	-0.004053963	0.000255866	0.657064958	0.03865088	EXTL1
Pancreas	T2DM_insulin	ENSG00000169248.8	0.001353192	2.35E-05	0.060260938	0.005478267	CXCL11
Pancreas	T2DM_insulin	ENSG00000177508.11	0.009364345	1.08E-09	2.78E-06	5.55E-07	IRX3
Pancreas	T2DM_insulin	ENSG00000179344.12	-0.003073255	7.74E-06	0.019877522	0.002208614	HLA-DQB1
Pancreas	T2DM_insulin	ENSG00000198502.5	-0.001748696	2.64E-10	6.79E-07	1.70E-07	HLA-DRB5
Pancreas	T2DM_insulin	ENSG00000204257.10	0.001495763	6.49E-06	0.016667122	0.00208339	HLA-DMA
Pancreas	T2DM_insulin	ENSG00000204301.5	-0.004171586	2.68E-08	6.88E-05	1.15E-05	NOTCH4
Pancreas	T2DM_insulin	ENSG00000204351.7	-0.001446573	1.79E-05	0.046073901	0.00460739	SKIV2L
Pancreas	T2DM_insulin	ENSG00000204540.6	-0.001441721	0.000111166	0.285475097	0.0202407	PSORS1C1
Pancreas	T2DM_insulin	ENSG00000204618.4	0.002611841	0.000280247	0.719673768	0.039981876	RNF39
Pancreas	T2DM_insulin	ENSG00000229391.3	0.003267528	5.59E-13	1.43E-09	1.43E-09	HLA-DRB6
Pancreas	T2DM_insulin	ENSG00000232629.4	0.002794741	3.69E-12	9.48E-09	4.74E-09	HLA-DQB2
Pancreas	T2DM_insulin	ENSG00000237541.3	0.004150398	1.81E-10	4.66E-07	1.55E-07	HLA-DQA2
Pancreas	T2DM_insulin	ENSG00000242142.1	0.002117592	0.000357618	0.918363457	0.048334919	SERBP1P3
Pancreas	T2DM_insulin	ENSG00000244731.3	-0.002611987	9.79E-08	0.000251466	3.59E-05	C4A
Pancreas	T2DM_insulin	ENSG00000251417.1	-0.004905942	0.00021283	0.546547838	0.03415924	RP11-1348G14.4
Pancreas	T2DM_insulin	ENSG00000256166.1	0.002186259	5.74E-05	0.147433792	0.011341061	XXbac-BPG248L24.13
SAT	CAD	ENSG00000001617.7	-0.014757532	5.22E-05	0.171185343	0.009510297	SEMA3F
SAT	CAD	ENSG00000004534.10	-0.002869433	0.000780112	1	0.049222079	RBM6
SAT	CAD	ENSG00000065060.12	0.007031212	8.23E-06	0.026991917	0.002453811	UHRF1BP1
SAT	CAD	ENSG00000075131.5	0.005546056	0.000164166	0.538627495	0.020332943	TIPIN
SAT	CAD	ENSG00000087301.4	-0.01651292	0.000196849	0.645862115	0.021008952	TXNDC16
SAT	CAD	ENSG00000089558.4	0.01354567	0.0006759	1	0.044352545	KCNH4
SAT	CAD	ENSG00000090621.9	-0.007792787	0.000618717	1	0.041428814	PABPC4
SAT	CAD	ENSG00000100997.14	0.014054407	0.000209189	0.686349333	0.021008952	ABHD12
SAT	CAD	ENSG00000101460.8	0.02217467	6.33E-06	0.020772867	0.002077287	MAP1LC3A
SAT	CAD	ENSG00000101464.6	0.011067404	0.000518914	1	0.037834601	PIGU
SAT	CAD	ENSG00000114023.11	-0.017405772	0.000401638	1	0.031912061	FAM162A
SAT	CAD	ENSG00000114200.5	-0.018311052	5.12E-05	0.168023676	0.009510297	BCHE
SAT	CAD	ENSG00000114491.9	0.018732502	0.000101289	0.332328498	0.015825167	UMPS
SAT	CAD	ENSG00000114554.7	0.012283305	0.000201054	0.659657844	0.021008952	PLXNA1
SAT	CAD	ENSG00000115486.7	0.009401774	1.23E-05	0.040309209	0.003359101	GGCX
SAT	CAD	ENSG00000119718.6	0.006710809	0.000398018	1	0.031912061	EIF2B2
SAT	CAD	ENSG00000124562.5	-0.008949368	5.83E-06	0.019112485	0.002077287	SNRPC
SAT	CAD	ENSG00000125388.15	0.004794537	0.00022184	0.727855892	0.021008952	GRK4
SAT	CAD	ENSG00000137054.11	0.008517123	0.000577705	1	0.040328707	POLR1E
SAT	CAD	ENSG00000138380.13	-0.016780187	1.74E-08	5.72E-05	4.20E-05	CARF
SAT	CAD	ENSG00000142046.10	0.011165307	0.000146644	0.481139752	0.019375329	TMEM91
SAT	CAD	ENSG00000143156.9	-0.022267702	5.16E-06	0.016939967	0.002077287	NME7
SAT	CAD	ENSG00000144426.14	-0.013521673	2.56E-08	8.40E-05	4.20E-05	NBEAL1
SAT	CAD	ENSG00000145020.10	-0.004954599	7.48E-05	0.245401176	0.012270059	AMT
SAT	CAD	ENSG00000145029.7	-0.008297714	7.13E-05	0.234070148	0.012270059	NICN1
SAT	CAD	ENSG00000149084.7	-0.003361131	0.00039596	1	0.031912061	HSD17B12
SAT	CAD	ENSG00000154309.7	0.010130475	0.000147633	0.484383225	0.019375329	DISP1
SAT	CAD	ENSG00000163431.11	-0.017129977	4.82E-05	0.158283524	0.009510297	LMOD1
SAT	CAD	ENSG00000163596.12	0.016834253	5.00E-08	0.000164174	5.47E-05	ICA1L
SAT	CAD	ENSG00000164062.8	-0.013190522	1.51E-06	0.00495548	0.000707926	APEH
SAT	CAD	ENSG00000164068.11	0.005461084	0.000221335	0.726200621	0.021008952	RNF123
SAT	CAD	ENSG00000169047.5	-0.013297668	5.00E-07	0.001639411	0.000327882	IRS1
SAT	CAD	ENSG00000175445.10	-0.020132155	8.84E-08	0.00028992	7.25E-05	LPL
SAT	CAD	ENSG00000176390.10	0.020520044	2.40E-05	0.078716095	0.006055084	CRLF3
SAT	CAD	ENSG00000181264.4	0.01218491	0.000266654	0.874891114	0.023645706	TMEM136
SAT	CAD	ENSG00000188895.7	-0.007167999	0.000229173	0.751916536	0.021008952	MSL1
SAT	CAD	ENSG00000196821.5	0.012407021	1.14E-06	0.003747179	0.00062453	C6orf106
SAT	CAD	ENSG00000196981.2	0.017052005	0.000408506	1	0.031912061	WDR5B
SAT	CAD	ENSG00000198270.8	-0.006554776	0.000230516	0.756322289	0.021008952	TMEM116
SAT	CAD	ENSG00000204978.2	-0.011016248	0.000134903	0.442615565	0.019244155	C19orf69
SAT	CAD	ENSG00000213277.3	-0.01436543	0.000219656	0.720691506	0.021008952	MARCKSL1P1
SAT	CAD	ENSG00000213689.5	0.007456711	0.000448786	1	0.033465169	TREX1
SAT	CAD	ENSG00000215452.3	-0.019187442	0.00033043	1	0.028529979	ZNF663P
SAT	CAD	ENSG00000249685.1	-0.011005016	0.000609622	1	0.041428814	RP11-360F5.3
SAT	CAD	ENSG00000256269.2	0.010887665	4.81E-05	0.157810392	0.009510297	HMBS
SAT	CAD	ENSG00000261143.1	-0.011409273	0.000128153	0.420471326	0.019112333	ADAMTS7P3
SAT	CAD	ENSG00000264538.2	0.005846046	0.000441234	1	0.033465169	SUZ12P
SAT	CAD	ENSG00000269001.1	-0.012157662	0.00021491	0.70512093	0.021008952	ZNF818P
SAT	CAD	ENSG00000269051.1	-0.019823445	0.000167324	0.548989452	0.020332943	CTD-2245F17.3
SAT	CAD	ENSG00000269202.1	-0.01010399	0.000775577	1	0.049222079	RP4-614O4.12
SAT	CAD	ENSG00000270018.1	0.014404178	0.000569487	1	0.040328707	RP3-462E2.5
SAT	CAD	ENSG00000271751.1	-0.018429148	4.29E-05	0.140719533	0.009510297	RP11-110I1.14
SAT	T2DM	ENSG00000001617.7	-0.01381935	7.46E-07	0.002447204	0.0001064	SEMA3F
SAT	T2DM	ENSG00000004534.10	-0.00357068	4.72E-08	0.000154812	1.19E-05	RBM6
SAT	T2DM	ENSG00000005955.8	-0.012528307	1.22E-06	0.003992826	0.000137684	GGNBP2
SAT	T2DM	ENSG00000011523.9	0.009709795	1.18E-09	3.87E-06	6.46E-07	CEP68
SAT	T2DM	ENSG00000037042.8	-0.008467234	7.67E-09	2.52E-05	2.80E-06	TUBG2
SAT	T2DM	ENSG00000055955.11	0.005059899	8.41E-06	0.027589044	0.000689726	ITIH4
SAT	T2DM	ENSG00000073578.12	0.007899305	0.001738269	1	0.044907563	SDHA
SAT	T2DM	ENSG00000077454.11	-0.00967636	0.000930098	1	0.027246889	LRCH4
SAT	T2DM	ENSG00000090621.9	-0.010218366	4.52E-09	1.48E-05	1.85E-06	PABPC4
SAT	T2DM	ENSG00000091542.8	-0.006521455	0.002000284	1	0.049345351	ALKBH5
SAT	T2DM	ENSG00000095485.12	-0.00414476	0.000116993	0.383854135	0.006618175	CWF19L1
SAT	T2DM	ENSG00000103479.10	0.005953016	0.000107304	0.352063407	0.006213443	RBL2
SAT	T2DM	ENSG00000104313.13	0.012667694	5.61E-05	0.184031207	0.003472287	EYA1
SAT	T2DM	ENSG00000105726.12	0.010044259	3.60E-07	0.001181578	6.22E-05	ATP13A1
SAT	T2DM	ENSG00000108272.9	-0.009583119	0.001686433	1	0.043914172	DHRS11
SAT	T2DM	ENSG00000108786.6	-0.006297286	4.16E-07	0.001363469	6.82E-05	HSD17B1
SAT	T2DM	ENSG00000111328.2	0.00583938	0.000140401	0.460654189	0.007718167	CDK2AP1
SAT	T2DM	ENSG00000111405.4	-0.010784648	0.001251031	1	0.034785013	ENDOU
SAT	T2DM	ENSG00000112276.9	-0.007489759	0.000818288	1	0.024700393	BVES
SAT	T2DM	ENSG00000115216.9	-0.012635055	8.70E-07	0.002854673	0.000109795	NRBP1
SAT	T2DM	ENSG00000115241.9	-0.017497646	4.51E-07	0.001479264	7.04E-05	PPM1G
SAT	T2DM	ENSG00000115561.10	-0.007094507	9.17E-06	0.030097647	0.000734089	CHMP3

SAT	T2DM	ENSG00000116157.5	-0.004077206	0.000543105	1	0.019368778	GPX7
SAT	T2DM	ENSG00000117280.8	-0.006528854	0.000784418	1	0.024351274	RAB7L1
SAT	T2DM	ENSG00000117877.6	-0.011219767	3.59E-05	0.117693459	0.002401907	CD3EAP
SAT	T2DM	ENSG00000118402.5	0.008916566	0.001651815	1	0.04335685	ELOVL4
SAT	T2DM	ENSG00000118997.9	-0.006637526	1.68E-05	0.055028986	0.001250659	DNAH7
SAT	T2DM	ENSG00000119242.4	-0.008684463	0.001120496	1	0.03169265	CCDC92
SAT	T2DM	ENSG00000120093.7	-0.010823248	4.23E-05	0.138943375	0.002749283	HOXB3
SAT	T2DM	ENSG00000120907.13	0.005156029	0.000181567	0.595721453	0.008583862	ADRA1A
SAT	T2DM	ENSG00000121101.11	0.01516347	0.000144956	0.475600177	0.00776917	TEX14
SAT	T2DM	ENSG00000125388.15	0.003432358	0.000553525	1	0.019528141	GRK4
SAT	T2DM	ENSG00000125510.11	-0.014209361	7.89E-06	0.025878571	0.000663553	OPRL1
SAT	T2DM	ENSG00000126464.9	0.006654648	0.000602382	1	0.02037543	PRR12
SAT	T2DM	ENSG00000127957.12	-0.007799487	9.84E-06	0.032271237	0.000768363	PMS2P3
SAT	T2DM	ENSG00000130299.12	-0.010153632	0.000748955	1	0.023857503	GTPBP3
SAT	T2DM	ENSG00000130921.3	0.0141348	0.000271393	0.890441137	0.011415912	C12orf65
SAT	T2DM	ENSG00000133059.12	-0.010468085	0.000791863	1	0.024351274	DSTYK
SAT	T2DM	ENSG00000135124.10	0.009671505	0.00186461	1	0.04742469	P2RX4
SAT	T2DM	ENSG00000135926.8	-0.008560775	0.001183505	1	0.033188714	TMBIM1
SAT	T2DM	ENSG00000137877.8	0.006913974	4.57E-05	0.149831054	0.002881366	SPTBN5
SAT	T2DM	ENSG00000138380.13	0.007705973	0.00072242	1	0.023237829	CARF
SAT	T2DM	ENSG00000140400.10	-0.00267677	2.84E-05	0.093085174	0.001980536	MAN2C1
SAT	T2DM	ENSG00000141252.15	-0.008527486	0.001477396	1	0.039732269	VPS53
SAT	T2DM	ENSG00000141452.5	-0.010261089	0.000141143	0.463090023	0.007718167	C18orf8
SAT	T2DM	ENSG00000141458.8	-0.016433383	7.27E-06	0.023857506	0.000627829	NPC1
SAT	T2DM	ENSG00000142082.10	0.011993739	0.000590798	1	0.020191744	SIRT3
SAT	T2DM	ENSG00000144426.14	0.006406391	0.000567287	1	0.019800723	NBEAL1
SAT	T2DM	ENSG00000144837.4	-0.010389887	0.002088159	1	0.049971446	PLA1A
SAT	T2DM	ENSG00000145692.10	-0.006711055	0.000153347	0.503132281	0.007957713	BHMT
SAT	T2DM	ENSG00000145725.15	-0.006594666	0.000103874	0.340811119	0.006196566	PPIP5K2
SAT	T2DM	ENSG00000147419.12	0.012799654	0.000517662	1	0.018677439	CCDC25
SAT	T2DM	ENSG00000149084.7	-0.004111705	1.50E-08	4.92E-05	4.10E-06	HSD17B12
SAT	T2DM	ENSG00000149089.8	-0.002683842	0.000165478	0.542934157	0.00820853	APIP
SAT	T2DM	ENSG00000149100.8	-0.010746473	0.000518027	1	0.018677439	EIF3M
SAT	T2DM	ENSG00000152219.4	-0.017520121	2.40E-07	0.000785911	4.37E-05	ARL14EP
SAT	T2DM	ENSG00000152359.10	-0.006039661	0.000224734	0.737353088	0.009831375	POC5
SAT	T2DM	ENSG00000153814.7	-0.030438495	8.69E-21	2.85E-17	1.42E-17	JAZF1
SAT	T2DM	ENSG00000154743.13	0.017860588	1.01E-06	0.003326096	0.000120866	TSEN2
SAT	T2DM	ENSG00000156170.8	-0.016574211	1.79E-06	0.005860807	0.000184017	NDUFAF6
SAT	T2DM	ENSG00000157322.12	-0.008678203	1.17E-08	3.84E-05	3.84E-06	CLEC18A
SAT	T2DM	ENSG00000157823.12	0.010120838	1.74E-10	5.71E-07	1.14E-07	AP3S2
SAT	T2DM	ENSG00000157895.7	0.008349819	0.002100165	1	0.049971446	C12orf43
SAT	T2DM	ENSG00000158022.6	0.008912887	0.001285858	1	0.03545294	TRIM63
SAT	T2DM	ENSG00000158805.7	-0.012003329	0.000633986	1	0.021225603	ZNF276
SAT	T2DM	ENSG00000160305.13	-0.003829623	0.000895113	1	0.02645824	DIP2A
SAT	T2DM	ENSG00000160752.10	0.008317422	0.001369688	1	0.03714006	FDPS
SAT	T2DM	ENSG00000163554.7	-0.004697565	0.002098839	1	0.049971446	SPTA1
SAT	T2DM	ENSG00000163596.12	-0.008032222	0.000679613	1	0.022298088	ICA1L
SAT	T2DM	ENSG00000163933.5	-0.013872735	3.10E-06	0.010154793	0.00029867	RFT1
SAT	T2DM	ENSG00000164039.10	-0.007877137	0.000229539	0.753118457	0.009909453	BDH2
SAT	T2DM	ENSG00000164068.11	0.005567757	8.70E-07	0.002854234	0.000109795	RNF123
SAT	T2DM	ENSG00000165178.8	0.008500072	0.0003751	1	0.014946268	NCF1C
SAT	T2DM	ENSG00000165476.8	-0.009984603	0.001043225	1	0.029763665	REEP3
SAT	T2DM	ENSG00000166166.8	-0.019052313	1.03E-06	0.003384238	0.000120866	TRMT61A
SAT	T2DM	ENSG00000166228.4	-0.005479857	0.000107945	0.354166225	0.006213443	PCBD1
SAT	T2DM	ENSG00000166278.10	0.01966209	2.08E-06	0.006826591	0.000206866	C2
SAT	T2DM	ENSG00000167397.10	-0.010582631	0.000697092	1	0.022645139	VKORC1
SAT	T2DM	ENSG00000167528.8	0.004292774	0.001998061	1	0.049345351	ZNF641
SAT	T2DM	ENSG00000167930.11	0.010005045	0.00149276	1	0.039819067	ITFG3
SAT	T2DM	ENSG00000168056.10	0.011064621	0.000208443	0.68390041	0.009368499	LTBP3
SAT	T2DM	ENSG00000168297.11	0.011804575	0.000155225	0.509293604	0.007957713	PXK
SAT	T2DM	ENSG00000168394.9	-0.021736455	1.78E-07	0.000582522	3.64E-05	TAP1
SAT	T2DM	ENSG00000169047.5	-0.021698235	8.68E-27	2.85E-23	2.85E-23	IRS1
SAT	T2DM	ENSG00000169592.10	-0.008776437	0.000642758	1	0.021301893	INO80E
SAT	T2DM	ENSG00000170092.10	0.009154091	0.001007324	1	0.029248051	AC018720.10
SAT	T2DM	ENSG00000170571.7	-0.011055356	0.000260389	0.854336845	0.011095284	EMB
SAT	T2DM	ENSG00000171700.9	-0.010305651	1.91E-07	0.000626829	3.69E-05	RGS19
SAT	T2DM	ENSG00000171723.11	-0.008564429	0.001354533	1	0.037035189	GPHN
SAT	T2DM	ENSG00000172037.9	-0.014680252	1.74E-05	0.057116562	0.001269257	LAMB2
SAT	T2DM	ENSG00000172247.3	-0.00357062	0.000186796	0.612876884	0.008632069	C1QTNF4
SAT	T2DM	ENSG00000173511.5	-0.010267639	6.96E-06	0.022826296	0.000616927	VEGFB
SAT	T2DM	ENSG00000173917.9	-0.010213979	3.31E-05	0.10844693	0.002259311	HOXB2
SAT	T2DM	ENSG00000175334.3	0.007415485	0.000496071	1	0.018287726	BANF1
SAT	T2DM	ENSG00000178750.2	0.013686705	4.27E-05	0.140213418	0.002749283	STX19
SAT	T2DM	ENSG00000179115.6	-0.018846323	1.58E-06	0.005183293	0.000172776	FARSA
SAT	T2DM	ENSG00000179218.9	0.016085737	2.62E-05	0.085805061	0.001865327	CALR
SAT	T2DM	ENSG00000182986.8	0.002406435	0.000887987	1	0.02645824	ZNF320
SAT	T2DM	ENSG00000183779.5	-0.013951765	6.77E-05	0.22220762	0.004114956	ZNF703
SAT	T2DM	ENSG00000184110.10	0.007364308	0.000483352	1	0.018228475	EIF3C
SAT	T2DM	ENSG00000185904.7	0.013950684	0.000416109	1	0.016061818	LINC00839
SAT	T2DM	ENSG00000187166.1	0.005316135	0.000791175	1	0.024351274	H1FNT
SAT	T2DM	ENSG00000187778.9	0.010189324	0.001547911	1	0.040957227	MCRS1
SAT	T2DM	ENSG00000189398.5	-0.011963901	0.002117047	1	0.049971446	OR7E12P
SAT	T2DM	ENSG00000196126.6	-0.016018717	1.11E-07	0.000365797	2.44E-05	HLA-DRB1
SAT	T2DM	ENSG00000196693.10	-0.005335639	0.000326552	1	0.013392728	ZNF33B
SAT	T2DM	ENSG00000198056.9	-0.011652568	0.000183136	0.600870316	0.008583862	PRIM1
SAT	T2DM	ENSG00000198453.8	0.011664832	0.000575495	1	0.019875797	ZNF568
SAT	T2DM	ENSG00000198502.5	-0.002858768	0.00033098	1	0.013406744	HLA-DRB5
SAT	T2DM	ENSG00000203896.5	-0.008794209	0.000405167	1	0.015825616	LIME1
SAT	T2DM	ENSG00000204237.4	0.011762554	0.001893709	1	0.047650044	OXLD1
SAT	T2DM	ENSG00000204287.9	0.014789878	2.87E-09	9.41E-06	1.34E-06	HLA-DRA
SAT	T2DM	ENSG00000204622.6	0.004299143	0.002105722	1	0.049971446	HLA-J
SAT	T2DM	ENSG00000205583.9	-0.004911484	0.000215074	0.705657617	0.009535914	STAG3L1
SAT	T2DM	ENSG00000205659.6	0.010944158	0.000146812	0.48168855	0.00776917	LIN52

SAT	T2DM	ENSG00000206344.6	0.004371075	6.79E-07	0.002227894	0.000101268	HCG27
SAT	T2DM	ENSG00000213221.4	0.00343402	0.000193347	0.63436993	0.008810693	DNLZ
SAT	T2DM	ENSG00000214530.3	0.005513693	8.07E-07	0.002647908	0.000109795	STARD10
SAT	T2DM	ENSG00000223756.2	-0.009024297	0.000453644	1	0.017307053	TSSC2
SAT	T2DM	ENSG00000226232.4	-0.005889907	1.79E-06	0.00588853	0.000184017	RP11-419C5.2
SAT	T2DM	ENSG00000229391.3	0.007004399	1.89E-11	6.22E-08	2.07E-08	HLA-DRB6
SAT	T2DM	ENSG00000230953.2	-0.012322511	0.000820586	1	0.024700393	RP4-631H13.6
SAT	T2DM	ENSG00000231852.2	0.006404531	4.68E-06	0.015356883	0.000430718	CYP21A2
SAT	T2DM	ENSG00000235081.1	0.013909923	0.000167623	0.549971522	0.00820853	AC010492.2
SAT	T2DM	ENSG00000237541.3	0.014020545	3.25E-11	1.07E-07	2.66E-08	HLA-DQA2
SAT	T2DM	ENSG00000237984.2	-0.01208302	0.001902516	1	0.047650044	PTENP1
SAT	T2DM	ENSG00000239557.1	0.013394316	0.001026754	1	0.029550704	RP11-168J18.6
SAT	T2DM	ENSG00000242600.2	0.00749259	0.000378098	1	0.014946268	MBL1P
SAT	T2DM	ENSG00000243708.4	0.007847131	6.40E-08	0.000210073	1.50E-05	PLA2G4B
SAT	T2DM	ENSG00000243789.6	0.012285133	1.50E-08	4.91E-05	4.10E-06	JMJD7
SAT	T2DM	ENSG00000246523.3	-0.009302668	0.000282444	0.926699671	0.011730376	RP11-736K20.6
SAT	T2DM	ENSG00000253813.1	0.009791969	0.000167324	0.548990588	0.00820853	COX6B1P4
SAT	T2DM	ENSG00000253816.2	-0.008605434	0.001801112	1	0.046167565	RP11-1415C14.3
SAT	T2DM	ENSG00000256269.2	0.009006153	1.12E-05	0.036810281	0.000856053	HMBS
SAT	T2DM	ENSG00000258602.1	0.008314567	0.002044449	1	0.049971446	RP11-7F17.7
SAT	T2DM	ENSG00000260274.1	-0.004337331	0.000794144	1	0.024351274	RP11-817O13.8
SAT	T2DM	ENSG00000269963.1	-0.010815618	0.000493927	1	0.018287726	RP11-73M18.9
SAT	T2DM	ENSG00000271751.1	-0.012907761	0.00018156	0.595698457	0.008583862	RP11-110I1.14
SAT	T2DM	ENSG00000272221.1	-0.003233973	4.73E-06	0.01550583	0.000430718	XXbac-BPG181B23.7
SAT	Obesity	ENSG00000001617.7	-0.042278276	7.59E-24	2.49E-20	3.11E-21	SEMA3F
SAT	Obesity	ENSG00000004534.10	-0.010277366	1.41E-25	4.63E-22	6.62E-23	RBM6
SAT	Obesity	ENSG00000005955.8	-0.025349744	6.61E-11	2.17E-07	6.57E-09	GGNBP2
SAT	Obesity	ENSG00000010704.14	-0.016780294	1.51E-07	0.000495766	8.70E-06	HFE
SAT	Obesity	ENSG00000011295.11	0.020785063	3.82E-05	0.125325841	0.001046683	TTC19
SAT	Obesity	ENSG00000013275.3	0.012990825	0.000773095	1	0.012176893	PSMC4
SAT	Obesity	ENSG00000026950.12	-0.006436415	0.001248371	1	0.017886049	BTN3A1
SAT	Obesity	ENSG00000029725.12	0.013705185	1.77E-05	0.058046705	0.00054761	RABEP1
SAT	Obesity	ENSG00000035115.17	-0.01384339	1.37E-05	0.044874705	0.000439948	SH3YL1
SAT	Obesity	ENSG00000041802.6	0.003202284	0.002899481	1	0.035691396	LSG1
SAT	Obesity	ENSG00000049449.4	-0.017530283	0.001091641	1	0.016106882	RCN1
SAT	Obesity	ENSG00000050130.13	0.010533917	0.003623772	1	0.042161685	JKAMP
SAT	Obesity	ENSG00000053900.6	0.016325778	0.000596746	1	0.009888499	ANAPC4
SAT	Obesity	ENSG00000054282.11	0.010568712	0.000253019	0.830156695	0.004854718	SDCCAG8
SAT	Obesity	ENSG00000055955.11	0.010930379	1.57E-10	5.15E-07	1.47E-08	ITIH4
SAT	Obesity	ENSG00000058063.11	0.008410033	0.002324537	1	0.02956126	ATP11B
SAT	Obesity	ENSG00000064995.12	-0.008993872	0.001731465	1	0.022999738	TAF11
SAT	Obesity	ENSG00000065060.12	0.017877663	7.04E-23	2.31E-19	2.31E-20	UHRF1BP1
SAT	Obesity	ENSG00000071794.11	-0.011929378	0.004136335	1	0.046318486	HLTF
SAT	Obesity	ENSG00000075131.5	0.007142579	2.48E-05	0.081257563	0.00073805	TIPIN
SAT	Obesity	ENSG00000076685.14	0.027327284	3.57E-06	0.011724467	0.000160609	NT5C2
SAT	Obesity	ENSG00000077380.11	0.024794871	4.63E-06	0.015187396	0.000192246	DYNC1I2
SAT	Obesity	ENSG00000082258.8	-0.009146466	6.69E-05	0.219488925	0.001602715	CCNT2
SAT	Obesity	ENSG00000083097.10	0.005179793	0.00429089	1	0.047531447	DOPEY1
SAT	Obesity	ENSG00000085365.13	0.017474778	0.000244244	0.80136467	0.00471391	SCAMP1
SAT	Obesity	ENSG00000087263.12	-0.020899752	5.74E-07	0.001884118	2.98E-05	OGFOD1
SAT	Obesity	ENSG00000090238.7	0.006676745	8.90E-09	2.92E-05	6.64E-07	YPEL3
SAT	Obesity	ENSG00000090621.9	-0.016852852	1.26E-10	4.15E-07	1.22E-08	PABPC4
SAT	Obesity	ENSG00000099956.13	0.00767829	0.001282303	1	0.018134645	SMARCB1
SAT	Obesity	ENSG00000100124.8	-0.012590642	8.06E-05	0.264521688	0.001862829	ANKRD54
SAT	Obesity	ENSG00000100225.13	0.013094019	0.00056832	1	0.009661437	FBXO7
SAT	Obesity	ENSG00000100418.7	0.009406871	2.48E-05	0.081304379	0.00073805	DESI1
SAT	Obesity	ENSG00000100697.10	0.009578081	0.000337454	1	0.006185402	DICER1
SAT	Obesity	ENSG00000100997.14	0.018638295	1.95E-05	0.063843665	0.00059667	ABHD12
SAT	Obesity	ENSG00000102595.14	-0.019278175	0.000893975	1	0.013579312	UGGT2
SAT	Obesity	ENSG00000102890.10	0.007148976	0.002061819	1	0.026738446	ELMO3
SAT	Obesity	ENSG00000103051.14	-0.020206062	1.48E-06	0.004853661	6.93E-05	COG4
SAT	Obesity	ENSG00000103351.8	0.009005263	0.0003845	1	0.00693156	CLUAP1
SAT	Obesity	ENSG00000103550.9	-0.017974194	4.37E-06	0.014325206	0.000186042	KNOP1
SAT	Obesity	ENSG00000104219.8	-0.011961943	8.71E-06	0.028562241	0.000299495	ZDHHC2
SAT	Obesity	ENSG00000104738.12	0.014983574	0.002942468	1	0.036023276	MCM4
SAT	Obesity	ENSG00000104852.10	-0.024042722	4.89E-05	0.16030374	0.001292772	SNRNP70
SAT	Obesity	ENSG00000104866.6	0.020159468	0.000490047	1	0.008507117	PPP1R37
SAT	Obesity	ENSG00000105656.8	-0.020595188	0.000712265	1	0.01139972	ELL
SAT	Obesity	ENSG00000105726.12	-0.013128225	9.77E-06	0.032061565	0.000323854	ATP13A1
SAT	Obesity	ENSG00000108384.10	-0.005753469	0.000421317	1	0.007477175	RAD51C
SAT	Obesity	ENSG00000108556.7	0.00785795	0.001099647	1	0.016106882	CHRNE
SAT	Obesity	ENSG00000110906.8	-0.007453852	0.001712482	1	0.022842502	KCTD10
SAT	Obesity	ENSG00000111364.11	0.010011036	9.08E-05	0.297799924	0.00202585	DDX55
SAT	Obesity	ENSG00000112053.9	-0.008512259	0.002371971	1	0.030048015	SLC26A8
SAT	Obesity	ENSG00000112812.11	0.017307355	3.78E-06	0.012414489	0.000167763	PRSS16
SAT	Obesity	ENSG00000113141.11	-0.011799974	0.000228853	0.750867837	0.004550714	IK
SAT	Obesity	ENSG00000113734.13	-0.005273578	0.001810209	1	0.023757177	BNIP1
SAT	Obesity	ENSG00000114302.11	0.013108288	0.003297945	1	0.039204918	PRKAR2A
SAT	Obesity	ENSG00000114378.12	-0.008814288	0.000409199	1	0.007336518	HYAL1
SAT	Obesity	ENSG00000114796.11	0.018763661	0.000639387	1	0.010446545	KLHL24
SAT	Obesity	ENSG00000115310.13	-0.014383428	0.003917062	1	0.044436554	RTN4
SAT	Obesity	ENSG00000115561.10	-0.012536593	1.88E-07	0.000616035	1.06E-05	CHMP3
SAT	Obesity	ENSG00000116138.8	-0.015460011	0.00083258	1	0.012764933	DNAJC16
SAT	Obesity	ENSG00000117122.9	-0.008629217	0.000775669	1	0.012176893	MFAP2
SAT	Obesity	ENSG00000117523.11	0.018647624	0.00059242	1	0.009866658	PRRC2C
SAT	Obesity	ENSG00000117601.9	0.021715825	5.63E-10	1.85E-06	5.00E-08	SERPINC1
SAT	Obesity	ENSG00000118004.13	-0.017575243	0.00372367	1	0.043018874	COLEC11
SAT	Obesity	ENSG00000119242.4	0.027390299	8.21E-12	2.69E-08	8.69E-10	CCDC92
SAT	Obesity	ENSG00000119599.12	0.016618221	8.71E-05	0.285612737	0.001973489	DCAF4
SAT	Obesity	ENSG00000119865.4	-0.013705844	0.001403582	1	0.019431029	CNRIP1
SAT	Obesity	ENSG00000119878.5	-0.020584036	0.000723481	1	0.011523022	CRIPT
SAT	Obesity	ENSG00000120093.7	0.017886924	6.84E-06	0.02243978	0.000249331	HOXB3
SAT	Obesity	ENSG00000120314.14	-0.021424112	0.000173122	0.568012556	0.003641106	WDR55

SAT	Obesity	ENSG00000120451.6	-0.017572975	3.82E-05	0.12529416	0.001046683	SNX19
SAT	Obesity	ENSG00000120907.13	0.00823833	6.98E-05	0.229108206	0.001660204	ADRA1A
SAT	Obesity	ENSG00000121101.11	0.019050009	0.001503146	1	0.020513962	TEX14
SAT	Obesity	ENSG00000121957.8	0.017593969	0.003691149	1	0.042793854	GPSM2
SAT	Obesity	ENSG00000124562.5	-0.02280671	1.09E-23	3.57E-20	3.96E-21	SNRPC
SAT	Obesity	ENSG00000124635.7	0.027951843	6.71E-07	0.002202038	3.34E-05	HIST1H2BJ
SAT	Obesity	ENSG00000125388.15	0.006154441	3.83E-05	0.12560192	0.001046683	GRK4
SAT	Obesity	ENSG00000125510.11	0.015497886	0.00119393	1	0.017256766	OPRL1
SAT	Obesity	ENSG00000127337.2	0.006090088	0.0017743	1	0.023473705	YEATS4
SAT	Obesity	ENSG00000127957.12	-0.024926563	5.78E-21	1.90E-17	1.46E-18	PMS2P3
SAT	Obesity	ENSG00000128408.7	0.013371095	0.000305384	1	0.005692976	RIBC2
SAT	Obesity	ENSG00000129197.10	-0.032204003	3.65E-08	0.000119856	2.45E-06	RPAIN
SAT	Obesity	ENSG00000130270.12	-0.01400295	1.47E-08	4.83E-05	1.03E-06	ATP8B3
SAT	Obesity	ENSG00000130517.9	0.009807601	0.001624761	1	0.021937612	PGPEP1
SAT	Obesity	ENSG00000130943.5	-0.008874988	0.001784837	1	0.02351828	PKDREJ
SAT	Obesity	ENSG00000132205.6	0.026821135	5.01E-07	0.001642551	2.65E-05	EMILIN2
SAT	Obesity	ENSG00000132740.4	0.008664672	1.17E-05	0.038497372	0.000381162	IGHMBP2
SAT	Obesity	ENSG00000133243.4	-0.015967862	0.002319207	1	0.02956126	BTBD2
SAT	Obesity	ENSG00000133706.13	-0.00508441	0.00022355	0.733468235	0.004472367	LARS
SAT	Obesity	ENSG00000134201.6	0.013463108	0.004302603	1	0.047531447	GSTM5
SAT	Obesity	ENSG00000134575.5	0.017574904	5.48E-05	0.17982338	0.001415932	ACP2
SAT	Obesity	ENSG00000135124.10	0.014759029	0.001597735	1	0.021661855	P2RX4
SAT	Obesity	ENSG00000135211.5	-0.01668482	1.50E-05	0.049339487	0.000479024	TMEM60
SAT	Obesity	ENSG00000135213.8	-0.046739279	1.65E-19	5.41E-16	3.61E-17	POM121C
SAT	Obesity	ENSG00000135913.6	0.01635167	7.54E-05	0.247256916	0.001778827	USP37
SAT	Obesity	ENSG00000135972.4	0.006534016	0.004170656	1	0.046543954	MRPS9
SAT	Obesity	ENSG00000136982.5	0.003482088	0.003226864	1	0.038781468	DSCC1
SAT	Obesity	ENSG00000137185.7	0.009808909	8.76E-05	0.287531101	0.001973489	ZSCAN9
SAT	Obesity	ENSG00000137216.14	-0.012363997	0.003313348	1	0.039245833	TMEM63B
SAT	Obesity	ENSG00000137404.10	0.015205567	0.002904481	1	0.035691396	NRM
SAT	Obesity	ENSG00000138380.13	0.015750905	4.30E-06	0.014119163	0.000185778	CARF
SAT	Obesity	ENSG00000139187.5	0.006733262	0.003389699	1	0.040005764	KLRG1
SAT	Obesity	ENSG00000139405.11	-0.007247464	0.002727848	1	0.034160566	RITA1
SAT	Obesity	ENSG00000140600.12	-0.018049471	0.000203285	0.666978035	0.004142721	SH3GL3
SAT	Obesity	ENSG00000141076.13	0.008823096	0.000643158	1	0.010446545	CIRH1A
SAT	Obesity	ENSG00000141452.5	-0.029765758	2.11E-13	6.94E-10	2.78E-11	C18orf8
SAT	Obesity	ENSG00000141458.8	-0.040230733	2.84E-13	9.32E-10	3.58E-11	NPC1
SAT	Obesity	ENSG00000141569.6	0.013012468	1.76E-05	0.057832235	0.00054761	TRIM65
SAT	Obesity	ENSG00000142534.2	0.013970509	0.002499151	1	0.031537368	RPS11
SAT	Obesity	ENSG00000143367.11	-0.01534896	0.000815486	1	0.012561551	TUFT1
SAT	Obesity	ENSG00000143575.10	0.01914416	0.000573508	1	0.009667596	HAX1
SAT	Obesity	ENSG00000143702.11	0.01722168	0.000360136	1	0.00654777	CEP170
SAT	Obesity	ENSG00000144218.14	-0.020145986	0.000809544	1	0.012532499	AFF3
SAT	Obesity	ENSG00000144426.14	0.011405036	4.48E-05	0.147047397	0.001205307	NBEAL1
SAT	Obesity	ENSG00000145020.10	-0.004643925	0.00126081	1	0.017985728	AMT
SAT	Obesity	ENSG00000145029.7	-0.007688364	0.001389118	1	0.019312276	NICN1
SAT	Obesity	ENSG00000145241.6	-0.014759239	8.36E-06	0.027435231	0.00029764	CENPC
SAT	Obesity	ENSG00000145416.9	-0.012797829	0.001277164	1	0.018134645	MARCH1
SAT	Obesity	ENSG00000145832.8	-0.013198781	0.000751391	1	0.011909726	SLC25A48
SAT	Obesity	ENSG00000145882.6	-0.012551354	0.000215879	0.70830057	0.004372226	PCYOX1L
SAT	Obesity	ENSG00000146109.3	0.012643625	0.000270544	0.887656266	0.005101473	ABT1
SAT	Obesity	ENSG00000148572.10	-0.010926754	0.000361215	1	0.00654777	NRBF2
SAT	Obesity	ENSG00000148824.14	0.006880983	0.000952249	1	0.014397835	MTG1
SAT	Obesity	ENSG00000149084.7	-0.007552758	4.68E-12	1.54E-08	5.12E-10	HSD17B12
SAT	Obesity	ENSG00000149292.12	0.005345496	6.25E-05	0.204985657	0.001541246	TTC12
SAT	Obesity	ENSG00000149548.10	-0.012312527	9.13E-06	0.029954828	0.000305662	CCDC15
SAT	Obesity	ENSG00000149922.6	0.01776672	5.99E-08	0.000196444	3.71E-06	TBX6
SAT	Obesity	ENSG00000151006.7	-0.023778637	1.16E-06	0.003818547	5.62E-05	PRSS53
SAT	Obesity	ENSG00000151640.8	0.005947517	0.000153636	0.504078468	0.003273237	DPYSL4
SAT	Obesity	ENSG00000151917.13	-0.016022363	0.001481122	1	0.020332893	BEND6
SAT	Obesity	ENSG00000152061.17	0.00993509	0.001712666	1	0.022842502	RABGAP1L
SAT	Obesity	ENSG00000152219.4	-0.022440645	1.08E-05	0.035524439	0.000355244	ARL14EP
SAT	Obesity	ENSG00000152359.10	-0.013535617	3.82E-08	0.000125486	2.51E-06	POC5
SAT	Obesity	ENSG00000153814.7	0.020498389	2.82E-05	0.092484676	0.000818448	JAZF1
SAT	Obesity	ENSG00000154240.12	0.015441913	0.003927644	1	0.044436554	CEP112
SAT	Obesity	ENSG00000154328.11	0.015725558	0.000238861	0.783703498	0.004690985	NEIL2
SAT	Obesity	ENSG00000154710.11	-0.004344335	0.003169214	1	0.03822864	RABGEF1
SAT	Obesity	ENSG00000157214.9	-0.011628705	0.000197743	0.648793464	0.004054959	STEAP2
SAT	Obesity	ENSG00000157322.12	-0.019597129	1.07E-17	3.51E-14	2.06E-15	CLEC18A
SAT	Obesity	ENSG00000158104.7	0.027885679	3.90E-06	0.012798118	0.000170642	HPD
SAT	Obesity	ENSG00000158623.10	-0.005595692	0.001306911	1	0.018403323	COPG2
SAT	Obesity	ENSG00000158865.8	-0.009120169	1.43E-07	0.000469438	8.38E-06	SLC5A11
SAT	Obesity	ENSG00000160049.7	0.012898403	0.00435504	1	0.047949285	DFFA
SAT	Obesity	ENSG00000160200.13	-0.010800855	0.002094583	1	0.027056402	CBS
SAT	Obesity	ENSG00000162341.11	0.016427474	0.004189299	1	0.046593526	TPCN2
SAT	Obesity	ENSG00000162994.11	0.002986389	0.003855174	1	0.043920336	CLHC1
SAT	Obesity	ENSG00000163297.12	0.024608233	3.11E-05	0.102058163	0.000895247	ANTXR2
SAT	Obesity	ENSG00000163406.6	-0.012292034	0.002569742	1	0.032303922	SLC15A2
SAT	Obesity	ENSG00000163431.11	-0.038194812	3.52E-15	1.16E-11	5.50E-13	LMOD1
SAT	Obesity	ENSG00000163596.12	-0.014307922	5.69E-05	0.186685093	0.001429286	ICA1L
SAT	Obesity	ENSG00000163933.5	-0.022945619	2.91E-07	0.000955264	1.59E-05	RFT1
SAT	Obesity	ENSG00000164062.8	-0.022516413	9.88E-13	3.24E-09	1.20E-10	APEH
SAT	Obesity	ENSG00000164068.11	0.015964241	6.72E-21	2.20E-17	1.57E-18	RNF123
SAT	Obesity	ENSG00000164078.8	0.021221784	5.81E-07	0.001905393	2.98E-05	MST1R
SAT	Obesity	ENSG00000164081.8	-0.00872573	0.000308979	1	0.005727464	TEX264
SAT	Obesity	ENSG00000164088.13	-0.01339176	4.99E-05	0.163870549	0.00130056	PPM1M
SAT	Obesity	ENSG00000164307.8	-0.012260691	5.64E-05	0.185177429	0.001429286	ERAP1
SAT	Obesity	ENSG00000164924.13	-0.010821797	0.000121501	0.398643746	0.002622656	YWHAZ
SAT	Obesity	ENSG00000165178.8	0.016283427	5.89E-06	0.019317811	0.000225872	NCF1C
SAT	Obesity	ENSG00000165476.8	-0.01650235	0.000313681	1	0.005781949	REEP3
SAT	Obesity	ENSG00000165916.4	0.009940708	0.003985623	1	0.044937557	PSMC3
SAT	Obesity	ENSG00000166166.8	-0.027505078	2.74E-06	0.008979114	0.00012471	TRMT61A
SAT	Obesity	ENSG00000166529.10	-0.006685048	0.002858769	1	0.035394795	ZSCAN21

SAT	Obesity	ENSG00000166532.11	-0.004663568	0.003842084	1	0.043920336	RIMKLB
SAT	Obesity	ENSG00000166913.8	-0.014732008	0.002302946	1	0.029515494	YWHAB
SAT	Obesity	ENSG00000167118.6	-0.018423772	0.000240469	0.788979083	0.004690985	URM1
SAT	Obesity	ENSG00000167395.10	0.036937091	2.40E-09	7.87E-06	1.92E-07	ZNF646
SAT	Obesity	ENSG00000167397.10	-0.028526597	1.22E-09	3.99E-06	1.05E-07	VKORC1
SAT	Obesity	ENSG00000167671.7	-0.021454305	6.41E-05	0.210389038	0.001558437	UBXN6
SAT	Obesity	ENSG00000167720.8	0.005885123	8.76E-06	0.028751567	0.000299495	SRR
SAT	Obesity	ENSG00000168090.5	-0.016659455	0.001031198	1	0.015449135	COPS6
SAT	Obesity	ENSG00000168268.6	-0.017166669	0.003602275	1	0.042161685	NT5DC2
SAT	Obesity	ENSG00000168405.10	0.023295629	4.43E-05	0.145510061	0.001202562	CMAHP
SAT	Obesity	ENSG00000169085.7	0.003778689	0.004081271	1	0.04585839	C8orf46
SAT	Obesity	ENSG00000169203.12	0.016424187	1.57E-13	5.14E-10	2.14E-11	RP11-231C14.4
SAT	Obesity	ENSG00000169592.10	-0.030717024	1.97E-15	6.47E-12	3.60E-13	INO80E
SAT	Obesity	ENSG00000169612.3	-0.011326705	0.000147915	0.485307476	0.003171944	FAM103A1
SAT	Obesity	ENSG00000170092.10	0.035993839	8.11E-18	2.66E-14	1.66E-15	AC018720.10
SAT	Obesity	ENSG00000170425.3	-0.01180501	9.26E-07	0.003038024	4.53E-05	ADORA2B
SAT	Obesity	ENSG00000171103.6	0.006621874	3.14E-05	0.103008686	0.000895728	TRMT61B
SAT	Obesity	ENSG00000172247.3	-0.011342034	2.98E-15	9.78E-12	4.89E-13	C1QTNF4
SAT	Obesity	ENSG00000172728.11	-0.008454642	5.71E-05	0.187236523	0.001429286	FUT10
SAT	Obesity	ENSG00000172780.12	0.01313391	0.000526879	1	0.009009799	RAB43
SAT	Obesity	ENSG00000173226.12	-0.006389418	0.000861686	1	0.013149728	IQCB1
SAT	Obesity	ENSG00000173531.11	-0.013773754	8.53E-06	0.027978132	0.00029764	MST1
SAT	Obesity	ENSG00000173540.8	0.031979962	8.01E-08	0.000262732	4.78E-06	GMPPB
SAT	Obesity	ENSG00000173917.9	0.010888814	0.003255678	1	0.038984968	HOXB2
SAT	Obesity	ENSG00000174775.12	0.018442261	0.002056266	1	0.026738446	HRAS
SAT	Obesity	ENSG00000174891.8	-0.007745753	0.001706672	1	0.022842502	RSRC1
SAT	Obesity	ENSG00000176095.7	0.013326345	0.000291662	0.956943251	0.005468247	IP6K1
SAT	Obesity	ENSG00000176155.14	-0.011091601	0.000221105	0.725444264	0.004450578	CCDC57
SAT	Obesity	ENSG00000176783.10	0.012548425	4.67E-05	0.153264522	0.001246053	RUFY1
SAT	Obesity	ENSG00000177156.6	-0.017488416	0.000999924	1	0.015049313	TALDO1
SAT	Obesity	ENSG00000177380.9	-0.021047357	0.000179414	0.588655802	0.0037494	PPFIA3
SAT	Obesity	ENSG00000177595.13	0.01415513	6.69E-06	0.021963429	0.00024678	PIDD
SAT	Obesity	ENSG00000177697.13	0.015511684	0.001156523	1	0.016790048	CD151
SAT	Obesity	ENSG00000177700.5	-0.017361272	0.001419596	1	0.019570148	POLR2L
SAT	Obesity	ENSG00000178188.10	-0.036534084	1.96E-31	6.44E-28	3.22E-28	SH2B1
SAT	Obesity	ENSG00000178467.13	-0.024224317	4.97E-05	0.162925248	0.00130056	P4HTM
SAT	Obesity	ENSG00000178718.5	-0.012410505	0.000467919	1	0.008253984	RPP25
SAT	Obesity	ENSG00000178750.2	0.025963157	2.44E-07	0.000799682	1.36E-05	STX19
SAT	Obesity	ENSG00000178809.7	-0.037929965	1.80E-12	5.91E-09	2.11E-10	TRIM73
SAT	Obesity	ENSG00000178952.4	0.02279601	8.03E-23	2.63E-19	2.39E-20	TUFM
SAT	Obesity	ENSG00000179115.6	-0.019354201	0.001044378	1	0.015575476	FARSA
SAT	Obesity	ENSG00000179673.3	0.008953975	0.000481406	1	0.008401553	RPRML
SAT	Obesity	ENSG00000180385.4	0.015022513	2.39E-05	0.078356152	0.00072552	EMC3-AS1
SAT	Obesity	ENSG00000181315.6	-0.01832876	4.55E-08	0.000149321	2.93E-06	ZNF322
SAT	Obesity	ENSG00000182533.6	-0.00964656	0.001506816	1	0.020513962	CAV3
SAT	Obesity	ENSG00000182685.3	-0.00977826	0.002277223	1	0.029300269	BRICD5
SAT	Obesity	ENSG00000182841.8	0.026747317	9.08E-06	0.029782437	0.000305662	RRP7B
SAT	Obesity	ENSG00000183060.11	0.006281201	0.003160748	1	0.03822864	LYSMD4
SAT	Obesity	ENSG00000183527.7	0.006590148	3.40E-05	0.111687929	0.000954598	PSMG1
SAT	Obesity	ENSG00000183604.10	-0.027129336	1.74E-14	5.72E-11	2.60E-12	RP11-347C12.2
SAT	Obesity	ENSG00000184110.10	0.034581887	1.16E-27	3.81E-24	6.35E-25	EIF3C
SAT	Obesity	ENSG00000184381.14	0.028566128	6.66E-07	0.002184204	3.34E-05	PLA2G6
SAT	Obesity	ENSG00000185245.6	0.024790052	7.54E-06	0.02472934	0.000271751	GP1BA
SAT	Obesity	ENSG00000186470.9	-0.005600123	5.80E-08	0.000190203	3.66E-06	BTN3A2
SAT	Obesity	ENSG00000186792.11	-0.008932908	5.69E-06	0.018657521	0.000222113	HYAL3
SAT	Obesity	ENSG00000188312.9	0.00826765	1.20E-06	0.003948386	5.72E-05	CENPP
SAT	Obesity	ENSG00000188878.12	0.006909775	0.000527242	1	0.009009799	FBF1
SAT	Obesity	ENSG00000189157.9	0.020440046	0.000473803	1	0.008313089	FAM47E
SAT	Obesity	ENSG00000196502.7	-0.02228088	7.37E-08	0.000241689	4.48E-06	SULT1A1
SAT	Obesity	ENSG00000196821.5	0.016928598	8.12E-09	2.66E-05	6.20E-07	C6orf106
SAT	Obesity	ENSG00000196968.6	0.017456293	0.000501354	1	0.008657585	FUT11
SAT	Obesity	ENSG00000197062.7	-0.007421931	0.000112433	0.368891926	0.002475785	RP5-874C20.3
SAT	Obesity	ENSG00000197119.8	0.015802193	0.003617774	1	0.042161685	SLC25A29
SAT	Obesity	ENSG00000197165.6	0.025056674	4.73E-29	1.55E-25	3.88E-26	SULT1A2
SAT	Obesity	ENSG00000197262.8	-0.022032746	5.92E-06	0.019424959	0.000225872	CCL4L2
SAT	Obesity	ENSG00000197279.3	-0.013316878	0.003458675	1	0.040673517	ZNF165
SAT	Obesity	ENSG00000197345.8	-0.00459975	0.000260086	0.853342504	0.004934546	MRPL21
SAT	Obesity	ENSG00000197728.5	-0.004951639	7.76E-05	0.254532644	0.00181809	RPS26
SAT	Obesity	ENSG00000197782.10	-0.005772295	6.58E-06	0.021573699	0.000245156	ZNF780A
SAT	Obesity	ENSG00000198093.6	-0.011036791	0.004524819	1	0.049651945	ZNF649
SAT	Obesity	ENSG00000198099.4	-0.012664397	6.69E-05	0.219571995	0.001602715	ADH4
SAT	Obesity	ENSG00000198315.6	0.024473063	4.94E-06	0.016193189	0.000199916	ZKSCAN8
SAT	Obesity	ENSG00000204316.8	-0.016294726	0.00064131	1	0.010446545	MRPL38
SAT	Obesity	ENSG00000204351.7	-0.004266343	6.31E-05	0.207024255	0.001544957	SKIV2L
SAT	Obesity	ENSG00000204356.7	0.008249118	0.000181971	0.597045615	0.00377877	NELFE
SAT	Obesity	ENSG00000204531.11	0.004670615	0.000629435	1	0.010377772	POU5F1
SAT	Obesity	ENSG00000204536.9	0.004687221	0.000241626	0.792776436	0.004690985	CCHCR1
SAT	Obesity	ENSG00000204574.8	0.010231213	0.003855244	1	0.043920336	ABCF1
SAT	Obesity	ENSG00000204592.5	0.022714046	1.39E-09	4.56E-06	1.17E-07	HLA-E
SAT	Obesity	ENSG00000204644.5	0.004463343	0.000109397	0.358933116	0.002425224	ZFP57
SAT	Obesity	ENSG00000204789.3	0.012733339	4.93E-06	0.016168856	0.000199916	ZNF204P
SAT	Obesity	ENSG00000205571.8	-0.006592112	0.003294321	1	0.039204918	SMN2
SAT	Obesity	ENSG00000205583.9	-0.018817342	4.17E-21	1.37E-17	1.14E-18	STAG3L1
SAT	Obesity	ENSG00000205710.3	0.007777583	0.000231813	0.760578827	0.0045818	C17orf107
SAT	Obesity	ENSG00000212694.4	-0.010187849	0.000187365	0.614743135	0.003866309	AC084018.1
SAT	Obesity	ENSG00000213199.3	0.014354611	5.79E-05	0.190112536	0.001440246	ASIC3
SAT	Obesity	ENSG00000213240.7	0.01155662	0.000795788	1	0.012433244	NOTCH2NL
SAT	Obesity	ENSG00000213277.3	0.017674233	7.88E-05	0.258687005	0.00183466	MARCKSL1P1
SAT	Obesity	ENSG00000213639.5	-0.020123905	2.50E-05	0.081923556	0.00073805	PPP1CB
SAT	Obesity	ENSG00000214279.8	-0.013128371	0.00193562	1	0.025301871	RP11-108K14.4
SAT	Obesity	ENSG00000214659.4	0.02253179	0.000115136	0.377760746	0.002501727	KRT8P26
SAT	Obesity	ENSG00000223496.1	-0.033906615	2.24E-15	7.35E-12	3.87E-13	EXOSC6
SAT	Obesity	ENSG00000224389.4	0.011035607	0.000578937	1	0.009691288	C4B

SAT	Obesity	ENSG00000224843.2	0.027332415	3.56E-07	0.001166758	1.91E-05	LINC00240
SAT	Obesity	ENSG00000224997.1	-0.01067946	8.47E-06	0.027775013	0.00029764	AL049840.1
SAT	Obesity	ENSG00000225726.1	-0.014695957	0.002756575	1	0.034389064	AC007000.10
SAT	Obesity	ENSG00000226232.4	-0.010804259	5.71E-09	1.87E-05	4.46E-07	RP11-419C5.2
SAT	Obesity	ENSG00000227370.1	-0.019186444	0.001106192	1	0.016130738	RP4-669P10.19
SAT	Obesity	ENSG00000230521.1	0.004482198	0.003829703	1	0.043920336	HCG4P7
SAT	Obesity	ENSG00000231500.2	0.008023035	0.000260188	0.853676482	0.004934546	RPS18
SAT	Obesity	ENSG00000231852.2	0.008473329	5.63E-05	0.184626332	0.001429286	CYP21A2
SAT	Obesity	ENSG00000232561.2	-0.030197708	3.72E-12	1.22E-08	4.21E-10	GTF2IP1
SAT	Obesity	ENSG00000233137.2	0.025925009	2.52E-05	0.082800682	0.000739292	RP11-220I1.1
SAT	Obesity	ENSG00000233609.3	0.017348264	0.000809781	1	0.012532499	RP11-62H7.2
SAT	Obesity	ENSG00000235109.3	0.00630703	0.002836556	1	0.035252799	ZSCAN31
SAT	Obesity	ENSG00000235821.1	-0.006112398	0.003086069	1	0.037501449	IFITM4P
SAT	Obesity	ENSG00000239557.1	0.03522512	9.39E-09	3.08E-05	6.85E-07	RP11-168J18.6
SAT	Obesity	ENSG00000245958.2	-0.007466345	0.001369965	1	0.019127047	RP11-33B1.1
SAT	Obesity	ENSG00000248290.1	-0.006363871	3.32E-05	0.109084671	0.000940385	TNXA
SAT	Obesity	ENSG00000248408.1	-0.026588763	5.52E-06	0.01812343	0.000218355	RP11-452C8.1
SAT	Obesity	ENSG00000250412.1	0.015571498	0.000574575	1	0.009667596	KLHL2P1
SAT	Obesity	ENSG00000251417.1	-0.022797794	4.83E-30	1.59E-26	5.29E-27	RP11-1348G14.4
SAT	Obesity	ENSG00000255298.2	-0.013003033	0.00122897	1	0.017685316	OR8G5
SAT	Obesity	ENSG00000256269.2	0.020655418	2.11E-11	6.91E-08	2.16E-09	HMBS
SAT	Obesity	ENSG00000256604.1	-0.012777939	0.003015237	1	0.036776926	AC093668.2
SAT	Obesity	ENSG00000259863.1	-0.012834804	0.000157677	0.517338	0.003337665	SH3RF3-AS1
SAT	Obesity	ENSG00000259982.1	0.030223046	3.88E-33	1.27E-29	1.27E-29	CDC37P1
SAT	Obesity	ENSG00000260388.1	-0.011396776	0.00066081	1	0.010628026	LINC00562
SAT	Obesity	ENSG00000260911.1	0.016932484	2.39E-08	7.84E-05	1.63E-06	RP11-196G11.2
SAT	Obesity	ENSG00000260947.1	-0.00530445	8.78E-05	0.288129407	0.001973489	RP11-384P7.7
SAT	Obesity	ENSG00000261556.4	-0.026703012	1.48E-13	4.86E-10	2.11E-11	RP11-296I10.6
SAT	Obesity	ENSG00000265055.1	0.016273156	4.51E-06	0.014813626	0.000189918	AC145343.2
SAT	Obesity	ENSG00000265798.2	-0.01151471	0.001096313	1	0.016106882	RP11-271K11.5
SAT	Obesity	ENSG00000266173.1	-0.016871158	0.000113659	0.372914451	0.002486096	STRADA
SAT	Obesity	ENSG00000267352.1	0.02820439	5.11E-06	0.016753551	0.000204312	SH3GL1P3
SAT	Obesity	ENSG00000269928.1	0.013509007	0.000421602	1	0.007477175	RP11-849F2.8
SAT	Obesity	ENSG00000269963.1	-0.026754741	1.00E-08	3.28E-05	7.14E-07	RP11-73M18.9
SAT	Obesity	ENSG00000269990.1	0.023598735	6.52E-06	0.021379575	0.000245156	CTD-3074O7.12
SAT	Obesity	ENSG00000270692.1	0.020520586	1.76E-05	0.057669744	0.00054761	RP11-89F17.5
SAT	Obesity	ENSG00000271623.1	-0.051129969	2.18E-28	7.15E-25	1.43E-25	RP11-435I10.5
SAT	Obesity	ENSG00000271751.1	-0.031308692	1.56E-09	5.13E-06	1.28E-07	RP11-110I1.14
SAT	Obesity	ENSG00000271780.1	-0.008895472	0.001061782	1	0.015763375	RP11-1017G21.5
SAT	Obesity	ENSG00000272047.1	0.013983929	0.000653312	1	0.010559198	GTF2H5
SAT	Obesity	ENSG00000272462.2	0.006008114	3.73E-10	1.22E-06	3.40E-08	U91328.19
SAT	Obesity	ENSG00000272810.1	0.012607958	1.58E-06	0.005186625	7.31E-05	U91328.22
SAT	Obesity	ENSG00000272849.1	-0.013163843	8.61E-05	0.282504754	0.001973489	RP11-347I19.8
SAT	Obesity	ENSG00000272983.1	0.006323255	0.001352385	1	0.01896229	RP11-508N22.12
SAT	HTN	ENSG00000001617.7	-0.024725353	2.55E-07	0.000836316	1.49E-05	SEMA3F
SAT	HTN	ENSG00000003056.3	-0.014748332	0.000175393	0.575464512	0.003585193	M6PR
SAT	HTN	ENSG00000004534.10	-0.005129629	4.95E-06	0.016234673	0.000186605	RBM6
SAT	HTN	ENSG00000005243.5	0.017019524	6.62E-05	0.21727313	0.001621441	COPZ2
SAT	HTN	ENSG00000005955.8	-0.015963459	0.000319207	1	0.00557084	GGNBP2
SAT	HTN	ENSG00000007312.8	-0.020285484	0.001894128	1	0.0223548	CD79B
SAT	HTN	ENSG00000007341.14	-0.005382131	0.004320172	1	0.040731275	ST7L
SAT	HTN	ENSG00000010704.14	-0.019455954	9.86E-08	0.000323437	6.88E-06	HFE
SAT	HTN	ENSG00000011523.9	0.014929688	5.21E-08	0.000170892	4.07E-06	CEP68
SAT	HTN	ENSG00000028528.10	0.013614597	0.000313311	1	0.005497191	SNX1
SAT	HTN	ENSG00000050405.9	0.015528929	0.000112615	0.369488452	0.002548196	LIMA1
SAT	HTN	ENSG00000053900.6	0.020535732	0.000156636	0.513923815	0.003315638	ANAPC4
SAT	HTN	ENSG00000054282.11	0.020938725	2.20E-10	7.23E-07	4.82E-08	SDCCAG8
SAT	HTN	ENSG00000055955.11	0.007016938	0.000323567	1	0.005617053	ITIH4
SAT	HTN	ENSG00000060642.6	-0.03512346	5.42E-07	0.001779741	2.78E-05	PIGV
SAT	HTN	ENSG00000063854.8	0.010984352	0.001279202	1	0.016552068	HAGH
SAT	HTN	ENSG00000065060.12	0.010163766	9.52E-07	0.003123699	4.53E-05	UHRF1BP1
SAT	HTN	ENSG00000068654.11	-0.006997956	0.000100095	0.328412008	0.002296587	POLR1A
SAT	HTN	ENSG00000073584.14	0.017514597	0.004242158	1	0.040343539	SMARCE1
SAT	HTN	ENSG00000074201.4	0.010995411	1.49E-05	0.048847232	0.000460823	CLNS1A
SAT	HTN	ENSG00000076685.14	0.019531652	0.003737122	1	0.037382609	NT5C2
SAT	HTN	ENSG00000082258.8	-0.009231772	0.0004251	1	0.0068707	CCNT2
SAT	HTN	ENSG00000085365.13	0.015609337	0.00412717	1	0.039725318	SCAMP1
SAT	HTN	ENSG00000086015.16	0.018917631	0.001649091	1	0.019892164	MAST2
SAT	HTN	ENSG00000086300.11	-0.007553247	4.93E-07	0.001618651	2.57E-05	SNX10
SAT	HTN	ENSG00000087884.10	-0.011124874	2.35E-05	0.077161004	0.000682841	AAMDC
SAT	HTN	ENSG00000090238.7	0.005156261	0.000101286	0.332320906	0.002307784	YPEL3
SAT	HTN	ENSG00000090621.9	-0.013853296	3.70E-06	0.012151461	0.000150018	PABPC4
SAT	HTN	ENSG00000091542.8	0.010360647	0.004268478	1	0.040476523	ALKBH5
SAT	HTN	ENSG00000092295.7	0.019496423	0.000180944	0.593676249	0.003619977	TGM1
SAT	HTN	ENSG00000095787.17	0.025705085	3.20E-05	0.105016618	0.000867906	WAC
SAT	HTN	ENSG00000100106.15	-0.008560302	0.004547328	1	0.042219581	TRIOBP
SAT	HTN	ENSG00000100116.12	0.019493943	0.001997123	1	0.023153921	GCAT
SAT	HTN	ENSG00000100731.11	0.008406302	0.000203554	0.667861435	0.003975366	PCNX
SAT	HTN	ENSG00000101457.8	-0.009135436	0.000131996	0.433079389	0.002873849	DNTTIP1
SAT	HTN	ENSG00000101751.6	-0.006201173	9.63E-05	0.315865668	0.002234893	POLI
SAT	HTN	ENSG00000102858.8	0.012405398	0.000439709	1	0.007003334	MGRN1
SAT	HTN	ENSG00000103196.7	0.021845417	0.000180824	0.593283879	0.003619977	CRISPLD2
SAT	HTN	ENSG00000103199.9	-0.010081731	0.004172775	1	0.040031798	ZNF500
SAT	HTN	ENSG00000103248.13	0.007554851	0.000728042	1	0.010472448	MTHFSD
SAT	HTN	ENSG00000103671.5	0.008892772	0.005381657	1	0.048776843	TRIP4
SAT	HTN	ENSG00000104231.6	0.012707267	0.001309759	1	0.016591972	ZFAND1
SAT	HTN	ENSG00000104313.13	0.022268515	3.75E-05	0.123109758	0.000992821	EYA1
SAT	HTN	ENSG00000105726.12	0.014676056	1.50E-05	0.049362984	0.000461336	ATP13A1
SAT	HTN	ENSG00000106290.10	-0.020268034	0.001091175	1	0.014494516	TAF6
SAT	HTN	ENSG00000107960.6	-0.03814924	9.77E-08	0.000320627	6.88E-06	OBFC1
SAT	HTN	ENSG00000108384.10	-0.007310663	8.77E-05	0.287714717	0.002055105	RAD51C
SAT	HTN	ENSG00000108639.3	-0.015873155	0.000755404	1	0.010637259	SYNGR2
SAT	HTN	ENSG00000108654.7	0.016376652	2.67E-05	0.087626082	0.000742594	DDX5

SAT	HTN	ENSG00000108669.12	0.022036613	0.000702286	1	0.010195574	CYTH1
SAT	HTN	ENSG00000110906.8	-0.010308762	0.000146464	0.480547383	0.003120438	KCTD10
SAT	HTN	ENSG00000111405.4	-0.017124075	0.002859541	1	0.030167694	ENDOU
SAT	HTN	ENSG00000111530.8	-0.017413605	4.90E-07	0.001608484	2.57E-05	CAND1
SAT	HTN	ENSG00000112877.6	0.015779019	0.000695415	1	0.010185969	CEP72
SAT	HTN	ENSG00000112992.12	-0.012979067	0.000789709	1	0.011072796	NNT
SAT	HTN	ENSG00000113971.14	0.00372186	0.003569222	1	0.036032666	NPHP3
SAT	HTN	ENSG00000114023.11	-0.021046116	0.001138794	1	0.014945536	FAM162A
SAT	HTN	ENSG00000114378.12	-0.008549243	0.00269345	1	0.028974455	HYAL1
SAT	HTN	ENSG00000115084.8	-0.011234598	0.003132345	1	0.032116323	SLC35F5
SAT	HTN	ENSG00000115310.13	-0.020337191	0.000356452	1	0.006123144	RTN4
SAT	HTN	ENSG00000115568.11	-0.018198099	0.000334844	1	0.005782235	ZNF142
SAT	HTN	ENSG00000116489.8	-0.020197665	0.001291475	1	0.016552068	CAPZA1
SAT	HTN	ENSG00000116667.8	-0.016096114	0.000479798	1	0.007532147	C1orf21
SAT	HTN	ENSG00000117450.9	0.017664502	0.003010834	1	0.031261227	PRDX1
SAT	HTN	ENSG00000117481.6	0.018062531	0.002538167	1	0.027986366	NSUN4
SAT	HTN	ENSG00000117595.6	-0.012764197	0.002669851	1	0.028815066	IRF6
SAT	HTN	ENSG00000117877.6	-0.013060575	0.005108722	1	0.046690022	CD3EAP
SAT	HTN	ENSG00000118197.9	-0.009474786	0.002704779	1	0.028984413	DDX59
SAT	HTN	ENSG00000118407.10	0.016554398	0.005413566	1	0.048930885	FILIP1
SAT	HTN	ENSG00000119431.5	-0.006120676	0.003954199	1	0.038497707	HDHD3
SAT	HTN	ENSG00000119969.10	-0.015576292	0.003821897	1	0.037884123	HELLS
SAT	HTN	ENSG00000120784.11	0.008265877	0.000755278	1	0.010637259	ZFP30
SAT	HTN	ENSG00000121101.11	0.042853366	4.10E-10	1.34E-06	8.40E-08	TEX14
SAT	HTN	ENSG00000122565.14	-0.026013402	4.84E-07	0.00158637	2.57E-05	CBX3
SAT	HTN	ENSG00000123545.5	0.007468869	0.004128721	1	0.039725318	NDUFAF4
SAT	HTN	ENSG00000124116.14	-0.004629516	0.004466909	1	0.041729241	WFDC3
SAT	HTN	ENSG00000124191.13	-0.006294108	0.004024871	1	0.039069831	TOX2
SAT	HTN	ENSG00000124508.12	0.007426189	0.001391382	1	0.017448468	BTN2A2
SAT	HTN	ENSG00000124562.5	-0.013184049	3.83E-07	0.00125572	2.13E-05	SNRPC
SAT	HTN	ENSG00000124575.5	0.029707316	2.31E-07	0.000758423	1.38E-05	HIST1H1D
SAT	HTN	ENSG00000124613.4	-0.010722599	6.15E-06	0.020162759	0.000224031	ZNF391
SAT	HTN	ENSG00000125388.15	0.006005631	0.000436206	1	0.006981431	GRK4
SAT	HTN	ENSG00000125510.11	-0.029883644	4.51E-08	0.00014783	3.61E-06	OPRL1
SAT	HTN	ENSG00000126602.6	0.005779148	7.34E-07	0.002407481	3.63E-05	TRAP1
SAT	HTN	ENSG00000126858.12	0.017778398	0.002360345	1	0.026466726	RHOT1
SAT	HTN	ENSG00000127957.12	-0.019512502	1.21E-10	3.98E-07	2.84E-08	PMS2P3
SAT	HTN	ENSG00000128829.7	-0.007856602	0.003362488	1	0.034155797	EIF2AK4
SAT	HTN	ENSG00000130177.10	0.013551523	3.37E-05	0.110524543	0.000905939	CDC16
SAT	HTN	ENSG00000130270.12	-0.009512404	0.000755286	1	0.010637259	ATP8B3
SAT	HTN	ENSG00000130517.9	0.01263956	0.000377823	1	0.006324679	PGPEP1
SAT	HTN	ENSG00000131165.10	0.021509591	1.27E-06	0.004169694	5.79E-05	CHMP1A
SAT	HTN	ENSG00000131435.8	0.035750399	1.22E-07	0.000401808	8.04E-06	PDLIM4
SAT	HTN	ENSG00000133731.5	0.00571594	0.001539978	1	0.018853233	IMPA1
SAT	HTN	ENSG00000134245.13	-0.026443864	3.36E-08	0.000110153	2.90E-06	WNT2B
SAT	HTN	ENSG00000134574.7	0.045911632	1.09E-11	3.58E-08	4.04E-09	DDB2
SAT	HTN	ENSG00000134575.5	0.049714348	1.69E-23	5.54E-20	5.54E-20	ACP2
SAT	HTN	ENSG00000134851.8	0.033051563	1.04E-07	0.00033991	7.08E-06	TMEM165
SAT	HTN	ENSG00000135213.8	-0.024117316	4.50E-05	0.147797145	0.001172993	POM121C
SAT	HTN	ENSG00000135744.7	0.007112811	0.000134662	0.441825993	0.00290675	AGT
SAT	HTN	ENSG00000135913.6	0.015258363	0.001222842	1	0.015921202	USP37
SAT	HTN	ENSG00000135968.15	0.010006925	0.002174492	1	0.024858915	GCC2
SAT	HTN	ENSG00000136425.8	-0.0173279	0.002794756	1	0.029675062	CIB2
SAT	HTN	ENSG00000136448.7	0.015703223	6.52E-10	2.14E-06	1.07E-07	NMT1
SAT	HTN	ENSG00000136856.13	0.015911073	0.00274977	1	0.029292189	SLC2A8
SAT	HTN	ENSG00000137411.12	0.00372299	0.002599218	1	0.028332336	VARS2
SAT	HTN	ENSG00000137944.12	-0.024764378	0.000386707	1	0.006408012	CCBL2
SAT	HTN	ENSG00000138036.14	0.017231602	0.000381063	1	0.006346531	DYNC2LI1
SAT	HTN	ENSG00000138175.8	0.021719004	7.91E-07	0.002595531	3.82E-05	ARL3
SAT	HTN	ENSG00000138757.10	0.013615797	0.000569228	1	0.008727282	G3BP2
SAT	HTN	ENSG00000138829.6	0.005630305	0.000230312	0.755652406	0.004297476	FBN2
SAT	HTN	ENSG00000139636.11	-0.017951079	0.00197106	1	0.02301441	LMBR1L
SAT	HTN	ENSG00000140995.12	0.017160074	3.86E-05	0.126664381	0.001013315	DEF8
SAT	HTN	ENSG00000141076.13	0.012713997	1.68E-05	0.054958636	0.000508876	CIRH1A
SAT	HTN	ENSG00000141252.15	-0.014723285	0.001398643	1	0.017448468	VPS53
SAT	HTN	ENSG00000141295.9	-0.009163468	0.000227008	0.74481305	0.004297476	SCRN2
SAT	HTN	ENSG00000141452.5	-0.020245865	1.24E-05	0.040532123	0.000401308	C18orf8
SAT	HTN	ENSG00000141458.8	-0.02993074	1.98E-06	0.006498719	8.33E-05	NPC1
SAT	HTN	ENSG00000141569.6	-0.012649129	0.000257874	0.846086136	0.004726738	TRIM65
SAT	HTN	ENSG00000142599.13	0.024103573	8.38E-05	0.274874119	0.001977512	RERE
SAT	HTN	ENSG00000143028.7	0.01619207	0.002541889	1	0.027986366	SYPL2
SAT	HTN	ENSG00000143156.9	-0.021484027	0.000826253	1	0.011487014	NME7
SAT	HTN	ENSG00000143248.8	-0.029395488	2.47E-05	0.081163813	0.000693708	RGS5
SAT	HTN	ENSG00000143437.16	-0.021409627	0.000265265	0.870335062	0.004782061	ARNT
SAT	HTN	ENSG00000143578.11	-0.017464012	2.39E-05	0.07854582	0.000683007	CREB3L4
SAT	HTN	ENSG00000143702.11	0.036247951	4.91E-11	1.61E-07	1.34E-08	CEP170
SAT	HTN	ENSG00000144677.10	-0.021331105	0.001343634	1	0.016955623	CTDSPL
SAT	HTN	ENSG00000145020.10	-0.005006805	0.002340835	1	0.026392712	AMT
SAT	HTN	ENSG00000145029.7	-0.008353365	0.002363533	1	0.026466726	NICN1
SAT	HTN	ENSG00000145241.6	-0.011371229	0.002660035	1	0.028803875	CENPC
SAT	HTN	ENSG00000145777.10	-0.016608027	0.003852015	1	0.038067659	TSLP
SAT	HTN	ENSG00000145990.6	0.01182034	0.005283333	1	0.048151705	GFOD1
SAT	HTN	ENSG00000146109.3	0.014008239	0.000411778	1	0.006721602	ABT1
SAT	HTN	ENSG00000146409.6	-0.008010662	0.000192284	0.63088224	0.003777738	SLC18B1
SAT	HTN	ENSG00000146556.10	-0.028580901	9.32E-06	0.030588524	0.000321984	WASH2P
SAT	HTN	ENSG00000147419.12	0.021396087	0.000730933	1	0.010472448	CCDC25
SAT	HTN	ENSG00000148572.10	-0.016184001	3.77E-06	0.012367156	0.000150819	NRBF2
SAT	HTN	ENSG00000148843.9	0.065679101	1.66E-22	5.45E-19	2.72E-19	PDCD11
SAT	HTN	ENSG00000149084.7	-0.007351138	3.82E-09	1.25E-05	4.32E-07	HSD17B12
SAT	HTN	ENSG00000149100.8	-0.016022636	0.002590911	1	0.028332336	EIF3M
SAT	HTN	ENSG00000149179.9	-0.021343736	0.001412147	1	0.017483974	C11orf49
SAT	HTN	ENSG00000149292.12	0.004365936	0.004206087	1	0.040173476	TTC12
SAT	HTN	ENSG00000152219.4	-0.023451201	5.71E-05	0.1871961	0.001428978	ARL14EP

SAT	HTN	ENSG00000152784.11	-0.023839423	0.000699009	1	0.010193108	PRDM8
SAT	HTN	ENSG00000153113.19	-0.010020763	0.000844023	1	0.011635455	CAST
SAT	HTN	ENSG00000153814.7	-0.018301822	0.001065844	1	0.014273607	JAZF1
SAT	HTN	ENSG00000154328.11	0.027123923	2.90E-08	9.51E-05	2.72E-06	NEIL2
SAT	HTN	ENSG00000155085.11	0.021832095	0.001073954	1	0.014323756	AK9
SAT	HTN	ENSG00000156170.8	-0.035283255	3.25E-09	1.07E-05	3.83E-07	NDUFAF6
SAT	HTN	ENSG00000156398.8	0.027534525	1.82E-05	0.059592552	0.000546721	SFXN2
SAT	HTN	ENSG00000156931.11	0.013108317	0.000220024	0.721897342	0.004271582	VPS8
SAT	HTN	ENSG00000157322.12	-0.011707892	7.49E-06	0.024585509	0.000261923	CLEC18A
SAT	HTN	ENSG00000158022.6	0.017157923	0.00030963	1	0.005461812	TRIM63
SAT	HTN	ENSG00000158805.7	0.026253204	1.36E-05	0.044732652	0.000430122	ZNF276
SAT	HTN	ENSG00000158865.8	-0.009692173	9.86E-07	0.0032355	4.62E-05	SLC5A11
SAT	HTN	ENSG00000159479.12	0.0257854	4.09E-06	0.013410438	0.000159648	MED8
SAT	HTN	ENSG00000160307.5	-0.009359372	5.90E-05	0.193638209	0.001466956	S100B
SAT	HTN	ENSG00000160752.10	0.014940592	0.000817375	1	0.011411947	FDPS
SAT	HTN	ENSG00000160783.15	0.016540556	0.000899106	1	0.012139779	PMF1
SAT	HTN	ENSG00000161204.7	0.014170824	1.89E-06	0.006187277	8.04E-05	ABCF3
SAT	HTN	ENSG00000161955.12	0.034166373	1.93E-07	0.000634652	1.18E-05	TNFSF13
SAT	HTN	ENSG00000163431.11	-0.034253391	6.44E-10	2.11E-06	1.07E-07	LMOD1
SAT	HTN	ENSG00000163933.5	-0.019082181	0.000188064	0.617038859	0.003717102	RFT1
SAT	HTN	ENSG00000163946.9	0.016315152	4.21E-06	0.013819791	0.000162586	FAM208A
SAT	HTN	ENSG00000164002.7	-0.020486811	2.11E-05	0.069345539	0.000619157	EXO5
SAT	HTN	ENSG00000164039.10	-0.0122778	0.000830657	1	0.011499513	BDH2
SAT	HTN	ENSG00000164045.7	-0.027088268	8.41E-11	2.76E-07	2.12E-08	CDC25A
SAT	HTN	ENSG00000164048.9	-0.010724119	1.32E-11	4.33E-08	4.33E-09	ZNF589
SAT	HTN	ENSG00000164062.8	-0.021557081	2.27E-09	7.45E-06	3.10E-07	APEH
SAT	HTN	ENSG00000164068.11	0.013241839	9.78E-12	3.21E-08	4.04E-09	RNF123
SAT	HTN	ENSG00000164307.8	-0.018727963	7.22E-08	0.000236849	5.38E-06	ERAP1
SAT	HTN	ENSG00000164308.12	0.002794442	0.001682424	1	0.020219897	ERAP2
SAT	HTN	ENSG00000164587.7	-0.020819257	0.001787916	1	0.021361157	RPS14
SAT	HTN	ENSG00000164695.4	-0.00982677	0.004212032	1	0.040173476	CHMP4C
SAT	HTN	ENSG00000165118.10	0.015284365	0.005509073	1	0.049385977	C9orf64
SAT	HTN	ENSG00000165178.8	0.014610602	0.000372526	1	0.006267988	NCF1C
SAT	HTN	ENSG00000165244.6	0.020100828	0.002498579	1	0.027695394	ZNF367
SAT	HTN	ENSG00000165476.8	-0.028113071	7.73E-08	0.000253571	5.63E-06	REEP3
SAT	HTN	ENSG00000165490.8	-0.017045303	0.005556178	1	0.049672537	C11orf82
SAT	HTN	ENSG00000166037.6	-0.014508221	0.000503241	1	0.007862535	CEP57
SAT	HTN	ENSG00000166166.8	-0.032214089	1.52E-06	0.004995927	6.75E-05	TRMT61A
SAT	HTN	ENSG00000166435.11	-0.007249154	0.000605426	1	0.009196305	XRRA1
SAT	HTN	ENSG00000166436.11	-0.004834316	3.46E-05	0.113639107	0.000923895	TRIM66
SAT	HTN	ENSG00000166532.11	-0.009600905	1.89E-07	0.000619154	1.17E-05	RIMKLB
SAT	HTN	ENSG00000167395.10	0.024064028	0.00066502	1	0.009917863	ZNF646
SAT	HTN	ENSG00000167397.10	-0.015480994	0.003887291	1	0.038072244	VKORC1
SAT	HTN	ENSG00000167528.8	0.007155712	0.002712043	1	0.028984413	ZNF641
SAT	HTN	ENSG00000167633.12	0.026542571	0.000120062	0.393923321	0.002698105	KIR3DL1
SAT	HTN	ENSG00000167720.8	0.005780621	0.000132262	0.433951171	0.002873849	SRR
SAT	HTN	ENSG00000167910.3	0.023350792	0.000421602	1	0.006847904	CYP7A1
SAT	HTN	ENSG00000168004.5	0.01246669	0.000578545	1	0.008828861	HRASLS5
SAT	HTN	ENSG00000168297.11	-0.017016637	0.001504431	1	0.018487036	PXK
SAT	HTN	ENSG00000168394.9	-0.021694434	0.002418657	1	0.026900389	TAP1
SAT	HTN	ENSG00000168702.12	-0.022536887	0.000402906	1	0.00660968	LRP1B
SAT	HTN	ENSG00000168884.10	-0.008691035	0.003444834	1	0.034884257	TNIP2
SAT	HTN	ENSG00000168936.6	0.010356987	0.003928514	1	0.038361473	TMEM129
SAT	HTN	ENSG00000169047.5	-0.020587958	3.27E-09	1.07E-05	3.83E-07	IRS1
SAT	HTN	ENSG00000169203.12	0.01143981	6.80E-06	0.02231238	0.000245191	RP11-231C14.4
SAT	HTN	ENSG00000169592.10	-0.026623454	1.68E-09	5.50E-06	2.39E-07	INO80E
SAT	HTN	ENSG00000169629.7	-0.004582065	0.004459906	1	0.041729241	RGPD8
SAT	HTN	ENSG00000170049.5	-0.015140591	0.002917253	1	0.030579894	KCNAB3
SAT	HTN	ENSG00000170092.10	0.021828856	5.01E-06	0.016432151	0.000186729	AC018720.10
SAT	HTN	ENSG00000170633.12	-0.017256325	0.000231836	0.760653289	0.004297476	RNF34
SAT	HTN	ENSG00000170955.9	0.01733567	7.30E-05	0.239584078	0.001774697	PRKCDBP
SAT	HTN	ENSG00000171421.8	0.020897637	0.002376693	1	0.026523566	MRPL36
SAT	HTN	ENSG00000171700.9	-0.025908773	2.52E-14	8.26E-11	1.38E-11	RGS19
SAT	HTN	ENSG00000172113.4	0.017943362	5.92E-10	1.94E-06	1.07E-07	NME6
SAT	HTN	ENSG00000172687.9	0.005910219	0.001405167	1	0.017463464	ZNF738
SAT	HTN	ENSG00000172789.3	0.0219391	0.001285255	1	0.016552068	HOXC5
SAT	HTN	ENSG00000172927.3	-0.016180647	0.002043549	1	0.023608743	MYEOV
SAT	HTN	ENSG00000172992.7	-0.008149312	0.000370583	1	0.006267431	DCAKD
SAT	HTN	ENSG00000173209.18	0.014235339	0.002987563	1	0.031118074	AHSA2
SAT	HTN	ENSG00000173511.5	-0.0233707	2.59E-09	8.51E-06	3.41E-07	VEGFB
SAT	HTN	ENSG00000173531.11	-0.016085402	5.37E-06	0.017622722	0.000198008	MST1
SAT	HTN	ENSG00000174004.5	0.015382697	0.001963171	1	0.023004153	NRROS
SAT	HTN	ENSG00000174177.8	-0.021717258	0.001641157	1	0.019869511	CTU2
SAT	HTN	ENSG00000174226.4	-0.026903752	3.09E-05	0.101233222	0.00084361	SNX31
SAT	HTN	ENSG00000174891.8	-0.01476271	1.66E-07	0.000545032	1.07E-05	RSRC1
SAT	HTN	ENSG00000175220.7	0.01778731	0.000170162	0.558302901	0.003511339	ARHGAP1
SAT	HTN	ENSG00000175334.3	0.010312241	0.004814434	1	0.044371228	BANF1
SAT	HTN	ENSG00000175470.14	-0.02015348	1.42E-05	0.046684881	0.000444618	PPP2R2D
SAT	HTN	ENSG00000175489.9	0.009821118	0.003224548	1	0.032958701	LRRC25
SAT	HTN	ENSG00000176390.10	0.023940506	0.000178765	0.586527662	0.003619977	CRLF3
SAT	HTN	ENSG00000177000.6	-0.054668639	3.95E-17	1.29E-13	3.24E-14	MTHFR
SAT	HTN	ENSG00000177311.6	0.019990895	0.000514385	1	0.007998565	ZBTB38
SAT	HTN	ENSG00000177380.9	-0.02259175	0.000430774	1	0.00692828	PPFIA3
SAT	HTN	ENSG00000177697.13	0.016151435	0.003055603	1	0.03162597	CD151
SAT	HTN	ENSG00000177700.5	-0.018292018	0.003251771	1	0.033133733	POLR2L
SAT	HTN	ENSG00000177839.4	0.014908018	0.001288495	1	0.016552068	PCDHB9
SAT	HTN	ENSG00000178188.10	-0.013482943	0.000164981	0.541304063	0.003447797	SH2B1
SAT	HTN	ENSG00000178467.13	-0.020786655	0.002309686	1	0.026131312	P4HTM
SAT	HTN	ENSG00000178718.5	0.024589834	1.30E-09	4.27E-06	1.94E-07	RPP25
SAT	HTN	ENSG00000178809.7	-0.021310413	0.000525392	1	0.008131178	TRIM73
SAT	HTN	ENSG00000178922.12	-0.015000191	2.84E-07	0.000932114	1.64E-05	HYI
SAT	HTN	ENSG00000178971.9	0.010463225	0.000261805	0.858981653	0.004769464	CTC1
SAT	HTN	ENSG00000180855.11	0.01984662	0.000139471	0.457603234	0.002990871	ZNF443

SAT	HTN	ENSG00000181885.14	-0.039664138	6.52E-10	2.14E-06	1.07E-07	CLDN7
SAT	HTN	ENSG00000182372.6	0.029691644	1.06E-07	0.000347083	7.08E-06	CLN8
SAT	HTN	ENSG00000182871.10	0.010930603	0.001548288	1	0.018884508	COL18A1
SAT	HTN	ENSG00000182986.8	0.004501667	0.000295536	0.969653396	0.005269855	ZNF320
SAT	HTN	ENSG00000183604.10	-0.013430969	0.000890578	1	0.012122569	RP11-347C12.2
SAT	HTN	ENSG00000183762.8	-0.008017406	0.000224702	0.737248672	0.004297476	KREMEN1
SAT	HTN	ENSG00000184110.10	0.017946603	7.41E-07	0.002431272	3.63E-05	EIF3C
SAT	HTN	ENSG00000184385.2	-0.009934233	0.000388661	1	0.006408027	C21orf128
SAT	HTN	ENSG00000184389.8	-0.014988451	1.33E-05	0.043766819	0.000424921	A3GALT2
SAT	HTN	ENSG00000184574.5	-0.017681058	0.000875585	1	0.011992756	LPAR5
SAT	HTN	ENSG00000184923.8	-0.018817736	0.004719878	1	0.043622307	NUTM2A
SAT	HTN	ENSG00000185163.5	0.009052041	0.00069333	1	0.010185969	DDX51
SAT	HTN	ENSG00000185324.17	-0.009445977	1.64E-06	0.005374204	7.07E-05	CDK10
SAT	HTN	ENSG00000185339.4	-0.007823014	0.001790405	1	0.021361157	TCN2
SAT	HTN	ENSG00000185829.11	-0.015682496	4.55E-05	0.14941435	0.001176491	ARL17A
SAT	HTN	ENSG00000186792.11	-0.006543891	0.00361868	1	0.036308532	HYAL3
SAT	HTN	ENSG00000187583.6	0.02089978	0.000686367	1	0.01015143	PLEKHN1
SAT	HTN	ENSG00000187778.9	0.03047864	3.56E-08	0.000116657	2.92E-06	MCRS1
SAT	HTN	ENSG00000188227.8	-0.008217238	0.000653723	1	0.009793908	ZNF793
SAT	HTN	ENSG00000188321.9	-0.003648849	0.002087143	1	0.024027772	ZNF559
SAT	HTN	ENSG00000188383.7	-0.016129133	0.00110814	1	0.014637359	AC018892.9
SAT	HTN	ENSG00000188629.7	-0.006637237	0.001882042	1	0.022292345	ZNF177
SAT	HTN	ENSG00000188878.12	-0.011550304	3.93E-07	0.001288415	2.15E-05	FBF1
SAT	HTN	ENSG00000188976.6	-0.006951196	0.001303591	1	0.016590986	NOC2L
SAT	HTN	ENSG00000196126.6	-0.031825735	8.40E-10	2.76E-06	1.31E-07	HLA-DRB1
SAT	HTN	ENSG00000196502.7	-0.017733121	0.000175927	0.577216082	0.003585193	SULT1A1
SAT	HTN	ENSG00000196821.5	0.016134623	1.50E-06	0.004925645	6.75E-05	C6orf106
SAT	HTN	ENSG00000196961.8	-0.013587551	0.004406645	1	0.041427517	AP2A1
SAT	HTN	ENSG00000196966.3	0.024088968	1.06E-08	3.49E-05	1.09E-06	HIST1H3E
SAT	HTN	ENSG00000196968.6	-0.021102971	0.000230554	0.756447477	0.004297476	FUT11
SAT	HTN	ENSG00000196981.2	0.02111026	0.000877251	1	0.011992756	WDR5B
SAT	HTN	ENSG00000197020.6	-0.008765391	1.19E-05	0.039040158	0.000394345	ZNF100
SAT	HTN	ENSG00000197062.7	0.008927332	4.77E-05	0.156577668	0.001223263	RP5-874C20.3
SAT	HTN	ENSG00000197165.6	0.010937042	1.92E-05	0.063035388	0.000572975	SULT1A2
SAT	HTN	ENSG00000197279.3	0.017284393	0.000894136	1	0.012122569	ZNF165
SAT	HTN	ENSG00000197375.8	0.02341602	1.33E-08	4.35E-05	1.32E-06	SLC22A5
SAT	HTN	ENSG00000197535.10	-0.017775822	0.005488053	1	0.049332333	MYO5A
SAT	HTN	ENSG00000197728.5	-0.006573244	4.39E-06	0.014402644	0.000167473	RPS26
SAT	HTN	ENSG00000198208.7	0.02037609	0.002570519	1	0.028206929	RPS6KL1
SAT	HTN	ENSG00000198270.8	-0.015802767	1.46E-11	4.79E-08	4.36E-09	TMEM116
SAT	HTN	ENSG00000198646.9	-0.017593379	0.003613698	1	0.036308532	NCOA6
SAT	HTN	ENSG00000198805.7	-0.005441425	0.002625827	1	0.028527611	PNP
SAT	HTN	ENSG00000198837.5	-0.037618236	6.43E-08	0.000211112	4.91E-06	DENND4B
SAT	HTN	ENSG00000203301.2	-0.01907133	0.000369931	1	0.006267431	AL590822.1
SAT	HTN	ENSG00000204228.3	-0.022113084	2.60E-06	0.008531918	0.000107999	HSD17B8
SAT	HTN	ENSG00000204252.8	-0.012935674	0.003793237	1	0.037713967	HLA-DOA
SAT	HTN	ENSG00000204287.9	0.012161025	0.004476895	1	0.041729241	HLA-DRA
SAT	HTN	ENSG00000204314.6	0.015864263	0.000231365	0.75910892	0.004297476	PRRT1
SAT	HTN	ENSG00000204316.8	0.017387823	0.001428342	1	0.017618005	MRPL38
SAT	HTN	ENSG00000204338.4	0.020866772	8.98E-15	2.95E-11	5.90E-12	CYP21A1P
SAT	HTN	ENSG00000204344.10	0.014356281	1.55E-06	0.005088711	6.78E-05	STK19
SAT	HTN	ENSG00000204387.8	0.014596686	2.88E-05	0.094393682	0.000793224	C6orf48
SAT	HTN	ENSG00000204438.6	0.018295014	9.89E-06	0.032435179	0.000334383	GPANK1
SAT	HTN	ENSG00000204463.8	0.011548621	1.09E-05	0.035620101	0.00036347	BAG6
SAT	HTN	ENSG00000204516.5	-0.004195103	0.004120911	1	0.039725318	MICB
SAT	HTN	ENSG00000204525.10	-0.008179527	0.000228991	0.751320646	0.004297476	HLA-C
SAT	HTN	ENSG00000204531.11	-0.004663311	0.002803926	1	0.029676388	POU5F1
SAT	HTN	ENSG00000204536.9	-0.005751687	8.04E-05	0.263683982	0.001918893	CCHCR1
SAT	HTN	ENSG00000204789.3	0.01346018	2.37E-05	0.077854105	0.000682931	ZNF204P
SAT	HTN	ENSG00000205583.9	-0.013540687	2.88E-09	9.43E-06	3.63E-07	STAG3L1
SAT	HTN	ENSG00000206077.6	0.016987818	0.003114828	1	0.032036838	ZDHHC11B
SAT	HTN	ENSG00000213240.7	0.012207423	0.001915064	1	0.02252088	NOTCH2NL
SAT	HTN	ENSG00000213246.2	0.023350234	0.000160524	0.526677971	0.003376141	SUPT4H1
SAT	HTN	ENSG00000213277.3	-0.046311774	1.32E-19	4.33E-16	1.44E-16	MARCKSL1P1
SAT	HTN	ENSG00000213347.6	-0.011579372	0.001397672	1	0.017448468	MXD3
SAT	HTN	ENSG00000213445.4	0.027571394	2.75E-08	9.02E-05	2.65E-06	SIPA1
SAT	HTN	ENSG00000213676.6	0.013173741	6.91E-09	2.27E-05	7.31E-07	ATF6B
SAT	HTN	ENSG00000213719.4	0.04350156	3.54E-08	0.000116308	2.92E-06	CLIC1
SAT	HTN	ENSG00000213903.4	-0.013586873	9.71E-06	0.031868696	0.000331966	LTB4R
SAT	HTN	ENSG00000214331.4	-0.014354367	0.003779971	1	0.037696311	RP11-252A24.2
SAT	HTN	ENSG00000214517.4	-0.009065405	0.002938107	1	0.030700413	PPME1
SAT	HTN	ENSG00000216901.1	-0.005755527	0.001304625	1	0.016590986	AL022393.7
SAT	HTN	ENSG00000218422.2	0.009341112	0.000470573	1	0.00742284	AC016773.1
SAT	HTN	ENSG00000219392.1	0.006301656	0.000737134	1	0.010515378	RP1-265C24.5
SAT	HTN	ENSG00000221838.5	-0.004671752	0.003877625	1	0.038072244	AP4M1
SAT	HTN	ENSG00000223496.1	-0.020875224	1.94E-05	0.063600177	0.000572975	EXOSC6
SAT	HTN	ENSG00000223718.2	0.017611431	0.004316934	1	0.040731275	AC093107.7
SAT	HTN	ENSG00000223865.6	0.014349701	0.001640129	1	0.019869511	HLA-DPB1
SAT	HTN	ENSG00000224389.4	-0.010576109	0.003886077	1	0.038072244	C4B
SAT	HTN	ENSG00000224997.1	-0.011093593	5.13E-05	0.168425256	0.001305622	AL049840.1
SAT	HTN	ENSG00000225398.2	0.013847765	8.07E-05	0.26480729	0.001918893	RP11-395L14.5
SAT	HTN	ENSG00000225851.1	0.009155689	0.000364728	1	0.006232678	HLA-S
SAT	HTN	ENSG00000226232.4	-0.007238731	0.00063537	1	0.009606677	RP11-419C5.2
SAT	HTN	ENSG00000228638.1	0.014252047	2.84E-06	0.009310949	0.000116387	FCF1P2
SAT	HTN	ENSG00000229391.3	0.006871832	0.000126107	0.413756274	0.002814669	HLA-DRB6
SAT	HTN	ENSG00000229759.1	-0.014069908	1.11E-11	3.64E-08	4.04E-09	MRPS18AP1
SAT	HTN	ENSG00000231130.1	0.006676282	0.005039457	1	0.046258571	HLA-T
SAT	HTN	ENSG00000232561.2	-0.015235432	0.002154753	1	0.024719383	GTF2IP1
SAT	HTN	ENSG00000233137.2	0.021873393	0.001859759	1	0.022108226	RP11-220I1.1
SAT	HTN	ENSG00000236397.2	0.010275443	7.50E-06	0.024620795	0.000261923	DDX11L2
SAT	HTN	ENSG00000237541.3	0.018590358	3.04E-07	0.00099886	1.72E-05	HLA-DQA2
SAT	HTN	ENSG00000238024.1	-0.024601528	0.000234573	0.769635591	0.004323795	DDX39BP2
SAT	HTN	ENSG00000239557.1	0.027326064	9.67E-05	0.317354766	0.002234893	RP11-168J18.6

SAT	HTN	ENSG00000240399.1	-0.025047249	7.43E-05	0.243622707	0.001791343	RP1-228P16.1
SAT	HTN	ENSG00000241170.2	0.011680517	0.002219304	1	0.025283117	RP11-147I3.1
SAT	HTN	ENSG00000242852.2	-0.01621878	0.004555237	1	0.042219581	ZNF709
SAT	HTN	ENSG00000243697.1	-0.02281377	0.001163228	1	0.015205383	RP11-463O9.1
SAT	HTN	ENSG00000243708.4	0.009320828	0.000185195	0.607623764	0.003682568	PLA2G4B
SAT	HTN	ENSG00000243789.6	0.012603156	0.000722235	1	0.010439001	JMJD7
SAT	HTN	ENSG00000248290.1	-0.006705231	0.000128516	0.421662093	0.002849068	TNXA
SAT	HTN	ENSG00000249740.1	0.018112597	0.003099877	1	0.031983321	CTD-2127H9.1
SAT	HTN	ENSG00000251417.1	-0.008446506	0.000222022	0.728452935	0.004285017	RP11-1348G14.4
SAT	HTN	ENSG00000251600.1	-0.013531364	0.002894746	1	0.030441221	RP11-673E1.1
SAT	HTN	ENSG00000253203.2	-0.013051401	0.001110851	1	0.014637359	GUSBP3
SAT	HTN	ENSG00000253320.1	0.013503547	0.000268763	0.881812692	0.004818649	KB-1507C5.2
SAT	HTN	ENSG00000253669.3	0.025048887	3.09E-08	0.000101338	2.81E-06	KB-1732A1.1
SAT	HTN	ENSG00000254851.1	0.009851543	1.78E-07	0.000585025	1.13E-05	RP11-109L13.1
SAT	HTN	ENSG00000256223.1	-0.017832451	3.91E-06	0.01281652	0.000154416	ZNF10
SAT	HTN	ENSG00000256269.2	0.015386624	1.25E-05	0.04117433	0.00040367	HMBS
SAT	HTN	ENSG00000256885.1	-0.008673306	0.000536282	1	0.00826075	AP001877.1
SAT	HTN	ENSG00000259295.2	0.01513138	0.000949996	1	0.012774334	CSPG4P12
SAT	HTN	ENSG00000259982.1	0.012156686	2.42E-05	0.079480759	0.000685179	CDC37P1
SAT	HTN	ENSG00000260075.1	0.011145285	0.005301518	1	0.0481836	NSFP1
SAT	HTN	ENSG00000260077.1	-0.013121778	0.001992879	1	0.023153921	RP11-254F7.2
SAT	HTN	ENSG00000260877.1	-0.021502211	1.17E-06	0.003847323	5.42E-05	RP11-211G23.2
SAT	HTN	ENSG00000261556.4	-0.016697628	5.25E-05	0.172303871	0.001325414	RP11-296I10.6
SAT	HTN	ENSG00000264538.2	0.008750856	6.34E-05	0.208043723	0.001564239	SUZ12P
SAT	HTN	ENSG00000266497.1	0.015301626	0.005440137	1	0.04903596	RP11-995C19.2
SAT	HTN	ENSG00000267592.1	0.01762604	0.005047415	1	0.046258571	CTC-507E2.2
SAT	HTN	ENSG00000268117.1	-0.010878449	0.000299234	0.981787206	0.005306958	VN1R84P
SAT	HTN	ENSG00000268225.1	0.01241561	0.000447546	1	0.00709371	CTD-2331H12.5
SAT	HTN	ENSG00000268535.1	-0.018618942	0.000646199	1	0.009725584	RP11-420K14.3
SAT	HTN	ENSG00000268555.1	-0.014587446	0.000130331	0.42761721	0.002869914	RP11-678G14.3
SAT	HTN	ENSG00000269963.1	-0.026641592	5.87E-07	0.001926583	2.96E-05	RP11-73M18.9
SAT	HTN	ENSG00000270018.1	0.030384429	3.26E-08	0.000106961	2.89E-06	RP3-462E2.5
SAT	HTN	ENSG00000270020.1	-0.008318399	0.000167471	0.54947322	0.003477679	RP11-463O9.9
SAT	HTN	ENSG00000270091.1	-0.016506116	0.000686869	1	0.01015143	RP11-78O7.2
SAT	HTN	ENSG00000271581.1	0.01143513	4.68E-09	1.54E-05	5.12E-07	XXbac-BPG248L24.12
SAT	HTN	ENSG00000271584.1	0.009297776	0.002297031	1	0.02607806	RP11-89C3.4
SAT	HTN	ENSG00000271623.1	-0.023676658	7.50E-06	0.024609466	0.000261923	RP11-435I10.5
SAT	HTN	ENSG00000271751.1	-0.025899965	1.23E-05	0.040377418	0.000401308	RP11-110I1.14
SAT	HTN	ENSG00000273117.1	0.014651574	0.000263113	0.863273011	0.004769464	AC144652.1
SAT	Heart_Failure	ENSG00000001617.7	-0.013173139	1.05E-06	0.003449752	0.000581811	SEMA3F
SAT	Heart_Failure	ENSG00000004534.10	-0.003315477	1.54E-07	0.000505253	0.000168418	RBM6
SAT	Heart_Failure	ENSG00000008838.13	-0.010686635	0.000182938	0.600219466	0.025009144	MED24
SAT	Heart_Failure	ENSG00000083720.8	-0.014315518	0.000100061	0.328301019	0.015633382	OXCT1
SAT	Heart_Failure	ENSG00000090621.9	-0.006657589	7.70E-05	0.252746332	0.012637317	PABPC4
SAT	Heart_Failure	ENSG00000114735.5	0.011309655	0.000265749	0.871922754	0.027247586	HEMK1
SAT	Heart_Failure	ENSG00000116883.8	-0.006508555	4.34E-05	0.142549737	0.009503316	RP11-268J15.5
SAT	Heart_Failure	ENSG00000127957.12	-0.007254743	2.09E-05	0.068565762	0.006233251	PMS2P3
SAT	Heart_Failure	ENSG00000131165.10	0.008755673	0.00045558	1	0.039533553	CHMP1A
SAT	Heart_Failure	ENSG00000131435.8	0.017314273	5.25E-06	0.017229775	0.002461396	PDLIM4
SAT	Heart_Failure	ENSG00000132563.11	-0.007308444	6.30E-05	0.206637533	0.011955478	REEP2
SAT	Heart_Failure	ENSG00000135213.8	-0.016235475	1.05E-06	0.003430974	0.000581811	POM121C
SAT	Heart_Failure	ENSG00000137312.10	0.009188357	6.79E-05	0.222937851	0.011955478	FLOT1
SAT	Heart_Failure	ENSG00000145692.10	-0.005991572	0.00046992	1	0.039533553	BHMT
SAT	Heart_Failure	ENSG00000145777.10	0.014444087	7.88E-06	0.025849598	0.00258496	TSLP
SAT	Heart_Failure	ENSG00000156170.8	-0.012287641	0.000248167	0.814234976	0.027247586	NDUFAF6
SAT	Heart_Failure	ENSG00000164068.11	0.004947231	6.10E-06	0.020018014	0.002502252	RNF123
SAT	Heart_Failure	ENSG00000170092.10	0.009495741	0.000415699	1	0.038968793	AC018720.10
SAT	Heart_Failure	ENSG00000172057.5	-0.019872989	7.34E-12	2.41E-08	2.41E-08	ORMDL3
SAT	Heart_Failure	ENSG00000172992.7	-0.005162731	6.08E-05	0.1994629	0.011955478	DCAKD
SAT	Heart_Failure	ENSG00000173540.8	0.013994653	0.00025701	0.843250484	0.027247586	GMPPB
SAT	Heart_Failure	ENSG00000173905.4	0.011184706	0.000337862	1	0.033591688	GOLIM4
SAT	Heart_Failure	ENSG00000174004.5	-0.009783529	0.000464676	1	0.039533553	NRROS
SAT	Heart_Failure	ENSG00000176715.11	-0.008858515	0.000210541	0.690786369	0.027247586	ACSF3
SAT	Heart_Failure	ENSG00000179344.12	-0.010040039	0.00025911	0.850138983	0.027247586	HLA-DQB1
SAT	Heart_Failure	ENSG00000182134.11	-0.010459075	0.000572017	1	0.046919683	TDRKH
SAT	Heart_Failure	ENSG00000183604.10	-0.007986835	0.000443093	1	0.039533553	RP11-347C12.2
SAT	Heart_Failure	ENSG00000185499.12	0.011829975	3.77E-05	0.123763084	0.009269033	MUC1
SAT	Heart_Failure	ENSG00000187778.9	0.012376657	6.92E-05	0.227154091	0.011955478	MCRS1
SAT	Heart_Failure	ENSG00000188895.7	-0.005474804	0.000142301	0.46688838	0.020801401	MSL1
SAT	Heart_Failure	ENSG00000197375.8	0.012305926	1.10E-07	0.000360941	0.000168418	SLC22A5
SAT	Heart_Failure	ENSG00000197728.5	0.00305817	0.000145819	0.478432218	0.020801401	RPS26
SAT	Heart_Failure	ENSG00000204438.6	-0.009692416	3.15E-05	0.103222284	0.008601857	GPANK1
SAT	Heart_Failure	ENSG00000204516.5	0.00338079	3.96E-05	0.129766464	0.009269033	MICB
SAT	Heart_Failure	ENSG00000205583.9	-0.006258194	1.06E-06	0.003490867	0.000581811	STAG3L1
SAT	Heart_Failure	ENSG00000214078.7	-0.002434439	0.000412456	1	0.038968793	CPNE1
SAT	Heart_Failure	ENSG00000225851.1	-0.005296477	0.000246642	0.809233072	0.027247586	HLA-S
SAT	Heart_Failure	ENSG00000232491.1	0.0133915	7.78E-06	0.025520246	0.00258496	RP4-756H11.1
SAT	Heart_Failure	ENSG00000243753.1	-0.002967725	0.000261247	0.857152676	0.027247586	HLA-L
SAT	Heart_Failure	ENSG00000267369.1	-0.013093376	0.00023909	0.784454911	0.027247586	RP11-1094M14.8
VAT	CAD	ENSG00000065060.12	0.009846128	1.82E-05	0.050297922	0.00628724	UHRF1BP1
VAT	CAD	ENSG00000075131.5	0.006277787	0.000441759	1	0.043413258	TIPIN
VAT	CAD	ENSG00000076258.5	0.025806204	9.11E-07	0.002514751	0.001257375	FMO4
VAT	CAD	ENSG00000096433.6	0.020058784	4.21E-05	0.116207024	0.0124879	ITPR3
VAT	CAD	ENSG00000099194.5	-0.018167814	0.000317871	0.877323535	0.038787669	SCD
VAT	CAD	ENSG00000115486.7	0.012781827	0.000297342	0.820663296	0.038787669	GGCX
VAT	CAD	ENSG00000124562.5	-0.014130006	4.71E-06	0.013007468	0.004335823	SNRPC
VAT	CAD	ENSG00000124678.13	0.009730688	0.00020691	0.571071418	0.032700425	TCP11
VAT	CAD	ENSG00000125388.15	0.004473991	0.000110826	0.305880846	0.025490071	GRK4
VAT	CAD	ENSG00000137700.12	0.01458388	0.000216117	0.596482349	0.032700425	SLC37A4
VAT	CAD	ENSG00000138303.13	-0.012234845	0.000425295	1	0.043413258	ASCC1
VAT	CAD	ENSG00000142046.10	0.006917361	0.000471883	1	0.043413258	TMEM91
VAT	CAD	ENSG00000143156.9	-0.01984382	1.10E-05	0.030430146	0.006086029	NME7
VAT	CAD	ENSG00000143437.16	-0.02122307	6.05E-07	0.001668724	0.001257375	ARNT

VAT	CAD	ENSG00000143951.11	0.01971114	0.000142036	0.392019033	0.03015531	WDPCP
VAT	CAD	ENSG00000145020.10	-0.004068616	0.000457551	1	0.043413258	AMT
VAT	CAD	ENSG00000145029.7	-0.010245957	1.01E-05	0.027853713	0.006086029	NICN1
VAT	CAD	ENSG00000147912.8	0.018442494	0.000198155	0.546908391	0.032700425	FBXO10
VAT	CAD	ENSG00000149084.7	-0.003180787	0.000323231	0.892116391	0.038787669	HSD17B12
VAT	CAD	ENSG00000164068.11	0.007463329	7.65E-05	0.211276889	0.01920699	RNF123
VAT	CAD	ENSG00000168038.6	-0.009214595	0.000458819	1	0.043413258	ULK4
VAT	CAD	ENSG00000172171.6	0.015175036	0.000410809	1	0.043413258	TEFM
VAT	CAD	ENSG00000176390.10	0.011282356	0.000219483	0.605772978	0.032700425	CRLF3
VAT	CAD	ENSG00000181092.5	-0.011737578	0.000338054	0.933027817	0.038876159	ADIPOQ
VAT	CAD	ENSG00000182511.7	-0.020917584	1.66E-05	0.045721642	0.00628724	FES
VAT	CAD	ENSG00000197081.8	-0.016782083	0.000225112	0.621308073	0.032700425	IGF2R
VAT	CAD	ENSG00000198270.8	-0.006122141	4.52E-05	0.124879004	0.0124879	TMEM116
VAT	CAD	ENSG00000213689.5	0.005765878	0.000157238	0.433977799	0.030998414	TREX1
VAT	CAD	ENSG00000264538.2	0.008847486	0.000280086	0.773038338	0.038651917	SUZ12P
VAT	CAD	ENSG00000271581.1	0.025678587	1.54E-05	0.042601867	0.00628724	XXbac-BPG248L24.12
VAT	T2DM	ENSG00000004534.10	-0.004332966	8.35E-09	2.31E-05	2.88E-06	RBM6
VAT	T2DM	ENSG00000011105.7	-0.007855696	0.001608371	1	0.045352953	TSPAN9
VAT	T2DM	ENSG00000011523.9	0.017150923	3.96E-11	1.09E-07	2.18E-08	CEP68
VAT	T2DM	ENSG00000037042.8	-0.018185417	1.75E-07	0.000483299	3.72E-05	TUBG2
VAT	T2DM	ENSG00000055955.11	0.004708028	7.06E-06	0.019493733	0.000749759	ITIH4
VAT	T2DM	ENSG00000065665.16	-0.015980757	2.44E-06	0.006744149	0.000293224	SEC61A2
VAT	T2DM	ENSG00000078618.15	0.011965055	0.000268002	0.739685085	0.012455159	NRD1
VAT	T2DM	ENSG00000082258.8	0.01172735	0.000873292	1	0.028693864	CCNT2
VAT	T2DM	ENSG00000086619.9	0.010205169	4.33E-06	0.011941327	0.000497555	ERO1LB
VAT	T2DM	ENSG00000090621.9	-0.021186856	1.68E-12	4.64E-09	1.16E-09	PABPC4
VAT	T2DM	ENSG00000091542.8	-0.013200608	8.26E-05	0.228005177	0.005066782	ALKBH5
VAT	T2DM	ENSG00000095485.12	-0.003234458	6.19E-05	0.170837118	0.004163595	CWF19L1
VAT	T2DM	ENSG00000099194.5	-0.012184002	0.001610358	1	0.045352953	SCD
VAT	T2DM	ENSG00000103226.13	-0.009507402	0.000629956	1	0.022877354	NOMO3
VAT	T2DM	ENSG00000103479.10	0.013294817	9.75E-05	0.269144509	0.005726479	RBL2
VAT	T2DM	ENSG00000108785.7	-0.016199637	4.07E-05	0.112285703	0.003034749	HSD17B1P1
VAT	T2DM	ENSG00000111215.7	0.005219074	0.00139763	1	0.041478058	PRR4
VAT	T2DM	ENSG00000112276.9	-0.006415103	0.000417895	1	0.017475613	BVES
VAT	T2DM	ENSG00000116157.5	-0.003528954	0.000819006	1	0.027234428	GPX7
VAT	T2DM	ENSG00000119242.4	-0.011382156	0.000475094	1	0.018940823	CCDC92
VAT	T2DM	ENSG00000120907.13	0.005038235	3.90E-05	0.10772573	0.002992381	ADRA1A
VAT	T2DM	ENSG00000124767.6	0.013392092	0.000985229	1	0.030900358	GLO1
VAT	T2DM	ENSG00000125388.15	0.00292367	0.000967516	1	0.030693597	GRK4
VAT	T2DM	ENSG00000126464.9	0.009820216	0.001381229	1	0.041436863	PRR12
VAT	T2DM	ENSG00000127220.5	-0.008076621	0.000594626	1	0.022177952	ABHD8
VAT	T2DM	ENSG00000127957.12	-0.012346798	2.65E-05	0.073082294	0.002149479	PMS2P3
VAT	T2DM	ENSG00000130921.3	0.009891253	0.000270764	0.747309516	0.012455159	C12orf65
VAT	T2DM	ENSG00000137265.10	0.006881096	0.000947059	1	0.030693597	IRF4
VAT	T2DM	ENSG00000137310.7	0.015733453	4.31E-05	0.118967945	0.003109431	TCF19
VAT	T2DM	ENSG00000137501.12	-0.012988865	0.000541869	1	0.020487111	SYTL2
VAT	T2DM	ENSG00000137700.12	0.01110075	0.000234761	0.647941435	0.011907029	SLC37A4
VAT	T2DM	ENSG00000137877.8	0.005685689	6.61E-05	0.182401528	0.004241896	SPTBN5
VAT	T2DM	ENSG00000140398.9	-0.0116514	2.93E-05	0.081000887	0.002314311	NEIL1
VAT	T2DM	ENSG00000140400.10	-0.002826645	1.04E-05	0.028831068	0.001029681	MAN2C1
VAT	T2DM	ENSG00000141140.12	0.016661083	1.11E-06	0.003071798	0.00015359	MYO19
VAT	T2DM	ENSG00000141252.15	-0.006793446	0.000230029	0.634880333	0.011907029	VPS53
VAT	T2DM	ENSG00000141452.5	-0.015369868	2.94E-07	0.000812085	5.41E-05	C18orf8
VAT	T2DM	ENSG00000141994.11	0.004915812	0.001111163	1	0.034458528	DUS3L
VAT	T2DM	ENSG00000149084.7	-0.003847924	1.33E-08	3.66E-05	4.07E-06	HSD17B12
VAT	T2DM	ENSG00000149089.8	-0.003930868	0.000170065	0.46937914	0.009203513	APIP
VAT	T2DM	ENSG00000152359.10	-0.004289249	0.000230042	0.634916231	0.011907029	POC5
VAT	T2DM	ENSG00000153814.7	-0.018729946	4.56E-21	1.26E-17	1.26E-17	JAZF1
VAT	T2DM	ENSG00000156931.11	0.013325172	0.001246879	1	0.038237629	VPS8
VAT	T2DM	ENSG00000157322.12	-0.010306535	3.56E-08	9.82E-05	8.92E-06	CLEC18A
VAT	T2DM	ENSG00000157992.8	0.009026748	1.14E-05	0.031349587	0.001044986	KRTCAP3
VAT	T2DM	ENSG00000163554.7	-0.006730558	0.000277531	0.765985439	0.012557138	SPTA1
VAT	T2DM	ENSG00000163933.5	-0.008955202	5.77E-07	0.001591183	9.94E-05	RFT1
VAT	T2DM	ENSG00000164068.11	0.006429384	8.56E-06	0.023631569	0.000875243	RNF123
VAT	T2DM	ENSG00000164078.8	0.018391917	7.57E-07	0.002088443	0.000121943	MST1R
VAT	T2DM	ENSG00000165917.5	-0.014135316	2.11E-05	0.058128376	0.001816512	RAPSN
VAT	T2DM	ENSG00000166825.9	0.006954768	0.000655704	1	0.023226937	ANPEP
VAT	T2DM	ENSG00000168612.4	0.004580254	0.000500891	1	0.019471269	ZSWIM1
VAT	T2DM	ENSG00000169047.5	-0.012800604	2.62E-07	0.000722378	5.16E-05	IRS1
VAT	T2DM	ENSG00000169592.10	-0.005985636	0.000247041	0.681832834	0.011907029	INO80E
VAT	T2DM	ENSG00000170092.10	0.010439489	0.000757196	1	0.025800768	AC018720.10
VAT	T2DM	ENSG00000170571.7	-0.013382621	0.000136639	0.377124602	0.007667187	EMB
VAT	T2DM	ENSG00000171700.9	-0.008129381	2.61E-05	0.072044667	0.002149479	RGS19
VAT	T2DM	ENSG00000172247.3	-0.00611146	0.000138898	0.383359364	0.007667187	C1QTNF4
VAT	T2DM	ENSG00000173917.9	-0.008600179	0.000808705	1	0.027219831	HOXB2
VAT	T2DM	ENSG00000175305.12	-0.01287363	7.95E-07	0.002194966	0.000121943	CCNE2
VAT	T2DM	ENSG00000178750.2	0.009638938	0.000656413	1	0.023226937	STX19
VAT	T2DM	ENSG00000178917.10	-0.010530923	0.00138109	1	0.041436863	ZNF852
VAT	T2DM	ENSG00000179344.12	-0.010906808	5.89E-09	1.63E-05	2.32E-06	HLA-DQB1
VAT	T2DM	ENSG00000182670.9	0.002819548	0.001485644	1	0.043621024	TTC3
VAT	T2DM	ENSG00000185245.6	0.011856977	0.001534186	1	0.044404773	GP1BA
VAT	T2DM	ENSG00000185800.7	-0.01187574	0.000101738	0.280797392	0.005849946	DMWD
VAT	T2DM	ENSG00000185989.9	-0.012188646	7.09E-05	0.195566885	0.004444702	RASA3
VAT	T2DM	ENSG00000196126.6	-0.014329638	3.23E-10	8.92E-07	1.49E-07	HLA-DRB1
VAT	T2DM	ENSG00000196301.3	0.00636131	2.05E-05	0.056686469	0.001816512	HLA-DRB9
VAT	T2DM	ENSG00000196735.7	-0.016587164	9.85E-07	0.002717469	0.000143025	HLA-DQA1
VAT	T2DM	ENSG00000198502.5	-0.003934515	4.39E-05	0.12126781	0.003109431	HLA-DRB5
VAT	T2DM	ENSG00000198909.3	-0.014350704	1.13E-05	0.031065747	0.001044986	MAP3K3
VAT	T2DM	ENSG00000204344.10	-0.010911555	0.00025022	0.690607666	0.011907029	STK19
VAT	T2DM	ENSG00000204525.10	-0.00392544	0.000707124	1	0.024704568	HLA-C
VAT	T2DM	ENSG00000204560.5	0.010150218	0.000474877	1	0.018940823	DHX16
VAT	T2DM	ENSG00000204622.6	0.003817344	0.000729002	1	0.025150583	HLA-J
VAT	T2DM	ENSG00000205583.9	-0.004657484	0.000615596	1	0.022653918	STAG3L1

VAT	T2DM	ENSG00000205659.6	0.013875109	6.34E-05	0.174870981	0.004163595	LIN52
VAT	T2DM	ENSG00000206344.6	0.006194087	1.60E-06	0.004405667	0.000200258	HCG27
VAT	T2DM	ENSG00000212127.5	0.006917457	0.000468892	1	0.018940823	TAS2R14
VAT	T2DM	ENSG00000213221.4	0.003317361	0.000249452	0.688488117	0.011907029	DNLZ
VAT	T2DM	ENSG00000214530.3	0.008081462	2.72E-08	7.52E-05	7.52E-06	STARD10
VAT	T2DM	ENSG00000214706.6	-0.007967616	0.001544514	1	0.044404773	IFRD2
VAT	T2DM	ENSG00000226232.4	-0.011696758	1.57E-06	0.00433226	0.000200258	RP11-419C5.2
VAT	T2DM	ENSG00000229391.3	0.009970144	8.93E-16	2.46E-12	1.23E-12	HLA-DRB6
VAT	T2DM	ENSG00000230224.1	-0.008905757	8.59E-05	0.237220907	0.005156976	PHBP9
VAT	T2DM	ENSG00000231742.1	-0.010218315	0.000408506	1	0.017475613	RP11-112L6.4
VAT	T2DM	ENSG00000232063.1	0.003214853	0.000966094	1	0.030693597	RP11-307E17.8
VAT	T2DM	ENSG00000233569.1	-0.014553418	0.000364413	1	0.015964759	RP11-500B12.1
VAT	T2DM	ENSG00000233609.3	0.010851893	0.000523609	1	0.020071668	RP11-62H7.2
VAT	T2DM	ENSG00000237541.3	0.009016539	3.46E-15	9.55E-12	3.18E-12	HLA-DQA2
VAT	T2DM	ENSG00000237854.3	0.009228254	0.000480383	1	0.018940823	LINC00674
VAT	T2DM	ENSG00000243789.6	0.00914322	1.21E-07	0.00033434	2.79E-05	JMJD7
VAT	T2DM	ENSG00000253813.1	0.007287578	5.29E-05	0.145903188	0.00364758	COX6B1P4
VAT	T2DM	ENSG00000261760.2	0.011217579	0.000245298	0.677023394	0.011907029	RP11-1223D19.1
VAT	T2DM	ENSG00000268879.1	-0.007729438	0.001767767	1	0.0492832	IGFL1P1
VAT	T2DM	ENSG00000269963.1	-0.014579698	0.00041611	1	0.017475613	RP11-73M18.9
VAT	T2DM	ENSG00000271581.1	0.020804375	4.77E-06	0.013171662	0.000526866	XXbac-BPG248L24.12
VAT	T2DM	ENSG00000272221.1	-0.002981503	0.00028465	0.785633404	0.012671507	XXbac-BPG181B23.7
VAT	Obesity	ENSG00000004534.10	-0.012003509	2.58E-26	7.13E-23	2.38E-23	RBM6
VAT	Obesity	ENSG00000011021.17	0.013500691	0.000885796	1	0.013658085	CLCN6
VAT	Obesity	ENSG00000015676.13	0.007905229	0.003327451	1	0.03573449	NUDCD3
VAT	Obesity	ENSG00000031691.6	-0.00386726	0.004294372	1	0.043575249	CENPQ
VAT	Obesity	ENSG00000033800.9	0.033988019	1.05E-07	0.000290608	6.46E-06	PIAS1
VAT	Obesity	ENSG00000035115.17	-0.026225758	2.47E-06	0.006811713	0.000111667	SH3YL1
VAT	Obesity	ENSG00000037280.11	-0.018298065	0.000481932	1	0.008365619	FLT4
VAT	Obesity	ENSG00000041802.6	0.00351383	0.002751449	1	0.031393572	LSG1
VAT	Obesity	ENSG00000049449.4	-0.016994951	0.000684696	1	0.010986495	RCN1
VAT	Obesity	ENSG00000053900.6	0.01925026	0.000688646	1	0.010986495	ANAPC4
VAT	Obesity	ENSG00000054282.11	0.007832158	0.0012334	1	0.017587781	SDCCAG8
VAT	Obesity	ENSG00000055955.11	0.010291055	6.65E-11	1.84E-07	5.74E-09	ITIH4
VAT	Obesity	ENSG00000058063.11	0.0176696	0.001732351	1	0.022553246	ATP11B
VAT	Obesity	ENSG00000065060.12	0.026202429	3.92E-23	1.08E-19	1.80E-20	UHRF1BP1
VAT	Obesity	ENSG00000066855.11	-0.0158627	0.002044103	1	0.02463635	MTFR1
VAT	Obesity	ENSG00000070540.8	-0.012133575	0.00298535	1	0.033090625	WIPI1
VAT	Obesity	ENSG00000073754.5	0.017280654	0.005173729	1	0.048735468	CD5L
VAT	Obesity	ENSG00000075131.5	0.006857447	0.000853405	1	0.013232578	TIPIN
VAT	Obesity	ENSG00000076685.14	0.009746773	0.000149162	0.41168648	0.003293492	NT5C2
VAT	Obesity	ENSG00000082258.8	-0.015736066	0.002982103	1	0.033090625	CCNT2
VAT	Obesity	ENSG00000083097.10	0.006290028	0.004378496	1	0.0439491	DOPEY1
VAT	Obesity	ENSG00000084207.11	0.013535305	2.61E-05	0.071910214	0.00080798	GSTP1
VAT	Obesity	ENSG00000085832.12	-0.011717602	0.000469143	1	0.00819516	EPS15
VAT	Obesity	ENSG00000086015.16	0.014842102	0.001143441	1	0.016436964	MAST2
VAT	Obesity	ENSG00000087263.12	-0.020889599	6.35E-06	0.017512614	0.000261382	OGFOD1
VAT	Obesity	ENSG00000088833.13	0.012381224	0.004378799	1	0.0439491	NSFL1C
VAT	Obesity	ENSG00000090238.7	0.014557413	2.79E-09	7.70E-06	2.08E-07	YPEL3
VAT	Obesity	ENSG00000090621.9	-0.029430877	6.96E-11	1.92E-07	5.82E-09	PABPC4
VAT	Obesity	ENSG00000092020.6	0.016177942	0.000134442	0.371058792	0.003016738	PPP2R3C
VAT	Obesity	ENSG00000096433.6	0.026003821	4.00E-06	0.011048227	0.000172629	ITPR3
VAT	Obesity	ENSG00000100124.8	-0.013321151	0.000161725	0.446360912	0.003514653	ANKRD54
VAT	Obesity	ENSG00000100162.10	0.018974267	4.14E-05	0.114208827	0.001165396	CENPM
VAT	Obesity	ENSG00000100997.14	0.023030399	1.48E-05	0.040738325	0.000515675	ABHD12
VAT	Obesity	ENSG00000102595.14	-0.017286049	0.001626071	1	0.021473469	UGGT2
VAT	Obesity	ENSG00000102890.10	0.009346793	0.003406491	1	0.036161216	ELMO3
VAT	Obesity	ENSG00000103051.14	-0.011816616	0.000818241	1	0.012759014	COG4
VAT	Obesity	ENSG00000103351.8	0.005819632	0.001246349	1	0.01764063	CLUAP1
VAT	Obesity	ENSG00000103472.5	-0.020124905	0.000567481	1	0.009378732	RRN3P2
VAT	Obesity	ENSG00000103932.7	-0.01914295	0.001489429	1	0.020451858	RPAP1
VAT	Obesity	ENSG00000104219.8	-0.019595967	0.000505744	1	0.008563512	ZDHHC2
VAT	Obesity	ENSG00000104714.9	-0.019871922	0.000337953	0.932749424	0.006218329	ERICH1
VAT	Obesity	ENSG00000106290.10	-0.008079442	0.004432314	1	0.044318348	TAF6
VAT	Obesity	ENSG00000106638.11	0.012038339	0.001948608	1	0.023938382	TBL2
VAT	Obesity	ENSG00000107521.14	-0.018214315	0.002335128	1	0.027779968	HPS1
VAT	Obesity	ENSG00000108384.10	-0.011455808	5.43E-05	0.149901575	0.001441361	RAD51C
VAT	Obesity	ENSG00000108556.7	0.005360431	0.000502248	1	0.008563512	CHRNE
VAT	Obesity	ENSG00000110906.8	-0.015803936	0.001068617	1	0.015746258	KCTD10
VAT	Obesity	ENSG00000111801.11	0.016884885	6.22E-08	0.000171758	3.90E-06	BTN3A3
VAT	Obesity	ENSG00000112041.8	-0.016852345	0.000368778	1	0.006696239	TULP1
VAT	Obesity	ENSG00000112514.11	-0.01119687	7.77E-05	0.214367329	0.001981293	CUTA
VAT	Obesity	ENSG00000112981.3	0.015932553	0.000190646	0.526181988	0.004057373	NME5
VAT	Obesity	ENSG00000113119.8	-0.017969029	0.000285796	0.788795859	0.005533516	TMCO6
VAT	Obesity	ENSG00000113141.11	-0.010831715	0.000327891	0.904978408	0.006073681	IK
VAT	Obesity	ENSG00000113734.13	-0.00636403	0.00446395	1	0.044318348	BNIP1
VAT	Obesity	ENSG00000114054.9	0.02095936	1.65E-06	0.004551112	7.59E-05	PCCB
VAT	Obesity	ENSG00000114904.8	-0.012848176	0.004768645	1	0.046019087	NEK4
VAT	Obesity	ENSG00000115561.10	-0.015518132	9.93E-07	0.002739306	4.72E-05	CHMP3
VAT	Obesity	ENSG00000117481.6	-0.018949557	3.82E-05	0.10542661	0.001109754	NSUN4
VAT	Obesity	ENSG00000117523.11	0.01663217	0.000519791	1	0.0087477	PRRC2C
VAT	Obesity	ENSG00000119242.4	0.022350386	5.05E-06	0.013927662	0.000214272	CCDC92
VAT	Obesity	ENSG00000119509.8	0.011394059	5.94E-07	0.001640224	2.98E-05	INVS
VAT	Obesity	ENSG00000119865.4	-0.014601175	0.001236243	1	0.017587781	CNRIP1
VAT	Obesity	ENSG00000120451.6	-0.019453561	4.02E-07	0.001110843	2.10E-05	SNX19
VAT	Obesity	ENSG00000120907.13	0.005940957	0.001259209	1	0.017731721	ADRA1A
VAT	Obesity	ENSG00000121101.11	0.02223548	3.06E-05	0.084422637	0.000927721	TEX14
VAT	Obesity	ENSG00000122696.8	0.007546505	0.002925596	1	0.032690866	SLC25A51
VAT	Obesity	ENSG00000123243.10	0.013920304	0.004620509	1	0.044966202	ITIH5
VAT	Obesity	ENSG00000124508.12	0.010322219	4.46E-05	0.123161055	0.001207461	BTN2A2
VAT	Obesity	ENSG00000124562.5	-0.036021943	3.86E-24	1.07E-20	2.13E-21	SNRPC
VAT	Obesity	ENSG00000124678.13	0.008563181	0.004562759	1	0.044966202	TCP11
VAT	Obesity	ENSG00000125388.15	0.005089653	0.000133419	0.368235569	0.003016738	GRK4

VAT	Obesity	ENSG00000127337.2	0.006435319	0.002997626	1	0.033093789	YEATS4
VAT	Obesity	ENSG00000127957.12	-0.042314049	1.02E-21	2.80E-18	3.51E-19	PMS2P3
VAT	Obesity	ENSG00000128408.7	0.014354065	0.000584391	1	0.009600702	RIBC2
VAT	Obesity	ENSG00000128829.7	0.018408673	0.001863703	1	0.023559873	EIF2AK4
VAT	Obesity	ENSG00000129197.10	-0.020264101	1.82E-05	0.050340929	0.000606517	RPAIN
VAT	Obesity	ENSG00000129354.7	-0.017183279	0.000709502	1	0.01125417	AP1M2
VAT	Obesity	ENSG00000130270.12	-0.018745558	3.93E-11	1.08E-07	3.50E-09	ATP8B3
VAT	Obesity	ENSG00000130517.9	0.008814609	0.001112362	1	0.016244016	PGPEP1
VAT	Obesity	ENSG00000130649.5	-0.011128478	0.001770611	1	0.02256353	CYP2E1
VAT	Obesity	ENSG00000130921.3	0.013453053	0.000990195	1	0.01501615	C12orf65
VAT	Obesity	ENSG00000130943.5	-0.009607804	0.003403407	1	0.036161216	PKDREJ
VAT	Obesity	ENSG00000131495.4	0.013137927	0.000127808	0.352748922	0.00291528	NDUFA2
VAT	Obesity	ENSG00000131558.10	0.019289287	0.000101199	0.279309184	0.002428776	EXOC4
VAT	Obesity	ENSG00000132207.13	-0.01004113	0.00025522	0.704408389	0.004995804	SLX1A
VAT	Obesity	ENSG00000132382.10	0.011151368	0.001702489	1	0.02237557	MYBBP1A
VAT	Obesity	ENSG00000132740.4	0.007165858	4.02E-05	0.110868798	0.001142977	IGHMBP2
VAT	Obesity	ENSG00000132781.13	0.013527145	0.003114806	1	0.034114543	MUTYH
VAT	Obesity	ENSG00000133706.13	-0.005728604	0.0002489	0.686965306	0.004942196	LARS
VAT	Obesity	ENSG00000134201.6	0.016020355	0.001869425	1	0.023559873	GSTM5
VAT	Obesity	ENSG00000135213.8	-0.03665955	9.92E-18	2.74E-14	2.11E-15	POM121C
VAT	Obesity	ENSG00000135596.13	0.011002853	0.00278482	1	0.031500426	MICAL1
VAT	Obesity	ENSG00000135913.6	0.011651197	0.00073972	1	0.011600152	USP37
VAT	Obesity	ENSG00000137700.12	0.024583661	6.05E-08	0.000166866	3.88E-06	SLC37A4
VAT	Obesity	ENSG00000137806.4	0.021405284	2.25E-05	0.062222594	0.000715202	NDUFAF1
VAT	Obesity	ENSG00000137876.5	-0.021077465	0.000117355	0.32389977	0.002744913	RSL24D1
VAT	Obesity	ENSG00000139187.5	0.00559315	0.003635112	1	0.037860038	KLRG1
VAT	Obesity	ENSG00000139719.5	0.020459415	8.39E-06	0.023154862	0.000340513	VPS33A
VAT	Obesity	ENSG00000139910.15	0.011640891	0.005013493	1	0.047714623	NOVA1
VAT	Obesity	ENSG00000141076.13	0.010079368	0.001936628	1	0.023938382	CIRH1A
VAT	Obesity	ENSG00000141140.12	0.034991488	1.03E-11	2.84E-08	1.06E-09	MYO19
VAT	Obesity	ENSG00000141452.5	-0.037094171	1.91E-16	5.27E-13	3.76E-14	C18orf8
VAT	Obesity	ENSG00000141570.6	0.013444641	0.00268575	1	0.031015357	CBX8
VAT	Obesity	ENSG00000142156.10	0.008914991	0.000524442	1	0.008772485	COL6A1
VAT	Obesity	ENSG00000144199.7	-0.015170688	0.001740586	1	0.022554068	FAHD2B
VAT	Obesity	ENSG00000144791.5	0.018998099	0.000169904	0.468935712	0.00366356	LIMD1
VAT	Obesity	ENSG00000145020.10	-0.003929861	0.003274455	1	0.035441163	AMT
VAT	Obesity	ENSG00000145029.7	-0.010973657	4.00E-05	0.110283331	0.001142977	NICN1
VAT	Obesity	ENSG00000146001.4	0.008686514	0.005038134	1	0.047784366	PCDHB18
VAT	Obesity	ENSG00000146109.3	0.009540872	0.001138869	1	0.016436964	ABT1
VAT	Obesity	ENSG00000146555.14	-0.011270494	0.003849018	1	0.03963914	SDK1
VAT	Obesity	ENSG00000147576.11	-0.010850738	0.00099774	1	0.015047886	ADHFE1
VAT	Obesity	ENSG00000148824.14	0.007243889	0.000466278	1	0.00819516	MTG1
VAT	Obesity	ENSG00000148843.9	0.016097028	0.004821483	1	0.046205883	PDCD11
VAT	Obesity	ENSG00000149084.7	-0.006785865	2.67E-11	7.38E-08	2.54E-09	HSD17B12
VAT	Obesity	ENSG00000149115.9	0.015175782	3.37E-07	0.000929397	1.79E-05	TNKS1BP1
VAT	Obesity	ENSG00000149292.12	0.012034421	4.82E-05	0.132935468	0.001290636	TTC12
VAT	Obesity	ENSG00000149922.6	0.013463047	2.53E-11	6.98E-08	2.49E-09	TBX6
VAT	Obesity	ENSG00000151640.8	0.004053569	0.002018203	1	0.024430883	DPYSL4
VAT	Obesity	ENSG00000152359.10	-0.008208809	2.75E-06	0.00759498	0.0001225	POC5
VAT	Obesity	ENSG00000152404.11	0.015522862	0.000898503	1	0.01370093	CWF19L2
VAT	Obesity	ENSG00000153246.7	0.008905953	0.002358135	1	0.027933277	PLA2R1
VAT	Obesity	ENSG00000153814.7	0.014463193	1.32E-06	0.00364871	6.18E-05	JAZF1
VAT	Obesity	ENSG00000154328.11	0.015737866	9.14E-05	0.252314893	0.002232875	NEIL2
VAT	Obesity	ENSG00000156103.11	-0.011650417	0.000314157	0.867072481	0.005858598	MMP16
VAT	Obesity	ENSG00000156253.2	-0.010235138	0.001951499	1	0.023938382	RWDD2B
VAT	Obesity	ENSG00000157214.9	-0.014480599	0.000390016	1	0.007035575	STEAP2
VAT	Obesity	ENSG00000157322.12	-0.024588954	2.22E-18	6.14E-15	5.11E-16	CLEC18A
VAT	Obesity	ENSG00000157557.7	0.009213634	0.001716884	1	0.022457821	ETS2
VAT	Obesity	ENSG00000158865.8	-0.011617253	2.33E-07	0.000643665	1.31E-05	SLC5A11
VAT	Obesity	ENSG00000160294.6	-0.00694216	0.001524038	1	0.020753408	MCM3AP
VAT	Obesity	ENSG00000160360.7	0.017604068	0.003300264	1	0.035580971	GPSM1
VAT	Obesity	ENSG00000161036.6	-0.012490064	0.002480624	1	0.029010686	LRWD1
VAT	Obesity	ENSG00000161204.7	0.010987081	0.001477941	1	0.020395589	ABCF3
VAT	Obesity	ENSG00000162994.11	0.00306279	0.003575483	1	0.037380051	CLHC1
VAT	Obesity	ENSG00000163297.12	0.021509946	1.30E-05	0.035924051	0.000466546	ANTXR2
VAT	Obesity	ENSG00000163728.6	-0.014450928	0.003169532	1	0.034440579	TTC14
VAT	Obesity	ENSG00000163933.5	-0.014200955	1.36E-07	0.000375284	7.88E-06	RFT1
VAT	Obesity	ENSG00000164068.11	0.019480678	3.06E-19	8.43E-16	8.43E-17	RNF123
VAT	Obesity	ENSG00000164078.8	0.054426081	2.18E-22	6.00E-19	8.58E-20	MST1R
VAT	Obesity	ENSG00000164081.8	-0.012301614	0.000291542	0.804656486	0.005549355	TEX264
VAT	Obesity	ENSG00000164144.10	0.017306391	0.003531265	1	0.037199588	ARFIP1
VAT	Obesity	ENSG00000164307.8	-0.018613439	5.22E-06	0.014406061	0.000218274	ERAP1
VAT	Obesity	ENSG00000164574.11	-0.025235181	1.52E-05	0.041869671	0.000523371	GALNT10
VAT	Obesity	ENSG00000164733.16	-0.009357891	0.005172656	1	0.048735468	CTSB
VAT	Obesity	ENSG00000164924.13	-0.015663421	7.89E-05	0.217643783	0.001981293	YWHAZ
VAT	Obesity	ENSG00000164967.5	-0.019022999	0.000408121	1	0.007220609	RPP25L
VAT	Obesity	ENSG00000165138.12	0.023991911	6.28E-05	0.173410635	0.00162066	ANKS6
VAT	Obesity	ENSG00000165917.5	-0.040591581	4.58E-16	1.26E-12	7.90E-14	RAPSN
VAT	Obesity	ENSG00000166529.10	-0.00753569	0.001980414	1	0.024079037	ZSCAN21
VAT	Obesity	ENSG00000166667.8	0.009434995	0.004492196	1	0.044438931	SPDYE6
VAT	Obesity	ENSG00000167720.8	0.006216213	1.92E-05	0.052932954	0.000626206	SRR
VAT	Obesity	ENSG00000168116.9	-0.012692844	0.00106302	1	0.015746258	KIAA1586
VAT	Obesity	ENSG00000168268.6	-0.0205891	3.50E-06	0.009673111	0.000153541	NT5DC2
VAT	Obesity	ENSG00000168405.10	0.010253168	0.001290601	1	0.017990194	CMAHP
VAT	Obesity	ENSG00000168522.8	0.012501451	6.24E-05	0.172290581	0.00162066	FNTA
VAT	Obesity	ENSG00000168653.6	0.014903049	0.00481777	1	0.046205883	NDUFS5
VAT	Obesity	ENSG00000168743.8	0.018508377	0.001310122	1	0.018170536	NPNT
VAT	Obesity	ENSG00000169085.7	0.006408825	0.001604268	1	0.021287397	C8orf46
VAT	Obesity	ENSG00000169203.12	0.03701837	5.21E-14	1.44E-10	7.19E-12	RP11-231C14.4
VAT	Obesity	ENSG00000169592.10	-0.019555013	1.68E-15	4.63E-12	2.72E-13	INO80E
VAT	Obesity	ENSG00000170092.10	0.041701703	3.66E-19	1.01E-15	9.19E-17	AC018720.10
VAT	Obesity	ENSG00000170289.8	0.019824786	0.000683629	1	0.010986495	CNGB3
VAT	Obesity	ENSG00000170425.3	-0.011051696	6.17E-07	0.001703607	3.04E-05	ADORA2B

VAT	Obesity	ENSG00000171045.10	0.01441411	1.13E-05	0.031313061	0.000417507	TSNARE1
VAT	Obesity	ENSG00000171103.6	0.016348216	2.55E-05	0.070366984	0.000799625	TRMT61B
VAT	Obesity	ENSG00000171700.9	0.008799469	0.002473166	1	0.029010686	RGS19
VAT	Obesity	ENSG00000171735.14	0.017679161	0.001774017	1	0.02256353	CAMTA1
VAT	Obesity	ENSG00000172247.3	-0.019118297	2.27E-15	6.25E-12	3.47E-13	C1QTNF4
VAT	Obesity	ENSG00000172650.8	-0.013083155	0.001526428	1	0.020753408	AGAP5
VAT	Obesity	ENSG00000172671.15	-0.012765018	0.002583273	1	0.030083688	ZFAND4
VAT	Obesity	ENSG00000172728.11	-0.004941714	0.000246046	0.67908709	0.004920921	FUT10
VAT	Obesity	ENSG00000172780.12	0.017862012	0.000161043	0.444477774	0.003514653	RAB43
VAT	Obesity	ENSG00000172935.8	0.020836133	3.32E-05	0.091525667	0.000984147	MRGPRF
VAT	Obesity	ENSG00000173226.12	-0.010749192	0.002321423	1	0.027736481	IQCB1
VAT	Obesity	ENSG00000173531.11	-0.01511439	0.000208654	0.575885908	0.004290423	MST1
VAT	Obesity	ENSG00000173917.9	0.013230121	0.00061136	1	0.009984341	HOXB2
VAT	Obesity	ENSG00000174780.11	0.005328571	0.004639881	1	0.044966202	SRP72
VAT	Obesity	ENSG00000174943.5	0.017536839	2.85E-07	0.000785888	1.54E-05	KCTD13
VAT	Obesity	ENSG00000175115.7	-0.015369721	0.000120833	0.333499937	0.00280252	PACS1
VAT	Obesity	ENSG00000175279.17	0.00686758	0.003530537	1	0.037199588	APITD1
VAT	Obesity	ENSG00000175938.6	0.028725649	1.04E-11	2.87E-08	1.06E-09	ORAI3
VAT	Obesity	ENSG00000176155.14	-0.009931938	0.001125781	1	0.016353444	CCDC57
VAT	Obesity	ENSG00000176422.10	-0.010975319	0.001757854	1	0.02256353	SPRYD4
VAT	Obesity	ENSG00000176623.7	0.007339807	0.002385276	1	0.028134026	RMDN1
VAT	Obesity	ENSG00000176783.10	0.013696833	1.36E-05	0.037411072	0.000479629	RUFY1
VAT	Obesity	ENSG00000177697.13	0.013533477	0.0029159	1	0.032690866	CD151
VAT	Obesity	ENSG00000178718.5	-0.01375462	0.000125888	0.347451702	0.002895431	RPP25
VAT	Obesity	ENSG00000178750.2	0.021002931	7.90E-07	0.002181146	3.83E-05	STX19
VAT	Obesity	ENSG00000178802.13	0.016566589	7.90E-05	0.217942244	0.001981293	MPI
VAT	Obesity	ENSG00000178809.7	-0.04038699	1.58E-13	4.36E-10	1.98E-11	TRIM73
VAT	Obesity	ENSG00000178917.10	-0.021790144	1.08E-05	0.029835669	0.00040653	ZNF852
VAT	Obesity	ENSG00000178952.4	0.021672909	3.26E-11	9.01E-08	3.00E-09	TUFM
VAT	Obesity	ENSG00000179021.5	0.0153587	3.90E-08	0.000107762	2.63E-06	C3orf38
VAT	Obesity	ENSG00000181019.8	-0.013665818	0.003714534	1	0.038541785	NQO1
VAT	Obesity	ENSG00000181315.6	-0.012943813	0.000294877	0.813859235	0.005574378	ZNF322
VAT	Obesity	ENSG00000181625.13	0.009859835	0.000191108	0.527458497	0.004057373	SLX1B
VAT	Obesity	ENSG00000182871.10	0.020748871	0.000209858	0.57920706	0.004290423	COL18A1
VAT	Obesity	ENSG00000183060.11	0.01255758	0.002882733	1	0.032474874	LYSMD4
VAT	Obesity	ENSG00000183250.7	-0.012185771	0.00460266	1	0.044966202	C21orf67
VAT	Obesity	ENSG00000183281.10	0.01237416	0.001033511	1	0.015502666	PLGLB1
VAT	Obesity	ENSG00000183336.7	-0.014480323	2.47E-12	6.82E-09	2.73E-10	BOLA2
VAT	Obesity	ENSG00000183506.12	-0.008746361	0.001060625	1	0.015746258	PI4KAP2
VAT	Obesity	ENSG00000183527.7	0.016017532	0.000310513	0.85701644	0.005830044	PSMG1
VAT	Obesity	ENSG00000184227.3	0.014981728	0.000116174	0.32064074	0.002740519	ACOT1
VAT	Obesity	ENSG00000184923.8	-0.014593362	1.04E-05	0.028754851	0.000403836	NUTM2A
VAT	Obesity	ENSG00000185022.7	-0.014628714	0.000255125	0.704144699	0.004995804	MAFF
VAT	Obesity	ENSG00000185245.6	0.028348483	4.72E-07	0.001303903	2.41E-05	GP1BA
VAT	Obesity	ENSG00000185344.9	-0.023173714	5.96E-05	0.164384688	0.001565568	ATP6V0A2
VAT	Obesity	ENSG00000186470.9	-0.004828131	2.76E-07	0.000760503	1.52E-05	BTN3A2
VAT	Obesity	ENSG00000186715.6	0.015239998	0.000738525	1	0.011600152	MST1L
VAT	Obesity	ENSG00000188312.9	0.009515507	1.03E-05	0.02850231	0.000403836	CENPP
VAT	Obesity	ENSG00000188878.12	0.015175032	0.000505567	1	0.008563512	FBF1
VAT	Obesity	ENSG00000189298.9	-0.015262222	1.03E-05	0.02831071	0.000403836	ZKSCAN3
VAT	Obesity	ENSG00000196458.6	0.013017935	0.000207675	0.57318292	0.004290423	ZNF605
VAT	Obesity	ENSG00000197062.7	-0.007990855	4.26E-05	0.117648929	0.001170502	RP5-874C20.3
VAT	Obesity	ENSG00000197165.6	0.062849037	7.74E-29	2.14E-25	1.07E-25	SULT1A2
VAT	Obesity	ENSG00000197345.8	-0.005043928	0.000289787	0.799810892	0.005549355	MRPL21
VAT	Obesity	ENSG00000197653.10	0.038775569	8.50E-13	2.35E-09	9.77E-11	DNAH10
VAT	Obesity	ENSG00000197728.5	-0.007396076	0.000346933	0.957535616	0.006341295	RPS26
VAT	Obesity	ENSG00000197782.10	-0.004433373	4.27E-05	0.117906757	0.001170502	ZNF780A
VAT	Obesity	ENSG00000198099.4	-0.012934222	0.004378986	1	0.0439491	ADH4
VAT	Obesity	ENSG00000198792.8	-0.014408282	0.00022285	0.61506588	0.004522543	TMEM184B
VAT	Obesity	ENSG00000198909.3	-0.026013503	1.20E-07	0.000332085	7.22E-06	MAP3K3
VAT	Obesity	ENSG00000204351.7	-0.006943576	1.75E-05	0.048301174	0.000589039	SKIV2L
VAT	Obesity	ENSG00000204438.6	-0.019733244	1.37E-07	0.00037817	7.88E-06	GPANK1
VAT	Obesity	ENSG00000204618.4	-0.018908854	0.001756312	1	0.02256353	RNF39
VAT	Obesity	ENSG00000204644.5	0.004078199	3.77E-05	0.103922864	0.001105562	ZFP57
VAT	Obesity	ENSG00000204709.4	0.011580548	2.70E-05	0.074453131	0.000827257	C6orf100
VAT	Obesity	ENSG00000204713.6	-0.010341312	0.001891382	1	0.023620883	TRIM27
VAT	Obesity	ENSG00000205583.9	-0.018842512	3.15E-20	8.68E-17	9.65E-18	STAG3L1
VAT	Obesity	ENSG00000205710.3	0.011234371	0.0002867	0.791292807	0.005533516	C17orf107
VAT	Obesity	ENSG00000212694.4	-0.011770043	8.19E-05	0.226036704	0.002018185	AC084018.1
VAT	Obesity	ENSG00000213199.3	0.014590844	0.003387617	1	0.036161216	ASIC3
VAT	Obesity	ENSG00000213402.2	0.007284576	0.00155021	1	0.020868421	PTPRCAP
VAT	Obesity	ENSG00000213639.5	-0.015846028	1.54E-05	0.042523325	0.000524979	PPP1CB
VAT	Obesity	ENSG00000213689.5	0.005209813	0.00301453	1	0.033147822	TREX1
VAT	Obesity	ENSG00000214063.6	0.014773845	0.004032406	1	0.041068046	TSPAN4
VAT	Obesity	ENSG00000214659.4	0.014177506	0.001072571	1	0.015746258	KRT8P26
VAT	Obesity	ENSG00000216901.1	0.004908492	0.00023558	0.650200703	0.004745991	AL022393.7
VAT	Obesity	ENSG00000223496.1	-0.039467672	7.61E-15	2.10E-11	1.11E-12	EXOSC6
VAT	Obesity	ENSG00000223861.1	-0.022070677	0.000141769	0.39128379	0.003155514	RP11-74C1.2
VAT	Obesity	ENSG00000224843.2	0.006041627	0.003900377	1	0.040018734	LINC00240
VAT	Obesity	ENSG00000224997.1	-0.028787917	4.70E-08	0.000129621	3.09E-06	AL049840.1
VAT	Obesity	ENSG00000225498.1	-0.019430182	0.001593722	1	0.021249624	AC002064.5
VAT	Obesity	ENSG00000226232.4	-0.021343988	5.63E-09	1.55E-05	3.98E-07	RP11-419C5.2
VAT	Obesity	ENSG00000228022.1	-0.013910012	0.003132945	1	0.03417758	HCG20
VAT	Obesity	ENSG00000228078.1	0.005851993	0.002134658	1	0.025615891	HLA-U
VAT	Obesity	ENSG00000230772.1	-0.013784895	0.001541298	1	0.020852861	VN1R108P
VAT	Obesity	ENSG00000231043.2	0.01697534	0.001976643	1	0.024079037	AC007238.1
VAT	Obesity	ENSG00000231925.7	0.013618858	0.000396691	1	0.007109524	TAPBP
VAT	Obesity	ENSG00000232075.1	0.018270716	0.000488716	1	0.008430344	MRPL35P2
VAT	Obesity	ENSG00000232112.3	0.011467787	0.004643249	1	0.044966202	TMA7
VAT	Obesity	ENSG00000232561.2	-0.01750575	3.88E-08	0.000107003	2.63E-06	GTF2IP1
VAT	Obesity	ENSG00000233569.1	-0.025122472	4.28E-05	0.118220734	0.001170502	RP11-500B12.1
VAT	Obesity	ENSG00000233609.3	0.016265015	0.000548708	1	0.009123097	RP11-62H7.2
VAT	Obesity	ENSG00000233633.1	0.047956137	2.62E-16	7.24E-13	4.83E-14	AC093326.3

VAT	Obesity	ENSG00000235109.3	0.006747713	1.93E-05	0.053308014	0.000626206	ZSCAN31
VAT	Obesity	ENSG00000235374.1	-0.012762803	0.000617721	1	0.01002889	SSR4P1
VAT	Obesity	ENSG00000237854.3	0.016517246	3.25E-05	0.089627377	0.000974211	LINC00674
VAT	Obesity	ENSG00000240065.3	0.005532217	0.00190308	1	0.023659912	PSMB9
VAT	Obesity	ENSG00000240682.5	-0.012017661	0.000400438	1	0.007130384	ISY1
VAT	Obesity	ENSG00000242193.5	-0.016776811	0.00010237	0.282541352	0.002435701	RP11-568K15.1
VAT	Obesity	ENSG00000244219.2	0.020286129	1.95E-05	0.053853684	0.000626206	GS1-259H13.2
VAT	Obesity	ENSG00000245958.2	-0.009238838	0.001884532	1	0.023620883	RP11-33B1.1
VAT	Obesity	ENSG00000251417.1	-0.035332435	3.46E-32	9.54E-29	9.54E-29	RP11-1348G14.4
VAT	Obesity	ENSG00000253704.1	-0.022984484	5.38E-09	1.49E-05	3.91E-07	RP11-267M23.4
VAT	Obesity	ENSG00000253813.1	0.008117002	0.002752625	1	0.031393572	COX6B1P4
VAT	Obesity	ENSG00000253981.1	0.037653507	1.85E-13	5.11E-10	2.22E-11	ALG1L13P
VAT	Obesity	ENSG00000254634.3	-0.021242474	3.14E-10	8.66E-07	2.47E-08	RP11-231C14.3
VAT	Obesity	ENSG00000254986.3	0.006169819	0.004452802	1	0.044318348	DPP3
VAT	Obesity	ENSG00000254999.2	-0.009747345	0.00010041	0.277131732	0.002428776	BRK1
VAT	Obesity	ENSG00000257595.2	0.014405593	0.001282352	1	0.017965945	RP3-473L9.4
VAT	Obesity	ENSG00000258512.1	0.012873274	0.004879288	1	0.046598047	RP11-796G6.2
VAT	Obesity	ENSG00000259982.1	0.027019652	1.35E-24	3.72E-21	9.29E-22	CDC37P1
VAT	Obesity	ENSG00000260911.1	0.012569447	3.77E-10	1.04E-06	2.89E-08	RP11-196G11.2
VAT	Obesity	ENSG00000260947.1	-0.005234709	1.16E-05	0.031990896	0.000420933	RP11-384P7.7
VAT	Obesity	ENSG00000261556.4	-0.017054478	7.31E-14	2.02E-10	9.61E-12	RP11-296I10.6
VAT	Obesity	ENSG00000261706.1	0.009476289	0.003780966	1	0.03908414	AP001505.9
VAT	Obesity	ENSG00000265055.1	0.0196158	1.09E-05	0.030083252	0.00040653	AC145343.2
VAT	Obesity	ENSG00000266173.1	-0.012274283	0.003573702	1	0.037380051	STRADA
VAT	Obesity	ENSG00000267731.1	0.008832746	0.002764996	1	0.031404892	RP11-147L13.8
VAT	Obesity	ENSG00000268171.1	0.014386346	0.002620104	1	0.030384397	AC068620.1
VAT	Obesity	ENSG00000269963.1	-0.027372986	1.05E-05	0.029076197	0.000403836	RP11-73M18.9
VAT	Obesity	ENSG00000270071.1	0.008374599	0.000897283	1	0.01370093	AP001172.2
VAT	Obesity	ENSG00000271751.1	-0.019079697	0.000195191	0.538727649	0.004112425	RP11-110I1.14
VAT	Obesity	ENSG00000272047.1	0.006412849	0.003997369	1	0.040861995	GTF2H5
VAT	Obesity	ENSG00000272275.1	-0.008568137	0.00462921	1	0.044966202	RP11-791G15.2
VAT	Obesity	ENSG00000272338.1	-0.020686037	0.000208345	0.575030975	0.004290423	RP11-722E23.2
VAT	Obesity	ENSG00000272462.2	0.00669793	1.25E-10	3.44E-07	1.01E-08	U91328.19
VAT	Obesity	ENSG00000272505.1	0.018175902	0.002725409	1	0.031342199	RP11-981G7.6
VAT	Obesity	ENSG00000272849.1	-0.01652107	8.09E-05	0.223329467	0.002011977	RP11-347I19.8
VAT	Obesity	ENSG00000272983.1	0.010873305	0.001557571	1	0.020868421	RP11-508N22.12
VAT	HTN	ENSG00000003056.3	-0.014919899	0.000411253	1	0.00822505	M6PR
VAT	HTN	ENSG00000004534.10	-0.005959478	3.98E-06	0.010983982	0.000183066	RBM6
VAT	HTN	ENSG00000007341.14	-0.006490431	0.003495248	1	0.038281288	ST7L
VAT	HTN	ENSG00000009950.11	-0.019846191	0.00263143	1	0.030644506	MLXIPL
VAT	HTN	ENSG00000011021.17	-0.015481276	0.000843934	1	0.013542191	CLCN6
VAT	HTN	ENSG00000011523.9	0.026018476	5.44E-09	1.50E-05	6.52E-07	CEP68
VAT	HTN	ENSG00000026297.11	-0.015855893	0.000614532	1	0.010600675	RNASET2
VAT	HTN	ENSG00000035115.17	-0.018036684	0.004561216	1	0.046861116	SH3YL1
VAT	HTN	ENSG00000053900.6	0.030408377	2.69E-06	0.007423149	0.000125816	ANAPC4
VAT	HTN	ENSG00000054282.11	0.016840477	1.19E-09	3.28E-06	1.93E-07	SDCCAG8
VAT	HTN	ENSG00000054523.12	0.013449302	0.001486974	1	0.020939026	KIF1B
VAT	HTN	ENSG00000055955.11	0.006195101	0.00058058	1	0.010205917	ITIH4
VAT	HTN	ENSG00000060642.6	-0.013759292	7.55E-06	0.020832632	0.000306731	PIGV
VAT	HTN	ENSG00000063854.8	0.012754888	0.004448854	1	0.046161039	HAGH
VAT	HTN	ENSG00000064703.7	0.015665492	0.003231204	1	0.036252527	DDX20
VAT	HTN	ENSG00000065060.12	0.015011965	6.75E-07	0.001862089	3.88E-05	UHRF1BP1
VAT	HTN	ENSG00000068654.11	-0.010460665	0.000140691	0.388308383	0.003463845	POLR1A
VAT	HTN	ENSG00000085788.9	0.023991964	0.00037489	1	0.007608054	DDHD2
VAT	HTN	ENSG00000086300.11	-0.025971325	4.88E-07	0.001346797	3.28E-05	SNX10
VAT	HTN	ENSG00000087884.10	-0.012506741	1.63E-05	0.044983217	0.000576708	AAMDC
VAT	HTN	ENSG00000090238.7	0.011233713	5.95E-05	0.164102622	0.001676064	YPEL3
VAT	HTN	ENSG00000090432.5	-0.011921128	0.001592175	1	0.022193958	MUL1
VAT	HTN	ENSG00000090621.9	-0.025704807	6.19E-07	0.001709597	3.64E-05	PABPC4
VAT	HTN	ENSG00000096433.6	0.021988685	0.000640344	1	0.01096636	ITPR3
VAT	HTN	ENSG00000099194.5	-0.032533335	9.47E-07	0.00261343	5.03E-05	SCD
VAT	HTN	ENSG00000099785.6	0.018580431	2.45E-06	0.006761129	0.000118124	MARCH2
VAT	HTN	ENSG00000099810.14	-0.021152367	0.00125882	1	0.018581684	MTAP
VAT	HTN	ENSG00000100296.9	-0.009097575	0.003172839	1	0.03594872	THOC5
VAT	HTN	ENSG00000100731.11	0.014266314	0.001945963	1	0.025575518	PCNX
VAT	HTN	ENSG00000101197.8	-0.008692573	0.000743136	1	0.012305555	BIRC7
VAT	HTN	ENSG00000101363.8	0.011510974	0.001292639	1	0.018581684	MANBAL
VAT	HTN	ENSG00000101457.8	-0.007879223	0.000549212	1	0.010098967	DNTTIP1
VAT	HTN	ENSG00000101751.6	-0.007145307	0.000114777	0.316783726	0.002960596	POLI
VAT	HTN	ENSG00000102858.8	0.028526491	1.05E-05	0.029020646	0.000397543	MGRN1
VAT	HTN	ENSG00000103248.13	0.007122091	0.000237708	0.656073133	0.005248585	MTHFSD
VAT	HTN	ENSG00000105376.4	-0.018148762	0.00228798	1	0.028307557	ICAM5
VAT	HTN	ENSG00000105404.6	0.019912277	0.000401054	1	0.008079637	RABAC1
VAT	HTN	ENSG00000106125.14	-0.015579308	0.000369529	1	0.007554817	FAM188B
VAT	HTN	ENSG00000106133.13	-0.007756566	0.00315018	1	0.035927668	NSUN5P2
VAT	HTN	ENSG00000106290.10	-0.011183109	0.000564757	1	0.010098967	TAF6
VAT	HTN	ENSG00000108384.10	-0.009884795	0.002297425	1	0.028307557	RAD51C
VAT	HTN	ENSG00000108823.11	0.009940583	0.00474155	1	0.048112786	SGCA
VAT	HTN	ENSG00000110851.7	0.01889672	0.002177712	1	0.027474564	PRDM4
VAT	HTN	ENSG00000111801.11	-0.010838049	0.002357226	1	0.02884247	BTN3A3
VAT	HTN	ENSG00000112981.3	0.014165448	0.003695866	1	0.040002317	NME5
VAT	HTN	ENSG00000113269.9	0.021451891	0.000556264	1	0.010098967	RNF130
VAT	HTN	ENSG00000113971.14	0.004635239	0.003954922	1	0.042308471	NPHP3
VAT	HTN	ENSG00000114054.9	0.027607456	3.29E-08	9.08E-05	3.24E-06	PCCB
VAT	HTN	ENSG00000114904.8	-0.015327133	0.003199378	1	0.036041975	NEK4
VAT	HTN	ENSG00000116151.9	-0.014851418	0.003178075	1	0.03594872	MORN1
VAT	HTN	ENSG00000117595.6	-0.010551096	0.004434898	1	0.046161039	IRF6
VAT	HTN	ENSG00000119673.10	0.007104652	0.001268536	1	0.018581684	ACOT2
VAT	HTN	ENSG00000120784.11	0.013377832	4.84E-05	0.133575816	0.001451911	ZFP30
VAT	HTN	ENSG00000121068.9	-0.03001438	3.52E-07	0.000972808	2.55E-05	TBX2
VAT	HTN	ENSG00000121101.11	0.03300021	6.10E-08	0.000168359	5.71E-06	TEX14
VAT	HTN	ENSG00000123374.6	-0.019071512	0.00367685	1	0.039953174	CDK2
VAT	HTN	ENSG00000124116.14	-0.006972357	0.000329	0.908040755	0.006869451	WFDC3

VAT	HTN	ENSG00000124508.12	0.012935719	7.56E-06	0.020857722	0.000306731	BTN2A2
VAT	HTN	ENSG00000124562.5	-0.017368984	1.88E-05	0.051980452	0.000641734	SNRPC
VAT	HTN	ENSG00000124613.4	-0.007007258	2.71E-05	0.074933901	0.000902818	ZNF391
VAT	HTN	ENSG00000125388.15	0.004801917	0.001606016	1	0.02227439	GRK4
VAT	HTN	ENSG00000125734.9	0.011865387	0.002751575	1	0.031775514	GPR108
VAT	HTN	ENSG00000126088.8	-0.017037331	0.0024555	1	0.02959467	UROD
VAT	HTN	ENSG00000126602.6	0.007726321	7.10E-07	0.001958312	3.92E-05	TRAP1
VAT	HTN	ENSG00000127220.5	-0.018191378	6.72E-06	0.018544774	0.000280981	ABHD8
VAT	HTN	ENSG00000127957.12	-0.031705797	3.37E-10	9.29E-07	9.29E-08	PMS2P3
VAT	HTN	ENSG00000128829.7	-0.033522651	7.06E-07	0.001948855	3.92E-05	EIF2AK4
VAT	HTN	ENSG00000130177.10	0.009688663	0.000213751	0.589951873	0.004835671	CDC16
VAT	HTN	ENSG00000130270.12	-0.011239855	0.000524744	1	0.009785759	ATP8B3
VAT	HTN	ENSG00000130517.9	0.010688188	0.000538294	1	0.009971089	PGPEP1
VAT	HTN	ENSG00000130529.11	-0.023118435	0.000149318	0.412116568	0.003552729	TRPM4
VAT	HTN	ENSG00000131351.10	-0.030883699	9.33E-09	2.58E-05	1.03E-06	HAUS8
VAT	HTN	ENSG00000131558.10	0.024846442	1.17E-05	0.032209103	0.000435258	EXOC4
VAT	HTN	ENSG00000132781.13	0.01526582	0.003494113	1	0.038281288	MUTYH
VAT	HTN	ENSG00000133731.5	0.007317355	0.000889237	1	0.014105132	IMPA1
VAT	HTN	ENSG00000134490.9	-0.014812412	0.003532448	1	0.038535799	TMEM241
VAT	HTN	ENSG00000135213.8	-0.015189366	0.001873194	1	0.02509716	POM121C
VAT	HTN	ENSG00000135744.7	0.014794462	0.000428479	1	0.008447166	AGT
VAT	HTN	ENSG00000135968.15	0.015347173	0.002027909	1	0.026401085	GCC2
VAT	HTN	ENSG00000136448.7	0.012507012	1.03E-07	0.000282914	8.57E-06	NMT1
VAT	HTN	ENSG00000137700.12	0.021209868	4.29E-05	0.118512663	0.001331603	SLC37A4
VAT	HTN	ENSG00000137944.12	-0.013915527	5.26E-05	0.145214526	0.001530313	CCBL2
VAT	HTN	ENSG00000138036.14	0.01678463	0.00093551	1	0.014754326	DYNC2LI1
VAT	HTN	ENSG00000138111.10	-0.004453934	0.004155056	1	0.04410752	TMEM180
VAT	HTN	ENSG00000138175.8	0.009614262	0.002361738	1	0.02884247	ARL3
VAT	HTN	ENSG00000138600.5	-0.019039935	5.27E-05	0.145379761	0.001530313	SPPL2A
VAT	HTN	ENSG00000138757.10	0.011789096	0.000869199	1	0.013866987	G3BP2
VAT	HTN	ENSG00000138829.6	0.009324679	4.81E-05	0.132857361	0.001451911	FBN2
VAT	HTN	ENSG00000139970.12	0.022602858	0.000298124	0.822821044	0.006329393	RTN1
VAT	HTN	ENSG00000140995.12	0.025940009	2.48E-06	0.006851187	0.000118124	DEF8
VAT	HTN	ENSG00000141002.14	0.020059327	0.000514019	1	0.009771271	TCF25
VAT	HTN	ENSG00000141076.13	0.01350772	0.000277123	0.764859141	0.005975462	CIRH1A
VAT	HTN	ENSG00000141140.12	0.021897048	0.000194458	0.536703269	0.004472527	MYO19
VAT	HTN	ENSG00000141252.15	-0.010920708	0.000567152	1	0.010098967	VPS53
VAT	HTN	ENSG00000141295.9	-0.009612055	0.000585838	1	0.010205917	SCRN2
VAT	HTN	ENSG00000141452.5	-0.02660741	2.38E-07	0.000658012	1.88E-05	C18orf8
VAT	HTN	ENSG00000143156.9	-0.020820691	0.000452738	1	0.008738169	NME7
VAT	HTN	ENSG00000143376.8	0.023751049	5.24E-05	0.144656132	0.001530313	SNX27
VAT	HTN	ENSG00000143437.16	-0.019035908	0.000666141	1	0.011210665	ARNT
VAT	HTN	ENSG00000143578.11	-0.033300218	5.73E-07	0.001580135	3.51E-05	CREB3L4
VAT	HTN	ENSG00000144026.7	-0.010839827	0.004002226	1	0.042649204	ZNF514
VAT	HTN	ENSG00000144134.14	-0.012308273	0.003073313	1	0.03519645	RABL2A
VAT	HTN	ENSG00000145029.7	-0.010775132	0.000414484	1	0.008230051	NICN1
VAT	HTN	ENSG00000146109.3	0.010266399	0.002180047	1	0.027474564	ABT1
VAT	HTN	ENSG00000146243.9	0.040514616	3.20E-09	8.82E-06	4.45E-07	IRAK1BP1
VAT	HTN	ENSG00000146409.6	-0.016244236	0.000517647	1	0.009771271	SLC18B1
VAT	HTN	ENSG00000146556.10	-0.019096743	5.94E-06	0.016393759	0.000252212	WASH2P
VAT	HTN	ENSG00000147576.11	-0.01213279	0.001274589	1	0.018581684	ADHFE1
VAT	HTN	ENSG00000149084.7	-0.007132402	8.71E-10	2.40E-06	1.60E-07	HSD17B12
VAT	HTN	ENSG00000149115.9	0.022034126	8.91E-11	2.46E-07	3.66E-08	TNKS1BP1
VAT	HTN	ENSG00000149292.12	0.010890964	0.001282493	1	0.018581684	TTC12
VAT	HTN	ENSG00000149743.9	-0.014109572	0.001800158	1	0.024236277	TRPT1
VAT	HTN	ENSG00000149922.6	0.007556028	0.001046632	1	0.016048355	TBX6
VAT	HTN	ENSG00000152404.11	0.026630832	6.13E-07	0.001691057	3.64E-05	CWF19L2
VAT	HTN	ENSG00000153113.19	-0.00999079	0.000744575	1	0.012305555	CAST
VAT	HTN	ENSG00000154328.11	0.026998523	4.21E-09	1.16E-05	5.29E-07	NEIL2
VAT	HTN	ENSG00000156103.11	-0.010395134	0.004885205	1	0.049388889	MMP16
VAT	HTN	ENSG00000156219.12	-0.015385284	0.002466677	1	0.029600124	ART3
VAT	HTN	ENSG00000156398.8	0.013865929	2.46E-08	6.79E-05	2.52E-06	SFXN2
VAT	HTN	ENSG00000156603.10	0.012466744	0.00209083	1	0.026978653	MED19
VAT	HTN	ENSG00000156931.11	0.025349329	0.000350872	0.968405953	0.00722691	VPS8
VAT	HTN	ENSG00000157322.12	-0.015574217	1.25E-06	0.003441552	6.37E-05	CLEC18A
VAT	HTN	ENSG00000158865.8	-0.012855734	5.47E-07	0.001510664	3.51E-05	SLC5A11
VAT	HTN	ENSG00000160307.5	-0.007833076	3.54E-05	0.097632917	0.001135266	S100B
VAT	HTN	ENSG00000160321.10	-0.026928126	5.98E-05	0.165182137	0.001676064	ZNF208
VAT	HTN	ENSG00000161204.7	0.021370919	6.21E-08	0.000171308	5.71E-06	ABCF3
VAT	HTN	ENSG00000162910.14	0.036422041	2.02E-12	5.58E-09	1.40E-09	MRPL55
VAT	HTN	ENSG00000163155.7	-0.01359099	3.45E-05	0.095173587	0.001119689	LYSMD1
VAT	HTN	ENSG00000163933.5	-0.01179084	0.000127485	0.351859582	0.00322807	RFT1
VAT	HTN	ENSG00000164048.9	-0.018465782	8.69E-10	2.40E-06	1.60E-07	ZNF589
VAT	HTN	ENSG00000164068.11	0.015964479	1.25E-10	3.45E-07	4.32E-08	RNF123
VAT	HTN	ENSG00000164078.8	0.026040659	4.57E-05	0.126141889	0.001401577	MST1R
VAT	HTN	ENSG00000164307.8	-0.028869176	6.11E-10	1.69E-06	1.30E-07	ERAP1
VAT	HTN	ENSG00000164308.12	0.002988645	0.001575253	1	0.022069535	ERAP2
VAT	HTN	ENSG00000164967.5	-0.021529835	0.000461586	1	0.008847074	RPP25L
VAT	HTN	ENSG00000166037.6	-0.017208976	0.000449318	1	0.008733218	CEP57
VAT	HTN	ENSG00000166275.11	-0.004383795	0.003431034	1	0.037878616	C10orf32
VAT	HTN	ENSG00000166394.10	0.01541718	0.002154223	1	0.027474564	CYB5R2
VAT	HTN	ENSG00000166435.11	-0.011762891	0.002446103	1	0.02959467	XRRA1
VAT	HTN	ENSG00000166436.11	-0.007144876	2.21E-05	0.061025527	0.000744214	TRIM66
VAT	HTN	ENSG00000166532.11	-0.018036485	9.29E-11	2.57E-07	3.66E-08	RIMKLB
VAT	HTN	ENSG00000166924.4	0.012405556	0.00419028	1	0.044311011	NYAP1
VAT	HTN	ENSG00000166997.3	0.017815928	0.002615291	1	0.030644506	CNPY4
VAT	HTN	ENSG00000167720.8	0.006317744	0.000143279	0.395449219	0.003468853	SRR
VAT	HTN	ENSG00000168268.6	-0.015564998	0.002142838	1	0.027474564	NT5DC2
VAT	HTN	ENSG00000169062.10	0.013600007	0.001290836	1	0.018581684	UPF3A
VAT	HTN	ENSG00000169085.7	0.007202816	0.001907316	1	0.025341822	C8orf46
VAT	HTN	ENSG00000169169.10	0.033613689	1.60E-07	0.000442463	1.30E-05	CPT1C
VAT	HTN	ENSG00000169203.12	0.016495278	0.003328411	1	0.037041994	RP11-231C14.4
VAT	HTN	ENSG00000169592.10	-0.017507831	4.35E-10	1.20E-06	1.00E-07	INO80E

VAT	HTN	ENSG00000169629.7	-0.004698666	0.000775424	1	0.012663725	RGPD8
VAT	HTN	ENSG00000170092.10	0.023544548	9.80E-06	0.027037163	0.000386245	AC018720.10
VAT	HTN	ENSG00000170464.5	-0.023156457	6.81E-05	0.187992018	0.001843059	DNAJC18
VAT	HTN	ENSG00000170915.8	-0.021779112	0.000831422	1	0.013419446	PAQR8
VAT	HTN	ENSG00000171421.8	0.007907723	0.001671174	1	0.022833865	MRPL36
VAT	HTN	ENSG00000171700.9	-0.019110622	8.70E-09	2.40E-05	1.00E-06	RGS19
VAT	HTN	ENSG00000172113.4	0.034405149	4.16E-10	1.15E-06	1.00E-07	NME6
VAT	HTN	ENSG00000172687.9	0.006137524	0.00199575	1	0.026105551	ZNF738
VAT	HTN	ENSG00000172746.6	-0.018830743	0.002585544	1	0.030627042	RP11-344H11.4
VAT	HTN	ENSG00000173531.11	-0.021166612	5.48E-06	0.015114334	0.000236161	MST1
VAT	HTN	ENSG00000174177.8	-0.013211707	0.002406173	1	0.029255675	CTU2
VAT	HTN	ENSG00000174226.4	-0.01448089	5.12E-06	0.014132487	0.000224325	SNX31
VAT	HTN	ENSG00000175305.12	-0.026414887	3.72E-09	1.03E-05	4.89E-07	CCNE2
VAT	HTN	ENSG00000175470.14	-0.018926511	0.000133152	0.367500698	0.003310817	PPP2R2D
VAT	HTN	ENSG00000175772.10	0.006596755	0.001919	1	0.025341822	LINC01106
VAT	HTN	ENSG00000175826.7	0.023690121	8.00E-05	0.220732361	0.002143033	CTDNEP1
VAT	HTN	ENSG00000175873.3	-0.023150149	0.00031646	0.873428867	0.006667396	AC004840.9
VAT	HTN	ENSG00000175938.6	0.024739225	2.93E-07	0.000809814	2.19E-05	ORAI3
VAT	HTN	ENSG00000176390.10	0.013701373	0.000643678	1	0.01096636	CRLF3
VAT	HTN	ENSG00000177989.9	0.010021384	0.002164701	1	0.027474564	ODF3B
VAT	HTN	ENSG00000178295.10	-0.006371754	0.001623851	1	0.022409137	GEN1
VAT	HTN	ENSG00000178718.5	0.019526007	1.90E-06	0.005245327	9.37E-05	RPP25
VAT	HTN	ENSG00000178802.13	-0.039096741	3.51E-16	9.69E-13	4.85E-13	MPI
VAT	HTN	ENSG00000178809.7	-0.02155279	0.00056573	1	0.010098967	TRIM73
VAT	HTN	ENSG00000178922.12	-0.015185959	2.77E-07	0.000763296	2.12E-05	HYI
VAT	HTN	ENSG00000178971.9	0.020207273	0.000130681	0.360678915	0.003278899	CTC1
VAT	HTN	ENSG00000181915.3	0.026755312	1.02E-09	2.82E-06	1.76E-07	ADO
VAT	HTN	ENSG00000182372.6	0.015439995	1.04E-05	0.028610072	0.000397362	CLN8
VAT	HTN	ENSG00000182511.7	-0.057726927	1.60E-19	4.40E-16	4.40E-16	FES
VAT	HTN	ENSG00000182986.8	0.00485425	0.000185348	0.511559856	0.004298822	ZNF320
VAT	HTN	ENSG00000183336.7	-0.007210534	0.002263462	1	0.028267668	BOLA2
VAT	HTN	ENSG00000184254.12	-0.018789251	0.001755846	1	0.023755566	ALDH1A3
VAT	HTN	ENSG00000184385.2	-0.00734495	0.000141817	0.391414429	0.003463845	C21orf128
VAT	HTN	ENSG00000184389.8	-0.014317631	6.24E-05	0.17231603	0.00172316	A3GALT2
VAT	HTN	ENSG00000184923.8	-0.014551063	0.000118929	0.328245316	0.003039308	NUTM2A
VAT	HTN	ENSG00000185324.17	-0.006119511	9.37E-05	0.25854422	0.002439096	CDK10
VAT	HTN	ENSG00000186409.10	0.012520681	0.001653737	1	0.022708031	CCDC30
VAT	HTN	ENSG00000186417.9	0.011377274	0.000998015	1	0.015650693	GLDN
VAT	HTN	ENSG00000187961.9	0.018659186	0.001291011	1	0.018581684	KLHL17
VAT	HTN	ENSG00000188321.9	-0.003730897	0.002653153	1	0.030767652	ZNF559
VAT	HTN	ENSG00000188629.7	-0.007576082	0.002603289	1	0.030644506	ZNF177
VAT	HTN	ENSG00000188820.8	-0.012959504	0.004214612	1	0.044398198	FAM26F
VAT	HTN	ENSG00000188878.12	-0.024923496	5.73E-07	0.001580927	3.51E-05	FBF1
VAT	HTN	ENSG00000188895.7	-0.008566505	0.002933341	1	0.033733423	MSL1
VAT	HTN	ENSG00000196126.6	-0.013586704	0.000520426	1	0.009771271	HLA-DRB1
VAT	HTN	ENSG00000196196.2	0.016311348	0.000689577	1	0.011534741	HRCT1
VAT	HTN	ENSG00000196966.3	0.017547845	9.62E-08	0.000265476	8.30E-06	HIST1H3E
VAT	HTN	ENSG00000197020.6	-0.013644606	1.03E-05	0.028399626	0.000397362	ZNF100
VAT	HTN	ENSG00000197165.6	0.027713692	1.70E-05	0.046990952	0.000594822	SULT1A2
VAT	HTN	ENSG00000197448.9	-0.013008176	0.002243486	1	0.028145547	GSTK1
VAT	HTN	ENSG00000197479.4	-0.008101643	0.004605358	1	0.047053283	PCDHB11
VAT	HTN	ENSG00000197728.5	-0.010540702	8.08E-06	0.022291892	0.000323071	RPS26
VAT	HTN	ENSG00000197935.6	0.012240733	0.004620087	1	0.047053283	ZNF311
VAT	HTN	ENSG00000198270.8	-0.014726784	8.58E-14	2.37E-10	7.90E-11	TMEM116
VAT	HTN	ENSG00000198502.5	-0.005686134	0.00058795	1	0.010205917	HLA-DRB5
VAT	HTN	ENSG00000198646.9	-0.010396593	0.003259267	1	0.036419338	NCOA6
VAT	HTN	ENSG00000198805.7	-0.005642425	0.002623473	1	0.030644506	PNP
VAT	HTN	ENSG00000203896.5	0.018836878	0.002529076	1	0.030217531	LIME1
VAT	HTN	ENSG00000204228.3	-0.019993031	4.14E-05	0.114384039	0.001299819	HSD17B8
VAT	HTN	ENSG00000204314.6	0.017060208	4.63E-06	0.012768436	0.000209319	PRRT1
VAT	HTN	ENSG00000204338.4	0.009463215	1.19E-05	0.032787313	0.000437164	CYP21A1P
VAT	HTN	ENSG00000204525.10	-0.01185149	2.65E-09	7.30E-06	4.06E-07	HLA-C
VAT	HTN	ENSG00000204531.11	-0.00413257	0.000224101	0.618518387	0.005028605	POU5F1
VAT	HTN	ENSG00000205269.4	0.017507615	0.004500833	1	0.046525467	TMEM170B
VAT	HTN	ENSG00000205583.9	-0.011889096	3.60E-07	0.000994519	2.55E-05	STAG3L1
VAT	HTN	ENSG00000213347.6	-0.014064225	0.001084872	1	0.016542793	MXD3
VAT	HTN	ENSG00000213676.6	0.02114734	3.23E-09	8.90E-06	4.45E-07	ATF6B
VAT	HTN	ENSG00000213889.6	0.016855429	0.002281503	1	0.028307557	PPM1N
VAT	HTN	ENSG00000214435.3	-0.00450098	0.001112069	1	0.016772188	AS3MT
VAT	HTN	ENSG00000214659.4	0.018023923	0.000272832	0.753016283	0.005929262	KRT8P26
VAT	HTN	ENSG00000215114.3	0.016932557	0.002091823	1	0.026978653	UBXN2B
VAT	HTN	ENSG00000215464.4	-0.021754908	0.000813093	1	0.013200804	AP000354.2
VAT	HTN	ENSG00000218422.2	0.017156351	0.000291116	0.803478988	0.006228519	AC016773.1
VAT	HTN	ENSG00000221838.5	-0.009193481	0.00043354	1	0.008486318	AP4M1
VAT	HTN	ENSG00000221955.6	0.021069758	0.000663512	1	0.011210665	SLC12A8
VAT	HTN	ENSG00000223496.1	-0.025385273	1.21E-05	0.03327451	0.000437822	EXOSC6
VAT	HTN	ENSG00000223891.1	0.010124805	0.003348943	1	0.037120812	OSER1-AS1
VAT	HTN	ENSG00000224997.1	-0.029228153	1.20E-06	0.003320986	6.27E-05	AL049840.1
VAT	HTN	ENSG00000225190.4	-0.025351329	0.000172703	0.476659174	0.004039485	PLEKHM1
VAT	HTN	ENSG00000225851.1	0.005595613	0.003883949	1	0.04171089	HLA-S
VAT	HTN	ENSG00000226232.4	-0.014415003	0.000571181	1	0.010105514	RP11-419C5.2
VAT	HTN	ENSG00000228998.3	-0.0219941	0.001043829	1	0.016048355	RP11-697E2.7
VAT	HTN	ENSG00000229391.3	0.008547019	6.01E-05	0.165930289	0.001676064	HLA-DRB6
VAT	HTN	ENSG00000229759.1	-0.020544178	1.81E-11	4.99E-08	9.99E-09	MRPS18AP1
VAT	HTN	ENSG00000231130.1	0.007436088	0.004396269	1	0.045960991	HLA-T
VAT	HTN	ENSG00000232075.1	0.027344422	4.92E-06	0.01358799	0.000219161	MRPL35P2
VAT	HTN	ENSG00000232112.3	0.017188242	0.000203697	0.562203004	0.004646306	TMA7
VAT	HTN	ENSG00000232561.2	-0.01195486	0.001018213	1	0.015810108	GTF2IP1
VAT	HTN	ENSG00000236397.2	0.008955716	6.50E-05	0.179533751	0.001777562	DDX11L2
VAT	HTN	ENSG00000237541.3	0.008486144	1.61E-05	0.044397706	0.000576594	HLA-DQA2
VAT	HTN	ENSG00000239697.6	0.030712299	6.93E-08	0.000191319	6.17E-06	TNFSF12
VAT	HTN	ENSG00000240399.1	-0.012143521	0.000331028	0.91363702	0.006869451	RP1-228P16.1
VAT	HTN	ENSG00000243789.6	0.00968732	0.001099957	1	0.016680674	JMJD7

VAT	HTN	ENSG00000244219.2	0.018730011	0.00055621	1	0.010098967	GS1-259H13.2
VAT	HTN	ENSG00000244291.1	-0.022701594	0.001019637	1	0.015810108	C7orf13
VAT	HTN	ENSG00000251417.1	-0.01466861	1.78E-05	0.04901568	0.000612696	RP11-1348G14.4
VAT	HTN	ENSG00000253981.1	0.029243817	5.65E-07	0.001559224	3.51E-05	ALG1L13P
VAT	HTN	ENSG00000254634.3	-0.010939008	0.004567261	1	0.046861116	RP11-231C14.3
VAT	HTN	ENSG00000254851.1	0.007205896	4.67E-07	0.001288313	3.22E-05	RP11-109L13.1
VAT	HTN	ENSG00000256885.1	-0.011771235	0.00126024	1	0.018581684	AP001877.1
VAT	HTN	ENSG00000257595.2	-0.03269804	1.59E-10	4.38E-07	4.87E-08	RP3-473L9.4
VAT	HTN	ENSG00000257877.1	-0.023318786	0.000262543	0.724618892	0.005750944	RP3-462E2.3
VAT	HTN	ENSG00000258408.1	0.014940924	0.001313977	1	0.018790547	NT5CP2
VAT	HTN	ENSG00000258512.1	0.016735002	0.001361483	1	0.019369546	RP11-796G6.2
VAT	HTN	ENSG00000258819.1	-0.021289961	0.001381589	1	0.019554794	RP11-7F17.3
VAT	HTN	ENSG00000258908.1	-0.018880547	0.002563092	1	0.030491956	RP11-203M5.8
VAT	HTN	ENSG00000259982.1	0.011369371	0.000162907	0.449623483	0.003842936	CDC37P1
VAT	HTN	ENSG00000260077.1	-0.019889877	0.001179756	1	0.017696346	RP11-254F7.2
VAT	HTN	ENSG00000260265.1	-0.016919347	0.003752784	1	0.040459698	RP11-44F21.5
VAT	HTN	ENSG00000260911.1	0.008962444	9.25E-05	0.255413501	0.00243251	RP11-196G11.2
VAT	HTN	ENSG00000261556.4	-0.010232066	8.53E-05	0.235331206	0.0022628	RP11-296I10.6
VAT	HTN	ENSG00000264538.2	0.013204862	3.74E-05	0.103156097	0.001185702	SUZ12P
VAT	HTN	ENSG00000266912.1	-0.011378551	0.00191062	1	0.025341822	CTC-325H20.2
VAT	HTN	ENSG00000266970.1	0.01543606	0.004347509	1	0.045624044	RP11-806H10.4
VAT	HTN	ENSG00000266992.1	-0.017329672	0.000751499	1	0.012346048	DHX40P1
VAT	HTN	ENSG00000268225.1	0.014209688	0.000148836	0.410786292	0.003552729	CTD-2331H12.5
VAT	HTN	ENSG00000268555.1	-0.01913086	0.001689173	1	0.02296609	RP11-678G14.3
VAT	HTN	ENSG00000269963.1	-0.034195482	1.44E-06	0.003982953	7.24E-05	RP11-73M18.9
VAT	HTN	ENSG00000270018.1	0.028315386	9.06E-07	0.002499754	4.90E-05	RP3-462E2.5
VAT	HTN	ENSG00000270020.1	-0.011914584	5.95E-05	0.164321217	0.001676064	RP11-463O9.9
VAT	HTN	ENSG00000271581.1	0.044446831	1.28E-08	3.53E-05	1.36E-06	XXbac-BPG248L24.12
VAT	HTN	ENSG00000271751.1	-0.024355655	3.15E-05	0.086968195	0.001035336	RP11-110I1.14
VAT	HTN	ENSG00000272505.1	0.025490349	0.000232852	0.642670646	0.005182828	RP11-981G7.6
VAT	Heart_Failure	ENSG00000004534.10	-0.00396061	5.06E-08	0.000139782	1.75E-05	RBM6
VAT	Heart_Failure	ENSG00000086015.16	0.011575077	7.90E-05	0.217930737	0.011470039	MAST2
VAT	Heart_Failure	ENSG00000088340.11	-0.00956845	0.000127609	0.352200569	0.016009117	FER1L4
VAT	Heart_Failure	ENSG00000090621.9	-0.011305629	9.72E-05	0.268293058	0.01277586	PABPC4
VAT	Heart_Failure	ENSG00000116883.8	-0.006517515	3.77E-05	0.104013135	0.006798613	RP11-268J15.5
VAT	Heart_Failure	ENSG00000123243.10	-0.011163945	0.000408276	1	0.037561413	ITIH5
VAT	Heart_Failure	ENSG00000127957.12	-0.012652278	8.38E-06	0.023115823	0.00165113	PMS2P3
VAT	Heart_Failure	ENSG00000135213.8	-0.00968636	0.000423427	1	0.037698636	POM121C
VAT	Heart_Failure	ENSG00000142694.6	0.011048087	5.87E-05	0.162053448	0.009002969	EVA1B
VAT	Heart_Failure	ENSG00000143631.10	-0.005208116	0.000548707	1	0.04577203	FLG
VAT	Heart_Failure	ENSG00000145777.10	0.008131126	2.23E-06	0.00615126	0.000559205	TSLP
VAT	Heart_Failure	ENSG00000164068.11	0.006439656	3.98E-06	0.010975386	0.000914615	RNF123
VAT	Heart_Failure	ENSG00000164078.8	0.019788337	3.66E-08	0.000100932	1.44E-05	MST1R
VAT	Heart_Failure	ENSG00000167914.6	-0.018985077	4.61E-11	1.27E-07	2.12E-08	GSDMA
VAT	Heart_Failure	ENSG00000170092.10	0.010967044	0.000251127	0.693110117	0.030135222	AC018720.10
VAT	Heart_Failure	ENSG00000179344.12	-0.017737781	1.19E-22	3.30E-19	3.30E-19	HLA-DQB1
VAT	Heart_Failure	ENSG00000179528.11	0.009003784	0.000607779	1	0.046596405	LBX2
VAT	Heart_Failure	ENSG00000183336.7	-0.004722502	0.000378371	1	0.036010503	BOLA2
VAT	Heart_Failure	ENSG00000187145.10	-0.004703193	0.000360208	0.994173902	0.036010503	MRPS21
VAT	Heart_Failure	ENSG00000188895.7	-0.005811648	0.000334166	0.922297947	0.036010503	MSL1
VAT	Heart_Failure	ENSG00000196301.3	0.00989992	6.96E-12	1.92E-08	3.84E-09	HLA-DRB9
VAT	Heart_Failure	ENSG00000196735.7	-0.029201905	4.80E-19	1.32E-15	6.62E-16	HLA-DQA1
VAT	Heart_Failure	ENSG00000197728.5	0.004571214	0.000580442	1	0.04577203	RPS26
VAT	Heart_Failure	ENSG00000204516.5	0.002793396	0.000577761	1	0.04577203	MICB
VAT	Heart_Failure	ENSG00000204520.8	0.006116252	6.79E-06	0.018742897	0.001441761	MICA
VAT	Heart_Failure	ENSG00000205583.9	-0.006316305	1.54E-06	0.004255017	0.00047278	STAG3L1
VAT	Heart_Failure	ENSG00000213676.6	0.007146307	0.000376644	1	0.036010503	ATF6B
VAT	Heart_Failure	ENSG00000214078.7	-0.002215688	0.000529886	1	0.045702697	CPNE1
VAT	Heart_Failure	ENSG00000214194.4	0.014245906	4.96E-05	0.137026576	0.008060387	LINC00998
VAT	Heart_Failure	ENSG00000229391.3	0.0049263	3.94E-05	0.108777811	0.006798613	HLA-DRB6
VAT	Heart_Failure	ENSG00000232629.4	0.008056206	2.35E-14	6.49E-11	2.16E-11	HLA-DQB2
VAT	Heart_Failure	ENSG00000237541.3	0.007866205	1.18E-12	3.27E-09	8.17E-10	HLA-DQA2
VAT	Heart_Failure	ENSG00000240065.3	0.00545849	1.87E-06	0.005156801	0.00051568	PSMB9
VAT	Heart_Failure	ENSG00000243725.2	-0.009904762	0.000267003	0.736928354	0.030705348	TTC4
VAT	Heart_Failure	ENSG00000248290.1	-0.0045749	0.000346877	0.957379669	0.036010503	TNXA
VAT	Heart_Failure	ENSG00000272221.1	0.003118532	8.58E-05	0.236867022	0.011843351	XXbac-BPG181B23.7
