# Supplementary material for: CausalCellInfer: Prioritizing Cell‐Type‐Specific Disease Associations From Genetically Predicted Transcriptomes in Biobank Cohorts
Source: Adv Sci (Weinh). 2026 Oct 14:e78197. Online ahead of print. doi: 10.1002/advs.78197 (PMC13669230; doi:10.1002/advs.78197)
Supplement: Supplementary file 2 [file ADVS-9999-e78197-s002.zip › advs78197-sup-0009-S8.pdf]

Table S8 Number of genes with significant coefficient changes after adjusting for estimated cell proportions

Tissue	Disorder	No. genes with significant coefficient change in the original bulk level TWAS analysis	No. significant genes from original bulk level TWAS analysis	No. significant genes(original TWAS analysis) with significant coefficient change	No. significant genes from adjusted(by estimated cell proportions) bulk level TWAS analysis	No. common significant genes	Proprotion of significant genes with significant change
Frontal_Cortex	Anxiety	174	0	0	0	0	
Frontal_Cortex	Bipolar	0	0	0	0	0	
Frontal_Cortex	Depression	6	3	0	3	3	0
Frontal_Cortex	Psychosis	109	0	0	0	0	
Pancreas	Prediabetes	24	0	0	0	0	
Pancreas	T1DM	683	57	34	56	53	0.596491228
Pancreas	T2DM_admission	368	135	23	132	130	0.17037037
Pancreas	T2DM_insulin	151	20	4	19	17	0.2
Pancreas	T2DM	333	88	18	85	84	0.204545455
SAT	CAD	99	55	5	52	52	0.090909091
SAT	Heart_Failure	1280	52	31	40	36	0.596153846
SAT	HTN	572	364	81	367	350	0.222527473
SAT	Obesity	1031	331	127	299	297	0.383685801
SAT	Stroke	28	0	0	0	0	
SAT	T2DM	1189	176	68	139	138	0.386363636
VAT	CAD	17	30	1	30	28	0.033333333
VAT	Heart_Failure	392	36	7	36	34	0.194444444
VAT	HTN	696	275	68	273	267	0.247272727
VAT	Obesity	966	326	152	293	291	0.466257669
VAT	Stroke	1	0	0	0	0	
VAT	T2DM	679	124	42	99	98	0.338709677

Note: No. genes with significant coefficient change in the original bulk level TWAS analysis indicates genes with significant coefficient changes, some genes with significant coefficient changes are not associated with the target trait based on the TWAS analysis results, here significant genes indicated those that have a adjusted pvalue(FDR) <=0.05
